# Disrupted *Tuzzerella* abundance and impaired l-glutamine levels induce Treg accumulation in ovarian endometriosis: a comprehensive multi-omics analysis

**DOI:** 10.1007/s11306-023-02072-0

**Published:** 2024-02-29

**Authors:** Yichen Chen, Lingfang Ye, Jue Zhu, Liang Chen, Huan Chen, Yuhui Sun, Yishen Rong, Jing Zhang

**Affiliations:** 1 Department of Gynaecology, Women and Children’s Hospital of Ningbo University, Ningbo, 315012 People’s Republic of China; 2 Medical School, Ningbo University, Ningbo, 315000 People’s Republic of China

**Keywords:** Endometriosis, Microbiome, Metabolomics, Proteomics, Immune infiltration

## Abstract

**Introduction:**

The microbial community plays a crucial role in the pathological microenvironment. However, the structure of the microbial community within endometriotic lesions and its impact on the microenvironment is still limited.

**Methods:**

All 55 tissue samples, including ovarian ectopic (OEMs) and normal (NE) endometrium, were subjected to 16S rRNA sequencing, metabolomic and proteomic analysis.

**Results:**

We found the abundance of *Tuzzerella* is significantly lower in OEMs compared to NE tissue (p < 0.01). We selected samples from these two groups that exhibited the most pronounced difference in *Tuzzerella* abundance for further metabolomic and proteomic analysis. Our findings indicated that endometriotic lesions were associated with a decrease in l-Glutamine levels. However, proteomic analysis revealed a significant upregulation of proteins related to the complement pathway, including C3, C7, C1S, CLU, and A2M. Subsequent metabolic and protein correlation predictions demonstrated a negative regulation between l-Glutamine and C7. In vitro experiments further confirmed that high concentrations of Glutamine significantly inhibit C7 protein expression. Additionally, immune cell infiltration analysis, multiplex immunofluorescence, and multifactorial testing demonstrated a positive correlation between C7 expression and the infiltration of regulatory T cells (Tregs) in ectopic lesions, while l-Glutamine was found to negatively regulate the expression of chemotactic factors for Tregs.

**Conclusion:**

In this study, we found a clear multi-omics pathway alteration, “*Tuzzerella* (microbe)—l-Glutamine (metabolite)—C7 (protein),” which affects the infiltration of Tregs in endometriotic lesions. Our findings provide insights into endometriosis classification and personalized treatment strategies based on microbial structures.

**Graphical abstract:**

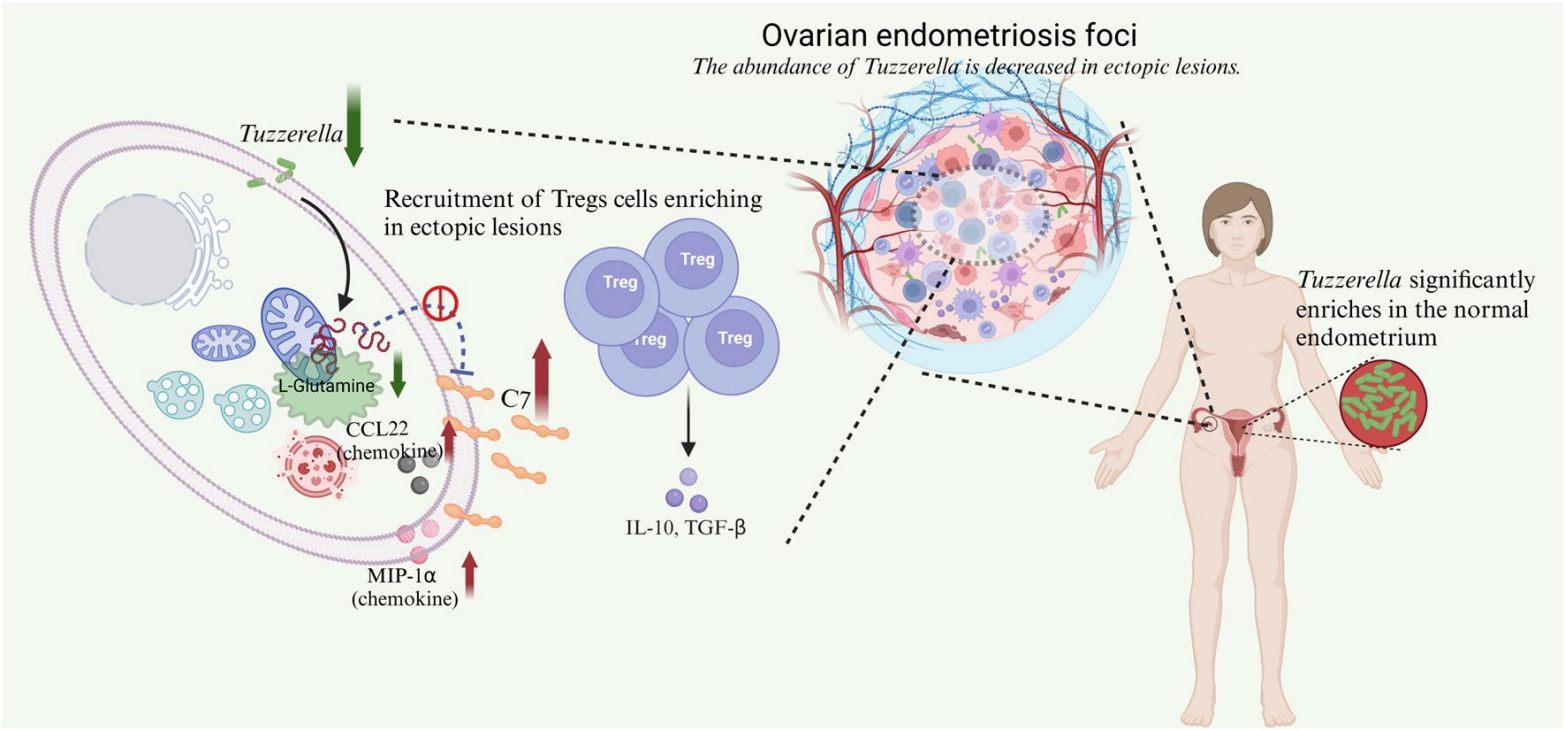

**Supplementary Information:**

The online version contains supplementary material available at 10.1007/s11306-023-02072-0.

## Introduction

Endometriosis is a chronic gynecological disease that is estrogen-dependent, characterized by the ectopic implantation and growth of endometrial stromal cells and glandular epithelial cells outside the uterine cavity (Saunders & Horne 2021; Vercellini et al., 2014). Although surgical removal of endometriotic lesions through hysteroscopic excision can greatly alleviate symptoms, the recurrence rate within 5 years is as high as 40–50% (Shakiba et al., 2008), (Guo, 2009), significantly affecting patients’ quality of life. Unfortunately, the underlying mechanisms of endometriosis remain unclear.

Research on the pathogenesis of endometriosis has shifted its focus from solely studying the ectopic endometrium itself to the microenvironment within the ectopic lesions. Microbes, metabolism, and immune cells are important components of the lesion microenvironment. Previous studies have shown that dysbiosis and depletion of *lactobacilli* in the cervicovaginal microbiota are associated with endometriosis and infertility (Mitchell & Marrazzo, 2014). Yu et al. found that the diversity of gut microbiota is reduced in patients with endometriosis (Chen et al., 2017). However, few research investigated the association between alterations in the microbial community structure in lesion tissues and abnormal immune microenvironment.

Regulatory T cells (Tregs) regulate abnormal/excessive immune responses to self and non-self antigens in order to maintain immune balance (Panduro et al., 2016). Some studies have confirmed the abnormal elevation of chemokines CCL17, CXCL12, and CX3CL1 in ectopic endometriotic lesions, which recruit Tregs from the thymus and lymph nodes to exert an inhibitory effect on immune cells (Hey-Cunningham et al., 2022). In this study, we conducted a linkage analysis of microbial structure, tissue metabolism, and proteins in ectopic endometrial tissue and normal endometrial tissue. Additionally, we analyzed the infiltration of immune cells using proteomics. Through this research, we aim to provide evidence for the microbial structure-induced changes in metabolite levels and protein expression and alterations in the immune environment of endometriotic tissue, in order to explore the pathogenesis of endometriosis and develop new therapeutic approaches.

## Methods and materials

### Patients

A total of 45 women with endometriosis (OEMs group, n = 23) and without endometriosis (Uterine fibroids patients, control group, n = 22) who were admitted to Ningbo Women & Children’s Hospital between November 2022 and May 2023 were recruited for this study (Sample information is presented in Table 1). All participants underwent laparoscopic surgery to determine the presence and staging of endometriosis. The specimen was confirmed by pathologists after surgery. None of the participants had received hormone therapy in the 6 months prior to surgery. Informed consent was obtained from all participating patients. Samples were collected in sterile centrifuge tube on ice, and then immediately transferred to the laboratory and frozen at − 80 °C for further analysis.

**Table 1:** Subject’s characteristics in women with and without ovarian endometriosis

Parameters	Endometriosis (n = 23)	No endometriosis (n = 22)	p value
Age(years)	34.78 ± 6.82	37.23 ± 8.23	0.28
Menstrual cycle phase			
Proliferative	22 (95.7%)	21 (95.4%)	
Secretory	1 (4.3%)	1 (4.6%)	
Ovarian endometriosis r-AFS staging score			
I (1–5)	0		
II (6–15)	0		
III (16–40)	9 (39.13%)		
IV (> 40)	14 (60.87%)		
Size of Cyst (cm^3^)			
< 64 cm^3^	4 (17.39%)		
< 216 cm^3^, ≥ 64 cm^3^	3 (13.04%)		
≥ 216 cm^3^	16 (69.57%)		
Tumour marker indicators			
CA125 (U/mL)	50.58 ± 59.74 (16/23)	15.52 ± 5.69 (17/22)	0.0217*
AFP (pg/mL)	2.00 ± 0.37 (15/23)	2.86 ± 1.4 (17/22)	0.028*
CA199 (U/mL)	11.08 ± 6.29 (15/23)	4.82 ± 2.56 (17/22)	0.0007****
CEA (pg/mL)	1.068 ± 0.69 (15/23)	0.88 ± 0.25 (17/22)	0.3022
VAS (> 5)	4 (17.39%)		

### Microbiota sequencing and bioinformatic analysis

Microbial DNA was isolated from endometrial samples using the MagPure Soil DNA LQ Kit (Magen, Guangdong, China) according to the manufacturer’s instructions. The amplicons were purified with Agencourt AMPure XP beads (Beckman Coulter Co., United States) and quantified using Qubit dsDNA assay kit and performed at Oebiotech Biotech Co., Ltd. (Shanghai, China) using the Illumina NovaSeq 6000 with 250 bp paired-end reads.

The library sequencing and data processing were conducted by OE biotech Co., Ltd. (Shanghai, China). The software then generated the representative reads and the abundance table for each ASV. The representative read for each ASV was selected using the QIIME2 package. To assess beta diversity, the unweighted Unifrac distance matrix was generated using an R package.

### LC–MS analysis

Samples were handled according to the standardization of the LC–MS assay. The chromatographic separations were carried out using a Thermo Scientific UltiMate 3000 HPLC system equipped with an ACQUITY UPLC BEH C18 column maintained at 35 °C for reverse phase separation. The original LC–MS data underwent several processing steps, including baseline filtering, peak identification, integration, retention time correction, peak alignment, and normalization, using Progenesis QI V2.3 software (Nonlinear Dynamics, Newcastle, United Kingdom). Compounds with scores below 36 (out of 60) points were also considered inaccurate and excluded. Tissue sample analyses were performed at Oebiotech Biotech Co., Ltd. (Shanghai, China).

### TMT quantitative proteomic analysis

A total of 15 endometrial tissues included normal endometrium group (n = 7) and ectopic endometrium group (n = 8) were subjected to tandem mass tags (TMT) proteomic analysis (Oebiotech Biotech Co., Ltd., China). The samples were extracted, lysed, and marked with TMT, and then prepared for Liquid chromatography-mass spectrometer (LC–MS) detection. The differentially expressed proteins were further used for enrichment analysis. Spectronaut (Version 15.3.210906.50606) was used to search all of the raw data thoroughly against the sample protein database.

### RNA isolation and real-time qPCR

Total RNA was extracted from tissues using TRIzolTM reagent (Life Technologies, USA), following the manufacturer’s instructions. The cDNA was synthesized using a transcription kit as per the manufacturer’s instructions (Takara, Japan). The qPCR analysis was conducted using TB Green Premix Ex Taq II ROX plus (Takara, Japan) on an Applied ABI Q1 Real-Time PCR System (Thermo, USA). After the initial denaturation step at 95 °C for 30 s, two-step amplification was performed (95 °C for 5 s, 60 °C for 30 s) for 40 cycles. The results were calculated with 2^−∆CT^. The primer sequences were displayed in Table 2.

**Table 2:** PCR primer sequences used in experiments

Primer name	Sequence 5′–3′
C3 F	CGCAACAAGTTCGTGACCG
C3 R	GATGCCTTCCGGGTTCTCAAT
C7 F	CTGAGTGGAAATGTCCTGTCC
C7 R	CGCTTCCGACTAGATGATGTGT
C1S F	TCCAAGTCCCATACAACAAACTC
C1S R	CAAACCCCGTAAAACGCTCT
CLU F	CTACTTCTGGATGAATGGTGACC
CLU R	CGGGTGAAGAACCTGTCCT
A2M F	CAGTGGACAGCTAAACAGCC
A2M R	GGGACGCCTTTCCCATCTAC

### Cell culture and l-glutamine treatment

Primary normal endometrial cells (NE), ectopic endometrial cells (EC) were isolated and incubated in DME/F12 medium (BI, USA) supplemented with 10% fetal bovine serum (FBS) (SERANA, Germany), 1% penicillin (100 U/mL) and streptomycin (100 μg/mL) at 37 °C and 5% CO_2_ in a humidified incubator. Glutamine powder was purchased from Yuanye Company(Shanghai) and l-glutamine concentrations of 20 and 40 mM were fed onto ectopic endometrial cells during 24 h. Glutaminase inhibitor-A-446 was obtained from MedChemExpress (MCE, USA). The supernatant from the cells was collected, and the cellular proteins were isolated.

### Western blot

The total proteins of cells were lysed with RIPA buffer supplemented with proteinase inhibitors (Biosharp, China), as per manufacturer’s protocols. Protein concentrations were determined using the BCA protein assay kit, as previously described. Protein samples were (25 μg) separated by 10% sodium dodecyl sulfate–polyacrylamide gel electrophoresis (SDS-PAGE), and transferred onto 0.22 μm polyvinylidene difluoride (PVDF) membranes. Primary antibodies were incubated overnight at 4 °C. The following primary antibodies were used in this blotting: anti-C7 (1:1000; Affinity, China), anti-beta-actin (1:3000; Affinity, China) followed by incubation with appropriate horseradish peroxidase-conjugated secondary antibodies at room temperature for 1 h. Subsequently, immune-reactive protein bands were visualized with chemiluminescence reagents (Biosharp, China), followed by imaging on an electrophoresis gel imaging analysis system (Tanon, Shanghai, China).

### Multiplex immunofluorescence (mIF)

Tissue sections were then permeabilized by 0.2% TritonX-100 (Sigma Aldrich) for 10 min. Unspecific bindings were blocked by using PBS + 5% Normal goat serum for 1 h at RT. Tissue sections were incubated overnight at 4 °C with the following anti-CD3 (1:100; rat, abcam, USA), anti-FOXP3 (1:100; mouse, abcam, USA), anti-C7 (1:100; rabbit, affinity, China). The second day, slides were washed 3 times in PBS and incubated with the secondary antibody: Goat anti-rabbit 647 antibody (1:500, abcam, USA), Dnk anti-rat 555 antibody (1:500, abcam, USA), Goat anti-mouse 488 antibody (1:500, abcam, USA) for 1 h at RT and slides were washed 3 times in PBS again. Finally, sections were mounted using Fluorescent Mounting Medium and visualized under the microscope (APX100, Olympus, Japan).

### Analyses of 40-plex cytokines/chemokines using the luminex system

The expression levels of 40 cytokines/chemokines (IL-1b, IL-2, IL-4, IL-6, IL-8, IL-10, IL-16, TNF-a, IFN-g, GM-CSF, CCL1/I-309, CCL2/MCP-1, CCL3, CCL7, CCL8, CCL11, CCL13, CCL15, CCL17, CCL19, CCL20, CCL21, CCL22, CCL23, CCL24, CCL25, CCL26, CCL27, CXCL1, CXCL2, CX3CL1, CXCL5, CXCL6, CXCL9, CXCL10, CXCL11, CXCL12, CXCL13, CXCL16, and MIF) were measured using the Bio-Plex Pro Human Chemokine Panel 40-plex kit (#1171AK99MR2) with the Bio-Plex™ 200 system (Bio-Rad, Hercules, CA, USA) by Jingxi Biosupport(Ningbo, China). Each experimental run included appropriate quality controls, which were performed in duplicate.

### Statistics

Statistical analysis was performed by using GraphPad Prism 8.0 (GraphPad Software, USA). All the results were expressed as the mean ± standard error of the mean (SEM). The normality of the data was tested with the Shapiro–Wilk test. An unpaired Student’s *t*-test (two-tailed) was used to identify statistically significant differences between the two groups. Pearson correlation was used to analyze the correlation. A p value of < 0.05 was considered statistically significant.

## Result

### Analysis of microbial abundance and diversity in ectopic endometrial tissue

**Fig. 1:**
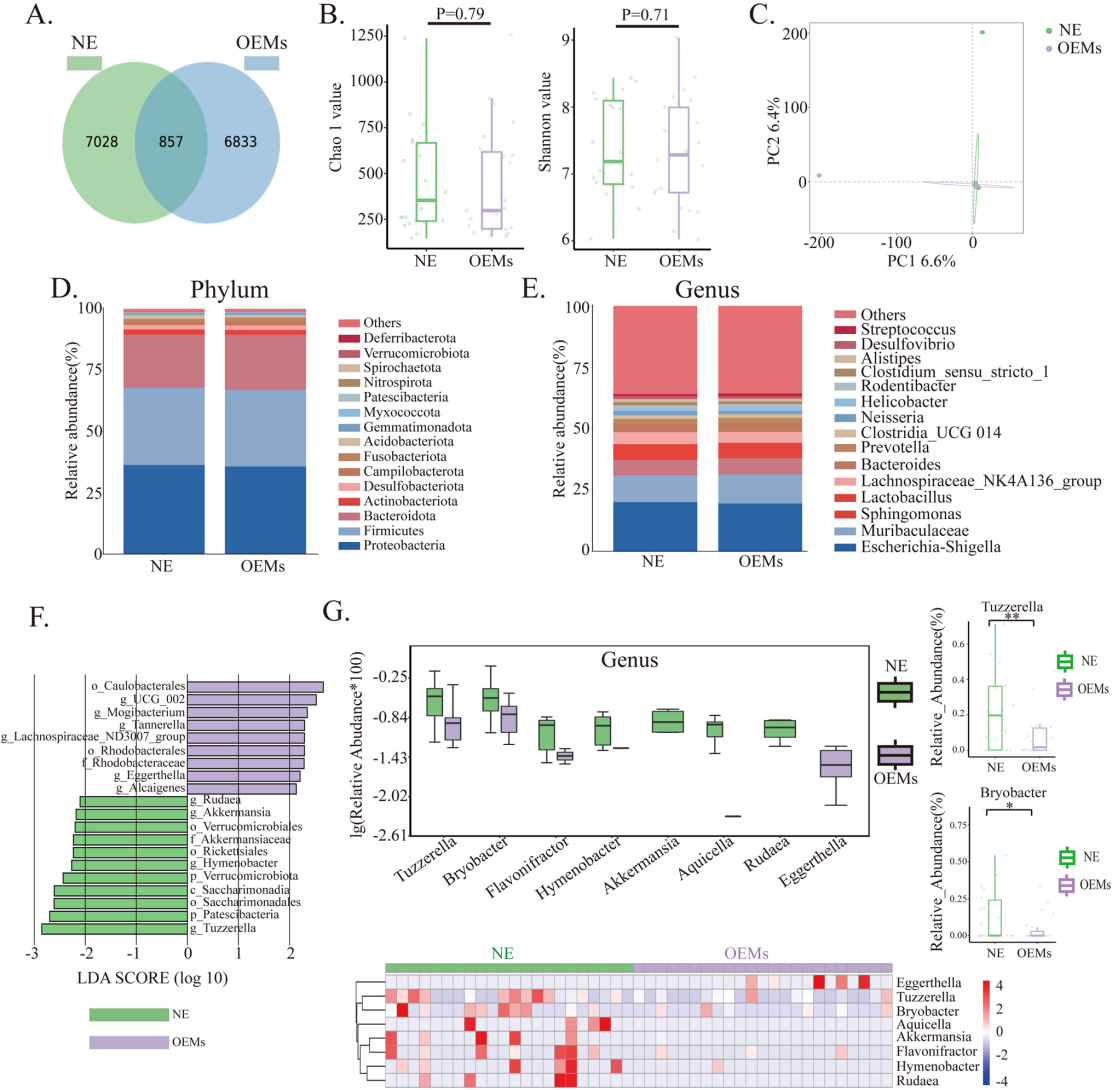
Overall microbiota composition in ectopic endometrial tissue and normal endometrial tissue. **A** Venn diagram. **B** The Chao1 and Shannon indices. **C** The principal component analysis (PCA). **D** The barplot of phylum community structure in NE group and OEMs group. **E** The barplot of phylum community structure in NE group and OEMs group. **F** The LEfe table of Meta Statistic. **G** Differential microbial genera in NE group and OEMs group. *NE* normal endometrium group, *OEMs* ovarian endometriosis. **p < 0.01; *p < 0.05

Based on pyrosequencing of barcoded 16S rRNA genes (V3-V4), we evaluated 45 endometrial samples and acquired 2,728,218 qualified sequences (median = 62,275) and 14,718 OTUs (Supplementary Table S1). A Venn diagram was used to identify the 857 out of 14,718 OTUs shared by the NE and OEM groups (Fig. 1A). The Chao1 and Shannon indices, which describe the richness and diversity of the microbiota and were used to profile the structure of endometrial tissue microbiota in OEMs, were not statistically significant (Chao1 value, p = 0.79 and Shannon value, p = 0.71, respectively (Fig. 1B). The microbial community structure of the two groups was quite comparable, according to principal component analysis (PCA) based on variance decomposition to represent the variations in composition (PC1:6.6%; PC2:6.4%) (Fig. 1C). The relative abundance of various species was used to analyze the spread of microbiota. In the NE and OEMs groups, the three largest phyla were *Proteobacteria*, *Firmicutes*, and *Bacteroidota*, which contributed for 89.36 and 89.44% of total OTUs, respectively (Fig. 1D). Figure 1E depicted the top 15 bacterial community compositions at the genus level. We also used Wilcoxon rank sum tests at the phylum and genus levels to gain a more precise picture of the differences in endometrial microorganisms between the two groups (Supplementary Table S2). *Tuzzerella* (genus), *Patescibacteria* (phylum), *Saccharimonadales* (order), and *Saccharimonadia* (class) were substantially overrepresented in the NE group (LDA scores (log10) − 2.5) (Figure F). The Next, we concentrated on distinct bacteria in the genus community structure. Compared to the NE group, *Tuzzerella*, *Bryobacter*,*Flavonifractor*, *Hymenobacter*, *Akkermansia*, *Aquicella*, *Rudae*a, *Eggerthella* were enriched in the OEMs group (all p < 0.05), most importantly, *Tuzzerella* was the bacteria with the most significant enrichment differences in the OEMs group (p < 0.01, Fig. 1G). The similarity of microbial community structure between ectopic endometrial tissue and normal endometrial tissue provides direct evidence for the hypothesis that ectopic lesions are formed by the migration and settlement of normal endometrium. However, due to the environmental factors within ectopic lesions, slight changes have occurred in the structure of some microorganisms.

### Global overview of metabolism in ectopic endometrial lesions with low abundance of *Tuzzerella*

**Fig. 2:**
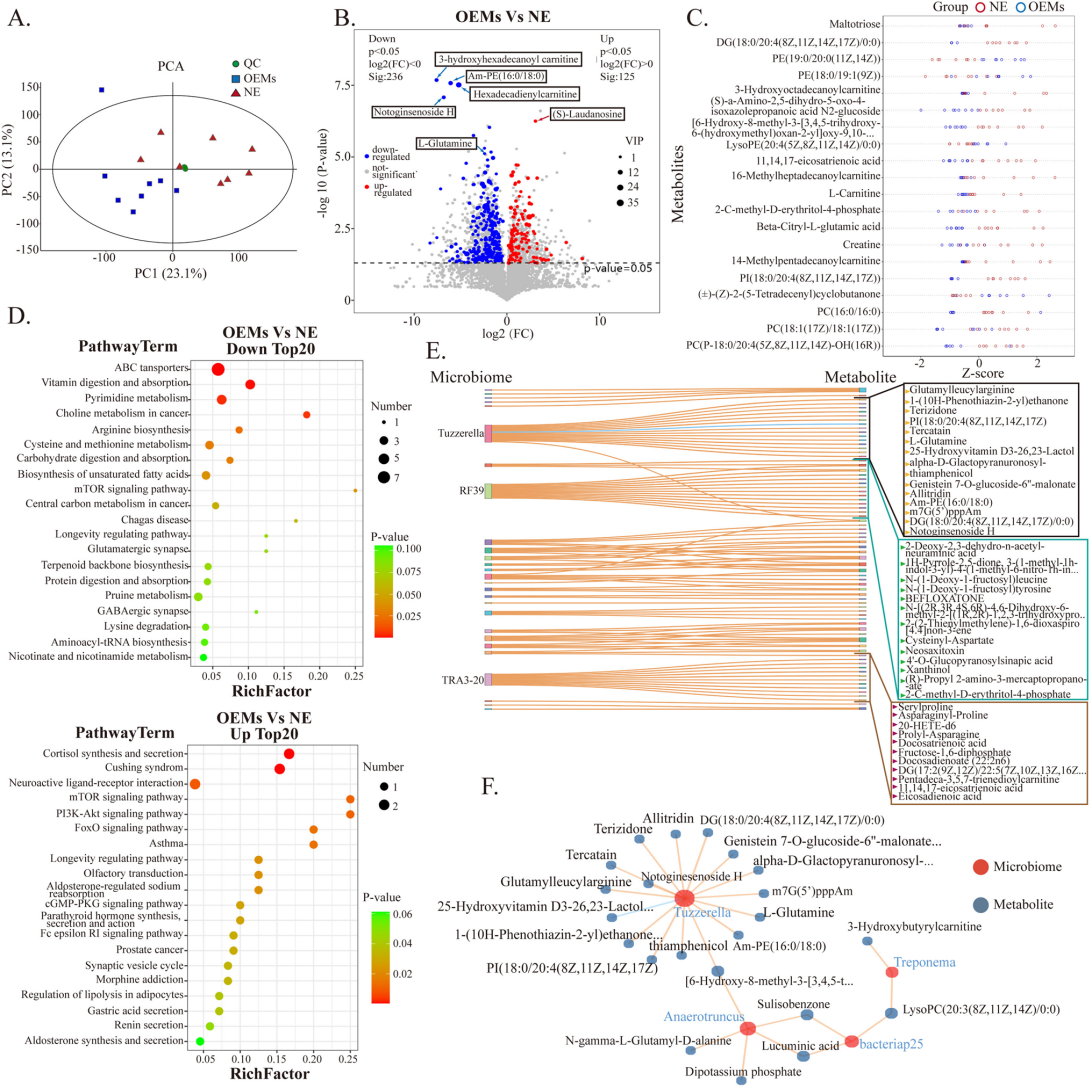
Overall analysis of differential metabolites in ectopic endometrium with low abundance of *Tuzzerella* and normal endometrium. **A** The principal component analysis (PCA). **B** Volcano plot of differential metabolites. **C** The unit-less Z-score values in two groups. **D** Top 20 upregulated and downregulated KEGG pathways (OEMs vs NE). **E** A Sankey diagram depicting the top 100 bacteria and metabolites. **F** Metabolites associated with *Tuzzerella*. *VIP* variable important in projection, *NE* normal endometrium group, *OEMs* ovarian endometriosis

In order to clarify whether there is also a certain similarity in metabolites between the two groups, we selected 15 samples (8 normal endometrial tissues and 7 ectopic endometrial tissues) with low abundance of *Tuzzerella* in pyrosequencing for metabolite analysis. Unlike the PCA results in the microbial community structure analysis, we found that there was a significant difference in the distribution of the two sample groups in PCA (PC1: 23.1%; PC2: 13.1%) (Fig. 2A). In the subsequent analysis, we initially classified the metabolites that were identified into two distinct categories: human metabolites and microbial metabolites. By doing so, we aimed to differentiate between the metabolites that originated from human cells and those that were produced by microorganisms present in the biological samples under investigation. We separately analyzed the unique metabolites of bacteria, short-chain fatty acids and secondary bile acids (Nicholson et al., 2012), but there was no significant difference between the two groups of metabolites (Supplementary Fig. S1, Table S3). According to the volcano plot, we found that in the comparison of the two sample groups (OEMs group vs NE group), there were 236 significantly down-regulated metabolites (log2(FC) < 0, p < 0.05) and 125 significantly up-regulated metabolites (log2(FC) > 0, p < 0.05). We noticed that compared with the NE group, there were more significantly down-regulated metabolites in the OEMs group, such as 3-hydroxyhexadecanoul carnitine, AM-PE(16:0/18:0), Hexadecadienylcarnitine, Notoginsenoside H, and l-Glutamine (Fig. 2B). At the same time, we converted the two sets of data into unit-less Z-score values using (x − μ)/δ, and then used Variable important in projection (VIP) to extract the top 20 different metabolites. The results indicated that the top 20 different metabolites were mainly concentrated in the NE group (Fig. 2C). We used KEGG to enrich the relevant pathways of different metabolites and showed that ABC transporters(the most significant enrichment, p < 0.0001, FDR correction = 0.003772561), Vitamin digestion and absorption, Pyrimidine metabolism, and Choline metabolism in cancer were the most significant pathways in down-regulated metabolism, while Cortisol synthesis secretion(the most pronounced enrichment, p = 0.000478233, FDR correction = 0.011850772), Cushing syndrome, mTOR signaling pathway, and FoxO signaling pathway were the most significant pathways in up-regulated metabolism (Fig. 2D). In order to further understand the relationship between different bacterial communities and different metabolites, we conducted a combined analysis of microbial diversity and metabolome. Firstly, we used the random forest method to analyze the importance of different tissues in microbial and metabolite communities for the current grouping. The higher the importance results showed that *Tuzzerella*, a different genus at the genus level, had the highest importance value of 0.014, with O_C: p value = 0.000999 (Supplementary Table S4). Under the premise of significance, we plotted the relationship between the microbiota and metabolites with the largest absolute correlation coefficient between different bacterial communities and different metabolites. The top 100 bacteria and metabolites were displayed in a Sankey diagram. The diagram marked the three microbiota that had the most correlations with different metabolites: *Tuzzerella*, *RF39*, and *TRA3-20*, and their respective correlated metabolites (Fig. 2E). Finally, we focused on *Tuzzerella*, the microbiota with the highest importance value, and its related metabolites. We found that most of the metabolites related to it were down-regulated in the OEMs group (Fig. 2F).

### Aberrant activation of complement signaling pathway in ectopic lesions with low abundance of *Tuzzerella*

**Fig. 3:**
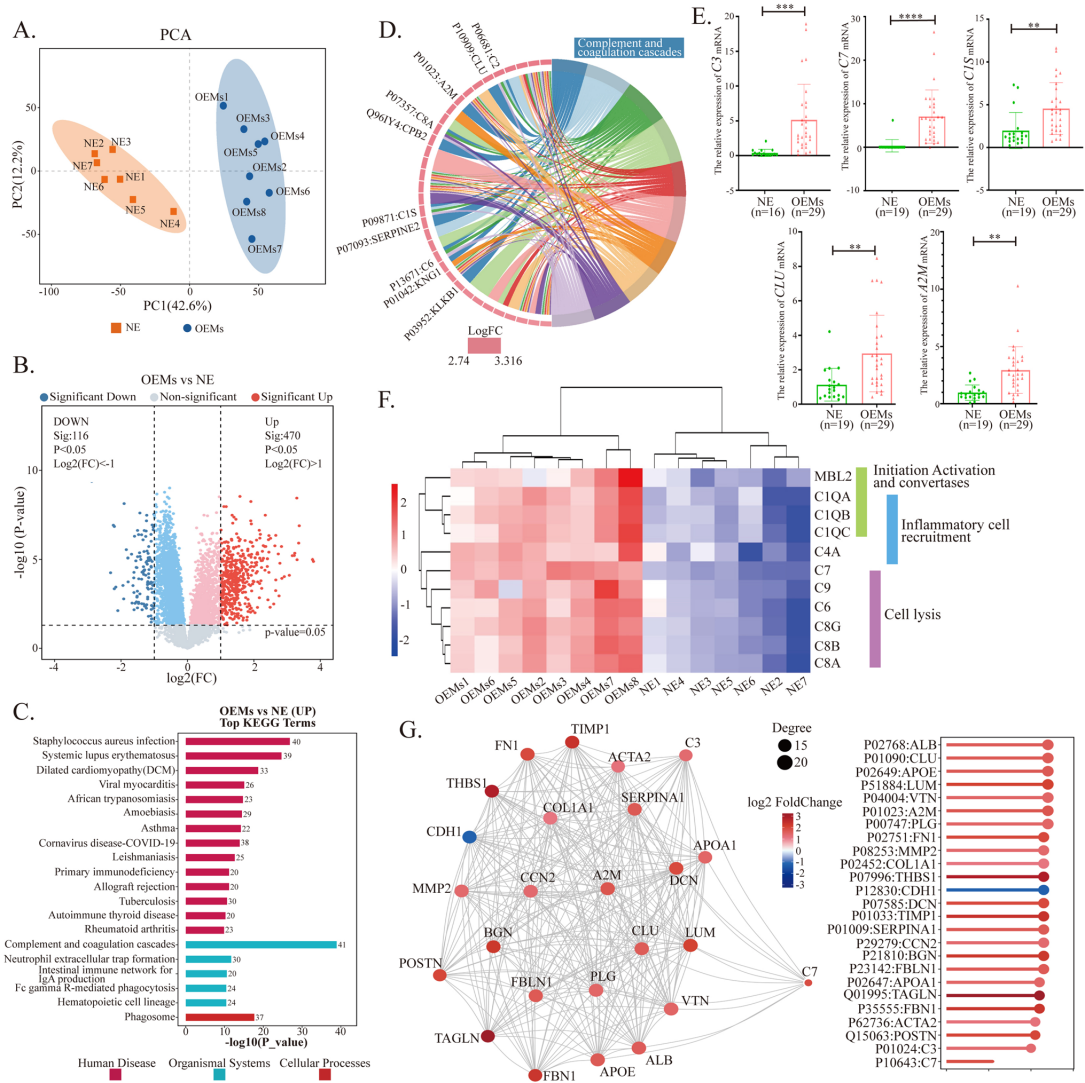
Complement-related proteins exhibit abnormally high expression in ectopic endometrial tissue with low abundance of *Tuzzerella*. **A** The principal component analysis (PCA). **B** Volcano plot of differential proteins. **C** Display of significantly upregulated KEGG pathways (OEMs group vs NE group). **D** The chord diagram generated from KEGG enrichment analysis. **E** qPCR validation of *C3*, *C7*, *C1S*, *CLU*, and *A2M* mRNA expression in ectopic and normal endometrium. The mRNA expression levels were calculated using 2^−∆CT^ formula. **p < 0.01, ***p < 0.001, ****p < 0.0001. **G** Heatmap representation of complement proteins expressed in tissues with different biological functions. **F** Protein–protein interaction (PPI) network visualization of the correlation between complement proteins and other proteins. *NE* normal endometrium group, *OEMs* ovarian endometriosis

It is well known that changes in biological behavior are the ultimate manifestation of differential protein expression. Therefore, we conducted proteomic profiling of two groups of tissues. We selected 15 samples, including 7 samples from the NE group and 8 samples from the OEMs group (low abundance of *Tuzzerella*). The PCA results indicate significant differences in protein expression between the two groups (Fig. 3A). The volcano plot shows that, compared to the normal endometrium group, there are 470 upregulated differentially expressed proteins (Log2(FC) < 1, p < 0.05) and 116 downregulated differentially expressed proteins (Log2(FC) > 1, p > 0.05) in the ectopic endometrium group (Fig. 3B). We performed KEGG pathway enrichment analysis on the differentially expressed proteins and found that the pathway with the most upregulated proteins (41 differentially expressed proteins) is the Complement and coagulation cascades pathway (Fig. 3C). Moreover, the upregulated and downregulated differentially expressed proteins, enriched in KEGG level 2 metabolic pathways, are most prominently associated with immunity (Supplementary Fig. S2). According to the results of Gene Set Enrichment Analysis (GSEA), the enrichment score (ES) for Complement activation, classical pathway is 0.817, absolute normalized enrichment score (NES) is 2.82, p < 0.001, and FDR < 0.001, indicating significant enrichment of differentially expressed proteins in this pathway (Supplementary Fig. S3). The chord diagram generated from KEGG enrichment analysis also clearly identifies the proteins enriched in the Complement and coagulation cascades pathway, including C2, CLU, A2M, C8A, CPB2, C1S, SERPINE2, C6, KNG1, and KLKB1 (selecting the top 10 entries with ListHits > 2 and the smallest p-value) (Fig. 3D). We validated the mRNA expression levels of several key proteins, such as C3, C7, C1S, CLU, and A2M, in the OEMs and NE tissue. The qPCR results showed that the mRNA expression levels of these genes were significantly higher in the OEMs group (n = 29) compared to the NE group (n = 16). Specifically, the expression of *C7* mRNA was the most significant (****, p < 0.0001) (Fig. 3E). Different complement-related proteins have different functions. We found that proteins associated with Initiation Activation and Convertases, such as MBL2 and C1Q, proteins associated with Inflammation cell recruitment, such as C1Q and C4A, and proteins associated with Cell lysis, such as C7, C9, C6, and C8, are highly expressed in the OEMS group samples (Fig. 3F). Furthermore, protein–protein interaction analysis using the STRING database (blast e-value: 1e-5) was performed on the differentially expressed proteins. The protein–protein interaction network diagram was generated by selecting the top 25 proteins based on connectivity. It revealed that C7 protein is associated with migration-related proteins and lipid metabolism-related proteins, suggesting that it may be a key node protein in this network (Fig. 3G).

### The “Tuzzerella-l-glutamine-C7” axis is present within ectopic lesions

**Fig. 4:**
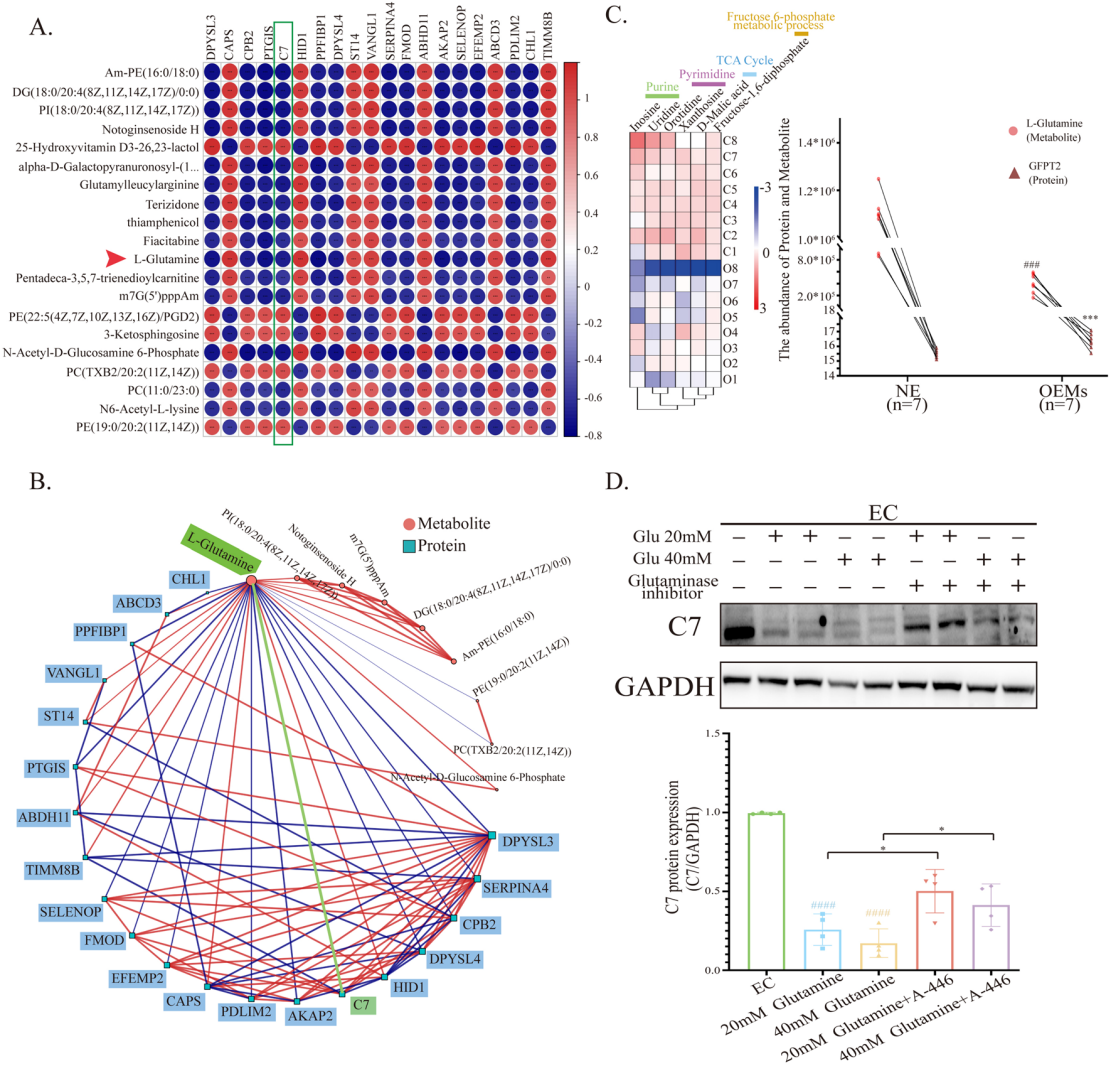
Glutamine negatively regulates the expression of C7 in ectopic endometrial tissue with low abundance of *Tuzzerella*. **A** Significant matrix plot depicting the connections between differential proteins and differential metabolites. **B** Cornetwork plot illustrating the relationships between differential proteins and differential metabolites. **C** Heatmap showing the downstream related metabolites of l-Glutamine, and the negative correlation between l-Glutamine and GFPT2. **F** Western blot analysis demonstrating the expression of C7 after treatment with high concentrations of glutamine. ####, p < 0.0001, vs EC; *, p < 0.05; A-446: glutaminase inhibitor

We conducted a correlation analysis on the top 20 differentially expressed proteins and metabolites in order to identify their associations. We found a negative correlation between protein C7 in the Complement activation, classical pathway and the metabolites related to *Tuzzerell* (Fig. 4A). In Fig. 4B, we observed that only l-Glutamine was connected to most of the correlated proteins among these differentially expressed metabolites, including C7 (Fig. 4B). Therefore, we selected l-Glutamine as the object of interest for further investigation. In proteomics, we found that the rate-limiting enzyme GFPT2, which is associated with l-Glutamine, had high expression in ectopic lesions, opposite to the level of l-Glutamine. However, in the downstream metabolic pathways related to l-Glutamine, such as the tricarboxylic acid cycle and Fructose-6-Phosphate metabolic process, only the levels of d-Malic acid and Fructose-1,6-diphosphate showed positive correlation with l-Glutamine, while the levels of other key metabolites showed no statistical difference between NE and OEMs groups. On the other hand, the downstream metabolites associated with l-Glutamine metabolism, such as Inosine, Uridine, Orotidine, and Xanthosine, were significantly elevated in the NE group. Based on these results, we speculate that the increased expression of the rate-limiting enzyme GFPT2 is compensatory and only serves to maintain normal cellular physiological function. Furthermore, we found that the expression of the transport proteins SLC1A5 and SLC7A5, which are associated with the intake and excretion of l-Glutamine, showed no statistical difference between the two groups (data not shown), indicating that the low levels of l-Glutamine in ectopic endometrial tissue may be caused by a deficiency in *Tuzzerell*, which can be taken up by cells (Fig. 4C). Next, to verify the relationship between glutamine and C7, we added high concentrations of glutamine (20 mM and 40 mM) to primary ectopic endometrial cells in an in vitro experiment. After 24 h, Western blot analysis showed that high concentrations of glutamine could reduce the expression but inhibitor could reverse this effect (Fig. 4D), confirming the negative correlation between glutamine and C7 expression.

### Enrichment of tregs in ectopic lesions may be caused by elevated expression of C7

**Fig. 5:**
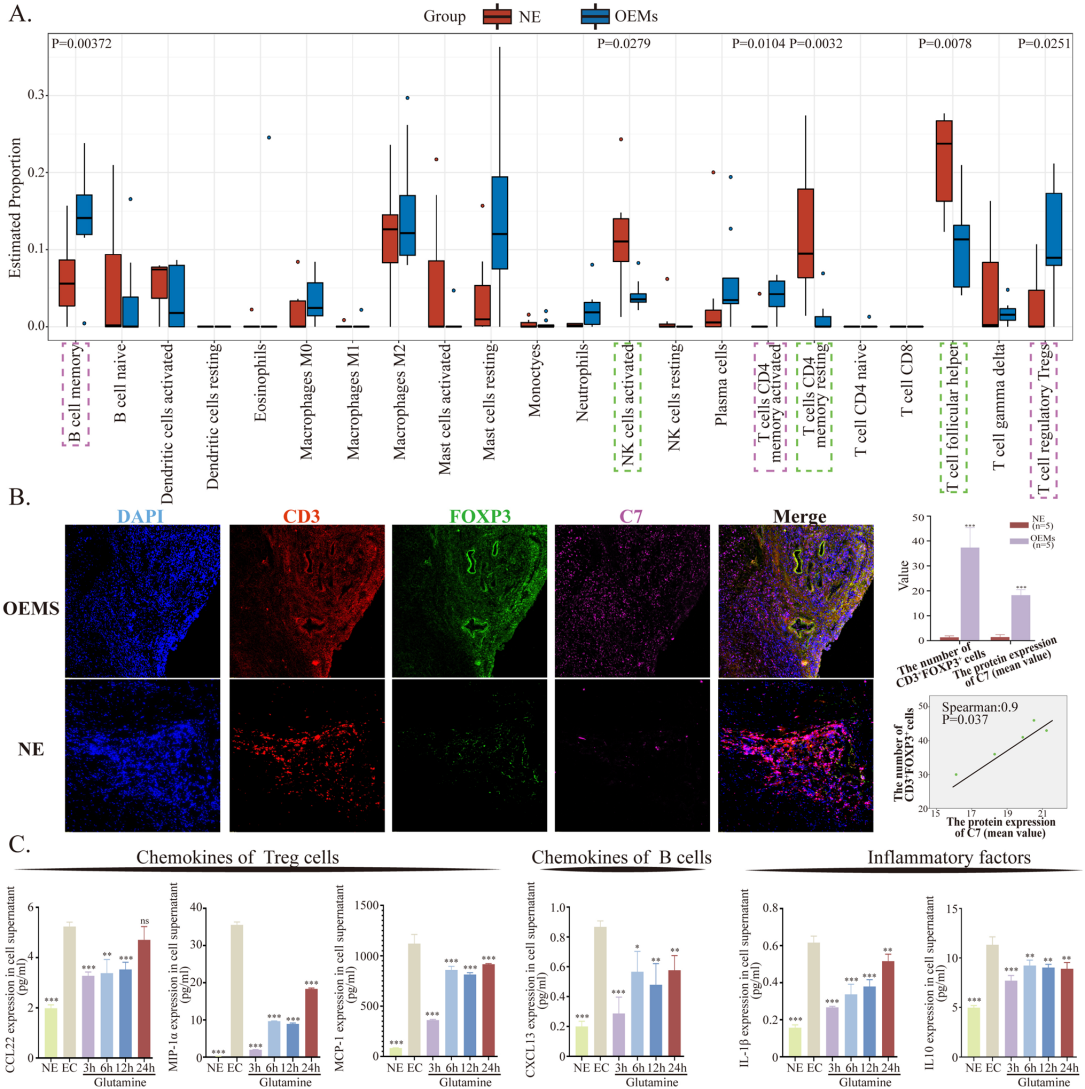
The levels of glutamine and high expression of C7 within ectopic lesions are correlated with the infiltration quantity of Tregs. **A** Analysis of immune cell infiltration. **B** Multiple fluorescence detection of FoxP3 + CD3 + Tregs, with C7 protein labeled with 647 fluorescence. The expression level of C7 protein is positively correlated with the infiltration quantity of Tregs. **C** Expression profile of chemokines. **, p < 0.01. ***, p < 0.001

In order to explore the influence of microbiota-metabolism-protein interplay on immune cell infiltration in endometriosis lesions, we employed proteomics to analyze immune cell infiltration. This analysis revealed that B memory cells, CD4^+^ T memory cells, and T regulatory cells (Tregs) exhibited significantly higher levels of infiltration in endometriosis lesions compared to normal tissue (p = 0.00372, p = 0.0104, and p = 0.0251, respectively). Conversely, NK activated cells, CD4^+^ memory resting cells, and T follicular helper cells showed decreased infiltration in endometriosis lesions (p = 0.0279, p = 0.0032, and p = 0.0078, respectively) (Fig. 5A). The immunosuppression of T cells has been a focal point of our research, and the increased number of Treg cells disrupts immune balance and inhibits the biological function of T effector cells. Therefore, we selected Treg cells for further validation to determine whether the infiltration number of Treg cells is associated with the protein C7 in the Complement and Coagulation Cascades pathway. Multicolor immunofluorescence staining(mIF) was performed on normal endometrial tissue (n = 5) and endometriosis lesions (n = 5). The results showed a significant increase in the expression of protein C7 and the number of CD3^+^FOXP3^+^ Treg cells in endometriosis lesions compared to normal endometrium (protein C7 expression, NE groups vs. OEMs groups, p < 0.001; CD3^+^FOXP3^+^ Treg cell number, NE groups vs. OEMs groups, p < 0.001). Importantly, within endometriosis lesions, the expression of protein C7 was positively correlated with the number of CD3^+^FOXP3^+^ Treg cells (Spearman: 0.9, p = 0.037) (Fig. 5B). Lastly, we investigated the potential association between the aggregation of Treg cells and reduced levels of glutamine in endometriosis lesions. Primary endometrial cells from endometriosis lesions were treated with high concentrations of glutamine at different time points, and the conditioned medium was collected for multiplex detection of chemotactic factors. Our results showed that after glutamine treatment, the expression of Treg cell-related chemokines CCL22, MIP-1α, and MCP-1 was significantly downregulated with statistical significance. Additionally, B cell-related chemokines CXCL13, the inflammatory factor IL-1β, and IL10 also exhibited decreased expression with p < 0.01 (Fig. 5C) (The expression of other relevant factors can be found in the supplementary Figure S4).

## Discussion

The microbiota is essential for metabolism and the maintenance of the body’s physiological functioning. Imbalances in the microbiota can have detrimental effects and contribute to the development of various diseases (Zhou et al., 2020). Recent research has provided evidence demonstrating that endometriosis can cause alterations in the microbiota, and that antibiotics can be effective in treating this condition. The microbiotas present in endometriosis patients have consistently shown reduced dominance of *Lactobacillus*, along with an increased presence of bacteria associated with bacterial vaginosis and other opportunistic pathogens (Itoh et al., 2011; Khodaverdi et al., 2019). Several potential explanations have been proposed to explain the implications of dysbiosis in endometriosis, including the bacterial contamination theory, immune activation (Laschke & Menger, 2016; Symons et al., 2018), impaired gut function due to cytokines (Bedaiwy et al., 2007; Plaza et al., 1997), altered metabolism and signaling of estrogen (Baj et al., 2019; Chen et al., 2015), and disrupted homeostasis of progenitor and stem cells (Hufnagel et al., 2015; Kwon et al., 2015). However, it is not difficult to see that the majority of research has focused on the connection between gut microbiota diversity and endometriosis. With the deepening research on host-microbe interactions, the concept of tumor-associated microbiota within tumor tissues has been proposed. In the field of endometriosis, Hernandes et al. (2020) found that compared to normal endometrium, there is greater microbial diversity in ectopic endometrium and endometriotic lesions (Hernandes et al., 2020). However, in our study, chao1 and Shannon indices also indicate no statistically significant differences in the overall microbial structure between the two groups, except for some differences at the genus level. The reason for the discrepancy with Hernandes et al.’s study might be that they had only 5 samples in each group, while we had more than 20 samples per group (Hernandes et al., 2020). On the other hand, Svensson et al.’s (2021) study also did not find any significant differences in the abundance of bacterial classes between patients with or without isolated ovarian endometriosis, involvement of the gastrointestinal tract, gastrointestinal symptoms, or hormonal treatment (Svensson et al., 2021). This conclusion is consistent with our research findings.

Through analysis of the genera, it cloud be observed that only *Eggerthella* shows higher abundance in ectopic tissues, but it was not statistically significant. The abundance of other differentiating bacteria showed a decreasing trend in endometriotic lesions, with *Tuzzerella* being the most markedly different. However, unlike what has been mentioned in other studies (Khodaverdi et al., 2019; Itoh et al., 2011) regarding endometriosis, in our study, there is no statistically significant difference in the abundance of *Lactobacillus* between the two groups. Currently, there is still debate as to whether *Tuzzerella* is a pathogen or a probiotic. In different models, *Tuzzerella* plays different roles. Yimeng Fan et al.’s (2023) study found that Pingwei San can treat Spleen-deficiency diarrhea (SDD), regulate imbalanced gut microbiota, and discover a significant increase in *Tuzzerella* in rat feces after treatment (Fan et al., 2023). On the other hand, another study found that dietary contamination caused by polystyrene microplastics (MPs) can lead to liver damage and dysbiosis of the gut microbiota, resulting in an increase in pathogenic *Tuzzerella* (Yang et al., 2023). In this study, we considered that the decrease in *Tuzzerella* abundance might be related to the colonization and development of ectopic endometrial lesions.

We selected samples with the largest differences in the abundance of *Tuzzerella* from the two groups of samples and conducted biological analyses of the metabolome and proteome. From the metabolomics analysis, it was found that low abundance of *Tuzzerella* leads to a significant downregulation of several metabolic pathways in endometriotic lesions, such as glutamatergic synapse, nicotinate and nicotinamide metabolism, and aldosterone synthesis and secretion. In contrast, there was an upregulation of the mTOR signaling pathway, and the abnormal mTOR pathway may be associated with a decrease in glutamine levels in endometriotic lesions. It has been reported that inhibiting mTOR signaling can reduce the expression of HIF-1a, thereby enhancing the flux of glutamine and glucose metabolism pathways (Araujo et al., 2017). At the protein level, we noted that the expression of the Complement and Coagulation Cascades pathway was abnormally active in endometriotic lesions with low abundance of *Tuzzerella*. We also validated this by performing qPCR on several complement-related mRNAs, confirming that complement-related mRNAs *(C3*, *C7*, *C1S*, *CLU*, *A2M*) were upregulated in endometriotic lesions. Finally, by combining the differential metabolites and differential proteins associated with *Tuzzerella* abundance, we found low abundance of *Tuzzerella* leads to a decrease in glutamine content in endometriotic lesions while stimulating the upregulation of complement protein C7. Although there are currently no direct studies reporting the relationship between low concentration of glutamine and protein C7, a study by A Spittler et al. in 1997 already reported that low concentrations of glutamine in cultured cells can increase the expression of complement receptor type 3 (Spittler et al., 1997). Western blot also confirmed this result, as the expression of protein C7 was significantly inhibited when high concentrations of glutamine were added to ectopic endometrial cells.

Treg cells are key cells in immune suppression and play an important role in the progression of many diseases. Numerous previous studies have confirmed that Tregs are increased in the lesions of patients with endometriosis (Basta et al., 2014; Braundmeier et al., 2012; Hey-Cunningham et al., 2022) and exhibit functional abnormalities (Tanaka et al., 2017). Using an immune infiltration profile, we also found a significant increase in the infiltration ratio of Tregs in the lesions of endometriosis with low abundance of *Tuzzerella*. Moreover, we observed a positive correlation between the increase in Tregs proportion and the expression of C7 by mIF. Although there is no literature reporting the relationship between protein C7 and the number of Tregs, as mentioned in previous studies, C3a and C5a in the complement pathway could specifically bind to C3aR and C5aR on Tregs during the inflammatory phase, playing a crucial role in regulating the induction, function, and stability of Tregs (Chen et al., 2018; Strainic et al., 2013; van der Touw et al., 2013). Finally, we made a simple identification of the relationship between glutamine and Tregs and found that the chemotactic factor of Tregs decreased when high concentrations of glutamine were added to primary ectopic endometrial cells, indirectly indicating that high concentrations of glutamine might reduce the enrichment of Tregs in the lesions.

In our future research endeavors, we will undertake further investigations to explore the effects of *Tuzzerella* on the levels of l-Glutamine, as well as elucidate the mechanisms through which l-Glutamine regulates C7 expression. However, it is important to acknowledge certain limitations in our current study. Due to constraints in sample size, we were unable to establish a correlation between the abundance of *Tuzzerella* and the staging of endometriosis. We intend to address this limitation by continuing this line of inquiry in subsequent studies. Additionally, the lack of in vivo experiments has resulted in insufficient evidence to support the hypothesis that *Tuzzerella* may act as a probiotic in the disease model of endometriosis. We have already initiated experiments to remedy this gap in knowledge.

Overall, by both expanding our understanding of the impact of *Tuzzerella* on l-Glutamine levels and examining its potential as a probiotic in endometriosis, we aim to address these limitations and contribute to the advancement of knowledge in the field.

## Conclusion

In this study, we combined three different levels of analytical techniques: microbial 16 s rRNA sequencing, metabolomics, and proteomics. We found a clear multi-omics pathway alteration, “*Tuzzerella* (microbe)—l-Glutamine (metabolite)—C7 (protein),” which affects the infiltration of Tregs in endometriotic lesions. This result may provide a microbial perspective for the classification of endometriosis and guide treatment strategies based on different microbial structures, for example, altering the abundance of *Tuzzerella* or supplementing appropriate glutamine to the lesion may reduce Tregs infiltration and immunosuppression, thereby enhancing the cytotoxicity of effector T cells against ectopic endometrial tissue.

### Supplementary Information

Below is the link to the electronic supplementary material.

Supplementary file1 (PDF 210 KB)

Supplementary file2 (PDF 46585 KB)

Supplementary file3 (PDF 122057 KB)

Supplementary file4 (PDF 14572 KB)

Supplementary file5 (TIFF 110 KB)

Supplementary file6 (TIF 2515 KB)

Supplementary file7 (TIF 3352 KB)

Supplementary file8 (DOCX 20 KB)

Supplementary file9 (DOCX 80 KB)

Supplementary file10 (DOCX 15 KB)

Supplementary file11 (DOCX 989 KB)

## Data Availability

All the data generated or analyzed during this study are included in this article and its supplementary files. The raw sequencing data can be obtained from the corresponding author or Yichen Chen (cyc_0605@hotmal.com) upon reasonable request.
