# Supplementary figures and images for: Disrupted Tuzzerella abundance and impaired l-glutamine levels induce Treg accumulation in ovarian endometriosis: a comprehensive multi-omics analysis
Source: Metabolomics. 2024 Feb 29;20(2):32. doi: 10.1007/s11306-023-02072-0 (PMC10904428; doi:10.1007/s11306-023-02072-0)

C7

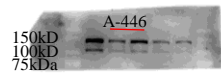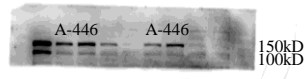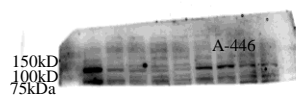

GAPDH

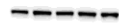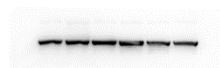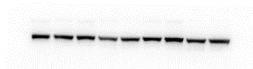

Supplement: Supplementary file 1 — Supplementary file1 (PDF 210 KB) [file 11306_2023_2072_MOESM1_ESM.pdf]

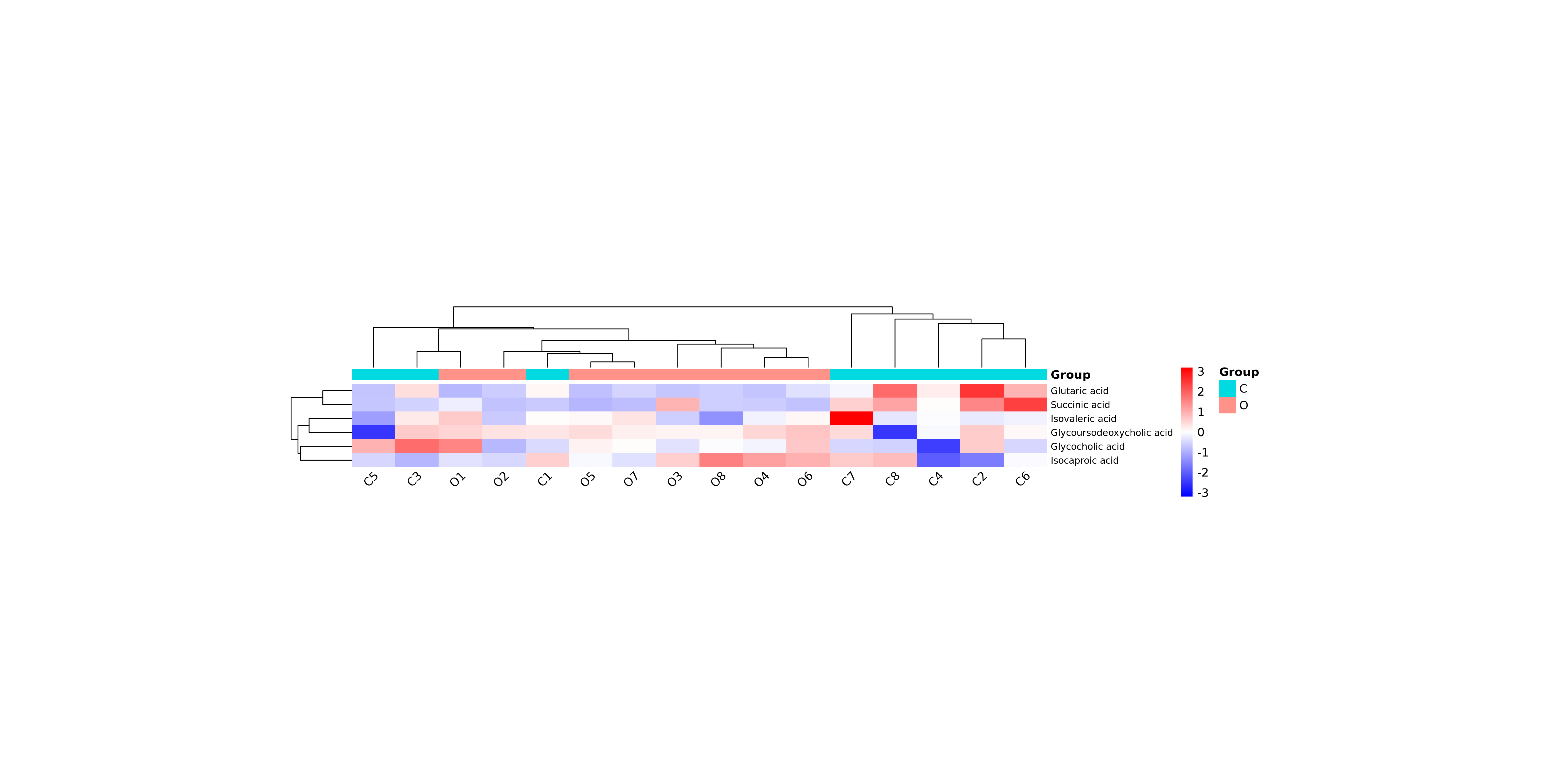

Supplement: Supplementary file 5 — Supplementary file5 (TIFF 110 KB) [file 11306_2023_2072_MOESM5_ESM.tiff]

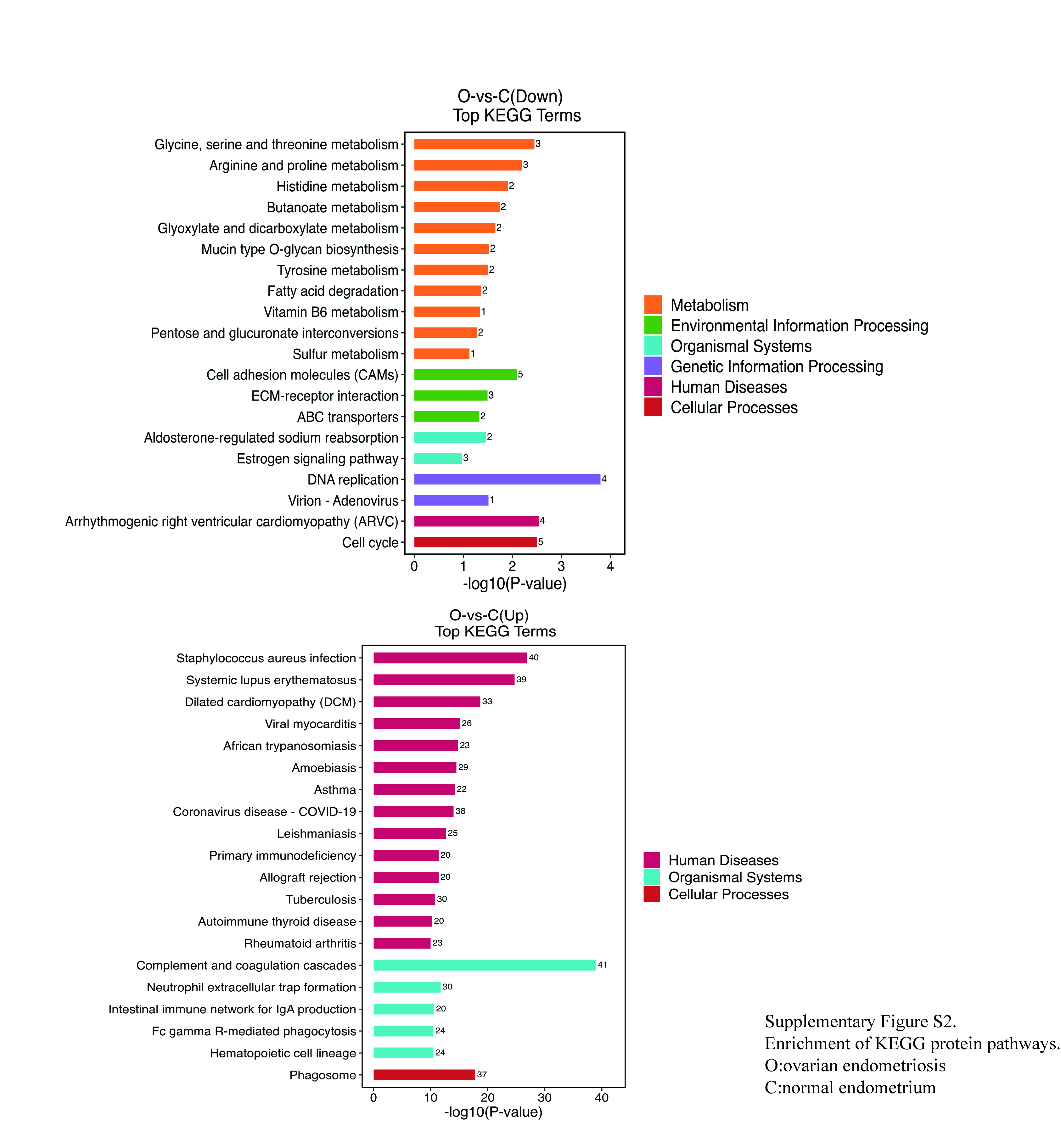

Supplement: Supplementary file 6 — Supplementary file6 (TIF 2515 KB) [file 11306_2023_2072_MOESM6_ESM.tif]

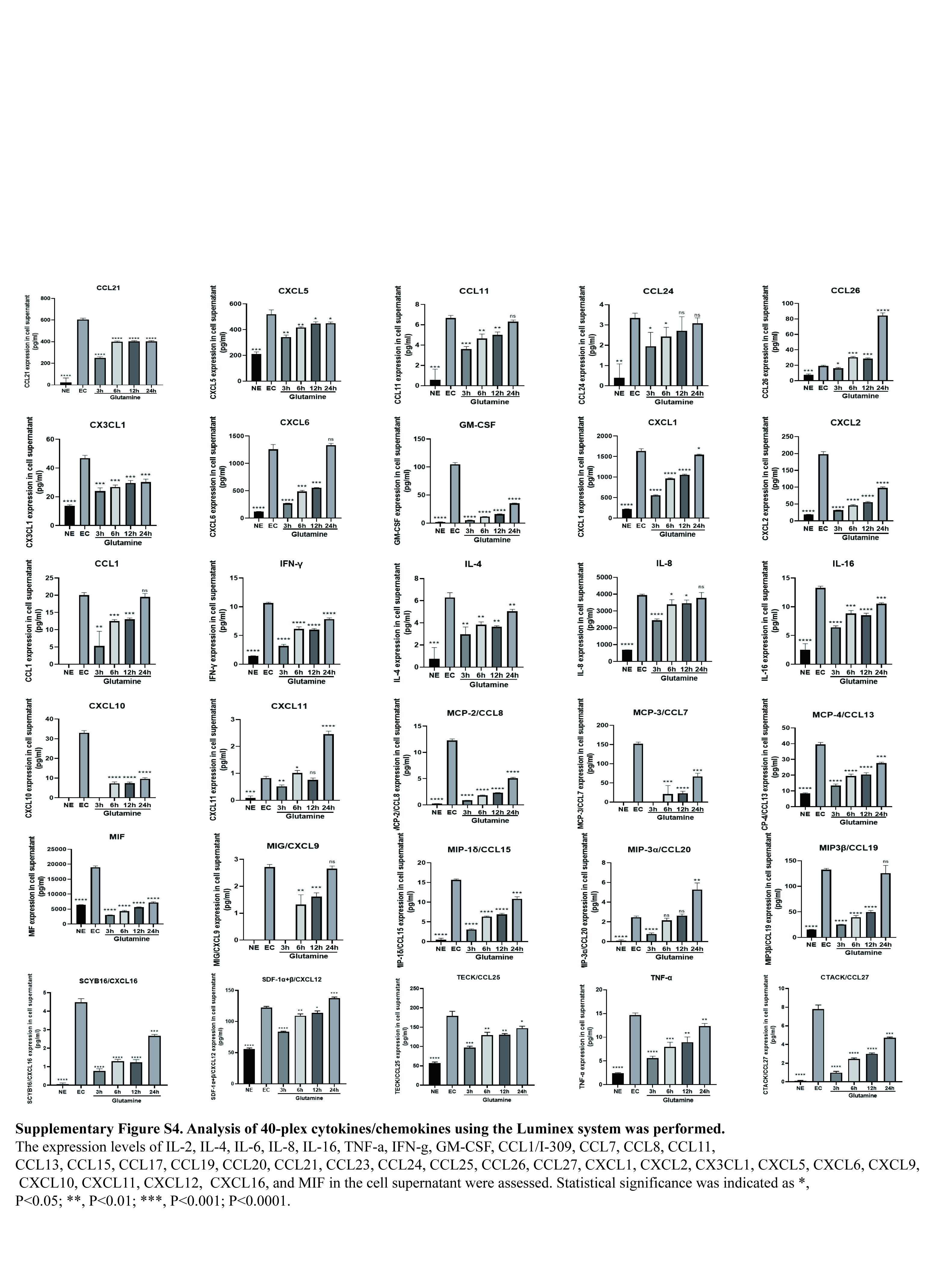

Supplement: Supplementary file 7 — Supplementary file7 (TIF 3352 KB) [file 11306_2023_2072_MOESM7_ESM.tif]
