# Supplementary material for: Disrupted Tuzzerella abundance and impaired l-glutamine levels induce Treg accumulation in ovarian endometriosis: a comprehensive multi-omics analysis
Source: Metabolomics. 2024 Feb 29;20(2):32. doi: 10.1007/s11306-023-02072-0 (PMC10904428; doi:10.1007/s11306-023-02072-0)
Supplement: Supplementary file 4 — Supplementary file4 (PDF 14572 KB) [file 11306_2023_2072_MOESM4_ESM.pdf]

Accession	Gene Name	Description	Coverage [%	# Peptides	# PSMs	# Unique Pe	# AAs	MW [kDa]	calc. pI	Score Sequ
Q09666	AHNAK	Neuroblast d	71	263	619	260	5890	628.7	6.15	1892.51
Q15149	PLEC	Plectin OS=H	58	265	524	258	4684	531.5	5.96	1616.98
P21333	FLNA	Filamin-A OS	65	141	505	127	2647	280.6	6.06	1681.44
P35579	MYH9	Myosin-9 OS	63	152	537	120	1960	226.4	5.6	1788.52
P12111	COL6A3	Collagen alpl	49	136	437	136	3177	343.5	6.68	1337.2
P35749	MYH11	Myosin-11 O	60	132	344	102	1972	227.2	5.5	1152.48
P02768	ALB	Albumin OS=	90	77	1947	77	609	69.3	6.28	5114.09
Q14204	DYNC1H1	Cytoplasmic	39	168	263	168	4646	532.1	6.4	834.13
P35555	FBN1	Fibrillin-1 OS	48	105	312	102	2871	312.1	4.93	1039.59
Q9Y490	TLN1	Talin-1 OS=H	60	119	268	104	2541	269.6	6.07	947.21
Q13813	SPTAN1	Spectrin alph	62	140	273	140	2472	284.4	5.35	853.94
P35580	MYH10	Myosin-10 O	56	125	283	92	1976	228.9	5.54	907.39
Q99715	COL12A1	Collagen alpl	47	121	270	121	3063	332.9	5.53	883.71
P02751	FN1	Fibronectin C	47	87	279	87	2477	272.2	5.5	1018.59
P01024	C3	Complement	66	105	269	105	1663	187	6.4	879.11
Q01082	SPTBN1	Spectrin beta	52	111	224	93	2364	274.4	5.57	756.92
P04114	APOB	Apolipoprote	33	137	216	137	4563	515.3	7.05	665.73
P78527	PRKDC	DNA-depend	32	132	221	132	4128	468.8	7.12	639.42
O75369	FLNB	Filamin-B OS	51	104	190	91	2602	278	5.73	602.18
P98160	HSPG2	Basement m	33	99	202	99	4391	468.5	6.51	644.53
P46940	IQGAP1	Ras GTPase	54	74	164	71	1657	189.1	6.48	601.18
P18206	VCL	Vinculin OS=	61	66	213	66	1134	123.7	5.66	701.85
P22105	TNXB	Tenascin-X C	34	100	166	100	4244	458.1	5.17	526.97
P02787	TF	Serotransferr	75	59	275	59	698	77	7.12	820.49
Q00610	CLTC	Clathrin heav	49	71	162	71	1675	191.5	5.69	602.55
P12814	ACTN1	Alpha-actinin	74	56	230	34	892	103	5.41	762.61
Q05707	COL14A1	Collagen alpl	45	59	179	59	1796	193.4	5.3	597.42
Q9NZM1	MYOF	Myoferlin OS	50	87	148	85	2061	234.6	6.18	473.13
Q9P2E9	RRBP1	Ribosome-bi	68	81	156	81	1410	152.4	8.6	477.75
O43707	ACTN4	Alpha-actinin	71	59	189	40	911	104.8	5.44	666.11
P08670	VIM	Vimentin OS	83	51	470	45	466	53.6	5.12	1266.49
P02675	FGB	Fibrinogen b	75	39	236	39	491	55.9	8.27	744.89

Q14980	NUMA1	Nuclear mito	47	88	141	87	2115	238.1	5.78	450.24
P11277	SPTB	Spectrin beta	45	85	130	74	2137	246.3	5.27	419.9
P0C0L4	C4A	Complement	41	61	134	3	1744	192.7	7.08	420.41
P01023	A2M	Alpha-2-mac	48	54	147	47	1474	163.2	6.46	501.23
Q14315	FLNC	Filamin-C OS	36	80	122	65	2725	290.8	5.97	403.71
P14618	PKM	Pyruvate kina	70	40	185	36	531	57.9	7.84	557.89
P11021	HSPA5	Endoplasmic	59	44	168	41	654	72.3	5.16	514.69
P02549	SPTA1	Spectrin alph	38	78	119	78	2419	279.8	5.05	387.33
P0C0L5	C4B_2	Complement	40	59	128	1	1744	192.6	7.27	394.84
P24821	TNC	Tenascin OS	35	57	113	57	2201	240.7	4.89	373.51
P08238	HSP90AB1	Heat shock p	59	45	160	24	724	83.2	5.03	540.7
P63261	ACTG1	Actin, cytopla	77	27	643	11	375	41.8	5.48	1805.66
P05787	KRT8	Keratin, type	74	47	154	38	483	53.7	5.59	474.12
P55072	VCP	Transitional e	71	48	129	48	806	89.3	5.26	416.7
P10809	HSPD1	60 kDa heat	75	36	142	36	573	61	5.87	484.79
P07900	HSP90AA1	Heat shock p	57	44	154	25	732	84.6	5.02	502.22
P11142	HSPA8	Heat shock c	67	42	146	29	646	70.9	5.52	472.91
P46939	UTRN	Utrophin OS=	27	81	111	80	3433	394.2	5.33	335.35
P02545	LMNA	Prelamin-A/C	66	49	164	47	664	74.1	7.02	519.38
P07996	THBS1	Thrombospo	44	45	114	42	1170	129.3	4.94	353.63
Q07954	LRP1	Prolow-densi	19	80	120	80	4544	504.3	5.39	347.63
P15924	DSP	Desmoplakin	32	82	103	82	2871	331.6	6.81	297.02
P06733	ENO1	Alpha-enolas	73	35	179	32	434	47.1	7.39	555.74
P12109	COL6A1	Collagen alp	41	38	127	38	1028	108.5	5.43	383.04
P22314	UBA1	Ubiquitin-like	49	39	112	39	1058	117.8	5.76	381.48
P62736	ACTA2	Actin, aortic s	71	25	454	3	377	42	5.39	1076.92
Q02952	AKAP12	A-kinase anc	47	58	90	58	1782	191.4	4.41	297.71
P13639	EEF2	Elongation fa	52	44	136	42	858	95.3	6.83	400.84
Q9UPN3	MACF1	Microtubule-a	12	80	100	78	7388	837.8	5.39	276.16
P14625	HSP90B1	Endoplasmin	51	46	223	44	803	92.4	4.84	638.3
P13667	PDIA4	Protein disulf	54	37	101	37	645	72.9	5.07	334.81
Q9Y4G6	TLN2	Talin-2 OS=H	30	64	100	50	2542	271.4	5.57	295.89
P0DMV9	HSPA1B	Heat shock 7	63	36	109	26	641	70	5.66	341.17

P02671	FGA	Fibrinogen a	42	40	158	39	866	94.9	6.01	434.86
P26038	MSN	Moesin OS=I	58	46	121	34	577	67.8	6.4	339.32
P04406	GAPDH	Glyceraldehy	67	23	307	23	335	36	8.46	1029.42
P01009	SERPINA1	Alpha-1-antit	58	30	373	30	418	46.7	5.59	937.53
P68133	ACTA1	Actin, alpha s	70	24	446	1	377	42	5.39	1033.07
Q00341	HDLBP	Vigilin OS=H	44	53	88	52	1268	141.4	6.87	280.91
P68104	EEF1A1	Elongation fa	60	24	146	24	462	50.1	9.01	430.64
P00450	CP	Ceruloplasm	40	36	86	36	1065	122.1	5.72	334.01
P02788	LTF	Lactotransfe	67	41	104	41	710	78.1	8.12	331.22
P08133	ANXA6	Annexin A6 (	67	46	109	46	673	75.8	5.6	324.91
P06576	ATP5F1B	ATP synthas	69	25	99	25	529	56.5	5.4	358.21
P27816	MAP4	Microtubule-a	51	49	91	49	1152	120.9	5.43	242.28
Q71U36	TUBA1A	Tubulin alpha	63	23	197	1	451	50.1	5.06	523.5
P12270	TPR	Nucleoprotei	31	68	91	68	2363	267.1	5.02	257.18
Q6P2Q9	PRPF8	Pre-mRNA-p	28	62	104	61	2335	273.4	8.84	289.35
P11047	LAMC1	Laminin subu	36	49	85	49	1609	177.5	5.12	259.29
P52272	HNRNPM	Heterogeneo	59	40	97	40	730	77.5	8.7	284.06
Q05682	CALD1	Caldesmon (	46	43	90	42	793	93.2	5.66	284.43
Q9BQE3	TUBA1C	Tubulin alpha	58	22	156	2	449	49.9	5.1	375.01
P02679	FGG	Fibrinogen g	67	28	126	28	453	51.5	5.62	394.85
P00558	PGK1	Phosphoglyc	85	32	94	26	417	44.6	8.1	295.02
P40939	HADHA	Trifunctional	54	34	81	34	763	82.9	9.04	268.5
P02452	COL1A1	Collagen alpl	27	34	122	34	1464	138.8	5.8	412.44
P34932	HSPA4	Heat shock 7	64	42	70	39	840	94.3	5.19	240.16
P04264	KRT1	Keratin, type	50	39	103	32	644	66	8.12	293
P12956	XRCC6	X-ray repair (	65	36	96	36	609	69.8	6.64	266.51
Q14764	MVP	Major vault p	50	36	72	35	893	99.3	5.48	250.66
P53621	COPA	Coatomer su	49	49	77	49	1224	138.3	7.66	232.21
P30101	PDIA3	Protein disulf	54	30	117	30	505	56.7	6.35	376.18
P35908	KRT2	Keratin, type	68	40	87	30	639	65.4	8	273.23
P08727	KRT19	Keratin, type	74	35	108	22	400	44.1	5.14	327.83
P09874	PARP1	Poly [ADP-rib	42	44	82	44	1014	113	8.88	237.86
Q86UP2	KTN1	Kinectin OS=	41	56	83	56	1357	156.2	5.64	239.72

P35221	CTNNA1	Catenin alph	51	37	70	30	906	100	6.29	274.84
Q07065	CKAP4	Cytoskeleton	66	42	90	42	602	66	5.92	281.84
P07355	ANXA2	Annexin A2 (	78	35	136	35	339	38.6	7.75	401.69
P06396	GSN	Gelsolin OS=	52	38	125	38	782	85.6	6.28	383.55
P42704	LRPPRC	Leucine-rich	44	55	81	55	1394	157.8	6.13	232.79
P50454	SERPINH1	Serpin H1 OS	59	23	89	23	418	46.4	8.69	335.73
O75643	SNRNP200	U5 small nuc	29	52	74	52	2136	244.4	6.06	225.9
P20700	LMNB1	Lamin-B1 OS	58	35	89	30	586	66.4	5.16	295.33
P13647	KRT5	Keratin, type	54	39	101	24	590	62.3	7.74	280.5
P68366	TUBA4A	Tubulin alpha	52	20	163	3	448	49.9	5.06	427.08
Q00839	HNRNPU	Heterogeneo	43	40	103	39	825	90.5	6	305.25
P02647	APOA1	Apolipoprote	79	31	140	31	267	30.8	5.76	415.44
P68371	TUBB4B	Tubulin beta-	68	22	141	3	445	49.8	4.89	395.58
Q03252	LMNB2	Lamin-B2 OS	60	42	96	37	620	69.9	5.59	259.23
P09493	TPM1	Tropomyosin	72	40	100	15	284	32.7	4.74	247.71
P07237	P4HB	Protein disulf	70	37	94	36	508	57.1	4.87	294.65
P08603	CFH	Complement	41	40	85	36	1231	139	6.61	266.96
P19338	NCL	Nucleolin OS	47	45	105	45	710	76.6	4.7	310.56
P00352	ALDH1A1	Aldehyde del	63	30	70	26	501	54.8	6.73	251.23
P02774	GC	Vitamin D-bir	63	30	77	29	474	52.9	5.45	269.35
Q14195	DPYSL3	Dihydropyrim	59	26	90	20	570	61.9	6.49	339.38
Q7Z6Z7	HUWE1	E3 ubiquitin-|	16	55	75	55	4374	481.6	5.22	203.3
P48643	CCT5	T-complex pr	67	32	74	31	541	59.6	5.66	234.65
Q9NYU2	UGGT1	UDP-glucose	35	41	62	40	1555	177.1	5.63	209.04
Q7Z406	MYH14	Myosin-14 O	23	44	77	29	1995	227.7	5.6	241.53
P05023	ATP1A1	Sodium/pota	42	35	81	35	1023	112.8	5.49	243.54
P07437	TUBB	Tubulin beta	68	21	148	4	444	49.6	4.89	414.15
P07942	LAMB1	Laminin subu	26	46	71	46	1786	197.9	4.94	223.74
P00734	F2	Prothrombin	57	28	68	28	622	70	5.9	268.62
P21980	TGM2	Protein-gluta	52	30	88	30	687	77.3	5.22	260.35
P08729	KRT7	Keratin, type	61	35	94	30	469	51.4	5.48	293.6
P13645	KRT10	Keratin, type	57	33	81	25	584	58.8	5.21	278.89
Q08211	DHX9	ATP-depend	35	37	71	37	1270	140.9	6.84	243.36

P27797	CALR	Calreticulin (	67	25	88	25	417	48.1	4.44	309.26
P53618	COPB1	Coatomer su	42	36	65	36	953	107.1	6.05	210.08
Q99798	ACO2	Aconitate hy	45	33	62	33	780	85.4	7.61	207.02
P04083	ANXA1	Annexin A1 (	76	26	73	26	346	38.7	7.02	273.59
P16615	ATP2A2	Sarcoplasmi	35	34	72	28	1042	114.7	5.34	249.05
P13796	LCP1	Plastin-2 OS	55	27	71	23	627	70.2	5.43	232.82
P68871	HBB	Hemoglobin	96	17	1176	10	147	16	7.28	2669.65
O43491	EPB41L2	Band 4.1-like	41	37	69	34	1005	112.5	5.44	236.71
P63010	AP2B1	AP-2 comple	39	37	71	19	937	104.5	5.38	233.99
Q86VP6	CAND1	Cullin-associ	33	38	66	38	1230	136.3	5.78	211.04
P05783	KRT18	Keratin, type	66	34	75	32	430	48	5.45	251.1
Q10567	AP1B1	AP-1 comple	39	35	68	17	949	104.5	5.06	219.93
P35442	THBS2	Thrombospo	30	28	57	25	1172	129.9	4.83	196.61
P00738	HP	Haptoglobin	58	27	88	14	406	45.2	6.58	256.28
P0DOX5		Immunoglobu	50	20	261	11	449	49.3	8.72	690.96
P12110	COL6A2	Collagen alp	31	28	90	28	1019	108.5	6.21	323.67
Q14789	GOLGB1	Golgin subfa	19	55	68	55	3259	375.8	5	176.62
Q92896	GLG1	Golgi appara	41	46	73	46	1179	134.5	6.9	211.05
O95782	AP2A1	AP-2 comple	39	38	61	25	977	107.5	7.03	178.99
Q92616	GCN1	eIF-2-alpha k	21	50	64	50	2671	292.5	7.43	188.75
P04040	CAT	Catalase OS	57	29	76	29	527	59.7	7.39	268.53
P49327	FASN	Fatty acid sy	22	46	60	46	2511	273.3	6.44	197.16
Q14152	EIF3A	Eukaryotic tr	40	53	75	53	1382	166.5	6.79	187.37
P07951	TPM2	Tropomyosin	65	36	106	15	284	32.8	4.7	265.51
P50395	GDI2	Rab GDP dis	64	30	73	22	445	50.6	6.47	217.38
Q01995	TAGLN	Transgelin O	81	20	114	20	201	22.6	8.84	291.42
P16157	ANK1	Ankyrin-1 OS	28	45	69	42	1881	206.1	6.01	206.92
Q9Y4L1	HYOU1	Hypoxia up-r	39	35	68	35	999	111.3	5.22	218.56
P07814	EPRS1	Bifunctional g	35	45	63	45	1512	170.5	7.33	180.12
P13010	XRCC5	X-ray repair (	49	27	78	27	732	82.7	5.81	256.38
P02538	KRT6A	Keratin, type	54	37	71	4	564	60	8	193.92
P54652	HSPA2	Heat shock-r	52	34	67	21	639	70	5.74	207.75
P25705	ATP5F1A	ATP synthas	60	30	91	30	553	59.7	9.13	245.69

P00747	PLG	Plasminogen	52	36	66	36	810	90.5	7.24	227.87
Q96KP4	CNDP2	Cytosolic nor	55	22	73	22	475	52.8	5.97	231.92
P38646	HSPA9	Stress-70 pro	57	36	67	35	679	73.6	6.16	196.88
P04275	VWF	von Willebrar	23	48	59	48	2813	309.1	5.48	186.51
Q14697	GANAB	Neutral alpha	33	31	73	31	944	106.8	6.14	225.16
P62258	YWHAE	14-3-3 protei	80	25	97	22	255	29.2	4.74	319.8
Q9Y6C2	EMILIN1	EMILIN-1 OS	36	29	61	29	1016	106.6	5.17	229.24
Q16555	DPYSL2	Dihydropyrim	65	25	60	19	572	62.3	6.38	227.51
P35606	COPB2	Coatomer su	40	33	61	33	906	102.4	5.27	190.58
Q13263	TRIM28	Transcription	39	24	64	24	835	88.5	5.77	228.27
Q15063	POSTN	Periostin OS	46	31	57	31	836	93.3	7.53	199.13
P17661	DES	Desmin OS=	69	34	86	29	470	53.5	5.27	242.17
O15230	LAMA5	Laminin subu	14	43	64	43	3695	399.5	7.02	185.34
Q8WUM4	PDCD6IP	Programmed	50	42	67	42	868	96	6.52	183.22
P14866	HNRNPL	Heterogeneo	51	23	60	22	589	64.1	8.22	199.29
P29401	TKT	Transketolas	50	24	69	24	623	67.8	7.66	241.93
P15311	EZR	Ezrin OS=Ho	54	37	86	24	586	69.4	6.27	220
P35527	KRT9	Keratin, type	47	28	74	26	623	62	5.24	249.57
Q13885	TUBB2A	Tubulin beta-	51	18	124	1	445	49.9	4.89	344.2
P01859	IGHG2	Immunoglobu	57	17	190	9	326	35.9	7.59	449.38
P55084	HADHB	Trifunctional	62	27	72	27	474	51.3	9.41	198.64
P04075	ALDOA	Fructose-bisp	70	25	105	19	364	39.4	8.09	337.21
O60506	SYNCRIP	Heterogeneo	48	29	63	21	623	69.6	8.59	226.42
P11586	MTHFD1	C-1-tetrahyd	43	32	60	31	935	101.5	7.3	168.46
P23142	FBLN1	Fibulin-1 OS	47	25	61	25	703	77.2	5.22	216.31
P48681	NES	Nestin OS=H	30	41	59	40	1621	177.3	4.36	182.53
P19367	HK1	Hexokinase-	36	32	62	29	917	102.4	6.8	189.48
P53396	ACLY	ATP-citrate s	40	41	63	41	1101	120.8	7.33	191.63
Q15075	EEA1	Early endoso	38	53	60	53	1411	162.4	5.68	155.57
P08758	ANXA5	Annexin A5 (	74	25	88	24	320	35.9	5.05	293.96
O75083	WDR1	WD repeat-c	47	25	59	25	606	66.2	6.65	196.05
P49368	CCT3	T-complex pr	56	29	63	29	545	60.5	6.49	197.36
P07384	CAPN1	Calpain-1 ca	45	33	56	33	714	81.8	5.67	178.44

P04259	KRT6B	Keratin, type	49	35	65	2	564	60	8	172.72
Q16891	IMMT	MICOS comp	49	35	60	35	758	83.6	6.48	173.17
P78371	CCT2	T-complex pr	63	29	56	29	535	57.5	6.46	176.41
Q8WXH0	SYNE2	Nesprin-2 OS	9	57	64	55	6885	795.9	5.36	164.49
O75874	IDH1	Isocitrate del	68	28	70	26	414	46.6	7.01	205.03
P02042	HBD	Hemoglobin	88	14	587	7	147	16	8.05	1387.65
Q13308	PTK7	Inactive tyros	38	28	52	28	1070	118.3	7.09	201.03
Q7KZF4	SND1	Staphylococc	42	34	62	34	910	101.9	7.17	203.49
P23246	SFPQ	Splicing facto	41	30	61	29	707	76.1	9.44	197.36
Q01518	CAP1	Adenylyl cycl	56	26	87	24	475	51.9	8.06	279.53
P67936	TPM4	Tropomyosin	79	31	97	13	248	28.5	4.69	247.43
O43390	HNRNPR	Heterogeneo	41	25	64	16	633	70.9	8.13	218.45
P50991	CCT4	T-complex pr	62	30	55	29	539	57.9	7.83	180.55
Q02880	TOP2B	DNA topoiso	31	41	52	31	1626	183.2	8	161.23
P17987	TCP1	T-complex pr	56	23	54	23	556	60.3	6.11	192.01
P01008	SERPINC1	Antithrombin	52	24	85	24	464	52.6	6.71	227.99
P69905	HBA2	Hemoglobin	92	11	511	10	142	15.2	8.68	1219.38
P06753	TPM3	Tropomyosin	61	32	78	7	285	32.9	4.72	196.03
O75533	SF3B1	Splicing facto	34	35	50	35	1304	145.7	7.09	160.81
P50990	CCT8	T-complex pr	51	26	57	26	548	59.6	5.6	177.77
P60174	TPI1	Triosephospl	85	17	75	17	249	26.7	6.9	253.28
P00488	F13A1	Coagulation	38	24	57	23	732	83.2	6	169.89
Q16363	LAMA4	Laminin subu	22	38	56	38	1823	202.4	6.28	156.42
P01860	IGHG3	Immunoglobu	55	17	143	6	377	41.3	7.9	427.19
Q9Y3Z3	SAMHD1	Deoxynucleo	60	32	59	32	626	72.2	7.14	192.82
P08123	COL1A2	Collagen alpl	21	27	82	27	1366	129.2	8.95	257.72
P21399	ACO1	Cytoplasmic	42	33	59	33	889	98.3	6.68	172.25
O00159	MYO1C	Unconventio	33	31	55	30	1063	121.6	9.41	173.01
P07339	CTSD	Cathepsin D	50	17	55	17	412	44.5	6.54	203.66
P55786	NPEPPS	Puromycin-se	41	36	58	36	919	103.2	5.72	169.57
Q99832	CCT7	T-complex pr	56	27	52	27	543	59.3	7.65	156.15
P51659	HSD17B4	Peroxisomal	55	32	58	32	736	79.6	8.84	187.63
P31948	STIP1	Stress-induce	58	35	60	35	543	62.6	6.8	161.62

P02730	SLC4A1	Band 3 anior	28	19	67	19	911	101.7	5.19	226.03
P37802	TAGLN2	Transgelin-2	75	15	70	15	199	22.4	8.25	209.85
P60842	EIF4A1	Eukaryotic in	57	23	53	13	406	46.1	5.48	190.73
Q14683	SMC1A	Structural ma	35	43	53	43	1233	143.1	7.64	157.81
Q5T4S7	UBR4	E3 ubiquitin-|	10	45	51	45	5183	573.5	6.04	130.8
P23528	CFL1	Cofilin-1 OS=	73	18	72	15	166	18.5	8.09	237.97
Q9NZN4	EHD2	EH domain-c	55	22	50	20	543	61.1	6.46	178.04
Q16531	DDB1	DNA damage	35	36	56	36	1140	126.9	5.26	169.79
Q15029	EFTUD2	116 kDa U5 :	35	28	51	27	972	109.4	5	164.14
Q04695	KRT17	Keratin, type	63	30	68	15	432	48.1	5.02	173.8
Q9UQE7	SMC3	Structural ma	33	40	53	40	1217	141.5	7.18	155.25
Q9NR99	MXRA5	Matrix-remoc	18	42	55	42	2828	312	8.32	147.98
Q15582	TGFBI	Transforming	39	25	70	25	683	74.6	7.71	194.29
Q15746	MYLK	Myosin light (	21	34	47	34	1914	210.6	6.15	133.81
P33176	KIF5B	Kinesin-1 hea	40	36	49	36	963	109.6	6.51	154.51
Q99460	PSMD1	26S proteasc	36	26	42	26	953	105.8	5.39	136.89
Q13509	TUBB3	Tubulin beta-	50	16	89	3	450	50.4	4.93	260.43
O00410	IPO5	Importin-5 O	36	29	43	28	1097	123.6	4.94	152.77
P00751	CFB	Complement	34	26	59	26	764	85.5	7.06	177.44
P27824	CANX	Calnexin OS	37	30	70	30	592	67.5	4.6	213.22
P12277	CKB	Creatine kina	69	19	55	19	381	42.6	5.59	217.92
P49588	AARS1	Alanine--tRN	35	30	45	30	968	106.7	5.53	127.68
P49419	ALDH7A1	Alpha-amino	48	22	39	22	539	58.5	7.99	144.49
Q12906	ILF3	Interleukin er	32	27	58	23	894	95.3	8.76	173.46
P55268	LAMB2	Laminin subu	24	36	53	36	1798	195.9	6.52	149.96
P05164	MPO	Myeloperoxic	35	24	47	22	745	83.8	8.97	145.25
P26640	VARS1	Valine--tRNA	29	32	51	32	1264	140.4	7.59	159.37
Q8IUX7	AEBP1	Adipocyte en	32	28	52	27	1158	130.8	5.11	166.47
Q12805	EFEMP1	EGF-contain	51	20	58	20	493	54.6	5.07	198.84
Q9Y678	COPG1	Coatomer su	42	29	51	27	874	97.7	5.47	156.35
P78347	GTF2I	General tran	38	33	54	33	998	112.3	6.39	163.21
Q13576	IQGAP2	Ras GTPase	27	39	54	36	1575	180.5	5.64	149.6
Q92499	DDX1	ATP-depend	48	33	52	33	740	82.4	7.23	153.83

P06748	NPM1	Nucleophosr	45	14	49	14	294	32.6	4.78	167.7
Q709C8	VPS13C	Intermembra	15	48	53	47	3753	422.1	6.83	128.9
P09211	GSTP1	Glutathione S	70	14	76	14	210	23.3	5.64	276.51
P80723	BASP1	Brain acid sc	89	16	42	16	227	22.7	4.63	159.13
P28331	NDUFS1	NADH-ubiqu	47	27	52	27	727	79.4	6.23	174.24
P30041	PRDX6	Peroxiredoxi	66	18	49	18	224	25	6.38	171.52
O00567	NOP56	Nucleolar pro	51	23	45	23	594	66	9.19	150.2
P17655	CAPN2	Calpain-2 ca	40	21	40	21	700	79.9	4.98	152.15
P13611	VCAN	Versican cor	9	32	62	32	3396	372.6	4.51	186.55
P35241	RDX	Radixin OS=	45	30	68	15	583	68.5	6.37	177.18
Q93052	LPP	Lipoma-prefe	47	20	35	20	612	65.7	7.37	129.99
P61978	HNRNPK	Heterogeneo	53	22	59	22	463	50.9	5.54	184.56
P02790	HPX	Hemopexin O	47	20	77	19	462	51.6	7.02	249.34
Q07157	TJP1	Tight junction	22	32	44	31	1748	195.3	6.7	141.28
P13489	RNH1	Ribonuclease	57	19	44	19	461	49.9	4.82	177.05
P00367	GLUD1	Glutamate de	48	25	50	25	558	61.4	7.8	156.54
P28838	LAP3	Cytosol amin	57	24	52	24	519	56.1	7.93	163.63
Q14203	DCTN1	Dynactin sub	34	36	44	36	1278	141.6	5.81	150.39
P11717	IGF2R	Cation-indep	18	40	47	40	2491	274.2	5.94	130.63
O75116	ROCK2	Rho-associat	28	39	50	31	1388	160.8	6.02	138.08
Q92841	DDX17	Probable AT	36	22	53	16	729	80.2	8.27	184.82
P04844	RPN2	Dolichyl-diph	47	19	41	19	631	69.2	5.69	163.46
P11216	PYGB	Glycogen ph	46	35	51	26	843	96.6	6.86	154.9
P55884	EIF3B	Eukaryotic tr	36	26	48	26	814	92.4	5	148.78
Q15393	SF3B3	Splicing facto	31	30	50	30	1217	135.5	5.26	139.19
P27348	YWHAQ	14-3-3 protei	69	18	75	11	245	27.7	4.78	240.73
Q96AC1	FERMT2	Fermitin fam	43	26	45	23	680	77.8	6.7	150.3
P21796	VDAC1	Voltage-depe	69	15	52	14	283	30.8	8.54	200.27
P14923	JUP	Junction plak	45	24	48	21	745	81.7	6.14	153.68
P30153	PPP2R1A	Serine/threor	49	28	55	19	589	65.3	5.11	153.61
Q14112	NID2	Nidogen-2 O	24	27	48	26	1375	151.2	5.29	176.93
Q15233	NONO	Non-POU do	50	23	72	21	471	54.2	8.95	206.8
P06737	PYGL	Glycogen ph	45	34	46	28	847	97.1	7.17	138.5

P09525	ANXA4	Annexin A4 (	68	24	57	23	319	35.9	6.13	172.85
P22626	HNRNPA2B1	Heterogenec	52	21	79	17	353	37.4	8.95	252.3
Q14624	ITIH4	Inter-alpha-tr	30	24	50	24	930	103.3	6.98	153.91
P17858	PFKL	ATP-depend	40	26	42	21	780	85	7.5	155.47
Q16181	SEPTIN7	Septin-7 OS=	51	23	50	22	437	50.6	8.63	176.76
P46821	MAP1B	Microtubule-a	19	34	43	32	2468	270.5	4.81	125.83
P09455	RBP1	Retinol-bindi	81	18	55	18	135	15.8	5.11	188.23
P22102	GART	Trifunctional	34	26	43	26	1010	107.7	6.7	147.87
Q8WX93	PALLD	Palladin OS=	25	26	46	26	1383	150.5	7.09	143.69
Q92945	KHSRP	Far upstrean	45	27	54	23	711	73.1	7.3	141.9
Q14974	KPNB1	Importin subu	34	23	37	23	876	97.1	4.78	130.33
P48147	PREP	Prolyl endope	53	29	43	29	710	80.6	5.86	128
P05455	SSB	Lupus La pro	55	28	52	28	408	46.8	7.12	124.68
Q9BSJ8	ESYT1	Extended syn	32	27	42	27	1104	122.8	5.83	137.35
P05091	ALDH2	Aldehyde deh	53	22	48	18	517	56.3	7.05	161.14
P13797	PLS3	Plastin-3 OS	42	23	55	18	630	70.8	5.6	160.15
P21810	BGN	Biglycan OS=	55	16	56	15	368	41.6	7.52	216.19
Q9Y230	RUVBL2	RuvB-like 2 (	57	24	40	24	463	51.1	5.64	131.39
Q92878	RAD50	DNA repair p	29	40	50	40	1312	153.8	6.89	117.21
P49411	TUFM	Elongation fa	49	20	39	20	455	49.8	7.61	129.7
P49792	RANBP2	E3 SUMO-pr	16	42	48	42	3224	358	6.2	131.01
O00468	AGRN	Agrin OS=Hc	21	33	43	33	2068	217.2	6.39	136.51
O94788	ALDH1A2	Retinal dehy	62	24	52	18	518	56.7	6.05	172.36
P01871	IGHM	Immunoglobu	48	20	55	7	453	49.4	6.77	172.38
P01031	C5	Complement	23	37	48	37	1676	188.2	6.52	128.3
P24043	LAMA2	Laminin subu	15	42	43	42	3122	343.7	6.4	111.82
P09651	HNRNPA1	Heterogenec	49	17	76	13	372	38.7	9.13	236.93
P49748	ACADVL	Very long-ch	41	22	43	22	655	70.3	8.75	155.42
Q15084	PDIA6	Protein disulf	51	20	48	19	440	48.1	5.08	161.38
P54136	RARS1	Arginine--tRN	46	29	46	29	660	75.3	6.68	151.31
Q16666	IFI16	Gamma-inter	38	27	43	26	785	88.2	9.28	138.91
P40926	MDH2	Malate dehyc	60	19	54	19	338	35.5	8.68	184.67
Q1KMD3	HNRNPUL2	Heterogenec	40	25	46	25	747	85.1	4.91	137.89

Q9HC84	MUC5B	Mucin-5B OS	10	33	44	31	5762	596	6.64	133.29
P06727	APOA4	Apolipoprote	65	24	42	24	396	45.3	5.38	130.01
P02533	KRT14	Keratin, type	51	28	55	6	472	51.5	5.16	140.26
Q02818	NUCB1	Nucleobindin	60	27	46	27	461	53.8	5.25	149.96
P01042	KNG1	Kininogen-1	31	21	48	21	644	71.9	6.81	173.28
P04843	RPN1	Dolichyl-diph	43	26	51	26	607	68.5	6.38	159.31
Q14839	CHD4	Chromodoma	18	25	35	21	1912	217.9	5.86	131.69
P09960	LTA4H	Leukotriene A	49	26	45	26	611	69.2	6.18	133.86
Q14103	HNRNPD	Heterogeneo	39	18	55	16	355	38.4	7.81	187.34
Q9HBL0	TNS1	Tensin-1 OS	21	34	47	30	1839	196.9	8.16	126.58
O60763	USO1	General vesi	35	27	42	27	962	107.8	4.91	137.56
Q16658	FSCN1	Fascin OS=H	52	23	41	23	493	54.5	7.24	134.38
P29144	TPP2	Tripeptidyl-pe	27	32	40	32	1249	138.3	6.32	124.61
P16403	H1-2	Histone H1.2	54	19	78	8	213	21.4	10.93	209.46
O43852	CALU	Calumenin C	71	19	39	19	315	37.1	4.64	135.53
Q12888	TP53BP1	TP53-binding	20	29	37	29	1972	213.4	4.7	110.7
P10412	H1-4	Histone H1.4	53	18	75	6	219	21.9	11.03	201.4
P23634	ATP2B4	Plasma mem	23	26	42	15	1241	137.8	6.6	129.62
Q12797	ASPH	Aspartyl/aspa	40	26	45	26	758	85.8	5.01	140.19
P19827	ITIH1	Inter-alpha-tr	23	15	37	15	911	101.3	6.79	138.2
P26641	EEF1G	Elongation fa	59	21	45	21	437	50.1	6.67	158.9
Q13228	SELENBP1	Methanethiol	50	21	42	21	472	52.4	6.37	139.44
O94973	AP2A2	AP-2 comple	34	28	40	15	939	103.9	6.96	117.51
P55060	CSE1L	Exportin-2 O	28	27	42	27	971	110.3	5.77	134.57
O75165	DNAJC13	DnaJ homolo	17	36	44	36	2243	254.3	6.74	120.19
P23284	PPIB	Peptidyl-prol	60	16	78	15	216	23.7	9.41	215.57
P45974	USP5	Ubiquitin car	35	26	38	24	858	95.7	5.03	115.48
P31939	ATIC	Bifunctional p	48	22	38	22	592	64.6	6.71	135.69
P23526	AHCY	Adenosylhom	46	20	42	20	432	47.7	6.34	135.58
Q04637	EIF4G1	Eukaryotic tr	23	34	46	30	1599	175.4	5.33	119.17
P61158	ACTR3	Actin-related	52	20	51	20	418	47.3	5.88	163
Q16881	TXNRD1	Thioredoxin r	37	22	35	21	649	70.9	7.39	115.39
O00203	AP3B1	AP-3 comple	32	27	41	26	1094	121.2	6.04	119.24

Q15424	SAFB	Scaffold atta	29	28	50	17	915	102.6	5.47	123.57
P36871	PGM1	Phosphogluc	43	24	53	24	562	61.4	6.76	141.57
Q96QK1	VPS35	Vacuolar pro	31	23	39	23	796	91.6	5.49	116.02
Q15019	SEPTIN2	Septin-2 OS=	65	18	42	18	361	41.5	6.6	153.11
P04792	HSPB1	Heat shock p	82	13	44	13	205	22.8	6.4	164.26
P36776	LONP1	Lon protease	28	23	36	23	959	106.4	6.39	122.89
O60701	UGDH	UDP-glucose	46	18	37	18	494	55	7.12	131.56
Q5VYK3	ECPAS	Proteasome	19	27	35	27	1845	204.2	7.12	107.88
P62424	RPL7A	60S ribosom	47	18	41	18	266	30	10.61	137.23
P63104	YWHAZ	14-3-3 protei	66	17	104	10	245	27.7	4.79	320.91
O60437	PPL	Periplakin OS	21	37	42	37	1756	204.6	5.6	108.45
P20810	CAST	Calpastatin C	48	28	47	28	708	76.5	5.07	127.75
P31150	GDI1	Rab GDP dis	49	18	43	10	447	50.6	5.14	128.57
P49257	LMAN1	Protein ERG	39	20	50	19	510	57.5	6.77	140.23
Q9BUJ2	HNRNPUL1	Heterogeneo	30	27	41	26	856	95.7	6.92	115.62
P31946	YWHAB	14-3-3 protei	66	16	67	7	246	28.1	4.83	209.16
P01876	IGHA1	Immunoglobu	57	15	48	9	353	37.6	6.51	185.69
Q06210	GFPT1	Glutamine--fr	43	26	41	23	699	78.8	7.11	105.19
P40227	CCT6A	T-complex pr	45	23	43	17	531	58	6.68	128.19
Q14767	LTBP2	Latent-transf	18	28	45	27	1821	194.9	5.19	130.12
P06744	GPI	Glucose-6-ph	35	21	49	20	558	63.1	8.32	132.38
P18669	PGAM1	Phosphoglyc	51	11	50	11	254	28.8	7.18	183.52
Q7L014	DDX46	Probable AT	26	26	37	26	1031	117.3	9.29	116.08
Q15293	RCN1	Reticulocalbi	56	17	43	17	331	38.9	5	155.66
P13646	KRT13	Keratin, type	46	24	48	8	458	49.6	4.96	139.99
P26639	TARS1	Threonine--tl	39	25	43	24	723	83.4	6.67	126.2
Q7L576	CYFIP1	Cytoplasmic	25	30	46	30	1253	145.1	6.9	116.86
Q02252	ALDH6A1	Methylmalon	46	22	45	22	535	57.8	8.5	158.88
O94979	SEC31A	Protein trans	21	24	43	24	1220	132.9	6.89	135.75
P09429	HMGB1	High mobility	44	16	51	12	215	24.9	5.74	157.3
P52209	PGD	6-phosphoglu	53	20	38	20	483	53.1	7.23	115.36
Q9Y265	RUVBL1	RuvB-like 1 (	66	21	36	21	456	50.2	6.42	121.4
P07195	LDHB	L-lactate deh	52	19	42	17	334	36.6	6.05	140.09

P11940	PABPC1	Polyadenylat	38	25	45	4	636	70.6	9.5	130.38
P14314	PRKCSH	Glucosidase	41	18	35	18	528	59.4	4.41	131.12
P61981	YWHAG	14-3-3 protei	61	16	60	9	247	28.3	4.89	176.05
Q02790	FKBP4	Peptidyl-proly	65	28	41	28	459	51.8	5.43	130.63
P35222	CTNNB1	Catenin beta	36	24	45	21	781	85.4	5.86	117.81
P46459	NSF	Vesicle-fusin	36	29	41	28	744	82.5	6.95	106.53
P16152	CBR1	Carbonyl red	73	17	40	14	277	30.4	8.32	126.74
P29590	PML	Protein PML	34	32	47	32	882	97.5	6.21	133.05
Q63ZY3	KANK2	KN motif and	32	21	35	20	851	91.1	5.63	115.27
Q06323	PSME1	Proteasome	67	19	37	19	249	28.7	6.02	117.35
P35998	PSMC2	26S proteaso	62	22	40	22	433	48.6	5.95	145.22
P17980	PSMC3	26S proteaso	54	19	35	19	439	49.2	5.24	109.63
O00151	PDLIM1	PDZ and LIM	62	16	37	16	329	36	7.02	139.07
P35609	ACTN2	Alpha-actinin	20	17	73	2	894	103.8	5.45	222
P43304	GPD2	Glycerol-3-ph	43	28	36	28	727	80.8	7.69	110.57
P00491	PNP	Purine nucle	68	15	36	15	289	32.1	6.95	132.45
O60313	OPA1	Dynamin-like	32	29	39	29	960	111.6	7.87	103.25
Q9Y3I0	RTCB	RNA-splicing	43	19	35	19	505	55.2	7.23	120.19
P14543	NID1	Nidogen-1 O	27	27	39	26	1247	136.3	5.29	110.58
Q14008	CKAP5	Cytoskeleton	18	33	40	33	2032	225.4	7.8	99.16
Q01813	PFKP	ATP-depend	42	27	37	22	784	85.5	7.55	102.13
Q9ULC5	ACSL5	Long-chain-f	40	21	37	20	683	75.9	6.92	118.98
P17844	DDX5	Probable AT	40	23	49	17	614	69.1	8.92	157.64
P21291	CSRP1	Cysteine and	64	13	53	13	193	20.6	8.57	192.67
Q8WVM8	SCFD1	Sec1 family d	37	18	33	18	642	72.3	6.27	116.14
Q99439	CNN2	Calponin-2 O	64	15	37	13	309	33.7	7.33	125.01
Q15436	SEC23A	Protein trans	30	17	35	16	765	86.1	7.08	128.46
P48444	ARCN1	Coatomer su	50	24	42	24	511	57.2	6.21	122.62
P07585	DCN	Decorin OS=	40	15	62	14	359	39.7	8.54	204.31
P54577	YARS1	Tyrosine--tRN	51	27	45	27	528	59.1	7.05	125.81
Q9UGI8	TES	Testin OS=H	52	21	41	21	421	48	7.68	111.58
Q63HN8	RNF213	E3 ubiquitin-p	8	38	40	38	5207	591	6.48	103.64
P62937	PPIA	Peptidyl-proly	87	17	91	16	165	18	7.81	204.23

Q9H4M9	EHD1	EH domain-c	54	22	36	14	534	60.6	6.83	129.14
P19823	ITIH2	Inter-alpha-tr	28	23	44	23	946	106.4	6.86	125.57
P14550	AKR1A1	Aldo-keto red	58	20	40	20	325	36.6	6.79	130.89
P36578	RPL4	60S ribosom	40	21	53	21	427	47.7	11.06	158.57
P43490	NAMPT	Nicotinamide	47	21	42	21	491	55.5	7.15	127.24
P47897	QARS1	Glutamine--tl	34	22	36	22	775	87.7	7.15	100.82
P14868	DARS1	Aspartate--tF	53	23	40	23	501	57.1	6.55	119.79
P00915	CA1	Carbonic anh	57	11	106	11	261	28.9	7.12	310.61
O15020	SPTBN2	Spectrin beta	12	29	44	16	2390	271.2	6.11	114.79
Q9UHB9	SRP68	Signal recog	46	26	35	26	627	70.7	8.56	116.32
Q9UMS6	SYNPO2	Synaptopodi	30	28	46	28	1093	117.4	8.57	133.98
Q9NTI5	PDS5B	Sister chrom	21	32	43	28	1447	164.6	8.47	106.02
Q99714	HSD17B10	3-hydroxyacy	74	14	36	14	261	26.9	7.78	124.34
P37837	TALDO1	Transaldolas	49	20	47	20	337	37.5	6.81	130.14
P98095	FBLN2	Fibulin-2 OS	25	20	31	20	1184	126.5	4.82	111.34
P55795	HNRNPH2	Heterogenec	40	16	47	5	449	49.2	6.3	141.94
P01861	IGHG4	Immunoglobu	51	12	69	5	327	35.9	7.36	216.31
Q15459	SF3A1	Splicing facto	27	18	37	18	793	88.8	5.22	122.17
P11413	G6PD	Glucose-6-pl	49	21	33	21	515	59.2	6.84	107.65
P61224	RAP1B	Ras-related p	69	15	39	7	184	20.8	5.78	153.4
Q9P2J5	LARS1	Leucine--tRN	26	27	37	27	1176	134.4	7.3	91.81
P27338	MAOB	Amine oxidas	43	21	38	17	520	58.7	7.5	119.35
Q6UWY5	OLFML1	Olfactomedir	34	16	36	16	402	45.9	8.09	114.46
P27708	CAD	CAD protein	18	29	34	29	2225	242.8	6.46	99.55
Q13200	PSMD2	26S proteasc	29	25	40	25	908	100.1	5.2	117.98
P23229	ITGA6	Integrin alpha	30	30	38	30	1130	126.5	6.61	102.07
P40925	MDH1	Malate dehyc	52	20	42	20	334	36.4	7.36	115.68
P26599	PTBP1	Polypyrimidir	45	18	40	14	557	59.6	9.16	143.41
P51991	HNRNPA3	Heterogenec	40	18	39	17	378	39.6	9.01	121.87
P23396	RPS3	40S ribosom	68	16	40	16	243	26.7	9.66	114.5
P10643	C7	Complement	37	24	41	23	843	93.5	6.48	120.04
Q13423	NNT	NAD(P) trans	27	24	36	24	1086	113.8	8.09	116.21
P08253	MMP2	72 kDa type	48	21	32	21	660	73.8	5.47	109.47

P48735	IDH2	Isocitrate del	42	21	48	19	452	50.9	8.69	148.06
P31943	HNRNPH1	Heterogeneo	40	16	58	4	449	49.2	6.3	145.18
Q9UBG0	MRC2	C-type mann	21	25	39	25	1479	166.6	5.83	124.1
Q14240	EIF4A2	Eukaryotic in	54	17	40	7	407	46.4	5.48	140.15
Q8N163	CCAR2	Cell cycle an	32	22	31	22	923	102.8	5.22	104.5
Q92900	UPF1	Regulator of	29	25	34	25	1129	124.3	6.61	120.23
P07910	HNRNPC	Heterogeneo	38	16	44	16	306	33.7	5.08	116.48
P51884	LUM	Lumican OS=	41	12	73	12	338	38.4	6.61	233.33
P06756	ITGAV	Integrin alpha	25	22	34	22	1048	116	5.68	93.77
P41252	IARS1	Isoleucine--tl	24	24	34	24	1262	144.4	6.15	107.62
P62195	PSMC5	26S proteaso	49	19	33	18	406	45.6	7.55	104.17
P16402	H1-3	Histone H1.3	46	17	65	7	221	22.3	11.02	182.24
P63244	RACK1	Receptor of a	60	16	37	16	317	35.1	7.69	144.72
P27694	RPA1	Replication p	38	22	33	22	616	68.1	7.21	105.08
Q9BUF5	TUBB6	Tubulin beta-	46	16	53	8	446	49.8	4.88	146.65
O14980	XPO1	Exportin-1 O	23	19	33	19	1071	123.3	6.06	112.71
Q13838	DDX39B	Spliceosome	37	15	41	8	428	49	5.67	131.09
Q13435	SF3B2	Splicing facto	30	24	39	24	895	100.2	5.67	104.65
P12268	IMPDH2	Inosine-5'-mo	40	16	30	15	514	55.8	6.9	104.19
P00338	LDHA	L-lactate deh	47	18	51	16	332	36.7	8.27	150.69
Q9Y2X3	NOP58	Nucleolar pro	44	18	33	18	529	59.5	8.92	106.83
O14617	AP3D1	AP-3 comple	24	24	37	24	1153	130.1	8.48	104.19
P46777	RPL5	60S ribosom	39	17	44	17	297	34.3	9.72	110.29
P02794	FTH1	Ferritin heavy	73	15	43	15	183	21.2	5.55	135.65
P38606	ATP6V1A	V-type proton	46	20	33	20	617	68.3	5.52	97.02
P27695	APEX1	DNA-(apurini	60	19	35	19	318	35.5	8.12	123.59
P07954	FH	Fumarate hy	42	19	37	18	510	54.6	8.76	111.95
O00571	DDX3X	ATP-depend	41	25	41	24	662	73.2	7.18	119.69
P22234	PAICS	Bifunctional p	47	22	35	22	425	47	7.23	105.39
O00299	CLIC1	Chloride intra	77	13	33	13	241	26.9	5.17	117.48
O43143	DHX15	ATP-depend	31	22	35	21	795	90.9	7.46	93.16
O94905	ERLIN2	Erlin-2 OS=H	50	16	34	13	339	37.8	5.62	111.66
Q6NZI2	CAVIN1	Caveolae-as	38	15	36	15	390	43.5	5.6	129.93

Q96AY3	FKBP10	Peptidyl-proly	40	21	38	20	582	64.2	5.62	114.24
Q9P0K7	RAI14	Ankycorbin C	23	24	40	23	980	110	6.21	108.18
Q12955	ANK3	Ankyrin-3 OS	8	26	37	20	4377	480.1	6.49	98.57
Q9NZ08	ERAP1	Endoplasmic	24	21	38	21	941	107.2	6.46	109.97
P31146	CORO1A	Coronin-1A C	38	18	41	17	461	51	6.68	138.61
Q13561	DCTN2	Dynactin sub	45	14	32	14	401	44.2	5.21	115.39
O15371	EIF3D	Eukaryotic tr	39	15	25	15	548	63.9	6.05	99.83
P46063	RECQL	ATP-depend	35	20	32	20	649	73.4	7.88	100.58
P21589	NT5E	5'-nucleotida	32	13	29	13	574	63.3	7.03	106
Q9UHG3	PCYOX1	Prenylcystein	36	15	31	15	505	56.6	6.18	101.95
P15880	RPS2	40S ribosom	46	14	40	14	293	31.3	10.24	99.37
Q9Y696	CLIC4	Chloride intra	62	12	28	12	253	28.8	5.59	106.12
Q9P0M6	MACROH2A	Core histone	50	16	35	14	372	40	9.69	116.41
P13798	APEH	Acylamino-ac	27	17	31	17	732	81.2	5.48	109.51
P50570	DNM2	Dynamin-2 C	35	29	40	19	870	98	7.44	102.69
O14950	MYL12B	Myosin regul	71	12	62	5	172	19.8	4.84	176.84
P54920	NAPA	Alpha-soluble	64	18	27	18	295	33.2	5.36	102.44
Q8N2S1	LTBP4	Latent-transf	17	25	36	24	1624	173.3	5.43	96.3
P39023	RPL3	60S ribosom	53	23	51	23	403	46.1	10.18	111.33
O43776	NARS1	Asparagine--	43	21	33	21	548	62.9	6.25	93.09
Q92598	HSPH1	Heat shock p	34	23	34	20	858	96.8	5.39	101.82
P00736	C1R	Complement	37	22	34	21	705	80.1	6.21	110.72
Q16643	DBN1	Drebrin OS=	39	18	27	18	649	71.4	4.45	97.48
Q15417	CNN3	Calponin-3 C	56	16	34	12	329	36.4	6.05	109.71
Q15365	PCBP1	Poly(rC)-binc	44	12	34	6	356	37.5	7.09	116.57
P04899	GNAI2	Guanine nuc	61	17	40	10	355	40.4	5.54	128.63
Q96HC4	PDLIM5	PDZ and LIM	42	21	33	21	596	63.9	8.21	101.06
P05556	ITGB1	Integrin beta	22	16	37	16	798	88.4	5.39	118.44
Q9NRN5	OLFML3	Olfactomedin	41	15	35	15	406	46	6.57	108.6
P51888	PRELP	Prolargin OS	45	15	43	15	382	43.8	9.38	134.16
Q99613	EIF3C	Eukaryotic tr	28	25	38	25	913	105.3	5.68	108.86
P61160	ACTR2	Actin-related	39	14	35	14	394	44.7	6.74	107.01
Q9C0C2	TNKS1BP1	182 kDa tank	17	24	29	24	1729	181.7	4.86	91.34

Q02218	OGDH	2-oxoglutara	27	22	32	22	1023	115.9	6.86	88.27
Q9UBT2	UBA2	SUMO-activa	41	21	31	21	640	71.2	5.29	92.28
P30740	SERPINB1	Leukocyte el	49	19	30	19	379	42.7	6.28	97.54
Q04917	YWHAH	14-3-3 protei	70	16	46	11	246	28.2	4.84	131.4
P31930	UQCRC1	Cytochrome	41	15	32	14	480	52.6	6.37	120.69
Q13724	MOGS	Mannosyl-oli	34	21	32	21	837	91.9	8.9	100.76
P12236	SLC25A6	ADP/ATP tra	49	18	40	5	298	32.8	9.74	103.46
Q99497	PARK7	Parkinson di:	80	14	45	14	189	19.9	6.79	139.61
P42167	TMPO	Lamina-asso	42	15	33	8	454	50.6	9.38	114.62
P43243	MATR3	Matrin-3 OS=	27	26	42	26	847	94.6	6.25	114.89
Q96TA1	NIBAN2	Protein Nibar	39	22	35	21	746	84.1	6.19	93.07
P26232	CTNNA2	Catenin alph	24	19	30	12	953	105.2	5.71	95.74
P62191	PSMC1	26S proteaso	53	21	37	20	440	49.2	6.21	111.26
Q9Y5B9	SUPT16H	FACT compl	22	25	33	24	1047	119.8	5.66	93.17
P11766	ADH5	Alcohol dehy	34	14	38	14	374	39.7	7.49	122.12
Q9NQT8	KIF13B	Kinesin-like p	15	25	30	24	1826	202.7	5.88	85.56
Q9UHD8	SEPTIN9	Septin-9 OS=	42	19	34	19	586	65.4	8.97	111.61
Q9NY33	DPP3	Dipeptidyl pe	34	19	33	19	737	82.5	5.1	112.41
P42765	ACAA2	3-ketoacyl-C	59	17	32	17	397	41.9	8.09	115.52
Q9NVA2	SEPTIN11	Septin-11 OS	42	19	36	8	429	49.4	6.81	103.35
P0DOX6		Immunoglobu	29	14	43	1	576	63.4	7.87	137.65
P00387	CYB5R3	NADH-cytocl	60	18	31	17	301	34.2	7.59	106.27
P20073	ANXA7	Annexin A7 (	33	16	39	15	488	52.7	5.68	96.83
Q96CW1	AP2M1	AP-2 comple	44	19	41	19	435	49.6	9.54	127.75
P13861	PRKAR2A	cAMP-depen	50	18	28	16	404	45.5	5.07	95.05
Q6WCQ1	MPRIP	Myosin phos	28	23	26	22	1025	116.5	6.21	84.37
P23381	WARS1	Tryptophan--	38	18	26	18	471	53.1	6.23	85.22
Q13310	PABPC4	Polyadenylat	31	21	35	13	644	70.7	9.26	93.59
P40763	STAT3	Signal transc	33	19	28	19	770	88	6.3	94.31
Q15046	KARS1	Lysine--tRNA	28	17	33	17	597	68	6.35	96.92
A5YKK6	CNOT1	CCR4-NOT t	12	27	31	27	2376	266.8	7.11	81.39
Q6NUK1	SLC25A24	Calcium-binc	42	18	32	18	477	53.3	6.33	95.59
Q12931	TRAP1	Heat shock p	37	21	30	20	704	80.1	8.21	103.41

P18583	SON	Protein SON	13	22	30	22	2426	263.7	5.64	84.57
O00429	DNM1L	Dynamin-1-li	39	22	30	22	736	81.8	6.81	91.44
P00390	GSR	Glutathione r	40	16	29	16	522	56.2	8.5	103.63
P62805	H4C16	Histone H4 C	52	8	163	8	103	11.4	11.36	362.19
P00505	GOT2	Aspartate am	46	19	37	19	430	47.5	9.01	99.19
P30837	ALDH1B1	Aldehyde del	44	19	35	15	517	57.2	6.99	102.49
P02749	APOH	Beta-2-glyco	50	14	41	14	345	38.3	7.97	143.16
Q9Y262	EIF3L	Eukaryotic tr	36	18	31	18	564	66.7	6.34	100.87
P05388	RPLP0	60S acidic rib	50	14	46	14	317	34.3	5.97	146.12
O75367	MACROH2A	Core histone	47	15	40	13	369	39.2	9.83	138.61
P13674	P4HA1	Prolyl 4-hydr	46	23	37	23	534	61	6.01	112.08
Q14914	PTGR1	Prostaglandi	55	15	29	15	329	35.8	8.29	102.78
P50995	ANXA11	Annexin A11	35	17	35	17	505	54.4	7.65	115.21
O60664	PLIN3	Perilipin-3 OS	60	20	29	20	434	47	5.44	97.82
P49189	ALDH9A1	4-trimethylan	39	17	38	17	494	53.8	5.87	115.79
P69892	HBG2	Hemoglobin	84	11	40	1	147	16.1	7.2	139.86
P00739	HPR	Haptoglobin-	31	15	45	2	348	39	7.09	119.91
P09622	DLD	Dihydrolipoyl	35	13	25	13	509	54.1	7.85	95.84
Q06830	PRDX1	Peroxiredoxi	71	13	35	10	199	22.1	8.13	109.15
Q9UH99	SUN2	SUN domain	38	21	28	21	717	80.3	6.73	105.44
Q02809	PLOD1	Procollagen-	29	19	29	18	727	83.5	6.95	96.09
P08779	KRT16	Keratin, type	44	20	41	6	473	51.2	5.05	103.78
P38919	EIF4A3	Eukaryotic in	39	16	32	13	411	46.8	6.73	100.36
Q9ULV4	CORO1C	Coronin-1C C	40	19	35	18	474	53.2	7.08	109.31
Q15181	PPA1	Inorganic pyr	65	15	30	13	289	32.6	5.86	98.81
P09382	LGALS1	Galectin-1 O	93	10	88	10	135	14.7	5.5	221.53
P05165	PCCA	Propionyl-Co	35	22	29	22	728	80	7.52	78.43
P41250	GARS1	Glycine--tRN	35	20	32	20	739	83.1	7.03	83.49
P30043	BLVRB	Flavin reduct	61	9	32	9	206	22.1	7.65	105.04
Q12905	ILF2	Interleukin en	46	12	26	12	390	43	5.26	97.21
Q12884	FAP	Prolyl endope	26	20	30	19	760	87.7	6.65	87.97
Q9NSE4	IARS2	Isoleucine--tl	23	21	30	21	1012	113.7	7.2	86.43
Q99873	PRMT1	Protein argin	39	15	27	15	371	42.4	5.35	82.12

P02461	COL3A1	Collagen alpl	17	24	37	24	1466	138.5	6.61	93.38
P02748	C9	Complement	35	17	38	16	559	63.1	5.59	103.6
P45880	VDAC2	Voltage-depe	52	12	36	12	294	31.5	7.56	134.96
P04632	CAPNS1	Calpain smal	51	7	20	7	268	28.3	5.2	95.7
P20908	COL5A1	Collagen alpl	12	15	28	13	1838	183.4	5.06	93.04
P24844	MYL9	Myosin regul	71	11	58	4	172	19.8	4.92	165
Q14847	LASP1	LIM and SH3	57	17	36	17	261	29.7	7.05	95.15
Q9Y2A7	NCKAP1	Nck-associat	24	24	31	23	1128	128.7	6.62	83.3
Q9UJS0	SLC25A13	Electrogenic	40	18	27	15	675	74.1	8.62	85.8
P08237	PFKM	ATP-depend	34	24	30	21	780	85.1	7.99	81.64
P52597	HNRNPF	Heterogeneo	34	12	32	9	415	45.6	5.58	99.83
P52566	ARHGDIB	Rho GDP-dis	67	11	27	11	201	23	5.21	83.17
Q93009	USP7	Ubiquitin car	26	22	27	22	1102	128.2	5.55	84.8
Q14126	DSG2	Desmoglein-	28	22	26	22	1118	122.2	5.24	78.17
P54578	USP14	Ubiquitin car	40	14	25	14	494	56	5.3	92.81
Q5SSJ5	HP1BP3	Heterochrom	36	20	36	20	553	61.2	9.67	99.08
P51911	CNN1	Calponin-1 C	53	15	36	12	297	33.2	9.07	80.94
Q96AG4	LRRC59	Leucine-rich	46	16	38	16	307	34.9	9.57	101.55
P07737	PFN1	Profilin-1 OS	65	11	41	11	140	15	8.27	119.94
P49321	NASP	Nuclear auto	39	22	33	22	788	85.2	4.3	96.24
Q9NQW7	XPNPEP1	Xaa-Pro ami	36	20	28	20	623	69.9	5.67	80.1
Q9UKV3	ACIN1	Apoptotic chi	21	26	34	25	1341	151.8	6.43	93.74
O00232	PSMD12	26S proteaso	33	15	29	15	456	52.9	7.65	86.99
O95831	AIFM1	Apoptosis-in	25	12	28	12	613	66.9	8.95	99.04
P51572	BCAP31	B-cell recept	50	17	41	17	246	28	8.44	104.49
P21397	MAOA	Amine oxidas	33	18	31	14	527	59.6	7.85	86.14
P61221	ABCE1	ATP-binding	33	18	27	18	599	67.3	8.34	83.95
P54886	ALDH18A1	Delta-1-pyrro	23	19	30	19	795	87.2	7.12	75.76
Q9UNH7	SNX6	Sorting nexir	46	21	34	19	406	46.6	6.16	101.86
O43252	PAPSS1	Bifunctional 3	38	21	29	20	624	70.8	6.86	82.67
Q14258	TRIM25	E3 ubiquitin/I	34	18	24	18	630	70.9	8.09	86.14
P39060	COL18A1	Collagen alpl	11	15	34	15	1754	178.1	6.01	108.64
P62333	PSMC6	26S proteaso	47	14	30	14	389	44.1	7.49	89.83

O15144	ARPC2	Actin-related	57	19	44	19	300	34.3	7.36	105.96
P47895	ALDH1A3	Aldehyde del	37	16	36	12	512	56.1	7.25	108.09
P17480	UBTF	Nucleolar tra	31	23	36	23	764	89.4	5.81	92.04
Q8TBC4	UBA3	NEDD8-activ	46	16	22	16	463	51.8	5.45	75.63
Q9UDY2	TJP2	Tight junctior	25	24	31	23	1190	133.9	7.4	89.48
Q14151	SAFB2	Scaffold atta	20	21	34	10	953	107.4	6.16	90.53
P05141	SLC25A5	ADP/ATP tra	39	15	33	5	298	32.8	9.69	86.9
Q07955	SRSF1	Serine/argini	53	17	45	17	248	27.7	10.36	129.58
P0DOX7		Immunoglobu	41	6	87	2	214	23.4	7.17	278.14
Q9HC38	GLOD4	Glyoxalase d	52	16	32	16	313	34.8	5.6	96.23
P29508	SERPINB3	Serpin B3 OS	46	17	29	9	390	44.5	6.81	81.76
P09871	C1S	Complement	32	18	30	17	688	76.6	4.96	91.08
P30084	ECHS1	Enoyl-CoA h	55	16	36	16	290	31.4	8.07	113.75
O60716	CTNND1	Catenin delta	25	24	29	24	968	108.1	6.23	86.69
Q9HD20	ATP13A1	Endoplasmic	19	20	27	20	1204	132.9	8.13	87.15
O94855	SEC24D	Protein trans	25	22	31	21	1032	112.9	7.25	93.3
Q86XP3	DDX42	ATP-depend	26	21	29	21	938	102.9	7.02	95.62
P69891	HBG1	Hemoglobin	84	11	37	1	147	16.1	7.2	126.2
P38159	RBMX	RNA-binding	51	23	39	9	391	42.3	10.05	100.58
P30086	PEBP1	Phosphatidyl	56	11	33	11	187	21	7.53	122.01
P49915	GMPS	GMP synthas	37	22	32	22	693	76.7	6.87	82.86
Q96G03	PGM2	Phosphopen	38	22	38	20	612	68.2	6.73	91.45
Q9Y266	NUDC	Nuclear migr	51	18	28	18	331	38.2	5.38	75.48
Q13428	TCOF1	Treacle prote	16	22	31	22	1488	152	9.04	81.34
Q9H0U4	RAB1B	Ras-related p	69	11	39	4	201	22.2	5.73	115.89
P21266	GSTM3	Glutathione S	55	15	37	13	225	26.5	5.54	113.51
Q13011	ECH1	Delta(3,5)-De	39	11	30	11	328	35.8	8	87.43
P34897	SHMT2	Serine hydro	38	18	30	16	504	56	8.53	84.75
Q13045	FLII	Protein flight	22	23	28	23	1269	144.7	6.05	86.39
Q9Y5S2	CDC42BPB	Serine/threor	16	26	31	23	1711	194.2	6.37	85.61
Q9UHX1	PUF60	Poly(U)-bindi	41	17	27	17	559	59.8	5.29	102.23
Q16851	UGP2	UTP--glucose	36	17	28	17	508	56.9	8.15	83.19
Q9NXG2	THUMPD1	THUMP dom	48	15	28	15	353	39.3	7.88	89.67

Q9H223	EHD4	EH domain-c	40	15	31	11	541	61.1	6.76	98.84
P18124	RPL7	60S ribosom	42	15	30	15	248	29.2	10.65	79.11
P13671	C6	Complement	26	22	33	22	934	104.7	6.76	89.43
A0FGR8	ESYT2	Extended syı	25	17	24	17	921	102.3	9.26	84.88
Q07960	ARHGAP1	Rho GTPase	42	15	27	15	439	50.4	6.29	93.93
P08572	COL4A2	Collagen alp	11	13	29	13	1712	167.4	8.66	99.11
Q13510	ASAH1	Acid ceramid	39	15	32	15	395	44.6	7.62	101.27
P01834	IGKC	Immunoglobu	64	5	121	1	107	11.8	6.52	309.68
P62879	GNB2	Guanine nuc	41	11	25	4	340	37.3	6	87.36
O00231	PSMD11	26S proteaso	47	18	29	18	422	47.4	6.48	93.85
P06401	PGR	Progesterone	27	15	23	15	933	98.9	6.49	80.67
Q9NTK5	OLA1	Obg-like ATF	51	17	31	17	396	44.7	7.81	78.31
P18085	ARF4	ADP-ribosyla	64	10	34	6	180	20.5	7.14	117.35
P10155	RO60	RNA-binding	39	19	31	19	538	60.6	8.03	101.95
O75390	CS	Citrate synth	31	14	32	14	466	51.7	8.32	77.16
Q8NBS9	TXNDC5	Thioredoxin (	39	17	33	17	432	47.6	5.97	96.5
P16401	H1-5	Histone H1.5	42	13	39	9	226	22.6	10.92	107.39
P47756	CAPZB	F-actin-capp	53	15	36	15	272	30.6	6	105.8
P31040	SDHA	Succinate de	31	17	26	17	664	72.6	7.39	94.62
Q13418	ILK	Integrin-linke	36	17	36	17	452	51.4	8.07	96.32
Q9NZB2	FAM120A	Constitutive (	19	19	29	18	1118	121.8	8.88	93.31
P62701	RPS4X	40S ribosom	57	17	42	17	263	29.6	10.15	104.29
P02786	TFRC	Transferrin re	27	16	25	16	760	84.8	6.61	82.63
Q9UHB6	LIMA1	LIM domain a	28	21	30	20	759	85.2	6.84	87.48
P32455	GBP1	Guanylate-bi	31	18	28	17	592	67.9	6.32	91.17
Q9HCC0	MCCC2	Methylcroton	42	18	25	18	563	61.3	7.68	83.09
P51531	SMARCA2	Probable glo	16	24	28	14	1590	181.2	7.2	72.98
P04003	C4BPA	C4b-binding	28	14	35	14	597	67	7.3	112.65
P35232	PHB1	Prohibitin 1 (	64	15	30	15	272	29.8	5.76	95.34
Q8N1G4	LRRC47	Leucine-rich	45	18	27	18	583	63.4	8.28	85.43
Q9P2B2	PTGFRN	Prostaglandi	27	23	30	23	879	98.5	6.61	82.43
Q86UU1	PHLDB1	Pleckstrin ho	16	19	29	19	1377	151.1	8.63	94.67
P48637	GSS	Glutathione s	49	20	31	20	474	52.4	5.92	105.83

Q9NYL9	TMOD3	Tropomoduli	46	15	25	14	352	39.6	5.19	80.58
Q15366	PCBP2	Poly(rC)-binc	44	12	29	5	365	38.6	6.79	92.76
P04004	VTN	Vitronectin C	32	12	36	12	478	54.3	5.8	96.1
P10515	DLAT	Dihydrolipoyl	28	13	26	13	647	69	7.84	93.4
P01011	SERPINA3	Alpha-1-antic	37	13	49	13	423	47.6	5.52	132.44
Q13740	ALCAM	CD166 antig	28	15	24	15	583	65.1	6.25	85.99
Q14766	LTBP1	Latent-transf	12	20	26	19	1721	186.7	5.96	80.02
P32119	PRDX2	Peroxiredoxi	42	12	41	11	198	21.9	5.97	119.73
Q16610	ECM1	Extracellular	38	17	27	17	540	60.6	6.71	84.97
P33778	H2BC3	Histone H2B	63	11	130	1	126	13.9	10.32	292.12
Q16698	DECR1	2,4-dienoyl-C	52	15	26	15	335	36	9.28	80.46
Q08378	GOLGA3	Golgin subfa	16	22	25	21	1498	167.3	5.44	75.66
P58107	EPPK1	Epiplakin OS	24	19	27	14	5088	555.3	5.62	79.43
P43034	PAFAH1B1	Platelet-activ	44	15	26	15	410	46.6	7.37	81.13
P56192	MARS1	Methionine--	28	19	25	19	900	101.1	6.16	86.51
P51858	HDGF	Hepatoma-de	67	15	26	14	240	26.8	4.73	90.86
O14974	PPP1R12A	Protein phos	23	20	26	20	1030	115.2	5.4	78.44
P60228	EIF3E	Eukaryotic tr	47	20	34	20	445	52.2	6.04	95.63
Q99880	H2BC13	Histone H2B	63	11	144	1	126	13.9	10.32	307.99
Q5JPE7	NOMO2	Nodal modul	20	20	27	20	1267	139.4	5.76	89.56
P08559	PDHA1	Pyruvate deh	44	18	29	18	390	43.3	8.06	79.83
P49591	SARS1	Serine--tRNA	53	22	27	22	514	58.7	6.43	79.73
Q9BXP5	SRRT	Serrate RNA	23	18	25	18	876	100.6	5.96	64.92
P04196	HRG	Histidine-rich	30	14	30	14	525	59.5	7.5	95.16
P36955	SERPINF1	Pigment epit	34	14	32	14	418	46.3	6.38	90.09
P28482	MAPK1	Mitogen-activ	53	16	34	12	360	41.4	6.98	108.63
P07099	EPHX1	Epoxide hydr	38	14	28	14	455	52.9	7.25	85.19
Q99879	H2BC14	Histone H2B	63	11	145	1	126	14	10.32	310.45
P35611	ADD1	Alpha-adduc	26	15	26	14	737	80.9	5.83	82.05
P51532	SMARCA4	Transcription	14	21	28	11	1647	184.5	7.88	78.49
P09972	ALDOC	Fructose-bis	47	15	29	9	364	39.4	6.87	102.09
Q5QNW6	H2BC18	Histone H2B	63	11	145	1	126	13.9	10.32	310.45
Q86UX7	FERMT3	Fermitin fam	33	16	24	16	667	75.9	6.98	76.12

P20020	ATP2B1	Plasma mem	18	18	28	7	1220	134.6	5.91	77.06
P55196	AFDN	Afadin OS=H	15	21	27	21	1824	206.7	6.47	74.4
Q8N257	H2BC26	Histone H2B	59	10	126	1	126	13.9	10.32	285.45
P08708	RPS17	40S ribosom	66	10	27	10	135	15.5	9.85	99.41
Q96ST3	SIN3A	Paired amph	19	18	25	18	1273	145.1	7.25	76.11
Q92538	GBF1	Golgi-specifi	14	22	25	22	1859	206.3	5.73	62.97
P26368	U2AF2	Splicing facto	38	13	21	13	475	53.5	9.09	82.26
O43242	PSMD3	26S proteaso	34	16	25	16	534	60.9	8.44	80.71
Q9NR45	NANS	Sialic acid sy	52	14	24	14	359	40.3	6.74	82.78
P23141	CES1	Liver carboxy	35	18	32	16	567	62.5	6.6	75.61
Q6XQN6	NAPRT	Nicotinate ph	42	16	24	16	538	57.5	5.68	85.77
P22392	NME2	Nucleoside d	69	10	34	5	152	17.3	8.41	108.69
P50502	ST13	Hsc70-intera	31	12	39	12	369	41.3	5.27	118.26
P00918	CA2	Carbonic anh	65	14	31	14	260	29.2	7.4	103.6
P43652	AFM	Afamin OS=H	29	18	31	18	599	69	5.9	79.65
O76021	RSL1D1	Ribosomal L	36	20	26	20	490	54.9	10.13	77.02
Q9H4A4	RNPEP	Aminopeptida	32	18	24	18	650	72.5	5.74	77.69
Q96HE7	ERO1A	ERO1-like pr	44	18	26	16	468	54.4	5.68	81.83
P13804	ETFA	Electron tran	47	11	22	11	333	35.1	8.38	86.07
P42224	STAT1	Signal transc	25	17	25	17	750	87.3	6.05	78
P51149	RAB7A	Ras-related p	73	13	32	13	207	23.5	6.7	106.44
P51610	HCFC1	Host cell fact	14	21	25	21	2035	208.6	7.46	72.47
Q9Y4F1	FARP1	FERM, ARHG	22	23	33	22	1045	118.6	8.15	81.82
O95479	H6PD	GDH/6PGL e	25	16	29	16	791	88.8	7.3	88.41
P60660	MYL6	Myosin light p	54	9	39	7	151	16.9	4.65	104.88
Q16401	PSMD5	26S proteaso	44	17	24	17	504	56.2	5.48	86.01
P11532	DMD	Dystrophin O	8	24	28	23	3685	426.5	5.88	73.41
P62753	RPS6	40S ribosom	40	12	31	12	249	28.7	10.84	97.65
P16435	POR	NADPH--cyto	29	17	25	17	677	76.6	5.58	72.83
P47755	CAPZA2	F-actin-capp	64	12	21	10	286	32.9	5.85	74.25
Q00796	SORD	Sorbitol dehy	45	12	27	12	357	38.3	7.97	77.25
P12081	HARS1	Histidine--tRI	47	20	27	16	509	57.4	5.88	72.33
P55809	OXCT1	Succinyl-CoA	42	15	20	15	520	56.1	7.46	71.5

O60264	SMARCA5	SWI/SNF-rel	20	23	31	14	1052	121.8	8.09	76.85
P01833	PIGR	Polymeric im	25	16	27	16	764	83.2	5.74	83.29
P61247	RPS3A	40S ribosom	61	19	58	19	264	29.9	9.73	142.07
P08473	MME	Neprilysin O	24	18	31	18	750	85.5	5.73	83.77
P49821	NDUFV1	NADH dehyd	47	17	25	17	464	50.8	8.21	83.75
Q92626	PXDN	Peroxidasin	18	21	27	21	1479	165.2	7.17	80.05
P52907	CAPZA1	F-actin-capp	58	10	21	8	286	32.9	5.69	81.2
Q9UJU6	DBNL	Drebrin-like	44	15	29	15	430	48.2	5.05	91.77
O60749	SNX2	Sorting nexir	35	17	28	15	519	58.4	5.12	79.17
P16144	ITGB4	Integrin beta	14	21	24	21	1822	202	6.09	66.98
Q9UKM9	RALY	RNA-binding	39	12	24	12	306	32.4	9.17	76.38
P25311	AZGP1	Zinc-alpha-2	53	16	31	16	298	34.2	6.05	87.15
P30044	PRDX5	Peroxiredoxi	50	8	24	8	214	22.1	8.7	92.6
P28289	TMOD1	Tropomoduli	51	15	25	14	359	40.5	5.1	87.78
P50453	SERPINB9	Serpin B9 O	48	17	25	16	376	42.4	5.86	79.64
Q9HC35	EML4	Echinoderm	20	18	24	18	981	108.8	6.4	71.29
Q8N766	EMC1	ER membrar	19	17	23	17	993	111.7	7.66	67.76
P49589	CARS1	Cysteine--tR	30	18	22	17	748	85.4	6.76	69.25
O95202	LETM1	Mitochondria	34	19	27	19	739	83.3	6.7	74.02
P62834	RAP1A	Ras-related	65	11	28	3	184	21	6.67	97.85
P22087	FBL	rRNA 2'-O-m	36	11	25	11	321	33.8	10.18	85.45
O94874	UFL1	E3 UFM1-pro	26	18	24	18	794	89.5	6.79	68.36
Q3LXA3	TKFC	Triokinase/F	34	17	26	17	575	58.9	7.49	88.83
P57053	H2BC12L	Histone H2B	67	10	140	2	126	13.9	10.37	299.2
O14776	TCERG1	Transcriptior	17	21	26	21	1098	123.8	8.65	66.26
Q5TZA2	CROCC	Rootletin OS	12	24	26	22	2017	228.3	5.49	63.87
Q7L266	ASRGL1	Isoaspartyl p	42	9	27	9	308	32	6.24	102.92
P56199	ITGA1	Integrin alpha	16	21	32	21	1179	130.8	6.29	80.74
Q9Y6N5	SQOR	Sulfide:quino	48	17	26	17	450	49.9	9.11	86.48
Q5JRX3	PITRM1	Presequence	26	25	28	25	1037	117.3	6.92	86.11
P10909	CLU	Clusterin OS	27	15	27	15	449	52.5	6.27	84.41
P22059	OSBP	Oxysterol-bir	24	21	28	21	807	89.4	7.3	77.16
Q86VB7	CD163	Scavenger re	20	19	29	19	1156	125.4	5.95	83.91

P62917	RPL8	60S ribosom	42	13	26	13	257	28	11.03	74.91
Q8TAQ2	SMARCC2	SWI/SNF co	17	20	26	13	1214	132.8	5.69	77.99
P05546	SERPIND1	Heparin cofa	34	16	33	16	499	57	6.9	90.61
Q9NR12	PDLIM7	PDZ and LIM	33	15	32	15	457	49.8	8.41	91.1
Q9BZZ5	API5	Apoptosis inl	34	15	24	15	524	59	7.34	86.18
P61204	ARF3	ADP-ribosyla	59	10	28	5	181	20.6	7.43	97.01
P05198	EIF2S1	Eukaryotic tr	55	15	28	15	315	36.1	5.08	85.72
P09104	ENO2	Gamma-eno	46	14	43	11	434	47.2	5.03	116.54
P78344	EIF4G2	Eukaryotic tr	24	22	26	22	907	102.3	7.14	70.95
P62136	PPP1CA	Serine/threo	47	12	22	4	330	37.5	6.33	78.37
P12429	ANXA3	Annexin A3 (	52	16	25	16	323	36.4	5.92	76.55
P29536	LMOD1	Leiomodin-1	33	21	27	21	600	67	9.33	65.46
P12235	SLC25A4	ADP/ATP tra	42	14	30	5	298	33	9.76	73.91
Q9UBC2	EPS15L1	Epidermal gr	30	19	23	19	864	94.2	5.11	61.36
Q14194	CRMP1	Dihydropyrim	36	16	26	11	572	62.1	7.03	87.2
Q9BS26	ERP44	Endoplasmic	36	14	24	14	406	46.9	5.26	79.55
P22695	UQCRC2	Cytochrome	38	15	26	15	453	48.4	8.63	83.71
O00764	PDXK	Pyridoxal kin	53	15	25	15	312	35.1	6.13	77.16
P62140	PPP1CB	Serine/threo	55	12	21	4	327	37.2	6.19	77.91
Q93034	CUL5	Cullin-5 OS=	25	17	26	17	780	90.9	7.94	66.01
Q96AE4	FUBP1	Far upstream	29	16	29	12	644	67.5	7.61	78.01
P00325	ADH1B	All-trans-reti	43	15	27	15	375	39.8	8.19	84.63
P43686	PSMC4	26S proteaso	52	17	22	16	418	47.3	5.21	75.47
O75396	SEC22B	Vesicle-traffi	57	11	25	11	215	24.7	8.51	81.88
Q16647	PTGIS	Prostacyclin	28	11	23	11	500	57.1	7.31	77.81
Q9UQ80	PA2G4	Proliferation-	38	14	27	14	394	43.8	6.55	81.05
P08865	RPSA	40S ribosom	41	11	25	11	295	32.8	4.87	95.63
O43795	MYO1B	Unconventio	19	19	24	18	1136	131.9	9.38	65.88
Q9UNM6	PSMD13	26S proteaso	46	16	26	16	376	42.9	5.81	81.7
Q0ZGT2	NEXN	Nexilin OS=H	32	22	32	22	675	80.6	5.33	72.19
P16949	STMN1	Stathmin OS	68	14	30	14	149	17.3	5.97	84.4
P08575	PTPRC	Receptor-typ	17	21	24	21	1306	147.4	6.15	60.11
Q9H0D6	XRN2	5'-3' exoribo	19	15	21	15	950	108.5	7.47	68.82

P62820	RAB1A	Ras-related p	61	12	39	5	205	22.7	6.21	107.73
Q9NQC3	RTN4	Reticulon-4 (	11	8	28	8	1192	129.9	4.5	92.47
P24928	POLR2A	DNA-directed	14	23	25	23	1970	217	7.37	62.58
P02765	AHSG	Alpha-2-HS-g	39	12	49	12	367	39.3	5.72	123.72
Q5JRA6	MIA3	Transport an	13	23	25	22	1907	213.6	4.84	68.06
Q14192	FHL2	Four and a h	58	15	30	15	279	32.2	7.55	85.86
P05107	ITGB2	Integrin beta-	27	17	23	17	769	84.7	6.95	69.18
Q14254	FLOT2	Flotillin-2 OS	45	18	24	18	428	47	5.25	70.6
Q9UEY8	ADD3	Gamma-add	22	14	23	14	706	79.1	6.32	79.99
Q13451	FKBP5	Peptidyl-prol	45	18	26	18	457	51.2	5.9	71.32
P62873	GNB1	Guanine nuc	49	12	26	6	340	37.4	6	82.04
P22307	SCP2	Sterol carrier	32	14	26	14	547	59	6.89	81.73
Q9UBS4	DNAJB11	DnaJ homolo	37	12	21	12	358	40.5	6.18	72.74
P61201	COPS2	COP9 signal	39	17	24	17	443	51.6	5.53	63.65
P0DOX2		Immunoglobu	39	13	30	6	455	48.9	6.67	93.01
P60900	PSMA6	Proteasome	54	13	24	13	246	27.4	6.76	84.31
P48506	GCLC	Glutamate--c	33	18	27	17	637	72.7	6.09	81.57
Q8NC51	SERBP1	Plasminogen	38	15	33	15	408	44.9	8.65	87.04
P02760	AMBP	Protein AMB	37	10	21	10	352	39	6.25	71.1
Q15477	SKIC2	SKI2 subunit	17	19	22	18	1246	137.7	6.06	62.34
Q9P0L0	VAPA	Vesicle-asso	41	10	24	9	249	27.9	8.62	68.64
P38117	ETFB	Electron tran	65	14	25	14	255	27.8	8.1	74.65
Q15404	RSU1	Ras suppres	43	11	28	11	277	31.5	8.65	83.48
Q9BQG0	MYBBP1A	Myb-binding	16	18	25	18	1328	148.8	9.28	74.19
P61011	SRP54	Signal recog	39	17	27	17	504	55.7	8.75	72.28
P20774	OGN	Mimecan OS	46	13	36	13	298	33.9	5.63	102.69
O14979	HNRNPDL	Heterogeneo	27	12	24	11	420	46.4	9.57	75.81
P19012	KRT15	Keratin, type	29	18	34	3	456	49.2	4.77	86.92
Q96I24	FUBP3	Far upstream	36	18	28	16	572	61.6	8.38	75.78
Q9Y3F4	STRAP	Serine-threo	49	13	26	13	350	38.4	5.12	86.28
Q8N1F7	NUP93	Nuclear pore	26	20	25	20	819	93.4	5.72	67.35
Q92597	NDRG1	Protein NDR	48	10	24	10	394	42.8	5.82	78.38
Q13557	CAMK2D	Calcium/caln	30	13	26	9	499	56.3	7.25	73.3

P60981	DSTN	Destrin OS=I	53	11	31	10	165	18.5	7.85	87.06
O15031	PLXNB2	Plexin-B2 OS	15	22	24	22	1838	205	6.24	64.01
P13716	ALAD	Delta-aminol	38	11	21	11	330	36.3	6.79	71.33
O75947	ATP5PD	ATP synthas	78	12	28	12	161	18.5	5.3	76.28
Q92621	NUP205	Nuclear pore	11	20	23	20	2012	227.8	6.19	67.79
Q13177	PAK2	Serine/threoı	33	14	21	11	524	58	5.96	70.19
O75131	CPNE3	Copine-3 OS	34	15	25	14	537	60.1	5.85	87.45
Q96D15	RCN3	Reticulocalbi	39	11	30	11	328	37.5	4.89	109.23
Q9UKV8	AGO2	Protein argoı	22	17	23	12	859	97.1	9.19	65.76
P11678	EPX	Eosinophil pe	22	16	30	14	715	81	10.29	71.8
O95302	FKBP9	Peptidyl-proly	28	15	24	14	570	63	5.08	74.76
Q9BZF9	UACA	Uveal autoar	16	22	24	22	1416	162.4	7.03	51.99
Q32P28	P3H1	Prolyl 3-hydr	23	15	27	14	736	83.3	5.14	77.67
Q6DD88	ATL3	Atlastin-3 OS	33	14	25	14	541	60.5	5.66	73.24
O75955	FLOT1	Flotillin-1 OS	52	18	21	18	427	47.3	7.49	61.55
Q9H3S7	PTPN23	Tyrosine-pro	14	19	25	19	1636	178.9	6.92	74.49
P25786	PSMA1	Proteasome	42	12	30	12	263	29.5	6.61	84.11
Q00325	SLC25A3	Phosphate ca	27	11	36	11	362	40.1	9.38	82.86
P50851	LRBA	Lipopolysacc	8	21	24	20	2863	318.9	5.6	55.44
P63241	EIF5A	Eukaryotic tr	56	11	28	5	154	16.8	5.24	86.7
Q9NQR4	NIT2	Omega-amic	64	14	22	14	276	30.6	7.21	70.94
P21964	COMT	Catechol O-r	47	13	23	13	271	30	5.47	65.22
P27487	DPP4	Dipeptidyl pe	26	21	28	21	766	88.2	6.04	70.19
Q08380	LGALS3BP	Galectin-3-bi	27	11	21	11	585	65.3	5.27	74.51
Q99536	VAT1	Synaptic ves	34	10	28	10	393	41.9	6.29	89.37
P11387	TOP1	DNA topoiso	22	17	25	17	765	90.7	9.31	67.6
Q9UQ35	SRRM2	Serine/argini	9	20	22	20	2752	299.4	12.06	62.94
Q13596	SNX1	Sorting nexir	30	14	22	12	522	59	5.15	62.33
P35556	FBN2	Fibrillin-2 OS	7	17	32	14	2912	314.6	4.86	54.2
P42166	TMPO	Lamina-asso	25	12	22	5	694	75.4	7.66	78.16
Q13642	FHL1	Four and a h	41	12	30	12	323	36.2	8.97	84
P54819	AK2	Adenylate kiı	51	10	27	10	239	26.5	7.81	78.82
Q15942	ZYX	Zyxin OS=Hc	32	13	21	13	572	61.2	6.67	70.75

P30626	SRI	Sorcin OS=H	57	9	22	9	198	21.7	5.59	71.84
Q99623	PHB2	Prohibitin-2 (	51	16	27	16	299	33.3	9.83	81.83
Q14247	CTTN	Src substrate	29	17	25	17	550	61.5	5.4	71.12
Q96E39	RBMXL1	RNA binding	39	17	27	3	390	42.1	9.89	74.98
P67809	YBX1	Y-box-bindin	39	9	15	8	324	35.9	9.88	66.36
Q14738	PPP2R5D	Serine/threor	29	16	25	13	602	69.9	8.13	75.36
Q29RF7	PDS5A	Sister chrom	17	22	25	18	1337	150.7	7.91	68.55
O00264	PGRMC1	Membrane-a	66	12	24	10	195	21.7	4.7	67.75
P48594	SERPINB4	Serpin B4 OS	38	14	26	6	390	44.8	6.21	73.02
P50479	PDLIM4	PDZ and LIM	47	13	20	13	330	35.4	7.91	65.67
O94760	DDAH1	N(G),N(G)-di	58	16	21	15	285	31.1	5.81	64.14
P10768	ESD	S-formylgluta	50	11	25	11	282	31.4	7.02	82.95
Q16836	HADH	Hydroxyacyl-	40	12	28	12	314	34.3	8.85	80.16
Q9Y2W1	THRAP3	Thyroid horm	22	21	26	20	955	108.6	10.15	73.62
O76094	SRP72	Signal recog	30	18	22	18	671	74.6	9.26	66.23
Q9NZU5	LMCD1	LIM and cyst	56	16	23	16	365	40.8	7.93	69.24
P15531	NME1	Nucleoside d	55	8	21	3	152	17.1	6.19	75.07
P09543	CNP	2',3'-cyclic-nu	35	15	23	14	421	47.5	9.07	73.36
O75351	VPS4B	Vacuolar pro	46	17	23	13	444	49.3	7.23	68.92
Q96I99	SUCLG2	Succinate--C	39	13	21	12	432	46.5	6.39	74.43
P62750	RPL23A	60S ribosom	53	12	44	12	156	17.7	10.45	89.75
P21281	ATP6V1B2	V-type protor	41	16	22	16	511	56.5	5.81	75.13
P24752	ACAT1	Acetyl-CoA a	36	11	22	11	427	45.2	8.85	80.53
P51148	RAB5C	Ras-related p	54	8	26	6	216	23.5	8.41	83.42
P30040	ERP29	Endoplasmic	47	12	26	12	261	29	7.31	72.38
O43488	AKR7A2	Aflatoxin B1	28	11	29	11	359	39.6	7.17	81.51
P35613	BSG	Basigin OS=	38	12	22	12	385	42.2	5.66	76.24
P09936	UCHL1	Ubiquitin car	53	10	21	10	223	24.8	5.48	78.07
P51648	ALDH3A2	Aldehyde del	28	13	21	13	485	54.8	7.88	66.96
P49747	COMP	Cartilage olig	28	13	18	12	757	82.8	4.6	63.37
Q9HCB6	SPON1	Spondin-1 O	24	14	23	14	807	90.9	6.11	68.15
Q12769	NUP160	Nuclear pore	14	16	19	16	1436	162	5.5	56.66
Q14166	TTLL12	Tubulin--tyro	34	15	17	15	644	74.4	5.53	58.07

P55209	NAP1L1	Nucleosome	36	10	21	9	391	45.3	4.46	85.25
P11171	EPB41	Protein 4.1 C	24	17	23	15	864	97	5.58	78.99
P31689	DNAJA1	DnaJ homolc	46	14	20	14	397	44.8	7.08	68.24
Q13464	ROCK1	Rho-associat	19	22	28	14	1354	158.1	5.9	60.3
P02792	FTL	Ferritin light	51	10	29	10	175	20	5.78	87.72
P24534	EEF1B2	Elongation fa	52	9	24	8	225	24.7	4.67	82.53
P19971	TYMP	Thymidine pl	33	11	18	11	482	49.9	5.53	64.86
P10253	GAA	Lysosomal a	17	13	20	13	952	105.3	6	74.47
P27635	RPL10	60S ribosom	45	11	32	11	214	24.6	10.08	105.47
Q9UIJ7	AK3	GTP:AMP ph	55	12	25	12	227	25.6	9.16	75.75
Q9UBE0	SAE1	SUMO-activa	53	13	19	13	346	38.4	5.3	52.68
Q15124	PGM5	Phosphogluc	35	18	25	18	567	62.2	7.21	62.79
P25788	PSMA3	Proteasome	38	12	23	12	255	28.4	5.33	62.05
P62906	RPL10A	60S ribosom	44	11	33	11	217	24.8	9.94	91.85
P12004	PCNA	Proliferating	51	11	22	11	261	28.8	4.69	68.17
Q9ULA0	DNPEP	Aspartyl amir	31	13	20	13	485	53.4	7.58	59.4
P51608	MECP2	Methyl-CpG-	32	14	19	14	486	52.4	9.95	65
Q9Y2J2	EPB41L3	Band 4.1-like	15	18	23	15	1087	120.6	5.19	64.59
Q9Y285	FARSA	Phenylalanin	32	13	21	13	508	57.5	7.8	73.4
P22061	PCMT1	Protein-L-iso	63	13	21	13	227	24.6	7.21	68.53
P05387	RPLP2	60S acidic rib	69	7	18	6	115	11.7	4.54	74.05
O75475	PSIP1	PC4 and SFI	32	17	26	16	530	60.1	9.13	67.48
Q13126	MTAP	S-methyl-5'-t	65	11	21	11	283	31.2	7.18	74.11
P05109	S100A8	Protein S100	71	11	56	11	93	10.8	7.03	129.74
O00469	PLOD2	Procollagen-	26	17	20	17	737	84.6	6.71	57.82
O94832	MYO1D	Unconventior	19	19	23	18	1006	116.1	9.39	61.68
P61586	RHOA	Transforming	54	11	29	5	193	21.8	6.1	84.12
Q12965	MYO1E	Unconventior	24	22	25	19	1108	127	8.92	50.88
P06702	S100A9	Protein S100	88	10	59	10	114	13.2	6.13	155.71
O75923	DYSF	Dysferlin OS	12	22	22	20	2080	237.1	5.64	51.73
P52948	NUP98	Nuclear pore	11	16	19	16	1817	197.5	6.4	59.43
P02763	ORM1	Alpha-1-acid	43	9	33	6	201	23.5	5.11	122.19
Q7L1Q6	BZW1	eIF5-mimic p	27	14	27	14	419	48	5.92	82

Q9H2G2	SLK	STE20-like s	18	19	22	18	1235	142.6	5.15	58.02
P07686	HEXB	Beta-hexosa	23	11	21	11	556	63.1	6.76	62.73
O95967	EFEMP2	EGF-contain	35	11	18	11	443	49.4	4.94	63.38
Q53GG5	PDLIM3	PDZ and LIM	42	11	23	11	364	39.2	6.89	71.52
P08754	GNAI3	Guanine nuc	37	11	24	4	354	40.5	5.69	72.88
P55265	ADAR	Double-stran	18	22	26	22	1226	136	8.65	64.89
P61019	RAB2A	Ras-related p	51	10	21	4	212	23.5	6.54	69
Q9BUQ8	DDX23	Probable AT	22	16	22	16	820	95.5	9.55	58
P17066	HSPA6	Heat shock 7	21	12	27	1	643	71	6.14	72.57
Q15020	SART3	Squamous c	18	15	19	15	963	109.9	5.57	60.89
P46060	RANGAP1	Ran GTPase	37	17	21	17	587	63.5	4.68	60.68
Q08AF3	SLFN5	Schlafen fam	24	21	25	19	891	101	8.22	61.52
P35637	FUS	RNA-binding	18	9	24	7	526	53.4	9.36	79.89
P28066	PSMA5	Proteasome	49	9	17	9	241	26.4	4.79	72.81
Q07866	KLC1	Kinesin light	28	14	19	9	573	65.3	6.2	60.32
A2RRP1	NBAS	NBAS subun	10	20	22	20	2371	268.4	5.96	61.64
Q01105	SET	Protein SET	34	9	17	9	290	33.5	4.32	71.76
Q9H361	PABPC3	Polyadenylat	26	16	28	1	631	70	9.67	76.69
P05155	SERPING1	Plasma prote	20	9	25	9	500	55.1	6.55	74.19
Q9UGP8	SEC63	Translocatio	28	17	21	17	760	87.9	5.31	62.87
Q8IZ83	ALDH16A1	Aldehyde de	22	12	18	12	802	85.1	6.79	69.59
Q9NR30	DDX21	Nucleolar RN	27	17	19	16	783	87.3	9.28	56.05
Q9UJ70	NAGK	N-acetyl-D-g	43	13	17	13	344	37.4	6.24	65.33
Q9NSD9	FARSB	Phenylalanin	27	16	27	16	589	66.1	6.84	75.42
P25789	PSMA4	Proteasome	45	11	21	11	261	29.5	7.72	66.83
O43301	HSPA12A	Heat shock 7	27	16	22	15	675	74.9	6.77	69.91
P02649	APOE	Apolipoprote	52	16	22	16	317	36.1	5.73	67.36
P61916	NPC2	NPC intracel	48	6	19	6	151	16.6	7.65	73.48
Q02878	RPL6	60S ribosom	45	18	29	18	288	32.7	10.58	66.96
O75534	CSDE1	Cold shock d	24	20	25	20	798	88.8	6.25	53.2
Q86UE4	MTDH	Protein LYRI	33	14	20	14	582	63.8	9.32	58.4
O43747	AP1G1	AP-1 comple	20	19	27	19	822	91.3	6.8	65.09
P62979	RPS27A	Ubiquitin-40S	73	13	51	5	156	18	9.64	132.96

P54289	CACNA2D1	Voltage-depe	20	16	18	16	1103	124.5	5.27	51.88
P68036	UBE2L3	Ubiquitin-cor	69	8	18	8	154	17.9	8.51	77.47
P04181	OAT	Ornithine am	29	12	21	12	439	48.5	7.03	64.87
Q9UPT5	EXOC7	Exocyst com	26	18	20	18	735	83.3	6.79	53.98
P48047	ATP5PO	ATP synthas	60	11	25	11	213	23.3	9.96	73.41
Q9BTV4	TMEM43	Transmembr	38	14	22	14	400	44.8	8.13	68.65
Q9UMX0	UBQLN1	Ubiquilin-1 C	30	10	18	5	589	62.5	5.11	63.27
Q9H3U1	UNC45A	Protein unc-4	22	17	21	17	944	103	6.07	58.39
Q9P2R3	ANKFY1	Rabankyrin-5	17	17	23	17	1169	128.3	6.1	55.5
Q93008	USP9X	Probable ubi	9	22	24	22	2554	290.3	5.8	65.16
P08621	SNRNP70	U1 small nuc	38	16	28	16	437	51.5	9.94	68.14
P53992	SEC24C	Protein trans	19	16	27	15	1094	118.2	7.06	77.98
O95336	PGLS	6-phosphoglu	48	9	20	9	258	27.5	6.05	75.83
O43684	BUB3	Mitotic check	40	11	19	11	328	37.1	6.84	64.71
P0DP25	CALM3	Calmodulin-3	49	9	66	9	149	16.8	4.22	180.7
Q10471	GALNT2	Polypeptide I	25	16	27	15	571	64.7	8.35	71.78
O43396	TXNL1	Thioredoxin-l	51	9	15	9	289	32.2	4.96	62.93
P00441	SOD1	Superoxide d	76	14	33	14	154	15.9	6.13	79.82
Q9Y6Y8	SEC23IP	SEC23-intera	19	18	22	17	1000	111	5.54	59.36
P28161	GSTM2	Glutathione S	57	13	22	6	218	25.7	6.37	57.71
Q96PK6	RBM14	RNA-binding	22	15	23	15	669	69.4	9.67	69.83
Q99733	NAP1L4	Nucleosome	29	9	17	8	375	42.8	4.69	63.02
Q9BX66	SORBS1	Sorbin and S	13	15	22	14	1292	142.4	6.84	60.53
Q13217	DNAJC3	DnaJ homolo	34	15	22	15	504	57.5	6.15	64.67
Q9H0A0	NAT10	RNA cytidine	16	13	19	13	1025	115.7	8.27	57.63
Q92599	SEPTIN8	Septin-8 OS=	32	14	23	9	483	55.7	6.28	63.11
Q13185	CBX3	Chromobox p	53	8	27	7	183	20.8	5.33	84.69
P84085	ARF5	ADP-ribosyla	64	10	23	5	180	20.5	6.79	81.29
O75521	ECI2	Enoyl-CoA d	34	10	18	10	394	43.6	9	60.34
P15144	ANPEP	Aminopeptida	20	16	21	16	967	109.5	5.48	57.8
Q9UNZ2	NSFL1C	NSFL1 cofac	43	11	21	11	370	40.5	5.1	75.48
P33121	ACSL1	Long-chain-f	21	14	19	13	698	77.9	7.15	49.95
P50552	VASP	Vasodilator-s	37	12	19	12	380	39.8	8.94	71.09

P29692	EEF1D	Elongation fa	48	12	27	11	281	31.1	5.01	84.15
P04217	A1BG	Alpha-1B-gly	26	10	23	10	495	54.2	5.86	86.35
P41091	EIF2S3	Eukaryotic tr	29	11	19	11	472	51.1	8.4	64.43
P31942	HNRNPH3	Heterogenec	40	10	26	9	346	36.9	6.87	86.52
P35573	AGL	Glycogen de	13	20	27	20	1532	174.7	6.76	62.71
P19652	ORM2	Alpha-1-acid	42	8	28	5	201	23.6	5.11	94.25
Q9Y4W6	AFG3L2	AFG3-like pr	22	17	20	16	797	88.5	8.66	55.36
P43121	MCAM	Cell surface	23	15	21	15	646	71.6	5.76	65.72
O75400	PRPF40A	Pre-mRNA-p	16	17	23	15	957	108.7	7.56	62.07
O95810	CAVIN2	Caveolae-as	32	12	18	12	425	47.1	5.21	57.45
P67775	PPP2CA	Serine/threor	46	12	23	2	309	35.6	5.54	67.91
Q9Y277	VDAC3	Voltage-depe	31	10	23	9	283	30.6	8.66	59.37
Q02388	COL7A1	Collagen alp	8	15	16	15	2944	295	6.27	45.31
Q14141	SEPTIN6	Septin-6 OS=	30	14	24	4	434	49.7	6.67	58.74
Q9NUQ9	CYRIB	CYFIP-relate	39	11	23	10	324	36.7	6.06	68.21
Q6YN16	HSDL2	Hydroxysterc	38	13	18	13	418	45.4	7.99	61.22
Q7Z4H8	POGLUT3	Protein O-glu	35	15	19	15	507	58.5	8.24	53.85
P60891	PRPS1	Ribose-phos	39	11	18	7	318	34.8	6.98	57.35
P27105	STOM	Stomatin OS	35	9	23	9	288	31.7	7.88	65.87
Q15717	ELAVL1	ELAV-like pr	36	11	20	11	326	36.1	9.17	57.4
Q9UM54	MYO6	Unconventio	16	20	25	20	1294	149.6	8.53	59.33
Q53EL6	PDCD4	Programmec	33	12	17	12	469	51.7	5.21	59.47
Q13148	TARDBP	TAR DNA-bir	32	9	22	9	414	44.7	6.19	79.58
P26373	RPL13	60S ribosom	46	12	37	12	211	24.2	11.65	110.04
P07203	GPX1	Glutathione p	65	10	20	10	203	22.1	6.55	69.63
Q8NBJ5	COLGALT1	Procollagen	26	16	21	16	622	71.6	7.31	55.76
O00584	RNASET2	Ribonucleas	34	8	22	8	256	29.5	7.08	62.65
P34949	MPI	Mannose-6-p	35	9	17	9	423	46.6	5.95	60.93
O00505	KPNA3	Importin subu	27	11	17	7	521	57.8	4.94	67.03
Q8IUD2	ERC1	ELKS/Rab6-i	16	19	23	19	1116	128	5.97	56.67
P09486	SPARC	SPARC OS=	42	12	20	12	303	34.6	4.84	62.03
P23368	ME2	NAD-depend	25	11	16	10	584	65.4	7.61	54
Q96FW1	OTUB1	Ubiquitin thic	50	11	19	11	271	31.3	4.94	60.88

Q7Z2W4	ZC3HAV1	Zinc finger C	19	15	22	15	902	101.4	8.4	55.88
P30520	ADSS2	Adenylosucc	34	13	21	13	456	50.1	6.55	63.31
Q13151	HNRNPA0	Heterogeneo	48	10	25	9	305	30.8	9.29	73.5
P29622	SERPINA4	Kallistatin OS	31	14	20	14	427	48.5	7.75	49.3
P35237	SERPINB6	Serpin B6 OS	45	15	24	14	376	42.6	5.27	66.47
Q08945	SSRP1	FACT compl	23	16	22	16	709	81	6.87	64.68
Q9BQS8	FYCO1	FYVE and co	14	18	20	18	1478	166.9	4.92	57.82
Q08257	CRYZ	Quinone oxid	50	14	21	14	329	35.2	8.44	66.81
O75477	ERLIN1	Erlin-1 OS=H	37	12	20	8	348	39.1	7.87	67.03
O14818	PSMA7	Proteasome	56	14	22	14	248	27.9	8.46	73.55
Q92688	ANP32B	Acidic leucin	26	8	22	3	251	28.8	4.06	73.25
P43246	MSH2	DNA mismat	22	15	17	15	934	104.7	5.77	50.49
P51812	RPS6KA3	Ribosomal p	25	17	21	9	740	83.7	6.89	53.37
Q7L2H7	EIF3M	Eukaryotic tr	29	11	17	11	374	42.5	5.63	55.98
Q9NVI7	ATAD3A	ATPase fami	28	16	19	16	634	71.3	8.98	48.39
Q6FHJ7	SFRP4	Secreted friz	29	9	24	9	346	39.8	8.82	75.01
Q9Y3A5	SBDS	Ribosome m	53	13	21	13	250	28.7	8.75	62.02
Q13459	MYO9B	Unconventio	11	19	22	19	2157	243.2	8.75	48.69
P08240	SRPRA	Signal recog	24	15	18	15	638	69.8	8.95	54.15
P63092	GNAS	Guanine nuc	39	13	24	12	394	45.6	5.82	67.85
Q5SZK8	FREM2	FRAS1-relat	7	17	20	17	3169	350.9	5.03	54.82
P61106	RAB14	Ras-related p	66	11	18	11	215	23.9	6.21	56.78
O00267	SUPT5H	Transcription	17	15	18	15	1087	120.9	5.06	49.78
O94875	SORBS2	Sorbin and S	15	12	17	11	1100	124	8.31	58.99
Q9UBQ7	GRHPR	Glyoxylate re	36	12	22	12	328	35.6	7.39	60.74
Q9NZN3	EHD3	EH domain-c	31	14	19	4	535	60.8	6.57	62.25
Q9HDC9	APMAP	Adipocyte pla	36	14	22	14	416	46.5	6.16	65.04
P17174	GOT1	Aspartate an	44	15	19	15	413	46.2	7.01	59.36
Q8TAT6	NPLOC4	Nuclear prote	35	15	20	15	608	68.1	6.38	57.13
Q13618	CUL3	Cullin-3 OS=	20	14	19	14	768	88.9	8.48	53.6
P06865	HEXA	Beta-hexosa	27	12	24	12	529	60.7	5.16	64.7
O95373	IPO7	Importin-7 O	14	13	22	13	1038	119.4	4.82	76.13
P26196	DDX6	Probable AT	24	12	18	12	483	54.4	8.66	52.73

O60610	DIAPH1	Protein diapł	14	16	19	15	1272	141.3	5.41	51.48
O14786	NRP1	Neuropilin-1	18	13	18	13	923	103.1	5.88	45.06
P62263	RPS14	40S ribosom	43	9	17	9	151	16.3	10.05	62.13
O75368	SH3BGRL	Adapter SH3	90	9	20	9	114	12.8	5.25	65.68
Q9Y625	GPC6	Glypican-6 C	37	15	21	13	555	62.7	5.41	62.72
Q99729	HNRNPAB	Heterogeneo	30	12	27	10	332	36.2	8.21	79.41
P40121	CAPG	Macrophage-	30	7	16	7	348	38.5	6.19	57.73
Q9HAU0	PLEKHA5	Pleckstrin ho	19	21	21	21	1116	127.4	7.53	43.72
P54727	RAD23B	UV excision r	29	10	22	9	409	43.1	4.84	67.69
P08174	CD55	Complement	29	12	20	12	381	41.4	7.59	64.37
Q14160	SCRIB	Protein scrib	11	13	15	10	1630	174.8	5.07	51.04
O14936	CASK	Peripheral pl	16	13	19	13	926	105.1	6.43	59.98
Q6IBS0	TWF2	Twinfilin-2 O	41	12	20	9	349	39.5	6.84	63.98
Q9UNF0	PACSIN2	Protein kinas	32	12	17	12	486	55.7	5.2	55.77
Q9NYF8	BCLAF1	Bcl-2-associa	25	22	26	20	920	106.1	9.98	59.36
Q8TDQ7	GNPDA2	Glucosamine	59	12	17	8	276	31.1	6.95	56.52
Q07021	C1QBP	Complement	32	6	17	6	282	31.3	4.84	64.61
P10644	PRKAR1A	cAMP-depen	36	14	17	14	381	43	5.35	54.57
Q92922	SMARCC1	SWI/SNF cor	14	16	24	9	1105	122.8	5.76	67.49
Q16795	NDUFA9	NADH dehyd	29	9	16	9	377	42.5	9.8	52.45
A0AVT1	UBA6	Ubiquitin-like	17	15	20	15	1052	117.9	6.14	61.87
Q8NE71	ABCF1	ATP-binding	19	14	18	12	845	95.9	6.8	56.15
P62241	RPS8	40S ribosom	46	9	20	9	208	24.2	10.32	70.72
P63208	SKP1	S-phase kina	50	8	19	8	163	18.6	4.54	60.96
P49959	MRE11	Double-stran	24	15	18	15	708	80.5	5.9	49.13
Q8IXB1	DNAJC10	DnaJ homolo	24	16	19	16	793	91	7.18	54.48
Q14344	GNA13	Guanine nuc	38	13	20	11	377	44	8	58.57
O14672	ADAM10	Disintegrin a	24	15	20	15	748	84.1	7.77	49.82
P25325	MPST	3-mercaptop	48	11	18	11	297	33.2	6.6	56.72
P22694	PRKACB	cAMP-depen	45	14	22	5	351	40.6	8.78	57.02
Q13442	PDAP1	28 kDa heat-	39	10	17	10	181	20.6	8.87	53.64
Q15907	RAB11B	Ras-related p	59	12	30	11	218	24.5	5.94	87.37
P15170	GSPT1	Eukaryotic p	29	14	18	14	499	55.7	5.62	53.42

Q13425	SNTB2	Beta-2-syntr	29	14	25	12	540	57.9	8.82	62.45
P40429	RPL13A	60S ribosom	40	12	33	12	203	23.6	10.93	86.82
Q96P70	IPO9	Importin-9 O	18	15	17	15	1041	115.9	4.81	55.47
Q9UJZ1	STOML2	Stomatin-like	42	10	13	10	356	38.5	7.39	51.08
Q8NF91	SYNE1	Nesprin-1 OS	3	22	23	20	8797	1010.5	5.53	45.81
O95865	DDAH2	N(G),N(G)-di	46	10	20	9	285	29.6	6.01	65.48
Q96CX2	KCTD12	BTB/POZ do	47	11	18	11	325	35.7	5.64	61.36
O15511	ARPC5	Actin-related	76	10	20	9	151	16.3	5.67	73.73
Q9Y281	CFL2	Cofilin-2 OS=	66	9	23	6	166	18.7	7.88	67.64
A0A0B4J2D5	GATD3B	Putative glut	46	10	18	10	268	28.1	8.27	59.53
P39656	DDOST	Dolichyl-diph	25	11	25	11	456	50.8	6.55	63.84
P26583	HMGB2	High mobility	43	12	26	9	209	24	7.81	58.52
P11908	PRPS2	Ribose-phos	32	9	16	5	318	34.7	6.61	53.71
Q9H307	PNN	Pinin OS=Ho	23	18	22	18	717	81.6	7.14	50.43
Q8WXF1	PSPC1	Paraspeckle	29	13	18	12	523	58.7	6.67	52.65
P55735	SEC13	Protein SEC1	42	10	20	10	322	35.5	5.48	73.29
P57740	NUP107	Nuclear pore	19	15	20	15	925	106.3	5.43	55.69
P46781	RPS9	40S ribosom	57	13	34	13	194	22.6	10.65	67.54
Q02539	H1-1	Histone H1.1	34	10	28	5	215	21.8	10.99	80.95
P07741	APRT	Adenine pho	63	12	23	12	180	19.6	6.02	64.29
O94906	PRPF6	Pre-mRNA-p	18	15	17	15	941	106.9	8.25	49.88
Q8TDL5	BPIFB1	BPI fold-cont	29	11	18	11	484	52.4	7.23	49.47
P06681	C2	Complement	22	15	18	15	752	83.2	7.42	59.8
P61225	RAP2B	Ras-related p	59	9	19	9	183	20.5	4.81	68.28
Q96JJ3	ELMO2	Engulfment a	20	13	18	10	720	82.6	5.9	49.88
Q9Y315	DERA	Deoxyribose-	43	13	17	13	318	35.2	8.94	51.5
P13591	NCAM1	Neural cell a	21	15	16	15	858	94.5	4.87	48.26
P04179	SOD2	Superoxide d	45	9	31	9	222	24.7	8.25	84.28
P0DOX8		Immunoglobu	42	8	40	3	216	22.8	6.76	112.56
P04439	HLA-A	HLA class I h	38	12	15	7	365	40.8	6	50.09
O43399	TPD52L2	Tumor protei	62	11	22	11	206	22.2	5.36	65.26
P26440	IVD	Isovaleryl-Co	30	13	21	13	426	46.6	8.05	56.57
P39019	RPS19	40S ribosom	55	10	25	10	145	16.1	10.32	64.52

P17612	PRKACA	cAMP-depen	42	15	26	6	351	40.6	8.79	65.43
Q99959	PKP2	Plakophilin-2	20	16	18	16	881	97.4	9.33	55.62
Q15691	MAPRE1	Microtubule-a	52	12	18	12	268	30	5.14	56.56
P36269	GGT5	Glutathione h	21	10	16	10	586	62.2	7.55	54.17
O94826	TOMM70	Mitochondria	21	12	20	12	608	67.4	7.12	59.5
P55287	CDH11	Cadherin-11	21	14	22	14	796	87.9	4.91	58.4
Q92973	TNPO1	Transportin-1	17	11	17	9	898	102.3	4.98	57.33
P78417	GSTO1	Glutathione S	34	11	25	11	241	27.5	6.6	61.72
P62714	PPP2CB	Serine/threor	46	12	20	2	309	35.6	5.43	64.33
P09110	ACAA1	3-ketoacyl-C	34	10	15	10	424	44.3	8.44	54.61
Q5T5P2	KIAA1217	Sickle tail pro	10	18	22	18	1943	214	7.06	47.08
Q8TEX9	IPO4	Importin-4 O	19	15	16	15	1081	118.6	4.96	43.85
O00291	HIP1	Huntingtin-in	17	16	20	16	1037	116.1	5.3	48.18
P61604	HSPE1	10 kDa heat	76	10	36	10	102	10.9	8.92	95.93
Q06828	FMOD	Fibromodulin	35	8	14	8	376	43.2	6.04	54.08
Q16762	TST	Thiosulfate s	47	12	20	12	297	33.4	7.25	67.59
O60547	GMDS	GDP-manno	44	15	19	15	372	41.9	7.31	46.54
P50895	BCAM	Basal cell ad	24	13	19	13	628	67.4	5.81	57.56
Q13619	CUL4A	Cullin-4A OS	25	20	25	10	759	87.6	8.13	56.02
Q96RQ3	MCCC1	Methylcroton	20	11	16	11	725	80.4	7.78	53.11
O15460	P4HA2	Prolyl 4-hydr	27	13	18	13	535	60.9	5.71	49.88
Q96C19	EFHD2	EF-hand don	44	11	16	9	240	26.7	5.2	44
P07602	PSAP	Prosaposin C	23	13	25	13	524	58.1	5.17	63.66
O75436	VPS26A	Vacuolar pro	41	13	22	13	327	38.1	6.57	58.37
P62495	ETF1	Eukaryotic pe	25	12	17	12	437	49	5.71	45.42
P08697	SERPINF2	Alpha-2-antip	22	10	21	10	491	54.5	6.29	56.23
Q9UBF2	COPG2	Coatomer su	19	13	17	11	871	97.6	5.81	55.42
P42566	EPS15	Epidermal gr	16	13	16	13	896	98.6	4.64	46.57
Q9UNF1	MAGED2	Melanoma-as	25	11	15	10	606	64.9	9.32	57.01
P07305	H1-0	Histone H1.0	44	10	32	10	194	20.9	10.84	84.78
Q9BWM7	SFXN3	Sideroflexin-3	43	11	15	11	321	35.5	9.1	48.5
O94776	MTA2	Metastasis-a	25	15	18	14	668	75	9.66	43.47
Q92888	ARHGEF1	Rho guanine	19	14	16	14	912	102.4	5.66	41.88

P49755	TMED10	Transmembr	46	10	28	10	219	25	7.44	79.13
P31937	HIBADH	3-hydroxyiso	42	10	17	10	336	35.3	8.13	63.13
Q9BRX8	PRXL2A	Peroxiredoxi	35	9	23	9	229	25.7	8.84	65.97
O43866	CD5L	CD5 antigen-	36	12	18	12	347	38.1	5.47	69.07
Q05193	DNM1	Dynamin-1 C	22	18	21	10	864	97.3	7.17	59.99
Q92520	FAM3C	Protein FAM3	60	11	14	11	227	24.7	8.29	50.04
Q14690	PDCD11	Protein RRP5	10	16	18	16	1871	208.6	8.87	54.76
Q6P2E9	EDC4	Enhancer of	14	15	16	15	1401	151.6	5.86	50.25
P25398	RPS12	40S ribosom	57	7	15	7	132	14.5	7.21	58.59
Q9BWF3	RBM4	RNA-binding	33	13	23	13	364	40.3	7.08	60.87
Q16543	CDC37	Hsp90 co-ch	31	11	26	11	378	44.4	5.25	78.88
Q92734	TFG	Protein TFG	30	8	15	8	400	43.4	5.1	62.66
Q9UKG1	APPL1	DCC-interact	22	12	14	11	709	79.6	5.41	46.76
P07093	SERPINE2	Glia-derived	25	11	17	11	398	44	9.29	40.23
Q8WUM0	NUP133	Nuclear pore	17	14	21	14	1156	128.9	5.1	61.86
P0DOY2	IGLC2	Immunoglobu	70	6	36	2	106	11.3	7.24	106.48
P53990	IST1	IST1 homolo	42	9	20	9	364	39.7	5.35	57.53
Q92804	TAF15	TATA-bindin	36	14	21	12	592	61.8	8.02	52.36
O00499	BIN1	Myc box-dep	23	14	19	14	593	64.7	5.06	61.06
P48739	PITPNB	Phosphatidyl	59	14	21	11	271	31.5	6.87	60.5
P11310	ACADM	Medium-chai	34	13	18	13	421	46.6	8.37	48.43
P32969	RPL9P9	60S ribosom	58	9	18	9	192	21.9	9.95	59
Q15363	TMED2	Transmembr	39	6	20	6	201	22.7	5.17	52.48
P09601	HMOX1	Heme oxyge	37	9	15	9	288	32.8	8.25	58.94
P40261	NNMT	Nicotinamide	38	10	18	10	264	29.6	5.74	58.13
Q9UBX5	FBLN5	Fibulin-5 OS=	21	9	22	9	448	50.1	4.73	63.64
Q9Y224	RTRAF	RNA transcri	40	11	16	11	244	28.1	6.65	53.15
O60341	KDM1A	Lysine-specif	22	16	19	15	852	92.8	6.52	49.86
Q86W92	PPFIBP1	Liprin-beta-1	17	14	17	13	1011	114	5.55	48.97
P40123	CAP2	Adenylyl cycl	28	12	19	10	477	52.8	6.37	49.98
O60504	SORBS3	Vinexin OS=	22	13	17	13	671	75.3	9.45	48.86
Q8IZL8	PELP1	Proline-, glut	15	13	16	13	1130	119.6	4.34	53.85
O00186	STXBP3	Syntaxin-bin	20	13	20	13	592	67.7	7.8	54.74

Q9BW30	TPPP3	Tubulin polyr	58	13	18	13	176	19	9.13	50.43
P12821	ACE	Angiotensin-(	12	14	17	14	1306	149.6	6.39	48.61
Q16630	CPSF6	Cleavage an	24	14	22	14	551	59.2	7.15	51.01
Q96AQ6	PBXIP1	Pre-B-cell leu	20	11	14	11	731	80.6	5.33	47.4
Q12907	LMAN2	Vesicular inte	37	11	20	11	356	40.2	6.95	61.88
Q9BT78	COPS4	COP9 signal	41	12	17	12	406	46.2	5.83	57.81
O76024	WFS1	Wolframin O	21	16	19	16	890	100.2	8.05	50.29
P07858	CTSB	Cathepsin B	30	7	20	7	339	37.8	6.3	62.84
Q16270	IGFBP7	Insulin-like g	43	10	16	10	282	29.1	7.9	58.61
Q52LJ0	FAM98B	Protein FAM	32	10	14	9	433	45.5	8.69	40.42
Q7Z3D6	DGLUCY	D-glutamate	25	14	17	14	616	66.4	6.79	48.53
Q14165	MLEC	Malectin OS=	48	12	20	12	292	32.2	5.41	57.97
O75937	DNAJC8	DnaJ homolo	48	11	19	11	253	29.8	9.06	60.88
P16278	GLB1	Beta-galacto	13	7	15	7	677	76	6.57	47.27
P33993	MCM7	DNA replicat	29	16	18	16	719	81.3	6.46	39.97
Q9Y6G9	DYNC1LI1	Cytoplasmic	37	14	19	13	523	56.5	6.42	51.26
Q9UIA9	XPO7	Exportin-7 O	16	15	19	15	1087	123.8	6.32	54.5
Q8N684	CPSF7	Cleavage an	28	11	18	11	471	52	8	49.42
O60234	GMFG	Glia maturati	64	7	21	5	142	16.8	5.26	72.27
P46108	CRK	Adapter mole	38	11	17	11	304	33.8	5.55	47.51
Q6DKJ4	NXN	Nucleoredox	38	12	12	12	435	48.4	4.97	45.06
Q96CS3	FAF2	FAS-associa	32	9	13	9	445	52.6	5.62	47.23
P83916	CBX1	Chromobox p	54	7	19	6	185	21.4	4.93	54.25
Q9HB71	CACYBP	Calcyclin-bin	48	10	16	10	228	26.2	8.25	50.19
P28799	GRN	Progranulin (	21	9	16	9	593	63.5	6.83	52.59
P00492	HPRT1	Hypoxanthin	56	9	18	8	218	24.6	6.68	57.71
O15231	ZNF185	Zinc finger pr	27	15	19	15	689	73.5	7.01	56.14
Q13283	G3BP1	Ras GTPase	27	10	18	9	466	52.1	5.52	57.87
O43294	TGFB1I1	Transforming	23	9	19	9	461	49.8	7.03	58.97
P57721	PCBP3	Poly(rC)-binc	22	7	19	1	371	39.4	8.07	57.3
Q9NX63	CHCHD3	MICOS comp	43	13	19	13	227	26.1	8.28	48.78
Q9NTZ6	RBM12	RNA-binding	15	14	20	14	932	97.3	8.63	51.01
P23193	TCEA1	Transcriptior	47	13	20	13	301	33.9	8.38	57.92

P07225	PROS1	Vitamin K-de	22	13	17	13	676	75.1	5.67	53.15
Q08209	PPP3CA	Protein phos	31	12	16	7	521	58.7	5.86	50
O00116	AGPS	Alkyldihydro›	27	12	15	12	658	72.9	7.34	43.71
O75489	NDUFS3	NADH dehyc	47	10	19	10	264	30.2	7.5	57.7
Q02543	RPL18A	60S ribosom	39	8	18	8	176	20.7	10.71	51.05
Q9UKK3	PARP4	Protein monc	9	13	17	13	1724	192.5	5.66	50.46
P08134	RHOC	Rho-related (	37	9	25	3	193	22	6.58	77.74
P62249	RPS16	40S ribosom	53	10	22	10	146	16.4	10.21	52.29
Q9P258	RCC2	Protein RCC	23	12	15	12	522	56	8.78	54.62
P15121	AKR1B1	Aldo-keto rec	41	10	17	10	316	35.8	6.98	55.78
Q9BVK6	TMED9	Transmembr	39	9	17	8	235	27.3	8.02	64.29
Q96JQ0	DCHS1	Protocadheri	6	11	14	11	3298	346	4.94	46.27
P52815	MRPL12	39S ribosom	40	6	15	6	198	21.3	8.87	45.84
Q12882	DPYD	Dihydropyrim	15	13	15	13	1025	111.3	7.05	51.56
P52565	ARHGDIA	Rho GDP-dis	36	9	23	9	204	23.2	5.11	67.56
Q9BR76	CORO1B	Coronin-1B (	26	13	25	13	489	54.2	5.88	56.91
P52630	STAT2	Signal transc	15	11	16	11	851	97.9	5.49	52.43
P15289	ARSA	Arylsulfatase	24	9	16	8	507	53.6	6.07	54.39
Q86VS8	HOOK3	Protein Hook	21	14	17	14	718	83.1	5.17	47.03
Q9NVP1	DDX18	ATP-depend	21	12	16	12	670	75.4	9.5	43.76
Q9Y5X1	SNX9	Sorting nexir	28	13	16	13	595	66.6	5.58	39.31
P11388	TOP2A	DNA topoiso	9	14	19	3	1531	174.3	8.72	53.01
Q2TAY7	SMU1	WD40 repea	26	12	17	12	513	57.5	7.18	44.73
P68400	CSNK2A1	Casein kinas	36	11	15	10	391	45.1	7.74	44
Q9UMS4	PRPF19	Pre-mRNA-p	30	11	26	11	504	55.1	6.61	77.18
O60488	ACSL4	Long-chain-f	25	13	16	12	711	79.1	8.38	49.85
P17252	PRKCA	Protein kinas	23	14	19	13	672	76.7	7.05	56.02
P13807	GYS1	Glycogen [st	21	12	15	12	737	83.7	6.18	53.45
Q15113	PCOLCE	Procollagen (	32	12	18	12	449	47.9	7.43	51.62
O43681	GET3	ATPase GET	31	9	17	9	348	38.8	4.91	55.91
Q9H2U2	PPA2	Inorganic pyı	37	12	22	10	334	37.9	7.39	63.17
Q99459	CDC5L	Cell division (	22	11	14	11	802	92.2	8.18	37.52
O60216	RAD21	Double-stran	19	9	19	9	631	71.6	4.65	69.68

Q13409	DYNC1I2	Cytoplasmic	22	9	13	9	638	71.4	5.2	50.06
Q9UFN0	NIPSNAP3A	Protein NipS	51	9	13	8	247	28.4	9.16	41.71
P23588	EIF4B	Eukaryotic tr	21	13	17	13	611	69.1	5.73	40.21
Q5JTV8	TOR1AIP1	Torsin-1A-int	21	11	19	10	583	66.2	8.18	55.94
P05106	ITGB3	Integrin beta	16	11	16	11	788	87	5.24	49.56
P35659	DEK	Protein DEK	23	12	19	12	375	42.6	8.56	51.48
Q01484	ANK2	Ankyrin-2 OS	4	14	18	8	3957	433.4	5.14	52.61
P30050	RPL12	60S ribosom	58	8	19	8	165	17.8	9.42	70.7
P17813	ENG	Endoglin OS	20	10	16	10	658	70.5	6.61	47.96
Q14157	UBAP2L	Ubiquitin-ass	14	11	17	11	1087	114.5	7.11	54.44
P53041	PPP5C	Serine/threoı	27	13	19	13	499	56.8	6.28	45.23
O15372	EIF3H	Eukaryotic tr	28	13	19	13	352	39.9	6.54	49.44
Q15811	ITSN1	Intersectin-1	12	15	18	15	1721	195.3	7.77	42.61
P49750	YLPM1	YLP motif-co	10	19	21	18	2146	241.5	6.55	48.4
Q93099	HGD	Homogentisa	29	11	17	11	445	49.9	6.96	46.37
Q13162	PRDX4	Peroxiredoxi	41	9	16	7	271	30.5	6.29	55.1
P53999	SUB1	Activated RN	50	8	23	8	127	14.4	9.6	70.34
O75306	NDUFS2	NADH dehyc	27	10	16	10	463	52.5	7.55	55.16
Q9Y383	LUC7L2	Putative RN/	30	11	20	8	392	46.5	10.01	59.11
Q14554	PDIA5	Protein disulf	25	14	20	14	519	59.6	7.91	54.23
O14745	SLC9A3R1	Na(+)/H(+) e	36	11	17	11	358	38.8	5.77	49.97
Q9Y680	FKBP7	Peptidyl-prol	42	10	23	10	222	25.8	6.32	56.52
Q07666	KHDRBS1	KH domain-c	27	12	16	12	443	48.2	8.66	51.92
P37108	SRP14	Signal recog	66	8	14	8	136	14.6	10.04	44.67
P20591	MX1	Interferon-inc	21	12	14	9	662	75.5	5.83	45.34
P07358	C8B	Complement	23	13	15	13	591	67	8.13	41.45
O95793	STAU1	Double-stran	24	14	16	13	577	63.1	9.44	46.29
Q9UMR2	DDX19B	ATP-depend	31	14	18	14	479	53.9	6.3	44.64
O95425	SVIL	Supervillin O	9	16	16	16	2214	247.6	6.98	43.88
P29350	PTPN6	Tyrosine-pro	25	14	17	14	595	67.5	7.78	49.4
Q9UL46	PSME2	Proteasome	44	10	16	10	239	27.4	5.73	46.51
O43290	SART1	U4/U6.U5 tri-	19	12	14	12	800	90.2	6.13	44.72
P29966	MARCKS	Myristoylatec	36	9	18	9	332	31.5	4.45	48.41

Q16853	AOC3	Membrane p	14	9	16	9	763	84.6	6.52	48.98
O43175	PHGDH	D-3-phospho	28	11	14	11	533	56.6	6.71	38.75
Q9Y6I3	EPN1	Epsin-1 OS=	32	12	15	11	576	60.3	4.83	47.45
Q00688	FKBP3	Peptidyl-proly	50	10	15	10	224	25.2	9.28	52.29
O76003	GLRX3	Glutaredoxin	31	9	16	9	335	37.4	5.39	48.42
Q13098	GPS1	COP9 signal	28	12	17	12	491	55.5	6.74	57.56
Q10570	CPSF1	Cleavage an	12	13	17	13	1443	160.8	6.4	47.12
P46109	CRKL	Crk-like prote	44	11	17	11	303	33.8	6.74	53.35
Q9NTJ5	SACM1L	Phosphatidyl	22	12	17	12	587	66.9	7.12	48.98
Q06124	PTPN11	Tyrosine-pro	27	13	21	13	593	68	7.3	62.27
P46783	RPS10	40S ribosom	65	14	30	13	165	18.9	10.15	73.39
O15355	PPM1G	Protein phos	23	11	18	11	546	59.2	4.36	55.18
Q9Y376	CAB39	Calcium-binc	41	14	16	14	341	39.8	6.89	48.02
Q96A33	CCDC47	PAT complex	25	12	21	12	483	55.8	4.87	61.69
Q92614	MYO18A	Unconvention	9	16	18	15	2054	233	6.3	36.32
Q13620	CUL4B	Cullin-4B OS	19	19	22	9	913	103.9	7.37	52.42
P98082	DAB2	Disabled hor	15	9	15	9	770	82.4	5.53	46.57
P08236	GUSB	Beta-glucuro	18	13	17	13	651	74.7	7.02	41.42
Q9H7Z7	PTGES2	Prostaglandi	38	11	13	11	377	41.9	9.16	43.89
P25205	MCM3	DNA replicat	21	13	16	13	808	90.9	5.77	43.54
O95292	VAPB	Vesicle-asso	53	10	17	9	243	27.2	7.3	49.91
Q9H8Y8	GORASP2	Golgi reasse	29	12	17	12	452	47.1	4.82	37.37
O14744	PRMT5	Protein argin	23	13	18	13	637	72.6	6.29	56.95
Q9BY32	ITPA	Inosine tripho	63	8	15	8	194	21.4	5.66	44.28
O00461	GOLIM4	Golgi integra	20	14	18	14	696	81.8	4.77	50.64
Q15008	PSMD6	26S proteaso	27	11	17	11	389	45.5	5.62	54.88
P51692	STAT5B	Signal transc	19	13	16	2	787	89.8	6.05	45.47
O75821	EIF3G	Eukaryotic tr	46	12	21	12	320	35.6	6.13	63.04
Q9Y2Q3	GSTK1	Glutathione S	46	9	19	9	226	25.5	8.41	53.66
O95340	PAPSS2	Bifunctional 3	23	13	16	12	614	69.5	8.03	48.99
Q7L5N1	COPS6	COP9 signal	37	10	14	10	327	36.1	5.73	51.79
O60271	SPAG9	C-Jun-amino	11	12	17	12	1321	146.1	5.15	44.98
Q9Y6W5	WASF2	Actin-binding	17	8	14	8	498	54.3	5.53	39.41

P27169	PON1	Serum parac	29	9	15	9	355	39.7	5.22	43.61
Q93084	ATP2A3	Sarcoplasmi	9	7	14	1	999	109.2	5.52	58.13
P62826	RAN	GTP-binding	40	8	21	8	216	24.4	7.49	65.61
Q14699	RFTN1	Raftlin OS=H	19	10	16	10	578	63.1	5.67	50.91
Q9UH65	SWAP70	Switch-assoc	28	13	17	13	585	69	5.87	51.48
P50897	PPT1	Palmitoyl-pro	32	8	15	8	306	34.2	6.52	54.53
O95671	ASMTL	Probable bifu	24	12	17	12	621	68.8	6.07	53.94
Q9Y5M8	SRPRB	Signal recog	46	10	15	10	271	29.7	9.04	46.31
Q9Y2T2	AP3M1	AP-3 comple	39	12	16	12	418	46.9	6.93	44.04
Q8IVL6	P3H3	Prolyl 3-hydr	25	12	14	12	736	81.8	6.32	47.43
O14964	HGS	Hepatocyte g	17	15	21	14	777	86.1	6.16	47.64
Q14573	ITPR3	Inositol 1,4,5	6	14	16	9	2671	303.9	6.48	44.24
O00471	EXOC5	Exocyst com	18	13	18	12	708	81.8	6.71	45.38
O15027	SEC16A	Protein trans	7	14	14	14	2357	251.7	5.8	42.22
Q6PCE3	PGM2L1	Glucose 1,6-	29	14	22	13	622	70.4	7.15	56.86
Q16204	CCDC6	Coiled-coil do	29	12	13	12	474	53.3	7.34	46.64
P42285	MTREX	Exosome RN	13	15	17	15	1042	117.7	6.52	42.9
P49756	RBM25	RNA-binding	17	11	14	11	843	100.1	6.32	42.11
P10321	HLA-C	HLA class I h	26	7	14	3	366	40.6	6.04	49.91
Q13573	SNW1	SNW domair	26	13	15	13	536	61.5	9.52	44.76
P36957	DLST	Dihydrolipoyl	26	11	16	11	453	48.7	8.95	52.15
P49790	NUP153	Nuclear pore	13	14	15	14	1475	153.8	8.73	41.38
P42126	ECI1	Enoyl-CoA d	31	8	16	8	302	32.8	8.54	50.99
P55145	MANF	Mesencepha	43	9	20	9	182	20.7	8.69	63.98
P20042	EIF2S2	Eukaryotic tr	40	10	18	10	333	38.4	5.8	47.67
P13693	TPT1	Translational	43	7	16	7	172	19.6	4.93	52.74
Q13630	GFUS	GDP-L-fucos	41	9	13	9	321	35.9	6.6	41.89
O43809	NUDT21	Cleavage an	42	11	19	11	227	26.2	8.82	56.1
Q13547	HDAC1	Histone deac	27	12	16	7	482	55.1	5.48	38.58
P18621	RPL17	60S ribosom	40	9	18	9	184	21.4	10.17	48.04
O14579	COPE	Coatomer su	41	11	16	11	308	34.5	5.12	53.88
A1L0T0	ILVBL	2-hydroxyacy	22	10	12	10	632	67.8	8.15	43.88
O75746	SLC25A12	Electrogenic	20	11	14	8	678	74.7	8.38	37.9

P60983	GMFB	Glia maturati	54	5	16	3	142	16.7	5.29	59.71
Q7Z2K6	ERMP1	Endoplasmic	13	11	15	11	904	100.2	7.52	42.23
Q15435	PPP1R7	Protein phos	37	10	12	10	360	41.5	4.91	38.57
Q12768	WASHC5	WASH comp	14	15	16	15	1159	134.2	6.98	39.39
P31947	SFN	14-3-3 protei	43	9	22	5	248	27.8	4.74	56.67
Q96JB5	CDK5RAP3	CDK5 regula	25	13	17	13	506	56.9	4.75	41.82
Q01433	AMPD2	AMP deamin	17	15	20	14	879	100.6	6.93	54.61
Q14571	ITPR2	Inositol 1,4,5	5	15	16	10	2701	307.9	6.43	43.44
P62081	RPS7	40S ribosom	45	10	27	10	194	22.1	10.1	68.9
Q9NRV9	HEBP1	Heme-bindin	65	9	18	9	189	21.1	5.8	60.28
O94901	SUN1	SUN domain	18	12	13	12	785	87.1	6.93	41.68
Q8WUD1	RAB2B	Ras-related p	39	8	14	2	216	24.2	7.83	48.94
P49593	PPM1F	Protein phos	31	10	11	10	454	49.8	5.1	42.46
O15143	ARPC1B	Actin-related	36	10	20	9	372	40.9	8.35	53.62
P49736	MCM2	DNA replicat	15	14	17	14	904	101.8	5.52	43.63
Q9UNS2	COPS3	COP9 signal	22	7	15	7	423	47.8	6.65	51.01
Q8TEM1	NUP210	Nuclear pore	9	14	15	14	1887	205	6.81	42.65
P14384	CPM	Carboxypept	25	9	16	9	443	50.5	7.36	37.5
P61020	RAB5B	Ras-related p	48	7	18	5	215	23.7	8.13	66.34
Q9BY44	EIF2A	Eukaryotic tr	23	12	17	12	585	64.9	8.87	45.73
Q8IYI6	EXOC8	Exocyst com	17	12	15	12	725	81.7	5.49	44.55
Q8NBX0	SCCPDH	Saccharopine	29	9	16	9	429	47.1	9.14	58.68
Q16629	SRSF7	Serine/argini	34	10	24	9	238	27.4	11.82	61.97
Q9Y5Z4	HEBP2	Heme-bindin	44	9	17	9	205	22.9	4.63	53.07
Q09028	RBBP4	Histone-bind	25	11	21	5	425	47.6	4.89	61.36
O75822	EIF3J	Eukaryotic tr	37	9	13	9	258	29	4.83	47.39
P00568	AK1	Adenylate kir	52	10	18	10	194	21.6	8.63	52.76
O95861	BPNT1	3'(2'),5'-bisph	28	9	17	9	308	33.4	5.69	54.51
Q5JTH9	RRP12	RRP12-like p	13	17	22	17	1297	143.6	8.75	47.94
Q8IX12	CCAR1	Cell division	12	13	17	13	1150	132.7	5.76	35.97
Q07020	RPL18	60S ribosom	45	8	24	8	188	21.6	11.72	70.23
P22033	MMUT	Methylmalon	19	14	18	13	750	83.1	6.93	44.73
Q15437	SEC23B	Protein trans	17	11	16	10	767	86.4	6.89	47.27

Q12792	TWF1	Twinfilin-1 O	33	10	16	8	350	40.3	6.96	43.54
Q08379	GOLGA2	Golgin subfa	17	14	14	14	1002	113	5.02	37.25
Q8NC56	LEMD2	LEM domain	27	13	18	13	503	56.9	9	47.17
P20339	RAB5A	Ras-related p	43	6	17	4	215	23.6	8.15	63.88
P29992	GNA11	Guanine nuc	30	10	18	6	359	42.1	5.69	42.47
Q9HAV0	GNB4	Guanine nuc	23	8	13	4	340	37.5	6	45.11
Q66K74	MAP1S	Microtubule-a	13	13	17	13	1059	112.1	7.3	48.52
Q96K76	USP47	Ubiquitin car	11	13	14	13	1375	157.2	5.08	41.64
O43615	TIMM44	Mitochondria	25	10	14	10	452	51.3	8.32	46.87
P61619	SEC61A1	Protein trans	20	9	15	9	476	52.2	8.06	42.14
P05156	CFI	Complement	19	10	15	10	583	65.7	7.5	45.89
Q12765	SCRN1	Secernin-1 C	30	12	16	11	414	46.4	4.75	46.71
Q13439	GOLGA4	Golgin subfa	7	14	17	13	2230	261	5.39	44.43
O15067	PFAS	Phosphoribo	10	12	16	12	1338	144.6	5.76	38.65
Q15185	PTGES3	Prostaglandi	38	7	17	7	160	18.7	4.54	57.92
O95571	ETHE1	Persulfide di	40	7	11	7	254	27.9	6.83	37.3
H7BZ55	CROCC2	Ciliary rootle	11	15	16	15	1653	185.5	5.5	37.61
P07357	C8A	Complement	18	8	14	8	584	65.1	6.47	49.21
P14735	IDE	Insulin-degra	15	13	14	13	1019	117.9	6.61	40.57
Q9UL18	AGO1	Protein argo	16	11	16	5	857	97.2	9.16	55.97
Q14515	SPARCL1	SPARC-like p	24	11	12	11	664	75.2	4.81	42.12
P18084	ITGB5	Integrin beta	20	13	16	13	799	88	6.06	45.87
Q99805	TM9SF2	Transmembr	20	10	17	10	663	75.7	7.44	58.53
Q8IYB3	SRRM1	Serine/argini	12	8	15	8	904	102.3	11.84	40.61
P57737	CORO7	Coronin-7 OS	15	11	14	11	925	100.5	5.8	48.07
P36542	ATP5F1C	ATP synthas	36	12	26	11	298	33	9.22	60.5
Q9BZF1	OSBPL8	Oxysterol-bir	18	13	13	13	889	101.1	6.96	44.52
Q9H9B4	SFXN1	Sideroflexin-	36	10	16	10	322	35.6	9.07	47.74
Q9H3P7	ACBD3	Golgi resider	29	9	12	9	528	60.6	5.06	40.95
P50281	MMP14	Matrix metall	26	13	15	13	582	65.9	7.77	44.3
P20742	PZP	Pregnancy z	7	10	21	3	1482	163.8	6.38	64.57
Q14520	HABP2	Hyaluronan-b	20	11	19	11	560	62.6	6.54	50.73
P43487	RANBP1	Ran-specific	49	8	20	8	201	23.3	5.29	49.37

Q9BWD1	ACAT2	Acetyl-CoA a	37	10	12	10	397	41.3	6.92	38.68
P16452	EPB42	Protein 4.2 C	20	13	21	12	691	77	8.09	47.9
P05166	PCCB	Propionyl-Co	27	11	17	11	539	58.2	7.64	41.61
Q01469	FABP5	Fatty acid-bi	61	8	16	8	135	15.2	7.01	55.66
Q12904	AIMP1	Aminoacyl tF	42	10	16	10	312	34.3	8.43	50.22
P27144	AK4	Adenylate ki	50	9	16	9	223	25.3	8.4	47.48
Q13765	NACA	Nascent poly	33	5	20	5	215	23.4	4.56	77.69
O43237	DYNC1LI2	Cytoplasmic	26	10	13	9	492	54.1	6.38	45.08
P39059	COL15A1	Collagen alp	8	9	16	9	1388	141.6	5	44.46
Q8WUP2	FBLIM1	Filamin-bindi	33	10	12	10	373	40.6	6.02	43.44
Q7Z7H5	TMED4	Transmembr	28	8	19	7	227	25.9	8.28	42.66
Q9Y570	PPME1	Protein phos	34	9	14	9	386	42.3	5.97	41.52
P63279	UBE2I	SUMO-conju	35	8	14	8	158	18	8.66	47.2
Q9H4B7	TUBB1	Tubulin beta-	19	8	25	3	451	50.3	5.17	65.87
Q14011	CIRBP	Cold-inducib	48	8	16	7	172	18.6	9.51	53.58
P02652	APOA2	Apolipoprote	42	6	22	6	100	11.2	6.62	69.34
P48059	LIMS1	LIM and sen	38	10	17	5	325	37.2	8.05	46.8
Q9ULC3	RAB23	Ras-related	35	7	11	7	237	26.6	6.6	36.26
P09661	SNRPA1	U2 small nuc	39	11	14	11	255	28.4	8.62	43.65
P10301	RRAS	Ras-related	38	7	13	5	218	23.5	6.93	40.35
Q8NI27	THOC2	THO comple	9	14	19	14	1593	182.7	8.44	37.59
Q96KR1	ZFR	Zinc finger R	14	13	16	13	1074	116.9	9.04	39.07
P22352	GPX3	Glutathione	38	8	19	8	226	25.5	8.13	56.9
Q9GZS3	SKIC8	SKI8 subunit	40	11	13	11	305	33.6	5.47	43.01
O00754	MAN2B1	Lysosomal a	16	15	17	15	1011	113.7	7.28	39.69
Q9Y2S2	CRYL1	Lambda-crys	31	8	14	8	319	35.4	6.18	49.68
Q9Y4E1	WASHC2C	WASH comp	14	11	13	3	1320	144.8	4.79	33.49
Q6ZMI0	PPP1R21	Protein phos	17	10	14	10	780	88.3	6.84	46.51
P61353	RPL27	60S ribosom	46	7	21	7	136	15.8	10.56	51.14
Q4V328	GRIPAP1	GRIP1-asso	21	14	15	14	841	95.9	5.11	36.48
P30048	PRDX3	Thioredoxin-	41	8	17	8	256	27.7	7.78	55.8
Q03519	TAP2	Antigen pept	18	9	12	8	686	75.6	8.02	37.15
O95487	SEC24B	Protein trans	12	12	13	10	1268	137.3	6.67	39.58

P45973	CBX5	Chromobox p	45	8	15	8	191	22.2	5.86	41.22
P62277	RPS13	40S ribosom	68	12	28	12	151	17.2	10.54	69.03
O95573	ACSL3	Fatty acid Co	19	11	13	10	720	80.4	8.38	45.08
P62847	RPS24	40S ribosom	40	6	18	6	133	15.4	10.78	48.02
Q96CN7	ISOC1	Isochorismat	31	8	13	8	298	32.2	7.39	41.79
P12931	SRC	Proto-oncoge	28	12	14	7	536	59.8	7.42	41.07
O00487	PSMD14	26S proteaso	34	9	12	9	310	34.6	6.52	45.96
Q15005	SPCS2	Signal peptic	48	9	20	9	226	25	8.47	49.02
Q01081	U2AF1	Splicing facto	34	8	15	8	240	27.9	8.81	41.16
P31323	PRKAR2B	cAMP-depen	22	7	11	5	418	46.3	4.92	43.9
P61086	UBE2K	Ubiquitin-cor	56	9	13	9	200	22.4	5.44	41.74
P05362	ICAM1	Intercellular a	25	12	15	12	532	57.8	7.99	41.36
Q8TCJ2	STT3B	Dolichyl-diph	15	13	19	11	826	93.6	8.91	51.87
P54709	ATP1B3	Sodium/pota	40	9	17	9	279	31.5	8.35	58.2
P05413	FABP3	Fatty acid-bi	57	9	15	9	133	14.8	6.8	43.96
Q8WU90	ZC3H15	Zinc finger C	31	11	12	11	426	48.6	5.31	38.5
Q03013	GSTM4	Glutathione S	46	10	15	2	218	25.5	5.9	38.7
P36543	ATP6V1E1	V-type protor	41	9	15	9	226	26.1	8	46.71
P05026	ATP1B1	Sodium/pota	34	9	17	9	303	35	8.53	44.04
P46778	RPL21	60S ribosom	50	10	23	10	160	18.6	10.49	53.75
P49903	SEPHS1	Selenide, wa	35	9	11	8	392	42.9	5.97	36.48
Q13131	PRKAA1	5'-AMP-activ	28	13	17	13	559	64	8.12	37.47
P61960	UFM1	Ubiquitin-folc	59	4	11	4	85	9.1	9.31	39.12
Q8IWE2	FAM114A1	Protein NOX	19	7	13	7	563	60.7	4.68	53.29
P39880	CUX1	Homeobox p	10	11	13	3	1505	164.1	5.9	37.39
P52789	HK2	Hexokinase-2	11	10	15	7	917	102.3	6.05	43.72
Q9Y512	SAMM50	Sorting and a	26	9	13	9	469	51.9	6.9	40.99
O60832	DKC1	H/ACA ribonu	23	14	15	14	514	57.6	9.42	36.94
P20340	RAB6A	Ras-related p	44	9	14	9	208	23.6	5.54	42.96
Q9Y4P3	TBL2	Transducin b	28	13	17	13	447	49.8	9.44	46.54
Q6PKG0	LARP1	La-related pr	14	15	17	14	1096	123.4	8.82	40.47
O14787	TNPO2	Transportin-2	17	11	16	9	897	101.3	5.01	43.31
O00303	EIF3F	Eukaryotic tr	30	10	17	10	357	37.5	5.45	45.45

P13073	COX4I1	Cytochrome	40	8	18	8	169	19.6	9.51	44.68
Q63HR2	TNS2	Tensin-2 OS	9	11	15	9	1409	152.5	8.35	43.38
Q12959	DLG1	Disks large h	9	8	13	6	904	100.4	5.76	39.73
P15586	GNS	N-acetylgluc	16	7	12	7	552	62	8.31	37.98
Q9H2D6	TRIOBP	TRIO and F-	5	13	15	12	2365	261.2	8.48	37.16
Q15257	PTPA	Serine/threo	32	10	22	10	358	40.6	5.94	57.15
P22570	FDXR	NADPH:adre	27	10	14	10	491	53.8	8.44	37.69
Q9UI12	ATP6V1H	V-type proto	25	9	11	9	483	55.8	6.48	41.1
P46100	ATRX	Transcriptio	6	13	14	13	2492	282.4	6.55	37.87
Q96BM9	ARL8A	ADP-ribosyla	54	9	12	4	186	21.4	7.77	36.56
P41218	MNDA	Myeloid cell r	29	13	18	13	407	45.8	9.76	44.63
P08514	ITGA2B	Integrin alpha	12	10	12	10	1039	113.3	5.38	42.05
Q15057	ACAP2	Arf-GAP with	19	14	17	14	778	88	6.8	42.43
P11215	ITGAM	Integrin alpha	13	12	15	11	1152	127.1	7.23	45.87
P00403	MT-CO2	Cytochrome	28	5	13	5	227	25.5	4.82	40.61
Q12860	CNTN1	Contactin-1 (	12	11	16	11	1018	113.2	5.9	39.04
P30038	ALDH4A1	Delta-1-pyrrc	17	9	13	9	563	61.7	8.07	43.2
Q14257	RCN2	Reticulocalbi	44	10	13	10	317	36.9	4.4	50.93
Q9HA64	FN3KRP	Ketosamine-	42	11	14	11	309	34.4	7.33	38.7
Q6UXG2	ELAPOR1	Endosome/ly	10	9	14	9	1013	111.3	6.55	49.24
O15173	PGRMC2	Membrane-a	32	7	16	5	223	23.8	4.88	64.6
O00160	MYO1F	Unconventio	13	14	17	11	1098	124.8	9.11	33.19
Q9Y263	PLAA	Phospholipa	18	11	12	11	795	87.1	6.37	36.36
P22897	MRC1	Macrophage	10	14	16	14	1456	165.9	6.54	37.97
O75828	CBR3	Carbonyl red	40	10	13	7	277	30.8	6.18	40.89
Q9BZK7	TBL1XR1	F-box-like/W	26	10	11	6	514	55.6	5.55	38.09
P27361	MAPK3	Mitogen-activ	34	12	27	8	379	43.1	6.74	71.81
P05186	ALPL	Alkaline phos	23	10	13	10	524	57.3	6.67	39.37
P30876	POLR2B	DNA-directe	13	13	16	13	1174	133.8	6.87	44.22
P09012	SNRPA	U1 small nuc	46	10	19	8	282	31.3	9.83	58.89
O75569	PRKRA	Interferon-in	39	10	15	9	313	34.4	8.41	41
O15269	SPTLC1	Serine palmi	28	11	14	11	473	52.7	6.01	32.76
P40222	TXLNA	Alpha-taxilin	20	13	18	13	546	61.9	6.52	47.87

P42229	STAT5A	Signal transc	16	12	15	1	794	90.6	6.39	39.73
P52888	THOP1	Thimet oligop	22	14	15	14	689	78.8	6.05	43.47
O95232	LUC7L3	Luc7-like pro	24	11	16	11	432	51.4	9.79	36.86
P53007	SLC25A1	Tricarboxylat	35	10	15	10	311	34	9.89	46.36
P46782	RPS5	40S ribosom	40	9	20	9	204	22.9	9.72	53.13
Q9Y305	ACOT9	Acyl-coenzyr	26	10	16	10	439	49.9	8.6	52.49
P07205	PGK2	Phosphoglyc	21	8	20	2	417	44.8	8.54	49.5
O00148	DDX39A	ATP-depend	22	10	19	3	427	49.1	5.68	54.74
O96000	NDUFB10	NADH dehyc	63	8	14	8	172	20.8	8.48	39.4
Q03591	CFHR1	Complement	27	9	19	2	330	37.6	7.39	52.58
Q15370	ELOB	Elongin-B OS	91	8	15	8	118	13.1	4.88	45.65
O43148	RNMT	mRNA cap g	23	12	14	12	476	54.8	6.61	35.14
P36915	GNL1	Guanine nuc	22	11	14	11	607	68.6	5.8	40.71
Q15631	TSN	Translin OS=	34	7	14	7	228	26.2	6.44	45.2
Q96QR8	PURB	Transcriptior	38	7	12	7	312	33.2	5.43	41.09
P20618	PSMB1	Proteasome	36	7	14	7	241	26.5	8.13	46.01
Q09161	NCBP1	Nuclear cap-	17	10	12	10	790	91.8	6.43	35.86
P23786	CPT2	Carnitine O-p	19	11	12	11	658	73.7	8.18	39.69
Q9BXX0	EMILIN2	EMILIN-2 OS	14	15	18	15	1053	115.6	6.46	43.21
Q15661	TPSAB1	Tryptase alpl	31	7	15	7	275	30.5	7.11	45.65
P10599	TXN	Thioredoxin (	51	6	30	6	105	11.7	4.92	84.87
Q9HAV4	XPO5	Exportin-5 O	13	13	16	13	1204	136.2	5.8	39.45
P62910	RPL32	60S ribosom	50	8	15	8	135	15.9	11.33	43.83
Q15642	TRIP10	Cdc42-intera	22	13	14	13	601	68.3	5.73	35.51
Q00169	PITPNA	Phosphatidyl	43	14	19	11	270	31.8	6.55	48.25
Q08170	SRSF4	Serine/argini	21	13	17	8	494	56.6	11.52	40.55
P25685	DNAJB1	DnaJ homolo	30	9	13	9	340	38	8.63	35.06
P42345	MTOR	Serine/threo	5	13	17	13	2549	288.7	7.17	47.03
Q12996	CSTF3	Cleavage sti	17	12	14	12	717	82.9	8.12	39.85
Q13938	CAPS	Calcyphosin	33	8	19	8	275	30.2	6.04	65.74
Q9Y2D5	AKAP2	A-kinase anc	17	11	11	11	859	94.6	5.11	34.59
O95747	OXSR1	Serine/threo	22	11	14	9	527	58	6.43	34.56
Q9Y5P6	GMPPB	Mannose-1-p	29	7	11	7	360	39.8	6.61	41.08

Q96PD5	PGLYRP2	N-acetylmura	20	7	12	7	576	62.2	7.55	42.72
P28074	PSMB5	Proteasome	32	8	13	8	263	28.5	6.92	45.53
P62244	RPS15A	40S ribosom	65	8	23	8	130	14.8	10.13	68.18
Q9BZQ8	NIBAN1	Protein Nibar	14	10	16	10	928	103.1	4.78	39.66
Q12873	CHD3	Chromodoma	7	14	18	10	2000	226.5	7.3	40.19
P28370	SMARCA1	Probable glo	12	13	17	4	1054	122.5	8.09	39.1
Q16706	MAN2A1	Alpha-manno	12	13	16	13	1144	131.1	7.58	39.36
Q8IZP0	ABI1	Abl interacto	20	10	16	7	508	55	7.06	47.05
P49590	HARS2	Histidine--tRI	20	10	16	6	506	56.9	8.24	37.93
P22413	ENPP1	Ectonucleotic	14	12	14	12	925	104.9	7.14	40.73
O14498	ISLR	Immunoglobu	21	6	10	6	428	46	5.15	38.4
P11182	DBT	Lipoamide ac	26	10	12	10	482	53.5	8.51	33.02
O95433	AHSA1	Activator of 9	32	9	12	9	338	38.3	5.53	36.03
P11172	UMPS	Uridine 5'-mo	26	12	14	12	480	52.2	7.24	33.08
P62987	UBA52	Ubiquitin-60S	59	9	45	1	128	14.7	9.83	103.08
Q15637	SF1	Splicing facto	17	7	13	7	639	68.3	8.98	43.27
P30154	PPP2R1B	Serine/threor	19	10	17	1	601	66.2	4.94	47.08
Q9Y295	DRG1	Developmen	31	10	14	9	367	40.5	8.9	43.66
P19174	PLCG1	1-phosphatid	12	13	13	13	1290	148.4	6.05	34.65
P19404	NDUFV2	NADH dehyc	40	8	11	8	249	27.4	8.06	39.58
Q14118	DAG1	Dystroglycan	16	11	14	11	895	97.4	8.56	45.9
P01903	HLA-DRA	HLA class II	38	9	12	8	254	28.6	5	43.09
P52701	MSH6	DNA mismat	10	11	14	11	1360	152.7	6.9	43.94
Q07075	ENPEP	Glutamyl am	13	12	14	12	957	109.2	5.47	34.58
Q96CT7	CCDC124	Coiled-coil de	51	12	13	12	223	25.8	9.54	35.38
Q9HCU5	PREB	Prolactin reg	29	7	10	7	417	45.4	7.88	45.06
Q9P2R7	SUCLA2	Succinate--C	27	12	15	11	463	50.3	7.42	36.41
Q9HB07	MYG1	MYG1 exonu	32	8	13	8	376	42.5	6.77	36.39
P06132	UROD	Uroporphyrin	28	7	12	7	367	40.8	6.14	38.34
Q13867	BLMH	Bleomycin hy	24	9	15	9	455	52.5	6.27	44.9
Q00013	MPP1	55 kDa eryth	29	11	12	11	466	52.3	7.37	39.38
P10606	COX5B	Cytochrome	36	7	20	7	129	13.7	8.81	57.37
Q02750	MAP2K1	Dual specific	28	11	14	6	393	43.4	6.62	38.53

P49916	LIG3	DNA ligase 3	12	12	14	12	1009	112.8	9.01	39.94
P62280	RPS11	40S ribosom	39	10	20	10	158	18.4	10.3	46.14
O00629	KPNA4	Importin sub	20	7	11	3	521	57.9	4.96	35.12
P62633	CNBP	CCHC-type z	48	8	19	8	177	19.5	7.71	46.81
Q9H1E3	NUCKS1	Nuclear ubiq	55	9	14	9	243	27.3	5.08	45.25
P55263	ADK	Adenosine ki	31	11	14	11	362	40.5	6.7	38.54
P61088	UBE2N	Ubiquitin-cor	55	7	16	7	152	17.1	6.57	47.22
P11177	PDHB	Pyruvate deh	27	8	15	8	359	39.2	6.65	46.23
Q9NTX5	ECHDC1	Ethylmalonyl	32	8	12	8	307	33.7	8.21	39.52
P35658	NUP214	Nuclear pore	8	12	13	12	2090	213.5	7.47	36.26
Q00059	TFAM	Transcription	39	12	20	12	246	29.1	9.72	42.5
O60684	KPNA6	Importin sub	22	9	13	6	536	60	4.98	42.17
Q16787	LAMA3	Laminin subu	5	11	12	11	3333	366.4	7.24	31.35
P31153	MAT2A	S-adenosylm	29	10	14	10	395	43.6	6.48	41.46
O00161	SNAP23	Synaptosoma	47	8	14	8	211	23.3	5.01	46.81
P12544	GZMA	Granzyme A	42	9	14	9	262	29	8.91	47.33
Q14155	ARHGEF7	Rho guanine	13	10	12	7	803	90	7.09	36.14
Q9UKK9	NUDT5	ADP-sugar p	46	8	11	8	219	24.3	4.94	38.2
Q9BTW9	TBCD	Tubulin-spec	11	10	11	10	1192	132.5	6.19	35.04
Q12874	SF3A3	Splicing facto	24	10	14	10	501	58.8	5.38	30.69
P42226	STAT6	Signal transc	18	10	13	10	847	94.1	6.23	47.38
Q13636	RAB31	Ras-related p	46	8	11	7	194	21.6	7.06	36.42
Q9BXS5	AP1M1	AP-1 comple	28	10	13	8	423	48.6	7.3	34.6
P16104	H2AX	Histone H2A	43	8	39	3	143	15.1	10.74	78.75
P46977	STT3A	Dolichyl-diph	14	11	18	9	705	80.5	8.07	53.92
P05452	CLEC3B	Tetranectin C	51	9	14	9	202	22.5	5.67	41.39
P08311	CTSG	Cathepsin G	38	9	15	9	255	28.8	11.19	55.85
A6NHR9	SMCHD1	Structural ma	8	15	17	15	2005	226.2	7.3	34.12
Q15418	RPS6KA1	Ribosomal pr	16	11	14	3	735	82.7	7.83	35.07
Q9GZT8	NIF3L1	NIF3-like pro	34	11	13	11	377	41.9	6.65	33.87
Q92905	COPS5	COP9 signal	28	8	13	8	334	37.6	6.54	33.75
Q13616	CUL1	Cullin-1 OS=	18	11	13	11	776	89.6	8	40.65
O43432	EIF4G3	Eukaryotic tr	8	14	16	9	1585	176.5	5.38	32.36

P78406	RAE1	mRNA expor	27	8	11	8	368	40.9	7.83	43.26
Q08752	PPID	Peptidyl-proly	23	10	13	10	370	40.7	7.21	37.1
Q6L8Q7	PDE12	2',5'-phospho	21	8	10	8	609	67.3	6.57	33.21
Q92769	HDAC2	Histone deac	23	12	16	7	488	55.3	5.91	32.35
Q99747	NAPG	Gamma-solu	30	10	14	10	312	34.7	5.41	43.7
O15260	SURF4	Surfeit locus	22	5	20	5	269	30.4	7.78	61.8
P00966	ASS1	Argininosucc	24	10	16	10	412	46.5	8.02	38.61
Q9UJW0	DCTN4	Dynactin sub	31	10	14	10	460	52.3	7.34	42.38
P39687	ANP32A	Acidic leucin	35	11	17	6	249	28.6	4.09	40.13
Q9BRF8	CPPED1	Serine/threo	26	8	15	8	314	35.5	6.2	45.11
Q8NCW5	NAXE	NAD(P)H-hy	38	8	12	8	288	31.7	7.66	39.02
P05386	RPLP1	60S acidic ril	67	3	10	2	114	11.5	4.32	51.39
Q9NQ48	LZTFL1	Leucine zipp	30	9	15	9	299	34.6	5.36	47.62
Q96SI9	STRBP	Spermatid pe	15	9	17	5	672	73.6	8.72	48.16
P17931	LGALS3	Galectin-3 O	42	10	20	10	250	26.1	8.56	47.49
Q53GQ0	HSD17B12	Very-long-ch	32	7	13	7	312	34.3	9.32	41.63
Q16527	CSRP2	Cysteine and	47	9	16	9	193	20.9	8.62	51.6
A5A3E0	POTEF	POTE ankyri	7	6	51	1	1075	121.4	6.2	145.81
O43493	TGOLN2	Trans-Golgi	27	10	14	10	437	45.9	5.63	39.03
Q9GZV4	EIF5A2	Eukaryotic tr	41	7	18	1	153	16.8	5.58	53.58
P07360	C8G	Complement	45	8	11	8	202	22.3	8.31	42.56
Q9NP81	SARS2	Serine--tRN	25	10	13	10	518	58.2	8.13	38.93
Q9P0V9	SEPTIN10	Septin-10 OS	20	8	16	6	454	52.6	6.8	45.87
O60568	PLOD3	Multifunction	16	12	15	12	738	84.7	6.05	43.05
Q9H9G7	AGO3	Protein argo	13	10	12	5	860	97.3	9.11	36.49
Q14185	DOCK1	Dedicator of	8	15	16	14	1865	215.2	7.56	36.69
Q96GQ5	RUSF1	RUS family r	22	9	13	9	468	51	6.93	38.17
Q49A26	GLYR1	Cytokine-like	22	9	11	9	553	60.5	9.17	36.76
Q92747	ARPC1A	Actin-related	31	10	13	9	370	41.5	8.18	36.58
Q99615	DNAJC7	DnaJ homolo	21	11	14	11	494	56.4	6.96	31.58
Q9NY15	STAB1	Stabilin-1 OS	5	11	16	11	2570	275.3	6.49	39.46
Q9UIG0	BAZ1B	Tyrosine-pro	11	16	18	16	1483	170.8	8.48	37.71
Q9NVD7	PARVA	Alpha-parvin	22	8	15	7	372	42.2	5.95	42.16

P61326	MAGOH	Protein mag	47	8	15	8	146	17.2	6.11	45.22
P50148	GNAQ	Guanine nuc	25	8	13	4	359	42.1	5.68	39.34
P28070	PSMB4	Proteasome	41	7	15	7	264	29.2	5.97	49.87
P25787	PSMA2	Proteasome	40	7	10	7	234	25.9	7.43	39.34
P51178	PLCD1	1-phosphatid	14	9	13	9	756	85.6	6.7	44.07
P28062	PSMB8	Proteasome	38	8	11	8	276	30.3	7.43	37.71
Q08J23	NSUN2	RNA cytosin	21	12	15	12	767	86.4	6.77	41.93
O75694	NUP155	Nuclear pore	9	11	14	11	1391	155.1	6.16	42.81
Q9UBB4	ATXN10	Ataxin-10 OS	21	8	12	8	475	53.5	5.25	37.81
P30622	CLIP1	CAP-Gly dor	9	11	11	8	1438	162.1	5.36	33.31
Q9NW15	ANO10	Anoctamin-1	16	8	14	8	660	76.3	7.4	44.11
Q96A65	EXOC4	Exocyst com	13	12	14	12	974	110.4	6.49	35.9
P84243	H3-3B	Histone H3.3	38	9	40	5	136	15.3	11.27	100.84
Q13948	CUX1	Protein CASI	19	9	10	1	678	77.4	5.44	32.89
Q8WYA6	CTNNBL1	Beta-catenin	21	12	17	12	563	65.1	5.05	41.68
P55010	EIF5	Eukaryotic tr	22	8	17	8	431	49.2	5.58	48.94
P05114	HMGN1	Non-histone	56	7	10	7	100	10.7	9.6	32.56
O95299	NDUFA10	NADH dehyd	27	13	17	13	355	40.7	8.48	36.55
Q13330	MTA1	Metastasis-a	18	12	13	11	715	80.7	9.26	31.8
Q8WWY3	PRPF31	U4/U6 small	21	10	12	10	499	55.4	5.78	37.67
Q96RW7	HMCN1	Hemicentin-1	3	11	13	11	5635	613	6.49	33.4
P46926	GNPDA1	Glucosamine	47	10	13	6	289	32.6	6.92	43.54
Q6VY07	PACS1	Phosphofurin	8	8	16	8	963	104.8	7.74	51.73
Q9NQG5	RPRD1B	Regulation o	24	8	13	8	326	36.9	5.97	44.92
Q9UBV2	SEL1L	Protein sel-1	18	9	11	9	794	88.7	5.39	33
P14324	FDPS	Farnesyl pyr	18	8	16	8	419	48.2	6.15	48.43
Q6PGP7	SKIC3	SKI3 subunit	9	12	13	12	1564	175.4	7.53	35.33
P24539	ATP5PB	ATP synthas	36	9	14	9	256	28.9	9.36	40.61
P42330	AKR1C3	Aldo-keto re	37	9	13	5	323	36.8	7.94	28.86
P30566	ADSL	Adenylosucc	31	10	13	10	484	54.9	7.11	38.59
P31483	TIA1	Cytotoxic gra	28	9	14	5	386	42.9	7.74	46.66
O00154	ACOT7	Cytosolic acy	27	9	14	9	380	41.8	8.54	38.73
P30419	NMT1	Glycylpeptide	27	11	12	11	496	56.8	7.8	39.06

P63096	GNAI1	Guanine nuc	38	11	17	4	354	40.3	5.97	37.7
Q14651	PLS1	Plastin-1 OS	14	6	12	4	629	70.2	5.41	41.5
Q03001	DST	Dystonin OS	2	16	16	14	7570	860.1	5.25	30.65
Q03518	TAP1	Antigen pept	14	7	13	7	748	80.9	7.31	43.42
P62330	ARF6	ADP-ribosyla	49	5	13	5	175	20.1	8.95	45.61
Q14558	PRPSAP1	Phosphoribo	28	7	9	6	356	39.4	7.2	35.25
O75339	CILP	Cartilage inte	11	11	16	11	1184	132.5	8.41	42.89
P51570	GALK1	Galactokinas	23	8	16	8	392	42.2	6.46	49.64
Q9BPX5	ARPC5L	Actin-related	65	7	11	6	153	16.9	6.6	35.37
Q14498	RBM39	RNA-binding	18	8	11	8	530	59.3	10.1	29.85
P16422	EPCAM	Epithelial cel	23	5	11	5	314	34.9	7.46	40.62
P51153	RAB13	Ras-related p	31	7	18	4	203	22.8	9.19	51.97
Q99426	TBCB	Tubulin-foldi	37	7	14	7	244	27.3	5.15	30.35
Q03135	CAV1	Caveolin-1 C	45	7	14	7	178	20.5	6.02	29.16
P07108	DBI	Acyl-CoA-bir	75	6	10	6	87	10	6.57	33.25
Q8NBQ5	HSD17B11	Estradiol 17-	28	9	14	9	300	32.9	9.07	39.6
Q9NVJ2	ARL8B	ADP-ribosyla	48	7	10	2	186	21.5	8.43	34.97
Q01085	TIAL1	Nucleolysin T	27	9	14	5	375	41.6	7.74	45.86
Q969V3	NCLN	Nicalin OS=H	16	9	15	9	563	62.9	6.89	52.17
Q15276	RABEP1	Rab GTPase	15	14	16	13	862	99.2	5.01	31.01
P63151	PPP2R2A	Serine/threo	30	11	13	7	447	51.7	6.2	30.79
P62269	RPS18	40S ribosom	45	10	26	10	152	17.7	10.99	72.51
O75915	ARL6IP5	PRA1 family	24	5	18	5	188	21.6	9.77	75.15
P35268	RPL22	60S ribosom	52	5	10	5	128	14.8	9.19	41.76
O60841	EIF5B	Eukaryotic tr	12	10	12	10	1220	138.7	5.49	28.97
Q16186	ADRM1	Proteasomal	15	7	16	7	407	42.1	5.07	37.64
Q9Y6K5	OAS3	2'-5'-oligoade	13	11	12	11	1087	121.1	8.4	35
Q0VF96	CGNL1	Cingulin-like	10	11	11	11	1302	149	5.67	36.92
P49720	PSMB3	Proteasome	32	6	10	6	205	22.9	6.55	37.7
Q8N4A0	GALNT4	Polypeptide I	20	11	14	10	578	66.6	7.61	38.91
P84098	RPL19	60S ribosom	36	8	24	8	196	23.5	11.47	46.77
Q9BWS9	CHID1	Chitinase do	22	10	15	10	393	44.9	8.63	43.34
Q66K14	TBC1D9B	TBC1 domai	10	10	12	10	1250	140.4	5.25	37.27

P30533	LRPAP1	Alpha-2-mac	25	11	16	11	357	41.4	8.78	47.46
Q10713	PMPCA	Mitochondria	20	10	14	10	525	58.2	6.92	40.4
Q9Y5K6	CD2AP	CD2-associa	21	11	14	11	639	71.4	6.4	31.49
Q5JTZ9	AARS2	Alanine--tRN	15	10	10	10	985	107.3	6.27	32.11
Q13586	STIM1	Stromal inter	13	9	16	9	685	77.4	6.67	45.88
Q9UNE7	STUB1	E3 ubiquitin-|	39	11	12	11	303	34.8	5.87	36.9
Q9NS69	TOMM22	Mitochondria	51	5	10	5	142	15.5	4.34	44.6
Q9H0W9	C11orf54	Ester hydrola	30	7	12	7	315	35.1	6.7	49.05
Q14677	CLINT1	Clathrin inter	16	9	11	9	625	68.2	6.42	34.34
P09917	ALOX5	Polyunsatura	18	9	11	9	674	77.9	5.77	35.94
Q02978	SLC25A11	Mitochondria	35	9	14	9	314	34	9.91	44.81
P13533	MYH6	Myosin-6 OS	6	12	14	9	1939	223.6	5.73	27.05
Q14696	MESD	LRP chaperc	43	9	14	9	234	26.1	7.78	37.5
P61964	WDR5	WD repeat-c	31	8	14	8	334	36.6	8.27	41.46
P19623	SRM	Spermidine s	27	6	12	6	302	33.8	5.49	42.11
O75439	PMPCB	Mitochondria	26	11	12	10	489	54.3	6.83	36.91
Q9H845	ACAD9	Complex I as	19	10	12	10	621	68.7	7.96	28.89
O75781	PALM	Paralemmin-	32	13	17	12	387	42.1	5	37.53
P08294	SOD3	Extracellular	40	6	9	6	240	25.8	6.61	34.67
Q9HCE1	MOV10	Helicase MO	13	10	12	10	1003	113.6	8.82	37.71
Q9Y617	PSAT1	Phosphoseri	30	12	15	11	370	40.4	7.66	45.31
Q5SWX8	ODR4	Protein odr-4	22	8	11	8	454	51.1	5.92	31.23
Q86TX2	ACOT1	Acyl-coenzyr	23	8	11	8	421	46.2	7.34	37.01
P60510	PPP4C	Serine/threoı	28	5	10	4	307	35.1	5.06	37.7
Q14137	BOP1	Ribosome bi	16	8	10	8	746	83.6	6.19	36.12
Q9Y2Z0	SUGT1	Protein SGT	28	9	12	9	365	41	5.16	34.3
P16219	ACADS	Short-chain s	26	7	9	7	412	44.3	7.99	30.98
P19525	EIF2AK2	Interferon-inc	19	9	12	9	551	62.1	8.4	24.75
P29373	CRABP2	Cellular retin	54	9	20	9	138	15.7	5.4	54.47
Q16576	RBBP7	Histone-bind	23	9	19	3	425	47.8	5.05	48.95
Q641Q2	WASHC2A	WASH comp	11	9	11	1	1341	147.1	4.81	29.53
Q9BX68	HINT2	Adenosine 5'	42	4	8	4	163	17.2	9.16	39.95
O96005	CLPTM1	Putative lipid	19	10	12	10	669	76	6.3	44.83

Q13523	PRPF4B	Serine/threor	10	9	11	9	1007	116.9	10.26	32.93
P62829	RPL23	60S ribosom	56	6	21	6	140	14.9	10.51	50.16
Q14019	COTL1	Coactosin-lik	63	8	14	8	142	15.9	5.67	39.59
Q9NX40	OCIAD1	OCIA domair	33	8	12	8	245	27.6	7.49	40.86
P36507	MAP2K2	Dual specific	23	9	11	4	400	44.4	6.55	34.17
Q9UHD9	UBQLN2	Ubiquilin-2 C	18	7	11	3	624	65.7	5.22	38.47
Q8TCS8	PNPT1	Polyribonucle	15	11	13	11	783	85.9	7.77	33.32
P56537	EIF6	Eukaryotic tr	33	5	8	5	245	26.6	4.68	38.8
O95394	PGM3	Phosphoace	15	9	14	9	542	59.8	6.25	35.13
Q9NV70	EXOC1	Exocyst com	12	12	15	12	894	101.9	6.61	35.26
O75347	TBCA	Tubulin-spec	44	6	15	6	108	12.8	5.29	44.78
Q96DI7	SNRNP40	U5 small nuc	19	7	11	7	357	39.3	8.1	38.49
Q96DG6	CMBL	Carboxymeth	28	8	13	8	245	28	7.18	32.4
P80303	NUCB2	Nucleobindin	22	8	11	8	420	50.2	5.12	36.19
Q9HCS7	XAB2	Pre-mRNA-s	15	13	15	13	855	99.9	6.23	37.29
Q96KP1	EXOC2	Exocyst com	12	10	14	10	924	104	6.9	33.75
Q15059	BRD3	Bromodomai	14	9	10	5	726	79.5	9.36	31.56
Q99584	S100A13	Protein S100	48	6	18	6	98	11.5	6.16	44.32
P52294	KPNA1	Importin subu	17	7	10	4	538	60.2	5.01	34.16
O00170	AIP	AH receptor-	29	9	15	9	330	37.6	6.42	34.32
Q70UQ0	IKBIP	Inhibitor of nu	27	11	12	11	350	39.3	9.17	28.58
O15145	ARPC3	Actin-related	34	7	20	7	178	20.5	8.59	41.61
Q8TD55	PLEKHO2	Pleckstrin ho	16	8	15	8	490	53.3	5.43	33.49
O00534	VWA5A	von Willebrar	14	9	12	9	786	86.4	6.58	34.37
Q9H269	VPS16	Vacuolar pro	15	10	11	10	839	94.6	6.77	29.19
P59998	ARPC4	Actin-related	47	8	18	8	168	19.7	8.43	52.07
Q00577	PURA	Transcription	35	7	12	7	322	34.9	6.44	53.3
O43865	AHCYL1	S-adenosylh	18	11	15	5	530	58.9	6.89	37.06
P35858	IGFALS	Insulin-like g	17	8	11	8	605	66	6.79	35.28
Q9BUH6	PAXX	Protein PAX	42	6	11	6	204	21.6	5.48	37.87
Q9NRX4	PHPT1	14 kDa phos	50	7	21	7	125	13.8	6.07	54.87
Q9NR31	SAR1A	GTP-binding	33	6	17	2	198	22.4	6.68	43.44
P39748	FEN1	Flap endonu	30	9	11	9	380	42.6	8.62	36.78

P33240	CSTF2	Cleavage sti	20	9	11	6	577	60.9	6.83	31.99
Q96ME7	ZNF512	Zinc finger pı	17	8	10	8	567	64.6	9.76	29.84
Q99719	SEPTIN5	Septin-5 OS=	23	8	12	7	369	42.8	6.67	27.8
Q68CZ2	TNS3	Tensin-3 OS	9	9	13	5	1445	155.2	6.81	31.58
Q9BVP2	GNL3	Guanine nuc	17	7	11	7	549	62	9.16	39.14
P53004	BLVRA	Biliverdin red	26	9	14	9	296	33.4	6.44	41.94
P40616	ARL1	ADP-ribosyla	39	5	11	5	181	20.4	5.72	37.6
P78559	MAP1A	Microtubule-a	4	11	13	10	2803	305.3	4.92	28.63
O95994	AGR2	Anterior grac	26	4	14	3	175	20	9	35
Q9Y371	SH3GLB1	Endophilin-B	19	7	15	6	365	40.8	6.04	47.46
P12830	CDH1	Cadherin-1 C	14	11	13	11	882	97.4	4.73	36.04
P61026	RAB10	Ras-related p	39	9	15	7	200	22.5	8.38	36.46
Q9H4A6	GOLPH3	Golgi phosph	26	7	14	7	298	33.8	6.44	38.7
P53597	SUCLG1	Succinate--C	21	6	15	6	346	36.2	8.79	44.67
Q14108	SCARB2	Lysosome m	14	7	14	7	478	54.3	5.14	39.87
P32456	GBP2	Guanylate-bi	16	8	12	7	591	67.2	5.71	36.87
P62888	RPL30	60S ribosom	40	5	13	5	115	12.8	9.63	38.01
O14841	OPLAH	5-oxoprolinas	10	9	10	9	1288	137.4	6.58	26.56
Q9Y240	CLEC11A	C-type lectin	27	9	11	9	323	35.7	5.16	32.5
O76031	CLPX	ATP-depend	18	11	11	11	633	69.2	7.58	27.57
Q53T59	HS1BP3	HCLS1-bindi	21	6	9	6	392	42.8	5.01	34.13
O60493	SNX3	Sorting nexir	43	7	17	6	162	18.8	8.66	45.82
Q86SF2	GALNT7	N-acetylgala	19	10	11	10	657	75.3	7.11	34.76
P22676	CALB2	Calretinin OS	34	9	13	9	271	31.5	5.15	37.43
P62318	SNRPD3	Small nuclea	48	4	13	4	126	13.9	10.32	48.01
O43719	HTATSF1	HIV Tat-spec	15	13	13	13	755	85.8	4.4	27.65
P62266	RPS23	40S ribosom	49	7	14	7	143	15.8	10.49	43.2
Q9UHV9	PFDN2	Prefoldin sub	53	7	11	7	154	16.6	6.58	32.04
O14976	GAK	Cyclin-G-ass	8	11	13	11	1311	143.1	5.73	32.14
P10619	CTSA	Lysosomal p	15	6	11	6	480	54.4	6.61	29.82
Q92797	SYMPK	Symplekin O	10	11	13	11	1274	141.1	6.13	37.56
P55769	SNU13	NHP2-like pr	45	6	13	6	128	14.2	8.46	35.14
Q15643	TRIP11	Thyroid rece	7	13	13	13	1979	227.4	5.26	27.29

Q96T51	RUFY1	RUN and FY	18	13	13	12	708	79.8	5.74	31.2
O95834	EML2	Echinoderm	11	6	9	5	649	70.6	6.32	34.31
P07738	BPGM	Bisphosphog	36	9	14	9	259	30	6.54	42.25
P50914	RPL14	60S ribosom	31	7	15	7	215	23.4	10.93	40.01
Q9BRK5	SDF4	45 kDa calciu	36	9	11	9	362	41.8	4.86	32.28
P54619	PRKAG1	5'-AMP-activ	34	9	10	9	331	37.6	6.92	29.26
P23497	SP100	Nuclear auto	10	10	17	6	879	100.4	8.22	31.33
O95817	BAG3	BAG family r	21	10	12	10	575	61.6	6.95	32.9
Q9UPU7	TBC1D2B	TBC1 domai	13	12	12	12	963	109.8	6.16	30.63
Q9Y394	DHRS7	Dehydrogena	30	8	12	8	339	38.3	8.32	45.01
Q92783	STAM	Signal transc	19	8	10	7	540	59.1	4.82	30.37
Q9Y3B7	MRPL11	39S ribosom	35	7	11	7	192	20.7	9.91	43.34
P05997	COL5A2	Collagen alp	8	9	12	9	1499	144.8	6.46	31.73
Q9UK41	VPS28	Vacuolar pro	38	7	10	7	221	25.4	5.54	27.34
Q86VN1	VPS36	Vacuolar pro	23	10	13	9	386	43.8	7.2	33.89
Q4G0N4	NADK2	NAD kinase 2	25	9	13	9	442	49.4	8.18	36.03
P60953	CDC42	Cell division	33	5	13	5	191	21.2	6.55	41.49
P09668	CTSH	Pro-cathepsi	19	6	12	6	335	37.4	8.07	27.02
Q9GZM7	TINAGL1	Tubulointers	24	8	11	8	467	52.4	6.99	30.57
P62070	RRAS2	Ras-related p	26	5	11	3	204	23.4	6.01	32.34
P02753	RBP4	Retinol-bindi	46	6	10	6	201	23	6.07	36.9
P02766	TTR	Transthyretir	50	5	13	5	147	15.9	5.76	56.21
Q9UQ16	DNM3	Dynamin-3 C	11	11	13	2	869	97.7	8.35	33.39
P04424	ASL	Argininosucc	18	8	10	8	464	51.6	6.48	27.27
Q96RP9	GFM1	Elongation fa	18	11	12	11	751	83.4	7.01	36.17
Q96P48	ARAP1	Arf-GAP with	9	10	11	10	1450	162.1	6.23	31.29
Q99829	CPNE1	Copine-1 OS	19	10	15	10	537	59	5.83	41.13
P21127	CDK11B	Cyclin-deper	14	11	12	11	795	92.6	5.54	30.67
P43155	CRAT	Carnitine O-a	15	9	14	9	626	70.8	8.44	38.75
Q96DB5	RMDN1	Regulator of	32	9	13	8	314	35.8	8.5	37.6
P14927	UQCRB	Cytochrome	67	8	12	8	111	13.5	8.78	35.81
P16885	PLCG2	1-phosphatic	9	10	11	10	1265	147.8	6.64	28.35
P46976	GYG1	Glycogenin-1	22	7	14	7	350	39.4	5.53	40.81

Q14BN4	SLMAP	Sarcolemma	10	8	11	8	828	95.1	5.47	31.19
Q9UBQ0	VPS29	Vacuolar pro	57	9	16	9	182	20.5	6.79	48.53
Q13347	EIF3I	Eukaryotic tr	30	8	10	8	325	36.5	5.64	28.62
Q06278	AOX1	Aldehyde oxi	9	12	13	11	1338	147.8	7.17	31.32
Q4ZHG4	FNDC1	Fibronectin ty	7	9	11	9	1894	205.4	9.32	37.66
Q14643	ITPR1	Inositol 1,4,5	5	14	14	8	2758	313.7	6.04	28.62
Q5TDH0	DDI2	Protein DDI1	29	8	10	8	399	44.5	5.05	29.7
Q96SM3	CPXM1	Probable car	13	8	13	8	734	81.6	6.67	38.17
Q53GS9	USP39	U4/U6.U5 tri-	22	11	12	11	565	65.3	8.91	32.25
Q8NFV4	ABHD11	Protein ABHI	21	6	10	6	315	34.7	9.48	34.44
P41227	NAA10	N-alpha-acet	37	8	13	8	235	26.4	5.64	33.11
P99999	CYCS	Cytochrome	61	7	13	7	105	11.7	9.57	38.4
O75718	CRTAP	Cartilage-ass	26	10	13	10	401	46.5	5.73	35.81
P08648	ITGA5	Integrin alpha	10	9	11	9	1049	114.5	5.77	34.12
Q6ZMP0	THSD4	Thrombospo	12	11	11	11	1018	112.4	7.65	30.06
P01893	HLA-H	Putative HLA	25	6	8	1	362	40.9	6.3	30.27
P49354	FNTA	Protein farne	22	7	11	7	379	44.4	5.08	39.74
Q9NPQ8	RIC8A	Synembryn-A	19	10	12	9	531	59.7	5.33	35.09
P61006	RAB8A	Ras-related p	37	8	19	2	207	23.7	9.07	48.49
O15382	BCAT2	Branched-ch	27	6	9	6	392	44.3	8.65	36.78
P46779	RPL28	60S ribosom	47	7	23	7	137	15.7	12.02	54.79
Q27J81	INF2	Inverted form	11	9	11	9	1249	135.5	5.38	30.02
P49585	PCYT1A	Choline-phos	29	9	11	7	367	41.7	7.25	32.29
P51114	FXR1	RNA-binding	14	9	13	7	621	69.7	6.15	33
Q9NX62	BPNT2	Golgi-resider	23	6	10	6	359	38.7	6.86	33.13
P41240	CSK	Tyrosine-pro	22	8	11	8	450	50.7	7.06	29.36
O95721	SNAP29	Synaptosom	47	10	12	10	258	29	5.81	33.09
Q8NBJ4	GOLM1	Golgi membr	21	10	15	10	401	45.3	4.97	31.24
Q9NPH2	ISYNA1	Inositol-3-ph	16	9	15	9	558	61	5.76	35.15
Q9P253	VPS18	Vacuolar pro	12	11	14	11	973	110.1	6.07	34.98
Q7Z434	MAVS	Mitochondria	24	8	12	8	540	56.5	5.52	31.26
P09467	FBP1	Fructose-1,6	30	8	10	8	338	36.8	6.99	27.33
Q9Y3U8	RPL36	60S ribosom	36	6	13	6	105	12.2	11.59	34.63

Q9H553	ALG2	Alpha-1,3/1,6	22	7	12	7	416	47.1	7.05	43.85
Q9C0B1	FTO	Alpha-ketogl	20	10	13	10	505	58.2	5.22	30.97
P00533	EGFR	Epidermal gr	6	6	9	5	1210	134.2	6.68	28.98
P19838	NFKB1	Nuclear facto	12	9	10	9	968	105.3	5.4	29.58
P02750	LRG1	Leucine-rich	30	10	14	10	347	38.2	6.95	41.92
Q9BTY2	FUCA2	Plasma alpha	21	10	11	10	467	54	6.25	29.57
P11217	PYGM	Glycogen ph	12	11	13	1	842	97	7.03	27.96
Q12929	EPS8	Epidermal gr	17	10	10	10	822	91.8	7.5	29.42
P61163	ACTR1A	Alpha-centra	22	7	12	4	376	42.6	6.64	38.22
Q9BYT8	NLN	Neurolysin, r	18	11	12	11	704	80.6	6.64	27.65
P46379	BAG6	Large proline	10	9	12	9	1132	119.3	5.6	35.14
Q8IW45	NAXD	ATP-depend	23	5	8	5	347	36.6	8.06	37.99
Q969H8	MYDGF	Myeloid-deriv	31	7	14	7	173	18.8	6.68	36.04
P98175	RBM10	RNA-binding	17	13	13	10	930	103.5	5.97	29.38
O60784	TOM1	Target of My	20	7	12	7	492	53.8	4.7	44.95
Q96A08	H2BC1	Histone H2B	45	6	82	1	127	14.2	10.32	140
P46439	GSTM5	Glutathione S	46	10	12	4	218	25.7	7.39	33.57
Q96N67	DOCK7	Dedicator of	6	13	13	10	2140	242.4	6.8	35.31
Q96S52	PIGS	GPI transam	14	7	10	7	555	61.6	6.49	35.12
O75531	BANF1	Barrier-to-au	54	5	12	5	89	10.1	6.09	40.64
P17301	ITGA2	Integrin alpha	10	8	10	8	1181	129.2	5.25	31.28
Q0JRZ9	FCHO2	F-BAR doma	15	9	9	9	810	88.9	6.86	24.11
Q96DZ1	ERLEC1	Endoplasmic	23	10	11	10	483	54.8	6.28	29.52
O94903	PLPBP	Pyridoxal pho	42	11	14	11	275	30.3	7.5	35.94
Q6UX71	PLXDC2	Plexin domai	19	9	13	9	529	59.5	6.46	31.6
Q8N201	INTS1	Integrator co	6	11	11	11	2190	244.1	6.13	29.25
P34896	SHMT1	Serine hydro	23	8	13	6	483	53	7.71	35.78
Q9BXJ9	NAA15	N-alpha-acet	12	10	15	9	866	101.2	7.42	34.78
P33992	MCM5	DNA replicat	16	11	13	11	734	82.2	8.37	32.45
P53634	CTSC	Dipeptidyl pe	13	5	11	5	463	51.8	6.99	44.92
Q460N5	PARP14	Protein mono	8	13	13	13	1801	202.7	7.18	24.57
P08195	SLC3A2	4F2 cell-surfa	19	9	12	9	630	68	5.01	29.94
P62316	SNRPD2	Small nuclea	53	7	14	7	118	13.5	9.91	42.35

Q9Y613	FHOD1	FH1/FH2 dor	9	9	11	9	1164	126.5	6.39	31.81
P30046	DDT	D-dopachron	57	6	12	6	118	12.7	7.3	42.6
Q9UHL4	DPP7	Dipeptidyl pe	26	10	11	10	492	54.3	6.32	27.54
Q9UN86	G3BP2	Ras GTPase	23	11	14	10	482	54.1	5.55	32.63
Q9BZJ0	CRNKL1	Crooked nec	10	8	11	8	848	100.4	8	29.31
Q01844	EWSR1	RNA-binding	9	7	10	7	656	68.4	9.33	26.98
Q9P0J0	NDUFA13	NADH dehyd	44	6	11	6	144	16.7	8.43	34.55
Q8TCT9	HM13	Minor histoco	20	7	10	7	377	41.5	6.43	29.42
P31949	S100A11	Protein S100	56	6	14	6	105	11.7	7.12	41.33
Q92522	H1-10	Histone H1.1	32	8	14	8	213	22.5	10.76	38.09
P60033	CD81	CD81 antige	31	5	10	5	236	25.8	5.29	33.25
O75695	RP2	Protein XRP	28	10	14	10	350	39.6	5.12	36.42
P21912	SDHB	Succinate de	31	10	11	10	280	31.6	8.76	34.21
P04080	CSTB	Cystatin-B O	77	5	14	5	98	11.1	7.56	49.58
Q96TC7	RMDN3	Regulator of	18	7	11	7	470	52.1	5.1	34.1
Q9BXN1	ASPN	Asporin OS=	24	10	13	10	380	43.4	7.08	28.87
P63027	VAMP2	Vesicle-asso	34	4	8	1	116	12.7	8.13	40.85
Q8TC12	RDH11	Retinol dehy	26	7	13	7	318	35.4	8.82	34.65
Q9NZ32	ACTR10	Actin-related	35	11	12	11	417	46.3	7.37	34.98
P43897	TSFM	Elongation fa	35	9	11	9	325	35.4	8.38	29.16
Q15121	PEA15	Astrocytic ph	57	6	10	6	130	15	5.02	33.04
O15254	ACOX3	Peroxisomal	15	8	9	8	700	77.6	7.25	29.05
Q08AM6	VAC14	Protein VAC	13	8	9	8	782	87.9	6.13	29.98
P42858	HTT	Huntingtin O	4	9	10	9	3142	347.4	6.2	31.69
P62328	TMSB4X	Thymosin be	73	7	13	5	44	5.1	5.06	35.06
Q9BUT1	BDH2	Dehydrogena	26	7	11	7	245	26.7	7.65	32.13
P62899	RPL31	60S ribosom	42	8	18	8	125	14.5	10.54	36.1
Q4KMQ2	ANO6	Anoctamin-6	14	12	12	12	910	106.1	7.77	34.14
Q99538	LGMN	Legumain OS	15	5	11	5	433	49.4	6.55	35.38
Q04446	GBE1	1,4-alpha-glu	16	10	11	10	702	80.4	6.32	31.58
P84090	ERH	Enhancer of	49	5	13	5	104	12.3	5.92	39.06
P50402	EMD	Emerin OS=I	35	7	11	7	254	29	5.5	30.55
Q9ULC4	MCTS1	Malignant T-	52	7	13	7	181	20.5	8.82	37.53

Q9NT62	ATG3	Ubiquitin-like	19	6	9	6	314	35.8	4.74	28.81
P07948	LYN	Tyrosine-pro	20	9	11	7	512	58.5	7.11	26.6
Q9H6R3	ACSS3	Acyl-CoA syr	16	10	11	10	686	74.7	8.63	31.26
Q13315	ATM	Serine-protei	4	11	12	11	3056	350.5	6.81	24.27
Q9BXK5	BCL2L13	Bcl-2-like prc	20	7	10	7	485	52.7	4.44	25.75
Q8WWM7	ATXN2L	Ataxin-2-like	8	10	13	10	1075	113.3	8.59	31.35
Q6P1N0	CC2D1A	Coiled-coil ar	11	10	11	10	951	104	8.09	32.21
Q9UN37	VPS4A	Vacuolar pro	21	10	12	6	437	48.9	7.8	36.28
O75832	PSMD10	26S proteaso	31	7	10	7	226	24.4	6.1	37.36
Q92785	DPF2	Zinc finger pr	27	7	9	7	391	44.1	6.33	33.38
Q13405	MRPL49	39S ribosom	44	7	11	7	166	19.2	9.45	32.27
P49458	SRP9	Signal recog	64	6	14	6	86	10.1	7.97	38.71
Q14508	WFDC2	WAP four-dis	39	3	9	3	124	13	4.84	37.11
Q9C0C9	UBE2O	(E3-indepenc	10	10	10	10	1292	141.2	5.12	26.95
Q6NUQ4	TMEM214	Transmembr	15	11	14	11	689	77.1	9.14	30.26
P36969	GPX4	Phospholipid	41	8	12	8	197	22.2	8.37	31.78
Q9HB40	SCPEP1	Retinoid-indu	20	7	10	7	452	50.8	5.81	32.48
P55290	CDH13	Cadherin-13	15	9	12	9	713	78.2	4.98	34.67
Q9H2H8	PPIL3	Peptidyl-proly	53	8	16	8	161	18.1	6.79	48.29
P48449	LSS	Lanosterol sy	12	8	11	8	732	83.3	6.61	36.02
Q86U86	PBRM1	Protein polyb	7	11	11	11	1689	192.8	6.89	26.1
Q99436	PSMB7	Proteasome	33	7	12	7	277	29.9	7.68	35.19
P01033	TIMP1	Metalloprotei	42	6	12	6	207	23.2	8.1	41.57
P84095	RHOG	Rho-related (	36	6	10	6	191	21.3	8.12	33.86
P46952	HAAO	3-hydroxyant	38	8	8	8	286	32.5	5.88	31.05
Q9UHD1	CHORDC1	Cysteine anc	37	8	12	8	332	37.5	7.87	32.02
Q9BVJ6	UTP14A	U3 small nuc	16	9	10	9	771	87.9	7.87	25.46
O14497	ARID1A	AT-rich intera	6	8	8	6	2285	241.9	6.7	25.37
P50416	CPT1A	Carnitine O-p	13	11	14	11	773	88.3	8.65	36.95
Q5T1M5	FKBP15	FK506-bindir	9	8	10	8	1219	133.5	5.2	30.39
Q9UI10	EIF2B4	Translation ir	20	7	10	7	523	57.5	9.38	36.48
P22830	FECH	Ferrochelata	24	8	10	8	423	47.8	8.73	28.41
Q8N2G8	GHDC	GH3 domain	23	8	9	8	530	57.5	7.88	25.71

P36405	ARL3	ADP-ribosyla	42	6	9	6	182	20.4	7.24	30.62
Q13363	CTBP1	C-terminal-bi	13	6	11	4	440	47.5	6.77	33.73
P18754	RCC1	Regulator of	20	7	12	7	421	44.9	7.52	34.83
P53367	ARFIP1	Arfaptin-1 OS	18	8	11	8	373	41.7	6.7	30.16
Q7L7L0	H2AC25	Histone H2A	32	7	44	3	130	14.1	11.05	75.03
A0M8Q6	IGLC7	Immunoglobu	56	5	25	2	106	11.2	8.29	78.02
A1L4H1	SSC5D	Soluble scav	13	10	11	10	1573	165.6	6.13	28.77
O00233	PSMD9	26S proteaso	30	7	11	7	223	24.7	6.95	31.82
Q9BW27	NUP85	Nuclear pore	16	8	8	8	656	75	5.55	28.06
Q14566	MCM6	DNA replicat	13	11	12	11	821	92.8	5.41	30.03
Q7Z478	DHX29	ATP-depend	8	10	10	10	1369	155.1	8.09	24.97
P18859	ATP5PF	ATP synthas	51	5	10	5	108	12.6	9.52	30.63
Q13976	PRKG1	cGMP-deper	14	9	11	9	671	76.3	6.04	28.65
Q9UIQ6	LNPEP	Leucyl-cystin	11	10	10	10	1025	117.3	5.73	25.18
O75223	GGCT	Gamma-gluta	39	7	11	7	188	21	5.14	29.07
P55039	DRG2	Developmen	32	9	10	8	364	40.7	8.88	27.56
P18031	PTPN1	Tyrosine-pro	20	8	10	8	435	49.9	6.27	28.44
P16989	YBX3	Y-box-bindin	19	5	7	4	372	40.1	9.77	29.1
Q9NUQ8	ABCF3	ATP-binding	14	8	11	8	709	79.7	6.34	28.55
P16284	PECAM1	Platelet endo	14	8	10	8	738	82.5	6.99	30.15
Q5T013	HYI	Putative hydı	34	7	12	6	277	30.4	5.5	31.56
Q13492	PICALM	Phosphatidyl	13	8	10	8	652	70.7	7.9	28.28
Q8IWB7	WDFY1	WD repeat a	24	7	8	7	410	46.3	7.33	27.11
Q68EM7	ARHGAP17	Rho GTPase	10	7	8	7	881	95.4	7.62	28.41
Q9H0U3	MAGT1	Magnesium t	21	8	16	7	335	38	9.63	46.49
P08567	PLEK	Pleckstrin OS	28	7	9	7	350	40.1	8.28	32.81
P35813	PPM1A	Protein phos	22	7	8	5	382	42.4	5.36	27.04
Q6P996	PDXDC1	Pyridoxal-de	16	12	12	12	788	86.7	5.38	29.26
Q13123	IK	Protein Red	21	9	10	9	557	65.6	6.64	30.74
Q8WX92	NELFB	Negative elo	16	9	11	8	580	65.7	6.13	28.35
O14639	ABLIM1	Actin-binding	12	7	9	7	778	87.6	8.59	31.57
O95466	FMNL1	Formin-like p	10	9	11	8	1100	121.8	5.72	28.21
P04839	CYBB	Cytochrome	12	8	12	8	570	65.3	8.63	29.06

P20592	MX2	Interferon-inc	13	8	9	5	715	82	8.76	28.04
Q9UBR2	CTSZ	Cathepsin Z	26	9	14	9	303	33.8	7.11	35.5
P10153	RNASE2	Non-secretor	20	3	8	3	161	18.3	8.73	31.79
Q99567	NUP88	Nuclear pore	13	9	12	9	741	83.5	5.69	29.08
P14678	SNRPB	Small nuclea	20	5	12	5	240	24.6	11.19	39.12
O95881	TXNDC12	Thioredoxin d	36	5	10	5	172	19.2	5.4	28.51
P63000	RAC1	Ras-related C	33	7	13	4	192	21.4	8.5	28.98
Q9NZL9	MAT2B	Methionine a	28	7	9	7	334	37.5	7.36	30.82
Q99598	TSNAX	Translin-asso	44	10	10	10	290	33.1	6.55	27.08
P51003	PAPOLA	Poly(A) polyr	14	8	9	8	745	82.8	7.37	27.16
Q9H9E3	COG4	Conserved o	14	9	11	9	785	89	5.19	25.03
Q8NEU8	APPL2	DCC-interact	16	8	9	7	664	74.4	4.94	23.14
P47985	UQCRFS1	Cytochrome	35	8	17	8	274	29.6	8.32	41.48
Q13247	SRSF6	Serine/argini	27	11	16	7	344	39.6	11.43	37.66
O15305	PMM2	Phosphomar	37	10	12	10	246	28.1	6.77	26.91
O94925	GLS	Glutaminase	15	8	14	8	669	73.4	7.77	34.79
Q9NXF1	TEX10	Testis-expres	11	9	9	9	929	105.6	9.36	29.31
P09619	PDGFRB	Platelet-deriv	9	9	12	8	1106	123.9	4.98	38.27
P56545	CTBP2	C-terminal-bi	14	7	9	5	445	48.9	6.95	28.52
Q9C0E8	LNPK	Endoplasmic	14	6	13	6	428	47.7	5.11	39.06
Q5XKP0	MICOS13	MICOS comp	47	4	9	4	118	13.1	9.42	38.7
Q96CV9	OPTN	Optineurin O	15	9	10	9	577	65.9	5.17	29.09
P83731	RPL24	60S ribosom	36	7	12	7	157	17.8	11.25	40.3
P33527	ABCC1	Multidrug res	7	10	10	10	1531	171.5	7.11	26.64
O15400	STX7	Syntaxin-7 O	30	7	9	7	261	29.8	5.55	27.9
Q9ULR3	PPM1H	Protein phos	12	6	11	6	514	56.4	6.6	27.28
Q9UKF6	CPSF3	Cleavage an	13	8	10	8	684	77.4	5.6	28.09
O43765	SGTA	Small glutam	31	8	9	8	313	34	4.87	27.89
Q53FA7	TP53I3	Quinone oxic	20	7	10	7	332	35.5	7.17	23.53
Q9BYX7	POTEKP	Putative beta	14	4	41	1	375	42	6.33	120.01
O95219	SNX4	Sorting nexir	24	10	11	10	450	51.9	5.99	26.12
Q96HY6	DDRGK1	DDRGK dom	29	6	10	6	314	35.6	5.12	40.24
Q13555	CAMK2G	Calcium/caln	18	8	11	4	558	62.6	7.83	26.07

P61421	ATP6V0D1	V-type protor	22	7	11	7	351	40.3	5	34.78
P03952	KLKB1	Plasma kallik	16	10	11	10	638	71.3	8.22	30.81
Q9Y2L1	DIS3	Exosome coı	11	8	9	8	958	108.9	7.14	27.29
O15347	HMGB3	High mobility	29	9	11	8	200	23	8.37	32.36
Q8IY81	FTSJ3	pre-rRNA 2'-	14	9	9	9	847	96.5	8.4	26.17
Q9NRY5	FAM114A2	Protein FAM	19	8	10	8	505	55.4	4.88	29.28
P51398	DAP3	28S ribosom	19	7	9	7	398	45.5	8.88	35.51
Q07812	BAX	Apoptosis re	29	4	7	4	192	21.2	5.22	26.9
O15294	OGT	UDP-N-acety	11	10	13	10	1046	116.9	6.7	37.4
O95816	BAG2	BAG family r	33	8	14	8	211	23.8	6.7	34.82
Q8N392	ARHGAP18	Rho GTPase	16	9	10	9	663	74.9	6.44	23.77
Q9Y2T3	GDA	Guanine dea	19	8	10	8	454	51	5.68	28.86
Q96IJ6	GMPPA	Mannose-1-p	20	6	10	6	420	46.3	7.21	29.78
Q3SY69	ALDH1L2	Mitochondria	12	11	12	10	923	101.7	6.52	25.68
P52788	SMS	Spermine syı	25	8	12	8	366	41.2	5.02	30.77
Q9BYD3	MRPL4	39S ribosom	33	6	11	6	311	34.9	9.72	36.95
Q05655	PRKCD	Protein kinas	16	12	14	11	676	77.5	7.75	27.7
Q96IU4	ABHD14B	Putative prot	33	5	8	5	210	22.3	6.4	29.57
Q8NDH3	NPEPL1	Probable am	17	7	8	7	523	55.8	6.87	25.75
Q04760	GLO1	Lactoylglutat	46	8	15	8	184	20.8	5.31	27.76
Q96GK7	FAHD2A	Fumarylacet	24	7	9	7	314	34.6	8.24	29.99
P00740	F9	Coagulation	16	7	11	7	461	51.7	5.47	35.07
Q13617	CUL2	Cullin-2 OS=	13	11	11	11	745	86.9	6.92	31.45
P01889	HLA-B	HLA class I h	29	8	12	3	362	40.4	5.85	29.64
Q99816	TSG101	Tumor susce	18	6	8	6	390	43.9	6.46	24.47
Q96F85	CNRIP1	CB1 cannabi	52	6	8	6	164	18.6	7.94	30.63
Q96EE3	SEH1L	Nucleoporin	26	7	8	7	360	39.6	8.09	27.03
P09497	CLTB	Clathrin light	31	9	12	9	229	25.2	4.64	33.07
O75608	LYPLA1	Acyl-protein	28	5	10	5	230	24.7	6.77	28.31
Q7Z4W1	DCXR	L-xylulose re	30	6	9	6	244	25.9	8.1	24.68
Q10588	BST1	ADP-ribosyl	24	7	9	7	318	35.7	7.8	26.91
P31749	AKT1	RAC-alpha s	22	10	14	6	480	55.7	6.07	38.57
P15927	RPA2	Replication p	32	6	9	6	270	29.2	6.15	26.58

P41226	UBA7	Ubiquitin-like	9	8	10	8	1012	111.6	6.04	29.89
Q15428	SF3A2	Splicing facto	14	5	8	5	464	49.2	9.64	25.95
Q8WXX5	DNAJC9	DnaJ homolo	31	8	9	8	260	29.9	5.73	23.67
Q32MZ4	LRRFIP1	Leucine-rich	10	7	9	7	808	89.2	4.65	27.32
O95758	PTBP3	Polypyrimidir	19	8	11	4	552	59.7	9.04	34.52
Q969G3	SMARCE1	SWI/SNF-rel	22	9	13	8	411	46.6	4.88	39.65
Q9H0E2	TOLLIP	Toll-interactir	24	6	9	6	274	30.3	5.97	25
P01112	HRAS	GTPase HRa	35	5	10	3	189	21.3	5.31	39.13
O43815	STRN	Striatin OS=H	16	9	9	9	780	86.1	5.27	25.36
Q9Y5L0	TNPO3	Transportin-3	11	8	10	8	923	104.1	5.57	35.46
P05154	SERPINA5	Plasma serir	17	6	10	6	406	45.6	9.26	32.72
P63167	DYNLL1	Dynein light c	63	4	10	2	89	10.4	7.4	40.47
Q9BV38	WDR18	WD repeat-c	17	7	11	7	432	47.4	6.7	28.81
Q8N8N7	PTGR2	Prostaglandi	19	4	9	4	351	38.5	5.41	33.13
Q8N8S7	ENAH	Protein enab	17	9	13	9	591	66.5	6.93	37.51
P33241	LSP1	Lymphocyte-	35	9	10	9	339	37.2	4.74	31.25
Q13144	EIF2B5	Translation ir	13	9	11	9	721	80.3	5.08	28.84
Q15334	LLGL1	Lethal(2) gia	9	8	8	8	1064	115.3	6.29	29.14
P08571	CD14	Monocyte dif	21	6	10	6	375	40.1	6.23	31.91
P52943	CRIP2	Cysteine-rich	39	5	8	5	208	22.5	8.72	33
Q92556	ELMO1	Engulfment a	11	8	10	5	727	83.8	6.28	29.27
P50135	HNMT	Histamine N-	23	5	12	5	292	33.3	5.34	35.7
Q9BZG1	RAB34	Ras-related p	29	7	10	7	259	29	7.88	31.82
P11279	LAMP1	Lysosome-as	15	7	13	7	417	44.9	8.75	33.77
Q9NR46	SH3GLB2	Endophilin-B	19	7	11	6	395	43.9	5.99	32.8
P42025	ACTR1B	Beta-centrac	28	8	10	5	376	42.3	6.4	31.51
P11233	RALA	Ras-related p	23	4	9	2	206	23.6	7.11	27.3
Q9HCU0	CD248	Endosialin O	13	8	15	8	757	80.8	5.35	32.74
Q7Z4I7	LIMS2	LIM and sen	21	7	13	2	341	38.9	8.05	27.3
P17050	NAGA	Alpha-N-ace	20	7	11	7	411	46.5	5.19	36.17
Q96PU8	QKI	KH domain-c	26	6	11	6	341	37.6	8.56	35
Q9BZV1	UBXN6	UBX domain	24	6	8	6	441	49.7	6.89	24.14
Q9UNX3	RPL26L1	60S ribosom	36	8	16	8	145	17.2	10.55	38.66

P01019	AGT	Angiotensinc	11	5	10	5	485	53.1	6.32	34.52
Q8NFD5	ARID1B	AT-rich intera	5	9	11	7	2319	243.8	6.9	27.32
Q7Z3U7	MON2	Protein MON	6	8	11	8	1717	190.2	6.06	34.95
P42356	PI4KA	Phosphatidyl	5	8	8	6	2102	236.7	7.06	22.85
Q5VW32	BROX	BRO1 domai	23	8	12	8	411	46.4	7.65	37.31
P52790	HK3	Hexokinase-:	6	5	9	4	923	99	5.4	26.77
O60762	DPM1	Dolichol-pho:	25	6	9	6	260	29.6	9.57	29.56
P46937	YAP1	Transcriptior	19	6	8	6	504	54.4	5.17	24.69
Q7Z460	CLASP1	CLIP-associa	6	7	10	6	1538	169.3	9.03	24.02
Q9Y2H6	FNDC3A	Fibronectin ty	10	11	12	11	1198	131.8	6.71	31.19
O43813	LANCL1	Glutathione S	15	5	9	5	399	45.3	7.75	28.55
O95825	CRYZL1	Quinone oxic	28	7	8	7	349	38.7	5.78	24.62
Q99848	EBNA1BP2	Probable rRN	32	9	11	9	306	34.8	10.1	20.81
Q8WU39	MZB1	Marginal zon	40	5	9	5	189	20.7	5.57	36.81
Q96AB3	ISOC2	Isochorismat	31	3	8	3	205	22.3	7.77	36.88
O95786	RIGI	Antiviral inna	9	8	10	8	925	106.5	6.4	26.83
P61313	RPL15	60S ribosom	34	9	14	9	204	24.1	11.62	23.53
O60826	CCDC22	Coiled-coil do	18	7	7	7	627	70.7	6.74	25.64
Q12802	AKAP13	A-kinase anc	4	10	11	10	2813	307.4	5.24	21.93
Q9BQE5	APOL2	Apolipoprote	22	7	11	7	337	37.1	6.74	30.67
P37235	HPCAL1	Hippocalcin-l	50	9	12	6	193	22.3	5.35	31.28
Q04837	SSBP1	Single-strand	44	5	9	5	148	17.2	9.6	28.87
Q9UHD2	TBK1	Serine/threor	13	8	11	8	729	83.6	6.79	28.11
Q9HD45	TM9SF3	Transmembr	11	7	9	7	589	67.8	7.21	30.71
O75935	DCTN3	Dynactin sub	37	7	11	7	186	21.1	5.47	32.48
Q9H4G0	EPB41L1	Band 4.1-like	11	9	12	7	881	98.4	5.62	35.17
P23919	DTYMK	Thymidylate	27	6	13	6	212	23.8	8.27	38.94
Q8WVV9	HNRNPLL	Heterogeneo	17	8	9	7	542	60	7.72	28.23
P11234	RALB	Ras-related p	23	6	9	4	206	23.4	6.62	28.2
P08579	SNRPB2	U2 small nuc	27	6	11	4	225	25.5	9.72	32.4
Q8IY67	RAVER1	Ribonucleopr	11	6	7	6	606	63.8	8.48	24.88
Q04206	RELA	Transcriptior	19	10	11	10	551	60.2	5.68	31.65
Q9Y646	CPQ	Carboxypept	16	7	11	7	472	51.9	6.18	33.46

P28072	PSMB6	Proteasome	25	7	13	7	239	25.3	4.92	35.93
Q5TEC6	H3-7	Histone H3-7	34	7	31	3	136	15.4	11.27	77.38
Q6NVY1	HIBCH	3-hydroxyiso	28	11	14	11	386	43.5	8.19	26.38
Q01130	SRSF2	Serine/argini	22	5	19	5	221	25.5	11.85	65.95
Q9H8H3	METTL7A	Putative met	22	4	9	4	244	28.3	8.38	34.11
Q92629	SGCD	Delta-sarcog	29	8	11	8	289	32.1	9.11	30.7
Q16822	PCK2	Phosphoeno	16	9	10	9	640	70.7	7.62	26.21
P09238	MMP10	Stromelysin-	17	8	10	8	476	54.1	5.8	22.97
Q8NI36	WDR36	WD repeat-c	11	10	10	10	951	105.3	7.53	27.67
O15042	U2SURP	U2 snRNP-a	9	8	8	8	1029	118.2	8.47	27.42
P16298	PPP3CB	Serine/threo	15	7	8	2	524	59	5.91	24.3
Q8TD19	NEK9	Serine/threo	10	11	13	11	979	107.1	5.74	29.14
O00422	SAP18	Histone deac	59	9	13	9	153	17.6	9.35	30.05
Q92925	SMARCD2	SWI/SNF-rel	15	7	9	5	531	58.9	9.64	24.77
P55212	CASP6	Caspase-6 C	28	8	11	8	293	33.3	6.93	28.64
P00748	F12	Coagulation	14	8	11	8	615	67.7	7.74	26.49
Q9H4G4	GLIPR2	Golgi-associ	40	5	9	5	154	17.2	9.41	28.69
Q7L775	EPM2AIP1	EPM2A-inter	18	10	11	10	607	70.3	6.11	25.78
O75380	NDUFS6	NADH dehyd	58	5	7	5	124	13.7	8.28	23.09
P35270	SPR	Sepiapterin r	32	7	9	7	261	28	8.05	29.21
O00273	DFFA	DNA fragmer	22	6	8	6	331	36.5	4.79	28.83
P53814	SMTN	Smoothelin C	12	11	12	11	917	99	9.07	21.83
O14880	MGST3	Microsomal g	31	4	8	4	152	16.5	9.38	28.36
P58546	MTPN	Myotrophin C	39	5	12	5	118	12.9	5.52	33.91
Q96M27	PRRC1	Protein PRR	18	8	9	8	445	46.7	5.83	27.94
Q15067	ACOX1	Peroxisomal	16	7	9	7	660	74.4	8.16	26.83
Q96HD1	CRELD1	Protein disulf	24	7	8	7	420	45.4	4.87	23.32
Q9BS40	LXN	Latexin OS=l	30	4	8	4	222	25.7	5.78	26.79
P12955	PEPD	Xaa-Pro dipe	17	8	10	8	493	54.5	6	27.29
Q8N3C0	ASCC3	Activating sig	4	11	12	11	2202	251.3	7.09	25.28
P84103	SRSF3	Serine/argini	34	5	14	4	164	19.3	11.65	39.25
Q9UBX3	SLC25A10	Mitochondria	40	9	10	9	287	31.3	9.54	25.39
Q9H074	PAIP1	Polyadenylat	16	8	11	8	479	53.5	4.81	24.17

Q8TE77	SSH3	Protein phos	10	6	9	6	659	73	5.3	27.87
P40424	PBX1	Pre-B-cell leu	15	6	8	6	430	46.6	7.03	23.52
Q9H6Z4	RANBP3	Ran-binding	20	9	9	9	567	60.2	4.78	25.16
Q9P035	HACD3	Very-long-ch	22	7	9	7	362	43.1	8.94	29.69
Q9NUQ6	SPATS2L	SPATS2-like	16	8	10	8	558	61.7	9.64	20.67
Q5H9R7	PPP6R3	Serine/threor	11	7	10	7	873	97.6	4.6	34.06
Q15154	PCM1	Pericentriolar	5	10	11	10	2024	228.4	5.02	29.76
P24666	ACP1	Low molecula	53	6	8	6	158	18	6.74	24.97
P09496	CLTA	Clathrin light	23	8	15	8	248	27.1	4.51	35.54
P61970	NUTF2	Nuclear trans	37	3	10	3	127	14.5	5.38	29.93
O94808	GFPT2	Glutamine--fr	14	8	9	5	682	76.9	7.37	20.76
Q92541	RTF1	RNA polyme	10	6	9	6	710	80.3	8.15	21.3
O43708	GSTZ1	Maleylacetoa	27	5	9	5	216	24.2	8.54	34.08
Q6UXV4	APOOL	MICOS comp	32	7	9	7	268	29.1	9.52	23.7
P50440	GATM	Glycine amid	23	8	10	8	423	48.4	8.05	29.9
P20851	C4BPB	C4b-binding	32	7	8	7	252	28.3	5.14	24.75
P20290	BTF3	Transcription	43	6	8	5	206	22.2	9.38	23.19
Q567U6	CCDC93	Coiled-coil do	13	9	12	9	631	73.2	8.15	33.37
O00339	MATN2	Matrilin-2 OS	11	8	8	8	956	106.8	6.27	23.99
Q7LBC6	KDM3B	Lysine-speci	8	9	9	9	1761	191.5	7.18	22.89
P62995	TRA2B	Transformer-	25	6	11	5	288	33.6	11.25	35.16
P06454	PTMA	Prothymosin	14	4	11	4	111	12.2	3.78	21.56
Q96C36	PYCR2	Pyrroline-5-c	15	5	8	4	320	33.6	7.77	30.02
Q56VL3	OCIAD2	OCIA domain	40	6	10	6	154	16.9	9.03	30.12
Q3YEC7	RABL6	Rab-like prot	13	8	11	8	729	79.5	5.22	24.14
Q12788	TBL3	Transducin b	11	6	7	6	808	89	6.9	25.02
P30519	HMOX2	Heme oxyge	21	6	8	6	316	36	5.41	28.58
P82650	MRPS22	28S ribosom	15	5	8	5	360	41.3	7.9	23.99
Q8NBF2	NHLRC2	NHL repeat-c	14	8	8	8	726	79.4	5.55	24.56
Q9H0B6	KLC2	Kinesin light	9	5	8	1	622	68.9	7.15	26.13
Q5T6V5	C9orf64	Queuosine s	27	8	9	8	341	39	5.88	27.94
P26885	FKBP2	Peptidyl-prol	49	6	11	6	142	15.6	9.13	31.4
O43633	CHMP2A	Charged mul	27	7	10	7	222	25.1	5.97	29.74

Q9H2M9	RAB3GAP2	Rab3 GTPas	7	8	9	8	1393	155.9	5.62	22.81
Q9UMY4	SNX12	Sorting nexir	43	7	11	6	162	18.9	8.44	28.63
Q5ZPR3	CD276	CD276 antig	15	4	6	4	534	57.2	4.91	27.81
Q8N3U4	STAG2	Cohesin sub	7	10	11	4	1231	141.2	5.43	30.91
Q9NXE4	SMPD4	Sphingomyel	10	8	9	8	866	97.7	8.27	29.14
Q86U38	NOP9	Nucleolar pro	13	7	9	6	636	69.4	7.28	21.49
O60885	BRD4	Bromodomai	7	9	9	6	1362	152.1	9.19	26.6
Q92552	MRPS27	28S ribosom	17	7	10	7	414	47.6	6.18	21.59
P20338	RAB4A	Ras-related p	34	6	9	5	218	24.4	6.07	28.23
O60884	DNAJA2	DnaJ homolo	17	7	11	7	412	45.7	6.48	31.15
Q86X10	RALGAPB	Ral GTPase-	7	8	10	8	1494	166.7	6.79	23.06
O43660	PLRG1	Pleiotropic re	15	8	11	8	514	57.2	9.17	30.62
P17812	CTPS1	CTP synthas	15	8	10	7	591	66.6	6.46	26.21
P55036	PSMD4	26S proteaso	26	8	11	8	377	40.7	4.79	39.85
P80188	LCN2	Neutrophil ge	36	5	11	5	198	22.6	8.91	30.66
P33316	DUT	Deoxyuridine	35	7	9	7	252	26.5	9.36	23.46
Q9NP61	ARFGAP3	ADP-ribosyla	19	9	9	9	516	56.9	7.36	20.43
P62942	FKBP1A	Peptidyl-proly	41	3	12	3	108	11.9	8.16	42.35
Q14161	GIT2	ARF GTPase	11	9	12	8	759	84.5	7.23	28.34
Q9NUV9	GIMAP4	GTPase IMA	23	7	14	6	329	37.5	7.81	38.31
Q9HAV7	GRPEL1	GrpE protein	40	8	11	8	217	24.3	8.12	30.63
Q13404	UBE2V1	Ubiquitin-cor	41	5	11	2	147	16.5	7.93	29.1
Q92930	RAB8B	Ras-related p	31	8	18	2	207	23.6	9.07	40.69
O96019	ACTL6A	Actin-like pro	20	7	8	7	429	47.4	5.6	25.25
P62851	RPS25	40S ribosom	38	6	13	6	125	13.7	10.11	35.31
P20962	PTMS	Parathymosi	24	4	26	4	102	11.5	4.16	29.82
Q96Q11	TRNT1	CCA tRNA n	24	10	11	10	434	50.1	8.1	31.28
P63173	RPL38	60S ribosom	50	5	11	5	70	8.2	10.1	32.77
Q8IYM9	TRIM22	E3 ubiquitin-	15	5	8	5	498	56.9	7.72	30.15
Q9NR28	DIABLO	Diablo IAP-b	31	8	11	8	239	27.1	5.9	31.62
Q9Y6Q5	AP1M2	AP-1 comple	18	8	11	6	423	48.1	8.22	28.63
Q06787	FMR1	Fragile X me	15	9	10	7	632	71.1	7.42	25.29
P43307	SSR1	Translocon-a	18	3	10	3	286	32.2	4.49	37.2

P35052	GPC1	Glypican-1 C	17	9	10	9	558	61.6	7.3	25.23
P28676	GCA	Grancalcin C	32	6	9	6	217	24	5.21	24.82
Q14956	GPNMB	Transmembr	6	3	10	3	572	63.9	6.64	33.18
Q7KZ85	SUPT6H	Transcription	6	8	8	8	1726	198.9	4.91	24.82
Q92743	HTRA1	Serine protea	22	8	9	8	480	51.3	7.83	25.84
Q9Y2B0	CNPY2	Protein cano	33	5	12	5	182	20.6	4.92	45.07
P54687	BCAT1	Branched-ch	15	5	7	5	386	42.9	5.3	26.01
Q9UKU9	ANGPTL2	Angiopoietin-	16	7	9	7	493	57.1	7.53	23.02
Q8WYP5	AHCTF1	Protein ELYS	4	7	8	7	2266	252.3	6.6	24.79
Q15836	VAMP3	Vesicle-asso	40	4	7	1	100	11.3	8.79	35.49
Q15291	RBBP5	Retinoblastor	22	7	8	7	538	59.1	5.1	22.66
Q8WVM7	STAG1	Cohesin subu	6	9	11	3	1258	144.3	5.59	28.99
P53582	METAP1	Methionine a	21	6	8	6	386	43.2	7.17	24.98
Q9Y333	LSM2	U6 snRNA-a	67	5	9	5	95	10.8	6.52	28.56
Q8IWJ2	GCC2	GRIP and co	7	9	10	9	1684	195.8	5.14	24.5
Q9GZP4	PITHD1	PITH domain	38	5	6	5	211	24.2	5.74	24.72
Q96SL4	GPX7	Glutathione p	47	6	7	6	187	21	8.27	26.42
Q8WTW3	COG1	Conserved o	11	8	10	8	980	108.9	7.31	15.7
Q96LJ7	DHRS1	Dehydrogena	25	6	9	6	313	33.9	7.83	30.22
P23743	DGKA	Diacylglycero	17	9	9	9	735	82.6	6.73	23.55
Q9NP72	RAB18	Ras-related p	36	6	9	6	206	23	5.24	27.58
P35914	HMGCL	Hydroxymeth	19	6	10	6	325	34.3	8.54	31.85
O95757	HSPA4L	Heat shock 7	6	5	12	1	839	94.5	5.88	33.56
P54725	RAD23A	UV excision r	15	7	12	6	363	39.6	4.58	30.49
Q96S66	CLCC1	Chloride cha	13	6	9	6	551	62	5.55	26.79
P67870	CSNK2B	Casein kinas	35	6	10	6	215	24.9	5.55	30.88
Q12857	NFIA	Nuclear facto	11	5	8	3	509	55.9	8.44	22.27
P50213	IDH3A	Isocitrate del	20	7	9	7	366	39.6	6.92	27.68
Q9NSK0	KLC4	Kinesin light	12	7	8	5	619	68.6	6.18	24.09
Q99996	AKAP9	A-kinase anc	3	10	10	10	3907	452.7	4.98	19.5
O75494	SRSF10	Serine/argini	16	6	12	6	262	31.3	11.27	32.98
O15357	INPPL1	Phosphatidyl	10	9	11	9	1258	138.5	6.54	22.33
Q96BW5	PTER	Phosphotries	24	8	9	8	349	39	6.52	26.77

Q92526	CCT6B	T-complex pı	12	7	12	1	530	57.8	7.24	32.82
Q9Y3E1	HDGFL3	Hepatoma-d	33	6	11	5	203	22.6	7.99	31.02
Q9GZT3	SLIRP	SRA stem-lo	74	9	10	9	109	12.3	10.24	24.42
P30613	PKLR	Pyruvate kin	11	6	13	3	574	61.8	7.74	32.59
P47813	EIF1AX	Eukaryotic tr	33	6	10	6	144	16.5	5.24	20.31
Q12824	SMARCB1	SWI/SNF-rel	25	7	7	7	385	44.1	6.23	24.8
O14773	TPP1	Tripeptidyl-p	14	6	10	6	563	61.2	6.48	37.84
O60888	CUTA	Protein CutA	23	4	8	4	179	19.1	5.5	25.97
P62993	GRB2	Growth facto	38	5	7	5	217	25.2	6.32	23.19
A0MZ66	SHTN1	Shootin-1 OS	16	9	10	9	631	71.6	5.33	21.66
Q8ND56	LSM14A	Protein LSM1	15	7	9	7	463	50.5	9.52	23.36
P61601	NCALD	Neurocalcin-	32	6	9	3	193	22.2	5.35	29.06
Q8IWX8	CHERP	Calcium hom	9	7	9	7	916	103.6	9.04	28.84
Q15056	EIF4H	Eukaryotic tr	24	6	11	6	248	27.4	7.23	32.08
Q9NWV4	CZIB	CXXC motif	40	5	10	5	160	18	5.01	28.86
P14780	MMP9	Matrix metall	14	9	10	9	707	78.4	6.06	28.29
Q9Y520	PRRC2C	Protein PRR	4	11	11	11	2896	316.7	9.13	28.55
Q8WVY7	UBLCP1	Ubiquitin-like	19	5	9	5	318	36.8	6.46	27.68
Q96FN4	CPNE2	Copine-2 OS	15	7	9	6	548	61.2	6.07	23.9
P61289	PSME3	Proteasome	22	5	7	5	254	29.5	5.95	23.75
P41208	CETN2	Centrin-2 OS	30	4	9	4	172	19.7	5	26.2
Q15274	QPRT	Nicotinate-nı	33	8	10	8	297	30.8	6.21	23.76
P49902	NT5C2	Cytosolic pur	12	6	8	6	561	64.9	6.14	24.07
Q12979	ABR	Active break	12	9	10	7	859	97.5	6.55	28.13
A1X283	SH3PXD2B	SH3 and PX	10	9	10	9	911	101.5	8.69	21.61
Q9NQ66	PLCB1	1-phosphatid	9	8	8	8	1216	138.5	6.23	23.44
P14854	COX6B1	Cytochrome	66	5	11	5	86	10.2	7.05	37.74
Q9UH62	ARMCX3	Armadillo rep	25	8	9	8	379	42.5	8.37	22.19
O43395	PRPF3	U4/U6 small	14	9	16	9	683	77.5	9.5	38.82
Q13085	ACACA	Acetyl-CoA c	4	8	9	8	2346	265.4	6.37	17.92
P51452	DUSP3	Dual specific	33	5	8	5	185	20.5	7.8	28.3
Q92643	PIGK	GPI-anchor t	17	5	7	5	395	45.2	6.16	22.11
Q9GZZ9	UBA5	Ubiquitin-like	24	7	7	7	404	44.8	4.84	22.29

Q15819	UBE2V2	Ubiquitin-cor	43	5	12	2	145	16.4	8.09	34.1
P17568	NDUFB7	NADH dehyc	56	6	11	6	137	16.4	8.92	30.78
Q15738	NSDHL	Sterol-4-alph	24	9	10	9	373	41.9	8.06	27.24
P55008	AIF1	Allograft infla	45	6	10	5	147	16.7	6.24	24.5
P42892	ECE1	Endothelin-co	11	7	7	7	770	87.1	5.88	18.97
Q9H267	VPS33B	Vacuolar pro	13	8	10	8	617	70.5	6.71	29.32
Q53H96	PYCR3	Pyrroline-5-c	26	5	7	5	274	28.6	7.72	29.19
Q8WZA0	LZIC	Protein LZIC	30	6	7	6	190	21.5	4.94	24.26
Q9NQ29	LUC7L	Putative RNA	19	7	9	4	371	43.7	9.92	28.44
Q4G0J3	LARP7	La-related pr	14	8	10	8	582	66.9	9.55	27.76
Q13242	SRSF9	Serine/argini	29	8	11	8	221	25.5	8.65	22.92
Q8TB45	DEPTOR	DEP domain	19	7	9	7	409	46.3	8.07	23.04
Q93050	ATP6V0A1	V-type protor	11	7	7	7	837	96.4	6.43	22.72
Q9UHQ9	CYB5R1	NADH-cytocl	30	8	9	8	305	34.1	9.38	25.85
Q9HCD5	NCOA5	Nuclear rece	17	9	10	9	579	65.5	9.6	27.59
Q99541	PLIN2	Perilipin-2 OS	18	7	8	7	437	48	6.8	20.76
Q8NE86	MCU	Calcium unip	26	7	8	7	351	39.8	8.65	25.49
O60687	SRPX2	Sushi repeat	17	7	8	7	465	52.9	7.25	24.36
Q13153	PAK1	Serine/threor	14	5	7	2	545	60.6	5.76	26.15
P22749	GNLY	Granulysin C	37	6	11	6	145	16.4	9.17	25.11
P37840	SNCA	Alpha-synucl	31	4	6	4	140	14.5	4.7	24.25
Q8TDB6	DTX3L	E3 ubiquitin-p	10	6	9	6	740	83.5	8.06	22.07
Q9BTT6	LRRC1	Leucine-rich	9	5	7	2	524	59.2	5.02	20.66
Q96N66	MBOAT7	Lysophosphc	15	6	8	6	472	52.7	8.97	25.44
Q96JJ7	TMX3	Protein disulf	14	7	11	6	454	51.8	4.91	25.44
Q9UI09	NDUFA12	NADH dehyc	43	5	8	5	145	17.1	9.63	27.88
O95218	ZRANB2	Zinc finger R	25	7	8	7	330	37.4	10.01	20.92
O95602	POLR1A	DNA-directed	6	9	9	9	1720	194.7	7.03	21.04
P83111	LACTB	Serine beta-l	16	8	9	8	547	60.7	8.53	25.86
P50225	SULT1A1	Sulfotransfer	29	7	9	4	295	34.1	6.62	26.84
O43837	IDH3B	Isocitrate del	22	8	9	8	385	42.2	8.46	25.5
Q9UI08	EVL	Ena/VASP-lil	17	6	10	6	416	44.6	8.84	25.99
Q8NB37	GATD1	Glutamine ar	30	4	6	4	220	23.3	6.61	23.1

P42766	RPL35	60S ribosom	40	6	12	6	123	14.5	11.05	24.05
P24468	NR2F2	COUP transc	17	7	10	2	414	45.5	8.28	27.31
Q6IAN0	DHRS7B	Dehydrogena	19	6	10	6	325	35.1	9.55	30.23
O75340	PDCD6	Programmed	29	5	10	5	191	21.9	5.4	25.8
P55011	SLC12A2	Solute carrie	8	7	8	7	1212	131.4	6.4	25.58
Q8TED1	GPX8	Probable glu	22	5	10	5	209	23.9	9.35	30.97
Q9BVJ7	DUSP23	Dual specific	56	7	9	7	150	16.6	8.21	30.35
Q969X5	ERGIC1	Endoplasmic	27	6	9	6	290	32.6	7.06	32.09
Q14318	FKBP8	Peptidyl-proly	16	5	8	5	412	44.5	4.84	28.34
Q9BUP0	EFHD1	EF-hand dom	25	5	10	3	239	26.9	5.39	25.84
P26927	MST1	Hepatocyte g	13	8	8	8	711	80.3	7.68	22.62
P01111	NRAS	GTPase NRa	23	3	8	1	189	21.2	5.17	30.52
Q8WXF7	ATL1	Atlastin-1 OS	11	6	8	5	558	63.5	6.18	18.54
Q16777	H2AC20	Histone H2A	26	6	32	1	129	14	10.9	66.26
P13984	GTF2F2	General trans	22	7	10	7	249	28.4	9.23	27.02
Q8TEQ6	GEMIN5	Gem-associa	6	8	9	8	1508	168.5	6.62	25.72
P18077	RPL35A	60S ribosom	35	6	16	6	110	12.5	11.06	34.87
Q14978	NOLC1	Nucleolar an	10	9	11	9	699	73.6	9.47	23.08
Q04724	TLE1	Transducin-li	8	5	7	5	770	83.1	7.24	21.67
O14561	NDUFAB1	Acyl carrier p	24	4	9	4	156	17.4	4.93	26.96
Q6P587	FAHD1	Acylpyruvase	37	6	8	6	224	24.8	7.39	25.71
O14558	HSPB6	Heat shock p	45	4	9	4	160	17.1	6.4	32.82
P62841	RPS15	40S ribosom	31	4	11	4	145	17	10.39	30.48
P34913	EPHX2	Bifunctional e	12	5	7	5	555	62.6	6.28	23.28
Q96T76	MMS19	MMS19 nucle	7	5	7	5	1030	113.2	6.35	17.9
Q9BVG4	PBDC1	Protein PBD	30	6	9	6	233	26	4.79	25.6
P06280	GLA	Alpha-galact	19	6	7	6	429	48.7	5.6	22.9
Q9NXC5	MIOS	GATOR com	12	8	8	8	875	98.5	6.73	17.96
O95155	UBE4B	Ubiquitin con	6	7	8	6	1302	146.1	6.55	21.32
Q8TC07	TBC1D15	TBC1 domai	8	5	7	5	691	79.4	5.67	22.27
Q9H1B7	IRF2BPL	Probable E3	12	6	7	5	796	82.6	8.24	21.64
P15941	MUC1	Mucin-1 OS=	4	4	8	4	1255	122	7.47	21.9
P19878	NCF2	Neutrophil cy	17	8	9	8	526	59.7	6.16	19.84

Q9NVG8	TBC1D13	TBC1 domai	18	6	8	6	400	46.5	5.24	21.51
Q9H488	POFUT1	GDP-fucose	21	5	6	5	388	43.9	8.53	23.75
Q99653	CHP1	Calcineurin E	37	6	8	6	195	22.4	5.1	19.4
P04216	THY1	Thy-1 memb	19	3	8	3	161	17.9	8.73	31.04
P21926	CD9	CD9 antigen	21	4	13	4	228	25.4	7.15	39.13
P04233	CD74	HLA class II	15	4	7	4	296	33.5	8.44	25.02
Q13188	STK3	Serine/threor	12	6	10	5	491	56.3	5.24	21.74
O15484	CAPN5	Calpain-5 OS	12	6	8	6	640	73.1	7.64	20.26
P16234	PDGFRA	Platelet-deriv	7	7	9	6	1089	122.6	5.17	25.78
O75884	RBBP9	Serine hydro	37	7	10	7	186	21	6.2	29.03
Q9Y4X5	ARIH1	E3 ubiquitin-p	15	8	8	8	557	64.1	5.08	22.13
Q9BVL2	NUP58	Nucleoporin	15	7	8	7	599	60.9	9.33	18.48
Q96DH6	MSI2	RNA-binding	29	7	9	5	328	35.2	8.48	29.99
Q9NRF8	CTPS2	CTP synthas	15	9	10	8	586	65.6	6.9	20.09
Q14669	TRIP12	E3 ubiquitin-p	5	8	8	8	1992	220.3	8.48	22.5
P16930	FAH	Fumarylacet	12	5	9	5	419	46.3	6.95	23.57
Q8WZA9	IRGQ	Immunity-rela	16	6	8	6	623	62.7	4.88	31.65
Q86U42	PABPN1	Polyadenylat	27	4	7	4	306	32.7	5.06	28.24
O75153	CLUH	Clustered mi	8	9	9	9	1309	146.6	6.13	20.78
Q5SY16	NOL9	Polynucleotic	17	9	9	9	702	79.3	9.13	21.12
Q9HB90	RRAGC	Ras-related (	21	6	7	6	399	44.2	5.1	22.22
Q9UKD2	MRTO4	mRNA turnov	26	6	8	6	239	27.5	8.29	19.23
Q9Y673	ALG5	Dolichyl-phos	31	8	9	8	324	36.9	9.28	18.94
Q13043	STK4	Serine/threor	14	6	8	5	487	55.6	5.07	23.39
Q9BTT0	ANP32E	Acidic leucin	18	3	8	3	268	30.7	3.85	32.71
Q9NWH9	SLTM	SAFB-like tra	9	9	9	9	1034	117.1	7.87	19.54
Q96T60	PNKP	Bifunctional p	15	6	8	6	521	57	8.46	26.07
P55327	TPD52	Tumor protei	33	5	6	5	224	24.3	4.83	18.48
Q13057	COASY	Bifunctional c	11	5	11	5	564	62.3	6.99	29.09
Q9Y3C6	PPIL1	Peptidyl-proly	27	4	11	4	166	18.2	7.99	18.93
Q9HAT2	SIAE	Sialate O-ac	12	6	10	6	523	58.3	7.33	26.8
Q15102	PAFAH1B3	Platelet-activ	27	6	11	6	231	25.7	6.84	29.96
Q71UI9	H2AZ2	Histone H2A	31	4	34	2	128	13.5	10.58	82.14

Q15286	RAB35	Ras-related p	33	7	7	6	201	23	8.29	23.19
O94804	STK10	Serine/threoi	8	7	8	6	968	112.1	6.95	18.26
Q99627	COPS8	COP9 signal	28	4	8	4	209	23.2	5.38	28.06
Q14444	CAPRIN1	Caprin-1 OS	9	7	12	7	709	78.3	5.25	32.71
P53680	AP2S1	AP-2 comple	30	5	9	5	142	17	6.18	24.41
Q9NT22	EMILIN3	EMILIN-3 OS	9	6	6	6	766	82.6	7.72	19.84
P41567	EIF1	Eukaryotic tr	61	5	7	5	113	12.7	7.44	26.13
A0A0C4DH3	IGHV5-51	Immunoglobu	47	4	11	2	117	12.7	8.27	36.84
Q16798	ME3	NADP-deper	13	7	8	6	604	67	7.97	23.36
P23219	PTGS1	Prostaglandi	17	7	7	7	599	68.6	7.23	24.44
P60468	SEC61B	Protein trans	47	5	11	5	96	10	11.56	30.4
Q8N4X5	AFAP1L2	Actin filamen	11	8	8	8	818	91.2	5.31	23.34
P60866	RPS20	40S ribosom	36	6	11	6	119	13.4	9.94	30.47
Q7Z3J2	VPS35L	VPS35 endo	9	9	10	9	963	109.5	7.21	18.33
Q7Z3B4	NUP54	Nucleoporin	11	5	7	5	507	55.4	7.02	19.86
Q06033	ITIH3	Inter-alpha-tr	8	8	11	8	890	99.8	5.74	19.35
P07947	YES1	Tyrosine-pro	18	9	9	3	543	60.8	6.74	22.3
Q9P2W9	STX18	Syntaxin-18	28	8	9	8	335	38.7	5.49	15.01
Q9NXV6	CDKN2AIP	CDKN2A-inte	15	7	9	7	580	61.1	9.01	22.24
P82909	MRPS36	Alpha-ketogl	42	4	7	4	103	11.5	9.99	25.74
Q92974	ARHGEF2	Rho guanine	10	9	9	9	986	111.5	7.27	23.42
Q92542	NCSTN	Nicastrin OS	10	6	8	6	709	78.4	5.99	24.02
Q9Y2D4	EXOC6B	Exocyst com	8	6	7	6	811	94.1	6.46	20.11
Q15369	ELOC	Elongin-C OS	46	4	10	4	112	12.5	4.78	33.72
Q9NVT9	ARMC1	Armadillo rep	30	6	7	6	282	31.3	5.74	19.31
Q96A49	SYAP1	Synapse-ass	24	5	7	5	352	39.9	4.53	23.73
P55001	MFAP2	Microfibrillar-	27	4	8	4	183	20.8	4.97	26.13
Q9UHY1	NRBP1	Nuclear rece	15	6	7	6	535	59.8	5.08	26.35
Q6GMV3	PTRHD1	Putative pep	49	6	9	6	140	15.8	9.1	25.83
Q9Y2W2	WBP11	WW domain-	12	7	9	7	641	70	8.38	23.91
O95470	SGPL1	Sphingosine-	16	9	10	9	568	63.5	9.16	22.54
Q9H583	HEATR1	HEAT repeat	3	7	10	7	2144	242.2	6.54	20.33
Q9Y5X2	SNX8	Sorting nexir	16	4	5	4	465	52.5	7.39	23.25

P53384	NUBP1	Cytosolic Fe-	22	5	11	5	320	34.5	5.33	29.57
Q8IWZ3	ANKHD1	Ankyrin repe	3	8	8	4	2542	269.3	5.73	17.66
Q14376	GALE	UDP-glucose	26	8	10	8	348	38.3	6.73	28.92
O43172	PRPF4	U4/U6 small	14	5	8	5	522	58.4	7.42	26.17
Q92530	PSMF1	Proteasome	23	5	6	5	271	29.8	5.74	20.62
Q9Y3T9	NOC2L	Nucleolar co	10	8	9	8	749	84.9	5.62	14.83
Q9BQ67	GRWD1	Glutamate-ri	12	4	7	4	446	49.4	4.92	26.04
Q9H0C8	ILKAP	Integrin-linke	20	8	11	8	392	42.9	7.09	26.2
Q96HN2	AHCYL2	Adenosylhon	10	8	10	2	611	66.7	7.36	20.79
Q8IVM0	CCDC50	Coiled-coil d	24	7	9	7	306	35.8	6.65	25.99
Q9BSJ2	TUBGCP2	Gamma-tubu	8	7	8	7	902	102.5	6.84	18.71
O14494	PLPP1	Phospholipid	21	5	9	5	284	32.1	7.97	32.69
P23258	TUBG1	Tubulin gam	19	6	7	6	451	51.1	6.14	25.63
O00139	KIF2A	Kinesin-like p	13	10	12	10	706	79.9	6.68	23.48
Q9HC78	ZBTB20	Zinc finger a	11	6	9	6	741	81	6.49	22.25
Q99961	SH3GL1	Endophilin-A	18	6	8	6	368	41.5	5.43	25.75
O95248	SBF1	Myotubularin	5	9	9	8	1868	208.3	6.9	18.19
Q9Y3L3	SH3BP1	SH3 domain-	12	7	8	7	701	75.7	6.77	22.32
Q9H3N1	TMX1	Thioredoxin-	22	8	9	8	280	31.8	4.98	22.64
Q15036	SNX17	Sorting nexir	14	5	11	5	470	52.9	7.46	32.66
Q7Z4V5	HDGFL2	Hepatoma-d	11	7	12	6	671	74.3	7.49	23.2
Q6Y7W6	GIGYF2	GRB10-inter	6	8	10	8	1299	150	5.54	23.39
Q8IWA5	SLC44A2	Choline trans	14	9	9	8	706	80.1	8.57	23.71
P14649	MYL6B	Myosin light	25	5	11	4	208	22.8	5.73	26.48
Q5T8P6	RBM26	RNA-binding	7	7	9	6	1007	113.5	9.16	20.84
Q9Y3I1	FBXO7	F-box only p	12	6	8	6	522	58.5	6.55	21.42
Q3ZCQ8	TIMM50	Mitochondria	24	7	8	7	353	39.6	8.37	19.89
Q01970	PLCB3	1-phosphatid	6	8	11	8	1234	138.7	5.9	21.78
Q2NL82	TSR1	Pre-rRNA-pr	11	7	8	7	804	91.8	7.42	19.77
Q13243	SRSF5	Serine/argini	20	5	8	4	272	31.2	11.59	22.31
Q6NYC8	PPP1R18	Phostensin (	15	7	8	7	613	67.9	5.4	23.15
Q16539	MAPK14	Mitogen-activ	22	7	9	7	360	41.3	5.78	20.7
P09758	TACSTD2	Tumor-assoc	16	4	7	4	323	35.7	8.87	14.89

Q7L2E3	DHX30	ATP-depend	7	8	9	8	1194	133.9	8.78	22.54
P08397	HMBS	Porphobilino	20	7	11	7	361	39.3	7.18	22.66
Q96JY6	PDLIM2	PDZ and LIM	20	6	8	6	352	37.4	8.72	18.34
Q8NFW8	CMAS	N-acylneurar	15	6	8	6	434	48.3	7.93	25.3
P51571	SSR4	Translocon-a	25	4	10	4	173	19	6.15	34.74
O95372	LYPLA2	Acyl-protein t	22	5	10	5	231	24.7	7.23	32.04
Q9P2T1	GMPR2	GMP reducta	24	7	8	7	348	37.9	7.23	22.12
P09466	PAEP	Glycodelin O	19	4	14	3	180	20.6	5.57	29.18
Q96HS1	PGAM5	Serine/threor	34	7	7	7	289	32	8.68	21.14
Q15847	ADIRF	Adipogenesis	51	2	5	2	76	7.9	5.31	29.92
Q05397	PTK2	Focal adhesi	8	8	9	7	1052	119.2	6.62	20.32
P26358	DNMT1	DNA (cytosir	5	8	10	8	1616	183.1	7.75	28.52
Q9H4A3	WNK1	Serine/threor	3	9	10	9	2382	250.6	6.34	20.9
O14562	UBFD1	Ubiquitin dor	22	6	10	6	309	33.4	5.77	24.03
P84157	MXRA7	Matrix-remoc	33	6	8	6	204	21.5	4.26	31.02
P08651	NFIC	Nuclear facto	14	8	9	7	508	55.6	8.38	15.37
Q00765	REEP5	Receptor exp	15	4	9	4	189	21.5	8.1	20.97
Q9Y3P9	RABGAP1	Rab GTPase	8	8	8	7	1069	121.7	5.25	21.62
Q15382	RHEB	GTP-binding	45	7	11	7	184	20.5	5.92	26.29
O15126	SCAMP1	Secretory ca	28	6	6	6	338	37.9	7.42	20.8
Q14005	IL16	Pro-interleuk	8	6	9	6	1332	141.7	8.06	29.06
Q9UJX2	CDC23	Cell division	14	7	8	7	597	68.8	7.02	20.09
Q9BW92	TARS2	Threonine--tl	11	7	8	7	718	81	7.3	15.8
O43920	NDUFS5	NADH dehyc	49	5	8	5	106	12.5	9.14	25.16
Q96KG9	SCYL1	N-terminal ki	10	7	10	7	808	89.6	6.3	26.34
Q92947	GCDH	Glutaryl-CoA	16	6	8	6	438	48.1	8.06	18.87
P35475	IDUA	Alpha-L-idurc	13	6	6	6	653	72.6	9.14	17.98
Q9UDT6	CLIP2	CAP-Gly dor	7	7	7	4	1046	115.8	6.73	18.95
Q96FQ6	S100A16	Protein S100	52	4	8	4	103	11.8	6.79	28.75
Q9NYB9	ABI2	Abl interacto	14	7	10	4	513	55.6	6.16	26.54
Q05209	PTPN12	Tyrosine-pro	11	7	7	7	780	88.1	5.62	20.68
Q9UDR5	AASS	Alpha-amino	12	8	8	8	926	102.1	6.64	20.96
P41221	WNT5A	Protein Wnt-	21	7	9	7	380	42.3	8.46	28.43

O75487	GPC4	Glypican-4 C	10	4	6	2	556	62.4	6.68	17.21
P33991	MCM4	DNA replicat	9	7	7	7	863	96.5	6.74	19.98
O60306	AQR	RNA helicase	6	7	8	7	1485	171.2	6.37	20.19
Q15126	PMVK	Phosphomev	34	6	8	6	192	22	5.73	25.57
P15090	FABP4	Fatty acid-bi	43	4	9	4	132	14.7	7.14	29.63
Q9NQ55	PPAN	Suppressor c	18	6	7	6	473	53.2	10.13	16.23
Q05086	UBE3A	Ubiquitin-pro	9	7	7	7	875	100.6	5.22	22.07
Q86TI2	DPP9	Dipeptidyl pe	8	7	9	7	863	98.2	6.46	22.54
Q6PI48	DARS2	Aspartate--tF	13	8	11	8	645	73.5	8.02	25.1
Q8NCM8	DYNC2H1	Cytoplasmic	2	7	8	7	4307	492.3	6.54	15.02
Q53H12	AGK	Acylglycerol	19	7	9	7	422	47.1	8.09	26.93
Q9BTM1	H2AJ	Histone H2A	26	6	31	1	129	14	10.9	64.11
Q9Y5X3	SNX5	Sorting nexir	16	7	9	5	404	46.8	6.76	20.49
Q13501	SQSTM1	Sequestosor	23	6	6	6	440	47.7	5.22	18
O75934	BCAS2	Pre-mRNA-s	25	6	8	6	225	26.1	5.66	16.34
Q8N6H7	ARFGAP2	ADP-ribosyla	16	7	7	7	521	56.7	7.99	20.51
Q9H6T3	RPAP3	RNA polyme	11	8	9	8	665	75.7	6.84	21.2
P16083	NQO2	Ribosyldihyd	35	6	6	6	231	25.9	6.29	21.48
Q6BCY4	CYB5R2	NADH-cytocl	24	6	8	6	276	31.4	8.5	23.37
Q7L5N7	LPCAT2	Lysophospha	10	5	7	5	544	60.2	6.55	23.17
Q14694	USP10	Ubiquitin car	11	7	8	7	798	87.1	5.31	26.48
P17936	IGFBP3	Insulin-like g	33	8	8	8	291	31.7	8.69	19.88
Q96MM6	HSPA12B	Heat shock 7	14	8	9	7	686	75.6	8.53	25.29
Q96JB2	COG3	Conserved o	12	7	7	7	828	94	5.57	19.11
Q9UHY7	ENOPH1	Enolase-pho	30	6	7	6	261	28.9	4.78	19.49
Q9Y6R7	FCGBP	IgGFc-bindin	4	8	8	8	5405	571.6	5.34	18.59
Q5T440	IBA57	Putative tran	25	5	6	5	356	38.1	9.83	22.31
P49023	PXN	Paxillin OS=I	12	6	9	6	591	64.5	6.19	27.76
Q16222	UAP1	UDP-N-acety	16	7	8	6	522	58.7	6.33	23.97
Q02318	CYP27A1	Sterol 26-hyd	17	8	9	8	531	60.2	8.9	22.51
Q9UBV8	PEF1	Peflin OS=H	19	6	10	6	284	30.4	6.54	25.2
P36551	CPOX	Oxygen-depe	18	6	7	6	454	50.1	8.25	18.77
P46776	RPL27A	60S ribosom	30	6	10	6	148	16.6	11	26.81

Q9BZE4	GTPBP4	GTP-binding	11	6	7	6	634	73.9	9.5	19.14
Q7Z5N4	SDK1	Protein sidek	4	9	11	9	2213	242	6.39	23.86
P49773	HINT1	Adenosine 5'	40	4	7	4	126	13.8	6.95	20.87
Q9Y282	ERGIC3	Endoplasmic	16	7	10	7	383	43.2	6.06	24.5
O43264	ZW10	Centromere/	12	7	8	7	779	88.8	6.27	25.39
P62314	SNRPD1	Small nuclea	37	4	6	4	119	13.3	11.56	21.89
Q96BJ3	AIDA	Axin interact	23	6	9	6	306	35	6.55	28.96
Q5VTR2	RNF20	E3 ubiquitin-	9	9	10	7	975	113.6	5.94	18.55
Q8NCA5	FAM98A	Protein FAM	14	5	7	4	518	55.2	8.95	25.91
Q15758	SLC1A5	Neutral amin	11	4	8	4	541	56.6	5.48	25.87
Q9NY12	GAR1	H/ACA ribon	27	6	11	6	217	22.3	10.92	31.13
Q8NCN5	PDPR	Pyruvate deh	9	8	10	8	879	99.3	6.35	25.08
Q96EK6	GNPNAT1	Glucosamine	42	6	6	6	184	20.7	7.99	22.45
O95436	SLC34A2	Sodium-depe	7	4	6	4	690	75.7	8.15	20.29
Q9NZ01	TECR	Very-long-ch	14	6	10	6	308	36	9.45	14.95
P78318	IGBP1	Immunoglob	19	6	7	6	339	39.2	5.38	18.36
Q04323	UBXN1	UBX domain	21	6	10	6	297	33.3	5.25	27.42
Q9NRR5	UBQLN4	Ubiquilin-4 C	10	4	6	2	601	63.8	5.22	20.48
Q16512	PKN1	Serine/threo	8	7	7	6	942	103.9	6.37	19.32
Q8TD16	BICD2	Protein bicau	9	7	8	7	824	93.5	5.44	20.92
Q8NDA8	MROH1	Maestro hea	5	8	9	8	1641	181.1	6.89	21.29
Q14232	EIF2B1	Translation i	22	6	9	6	305	33.7	7.33	22.6
Q9NUY8	TBC1D23	TBC1 domai	8	4	7	4	699	78.3	5.41	25.18
Q5SW79	CEP170	Centrosomal	5	8	9	6	1584	175.2	7.11	22.09
Q8N2F6	ARMC10	Armadillo rep	18	5	8	5	343	37.5	6.61	15.02
P52564	MAP2K6	Dual specific	20	5	7	5	334	37.5	7.39	21.32
P18428	LBP	Lipopolysacc	11	4	6	4	481	53.4	6.7	20.63
P51649	ALDH5A1	Succinate-se	11	4	6	4	535	57.2	8.28	20.83
Q8IV08	PLD3	5'-3' exonucl	15	7	7	7	490	54.7	6.47	20.92
Q9H9A5	CNOT10	CCR4-NOT t	9	7	8	7	744	82.3	7.78	20.7
Q15555	MAPRE2	Microtubule-a	17	5	6	4	327	37	5.57	21.5
Q96T23	RSF1	Remodeling	7	8	8	8	1441	163.7	5.01	18.71
Q9Y624	F11R	Junctional ac	26	6	7	6	299	32.6	7.9	25.29

P05067	APP	Amyloid-beta	8	7	8	6	770	86.9	4.82	22.63
Q86V48	LUZP1	Leucine zipp	9	9	9	9	1076	120.2	8.5	17.82
Q86V81	ALYREF	THO comple	23	5	9	5	257	26.9	11.15	26
Q9H3Z4	DNAJC5	DnaJ homolo	28	4	5	4	198	22.1	5.07	16.32
O95104	SCAF4	SR-related a	9	7	8	5	1147	125.8	9.55	24.66
P08574	CYC1	Cytochrome	28	6	8	6	325	35.4	9	23.95
O00330	PDHX	Pyruvate deh	12	6	12	6	501	54.1	8.66	28.54
Q15050	RRS1	Ribosome bi	18	5	6	5	365	41.2	10.7	19.68
P61764	STXBP1	Syntaxin-bind	14	8	8	7	594	67.5	6.96	19.98
Q86VM9	ZC3H18	Zinc finger C	8	6	6	6	953	106.3	8.32	17.05
P13747	HLA-E	HLA class I h	21	6	6	4	358	40	5.83	16.35
O14929	HAT1	Histone acety	20	6	7	6	419	49.5	5.69	22.07
P02743	APCS	Serum amylo	22	5	9	5	223	25.4	6.54	26.86
Q9Y4W2	LAS1L	Ribosomal bi	13	7	10	7	734	83	4.73	17.98
P01619	IGKV3-20	Immunoglobu	33	2	7	1	116	12.5	4.96	34.18
P49247	RPIA	Ribose-5-pho	24	6	7	6	311	33.2	8.54	20.88
P52756	RBM5	RNA-binding	10	8	9	5	815	92.1	6.28	20.6
P48723	HSPA13	Heat shock 7	17	7	8	7	471	51.9	5.76	19.68
O60524	NEMF	Ribosome qu	6	7	11	7	1076	122.9	6.35	18.04
P47989	XDH	Xanthine deh	6	6	7	5	1333	146.3	7.66	19.65
Q6YHK3	CD109	CD109 antige	5	7	7	7	1445	161.6	5.85	19.32
Q9NRW7	VPS45	Vacuolar pro	12	7	8	7	570	65	8.24	19.51
Q9H270	VPS11	Vacuolar pro	8	7	7	7	941	107.8	7.05	20.8
Q9UKS6	PACSIN3	Protein kinas	17	5	6	5	424	48.5	6.18	19.26
Q6UW63	POGLUT2	Protein O-glu	17	7	7	7	502	58	7.71	21.7
Q9UN36	NDRG2	Protein NDR	19	5	10	5	371	40.8	5.21	29.76
Q9NZ45	CISD1	CDGSH iron-	44	4	12	4	108	12.2	9.09	35.65
Q8NBJ7	SUMF2	Inactive C-alp	21	7	9	7	301	33.8	8	23.15
P29728	OAS2	2'-5'-oligoade	10	6	6	6	719	82.4	8.25	16.45
Q9H9J2	MRPL44	39S ribosom	27	5	8	5	332	37.5	8.4	23.15
Q14139	UBE4A	Ubiquitin con	7	6	6	6	1066	122.5	5.24	21.98
Q96CG8	CTHRC1	Collagen trip	23	5	10	5	243	26.2	7.99	27.54
Q14C86	GAPVD1	GTPase-activ	5	7	8	7	1478	164.9	5.22	21.2

Q8IY17	PNPLA6	Patatin-like p	7	8	8	8	1375	150.9	7.74	16.86
Q8WWQ0	PHIP	PH-interactin	5	7	7	7	1821	206.6	8.85	19.51
Q13084	MRPL28	39S ribosom	35	7	9	7	256	30.1	8.29	16.51
O75886	STAM2	Signal transc	13	6	7	5	525	58.1	5.07	16.38
Q9UP83	COG5	Conserved o	8	6	8	6	839	92.7	6.6	21.19
Q86X55	CARM1	Histone-argir	10	7	8	7	608	65.8	6.73	22.51
Q9H6R4	NOL6	Nucleolar prc	6	6	6	6	1146	127.5	7.64	16.54
O95716	RAB3D	Ras-related p	23	5	8	3	219	24.3	4.93	22.9
P50583	NUDT2	Bis(5'-nucleo	41	5	8	5	147	16.8	5.35	22.57
P11166	SLC2A1	Solute carrie	9	5	8	5	492	54	8.72	24.56
Q9H9A6	LRRC40	Leucine-rich	15	8	8	8	602	68.2	6.43	19.44
Q8WXA9	SREK1	Splicing regu	13	5	7	5	508	59.3	10.39	21.57
Q9UPT8	ZC3H4	Zinc finger C	8	7	8	6	1303	140.2	6.27	21.58
O60825	PFKFB2	6-phosphofru	15	8	9	6	505	58.4	8.38	22.08
Q9GZL7	WDR12	Ribosome bi	17	6	7	6	423	47.7	5.9	22.96
P02776	PF4	Platelet facto	36	4	6	4	101	10.8	8.62	21.38
Q9Y4E8	USP15	Ubiquitin car	6	7	9	7	981	112.3	5.22	19.05
Q9H299	SH3BGRL3	SH3 domain-	47	5	10	5	93	10.4	4.93	36.72
Q9Y2V2	CARHSP1	Calcium-regu	24	3	7	3	147	15.9	8.21	22.49
Q92696	RABGGTA	Geranylgerar	13	6	6	6	567	65	5.67	18.35
P24347	MMP11	Stromelysin-3	12	5	7	5	488	54.6	6.87	19.42
Q9UKU7	ACAD8	Isobutyryl-Co	20	7	7	7	415	45	7.85	18.19
Q7RTV0	PHF5A	PHD finger-li	55	6	9	6	110	12.4	8.41	21.55
Q06136	KDSR	3-ketodihydro	19	5	7	5	332	36.2	7.12	18.32
P17302	GJA1	Gap junction	20	7	7	7	382	43	8.76	20.63
P02656	APOC3	Apolipoprote	34	3	9	3	99	10.8	5.41	30.85
Q7L9L4	MOB1B	MOB kinase	25	5	8	5	216	25.1	6.73	21.15
P04062	GBA1	Lysosomal a	9	5	7	5	536	59.7	7.61	19.36
P14174	MIF	Macrophage	43	4	17	4	115	12.5	7.88	36.2
P61081	UBE2M	NEDD8-conju	31	5	9	5	183	20.9	7.69	19.39
O75312	ZPR1	Zinc finger pr	15	6	7	6	459	50.9	4.73	19.1
O95819	MAP4K4	Mitogen-activ	7	7	7	4	1239	142	7.46	18.39
O00743	PPP6C	Serine/threor	20	6	8	6	305	35.1	5.69	22.28

Q92879	CELF1	CUGBP Elav	15	8	9	7	486	52	8.46	25.7
P45954	ACADSB	Short/branch	17	5	5	5	432	47.5	6.99	19.86
Q9Y6M9	NDUFB9	NADH dehyd	40	5	7	5	179	21.8	8.38	26.24
O75251	NDUFS7	NADH dehyd	23	6	8	6	213	23.5	9.99	18.28
Q9Y2R9	MRPS7	28S ribosom	24	5	6	5	242	28.1	9.99	15.32
Q9Y3D6	FIS1	Mitochondria	20	4	8	4	152	16.9	8.79	28.31
Q16513	PKN2	Serine/threor	9	6	6	5	984	112	6.3	20.53
Q15750	TAB1	TGF-beta-ac	14	5	8	5	504	54.6	5.52	24.82
Q7Z794	KRT77	Keratin, type	6	5	12	0	578	61.9	5.99	31.35
Q13427	PPIG	Peptidyl-prol	11	8	10	8	754	88.6	10.29	17.66
Q9P2I0	CPSF2	Cleavage an	8	5	7	5	782	88.4	5.11	19.37
P28065	PSMB9	Proteasome	21	4	6	4	219	23.3	5.03	19.68
Q9BWH2	FUNDC2	FUN14 doma	18	5	6	5	189	20.7	9.73	18.69
Q9NQT5	EXOSC3	Exosome cor	35	6	6	6	275	29.6	8.1	20.97
Q8IU81	IRF2BP1	Interferon re	12	7	7	7	584	61.6	8.18	15.55
P49207	RPL34	60S ribosom	34	5	13	5	117	13.3	11.47	35.36
Q9BY67	CADM1	Cell adhesion	21	5	6	5	442	48.5	5.07	21.03
Q9H3K2	GHITM	Growth horm	15	4	7	4	345	37.2	9.94	21.14
Q96C23	GALM	Galactose m	20	5	6	5	342	37.7	6.65	17.41
Q5T0N5	FNBP1L	Formin-bindi	12	8	8	8	605	70	6.64	18.34
O95486	SEC24A	Protein trans	6	5	6	3	1093	119.7	7.66	18.3
Q6FI81	CIAPIN1	Anamorsin C	13	5	10	5	312	33.6	5.62	18.3
O15498	YKT6	Synaptobrev	40	8	10	8	198	22.4	6.92	24.59
Q969N2	PIGT	GPI transam	12	7	9	7	578	65.7	8.38	26.13
P36980	CFHR2	Complement	23	6	9	2	270	30.6	6.38	22.02
Q9BZE1	MRPL37	39S ribosom	15	6	8	6	423	48.1	8.59	20.08
Q7Z3K3	POGZ	Pogo transpo	6	7	7	7	1410	155.2	7.4	18.16
P21741	MDK	Midkine OS=	39	6	10	6	143	15.6	9.79	22.89
P30085	CMPK1	UMP-CMP ki	21	3	7	3	196	22.2	5.57	26.45
Q9NPF4	OSGEP	tRNA N6-ade	20	6	8	6	335	36.4	6.35	16.94
Q92544	TM9SF4	Transmembr	12	7	7	7	642	74.5	6.54	18.03
O00178	GTPBP1	GTP-binding	10	5	5	5	669	72.4	8.34	16.5
Q14156	EFR3A	Protein EFR3	8	6	7	6	821	92.9	6.7	15.26

P20645	M6PR	Cation-deper	29	7	8	7	277	31	5.83	22.22
Q9UKY7	CDV3	Protein CDV3	30	7	7	7	258	27.3	6.4	16.6
Q15833	STXBP2	Syntaxin-bind	16	7	7	6	593	66.4	6.55	17.1
P60903	S100A10	Protein S100	28	4	7	4	97	11.2	7.37	22.59
Q5GLZ8	HERC4	Probable E3	9	9	9	9	1057	118.5	6.19	16.54
P56182	RRP1	Ribosomal R	18	8	8	8	461	52.8	9.33	17.34
Q86UV5	USP48	Ubiquitin car	9	7	7	7	1035	119	6.05	15.74
O00391	QSOX1	Sulfhydryl ox	11	6	6	6	747	82.5	8.92	19.85
Q8TCD5	NT5C	5'(3')-deoxyri	32	6	8	5	201	23.4	6.64	25.46
Q6UB35	MTHFD1L	Monofunction	8	8	11	7	978	105.7	8.06	21.32
O95182	NDUFA7	NADH dehyc	50	8	11	8	113	12.5	10.18	30.09
P61923	COPZ1	Coatomer su	32	4	9	4	177	20.2	4.81	22.07
Q96PY5	FMNL2	Formin-like p	7	8	9	4	1086	123.2	7.4	23.96
P35612	ADD2	Beta-adducir	11	8	9	7	726	80.8	5.92	13.47
O43324	EEF1E1	Eukaryotic tr	29	6	9	6	174	19.8	8.54	20.15
Q9UL15	BAG5	BAG family r	13	5	6	5	447	51.2	6.05	19.11
Q9Y446	PKP3	Plakophilin-3	10	8	8	8	797	87	9.32	17.55
Q5VWZ2	LYPLAL1	Lysophospho	28	6	6	6	237	26.3	7.84	17.6
Q9UK45	LSM7	U6 snRNA-a	43	4	9	4	103	11.6	5.27	25.4
Q2PZI1	DPY19L1	Probable C-r	10	5	6	5	675	77.3	8.95	19.16
P83436	COG7	Conserved o	9	6	6	6	770	86.3	5.47	21.23
Q8NBL1	POGLUT1	Protein O-glu	15	6	8	6	392	46.2	8.72	16.79
P55081	MFAP1	Microfibrillar-	9	3	5	3	439	51.9	4.98	15.3
P28288	ABCD3	ATP-binding	10	7	7	7	659	75.4	9.36	20.35
Q2M389	WASHC4	WASH comp	5	6	7	6	1173	136.3	7.44	18.88
P05204	HMGN2	Non-histone	54	5	10	4	90	9.4	9.99	19.2
O43598	DNPH1	2'-deoxynucl	48	6	8	6	174	19.1	5.05	23.23
Q96EY7	PTCD3	Pentatricope	11	7	7	7	689	78.5	6.42	19.09
Q9H0L4	CSTF2T	Cleavage stir	14	6	6	3	616	64.4	7.25	16.07
Q15269	PWP2	Periodic tryp	9	5	7	5	919	102.4	6.15	18.57
Q9Y6D9	MAD1L1	Mitotic spindl	8	6	7	6	718	83	5.92	19.19
Q96I25	RBM17	Splicing facto	15	5	6	5	401	44.9	5.97	19.54
Q9BXD5	NPL	N-acetylneur	20	6	8	6	320	35.1	5.57	17.62

Q9NYL2	MAP3K20	Mitogen-activ	7	5	6	5	800	91.1	7.87	18.33
Q92882	OSTF1	Osteoclast-s	26	4	6	4	214	23.8	5.68	14.93
O75688	PPM1B	Protein phos	11	5	6	3	479	52.6	5.05	17.94
Q9Y5K5	UCHL5	Ubiquitin carl	19	4	5	4	329	37.6	5.33	17.1
P48507	GCLM	Glutamate--c	20	5	7	5	274	30.7	6.02	25.63
O75431	MTX2	Metaxin-2 OS	20	4	6	4	263	29.7	6.29	18.76
P15559	NQO1	NAD(P)H del	15	5	8	5	274	30.8	8.88	19.36
Q00653	NFKB2	Nuclear facto	8	7	7	6	900	96.7	6.25	18.3
Q96L92	SNX27	Sorting nexir	13	7	7	7	541	61.2	6.49	16.76
Q96JM3	CHAMP1	Chromosome	11	7	7	7	812	89	8.44	17.41
O00217	NDUFS8	NADH dehyc	27	5	7	5	210	23.7	6.34	22.15
O15270	SPTLC2	Serine palmi	13	6	7	6	562	62.9	7.78	20.91
Q9UBI6	GNG12	Guanine nuc	67	4	6	4	72	8	8.97	16.15
P03951	F11	Coagulation	10	6	7	6	625	70.1	8.1	18.85
Q99417	MYCBP	c-Myc-bindin	49	4	6	4	103	12	5.91	16.59
Q99700	ATXN2	Ataxin-2 OS=	6	8	8	8	1313	140.2	9.57	15.76
O43678	NDUFA2	NADH dehyc	56	6	7	6	99	10.9	9.57	20.72
P04066	FUCA1	Tissue alpha	12	4	6	4	466	53.7	6.84	20.56
Q96PE2	ARHGEF17	Rho guanine	3	6	8	6	2063	221.5	6.29	11.65
P51665	PSMD7	26S proteasc	13	4	7	4	324	37	6.77	16.56
Q9NVZ3	NECAP2	Adaptin ear-l	20	6	8	6	263	28.3	8.38	22.1
Q9H9Q2	COPS7B	COP9 signal	31	6	7	6	264	29.6	6.15	17.47
Q9BRA2	TXNDC17	Thioredoxin	51	5	9	5	123	13.9	5.52	22.37
P80404	ABAT	4-aminobuty	14	6	6	6	500	56.4	7.96	16.76
P48426	PIP4K2A	Phosphatidyl	15	6	7	3	406	46.2	6.99	17.78
O94769	ECM2	Extracellular	14	7	8	7	699	79.7	5.41	19.04
Q9NVH1	DNAJC11	DnaJ homolc	10	5	6	5	559	63.2	8.4	20.23
Q53EP0	FNDC3B	Fibronectin t	6	6	6	6	1204	132.8	5.95	18.54
O43447	PPIH	Peptidyl-prol	24	5	13	4	177	19.2	8.07	22.6
Q13362	PPP2R5C	Serine/threoı	15	7	9	4	524	61	6.87	27.43
Q8TD06	AGR3	Anterior grac	22	3	8	2	166	19.2	7.99	16.87
P09669	COX6C	Cytochrome	41	4	10	4	75	8.8	10.39	27.99
P61626	LYZ	Lysozyme C	39	6	11	6	148	16.5	9.16	28.8

P56134	ATP5MF	ATP synthas	26	2	9	2	94	10.9	9.67	26.84
Q8TDZ2	MICAL1	[F-actin]-mor	8	7	9	7	1067	117.8	6.4	20.25
Q9BSD7	NTPCR	Cancer-relat	37	6	7	6	190	20.7	9.54	21
Q6UVY6	MOXD1	DBH-like mo	11	8	10	8	613	69.6	6.43	22.48
O43670	ZNF207	BUB3-intera	13	5	6	5	478	50.7	9.1	17.48
Q9Y6Q1	CAPN6	Calpain-6 OS	11	6	7	6	641	74.5	7.05	17.38
P46087	NOP2	Probable 28S	10	7	8	7	812	89.2	9.23	18.79
Q9Y5K8	ATP6V1D	V-type protor	28	6	6	6	247	28.2	9.36	17.94
P15104	GLUL	Glutamine sy	18	6	8	6	373	42	6.89	23.44
Q9UBU9	NXF1	Nuclear RNA	10	5	7	5	619	70.1	8.51	18.57
Q96EI5	TCEAL4	Transcription	20	4	7	4	215	24.6	5.2	18.52
Q6PIU2	NCEH1	Neutral chole	19	5	6	5	408	45.8	7.23	14.34
Q9BZH6	WDR11	WD repeat-c	5	6	7	6	1224	136.6	6.92	19.11
P52306	RAP1GDS1	Rap1 GTPas	12	6	7	6	607	66.3	5.31	15.36
P23921	RRM1	Ribonucleosi	9	7	7	7	792	90	7.15	15.14
P53985	SLC16A1	Monocarboxy	12	5	6	5	500	53.9	8.66	19.45
Q15043	SLC39A14	Metal cation	12	5	6	5	492	54.2	5.33	19.65
Q9P000	COMMD9	COMM doma	26	4	6	4	198	21.8	5.88	20.51
Q9Y6E0	STK24	Serine/threor	15	6	8	4	443	49.3	5.69	15.88
Q16678	CYP1B1	Cytochrome	11	6	8	6	543	60.8	8.98	19.52
P29218	IMPA1	Inositol mon	17	5	8	5	277	30.2	5.26	28.87
P07919	UQCRH	Cytochrome	64	4	7	4	91	10.7	4.44	25.48
Q9BZE9	ASPSCR1	Tether conta	12	5	5	5	553	60.1	6.64	19.74
Q6P1J9	CDC73	Parafibromin	13	7	7	7	531	60.5	9.61	14.26
Q8N6R0	METTL13	eEF1A lysine	9	5	7	5	699	78.7	6.73	19.16
Q13751	LAMB3	Laminin subu	7	6	9	6	1172	129.5	7.21	22.14
Q9BQA1	WDR77	Methylosome	11	3	8	3	342	36.7	5.17	26.77
O75179	ANKRD17	Ankyrin repe	3	7	7	3	2603	274.1	6.52	15.66
Q13769	THOC5	THO comple	10	8	9	8	683	78.5	6.87	17.98
Q9BYG3	NIFK	MKI67 FHA d	25	5	6	5	293	34.2	9.88	22.42
P35249	RFC4	Replication fa	18	5	5	5	363	39.7	8.02	18.28
Q01780	EXOSC10	Exosome co	8	6	6	6	885	100.8	8.46	21.13
Q6PCB0	VWA1	von Willebra	12	5	8	5	445	46.8	7.68	18.95

Q9UBI1	COMMD3	COMM doma	29	4	5	4	195	22.1	5.99	17.23
Q9H7D0	DOCK5	Dedicator of	4	8	8	6	1870	215.2	7.96	19.8
P01880	IGHD	Immunoglobu	16	5	7	5	384	42.3	8.12	16.64
Q9NUJ1	ABHD10	Palmitoyl-prc	25	7	9	7	306	33.9	8.57	19.82
Q8N0X7	SPART	Spartin OS=I	10	6	8	6	666	72.8	5.91	21.03
O00541	PES1	Pescadillo hc	11	8	9	8	588	68	7.33	18.73
P35269	GTF2F1	General tran:	16	6	7	6	517	58.2	7.49	18.16
Q14012	CAMK1	Calcium/caln	21	5	5	3	370	41.3	5.29	15.95
Q96C86	DCPS	m7GpppX di	17	4	6	4	337	38.6	6.38	22.56
Q9UK59	DBR1	Lariat debrar	11	4	6	4	544	61.5	5.47	20.97
Q9NS86	LANCL2	LanC-like prc	13	5	6	5	450	50.8	7.43	15.82
Q8IVF2	AHNAK2	Protein AHN/	6	6	8	4	5795	616.2	5.36	19.23
P26447	S100A4	Protein S10C	37	5	11	5	101	11.7	6.11	31.07
Q5VZ89	DENND4C	DENN doma	4	7	7	7	1909	212.6	6.83	16.85
O75844	ZMPSTE24	CAAX prenyl	15	6	7	6	475	54.8	7.49	13.3
O00483	NDUFA4	Cytochrome	46	4	8	4	81	9.4	9.38	23.69
P16333	NCK1	Cytoplasmic	17	6	7	5	377	42.8	6.47	16.25
Q7L5D6	GET4	Golgi to ER t	20	6	8	6	327	36.5	5.41	19.18
Q9UPU5	USP24	Ubiquitin car	3	7	7	7	2620	294.2	6.14	16.29
P23946	CMA1	Chymase OS	30	5	6	5	247	27.3	9.29	15.2
Q96FV2	SCRN2	Secernin-2 C	17	5	5	5	425	46.6	5.67	14.8
Q5VT66	MTARC1	Mitochondria	17	5	6	4	337	37.5	8.88	17.07
Q8N5K1	CISD2	CDGSH iron-	27	3	5	3	135	15.3	9.61	20.23
Q96HP0	DOCK6	Dedicator of	4	7	7	5	2047	229.4	6.74	16.44
P00167	CYB5A	Cytochrome	32	3	7	3	134	15.3	4.96	27.22
Q04828	AKR1C1	Aldo-keto rec	16	5	8	1	323	36.8	7.88	15.76
P01768	IGHV3-30	Immunoglobu	28	4	13	3	117	12.9	8.92	28.93
P06213	INSR	Insulin recep	4	6	6	4	1382	156.2	6.2	16.15
Q99523	SORT1	Sortilin OS=I	8	6	6	5	831	92	5.74	18.75
Q9NWU5	MRPL22	39S ribosom	26	5	6	5	206	23.6	9.94	16.18
O60220	TIMM8A	Mitochondria	68	4	5	4	97	11	5.16	18.96
Q13303	KCNAB2	Voltage-gate	26	5	5	5	367	41	9	15.08
Q9UMX5	NENF	Neudesin OS	35	4	6	4	172	18.8	5.69	23.54

P32322	PYCR1	Pyrroline-5-c	11	3	5	2	319	33.3	7.61	18.58
P41214	EIF2D	Eukaryotic tr	12	5	5	5	584	64.7	7.65	17.19
Q9Y6C9	MTCH2	Mitochondria	21	5	6	5	303	33.3	7.97	21.74
Q8IUE6	H2AC21	Histone H2A	42	5	18	2	130	14	10.89	39.57
P61758	VBP1	Prefoldin sub	25	5	12	5	197	22.6	7.11	16.8
P42331	ARHGAP25	Rho GTPase	12	6	7	6	645	73.4	6.37	14.99
Q9ULT8	HECTD1	E3 ubiquitin-|	3	7	7	7	2610	289.2	5.35	16.11
Q9GZZ1	NAA50	N-alpha-acet	33	6	7	6	169	19.4	8.81	15.48
Q66LE6	PPP2R2D	Serine/threo	19	7	8	3	453	52	6.39	17.48
P16070	CD44	CD44 antige	7	5	10	5	742	81.5	5.33	30.27
Q5VT79	ANXA8L1	Annexin A8-l	17	5	7	1	327	36.9	5.78	17.15
Q9H2P0	ADNP	Activity-depe	7	7	8	7	1102	123.5	7.34	16.58
Q9Y6K9	IKBKG	NF-kappa-B	14	6	8	6	419	48.2	5.71	19.73
P13987	CD59	CD59 glycop	23	3	13	3	128	14.2	6.48	43.91
P11498	PC	Pyruvate car	7	7	7	7	1178	129.6	6.84	15.61
P01591	JCHAIN	Immunoglobu	32	5	8	5	159	18.1	5.24	21.17
Q9UBB5	MBD2	Methyl-CpG-	17	7	7	6	411	43.2	10.04	20.17
Q08431	MFGE8	Lactadherin	13	4	6	4	387	43.1	8.15	22.69
P35244	RPA3	Replication p	45	4	6	4	121	13.6	5.08	20.6
O95163	ELP1	Elongator co	6	6	6	6	1332	150.2	5.94	17.47
O95400	CD2BP2	CD2 antigen	20	5	5	5	341	37.6	4.61	16.17
O60502	OGA	Protein O-Gl	7	6	8	6	916	102.8	4.91	18.03
Q99471	PFDN5	Prefoldin sub	42	5	6	5	154	17.3	6.33	17.96
Q9NXR7	BABAM2	BRISC and E	18	7	7	7	383	43.5	5.81	17.94
P62854	RPS26	40S ribosom	43	5	10	5	115	13	11	25.97
O95674	CDS2	Phosphatida	11	4	5	4	445	51.4	7.09	17.49
O14908	GIPC1	PDZ domain-	20	5	5	5	333	36	6.28	16.14
P15374	UCHL3	Ubiquitin car	23	4	5	4	230	26.2	4.92	17.34
Q86Y82	STX12	Syntaxin-12	20	5	6	5	276	31.6	5.59	18.73
Q96GX9	APIP	Methylthiorib	20	3	5	3	242	27.1	7.12	19.85
Q9Y3A3	MOB4	MOB-like pro	36	6	6	6	225	26	5.78	19.69
Q9NTJ4	MAN2C1	Alpha-mann	7	5	6	5	1040	115.8	6.57	19.07
P51116	FXR2	RNA-binding	8	5	6	2	673	74.2	6.23	16.6

Q9P265	DIP2B	Disco-interac	4	7	8	7	1576	171.4	8.09	17.94
A0A0C4DH3	IGHV1-18	Immunoglobu	30	3	7	1	117	12.8	8.84	22.6
P50579	METAP2	Methionine a	16	7	8	7	478	52.9	5.82	22.94
O14907	TAX1BP3	Tax1-binding	34	3	8	3	124	13.7	8.48	37.04
O94911	ABCA8	ABC-type org	5	6	7	6	1621	183.6	7.25	17.05
Q9BRK3	MXRA8	Matrix remod	17	7	8	7	442	49.1	7.23	20.54
O75964	ATP5MG	ATP synthas	24	3	6	3	103	11.4	9.64	18.75
O15439	ABCC4	ATP-binding	6	7	9	7	1325	149.4	8.19	14.78
O14638	ENPP3	Ectonucleotid	10	6	6	6	875	100.1	6.57	19.13
O95989	NUDT3	Diphosphoino	37	5	6	4	172	19.5	6.34	18.21
O43504	LAMTOR5	Ragulator co	86	4	4	4	91	9.6	4.87	16.12
Q8WVC6	DCAKD	Dephospho-C	22	5	6	5	231	26.5	9.58	15.79
P10589	NR2F1	COUP transc	13	6	8	1	423	46.1	8.25	22.83
Q96RF0	SNX18	Sorting nexin	13	8	8	8	628	68.9	5.68	20.43
Q9BRP8	PYM1	Partner of Y1	25	5	5	5	204	22.6	9.45	15.13
Q2TAA2	IAH1	Isoamyl acet	22	4	7	4	248	27.6	5.3	22.5
P01780	IGHV3-7	Immunoglobu	38	5	8	3	117	12.9	6.57	24.18
P54803	GALC	Galactocereb	7	4	6	4	685	77	6.64	15.83
Q9UBQ5	EIF3K	Eukaryotic tr	28	5	8	5	218	25	4.93	19.63
Q9UL25	RAB21	Ras-related p	15	3	5	3	225	24.3	7.94	17.11
Q13595	TRA2A	Transformer-	15	5	9	4	282	32.7	11.27	24.04
Q96CM8	ACSF2	Medium-chai	11	5	6	5	615	68.1	7.55	12.84
Q9H4Z3	PCIF1	mRNA (2'-O-	7	5	7	5	704	80.6	7.42	18.02
P29466	CASP1	Caspase-1 C	19	6	7	5	404	45.1	5.91	12.71
P49770	EIF2B2	Translation in	21	6	7	6	351	39	6.16	18.48
P61513	RPL37A	60S ribosom	49	4	7	4	92	10.3	10.43	19.22
P04626	ERBB2	Receptor tyro	4	5	7	4	1255	137.8	5.91	15.21
P62857	RPS28	40S ribosom	46	3	11	3	69	7.8	10.7	36.32
O60343	TBC1D4	TBC1 domai	5	5	6	5	1298	146.5	7.01	16.89
P19474	TRIM21	E3 ubiquitin-p	13	6	6	6	475	54.1	6.38	16.17
Q9HBH5	RDH14	Retinol dehy	21	6	7	6	336	36.8	8.79	20.28
Q5JVF3	PCID2	PCI domain-c	12	6	8	6	399	46	8.53	21.14
Q96A26	FAM162A	Protein FAM1	25	4	5	4	154	17.3	9.77	16.2

O14791	APOL1	Apolipoprote	18	7	7	7	398	43.9	5.81	19.87
Q7Z7G0	ABI3BP	Target of Ne	7	5	6	5	1068	117.8	9.42	19.95
Q9H444	CHMP4B	Charged mul	36	6	8	6	224	24.9	4.82	17.97
Q96FJ2	DYNLL2	Dynein light	57	4	7	2	89	10.3	7.37	23.46
Q9Y6B6	SAR1B	GTP-binding	21	5	11	1	198	22.4	6.11	27.1
Q96NY7	CLIC6	Chloride intra	9	6	6	6	704	73	4.37	16.98
Q96RS6	NUDCD1	NudC domai	11	6	8	6	583	66.7	5.11	18.3
Q6P9B6	MEAK7	MTOR-assoc	17	5	5	5	456	51	6.24	17.11
Q06546	GABPA	GA-binding p	12	5	8	5	454	51.3	4.97	21.7
P18065	IGFBP2	Insulin-like g	18	4	5	4	325	34.8	7.5	17.57
P52594	AGFG1	Arf-GAP dom	14	6	9	5	562	58.2	8.63	21.15
P67812	SEC11A	Signal peptic	28	6	10	6	179	20.6	9.48	19.52
Q8WUA4	GTF3C2	General tran	7	5	5	5	911	100.6	7.31	17.17
Q03154	ACY1	Aminoacylas	15	5	6	5	408	45.9	6.18	10.29
O94919	ENDOD1	Endonucleas	22	7	9	7	500	55	5.71	12.96
P60484	PTEN	Phosphatidyl	12	4	6	4	403	47.1	6.37	18.57
Q9P2N5	RBM27	RNA-binding	5	5	5	4	1060	118.6	9.19	14.76
Q9H832	UBE2Z	Ubiquitin-cor	16	6	7	6	354	38.2	5.62	17.78
P51970	NDUFA8	NADH dehyc	31	5	9	5	172	20.1	7.65	19.5
Q7Z7H8	MRPL10	39S ribosom	25	4	5	4	261	29.3	9.58	17.73
Q14790	CASP8	Caspase-8 C	14	4	5	4	479	55.4	5.1	15.24
Q9Y3Y2	CHTOP	Chromatin ta	21	4	7	4	248	26.4	12.23	27.53
Q15006	EMC2	ER membrar	22	5	6	5	297	34.8	6.57	16.34
P43251	BTD	Biotinidase C	13	5	6	5	543	61.1	6.25	15.27
Q9P013	CWC15	Spliceosome	22	4	5	4	229	26.6	5.71	17.55
P32320	CDA	Cytidine dea	40	3	5	3	146	16.2	6.92	21.09
Q9UBB9	TFIP11	Tuftelin-inter	9	6	6	6	837	96.8	5.67	15.01
Q9P016	THYN1	Thymocyte n	28	6	6	6	225	25.7	9.25	16.2
Q00978	IRF9	Interferon reg	17	6	6	6	393	43.7	5.77	17.62
P15428	HPGD	15-hydroxypr	24	6	6	6	266	29	5.86	17.22
Q8N573	OXR1	Oxidation res	7	6	6	5	874	97.9	5.47	13.08
Q5SRE5	NUP188	Nucleoporin	4	6	6	6	1749	195.9	6.73	17.54
Q9H0Q0	CYRIA	CYFIP-relate	15	3	7	2	323	37.3	6.01	22.74

Q9BXV9	GON7	EKC/KEOPS	41	2	6	2	100	10.9	4.27	26.53
Q9UEW8	STK39	STE20/SPS1	15	7	9	5	545	59.4	6.29	20.44
Q76M96	CCDC80	Coiled-coil d	7	5	5	5	950	108.1	9.72	13.53
Q15262	PTPRK	Receptor-typ	6	6	6	6	1439	162	5.9	12.03
P03973	SLPI	Antileukopro	39	7	10	7	132	14.3	8.75	19.22
Q96LR5	UBE2E2	Ubiquitin-cor	18	3	5	1	201	22.2	7.71	16.89
P08962	CD63	CD63 antige	10	3	12	3	238	25.6	7.81	26.51
Q9NUQ7	UFSP2	Ufm1-specifi	11	5	6	5	469	53.2	7.01	19.23
O75150	RNF40	E3 ubiquitin-	7	6	6	4	1001	113.6	6.29	17.17
Q8N3V7	SYNPO	Synaptopodi	8	5	7	5	929	99.4	8.72	19.98
Q13485	SMAD4	Mothers aga	11	6	7	6	552	60.4	6.99	16.67
O60907	TBL1X	F-box-like/W	10	5	6	1	577	62.5	6.55	11.77
P49754	VPS41	Vacuolar pro	8	6	6	6	854	98.5	5.85	14.09
O43847	NRDC	Nardilysin O	4	5	5	5	1151	131.6	4.98	16.24
Q9P2B4	CTTNBP2NL	CTTNBP2 N	12	7	7	7	639	70.1	8.06	14.71
O00442	RTCA	RNA 3'-termi	20	5	5	5	366	39.3	7.85	14.69
Q16134	ETFDH	Electron tran	9	5	6	5	617	68.5	7.55	14.5
Q13643	FHL3	Four and a h	19	5	8	5	280	31.2	6.2	19.81
P57772	EEFSEC	Selenocystei	11	6	7	6	596	65.3	8.35	20.21
P62308	SNRPG	Small nuclea	34	3	8	3	76	8.5	8.88	21.08
P06312	IGKV4-1	Immunoglobu	28	3	8	3	121	13.4	5.25	28.71
P31751	AKT2	RAC-beta se	15	8	8	4	481	55.7	6.37	18.11
P51687	SUOX	Sulfite oxidas	14	7	7	7	545	60.2	6.11	17.15
Q4L180	FILIP1L	Filamin A-int	4	6	7	6	1135	130.3	6.57	20.66
Q5VT25	CDC42BPA	Serine/threo	3	5	6	2	1732	197.2	6.58	14.69
Q14671	PUM1	Pumilio hom	5	6	8	3	1186	126.4	6.84	15.65
Q9Y450	HBS1L	HBS1-like pr	10	6	10	6	684	75.4	6.61	21.86
Q9Y3B4	SF3B6	Splicing fact	34	4	6	4	125	14.6	9.38	15.18
Q9NZJ9	NUDT4	Diphosphoin	36	5	5	3	180	20.3	6.35	14.89
Q13438	OS9	Protein OS-9	12	5	5	5	667	75.5	4.87	11.86
Q7LBR1	CHMP1B	Charged mul	23	6	8	6	199	22.1	8.1	18.91
Q5T9L3	WLS	Protein wntle	11	6	7	6	541	62.2	7.36	21.28
Q96C90	PPP1R14B	Protein phos	31	3	5	3	147	15.9	4.86	19.71

Q96B97	SH3KBP1	SH3 domain-	11	6	7	6	665	73.1	6.62	16.28
O95777	LSM8	U6 snRNA-a	59	4	6	4	96	10.4	4.48	17.77
Q92466	DDB2	DNA damage	15	4	4	4	427	47.8	9.47	16.73
Q9UBW8	COPS7A	COP9 signal	20	5	6	5	275	30.3	8.22	14.11
Q9NRG7	SDR39U1	Epimerase fa	22	4	5	4	293	31.1	9.04	18.83
Q9Y5S9	RBM8A	RNA-binding	22	3	6	3	174	19.9	5.72	22.12
P49961	ENTPD1	Ectonucleosi	10	5	6	5	510	57.9	6.34	15.31
P02654	APOC1	Apolipoprote	35	5	10	5	83	9.3	8.47	25.62
P11117	ACP2	Lysosomal a	16	6	8	6	423	48.3	6.74	19.28
Q7L0Y3	TRMT10C	tRNA methyl	15	5	6	5	403	47.3	9.36	15.6
P32929	CTH	Cystathionine	18	5	6	5	405	44.5	6.7	19.3
Q658Y4	FAM91A1	Protein FAM9	8	8	8	8	838	93.9	6.39	17.06
Q96T37	RBM15	RNA-binding	6	5	8	5	977	107.1	10.08	16.54
O43278	SPINT1	Kunitz-type p	10	5	6	5	529	58.4	6.29	15.08
Q15654	TRIP6	Thyroid rece	16	6	6	6	476	50.3	7.37	18.88
Q96PC5	MIA2	Melanoma in	5	7	8	7	1412	159.7	4.69	16.08
Q9BQB6	VKORC1	Vitamin K ep	20	4	6	4	163	18.2	9.36	19.07
P23083	IGHV1-2	Immunoglobu	26	3	7	1	117	13.1	9.13	20.14
P48729	CSNK1A1	Casein kinas	17	6	6	6	337	38.9	9.57	17.92
O95453	PARN	Poly(A)-spec	14	9	10	9	639	73.4	6.2	13.25
Q9H5N1	RABEP2	Rab GTPase	10	5	6	5	569	63.5	4.78	18.28
Q01459	CTBS	Di-N-acetylch	15	5	6	5	385	43.7	6.64	14.51
O95140	MFN2	Mitofusin-2 O	11	7	7	7	757	86.3	6.98	15.18
Q9NR09	BIRC6	Baculoviral IA	2	6	7	6	4857	529.9	6.05	18.34
Q8N2K0	ABHD12	Lysophospha	12	4	5	4	398	45.1	8.65	14.53
Q9UG63	ABCF2	ATP-binding	10	5	6	5	623	71.2	7.37	20.95
P35754	GLRX	Glutaredoxin	31	3	7	3	106	11.8	8.09	34.38
P25774	CTSS	Cathepsin S	15	5	7	5	331	37.5	8.34	23.49
Q9UHA4	LAMTOR3	Ragulator co	44	3	5	3	124	13.6	7.34	20.94
Q96H20	SNF8	Vacuolar-sor	19	5	6	5	258	28.8	6.65	18.52
P14222	PRF1	Perforin-1 OS	11	5	9	5	555	61.3	7.83	14.53
Q9Y5L4	TIMM13	Mitochondria	26	2	5	2	95	10.5	8.18	12.55
Q75QN2	INTS8	Integrator co	8	6	6	6	995	113	7.05	14.11

A1A4S6	ARHGAP10	Rho GTPase	8	6	7	5	786	89.3	7.18	15.54
Q9NZN5	ARHGEF12	Rho guanine	4	6	6	6	1544	173.1	5.74	16.35
O00193	SMAP	Small acidic	30	4	8	4	183	20.3	4.72	20.84
P09471	GNAO1	Guanine nuc	18	5	6	4	354	40	5.53	16.42
Q96AM1	MRGPRF	Mas-related	6	2	7	2	343	38.1	8.56	24.47
Q9UGV2	NDRG3	Protein NDR	14	4	5	4	375	41.4	5.31	16.19
Q9H8L6	MMRN2	Multimerin-2	6	6	7	6	949	104.3	5.86	15.14
Q9Y6E2	BZW2	eIF5-mimic p	12	5	9	5	419	48.1	6.68	18.47
Q9Y606	PUS1	Pseudouridyl	15	6	6	6	427	47.4	8.41	16.2
Q9Y6F6	IRAG1	Inositol 1,4,5	8	6	7	6	904	97.9	5.63	15.82
P52735	VAV2	Guanine nuc	9	6	6	6	878	101.2	7.08	15.05
P56385	ATP5ME	ATP synthas	32	2	7	2	69	7.9	9.35	24.41
P24941	CDK2	Cyclin-deper	19	5	5	3	298	33.9	8.68	15.36
Q6PJT7	ZC3H14	Zinc finger C	8	5	5	5	736	82.8	7.31	13.8
P55083	MFAP4	Microfibril-as	12	3	6	3	255	28.6	5.63	21.34
Q9Y2S7	POLDIP2	Polymerase	17	6	6	6	368	42	8.63	16.69
Q96B26	EXOSC8	Exosome cor	15	5	6	5	276	30	5.3	17.1
Q9H425	C1orf198	Uncharacteri	20	5	5	5	327	36.3	5.72	14.76
Q5QJ74	TBCEL	Tubulin-spec	17	6	6	6	424	48.2	5.38	14
Q02338	BDH1	D-beta-hydrc	13	3	4	3	343	38.1	8.95	15.32
Q8IYU8	MICU2	Calcium upta	11	3	5	3	434	49.6	9.09	19.62
P49746	THBS3	Thrombospo	6	6	7	6	956	104.1	4.65	10.96
Q96AX1	VPS33A	Vacuolar pro	9	5	7	5	596	67.6	6.96	19.64
Q8WUA7	TBC1D22A	TBC1 domai	10	5	5	5	517	59.1	5.97	16.14
P26572	MGAT1	Alpha-1,3-ma	11	5	6	5	445	50.8	9.16	17.15
P14207	FOLR2	Folate recep	15	4	5	4	255	29.3	7.53	16.31
P49407	ARRB1	Beta-arrestin	14	4	5	4	418	47	6.2	15.13
O95870	ABHD16A	Phosphatidyl	11	5	6	5	558	63.2	8.13	20.05
Q6P4E1	GOLM2	Protein GOL	13	4	5	4	436	49.5	5.29	20.53
Q6ZXV5	TMTC3	Protein O-ma	7	5	6	4	915	103.9	8.87	10.31
O00115	DNASE2	Deoxyribonu	16	7	9	7	360	39.6	8.05	18.43
Q9Y3B3	TMED7	Transmembr	18	4	5	4	224	25.2	6.89	17.51
Q6UX06	OLFM4	Olfactomedir	13	5	5	5	510	57.2	5.69	16.51

P16455	MGMT	Methylated-D	27	4	5	4	207	21.6	8.1	20.6
Q96SB3	PPP1R9B	Neurabin-2 C	7	5	8	5	817	89.3	4.97	26.15
O60879	DIAPH2	Protein diaph	7	8	9	7	1101	125.5	6.58	20.08
Q8NBN3	TMEM87A	Transmembr	12	6	6	6	555	63.4	6.74	17.87
P49840	GSK3A	Glycogen syı	10	4	6	2	483	50.9	8.75	14.07
P15529	CD46	Membrane co	10	4	8	4	392	43.7	6.74	23.17
Q9NR19	ACSS2	Acetyl-coenz	9	6	7	6	701	78.5	6.46	17.96
Q9BPW8	NIPSNAP1	Protein NipS	21	5	7	4	284	33.3	9.31	18.53
P80365	HSD11B2	11-beta-hydr	13	4	6	4	405	44.1	9.28	15.65
Q5JSL3	DOCK11	Dedicator of	3	6	7	5	2073	237.5	7.74	11.68
Q9UDY4	DNAJB4	DnaJ homolo	19	4	5	4	337	37.8	8.5	18.34
Q6UY14	ADAMTSL4	ADAMTS-like	6	5	5	5	1074	116.5	8.34	12.11
P15088	CPA3	Mast cell car	17	7	9	7	417	48.6	9	19.9
P61457	PCBD1	Pterin-4-alph	48	5	7	4	104	12	6.8	19.06
Q96E11	MRRF	Ribosome-re	19	3	4	3	262	29.3	9.79	15.01
Q9NP79	VTA1	Vacuolar pro	18	6	7	6	307	33.9	6.29	17.32
P18887	XRCC1	DNA repair p	10	5	5	5	633	69.5	6.28	14.25
P82979	SARNP	SAP domain-	24	5	6	5	210	23.7	6.42	16.26
O75663	TIPRL	TIP41-like pr	19	6	7	6	272	31.4	5.91	16.74
P51159	RAB27A	Ras-related p	21	4	6	4	221	24.9	5.22	22.93
Q15018	ABRAXAS2	BRISC comp	14	5	7	5	415	46.9	6.21	17.02
Q9HD26	GOPC	Golgi-associ	15	6	9	6	462	50.5	5.92	18.34
O15258	RER1	Protein RER	38	6	7	6	196	22.9	9.54	18.48
Q6IN85	PPP4R3A	Serine/threoı	9	6	6	6	833	95.3	4.94	13.84
Q8IXH7	NELFCD	Negative elo	11	6	6	6	590	66.2	5.1	12.17
O15321	TM9SF1	Transmembr	9	5	5	5	606	68.8	7.17	16.38
O60256	PRPSAP2	Phosphoribo	19	5	5	4	369	40.9	7.44	12.26
O00625	PIR	Pirin OS=Ho	17	5	7	5	290	32.1	6.92	18.89
Q9H773	DCTPP1	dCTP pyroph	24	4	7	4	170	18.7	5.03	13.82
O43772	SLC25A20	Mitochondria	15	5	7	5	301	32.9	9.41	18.95
Q9Y3A6	TMED5	Transmembr	15	4	7	4	229	26	4.84	21.49
P33151	CDH5	Cadherin-5 C	8	5	6	5	784	87.5	5.43	11.39
Q7Z5L7	PODN	Podocan OS	12	7	7	7	613	68.9	6.99	18.02

Q9H6S3	EPS8L2	Epidermal gr	10	6	6	6	715	80.6	6.84	12.4
Q6ICL3	TANGO2	Transport an	26	5	5	5	276	30.9	5.17	14.62
Q9P0J1	PDP1	[Pyruvate de	15	5	6	5	537	61	6.67	15.76
Q6IQ22	RAB12	Ras-related p	21	5	6	5	244	27.2	8.41	14.45
Q4V9L6	TMEM119	Transmembr	18	5	6	5	283	29.2	4.58	15.93
O14874	BCKDK	[3-methyl-2-c	13	4	4	4	412	46.3	8.82	14.35
Q8WTT2	NOC3L	Nucleolar co	9	5	5	5	800	92.5	9.17	15.49
Q9Y2V7	COG6	Conserved o	7	5	6	5	657	73.2	5.76	13.39
Q9BVC6	TMEM109	Transmembr	13	4	7	4	243	26.2	10.48	22.73
Q96EP5	DAZAP1	DAZ-associa	10	3	5	3	407	43.4	8.56	17.11
P25445	FAS	Tumor necro	11	5	6	5	335	37.7	7.94	14.07
P54760	EPHB4	Ephrin type-E	4	3	5	2	987	108.2	6.9	16.4
O43181	NDUFS4	NADH dehyc	26	4	5	4	175	20.1	10.3	16.51
Q9P270	SLAIN2	SLAIN motif-	8	2	3	2	581	62.5	9.45	13.65
Q92665	MRPS31	28S ribosom	16	6	8	6	395	45.3	9.29	17.38
Q9NNW7	TXNRD2	Thioredoxin r	10	5	6	4	524	56.5	7.5	16.42
P01034	CST3	Cystatin-C O	29	3	4	3	146	15.8	8.75	18.08
P83881	RPL36A	60S ribosom	26	5	8	2	106	12.4	10.58	20.1
O43676	NDUFB3	NADH dehyc	29	3	6	3	98	11.4	9.2	17.31
Q9NZW5	PALS2	Protein PALS	11	5	5	5	540	61.1	6.18	13.2
Q8N474	SFRP1	Secreted friz	14	4	5	4	314	35.4	8.85	17.22
Q08623	PUDP	Pseudouridir	23	4	7	4	228	25.2	5.31	19.87
P05090	APOD	Apolipoprote	22	4	7	4	189	21.3	5.15	22.66
Q14966	ZNF638	Zinc finger pr	3	6	6	6	1978	220.5	6.38	13.02
Q96GQ7	DDX27	Probable AT	8	5	5	5	796	89.8	9.28	13.63
P81605	DCD	Dermcidin O	35	5	7	5	110	11.3	6.54	18.5
Q9BUR5	APOO	MICOS comp	18	3	7	3	198	22.3	9.13	19.01
Q15061	WDR43	WD repeat-c	10	5	5	5	677	74.8	5.57	17.87
P82970	HMGN5	High mobility	24	5	5	5	282	31.5	4.55	15.74
Q16537	PPP2R5E	Serine/threor	9	4	6	3	467	54.7	6.95	16.56
Q4G0F5	VPS26B	Vacuolar pro	18	5	5	5	336	39.1	7.36	12.21
Q8N0W3	FCSK	L-fucose kina	6	4	4	4	1084	117.5	6.25	16.64
O14495	PLPP3	Phospholipid	21	5	5	5	311	35.1	9.14	14.4

A0A0J9YXX:	IGHV5-10-1	Immunoglobu	26	3	11	1	117	12.8	8.28	32.64
Q14684	RRP1B	Ribosomal R	6	3	4	3	758	84.4	9.76	13.74
Q9H6X2	ANTXR1	Anthrax toxir	7	4	6	4	564	62.7	7.61	18.28
P61927	RPL37	60S ribosom	42	7	12	7	97	11.1	11.74	25.21
Q13505	MTX1	Metaxin-1 OS	15	6	7	6	466	51.4	9.79	15.08
Q86SZ2	TRAPPC6B	Trafficking pı	24	4	7	4	158	18	8.68	12.44
Q9BW83	IFT27	Intraflagellar	20	3	5	3	186	20.5	5.41	16.84
Q14938	NFIX	Nuclear facto	8	4	5	2	502	55.1	8.68	13.72
Q9Y5K3	PCYT1B	Choline-phos	11	3	4	1	369	41.9	6.4	14.25
Q2M2I8	AAK1	AP2-associa	6	5	6	5	961	103.8	6.6	15.13
O95980	RECK	Reversion-in	6	6	8	6	971	106.4	6.74	15.22
Q8WUF5	PPP1R13L	RelA-associa	8	5	5	5	828	89	6.81	14.1
Q96RQ1	ERGIC2	Endoplasmic	19	5	6	5	377	42.5	6.77	16.26
Q9BT22	ALG1	Chitobiosyldi	13	5	5	5	464	52.5	7.23	13.78
O14737	PDCD5	Programmec	23	3	5	3	125	14.3	6.04	16.2
P15848	ARSB	Arylsulfatase	13	6	6	6	533	59.6	8.21	17.47
Q9NUQ2	AGPAT5	1-acyl-sn-gly	15	4	4	4	364	42	9.1	14.47
Q9H3H3	C11orf68	UPF0696 prc	18	5	5	5	292	31.4	6.32	14.79
Q01968	OCRL	Inositol polyp	8	6	7	6	901	104.1	6.55	14.67
Q15843	NEDD8	NEDD8 OS=	51	6	13	6	81	9.1	8.43	26.66
P98088	MUC5AC	Mucin-5AC C	2	5	7	3	5654	585.2	7.02	17.55
Q15785	TOMM34	Mitochondria	18	5	5	5	309	34.5	8.98	14.96
P30049	ATP5F1D	ATP synthas	14	2	9	2	168	17.5	5.49	32.84
Q9NWU2	GID8	Glucose-indu	28	5	6	5	228	26.7	4.97	15.49
Q9H7C9	AAMDC	Mth938 dom	36	5	6	5	122	13.3	8.46	15
A6NI72	NCF1B	Putative neu	13	5	5	5	391	44.8	9.13	15.27
Q8NB49	ATP11C	Phospholipid	4	5	6	4	1132	129.4	6.67	14.33
Q00403	GTF2B	Transcriptior	20	5	5	5	316	34.8	8.35	14.44
Q14314	FGL2	Fibroleukin C	9	5	7	4	439	50.2	7.39	18.74
Q8TDN6	BRIX1	Ribosome bi	17	7	8	7	353	41.4	9.92	19.53
P62913	RPL11	60S ribosom	19	3	6	3	178	20.2	9.6	15.84
P13686	ACP5	Tartrate-resis	15	4	6	4	325	36.6	8.7	15.49
P21283	ATP6V1C1	V-type protor	13	4	5	4	382	43.9	7.46	15.92

Q9BY50	SEC11C	Signal peptic	29	5	5	5	192	21.5	9.2	13.37
P19784	CSNK2A2	Casein kinas	17	4	4	3	350	41.2	8.56	14.71
P13284	IFI30	Gamma-inter	8	2	7	2	250	27.9	4.88	19.52
Q6P1N9	TATDN1	Deoxyribonu	18	5	5	5	297	33.6	6.96	13.33
Q13951	CBFB	Core-binding	29	5	6	5	182	21.5	6.6	15.91
Q9NYK5	MRPL39	39S ribosom	19	6	6	6	338	38.7	7.65	16.54
Q9UNK0	STX8	Syntaxin-8 O	27	4	5	4	236	26.9	4.98	17.71
Q13232	NME3	Nucleoside d	37	6	6	6	169	19	7.84	16.35
Q9UBP9	GULP1	PTB domain-	16	5	5	5	304	34.5	7.9	13.87
O95298	NDUFC2	NADH dehyc	32	6	10	6	119	14.2	8.98	21.67
O00533	CHL1	Neural cell a	7	6	6	6	1208	135	5.76	16.14
P41223	BUD31	Protein BUD	42	5	5	5	144	17	8.82	13.3
Q13190	STX5	Syntaxin-5 O	15	6	6	6	355	39.6	9.16	12.79
Q9Y4A5	TRRAP	Transformati	2	7	8	7	3859	437.3	8.19	18.33
Q15042	RAB3GAP1	Rab3 GTPas	7	6	7	6	981	110.5	5.55	12.44
P05161	ISG15	Ubiquitin-like	21	3	6	3	165	17.9	7.44	20.5
P62487	POLR2G	DNA-directe	35	5	5	5	172	19.3	5.54	15.61
Q9NRL3	STRN4	Striatin-4 OS	6	5	8	4	753	80.5	5.4	21.07
P49721	PSMB2	Proteasome	15	4	6	4	201	22.8	7.02	15.22
P17540	CKMT2	Creatine kina	11	3	4	2	419	47.5	8.19	14.05
Q9UNP9	PPIE	Peptidyl-prol	23	7	7	6	301	33.4	5.6	10.38
Q8IY21	DDX60	Probable AT	4	5	6	5	1712	197.7	7.59	8.41
Q9P032	NDUFAF4	NADH dehyc	25	4	6	4	175	20.3	8.82	13.76
Q8WUW1	BRK1	Protein BRIC	53	4	6	4	75	8.7	5.45	17.34
Q6N069	NAA16	N-alpha-acet	6	4	5	3	864	101.4	7.87	15.82
Q13564	NAE1	NEDD8-activ	10	5	6	5	534	60.2	5.4	17.37
Q13042	CDC16	Cell division	8	5	6	5	620	71.6	5.85	16.16
Q8TEA8	DTD1	D-aminoacyl-	19	4	5	4	209	23.4	8.24	17.05
Q969M7	UBE2F	NEDD8-conj	31	4	5	4	185	21.1	6.79	17.39
O15160	POLR1C	DNA-directe	14	4	5	4	346	39.2	5.5	14.22
Q03169	TNFAIP2	Tumor necro	10	5	6	5	654	72.6	6.46	15.54
O60831	PRAF2	PRA1 family	21	4	6	4	178	19.2	9.19	14.96
P62745	RHOB	Rho-related	19	3	5	2	196	22.1	5.24	19.82

Q9H7D7	WDR26	WD repeat-c	9	4	4	4	661	72.1	6.16	14.01
Q15052	ARHGEF6	Rho guanine	6	4	4	1	776	87.4	6.05	13.09
Q15599	SLC9A3R2	Na(+)/H(+) e	17	5	6	5	337	37.4	7.93	17.17
Q9HBI1	PARVB	Beta-parvin (	15	4	4	3	364	41.7	6.73	15.56
O95070	YIF1A	Protein YIF1.	11	3	5	3	293	32	8.95	14.26
P20674	COX5A	Cytochrome	27	5	6	5	150	16.8	6.79	20.49
Q9Y2U8	LEMD3	Inner nuclear	8	6	7	6	911	99.9	7.55	17.68
Q8WW22	DNAJA4	DnaJ homolo	12	4	6	4	397	44.8	7.59	15.69
P48454	PPP3CC	Serine/threor	11	5	5	2	512	58.1	6.98	13.94
P12694	BCKDHA	2-oxoisovale	13	5	5	5	445	50.4	8.27	18.6
P05543	SERPINA7	Thyroxine-bi	11	5	6	5	415	46.3	6.3	15.98
Q9H2U1	DHX36	ATP-depend	6	6	6	6	1008	114.7	7.68	15.11
O14548	COX7A2L	Cytochrome	48	3	4	3	114	12.6	9.42	14.95
Q9NZL4	HSPBP1	Hsp70-bindir	12	3	5	3	359	39.3	5.21	21.86
Q99933	BAG1	BAG family r	16	4	5	4	345	38.8	7.81	13.65
Q6P1M3	LLGL2	LLGL scribbl	7	6	6	6	1020	113.4	7.52	12.11
Q9HAB8	PPCS	Phosphopan	14	5	6	5	311	34	6.71	14.84
P02462	COL4A1	Collagen alp	2	3	6	3	1669	160.5	8.28	23.25
Q69YQ0	SPECC1L	Cytospin-A (	5	6	6	5	1117	124.5	5.76	14.77
Q03113	GNA12	Guanine nuc	9	4	7	2	381	44.3	9.83	19.49
Q9BV20	MRI1	Methylthiorib	12	4	6	4	369	39.1	6.3	10.92
P18433	PTPRA	Receptor-typ	8	5	5	5	802	90.7	6.65	12.74
Q9UJ41	RABGEF1	Rab5 GDP/G	9	4	5	4	491	56.9	7.02	16.32
Q96AY4	TTC28	Tetratricopep	3	6	7	6	2481	270.7	6.89	16.03
O15247	CLIC2	Chloride intra	23	3	3	3	247	28.3	5.59	14.17
Q9H857	NT5DC2	5'-nucleotida	10	5	5	5	520	60.7	6.77	14.69
Q9ULZ3	PYCARD	Apoptosis-as	22	3	4	3	195	21.6	6.34	13.19
Q8N6T3	ARFGAP1	ADP-ribosyla	17	5	5	5	406	44.6	5.66	14.39
Q8NF37	LPCAT1	Lysophospha	10	4	4	4	534	59.1	6.02	13.85
O43293	DAPK3	Death-assoc	12	5	5	5	454	52.5	6.89	16.8
Q9Y5Y2	NUBP2	Cytosolic Fe-	16	3	6	3	271	28.8	5.83	19.44
Q9NPJ3	ACOT13	Acyl-coenzyr	25	3	4	3	140	15	9.14	13.53
Q9NPF5	DMAP1	DNA methylt	11	4	4	4	467	53	9.5	15.14

Q96FZ7	CHMP6	Charged mul	20	4	5	4	201	23.5	5.31	16.07
Q12849	GRSF1	G-rich seque	13	6	7	6	480	53.1	6.19	15.12
Q7Z4H3	HDDC2	5'-deoxynucl	34	4	5	4	204	23.4	5.49	19.62
Q9BV57	ADI1	Acireductone	27	4	5	4	179	21.5	5.68	14.75
P09914	IFIT1	Interferon-inc	11	4	5	3	478	55.3	7.2	13.32
Q9H479	FN3K	Fructosamin	15	4	5	4	309	35.1	7.55	16.27
P11169	SLC2A3	Solute carrie	11	5	6	5	496	53.9	7.2	16.1
O75323	NIPSNAP2	Protein NipS	21	7	9	6	286	33.7	9.36	22.76
Q9Y2P8	RCL1	RNA 3'-termi	18	5	6	5	373	40.8	9.26	15.28
Q13033	STRN3	Striatin-3 OS	9	6	8	5	797	87.2	5.36	22.59
Q13155	AIMP2	Aminoacyl tF	20	4	5	4	320	35.3	8.22	15.34
O43583	DENR	Density-regu	28	5	5	5	198	22.1	5.3	14.08
Q96GM5	SMARCD1	SWI/SNF-rel	7	3	5	2	515	58.2	9.25	12.94
Q9Y6D6	ARFGEF1	Brefeldin A-i	4	8	9	4	1849	208.6	5.85	13.69
P47914	RPL29	60S ribosom	23	4	7	4	159	17.7	11.66	16.45
Q9Y2X7	GIT1	ARF GTPase	7	5	7	4	761	84.3	6.8	18.47
P61803	DAD1	Dolichyl-diph	27	3	5	3	113	12.5	7.08	15.64
Q9UJA5	TRMT6	tRNA (adenir	12	5	5	5	497	55.8	7.55	14.85
Q9UPN7	PPP6R1	Serine/threor	7	5	6	5	881	96.7	4.55	16.28
P06331	IGHV4-34	Immunoglobu	20	2	4	2	123	13.8	9.33	15.5
P20936	RASA1	Ras GTPase	7	7	7	7	1047	116.3	6.54	16.46
Q9Y2I8	WDR37	WD repeat-c	9	5	6	5	494	54.6	7.23	17.12
Q9H173	SIL1	Nucleotide e:	13	5	5	5	461	52.1	5.36	12.63
P09417	QDPR	Dihydropteric	17	4	5	4	244	25.8	7.37	15.69
Q9Y316	MEMO1	Protein MEM	15	3	4	3	297	33.7	7.14	13.88
Q9NXU5	ARL15	ADP-ribosyla	15	3	4	3	204	22.9	5.63	14.05
Q5VSL9	STRIP1	Striatin-intera	6	5	5	5	837	95.5	6.29	13.3
Q8WVT3	TRAPPC12	Trafficking pr	6	3	4	3	735	79.3	4.91	13.19
Q9NVE7	PANK4	4'-phosphopa	5	4	5	3	773	85.9	6.28	14.55
O95260	ATE1	Arginyl-tRNA	10	4	5	4	518	59.1	7.93	12.93
O94888	UBXN7	UBX domain	12	5	6	5	489	54.8	5.16	11.26
P14317	HCLS1	Hematopoiet	12	5	6	5	486	54	4.81	16.56
Q96LD4	TRIM47	E3 ubiquitin-|	7	5	6	4	638	69.5	6.44	17.99

Q13325	IFIT5	Interferon-inc	11	4	6	3	482	55.8	7.4	21
P45985	MAP2K4	Dual specific	13	4	5	4	399	44.3	8.07	11.77
P63098	PPP3R1	Calcineurin s	32	4	5	4	170	19.3	4.81	12.77
Q12974	PTP4A2	Protein tyros	26	4	5	1	167	19.1	8.37	12.14
Q14919	DRAP1	Dr1-associat	20	4	6	4	205	22.3	5.17	18.24
Q99447	PCYT2	Ethanolamine	12	3	4	3	389	43.8	6.92	12.78
P04921	GYPC	Glycophorin-	27	2	3	2	128	13.8	4.84	14.82
Q8IXM2	BAP18	Chromatin co	34	4	5	4	172	17.9	7.33	12.2
O14641	DVL2	Segment pol	8	5	5	2	736	78.9	6.02	11.36
P37198	NUP62	Nuclear pore	7	4	5	4	522	53.2	5.31	13.1
A0A0C4DH2	IGHV1-3	Immunoglobu	28	3	8	1	117	13	9.55	22.87
P61009	SPCS3	Signal peptic	16	3	7	3	180	20.3	8.62	20.52
P62760	VSNL1	Visinin-like p	32	6	7	6	191	22.1	5.15	12.27
Q5SRE7	PHYHD1	Phytanoyl-Co	13	3	4	3	291	32.4	6.32	15.17
Q8WUA2	PPIL4	Peptidyl-prol	11	4	4	4	492	57.2	5.92	14.66
O00515	LAD1	Ladinin-1 OS	7	5	5	5	517	57.1	9.67	13.75
Q9NY27	PPP4R2	Serine/threor	13	5	5	5	417	46.9	4.54	15.07
O60507	TPST1	Protein-tyros	11	3	4	3	370	42.2	9.09	13.61
Q16718	NDUFA5	NADH dehyc	22	2	5	2	116	13.5	5.99	17.12
Q9BYN8	MRPS26	28S ribosom	23	4	6	4	205	24.2	10.39	14.26
Q9Y657	SPIN1	Spindlin-1 OS	17	3	5	3	262	29.6	6.96	16.71
Q92997	DVL3	Segment pol	8	5	5	2	716	78	6.65	12.44
O60869	EDF1	Endothelial d	35	5	6	5	148	16.4	9.95	13.64
Q13201	MMRN1	Multimerin-1	5	5	7	5	1228	138	7.93	20.87
Q9BTZ2	DHRS4	Dehydrogena	14	5	6	5	278	29.5	8.56	16.3
Q9Y3E7	CHMP3	Charged mul	18	5	7	5	222	25.1	5.12	15.31
Q86W42	THOC6	THO comple	18	4	5	4	341	37.5	7.43	10.71
A4D1P6	WDR91	WD repeat-c	8	5	7	5	747	83.3	6.58	23.3
Q99707	MTR	Methionine s	5	5	5	5	1265	140.4	5.58	13.7
Q9BTD8	RBM42	RNA-binding	9	4	6	4	480	50.4	9.63	12.91
P27918	CFP	Properdin OS	10	4	7	4	469	51.2	7.9	19.83
Q5JSH3	WDR44	WD repeat-c	7	5	5	5	913	101.3	5.45	10.09
Q9NZT2	OGFR	Opioid growt	8	5	6	5	677	73.3	4.84	12.19

Q8N1B4	VPS52	Vacuolar pro	7	6	6	6	723	82.2	5.99	14.51
Q6UW02	CYP20A1	Cytochrome	11	4	5	4	462	52.4	6.21	11.35
O43639	NCK2	Cytoplasmic	12	5	6	4	380	42.9	6.95	6.79
Q14999	CUL7	Cullin-7 OS=	4	5	5	5	1698	191	5.87	12.22
O43414	ERI3	ERI1 exoribo	14	5	6	5	337	37.2	8.07	16.4
Q6SZW1	SARM1	NAD(+) hydr	7	4	5	4	724	79.3	6.55	15.22
P29034	S100A2	Protein S100	17	4	7	4	98	11.1	4.78	9.5
Q9H008	LHPP	Phospholysir	20	4	4	4	270	29.1	6.15	14.51
Q86WG5	SBF2	Myotubularin	4	5	5	4	1849	208.3	7.06	9.5
Q7RTS7	KRT74	Keratin, type	7	6	8	1	529	57.8	7.71	16.07
P26006	ITGA3	Integrin alpha	4	4	6	4	1051	116.5	6.77	13.46
Q93062	RBPMS	RNA-binding	21	3	6	3	196	21.8	8.07	14.59
Q7Z7K6	CENPV	Centromere	20	5	5	5	275	29.9	9.73	15.88
Q00534	CDK6	Cyclin-deper	13	3	4	3	326	36.9	6.46	13.9
O14981	BTAF1	TATA-bindin	3	5	5	5	1849	206.8	6.52	14.47
P41236	PPP1R2	Protein phos	26	4	4	4	205	23	4.74	10.6
O76070	SNCG	Gamma-synu	30	2	3	2	127	13.3	4.86	13.15
P24593	IGFBP5	Insulin-like g	18	5	6	5	272	30.6	8.21	17.12
Q15814	TBCC	Tubulin-spec	15	4	5	4	346	39.2	5.71	13.17
Q15287	RNPS1	RNA-binding	14	4	7	4	305	34.2	11.84	19.08
P61962	DCAF7	DDB1- and C	11	3	4	3	342	38.9	5.52	12.56
Q15397	PUM3	Pumilio home	10	7	7	7	648	73.5	9.64	10.34
P06703	S100A6	Protein S100	27	3	8	3	90	10.2	5.48	18.95
P59190	RAB15	Ras-related p	10	2	6	1	212	24.4	5.71	16.98
Q8WXI9	GATAD2B	Transcription	7	3	4	2	593	65.2	9.7	11.4
Q9GZY8	MFF	Mitochondria	17	4	4	4	342	38.4	8.95	14.66
P13928	ANXA8	Annexin A8 (	17	5	6	1	327	36.9	5.78	16.26
Q93096	PTP4A1	Protein tyros	25	4	5	1	173	19.8	8.97	11.75
Q96SU4	OSBPL9	Oxysterol-bir	7	5	6	5	736	83.1	6.18	12.82
Q9BW91	NUDT9	ADP-ribose p	14	5	5	5	350	39.1	8.22	14.16
P40818	USP8	Ubiquitin car	6	6	6	6	1118	127.4	8.51	13.23
Q712K3	UBE2R2	Ubiquitin-cor	18	4	5	4	238	27.1	4.42	14.78
Q15208	STK38	Serine/threor	7	3	4	3	465	54.2	7.15	13.62

Q9Y296	TRAPPC4	Trafficking pı	23	4	4	4	219	24.3	6.21	13.03
Q9HCE5	METTL14	N6-adenosin	11	4	5	4	456	52.1	6.21	13.46
Q8NBN7	RDH13	Retinol dehy	15	4	5	4	331	35.9	8.1	13.38
Q9BTE3	MCMBP	Mini-chromos	9	5	5	5	642	72.9	5.87	15.36
Q92608	DOCK2	Dedicator of	3	5	6	4	1830	211.8	6.87	11.29
Q9NRY6	PLSCR3	Phospholipid	14	3	4	3	295	31.6	6.65	13.62
Q07092	COL16A1	Collagen alpl	3	4	4	4	1604	157.7	7.84	15.81
P51151	RAB9A	Ras-related ı	22	4	5	4	201	22.8	5.47	12.58
Q9BY43	CHMP4A	Charged mul	25	4	4	4	222	25.1	4.7	15.8
P29353	SHC1	SHC-transfoı	12	5	5	5	583	62.8	6.44	14.64
Q9NW64	RBM22	Pre-mRNA-s	14	5	5	5	420	46.9	8.54	15.02
Q8IX01	SUGP2	SURP and G	6	6	7	6	1082	120.1	7.28	11.53
Q9Y6X3	MAU2	MAU2 chrom	9	3	4	3	613	69	7.25	17.43
Q6UXH1	CRELD2	Protein disulf	19	6	6	6	353	38.2	4.59	9.44
O14920	IKBKB	Inhibitor of nı	9	6	6	6	756	86.5	5.78	11.25
Q96NB2	SFXN2	Sideroflexin-:	17	5	5	5	322	36.2	9.41	13.36
Q9Y237	PIN4	Peptidyl-proly	32	5	6	5	131	13.8	9.77	15.39
Q9NR56	MBNL1	Muscleblind-	14	6	6	6	388	41.8	8.9	13.41
P43405	SYK	Tyrosine-pro	6	3	4	3	635	72	8.25	12.45
Q92979	EMG1	Ribosomal R	21	4	6	4	244	26.7	9.17	16.09
Q86U28	ISCA2	Iron-sulfur clı	23	3	4	3	154	16.5	5.25	10.52
Q92572	AP3S1	AP-3 comple	16	3	5	3	193	21.7	5.39	12.64
Q16775	HAGH	Hydroxyacylg	16	6	6	6	308	33.8	8.12	17.37
Q5EBL4	RILPL1	RILP-like prc	13	5	6	5	403	47.1	5.21	11.92
Q9NZD8	SPG21	Maspardin O	10	3	4	3	308	34.9	6.28	12.38
Q9H3U7	SMOC2	SPARC-relat	12	5	7	5	446	49.6	8.46	13.11
Q9ULL0	KIAA1210	Acrosomal p	4	6	6	6	1709	186.9	8.51	11.3
P36952	SERPINB5	Serpin B5 OS	12	5	6	5	375	42.1	6.05	13.88
O43818	RRP9	U3 small nuc	11	5	6	5	475	51.8	7.85	13.55
O00560	SDCBP	Syntenin-1 C	20	4	4	4	298	32.4	7.53	14.85
P98172	EFNB1	Ephrin-B1 OS	16	4	4	4	346	38	8.94	12.13
O43149	ZZEF1	Zinc finger Z	2	5	7	5	2961	330.9	5.95	11.77
Q06203	PPAT	Amidophospl	11	5	5	5	517	57.4	6.76	13.68

Q9UJY5	GGA1	ADP-ribosyla	11	6	7	5	639	70.3	5.29	14.27
Q96C01	FAM136A	Protein FAM	27	4	5	4	138	15.6	7.61	13.61
O43169	CYB5B	Cytochrome	23	4	6	4	150	16.7	4.97	17.17
Q9H0A8	COMMD4	COMM doma	18	4	7	4	199	21.8	7.31	18.45
P63172	DYNLT1	Dynein light	30	2	3	2	113	12.4	5.08	14.22
Q9NQT4	EXOSC5	Exosome co	15	2	3	2	235	25.2	7.59	13.6
P82933	MRPS9	28S ribosom	13	4	5	4	396	45.8	9.51	16.84
P34059	GALNS	N-acetylgala	12	5	5	4	522	58	6.74	16
Q9UKL0	RCOR1	REST corepr	14	5	6	5	485	53.3	7.03	13.57
P54753	EPHB3	Ephrin type-E	4	3	4	2	998	110.3	6.32	14.18
Q9H993	ARMT1	Damage-con	10	5	9	5	441	51.1	5.76	17.05
P48556	PSMD8	26S proteas	14	3	4	3	350	39.6	9.7	16.18
Q92692	NECTIN2	Nectin-2 OS=	7	5	9	5	538	57.7	4.82	15.12
Q96EY1	DNAJA3	DnaJ homolo	9	3	4	3	480	52.5	9.26	13.35
Q5W0V3	FHIP2A	FHF complex	6	4	4	4	765	86.5	5.29	12.43
Q9C075	KRT23	Keratin, type	12	4	9	2	422	48.1	6.54	20.76
Q92793	CREBBP	CREB-bindin	2	6	6	1	2442	265.2	8.53	10.98
Q9Y276	BCS1L	Mitochondria	15	5	6	5	419	47.5	8.5	15.66
Q10472	GALNT1	Polypeptide I	7	3	5	3	559	64.2	7.72	16.65
Q9Y5A9	YTHDF2	YTH domain-	9	5	7	2	579	62.3	8.79	19.97
Q9UBN7	HDAC6	Histone deac	4	5	6	5	1215	131.3	5.3	16.07
Q9HBL8	NMRAL1	NmrA-like fa	17	6	6	6	299	33.3	7.52	14.66
Q96RE7	NACC1	Nucleus accu	9	4	5	4	527	57.2	5.74	14.82
O75556	SCGB2A1	Mammaglobi	27	2	7	2	95	10.9	5.78	21.76
Q96S59	RANBP9	Ran-binding	9	4	4	4	729	77.8	6.79	11.89
Q9UQ03	CORO2B	Coronin-2B (	10	4	5	4	480	54.9	8.27	13.44
Q13641	TPBG	Trophoblast	7	3	5	3	420	46	6.83	12.31
Q92619	ARHGAP45	Rho GTPase	6	6	7	6	1136	124.5	6.1	16.44
Q92820	GGH	Gamma-glut	13	5	7	5	318	35.9	7.11	14.96
P37059	HSD17B2	17-beta-hydr	15	4	4	4	387	42.8	8.5	15.08
Q9NZQ3	NCKIPSD	NCK-interact	7	5	6	5	722	78.9	6.38	11.81
Q9H3G5	CPVL	Probable ser	11	5	5	5	476	54.1	5.62	12.82
Q9UHQ4	BCAP29	B-cell recept	16	5	7	5	241	28.3	9.54	18.56

Q6QNY0	BLOC1S3	Biogenesis o	30	5	5	5	202	21.2	5.15	13.27
Q96EM0	L3HYPDH	Trans-3-hydr	14	4	5	4	354	38.1	6.68	16.14
O14828	SCAMP3	Secretory ca	13	2	3	2	347	38.3	7.64	8.66
Q9NU22	MDN1	Midasin OS=	1	6	6	6	5596	632.4	5.68	13.31
Q9Y3D9	MRPS23	28S ribosom	16	3	5	3	190	21.8	8.9	10.38
Q96GG9	DCUN1D1	DCN1-like pr	22	6	9	6	259	30.1	5.34	18.59
Q6IAA8	LAMTOR1	Ragulator co	30	3	4	3	161	17.7	5.15	8.08
Q6VEQ5	WASH2P	WAS protein	12	4	4	4	465	50.3	5.71	13.43
Q96RU3	FNBP1	Formin-bindi	9	7	7	7	617	71.3	5.72	9.5
Q9BUL8	PDCD10	Programmed	22	5	6	5	212	24.7	8.19	15.79
Q9NVH6	TMLHE	Trimethyllysi	14	5	5	5	421	49.5	7.72	12.53
P04732	MT1E	Metallothione	33	2	4	1	61	6	7.96	13.17
Q9UK76	JPT1	Jupiter micro	30	4	4	4	154	16	5.6	10.79
Q9NP97	DYNLRB1	Dynein light	52	3	5	3	96	10.9	7.25	11.09
Q9NS15	LTBP3	Latent-transf	3	4	5	3	1303	139.3	6.07	11.94
Q9H3Q1	CDC42EP4	Cdc42 effect	14	3	3	3	356	38	5.19	13.77
Q9BXW7	HDHD5	Haloacid deh	14	4	4	4	423	46.3	8.13	13.8
O75122	CLASP2	CLIP-associa	3	3	4	2	1294	141	8.4	13.06
Q5VIR6	VPS53	Vacuolar pro	8	8	8	8	832	94.3	6.7	14.73
Q8NHH9	ATL2	Atlastin-2 OS	6	3	4	2	583	66.2	5.48	11.7
Q13637	RAB32	Ras-related p	25	6	6	6	225	25	6.54	14.09
Q9UNX4	WDR3	WD repeat-c	6	4	4	4	943	106	6.64	8.81
Q12841	FSTL1	Follistatin-rel	17	5	5	5	308	35	5.52	9.94
Q6STE5	SMARCD3	SWI/SNF-rel	7	3	5	1	483	55	9.35	11.83
Q13526	PIN1	Peptidyl-proly	42	5	5	5	163	18.2	8.82	8.71
O14656	TOR1A	Torsin-1A OS	14	4	6	4	332	37.8	6.99	19.7
Q7L7X3	TAOK1	Serine/threor	5	5	5	3	1001	116	7.65	14.55
Q30154	HLA-DRB5	HLA class II	13	3	4	1	266	30	6.93	14.63
Q99614	TTC1	Tetratricopep	17	3	5	3	292	33.5	4.84	10.35
Q8N436	CPXM2	Inactive carb	6	5	6	4	756	85.8	6.87	14.47
O00470	MEIS1	Homeobox p	13	4	5	4	390	43	6.33	16.5
Q96HQ2	CDKN2AIPN	CDKN2AIP N	42	4	4	4	116	13.2	5	12.97
L0R6Q1	SLC35A4	SLC35A4 up	45	4	4	4	103	11.1	8.1	14.18

Q9Y639	NPTN	Neuroplastin	9	3	6	3	398	44.4	7.99	18.28
Q68E01	INTS3	Integrator co	5	4	4	4	1043	118	5.8	12.17
P01743	IGHV1-46	Immunoglob	28	3	7	1	117	12.9	8.92	20.17
Q8WWI5	SLC44A1	Choline trans	8	4	6	4	657	73.3	8.6	14.14
Q9UNW1	MINPP1	Multiple inosi	10	4	5	4	487	55	7.81	14.78
Q7Z5K2	WAPL	Wings apart-	5	5	5	4	1190	132.9	5.44	11.97
Q9BZL6	PRKD2	Serine/threo	6	3	3	1	878	96.7	6.84	11.98
P17096	HMGA1	High mobility	38	4	5	4	107	11.7	10.32	14.07
P02775	PPBP	Platelet basi	27	3	4	3	128	13.9	8.79	14.32
Q53H82	LACTB2	Endoribonuc	14	4	4	4	288	32.8	6.8	13.5
Q86Y39	NDUFA11	NADH dehyc	27	2	4	2	141	14.8	8.72	17.46
Q8NHP8	PLBD2	Putative pho	8	4	5	4	589	65.4	6.8	16.71
Q14739	LBR	Delta(14)-ste	9	6	7	5	615	70.7	9.36	7.25
Q07507	DPT	Dermatopon	19	4	7	4	201	24	4.82	17.92
Q6WKZ4	RAB11FIP1	Rab11 family	3	4	6	4	1283	137.1	5.43	14.8
P53365	ARFIP2	Arfaptin-2 OS	16	5	5	5	341	37.8	6.04	9.64
Q8IYQ7	THNSL1	Threonine sy	7	4	4	4	743	83	7.12	12.31
Q32P44	EML3	Echinoderm	5	4	4	4	896	95.1	7.12	11.36
P09234	SNRPC	U1 small nuc	17	3	4	3	159	17.4	9.67	12.8
Q99569	PKP4	Plakophilin-4	4	5	8	5	1192	131.8	8.94	13.07
Q16585	SGCB	Beta-sarcogl	13	3	4	3	318	34.8	8.62	11.99
Q86YZ3	HRNR	Hornerin OS	8	4	6	4	2850	282.2	10.04	14.98
P49711	CTCF	Transcriptior	7	6	6	6	727	82.7	6.96	11
P59768	GNG2	Guanine nuc	42	2	3	2	71	7.8	7.99	11.26
Q96EK5	KIFBP	KIF-binding p	10	5	5	4	621	71.8	5.49	14.16
Q9HCN8	SDF2L1	Stromal cell-	24	4	4	4	221	23.6	7.03	15.47
P17152	TMEM11	Transmembr	19	4	6	4	192	21.5	7.36	14.61
Q9BSH5	HDHD3	Haloacid deh	22	3	3	3	251	28	6.71	13.41
P35542	SAA4	Serum amylo	33	5	7	5	130	14.7	9.07	14.37
Q9Y6G5	COMMD10	COMM doma	26	4	4	4	202	23	6.54	13.34
O95352	ATG7	Ubiquitin-like	7	5	5	5	703	77.9	6.24	10.83
P60604	UBE2G2	Ubiquitin-cor	34	4	5	4	165	18.6	4.7	7.86
Q6PD62	CTR9	RNA polyme	5	6	6	6	1173	133.4	6.77	13.47

Q9H2K8	TAOK3	Serine/threor	5	5	6	3	898	105.3	7.3	16.42
Q05048	CSTF1	Cleavage stir	12	5	7	5	431	48.3	6.58	11.46
Q96MW5	COG8	Conserved o	9	5	5	5	612	68.4	5.2	14.36
Q969Z3	MTARC2	Mitochondria	10	3	4	2	335	38	9.16	13.09
Q9NQP4	PFDN4	Prefoldin sub	37	4	5	4	134	15.3	4.53	14.63
O95749	GGPS1	Geranylgerai	12	4	6	4	300	34.8	6.14	12.88
Q6P1X6	C8orf82	UPF0598 pro	23	4	4	4	216	23.9	9.14	11.24
Q99685	MGLL	Monoglyceric	11	3	6	3	303	33.2	6.99	18.22
P06730	EIF4E	Eukaryotic tr	15	3	5	3	217	25.1	6.15	15.38
Q96SZ5	ADO	2-aminoetha	23	5	5	5	270	29.7	6.04	10.82
O43592	XPOT	Exportin-T O	4	3	4	3	962	109.9	5.39	13.43
O75882	ATRN	Attractin OS=	3	4	4	4	1429	158.4	7.31	13.4
Q5R3I4	TTC38	Tetratricopep	10	4	5	4	469	52.8	5.99	12.87
Q9UKM7	MAN1B1	Endoplasmic	7	4	4	4	699	79.5	7.72	12.89
P36404	ARL2	ADP-ribosyla	23	4	5	4	184	20.9	6.34	11.87
Q13107	USP4	Ubiquitin car	6	5	5	5	963	108.5	5.71	8.56
P35900	KRT20	Keratin, type	7	5	8	1	424	48.5	5.69	16.87
Q9NZN8	CNOT2	CCR4-NOT t	9	5	5	5	540	59.7	7.66	11.77
Q9NVM9	INTS13	Integrator co	8	5	5	5	706	80.2	6.7	11.52
Q14657	LAGE3	EKC/KEOPS	37	4	4	4	143	14.8	8.63	13.3
Q86WV6	STING1	Stimulator of	18	5	5	5	379	42.2	7.05	13.1
O14972	VPS26C	Vacuolar pro	14	3	3	3	297	33	7.68	12.69
Q12846	STX4	Syntaxin-4 O	18	5	6	5	297	34.2	6.28	15.32
O43556	SGCE	Epsilon-sarc	9	3	4	3	437	49.8	6.55	11.55
Q9UNL2	SSR3	Translocon-a	16	3	5	3	185	21.1	9.61	17.47
Q6P1A2	LPCAT3	Lysophospho	8	4	4	4	487	56	8.69	10.76
A0A0A0MS1	IGHV3-49	Immunoglobu	37	4	5	4	119	13	8.62	16.7
Q13884	SNTB1	Beta-1-syntro	6	4	12	2	538	58	8.63	18.68
P51809	VAMP7	Vesicle-asso	16	4	5	4	220	24.9	8.6	12.34
Q9Y2E5	MAN2B2	Epididymis-s	6	5	5	5	1009	113.9	7.24	13.24
O15117	FYB1	FYN-binding	7	5	5	5	783	85.3	6.48	10.76
Q9C0E2	XPO4	Exportin-4 O	4	5	5	5	1151	130.1	5.05	13.3
Q9NX58	LYAR	Cell growth-r	11	4	4	4	379	43.6	9.57	12.38

O75175	CNOT3	CCR4-NOT t	5	4	4	4	753	81.8	6.2	12.31
P35250	RFC2	Replication fa	16	4	6	4	354	39.1	6.44	14.66
O43292	GPAA1	Glycosylphos	10	5	5	5	621	67.6	8.06	15.15
P82932	MRPS6	28S ribosom	23	3	5	3	125	14.2	9.26	13.5
Q8WUZ0	BCL7C	B-cell CLL/ly	18	3	4	3	217	23.5	5.2	10.54
Q8N7H5	PAF1	RNA polyme	10	4	4	4	531	59.9	4.63	11.32
Q9NR50	EIF2B3	Translation in	9	3	4	3	452	50.2	6.47	10.67
P62256	UBE2H	Ubiquitin-con	22	4	5	4	183	20.6	4.67	13.72
Q8N5N7	MRPL50	39S ribosom	23	3	4	3	158	18.3	7.88	11.45
Q8IZH2	XRN1	5'-3' exoribon	3	6	6	6	1706	194	7.21	12.93
Q9H910	JPT2	Jupiter micro	21	4	5	4	190	20.1	9.26	9.17
Q9UQB8	BAIAP2	Brain-specifi	8	4	4	4	552	60.8	8.9	11.82
Q8N357	SLC35F6	Solute carrie	9	3	10	3	371	40.2	6.93	26.4
Q8IWA0	WDR75	WD repeat-c	7	4	4	4	830	94.4	5.96	11.27
Q8WTS6	SETD7	Histone-lysin	9	3	6	3	366	40.7	4.63	19.15
Q8NG11	TSPAN14	Tetraspanin-	11	3	4	3	270	30.7	6.84	11
Q8N0X4	CLYBL	Citramalyl-Co	16	5	5	5	340	37.3	8.73	15.8
P07197	NEFM	Neurofilamen	3	3	8	2	916	102.4	4.91	13.95
P29323	EPHB2	Ephrin type-E	4	3	3	3	1055	117.4	6.55	13.38
Q8TBA6	GOLGA5	Golgin subfa	6	4	4	4	731	83	5.83	9.75
P42785	PRCP	Lysosomal P	7	4	5	4	496	55.8	7.21	12.36
Q15700	DLG2	Disks large h	4	3	4	1	870	97.5	6.47	8.92
Q9HD89	RETN	Resistin OS=	38	3	3	3	108	11.4	6.86	11.49
Q96IX5	ATP5MK	ATP synthas	45	2	4	2	58	6.5	9.76	12.59
Q9H061	TMEM126A	Transmembr	15	2	4	2	195	21.5	9.26	9.97
Q13361	MFAP5	Microfibrillar-	16	4	6	3	173	19.6	5.94	15.74
O95249	GOSR1	Golgi SNAP	20	6	7	6	250	28.6	9.42	12.42
Q8N4H5	TOMM5	Mitochondria	63	6	8	6	51	6	9.7	18.55
P02100	HBE1	Hemoglobin	18	3	8	1	147	16.2	8.63	23.03
P19388	POLR2E	DNA-directe	23	4	4	4	210	24.5	5.95	13.64
Q5HYK3	COQ5	2-methoxy-6-	12	4	5	4	327	37.1	6.95	15.34
Q4J6C6	PREPL	Prolyl endop	6	4	5	4	727	83.9	6.38	10.62
Q9NW13	RBM28	RNA-binding	7	6	7	6	759	85.7	9.22	7.34

P25686	DNAJB2	DnaJ homolo	18	5	6	4	324	35.6	5.95	7.54
P29083	GTF2E1	General tran	10	3	4	3	439	49.4	4.82	10.83
P49137	MAPKAPK2	MAP kinase-	9	3	5	2	400	45.5	8.68	11.18
Q14692	BMS1	Ribosome bi	4	5	5	5	1282	145.7	6.44	9.5
Q9Y6W3	CAPN7	Calpain-7 OS	6	4	5	4	813	92.6	7.65	13.79
Q9NZZ3	CHMP5	Charged mul	21	4	6	4	219	24.6	4.83	12.25
Q15007	WTAP	Pre-mRNA-s	12	4	4	4	396	44.2	5.19	12.14
Q9BTY7	HGH1	Protein HGH	14	4	5	4	390	42.1	4.81	17.86
P15502	ELN	Elastin OS=H	10	3	4	3	786	68.4	10.4	17.04
Q9P003	CNIH4	Protein corni	14	1	4	1	139	16.1	6.65	18.53
O43427	FIBP	Acidic fibrobl	14	4	4	4	364	41.9	6.48	12.95
Q969E4	TCEAL3	Transcription	29	5	6	1	200	22.5	4.92	15.53
P78356	PIP4K2B	Phosphatidyl	12	5	5	3	416	47.3	7.33	6.73
Q8TAF3	WDR48	WD repeat-c	6	5	5	5	677	76.2	7.03	11.71
Q9NP92	MRPS30	39S ribosom	15	4	4	4	439	50.3	7.97	11.94
Q8IVH4	MMAA	Methylmalon	13	5	6	5	418	46.5	9.29	12.44
Q8IUR7	ARMC8	Armadillo rep	9	5	5	5	673	75.5	6.73	12.34
Q96IY4	CPB2	Carboxypept	6	3	5	3	423	48.4	7.71	15.61
P34741	SDC2	Syndecan-2	17	3	4	3	201	22.1	4.86	10.7
P61966	AP1S1	AP-1 comple	27	4	6	3	158	18.7	5.73	14.9
Q9Y6Q2	STON1	Stonin-1 OS=	7	4	4	4	735	83.1	6.2	11.2
Q08495	DMTN	Dematin OS=	12	4	4	4	405	45.5	8.88	8.14
O94992	HEXIM1	Protein HEXI	15	4	4	4	359	40.6	4.89	13.55
Q9NRZ7	AGPAT3	1-acyl-sn-gly	14	5	5	5	376	43.4	8.72	11.51
Q7LG56	RRM2B	Ribonucleosi	9	3	4	3	351	40.7	4.97	12.72
P30405	PPIF	Peptidyl-proly	15	4	5	3	207	22	9.38	12.99
Q96K17	BTF3L4	Transcription	25	5	7	4	158	17.3	6.35	9.38
Q9UKN8	GTF3C4	General tran	7	4	4	4	822	91.9	6.65	9.78
Q9UEE9	CFDP1	Craniofacial	16	5	5	5	299	33.6	4.81	8.03
Q9Y5Q8	GTF3C5	General tran	15	6	7	6	519	59.5	6.9	11.9
P82094	TMF1	TATA eleme	4	4	4	4	1093	122.8	4.92	12.29
Q9UI30	TRMT112	Multifunction	22	2	3	2	125	14.2	5.26	11.84
P56202	CTSW	Cathepsin W	14	3	3	3	376	42.1	7.42	13.02

Q09472	EP300	Histone acet	3	6	6	1	2414	264	8.5	6.98
Q16832	DDR2	Discoidin dor	7	4	5	4	855	96.7	5.36	13.03
Q04759	PRKCQ	Protein kinas	7	5	5	4	706	81.8	7.61	9.03
Q7Z2Z2	EFL1	Elongation fa	5	5	6	5	1120	125.4	5.91	11.59
P49441	INPP1	Inositol polyp	8	3	5	3	399	44	5.26	12.55
Q9BTE6	AARSD1	Alanyl-tRNA	12	4	6	4	412	45.5	6.42	15.16
Q0VDF9	HSPA14	Heat shock 7	8	4	5	4	509	54.8	5.59	11.82
Q9UGM5	FETUB	Fetuin-B OS=	11	4	6	4	382	42	6.83	7.93
A6NDG6	PGP	Glycerol-3-pl	14	3	3	3	321	34	6.14	11.64
Q8TEW0	PARD3	Partitioning c	3	5	5	5	1356	151.3	7.68	13.15
Q9BRG1	VPS25	Vacuolar pro	18	3	4	3	176	20.7	6.34	14.4
Q12800	TFCP2	Alpha-globin	9	3	4	1	502	57.2	5.8	16.34
O75146	HIP1R	Huntingtin-in	4	3	4	3	1068	119.3	6.67	14.46
Q8IXT5	RBM12B	RNA-binding	6	6	6	6	1001	118	6.81	13.14
Q16740	CLPP	ATP-depend	13	3	4	3	277	30.2	8.09	12.78
O96008	TOMM40	Mitochondria	17	3	4	3	361	37.9	7.25	15.05
Q04721	NOTCH2	Neurogenic l	2	4	5	4	2471	265.2	5.14	15.23
O95168	NDUFB4	NADH dehyc	22	2	3	2	129	15.2	9.85	12.11
Q92673	SORL1	Sortilin-relate	3	4	4	4	2214	248.3	5.55	8.67
O14879	IFIT3	Interferon-inc	9	4	4	4	490	56	5.2	14.58
Q15139	PRKD1	Serine/threoi	5	3	3	1	912	101.6	6.62	11.44
Q15628	TRADD	Tumor necro	15	3	3	3	312	34.2	6.27	11.85
P23470	PTPRG	Receptor-typ	4	5	5	5	1445	161.9	6.42	8.28
P48960	ADGRE5	Adhesion G	5	3	4	3	835	91.8	6.87	17.48
Q6NXG1	ESRP1	Epithelial spl	7	4	4	4	681	75.5	6.68	12.24
Q8N4T8	CBR4	3-oxoacyl-[ac	21	4	5	4	237	25.3	9.33	9.82
O75190	DNAJB6	DnaJ homolc	13	4	5	3	326	36.1	9.16	9.86
Q86U44	METTL3	N6-adenosin	9	4	4	4	580	64.4	6.42	9.58
P10620	MGST1	Microsomal g	17	3	5	3	155	17.6	9.39	12.28
Q8TED0	UTP15	U3 small nuc	8	4	5	4	518	58.4	9.11	11.5
O00401	WASL	Actin nucleat	13	5	6	5	505	54.8	7.93	12.85
Q99442	SEC62	Translocatioi	7	3	5	3	399	45.8	7.12	11.26
A0A0C4DH2	IGKV3D-20	Immunoglobu	22	2	5	1	116	12.5	4.59	21.09

Q7Z5L9	IRF2BP2	Interferon re	7	4	4	3	587	61	8.69	12.17
Q9NRW3	APOBEC3C	DNA dC->dU	30	4	5	4	190	22.8	7.59	9.26
Q9NPA8	ENY2	Transcription	40	5	5	5	101	11.5	9.33	13.96
Q9GZN8	C20orf27	UPF0687 pro	27	3	4	3	174	19.3	6.84	16.79
Q16719	KYNU	Kynureninas	7	3	4	3	465	52.3	7.03	11.3
A0A0B4J1X5	IGHV3-74	Immunoglobu	25	3	7	1	117	12.8	8.66	15.65
Q9NYH9	UTP6	U3 small nuc	8	5	5	5	597	70.1	7.28	12.79
Q02153	GUCY1B1	Guanylate cy	6	4	4	4	619	70.5	5.35	9.79
Q8WYA0	IFT81	Intraflagellar	7	5	5	5	676	79.7	8.82	13.15
Q9Y6M1	IGF2BP2	Insulin-like g	8	4	4	4	599	66.1	8.46	13.77
O00566	MPHOSPH1	U3 small nuc	8	3	4	3	681	78.8	4.86	11.62
Q96FS4	SIPA1	Signal-induc	6	4	4	4	1042	112.1	6.6	11.11
Q9ULU4	ZMYND8	Protein kinas	4	5	6	5	1186	131.6	7.2	9.92
O60476	MAN1A2	Mannosyl-oli	6	4	6	3	641	73	7.61	12.26
Q9GZS1	POLR1E	DNA-directe	9	3	4	3	419	47.2	8.94	12.62
Q96KA5	CLPTM1L	Lipid scramb	11	5	5	5	538	62.2	8.56	12.29
P02724	GYPA	Glycophorin-	21	2	3	2	150	16.3	5.47	13.66
P10588	NR2F6	Nuclear rece	11	5	5	3	404	43	7.78	13.09
O43823	AKAP8	A-kinase anc	7	4	4	4	692	76.1	5.15	11.99
Q96RQ9	IL4I1	L-amino-acid	8	5	5	5	567	62.8	8.68	10.32
O60925	PFDN1	Prefoldin sub	29	4	7	4	122	14.2	6.81	18.15
P53611	RABGGTB	Geranylgera	13	5	6	5	331	36.9	5.03	13.74
Q9UJV9	DDX41	Probable AT	7	4	6	4	622	69.8	6.84	14.36
Q9Y2R4	DDX52	Probable AT	8	4	4	4	599	67.4	9.67	7.12
Q9Y5V3	MAGED1	Melanoma-as	4	3	7	2	778	86.1	5.83	21.99
Q9NZD4	AHSP	Alpha-hemog	30	3	4	3	102	11.8	5	13.82
P19387	POLR2C	DNA-directe	14	4	4	4	275	31.4	4.92	8.86
Q969G5	CAVIN3	Caveolae-as	16	4	6	4	261	27.7	6.43	20.43
O75506	HSBP1	Heat shock f	72	3	3	3	76	8.5	4.36	8.85
P01706	IGLV2-11	Immunoglobu	13	2	8	1	119	12.6	7.24	19.33
Q86YS7	C2CD5	C2 domain-c	5	4	4	4	1000	110.4	5.69	8.5
P11441	UBL4A	Ubiquitin-like	26	4	4	4	157	17.8	8.66	12.69
O60613	SELENOF	Selenoprotei	22	3	6	3	165	18.1	5.03	17.73

Q9NYL4	FKBP11	Peptidyl-proly	14	4	4	4	201	22.2	9.39	9.96
P13727	PRG2	Bone marrov	23	4	4	4	222	25.2	6.76	13.12
Q9NX24	NHP2	H/ACA ribon	25	3	3	3	153	17.2	8.22	10.4
Q15080	NCF4	Neutrophil cy	15	4	6	4	339	39	6.83	12.76
C9JLW8	MCRIP1	Mapk-regula	32	3	4	3	97	10.9	9.41	11.04
O60645	EXOC3	Exocyst com	6	5	5	5	745	85.5	6.29	12.51
Q8IU85	CAMK1D	Calcium/caln	12	4	4	2	385	42.9	7.21	10.21
Q02487	DSC2	Desmocollin-	5	4	4	4	901	99.9	5.34	11.22
Q9Y3Q3	TMED3	Transmembr	20	5	5	5	217	24.8	5.6	13.44
P06493	CDK1	Cyclin-deper	13	3	3	2	297	34.1	8.4	10.4
P35080	PFN2	Profilin-2 OS	20	2	3	2	140	15	6.99	10.86
Q6PJG6	BRAT1	BRCA1-asso	5	3	3	3	821	88.1	5.27	12.35
Q9H078	CLPB	Caseinolytic	6	4	6	4	707	78.7	9.01	17.05
P50452	SERPINB8	Serpin B8 OS	12	4	5	2	374	42.7	5.57	18.38
Q9UP95	SLC12A4	Solute carrie	3	4	5	3	1085	120.6	6.44	13.76
Q9Y6N6	LAMC3	Laminin subu	2	2	3	2	1575	171.1	6.58	10.66
P14406	COX7A2	Cytochrome	28	2	5	2	83	9.4	9.76	9.71
Q9Y259	CHKB	Choline/etha	12	4	4	4	395	45.2	5.49	13.3
Q5T653	MRPL2	39S ribosom	15	3	3	3	305	33.3	11.3	8.72
P50151	GNG10	Guanine nuc	26	2	4	2	68	7.2	7.85	16.83
Q8N3D4	EHBP1L1	EH domain-b	3	4	4	4	1523	161.8	4.83	6.31
P23458	JAK1	Tyrosine-pro	5	5	5	5	1154	133.2	7.55	8.75
Q14642	INPP5A	Inositol polyp	10	5	5	5	412	47.8	7.03	8.09
P31270	HOXA11	Homeobox p	11	3	4	3	313	34.5	8.7	11.33
Q8WUN7	UBTD2	Ubiquitin dor	14	3	3	3	234	26.2	5.67	10.72
O95319	CELF2	CUGBP Elav	8	4	5	3	508	54.3	8.76	11.77
Q96IZ0	PAWR	PRKC apopt	12	3	3	3	340	36.5	5.41	11.22
O94985	CLSTN1	Calsyntenin-	5	5	5	5	981	109.7	4.91	10.79
Q92609	TBC1D5	TBC1 domai	5	3	5	3	795	88.9	6.54	10.96
Q9NZP8	C1RL	Complement	9	4	4	3	487	53.5	7.2	12.98
Q03701	CEBPZ	CCAAT/enha	5	4	4	4	1054	120.9	5.94	9.03
Q99496	RNF2	E3 ubiquitin-	15	4	4	2	336	37.6	6.84	11.38
Q92890	UFD1	Ubiquitin rec	19	4	5	4	307	34.5	6.7	18.65

Q8N335	GPD1L	Glycerol-3-pl	9	3	4	3	351	38.4	7.02	12.31
P57764	GSDMD	Gasdermin-D	7	3	4	3	484	52.8	5.08	11.46
P10109	FDX1	Adrenodoxin	14	3	6	3	184	19.4	5.83	11.85
Q7Z2W9	MRPL21	39S ribosom	25	3	4	3	205	22.8	9.89	15.56
Q9NSC7	ST6GALNAC	Alpha-N-acet	7	5	5	5	600	68.5	9.92	12.41
Q5H9L2	TCEAL5	Transcription	21	5	6	1	206	23.3	4.81	14.37
Q9NUL3	STAU2	Double-stran	8	4	5	3	570	62.6	9.61	18.24
Q8WU76	SCFD2	Sec1 family (	5	4	4	4	684	75.1	6.68	12.26
Q5T0F9	CC2D1B	Coiled-coil ar	4	4	6	4	858	94.2	5.26	11.79
Q9NWB6	ARGLU1	Arginine and	11	5	5	5	273	33.2	10.35	11.49
Q8NEW0	SLC30A7	Zinc transpor	11	4	4	4	376	41.6	6.95	11.8
Q06265	EXOSC9	Exosome cor	7	3	4	3	439	48.9	5.29	11.25
P11802	CDK4	Cyclin-deper	14	3	3	3	303	33.7	7.01	12.8
O95985	TOP3B	DNA topoiso	6	4	4	4	862	96.6	8.12	6.05
Q9HD42	CHMP1A	Charged mul	20	5	5	5	196	21.7	8.06	13.81
Q9Y6U3	SCIN	Scinderin OS	7	4	4	4	715	80.4	5.71	10.34
Q9UNA1	ARHGAP26	Rho GTPase	5	5	6	4	814	92.2	6.64	11.38
Q9H7B4	SMYD3	Histone-lysin	8	2	3	2	428	49.1	7.25	12.09
Q92600	CNOT9	CCR4-NOT t	11	3	4	3	299	33.6	8.03	11.82
Q6EMK4	VASN	Vasorin OS=	7	4	4	4	673	71.7	7.39	11.09
Q96D46	NMD3	60S ribosom	7	3	4	3	503	57.6	7.14	10.17
P27658	COL8A1	Collagen alpl	7	4	5	4	744	73.3	9.61	12.83
P0DMN0	SULT1A4	Sulfotransfer	14	4	4	1	295	34.2	6.01	12.51
P51553	IDH3G	Isocitrate del	9	3	4	3	393	42.8	8.5	9.05
Q96RT1	ERBIN	Erbin OS=Hc	3	4	4	4	1412	158.2	5.5	9.1
Q9BV79	MECR	Enoyl-[acyl-c	8	3	4	3	373	40.4	8.76	11
Q96JH7	VCPIP1	Deubiquitina	4	5	5	5	1222	134.2	7.2	12.12
Q8NE01	CNNM3	Metal transp	6	4	4	3	707	76.1	6.09	10.31
Q8NFQ8	TOR1AIP2	Torsin-1A-int	11	4	5	3	470	51.2	4.96	9.04
P63220	RPS21	40S ribosom	47	4	8	4	83	9.1	8.5	19.08
P01700	IGLV1-47	Immunoglobu	25	2	3	2	117	12.3	5.91	11.74
P52758	RIDA	2-iminobutan	26	2	3	2	137	14.5	8.68	9.78
Q3MHD2	LSM12	Protein LSM1	18	3	3	3	195	21.7	7.74	11.85

Q16563	SYPL1	Synaptophys	10	2	4	2	259	28.5	8.43	15.16
P15153	RAC2	Ras-related (	22	5	7	2	192	21.4	7.61	12.77
Q96S97	MYADM	Myeloid-asso	10	2	4	2	322	35.3	8.15	17.04
Q9Y2H0	DLGAP4	Disks large-a	4	4	4	4	992	107.9	7.08	10.67
Q9H9Q4	NHEJ1	Non-homolog	14	3	4	3	299	33.3	5.97	12.63
P82675	MRPS5	28S ribosom	11	5	5	5	430	48	9.92	9.67
Q9BVM2	DPCD	Protein DPC	25	3	3	3	203	23.2	9.03	7.49
Q9Y4P1	ATG4B	Cysteine pro	13	3	3	3	393	44.3	5.07	6.83
Q9UL26	RAB22A	Ras-related p	10	2	4	1	194	21.8	8.15	8.84
Q86YP4	GATAD2A	Transcription	5	3	4	2	633	68	9.94	11.11
Q9ULX6	AKAP8L	A-kinase anc	5	3	4	3	646	71.6	5.05	12.5
P20702	ITGAX	Integrin alph	2	3	4	2	1163	127.7	6.64	10.35
Q8IVF7	FMNL3	Formin-like p	4	5	5	1	1028	117.1	6.65	13.07
Q9UNN5	FAF1	FAS-associa	9	6	6	6	650	73.9	4.88	12.89
P09132	SRP19	Signal recog	26	2	3	2	144	16.1	9.85	12.34
O60462	NRP2	Neuropilin-2	5	4	4	4	931	104.8	5.17	12.42
Q14653	IRF3	Interferon re	10	3	3	3	427	47.2	5.34	10.79
P29372	MPG	DNA-3-meth	14	3	3	3	298	32.8	9.57	10.22
O76074	PDE5A	cGMP-specif	5	4	4	4	875	99.9	6.09	11.52
Q9Y5P4	CERT1	Ceramide tra	7	5	5	5	624	70.8	5.48	12.75
P23434	GCSH	Glycine clea	17	2	3	2	173	18.9	4.88	10.65
Q8IXQ6	PARP9	Protein mon	5	5	7	5	854	96.3	7.91	14.16
Q9HA77	CARS2	Probable cys	9	6	6	6	564	62.2	8.34	13.51
P40855	PEX19	Peroxisomal	9	3	5	3	299	32.8	4.34	10.78
Q8N183	NDUFAF2	NADH dehyd	23	4	4	4	169	19.8	8.97	12.51
Q9BZ67	FRMD8	FERM doma	10	3	4	3	464	51.2	6.23	12.81
P80297	MT1X	Metallothione	33	2	4	1	61	6.1	7.96	10.87
Q9Y6X4	FAM169A	Soluble lami	7	3	3	3	670	74.9	4.6	10.71
Q99470	SDF2	Stromal cell-	21	5	7	5	211	23	7.33	13.15
Q86VR2	RETREG3	Reticulophag	13	4	4	4	466	51.4	4.97	9.67
Q8IUZ5	PHYKPL	5-phosphohy	9	4	4	4	450	49.7	6.76	11.43
Q92575	UBXN4	UBX domain	8	4	4	4	508	56.7	6.38	11.12
P48509	CD151	CD151 antig	13	4	6	4	253	28.3	7.47	15.59

Q6UXN9	WDR82	WD repeat-c	15	5	6	5	313	35.1	7.69	4.79
Q9BQA9	CYBC1	Cytochrome	19	4	5	4	187	20.8	6.8	13.58
Q8TAE8	GADD45GIP	Growth arres	18	3	4	3	222	25.4	10.02	16.25
Q05519	SRSF11	Serine/argini	9	4	5	4	484	53.5	10.52	9.94
Q96IV0	NGLY1	Peptide-N(4)	5	3	4	3	654	74.3	6.89	12.25
O75607	NPM3	Nucleoplasm	29	4	4	4	178	19.3	4.63	13.66
Q96I15	SCLY	Selenocystei	12	3	3	3	445	48.1	7.12	11.96
Q96G23	CERS2	Ceramide sy	11	4	4	4	380	44.8	8.98	12.46
Q9H1A4	ANAPC1	Anaphase-pr	2	4	5	4	1944	216.4	6.3	8.48
O43617	TRAPPC3	Trafficking pr	21	4	4	4	180	20.3	4.96	10.56
O15533	TAPBP	Tapasin OS=	7	3	4	3	448	47.5	6.99	8.72
Q96PZ0	PUS7	Pseudouridyl	6	3	3	3	661	75	6.37	10.11
Q5UCC4	EMC10	ER membrar	15	5	5	5	262	27.3	6.13	10.79
Q8IWA4	MFN1	Mitofusin-1 C	6	4	4	4	741	84.1	6.33	9.27
O15229	KMO	Kynurenine 3	8	4	4	4	486	55.8	9.03	8.67
Q9NUI1	DECR2	Peroxisomal	14	4	4	4	292	30.8	9.22	11.51
Q8N5M9	JAGN1	Protein jagur	13	2	3	2	183	21.1	9.73	12.49
Q9UPY8	MAPRE3	Microtubule-a	13	4	5	3	281	32	5.54	11.07
O75676	RPS6KA4	Ribosomal pr	6	4	4	2	772	85.6	8.28	10.75
P51965	UBE2E1	Ubiquitin-cor	13	3	4	1	193	21.4	8.53	10.81
P48634	PRRC2A	Protein PRR	3	5	5	5	2157	228.7	9.45	7.66
P46199	MTIF2	Translation ir	7	4	4	4	727	81.3	7.15	12.42
Q13796	SHROOM2	Protein Shro	3	5	5	4	1616	176.3	7.09	9.36
P01906	HLA-DQA2	HLA class II	11	2	3	1	255	28	4.88	11.94
O15514	POLR2D	DNA-directe	29	3	3	3	142	16.3	4.79	10.74
P49841	GSK3B	Glycogen syr	10	3	4	1	420	46.7	8.78	9.22
Q86VY4	TSPYL5	Testis-specif	12	3	3	3	417	45.1	9.54	8.31
Q92504	SLC39A7	Zinc transpor	7	4	4	4	469	50.1	6.87	7.66
Q96BP3	PPWD1	Peptidylproly	6	4	4	4	646	73.5	7.15	8.61
Q96MU7	YTHDC1	YTH domain-	8	7	7	7	727	84.6	6.23	13.87
Q9Y618	NCOR2	Nuclear rece	3	4	4	4	2514	273.5	7.59	9.38
P22692	IGFBP4	Insulin-like g	16	4	5	4	258	27.9	7.15	15.73
Q9Y4Z0	LSM4	U6 snRNA-a	21	3	6	3	139	15.3	9.99	15.32

Q13488	TCIRG1	V-type protor	6	5	5	5	830	92.9	7.12	11.93
P27449	ATP6V0C	V-type protor	16	2	6	2	155	15.7	8.44	22.02
Q9UBL3	ASH2L	Set1/Ash2 hi	7	4	5	4	628	68.7	5.69	15.47
P57735	RAB25	Ras-related p	17	4	6	3	213	23.5	5.96	17.46
P18858	LIG1	DNA ligase 1	5	4	4	3	919	101.7	5.62	10.83
Q969Z0	TBRG4	FAST kinase	7	5	5	5	631	70.7	7.42	11.86
O43402	EMC8	ER membrar	20	3	3	3	210	23.8	6.4	10.05
O95983	MBD3	Methyl-CpG-	14	4	4	3	291	32.8	5.34	11.22
O76071	CIAO1	Probable cyt	13	4	4	4	339	37.8	4.97	8.64
Q9UHR5	SAP30BP	SAP30-bindi	11	3	4	3	308	33.9	4.84	8.06
Q3KQV9	UAP1L1	UDP-N-acety	7	4	5	3	507	57	6.32	13.12
Q9NRG9	AAAS	Aladin OS=H	7	3	3	3	546	59.5	7.5	10.93
P82673	MRPS35	28S ribosom	12	4	5	4	323	36.8	8.24	12.44
Q6NW29	RWDD4	RWD domair	15	3	4	3	188	21.2	5.31	12.42
P21246	PTN	Pleiotrophin	31	5	5	5	168	18.9	9.6	9.8
Q96AT9	RPE	Ribulose-pho	16	3	4	3	228	24.9	5.58	14.05
Q13868	EXOSC2	Exosome cor	16	3	4	3	293	32.8	7.5	11.89
Q8N4C8	MINK1	Misshapen-li	4	5	5	2	1332	149.7	7.85	7.41
O75525	KHDRBS3	KH domain-c	8	3	5	3	346	38.8	7.61	14.64
Q5BJF6	ODF2	Outer dense	4	3	4	3	829	95.3	7.62	9
Q9UEU0	VTI1B	Vesicle trans	19	4	4	4	232	26.7	9.04	12.89
Q01831	XPC	DNA repair p	6	6	6	6	940	105.9	8.9	7.85
Q13753	LAMC2	Laminin subu	3	3	3	3	1193	130.9	6.19	9.31
O75381	PEX14	Peroxisomal	9	3	3	3	377	41.2	4.94	11.03
Q8N1G2	CMTR1	Cap-specific	6	5	7	5	835	95.3	7.05	14.06
Q7Z3T8	ZFYVE16	Zinc finger F	3	4	4	4	1539	168.8	4.82	10.11
Q16774	GUK1	Guanylate ki	10	2	4	2	197	21.7	6.55	12.75
O60237	PPP1R12B	Protein phos	3	4	7	4	982	110.3	5.67	21.63
Q96S55	WRNIP1	ATPase WR	6	3	3	3	665	72.1	6.1	10.26
P14209	CD99	CD99 antige	11	2	8	2	185	18.8	4.75	21.56
O14802	POLR3A	DNA-directe	5	6	6	6	1390	155.5	8.48	11.94
P46736	BRCC3	Lys-63-speci	14	4	4	4	316	36	5.92	10.92
O95295	SNAPIN	SNARE-assc	19	2	3	2	136	14.9	9.31	8.62

Q8IUI8	CRLF3	Cytokine rec	7	4	5	4	442	49.7	5.14	10.83
Q9NX20	MRPL16	39S ribosom	18	3	3	3	251	28.4	10.13	9.16
Q15427	SF3B4	Splicing facto	10	3	3	3	424	44.4	8.56	11.74
Q99943	AGPAT1	1-acyl-sn-gly	16	4	4	4	283	31.7	9.38	11.42
Q9BYD1	MRPL13	39S ribosom	21	3	3	3	178	20.7	9.16	12.64
Q9NSA3	CTNNBIP1	Beta-catenin	30	2	4	2	81	9.2	5.41	11.73
Q8WWX9	SELENOM	Selenoprotei	26	3	4	3	145	16.2	5.54	9.11
O95167	NDUFA3	NADH dehyd	50	3	4	3	84	9.3	8.46	11.74
Q9UMS0	NFU1	NFU1 iron-su	19	4	4	4	254	28.4	5.07	8.33
Q96CW5	TUBGCP3	Gamma-tubu	5	4	4	4	907	103.5	8.12	11.72
P98194	ATP2C1	Calcium-tran	5	3	3	3	919	100.5	6.74	10.34
Q6UXH9	PAMR1	Inactive serin	7	4	5	4	720	80.1	7.46	14.03
Q9Y4K0	LOXL2	Lysyl oxidase	6	4	4	3	774	86.7	6.38	12.24
Q9NVC6	MED17	Mediator of F	7	4	4	4	651	72.8	7.44	9.37
Q5J8M3	EMC4	ER membran	16	2	3	2	183	20.1	8.62	9.48
O60508	CDC40	Pre-mRNA-p	11	4	4	4	579	65.5	7.06	11.5
Q13685	AAMP	Angio-associ	7	3	3	3	434	46.7	4.42	9.05
Q8NFH4	NUP37	Nucleoporin	13	3	3	3	326	36.7	5.92	10.31
Q06587	RING1	E3 ubiquitin-	13	4	4	2	406	42.4	5.62	8.72
Q9NX46	ADPRS	ADP-ribosylh	12	3	3	3	363	38.9	5.07	7.65
P04156	PRNP	Major prion p	11	3	7	3	253	27.6	9	20
Q9BXR0	QTRT1	Queuine tRN	11	4	4	4	403	44	7.23	10.76
Q9NP66	HMG20A	High mobility	11	3	3	3	347	40.1	6.49	11.9
Q9P2K5	MYEF2	Myelin expre	8	5	5	5	600	64.1	8.75	6.2
O00506	STK25	Serine/threo	10	3	3	1	426	48.1	6.74	8.69
Q9NWT6	HIF1AN	Hypoxia-indu	15	4	4	4	349	40.3	5.57	10.57
Q9NRY4	ARHGAP35	Rho GTPase	4	4	4	4	1499	170.4	6.64	8.31
Q9Y2X0	MED16	Mediator of F	5	3	4	3	877	96.7	7.37	12.28
P83876	TXNL4A	Thioredoxin-l	16	2	3	2	142	16.8	5.85	10.45
O00479	HMGN4	High mobility	27	3	6	2	90	9.5	10.48	10.53
P98196	ATP11A	Phospholipid	3	4	5	3	1134	129.7	6.6	7.72
Q9NP58	ABCB6	ATP-binding	5	3	3	3	842	93.8	8.48	10.08
P22792	CPN2	Carboxypept	8	4	5	4	545	60.5	5.99	11.91

Q8N9N7	LRRC57	Leucine-rich	15	4	4	4	239	26.7	8.43	11.11
A0AV96	RBM47	RNA-binding	10	3	4	3	593	64.1	7.68	7.37
O75151	PHF2	Lysine-speci	4	4	4	4	1096	120.7	9.17	12.02
Q9Y4B6	DCAF1	DDB1- and C	3	4	4	4	1507	168.9	5.06	10.2
Q6P1M0	SLC27A4	Long-chain fa	6	3	4	3	643	72	8.47	13.32
P28347	TEAD1	Transcription	12	3	4	3	426	47.9	8.15	11.24
Q9H1E5	TMX4	Thioredoxin-r	8	2	3	2	349	38.9	4.37	9.89
P20036	HLA-DPA1	HLA class II	18	3	3	3	260	29.4	5.21	10.28
P25440	BRD2	Bromodomai	5	4	4	3	801	88	9.09	8.84
Q7L523	RRAGA	Ras-related (	15	4	5	4	313	36.5	7.72	11.56
P52298	NCBP2	Nuclear cap-	17	3	4	3	156	18	8.21	11.84
Q96A00	PPP1R14A	Protein phos	18	2	3	2	147	16.7	9.38	9.41
Q5VTL8	PRPF38B	Pre-mRNA-s	9	5	6	5	546	64.4	10.54	11.32
Q9HAN9	NMNAT1	Nicotinamide	20	6	6	6	279	31.9	8.87	11.42
Q6DKI1	RPL7L1	60S ribosom	14	3	4	3	255	29.7	10.52	13.97
P36222	CHI3L1	Chitinase-3-l	10	4	4	4	383	42.6	8.46	9.07
Q92791	P3H4	Endoplasmic	9	3	3	3	437	50.3	4.77	10.86
P22670	RFX1	MHC class II	5	4	4	4	979	104.7	6.29	9.77
P38571	LIPA	Lysosomal a	6	2	3	2	399	45.4	6.92	10.15
Q96EP0	RNF31	E3 ubiquitin-j	5	5	5	5	1072	119.6	6.57	4.99
P61769	B2M	Beta-2-micro	41	4	5	4	119	13.7	6.52	10.76
Q5XPI4	RNF123	E3 ubiquitin-j	3	4	6	4	1314	148.4	6.74	13.25
Q13683	ITGA7	Integrin alpha	5	5	5	5	1181	128.9	5.73	13.31
O95785	WIZ	Protein Wiz (	4	4	4	4	1651	178.6	6.86	8.34
P17900	GM2A	Ganglioside (	13	3	5	3	193	20.8	5.31	8.11
Q6NZY4	ZCCHC8	Zinc finger C	7	4	4	4	707	78.5	4.87	10.71
Q12789	GTF3C1	General tran	1	3	3	3	2109	238.7	7.3	10.3
P52434	POLR2H	DNA-directed	23	3	3	3	150	17.1	4.68	9.4
P78537	BLOC1S1	Biogenesis o	30	4	5	4	153	17.3	9.33	11.64
Q9NPA0	EMC7	ER membran	15	3	3	3	242	26.5	9.25	10.78
P36959	GMPR	GMP reducta	10	3	3	3	345	37.4	7.06	10.71
Q99757	TXN2	Thioredoxin,	13	1	2	1	166	18.4	8.29	11.31
Q9Y294	ASF1A	Histone chap	14	1	2	1	204	23	4.41	10.82

Q9UPN6	SCAF8	SR-related a	5	4	5	2	1271	140.4	8.29	13.09
Q9GZP8	IMUP	Immortalizati	16	2	4	2	106	10.9	9.73	6.78
P63218	GNG5	Guanine nuc	63	3	3	3	68	7.3	9.85	12.37
Q6P179	ERAP2	Endoplasmic	4	4	7	4	960	110.4	6.71	15.38
P55058	PLTP	Phospholipid	5	2	3	2	493	54.7	7.01	10.22
P55160	NCKAP1L	Nck-associat	4	4	5	3	1127	128.1	6.86	8.81
Q9HBL7	PLGRKT	Plasminogen	14	2	5	2	147	17.2	9.58	12.6
Q13332	PTPRS	Receptor-typ	3	4	4	3	1948	216.9	6.46	10.01
P02746	C1QB	Complement	8	2	6	2	253	26.7	8.63	26.18
Q9H3P2	NELFA	Negative elo	8	3	3	3	528	57.2	9.03	9.51
Q86YR5	GPSM1	G-protein-sig	6	3	4	3	675	74.5	6.54	14.01
Q96JC1	VPS39	Vam6/Vps39	4	3	4	3	886	101.7	6.99	15.23
Q96QD9	FYTTD1	UAP56-intera	14	4	5	4	318	35.8	11.78	6.36
Q96HY7	DHTKD1	2-oxoadipate	5	4	4	4	919	103	6.93	6.94
P39210	MPV17	Protein Mpv1	22	3	4	3	176	19.7	9.47	12.28
P08519	LPA	Apolipoprote	9	2	3	2	2040	226.4	6.07	10.05
Q9UKY3	CES1P1	Putative inac	12	3	3	1	287	30.7	8.03	9.94
P01920	HLA-DQB1	HLA class II	8	2	4	1	261	30	7.25	13.56
P13473	LAMP2	Lysosome-as	5	2	3	2	410	44.9	5.63	6.49
O14949	UQCRQ	Cytochrome	30	3	4	3	82	9.9	10.08	10.76
Q9NRP0	OSTC	Oligosacchar	8	1	3	1	149	16.8	9.13	10.07
Q8IYB8	SUPV3L1	ATP-depend	5	3	3	3	786	87.9	7.99	10.52
Q6YP21	KYAT3	Kynurenine--	9	4	4	4	454	51.4	8.19	8.36
Q9H936	SLC25A22	Mitochondria	11	3	3	3	323	34.4	9.29	9.08
Q7Z6K5	ARPIN	Arpin OS=Hc	16	3	4	3	226	24.9	5.83	9.94
O00399	DCTN6	Dynactin sub	22	4	6	4	190	20.7	6.32	11.27
P82930	MRPS34	28S ribosom	18	3	3	3	218	25.6	9.98	8.83
P16444	DPEP1	Dipeptidase	10	4	4	4	411	45.6	6.15	7.98
P0DP04	IGHV3-43D	Immunoglobu	19	2	8	1	118	13	5.41	19.66
P54826	GAS1	Growth arres	12	4	4	4	345	35.7	5.55	10.09
Q05315	CLC	Galectin-10 (	28	4	6	4	142	16.4	7.37	10.31
Q13287	NMI	N-myc-intera	12	4	4	4	307	35	5.34	11.69
O75636	FCN3	Ficolin-3 OS=	17	4	4	3	299	32.9	6.67	12.25

Q8N584	TTC39C	Tetratricopep	9	4	5	4	583	65.8	6.99	10.87
Q96PE3	INPP4A	Inositol polyp	4	3	3	3	977	109.9	6.96	9.68
Q9P2M7	CGN	Cingulin OS=	3	4	4	3	1203	137	5.52	9.94
Q3KQU3	MAP7D1	MAP7 domai	3	3	4	3	841	92.8	10.11	10.73
Q9ULK4	MED23	Mediator of F	3	4	4	4	1368	156.4	7.4	10.57
P08263	GSTA1	Glutathione S	14	4	6	4	222	25.6	8.88	12.86
P06310	IGKV2-30	Immunoglobu	17	2	8	2	120	13.2	8.51	22.03
Q96PE7	MCEE	Methylmalon	13	2	3	2	176	18.7	9.09	9.51
P24557	TBXAS1	Thromboxan	6	3	3	3	533	60.5	7.62	8.92
Q8WVJ2	NUDCD2	NudC domai	28	2	2	2	157	17.7	5.07	8.62
A0A075B6K4	IGLV3-10	Immunoglobu	26	3	4	3	115	12.4	4.83	11.67
P08069	IGF1R	Insulin-like g	3	4	4	2	1367	154.7	5.8	7.05
P35219	CA8	Carbonic anh	19	4	4	4	290	33	4.86	7.34
P22415	USF1	Upstream sti	13	3	5	3	310	33.5	5.54	15.02
Q9BQ69	MACROD1	ADP-ribose g	11	3	3	3	325	35.5	9.51	9.09
P51580	TPMT	Thiopurine S	12	3	4	3	245	28.2	6.23	8.13
Q9BWU0	SLC4A1AP	Kanadaptin C	5	4	4	4	796	88.8	5.19	9.9
P56377	AP1S2	AP-1 comple	22	4	5	3	157	18.6	5.47	8.58
Q9H300	PARL	Presenilins-a	12	4	4	4	379	42.2	9.79	11.01
Q99704	DOK1	Docking prot	8	3	3	3	481	52.4	6.47	10.49
Q13613	MTMR1	Myotubularin	5	3	4	3	665	74.6	7.14	5.14
Q96TA2	YME1L1	ATP-depend	4	3	4	3	773	86.4	8.76	12.47
Q8NEN9	PDZD8	PDZ domain-	4	3	3	3	1154	128.5	6.09	9.73
Q16854	DGUOK	Deoxyguano	9	2	3	2	277	32	8.66	10.28
Q9Y619	SLC25A15	Mitochondria	16	3	3	3	301	32.7	9.13	9.43
Q8WWI1	LMO7	LIM domain c	2	4	4	4	1683	192.6	8.09	7.76
P33908	MAN1A1	Mannosyl-oli	6	4	5	3	653	72.9	6.47	10.19
Q69YN4	VIRMA	Protein viriliz	4	4	4	4	1812	201.9	5.01	5.74
Q86WI1	PKHD1L1	Fibrocystin-L	1	4	5	4	4243	465.4	6.11	6.87
Q99622	C12orf57	Protein C10 (	36	3	3	3	126	13.2	5.14	8.81
Q9NP74	PALMD	Palmdelphin	8	3	4	2	551	62.7	5.44	11.85
Q9Y3S2	ZNF330	Zinc finger pr	11	4	4	4	320	36.2	6.16	8.22
Q8NFP7	NUDT10	Diphosphoin	23	4	4	2	164	18.5	5.68	6.04

Q8NDT2	RBM15B	Putative RN/	4	3	3	3	890	97.1	9.85	8.73
P16220	CREB1	Cyclic AMP-r	6	2	4	2	327	35.1	5.27	11.03
Q9BQI0	AIF1L	Allograft infla	15	3	6	2	150	17.1	7.2	11.48
Q8N3P4	VPS8	Vacuolar pro	3	3	3	3	1428	161.7	5.64	7.33
P10398	ARAF	Serine/threor	6	4	5	2	606	67.5	9.01	10.98
Q14202	ZMYM3	Zinc finger M	2	4	6	2	1370	152.3	6.35	13.35
Q9UFG5	C19orf25	UPF0449 pro	36	2	2	2	118	12.9	5.07	8.89
Q8IYB7	DIS3L2	DIS3-like exo	5	4	4	4	885	99.2	6.1	10.2
P07451	CA3	Carbonic anh	16	4	4	4	260	29.5	7.34	8.36
Q9H477	RBKS	Ribokinase C	14	3	3	3	322	34.1	5.05	7.37
Q5XUX1	FBXW9	F-box/WD re	5	3	6	3	458	50.7	6.48	10.23
P08246	ELANE	Neutrophil el	9	3	5	3	267	28.5	9.35	14.93
O95456	PSMG1	Proteasome	14	4	4	4	288	32.8	7.17	8.79
P61244	MAX	Protein max	18	3	4	3	160	18.3	6.3	6.62
Q9UBB6	NCDN	Neurochondr	3	2	3	2	729	78.8	5.48	10.28
Q9H330	TMEM245	Transmembr	4	3	4	3	879	97.3	8.91	11.43
Q8IWR0	ZC3H7A	Zinc finger C	3	4	5	4	971	110.5	7.3	8.73
O43822	CFAP410	Cilia- and fla	16	3	3	3	256	28.3	7.34	7.94
P45877	PPIC	Peptidyl-prol	17	4	10	3	212	22.7	8.4	13.49
Q9Y697	NFS1	Cysteine des	8	4	4	4	457	50.2	8.31	8.97
Q96Q05	TRAPPC9	Trafficking pr	4	3	3	3	1148	128.4	6.62	7.83
Q9BS92	NIPSNAP3B	Protein NipS	16	3	3	2	247	28.3	9.28	8.9
O00400	SLC33A1	Acetyl-coenz	7	3	3	3	549	60.9	7.33	9.82
P07711	CTSL	Procathepsir	7	3	5	3	333	37.5	5.45	11.76
Q8TB72	PUM2	Pumilio hom	4	5	7	2	1066	114.1	7.08	9.5
O15504	NUP42	Nucleoporin	11	3	3	3	423	44.8	9.19	9.36
Q03426	MVK	Mevalonate k	12	4	4	4	396	42.4	6.46	8.52
Q8TDX7	NEK7	Serine/threor	13	3	3	1	302	34.5	8.25	9.17
Q969Q0	RPL36AL	60S ribosom	25	4	6	1	106	12.5	10.65	12.03
Q01432	AMPD3	AMP deamin	5	4	5	3	767	88.8	6.98	10.38
P42768	WAS	Actin nucleat	8	3	4	3	502	52.9	6.64	13.68
Q92620	DHX38	Pre-mRNA-s	3	4	4	3	1227	140.4	6.54	7.7
Q14678	KANK1	KN motif anc	2	2	3	1	1352	147.2	5.3	11.58

Q15813	TBCE	Tubulin-spec	12	5	5	5	527	59.3	6.76	5.79
O43318	MAP3K7	Mitogen-activ	5	2	3	2	606	67.2	7.11	9.51
O75170	PPP6R2	Serine/threor	3	3	4	3	966	104.9	4.87	9.91
P04070	PROC	Vitamin K-de	7	3	3	3	461	52	6.28	8.23
A6NIH7	UNC119B	Protein unc-1	17	4	5	4	251	28.1	5.68	14.26
O95139	NDUFB6	NADH dehyd	33	4	6	4	128	15.5	9.63	15.56
Q9H0W5	CCDC8	Coiled-coil do	11	5	5	5	538	59.3	8.63	8.82
Q86X76	NIT1	Deaminated	7	3	6	3	327	35.9	7.74	12.16
Q8TAA9	VANGL1	Vang-like pro	6	3	3	3	524	59.9	8.81	8.84
Q13601	KRR1	KRR1 small s	10	4	4	4	381	43.6	9.77	9.55
P54802	NAGLU	Alpha-N-acet	5	3	4	3	743	82.2	6.65	13.59
P14555	PLA2G2A	Phospholipas	18	2	3	2	144	16.1	9.23	10.43
Q99717	SMAD5	Mothers aga	6	3	3	3	465	52.2	7.71	10.04
Q5K651	SAMD9	Sterile alpha	2	4	5	3	1589	184.2	7.83	13.46
Q53HC9	EIPR1	EARP and G	13	4	5	4	387	43.6	5.06	8.77
P12259	F5	Coagulation	2	3	3	3	2224	251.5	6.05	10.49
Q9UGM6	WARS2	Tryptophan--	12	4	4	4	360	40.1	9.28	6.61
Q9Y3C8	UFC1	Ubiquitin-fold	15	3	4	3	167	19.4	7.4	10.75
Q9NQ88	TIGAR	Fructose-2,6	20	3	3	3	270	30	7.69	6.3
Q96S19	METTL26	Methyltransfe	21	3	3	3	204	22.6	7.83	7.18
Q9BYJ9	YTHDF1	YTH domain-	6	4	6	1	559	60.8	8.79	15.96
Q5KU26	COLEC12	Collectin-12	4	3	3	3	742	81.5	5.69	9.04
Q13449	LSAMP	Limbic syster	10	4	4	4	338	37.4	6.98	10.61
Q01974	ROR2	Tyrosine-pro	3	3	4	3	943	104.7	6.55	5.45
Q99081	TCF12	Transcription	5	3	3	1	682	72.9	7.02	9.47
Q96H79	ZC3HAV1L	Zinc finger C	11	3	4	3	300	32.9	8.13	12.66
Q9H3K6	BOLA2B	BolA-like pro	37	3	6	3	86	10.1	6.52	15.32
Q96D96	HVCN1	Voltage-gate	12	3	4	3	273	31.7	6.8	9.8
P52435	POLR2J	DNA-directed	20	2	3	2	117	13.3	5.86	10.75
Q14061	COX17	Cytochrome	59	3	3	3	63	6.9	7.24	9.42
P46934	NEDD4	E3 ubiquitin-	3	3	4	3	1319	149	6.58	15.23
P98179	RBM3	RNA-binding	27	4	5	3	157	17.2	8.91	9.55
O95071	UBR5	E3 ubiquitin-	1	3	3	3	2799	309.2	5.85	7.85

Q9P2L0	WDR35	WD repeat-c	4	3	3	3	1181	133.5	6.38	4.96
Q9NXH8	TOR4A	Torsin-4A OS	7	3	4	3	423	46.9	9.94	10
P42575	CASP2	Caspase-2 C	8	3	3	3	452	50.7	6.81	9.04
P08185	SERPINA6	Corticosteroi	9	3	4	3	405	45.1	6.04	11.19
O95208	EPN2	Epsin-2 OS=	5	4	4	3	641	68.4	7.52	9.14
Q8IWV7	UBR1	E3 ubiquitin-|	2	2	2	2	1749	200.1	6.01	10.6
Q86XX4	FRAS1	Extracellular	1	4	4	4	4008	442.9	5.57	6.69
P48730	CSNK1D	Casein kinas	6	2	3	2	415	47.3	9.74	9.52
Q13797	ITGA9	Integrin alpha	4	5	5	5	1035	114.4	6.14	8.86
Q3KQZ1	SLC25A35	Solute carrie	8	2	3	2	300	32.4	9.11	7.44
Q9NUB1	ACSS1	Acetyl-coenz	6	3	4	3	689	74.8	7.11	11.65
O00182	LGALS9	Galectin-9 O	10	3	3	3	355	39.5	9.17	8.39
Q9NXR1	NDE1	Nuclear distr	11	4	4	2	335	37.7	5.15	9.85
Q9NX47	MARCHF5	E3 ubiquitin-|	14	4	4	4	278	31.2	8.7	8.89
O00622	CCN1	CCN family r	11	4	4	4	381	42	8.21	6.49
Q9GZR7	DDX24	ATP-depend	4	4	4	4	859	96.3	9.06	8.38
Q8IUX4	APOBEC3F	DNA dC->dU	10	3	3	2	373	45	7.23	9.39
Q9HBG6	IFT122	Intraflagellar	4	3	3	3	1241	141.7	6.49	8.56
O14618	CCS	Copper chap	8	2	3	2	274	29	5.58	10.16
P32856	STX2	Syntaxin-2 O	14	2	3	2	288	33.3	6.28	8.29
Q86TV6	TTC7B	Tetratricopep	4	3	3	3	843	94.1	6.89	8.77
Q9Y3B9	RRP15	RRP15-like p	11	4	4	4	282	31.5	5.52	8.12
O15379	HDAC3	Histone deac	10	3	4	3	428	48.8	5.16	14.96
P53602	MVD	Diphosphom	10	3	3	3	400	43.4	7.23	9.24
Q9Y287	ITM2B	Integral mem	18	3	3	3	266	30.3	5.14	9.77
Q9BRR6	ADPGK	ADP-depend	8	3	3	3	497	54.1	6.2	8.06
Q9BZL4	PPP1R12C	Protein phos	5	4	4	4	782	84.8	5.57	7.77
Q15650	TRIP4	Activating sig	7	4	4	4	581	66.1	7.85	6.22
O15061	SYNM	Synemin OS	3	4	5	4	1565	172.8	5.15	6.79
P03886	MT-ND1	NADH-ubiqu	6	2	2	2	318	35.6	6.55	8.2
P48651	PTDSS1	Phosphatidyl	8	3	3	3	473	55.5	8.43	7.44
P48163	ME1	NADP-deper	10	5	5	5	572	64.1	6.13	8.8
P05976	MYL1	Myosin light	13	2	4	1	194	21.1	5.03	8.61

O75592	MYCBP2	E3 ubiquitin-|	1	3	3	3	4678	513.3	7.02	8.59
P28845	HSD11B1	11-beta-hydr	9	3	4	3	292	32.4	8.56	10.58
P51688	SGSH	N-sulphogluc	7	2	3	2	502	56.7	6.95	8.74
Q8WWC4	MAIP1	m-AAA prote	11	3	3	3	291	32.5	9.17	8.2
O00423	EML1	Echinoderm	4	4	4	3	815	89.8	7.06	7.55
Q8WVQ1	CANT1	Soluble calci	10	3	3	3	401	44.8	6.09	8.77
Q9H930	SP140L	Nuclear body	3	2	3	1	580	67	8.48	6.65
P80748	IGLV3-21	Immunoglobu	14	1	4	1	117	12.4	5.29	17.73
Q9BQ24	ZFYVE21	Zinc finger F	16	3	3	3	234	26.5	8.41	10.61
Q9BYI3	HYCC1	Hyccin OS=H	8	3	3	3	521	57.6	8.16	9.45
Q9BQL6	FERMT1	Fermitin fam	6	5	5	2	677	77.4	6.28	10.79
P49356	FNTB	Protein farne	12	3	3	3	437	48.7	5.82	10.85
Q9BSL1	UBAC1	Ubiquitin-ass	8	3	4	3	405	45.3	4.92	4.63
Q8IXM6	NRM	Nurim OS=H	16	4	4	4	262	29.4	8.63	11.06
Q9Y2Q5	LAMTOR2	Ragulator co	19	2	3	2	125	13.5	5.4	9.27
Q8N5C6	SRBD1	S1 RNA-binc	4	4	4	4	995	111.7	8.72	8.52
Q16799	RTN1	Reticulon-1 (	3	2	4	2	776	83.6	4.69	15.82
O00478	BTN3A3	Butyrophilin s	7	3	3	3	584	65	5.53	7.26
O43314	PPIP5K2	Inositol hexa	3	3	3	3	1243	140.3	8.22	8.31
P21359	NF1	Neurofibromi	1	3	3	3	2839	319.2	7.39	9.54
P62072	TIMM10	Mitochondria	30	4	4	4	90	10.3	6.29	8.93
P25391	LAMA1	Laminin subu	1	4	4	4	3075	336.9	6.35	8.19
Q9HBR0	SLC38A10	Putative sodi	3	3	4	3	1119	119.7	5.73	8.41
Q08722	CD47	Leukocyte su	7	3	4	3	323	35.2	7.21	9.51
O14531	DPYSL4	Dihydropyrim	9	4	5	4	572	61.8	7.09	6.55
Q9P015	MRPL15	39S ribosom	13	3	3	3	296	33.4	10.01	5.73
P56378	ATP5MJ	ATP synthas	28	2	5	2	58	6.7	10.08	13.64
Q6KC79	NIPBL	Nipped-B-like	1	4	5	4	2804	315.9	7.91	2.64
A0A075B6R!	IGKV2D-24	Probable nor	17	2	4	2	120	13.1	8.87	9.98
Q8IZ81	ELMOD2	ELMO doma	11	3	5	3	293	34.9	8.05	11.53
Q14435	GALNT3	Polypeptide I	6	3	4	3	633	72.6	7.99	9.57
P56705	WNT4	Protein Wnt-	10	3	3	3	351	39	8.51	7.92
O95396	MOCS3	Adenylyltrans	9	3	3	3	460	49.6	6.21	9.1

Q8TBX8	PIP4K2C	Phosphatidyl	9	4	4	3	421	47.3	6.84	8.45
P49908	SELENOP	Selenoprotei	9	4	4	4	381	43.2	7.87	10.08
Q01415	GALK2	N-acetylgala	9	3	3	3	458	50.3	6.61	9.78
Q6UN15	FIP1L1	Pre-mRNA 3	8	3	3	3	594	66.5	5.59	7.17
Q9Y6M5	SLC30A1	Proton-coupl	6	2	3	2	507	55.3	6.48	6.51
P25815	S100P	Protein S10C	24	2	3	2	95	10.4	4.88	8.22
Q14746	COG2	Conserved o	6	4	4	4	738	83.2	6.62	5.08
Q8NBU5	ATAD1	Outer mitoch	8	2	3	2	361	40.7	6.9	9.64
Q9NZI7	UBP1	Upstream-bi	6	2	3	1	540	60.5	6.35	12.21
Q9P2C4	TMEM181	Transmembr	4	2	3	2	612	69.3	8.92	9.13
O95379	TNFAIP8	Tumor necro	16	3	3	3	198	23	7.93	8.08
Q96DA6	DNAJC19	Mitochondria	32	4	4	4	116	12.5	10.1	2.5
Q13952	NFYC	Nuclear trans	5	2	3	2	458	50.3	6.1	7.57
Q96S44	TP53RK	EKC/KEOPS	11	3	4	3	253	28.1	9.54	7.82
P15735	PHKG2	Phosphoryla	11	4	4	4	406	46.4	6.38	9.47
Q9Y2S6	TMA7	Translation n	30	3	4	3	64	7.1	9.99	9.58
O43347	MSI1	RNA-binding	10	3	3	1	362	39.1	7.85	8.41
Q9Y312	AAR2	Protein AAR	10	2	2	2	384	43.4	5.96	7.93
P01742	IGHV1-69	Immunoglobu	15	2	6	1	117	12.7	9.17	13.4
Q8WUD4	CCDC12	Coiled-coil d	23	3	3	3	166	19.2	7.34	9.7
Q71UM5	RPS27L	40S ribosom	25	2	4	2	84	9.5	9.45	13.09
P01911	HLA-DRB1	HLA class II	12	3	5	0	266	29.9	7.74	14.09
Q7Z7L1	SLFN11	Schlafen fam	4	3	3	1	901	102.8	7.77	9.08
Q9P0I2	EMC3	ER membrar	17	3	4	3	261	29.9	6.81	10.44
P13224	GP1BB	Platelet glyco	11	2	5	2	206	21.7	9.31	12.75
Q96A35	MRPL24	39S ribosom	21	4	4	4	216	24.9	9.29	9.09
Q9H0V9	LMAN2L	VIP36-like pr	10	3	4	3	348	39.7	8.38	13.16
Q9NTG7	SIRT3	NAD-depend	6	2	4	2	399	43.5	8.7	8.71
P12532	CKMT1B	Creatine kina	7	3	3	2	417	47	8.34	8.24
Q96JG6	VPS50	Syndetin OS	4	3	3	3	964	111.1	6.2	6.18
Q9UK99	FBXO3	F-box only pr	11	5	5	5	471	54.5	5	5.18
Q53FZ2	ACSM3	Acyl-coenzyr	6	3	3	3	586	66.1	9.04	7.92
Q8TB36	GDAP1	Ganglioside-	9	3	3	3	358	41.3	8.34	5.9

Q13895	BYSL	Bystin OS=H	8	3	4	3	437	49.6	8.12	11.67
P28906	CD34	Hematopoiet	9	4	5	4	385	40.7	7.37	8.78
O95822	MLYCD	Malonyl-CoA	8	3	4	3	493	55	8.95	11.27
Q5QJE6	DNTTIP2	Deoxynucleo	3	2	3	2	756	84.4	6.16	7.99
Q96G21	IMP4	U3 small nuc	11	4	5	4	291	33.7	9.47	11.87
P59666	DEFA3	Neutrophil de	28	3	6	3	94	10.2	5.99	15.72
Q4G176	ACSF3	Malonate--Co	8	4	4	4	576	64.1	8.37	8.9
Q9NVH2	INTS7	Integrator co	4	3	3	3	962	106.8	8.02	8.33
Q8NAV1	PRPF38A	Pre-mRNA-s	8	2	3	2	312	37.5	9.96	5.99
Q9NX05	FAM120C	Constitutive c	5	4	4	3	1096	120.5	9.03	7.4
Q9Y5Q9	GTF3C3	General trans	4	4	4	4	886	101.2	5.07	9.93
Q96LB3	IFT74	Intraflagellar	7	3	3	3	600	69.2	6	5.77
Q9Y676	MRPS18B	28S ribosom	15	3	4	3	258	29.4	9.38	14.21
Q9UK61	TASOR	Protein TASC	2	4	5	4	1670	188.9	5.8	10.8
Q9H1Y0	ATG5	Autophagy p	14	3	3	3	275	32.4	5.77	8.41
P50150	GNG4	Guanine nuc	29	2	5	2	75	8.4	7.08	15.87
Q9BYC9	MRPL20	39S ribosom	14	2	3	2	149	17.4	10.86	8.11
P23511	NFYA	Nuclear trans	5	1	2	1	347	36.9	8.94	7.58
P22681	CBL	E3 ubiquitin-p	5	3	4	2	906	99.6	6.54	10.23
Q9BUP3	HTATIP2	Oxidoreducta	14	3	3	3	242	27	8.38	8.05
P52655	GTF2A1	Transcription	9	3	3	3	376	41.5	4.55	7.5
Q9NUP9	LIN7C	Protein lin-7	18	4	6	4	197	21.8	8.43	14.09
Q8WW59	SPRYD4	SPRY domai	15	3	3	3	207	23.1	6.93	6.1
Q9Y320	TMX2	Thioredoxin-r	12	4	4	4	296	34	8.69	10.4
Q9BQS7	HEPH	Hephaestin C	4	4	5	4	1158	130.4	5.99	11.95
P80217	IFI35	Interferon-ind	11	3	4	3	286	31.5	6.09	12.9
P29558	RBMS1	RNA-binding	9	3	3	1	406	44.5	8.79	9.48
Q8NI22	MCFD2	Multiple coag	12	1	3	1	146	16.4	4.63	12.36
Q9UII2	ATP5IF1	ATPase inhib	15	4	6	4	106	12.2	9.35	10.09
O60518	RANBP6	Ran-binding	2	2	4	1	1105	124.6	5.01	11.85
P04278	SHBG	Sex hormone	10	3	3	3	402	43.8	6.71	7.78
P63313	TMSB10	Thymosin be	45	3	5	1	44	5	5.36	13.36
P49863	GZMK	Granzyme K	13	3	4	3	264	28.9	9.39	8.6

O43157	PLXNB1	Plexin-B1 OS	2	3	3	3	2135	232.2	5.49	8.43
Q08426	EHHADH	Peroxisomal	6	3	4	3	723	79.4	9.14	7.28
Q9H0P0	NT5C3A	Cytosolic 5'-r	10	3	3	3	336	37.9	7.12	9.18
Q9Y3B8	REXO2	Oligoribonuc	22	4	4	4	237	26.8	6.87	7.81
P29279	CCN2	CCN family r	8	3	4	3	349	38.1	8	10.61
O15118	NPC1	NPC intracel	3	3	3	3	1278	142.1	5.36	7.57
Q8N129	CNPY4	Protein cano	13	3	3	3	248	28.3	4.64	9.91
O15013	ARHGEF10	Rho guanine	3	3	3	3	1369	151.5	5.68	9.37
O43490	PROM1	Prominin-1 C	5	3	4	3	865	97.1	7.27	4.76
O14896	IRF6	Interferon re	7	3	4	3	467	53.1	5.33	10.2
P03950	ANG	Angiogenin (	14	3	3	3	147	16.5	9.64	8.55
Q9NXH9	TRMT1	tRNA (guanir	4	3	3	3	659	72.2	7.64	8.4
Q9UKX5	ITGA11	Integrin alpha	3	4	5	4	1188	133.4	6.7	11.95
Q9NQ50	MRPL40	39S ribosom	16	2	2	2	206	24.5	9.63	9.36
Q13356	PPIL2	RING-type E	4	1	2	1	520	58.8	8.78	2.91
Q8IWU6	SULF1	Extracellular	4	4	5	3	871	101	9.09	10.71
P02655	APOC2	Apolipoprote	30	2	2	2	101	11.3	4.72	7.56
Q96EY5	MVB12A	Multivesicula	12	2	2	2	273	28.8	8.91	7.81
P56556	NDUFA6	NADH dehyc	19	3	4	3	128	15.1	9.98	9.8
Q16864	ATP6V1F	V-type protor	32	4	5	4	119	13.4	5.52	8.48
Q6UWP2	DHRS11	Dehydrogena	14	3	3	3	260	28.3	6.64	8.95
Q9Y608	LRRFIP2	Leucine-rich	3	2	3	2	721	82.1	6.95	6.82
P15954	COX7C	Cytochrome	29	2	3	2	63	7.2	10.27	9.25
Q96ST2	IWS1	Protein IWS1	3	2	3	2	819	91.9	4.69	9.48
O95837	GNA14	Guanine nuc	8	3	6	1	355	41.5	6.07	12.41
Q99766	DMAC2L	ATP synthas	15	3	3	3	215	24.9	7.56	5.11
A0A075B6I9	IGLV7-46	Immunoglobu	21	3	4	3	117	12.5	7.2	6.8
Q96J02	ITCH	E3 ubiquitin-	6	5	5	5	903	102.7	6.3	9.94
Q9H1P3	OSBPL2	Oxysterol-bir	9	3	3	3	480	55.2	6.35	7.79
Q9BRX2	PELO	Protein pelot	11	6	6	6	385	43.3	6.34	8.68
Q9NWS0	PIH1D1	PIH1 domair	10	2	2	2	290	32.3	5.14	7.91
O15212	PFDN6	Prefoldin sub	23	3	5	3	129	14.6	8.88	11.75
Q96J84	KIRREL1	Kin of IRRE-	5	2	2	2	757	83.5	5.73	8.39

A0A0B4J1X8	IGHV3-43	Immunoglobu	19	2	3	1	118	13.1	5.41	5.87
Q96R05	RBP7	Retinoid-binc	28	4	4	4	134	15.5	7.8	8.59
Q9BT40	INPP5K	Inositol polyp	7	3	3	3	448	51.1	6.54	6.93
P42574	CASP3	Caspase-3 C	13	4	4	4	277	31.6	6.54	10.29
Q9BX59	TAPBPL	Tapasin-rela	7	3	3	3	468	50.2	5.26	8.45
Q12972	PPP1R8	Nuclear inhib	11	2	2	2	351	38.5	7.37	8.55
O75880	SCO1	Protein SCO	8	3	3	3	301	33.8	8.88	6.56
Q9UHJ6	SHPK	Sedoheptulo	6	3	3	3	478	51.5	6.83	6.54
P00813	ADA	Adenosine de	9	3	3	3	363	40.7	5.95	6.22
O75027	ABCB7	Iron-sulfur clu	4	3	5	3	752	82.6	9.33	13.29
P09105	HBQ1	Hemoglobin	25	3	3	3	142	15.5	7.62	7.76
Q10589	BST2	Bone marrov	17	3	6	3	180	19.8	5.6	19.58
O75582	RPS6KA5	Ribosomal pr	5	4	4	2	802	89.8	7.11	7.14
P57088	TMEM33	Transmembr	11	3	5	3	247	28	9.7	14.06
Q12999	TSPAN31	Tetraspanin-	9	2	4	2	210	23	7.97	10.52
Q9P0J7	KCMF1	E3 ubiquitin-p	10	4	6	4	381	41.9	5.66	6.55
O95471	CLDN7	Claudin-7 OS	16	2	3	2	211	22.4	8.6	13.38
A6NGB9	WIPF3	WAS/WASL-	8	2	2	2	483	49.4	10.15	7.57
Q3ZCW2	LGALSL	Galectin-rela	16	3	5	3	172	19	5.35	12.18
P43378	PTPN9	Tyrosine-pro	6	3	3	3	593	68	8.02	8.91
Q9Y4E6	WDR7	WD repeat-c	2	3	3	3	1490	163.7	6.92	7.26
Q9HCN4	GPN1	GPN-loop GT	9	3	3	3	374	41.7	4.92	7.07
Q66PJ3	ARL6IP4	ADP-ribosyla	15	3	3	3	237	26.4	10.86	4.2
P15169	CPN1	Carboxypept	7	3	4	3	458	52.3	7.34	10.04
O14681	EI24	Etoposide-ind	5	2	3	2	340	38.9	9.72	8.38
P62341	SELENOT	Thioredoxin r	12	2	3	2	195	22.3	8.6	7.74
Q9BT73	PSMG3	Proteasome	32	2	2	2	122	13.1	7.88	8.01
P05108	CYP11A1	Cholesterol s	4	2	4	2	521	60.1	8.84	9.19
Q9BU23	LMF2	Lipase matur	5	3	5	3	707	79.6	10.1	11.92
Q9ULH1	ASAP1	Arf-GAP with	4	4	4	4	1129	125.4	7.31	8.07
Q8IXS6	PALM2	Paralemmin-	8	3	3	2	379	42.2	5.1	7.65
O75152	ZC3H11A	Zinc finger C	5	4	4	4	810	89.1	8.37	8.79
P55854	SUMO3	Small ubiquit	30	4	4	2	103	11.6	5.49	2.22

P17676	CEBPB	CCAAT/enha	8	2	2	2	345	36.1	8.31	7.6
Q6UW68	TMEM205	Transmembr	16	2	4	2	189	21.2	8.62	14.85
P02511	CRYAB	Alpha-crystal	15	4	4	4	175	20.1	7.33	8.86
A0AVF1	TTC26	Intraflagellar	6	3	3	3	554	64.1	6.93	8.88
Q8TE68	EPS8L1	Epidermal gr	4	3	4	3	723	80.2	6.04	7.68
Q96KM6	ZNF512B	Zinc finger pr	3	2	3	2	892	97.2	9.83	5.47
Q96PP9	GBP4	Guanylate-bi	5	2	2	2	640	73.1	6.02	9.32
Q4LE39	ARID4B	AT-rich intera	3	3	3	3	1312	147.7	5.12	6.1
Q86WA6	BPHL	Valacyclovir	12	3	3	3	291	32.5	9.14	6.91
Q9Y2Y8	PRG3	Proteoglycar	8	2	3	2	225	25.4	4.81	8.63
Q86TP1	PRUNE1	Exopolyphos	9	3	3	3	453	50.2	5.5	7.52
Q9BXF6	RAB11FIP5	Rab11 family	5	3	3	3	653	70.4	9.23	8.55
Q96I59	NARS2	Probable asp	7	4	5	4	477	54.1	7.24	9.78
Q16875	PFKFB3	6-phosphofru	5	3	3	1	520	59.6	8.21	7.86
Q9UL03	INTS6	Integrator co	4	2	2	2	887	100.3	8.62	8.6
P49006	MARCKSL1	MARCKS-rel	15	3	5	3	195	19.5	4.67	11.06
Q9UI26	IPO11	Importin-11 (	3	2	2	2	975	112.5	5.25	7.61
Q13094	LCP2	Lymphocyte	7	3	3	3	533	60.2	6.27	7.98
O43657	TSPAN6	Tetraspanin-	12	3	3	3	245	27.5	8.1	7.91
Q9NX14	NDUFB11	NADH dehyc	22	2	2	2	153	17.3	5.22	8.09
Q6PK18	OGFOD3	2-oxoglutara	11	4	5	4	319	35.6	8.18	8.73
Q8WU79	SMAP2	Stromal mem	9	3	3	3	429	46.8	8.87	9.06
P12107	COL11A1	Collagen alpl	1	3	4	1	1806	181	5.17	9.25
P07311	ACYP1	Acylphospha	29	3	5	3	99	11.3	9.31	11.27
Q9H0R4	HDHD2	Haloacid deh	14	2	2	2	259	28.5	6.24	7.97
Q5JPH6	EARS2	Probable glu	7	3	4	3	523	58.7	8.76	9.68
Q8IYS2	KIAA2013	Uncharacteri	4	3	3	3	634	69.1	8.19	8.62
P46734	MAP2K3	Dual specific	12	3	3	3	347	39.3	7.43	7.23
O43353	RIPK2	Receptor-inte	7	3	3	3	540	61.2	7.09	8.64
Q7L2J0	MEPCE	7SK snRNA	6	4	5	4	689	74.3	9.57	5.23
Q9NUM4	TMEM106B	Transmembr	14	3	4	3	274	31.1	6.99	10.77
P51636	CAV2	Caveolin-2 C	12	2	3	2	162	18.3	5.27	7.93
Q9BVM4	GGACT	Gamma-gluta	24	3	3	2	153	17.3	6.87	9.44

Q9H1K0	RBSN	Rabenosyn-5	3	3	3	3	784	88.8	5.5	8.11
Q86VW0	SESTD1	SEC14 doma	5	3	3	3	696	79.3	5.1	6.38
Q9UM22	EPDR1	Mammalian e	15	3	4	3	224	25.4	6.6	7.86
Q96EB1	ELP4	Elongator co	7	2	2	2	424	46.6	8.51	7.18
Q99828	CIB1	Calcium and	17	3	3	3	191	21.7	4.78	8.92
Q99570	PIK3R4	Phosphoinos	2	3	3	3	1358	153	7.17	5.95
Q8NI08	NCOA7	Nuclear rece	5	2	2	2	942	106.1	5.59	7.96
Q5NDL2	EOGT	EGF domain	6	3	3	3	527	62	7.05	7.17
Q687X5	STEAP4	Metalloreduc	7	2	2	2	459	51.9	9.29	9.94
Q9UK55	SERPINA10	Protein Z-dep	7	3	3	3	444	50.7	8.27	7.95
Q0VDG4	SCRN3	Secernin-3 C	8	3	3	2	424	48.5	5.55	9.81
Q9BV86	NTMT1	N-terminal X	13	2	2	2	223	25.4	5.52	7.84
P08243	ASNS	Asparagine s	7	3	3	3	561	64.3	6.86	8.48
Q8N668	COMMD1	COMM doma	21	3	3	2	190	21.2	6.2	6.14
Q8IY31	IFT20	Intraflagellar	23	2	2	2	132	15.3	5.12	8.18
Q9BY77	POLDIP3	Polymerase (	9	2	2	2	421	46.1	9.99	8.73
Q9BRZ2	TRIM56	E3 ubiquitin-p	4	2	2	2	755	81.4	7.74	3.57
P55210	CASP7	Caspase-7 C	10	4	4	4	303	34.3	6.07	6.64
P17275	JUNB	Transcription	8	3	3	2	347	35.9	9.22	8.15
P16671	CD36	Platelet glyco	7	2	2	2	472	53	7.96	8.37
Q6PCB7	SLC27A1	Long-chain fa	5	2	2	2	646	71.1	8.53	8.23
P51668	UBE2D1	Ubiquitin-con	12	2	3	2	147	16.6	7.42	9.17
Q9NPI1	BRD7	Bromodomai	3	1	4	1	651	74.1	6.39	14.99
Q9BZI7	UPF3B	Regulator of	9	4	4	4	483	57.7	9.48	7.82
P12724	RNASE3	Eosinophil ca	12	3	5	3	160	18.4	10.02	11.06
Q5TFE4	NT5DC1	5'-nucleotida	9	3	3	3	455	51.8	6.35	4.98
Q07352	ZFP36L1	mRNA decay	9	3	3	2	338	36.3	7.96	5.64
Q9NWT1	PAK1IP1	p21-activated	8	2	2	2	392	43.9	8.91	7.67
Q5TBB1	RNASEH2B	Ribonuclease	13	3	3	3	312	35.1	9.13	3.95
O43674	NDUFB5	NADH dehyd	10	2	4	2	189	21.7	9.63	10.69
Q9H2J4	PDCL3	Phosducin-lik	14	3	3	3	239	27.6	4.84	6.2
P53701	HCCS	Holocytochro	12	3	3	3	268	30.6	6.68	8.04
Q96J01	THOC3	THO comple	11	4	6	4	351	38.7	6.09	11.62

Q8NF50	DOCK8	Dedicator of	2	3	3	2	2099	238.4	6.87	8.48
O75410	TACC1	Transforming	4	2	2	1	805	87.7	4.88	7.46
Q7LGA3	HS2ST1	Heparan sulf	7	2	3	2	356	41.9	8.69	7.83
Q8NE62	CHDH	Choline dehy	7	3	3	3	594	65.3	8.28	5.99
Q9UQ90	SPG7	Paraplegin C	4	3	3	3	795	88.2	8.69	7.06
P10586	PTPRF	Receptor-typ	2	3	3	2	1907	212.7	6.3	8.06
Q7Z739	YTHDF3	YTH domain-	5	3	5	1	585	63.8	9.04	14.13
Q8TCE6	DENND10	DENN doma	10	3	3	3	357	40.5	6.61	8.16
Q9NVS2	MRPS18A	39S ribosom	20	4	4	4	196	22.2	10.33	7.61
Q14320	FAM50A	Protein FAM5	9	3	3	3	339	40.2	6.83	10.21
Q86UY8	NT5DC3	5'-nucleotida	5	3	4	3	548	63.4	8.31	5.48
O43760	SYNGR2	Synaptogyrin	13	3	3	3	224	24.8	4.94	7.57
Q12756	KIF1A	Kinesin-like p	2	4	4	3	1690	190.9	6.21	8.43
Q9Y2G5	POFUT2	GDP-fucose	9	4	7	4	429	49.9	6.6	12.12
Q03405	PLAUR	Urokinase pl	12	3	3	3	335	37	6.65	8.1
Q9NUL5	SHFL	Shiftless anti	10	2	2	2	291	33.1	7.25	7.93
Q9Y5A7	NUB1	NEDD8 ultim	6	4	5	4	615	70.5	5.96	10.18
Q92733	PRCC	Proline-rich p	8	4	4	4	491	52.4	5.1	3.28
Q8N806	UBR7	Putative E3 u	7	3	3	3	425	48	4.81	5.4
Q9H9Y6	POLR1B	DNA-directed	3	3	3	3	1135	128.1	7.83	5.09
P05160	F13B	Coagulation	7	3	3	3	661	75.5	6.39	6.05
Q96FV9	THOC1	THO comple	5	3	3	3	657	75.6	4.98	8.35
P78330	PSPH	Phosphoserin	9	2	3	2	225	25	5.69	7.9
Q8IWZ8	SUGP1	SURP and G	4	2	3	2	645	72.4	7.61	8.2
Q9H8H0	NOL11	Nucleolar pro	4	3	3	3	719	81.1	6.07	8.71
Q7L5Y1	ENOSF1	Mitochondria	7	2	3	2	443	49.8	6.48	11.24
P04180	LCAT	Phosphatidyl	8	3	3	3	440	49.5	6.11	5.48
Q9UIS9	MBD1	Methyl-CpG-	5	3	3	3	605	66.6	9.04	5.59
P51808	DYNLT3	Dynein light c	23	2	2	2	116	13.1	5.66	8.16
P78346	RPP30	Ribonuclease	15	3	3	3	268	29.3	8.91	6.26
Q9BWJ5	SF3B5	Splicing facto	40	3	5	3	86	10.1	6.35	9.43
Q8NFX7	STXBP6	Syntaxin-bind	13	3	3	3	210	23.5	9.04	7.14
Q96SY0	INTS14	Integrator co	11	3	3	3	518	57.4	5.12	5.85

A4QPH2	PI4KAP2	Putative phos	6	3	4	1	592	66.9	7.05	9.12
Q9BVV7	TIMM21	Mitochondria	13	3	3	3	248	28.2	9.7	8.82
Q7Z3B1	NEGR1	Neuronal grc	9	3	3	3	354	38.7	6.21	7.03
Q99985	SEMA3C	Semaphorin-	4	3	3	3	751	85.2	8.69	6.02
Q96DM3	RMC1	Regulator of	4	2	2	2	657	74.9	7.83	7.18
Q7Z417	NUFIP2	FMR1-intera	5	4	4	4	695	76.1	8.7	10.36
Q15031	LARS2	Leucine--tRN	4	2	2	2	903	101.9	8.22	8.07
Q8WWP7	GIMAP1	GTPase IMA	9	3	3	3	306	34.3	8.87	8.15
Q69YN2	CWF19L1	CWF19-like	6	3	3	3	538	60.6	7.24	8.44
Q9BRT2	UQCC2	Ubiquinol-cyt	35	4	4	4	126	14.9	7.37	6.73
Q92574	TSC1	Hamartin OS	3	3	4	3	1164	129.7	6.47	7.48
Q8NEZ5	FBXO22	F-box only pr	6	2	3	2	403	44.5	7.03	11.39
P45984	MAPK9	Mitogen-activ	8	4	5	1	424	48.1	5.63	12.24
Q14728	MFSD10	Major facilita	7	3	4	3	455	48.3	9.6	5.55
P28907	CD38	ADP-ribosyl	9	2	2	2	300	34.3	7.66	8.64
O75208	COQ9	Ubiquinone b	8	3	4	3	318	35.5	5.94	12.64
P51784	USP11	Ubiquitin car	3	3	4	3	963	109.7	5.45	11.17
Q6I9Y2	THOC7	THO comple	15	3	3	3	204	23.7	5.67	9.18
O15127	SCAMP2	Secretory ca	10	3	4	3	329	36.6	6.1	7.44
O00592	PODXL	Podocalyxin	2	1	2	1	558	58.6	5.49	7.17
P53779	MAPK10	Mitogen-activ	7	4	5	1	464	52.6	6.79	12.35
Q5EB52	MEST	Mesoderm-sp	11	2	2	2	335	38.8	9.74	5.93
Q9NYJ1	COA4	Cytochrome	40	3	3	3	87	10.1	6.04	7.35
Q9NV06	DCAF13	DDB1- and C	7	2	2	2	445	51.4	9.19	8.06
O43505	B4GAT1	Beta-1,4-gluc	8	3	3	3	415	47.1	7.2	5.97
Q15386	UBE3C	Ubiquitin-pro	3	3	4	3	1083	123.8	6.71	9.61
P07998	RNASE1	Ribonuclease	30	2	2	2	156	17.6	8.79	8.15
Q14CX7	NAA25	N-alpha-acet	3	2	3	2	972	112.2	6.64	11.66
Q9HC98	NEK6	Serine/threor	11	3	3	1	313	35.7	8.03	6.42
Q01740	FMO1	Flavin-contai	7	3	3	3	532	60.3	7.2	6.29
O60934	NBN	Nibrin OS=H	5	4	4	4	754	84.9	6.9	4.89
Q96RK0	CIC	Protein capic	2	3	3	3	1608	163.7	8.56	6.75
Q8WUY8	NAT14	Probable N-a	15	2	2	2	206	21.6	10.74	7.51

P13498	CYBA	Cytochrome	24	2	2	2	195	21	9.54	7.01
Q9H9T3	ELP3	Elongator co	5	3	3	3	547	62.2	8.88	7.75
Q8TBN0	RAB3IL1	Guanine nuc	6	1	2	1	382	42.6	6.47	3.47
Q9Y666	SLC12A7	Solute carrie	4	4	4	4	1083	119	6.71	6.68
Q7Z422	SZRD1	SUZ domain-	19	2	3	2	152	17	8.95	12.1
O95376	ARIH2	E3 ubiquitin-|	3	1	2	1	493	57.8	5.63	8.38
O95445	APOM	Apolipoprote	18	4	5	4	188	21.2	6.01	9.91
Q6UWE0	LRSAM1	E3 ubiquitin-|	4	3	3	3	723	83.5	5.94	6.34
Q562E7	WDR81	WD repeat-c	2	4	4	4	1941	211.6	5.58	4.49
Q9NXW2	DNAJB12	DnaJ homolo	8	3	3	3	375	41.8	8.69	4.79
Q8TDR0	TRAF3IP1	TRAF3-intera	4	3	3	3	691	78.6	7.93	7.91
Q8TDH9	BLOC1S5	Biogenesis o	17	3	4	3	187	21.6	7.59	6.06
Q8N142	ADSS1	Adenylosucc	5	2	3	2	457	50.2	8.59	7.92
P19320	VCAM1	Vascular cell	5	3	3	3	739	81.2	5.22	5.63
Q6UXI9	NPNT	Nephronectir	6	3	3	3	565	61.9	8.34	3.89
Q7Z7F7	MRPL55	39S ribosom	13	1	2	1	128	15.1	11.15	7.55
O95613	PCNT	Pericentrin C	1	3	3	3	3336	377.8	5.55	4.71
Q15172	PPP2R5A	Serine/threor	7	3	3	3	486	56.2	6.71	9.46
P40938	RFC3	Replication fa	8	3	3	3	356	40.5	8.34	9.33
Q13421	MSLN	Mesothelin C	5	2	2	2	630	68.9	6.38	7.23
P40937	RFC5	Replication fa	9	2	2	2	340	38.5	7.2	7.32
Q9BQ95	ECSIT	Evolutionarily	8	3	3	3	431	49.1	6.29	6.32
P56381	ATP5F1E	ATP synthas	43	3	5	3	51	5.8	9.92	11.39
Q9BXJ0	C1QTNF5	Complement	14	3	3	3	243	25.3	6.54	9.29
Q96Q06	PLIN4	Perilipin-4 OS	9	3	3	3	1357	134.3	8.73	6.55
Q96CP2	FLYWCH2	FLYWCH far	21	2	2	2	140	14.6	8.46	8.48
Q9BRJ6	C7orf50	Uncharacteri	12	2	2	2	194	22.1	9.64	7.12
Q6NWY9	PRPF40B	Pre-mRNA-p	5	4	4	2	871	99.3	6.83	4.66
Q6IA69	NADSYN1	Glutamine-de	3	2	2	2	706	79.2	6.44	6.3
Q9H098	FAM107B	Protein FAM	13	2	4	2	131	15.5	8.29	10.78
O75683	SURF6	Surfeit locus	8	3	3	3	361	41.4	10.64	7.25
Q96BX8	MOB3A	MOB kinase	16	4	5	4	217	25.4	8.63	11.87
Q9UBK7	RABL2A	Rab-like prot	20	3	4	3	228	26.1	4.89	5.44

Q14894	CRYM	Ketimine red	9	2	2	2	314	33.8	5.14	7.26
Q05084	ICA1	Islet cell autc	6	3	3	3	483	54.6	5.81	6.08
Q5RI15	COX20	Cytochrome	12	1	2	1	118	13.3	8.76	7.88
P25490	YY1	Transcriptior	7	3	3	3	414	44.7	6.25	7.75
Q5T200	ZC3H13	Zinc finger C	2	4	4	4	1668	196.5	9.42	2.52
Q9BQ52	ELAC2	Zinc phospho	3	2	2	2	826	92.2	7.9	7.11
P38435	GGCX	Vitamin K-de	4	2	3	2	758	87.5	8.02	7.86
Q9UP38	FZD1	Frizzled-1 OS	4	3	3	3	647	71.1	7.99	8.35
P02747	C1QC	Complement	10	2	3	2	245	25.8	8.41	11.17
Q86YB8	ERO1B	ERO1-like pr	6	3	4	1	467	53.5	7.99	8.78
Q4VC31	MIX23	Protein MIX2	22	2	2	2	144	16.6	7.81	8.21
Q16850	CYP51A1	Lanosterol 14	7	4	5	4	509	57.2	8.53	9.23
Q9Y3C4	TPRKB	EKC/KEOPS	15	2	2	2	175	19.6	6.79	7.78
P82914	MRPS15	28S ribosom	10	3	4	3	257	29.8	10.48	6.82
Q9P2E5	CHPF2	Chondroitin s	4	2	2	2	772	85.9	7.83	7.02
P48060	GLIPR1	Glioma patho	13	2	2	2	266	30.3	8.48	6.82
A0A075B6I0	IGLV8-61	Immunoglobu	15	2	3	2	122	12.8	4.55	5.88
Q16644	MAPKAPK3	MAP kinase-	5	2	3	1	382	43	7.28	6.84
Q9Y547	HSPB11	Intraflagellar	28	2	2	2	144	16.3	5.03	5.53
Q15545	TAF7	Transcriptior	5	2	3	2	349	40.2	5.2	8.05
O43688	PLPP2	Phospholipid	9	2	2	2	288	32.6	8.35	6.51
Q8TB22	SPATA20	Spermatoger	4	3	3	3	786	87.8	7.43	4.94
Q14534	SQLE	Squalene mc	5	2	2	2	574	63.9	8.63	7
Q6P4Q7	CNNM4	Metal transp	4	3	3	2	775	86.6	6.07	5.62
Q9P260	RELCH	RAB11-bindi	3	4	5	4	1216	134.5	5.45	9.93
Q9HD15	SRA1	Steroid recep	14	3	3	3	224	24.4	7.5	5.75
Q9Y3C0	WASHC3	WASH comp	11	2	3	2	194	21.2	4.46	7.05
Q9Y243	AKT3	RAC-gamma	9	4	4	2	479	55.7	6.02	6.38
P0DPB6	POLR1D	DNA-directed	25	3	3	3	133	15.2	5.8	4.28
Q9Y6D5	ARFGEF2	Brefeldin A-ii	2	5	5	1	1785	201.9	6.33	7.51
Q96BY6	DOCK10	Dedicator of	1	3	3	2	2186	249.4	7.14	5.98
Q15165	PON2	Serum parac	5	1	2	1	354	39.4	5.6	7.91
Q9NTJ3	SMC4	Structural ma	2	3	3	3	1288	147.1	6.79	8.07

Q9Y2C4	EXOG	Nuclease EX	8	2	2	2	368	41.1	8.27	6.97
O14653	GOSR2	Golgi SNAP	11	3	5	3	212	24.8	8.06	10.66
Q9HB19	PLEKHA2	Pleckstrin ho	7	3	3	3	425	47.2	8.66	8.14
Q86SX6	GLRX5	Glutaredoxin	14	3	4	3	157	16.6	6.79	9
Q8WY22	BRI3BP	BRI3-binding	14	2	2	2	251	27.8	9.44	8.4
Q8NFH5	NUP35	Nucleoporin	11	2	2	2	326	34.8	9.09	6.87
Q9H7E9	C8orf33	UPF0488 pro	7	1	3	1	229	25	9.95	10.19
Q9H6K4	OPA3	Optic atrophy	9	2	4	2	179	20	8.91	5.75
Q5VZK9	CARMIL1	F-actin-unca	2	3	3	3	1371	151.5	7.85	8.02
Q13546	RIPK1	Receptor-inte	5	4	4	4	671	75.9	6.33	6.48
Q53TN4	CYBRD1	Plasma mem	8	2	4	2	286	31.6	8.76	9.75
O75976	CPD	Carboxypept	2	3	3	3	1380	152.8	6.05	4.81
Q13049	TRIM32	E3 ubiquitin-p	4	2	2	2	653	71.9	6.98	6.76
Q9UIV1	CNOT7	CCR4-NOT t	8	2	2	2	285	32.7	4.84	7.67
O75940	SMNDC1	Survival of m	12	3	3	3	238	26.7	7.24	8.13
Q8N3R9	PALS1	Protein PALS	7	3	3	3	675	77.2	6.14	9.01
Q9NR34	MAN1C1	Mannosyl-oli	6	3	4	3	630	70.9	7.46	7.71
O75157	TSC22D2	TSC22 doma	3	3	3	1	780	79.2	5.02	6.89
Q5W111	SPRYD7	SPRY domai	16	3	3	3	196	21.7	6.7	7.26
Q9NZJ7	MTCH1	Mitochondria	10	4	4	4	389	41.5	9.32	9.7
Q6UWU4	C6orf89	Bombesin re	8	2	4	2	347	39.8	6.83	2.73
Q13443	ADAM9	Disintegrin a	4	2	4	2	819	90.5	7.52	11.92
P30047	GCHFR	GTP cyclohy	49	2	2	2	84	9.7	6.54	6.68
P86790	CCZ1B	Vacuolar fus	7	3	3	3	482	55.8	6.48	7.27
Q9BRJ2	MRPL45	39S ribosom	11	4	4	4	306	35.3	9.03	9.63
O15211	RGL2	Ral guanine	4	3	4	3	777	83.5	6.18	6.8
Q2TAL8	QRICH1	Transcription	4	3	3	3	776	86.4	5.87	5.27
O15264	MAPK13	Mitogen-activ	7	3	3	3	365	42.1	8.38	8.75
Q9HA65	TBC1D17	TBC1 domai	4	3	4	3	648	72.6	5.21	10.1
Q86SK9	SCD5	Stearoyl-CoA	8	2	2	2	330	37.6	9.61	7.7
Q6NY19	KANK3	KN motif anc	3	3	3	3	821	85.8	5.02	7.27
Q8TF42	UBASH3B	Ubiquitin-ass	3	2	3	2	649	72.6	6.93	7.39
O75909	CCNK	Cyclin-K OS=	6	3	3	3	580	64.2	8.41	6.23

P56211	ARPP19	cAMP-regula	33	2	3	2	112	12.3	9.09	11.19
Q9BX79	STRA6	Receptor for	5	2	2	2	667	73.5	8.98	5.78
Q02127	DHODH	Dihydroorota	8	2	3	2	395	42.8	9.67	8.17
Q6UWP7	LCLAT1	Lysocardiolip	7	3	3	3	414	48.9	8.62	6.63
Q9UBU8	MORF4L1	Mortality fact	7	3	3	3	362	41.4	9.28	5.5
Q8N6S5	ARL6IP6	ADP-ribosyla	5	1	2	1	226	24.7	6.33	7.19
Q53LP3	SOWAHC	Ankyrin repe	6	2	2	2	525	55.6	7.03	6.7
Q6XE24	RBMS3	RNA-binding	8	3	3	1	437	47.8	8.03	8.64
Q96EY4	TMA16	Translation r	14	2	2	2	203	23.8	9.26	7.38
Q8TB61	SLC35B2	Adenosine 3'	6	2	2	2	432	47.5	9.16	7.41
Q14197	MRPL58	Peptidyl-tRN	13	3	3	3	206	23.6	10.07	7.78
Q9H6U6	BCAS3	BCAS3 micro	3	2	3	2	928	101.2	6.7	9.72
O95169	NDUFB8	NADH dehyd	17	2	2	2	186	21.8	6.8	7.7
Q1ED39	KNOP1	Lysine-rich n	6	3	3	3	458	51.6	9.86	6.48
P49815	TSC2	Tuberin OS=	2	3	3	3	1807	200.5	7.31	4.59
P19022	CDH2	Cadherin-2 C	5	2	2	2	906	99.7	4.81	8.09
Q6RFH5	WDR74	WD repeat-c	8	2	2	2	385	42.4	8.32	4.93
Q96B54	ZNF428	Zinc finger pr	16	2	3	2	188	20.5	4.17	5.87
P61077	UBE2D3	Ubiquitin-cor	7	1	3	1	147	16.7	7.8	10.25
Q6IA86	ELP2	Elongator co	5	3	3	3	826	92.4	5.96	5.5
P78536	ADAM17	Disintegrin a	3	3	5	3	824	93	5.76	11.05
Q9Y2Z4	YARS2	Tyrosine--tRI	6	2	3	2	477	53.2	8.98	10.36
Q13610	PWP1	Periodic tryp	5	3	3	3	501	55.8	4.77	6.58
Q8IXK0	PHC2	Polyhomeoti	3	3	3	3	858	90.7	8.69	6.39
P78539	SRPX	Sushi repeat	5	2	2	2	464	51.5	8.66	6.89
Q8IVD9	NUDCD3	NudC domai	6	3	4	3	361	40.8	5.25	6.85
Q969X6	UTP4	U3 small nuc	4	3	3	3	686	76.8	8.85	8.01
Q9BYD2	MRPL9	39S ribosom	7	2	3	2	267	30.2	10.08	5.16
Q01658	DR1	Protein Dr1 C	19	4	5	4	176	19.4	4.75	11.2
P12314	FCGR1A	High affinity i	8	2	2	2	374	42.6	7.97	6.74
Q8NDZ4	DIPK2A	Divergent pro	7	3	3	3	430	49.5	8.53	5.43
Q9Y5Y6	ST14	Suppressor c	4	3	3	3	855	94.7	6.55	7.94
Q8WUH6	TMEM263	Transmembr	28	2	2	2	116	11.7	9.32	5.89

Q9UPN4	CEP131	Centrosomal	2	2	2	2	1083	122.1	8.69	7
Q9BY89	KIAA1671	Uncharacteri	2	3	3	3	1806	196.6	8.47	7.12
P30273	FCER1G	High affinity i	23	2	2	2	86	9.7	7.12	6.5
P62310	LSM3	U6 snRNA-a	12	1	2	1	102	11.8	4.7	7.27
Q8IXJ6	SIRT2	NAD-depend	8	3	3	3	389	43.2	5.36	7.39
Q8WYJ6	SEPTIN1	Septin-1 OS=	10	3	3	2	372	42.4	5.8	8.45
Q9GZU7	CTDSP1	Carboxy-tern	11	3	3	3	261	29.2	6	5.18
P21953	BCKDHB	2-oxoisovale	8	3	3	3	392	43.1	6.29	8.08
P78345	RPP38	Ribonucleas	7	2	3	2	283	31.8	9.92	4.64
P61018	RAB4B	Ras-related p	12	2	2	1	213	23.6	6.06	6
Q13158	FADD	FAS-associa	12	2	2	2	208	23.3	5.69	9.05
P20585	MSH3	DNA mismat	3	3	3	3	1137	127.3	8.02	4.94
Q5EBL8	PDZD11	PDZ domain-	25	2	2	2	140	16.1	7.21	6.66
Q6ZS17	RIPOR1	Rho family-ir	2	2	2	2	1223	132.2	6.28	6.51
Q06330	RBPJ	Recombining	5	3	3	3	500	55.6	7.18	4.5
Q15223	NECTIN1	Nectin-1 OS=	6	2	2	2	517	57.1	6.1	5.53
O15530	PDPK1	3-phosphoin	5	3	3	3	556	63.1	7.36	4.55
P14621	ACYP2	Acylphospha	30	3	4	3	99	11.1	9.5	5.31
P01624	IGKV3-15	Immunoglobu	24	2	2	1	115	12.5	5.19	7.41
P15884	TCF4	Transcription	3	3	3	1	667	71.3	7.01	7.95
Q14562	DHX8	ATP-depend	3	3	3	1	1220	139.2	8.32	5.21
Q5EG05	CARD16	Caspase rec	9	2	3	1	197	22.6	8.44	6.78
Q9Y3R5	DOP1B	Protein dope	1	2	2	2	2298	258.1	6.29	7.11
Q15796	SMAD2	Mothers aga	5	2	3	1	467	52.3	6.58	13
P24310	COX7A1	Cytochrome	29	2	2	2	79	9.1	10.11	5.65
Q5BJH7	YIF1B	Protein YIF1	4	1	2	1	314	34.4	9.16	5.95
Q8IXI2	RHOT1	Mitochondria	5	3	3	2	618	70.7	6.27	9.12
Q8N5M4	TTC9C	Tetratricope	14	2	2	2	171	20	8.92	7.37
Q9GZQ3	COMMD5	COMM doma	12	3	4	3	224	24.7	7.02	10.12
P68402	PAFAH1B2	Platelet-activ	14	3	4	2	229	25.6	5.92	9.67
P20160	AZU1	Azurocidin O	8	2	6	2	251	26.9	9.5	22.62
P01766	IGHV3-13	Immunoglobu	19	2	3	1	116	12.5	7.08	10.13
Q9BPY3	FAM118B	Protein FAM	9	3	4	2	351	39.5	5.99	10.71

P41217	CD200	OX-2 membr	9	2	2	2	278	31.2	8.51	7.18
Q9Y6H1	CHCHD2	Coiled-coil-h	15	2	2	2	151	15.5	9.22	8.05
O00443	PIK3C2A	Phosphatidyl	2	3	3	3	1686	190.6	8.02	3.77
Q9H237	PORCN	Protein-serin	4	2	3	2	461	52.3	8.84	5.15
Q8NEB9	PIK3C3	Phosphatidyl	3	3	3	3	887	101.5	6.81	8.11
Q86Y56	DNAAF5	Dynein axon	3	2	2	2	855	93.5	6.42	8.36
P14902	IDO1	Indoleamine	10	4	4	4	403	45.3	7.3	7.39
O43251	RBFOX2	RNA binding	6	2	2	2	390	41.3	7.27	7.05
O14772	FPGT	Fucose-1-ph	5	2	2	2	607	68	6.87	5.76
O43768	ENSA	Alpha-endos	24	3	3	3	121	13.4	7.24	6.2
O14662	STX16	Syntaxin-16	7	3	3	3	325	37	6.11	7.81
Q9Y2R5	MRPS17	28S ribosom	29	2	2	2	130	14.5	9.85	6.35
Q8N9T8	KRI1	Protein KRI1	7	4	4	4	703	82.5	5.14	4.52
Q5EBM0	CMPK2	UMP-CMP ki	6	2	2	2	449	49.4	7.01	6.8
Q9NY93	DDX56	Probable AT	5	3	3	3	547	61.6	9.26	7.14
Q9UDW1	UQCR10	Cytochrome	41	2	2	2	63	7.3	9.47	7.07
P49662	CASP4	Caspase-4 C	7	3	4	3	377	43.2	6	9.7
Q9BT23	LIMD2	LIM domain-	19	3	5	2	127	14.1	9.03	6.77
Q96EY8	MMAB	Corrinoid ade	9	2	2	2	250	27.4	8.6	6.82
Q9BX40	LSM14B	Protein LSM	9	2	2	2	385	42	9.69	7.33
Q8ND04	SMG8	Nonsense-m	3	3	4	3	991	109.6	7.68	6.25
P49913	CAMP	Cathelicidin a	12	2	4	2	170	19.3	9.41	4.57
P18074	ERCC2	General tran	3	2	2	2	760	86.9	7.15	6.78
Q96CD0	FBXL8	F-box/LRR-r	10	3	3	3	374	40.5	7.31	6.93
O43739	CYTH3	Cytohesin-3	8	3	4	3	400	46.3	5.47	5.75
Q9BV44	THUMPD3	tRNA (guani	6	3	4	3	507	57	6.37	9.46
P01709	IGLV2-8	Immunoglob	14	2	7	1	118	12.4	5.91	13.68
Q9Y4F5	CEP170B	Centrosomal	2	4	4	2	1589	171.6	6.84	6.86
Q9BX67	JAM3	Junctional ac	8	3	3	3	310	35	7.59	5.08
Q9NZ09	UBAP1	Ubiquitin-ass	10	3	3	3	502	55	5.11	5.86
Q16890	TPD52L1	Tumor protei	5	1	2	1	204	22.4	5.62	6.59
Q8N9R8	SCAI	Protein SCAI	5	3	3	3	606	70.4	8.6	7.47
P18615	NELFE	Negative elo	8	2	2	2	380	43.2	9.33	6.87

Q96GD0	PDXP	Chronophin (	9	2	2	2	296	31.7	6.55	5.84
Q13445	TMED1	Transmembr	11	2	3	2	227	25.2	4.48	6.56
Q9Y5U9	IER3IP1	Immediate ea	34	2	2	2	82	9	8.22	7.75
Q9H944	MED20	Mediator of F	12	2	2	2	212	23.2	6.87	6.12
Q9Y3D7	PAM16	Mitochondria	19	3	4	3	125	13.8	9.7	7.14
Q8NHP6	MOSPD2	Motile sperm	4	2	2	2	518	59.7	5.96	8.05
Q99643	SDHC	Succinate de	12	2	2	2	169	18.6	9.69	6.69
Q86WQ0	NR2C2AP	Nuclear rece	22	2	2	2	139	15.9	6.16	7.84
Q7Z3E5	ARMC9	LisH domain-	4	3	3	3	818	91.8	6.2	8.23
Q8IWV8	UBR2	E3 ubiquitin-		2	3	3	3	1755	200.4	6.24	2.5
Q9H6I2	SOX17	Transcriptior	10	2	2	2	414	44.1	6.48	6.06
Q9NP77	SSU72	RNA polyme	6	1	2	1	194	22.6	5.33	6.42
Q9Y385	UBE2J1	Ubiquitin-cor	9	3	3	3	318	35.2	6.74	3.64
Q6ZMZ3	SYNE3	Nesprin-3 OS	3	2	2	2	975	112.1	6.23	6.83
A0A0C4DH3	IGHV3-35	Probable nor	9	1	2	1	117	12.8	7.88	7.12
Q9UL40	ZNF346	Zinc finger pr	4	1	2	1	294	32.9	9.09	6.84
O60443	GSDME	Gasdermin-E	5	2	2	2	496	54.5	5.17	5.01
P00742	F10	Coagulation	6	2	2	2	488	54.7	5.94	7.06
O43310	CTIF	CBP80/20-de	5	2	3	2	598	67.5	6.54	8.29
Q02040	AKAP17A	A-kinase anc	4	4	4	4	695	80.7	9.73	8.01
Q8TCD1	C18orf32	UPF0729 prc	24	2	3	2	76	8.7	9.13	7.84
P56589	PEX3	Peroxisomal	7	2	2	2	373	42.1	8.15	6.53
Q96II8	LRCH3	DISP comple	3	2	2	2	777	86	6.71	7.54
Q6UVK1	CSPG4	Chondroitin s	1	2	2	2	2322	250.4	5.47	7.09
Q8ND76	CCNY	Cyclin-Y OS=	7	2	2	2	341	39.3	7.2	5.66
Q92765	FRZB	Secreted friz	9	2	2	2	325	36.2	8.46	7.62
Q12981	BNIP1	Vesicle trans	14	2	2	2	228	26.1	8.95	2.73
Q86V88	MDP1	Magnesium-(	15	3	3	3	176	20.1	6.39	6.93
Q9BXW6	OSBPL1A	Oxysterol-bir	3	3	4	3	950	108.4	6.38	6.8
Q9BTC0	DIDO1	Death-induce	1	3	3	3	2240	243.7	7.88	5.08
O43924	PDE6D	Retinal rod rl	16	2	3	2	150	17.4	5.67	10.63
Q5VZL5	ZMYM4	Zinc finger M	2	3	4	1	1548	172.7	6.84	9.62
P61956	SUMO2	Small ubiquit	29	3	3	1	95	10.9	5.5	5.19

Q9UJC3	HOOK1	Protein Hook	6	3	3	3	728	84.6	5.15	7.21
Q8IV36	HID1	Protein HID1	3	2	2	2	788	88.7	6.06	6.49
Q9H1Z4	WDR13	WD repeat-c	7	2	2	2	485	53.7	9.14	5.57
Q9Y3E5	PTRH2	Peptidyl-tRN	15	3	3	3	179	19.2	8.73	8.07
Q6Y1H2	HACD2	Very-long-ch	9	2	2	2	254	28.4	9.55	6.28
O43929	ORC4	Origin recogi	2	1	2	1	436	50.3	8	5.58
P78368	CSNK1G2	Casein kinas	9	3	3	3	415	47.4	9.06	2.16
Q9H0U6	MRPL18	39S ribosom	13	2	2	2	180	20.6	9.54	6.77
Q7Z4R8	C6orf120	UPF0669 pro	17	2	2	2	191	20.8	4.84	7.99
P42773	CDKN2C	Cyclin-deper	15	3	3	3	168	18.1	6.52	7.77
Q9BZ29	DOCK9	Dedicator of	1	3	3	2	2069	236.3	7.49	8.26
O94851	MICAL2	[F-actin]-mor	1	2	2	2	1957	218.9	8.34	5.49
Q9Y5B0	CTDP1	RNA polyme	3	2	3	2	961	104.3	5.27	8.46
Q8WW12	PCNP	PEST proteo	16	2	2	2	178	18.9	7.49	5.71
Q6PML9	SLC30A9	Zinc transpoi	5	3	3	3	568	63.5	8.32	8.08
Q99983	OMD	Osteomoduli	5	2	2	2	421	49.5	5.59	4.17
P52823	STC1	Stanniocalcir	9	2	2	2	247	27.6	7.99	5.72
O75348	ATP6V1G1	V-type protoi	19	2	3	2	118	13.7	8.79	6.83
Q8IYB5	SMAP1	Stromal mem	4	2	3	2	467	50.4	8.75	8.7
Q9H7B2	RPF2	Ribosome pr	8	3	4	3	306	35.6	9.99	11.06
Q9NW68	BSDC1	BSD domain	5	2	2	2	430	47.1	4.49	7.14
Q86U70	LDB1	LIM domain-l	8	3	3	3	411	46.5	6.96	6.3
Q9UJY4	GGA2	ADP-ribosyla	5	3	3	2	613	67.1	6.55	6.74
Q86TU7	SETD3	Actin-histidin	5	3	3	3	594	67.2	5.96	2.42
Q96FK6	WDR89	WD repeat-c	6	2	2	2	387	43.2	6.13	5.77
Q8IZQ5	SELENOH	Selenoprotei	20	2	2	2	122	13.4	9.74	6.73
Q9Y5T5	USP16	Ubiquitin car	3	2	2	2	823	93.5	6.93	5.66
Q96RK4	BBS4	Bardet-Biedl	4	2	2	2	519	58.2	7.31	6.58
Q969E2	SCAMP4	Secretory ca	5	1	2	1	229	25.7	8.82	6.93
O14807	MRAS	Ras-related p	13	3	3	3	208	23.8	8.79	8.38
Q15532	SS18	Protein SSX	3	1	2	1	418	45.9	6.46	7.22
Q00722	PLCB2	1-phosphatic	2	2	2	2	1185	133.9	6.29	3.42
P47974	ZFP36L2	mRNA decay	5	2	2	1	494	51	8.16	5.99

Q96RN5	MED15	Mediator of F	5	2	2	2	788	86.7	9.42	5
P29972	AQP1	Aquaporin-1	10	2	2	2	269	28.5	7.42	7.84
P54105	CLNS1A	Methylosome	11	2	2	2	237	26.2	4.11	6.46
Q8IVI9	NOSTRIN	Nostrin OS=I	5	2	2	2	506	57.6	8.97	5.83
P13762	HLA-DRB4	HLA class II	9	3	5	1	266	29.9	7.33	11.07
Q99836	MYD88	Myeloid diffe	5	2	3	2	296	33.2	6.15	7.24
Q8N8A2	ANKRD44	Serine/threoı	3	2	3	2	993	107.5	6.3	6.34
P03897	MT-ND3	NADH-ubiqu	13	1	2	1	115	13.2	4.44	7.13
Q9UQN3	CHMP2B	Charged mul	14	4	4	4	213	23.9	8.76	7.67
Q5JPI3	C3orf38	Uncharacteri	8	2	2	2	329	37.5	6.47	7.07
Q9HAS0	C17orf75	Protein Njmu	8	3	3	3	396	44.6	5.03	4.18
Q8IWS0	PHF6	PHD finger p	6	3	4	3	365	41.3	8.68	5.86
O75382	TRIM3	Tripartite mo	3	2	2	2	744	80.8	7.83	6.85
O60828	PQBP1	Polyglutamin	6	2	3	2	265	30.5	6.33	7.8
Q9UM00	TMCO1	Calcium load	10	2	2	2	239	27.1	10.26	6.19
Q99807	COQ7	5-demethoxy	11	2	3	2	217	24.3	8.59	1.79
P20933	AGA	N(4)-(beta-N	8	2	2	2	346	37.2	6.28	8
Q96BW9	TAMM41	Phosphatida	6	2	2	2	452	51	7.94	5
Q9NQX3	GPHN	Gephyrin OS	3	3	3	3	736	79.7	5.43	7.01
P04049	RAF1	RAF proto-oı	4	3	3	1	648	73	9.2	5.19
Q587I9	SFT2D3	Vesicle trans	14	2	2	2	215	21.8	9.89	6.34
Q05481	ZNF91	Zinc finger pı	5	3	4	3	1191	137.1	9.32	5.2
Q9UJ68	MSRA	Mitochondria	9	3	3	3	235	26.1	8.09	7.87
P02745	C1QA	Complement	9	2	2	2	245	26	9.11	5.99
Q8ND94	LRRN4CL	LRRN4 C-teı	12	2	2	2	238	25.2	6.51	7.05
Q9UKX7	NUP50	Nuclear pore	7	3	3	3	468	50.1	7.06	6.8
Q96L93	KIF16B	Kinesin-like p	2	2	2	1	1317	151.9	6.16	7.34
Q96P44	COL21A1	Collagen alpl	3	3	4	3	957	99.3	8.32	8.54
Q9Y4K3	TRAF6	TNF recepto	4	2	2	2	522	59.5	6.44	6.12
P62068	USP46	Ubiquitin carl	7	2	2	2	366	42.4	6.83	7.64
Q15047	SETDB1	Histone-lysin	2	3	3	3	1291	143.1	6.02	7.56
Q5HYI8	RABL3	Rab-like prot	11	3	3	3	236	26.4	7.11	7.51
O75954	TSPAN9	Tetraspanin-	5	1	2	1	239	26.8	7.68	6.16

Q15554	TERF2	Telomeric re	5	2	2	2	542	59.6	9.35	6.8
P49005	POLD2	DNA polyme	9	2	2	2	469	51.3	5.58	4.65
Q96CU9	FOXRED1	FAD-depend	3	1	4	1	486	53.8	7.78	10.95
Q96BR1	SGK3	Serine/threor	2	1	2	1	496	57.1	6.93	6.06
Q9GZU8	PSME3IP1	PSME3-inter	10	2	2	2	254	28.9	5.45	5.88
O15226	NKRF	NF-kappa-B-	4	2	2	2	690	77.6	8.79	6.23
Q70J99	UNC13D	Protein unc-1	2	2	2	2	1090	123.2	6.65	5.67
P47712	PLA2G4A	Cytosolic pho	3	3	3	3	749	85.2	5.38	5.22
P58335	ANTXR2	Anthrax toxir	4	2	2	2	489	53.6	7.46	6.55
P08582	MELTF	Melanotransf	4	3	3	3	738	80.2	5.94	4.97
Q92506	HSD17B8	(3R)-3-hydro	9	2	2	2	261	27	6.54	6.44
Q8WXI4	ACOT11	Acyl-coenzyr	4	2	2	2	607	68.4	8.35	7.05
P54821	PRRX1	Paired meso	11	2	2	2	245	27.3	9.47	8.1
Q9H1I8	ASCC2	Activating sig	5	3	3	3	757	86.3	5.16	4.84
O96011	PEX11B	Peroxisomal	9	2	2	2	259	28.4	9.85	7.35
O75448	MED24	Mediator of F	3	3	3	3	989	110.2	6.95	4.47
Q9H446	RWDD1	RWD domair	4	1	2	1	243	27.9	4.2	5.11
Q08345	DDR1	Epithelial dis	3	2	3	2	913	101.1	6.83	13.58
P30711	GSTT1	Glutathione S	15	3	3	3	240	27.3	7.49	7.49
P57105	SYNJ2BP	Synaptojanin	14	2	2	2	145	15.9	6.3	6.41
O15162	PLSCR1	Phospholipid	3	1	2	1	318	35	4.94	6.05
P54922	ADPRH	ADP-ribosylh	8	2	2	2	357	39.5	6.52	6.3
Q9UJX3	ANAPC7	Anaphase-pr	5	3	3	3	565	63.1	5.72	2.92
Q8IX05	CD302	CD302 antig	8	1	2	1	232	26.2	4.61	4.82
Q96SK2	TMEM209	Transmembr	5	2	2	2	561	62.9	8.63	4.95
O60513	B4GALT4	Beta-1,4-gala	8	3	3	3	344	40	9.07	7.48
Q8ND30	PPFIBP2	Liprin-beta-2	3	2	2	1	876	98.5	6.27	6.8
Q5PRF9	SAMD4B	Protein Smau	4	2	2	1	694	75.4	6.83	4.28
Q96MX0	CMTM3	CKLF-like MA	8	2	3	2	182	19.7	4.56	5.2
O43665	RGS10	Regulator of	15	3	3	3	181	21.2	7.49	7.55
Q9P2D3	HEATR5B	HEAT repeat	1	3	4	3	2071	224.2	7.17	4.89
O60487	MPZL2	Myelin protei	10	2	2	2	215	24.5	7.02	5.28
Q9Y399	MRPS2	28S ribosom	8	3	3	3	296	33.2	9.26	8.87

Q9Y3Q8	TSC22D4	TSC22 doma	6	2	2	1	395	41	7.21	7.52
O14545	TRAFD1	TRAF-type z	5	2	2	2	582	64.8	5.29	7.34
Q7Z304	MAMDC2	MAM domair	4	2	2	2	686	77.5	5.16	5.68
O00244	ATOX1	Copper trans	38	2	2	2	68	7.4	7.24	6.14
Q86W50	METTL16	RNA N6-ade	4	2	2	2	562	63.6	7.85	5.11
Q6P6C2	ALKBH5	RNA demeth	2	1	2	1	394	44.2	9.09	5.58
P51397	DAP	Death-assoc	16	2	5	2	102	11.2	9.32	4.86
Q9NYU1	UGGT2	UDP-glucose	1	2	2	1	1516	174.6	6.89	7.77
O15427	SLC16A3	Monocarboxy	5	3	3	3	465	49.4	7.96	6.75
Q99575	POP1	Ribonuclease	2	2	2	2	1024	114.6	9.22	3.57
Q9P2X0	DPM3	Dolichol-pho:	24	2	2	2	92	10.1	5.94	5.46
P49184	DNASE1L1	Deoxyribonu	8	3	3	3	302	33.9	5.74	5.47
Q9BZ95	NSD3	Histone-lysin	2	2	2	2	1437	161.5	8.21	3.76
Q8NHV1	GIMAP7	GTPase IMA	8	3	3	1	300	34.5	6.46	8.35
Q9NRD9	DUOX1	Dual oxidase	3	4	4	4	1551	177.1	7.9	6.88
Q8IXI1	RHOT2	Mitochondria	4	2	2	1	618	68.1	5.86	6.42
A3KMH1	VWA8	von Willebrar	1	3	3	3	1905	214.7	7.4	4.38
Q9Y6X5	ENPP4	Bis(5'-adeno	6	3	3	3	453	51.6	6.15	4.09
Q96MH6	TMEM68	Transmembr	10	2	2	2	324	37.4	7.85	3.51
P18440	NAT1	Arylamine N-	11	3	3	3	290	33.9	6.54	4.73
Q16595	FXN	Frataxin, mit	11	2	2	2	210	23.1	8.69	7.24
Q8IVN8	SBSPON	Somatomedi	14	2	2	2	264	29.6	7.5	5.13
Q9H2W6	MRPL46	39S ribosom	9	2	2	2	279	31.7	7.05	5.78
O95251	KAT7	Histone acet	5	3	3	3	611	70.6	8.85	4.78
O43464	HTRA2	Serine protea	5	2	2	2	458	48.8	10.07	5.37
Q9BTC8	MTA3	Metastasis-a	5	3	3	2	594	67.5	8.57	7.23
P50749	RASSF2	Ras associat	9	3	3	3	326	37.8	8.84	6.37
O43236	SEPTIN4	Septin-4 OS=	6	3	3	1	478	55.1	6.11	7.58
P10144	GZMB	Granzyme B	9	2	2	2	247	27.7	9.57	6.51
Q13454	TUSC3	Tumor suppr	5	2	3	1	348	39.7	9.91	7.68
Q7LGC8	CHST3	Carbohydrat	6	2	2	2	479	54.7	8.59	5.35
Q66K79	CPZ	Carboxypept	4	2	2	2	652	73.6	7.97	6.54
Q7Z2E3	APTX	Aprataxin OS	7	2	2	2	356	40.7	9.17	3.75

O43570	CA12	Carbonic anl	8	2	2	2	354	39.4	7.23	3.64
Q9Y692	GMEB1	Glucocortico	3	2	2	1	573	62.6	4.83	5.63
A0A0A0MRZ	IGKV3D-11	Immunoglobu	14	2	4	1	115	12.6	5.29	9.11
Q9BV19	C1orf50	Uncharacteri	11	2	2	2	199	21.9	5.88	9.03
Q8NDI1	EHBP1	EH domain-b	3	3	3	3	1231	139.9	5.35	4.27
Q9Y5B8	NME7	Nucleoside d	6	2	2	2	376	42.5	6.47	4.98
Q8TDP1	RNASEH2C	Ribonucleas	16	2	2	2	164	17.8	5.03	7.02
Q9NPE3	NOP10	H/ACA ribon	34	2	2	2	64	7.7	9.99	6.44
P33402	GUCY1A2	Guanylate cy	3	2	2	2	732	81.7	7.66	4.96
O95159	ZFPL1	Zinc finger p	8	2	2	2	310	34.1	8.07	6.22
O95630	STAMBP	STAM-bindin	6	2	2	2	424	48	6.29	8.63
Q8WUY1	THEM6	Protein THE	14	4	5	4	208	23.9	9.55	11
Q9NQH7	XPNPEP3	Xaa-Pro ami	6	2	2	2	507	57	6.83	3.48
Q9UHW9	SLC12A6	Solute carrie	1	2	3	1	1150	127.5	7.08	7.61
Q96BY7	ATG2B	Autophagy-re	1	3	4	3	2078	232.6	5.76	6.98
Q8WUH2	TGFBRAP1	Transforming	4	4	4	4	860	97.1	6.55	2.11
Q9P0U3	SENP1	Sentrin-spec	4	2	2	2	644	73.4	8.47	6.31
P98171	ARHGAP4	Rho GTPase	3	3	3	3	946	105	6.34	6.79
P43353	ALDH3B1	Aldehyde de	5	2	2	2	468	51.8	7.62	5.85
O00165	HAX1	HCLS1-asso	8	2	2	2	279	31.6	4.92	5.17
Q5VT52	RPRD2	Regulation o	2	2	3	2	1461	155.9	7.42	8.47
Q96AG3	SLC25A46	Mitochondria	7	2	2	2	418	46.1	7.43	7.05
O60870	KIN	DNA/RNA-bi	6	2	2	2	393	45.3	8.95	3.55
Q9P0V3	SH3BP4	SH3 domain-	3	2	2	2	963	107.4	7.71	4.93
Q96BQ5	CCDC127	Coiled-coil d	9	3	3	3	260	30.8	9.2	6.98
Q9Y221	NIP7	60S ribosom	22	2	2	2	180	20.4	8.51	4.14
Q6P3W7	SCYL2	SCY1-like pr	3	3	3	3	929	103.6	8.22	4.7
P07359	GP1BA	Platelet glyc	3	2	2	2	652	71.5	6.29	5.32
P51690	ARSL	Arylsulfatase	6	3	3	2	589	65.6	6.96	7.23
Q9H4I9	SMDT1	Essential MC	29	2	2	2	107	11.4	7.27	5.91
Q9Y5U2	TSSC4	Protein TSS	7	2	2	2	329	34.3	5.19	6.15
Q15648	MED1	Mediator of F	2	3	3	3	1581	168.4	8.73	0
Q9BU02	THTPA	Thiamine-trip	14	3	3	3	230	25.6	4.82	4.48

P57076	CFAP298	Cilia- and fla	8	3	3	3	290	33.2	7.44	5.11
Q9BQC6	MRPL57	Ribosomal pı	20	2	2	2	102	12.3	11.44	7.27
Q96D71	REPS1	RalBP1-assc	3	2	2	2	796	86.6	5.69	6.74
P02008	HBZ	Hemoglobin	13	2	73	1	142	15.6	8.21	71.61
Q9BW61	DDA1	DET1- and D	20	2	2	2	102	11.8	8.68	6.1
P0CG08	GPR89B	Golgi pH reg	6	1	1	1	455	52.9	9.28	7.73
Q92995	USP13	Ubiquitin car	3	2	4	1	863	97.3	5.53	2.57
P00846	MT-ATP6	ATP synthas	4	1	4	1	226	24.8	10.1	14.55
Q5SNT2	TMEM201	Transmembr	3	2	3	2	666	72.2	9.22	9.78
Q9NPY3	CD93	Complement	5	2	2	2	652	68.5	5.44	6.13
Q96AP7	ESAM	Endothelial c	6	2	2	2	390	41.2	9.32	6.16
Q9UNH6	SNX7	Sorting nexir	7	3	3	3	387	45.3	5.11	4.56
Q15773	MLF2	Myeloid leuk	12	3	3	3	248	28.1	6.9	6.22
O00192	ARVCF	Splicing regu	3	2	2	2	962	104.6	6.81	4.94
Q9H6U8	ALG9	Alpha-1,2-ma	4	2	2	2	611	69.8	8.68	6.96
P15291	B4GALT1	Beta-1,4-gala	7	2	2	2	398	43.9	8.65	6.78
Q9HAD4	WDR41	WD repeat-c	5	2	2	2	459	51.7	5.44	2.55
Q15054	POLD3	DNA polyme	5	2	2	2	466	51.4	9.35	4.83
P07332	FES	Tyrosine-pro	3	2	2	2	822	93.4	6.73	5.26
P31431	SDC4	Syndecan-4	13	2	2	2	198	21.6	4.5	5.85
Q5T2E6	ARMH3	Armadillo-like	3	2	2	2	689	78.7	6.6	5.96
P41222	PTGDS	Prostaglandi	16	3	5	3	190	21	7.8	9.99
P01701	IGLV1-51	Immunoglobu	14	2	4	2	117	12.2	7.03	10.11
Q8TBF4	ZCRB1	Zinc finger C	11	2	2	2	217	24.6	8.53	3.34
P54108	CRISP3	Cysteine-rich	6	2	3	2	245	27.6	7.8	6.29
P85037	FOXK1	Forkhead bo	4	4	4	4	733	75.4	9.32	4.23
Q9NX08	COMMD8	COMM doma	12	2	2	2	183	21.1	5.43	7.16
Q9Y5J7	TIMM9	Mitochondria	17	1	2	1	89	10.4	7.21	4.05
Q9C0B0	UNK	RING finger	3	3	3	3	810	88	6.86	4.42
Q15714	TSC22D1	TSC22 doma	3	3	3	1	1073	109.6	5.64	4.6
Q08397	LOXL1	Lysyl oxidase	5	3	3	2	574	63.1	7.52	5.4
Q63HM9	PLCXD3	PI-PLC X do	9	2	2	2	321	36.3	6.29	5.78
Q2TB10	ZNF800	Zinc finger pı	4	3	3	3	664	75.2	9.47	4.99

P32926	DSG3	Desmoglein-	2	2	2	2	999	107.5	5	5.29
Q9GZM8	NDEL1	Nuclear distr	6	2	2	1	345	38.4	5.24	5.05
Q9NWV8	BABAM1	BRISC and E	7	2	2	2	329	36.5	4.64	4.17
P83110	HTRA3	Serine protea	5	2	2	2	453	48.6	7.09	5.3
Q9BYM8	RBCK1	RanBP-type	4	2	2	2	510	57.5	5.67	4.69
Q96GC5	MRPL48	39S ribosom	11	2	2	2	212	23.9	8.98	5.9
Q99720	SIGMAR1	Sigma non-o	9	2	3	2	223	25.1	5.96	7.49
Q9H6A0	DENND2D	DENN doma	5	2	2	2	471	53.6	7.64	5.6
Q6PD74	AAGAB	Alpha- and g	7	2	2	2	315	34.6	4.64	6.83
P31997	CEACAM8	Carcinoembr	6	2	2	2	349	38.1	7.39	5.76
Q96F10	SAT2	Thialysine N-	18	2	2	2	170	19.1	6.04	4.32
Q8N1N4	KRT78	Keratin, type	4	3	5	1	520	56.8	6.02	11.16
Q9UID3	VPS51	Vacuolar pro	3	2	2	2	782	86	6.47	3.88
Q9BXB5	OSBPL10	Oxysterol-bir	5	3	3	2	764	83.9	8.31	5.21
P37268	FDFT1	Squalene syr	5	2	2	2	417	48.1	6.54	5.51
P62861	FAU	FAU ubiquitir	8	1	4	1	133	14.4	10.17	11.98
Q9P2X3	IMPACT	Protein IMPA	8	2	2	2	320	36.5	4.97	6.32
Q86TM6	SYVN1	E3 ubiquitin-p	4	3	4	3	617	67.6	6.95	6.04
Q15041	ARL6IP1	ADP-ribosyla	12	3	4	2	203	23.3	9.32	4.31
Q9Y4K4	MAP4K5	Mitogen-activ	3	2	2	2	846	95	7.83	5.5
Q96EK7	FAM120B	Constitutive c	4	3	4	3	910	103.7	5.69	7.49
Q9UPN9	TRIM33	E3 ubiquitin-p	2	3	3	3	1127	122.5	6.67	4.84
Q9UPP1	PHF8	Histone lysin	2	2	2	2	1060	117.8	8.72	5.07
Q86WJ1	CHD1L	Chromodoma	3	3	3	3	897	100.9	6.9	4.47
O43716	GATC	Glutamyl-tRN	20	2	2	2	136	15.1	5.05	5.14
Q676U5	ATG16L1	Autophagy-re	4	2	2	2	607	68.2	6.64	5.5
O60231	DHX16	Pre-mRNA-s	2	2	3	1	1041	119.2	6.8	5.47
Q8IWB1	ITPRIP	Inositol 1,4,5	4	2	2	2	547	62	5.88	6.58
Q5W0Z9	ZDHHC20	Palmitoyltran	5	2	2	2	365	42.3	7.71	5.27
Q7Z4S6	KIF21A	Kinesin-like p	1	2	2	2	1674	187.1	6.42	5.64
Q8NEY8	PPHLN1	Periphilin-1 C	6	3	3	3	458	52.7	9.11	5.55
Q9H9P8	L2HGDH	L-2-hydroxyg	5	2	2	2	463	50.3	8.15	5.46
Q6NUM9	RETSAT	All-trans-retir	4	2	2	2	610	66.8	8.28	5.92

Q9HAU5	UPF2	Regulator of	2	3	3	3	1272	147.7	5.69	3.98
Q9BTX1	NDC1	Nucleoporin	4	2	2	2	674	76.3	9.09	5.83
P78324	SIRPA	Tyrosine-pro	5	2	2	2	504	54.9	6.98	6.45
Q9UL54	TAOK2	Serine/threor	2	2	2	1	1235	138.2	7.27	5.05
O00459	PIK3R2	Phosphatidyl	4	3	3	3	728	81.5	6.43	5.14
Q9NQY0	BIN3	Bridging inte	8	2	2	2	253	29.6	7.47	6.27
Q8IVL5	P3H2	Prolyl 3-hydr	4	3	4	2	708	80.9	5.71	5.72
Q96A57	TMEM230	Transmembr	18	2	2	2	120	13.2	9.31	5.78
Q9BQ39	DDX50	ATP-depend	3	2	2	1	737	82.5	9.17	5.77
Q12872	SFSWAP	Splicing facto	3	3	3	3	951	104.8	8.05	6.82
Q6UWR7	ENPP6	Glycerophos	6	2	2	2	440	50.2	7.99	6.05
Q96FX7	TRMT61A	tRNA (adenir	9	2	2	2	289	31.4	7.36	6.57
Q7Z7N9	TMEM179B	Transmembr	12	2	2	2	219	23.5	7.85	6.17
Q00535	CDK5	Cyclin-deper	13	2	2	2	292	33.3	7.66	5.87
O95801	TTC4	Tetratricopep	5	2	2	2	387	44.7	5.6	4.7
O15066	KIF3B	Kinesin-like p	3	2	2	2	747	85.1	7.69	5.29
Q5U651	RASIP1	Ras-interacti	3	3	4	3	963	103.4	7.96	5.88
Q969U7	PSMG2	Proteasome	8	2	2	2	264	29.4	6.98	6.57
Q9Y679	AUP1	Lipid droplet-	5	3	3	3	410	45.8	8.65	3.92
Q9P287	BCCIP	BRCA2 and	9	3	3	3	314	36	4.61	5.26
O60830	TIMM17B	Mitochondria	13	1	1	1	172	18.3	9.03	6.17
Q9NVM6	DNAJC17	DnaJ homolo	8	2	2	2	304	34.7	8.53	5.45
P35443	THBS4	Thrombospo	2	2	2	1	961	105.8	4.68	5.08
P78524	DENND2B	DENN doma	2	2	2	2	1137	126.4	9.25	4.89
Q96KC8	DNAJC1	DnaJ homolo	3	2	2	2	554	63.8	8.63	5.37
O60496	DOK2	Docking prot	6	2	2	2	412	45.4	6.02	3.06
Q96HU1	SGSM3	Small G prot	3	2	2	2	749	85.3	6	5.17
O95864	FADS2	Acyl-CoA 6-c	6	3	4	3	444	52.2	8.82	9.66
Q5BJF2	TMEM97	Sigma intrac	14	2	2	2	176	20.8	9.38	5.14
Q4KMP7	TBC1D10B	TBC1 domai	3	3	3	3	808	87.1	9.19	5.89
Q9P0T7	TMEM9	Proton-trans	15	1	1	1	183	20.6	6.65	4.53
P08637	FCGR3A	Low affinity i	9	2	3	2	254	29.1	8.07	2.8
Q5HYI7	MTX3	Metaxin-3 OS	7	2	2	2	312	35.1	7.8	4.95

Q05823	RNASEL	2-5A-depend	3	2	2	2	741	83.5	6.65	4.7
A0A0C4DH3	IGHV4-28	Immunoglobu	8	1	2	1	117	13.1	9.29	6
P84022	SMAD3	Mothers aga	5	2	3	1	425	48.1	7.15	10.54
O94886	TMEM63A	CSC1-like pr	3	2	2	2	807	92.1	7.27	5.42
Q9BV73	CEP250	Centrosome-	1	3	3	1	2442	281	5.02	4.58
Q9BQC3	DPH2	2-(3-amino-3	8	3	3	3	489	52.1	5.53	4.56
Q5T447	HECTD3	E3 ubiquitin-	5	2	2	2	861	97.1	5.64	5.72
Q9NRG1	PRTFDC1	Phosphoribo	9	2	3	1	225	25.7	6.15	4.11
Q8NFC6	BOD1L1	Biorientation	1	3	3	2	3051	330.3	5.08	5
Q9UNQ2	DIMT1	Probable dim	7	2	2	2	313	35.2	9.99	5.11
Q92903	CDS1	Phosphatida	5	2	2	2	461	53.3	8.09	6.65
P53609	PGGT1B	Geranylgera	5	2	3	2	377	42.3	6.83	9.37
Q9BPX6	MICU1	Calcium upta	6	3	3	3	476	54.3	8.41	3.86
Q9BZL1	UBL5	Ubiquitin-like	29	2	2	2	73	8.5	8.44	4.95
P11274	BCR	Breakpoint c	2	3	3	1	1271	142.7	7.03	5.58
Q5SGD2	PPM1L	Protein phos	6	2	2	2	360	41	5.87	4.83
A0A0J9YX3	IGHV3-64D	Immunoglobu	19	2	2	2	117	12.8	7.85	2.79
Q8IWE4	DCUN1D3	DCN1-like pr	4	1	2	1	304	34.3	5.12	5.67
P07902	GALT	Galactose-1-	6	2	2	2	379	43.3	6.99	5.62
Q8IVT5	KSR1	Kinase suppr	2	2	2	2	923	102.1	8.66	4.65
Q9NVU7	SDAD1	Protein SDA	3	2	2	2	687	79.8	9.25	3.02
P19075	TSPAN8	Tetraspanin-	10	2	2	2	237	26	5.6	3.51
Q6QNY1	BLOC1S2	Biogenesis o	16	2	2	2	142	16	4.86	7.13
Q13795	ARFRP1	ADP-ribosyla	13	2	2	2	201	22.6	7.56	4.41
Q9P086	MED11	Mediator of F	21	2	2	2	117	13.1	5.96	4.55
Q9NZC3	GDE1	Glycerophos	7	2	2	2	331	37.7	6.71	3.01
P62304	SNRPE	Small nuclea	26	2	3	2	92	10.8	9.44	7.18
Q96PU4	UHRF2	E3 ubiquitin-	3	2	2	2	802	89.9	8.21	6.18
P17026	ZNF22	Zinc finger pr	9	2	3	2	224	25.9	10.04	8
Q53QV2	LBH	Protein LBH	16	1	1	1	105	12.2	4.41	4.46
Q96IG2	FBXL20	F-box/LRR-r	6	1	1	1	436	48.4	7.49	5.34
Q3V6T2	CCDC88A	Girdin OS=H	1	2	2	2	1871	215.9	6.21	4.89
Q9NZD2	GLTP	Glycolipid tra	10	2	2	2	209	23.8	7.39	6.73

Q969G6	RFK	Riboflavin kir	13	2	2	2	155	17.6	8.13	5.67
O95347	SMC2	Structural ma	2	2	2	2	1197	135.6	8.43	5.21
Q9UM07	PADI4	Protein-argin	3	2	2	2	663	74	6.58	2.71
Q6GMV2	SMYD5	Histone-lysin	5	3	3	3	418	47.3	5.05	7.06
Q9BQI9	NRIP2	Nuclear rece	10	2	2	2	281	31.3	8.4	3.39
Q15493	RGN	Regucalcin (	8	2	2	2	299	33.2	6.25	8.13
Q9GZQ8	MAP1LC3B	Microtubule-a	17	2	2	2	125	14.7	8.94	6.05
Q96F63	CCDC97	Coiled-coil do	6	2	2	2	343	38.9	4.59	1.87
Q53GS7	GLE1	mRNA expor	3	2	2	2	698	79.8	7.43	4.47
Q16831	UPP1	Uridine phos	6	2	2	2	310	33.9	7.88	5.4
Q8WUB8	PHF10	PHD finger p	4	2	2	2	498	56	6.62	5.13
O43854	EDIL3	EGF-like rep	4	2	2	2	480	53.7	7.28	5.3
P51828	ADCY7	Adenylate cy	2	2	2	2	1080	120.2	8.12	6.1
Q96F86	EDC3	Enhancer of	4	2	3	2	508	56	7.11	5.28
Q8TCA0	LRRC20	Leucine-rich	10	2	2	2	184	20.5	6.55	6.03
A5D8V6	VPS37C	Vacuolar pro	5	2	2	2	355	38.6	5.21	3.44
Q96HR9	REEP6	Receptor exp	9	2	2	2	211	23.4	8.56	5.2
Q2TAA5	ALG11	GDP-Man:Ma	5	2	2	2	492	55.6	8.48	5.14
Q6KB66	KRT80	Keratin, type	6	2	2	2	452	50.5	5.67	2.94
Q8N5G0	SMIM20	Small integra	33	2	2	2	67	7.7	9.86	5.62
O00585	CCL21	C-C motif ch	13	1	3	1	134	14.6	10.1	11.59
Q92633	LPAR1	Lysophospha	6	2	2	2	364	41.1	8.53	4.42
B7ZBB8	PPP1R3G	Protein phos	7	1	1	1	358	38	4.93	6.49
Q15070	OXA1L	Mitochondria	5	2	2	2	435	48.5	9.45	6.38
Q99969	RARRES2	Retinoic acid	12	2	3	2	163	18.6	9.09	10.33
Q8TDM6	DLG5	Disks large h	2	3	3	3	1919	213.7	7.42	3.27
P78310	CXADR	Coxsackievir	5	2	2	2	365	40	7.56	5.12
Q9UFW8	CGGBP1	CGG triplet r	5	1	2	1	167	18.8	8.95	4.95
Q9UIC8	LCMT1	Leucine carb	5	2	2	2	334	38.4	6.04	5.55
Q8TAD4	SLC30A5	Proton-coupl	2	2	2	2	765	84	7.31	5.81
Q13825	AUH	Methylglutac	6	1	1	1	339	35.6	9.48	4.84
O94817	ATG12	Ubiquitin-like	7	1	2	1	140	15.1	5.1	6.52
Q86YE8	ZNF573	Zinc finger pr	3	2	2	1	665	78.1	9.16	4.99

P51689	ARSD	Arylsulfatase	5	3	3	2	593	64.8	7.23	7.31
Q9BXY0	MAK16	Protein MAK	7	2	3	2	300	35.3	5.38	6.28
P04440	HLA-DPB1	HLA class II	10	2	2	1	258	29.1	8.57	6.72
Q96DE0	NUDT16	U8 snoRNA-	11	2	2	2	195	21.3	6.89	3.89
Q9H6Y2	WDR55	WD repeat-c	6	2	2	2	383	42	4.92	3.52
Q9P1F3	ABRACL	Costars fami	20	2	2	2	81	9.1	6.29	6.11
Q6UUV7	CRTC3	CREB-regula	4	2	2	2	619	66.9	6.84	2.77
Q5JS54	PSMG4	Proteasome	20	1	1	1	123	13.8	6.52	5.65
Q9NRX1	PNO1	RNA-binding	9	2	2	2	252	27.9	9.73	5.27
Q9Y2D2	SLC35A3	UDP-N-acety	7	2	2	2	325	36	9.16	4.05
Q8NC96	NECAP1	Adaptin ear-l	10	1	2	1	275	29.7	6.8	9.74
P0C7P0	CISD3	CDGSH iron-	16	2	2	2	127	14.2	10.55	5.88
P48307	TFPI2	Tissue factor	8	2	2	2	235	26.9	8.53	4.85
Q8NCE2	MTMR14	Myotubularin	5	1	2	1	650	72.2	6.24	10.98
L0R819	ASDURF	ASNSD1 ups	24	2	2	2	96	11.2	8.88	5.34
Q96S99	PLEKHF1	Pleckstrin ho	7	2	2	2	279	31.2	8.24	6.42
P06746	POLB	DNA polyme	6	2	2	2	335	38.2	8.95	5.63
Q9UPU9	SAMD4A	Protein Smau	4	2	2	1	718	79.4	8.32	4.74
Q96KN1	LRATD2	Protein LRAT	8	1	1	1	310	34.5	5.54	4.82
Q9H490	PIGU	Phosphatidyl	5	2	2	2	435	50	7.72	4.86
Q9Y2R0	COA3	Cytochrome	18	2	3	2	106	11.7	9.6	6.62
Q8TEQ0	SNX29	Sorting nexir	2	2	2	2	813	91.2	6.21	5.29
P54253	ATXN1	Ataxin-1 OS=	3	3	3	3	815	86.9	8.35	3.86
P62875	POLR2L	DNA-directe	13	1	2	1	67	7.6	7.77	5.35
Q7Z4L5	TTC21B	Tetratricopep	2	2	2	2	1316	150.8	6.96	2.3
Q9C0J8	WDR33	pre-mRNA 3'	2	2	2	2	1336	145.8	9.17	2.06
P54252	ATXN3	Ataxin-3 OS=	5	2	2	2	361	41.2	4.78	3.28
Q96P11	NSUN5	28S rRNA (c	5	2	2	2	429	46.7	8.62	5.69
Q9Y2U5	MAP3K2	Mitogen-activ	4	2	2	1	619	69.7	8	4.48
Q8N5M1	ATPAF2	ATP synthas	7	2	2	2	289	32.8	7.09	5.24
Q8WYQ3	CHCHD10	Coiled-coil-h	16	1	1	1	142	14.1	7.84	5.04
P49757	NUMB	Protein numb	3	2	3	2	651	70.8	8.51	7.97
Q13907	IDI1	Isopentenyl-c	19	3	3	3	227	26.3	6.34	0

Q8IWT0	ZBTB8OS	Protein arche	11	2	2	2	167	19.5	4.49	2.83
Q9Y2L5	TRAPPC8	Trafficking pı	2	2	2	2	1435	160.9	6.87	2.5
Q9H9C1	VIPAS39	Spermatogeı	4	3	4	3	493	57	7.4	10.3
Q5GJ75	TNFAIP8L3	Tumor necro	6	2	2	2	292	32.6	8.59	5.41
Q15125	EBP	3-beta-hydro	12	2	2	2	230	26.3	7.9	5.08
Q9UQ13	SHOC2	Leucine-rich	5	2	2	2	582	64.8	8.46	2.8
P08631	HCK	Tyrosine-pro	5	3	3	1	526	59.6	6.7	4.97
Q969M3	YIPF5	Protein YIPF	8	2	2	2	257	28	4.36	5.1
P22748	CA4	Carbonic anł	6	2	2	2	312	35	7.83	4.92
Q9NVV5	AIG1	Androgen-in	8	2	2	2	238	27.4	7.17	5.44
Q9NWS6	FAM118A	Protein FAM	6	2	2	2	357	40.2	6.09	4.12
Q8IZ07	ANKRD13A	Ankyrin repe	3	2	3	2	590	67.6	5.02	11.91
P10746	UROS	Uroporphyrin	10	1	1	1	265	28.6	5.48	5.16
Q6XZF7	DNMBP	Dynamin-bin	2	3	3	3	1577	177.2	5.39	5.74
Q6NUK4	REEP3	Receptor exp	7	2	2	2	255	29.2	9.57	4.41
O60244	MED14	Mediator of F	2	2	2	2	1454	160.5	8.73	5.59
Q9UPY3	DICER1	Endoribonuc	1	2	2	2	1922	218.5	5.68	4.91
Q15771	RAB30	Ras-related p	7	2	2	1	203	23	4.97	5.42
Q9NUU6	OTULINL	Inactive ubiq	6	2	2	2	356	42.2	9.29	4.72
Q9Y4D8	HECTD4	Probable E3	1	2	2	2	3996	439.1	6.19	3.27
O75446	SAP30	Histone deac	10	2	2	2	220	23.3	9.17	4.61
Q96AZ6	ISG20	Interferon-sti	13	2	2	2	181	20.4	8.92	3.4
Q9H0N5	PCBD2	Pterin-4-alph	13	2	2	1	130	14.4	9.11	4.82
Q9BRT3	MIEN1	Migration and	16	2	4	2	115	12.4	4.37	10.04
Q9H7N4	SCAF1	Splicing facto	1	2	2	2	1312	139.2	9.25	4.47
A5PLL7	PEDS1	Plasmanyletł	9	2	2	2	270	31.1	6.83	6.05
P06239	LCK	Tyrosine-pro	6	2	2	1	509	58	5.36	4.19
P78362	SRPK2	SRSF proteir	4	2	2	2	688	77.5	4.97	2.23
O75147	OBSL1	Obscurin-like	1	2	2	2	1896	206.8	5.63	5.21
Q9HCG8	CWC22	Pre-mRNA-s	3	2	2	2	908	105.4	7.03	4.22
Q99759	MAP3K3	Mitogen-acti	4	2	2	1	626	70.9	8.84	4.9
P51606	RENBP	N-acylglucos	6	3	3	3	417	47.7	6.25	6.21
Q92870	APBB2	Amyloid beta	3	2	2	2	758	83.3	6.1	3.59

Q9BRJ7	NUDT16L1	Tudor-interac	9	2	2	2	211	23.3	8.91	4.31
Q9H0X9	OSBPL5	Oxysterol-bir	2	2	2	2	879	98.6	7.47	3.94
Q9BYV8	CEP41	Centrosomal	6	2	2	2	373	41.3	8.32	2.51
Q8WXA3	RUFY2	RUN and FY	3	2	2	1	606	70	5.83	5.18
Q86YT6	MIB1	E3 ubiquitin-	3	2	2	2	1006	110.1	6.92	5.92
Q9H6V9	LDAH	Lipid droplet-	6	2	2	2	325	37.3	6.54	5.9
Q92889	ERCC4	DNA repair e	2	2	2	2	916	104.4	6.93	5.56
O14521	SDHD	Succinate de	4	1	7	1	159	17	8.63	12.39
Q9NWX6	THG1L	Probable tRN	6	1	1	1	298	34.8	8	5.77
Q8ND24	RNF214	RING finger	2	2	2	2	703	77.6	6.95	5.41
O95359	TACC2	Transforming	1	2	2	1	2948	309.2	4.79	5.24
P29374	ARID4A	AT-rich intera	2	2	2	2	1257	142.7	5.1	4.95
Q92625	ANKS1A	Ankyrin repe	2	2	3	2	1134	123	6.38	8.66
A6NHQ2	FBLL1	rRNA/tRNA 2	5	1	1	1	334	34.8	10.35	5.92
Q8TDJ6	DMXL2	DmX-like pro	1	3	4	2	3036	339.4	6.38	7.48
P35625	TIMP3	Metalloprotei	15	2	2	2	211	24.1	8.72	3.22
Q86TN4	TRPT1	tRNA 2'-phos	8	2	2	2	253	27.7	9.98	4.66
P30825	SLC7A1	High affinity	3	2	2	2	629	67.6	5.43	3.59
O14925	TIMM23	Mitochondria	10	1	1	1	209	21.9	8.6	4.17
O96013	PAK4	Serine/threor	3	2	2	2	591	64	9.73	4.51
O00214	LGALS8	Galectin-8 O	7	2	2	2	317	35.8	8.25	5.94
Q9NXK8	FBXL12	F-box/LRR-re	6	1	1	1	326	37	8.79	5.91
O75600	GCAT	2-amino-3-ke	8	2	2	2	419	45.3	8.05	3.01
Q9NZC9	SMARCAL1	SWI/SNF-rel	3	2	2	2	954	105.9	9.06	2.98
Q9ULF5	SLC39A10	Zinc transpor	2	2	2	2	831	94.1	6.76	5.05
Q8NFP9	NBEA	Neurobeachi	1	2	2	1	2946	327.6	6.18	2.59
Q4ZIN3	TMEM259	Membralin O	3	2	2	2	620	67.8	5.3	5.29
Q9Y4D1	DAAM1	Disheveled-a	2	2	2	2	1078	123.4	7.23	2.07
Q14244	MAP7	Ensconsin O	4	4	4	4	749	84	9.61	0
Q9BUB7	TMEM70	Transmembr	7	2	2	2	260	29	8.91	4.39
Q99986	VRK1	Serine/threor	6	2	2	2	396	45.4	8.91	6.52
O00194	RAB27B	Ras-related p	10	2	2	2	218	24.6	5.52	5.12
P48740	MASP1	Mannan-binc	3	2	3	1	699	79.2	5.49	7.23

Q9P299	COPZ2	Coatomer su	10	2	2	2	210	23.5	5.17	5.46
Q9GZP9	DERL2	Derlin-2 OS=	13	1	1	1	239	27.5	7.28	5.03
Q7KZI7	MARK2	Serine/threor	3	3	3	3	788	87.9	9.72	4.59
Q9BSF0	C2orf88	Small memb	20	1	2	1	95	11	4.87	6.28
Q8IWU5	SULF2	Extracellular	3	3	3	2	870	100.4	9.17	7.4
A0A087WSZ	IGKV1D-8	Immunoglobu	16	1	2	1	117	12.8	7.74	9.97
P48728	AMT	Aminomethyl	4	2	2	2	403	43.9	8.57	5.32
Q9NZT1	CALML5	Calmodulin-li	16	1	1	1	146	15.9	4.44	5.51
O95081	AGFG2	Arf-GAP dom	4	2	2	1	481	48.9	9.11	5.01
Q96BJ8	ELMO3	Engulfment a	4	2	2	2	720	81.4	6.3	2.33
Q14332	FZD2	Frizzled-2 OS	4	3	3	3	565	63.5	8.1	6.75
Q5K4L6	SLC27A3	Long-chain fa	4	2	2	2	683	73.5	7.09	2.82
Q9NSC5	HOMER3	Homer prote	5	2	2	2	361	39.8	5.45	5.27
O00142	TK2	Thymidine ki	11	2	2	2	265	31	8.46	6.04
Q9UGM3	DMBT1	Deleted in m	7	1	1	1	2413	260.6	5.44	3.66
Q5VW36	FOCAD	Focadhesin (	1	2	2	2	1801	199.9	6.62	5.46
P82663	MRPS25	28S ribosom	10	2	2	2	173	20.1	8.82	4.97
P14373	TRIM27	Zinc finger pr	4	2	2	2	513	58.5	6.21	4.9
Q92990	GLMN	Glomulin OS	3	2	2	2	594	68.2	5.33	5.51
Q5JU69	TOR2A	Torsin-2A OS	8	2	2	2	321	35.7	7.97	3.4
Q8TCU6	PREX1	Phosphatidyl	2	3	3	3	1659	186.1	6.44	4.45
Q8N1A6	C4orf33	UPF0462 pro	10	2	2	2	199	23.5	5.43	5.07
Q5M775	SPECC1	Cytospin-B C	2	3	3	3	1068	118.5	6.7	2.61
Q504Q3	PAN2	PAN2-PAN3	2	1	1	1	1202	135.3	5.99	5.59
Q8TDB4	MGARP	Protein MGA	8	2	2	2	240	25.4	4.44	6.63
O75427	LRCH4	Leucine-rich	3	2	2	2	683	73.4	8.22	5.54
Q9NYI0	PSD3	PH and SEC	3	2	2	2	1048	116	5.99	4.23
P31152	MAPK4	Mitogen-activ	4	2	4	1	587	65.9	5.45	2.93
O94842	TOX4	TOX high mo	3	2	2	2	621	66.2	5.06	5.25
Q8N983	MRPL43	39S ribosom	8	2	2	2	215	23.4	8.65	4.6
Q9H792	PEAK1	Inactive tyros	2	3	3	3	1746	193	6.89	6.94
Q96HF1	SFRP2	Secreted friz	8	1	1	1	295	33.5	7.42	4.4
P50336	PPOX	Protoporphyr	4	2	3	2	477	50.7	8.16	6.22

O60287	URB1	Nucleolar pre	1	2	2	2	2271	254.2	6.47	5.31
Q9C035	TRIM5	Tripartite mo	4	2	2	2	493	56.3	6.05	3.23
P33947	KDELR2	ER lumen pr	4	1	2	1	212	24.4	8.72	5.5
O15063	GARRE1	Granule ass	2	2	2	2	1070	115.9	7.15	4.05
Q5RKV6	EXOSC6	Exosome co	9	2	2	2	272	28.2	6.28	4.46
Q9UGN4	CD300A	CMRF35-like	6	1	1	1	299	33.2	5.49	4.25
Q9GZT9	EGLN1	Egl nine hon	7	3	3	3	426	46	8.53	2.18
Q9H3H5	DPAGT1	UDP-N-acety	5	3	3	3	408	46.1	8	2.58
P52926	HMGA2	High mobility	16	2	2	2	109	11.8	10.62	2.65
Q9BPY8	HOPX	Homeodoma	14	2	2	2	73	8.3	4.81	5.27
Q7L3T8	PARS2	Probable pro	4	2	2	2	475	53.2	8.1	5
Q8N556	AFAP1	Actin filamen	3	2	2	2	730	80.7	8.68	5.9
Q13277	STX3	Syntaxin-3 O	8	2	2	2	289	33.1	5.44	5.16
P20292	ALOX5AP	Arachidonate	14	2	2	2	161	18.1	8.44	4.87
O60573	EIF4E2	Eukaryotic tr	7	2	2	2	245	28.3	8.88	5.47
P19634	SLC9A1	Sodium/hydr	2	2	2	2	815	90.7	7.21	5.45
Q5JTJ3	COA6	Cytochrome	16	2	2	2	125	14.1	8.25	6.2
Q9BYC5	FUT8	Alpha-(1,6)-f	3	2	2	2	575	66.5	7.66	4.99
Q9UKD1	GMEB2	Glucocortico	3	2	2	1	530	56.4	5.6	5.15
Q06547	GABPB1	GA-binding p	4	2	2	2	395	42.5	4.86	5.54
Q6RW13	AGTRAP	Type-1 angio	14	1	1	1	159	17.4	6.14	4.99
Q9BTL3	RAMAC	RNA guanine	13	1	1	1	118	14.4	8.94	4.37
O15213	WDR46	WD repeat-c	4	3	3	3	610	68	9.67	2.06
Q15434	RBMS2	RNA-binding	5	2	2	1	407	43.9	9.07	5.28
Q7Z5G4	GOLGA7	Golgin subfa	16	2	2	2	137	15.8	7.05	5.88
Q8N1Q1	CA13	Carbonic anh	9	2	3	2	262	29.4	6.96	8.09
Q92759	GTF2H4	General tran	4	2	2	2	462	52.2	9.04	5.56
Q9GZX9	TWSG1	Twisted gast	7	1	1	1	223	25	5.34	4.37
Q15392	DHCR24	Delta(24)-ste	4	2	2	2	516	60.1	8.16	2.99
O75063	FAM20B	Glycosamino	5	2	2	2	409	46.4	6.87	2.41
Q92823	NRCAM	Neuronal cel	2	2	2	2	1304	143.8	5.66	4.95
P51157	RAB28	Ras-related p	9	2	2	2	221	24.8	5.97	2.79
Q9H0E3	SAP130	Histone deac	2	2	2	2	1048	110.3	9.83	5.33

Q8TER5	ARHGEF40	Rho guanine	1	2	2	2	1519	164.6	6.15	5.78
Q9HBE1	PATZ1	POZ-, AT ho	3	2	2	1	687	74	8.35	5.71
Q92805	GOLGA1	Golgin subfa	3	2	2	2	767	88.1	5.27	5.6
P53801	PTTG1IP	Pituitary tum	10	1	1	1	180	20.3	8.79	3.99
Q6P4A8	PLBD1	Phospholipas	4	2	2	2	553	63.2	9.06	4.65
O60941	DTNB	Dystrobrevin	3	2	2	2	627	71.3	7.91	5.93
Q8TBC5	ZSCAN18	Zinc finger a	5	2	2	2	510	54.8	4.82	4.28
Q6P2C8	MED27	Mediator of F	6	1	1	1	311	35.4	9.31	4.18
O14933	UBE2L6	Ubiquitin/ISG	10	1	1	1	153	17.8	7.88	3.8
O14795	UNC13B	Protein unc-1	1	2	2	2	1591	180.6	5.99	5.15
Q9NV31	IMP3	U3 small nuc	13	2	2	2	184	21.8	9.5	5.1
Q4KWH8	PLCH1	1-phosphatic	0	1	3	1	1693	189.1	7.74	4.88
P25208	NFYB	Nuclear trans	10	2	2	2	207	22.8	4.59	2.5
Q9Y4P8	WIPI2	WD repeat d	4	2	2	2	454	49.4	6.46	6.21
O75503	CLN5	Ceroid-lipofu	5	2	2	2	358	41.5	7.4	5.57
Q9UPQ0	LIMCH1	LIM and calp	1	2	2	2	1083	121.8	6.47	5.53
Q7Z5R6	APBB1IP	Amyloid beta	1	1	2	1	666	73.1	5.59	5.94
Q9BT09	CNPY3	Protein cano	6	2	2	2	278	30.7	5.49	4.94
Q9P2K8	EIF2AK4	eIF-2-alpha k	2	2	2	2	1649	186.8	6.28	2.55
P78540	ARG2	Arginase-2, r	4	1	1	1	354	38.6	6.47	4.37
Q86UT6	NLRX1	NLR family n	2	2	2	2	975	107.5	7.37	3.08
Q8WWM9	CYGB	Cytoglobin C	9	2	2	2	190	21.4	6.8	2.5
Q96RL7	VPS13A	Intermembra	1	2	3	2	3174	360	6.33	2.16
Q96I51	RCC1L	RCC1-like G	5	1	1	1	464	49.9	8.29	4.86
Q7KZN9	COX15	Cytochrome	4	1	1	1	410	46	9.82	4.59
Q01629	IFITM2	Interferon-inc	12	1	2	1	132	14.6	7.42	10.23
Q9NWU1	OXSM	3-oxoacyl-[ac	4	1	1	1	459	48.8	7.66	4.04
Q9H082	RAB33B	Ras-related p	9	2	2	2	229	25.7	7.18	4.37
Q8TB37	NUBPL	Iron-sulfur pr	6	2	2	2	319	34.1	9.04	5.78
B7ZAP0	RABGAP1L	Rab GTPase	8	2	4	1	253	29	5.33	6.37
Q13888	GTF2H2	General tran	5	2	2	2	395	44.4	6.76	6.05
Q7Z4Q2	HEATR3	HEAT repeat	3	2	2	2	680	74.5	5.11	5.55
Q2PPJ7	RALGAPA2	Ral GTPase-	1	2	2	2	1873	210.6	6.07	4.27

E9PRG8	C11orf98	Uncharacteri	16	2	2	2	123	14.2	11.53	2.04
Q8N465	D2HGDH	D-2-hydroxy(	3	2	2	2	521	56.4	7.99	4.92
A8CG34	POM121C	Nuclear enve	2	3	3	3	1229	125	10.37	5.89
Q969P0	IGSF8	Immunoglobu	5	2	2	2	613	65	8	0
P09038	FGF2	Fibroblast gr	6	2	3	2	288	30.8	11.18	8.33
P00746	CFD	Complement	10	1	1	1	253	27	7.71	4.34
Q9NXG6	P4HTM	Transmembr	3	1	1	1	502	56.6	6.06	4.24
O43677	NDUFC1	NADH dehyc	14	1	2	1	76	8.7	10.2	2.86
Q9NRK6	ABCB10	ATP-binding	3	2	2	1	738	79.1	9.85	4.44
Q5JTD0	TJAP1	Tight junctior	3	2	2	2	557	61.8	5.97	5.81
P46019	PHKA2	Phosphoryla:	2	3	4	3	1235	138.3	6.44	4.43
Q7RTS9	DYM	Dymeclin OS	2	2	3	2	669	75.9	5.86	4.32
Q6JQN1	ACAD10	Acyl-CoA del	2	2	3	2	1059	118.8	8.06	5.99
O94952	FBXO21	F-box only pi	3	1	1	1	628	72.2	6.09	4.27
Q99797	MIPEP	Mitochondria	3	2	2	2	713	80.6	7.05	3.69
Q96G25	MED8	Mediator of F	7	2	2	2	268	29.1	7.44	3.07
Q9H0R3	TMEM222	Transmembr	13	1	1	1	208	23.2	6.51	3.17
Q96SN8	CDK5RAP2	CDK5 regula	2	3	3	3	1893	214.9	5.58	4.07
O75891	ALDH1L1	Cytosolic 10-	2	2	2	1	902	98.8	5.94	4.48
Q9UKL6	PCTP	Phosphatidyl	11	3	4	3	214	24.8	5.78	4.56
Q7L4I2	RSRC2	Arginine/seri	5	1	1	1	434	50.5	11.33	4.19
P56937	HSD17B7	3-keto-steroi	4	1	1	1	341	38.2	8.1	3.14
Q96EL3	MRPL53	39S ribosom	12	1	1	1	112	12.1	8.76	3.81
Q14289	PTK2B	Protein-tyros	2	2	2	1	1009	115.8	6.25	4.99
Q5T160	RARS2	Probable arg	2	1	1	1	578	65.5	8.21	4.53
P31995	FCGR2C	Low affinity ii	6	1	1	1	323	35.6	6.9	5.26
O14613	CDC42EP2	Cdc42 effect	10	1	1	1	210	22.5	5.12	5.33
Q96DV4	MRPL38	39S ribosom	4	2	2	2	380	44.6	7.53	5.01
Q9Y5R8	TRAPPC1	Trafficking pi	15	2	2	2	145	16.8	9.16	5.74
Q9Y4C2	TCAF1	TRPM8 char	2	2	2	2	921	102.1	6.54	5.62
Q9BQP9	BPIFA3	BPI fold-cont	3	1	2	1	254	28.4	6.65	4.82
Q9ULN7	PNMA8B	Paraneoplas	3	1	1	1	635	68.6	5.45	3.46
Q6B0K9	HBM	Hemoglobin	11	1	1	1	141	15.6	6.62	5.29

A0A075B6S!	IGKV1-27	Immunoglobu	15	1	2	1	117	12.7	8.29	9.19
O75912	DGKI	Diacylglycero	2	2	2	2	1065	116.9	7.77	4.59
Q9BQ70	TCF25	Transcriptior	3	1	1	1	676	76.6	6.35	3.59
A8MXV4	NUDT19	Acyl-coenzyr	8	1	1	1	375	42.2	7.64	5.02
Q8TDD1	DDX54	ATP-depend	2	2	2	2	881	98.5	10.02	5.81
O15234	CASC3	Protein CAS(	2	1	1	1	703	76.2	6.48	3.26
Q9H0H0	INTS2	Integrator co	1	1	1	1	1204	134.2	6.05	3.95
Q9UKT5	FBXO4	F-box only pı	3	1	2	1	387	44.1	6.16	5.04
Q8N6L1	KRTCAP2	Keratinocyte-	13	1	1	1	136	14.7	9.61	4.32
Q7RTN6	STRADA	STE20-relate	5	1	1	1	431	48.3	6.48	4.82
O75113	N4BP1	NEDD4-bind	2	2	2	2	896	100.3	5.36	3.13
Q9H147	DNTTIP1	Deoxynucleo	5	2	2	2	329	37	8.97	5.88
Q04864	REL	Proto-oncoge	3	2	2	1	619	68.5	5.86	5.22
Q9NV96	TMEM30A	Cell cycle co	5	1	1	1	361	40.7	8.59	4.33
Q7Z2T5	TRMT1L	TRMT1-like p	4	2	2	2	733	81.7	7.88	4.44
Q9HC16	APOBEC3G	DNA dC->dU	7	2	2	1	384	46.4	8	5.08
Q96GF1	RNF185	E3 ubiquitin-|	17	3	3	3	192	20.4	6.52	4.09
Q6ZU35	CRACD	Capping prot	1	2	2	1	1233	136.7	5.6	4.83
Q9UBW7	ZMYM2	Zinc finger M	2	2	2	1	1377	154.8	6.34	2.38
Q9H0C5	BTBD1	BTB/POZ do	4	2	2	2	482	52.7	6.1	0
Q9H089	LSG1	Large subun	2	2	2	2	658	75.2	6.38	4.68
Q96CC6	RHBDF1	Inactive rhon	2	2	2	2	855	97.3	8.44	5.07
Q13137	CALCOCO2	Calcium-binc	5	2	2	2	446	52.2	5.02	2.48
Q96ET8	TVP23C	Golgi appara	6	2	3	2	276	31.1	9.29	5.3
Q15848	ADIPOQ	Adiponectin (	6	1	1	1	244	26.4	5.74	4.52
Q6UWH4	GASK1B	Golgi-associ	3	2	2	2	519	57.5	9.7	5.59
Q6IQ23	PLEKHA7	Pleckstrin ho	2	2	2	2	1121	127.1	9.35	1.93
Q6PJF5	RHBDF2	Inactive rhon	2	2	2	2	856	96.6	8.82	2.2
Q9UI14	RABAC1	Prenylated R	12	2	2	2	185	20.6	7.34	3.65
P47929	LGALS7B	Galectin-7 O	12	1	1	1	136	15.1	7.62	3.59
Q9Y6M7	SLC4A7	Sodium bicaı	1	2	2	2	1214	136	6.71	4.63
O75391	SPAG7	Sperm-asso(	8	2	2	1	227	26	7.91	6.12
Q9C040	TRIM2	Tripartite mo	2	1	2	1	744	81.5	6.96	9.25

Q96FC7	PHYHIPL	Phytanoyl-Co	5	1	1	1	376	42.5	6.42	4.09
Q9NUQ3	TXLNG	Gamma-taxil	3	1	1	1	528	60.5	7.52	4.13
O60499	STX10	Syntaxin-10 (	7	2	2	2	249	28.1	4.89	3.98
Q9Y4I1	MYO5A	Unconventior	1	2	2	1	1855	215.3	8.48	4.8
Q9Y3D3	MRPS16	28S ribosom	13	1	1	1	137	15.3	9.5	4.22
Q15311	RALBP1	RalA-binding	6	2	2	2	655	76	5.88	4.43
Q8IX04	UEVLD	Ubiquitin-cor	4	2	2	2	471	52.2	7.09	2.69
P50542	PEX5	Peroxisomal	4	2	2	2	639	70.8	4.54	4.47
A9UHW6	MIF4GD	MIF4G doma	9	2	2	2	222	25.4	5.33	2.24
Q86X52	CHSY1	Chondroitin s	4	2	2	2	802	91.7	9.23	2.04
Q70IA6	MOB2	MOB kinase	8	2	3	2	237	26.9	6.79	6.52
P03372	ESR1	Estrogen rec	3	2	2	2	595	66.2	8.06	4.7
P78316	NOP14	Nucleolar pro	2	2	2	2	857	97.6	7.58	2.29
O00519	FAAH	Fatty-acid an	4	1	2	1	579	63	7.66	3.69
Q15326	ZMYND11	Zinc finger M	4	2	2	2	602	70.9	8.53	2.13
Q9UFC0	LRWD1	Leucine-rich	3	2	2	2	647	70.8	7.21	5.37
P20839	IMPDH1	Inosine-5'-mo	3	2	3	1	514	55.4	6.9	5.8
P00973	OAS1	2'-5'-oligoade	5	2	2	2	400	46	8.22	5.46
O94885	SASH1	SAM and SH	1	1	1	1	1247	136.6	6.09	5.14
Q9Y217	MTMR6	Myotubularin	3	2	2	2	621	71.9	7.66	2.96
Q9H7X7	IFT22	Intraflagellar	8	1	1	1	185	20.8	5.15	4.49
Q92828	CORO2A	Coronin-2A (	4	1	1	1	525	59.7	8.05	4.27
Q9BV40	VAMP8	Vesicle-asso	17	2	3	2	100	11.4	7.34	5.24
Q9BY31	ZNF717	Zinc finger pr	3	2	2	1	914	106.5	8.59	4.64
Q96LZ7	RMDN2	Regulator of	5	2	2	2	410	47.4	6.54	5.13
Q8IY37	DHX37	Probable AT	1	1	1	1	1157	129.5	8.1	3.94
A0A0A0MT8	IGKJ1	Immunoglobu	67	1	2	1	12	1.4	8.84	2.16
Q9NRM1	ENAM	Enamelin OS	1	2	3	1	1142	128.7	6.83	7.67
Q6ZRP7	QSOX2	Sulfhydryl ox	4	2	2	2	698	77.5	7.72	5.49
Q99595	TIMM17A	Mitochondria	13	1	1	1	171	18	7.87	3.68
Q9UK53	ING1	Inhibitor of gr	3	1	1	1	422	46.7	9.06	3.8
Q9BUE6	ISCA1	Iron-sulfur clu	18	2	2	2	129	14.2	9.07	2.22
Q8NBM8	PCYOX1L	Prenylcystein	4	2	2	2	494	54.6	7.31	4.63

Q96BI3	APH1A	Gamma-secı	8	2	2	2	265	29	7.9	3.01
A2RTX5	TARS3	Threonine--tl	2	2	2	1	802	92.6	6.05	4.59
P19532	TFE3	Transcriptior	3	2	2	2	575	61.5	5.58	4.49
Q9NZR1	TMOD2	Tropomoduli	6	1	1	1	351	39.6	5.27	4.19
Q9BXP2	SLC12A9	Solute carrie	2	2	2	2	914	96	8.07	4.38
Q9BX97	PLVAP	Plasmalemm	5	3	3	3	442	50.6	8.79	5.15
Q9Y4D7	PLXND1	Plexin-D1 OS	1	1	1	1	1925	211.9	7.15	2.85
Q8N8J7	FAM241A	Uncharacteri	13	1	1	1	132	14.6	4.7	3.35
Q96PV6	LENG8	Leukocyte re	2	2	2	2	800	88.1	9.11	4.37
Q5VWC8	HACD4	Very-long-ch	4	2	2	2	232	27.5	8.57	5.11
Q9NUJ3	TCP11L1	T-complex pı	5	2	2	2	509	57	5.59	3.05
Q9UG01	IFT172	Intraflagellar	1	2	2	2	1749	197.5	6.13	2.82
Q14934	NFATC4	Nuclear facto	2	1	1	1	902	95.4	5.38	3.02
P51617	IRAK1	Interleukin-1	3	2	2	2	712	76.5	6.62	1.67
Q6GQQ9	OTUD7B	OTU domain	2	2	2	2	843	92.5	6.71	4.42
Q9NVX2	NLE1	Notchless pr	5	1	1	1	485	53.3	7.34	4.1
Q9NQ34	TMEM9B	Transmembr	9	2	2	2	198	22.5	8.06	4.73
Q9HCE6	ARHGEF10L	Rho guanine	2	2	2	2	1279	140.3	5.94	4.72
Q5T619	ZNF648	Zinc finger pı	2	1	26	1	568	62.3	8.62	48.22
Q15012	LAPTM4A	Lysosomal-a	12	1	1	1	233	26.8	6.49	4.51
Q9BUE0	MED18	Mediator of F	9	2	2	2	208	23.6	6.54	5.25
Q9Y2H1	STK38L	Serine/threor	4	2	2	2	464	54	6.81	2.74
Q8N5L8	RPP25L	Ribonuclease	14	1	1	1	163	17.6	10.3	4.07
Q86VU5	COMTD1	Catechol O-r	10	1	1	1	262	28.8	8.38	4.78
O60245	PCDH7	Protocadheri	1	1	1	1	1069	116	5.19	3.77
P60059	SEC61G	Protein trans	19	1	2	1	68	7.7	9.99	4.7
Q96PE1	ADGRA2	Adhesion G ı	1	1	1	1	1338	142.6	8.44	5.29
Q9Y365	STARD10	START doma	5	1	1	1	291	33	7.12	3.77
Q96M96	FGD4	FYVE, RhoG	3	2	2	2	766	86.6	6.13	2.62
Q8NF64	ZMIZ2	Zinc finger M	1	1	2	1	920	96.5	7.12	2.61
Q9BTT4	MED10	Mediator of F	16	2	2	2	135	15.7	6.19	3.74
Q6GYQ0	RALGAPA1	Ral GTPase-	1	1	2	1	2036	229.7	6.19	2.7
Q9HCM4	EPB41L5	Band 4.1-like	3	1	1	1	733	81.8	6.58	4.89

Q16656	NRF1	Nuclear resp	3	2	2	2	503	53.5	5.05	5.19
Q86WB0	ZC3HC1	Zinc finger C	3	1	1	1	502	55.2	5.62	4.28
Q12933	TRAF2	TNF recepto	3	2	2	2	501	55.8	7.53	2.58
Q5JSZ5	PRRC2B	Protein PRR	1	2	2	2	2229	242.8	8.34	2.41
P63165	SUMO1	Small ubiquit	22	3	3	3	101	11.6	5.52	7.62
Q6ZRV2	FAM83H	Protein FAM	2	1	1	1	1179	127	6.98	4.39
P41229	KDM5C	Lysine-speci	1	1	1	1	1560	175.6	5.58	4.2
Q9H1K1	ISCU	Iron-sulfur cl	10	2	2	2	167	18	9.48	3.28
Q14409	GK3P	Glycerol kina	3	2	2	2	553	60.6	6.39	4.12
P22532	SPRR2D	Small proline	18	1	1	1	72	7.9	8.37	3.2
O14730	RIOK3	Serine/threo	4	2	2	2	519	59.1	5.76	0
P28340	POLD1	DNA polyme	2	2	2	2	1107	123.6	7.03	2.07
O00168	FXYD1	Phospholem	13	1	1	1	92	10.4	9.14	3.37
P43250	GRK6	G protein-co	3	2	2	1	576	65.9	8	0
P17706	PTPN2	Tyrosine-pro	5	1	1	1	415	48.4	8.29	3.48
Q9UNN8	PROCR	Endothelial p	8	2	2	2	238	26.7	7.18	2.25
P55211	CASP9	Caspase-9 C	7	1	1	1	416	46.3	6.05	4.21
P17535	JUND	Transcription	4	2	2	1	347	35.2	7.37	4.23
Q8NFH3	NUP43	Nucleoporin	5	2	2	2	380	42.1	5.63	5.01
Q15173	PPP2R5B	Serine/threo	3	2	2	1	497	57.4	6.71	4.51
Q96A19	CCDC102A	Coiled-coil d	2	1	1	1	550	62.6	5.58	3.39
Q8N4Q0	PTGR3	Prostaglandi	4	1	1	1	377	40.1	8.18	3.71
Q96EC8	YIPF6	Protein YIPF	5	1	1	1	236	26.2	5.64	3.12
Q969X1	TMBIM1	Protein lifegu	5	1	1	1	311	34.6	7.72	4.61
Q06481	APLP2	Amyloid beta	2	2	2	1	763	86.9	4.79	2.27
Q96DZ9	CMTM5	CKLF-like M/	9	1	1	1	223	24.6	6.62	4.12
Q13823	GNL2	Nucleolar GT	2	1	2	1	731	83.6	9.25	8.42
Q9Y3C1	NOP16	Nucleolar pro	10	2	2	2	178	21.2	9.94	5.53
Q7LDG7	RASGRP2	RAS guanyl-	2	1	1	1	609	69.2	7.8	3.9
P27701	CD82	CD82 antige	5	1	1	1	267	29.6	5.24	4.47
Q8TD43	TRPM4	Transient rec	2	2	2	2	1214	134.2	8.15	0
Q5XXA6	ANO1	Anoctamin-1	2	2	2	2	986	114	8.54	2.43
Q9Y223	GNE	Bifunctional I	2	1	1	1	722	79.2	6.8	4.04

Q92917	GPKOW	G-patch dom	3	2	2	2	476	52.2	6.15	4.52
Q86UW6	N4BP2	NEDD4-bind	1	2	3	1	1770	198.7	5.21	6.4
Q9NNW5	WDR6	WD repeat-c	1	2	2	2	1121	121.6	6.87	4.9
O94766	B3GAT3	Galactosylga	5	2	2	2	335	37.1	8.27	3.94
Q6P9B9	INTS5	Integrator co	2	1	1	1	1019	107.9	7.05	4.01
Q658P3	STEAP3	Metalloreduc	4	1	1	1	488	54.6	8.6	3.21
P40189	IL6ST	Interleukin-6	2	2	2	2	918	103.5	5.95	5.54
P11137	MAP2	Microtubule-a	1	1	1	1	1827	199.4	4.91	3.64
P34947	GRK5	G protein-cou	3	2	2	1	590	67.7	8.1	2.11
Q8IYS1	PM20D2	Xaa-Arg dipe	3	2	2	2	436	47.7	5.85	2.35
Q4G0X4	KCTD21	BTB/POZ do	7	1	1	1	260	29.6	6.54	3.98
O94921	CDK14	Cyclin-deper	4	1	1	1	469	53	8.92	4.13
Q96IK1	BOD1	Biorientation	11	2	2	1	185	19.2	6.33	2.83
P09237	MMP7	Matrilysin OS	6	2	3	2	267	29.7	7.91	7.37
Q9C002	NMES1	Normal muco	20	1	1	1	83	9.6	9.47	3.92
P41743	PRKCI	Protein kinas	3	1	1	1	596	68.2	5.85	3.35
Q96FN9	DTD2	D-aminoacyl-	11	1	1	1	168	18.6	7.84	3.8
Q96P47	AGAP3	Arf-GAP with	2	2	2	2	875	95	7.97	3.17
Q9Y3A2	UTP11	Probable U3	6	2	2	2	253	30.4	10.15	2.93
Q8NHZ8	CDC26	Anaphase-pr	14	1	1	1	85	9.8	6.81	4.87
P62306	SNRPF	Small nuclea	19	2	2	2	86	9.7	4.67	2.5
Q765P7	MTSS2	Protein MTS	2	1	1	1	747	79.9	7.47	3.5
Q96B36	AKT1S1	Proline-rich A	6	1	1	1	256	27.4	4.75	2.72
Q8WUU5	GATAD1	GATA zinc fi	6	1	1	1	269	28.7	9.41	3.43
Q96GY0	ZC2HC1A	Zinc finger C	6	2	2	2	325	35.1	9.82	2.32
Q8NBZ7	UXS1	UDP-glucuro	4	1	1	1	420	47.5	8.94	4.04
O94830	DDHD2	Phospholipas	2	2	2	2	711	81	5.39	2.03
Q12899	TRIM26	Tripartite mo	2	1	2	1	539	62.1	5.03	6.66
Q13530	SERINC3	Serine incorp	4	2	2	2	473	52.5	7.46	5.87
Q71SY5	MED25	Mediator of F	2	1	1	1	747	78.1	8.34	3.61
Q86YQ8	CPNE8	Copine-8 OS	3	2	3	1	564	63.1	5.96	2.49
Q6NUQ1	RINT1	RAD50-intera	2	1	1	1	792	90.6	5.45	3.85
Q99543	DNAJC2	DnaJ homolo	3	1	1	1	621	72	8.7	4.24

Q92604	LPGAT1	Acyl-CoA:lys	2	1	2	1	370	43.1	8.92	4.86
P19544	WT1	Wilms tumor	2	1	1	1	449	49.2	9	2.65
Q53GT1	KLHL22	Kelch-like pr	3	2	2	2	634	71.6	5.49	5.12
P13995	MTHFD2	Bifunctional r	3	1	1	1	350	37.9	8.73	3.19
P57081	WDR4	tRNA (guanir	6	1	1	1	412	45.5	7.11	3.76
P13688	CEACAM1	Carcinoembr	3	1	1	1	526	57.5	5.97	3.72
Q8TBP6	SLC25A40	Probable mit	4	1	1	1	338	38.1	9.35	4.43
Q6ZVM7	TOM1L2	TOM1-like pr	7	2	2	2	507	55.5	4.79	4.51
Q14119	VEZF1	Vascular enc	3	2	2	1	521	56.9	9.52	4.96
Q9NZE8	MRPL35	39S ribosom	8	2	3	2	188	21.5	11.3	8.34
Q93074	MED12	Mediator of F	2	3	3	3	2177	242.9	7.05	4.1
P31350	RRM2	Ribonucleosi	5	2	2	2	389	44.8	5.38	2.19
A0A0C4DH6	IGKV1-9	Immunoglobu	15	1	1	1	117	12.7	8.29	4.34
O94966	USP19	Ubiquitin car	1	2	3	2	1318	145.6	6.28	4.18
O94880	PHF14	PHD finger p	2	2	2	2	948	106.9	5.34	4.34
Q9NRL2	BAZ1A	Bromodomai	1	1	1	1	1556	178.6	6.6	3.85
Q9HAF1	MEAF6	Chromatin m	9	2	3	2	191	21.6	9.32	6.48
Q9P291	ARMCX1	Armadillo rep	3	1	1	1	453	49.2	9.22	3.24
P18827	SDC1	Syndecan-1	5	1	1	1	310	32.4	4.63	3.57
Q9NYM9	BET1L	BET1-like pr	14	1	1	1	111	12.4	8.16	3.26
Q9NSI2	SLX9	Ribosome bi	6	1	1	1	230	25.4	11.08	3.22
Q8N4L2	PIP4P2	Type 2 phos	7	2	2	2	257	28.1	8.68	2.99
O14735	CDIPT	CDP-diacylgl	8	2	2	2	213	23.5	8.03	4.72
Q9BWH6	RPAP1	RNA polyme	1	1	1	1	1393	152.7	6.38	4.23
Q9NRG0	CHRAC1	Chromatin ac	14	1	1	1	131	14.7	5.1	4.4
P57678	GEMIN4	Gem-associa	3	2	2	2	1058	120	6.04	2.11
Q15170	TCEAL1	Transcriptior	8	1	1	1	159	18.6	5.02	2.43
P15309	ACP3	Prostatic aci	4	1	1	1	386	44.5	6.24	4.31
Q9C0H2	TTYH3	Protein twee	3	1	1	1	523	57.5	5.39	3.69
Q86UA1	PRPF39	Pre-mRNA-p	2	1	1	1	669	78.4	5.4	2.84
Q9UBP6	METTL1	tRNA (guanir	6	2	2	2	276	31.5	7.64	5.17
O75071	EFCAB14	EF-hand calc	5	1	1	1	495	55	6.32	2.83
P05187	ALPP	Alkaline phos	3	1	1	1	535	57.9	6.29	3.18

Q9NY61	AATF	Protein AATF	3	1	2	1	560	63.1	4.94	8.77
Q9HD34	LYRM4	LYR motif-cc	16	2	2	2	91	10.8	10.73	5.08
Q9Y2D0	CA5B	Carbonic anh	5	1	1	1	317	36.4	7.81	4.07
O60486	PLXNC1	Plexin-C1 OS	1	2	2	2	1568	175.6	7.61	1.97
O14657	TOR1B	Torsin-1B OS	3	1	1	1	336	38	8.54	2.66
P28067	HLA-DMA	HLA class II	8	1	1	1	261	29.2	4.64	3.68
Q9Y4R8	TELO2	Telomere ler	3	2	2	2	837	91.7	5.76	0
Q9UBM1	PEMT	Phosphatidyl	7	1	1	1	199	22.1	8.76	4.72
O96007	MOCS2	Molybdopteri	8	1	1	1	188	20.9	5.44	4.2
Q15119	PDK2	[Pyruvate de	5	1	1	1	407	46.1	6.61	3.81
A5PLN9	TRAPPC13	Trafficking pr	5	2	2	2	417	46.5	5.6	2.35
P01040	CSTA	Cystatin-A O	18	1	1	1	98	11	5.5	3.88
P31260	HOXA10	Homeobox p	3	1	1	1	410	42.4	8.34	4.79
Q9UJF2	RASAL2	Ras GTPase	1	2	2	2	1139	128.5	8.24	2.26
Q4ZG55	GREB1	Protein GRE	1	2	3	2	1949	216.3	6.95	4.54
Q8IZ52	CHPF	Chondroitin s	3	2	2	2	775	85.4	6.93	2.09
O43182	ARHGAP6	Rho GTPase	2	1	1	1	974	105.9	7.36	3.25
Q16626	MEA1	Male-enhanc	7	1	1	1	185	19.9	4.22	3.81
Q9UMX3	BOK	Bcl-2-related	7	1	1	1	212	23.3	9.11	4.21
Q96GA3	LTV1	Protein LTV1	4	1	1	1	475	54.8	4.91	4.01
P62380	TBPL1	TATA box-bi	9	1	1	1	186	20.9	9.54	3.88
O95365	ZBTB7A	Zinc finger ar	3	1	1	1	584	61.4	5.19	3.27
Q9Y342	PLLP	Plasmolipin (	13	1	1	1	182	20	9.36	3.01
P42771	CDKN2A	Cyclin-deper	8	1	1	1	156	16.5	5.81	4.31
P05019	IGF1	Insulin-like g	7	1	2	1	195	21.8	9.72	8.53
O75594	PGLYRP1	Peptidoglyca	8	1	1	1	196	21.7	8.59	4.53
Q9UQL6	HDAC5	Histone deac	1	1	1	1	1122	121.9	6.24	4.26
Q12983	BNIP3	BCL2/adenov	10	2	2	2	194	21.5	6.8	0
P01137	TGFB1	Transforming	4	2	2	2	390	44.3	8.53	4.6
Q96S82	UBL7	Ubiquitin-like	5	1	2	1	380	40.5	5.07	9.7
Q8WZ42	TTN	Titin OS=Hor	0	3	4	3	34350	3813.7	6.35	4.66
Q8N6M0	OTUD6B	Deubiquitinas	10	1	1	1	293	33.8	6.05	2.94
P49366	DHPS	Deoxyhypusi	5	1	1	1	369	40.9	5.36	4.07

Q05D32	CTDSPL2	CTD small pl	2	1	2	1	466	53	6.4	2.41
O00757	FBP2	Fructose-1,6	3	1	1	1	339	36.7	7.23	3.84
Q96CB9	NSUN4	5-methylcyto	5	2	2	2	384	43.1	8.18	4.64
Q99735	MGST2	Microsomal g	10	1	2	1	147	16.6	9.55	6.76
Q6NXE6	ARMC6	Armadillo rep	4	1	1	1	501	54.1	6.24	2.98
Q9H0S4	DDX47	Probable AT	5	2	2	2	455	50.6	9.1	2.22
Q15651	HMGN3	High mobility	22	2	2	2	99	10.7	9.66	2.67
Q6P5R6	RPL22L1	60S ribosom	10	1	1	1	122	14.6	9.38	3.9
Q96F45	ZNF503	Zinc finger pr	2	1	1	1	646	62.5	8.7	3.99
O95210	STBD1	Starch-bindir	5	2	2	2	358	39	5.73	5.35
Q8IUL8	CILP2	Cartilage inte	1	1	1	1	1156	126.2	8.22	3.04
P42658	DPP6	Dipeptidyl an	3	2	2	2	865	97.5	6.37	3.33
Q86VE9	SERINC5	Serine incorp	4	2	4	2	423	47	7.62	10.15
Q9UJX5	ANAPC4	Anaphase-pr	2	1	1	1	808	92.1	5.53	3.22
A0A075B6Q	IGHV3-64	Immunoglobu	9	1	1	1	118	12.9	7.85	3.12
Q9GZM5	YIPF3	Protein YIPF	5	2	2	2	350	38.2	5.76	4.84
O95428	PAPLN	Papilin OS=H	2	2	2	2	1278	137.6	6.89	4.44
Q6R327	RICTOR	Rapamycin-in	2	2	2	2	1708	192.1	7.47	2.11
Q14814	MEF2D	Myocyte-spe	4	2	2	2	521	55.9	7.88	3.31
Q8IV48	ERI1	3'-5' exoribor	5	2	2	2	349	40	6.7	4.74
Q5BKZ1	ZNF326	DBIRD comp	3	1	1	1	582	65.6	5.15	3.9
Q96RT7	TUBGCP6	Gamma-tubu	1	1	1	1	1819	200.4	6.32	2.24
O00562	PITPNM1	Membrane-a	1	1	1	1	1244	134.8	5.95	3.71
Q9NXA8	SIRT5	NAD-depend	5	1	1	1	310	33.9	8.47	3.45
Q9Y580	RBM7	RNA-binding	5	1	1	1	266	30.5	9.57	3.79
Q9H4L4	SENP3	Sentrin-spec	3	1	1	1	574	65	8.56	3.26
O75616	ERAL1	GTPase Era,	4	1	1	1	437	48.3	8.84	3.18
Q9BVA0	KATNB1	Katanin p80	2	1	1	1	655	72.3	7.56	3.25
Q9P2A4	ABI3	ABI gene far	4	1	1	1	366	39	5.08	2.85
Q9Y5J6	TIMM10B	Mitochondria	24	1	1	1	103	11.6	7.43	4.04
Q9UKG9	CROT	Peroxisomal	3	1	2	1	612	70.1	7.08	5.28
P25942	CD40	Tumor necro	3	1	2	1	277	30.6	5.76	3.75
P07199	CENPB	Major centro	3	2	2	2	599	65.1	4.55	3.93

Q13393	PLD1	Phospholipas	2	1	1	1	1074	124.1	8.78	2.69
Q9H694	BICC1	Protein bicau	2	2	2	2	974	104.8	8.54	2.09
O75110	ATP9A	Probable pho	2	2	2	2	1047	118.5	7.77	0
P08047	SP1	Transcriptior	2	2	2	1	785	80.6	7.34	5.4
Q96JK2	DCAF5	DDB1- and C	1	1	1	1	942	103.9	5.76	3.99
P29084	GTF2E2	Transcriptior	7	2	2	2	291	33	9.66	2.17
Q8N1F8	STK11IP	Serine/threoı	1	1	1	1	1088	120.2	5.36	3.3
Q9HD33	MRPL47	39S ribosom	8	2	2	2	250	29.4	10.37	2.65
O60218	AKR1B10	Aldo-keto rec	5	2	2	2	316	36	7.84	3.45
Q9H5K3	POMK	Protein O-ma	4	2	2	2	350	40	6.1	3.03
O75438	NDUFB1	NADH dehyc	19	1	1	1	58	7	8.92	3.01
Q9H0V1	TMEM168	Transmembr	2	1	1	1	697	79.7	8.03	2.94
A0A075B6H7	IGKV3-7	Probable nor	16	1	3	1	116	12.8	5.25	6.3
Q66GS9	CEP135	Centrosomal	1	2	2	2	1140	133.4	6.21	2.44
Q9NYB0	TERF2IP	Telomeric re	5	2	2	2	399	44.2	4.73	3.3
Q15024	EXOSC7	Exosome coı	4	1	1	1	291	31.8	5.19	3.7
P00750	PLAT	Tissue-type ı	3	1	1	1	562	62.9	7.8	2.81
Q96HH9	GRAMD2B	GRAM doma	6	2	2	2	432	47.8	7.9	3.31
P55789	GFER	FAD-linked s	6	1	1	1	205	23.4	7.62	2.95
Q9BSH4	TACO1	Translational	7	1	1	1	297	32.5	8.13	2.36
Q96ER9	CCDC51	Mitochondria	3	1	1	1	411	45.8	8.19	2.96
Q00613	HSF1	Heat shock f	2	1	1	1	529	57.2	5.19	2.89
Q92576	PHF3	PHD finger p	1	2	2	2	2039	229.3	6.96	2.05
Q8N9V3	WDSUB1	WD repeat, S	3	2	2	2	476	52.8	6.37	4.77
Q13191	CBLB	E3 ubiquitin-ı	2	2	3	1	982	109.4	7.88	4.85
Q969Q5	RAB24	Ras-related ı	4	1	1	1	203	23.1	6.23	3.63
Q9BVI4	NOC4L	Nucleolar coı	3	1	1	1	516	58.4	7.49	4.22
Q75N03	CBLL1	E3 ubiquitin-ı	5	2	2	2	491	54.5	8.29	5.69
O75911	DHRS3	Short-chain c	6	1	1	1	302	33.5	8.84	3.4
Q8IV56	PRR15	Proline-rich p	12	1	1	1	129	13.7	9.64	4.12
Q6ZSZ5	ARHGEF18	Rho guanine	2	2	2	1	1361	151.5	6.33	0
Q92615	LARP4B	La-related pr	3	3	3	3	738	80.5	6.92	4.15
Q92817	EVPL	Envoplakin C	1	1	1	1	2033	231.5	6.96	2.75

O95639	CPSF4	Cleavage an	9	2	2	2	269	30.2	8.31	0
Q96QC0	PPP1R10	Serine/threor	4	2	2	2	940	99	9.17	2.86
Q96GS4	BORCS6	BLOC-1-rela	4	1	1	1	357	37.2	5.76	2.15
Q7Z7E8	UBE2Q1	Ubiquitin-cor	4	2	2	1	422	46.1	5.1	3.83
Q13136	PPFIA1	Liprin-alpha-	1	3	3	3	1202	135.7	6.29	4.95
Q9BWG4	SSBP4	Single-strand	4	1	1	1	385	39.4	6.8	3.27
Q9NUD5	ZCCHC3	Zinc finger C	4	2	2	2	403	43.5	8.53	3.78
P24588	AKAP5	A-kinase anc	4	1	1	1	427	47.1	4.93	3.54
Q96LW7	CARD19	Caspase rec	4	1	2	1	228	25.6	9.2	7.81
P30260	CDC27	Cell division	2	2	2	2	824	91.8	7.02	0
Q9UHW5	GPN3	GPN-loop G	6	1	1	1	284	32.7	4.5	3.75
Q9HBI0	PARVG	Gamma-parv	4	1	1	1	331	37.5	5.49	3.34
Q9NWS8	RMND1	Required for	4	2	2	2	449	51.6	8.69	4.7
Q9NRY2	INIP	SOSS compl	13	1	1	1	104	11.4	9.25	5.3
P06400	RB1	Retinoblasto	2	2	2	2	928	106.1	7.94	5.25
Q04941	PLP2	Proteolipid p	9	1	1	1	152	16.7	7.24	3.23
Q15198	PDGFRL	Platelet-deriv	3	1	1	1	375	41.8	8.5	3.2
Q969J3	BORCS5	BLOC-1-rela	11	2	2	2	196	22.2	6.35	1.97
P50613	CDK7	Cyclin-deper	4	1	1	1	346	39	8.47	3.83
O43709	BUD23	Probable 18S	6	1	1	1	281	31.9	8.73	3.73
Q969Y2	GTPBP3	tRNA modific	4	1	2	1	492	52	6.48	6.78
Q9H4I3	TRABD	TraB domain	3	1	1	1	376	42.3	8	2.85
Q9UG56	PISD	Phosphatidyl	3	1	1	1	409	46.6	9.42	3.88
Q8N488	RYBP	RING1 and Y	8	1	1	1	228	24.8	9.63	3.52
Q9Y6N7	ROBO1	Roundabout	1	2	2	1	1651	180.8	6.04	3.28
Q8TE73	DNAH5	Dynein axon	0	1	2	1	4624	528.7	6.1	4.68
Q9BRP4	PAAF1	Proteasomal	3	1	1	1	392	42.2	6.32	3.82
Q5SYB0	FRMPD1	FERM and P	0	1	2	1	1578	173.3	5.25	4.32
Q5VV42	CDKAL1	Threonylcarb	3	2	2	2	579	65.1	7.46	4.77
Q86WR0	CCDC25	Coiled-coil d	6	1	1	1	208	24.5	6.8	4.2
Q9Y6X9	MORC2	ATPase MOI	2	2	2	1	1032	117.8	8.38	5.05
O60563	CCNT1	Cyclin-T1 OS	2	2	2	2	726	80.6	8.78	2.55
P62166	NCS1	Neuronal cal	6	1	1	1	190	21.9	4.83	2.66

Q9ULX3	NOB1	RNA-binding	5	2	2	2	412	46.6	7.18	2.76
Q03692	COL10A1	Collagen alpl	2	1	1	1	680	66.1	9.67	3.58
Q96G97	BSCL2	Seipin OS=H	4	2	2	2	398	44.4	5.17	1.87
P26012	ITGB8	Integrin beta	2	2	2	2	769	85.6	7.25	3.52
Q8N128	FAM177A1	Protein FAM	5	1	2	1	213	23.7	4.45	6.44
Q10469	MGAT2	Alpha-1,6-ma	3	2	2	2	447	51.5	8.76	4.8
P49427	CDC34	Ubiquitin-cor	5	1	1	1	236	26.7	4.54	3.24
Q53GL0	PLEKHO1	Pleckstrin ho	3	1	1	1	409	46.2	8.82	2.91
O75563	SKAP2	Src kinase-a	3	1	2	1	359	41.2	4.69	3.03
Q9NY35	CLDND1	Claudin dom	6	1	1	1	253	28.6	5.82	2.84
P15328	FOLR1	Folate recep	5	1	1	1	257	29.8	7.97	2.85
Q02447	SP3	Transcriptior	2	2	2	1	781	81.9	5.26	5.45
P28300	LOX	Protein-lysin	3	2	2	1	417	46.9	8.09	4.48
Q8N0U7	C1orf87	Uncharacteri	3	1	1	1	546	62	8.65	2.83
Q9BQ13	KCTD14	BTB/POZ do	4	1	2	1	255	29.6	8.59	2.32
Q8TC71	SPATA18	Mitochondria	3	1	1	1	538	61.1	8.63	2.28
P19883	FST	Follistatin OS	4	1	1	1	344	38	5.67	2.9
Q9BV81	EMC6	ER membrar	9	1	1	1	110	12	10.07	2.68
O60942	RNGTT	mRNA-cappi	3	1	1	1	597	68.5	8.13	2.8
Q5VT06	CEP350	Centrosome-	0	2	3	1	3117	350.7	6.33	3.57
Q9NSY2	STARD5	StAR-related	4	1	2	1	213	23.8	6.67	0
Q13480	GAB1	GRB2-assoc	2	1	1	1	694	76.6	5.96	3.02
Q01581	HMGCS1	Hydroxymeth	2	1	1	1	520	57.3	5.41	2.84
A0A075B6I1	IGLV4-60	Immunoglobu	8	1	2	1	120	13	6.25	5.24
P24158	PRTN3	Myeloblastin	3	1	2	1	256	27.8	8.35	2.16
Q9BX69	CARD6	Caspase rec	1	1	1	1	1037	116.4	6.37	2.83
O95696	BRD1	Bromodomai	1	1	1	1	1058	119.4	8.6	3.24
P48382	RFX5	DNA-binding	2	1	1	1	616	65.3	9.29	3.4
Q9H7L9	SUDS3	Sin3 histone	5	2	2	2	328	38.1	5.66	0
Q8N283	ANKRD35	Ankyrin repe	1	2	2	2	1001	109.9	6.02	4.82
Q13017	ARHGAP5	Rho GTPase	1	2	2	2	1502	172.4	6.62	2.38
Q9Y6Y0	IVNS1ABP	Influenza viru	3	2	3	2	642	71.7	5.53	6.65
Q9BTE1	DCTN5	Dynactin sub	5	1	1	1	182	20.1	8.02	2.6

Q9NXW9	ALKBH4	Alpha-ketogl	6	1	1	1	302	33.8	6.67	3.08
Q969L2	MAL2	Protein MAL2	6	1	1	1	176	19.1	6.24	2.53
Q9UEG4	ZNF629	Zinc finger pr	1	1	1	1	869	96.6	7.93	3.42
P09769	FGR	Tyrosine-pro	4	2	2	1	529	59.4	5.59	2
Q969S9	GFM2	Ribosome-re	2	1	1	1	779	86.5	6.51	3.38
Q8NFI3	ENGASE	Cytosolic enc	3	1	1	1	743	83.9	6.79	3.8
Q8IUX1	TMEM126B	Complex I as	6	1	1	1	230	25.9	8.81	2.41
Q9HBM6	TAF9B	Transcription	4	1	1	1	251	27.6	9.55	2.78
Q96C24	SYTL4	Synaptotagm	2	2	2	2	671	76	8.98	2.2
Q9Y6N1	COX11	Cytochrome	4	1	1	1	276	31.4	9.06	2.9
Q9GZT6	CCDC90B	Coiled-coil de	3	1	2	1	254	29.5	7.55	5.34
Q8IZQ1	WDFY3	WD repeat a	0	1	1	1	3526	395	6.76	3.51
Q86TB9	PATL1	Protein PAT1	2	2	2	2	770	86.8	6.67	1.89
O43715	TRIAP1	TP53-regulat	17	1	1	1	76	8.8	5.48	2.2
Q8WVM0	TFB1M	Dimethylader	4	1	1	1	346	39.5	9.26	3.41
Q9BPX3	NCAPG	Condensin co	1	1	1	1	1015	114.3	5.59	3.74
P53794	SLC5A3	Sodium/myo-	1	1	1	1	718	79.6	7.27	3.25
O75962	TRIO	Triple functio	0	1	1	1	3097	346.7	6.37	3.74
O60678	PRMT3	Protein argin	3	1	1	1	531	59.9	5.35	2.53
Q9UJC5	SH3BGRL2	SH3 domain-	11	1	2	1	107	12.3	6.71	6.16
Q96FL8	SLC47A1	Multidrug and	2	1	1	1	570	61.9	7.58	3.44
P78545	ELF3	ETS-related	4	1	1	1	371	41.4	5.77	3.69
P0CW20	LIMS4	LIM and sen	12	1	1	1	117	13.2	5.49	2.81
Q96HW7	INTS4	Integrator co	1	1	1	1	963	108.1	6.44	3.11
Q9BTU6	PI4K2A	Phosphatidyl	3	1	1	1	479	54	8.29	4.02
Q9H2K0	MTIF3	Translation in	5	2	3	2	278	31.7	9.69	4.81
Q5TAQ9	DCAF8	DDB1- and C	3	2	2	2	597	66.8	5.39	4.16
Q9NZ63	C9orf78	Splicing facto	4	1	1	1	289	33.7	6.74	3.06
P62312	LSM6	U6 snRNA-a	14	1	1	1	80	9.1	9.58	3.03
P50238	CRIP1	Cysteine-rich	19	2	3	2	77	8.5	8.75	7.42
Q9Y5J1	UTP18	U3 small nuc	3	1	1	1	556	62	8.76	3.37
Q9BXB4	OSBPL11	Oxysterol-bir	3	2	2	1	747	83.6	7.06	2.17
Q9BYX2	TBC1D2	TBC1 domai	2	1	1	1	928	105.3	6.58	2.63

P54132	BLM	RecQ-like DI	1	1	2	1	1417	158.9	7.49	6.32
Q9Y6I4	USP3	Ubiquitin car	2	1	1	1	520	58.9	8.18	3.31
Q9BTX3	TMEM208	Transmembr	6	1	1	1	173	19.6	9.26	2.8
A0A075B6H!	IGLV4-69	Immunoglobu	8	1	1	1	119	12.8	6.51	2.81
Q8WUI4	HDAC7	Histone deac	1	1	1	1	952	102.9	7.58	3.46
O60262	GNG7	Guanine nuc	24	1	1	1	68	7.5	8.51	2.59
P52292	KPNA2	Importin subu	5	2	2	2	529	57.8	5.4	5.03
Q9H0X4	FAM234A	Protein FAM:	2	1	1	1	552	59.6	6.28	2.97
P04432	IGKV1D-39	Immunoglobu	15	1	1	1	117	12.7	8.66	2.59
Q9Y3A4	RRP7A	Ribosomal R	5	1	1	1	280	32.3	9.58	2.79
O60711	LPXN	Leupaxin OS	3	1	1	1	386	43.3	6.01	2.62
P16383	GCFC2	Intron Large	2	1	1	1	781	89.3	5.99	2.92
Q9BU89	DOHH	Deoxyhypusi	4	1	1	1	302	32.9	4.83	2.68
Q8WV41	SNX33	Sorting nexir	3	1	1	1	574	65.2	6.79	2.66
Q9BUR4	WRAP53	Telomerase	3	1	1	1	548	59.3	4.58	2.09
Q9NZ43	USE1	Vesicle trans	5	1	1	1	259	29.4	9.07	3.84
A0A0B4J1Y!	IGHV3-72	Immunoglobu	15	2	3	2	119	13.2	7.85	6.75
Q9P1U0	POLR1H	DNA-directe	13	1	1	1	126	13.9	5.06	2.46
P36268	GGT2P	Inactive glut	2	1	1	1	569	61.7	7.58	2.81
P03891	MT-ND2	NADH-ubiqu	3	1	1	1	347	38.9	9.83	3.43
O15397	IPO8	Importin-8 O	1	1	1	1	1037	119.9	5.16	3.52
P78332	RBM6	RNA-binding	2	2	2	2	1123	128.6	6.32	2.17
O43752	STX6	Syntaxin-6 O	5	1	1	1	255	29.2	4.93	2.18
Q9NVE5	USP40	Ubiquitin car	1	1	1	1	1235	140	5.67	2.36
Q86X83	COMMD2	COMM doma	7	2	4	2	199	22.7	6.73	8.41
A0A0C4DH7	IGKV1-6	Immunoglobu	10	1	1	1	117	12.7	8.29	2.76
Q14687	GSE1	Genetic supp	1	1	1	1	1217	136.1	7.74	3.42
Q96KQ7	EHMT2	Histone-lysin	1	2	2	2	1210	132.3	5.45	2
Q96QZ7	MAGI1	Membrane-a	1	1	1	1	1491	164.5	7.58	4.09
O43295	SRGAP3	SLIT-ROBO	2	2	2	1	1099	124.4	6.68	2.24
Q9NR16	CD163L1	Scavenger re	1	1	1	1	1453	159.1	5.76	3.25
Q8TD22	SFXN5	Sideroflexin-	6	2	2	2	340	37.1	9.33	2.54
Q86VX2	COMMD7	COMM doma	8	1	1	1	200	22.5	5.92	2.4

Q9P0P0	RNF181	E3 ubiquitin-		10	2	2	2	153	17.9	5.06	2.21
Q9NWM8	FKBP14	Peptidyl-proly	6	1	1	1	211	24.2	6.07	2.52
Q9NRF2	SH2B1	SH2B adapte	1	1	1	1	756	79.3	5.38	2.95
Q9Y3T6	R3HCC1	R3H and coil	3	1	1	1	440	49.1	5.63	2.87
O95363	FARS2	Phenylalanin	2	1	1	1	451	52.3	7.46	3.48
Q9P2K2	TXNDC16	Thioredoxin (	2	1	1	1	825	93.5	5.21	3.37
O95628	CNOT4	CCR4-NOT t	3	2	2	2	575	63.5	7.03	0
P31151	S100A7	Protein S100	13	1	1	1	101	11.5	6.77	2.22
O94822	LTN1	E3 ubiquitin-		1	1	2	1	1766	200.4	6.25	6.06
O75044	SRGAP2	SLIT-ROBO	2	3	3	2	1071	120.8	6.7	1.76
Q12912	IRAG2	Inositol 1,4,5	3	1	5	1	555	62.1	5.85	2.94
P05121	SERPINE1	Plasminogen	2	1	1	1	402	45	7.2	2.9
Q9P0S9	TMEM14C	Transmembr	14	2	2	2	112	11.6	9.88	2.86
Q96MW1	CCDC43	Coiled-coil d	6	1	1	1	224	25.2	4.92	2.41
A0A0B4J1V7	IGHV7-81	Probable nor	6	1	3	1	117	12.9	7.11	1.68
Q92618	ZNF516	Zinc finger pr	1	1	1	1	1163	124.2	8.72	2.51
Q86Y79	PTRH1	Probable pep	5	1	1	1	214	22.9	10.56	3.29
P84101	SERF2	Small EDRK-	31	2	2	2	59	6.9	10.45	5.22
Q9H3S4	TPK1	Thiamin pyro	4	1	1	1	243	27.2	5.2	0
Q96BZ9	TBC1D20	TBC1 domai	2	1	1	1	403	45.8	6.86	2.83
Q96IZ7	RSRC1	Serine/Argini	4	1	1	1	334	38.7	11.08	2.73
Q14997	PSME4	Proteasome	1	1	1	1	1843	211.2	6.9	3.45
Q5QP82	DCAF10	DDB1- and C	2	1	1	1	559	60.5	7.5	3.18
Q86YM7	HOMER1	Homer prote	4	1	1	1	354	40.3	5.44	2.41
Q8N5I2	ARRDC1	Arrestin dom	7	1	1	1	433	46	7.02	3.63
Q8N2M8	CLASRP	CLK4-associ	2	2	2	2	674	77.1	10.45	4.11
P47224	RABIF	Guanine nuc	9	1	2	1	123	13.8	5.52	5.04
Q6ISB3	GRHL2	Grainyhead-l	2	1	1	1	625	71.1	6.44	2.06
Q96JX3	SERAC1	Protein SER/	2	1	1	1	654	74.1	7.68	2.77
O15446	POLR1G	DNA-directe	6	1	2	1	510	55	8.51	5.3
O95084	PRSS23	Serine prote	5	2	2	2	383	43	9.42	0
Q9NP59	SLC40A1	Solute carrie	3	2	2	2	571	62.5	6.52	4.07
P02686	MBP	Myelin basic	3	1	1	1	304	33.1	9.79	2.29

Q8IXS8	HYCC2	Hyccin 2 OS	3	1	1	1	530	58.6	7.9	3.07
P49459	UBE2A	Ubiquitin-cor	7	1	1	1	152	17.3	5.15	2.57
Q8TCC3	MRPL30	39S ribosom	6	1	1	1	161	18.5	9.99	3.11
Q5TAX3	TUT4	Terminal uric	1	1	1	1	1644	185	7.97	3.08
Q7Z3E2	CCDC186	Coiled-coil d	1	1	1	1	898	103.6	6.27	2.47
B0I1T2	MYO1G	Unconvention	1	1	1	1	1018	116.4	8.73	3.38
Q9H6X4	TMEM134	Transmembr	4	1	1	1	195	21.6	6.54	2.54
Q86UY6	NAA40	N-alpha-acet	5	1	1	1	237	27.2	7.39	2.77
P16035	TIMP2	Metalloprotei	14	2	2	2	220	24.4	7.49	2.3
Q9BUL5	PHF23	PHD finger p	3	1	1	1	403	43.8	5.78	3.87
Q6P1L8	MRPL14	39S ribosom	7	1	1	1	145	15.9	10.24	3.04
Q9UM47	NOTCH3	Neurogenic l	1	1	1	1	2321	243.5	5.39	3.15
Q9UBS0	RPS6KB2	Ribosomal p	2	1	1	1	482	53.4	7.34	2.39
O15327	INPP4B	Inositol polyp	1	1	1	1	924	104.7	6.27	2.79
Q9Y5B6	PAXBP1	PAX3- and P	1	1	1	1	917	104.7	5.68	3.28
O75376	NCOR1	Nuclear rece	1	2	2	2	2440	270	7.11	1.73
Q9UKF7	PITPNC1	Cytoplasmic	5	2	2	2	332	38.4	6.4	3.03
Q8NBK3	SUMF1	Formylglycin	4	1	1	1	374	40.5	6.65	2.71
P35251	RFC1	Replication f	1	1	1	1	1148	128.2	9.36	2.66
Q9HD47	RANGRF	Ran guanine	5	1	1	1	186	20.4	4.94	2.9
O00767	SCD	Stearoyl-CoA	4	1	1	1	359	41.5	9	2.53
Q12767	TMEM94	Transmembr	1	1	1	1	1356	151.1	6.44	3.28
Q08554	DSC1	Desmocollin-	2	1	1	1	894	99.9	5.43	2.93
Q7Z6M1	RABEPK	Rab9 effecto	3	1	1	1	372	40.5	6.25	3.4
Q9BXI6	TBC1D10A	TBC1 domai	2	1	1	1	508	57.1	8.44	3.47
P28068	HLA-DMB	HLA class II	5	1	1	1	263	28.9	7.44	2.86
O14827	RASGRF2	Ras-specific	1	1	1	1	1237	140.7	7.53	2.5
O60318	MCM3AP	Germinal-cer	0	1	1	1	1980	218.3	6.39	2.93
Q9Y2P4	SLC27A6	Long-chain f	2	1	1	1	619	70.1	8.51	4.31
Q6UXB8	PI16	Peptidase inl	2	1	1	1	463	49.4	5.39	2.81
Q8WVN8	UBE2Q2	Ubiquitin-cor	4	2	2	1	375	42.8	4.96	3.99
Q9H7Z6	KAT8	Histone acet	2	1	1	1	458	52.4	8.27	3.55
Q8WUH1	CHURC1	Protein Chur	16	1	1	1	112	12.9	4.84	2.52

Q96QU8	XPO6	Exportin-6 O	1	1	1	1	1125	128.8	6.35	2.36
P24311	COX7B	Cytochrome	10	2	2	2	80	9.2	10.27	2.89
Q8IVS2	MCAT	Malonyl-CoA	3	1	1	1	390	42.9	8.72	3.15
Q9UNL4	ING4	Inhibitor of g	8	2	2	2	249	28.5	7.62	2.42
Q8IWZ6	BBS7	Bardet-Biedl	2	1	1	1	715	80.3	6.05	2.81
O95969	SCGB1D2	Secretoglobi	12	1	1	1	90	9.9	8.25	2.77
Q9NVX7	KBTBD4	Kelch repeat	3	2	2	2	518	58.1	5.67	2.02
Q9BYD6	MRPL1	39S ribosom	3	1	1	1	325	36.9	8.78	3.24
O14733	MAP2K7	Dual specific	3	1	1	1	419	47.5	9.16	2.84
Q9Y375	NDUFAF1	Complex I int	3	1	1	1	327	37.7	7.64	3.71
Q14865	ARID5B	AT-rich intera	1	1	1	1	1188	132.3	8.72	3
Q8TEH3	DENND1A	DENN doma	1	1	1	1	1009	110.5	6.96	1.65
Q9UPY6	WASF3	Actin-binding	3	1	1	1	502	55.3	6.43	2.7
O15121	DEGS1	Sphingolipid	6	1	1	1	323	37.8	7.46	0
P25067	COL8A2	Collagen alp	1	1	1	1	703	67.2	8.98	3.01
Q5JUR7	TEX30	Testis-expres	7	2	2	2	227	25.6	8.66	1.94
O00602	FCN1	Ficolin-1 OS=	3	1	1	1	326	35.1	6.86	3.22
P52799	EFNB2	Ephrin-B2 OS	7	1	1	1	333	36.9	8.85	3.26
Q9H871	RMND5A	E3 ubiquitin-p	3	1	1	1	391	44	6.06	2.44
Q9H1N7	SLC35B3	Adenosine 3'	2	1	1	1	401	44.6	9.23	2.93
Q9UDX5	MTFP1	Mitochondria	8	1	1	1	166	18	9.31	2.15
Q8N4P3	HDDC3	Guanosine-3	7	1	1	1	179	20.3	6.74	2.46
Q96CQ1	SLC25A36	Solute carrie	4	1	1	1	311	34.3	8.57	3.4
Q8IVB5	LIX1L	LIX1-like pro	3	1	1	1	337	36.5	8.56	0
Q5T4B2	CERCAM	Inactive glyco	2	1	1	1	595	67.5	6.06	2.9
O75792	RNASEH2A	Ribonuclease	2	2	2	2	299	33.4	5.25	5.57
Q7Z4G1	COMMD6	COMM doma	11	1	1	1	85	9.6	6	2.38
P48200	IREB2	Iron-respons	1	1	1	1	963	105	7.05	3.05
Q53SF7	COBLL1	Cordon-bleu	1	1	1	1	1128	123.8	6.58	2.57
O14966	RAB29	Ras-related p	5	1	1	1	203	23.1	7.18	2.17
Q9NSU2	TREX1	Three-prime	4	1	1	1	314	33.2	7.93	3.39
Q9HA82	CERS4	Ceramide sy	5	1	1	1	394	46.4	9.07	0
Q9BZM1	PLA2G12A	Group XIIA s	8	1	1	1	189	21.1	7.24	2.27

Q9UQR1	ZNF148	Zinc finger pı	2	1	1	1	794	88.9	6.48	2.31
Q7Z6E9	RBBP6	E3 ubiquitin-ı	1	2	2	2	1792	201.4	9.64	4.45
Q96HJ9	FMC1	Protein FMC	11	1	1	1	113	12.7	10.07	2.71
Q96S86	HAPLN3	Hyaluronan a	3	1	1	1	360	40.9	6.52	2.83
Q9UKI8	TLK1	Serine/threor	2	2	3	2	766	86.6	8.72	5.51
Q9H1D9	POLR3F	DNA-directed	4	1	1	1	316	35.7	6.11	2.71
Q13433	SLC39A6	Zinc transpor	1	1	1	1	755	85	6.95	2.92
O15551	CLDN3	Claudin-3 OS	5	1	1	1	220	23.3	8.05	2.57
Q8N5D0	WDTC1	WD and tetra	2	1	1	1	677	75.9	7.27	2.75
Q969T9	WBP2	WW domain-	4	1	1	1	261	28.1	5.91	2.45
O75387	SLC43A1	Large neutra	5	2	2	2	559	61.4	7.68	1.86
P15336	ATF2	Cyclic AMP-c	2	1	1	1	505	54.5	7.88	3.58
Q6ZVK8	NUDT18	8-oxo-dGDP	4	1	1	1	323	35.5	6.23	3.26
Q15147	PLCB4	1-phosphatic	1	1	1	1	1175	134.4	6.9	3.14
Q5VTE6	ANGEL2	Protein ange	2	1	1	1	544	62.3	7.81	3.01
Q8NC44	RETREG2	Reticulophag	5	1	1	1	543	57.8	4.45	3
Q9BU61	NDUFAF3	NADH dehyc	5	1	2	1	184	20.3	8.22	4.98
Q96B45	BORCS7	BLOC-1-rela	12	1	1	1	106	11.7	6.79	2.77
Q9NPL8	TIMMDC1	Complex I as	4	1	2	1	285	32.2	8.5	4.87
O60353	FZD6	Frizzled-6 OS	2	1	1	1	706	79.2	7.96	2.39
P48061	CXCL12	Stromal cell-c	15	1	1	1	93	10.7	9.88	3.75
O14678	ABCD4	Lysosomal cc	2	1	1	1	606	68.6	6.55	3.21
Q9BR61	ACBD6	Acyl-CoA-bir	4	1	1	1	282	31.1	5.11	3.55
Q8N9U0	TC2N	Tandem C2 c	3	1	2	1	490	55.2	9.19	5.31
Q9Y2H5	PLEKHA6	Pleckstrin ho	1	1	1	1	1048	117.1	9.1	3.28
Q9NRX2	MRPL17	39S ribosom	5	1	2	1	175	20	10.11	3.98
Q9Y6A9	SPCS1	Signal peptic	7	1	1	1	169	18.3	8.72	2.77
Q9H2X8	IFI27L2	Interferon alp	11	1	1	1	130	12.4	5.34	2.2
Q96L91	EP400	E1A-binding	0	1	1	1	3159	343.3	9.19	2.95
O95622	ADCY5	Adenylate cy	1	1	1	1	1261	138.8	7.24	3.24
Q15542	TAF5	Transcriptior	1	1	1	1	800	86.8	5.64	2.43
Q9Y6I9	TEX264	Testis-expres	4	1	1	1	313	34.2	4.86	2.59
Q14181	POLA2	DNA polyme	3	2	2	1	598	65.9	5.24	0

O43379	WDR62	WD repeat-c	2	2	2	2	1518	165.8	5.91	0
P53355	DAPK1	Death-assoc	1	1	1	1	1430	159.9	6.83	3.38
Q8N122	RPTOR	Regulatory-a	1	2	2	2	1335	148.9	6.89	1.62
O94915	FRYL	Protein furry	0	1	1	1	3013	339.4	5.58	3.18
Q5TGL8	PXDC1	PX domain-c	4	1	1	1	231	26.5	5.03	1.71
Q8WWH5	TRUB1	Pseudouridyl	3	1	1	1	349	37.2	8.25	2.37
Q6IQ32	ADNP2	Activity-depe	1	1	1	1	1131	122.8	9.16	3.25
Q9BSR8	YIPF4	Protein YIPF	4	1	1	1	244	27.1	4.65	2.55
Q8IWW6	ARHGAP12	Rho GTPase	1	1	2	1	846	96.2	7.61	4.56
P82664	MRPS10	28S ribosom	9	1	1	1	201	23	8	2.63
Q96B96	LDAF1	Lipid droplet	8	1	1	1	161	17.5	5.06	3.46
Q86V21	AACS	Acetoacetyl-(	1	1	1	1	672	75.1	6.24	2.82
Q9NRF9	POLE3	DNA polyme	7	1	1	1	147	16.8	4.74	3.01
Q16514	TAF12	Transcriptior	5	1	1	1	161	17.9	7.99	3.47
Q8N108	MIER1	Mesoderm in	2	1	1	1	512	57.9	4.42	2.4
Q93091	RNASE6	Ribonucleas	7	1	1	1	150	17.2	8.76	3.57
Q7Z6B0	CCDC91	Coiled-coil d	5	2	2	2	441	49.9	5.07	2.02
Q9NZM3	ITSN2	Intersectin-2	1	1	1	1	1697	193.3	8.12	2.03
P01718	IGLV3-27	Immunoglob	10	1	1	1	113	12.2	5.01	3.67
Q8IY95	TMEM192	Transmembr	4	1	2	1	271	30.9	7.99	6.34
O75182	SIN3B	Paired amph	1	1	1	1	1162	133	6.93	3.26
Q659C4	LARP1B	La-related pr	2	2	2	1	914	105.3	7.61	1.75
Q7Z7F0	KHDC4	KH homolog	1	1	1	1	614	64.8	8.73	2.33
Q8IYT4	KATNAL2	Katanin p60	2	1	1	1	538	61.2	7.56	2.97
O43516	WIPF1	WAS/WASL-	2	1	1	1	503	51.2	11.47	2.82
A0A0B4J1Y8	IGLV9-49	Immunoglob	8	1	2	1	123	13	7.28	5.18
Q03167	TGFBR3	Transforming	1	1	1	1	851	93.4	5.71	2.88
Q9P0U1	TOMM7	Mitochondria	55	3	4	3	55	6.2	10.29	6.07
A1L157	TSPAN11	Tetraspanin-	3	1	2	1	253	28.2	7.58	5.41
P19224	UGT1A6	UDP-glucuro	2	1	1	1	532	60.7	8.41	2.25
Q8TE04	PANK1	Pantothenate	2	1	1	1	598	64.3	7.56	2.76
Q9UBM7	DHCR7	7-dehydroch	3	2	2	2	475	54.5	8.7	2.82
Q15629	TRAM1	Translocating	3	1	1	1	374	43	9.63	2.23

Q86WN1	FCHSD1	F-BAR and d	2	1	1	1	690	76.9	5.34	2.7
Q03393	PTS	6-pyruvoyl te	6	1	1	1	145	16.4	6.68	2.24
Q9Y248	GINS2	DNA replicat	7	1	1	1	185	21.4	5.44	2.75
Q86X27	RALGPS2	Ras-specific	2	1	1	1	583	65.1	8.73	2.38
Q2VPK5	CTU2	Cytoplasmic	3	1	1	1	515	56.1	6.32	3.05
Q16611	BAK1	Bcl-2 homolc	5	1	1	1	211	23.4	6.01	3.04
P28702	RXRB	Retinoic acid	2	1	1	1	533	56.9	8.18	3
P49281	SLC11A2	Natural resis	3	1	1	1	568	62.2	6.09	0
Q6NXT4	SLC30A6	Zinc transpo	2	1	1	1	461	51.1	9.16	3.37
Q08AE8	SPIRE1	Protein spire	1	1	1	1	756	85.5	8.62	1.87
Q99487	PAFAH2	Platelet-activ	4	2	2	2	392	44	6.89	2.69
Q16602	CALCRL	Calcitonin ge	2	1	1	1	461	52.9	6.74	2.6
Q9P0R6	GSKIP	GSK3B-inter	8	1	1	1	139	15.6	4.48	2.56
Q6ZWT7	MBOAT2	Lysophosphc	3	1	1	1	520	59.5	8.03	2.21
Q9BRQ0	PYGO2	Pygopus hor	4	1	1	1	406	41.2	7.28	3.84
P55056	APOC4	Apolipoprote	9	1	1	1	127	14.5	8.92	3.48
Q96K37	SLC35E1	Solute carrie	3	1	1	1	410	44.7	9.79	3.24
P27707	DCK	Deoxycytidin	4	1	1	1	260	30.5	5.21	3.36
Q68BL8	OLFML2B	Olfactomedir	3	2	2	2	750	83.9	5.2	5.11
Q9ULH0	KIDINS220	Kinase D-inte	0	1	1	1	1771	196.4	6.62	3.06
Q9Y5S1	TRPV2	Transient rec	1	1	1	1	764	85.9	5.83	2.53
Q9UMD9	COL17A1	Collagen alpl	1	1	1	1	1497	150.3	8.79	2.43
Q96AB6	NTAN1	Protein N-ter	5	2	2	2	310	34.7	6.27	4.75
O14647	CHD2	Chromodoma	1	1	1	1	1828	211.2	8.1	3.93
Q96B49	TOMM6	Mitochondria	19	1	1	1	74	8	4.89	2.59
Q8WWV3	RTN4IP1	Reticulon-4-i	3	1	2	1	396	43.6	9.11	4.69
O76076	CCN5	CCN family r	6	2	2	2	250	26.8	7.88	5.2
P36406	TRIM23	E3 ubiquitin-r	2	1	2	1	574	64	6.38	2.79
Q9Y487	ATP6V0A2	V-type protor	2	1	1	1	856	98	6.61	2.11
Q96F24	NRBF2	Nuclear rece	3	1	1	1	287	32.4	5.87	2.66
Q96CS2	HAUS1	HAUS augmi	6	2	2	2	278	31.8	5.53	2.03
Q8N5C1	CALHM5	Calcium hom	5	1	1	1	309	35.1	8.13	3.11
Q9BQE4	SELENOS	Selenoprotei	5	1	1	1	189	21.2	9.7	2.88

O15085	ARHGEF11	Rho guanine	1	1	2	1	1522	167.6	5.5	4.91
O43513	MED7	Mediator of F	4	1	1	1	233	27.2	5.78	2.5
Q8TBP5	FAM174A	Membrane p	5	1	2	1	190	19.9	6.33	5
Q9NWY4	HPF1	Histone PAR	6	2	2	2	346	39.4	6.8	2.25
Q6ZS30	NBEAL1	Neurobeachi	0	1	1	1	2694	307	6.44	2.13
O95059	RPP14	Ribonuclease	6	1	1	1	124	13.7	7.75	3.05
Q9P0P8	MTRES1	Mitochondria	9	1	1	1	240	27.9	9.29	2.15
P49810	PSEN2	Presenilin-2 (	2	1	1	1	448	50.1	4.59	2.54
Q9Y3B2	EXOSC1	Exosome cor	9	1	1	1	195	21.4	8.24	0
Q16625	OCLN	Occludin OS	2	1	1	1	522	59.1	6.09	2.58
O75843	AP1G2	AP-1 comple	1	1	2	1	785	87.1	6.55	4.65
Q5SW96	LDLRAP1	Low density l	4	1	1	1	308	33.9	6.7	2.72
Q8TF01	PNISR	Arginine/seri	2	2	2	2	805	92.5	10.02	2.49
Q8WUF8	FAM172A	Cotranscripti	4	1	1	1	416	47.9	6.09	2
Q147X3	NAA30	N-alpha-acet	3	1	1	1	362	39.3	5.52	2.45
O15235	MRPS12	28S ribosom	7	1	1	1	138	15.2	10.29	2.63
P01344	IGF2	Insulin-like g	5	1	2	1	180	20.1	9.32	4.4
Q9H1C3	GLT8D2	Glycosyltrans	3	1	1	1	349	40	7.06	2.57
Q9BSY4	CHCHD5	Coiled-coil-h	16	1	1	1	110	12.4	6.73	2.32
Q8IUF8	RIOX2	Ribosomal o:	2	1	1	1	465	52.8	6.7	3.15
Q96P16	RPRD1A	Regulation o	4	1	1	1	312	35.7	7.55	2.31
O15344	MID1	E3 ubiquitin-|	2	1	1	1	667	75.2	6.8	3.15
Q8WUR7	C15orf40	UPF0235 pro	11	1	1	1	153	16.3	9.83	2.58
A0A0B4J2F(	PIGBOS1	Protein PIGE	19	1	1	1	54	6.3	8.5	3
Q96NE9	FRMD6	FERM doma	2	1	1	1	622	72	7.46	3.5
Q15697	ZNF174	Zinc finger pr	3	1	1	1	407	46.4	9.58	2.78
Q14210	LY6D	Lymphocyte	7	1	1	1	128	13.3	8.21	2.22
Q92729	PTPRU	Receptor-typ	1	2	2	2	1446	162.3	6.92	1.85
O14727	APAF1	Apoptotic pro	1	1	1	1	1248	141.7	6.4	3.11
Q6ZPD9	DPY19L3	Probable C-r	1	1	1	1	716	83.1	8.72	1.75
Q8WY36	BBX	HMG box tra	1	2	2	2	941	105.1	8.79	3.76
Q9NVC3	SLC38A7	Sodium-coup	3	1	1	1	462	49.9	5.08	3.08
Q8N3Y1	FBXW8	F-box/WD re	2	1	1	1	598	67.4	5.67	2.09

P35226	BMI1	Polycomb co	2	1	1	1	326	36.9	8.63	3.31
Q9Y244	POMP	Proteasome	7	1	1	1	141	15.8	5.11	2.96
Q9UJX4	ANAPC5	Anaphase-pr	2	1	1	1	755	85	6.87	2.67
Q9NUP1	BLOC1S4	Biogenesis o	6	1	1	1	217	23.3	4.98	2.47
Q8NFF5	FLAD1	FAD synthas	5	2	2	2	587	65.2	6.93	0
Q15345	LRRC41	Leucine-rich	1	1	1	1	812	88.6	8.38	1.75
Q12778	FOXO1	Forkhead bo	1	1	2	1	655	69.6	6.77	5.77
P10176	COX8A	Cytochrome	13	1	1	1	69	7.6	10.24	3.05
Q8N0U8	VKORC1L1	Vitamin K ep	6	1	1	1	176	19.8	9.13	2.81
Q8IUH4	ZDHHC13	Palmitoyltran	1	1	1	1	622	70.8	8.07	2.33
P13598	ICAM2	Intercellular a	6	1	1	1	275	30.6	7.43	3.02
P01714	IGLV3-19	Immunoglobu	9	1	1	1	112	12	4.96	2.49
Q92545	TMEM131	Transmembr	1	1	1	1	1883	205	8.59	2.92
Q8IZ69	TRMT2A	tRNA (uracil-	2	1	1	1	625	68.7	7.94	2.81
Q8IUF1	ZNG1B	Zinc-regulate	4	1	1	1	395	44	4.89	0
Q68CQ7	GLT8D1	Glycosyltrans	3	1	1	1	371	41.9	9.35	2.74
Q96F46	IL17RA	Interleukin-1	1	1	1	1	866	96.1	5.21	2.21
Q8IZD4	DCP1B	mRNA-decap	6	1	1	1	617	67.7	8.56	3.38
Q8TBQ9	TMEM167A	Protein kish-	13	1	1	1	72	8.1	8.95	2.77
P50750	CDK9	Cyclin-deper	2	1	1	1	372	42.8	8.79	2.5
Q9ULR0	ISY1	Pre-mRNA-s	4	2	2	2	285	33	5.17	2.19
Q96Q15	SMG1	Serine/threo	0	1	1	1	3661	410.2	6.46	2.7
Q92854	SEMA4D	Semaphorin-	2	1	1	1	862	96.1	7.96	2.44
P17213	BPI	Bactericidal p	2	1	1	1	487	53.9	9.38	3.18
Q5TGZ0	MICOS10	MICOS comp	10	1	1	1	78	8.8	8.5	2.45
Q9Y2L9	LRCH1	Leucine-rich	3	2	3	2	728	80.8	5.94	4.49
P23443	RPS6KB1	Ribosomal pr	2	1	1	1	525	59.1	6.65	2.2
Q5T749	KPRP	Keratinocyte	2	1	1	1	579	64.1	8.27	2.64
Q8TDW0	LRRC8C	Volume-regu	1	1	1	1	803	92.4	7.62	2.86
O95714	HERC2	E3 ubiquitin-p	0	1	1	1	4834	526.9	6.28	3.06
P05089	ARG1	Arginase-1 C	3	1	1	1	322	34.7	7.21	2.45
Q86YV9	HPS6	BLOC-2 com	1	1	1	1	775	82.9	6.28	3.2
Q96CD2	PPCDC	Phosphopan	6	1	1	1	204	22.4	6.1	2.84

A0A075B6J9	IGLV2-18	Immunoglobu	14	1	1	1	118	12.4	4.82	0
Q9Y6A4	CFAP20	Cilia- and fla	5	1	1	1	193	22.8	9.76	2.57
Q96BN8	OTULIN	Ubiquitin thic	3	1	1	1	352	40.2	5.47	2.1
O95563	MPC2	Mitochondria	9	1	1	1	127	14.3	10.43	3.13
Q8NBI5	SLC43A3	Equilibrative	2	1	1	1	491	54.5	8.53	3.67
Q6ICB4	PHETA2	Sesquipedali	4	1	1	1	259	28.3	7.11	2.71
Q5T0D9	TPRG1L	Tumor protei	4	1	1	1	272	30.2	7.34	2.89
Q6PJI9	WDR59	GATOR com	1	1	1	1	974	109.7	7.91	2.73
P29597	TYK2	Non-receptor	1	1	1	1	1187	133.6	7.15	2.7
Q8NBI6	XXYLT1	Xyloside xylo	3	1	1	1	393	43.8	8.13	2.57
Q96EL2	MRPS24	28S ribosom	7	1	1	1	167	19	9.38	2.32
Q9H4L5	OSBPL3	Oxysterol-bir	1	1	1	1	887	101.2	6.87	2.98
Q9UNI6	DUSP12	Dual specific	3	1	1	1	340	37.7	6.84	2.77
Q8NHV4	NEDD1	Protein NED	3	2	2	2	660	71.9	7.97	0
Q99732	LITAF	Lipopolysacc	4	1	2	1	161	17.1	6.44	5.11
Q8WUQ7	CACTIN	Splicing facto	1	1	1	1	758	88.6	9.14	2.6
Q8IZU0	FAM9B	Protein FAM9	3	1	62	1	186	22.4	5.41	80.39
P23508	MCC	Colorectal m	1	1	1	1	829	93	5.52	2.93
A7KAX9	ARHGAP32	Rho GTPase	1	1	1	1	2087	230.4	6.74	2.62
Q96EQ0	SGTB	Small glutam	3	1	1	1	304	33.4	4.92	2.88
O76062	TM7SF2	Delta(14)-ste	3	2	2	1	418	46.4	8.87	3.97
Q14031	COL4A6	Collagen alp	1	1	1	1	1691	163.7	9.2	0
O95166	GABARAP	Gamma-amir	9	1	1	1	117	13.9	8.79	3.23
Q9Y279	VSIG4	V-set and im	4	1	1	1	399	44	6.35	2
Q9BWW4	SSBP3	Single-strand	3	1	1	1	388	40.4	6.9	2.48
Q5JYT7	KIAA1755	Uncharacteri	1	2	2	2	1200	130.8	6.37	4.42
Q14249	ENDOG	Endonucleas	6	2	2	2	297	32.6	9.5	1.61
P46527	CDKN1B	Cyclin-deper	8	1	1	1	198	22.1	7.02	2.74
Q5VVQ6	YOD1	Ubiquitin thic	3	1	1	1	348	38.3	6.11	1.92
Q9H5X1	CIAO2A	Cytosolic iron	7	1	1	1	160	18.3	4.88	3.3
Q9Y605	MRFAP1	MORF4 fami	8	1	1	1	127	14.6	4.7	2.69
Q14191	WRN	Bifunctional 3	1	1	1	1	1432	162.4	6.34	2.7
Q5JTB6	PLAC9	Placenta-spe	10	1	1	1	97	10.3	4.92	2.57

O75953	DNAJB5	DnaJ homolo	5	1	2	1	348	39.1	9.04	5.08
P60520	GABARAPL2	Gamma-amii	9	1	1	1	117	13.7	8.1	2.17
O15047	SETD1A	Histone-lysin	1	2	2	2	1707	185.9	5.14	0
Q7Z392	TRAPPC11	Trafficking pr	1	1	1	1	1133	128.8	7.14	2.54
Q9BW66	CINP	Cyclin-deper	6	1	1	1	212	24.3	6.28	2.54
Q6ZU80	CEP128	Centrosomal	2	1	1	1	1094	127.9	6.52	2.34
Q68CR1	SEL1L3	Protein sel-1	1	1	2	1	1132	128.5	7.28	2.03
Q9NNX6	CD209	CD209 antig	13	1	2	1	404	45.7	5.47	2.85
P30536	TSPO	Translocator	8	2	2	2	169	18.8	9.36	2.6
Q7Z589	EMSY	BRCA2-inter	2	2	2	2	1322	141.4	9.33	4
Q92835	INPP5D	Phosphatidyl	1	1	1	1	1189	133.2	7.59	3.19
Q9UJY1	HSPB8	Heat shock p	6	1	1	1	196	21.6	5.12	2.21
Q9UGR2	ZC3H7B	Zinc finger C	1	1	1	1	977	109.8	7.25	2.71
Q9Y2K7	KDM2A	Lysine-speci	1	1	1	1	1162	132.7	7.58	2.56
Q8N9B5	JMY	Junction-mec	2	2	2	2	988	111.4	6.18	0
P62699	YPEL5	Protein yippe	8	1	1	1	121	13.8	7.31	2.94
Q8N490	PNKD	Probable hyc	3	1	1	1	385	42.8	9.09	3.22
Q6UXD5	SEZ6L2	Seizure 6-like	1	1	1	1	910	97.5	4.89	2.8
Q96KN2	CNDP1	Beta-Ala-His	2	1	1	1	507	56.7	5.3	2.73
Q9H939	PSTPIP2	Proline-serin	5	2	2	2	334	38.8	8.48	2.07
Q8IW41	MAPKAPK5	MAP kinase-	2	1	1	1	473	54.2	7.78	2.74
P11226	MBL2	Mannose-bin	4	1	1	1	248	26.1	5.49	2.36
P49406	MRPL19	39S ribosom	4	2	2	1	292	33.5	9.5	4.33
Q9NVN8	GNL3L	Guanine nuc	1	1	1	1	582	65.5	8.44	2.91
Q5VST9	OBSCN	Obscurin OS	0	2	2	2	7968	867.9	5.99	0
Q8NFZ8	CADM4	Cell adhesion	2	1	1	1	388	42.8	6.3	2.72
P40692	MLH1	DNA mismat	1	1	1	1	756	84.5	5.72	2.35
Q9P021	CRIPT	Cysteine-rich	8	1	1	1	101	11.2	9.47	2.28
P0DI82	TRAPPC2B	Trafficking pr	6	1	1	1	140	16.4	6.52	2.67
Q8TDY4	ASAP3	Arf-GAP with	2	1	1	1	903	99.1	6.4	2.35
Q9BUI4	POLR3C	DNA-directed	3	2	2	2	534	60.6	7.31	2.09
Q9BPZ7	MAPKAP1	Target of rap	2	1	1	1	522	59.1	7.55	2.93
Q53S33	BOLA3	BolA-like pro	14	1	1	1	107	12.1	9.64	0

Q53ET0	CRTC2	CREB-regula	2	1	1	1	693	73.3	7.11	2.72
Q96LD8	SENP8	Sentrin-spec	7	2	2	2	212	24.1	5.97	2.01
Q9H8W4	PLEKHF2	Pleckstrin ho	5	1	1	1	249	27.8	8.21	0
Q9H8H2	DDX31	Probable AT	1	1	1	1	851	94	9.99	2.96
Q01804	OTUD4	OTU domain	1	1	1	1	1114	124	6.71	2.13
Q86XA9	HEATR5A	HEAT repeat	1	1	1	1	2040	221.9	6.58	0
Q9UK22	FBXO2	F-box only pr	4	1	1	1	296	33.3	4.37	2.2
Q5TA45	INTS11	Integrator co	2	1	1	1	600	67.6	8.06	0
A0A075B6I4	IGLV10-54	Immunoglobu	9	1	2	1	117	12.4	8.03	4.54
P09001	MRPL3	39S ribosom	3	1	1	1	348	38.6	9.48	2.44
P19021	PAM	Peptidyl-glyc	1	1	1	1	973	108.3	6.42	2.2
Q9BRX5	GINS3	DNA replicat	11	1	1	1	216	24.5	5.34	2.57
Q9NP73	ALG13	Putative bifu	1	1	1	1	1137	126	6.74	2.19
Q9NX02	NLRP2	NACHT, LRF	1	1	1	1	1062	120.4	6.07	2.04
Q8IYJ3	SYTL1	Synaptotagm	2	1	2	1	562	61.8	5.48	2.62
Q96GA7	SDSL	Serine dehyc	4	1	1	1	329	34.7	6.89	2.25
Q6A555	TXNDC8	Thioredoxin c	5	1	2	1	127	14.6	9.47	4.41
Q9NRD5	PICK1	PRKCA-bind	4	1	1	1	415	46.6	5.3	0
O94829	IPO13	Importin-13 (	1	1	1	1	963	108.1	5.3	2.58
Q14241	ELOA	Elongin-A OS	1	1	1	1	798	89.9	9.57	0
Q9NPI9	KCNJ16	Inward rectifi	4	1	3	1	418	47.9	7.87	4.22
P78357	CNTNAP1	Contactin-as	1	1	1	1	1384	156.2	7.05	2.63
Q9UKK6	NXT1	NTF2-relatec	6	1	1	1	140	15.8	5.03	2.3
P26678	PLN	Cardiac phos	21	1	1	1	52	6.1	9.28	2.86
Q15388	TOMM20	Mitochondria	9	1	2	1	145	16.3	8.6	5.88
O75355	ENTPD3	Ectonucleosi	2	1	1	1	529	59.1	6.42	2.92
Q86YJ6	THNSL2	Threonine sy	3	1	1	1	484	54.1	6.52	2.72
Q9BSC4	NOL10	Nucleolar prc	1	1	1	1	688	80.3	8.46	2.65
Q8IV38	ANKMY2	Ankyrin repe	2	1	1	1	441	49.3	6.25	3.14
O60449	LY75	Lymphocyte	1	1	1	1	1722	198.2	6.67	0
Q8N1I0	DOCK4	Dedicator of	1	1	1	1	1966	225.1	7.65	0
P30511	HLA-F	HLA class I h	5	2	2	2	346	39	5.57	1.88
Q02548	PAX5	Paired box p	4	1	1	1	391	42.1	9	0

O43166	SIPA1L1	Signal-induc	0	1	1	1	1804	199.9	8.19	2.41
Q86WA8	LONP2	Lon protease	2	2	2	2	852	94.6	7.3	4.3
Q9NUW8	TDP1	Tyrosyl-DNA	1	1	1	1	608	68.4	7.65	2.6
Q9H4L7	SMARCAD1	SWI/SNF-rel	1	1	1	1	1026	117.3	5.55	1.96
Q9NVS9	PNPO	Pyridoxine-5'	3	1	1	1	261	30	7.06	2.57
Q9NVH0	EXD2	Exonuclease	2	2	4	1	621	70.3	8.32	0
Q8NBM4	UBAC2	Ubiquitin-ass	2	1	1	1	344	38.9	9.01	1.87
O95866	MPIG6B	Megakaryocy	4	1	1	1	241	26.1	9.61	3.56
Q9UBU6	FAM8A1	Protein FAM8	3	1	1	1	413	44.1	7.21	1.77
Q92485	SMPDL3B	Acid sphingo	2	1	1	1	455	50.8	5.64	2.03
P16050	ALOX15	Polyunsatura	1	1	1	1	662	74.8	6.58	1.87
P55347	PKNOX1	Homeobox p	2	1	1	1	436	47.6	4.91	2.69
Q9UJX6	ANAPC2	Anaphase-pr	1	1	1	1	822	93.8	5.22	2.74
Q53F39	MPPE1	Metallophosp	3	1	1	1	396	45.1	7.15	2.04
O75751	SLC22A3	Solute carrie	2	1	1	1	556	61.2	8.57	3.16
Q8N6Q3	CD177	CD177 antig	2	1	1	1	437	46.3	6.29	2.46
Q9Y2Q9	MRPS28	28S ribosom	7	2	2	2	187	20.8	9.1	0
Q7Z3C6	ATG9A	Autophagy-re	1	1	1	1	839	94.4	6.67	0
Q6ZUK4	TMEM26	Transmembr	3	1	1	1	368	41.6	6.87	2.58
Q96E52	OMA1	Metalloendop	3	2	2	2	524	60.1	9.25	1.91
Q07617	SPAG1	Sperm-assoc	1	1	1	1	926	103.6	6.86	2.86
Q9UGT4	SUSD2	Sushi domain	2	1	1	1	822	90.2	6.28	2.1
Q8IY22	CMIP	C-Maf-induci	1	1	1	1	773	86.3	6.7	2.33
Q8IUR0	TRAPPC5	Trafficking pr	4	1	1	1	188	20.8	9.66	2.5
Q96AQ8	MCUR1	Mitochondria	2	1	2	1	359	39.7	9.63	1.93
Q04656	ATP7A	Copper-trans	1	1	1	1	1500	163.3	6.33	2.16
Q9Y241	HIGD1A	HIG1 domain	9	1	1	1	93	10.1	9.79	2.15
A0A0G2JS0	IGLV5-39	Immunoglobu	7	1	2	1	123	13.4	8.63	1.72
Q96BH1	RNF25	E3 ubiquitin-|	2	1	1	1	459	51.2	6.54	2.2
Q9HC52	CBX8	Chromobox p	5	1	1	1	389	43.4	9.91	0
P19256	CD58	Lymphocyte	4	1	1	1	250	28.1	6.76	2.51
A4FU01	MTMR11	Myotubularin	1	1	3	1	709	79.5	7.03	6.4
Q96MG7	NSMCE3	Non-structura	3	1	1	1	304	34.3	9.28	2.3

O75528	TADA3	Transcription	3	1	1	1	432	48.9	6.27	1.74
P49643	PRIM2	DNA primase	2	1	2	1	509	58.8	7.91	5.8
P32780	GTF2H1	General tran:	2	1	1	1	548	62	8.66	2.45
Q5T8I3	FAM102B	Protein FAM:	4	1	1	1	360	39.3	7.02	2.25
Q92824	PCSK5	Proprotein co	1	1	1	1	1860	206.8	6.1	2.11
Q96IK0	TMEM101	Transmembr	4	1	1	1	257	28.8	9.57	3.58
P48553	TRAPPC10	Trafficking pr	1	1	1	1	1259	142.1	6.04	2.47
O75460	ERN1	Serine/threor	1	1	1	1	977	109.7	6.42	2.42
O95926	SYF2	Pre-mRNA-s	4	1	1	1	243	28.7	8.4	2.29
P14151	SELL	L-selectin OS	2	1	1	1	372	42.2	6.6	2.37
Q8TF05	PPP4R1	Serine/threor	1	1	1	1	950	106.9	4.77	2.12
Q96CP7	TLCD1	TLC domain-	3	1	2	1	247	28.5	9.47	2.19
Q15283	RASA2	Ras GTPase	2	1	1	1	850	96.6	7.2	2.13
Q15742	NAB2	NGFI-A-bind	2	1	1	1	525	56.6	6.96	3.23
Q96F15	GIMAP5	GTPase IMA	4	2	2	2	307	34.8	7.68	3.64
O75841	UPK1B	Uroplakin-1b	3	1	1	1	260	29.6	5.35	2.69
O15037	KHNYN	Protein KHN	1	1	1	1	678	74.5	7.02	2.81
P43007	SLC1A4	Neutral amin	2	1	1	1	532	55.7	6.25	2.23
Q8IWB9	TEX2	Testis-expres	1	1	1	1	1127	125.2	6.01	2.28
P23610	F8A3	40-kDa hunti	4	1	1	1	371	39.1	6.84	2.55
P28290	ITPRID2	Protein ITPR	1	1	1	1	1259	138.3	5.19	2.85
O95498	VNN2	Pantetheine	3	1	1	1	520	58.5	6.52	2.63
Q13426	XRCC4	DNA repair p	2	1	1	1	336	38.3	4.98	2.01
Q8N4V1	MMGT1	ER membrar	7	1	1	1	131	14.7	9.16	2.2
Q8N9L9	ACOT4	Peroxisomal	2	1	2	1	421	46.3	8.07	2.16
Q02083	NAAA	N-acylethanc	2	1	1	1	359	40	8.75	2.19
Q9UKR5	ERG28	Ergosterol bi	6	1	1	1	140	15.9	9.83	2.9
Q8WV22	NSMCE1	Non-structura	3	1	1	1	266	30.8	7.47	2.45
Q9NRA2	SLC17A5	Sialin OS=Ho	2	1	1	1	495	54.6	8.27	2.56
Q5VWJ9	SNX30	Sorting nexir	2	1	1	1	437	49.6	5.35	0
Q9UKY1	ZHX1	Zinc fingers a	2	2	2	2	873	98	6.05	1.67
Q14353	GAMT	Guanidinoac	6	1	1	1	236	26.3	6.14	2.16
Q8NI60	COQ8A	Atypical kina	2	1	1	1	647	71.9	6.99	1.95

Q7L9B9	EEPD1	Endonucleas	2	1	1	1	569	62.4	8.41	2.16
Q6P2P2	PRMT9	Protein argin	1	1	1	1	845	94.4	5.25	2.52
P10600	TGFB3	Transforming	4	1	1	1	412	47.3	8.03	0
Q9UJQ1	LAMP5	Lysosome-as	4	1	1	1	280	31.5	6.46	2.77
P0C870	JMJD7	Bifunctional p	5	1	1	1	316	35.9	5.38	2.85
Q14116	IL18	Interleukin-18	5	1	1	1	193	22.3	4.67	3.18
O95619	YEATS4	YEATS doma	3	1	1	1	227	26.5	8.41	2.29
Q8WWX0	ASB5	Ankyrin repe	3	1	2	1	329	36.3	6.81	0
Q9Y6D0	SELENOK	Selenoprotei	10	1	1	1	94	10.6	10.86	0
P06307	CCK	Cholecystoki	8	1	1	1	115	12.7	9.01	2.79
O15541	RNF113A	E3 ubiquitin-p	2	1	1	1	343	38.8	5.69	2.06
Q9H9H4	VPS37B	Vacuolar pro	4	1	1	1	285	31.3	7.34	0
Q9BXC9	BBS2	Bardet-Biedl	1	1	1	1	721	79.8	6.15	0
P53708	ITGA8	Integrin alpha	1	1	1	1	1063	117.4	5.58	2.52
Q8NFJ9	BBS1	Bardet-Biedl	3	1	1	1	593	65	7.93	2.55
Q99611	SEPHS2	Selenide, wa	3	2	2	1	448	47.3	5.88	0
Q6NTF9	RHBDD2	Rhomboid do	2	1	1	1	364	39.2	9.32	2.4
Q9UHQ1	NARF	Nuclear prela	2	1	1	1	456	51.1	7.08	0
Q9NWT8	AURKAIP1	Aurora kinas	5	1	1	1	199	22.3	10.76	2.47
Q86UW7	CADPS2	Calcium-dep	1	1	1	1	1296	147.6	6.19	2.76
Q9UIW2	PLXNA1	Plexin-A1 OS	1	2	2	2	1896	210.9	6.92	1.85
P04035	HMGCR	3-hydroxy-3-	2	1	1	1	888	97.4	6.74	0
Q674X7	KAZN	Kazrin OS=H	1	1	1	1	775	86.3	7.03	2.75
Q9NQ36	SCUBE2	Signal peptid	1	1	1	1	999	109.9	6.64	2.34
Q8N4F7	RNF175	RING finger	2	1	1	1	328	38.2	8.85	2.18
Q14714	SSPN	Sarcospan O	3	1	1	1	243	26.6	7.8	2.79
O43913	ORC5	Origin recogi	2	1	1	1	435	50.3	7.74	2.3
Q7L311	ARMCX2	Armadillo rep	2	1	1	1	632	65.6	8.53	2.85
Q96JP5	ZFP91	E3 ubiquitin-p	2	1	1	1	570	63.4	7.36	2.22
Q6UX04	CWC27	Spliceosome	1	1	1	1	472	53.8	5.8	1.7
P13497	BMP1	Bone morpho	1	1	1	1	986	111.2	6.9	2.53
Q9H5Q4	TFB2M	Dimethyladen	2	1	1	1	396	45.3	9.19	0
Q13901	C1D	Nuclear nucl	7	1	1	1	141	16	9.03	0

Q6ZN54	DEF8	Differentially	2	1	1	1	512	58.7	6.52	2.39
O60331	PIP5K1C	Phosphatidyl	1	1	1	1	668	73.2	5.29	2.24
Q9Y6J9	TAF6L	TAF6-like RN	1	1	1	1	622	67.8	8.97	1.75
P49069	CAMLG	Guided entry	3	1	1	1	296	32.9	8.05	2.14
Q9Y303	AMDHD2	N-acetylgluc	2	1	1	1	409	43.7	6.7	2.42
Q9NWW6	NMRK1	Nicotinamide	7	1	1	1	199	23.2	4.92	1.87
O95478	NSA2	Ribosome bi	3	1	1	1	260	30	10.27	2.51
Q6NUS6	TCTN3	Tectonic-3 O	2	1	1	1	607	66.1	8.21	2.59
Q7Z6V5	ADAT2	tRNA-specifi	5	1	1	1	191	21	6.74	1.73
Q9BUD6	SPON2	Spondin-2 O	2	1	1	1	331	35.8	5.68	2.2
O75691	UTP20	Small subuni	0	1	1	1	2785	318.2	7.39	2.46
Q96DE5	ANAPC16	Anaphase-pr	14	1	1	1	110	11.7	4.97	2.58
Q5VYX0	RNLS	Renalase OS	2	1	1	1	342	37.8	6.52	2.03
Q8NHG7	SVIP	Small VCP/p	14	1	1	1	77	8.4	8.91	2.38
Q8NE09	RGS22	Regulator of	1	1	1	1	1264	147.1	7.9	2.25
A0A0C4DH3	IGHV3-38	Probable nor	9	1	1	1	116	12.8	9.25	1.81
Q32MK0	MYLK3	Myosin light	1	1	1	1	819	88.3	6.11	2.17
Q9Y6K1	DNMT3A	DNA (cytosir	1	1	1	1	912	101.8	6.57	1.82
Q14526	HIC1	Hypermethyl	1	1	1	1	733	76.5	6.83	3.07
P78369	CLDN10	Claudin-10 C	4	1	1	1	228	24.5	8.03	2.17
Q14676	MDC1	Mediator of D	1	1	1	1	2089	226.5	5.47	2.09
P36954	POLR2I	DNA-directed	6	1	1	1	125	14.5	5.14	2.47
Q8IZA0	KIAA0319L	Dyslexia-ass	1	1	1	1	1049	115.6	6.05	2.3
Q92610	ZNF592	Zinc finger pr	1	1	1	1	1267	137.4	7.84	2
Q9UBI9	HECA	Headcase pr	2	1	1	1	543	58.8	8.54	2.79
Q6ULP2	AFTPH	Aftiphilin OS	1	1	1	1	936	102.1	4.54	2.81
Q96T58	SPEN	Msx2-interac	0	1	1	1	3664	402	7.64	1.84
Q9HAC7	SUGCT	Succinate--h	3	1	1	1	445	48.4	8.35	0
Q9HBH1	PDF	Peptide defo	4	1	1	1	243	27	9.16	2.12
Q2TAK8	PWWP3A	PWWP doma	1	1	1	1	710	78.6	8.54	3.31
P61952	GNG11	Guanine nuc	11	1	1	1	73	8.5	5.54	2.48
Q9BW85	YJU2	Splicing facto	4	1	1	1	323	37.1	5.92	0
Q53H47	SETMAR	Histone-lysin	1	1	1	1	684	78	7.14	2.34

Q8NG68	TTL	Tubulin--tyro	2	1	1	1	377	43.2	6.74	2
Q9UKB1	FBXW11	F-box/WD re	1	1	1	1	542	62.1	7.12	2.54
Q12913	PTPRJ	Receptor-typ	1	1	1	1	1337	145.9	5.58	2.33
Q13489	BIRC3	Baculoviral IA	2	1	1	1	604	68.3	6.04	0
Q6PIJ6	FBXO38	F-box only pr	1	1	1	1	1188	133.9	6.33	1.78
Q7L3B6	CDC37L1	Hsp90 co-ch	3	1	1	1	337	38.8	5.34	2.1
Q9HCL0	PCDH18	Protocadheri	1	1	1	1	1135	126.1	5.15	0
P61165	TMEM258	Transmembr	10	1	1	1	79	9.1	5.83	2.34
Q13227	GPS2	G protein pat	2	1	1	1	327	36.7	9.52	2.3
Q96RR4	CAMKK2	Calcium/calm	4	1	1	1	588	64.7	6.68	0
Q9UL63	MKLN1	Muskelin OS	2	1	1	1	735	84.7	6.34	2.41
Q9BQQ3	GORASP1	Golgi reasse	2	1	1	1	440	46.5	4.5	2.5
Q9NR64	KLHL1	Kelch-like pro	1	1	1	1	748	82.6	6.39	2.67
Q6ZN30	BNC2	Zinc finger pr	1	1	2	1	1099	122.3	6.52	1.79
Q13542	EIF4EBP2	Eukaryotic tra	11	1	1	1	120	12.9	6.67	2.09
Q8N6G6	ADAMTSL1	ADAMTS-like	0	1	1	1	1762	193.3	7.8	2.98
Q96DC8	ECHDC3	Enoyl-CoA hy	3	1	1	1	303	32.6	8.65	0
Q9UNW9	NOVA2	RNA-binding	2	1	4	1	492	49	8.16	7.16
Q8IVP5	FUNDC1	FUN14 doma	11	1	1	1	155	17.2	8.62	0
O75558	STX11	Syntaxin-11 (	4	1	1	1	287	33.2	6.55	2.58
O95050	INMT	Indolethylam	3	1	1	1	263	28.9	5.27	2.52
O95715	CXCL14	C-X-C motif c	6	1	1	1	111	13.1	10.08	2.24
P35568	IRS1	Insulin recep	1	1	1	1	1242	131.5	8.54	1.8
O75689	ADAP1	Arf-GAP with	3	1	1	1	374	43.4	8.97	2.43
P31321	PRKAR1B	cAMP-depen	4	1	1	1	381	43	5.71	2.55
Q8NCH0	CHST14	Carbohydrate	3	1	2	1	376	43	9.48	4.5
O94819	KBTBD11	Kelch repeat	2	1	1	1	623	65.7	6.07	0
P62877	RBX1	E3 ubiquitin-p	7	1	3	1	108	12.3	6.96	7.03
Q9UIL1	SCOC	Short coiled-	16	1	1	1	159	18	8.85	0
O14817	TSPAN4	Tetraspanin-	3	1	1	1	238	26.1	6.47	2.51
Q9Y5Q0	FADS3	Fatty acid de	2	1	1	1	445	51.1	7.77	2.41
Q14186	TFDP1	Transcription	4	1	1	1	410	45	6.05	0
P82921	MRPS21	28S ribosom	9	1	1	1	87	10.7	9.92	2.04

Q5T5Y3	CAMSAP1	Calmodulin-r	1	1	1	1	1602	177.9	6.73	0
Q9P1Z0	ZBTB4	Zinc finger ar	1	1	1	1	1013	105	8.37	2.34
P61599	NAA20	N-alpha-acet	6	1	1	1	178	20.4	5.03	0
Q6PL24	TMED8	Protein TME	2	1	1	1	325	35.7	4.74	2.76
Q96RD7	PANX1	Pannexin-1 (	3	1	1	1	426	48	6.01	0
Q96BW1	UPRT	Uracil phospl	4	1	1	1	309	33.8	6.04	2.22
Q7Z7A1	CNTRL	Centriolin OS	1	2	2	1	2325	268.7	5.55	2.29
Q9H0D2	ZNF541	Zinc finger pı	1	1	2	1	1346	145.5	7.96	3.33
Q15438	CYTH1	Cytohesin-1	3	1	1	1	398	46.4	5.54	0
P17028	ZNF24	Zinc finger pı	3	1	1	1	368	42.1	6.21	2.56
Q8N9N2	ASCC1	Activating sig	2	1	1	1	400	45.5	5.54	2.57
Q9BSU1	PHAF1	Phagosome	2	1	1	1	422	47.5	7.72	1.65
P0C7T5	ATXN1L	Ataxin-1-like	2	1	1	1	689	73.3	6.6	2.18
Q9Y508	RNF114	E3 ubiquitin-|	5	1	1	1	228	25.7	7.25	0
Q8WXW3	PIBF1	Progesterone	2	2	2	1	757	89.8	6.02	2.09
Q96D53	COQ8B	Atypical kina	2	1	1	1	544	60	7.23	1.81
Q9NZK5	ADA2	Adenosine d	2	1	1	1	511	58.9	7.91	2.07
Q9NZL6	RGL1	Ral guanine	1	1	1	1	768	86.6	6.09	2
Q9P0M9	MRPL27	39S ribosom	5	1	1	1	148	16.1	10.42	2.08
O95429	BAG4	BAG family r	2	1	1	1	457	49.6	5.12	1.92
O75298	RTN2	Reticulon-2 (	1	1	1	1	545	59.2	5.31	2.47
Q9H7M9	VSIR	V-type immu	2	1	1	1	311	33.9	6.95	2.12
Q6JBY9	RCSD1	CapZ-interac	2	1	1	1	416	44.5	5.4	2.08
Q6NXT6	TAPT1	Transmembr	2	1	1	1	567	64.2	8.28	3
Q6P1K1	SLC48A1	Heme transp	8	1	1	1	146	16.4	9.64	1.85
Q96LA8	PRMT6	Protein argin	4	1	1	1	375	41.9	5.44	2.03
P42696	RBM34	RNA-binding	2	1	1	1	430	48.5	10.11	0
Q9NVR2	INTS10	Integrator co	1	1	1	1	710	82.2	7.44	0
O94763	URI1	Unconventior	5	2	2	2	535	59.8	5.05	0
Q99590	SCAF11	Protein SCAI	0	1	1	1	1463	164.6	8.41	1.69
Q9NPR9	GPR108	Protein GPR	2	1	1	1	543	60.6	8.69	2.17
O75191	XYLB	Xylulose kina	2	1	1	1	536	58.3	5.86	1.95
Q01650	SLC7A5	Large neutra	1	1	3	1	507	55	7.72	4.32

Q96E29	MTERF3	Transcriptior	4	1	1	1	417	47.9	8.53	0
Q92508	PIEZO1	Piezo-type m	0	1	2	1	2521	286.6	7.47	0
P42694	HELZ	Probable hel	1	1	1	1	1942	218.8	7.42	1.75
Q53FT3	HIKESHI	Protein Hikes	5	1	1	1	197	21.6	5.45	0
Q86UK7	ZNF598	E3 ubiquitin-	1	1	1	1	904	98.6	8.4	0
Q9NQS1	AVEN	Cell death re	3	1	1	1	362	38.5	4.98	0
Q12923	PTPN13	Tyrosine-pro	0	1	1	1	2485	276.7	6.42	0
O15431	SLC31A1	High affinity	6	1	1	1	190	21.1	7.42	2.47
P23378	GLDC	Glycine dehy	1	1	1	1	1020	112.7	7.11	0
Q11206	ST3GAL4	CMP-N-acety	3	1	1	1	333	38	9.41	2.19
Q15560	TCEA2	Transcriptior	3	1	1	1	299	33.6	9.13	1.96
Q14432	PDE3A	cGMP-inhibit	1	1	1	1	1141	124.9	6	2.45
Q04756	HGFAC	Hepatocyte g	2	2	2	2	655	70.6	7.24	1.61
O00213	APBB1	Amyloid beta	3	2	2	1	710	77.2	5.06	2.51
O60315	ZEB2	Zinc finger E	1	1	1	1	1214	136.4	6.32	2.23
Q96HH6	TMEM19	Transmembr	2	1	1	1	336	36.4	8.5	2.7
O75167	PHACTR2	Phosphatase	2	1	1	1	634	69.7	8.16	2.2
O95294	RASAL1	RasGAP-act	2	1	1	1	804	90	6.51	0
Q8WTV0	SCARB1	Scavenger re	1	1	1	1	552	60.8	8.24	2.56
Q8NB25	FAM184A	Protein FAM	1	1	1	1	1140	132.9	5.83	1.98
Q9NYQ6	CELSR1	Cadherin EG	0	1	1	1	3014	329.3	5.92	2.1
Q75T13	PGAP1	GPI inositol-	1	1	1	1	922	105.3	9.01	2.02
Q9BSA9	TMEM175	Endosomal/ly	2	1	1	1	504	55.6	7.66	2.49
Q8TEA7	TBCK	TBC domain-	2	1	1	1	893	100.6	6.58	2.13
Q9BVT8	TMUB1	Transmembr	4	1	1	1	246	26.2	5.72	1.84
O00308	WWP2	NEDD4-like	2	1	1	1	870	98.9	7.12	0
Q8N5J2	MINDY1	Ubiquitin car	1	1	1	1	469	51.7	4.86	1.9
Q6NZ67	MZT2B	Mitotic-spind	4	1	1	1	158	16.2	10.15	2.5
Q9NX07	TRNAU1AP	tRNA seleno	2	1	1	1	287	32.5	4.74	2.44
Q96JQ2	CLMN	Calmin OS=I	1	1	1	1	1002	111.6	4.94	1.99
Q8IXM3	MRPL41	39S ribosom	8	1	1	1	137	15.4	9.57	2.17
O75970	MPDZ	Multiple PDZ	0	1	1	1	2070	221.5	5.06	1.83
Q9UBZ9	REV1	DNA repair p	1	1	2	1	1251	138.2	8.51	5.02

Q9NXN4	GDAP2	Ganglioside-	2	1	1	1	497	56.2	5.74	2.57
Q32P41	TRMT5	tRNA (guanii	2	1	1	1	509	58.2	8.62	0
Q96SQ9	CYP2S1	Cytochrome	2	1	1	1	504	55.8	8.62	0
Q6AZY7	SCARA3	Scavenger re	2	1	1	1	606	65.1	6.54	0
Q9Y5J9	TIMM8B	Mitochondria	8	1	1	1	83	9.3	5.12	2.38
Q15003	NCAPH	Condensin c	1	1	1	1	741	82.5	5.06	0
O43914	TYROBP	TYRO protei	7	1	1	1	113	12.2	8.31	1.81
Q7RTU4	BHLHA9	Class A basi	3	1	1	1	235	24.1	11.21	2.29
Q9Y6R0	NUMBL	Numb-like pr	1	1	1	1	609	64.9	8.85	0
P40306	PSMB10	Proteasome	3	1	2	1	273	28.9	7.81	0
Q8TF68	ZNF384	Zinc finger pi	2	1	1	1	577	63.2	8.95	0
O95361	TRIM16	Tripartite mo	2	1	1	1	564	63.9	5.49	0
Q96K49	TMEM87B	Transmembr	2	2	2	2	555	63.5	7.44	3.99
Q7L7V1	DHX32	Putative pre-	1	1	1	1	743	84.4	4.97	2.11
O94916	NFAT5	Nuclear facto	1	1	1	1	1531	165.7	5.24	0
Q6AWC2	WWC2	Protein WW(	1	1	1	1	1192	133.8	5.53	2.01
Q16637	SMN2	Survival mot	3	1	1	1	294	31.8	6.55	2.1
Q9NXD2	MTMR10	Myotubularin	3	1	1	1	777	88.2	8.53	0
Q8IZV5	RDH10	Retinol dehy	11	1	1	1	341	38.1	7.4	0
A6NDU8	RIMOC1	RAB7A-intera	2	1	1	1	294	33.6	5.26	1.92
Q9C037	TRIM4	E3 ubiquitin-j	3	1	1	1	500	57.4	8.1	0
Q96MX6	DNAAF10	Dynein axon	2	1	1	1	357	39.7	8.09	2.63
Q3L8U1	CHD9	Chromodoma	0	1	1	1	2897	325.8	7.01	2.29
Q9H8M7	MINDY3	Ubiquitin car	3	1	1	1	445	49.7	4.77	0
Q86TH1	ADAMTSL2	ADAMTS-like	1	1	1	1	951	104.6	6.42	0
Q9NPD3	EXOSC4	Exosome coi	3	1	2	1	245	26.4	6.52	4.29
Q6ZMJ2	SCARA5	Scavenger re	1	1	1	1	495	54	7.09	0
Q12982	BNIP2	BCL2/adeno	2	1	1	1	314	36	4.81	2.71
Q68CQ4	UTP25	U3 small nuc	1	1	1	1	756	87	5.88	2.21
Q9NRX5	SERINC1	Serine incorp	2	1	1	1	453	50.5	5.85	2.2
Q6NZY7	CDC42EP5	Cdc42 effect	5	1	1	1	148	15.2	7.52	1.83
P10646	TFPI	Tissue factor	3	1	1	1	304	35	8.25	0
Q96NC0	ZMAT2	Zinc finger m	4	1	1	1	199	23.6	9.01	0

Q2VPB7	AP5B1	AP-5 comple	2	1	1	1	878	93.9	5.92	2.15
Q8TF44	C2CD4C	C2 calcium-c	3	1	1	1	421	44.5	9.73	2.23
P50747	HLCS	Biotin--protei	1	1	1	1	726	80.7	5.62	0
Q9H257	CARD9	Caspase rec	1	1	1	1	536	62.2	6.07	2.46
O43829	ZBTB14	Zinc finger ar	4	1	1	1	449	50.9	6.04	0
Q969E8	TSR2	Pre-rRNA-pr	5	1	1	1	191	20.9	4.39	0
Q3SXM5	HSDL1	Inactive hydr	2	1	2	1	330	37	8.72	4.37
O00462	MANBA	Beta-mannos	1	1	1	1	879	100.8	5.52	2.01
Q9NRE2	TSHZ2	Teashirt hom	1	1	1	1	1034	114.9	7.83	0
Q8N4P2	TTC30B	Tetratricopep	2	1	1	1	665	76.1	5.2	0
Q6V1X1	DPP8	Dipeptidyl pe	2	1	1	1	898	103.3	5.8	0
Q99608	NDN	Necdin OS=H	3	1	1	1	321	36.1	8.78	1.69
Q5VYS8	TUT7	Terminal uric	1	1	1	1	1495	171.1	6.83	2.17
Q5VTQ0	TTC39B	Tetratricopep	2	1	1	1	682	76.9	7.39	0
Q9UKA4	AKAP11	A-kinase anc	0	1	1	1	1901	210.4	5.39	0
Q8TCT8	SPPL2A	Signal peptic	1	1	1	1	520	58.1	8.32	2.7
Q8TAD8	SNIP1	Smad nuclea	4	1	1	1	396	45.8	9.99	0
Q96I36	COX14	Cytochrome	12	1	1	1	57	6.6	9.55	2.78
Q99871	HAUS7	HAUS augmi	4	1	1	1	358	39.8	4.75	0
Q9BZ72	PITPNM2	Membrane-a	1	1	1	1	1349	148.8	7.17	2.09
Q9BW71	HIRIP3	HIRA-interac	1	1	1	1	556	61.9	8.54	2.21
Q6ZVS7	FAM183BP	Protein FAM	4	1	1	1	135	16.2	8.43	2.19
Q9ULV3	CIZ1	Cip1-interact	1	1	1	1	898	100	6.11	1.61
A0A0A0MS1	IGHV1-45	Immunoglobu	5	1	1	1	117	13.5	9.1	2.17
Q9UMY1	NOL7	Nucleolar pro	3	1	1	1	257	29.4	9.67	1.96
Q9BY12	SCAPER	S phase cycl	1	1	1	1	1400	158.2	7.44	0
Q96JG8	MAGED4B	Melanoma-as	1	1	1	1	741	81.3	6.79	2.25
Q9NTN3	SLC35D1	UDP-glucuro	2	1	1	1	355	39.2	8.97	2.18
Q6P4A7	SFXN4	Sideroflexin-4	4	1	1	1	337	38	9.19	2.19
Q9NVU0	POLR3E	DNA-directed	1	1	1	1	708	79.8	6.47	2.33
Q9Y2E4	DIP2C	Disco-interac	1	1	1	1	1556	170.7	7.39	2.32
O60861	GAS7	Growth arres	2	1	1	1	476	54.3	7.34	2.15
Q86XL3	ANKLE2	Ankyrin repe	1	1	1	1	938	104.1	7.09	1.77

Q6NW34	NEPRO	Nucleolus an	2	1	1	1	567	64.5	9.74	0
Q9H4M3	FBXO44	F-box only pı	3	1	1	1	255	29.7	5.82	1.9
O43150	ASAP2	Arf-GAP with	1	1	1	1	1006	111.6	6.68	2.38
O43805	SSNA1	Microtubule ı	6	1	1	1	119	13.6	5.38	1.77
Q96NT0	CCDC115	Coiled-coil d	6	1	1	1	180	19.7	6.95	0
Q96HI0	SENP5	Sentrin-spec	1	1	2	1	755	86.6	9.17	1.96
Q6P1Q0	LETMD1	LETM1 doma	2	1	1	1	360	41.8	10.32	2.81
O00635	TRIM38	E3 ubiquitin-	3	1	1	1	465	53.4	7.02	2.69
Q96RL1	UIMC1	BRCA1-A co	2	1	1	1	719	79.7	5.45	1.75
O75081	CBFA2T3	Protein CBF/	1	1	1	1	653	71.1	8.18	0
P20061	TCN1	Transcobala	2	1	1	1	433	48.2	5.03	0
Q96PZ2	FAM111A	Serine prote	1	1	1	1	611	70.2	8.53	1.77
Q8J025	APCDD1	Protein APCI	2	1	1	1	514	58.8	8.15	0
P51798	CLCN7	H(+)/Cl(-) ex	1	1	1	1	805	88.6	8.43	2.22
Q9Y4G8	RAPGEF2	Rap guanine	1	1	1	1	1499	167.3	6.67	2.17
Q9H0R8	GABARAPL1	Gamma-ami	7	1	1	1	117	14	8.73	2.27
Q9NRZ5	AGPAT4	1-acyl-sn-gly	2	1	1	1	378	44	8.72	0
Q9Y496	KIF3A	Kinesin-like	1	1	1	1	699	80	6.54	0
P24386	CHM	Rab proteins	1	1	1	1	653	73.4	4.75	2.02
Q5BKT4	ALG10	Dol-P-Glc:Gl	1	1	1	1	473	55.6	9.29	1.85
Q96QG7	MTMR9	Myotubularin	1	1	1	1	549	63.4	6.39	2.07
Q9H6L2	TMEM231	Transmembr	2	1	1	1	316	36	7.94	2.11
P30039	PBLD	Phenazine bi	4	1	1	1	288	31.8	6.52	0
Q9UID6	ZNF639	Zinc finger pı	2	1	1	1	485	56	5.57	0
Q9P0U4	CXXC1	CXXC-type z	1	1	1	1	656	75.7	8.24	2.5
Q7LC44	ARC	Activity-regul	2	1	2	1	396	45.3	5.63	3.88
Q9NRE1	MMP26	Matrix metall	4	1	1	1	261	29.7	6.47	2.09
P58012	FOXL2	Forkhead bo	2	1	1	1	376	38.7	9.16	0
Q12968	NFATC3	Nuclear facto	2	1	1	1	1075	115.5	6.37	0
Q14667	BLTP2	Bridge-like lip	0	1	1	1	2235	253.5	7.14	2.43
Q86Z02	HIPK1	Homeodoma	1	1	2	1	1210	130.8	8.22	3.58
Q99743	NPAS2	Neuronal PA	1	1	1	1	824	91.7	6.81	2.41
O14523	C2CD2L	Phospholipid	1	1	1	1	706	76.1	7.69	0

Q8N3F8	MICALL1	MICAL-like p	1	1	1	1	863	93.4	7.25	0
A4D1E9	GTPBP10	GTP-binding	2	1	1	1	387	42.9	9.03	0
Q9P2R6	RERE	Arginine-glut	0	1	1	1	1566	172.3	7.69	2.16
Q9UET6	FTSJ1	Putative tRN	4	1	1	1	329	36.1	5.69	0
Q9BXT8	RNF17	RING finger	0	1	1	1	1623	184.5	5.4	1.91
O60637	TSPAN3	Tetraspanin-	3	1	2	1	253	28	5.81	4.47
P16591	FER	Tyrosine-pro	1	1	1	1	822	94.6	7.14	2.16
Q8NHY3	GAS2L2	GAS2-like pr	2	1	1	1	880	96.5	9.13	0
P51790	CLCN3	H(+)/Cl(-) ex	2	1	1	1	818	90.9	6.28	2.08
Q9NRN7	AASDHPPT	L-aminoadip	2	1	2	1	309	35.8	6.8	3.89
Q9H6B4	CLMP	CXADR-like	2	1	1	1	373	41.3	7.99	2.87
Q8WUK0	PTPMT1	Phosphatidyl	6	1	1	1	201	22.8	9.77	1.74
Q5VUA4	ZNF318	Zinc finger p	1	1	1	1	2279	251	7.2	2.13
P56277	CMC4	Cx9C motif-c	10	1	1	1	68	7.7	8.18	2.2
Q86SQ0	PHLDB2	Pleckstrin ho	1	1	1	1	1253	142.1	7.43	0
Q92994	BRF1	Transcription	1	1	1	1	677	73.8	5.43	0
Q9Y6A1	POMT1	Protein O-ma	1	1	1	1	747	84.8	8.4	0
Q9BW62	KATNAL1	Katanin p60	2	1	1	1	490	55.4	6.74	0
Q9UBN4	TRPC4	Short transie	1	1	1	1	977	112	7.75	2.23
O94762	RECQL5	ATP-depend	1	1	1	1	991	108.8	8.56	0
P60602	ROMO1	Reactive oxy	10	1	1	1	79	8.2	9.33	1.88
Q9HAK2	EBF2	Transcription	1	1	1	1	575	62.6	9.06	0
Q9BY08	EBPL	Emopamil-bi	3	1	1	1	206	23.2	6.29	2.22
Q8NFZ5	TNIP2	TNFAIP3-int	3	1	1	1	429	48.7	6.44	2.33
Q9NW81	DMAC2	Distal memb	3	1	2	1	257	29.2	6.43	1.76
Q16873	LTC4S	Leukotriene	9	1	1	1	150	16.6	10.18	0
Q86WW8	COA5	Cytochrome	9	1	1	1	74	8.4	8.68	1.74
Q14331	FRG1	Protein FRG	3	1	1	1	258	29.2	9.01	2.25
P63302	SELENOW	Selenoprotei	9	1	1	1	87	9.4	9.23	2.16
Q9NWZ5	UCKL1	Uridine-cytidi	2	1	1	1	548	61.1	7.36	0
Q6P158	DHX57	Putative ATF	1	1	1	1	1386	155.5	7.71	2.12
Q5MNZ9	WIPI1	WD repeat d	2	1	1	1	446	48.6	6.6	2.37
Q76N32	CEP68	Centrosomal	1	1	2	1	757	81.1	5.16	0

Q92539	LPIN2	Phosphatida	1	1	1	1	896	99.3	5.33	2.43
Q96CN9	GCC1	GRIP and co	1	1	1	1	775	87.8	5.45	1.99
Q15906	VPS72	Vacuolar pro	4	1	1	1	364	40.6	6.48	0
Q969S2	NEIL2	Endonucleas	3	1	1	1	332	36.8	6.79	1.89
P04198	MYCN	N-myc proto-	2	1	1	1	464	49.5	5.66	0
Q06187	BTK	Tyrosine-pro	2	1	1	1	659	76.2	7.77	0
P32418	SLC8A1	Sodium/calci	1	1	1	1	973	108.5	5	2.42
Q9NPJ6	MED4	Mediator of F	4	1	1	1	270	29.7	5.1	2.44
Q99571	P2RX4	P2X purinoce	2	1	1	1	388	43.3	7.99	1.8
P40967	PMEL	Melanocyte p	1	1	2	1	661	70.2	5.58	4.72
Q6VN20	RANBP10	Ran-binding	2	1	1	1	620	67.2	6.77	2.06
O95164	UBL3	Ubiquitin-like	6	1	1	1	117	13.1	6.77	2.18
A0PJZ3	GXYLT2	Glucoside xy	2	1	1	1	443	51	9.77	0
Q96MF7	NSMCE2	E3 SUMO-pr	3	1	1	1	247	27.9	7.74	2
Q8WVF1	OSCP1	Protein OSC	3	1	1	1	389	44.6	5.83	1.88
Q15021	NCAPD2	Condensin c	1	1	1	1	1401	157.1	6.61	2.31
P78563	ADARB1	Double-stran	1	1	1	1	741	80.7	9.01	0
Q8NG27	PJA1	E3 ubiquitin-p	1	1	1	1	643	71	5.17	1.68
O15116	LSM1	U6 snRNA-a	5	1	1	1	133	15.2	5.22	2.08
P48788	TNNI2	Troponin I, fa	4	1	1	1	182	21.3	8.73	0
Q9C0C6	CIPC	CLOCK-inter	2	1	1	1	399	42.7	8.95	0
Q8WVB3	HEXD	Hexosaminid	1	1	1	1	486	53.8	5.97	2.62
Q96AA3	RFT1	Protein RFT1	2	1	1	1	541	60.3	8.85	2.02
Q9BUZ4	TRAF4	TNF recepto	2	1	1	1	470	53.5	8.15	0
Q8WUX9	CHMP7	Charged mul	2	1	1	1	453	50.9	5.35	2.31
O94856	NFASC	Neurofascin	1	1	1	1	1347	149.9	6.65	0
Q0VGL1	LAMTOR4	Ragulator co	7	1	1	1	99	10.7	6.54	2.18
Q4LDE5	SVEP1	Sushi, von W	0	1	1	1	3571	389.9	5.5	2.29
O76054	SEC14L2	SEC14-like p	2	1	1	1	403	46.1	7.84	1.93
Q9UGH3	SLC23A2	Solute carrie	2	1	1	1	650	70.3	7.68	1.93
Q16651	PRSS8	Prostasin OS	2	1	1	1	343	36.4	5.85	1.85
Q6MZQ0	PRR5L	Proline-rich p	3	1	1	1	368	40.8	6.73	0
Q9BYG4	PARD6G	Partitioning c	2	1	1	1	376	40.9	8.22	2.51

Q86UP0	CDH24	Cadherin-24	1	1	1	1	819	87.7	4.79	2.48
Q8N5G2	MACO1	Macoilin OS=	1	1	1	1	664	76.1	9.07	1.93
Q96CB8	INTS12	Integrator co	2	1	1	1	462	48.8	9.69	2.21
Q5TC82	RC3H1	Roquin-1 OS	1	1	1	1	1133	125.7	7.27	1.78
Q9NRX6	TMEM167B	Protein kish-	9	1	1	1	74	8.3	9.61	0
Q9Y530	OARD1	ADP-ribose g	5	1	1	1	152	17	8.31	2.12
Q8IWC1	MAP7D3	MAP7 domai	1	1	2	1	876	98.4	9.32	3.74
Q96SB4	SRPK1	SRSF protei	1	1	1	1	655	74.3	6.16	1.78
Q53F19	NCBP3	Nuclear cap-	1	1	1	1	620	70.5	5.73	2.43
Q8IVU3	HERC6	Probable E3	1	1	1	1	1022	115.1	7.96	2.9
P11597	CETP	Cholesteryl e	2	1	1	1	493	54.7	6.09	1.69
Q69YL0	NCBP2AS2	Protein NCB	9	1	1	1	99	10.9	12	1.85
Q92968	PEX13	Peroxisomal	5	1	1	1	403	44.1	8.05	0
Q8NFH8	REPS2	RalBP1-asso	1	1	3	1	660	71.5	7.65	2.05
Q8N5A5	ZGPAT	Zinc finger C	2	1	1	1	531	57.3	5.43	2.04
Q5JS37	NHLRC3	NHL repeat-c	2	1	1	1	347	38.3	6.43	2.28
Q6EEV4	POLR2M	DNA-directec	9	1	1	1	148	15.1	8.37	0
P25098	GRK2	Beta-adrene	1	1	1	1	689	79.5	7.28	2.14
Q4ADV7	RIC1	Guanine nuc	1	1	1	1	1423	159.2	6.38	2.44
Q6ZU65	UBN2	Ubinuclein-2	1	1	1	1	1347	146	9.19	0
Q8N139	ABCA6	ATP-binding	0	1	1	1	1617	184.2	7.36	1.87
Q96KB5	PBK	Lymphokine-	2	1	1	1	322	36.1	5.12	2.66
Q13496	MTM1	Myotubularin	1	1	1	1	603	69.9	8.18	2.03
P35240	NF2	Merlin OS=H	2	1	1	1	595	69.6	6.47	0
Q96KX1	C4orf36	Uncharacteri	6	1	1	1	117	13.3	9.47	2.22
Q96FZ2	HMCES	Abasic site p	2	1	1	1	354	40.5	8.15	2.01
P07477	PRSS1	Serine protea	8	1	1	1	247	26.5	6.51	0
Q15904	ATP6AP1	V-type protor	1	1	1	1	470	52	6.14	0
Q8N6Y2	LRRC17	Leucine-rich	2	1	1	1	441	51.8	8.24	0
Q5VWN6	TASOR2	Protein TASC	0	1	1	1	2430	268.7	5.9	0
Q5VWQ0	RSBN1	Lysine-speci	1	1	1	1	802	90	8.6	1.9
O75530	EED	Polycomb pr	3	1	1	1	441	50.2	7.03	0
Q9P2H3	IFT80	Intraflagellar	2	1	1	1	777	88	7.69	0

Q53GL7	PARP10	Protein mono	1	1	1	1	1025	109.9	4.97	2.6
Q8N819	PPM1N	Probable pro	2	1	1	1	430	46.1	6.62	0
Q7Z404	TMC4	Transmembr	1	1	1	1	712	79.2	8.94	1.84
O95497	VNN1	Pantetheinas	4	1	1	1	513	57	5.55	0
Q6DKK2	TTC19	Tetratricoper	2	1	1	1	380	42.4	5.77	0
Q9BRR9	ARHGAP9	Rho GTPase	2	1	1	1	750	83.2	8.34	0
Q9NV66	TYW1	S-adenosyl-L	1	1	1	1	732	83.6	6.87	0
O75818	RPP40	Ribonucleas	2	1	1	1	363	41.8	6.67	2.27
Q13296	SCGB2A2	Mammaglobi	9	1	1	1	93	10.5	4.3	0
Q8NA72	POC5	Centrosomal	1	1	1	1	575	63.3	7.42	0
Q9UBC9	SPRR3	Small proline	24	1	1	1	169	18.1	8.57	1.98
Q8N130	SLC34A3	Sodium-depe	1	1	1	1	599	63.5	8.27	2.52
Q9HCG7	GBA2	Non-lysosom	1	1	1	1	927	104.6	5.92	1.92
O95170	FBXW10B	F-box and W	1	1	1	1	752	85.3	9.04	2.27
Q5T7V8	GORAB	RAB6-intera	2	1	1	1	369	42.2	6.48	0
Q86YB7	ECHDC2	Enoyl-CoA h	2	1	1	1	292	31.1	8.72	1.8
Q9NQ79	CRTAC1	Cartilage aci	1	1	1	1	661	71.4	5.12	1.86
Q96JD6	AKR1E2	1,5-anhydro-	3	1	1	1	320	36.6	7.49	2.06
O95183	VAMP5	Vesicle-asso	7	1	1	1	116	12.8	7.85	1.96
Q96BR5	COA7	Cytochrome	3	1	1	1	231	25.7	6.02	2.59
Q86WU2	LDHD	Probable D-l	1	1	1	1	507	54.8	6.65	2.07
P56962	STX17	Syntaxin-17	3	1	1	1	302	33.4	6.57	3.25
Q07654	TFF3	Trefoil factor	9	1	1	1	80	8.6	5.92	1.82
P10916	MYL2	Myosin regul	5	1	1	1	166	18.8	5	1.63
Q8IY18	SMC5	Structural ma	1	1	1	1	1101	128.7	8.38	0
Q9ULT0	TTC7A	Tetratricoper	2	1	1	1	858	96.1	6.46	0
O95147	DUSP14	Dual specific	4	1	1	1	198	22.2	9.57	0
P51530	DNA2	DNA replicat	1	1	1	1	1060	120.3	7.74	2.07
Q6NXR0	IRGC	Interferon-in	3	1	1	1	463	50.3	5.35	0
O00258	GET1	Guided entry	6	1	1	1	174	19.8	9.67	0
Q9H9S5	FKRP	Ribitol 5-pho	5	1	1	1	495	54.5	7.4	0
Q96RS0	TGS1	Trimethylgua	1	1	1	1	853	96.6	4.94	2.18
O60516	EIF4EBP3	Eukaryotic tr	6	1	1	1	100	10.9	4.48	2.52

Q8TBF2	PRXL2B	Prostamide/p	4	1	1	1	198	21.2	6.67	2.06
Q15391	P2RY14	P2Y purinoce	2	1	1	1	338	38.9	9.47	2.08
Q8N7R7	CCNYL1	Cyclin-Y-like	2	1	1	1	359	40.7	6.07	0
A3KN83	SBNO1	Protein straw	1	1	1	1	1393	154.2	7.88	0
A0A0B4J1U	IGHV6-1	Immunoglobu	6	1	2	1	121	13.5	9.2	4.24
Q96KS0	EGLN2	Prolyl hydrox	2	1	1	1	407	43.6	7.91	2.55
Q9H6W3	RIOX1	Ribosomal o	1	1	1	1	641	71	6.46	0
P35610	SOAT1	Sterol O-acyl	1	1	1	1	550	64.7	8.94	1.96
Q9BXG8	SPZ1	Spermatoger	1	1	7	1	430	49.4	7.59	14.98
Q9HAZ2	PRDM16	Histone-lysin	1	1	1	1	1276	140.2	6.21	0
Q8N2H3	PYROXD2	Pyridine nucl	2	1	1	1	581	63	6.95	0
Q86W56	PARG	Poly(ADP-rib	1	1	1	1	976	111	6.43	0
Q8WXE1	ATRIP	ATR-interact	1	1	1	1	791	85.8	6.32	2.26
P62273	RPS29	40S ribosom	13	1	1	1	56	6.7	10.13	0
P17405	SMPD1	Sphingomyel	1	1	1	1	631	69.9	7.28	1.8
Q9NVF7	FBXO28	F-box only pr	2	1	1	1	368	41.1	9.55	1.95
Q96PX6	CCDC85A	Coiled-coil de	1	1	1	1	553	59.9	8.79	1.91
A6NI61	MYMK	Protein myor	3	1	1	1	221	24.7	8.72	1.84
Q9Y548	YIPF1	Protein YIPF	3	1	1	1	306	34.3	5.31	2.18
Q99549	MPHOSPH8	M-phase pho	1	1	1	1	860	97.1	6.06	2.02
Q9BRR8	GPATCH1	G patch dom	1	1	1	1	931	103.3	7.05	0
P32321	DCTD	Deoxycytidyl	4	1	1	1	178	20	7.56	0
O60293	ZFC3H1	Zinc finger C	0	1	1	1	1989	226.2	8.13	0
Q9Y6H3	ATP23	Mitochondria	2	1	1	1	246	28.1	8.03	2.4
A7E2V4	ZSWIM8	Zinc finger S	0	1	1	1	1837	197.2	6.8	1.72
Q9H3U5	MFSD1	Major facilita	1	1	1	1	465	51.2	6.74	2.03
O43189	PHF1	PHD finger p	1	1	1	1	567	62.1	9.01	1.83
Q86VP1	TAX1BP1	Tax1-binding	1	1	1	1	789	90.8	5.43	2.21
Q9GZU2	PEG3	Paternally-ex	1	1	1	1	1588	180.7	5.48	1.99
P23409	MYF6	Myogenic fac	3	1	1	1	242	26.9	5.96	2.58
Q9BPZ3	PAIP2	Polyadenylat	6	1	1	1	127	15	4.12	2.15
Q14517	FAT1	Protocadheri	0	1	1	1	4588	506	5	1.7
Q9Y572	RIPK3	Receptor-inte	2	1	1	1	518	56.9	6.47	0

O43819	SCO2	Protein SCO	6	1	1	1	266	29.8	8.85	0
Q9BXI9	C1QTNF6	Complement	3	1	1	1	278	30.8	6.34	0
P61218	POLR2F	DNA-directed	6	1	1	1	127	14.5	4.22	0
O95858	TSPAN15	Tetraspanin-	2	1	1	1	294	33.1	5.48	1.77
P21730	C5AR1	C5a anaphyl	3	1	1	1	350	39.3	9.01	0
Q9HB21	PLEKHA1	Pleckstrin ho	1	1	1	1	404	45.5	8.56	1.84
Q75N90	FBN3	Fibrillin-3 OS	0	1	1	1	2809	300.1	5.07	1.89
Q96KN7	RPGRIP1	X-linked retir	1	1	1	1	1286	146.6	5.68	1.81
Q96RY7	IFT140	Intraflagellar	1	1	1	1	1462	165.1	6.04	0
Q8NCF5	NFATC2IP	NFATC2-inte	2	1	1	1	419	45.8	6.6	0
Q9H6F5	CCDC86	Coiled-coil do	2	1	1	1	360	40.2	10.33	0
Q8NBF6	AVL9	Late secretor	1	1	1	1	648	71.9	6.21	0
Q6IN84	MRM1	rRNA methyl	7	1	1	1	353	38.6	7.94	0
Q5VW38	GPR107	Protein GPR	1	1	1	1	600	66.9	7.24	1.72
Q9H2V7	SPNS1	Protein spins	3	1	1	1	528	56.6	6.64	0
Q9BXJ4	C1QTNF3	Complement	3	1	1	1	246	27	6.52	2.22
Q13627	DYRK1A	Dual specific	1	1	1	1	763	85.5	8.75	1.63
O94887	FARP2	FERM, ARH(	1	1	1	1	1054	119.8	8.79	2.47
Q96GC9	VMP1	Vacuole mer	1	1	1	1	406	46.2	6.95	2.27
Q96CN4	EVI5L	EVI5-like pro	1	1	1	1	794	91.3	5.34	1.69
Q53EU6	GPAT3	Glycerol-3-pl	2	1	1	1	434	48.7	8.87	2.45
Q9C0B7	TANGO6	Transport an	1	1	1	1	1094	120.7	6.11	0
Q01955	COL4A3	Collagen alpl	1	1	1	1	1670	161.7	9.16	0
Q99942	RNF5	E3 ubiquitin-	4	1	1	1	180	19.9	6.65	2.07
Q15517	CDSN	Corneodesm	2	1	1	1	529	51.5	8.35	1.88
Q9UKT9	IKZF3	Zinc finger pr	2	1	1	1	509	58	6.55	0
Q9UHJ3	SFMBT1	Scm-like with	2	1	1	1	866	98.1	6.21	0
P60022	DEFB1	Beta-defensi	10	1	1	1	68	7.4	8.6	1.67
Q92954	PRG4	Proteoglycar	1	1	1	1	1404	151	9.51	0
Q9H000	MKRN2	E3 ubiquitin-	2	1	1	1	416	46.9	7.61	1.97
Q8TF74	WIPF2	WAS/WASL-	3	1	1	1	440	46.3	10.93	0
P52747	ZNF143	Zinc finger pr	1	1	1	1	638	68.9	6.05	1.81
Q969H0	FBXW7	F-box/WD re	2	1	1	1	707	79.6	5.8	0

O75319	DUSP11	RNA/RNP cc	2	1	1	1	377	43.7	9.29	2.08
P82251	SLC7A9	b(0,+)-type a	1	1	1	1	487	53.4	8.12	2.21
Q5VST6	ABHD17B	Alpha/beta h	3	1	1	1	288	32.2	6.29	1.82
Q9UKJ3	GPATCH8	G patch dom	0	1	1	1	1502	164.1	8.66	0
Q8TAV4	STOML3	Stomatin-like	2	1	1	1	291	32.1	8.63	1.87
Q9Y3D2	MSRB2	Methionine-F	6	1	1	1	182	19.5	8.63	0
Q9NV88	INTS9	Integrator co	1	1	1	1	658	73.8	6.33	0
Q9BV68	RNF126	E3 ubiquitin-		3	1	1	1	311	33.8	5.47	0
P58499	FAM3B	Protein FAM	3	1	1	1	235	26	8.75	0
O60706	ABCC9	ATP-binding	0	1	1	1	1549	174.1	7.37	0
Q5S007	LRRK2	Leucine-rich	0	1	1	1	2527	285.9	6.8	0
Q5VZE5	NAA35	N-alpha-acet	1	1	1	1	725	83.6	7.05	1.74
Q9Y3M8	STARD13	StAR-related	1	1	1	1	1113	124.9	7.02	0
Q9UER7	DAXX	Death domai	1	1	1	1	740	81.3	4.87	2.2
Q9HBU6	ETNK1	Ethanolamin	2	1	1	1	452	50.9	6.54	1.7
Q96BY2	MOAP1	Modulator of	3	1	4	1	351	39.5	5.27	0
Q9Y5F7	PCDHGC4	Protocadheri	1	1	1	1	938	101.2	5.35	2.27
Q8NCC5	SLC37A3	Sugar phosp	2	1	1	1	494	54.5	5.66	1.84
Q96I23	PYURF	Protein preY	7	1	1	1	114	12.6	9.33	2.05
P13612	ITGA4	Integrin alph	1	1	1	1	1032	114.8	6.48	0
Q9NVV4	MTPAP	Poly(A) RNA	1	1	1	1	582	66.1	9.04	0
Q86YR7	MCF2L2	Probable gua	1	1	1	1	1114	126.9	6.42	0
O75508	CLDN11	Claudin-11 C	4	1	1	1	207	22	7.9	2.16
P16109	SELP	P-selectin OS	1	1	1	1	830	90.8	6.6	0
Q93033	CD101	Immunoglobu	1	1	1	1	1021	115	6.96	0
Q9Y6G3	MRPL42	39S ribosom	4	1	2	1	142	16.7	8.35	4.79
P0C671	BNIP5	Protein BNIF	2	1	2	1	652	71.9	8.47	1.63
Q6ZN66	GBP6	Guanylate-bi	1	1	1	1	633	72.4	6.37	1.94
Q8TBY8	PMFBP1	Polyamine-m	1	1	1	1	1007	117.4	6.29	0
O00255	MEN1	Menin OS=H	2	1	1	1	615	68	6.55	0
Q685J3	MUC17	Mucin-17 OS	0	1	1	1	4493	451.5	4.11	2.12
Q9UNH5	CDC14A	Dual specific	1	1	1	1	594	66.5	9.1	1.89
Q9BSV6	TSEN34	tRNA-splicin	6	1	1	1	310	33.6	8.43	0

Q13822	ENPP2	Ectonucleoti	3	1	1	1	863	98.9	7.37	0
Q9H4A5	GOLPH3L	Golgi phosph	4	1	1	1	285	32.7	5.83	0
Q16534	HLF	Hepatic leuk	5	1	1	1	295	33.2	7.97	2.11
Q9ULG1	INO80	Chromatin-re	0	1	1	1	1556	176.6	9.5	0
Q96CN5	LRRC45	Leucine-rich	2	1	1	1	670	75.9	6.23	0
Q8NCN4	RNF169	E3 ubiquitin-	1	1	2	1	708	77.1	9.1	2.12
Q9BX74	TM2D1	TM2 domain-	3	1	1	1	207	22.3	6.51	2.32
Q08174	PCDH1	Protocadheri	1	1	1	1	1060	114.7	5.03	0
Q8N8A6	DDX51	ATP-depend	1	1	1	1	666	72.4	8.16	2.25
Q9BT17	MTG1	Mitochondria	3	1	1	1	334	37.2	9.47	0
Q99808	SLC29A1	Equilibrative	1	1	1	1	456	50.2	8.29	0
Q5THJ4	VPS13D	Intermembra	0	1	1	1	4388	491.6	6.58	0
Q9Y291	MRPS33	28S ribosom	8	1	1	1	106	12.6	10.11	0
Q6P1R3	MSANTD2	Myb/SANT-li	2	1	1	1	559	61.3	6.19	0
Q86Y22	COL23A1	Collagen alp	2	1	1	1	540	51.9	7.25	0
Q86WI3	NLRC5	Protein NLR	1	1	1	1	1866	204.5	6.44	0
Q9BTE7	DCUN1D5	DCN1-like pr	3	1	2	1	237	27.5	5.58	5.55
Q9NX76	CMTM6	CKLF-like M	4	1	1	1	183	20.4	5.29	3.02
Q9NYB5	SLCO1C1	Solute carrie	1	1	1	1	712	78.6	8.5	2.08
Q15678	PTPN14	Tyrosine-pro	1	1	1	1	1187	135.2	8.31	2.19
O75094	SLIT3	Slit homolog	0	1	1	1	1523	167.6	7.65	2.02
Q3SXM0	DCAF4L1	DDB1- and C	2	1	1	1	396	44.2	8.29	2.36
Q9NWB7	IFT57	Intraflagellar	3	1	1	1	429	49.1	4.98	0
Q8N3J3	HROB	Homologous	1	1	2	1	647	69.7	6.23	5.35
Q9BXT6	MOV10L1	RNA helicas	1	1	1	1	1211	135.2	6.46	0
Q8WYP3	RIN2	Ras and Rab	2	1	2	1	895	100.1	6.58	0
Q92982	NINJ1	Ninjurin-1 OS	13	1	1	1	152	16.3	6.28	0
Q9Y4Y9	LSM5	U6 snRNA-a	7	1	1	1	91	9.9	4.54	2.27
Q9NWR8	MCUB	Calcium unip	2	1	1	1	336	39.1	9.16	0
O43808	SLC25A17	Peroxisomal	2	1	2	1	307	34.5	10.08	4.52
Q8NEM7	SUPT20H	Transcription	1	1	1	1	779	85.7	8.48	0
P09544	WNT2	Protein Wnt-	2	1	1	1	360	40.4	8.7	2.01
Q8NHG8	ZNRF2	E3 ubiquitin-	5	1	1	1	242	24.1	7.09	0

Q9Y6Q9	NCOA3	Nuclear rece	1	1	1	1	1424	155.2	7.47	0
Q0VDI3	TMEM267	Transmembr	3	1	1	1	215	24.2	8.59	2.35
Q96R84	OR1F2P	Putative olfa	2	1	1	1	312	34.9	8.6	1.86
Q6UWU2	GLB1L	Beta-galacto	2	1	1	1	654	74.1	8.92	0
Q8TCG1	CIP2A	Protein CIP2	1	1	1	1	905	102.1	6.23	0
Q9HD40	SEPSECS	O-phosphos	1	1	1	1	501	55.7	8.05	1.65
Q02108	GUCY1A1	Guanylate cy	1	1	1	1	690	77.4	7.11	0
Q9Y6R9	CCDC61	Centrosomal	3	1	1	1	512	57.3	10.27	0
Q86US8	SMG6	Telomerase-	1	1	1	1	1419	160.4	7.05	0
P98174	FGD1	FYVE, RhoG	1	1	1	1	961	106.5	6.58	0
Q86X29	LSR	Lipolysis-stin	1	1	1	1	649	71.4	7.97	0
Q9H4B0	OSGEPL1	tRNA N6-ade	1	1	2	1	414	45.1	8.56	4.74
Q5JVG2	ZNF484	Zinc finger pı	1	1	1	1	852	98.2	8.32	0
O00418	EEF2K	Eukaryotic el	1	1	1	1	725	82.1	5.33	0
Q8TE02	ELP5	Elongator co	3	1	1	1	316	34.8	4.97	0
F2Z3M2	LINC02876	Uncharacteri	5	1	1	1	114	12.7	5.97	2.25
Q9NVI1	FANCI	Fanconi aner	1	1	1	1	1328	149.2	6.74	0
C9J798	RASA4B	Ras GTPase	2	1	1	1	803	90.4	7.74	0
Q9UKY0	PRND	Prion-like prc	7	1	1	1	176	20.3	9.03	2.29
P08709	F7	Coagulation	2	1	1	1	466	51.6	7.23	2.38
Q9H0H5	RACGAP1	Rac GTPase	1	1	1	1	632	71	8.88	0
Q96KR4	LMLN	Leishmanoly	1	1	1	1	655	73.5	6.93	0
Q56P03	EAPP	E2F-associa	2	1	1	1	285	32.7	5.12	1.87
Q99504	EYA3	Eyes absent	1	1	1	1	573	62.6	5.21	1.8
Q86VP3	PACS2	Phosphofurir	1	1	1	1	889	97.6	6.6	0
P04150	NR3C1	Glucocortico	1	1	1	1	777	85.6	6.38	0
Q9C019	TRIM15	Tripartite mo	4	1	1	1	465	52.1	5.57	0
Q9UHF7	TRPS1	Zinc finger tr	1	1	1	1	1281	141.4	7.58	0
Q969U6	FBXW5	F-box/WD re	1	1	1	1	566	63.9	6.33	1.8
O00555	CACNA1A	Voltage-depe	1	1	1	1	2506	282.4	8.84	0
Q02410	APBA1	Amyloid-beta	1	1	1	1	837	92.8	4.93	0
Q9C005	DPY30	Protein dpy-3	7	1	1	1	99	11.2	4.88	2.07
Q8WXG6	MADD	MAP kinase-	0	1	1	1	1647	183.2	6.04	0

Q6ZMG9	CERS6	Ceramide sy	2	1	1	1	384	44.9	7.65	1.69
Q96M86	DNHD1	Dynein heav	0	1	1	1	4753	533.3	6.71	0
Q5H9J7	BEX5	Protein BEX5	5	1	1	1	111	12.6	4.84	1.79
O75871	CEACAM4	Carcinoembr	4	1	1	1	244	25.9	6.52	2.03
Q7Z4P5	GDF7	Growth/differ	2	1	1	1	450	46.9	9.8	0
Q00975	CACNA1B	Voltage-depe	0	1	1	1	2339	262.3	8.51	0
Q6ZYL4	GTF2H5	General tran	8	1	1	1	71	8	4.65	2.23
Q9H5V9	STEEP1	STING ER e	3	1	1	1	222	25.6	8.73	2
A6NGC4	TLCD2	TLC domain-	3	1	1	1	264	28.7	10.1	0
Q495W5	FUT11	Alpha-(1,3)-f	2	1	1	1	492	55.8	5.94	0
P05093	CYP17A1	Steroid 17-al	2	1	1	1	508	57.3	8.57	0
O96033	MOCS2	Molybdopteri	7	1	1	1	88	9.7	4.72	2.44
O14798	TNFRSF10C	Tumor necro	4	1	1	1	259	27.4	4.82	0
Q8N5C7	DTWD1	tRNA-uridine	3	1	1	1	304	35.2	8.38	0
Q8IVW6	ARID3B	AT-rich intera	1	1	1	1	561	60.6	7.52	2.07
P31944	CASP14	Caspase-14	6	1	1	1	242	27.7	5.58	0
Q96B01	RAD51AP1	RAD51-asso	2	1	2	1	352	38.4	9.11	4.29
Q9P2G4	MAP10	Microtubule-a	1	1	1	1	905	100.3	7.17	2.22
P37023	ACVRL1	Serine/threo	1	1	1	1	503	56.1	7.61	0
Q9BUK6	MSTO1	Protein misa	1	1	1	1	570	61.8	6.11	2.02
Q09328	MGAT5	Alpha-1,6-ma	1	1	1	1	741	84.5	8.12	0
Q9P0K1	ADAM22	Disintegrin a	1	1	1	1	906	100.4	7.2	2.22
Q14644	RASA3	Ras GTPase	1	1	1	1	834	95.6	7.15	0
Q02846	GUCY2D	Retinal guan	1	1	1	1	1103	120	7.44	0
O75716	STK16	Serine/threo	3	1	1	1	305	34.6	6.9	0
P19397	CD53	Leukocyte su	4	1	1	1	219	24.3	7.52	0
O60725	ICMT	Protein-S-iso	3	1	1	1	284	31.9	7.96	1.83
Q6P3X3	TTC27	Tetratricope	1	1	1	1	843	96.6	5.59	2.19
A6NHN0	OTOL1	Otolin-1 OS=	4	1	1	1	477	49.4	8.34	0
O60783	MRPS14	28S ribosom	5	1	1	1	128	15.1	11.41	1.74
Q9Y546	LRRC42	Leucine-rich	3	1	1	1	428	48.5	7.46	0
Q4G0P3	HYDIN	Hydrocephal	0	1	1	1	5121	575.5	6.06	1.78
Q8IV33	KIAA0825	Uncharacteri	1	1	1	1	1275	147.6	6.61	2.02

P49795	RGS19	Regulator of	4	1	1	1	217	24.6	5.62	1.94
Q6ZN28	MACC1	Metastasis-a	1	1	1	1	852	96.6	6.9	0
Q99683	MAP3K5	Mitogen-activ	1	1	1	1	1374	154.4	5.78	0
Q7L591	DOK3	Docking prot	1	1	1	1	496	53.3	7.77	0
Q9UPM9	B9D1	B9 domain-c	4	1	1	1	204	22.8	7.28	0
Q9NVQ4	FAIM	Fas apoptoti	3	1	1	1	179	20.2	5.83	2.03
Q9C0C4	SEMA4C	Semaphorin-	1	1	1	1	833	92.6	7.28	0
Q9NX61	TMEM161A	Transmembr	2	1	1	1	479	53.6	8.44	0
Q8TET4	GANC	Neutral alpha	1	1	2	1	914	104.3	6.23	4.74
P32519	ELF1	ETS-related	1	1	1	1	619	67.5	5.21	0
Q15878	CACNA1E	Voltage-depe	1	1	1	1	2313	261.6	8.24	0
Q6ZS27	ZNF662	Zinc finger pr	1	1	1	1	426	48.5	7.66	2.28
Q9UHY8	FEZ2	Fasciculation	3	1	1	1	353	39.6	4.6	0
Q96T21	SECISBP2	Selenocystei	1	1	1	1	854	95.4	8.12	0
Q8IXL7	MSRB3	Methionine-R	3	1	1	1	192	20.7	7.33	1.6
Q6NSJ2	PHLDB3	Pleckstrin ho	1	1	1	1	640	71.9	6.57	0
P49454	CENPF	Centromere	0	1	1	1	3114	357.3	5.1	2.6
O15014	ZNF609	Zinc finger pr	1	1	1	1	1411	151.1	8.03	0
A8MVM7		Putative uncl	1	1	1	1	634	73.4	9.79	2.33
Q9NXS2	QPCTL	Glutaminyl-p	2	1	2	1	382	42.9	9.82	3.97
Q86UK5	EVC2	Limbin OS=H	0	1	1	1	1308	147.9	6.96	2.14
P34096	RNASE4	Ribonuclease	6	1	1	1	147	16.8	9.03	0
P49450	CENPA	Histone H3-li	4	1	1	1	140	16	11.71	1.77
Q9UKP5	ADAMTS6	A disintegrin	1	1	1	1	1117	125.2	6.68	2.25
P50443	SLC26A2	Sulfate trans	1	1	1	1	739	81.6	8.38	1.67
Q8IWV2	CNTN4	Contactin-4 (	1	1	1	1	1026	113.4	7.47	2.02
Q9P219	CCDC88C	Protein Daple	0	1	1	1	2028	228.1	6.23	0
Q96A73	KIAA1191	Putative mon	2	1	1	1	305	33.2	9.39	0
Q9NVR5	DNAAF2	Protein kinto	1	1	1	1	837	91.1	5.2	1.97
P54851	EMP2	Epithelial me	4	1	1	1	167	19.2	7.62	0
Q9P0B6	CCDC167	Coiled-coil do	6	1	1	1	97	11.5	9.5	2.17
Q8NGR3	OR1K1	Olfactory rec	2	1	2	1	316	34.2	7.93	3.66
Q6P995	FAM171B	Protein FAM1	1	1	1	1	826	92.1	8.65	2.35

Q96PL5	ERMAP	Erythroid me	2	1	1	1	475	52.6	8.47	0
Q96C12	ARMC5	Armadillo rep	1	1	1	1	935	97.6	7.11	0
O43692	PI15	Peptidase inl	4	1	1	1	258	29	8.03	2.18
Q9NW82	WDR70	WD repeat-c	1	1	1	1	654	73.2	6.33	2.02
Q6SJ93	FAM111B	Serine protea	1	1	1	1	734	84.6	8.59	0
Q8IWW8	ADHFE1	Hydroxyacid-	1	1	1	1	467	50.3	7.77	0
Q7L5Y9	MAEA	E3 ubiquitin-|	2	1	1	1	396	45.3	8.69	1.9
Q14164	IKBKE	Inhibitor of nu	2	1	1	1	716	80.4	7.84	0
P53674	CRYBB1	Beta-crystalli	2	1	1	1	252	28	8.56	2.34
P54284	CACNB3	Voltage-depe	1	1	1	1	484	54.5	6.35	2.23
Q9NPF2	CHST11	Carbohydrate	2	1	1	1	352	41.5	8.85	0
Q9Y3X0	CCDC9	Coiled-coil do	2	1	2	1	531	59.7	5.4	0
O75352	MPDU1	Mannose-P-d	2	1	1	1	247	26.6	8.94	1.63
Q8NEJ9	NGDN	Neuroguidin	2	1	1	1	315	35.9	9.57	0
Q8N4U5	TCP11L2	T-complex pr	1	1	1	1	519	58.1	4.96	1.75
O77932	DXO	Decapping a	2	1	1	1	396	44.9	7.69	1.92
Q53FP2	TMEM35A	Novel acetylc	4	1	1	1	167	18.4	10.08	2.63
Q8N697	SLC15A4	Solute carrie	1	1	1	1	577	62	9	1.85
P28324	ELK4	ETS domain-	2	1	1	1	431	46.9	7.91	0
Q9Y231	FUT9	4-galactosyl-	2	1	1	1	359	42	7.71	2.17
Q9NVA1	UQCC1	Ubiquinol-cyt	2	1	1	1	299	34.6	8.91	2.08
Q24JP5	TMEM132A	Transmembr	1	1	1	1	1023	110	5.62	2
Q495C1	RNF212	Probable E3	2	1	1	1	297	33.3	8.68	2.49
O75143	ATG13	Autophagy-re	1	1	1	1	517	56.5	5.12	0
A6ND36	FAM83G	Protein FAM8	1	1	1	1	823	90.8	6.39	2.12
Q96DB2	HDAC11	Histone deac	2	1	1	1	347	39.2	7.65	0
Q5SXM2	SNAPC4	snRNA-activa	1	1	1	1	1469	159.3	8.28	0
Q8WZ64	ARAP2	Arf-GAP with	1	1	1	1	1704	193.3	7.39	2.02

O1	O2	O3	O4	O5	O6	O7	O8	C1	C2	C3
146620	59835.1	117510.4	90479.9	104013.9	115411.4	76460.8	61661.9	60398.6	137961.3	103287.5
55929.9	56845.4	76876.2	64149.3	51832.3	108146.1	97286.8	67605.1	43265.7	64416.3	68823.7
56817.8	88796.2	104085.5	83017.5	80689.5	150767.8	71291.7	76325.7	58061.7	39194.9	74422.4
56115.3	105381.7	80380.9	80863.2	69133.2	158404.2	96494.9	95071.2	72003	62558.6	78649.3
89064.7	105782.5	97257.9	74438.2	32575.8	55590.5	52299	61352	29898.6	39331.6	38476.7
32333.2	68799.6	82235.9	74010.4	38176.3	73788.6	19273	15439.3	9623.3	9977.2	31295.3
214017.4	201327.8	228345.3	236192.6	246587.6	190455.1	217643.6	219999.9	170412.1	97818.9	156821.6
26827.7	28003	30498.1	21722.6	21522.9	29765.5	32491	28381	43140.3	43310.6	57865.4
54511.6	81378.5	97722.4	82279.1	55348.7	88076.6	31749.8	61995.6	16006.5	28704.3	27787.5
43211	41977.5	43747.5	33842.5	34833.8	48189.5	42893.2	37797.6	29912.3	29324.7	41503.3
72851.4	34784.2	48468.5	33238.3	29724.7	20863.1	31422.3	34098	31244.8	52961.8	54549.9
20463.5	34941.8	37324.8	40564.4	35189.5	66808.4	26677.5	31632.8	17419.6	15221.7	18245.3
87169	53226.8	33528.7	26147.8	31944.6	134704.1	75173.5	38184.6	18809.3	17674	20277.9
23701.1	61277.6	25178.1	32485.9	30516.3	80973.2	118615.5	105761.2	13447.2	19782	18898.9
43434.3	43226.6	43346	44283	50373.8	39140.8	51856.2	54050.2	36940.2	22522.8	39444
64924.2	30032	40923.6	27472.3	25691.4	17400.9	26382.5	30062.3	25496.3	44345.3	45397.7
25146.9	28683.6	40279.5	24752.8	45907.6	23995.3	38481.7	43148	33518.4	16178.3	35515.1
22829.3	16241.6	19622	12640.9	13798.4	14978.2	17802.7	18388.2	39645.8	53646.6	53700.3
25541.7	23617.5	16853.3	13081.7	17155.3	22876.8	26576	17114.7	17785.5	34651.7	28944
23143.3	30559.7	30229.8	30006.4	15141.7	26649	24921	25177.2	9501.7	19315.2	16794.4
18406.3	20264.8	23789.1	17003.6	14356.4	23287.7	28251.9	19725.6	19748.5	30816.3	27245
28367.7	33101	38378.4	38423.4	26919.1	44682.4	26815.8	24068.7	21570.6	25122.7	29451.6
31482.7	20452.4	40875.2	24761.9	13060.6	11030	20458.7	10786.4	25125.5	26013.9	22917.6
33884.8	40534.3	59778.1	54497.3	60284	45400.5	45797.5	53318.4	37936.3	22306	34842.3
17060	17248.3	18077.3	13598.9	11727	16260.6	21261.7	16917.8	25222.4	35292.2	35723.7
15983.3	36803.8	33326.3	33666.8	22521.2	51798.9	25374.3	32923.1	15297.8	14670.3	22244.1
41477.4	21235.6	77783.4	34669.8	22350.8	15414.7	11386.2	18506.1	12781.1	16987.3	14350.6
11480.2	16067.1	17898.3	18433.9	13282.1	19264.2	21054.2	16163.9	23082.2	23578.6	24658.6
11278.3	17771.3	16809.2	14148.1	10326.6	15989.5	16932.6	22424.6	29404.5	23446	32392
21906.2	31176.7	30329	32544.4	19451.7	37201.4	23228.8	21772.4	22380.4	27550	30430
67720.9	49763.3	67610.1	47629.5	39624.3	40200.7	57293.4	38020	51452.9	56618.1	65976.4
34730.9	42842.1	14579.3	17421.1	27824.4	12710.3	86173	33660.2	20413.5	15735.8	22456.2

18740.7	10680.5	14803.2	8223.9	9050.1	10314.3	9126.3	10367	20938	38562.1	35986.4
17865.5	15831.4	10195.8	7587.1	17173.9	9609.2	19510.4	12524.3	6984.1	9850.5	7836.8
23131.6	21163	19085.8	16369.3	20117.9	19710	18213.2	33013.9	19395.7	11823.4	23038.4
26107	21938.9	41812.1	22686.7	40328.1	19166.9	42561.1	41702.1	18891.6	10677.6	14839.2
6818.6	10374.5	10618.6	9333.3	6838.6	11423.7	15618.8	9118.7	8543.2	9412.9	12904.6
16663	17241.4	23041.4	20053.4	14410.9	30134.8	32235.5	18388	17974.4	20605.1	30229.7
19574.9	22914	21475.9	17893	15430.6	18737.4	24634	19197	37726.3	37393.8	44783.3
17933.8	15958.6	9860.4	7902.4	17458.8	9273.5	19315	12749.3	6779.9	10052.7	7856.5
27.9	26.6	23.7	20.8	26.7	27.5	34.1	24	35.1	28.8	39.7
4819.6	33379.4	6919.4	5081.1	6047.2	11698.6	46533.5	21277.6	18473	5668.9	21340.2
22411	18103.6	21505	15522.4	16296.5	18588.9	15645.2	15101.1	37839.3	35918.3	52767.7
34872.9	52464.9	48656.9	55844.6	41143.7	75909	46655	43963.1	41032.6	39604.6	49220.6
5410.9	5073	8982.4	9343.6	6980.1	11502.3	33635.4	5397	13756.2	72442.2	34830.9
13234.8	14157.7	14118.5	10393.1	10951.3	12594.5	12686.7	9310.9	18529.8	24412.6	26930
13883.4	10525.5	11337.3	8870.6	8208.8	8458.8	10943.7	8214.3	20680.7	27952.1	27850.2
8961.4	6709.2	8338.1	6241.6	6371.9	7132.5	6425.5	6142.1	11987.1	14844.6	19139.8
17112.3	18352.7	19635.5	14285.9	12867.2	16394.9	15049.4	15144.6	29554.3	34211.6	39847
12830.9	10807.7	16152.8	11015.6	9235.8	10847	8742.4	9945.3	11417.1	14556.2	18623.9
50640	19656.3	42586	33241.4	25680.5	27290.2	18281.2	15565.7	14476.5	24706.3	33490.8
5355.7	18667.2	5113.6	18524.1	12927.7	90824.1	19451.4	15776.3	2341.2	3090.6	3218.3
16589.3	18799.9	18920.3	17282.5	12376.9	17885.6	15612.1	16206.6	16397.4	13438.6	18263.9
5376.3	4947.9	6680.6	5173.5	4551.7	5324.5	6810	4958	11121.4	42656.3	18874.7
20981.9	20629.6	27118.4	22164.6	18744.7	25541.4	25395.2	17074.3	19198.2	28195.8	33056.4
31030.3	36629.6	32913.2	23737.4	10155.4	19923.9	15955.7	20579	10909.1	13803.4	13659.3
13617.6	11154.6	13528.4	10211.6	9848.1	9836.8	9286.7	9791.5	17413.4	24948.1	27632.4
17248.4	33011.8	30174	41236.9	22369.8	47964.7	21262.8	22834.3	15744.4	15841.4	21142.4
21521.7	15078.4	27702.7	11921.9	20692.8	8476.5	6421.5	12998.7	3906.9	4770.8	6340.8
15221.6	16358.6	15198	11268	11647	14213.1	16218.2	13950.6	25339.7	25659.7	33958.9
8843.8	10391.8	9636.3	7989.9	7170	10224.1	10625.8	9475.5	11627.4	13997.2	14860.4
20188.4	21118.7	21406.6	17868.8	14762.9	18198.5	22297.1	18047.8	34868.4	36870.5	46439
10862.5	10978.3	10705.1	9290.1	7628.4	8614.6	10823.7	10132.5	21332.4	20796.9	25693.5
6871	6899.6	8591.2	5214.7	5364.8	6398.5	4923.3	5536	6248.8	8166.8	10954.1
17965.3	8833.7	14159.7	10110.2	12375.2	9930.8	8524	6526.8	8904.9	17553.2	19104.6

24073.4	26917.6	10138.1	12712.3	17572.9	9361.5	59507.9	33439.4	11344.2	10635.5	13789.8
14898	15830.3	17586.7	16090.7	11686.7	19260.8	17301.2	13764.2	13983	12634.3	18673.1
19820.9	23066	26285.8	21068.9	19744.8	29140.5	31234	18102	22330.4	32185.1	36221.8
40299.6	47448.8	43756	52544.5	46717.9	45353.4	42466	42065.4	35549.6	20184.7	26668.7
32.3	91.5	52	67.7	40.9	72.6	52.8	47.3	32.9	32.8	37.8
8750.7	11515	9879.6	8265.2	6282.7	10306.1	12473.2	14451.6	16453.5	14534	17026
10135.8	14004.6	12561.5	9646.4	7606.3	10554.8	13328.8	12974.1	19980.5	18774.2	23684.7
14159.2	12148.6	14455.5	13903.6	15526.3	12353.8	21242.3	16014.7	14408.2	10397.7	11760.7
11653	19582.7	19923.8	6848.3	9446.5	24719.4	39692.6	27288.7	1930.8	2653.7	3232.8
29451.2	19251.6	23363.6	26493.4	13578.2	20273.6	15040.4	15029.5	16584.3	11082.5	17091.6
11859.7	9800.8	12154.7	10350.9	9096.3	10306.6	11416.6	7630.4	15522.5	21273.5	21262.2
9715.1	8344.9	8049.7	7632.1	7018.7	8449.8	7222.4	10171.2	12803.7	9793.1	12190.9
15589.4	14971.7	14894.3	11675.7	9666.8	14086.6	14379.6	13382.6	27058.1	23546.2	30333.5
8814.3	6529.5	8272.8	5291	5400.4	6038.2	5895.1	6149.3	11111.7	18868.4	16104.8
10455.4	6075.8	7888.7	6139.1	6602.6	6870.3	7462	8063.1	16634.3	18917.2	18224.5
18633.5	19248.5	16449.2	10193.4	5395.8	7852.9	7912.3	10123.3	10394.3	15736.1	15591.6
10391.7	8233.8	8627.3	6071.4	5106.4	6194.6	6739.9	8668.5	16458.5	26026.8	24184.3
15930.7	28603.8	21226.8	39811.7	15313.3	29782	13886.1	25205.3	5790.9	4280.2	6801.7
468.5	541.9	531.8	520.9	418.1	703.5	706.2	612.5	884.7	1209.3	1211.1
34634.4	41468.5	12971.5	16369.8	25402.6	10896.6	82947.9	36189.1	15051.9	13287.5	19081.8
17113.9	13952.5	18072.6	14367	13643.9	17412.9	19426.7	11853.4	13324.5	19814.9	23629.6
9229.3	7943.4	9701.2	6419.8	5503.3	6350.9	7732.8	6120.7	10671.1	19040.8	16516.8
14408	20068.3	25368.6	20782.1	14196.4	23342.7	26724.5	24202.9	41119.4	14025.3	8905.9
9061.3	8025	9584.5	6801.9	6657.6	7196.2	7202	6599.4	13435.8	17808.5	20592.9
11054.3	17336.2	28034.5	8473.5	11266.2	15968.3	10900.2	12145.5	13062.8	16770.1	9928.9
12651.5	7519.3	9842.1	6810.2	7514.1	6464.5	7198.1	7123.5	13984.3	20897.8	23177.8
6416.3	12992.6	13179.7	9364.6	7169.1	7978.1	9361	10387.4	4581.5	13416.3	7077.5
6041.1	7198.3	7112.4	5700	4841.4	7099.4	8882.7	7843.7	11435.2	14181	14798.7
16829.5	14019.5	14630.5	14268.6	10856.9	11688.3	16782.5	14310.7	26489.7	26842.9	35341.2
10902.4	12857.6	13363.2	7978.9	11365.8	13195.1	7199.7	10011.1	11024.8	13641.6	7729.7
4134.1	3534.7	5437.9	5232.8	3757.4	5058.9	15658	4139.2	6275.5	23322.7	12766.6
10122.3	6436.9	7533.4	5549	6115.2	5459.5	5390.3	5357.7	19618.6	21487.5	25355.1
7776.6	8611.5	9234.3	6747.1	6151	7476.1	8038.6	6907.2	9578.8	13771.5	15478.6

6705.3	6164.3	7265.3	5519.7	4971.5	5720.4	5230.9	6135.3	8771.9	13120	14170.9
10578.2	13958	12038.5	10703.7	6964	10115.2	17274.1	20268.6	15941.6	19282.2	18488.7
40079.9	19003.4	30007.3	28522.6	19443.2	31751.6	40504.8	17419.5	13283.9	29456	15422
19071.6	18262.1	24122.9	22187.9	15840.2	21126.1	16479.9	17751.1	15284.3	28875.3	22566.1
9141	7627.4	8655.5	5801.9	5981.5	5433.6	7210.6	6698.7	14984	19202.3	11697.4
9832.2	11838.8	9932.6	8573.9	5786.8	7833.8	14079.8	14863.6	28260.9	12795.9	17691.8
6855.9	4655.9	5683	4249.4	4151.3	4354.7	4954.5	4787.4	9784.7	13484.1	13788.6
11328.7	7479.1	7352.9	4679.8	5012.3	5815.7	6757	6081.1	13798.2	22612.1	18872.5
2507.1	3127.8	4309.9	2434.9	2904.7	3208.7	3306.1	2504.4	2829.2	4221.2	3116
2438.7	2102	2245.3	1598.9	1348.7	1921.9	2240.1	1803	3510.5	3786.1	4256.3
17770.5	12534.4	13641.1	9523.8	8439	9323.5	9122.9	11487.8	24765.4	28554.1	29091.3
27712.2	28782.7	35124.9	33311.2	46226.5	21073	34558.8	35054	31315	13560	25423.1
14171.1	13552.1	14042.5	10850	9290.6	13091.8	13903.5	12269.3	23884.1	25561.8	28418.3
21032.6	7485.9	13735.5	9446.6	9653	7779.1	5703.5	6294.6	12082.9	19363.9	22462.9
13399.8	34783.8	32603.7	53416.2	28913.8	62372.3	18901.6	25424.1	8634.5	5406.9	12172.6
10244.2	15124.5	12183.8	11117.2	8360.3	11980.9	23261.5	13629.2	22484	21678.8	25550.2
10233.5	10992.6	10780.8	10687.6	9842.5	11568.5	11336	15294	7969.9	6149.4	10084.6
17577.1	10411.2	12879.6	11233.2	10726.8	8423.9	7395.1	10736.3	19249.2	25542.8	30211
16929.5	6187.6	12836.2	6365	9554.6	4828.8	5538	3815.3	20089.6	16008.9	22652.6
18006	19859.2	18426	18727.9	16214	15248.6	16383.7	23177.3	12464.4	7566.6	10714.5
16666.3	19848.3	18429.3	21671.7	13549.6	20345.4	15152.4	19209.8	4747.6	4504.8	6496.6
6929.6	5987.4	6305.9	4390.4	5090.9	5307.8	5905.5	5092.7	9383.8	9895.5	13759.2
8732.4	7360.3	7542.3	6186.1	5696.1	6795.5	7680	5614.6	12699.3	15423.2	16914
5503.6	5532.5	5105.2	4480.7	4028.5	4793.7	7306.5	4230.1	9613.5	9355.2	11708
2010.9	1986.4	2199.5	2536	1783.1	2066.6	2138.5	2209.5	4510.1	8923.8	5590.7
9413.6	6330	8067.7	6870.3	5176.2	5873.2	5995.7	5292.3	6287.2	15414.1	9909.1
4355	4447.6	3934.1	3086.3	2795.1	4149.6	4424.3	4089.1	7981.8	5820	8613.7
8679.5	17155.5	9947.8	8080.6	4808.7	8618.6	7623.1	11292.7	9376.6	12922.9	13540.5
17779.6	10623.9	9496.1	11909.3	11712.8	7611.8	14464	16955.4	9436.9	5538.4	7471.6
3902.7	8703.8	5896.1	3780.5	5474.3	6935.5	20222.8	5805.5	16685.7	14512.1	26550.9
4461.1	4250.1	9519.1	7672.7	3592.9	4964.6	16162.1	4471.7	6634.8	11220.6	9938.7
12537	16307.6	16540.8	9199	12001.1	15123.8	10020.9	11343.9	13729.9	15579	9597.3
7468	5139.5	6386.5	4438.7	4111.5	4629.7	4771.6	4835.6	11313.9	15909.7	16696.1

15956.7	16556.4	16945	16412.4	11626	14354.9	17000	14342	23411.4	22422	26273.9
5197	6597.7	5732.7	4804.5	4155.9	6235.4	7990.9	7145.5	9646.3	11196.2	12084.7
5847.1	5156.4	5902.2	4670.8	3735.4	4560.1	4531	3868.8	7944.3	11670.8	12672.7
10006.4	8726.4	11462.3	13083.4	7506.8	13383.5	16408.6	9851.9	6534.8	13956.1	10966.5
8518.2	8965.1	8217.4	7947.1	5883.6	6560.8	8948	8033.6	12408.7	20769.9	15012.3
7301.2	10703.9	10083.3	7475.1	6819.6	16752.3	13740.7	9449.4	6735.6	8833	10905.4
70288.8	72938.1	43899.6	43000.1	113101.8	63685.9	112071.9	67439.4	34558.9	46425.8	37521.7
14001.5	7395.1	12256.9	8533	8352.6	6931.6	4260.2	9879.6	8825.7	9504.3	11130.8
7940.9	8110.2	9016	7131.3	6136	7512.4	7979.9	6974.9	10112.8	12772.3	13365.7
7010.8	6764.8	8256.8	5665.6	5852.2	7240.4	6946.6	6687.2	8414.9	10474.2	12495.9
2676.1	2713.5	3694.4	3669.2	3278.5	5040.3	11069.5	2910.8	5150	24155.2	11521.2
2915	3488.3	3414	2471.5	2628.5	3505.6	4107.1	2656.5	4055.4	7148.2	6227.1
1266.9	5883	1636.4	6010.1	3726.2	39497.3	13396.5	16060.1	907.5	1414.1	1563.7
14418.3	11881.9	18997.9	18566.2	22661.4	9470.7	19152.9	20143	18099.7	7577.6	13818.1
25513.4	27951.9	34010	34292.1	33219.8	33911.2	27043.6	34748	29867.9	16865.1	25241
15864	19870.7	18566.6	12508.6	5213.7	10658.3	8459.4	11224.3	6416.9	8378.9	7731.1
5241	5992.4	5982.9	4524.1	4355.9	5273.4	6797.8	5733.1	9375.4	13802.8	11858.9
8282.3	7548.5	6716	6696.3	4856.3	6337.8	7632.9	6962	12010.8	12010.6	13598.1
7457.9	6794.7	8186.2	6614	5923.5	7501.6	7936.8	7344.2	9995.4	10116	12035.7
5240.1	4158	4949.1	3811.9	3520.6	4515.9	5455	5550.2	9961.2	9262.3	11518.7
7031.2	6844.5	4419	4148.2	9026.2	6837.7	11684.8	5185.3	5055.6	5827.5	5686.6
4278.3	4335.1	4064	3493	3633.8	4232.3	4598.6	4187	9293.4	12358.4	11256.7
8303	7143.4	7905	5796.6	5599.2	6069	6736.5	6714.6	11086.2	12270.2	15651.7
9893.9	21207.6	21323.8	30179	14628.3	36725.2	14410.4	14860.8	9090.6	6941.9	11547.4
10910	11224.4	12541	10420.7	8809.4	10826.6	9806.5	10238.5	12903.7	14171.1	16908
20818.8	39488	32410.7	85561.5	25640.4	54767.1	20520	26181.4	6363.5	3181	7383.7
9483.5	7990.4	5703.4	4351.3	9408.7	5653.8	9939.3	6584.8	4357.9	6287.7	5190.1
5433.2	6453.5	5694	4694	4281.4	5524.4	6809	5437.1	15594.2	12892.6	17803
5730.1	6170.9	6492.6	4882.9	4410.2	5426.7	6266.9	5594.2	9141.5	11714.1	13591.3
10561.5	6465.9	8388.7	5405.6	6008.7	5339.9	5616.3	5640.8	11502	18271.8	19036.7
1193.8	2105.8	2838.9	1627	1162.4	2129.1	1483.7	1474.9	1976.4	1826.1	1608.2
2369.1	5448.5	3974.6	3129.7	2223.8	3601	2785.6	2593.7	5744.6	7851.5	6053.5
8358.4	7217.6	8940.9	6607.5	5582.8	6389	8088.6	5891.2	12016.9	16187.5	16124.5

16115.5	10697.7	7793	8951.6	11171.6	6126.7	19035.7	15394.5	9020.5	5181.7	6140.1
4239.6	4037.5	4812	3677.7	3211.7	4569.9	4366.2	3216.1	7936.2	31718.2	13372.7
6239.1	5842.6	5913.8	4482.3	4174.7	4633.7	5886.4	4517.3	9938	14001.1	13779.2
9972	6582.1	7509.9	4916.5	4051.1	4288.6	5436.6	5142.6	6784.1	5060.2	10154.7
11242.5	8239	8682.3	6956.1	7073.1	6424.3	9087.3	7517.3	14917.6	15116.9	18167.6
14337.6	11072.9	13878.4	10344.3	10767.5	10771.9	10553.9	9245.4	15412.4	22470.1	24917.9
9057.2	9220.4	12014.1	5755.2	7497	6741.1	5076	10600.8	3162.6	3957.3	5735.5
7660.8	6616.1	7924	6224	5398.1	5554.8	5962.8	4535.7	6048.9	5410.8	7229.6
5573.5	7184.9	6600.6	5493.6	4519.9	6370.1	7949.5	7058.2	11296.1	13509.3	14486.7
6415.2	4854	6187.2	3624.6	3836.3	4032	3768.9	4605.3	12592	13812.7	18609.4
3359.4	18126	3822.4	2199.5	4932.2	57585.5	8693.1	12885.9	2112.9	2171.7	4980.4
5013.5	15169.4	19514.9	12399.9	6082.5	5299.8	6255.9	3299	1766.5	3127.9	8369.6
7247.5	7170.4	10485.9	5213.6	3881.9	4161.9	5009.5	4023.1	5740.6	8743.6	8379.6
8615.7	7895.2	9405.6	6780.6	5678.7	6769.4	7842.8	6965	9575.3	12108.1	13152.9
6069.2	4512.5	5541.2	3830.2	3353.7	3584	3816.2	4371.2	8427.2	12981.2	13209.9
13939.4	10731.9	16040.2	10561.2	10260.2	8024.4	8623.1	6639.3	9290	12917.9	19066.7
8027	5056.1	5765.4	5905.2	4881.5	4637.9	10464.3	4166.7	6937.3	11904.7	11519.6
5502	9606.6	20386.1	4423.7	5984.1	8609.7	5006.6	6154.5	6441.4	10397.8	6216.1
953.7	1022.9	982.7	809.4	595.6	890.2	1086.4	982.5	1247.5	1217.7	1156.1
13196	11432.3	10838.9	9582.2	11554.9	8467.5	9393.5	13739.3	6681.5	6801.8	9959.3
8054.3	6571	7837.7	5758.9	4650.6	5836.9	6206.4	5034.5	9224	16826.8	14075.1
7695.5	7583.9	9217	7176.9	6734.3	8299.4	9691.3	6366.8	5842.5	7140.6	8878.2
6750.8	6177.6	7098.8	5250.6	4661.1	5460.8	5651.8	6208.2	11914.9	13894.1	16403.2
6439.8	5278.4	5793.5	4896.2	4301.9	4592.2	4964.8	5055.2	10022.5	11093.5	13378
3292.3	9192.3	7704.5	9521.6	8616.3	21266.7	7937.1	11668.2	4178	3384.9	5297
4690.7	3118.9	4693.7	2961.7	3300.9	2953.4	3383.9	3605.5	17932.5	8878.7	16775.1
4816.4	5261.3	5857.2	4272	3986.5	5120.6	7462.5	4743.4	7974.7	8222.5	10554.5
7986.7	6534.1	6240.3	5391.5	5833	7390.5	8881.8	7188	8964.4	16188.8	10836.3
5928.6	6542.9	8468.5	6302.6	5432.8	7384.1	6721.3	6093.5	6448.5	7742.7	9589.7
12408.3	9274.5	13013.4	15133.1	7640	8901.3	11620.2	9520.1	13754.2	12452.1	17706.2
9324	9549.6	10161	9530.2	7596.5	12872.5	8223.7	7085.6	7954.4	9024.9	10973.3
7076.4	5995.3	5735.1	4850.1	4633.5	5108.2	6096.1	4746.1	10317.8	11162.2	13239.5
6951.6	5660.4	6917.4	5184.2	5280.9	6364.2	6553.9	5143.8	7331.8	11334.1	12119.5

238	388.4	631.1	268.2	311.3	498.3	303.7	230.6	326.3	278.3	265.5
5790.7	5258.3	5905.7	4184.5	3949	5076.2	6106.5	4363.2	8055.4	10294.7	11419.3
5849.4	5097.4	4969.6	4335.1	4616.8	4720.1	5422.3	4033.7	8844.9	9896.5	11065.6
7799.7	5499	6186.7	4411.4	4932.8	4694.1	4904.3	4653.2	6783.2	8520.4	8326.7
6827.8	6091.7	8955.1	4869.3	5515.1	5582.3	6305.3	3847.4	5982.2	45659.5	11270.1
3758.6	4519.8	2494.6	2562.5	6308.4	3808.5	6595.8	4162.8	2046.8	2935	2214.6
4684.2	4896.2	6301	4089.9	3630.9	4639	3247.3	4627.5	8286.9	7068.9	8993.6
5683.1	6696.3	6072.1	4957.5	3737.7	5154	6570.4	5854.3	11923.1	11439.5	15488.3
5401.5	4163.5	5108.7	3699.3	3128.6	3471.2	3609.4	4334.3	10266.5	13097.5	13528.9
11095	14025.2	13037.1	11747	9755.2	18670.7	15069.3	11417.9	10934.3	16848.3	15725.3
3754	6031.6	6712.3	6079.3	3404.9	12533.7	5424.1	6300.5	5896.8	4947.8	6232.2
5248.9	3441.1	4463.4	3093.2	3109	2915.3	2865.3	3048.3	7569.4	11837	11771.8
5888	5132.1	4826.3	3967.8	3835.6	4086.1	5218.9	3795.2	8248.8	10738.6	11266.6
5310.7	3750.4	4531.2	3346.9	3309.9	3493.1	3557.9	3777	11172.9	11345.7	13989.8
6053.6	5065.6	5086.1	4070.3	3525.7	4197.6	5116.9	3929.8	8859.7	10728.1	11766.6
32084.9	13768.8	20127.6	26048.4	16657.6	7816.7	11044.7	13138.2	8722.1	6255.9	8940.2
28613.8	29745.9	18474.8	17471.9	46158.4	25332.7	45393.2	28379.3	15402.8	19907.8	16201.1
2392.9	3134.9	3693.6	2822.5	2195.2	4604.5	3845.3	2975.3	3970.6	4964.2	5497.8
4914.3	3647.1	4135	3205.7	3023.6	3458.5	3611	3823.3	8960.1	11093.6	11992.6
6528.4	5260.1	5634.7	4859.5	4631.7	4663	5719.9	4356.4	9072.2	10772.1	12184.1
11689	8647.2	13876.5	9963.2	9020.2	11234.1	9506.4	7400	11278.2	19023.5	21666.4
6028.5	9743.5	8954.3	7899	8278.1	12540.4	7806.9	5888.4	13652.6	6336.1	6740
9931.7	10036.3	8008.9	6345.8	3967.4	5703.4	5127.8	7202.6	5309	6057	7106.6
11522.1	14175.4	14440.6	9228.9	8828.3	12421.7	10237.6	16449.4	9135.1	6513.8	12366.3
4261.2	6362.5	6036.1	3230	3230.5	5038.4	7174.2	3771.9	8660.6	17639	11699.1
5634.4	8714.4	10600.4	8445.2	6979.8	11429.1	12651.7	11676.5	20005.1	14969.8	5416.8
6629.7	10590.9	10648.7	7104.5	6126.8	6847.5	6214.1	8366.4	7443	8426.2	9939.2
11367.7	4804.8	8551.3	7038.2	6293.1	5560.9	6511.7	5588.3	6957.4	8548.1	9936.7
9673.6	11375.2	10916	4593.5	6099	17336.9	13998.4	4109.1	5532.4	7413.2	7804.6
9131.5	7867.2	11819.9	7723.6	6909.7	7225.8	7178.7	6376.6	8091.6	9917.5	11911.3
6001.1	5051.9	5287.7	4573	4030.2	4282.6	5088.5	3534.8	8547.4	10450.1	11123.4
3692	4659.9	3933.8	2842.1	2724.6	3663.9	6298	3355.4	6883.2	9532.1	8653.9
9348.4	6572	8258.2	5799.3	7118.8	6554.9	5942.9	5132.5	10747.5	14113.6	17785.3

10821	9132.4	5866	4427.1	11427.1	7569.9	11979.5	6840.8	3922.2	6183.6	4753.8
9088.6	10718	11888.8	16303	9182.1	26503	14402.3	11209.6	6725.2	9847.5	9573.2
5854.8	6043.2	5842.2	4628.6	4540.2	5204	5749	5062.9	9336.9	8527.5	11650.9
7226.9	4127.6	5214.1	3649.8	3579.4	3849.6	3892.3	3556.9	8279.1	10980.6	11994.7
4063.2	4094.4	4342.3	3290.8	3487.6	4177.1	4800.4	4106.8	5220.5	6721.8	7256
9136	11591.8	10372.7	8785.8	6691	12971	10695.5	8274.9	12475.2	15007.5	16642.1
22026.2	7931.6	13613.6	14255.5	10841.8	4086.9	5395.9	3293.6	4398.3	6172.3	9187.5
6552.7	5477.8	6396.8	4763.2	4960.1	4846.2	5073.6	4232.7	8122.1	11895.6	12868.1
5001.4	3778.5	4525.8	3420.6	2958.2	3553.8	3968.3	3366.6	7306.2	10099.9	10256.9
1163.1	1851.6	1757	2789.9	1369.4	1500.2	1683.7	1648	1839.8	2104.4	1659.5
7195.3	4447.8	5846.7	4212.9	3970.8	4377.2	4174.2	4076.3	7852.6	10008.2	11033.9
3438.5	11955.9	5861.8	6366	6311.9	14839.7	17228.8	12355.8	2804.9	3068.2	3853.5
4881.2	18924.4	9270.7	9430.4	4108	20787.8	19707.1	9957.4	8559.6	4971.4	7487.5
7019.7	7979.9	9270	9800.6	6765	10557.2	5513.3	4140.6	4197	3107.9	5522.4
3871.4	4983.2	4629.4	3795	2974.2	4952.5	4788.4	4771	6479.1	7070.2	8135.2
3320.5	3435.1	3575.4	2728.4	2607.4	3057.1	3740.3	2822.4	4751.1	6031.8	7067.1
256.2	714.8	542.6	398.7	222.8	386	384.3	632.8	1026.5	468.9	827.3
2949.6	3629.1	3134	2300.8	2546.2	2985.3	3317.3	3194.6	4836.3	4943.9	6239.1
11350.8	11990.6	11865.5	12063.7	8432.6	9468.1	9951.2	14611.4	9238.8	5294.1	9832.5
11329.8	9441.8	10381.9	8021.5	6421.3	7627.4	10340	7296	16591.7	18166.3	23314.2
4103.3	2248.9	3488.9	3298.7	2918.7	1774.3	1827.1	1590.7	10844.5	21625.4	13008.7
5131.1	4991.2	5554.2	4151.8	3477.2	4604.8	4037.5	4070.8	8125.4	6112.8	10612
10964.2	3476.8	3767.6	3731.2	4479.9	2658.2	2887.3	2836.1	4523.1	8818.4	9864.9
6423.8	5214.4	6243.8	4126.6	4055.3	4492.9	4351.6	4810.7	10601	15622.3	16167.5
12019.9	5872.4	9196.7	4444.5	2938.9	2661.1	3412	2396.4	4072	5748.9	5697.3
5521.9	3781.7	3446.2	3300.8	3403.9	9415.4	35341.7	7337.1	816.5	1539.5	1754.4
4116.6	3664.1	4433.3	3270.6	3150.7	3739.5	3937.7	3835.6	5319.2	6187.5	8148.9
5433.5	6815.2	5627.2	14651.6	4450.3	19333.8	10048.6	9611.5	3244	2754.6	3147.2
4120.1	13397.9	7446.6	20931.9	8855.7	13242.5	7004.8	9906.7	1976.2	2029.4	3209
3720.1	4998.9	4714.8	3716.6	3085.5	4784.8	5935.8	5963.4	7468.3	9588.4	9943.4
6020	3582.4	4617.8	3277.6	3012.4	3317.6	3146.5	3464.1	10520.6	8398	13415.1
4066.4	4216.5	3818.4	2581.3	2577.8	3879.2	5672.5	2859.7	5020.3	9837.6	8406.7
7470.9	7014.5	7688.5	5534.6	4474.7	5414.7	5399	6084.9	9385.3	13337.3	14611.8

6005.3	5127.3	5989.8	3885.2	4494.8	3971.1	4067.9	5096.2	9594.8	13049.3	13695.6
4482.7	4447.1	5581	4450.7	4008.9	4869.1	4995.7	4270.5	5577.1	7243.2	8831.9
12550.4	8458.8	12490.5	11675.4	7122.8	9042.1	6388.3	7136	11777.3	13414.3	17591.2
9357.2	10950.7	15252.7	6244.5	8782.3	11878	5855	12861.3	7710.1	12869.6	12123.1
4523.9	4261.7	4864.1	3658.2	2705.6	3404.5	4375	3370.1	6778.5	9008.1	8758.1
9909.1	6568.8	9443.5	6399	7848.5	6539.2	7190.5	6428.4	6966.8	10226.7	11292.7
5036.4	3207.3	3893.1	2469.8	2841.6	2584.6	2719.4	2860.4	5649.6	7866.7	9336
6499.6	7414	7860.2	7207.5	5577.8	8151.5	7005.6	4518.8	3979.5	4656.4	4628.8
3243.4	16454.1	10169.2	12594.2	7552.3	18462.4	6705.7	19550.7	7169	3147.9	7377.9
4615.7	4477.2	4313	3266.3	2823.5	2815.8	2741.2	2816.7	4494.6	4496.9	6369.2
4074.5	5148.9	5192.1	5265.9	3361.8	6586.5	2699	3182.8	2124.3	2508	3317.4
7538.6	5367.3	5898.3	4599.7	4884	4422.4	4260.1	5675.5	11696.2	14007.9	15103.4
12712.5	9797	12317.4	15910.4	13600.5	7793.4	13114.5	14197.2	13832.4	7942.8	11527.6
4867.5	4252.3	4981.2	4066.7	3613.5	4641.8	3408	4121.3	7575.9	9355.4	9086.1
8166.7	3568	8169.7	5674.4	5529.3	5659.5	5956.7	4089.5	4801	6365	7205.5
7196.7	5872	7166	5463.8	4669.8	6413.3	6997.5	4445.3	7252.3	10778.8	11436.4
5559.6	6589.9	7111.3	4897.8	4202.1	6767.6	6385.1	4073.8	5927.3	6507.3	9293.9
5906.8	5654.6	6211.8	4905.4	3864	5171.1	5347.5	4730.4	6582.6	7500.9	8876
4049.2	5849.2	5393.5	4154.2	3119.5	4089.2	5409.2	4476	7613	7204.6	8260.2
7458.8	5717.2	7467.7	5470.2	5345.9	5949.8	5066.1	5082.3	6028.4	8549.5	8663
3709.1	2465.3	3079.7	2079.2	2060.7	2117.1	2490.1	2612.9	5826.7	8080.8	8008.5
5523.4	4488.1	4543.2	3699.5	3338.1	3792	5496.9	4535.8	8713	9119.9	10243.6
6228.1	5148.9	6356.5	5106.3	4809.3	5121.5	5497.7	4083.7	5979.6	7138.7	8583.5
5792.8	5361.1	5857.9	4358.4	3984	4451.1	4760.7	4254.4	7287.2	8052.9	10315.6
4028.2	3225.4	3754.2	2771.1	2557.6	2833.6	3020.1	2942	5941.3	7998.7	8470.9
11537.2	9015.6	11658.2	8651.2	8518.9	8819.6	7247.4	7297.8	11906.4	11966.3	17392.8
7232.6	7491.6	8395.2	7268.7	5982	7025	4951.4	4887	4145.7	5100.9	6644.6
4893.6	5402.1	5085.7	3713.5	3916.5	4792.3	8633	4204.2	9441	16836.8	12211.3
2686.4	2586.1	3236.7	2633.6	2156.4	2759	2707.7	2380	3851	15373.6	7394.2
6763.3	6179.8	7047	4979	4948.1	5212.7	5341.3	5035.5	8101.8	10464.9	11649.3
9559.4	8643.7	6631.4	4618.2	3021.8	5393.8	5256.8	6251.1	2283.4	3341.1	3752.7
7391.2	5315.4	6457.9	4746.5	4312.3	4688.7	4862.7	5418.2	11846.8	14109	14977.9
5380.3	4608.3	5679.8	4311.1	4269.9	6314	8434.3	4913	3401	4827.7	5444.5

6601.8	4196.1	7308.4	7321.4	3711.3	5662.6	6269.7	3731.3	6784.3	9256.1	13548.6
12322.2	8957.3	10194.8	7841.1	7939.4	7776.1	6923.4	9193.3	17754.6	21128.6	23680.7
6610.9	6155.4	5489	6455	7522.3	4300.5	6371.3	7959.6	4953	3385.1	4529.9
3137.1	2521.8	2785.8	2000.1	2379.4	2773	3521.4	2204.8	4125.4	5917.9	5744
8657.4	6966	10165.5	8585.6	5921.8	6783.5	5256.3	5943.3	7099.8	9284.5	10241.6
5595	6136.3	5412.2	4458.6	4030.5	3161.3	4952.6	4786.3	8263.2	4902	8100.2
13015.7	15506.8	16016.7	4252	12455	2305.6	4763.3	4386.3	8207.7	5141.1	8731.4
4456.8	4000.9	4726.6	3149.6	3461.2	4015.9	3813.1	3319.2	6844.7	6899.9	9742.4
3094.8	6083.6	5044.2	5944.8	3621.4	7904.6	3804.5	9290.6	6557.3	3088.2	5949.8
6185.3	4527.2	5607.2	3563.8	4205.9	3923.1	3892.7	3918.7	9615	12521.8	14083.9
4760.5	4481.1	4788.1	3619.5	3533.6	3790.7	4232.2	4026	5904.6	7179	7918.5
3756.3	3454.6	4264	3338.5	2655.9	3228.8	2860.3	3004.9	4635.2	9894.3	8425.1
10711.6	7585.5	9398.1	7175.9	7278.8	6721.7	5631.7	6279.4	12573.4	15155.2	19498.5
5379.5	4473.4	5440	4238.3	3231.3	3618.9	4218.4	3631.6	6351.8	5371.5	6630.7
10642.9	3245.5	9350.3	2837.1	4894.5	6440.4	6650.8	4624.9	3829.3	11214.8	4966.6
5602.8	7396.2	7182.7	9067.6	4705.8	11572.8	6896.9	7547.9	3750.9	5676.3	5240.1
3707.2	10105.7	10483.8	13385.7	3928.7	17277.8	8207.4	7070.4	2804.9	2570.6	3541.6
4505	3607.1	4142.8	3098	3128.7	3438.8	3346.2	3057.8	6961	8966.8	10008.8
5673.8	4845.4	5982.9	3583.3	3830.1	4051.4	4354.4	4638.3	8674.3	11712.1	12071.1
3652.2	2943.2	3347.1	2355.6	2253.3	2429.6	2855.6	2779.7	5468.6	7964.5	7848.1
4853.5	3726.4	4583.9	2968.1	3020.7	3419.7	3580.1	3781.7	6370.4	10214.3	9212.8
3050.4	4287.2	3521.5	3023.1	1653.6	2266.9	2921.7	2158.2	4055.3	5207	6956.3
1858.1	1554	2046.9	1627.6	1553.5	2404.7	2725.9	1612	12253.9	8285.6	17383.6
8913.9	6169.7	9895.3	12135.2	15687.6	8698.3	9179.5	9617.4	9087.4	3627.8	7112.8
5850.6	6519.1	5564.4	5730	6289.7	5016.4	12004.1	8343.9	5745.4	3605.4	6078.8
6673.7	5780.1	3967.6	2630.9	2113.6	2202.4	2431	2602.7	2919.1	10981	5154
4137	3283	3424.1	2645.4	3052.7	2942.6	3203	4040.8	7222.4	7708.9	8837.9
3719.7	3423.4	4242.8	2915.5	2709.7	2732.9	3878.4	3722.2	5724	6897.7	9938.7
4235.2	4175	4014.4	3359.5	2728.5	3132.4	4318.7	3456	8697.2	8247.3	9878.8
3899	4273.7	4503.1	3324.6	2834.9	3678.3	4726.7	4099.1	6284.1	9694.5	8996.6
5541.6	4561.2	5404	3117.5	3376.6	3582.6	3848.3	4263.1	6129.5	8219.5	8499.3
6332.7	4932.1	6125.1	5032.5	4324.5	4875.4	4845.4	4167.2	7863.3	13357.8	12179
4657.3	4037.6	5341.3	3203.6	3028.4	3564.6	3698.2	3828.5	6579.9	11777.7	10635.4

1095.5	1279.4	3025.2	1505.8	1040.9	1247.2	2752.2	1544.5	1467.7	1846.1	4646.2
5254.9	6272.3	7820.2	12248.8	4335.8	5873.2	7004.2	8968.3	4993.2	4033.9	5373.1
1319.5	1999.9	4217.3	1210.6	1317	1803.9	1342.5	1455.6	2008.1	3341.5	1748.4
3225.4	3482.5	3243.2	3297.4	2881.7	3384.5	2864.9	3537.4	4812.8	6568	5824.4
9206.5	9005.4	8857.9	9951.8	8850.1	6772.8	8696	10553.7	8201.3	4735.6	6544.3
6545.1	5478.2	5441.1	4490.2	3981.6	4673.6	6215.1	5508.7	11012.2	11034	12948.4
3090.6	2316	2681.8	1768.9	1962.1	2176.2	2094	2296.4	5683.5	6385.6	7888.5
8352.3	5265.1	7601.4	5336.2	5561.1	6448.7	6648.1	3576.2	3919.6	5946.8	7844.4
7894.3	6136.2	7733	4925.8	5628.3	4974.8	5161.9	5099.2	11147.6	16965.3	18288.8
9931	7342.9	9528	8014.3	7055.4	8018.7	4876.7	5966.1	4268.1	5077.2	7045.5
3513.7	4293.1	4151.3	3222	2721.6	4101	4796.2	4433.8	5518.6	8021.8	8199.9
4943.1	6677.1	6226.4	3792.3	3022.7	4458.1	4564.1	5347	5739.1	4092.1	5883.8
3849.9	3598	3794.3	2970.1	3112.1	3451.6	4062.6	3225.9	5768	5196.5	6894.8
24356.3	7795.1	10598.7	7244.7	6811.5	4574.9	5102.6	7215.8	14680.4	27168.5	24870.4
5081.8	6052.7	5331	6238.8	4532.9	5802.3	5166.7	5962.9	8970.6	6863.3	8696.9
2897.7	2950	3556.1	2216.4	2312.2	2675.7	2698.8	3056.5	5934	6109	8203.6
3923	1278.9	2163.8	1134.7	1168.1	729.6	759	1127.2	2432.3	4254.9	4470.3
4691.8	3701.1	4258.1	3405.8	2804.8	3201.3	3829.1	2732	6636.6	9312.2	9837.8
5648.1	5668.4	6568.4	5073.3	4377.6	4903.5	6018.6	5975	4852.2	4498	6446.6
3842.5	3827.9	3737.3	3720.6	4856.4	3654.7	4429.6	7511.3	3630.5	1953.8	3531.2
4113.5	4923.2	5308.4	3521.3	3231.2	4360	4757.3	4763.3	7236.4	8457.4	10434.6
7315.8	3637	6112.1	3600.5	6507.7	3944.4	4400.2	3009.1	3191.4	4733.6	6462.7
1915.3	1974.7	2081.1	1755.6	1575	2084.3	2465	1736.2	2336.7	3582.7	3296.3
5137.7	3517.3	4195.1	3184.9	3573.2	3482.6	3859.9	3870.5	8588.8	8961.3	10801
3205.9	3045.4	3343.5	2628.1	2735.3	3237.3	4228	3254.1	4175.3	4922.2	5425.3
10749.5	12637.3	11347.7	9625	7669.5	9028.7	12992.8	11358.4	20979.7	20726.7	25169.1
3086.8	2869.6	3413.1	2259	2577.1	2711.7	2742	2326.9	3860.6	4882.8	5900.1
3551.9	2780.6	4002.9	2836.4	2478	2942.5	2541.5	2445.9	4274	6040.2	6889.4
5130.2	4426.4	5427	3580.3	4556.5	4330.4	4375.2	3247.4	6876.2	11958.6	12163.2
3841.2	4310.7	3766.1	3371.1	2957.5	3534.8	4457	4503.7	7329.2	6459.2	7868.4
5608.3	7306.8	6933.9	6090	4738.4	9640.4	8160.2	5543	6650.6	8876.2	9716.7
4467.9	5114.4	5842.9	5278.1	3665.3	5177.2	4124.5	4021	3483.6	2996.9	4963.1
3143.2	3902.6	4107.4	3271.1	2665.1	3364.4	4036.7	3491.8	5338.5	6472.5	6875

3878.1	3099.7	3951.1	2411.2	2576	2846.1	2502.6	2905.8	6949.6	9357.2	10483.9
9478	8984.9	18506.2	12181.9	7228.5	7929.7	7576.1	9510.4	3786.7	4871.9	6266.8
5041.8	4871.2	5310.3	3846.2	3580.7	4404.9	5025.8	4102	5551	7625	8052.4
7031	5424.7	7793.6	6730.8	5212.1	5806	4421.7	4691.2	5533.3	6744.1	7670.3
11635.2	7095.5	9312.7	9605.8	7302	8195.1	6991.5	4825.4	10272.9	7775.2	12530.5
3546.2	3244	3192.7	2472.5	2657.2	2949.9	3804.8	2657.8	4669	7949.6	7002
3859.3	3843	3913.3	3218.6	2734.5	3468.4	4608.5	3873.6	7541.7	9917	11893.5
2662	2401.7	2672.6	1986.5	1981.6	2395.3	2967.5	2587.1	3997.9	4260.6	5181.9
5544.6	5119.7	5461	4090.4	3662.9	4507.6	5388.5	4043.1	9282.7	10467.7	12861.6
15587.4	11606.2	14848.1	12681.4	11725.9	12665.7	10510	8396.8	12134.2	17399.2	19306.9
3111.7	2116.7	3721.1	3270	2466.3	2845.4	3587.4	2040.9	3201.6	17181.2	5615.3
5700.9	5462.8	6205.9	5125.4	5968.1	6713	5049.5	4908.5	4479.5	6234.3	5743.3
4176	3087.4	3983.5	3644.7	2945.1	3028.6	2334.2	2164.8	2676.5	2939.6	3801
5579.4	5897.3	5420.4	4508.8	3833.1	4867.9	9688.3	6130.6	10234.1	11939.3	13275.8
3931.6	2935.6	3689.3	2780.7	2401.9	2339	2740.4	2984.3	5530.7	7931.5	7625.1
4676.3	3913.6	4681.7	3863.5	3462.2	3650.2	3426.1	3252.7	4566	7086.1	7693.6
8527.2	9122.8	7449.7	9707.4	8097.1	6017.9	12418.2	7277.9	5343.9	4323.3	11154.6
3104.6	3507.7	3201.6	3086.3	2288.9	3109.7	3935.8	3574.9	4832.2	8246.5	8539.1
5869.9	4808.2	4737.5	3942.2	3564.4	4141.7	4938.2	3861.6	8201.8	9695.1	11153.4
3721.9	8613.2	7424.4	20257	5937	10509.6	5145.9	9804.1	871	1141.5	1436.4
7273.9	6294.5	8760.1	8152.3	5439.4	7889.9	7845.4	6218.1	6118.2	9116.4	10285.8
7776.6	6045.9	9013.9	7772.3	5815.9	6670.1	6231.3	4695.8	7122.8	8796.4	10729.4
3624.7	3108.5	3422.8	2482	2271.1	2715.8	3051.2	3026.9	6000.1	7838.2	8367.4
4888.9	4235.7	4712.9	3911.9	3973.4	3457.5	3626.4	4141.2	8804.8	20588.6	10944.6
702	618	670.1	572.3	468.3	459.6	550.5	479	1100.1	858.2	839.3
3678	4381.6	4139.3	3329.4	3299.5	4195.7	4070.2	3943.6	4590.8	6295.5	8095.7
4651.1	3727.7	5136.3	3914.5	3604.2	4038	4736.4	3986.6	5597.3	6633.4	7226.5
3698.2	2110.9	2808.8	2262.7	2079	1984.8	1777.2	1879.7	3808.7	14848.9	7582.8
3105.6	4404.3	3868.1	3214.8	2592.6	3838.1	4437.8	4388.4	6370.2	5405.7	6745.6
10469.2	4655	5341.1	4877.7	5764.1	4158.8	4252.9	4165.5	10205.4	15014.2	18130.5
3662.2	4397.5	4102.5	2606.4	2872	4412.3	5406.5	3802.4	4260.2	4685.9	6124.9
4081.6	3477.6	3810.8	2818.8	3192.3	3269.3	3406.3	2943.6	6406	8541.5	9354.7
6731.7	5255.5	7306.6	5556.9	5370.2	5323.8	4485.6	4075.8	7614.6	15449.5	12940

4860.2	5354.4	4936.2	4017.1	3436	4707.9	5320.2	4709.4	9677.5	9130	11482.5
6831.9	4978.9	5451.7	4224.9	4833.4	4193.2	4604.1	4552.8	8803.6	9025.3	10934.9
6411.3	5960	7707.5	5690	4792	5631.7	5323.2	4592.1	6203.7	8336.9	9320.5
4026.3	2719.7	3323.8	2503.2	2475.2	2400.4	2469.8	2461.6	6984.2	8510.5	12430.1
3389.6	3261.7	3958.1	3128.6	2647.6	3209.6	2934.4	3237.5	5499.5	9529.6	8759.8
4157.2	4516.7	4280.8	3083.5	2749.6	3965.3	4668.8	3937.7	7292.7	7325.2	9503.7
6436.1	4311.8	7107.1	5400.6	4355.4	4129.3	3532	3635.2	4589.2	6519.1	8323.4
4506.7	5128.3	6725.4	3924.3	3857.2	6252.9	5587.6	6063.2	5192.3	6916.2	7425.9
5841.2	4240.7	6304.1	3979.3	3543.7	3693.9	2338.1	3349.9	3714.3	4401.9	6169.3
3622.8	3623.6	4033.5	2607.8	2777.9	3385.7	3561.7	2918.5	5384.8	6571.5	9076.7
3612.1	3862.6	3872.7	2748.4	2777.4	3441.3	4154	3190.9	5269.1	6110.1	7573.7
3680.4	3687.2	3622.7	2707.8	2920.3	3384.4	4075.2	3313.3	5359.2	5986.1	7254.1
1874.7	3852.6	2719	2192.7	1948.9	2942.6	3400	2781.5	4823.1	4469.6	5372.2
226.2	239.3	239	267.9	122.6	193.5	159.6	125.3	193.3	245.2	226.1
2692.2	1998.5	2335.2	2011.5	1732.4	1910	3055.3	1867.6	4167.1	6306.5	5880.2
2400.1	3050.1	2380.8	1775.5	4040.6	2817.3	4281.9	2673.9	2539.7	3531.4	4705.9
3954.7	3281.8	3783.8	3057.6	2726.6	3154.4	3712.1	3070.1	4689.3	6551.2	6961.8
4666.6	4405.7	4831.7	3423.9	2975.3	3614.8	3616.9	3826.2	6590.5	9053.4	10222.8
3411.5	4397.8	3523.3	2540.4	1582.3	1646.7	1759.8	2229.9	2473.7	4425.2	3873.5
3186.9	2881.7	3074	2462.3	2508.8	2434.1	2856	2674.4	3771	4623.9	4751.1
3605.2	3973.2	4019.7	3580.5	3040.5	2447.7	4145.3	2493.6	3321.8	4182.3	4416.7
1007.1	1109.2	1148.5	1046.4	1044.1	1034.5	1447.6	1188.2	2178.2	14813.4	4097.3
4173.3	3769.2	3778.6	2857.1	2643.9	3266	3591.4	3872.2	9267.5	9056.6	11050.7
12517.2	16749.6	16441.8	27577.4	15143.4	32697.7	10772.7	10601.8	4268.3	5378.3	6810.1
3190.8	3682.3	3462.1	2746.4	2421.6	3041.9	3426.5	3317.6	5397.7	6796.6	7506.3
4680.7	8590.7	7114.3	10205	6716.7	16983	5965.6	14221	3762.7	2118.3	3238.9
2406.5	2848.9	2842.3	2274.5	1926	2794.7	3626.6	3466.6	4952.7	3996.3	5338.1
4599.4	6054.1	5519	5083.7	3447.8	5200.8	5919.1	5989.2	8781.1	10951.1	11316.4
9756.6	8587.1	13067.8	13715.9	6321.7	16973.8	6725.1	6864.5	4381.7	5342.1	5347.6
5966.3	6661.7	6597.6	5193.4	4641.2	5259.1	5849.7	5610.7	6451.5	6638.5	8927.5
2985.4	4468	3703.9	2793.4	2378.3	6398.6	4871.7	3271.2	5982.7	6087.1	7948.8
3262.7	3410.5	3529.2	2818.9	2363.7	3200.3	3989.1	3426.3	4331.5	4360.1	5189.8
17553.5	13450	17826.4	15233	12096.7	13516.2	10434	10262	15907.4	23131.2	25783.5

5902.8	5476.3	8262.3	4888.3	4608.1	4424.1	5036.5	4750.9	3205.2	3743.3	4731
6979.2	7229.5	6262.6	6753.5	7732.7	5573.7	7791.7	10327.7	6276.9	4099.7	6527
8007	6313.4	8776.8	6763.8	5202.5	6991.2	4927.9	4995.3	6572.4	9293.5	11211.3
6701.3	5625.6	6155	4409.3	4080.2	4800.6	6763.4	5468.1	12604.1	13295.1	15977.3
3322.9	6707.7	5157.1	3187.6	4691.5	5546	8700.1	6716.6	3997.2	6222.3	5006.6
3121.6	3122.5	3151.8	2524.1	2275.7	2834.6	3616.2	3251.7	5966.7	6980.3	8079.9
4103.4	3944.9	3805.3	2937.6	2977.6	3557.2	4373.9	3454.7	5946.7	7023.1	8304.1
9080.8	10225.6	6141.9	4941.2	15577.1	8110.3	13899.4	8116.1	5099.2	4574.1	4812
1040.8	937.3	1118.6	987.2	973	978.4	1074.7	1008.9	1534.1	1904.6	1790
2878.6	2668.1	3042.4	2235.9	1937.8	2342.9	3017.9	2982.3	5113.5	5410.9	6523
6044	11923.2	11043.1	12084	6109.8	8108	4082.2	8372.7	3254.4	3472.8	6155.2
5408.7	3142.8	3805.2	2752.7	3243	2975.3	3221.2	3253.5	6666.6	8915.1	10490
3077.3	2812.7	2998.1	2492	2372.4	2560.7	2839.9	2415.9	4762.8	5494.5	5818.3
12312.4	6886.1	9460.4	6486.8	8101.6	7788.3	6959.6	5497.3	8024.7	15968	15325.8
1714.6	9654.2	4915.5	6303.3	3664	5481.5	5485.5	8160.3	1830.2	3262.5	2423.7
1945.2	1723.5	2285.5	1398.3	1191.8	1365.8	1651	1749.7	3521.8	5647.9	5047.4
1204.4	1415	4214.9	4136.1	1842.4	4550.8	1140.4	2419.9	2667.3	2148.6	1462.6
3897.9	3010.4	3601	2570.5	2494.7	2708.3	2565.5	2742.3	6163	8296.4	9086.9
3787.4	3772.8	4707	2836.5	2832.4	4401.4	5795.5	3386.3	3839.2	4066	5807.7
4869.3	3942.7	5038.8	3713.9	3971.2	4445.7	4416.3	3130.3	3485	4998.2	5430.3
3389.1	3329.7	3496.3	2492.4	2524.8	2962.6	3652.4	3522.3	5466.1	6539.2	8322.6
11141.4	4469.6	7651.2	5918.1	4613.4	2311	2410.2	2079.5	4356.2	5533.6	7865.8
18033.4	6927.3	15403.1	10709.9	6992.7	5009.9	2549	4770.3	1692.6	1640.9	1871.6
2984.8	2517.2	3015.6	2110.2	2428.6	3006.2	3185.2	2819.8	4536.5	4453.7	5662.5
4456.4	4686.3	4821.3	3475.6	3919.6	4475	5416.8	4101	7063.6	8003.5	9926.2
3426.2	1961	2191.7	1783.4	1653.6	1547.6	1752.1	1583.8	7411.3	8159.7	7467.8
7044	5560.7	8281.8	6066.9	5876.9	6830.2	4862.7	4052.6	6251.8	9635.1	9939.7
4269.9	3248	3865.1	2731.6	2902.9	2769	2932.5	2796	5991.3	8341.8	8224.4
5705.8	4090.8	4750.8	3474.3	3587	3514	3482	4383.6	9250.5	10902.8	12381.8
5187.2	4774	4708.1	3508	3209	4380.3	5277.5	3994.7	7769	7822.3	9993.2
5747.7	5234.2	8299.6	6291.2	4821.5	4671.2	6566.7	5095.2	2722.3	1938.7	2821.9
2719.3	2523.8	2633.9	2137.6	2102.9	2511.9	3587.2	2861.4	5151.6	7721.2	7702.9
2470	5275.7	3584.5	4532	2671.5	7443.2	3697	5735.6	2271.5	2988	3208.6

3959.1	5330	4723.8	4186.2	3407.7	4658.9	5556.7	4543.8	8391.8	8197	11103.4
4784.4	3291.5	4028.1	2660.6	2861.8	2862.8	3120.3	3267.3	8066.2	9551.2	10405.9
4129.1	5001.7	5562.2	4994.8	3181.7	4680.9	4668.9	4030	4635.3	2939.7	4911.1
1861.7	1563.9	2025.6	2023.6	1389.6	1753.3	1491.3	1381	1747.9	2031.7	2523.9
3614.4	2701.1	3088.4	2063.3	2128.9	2219.8	2425.4	2710.1	6179.9	7748.9	8633.7
2889.3	2739.6	2791.5	2427.3	2322.7	2640.7	2765.5	2161.8	4340.3	5827.8	5449
4638.1	3293.9	4113.2	2614.8	2958.8	2898.1	2788.4	3103	7431.5	10536	11096.5
8462.5	14985.2	17474.9	18036.1	8523.8	27767.2	12042.1	9221.1	5838.4	6170.3	6502.4
2990.1	3399.1	3050.5	2873.1	2582.5	4060.2	4100.3	2701.2	4016.1	4023.8	5078.6
2941.1	2824.5	2974	2322	1986.3	2827.7	3318.8	3157.3	5822	5471.4	8333.5
3066.5	3226.5	3316.7	2426.4	2447.5	2958.9	3499.9	2640.5	4734.1	5423.7	6528.1
5182.4	1730.8	1809.1	1086.8	1175.7	1118.9	1540.9	2532.2	4238.5	7203.7	6063.4
5057.9	4624.6	5043.5	3823.6	2686.7	3897.4	4558.1	3770.9	7602.7	8498.2	10633.4
4610.8	2992.3	4205.5	2971.9	3480.2	3210.8	2932.8	2638.2	6159.6	7718.9	8168
2219.6	2984.9	2538.9	2461	1719.8	2548.5	2389.6	3429	2966.3	1709.2	2652.1
3684.4	2409.5	3233.5	2182.2	2365.6	2592.5	2694.3	2818.8	4784.1	6929.3	7425.3
4134.5	3184.6	3885.6	2631.1	2878.9	2775.3	2820.9	2773.1	5900.4	8674.3	9581.7
3984.3	3110.1	3504.8	2584.7	2187.3	2673.8	2682.8	3277.8	6731.3	8218.6	9315.8
3504.6	2792.7	3353.2	2265.8	2224	2580.3	3123.4	2742.5	6317.4	4863.2	8242.1
5427.1	7788.7	7741.8	7478.8	5447.3	11727.9	9318.1	7069.3	4751.8	10156.5	8749.2
3949.9	2699.5	3191.8	2112.1	2368.7	2261.4	2415.8	2468.7	4560	7098.1	7599.4
3073.1	3483.6	3463.1	3149.2	2725.2	3263.2	3690.5	3736.4	4847.8	5148.9	5718
4077	3595.5	3957.5	3345.7	2672.9	3506.9	4122.6	3591.3	6646.7	7068.6	9024.7
5597.5	10489.2	6347.2	3386.8	3107.2	14465.7	15331.8	5509.8	2132.4	2631.8	2690.6
3042.1	3747.4	3684.7	2958.2	2523.3	4094.3	4948.4	2633	3412.3	4242.1	4764.7
7318.6	3795.7	4745.1	3263.8	4031	2460.1	2560	2935	6306.7	11229.1	13393.7
3807.5	3795.8	4128.8	3406.4	2865.2	3646.8	3882.8	3103.1	7154.6	8887	9835.7
3483.1	3654.7	3394.1	3052.8	2370	3365.7	4180.2	4297	6664.3	6233.8	7131
3809.7	3506.3	3678.8	2613.4	2674.8	3092.1	3623.5	2676.2	7153.7	7271	9375.8
4097.6	4007.7	4178.2	3699.4	2789.4	4645.1	4249.4	3011.9	3645.1	5488.2	5730.7
3643.1	2726	3290.2	2300	2165.9	2344.7	2731.5	2720.2	5543.8	7976.3	7853.1
8413.1	3663.3	5318.1	4025.5	4166.4	2875.6	3313.7	2958.1	5002.4	6335.1	7756.3
20356.6	8710.2	11590.6	13452.6	9437.1	5029.8	5702.9	3536.7	3430.2	4600.1	6448.7

3731.7	4784	4290.3	4155.9	2972	4415.3	8241.2	7624.5	12727.7	4786.4	9783.9
3321	5777.3	6442.3	4823.1	3703.4	5373	4640.6	6496.5	7195.9	5679.9	6908.5
2119.5	1550	1900.3	1599.3	1273.5	1322.3	1382.2	1637.5	2874.2	6519.6	4420.6
3955.2	3269.3	3119.7	2618.7	2265.3	3897.6	4407.1	3099.4	5059	6407.3	6875.4
3652.5	5279.1	5228.5	3261.2	3305.4	7062	8488	5435.6	3761.6	5124.1	6768.1
3588.9	3387.5	3840.1	3293.6	2583.8	3114.9	2983.3	2720.1	3912.8	4334.9	5118.8
2561.7	2401	2537.3	1916.5	1586.1	1801.8	1926.9	1976.6	3170.6	3640	4533
3082.3	2906.7	3562	2425.3	2388.9	3179.2	3423.7	3314.3	3550.8	5367.5	5877.9
4845.7	2739.9	2957.6	2514.6	2906	3246.4	2390.4	2128	2554.1	3976.8	4268.9
7888	3536.2	5535.1	4929	3197.6	2502.3	3365.4	2623.5	6412.8	6776.8	9150.1
4732	4357.6	4385.3	3216.6	2699.4	3485.2	4536.1	3662.8	7634.1	8325	10198.5
3640.7	4538.6	4350.6	5072.7	3097.9	6258.3	4813	4722.6	4223.8	4840.5	5791.1
4162.9	2398	3094.7	2033.6	2135	2070.2	1776.8	1926	5170.5	7825	9829.8
3046.7	3309.2	3203.9	2235	3726	3162.2	4010.6	2640.5	3849.6	6087.1	6241.2
3765.3	3594.9	3721.2	2918.5	2816.5	3456.1	4240.1	3178.5	5284.9	7200.6	6644.9
6351.3	10711.6	9857.8	12620.2	7892	15736.5	8008	7597.6	5713.5	5158.6	6960.8
3561.9	3605.4	3452.9	2940.9	2549.4	2872.8	3483.8	2553.6	4147.7	5548.7	5184.8
6494.1	3870.9	5472.5	6536	3399.4	1592.6	2166.6	2159.7	3201	6347.4	4841.3
5620.5	5044.9	5258.5	3609.6	3221.6	4004	5364.6	4747.2	9205.7	9732.7	12072.4
2853.9	2904.7	3095.6	2415.9	1983.2	2585	2631.9	2503.7	3904.9	4355.1	5828.2
3136.6	2801	3151.9	2405.7	2210.3	2717.5	3186.3	2569.7	7164	4918.5	8823
2934	4822.7	4459.7	4332.5	3594.9	4601.3	5090.9	7100.6	2122.7	2080.4	3087.1
1715	2218.1	1926.1	1540.5	1151.2	2154	1406.8	2255.1	6031.3	2019.8	5181.4
2828.4	4868.3	3288.1	9066.5	4301.8	13131.5	4227.4	8027.2	2567.5	1694.1	2742.8
1727.7	1441.8	1571.3	1115.9	1051	1272.5	1345.8	1383.7	2623.8	2903	3150
6181.7	4905.9	6251.9	4398.8	3261.3	4400.1	5246.3	4254.2	4528.5	5290.4	6944.6
2993.2	6105.9	4528.3	6155.1	4628.6	10872.4	4245.4	10487.8	1153.4	1642.5	2063.6
7642.9	8314.3	8393.5	8002.8	5833.3	7841.5	6530.7	6255.5	7374.1	9310	9414.5
12417.2	5940.8	11241.7	8096.1	5044.4	5200.1	2425.3	4630	3826.2	2027.4	3235.7
7624.1	5754.8	10440.1	15466.3	5450.3	18969.5	3015.2	2603.1	1236.3	1420.2	2397.6
3991.1	3429.6	3859.6	2712.2	2365.4	2747.4	3086.2	3156.9	5189.4	5814.7	7391.5
2665.3	3384.3	3396	2892.1	2300.3	4581	3945.2	2947	3460.1	4068.4	4636.5
2994.3	2924.1	2626.2	2310.9	1747.4	2186.2	2090.5	2818.5	3322.4	3189.8	3612.5

3413.6	3158.3	3363.7	2919.6	2311.3	2866	3106.3	2642	3926.7	5326.6	5885.9
3595.2	2739.9	3515	2555.8	2630.5	2674.7	2288.3	2392.9	4640.4	4979.2	7016.7
4999.3	5485	6711.4	4497.3	4171.7	6976.2	6944.9	4252.3	3254.3	4401.9	6166.4
4955.6	4313.1	5349.8	4506.1	3544.9	5059.1	4505.6	4122.7	5351.7	5881.6	6713.7
3258.9	3143.3	3301.9	2440.3	2127.1	2609.7	3263.6	2320.3	4919.5	6329.4	6917.8
3422.3	2895.1	2943.7	2291.8	2304.5	2470.8	3662.6	2743.5	4653.2	5321.9	6415.5
4200.9	4056.4	4052.3	3485.2	3054.3	3890.7	4402.7	3642.6	6949.2	7516.4	8571.1
9542.4	7141.2	10249.5	8151.1	7656.7	6827.6	5454.3	4442.6	7462.8	9580.1	12139.2
7868.2	2705.7	4760.3	3233.9	3714.7	2107	2161.6	2484.2	4797.7	7635.9	7333.4
4712.4	3361.4	4164.1	2649.9	2600.3	2597.3	2786.6	3328.6	7633	10487	10933.8
3396.4	2909.5	3289.6	3018.5	2204.1	2959.1	4172.4	2787.3	2979.8	11806.1	4974.5
702	757	751.6	714.6	606.8	657.9	739.5	645.5	1093.5	5144.1	2057.1
3335	3622.4	3414.8	2528.9	2572.3	3085.7	3752.6	3033.9	4993.9	5467.9	6914.3
4180.9	2515.5	3139.7	2138.5	2533.1	2174.5	2497.5	2283.3	5522.2	6836	7709.9
9602.4	5183.3	9370.1	7399.4	5699.8	4581	3618.9	3284	3844.1	10008.2	11117.7
1706.8	2008.8	2227.3	1797	1434.8	1742.2	1905.5	1872.4	2753.2	6219.7	4369
3465.5	3009	3317.2	2670.9	2333.4	2608	2849.3	2701.2	4157.6	4955.5	5766.9
2767.2	2768.2	3070.6	2240	2577.1	3272.3	3140.7	2560.6	4695.9	6111.9	7146.4
4423.6	3357.6	3998.3	2550	2705.6	3201.8	3861.7	2717.5	4588.9	4967.7	5925.5
6031.4	5461.7	7961.2	6357.4	4378.3	5386.5	4383.5	5041.6	5590.9	7450.5	8214.2
186.2	161.3	170.5	285.5	368.1	5.1	218	267.2	305.7	73.2	217
5634.5	5083.5	5619.1	4678.6	3547.7	4577.9	4986.9	3921.5	5043.6	5503.8	6450.8
4503.7	3636.5	4774.1	4637.2	2773.1	3614	4120.5	3067.4	5206.6	5783.4	6150.1
4613.1	4858.7	5280.4	4148.7	3528.5	4569.8	5033.8	4275	5994.7	7261.1	7738.9
5079.8	3507.9	4391.1	3075.9	4099.2	2967.1	2726.9	2818.9	3166.1	5069.1	5570
2044	2682.5	3036	2556.3	2039.7	3351.2	2190.7	2989.2	2730.4	2239	2957.6
2324.1	2841.6	2746.3	2004.2	1921.9	2799.7	3886.9	1869.3	3235.4	5559.3	4488.8
2409.9	2642	2438.6	1927.6	1734.2	2026.9	2268.5	2255.6	4322.9	3977.5	5351.4
2581.5	2848.8	2874.6	1818.8	2049	1951.5	2292.3	2738.8	2953.8	3844.5	4323.1
3812.1	3653	4026.3	3116.6	2840.6	3456.7	3581.2	3026.9	5733.9	6297.5	8598.9
2239.3	1884.8	2134	1648.8	1780.5	2135.8	2325.7	2104.1	4120.8	3789.9	5010.7
3815.2	3254.5	3797.1	2928.8	2768.4	3377.3	5269.2	2498.8	5337.1	8555.2	8445
2746.8	2292.8	2602.2	2165.1	2044	2052.2	2207.6	2199.5	5161.6	6840.8	6964

3388.8	2832.1	3296.3	2359.3	2291.7	2563.1	2687.2	2685.5	5291.6	6176.5	7014.6
2727.8	2527.9	2686	2119.2	2035.6	2227.9	2572.6	2083.9	2979.6	3537.4	4340
3706.9	2874	4026	2700.1	2346.5	2121.6	2584.3	1769	2581.9	5387.8	5210.2
26078.1	9664.1	13098.6	9859.3	12176.8	11402.4	12582.8	8521.3	20825	30370.4	33688.6
4059.4	3155.8	4101.9	3680.2	2935.7	2902.7	3048.1	2592.4	4395.3	7197.4	7554.6
2118.5	4290.4	2323.8	2491.7	4844.2	7096.1	5837.2	5066.1	2218.8	3546.9	3392.4
7399.1	6082.7	5528.1	6682.8	5025.1	4066	5710.1	8410.9	5506	4862.1	3781.1
2756.6	2191.6	2328.2	1848.3	1839.6	1953.6	2367.4	2304.9	3865.9	3989	5316.2
4798.9	4717	4874.1	3940.5	3342.4	4277.8	4632.5	3697.8	7597.5	8269.9	10550.6
4815.1	3087	3858.7	2888.5	2822.5	2909.7	2999.4	2525.4	4597.2	7911.6	8490.9
2780.3	3349	2992.2	2686.4	2211.4	3385.1	9938.8	4740.1	6226.9	5538.9	5892
1869.7	2067.3	5132.9	2223.6	2984.9	2822.6	2700.1	2609.7	5166.8	7602.3	6125.3
4627.7	3806	4880.7	5272.3	3113.2	4868.3	4456.3	3808.4	4241.2	7013.1	5903.2
4913.1	5263.5	5421.9	4689.9	4041.4	4721.1	3567.4	5248.8	3540.3	4259.9	5188.8
7703	4540.2	6106.2	4169.7	4992.5	4429.4	3829.6	3423	7142.3	7106.5	12039
316.4	126.1	205.8	151.1	223.1	326.1	179.1	71.7	224.2	161.2	129
188.8	81.9	140.5	204.9	215.6	184.1	237.2	360.2	232.7	80.8	158
2912.2	2819.5	3092.2	2640.4	2174.7	2267.3	2745.8	2048.9	3911.5	6576.5	5753.5
5079.7	4723.2	6059.8	4160.8	3622.9	5272.4	5317.6	3649.5	4396.7	5658.4	7594
3979.7	2608.3	3678.3	2938.5	3021.8	3169.6	2947.6	2419.5	3552.9	5111	4361.1
2186.1	3042.6	2166.4	2166.2	1734.7	2341.9	4604.3	3322.6	5055.5	2905.2	4456.4
297.3	505.5	699.5	253.5	284.5	301.9	326.6	256.6	494	782	511.9
2673.6	1992.2	2273.2	1702.2	1595	1721.1	1918.9	1877.1	3916.6	5233.7	5587.1
3919.8	5932.1	5609.5	6624.2	3572.4	7461.3	5706.8	4542.4	4071.1	3849.2	5253.3
2453	3482.2	2931.5	2356.9	1920.3	2328.7	2473.4	2200	5780.2	10142	7592.1
6625.6	7506.3	8890.6	6625.5	5978.8	9710.2	7536.8	6849.4	10395.9	7915.7	10513.7
3477.1	2607.4	4139.3	2380.7	2379.4	2594.7	2816.9	2120.2	4450.4	7278.7	6397.8
2419.7	3016	2938	2490.9	2188.6	3429	3901.3	2863.2	3531.2	3244.6	4489.8
3201	2974.7	2296.2	2057.5	4366.9	2356.8	3684.5	2313.7	1671.7	2121.3	1972
2086.9	1746.5	2121.7	1485.1	1418	1591.2	1521.1	1661.4	3442.6	4913	5130.8
1348.7	3894.4	4063.5	3940.7	3095	8947.9	9295.7	7386	2494.4	2440.9	3720.9
3299.2	2846.8	3436.8	2537.7	2445.7	2766.6	3027.5	2385.9	3930	5983.9	6285.2
3340.3	3698.9	4021.3	2537.1	2354.7	3002.6	2876.5	2909.3	5797.4	6796	8998.9

3451.5	4722.8	5044.6	4919.6	3359.6	5367.6	7233.1	4446.3	11724.6	5671.7	5159
5860.8	6589.9	5689.1	5927.7	2454.7	6989.4	16021	7369.4	4930.8	2520.6	3917.7
4240.2	3987.1	4299.7	3158.9	3145	3528.5	5175	2984.7	5975.6	9702.1	8697.3
2146.7	2109.4	2284.1	2101.9	2111.3	2251.3	2316.4	1599.6	1755.2	2457.4	2364.9
1340.3	2831.3	1793.9	1909.7	1754.5	5027.1	6332.3	4028.5	6373.8	2191	2979.6
1076.8	2223.5	2490.6	3260.4	1578.5	3837.2	1343.4	1143.3	725.2	653.6	1126.9
5251.9	5936.8	5550.6	12001.8	3954.5	6489.1	4144.5	4018.2	3749.5	3429.9	3735.4
3540	2497.4	3028.9	2445.2	2293.4	2534.6	2634.8	2820	4136.2	5780.3	5877.4
1769.7	1836.1	1805.3	1545.7	1489.3	1569.5	2025.9	1637.2	2626	4274.1	4213.4
5168.7	3538.7	5049	3591.6	3311.5	2588.7	2838.5	2540.3	3814.9	2586.1	4967
2193.7	1816.1	2182.6	1549.9	1518.3	1484.8	1639.7	1885.5	3888.3	5454.2	5163
3670.9	4077.2	4683.4	3603.3	3299.1	7295.6	5592.7	3760.3	2375.5	2598.3	4362.1
2698.1	2110.5	2605.8	1885.7	1810.8	1932.3	2146.8	2075.3	3407.7	5018.4	5328.3
1342.4	1537.5	1807.7	1592.4	1673.6	1696.1	2086.1	1336.6	1982	7509.2	3441
3643.2	3514	4186	3502.4	3483.2	3163	3539.5	3136.8	3932.1	5834.8	5752.3
9915.6	4714.9	6275.2	4771.2	4129	3174.9	2966.8	4339.9	6167.7	11060.4	11594.4
2379.1	6134.9	3543.8	15238.1	3644.4	10305.7	4051.4	5266.5	875.5	864.8	2597.6
2078.4	3174.6	2767.2	2411.4	1802.8	2879	3847.8	3366.8	5517	6587.5	6681.3
3954	4467.2	4808.7	4216.3	3504.9	4843	4930.3	3406.9	5089.9	5969.2	6960.4
3029.7	2237.2	2761	1848.7	2410.5	2022.1	1902.4	2201.2	9151.8	6914.1	11511.9
2126.2	2161.7	2535.4	1953.4	1721.4	2087.1	2228.6	1843.9	3084.9	4051.4	4379.1
3357.2	2718.7	3016	2154.9	2107.5	2462.5	2301.3	2441.1	4779.1	6746.9	7213.9
2539.8	2610	2758.1	1928.7	1940.3	2422.6	2880.5	2166.6	3929.8	4772	5709.7
2481.3	2326	2813.4	2036	1830.1	2063.2	2356.3	1859.3	5013.9	8909.3	8426.4
8655	6254.7	6582.5	5104.5	5686.6	6679.5	9409.4	4615.5	8843.3	12977	12078.4
2038.1	1290	1294.3	1212.2	1134.3	1180.9	1364.7	920.6	2168.1	3506.1	5736.9
2314.2	2338.2	2307.6	1899.4	1557.2	2453.1	2216.7	1901.4	3648.8	3404.9	4883.4
1754.4	2400.7	1949.3	1720.8	1546.2	2025.8	3199.1	2464.7	4400.3	3767.1	5437.8
4316.4	5111.6	5251.6	3918.8	3109.5	4286.7	4444.1	4077.2	4911.4	5660.6	6764.6
1464.1	1571.6	1529.5	1351.8	1099.3	1325.3	1606.5	1492.4	3181.9	11800.7	4275.4
3110.7	3001.6	3300	2307.3	2065	2416.5	2981	2569.1	4995.7	4970.3	6147.4
5635.3	5562.2	5405.8	5618.6	2537.7	2997.9	2658.3	3467.3	2112.8	4695.3	3575.2
2350.5	2450.5	2356.9	1708.9	1852.4	2263.4	2747	2202.7	3315.8	3683	4592

4324.7	5741.9	5505.3	4833.5	3943.2	7690.1	6678.2	4602	5329.3	6996.4	7351
1003.3	1234.2	1428.8	1490.4	1263.5	2822.4	4449.5	1099.2	882.6	3554.5	1650.1
4762.7	3041.6	3929.5	2732.8	2553.8	2977.9	2399.5	2978	6442.5	8365.6	9605.7
2146.6	1724.9	2097.9	1599.1	1656.6	1595.2	1644.3	1458.7	2156.9	2780.8	3278.7
2878.7	2167.2	2810.9	2477.1	2297.9	1947.5	2211.6	1796.7	3189	6745.7	4417.7
1086	898.9	1066.4	674.6	596.7	747.6	755.2	754.3	1796.8	3318.3	2628.6
1663.2	1551.9	1626.8	1223.6	1163.7	1517.1	1846.7	1399.6	3935.6	4719.6	4770.8
4568.4	3445.1	4108.4	3162.1	2844.5	3356.1	2832.7	3323.9	7150.5	9480.3	10060.1
7407.3	7305.4	8246.7	9237.5	6788.2	9853.4	7176	10126.8	5217.2	4193.9	6883
7659.8	4918.3	8249.4	5706.4	5280.4	4388.8	3355.1	3719.2	4528.1	7167.1	8882.5
883	1074.5	1024	1014.9	866.5	888	1138.8	914.5	1044.5	1188.2	1336.7
2636.2	3916.1	3657.3	3178.6	2713.6	3325.8	3689.9	5636.6	2010.2	1943	2538
3301.5	2877.4	3256.8	2522.9	2100.9	2099.8	2614.4	2284.2	4759.9	10254.5	7322.3
2092.8	1882.1	2000.6	1787.6	1577	1807.7	1705.7	1960.3	2891	4924.6	4020
2403	2130.4	2514.6	1847.9	1640.7	2180.2	2481.4	2063.8	4024.5	4274.9	5286.7
2432	3596.5	3313.9	2993.3	2396.2	3598.9	4096.3	4108.4	5351	4748	5964.4
2473.4	2051.3	2410.9	1914.8	1635.3	1807.9	2021.4	2058.2	4054	4365.6	5495.9
8372.9	3777.6	3675.1	4329.4	7308.5	2916.7	5225.6	2589.3	1871	3599.1	1524.2
5535.5	3912	4826.5	3555.2	3870.7	3379.4	3115.2	4492.8	9714	11073.8	13143.9
9072.1	4014.6	8718.5	7498.2	6132.3	4439.5	2857.2	2247.1	3340.7	6362.6	7376.3
2886.2	2513.7	3006.4	2305.1	2043.8	2272.7	2304.3	2439.1	4928.8	5314.7	6452.8
4010.1	4615.3	5355.2	3532.4	4075.7	5052.1	3190.2	3047.4	3723.3	5878.4	7108.9
2926.9	2385.2	2499.6	2045.3	2071.5	2367	2034.4	2083.7	5120.1	4282.7	6481.5
3996.5	3351	3666.5	2558.3	3172	3137.1	2384.4	3515.2	4913.2	6231.8	5966.8
1537.4	1418.7	1628.4	1324.5	1170.3	1129.8	1397.5	996.2	2203.4	3672.7	3228.9
9829.7	5955.6	7771.9	4664.5	4753.2	2876.7	3151.7	3411.7	3152.6	3610.3	4377.6
4203.2	2893.8	3455.4	2723	2491.6	2623.7	2625.7	2279.6	4430.2	7908.6	7241.7
2070	2208.7	2247.9	1790.3	1767	2306.8	3015.1	2153.2	4068.7	4298.3	5299.7
2226.2	2505.9	2637.8	2100.7	1754.4	2610.5	3944.1	1972.4	3328.8	3871.1	4637.6
3014.9	3190.7	3409.1	2559.3	2226.3	2801.7	2710.1	2949.1	3564	4200.5	4875.7
2275	1895.5	2269.7	1656.9	1721.4	1825.3	1597.3	1609.8	3664	4661	5307.1
3528.6	4213.2	4497.5	3471.9	3154.9	4626.8	5635.5	3966	2791	3609.1	4444.9
2617.1	1882.2	3103.7	1857.5	1776.9	1856	1777.6	2029.3	3412.3	7908.2	6688.3

3460.5	2199.9	3313.6	2089.8	2227	2439.1	2537.7	2131.4	2018.8	3052.1	3123.4
3740.2	2836.3	3279.7	2520.1	2429.3	3010.2	3971.5	3070.2	6694.9	6703.8	8732.1
3411.1	4429.9	3419.5	3997.4	3411.2	3007.9	6038	5188.1	3730.3	1940.3	3181.9
3328	2776	3427.8	2863.8	2243	2655.1	2526.7	2498.2	2295.8	3290.1	3480.7
3441.3	6267.6	4083.3	5360.4	2935.8	3600.3	3460.8	3651	3426.7	3741.6	4141.4
3647.2	5332	3904.1	2621.8	1918.8	3286.7	2501.2	3588.2	2481.7	4228.2	3642.1
3844.6	4709.8	4525.9	2888.6	2258.6	3543.4	5015.6	1912	3338.2	5349	7087
2221.3	2398.3	2802.9	2671	2414.7	2556.5	2162.3	2630.5	1749.5	1443.8	2218.4
2745.5	1828.4	2570.3	1826.4	1231.2	1359	1414.4	1054.3	1649.7	2972.6	2931.1
2897	3065.3	3250.1	2444.9	2331	2828.1	3307.2	2523.8	4486.3	5143.8	6238.6
1203.5	893.1	1118	970.7	914.6	950.4	1032.5	1011	4872.6	2182	5778.6
3308	3426	3664.6	3172	2729.9	3128.4	3223.6	3035.3	5108.9	5554.4	6899.6
2827.1	4495.3	3639	3029.6	2322.3	3155.1	4629.4	4800.2	6035	5707.7	6013.4
4328.4	3746.7	4586	3142.9	2887.4	3321.4	2984.6	2584	4562.3	6352.1	7505.4
2839.3	2221.6	2592.6	2025.8	1891.6	2125.8	2618	2256.1	5399.7	13328.7	7650.3
4028.5	7005	4634.5	4206.5	3062.8	3837.5	3926.5	4912.3	6126.1	4495.8	6204.6
3031.5	2002.3	1524.6	837.7	839.7	840.4	1993.1	3000.4	4313.7	10969.3	6033
4405.7	5178.8	5542.4	3888.9	3650.9	5139.2	5190.5	4381.1	5468.4	6682.7	7556.4
2745.6	2399	2926.1	2157.4	1945	2175	2528.3	1735.4	3196.7	4212.7	4999.8
5460.3	5179.9	5912.1	5471.3	4046.3	5386.7	4185.2	4754.8	3793.4	4343.5	5938.3
2657.8	2354.1	2509.1	1965.6	1937.8	2214.3	2516.8	2372.3	4172.1	4371.6	4931.3
4135.8	3645.6	3839.8	2764.8	2620.6	3105.3	4256.3	3472	6661.6	7244	8620.2
2768.8	2764.9	2527.9	2118.6	2686.6	1449.3	2434	1805.1	6482.9	3455.2	4866.5
2052.6	3411.8	3058.8	2790.6	2177.5	3669.1	2978.6	4973.1	3293.8	3554.2	3344.7
3337.9	4897.6	4419.1	3322.4	2663.6	4775.4	5271.4	3442.3	4933.2	4009.3	4924.4
1248.4	1158.6	1258.8	1021.6	852.3	958.6	1244.9	1018.6	2672.5	6324.5	4839.8
2837.9	1988.8	2446.9	1782.6	1955.7	1832.9	2050.4	1906.6	2941	3888.5	4428.3
6231.8	4499.3	4123	3984.4	5851.9	4359.4	6547.7	8602.3	6393.9	2956.5	4154.7
3670	2967.7	3153.2	2549.4	2462.5	2892	3550	2492.4	5523.5	10254.5	7869.8
3442.1	2432.6	2968.1	2287.4	2213.1	2160.3	2043.8	1990.7	3326.1	4157.3	5248.9
2917.5	3986.6	8177.1	4762.8	3590.4	4420.1	2339.7	3648.4	2647.6	3567.8	4605.7
2303.3	2162.8	2376.6	1971.2	1769.8	2317	2177.7	2755.9	3709	3361.3	3531
2950.6	2685.8	3516.3	2661.8	2559.2	2879.1	2679.7	2210.6	3456.2	4462.4	5027.9

3133.8	2801.6	3160.2	2605	2488.8	3447.4	3663.9	3136.2	3982.4	4844.8	4566.3
4237.5	3446.4	3667.7	2485.5	2516.5	2751.4	2896	3230	7031.8	6725.3	7951.7
7047.6	4789.5	3155.4	4381.7	5116.5	3759.2	10524.7	4765.4	3621.8	2444.4	2953.1
2203.6	2018.4	2087	1638.3	1579	1859.6	2107.8	1578.5	2838.7	4846.2	3949
7925.2	6958.3	4005.1	5062.8	5482.8	5214.9	8460.3	10134.4	8835.1	3859.1	6113.7
1191.5	1300.9	1371.8	1136.5	1330.7	1711.7	1433.1	814.6	2390.7	14648.6	4659
3072	9664.8	4767.9	6328.5	5199.8	5916.6	3571.6	9957.1	2073	1502.4	2673.7
6332.3	5857.2	4941.7	4223.7	9289.4	5260.6	8475.6	5066.3	4310.5	5001.9	5847.8
1772.1	2098.2	2066.4	2242.5	1986.4	2747.3	3088.1	3206.6	5975.4	1774.3	3758.7
24776.1	9334.6	12360.6	9078.9	11346.4	10158.3	9379.4	8443.2	18557.8	26943.9	30811.4
2019.4	1897.8	1950.6	987.8	1381.8	1531.9	1898.2	1453.5	2785	3812.2	4041.5
1591.9	1810.8	1829.6	1579.5	1525.7	1962.2	2104	1700.5	2762.7	3093.1	2990.9
660	559.4	724.5	696.6	501.5	471	637.2	670.7	931.1	1741.9	1155.9
3355.5	2896.9	3651.9	2901.4	2726	3319.4	3011.3	2453.5	3086.4	3811.9	4396.3
1983.3	2011.2	2038.1	1631.2	1408.6	1939.8	2118.5	1814.7	2889.1	3495.7	4483.5
6204.7	4351.5	6283.6	4178.3	4182.7	4531.2	3117.9	3794.2	7498.6	10775.5	14019.9
3170.7	3318.3	3386.3	3759.2	2674.4	3404.3	2553.4	3339.1	3211.2	3210.6	3769.4
3832.6	2738	3148.4	2466.9	2533.9	2751.7	3607.1	3072	4948.8	5503.2	6472.5
301.6	131.3	160.4	104.4	166.8	148.3	191.7	172.1	494	526.9	501.4
2476.1	2654.4	2266	2068.1	1735.6	2533.1	2799.4	2242.8	4195	4592.9	4930
3618.3	2981.6	3384	2888.5	2154.8	2340.7	2803.5	2399	4402	5193.3	6444.3
2484.3	2824.7	3107.6	2552	1912.4	2618.4	2353.4	2402.5	3201	3327.5	4759.9
2556.4	1975.6	2271.9	1961.2	1640.2	1586.8	1895.8	1853.2	3515.9	5006.9	5163.1
9347	6185.1	4074.3	5588.4	4708.6	3416.2	5854.1	5475.9	3497.8	2849	3389.9
6160.9	10137.1	7524.3	12575	5993.9	15669.7	7219.1	12921.4	4643.2	3080.4	5169.9
4082.8	3213.1	3925.1	3009.3	2950.5	3451.6	3428.5	2719.7	4277.1	5513.8	5644.5
10316.2	5814.9	6185.3	3570.4	3593.1	2202.6	2935	2640.8	3909.1	5226.3	5865.8
79.7	61	51.4	36.8	29.8	37.3	59.9	66.1	168.7	284	181.6
5329.2	2818.9	3304.7	2018.2	2792.8	1602.8	2509.9	2902.5	3558.6	5365.4	5078.3
937.3	868.9	847.3	745.9	838.3	845.4	929.4	783.4	1645.9	1558.2	2169
3701.4	1949.9	3393.6	2688.7	2715.6	2740.4	2667.8	1856	1541.2	1464.6	2404
3776.9	1469.3	1905.7	1374.4	1549.7	1432.2	1508.1	1253.6	3105.1	4807.3	5084.1
3280.9	2532	2107.4	1830.9	2333.2	2991	4922	2622.3	1600	1800.4	2004.6

573	547	580.4	534.4	395.6	482.7	595.2	526	992.4	1259.3	1394
1986.4	1606.2	1844.1	1662	1591.6	1770.7	1904.9	1608.1	3023.3	4831.7	3957.8
153.3	92.2	131.2	115.3	113	140.1	131.1	77.6	285.8	446.5	421.3
3422.8	3148	3057.8	2322.4	2144.5	2656.2	3444.7	2617	5362.4	5672.2	7235.9
2508.2	1688	1975.7	1314.7	1883.2	1511	1897.7	1512.9	2800	3800.6	4165.1
1594.4	1680.3	1695.3	1431.5	1332.7	1838.3	2039	1884.2	2806.1	2587.6	3106.9
2517.8	1944.3	2205.5	1518.6	1593.2	1384.8	1426.8	1683.3	4100.9	5027	5765.7
2426.3	2571	2702.9	2321.7	2146.8	2868.6	3041.2	2356.8	3950	4175.7	5364.5
1946.1	2302.7	2586.1	1714.8	1677.4	2029.9	1905.6	1905.7	2802.2	4254.8	5329.1
4249.7	9005.7	2424.8	6257.7	3732.3	4915.1	1857.7	4109.5	3207.4	1398.5	4568.4
2153.2	1538.9	1694.1	1344.8	2580.3	1525.7	1431.6	1108.8	1861.2	2716.7	1829.5
4457.6	4972.4	4967.7	4200.8	3875.7	5437.9	5072.1	4671.5	6490.4	7138.4	9074.4
10630.6	5052	7651.6	5474.4	7474.8	4633.5	4095	3879.6	6070.5	7307.4	10809.5
5003.4	4751.2	3030.9	3007.1	8378.8	3743.9	7109.8	4528.5	2385.7	2882.3	2617.3
4396.8	4550.8	4526.9	5050.8	4502.1	3479.5	4584.8	5088	4016.8	2349	3944.2
2951	2075.6	2259.3	1560.6	1742.8	1737.9	2023.1	2164	4451.6	6485.9	6287.3
2748.5	2022.4	2914.2	1898.7	1738.9	2411.5	2376.4	1887.7	2275.8	2794.8	4581.1
2542.5	2838.1	2789.4	2375.4	1967	3216.2	7664.1	2181.7	2437	3923.6	3875.8
2830.6	2747.5	3143.3	2263.5	2037.9	2246.8	2570.8	2214.5	3866	6279	5826.4
4077.2	4029.4	3675.2	2849.6	2470.5	3053.6	4337.7	3728.5	4646.4	2609.3	4991.1
4550.7	3854.3	4473.3	3561.4	3513.3	3603.7	4101.5	2739.3	3657.4	4596.6	5158
2591.2	1912.5	2043.4	1577.3	1846.7	1773.9	1908.9	1779.1	2700.3	3248.3	3736.7
2050.5	2702.7	3106.4	2044.6	1817.4	2274.8	1689.9	2497.6	5203.6	5126.5	5999.8
2641.6	2151.5	2003.4	1842.9	1926.6	1635.8	1812.9	2115.1	2740	3352.2	4015.4
6297.7	11879.9	11070	13271.7	8159.1	16816.3	8739.8	8293.6	5828.5	5875.5	7713.2
2475.9	1985.6	2314.3	1379.1	1872	1840.4	2077.6	2095.1	2797.2	3335.1	4291.9
2851.4	2668.5	3924.6	2443.4	1944.7	2057	1559.3	1530.8	1344.4	1655.4	2030.6
3967.8	3308.8	3582.8	2596.4	2285	2791.8	3856.7	3025.9	6282	6958.6	8158.4
3083.6	2392.4	2470.1	1760.6	1676.3	1679.3	2555.4	1753	2677.3	2762.4	3633.6
2585.8	2890.5	3316.4	2562.4	2299.2	3052.5	2861.7	2160.8	2674.9	3936.3	4060.3
1277.8	1403.1	1436.6	1109.2	1194.4	1217.6	1435.1	1106.1	2920.2	7839.1	9287.6
2975.5	3170.2	3224.6	2618.2	2105	2622.3	2880.9	2902.1	4560.3	3556.4	5954.4
2003.3	1678	2529.6	2241.2	1652.2	2016.8	1802.9	1586.9	2329.4	6264.1	4084.5

2935.2	2052.3	2506.9	1752.2	1615.9	1912.6	1914	2042.4	4895.9	5592.9	6718.2
892.1	917.6	1392.4	1027.7	908.3	1160.6	1597.3	973	2042.3	9736	6793.7
4498	4207.1	4394.4	3094.1	2731.1	3507	4287.4	3666.1	7481.1	7533.9	9191.4
1274.2	1630.6	2583.6	1738.7	1444.9	1609.8	2171.4	1386.8	3362.4	13685.8	5863.2
3102.1	3041.5	3682.9	3129.5	2511.3	2914.7	3204.7	2707.7	4700	5506.4	5802.4
2330.7	4004.2	2072.1	2151.3	1422.3	2535.3	2620.3	3296.2	2776.5	4485.5	3452.9
1423.3	1732.5	1808.7	1294.2	1149.6	1910.7	1885.7	1518	1934.1	2218	2641.4
2670.9	2648.9	2532.7	2673.5	2073.1	2595.1	2676.5	2936	3161.1	3659.9	3655.6
6183.3	5616.6	6274.6	4675.9	4208	4523	5165	4515.7	4974.1	6602.6	7078.3
1571	1238.5	1737.6	1224.6	1047	1099.6	1273.4	1254.2	2255.2	6519.5	2767.7
4044	2916.4	3144.5	2337.3	2288.5	2224.9	2255.7	2426.7	6714.3	8643.3	8819.5
5370.4	5665.1	6722.3	8120	5186.6	5397.7	4971.1	6008.2	5371.4	3544	4711.1
2850	2558.4	3392.7	2742.7	2104.7	2260.7	2299.8	1941.9	3812.6	5652.8	6677.7
2867.9	2738	2892.4	2220.2	2798.6	1711.4	1589.7	1787	1642.7	2990.9	2850.7
1724.9	2456.4	2534.9	2271.1	1623.4	3043.6	2606.9	1662.6	1999.3	5774.4	5509.6
2566.5	2576	2777.2	2093.4	1867.3	2559.9	2504.2	2338.8	2394	3142.5	3795.4
1695	1670.5	1702.8	1542.5	1300.3	1698.1	2231.9	1586.2	2841	3045.6	3543.4
1721.5	1902.6	2063.8	1647.2	1512.1	2124.2	1940.4	1604.7	2086.2	2401.8	3060.6
1699	1764.6	1855.1	1488.9	1344.4	1550.7	2172.8	1578	2940.5	4080.9	3956
1273.8	1178.6	1470	1171.6	1018.8	1210.4	1026.6	807.5	705.4	1319.8	1331.2
2778.8	1950.9	2299.2	1527.5	1382.4	1298.4	1452.5	1722.6	3484.3	4539.3	5197.1
2163.5	2102.2	2453.4	1816.8	1443.1	1818.1	1987.1	1972.2	2640.9	4020.5	3968.4
1519.6	1744.4	2349.6	1552.6	1979.3	1649.8	1704.7	1379.3	2697.8	5305.8	5865.7
279.8	77.1	132.6	113.1	94.4	95.2	83.1	64.7	182	259.1	294.2
3054.6	2472.7	3020	2219.6	2020.7	2363	2449.2	2581.1	4946.8	5823.9	6587
2248.1	1501.9	2010.8	1185.6	1348.3	1348.4	1300.8	1256.3	2896.5	3551.7	3982.5
1021.6	1035.8	1241.6	1178.9	869	1119	1064.7	1036.9	2564.4	10168.4	8023
3006.7	4076.4	6867.7	4528.4	4093.2	5897.5	2015.9	3642.5	1857.4	4725.1	2952.6
2589.5	2738.9	2739.5	2614.7	1914.9	3087.6	3664.8	1986.8	1986.7	3568.4	3277.7
1944.2	2040.9	2416	2108.8	1505.1	1960.2	2072.9	2118.1	3138.8	3949.1	4143.9
6367.8	6204.9	4849.8	5478.8	5704.2	5285.6	7845.2	7859	3379	2389.7	3837.2
2356.5	2426.6	2633.3	1960.3	1757	2131	2186.4	2206	3275.4	4645.3	5081.9
2069.4	4278	5373.4	2363.6	2756	6310	8103.5	4603.2	1547.7	1387.2	2000

2570.4	2352.4	2550	1882	1714.1	2113.4	2338.9	2024.8	4529.3	4691.8	6008.8
2590.7	1730.9	2360.2	1597.1	1717.3	1678.7	1739	1697.8	3562.2	4915.8	5385.9
8182.5	6007.6	4605.3	6397.3	6116.7	3448.4	8114.4	8735.7	4727.1	2465.5	3939.5
3167.3	5361.3	5553.7	6651.6	3856.6	7314.2	3409.5	4058.4	2199.6	1673.8	3127.3
2613.6	2016.3	2506.3	1779.5	1875.9	1571.9	1700.9	1797.1	3968.6	5883.5	6485.1
3955.2	4225.7	3884	3173.3	3131.7	3534.8	3947.3	3559	4931	6891.6	6931.7
2632.4	2796.3	2728.6	2152.6	1986.3	2413	2715	2463.1	3736.6	4315.4	4872.2
1523	1354.8	2752.4	2635.8	1615.4	2686.3	2105	1181.1	656.6	737.3	1194
2749.7	2529.7	2622	2183.7	1834.1	2422.8	2779.3	2742	4735.4	3435.3	4757.9
2317.7	2304.8	2381.8	1919.3	1842	2542.2	2387	1913.2	2679.4	3930.7	3778
2694.5	1737	1888.6	3004.7	2215	4003.3	6425.7	1352.5	2622.1	5967.5	4394.6
4191.1	7120.4	6401.8	7979.9	3675	6615	2804.9	2885.1	1699.2	2467.6	3045.3
854.2	729.5	932.3	955.8	628.4	808.2	514	381.6	485.3	860.8	890.4
1723.3	1633	1654	1478	1405.1	1549.7	1570	1434	1830.3	1947.3	2263.2
1028.9	1168.2	1072.1	804.3	777.3	722	723.5	1120.2	2694	2241.4	2596.4
1809.9	1965.8	1935.8	1681.9	1304.7	1567.6	2187.4	1500.5	2800.7	3278.3	3487.9
2121.1	2021	2257.7	1685.8	1399.5	1791.1	2229.5	1689.8	3415.5	4180.2	4664.4
2873.3	2408.7	3482	2878.7	2102.2	3152.6	2118.6	2244.7	2226	2980.6	3227.3
234	246.5	314.3	255.6	212.9	244	223.7	200.1	270.7	413.7	413.4
2650.6	2202.3	2830.6	2070.9	2130.9	2098.8	2061.6	1885.2	2922.1	4374.4	4759.3
2031.6	1950.1	2572.6	1989.8	1894.4	2091.1	1870.7	1941.1	3983.5	4100.3	4502.9
11344.3	2824.1	8442.8	8658.5	3669.7	1860	1789.7	1224.2	1600.7	1408.1	3698.7
2435.4	2725.3	2690.6	1893.7	1861.6	2323.8	2759.2	2385.2	3793	4153.2	5328
2552.3	2754.3	2630.7	2110.1	1738.9	2125.1	2660.8	2675.7	3921.4	4375.7	4611.1
3715.5	3805.4	5169.2	4326.7	4294	5324.7	8047.9	7337.9	411.1	597.1	1235.6
3278.2	2485.3	2854.4	2295.9	1985.8	2567.5	2396.7	2061.4	4214	5732.5	6294.3
3627.7	3345.2	3634.6	2723.5	2130.9	2955.5	3358.4	2844	5276.1	5574.9	7277.8
2253	1480.9	1852.8	1514.1	1549.6	1860.2	2231	1991.2	3297.8	2411.1	3583.1
2381.9	2615.8	2514.4	1863.6	1927.1	2323.8	2784.2	2134.9	3230.7	3836.4	4522.1
3862.7	7265	6115.2	9320.1	5890	11055.4	4672	5490.7	1426.1	1332.6	2050.4
3131.6	2094.8	2361.2	1819.8	1962.2	1571.8	1410.1	1488.6	6383	4053.2	6352.2
2143.1	3083.5	2813.8	1875.7	1995.1	3167.4	5375.3	2431	2189.7	3278.5	3631.8
1667.9	1459.8	1655.3	1155.7	1257.3	1335	1498.1	1440.6	2660	3332.2	3872

3621.1	3707.7	3919.1	3211.3	2862.1	3553.7	4099.8	2909.4	4742.8	6620.5	6747.9
2017.9	2502.8	2323.1	2111.4	1818.5	2200.6	2704.4	2577.8	1781.1	1719.9	2011.7
2463.9	1847.1	2108.1	1586.1	1556.3	1773.8	1865.2	1856.8	3723.6	4172.8	4774.6
4896.7	4703.4	4564.7	6253.4	6058.3	4163.6	5022.6	5173.4	3544.3	2465	3330.5
1806.5	1957.9	1912.1	1707.1	1478.8	1873	2100.5	2129.2	3844	3284	4106.6
5503.5	7579.6	6321.7	7308.9	4360.7	9781.1	3825.1	6116.4	2091.6	1354.8	2159.2
2101.3	2360	1841.9	1229.2	1575.1	3059.3	6227.3	1883.2	1302.7	1539.6	1995.8
2335.5	1936.7	2183.2	1903.2	1640.4	1859.2	2243.1	1382.4	1909.5	3671.1	2876
3666.9	1800.6	2699	1595.3	1882	1284.6	1624.2	1975.9	2478.2	4417.3	3940.5
3478.6	2606.5	2929.5	1816.2	2005.7	2055.8	1796.7	2036.5	1826.5	5684.7	4089.2
3820	2608.6	3719.3	2770.9	2017.9	2391.9	2311	2084	2657	3697.9	4159.1
2107.2	2395.2	1927.2	1601.9	1329.6	1855.4	2596.6	1776.8	3791.6	7402	5348.5
1729.6	2091.6	1784.1	1487.6	1421.5	2008.7	2491.9	1750.7	3793.9	3489.5	3994.4
3053.7	2791.1	3198.1	2268.5	2446	2637.9	2739.9	2702.9	3795	4650.3	5889.3
1857	2502.7	1384.9	2000	1830.7	1577.4	1368	1298.4	2022.7	925.1	1302.3
2461.1	2414.9	2594.4	1918.6	2221.3	2606.5	2803.4	2002.9	3209.8	4203.4	4948.6
2034.9	1971.6	1969.6	1522.2	2248.1	1996.5	2159	1829.7	5260	3778.6	10623
4431.8	3525.7	3782.1	3500.2	2622.4	2970.5	2697.8	3722.7	6189.5	6001.4	8334.2
3257.4	2750.9	2344.8	4571.8	3313.4	2271.5	2746.4	3973.4	2254.9	1167	2028.4
1476.7	1561.9	1812.5	1443.2	1237.3	1569.1	1727.3	1767.7	1903.3	2349	2646.8
3696.2	3192.6	3668	2659.1	2575.8	2740.3	2737.8	2547.5	3586.3	4389.9	5004
3265.5	3056.7	3408.2	2642.6	2023.6	2430.4	3024.1	2527.5	4942.3	7700.4	6914.8
3956.9	3865.1	4199.5	5367.8	3765.1	4278	3113.3	2834.9	2537.3	3048.5	4357.9
1622.3	1357	1395.3	1071.8	1081.6	1214.3	1427	1443.5	3316.5	3894.3	4347.5
2316.1	2302.9	2483.3	1835.6	1527.1	1828.9	2301.6	2034.2	3848.9	4387.4	5058
10441.8	5702.6	10774	12169.6	3683.8	1595.5	3296.3	1638.8	5270	5446.7	6098.5
2449.7	1835	2162.5	1568.1	1525.6	1406.9	1419.4	1738.4	3823.5	4526.4	5620.5
100	96.1	113.9	96.3	81.8	117.8	115.1	112.5	128.1	132.3	161
2303.7	2063.9	2370.7	1799.6	1522.7	1731	1783.9	2309	4023.8	4391	4838.5
2402.1	2623.9	2413.2	2208.9	1787.7	2443.4	2561.3	2092.6	3991.4	3828.9	4916.6
2444.3	1816.8	2106.7	1631.3	1491.7	1574.6	1886.2	1954.9	3166.5	4636.1	4491.1
4675.2	2522.7	3295	3058.3	2307.2	2145.6	2705.3	2323.8	1351.6	2861.5	1932.2
2340.5	2309.4	3194.6	1829.2	2069.8	2519	2093.4	1993.5	2915.6	4360.1	3911.8

3290.5	3537.8	3852.3	3789.8	2267.6	3596.5	2399.6	2126.5	2257.1	2459.9	2913.2
3416.3	2734.6	3298.7	2336	2001.7	2915.6	2921.9	2269.6	2316.7	3007.6	3517.7
2966.6	2226	1746.5	1531.6	4445.2	2775.3	3263.8	1963.8	1423.9	2183.5	1842.6
3371.4	3280.5	4022.7	3012.6	2403.6	2952.5	3561.4	2210.1	5647.6	7914.9	7918.7
2048.9	1332.7	1682.6	1323.2	1282.6	1497.1	1592.7	1626.1	2649.4	3750	3216.7
2043.6	2271.9	2067.2	1757.3	1563.7	1993	2067.3	2007.5	2984.9	3163.3	3826.8
2678.5	1831.9	2373.7	1925.3	1554.3	2216.8	2528.4	1475.6	2184.9	3699.4	3409.3
2837.9	3822.4	3779.3	3218.5	2722.8	3775.2	4735.3	6767.5	4549.6	2902.9	5103.6
2528.5	1859.2	2390.2	1962	2003.1	2171.7	2078.4	1876.3	2331.1	3056	3606.5
913.2	2096.2	1550.6	2071	931.4	1562.2	2891	26399.2	274.3	442.5	922.4
2981.6	3896.9	3880.6	3813.1	2267.4	3363.8	3309.3	3924.6	4853.5	2983.6	4045.8
3548.5	2057	3175.8	2240.7	2212.4	2385	2289.9	2074.3	2782.4	4012.2	3666.4
1771.8	2561.6	2119.3	2024.1	1337.9	2108.7	3596.8	3366	6170.6	2992.7	4569.6
2710.3	3119.7	3095.4	3161	2206.3	2688.4	3111.1	2549.5	2574.3	3277.5	3316.5
1842.3	1585.2	1647.2	1557.6	1429.1	1512.5	1842.8	1160.6	1496.3	2709.3	2045.1
2534.2	2135	2763.1	1912.5	1712.9	2049.5	1928.8	2071.2	2860.7	3565.1	4313
2678.5	2539.5	2984.6	2094.2	2252.8	2578.1	2833.1	2129.6	3653.1	4685	5359.4
4115.6	4061.3	3918.6	3315.2	3038.7	3864	5302	3432.5	5987.5	7340.7	7873
1801.2	1501.5	1819.2	1536.2	1454.5	1609.4	1690.1	1529.7	2398.1	5038.1	3593.8
3639	3199.4	3477.2	2943.3	2641	2902	3058.8	2612.5	4745.5	5479	6500.7
2764.7	2310.9	3010.9	2443.3	1933.3	2234.7	1913.2	1588.6	2986.8	3871.7	4682.2
3690.1	3345.8	2749.6	2313.5	1985.7	2316.7	3515.9	1603.6	2751	4327.4	4222.8
9244.9	2318.5	3388.9	2077.4	3901.3	1595.5	3613.7	1314.8	1565.5	1887.7	3028.4
1892.9	2348	3448.5	2101.2	1615.9	1986.9	1751.2	1479.2	3779.2	3475.2	4155.6
4589.2	3299.7	4434.3	3437.3	3363.2	2281.2	2530.6	2615.6	1852.2	2182.6	2667.6
2890.1	2133.1	2152.5	1715.4	1621.8	1691.2	1832.3	1956.6	5357.5	6057	6740.3
2167.9	1842.3	1784.9	1309.4	1307	1531.2	1468.2	1715.6	3419.2	3225.9	3464.8
1904.4	1979.1	2017.5	1744	1579.3	1730.1	1678.8	1737	2113	1803.7	2119.5
2759.1	3555.4	2645.2	1769.9	1004.4	1702.1	1014.4	2720.2	1572.3	1691.7	2099.2
540.9	379.9	437	361.2	432.3	460	466.3	405.2	629.4	715.3	682.6
8256	3327.3	5917.9	6291.5	4553.1	4315.4	2481.9	1670.8	3718.9	3900.1	5007.5
2516.4	2179.4	2672.8	2039.5	1976.3	2139	2415.4	1877.1	3483	5556.4	5846.5
2803.3	4070	2864.7	4529.2	2395.4	4624.9	3186.1	4211.4	3442.3	2932.6	3201.8

3662.7	2832.8	3093.3	2809.9	3264.8	3433.4	3143.7	2441.6	2687.7	4027.8	3895.3
3504.5	2975	3084.5	2416.2	2446.7	2744.6	3359.5	2402.4	5167.6	9319.6	7300.6
3079.4	4207	2276.7	4434	1817.6	2693.1	2413.8	5253.2	3352.8	2832.6	2454
761.7	518	858.9	514.3	686.4	599.8	458.6	495.2	787.5	1594.1	1615.9
826.1	855.7	831.9	642.7	602.7	864	888.4	766.5	2162.4	1643.3	2232.8
2948.2	2325.6	2623.8	2147.4	2015.9	2216.1	2273.3	2425.2	3539	4114.2	4753.9
1707.8	1239.5	1427	1043.3	1170.6	1222.6	1406.2	1224.9	2319.9	2942.9	3268.4
2092.5	1085	1421.8	1388.1	1164.5	1179.8	1183.4	1152.3	7337.3	4544.7	6584.1
253.8	336.6	260.7	225.1	277.1	258.7	322.3	245.8	298	371.5	359
1751.3	2915.1	2881.3	3077.1	1985.4	3628.3	2214.2	3039.1	2737.5	2127.9	3280.2
2630.7	1498.1	2336.2	1991.9	1656.3	1593.9	1075.4	1184.5	2346.7	4585.8	4481.1
3691.5	2380.5	4339.5	2880	3367.8	3242.8	1808.3	1989	1769.9	3506.2	3153.4
4088.1	2446.5	3429.2	2560.8	2419.3	2259	2038.6	1847.8	5501.4	9919.1	8769.2
3546.6	3067.6	3250.8	2511.9	2401.5	2498.9	2390.3	2879.2	5848	7997.6	8181.2
1976.5	2063.8	2270.8	1685.4	1380.1	1744.4	2037	1769.3	3377	3611	4442.1
4065.9	3290.5	5361.5	4970.7	3063.4	6158.9	3052.8	4512.4	1271.5	1478.8	2158.6
390.8	458.2	392.5	328.7	402.2	463.2	438.2	390	692.9	803.1	944.2
3334.4	2333.2	3034.3	2083.4	1825.5	1894.1	1975.2	2258.9	3044.2	3347.5	3832
2439.2	2182.5	2334.9	1853.8	1584	2059.3	2381.8	2026.8	2939.4	4264.7	4196.7
3017.3	2569.5	2897.4	2310.8	2303.9	2132.7	2655.7	1999.7	3355.5	6138.1	5541.7
4275	3987.8	3807.3	2833.4	2426.6	3001.7	3660.7	3741.8	7205.3	7263.7	9203.8
1806	2393.9	2454.3	1883.3	1629.9	2523.5	2956	1640.3	2251.3	2669	2912.9
2432	1839.5	2616.8	1835.9	1500.6	1392	1430	1213.1	2537.1	3776	3833.5
2148.1	1918.6	2189.9	1652.5	1647.9	1872.2	2160.3	1431.3	2143.6	2603.2	3035.6
2254.1	2035.3	2165	1781.6	1687.8	1760.2	2292.8	1538.9	3844	5107.8	5412.6
4318.9	3047.9	3867.9	2700.5	2590.1	2199.1	3317.7	2610.8	3133.2	3961.7	5481.5
1104.2	1327	1256.7	924	1066	1174.4	1893	1160.2	1832.6	3664.7	2316.8
5642.5	4161.8	7766	5277.7	1933.3	3109.1	2957	3781.1	1564.7	1428.9	1424.6
2614.6	1645.9	1439.9	1138.3	1199.9	1169.5	1894.7	1090	3334.9	4904.6	6543.8
653.6	807.9	1051.9	1946.7	1501.5	9595.1	949.1	2360.8	470.3	512.7	679.2
2251.6	2981.9	4115.4	5503.7	3239	6275.9	1351	4688.1	1843.2	1590.5	2214.9
1908.3	1244.9	1477.7	1084.9	1215.9	1331.4	1375.5	1516.9	2537.9	3607.4	3145.4
1698.3	1471.4	1638.1	1035.9	889.7	1084.1	1331.4	1229.8	1381.9	2421.5	2463.9

3079.5	3426.3	3313.1	2708.3	2299.2	2934.7	3289.3	3140.3	5587.4	3886.9	6147.7
3991.2	3186.2	2131.9	1667.7	3783.6	2113.3	3933.1	2552.6	1555.1	2675.6	2050.8
1804.2	1623.9	1687	1240.8	1182.9	1452.2	1601.1	1477.5	3530.1	3016.4	4277.1
1506.4	1446.8	1853.1	1335.4	1148.9	1538.3	1474.2	1301.4	1786.7	2097.9	2716.7
7820.2	22951.4	8446.6	2248.7	3597.9	23656	11466.1	5095.4	1248.7	1243.9	1395.4
2124.9	2681.9	2744.1	1885.7	1685.4	2063.7	2232.5	2425.7	3564.6	4099.4	5373.3
1212.8	2217.7	1873.6	1105.6	1238.4	4206.2	3761.8	1702.8	1046.3	1068	1580.6
1353.9	1441.8	1605	1702.9	1507.8	1557.4	2169.7	1746.4	1820	3858.4	2383.1
2693.2	2390	2496.2	1776.5	1651.7	1992.4	2665.3	2309.9	4550.5	4694	5849.4
3583.1	2352.4	2822.4	2749.1	2329.6	2005.4	2025.2	1430.9	2787	4056.8	4461.7
2205	1666.1	1962.6	1487.5	1459.6	1356.6	1181.8	1273.7	2892.4	3099.9	4380.7
4481.5	3187	5223.4	5248	4097.7	4451.8	1970.9	1495.7	1584.6	1831	4133.1
2219.3	2220.1	2399.5	1744.9	2022.5	2237.1	2325	1838.4	2963.4	3575	4367.6
5050.5	4795.5	4874.2	3685.1	2965	3862.3	4599.7	4164	8156.4	8843.6	11383.7
2063.8	1559.4	1959.6	1450	1604.9	1622.3	1629	1407.8	4383.1	2980	5500.7
2933.9	1616.5	2821.8	1704.8	2336.7	1850	1645.9	1142.4	2232	3469.2	6205.4
5738.9	1914.6	3169.9	2608.4	2674.9	1693.2	1186	1499	2070.5	3190.9	5167
1565.6	1922.7	2329.7	1356.4	1618.2	1803.5	2138.4	1534	2219	2368.7	2713
2815.8	2107.1	2491.1	1968	1758.1	1732.3	1775.8	1704.7	3247.1	4362.4	4606.6
3536.1	2459.4	3196.8	2317.4	2717.2	2417.2	2641	2144.1	2899	3659.5	4256.5
2262.9	1984.8	2113	1442.1	1772.8	2217.5	1989.5	2133.1	4692.6	4698.7	5217.8
7030.6	2272	3923.6	2620.2	3699.5	2176.5	1654.8	1912.5	5677.2	5952.8	8182.4
2626	1861.1	2778.8	1975.4	2301.5	2433.9	1707.2	1285.1	2102	2963.2	3641.1
12325.5	6498.4	5334	4725.5	6536.6	25499.2	18102.2	9350.1	2132.2	2119.3	2419.1
1338.1	2605.5	1299.5	1500.1	1281.2	2653.6	15567.4	2557.2	540	953.3	1394.3
1839.5	1379.1	2451.4	2509.6	2409.3	3728.8	1693.7	2410.4	1091.5	2008.6	1776.9
5730.9	5086.1	5751	4708.9	3836.4	5255.4	4835.5	3574.8	4726.5	5724.3	7205.7
2111.7	2493.6	2376.7	2274.5	1782	2580.6	2791.7	2597.8	2850.8	3073.2	3570.3
9318.9	5938	4812.5	4734	5641.3	19900.5	13846.9	8621	2515.8	2528.3	2883.9
2089.4	1655.1	1780.1	1282.9	1080.7	1588.9	2426.4	1215.6	1635.4	2140.3	2422.5
1852.1	1420.6	1884.7	1260.3	1190.6	1311.5	1351.6	1421.2	2500.7	3996.7	3499.7
7915.9	8051.5	6845.1	7709.3	7934.9	11167.8	11436.4	11896.5	7789.5	3930.4	5534.2
3595.4	4130.4	3968.8	3367.7	2564.3	3935.1	4406.3	4526.7	5340	4543.5	6893.5

2523	2225.5	2637.9	2048.1	1853.7	1978.1	1973.5	1993.5	2691.1	3370.1	3540.9
2259.6	2578.9	2950.6	2799.1	1741.3	3301.7	3049.7	1860.1	3573.3	3700.1	5151
2407.9	2766	3070.2	4373.7	2267.7	3069.4	2077.1	2950.1	1234.4	1131.1	1651.7
1533.6	4002.1	2845.7	5877.3	2610.8	8468.1	2635.9	5362.5	361.8	389.3	754.3
1217.8	1000.4	1307.1	899	698.4	866.3	970.6	960.9	1130.4	1292	1570.7
3248.6	2761.3	3105.5	1968	1952.6	2144.7	2397.3	2538	5356.4	6590.4	7354.6
2681.5	2507.9	2698.6	2414	2074.7	2454.6	2905.8	2037.2	2868.1	4655.8	4102.8
2216.7	1593.3	1917.6	1380	1265	1485.9	1514.8	1608.6	3032	4268.4	4546.1
2189.6	2162	2137.9	1752.5	1641.7	1826.4	1774.5	2084	2381.2	3408.4	4059.7
1923	1334.4	1755.8	1257.4	1188.8	1284.1	1369.7	1408.5	2297.5	3100.1	3299.4
2158.4	1584.6	1806.7	1458.1	1461.2	1638.1	1688.6	1634.9	4316.2	3078.5	5275.7
1615.6	1061.8	1397.2	1047	881.9	847.2	772.1	1032.7	3462.8	3019.3	3889.2
2317.6	2241.6	2458.1	1904.1	2102.6	2642.5	2329.2	1958.8	3068	3569.8	4543.4
1640.7	2043.2	2013.4	1629.7	1251.4	1965.2	1933.2	1981.6	2504	2316.7	2961.3
1413.3	1217.3	1377.9	1037.6	1116	1158.8	1619.8	1408.8	2069.7	2382.7	2646.7
1948.9	1606.3	2071.8	1424.2	1433	1524.6	1273.7	1568	3254.4	4472.8	5532
102.4	110.3	93.9	78.5	65.6	84.5	94.9	101.2	231.2	188.9	249.1
3056.1	3560.5	3822.8	3550.7	3526.7	3759.3	3715.1	4257.8	3029.1	2229	3005.3
2452.4	2474.8	2353.4	1729.9	1588.3	2066.5	2543.9	2404.9	4308	3264.5	4802.6
1219.5	1078.1	1309.5	827.7	977.9	1246.4	1211.7	841.8	1160.6	1980.9	2570
1607.1	1416.9	1401.1	1215.6	1331.2	1517.8	1531.9	1286	3078	2893.3	3780.3
3003.5	3617.8	3591.7	2240.6	2014	3491.9	3492.7	2498.8	3510.6	3366.9	4899.6
3005.8	2103.6	2757.4	2252.4	1744.2	1790.1	1919.5	1698.4	3541.1	5613.1	5432.7
2094.3	2058.4	2220.5	1509.6	1707.8	2107.8	2134.9	1545.7	2830.4	3526.9	4397.3
7398.2	2869.2	5298.8	3308.1	3797.8	1983.8	1786.9	2369.6	1858.2	1900.8	2592.6
3335.5	3167.2	2921.5	3388	4117.1	3319.6	3880.3	5060	2518.5	1351.6	1930.3
1763.2	2740.5	3897.6	1944.7	1710.1	3043.4	3319.7	1786.2	2461.3	2631.7	2870.8
3463.4	3348	3469.1	2411.2	2036.1	2601.4	3252.3	2922.6	6470.7	6658.8	8681.5
2209.1	2781.2	2547.9	2555.4	2059.2	3155.8	2679.4	2537.4	4056.6	3116.7	4501
2803.7	2283.7	2435.1	2450.2	1968.9	2171.5	1894.6	2534.4	3713.3	2969	3895.9
1813.1	1905.7	1932.1	1501.6	1415.3	1897.5	2439.3	1768	3295.5	5834.5	4722.6
7469.6	5466	6657.7	5236	6152.2	5163.6	5175	4793.3	6984.2	9904	10022.5

3968.3	1761.7	2312.1	1880.5	1522.4	1488.1	2035.9	1073.3	1032	1244	1359.3
2986.8	3087.9	3430	2577.6	2619.8	3056.2	2677.2	2160	3856.5	4848	5356.6
1886.6	1737.8	1727.1	1639.2	1329.8	1359.2	2357.3	1446.8	2491.9	2032.2	2607.6
2155.2	2120.6	2219.2	1808.7	1462.4	1800.7	2116.3	1895.2	2592.5	2970.9	3448.7
3040.9	2752.8	3202.6	2418.8	2062.8	2375.6	2919.5	2128.4	4410.2	6070	6217
4534.7	2331.1	3216.5	2291.9	2305.7	2313.6	2455.3	2391.4	3165.4	3568.3	4420.5
1395.9	1150.9	1326.9	1148.7	1156.9	1199.9	1149.3	1052.3	1855.4	1803.8	2326.2
1804.7	1640.6	1818.6	1437.6	1298.4	1618	1759	1403.8	2051.4	2660.7	3130.3
2831.6	2418.9	2761	2032.3	1716.1	2415.9	2355.6	2453.9	3032	3246.7	4230.2
2149	2098.3	2356.5	2013.7	2017.3	2633.7	3029.7	2491.6	2508.3	3360.4	3235.2
2405.3	2024.7	2390.5	1637.5	1573.4	1760.3	1718.8	1707.9	4584.9	5810.4	6073.1
1905.5	2083.2	2189.7	1491.1	1464.9	1886.5	2334.3	1739.5	4216.5	5081.4	5435.1
1855.1	1793.9	2233.6	1578.9	1489.1	1634.8	1468.1	1251.4	2003.8	2387.3	2907.5
2079.7	1514.3	2001.4	1227.9	1372.7	1311.9	1499.5	1246.4	4263.2	4968.6	5327
5380.7	4001.1	5391.7	6130.2	5036.7	5337.8	3896.8	3351	3940.6	4915.2	5548.6
2921.4	3996.8	2686.8	2590	1946.5	2417.8	3697.4	3009.8	4820.9	5968.1	4967.8
2785.5	2396	2784.4	2151.2	1815.3	1809.9	1628	1989.4	3555.2	4337.9	5365
6589	3353.4	6574	4780.7	5845.3	3872.9	2598.1	2195	2890.6	3935.2	4747.2
1905.2	2284.8	2113.4	1634.3	1450.3	1814.7	2681	2459.3	3541.9	3544.7	4373.3
6729.6	2981	4606	2994.1	3210.7	2471.9	1284.7	2200.6	2864.7	3124.5	2921.7
3244	2146.4	2144.1	1817.4	1640.6	2127.8	2106.7	2406.7	4744.2	5504.6	5513.3
1848.3	1709.9	1845.4	1474.5	1383.8	1459.7	1421.8	1412.3	3123.6	3228.6	3945.7
2567.7	4720.4	4725.9	5341.3	2363.5	4772.4	2457.1	2971.8	1743.5	1911.4	3522.6
1703	2054.1	1957.1	1659.7	1367.7	1745.5	2287.2	1803.4	3341.4	3082.3	3970.9
1527.7	1090.6	1265.2	905.2	1051.6	1129.4	1247.7	1105	2524.7	2632	3235.2
1788.4	1545.2	2044.9	2053.6	1275.9	1573.4	1070.7	1034.3	1252.7	1394.4	1789.3
2603.2	1871.1	2276.2	1455.3	1525.2	1654.5	1661.3	1766.4	4005.2	5407.9	6035
552	568.5	463.3	363.2	362	401.3	564.9	408.2	1040.6	1350.8	1190.2
1246	1168.9	1287.2	1037.6	875.9	966.7	1027.2	958.3	2299.7	4891.8	2973
1270.1	1765	1570.4	1232.8	1208.6	2657.9	5090.2	1564.8	2929.9	4301.3	3576.6
3053.6	2273	2562.6	2373.8	2421.6	2160.6	2002.3	1950.3	2827.2	3372.6	4112
1232.8	1712.4	1382.6	964.9	934.1	1597.3	2266.2	1592.1	1341.4	1677.4	1918.2
3275.3	4082.7	3332.3	3481.8	2965.4	4296.9	4353	3586.2	3374	4401.7	4757.6

2622	2984.7	3294	2370.3	2257.3	2727.6	3016.9	2808.8	4436.3	4090.6	5557.7
3569	3856.3	4123.4	3631.1	4505.2	3057.6	4014.1	3706.8	3874.5	2347	3319.2
1883	2203.1	2243.4	1855.8	1448.8	2120.9	2218.3	1504.9	2956.2	3584.7	4245.6
4919.4	2643.8	3841.1	2505	2539.4	2238.1	1993.9	2166.7	5667.9	6576.8	7953.4
2221.9	1953.2	2208.2	1855	1960.8	2093.2	2719.6	2003.8	3431	3847.1	4058.7
2579.1	3281.7	3221.3	3410.8	1924.9	3210.9	2655.3	3722.7	2352.8	1717.2	2532.9
2414.2	2080.2	2155.4	1690.7	1361.4	1618.8	2019.4	1646.7	3157.9	4613.1	4976.2
3991.8	5922.3	4655.9	5456.6	2259.8	2197.1	1727.8	2339.1	1881.8	1833	2442
2668.1	2266	2473.6	1619	1754.2	1974.4	2282.2	1976.3	4471.5	6017.7	6496.7
5049.8	2356.8	3941.1	3873.8	2987.2	1321.4	1492.3	1290.8	1047.3	1428.1	1347.5
348.5	366.9	345.5	267.6	285.9	379.7	418.3	305.7	482.5	566.7	731
2743.6	2426.8	2509.6	1767.6	1848.5	2107.4	3160.4	2084.6	4097.1	5406.2	5224.6
1041.2	1205.2	1267.6	784.4	845.8	1328.2	1192.2	1297.8	1890.8	1798	1916.2
581.6	1058	525.5	344.9	532.5	503.6	505.3	800.5	423.5	624.2	870.9
2305.3	2782.1	2917	2061.1	2141.1	3691	3388.2	2546.7	2460.8	3030.7	3677.7
3484.4	1786	2058	1647.7	1341.7	1110.5	1279.1	1151.3	2523.5	3840.6	4154.2
3160.2	1792.9	2664.2	2608.1	2133.3	1796.8	1822.7	1899.5	3134.2	2265.5	3540
2834.5	1891.4	1902.7	1424.1	1801.4	1668.4	2102	1398.1	2320	2308.1	3241.8
5142.7	3350.6	3095.2	2591	3678.9	4306.8	5470.5	2651.8	1838.4	2597.2	2582.3
1705.4	1331.2	1698.1	1091.3	1018	1094.8	1022.8	1222	2855.9	3886	3943.4
1604.4	1738	1950.3	1326.4	1301.4	1829.4	1589.3	1663.5	2920.5	4234.8	4679.3
2609.2	1362.9	2273.1	1177.1	1491.1	946.4	1039	1147.3	2235.5	2762.9	4172.8
2794.7	1836	2276.6	1398.6	1249.1	1511	1458.4	1914.1	3804.2	4940.6	5135
5627.8	5420.4	5450.6	3894.6	3113.1	3850.8	4872.4	4568.8	8946.6	9119.9	11468.8
2202.5	2601.6	2886.1	1867.4	1836.1	3544.8	3108.3	1855.6	2236.6	3801	3497.5
1802.7	2362.6	1859.3	1629.8	1281.6	2147.5	3991.4	2486.9	3867.9	2417	3369
1563	1618.1	1947.1	1591.1	1352.6	2125.8	2276.9	1556.3	3237	15572.9	5429.5
1664.3	1424.8	2113.2	1400.2	1287.3	1346.3	1452.1	1856.4	1216.8	2254.4	2535.5
1021.3	1099.4	1123.1	914.1	735.3	1002.6	1100	1017.8	1801.9	2172.8	2463.6
1545.7	1629.2	1902.2	1688.5	1332.1	1760.8	1688.2	1698.4	2426	2810.1	3195.1
1920.8	3122.8	1513.4	2454.5	1597.3	2263	3848	3946	2993.8	1826.6	1841.7
1974.3	2325.5	2183.7	1577.6	1421	2252	2457.3	1721	2668.8	3093.6	3824.3
2374.2	1969.3	2461.1	1957.9	1581.1	1859.3	1722.2	1567.7	2686.5	3002.2	4248.3

1485.4	1249	1313.6	1069.2	1041	1244.4	1238.2	1456.8	1873.6	2389.1	2055.7
2051.6	2083.1	1993.6	1975.3	1552	2102.5	2023.1	1713.1	2630.2	2842.2	3665.6
2212.2	1318.5	1161.6	1066.7	1041.6	1100	1008.9	1417.5	2823.5	1777.4	2391.4
3359.2	3515.5	3416.5	3647	2693	2825.3	3410.9	3888.8	2150.5	1792.2	2606.1
4911.4	3663.8	4242.2	4336.8	2721.8	3020.8	3218	2808.6	4076.1	1771.9	3129.7
2477.4	1440.7	1830.9	1503	1429.5	1364.7	1294.8	1313.6	3404.2	4060.6	4719.2
2343.2	2011.9	2532.7	2124.7	1790.2	1677.5	1474.4	1856.7	2072.5	1752.3	2730.2
2894.2	1883.1	2906.6	2451.3	1203.8	1902.8	1582.3	1476.8	2740.8	4339.6	4260
2101.7	975.8	1419	1044.3	1016	803.6	1009.8	746.8	1532.6	2045.3	2151.2
3387.9	3219.2	3434	2350.8	2982	3446.9	3506.1	2506.6	4434.2	5480.9	6574.1
1351.7	1138.4	1261.2	865.8	965.3	898	913.2	1017	1881.3	2775	2907
1681.8	1206.8	1460.6	916.9	1046.6	1055.1	1162.2	1037.5	2465.5	2797.9	2955
2588.7	2510.2	2573.6	1744.9	1984.4	2700.3	3378.1	2278.6	3180.6	3945.1	4127.8
2413.3	2356.5	2377.5	1947.7	1506.4	1858.3	2138.2	1813.4	2896.5	3185.6	4154.7
1895.1	1865.3	1941.4	1592.1	1484.6	1666	2142.9	1688.5	3659.3	4427.7	4553.8
1093.6	3015.2	3618.8	9957.2	1520.9	4447.6	1325.6	9093.2	6567.9	842.7	5862.1
2747.5	2225.6	2525.5	2575.5	1892.7	2234.2	2219	1764.9	1811.2	2616.1	2673.8
1793	2394.2	1879.9	1508.8	1695.4	2378.2	2660.5	2135	2708.6	2401.4	2789.4
1290.4	1438.8	1315.5	1193.8	1011.1	1315.6	1690.9	1414.5	2102.4	2295	2613.3
3073.6	1767.8	2847.7	2190.4	1820.9	2010.4	2014.5	1558.9	2215	3958.9	3517.7
1137.4	1132.9	1099.3	1077.6	1155.3	1105.6	1402.8	1063.1	2330.4	5679	4736.3
2621.5	2527.9	2562.4	2014.6	1927.2	2339	2420.8	2175.2	2714.6	4248.1	4087.5
1953.3	1722.5	1915.2	1411.2	1474.2	2414.3	2018.5	1698.3	2539.4	2764.6	3144.9
1973.1	3533.7	2875.2	3455.1	1417.8	2138.2	1092.7	1709	701.6	1869.8	1399.5
2784.6	2176.1	2644.6	2404.4	1878.5	2159.4	1946.9	1740.4	2333.6	3225.6	3641.1
860.9	914.6	1170.4	787.6	1008.9	878.1	909.6	829.4	542.2	579	678.5
2801.6	2085.4	2090.4	1715.9	1551.5	2200.4	4035.2	1655.4	2726	2820.4	4045.6
2300	1946.5	2341	1944.8	2176.6	1900.5	1893	1397	2430	3595.6	3585
1496.5	1571.4	1833.8	1390.4	1173	1448.1	1488.7	1318.3	1882.5	2356.9	2764.1
1950.9	1996	1984.1	1614.6	1591	1757.8	1878.9	1678.5	2463.9	2875.2	3671.1
2381	2571.2	2899.8	3032.4	1748.2	3002.6	2910.4	2068	4215	4890.6	5703.4
2002.5	1519	1857.3	1863.1	1737.4	2181.6	2254.8	2151.1	3532.5	2921.3	4062.1
1457.1	1179.1	1283.2	1009.9	974.4	1084.2	1056.9	1243.5	2293.5	2264.7	2817.9

1444.6	1382.8	1428.3	1170.3	1231	1488.2	1896.7	1496.8	1811.8	2085.1	2639
2519	2510.4	1855.3	1770.1	1309	1859.6	1685.8	1649	3039.2	3289.8	4313.2
1657.8	1567.2	1710	1221.9	1062.1	1276.6	1656.5	1322.9	3021.3	3177.1	3762
2566.7	2486.3	3134	2889.2	2452	2818.1	1617	1556.1	2407.7	2168.2	3318.7
4494.1	3423.9	2573.6	3345.9	1968.4	2531.7	1491.5	5007	949.7	1175.5	1451.4
3077.9	2762.1	2786.6	2204.6	2235.9	2222.2	2217.2	2206.8	6261.9	7406.1	8758.4
2211	2035.9	1918.3	1019.5	1366.2	5829.2	3093.1	1206.7	1647.6	2057.1	2070.8
1919.4	1429.8	1675.2	1271.6	1182	1344.2	1320.9	1578.4	3497	3837.7	3769.4
2762	2409.5	2715.6	2385	2241.2	2033.1	2023.9	1717.8	3368.5	4489.3	4843.5
4054.9	4259.9	4441.7	6626.8	3914.6	6283	2306.4	4127.7	1015.7	2273.1	1632.2
1520.9	1165.8	1451	1018.9	958.3	1146.2	1076.7	1146.2	2117.8	4133.6	3065.7
1387.6	2051.4	2521.9	1739.3	1440.1	1850.1	1500.5	1959.8	1471.3	2973.5	2741
1936.2	1911.2	2139	1632.5	1515.2	2254	2305	1594.1	2254.5	3526	3097.5
2483.3	2151	2389.7	2052.4	1483.3	1713	1995.1	1681.6	2396.5	4342.8	3532.5
2450.9	1910	2151	1671.5	1304.8	1514.1	1649.6	2080.4	3436.9	4448.3	4798.6
1782.6	1589.5	2113.7	1623.6	1567	1635.1	1442.4	1216.3	1269.3	1483.4	2084.7
2176.6	1939.2	2026	1657.1	1452.3	1552.7	1630.1	1665.4	3556.3	4487.3	5168.9
1900.9	1474.3	1732.5	1407.4	1149.8	1071.1	1412.9	1250.3	1932.6	3454.5	2578.9
983.7	865.2	911.7	630.8	671.5	643.1	801.3	847.9	1841.9	1977.4	2497.5
2056.9	1927.3	2055.5	1626	1618.1	1643.1	2296.4	1659	3160.8	3936.2	3919
2382.9	2179.1	2375.3	1584.9	1560.9	2292.9	3103.3	1956	2517.5	3052	4027.7
1252.1	1306.6	1150.9	1105.3	893.1	1241.1	1236.8	1391.5	1952.5	1817.3	2255
2000.7	1870.6	1941.5	1305.4	1223	1530.5	2018.6	1501.5	3419.5	3640.1	4564.7
1976.3	1657.4	1883.7	1529.6	1427.8	1327.1	1483.9	1162.5	2400.2	2958.7	3127.2
1797.8	1551.7	1767.6	1355	1191.1	1488.5	1545	1393.6	2261.6	3296.7	3157.3
913.1	877.9	874.5	830.9	694.9	823.9	1011	847.3	2916	2318.7	3911.4
3591.3	2137.9	2697.3	2009.3	1876.5	1758.6	2019.9	1653.7	2364.3	3966.4	3775.7
1482.4	1599.3	1647.3	1292.9	1235.5	1287.6	1754.2	1359.5	2338.7	3698.6	3409.1
1839.4	1351.7	1618	1218.1	1288	1160.2	1118.6	1369.8	2079.2	2596.3	3299.9
1630.4	1201.4	1831.9	1023	1414.6	890.8	917.5	1046.9	1306.9	1287.7	1960
1858.7	1819	1656.4	1622.4	1221.9	1992.1	1429.2	1993.8	2676.6	2481.9	3279.1
4375.8	4217.8	4455.8	3795	3462.4	4025.5	4363.2	3237.1	5228.9	6280.6	6803.7
1850.9	1905.4	2005.4	1454.7	1499.8	1649.1	1966.5	1637.4	2659.6	3145.5	3815.5

3582.4	2631.4	3819.7	3062.2	2207.9	2494.6	1875.6	2195.2	2386	3410	4021.9
3717.8	3372.6	3679.3	2735.3	2347	3258.8	3881.5	3063.9	7137.5	6935.4	8584
942.2	815.6	916.6	633.5	806	899.5	1008.4	966.4	1723.4	1438.7	2008.1
1035.4	927.7	947.9	794	757.9	866.9	1116.6	801.1	1914.9	2471	2652.6
1660.7	1322.6	1800.8	1211.7	1330.9	1410.4	1586.2	1356	1627.8	1754.6	2165.8
3181.1	1676.4	2698.5	2080.8	2416.1	1848.9	1424.7	1378.3	1915.6	2188.9	3309.2
4461.1	3229.2	4331	2958.6	2323.3	2915.6	3450.1	2112.9	1904.1	2930	4704.8
1732.6	2393.9	2233.5	2026.9	1564	3223.4	2800.1	2261.2	2172	2679.8	3054.9
2440.9	1762	1846.8	1987.9	1578.8	1345.7	1442.4	754.7	1248	1171.8	1597.5
2644	1798.1	2523.3	1759.7	1815.2	1873.8	1899.5	1469.9	2601.3	4362.4	3908.5
3742	3176.9	3164.1	2502	2271.9	2802.7	3627.6	2972.1	5792.8	6026.3	7006.5
4866.6	1454.5	1426.3	1477.9	2030.4	1202.8	1477	1461.1	3738.2	5346.5	4886.2
1599.7	985.6	722.4	539.7	603.5	651.6	773.5	512.5	1642.6	1864.4	2343.9
2092.9	1533.8	1817.5	1538	1348.8	1499.9	1505.8	1710.6	2980.9	3799.2	4158.2
1710.2	1351	1558	1140.2	1044.1	994.4	1179.4	1316.1	3389.4	4720.8	4738
1620.8	2070.6	1971.6	1559.8	1153.8	1737.3	1998.5	1754.2	3054.2	3691.9	3897.5
1573	1267.7	1545.6	1079.4	1029.1	1130.1	1282.6	1183.4	2011.1	2983	2811.1
3582.7	3244.9	3419.4	2471.3	2390.3	2903.2	3743.9	2971	6179.8	6243.1	7446.5
1969.1	460.9	479.1	507.2	379.8	218.2	233.6	651.2	738.7	2091.4	1166.1
1997.3	1824.1	2365.3	1611.3	1579.7	2118.4	1636.6	1507.9	2025	3247.9	3725.3
1727.9	1153.7	1529.8	1164.8	952.3	1201.4	1241.3	1196.8	2381.1	3136	3446.9
526.1	492.8	563.9	440.6	542	520.2	671.4	511.7	544.3	826.4	1073.3
2443.1	3224.3	3138.1	3451.5	1966.7	2590.1	2321.6	3981.6	2072.8	1431	2401.8
2556	2369.2	2469.2	1397.4	1619.2	2061	3089.6	1875.9	1675.9	2280.6	2798.4
1777.1	1688.9	1886.8	1650.7	1266.6	1716.7	1765.2	1567.2	2189.7	1848.1	2816.6
1246.6	1248.5	1326.2	1095.7	1123.1	1309.4	1469.6	1007.5	1446.8	2082.9	2312.6
5297.3	2490.4	6051.1	5444.3	2534.2	2197.2	1493.4	1288.7	1547	1602.7	2168.3
2942.5	3463.4	4828.8	2687.7	3251.3	4754.9	4586	3755.1	1794.5	2956.8	4088.6
10783.7	8539.9	9537.4	10848	11435.3	8151	8154.4	12322.3	10052.8	5075	9472.5
1284.8	1475.5	2101.5	970.4	1704.9	1702.1	2496.2	1150.6	1579.8	3439.2	2157.5
2030.7	2297.2	2092.4	2419.2	1800.3	2122	1814.9	1827.5	2033.7	2437.2	2715.8
1832.8	1711.5	1991.3	1373.3	1527	1577.1	1320.4	1279.8	2550.3	3421.4	3271
3807.9	3585.9	3393.8	2668.9	2050.2	2885.2	3313.4	3161.6	6145.5	6229.7	7967.7

4271.5	2695.8	4463.7	2484.3	3888.6	1882.7	2208	2127.1	2639.7	2890.7	3702
1034.6	1322.2	1386.7	1201.7	996.2	703.8	914.5	1184.9	1548.4	7092.2	2702.8
1391.4	1692.7	1581.1	1831.4	1235	1679.2	1576.9	1277.1	2014.9	2132.7	2481.4
1365.6	3292	3077.2	3107.9	2189.9	2787.2	835.5	2189	723.1	697.9	1538.2
2202.8	1672.5	1939.6	1500.8	1077.4	1275.2	1637.3	1464.6	2949.8	4637.2	4615.2
2637.2	3154.2	3350.7	2984	2704.8	3922.7	2124.1	2744.1	2771.8	1985.7	3146
1497.8	1355.8	1496.8	1161.7	985.2	1264.4	1355.5	1252.2	1769	2372.2	2626
4642.5	4312.8	5328.6	3753.4	4245.5	4984.9	4755.7	3409.9	3933.5	5027.6	6097.6
2084.1	1853.6	2096	1616	1536.9	1649.7	1605.6	1384	2157.6	2984.2	3206.7
1021	1156.2	969.4	745.3	771	962.9	1364.9	938.8	1653.8	2497.1	1857
1530.1	2699.3	2606.7	2559.5	2212.7	3695	1823.8	4189.4	1529	2451.7	2315.7
856.6	817.4	849.7	714.1	746.3	864.4	953.9	872.3	1544.9	1411.5	2166.5
1766.9	1624.1	1788	1702.3	1217.1	2088.8	1454.4	1485.5	2404.1	2643.2	2947
5454	4520.6	5316.9	4345.8	4712.2	4605.8	4514.4	4132.3	9533.8	12348.9	12419
3100.4	2013.4	2977.3	3939.2	1351.5	1466.3	1810.5	2430.9	656.7	636.3	801.1
1275.7	1025.3	1143	850.2	866.9	794.4	1030	1081.1	2315	2943	3256.7
1432.6	1550.2	1702.8	1412.8	1164.1	1672.2	1473.4	1515	2196.7	3151.6	3582.3
3465.1	2121.1	4603	3131.1	1130.9	924	1045.8	897.6	1672.1	2243.7	3560.5
2235.7	2245	2396.3	1666.2	1646	1922.2	2139	1996.5	3615.4	4401.9	5563.1
1078.2	874.2	1013.5	798.8	844	789.3	1055.3	783.4	1286.7	3631.7	2290
985.7	1837.8	1361.3	1282.1	1085.3	1859.6	4947.1	2322.8	5108.4	2989.2	3349.2
1838	1996.6	2349	1747.9	1409.4	2051.3	2373.7	1603	1726.7	1770.2	2160.5
5579.5	5601.6	7341.7	3683	3785.6	8004.1	6940.6	3318.9	3126.2	4063.3	4373.4
2435.3	2202.8	2684.6	2033.3	1972.1	2135.7	2422.7	1750	2739	3583.6	3838.5
1423.1	1630	1532.5	1345	1129.7	1431.7	1553.7	1534.9	2532.4	2672.3	3404.9
2270.3	2275.3	2013	2443.5	2254.4	1460.1	2845.6	2613.1	1692.7	1000.6	1501.1
1141.7	1144.3	1118	863.3	868.5	922.6	1125.7	639.8	2242	2069.4	2564
1551.8	1408.3	1685.9	1232.8	1251.5	1501.2	1495.2	1319.8	1274.7	2279.1	1979.6
1632.7	1577.7	1330.8	1057.1	888.8	1149.8	1277	1452.7	2835.4	1621	2923.3
15525.2	2558.5	5262.5	3209.6	4112.6	2643.8	1554.5	2047.7	5304.1	8501.5	16285.8
1574.8	1860.4	2134.4	1882.9	1411.1	2408.1	2612.7	1810.7	2101.9	2033.8	2719
1122.2	923.5	1066.2	717.8	730.8	886.3	1003.8	895.8	2114.4	3003.5	3022.1
1158.9	1207	1124.7	877.1	820.9	1081.9	1319	1114.7	1380	1513.5	1841.7

2354.4	2612.7	2344	2069.4	1710.8	2452.2	2889.1	2284	3901.2	4475.3	4810.7
1794	1332.7	1495.1	1270.7	1257.7	1166.2	1164.9	1010.3	1931.2	2621.4	3162.3
2083.4	3022.3	3378.7	2622.2	1979.8	1788.4	1903.1	2247.2	4428.7	10688.2	5448.5
2313.7	1640.6	2239	2928.6	3923.2	2331.2	2269.4	2721	2207	999.6	1745.6
746.8	968.5	1416.1	1052.2	689.8	1118.8	813.5	1459	2641.1	2177.6	3365.7
1155	1398.3	1438.8	1396.1	1039.8	1557.5	1271.9	1526.2	1849.2	4070.1	2982.2
1189.9	986.8	1123.6	845.7	791.6	975	1018.2	963.2	2147.8	2393.8	2739.9
1443.6	1230.9	1424.6	940.1	979.3	1112.8	1142.3	1215.2	2398.7	2458.9	2870.6
2488.2	2278.5	2270.7	1754.6	1560.7	2189.3	2319.1	2070.5	3407.7	3626.2	4381.1
2027.2	1587	1786	1233	1263	1344.7	1409.3	1497.2	3421.5	3984.3	4703.6
3288.2	2905.2	3312	2437.7	2203.7	2705.7	2513.4	2407.9	3951.4	4893.1	6302.7
1410.3	1614.5	1677.2	1325.1	1168.9	1465.2	1620	1652.5	2015.5	2094.4	2145.8
1979.5	1440	1896.5	1463.3	1490.9	1512.8	1541.3	1268.4	1656.6	2150.6	2471
3177.6	5406.1	4049.3	6817.3	1652.9	1381.9	3036.4	2941	858.6	976.3	1152.3
1763.5	1376	1660.6	1249.8	1148.1	1289	1376.9	1308.5	2126.9	3136	2985.2
2742.8	2435.3	1907.1	1938.1	3198.4	1856.5	2417.7	2776.4	2571.7	1123.3	2099.2
2067.3	1948.9	2197.4	1731.5	1667.4	1736.4	1919.7	1887	2804	3714.5	3980
1952.6	1799.3	1880.1	1773.8	1133.8	1484.7	1539.7	1940	4752	6662.5	5694.7
2051	1625	1901.4	1459.4	1357.3	1260.2	1415.1	1103.6	2793.4	2777.2	4219.1
1946.2	1682.2	2290.4	2261.3	1548.7	1619.2	1586.7	1725.3	2340.4	2207.1	3135.1
2526	2451.1	2715.7	1923.3	1842.5	2053.1	2002.5	2036.1	2905.6	3398.4	4637.2
1983.9	1709.3	1801.3	1250.5	1120.3	1520.3	1790.9	1526.6	2882.9	3108.4	3923
1505.8	1699.8	1434.3	1150.2	1234.1	1373.9	2040	1508.1	2632.6	3151.6	3170.2
1102.5	1871.9	2668.8	618.5	986.8	2026.8	4174.7	2210.4	592.9	602.7	783
1191.7	3988.3	2509.4	2869.7	2473.7	9492.8	5352.5	7269.3	300.6	489.6	837.3
3880.1	2947.1	5068	12568.2	1474.6	790.6	1362	1771.5	1489.1	2157.5	2387.1
1608.3	1515.8	1718.6	1334	1217.7	1306.9	1251.6	1217.7	2263.9	3077.9	3425.3
2170.7	1456.8	1873.8	1317.7	1317.6	1429.2	1405.4	1348.5	3429.5	3931.5	4791.4
1822.1	1846.3	1978.3	1391.4	1309.7	1845	1390.4	1892.6	1074.3	1239.3	1234.6
1350.2	1594.3	1881.1	2292.6	1240.3	2091.7	1438	1510.5	2864.1	1120.8	2314.9
2065.4	1768.6	3050.5	2214.9	1743.1	2317.9	1448.4	1575.5	1613.5	1963.7	2500.3
1007.2	878.6	992.7	741.2	741.1	849.7	955.7	947.7	1723.4	2411.9	2434
2854.5	1989.6	2363.1	1805.5	1468.3	1800	1984.1	1595.2	2508.7	4088.3	4294.4

1210.9	1120.2	1594.6	1417.5	631.1	570.4	702.9	704.1	4297.9	4311.5	6779.2
1294.5	1465.5	3245.2	1741.3	1588.2	1347	1738.9	1538.1	1906.3	4822.9	3525
2814.2	2405.6	2633.7	1882	2046.6	2436	2227.1	2294	4329.4	5689	5977.8
2030.4	1736.7	1461.5	1427.1	1200.8	1250.3	1227.1	1212.7	1463.7	1937.8	2265.1
1732.5	1944.5	1798.3	1523.8	1429.9	1762.2	2008.4	1594.3	2839.6	3347.8	3680.9
1797.4	1610.5	1909.7	1421.8	1629.4	1823.6	1543.1	1391.6	2055.4	2640.7	3080.8
4223.4	1763.1	2093	1792.3	1983.2	1488.5	1286.2	1047.9	4969.1	5823.7	5653.7
1016	1580.1	1739	875.8	735.4	2029.9	2818.1	1332.8	987.4	1542.5	1845.8
1563.7	3090.9	2020.8	2932.4	1331.1	2819.9	1948.5	2800	1622.7	2046.7	1962.4
1064.5	891.4	1073.8	721.1	797.2	858.1	749.3	690.3	1241.3	1965.8	2196.9
1522.6	883.2	1338.4	926	882.4	739.9	854.1	357	1549.2	2938.6	3141.6
2412.5	2457	2531.1	1729.6	1546.5	1869.7	2375.5	1917.3	3989.6	4380.3	5133.3
3164.4	1979.2	2822.5	2009.3	2288	1939.8	1606.1	1688.8	3862.6	4695.2	6453.6
1277	1787.3	1472.9	1552.2	842.4	1100.2	1323.4	1400.6	1954.1	2319.5	2893.1
1249.4	910	1030.9	814.1	816.5	919.9	1005.7	925.1	3874	2532.2	3856.1
1935.6	1870.3	1864.1	1401.2	1401.1	1593.6	1924.8	1734.9	2629.8	2776.1	3543.3
1843.8	1239.5	1455.9	1213.4	1510.1	1553.6	1756.7	1683.1	2117.7	2730.5	2905.5
1306.9	952	1127.2	796.6	886.6	879.1	967.1	957.7	2189	2578.9	2875.5
2150.7	2550.9	2058	1619.5	1659.7	3004.6	3355.5	2061.5	1647.4	2206.7	2915.9
1261	1075.3	1351.4	1099.5	988.4	1081	878.2	896.3	1411.7	1822.2	2046.7
615.6	718.1	606.4	591.5	571.5	756	616.8	679.8	1429.9	986	1686.5
813.7	744.3	828.4	683.1	644	723.9	868.7	701	1278	1332.4	1555.8
1076.3	530.5	754.6	546.4	495.3	472.8	400.8	425	1076.5	1592	1854.6
2314.8	1591.9	1978.7	1478.5	1514.1	1821.9	1642.9	1410	3056.6	3939.5	4672.3
1211.6	1304.1	1367.8	896.9	644.6	1406.8	1496.4	1022.6	1368.2	2005.5	1487.6
1433.7	1358.7	1441.9	1050.6	1638.8	1328.2	1470.6	1083.5	1859.3	6143	2848.9
1302	1173	1138.9	1083.3	996.9	1108.4	1114.8	1039.7	4733.9	3531.4	3061.7
1456.1	1448.4	1335.4	1189.6	1095.3	1117.8	1216	1365.2	2583.7	2137.8	2730.3
3302.7	3477	3685.5	3927.8	3041.6	6637.7	2749.7	3252.8	1822.4	1456.1	2544.5
56.2	81.2	90.6	62.3	81.9	70.5	96.1	67.9	193.4	142.3	197
1927.5	1758.7	1834.8	1392.8	1317.6	1651.9	1976	1645.9	2660.5	3390.5	3684.6
1716.7	1306.8	1652.4	1037.4	1279.5	1145.4	1310.3	1308.5	2681.8	3030.2	3433.2
2090.7	1198.6	1560.5	1154.7	1276.9	1203.7	939.5	1098.9	2479.2	3453.8	4214.4

3889.3	2864.1	2412.7	2686.2	3478.6	2083.5	3710.4	4169.1	3365.3	1599.3	2332
2178.3	1840	2173.9	1715.9	1568.1	2061.8	1658.2	1712.6	1825.9	2144.8	2474.3
1139.7	1407.2	1117.4	1027.4	1133.9	1305	1595.4	1178.5	1875.8	1996	2414.9
1548.3	1588.7	1647.4	1288.1	1116.6	1362.9	1719.9	1261	2407.4	2926.5	2942.5
1825.9	1641.8	1674.8	1216.2	1097.2	1347.6	1881.2	1431.7	2906.1	3154.8	4300.3
1190.8	1608.7	1704.1	1154.4	1162.7	1320.4	1701.6	1398.5	1224.3	2393.5	1661.8
518.3	591	642.9	524.1	402.9	491.4	532.1	458.5	621.5	590.9	759.1
4593.3	4005.8	4010	2917.9	2529	3431.4	4039.9	3361.4	6896.8	6918.3	8535.3
2599	1859.9	2276.3	1807.5	1787.4	1792.7	1884.4	2001.6	4300.3	4756.9	6476.2
4779.1	2342.3	3943.3	2605.6	2726.7	2931.7	2638.1	1390.5	1900.4	1946.9	2689
1309.9	1713.8	1519.2	1252.1	1180	1364.3	2244.4	1578.2	2792.8	2951.9	3286.2
959.2	911.1	1084	840.5	803.2	813.3	823.5	836.2	1265	1149.6	1355.5
714	849.2	677.3	707.3	660.7	635.5	740.3	673.9	1601.1	2083.8	2074.1
1687.2	1716.2	2425.2	1307.4	1697.5	2275.2	2318.5	1624.1	986.4	1321.4	1582.1
5012.7	4722.6	6206.8	5144.7	4460.2	5445.2	4101.6	3899.5	4723.4	5864.5	6748
2536.3	2812.9	3439.4	2758.4	2169.8	3062.4	2666.6	2146.2	3599.4	6201.4	5773.6
1213.3	1688.4	1569.8	1091.2	1082.2	1557	1610.8	1728	1686.3	1364.7	1910.9
1022.3	1171.5	1272.4	1091.6	860.2	1474.4	1613.9	1055.3	1397.4	3223.2	2232.2
1707.4	1591.7	1781.4	1533.9	1202.4	1545.9	1565.2	1706	1868.5	1608.5	2217.3
1387	1015.8	1185.4	901	971.8	1033.4	1068.2	984.8	2252.9	2710.5	3063.4
1426.8	2009.7	1991.3	1859	1334.4	2413.6	1586.8	2117.1	1611.9	3121.8	2059.9
374.7	280.3	293.9	276.4	327.2	348.4	305.7	290.9	1073.8	450.2	1002.6
1718.2	1311	1703.3	1195.5	1236.5	1396.2	1310.4	1267.9	2429.4	3339.2	3449.6
1502.8	1200.7	1339.1	1122.5	1079.5	1179	1370.6	1219	2330	2471.5	2802.2
3806.8	2906	3509	2216	2332.9	2344	2651.5	2470	5943.4	8194.7	8545.1
1777.6	1450.4	1657.1	1456.9	1124.4	1443.9	2044.8	1521.1	1747.9	6128.7	2646.7
2383.8	2108.7	2685	2075.7	1603.7	1858.7	1785.7	1802.1	1990.8	2176.2	2895.9
1315.9	1042.3	1110.7	899.2	968.3	1043.7	1773.1	976.2	1512.9	4174.7	2011.5
1446.6	2943.1	2756.6	3062	1621.9	2986.4	2560.1	4006.7	3594.5	958.1	2259.6
1491.4	1318.4	1457.3	1128.6	1108.7	1303.5	1257	1009.9	1834.7	1968.9	2679.9
1574.1	1584.4	2033.4	1507.4	1244.3	1330.1	1397.2	1242.9	2370.8	4034.7	4050.2
1014	971.1	1050.4	887.1	698.9	1014.4	823.2	825	1355.6	1613.7	1756.2
2036.2	1246.6	1592.8	1192.2	1217.6	1220.3	1168.1	1236.3	2128.8	2649.2	3019.9

1569.2	1852	1941.5	1524.7	1271.3	1870.9	1706	1560.1	2418.7	2287.4	3156.1
946.3	850.3	1088.2	970.5	614.7	839.9	1180	796	1765.9	2512.8	1973.3
1100	955.5	1070.5	971.2	832.7	908.5	717.8	909.7	1621.5	1473.4	1861.2
1736.3	1482.1	1950.7	1505.1	1173.4	1302.9	1507.3	1307.8	1678.8	2284.2	2410.5
3871	1794.4	1154.8	1797.8	2098.3	1251	3522.9	1876.6	1732.5	1680.2	1910.4
4230.5	2134.8	2495.2	1975.9	2469	1800.7	1899.7	1953.9	4377.9	6592	7061.1
1932.9	920.1	1271.1	888.6	626.5	591.8	580.9	734	889.3	575.8	939.4
1841.6	1771.6	1924	1381.3	1283.5	1656.7	1862.1	1546.7	2918.4	3162.7	3865
2340.1	2015.2	2194.6	1867.8	1404.3	1107.3	859.3	1315.1	1154.5	1396	1516.2
1729.8	1479.6	1663.2	1685.5	1238.6	1252.4	1236.5	1908.7	2256.3	1869.4	2125.7
1707	1455.5	1673.5	1432.9	1299.1	1505.9	1339.8	1262.1	2207.5	2793.2	3229.5
2410.1	2188.2	2136.5	1654.7	1385.7	2004.2	2258.3	1842	3143.1	3335.7	4168
2016.3	1796.9	2114.7	1691.8	1586.6	1590.4	1453.8	1492.9	1766.3	2015.9	2333.7
1715.3	1414.7	1613.3	1252.7	1123.6	1342.8	1331.1	1659.2	2650.1	2968	3536.7
479.2	487.2	598	572.1	411.2	546.8	526.7	439.3	953.3	5759	1796.8
915.3	957.5	804	756.1	665.4	695.8	1110.9	839.8	1024	1426.7	1396.3
3384.9	2720.4	3105.6	2291	2527.6	2201.4	2228.4	2280.6	4178.7	6112.8	7742.6
1242.9	1230.4	1427.7	1163.2	982.9	1165.1	1419.9	1110.2	1940.6	2319.9	2518.6
1687.6	1275.7	1603.6	1194.7	1190.3	1704.5	1495.1	1331.1	2509	3568	3460
2377.2	2801.8	2739.6	2500.7	1589.2	1913	2525.9	2707	3003.7	4562.6	4951.6
1255	1294.9	1407.3	1149.2	915.2	1311.8	1668.8	878.6	2927.4	2597	6246.8
2759.2	1976.3	2226.3	2417.8	2104.9	1594.7	1688.3	2077	3377.8	2441.2	3299.9
2433.8	1656.6	2017.8	1392.2	1435.4	1381.9	1323.7	1957.1	3904.2	4047.7	4902.4
2203.3	1457.7	1684.4	1230.7	1084.6	1043.3	1008.3	1084.9	3135.1	4220.5	4941.3
3068.7	2244.1	1109.6	1073.2	1081.4	1023.2	1114.9	831	2513.2	1076.6	1566.2
1595.8	2094.6	1695.4	1722.2	1298.9	1229	2819.1	2271.7	1228.6	893.6	1315.6
1331.8	1380.6	1346.4	1184.4	984.3	1378.2	1439.5	1326.6	2815	2688.7	3404
2236.6	2196.6	2190.4	1830.7	1563.4	1839.1	2058.8	2086.8	3881.4	3530.2	4059.6
2398.3	1642.7	3092.2	1792.1	1799.6	1427.5	1235.6	1596.6	1386	1516.7	1848.6
1370.7	1358.5	1334.7	921.6	864.6	1714.2	2799.7	1272.2	1271.1	2108.5	2226.7
1662.8	1760.8	1915.3	1374.8	1426.8	1666.8	1875.1	1339.2	2799	2842.5	4241.8
1340.8	1002.8	1090.8	821	738	849.5	786.1	1009.8	1736.3	2478.1	2588.6
2279.3	1438.1	1686	1490	1190	1814.3	1253.5	2465.3	2844.4	1323.1	2403.3

2896	2975.4	4621.1	3166.7	1342.4	1176.1	1004.6	851.3	674.6	822.3	1680.7
1643.6	891.9	1251.7	811.7	964.8	787.8	838.7	693.3	1485.3	1358.5	1768.6
2560.5	2059.5	2421.6	2096.4	1920.7	1922.4	1953.1	1887	2366.5	2730.6	3169.7
3283.6	2989.7	3060.3	2297.9	2355.3	2424.4	2040.3	2487	4347.1	4730.9	5897.5
1449.9	1475.4	1400.7	1176	1218.9	1520.8	1544.2	1259	2189.4	2073.7	2852.5
2097.3	1784	2087.3	1530.6	1563.9	1721.6	1695.2	1575.8	2483.6	2929.8	3420
1175	1028.2	1143.1	868.9	877.5	1061.6	1156.5	1008.8	1845.1	2234.7	2384.3
1530.7	1122.6	1492.8	1311.9	1274.6	1145.9	1135.1	1200.4	1709.4	1831.7	2472.2
1981.5	1556.8	1732.4	1479.2	1216.1	1415.5	2005.1	1551.1	2491.4	3330.2	3361.7
2546.2	2524.9	2798.3	2233.5	1902.5	2410.5	2072.8	1957.1	2840	3672.8	4137.4
4804.4	4366	4185.6	3328.7	2850.3	3250.6	3850.6	3733.9	7144.8	7461.1	9062.9
1937.6	1759.2	1762.7	1278.7	1109.4	1404	1211	1473.3	3509.8	4874.1	4889.2
2442.7	2568	2645.2	2276.1	2132	2958.4	2451.6	2065.9	2610.9	3257.7	3755.3
1634.8	1665	1707.1	1518.5	1151.1	1552.3	1797.7	1473.7	2860.2	3088	3732.6
1389.9	1169.8	1428.6	1106.5	1041.3	1355	1553.1	1080.9	1644.7	1650.6	2096.6
861.8	891.9	948.9	716.8	745.7	849.5	843.7	813.2	1353.3	1328.9	2195.4
1476.1	1618.4	1431.4	1154.7	977	1378.5	1508.1	1335.4	2676	1949.5	1947.1
948.2	996.4	955.5	988.7	864.5	860.1	1387	950.8	3242.8	5312.9	4761.1
1251.6	1238.7	1162.5	1035.6	1063.4	1066.4	1235.4	949.1	1706.3	2095.2	2549.6
1159.3	639	791.9	576.9	665.9	718.1	699.9	729.3	4058.4	2365.3	3889.1
1651.5	1324.6	1546.9	1262.2	1122.1	1139.2	1364.1	1501.6	1752	2405.7	2474.3
1813.7	1837.1	1987.2	1574.3	1222.4	1621.5	1598.8	1636.9	2913.2	3416.2	3993.1
1722.7	1910.5	1887.4	1437.8	1552.2	1693.3	1807.4	1575.4	2819.6	3582.1	4374.7
2096.1	1659.8	2626.8	1419.9	1623.1	2110.9	1708.6	1251.2	2040.8	3314.4	3870.1
1499.3	1775.8	1666	1493.1	1282.4	1798.2	1998.5	2093.1	2784.7	2353.3	3535.8
1112.9	740.1	971.7	850.2	973.4	987.8	1343.8	1131.1	1671.4	1702.6	2127.4
2368.2	1615.9	2184.1	1485.3	1694.7	1735.2	1465	1440.4	1345.8	1494.9	2121.1
2227	2087.8	2219.8	1664.2	1387.5	1720.7	1649.3	1761.2	3143	3413.9	4365.9
2100.6	2281.8	2344.9	1745.8	1826.8	2008.4	2759.8	1937.3	3438.7	6348.7	5263.7
2155.2	2184.7	1503.1	1652	1101.3	1118.5	1937	1952.2	1654.8	928.1	1670.1
1675.3	1559.3	1750.8	1239.7	1456.2	1467.1	1546.7	1263.3	1986.6	2602	2892.2
1406.6	1271.1	1375	1085.9	1152.8	1377.4	1252.1	1026.4	1356.2	1490	1702.5
1582.1	1210.2	1540.8	1060.6	908.7	1103.6	1127.3	986.5	1478.4	2152.5	2073.7

2742.9	2341	2356.8	2240.2	2721.4	1622.5	3665.8	3873.3	1859.7	1090.2	1942.6
62.1	74.8	57	45.3	47.9	47.2	104.9	64.6	38.5	45	63.2
2867.1	2442.3	2567.6	1760.2	2258.7	1950.3	2383.4	1705.7	3460.5	5100.8	5269.5
2207.8	1950.7	2175.8	1816.3	1275.9	1422.5	1149	1768.2	2116.1	3479.1	3393.9
1920.9	1951.1	2095.6	1867	1584.2	2295.9	2082.3	1770.2	1738	2264.8	2448.9
1517	1343	1565.6	1120.5	1270.9	1215.6	1449.9	1096.4	2480.9	2687.9	3214.5
1923	1810.4	2225	1460.9	1222.4	1650.5	1364.6	1292.9	2627.4	2645.4	3441.6
1164.9	1541.6	1472.4	1199.2	1082.2	1502.4	1750.5	1498.2	2603.9	2078.2	2876.9
1135.3	1458.3	1620.3	1481.7	1077.1	1558.1	1386.6	1312.6	1738.7	2201.3	2257.4
735.9	1292	970	1078.9	633.7	998.9	1760.6	1849.3	2071	881.6	1689.4
2601.5	2430.5	2757.3	2268.5	2008.4	2409	2405.2	2367.3	3127.5	3585.6	3972.7
511.2	447.6	448.6	410	407.9	374.6	626.9	425.8	679.4	1510.6	905
1398.1	1259.8	1448.7	1046.3	1127.2	1234.5	1384.2	1137.4	1412.9	1881.6	1913
1098.9	1262.1	1269.5	871.7	914.3	1025.5	1156.6	1310.5	2368.8	3270.3	2806.8
1251.2	2056.4	1856	1698.4	1474.4	2196.8	2055	1704.8	2195.3	1639.3	2719.1
1549.6	1500.3	1590.3	1354.4	1085.9	1621	1459.1	1425.9	2194.3	1915	2988.6
1597	1243.4	1551.5	1196.2	1340.4	1288.9	1454.6	1125	2205.7	3125.8	3421.8
1178	898.9	1104.3	746.5	685	782.8	837	807.5	1887.6	2544.7	2920.5
590.5	835.9	443	714.4	371.1	769.9	731.2	421.5	835.4	1039.3	1089.9
984.9	837.9	924.2	721	592.3	719.5	759	865.2	1807.6	2100.5	2382.1
1506.3	1445.5	1599.4	1254.3	1268.7	1420.2	1560.1	1012.7	2164.3	2961	2983.2
1199.3	944	1177.6	800.3	721.5	890.1	857.9	974	1598.5	3278	1990
1023.5	742.7	979.2	722.8	732.8	763.5	791.5	607.6	2212.7	3625.1	3505.2
2232.1	2921	2573.7	2325.4	1960.6	2549.6	2381.9	2472.5	4939.9	5091.7	5575
1649.8	1758.3	1664.3	1343.6	1213.2	1573	1763.3	1660.5	2297.8	2402.6	2727.9
1637.7	2240.4	1887.2	1922.4	1530	2067.8	2142	1676.4	2436.6	2354.4	3071.6
931.9	920.2	974.2	806.5	1116.4	846.1	1031.2	768.5	1026.8	1702	1666.3
1893.9	1421.2	1586.9	1133.2	1289.6	1227.2	1432.1	1267.9	3029.2	3923.4	4344.6
1581.3	1116.6	1489.2	928	1037.8	1019.8	917.2	1028.4	2598.1	3622.6	3674.3
2583.2	2297.6	2626.6	1770.9	1438.5	1842.7	2336.5	2177.7	4569.4	4710.2	5869.8
1262.4	1666.2	1560.8	1392.4	1116.4	1482.8	1827.8	1768.5	2691.8	3045.3	3516.1
1746.3	1590.2	1216.2	1286.4	1222.1	1084.6	1654.6	1140.8	1962.4	2138.6	2496
639.6	582.3	737.5	600	518.5	704	597.3	560.7	526.8	615.2	824.2

518.2	476.3	506.7	369.7	389.9	505.3	578.4	400.2	539.5	789	917
1448.4	1276.2	1536.6	1482.5	1321.6	1258.1	1116.8	1299.4	2919.2	2275	3756.5
1808.3	1436.2	1978.5	1548.7	1440.6	1424.3	1257.1	1246.1	1890.3	2440.1	2720.3
995.7	865.1	1093.1	965.9	859.8	1213.1	1392.8	1097.5	1243	1334.6	1212.9
398.6	360.8	429.9	465.7	434.2	483.9	418	372.4	993.2	2430.5	1438.4
1284.3	1273.5	1498.8	1063.9	819.2	958	1120.8	1051.3	1570	2573.3	2392.1
1838	1866.9	2321.1	1588.5	1389.7	2125.5	2447.8	1931.3	2524.4	2531.9	3630.8
1313.3	1396.9	1491.9	1133.1	1049.3	1220.5	1666	1525.5	2408	2029.3	2203.1
3266.3	2887.7	3026.6	1912	1925	2299	2907.4	2722.7	5346.9	5333.1	6765.1
4585.8	3419.4	4183.5	3203.4	2914.5	2544.9	2775.4	1960.7	2021.4	2806.8	3133.8
2965	1027.6	1846.5	1380.1	1404.2	1241.3	1234.6	1124.9	1780	2385.6	2722
65.2	70.4	78.9	51.6	46	56.8	63.2	51.6	57.1	89.3	73.6
1088.3	961.5	1125.8	791.5	805	1022.8	923.7	735.2	974.9	896.2	1194
1523.1	2519.6	2573.4	2197.1	1558.7	3540.1	3209	2037.9	2280	2758.4	3147.7
887.2	581.3	692	498.4	628.1	719	688.5	650.7	2916.7	1854.4	3299.4
1002	929.1	1056.3	786.1	862.9	919.2	967.4	901.4	1280.4	1702.9	1960.8
964.9	867.4	833	627	811	818.3	1243.8	703.1	1691.1	3226.7	2483.6
593.2	587.4	485.3	413	397.8	593.7	473.6	402.5	2836.9	1451.4	7016.3
924.6	693.6	898.1	677.7	729.4	638.5	776.5	567.5	915	1202.7	1268.1
1032.4	1100.6	1086.3	1024.5	810.8	954.7	1120.9	1038.3	1745.3	2017.9	2376.2
1260	1175.2	1298.9	965.3	856.2	1141.5	1150.7	1063.8	1363.3	1781.9	1964.4
1737.3	1863.4	1797.8	1291.5	1045.8	1249.6	1932	1196.1	2573.8	3719	4167.8
2113.9	1668.1	1857.6	1450.5	1297	1446.4	1702.2	1574.4	3506.7	4950.5	4937.4
2196.4	1907.5	2833.5	2366.2	1809.4	2163.9	1686	1483.3	2188.7	3023.4	4132.7
2305.7	1528.8	1979.4	1487.4	1424.3	1504	1307.5	1381.9	4212.8	4728.7	5842.6
1636.1	1946.1	1623.2	1517.3	1357.2	1814	1749.3	1870.7	2442.3	2707.8	3441
4995.7	3036.5	5136.3	3127.9	4001.1	2434.2	2520.3	2137.1	1923.4	2354.9	2999.9
1879.7	1474.3	2129.1	1464.4	1377.1	1328.9	1196	969	1615.4	2367.1	1845.4
1696	1331.6	1372.5	1052.3	1250.7	1296.4	1545	1418.6	2518.5	3403.7	3115
1163.1	935.7	1415.5	814.4	881.7	885.4	1050.4	885.3	1805.8	2390.1	2634.9
2115.4	1924.8	2088.7	1574	1611.6	1791	2357.9	1959.2	3525.9	3989.5	4470
2101.4	1975.7	2264	1732.9	1638.6	1632.3	1870.1	2324.6	2496.3	2989.8	3380.5
779.2	857.7	812.2	692	836.2	850.5	1053.3	848.9	1496.1	2630.5	2165.3

830.6	1026.1	1093.2	815.9	647.1	978.5	1012.4	959.9	1687	1748.2	2410.5
750.2	917.2	887.4	713.2	665.5	1005.3	1162.4	981.5	1493.6	1972.4	1588.7
5072.9	1907.4	3610.5	2831.6	2365.4	2327.3	1554.6	1893.6	1963.2	2553.9	3630.4
647.2	701.9	691.2	600.1	504.3	603.9	706.4	506.6	865.9	1212.6	1315.1
2529	1652.7	2143.7	1669.4	1339.7	1355.8	1191.6	1275	1665.4	2334.1	2659.3
509.7	489.9	670.1	627.6	338.3	404.8	494.7	460.6	367.4	390	482.2
2291.5	1881.3	2375	1639.9	1705.4	1523	1940.8	1706.8	2099	2776.7	3058.2
1178.3	1093.2	1217.7	861.9	901.2	1062.9	1068.6	898.4	1532.9	2084.3	2211
1749.5	1697.4	1795.7	1397.9	1402.1	1627	1684.8	1592.5	2912.7	3396.5	4004.9
1016.9	1095	1056	862.4	801.2	1164.3	1664.8	1266	2674.2	2162	2606.8
1847.9	2356.1	2052	2048	2054.5	1953.7	2136.9	2371.7	2120	1328.9	2244.2
2493.7	2039.9	3491.5	2849.1	1985.1	2259.7	1494.6	1565.8	1278.4	1208.2	1793.5
1160.6	1333.7	1237.1	1239.2	984.5	1314.1	1466	1373	2184.8	2539.1	2304.2
1288	1080.7	1299.9	934.9	1195	1114.1	1016.7	927.5	1975.2	2138.8	3197.7
2412.8	1501.6	2268.9	1548.8	1441.1	1486.5	1386	1379.3	2570.8	3412.8	3900.1
749.4	685.3	676.4	677.5	635.2	761.9	896.9	660.2	970.9	1291.8	1188.7
853.5	781.5	914.8	741	805	834.3	877.9	728.3	1621.5	2045.3	2110.1
2660.9	3301.4	2711.7	2876.1	2248.7	2058	3912	3991.2	2242.2	1395.5	2285.5
858.8	1015.9	896.5	1084.7	798.1	996.2	1041.6	909.4	1275.8	1440	1643.1
479.1	323.8	472.3	374.1	406.3	436.3	437.1	385.3	460.2	640.9	794.9
928.1	1722.2	1183.4	832.2	680.9	967.6	726.9	884.6	809.1	931.3	1119.1
1304.8	1939.9	1978.6	1960.2	1596.1	2703.9	2047.5	1770.6	2551.4	2035	2857.8
1032.6	1174.2	1059.8	986.6	726.1	1018.3	1172.9	1335.7	2394.2	2684	2480.2
1841	1456.4	1616.9	1189.1	1069.7	1211	1210.4	1230.6	3027.6	4797.5	4594.7
1732.5	2336.8	1834.4	1491	1393.3	2222.5	2937.9	1792.2	2296.3	2083.4	2810.6
3334.9	2959.7	3373.2	2570.6	2412.6	2513.4	3422.4	2327.1	4715.5	6797.5	6550.2
1239.5	1228.9	1296.6	990.8	993.4	1355.3	1702.9	1406	1652.6	1921.7	2080.1
1005.1	1147.1	1216	983.5	899.8	1210.9	1764.4	1272.8	1320.6	1907.9	1783.5
705.1	812.4	784.6	636.8	539	683.7	899.2	826.6	1283	2108.8	1652.9
1139.1	1669.6	1207.5	1082.9	861.3	1605.2	1710.4	1470.1	2173.1	1886.1	2375.4
225.6	232.5	257.3	262.3	494.1	277.3	394.2	373.1	387.2	298.5	521.8
3537.3	2589.2	2409.3	2548.8	2306.5	2103.4	2930.6	2824.9	2028.4	2039.4	2633.4
1896	1727.9	1876	1572.1	1353.1	1514	1372	1516.3	3171.2	2959.9	4168.1

1480	1030.8	1285.2	966.6	1246.3	1009.2	1045.2	807.7	1367.9	1861.7	1707.4
3250.3	2623.5	1735.8	1411.7	3116.8	2010.2	3536.2	2350.9	1234	1680.6	1249.9
1324.4	1259.6	1237.8	1020.7	1011.5	1017.7	1286.8	1054.2	1579.2	2449.4	2398.1
3666.9	3352.4	4704.2	3086.9	1937.6	3748	2441.3	2506.3	1605.5	2453	2288
1765.3	1795.8	1776	1447.6	1200.7	1500.1	1721.6	1530.1	2700.6	3662.3	3983.9
1091.8	1328.4	1362.4	1120.8	1114.9	1217.4	2355	1261.8	1388.3	5066.7	2812.3
1991.9	1768.8	1830.6	1573.1	1476.6	1481	1549	1369.3	3050.1	3223.5	3839.7
809	986.6	863.9	897.1	669.3	858.1	774.2	1072.8	1052.8	960.8	1278.1
2026.4	3224.3	1730.6	2315.8	1366.4	1821.9	1390.6	1591.1	1159.8	2462.5	2546.6
999	1726.2	1372.4	1413.8	1120	2120.2	1262	1417.7	1480.9	920	1357.7
1302.2	1156.6	1125.3	1021.1	951.9	932.3	1077.7	866.8	1687.1	2278.8	2383.7
1237.7	1154.7	1361.8	1086.6	1035.5	1039	1103.4	987.8	1977.5	1923.8	2360.8
2511.8	1696.3	2448.5	1632	1730	1577.3	1426.5	1416.7	2360.4	3648.9	4343
680.6	282.2	218.5	376	558.4	282	691.6	396.8	279.1	336.6	293.3
1655.6	1267.8	1628.4	1248.9	1278.1	965.5	894.3	1499.5	1901.5	2763.4	2995
4744.3	3563.3	3762.5	4760.8	7153.7	2893.4	4772.8	6731.9	5292.1	1289.1	3604.6
2636.9	2455.1	2854.4	2595	2085.9	2700.4	1944.3	1949.4	1653.3	2134.9	2916.3
1270.2	1542.6	1705.8	1813.1	1076.6	1336.3	687.4	1381.7	971.6	1138	1374
1376.3	1184.8	1370.6	1000.2	1058.3	1060.2	1047.5	1089.8	2252.5	2985.5	2956.3
2160.1	1677.2	2806.5	2345.9	1703.4	1899.4	1732	1576.9	1186	1703.3	1797.3
1106.6	948	1050.4	775.9	801.7	902.7	966.7	912.3	1630.1	2027.8	2172.4
1401.9	1127.3	1206.5	930.7	969.3	1125.3	1055.8	1266.3	1903.8	2028	2299
2927.6	3031.2	5801.1	9663.4	3913.3	4792	2146.9	2488.8	643.9	786.7	952.7
1412.7	1307.6	1650.8	1317.4	1007.2	1289	1304.8	1183.4	1935.8	2482.3	2807.1
1041.1	1428	1309.8	1145.9	943.4	1845.6	1559.7	1268.1	2234.2	1735.6	2319.6
1408.7	1118.7	1729.1	1102.5	1040.4	1188.4	954.2	804.7	1166.5	1679.1	2463.2
893.9	1045.8	928.6	882.5	806.3	995.7	1041.2	996.5	1008.1	1037.7	1171.7
1081.7	1004.9	1127	814.1	789.2	956.6	1000.3	906.4	1215.1	1476	1729.9
2509.3	2350.6	2555.2	1732.6	1503.2	2114.5	2475.4	2129.8	4564.4	4791.1	6026.3
1367.9	1366.6	1672.3	1226.9	1226.6	1360.4	1561.9	1517	1815.4	2330.9	2227.4
1547.8	1636.9	1752	1423.5	1078.1	1373.7	1502.3	1094.6	2161.3	3493.8	3368.4
877.3	817.2	922.4	727.1	739.6	880.3	1295.2	876.3	1367	1609.5	1485.8
1060.3	1057.5	1067.5	868.9	936.9	1118.7	1299.7	998	1459	1848.2	1848.8

1715.2	870.9	1333	851.6	837.9	778.8	715.6	848.3	2235.4	2618.9	2969.7
2560.1	2214.8	2291.7	1635.8	1580.5	1870.5	2436.9	2158.9	4102.6	4120.4	5155.5
1063.1	970.4	1102.5	942.8	916.3	1032.5	1403.2	1046.1	1240.5	3474.5	1709
2892.1	2666.4	2752.4	1885	1816.4	1963.9	2792.7	2303	4753.5	5341.6	5942.2
1323.6	883.7	1577.7	659.2	945	585.2	604.3	608.4	2514.3	1727.8	3170.5
1706	1426.6	1388.9	1254.7	1166.3	1115.5	1328.9	1020.9	1848.3	1953.8	2136.3
1022.1	1149.8	1127.7	882.9	902.1	1061.5	1303.7	892.8	1574.1	1757.5	2220.4
962.7	1265	1197.7	905	786.1	1046.3	1282.5	1004.2	2061.2	2398.1	2636.3
1737.1	1177.4	1387.4	875	774.1	904.5	1032.7	1050.6	2517.1	3208.7	3591.2
2206.6	1215.9	2489.5	798.6	2924.1	478.2	695.3	708.1	293.1	403.4	578.3
1168	1149.4	1157.5	1075.6	910.5	1113.5	1064.6	1020.5	1355.9	1827.2	2005.7
956	1454.8	2036.2	1110.6	1350.6	1453.8	3367	1737.2	617.8	847.2	1034.9
1695.4	815.3	1092.3	1396.6	1288.1	1234.7	1654.3	1824.4	3883.4	2108.3	3538.2
2374.3	2737	2246.4	2074.3	1932.8	2873.5	2273.9	2356.1	2933.5	5394.4	3681.5
4611.3	3377.4	5446	8360.4	2932.9	1603.4	1911.2	804.9	1182.2	773.7	1546.9
1319.4	1296.3	1153.8	1021.2	829.3	1181.4	1308	1366.6	2490.3	2255.8	3266.8
310.9	399.4	276.4	239.2	285.6	314.3	246.4	304.2	270.7	352.1	264.5
1339.3	1709	1582.2	1296.4	1147.7	1794.9	2121.8	1329.5	1548.2	1790.8	2097.8
2488.1	1187.6	2062.6	1723.7	1334.6	996.7	1171.7	754.2	1008	3505.2	1953.8
2787.2	2552.8	2688.1	1926.8	1882.2	2138.5	2631.1	2560.2	4754.9	4565.4	5768.7
1225.3	921.1	1144.2	791	898.2	823.6	844.9	706.9	1453.2	2033.2	2342.6
1565.5	1797.4	1756.1	1617.9	1381.4	1987.6	2154.9	1648.2	2051.5	2364.2	2699.1
895.3	912.5	878.3	580.3	746.7	860.8	767	775.7	1066.7	1341	1545.4
1848.6	2150.2	2278.5	1839.2	1354.5	1803.4	1846.3	2137.4	2207.1	2069.2	2319.6
782.3	791	750.1	619.3	624.5	783.2	867.9	765.9	1511.3	1830.6	1953.8
566.6	779.7	524.7	491.8	570.4	1257.6	2052.4	533.8	809.1	947.5	1001.7
1439.8	1441	1539.9	1251.6	1049.8	1429.4	1661.1	1194.3	1959.7	3845.6	2746.9
1539	1288.9	1492.4	959.3	988.1	1087.9	1201.5	1173.8	1934.4	3007	3147.3
1922.6	1964	2029.5	1815.8	1565.7	1889.8	2057.8	1573.7	1939	3027.6	2642.7
1769.6	1690.8	1699.9	1224.3	1019.5	1336.7	1904	1694.7	3096.2	3374.5	4166.8
1890.1	1816.7	1818.4	1408.6	1320.8	1540	1623	1797.4	3019.6	2765.6	3812.9
972	769.4	916	653.9	685.1	748.2	790	728.9	1164.4	1408.7	1554
1963.3	1685	1805.5	1388.5	1269.8	1332.4	1690.1	1389.5	2236.2	2625.3	3217.4

1513.5	1663	1587.4	1247.6	1271.1	1515	1987.3	1323.3	3236.6	4382.8	4134.4
1653.2	1152.3	1825.2	1302.7	1420.4	1205.1	943.3	959.1	903.3	1283.6	1434.4
1005.4	974.4	1231	1046.6	803.7	860	943	1049.4	1373.1	2304.6	2082.1
1535.3	1491.9	1689.4	1309.2	1223.8	1045.8	1446.9	1159.8	1382.6	1391.3	1633.6
2561.4	2062.4	2435.8	2044.1	1571.8	1524.3	1593	1905.3	2943.4	3374.2	3167.3
2615.4	2005.3	2890.5	2395.7	2289.5	2516	1969.2	2128.4	2693.9	3764.8	4084.9
1132.1	1056.5	1158.6	818.2	984.7	728.5	625.1	733	1280.7	2076.2	1859.1
881.5	1076.1	1046.3	842.2	806.9	1075.5	1438.6	900.6	1096.7	1164.6	1370.6
1023.8	785.6	896.3	615.1	736.5	726.7	816.3	703	1583.8	1676.1	2283.1
1532.1	1509.1	1753.7	1387.8	1085.2	1307.2	1665.4	1048.2	1666.4	2174.7	2335.3
1694.7	2032.7	1248.6	1483.9	1372.9	2935.7	5068.2	2021.9	936	1007.8	1234.2
2027.4	740.4	484.1	956.8	1228.8	587.2	1796.6	998.9	569.2	716.2	555.3
2883.1	2240.4	2897.6	2378.2	1953.2	2462	2375.1	2143.5	2394.4	2549.5	2988.8
1319.3	1346.4	1159.3	1013.2	1101.1	2396.8	5546.5	1510.9	790.2	845.2	984.9
1334.4	1434.7	1365.5	1068.8	1084	1439.5	1784.8	1167.4	2514.6	3398	3265.1
2133.6	1877.4	3313.6	2886.7	1719.1	3020.1	1096.3	1417.5	1396	959.2	1506.6
1119.9	821.3	978.1	802.8	663	695	799.5	662.6	1432.5	3081.7	2010.6
899.8	796.1	806.7	736.6	576.8	673.6	668.6	690.8	1395.7	1571.1	1697.5
1973.2	1316	1787.3	1580.7	1805.4	1375.8	972.2	1181.7	1460.4	1872.4	2233.1
660.3	667.2	687.5	624.1	592.6	608.8	724.4	630	2376	6082.4	3772.6
2917.3	2029.2	1978.6	1746.6	1860	1592.1	2328.4	1480.7	2501.5	3954.7	3753.6
952.8	1094.8	943.2	744.1	784.9	1373.2	2867.8	1347.4	741.4	738.8	989.3
1016.3	996.4	1003	785	770.6	967.8	1027.4	1001.8	1427.4	1713	2148.2
1016.4	1899.5	1275.5	1063.8	861.6	1163	1987.5	1045.6	1062.3	1374.6	1208.6
581.3	418.7	681.3	526.5	528.3	462.9	456.8	664	730.3	1280	1275.8
1350.9	992.6	1283.9	832.5	873.4	928.5	934.1	849.9	1818.1	2259.3	2635.3
1470.8	1218.7	1608.6	1056.1	1086.5	1138.8	1040.4	983.5	1134.5	1302.4	1385.5
1021.7	3879.5	970.1	1124.5	877.1	874.6	918.4	953.7	1398.2	5323.6	3058.9
1233.5	926.9	1113.2	809.4	860.2	974	1025.5	983.2	1765.3	1983.7	2398.4
1740.2	1594.8	1610.1	1255.9	1258.5	1165.9	1210.2	1284.2	2795.8	3808.1	3855.1
1697.7	1508.9	1677.6	1347.7	973.6	1325.9	1383.3	1491.5	2824.3	3297.4	3855.7
976.5	803.5	871.6	692.5	604.5	752	923.4	806.9	1757.3	1702.4	1947.6
1535.1	1518.9	1556.9	1169.6	1120	1402.3	1448.5	1479.1	2844.9	2309.9	2825.8

173.1	147.6	209.1	110.7	111.4	107.8	147.2	115.7	140.3	181.5	218.9
1239.5	1215.9	1240.1	961.1	1049.6	1360.2	1299.4	1014.7	1972	2285.9	2447.7
1375.7	1221	1350.2	991.1	942.2	1108.9	1038.6	1027	2213.3	2872.5	3215.4
1075.7	875.3	974	846	733.9	783.6	809	792.9	1824.8	3453.4	2131.5
2430.3	2213.4	2217	1693.4	1387.7	1846.3	2169.4	1794.2	3388.3	4175.7	5142.3
1701.5	1626.8	1621.8	1472.7	1282.6	1572.3	1887.3	1561.3	1972.7	2124.1	2246.6
298.4	235.8	257.5	219.6	227.2	291.8	338.1	162.4	206.7	308.4	378.9
131.6	121.3	119.7	84.4	98.4	132.6	151.6	125.2	296.4	241.7	261.4
1261.5	1350.5	1402.6	1078.2	934.5	1231.1	1543.8	1167.2	2322	2843.7	2710.8
1138.6	1109	1093.8	774.7	783.9	775.1	1124.8	1412.5	768.6	746.8	933.9
1836.8	1922	2165.2	2092.8	1498.4	2183.4	1632.8	1443.9	2405.6	3224.8	3393.1
1288.7	1154.4	1410.1	978.7	993.3	1083.5	1088.1	1209.7	1718.5	2090.3	2659
1478.7	1090.5	1334.5	982.9	1133.3	995.5	988.5	1077.1	1386.6	1538.9	2071.7
2956.4	2148.7	2774.5	1949	2253.5	2576.8	2177.1	1685	2693.9	3161.3	4367
983.9	831.3	903.2	740.5	720.5	751.7	876.7	586.5	1128	1371.5	1643.1
794.4	857.1	944.5	678.4	732.4	860.3	906.4	730.8	1216.8	1534.6	1912
1063.4	827	922.5	658.7	733.7	814.8	858.9	742.9	1552.2	2060.5	2333.2
1062.2	1051.8	1204.8	966.5	690.5	817	1275.1	981.8	1663.5	4146.6	2641.4
1090.4	5239.6	2861.7	1513.3	1697.6	2215.6	3589.3	1868.5	1724.1	2858.1	2324.9
688.1	421.2	1883.3	865.2	513.1	509.5	547	455.5	826.5	2415.6	4629
3692.8	3206.6	4147.4	2930.4	2961.1	3435.5	3496.7	2409.3	9100.4	13130.3	10687.6
1098.6	973	982.1	761.9	907.3	872	1023.8	936.7	1584.3	1660.2	2136
1427.6	1368.6	913	876.7	847.6	1235.7	1497.7	1246.4	2608	2392.6	3181.2
2282.7	1860.6	2688.3	2169.5	1696.9	1824.3	1475.9	1747.3	1486.4	1831.6	2063.3
2937.2	2692.9	2971.7	2594.2	2192.8	2759	2390	2382.4	3148.1	3843.7	4853.7
2680.4	1965.6	2498.9	1528.5	1549.9	1621.8	1510.2	1764.7	3013.8	4729.6	4686.6
1295.4	848.1	915.8	668.1	717.9	615.9	812.8	778.8	1139.2	1650.4	1776.8
876.8	649.1	800.9	705.5	637.2	752.9	859.5	775.4	1158.9	1390.7	1498.5
1078.2	1014.5	1145.2	825.3	871.5	953.5	992.2	1077.3	1982.8	2351.9	2559.4
370.1	403.4	662.6	431.3	380.3	407.9	557.5	389.4	4031.8	5971.1	8650.1
1821.2	1948.3	2008.5	2184.6	1485.2	2245.1	1716.1	2175.6	1031.8	1051.8	1253.3
1940.4	1529.4	1651.9	1220.5	1413	1665.2	1744.1	1429	1646.7	1993.6	2350.4
768.3	954.1	942	802.8	679.9	1165.6	974.9	869.8	1379.3	1750.9	2093.6

1247.4	1329.4	979.4	1439.1	1247.1	824.3	1280.4	1114.8	1020.9	836.6	848.9
1031.8	1043.6	1115.1	943.7	1077.5	1116.2	1201.3	921.2	1489.2	1550.1	2005.2
2184.8	2022.5	2344.9	1679.3	1400.3	1630.3	2180.7	2117.7	3536.6	3731.8	4449.7
2289.2	2348.2	2374.6	2296.7	1842.7	2840.8	2281.2	1444.9	1188.5	1348.9	1813.8
454.9	358.2	433.1	318.2	371.3	341.8	370.8	350.2	812.6	758	1122.4
376.3	231.5	272.9	207.3	228	200.1	195.7	227.7	479.1	419.8	644
1079.6	1247.5	1231.1	948.7	950.6	1168.3	1463.3	1249.6	2438.1	2612.2	2803.5
1408.7	1126.4	1250.9	1058.1	955.1	1174.6	1083.3	1016.2	1411.5	1956.5	2066.5
658.7	652.6	650.2	571.8	507.7	652.5	647.2	556.7	874.5	851.2	1145.4
928.4	1565.7	1983	2081.7	2692.6	4735.3	1800.7	1888.3	1009.4	1455.4	1596.8
770.8	1279.9	1528.9	1769.4	896.6	1530.1	532.8	1844.4	730.2	555.5	742.5
1132.5	865.3	1054.2	832.5	751.2	740.4	923.5	727.5	1428.4	2847.9	1992.3
1009.5	930.3	1017.2	814	756.7	881.5	790.3	796.1	1704.4	1909.5	2458.1
1206.7	1231	1216.3	889.7	1009.1	991.5	1084.2	1007.7	1918.4	2148.1	2706.9
70.4	56	60.5	52.9	27.2	42.1	39.5	53.6	115.1	143.2	160.8
991.7	811.9	1026	769.2	816	770.1	707.3	767.5	1670.7	2020.9	2276
58.7	55.2	56	34.9	53.7	43.5	43.3	41.2	54.3	52.6	63.7
1187.6	1088.8	1262.2	1050.3	887	1142.1	1151.9	1122.3	1850.6	1887.1	2577.1
1481.8	1480.9	1599.7	1191.1	1091.2	1403.4	1418.9	1500.2	2627.4	1974	2808.9
1112.8	1087.4	1272	946.1	768.5	897.6	1070.9	910	1757.2	2355.7	2306.4
2464.4	1699.1	2406.9	2802.2	1410.2	1520.8	917.5	999.7	1058.9	1234.1	1834.2
1637.2	3430.4	2358.7	1729.6	1462.5	2468.8	4200.9	1027.3	1028.1	1414.4	2231.6
1595.6	804	1050.3	717.1	863.5	768.9	843.8	852.9	1989.8	1970.7	2088.1
544.8	858.7	699.1	578.3	516.4	539.9	611.5	564.2	1384.2	1935.8	2843.3
1364.3	1181	1152.5	1010	828	1238.8	981.7	1139.8	1873.3	1921.9	2400.7
778.1	749.7	730.6	669.6	599.9	640.2	718.3	710.6	923.1	1289.8	1204.3
2840.7	2171.8	2782.5	2367.8	1461.5	1931.4	1930.2	1740.7	3698.3	5711	4680.6
1312.9	1055.8	1397.4	1104.2	1029.9	1019.6	1038.4	1036.1	1860.9	1884.8	2697.9
862.5	844.2	863.9	690.1	710.8	730.3	874.1	741.5	1129.5	1097.4	1426.8
1299.7	1483.1	1597.2	1357.5	1182.7	1644.1	1403.9	1374.2	1988.4	1733.8	2676.6
1451.3	1374.5	1216.3	797.2	1359	1119.8	1826.1	1180.2	1012.6	1137.4	1128.7
1964	2114.2	2040.4	1513.1	1471.2	1864.9	2287.9	1762	3829	5378	5119.3
1826.7	1708.7	1922.9	1544.8	1258.9	1663	2208.6	1457.5	1898.8	1939.4	2544.7

1537.9	1043.6	1285.1	946.9	999	979	1038.4	1052.8	2335.1	2996.9	3334
2658.4	2306.1	2466.2	1509.2	1388.9	1698.7	2541.2	2008	4674.6	4770.7	6112.7
393.6	385	351.3	273.4	352.1	382.7	422	335.8	435.6	508.9	602.3
1405.1	1179.5	1302.9	966.6	966	1228.2	889.5	819.2	2244	2164	2451.2
1858.6	936.9	1621.2	1178.7	1210.6	1056.5	773.4	862.7	1782.1	2754.4	3117
1649.3	1245.8	1814	1198.8	1122.1	1262.4	1138.1	985.2	1740	3736.5	3341.6
1803.2	1619.9	1997	1626.3	1575.9	1732.2	1513.8	1434.4	2244.8	2625.9	3243.1
1252.7	1096.5	1248.4	1054.9	804.5	926.1	1025.3	893	1492	1728.4	2240.8
1664.3	1817.3	1995.6	1347.2	1522.2	1704.8	1962.7	1302.4	1856.5	2216.8	2717.7
847.6	714.2	845.4	588.2	652.7	674.8	829.1	733.2	1021.7	1484.7	1463.7
3330.4	1752.4	2308.9	1721.7	1873.1	1503.2	1768.8	1449.7	3201.2	4243.5	5037.6
994.3	935	930.3	766.3	790.2	863.3	1090.2	902.4	1321	1466.6	1752.3
1017.8	853.3	1534.3	1066.2	733.4	737.4	941.9	1085.1	904	946.5	1168.1
987.1	920.5	1020.6	853.9	804.4	938.9	885.5	739.6	1463.1	1591.6	2066.9
828.7	824.9	796.4	662	661.1	855.4	901.3	833.4	919.2	1355.5	1390.6
538.5	709.5	535	436.2	489.9	602.4	750.7	669.3	1169.2	897.1	1555.5
1542.1	1415.5	1670.8	1278.2	1081.3	1319.4	1395.8	1342.8	1748.1	2045.6	2446.5
1110.3	1141	1278.5	1111	988.9	1152.8	1250.5	1038.8	1452	1468.6	1836.3
786.2	676.1	794	647.5	681.4	763.9	775.6	804.3	1256.6	1045.7	1407.4
1373.3	1077.8	1257.3	972.4	877.7	992.7	1002.4	1009.8	2279.9	2918	3201.8
1114.2	855.9	1064.2	765.2	956.1	797.8	992.4	766	947.9	1160.2	1290.2
955.4	1568.9	1519	1101.6	1120.6	1626.5	1389.9	1455.2	1165.2	929.6	1608.5
1174.2	1311.6	1198.4	946.8	913.7	1184.4	1482.9	1115	1610.9	2254.2	2300.5
7818.5	3203	4323.4	3088.2	3625.5	3349.3	3304.9	2684.3	6425.9	9779.5	9687.6
2448.8	1132.9	1442.8	1879.2	1905.8	1800.6	3057.1	2719.4	5886.4	4284.6	4989.8
2073.4	3351.5	2110.5	3391.9	1313.9	1545.4	2224.5	3189.3	819.6	797.9	952
1377.4	1034.4	1782.4	1768.3	1258.6	1304.9	4403	1457.1	337.9	715.9	550
1897.1	1702.3	1744.9	1437.7	1226.6	1460.1	1673.1	1342.3	2239.8	2919.4	2952.3
238.4	280.4	231.3	178.5	182.4	272.6	406.4	285.9	509.2	1022.5	832.2
1324.4	1071.5	1456.5	1025.4	1274	1182.6	1211	830.5	1267.5	1765.7	2029.3
1579	1512.6	1709.1	1256.8	1391.3	1437	1380.8	1234.6	1974	2463.8	2930.5
1865.9	1804.6	1801.1	1484.5	1495.2	1644.5	1773.7	1619.1	2057.4	2484.5	2750.8
661.3	786.2	836.2	697.6	530.7	810	743.6	758.4	1034.5	896.5	1194.1

1458.5	1067.8	1389.2	894.1	818.6	925.4	937.4	851.7	1769	3062.8	2820.6
1674	1403	1510.5	1472	1083.1	1406.7	1414.3	1559.2	2783.4	2504.2	4070.1
764.4	660.9	769.8	623.9	583.9	586.9	641.2	650.2	914.5	1009.4	1164.4
1196.6	835.2	1117.6	811.3	731.2	795.7	803.2	797.4	1969.9	2426.9	2969.5
1377.9	1395.5	1750.5	1471.4	963.8	1386.6	1490	1307.1	1948.5	2392.2	2349.7
1302.1	1308.5	1317.9	1062.1	993.2	1184.4	1629	1618.5	2696.8	2515.8	2610
2192.2	2118.3	2589.7	2337.7	1644.6	1217.9	1846.7	1167.3	1593.2	1105	2235.8
1235.9	1228.5	1357.2	1166.9	989.3	1244.3	1208.1	985.6	1493.2	1793	2047.6
4707	4080.2	5340	3448.2	3368	3445.4	2705.9	3146.1	6833.6	11022.9	12642.5
2205.3	2824	2941.2	2393.9	1622.3	2570.4	2482.1	1667.3	1974.6	3019.5	2469.6
1391.8	948	1372.8	1150.5	1055.3	1106.4	820.6	650.6	1169.1	1399.3	2049.4
1051.3	1026.7	1016.6	788	677.7	938.1	976.1	846.4	1696.1	1800.6	2232
1746.8	1507.3	1858.8	1668.8	1380.8	1631.7	1420.4	1448.2	2435.4	2848.7	4084.6
466.2	496.3	521.2	399.3	425.6	544.7	479.5	339.2	685.8	1288.1	1059.8
3780.6	2633.1	3174.7	2236.1	2354.4	2133.5	1948.2	1334.6	2997.6	5115.4	4952.9
1193.9	920.4	1145.8	958.4	809.1	939.2	1389.8	809.2	1272.6	1488.5	1652.2
1411.8	2121.9	2688.9	4376.8	1198.4	2418.4	1889.9	1289.2	1832.3	1575.4	1452.5
43	93.6	79.1	98.3	86	128.4	83.1	59.9	31	31.9	56.2
1007.7	1225.8	1105	1139.2	862.5	978.3	1239.5	1332.7	1853.6	1374.6	1449.1
122	173.6	169.1	179.3	118.6	264.3	171	163.5	108.1	89.6	153.8
1097.7	1681.1	1338.5	1221.6	1112.3	1110.9	2021	1561.6	845	531.4	874.2
1007.9	1051.3	1135.6	1169.3	919.2	1097.4	1326.6	964.7	1537.9	1799.3	1781.2
2526.4	1307.2	1901.8	1337.1	1433.8	950.4	997.2	784.8	1171.4	1567.5	1894.5
1607.3	2111.7	1681.2	1577.1	1088.2	1671.7	3223.3	2022.1	3566.4	2782.9	3128.4
848.6	529.6	715	561	545	718.4	622.9	606	537.1	657.9	891.5
1304.3	1009.6	1264.6	962.4	986	1055.5	1089.3	1137.1	1831.5	1631.8	2280.6
923.9	910.4	1015.5	836.3	681.9	821.4	924.5	794.5	1727.2	1869.7	2242.7
1403.6	876.2	994.4	764.1	729.5	750.1	802.6	813.4	1438.1	1709.9	1743.2
1798.7	1682.6	1659.3	1386.2	1304.8	1141.8	1130.2	886.1	1716.1	2963.6	2714.1
921.5	1041.2	954.8	736.8	691.2	980.6	1073.1	946.7	1869	1707.1	2420.1
730.9	1239.3	1070.8	771.1	826.9	1869.8	1038.3	981	1112.7	1055.7	983.8
2264.1	1542.4	1859.3	1420.5	1305.3	1417.6	1456.8	1630.7	3024.7	2771.6	3774.5
2038.3	2002.9	2473.6	2585.5	1939.5	2263	1339.8	1598.7	1440.6	1671	2310.8

2256.6	1549.3	1981.2	1335.4	1370	1441.4	1451.3	1314.7	3348.2	5006.6	4755.4
1032.9	614.1	791.4	737.7	463.6	530.7	423.5	425.2	482.3	872.6	840.7
953.8	867.5	1131.4	722.2	881.7	925.3	1059.4	675.1	1532.5	1724.8	2207.4
791.8	719.8	741.6	564.8	735.4	834.1	870.6	535.4	1111.6	1375.2	1651.5
2791.2	1282.1	2173.1	1581.3	1786.7	1279.2	844.9	1127.6	1529.1	1863.3	2242.3
939.1	893	1032.3	729.9	727.9	938.7	953	786.6	1333.1	1748.3	2035.4
1009.2	966.4	934.8	687.6	751.1	930.3	951.3	860.9	1355	1652	1989.4
1601.1	1285.4	1461.8	1206	1376.3	1306.6	1582.3	1700.3	2432.9	2344.4	2417.5
793.8	595.4	664.8	486.4	518.2	523.5	519.5	572.7	1006.3	974.9	1284.1
1059.3	1080	1205.4	985.5	886	1226.2	1042.6	1010.8	1268.6	1486.1	1494.3
910.8	678.3	816.6	761.4	694.7	778.9	1126.1	865.9	1819.2	2553.2	2027.2
1730.2	1670.8	1891.2	1394.6	1284.4	1454.6	1622.7	1603.8	1970.7	2577.8	2707.8
6039.3	3392	4646.8	3822.4	4413.4	3897.2	3773.6	3317.1	5493.3	7383.1	8028.6

1580.1	1266.2	1455.2	1217.4	999.4	1220	1264.5	1233.2	1958.9	2646.4	2850.1
2121.5	2229.6	2342.7	1837.7	1714.8	1972.1	2074.3	2014.1	2064.5	3004.4	2834.3
2904.2	676.4	1335.3	1059	2898.1	1250.7	557.8	757.3	2101.8	2870.7	4155.3
1393.5	1485	1655.9	1274.5	955	1346.2	1502.4	1233.4	2559.5	3027.5	2968.3
1031.8	599.1	772.8	555.8	616.1	654.2	639.3	615.7	1583.6	1651	2214.6
1494.3	1198.7	1404.2	1017	992.7	1076.8	1073.8	1230.4	2084.5	2839.1	3107.7
644.9	1227.5	1021.5	1154.2	848	1019.8	802.6	1709.3	636.3	656	766.6
811.1	656.4	1035.8	725.6	609.4	719.7	627.6	468.4	656.9	617.7	977.5
1859.9	2204.7	1981.8	2111.4	1390.1	2012.9	1552.1	2284.8	1578.6	1587.4	2097.5
1629.5	1028.6	1692.4	941.8	1081.1	1014.2	844.3	1045.5	1933.4	2804	3461.9
526.5	651.8	635.7	619.1	516.3	646.1	712.2	520.4	953.5	849.4	1115.4
3033.6	2343.9	2891.6	2637.5	2348.2	2473.8	2271.1	2181.9	3728.7	5137.2	5505.6
907.1	924.4	1107.2	915	715.1	1004.2	1019.5	1068.5	1291	1503.8	1794.5
1110.4	1010.6	1155.1	1033.9	838.4	1029.6	1271.6	885.9	1654.2	2117	2277.3
1704.1	1168.1	2480.6	1594.1	1306.7	1085.7	1045	754.9	1012.2	2821.3	1368
1527.3	1663.8	1577.5	1112.1	1470.5	1540.5	1793	1335.1	1877.8	2318	2617.5
401.6	337.6	375.2	306.6	267.5	297.9	273.5	352.4	915.2	725.5	924.6
982.5	844.9	707	780.3	687.7	803.1	910.4	729.9	2088.5	1448.2	1562.6
1096.7	1061.1	1083.5	947	826.9	954.8	1115.6	987.5	1591.6	1634.9	1896.7

407.9	401.4	579	411.5	285.2	242.3	286.3	305.3	295.2	427.8	396.9
234.8	238.4	225.2	228	223.2	213.2	269.1	243.5	488.2	733.7	819.8
1722	1353.5	1535	1214.5	1183.3	1212.8	1503.5	1304	2221.1	1762.3	2148.9
743.1	748.1	787.1	592	571.4	748.3	1193.7	713.1	1215.3	1593.1	1460.6
2037	1777	2024.9	1457.7	1238.1	1465.4	1985.4	1500.4	2141.2	3625.7	2919.8
679.6	537	589.8	468	535.7	624.4	654.2	572.9	896.9	895.9	1189.7
593.1	1487.1	1074.6	745.4	972.2	2166.2	2400.8	7237.2	258.8	1132.5	813.1
1435.8	1266.1	1462.1	1103.4	999.5	1155	1322.8	1220.6	1539.7	2025	2250.1
609.4	733.6	763.5	576.2	492.4	687	707.3	577.1	922.5	1413.2	1346.6
806.5	657.8	770	554.8	498.8	545	580.8	600.4	1288.9	1496	1588.3
355.5	344.4	418.4	433.7	369.1	353.9	381.5	375	1654.4	5268.7	2935.6
997.1	987.8	1191.1	806.7	721.3	869.7	1089.5	872.3	1424.2	1738	2046.2
1606.6	1270	1467.8	1210.1	1036	1032.6	1136.3	1133.7	1299	1624.8	1911.5
5046.3	1802.3	2734.1	2655.8	1900.6	1107.7	1789.3	1070	1471.7	1351.2	2453.7
2736.8	2139.7	3065.7	2637.6	2132.9	2890.2	1550.7	1478.7	1884.7	2680.7	3579.5
1597	2311	1735.7	1035.6	1317.9	1629	1971	1301.5	1727.5	1745.4	2074.1
590.7	554	774.3	563.1	457.8	592.1	668.4	391.1	555.3	760.4	813.2
2099.2	1712.5	1906.4	2037.6	1467.5	1513.9	1385.2	1510.6	3706.7	3414.6	4171.2
1052.3	1077.2	898.5	806	788.2	955.4	1183.1	972.8	1979.7	2223.1	2279.7
1396.5	1415.2	1612.9	1144.9	1112.3	1340.6	1280.2	1378.1	1866.3	1629.2	2425.1
1253.8	1039.2	1263	838.4	838.4	913.2	1086.1	836.9	1206.2	1456.2	1875.6
2540.3	2308.6	2414.9	1892.6	1945.2	2228.5	2567.1	2309.6	3957.9	3976	4897.6
1640.4	1282.4	1787.3	1459.1	1242.8	1667	1878.3	1680.2	1522.2	2442.6	2119.5
934.9	908.3	936.5	611.4	578.4	712.5	955.6	757.3	1623.2	1555.4	1973.2
714.4	762.6	737	621.9	593.2	695.7	803.6	679.1	1053.7	1057.8	1366.1
1386.7	1431.6	1438.8	1152.5	1108.6	1265.1	1392.2	1235.2	2142.4	2386.9	2744.3
1078.6	863.9	805.1	771.6	537.1	448.4	827.3	613.3	1374.8	803.5	1269.2
895.3	712.7	934.4	680.6	682.8	733.5	858.4	716.4	1696.2	1301.4	2614.7
964.2	982.6	1043.3	732.4	890.9	913.7	1087.9	859	1245.6	1704.7	1888.1
594.4	522	526.4	480.2	472.3	565.1	560.9	497.1	1745.7	3238.3	2276.2
2503.5	2213.7	2304.6	1701	1486	1803.1	2400.5	2077.4	4518.5	4504.2	5544.8
1663.4	1009	1406.9	1166.7	1034.8	908	1108.7	811.7	1589.2	2263.3	2413
1226.7	971.4	1070.1	765.1	791.2	907.9	981.6	905.2	1381.5	1498.4	1777.5

1931.9	1877.5	2161.4	1987.3	1533.2	1688.7	1833.4	2019	2223	2036.9	2355.7
1040.4	1054.9	1092.9	885.7	789.8	926.2	1061.7	934.9	1990.1	2536.7	2465.2
1224.2	1432.8	1475.2	1385.6	1060.1	1381.6	1428.6	1317.4	1709.5	2882.1	2623.4
571	462.3	547.3	432.9	404.7	441.6	540.6	502.2	829.9	1438.1	1049.4
1297.3	1001	935.1	754.4	891.4	810.4	1007.1	769.8	2409.9	3672.5	2306.3
1492.8	1048.9	1288.6	996.7	856.7	923.4	907.6	879.8	1504.1	2246.7	2313.2
791.5	767.3	693.6	643.5	621.1	771.9	812.9	704.6	1798.7	1955.2	2016
1532.9	964.7	2284.1	857.6	1003.4	983.9	766.2	663.6	1369.1	1700.9	2233.1
1049.6	1199.1	1107.9	1000.7	753.4	1014.1	1200.3	1137.7	1506.2	2693.5	2355.4
665.6	691.9	652.1	645.7	548.8	973.4	1943.6	661.3	578.5	818.2	781.8
1965.4	1687.7	1656.3	1413.7	1340.4	1582.7	1930	1097.2	2810.3	3633.9	3704.9
4578.2	431.7	344.9	628.3	589	578.2	532.9	427.4	560	593.7	710.4
2731.4	2436.6	2381.9	2042.7	1792.9	2037	2429.6	2100.9	3134.1	3117.4	3968.4
1502.7	1090.4	1373.7	905.8	983.6	1059.3	1045.6	1009.2	2320.2	3157.2	3579.5
1670.9	1548	1568.4	1235.9	1097.7	1330.8	1273.7	1327.3	1856.4	1657.7	2513.7
800.3	921.9	934	750.6	585	778.6	891.6	827.3	1681.7	2061.7	2024.9
996.1	861.1	1081.4	981.9	721.2	694.5	785.6	746.8	1766.4	1839.2	2412.5
3346.4	1808.3	2366.9	2254.7	2190.5	1278.9	1111.6	1849.7	1856.9	2159.3	2419.7
596.3	546.3	909.5	1260.5	449.6	900.1	397.5	387.1	333.7	573.1	537.2
1262.2	1044.4	1181.4	912.8	978	876.8	1150.4	1029.9	2076.5	1739.4	2385.7
806.5	785.3	900.1	816.4	724.8	799.6	817.5	678.5	1411.4	9371	2198.1
931.1	851	964.8	711.7	706.9	811.7	942.7	652.2	1157.8	1603.6	1694.7
1321.6	1205.5	1266.7	999.8	880.8	856.5	927.3	1012	1119.8	1528.7	1674.9
511.1	474.6	528.4	379.6	391.1	473.5	484	432.2	837.3	949.8	1172.5
669.6	626.5	703.5	448.3	504.6	536.8	634.7	667.1	1238.1	1711.1	1594.3
1595.8	1436.7	1607.3	1464.7	1178.6	1576.4	1436.4	1381.8	2388.6	2119	2597.9
881.9	693.7	768.5	677.2	680.1	631.1	811.9	593.6	1185	1454.8	2116.8
1119.6	927.1	780.9	649.5	558.7	655.7	766.1	769.5	1453.1	1120.4	1697.8
816.6	737	988.3	592.8	544.4	1069.5	1155.3	675.5	8893.2	3394.4	9529.2
581.7	432.3	474.1	370.9	422.4	391	427.2	419.8	874.4	706	1217.5
35.4	46.8	35	42	28.4	32.8	36	33.3	31	36.5	41.3
623.8	473.5	718.7	615.8	497.3	486.5	484.7	396	1088.6	1386.1	1553.3
744.2	674.2	779.4	554.2	518	641.6	753.1	525.2	1449.6	1369.6	1630.9

858.8	683.3	764.3	525.1	480.3	608.4	589.5	590.2	1360.9	1856.5	1834.2
2774	2423.1	2459.9	1586	1467.1	1855.4	2336.3	2115.7	4075.4	4529.7	5503.9
1905.6	2763.5	2752.6	1577.7	1657.5	2595.2	2874.6	2700.9	2985.6	2514.3	4290.7
933.3	819.3	818.3	667.1	611.6	690	764	714	1353.1	1757.9	1936.2
568.7	535.6	593.3	456.9	408.5	487	432.9	456.2	694.1	666.4	944.1
214.7	213.4	250.3	220.9	273.6	215.2	259.1	304.8	282.1	236	325.3
1164.2	1132.1	1170.6	986.9	914.7	1270.6	1236.8	1142.2	1946	2276.6	2739.3
1243.6	943.4	1108.2	836.5	740.3	883.4	965.5	730.2	1460	1911.7	2146.9
1063	1233.1	1362	941.7	721.2	988	1350.5	1232.7	1365.5	1741.3	1973.2
1781.2	1752.1	1983.8	1430.1	1262.8	1562.7	1717.9	1483.3	1981.2	2637.6	2742.1
2603.7	2172.7	2520.8	2560.4	1960.6	2055.8	1474.1	2876.3	2947.5	3082.6	4271.1
1555.3	1183.6	1372.4	1116.1	904.9	1075.4	1155.3	1178	2113.5	2587.6	2739.3
3790	2251.5	3882	2134.6	2425.5	1802.4	1592.9	1908.1	1738.5	2804	3100.5
819.7	953.3	813	877.3	788.8	727.4	1035.7	941.6	1677.3	1513.1	1759.5
1607.1	1342.8	1422.4	1202.2	1177.7	1307	1342.8	1331.1	2114.5	2301.8	2820.7
1008	930.9	1103	864.3	757	881.8	1012.9	873.5	1136.2	1396.8	1562.5
896.7	659.7	672.3	671.8	612.1	631.5	534.9	655.7	1185.6	1151.7	1506.1
2357	1231.2	1407	1168.7	1529.7	1428.6	1276.3	1059.7	3048.7	3372.2	3562.6
374.1	405.4	394.2	313.4	259.3	383.1	420.4	341.1	492.4	605.8	678.2
1432.8	1223.4	1604.8	1176.8	966.8	1020.7	1027.7	1086.2	1654.2	1745.6	2348.9
871.1	1482.5	1307.6	1020.6	793	1196.8	3155	1928.7	2045.3	1834.5	1941.4
1918.7	2542.2	2482.7	2220.9	1638.9	3654.8	2924	1938.9	2431.1	2949.7	3253.1
1515	1892.6	1515.4	1105.7	1034.2	1787.9	2107.9	1479	1131.5	1254	1602
1401.7	1648.9	2012.6	1447.1	1306	1639.1	1395.9	1509.2	1176.7	1648.2	2410.8
819.6	896.2	1008.3	744.3	614	857.9	965.7	882.9	1072.7	1184.9	1458.8
1396.3	1925.9	1819.2	1581.4	1283.2	2750	2171	1555	1749.7	2183.9	2393.5
1016.1	676.5	873.8	794.7	630.6	686.6	609	497.6	549.7	730	1014.4
2436.2	2369.4	2682.5	2028.8	1823	2098.8	1926.6	1547.2	2629.4	4794.3	4656.4
1156.9	1205.2	884.1	1127.1	1056.6	808.6	1396.8	1485	758.9	599.5	1022.1
821.1	769.7	943.6	686.4	696.1	820.1	790.5	659.7	1197.6	1744	1944.6
3165	2753.8	3809.5	3341.2	2848.6	2856.3	1964.8	2028.6	2942.8	3030.7	4703.2
2567.8	2295.4	2167.7	1802.3	1712	1909	2378.4	2128.3	3637.7	3096.5	3929.1
823.4	818.3	791.7	801.7	629.6	924.1	879	818.2	2503.3	2554.2	2757.1

983.5	790.8	773.9	697.3	837.2	665.9	718.9	710.1	1107.2	1311.2	1374.1
1494.6	717.3	962.3	665.7	686	705.4	597.3	706.5	1277.5	1714.9	2037.4
767	819.2	882	733.8	555	525.8	645.7	815.6	1137.1	1427.7	2045
439.2	462.7	472.4	368	356.1	439.6	353.9	408.4	380.3	401.9	457.1
999.8	820.4	839.8	627.5	586	618.8	780.7	806.3	1383.3	1969.3	1858.4
1694.2	1883.7	1829.6	1420.4	1492.9	1918.4	1659.4	1505.4	1342	1913.2	2052.2
1036.6	1137.8	949.9	864.7	847.3	909.6	1319.5	1169.9	1838.2	2450.7	1943.5
1127.9	1310.7	1474.6	1336.6	1122.1	1321.2	1179.8	1246.9	1257.9	976.9	1638.3
419	500.2	649	561.5	392.4	480.1	498.2	477.2	1037.2	1670.5	2580.1
1341	1456.8	1292.7	1208.1	906.8	1522.9	1874.9	1514.7	1655.5	2003.9	2053
467.4	456.1	567.4	498.9	382.1	516.3	569.3	499.7	1057.6	2619.6	2185.7
1341	1137.7	1221.9	923.1	946.3	1129.7	1425.9	862.3	1268.1	1760.9	1820.4
1097.1	1053.2	934.8	944.7	778.7	878.7	995.5	995	1389.3	3169.6	1864.3
1783	1467.7	1906.9	1511.7	1285.4	1493.6	1197.8	1192.5	1947.4	3592.9	3026.4
3769.9	2923.5	4404.8	3230.4	2216.6	2693	4175.1	2158.1	3579.2	4892.3	4517.6
1361.8	1496.6	1504.2	1165.5	1069	1717.1	1199.8	1300.6	1172.9	1824.9	1750.1
1799.8	1559.4	1588.7	1220.6	1149.4	1337.7	1699.3	1301.5	2319	2595	3213.3
360.9	346.3	371.5	285.4	335.3	378.7	410.9	337.3	531.9	772.5	786.7
677.1	1103.8	873.8	1804.7	813.8	3339.9	1062.2	1286.5	1489.7	507.1	957.7
748.4	734	714	564	542.1	628.2	758.3	758.4	1475.6	1597	1580
808.9	960.6	989	801	639.6	799.4	766.1	734	887.4	1036.8	1095.1
2267.2	2313.2	2170.1	2010	1304.2	2007.7	1907.1	2404	2426.3	2120.6	2806.3
844.5	968.4	1091.9	899.4	751.6	997	1223.5	979.9	1844.3	2470.8	2918.1
716.7	5382.4	1544	549.3	1721.6	3507.7	3575.9	1250.5	572.1	2027	756
1749.3	1255.9	1513.1	1130.7	1156.8	1136.3	1324.4	1133.2	2810.7	3640.4	3928.8
1595.3	1262.8	1463.7	1074.9	1135.6	1163.8	1094.3	1110.7	1839.3	2652.2	2778.4
1873.1	1665.1	1837.7	1197.7	1144.8	1264.3	1878.1	1663.1	3177.1	3330.7	4009.8
1120.9	962.5	1054.3	863.8	951.6	975.9	1035.7	846.8	1457.1	1387.3	1978.3
1259.9	1260.7	1392.7	939.9	1029.8	1204.7	1407.9	1128.2	2230.1	2872.7	2982.7
1031.4	1550.6	1697.2	1010.4	742.4	1285.1	1820.5	1032.8	1289.9	1553.9	1585.5
1041	869	943.9	639.6	821.1	793	964.3	844.3	1503.8	1778.6	1975.7
1979.5	1378.7	1739	1129.7	1211.3	1076.1	1121.1	1156.5	2180.5	3728.5	3823.3
1187.9	1278.7	1485	1390.7	1047.2	1176.4	1397.1	1303.4	1819.3	1872.6	2051.9

1082.1	1134	1378.7	962.8	852.2	1202.3	1282.7	1056	1290.2	1418.8	2123.2
891.5	680.7	1136.2	1049.7	819.8	1021.9	962	548.4	741.4	1639.4	1639.2
1341.7	1244.1	992	882.5	2206.3	1115.5	1895.3	1026	700.5	1025	821.9
1532.3	1488.8	1340	1195.1	1114.6	1250.6	1602.8	1383.3	1964.7	2067.4	2508.2
949.6	922.2	995.1	984.3	608.1	909.7	927.1	816	1585.8	1668.5	1896.9
1272.7	1258.8	1240.7	1112.5	1084.3	1401.5	1385	1138.9	1800.8	2236	2479
965	936.9	1114.8	692.2	650.1	795.4	813.6	855.6	830.1	947.1	1182.1
1032.3	1330.7	1232.2	1182.9	888.7	1267.1	1214.8	1213.8	1075.4	1045.3	1255.9
1281.2	1336.9	1448.5	1173.7	1027.2	1217.6	1168.5	1463.9	1250.8	1270.4	1608.1
1643.9	1342	1697.6	1301.5	932.4	1508.8	2091.5	1243.5	1720.1	4270.3	2472.9
1260.3	1120.1	1426.7	1216.4	1085.5	1127.1	1190.4	1194.6	1541.8	1606.9	1975.2
623.7	669.1	664.7	557.1	465.9	594.2	592.5	553.2	1241.2	1452.2	1569.3
472.5	820.3	695.2	744.4	809.7	2577.1	2424.1	1311.5	2198.3	959.7	913.7
775.3	811.4	832	756.4	644.4	759.4	821.3	757.4	1040.3	1528	1440.6
1030.9	1081.7	1122.8	927.1	1042.5	1178.5	1224.5	1027.1	1131.9	1676.1	1668.7
1520.1	1261.1	1445.5	1199.7	1152.3	1575	1255.9	1261.7	1497.2	2100.7	2217.8
1858.6	1728.7	1979.5	1530.9	1394.8	1696.9	1871.5	1472	1709.9	2650.4	2481.3
862.8	951	1090.1	961.3	660.6	701.4	1097.2	705.5	1452	1444.1	2460.7
1073.3	1267.3	1658.2	1473.6	697.4	805.5	942	735.6	673.7	880.6	1147.9
1089.2	745.6	954.4	691.2	733.3	707.3	598.8	606.6	781.8	863.5	1072.7
2594.2	2152.1	1703.2	2634.7	2200.6	1226.5	1760.7	3360.3	1435.2	979.7	1638.8
2009	1744.7	1606	2194.4	2167.3	1480.7	1838.3	1625.3	1673.4	1193	1572.1
196.5	196.9	193.3	162.7	184.9	167.8	233.4	169.5	233.8	310.9	319.1
732.7	616.5	723.2	643.7	653.5	703.3	710.9	576.3	1182.4	1972.3	1853.4
1336.4	1078.8	1254.7	970.4	874.9	986.8	1055.1	906.1	2005.8	2383.6	3037
795	815	944.2	735.9	696.6	834.5	1050.9	744.8	975.3	788.4	1166.2
2261.7	2046.1	2486.4	1920.1	1462.2	1760.6	2054.9	1866.6	3167.2	3128.3	3907.6
1198.1	1000.8	1028	813.3	828.1	937.6	989.6	1017.9	1558.7	2009	2209.4
1041.6	934.7	1088.7	878.4	752.4	1064.1	974.6	997.7	1337.1	1690.1	1714.5
1032.8	1234	1242.3	864.9	859.4	1022.7	1554.4	1014.6	1397.1	2437.2	2299.3
1653.5	1389.5	1656.8	1212.9	1078.2	1175.9	1478.8	1087.5	2675.7	3270.1	3550.7
889.2	833.2	877.5	676	622.4	933.4	1139.9	711.2	1145.7	1108.5	1624.8
1929.9	1355.2	1803	1548	1466.1	1519.2	1305.1	950.6	1123.9	1239.1	1620.8

1062.8	2275.2	1714.8	1859.3	1185.8	2240.3	1502.3	872.3	784.4	1019.6	1273.6
2357.7	2147.6	2527.7	2036.6	1645.8	1997.3	1971.2	1483.7	2367.4	3160.8	3489.2
713	620.4	677.6	522.4	429.4	479.4	524.2	492.5	891.3	987.2	1283.2
2779	1952.5	3270.1	2068.3	2111.9	1532	1575.7	1346.4	810.6	1256.9	1079.2
424.1	1937.4	580.3	1235.7	598.8	2039.4	1052.3	3004.6	202.4	326.9	426.8
1702.8	1334.7	1589	1071	906.1	791.1	850.2	761.2	769.7	930.3	1105.9
987.7	909.2	862	791.7	1303.3	818	1336.3	1007.3	1036.3	1216.3	1247.3
1468.7	2478.3	1572.4	1586.1	870.3	1342.7	1428.9	2579.2	2616.8	1303.1	2368.5
1414.2	791.4	834.1	730.1	693.8	710	772.8	694.5	1253.1	1731.5	1917.1
547.4	457.3	559.2	452.7	430.7	411.8	552.6	484.5	1230.3	2613.6	1738.1
794.8	736.9	731.7	589	570.2	738.5	703.7	700	1341	1294.8	1786.8
1020.4	1078.2	1057.1	909.7	888.7	1040.7	1214.8	838.9	954	1718.4	1882.7
1057.2	1324.8	1166.3	1055.6	797.5	1094.7	1868.3	1869.5	3129.2	1512.2	2313.6
1610.4	1934.8	1362.3	1124.3	1463.3	1405.3	1733.8	1276.3	1081.2	1169.1	1559.5
880.1	1257.4	1385.5	2010.1	1285.4	1840.9	1128.3	2005.5	835	980.5	1213.6
164.3	135.6	157.6	155.3	140.3	143.7	143.7	135.6	159.7	101.8	150.5
928.2	968.5	1112.3	825.8	781.6	1151.4	903.6	822.8	1073.3	1333	1681.1
1428.1	1443.7	1609.3	1187.8	1133.2	1427.1	1397.4	1295.8	1746.1	1483.3	2055.1
579.6	547.4	605.9	454.1	471.3	551.3	610	485.5	701.5	799.6	968.6
1219.5	815.5	1026.5	780.2	845.5	830.6	890.8	614.2	1445.2	2779.4	2272.3
2777.2	2527.2	2198.7	1578.8	1611.9	2036.9	2962	2426.8	5277.4	5383.4	6402.9
967.9	943	983.4	887.6	496.6	912.5	1080.2	772.5	1133.6	1036.2	1427.1
1301.6	1160.8	1302.1	911.2	925.9	1112.9	1262.4	1202.3	1845.3	2039.5	2446.3
1253.5	1075.2	1119.2	761.4	786.9	891.1	1206	991.9	2100.2	2053.7	2475.4
677.8	837	638.5	689	543.1	709.7	863.9	712.3	1839.8	1601.2	1931.2
1023.8	1028.7	1225.9	864.2	795	1327.6	1171.5	865.4	1294.3	1352.5	1948.9
1061	1188	1175.7	996.7	778.5	1045.2	988.4	894	1381.7	1520.5	1771.5
811.8	935.1	689.3	621.1	590	670.5	745.6	918.5	1972.9	1696.3	2828.9
3476.5	1955	3591.4	2245.2	2775.9	1623.3	1289.8	994.9	1817.8	1460.9	3126.5
1101.6	1092.7	1471.1	1095.5	926.7	1214.9	1275.1	1207.6	1334.4	1510	1860.3
820.1	739.6	676.1	576.8	572	639.8	629	652.3	1025.8	1291.5	1297.8
819.9	972.2	876.6	808.3	792.6	1829.3	2275.1	760.1	825	1225.3	2321.4
1950.3	1646.8	1746.1	1213.6	1031.9	1445	1620.9	1545.4	3325.2	3182.4	4017.1

1311.4	1174.9	1249.2	954.1	866	1068.4	1350.1	1129	2271.1	2181.9	2984.4
1194.1	951	1160.1	900	963.7	1043.6	1012.2	831.3	1693.4	1681.7	2366
788.5	474.8	634.4	466.5	403.6	427.4	579.9	484.8	1341.5	1360.3	2274
1477.1	1072.3	1409.2	1001.9	990	1285.8	1297.4	1113.5	1567.2	1707.5	2307.3
1069.3	1434.9	1404.9	1500	1356.8	1317	1159.8	1517.3	1352	1012.8	1122.9
1090.2	1123.9	1004.4	927.9	881.8	1036.5	997.6	845.4	1477.2	1811.8	1832.6
87.5	86.3	108.7	69.4	63.6	75.4	70.9	52.5	82.9	106.1	117
1277.9	989.9	1020.4	996.3	846	784.1	821.8	662.3	732.7	1479.2	1087.8
975.6	1058.9	1041.9	950.6	719.4	1089.7	952.5	748.1	1032	1146.6	1392.8
1362.3	1231.3	1318	999.7	786.9	1038.8	1310.7	968.9	2266.6	2934.5	3763.8
1127.6	1041.4	1154.4	855.3	904.4	1018.5	1008.2	904.4	1578.7	1641.8	1965.7
730	716.3	979.2	710.3	760.3	508.3	525.6	433.1	530	861.1	860.1
2801.6	2778.1	2657	2824.6	1992	2344.8	3292.1	2115.2	3281.2	3829.1	4486.7
1106.8	823	999.5	845.2	729.4	898.1	793.3	940.4	1678.7	2343.6	2456.7
736.3	953.3	1052.7	954.6	663.8	1399.2	1318.8	1248.8	883.3	1250.1	1464.3
295.3	178	174.9	132.1	157.3	144.1	143.9	132.6	229.2	351.1	395.6
880.7	1070	572.3	364.8	444.2	682.2	267.1	642.7	979.7	1108.3	533.6
1296.1	1142.1	1242.4	920.9	955.3	1159.3	1294.7	1192.8	1876.8	2211.1	2359.8
612.2	501.5	562.2	483.7	454.5	582.7	569.4	551.9	959.6	1039.3	1200.7
4810.5	1611	2955.1	2325	2141.2	1575.8	1133.1	1252.1	2758.4	3661.9	4704.1
393.1	397.2	402.9	402.6	408.9	610.9	614.9	496.7	594.6	753.6	780.2
624.7	686.5	745.7	539.5	735.5	754.7	802.4	513.4	631	1069.9	905.5
704.6	951.7	823.7	739.3	569.9	798	770.5	627.4	1251	1364.4	1539.7
1683.4	1303.5	1686.1	1260.7	1061.4	1148.8	1015.5	1073.3	1433.5	1718.3	2261.1
896.8	1354.1	1364	1316.8	1034.6	2243.3	1145.6	1138.1	823.6	659.5	911.5
889.1	688.3	797.7	627.9	680.5	658.5	876.3	744.6	1121.8	1318.2	1408.2
580	460.6	522.4	511.2	479.2	478	576.1	367	610	1244.6	1068.8
1353.3	1152.4	1207	1003.7	1058.7	1181.5	1516.4	1246	2166.2	2283.3	2796.8
829.4	547.9	639.3	474.8	543.4	647.9	610.5	579	2422.9	1402.7	2309.4
879.7	1493.3	1361.8	799.9	562.9	943.6	1465.1	721.8	2055.1	2806.5	2615.7
1011.7	989.1	998.4	815.1	748.1	1063.2	1182.6	990.2	1393	1253	1543.9
725.6	1104.3	787.3	584.6	630.8	792.3	1325.6	707	905.4	891	1308.7
1088.9	790.7	976.9	668.2	727.1	754.9	905.2	769.2	1780.9	2295	2510.5

1191.3	982.1	1194	920.2	787.4	913.6	1101.1	843.7	1178	1079.7	1453
4141	2688.1	3712.3	3129.7	4021.2	2890.9	2653.7	1850	2740.3	5209.6	5160.3
966.9	666.5	1119.4	1703.5	811.5	1086.8	1422.4	877.9	1009.5	1481.2	1700.9
1011.8	1143.8	1041	897.9	959.2	1094.9	1147.5	1151.1	1790	1662.9	2034.5
812.5	552.9	683.6	448.9	456	556.4	623.3	599.2	1136.8	1505.3	1650.8
1237.9	969.6	1234.7	843.5	958.5	768.1	861.6	820.9	2317.1	2842.3	2911.7
898.4	892.3	885.5	696.7	763.9	838.5	1006.6	765.2	1384.3	1595.4	1651.4
609	764.5	608.5	488.3	426.8	674.2	969.8	633.7	1321.3	1582.5	1699.3
1443.1	2016.3	2253	2032.6	1521.1	2998.5	3643.2	1797.3	1660.8	3496.6	2520.8
6582.5	1682.8	1902.4	1935.4	1785.1	1370.3	1209.4	1666.1	2330.4	5394	4945.4
2516.1	1340.8	2024.1	1526.5	1040.8	832.1	720.5	642.7	1592.5	3258.4	2811.9
1551	1477.4	1751.5	1483.3	1030.7	1314.2	1399.7	1290.5	1306.3	1777.3	1865.4
1702.9	1422.4	1608.3	1165.7	1030.4	1254.8	1637.6	1191.6	2021.5	2638.1	3131.4
1632.3	1047.8	1621.8	1302.5	969.5	2392.6	1034.7	782.2	795.4	1290.4	1231.6
966.1	887.3	890.9	658.8	614.9	841.2	842.2	704.5	1179.3	1724.2	1621.5
1885.1	4260.6	2221.4	3229.5	1397.7	6630.7	4274.1	685.3	841.5	1478.6	3255.7
1620.4	959.1	1022.1	1230.2	1052	820.8	835.9	916.6	1024.3	1355.5	1363.3
1356	1020.4	1001.5	846	719.3	805.4	1175.5	809.4	1620.3	1606.1	2154.1
1397.5	1279.5	1481.8	1196.5	1032.8	1162.7	1200.9	968.6	1589.5	2188.3	2308.1
666	628.1	758.6	575.7	526.2	600.7	754.5	591.9	1131.7	1288.5	1308.3
1366.7	1076.3	1398.5	1751.2	977	1358.1	964.3	962.1	911.5	1840.2	1314.3
348.8	385.5	433.6	378	286.4	408.2	462.5	352.4	625.5	2163.6	823.2
912	869	931.6	756.6	674.2	976.5	1014	813.6	1044.1	1169.9	1539.2
409.1	438.4	407.9	353	426.2	457.9	535.9	420.9	536.1	539.8	633.8
3089.6	1531.3	3547.7	1881.1	2774.3	3480.8	2032.4	1198.7	3402.3	4748.2	4814.1
1319.4	1242	1801.3	1009.9	1122.5	1020.4	961.6	806.2	1297	1510.4	2146.1
2162	1836.9	1849.6	1357.2	1189.2	1351.5	1836.6	1624.1	3500.2	3462	4431.1
3195.4	1748.4	2426.3	2294.7	1584.9	1932	1797.5	1508.1	1388.6	1676.5	2159.4
1364.7	2180.5	1517.4	903.1	763.6	1403	1802.7	1125.2	1367.7	2825.3	2818.4
1342.5	876.2	1184.8	1129.7	989.7	1135.7	1243.6	992.4	1230.2	1695.8	2395.5
1300.9	956.3	1151.8	779	1015.6	873.2	942.6	787	1849.4	2787.9	2747.4
1777.6	1079.6	1496.1	1138.3	949.5	1019.6	771	940.9	1393.8	2008.6	2402.4
1056.9	1161.4	1194.8	986.8	895.8	1036.8	1120	933.1	1384.4	1683.2	2012.9

912.9	961.4	951.4	674	707.6	984.6	1144.6	767.3	1079.8	1149.2	1423.1
871.7	834.8	865.6	643.4	651.8	825.2	1322.8	720.6	1010	1121.1	1230
1683.1	1158.9	1717.3	1172	1378.1	1015	767.4	1046.3	1201.2	2023.7	1807
936.6	749.2	996.2	641.9	704.6	748.1	899.2	834.5	1277	1484.7	1905.9
1004.9	811	905.6	702.6	774.1	884	1111.2	738.3	1153.8	2209.6	1746.8
1277.2	1370.5	1175.1	1062.9	948	1171.1	1342.3	1550.9	2463.8	1925.7	2283.8
821.8	884.2	882.6	829.9	742.5	835.9	911.5	859.6	1277.6	1290.5	1440.1
999.8	866.6	971.3	796.2	617.7	776.6	860.6	841.7	1196.6	1838.5	1707.7
1417.4	1233.6	1389.2	1058.7	970.8	1123.7	1166.8	973.9	1501.7	1970.9	2377.3
827	672.6	784.4	620.8	548.2	668.2	634.6	703.8	1262	1513.3	1846.4
684.9	683	760.4	554.8	568.9	635.2	703.1	621.3	1325.8	1534.2	1583.7
888.8	543.9	661.2	431.3	488	413.6	497.6	468	1216.2	1906.7	1985.5
112.1	136.1	198.2	137.6	93.3	178	140.9	153.8	413.7	1699.6	1364.9
925.7	771.6	804.9	607.5	699	579.1	801	618.9	974.2	999.7	1279.1
1204.9	1309.4	1247.9	1116.4	921.8	1260.3	1604.8	1784.9	2373.4	2229.6	2557.3
1685	1414.8	1872.2	1178.8	1170.4	1389.6	1882.1	1143.2	1958.4	2826.2	2713.4
658.7	719.3	962.6	481.8	501.1	578.4	876	554.8	800.7	798.4	1046.1
827.1	755.1	1279.9	1779.1	779.3	2009.6	2215.3	635.3	1508.3	2358.9	4055.3
2590.4	2243.3	2874.8	2164.8	1909.8	2317.8	1998.9	2203.2	3065	3765.1	4460.7
1095.3	531.1	658.3	589.3	497.8	462.5	786	618.8	1103	1523	1278.4
1083.5	814	967.7	713.4	718	805.7	993.8	877.4	2286.5	1681	2147.9
1479.4	1491.5	1518	1097.1	1308.6	1381.6	1604.4	1072.3	2040	2533.7	3088.9
867.2	3030.1	1249.9	2264.7	846.8	1988.7	2643.2	2690.8	305.4	378	522.5
1203.2	1079.1	1280.4	860.1	809.3	1184	1311.9	965.7	1052.6	1161.8	1404.1
1269.9	692.3	1259.5	994.3	930.8	748.8	550.9	531.5	1146.3	1086.9	1877
1100.7	1036.9	1133.9	894.8	832.4	1033.8	1025.2	917.1	1639	2122.9	2038.4
649.2	597.8	557.8	485	580.3	608.7	628.9	559.1	828.3	960.5	1129.5
370.9	340.4	388.4	301.2	326.9	425.9	436.4	326	620.3	639.6	771.5
1352.6	891.1	1026.1	937.1	730.6	906.8	1011.8	1043.6	1783.9	4543.5	2636.3
732.6	724.2	763.1	516.1	545.8	708.5	1001	669.2	932	973.7	1138.3
812.4	848.4	891.3	806.5	712.9	879.5	944.2	873.7	1285.5	1167.2	1414.7
676.6	774.1	894.4	527.9	556.1	766.2	775.2	480	913.1	1380.3	1070.8
579.9	625.3	709.2	575.6	537.3	552.7	761.9	545.4	451.2	878.8	1174.9

1038.8	1200.5	1142.1	936	857.8	802.2	762.8	724.6	1434.1	1126.2	1843.7
477.8	357.4	492.6	355	317.1	331.1	335.6	345.8	712.1	914	1062.4
1325.1	1085.5	1255.4	974.2	869.3	940.3	1046.5	1070.2	2139.5	2863.8	2896.5
851.1	862.6	811	696.5	644.3	799.5	879.8	779.5	1327.7	2413.1	1803.3
2607.9	997.3	1405.6	926.1	1155.7	1085	863.4	1074.2	2637.3	3685.5	3556.5
75.9	90.3	175.2	152.8	95.9	108.1	103.7	58	233.1	138.5	413.8
601.8	832.4	1380	2553.9	498.4	1072.6	759.3	499.4	421.8	379.8	405.5
1056.1	1110.2	990.2	866.1	966.6	978.9	987.2	926.7	1256.5	1352.8	1674.1
658.9	506.3	627	468.4	404.6	468.3	551.5	513.1	923.7	1217.5	1149
900.4	598.3	1021.5	603.5	778.8	726.3	687.1	662.4	3401.7	1836.4	3677.2
668.1	795.6	778.3	669.1	490.2	699.1	703.8	722.9	1198.3	1044.7	1394.2
1152.2	1183.5	1485.7	1178.5	834.5	1060.2	939.6	844.1	2198.2	3042	3022
925.3	1040.7	1574	1508.3	978.1	1869.3	813.5	928.4	502.6	574.3	771.6
1071.4	935.7	1057.2	853.5	766.1	1053.7	1177.8	777.2	1185.8	1310.1	1534
1064	1171.6	1413.5	963.1	848.6	923.4	1061.7	962.9	1267	3151.6	2798.7
762.9	732.4	753.8	647.6	592.8	768.7	784.6	682	891.4	897.4	1152.8
692.5	930.9	766.3	644.6	549.2	750.5	873.2	749.1	937.7	1023.9	1129.6
240.3	226.5	241.6	235.4	199.4	209.9	249.5	256.6	332	389.6	377.5
624.5	619.6	652.1	481.9	562	643.5	740.6	649.5	1151.4	1116.9	1314.6
865.8	987.8	851.4	669.8	581.7	736.4	962	658.1	1107.8	1411	1306.7
910.8	475.5	646.3	671.9	729.7	553.9	471	337.6	614.6	677.5	912.6
1276.2	1257.4	1376.9	1344.2	983.5	1352.8	1389.2	1328.6	1323.3	1369.7	1518.5
728.3	890.4	956.6	785	596	937	833.5	833.4	773.2	859.6	1106.1
905.3	831.7	982.6	749.7	519.7	754.3	865.6	700.9	1204	1533.4	1660.5
2172.1	1994.4	2166.4	1691.6	1486.6	1681.8	1968.6	1570.1	3192.5	3070.6	4041.5
950.7	780.1	656.4	644	770.2	778.5	1362.1	859.7	556.3	579.6	722.7
696.1	912.7	802.4	676.9	528.8	711	646.9	630.4	833.1	1043.8	1167.3
753.3	675.5	1243.7	812	573.9	616.2	827.7	848.5	1380.8	2075.4	1632.9
1062	760.3	860	619.2	527.7	633	644.5	765.3	1455.5	1894.9	2161
1105.8	796.8	937.4	725.6	725.3	734.4	778.8	787.3	1377.7	1719.6	2029.2
2266.9	954.3	954.6	1405.5	620.2	436.1	505.7	621.3	473.8	443.9	605.3
793.8	1206.3	1018.9	768.6	714.1	1393.2	2044.8	1052.9	744.8	766.8	1207
1043.7	1285.8	947.1	654.5	802.4	1454.2	3044.6	1244.1	856.3	1055.1	1116.7

524.6	509.7	580.3	399.8	497.5	409.7	450.4	397	523.2	460	642.1
940.7	1503.7	1522.6	908.7	717.9	1827.6	2228	958.6	989.4	1287.9	1505.4
261.3	584.2	463.4	425.2	230.8	503.5	498	2444	244.7	162.9	434.5
967.5	947.3	1081	692.3	711.6	798.9	941.4	971.9	1517.2	2250.7	2212.5
723.7	574.1	712.8	457.2	535.7	553.8	649.3	714.6	1117.2	1490.9	1431.3
1222.4	1098.8	983.6	831.6	1050	869.4	1275.2	1047.7	1563.7	1550.7	1795.5
1437.8	1455.3	1676.6	1334.7	1093.4	1607.1	1538.6	1094.3	1349.5	1587.8	1952.8
1160.6	970.5	1104.9	837.8	839.4	907.9	899	1042.4	1187.5	1465.7	1851.9
1050.7	758.4	968.4	759.3	742	726.3	765.3	625.6	1102.8	1241.6	1698.3
741.1	643.2	727.4	509.3	556.2	590.5	710	551.5	1136	1208.6	1584.9
607.8	633.4	596.9	514.9	494.2	578.3	748.9	607.5	1023.4	1328.8	1291.9
546.1	585	619.1	580.5	487.3	695.2	732.7	519.2	656.1	641	791.7
1331	1305.8	1340.7	1070	880.8	1325	1334.8	1149.7	2144.6	2352.9	2641.4
827.9	678.6	689.3	510.7	415.6	515.6	604	593.3	2197	2885.2	2794
700	772.7	920.4	764.6	586.1	855.5	864.2	777.1	1134.2	1914.6	2110.4
1265.1	1307.4	1369.1	1556.3	1003.9	1206.9	1498	985.4	1005.4	799.4	1215.2
786	812.6	819.8	1221.2	716	1096.4	761.7	957.2	1332.8	1674.4	1681.1
1126.4	687.5	862.7	698.2	581.8	639.2	494	564.3	987	870.5	1317.1
1155.6	640.9	915.1	647.4	617.2	567	666	660.8	1631.5	2395.2	2287.4
1879.5	1427.8	1726.3	1413.4	1171.3	1517.6	1888.6	1253.7	2125.9	2259.9	2720.9
642.3	605.5	681.1	544.1	518.6	562.1	631.5	489.3	935.5	1177.1	1351.1
2045.3	1413.5	1840.8	1201.5	1282.6	1191.9	1361.7	1322.5	1004.1	1119.9	1414.6
1970.6	1760.2	1555	1112.2	854.5	1059.1	1530.7	1740.5	3374.8	3668	4041.4
1039.4	957.5	961.1	838.7	963.7	877.2	1633.1	1174.1	1563.3	1548.1	1801.7
899.5	1062	1018.8	944.9	778	992.7	893.3	954.6	765.4	997.1	814.3
397.1	420.9	394.2	383.5	345.1	375.1	479.8	386.3	851.9	4586.3	1643.4
1022.1	888	1019.3	774.4	656.6	895.2	969.3	845.3	1525.6	1914.9	2105.3
746.9	655	669.9	559.2	616.3	584.1	768.8	613.2	1092.7	1142.4	1374.2
762.7	949.8	976.8	949.6	559.9	706.5	772.3	1029.3	1424	3201.7	2165.1
14.1	13.6	58.4	21.9	37.7	44.2	38.3	16.1	12.9	14.5	20.7
974.8	1119.5	1100.1	1024	822.5	1296.9	1074.7	1146.4	1338.1	1523.8	1605.9
529.3	474.2	472.7	379.1	522.1	454.1	533	399.6	598.2	842.6	808.3
235.3	247	287.1	216.1	180	303.7	264.4	237.9	317.9	318.5	400.1

784.7	975.6	887.8	728.1	663.4	890.9	1561.7	758.5	1232.7	1547.5	1372.6
2482.8	2354.1	2102	2214.9	2171.8	1586.8	2409.5	3268.4	2122.8	1328.6	2067.6
618.8	472.1	591.9	409.4	419.4	494.6	512.8	479	774	957.9	1080.9
870.1	673.5	742.5	563.7	575.2	546.6	731.6	890.3	2517.6	4528.1	3198.1
638.2	577.4	595.2	501	484	553.3	678.1	658.3	983.2	1273	1411.5
744.6	811.3	753.6	858.9	724.3	799.5	790.5	748.4	868.6	771.7	935.5
715.4	655.3	720.8	611.7	559.7	611.1	650.3	554.5	1102.4	1225.3	1373.9
623.2	703.8	788.3	515.6	460.2	833.5	695.7	593.2	1225	1269.4	1591.6
998.4	919.8	1155.7	834.2	914.7	1027.2	917.3	825.3	1461.4	1765.2	1966.3
1472.4	2062.5	1609.2	1415.8	961.3	1307.6	742.3	1561.9	1454.2	899.3	1419.7
871.7	965	1147.7	944.9	839.3	1149.8	1028.1	991.9	1082	1225.9	1424.5
584.1	493.1	747.3	565.7	359	430.7	506	450	1129.8	1867.9	1529.6
634.1	768.4	833.6	672.3	581.1	850.1	879.5	739.9	1142.6	1611.5	1681.4
831.2	1203.9	1140.9	963.4	822.2	1042.1	1328.4	1441.7	1219	1217.5	1310.8
1417.6	1040.5	1118.8	851.3	946.9	1000.5	933	766.1	1144.8	2266.9	1492.2
312	282.7	345.7	278.9	318.3	321.2	347.1	267.2	502	582.4	582.6
873.8	882.3	833.1	643.4	705.5	942.6	1417.8	790.6	972.3	1204.9	1559.8
1030.5	510.2	805	711.7	681	459.4	448.1	426.5	576.8	1017.8	1223.1
859	929.9	1087.5	869.7	780.1	876	939.8	602.9	841.6	1008.1	1626.7
2732.3	1674.3	2875.8	1805.5	2209.9	1602.5	1556.6	1275.4	1550.3	2483.3	2432.8
1270.7	667.1	1066.8	784	836.2	712.5	730.3	627	1214.5	1967	1894
2458.3	1888.6	2656.4	3595.7	1149.3	587.8	1369	1458.3	689.8	540.8	832.2
1270.7	1098.8	1268.1	1010.7	996.2	1365.6	1329.7	1286.7	1535.4	1893.6	2267.8
852.3	780.8	705.7	570.7	812.9	1174.8	2330.5	647.3	821.9	1085.7	1906.7
706	776.7	1183.3	1041	790.2	891.6	786.8	713.1	966.2	1580.7	1407.6
1360.5	805.6	1415.4	727.6	878.5	574	572.8	797	579.6	635.9	861.5
1053	768.7	1037.6	606.5	613.8	625.5	709.8	745.5	1282.1	2178	1794.5
1256.1	1114.5	1179	1045.1	892.3	1070.7	1081.9	739.6	1279.2	1960.6	1829.5
624	666.4	694.2	566.2	505.5	680.1	637.5	495.8	863.1	1730.4	1592.4
950.8	638.7	725.6	759.3	660.6	1284.3	778.6	674.7	836	2054.9	1181
2731.4	1618.1	1841.7	1992.3	1032.3	1831	1644.4	1201.7	801.2	953.2	1515.4
989.7	909.3	915.3	844.7	727.7	989.9	837.2	899.8	1418.8	1338.5	1789.1
783.5	520.6	664.8	519.1	661.2	606.4	589.2	449.2	806.7	1098.5	1129.1

924.6	892.2	967.3	790.2	705.5	869.2	769.5	844.4	804.9	839.9	1078
893.8	691.1	858.9	689	522.9	649.4	582.6	659.9	1385.4	1960	2040.2
1103.9	626.7	861.1	730.5	794.4	654.1	700.7	650.6	1390.5	1445.8	1765
599	859.7	685.5	554.9	463.6	898.4	1050.8	596.4	957.5	1116.4	1367.7
183.5	224.5	223.9	193.1	170.4	221.5	262	249.4	320.2	521.1	415.2
1896.9	1185.4	1569.3	1046.8	1080.5	1176.4	1123.5	1162.9	3105.5	3615.4	4563.6
942.7	830.5	1012.9	830.4	720.1	979.5	1060	813.3	944.9	1227.3	1318.8
1216.1	866.4	1107	810.1	879.6	853	853.1	826.9	1026.7	1483.2	1564.6
957.7	894.2	959.9	785.1	688.4	782.8	913.1	711.6	1210.1	1557.8	1643
647.6	591.8	649.8	520.6	445.3	597.1	714.8	595.2	1023.5	1148.6	1356.6
1172.2	1299.3	1181	1166.8	980.7	617.3	903.8	1264.3	1125.3	2283.8	1171.4
471.9	458.3	753.3	496.4	313	421.7	414.9	344.9	903.9	1248.8	1155.2
827.1	736.6	781.3	603.6	538.3	616.6	732.4	699.5	1477.1	1898.9	1970.6
1212.1	653.3	1048.5	816.7	672	622.4	600	418	869.1	1625	1727
1843.4	2845.8	2113.6	1831.1	1499.4	2042.4	1758.4	2218.1	1760.7	2737.3	2345.4
384.1	707	489.9	400.7	345.2	825.4	830.4	566.9	466.3	444.9	607.6
890.3	964	1045.2	769.9	730.7	860.9	941.3	866.8	1424.5	1509.4	1644.9
852.7	702.4	888.2	608.9	632.9	642.8	590.6	781.2	1446.8	937.5	1521.8
1079.4	1596.7	1497.4	894.9	928.6	1742.6	2001.3	1240.6	511.8	651.5	812
2801	1989.8	2020	3279.2	1503.3	3054	2099.1	1486	1907.9	1725.7	1451.4
929	991.1	1027.3	776	768.2	1082.5	1224.5	799.3	877.3	801.4	1145.4
1062.1	799.9	1394.6	851.1	804.3	1033.9	646.7	552.1	683.3	1059.2	1443.9
1069.6	847.5	1107	763.6	832.2	803	781.8	750.4	1003.1	872.6	1436.2
1244	1366.5	1614.6	1423.2	976.6	1288.1	1496.3	1014.5	1258.9	1289.2	1546.4
787.8	520.8	558	567.2	548.6	595.4	485.8	411.1	782.7	1234.6	1059.6
1835.5	1668.9	1793.5	1670.1	1370.8	1584.3	1533.6	1235.6	1848.9	2421.7	2674.6
1613.2	1166.9	1184.3	891.7	1008.2	963.2	1048.5	784.1	974.5	2108.5	1691.7
1441.1	1688.6	1545.8	1123	982.7	1156.4	1402.3	1585.2	3668.2	3997.7	5297.7
260.7	278.2	283.4	307.9	206.8	371.6	158.9	149.5	137.1	141.6	215.3
943	904.8	947.6	857.1	726.1	852.1	904.6	853.8	1460.7	1468.5	1619
772.3	641.9	759.8	549.6	597.7	667.1	681.5	613	1079.9	1113.9	1276.4
653	540.9	582.9	477	419.8	524.4	498.7	509.9	472.5	580.5	703.3
1741.1	1683.6	1737.3	1212.3	1066.3	1294.4	1577.6	1339.8	2969	2983.3	3828.2

3665.2	4466.1	3031.2	3632.2	3004.9	2737.9	3033	3244.2	2587.7	1525.4	2247.8
1012.9	840.4	973.3	761.8	808.2	793.6	895.5	754.3	1285	1530.1	1721.5
750.5	701.3	703.7	626.7	615	668.8	863.6	725	1097.2	1148.7	1315.2
721	510.1	622	539.5	504.1	586.4	677.7	652	859.3	1016.6	1085.8
1914.4	1500	1790.1	1575.8	1412.6	1485	1626.8	1343.7	1796.1	3116.9	3053.1
371.5	510.8	478.3	295.1	364.5	852.8	1166.1	564.3	297.7	259.4	333.6
1632.6	1345.8	1381.3	1079.2	978.5	1147.1	1606.7	1203.1	2570.3	3053	3534.4
1259.2	1199.2	1090.5	1443.3	855.8	945.2	693.2	985.8	1095.4	1198.3	1323.7
602.9	601.1	640.9	505.1	484.4	598.9	584.4	556.1	977.1	1220.1	1182.7
1004.2	1092.3	1150.7	891.5	676.4	861.2	965.4	1027.5	1591.7	1738.3	2083.7
1064.2	543.8	891.7	717.7	740.1	1013.8	666.3	573.5	827.7	1233.9	1353.3
576.4	563.2	679	504.8	491.5	546.2	592.4	475.5	632	788.7	973.5
1225.7	852.9	856.7	778.3	839.6	764.5	852.7	927	1338.9	1729.2	1653.1
300.4	3029.3	1039.8	438.3	388.3	3056.1	283.7	307.8	346.4	282.4	495.2
416.3	345	392	326.6	332.8	347.3	483.7	321.4	478.7	738.9	692.5
1062.2	1064.9	992.8	701.1	627.4	775.9	902.4	792.8	944.5	981.5	1506
1025.1	888	858.6	728.9	715	844.2	1173.8	983.5	1968.6	1853.9	2063.6
495.6	459.4	558.8	407.7	357.3	451.4	555.1	459.3	498.5	560.4	637.9
859.5	1035.8	1008.7	909.2	828.9	1206.7	1080.1	1042.2	1107.8	2036.1	1496.1
622.9	744.8	828.9	610	573.5	793.5	1017.1	769.6	798.3	1865.7	988.1
1277.3	995.5	1389	970.2	863.2	946.2	1063.1	718.3	1326.3	1648.3	1878.1
839.9	711.7	737.3	687.8	777.1	593.8	1467.5	1012.6	2697.3	2132.4	3359.9
1001	968.9	1060.6	806.4	761.2	995.7	1146.1	1001.5	1180.7	1329.1	1460.7
994.2	873.6	978.7	892.4	763.7	883.4	1118.8	964.3	2189.7	3105.3	2423.3
1667.8	1543.5	1613.2	1439.5	1206.4	1546.3	1438.5	1300.7	1743	2010.1	2423.6
1112.9	638.5	791	677.1	569.1	614.5	551.1	585.9	1003.9	907.4	1407.1
1063.4	915.7	887.6	827.1	717.7	859.1	1136.8	1033.4	2567	2455	2445
472	372.3	425.4	307	362.9	299	359	335	554	948.9	889.4
1618.7	1279.6	1531.6	1183.4	1088.6	1298	1441.8	1317.9	1122.3	2157.6	1908.9
757.4	557.2	666.4	474	469.5	524.2	518	537.4	1236.7	1776.5	1805.2
754.2	623.2	778.8	574.3	541.4	673.6	640	597	1102.6	1436.8	1675.3
960.8	868.8	1018.9	700.6	750.4	1109.2	870.4	778.3	1183.5	1362.7	1559.2
848.5	715.7	854.7	1010.5	667.3	544.6	568.3	514.4	969.1	1098.6	1230.1

926.3	900.6	935.4	689.5	829.2	739.1	938.1	675.5	1662.9	1901.7	2196.1
2788	1157.2	1514.9	1053	1286.7	1186.8	1309.1	997.7	3146.4	4425.8	4452.1
1033.4	929.8	1267.3	1159.6	805.6	1033.9	791.3	865.2	1536.5	2447.8	3056.4
2391.8	1854.4	2101.7	1653.3	1445.9	1511.2	1362.9	1610.1	3739.3	5060.5	5046.1
2247	1400.5	1967.1	1202.1	1497.4	2153.8	1025.8	1009.6	1596.3	2635.5	2428.8
2521.7	1740.7	2663.2	2716	1380.8	1278.3	826.8	1114.2	512.2	498.2	773.2
599.1	684.3	691.2	486.6	585	630.2	1144.2	513.7	753.9	1318.4	1112
547	2032.9	1261	890.5	844.6	2325.2	2248.5	1356.2	680.7	1212.3	757.9
763.6	663	692.3	530	551.3	629.2	762.4	651.4	1058.9	1330	1471.6
886.8	733.7	745.7	570.3	713.6	649.4	919.2	742.3	1301	1656.1	1906.2
206.8	203.2	271.3	219	215.8	225.4	177.5	179.8	214.2	188.6	263
1118.9	931.4	974.2	913.6	849.5	946.6	1166.3	1928.4	1041.5	992.3	1422.2
1616.2	1194.1	1279.3	933.5	1011.3	1046	1180.9	1146.5	2364.6	3243.4	3296.6
701.4	527.3	588.9	453.9	564.4	542.6	671.6	516.7	1004.5	1305.7	1417.6
642.8	830.4	844.3	566.4	582.4	715.4	807.4	674	1167.4	1478.9	2041.7
1560	1026.7	1250.9	953.9	1942.4	1044.9	1009.1	1910.5	1568.9	571.6	874.7
724.7	909	912.1	862.7	474.2	685.5	770.9	753.3	950.7	1038.9	1500.3
1010	848	1076.2	784.4	814.4	769.3	753.3	814.6	1490.9	1398.3	2130.2
540.3	596.6	628.7	477	346	462	547	472	1066.6	1175.8	1165.7
698.3	595.9	498.1	626.8	611.1	705	625.4	517	606.2	964.6	1191.4
1330.8	913.9	1242.3	817.3	1034.4	895.8	723.9	697.7	1166.4	1556.2	1753.1
842.3	1907.5	1321.2	1660.3	933.9	1743.4	1200.8	1172.4	943	729.1	1118
1371.8	1434.1	1714.9	1322.2	1258.3	1296.1	1095.2	1055.5	1258	1570.7	1868.5
2393.7	2244.9	3010.3	2434.6	1731.3	1874.3	1815.8	1894.2	2899.2	4531.2	4503.9
833.1	967.6	815.3	847.5	561.7	728.4	1051.6	934.7	1443.2	2133.6	1955.2
804.8	710.3	649.3	620.1	593.2	711.7	997	660.9	1256.3	2199.2	1692.5
430.3	1088.8	767.3	619.5	403.7	1032.9	670.8	698.8	844.8	799.4	1270.5
438.2	1155.2	1843.5	563.5	701.4	792.4	687.5	1263.3	260.9	318.8	414.4
931	1190.1	1254.6	993.1	858.4	1213.8	930.5	859.2	998.2	723.1	1176.9
720.2	694.2	754.9	629.8	549	697.4	864.3	827.6	1485.4	1172.9	1636.7
1263.2	872.7	966.4	629.4	742.1	631.7	785.5	656.4	1839.8	2857	2576.2
624.1	502.4	590.8	493.9	497.3	522.2	668.4	510.6	1258.1	2166.3	1862.3
868.6	895.6	955.9	761.9	658.7	795.1	796.7	814.8	1464.6	1559.6	1971.6

866.7	646	899.3	650.3	623.8	654.5	700.3	512.7	822.6	1265.6	1238.8
755.9	498.6	627.2	402.5	548.6	454.6	450.9	457.7	1218.9	1251.8	1652.8
1144.3	993.1	1272.2	911.6	964.2	884.6	884.6	825.6	1376.2	1636.8	1896.9
1762.8	714.8	1065.4	908.6	911.5	771.7	943.9	988.3	1802.4	2089.1	2239.2
793.4	884.1	1035.3	692.3	714.4	988.9	903.2	1131.7	1121.5	784.2	1094.4
643.8	650.8	698.9	553.9	544.7	642.6	660.3	606.6	892	968.1	1100
645.7	553.6	601.2	443.2	506.9	561.6	669.1	564.7	1123.2	1058.5	1225.1
460.1	449.4	422.5	343.6	435.9	425.5	511.9	338.1	485	548.2	659.9
1437	1988.6	1909.1	1529.7	1097.8	1705.5	1831.8	1424.1	2764.7	3668.6	3647.5
1612.8	1162.2	1560.1	1321	1268.9	1131.6	1136.2	950.3	1328.4	1794.7	2012.4
195.1	358.6	254.4	252.1	255.1	423.3	535.1	806.7	262.9	206.7	293.3
574.2	483.7	557.5	412	442.9	495.1	464.5	551.4	917.6	984.1	1138.6
523.8	527.1	618.6	473.3	537.2	528.3	524.4	464	956.3	1434.6	1364.7
1068	890.1	1168.7	882.4	879.7	910.7	911.2	775.7	1205.4	1670.4	1844.6
1783.7	775.3	949.6	757.3	774.5	522.1	598.4	435.5	432.8	451.8	648.3
1700.6	1204.5	1040.5	1058	1496.9	880.6	1541	2273.8	1720.7	739.4	1056.4
679	553.1	611.4	562.5	393	483	381.2	518.3	927	953.8	1143
1582	1481	1701.1	1343.3	1181	1473.4	1506.3	1322.3	1571.9	1888.8	2139.2
1992	1582.1	1759.5	2200.8	887.8	763.9	1270.6	1144.9	1124.3	1456.5	1829.7
705.2	750.5	671.3	651.8	634.6	1307.7	698.2	576.5	919.5	951.8	1224.6
1016.7	788.4	988.8	685.2	623.5	704.8	641.5	636.8	1604.8	2302.3	2317.3
697.7	456.2	713.5	509.7	497.8	537.4	312	235.1	1257.4	1172.2	2267.8
568.2	602.1	586.9	503	504.3	552.7	736.1	626.4	1128	951.5	1238.5
426	411	443.9	400.8	372	403.4	571	343.1	1245.9	2651.3	2313.5
956	948.7	1035.9	810.1	912	844.1	968.1	810.9	1382.7	1593.6	1863
336.7	289.9	307.7	248.7	247.6	283.8	312	299.7	489.4	583.4	652.7
612.3	617.4	575.2	541	480.2	558.5	642.2	557.4	969.4	1547.7	1343.7
605.5	603.8	647.8	558.8	409.8	468.4	597.6	616.1	1313.3	1412.2	1678.8
814.4	716.5	923.8	682.9	665.1	759.2	606.5	548	984.7	1109.2	1423.6
65.1	77.1	73.9	61.4	70.8	69.3	84.1	50.2	69.4	130.3	113.5
921.8	732.8	903.7	547.4	628.6	800.5	648.1	583.9	872.9	1252.5	1432.2
892.2	745.1	776.8	805.7	646.6	807.7	675.9	520.5	1144.5	1462.6	1800.1
628.3	644.9	567.3	556.4	513.7	618.2	688.3	548	837.3	1452.6	1200.4

863	758.8	889.7	707.4	764.4	785.3	884.1	841.8	1101.1	1057.2	1360.9
1428.3	1392.8	1354.1	1143	877.9	1210.2	1130.1	1252.1	1416.5	1489.1	1644.3
515.2	720.8	562	502.8	411.7	535.1	529.2	597.8	839.5	601.1	865.2
1556.3	814.5	1112.8	783.8	836.5	854.5	841.8	861.8	1792.7	2516.8	2656.4
597.6	432.1	534.4	457.2	436.9	499.8	494.1	496.6	784.4	943.6	945.3
456.5	313.1	356	317.1	342.8	372.8	370.2	346.4	565.9	535.9	729.6
588.6	455.7	558	419.4	434.7	430.8	371.3	432.5	659.2	798.9	975.8
712.3	646.2	739.1	599.5	515.2	535.7	653.5	663.4	1376.1	1602.4	1844.7
745.7	703	754.6	700.7	747.4	819	749.6	660.5	717.1	1162.9	983.9
1007.1	840.7	859.2	725.7	751.2	762.9	852.4	750	1318.9	1592.8	1725.4
823.4	790.5	809.2	741.3	696.5	661.9	831.2	603	907.3	1244.8	1193.5
1265.1	1028.8	1167.7	818.1	801.4	833.3	985.2	1046.8	2006.6	2424.2	2618.7
1087.6	1132.5	1003.5	864.2	946.4	952.7	1081.1	853.4	1479.6	1508.7	1789.6
1058	1271.2	1272	1059.2	825.9	1158.7	1164.9	1301.8	1635.2	1796.6	2191.9
1454.3	1199.1	922.8	1055.9	956	1947.3	2195.2	1275.4	532.5	634.4	959.2
802.5	622.1	752.5	595.4	647.8	608	651.8	462.1	1161	1080.1	1324.8
783.7	1039.9	873.6	755.2	684.5	792.3	1144.2	1090.8	1813.8	1837.9	1969.2
1174.6	1071.4	1258.6	1182.2	953.1	1034	787.1	797.1	1177.5	1227.1	1615.1
1586.8	1392.9	1544.5	1280.8	927.8	1377.2	1322.4	1408.2	1525.6	1574.6	1686.3
1337.7	1333.5	1668.9	1021.2	925.9	1479.2	1602.1	951.6	1018.8	1432.7	2054.3
1046.6	923.7	792.2	769.8	682	743.3	1017.8	694.3	1580.5	1816.1	1957.6
1513.8	1198	1170.6	1387	986.4	1034.5	1023.5	980.8	1136.5	1370.1	1555.9
1464.4	906.3	1043.4	722.6	843.2	1380.4	1398.2	646.4	1199.1	1265.6	1602.1
796.4	522.3	669.6	484.1	458.1	459	455.1	508.3	1149.2	1437.2	1712.9
882.8	805.6	816.9	671	498.4	673.4	806	773.7	1469.5	1406.4	1945
1862.3	802.6	1727.9	965.8	1288.2	1269.3	641.9	580.9	1808.3	1685	2920.2
1445.5	1296	1368.5	1044.7	984.8	1256.3	1126.3	1245.2	1796.8	2146.1	2494.3
2724.3	2266.3	2398.6	1671.9	1638.7	1656.6	2350.8	2169.1	4168.4	4472.2	5369.9
647.1	602.8	691.9	391.8	467.7	541.1	532.7	597.2	944.7	982.7	1490.1
930.3	795.1	983.5	728.9	711	746.4	863.5	652.2	1103.5	1621.2	1800.4
372.3	345.9	415.9	375	357.7	343.1	438.6	422.1	802.2	2245.4	1329.8
623.3	580.6	628.8	477.3	489.8	531.9	600.3	552.8	942.9	998.5	1177.7
1108.5	1286.5	1138.5	905.9	871.5	1238.5	1685.4	1302.2	2183.6	1909.2	2464.1

1443.9	931.1	1127.3	1118.7	746.6	762.3	750.9	913.3	779.6	671.9	1070.1
1249.8	1237.9	916.2	569.4	1076.4	1123.9	3413	1178.5	1046.2	1368.1	1485.8
3790	4025	3674.5	793	1652.4	5612.7	5179.6	545.3	586.6	1522.5	1432
741.1	564.4	719.9	480.8	459	511.8	550.8	487.7	909.5	1046.8	1247
492.7	1101.9	670.2	1070.9	872.4	1720.6	662.2	1233.7	362.5	382.5	468.5
1486.1	1250.9	1365.8	1219.6	1092.9	1070.9	1003	1064.5	1648.8	2246.7	2240.5
698.8	885.9	672.1	545	560.4	1697.6	1828	842.9	671.7	1171	1066.9
746	1053.3	809	1084	600.5	1381.5	818.8	1191.5	1373.5	756.9	843.6
512.3	345.8	485.4	311.1	347.7	367	358.7	345.9	525.3	700.6	725.8
141.9	83.6	77.3	99.3	99.7	85.1	82.3	134.2	108.4	96.4	125.8
708.2	544.8	648.5	513.4	472.6	548.6	529	469.6	989.1	1306.9	1468.6
390.7	295.5	344.6	275.7	283.7	268.1	303.9	241.9	458.3	650	627.8
552.5	521.1	510.8	478.7	500.4	565.1	504.6	442.9	926.4	1730.7	1552.8
673.6	550.3	735.8	561.3	586.3	668.9	511.9	456.5	874.8	1103.3	1346.2
487.3	467.5	527.2	344	469.7	500.6	597.7	465	763.7	1122.2	1060.6
1190	1024.2	1172.4	814.3	1115.8	1060.9	1215.7	757.6	1153	1333.7	1561
414.2	492.1	424.5	357	322.8	410.8	812.8	528.4	1271.9	1031	1260.1
485	532.5	518.7	417	447.1	554.6	586.3	525.9	596.1	787.1	744.4
535.4	433.2	505.2	453.9	396	310.9	575.2	419.8	787.2	1240.9	995.5
843	825.2	851.8	739.6	778.7	828	896.2	665.4	877.6	913.1	1200
1112.1	1048.8	1085.6	910.8	857.5	1044.4	1114.6	724	1390.3	1328.2	1658.3
688.2	718	778.7	567	512.8	603.6	865.5	528.1	1068.7	2349.1	1696
404.6	382.2	383.1	276	314	322.9	405	331.8	626.8	587.7	732.2
1162.8	929.9	1140.2	914.2	1257.8	988.5	951.9	905.2	1090.7	1391.2	1509.1
824.9	797.5	1189.9	974.1	734.7	907	789.2	648.2	1433.4	1253.2	1755.7
561.3	568.5	603.6	445	461	507.2	508.9	494.9	770.1	819.9	1003
1659.2	487.2	930.1	577.7	680.5	494.3	447.6	431.2	1025	1752.3	1760.7
681.7	705.2	704	499.5	488.8	576.9	664.4	495.9	828.3	1258.5	1237.9
441	505.6	565	420.8	340.5	505.7	517.6	429.3	662.2	953.6	931.6
749.3	735.8	733.4	618	635	981.9	841.6	701.5	904.9	1182.3	1238.4
878.4	664.1	770.6	576.8	549.6	677.2	659.6	588.9	1511	1969.8	2044.7
800	919.2	890	753.1	718.9	858.1	862.2	964.6	1163.1	1335.4	1383.2
665.3	609.8	830.1	438.2	481.1	498.3	553.1	507.7	761	1570	2190.1

237	248.3	219.8	216.4	156.9	188.5	240.4	192.2	514.6	538.5	647.6
2025	784	1358.7	1119.1	1104.1	793.6	562	732.4	1092.8	1686.5	2465.8
862.3	758.4	835.8	667.8	553.1	643.4	729.4	662.4	1441.5	1857.3	1322.4
207.1	188.8	173.5	147	236.9	172.7	239.4	179.7	219.3	215.1	231.9
1931.7	1318.8	1397.1	1210.4	1138.8	990.9	1104.3	1108.6	2085.1	2445.5	3041.9
656	519.1	617	449.4	403.8	422.9	476.4	488.1	979.5	1336.2	1456.5
868.6	1447.8	1474.3	883.9	924.3	1051.9	1625.6	1048.5	1188.5	1609	2112.6
809.9	714.6	969.3	955.7	774.7	853.2	639.9	590.1	825.5	1059.9	1247.7
553.5	492	534.7	450.6	388.1	563.9	568.2	368.4	529.3	821.3	817.1
807.5	655.2	693.9	575.8	603	837.2	772.5	564.6	914.1	1802	1599.3
871.4	689.9	739	715.3	610.9	649.9	704	815.1	1098.7	1024.7	1221.2
1189.1	753	1314.1	930.7	667.2	368.6	396.2	404.9	703.2	1396	857.8
637.5	522.1	599.8	427.2	419.5	454.4	566.7	529.8	1152.9	1416.5	1562.3
2100.4	1808.2	1597.9	2185.9	1758.8	1929.8	1437.4	1714	2139.2	2180.4	2689.4
1221	983.6	1302.5	1222.1	876.8	949.6	698.2	799.6	1216.5	1178.6	1713.8
1410.3	1099.6	863.1	1027.2	897.2	1595	2098.4	1158.9	770.8	730.6	910.1
1377.2	1625.9	1322.8	1449.4	1153.2	1457.7	1254.9	1612.7	1977.4	1831.3	1995.4
1212.5	1005.2	1161	860.6	824.8	977.2	901.6	1089.6	1802.9	2058	2802.6
570.3	614.6	602.7	536.3	426.5	563.8	655.4	493.7	987	1563.8	1420.1
486.4	556.3	476.4	410.5	396.4	510.8	538.9	431.8	1240	1482.2	1604.8
1194.2	678.1	957.4	1400.8	842.5	834.6	607.7	643.7	1428.6	1428.6	1903.5
1496.2	896.4	1716.8	1414.7	1135.4	803.6	808.9	783.4	2075.8	1372.9	1478.5
844.8	904.8	831.7	698.2	830.1	783.3	1105.9	740.7	829.2	849.6	1026.8
1021	1128.9	1208	920.9	788.6	1181.4	1533.8	1000.3	1212	1358.7	1633.5
837.4	893.9	892.8	775.2	681.8	892.3	663.2	1272.3	1199.6	802.8	1284
556.8	595.6	681.9	611.6	961.5	631.1	1041.2	701.5	1164.9	1873	2084
977.3	1095.4	1076.9	857.8	699.5	979.7	1230.9	833	1982.6	3046.9	2562.7
596.5	756.6	670	581.1	482	655.1	697.4	637	1095.5	1384.3	1382.6
1134.6	971.3	1007.8	887.2	697.8	722.5	941.5	935.4	1537.6	1716.7	2027.1
683.2	523.4	598.1	503.8	564.2	630.3	693.2	568	918	1064.7	1148
491.7	639.1	527.4	570.3	373.3	603.6	539.9	507.2	559.7	593.3	690.3
598.6	489.1	546	429.8	464.6	483.5	595.7	484.5	1099.3	1232.2	1272.5
628.5	668.4	675.7	586.4	513.3	672.1	587.9	591.9	782.2	734.6	939.7

324.6	269.9	308.2	282.3	272.4	284.7	239.3	222.8	352.5	435.4	497.8
867.7	939.2	973.7	741.8	796.6	812.5	1002.3	881.7	1319.8	1443.7	1535.1
778.3	718.7	662.8	788.7	494.8	592.7	875.4	660.4	1731.3	2573.9	1833.7
1272.8	2555.7	2249.7	1467.7	1575.2	3720.4	2952.4	1428.7	1319.9	813.5	1590.1
625.6	581.9	520.3	439	391.5	391.3	415.6	402.6	691.1	930.9	890.7
746.1	789	854.8	667.9	745.5	760.3	972.2	831.4	909.8	1205.6	1184.2
503.6	537.1	506.1	347	595.3	460.1	472.5	493	956.5	865.2	859.5
1777.2	1141.9	1752	1321.9	1428.6	1152.9	931.3	810.9	1476.2	1666.9	2180.3
238.7	224.7	244.8	173.2	180.6	194.6	209.3	176.2	368.5	399.6	533.4
1054.1	1002.6	1023.6	785	785.5	828.2	990.6	923.6	1537.8	1976.2	2335.8
1126.6	879.8	1077.9	902.7	930.2	827.2	857	828.7	1626.5	1897.9	2136.7
815.3	659.9	719.3	683.1	615.8	1005.6	578.8	840.4	683.4	871	940.8
443.9	415.7	431.4	416.4	349	462.3	660.1	450.4	534.8	735.2	687.7
713.4	750.9	905.4	678.2	586.5	842.4	1003.5	638.8	955.6	1632.9	1911.3
873.5	719.1	846.5	602.2	583.7	699.3	747.5	774.8	1593.4	1827.1	2138.9
708.5	1979.3	805.9	495	704.7	1104.2	895.3	607.1	1057	1166.3	1192.7
562.7	712.2	823.4	735.7	556.4	758.1	709.8	813.7	701.4	930	1024.2
426.4	1116.3	505.3	989.7	439.8	1661.5	721.9	987.8	280.7	275.7	325.2
146.4	125	147.8	102.6	105.2	111.1	103.5	104.7	219.8	233.5	322.4
262.7	428.7	280.6	250.9	249.6	288.9	411.8	471.4	1198	1858.1	1544.1
1118.7	792.7	340.7	408.9	1373.8	446	638.7	999.3	398.7	490.4	384.6
665.8	697.7	707.2	527.2	586	726	807.9	694.2	926.5	960.5	1059.1
68.8	73.4	81.2	79.4	63.8	72	91.9	67.6	132	612	227.8
653.5	383.1	525.5	532.2	561.3	518.1	760	681.9	1278.4	671.4	837.4
924.9	887.2	740	699.8	608.6	738.5	1229.8	770.5	1429.9	1256	1569
941.9	891.6	923.7	769.2	694.1	772.4	1029.1	776.6	1642.7	2020.9	1904
870.1	782.8	697.8	686.9	531.4	675.8	663.6	746.2	1052.4	1015.4	1385
627.2	528.9	636.1	486.2	496.6	627.5	561.5	505.8	997.3	1100.9	1264.9
779.2	1350.9	989.5	728.6	609.7	1218.6	1913.7	1017	759.6	905	1137.2
1339.8	938.7	888.1	570.8	684.3	1296.7	1109.7	757	685.2	637.3	1080.4
1041.8	966.3	1052.6	828.4	788.2	886.5	988.6	843.6	1275.9	1546.6	1798
501.3	570.4	639.1	489.3	466.7	756.2	722.4	562.2	769.7	729.4	993.8
544.7	445.5	613.8	479.1	392.6	482.2	473.4	391.9	522.6	695.7	819.6

1659.4	1397.1	1621.2	992.2	923	1054.1	1358.4	1268.7	2819.8	2712.6	3536.7
1451.4	640.3	886.7	662.6	548	515.6	448.3	645.2	1928.3	1761.1	2046.1
958.7	889	816.8	657.2	496.3	550	750.1	546.9	1380.1	2285.9	2018.4
1481	1048.7	1245.2	1067.1	1076.8	1011.4	1224.9	807.7	1461.9	2277	2357.4
406.5	428.6	393.6	348.7	295.5	333.7	375.5	358.5	638.9	732.7	890.8
966	1395.7	1228.8	1140.2	881.5	1173	2056.5	2190	2643.3	1493.2	1978.1
679.3	615.3	599.7	439.7	459.6	749.1	716.8	624.8	857.1	1207.2	1247.4
518.4	579.9	506.2	552.5	439.2	482.1	541.8	467.1	740.2	1041.3	1127.3
851.5	678.4	676.7	648.6	495.1	621.9	757.4	884.4	1035.4	1294.3	1242.9
391.2	314.6	530.3	376.3	253.2	254.2	247.8	196.2	307	555	482.9
1261.1	1363.3	1175.4	1245.5	1057.8	725.3	1209.8	1300	762.9	723.1	770.8
61.9	59.3	73.4	61.7	45.6	67.6	74.2	53.2	110.7	118.3	142.8
1086.4	844.1	1024.9	920.8	702.2	705.3	746.2	708.9	1024.9	874.8	1168.5
2123.3	515.1	1151.2	756.7	896.8	660	363.3	429.5	1253.9	1858.3	1884.1
725.3	706	796.7	679	598	676.5	645.2	715	1416	1677.4	1975.1
665.2	585	675.3	454.7	474.7	555.2	551.8	566	1042.6	823.5	1301.8
1446	1376	1382.9	995.7	993.4	1255.2	1477.8	915.7	2560	2683.5	3252
1004.6	877.7	974.2	738.8	671.3	654.5	585	823.1	1903.9	2277	2787.3
546.6	431.3	458.7	350.6	417.9	467.5	434.6	416.8	903.8	1661.9	1429.7
1367.5	1176.4	1420.8	1026.1	976.6	1101.1	1217.9	769.9	1928.6	2811.8	2831.4
1091.2	1092.8	1112.6	1099.8	747.1	815	959.1	739	1229.6	1773.4	1961.9
790.1	630.8	862.6	1271.8	691	478.8	389.6	515.4	428.1	516.8	489.7
1330	1162.3	1195.7	892.4	847.5	1123.4	1281.8	1061.9	2286	2280.6	2961.8
396.7	360.4	903.8	565.4	653.1	354.7	348.5	331.4	622.5	619.2	968.8
374.9	364.1	373.8	299.1	337.7	385.7	425.7	387.3	529.3	477.2	618.6
1141	981.3	1162.3	937.1	822.8	904.9	893.7	812	1428.5	1527.2	2216.3
393	489.5	440.6	366.1	401.1	432.9	464.4	332	1128.5	983.2	1033.6
640.9	655	682.6	488.5	449.9	537.6	611.1	542.7	678.9	950.3	933.8
541.4	495.1	533	473.6	445.2	553.9	553.4	524.9	1092.5	1086.5	1375.5
642.4	596.6	702.7	488.9	458.8	551.5	653.4	537.8	787	1039.6	1117.8
667.9	438.9	578.3	333.6	452	444.7	365.3	357.2	578.1	604.1	775.3
384.3	554.8	399.1	349.7	504.4	476.8	1636.6	385.7	464.7	3036.9	1640.1
651.5	655.9	610.1	541.4	544.9	937.8	1758.5	695.6	508	516.9	601.5

556	559.3	649.5	451.9	388.5	529.1	473.5	483.7	641.1	630.8	833.7
433.5	390.5	473.6	403.8	316	336	412.3	363.9	551.6	670.1	734.7
1092.7	915.5	1059.1	949.6	731.8	869.2	1094.8	770.9	1581.8	1936.2	2029.7
995.8	1243.3	1039.9	1122	901	1120.8	1353.4	931.8	1429.6	1284.4	1462.6
1315.4	1064.8	1697.2	1899	1968.8	3692.6	1719.6	1352.4	958.4	3875	2058.8
769.6	1057.4	909.6	674.1	636.2	1091.6	1664.1	698.7	707.5	770.6	1320.8
980	967.3	1114.4	945.9	849.6	1055	797.8	650.1	1265.3	1184.5	1422
886.1	670.7	836.7	650.2	533.6	468	551.6	752.7	838.9	928.5	1163.2
679.7	429.8	599.9	505.3	396.8	412.8	358.8	390.4	471.8	655.2	614.7
1808.5	1718.2	2338.2	1600.2	1374.3	1237	1037.3	1239.8	1776.5	2005.9	3207.1
1091	1006.3	1087.1	816.2	943.9	1057.9	1041.4	858.5	1057	1162.2	1452.7
835.9	711	780.1	567.8	545.9	619.1	940.2	717.8	1162.1	1730.3	1482.3
1290.5	1214.3	1197	866	859.3	787.5	698.3	833.2	1337.2	1408.5	2026.4
1106.2	930.5	1057.8	811.7	876.5	840.1	985.2	793.5	1271.5	1539	1982.6
516.8	454.5	559.6	388	452.7	489.4	604	502.4	684.9	850.9	937
1357.1	1280.9	1667.1	723.9	1102.7	1536.6	1489.3	440.8	937.9	1494.5	1682.3
530.3	619.9	588.2	495	424	584.4	524.6	588.9	887.3	782.2	1030
773.1	516.1	680.3	537.5	524.5	460.9	441.7	481.4	1175	1500.9	1839.9
619.6	550.9	521.1	438.1	491.2	522.6	585.7	508.1	1030.2	1203	1301.4
483.6	459.3	476.3	418.1	383.6	463.1	531.4	556.3	779	991.9	1013.8
475.4	503.2	611.7	485	499.3	499.9	595.4	409.3	436.5	508.4	591.7
792.4	552	630.3	514.3	497.3	553.9	509.5	530.1	1092	1230.8	1531.8
679.7	794.4	764.1	598.2	570.7	594.1	826.7	619.1	995.6	1129.1	1287.6
489.7	577.4	590.7	406.8	406.8	673.1	677	511.8	687.3	668.7	814
774.6	555	835.3	590.7	470.3	563.2	487	476.9	1005.4	1517.6	1741.6
856.7	871.1	941.9	777.4	673.4	754.3	793.2	991.8	1415.3	1323.2	1493.8
840.4	575.8	774.2	539.4	587.3	613.8	632.2	618.8	1153.7	1577	1737.6
252.2	389.9	258.1	295.7	259.9	377.5	330.8	211.8	745	1511.1	1085.7
1616.2	978.9	1170.7	1152	905	852.6	912.6	782.1	1141.4	1672	1680.9
1238.3	1086.7	1394.1	1040.5	948	994.5	873.9	963	1558.7	1805.6	2626.7
525.5	518.7	718.6	631.6	458	552.9	839.9	576.7	1203	1915.7	1919.3
905.6	554.9	812.1	560.7	697.9	440.5	528.9	461.1	793.5	778.6	1177.9
2238.6	780.4	1000.4	807.4	842.7	792.5	730.6	663.3	1505.3	2124.9	2491.5

1393.1	1170.9	1472.9	1033.9	1025.3	1127.1	1131.3	935.2	1186.2	1864.7	1989.3
1063.4	1163.8	1006.5	855.3	856	1171.1	1877.8	1039	967.4	1058.2	1223.9
1010.5	833.4	1014.4	734.7	821.6	827.4	757.6	701.8	1301.1	1540.9	1949.3
769.8	804.7	775.3	618.9	597.5	767.1	796.8	750.8	1720.1	1469.9	1738.3
818.6	883.9	973.9	766.3	644.9	823	881.6	808.1	1118.7	1151.6	1531
175.8	148.1	165.5	236.7	230.9	152	189	132.1	207.2	1132.4	347.6
545.1	566.6	565.6	494.6	445.4	473.2	563.9	479.7	727.9	980.1	887.3
1209.5	2383.6	1952.2	1631.8	2623.6	1638.5	1505.9	2613.2	1599	1004.7	1335.5
1080.8	872.2	922.1	786	722.2	728.6	626.9	545	973.6	809	1043.6
443.6	394.1	499.1	457.5	388.2	503	1127.1	407.6	424.6	1035.8	581.3
1106.8	1315.3	1436.7	1054.7	949.2	1004.5	1631.3	1381.7	2931.2	2198.6	2962.2
617.7	446.7	500.2	374.7	378.7	324.1	426.8	386.2	904.3	524	764.2
1160.3	1047.2	1118	904	677.5	909.1	1114.5	988.5	1586.1	1880.8	2303.5
825	735.7	785.4	664.3	613.6	780.7	899.9	695.1	846.6	917.3	1181.8
413.7	322.4	422	299.7	257.6	288.6	347.2	318.5	564.7	854	764.3
976.3	1143.4	1086	1147.8	1384.8	1213.4	1063	1921.6	1044.6	700.1	835
511.9	382.9	487.4	315.1	309	335.6	357.1	340.2	600	574.9	724.9
577.9	528.7	569.5	440	414.9	518.1	562.6	548.8	1136.4	1086.5	1218.6
598.4	510.2	583.2	419.2	484	430.1	495.7	438.7	861.3	1036.8	1151
730.6	771.6	795.6	690.1	609.3	748.4	791.8	561.8	1083	1556.3	1466.5
796.4	840.9	905.2	640.5	620.9	838.4	1156.3	820.2	1150.3	1176.7	1459.5
864.4	903.9	859.7	727.1	659.9	894.4	1108.4	658.1	1222.6	1481.4	1758.6
675.2	607.6	675.5	535.4	439.4	570.7	580.8	535.3	763	766.8	941.7
968.4	968.8	1069.4	951.3	710.8	856.4	857.4	768.4	1152.9	1886.3	1981
531.7	458.6	547.3	350.2	374.7	348.5	448.3	375.3	633.6	785	914.8
653.8	609.9	538.6	542.2	548.2	502.9	627.2	551.4	1005	965.8	1180.1
764.8	1150.6	1841.5	1285.9	1122.4	1767.3	819.4	1219.8	346.4	582.1	693.6
396	411.5	424	313.8	384.6	433.2	442.7	350.7	523.9	453.2	599.9
895.2	835.3	1060.7	857.5	631.8	644.6	802	755.8	1112.5	818.8	1159
1090.1	995.5	1034.1	903.5	640	706.8	718.4	1187.2	1594.8	1915.6	2171.4
956.5	1103.5	1141.3	787.1	665	1065.9	1385.1	1141.8	1853.2	2629	2466
653.3	495.6	513.6	407.4	464.6	526.2	597.7	517.5	934.9	1008.2	1173.1
403.3	396.1	417.7	307.6	311	377.2	467.2	365.1	600.8	464.8	564

613.2	529.7	590.7	400.7	366.2	529.2	491.1	417.2	761.7	939.4	1091.7
736.8	681.9	660.4	552.7	647.5	790.4	790.4	733.3	1085.9	1012.3	1365.8
786.2	716.1	756.5	592.3	551.7	659	1010.7	618.3	1335.5	1884.3	2107.7
914.5	678.2	829.3	631.1	558.5	707.8	711	630.9	1121	1534.6	1632.5
437.4	389.3	342	349.2	411.8	320.4	423.4	393.1	377.1	392.2	490.9
566.4	473.6	521.5	428.3	424.2	467.1	564.1	545.7	884.2	1074.7	1242.4
844.4	723.5	773.6	515	577.1	572.6	618.1	583.1	1134.5	1198.8	1490.7
814.6	748.6	736.4	601.9	592.7	653.1	680.3	852.4	1140.5	1243.9	1398.1
296.6	222.3	306.8	249.6	189.9	276.8	208	199.3	278.2	606.5	487.3
1515.9	1306.9	1020.8	1342.4	810.2	1166.4	797.9	1041.8	992.7	1130.4	1223
820.6	779.1	882.8	709.1	702.1	740.1	807	746.1	1065.9	1193.9	1399.7
445.8	430.7	607	565.1	444.6	642.7	488.5	472.2	1296.3	934.9	1476.6
660.3	593.1	689.7	564.2	477.4	582.2	574.2	544.6	865.2	876.6	1037.9
1225.9	1040.4	1134.8	998.8	858.6	1045.6	1327.5	1058	1804.3	1868.7	2130.8
411.7	307.8	377	291.9	301.9	318.3	318.8	278.7	490.6	1101.1	730.7
1716.4	1538.5	1631.8	1386.5	1061.6	1365	1403.4	1729.7	2437.7	1896.2	2688.5
773.5	675.2	727.5	587.9	607.5	614.3	767.9	627.1	780.4	913.1	987.2
471.1	485.2	568.7	451.3	395.5	548.6	599.1	1042	612.1	594.6	901.9
1594.2	1447.8	1292.6	1098.5	1032.3	1150.7	1597	1077.4	1970.7	2188.6	2386.8
1057.4	1060.5	1120.7	923.8	740.7	991.7	1070.3	1104.1	1594	1110.9	1483.4
734.9	505	639.9	404.4	413.7	418.6	402.8	449.1	978.9	1238.9	1537.7
894.6	778.6	756.3	680.4	661.9	781.3	809.2	887.8	1678.6	1641.9	1588
2273.2	1180.3	1612.6	1179.6	1010.1	1312.2	1500.2	894.9	1610.5	3039	2564.2
251.7	247.7	331.9	283.3	249.2	242	211.9	243.4	290.9	263.4	379.4
947.5	777.1	921.4	632.5	649.5	685.8	713.1	728.6	1400.1	1668.5	1917.7
1256.2	1067.8	762.4	609.1	1217.8	957.6	1623.4	925	873.9	869.2	1160.6
655.7	620	580.2	522.5	375.5	525.9	549.7	475.9	1102.5	1467.7	1520.6
1525.1	1352.6	1407.3	1060	1066.5	1155.2	1137.9	987.9	1872.8	1634	2297.4
604.5	509.7	596.1	480.6	474.7	562.6	567.3	467.9	882.3	804.1	1055.1
573.4	408.9	454.2	361.5	327.4	353.3	339.8	441.1	889.7	1064.2	1302
667.5	1192.8	805.9	888.9	498.5	994.4	952.5	1582.2	1192.2	710	967.7
842.8	844.5	984.3	743.4	745.5	839.2	853.7	774.1	1050.6	1159.7	1422.4
353.5	480	429.3	382.7	270.6	303.5	341.9	394.2	653.3	1061.1	1021.2

713.6	580.8	672.7	538.2	483.5	589.9	544.8	623.3	1257.7	1083.7	1519.4
774.1	815.3	667	710.9	767.7	707.7	988.6	779.4	989.7	1162.5	1346
799.5	715.3	628	1005	565.5	831.3	677.4	805.8	420.5	374	466.1
1554.5	856.3	1054.3	712.8	908.5	802.3	778.8	758.2	809.3	1950.4	1841.5
701	811.5	725.6	601	514.3	768.6	1063.8	785.7	1370.9	1272.3	1613.6
1125.2	1165	1391.4	975.2	769	1083.8	1018.9	923.4	1517.4	2114.4	2592.1
832.9	668.1	927.1	695.6	733.9	719.2	705.3	444.9	855.4	1379.4	1648.3
303.9	300.7	376.9	304.8	299.2	388.9	541.4	306.7	378	518.2	669.6
347.3	352.4	359.5	338.7	308.9	365.7	379.9	329.1	697.4	791.1	878.3
1207.4	171.2	593.9	1286.9	467.3	160.5	151.5	117.3	91.3	122.1	139.6
804.7	625.6	782	599.7	505.6	590.4	623.7	611.5	957	1385.9	1320.1
585.1	504	559.2	472.3	523	603.9	565.8	469.7	1171	1021.2	1422.7
785.8	854.3	798	705.9	779.2	922.9	808	862.9	869.5	958.8	1070.3
1172.5	1228.3	1208.5	1070.1	1090	1160.1	1112.1	1231.9	1876.6	1511.8	1841.4
1122.8	650.8	780.4	926.5	489.5	629.6	482.3	914.9	863.2	1166.8	936.2
1449.3	664.5	1086.9	741.1	845.5	660.1	732.2	587.8	1551.6	1901.9	2477.9
1327.9	1150.2	1216.5	1027	833.9	886.8	1313.6	1013.1	1116	1821.5	1737.8
675.9	646.6	732.4	491.8	566.5	626.9	622.9	587.3	948.7	1140.7	1185.9
1384.4	1512.7	1185.8	1192.5	1136.7	1776.8	1447.7	1278.5	1992.2	1439.7	2189.2
483.8	403.2	478.5	453.5	342.3	368	414.2	422.9	636.9	838.5	937.4
391.3	476.2	408.4	356.8	359.8	690.2	674.8	599.7	419.7	430.6	504.8
553.9	462.6	551.5	446.1	438.1	501.4	512.1	519.3	848.6	877.5	1116.5
587.5	624.3	689.5	571	527.6	657.4	732.9	659.9	1461.5	1552.3	1711.6
974	991.6	1101.3	821.1	773.8	861.7	1127.6	871.9	1581.2	1913.1	1821.9
925	849.4	863.1	700.8	637.9	767.5	1022.8	829.3	1404.7	1438.4	1711.6
858.7	604.7	635.4	520.1	469.8	548.4	438.6	525.7	930.2	1685.6	1614.9
541.3	451.5	563.9	472	434.3	441.1	436.7	446.4	693.7	899	1131
464.3	435.2	533.4	401.5	353.1	455.4	405.7	384.5	523.6	514.5	773.8
955.6	841	848.5	742.4	773.3	967	987.5	769.1	1019	1416.1	1300.7
718.1	561.8	796.8	642.6	501.6	544.3	464.6	534.4	803.1	840.8	1109.2
588.2	708.8	674.1	528.7	472.2	696.8	784.4	708.4	864.3	908.8	974.9
1061.2	691.4	842.6	606.2	744	621.2	767	623.7	823.6	1555.4	1302
780.7	1202.9	874.1	926.5	482.9	659.6	487.4	1028.3	969.8	653.1	856.1

215.7	195.6	172.4	177.8	154.7	290.3	191.3	220	173.5	164.2	266.6
583.4	380.7	444.1	368.1	510.8	443	504.2	376.2	1412.5	1048.7	1638
738.3	560.6	650.4	494.2	536.7	506.3	588.1	540.8	947	1178.7	1257.5
1105.1	865.8	1044.4	909.2	576.7	782.4	713.4	690	1401.6	1375.8	1650
1207.8	707.3	1245.2	701.7	615.8	547.1	787.6	691.8	708.6	560.7	628.6
412.6	334.4	312.1	272.3	319.7	303.1	376.1	271.9	519.4	612.1	657.6
619.4	574.4	605.7	441.1	452.9	495.4	577.4	486.9	818.4	1010.5	1140.5
895.4	1076.3	1153.9	909.7	1152.6	1240.2	1054.3	1151.6	1092.9	1216.8	1533.7
1232.7	1296	1190.3	1253.3	933	1008.9	1134.7	985.9	1457.9	2004	2259.3
310.5	274	351.1	241.8	261.6	290.4	295.4	270.2	459.6	435.1	564.1
650.5	670.7	677.8	488.7	447.2	519.4	648.3	586.7	970.9	1532.4	1420
149.1	60.1	104.2	113.2	78.6	101.7	39.4	42.6	61.3	114.8	177.4
828.5	704.1	889.3	681.8	536.3	585.7	545.6	602	617.5	713.3	881.9
277.6	323.8	301.2	251.1	244.8	275.3	336	322	349.1	494	489.1
636.1	544.4	609.2	502.3	488.5	525.1	531.7	586.7	978.4	1116.7	1245.8
724.2	643	574.8	636.5	511.9	561.3	601.7	808.4	1147.7	1129.8	1093
1001.3	888.1	1086.7	752.1	803.5	724.1	867.9	775.5	1487	1152.2	1840.1
717	1002.7	683.8	1291.5	520.9	544.5	475.3	724.7	555.6	898.8	824.1
948.1	777.7	1221.8	932.9	691.4	989.6	702.7	807.9	1557.1	1324.2	1897.3
873.5	571.2	755.5	612.8	508.8	774.3	1026.3	594.8	504.9	819.2	1121.6
644.9	626.2	580.9	441.3	471.6	572.6	653.9	548.3	1387.6	984.3	1692.5
498	1245.8	842.9	847	690.3	1071.4	1289.7	1246.1	769.7	750.8	744.8
794.3	468.8	635.3	386.9	390.8	344.4	343.7	450.8	603.5	605.9	745.2
309.3	332.9	292.6	299.9	275.7	300.5	345.4	281	411.5	519.1	593.5
1184.6	861.1	1263	1058.9	752.8	817.4	619.1	683.1	870.9	1135.3	1465.5
260.2	254	415.9	246	342.2	793	402.6	348.2	1438	401.1	1507.4
245.8	194.5	239.5	207.8	195.7	193.4	231.2	214.2	336.4	430.6	512.5
1358.3	1197.9	1208.2	890	856	1270.7	1457.4	912.6	1216	2163.8	1933.5
396	570.8	574	498.3	429.5	501.8	645.7	690.5	452.5	623	677.7
796.4	865.3	958.2	601	513.8	898.3	1742.6	651.3	577.6	1188.6	716.3
1305.7	1066.9	1314	1029.8	919.3	1028.5	1043.8	870.2	1434.4	1812.4	2041.9
579.9	587.4	508.4	360.9	451.3	544.4	773	498.3	506.3	845.6	918.5
869	801.6	867.5	547.7	502.3	585.7	838.2	708.5	1554.7	1542.9	1950.9

462.1	407.4	413	320.9	288.9	320.7	394.2	383.1	739.1	835.3	887.8
600.7	613.7	688.9	533.8	536.2	611	666.2	569.5	2240.6	2093.6	2848.7
799.9	542.1	764.3	709.5	573.9	652.7	539.1	346.8	712.2	1374.5	1161.6
836	896.9	836.2	780.6	677.2	729.2	943.1	765.4	1465.4	1538.9	1522.5
515.4	526.2	522.3	422.7	406	471.8	557.3	529.3	782.7	891.2	980.7
741.8	603.5	642.5	457.6	543.6	492.1	595.3	552.1	1198.1	1436.5	1659.8
1086.9	1168	1484.8	1166.8	873.7	952.9	772.5	1058.4	952.1	1367.1	1401.8
925.8	756.6	888.8	642.6	603.8	709.5	748.9	782.3	1433.5	1726.5	2077.7
586	695.5	656.9	583.8	459.5	557.2	634.9	692.6	812.3	734	736.1
758.1	879.4	696.7	551.6	519.4	564.5	674.3	668.1	1019.6	570.8	865.9
1078.3	816.6	976.2	658.7	615.4	694.6	730.1	738	1301.9	2295.8	2320.1
926.2	753	925.4	792	664.6	772.4	769.1	743.7	1191.2	1419.7	1496.1
640.8	622.3	678.5	519.4	453.1	596.6	544.6	546.6	1017	2148.3	2253
326.6	188.8	344.4	233.1	166.3	187.3	1129.8	177.2	570.4	3125.1	991.3
1124	606.4	880.6	735.8	717	801.1	874.6	719.8	1654.4	1524.5	1571.8
644	588	641.3	503.2	501	569.9	693.7	560.3	813.9	933.8	1036.2
1119	976.7	968.7	798.5	774.2	941.7	961.1	838	1682.4	1626.5	2224.9
167.4	152.9	181.8	130.1	143.8	171.4	161	137.8	292.5	261.4	321.9
742.4	760.2	705.1	594.8	567.9	723.5	919	681.1	872.1	969.9	1081.3
499	468.5	604.6	539	1068.4	575.3	503.7	452.7	564.8	678.8	727.6
364.7	330.1	346.2	307.4	330.9	364.1	415.4	338	385.8	564.8	513.1
975.9	965.3	1064	857.1	776.6	960.4	897.9	786	1318.2	1458.1	1773.6
369.6	481.4	503.2	391.3	367.2	463.7	492.3	424.4	499.2	754.1	697.9
1332.8	1152.9	1295.3	1058.1	973.2	1098.5	1147.3	1472	1492.8	1157	1676.9
774.8	706.5	849.5	700.6	624.6	680.5	680	553	813.2	1110.2	1285.5
707.9	621.8	636.2	458.2	547.7	424.4	514.7	498.9	982.9	1707.2	1441.2
627	706.1	596.4	631.9	429.5	366.2	754.1	582.7	407.6	333.3	436.8
570.5	437.7	615.9	383.3	330.7	313.1	345.6	300.1	806.1	1204.1	1242.2
646	793.7	940.8	414.5	379.7	719.6	820	330.9	495.3	503.2	594.1
664	622	718.8	539.6	556.2	610.4	716.2	593.4	991.8	1149.5	1346.3
1109.7	1229.2	1757.8	1449.7	795.6	946.9	1000.6	1210.6	786.2	1274.9	1057.1
588.3	547.1	566.3	458.4	471.2	557.1	534.9	640	748	882.2	1111.5
678.3	489.5	457.5	387.7	340	358.1	693.9	301.6	723.5	1884.3	1363.4

1017.3	698.7	764.2	736.4	600.8	657.6	645	570.4	783	953.2	977.2
781.3	961.3	848.6	828.4	667.6	1079.7	869.9	1158.4	1395.2	1034.5	1362.9
433.5	312.3	346.3	301.8	268.1	310.9	226.8	417.1	545	645.5	774.6
459.8	433.3	480	379.2	314.8	449.8	574.1	369.8	427	738.2	601.2
733.4	615.9	721	552	629.1	623.2	582.9	617.9	990.2	1172.4	1437
572.3	521.1	540.6	454.7	408.9	465.6	567.9	388.3	719	874.4	1037.6
937.6	871.4	905.7	830.2	705.1	818.1	1002.2	780.1	1292.2	2168.6	1942.2
396.7	339.6	354.1	315.5	285.8	250.8	340.2	347.4	573.9	661.9	818.1
1409.9	980.7	1543.8	1448.2	999.7	989.4	1187.2	912.2	937.9	1528.4	1361.1
763	618.6	706.9	471.7	457.5	544.8	644.4	567.6	1163.4	1683.1	1684.9
207.6	190.8	245.4	179.2	174.2	217.1	213	183.7	204.7	390.5	297.8
462.7	467.5	468.2	447.7	415.9	495.9	497.6	435.8	970.1	724.8	1294.6
1411.5	883.6	1354.5	1525	1345.9	1993.7	1207.1	1407.4	713.8	381	618.3
931.8	853.5	901.1	705.9	719.5	681.2	683	648.6	1292.1	1524.6	1843
216.6	270.3	275.7	371.6	337.4	204.5	228.3	462.2	173.1	118.6	171
742.9	691.3	688.5	866.4	985	902.6	966.6	621.5	1000.8	1521.6	1648.8
649.4	475	568.9	464.3	405.1	525	514.5	480.2	964.5	1202.6	1231.8
371.2	409.7	396.8	373.7	331.8	401.8	447.9	394.8	600.8	563.4	693.2
727.7	837.6	808.5	754.3	630.4	798.1	806	714.2	1088.8	1107	1349.6
263.8	245.2	313	250.4	237.9	206	243	226.8	223	527	246.7
459.1	553	808.9	467.7	491.7	1280.3	953.6	489.1	462.6	604.8	691.5
727.8	737.5	760.1	613	632.4	641.2	786.2	686.5	825.1	1092.9	1157.9
524.5	473.8	547	370.3	371.3	470.9	515.7	467.3	672.8	749.3	810.7
714	450.4	714.2	549.2	443.5	413.3	348.9	323.7	532.3	734.3	953.4
633.3	732.1	768.8	718.6	474.2	622.6	879.7	897.7	1159.9	708	1077.4
1666.2	693.8	1284.4	782.8	955.8	530.5	562.9	515.3	802.3	2092.9	1468.3
998	1209.2	1296.4	1064.4	1001	1284.7	1158.1	994.9	1230	1577.1	1901.1
794.5	675.3	778	601	501.9	517.8	758	438.4	1331.4	1914.9	1882.9
484.2	595.6	322.4	253	275.7	415.5	556.7	323.9	582.1	285.1	538.4
427.2	433.1	455.9	352.7	344.2	380	459.9	415.1	808.6	979.5	1061.5
552.7	458.9	574.9	476.7	491.8	511	553.7	512.8	792.9	1003.2	1151.2
533.7	1579.8	1202	1619.3	1196.6	3852.3	1661.9	2928.7	448.2	616.9	712.9
708.9	752.4	663.8	583.9	636.7	761	906.2	818.8	943.7	1011.1	1115

456.9	484.5	485.5	447.2	436.2	471.6	628	477.6	677	820.7	849
541.2	336.5	413.8	335.2	312.4	328.8	333.4	355.6	643.9	786.4	780
532.7	548.7	562.7	427.4	387	407.8	505.8	438.4	934.9	1158.4	1217.2
417.1	375.4	431.8	378.6	318.6	373.9	387.3	395.6	497.2	554.6	646
297.9	322.2	299	239.5	305	318.7	411.6	316.9	567.1	653.6	675.7
588.6	652.9	705.2	470.4	507.9	538.3	636.6	719.5	1058.6	966.4	1460.3
364.3	249.8	286.2	238.5	259	319.5	339.7	428.7	740.4	830.3	898.2
323	271.1	287	226.6	228.7	262.6	390.1	219.5	469.7	720.6	719.2
570	590.9	466.1	509.8	465.8	603.8	566.9	465.9	516	671.7	899.6
1496.3	1362.9	830.8	600.5	1913.6	1314	2959.4	908.9	699	1094	682.4
1029.8	903.1	1062.2	868.4	693.6	887.6	838.7	799	1348.6	1371.4	1901.3
468.5	410.8	447.5	350.3	321.2	353.8	343.9	525.6	735.3	883	1094.7
389	326.6	316.2	264.9	290.3	269.6	228.6	319.8	595.1	627.9	799.8
978.7	964.8	1036.6	891.6	810.3	1006.7	1140.5	923.7	1235.9	2096.8	1921.2
569.6	522.2	537.1	387	425.1	464.7	451.1	474	942.8	1138.4	1313.8
1397.6	436.5	377.1	766.3	907.8	338.3	952	775.3	466.2	487.9	414.1
833.9	876.6	894.7	731.2	852.5	897.9	998.2	870.1	793.7	922.1	1105.3
1487	1572.8	1495.3	1470.3	1113.7	2233.2	1761.8	1384.6	1615.8	2068.2	2016.9
945.3	1226.7	992.1	919.9	748.1	880.6	784.6	1152.6	979.4	853.8	994.5
341.8	349.7	391.2	312.7	261.6	379.7	413.3	312.2	375	411.2	434.2
344.5	346.3	408.4	391.9	317.7	616.2	524.7	459.3	996	466.6	713.1
522.9	503.1	468.5	299.8	362.2	395.1	471.2	494.6	731.3	964.1	1052.8
1062.5	807.2	964.1	715.3	567.4	716.9	769.7	664.2	1524.8	2185.6	2361.4
960.7	663.2	945	663.9	564.6	664.8	683.1	589.1	686	920.7	1050.2
369.2	456.3	420.5	439.5	373.9	513.1	618.2	700.3	2145.1	1017.9	2256
1416.8	1311.7	1306.3	1547.4	2728.9	849.4	1546.6	2439.1	1716.2	597.5	949.3
820.3	675.2	810.7	641.8	666.6	715.7	708.1	596.5	922.9	924.4	1291.9
584.4	654.4	656.1	634.5	585.4	641.6	760.7	618.4	936.5	1562.9	1366.5
1064.5	1216	1238	1052.9	1175.7	1103.9	1015.4	712	851.8	1481.7	1450.4
772.2	690.4	803.5	682.4	556.5	612	518.3	622.2	867.5	1052.7	1298
574.1	558.5	613.8	543.8	510.5	659.3	675.7	535.7	767.1	744.2	911
523.4	456.1	493.6	457.9	451.7	528	495.3	518.7	632.4	554.7	714.2
832.9	906.8	869	669.7	679.2	944.6	966.7	703	1210.1	1281.4	1647.8

883.5	855.5	945.5	648.9	621.5	716.8	784	734.6	1457.7	1874.5	1987.6
506.2	401.7	502.8	393.4	318.5	350.7	347.5	311.5	464.7	560.6	818.1
545.6	623.9	690.3	368.3	439	557.6	730.4	558.3	1107	1323.4	1417.9
460.7	447.4	501.9	406.4	342	439.8	453.1	431.4	729.1	845.3	876.4
498.8	495.3	531.8	402.9	368.1	437.8	446.7	486.9	957.4	1066.1	1292.8
1118.9	1037	912.5	838.7	920.6	958.2	1075.3	773.3	1166.2	1974.7	1919.6
358.9	317.6	352.5	287.3	318.9	328.7	327.9	324	411	427.3	552.2
744.7	633.4	766.8	560.5	527.5	632.4	647.6	573.1	1074.6	923.7	1323.7
788.1	623.4	763.7	555.6	521.9	547.9	606.7	582.7	1238.3	1506.4	1861.7
388.6	331.8	394.4	334.5	280.6	402.9	385.2	365.5	541.8	661.3	707.8
703.6	775.9	966.3	574.8	672.3	968	932.2	587.5	743.1	963.3	1187.2
1189.1	1008.1	1117.1	948.1	830	949	1127.5	831.3	1083.5	1537	1722.6
385.8	307.4	347.1	276.9	275.2	386.9	340.1	269.5	556.8	742.6	759.2
802.9	388.4	501.5	331.9	444.5	375.4	371.6	362.8	749.9	782.3	1082.1
1408.6	1211.8	1065.9	804.7	723.2	1008.4	1310.3	1262.2	2714.9	2609.3	3407
360.6	385.3	400.6	362.1	393.2	678.9	516.3	422.3	716.1	842.3	1293.1
730.9	574	650.1	521.3	585	598	826.5	525.7	850.2	1037.6	1197.7
798.3	639.7	982.9	607.3	596.9	821	562.1	528.3	571.5	602.6	1153.5
711.9	706.6	842.7	712.4	596.6	890.8	681.5	673.1	1141.4	1910.3	1770.3
251.7	338.8	314.9	284	243.1	325.3	403.7	325.5	514	479.5	550.4
805.8	842.3	863.9	765.7	641.4	1038.4	816	1114.6	1537	1138.5	1593.3
688.9	759	700.8	699.6	626.8	987	794.7	687.3	896.4	855.9	1081.9
605.3	427.7	499.4	388	380	423	462.2	437.5	1022.5	1164.2	1412.1
396.1	394.3	375.6	739.5	312.4	314.4	913.4	1009.6	523.7	386	630
661.2	657.4	720.3	615.7	528.8	510.8	673.3	629.3	1311.4	1515	1675.3
410.6	375.3	450.1	350	290.5	383.7	335.4	366.4	541.3	647.5	749.9
1855.2	3161.7	1807.8	2527.1	1219.7	1113.1	1170	3157.3	2868	756.4	3228.5
840.2	1060.7	1127.1	881.6	642	899.6	996.4	1314.5	1205.2	2053.5	1982.3
624.2	626.5	737.1	549.6	496	580.1	674.6	558.6	973.8	1366.2	1466
446.2	301.1	378.2	418.1	360.1	512.8	541.1	595	1461.8	651.4	1031.2
510.5	452.8	506.4	375.4	355.8	414.6	401	339.2	755	822.2	987.8
622.8	569.7	505.4	487.4	551.7	575.3	669.8	555.7	502.8	707.9	672.9

723.3	791.4	878.6	724.2	631.5	1047.5	1051.9	667.2	892.9	1288	1188.6
447.2	425.2	333.9	297.8	305	481.9	380.6	520.6	452.7	359.9	504.6
317.9	368.8	355.1	304.5	286.8	326	540.4	367	420.2	718.8	556.5
3078.7	950.4	2283.9	1784.3	1221.8	1620.1	2810.5	1234.4	251.2	1442.8	472.5
866.1	820	946.7	714.7	727.2	832.1	854.3	820.5	1039.1	1401.8	1621.6
747.3	611.4	610.7	498.6	522.2	594.9	624	564	1248.9	1543.6	1643.7
533.4	384.6	484.8	353.4	442.5	339.6	425.9	349.4	590.1	616.6	746.6
426.5	526.3	455.9	466.6	354.9	583.6	566.1	484.3	336.3	392.9	451.6
670.2	508.1	699.7	580.1	561	580.6	533.5	418.6	657.9	805.6	1076.4
810.1	980.6	820.9	700.2	784.2	769	1097.6	1010.8	983.7	1613.7	1462
1193.6	1136	1372.6	1146.3	925.4	1004.4	1183.8	1009.2	1996.1	2314.9	2446.2
634.6	752	797.4	616.4	651	732.1	864.6	685.5	1156.2	1413.7	1590.9
1027.7	932.6	780.5	725.9	544.6	549.7	795.6	722.4	606.2	589.2	924.8
442.3	433	378.6	260.6	487.2	262.3	563.6	463.8	297.9	339.7	326.8
711.7	703.6	667.4	523.9	534.6	641.2	684.2	591.6	1072	1314.2	1632.8
444.9	408.1	458.6	366.8	355.8	362.1	421.4	361.7	564.3	1389	770.6
472.3	410.5	534.3	471.5	420.2	460.5	495.5	456.4	813.6	1884.1	1042.7
758.5	751.3	804.9	751.6	532.5	553.7	537.2	483.4	571.6	754.9	954.8
489.6	434.9	522.2	382.1	415.3	379.4	320.8	323	578.1	833.5	977.6
342	363.1	374.9	340.3	393.4	352.9	417.6	403.3	970.6	559.8	814.3
460.8	511.1	475.9	408.1	331.4	432.5	537.6	535.4	822	1188.4	1049
1053.7	995.7	1004.9	820.5	718.1	770	852.5	752.5	1244.5	1247.3	1999
461.3	382	466	332.4	247.1	288.2	308.8	339.9	730.1	1066.1	1121.6
322	351.6	330.3	315.6	231.4	289.2	458.6	359.1	867.4	1485.2	1147.1
422.7	425	480.6	348.4	352.7	402.3	545.5	417	486.4	630.7	637.2
3795.6	838.5	1386.7	1091.9	2151.4	987.3	644.4	765.4	2564.6	3857.7	4099
674.9	499.9	669.4	552.5	514.2	618.2	464	433.9	759.9	969.5	1308.4
421.7	428.8	457.9	319.7	363.2	367.6	473.7	405.6	736.4	887.9	977.9
155.7	152.6	185	141.3	143.4	146	150.4	142	227.1	206.5	216.2
406.7	322.8	394.5	327.2	274.6	363.5	355	314.8	530.4	639.4	739.7
628.6	410.5	545.3	398.6	362.1	403.6	379.4	408.5	793.8	1286.5	1166.8
712.1	545.2	633.4	464.2	522.3	539	532.7	594	1058.8	1180.7	1255.9
766.3	977.6	1010.4	447.1	765.8	1259	974.7	470.6	401.8	385.9	467.3

398.1	315.6	474.1	293.2	298.7	270.8	287.8	290.3	448.1	427.4	740.2
399.2	562.1	482.5	343.8	314.5	488.8	582.1	440.4	571	631.3	809.8
232	215.1	264.7	215.8	182.5	191.8	221.9	217.6	302.5	450.2	430.8
334.8	299	373.8	266.7	237.7	280.3	276.6	252.4	433.3	629.8	707.3
1026.2	1015	934	747	959.7	1121.6	1821.6	934.2	680.6	872.9	861.1
415.9	407.4	384.5	335.7	298.2	319.7	425	327.4	627.4	848.6	825.2
356.8	795.8	1380.7	365.1	373.3	1015	980.3	504	751.8	2994.4	1634.7
710.8	689.5	686	499.9	481.6	680.3	922	766	850.9	952	1263.8
1001.3	868.1	1130.7	794.5	721.9	749.4	860.6	817.8	993.1	939.1	1202.9
948.4	622.1	739.1	531.5	593.6	580.4	481.5	590.8	1426.3	1517.4	2024.2
1150.7	1120.8	1191.9	880.3	679.4	821.3	1049.2	903	1912.9	2259.7	2384.6
531.4	495.8	484	407	425.9	518.4	604.7	493.9	1382.4	960	1244.5
601.5	432.9	634.6	506	327.1	335.8	291.9	353.9	247.5	439.2	575.1
645.4	631.9	590.3	602.8	378.8	425	506.3	707.6	397.5	373.5	494.9
573.3	542	598.7	503.6	465.6	511.2	653.8	423.3	1076.4	1425.5	1577.2
714.8	694.5	692	601.9	501.1	723.4	654	850.9	1125.1	896.9	1143.9
306.2	298.3	313.3	252.7	193.2	262.2	313.1	258.4	487.1	617	575.1
783.2	795.9	842.8	721.1	454.4	1095.4	570.2	390	511.8	806.4	1123.8
440.1	461.1	558.9	421.4	375.8	648.4	468.7	423.8	669.9	717.4	782
603	694.2	699.6	582.5	572	776.3	710.3	627.8	813.1	846.5	1046
805.8	957.8	903.8	729.4	627.6	812.8	859.6	802.8	819.2	804.7	913.5
365.9	333.6	344.2	317.2	292.3	321.2	326.6	314.6	473.1	509.4	607.8
1197.7	1136.9	1267.7	1166.2	990.9	1055.2	983.2	970.9	1689.4	2266.7	2712.3
299.2	393.5	337.1	283.4	272.8	220.1	277.3	225	240.3	660.3	500.8
733.6	746.1	709.8	559.8	656.5	750.1	773.8	612.2	769.6	1040.1	1106.2
1379.7	1282.8	840.6	984.9	840.1	1140.7	1384	1034.6	1223.3	937.7	1017.6
693.2	570.8	640.7	496.2	451.5	544.1	602.4	486.8	837.2	1092.7	1251.8
484	570	491.8	424.6	394.9	471.4	686.4	574.7	696.9	664.6	848.9
866.7	650.5	795.2	751.1	594	594.4	606.1	568.9	1089.1	1362.8	1665.6
304.2	271.4	270.7	247.5	252.3	229.1	253.1	274.3	355.8	462.3	522.2
41.1	37.8	109.5	53.9	36.8	39.2	51.8	51.8	291.9	399.1	393.9
1495.3	1515.2	1568.8	1274.9	1022.7	1313.1	1830.3	1290.1	3489.2	4768.2	4236.1
1494.6	1155.6	657.6	1046.6	839.5	3094.4	3207.6	1247.5	380.9	487.6	575.2

901.2	695.9	924.9	633.1	672.7	782.2	1023.1	682.3	1548.4	2041.2	2070.9
642.2	680.5	808.6	454	489.8	651.6	801.7	435.5	1294.9	965.6	1461.8
909.8	620.8	637.3	466.7	460.4	410.7	463.7	567.5	1637.8	1467.2	2165.8
538.5	755.8	1151.6	921.6	777.1	1304.8	623.6	1402.7	1718.7	2939.2	3147.9
638.7	466	561.2	349.3	377.4	385.9	432.6	381.4	1264.7	1479.2	1515.8
483.8	491.1	495.9	470.7	378.3	506.2	593.9	432.7	492.2	2176.4	577.4
780.7	702.2	666.9	644.1	634.6	827.2	690.6	732.1	1234.3	1177.8	1335.4
451.9	513.9	516.4	418.7	349.9	518.5	651.5	408.2	566.2	663.8	787.9
569	907.6	639.5	446.1	516.1	1285.8	1167.7	519.5	574.8	991.2	1008.5
567.9	411.3	496.6	392.9	373.8	422.1	393	467.2	773	1059.3	1060.5
652.6	506.3	503.5	357.5	627.9	349.4	391.7	289.6	394.5	386.7	525.3
691.9	711.9	667.2	547	499.7	815.2	1630.3	540.6	596.4	779.4	895.4
561.5	568.6	576.7	469.8	515.2	526.4	687.5	580.7	795	960.9	977.8
869.7	858.6	759.3	610.8	944.6	768.1	991.2	752.4	805.6	909.3	1002.8
744.8	663.9	611.9	514	755.4	680.1	1065.5	628.7	1584.7	899.8	1579.8
307.8	419.1	510.6	214.9	309.9	347.2	358.1	312.5	467.4	1558.3	543
302.9	442.2	340.4	383.1	294.2	397.5	592.8	526.3	322.7	1126.4	413.8
642.1	674.5	679.3	566.9	562.6	580.5	652.6	559.5	647.4	676.8	829.8
685.6	673.6	791.2	689.7	578.1	733.3	746.8	624.2	827.4	1037.3	1221.7
401.4	468.5	463.8	414.9	609.2	1410.5	1202.4	756	1397.1	726.9	2016.1
1405.3	1523.6	2125.9	1453.7	1283.8	1697.5	1326.4	828.8	1164.6	2019.6	1695.7
488.3	374.7	435.2	298.3	282.2	354.6	444.7	269.5	676.4	823.7	898.9
358.2	414.5	405.3	363.3	333.6	476.3	462.3	396.5	485.3	550.4	658.7
615.8	479.7	563.7	422.8	451.5	488.4	530.5	453.8	900.5	997.4	1195.8
455.5	382.6	420.2	371.4	291.6	327.2	368.8	358.6	583.5	615.3	782.4
327.6	362	527.1	550.3	432.9	398.1	515.9	638.3	344.9	459.7	447.5
574.4	542	590.7	428.8	526.9	500.3	577.9	420	840.1	1153.7	1397.9
223	200.8	212	169.3	196.8	222.9	238.3	196.1	281.3	271.8	358.9
654.6	596.3	557.7	451	477.9	521.5	753.2	513	878.4	1147.7	1238.5
523.2	402.8	451.2	385.1	396.6	402.3	498.2	407.6	796.8	848.2	937
601.3	349.7	426.3	311.2	314.4	340.4	350.1	351.2	1060.1	938.3	1241.4
546.6	458.5	584.6	471.8	442.8	419.3	482.3	426.5	849.5	956.8	1101.9
1059	1474.6	1615.7	1592.7	923.4	1390	944.4	1598.5	510.9	1012	925.9

328.2	317.6	326.9	277.6	242	303	373.1	236.5	342.6	417.1	474.9
1158.4	1100	997	1103.3	884.3	1092.8	1346.5	980.9	1055.2	1371.4	1755.1
1054.5	642.6	609.8	394.7	637.8	1170.1	308.2	503.6	351	683.8	740.7
1108.6	697.9	921.1	786.5	761.7	643	766.8	653.4	956.7	1329.1	1421.1
764.7	678.1	613.9	528.8	500.2	503.3	607.9	559.7	630.4	813.7	806.4
1160.8	875.1	943.7	707.8	723.6	818.9	1028.1	824.5	1910.9	2258.6	2389.5
693.4	629.7	588	583.7	475.2	582.5	563.9	661.2	1188.7	988.4	1088.6
644.1	524.2	632.1	495.6	408.6	480	545.9	498.2	590.1	531.1	827.4
769.3	523.7	794	518.2	517.5	497.6	422.3	432.4	869.8	1064.8	1408.1
588.1	551.5	627.4	406.5	436.3	439.3	524.3	478.5	923	1157	1263.8
455.2	415.4	487.6	425.8	399.6	387.2	511.8	372.8	447.3	617.6	657.7
372.7	289.3	330.2	462.9	314.9	650.8	720.7	449.9	502	563.4	853.5
1341.1	1523.7	1778.9	1168.6	1739	2463.8	3192.4	1192.3	945.2	1189.1	1776.7
606.8	523.6	582.8	449.5	439.8	512.4	540	525.5	724.7	823	861.2
558.8	569.5	648.2	500.1	466.4	606	580.3	522.3	1136.2	1090.6	1385.1
714.7	715.6	626.1	581.2	448.8	549.7	673.7	644.5	1589.6	2163.8	2118.1
640.8	593.3	741.1	594.5	481.9	554.8	590.3	558.4	666.5	854.5	912.9
845	732.5	708.1	573.3	706.6	969.5	748	582.3	916.7	1343.4	1262.2
473.7	369.9	452.1	320.1	345.6	379.4	430.8	321.8	538.1	654.5	740.9
720.4	336	2209.6	1104.6	1157.4	315.7	448.3	287.7	391.4	556	537
737.7	440.3	710	554.1	484.6	527.5	443.8	408.2	518.1	777.7	1038.7
572	392.2	561.7	362.2	383.1	360.5	358.5	389.3	422.8	960	737.6
435	420	422	262.2	247.2	366.8	568.6	342.4	666.8	724.8	833.4
312.1	277.7	293.9	212.5	230.5	238.7	301	224.4	420.3	476.4	535.3
1171.4	685.8	633.2	577.8	695.6	488.1	563.5	487.6	526.5	3497	1054.2
64.1	31.5	63	53.6	36.9	29.7	28.2	23.5	27.2	29.8	38.4
2213.5	2542	1969.6	2655.7	2613.3	1649.4	2514.7	2905.7	1624.6	1629.2	1911.5
635.3	872.4	854.1	762.8	548.9	597.1	486.8	571	555.8	1049.7	861.1
256.3	372.2	393.8	412.3	321.1	641.9	460	332.9	465.2	1011.2	718.1
447.5	469.8	502.1	393.1	373	484.2	593.7	448.6	855.4	1074.2	1105.3
305.2	221.2	267.2	217.5	260	256.7	236.3	185.6	345.6	614.5	536.1
304.6	375.3	366.5	280.2	285.6	572.2	528.1	271.3	345.6	389.4	438.8
1163.2	735.1	1089.4	1186.4	916.6	786.4	579.1	661.1	1284.4	891.8	1441.1

192.5	200.1	168.2	164.2	162.9	176.9	369.9	354.7	446.8	479.9	506.5
542.8	486	564.2	483.8	345.6	504.9	481.8	447.9	596.6	654.6	884.6
473.3	519.1	570.8	464.9	367.4	397.8	627.5	486.9	707.5	1097.6	1070.7
475.2	160.5	285	182.2	223	146.1	197.6	157.8	479.5	713.6	753.7
1193.1	1018.7	1139.8	912.8	899.9	946.5	932.9	758.2	1689.7	1717.3	2223.4
581	752	866.5	527.7	418.3	585.5	713.5	780.3	381.8	330.7	535.6
480.8	517.6	510.4	401	371.2	576.7	547.3	577.2	666.3	683.4	756.6
643.6	585.8	605	501.8	476	544	655.9	535.4	1066.9	1075.6	1415.9
273.4	240.8	309.7	179.5	183.4	184.4	179	182.6	312.4	376.9	446.7
688.3	894.7	825.5	816.4	707.7	1240.3	2065	711.7	1374.8	1535.9	1411.1
51.4	40.7	51.5	54.1	39.4	62.4	117.5	32.9	31.2	32.1	46.8
926.9	648.6	737.2	623.2	513	554.4	588.9	583.2	1446.1	1388.8	1830.5
708.9	758.7	638.6	504.8	661.3	713	931	666.6	738.2	782.9	856.1
4023.7	2929.4	2870.1	2617.3	2215.5	1934.1	1971.4	1502.4	1269.2	1891.5	2343.7
413.8	476	401.4	325.7	354.9	410.6	485.8	418.7	442.3	612.5	593.4
1203.5	996	1100.7	1243.4	1599.6	1224.2	1375.8	1058.5	935.6	741.5	1175.8
767.7	620.7	709.9	521.5	498.9	579	598.7	657.5	1171.2	1653.9	1639.7
357	773.1	483.9	655.7	346.4	2090.3	742.6	452.6	309.6	331.8	400.4
741.4	493.6	706	518	590.2	559.4	466.4	437	862.9	1158.9	1201.4
486.8	459.8	457.7	346.8	496.8	400.9	536.3	363.5	575.3	584.5	765.3
698	509.9	579.8	466.7	379.7	431	397.5	465.2	960.1	1111.4	1360.4
907.1	742.2	881.1	722.2	675.4	885.1	749.8	703.8	1184.3	1323.7	1668.4
882.8	705.4	821.6	637.5	707.8	742.2	708	571.2	1058.4	1030.6	1376.2
697.2	692.6	770.2	568.1	546.4	672	773.9	590.6	860.9	1267.6	1229.3
725.9	648.6	510.8	512	475.3	580.4	748.4	599.2	1096.1	1119.3	1406.6
451.6	277.6	407.4	334.1	320.4	346.9	377.6	325.1	574.7	700.3	669.7
446.7	427.5	455.7	438.6	307.5	473	473.3	419.6	596.7	861	782.2
504.1	515	579.8	484.3	429.8	630.6	525.5	444.6	561.8	831.6	948.8
506.6	597	589.7	509.2	439.3	609.5	637	459.6	624.3	933.4	748.5
556.3	625.1	655.8	465.6	468.5	572.2	550	425	878.4	1280.9	1523.1
560	530	590.7	474.7	394.9	486.2	533.3	439.9	662.5	823.5	926.9
577.1	499.1	655.1	480.2	515.2	597.3	484.9	455.7	616.9	697.7	878.4
266.8	223.6	252.7	173.6	234.3	244.7	146.4	209.7	394.9	461	517

528.3	533.1	611.3	489.1	477.5	554.5	625.8	555.5	753	911.3	873.2
173	241.4	195.4	260.2	166.6	165.5	225.9	177.6	116.7	72.7	161.6
574.4	708.9	618.4	489.8	598.5	500.3	705.1	526.2	828.6	1016.5	1031
1851.9	1939	1899	1764.8	1244.1	1378.3	1536.2	1383	2115.8	1826.2	2626.3
786	503.7	763.4	523.3	414.5	372.1	376.1	470.8	390.7	522.8	488.3
627.8	815.1	867.2	642.4	669.4	1251.6	573	624.1	620.7	764.3	856.9
556.6	471.5	537.3	507.9	381.5	422.7	492.9	371.6	664.1	862.4	966.6
593.2	558.5	617.5	509.9	621.5	495.2	636.2	610.9	560.4	770.4	761.2
262.3	223.1	264.2	255.6	221.8	266.3	349.1	246.7	290.2	1070.3	287.7
492.7	406.1	520.7	479	396.1	470	366	348.1	515.4	555.2	746.9
308.8	303.9	399.5	352.9	259.7	350.5	333.1	294.1	366.7	387.6	431.3
351.7	240.5	259.3	224.9	220.6	243.8	234.9	255.8	408.3	553.1	634.2
30.6	14.9	24.4	13.1	18.8	20.8	9.6	15.3	47.1	48	54.2
1457.1	1093.9	1101.4	1318.6	731.2	836	771.5	915.8	825	619	817.4
380.6	418.6	438.4	374	297.6	388.8	395.2	424.8	695.3	1053.7	945
778.7	616.4	968.5	692.3	615.8	703.4	559.7	441.5	600.2	877	1013.7
1165.7	1054.7	1135.4	991.3	984.3	756.7	1081.4	1453.2	760.9	606.6	907.7
637.3	578	632.2	449	378.5	368.2	673.9	405.9	1102.5	970.4	1029.3
655.3	574	607	481.4	402.9	455.1	519.9	492.6	790	898.4	1094
791.5	686.6	866.5	632.7	665.5	717.2	693.5	502.9	709.5	1017.2	1073.9
1466.1	1620.9	1437.9	1223.5	1710.1	1290.4	1748.6	1480.5	1618.9	1961.3	2045.8
474	377.5	525.8	351.5	336.9	276.6	391	282.2	532.8	681.8	628.9
731.6	435.9	570.1	383.4	438	412.8	424.4	392.2	674.2	907.7	1133.3
632.9	635.2	657.5	529.5	480.3	622.1	692.6	591.3	544.6	453.3	640.7
341.6	371.2	375.3	309.6	257.5	336.9	348.9	313.1	541.1	527	705.1
511.5	575.6	400.1	457	421.7	568.2	686.5	518.9	959.8	994.3	1280
442	337.9	445.7	385.7	333	355.8	416.5	338.9	540.8	739.3	646.5
1579.5	1576.2	1600.6	1217.7	782.3	1327.6	1324	1271.7	2811.3	3195.2	3728
352.4	299.3	351.4	221.1	239.9	251.2	243	367.7	316.9	377.9	381.6
862.4	675.1	853.9	559	737.4	665.2	750.6	659.8	702	762	1054.5
1401.2	955.5	1308.9	1026.2	843.5	945.9	984	987.7	1283.3	1903.8	1979.1
958.5	732.1	924.4	717	673.2	669.7	700.4	704.6	1221.9	1539.6	1691.7
1092.9	769	1041.9	775	733.1	708.4	882.2	518.8	747.5	1128.8	1293.1

786	685.2	950	931.7	1155.6	696.3	1160.9	1221.2	957.5	650.2	920.1
1051.2	1594.5	1686.5	1433.9	679.1	599.2	553.8	678.4	576.5	726.4	1055.5
1999.4	1095.4	1220.9	1022.6	963.5	1094.8	864.2	896.2	1911	2680.3	2316.2
718.9	542	677.2	498.7	547.6	560.3	493.2	481.6	841.6	1128.4	1345.7
59.5	53.1	47.5	40.3	40.4	49.5	62.2	45.3	103.7	107.4	140.7
668.7	536.4	906	581	588.7	600.3	356.1	624.8	418.1	475.2	1803.6
932.1	788.1	1095.1	819.9	621.4	745.7	1054.4	753.9	1442.9	1736.3	1980.5
792.6	620.4	721.6	565.3	462.6	546.1	549.2	526.2	665.8	767.4	879.1
865.5	532.9	674.7	462.9	572.3	583	583	486.5	938.3	981	1351.3
274.2	509	307.2	441.5	275.1	802.4	358.5	363.9	330.1	228.5	263.4
666.4	670.7	640	657.2	484.4	736.5	706.9	714.5	812.7	930.4	998.4
679.2	728.9	675.7	518.3	503.5	685.9	879.4	648.6	1218.9	1293	1586.4
383.9	298.5	327.2	254.1	308.8	367.5	312.1	281.1	545.3	674.7	722.4
329.5	305.7	389	277.3	284.9	338.6	319.2	244.9	411.5	629.1	723.4
545.9	568.4	576.3	509.7	478.3	431.7	593.8	432.2	488.9	551.2	616.2
540.2	558.8	603.3	462.4	381.5	519.9	426.7	530.6	888	751.3	966.2
107.3	79.1	79.3	66.6	93.4	84.6	101.4	83.5	113.8	116.3	143.5
579.8	469	560.9	489.3	372.7	473.1	466.8	419.1	678.9	981.8	1162.4
844.9	1001.6	1130.9	1017.2	725.4	875.3	1048.1	714	1426.1	1889.6	1796.3
259.1	254.2	251.2	201.3	205.9	193.8	244.7	211.1	490.3	638.5	622.2
436.9	414.5	470.7	334.5	305.9	383.5	406.7	323.6	463	518.5	749.2
518.9	400.4	424.9	340.5	234.5	280.5	302.5	470.5	696.6	927.2	1194.8
284	313.1	285.9	258.9	231	298.1	391.9	285.3	478.1	532.1	568.7
380.9	343.2	329.5	310.1	276.5	301.4	340.5	293.1	346.9	322.2	431.9
424.7	337	338.5	325.3	199.2	274.1	240.6	355.4	493.8	618.5	670.2
234.7	259.1	242.3	231.8	203.3	317.2	486.8	244.5	172.7	228	277.1
434.5	383.5	454.7	326.2	345.6	341.9	417.8	360.9	686.3	802.8	859.7
764	462.4	645.4	440.2	510.3	364.9	380.5	377.7	754.4	1344.7	1486.3
832.7	546.8	580.4	406.7	395.2	462.4	469.2	433.1	876.1	835.1	1069.6
199.1	216.4	238.3	195.5	213.5	207	212	197.7	280	548.9	1113.3
788	576.4	764.4	590.4	585.6	577.1	480.4	470.7	1127.9	1138	1804.6
373	228.9	296.2	225.5	249	243.1	312.9	319.3	580.7	787.8	727.8
104.5	99.7	134	69.4	90.2	116.1	110.1	99.5	107.8	75.9	99.4

307.7	319.4	433.9	307.5	374	352.6	298	285	429	523.4	611.3
410.2	439.5	478	461.1	423	543.7	541.5	599.4	742.3	837.3	799.2
229.7	487.3	253.9	393.4	228.1	615.8	1034.5	967.7	167.1	178.9	250.8
335.1	277.3	365.1	310.4	261.5	292.7	274.3	312	338.1	657.5	589
523.1	566.8	623.7	763.8	543.3	781.4	2180.3	868.9	756.6	1241.2	1690.8
49.8	34	43.8	36.2	38.9	42.6	30.3	36.8	52	69.1	89.1
1298.9	1769.7	1765.9	1633.5	1254.9	1809.7	2773.2	1519.9	1392.1	1265.7	1754.2
439.6	444	473.3	381.2	334.8	432.7	474.6	710.6	546.2	736.1	785.7
352.8	323.4	365.7	314.5	254	254	287.9	297.6	351.5	386.5	488.5
563.7	823.2	938	992.1	471.8	607.9	616.7	805.8	329.2	356.2	435.6
1029.5	635.5	845.8	589.8	589.7	630.4	517.6	459	1054.6	1351	1649.9
31.7	31.5	36.8	24.6	33.9	34.9	38.8	38.7	47.2	51.5	61.5
525.2	673	673	554.5	573	704.5	769.8	559.6	705.9	766.1	868.1
258.6	294.9	284.8	245.7	209.7	321.4	288.2	270	541.2	461.8	628
720	944.5	859.6	669.7	560.7	845.8	741.9	821.7	1228.7	1500.9	1509.7
220.4	249.7	255.6	188.5	187.1	244	266.6	203.3	239.9	393.1	422.7
391.4	320.2	389	302.4	247.4	309.8	324.1	281.4	460.9	604.3	645.9
888.6	1653	1325.2	1493.5	1071.7	4323.8	1595.9	1558.8	879.4	568.8	929.8
683.7	443.1	679.2	487.3	440	536.8	558.6	571.8	1019.1	874.3	1153.5
600.2	387.5	470.6	352.9	438.8	380.5	448	404.8	969.6	1241.9	1342.5
1821.7	3078	2789.8	2364.8	2361.2	1704.2	2000.8	3055.9	1325.7	877.8	1563.9
443.2	430.2	490.4	366.6	380	439.6	425.1	387	556.3	575.5	689.4
630.5	551.9	694.8	563	558.8	556.2	497.5	577.2	1018.8	1292	1540.9
1072.1	1760.3	1517.3	1344.1	1082.3	1537.7	1135	1026.9	680.5	612.2	749.6
680.1	523.4	411	450.3	1077.5	981.3	788.7	694.7	350.3	235.8	353
575.1	495.8	558.8	425.4	411.8	488.1	458.3	520.2	953.8	963	1137.9
923.5	907.1	974.8	742.7	684.5	789.8	814.2	872.3	1484.4	1202	1786.8
575.2	458.1	501.1	367	356.3	432.8	423.2	410.6	956.6	1259.3	1362.9
719.9	450	710.8	436.9	431.9	456.7	411.6	387.5	575.7	488.1	746.5
230.8	232.5	225	179.7	164.7	219.1	221.8	194.4	437.2	314.9	474.3
749	904.6	690.7	628.7	546.8	656.8	774.3	851	922.8	1287.2	1444.8
509.2	473.5	496.8	435	348.6	420	354.7	415.7	1568.6	907.7	1559.6
228.5	255.8	269.8	205.7	227.2	282.9	288	211.3	288	232.2	324.9

489.5	544.5	580.3	458.8	474.9	611.3	552.3	472.4	569.1	587.9	784.4
604.8	704.8	773.7	746	702.1	951.4	458.4	678.1	719.6	1087.2	1233.4
330.6	221.6	308.5	211.3	289.1	269.5	277.3	238.7	349.6	614.5	573.2
997.6	790.7	975	774.1	838.3	820.5	782.5	805.2	1052	1341.7	1631.2
287.8	289.6	311.4	251.2	231.8	282.6	207.2	228.6	462.4	901.9	795.1
772.5	530.5	641.4	435.8	459.6	511.4	445.1	463.4	1132.7	1601.7	1720.2
548.9	692.8	881.4	1055.8	462.4	706.3	576.3	408.5	549	695.9	857
2685	1770.9	1365.8	2745.7	3664.6	1540.7	2826.3	4400.5	2906.4	916	1534
580.2	707.4	737.8	530.9	550.3	700.2	877.1	590.8	786	837	972.2
499.7	531.5	560.2	481.5	494.2	589	588	574.6	932	1067.4	1108.9
444.5	449.2	421.1	407.6	519.4	496	647.9	459.9	524	1130.7	645.5
515.3	512.2	556.8	421.7	431	471.4	655.8	509.7	736.7	1002.5	1006.6
475	369.3	344.8	296.8	324.9	383.7	397.4	406.6	710	824.2	873.8
468.6	523.3	619.1	516.1	501.8	505.4	563.6	519.9	694.8	1756	1083.9
869.6	725.8	826.8	586.4	600.4	808.3	604.9	691.2	804.6	815.6	1042.3
1000.3	1139.6	1132.3	635.6	1084.7	829.2	1126.9	748.2	1091.8	1326.3	1382.7
1002.8	593.4	740	715	638.2	691.2	1371.8	1003.8	1146	1081	1236.8
1751.7	1796.1	2353.9	2486.2	2000.4	1753.4	1813.1	2559.6	1148.2	873.5	1630.1
530.4	483.6	446.6	419.3	413.7	436.8	526.5	469.9	810	971	974.3
803.2	763.5	811.3	610.4	593.2	765.8	713.3	784.1	1261.3	1934.2	1730
423.3	436.7	540.3	435.8	367.3	517.7	487.8	458.2	643.7	647.8	795.4
554.9	729.8	741.3	685.2	564.7	810.9	844.5	671.9	919.7	849.8	996.1
625.1	526.9	620.5	466.5	423.7	490.2	558.7	515.9	765.7	989.8	1132.3
328.5	356.2	310.5	250.6	273.6	299.4	383.3	293.9	441.4	536.6	616.5
350.7	332.2	321.6	272.2	292.7	313.3	438.1	282.2	477.1	521.2	607.3
594.4	554.7	584.7	450.3	471.7	570.3	616.7	473.7	1083.9	880	1063.9
609.5	839.2	733.4	570.2	521.6	907.8	858	592.4	506.8	664.7	721.6
1262.3	2021.6	2115.1	695.3	596.8	2533.9	2556.3	450.2	495.1	515.1	865.8
522.5	508.9	590.1	453.2	399	500.3	594	487.3	526	685.2	709.2
652.1	654.5	755	691.9	528.6	827.5	661.3	583.8	856.3	1234.3	1260.4
192.9	215.7	196.1	173.6	185.3	213	263.7	223	579.7	616.2	623.9
400.5	329.3	390.4	315	321.6	354.1	368	305.9	559.8	859.6	877.3
467.2	377.1	468.1	356.5	350.2	393.3	438.6	399.9	546.8	658.9	731.6

747.6	681.3	688.2	541.2	554.8	448.9	796.3	499.5	1091.8	1125.1	1184.9
527.3	455.6	542.1	371.6	356.6	400.4	414.8	405.6	713.3	1059.9	1005.3
1156.4	830.7	698.2	907.9	833.8	755.2	530	870.2	1119.9	911.7	923
303.4	255.2	283.9	325	241.9	281.5	239.2	249.7	440.5	714.3	823.5
1233.1	762.8	1163.6	1142.7	801.2	589	438.9	548.2	627.3	643.8	1008.6
527.2	445.4	554.7	436.2	374	466.6	396.3	393.4	760.7	778.4	969.3
972.9	675.5	653.5	431.4	377.1	360.9	386.2	420.7	772.4	926.4	1196.5
565.2	615	583.1	432.6	379.8	465	521	610.5	963.5	1053.5	1413.9
503.6	427	465.8	362.7	332.1	363.3	416.2	406.7	763	1246.8	1190.2
321.6	503.8	633.6	659.4	393.5	679.1	264.3	409.2	413.4	263	578.7
329.3	321.2	407.5	343.9	252.4	348.3	433	339.6	649.9	646.3	945.6
835.5	760.4	659.8	549.4	614.3	770.1	921.6	649.4	1413.1	1654.6	1792.3
434.3	282.7	368.5	275.6	333.7	311.9	271.2	260.3	666.8	426.6	814.6
216	183.6	179.4	164.4	126.3	145.8	146.6	208.7	346.9	466.7	463.2
1699.3	667.4	4477.5	4996.4	1116.2	1666.3	499.5	591.2	409.2	609.8	1208.8
444.2	417.4	378.7	290.7	299.8	340.5	396.4	372.8	714.1	691.6	780.4
422.7	365	414.4	308.5	302.5	339.1	342.5	297.6	608.3	841	925.9
663.2	661.4	525.6	1183.5	475.2	911.7	564.6	368.6	703.3	322.4	471.1
390.8	378.2	399.2	356.4	269	399.4	269.3	327.2	365.7	386.2	445.9
173.3	140.6	166.1	105.6	91.2	107.9	147.5	103.2	255.9	1033.9	710.8
320.7	355.1	396.2	322.1	310.6	420.7	403.6	340.9	362	469	478.8
691.4	869.7	858.4	951.1	493.5	1080.7	1205.7	1009.4	798.4	500.6	721.8
388.9	392.9	453.1	377.8	330.6	413	483.2	385.2	489.1	521.6	631.1
442.8	482.4	511.3	384	385.8	558.5	620.1	423.8	512.4	674.4	687.8
396.5	482.8	520.4	423.2	350.1	467.9	620.8	421	521.8	753.3	719
665.9	914.7	802.9	531.4	612.4	631.9	521.4	427.8	470.4	612.1	593.8
485.7	310	307.7	230.5	201.4	362.9	331.6	222.7	284.1	344.1	382.6
442.4	349	448.3	364.9	371.3	389.1	465.7	388	752.9	769.4	1030.3
307.2	312.7	357.5	289.5	252.8	278.2	342.4	320.8	533.6	452	500.1
315.3	306.7	311.6	273.7	260.6	266.8	337.6	343.2	432.2	527.4	478.9
1185.5	1117.8	1722.2	786.6	787.5	1199.3	1296.9	802.8	1135.4	1539.3	1582.9
540.4	588.6	534.4	486.7	397.4	493	725.6	556.2	907.7	1057.2	1186.9
364.1	622.4	538.6	565.4	320.4	1128.6	863.2	501.4	454.5	365.6	518.9

935.6	361.4	504.5	448.7	458.4	323.5	280.6	358.7	368.2	588.8	573.5
414.4	402	478.8	339.8	324	433.9	424.2	438.4	532.6	662.6	650.4
1093.7	946.9	1062.4	725	798.9	880.9	1064.1	808.5	820.7	1116.7	1294.7
392.2	556.6	447.2	398.7	365.7	501.1	537.8	576.5	669.6	996	856.8
380.5	374.3	459.2	390.6	376.5	433	427.3	336	537.8	520	703.8
1168.3	937.3	857.1	824.1	683	903.6	848.7	724.7	983.4	2831.8	1900.5
658.2	667.4	685.4	560.8	613.8	617.1	734.2	627.2	770.4	852.4	1017.7
592.5	424.2	535.3	387.9	348.6	391.8	465.1	447.4	1019.8	1237.8	1384.4
176.7	166.3	185	160.5	139.3	152.6	173.4	157	545.9	1612.4	845.5
379.3	413.7	436.2	353.8	415.4	554.7	661.6	448.9	360.5	439.2	476.7
462.6	468.9	475.7	448.1	371.2	509.4	553.2	416.7	340.9	382.5	370.6
587.6	585.3	598.7	430.5	354.5	258.1	564.8	270.9	346.1	682.9	432.3
1126.6	534.6	3672.2	1824.3	1971.9	431.6	560.2	383.3	585.9	872.3	824.7
510.2	370.9	587.6	528.4	426.7	454.4	336.5	260.2	663.7	1104	1156.9
256.4	241	259.6	246.5	213.5	219.1	241.5	256.9	331	415.7	456.1
877	931.9	1047.6	777.4	763	866	935.5	794.9	1138.7	1456	1613
973.9	646.3	739.3	652.5	622.9	644.7	530.9	673	1397.7	1522.4	1925
744.6	504.1	453.1	644.4	396.9	377.6	415.3	814.5	570.2	754.2	881.9
817.3	713.3	749.9	596.6	536.8	582.4	525.6	621.3	993	771.9	1111.7
580.6	764.6	663	575.4	550.1	832.7	1223.8	714.7	604.4	955.6	839.3
1006.6	793.9	834.3	664.9	1208	988.4	1052.4	670	843.3	1134.8	1186
731	824.9	671.2	776.3	552.7	899.3	814.7	932.5	808.1	771.6	841.3
1036.5	735.2	799.7	800.4	796.2	821.5	1247.4	1069.5	2075.1	1998.7	2101.8
420.5	318.6	345.4	251.3	342.8	291.8	311.3	301.2	508.6	707.3	807.8
524.5	365.2	444.7	354.3	366.3	403.8	390.5	366.4	766.2	1094.8	1128.4
256	262	288	273.9	224.5	263.2	332.8	286.1	607.2	712.1	755.5
367	256.5	232.3	199.3	270.4	284.4	290	216.6	394	371.1	495.6
801.6	588.1	831.9	596.7	548.3	501.2	487.2	327.3	655.6	1567.2	1153.7
243.8	233	248.2	211.9	213.4	267.6	218.4	222	458	519.6	548.4
982.9	939.9	972.7	784.8	620.1	958.5	1167.4	796.8	1429.2	1892.1	1880.7
1244.1	1548	1233.7	814.6	795.5	1034.7	1825.7	1009	1367.8	1806.5	1742.1
872.4	587.8	845.7	753.2	673.5	510.1	507.2	465.6	702	781.8	868.9
954	695.1	944.3	1396.3	599.7	858.7	597.1	537.5	696.1	585.8	793.6

422.5	301.1	407.2	421.7	343.2	377.3	487.1	310.1	453.9	1013.2	780.8
331.5	274.8	322.6	250.2	326.7	311.1	434.3	286.4	290	341.3	326.1
492.6	470.4	539.6	456.8	439.3	475.7	467.9	415.9	560.4	737.3	768.4
525.1	455.8	512.6	513.7	339.5	417.4	434.5	349.7	409.8	440	514.2
400.5	383.6	616.4	585	284.8	1086.7	218.5	244.1	1164.9	837.3	1580.5
310.3	289.5	315.1	257.1	223.5	244.5	388	273.8	387.1	732	559
417.3	365.1	381.5	297.3	307.3	338.3	379.2	414.5	666.1	850.7	963.3
412.4	430.5	356.4	292.7	340.2	357.4	520.6	624.8	535.6	686.7	704.4
4308.5	1732.6	2443.5	2130.7	2172.5	1793.5	1752.2	1540.7	2263.9	2661.4	3311.3
731.4	677.8	798.3	565.3	557.1	508.7	659	588.9	1390	2105.8	2093.7
804.2	788.9	833.4	630	585.8	691	625.8	720.6	640.4	835.2	857.1
351.6	273.6	352.5	292.3	256	243	269.5	255.9	514.6	503	627.4
430.2	429.3	462.8	390.4	263.5	390.4	383	385.7	733	913	941.4
77.8	67.3	69.1	60.7	53.7	77.3	78	69	119.6	123.7	137.6
546.8	550.4	593.8	523.2	459.2	396.3	528.1	533.3	1177.1	1095.1	1244.1
355.3	335.7	424.8	337.5	262.6	276.9	281.6	297.6	432.4	658	664.7
330.5	324.1	350.3	399.2	325.4	412.9	362.4	275	424.2	934.9	635.5
1085.7	912.9	949.3	662	529.3	674.5	920.9	863.1	2076.5	2258.3	2787.6
765.4	773.2	820.2	672.4	546.8	732	944.4	646.2	1419.4	1616.2	1646.7
384.4	361.6	394.7	310.9	274.8	349.8	313.6	351.6	299.3	340.5	383
766.1	1313.4	410.5	1536.9	283.4	407.4	689.7	730.8	1399	308	744.6
268.8	253.9	245.9	224.3	354.7	256.8	405.5	231.4	184.8	250.8	283.4
945.8	806.4	1017.1	769.1	1063	714.6	1602.7	919.2	983.6	725.7	788.8
402.2	331.8	353.7	272.3	255.8	271.2	270.5	317.9	593.1	779.4	982.5
688.7	472.9	515.3	367.2	440.6	460.1	552.9	538.3	970.9	1313.5	1212.7
1146.5	1330.8	901.7	1009.1	1122.6	1224.4	780.7	1095.8	1454.3	1607.8	698.1
507.2	433.6	446.3	324.1	324.3	368.1	423.1	356.9	675.8	1061.3	1056.1
465.7	373.3	405	288.3	264	336.9	337.4	340.5	762	954.7	1080.6
862.5	446.9	749.8	553.1	719.4	520.1	382.5	372	861.7	1667.9	1390.6
342.4	385.4	424.8	345.1	283	360.8	314.3	335.7	603.4	851.6	843.5
559.2	602.6	644.2	501.3	385.2	442.2	486.5	535.2	633.4	701.1	738.2
315.4	299.5	360.3	243.4	290.1	303.6	335.2	251.8	375.9	589.6	591.2
645.3	406.3	487.2	421.1	394.6	359.2	319	305.9	462.8	656.7	922.5

173.8	836.9	178.9	728.6	1078.8	562.2	116.3	486.6	582.5	272.9	442
314.5	258.5	265	209.4	251.6	238.4	275.5	297.5	485.4	437.3	574.2
577	846.6	675.3	735.2	588.5	1518.9	709.2	878.8	585	554.1	668.7
2363.9	1979.2	1993.8	1133.7	1023.8	1306.4	2021.3	1781.5	4255.3	4280.2	5623.4
306	282.8	322.7	250	202.2	263.6	311.1	227.7	448.9	619.5	720.4
707	726.1	644.7	547	668.3	579.2	793	597.8	651.8	833	856.9
442.2	431.4	441.4	427.4	304.7	411	341.9	364.1	770.4	747.3	1054.4
535.2	167.4	330.2	198.3	219.8	207	139.4	248.8	300.1	291.1	375.8
109	103.5	95.8	84.1	72.8	65.6	95.6	95.8	127.7	152.4	188.4
504.1	627.6	688	500.8	409	627.1	611.4	580.4	698.5	661.7	885.7
874.4	627.8	909.7	763.2	600.3	739.1	580.9	579.6	576.2	708.7	949.1
455.1	600.5	444.4	602.6	425.5	677.2	628	818	470.3	810.2	589.7
620	722	662.4	596.7	545.7	612.8	837.9	637.1	1040.3	1146.4	1252.8
355.1	416.1	394.3	365.6	307.5	403.9	446	384.8	576.4	637.6	712.9
838.5	740.6	732.4	739.6	731	662.3	521.5	718.3	1044.1	1052.7	1244
440	643.5	509.7	626.6	406.5	628.6	861.2	657.8	469.7	545.7	708.1
234.1	217.6	272.1	202	192.6	219.6	212.4	221.2	335.1	689.2	592.5
359.2	278.4	341.1	329.2	283.1	322.2	255.8	270	311.9	411.7	472.8
742.1	624.8	691.3	617.5	573.1	692.1	771.3	617	928.2	1024.8	1201.8
1319	1058.7	1286.1	1187.1	973.5	1056.3	1003.4	925.2	1375	1574.6	1976.5
156.5	144.6	114.4	96.9	95.2	121.7	117.4	107.2	130.3	209.6	199
421.3	398.4	389.8	384.7	338.1	281.1	327.1	367.5	766.9	639	962.8
1371	1154.4	1379	1010.2	1082.9	1155.3	1148.6	864.5	1968.7	2595.4	2670.5
470.9	435.3	432.9	404.2	373.9	405.6	489.4	397.2	556.4	602	699
1274.8	938.3	1045.1	705.8	896.5	528.4	525.6	575	789.9	1106.2	1117.5
443.5	425.9	388.8	345.3	357.4	661.3	1757.2	516.5	245.4	313.9	321.1
340.8	321.5	350.2	340.9	304.9	276.9	357.3	388.7	529.3	563	642.7
446.9	403.5	441.8	353.9	370.3	467.7	338	380.7	551.5	662.6	913.7
1209.9	1557.7	1845.3	1046	645.7	1827.1	1207.5	1170.9	472	594	658.3
1210.2	916.9	897.3	744.5	988.4	770.2	1037.8	959.5	1445.5	2131.1	1985.8
502.2	467.2	508.3	332.4	329.5	415.2	462.1	435.6	780.1	846.7	1063.1
1315.9	1251.7	1422.8	580	565.1	1243.8	955.8	479	530.1	535	680
664.1	796.5	794.7	561.7	539.6	919.3	1058.4	715.4	789.2	759.1	1082.5

283	1622.1	693.3	365.6	385.3	1189.7	468.4	416	678.1	917.6	1097.4
192.1	176.3	193	165.4	174.1	196.2	230.1	177.6	328.4	368.1	419
370.2	509.2	409.5	290.7	320.4	614	821.5	363.2	328.3	330	510.3
428.5	318.9	418.4	329.5	356.1	358.1	289.4	272.8	382.6	501.4	632.8
388.5	445	518.7	365.4	350.1	551.8	484.5	447.2	612.7	604.8	814.4
441	417.4	465.2	343.7	343.2	350.4	431	367.8	852.8	871.5	1093.9
295	355.3	352.7	304.2	252.1	316	394.7	326	395.2	494.5	412.2
497.9	405.5	514.7	382.4	362.2	402.3	374.7	314	603.9	976.3	1115.1
576.3	294.1	518	417.5	319.2	479.6	307.7	370.7	514.6	617.5	867.5
797.4	841.3	951.3	724	574.8	773.6	964	726.6	1447.5	1658.3	1756.3
903.6	885	1394	679.5	882.5	599.9	482.1	563.8	281	297.3	340.9
685.3	566.1	491.8	382.9	379.4	449.2	445.5	465.6	753.4	1210.1	1131
547.8	554.3	532	451.1	337.8	489.7	588.3	575.7	1011.3	1300	1324.2
636.3	531.1	629.4	568.5	611.3	670.7	649.2	642.5	834.7	852.1	998.2
515.2	520.7	477.2	390.3	391.8	465	586.3	497.3	646.9	651.3	820
955.5	947.3	489	474.8	390.2	452.7	569.2	535.1	1772.7	638.8	1201.3
346.6	287	300.3	242.5	271.2	276.5	289	248.8	442.1	529.6	610.3
632.5	631.7	599	403.6	435.5	516.8	525.2	555	862.8	918.4	1086.2
896.7	866.4	969.3	661.6	767.1	901.7	965.9	617.4	1262.1	1548.5	1691.7
384.7	118.8	104.3	125.6	95.2	71.6	69.3	81	100	506.8	139.7
722.5	586.7	649.3	530.6	462	503.4	621.7	548.1	963.9	1149.4	1280.7
448.2	379.1	351.3	359.1	358.9	305.6	557.8	357.7	383.6	441.6	558.2
470.4	394.1	468.5	350.7	358.7	387.2	396.5	336.1	520.7	633.5	668.7
1009.1	793.3	907.3	776.4	742.1	837	882	694.1	987.3	1324.9	1456.8
229.7	196.9	227.2	192.6	150.1	199.4	210.9	176.1	316.2	242.6	335.5
745	533.6	695	489.6	489.3	516.8	466.3	447.3	723.3	977.8	1181.5
518.9	473.1	533	398.2	456	563.8	617.7	527	684.2	705.7	901.6
700.7	700.7	485.9	737.3	617.4	617.3	599.9	682.8	766.1	704.9	792.4
348	287.1	375.6	314.4	269	298.2	331.7	243	353.7	394.3	474.9
272.9	262.7	272.6	185.6	208.8	332.6	245.8	221.5	460.7	582.8	636.9
336.1	413.9	651.8	331.6	267.4	335.5	572.5	461.8	467.4	643.4	603.8
280.6	253	302.8	259.7	210.1	228.3	328.6	373.1	579.8	377.1	578.1
193.3	193.2	185.8	136.1	132.3	119.2	91.9	118.4	252.6	421.1	250

236.6	228.8	260.9	228.3	183.7	221.3	263.2	229.9	297.3	369	402.8
29.6	25.6	25.6	18.7	21.5	20.5	28.4	21.8	22	21.1	34.2
553.1	340.9	441.4	386.1	348.5	310	343.3	290.5	590.9	1354.1	946.8
650.4	241.7	207.6	317.2	435.9	261.2	581.4	373.8	244.9	288.9	286.7
462.3	488.4	492.1	456.6	451.3	467.6	740	703.9	845.1	671.5	629.1
665.2	648.6	700.4	511.9	476.5	617.6	811.3	443.5	1499.8	2175	1981.6
890.9	573.5	765.5	586.4	563.6	597.3	538.8	595.4	842.4	1003.9	1164.6
332.8	317.7	294.3	230.5	347.8	328.3	407.9	268.8	521.2	512.9	789.6
116	108.4	115.1	119.9	105.5	130.4	148	124.1	136.7	143.8	173.6
433.3	296.7	355.3	296.8	273.8	269.9	301.4	254.1	554.8	933.7	896.1
661.7	716.4	584.7	641.7	697.3	663.5	754.4	855.9	679	345.2	588.7
590.3	574.6	636.5	483	471.1	532.2	663.7	630.4	823.2	886.1	1030.6
326.3	378.7	334.2	264.5	302.5	428.3	452.8	368.7	720.1	761	778.2
425.1	314.4	357.6	291.3	334.9	312.4	338.2	308.7	461.2	408.5	550
393.3	369.4	411.6	338.4	319.9	351.5	376.7	363.5	486.9	631.5	788.6
307.9	277.8	309.4	342	240.8	413.7	315.4	339.9	448.4	1200.5	518.8
699.4	698.5	758.4	643.9	574	812.9	794.7	711.4	917.3	1039.8	1404.7
631.6	823	583.9	398.8	292	516.5	349.5	627.7	417.7	662.1	587.5
246.7	327.5	325.4	290.3	256.6	384.6	306.3	372.2	313.5	290.9	388.7
337.8	217	253	217.8	214.2	191.4	211.4	216.7	340.5	236.3	298.1
480.8	301.6	427.3	370.3	375.1	370.3	380.3	368.2	576.8	616.8	742.7
326.4	228.3	303.3	212.8	211.8	239.4	213.9	179	285.9	320.1	389.9
314.7	328.3	394.6	290.3	281.4	372.9	322.9	296.2	452.7	420.1	560.7
554.7	450.7	528.2	404.5	418.4	504.6	553.4	534.8	694.9	727.8	878
242.1	148.2	376.2	175.3	215.1	115.3	128.8	114.6	143.4	177.9	227.1
440	540.1	514	556.9	496.1	670.8	619.1	774.8	1295.7	670	919.9
410	379.5	386.2	302.7	272.7	445.1	450.9	286	437.2	306.2	579.4
427.3	471.3	436.8	344.3	287.5	415.1	490.8	559.5	797	819.5	867.3
194.8	205.7	168.9	149.9	143.8	162.4	243.8	145.7	270.2	281.8	300.3
749.8	1101	842.3	942.5	705.9	1023	863.2	1006.4	891.6	751.5	826.7
220.7	211.7	217	152.6	154	213.1	208.8	198.4	331.8	332	425.7
537.3	568.4	653	500	430.8	542.9	566.9	373.1	733.1	940.1	1115
235.5	182.3	224.7	186.7	207.7	202.9	247.7	180.3	270	328.2	384.1

538.5	411	532.2	421.2	378.4	405.5	407.4	407.1	522.4	702.6	856.1
633	584.2	573.7	511.4	454.3	493.1	631.8	683.1	1134.9	918.1	1143.7
239.2	274.7	257.5	248.9	278.5	260.9	264.2	204.3	382.8	453.7	614.6
598.7	602.1	613.2	607.1	403.2	621.2	515.2	412.8	604.3	559	878.1
708.3	494.6	467.7	371.9	296.9	385.6	370.1	300.2	577.5	367.2	641.5
673.2	454.8	596	470.9	627	449.2	389.4	435.3	505.3	788.7	896.5
709.9	417.1	407	411.4	506.2	677.2	2385.3	489.9	549.1	620.8	676.6
1184.1	812.2	994.2	765.6	688.9	757.1	817.1	675.3	1470.4	2511.4	2369.8
244.4	238.3	244.7	223.4	226.9	254.1	239.7	228	374	413.5	471.8
576.9	556.9	654.2	517.1	437.3	604.3	540.7	461.4	761.6	763.2	1043
473.3	506.2	455.3	325.8	313.2	406.7	520.1	417.3	600.1	876.9	892.9
362.1	421.1	421.5	330.2	290.1	361.4	391.9	336.3	509.7	619.9	697.9
174.8	140.3	136.3	96.3	142.4	133.9	162.5	154.3	266.6	242	258.3
567.3	666.1	673.2	569.7	500.3	599	854.8	656.3	1034.4	1365.2	1206.3
601.9	447.4	507	355.7	264.7	317.6	349.3	372.5	844.5	891.6	1010.3
372	293.9	345.4	278.4	297.7	371	317.2	305.5	523.8	479.5	571.4
853.4	752.4	656.2	540.2	506.2	659.1	743.3	735.9	1340.2	1220.7	1488.4
350.7	325.4	345.6	259.6	397.6	304.6	424.6	276.6	568.2	538.8	707.2
306.5	330.4	307.5	225.1	265.3	333.5	433.2	280.1	364.1	334.3	463.9
739.8	950.6	807.1	776.7	1245.6	722	729.5	869.9	654.6	277.9	465.3
611.8	514.2	561.6	445.8	420.3	541.1	541.3	515.1	716.9	782.8	992.8
624.6	525.7	633.3	460.6	498.6	476.3	551.8	481.5	561.4	666.4	775.6
511.3	529.4	467.5	493.4	464.9	462.5	649.1	491.2	698.1	701.2	877.5
853.3	533.5	850.8	627.1	614.3	520.7	470	348.2	679.6	895.3	1073
205.8	230.1	207.1	196.2	156.3	224.5	261.3	218.9	332.8	383.6	486.7
206.3	218.5	220	240.6	174.7	182.8	184.2	157.5	426.2	287.4	409.8
753.7	662	795.2	601.7	559.7	725	679.4	631	1014.9	993.5	1216.9
379.8	366.1	492.3	362.7	366.1	376.4	396.3	471.6	421.4	397.6	501.2
668.7	776	1035.6	742.1	650.4	838.4	1015.5	624.6	1286.3	837.7	1155.6
424.2	316.1	366.8	315.6	287.9	306.5	366.5	325.5	397.4	488.4	552.7
555.7	425.6	462.4	332.8	509.2	381.7	485.6	377.7	566.6	676.2	834.9
188.8	261.9	157.6	206.9	164.1	283.3	240.7	370.8	178.5	156.9	183.2
952.6	703.8	1150.8	528.6	528.5	532.6	830.8	694.7	853.9	937.3	1187.3

480.9	469.6	431.1	332.1	237.4	396.4	313.7	303.4	493.6	421.6	695
432.5	413.4	434.6	329.9	285.5	385.9	408.8	413.6	508.6	552.8	678.3
865.9	779.8	902.6	789.2	682.1	934.2	663.2	566.4	655	754.1	947.2
251.1	227.4	259.9	202.8	220.3	193.2	179	99.9	159.4	255.8	246.8
956.5	692.5	757	615.1	611	679.5	606.2	573.6	1146.1	1247.6	1464.8
490.1	343.7	389	302.8	301.4	292.1	255.8	297	424.6	746.1	603.7
639.9	403.3	435.5	319.7	606.2	242	306.7	406.5	275.7	263.9	310.6
416.9	503	513.8	292.5	412.5	453.6	537	472.1	629.8	906.4	941.8
185.1	148.2	208.3	142.1	123.6	130.5	151.7	142.9	240	228.3	302.2
484.4	403.7	442.4	334.8	319.2	349	370	365.5	618.3	853.9	880.7
882.1	1139.3	1086.1	904.1	1008.1	841	523.6	919.1	698.8	381.5	631.9
443.4	631.3	567.9	416.4	373	469.3	665.8	465.1	915.4	1223.5	1212.9
542.4	711.5	965.4	973.3	627.3	674.2	540.6	1281.3	350.5	401.9	460.7
302.4	179.9	372.8	211	224.4	180.3	205.6	180.8	310.1	808.3	505
494.2	370.2	453.2	300.4	346.2	363.8	342.3	304.5	590.5	796.5	905.5
93.3	98.7	114.2	164.8	67.5	113.7	114	127.5	360.2	534.2	440.6
387.7	347.1	383.4	275.7	339.3	338.4	350	306.3	516.4	724.2	738.5
223.7	300.4	253.3	246.3	188.3	215.4	239.9	302.1	767.1	507.5	773.8
1169.4	1211.3	1413.3	1023.2	802.1	1095.8	1178.6	893.1	2098.1	2372.1	2530.4
336	367	381.7	332.5	332.8	352.5	411.8	343.9	584	572.1	659.8
654.6	340.2	492.8	335.8	372.3	279.4	252	275.1	826	884.5	1276.1
366	365.4	352.5	299.8	284.1	351.7	344.6	398	608.8	447	610
612	457.4	384.9	475.9	344.3	458.8	368.4	697.1	777.4	885	937.7
1538.1	656.5	642.2	666.3	795.4	432.7	1229.6	767.7	704.6	934.7	881.5
770.7	752.1	673.5	567.1	497.2	456.6	564.6	483.3	978.1	1049.2	1246
655.1	628.2	683.5	652.5	470.8	738.8	647.6	576.3	946.4	1312.1	1270.7
531.7	555.7	580.7	508.1	362.5	516.2	452.5	456.9	589.9	772	725.5
594.2	713.9	721	490.2	596.7	587.4	593.3	681.6	606.9	762.4	891.8
312.2	293.3	403.1	277.9	229.4	255.6	230.1	223.6	274.2	415.3	369.2
433.5	352.6	329.1	269.1	260.8	307.4	281.2	318.7	534	668.1	751.5
1048.5	1004.5	864.9	1027.5	1023.1	995.7	1180.7	1698.2	769	568.6	989.7
466.8	437.3	522	438	395.9	497.6	522.1	432.9	485.6	605.7	687.2
504.8	474.2	629.2	363.9	404.6	438.6	501.4	471.9	790.2	771.8	965.7

729	711.9	737.7	535.4	705	654.8	804.3	637.6	954.7	1185.3	1180.2
486.8	353.4	439	345.9	286.3	310.3	430.4	335.4	466.3	512.9	574.9
443	399.4	540.2	377.2	408.7	469.1	483.8	372.3	463.2	463.1	651.1
319.5	280.6	305.7	216.9	259.4	231.6	313.4	296.6	423.8	351.2	523.7
821.1	629.8	843.1	589.7	538.6	550.3	519.4	529.4	847.9	1386.2	1444.8
375.2	454.7	396.2	371	397.9	467.8	526.6	362.6	485.3	422.4	643.8
145.6	264.1	192.1	188.1	208	202.8	204.5	148.6	214.6	201.2	248.5
454.4	434.3	592.5	452.2	344	423.9	355.9	263.1	402.5	525.4	783.7
318.6	254.5	281.9	230.1	225.1	226.2	258	240.5	322.9	402.2	417.7
176.2	208.4	229.9	161	184	204.7	215.1	201.1	207.4	193.5	202.5
588.9	509.8	770.7	640.6	493.9	664.4	1181.4	352	553	865	767.5
569.9	491.3	464.5	442.7	409.6	424.5	383.9	360.9	677.4	701.5	790.2
778.3	412	508.1	352.3	343	382.8	264	402.6	621.3	1085.5	1465
266.2	426.4	444.1	349.7	256.2	330.2	265.3	433.6	373.9	381.7	413.1
395.6	354.2	387.4	304.5	299.4	458	471.1	404.9	693.5	826	732.3
568.1	710.9	702.9	656	491.1	667.1	745	581.7	675.2	619.6	835.2
610.4	265.5	927	410.5	462.6	190.1	236.5	147.8	177.4	267.3	264.5
877.8	1596.8	935.3	872.8	558.7	1165.2	932.1	1245.7	1238	268.2	551.2
454.6	463.9	511.4	428.6	348.3	430.8	372.1	400.8	735	736.1	944.9
807.5	613.3	680.3	445.1	439.6	624.8	523	530.2	1240.1	2004.3	1926.6
238.4	225.3	244.9	195.8	216.9	218.9	259.3	251.7	368.1	364.2	502.6
402	327.7	312.3	277.6	226.3	289.2	299.2	366.5	577.6	776.1	767.1
1295.5	709.5	1333.8	1661.9	1149.8	1775.6	1828.5	536	519.4	586.1	1000
193.9	186.4	220.1	187.2	150.5	234.1	222.1	138.6	200.2	307.4	301.3
226.8	176.4	181.3	165.9	123.5	108.9	111.6	162.5	290.9	317.1	430
253.7	197.5	208.6	175.1	183.7	179.3	231.1	256	346.2	346.9	365.6
220	146.8	257.5	274.7	160.1	413.4	1927.3	94.3	55.9	109.6	167
258.9	245.5	256.2	196.1	209.8	230	219.9	177.3	233	389.7	356.4
568.6	624.8	631.2	612.2	412.6	573.5	525.3	557	890.6	817.2	1055.9
548.8	507.3	616.2	472	485	571.7	465.2	444.7	769.8	895.1	1066.9
586.4	639.1	666.6	498.6	467	641.5	624.4	580.6	897.6	761.3	1114.6
552.2	504.5	558.7	432	408.7	483.9	452.5	505.6	793.4	779.4	1082.1
619.1	587.7	607.8	428.6	374.1	556	521.5	484.9	491.1	731.1	693.2

246.3	257.9	268.4	247	240.4	256.8	271.5	179.5	285.7	377.1	363
366.9	296.2	340.2	235.4	268	281.5	279.7	241.3	455.5	647.1	703.8
262.6	272.7	352.6	273.9	180.3	249.3	362.9	254.6	376.5	749.2	772.3
436.5	355.8	411.7	295.2	283.9	322.6	329.7	311.8	500.9	579.7	625.4
364.2	393.1	419.6	318	395.1	520.6	755.9	472	587.4	447.4	1145.4
382	345.4	367.2	307.5	293.2	307.6	356.8	273.1	433.9	500.9	528.5
598.1	539.3	802.5	1105.6	371.3	585.4	439	538.4	394.9	378.3	454.5
377	388.9	403	280.7	266.5	314.1	393.9	276.1	462.5	604.4	624.2
196.2	248.6	216.5	189.8	166.3	265.5	257.8	194.5	425	492.6	524.7
390	404.5	448.9	343	341.1	498.2	456.6	390.9	506.6	544.4	663.1
417.5	330.5	405.3	293.8	271.5	302.9	318.8	293.2	666.2	849.1	915.2
608.8	429.1	550.5	408.2	409.8	344.3	460.4	510.5	1049.9	1146	1341.3
190.5	180.3	169.5	133.7	145.6	157.3	183	146.9	204.3	268.4	294.6
314.1	413.2	442.2	335.2	274.2	414.1	349.8	367	757.7	619.2	823.9
527.9	426.3	476.7	341.3	362.2	427.4	600.2	377	604.8	583.5	777.2
202.3	200.1	212.1	165.9	187	209.3	265	195.4	671.4	1434.7	1140.7
663.6	495.3	642.1	596.8	590.7	561.1	486.3	347.3	954	1019.8	1403.8
633.9	539	636.3	462.7	460.3	528	464.6	524.2	690.4	615.1	685.9
385.6	432.6	321.7	288.5	524.1	416.2	835	430.6	379.9	431.1	438.2
657.1	459.2	470.1	416.9	459.8	437.1	524.2	432.1	894.6	1115	1214.8
261.2	263.7	247.5	193.7	217.6	210.3	318.9	235.4	333.1	343.5	408.8
255.6	311.2	356.4	293.4	193	299.4	316.5	277.5	440.8	503.1	603.3
933.6	851.2	941.7	1022.7	1125.7	1056.4	1057.1	508.5	646.9	1108.4	946
430.4	414.3	418.3	401.9	349.9	458.8	371.5	434.3	556.5	489.7	635.1
376.4	349.8	375.8	319.8	242.5	296.6	415.6	305.6	500.7	445.1	667.3
913.4	1093.4	755.7	2953.7	682.3	1900.1	798.5	635.4	532.4	436.9	490.4
316.1	258.4	363	282.5	278.7	313.8	301.4	282.8	794.4	762.8	644.6
376	363.8	380.3	348	333.1	388	457.2	343.5	512.3	540.1	755.1
511.1	478.4	381.6	318.8	311.9	340.6	489	434	763.8	1099.4	1043.2
319.3	334.1	349.1	240.3	218.2	386.6	465.5	248.6	245.1	396	405.7
470.1	300.2	329.4	271.3	321.4	238.8	305	260.6	461.7	327.7	509
354.7	372.8	412.1	280.2	278	373	397.9	376.9	488.5	540.3	631
477.6	475.7	666.8	472.2	543	480.3	470.3	390	544.5	546.9	794.2

268.3	238.4	260.3	231.3	240.3	234.4	296.8	217.5	295.1	354	432.3
497.1	496.8	508.9	388.6	381.7	441.7	474.6	336.3	877.2	1540.3	1381
376	303.8	338	263.8	282.5	291	394.6	312.1	639.2	725.2	806.2
792	796.6	792.1	652.9	624.8	785.3	918.6	653.7	834.4	959.6	1131.6
317.4	286.5	304.2	218.6	248.5	258.2	282.2	256	432.6	437.6	647.5
231.2	193.9	189.5	148.9	141.7	168.7	162.7	149.7	287.7	435.8	454.6
438.7	405.3	468.4	352.2	317	351.6	385.9	379	885.8	1031.4	1088.1
277.5	319.8	319.7	275.1	251	299.5	325.9	276.7	378.9	506.6	568.9
550.8	481.6	470.9	358.2	408.7	483.1	616.9	437.2	820.2	1004.3	1074.7
132.4	118	106.3	116.7	119.5	95.3	160.9	132.2	182.4	217.9	189.9
539	635.8	652.7	613.1	451.4	583.3	465.7	479.8	702.4	1109.8	1126.2
350.9	371.1	292.9	246.6	255.4	356	391.7	321.4	556.2	584.2	763
857.3	874.6	760.9	655.4	602.6	641	656.2	622.1	1346.7	1491	1839.6
245.6	258.3	299.1	234.1	216.6	261	263.2	230.9	344.7	343.6	412
414.2	413.4	380.5	293.2	304.8	339.4	423.7	358.1	376	465.2	454
55.2	69.3	80.4	68.7	65.5	73.9	68.2	53.9	74.8	110	113.4
582.6	420	463.8	344.5	401.1	445.8	392.6	435.3	947.5	972.7	1180
449.3	344.8	367.3	324.6	358.3	357.3	393	429.5	499.8	651.9	802.2
254.4	342	316.4	275.7	248.9	483.4	471.6	408.9	559.8	791.1	650.3
350.2	329.3	386.5	366.7	299.4	428.3	363.5	379.7	615.7	497.3	624.5
585.3	513.6	556	471.6	441.5	487.1	515.9	444.2	885.1	922.9	1121.3
794.5	458.3	751.8	501.5	518.4	568	478.1	479	996.4	1260.2	1434.9
387.8	340.2	369.8	280.5	275.2	357.8	386.5	325.2	894	860.4	986.4
249.2	267.1	262.6	264.2	199.2	276.5	267	246	374.1	2416.5	563.9
312.6	308.6	313.2	253.6	218.2	320.8	360.1	271.2	427.4	513.7	561.8
736	610.4	914.2	487.4	513.3	354	450.5	374.6	465.1	553.8	681.3
178.1	226.6	227.7	219.2	247.2	281.6	299.8	228.9	358.3	273.3	443.1
647.2	742.1	670.5	465.5	621.3	796.6	1489.3	628.6	596.8	623.2	799.3
680.4	717.8	823.9	621.9	631.6	690.6	1426.3	914.4	1506.4	1401.4	1582.7
244.1	188	218.4	266.8	202.6	194	209.1	188.8	217.6	1119.8	284.1
507.5	458.3	540.3	405	456.4	422.4	499.2	416.8	427.7	546.8	537.2
768.6	1420	1631.4	964.7	824.3	1182.2	1298.5	845.5	600.6	608.1	923.8
901.8	736	851.5	615	508.7	677.1	961.5	684	1454.9	2405.7	2114.9

259.9	299.6	395.2	344.7	193.5	248.8	227	253.2	360.1	383	447.1
320.3	420.5	535.9	357	297.8	408.6	345.9	352	271.7	244.7	399.3
107.8	127.7	114.8	115	90.6	124.1	131.9	125.5	196.6	290.4	253.5
538.7	418.2	444.4	375.2	495.8	616.4	383.1	423	988.2	401	480.1
313.2	316.1	351.5	274.2	279.4	272	320.4	279.7	607.4	690.6	804.3
1247.1	1169.7	1384	1055.9	1131.7	1192.7	1218.5	1183.9	1393.8	1820.1	2139.6
269.5	222.4	306.7	238	193.6	221	286.9	232.6	300.2	425.1	380
551.8	609.8	443.3	429.6	750	451.6	772.6	479.2	396.9	452.5	389.2
753.9	726.5	834.8	558.7	542.7	826.9	706.4	572.1	816.1	950.6	1244.2
591	522.8	578.5	535.1	487.1	578.8	610.1	500	663.8	882.2	960.9
272.5	350.8	271.6	242.1	267.6	230.2	219.1	233.8	295.8	428.5	442.6
611.5	1295.6	1837.6	717.9	537.6	950.9	639.1	2665.6	475.5	3675.3	815.3
199.1	184.3	160	157.2	122.8	201.6	157.7	272.7	469.8	322.1	378
467.6	458.3	442.1	399.9	499.6	528.1	510.8	376.4	486.3	513	643.4
314	691.7	518.5	554.3	341.5	599.4	447.5	526.2	330.3	236.3	300.9
256.6	123.8	163.6	154.1	180.7	97.9	124	94.4	252.2	232.5	277.5
400.4	395.7	378.3	324.7	325.3	338.6	475.4	389.1	597.5	687.8	729.4
185	149.1	178.3	149.2	93.3	133.8	134	147.2	244.6	250.8	296.2
946.9	890.2	910.7	717.4	633.7	824.5	889.6	728	1315.4	1702.2	1653.4
171.6	159.3	185.1	141.7	114.3	121.2	129.1	138.7	226.2	319.3	288.9
780.8	638	752.4	720.3	532.3	1085.3	1239.2	701.5	747.4	495.8	740.2
352.1	284.5	299.5	266.5	260.1	318	259.5	307.3	505.6	576.3	657.6
438.5	667.4	461.1	580.7	361.5	693.4	608.1	584.8	950.1	797.3	936.7
59	36.5	39.6	35.6	38.8	35.3	26.9	38.4	67	50.5	82
536.7	494.1	537	478	416.2	530.2	393.3	334.4	618.4	561.8	757.4
498.8	424	382.3	271.5	333.7	336	478.6	366.2	553.4	555.8	703.9
295.9	302	287.7	230.4	218.8	279.4	293.1	283.6	352.6	422.8	441.8
207.3	426.2	315.6	274.2	188.6	283.9	479.6	146.2	89.1	139.3	304.8
270.4	266.3	277.3	223	219.8	232.3	252.9	201.7	302.8	270.5	408.6
309	379.5	313.5	957.5	218.3	267.1	271.1	263.1	188.7	196.1	233.1
342.2	239	336.2	232.6	297.3	277.8	278.5	246.4	573	637.5	800
181	214.3	210	149.7	185.8	198.4	186.4	241.1	374.2	328.9	466.2
537.5	434.8	506.5	407	413.6	401.1	439.6	315.6	542.9	923.1	884.8

702.2	554.1	601	561.6	501.8	576.1	550.1	401.2	669.1	1110.7	1133.6
229.2	206.8	229.3	150.1	163.8	210.4	189.9	194.3	452.4	515.1	580.8
72.6	68.5	64.7	82.6	72.2	82.3	76.4	65.6	74.8	52	66.7
525.2	1434.3	729.2	532.7	521	1673.4	1492.2	826.1	530.8	566.1	624
369.7	427.3	479.8	390.4	325.8	399	441.8	354.9	558.7	717.8	493.6
274.8	268.9	247.9	214.5	233.1	250.9	253.6	242.7	358	401.8	485.2
137.5	141.5	100.6	79.6	101.8	105.7	106.2	101.7	107.4	146.8	174.1
143	102.8	133.2	102.6	115.5	184.4	143	158.8	306	620	632
784.4	542.7	189	608.2	840.1	255.8	1105.4	1343.9	416.1	448	302.1
326.9	319.2	396.5	264.3	315.7	286.2	315.1	219.8	390	528.2	518.2
551.8	536.3	579.3	451.3	412.3	523.8	629.9	442.6	883.1	1053.8	1083.3
814	662.2	1042.3	572.6	474.9	648.7	905.7	509.9	618.9	558.7	681.3
550.2	330.3	374.3	442.4	434.1	482	705.4	723.4	1562.6	1043.8	1003.1
1212.7	905.8	1002.2	835.9	458	517.7	494.6	520.2	847	897.7	979.2
332.7	501.3	421.5	347.4	401.2	473.6	795.4	415.2	382.5	788	509.4
345.9	397.4	401.6	306.1	261.6	355.7	382.2	355.8	678.6	1032.6	978.1
155.2	110.2	159.8	117	139.4	106.5	153.3	117.2	165.1	230.7	258.9
417.2	366.5	457.5	308	290.7	328.9	322.6	261	392.7	550.3	610.7
370.2	293.5	353.4	241.8	205	240.7	240.4	246.9	671.2	856	898.5
761.8	461.8	645	498.5	524.6	482.9	440.5	427.5	855.8	1125.8	1875.8
546.1	385.8	589.8	504	373.7	348.9	309.4	354.4	259.4	293.2	338.1
317.3	509.1	614.2	242.1	342.3	498.5	255.1	299.6	378.3	664.1	381.1
761.5	366	558.2	381.2	365.9	385.7	371.2	322	1025.1	1287.3	1394.5
569.8	431.6	597.6	308.6	367.3	340.6	384.8	407.8	278.1	253	385.8
292.4	286.8	295.4	302.5	234.3	268.2	318.8	292.8	364.7	374.6	454.2
244.3	279	382.9	347.6	269.3	285.2	351.2	300	603.1	463	579.9
755.9	657.4	688	526.5	528.6	666.6	707.4	586.5	1075.6	1594.7	1505.3
149.7	137.2	156.9	117.2	118.6	139.3	141.8	109.1	144.6	205.6	232.3
1626.4	1043.7	824.3	1108.1	1595.3	723.2	1421.5	2270.4	1710	453.4	1030.5
417.6	428.7	441.4	371.3	338.5	431.5	426.1	411.1	409	486.7	622.4
332.3	313.3	398.5	262.2	282.9	390.2	438.1	303.2	424.8	609.3	627.7
265.9	292.8	273.1	249.6	184.6	245.5	289.6	206.8	340.2	428.4	467.3
541.8	388.4	445.2	343.3	332.7	458.7	520.3	394	785.4	961.7	1054.7

267.6	218.3	181.3	174.2	219.1	232.2	286.2	230	233.2	263.5	263.4
624.9	515.9	590.4	410.3	448.9	465.2	495.1	487	981.3	1294.2	1351.9
330.7	400.6	353.2	330.3	298	434.1	448.6	407	558.9	741.4	684.3
382.6	216.3	335.9	208.3	192.3	160.4	135.9	230.7	167.2	232.2	297.2
586.8	551.2	622.5	519.2	438.8	552.3	456.4	385.9	695	812.2	1027.1
619.9	524.2	725	547.4	479.6	537.8	519.7	512.4	872.6	966	1213.9
311.3	235.1	340.1	270.6	257.7	242.1	276.2	224.2	303	547.2	488.3
827.2	908.2	664	549.7	604.9	752.2	804.9	450.7	887	1568.5	1221.7
426.6	425.4	487.7	412.5	336.5	385.2	385.5	392.6	673.5	746.9	926.7
369.9	311.3	405.4	319.4	324.2	337.9	346.5	297.4	419.4	461.3	461.9
331.4	317.9	329.6	245.3	222	308	330.5	341.8	432.6	470.7	569.2
619.2	599.7	765.8	653.5	755	613.2	713	762.9	833.1	610.5	832.9
362.2	311	364.7	245.2	271.2	262.1	420.9	420.7	414.1	540.8	593.2
265.5	311.4	273.8	247.1	245.1	289.2	387.7	319.3	531.6	632.4	663.6
340.3	304.8	363.3	275.1	224.5	339.8	313	277.2	370.8	365.2	516
362.8	328.4	329	252.9	237.8	324.7	344.4	304.5	525	681.4	731
339.6	209.7	175.3	190	263.3	143.7	368.4	324.8	223.6	147.2	156.6
240.4	199.9	219	177.3	159.7	214.3	189.5	231.4	473.1	447.9	582.8
457.5	402.3	454.9	363.8	377.4	439.5	460.1	432	560.6	693.1	775.8
342.7	353.7	376.7	311	282.5	412.4	725.7	320.2	511.6	536.2	589.1
486.8	296.5	361.8	184.8	275.9	282.9	611.2	374	549.6	450.7	289
388.8	359.5	421.5	320.9	240.7	284.2	382.1	347.4	408.2	589.1	603.2
607.9	458.8	509.5	403.8	372.1	368.2	474.3	367.8	576.1	835.6	883.5
1340.5	825.7	1293.8	1442.5	691	628.6	384.5	580.2	194.8	186.8	313.5
469.2	556.3	546.7	526	395.2	630.7	758.3	586.5	1167.7	926.6	1217.9
411.7	177	298.8	251.9	311.3	207	303.8	301.2	683.8	583.9	561.8
404.9	438.3	549.2	600.5	366.7	331.7	311.3	738.3	356.9	194.1	463.1
212.7	262.1	308.4	282.9	180	350.1	273	273.9	406.1	289.5	349
502.3	575	547.1	496.5	432.3	522.3	622.8	556.6	722.7	733.9	814.7
366.3	362.7	335	323.3	300.1	321.8	383.1	301.8	427	688.2	644.5
384.5	396.1	454.4	350.3	328.6	527.8	635.5	431	346.4	405.9	512.1
618.4	585.1	481.9	398.9	517.5	474.1	894.3	605.1	811.1	918.6	1164.7
338.3	217.1	224.3	193.6	175.8	201.8	223.6	208	417.2	511.5	623.1

493.7	444.7	454.9	355.7	431.9	486.7	436	422.9	760.1	668.8	971.2
331.5	219.4	242.7	164.5	223.3	222	233.2	224.7	587	510.2	702.1
345.9	220.5	274.1	236	300	295	285.1	291.6	574.9	538.1	607.1
346.5	344.8	350.5	280.3	245.7	236.4	329.3	300.7	764.9	819.1	976.8
289.2	223.9	260.7	195.6	203.3	251.6	244	214.3	388.1	506.9	534.4
296.4	257.2	320.3	271.1	222.6	240	240	237.2	453.1	556.5	617.2
224.5	235.8	264.1	192.7	169.8	237.6	237.8	190.9	322.2	389	425.7
639.2	596.3	719.9	726.8	513.5	532.2	502.8	480.6	933.5	885.5	1220.8
233.4	231.5	257.9	212.6	170.5	201.7	235.4	204.6	435.4	516.2	511.4
411.8	347.5	384.3	296.1	286.8	317	425.2	470.2	617.1	537	648
299.6	287	195.4	252.4	183.4	208.7	231.9	351.4	594.9	468.1	453.2
292.1	325.8	461.7	331.9	330.9	514.2	511.8	385.6	615	528.7	420
586.7	726.7	792.9	513.9	465.3	644.4	794.8	598	1121.9	1082.1	1168
355.3	346.3	243.2	240.8	296.9	276	392.8	328	429.1	469.7	571.2
767.6	726.5	988.1	937.8	556.6	903.6	677.2	664.2	845.8	962.1	1354.8
349.9	334.5	422.7	399	293.1	367.3	388.9	295.3	594.8	877.8	789.5
432	310.6	489.3	272	291.6	330.8	299.8	270.8	574.9	811.9	1023
165.5	98.9	124.6	87.3	94.3	119.9	112.1	97.7	252.7	160.5	281.2
424.6	260.9	447.2	307.7	207.5	178.7	243.3	236.8	228.5	169.6	282.9
440.9	475.1	505.9	441.5	390.2	429	591.2	487.2	726.9	1225.1	908.6
622.4	477.6	645.4	411.7	444.6	470.6	603.4	446.7	809.3	729.3	893.9
52.6	69.6	84.8	86.3	68.1	80.1	63.3	73.3	51.6	44	58.7
251.4	173.7	347	173.7	414.1	340.7	703.3	264.1	102.4	99.2	145.9
697.1	650.4	715	538.1	458.7	566.6	713.9	521.3	1022.8	1410.5	1444.3
150	153.8	169.7	137.8	127.4	153.3	171.8	160.7	208.5	252.1	310.4
753.5	775.9	999.6	1565.8	1045.5	2216.7	476.3	729.2	243.6	271.7	316.4
490.1	480.8	440.5	399.7	404.5	399.3	582.6	480.4	814.6	1084	943.8
822.8	828.4	810.7	604.6	631.6	681.8	884.7	706	1920.9	2031.2	2133.2
91.8	68	56.8	72.6	103	797	179.8	74	49.4	89.4	76
827.2	664.3	720.2	550.6	560	633.5	536.5	565.3	1324.8	1504.8	1855.1
678.1	488.9	634.5	542.9	445.5	408.1	439.6	428	701.2	990.8	1027.6
305.5	279.5	352.7	291.2	282.1	310.9	286	273.7	415	391.5	539.3
540	344.5	354.6	277.5	369.9	375.5	376.4	372.4	578.1	680	865.7

437.7	282.5	377.9	314.3	343.9	285.8	329.5	250.3	402	500.3	529.6
154.2	128.6	126.2	134.5	100.6	133	124.9	146	184.2	190.9	233.2
494.3	657.6	693.5	519.8	424.6	550.2	524.1	611.8	654.1	659.3	935.3
491.4	421.4	494.5	363.7	420.3	439.5	409.1	431.1	728.1	890.3	950.2
409.2	438.1	479.5	419.7	374.4	484	538.4	449	587.2	875.1	886.2
518.1	536.9	512	479	314.1	516.3	484.3	459.1	713.6	950.6	831.9
329.7	259	309.3	243	211.5	259.4	251.8	250.6	491	615.7	711
199.9	202.2	186.2	173.8	169.5	197.6	205.3	195.7	334.3	278.5	366.7
393.9	212.2	257.7	210.8	146.5	120.6	140.2	128.9	218.7	369.3	456.5
253.5	223.7	263.9	210.4	205.7	269.5	302.4	205.5	314.3	305.7	370.3
191.8	214.8	196.4	174.7	170.6	195.4	218.1	184.9	235	273.8	356.7
317.5	198.5	272.3	188.5	144.8	158.1	151.2	181.2	381.3	390.5	485.6
650.8	559.2	672.3	511.5	463	528.8	498.5	475.5	825.5	903	1149.4
265.6	292.1	297.8	238.6	209.6	256.3	268.7	420.8	503.9	658.6	697.7
273.3	295.1	321.5	334.6	230.4	245.6	309.4	300.4	363.4	518.2	538.2
278.5	257.1	265.1	236.2	200.5	229.8	257.8	267.9	370.5	336.1	449.6
1017	1143.5	912.8	985.5	967	824.3	1202.9	1070.9	543.8	462.4	640.7
951.1	774.6	970.1	851	572.7	386.3	225.9	491.9	1193.3	528.5	877.5
340.4	298.3	276.3	256.2	225.3	258.2	287.6	307.1	461.2	617.3	589.7
381.1	393.8	438	362.7	311.7	385.3	296.7	317.3	262.9	312.9	332
294.1	316.6	269.5	196	319.7	218.2	333.2	327.4	368.5	259.1	279.1
189.5	194	203.2	154.5	148.6	177.9	184.7	165.2	481.5	629.9	666.9
867.6	658.4	836.1	603.6	575.9	577.9	725.4	704.6	1064.1	1346.1	1480.5
334.4	327.7	382.4	377.6	349	564.6	392.4	238.7	449.9	591	677.6
277.9	247.1	241.4	168	177.3	180.8	256.6	229.6	287.5	283.8	336.3
834.1	730.1	829.5	693.2	598	683	566.8	622.3	1062	1126.6	1559.8
241.2	196.7	208.7	149.3	180.7	148.2	219.9	163.4	316.2	389.7	447.9
721.2	512.1	528.8	486	458.2	412.6	302.8	330.5	838.9	1119.4	1150.7
388.2	349.7	406	314.3	282.5	405.2	383.5	424.4	573.9	575.4	626.9
277.6	346.8	328	278	256.3	337.6	431	311.4	603.6	787.1	722.3
458	362.3	428.3	340.2	345.4	374.2	327.4	295.4	726	594.1	915.7
148.8	166.4	178.2	140.4	152.6	170.3	204.5	141.1	218.3	233.3	281.6

23	21.4	17.8	19.1	27.6	25.2	23.2	20.7	26	27.5	24.7
127.1	150.2	164.9	138.9	111.2	141.2	123	161.2	189.2	153.7	210.8
135.4	127.4	132.5	100.6	121	118.2	169.3	131	275.8	565.9	440.5
342.4	393.9	423.3	378.4	337.3	407	420.8	552	510.3	675.7	693.9
659.4	595.2	769.2	623.5	522.7	583.1	543	528.2	994.9	1065.7	1475.6
396.8	321.6	342.4	291.6	274.1	292.6	314.7	318.9	569.6	618.3	725.1
372.3	332.9	341.3	276.7	249.1	277.4	366.8	343.3	731.7	1073	959.9
717.3	722.6	807	827.9	684.9	604.9	1039.8	754.3	570.8	628.2	740.7
196.8	201.1	201.5	176.5	200.9	500.6	245.5	149	260.1	321.2	360.4
476	357.2	445.4	322.7	303	347.4	329.1	367.8	510.9	510.5	614.6
359.4	354.8	375.2	408.8	337.1	449.2	370	375	452.4	584.6	630.1
148.1	170.6	208	185.9	149.5	216.2	171	178	229.4	189.6	230.4
95.9	87.7	106.9	103.1	90.7	83.8	115.4	79.2	188.5	601	277.9
553.5	435.8	496.1	381.3	443.8	411.2	438.9	463.3	736.7	798.1	1058.9
301.8	308.1	302.3	250.5	303.5	298.2	421.6	246.1	373.2	613.2	534.5
353.8	347.4	365.7	270.7	277.2	319	385.6	298.3	603.9	741.2	810.9
484	526.9	543.3	422.5	451.7	543.5	437.6	476.2	319.3	398.4	414
194.8	186.1	225.3	174.2	145	202.8	243.5	196.9	340.5	383	419.7
394.3	398.4	309	310.6	309.3	341.3	604	375.3	460.9	359	670.2
746.9	516.8	512.4	356.2	328	335.9	447.4	329.2	575.6	488	546
294.3	448.7	425.9	392	340.8	514	294	342.5	351	382.2	480.1
132.3	157	152.5	129	114.5	149.5	183.3	150.2	180.3	233.2	245.2
268.3	224	270.2	212.1	213.7	263.5	227.6	229.2	373.5	450	405.7
172.4	226.7	184.4	188.5	244.7	237.6	320.1	188	173.2	200.4	222.5
317.6	493	482.7	650.9	452.9	653.4	410.7	293.8	459.3	944	773.3
269.6	260.8	229.7	231.3	219.5	220.7	219	199.1	383.8	691.2	714.3
225.3	198.7	188.3	168.3	183	190.4	190.5	168.8	216.3	304.6	320.1
505.5	349.2	474.2	303.9	354.2	375.4	338	356.5	578.6	766.3	886.4
288.8	916.1	473.9	256.2	323.3	370	743.3	328.4	615.3	2242.2	1032
665.8	434.5	437.2	393.1	403.3	377.7	426.1	352.4	1013	967.6	1077.3
591.9	593	606.9	606.1	489.2	625.2	438.5	587.1	617.5	552.9	639.2
885.9	784.7	637.1	502	491.2	650.1	941.6	569.3	1092.6	1513.5	1484.9
1058	1097.7	1308.7	1181.4	1055.3	830.2	871.3	1303.2	840.6	639.6	945.9

348.4	241.9	270	228.5	200.6	215.9	213	244.3	437.2	503.6	628.1
251.6	284.7	282.1	271	221.5	279.8	262	213.6	299.6	337	380
658.5	465.6	588.8	441.9	399.9	518.6	371.3	384.8	914.9	1121.8	1303.3
697.3	416.8	429.4	356	470.2	282.2	511.1	309.3	546.1	674.1	697.8
232.1	355.2	287.3	250.9	177.1	404	466.6	229.4	180.8	533.2	355.1
102.4	91.1	94.1	81.8	78.7	76.6	78.8	98.8	106.5	112.3	127.3
459.3	382	432.3	342.5	337.5	367.1	398.8	353.2	615.9	720.1	826.3
189	176.4	243.3	154.1	139.9	145.4	139.9	146.4	166.4	186.7	283.8
460.6	417.5	511	388.1	318.9	391.7	424.8	382.8	673.3	609	836.6
198.1	261.5	453.1	190.9	250.6	274	315	331.9	160.7	204.3	199.1
385.8	344.9	381.1	316.4	272.8	337.5	353.6	325.6	511.3	658.3	711.1
198.5	226.4	245.5	169.7	168.4	202	292.5	214.3	276.9	283.1	346.3
542.8	428.7	446.1	361.5	343.5	392	415.5	437.9	809.9	907.4	1034.9
401.4	387	409.6	353.9	333.3	390.2	445.8	392	625.9	797.5	804.3
313.9	279.5	312.4	241	257.3	285.5	282.1	286.6	561.4	692.2	755.9
494.9	461.6	501	403.9	354.4	463.3	622.5	487.7	680.4	684.8	901.1
431.8	361.7	217.3	122.7	636.7	249.8	410.8	357.4	135.6	209.2	148.1
178	133.6	157.2	120.4	151.3	130.1	156.3	135.4	312.6	543.7	448.1
316.5	283.4	292.8	220.4	244.5	268.6	294.4	255.9	568.6	727.6	827.6
228.5	169.4	257.8	239.3	202.5	291.4	1056.8	185.7	213.2	278.7	448.6
817.3	677.8	855.3	651.6	721	756.5	712.1	541.8	1002.4	1076.7	1449.4
559.7	654	631.5	560.1	586.4	756.6	745.4	603.6	887.3	701.8	900.6
413.8	365.1	418.7	296.1	345.7	345.7	389.1	375.1	576.9	701.4	814.1
341.3	337.7	300.2	275	244	281.5	310.9	306.7	603.5	920.3	738.9
189	275.5	187.3	182.8	136.2	204.7	216.5	291.7	651.6	327.8	628.2
591.8	769	357.5	386.4	415.7	395.5	738.4	565	402.5	412.8	703.4
404.3	316.9	366.2	269.9	249.1	287.4	289	280.9	621.7	716.9	844.3
634.7	267.4	390.8	449.7	320.9	197.6	240.7	128.8	161	191.8	332.6
288.4	338.8	345.2	308	318.1	366.1	250.5	368.3	468.5	473.3	469.1
550.9	1219.5	475.2	529	1212	629.8	602	622.9	635.8	388	457.7
170	200.3	219.8	162.5	163.1	189	193.7	178.4	233.5	289.3	342.6
480.1	364.3	374.8	331.4	391.2	346.7	371.6	329.1	503.2	617.3	662.5
646.4	710.9	638.2	498.3	438	584.3	788.7	537.2	1146.3	1094.7	1287.1

151.5	288.4	265.5	187.9	155	229.2	431.1	284.3	614.4	528.6	707.3
176.2	201.2	188.4	197.4	134	157.8	407.3	1248	170.3	187.3	233.2
235.5	236.3	249.3	193.7	154.7	180.3	201.9	211.3	326.4	598.1	547.7
435.8	556.5	453	405.3	359.9	719.1	1256.2	576.5	364.3	321.2	453.6
472.1	462.7	486.9	366.3	296.7	375.9	360	407.2	858.6	742.3	1024.9
472	473.7	557.9	480.4	342.2	449.4	442.4	441.8	532.2	678.3	728.8
160.2	187.5	212.6	237.9	85.4	209	163.4	175.9	117.7	124.5	155.3
98.3	110.8	156.5	144.4	106	153.2	170.3	116.6	241.9	830.1	669.1
255.3	339.2	329.6	284.9	203.9	308.7	330	409.9	664.4	923.3	808.1
90.6	103.8	146.8	125.3	122.9	190.2	106.1	110.8	356.9	126.3	315.3
641	407.3	558.1	419.4	348.7	349	299.5	336.4	494.3	265.7	666.6
204.9	171.7	183.1	151.9	154.7	139.7	203	430	264.2	301.4	360.1
468.9	475.4	468.2	401.2	338.7	404.6	555.8	416.2	827.7	871.9	1039.3
102.8	120.7	143.7	117.1	103.7	190.5	174	137.1	116.1	112.7	121.6
226.2	250.4	239.3	244.5	214.7	254	291.5	306.4	442.5	487.6	489.3
189	467.7	186.1	142.4	145	159.2	212.1	165.8	212.9	642.9	288.6
252.1	263	271.1	231.7	205.7	296.7	323.1	248.4	427.7	693.3	630.9
212.2	199.7	282.7	202.9	171.9	267.8	187.5	145.7	312.8	321	413.7
174.1	193.2	206	253.9	140.1	207.9	157.9	150.9	270	330.4	308.2
342.2	351.6	389.9	283.3	331.9	366.1	416	285.5	617.5	719.3	782.5
337.2	327.5	370	285.1	267.4	463.2	325.3	332.5	289.1	281.9	332.6
361.9	289.7	364.4	277.8	259.9	309.1	304.3	338.7	321	414.3	452.8
500.9	457.4	571.1	574.6	428.7	455.4	418.3	400.3	564.9	710.1	825.6
87.3	112.1	140.8	124.7	80.9	124.2	100.6	136.8	567.5	317.7	510.9
216.4	189.2	200.5	171.3	161.6	199.1	205.1	159.5	237.8	276.6	359.5
273	168.6	197.1	173.4	165	156.1	213.5	128.8	194.1	285.3	278.1
640.2	650.6	464.7	686	363.3	506.9	421.3	540.3	382.2	383	369.2
504.8	1020.8	610.9	765.2	458.5	1013.6	580.5	892.2	699.1	613.8	809.5
377.1	364.9	387.5	236.1	279.1	284.8	342.2	297.3	428.3	472.2	516.3
150	166.6	163	158.1	182.1	148.7	185.4	156.7	151.4	128.4	153.9
209.5	222.1	228.2	189.8	218.2	270.9	247	221.9	256.3	270	299.7
208.9	193.5	217.6	147.3	139.9	171.1	146.5	156.8	368.3	275.2	413.9
819.3	842.3	736.8	677.1	865.5	693.5	894.7	803	787.3	1085	1093.1

792.7	585.7	775.3	617.9	408.1	394.5	417.4	470.4	594.3	960.1	1190.8
306.8	310.7	357.8	229.8	227.8	273.9	358.5	240.8	364.8	362.5	583.5
1034.4	972.8	766	572.3	619.2	794.3	633.2	463	520.4	913.6	737.3
165.8	185	188	146.3	133.7	169.7	200.3	151.1	245.5	301.3	311.8
193.4	166	211.6	188.4	178.9	181.7	212.4	164.4	280.6	1782.4	412.3
1268.9	790.9	1206.9	813.5	753.4	591.4	504.6	576.7	802	839.2	1365.7
258.1	247.5	279.1	216.3	224.9	264	295.4	270	452.3	362.7	391.8
340.8	308.6	319.3	266.9	233.7	296.5	302.8	308.7	384.8	446.3	545.2
616.6	595.5	666.3	466.1	494.3	559	627.8	545.3	655.6	843.9	814.8
797.5	602.9	768.2	575.5	656.9	607.7	601.9	885.6	1291.8	1956.2	1933.8
276.9	306.3	292.1	266	217.5	294.5	361.1	326.6	422.5	495.1	531.3
265	225.9	260.1	164.2	214.4	231.9	251.8	194	394.3	559.9	568.8
185.9	131.8	168	118.7	136.2	138.8	105.3	105.3	158.5	123.3	142.8
155.9	129.7	155	139.3	136.7	155.7	267.6	139	212.6	230.9	277
726.6	804.2	801.4	687.6	576.3	720.7	622.8	713.4	1182.3	1631	1493.2
447.8	410.1	574.8	473	345.2	495	494.3	345.8	811.8	664.2	856.1
328.7	279.1	301.4	252.6	248.5	282.9	392.1	253	468.9	789.6	919
167.3	121.3	135.6	113.2	123	106.1	129.2	122.4	232	261.1	285.4
217.4	236.2	259	232.4	191.6	227.5	245.3	255.1	412	326	556.1
305.8	299.7	368.3	309.5	226.7	255.7	224.1	267.4	294.3	287.2	387.7
352.7	362.2	386.5	325.1	323.4	335.4	420.1	287.5	435.7	423.1	552.8
332	543.1	696.8	928.3	1184.1	1764.3	820.2	711.6	196.3	243.7	305.1
113.3	83.6	111.3	74.8	71.3	80	145.3	66.1	73	49.9	84.4
306.2	328.9	268.2	236.7	235.3	238.3	286.5	295.6	344.7	386.3	450.8
255.5	226.3	237.1	182.9	177.5	214.3	231.2	185.3	319.3	378.4	474.1
374.8	351.6	410.7	325.4	317.3	392.8	349.4	289.8	622.7	1008.7	910.9
409.7	373.6	397.3	293.9	313.8	369.9	390	373.4	481.1	522.2	596.9
254.3	201.2	246.9	189.3	189.3	212.5	246.7	173.9	337.6	653.7	504.7
292.3	254.9	269.5	256.3	214.4	232	278.7	275.5	345.9	459.7	450.7
821.1	864.7	954.2	729.6	518.6	680.7	768.2	769.7	1325.1	1616.7	1727.7
336.8	281.6	266.5	252	371.7	189	343.5	452.3	268.7	157.6	282.6
169.9	125.3	170.5	151.3	122.8	125	121	107.5	166.1	199.2	240.6
144.7	132.8	135.4	125	98.4	116.5	133.9	237.7	240.5	217.8	267.5

408.4	335.5	390	324.9	316.2	253.7	325.6	273.8	311.5	539.1	504.9
206.6	267.6	218.7	167.3	191.9	394.1	623.9	299.4	135	153	257.7
205.3	183.4	198.5	177.3	178.6	218.9	252.6	162.8	197.8	199.1	186.5
560	485.7	474.8	364.3	403.3	395.2	463.9	406.5	619.8	692.3	900.2
323.6	259.9	251.7	186.1	226.6	185.3	206.8	189.1	298.6	399.9	466.7
292.4	302.5	295.7	223.3	215.8	284.4	313.2	252	426.7	482	540.1
206.8	204.2	175.5	169.6	200.3	175.5	259.3	196.7	284.7	349.2	385.5
190	174.2	195.3	152.9	150.3	175	200.2	164.2	233	230.3	261.9
249	185.2	206.7	167.1	184	173.7	185.6	185.2	211.3	241.9	298.1
533.4	302	383.8	264.8	211.6	200.8	209	295.7	461.8	562.8	648.6
482	340.6	382.2	276.9	256.3	273.5	351.8	333.3	751.5	885.8	752.4
303.7	485	310	140.1	163.5	578.9	1116.3	209.7	190.8	250.7	325
24	17.9	19.8	22.8	9.1	12.4	10.7	16.3	13.5	7.9	19.9
699.9	592.4	666.1	535.7	579.6	635.3	547.8	504.7	942.7	1036.2	1312.8
86.6	81.1	91.2	61.3	57.6	76.5	71.8	61.4	100.3	106.8	112.8
374.8	455.8	438.8	410.7	341.6	356.6	352.6	415.2	328.4	287.6	295.9
597.8	453	663.2	462.9	460.9	505.7	482.8	426.9	716.8	627.1	980.4
308.8	213.1	235.4	198.5	220.6	240.2	242.3	212.2	385.6	459.2	486.3
236.6	158.1	221.2	175.7	141.3	170.9	177.8	207.9	342.9	239.8	462.2
298.2	308.4	354.7	271.7	256.9	274	311.8	279.3	356.2	552.5	595.4
444.4	258.5	340	345	288	226.2	210.4	195.2	929.1	1129.7	1318
820	883.6	829.1	649.9	578.4	841.7	1086.1	825.5	1254.9	1083.2	1305.7
514.4	517.2	447.9	433.9	469.8	548.7	704.4	494.6	707.1	1026.5	1107.3
414.4	386.1	445	342.5	335.6	398.9	336.2	303.2	517.9	619.4	705.7
340.6	314.9	372.3	280.6	277.9	279.5	344.8	263	463.6	574.6	689.1
270.7	283.8	312	259.7	219.5	246.4	292.5	281.4	407.2	387.7	451.2
70.9	165.3	151	68.7	68	76.2	93.1	240.3	85.9	269.8	124.5
344.5	267	324	241.8	284.2	274.8	259.1	230.2	461.9	783.8	751.2
336.5	306.9	321.3	278.9	230.4	273.6	333.6	291	719	582.7	713.8
576.4	524.8	527	405	384.1	497.6	687.4	595.2	847.1	1134.2	1012.2
294.9	212.5	342.6	220.3	221.9	278.9	262.7	180.3	197.4	227.4	328.9
585.5	497.1	557.6	399	341.7	367.7	395.1	466.8	989.1	1205.3	1264.9
918.4	703.4	656.7	609.6	546.8	588.4	782.6	361	598.2	1301.1	832.7

425.4	309.2	389.8	285.3	271.6	282.6	262.4	262.3	620.6	907.5	904.2
332.5	356.6	369.8	243.6	228.4	280.1	406.6	295.1	397.4	495.4	566
293.6	272.7	282	214.6	165	195.4	221.2	245	567.4	629	739.3
506.6	431.8	512.4	378.5	391.8	403.4	359	376.8	850.8	1014.9	1198.5
339.2	346.3	385	260.2	254.4	310.4	384.4	318	434.3	421.3	589.9
362.5	415.3	374.7	276.2	299.8	280.5	350.2	367.2	692.4	726.8	828.8
186.4	167.2	199.1	160.9	191	164.3	161.6	142.6	173.3	207.8	298.3
386.7	308.6	314.8	284.6	277.7	281.7	411.9	381.9	785.5	765.8	831.4
420.5	408	430.9	348.1	373.9	385.3	561.1	432.1	643.9	664.8	826.3
320.9	376.5	360.7	356.2	243.5	413.5	360.5	287.4	431.9	526	577.3
229.9	205.1	219.5	168.1	159.4	183.4	287.3	165.2	297.6	446.3	401.8
263.3	250.6	259.3	196.8	203.4	210.8	246.4	218.8	446.1	427.9	615.2
902.3	847.5	671.5	647.2	669.7	734.1	962.8	675.3	1312.9	1335.3	1629.3
204.7	179.9	206.1	154.6	162	158.3	219.7	152.6	309.6	364.5	407.4
154	140.4	158.5	140.5	151.4	137.7	207.7	120.9	554.5	486.2	698.7
392.4	348	408.4	390.7	375.7	366.8	496.4	328.9	818.7	753.8	800.5
650.2	401.9	453.2	413.9	477.3	482.2	548.2	555.3	875.3	845.2	1065.5
509.5	344	455.6	401.1	417	314.2	326.2	283.3	394.6	403.9	606.8
221.6	192.9	213	158.8	153.1	186.6	198.9	187.4	348.5	307.8	478.8
557.1	453	512.2	388.3	352.2	369.5	393.4	409.9	647.4	785.8	1080.7
198	176.5	199.4	205	193.3	167.9	187.9	210	264.6	237.2	250.9
592.8	594.7	591.1	458.4	647.3	493.3	764.5	518.3	689.3	954.5	842.5
238.9	245.5	232.7	208.8	177.2	382.5	181.1	233.9	253.1	208.1	269.3
87.1	37.4	39.7	110.9	78.2	109	270.3	83.6	68.4	103.2	94
287.7	221.6	260.1	192	205.3	219.7	251.1	180.1	359.2	473.1	530.7
48.8	58.4	53.6	45	50.5	62	54.4	54.6	79.8	65	104
393.5	360.7	440.1	405	324.3	396	294.3	376.6	398.1	304.6	346.9
425.6	460	542.3	479.4	337.3	502.5	519.5	516.2	1063.6	1179.6	1316.9
305.7	231.1	299.2	225.1	225.8	251	272	218.9	389.2	547.3	592.1
549	407.2	498	327.8	309.3	341.3	319.2	391.3	887.5	1087.1	1113.3
639.3	473.1	521.8	408.9	472.9	456.1	582.2	388.3	740.8	729.3	954.1
485.8	1135.6	524.2	859.6	517.7	399.2	701.4	1132.3	698.4	418.9	975
652.9	541.4	598.3	464.5	526.8	474.7	454.5	402.8	791.8	1050.1	1280.6

369.7	386.6	451.9	383.3	340.1	447	987	480.9	465.2	398.4	514.6
654	824.6	795.7	604	611.5	747.7	1409.9	515.3	821.8	948.2	967.6
425.1	341	389.8	312.2	291.2	296	339.7	311.3	479.6	625.4	698.3
263.4	183.8	308.8	268.5	277.9	364.6	257.1	195.9	635.6	1607.1	1125.7
163.9	124.9	221.6	119.2	131.6	151.6	150.7	120.9	261.1	195	276.8
500.9	371.3	427.3	358.2	338.3	405.6	443.9	318.6	690.6	822.8	806.9
175.4	193.3	206.7	156.6	132.9	252.3	238.5	179.8	321	314.1	354.4
268.6	161.2	189.2	155.2	151.1	159.2	156.7	150.7	554	626.9	768.3
271.7	263.4	283.4	228.5	213.4	292.7	257.9	212.4	319.4	353.1	475.4
299.7	272.1	275.8	274.3	219.7	270	253.8	364.7	439.6	513.7	623.1
192.6	324.8	400.9	207.9	199.7	317.8	253	262.2	372.2	352.4	477.5
297.1	236.5	283	224.3	207	239.3	252.8	239.1	446	547	579.6
388.7	378.7	430.8	355.5	300.8	356.6	378.7	347.1	611.7	807.2	723.8
604.7	484	492.1	475.2	410.8	404.4	415.8	428.6	581.2	558.6	770.9
367.9	436.1	646.2	528.2	227.2	422.1	347.5	377	509.7	708.1	730.5
655.9	502.6	699	626.3	503	478.2	453.4	360.8	615.4	722.3	928
269.6	246.8	243	193.2	227.1	228.2	230.9	188.9	367.4	495.5	500.2
134.3	61.2	68.9	66.4	61.3	45.7	49.8	59.8	86	95.7	66.2
455.1	355.9	447.7	447.8	403.2	329.7	385.4	483.6	871.9	3099.6	1421
214.5	149.1	193.2	142.6	136.2	157.7	170.7	166.6	292.2	404.2	510.1
292.9	349.4	311.6	242.7	246.2	312.7	338.7	346.6	409.7	454.5	486.2
1159.5	619.1	989.8	625.2	627.4	596	627.1	794.3	944	1417.6	1412.7
115.2	161.4	236.1	283.8	183.5	206.8	283.9	365.1	163.3	181.1	207.2
125.8	124.7	127.5	116.7	111.9	141.3	162.3	133.6	204.5	229.2	234.4
684.3	533.1	656.7	494.6	556.5	579.5	605.8	576.5	870.5	828.9	1059.2
330.7	374.9	333.5	266	288.6	315.2	358.4	319.8	377.4	372.8	527.7
813.5	716.4	786.4	798.9	621.6	617.3	597	600.5	751	1020.4	1114.9
1261	1219.2	1467	1639.4	1156.5	1356.2	778.5	674.3	440.5	554.4	825.4
274.4	343.8	250.4	203.5	175.5	213	448.9	261.2	436.2	428.7	549.4
460.4	353.5	430.4	453.2	509.2	327.2	273.3	514	287.9	443.5	407.4
400.3	391.8	426.7	341.8	368.1	373.4	446.8	375	503.2	552.3	696
555.2	566.3	579.7	470.4	417.9	883.6	625.1	467.3	546.5	734.6	770.3
158.1	191.2	223.4	146.4	169.7	191.4	181.1	141.4	166.4	220.1	232.9

359	362.8	358.9	278.3	300	396.8	457.4	335.2	419	424.5	544.1
148.9	132.8	158.7	120.3	104.7	129.1	154.6	138.1	293	386.5	372.5
147.2	109	115.2	102.8	98.1	99.8	103.4	110.9	239.3	347.5	356.3
519.1	378.2	462.3	348.6	280.9	336.1	306.1	329.8	597.3	585.3	789.2
258.1	236.6	228.3	180.2	153.3	269.6	261.4	193.2	462.7	581.3	583.9
392.5	317.9	477.6	363.1	398.1	413.6	398.7	303.9	593.2	966.7	735
370.8	472.4	457.3	537.5	419.3	433.3	389.8	386.6	360.7	288.4	456.4
275.1	254.4	179.5	183.6	192	241.8	314.2	260.7	475.7	531.5	605.6
413.8	409.9	427.2	376.9	386.6	333.5	385	279.8	588.4	604.1	741.3
466.8	343.6	425.1	334.4	361.6	454.2	508.7	385.7	587	703.1	803.8
170.3	188.2	202.8	183.6	160.6	178.1	234.8	192.9	313.8	439	401.7
313.9	504.5	450.7	387.6	314	608.1	389.1	458.1	707.8	782	804.7
143.3	341.8	204.5	329.2	158.8	392.1	995.3	322.8	182.7	165.7	172.8
163.7	161.5	194.2	125	119.6	137.2	156.8	190.1	239.2	276.6	299.4
235.5	244.8	264.7	203.6	171	220.5	273.3	230.1	433	426.4	504.7
282.7	232.2	254.9	196.7	169	184.4	214.5	173.6	494.7	654.3	752.4
325.6	249.4	355.8	217.1	234.4	238.4	262.6	223.5	325.7	347.4	460.7
528.6	416.2	530.2	394.6	342.1	362.4	385.9	334.4	687.5	1083.2	949.7
447.5	327.9	437.3	366.5	323.3	364.6	349.3	329.4	478.3	573.5	734.2
179	148.2	168.6	129.1	136.8	139	152.8	148.8	174.2	216.2	241.3
2785.1	1123.2	1606.9	1283.6	1669.3	1230.9	993.4	776.9	758.1	830.9	1141.5
307.6	228.2	279.6	217.4	226.2	215.3	235	215.8	366.4	505.7	599.6
304.4	271.7	314.1	227.6	236.4	266.6	259.7	235.8	451.2	501.9	663.8
345.7	256.7	405.8	318.6	278.8	385.9	459.5	220.4	523.8	1299.1	1003.2
86.2	94.8	89.4	78.7	64.2	116.5	85.8	101.2	100.5	106.9	118.2
217.3	218	243	180.1	177.7	220.9	195.3	196.2	327.4	263.8	374
170.9	163.8	196.1	152.9	152.7	212.7	173.1	163.5	238.5	217.7	297.9
342.9	311.6	323.7	295.4	335.6	325.8	427.6	300.4	441.6	482.7	531.1
263	224	244.6	200.4	176.2	195.2	185.2	187.1	330.1	465.1	463.3
967.6	111.4	364.1	163	340.9	243.1	82.6	196.5	271.6	496.4	947
237.9	209.3	272	211.6	207.8	211.9	283.6	256.3	302.3	326.6	358.2
257.7	149.2	271.7	212.3	215.7	183	290.7	201.1	329.9	321.4	334.5
1127.6	1071.2	882.4	1051.6	1051.1	724	1047.8	1237.4	1008.3	782.3	1420.4

457.5	346.2	358.3	274.5	325.3	278.5	395.7	294.4	437.8	538.1	552
104.4	101.4	112.8	98.4	94.9	106.3	143.5	114.3	166.5	237.6	227.4
490.6	328.3	345.8	281.6	331.1	281.2	273.4	317.4	555.2	696	795.2
236.6	220.7	259	206.5	184.3	212.2	215.3	202.2	312.4	440.3	403.9
143.8	131.8	117.4	109.1	117	120.8	192	154.9	201.1	204.6	234.5
650.7	335	388.1	308.8	311.9	334.5	255.7	349.3	699.4	804	1015.6
269.6	165.5	234.2	153.7	195.4	160.4	159.9	131.2	294.1	217.4	311
159.3	334.4	428.1	222.5	225	340.1	309.1	138.8	197.5	197.1	339.6
360.3	274.5	331.2	288.9	260.5	374.5	257.1	260.6	415.4	480.8	544.3
333.3	334.3	369.5	278	301.1	346.1	397.2	277.9	299.8	436.6	403.3
415.4	353.9	361.9	262.7	294.7	316.4	305.8	409.6	758.8	827.4	983.6
476.5	445.1	434.3	582.8	420.9	220	249.2	215.2	204.7	246.4	248.4
435.8	342.7	361.4	291.5	271.2	340.9	375	382.1	667	883.6	941.3
413.5	324.7	407.4	294.9	285.7	363.1	389.9	319.1	596.3	893.4	884.4
659.3	540.6	485.1	425.6	387.3	447.9	502.2	468.8	1063	1423.4	1450.3
361	452.1	368.9	228.8	278.7	2749.2	534.3	364.8	155.7	224.5	229.5
135.7	218.1	186.5	189.5	129.4	195.7	334.9	286.1	376.5	189.6	364.1
298.1	245	225	193.3	269.3	201.8	353	236.8	328.6	416.4	456.3
732.7	734	776.4	259.7	447.2	1000	1022.2	207.6	579.4	496.4	796.1
220.1	205	255.6	187.5	210.5	215.8	226.2	212.2	297.3	309.8	392.2
432	506.1	554.2	405.9	451.3	732.2	615.5	559.7	561	661.1	717.7
651.2	557.2	575.7	469.9	675.6	557.6	745.5	570.7	825.8	913.1	1027.7
529.1	639.3	844.5	752.5	353.4	331.2	580.3	363.7	337.7	347.4	532.4
415.1	317.7	415.9	323.2	279.3	370	311.1	311.1	713.6	676.3	788.9
822.2	587	916.2	697.3	536.1	1328	704.8	412.6	618.7	717.9	963.7
318.4	314	327.8	240.9	270.1	371.7	311.4	286.6	496	539.1	670.3
225.6	209.3	163.7	171.9	162.3	591.2	241.4	207.9	441	454	510.2
92.4	79.8	89.7	80.8	69.9	78.5	60.9	65.3	158.5	189	241.7
358.7	338.5	359	318.9	287.8	334.6	347.8	330	553.2	608.7	645.8
370.9	397.5	370.8	331.7	292.5	386.7	522.2	324.4	754.9	794.8	907.5
157.3	136.4	118.6	108.5	160	93.7	142.4	77.1	98.1	160.5	161.6
297.7	265.4	292.8	286.1	259.1	244	252.1	215.6	371.2	500.2	495.3
33.3	32.2	35.7	29.4	33.4	36.4	35.8	34.5	44.1	46.5	56.7

354.6	187.2	282.3	207.3	248.4	224.2	170.9	153.6	278.7	398.9	482.2
60.4	41.1	71.6	102	56.6	79.5	96.8	62.5	143	364.1	210.9
180.6	151.4	151.7	121.2	83.2	165.6	213.3	112.4	325.3	450.9	407.2
171.2	477.3	1023.9	452.2	625.1	216.5	763.8	169.8	196.9	205.1	1408.9
112.7	177.3	147.2	186.1	139.6	230.2	145.9	170.8	173.7	110.9	180.3
548.1	567.1	473.4	390.6	463.5	656.7	1128.2	497.9	452.7	460.7	583.6
666.6	1066.4	815.8	743.4	663.7	804.4	975.2	931	2226.5	1895.7	2493.5
276.7	236.9	261.2	198.9	223.3	249.5	266.3	265.3	336.3	362.9	391.5
1356.5	1726.2	1631.3	1226.2	1376.9	1478.1	2067.2	2660.1	955.4	699.5	1423.9
277.6	209.3	219	159.6	191.4	189.2	164.4	244.1	349.2	404.7	457.4
251.7	231.8	249.8	192.8	144.4	194	231.9	262.8	393	336.6	448
334.6	332.9	329.4	253.6	318.3	307.6	445.6	300.7	362.3	457.1	442.3
359.7	344.2	345.9	286.4	271.9	327.3	309.4	333.3	464.8	542	635.3
325.9	256.5	276.8	216.4	236.4	231	235.7	211.1	424.7	623	551.5
386.8	418.8	434.5	303.5	285.3	488.6	395.9	425	426.8	529.8	651.4
121.8	250.6	441	347	150.3	689.1	86.8	317.7	351.3	439.6	377.4
466.3	1096.3	204.8	669.9	392.6	542.5	126.4	404.6	332.4	82.8	460.2
194.5	204.2	217.3	166.5	177.6	216.1	376.2	188.4	190.7	194.7	284.8
272.4	288.8	301.2	220.9	236.6	277.9	417.6	221	251	343.4	378.9
512.5	434.8	493.2	353.4	361.8	386.2	568.7	401.8	1019.8	1132.9	1213.3
333.7	284.2	245.6	197.2	159.1	275.3	348.1	254.4	583.2	698	810.4
201.5	210.8	181.5	156.8	166	148.7	175.9	182.8	210	276.7	330.6
660.9	369	676.9	500.9	381.7	473.4	555.6	552.7	574.1	437.3	1008.7
266.9	267	282.4	201.3	243.1	237.5	305.2	265	341.5	649.3	471.8
471.3	495.2	468.3	410.8	367.6	519.6	413.5	532.6	492.9	351.3	549.1
358.2	352.9	392.2	342	292.7	363.9	325.3	287.1	429.9	454.7	563.3
125.8	169.7	197.5	125.6	125.7	131.3	198	128.3	233.3	551.5	431.4
292.7	340.9	372	501.6	232.7	277.6	231.4	213.6	461.6	669.8	952.9
1494.4	986.8	1637.7	1419.8	1428.9	675.7	1723.2	1393.1	1047.4	679.7	1204.3
427.9	367.8	427.7	402.8	523	399.3	325.1	372.3	232.3	234.1	240.1
270.6	1208.2	549.7	718.9	310.5	763.7	616.9	3718.4	55.3	107.4	148.7
421.8	494.8	439.8	364.3	410.4	559.9	812.1	447.3	479.5	559.9	664.7
211.2	191.7	182.9	200.1	171.9	206.6	327.5	271.8	238.4	347.1	271.6

536.3	644.3	605.9	650	639.4	617.5	579.3	749.8	609.1	504.7	659.7
196.5	199.6	210.3	168.2	157.3	178.3	191.3	175.1	213.6	219.4	258
158.1	117.3	149.1	145.6	154.6	173.7	159.4	97.2	217.9	684.9	299.6
244.9	307.2	261.2	198	218.8	335.9	333.3	289.1	395.3	498.6	408.5
143.6	97.5	119	110.5	106.4	113	123.5	130	232.7	207.7	214.5
3175.6	2885.8	193.9	366.3	1295.1	205.3	359	356.4	304.2	233.9	415.8
1693.9	1981.7	1977.3	1820.9	1174.9	1308.8	1332.4	3406.4	1424.1	1210.1	1166.4
130.7	139.3	154.7	150.9	113.4	142	141.1	156.2	154.4	357.8	279.9
513	719.2	649.7	406.8	425.1	714.7	1333	489.8	345.6	400.6	499.9
138.8	111.6	86.5	111.8	87.3	87.3	81.9	153.9	93.7	67	88.2
531.7	599.9	399.4	393.2	448.2	312.3	423.1	563.3	378.1	193.8	396.9
143.8	169.6	137.7	118.9	75.5	197	109.2	141.5	149.5	261.2	269.9
265	314.5	318.6	304.6	305.7	321.5	319.3	285.9	371.5	626.6	852.1
191.8	155.2	160.2	133.7	136.2	135	144.2	128.6	268.1	241.2	310.4
335.3	189.1	290.5	239	187.3	167.4	173.5	159.1	405.4	466.9	591.9
251.6	212.5	269.9	195.7	241.7	312.8	402.9	205.9	314	588.9	533.4
246.5	271.9	240.4	177.5	208.2	216.6	290.3	226.4	470.1	491.8	609.1
362.4	391.5	369	335.8	303.8	378.7	454.6	349.9	526.8	799.6	750.3
265.1	223.2	265.6	206.8	214.2	239.9	259.2	215.8	361.6	421.9	532.2
231	217.2	193.6	159.4	172.6	257.1	240.3	262.5	323.3	392.5	384.2
267.5	210.7	233.1	189.2	191.8	244	212	235.3	324.6	327.6	378
251.3	267	266.3	221.2	206.4	236.3	316.6	256.9	392.4	506.5	587.5
184.5	131.5	159	133.4	145.7	143.8	169.7	139.3	258.2	322.5	316.9
318.3	244.8	310.1	262.4	255.7	253.9	284.7	222.3	367.5	524.2	534.7
137.9	117.5	109.3	78.5	97.3	101.9	144.9	110.7	239.7	297.4	314.1
208.9	255.2	265.4	496.5	234.7	633.6	338.5	557.2	240.4	643.9	258.6
227	292.2	302.3	259.5	229	326.1	365.7	292.9	485.5	406.9	340.9
122.4	126.9	121.2	101.8	81.5	118.8	118.8	96.3	137.4	161.1	217.5
162.4	155.7	177.3	157.1	159	180.7	208.5	166.5	270.5	1039	319.6
153.8	155.4	152.5	169.7	129.4	155.5	158.4	162.6	215.8	133.4	235.7
109.3	120.2	126.4	128.6	99	119.9	100.4	116.6	132.1	172.6	160.5
274.3	248.5	261.4	234.9	196.7	301.5	239.8	236.8	309.3	298.4	359.1
215.7	170.3	163.7	115.1	151.7	239.5	150.4	154	296.5	120.4	243.3

172.4	153.8	166.9	126.5	136.7	129	140.3	159.4	310.2	319.1	380.5
340.2	225.5	277	196.6	204.6	267.8	233.6	237.3	458.7	549.6	605
436.1	301.8	364.4	286.7	206.2	204.6	220.2	148.8	592.7	619	1023
104.9	101.4	106.5	89.6	92.6	138.2	136.2	114.4	121.3	158.2	158.4
366.1	309.6	358.9	270.5	287.6	293.5	334.6	279.1	465.7	457.2	544.3
362.6	248.6	286.5	178.1	209.5	208.4	244.4	241.7	505.4	504	593
103.7	90.2	97.5	73.5	63.8	84.3	94.8	77.9	154.9	167.4	182.7
183.8	170.5	191.5	179.6	141	185.6	179	202.8	286.4	302	377.6
655.6	830.1	632.2	476.6	899.6	519.4	852.3	480	395.5	296.3	345.9
55.4	41.1	70.5	37.6	52	57.4	51.1	24.1	37.8	116.8	145.5
494.1	485.6	525	442	389.5	449.7	450.1	415.2	838.7	1202.9	1273.6
893.5	918.8	462.5	886.1	778.7	1874.1	2945.9	1280.6	155.2	249	261.3
446	398.9	408.6	326.7	382.3	383.7	427.4	341.4	614.8	640.7	796
485.3	511.1	508.8	459.8	458.8	468.9	469.9	480.9	698.6	723.2	982.8
231.1	244.6	260.2	208.8	178.7	220.2	234.4	232.4	308.1	242.8	398.7
281.7	199.8	261.7	233.7	245.2	204.3	233.5	202	266.2	286.3	354.8
199.4	211.6	249.9	206.1	166.3	245.1	250.1	231.5	362.5	372.4	361.2
141.4	139.1	152.9	116	107.7	126.6	124.7	149.6	236.1	185	250
174.1	279.9	294.5	294.9	208.7	338.3	458.9	360.9	665.3	373.5	423.9
191.2	178.4	202.7	158.8	138.3	153.8	168.4	141.6	241.8	233.1	341.8
256.9	271.7	252	202.6	268.1	227.2	296.1	170.2	302.5	305.3	333.2
50.9	61.3	98.3	79.4	51.6	85.2	51.2	53	115.3	144.8	168.3
207.9	228.5	221.1	196.3	196	201.4	236.3	241.4	448.6	427.4	454.9
669.4	1276.5	1462.3	560.6	491.3	1161.6	2352.4	666.6	561	701.3	1162.7
177.4	183.8	182.4	158.3	145.8	174.2	184	160.6	235.5	263	295.7
233.2	190.6	205.2	185.7	135.1	165.8	143.6	189.4	278	411	371
261.9	250.9	238.1	215	158.1	201.8	199.4	173.2	342.3	283.9	299.5
159.2	121.6	127.7	114	108.9	121.1	117.6	102	143.7	163.6	208.4
486.2	406.6	370.1	242	163.8	254.1	421.7	470.4	771.2	839.7	1050
169.8	201.8	163.1	144	148.2	254.4	449	186.1	135.3	163.5	236.8
271	387.2	352.2	267.3	295.3	449.4	560.6	331.8	273.7	303.8	360
286.4	233.9	244.8	186.8	226.3	249.2	262.5	228.6	359.5	431.6	454.3
683	500.6	704.1	419.3	386.1	352.4	198.4	337.8	388.2	497.3	705.3

196.8	208.4	240.8	226.9	202.5	244.2	228.6	214.6	277.8	263.6	294.3
175.8	195.5	174.6	148.3	163.7	180.3	201.2	144	256.2	269.3	331.9
294.4	336.5	377.4	304.1	307.9	336.5	365.7	302.8	422.8	440.1	564.4
1070.8	626.3	637.8	823.4	807.4	549.7	782.1	1027.6	560.1	402	631.7
723	502.8	774.5	522.6	518.1	588.7	479.5	521.5	740	711.3	1080.3
452.7	523	517.6	465.8	402.4	579.3	553.1	466.4	771.1	831.3	909.4
553.7	257	456.4	323.5	295.6	240.4	228.5	351.5	372.6	206.9	482.9
1082.6	827.4	1093.8	724.6	818.9	866.5	841.6	631.7	982.6	1647.9	1887.4
180	147.7	162	149.4	142.4	175	165.5	143.6	486.4	577.8	576.1
445.1	327.1	385.9	309.6	324.1	320.7	392.2	358	571.3	700.4	875.8
256.9	205.9	287.5	271.6	189.1	184	249.4	242.8	351.8	392.3	427.7
189	493.2	553.3	1455.2	258.3	476.8	412.9	610.3	149.8	132.1	171.3
470.5	312.2	350.9	285.5	291.6	306.5	318.8	330.9	552.3	602.6	661.5
227	200.3	249.9	179.5	178.5	206	264.2	195.1	310.6	337.7	388.8
392.8	372.9	404.8	360.3	279.9	447.4	381.4	292.7	499.8	645.7	664.2
395.9	252.9	220.7	255.1	327.4	190.5	407.7	312.5	274.3	443	303.5
288.1	251.7	279.5	221.2	175	250.1	214.8	239	303.8	595.9	485.3
348.8	342.2	393.8	290	270.3	281.8	279.3	263.7	408.9	519.7	668.7
104.8	114.3	124.9	113.8	124.7	127.6	159.1	106.3	134.6	140.3	167.9
263.4	267.7	270.2	210.9	238.5	231.7	258.5	263.2	282.4	261.7	354.4
143.9	110.1	118.8	98.2	69.7	113.2	85.5	95.1	225.4	212.8	303.3
341.3	535.3	524.3	367.4	419.4	790.5	1067.5	469.5	573.3	473.3	964.2
582.6	262.9	428.5	306.8	341.2	281.2	217.5	249	283.6	411.7	582.4
204.6	261.5	222.1	189.6	189.2	241	221	236.6	289	252	501.2
51.2	63.1	76	59.9	51.6	85.3	71.6	60.1	147.6	66	95
197.1	158.8	228.3	173.8	146.3	154	156.7	140.6	267.6	256	360
751.3	734.4	712.4	544.6	676.9	822.1	790.3	670.7	1070.4	1218.2	1388.6
308.4	327.8	302.8	240.4	243.8	332.6	432.9	287.8	421.3	323.2	469.8
396.7	343.1	376.3	234.8	286.7	287.6	318.6	307.7	593.5	678.3	821.4
269.6	292.3	322.8	325.5	242.8	280.5	289.3	189.9	333.4	561	496
314.4	295.2	347	314.1	271.8	333.1	353.6	336.8	362.8	338.1	462.1
191.7	158.1	155.7	181.9	143.4	143	155.2	332.8	179.4	132.9	138.9
186.5	191.7	181.9	183.5	179.7	219.5	197.6	179.3	273.4	267.6	330.7

80.1	78.2	62.7	53.1	102.4	64.6	111.4	77.5	85	76.2	74.4
316.4	284.5	326.3	235.6	269	250.9	349.1	333.5	260.4	295.5	378.7
133	101.9	117.9	76.9	89.1	95.9	105.8	101.2	159.3	126.1	198.5
484.1	442.1	518.6	562.9	569.8	372	479.1	645.3	405	250	360.6
352.9	320.3	406	489.2	294.3	291	288.7	210.2	480.8	488.2	581.5
94.6	103.4	103	73.1	104.3	110.8	127.4	79.9	107.1	135.2	167
169.4	191.2	186.1	186.2	171.3	233.8	249.5	195.2	472.9	1198.2	1118.3
367.6	443.2	306.8	234.1	269.3	250.7	296.3	358.9	556.4	914.4	631.7
717	411.6	666.3	568.9	264.2	246.6	268.9	226.2	450.9	548.2	784.3
79.1	66.4	69.2	71.1	57.4	73	95.2	63.1	391.9	675.9	571.5
315.5	300.8	287	244.9	197.5	245.8	268.2	229.5	349.6	618.2	584
277.6	315.8	410.5	210.5	152.1	220.6	218.5	278.4	821	412.9	736.6
284.5	316.2	334.6	339.9	222.1	380.1	323.5	317.3	485.2	527.6	555.5
323.1	281.6	309.1	267.6	390.1	273.9	349.4	328.2	437.4	488.9	568.8
149.3	202.5	154.6	169.8	147.9	396.3	609.5	302	104.9	110.9	124.7
424.6	345.2	454.5	298	515	476.3	357.8	279.4	696.3	1346.3	1186.4
189.6	150.8	199.4	165.1	154.2	152	169.6	142.8	280.5	317.3	361.8
227.1	260.6	299.7	225	200.2	251.3	302.6	221.3	449.3	547.5	633.3
387.1	301.7	307.9	249.5	341.5	229.3	308.7	252.2	291.2	350.2	436.9
415.9	261.6	331.7	273.3	192.1	222.9	192.3	186.4	240.8	264.8	315.8
215.9	186.9	203.1	181.4	141.4	162.1	169	151.4	230.1	288.1	278.2
260	206.2	221.6	181.8	161.8	181.7	214.1	190.7	459.5	514.3	540.7
283.9	288.5	267	247.4	251.8	278.1	382.1	254.6	334.1	328.4	437.6
340	278	279.2	312.6	298	294.4	372.9	273.5	348.7	368.2	456.6
266.4	263.1	274.8	238.8	210.2	223.6	239.2	250.8	396.9	416.9	455.3
285.4	348.5	277.5	187.1	184.2	225.5	428.4	285.8	415.9	497.6	504.4
277.9	283.5	292.4	279.6	202.1	360.8	250.5	346	356.6	417	462
438.2	427.6	485.5	373.9	434.9	404.1	434.8	420.1	685.8	613.7	873.2
398.4	563.8	831.2	646.6	360.8	391.2	371.7	299.5	356.7	307.6	461
168.4	165.6	161.2	135.5	135.2	162.1	205.1	156.1	278.7	317.6	342.6
257.4	218.7	203.4	206.9	163.2	214.8	215.7	199.6	344.3	425.4	389.2
959	1058.6	1555	1005.1	768.3	1362.1	1089.9	534.4	910.6	736.5	1003.8
219.4	395.5	406.1	504.2	284	549.3	302	267.9	209.6	227.7	292.7

374.2	322.2	339.3	252.8	237.9	321.4	325.6	280	378.9	415.3	544.2
420.6	1091.3	1272.8	1034	669.2	400.8	431	631.1	206.5	242.8	271
113.6	97.6	93.4	66.2	67.1	68.4	91.8	89.3	153.7	237.1	199
341.1	487.7	392	353.5	316.7	512.9	545.4	407.5	543.8	755.1	818.6
326.4	263.4	321.3	261.3	219.9	233.1	216.8	231.4	498.6	516.4	584.1
112.4	91.5	109	92	84.7	108.8	108.4	79.8	150.7	283.1	191.7
184.7	129.7	84.3	56.8	53.7	39	135	102.8	233.2	629.4	299.6
323	291.2	275.7	570.8	422.4	236.8	324.5	354.6	390.5	262.6	437.2
518.6	332.6	418.6	349.3	283.9	333.8	307.4	290.6	427.3	445.9	505.7
183.3	172.4	177	152.4	132.8	179.5	166.7	149.7	168.2	187.7	206.8
184.4	158.6	206.9	169.2	169.2	184.5	214.8	179.6	218.5	206.4	267.9
164.3	185.9	210.3	162.3	141.3	225.2	162.5	177.9	253.4	288.8	337.1
342	363.1	348.6	293.4	464.8	332.8	489.5	325	338.9	368.8	403.4
727.2	348.5	535	371.7	382.2	438	475.2	322.8	548.3	606.8	660.2
324.9	330.6	416	322.9	285.3	359.2	409.1	279.6	324.4	444.3	454.7
323.1	316.8	402.1	427.3	324.2	364.6	379.2	305.2	458.8	444.4	637.4
302.5	285.1	318.1	287.7	268.4	352.1	610.6	241.7	320.4	410.3	449.5
339.8	305.5	414	258.9	233.6	237.1	333.2	291.1	364.9	439.6	514.8
264.7	245.5	254	205.4	174.4	224.8	282.2	276.3	473.3	425.9	557.1
281.3	302.7	256	239.3	257.2	286.7	383.5	269.6	336.6	357	421.4
423.1	390.8	416.5	350.7	342.8	401.9	417.4	342.4	575.5	830	819
126.3	233.9	91.4	90.2	78.4	113.8	121.1	97.2	149.5	123.6	224.6
250.4	252.5	256.3	259.8	191.9	225.9	254.7	225.7	356.9	297.5	294.5
754.5	678.9	723.5	466.3	547.7	425.2	472.9	349.6	260.5	310.9	417
334.3	357	358.3	388.3	309.8	383.8	337.7	338.3	236.4	193.3	280.6
234.4	231	263.8	166.9	150.4	162.9	248.7	198.4	521.4	656.1	670.7
332.3	300	341.6	239.7	259.9	260.6	355	271.9	571.7	697.4	724.6
288.6	204.1	220.6	189.6	178	219.1	172.6	221.8	367.6	485.5	592
593.9	670.2	898.4	924.8	310.9	601.8	664.4	888.8	344.9	369.2	784.5
448.9	418.8	474.8	337.9	299.3	443.9	530.7	412.7	802.2	938.6	1028
103.2	107	125	139.8	130.4	116.6	121.1	96.4	190	467.8	390.3
210	331.9	388.7	262.3	211.6	246.8	217.6	295.7	242.1	236.9	281.3
276.4	199.7	262.4	196.4	180.5	208.2	216.2	235.6	354.1	350.4	448.7

385.6	319.7	382.8	350.2	271.5	350.4	403.4	277.5	506.4	744.7	681.3
389.1	325.2	355.6	351.7	299.2	226	301.5	390	205.2	153.1	200.3
209.7	203.6	219.5	180.7	164	214	193.6	190.1	165.3	253.1	278.7
155.5	137.1	144.1	106.6	86.5	130.6	112.1	128.5	233.5	296.4	353.6
198.6	243.2	295	176.4	315.3	269.5	493.3	269.3	210.9	248	194.5
91.3	116.5	61.4	91	92.8	102.1	380.7	144.1	90.9	123.1	78.2
403.8	422.3	395.3	372.5	347.9	371.8	462.6	406.9	656.7	866.1	813.4
146.2	134.8	134.4	109.5	113.5	115.3	111.1	133.6	169.8	233.2	228.4
134.7	110.9	125.6	106.1	78.2	118.7	105	139.1	244.3	224.6	277.4
415.2	505.7	447.3	393.6	309.6	428.2	482	526.4	634.4	908.6	800.6
243.1	290.9	275.2	216.7	204.2	349.7	396.4	301.2	281.4	295.3	393.8
205.5	197.1	175	152.6	162.2	183.1	207.8	160.7	271	326.1	354.5
206.2	176	202.5	138.6	152.8	167.9	156.1	152.9	284.5	291.7	367.7
425.3	397.7	471.5	338.8	303.7	352.7	399.8	360.2	579.4	802.8	984.8
221.6	212.2	231.1	158	175.4	199.2	259.4	237	297.2	324.2	351.8
1410	608.7	679.6	519.5	739.7	724	469.9	797.6	1270.7	1729	1958.3
45.7	40.1	53.8	49.3	42.6	35.4	34.8	41.6	76.5	66.7	116.2
232.8	138.4	180.8	141.4	142.1	151.2	140	131.8	191.6	280.8	278.9
357.9	592	522.5	1055.9	544.4	937.7	356.8	1174.7	572.5	251	326
134.1	79.8	88.1	68.8	78.2	68.3	78.6	80.3	170.1	218.8	227.6
733.6	634.8	598.4	443.7	381.6	539.6	645.6	475.1	1320.8	1289.2	1544.6
24.8	30.1	1338.8	312.4	242.9	23.9	24.1	241.7	210.6	20	18.5
220	238.7	255.2	210.6	159.7	213.6	298.6	222.4	392.7	425.2	514.4
696.3	287.3	247.3	352.5	471.6	245.8	614.9	359.7	241	247.4	266.1
229.7	271.4	263	232.4	238.4	244	290.2	256.8	475.9	475.8	493.9
498.9	389.9	408.5	319.1	254.9	258.9	322.1	276.3	522.7	538.7	611.9
382.3	344.7	425.6	393.2	368.2	384.1	369.4	395	441.6	495.2	522.3
308	165.6	270.2	230	169.9	138.7	186.3	174.9	257.1	434.3	332.9
232.9	264.7	278.6	222	316.6	270.4	346.8	258.6	282.1	339.3	369.2
637.5	526.2	568.2	492.5	424.7	550.7	633.4	497.9	949.5	1031.7	1285.4
218.7	197.9	233.3	181	162.6	182.3	206.4	191.2	411.9	1009.8	741.4
273.3	225.4	318.5	244	224.2	212.3	203.1	218.4	380.7	822.8	552.3

365.9	308	304.7	234.7	275.9	281.8	341.2	263.8	595.1	553.1	707.1
1293.1	349.8	685.4	439	435.7	103.8	119.3	154.6	544.8	532.3	660.6
264.7	215.8	229.7	210.8	201.4	209.3	245.2	214.6	294.1	462.6	398.8
111.1	111.7	108.3	94.6	166.7	92.2	115.7	116.2	131	130.1	159.9
587.4	520.2	558.5	431.2	471.3	520	558.2	509.5	1033.3	1256.4	1364.7
1029.6	910.2	563.7	463.5	527.2	1704.6	3373.1	723.1	232.1	549.9	325.4
237.3	250.5	293.9	191.6	221.1	245.8	322.1	269.7	342.4	526.2	676
232.2	214.5	212.1	168	184.4	180.9	220.8	192.3	263	342.6	384.6
124.3	121.9	140.7	118.9	128.3	103.4	126.3	111.7	182.9	195.3	218.9
357.7	283	396.9	368.3	225.7	440.1	256.2	245.3	289.5	360.2	351.4
338.7	256.3	306.9	209.7	238.1	297.6	291.4	269.6	556.7	532.7	689.6
177.3	167.4	173.7	141.4	137	160.1	156.4	144.3	262.6	282.9	327.6
150.8	155.8	177.7	141.6	102.6	119.4	141.9	155.6	342.4	351.3	400.7
573.5	459.2	507.3	418.8	401.6	478.7	499.5	433.5	843.2	1207.3	1190.7
198.4	204	228.6	179.7	163	202.9	228.5	174.5	200.7	269.4	257.4
894.2	693.5	935.2	744.2	652.4	447.8	356	545.2	333.2	385.6	587.8
195.2	209.7	179.1	163.5	200.4	169.5	230.6	180.6	344.7	395.9	460.2
138.1	72.2	92.7	87.1	86.5	81.5	69.1	85.9	178.9	214.3	212.7
359.3	370.4	378.3	257.6	276.5	330.1	383.4	364.5	418.7	379.2	442.6
203	256.2	255	193	156.4	263.6	382.8	220.1	217.2	392	330.6
153.8	125.4	159.6	122.5	147.7	117.5	168.6	137.2	238.9	249.7	278.6
1216.5	1405.4	1507.5	1119.2	1389.8	1207.2	1284.3	1318.1	886.5	1752.6	1532.1
218.5	223.3	226.3	177.1	149.9	152.4	196.7	148.8	217.2	370.4	373
435	393.8	448.4	377.6	274.9	365	382.6	389.1	799.3	803.9	967.9
502.1	678.3	632.1	588.4	530	822	429.7	552.9	578.7	455.2	675.1
450.6	505.9	479.6	367.9	355.8	596.6	743.3	420.2	486.9	663.1	771.2
66.9	74.5	70.6	66.6	58.8	58	69.8	62.2	102.2	118.4	126.8
561.5	579.5	477.1	539.4	243.4	426.8	398.9	615.7	479.7	589.7	663.1
397.8	304.3	304.6	367.8	244.8	257.6	251.3	266.8	766.7	1058.9	726.2
49.6	38.6	42.4	27.7	31	36.5	41.7	40.3	88.9	70.9	130.8
331.1	311.5	380.8	390.3	338.8	278	297.6	220.5	238.1	259.4	247.9
228.1	179.4	441.4	172	241.1	255.6	208.7	239.3	423.1	575.3	592.8
228	265.1	352.3	250.1	202.2	264.7	315.8	251.2	395.7	567.9	743.3

331.8	270.4	250	253.3	288.4	244.7	311.5	236.8	244.5	300.5	316.9
262	258.1	234.3	238.6	216.6	234.4	286.4	282.6	408.8	501.2	518.3
245.7	263.3	210.2	162.2	191.9	207.4	221.6	217.1	261.4	356.7	405.4
391.3	401.1	411.3	465.9	361.6	381	359.9	307.3	336.8	376.7	458.5
369	702.1	518.8	891.9	470.7	1613.6	754.9	659.4	332.8	627.5	339.4
298.2	269.3	192.3	191.7	216.6	224.4	442	184.2	256.7	324.3	303.1
492.7	268	371.9	374.3	241.7	341.7	210.7	434.6	494	454.1	557.6
152	148.1	140.4	113.9	122.7	165.4	196.5	137.6	212	148.8	203.9
511.3	393.1	319.2	267.4	369.1	224.5	1117	417.1	372.2	417	542.1
204.7	163.4	200.4	177.4	150.7	192.7	179.2	186.2	446.3	1044.7	744.8
474	510.6	480.4	652.1	321.7	532.4	400.2	973.7	249.8	285	393.6
178.5	146.3	168.2	128.5	136	134.7	151.7	144.1	293.8	331.1	413.5
511.2	1454.7	672.6	1339.5	847.8	1391.2	717.5	778.3	461.6	431.6	530.8
77.2	80.8	93.2	79.7	61.5	75.5	86.4	58.3	150.9	158.7	191.2
46	39.4	43.1	28.2	37.8	38.8	40.4	39.5	68.2	77.7	78
514.6	799.3	598.5	817.3	704.1	2133.4	1211.2	1969.9	488.8	481.8	581.8
610.6	598	489.5	804.2	1160.7	345.6	436	1407.1	1101.3	167.4	333.2
148.4	174.8	130.6	130.2	183.2	150.5	196.1	135	156.1	239.4	199.1
764.3	715.1	770.5	594	528	663.9	820.7	547.8	1210.9	1449	1478.8
487.2	537	419.3	546.2	328.4	442.3	522.8	358.8	488.6	551.3	533.1
253	206.9	245.5	195.3	205.4	217.3	205.8	149.1	298.3	603.7	504.1
332.1	362.4	388.5	295.6	228.6	389.6	355.7	268.4	453.7	583.4	590.1
281.1	287.5	271.8	198.1	161.3	218.4	354.8	267.7	570.1	956.1	766.2
213.3	203.1	179.5	149.6	126.6	171.9	144	199.7	348.7	350.9	424.8
379	334	488.5	327.3	246.4	239.4	231.8	246.6	326.7	381.7	452.9
258.9	236	229	228	198.3	219.6	249	198.5	344.4	495.4	489.6
317.8	353	198.4	217	333.7	224.9	258.7	321.3	305.6	207.9	313.6
515.8	430.8	453.5	368.7	384.1	458.2	506.6	395.1	680	747.7	701.7
226.9	208.5	213.2	197.6	178	211.5	229.4	206.8	276	452	376.4
550.3	528.5	554.6	454.5	398.6	549	466.5	611.9	847.3	781.5	1007
141.8	166.6	189.5	163.4	122.2	177.1	142.6	146.7	222.4	176.6	283.6
331	294.7	310.9	236	253.7	278	263.5	252.7	439.4	432.9	569.4
165.5	156.7	149	116.7	147.3	161.7	194	157.4	155.4	172.3	175.1

163.9	223.3	169.6	242.9	136	83.4	101.9	177.4	151.7	91.1	153.2
621.7	375.1	368.2	478.6	356.5	368.6	354.1	388.5	1171.8	763.6	1608.6
228.4	193.2	237.4	170.7	194.9	196	288.2	188.3	323.2	415.9	389.4
157.1	325.9	281.7	201.5	183.2	309.3	302.3	278.5	485.1	462	572.8
395	426.9	394.1	303.8	280.1	298.8	390.7	289.6	558.4	474.3	457.9
133.5	125.2	121.3	108.3	100.6	112.7	118.4	133.7	267.6	271.9	286.8
236	255	274.3	178.3	178.4	215	283	221.7	356.1	496	524.8
415.7	387	427.3	385.9	372.1	284.7	300.9	281.7	461.2	456.1	578.2
198.9	270.8	235.8	163.5	172.6	235.3	272.2	210	364.1	304.1	382.9
187.3	170.9	201.3	184.4	187.4	173	322.9	177.1	208.6	203	306.4
257.6	170.5	133.2	125.4	433.1	153.6	261.8	177.1	115.7	121.6	136.3
3065.4	1377	1688.2	1325	1087.3	481.4	411.2	664.8	353.4	313.7	655.6
170.6	137.4	151.2	115.1	148.8	146.8	156.9	150.2	274.8	689.8	446.1
318.9	242.5	281.2	209.9	220.8	297.3	372.5	279.7	493.8	554.8	570.7
280.5	283.3	425.1	312.1	284.6	439.8	421.8	392	713.4	750.4	691.5
287.5	304.7	287.8	215	241.9	222.6	312.1	261.8	358.3	420.7	415.9
111.4	103.9	103.4	108.1	98.2	95.6	130.5	90.3	198.9	598.7	367.4
158.4	158.3	128.6	167.7	106.2	106	97.2	125.5	84	76.8	120.5
277.9	175.3	219.1	230.4	162	131.7	216.2	171.4	265.3	494.5	469.5
91.7	87.6	82.1	76.3	77	71.6	94.8	83.6	115.1	129.4	137.8
221.7	238.3	260.3	178.1	186.6	301.4	350.7	218.3	293.2	306.7	378.9
136.7	126	125.7	132.5	97.7	208.5	96.2	108.9	134.5	152.4	173.9
213	182.5	159.1	166.1	125.3	120.4	128.5	185.1	339.1	337.4	420
534	612.4	397.3	561.2	547.1	426.1	661.6	817.7	533.1	290.7	465.2
362.7	280.5	503.2	361.6	240.3	285	284.2	292	350.4	397.7	459.3
80.8	83.1	91.2	80	69.9	100.1	122.5	104.6	103.5	112.8	152.9
266.2	258.2	266.9	264.4	258.1	285.4	294.8	226.1	307	388.3	436.3
921.6	1056.1	298.2	798.4	363.9	545.7	268.5	744	190.8	233.8	267.2
299.1	205.1	216.1	262	302.7	249.6	401.8	342.8	538.9	323.9	352.7
536.7	564.8	514.9	453.8	386	607.3	582.4	457.1	356.7	385.9	491.4
216.3	268.8	269.4	366.2	196.6	248.3	180.2	213	134.8	104.6	138.7
288.5	402.5	321.3	257.8	246.4	312.8	294.4	238.6	451.2	450.8	574.5
358.4	264.9	430.5	297.8	330.4	329.9	226.1	268.2	427.5	555.7	719.9

129	115.6	181.1	107.5	125.9	129.3	205.1	143.9	144.4	236.7	186.2
195.7	175.2	179.6	120.9	167.6	138	268.2	135.3	269	473.4	406.5
799.5	617	773.8	1043.6	525	636.6	679.4	336.4	329.3	359	513.8
157.1	140.9	171.4	142.7	138.3	158.9	168.8	154.4	285.6	292.7	335
256.8	281.5	333.2	299.3	288.6	309.5	494.5	296.4	511.9	673	754.5
232.3	118.2	183.2	101.2	150.4	111.6	106.7	129.5	327.1	454.8	516
177.9	134.9	207.1	121.5	101.8	105.3	128.4	114.1	165.7	137.3	208.6
245.6	183.1	188.7	160	195.9	199.6	188.4	177.7	275.7	334.8	431
329.6	272.2	265.8	231.4	333.7	207.4	321.5	310.4	318.8	336	371.5
38.1	121.7	87.9	112.6	41.4	74.5	206	1259.2	8.4	40.9	41.5
174	134	168.5	126	146	140.2	143.1	139	207.9	195.6	292.5
176.6	180.9	181.8	168.5	123.8	196.6	184.2	187.9	160	146.4	171.1
305	248.4	423.8	217.2	326.2	230.7	257.5	231.1	402.1	661.4	603.2
92.4	70.7	91	107.4	121	65.6	129.1	84.1	118.5	127.1	161.1
117.2	127	110.4	95.2	87.7	94.7	107.8	121	147.9	193.3	240.3
1727.1	973.5	623	580.4	513.1	283.4	304.4	920.9	1919.9	508	876.4
93	89.8	87.2	66.9	91	94.1	109.5	99.3	153.8	151.5	184.2
190.5	245.2	217.6	166.8	171.7	274.8	364.7	193.7	251.9	227.6	293.2
221.7	226.8	273.5	197.1	151.4	148.5	164.1	190.7	278	265.9	433.5
175.9	176	173.3	142.6	119.2	154	178.1	167.8	356.7	425.7	420.6
314.1	293.5	286.5	263.3	251.6	316.9	321.8	288.7	398.3	461.6	498.4
206.9	238.9	272.5	215.7	198.8	281.2	297.1	210.3	286.4	317.1	361
23.2	45	59.1	58.3	93.5	1136.4	460.5	138	31.6	26.9	27.1
480.7	416.7	423.2	403.4	533.3	472.6	649.8	519.4	499.1	790.9	808
81.3	71.9	75.8	52.9	73.7	67.2	64.4	41.2	55.5	73.5	97.3
261.9	236.5	234.9	222.3	215.4	222.7	281.9	289.3	432.6	499.8	557.3
120.8	128.9	102.8	97.5	90.6	128.2	133	134.4	270.3	644.8	340.8
127	131	135.9	107.5	104.1	136.7	159.1	145.9	165.5	201.1	250.8
87.2	103.6	107.4	86.8	72.4	122.6	135.2	120.2	225.7	251.7	349.6
411.2	350.7	360.4	275.9	319.1	263.7	428.4	388.9	519.3	614.1	719.5
645.3	555.6	851.4	548.5	512.3	661.7	662.9	404.7	878	724.1	915.4
434.3	300.2	315.2	393.1	208.8	171.5	227.4	179.7	194.8	167.9	255.4
155.9	188.7	135.7	131.8	119.8	244	187.2	177.4	215.1	316.3	402.4

442.9	354.1	475.9	364.8	314.7	365	322.4	334.3	474	417.8	572.7
337.7	242.1	231.5	271.5	210.6	344.4	249	241	336.5	436.5	372.7
184.1	211	272.9	205.7	164.3	194.5	185.1	179.8	272.6	303.7	919.8
122.2	110.6	104.4	91.4	94.3	122.4	102.9	95.3	228.9	118.1	219.4
229.4	213.3	226.2	194	192.7	225.9	468.2	208.9	419.5	1045.5	647.2
186.8	185.3	196.9	187	168.4	193.4	242.3	178	258.5	244.9	301.6
153.1	171.7	156.9	139	130.1	150.1	163.4	141.8	145.5	134.3	177.3
150.3	201.7	122.6	188.4	137.7	246.1	242.5	177.7	191.7	226.8	216.1
160.2	215	186.3	148.4	223.7	325.6	231.3	113	103.3	782.5	147.7
299.8	330.9	279.1	296.1	283	231.8	475.2	425.6	257.2	206.7	349.7
63	82.9	91.6	90.6	68.7	89.4	76.3	67.9	105.4	125.8	131.4
125.9	132.6	108.5	95.6	116.5	124.7	137.1	118.5	144.1	158.4	184.8
61.6	71.6	65.6	55.6	65.3	82.7	65.6	56.6	94.7	71.4	124.7
104.3	134.5	116.1	107.6	135.4	133.6	164.1	135.2	144.7	113.2	148
117.7	105	120.4	103.8	99.7	108.2	118.1	131.6	188.8	173.8	239.6
157.2	127.5	145.9	122.6	110.3	119.8	122.3	114.5	198.4	222.5	225.7
92.5	104	97.3	71.7	58.8	86.4	103.8	90.1	96.4	156.6	132.5
525.9	490.5	513.7	333.3	381.3	508.7	536.2	666.9	798.6	851.3	940.8
256.6	246	293.9	256.4	207.1	284	291.1	342	222.5	532.6	246.6
483.2	236.6	204.7	149.6	166	157	255	165.5	190.1	707.3	87.3
137.8	117.5	120.8	113.1	119.6	104.3	129.6	109.3	109.2	146.6	155.2
192.2	226.3	219.1	190.6	132	168.6	213	163.4	277.1	285.8	379.6
170.5	122.4	160.7	125.8	123.9	127.7	163.7	143.9	285.7	311.7	399.6
268.3	261.1	277.8	195.5	236.2	216.6	227.6	222.8	307	317.9	382.1
334.4	226.8	215.6	275	227.2	234.9	556.6	1821.7	50.4	100.5	132.2
125.7	123.1	128.6	109.9	122.4	134.4	156.6	126.2	118.7	155.7	180.1
202.7	157.6	143.7	144.7	136.5	129.2	183.6	163.4	254.5	244	195.7
89.8	95.1	74.5	71.5	76	76.7	84.8	76.3	84.9	122.3	122.4
131.5	130.4	147.7	131.4	104.3	145.8	138.4	153.8	216.2	204	299.7
312.9	330.7	373.4	308.4	256.9	310	407.6	305.3	585.4	798.2	736
164.4	191.5	162.7	158.1	156.2	195.7	195.2	153.3	265.1	181.9	275.6
296.2	331.9	293.7	258.8	245.8	323.7	347.8	284.2	421	435.6	565.8
567.9	526.2	616	442.4	385.2	483.1	536	518.6	994.5	1337.8	1349.7

139.2	120.5	106.3	105.2	123.1	145.9	188.4	109.6	104	111	114.7
47.7	37.7	45.7	38.4	38.8	38.4	37.9	36.9	49.6	43.4	52.9
148.7	142.4	134.7	137	140.8	146.8	157	145	164.7	148.1	205.1
148.5	131.2	169.8	122.1	139.4	158.1	150.3	129.3	266.6	245.7	332.6
174.4	321	172.8	86.6	97.1	285.8	219.4	121.6	103.9	148.6	166.6
94	76.8	93.5	57.4	136	93.6	84.4	71.9	76.1	160.8	112.9
81.8	84.8	80.3	91.4	73.8	111.5	93.4	103.2	117.2	102.5	102.5
338.3	297.5	338.7	297.9	261.4	299.3	335.4	277.9	309.8	363.4	487.5
301.8	311.8	374.2	273.5	227.9	279.8	306.9	300.8	658.4	761.6	856.8
304.1	305	262.6	234.9	205.9	356.5	243.1	260.5	567.2	725.2	734.3
487.3	352.6	499.1	379.6	324.2	418.7	354.7	488.5	565.7	1162.3	1078.7
238.6	279.5	239	200	176.8	234.5	463.7	152.8	392.9	870.6	679.6
132.4	158.1	164.5	141.2	98.4	137.2	147	170	222.5	222.7	243.8
902.1	734.8	828.5	755.5	599.5	637.6	761.4	722.8	1143.5	1113.2	1285.2
261.8	282.7	223.1	166.5	152.6	242.3	302.5	199.4	159.5	217.3	273.7
109.3	115.1	140.5	99.5	84.9	90	106.7	86.4	137.4	107.3	174.8
541.6	453.9	601.6	519.2	397.5	569.1	588.1	501.8	606.3	671.4	835.6
446.5	335.9	320	327.9	250.7	286.8	337.1	424.4	504.3	588.1	589.7
155.3	171.3	212	192	165.3	228.2	143.6	151.6	249.4	211.8	291.4
150.4	165.2	181.5	170.1	150.3	178.6	174	189.8	258.8	242.7	293.4
167.4	169.5	188.6	148.9	171.7	163.9	266.5	180.4	240.6	231.4	248.1
228.6	197.5	224.6	172.1	153.7	181.7	229.1	202	364.2	468.7	474.8
300.8	274.1	312.3	239.4	243.6	272.5	271.5	201.5	486	703.5	541
301.1	211.7	239.2	196.5	172.5	226	222	232.2	384	380.3	519.7
494.5	845.7	472.6	226.5	304.7	778.7	497.3	321.9	474.8	550.9	644.1
170.9	167.1	189.2	200.7	189.6	170.2	211.1	125.2	238.7	242.5	369
351.8	307.3	300.8	343.1	353	270.4	367.3	401.7	383.3	290.2	432.3
486	245.4	383.3	298.9	258.7	208.4	230.3	222.5	410.9	551.6	596.7
309.2	365.4	415.5	319.3	287.3	436.5	390.2	306.5	309.6	487.4	401.8
281.4	257.8	288.5	190.1	237.8	233.1	221.9	240.7	375.6	489.4	550.1
734.2	552	613.4	434.9	381	432.1	449.7	493.7	1055.5	1519.1	1513.4
220.8	247.7	249.1	213.5	259.4	334.4	365	197	506.9	924.6	738.6
86.3	77	85.5	76	83.7	84.5	98.9	89.4	105.3	124.7	143.1

212.1	313.8	235.9	173.8	114.8	162.5	228.4	170.4	158.7	282.2	264.9
240	258.3	308.4	210.2	222.2	241.2	281.5	226.7	392.7	512.2	521.8
1864.7	236.8	485.8	600.7	317.8	198.9	224	176.5	171.4	267.6	256.5
259.4	362.4	366.2	390.2	264.7	739.4	522.7	666.7	577.9	341	355.5
148.3	131.4	184.7	105.5	133	162.2	184.8	146.6	197.1	234.7	249.8
393.6	508.2	378.2	401	326.3	417.3	479.1	539.6	791.2	698	751
91.9	90.1	117.2	74.9	64.7	87.8	70.1	72.1	84.7	93.7	103.1
541.1	445.7	554.5	389.5	267.5	363.4	549.9	413.1	396.7	517.7	646.3
312.4	256.4	302.6	191.5	220.6	214.9	268.4	225.6	473.5	620.7	711.3
292.6	223.7	205.9	195.9	167	179.5	197.4	205	376.2	489.2	458.5
177.1	162.2	164	120.1	149.7	151.3	197.9	157.7	207.8	198.1	249.8
293	237.5	261.3	225.9	202.2	282.8	213.5	258.5	365	473.7	533.6
110	106.9	129.8	88.8	80.9	100.1	93.1	91.1	140.3	169.7	213.8
632.1	243.3	388.8	284.7	316	316.9	346.8	280.4	328.2	502.2	609.1
177.5	373.3	234.4	160	178.6	238.3	226.4	173.3	211.3	239.2	288.2
710.4	509.3	674.6	542.1	620	437.7	492.8	427.2	710.3	877.1	1029.4
227.7	216.2	264	164.3	192.2	270.7	255	239.6	322.5	264.6	381.4
455.9	416.7	457.2	332.2	296.5	335.6	363.6	368.9	787.4	1074.1	1090.2
208.2	233.5	236.9	265.9	205.1	246.6	302	365.2	286.2	316.7	290.8
198	112.9	147.4	121.7	91.2	112.1	183.1	86.6	285.5	445.8	307
812.5	805.5	967.2	679.8	570.6	780.8	698.7	690.2	986.7	1201.1	1327.5
80.3	65.6	60.2	56.4	62.5	62.3	96	70.9	126.1	74.5	118.2
52.5	37.6	46.6	51	46	39.4	25.9	32.6	99.9	111.7	142.8
169.9	163.6	140	132.3	135.3	126.7	226.3	137.2	165.6	187.5	219.2
282.8	227.9	264.2	234.3	263.9	205.8	229	247.8	388.3	382.5	447.8
383.8	355	399.8	333.3	312.3	396	518.2	383	546.1	536.1	671.2
115.7	135.3	129.7	151	94	160.3	128.6	108.7	110	101.8	121.4
326.9	279.1	285.4	247.3	333.7	244.4	388.9	267.8	384.4	504.6	598.8
223.4	231.9	208.2	183.5	159.6	232	242.6	248.8	166.1	240.4	228.3
328.9	397.2	386	403.4	246.7	127.8	211.8	440.6	177.4	122.9	145.4
292.1	253.7	267.1	239.4	189.5	254.4	252.4	209.6	312.1	428.1	517.3
213	186.1	227	125.4	177	186.8	157.9	163.2	317.1	319.7	420.1
198.5	149.3	156	122.8	126.2	134.4	171.5	137.2	279	272.1	406.1

131.8	184.2	225.7	109.8	178.8	173.2	225.9	143.7	309.9	153.7	149.8
422.6	367.6	437.7	342.7	281.8	386.1	434.1	385	624.4	753.6	884.9
111.4	101.8	133.7	88.1	114.7	101.5	86.1	104.1	169.4	131.9	205.1
269.8	223.3	223.3	183	217.8	203	223.6	225.4	293.2	415.3	338.1
185.9	213.2	195.4	173.8	178.7	246.2	182.3	188.6	303	351	456.1
70.5	71.7	66.8	68.3	65.7	78.8	79.7	66.8	103	83.5	124.7
1090.8	831.7	889	941.1	1264.4	777.1	1223.6	1489.9	1223.1	624	836
213.9	242.2	239.1	217.5	156.9	270.1	348.9	272.1	235.7	229	284.5
237.7	125.1	195.7	114.7	106	126.8	127.9	108.2	115.1	156.3	190.2
339.4	294.2	330.4	300.2	290.3	267.5	287.5	267.8	370.9	388.8	442.9
228.7	185.5	194.3	165.4	152.3	171.3	217.8	185.6	353	295.7	397.3
302.8	300.8	358.2	273.2	230.7	312.4	339.7	449.7	584.3	445	467
115.9	90	89.8	98.9	106.9	105.8	110.6	79.7	155	166.4	226.1
148.1	144.9	136.1	171.6	134.9	277.7	191.7	117.4	125.4	118	149.1
152.9	188.6	204.2	117.4	182.3	491.6	226.3	112.7	179.5	170.3	272
58	63.9	63.1	47.3	38.1	50.1	63.5	50	117.9	139.4	146.7
206.9	240.9	228	177.5	178.8	303.8	287.9	267.4	322.6	301.9	324.2
279.1	270.2	331.5	257.9	202.7	262.9	255	190.8	299	598.7	512.6
421.9	302.5	342.1	260.9	312.2	260	360.6	268.3	681.7	710.1	814.1
185	146.1	175.2	206.2	233.3	347	489.5	140.6	122.9	196.9	272.9
182.3	158.7	163.5	123	145.7	153.4	142.8	129.1	232.8	245.4	267.1
90	84.9	81.1	79.1	76.1	84.6	84.9	67.7	109.1	103.1	146.5
906.1	799.3	873.7	626.9	543.2	583.6	861.1	656.5	1482.9	2407.5	2128.4
236.7	683.7	295	568.4	189.3	785.7	380.1	502.5	284.1	171.8	258.5
575.8	735.3	618	402.1	517.4	173.5	340.1	219	228.4	212.4	376.8
247.2	141.7	165.8	147.7	160.8	159.5	141.7	174.8	283.2	232.7	265.9
97.2	76.1	74.8	60.5	69.2	121.8	82.8	74.7	119.6	139	159.6
134.4	135.9	153.7	127.6	124.4	130.3	145.5	118.8	170.4	161.7	249
211.4	214.6	244.5	173.2	170.6	225.3	295.9	205.6	296.6	427.5	493.4
309.2	301.6	340.6	321.6	286.5	608.7	430.9	334.6	454.8	837.7	628.4
262	211	213	162.7	177.2	187.6	208.6	206.2	375.4	507.9	481.9
683.9	556.8	550.3	432.6	440.7	621	591.9	464.1	723.8	780.6	1045.8
105.8	56.3	69.2	50.5	66.2	76.7	55.3	56.2	133.2	109.3	150.3

34.2	39.3	30.6	21.6	29.6	22.9	24.6	22.1	34	48.3	63.4
194.2	188	203.9	212.9	214.5	217.3	270.1	183.3	287.4	683.6	429.6
199.8	191	210.3	137.4	139.3	153.1	234.8	151.6	346.6	444.1	442.8
188	107.7	103.4	103.7	116.2	93.3	93.1	114.5	248.6	300.1	302.1
406.3	372	443.8	331.2	309.6	390.5	382.6	366.4	574.2	709.9	787.6
180.1	127.6	158.6	132.7	124.4	219.3	117.2	110.8	187.1	204.6	300.6
187.5	186.4	148.4	139.9	231.2	167.2	231.7	177.4	210.5	213.6	245.2
912.4	408.3	710.3	760.7	672.3	1035.6	396.3	761.2	761.1	1013.8	795.6
220.6	301.7	273.8	214.4	241.6	211.1	338.3	447.5	197.5	155.2	258.5
138.8	128.6	159.1	117.7	86.4	199.7	168.3	110.9	331.3	168.4	180.7
152.8	148.8	140.8	124.5	156.9	167.7	156	115.8	201.6	249.6	285.4
813.3	618.5	636.9	521	503.7	517.4	599.6	589.4	843.9	968.4	1065.9
167.5	176.3	185.8	167.3	137.1	176.3	220.1	137.3	238.6	280.2	330.5
333.9	282.5	341.3	266.8	258.1	272.8	303.5	294	598.8	709.4	815.8
50.8	56.5	49.4	46	34.3	44.4	55.3	62.8	63.2	64.6	64.4
146.8	193.1	200.6	157.9	167.4	207.3	214.7	202.6	231.5	150.7	240.2
1335	845.1	475.1	327.7	1073.2	372.5	62.2	1615.2	1137.3	270.3	272.9
49.8	41.3	42.7	32.3	29.6	42.3	49.5	35.8	47.8	45.3	62.8
115.9	123.1	125.9	126.1	125.6	116.7	136.9	132.4	109.7	106.5	165
278.4	210.7	241.3	205.1	213.2	215.7	235.2	193.4	335.4	418.2	511.9
73.3	135.3	134	94.8	180.3	102.3	106	121.4	256.6	527	441.9
280.4	293	340.4	234.4	239.6	269	323.3	299	378.2	449.4	601.2
190.5	143	105.8	102.4	117.7	96.7	165.1	128.2	222.5	429	248.2
154.1	137	159.2	122.8	115.3	128.6	191.6	128.7	280.1	278.6	308.9
445.2	404.8	448.9	344.5	319.2	427.4	440.8	401.7	643.2	900.7	965.5
235.4	231.6	213.6	230	216	278.8	290.3	297.5	357.1	330.3	418.6
297.4	348	371.8	292.5	288.3	344	415.2	306.1	337.2	401.6	455.7
171.3	191.2	194.1	164.4	174	182.6	178.7	166.7	178.2	173.3	239.5
219.6	207.3	225.7	174.5	183	224.3	197.9	217.1	313.9	368.8	399.5
23.7	27.7	23.7	18.6	19.8	16.5	31.3	25.6	31.3	42.4	43.4
139.2	113.7	165.1	94	94.8	149.2	137.6	103.2	143.5	140.4	223.7
239.9	224.5	208.8	173.1	148.5	173.5	235.3	159.5	298	340.8	404.6
181.5	136.4	127.9	118	142.3	188.1	205.3	157.7	719.6	312.6	555

70.7	57.3	68.2	71.9	58.9	59.5	84.9	64.6	84.3	95.4	108.6
366.2	425.7	377.6	323.5	281.8	322.5	419.4	397	693.7	646.2	801
196.3	182.1	242.6	160.5	170.2	201.2	218	164.8	278	284.4	408.7
591.3	408.6	434.9	419	391.3	366.1	391.3	378.2	663.1	665.3	896.9
79.6	70.9	88.6	58.3	70.7	65	86.3	71.1	118.5	175.1	175.7
172.5	120.4	134.6	125.3	117.7	121.9	145.1	117.1	128.8	201.1	174.8
106.4	97.5	94.7	78	90.1	84	84.2	95.9	118	112.1	109.7
321.3	284.9	306.6	222	231.7	260.5	339.9	241.7	389.4	512.1	552.3
265	299.5	350.6	242.7	214.5	264.7	282.6	226.9	443.1	495.1	458
140.5	139.8	114	92.7	83.5	120.8	151.9	164.9	200.5	185	203.4
250	1021	2103.1	968.8	1296.9	1107.1	766.3	1426.6	285.5	473.1	864.7
240.2	246.6	253.1	214.9	229.3	259.7	285.9	236.4	317.2	454.9	428.1
138.4	131.3	130.3	107.7	103.5	116.9	123.8	119.6	193	237.2	272.7
252.5	230.1	243.8	174.7	206.3	227.6	238.2	197.7	437.6	385.4	482.2
416.5	337.5	366.4	304.1	320.1	337.3	301.2	354.6	546.3	605	734.4
173.1	185	181.2	156	169.5	160.5	204.1	131.8	209.6	243.9	247.2
304.1	352.3	319	324.3	253.7	289	298.3	307.8	456.1	368.6	530.2
94.2	76.9	95.7	68.6	51.3	95.3	86.9	73.9	124.4	82.8	134.9
377	413.4	353.8	346	338.9	375.2	435.2	366.9	446.3	608.7	526.6
611.9	430.7	465.5	406.7	334.4	343.5	439.6	494.1	700.8	1211.7	1000.2
171.7	174.5	187.7	197.1	168.7	174.8	165.8	154.7	233.4	193.7	230.7
332.4	371.3	338.1	331.7	293.8	317.8	395.8	308.8	395.7	515.8	478.9
84.7	84	98.5	82.5	81.5	127.4	95	63.6	84.6	112.3	125.7
334.8	321.4	405.6	387.3	277	333.8	353.5	329.2	424.5	437.3	565.8
213.5	214.1	225.3	161.7	133.7	174.7	199.1	187.3	373	500.1	500
334.9	304.5	351.8	287.2	242	317.6	327.4	304.9	382	433.6	504.1
340.1	264.5	229.2	195.5	257.8	207.5	271.3	258.8	390.2	362.4	417.6
262.7	276.6	414.8	429	277.1	375.8	398.8	331.1	439.4	702.4	861.1
574.4	422	571.4	369.8	376	365.6	362.7	343.7	553.2	863.1	739.2
401.6	311.7	343.7	248.4	210.9	145.4	156.3	300	426.6	323.6	507.1
421.9	282.5	302.7	226.5	211.5	197.8	162.4	237.1	255.2	344.1	444.6
418.2	347.8	388.3	283.9	293.5	398.7	443.5	367.7	626.8	617.7	792.5
195.7	156.6	171.5	134.5	162	133.3	218	151.7	287.4	368.1	366.8

483.6	445.4	518.5	364.9	454.8	571.4	305.3	423.7	455.4	421.8	535.6
34	38.3	74.3	48.5	36.8	49.6	96.7	49.7	173.4	93	147.3
110.9	107.9	115.6	117.5	116.2	123	119.8	118.5	121.2	124.4	153.6
251.3	227	279.2	204	177.5	243.3	275.5	225.5	442.6	495.1	465
398.3	288.2	363.5	254.7	273.4	254.5	272	267.1	581.7	684.6	795
297.1	307.3	364.3	462.4	237.1	290.4	166.1	186	207.4	217.6	305
81.6	60.4	86.4	62.5	68.1	50.7	49.5	51.7	79.3	75.3	89.7
214.5	190.5	214.5	183.7	157.1	142.1	173	165.3	252	289.3	340.2
388.3	228.2	169	142.9	163.6	180.8	161.1	139.7	294.7	432.6	551.2
99.8	150.3	93.8	87.7	67.6	102.5	161.9	143	345.6	445.9	360.8
262.4	256.3	281.8	245.9	205.1	216.6	277.2	237.3	482.5	568.3	618.6
286	257.9	386.5	316.4	334.2	356.9	275.1	240.4	270.6	291.4	317.8
269.8	260.9	291.5	230	219.7	217.4	313.5	233.4	448.8	543.1	557
167.6	167.1	179.8	136.4	159.9	181.7	189.6	192.7	408.1	324.7	383.4
293.3	300.5	344.2	286.6	241.6	337.7	410.3	391.3	394.2	363.3	472.5
109.9	96.2	92.1	95.6	86.5	121.7	119.7	94.5	131.5	110.1	196.8
99.8	114.9	110	102.1	94.9	106.5	120.2	113.4	157.3	190.7	233.7
197.5	194.2	206.3	158.9	158	193.8	179.6	168.1	302.6	207	312.9
699.7	659.2	636	601.5	527.1	594	837.7	554.5	767.2	1025.1	1102.6
194.8	163.3	232.2	119.8	168	147.7	159	172.8	223.9	229.6	352.5
238	293.2	326.1	235.8	190.6	242.9	264.9	239.4	316.9	393.9	477.7
218.1	185.5	234.5	172.3	177.9	164	219.3	203	391	345.7	500.6
462.3	376.3	375.5	309.5	260.8	316.6	350.8	361.2	609.7	632.3	765.6
211.4	177.2	222.5	223.7	186	214.9	179.4	204	278.7	267.7	352.2
305.6	282.2	205.6	309	125.1	397.3	131	207.9	327	153.1	185.9
361.8	274.8	376.7	315.2	279.9	314.9	250.3	263.6	427.1	454.9	568.3
307.4	247	272.9	245.7	227.1	253.4	330.1	269.4	451.8	522.9	530.1
391.7	370.4	381.7	302.1	278.9	332.8	530.5	292.3	811.6	874.3	959.3
644.5	515.2	540.1	467.1	395.3	445.5	435.2	449.2	960.7	1015	1238.2
90.2	214.7	188.1	100.7	117.8	266.2	260.5	106.7	61.9	73.3	101.8
151.9	157.9	185.5	145.5	205.8	142.9	240.2	150.4	249.4	190.4	258.3
156.8	135	166.4	140	121.6	161.3	146.1	143.4	437.4	736.4	678.3
175.8	150.2	296.9	240.2	149.1	141.7	150.6	198.7	157.7	114.5	115

281.3	203.9	206.2	144.4	124.7	149.2	229.5	162.8	313.7	266.4	341.6
254.4	211.3	280.2	328.7	182.8	293.4	207	209.9	211.9	399.7	316.4
137.1	469.7	205	158.9	246.5	348.9	734.4	353.4	166.7	124.2	168
189.5	144.8	200.6	154.4	161.3	165.8	146.5	144.7	267.2	349.1	379.9
272.8	235.6	250.1	232.8	201.4	209.6	262.4	224.1	269.7	277.3	343.6
103.1	112.5	124	101.5	100.3	105.3	147.6	99.3	149.4	200.4	171.5
101	83.9	130.9	88.6	83	98	83.7	95.8	112.5	140.9	167.9
413.3	320.9	357.9	291.7	222.7	222.2	258.5	247.7	526	1183.4	927.5
153.1	164.1	168.2	133.2	120.2	174.5	178.2	159	273.6	339	364.5
53.1	64.6	68.8	51.7	54.1	55	72.4	51.8	52.6	95	83.7
166.2	178.4	193.9	146.1	124.5	146.9	155.6	136	194.9	231.3	277.6
152.5	140.6	176.5	147.5	139.8	127.9	166.8	142	199.2	265.7	252.6
61.1	53.8	62.4	45.3	57.2	56.3	60.8	51.5	91.1	105.5	119.9
191.3	199	192.9	187.7	189.3	225.1	234.7	201.6	208.8	231.8	278.4
343	214.7	291.4	186.2	207.3	265	254.6	173.3	392.5	583.3	660.8
175.1	166.9	189.8	132.1	151.2	192.4	173.4	155.3	228.1	204.9	302.7
281.9	290.5	316.6	254.3	211.7	318.1	255	253.1	309.4	331.2	389.7
165.5	162.7	203.5	176.6	158.8	166.8	182	147.8	188.6	184.1	245.3
29	55.3	45.3	50.1	34.8	26.6	32.8	75.4	23	17.5	23.3
257.8	178.3	234.2	176.5	176.1	194.4	150.2	215.4	658.2	529	790
26.8	33.9	31.6	26	32.4	32.4	32.8	28.2	31.6	48.1	45.7
320.3	261.2	285.6	248.9	177.5	192.6	236.8	257.5	245.7	140.5	170.7
106.6	123.8	106.3	125.3	114.6	122.7	149	112.5	136	126.7	143.2
175.2	157.2	143	112.1	97.4	139.5	143	175.1	181.5	180.3	197.1
225.5	170.8	331.7	260	193.8	204	205.1	172.5	475.8	472.9	498.7
85.8	93.6	92.5	72.4	88.9	90.1	135.7	88.5	123	122.6	137.6
197	149.4	162.5	118	110.2	136.8	151.2	140.4	310.3	378.5	387.4
105.6	95.2	108.3	80.1	83.9	79.5	85.4	102.9	175	150	199.7
733.9	694.6	778.4	618.8	521.9	720.5	745.4	658.7	787	881.5	1081.6
517.5	419.7	535	338.8	348.9	394	386.3	315.4	614.8	814.9	895.1
738.5	415	409.6	378.9	414.3	718	2414.2	765.4	289.5	569.5	523.1
305.8	468.3	347	361.2	297.5	229	388.4	574.7	260.2	220.7	277.6
301.7	286.1	350.4	202.8	168.2	236.4	211.3	251.1	403.5	394.2	466.4

316.6	318.2	218.7	225.7	328.6	241.7	331.1	201.8	141.9	168.1	181.2
298	257.4	283	247.9	216.9	198.1	188.9	153	359.2	631.8	582.6
177.2	163.9	177.6	148.4	196.9	161.3	244.3	178.6	222.7	285	297.5
259.5	242.8	216	180.5	302	271.7	372.3	252.3	400.1	348.2	398.8
207.6	212.9	218.8	175.5	133.1	215.2	255.2	212.1	267.7	255	322.5
86.9	79.2	88.5	74.2	68.7	78	95.9	79.8	136.6	125.6	168.8
338.4	371.6	409.1	341.1	334.7	313.2	362.5	375	325.9	699.7	427
368.1	212.7	289.1	216	263.7	224.4	184.3	247.8	597.9	527.6	734.5
23.9	22.7	27.1	26.6	22.7	32.2	32.8	19.9	27.8	44.3	34
249.3	128.5	228	123.1	196.1	188.5	153	146	224.9	248.2	295
208.5	320.1	232.5	198.1	152.4	214.7	243.9	243.1	385	423.5	445.6
100.1	117.9	108.9	86.1	84.5	93.2	113	93.2	191.5	204.2	224.8
318	309.4	325.6	253.6	245.8	336.5	361.5	283.7	486.1	512.4	627.3
189.8	182.5	194.3	169.1	197.3	154.8	184.4	130.1	142	133.7	181.8
277	232	251.5	175.3	211.9	227.9	279.9	220.6	409.2	482.4	538.7
191.6	161.2	166.3	129.1	119.9	147.1	175.2	117.5	291.5	368.5	392.7
363.2	436.5	440.1	336.4	312.6	451.6	519.5	401.5	312.8	370.6	400.2
156.1	201.9	163.4	179.1	172.2	255.7	242.1	297.6	189.8	175.5	197.1
193.9	158.8	191.2	121.5	156.8	134.2	151.8	133.3	230.3	202.6	253.4
125.7	99.2	104.7	87.8	111.6	101.8	95	97.2	181.8	156.1	240
257.5	248.1	230.1	183.9	177	196.5	289.4	232	348.7	324.5	450.8
263.4	249	158.3	243.7	248.8	612.6	590.9	262.9	118.4	112.8	117
183.1	137.5	171.2	147.6	133.2	151.3	174.1	176	222.5	238.9	263.6
379.3	340	258.4	305.1	296.5	290.1	404.1	281.8	340.6	491.6	487.7
335.9	231.6	250.9	223.2	361.8	294.4	441.2	271.8	330.6	413.1	445.3
282.9	307.2	316.4	291.1	259.4	326.5	360.3	319.2	470.8	452.2	636.4
216.2	366.2	170.6	260.8	314.3	179.8	180.4	257.6	115.2	96.5	213.7
26.1	32.5	35.8	31.5	23.8	33.9	35	31.3	89.4	96.7	101.9
495.4	355	438.4	392.7	317.9	283	202.3	341.7	366.3	280.2	391.3
141.3	159.7	147.4	143	132.5	138	183	155	163.3	153.9	195
45.9	57.5	78.8	84.7	53.7	73.4	75.3	51.1	240.5	1400.4	568.3
275.7	197.7	228	185.1	240.2	205.4	228	202.9	259.7	367.8	387.5
174	156.9	133.5	136.3	95.1	117.5	112.4	180.2	234.2	191.8	247.1

130.5	103.5	133.8	98.8	118.1	118.9	118.3	112.2	158.1	180.6	213
277.4	277.2	293.5	207	201	205.4	268.2	232.4	464.8	452.9	608.2
168.6	225.6	203.1	161	127	161.3	363.1	216.8	374.7	520.2	413.2
170.8	155.9	202.1	150.1	159.9	170.8	220.1	198.1	315.1	291.6	318.8
440.6	413.4	361	335.9	305.1	374	369.1	330.7	740.1	966.8	923.2
129.5	124.7	132.6	111.8	86.5	109.3	159.8	99.3	143.4	215.2	176.3
144.1	141.8	129.6	104.7	129.7	133	176.1	95.5	195.2	224.6	257.6
62.5	48.4	57.3	48.5	46.5	43.7	53.1	39.4	73.4	86.2	98.6
173.5	183	193.3	167.6	161.7	225.8	205.9	195.8	305.9	256.9	316
89	85.2	103.3	93.7	81.7	94.3	106.4	86.2	126	141.8	151
60.4	64.2	53.2	49.6	45.6	66	46.3	60.5	108.1	93.1	103
124.4	128.1	117.4	90.3	114.8	122.2	142.2	121.2	150.6	189.3	237.9
192	210.9	145.2	157.4	145.8	189.9	207.6	182.3	248.6	321.5	335
287.4	235.3	369.5	286.1	234.9	284.4	248.1	209	277.9	328.8	344.8
419.4	332.3	380.1	251.7	430.5	356.1	479.2	426.7	238.7	152	275.1
113.9	130.2	108.1	126	108.7	139.5	120.1	127.4	131.8	152.6	190.8
289	306	332.4	348.8	172.1	298	348.7	152.6	278.9	210.3	318.1
309.5	219.1	165.9	197.2	210.4	143.8	311.6	319.3	239.3	156.5	218.8
327.9	302.6	332.5	298.4	233.8	301.9	214.7	219.9	231.5	200.6	260.1
602.4	460.2	503.8	349.5	360.3	403.3	405.2	393	928.5	952.8	1185.1
219.2	246.7	186.5	170.1	144.1	182.1	162.7	201.5	272.2	317.2	378.5
83.4	95.9	99.9	77.3	63.1	95	103.5	90.8	125	181.6	168.1
124.7	99.5	111.8	85.3	105.9	106.5	129	102.8	159	145.1	168.3
166.7	172.2	196.6	131	105.8	143.3	215.3	144.3	224.3	216.2	294.1
205	182.7	200.7	165.9	153.6	184.3	186.9	186.6	227.6	261.1	328.7
55.5	66.7	83.8	63.5	76.8	74.7	68	38.6	71.4	191.6	280.8
328.6	550.3	616.2	623.5	325.3	995	444.3	394.4	311.5	346.4	396.7
303.7	258.4	353.5	321.2	260.4	349.3	235.1	205.2	324.3	411.9	492.9
242.7	230.1	275.7	172.9	169.8	170.9	187.2	142.2	213.4	166	239.9
389.4	264.1	331.2	227.3	245	260.4	232.2	260.7	463.3	540.3	631.9
551.3	352.4	458.3	288.8	334.7	426.1	321.9	279.7	443.5	505.6	735.6
86.6	85	89.9	74.6	66.3	87.3	81.2	61	151.5	149.2	194.9
40.8	50	51.4	32.7	39.7	43.4	93.2	36.1	77.4	85.2	111.3

200.8	159.5	193.9	167.9	160.9	158.7	190.1	184.5	284.1	490.8	392.1
85	80.9	83.2	67.9	61.5	59.9	68.2	68.1	252.9	591.2	512.1
111.4	126.6	106.4	83.2	70.5	94.1	76	101.8	110.9	138.5	166
187.9	187.1	212.1	163.7	126.4	168.8	215.1	180.7	315.2	426.6	416.7
234.2	49.1	124.6	94.8	113.1	93.6	99.6	105.2	349.8	214.9	210
175.9	105.6	139.9	82.4	98.9	103.8	103.3	82.7	271.6	259.5	361.4
313.6	253.2	296.9	264.4	225.2	227.5	232.1	214.6	264.1	318.8	367.7
216	185.9	242.6	162.3	149.6	160.8	167.7	143.4	256.4	296.3	324.7
312.7	132.6	209	159.9	139.6	109.7	121.7	123.4	200	213.4	293.9
501.5	349.2	452.1	326.1	350	317.3	275.1	348.1	386.1	480.2	520.6
308	221.5	293.5	210.1	219.1	246.8	240.5	223.2	302.6	436.5	403
54.6	64.6	49	60.9	81.1	80.7	86.9	59.1	69	53.6	71.6
208.8	234.2	224.4	176.4	144.2	216.6	222.7	240.9	366.2	330.6	433.2
260.9	128.5	233.3	144.3	198.6	163.2	106.5	143.9	234.8	323.3	378.2
216	180.4	205.3	185.6	169	138.1	179.4	158.3	250.9	336.9	370.7
321	272.3	418.7	333.1	166.4	283.4	296.7	216.6	433.8	241.1	296.1
55.8	87	69.3	64	64.2	82.1	94.1	85.8	60.7	291.9	76.1
454.9	657.2	664.9	493.8	417.6	786.5	731	365.1	632.3	736.4	853.4
136.7	156.5	135	118	142.1	120.5	146.9	147.2	195	222.2	225.1
735.2	589.4	637	492.3	473.7	425	543.4	603.2	1131.7	1536	1658.5
89	89.4	82.8	74.9	73.9	74.6	90.6	68.7	154.2	222.6	184.3
430.4	254.9	329.4	232.8	260.2	247.3	238.3	191.7	575.1	548.2	764.4
111.4	111.1	123.8	92.9	96.3	164.5	114.5	112.4	172.9	142.5	192.1
114.3	131.1	144.1	99.2	135.2	137.3	159.7	128.4	153.8	164.6	200.1
207	183.5	165	131.7	177.2	127.6	332.6	201.9	188.6	232.7	311.8
261.1	119	195.5	140.3	208.1	113.2	106.2	154.9	242.2	240	376.3
121.7	101.4	118.6	84.8	83.9	91.4	116.2	74.2	164.6	232	261
152.2	168.8	199.5	150.7	134.3	168.3	146.8	153.2	216.3	196.7	280.9
165.2	131.6	144.8	115.6	106.3	137	150	135.2	261.3	368.6	350.6
159.8	202	191.1	162.2	175.6	159.2	166.5	143	145.1	143.1	169.4
216.1	129.8	182.1	142.1	111.2	140.9	122.5	142.1	359.9	353.5	463
74.4	93.6	76.3	66.3	77.8	80.3	176.3	100.8	66.8	55.1	90
75.8	60.9	63.8	51.8	50.5	54.6	58.7	60.3	106.7	141.4	116

87.6	79.7	99	78.2	95.5	75.2	99.5	102.8	131.2	119	140.5
480.9	376.6	204.6	153.8	339.6	569.6	325.2	150.1	121.3	227.8	226.6
212.9	207.3	243.3	178	170.8	209.5	221.4	175.1	329.6	335.7	412.2
285.2	153.1	158.3	134.6	113.1	93.4	123.6	104.6	250.5	944.9	334.1
413.6	848.4	822.4	428.2	437.5	656	1245.5	361.5	436.3	379.3	578.1
284.6	287.6	273.7	217.1	199.6	271.8	307.4	262.9	385.4	450.5	493.7
193.7	227.4	211.1	164.3	187.1	229.7	262.1	206.2	237.5	237	315.1
134.6	134.6	161.3	116.2	105.4	118.6	128.9	114.1	233.9	247.7	280.9
526.3	486.7	524.9	709.2	401.7	430.4	455.7	687.2	576.6	534.1	493.8
139.4	127.5	156.2	110.6	122.5	117.2	151.8	119.6	186.6	226.9	275.2
347	365.6	360.6	326.9	281.4	339.4	403.3	320.3	521.6	812.1	681.5
479.3	317.7	370.1	234.7	299.9	280.8	260.6	268.6	627.2	594.9	783
165.1	187.7	187.9	168.2	132.6	207.6	206.2	199.6	257.9	272.5	333.1
406.5	333.3	282.8	277.3	207.5	275.8	217.4	340.2	595.1	586.1	612.9
138.4	164.3	142.3	116.5	110	129	247.2	165.3	263.8	296.2	337.7
215.3	153.8	234.6	154.9	145	145.9	144.5	132	191.1	285	312.8
142.4	182.6	191	195.2	134.7	142.6	197.2	158	226.4	420.8	308.8
78.6	62.5	78.3	61.6	35.9	44.3	40.2	44.1	86.1	154	152.7
148.1	143.9	154.1	129.2	112.9	135.7	129.6	108.5	214.6	331.9	376.4
81	91.1	93.8	66.7	81.9	91.1	92.5	78.2	112.6	117.4	129.2
131.8	113.8	149.1	105.9	122.5	104.6	128.5	99.5	211.3	179.3	294
466.9	383.1	387.3	279.6	299.4	324.3	349.7	378.5	849.4	948.8	1152.3
370.4	312.1	364.9	322.6	211.5	315.4	328.3	273.6	429.9	448.1	597
119	178.1	166	115.9	121.7	134.6	185.1	239.1	96.9	73.4	135.5
353.9	271.7	349.8	306.4	274.6	307	202.9	242.6	341.4	522.7	502.1
192	160.7	199.4	155.7	159.2	208.5	173.9	159.3	259.4	308.8	351
29.9	27.9	33.6	32.8	23.4	32.2	27.9	31.8	35.4	42	39.5
406.4	198.4	211.6	167.5	221	275.9	226.8	150.4	1545.9	468.8	636.4
137.1	133.2	151.7	135.7	134	122.7	154.1	120.5	151.9	180.4	219.4
187.1	178.5	185.3	131.4	127.5	162.1	168.4	128.7	186.5	229	269.6
236	274.4	246.2	173.4	142.6	216	258.6	206.8	395.3	408.2	496.4
245.1	200	205.6	152.8	150.4	166.4	193.2	165.1	302.8	372.6	475.7
199.9	179.1	232.2	156.2	153.2	163.3	147.8	234.1	137.4	227.6	221.8

208.6	180.7	209.3	172.9	210.2	204.6	235.6	179.1	221.2	306	296.2
84	80.2	64.5	63.7	65.1	84.8	105.3	136.1	115.2	123.5	160.4
88.6	122.1	93.1	86.2	95.3	78.5	146.5	110	182.9	244.9	206.5
71.2	75	74.7	56.5	73.7	85.8	99.1	59.3	87.6	73.1	103.9
168.7	172.1	174.3	121.5	118.8	144.3	109.1	114.6	358.3	411.4	582.3
74.8	63	61.4	59.6	64.6	66.1	80.8	52.9	106.6	134.4	156.7
130.3	81.6	127.4	77.5	113.9	102.5	104.8	102.6	100.8	111.3	163.9
138.4	132.1	171.6	158.1	126.6	151	181.6	147.5	193.4	307.3	272.2
271.3	304.6	271.1	253.2	272.8	320.3	273.7	313.9	261.4	199.9	233
92.5	124	138.4	141.1	99.7	124.5	569.4	119.7	130.2	154.3	174.9
185	170.5	144.2	125.5	139	147.7	185.8	141	205.9	423.4	421.8
98.8	82	93.6	77.9	72.5	124.2	143.6	129.3	103.6	375.6	208.3
118.7	219.4	127.8	113.7	169.2	148	114.8	150.4	195.4	185	294
236.3	191.3	185.7	160.1	149	160.7	205.7	163.5	285.4	296.6	375.3
35.2	35	45	35.2	40.7	46.9	70.8	49	91.2	164.9	113.5
115.8	96.8	82.9	68	69.3	81.7	93.9	106.5	191.5	194.6	245.4
208	221.9	249.9	210.5	183.4	219.1	215.7	209	242.3	249.6	321
136.4	150	168	161.6	136.1	204.9	193.6	146.9	220.3	384.1	309.6
218.4	232.3	212.2	195.4	1357.5	785.4	386.1	216.3	847.8	208.4	167.1
129.1	130.9	143.1	110.6	115.1	117.3	137.3	111.3	227.5	417.5	245.9
337.6	300.2	303.8	210.2	260.1	288.9	385.2	207.1	226.7	210.3	229.6
89.1	106.4	110	111.2	74.4	128.4	80	80.4	64.9	80.7	96.5
175.2	156.1	208.3	150.1	151.3	165.1	159.5	158.2	310.7	317.7	413.1
342.3	251.5	318.1	277.9	238.8	203.8	187.4	201.4	201.5	195.8	202.5
95.6	72.4	100.5	60	68.9	92.4	86.8	79.9	137.6	182.4	194.7
352.8	437.4	256.4	374.7	189.2	340.5	671.6	766.7	525.7	1398.9	495.7
37.1	45.1	59.8	41.9	35.1	41.5	35.1	47.4	51.4	74.6	63.3
211.6	201.7	216.9	176.3	159.5	215.2	182.3	184.3	268.9	280.4	324.3
208.4	382.4	354.5	265.2	252.2	535.1	328.2	388.5	457.5	369.2	586.8
312.3	291.3	345.4	352.7	250	312.2	346.3	320.9	397	360	537.5
368.8	274.8	323	249.6	269.2	301.8	373.9	295.3	497.6	736.6	712
223.6	176.1	232.4	222.3	176.9	200.7	210.6	230.3	341.7	1524.4	385
390	349.7	392.8	313.8	246.9	309	309.8	311.7	746.1	899.8	916.6

60.3	53.9	71	56.1	57.9	55.7	61.3	78.2	67.6	45	67.1
57	57	50.2	44.2	65.5	52.7	91.4	49.1	73.1	47.5	73.3
365.8	161.6	179.1	328.8	97.1	307.9	110.7	62.6	77.7	95.4	122.1
167.3	181.5	206.7	154	155	191.9	142.3	110	194.4	166.2	245.7
275	197.9	245.6	149.3	198.9	204.9	188.3	171.7	244.6	314.7	435.8
224.5	178.4	233.3	175.8	157	182.6	181.5	156.8	247.2	386.3	435.9
225.8	192.7	172.7	186.8	183.8	252.1	90.3	204.5	189.8	303.8	297
67.5	68	69.4	65.2	51.8	65.6	95.1	70.6	108.9	98.5	96.2
150.3	218.5	143.9	172.4	158.7	270	581.3	212.6	160	249.3	205.9
260.1	197.6	236.4	169.3	159	203.2	190.6	199.4	411.2	484.5	579.2
811.5	717.3	714.5	474.4	417.8	506.1	739.6	625.8	1273	1552.1	1552.6
234.5	182.3	242.3	189.3	172.5	244.1	258	159.7	238.5	421.6	443.5
193.7	144	172.1	131.7	171.7	178.5	195.4	148.3	269	275.9	377.7
85	90.8	92.3	62.1	62	54.2	53.5	50.4	54.7	70.1	97.3
185.6	176.9	202.3	173.6	230.2	346.7	222.3	178.6	206.9	419.4	257.1
75.3	73	77.2	63.9	60.7	62.8	72.3	82.8	118	137.5	161.7
316	228.4	274.2	272.4	235.8	288.8	310.7	256.8	574.8	570.2	710.2
105.4	133	118.6	121.4	119.8	158.3	143.1	91.4	132.4	222.8	225.1
157.7	112.5	138.1	100.4	102.6	103.1	144.9	98.5	158.1	246.8	242.7
110.7	154.6	139.1	125.3	113.1	138.3	173.3	126.6	189.3	218.3	254.4
258.5	207.8	257.3	227.3	218.5	168.1	189.2	135.1	310.9	379.1	429.5
770.9	342.7	462.1	546.3	139.1	183.3	160.2	131.3	125.1	188.9	422.6
270.2	246.1	273.3	231.9	183.8	181.9	228	216.5	489.3	630.7	683
93.8	67.5	81.3	48.1	34.5	60.3	61.1	58.4	190.1	203	236.9
199	165.5	213.6	159	162.7	168.4	139.4	121.8	189.7	264.3	297.8
126.8	102.4	115	91.9	86.1	78.6	93.8	95	243.1	333.4	355.6
382.3	359.7	304.6	266.7	327.7	345.7	427.4	301.3	414.9	268.4	445.9
43.3	33.2	35.8	32.2	44.8	39.3	37.4	37.7	27.9	34.3	25.2
101.5	97.7	85.8	80	82.9	74.2	97.7	96.8	130.9	152.1	142.6
102	109.8	102.4	110.6	82.8	92.1	81.7	105.2	148.3	112.5	141.6
112.2	168.8	137.3	156.5	122	138	140.4	133.9	150.4	192.6	159.6
192.3	216.6	165.3	224.6	171.2	242.8	310.2	246.1	209.1	240.2	258.5
91.6	80	76.1	80.2	73.4	73.1	91.6	72.5	92.8	126.9	135.7

76.9	86.1	68.2	64.7	74.2	56.2	262.1	83.2	323.7	154.3	423.8
248.9	166.7	209.2	164.8	161.7	183.5	164.1	147.9	276.1	339.6	433.5
521.2	508	562.4	498.4	524.5	481.7	510.6	786.5	570.2	381.8	502.4
170.6	190.6	184	129.6	146.5	162	180.4	142	220	236	342.1
644.8	429.2	520.7	414.7	532.5	584.9	462	391.3	774.4	827.3	1072.3
206.5	191.2	234.1	157.2	158.6	214.8	181.9	177.8	327.5	409	437.3
111.1	105.4	133	90.2	92.8	121.8	120	105.5	163.2	140.6	231.4
259	213.7	254	184	156.6	174.5	248.8	192.2	351.9	625.9	560.6
229.7	202.8	245	200.3	198.1	179.7	209.3	194.5	348.8	288.8	469.4
114	121.9	138.3	113.4	107.1	148	157.6	152.1	212.3	231	202.1
259.7	262.9	299.8	320.9	211	304.2	250.3	238	279.9	448.1	430.4
536.2	390.2	470.3	337.2	350.9	382.4	441.8	344.8	743.4	924	970.2
66.7	67.7	64.3	74.7	51.8	88.6	77.9	70.8	66.2	65.5	75.3
123.3	209.1	119.3	109	69.5	97.4	93.6	251.9	80.6	69.1	59.2
182.4	159.6	141.4	110.8	138.5	147.8	148	143	209.1	224.8	248.8
308.4	319.2	371.4	320.5	284.3	396.5	404.5	359.5	356.7	426	552.5
142.8	105.2	147.4	108.7	85.4	117.2	111	110.6	206.9	210.7	244.8
513.5	487.9	376.8	484.2	400.4	331.1	483.8	354.6	322.9	342.3	356.6
121	144.1	138.3	111.1	118.6	151.6	200.8	133.8	149	154.4	174.5
268.2	274.8	266.5	206.6	182.6	260.1	276.3	218.2	401.7	489.3	551
293.1	245.4	296.8	239.6	239.3	273.4	241.6	219.2	445.1	495.9	559.1
84.6	86	79.1	69.2	59.2	72.5	78.7	84.4	110.9	165.8	179.7
345.4	331.6	352	256.4	281.2	266.9	333	207.4	362.1	427.2	480.4
35.9	38	32.4	30.2	37.7	35.7	39.2	37.3	27.2	29.2	34.5
383.5	308.1	325.1	278.5	245.6	262.8	333.7	242.8	383.6	594.8	635.1
110.3	88.3	105.5	97.1	81.9	96.6	114.9	105.2	197	187.9	227.2
295.1	293.9	254.2	216.9	235.4	266.9	296.9	252.6	408.8	468.2	479.9
138.3	69.3	57.8	78.2	117.8	50	118.5	96.5	59.7	75.3	65.7
112	154.3	223.1	153.9	128.1	187.8	131.5	167.8	158.5	165.8	213.8
365.7	424.7	559.9	424.2	324.4	425.4	430	352.6	467.4	668.6	650.3
183.7	172.9	173.4	155	164.7	199.3	214.8	171.5	229.6	242.2	259.9
70.8	49.2	60.3	45.6	50.8	54.9	53.6	39.1	108.2	106	108.6
333.9	307.2	368.4	301.9	336.9	298.8	308.8	354	393.4	409.2	593.8

379.7	321.5	428.5	359.9	284	297	265	301.1	848.8	562.4	1155.3
290	300.8	287.7	226.5	213.2	256.3	347.2	260.3	592.8	701.5	757.9
382.3	334.2	454.2	332.3	315.7	295.6	234.5	354	443.4	767.1	703.9
247	149.1	275.7	445	221.4	546.8	81	68.9	21.2	28.9	50.9
128.9	103.3	127.6	94.2	107	104.3	88.9	96.1	169.5	224	261.9
16.9	20	17.3	17.7	29.9	15.3	23	17	22	17.3	21.7
77.9	57.9	74.8	59.7	64.9	65.3	68.6	64.9	131.4	87.9	118.6
771.8	668.2	831.8	565.2	493.7	624.2	776.5	447.6	1223.2	1560.2	1722.6
836.4	556.5	641	401.8	367.5	403.7	328.5	494.9	895.5	1342.3	1248.9
105.5	118.7	117	122.3	87.4	116	116.2	103.2	127	137.9	211
647.9	368.4	412.8	426.6	269.3	259.6	292.9	283.6	452.5	510	655.6
344.6	273.2	282.7	252.2	224.7	398.6	357.3	287.7	359.4	368.5	413.5
176.4	139.2	128.7	99.6	93	109.8	157	188.7	294.7	362.2	375.5
96.1	76.5	111.5	72.1	75.4	101.3	91.1	71.7	131.2	156.6	181.8
197.5	121.6	154.8	153.4	121.2	158.6	164.1	147.8	276.6	248.4	300.4
82.2	136	114.4	122.4	66	99.1	138.6	181.8	199.2	291.1	294.1
32	27.8	27	29.6	24.6	18	39.8	25.7	37.9	32.6	34
303.2	218.3	234.1	183.2	221.4	218.9	241.9	202.2	556.6	433.7	596.4
143.7	200.3	210.4	189.3	168.6	216.3	252.3	224	158.5	168.7	191
209.1	309.9	244.7	200.4	239.4	237.9	247.2	175.6	298.1	404.6	367.1
198.4	238.8	223.2	162.1	170.4	214.7	250.1	182.4	340	342.3	435.6
688.8	564.2	780.7	1162.6	549.1	716.7	718.8	520.2	870.5	723.2	778.4
515.2	518.3	448.5	468.1	677.5	454.5	437.8	764.1	459.6	289.6	522.6
109.2	98.3	94.1	87.8	88.5	81.9	77.3	103	215.2	250.4	294.6
355.5	277.6	328.9	359.8	359.2	444.5	527	389.2	228.6	270.3	260.9
645.6	547.7	711.3	449.9	437.8	554.4	468.6	590	708.2	752.2	1057.6
109.2	108.1	121.3	97.7	84.4	104.3	123.7	98.2	112.2	203.8	164.7
196.4	224.6	173.5	147.7	144.8	206.4	184.6	149.7	233	300	354.5
317.6	213.9	299.2	186.5	210.6	211.1	201.8	191.3	410	417	539.8
236	164.3	203.6	143.8	161.3	143.2	149.9	165.3	200.7	175.2	290.4
85.2	163	155.6	274.7	139.5	278.4	164.7	204.7	79.3	85.7	102.4
295.6	168.7	314.2	200.8	195.7	141.8	222.1	162.8	212.7	170.1	149.6
271.2	228	246.2	211.3	153.3	194.3	231.5	244.4	342.7	361	407.1

32.9	33.7	36.5	35.8	27.7	52	46.9	40.7	53.5	41.2	47.2
119.8	128.1	127.3	107.9	122.6	111.1	114.5	118.5	142.3	120	159.9
155.5	202.8	158.4	142.7	176.6	326.7	348.9	230.5	174.6	163.5	157.2
235.7	221.7	243.4	194.2	171.8	227.8	204.2	230.9	304.6	326.8	395.7
66.2	53	66.1	74.6	76.4	58.6	69.8	49	72	45.1	74.7
173.5	125.4	183	164.7	146.3	124.7	164.2	148.2	221.5	225.9	240.5
93.3	121.5	139.9	118.9	82.9	109.9	125.6	98	253.3	394.8	482.7
147.8	170.3	146.2	119.7	128.7	150.7	168.1	140.1	225.6	237.3	266.2
78	92.3	65.9	79.5	71.3	214.5	260.9	118.1	42.7	48.7	58
120.8	119.6	78.4	113.3	107.4	115	108.7	102.7	108.6	138.4	199.8
83.1	101.9	150.3	60	79.6	95.7	63.4	83.5	102.4	158.7	79.6
222.1	199.3	199.9	130.5	149.4	163.9	184.7	160.8	274.8	386.5	390
86.5	85.5	67.1	72	90.8	97.2	121	156	103.7	88.3	101.8
195.6	135.6	121.9	100.6	125.1	125	118.2	155.1	289.7	421.9	325.4
383.4	340.5	410	104.7	118.5	162.9	199.5	337.6	825.2	908.3	1111.9
121.6	121.5	134.3	109.6	104.6	104.2	129.5	144.7	135.4	143.1	176.3
202.4	197.8	187.7	183.9	182.1	187.9	246.5	189.6	430.8	470.2	602.6
331.9	300.7	339.3	264.6	242.2	260.9	469.1	266.9	587	809.8	610.9
172	198.1	178.1	167.1	151.2	234.3	214.2	218.9	218.2	244.4	293.6
234	222.4	269.3	176.9	224.7	256	254.1	250.5	359.9	447.2	441.9
277.1	321.1	310.4	323.3	195.8	364.5	387.5	399.8	460.9	493.1	510.2
101	66.8	99	75.3	72.3	68.1	85.2	70.9	126.2	254.4	179.1
199.2	179	229.8	169.7	131.2	181.4	183.2	160.5	373.8	398.6	513.8
111.8	119.3	119.9	89.9	107	113.6	129.7	104.3	180.3	197.4	222.4
155	138.7	167.2	129.4	126.8	131.1	140.2	123.3	153.2	234	220.3
82.2	101.3	94.4	87.2	85.5	114.7	96.9	85.8	110.9	140	121.7
89.1	90.7	101	81.1	79.4	100.3	98.9	120.1	102.5	138.2	109
207.4	219.5	229.2	204.4	162	213.6	252.2	299.5	293.6	303.9	349.4
124	142.5	153.2	100.5	118.2	128.6	150.7	127.2	237.6	167.4	283.6
147.5	122.9	147.6	198.1	90.8	108.5	117.6	120.3	228.3	292	283.9
107.7	101.7	124.6	86.1	91.8	110.4	108.4	91.2	136.5	153.6	189
201.6	231	197.5	220.5	187.8	265.5	260.2	222.3	204.1	266.5	305.8

379.4	572.4	465.3	384.3	355.6	854.6	519	424.1	507.3	458.7	643
148.7	129.3	141.6	128.1	131.9	124.6	171.3	145.5	223.7	274.6	279.1
437.6	384.5	457.4	291.7	349.8	496.5	541.3	336.7	590.6	740.8	788.8
117.8	110.3	83	77.1	120.3	104.4	133.6	89.1	93.6	87.1	117.7
347.7	328.2	360.1	298.8	265.3	341.7	316.2	329.7	478.9	558	560
386.7	244.4	416.2	405.9	233.8	227.7	295.1	198.7	492.4	507.3	725
149	159.1	184.4	145.6	143.2	119.1	199.5	157	336	219.9	298.6
270.2	268.2	228.2	189	175.8	212.4	235.4	187.4	363.5	403.7	518
135.9	95.3	96.2	63.7	88.1	83.3	97.5	98	191.3	149.2	228.2
295.2	214.1	251.2	180.2	217.2	206.7	193.4	176.6	406	493.1	595.9
1163.7	455	669.2	320.6	249.3	140.9	227	232.8	168.6	154.9	214.1
87.6	82.7	78.7	64.7	105.3	89.2	118.8	96.5	157.1	157.8	228.4
146.6	119.3	143.2	114.3	103.6	125.9	139.3	96	159.6	168.2	187.9
64.3	96.2	97.2	69.8	52.9	87	72.5	90.2	196.4	210.2	256.7
174.4	168.3	162.1	176.8	148.5	127.8	208.9	169	216.4	175	277
110.5	128.8	123.1	113.7	85.7	119.6	119.5	209.7	139	135.9	193.7
342.3	338.1	247.7	239.7	196	178.6	206.2	204.2	305.6	354.2	361.6
218.6	242	228.9	233.4	188.8	214.5	223.6	179.5	362.1	347.9	471.5
209.8	217.7	195.9	188.5	169.3	190.8	224.9	243.3	251.5	297.7	339.9
299.8	250.6	245.8	203.9	263	204.5	291.2	196.3	435.9	553.9	665.2
126.7	123.7	118.7	97.6	81.3	121.4	124.9	96.3	280.5	393.8	352.8
124.3	112.8	127.2	83.9	80.7	83.7	90.1	83	161.4	200.6	231.6
195.7	392.7	94.3	241.9	165.1	65.8	205.4	155.2	79.2	92.6	79.2
239	289.6	350.1	277.7	257.5	285.9	249.4	297	267.2	248.4	287.7
47.2	80.2	63.7	61.5	54.3	79.3	91.9	82.8	118.5	139.5	128.5
175.2	176	186.6	136.9	147.6	196.8	243	143.9	174.5	158.3	232
172.6	163.1	180.1	131.2	167.2	153.4	165	128.8	197.7	266.3	299
179.9	145.8	123.6	167.6	140.4	145.4	251.1	220.3	744	281.1	460.8
200.1	80.7	70.8	51.4	65.4	50.7	50.7	53.1	199.9	206.7	236
85.1	64.5	97.5	65.1	72.6	86.6	97.8	70.8	100	88.4	137.7
28.3	23.9	23.5	24.1	22.8	16.8	25.6	18.2	32.7	44.4	40.3
124.3	137.6	107.7	105.8	148.2	136.2	178.7	114.3	126.5	125.4	144.8
298.4	245.7	264.6	163.8	196	191	223.7	278.6	495.4	469.9	546.2

51.3	51.9	63.4	65.6	51.7	59.2	67.9	78.1	80.1	81.6	88.5
303.9	598.7	403.5	373.4	502.6	329.9	600.5	466.9	311.8	140.1	332.1
283.1	207.4	197.8	162.2	221.8	177.6	242.7	213.3	287.1	279.1	257.6
139.4	129.2	121.3	119.8	114.3	246	167	113.7	140	261.2	187.8
47.1	44.7	37.6	36.1	46.2	38.8	59.2	57.2	70.1	54.6	55.5
113.9	105.1	136.6	114.8	129.4	153.4	151.6	127.5	268.8	280.4	325.4
58.6	64.2	58.2	49.7	46.3	51.5	47.3	53.4	68.5	68.3	89.6
29.4	31	50.8	33.1	27.9	37.3	26.7	24.9	48.1	29.3	48.8
168.4	163.5	193.8	167.8	160.4	154.8	129.3	163	274.6	242.3	361.9
207.5	224.4	177.7	160.6	257.2	190.8	234.3	159.5	187.4	271.4	250.9
134	107	118.5	96.9	121.6	123.1	134.9	143.1	245.2	608.8	352.7
298.1	364.5	406.8	297.3	241.2	472.4	389.4	302.5	423.3	460.6	689.7
225.2	284.6	284	272.4	178.7	252.6	226.1	312.4	368.6	372.5	432.8
191.6	172.5	158	159.4	126.6	127.8	151.1	140.5	229.7	336.4	307.4
11.4	10.9	12.7	10	9.5	7.9	11.5	12.1	22.1	27.6	31.9
120.7	161.7	137.9	109.6	112.8	165.7	137.3	146.4	205	275.4	240.9
194	279.6	253.3	235.8	279	177.4	274.6	292.5	255.4	184.2	242.8
131.6	133.4	125.5	114.3	103	128.9	162.5	133.3	139.4	157.3	174.8
295.7	194.1	304.8	269.3	200	213.9	164.4	161.5	212.8	316	268
57.7	132.2	83.4	85.4	67.7	73.8	74.8	126.2	99.5	81.3	78.4
68	50.7	60.1	51.7	42.5	56.2	55.3	51.9	100.5	102	142.1
191.9	384.3	571.3	441.3	247.3	365.7	236.9	220.8	98.6	114.5	176.6
199.6	242.8	316.6	223.6	164.1	234	257.5	203.1	278.7	409.5	383
240.4	224	292.7	222.8	201.4	267.3	246	218.9	349.8	395.7	425.4
133.3	136.9	144.1	124.4	120.7	134.2	107.3	117.4	207.9	224.8	249.6
234.8	415.9	437.1	437.3	293.8	452.9	347.7	322	262	262.3	339.5
75	56.4	62.7	53.6	44.7	56.1	61.4	53.1	123.6	152.3	169.3
173.4	117.6	158.3	119.2	136.7	135.4	171.8	135	197.4	273.5	295.1
458.4	153.3	238.2	131.1	152.1	115.7	139.2	129.9	338.4	604.4	616.5
199.5	165.5	195.4	218.8	120.6	226.9	123.2	143.5	140	54.3	103.8
13.6	11.8	12.6	8.5	12	12.7	12.2	6.9	9.7	13.2	17.7
74.1	73.7	82.5	58.9	66.7	94.2	99.3	82.3	102.1	78.2	116.6
104.3	95.6	113	94.4	85.2	87	111.6	83.6	148.8	313.5	257.3

161.5	152.3	181.1	183.6	106.2	128.6	125.9	140.4	220.3	281	359.6
119.5	110.5	93.2	72.9	111.1	98.2	135.3	96.9	282.2	167.4	249.7
17.8	98.4	48.1	46.1	100.2	173.1	9.9	131.3	27.3	15.8	20.1
211.4	189.9	198.6	180	167.1	185.2	151	149.4	233.9	202.9	286.4
93.3	82.9	108.5	89.2	77.1	77.1	78.1	74	100.4	87.5	133.1
193.3	179.8	287	184.4	242.6	183.8	212.1	159.2	191.8	205.5	253.9
215.7	258.5	211.1	256.6	173.1	196.1	281.1	244.3	317.8	268.9	302.6
136.6	148.5	143	135.1	96.1	118	146.1	125.4	237.1	274.7	213.7
83.4	69.8	122.7	72.2	140.3	113.7	103	106.1	119	159.5	155.7
88.4	104.7	111.8	88.6	75.1	163.5	219.7	69.9	105.7	114.2	130.6
154.6	110.6	139.9	110.3	113.8	110.7	114.4	116	163.5	183.2	215.4
85.9	223.8	110.5	199	122.6	321.2	242.1	176.6	115.7	93.5	109.7
120.6	103.3	94.9	134.3	69.5	152.3	110.8	105.5	129.1	154.8	166.2
186.2	144	142.8	123.3	120.1	124.3	151.2	166.4	248.2	609.3	281.4
117.4	101.2	120	96.7	91.2	81.6	78.4	99.7	131.9	110.9	174.4
185.9	201.2	235	216.7	151.7	221.4	212.4	346.3	237.6	256.7	321
180.8	132.5	117.2	83.1	133.8	158	139.2	137.3	390.6	268.3	400.5
61.9	60	64.1	70.4	66.3	65.4	70.8	63.4	94.8	94.1	111.6
78.8	84.7	97.3	83.6	110	93.5	166.4	87.4	88.1	147.8	94.5
189	210.7	223.2	150.1	149	176.8	135.1	183.5	208.5	288.6	310.3
230.8	353.3	251	271.5	241.5	207.2	151.8	470.4	129.7	113.8	216.2
149.9	115.1	129	90.9	78.7	81.2	93	127.1	410.3	500.3	829.9
23.6	24.8	17.9	13	17.2	25.1	31.5	47.8	17.6	48.8	30.7
155	179.5	165.5	151.5	135.9	144.4	191.2	167.8	336.6	424.4	453.8
499	648.7	512.7	850.3	452.6	1380.3	603.3	1343.5	354.2	272.5	435.6
307.9	263.7	295	225.3	318.1	365.8	235	226.3	332.6	412.7	434.5
104.1	82.3	110.9	100.2	87.8	85.1	141.1	75.5	277.1	650.6	620
217.1	205.1	236.1	225.6	214.4	281.2	201.2	187	217.7	259.6	351.7
253.1	233.2	254.6	203.2	173.6	204.3	200.6	162.5	287.7	350.1	447.5
84.2	93.8	102.8	98.6	74.5	95.7	127.1	125.8	190.9	237	217.5
130.3	170.5	161.1	198.6	161.2	166.2	179.9	135.9	162.8	194.4	178.4
80.2	55.5	71.3	40.9	33.6	51.2	43.3	41.7	175.5	198	224.8

314	251.2	292.8	258.2	350.3	334.2	378.8	314.1	494.5	371.5	519.7
264.2	244.5	296.3	176.7	237.7	206.4	268.3	262.2	405.2	557.5	503.9
46.4	39.5	45.5	32.8	34.5	38.1	50.2	36.3	48	43.4	60
115.3	114.3	114.8	109.6	102.2	132.2	118.7	111.6	138.9	219.1	161.4
196.6	159.8	219.8	173.6	142.2	161.1	160	131.9	226.2	255.8	344.2
117.7	121.8	152.7	118.7	112	152.6	138.8	123.1	121.6	198.7	177
242.2	148.8	127.3	159.3	91.5	75.4	88.4	215.2	240.3	122.3	128.2
33.9	34.9	40.7	33.9	32.3	40.2	32.8	29.9	35.2	47	38.2
212.7	162.7	176.9	125.6	136.4	146.1	162.3	150.8	356.9	348	460
202.6	185	221.6	175.2	161.6	212.9	259.7	162.1	364.2	741.1	586.7
52.2	48.1	49	54.2	45.4	49.7	50.6	37.4	50	70	70
181.6	184	194.1	149.2	131.1	194.5	178.1	165.1	247.6	324.5	376.2
121.9	103	101.1	152.6	84.9	134.6	371.8	106.5	109.2	251.2	124.6
30	34.5	28.2	31	28.3	32.6	41.1	23.8	31.9	47.6	44.2
156.5	161.5	148.4	151.7	108.5	130.1	120.2	116.3	170.8	130.3	208.4
138.4	212.6	216.5	182.8	157.7	275.4	182.9	259.5	223	265.9	273.4
341.5	298.8	303.4	243.8	242.9	220.8	268.3	296.6	450.3	468.3	568.5
53.9	42.7	34.2	32.4	37	43.4	55.7	37.1	36	49.6	55.1
42.2	28.6	39.4	37.3	33.7	29.9	34.6	24.1	51.9	98.7	79
130.8	101.4	156.5	121.9	109.7	128	135.7	146.3	222.6	194.3	199.9
123.5	120.6	119.1	93.6	106.1	92.2	133.1	109	181.6	236.2	231.8
238.3	247.7	263.7	187.1	198	315.1	297.1	250	318.5	368.4	359.4
314.1	354.6	488.7	617	480.7	377.5	319.6	512.4	979.9	554.6	661.7
185.1	136.2	142.9	103.6	108.1	142.1	122.5	131.3	261	332.9	366.3
80.7	72.8	82.2	67	69.6	76.3	81.7	90.3	119.7	134.7	137.7
170.7	148.2	206.1	132.2	141.4	133.9	165.1	151.2	247.2	340.5	347.4
118.9	109.3	104.9	84.9	59.7	83	87.1	87.1	135.6	171.5	193.8
213.6	137.5	220.2	151.1	123.7	138.8	139	131.6	219.5	234.1	279.7
51.4	43.3	48.9	38.6	38.5	57.2	52.2	48.6	48.1	65.2	74.5
248.7	207	231.1	211.2	170.5	229.3	221.7	204.3	365.3	501.6	546.1
24.3	27.8	29.4	27.3	21.5	28.3	27.7	24.1	72.1	52.9	85.7
394.4	332.1	307.6	298.1	222.4	379.6	391.2	322.1	365.8	465.6	592.6
171.6	148.5	151.4	158.6	185.4	152.4	179.9	115.7	231.1	222.8	246.8

148.4	146.8	144.2	172.3	134.8	145.4	190.1	188	195.4	288.7	256.9
152.3	127.4	154.2	122.9	105.9	116.1	139.5	129.4	162	209.5	258.4
247.6	240.6	252.7	207.4	164.6	199.9	250.1	234.9	319.7	441.6	385.4
210.5	192.4	225.8	217.9	171.9	189.7	201.2	356.7	150.5	139.6	202.7
41.1	31.1	70.3	47.9	38.8	22.3	34.5	24.8	38.3	53.8	44.4
144.8	119.7	151.4	143.2	130.3	144.5	144.2	131.9	185.3	190.5	240.5
58.5	50.8	46.4	33.1	39.1	50.8	77.7	45.9	46.3	58.7	75.1
106.5	171.3	93.9	92	55.6	101.2	121.4	196.2	201	202	145
228.3	86.9	102.1	115	92.1	102.6	162.8	94.8	125.7	229.1	207.2
134.3	52	90.4	64.9	74.1	62.1	68.9	63.3	181.4	187.4	138.5
20.5	13.7	16.5	14.2	16.3	17.5	18.7	20.8	15.7	16.1	18.9
141.7	130.7	126.8	130.4	137	129.1	140	95.8	113	116.8	120.5
24.2	21.5	32.2	26	24.1	37.8	32.5	27.2	27.2	34.1	46
319.1	239	283.7	245.5	231	241.1	271.4	240.8	347.3	384.5	523
120.4	108.3	108.7	117.1	97.7	109.8	147.2	229.9	259.5	166.7	255
166.3	139.5	177.5	161.4	134.6	202.6	198.1	166.6	198.4	222.5	260.5
103.2	147.1	160.6	117.6	120.7	219.4	167.8	139.7	188.7	172.3	265.2
74.9	68.5	79.8	55.2	56.9	64.3	57.7	60.1	97.8	78.3	131.6
47.7	75	68.3	61.1	57.9	88.5	126.6	70	78.2	88.8	84.3
85.7	94.5	92.4	67.2	80.3	98.9	120.9	93.1	118	145.5	114.8
282.1	218.6	235.9	169.5	176.1	187.9	266.8	197.8	431.7	413.2	503.6
159.7	185.3	141.2	120.6	134.9	160.8	198.2	140.7	171.2	423.6	239.3
95.7	80.6	98	74.9	89.8	90.8	75.3	56.6	90.8	107.1	135.2
581.5	568.6	538.3	482.2	531.7	517.4	575.5	479	696.9	1056.3	952.9
397	303.6	241.5	260.3	184	223.5	241.4	276.5	266.5	307.7	281.4
179.4	195.4	133.5	143.4	190.1	149.2	239.1	194.2	235	247.8	207.6
19.9	23.4	31.2	26.4	21.2	27.1	34.2	25.4	23.5	25	34.2
109.7	92	104.1	73	59.2	86.1	85.2	79.8	167.9	176.9	193.7
326.1	217.1	237.5	163.7	165.2	157.3	179.5	194.8	300.6	237.2	374
151.5	103.7	124	83.2	91.9	139.9	105.7	105.8	221.1	221	292
174.1	174.6	184.6	185.8	150.6	225.4	208.7	158.9	206.5	210.4	256.7
530	599.7	509.9	336.9	380.1	766.6	839.1	399.2	389.3	427.8	547.2
272.2	229.4	310.6	203.6	184.7	246.3	222.3	176.4	218.5	259.5	310.5

45.8	25.2	28.4	22.4	27.7	24	29.8	27.4	59.7	67.9	90.8
119.2	124.6	140.2	81	79.4	116.8	117.7	123.6	226.7	267.2	276.6
124.4	141	138.3	110.5	140.4	129.2	207.7	132.8	120.6	141.6	182.1
90.7	83.9	86.7	70.8	62	69	75.2	92.3	134	137.5	170.5
101.6	99.9	118.4	87.9	86.1	96.1	99.6	83.8	143.9	183.5	198.7
134.8	109.6	119.4	117	137	161.4	156.3	112.4	154	221.9	211.2
123.3	101.8	97.9	88.1	76.4	94.8	110	107.9	179.8	190.8	229.7
815.9	679.4	582.3	546.3	451.9	659.9	719.5	531.5	839.9	1141.5	1413.7
43.9	52.2	50	31.7	49.1	45.7	46.8	43.4	55.4	59.6	63.6
290	227.1	266.9	164.3	159.6	224.2	203.7	228.6	432.9	341.3	506.7
259.5	230.1	251.6	168.1	214.8	213.3	234	159.2	302.2	392.8	389.7
239.6	139.1	203.9	127.2	147.6	131.9	146.9	115	247.8	359.3	423.4
309.6	281.4	314	198.6	211.7	226.2	239.2	226	390.1	629.4	491
52	88.6	79.2	41.1	63.4	52.3	52.7	44.7	123	300.4	244.3
72.1	76.7	69.8	65.6	59.4	85.7	85.8	80.3	83.7	90	89.6
89.1	95.2	92	65.5	90.2	132.4	150.4	90.6	105.7	118.8	125.3
229	174.5	210.2	163.4	191.3	211	160.2	172.1	268.3	345.4	420.3
55.7	67	56	65.9	59.3	42.8	103.7	53.8	180.7	406.9	158.3
40.6	61.7	53.2	36.9	49.2	51.4	54.4	57.9	99.9	106.2	116.2
86.1	42	84.7	96.8	195	45.8	53.3	54.5	147.3	162.7	177.3
96.1	107	113.1	90.9	88.8	110.2	94.3	86.5	126.6	94	104.6
44.3	26.9	42.9	32.3	39.4	30.7	32.4	27	40.6	43.4	60.8
103.2	94.1	83.9	73.4	77.4	78.9	103.6	78.2	100.9	108.2	209.1
95.4	90.5	92.4	65.9	78.6	100.6	99.5	90.1	115.8	115	144.3
162.7	227.1	240.8	203.2	152	209.7	216.3	178.1	316.4	289.6	419
93.4	67.6	92.7	70.1	86.6	77.7	95.3	144.6	79.5	84.1	93.1
121.6	127.9	124.5	111.6	105	121.5	112.6	111.3	189.5	287	382.4
406.2	270.5	228.1	188.8	203.1	440	305	202	281.6	346.7	352.1
120.6	127	138.3	126.1	98.6	127.4	205.5	180.3	226.9	562.3	353.1
155.1	169.9	161.8	140.4	129.3	164.1	182.7	159.4	225.7	242.8	283.9
372.8	242.8	280.5	222.5	243.6	256	269.8	234.1	682.7	806.5	878.4
214.7	108.5	147	124	150.1	131.5	131.9	102	108.8	251.9	192.4
89.8	87.1	117.5	95.2	95.8	84.7	119.6	75.7	128.6	164.1	152.1

141.5	176.8	149.3	183.9	116	190.4	264.9	225.9	199.1	154.6	170.4
47.5	28.3	35.7	32.1	35.6	35.7	45.8	44.4	82.5	57.7	74
301.6	308.2	262.9	250.2	236.6	271.5	327.1	295.6	468.9	568.2	557.2
59.1	57	64.5	72.2	51.2	52.5	62.5	48.7	57.8	142.7	75.2
190	277.1	146.1	258.2	109.3	152	198.2	223.3	270	153.8	227.1
79.8	88.5	74.3	76	106.7	77.9	96.4	75.2	72.6	100.3	102.7
211.2	144.1	165.7	119.1	151.7	104.2	182.7	150.8	282.8	506.9	479.1
216.2	219.5	242.5	230.6	228	210.2	200.3	215.4	188.5	144.1	188.2
96	31.2	66.9	47.3	32.4	32.3	19.6	27.9	62.7	72.7	83.5
122.6	130.3	86.5	81.3	151.1	91.2	164.8	90.1	99.8	165.2	151.4
470.9	301	454.9	320	372.5	371.4	359.8	339.3	641.1	513.1	985.9
58.8	62.4	81.3	52.4	71.1	61.7	91.3	64.1	76.8	89.8	96.4
89.7	74.7	60.9	49.6	48.7	102.6	74.1	72.8	103	84.5	118
97.7	90.8	98.4	75	167.4	82.7	113.3	66.4	75.2	95.9	97.9
43.4	48.4	48	39.1	31.1	53.4	50.7	56.8	212.8	136.9	510
99.3	95.4	95.6	101.4	79.6	89.2	130.9	140.1	198.1	191.4	248
125.7	94.9	142.5	80.1	100.4	170.8	182.8	111.4	144.5	161	195.6
170.9	122.8	138	91.8	83.3	128.2	108.1	117.7	289.4	238.5	311.3
188	172.4	168.3	145.5	142.2	185.1	169.7	147.4	263.4	265	333.7
197	185.8	170.8	134.8	157	194	193.8	161.1	236.1	245.3	335.4
254.7	276.6	282.2	229	226.2	322.5	374.1	272.4	413.2	404.7	522.6
192.2	194.3	166.8	121.2	293	148.5	301.7	224.2	186.5	307.7	246.1
288.6	274.9	366.3	248.1	237.3	262.4	255	248.1	305.1	336.6	396.8
22.7	29.1	20.5	16.9	14.6	22	29.3	23.8	34.2	33.7	32.5
569.1	376.9	264.4	276.4	413.1	374.3	245.6	150.7	73.1	105.4	100.1
84.5	71.2	66.9	46.2	50.9	69.8	73.4	55.1	92.3	96.8	107.5
239.1	199.4	184	137.1	166.8	142.4	139.4	229.2	258.1	287.5	318
375.1	125.8	152.7	120.4	154.5	144.2	140.4	120	288.7	449.9	498.1
132.3	114.7	136.4	93.9	102.5	90.6	118.6	101.2	203.3	239	289.3
170.5	164.1	167.5	144.1	137.6	140.5	170.3	135.1	290.5	405.6	395
245.2	247.9	248.6	188.8	222.7	315.9	266.2	216.8	239.6	239.3	276.7
10.8	14.8	21.7	9.2	65.7	515.6		36.8	13.6	13.2	16
338.1	329.2	408.7	289.1	260.9	308.4	319.5	331.6	530.4	710	852.9

146.5	150.2	136.3	199.1	122	145.5	139.5	257.4	285.7	225.2	270.9
227.4	250.1	274.5	172.8	254.3	225	245.8	216.1	388.4	296.4	313.8
152.5	81.4	137.1	190.5	139.7	131.9	233.4	274.7	437	299	293.7
165.7	153.1	162.3	165.8	132.4	190.8	177.8	192	272.8	302	305.8
70.6	63.9	66.7	48.8	52.8	72.4	62.4	48.5	106.6	124.1	158.1
92.9	93.4	130.8	107.6	69.9	105.7	208.6	91.1	123.9	104.2	156.6
191.3	191.9	219.6	188.3	208.7	200.7	223.1	221	214.3	218.4	264
192.4	154.7	190	116.1	132.2	155.2	181.4	161	321.9	376.3	351.2
367.5	335	640.1	271	453.7	273	208	603.6	433.1	850.5	714.5
96.3	180.9	525.5	321.6	172.5	443.5	91.8	294.8	13.2	21.1	63.4
47.6	37.6	52.5	50.5	37.1	37.1	41.2	41.5	49	56.3	66.4
88.6	183.4	131.6	134.9	123.6	253.6	192.4	251.4	123.5	122.3	137.9
189.2	185.5	220	156.9	125.1	160.2	237.2	171.6	243.6	370.4	370.3
84.9	102.6	93.7	67.4	85.5	171.2	348.2	106.4	54.2	73.7	74.5
418.6	385.6	395.8	274.6	249	290.3	339.8	354.5	820	839.1	985
220.7	189.3	219	181	134	132.5	169.1	166.8	298.6	259.2	322.2
304.1	251.3	315.7	239.8	203.7	211.4	278.7	212.6	306.2	548.7	552
316	399	374.9	368.3	311.4	343.4	392.5	476.7	566.3	302	575.5
62.7	50.3	65.3	48.5	35.3	62.7	57.1	49	82.4	95.3	136.8
269.5	159.1	199.6	139.7	167	202.9	183.4	145.8	334.6	413.8	496.9
32.5	30.5	28.1	24.9	20.2	35.5	36.3	31.3	30	24.8	36.8
65.6	53.6	65.6	50.3	47.5	51.8	61.3	52.5	77.1	79.3	108.6
164.1	133.8	160.9	120.1	99.4	121.3	142.3	142.4	279.8	277.6	355.1
115.2	113	91.4	108.9	77.3	132.2	109.7	147.3	192	144.7	176.1
127.7	121.3	90.6	111.4	96.8	116.3	126.8	102.8	172.4	247.7	239.8
184.4	214.7	273.7	213.7	231	249.6	258.4	224.8	259.8	467	365
99.2	88.2	93.4	74.2	77.5	85.9	95.8	79.7	130.8	145.7	155.9
48.1	40.2	44.9	42.4	29.5	49.6	38.7	47.3	110.8	50.6	83.5
150.2	128.1	120.7	115.6	101.3	129	143.7	143.9	185.2	223.6	213.4
75.7	70.5	101.2	60.3	61.8	85	68.2	68.5	179.9	189.1	416.4
140.8	148.5	124.8	113.1	120.5	117.2	139.9	111.7	134.7	159	164
165.6	121.2	167.9	119.8	126	130.3	103	98.7	289.5	314.9	410

228.9	260.9	268.5	236.9	270	281.5	296.8	277.5	222.2	263.6	252.6
92.5	219.2	371.2	131.9	135.7	214.8	333.5	110.9	184.9	277.1	253.3
122.8	108.3	107.2	79.8	110.3	180.9	273.9	85.3	78.2	126.6	98.2
146.5	179.7	211	148	116.8	239.5	787.3	193.9	189	115.3	174.2
94.4	92.4	144.3	75.7	69.6	80.2	76.9	56	95.9	177.6	188.1
142.8	117.3	160.3	102.7	112	106.5	109.2	103.7	172.8	193.5	286.9
44.3	55.7	52.2	60.9	49.8	45.9	52.1	46.8	63.7	66	97.3
100	117.7	120.8	66.2	52	80.4	80.8	108.2	158	121.4	176.2
89.4	82.5	97.4	84.3	79.5	91.1	98	80.7	148.3	163.7	177.6
151.4	127.1	126.6	112.3	122.7	130.8	152.7	131.4	219.6	266.2	286.3
242.4	280.9	267.6	217	186.6	265.5	233.3	201.6	254	298.4	340.1
179.4	160.4	173.3	146.9	132.4	172.9	149.9	166.7	256.7	278.5	351.7
317.1	237.5	327.2	212.4	202.7	283.8	217.2	256.5	459.9	318	537.4
97.5	88.4	126.4	110.2	78.1	110.8	105.4	78.3	145.1	189.5	186.2
174.2	154.1	182.6	179.1	143.4	266.8	165.7	102.6	405.3	387.2	447.1
133.9	165.3	133.2	97	139	205.1	328.3	152.8	253.9	217	560
225.5	164.4	212.3	181.4	179.7	202.7	255.6	146.3	253.1	249	327.5
140.2	150.2	133.7	117.3	112.6	148.4	135	112	263.2	208.7	301.5
48	72.8	50.3	40.9	46.9	47.7	49.9	42.7	61.2	424.1	105.1
140.3	140.6	218.4	183.7	122.3	143.8	300.7	148	172.5	279.9	278.3
214.9	105.8	272.3	200.8	135.1	113.2	83.2	93.5	195.1	138.9	296.3
299.1	295.6	400.1	256.6	260.7	278.4	293.3	305.3	395.5	481	530.7
69.7	57.4	67.2	67.5	54.3	60.5	58.3	50.4	86.5	121.3	141
136.3	123.7	118.1	98.4	80.5	125.7	154.5	86.1	171.5	193.2	267.3
191.9	216.6	165.4	178	148.2	214.1	494.5	135.6	293.8	151.8	280.3
113.6	99	117.9	116.6	83.5	83.4	96.1	78.4	151.6	196.7	236.3
245.7	213.7	238.4	220.1	169.2	241.6	247.9	191.8	417.4	287.6	349.2
450.2	297	371.1	287.9	254.9	219.6	204.3	237.3	445.4	677.2	727.4
456.8	274.3	331.4	225.4	241.8	247.5	289.4	217.5	546.4	836.4	793.4
230.9	204.3	250.4	194.6	170.9	180.7	196.7	211.5	310.2	392.6	412.5
185.4	130.8	142.6	114.7	93.1	120.1	137.5	130	214.7	279.9	297.1
133.4	166	129.7	106.1	91.8	108.7	136.5	132.2	186.2	384.1	237

211.1	150.5	182.9	145	125.7	131	190.7	193.4	220.5	252.4	301.8
147.9	127.5	141.6	120.2	117.1	103.7	116.9	119.5	226.4	253	407.8
414.5	296.1	335.5	262.4	239.3	266.4	252.7	368.9	422.3	423.7	465.9
172.1	116.7	89.5	101.2	113.3	97	116.2	98.5	161.8	202.5	247.5
411.9	184.9	304.6	204.7	229.3	184.2	177.1	161.8	393.9	583	681
107.2	89.1	116.8	111	62.3	76.1	76.1	74.9	79.2	60.6	90.1
18.5	16.6	21.8	21	18.9	18.8	27.5	20.6	36.9	53.7	48.3
233.5	229.7	236.4	217.1	198.8	235.3	231.7	221	302.7	337.4	353
68.4	57.7	94.9	59.1	50.2	43.7	61.1	42.8	85.6	95.6	116.7
259	225.3	232.9	185	164.8	190	224	207.7	389.9	326.4	440.5
558.1	526.4	509.9	351.9	576.7	537.8	792	505.7	552.3	827.8	756.2
270.5	245.4	284.8	222	208.6	246.7	265.4	229.4	440.4	467.3	477.4
156.5	143	155.4	130.1	136.5	155.8	175.6	143.9	220.8	215.5	264.2
83	77.4	85.5	62.9	89.6	68	86.2	68.4	79.1	124.8	98
97.5	92.1	121.6	98.3	99.5	116.1	115	121.2	208.5	193.9	238.5
177.8	168.3	191.1	188.1	133.5	217.3	167.5	157.7	350	365.4	455.3
100.3	236.4	230.7	347.3	121.8	236.9	111.1	96.4	97.5	88.4	133.2
247.9	309.7	518.4	284.9	620.9	336.7	243.6	352.8	385.5	304.4	494.1
79.4	71.1	82.7	52.3	75.9	67.8	55.5	69.3	85.2	122.5	154.2
471.2	480.1	480.7	475.9	448	519.4	514.5	382.1	572.8	1551	1032.5
89.4	81.1	73.5	72.2	49.7	75	68.1	62.1	137.7	153.2	172.1
64.9	73.6	72	60.8	57	67.2	78.5	55	130.5	203.3	161.6
61.2	63.1	75.4	54.9	45.3	83.8	105.8	62.4	73.3	75.1	102.1
53.5	54.1	40.1	47.4	52.6	34.6	54.9	49.2	101.9	69.4	158
35.7	51.1	51.1	25.9	33.7	64.2	196.3	28.3	19.5	19.5	32.3
77.1	51.7	61.4	69.6	55.1	54.5	55.9	57.3	69.5	52.7	62.6
215.4	148	337	181.5	215.1	147.8	163.1	136.3	259.7	457.2	447
73.8	64.4	71	45.1	58.4	80.4	64.6	55.8	70.7	90.3	102.8
138	115.5	143	100.4	123.6	126.4	150.9	128.6	165.5	126.3	208.2
108.3	87.5	94.9	80.6	81.6	85.8	100.4	86.4	181.5	208.1	278.6
33.5	41.9	34.5	30.2	35.6	36.1	35.4	36.7	36.2	30.9	54
50.7	55.4	44.5	40.3	121.5	57.9	71	54.8	40.7	52.1	41.8

140.7	189.2	128.9	150.9	124.9	709	71.9	180.2	107.3	85.9	140
99.3	101.8	115.6	88.5	84.2	137.2	149.7	101.7	125.4	129.8	180.4
96.3	115.1	85.6	90.6	52	66.1	78	183.2	194.1	127.3	120.9
28.2	27.7	30.9	20.9	21.1	21.1	29.6	22	31.3	80.5	56.4
150.4	98.5	130.4	83	84.5	94.8	97.6	109.7	205.5	323.8	283.4
85.2	132.6	90.1	74.7	70.8	79.2	77.9	63.7	135.5	176.3	229.4
76	78.8	68.9	48.6	64.1	58.2	119.6	79.1	66.5	70	83.5
150.1	181.2	191.6	142.7	147	229.6	182.5	190.1	187.1	272.6	298.5
102.2	129	121.1	107.7	70.2	144.6	144.1	139.9	182.8	187.5	210.5
66.2	58.2	59.6	43.5	45.2	41.5	51.8	31	89.3	81.9	111.9
94.5	83.5	92.9	67.3	57.1	67.1	92	74.5	133.3	138.6	168.2
130.2	81.6	101.3	83.9	85.9	93.1	73.9	98	140.4	160.6	183
59.6	67.2	77	43.2	58.1	70.6	88.8	74.8	97.6	126.6	147.9
110.9	119.4	122.3	100.3	83.2	131.4	134.4	103.7	167.5	197.4	235.8
98.5	96.8	101.8	74.7	68.9	88.9	100	100.2	177	97.7	140.8
133.2	136.9	156.6	98.8	91.7	117.8	129.8	115.7	209.5	225.1	300.1
308.9	234.7	294.6	207.7	190.3	225.5	242.8	207.3	541.8	583	585.8
37	47.7	46	41.1	23.4	48.1	33	47.7	100.4	81.3	49.3
43.9	43	51.2	33	39.2	45.8	53.3	42.8	57	59.6	76
173.9	122.6	161.5	115.6	94.1	122.3	105	129	224.5	190.2	277.8
183.8	173.6	205.8	160.8	146.7	162.5	172.8	153.3	297.9	342.6	376.5
102.9	186.2	136.7	148.1	131.6	126.6	140.9	163.5	121.3	181.4	162.9
81.6	101	89.9	79.6	65.4	93.6	94.3	87.8	124.6	141.5	146.4
354.3	288.2	302.5	343.8	285.2	274.4	604.9	417	622.5	883.1	770.9
208.4	156.5	310.2	364.9	214.3	249.8	530.3	249.3	203.1	252	542.6
93.5	88.2	90	71.1	74.7	126.6	84.9	99.8	150.4	113.4	140.8
79.8	54.8	46.3	51.9	46.9	43.5	50	43.6	85.4	369.3	63
231.3	237.8	269.7	233.9	200.7	253.7	344	233.9	256.3	245.4	310.6
112.5	98.5	75.7	77.5	60.6	96.9	125.6	108.5	185.1	274.4	224.3
31.2	39	40.1	34.5	25	28.7	26.1	30.4	32.2	32.9	36.4
64.3	61.3	73.3	53.4	94.7	79.5	58	124.9	139.7	97.3	92.2
49.9	46.2	53.8	38.6	42.5	41.8	45.5	40.2	85	124.2	131.8
85.5	83.1	92.1	82.1	66.7	84.5	65.7	103	116.6	138.1	156.7

26.8	23.9	41.9	48	28.2	26.6	28.7	23.9	36.1	124.8	38
24.4	31	29.8	22.7	20.4	26.7	23.2	25.6	29	30.8	40.8
117.3	119.6	110.3	103.2	103.8	139.8	129.6	131.5	165.3	190.2	219.4
29.4	23.7	26.9	20.9	16.7	25.1	40.5	23.4	27.2	36.1	41.2
167.6	185.5	91.1	100.8	127.8	61.8	287.9	262.5	151.1	152.9	190.7
133.6	147.3	163.8	130.3	128.1	140.6	245	137.4	152.6	145.6	188.2
231.3	235.3	245.9	207.2	187.5	189.7	214.3	196.6	246.9	308.6	371.4
165.2	143.4	112.3	103.1	101.9	105.2	207.4	119.3	166.9	126.4	195.9
203.6	190.1	208.6	217.7	93.9	164.1	134.8	148.5	167.7	160.4	216
28.3	34.2	35.1	32.1	26.5	27.6	31.2	31.2	40	32.5	45.1
338.7	280.7	351.8	247.4	247	293	273.9	300.2	327.6	500.4	527.3
94.3	85.1	88.6	75.6	71.9	64.7	85.1	72.2	350	316.7	561.2
104.7	99.2	95.4	113.7	65	98.4	89.7	103.8	135.6	147.3	221.8
53.6	64.8	67.6	58.5	54.8	100.8	92.6	57.2	82.7	70.8	105
192.9	151.6	201.9	159.2	138.5	174.3	158.3	164.4	207.7	235.1	286.7
171.8	157.8	158	127.5	128.5	131.1	144.8	146.4	204.9	197.2	319.1
178	178.4	193.6	161.4	163.7	210.8	278.6	208.1	294.3	276.4	307.3
277.7	231.6	103.4	105.4	132.9	166.7	236.4	135.3	131.9	93.1	138
78.8	87.2	89	84.2	49.1	83.2	84.6	71.1	80.6	109.8	113.9
92.6	94	134.5	104.9	111.9	92.8	103.9	81.3	87.1	108	100.1
53.9	49.7	67.5	57.8	47.1	60.6	67	59.5	80.1	88.4	126.3
40.7	61.4	58.3	77.2	36	49.7	57.7	33.4	52.8	110.6	92.6
116.9	110.7	92.5	86	87.5	108.4	187.8	100.6	129.7	221.1	176.3
275.1	145.8	192.2	153.2	187	150.4	169.7	121.9	293	411.9	446.5
121.7	115.1	163.7	133.9	80.9	118.7	127.8	148.8	163.7	269.5	287.4
44.5	25.8	31.7	22.3	21.1	32	26.9	29.6	65.3	98.4	96.3
226.7	294.9	342.5	395.6	215.5	279.9	306	367	241.8	124.2	217.2
246.1	677.2	533.7	821.2	399.5	1118.3	220.3	121	28.9	38	102.6
154.4	123.7	137.6	109.4	85.1	124	160	94.5	174.6	222.6	224
27.6	25.3	24.9	20.4	25.7	22	31.1	31.6	35	36.7	43.2
62.2	48.3	54.4	35	83.5	44.9	65.5	50.5	57.8	66.8	76
404.3	464.2	510.3	620.3	372.7	968.1	552.6	415.6	384	485.3	515
398.5	245.9	317.4	203.1	218.8	220.9	220	182.2	294.3	392.2	466.5

104.7	101.1	116.7	89.9	92.6	117.8	138.2	90.2	205.1	232.3	246.2
70.2	47.6	53.7	40.3	66.5	58.3	55.1	38.7	50.6	52.1	88.3
126.6	125.6	96.6	99.5	92.4	116.4	114.4	134.8	159.4	155.2	154
38.1	25.5	25	31.5	34.5	31.5	38.5	21.9	31.9	36.1	39.2
167.6	157.1	171.2	129.2	134.1	164.1	194.9	160.9	226.1	250.1	257.2
238.1	225	319.1	197	251.6	344.2	289.3	182.8	404.5	467.4	620.3
56.5	45.2	38.9	38.4	56.3	47.6	65.5	51.5	39.9	48.7	53
91	65.3	67.2	64.7	35	41.4	41.7	36.8	56	78.8	76.7
121.2	111.7	102.8	76.6	158.6	97	154.4	102.3	121.2	133	125.6
178	102.1	189.9	195.8	160.4	173.3	216.2	155.7	280	147.1	215.6
112	120.4	119.9	110.5	85.1	131.6	133.4	119.3	114.4	111.1	120.5
106.4	76.1	90.8	84.9	64.5	70.5	85.7	81.8	217.4	198.7	256.4
27.3	35.9	33.2	28.1	19.2	27.5	30.8	31	37.1	56.7	48.2
118.5	115.9	122.6	115.2	111.8	111.6	132.5	101.2	139.7	149.3	154.6
223.5	169.9	213	142.6	138.1	161.6	159.3	177.8	357.8	484	454.6
37	40.9	44.8	35.8	28.3	45.4	45.7	29.9	46.7	44.1	67
139.2	175	180.8	133.7	125.6	145.8	193.1	149.5	185	282.9	264.9
84.5	77.9	84.4	57.2	58.3	83.8	113.5	72.2	76.3	81.7	107.6
3955.9	4161.8	2134.5	2219.4	6299.3	3235.7	5983.2	2981.8	1868.8	2545.2	2006.1
30.7	32.1	30	26	31	28.5	40.4	23	68.6	43.7	67.6
151.5	138.1	144.1	139.5	121.6	155.7	136.7	136.4	256.4	264.6	319.6
69.2	58	71.4	69.2	59.3	78.6	71.4	69	55.9	79.7	92.6
38.1	36.6	36.8	26.5	30.7	32.2	38.5	26	58.1	72	66.1
17.5	19.1	13.8	23.3	21	34.4	24	21.8	19.3	26.1	25.9
48.6	94.3	75.7	101.1	97.3	169.5	65.5	130.1	89.9	126.1	138.9
27.1	41.5	41.3	26.9	33.3	35.5	57.8	42.6	71.1	37.8	59.3
49.9	41.8	43.9	47.6	40.5	50.9	47.2	41	56.8	55.2	73.5
19.8	11.7	16.2	13.1	14	21.5	23.2	17.1	19.3	26.8	22.4
131.3	92.4	117	82	95.4	91	124.4	112	206	230.3	300.5
222.4	205.2	153.1	133.7	256.6	172	238.3	139.5	224.1	291.7	295
32.5	31.2	29.7	31.6	31.5	35	40.3	52.4	31.7	32.4	27
79.7	50.4	56	39.3	42.5	39.8	39.6	35.2	93.8	143.6	134.9

357.9	191.2	260.3	268.5	188.2	194.5	196.5	267.9	424.3	433.3	550.5
55.1	38.7	56.2	38.7	39.8	35.7	27.7	47.5	68.5	109.5	111.9
78.8	66.2	57.9	58.5	60.1	77.2	72.4	66.4	92.5	149.7	137.1
99.9	94.6	106.8	105.4	87.2	96	128	122	176.8	147.9	196.8
518.6	419.6	520	370.5	426.1	406.6	412.8	385.1	647.8	883.8	984.4
140.8	211.9	135.8	128.3	128.7	116.1	143.7	127.9	132.2	132.6	149.7
12.3	15.2	6.6	12.8	9.1	16.9	12.9	15.6	16	17.1	21
198.8	159.3	179.6	158	145	169	158.9	148.2	204.8	194.9	263.7
221.1	347	248.7	206.5	221	394.1	453.3	251.3	233.8	214	407.2
61.6	89.2	161.5	55	49.8	76.1	78.1	76.5	131.1	66.9	98.2
246.7	272.9	275.9	304.6	224.3	346.2	300.7	338.4	318.4	505.5	359.4
442.4	231	347	221.1	227.7	140.8	241.7	246.1	423.3	538.8	596.3
675.7	189	417	299.5	282.4	68.6	73.8	101.1	81	112.9	155.6
142.1	105.6	99.1	96.5	71.6	105.3	128.7	123.6	134.4	139	185.5
50.3	48.5	61.2	47.4	49.5	58.3	78.2	50.4	79.2	62.6	85.4
189.8	130	178.6	134.5	158.6	284.6	185.5	90.2	148.8	187.1	174.4
39.1	49.7	59.9	43.3	52	41.9	56.2	46.4	75.3	85.2	87.3
96.8	63.4	87.5	57.3	76.9	69.9	51	67.7	69.8	172	116.6
71.4	49.9	61.7	46	39.4	43	46.4	43.1	69.7	102.6	112.1
122.6	100.6	137.2	126.2	94.4	117.5	139.2	121.4	186.8	239.6	230
95.6	69.2	92.3	86.3	79.3	83.5	73.2	82.3	136.9	102.9	159.5
85.2	63	69.2	66.4	35.6	45	48.8	33.5	82.5	170.4	142.8
35.7	45.8	44	49.2	38.8	42.1	60.4	57.6	79.8	112.6	97.3
149.6	154.8	165.1	126.4	164	163.2	183	118.8	113.6	216	128
114	95	117	102.4	138.4	115.6	112.6	89.7	110	105.7	161.8
141.1	66.5	65.1	80.7	105.9	68.9	142.8	76.9	73.2	134.5	102.2
51	58.9	59.3	44.6	53.2	67.7	65	45.6	64.4	78.4	89
195.3	150.1	172.1	146.5	139.6	141.1	177.7	160.9	309.3	401.9	409
136.5	101.9	113	103.8	112	115.4	168.7	100.9	90.5	119.4	139.4
217.5	168.2	194.3	137	112	143.5	597.7	116.7	68	120.9	202.7
11.8	11.5	19.7	13.6	11.4	20.7	19.7	17	29.1	41.1	43.3
48.4	67	67.8	69.6	64.9	57.5	52	70	112.1	112.3	173.3
104.6	53.4	65.9	44.8	75	58.5	74.9	49.7	59.1	58.6	80.9

96.8	134.4	103.6	86.8	72.5	121.8	103.6	139.8	200.9	227.2	252.4
48.9	52.1	27	43.5	43.8	54.7	44.7	56.6	48.5	46.1	69.7
151.9	125.5	157.3	137.4	121.5	147	163.1	154.2	218.2	193.9	261.5
96.3	105.5	104.7	87.9	70.4	88.9	109.7	85.9	170.8	217.8	189
83.7	58.3	82.6	56.5	66.5	70.8	72.7	62.5	92.1	77.8	104
40.5	39.8	48.4	35.2	37.5	49.8	46.3	36.7	55	58.9	73.8
240.6	185.2	259.1	162.4	185.1	151.5	212.1	155.1	248.8	426.9	261.1
88.7	45.2	45	55.6	68.3	31.3	31.5	18.4	29.7	30.7	46.3
234.8	217.4	203.2	139.8	145.2	137.3	140.3	164.4	198.1	197.8	252.3
171.7	162.3	151.7	119.1	125.2	151.6	149	128.1	167.1	146.8	192.9
85.8	105.9	107.8	80.3	64.7	82	80.6	95.2	97	108.4	122.4
32.4	35.3	27.3	22.5	33.6	25.4	33.5	30.5	21.5	20	36
57.1	75.9	58.8	54.7	48	83.8	54.4	72.4	66.9	72.4	100.3
75.9	80.3	83.6	68.1	71.2	250.8	107.5	83.7	129.6	117.8	173
56.1	63.9	46.7	44.8	45.1	72.3	137.4	32.9	58.4	50.5	95.1
83.9	53.4	91.9	81.7	83.5	68.6	75.4	80.7	73.4	97.5	93.9
94.6	99.8	106.2	107.7	85	123.5	99.5	83.7	101.2	100.1	144.2
341.5	266.6	358.9	267.3	262.7	313.8	324.7	293	406.5	387.1	444.2
157.2	118.9	104.1	106.6	145.2	114	175.7	102.5	165	219.8	216.8
147	146.8	158.6	119.9	99.9	146.3	115.8	121.3	291.8	276.9	382.3
158.3	143	192.7	129.1	150.4	131.7	179.1	106.5	356.9	487.4	446
46.4	47.5	43.4	32.6	39.6	32.8	45.3	31.3	51.8	54	70.1
141.3	106.5	120.3	97.5	83.3	100	83.3	97.5	158.5	193	235.5
46.8	44.2	44.1	35.4	33.8	34.3	43.5	48.2	67.6	66.9	89.4
169.7	117.2	143.6	147.3	129.2	137.9	148.7	131.1	227.2	175.8	166.4
72.8	70.4	64	52.5	46.6	51	80.1	65.6	123.7	113.9	154.8
285.2	255.4	261.7	238.5	189.7	201.1	271.5	240.6	278.5	304	377.3
148.5	158.7	242.3	268.1	159.9	174.3	158.1	169.3	234.3	164.5	304.5
276.6	278.8	321.7	316.3	225.7	377.8	327.8	281.8	269	350.6	351.8
82.2	88.9	93.2	82.2	58.3	97.5	87.7	70.6	114.9	141.8	167.6
57.6	69.6	91.1	56.3	80.9	47.5	56.8	61.9	47.1	58.4	60.7
53.8	56.6	61.2	39.3	40.1	45.9	50.8	44.8	85.9	118.1	104.6
55.8	67.7	44.5	34.9	35.7	50.6	57.6	53.3	117.4	113.7	141.6

125.1	124.8	108.1	105.5	97.1	122.6	150	131.8	148.2	138.8	183.4
111.8	90.8	105.5	82.9	94.8	77.7	91	94.7	211.6	171.5	131.4
199.3	197.2	204.8	145.7	142.1	165.6	183.9	208.6	220.4	181	249.8
57.3	66.6	67	55.7	51.3	50	86.1	60.2	119.4	101.6	121

98.5	102.2	123.3	102.7	90.3	109.6	140.6	116	141.9	199.8	210.9
135.4	99.1	130	119.9	83.1	103.5	102.9	87.3	118.9	177.6	160.2
163.2	55.3	85.4	64.5	66.1	45.2	58	74.3	205.2	170.7	239.6
286.3	236.5	273.1	199.4	192.9	204.1	197.6	179.3	428.2	560.1	578.1
175.5	166.8	199.4	137.4	156.2	143.5	139.7	175.1	253.9	303	311
125.7	119.6	131.6	95.1	119.1	124	147	122.8	385.2	144.2	325.3
46.9	48.8	23.3	44.4	40.1	121.7	30.6	48.6	42.8	25.7	45.3
241.3	213.6	247.8	174.9	195.4	204.1	240.1	241.5	352.5	458.4	461.9
125.9	113	147.3	106	94.6	111.8	93.9	103.5	227.9	212	271.6
44.4	47.5	50.8	43.5	37.4	42.5	54.5	47.2	85.5	79.9	79.5
303.6	331.9	294.8	381	329.4	504.3	220.8	287.5	291.5	345.3	441.4
158.1	141.7	179.2	95.5	82.8	92.1	103.5	124.9	192.3	147.3	243
128	208.9	142.1	140	78.4	204.9	61.4	189.4	75.2	67.1	94
55.8	70.2	46.5	58.7	34.8	41.3	55.3	70.8	103.1	93.1	90.7
22.7	14.2	15.5	14.1	16.2	15.7	19.8	14.7	18	24.8	23.6
144.1	144.6	162	133	163.3	137.5	135.7	130.7	156.4	197.4	193.5
98.3	62.2	89.5	58.4	48.5	50.9	55.8	57.7	85.5	92.9	95.4
56.8	41.4	30.9	29.7	44.6	51.3	50.2	47.4	74.3	70.1	101.9
40.3	49.2	41.1	42.5	36.4	64.4	60.4	31.7	66.6	64.1	85.3
88.5	86.6	89.6	83.3	79.4	89.5	97.2	80.7	130.5	117.1	156.9
260.4	113.9	222.5	167.8	255.2	99	66.3	72.5	120.2	110.7	197.8
61.1	78.9	60.9	50.7	55.6	83.2	115.4	51.1	61.9	472.9	97
35.3	33	34.9	24.3	22.8	30.6	43.7	32.9	58.4	44.1	64.1
106.8	77.4	88.4	59	81.4	81.4	72.8	72.1	137.1	145.5	213.5
154.6	150.1	119.5	94.8	145.4	110.6	194.9	129.5	211.4	258.7	326.5
35.3	32.3	29.5	28.1	25	34.3	39.1	26.4	59.3	49.5	68.8
36.9	36.9	35.2	27.5	29.6	28.6	140.7	31.8	51.3	51.1	78

115.5	96.5	103.2	84	104.5	79.9	116.2	78	130.4	170.7	178.3
340.3	192.8	444.2	282.2	281.8	199.8	265.1	169.4	437.4	466.7	391.2
70.2	56.6	68.9	46.2	80.1	70.2	70.3	66.1	65.4	69	78.9
226	201.3	243.3	148.3	157.2	228.1	199.5	140.8	235.3	248.7	297.9
106.1	142.7	110	98.9	77.3	130.4	160.3	121.6	164.8	172.1	169.1
30.4	33.8	20.7	23	25.9	41.5	70.1	26.2	26.5	27.3	35.5
151.5	112.3	124.4	99.2	103.4	123.1	125.8	117.6	267.8	228.2	312.3
57.5	46.9	61.5	48.1	54.4	47.7	71.6	51.9	209.8	189.5	150.4
112.8	90.7	107	90.4	73.5	66.2	98.9	51.5	101.3	117.3	146.1
82.9	68.9	74	49.1	59.8	77.5	51.4	50.2	71.4	74.7	89.2
122.7	126.1	119.4	95.5	85.8	108.1	125	104	161	193	190.7
199.7	203.3	316.1	224.7	181.4	201.2	141.3	161.9	232.2	206.8	232.2
30.4	26.1	33.8	36.1	28.3	39.7	37.5	46.4	68	40.7	55.3
415.7	387.2	354.4	329.9	254.6	270.6	254.3	358.2	454.8	292.3	348.7
935.8	666	475.7	328.8	395	233.2	229.2	339.6	590.7	500.4	786.5
52.1	48.8	54.1	46.1	50.3	74.2	104.8	58.6	64.5	48.6	65.1
64.9	56.1	51.4	41.9	41.8	36.2	46.6	36.6	35.3	30.9	44.9
91	102.1	116.5	104.4	75.3	118.1	91.7	95.7	110.5	120.1	132.9
30.3	34.9	27.3	23	38.2	19.7	43.7	29.2	49.4	35.2	42.4
64.7	50.9	47.7	40.4	40.9	57.5	66.6	60.8	73	46.2	85.7
27.8	27.6	22.4	16.5	27.3	19.5	37.7	29.4	26.6	23.8	29.9
40.8	58.5	43	44.2	51.2	56.4	54.3	48.7	69.1	272	78.8
55.4	55.2	39.6	51.6	55.7	60.7	63.5	52.6	50	42.8	72.1
104.6	140.5	109.9	137.2	115.4	165.1	123.5	133.8	276.8	110.2	168.1
213.1	165.1	183.4	201.1	162.4	211.8	164.4	160.5	148	199	191.2
80.3	64.1	67	58.3	55.5	62.9	52.3	65.2	94.3	114.9	97.8
158	132.9	156.1	121.8	116.6	104.9	117.9	127.9	160.1	305	176
128.2	168	101	189.3	135.6	110.1	173.7	145.8	89.1	108.8	128.6
97.2	79.3	93.9	76.4	72.2	88.5	88.9	80.4	99.7	108.9	145.4
871.8	1311.6	1002.7	1393.5	690.5	1544.7	931.8	1382.2	888.3	1005.9	1046.9
61	73.3	70.4	57.6	53.1	57.4	81.2	64.9	59.9	52.1	62.1
22.5	19	17.3	17	20.8	24.8	23.4	20.7	30.6	24.8	35.6

209.6	103	148.3	114.8	128.2	116.8	141.1	103.6	282.5	241	347.6
92.7	129.7	107	87.5	87.6	268.6	422.5	111	110.2	189.4	358.6
156.3	149.3	189.5	130	116.9	154.5	186.9	137	256.9	269.5	314
177.2	178.8	182.7	133	138.4	140.1	246.6	151.8	328.6	769.2	482.9
55.1	57.1	51	44.6	39.7	53.8	53.8	54.4	77.2	81.5	114.6
179.6	136	131.8	106.3	109.6	121.7	123.2	127.1	192.7	218.9	239.2
221.7	62.4	169.9	72.8	165.4	129.3	80.8	53.3	172.6	266.7	285.9
125.4	91.9	47.4	40.5	97.8	156.6	209.9	105.9	263.2	128.7	366.4
56.9	60.4	48.9	41.3	78.6	69.1	87.7	56.4	64.6	62.2	68.5
216.2	187	188.2	194.9	173.9	220.4	265.9	186.7	173.6	663.3	180.2
146	295.4	373.1	106.1	223.4	527.6	175.9	213.6	45	54.3	67.7
114.8	88.1	60.9	68.1	125.5	80.1	114.3	77.3	129.2	77.3	152.4
323.2	435.8	600.9	412.7	287.3	379.9	224	436.7	332.6	1466.9	576.7
43.2	52.1	34.6	36.8	50.2	46.6	55.2	41.1	54.2	60.6	77.7
95.7	177.8	90.4	101.7	113.1	47.3	102.7	100.3	77.2	34.9	72.3
634.9	239.8	355.8	269.1	192.1	148.6	162.8	198.8	182.1	232.7	234.7
65.9	117.6	136.2	57.1	55.3	56.7	80.2	91.4	105.3	293.3	100.9
192.1	186.4	319.3	270.2	192.9	236.1	202.6	177.6	240.5	284.5	341.7
267.9	218.1	237.2	210.4	225.1	207.7	225.4	194.7	307.3	351.1	340.9
233.2	199.9	185.2	194.4	148.7	168	206.5	219.2	427	377.3	482.9
198.9	183.9	217.6	151.8	127.9	157.8	154.1	147.7	398.8	548.5	563.8
51.6	40	54.4	38	40.4	42.7	43.9	43.3	64.4	81.1	97.4
16.5	15.6	14.2	10.3	11.2	13.3	15.8	15.3	21.9	24.9	25.1
77.3	74	89.2	60.3	60.3	90.9	78.1	62.4	96.3	242	143
111.3	119.5	152.3	154.4	101.6	109.7	141.9	133.6	172.6	123.7	176
59.4	66.6	68.1	57.4	57.9	79.3	70.9	59.4	87.1	88.6	104.1
86.2	48.6	58.3	47.1	51.1	53.9	78.3	58.6	91.9	85.7	120.7
53.2	56.4	64.9	39	64.1	70.3	58	55.6	80.6	83.5	87.3
26.6	29.2	35.2	22.3	22.2	26.1	33.2	32.9	49.8	47.9	56.7
45.7	35.9	35.6	41.4	33.1	39.3	39.9	30.8	31.7	53.5	64.1
71.6	63.5	71.5	65.8	67.1	73.7	66.4	53.9	77.2	101	107
109.8	116.1	130.9	122.9	73.4	133	223.7	113	116.9	122	135.5
246.8	184.1	181	157.8	211.7	174.5	226.4	192.6	194.6	285.1	282

51.6	36	59.7	49.8	56.9	38.6	40.8	97.6	26.1	28.1	41.2
114.1	122.3	131.9	124	102	134.7	124	206.3	90.2	69.2	80.2
549.9	264.4	301.9	221.1	267.1	256.9	225.5	295.4	705.4	887.7	881.9
87.6	82.7	83.7	72.4	101.1	100.9	87	90.6	108.9	109.7	134.2
95.3	86.5	97.6	83.4	79.1	86.9	67	92.4	132.8	126.9	154.1
13.4	17.8	9.2	12.5	11.6	21.3	16.1	21.3	16.9	19.1	20.3
149.9	149.8	124.7	89	104.4	108.9	150.7	107.1	165.8	153.6	184
115	95	178.1	118.3	109.5	119.6	144.1	125.9	132.5	93.7	172.7
83.3	57.2	80.3	49.8	43.1	57.7	63.6	69.4	165.1	141.6	193.1
110	108.4	129.4	92.2	72.4	89.6	121.8	82.6	215.7	493.3	242.1
84.7	110.1	116.4	143.5	83	168.3	91.9	106.3	122.1	118.7	138.9
223.1	212.9	304.6	235.8	518.4	196.7	221.7	433	265.7	183.2	254.7
188.8	122.1	139	103.1	126.6	135	131.9	125.3	247.9	248.6	316.9
295.6	242.3	269.9	244.6	208.1	238.7	239.7	228.4	382.9	528.2	517
83.8	67.8	101.8	69.5	105.7	135.1	138.7	65.2	125.2	207.2	171.3
48.7	61	58.9	46.3	42.2	65.1	56.7	56.9	71.2	89.4	92.6
468.4	257.9	334.8	246.3	257.5	165.8	258.8	230.4	248.9	460.6	420.7
97.5	88.9	130	95.8	78	100.9	79.2	56.2	151.6	195	240.6
20.7	16.2	27.8	20.5	29.1	22.5	18.6	28.6	33.7	31.5	32.8
69.1	94.1	82	80.5	92.8	90.8	105.2	81	73.7	113.9	103.6
266.5	213.4	257	192.1	223.1	200.3	188.6	168.7	262.1	290.5	331.5
96.9	82.1	101.5	49.9	67.5	67.6	79.7	76.8	147.8	199.1	218.7
154.1	127.4	148.9	108.4	114.4	124.6	139.5	106.2	153.9	320.2	226.7
61	44.8	51.9	51	43.7	69.4	48.3	48.4	53.5	53.7	68.4
171.6	177.9	147.3	130.2	134.8	148.6	207.3	155.4	189.4	236.6	236.3
74.9	76.3	72.6	71.3	77.9	97	92.2	71.7	97.5	71.7	94.2
113.1	93.8	116.9	98.4	80.9	98.1	112.3	112.5	203.4	223.4	272.8
101.7	175.6	99	70.2	109	153.9	157.6	83.2	54.9	72.9	93.9
106.9	58.6	29.6	81.5	60.2	88.9	98.5	52.5	198.2	273.4	151.8
40.4	36.7	30.1	30.3	35.2	36.4	37.5	32.1	38.1	34.3	51.1
265.2	279.9	283.9	296.3	228	320	338.1	254.7	323.2	435.3	475.2
49.4	43.5	67.7	66.3	49.6	54.9	66.8	57.2	81.3	167.7	74.2

159.6	125.8	139.7	106.2	103	105.8	130.6	106	236.3	268.8	293.6
127.2	108.1	119.7	80.1	111.7	98.7	100.9	123.2	230.4	308.9	332.2
51	55.5	41.8	52.4	45.5	52.1	53.3	46.9	45.8	60.6	50
146.8	116.1	119.7	91.6	66.1	103.2	175.6	318.2	116.2	123.7	168.5
275.1	401.4	411.7	365.2	240.6	310.4	320.6	366.6	418.3	444.7	537.8
41	54.8	56.3	60.7	58.8	63.4	52.8	45.1	53.2	65.3	60.4
154.4	88.6	102.5	83.6	74.3	68.8	72.2	103.5	223.9	156.1	254.4
30.6	70.1	62	54.3	42.7	80.9	32.5	57.2	56.3	28.1	25.3
119.3	90.4	99.8	74.2	127.4	114.6	125.5	87.2	92.1	118.1	144.8
148.9	151.8	146.4	125.3	147.2	146.6	189.3	156.9	237.9	250.3	405.6
26.2	32.4	37.2	25.1	29.9	31.4	34.7	26.7	40.2	34	51.5
180.5	181.1	211.4	158.3	152.5	152.2	195.7	134.2	387.7	613.5	538.3
24.1	15.8	23	15.9	13.7	16.9	12.9	21.5	49.6	55.5	54.8
152.1	160.9	146.4	129.4	121	132	167.3	157.5	385.1	285.7	414
105	74.7	131.8	101	94.1	109.6	153.9	124.7	87.8	125.1	108.4
92.6	157.2	129.2	197.3	112.5	276.6	187.1	277.7	94.6	94.4	127.4
175.3	192.7	171.2	146.8	242.5	190.1	236	171.5	161	221.4	236.8
60	50	53.1	49.4	49	56.1	60.3	56.4	88.9	110.6	121.8
69.1	74	74.2	54.3	85.5	80.1	91.1	78.4	56	71.7	76.4
60.1	81.2	99.7	84.1	69.8	92.6	93.1	64.7	123.1	184.8	154.1
62.3	56.9	53.8	58.9	87.1	71.1	87.5	61.3	48.6	66.3	63.9
87.4	64.4	48.2	61	48.2	53.6	82.3	97.2	99.2	120.4	136.8
137.2	140.6	166.8	115.9	111.4	111.4	114.5	120.4	158.2	178.7	218.3
196.1	185.4	183.9	197.1	124.9	165	227.2	234.3	337	286.6	341.4
111.3	61.6	57.9	68.7	147.7	87.3	87.7	74.4	125.1	138.3	187.6
186.8	752.8	592.4	494.9	414.9	800.2	389.6	540.1	349	225	496.2
107.1	92.4	87.2	69.5	147.1	79.9	146.4	92.4	101.3	108.8	110.9
103	100.1	118.3	97.7	76.1	94.8	101.9	109.8	138	166.6	184.7
116.8	100.8	106.9	102.1	84.9	94.4	89.1	112.7	115.2	120.1	176.2
89.7	86.6	93.7	74.1	73.4	91	83.9	70.6	132.4	180.3	207.4
114.9	117.5	110.4	132.4	80.2	89.9	96.1	97.6	130.3	136.2	179.6

310.6	345.6	312.8	262.7	264.4	341.5	352.4	291.1	558.4	444	663.6
29.8	50.1	73.5	179.8	208.7	403.2	57	118.3	19.1	16.8	24.1
213.5	134.4	148.9	128.2	156.8	169	162.5	148.4	277.7	276.9	310.6
61.4	48.9	57.5	62	31.7	39.1	58.5	49.8	97.2	490.9	111.1
115.7	144.1	116.1	143	107.9	130.6	147	140.6	153.5	531.6	213.1
181.5	224.6	160.1	146.9	131.7	156.2	255.9	203.3	359.2	322	391.8
107.8	119.5	109.5	89.9	88.1	104.8	90.7	77.3	130.9	153	165.8
48.3	38.3	50.4	34	42.9	49.9	57	35.1	44.7	56.5	68.7
222.9	259.4	202.2	179	187.7	278.9	418.4	225.6	144.1	178	240.1
73.3	83.9	79.5	64.5	71.4	79.9	101.7	64	81.8	131	126.9
31.3	45.3	42.9	38.3	39.4	54	61.1	38.8	53.7	156.2	70.2
131.3	83.6	101.8	85.2	83.6	85.3	66.4	79.7	278.3	309.1	411.1
129.1	176.8	172.9	164.4	137.4	192	410.1	190.1	178.9	200.9	225.4
26.4	22	25.4	22.1	28.8	30.3	27.1	23.2	16.6	22.7	30.5
56.1	67.6	83.3	51.1	58.1	63.6	78.7	49.2	71.2	151.3	148.6
26.3	63.3	58.8	56.4	34.7	82.2	39.3	37.6	49.7	82.6	78.5
84.3	178.9	76.5	172.3	105.2	317.3	116.7	98.6	65.4	81.7	75
153.7	146.1	117.2	111	122.8	135.5	198.6	107.3	158.9	276.9	178.3
48.1	53.6	43.8	33.8	37.7	38.1	49	34.1	66.6	83.8	77.6
107.1	85.6	93	63.6	60.6	80.4	123	96.6	143.5	175.8	249.2
70.9	83.7	74.6	75.6	62.6	66.9	79.8	74.4	71.5	75.7	81.9
65.9	40.7	51.5	37.4	44.4	41.1	26.6	32.1	38.8	37.4	54.9
46.7	34.4	41.8	36.3	32.9	36	44.6	33.9	68.9	142.4	95.3
261.1	40.9	199.4	88.4	39	48.2	253.8	353	320.8	127.9	249.3
111	122.1	63.9	85.9	70.8	179.6	290.1	134.7	16.5	24.5	31.8
26	21.5	20.3	21.1	35.7	28.1	34.6	26.4	21.9	36.1	29
84.2	55.5	76	56.1	50.1	46.8	80.1	61.6	75.7	98.5	134.9
42.8	35.3	55	40.4	48.1	43.3	48.7	39	56.1	65.4	79.4
248.8	163.9	214.1	150.6	147.7	133.3	153.8	135.4	351.5	475.7	504.5
50.2	44.8	63.5	40.4	47.3	60.6	54.5	46.5	53.7	183.2	56.2
259.1	199.3	209.5	167.5	128.8	172.1	209.7	215.2	258.7	295.6	359.5
417.2	427.1	551.4	362.6	462.2	501	725.8	741.4	491.4	655.1	693.3
133.5	134.4	140.1	120.1	127.2	136.9	141.5	110.2	119.7	151.5	168.4

78.6	93.6	94.6	76	76.6	108.6	98.7	89	101.4	113.2	145
73.6	58.6	77.2	98.2	74.2	62.8	110.5	56.2	166.7	424.4	331
105	81.4	106.4	77.9	74.6	94.3	103.3	103.2	93.7	90.2	135.6
107.5	120.5	93.5	79.9	111.3	168.9	240.5	127.8	115	136.7	152.4
56	40.9	59.2	55.6	43.7	50.8	58.1	39	78.5	100.4	124.5
36.1	24.8	33.8	29	21.2	24.8	29.7	18.4	25.5	38.6	52
41.9	31.8	34.7	27.5	27.8	34.5	29	22.2	40.3	51.4	67
85.4	74.9	78.5	57.7	55.7	56.6	50.1	79.5	121.6	125.3	143
233.4	217.9	238.3	156.5	238.6	176	236.3	197.1	249.2	280.3	283.1
94.5	118.8	92.1	100.5	93.7	147.7	179.3	106.6	138.3	150.3	179.9
157.8	176.8	198.2	166.7	132.2	188.7	228.5	232.3	262.9	338.3	360.6
49.1	66.9	93	51.6	60.3	61.6	59.9	61.2	65.9	73.6	78
156.1	161	177.4	145.1	126.2	163.7	192.7	166.6	301.2	315.8	381.2
93.2	80.9	105.2	101	75.3	86	82.6	101.4	191.7	182.2	195.9
43.4	35.2	29.5	26.9	36.4	37.1	42	59.3	68.6	39.4	52.1
41.8	68.9	60.1	40.3	29.6	47.7	105.8	53.4	273.7	69.6	191.5
77.7	63.6	65	61.8	53.3	68.3	66.1	69.7	87.9	71.2	114.5
24.6	33.7	34	24	22.3	31.8	29.5	29.7	37.8	32.8	51.1
209.4	187.1	239.6	204.5	166.9	205.4	213.6	169.3	192	468.2	344.2
27.5	49.2	28.2	28.9	38.2	34.8	36.8	43.4	186.3	50.1	130.5
47.5	47.2	28	30.8	52.5	33.8	61.8	45.4	34.1	93.3	57.8
33.4	48.6	44.9	47.4	36	48.6	43.2	35.1	49.2	51.1	68.2
75.4	74.7	72	66.6	59.1	76.5	80.4	73	137	164.6	170.2
80.6	106.6	97.7	91.5	60.8	104.9	136.6	89	100.8	100	117.2
264	256.4	274.7	245.1	185.8	172.5	319	263	328	432.1	468.3
196.6	178	193.9	158.8	142.5	158.9	156.3	153.5	276.2	274.3	419.5
113.7	79.8	106.4	88	97.7	98.8	111.4	105.8	118.8	112.1	128.8
34.1	28.1	23.4	17.5	23.1	21.6	25	17.6	30.2	45.5	63.8
565.8	415.5	639.2	727.9	341.3	268	500.1	154.3	649.2	721	691.5
38.7	35.7	43.6	32.3	39.1	32.4	44.7	36.3	43.7	40.4	58.2
84.4	82.9	91.7	69.3	53.6	82.8	153	96.1	101.5	129.6	137.2

41.5	51.7	54.6	38.5	33	58.5	55.4	57.5	58.3	68.6	95.6
146.1	131.6	127.7	112	109.1	153.9	111.3	101.2	158.5	182.1	217.1
112.4	114.5	144.1	95	141.7	122.1	246.1	96.3	218.2	189.5	217.9
120.2	105.7	82.6	25.3	115	65.3	134.4	137.6	132.1	36.5	73.9
49.3	57.7	64.7	64	49.8	59.4	59.7	47.5	53.6	65	78.5
89	92.7	83.7	73.6	78	80.5	92.5	91.9	263.5	181.5	264.9
53	43.5	40.4	39.4	25	33.8	30.7	32.3	88.7	86.3	94.1
56.4	71.6	66	79.7	68.6	76	74.3	67.9	59.4	63.6	78
61.4	56.1	57.3	42.9	53.5	48.5	47.4	49	72	85.7	101.3
150.3	210.2	194.5	157.8	127.6	226.3	248.6	151.4	193	277.8	269.7
30.2	34.8	32.9	24	19.2	31.5	32.5	43.1	55	37.5	66
39.2	33.7	36.9	33.7	38.7	34.6	51.9	27.6	35.1	37.6	39.8
45	30.2	30.2	30.5	36	32.1	40.6	36.5	39.4	58.1	66.1
26.3	29.1	29.6	18.5	27.9	26.4	30.4	24.7	45.5	49.9	65.1
124.4	127.3	124.4	84.9	77.3	84.6	105.3	121	205.1	247.2	214.5
976.7	1031.9	1196.1	933	866.6	981.3	848.6	1211.7	732	319	880.8
32.3	43.6	41.8	36.6	42.3	44.3	58.3	36.8	47	44.1	54.9
35.4	22.1	25.6	22.8	28.1	30.3	27.1	25.3	30.1	45.1	38.1
98.8	101.2	105.8	85.5	91.1	95.4	141.2	86.6	232.2	187.4	194.1
89.6	63.2	71.1	62.5	78.8	69.5	100.8	82	111.3	129.3	147.7
141.2	123	147.4	121.8	99	116.3	139.7	128.2	204.1	223.4	302.5
53.9	66.1	64.5	61.1	46	67.3	86.3	62.8	77.3	79.7	85.1
27.9	24.8	24.8	24.8	23	20.2	33.6	28.3	37.7	48.8	65.5
465	372	411.4	339.2	321.8	392.5	467.7	403.7	535	547.5	687.9
157	245.6	178.7	286	101	124.7	197.5	87.2	113.5	72.3	198.9
48.7	45	47.4	39	40.3	48.8	39.9	36.5	37.3	33	45.1
108.2	86.9	101.2	59.6	64	79.8	65.8	104	257.1	245.8	313.6
116.5	81.8	109.7	72.9	105.9	68.8	75.5	77.1	132	165.9	203.8
66.2	70.6	75.1	64.3	69.5	78.4	79.7	69.3	109	117.1	135.1
77.1	106.1	112.8	89.2	84.8	114.3	87.1	97.7	92.9	99.7	110.9
401.8	204.7	240.5	174.9	205.4	122.9	208.2	206.9	168.1	257	274.6

118.3	150.3	159.6	153.5	118.2	147.4	148.5	131.2	186.2	154.5	174.3
59.3	70.6	65.5	56.6	48.2	57.2	89.1	81.2	143.5	101.6	119.3
100.5	50.5	77.7	56.8	58.1	40.2	27	42.3	73.6	99	113
53.3	53.9	58.4	48.1	46.5	55.5	52.3	48.1	61.1	59.8	75.7
52.3	53.6	47.2	46.2	47.5	45.6	49.8	50.5	74.5	98	111.1
83.1	62.6	72.3	51.9	33.1	42.3	26.5	46.5	109.8	113.6	120.6
134.5	123.8	142.2	108	98.7	141.9	124.2	133.2	196.8	206.8	223.1
11.6	14.4	15.2	10.5	9.7	16.6	9.9	15.5	22.3	6.1	12.6
78.6	86.2	73.5	61.4	67.8	76.6	93	70.2	92.6	113.5	129.8
620.7	505.9	347	517.5	361.5	228.4	378.5	545.7	413.8	363.3	415
1031.9	2051.2	444.7	514.6	2155	726.2	1600.3	1820.4	784.9	1104.3	710.9

209	205.9	147.7	145.3	126.8	172.2	201.6	163.4	279.7	298.3	464
55.6	64.8	53.3	55.3	61.4	54.6	67.4	61.7	52	44.8	45.1
645.6	211.2	410.3	3437.8	54.3	165	363.6	217.9	242.8	234.7	312.9
24.4	21	16.9	15.2	13.7	22.9	12.5	20	74.1	51.9	84.8
26.2	23.1	22.3	20.8	16.7	20.7	29.7	32	58.7	60.9	64.2
107.6	82.6	79.2	70.5	78.3	93.8	98.5	154.2	139.3	163.8	156.8
123.7	81	125.4	98.2	96.9	118.8	66.6	42.9	36.7	63.7	76.9
113.2	121.5	136.4	115.1	84.4	105.8	110.7	120.1	148.7	192	199.8
73.6	56	76.5	60.4	63.8	83.3	80	51	71.8	93.9	110.7
56.4	52.2	51.9	48.2	36.3	42.4	69	52.5	79.5	82.5	92.6
82	68.9	83.7	50.8	47	67.4	71.9	186.1	73.6	70.4	81.6
27.3	28.3	31.8	23.5	20	23	33.8	24.4	36.9	42.9	43.5
28.5	30.1	29.9	25.8	35.8	30.2	38.8	31.6	29.3	38.4	37.8
140.6	143.7	135.7	113	134.4	135.5	179.6	137.3	157.5	230.1	229.8
147.3	145.3	147.5	125.6	80.3	141.2	175.7	149.7	198.8	174.8	254.1
8.6	7.9	11.8	9.5	5.3	8.7	8.9	5.1	15.8	23.3	25.4
47	48.9	53.2	37.2	28.4	45.1	45.8	33.1	50	86.3	84
41.6	35.9	37.3	33.4	35.3	35.3	39.1	37.3	44.9	53.6	62.1
58.4	127.6	74.2	54.6	63.9	189.4	212	83.4	89.4	87.1	97.6
205.9	531.8	514.4	144.9	198.5	279.9	212.9	272.7	187.9	299.7	264.2
52.3	24.5	38.2	73.5	36.2	37.5	43.3	33.4	63.6	167.2	85.4

46	38.8	40.6	34.8	40	34.8	53.7	34.2	51.6	54.8	58
192.4	132.9	157.1	129.6	109.2	132.7	170.4	143.3	260	298	364.5
212.6	231.5	240.8	167.7	131.1	170.4	170.9	176.8	525.2	609	675.7
32.4	37.7	42.7	37.9	45.6	42.2	38.1	41.8	62.6	43.4	76.6
43.7	50.6	48.1	35.4	44.5	46.6	61.7	52.1	58	68.4	80.7
53.5	50.5	67.4	49.9	55.1	59.3	92.4	56	64.5	72.1	74
22.7	16.7	13.6	18.3	9.8	13.3	13.7	16.1	22.2	49.5	32
42.5	40.4	39.3	30.4	33.9	31.2	54.2	41.8	101.8	82.3	141
146.4	322.9	200.6	269.8	161.3	482.8	204.9	317	138.2	144.7	164.6
88.8	93.3	89	81.1	86.5	103.2	99.6	126.9	166.8	146.9	175.2
52.9	59.5	38.5	46.5	40.9	50.5	70.6	81.1	86.1	116.6	106.3
99	89.7	129.2	102.2	101.8	108.9	154.2	139.4	115.3	104.2	138.8
37.3	43.8	95.4	35.1	62	56.9	96.4	36.8	52.7	70.2	73.9
101	92.5	85.3	64.6	84.8	83	108.6	88.9	105.5	128	132.3
34.4	39.1	35.3	34.7	34.7	47.2	46.5	43.1	57.2	59.7	65.8
58.2	50.3	47.6	27	25	38.1	46.4	58.3	80	108.6	107.9
96.4	93.5	82.8	66.8	77.1	76.3	91.4	107.3	113.7	146.2	160.1
47.8	67.7	62.3	50.2	35.4	47.8	73.1	66.8	74.2	52.2	64.6
76.2	60.9	64.2	48.6	69.1	61.5	71	59.5	87.4	78.7	90.6
66.5	65.2	52	48.7	42.6	55.3	71	122.3	56.2	100.3	105.7
101.4	86.2	92.1	97.7	75.2	97.9	95.3	80.4	110.1	144.5	149.5
58.1	66.6	51.1	43.5	64.1	63	68.3	59	83.1	95.6	96.2
31.3	32.8	32.3	20.1	18.3	21.6	32	30.4	41.3	43	53.5
95.3	109.4	114	109.6	121.1	95.8	119	91.4	112.9	129	150.4
78.3	59	66.8	63.7	58.2	90.6	66.4	56.4	68.8	64.8	82
73.7	105.4	82.6	57.9	51	85.5	214	48	52.1	245.4	120
85.5	106.6	126.1	100.9	88.7	102.5	99.4	103.5	113.8	136.2	149.9
163.3	117.2	129.3	127.8	114.1	122.9	142.8	155.7	191.5	272.1	260.2
102.2	76.3	145.8	56.5	82	53.9	45.4	84.3	102.2	91	120.4
68.9	45.8	46.6	67.2	49.7	44.9	68.5	42.3	44.7	47.5	43.3
457.5	417.4	630.9	543.8	625.2	811.9	483.9	336.1	551.5	683.8	728.9
112	96.1	101.3	66.9	75.2	88.7	118.2	79	126.8	158	175.8
42.6	47.7	49.9	39.9	36.2	63.6	43.7	39.1	68.6	63.9	81.6

95	77.6	75	63.3	69.7	80.2	100.5	86.1	121.9	122.1	138.5
134.5	145.3	128.8	107.7	96.2	136.2	186.4	121.4	307.3	439.9	407.9
62.9	69	72.1	55.7	54.2	77.4	83.3	65.6	81	100.5	99.5
139.6	146.3	176.2	118.4	105.2	202.4	136.9	104.8	252.9	290.5	323.2
90.8	88.1	113.4	90.8	106.9	101.2	101.3	105.8	111.1	86.5	122.3
61.5	60.8	65.5	55.7	60	76.7	67.5	62.1	85.8	67.9	144.2
134.2	120.6	132.5	120.2	138.1	121.7	166.6	154.4	180.7	213.8	243.8
67.4	64.8	80.4	59.8	52.9	67	68.5	65.6	136.3	161.5	197.6
113.7	79.8	104.8	82.2	77.3	113.5	83.4	86.9	150.3	209	194.3
72.2	64.3	71.1	53.1	51.1	64.3	56.5	52	124.3	91.4	150.3
48.3	42.1	55.3	45.5	46.1	57.7	37.8	61.5	58.4	160.5	102.2
59.4	56.2	62.5	37.6	39.8	52.5	40.5	40.7	72.5	85.5	109.5
93	90.8	91.2	70.9	73.7	68.4	84.7	80.6	65.1	82.9	114.8

43.4	75.4	154.4	208.2	185.8	332.9	66.6	80.6	38.2	42.3	68.9
167.8	192.1	183.6	166.1	107.3	127.9	180.1	162.2	307.8	352.9	382.9
111.9	108.1	127.8	92.3	125.2	178.4	266.2	144	67.9	53.9	127.1
39.5	40	47.1	40.2	50.7	37.3	53.3	51.7	39.2	38.3	49.9
26.4	32.3	22.4	31.2	41.3	30.4	46.6	37.6	28.1	27	44.5
41.6	33.7	52.1	40.2	33.9	44.5	76.3	52.5	92.8	91	94.1
55.6	61.2	77.8	65.5	63.6	71.5	82.5	77.2	119.7	139.6	132.2
45.1	35	38.3	37.7	37.7	40.3	49.6	32.1	48.6	62.9	76.2
54.8	41.3	60.7	37	43.3	48.3	48.7	41	67.8	93.9	125.9
104.5	66.3	92.5	81.2	59.6	86.8	62.9	67.2	80.7	100.4	98.8
110.7	130.1	121.3	91	140.3	123.4	181.6	163.2	127.6	84.3	129.2
269.1	241.2	298.4	204.2	226.2	275.5	270.5	219.6	433.5	398.6	623.2
70.6	75.8	64	52.5	48.4	74	73.7	54.9	80.6	92.5	108.6
54.5	60.2	52.7	36.8	54.1	53.2	62.1	44.1	54.3	54.2	71.1
63.1	75.2	96.5	67.1	57.9	89.8	106.9	65.1	55.9	107.7	82
141.9	90.1	133.8	134.5	79.9	95.1	78.9	74.1	72.3	88.4	113.2
48.6	46.4	45.7	35.2	52.5	45.4	62	44	49.3	55.2	64.5
68.3	69.9	70.2	54.8	71.2	64.6	83.9	56.3	96.5	98.6	115.6
52.1	51.5	65.2	66.4	49.9	31.7	54.9	54.1	73.6	82	129.9

79.5	49.1	75.5	55.7	76.8	134.4	101.6	67.2	43.1	48.8	56.3
102.7	100.4	116.4	87.6	82.6	78.5	85.5	91.5	187.6	191.9	222.4
78.7	77.4	75.7	63.5	62.5	74.4	64.2	64.4	135.4	182.8	168.9
73.3	87.6	89.9	122.6	120.8	230.4	83.8	142.8	111.5	92.2	143.7
433.2	362.6	457.1	368.9	339.2	409	403.3	281.9	425.4	667	573.6
46.2	51.4	51.5	38.5	72.7	52	78.2	42.2	50.1	57.2	74.5
110	119.4	155.1	133.9	84	112.4	96.4	92.3	162.5	133.9	200.2
90.3	170.7	121.6	119.3	189.6	370.7	170.5	188.9	102.5	229.3	156.2
42.3	47.4	47.2	38.5	36	35.4	53.6	28.8	46.6	66.7	62.6
85.1	90.7	103.1	75.7	84.3	96.5	111.4	54.5	60.1	130.2	80
38.7	29.4	36.4	36.4	30.8	36.1	38.1	36.8	56.4	75.6	65.8
88.1	57.1	81.4	40.7	65.3	57	41.4	52.8	93.4	141.1	148.4
81.8	83.7	81.3	72	91.2	85.2	98.8	103.3	84.3	93.2	123.1
73.9	64.1	80.5	70.7	67.3	64.5	57.9	52.5	117.4	206.6	182.7
96.1	47.6	83.1	57.5	44.2	47.7	51.4	72	148	183.3	207.6
31.9	18.7	26.2	22.5	21.3	39.9	37	28.9	31	29.2	53.2
200.1	198.8	206.8	171.1	180.4	187.1	187.6	188.1	251.5	270.4	309.4
80.5	78.9	89.3	80.8	58.3	80	71.7	76.1	98.9	125.5	130.2
244.1	238.1	212.4	181.7	175.9	179.4	205.8	163.4	292.3	308.4	431.2
13.3	18	25.4	15.3	19.7	25.7	27.7	21	50.4	113.7	76.1
57.7	53	60.5	52.9	66.9	115.3	90.4	62.8	60	66	84.8
70.6	52.5	77.7	51.9	54	58.8	81.6	80.2	105.3	128.8	149.5
30.7	26.7	31.9	23	25.1	33.1	34.9	41.9	40.3	21.6	38.7
56.8	46.9	50.7	42.2	64.2	54.5	70.2	60.1	54.5	131.1	96.8
105.1	72	91	75.9	95.6	225.1	94.7	67.5	57.8	67.1	120.3
154.4	151.5	154.6	141.6	135.8	151.5	162.6	130.3	309.7	302	343.8
76.4	73.7	81.2	64.5	66.5	83	93.9	75.5	156.7	144.7	177
53.7	40.5	60	34.4	36.4	41.2	44.9	45.5	94.5	59.8	104.6
60.2	41.7	61.4	59.9	36.6	49.8	57.9	61.2	73	113.2	108.3
183	152.8	167.9	130.6	127.2	147.9	190.9	187.4	320.8	353	411.6
95.4	140	167.8	115.3	127.9	116	106.4	141.6	328.4	204	326.1
10.4	10.6	15.4	11.1	14.6	16.4	14.2	10.2	77.9	26.2	36.8

15.9	19.7	17.9	16.9	9.5	25.1	22.1	22.6	52.2	21.6	21.6
130.8	203	155.7	181.9	127	201.8	150.2	163.3	144.8	111.7	148.5
147	147.5	175.7	137.5	122.7	152.8	159.1	144.4	184.2	217.8	229.2
35.6	36.9	38.6	32.8	29.4	48	59.3	42.8	83.1	86.6	95.3
105.5	77	88.1	73.9	67.3	55.8	55.1	75	155	153.9	216
69.8	36.6	76.1	41.3	41.7	57.4	63.6	59.6	137.8	134.4	173.4
55	60.6	57.7	48.4	40.9	55.3	64.8	61.7	135.1	134.8	145.5
165.6	127.5	166.7	138.4	127.3	158.9	160.3	136.4	136	183.4	185
56.7	75.8	63.7	51.5	55.3	63.7	61.1	76.6	63.8	78.4	97.9
93.6	77.5	71.2	58.7	49.6	51.3	67.2	68.8	95.3	88.4	98.6
67.6	54	60	46.7	56	65.1	86.9	76.5	121.7	106.7	177.4
81.7	73.4	74.8	74.3	53.7	79	68.3	62	164.5	142.9	206.8
142.9	82	114	90.9	64.4	97	76.7	93.6	217.2	254.3	298.8
92.2	68.7	89.8	58.4	68.1	75.9	59.6	59.2	95.2	137.3	151.3
94.6	144.9	154.8	96.5	142	169.5	292.2	89	74.9	71.9	139
39.8	31.6	33.6	30.8	28.6	48.9	36.6	39.2	32.2	44.2	49.7
89.8	103.6	116.8	82.6	80.5	112.6	123	79.8	135.4	160.4	173.1
143.1	126.7	67.9	202.9	243.8	35.5	136.4	262.8	145.5	93.4	135.3
128.5	117.7	137.3	112.9	139.5	117.4	172.6	143.4	192.2	274.7	212.2
72	48.9	70.2	45.3	39.3	51.5	50.9	52.4	104.4	122.3	168.2
123.9	86.3	113.4	73.8	81.1	78.2	76.3	84.3	200.4	266.9	284.2
57.6	52.8	57	42	63.8	52.7	64.9	43.5	64.8	83.9	100.7
97.8	141.6	81.1	95.8	68.4	112.7	89.6	166.6	138.3	113.7	189.4
156	379.8	180.2	144.6	395.2	112.6	244.2	260.6	477.4	184.4	161.2
125.7	162.4	78.8	45.8	122.6	32.5	28.3	40.3	27.7	50.2	49.1
265.9	277.8	274.9	221.1	187.6	229.6	273.7	210.9	555.8	616.7	640.7
333.6	308.4	251.8	249.6	218.4	233	289.2	187.3	268.2	472.2	380.6
80.2	67.1	86.2	117.8	78	64.3	75.5	63.5	72.6	76.4	108.2
78.1	298.9	78.5	75	56.9	82.1	82.1	61.6	113	106.7	155
470.8	158.6	166.9	213.9	225.2	138.9	222.8	276.5	463.2	250.4	349
80	92.8	91.8	96.3	83.8	97.9	110.3	92.3	123.7	75.8	98.1

25.1	24.8	27.9	23.4	39.2	26.3	37.4	26.5	27.4	31	28.7
49.3	93.4	58.7	69	63.3	146.3	100.3	93.9	52.6	123.6	61.3
34.2	36.5	33.3	24.6	63.3	44.4	57.9	49.5	104.8	50.8	88.5
78.5	80.9	63.7	52.1	69.7	52	70.5	71.7	82.6	45.6	84.8
52.9	48.2	61.7	57.5	42.6	44.3	37.9	33.2	70	62.4	95.1
30.3	27.9	32.9	24.5	25.9	34.1	36.9	19.4	31	30.4	39.7
69.9	93.7	110.8	43.5	56	71.2	60.1	60.9	103.8	110.4	137.3
35	41.4	35.2	30.9	41.6	44.8	55.8	37.9	35.5	39.6	54.2
76.6	71	65.8	65.2	59.6	62.1	82.5	76.9	127.4	142.5	149.4
57.8	45.5	49	41.8	39.8	54.4	57.1	43.1	70.6	56.2	67.6
262.3	135.6	201.5	163.7	137.4	142.2	129.7	123.2	154.1	316	226
81.9	81	79.8	59.4	69.4	65.9	73.1	54.8	69.6	88	112.9
85.3	114.7	79.8	64.2	66.5	89.9	93.7	72.5	100.7	94.9	125.8
58	48.6	51.9	47.7	63.8	73.6	70.8	55.7	61.6	72.1	67.3
185.8	151	237	159.9	136	130.6	114.5	149.5	229.8	253.2	325.3
56.2	66.5	70.3	83.1	88.8	76.5	61.3	81.1	113.3	71.5	81.5
79.1	62.7	75.1	65	54.6	67.3	79.5	63.2	138.2	183.1	167.9
77.5	75.2	79.6	82.5	79.6	75.8	114.7	69.1	129.9	115.6	164
633.2	412.2	567.5	447.8	543.5	414.4	497.2	461.8	753.9	797.7	927.8
94.9	77.9	87.3	64.7	57.9	81	88.1	80.6	125.1	160.2	154.6
52.4	53.8	56.3	56.8	45.8	53.6	44.4	41.2	39.7	42.8	64
43.8	88.2	56	39.1	61.7	158.4	77.1	64.4	46.1	46.4	74.4
133.5	72.1	96.3	131.2	56.4	67.3	56	55.6	88.7	77.3	129.1
91.9	63.1	77.1	53	52.8	58.7	83.2	68.8	127.8	150.4	154.7
58.3	65.6	57.7	61.6	59.3	70.5	72.8	59.7	88.4	77.3	86.3
186.6	177.5	205.3	165.2	164.7	154.7	202.2	146.1	326.9	459.7	429.4
193.3	156.6	197.4	140.5	136.1	164.8	152.9	120.8	196.5	214.8	249.5
321.5	278.5	326.3	234.5	221.6	260	375.7	249.2	296	595.5	490.4
24.4	23.4	19.3	17.7	23	24.8	28.4	22.2	26.6	37.7	27.9
54.4	81.2	56.1	50	51	68.8	88	120.1	79.7	78.7	83.3
62.9	56.4	74.5	59.8	50.3	54.3	79.9	68.3	100.9	83.7	102.3
64.7	41.8	49.4	42	38.5	44.3	44.5	27	54	119.2	87.1

101	103.9	106.5	69.4	88.2	96.4	111.7	91.4	106.3	120.1	160.3
60.7	45.3	61.2	37.2	34.4	53.6	43	56.6	112.2	115.4	132.8
113.4	133.8	142.5	123.7	94.4	120.5	124.3	133.3	228.8	429.9	270.9
208.8	156.4	200.7	139.7	117.1	127.4	190.4	145.1	280.9	289.1	375.6
46.8	115.3	67.4	71.9	40.6	84.3	73.1	246.6	74	91	94.1
64.7	57.1	70.6	52.4	47.6	57.8	66.2	47.4	83.2	111.5	120.8
64.8	69.9	78.8	66	45.4	70.1	67.8	65.5	118.9	124.1	138.1
52.5	51.7	43.3	45.9	47.4	51.9	54.2	55.8	74.2	74.8	94.2
103.2	111.3	115.9	102.5	108	104.5	150.1	108.2	159.7	190.4	246.8
59.6	56.8	62.2	46.4	70.5	56.5	76.1	50.5	64.1	81.3	91.8
166.3	127.2	153.2	133.8	110.9	116.6	115.9	111.8	221.2	262	352.8
94.3	61.8	88.1	73.7	64	51.8	70	62.6	82.4	90.6	119.1
84.2	67.7	61.7	56	51.5	57.2	71.8	44.6	78.4	93.6	123.9
93.3	100.1	101.2	79.4	62.7	86.9	89.2	69.5	166.5	189.1	214.4
173.4	228.7	180.2	193.4	179.6	169.8	223	345.4	227.8	304.2	181.1
39.4	43.1	55.3	33.2	38.5	65.6	46.6	50.9	52.2	33.5	53.1
40	39.7	43.3	25.4	35.4	47.9	36.8	24.6	40.9	51	75.6
49.1	39.5	65.6	39.3	36.1	37.6	38.9	41.9	52.4	55.5	60.5
81.7	52.9	90.4	96.2	61.2	75.9	54.5	48	59.2	97.3	84.6
104	118.7	122.5	93.8	126.2	95.7	146.3	120.1	128.9	178.1	187.4
25.6	25.1	23.3	21.5	23.7	31.6	26.3	26.2	31	46.1	37.4
6.4	7.9	14.4	15.9	10.9	12.9	12.5	9.1	10.9	4.3	8.8
49.8	44.9	65.4	40.4	51.8	61.8	47.8	66.7	91.9	190.2	150
92.4	111.7	129.8	100.6	82.5	104.4	108.6	87.2	102.1	105.5	121.3
158.6	106.5	110.6	131	78.4	89.7	121.9	347.3	164.8	184.9	167.1
414.4	244.6	354.9	240.3	246.2	243.3	224.3	212.8	365.8	426.7	621.1
34	48.5	57.5	40.4	40.9	56.8	69.2	67.9	79.8	52.9	66.4
74.2	54.4	58.2	57.4	40.7	42.5	48	71.6	84.9	46.7	77.8

168.5	110.3	146.6	101.1	93.6	103.7	86.8	91.8	258.6	235.9	382.2
41.7	42.8	45.3	43.6	52.9	43.7	60.2	44.2	63.7	62.6	77
40.4	40.4	37.7	30	42.2	44.8	54.2	34.4	28.8	39.9	35.6
229	514.2	436.2	702.5	209	459.8	206.9	187.9	254.9	273.7	384.7
153	95.9	127.5	79.3	90.6	79.9	97.9	92.2	176.5	282.2	306.3
353.9	217.3	236.4	159.9	173.7	198.9	171.2	192.6	264.6	719.7	426.5
51.6	71	50.1	69.5	58.6	78.2	75.5	58.1	98.9	139.9	139.9
194.5	81	95.7	99.9	102.8	87.9	125.6	119.6	178.3	189.6	196.8
73.2	93.1	75.8	61.9	88.9	78.3	101.1	75.8	81.7	128.7	95.3
20.1	21.3	21.5	17.7	27.9	19.2	30.8	22	18.9	15.1	19.9
194.1	249.2	127.9	156	265.6	82.8	103.9	179.1	205.4	92.2	101.1
48.6	60	52.2	46	45.8	54.1	61.5	60.8	75.7	67.9	96.2
63.8	54.3	65.1	34.9	43.1	51.6	86.3	90.4	105.8	116.2	148.8
37	24.6	33.5	30.1	21.7	25.4	22.6	23.4	39.1	52.5	52.3
106.5	104.5	114.9	110.5	80.8	79.2	102.3	115.5	201.8	180.3	188.7
58.3	51.2	49.2	48.7	59.1	56.7	75.9	60	72.5	74.5	96.4
20.8	15.4	16.1	16	16.6	20.8	18.2	22	15.7	15.6	23.1
161.4	177.8	146.1	140.3	137.5	154	322.1	168.2	277.9	265	272.8
136.4	156.9	148.6	115.9	99.1	187.4	149.4	140	204.7	266.3	265.6
188.8	89.5	113.4	102.5	68.7	85.3	88.8	88.2	240.3	293.6	363.8
20.7	29.1	24.3	29.7	20.5	26.4	23.4	20.3	20.2	22.6	25.7
33.9	37	38.3	32	39.6	45.5	44.9	35.5	38.9	41.6	44.4
338.6	123.2	40.2	157.1	118.7	189.1	895.8	100.6	24.1	40.5	38.9
54.1	62.1	69.5	50.6	35.7	73.3	74.1	66.4	63.6	79.3	86.3
318.7	547.6	344.1	335.7	364.9	612.3	1028.2	790.1	339.6	378.2	424
25.9	32.7	31.6	21.5	28.8	31.5	38.5	26.5	27.7	31.4	28.1
89.4	130.9	146	66.9	90	112.8	92.4	119.7	110.4	113.1	98.7
59.6	70.9	55.5	49	81.7	78.1	111.9	68.2	79.8	65.7	78.5
119	108.8	95.7	87.3	89.6	115.4	142.4	88.5	110.2	114.2	146.1
60.7	37.7	54.8	45.7	59	58.3	56.9	42.9	48.9	34.8	43.3
52	52.4	66.3	56	67.6	51.9	68	41.7	57.3	71.5	106.2

93.5	107.1	100.9	212.4	92.4	96.3	100.1	113.5	111.4	106.2	132.3
304.1	178.1	120.3	115.5	148.6	194.3	188.7	206.5	420.7	372.8	550.7
75.7	78.3	78.3	52.6	57.5	85.3	94.9	56.6	101.4	92.4	112.5
38.5	55.6	71.5	52.9	51.5	71.9	75	45.5	60.3	128.8	130.6
63	49.8	69.9	65.8	52.5	74.6	125.3	71.5	60.9	54.9	64.5
37.7	53.1	44	39.1	28.8	56.6	47	48.9	94.1	67.5	85.5
95.1	106.8	134.2	104.4	84.9	112.6	114.4	101.9	118.7	202.4	170.2
85.7	66.1	81.3	50.1	42.9	62.4	57.1	65	103.8	154.6	144.2
55.5	46.5	51.6	40.4	45.2	47.8	56.7	38.5	54.5	55.9	69.9
79.5	78.7	93.7	74.9	66.3	93.2	95.6	75.1	83.8	107.9	140.9
125.9	108.1	148.9	114	79.9	133.1	122.3	100.8	193.9	187.5	228.3
25.5	17.9	20.7	12.6	14.8	21.2	17.6	25.7	26.6	26.3	36.5
168.6	261.6	226	157.3	169.6	306	418.6	208	174.5	229.5	264.3
86	77.6	95.2	70.1	80.9	100.9	115.6	103.7	121.3	141	160.2
4260	4392.6	2636.8	2544.5	6662.3	3379.6	6497.6	4085	2313	3166.4	2668.6
49.3	28.1	28.6	17.3	29.9	28	21.7	24.6	66.6	47.5	62.7
51.6	52.2	63.3	45.4	43.9	59.9	53.1	51.6	66.5	84.8	72.7
42.3	42	45.2	32.1	27	46.4	34	26.1	41.6	42.8	50.3
85.6	92	122	47.8	80.6	173.7	125.4	68.3	51.4	55.7	118.4
132.2	151.4	113.5	125.2	98.3	144.7	134.3	107.1	253.4	195.8	286
36.3	46.3	28.5	31.8	28.1	36.5	53.9	40.8	34.8	35.7	33.1
86.6	30.6	34.5	29.8	44.7	37.9	20.3	17.7	118.8	73.1	155
266.7	278.5	350.1	255.9	109.9	150.9	228.8	196.1	232.6	539.6	318.9
150	136.1	138.3	139.7	176.5	152.6	148	103.9	200	536.9	290
213.5	122.3	155.3	122.9	128.9	122.9	111.7	99	76.3	174.1	165.4
121.2	91.4	70.1	52.2	138.6	83.5	133.9	78.3	57.7	80.1	77.1
72.3	72.9	86.6	59.6	78.6	89.9	93.1	68.6	85.1	88.4	117.3
79	73.2	53	48	75.4	46.8	88.8	66.2	89.2	69	99.4
53.4	48.4	55.1	34.5	38.3	47.2	61.5	45	81.4	120.2	124.2
122	128.5	140.1	113.8	89.7	108.4	105.2	98.2	156.2	321.6	223

71.9	83.5	78.1	70.6	74.2	69	80.9	80.8	94.8	73.9	119.5
64.6	61.5	50.3	52.7	53.5	70.5	66.3	55.4	77.3	82.5	85.5
72.4	42	50.2	53.1	46.5	50.3	57.1	49.4	87.6	92.6	108.3
91.9	108.6	88.2	70.1	63.4	106.8	108.9	69.6	146.5	197.4	206.9
45.3	48.2	41.8	48.8	34.9	61.6	52.7	49.1	73.5	65.3	80.4
22.2	38.2	26.8	38.1	21.3	39.7	53.7	47.9	82.9	60.9	108.1
138.7	301	155	173.1	197.4	151.7	179.1	178.5	133.1	126.9	163.6
84.2	82.3	77.1	39.8	38.3	53.7	92.1	59.9	39.9	85.8	100.5
72.9	64.9	79.2	56.8	56.1	83.4	91.7	78.4	155.5	100.6	128.6
91.9	119.5	111.6	111.3	100.2	174.8	191.8	90.4	116.6	113.9	188.3
49.8	71.5	72.1	56.8	55	68.2	78.5	62.7	60.4	41	61.9
68.6	54.1	59.9	41.1	34.8	50.8	60.4	58.6	127.7	86	118.7
163.1	153.7	175.8	157.1	137.4	218.6	138.6	110.8	168.5	184.3	215.2
64.8	62.4	71.2	51.5	62.2	72.6	75.5	61	91.3	104.2	103.9
43.1	70.1	62.5	46.5	32	52.2	90.6	52.7	89.6	211.4	130.7
45.9	44.8	46.4	41.6	31.1	33	51.8	56.6	57.8	73.5	70
25.9	30.2	30.7	29.3	34.8	30.8	36.9	38.8	34.5	29.3	41.7
148.9	107.5	113.6	103.7	99.9	115.1	189	140.1	157.6	244.7	186.8
222.3	186.2	212.3	146.2	149.3	197.2	233.5	150.2	252.3	412.1	377.1
96.5	70.8	86.4	87.9	73.4	88.1	129.5	198.4	93.1	66.5	82.1
69	74.8	98.5	80.7	55.2	71.5	98.5	74.8	116	126.4	151.9
158.7	108.6	126.7	86.4	82.8	96.5	109.2	123.1	239.4	343.3	289.1
219.2	267.2	266.4	179.1	163.5	256	213.2	263.7	300.1	486.1	443
182.9	151.2	253	126.9	144.2	98.6	85.1	108.6	132.7	116.1	213.5
178.6	138.3	157.8	100.1	115.7	114	123.8	130.7	386.1	419.3	448.3
62.5	40.9	27.1	51.3	27.9	34.9	38.2	62.4	90.7	85.9	48
54.7	40.8	38.7	42.1	46.2	45.3	49.8	37.7	57.2	74.8	81.2
157.7	130.3	171.4	99.4	95.8	136.5	94.1	139	83.8	85.1	97.2
45.5	68.1	45.5	50.4	37.2	35	40.1	56.1	61.3	58	59.5
57.3	68.3	56.3	49.8	70.8	60.1	89.8	59.7	64.6	76	90.7
204	207	187.5	207.6	170.4	176.9	176	198.4	160.2	136	178.5

60	41.4	60.3	43.9	48.4	42.4	50.3	49.5	56.7	80.2	96.6
192.7	158.9	185.1	132.8	207.7	131.2	188.9	119.4	201.2	259.3	299.1

27.4	20	23.9	18.9	22.9	19.2	24.3	21.4	34.9	53	53.8
59.1	53.9	51.7	29.4	36.8	42.7	50.7	46.1	88.8	106.1	86.8

34.9	30.9	47	39.1	30.6	28.2	70.1	24	27.6	34	37.9
132.8	77.4	114.8	81.2	73.9	82.9	89.8	114.3	149.8	187.2	206.2
154.8	180.1	139.4	87.7	31.4	71.4	186.6	182	53.5	86.7	114
62.1	60.5	76.6	48	49.5	55.2	57	67.1	102.3	127.3	126.6
116.8	125.2	116.2	141.8	89.5	165.7	103.5	117.3	235	755.8	303.5
8.9	9.1	11.3	8.1	14.6	14	17.4	14.7	22.6	21.8	22.7

52.8	52.5	55.1	48.1	47.7	62	59	58.1	81	144.1	139.8
27.9	26.7	25.5	34.1	17.5	19.1	18.1	16.8	31.3	47.7	50.1
39.7	44.1	48.5	45.4	43	70.7	46.8	37.8	40.4	27.5	60.9
55.9	89.1	93	60.6	44.6	76.6	90.2	57	96	81.7	116.4
52.9	50.6	38.6	43.7	74	43.5	75.3	38.2	54.1	100.1	55.7
56.2	55.9	68.3	43.7	61.2	93.6	63.3	61.6	76.3	82	98.3
62.4	57.9	57.9	59	40.9	36.9	38.4	62.4	77.8	84.5	81.3
538	369.6	579.4	534.1	341.3	452.3	422.6	303	358.7	232.8	375.4
34.2	33.1	39.9	32.8	43.4	53.1	44.2	43.4	50.8	41.9	60.8
53.4	50.2	50.6	39.8	39.8	33.1	38.1	35.1	56.5	99.7	85.4
189.8	275.2	204.3	146.6	140.3	281.6	99.3	215.4	75.6	79.4	103.7
102.3	94.5	85.4	63.4	59.4	78.8	99.8	85	146.2	169.3	173.5
100.7	169.7	141.8	214.7	125.9	97.7	134.8	130.7	92.8	577.7	201.5
32	29.3	37.7	38	35.4	37.4	45.6	38.1	28.4	35.2	39.2
47.2	31.4	39.8	25	31.5	37.6	35	39.6	62.3	67.3	81.3
170.9	103	148.7	90.2	82.5	92.1	90.3	110.9	156.5	213.7	254
46.9	58.4	47.8	47.2	53.9	57.7	71.9	59.1	76.1	71	85.6
40	31.7	23.7	30.1	31.1	31.1	37.5	33	28.3	33.3	46.9
112.9	134	132.4	106.6	97.3	263.2	147.8	109.1	101.3	102.6	178.1

31.3	27.6	28.3	31.1	17.4	36.8	33.6	37	35.6	32.3	35.2
165.2	151.7	143	159.2	135.5	197.5	174.1	146.4	234	310.9	282.2
83.7	68.1	98.1	74.7	72.6	94.6	71.6	77.6	133.9	156.5	169.2
72.2	81.9	70.7	53.2	56.7	55.8	61.6	57	149	137.6	198.1
101.5	83.7	110.4	100.7	76.7	91.6	67.6	63.5	106.5	134.7	164.9
79.8	65	83	57.6	65	67	62.5	66.2	102.4	137.1	155.6
91.9	66.2	75.3	59.8	57.6	72.8	65.8	61	127.1	103.8	145.2
101.4	42.8	32	52.7	76.9	30.6	77.5	52.7	36.5	43.4	44.4
138.7	144.5	149.2	112.5	74.9	108.6	80.3	103.3	138.8	131.4	174.9
32.1	64.2	48.6	44.5	40	94.6	64.6	37	111.7	98.8	147.4
78.3	103.8	70.6	71.6	64.3	81.5	113.6	302.9	81.9	69.2	105.1
106.8	63	79	50	60.4	35.2	59.6	86	148.7	127.2	176.2
115.7	94.5	115.2	94	81.9	107.6	99.4	103.9	190.6	185.5	232.5
12.4	20.5	16.7	13.4	13.6	10	19.1	17.3	27	45.1	48
88.7	79.9	109.9	88.7	81.3	91.6	177.7	77.3	139.1	172.2	184.4
102.3	98.6	93	94.6	90.3	184.3	390.3	148.2	68.1	79.5	99
118.4	111.2	118.5	101	74.1	86.5	129.7	107	313	304.5	329.9
13.1	19.6	11.8	14.3	14.9	16.5	16.2	12.4	28.6	30.9	20.7
10	6.2	12.3	5.4	3.2	6.9	6.8	11.8	65.2	46.1	34.2
198.4	182.7	194.9	157.3	173.8	176.1	235.3	162.1	245.8	413.7	402
30.3	30.3	31	29.5	27.8	28.7	33.3	26.8	45.8	48.2	66.9
42.5	48	57.4	41.2	40	50.8	49.9	49.1	66.3	79.5	83.3
153.1	121.5	157	95.5	96.9	96.8	145.9	145.9	182.6	181.4	343.8
99.2	95.7	80.2	73.1	83.4	77.1	104.5	87.7	120.4	149.3	145.1
155.8	190.3	199	180.1	150.2	230.7	223.2	187.4	324	436.7	397.9
50.6	49	51.4	37.9	40.8	46.4	55.2	38.9	54.3	86.5	79.7
150.4	171	135.5	124.7	109.7	131.8	182.6	86.6	354.1	301.1	343.9
108.4	112.4	235.3	148.4	173.8	117.9	127.3	175.8	136	77.6	167
25	27.2	33.1	22.5	14.6	19	19.4	26.1	47.6	34.5	53.5
74.8	69.5	49.6	73.4	48.6	72	51.1	38.3	64.4	54.5	61.5
115.7	119.9	111	74.5	105.2	111.5	143	91.5	103.7	123.4	118.8
34.5	63.1	136	166.9	131.9	165.6	179.6	76	144.3	811.2	244.7
69.6	39.3	48.4	47.4	47.8	51.5	53.5	53.1	91.4	107	116.2

14.4	12.2	12.5	9.4	13.1	12	9.7	11.5	19.7	30.4	30.1
560.7	391.6	254.2	237.6	314.4	1271.7	965.1	422.8	100.4	92.6	98.3
83.8	73.5	86.8	74.8	99.1	85.3	121.8	93.3	104.3	89.5	146
74.7	65.3	83.1	51	78.7	53.4	79	64.9	64.2	62.2	79.2
86.7	83.8	80.7	72.9	64.6	99.6	114.4	89.2	154.1	75.4	121.9
67.6	41.2	60.1	54.6	34	52.7	60.5	49.9	146.2	276.3	126.6
84.9	75.6	79	57.7	46.8	86.6	90.1	76.8	102.2	161.7	155.9
40	43.5	56.2	41.9	67.3	66.1	44.7	45.5	73.8	74.1	120.4
178.4	112.8	136.1	105.1	90.3	95.4	109	92.9	260.2	366.9	366.3
141.5	146.7	164	171.6	131.5	163.1	132	151.4	109.3	82.7	115
42.7	37.3	31.4	40.1	40.1	38.1	44.4	44.7	32.3	39.7	48
21.5	23.1	24.6	18.7	14.9	17.2	19.4	26	33.6	38.7	30
59.3	45.3	49.8	43.9	39.3	41.8	45.1	43.7	61.9	50.7	82.6
175	187.4	206.1	139.6	108.4	174.3	186.5	137.6	178.1	171.4	240.6
33.8	31	46.2	38.6	35.5	47.6	60.1	40.9	39.5	412.9	61.7
49.3	36.2	44.2	33.3	31.7	48.1	40.5	31.6	53	74	58.5
159.5	139.3	190.8	141.8	112.9	149.7	147.2	131.9	166.1	173.5	243.2
87.5	130.8	112.4	74.9	159.5	70.5	138.9	129.9	147.2	127	224.3
74.1	75.4	75.1	86.5	45	56.9	72.4	68.6	141.5	236.7	162
45.2	52.4	73.5	40.6	40.1	80.4	81.6	38.7	21.1	32.6	35.3
59	72.1	53.6	51.1	56.2	70.7	79	55.7	105.5	135.8	131.6
94	98.1	151.9	153.4	91.5	145.7	99.7	82.6	119.8	109.7	131.7
187.2	155.2	145.4	149.6	224.6	173	241.2	227.6	171.5	130.1	150.7
23.5	24.7	34.4	22.5	30.8	38.4	33.4	29.2	38.9	57.7	45.5
110.1	59.6	71.1	55.7	54.5	50.3	50	47.7	126.3	110.3	149.7
55.8	48.5	74.9	55.5	43.7	63.8	59.6	50.5	82.9	90.5	123.4
58.2	80	85.9	46.4	27.7	62.9	163	62.5	131.4	161	114.3
31.6	38.8	34.5	31.6	29.1	33	32	28.1	36.4	32.6	49.7
163.1	160.9	162.6	132.2	139.6	178.9	183	165.6	232.7	234.7	331.3
91.8	100.8	111.1	83	99.3	90.7	99.3	68.1	69	117.6	116.9

62.5	68	64.6	59.8	86	49.3	93.7	76.8	84.8	100.6	102
73.7	68.7	71	47.8	56.7	60.1	65	63.7	100.1	94	152.7
33.1	52.2	37	43.3	59.5	44.7	69.7	47	48.4	33.4	64.3
75.3	165.7	161	288.6	130.4	148	79.5	102.6	67.3	42.9	68.7
30.5	33.9	26.8	20.1	38.4	22.4	32.4	27.9	26.9	34.9	38.5
116	86.2	130.2	107.2	85.7	202.7	206.5	69	133.3	123.5	164.6
109.7	75.3	115.9	84.8	58.8	66.3	85.1	73.5	151.4	175.9	214.6
167.2	138.2	227	141.9	122.2	130	143.7	117.2	243.7	263.1	291.5
54.3	78.1	52.1	55.8	45.9	92.5	59.8	77.1	114.5	144.5	137.2
51.2	39.2	39.7	31.2	27.7	52.2	33.8	37.7	49.4	76.3	85.6
167.9	140.6	167.2	108	126	126.5	121	128.7	209.7	217.9	304.2
65.8	53.8	58	48.7	45.4	58.7	73.2	51.9	76.8	111.8	115.6
123.2	104.8	112.1	104.3	79.2	80.9	106.2	104.8	162.7	197.4	213.7
51.1	104.1	107.9	173.4	72.8	75.3	47.7	51.6	50.4	41.8	55.5
42.6	44.6	60.1	35.4	48.7	45.5	63.3	45.4	55.7	59.8	74.9
208.2	229.5	225.7	183.1	182	224.5	220.5	193.1	285.8	336.1	382.4
105.4	72.7	86.4	71	76	94.5	122	96.9	232.2	153.2	173.8
92.4	91.3	104.6	91.3	96.8	116.1	134.5	96	122.4	123.9	138
90.4	87.2	88.1	67.4	70.2	77.5	106.5	83	120.4	145.6	179.3

112.4	108.3	106.8	88.7	88.5	76.8	111	75.7	97.6	122.4	138.1
65.6	36.8	51.6	34.4	50	47.2	47.7	37.2	76.3	117.2	137.5
45.9	46.2	57.7	40.2	54.1	48.4	49.4	39.9	65.1	82.7	88.2
6.5	23.1	12.4	36.3	18.6	30.5	14.8	30.3	182.9	36.3	96.2
55.8	62.3	70.2	50	39.2	70.5	84.7	70.3	114.8	144.7	155.6
225.9	168	297.3	237.2	99.4	91.8	48.3	87.6	31.5	12.7	55.6
67.3	35.5	49.8	34	43.8	42.7	51.2	41.6	77.3	81.3	104.2
110.5	99.9	116	83.4	68.3	76.1	83.1	82.4	148.3	112.7	171.3
55.9	42.9	46.1	33.3	44.6	37.9	56.1	42.1	56.2	71.6	70.4
74.6	49.5	55.8	61.2	39.3	42.4	42.6	53	101	96.5	143.7
35	70.1	36.5	53.8	35.4	80.9	192.5	65.7	67	31.1	56
70.2	52.9	48.1	40.9	51.7	43.7	75.3	62.7	85.5	101.4	88.3
93	90.9	104.3	75.7	65.1	78.8	80.7	72.2	124.7	161.1	164.5

69.6	76.2	97.2	58.9	68.6	150.7	100.6	61.8	104.2	130.1	158.3
46.3	33.6	42.1	32.6	26.8	32.2	18.3	44.6	41.8	34	52.8
105.2	75.1	88.7	63.8	53.6	76	77.2	79	239.3	226.3	277.7
119.8	99.6	114.1	109.3	65.2	101.5	115	148.3	169.1	162.8	203.8
136.6	146	212.9	137.1	102.9	158.9	135.1	114.7	141.2	150.8	228.7
40.1	38.3	49.5	35.7	38.4	48.8	42.2	43.2	71.7	76	86.5
59.6	50.3	49	31.9	28.2	45.8	40.6	51.6	138.1	149.2	158.2
86.2	73	77.6	57.2	55.2	70.2	80.7	81.2	122.8	129.8	157.6
123.5	238.6	124.3	137.5	104.7	203.3	225.2	292.7	141.6	133.7	133.5
91.6	62	88.5	70.6	59.8	88.9	103.1	68	124	169.5	172.3
42.4	45.1	44.1	38.8	33.2	46.9	45.5	41.4	46.4	52.9	61.5
203.5	251.2	399.3	266.6	222	298.4	182.3	219.5	229.4	204.6	342.3
70.3	111.8	31.6	121.4	66.5	32.3	176.7	111.3	163.4	24.6	213.8
34.3	32.7	32.4	39.8	37.8	31.4	42.2	93.6	28.8	37	37
173.9	190.7	212.7	180.3	184.7	257.1	149.8	99	88.2	59.1	137.6
57.2	48.9	42.7	39.5	39.7	47.5	73.9	52.1	84.8	68.6	101.2
86.2	67.8	114.8	64	65.6	74.7	73.7	93.2	202.9	157.8	243
100.2	142.1	106.9	120.5	86	105.7	161.6	124.2	174.5	189.6	224.7
50.3	22.4	32.7	32.8	28.9	27.3	23.7	21.7	66.1	64	71.2
148.8	87.6	154.6	83.8	80.6	78.6	62.4	83.8	164.8	217.3	244.6
48.3	50.3	53.2	43.8	33.9	44.1	57.2	47	100.5	118.9	149.3
20.6	12.9	17.8	5.8	8.8	9.8	9.7	9.5	29	48.9	62.5
69.8	58.6	70.4	45	66.7	69.8	74.5	66.1	84.3	79	109.2
70.7	72.5	79.4	52.8	74.1	66.3	68.1	53.6	85.9	116.5	124.5
24.1	23.4	35.6	28.8	31.9	27.7	28.2	24.9	63.7	75.1	63.3
83.3	78.1	120.8	97.5	106.8	121.5	130.5	89.7	61.5	100.5	88
179.5	183.5	227.3	145.6	148	174.5	138.1	181.3	211.3	210.7	247.9
57.6	36.5	32.8	23.1	30.8	33.3	28.8	23.7	64.4	60.9	73
264.9	115.3	193.8	151	128.8	106.7	82.5	99.3	245.6	385.2	290.1
35.2	32.4	36.2	28.5	28.4	40.1	41	26.1	31	40.3	44.2

64	64.8	61.4	57.4	45	64.7	75.7	68.7	107	77.5	104.7
133.5	124.5	134.8	123	155.3	140.8	163.1	154	120.3	121.9	154.5
123	98	125.4	104.5	99.6	98.8	94.5	100.3	155.6	212.3	193.5
79.5	115.9	85.4	75.1	64.4	89.1	81.8	82.1	110.5	75.2	94.6
39.7	32.8	32.2	40.1	25.2	28.7	38.8	41.4	39.5	24	26.4
79.5	66.3	48.9	48.5	45.7	56.5	55.6	59.9	129.1	132.3	185.1
39.5	39.2	34.6	34.4	40.3	39.7	36.5	33.9	47.5	54.2	58.6
13.3	12.7	12.1	15.7	13.1	10.7	19	16.1	15.9	11.3	20
71.3	68.6	78.4	54.4	46.6	51	56.4	57.9	95	93.4	121.5
117.5	131.1	142.1	112.2	78.4	123.4	149.1	124	245.6	407.2	314.3
37.1	38	46.4	26.3	35.3	28.5	32.1	35.3	36.1	34.9	46.7
419.1	447.3	312.1	398.7	386.5	337.8	500.8	558.7	204.1	122.6	249.5
46.4	46.9	50.1	54.6	34.1	41.7	50.1	52	68.7	46	42.5
46.4	212.3	86.6	168.8	176.9	535.8	187.9	466.6	45.9	54.2	59
47.1	53.5	60.7	48	37.7	49.1	117.5	49.6	45.1	43.7	45.7
2232.3	394.8	693.8	444.1	713.9	355.8	286.9	361.5	855.5	1020.5	1490.2
15.1	23.1	26.1	18.5	17.6	19.9	20.7	25	19.8	20.4	22.8
122	94.4	99.5	109.9	105	176.6	195	132.7	259.5	170.7	171.4
172	225.4	21.3	22.3	19	147.7	109.8	18.4	18.4	37.2	22.4
35.3	29.7	52.6	50.5	31	119.4	46.4	33.6	50.1	58.7	71.3
43.4	44.7	70	38.8	34.4	143	34.4	44.8	42.1	31.1	37.4
27.1	24.4	29.3	28.9	23.2	33.9	35.5	25.4	26.1	30.5	27.8
62.7	43.7	62.7	59.5	40.6	40.2	39.6	41.5	71.9	118.7	92.5
184.8	173.6	223.6	190.2	152.9	208.6	196.9	222.9	289.9	216.6	292.9
92.8	120.2	122.5	131.3	87.6	161.3	117.8	109.6	88.8	85.3	95.5
424.9	396.7	446.4	313.8	326.4	358.8	354.9	300.6	448	685.7	845.4
51.4	32.3	40.2	43.3	50.6	39.7	50.7	44.4	55.8	39.1	57.1
354.5	247.9	386.4	352.6	223.7	294.3	252.5	184.9	131.8	182.1	190.9
85.9	83.7	95.4	92.9	70.4	95.9	87.4	96.7	128.9	142.6	171.1
35.1	28.1	32.9	32.4	36.6	36.5	50.1	29.8	43.2	35	49.3
137.2	142.3	140.4	89.8	123.1	113.6	148.3	125.8	212.6	231.9	257.3

35.7	40.2	33.6	27.7	33.5	46.2	39.1	30.9	37.7	42.6	44.6
96.3	69.1	95.9	61.6	56.2	71.9	82.9	75.4	153.3	139.2	173.6
14.6	25.9	21.3	13.9	21.8	19.2	21.3	23.4	8.1	11.2	12.3
157	196.1	209.1	132.9	139.5	199.1	183.7	183.1	200.8	223.3	312.5
39	41.8	37.1	31.3	59.7	35	61.7	31.9	36.7	33.8	30
75	86	88.8	150	77.8	122.6	65.4	90.9	55.6	37.4	43.3
95.6	78.4	128	81.7	80.5	67.7	86.1	76	145.5	209.7	220.4
93.9	83.7	96	80.3	73.7	90.6	82.2	70.4	99.1	121.6	132.4
52.1	42.4	62.4	38.5	54.9	52	50.1	51	93.4	99.6	126.4
84.3	73.4	79.8	58.5	70.7	94.4	73.2	86.3	107.5	125.5	127.5
205.9	248.9	231.4	273.2	181.3	316.7	209.4	202.3	212	216	261.1
82.8	92.1	120	98.3	53.4	71.8	84	60.9	132.6	108.6	147.5
35.2	29.1	39.2	27.7	30.1	125.7	60.4	36.5	76.7	82.6	95
50.7	55	68.1	56.9	46	64.1	66.5	49.3	56.5	64.7	88.8
54.5	59.9	73.6	55.6	49.2	56.2	58.1	45	52.4	58.7	58.1
32.5	42.3	46.4	31.6	26.1	44.1	44.7	31.1	54.9	61.2	75.3
41	35.4	39.3	38.6	36	47.5	50.5	61.1	84.7	68.4	57.9
62.2	59	68.2	48.1	65.1	82.3	86.2	59	80.9	98.3	99.3
177.4	140.7	122.1	173.1	131.8	201.1	271	124.6	80.3	92	92.6
109.4	138.1	86.4	85	53.7	122.2	107.1	124.2	112.2	67.4	105.5
125.4	104	114.3	112.2	92.6	128.7	130	105.8	195.7	232.5	223.6
27.5	26.8	26.8	20.9	37.1	28.9	57.8	27.1	21.3	25.4	19.6
146.8	145.9	156.2	149.5	145.2	157.7	134.1	190.2	240.7	297.4	283.9
28	14.4	19.2	11.2	11.1	7.6	18.8	32.6	75.6	59.7	82
34.5	31.8	55.1	37	42.2	35.5	42.3	35.8	58.2	83.9	83.5
145	178.5	178.2	129.9	136.4	146.6	195.3	125.1	217.4	238.4	302.2
156.6	145	174.4	112.6	129	142	122.2	123.4	246	264.3	378.4
143.8	148.3	174.6	131	117.8	122.7	165.8	127	198.8	353.4	271.4
84.3	81.4	102	74.5	79.1	88.9	79.5	70.6	90.5	129.8	137.4
359.7	222.5	343.2	300.1	277.3	343.1	380.2	299.7	298	277.7	359.6

19	23.2	14.6	15.8	11.2	16.2	23.1	20.5	25.6	28.5	32.5
64.4	46.5	52.6	40.3	49.9	85.2	77.7	81	60.4	55.1	68
32.3	35.4	38.2	32.2	28.7	34.1	46.4	36	63.6	76.5	68.1
35.5	30.4	24.3	35.3	43.1	172.1	31	21.7	30.6	26.9	38.1
44.8	43.8	51.3	47.5	54.9	60.7	45.4	51.5	113.4	76.3	123.6
23.8	25.2	30.6	20.2	21.9	39.8	28.8	27.6	25.5	27.8	47.6
82.8	85.2	106.3	72	86.4	87.2	111.5	95	107.8	106.6	125.7
186.1	176.4	218	164.4	142.9	203.6	195.7	126	155.3	161.7	197.7
60	63.1	45.1	45.1	81.2	54.7	96.9	64.3	58.9	54.7	68.2
77.1	108.2	67.6	57	89	61.5	77.7	80.1	75.4	97.6	69.5
33.8	25.5	25.7	22.8	27.8	28.9	22.9	25.2	41.2	41.8	53
72.9	62.6	126.1	64.9	64.2	82.1	58.3	63.4	65.4	70.3	98.9
124.3	107.9	146	168.1	155.7	121.3	116.9	249.2	159.2	115.6	169.8
47	42.7	35.1	37.3	37.1	43.6	61.3	47	44.5	75.6	132.8
106.4	72.5	94.3	77.8	63.5	75.1	73	84.5	125.6	72.6	141.9
47.5	33.5	51	42.9	24.6	30.1	43.9	25.5	50.8	76.3	74.2
42.9	52.7	35.9	32.2	45.4	41.5	59.9	42.9	51.3	57.1	60
443.8	294	80.5	64.5	48.8	45.8	73	49.8	75.8	32.9	83.2
24.7	25.4	28.2	30.5	29.2	38.2	38.8	26.8	38.2	56.7	49
59.4	37.2	60	39	43	47.2	46.7	45.6	64	53.7	66.6
39	35.9	44.8	37.5	33.9	42.3	45.4	33.3	49	42.5	57.6
74.1	55.5	61.3	43.6	41.7	48.5	50	62.6	92	89.9	120.5
108.6	102.1	114.6	123.2	83.9	123.1	144.8	118.9	168.9	138	184.9
39.2	45.9	51.7	44.8	34.4	44.7	58.6	37.5	35.4	46.6	57.3
151.4	98.1	165.1	65.2	97.4	74.3	84.6	88	93.7	133	218.9
130	107.9	121.8	93.4	133.7	104.1	128	94.6	153.9	161.2	199.5
130.8	108.6	116	72.2	99	106.5	82.2	96.9	196.8	228.2	295.8
59.7	59.7	85.6	61.8	64	88.4	100.2	71	141.8	370.6	218
244.2	130.5	413.7	237.1	848.1	182.6	158.6	99.6	147.5	184.7	216.1
203	197.3	182.7	158.4	184.7	173.8	233.7	183.6	412.4	488.7	496.2

61	49.5	66.2	45	49.4	58.8	68.7	52.1	73.3	79.3	83
36.4	27.8	27.2	23.8	30.8	27.5	40.3	41.1	37.8	38.5	43
34.6	49.3	40.9	32.7	60.5	43.7	60.5	41.7	41.4	57.2	44
27.1	26.1	35.6	32.5	23.5	46.8	31.8	35.7	45	34.8	44.2
152.2	143.6	107.4	94.2	98.7	102.8	109.9	99.5	284.4	398.3	384.8
82.3	90.6	95.4	63	104.3	89.3	125.6	65.6	85.4	72.2	75.6
43.7	53.7	42.9	26.1	34.2	110.4	198.8	35.7	37.6	31.3	48.6
110	64.1	86.6	68	71	61.2	58	87.4	144.5	179.9	199.9
140.6	109.3	145.6	118.4	79.5	76.3	95.1	97.1	161.6	167.4	225.2
102.5	115.2	143.8	96.1	107	134.1	140.5	111.8	117.3	153.7	183.8
42.1	32.5	40.9	24.3	23.9	28	22.8	32.7	62.9	72.4	83.7
68.8	75	64.2	60.9	65.4	64.2	85.3	84.1	89.1	126.9	119.4
208.4	293.6	194.3	269	170.2	205.4	307.9	403	529.6	222.2	264.7
119	100.2	120.6	95.3	99.3	98.3	111.6	95.7	125.1	142.4	176.2
53.4	45.6	48.1	44.3	54.2	48.7	60.6	46.9	61.1	69.6	68.9
98.7	81.3	68.6	69.1	76.1	91.7	93.9	91.3	90.2	53.9	60.7
275.7	209	182.4	157.4	114.1	133.3	149.9	249.5	494.8	524.8	536.1
36.9	30.9	45.2	28.5	32.2	33.6	42	46.3	55.6	52.1	70.5
24.3	24.6	23.7	18.6	18.8	25	28.3	22.9	30	25	35.5
113.6	92.8	123	93.9	83.6	116.5	103.9	86.3	91.4	116	126.2
46.3	49.6	50.8	44.1	46.9	38.3	58.9	48.8	74.5	60.2	97.2
47	36.9	43.6	21	28.5	26.2	32.4	30.1	55.8	45.5	44.2
44.3	29.9	47	33.9	30.7	44.3	51.5	69.7	87.3	94.2	116.8
165.7	109.8	153.3	119.8	118.1	223	121.3	105.6	77.4	74	168.7
120.1	159.8	153.9	231.7	171.2	277.3	199.1	159	151.4	88.8	93.9
71	67.8	78.7	61.4	69.1	62.8	73.1	70.4	84.7	95	116.5
66.5	48.8	58.8	45.2	56	58.2	53.2	45.4	70.7	80.7	93.7
216.9	211.4	198.3	165.2	142.5	219	289.2	155.4	244.8	223.7	330
88.3	73.4	77.2	62.4	76.1	73.8	100.7	78.5	128.9	122.9	134.4
76	98.3	110.3	99.2	84.7	141.8	183.5	92.6	114.8	150.4	135.9
118.2	75.9	84.5	112.2	87.2	105.3	81.7	103.4	121.1	87	136.1
276.7	443.1	309.1	277.1	261.4	221.4	294.1	307.2	163.4	201.9	247.1
133.9	102.7	92.8	84.9	56.8	82.9	78.8	101.8	177	243.4	279.8

17.6	11.8	17.3	11	21.5	9.3	29.1	12.1	34.9	17.7	42.1
144.3	163.5	194.1	137.7	120.7	176.6	155.7	150.8	351.5	298.8	514.5
62.6	66.5	67.6	61.5	57.5	72	82.5	68.2	119.4	116.5	159.6
47.9	45.2	41.7	44.7	51.8	37.4	48.5	39.5	46.7	50	55.8
70.2	61.8	68.9	51.6	60	63.1	47.9	50.5	64.3	67.6	88.6
86.1	62.5	65.6	50.9	53.8	39.2	67	66	148.2	151	170.7
66.4	59.9	78.3	65.4	65.9	70.4	88	56.8	70	113.2	118.5
63.7	53.5	48.2	34.4	69.3	53.5	66	50.9	61.6	52.4	84.1
61	59.9	60.8	54.6	72.6	57.5	97.6	72.9	57.7	54.3	79.1
106.7	109.2	115.6	161.2	90.2	163.3	99.3	125.5	105.6	68.8	92.9
95	80.6	95	67.4	59.5	97.8	108.1	63.4	93.6	93.5	154.2
112.5	133.6	141.6	68.8	97.5	143.1	184.5	126.1	206.4	163.5	206.1
13.7	10.7	8.6	9.8	10.8	11.4	9.8	9.6	28.6	28.6	16.8
181	176.3	177.9	122.6	114.6	117.8	148.7	171.5	290.9	324.5	337
6.3	6.1	9.6	4.6	3.5	7.9	6.2	8.8	23.2	11.8	15.5
21	10.8	16.8	10.4	13.4	11.4	15.1	13.4	11.4	33.2	21.1
428.6	231.1	377.8	226.9	241.1	242.7	192.4	225.8	624.5	601.4	974.4
126.6	124.5	109.8	74.1	103.2	95.1	129.2	100.9	183.7	205.7	216
23.6	16.2	20.9	10.9	19.6	14	14.6	17.8	66.5	61.2	69.2
61	62.5	62.5	171.2	62.7	60.5	102.4	77.9	44.5	16	60.8
131.7	98.8	105.6	87.5	60.7	90.3	94.8	76.5	169.4	236.1	235.5
88.5	91.3	107.3	68.2	66.5	98.8	78.5	76.2	114	120.8	127.8
30.7	30.3	30.7	21.6	22.8	25.1	29.9	28.5	46.7	35.8	47.2
31.4	82.7	59.3	47.9	42	80.1	93.3	98.1	88.7	175.2	117.8
34.5	46	46.4	30.8	34.6	42.6	54.6	37.4	48.6	58.7	87.8
55.9	76.1	54.6	34.5	55.1	41.4	70.4	92.5	38.3	52	51.5
65.8	51.4	59.1	43.6	43.3	37.2	56.9	56.8	86.1	63.7	89.4
32.7	33.8	33.5	27.7	23.1	35.8	46.1	46.6	62.3	39.5	43.5

50.6	67.6	60.2	46.5	55.7	92.1	79	62.5	87.5	67.3	61.1
191.8	131.9	160.7	118.6	130.4	136.1	158.4	113	177.1	232.4	267.1
133.2	98.6	150.1	106.3	95.5	111.4	136.3	118.2	212.4	242.5	328.5
70.1	65.1	78.1	52.8	41.4	62.4	64.2	45.3	105.6	129.2	126.4
71.2	90.4	80.8	83.8	79	92.3	95.8	71.2	90.9	132.4	145.8
92.8	81.5	105.5	77.3	63.8	121.2	94.1	77.7	67.4	71.1	103.2
67.9	52.4	71.1	57.5	49.2	67.9	59.9	47.8	98.1	129.2	132.1
145.9	174.5	181.7	153.3	163.3	274.7	269.8	230.9	141.4	150.4	132.1
24.2	23.1	25.3	21.6	15.2	19.3	15.8	24	34.1	34.2	36.9
25.1	21.8	20.2	20.5	22.1	21	19.6	21.9	38.2	31.6	35.9
47.8	74.1	56.6	60.7	49.1	89.2	81.5	71.1	83.6	98.4	115.7
16.9	28.3	16.4	11.1	7.7	8.1	10.9	19.2	47.6	31.8	56.8

64.9	72.6	71.1	56.1	64.1	61.7	76.9	53.5	111.7	86.3	111.4
58.5	46.9	50.5	34.4	54.5	42.6	68.6	42.4	59.5	54.5	83.4
40.8	55	63.8	50.1	44.2	66.5	67.8	70.9	76.7	292.3	109.3
90.4	97.6	80.7	70.1	105.5	74.9	127.1	89.4	87.9	106.5	106.8
62.4	69.4	62.7	50.8	39.3	59	68.5	61.3	107	133.2	146.9
90.5	82.1	95.7	82.2	88.5	97.1	85.1	59.2	138.2	102.6	147
60.8	18.5	31	54.8	55.2	31	41.1	70.5	180.1	37.3	44.5
105.9	161.1	119.1	111.6	91.4	109.8	200.5	144.9	157.8	121.2	181.7
66.6	41.2	64.3	54.1	49.1	40	56.6	48.3	113.7	83.9	113.2
131.7	115.3	127.1	96.3	113	108.8	124.9	103.6	124	124.5	177.3
46.1	42.7	47	36.5	32.4	37.2	42.2	33.1	48.1	53	72.9
153.6	99.8	131.9	89.5	101.9	107.7	118.7	111.4	186.3	241.6	303.8
482.2	155.4	206.4	136.2	144.3	134.6	178.4	172.5	349.5	520.8	384.2
88.6	88.3	97.5	98.3	95.5	107.5	85.2	71.5	93.2	117.6	133.2
234.4	67.1	121.5	81.6	85	47.2	60.7	69.1	457.8	210.8	529.4
32.6	29.4	41.2	41.7	28.8	42.3	44.7	28.3	39.2	38.1	53
48.4	43.8	60	35.2	33.8	40.1	44.9	43.6	51.6	56.7	68.9
45.2	45.2	43.1	34.2	43.4	36.1	35.7	41.5	99.6	91.3	107
38.9	21.7	29.3	21.1	22.4	23.4	22.6	18.1	43.7	61.8	67.8
58.8	47.8	72.1	44.6	75.1	62.1	65.8	51.9	84.8	101.1	78.5

44.4	31.7	36.9	23.2	34.5	30.1	41.8	36.4	60	59.6	54.8
78.9	62.8	88.5	50.1	58.4	77.3	46.4	43.4	137.6	231	257.1
70.6	92.4	80.6	51.7	57.6	70.2	70.3	66.5	63.7	81.9	96.4
73.6	100.4	114	112.1	76.8	124.1	104.8	130.5	350.5	182	277.9
377.3	234.5	368.6	264.3	222.6	307	352.7	223.3	534.4	319.6	588.3
137.3	124.7	127.8	97.9	89.8	118.7	127.7	118.7	120	126.1	146.9
40.9	35.5	48.4	42.2	38.6	55.5	55.3	43.6	57.2	44.2	64.3
87.2	87.5	80.1	89	57.6	80.1	101.4	105.1	147.7	127.4	169
131.9	122.8	124.6	109.7	74.8	103.9	117.3	95.9	205.2	192.5	279.4
118.9	134	102.3	94.6	116.7	119.1	78.5	126	148.9	86.7	192.1
39.5	44.3	35.3	36.7	31.7	36.4	42.4	38.6	57	70.8	63.3
69.7	88.6	105.4	96	115.5	126.5	135.5	111.4	170.6	121.4	122
62.7	53.7	99.8	49.4	80.7	60.5	43.2	30.9	93.5	123.1	199.9
56.8	48.6	53.6	54.2	41.2	72.4	58.6	44.6	84.5	136.6	111
24.2	18.6	23.9	26.2	22.2	18.7	28.5	33.3	83.9	76.1	59.6
27.4	36.7	27.1	29.5	29.3	35.2	42.6	29.4	39.5	35	54.9
92.4	44.9	89.1	30.9	50.9	35	39.9	36	65.9	92.7	128.8
88.5	101.6	85.3	62.6	56.4	91.8	96.4	72.7	108.7	128.6	144.5
196.9	161	163.7	94.7	96.9	152.9	133	93.4	348.9	281.1	345.1
28.4	31.2	36.4	28.8	43.7	50.4	56.1	31.1	39.7	44.6	55.6
63	55.5	63.7	50.8	36.5	52.2	59.6	64	154.3	215.7	126.3
93.9	59.4	87.1	60.1	74	86.3	75	65.3	99.7	118.2	134
138.3	144.5	144.6	197.9	94.7	89.4	109.8	121	126.8	208.4	221.4
83.8	52.4	70.7	53	57.8	48.8	45.5	45.7	121.4	136.3	141.4
180.2	117.1	185.1	143.3	131.1	144.7	156.4	101.6	153.4	173	202.1
219.7	196.2	206.1	177.1	161.7	185.5	199.7	132.8	210.6	255.2	315.3
130.9	108.1	124.2	74	94.1	105	88	96.2	170.6	160.7	244.1
49.5	58.9	64.4	52.3	46	69.1	81.3	62.1	80.4	103.1	85
66.6	52	57.8	45.3	38.8	49.8	47.6	43.5	82.1	128.1	98

148.6	121.4	129.6	91.7	117	101.2	126.8	117.6	214.3	224.6	226.7
10	12.7	15.2	9.9	5.4	9.5	11.5	12.6	17.5	43.9	22.6
174.2	113.1	152.5	122.4	127.3	179.2	96.7	91.3	154.7	167.8	225.3
29.6	25.9	29.6	18.3	17.8	24.8	18.7	24.2	50.1	49.8	79.2
37.6	42.6	54.7	50.6	35.5	66.2	40.2	43.8	100.9	137.2	123.1
17.7	16.3	16.7	14.1	16.6	24.8	25.9	18.1	14.8	14.2	16.9
98.6	119.2	112	113.5	66.9	101	132.8	125.8	210.7	177.8	364.9
69.7	55.5	77.6	60.6	51.7	72.9	58.4	53.7	95.4	91.5	126.3
86.2	123.4	125	121.8	100.3	117.7	193.1	85.9	113.1	149.2	179
290.7	931.8	386	561.6	332.3	241.3	331.7	354.7	414	384.5	418
25.2	33.2	29.8	37	34.2	105.5	43.3	43.4	42.5	40.4	47.6
73	70	79.5	55.5	66.6	70.3	70.5	67.7	61.5	103.3	95.2
29.6	40.8	48.1	36.9	43.5	42.9	39.1	37.7	39	53.4	80.2
66.3	59.8	73.2	53.6	61.6	66.7	71.1	67	96.5	117.9	127.2
78.9	93.9	88	74.3	69.2	167.7	99.5	81.4	101	72.6	108.6
22.1	18.3	7.7	7.5	17.7	10	13.6	12.1	45.9	22.6	29.2
125.2	58.4	113.4	79.9	92.4	158.8	67.3	83.4	54.4	45	70.2
150.6	182.3	120.9	71.6	69.1	96.8	107.1	145.9	240.2	188.1	277.4
9.8	6.6	14.9	10.7	10.1	8.9	4.7	12.5	14.1	17.4	18.1

67	32	65.6	48.3	53.2	43	42	49.1	37.4	75.9	84.5
57.9	48.2	36.8	33.4	56.9	47.3	65	48.6	68.6	59.4	54.2
72.1	57.2	71.7	58.9	60.9	61.2	63.1	69.4	104.9	128.3	130.8
160.1	162.2	161.8	151.8	122	158.3	157.5	142.5	285.5	272.1	331.7
175.1	73.5	89.5	87.4	73.9	69.4	67.3	51.1	69.6	87	113.1
258.6	259.7	277.2	247.1	200	295.8	291.5	243.4	251.5	308.8	342.9
29.8	29.4	36.6	34.6	38.4	80.2	55.1	48	42.5	55.7	41.3
83.8	94.1	105.4	90.2	76.5	83.7	137.6	95.3	101.3	152.7	179.7
31.3	20.5	25.1	24.3	18.1	15.3	14.5	23.4	26.1	17.1	24.7
60.6	47.2	67.9	52.9	42.8	50.5	54	46.3	105.2	287.2	181.6
27.5	33	28.2	22.2	33.7	32	31.1	24.7	31.5	26.6	44.4
32.8	29.9	29.6	21.2	25.3	21.9	27.8	46.8	40.2	24.1	40.3

239.5	278.4	292.2	230.5	175.6	278.8	170.2	231.4	248.3	204	244.6
247.8	201	167.1	178.8	169.7	298.1	411.4	346.1	215.4	203.5	161.2
80	87.6	83.8	67.6	61.2	71.2	67.3	70.4	151.1	188.7	202.8
89.8	80.7	72.8	57.2	58.2	62.4	77.9	98.1	97.1	132.2	117.7
71.7	83.5	88.3	72.1	53.3	97.2	83.1	81.9	123.9	137.4	148.1
71.2	53.8	78.9	43.2	49.7	48.2	59.2	51.2	83.6	112.3	145.5
188.7	143.4	185.3	163.1	165.7	139.4	131.9	130.4	275.7	237.1	297.2
82.2	85.7	106.4	96.4	108.8	129.6	84.9	80.9	110	117	177.1
83.3	75.6	57.4	74.9	41.7	46.7	55.1	109.2	137.2	142.8	200.3
75.4	112.9	89.3	95.7	76.7	226.9	105.2	158.6	79.6	70.5	91.3
146.8	116.4	138.2	119.9	140.5	114.9	130.3	137.4	192.3	233.6	209.2
28.4	26.8	26	22.4	18.6	24.8	26.9	19.9	43.4	64.5	53
42.8	58.1	39.6	51.8	53.5	49.2	57.8	45.8	51.7	74.6	57.7
107.2	107.5	80.8	81.9	68.5	84.3	79.8	86.4	105.6	135.8	143.2
45.2	37.9	34.6	30.3	38.9	34.2	56.4	44.8	58.3	53.8	69.1
156.8	159	159.2	189.8	115.5	151	160.9	128.2	176.6	302.9	376.9
44.8	49.3	38.7	33.5	63	54.6	65.1	41.5	48.5	38.8	45.2
92.5	92.2	115.8	101.5	79.8	136.9	123.6	77.1	112.5	107	155.1
73.5	74.9	72.7	62.6	56.3	85.2	100.2	57.2	95.3	126.8	151.5
102.8	94.6	105.8	90.4	71.1	78	71.1	83	81.6	89.9	107.4
33.3	59.6	125.9	100	76	144.1	45.6	187.8	188.2	358.9	419.2
99.3	79.9	97.9	77.6	82.8	77.2	94.7	81.9	69.9	119.9	127.9
48.3	33.7	46.2	41.2	41.8	60.1	49.9	42.1	52.6	50.6	71.4
259.3	179.7	231.9	152	162.8	147.7	146.8	123.6	133.4	204.4	200.7
105.5	61.7	64	50.3	53.7	50.6	38	48.3	92.2	141	154.9
101.8	83.1	61.5	93.2	67.2	53.7	65.1	50.2	78.1	76.9	73.9
294.5	152.9	186.9	108.1	109.2	75.6	152.8	135.8	125.2	137.9	152.8
135.7	67.6	88	70.1	74.6	66.6	85.5	66.6	116	181	199.1
68.6	64.3	65.9	54.3	41.8	58.8	62.8	62.1	108.9	93.9	141.3
47.6	49.3	62.1	46.7	53.1	60.7	52.7	42.8	46.9	50.3	57.5

50.5	32.5	43.5	30.5	65.6	64.2	71.3	71.1	109.7	86.6	69.1
95.1	96.5	108.5	90	71.8	107	85.8	83.1	152.5	117.1	122.6
16.3	12.1	12	19.3	13.2	27.5	80.5	17.1	98.8	221.6	67.9
38.9	28	27.4	28.7	20.3	25.4	31.5	26.3	29.9	43.2	47
55.3	54	52.7	44.9	40.9	52.7	46.6	40.6	76.5	105.5	109.6
33.6	28.2	28.7	26.3	23.7	22.5	26.3	18.7	23.4	22.1	25.3
52.1	29.7	43.3	28.8	29.9	42.3	41.3	36.9	78.7	77.4	102.8
159.8	166.6	185.2	165.1	155.3	249.7	187.5	179.8	154.2	238.9	222.8
42.8	46.5	48.9	42.9	39.3	48.9	42	53.2	59.3	44.4	40.5
39.6	24.2	36.3	27.4	27.2	31.6	36.6	28.5	48.4	59.6	74.3
120.2	74.6	78	72.9	31.4	62.6	54.8	52.4	406.4	155.2	298.7
99.5	198.5	100.8	120.2	123.6	285.9	317.2	286	349	109.1	214.9
72.8	48.2	76.7	62.6	86.8	60	52.1	50.3	110.2	130	145.9
89.1	67.7	82.9	70.7	87.6	70.1	80.8	59.9	93.4	165.5	186.3
92.4	98.6	116.1	74.8	89	123.2	77.1	86	67.5	58.3	70.3
140.5	130.8	166.4	119	108.7	167.8	111.7	119.2	142.5	181.9	226.4
60.1	78.7	77.7	40.3	50.9	39.5	70.6	62.5	258.4	250.6	308.2
41.5	18.7	30.7	21.9	21.8	21.1	14.7	28.1	32.3	55.5	54.6
234.3	209.5	189.9	148.6	183.1	183.7	315.4	188.9	274.6	241.3	289.8
5.9	5.5	6.4	5.8	4.4	4.4	4.4	2.5	31.6	31	243.9
598.7	70.4	24.8	61.6	55.3	51.7	80.4	114.2	84.2	88	81.5
71.4	74.8	73.7	56.9	56.3	61.6	60.3	62.8	90.4	88.7	103.4
53.8	55.2	56.1	55.8	46.9	52.8	68.3	51	85.1	158.1	98.6
172.5	158	177.9	132.7	127.1	150.8	154.3	126.4	310.4	430.9	372.2
99.5	99.6	101.2	92.7	79.8	90.5	131.8	86.8	97.3	90.4	113.1
33	21.8	24.8	15.9	31.5	29.2	30.1	27.3	29.5	27.9	30.6
22.3	28.1	25.7	24.8	27.8	25.1	33.3	26.3	32	39	42.3
61.9	167.9	135	224.5	97.5	262.2	58.8	39.5	6.6	8.7	27.9
151.8	119	234.6	138.7	121.9	102.7	84.9	92.7	82	167.4	162.5

147.4	109.2	98.6	89.6	88.6	80.2	87	75	120.7	150	164.1
105.4	25.3	21	14.7	27.5	29.7	39.7	27.7	75.1	64.5	97.9
67.3	68	69.1	53.7	53.3	61.4	76.8	57	95.7	134.2	117.9
437.7	334.7	645.7	382.2	281.5	686.8	411.4	348.1	216.1	182.8	338.6
76.7	86.5	52	47	105.4	72.6	108.2	63.2	58.8	49	67.5
85.6	80.7	102	73.4	90.3	93.1	80.7	67.5	168.9	212.2	238.4
142.6	65.7	89.1	65.7	66.9	89.9	76.4	94.8	180.3	97.4	125
1470	1428.8	1541.3	1485.9	1694.6	1284	1527.4	1387.2	1220.9	911.3	1266
49.5	37	48.4	33.7	26.2	32.6	33.1	50	59.9	74.5	83
29.4	24.4	21.4	20.2	24.1	23.2	32.1	18.3	31.4	19	52
106	94.6	99.1	77.9	94.2	89.6	91.3	78.4	132.7	148.6	163.5
110.2	111.2	105.7	70.5	77	87.8	126.8	82.4	175	170.1	246.5
84.4	85.6	99.9	86.1	55.2	58	91	74.7	133	190.4	201.6
46.8	59.4	64	65.8	48.1	64	58.9	42	59.9	61.9	71.4
37.6	21	31	31.4	32.1	26.1	25.6	19.1	57.1	65.4	81.2
82.3	72	82.2	69.7	96.3	56.4	67.1	104.5	109.5	126.1	127.7
189.6	165.1	153.4	124.7	110.8	146.2	229.2	231.3	234.5	257.1	293.5
21.3	21.9	18.4	17.2	23.8	27.3	35.7	23.4	43.4	44	45.5
57.3	36.8	52.1	40.2	34.3	36	59	66.2	103.6	134	129.3
89.9	71.1	71.5	60.1	83.1	75.6	64.8	68.2	99.2	130.6	158.6
79.7	89	92.2	59.9	53.1	67.6	81.3	60.3	91.4	63.6	101.3
48.1	35.6	57.1	40.6	37.1	49.1	41.1	43.5	50.9	65.5	78.5
112.8	87	94.6	70.2	76.6	76.9	90	95.6	160.2	147.6	200.6
27.3	26	24.1	17.6	19.2	27.4	27.7	30.1	38.9	50.4	47.7
133.2	141.7	156.4	117	104	146.9	281.2	144.5	124.5	108.9	123.6
189.2	102.4	146.9	112	107.6	107.6	80.4	87.6	119.2	175.3	242.3
168.8	146.5	124.2	107.9	120.2	119	143.3	114.5	217.2	318.6	284.8
337.9	52.5	37.7	32.1	38.2	13.1	25.4	25	26	53	41.8
99.4	74.9	91.7	69.2	82	92.2	125.6	88	101.5	125.5	154.2
149	161.9	155.8	112.1	104.4	128.6	156.6	143.2	259.4	205.5	285.9
15.6	24.8	25.5	30.4	22.6	45.4	23.5	37.9	38.2	29.1	37.8

57.4	59	45.1	42.5	41.9	51.9	63.3	57.6	90.8	86	123.6
16.4	64.5	20.7	11.7	7.5	22.9	45.3	29.7	66.3	27.1	28.9
27.6	25.5	34	18.2	20.6	25.8	28.9	22.4	41.2	44.6	60.7
23	31.4	34.3	32.4	22.5	28.5	31	21.7	58.3	59.7	93.4
82	97.6	84.5	89.7	73.5	97.3	121.6	116.7	85.9	86.6	95.7
80	69.2	96.5	65.8	63.9	72.6	81.3	68.6	158.9	280.9	207.5
89.2	55.3	81.5	73.4	65.4	63.2	78.6	57	257	218.3	228.7
21.9	19.8	24.1	22.2	17	24.9	23.3	18.2	66.7	119.6	89.7
164.1	131.9	146.6	119.7	100.5	99.3	93.1	85.8	170.3	204.3	275.9
55.4	38.7	32.9	31.2	37	46.8	42.7	44.1	71.4	47	83
17.1	21.6	19.1	14.2	12.1	21.9	14.8	11.8	27	33.7	30.2
25	24.2	19.8	22.5	24.3	29.9	27.3	31.3	24.4	39.2	46.4
42.8	60.2	65.2	56.2	38.5	50.3	44.7	67.4	130.5	188.7	142.3
87	114.3	96.1	136.2	141.1	140	170.1	100.6	104.9	87	74.2
68.3	88.3	87.6	76	98.8	458	84	35.5	68.6	83.5	114.1
100.9	95	114.3	78.9	84.6	96.6	99	102.5	175.8	146.5	194.7
98.1	93.7	89.9	76.9	60.4	81.6	81.2	82.2	111.6	151.9	151.4
199.1	145.4	198.7	159.5	189.7	206.1	254.3	192.3	201.6	256.9	279.3
144.2	132.6	147.7	143.7	138.2	115.9	155.8	159.2	162	130.6	163.1
274.9	229.3	116	87.8	253.3	133.4	302.1	223.3	83.6	115.7	83.2
37.4	56	38.4	33.2	41.2	40.7	43.5	31.2	32.2	32.3	50.1
49	58.4	37.3	28.4	22	38.8	36.6	44.8	21.7	40.6	34.9
85.8	73.3	64.8	63	55.5	59.8	63.3	62.2	141.6	184.2	181.8
53	128.6	129.5	83	73.2	165.2	101.8	84	140.2	68.6	65
34.7	33.1	53.1	48	43.8	32.9	45.8	49.3	42.9	48.1	69.5
141.1	124.5	177.4	134.3	122	128.6	237.7	143.6	239.5	628.2	349.2
81.8	77.9	109.6	77.5	81.1	79.9	86	80.8	80.8	69.9	92.1
137.8	148.1	149.8	103.5	86.3	140.2	133.3	127.9	174.6	207.8	227
127	124.5	112.6	115.8	98.2	123.6	113.3	107	149.6	150.5	170.1
101.5	33.6	46.8	47.9	45.3	35	34.1	34.5	50.5	48.2	79.6
22.3	22.4	19	14.2	18.6	23.3	21.9	21.5	30.7	40.4	25.2

118	82.1	83.6	61.3	68	69	56.4	63.4	94.9	111	121.3
65.7	61.2	81.7	57.2	48.6	68.9	63.9	53.6	84.2	105.3	106.2
38.2	31.3	33.9	28.3	24.9	38.8	23.8	41.6	56.7	45.5	65.9
278.3	146.8	125	103.9	166.4	197.7	263.7	105.4	75	135.4	101.4
81.2	96.2	100.7	96.4	75.6	126.7	80.7	104.3	98.4	98.6	150.3
80.2	79.8	96	71.1	117.3	90.7	91.2	109.5	76.5	110.4	121.5
66.5	52.5	61.4	72.5	50.2	64.3	53	63	79	99.2	95.9
15	12	12.2	13.4	18.6	17.3	22.6	27.6	16.2	14.3	15
45.3	36.5	52.9	40.9	55.2	53.2	56.1	49.4	58.2	57.1	70.8
47.5	33.3	55	33.9	46.5	65.5	36.5	51.1	52.3	50.2	66.3
42.6	58	53.5	29.3	29.3	56.2	42.5	43.6	55.2	51.7	65.7
80.3	79.6	75	50.7	57.1	74.6	90.5	69.3	96.3	125.4	135.9
86.6	88.1	94.2	53.4	76.1	104.1	134.3	87.4	138.6	147.5	179.5
155.9	104.6	153.9	152.5	98	83.7	51.6	103.1	211.8	244	213.4
28.2	29.1	41.8	30.5	31.1	30.4	30.7	24	51.9	63	58
61.8	65	71.4	65.7	42.6	66.8	59.3	81.1	128.5	136.2	127.8
117.8	113.3	75	71.5	154.1	91.7	146.7	113.8	82.1	90.9	78.7
184.9	138.7	178.2	115.5	88.4	91.1	65.4	78.3	59.6	57.2	92.3
146.3	87.8	101.2	98.9	99.8	84.9	98.5	129.9	68.6	63.1	78.8
250.9	181.5	176.2	178.2	161.8	212.5	138.9	184.8	239.4	201.7	303.6
187.3	200.9	287	172.6	181.9	225.4	231.9	183	436.2	553	552.4
126.9	108.7	208.6	260.3	105.3	71.3	140.7	107.5	302.7	105	277.5
127.2	108	153.9	220	75.5	111.5	209.1	124.5	135.3	138	249.1
82	87.5	67.7	49.6	52.4	65.9	76	61.3	144.8	132.4	199
138.9	86.1	118.3	101.8	104.5	103.1	118.2	94.6	195.5	186.5	198.4
82.9	102.3	47.8	62.9	75.7	127.6	279.7	82.5	20.2	29.1	27.2
37.8	41.5	49.9	33.4	38.7	44.9	42	43.1	74.4	96.8	73.6

44.1	59.8	59.3	48.9	55.2	54.1	71.3	47.1	44.7	37.4	66.6
35.2	38	42.5	40.8	34.3	46.6	41.3	37.7	54.6	60	88.6
363	324	317.6	333.9	397.7	321.7	357.6	263.8	182.3	122.7	146.1
9.7	9.4	10.5	8.5	9.3	11.5	13.5	9.2	15.1	19	22.5
75.3	70.7	75.5	65.6	69.9	99.5	79.7	73.7	86	156.5	115.5
131.7	95.6	108	91.3	93.6	118.9	67.8	76.3	112.4	116.5	189.8
131.8	94.2	108.8	88	77.1	69.1	102.7	81.4	125.2	147.2	153.3
80.5	69.2	84.9	74.2	66.6	80.2	77.4	77.6	102.8	125.5	121.8
68.3	58.1	57.8	51.2	48.3	58.5	54.7	51.5	73.7	76.8	94.7
72.9	69.2	68.4	53.8	48.3	62.8	69.4	74.4	177.1	126	153.2
65.3	40	36.5	20.4	28.8	32.5	43.2	37.3	50.8	58.4	70.4
89.4	66.4	77.6	73.2	57.8	77.6	88.3	72.1	60.6	120	124.3
35	46.3	37.3	46.3	62.9	59.6	51.3	51.4	56.3	39.9	53.8
80.2	71.2	72.4	54.4	66.8	74.8	76.6	73.1	124.8	133.2	162.4
10.6	6.3	9.5	7.7	6.8	8.1	15.2	8.1	39.6	16	65.1
487.4	451.2	495.9	363.7	326.8	393.8	507.5	328	741.4	953.8	1041.3
67.7	74.7	67.7	68.1	63.4	89.9	133	85.4	146.5	141.8	162.7
158.1	155.2	161.7	101.8	100.9	113.9	205.5	163.8	252.2	306.7	292.5
58.5	57.5	74	53.9	50.2	90.6	74.6	75.2	113.1	106.5	129
83.9	93.7	129.2	178.1	95.7	158.2	102.8	116.6	66.2	70.9	103.2
29	31	34.8	23.2	25	27.6	36	28.1	40.8	47.1	36.3
1040.8	1132.1	1234.7	1230.9	1215.5	930.2	1213.6	792.1	929.2	743.2	984.6
44.4	34.1	43.5	33.5	29.6	38	35.1	33.3	51.5	64.8	81.1
75.9	105.1	122.2	128.2	85.3	129.7	84.8	92.4	100	110.9	133
16.5	17.5	19.7	15.1	18.5	28.8	21.8	17.6	21.1	20.7	23.3
53.1	43.6	64.9	42.3	42.1	31.9	33.9	29.8	72.2	105.8	120.2
43.7	36.6	49.3	37.4	37.6	48.5	52.8	38.8	52.3	62.5	74
239.9	200.5	209.5	141.8	202.6	181.6	218	171.2	351	418.8	386.4
108.8	119.9	80.6	54.1	60	58.4	66.9	60.7	112.2	100.9	124.1
13.9	29.7	51.2	50.8	23.3	38.8	24.6	82.5	95.7	85	168
141.1	227	180.9	185.8	144.1	243.2	193.1	153	360.7	254.7	230.9

44	59.9	48.6	48.1	42.8	40.7	70.3	53.3	43.4	32.6	37.6
385.8	95.9	122.3	161.7	132.4	160.9	180.2	247.1	286.8	218.1	313.3
26.3	33.6	38.3	33.2	35	77.2	37.7	39.6	115.6	212.2	169
51.1	52.5	50.2	61.3	62.1	60	76.7	54.7	75.3	62.2	73.5
35.7	29.9	31.1	31.2	29.1	29.1	35.8	25.6	81.3	35.4	81.5
83.3	58.7	82.6	47.3	45.8	50.9	62.9	52.2	116.8	140.4	142.6
79.1	83.9	110.3	80.6	82	78.2	68.8	70.2	100.4	121	158.3
40.1	29.9	32.7	26.9	36.5	30.9	30.8	28.3	48	44.8	59.2
85.2	59.2	137	111.2	92.7	84.9	66.6	72.7	125.3	94.3	124.4
29	31.6	25.3	26.6	20.5	35.5	34.7	33.1	30	29.1	46.2
95	100.8	113.1	118.4	116.3	103.8	121.4	119.1	146.2	254.5	201.3
545.7	391.2	438.6	314.4	461.2	438.6	420.8	414.3	573	532.9	639.6
52.5	35.2	61	46	30.4	41.8	33.3	34.2	38.6	39.5	49.9
36.9	29.4	37.5	29.8	28.8	35.9	37.2	43.5	68.5	85.3	74.1
75.3	85.5	88.2	73.8	66.3	100.1	85.1	86.9	160.4	82.5	128.5
61.6	92.8	100.4	57.1	48.3	77.1	110.6	65.9	71.2	71.9	101.1
24.5	18	19.8	32.8	22.3	20.1	18	13.9	51.3	21.6	40.6
65.6	59.5	62.5	39.7	43	60.6	77.9	48.6	69.2	72.1	92.7
100.1	84.2	56.6	38.4	64.6	57.5	94.1	66.3	153.9	128.7	194.4
106.7	96.7	119.6	100.8	105	127.9	138.6	100.3	207.2	283.8	244.9
54.5	53.7	56	41	34.5	57.2	51.7	44.9	56	58.6	75.7
156.5	117.1	134.7	86.6	70.2	93.6	75.4	95.6	186.9	222.6	292.1
73.5	60.4	67.1	47.4	32.4	44.6	65.6	72.3	114.6	98.1	143.7
35.2	23.6	31.4	19.7	23.4	23	21.5	19.3	36.9	39.9	45.6
120.4	50.3	355.3	151	281.3	127.5	62.6	134.8	65.4	94.9	166.8
43.2	30.6	29.3	32.6	32.7	41.5	63	33.4	34.4	54.3	50.2
29.5	32.2	110.5	46.5	115.1	47.9	100.1	38.2	58.5	42.7	70.7
71.6	83.1	90.5	76.4	74.9	75.4	84.8	79.5	76.5	84	86.9
147.6	141.7	184.3	108.9	107.6	148.6	128.4	98.8	256.8	275.6	390.3
68.9	66.1	75.2	80.9	54.7	94.2	109.7	61.6	43.3	95.7	74.2
526.3	390.7	477.7	292.6	331	375.4	322	242.9	828.5	1056.2	1211.5

159.9	127.5	215	111.6	196.5	123	150.2	169.7	196.1	157.7	191.3
166.5	262.6	210.5	220.3	149.3	349.4	163.1	288.7	143.4	199	195.4
3.1	5.5	11.1	5.2	4.2	6.1	8.6	16	124	17.8	7.5
109.4	114.3	153.7	88.7	129.8	108.8	112.5	125.2	180.4	194.8	192.7
48.6	35.3	36.7	25.2	28.8	33.6	30.7	28	78.6	90.9	102.4
12.4	12.8	13.8	11	7.2	12.9	13.8	10.9	11.5	11.1	17.3
101.6	80.9	93.8	103	100.3	93.3	101.3	90.2	136.5	112.9	169.4
218	179.1	217.9	168.8	178.3	164.8	124.5	144.4	190.2	227.7	290.5
9	7.9	11.1	10.5	9.6	10.9	10.4	11.6	16	14.7	11.3
68.3	91.6	50.5	88.3	37.8	57.2	26.9	40.1	86.9	88.9	136.1
80.6	75.2	87.6	67.7	53.7	78.3	65.8	69	116.4	115.3	129
24.7	22.4	15.2	20.6	28.6	23.5	32.7	21.1	28.4	33.5	26.8
442.6	286.9	290.8	230.7	178.3	224.3	220.4	292.9	595.4	659	842.5
149.9	193.8	343.1	243.4	176.1	330.9	176.9	207.4	117.3	94.3	178
23.6	23.2	17.7	19.4	20.4	19.2	23	25.5	34.2	31.8	34.1
30	25	19.7	14.6	26.1	21.8	29	30	27	32.5	35.1
42.1	50.9	36.8	34.6	46.3	48.9	67.6	44.6	52.4	58.8	65.3
39	51.1	65.9	45.8	51.4	79.3	29.4	52.3	53.3	55.7	71.7
31.8	36.5	34.3	33.7	26.1	50.2	51.3	50.5	46.6	40	43.5
90.6	71.5	87.8	72	83.1	86.4	115.6	90.7	104.6	101.3	136.5
77.5	77.9	85	63.6	67.7	73.2	84.3	72.3	81.5	104.4	107.8
97.2	86.5	92.6	67.5	81.6	122.4	194.8	89.8	75.4	109.2	122.7
69	59.9	68.7	51.3	51.7	68.8	61.7	46.5	124.8	116.4	140.8
42.4	41.1	33.4	35.2	50.2	43.3	43.8	41.6	81.7	181.5	106.2
74.5	79.8	66.7	67.6	64.6	62.6	80.8	73.2	75.5	55.8	74.8
52.7	43.1	62.6	37.4	38.5	39.5	50.3	45.6	106.6	119.9	131.7
58.4	71.3	64.9	78.9	56.3	63.3	86.9	86.1	88.9	68.2	111
178.4	150.3	163.8	160.3	167.7	144.1	173.3	212.2	119.9	72.4	140.7
79.7	82.1	89.4	58.3	59.5	76.1	85.7	64.3	87	108.4	144.7

111.2	95.5	95.1	71	67.8	87.3	104.9	75.9	87.8	106.6	119.4
65.6	55.8	57.2	56.4	63	58.3	59.5	62.8	68.7	72.9	90.2
44.5	92.1	101.8	133.6	81.7	155	68.9	77.8	55.3	40.6	62.9
65.4	80.3	72.6	62.2	62	75	134.7	64.5	59	86.5	77.5
214.3	177.5	239.4	189.3	166.9	161.4	188.6	207.6	295.3	316.3	398.7
89	79.9	72.5	73.6	52.6	65.7	59.3	75.6	205.8	213.7	228.9
71.2	61.9	50.3	62.6	75.6	56.3	71.6	78.7	57.4	39	46.6
293.5	268.1	327.9	208	198.3	214.6	232.3	240.6	510.3	417	574.1
221.2	154.6	162.2	122.7	203.2	157.1	224.7	178.1	251.2	306.9	317.9
89.2	64.1	57.3	49.2	139.8	74.3	217.6	69.9	61.8	87.9	97
286.9	245	273.5	221.9	230.4	250.1	220	224.4	301.7	373.3	475.6
31.7	32.1	29.1	25.8	16.9	26	39.8	40	48.8	39	44.1
216.1	259.9	275.5	395.7	190.8	403.8	191.6	321.8	114.7	88.9	137.1
22.5	36.1	27.4	25.7	19.5	14.6	16.3	18.2	28.2	29.3	42.1
431	194.8	319.4	242.1	186.4	212	179	196.4	385.3	507.3	585.3
16.1	10.8	15	12.4	10.3	10.6	14.5	16.8	31.9	24.7	20.7
126.2	89	127.7	100.8	90.2	93.4	88.8	81.2	191.5	239.6	290.3
219.1	215.6	230.7	266.5	182.3	206.6	279.4	216.7	347	442.5	495.5
139.6	128.5	94.5	102.6	58.1	79.8	129.2	472.3	158.7	168	236.9
79.1	70.9	97.6	78.2	67.6	92.3	87.4	77.5	97.6	99.4	143.5
133.1	49	66.8	44	60.3	51.7	51.3	43.1	101.4	144.2	121.7
215.2	149.5	171.8	104	99.9	122.7	128.6	137.9	279.1	391.7	424.5
59.2	57.8	63.9	47.3	43.3	52	53.6	49.7	138.3	549.5	203.3
86.1	83.6	104.4	72.3	55.6	95.2	108.6	91.5	156.2	223	230.7
70.3	70.9	71	61.3	47.5	81.7	67.9	63.8	106.8	107.2	162.8
370.1	59.4	121.1	62	109.6	59.4	43.3	40.3	61	106.4	108.6
135.1	146.5	144.3	101.9	111.6	118.9	116.9	119.6	432.1	356.9	465.5
157.8	202.1	228.2	253.1	152.3	200.2	233.6	228.4	169	151.7	177.9
450.1	518.5	157.8	184.9	391.1	111.8	1140.8	290.9	262.9	184.1	216.3

57.7	55.7	49.6	38.3	43.4	54.1	45.1	37	67.6	71	88.4
250.6	271.9	254.9	175.4	110.9	160	157.5	271	155.8	186.4	180.6
56.5	74.2	78.7	60.3	52.9	81.1	100.8	75.6	82.2	85.2	87.1
199	132.1	156.3	81.9	97.3	103.8	83.5	83.6	266.1	270.8	340.6
93.9	65.3	70.9	66.5	65.6	68.2	87.9	85	121.8	159.9	154.4
102.1	98.9	96.9	145.1	78.4	66.9	76.4	65.6	99.4	113.9	184.4
386.7	158.6	227.5	140.5	103	99.5	103.7	144	272.5	491.8	564.8
75.8	49.5	76.3	41.4	35.7	49.7	72.2	52.4	129.9	192.6	231.9
28.8	47.4	50.6	54.1	37.1	53.2	53.6	71.1	29.3	28.6	44.4
126.7	99	82.7	76.9	42.3	45.3	76.2	128.2	218.2	210	191.4
132.2	135	148.9	100.9	126.7	121.7	227.3	133.6	216.6	248.6	259.2
181.1	110.1	132.4	100.8	117.4	112.7	136	101.9	245.5	282.8	296
61.3	56.8	63	82.3	51.8	127.1	106.3	119.6	366.8	71.1	92.6
71	58.2	60.9	49.5	63.1	55.2	69.9	59.6	102.2	147	153.2
147	81.7	117.7	95	69.8	79	82	83.8	258.8	195	292
115.1	88.8	117.9	104.4	84.5	107.6	156.8	114.3	124.6	127.2	160.9
184.8	155	98.5	130.3	116.5	102.7	90.1	200.8	242.7	137.6	114.5
202.4	159	193.1	155.1	149.7	194.1	158	150.3	329.9	267.6	416.7
69.7	112.3	134.3	281.6	259.6	519.2	97.8	230.6	54.2	89.7	72
103.8	75.5	96.2	80	85.5	115.5	82.2	93	100.4	90.4	142.9
48.6	47.7	56	36.2	29.9	32.8	54.9	53.6	71.2	102.4	116.4
153.3	143.8	140.1	111	117	111	114.5	100.3	231.9	261.1	298.1

C4	C5	C6	C7
87778.8	95177.7	91274	131139.9
80176.5	58214.8	65621.8	60240
75338.2	42049	76499	42186.1
64521.1	74559.7	107176.1	80029.7
52761.1	23547.3	32161.2	26838.8
98512	6598	10449.5	11138.6
114029.3	113378.7	103224.7	57758.9
35163.4	40464	63887.3	53148.4
29320.5	17341.9	14735.5	16166.3
37562.4	43449.1	53860.1	38647.5
33369.4	37297.3	43024.1	45619.7
10158.4	10670.1	19516	20931.8
16500.8	8722.7	18781.1	10694.3
19449.2	17932	23406.1	28867.1
34893.5	30011	24695.5	16243.1
23679.4	35131.8	34659.3	39457.6
29436.2	20147.2	28337.2	15849.2
27312.8	36991.6	55097.8	58208.9
22811.2	25558.5	26527.2	25834.2
17935	13535.9	13146.4	18602.6
27736.1	29606	28992.5	34277.4
27614.5	21372.1	30778.3	28221.6
15330.3	8167.8	13795.5	10092.7
28695.7	26139.9	26211.5	15710.5
24584.1	31708.2	39724.4	36567.2
20603.5	11903.4	24138.3	16769.8
15043.8	8864.5	10742.2	11911.1
19832.6	20384.1	28492.2	19382.7
23033	19816.3	39432.4	26520.1
27923.5	25081.5	33257.1	25865
31067.7	38701.3	55582.4	66391.2
43912.9	37022.5	50575	18538.5

12225.8	18972.8	30637.1	43786.7
12171.7	17582	14626.9	36643.5
10617.3	13572.6	9231	10521.2
16178.2	16115.3	18584.9	8232.7
16492.6	4912.3	11333.5	7650.4
42164.7	24620.1	32875.9	26333.7
34374.9	33277.8	49066.8	43817
12469.6	18055.2	14552.4	37766.5
38.8	38.3	30.8	48.8
28482.7	4410.7	27644.4	5372
26838.3	38910.3	60893.4	50309.5
39453.7	42563.1	56737.8	48982.2
42574.6	30683.5	27785	54511.7
18014.1	21278.3	28664.9	27999.4
15994.7	21733.4	31078.9	29560.8
9705.7	12877.3	17579	18164.4
23720.6	31363.1	44927.4	40578.5
9478	12852.3	15371.6	14088.5
18096.9	13801.3	22997.8	19857.8
3621.8	2925.5	5568.3	3868.2
9911.5	15701.7	19118	15138.4
27152.6	17877.2	17954.4	32088.9
24421	33833.6	31288.7	34534.7
20450.8	8244.5	11218.6	9078.1
14282.7	18144.2	24406.8	28379.9
24746	15757.9	22166.2	17699.4
3362.8	5463.2	5737	7725.7
22840	29804.4	40258	32849.2
10197.6	12844.4	16065.6	13390.6
29380.7	29592.4	48644.7	41427.7
18546	15178.2	27866.5	21079.4
4931.9	5703.3	9178.9	8059.7
12018.1	13293.5	14377.5	18551.7

33384.6	21562.4	31906.4	11559
15408.6	18733.2	22555.7	15316.6
25185.6	35601.9	36529.7	40063
23938	20339.5	20931.7	12879.6
56.6	28.1	41.1	68.3
12251.4	13606.6	22895.9	19065.1
15198.6	17932.8	30672.6	25216.3
12759.7	18394.6	8001.6	4905.6
41285.4	4018.9	4987.5	6166.6
16454.6	14955.8	20180.2	14991.6
15179.7	17488.3	22292.8	22809.9
7578.4	12697.8	18770.9	18576
15551.5	21909.4	36574.9	30497
8245.5	11834.1	15984.4	18599.6
12028.1	16067.2	23145.8	22794.7
12869.7	12578.1	11181	9397.9
8500.1	14578	22887	27832.8
8616.9	5297.2	8231.4	6977.2
790.4	932.6	1225.9	1220.6
44143	33528	49022.3	15111.2
19786.4	23738.8	22159.9	24283.9
9679.3	12454.5	15316.7	19985.6
10163.4	9695	24806.5	10876.7
11239.3	13100.4	20360.6	21369.2
10973.5	39425.7	14406.1	10717.6
9792.5	14271.3	20506.1	20321.3
10434.9	13130	8760.9	11496.6
10708.3	12002.8	16267.4	15629.1
20300.3	21032.7	33833.6	29539.3
38386.7	25698.2	9963.5	7563.5
61942.8	15760.5	13613.1	18876.6
11825.9	13126.8	24277.1	24734.4
10669.6	11181.8	15612.6	14099.3

10177.9	9824.7	15077.7	11768.2
9988.4	11989.3	21895.7	21441.9
31042	34050.2	24172.7	22537.4
14677	17920.7	22421.3	33932.2
12528.2	15212	25730.8	20695.6
8222.1	13295.8	32023.9	14693.9
7177.4	9759.3	14585.9	13820.6
9434.5	13003.6	19242.9	24582.9
162231.6	4631.6	2141.4	3553.9
2683.2	3639.3	5467.7	5220.3
12562.7	23228.7	39794.9	41218.7
20682.2	18304.2	18055.7	9100.4
15857.3	23182.5	32487.7	32666.5
7790.6	10444.9	16980	18597.4
20935.5	5718.6	8778	6271.1
18942.1	18891.3	28008.3	23698.4
8620.6	7342.6	7581.8	4593.5
13696.7	29638.8	37245.6	41712
11017.6	11598.8	23981.3	22081.8
10117.8	9111.9	7759.7	4023.2
4648.3	4213.7	6131.7	5231
7295.5	8927.4	13362.5	12286.7
9223.5	12353.5	17907.5	19094
8356.8	7914	12702.2	10917.6
5971.4	6011.6	7281.5	8849.5
12868.2	8448.4	9103.8	14315.9
4130.6	6117.9	11011.7	8960.9
10021.7	14529.4	12153.3	12072.6
10518	6612.9	8062.1	4757.3
12227.8	31840.9	24258.7	16680
37388.1	21586.7	26128.6	7050.8
34137.8	31855.5	13289.6	9972.4
8119.9	11698.9	17101.3	16657.9

21229.8	21833.2	34650.7	24209.3
8613.3	9635.6	13207.3	13148.8
8771.8	8980.8	12337.7	11686.4
14327.2	9630.1	11765.2	12256.2
12493.9	16273.9	17045.5	19100.9
13403.4	23234.9	9629.3	12013.2
54707.3	103931.5	68433.6	163726.8
5703.4	5250.8	12586	16878.2
7914.5	12027.7	14432	13921
7842	9138.4	12992.6	14068.7
10128.1	9534.9	10015.4	17937
4666.4	6174.8	6331.1	7534.1
1411	1574.3	1703.4	1800.2
7790.4	12479.1	7355	3816.9
28481	17475.2	18131.8	11475.2
10745.6	4654.8	6063.1	5424
8339.6	8837	12845.2	14567.9
9034.2	9278.3	17130.4	12799
7465.4	11656.2	14343.2	12803.5
7283.3	8804.1	12911.1	12245.3
6724.7	10364	7433	16236.4
7511.1	10893.8	13737.7	17471.4
9760.1	13735.5	17604.6	15648.1
13449	6989.2	11498.2	7555.7
11305.5	13671.5	17974.3	18241.3
14771.1	4158.8	5616.3	3628.2
6701.6	9644.4	8283.7	20570.4
12006.8	12789.2	20346.9	15221.5
9002.8	10292.6	13780.3	13862.8
8485.4	11583.1	16764.4	17527.6
33488.7	7929.6	2468.6	1474.1
4063.1	7722.8	7354.2	10832.1
10963.2	12729.7	16297	16701.6

6528.3	6756	6714.5	5059
7040.4	15633.6	12455.9	27810.6
8707.8	11966.5	15266.2	14884.2
4446.4	3630.4	7380.9	5516.7
9804.8	11505.6	19060.4	17152.2
13419.6	18508.1	22792.3	26723.3
4215.9	2746	3558.7	3488.5
4067.9	8130.8	10019.9	6864.2
10039.5	11069.2	15400.1	15293.4
6647.5	9731.1	18652.4	16578.8
9249.5	1548.8	2965.6	2596
33430.3	1255.4	1668.4	1986.3
8456.5	8975.2	7064.1	7839.1
9421.5	10952.3	12332.5	14570.7
6093.2	8495	12678.2	13640.2
13195.5	12176.9	15524.5	15084
13645.2	11496	11027.8	12989.3
5272.7	20295.1	8525.8	7486.7
883.1	1067.4	1379.1	1311.5
5720.9	7002.5	5752.5	4794.8
7793.1	10107.9	13177.3	16967.2
8228.9	10733.9	9196.5	9629.4
8243.4	11537	19140.3	16134.7
6827.3	8974.4	13236.7	16129
4505.1	4269.3	5562.5	3716.8
6569.7	7375.1	20847.5	12135.5
8091.6	9382.6	10886.6	8304.3
7478.2	12969.8	12633.9	20867.1
6243.6	7867.2	9438.1	7767.8
8340.4	10767.1	16874.2	13438
8142.7	11111.4	12320.3	10810.4
7244.5	10037.4	14569.7	15303.8
10286.7	9771.5	11861	13447.2

534.4	1265.3	379.4	219.8
7002.3	8140.6	11665.1	11024.2
6164.2	7924	11981.3	12532
7595.7	8417.8	8796.3	7178
4537.6	16153.6	9403.1	49523.8
3351.6	7489	3959.4	11838.4
4510.8	4519.9	13322.2	9937.7
9251.5	9466.1	15163.2	13212.8
5940.6	9362.8	14264.5	14558
12433.2	17434.9	18263.6	20780.6
3705.5	4977	8518.5	5301.9
4337.3	6987.4	10600.1	12180.4
5887.3	8638	12732.3	14325.2
6750.7	8255.8	15962.5	11802.9
5802.6	8263.6	12492.1	13417.8
7073.1	6225.1	6981.9	4515.5
22378.6	43999.1	28491	69044.8
3885.9	4316.6	5530.8	5479.3
5570	8270.1	13474.8	12500.4
6532.5	8773.5	12456.1	13102.1
10687.8	16794.9	19012	21750
9306.4	8920	9429.5	10946.1
6247.9	4124.1	5066.2	4342.5
8079.7	8204	5505.8	4329.3
6015.8	9441.3	12174.5	22938
4968.9	9708.6	11722.5	19110.1
5272.7	6874.6	9526.4	9920
7841.3	7157.6	9104.9	9701.4
9417.8	6634.6	8287.3	7206.1
8189.5	9609.3	12054.1	11779.2
6235.1	8058.3	11567.2	12950.5
6969.6	8452.6	10864.9	10820.2
8906.7	13620.5	18079.3	16957.5

7357.4	10293.8	9027.4	23900.6
9085.8	10161.2	10762.4	9299.2
7526.9	10953.2	14129	10726.5
5496.5	7798.8	11479.7	11886.4
5961.4	6526.6	8188.8	8872.4
10621.1	14059.1	20272.6	18768.3
8123.3	4504.1	5829.1	4057.2
7349.4	9413.6	12173.1	11995.2
5152.5	7576.5	10051	10353.6
63096.9	2971.9	1425.2	1703.3
5424	7054.4	10447.2	10850.7
3696.6	2914	4404.9	3875.9
9845.6	2836.9	9464.8	2702.2
6797.5	2580.8	5051.2	3623.8
5273.5	6611.5	9539.1	8267.6
4156.1	5224.3	7175.4	7065.8
501.9	560.5	1776.7	685.6
4160.3	4717.3	7162.6	5860.5
8449.6	6390.1	6422.5	3142
12139	14368.6	20459	20735.5
6122	10673.1	7875.3	24443.3
6080	7192.2	11105.3	8601.4
4991.9	4965.4	8497.5	9291
6889	10657.5	16503.4	16528.7
6571.1	3364.6	3697.8	3473.7
12542.3	2341	2760.1	3020
5921.7	6538.4	8351.6	6819.7
2904.2	2443.1	3447.2	2669.2
2700.7	2401.3	2716.3	2173
7005.1	8500.8	10095	10520
6119.8	7591.3	14668.9	11693
8002.5	5896	6532.5	9039.9
7528.6	8289.9	14471.2	14818.9

6113.3	9368.3	14602.1	15252.5
6384	6993.7	7466.7	8012.2
11893.9	13877.6	17719.3	16387.8
5002.1	10226	16174.8	18562.4
6281.3	7037.3	10002.9	9240
6263.9	8722.8	9807.7	13876.6
3716.9	5815.9	8096.7	7950.7
5031.8	5888.7	5800.8	4599.6
3614.9	6396.4	15074.5	4084.6
3552.4	3777.4	6316.4	5928.3
3600.5	2104.5	2827.2	2846
6349.6	12076.4	18290.3	19608
9937.3	8250.5	8071.7	5829.3
4725.8	6021.4	9378.2	7710.1
4809.7	8442.8	7644.3	8387.9
8678.7	11348.1	12453.3	10869
6521.4	6760.3	8182.1	7950.5
6174.5	7001.4	10150.6	9046.2
4665.7	7909.3	11683.3	7507.9
6309	8372.7	8582.2	10320.6
3561.3	5603.5	8100.7	9122.4
6771	6629.5	9936.7	10772.5
7710.4	11358.8	10137.6	8133.7
6502.6	8759.1	10868.9	9701.8
4515.5	5941.5	8450.4	8068.8
9964.9	10785.4	18610.1	14607.8
5000.7	5834	6197.8	5491.1
7946.7	13037.6	13425.1	15444.3
10248.6	7309.1	6667.2	11513
7047.2	9874.7	12677.3	12765.1
4361.9	2519.4	2817.5	2771.3
7152.1	10036.4	16415	16158.8
4854.7	5393.9	6063.2	6102.2

7898.8	25250.9	17735.7	8805
9468	17544.6	27343.4	28791.6
3597.5	4215.2	4068.7	3157.2
3496.2	6162.4	6144.5	8456.3
5385.7	6875.9	9345.1	10130.2
4410.8	5496.1	12839.4	6623.2
4046	8538.4	12291	7768.1
5260.6	7048.9	10387.1	9049.3
3868.8	2317.3	7600.1	3521.1
5640.9	8449.9	13671.4	14058
4831.7	6767.4	8733.9	8494.2
3830.2	6479.2	6943.2	10863
9199.6	13550.7	19408.1	18498.3
5305.3	6551.9	7621.7	5853.6
3942.7	6719.4	5913.3	4171.8
4301.3	4151.3	5152.9	4798
2795.5	2024.9	2909.8	2167.8
4469	6779.1	9664.3	11156.2
5980	7875.2	11455.3	12998.3
5333.8	5751.4	8364.4	7599.2
5045.7	6933.3	9171.5	10197
4037.8	3984.4	4700.8	4302.6
4340.6	6115.6	15759.2	9520.9
6908.1	6675.9	4813.7	3476.5
4731.5	4075.4	3842.3	3272
3984.4	12366.4	4730.3	7477.2
4277.5	7790.7	12830	11252.4
7849.1	5942.6	8971.9	8407.7
6057.5	7418.2	10425.4	9227.3
6229.9	7637.9	9693.5	11488.7
5648.8	8610.8	9716.7	9276.6
7946	9652.5	12344.3	12354.8
4714.2	7797	10413.6	11914.6

43669.7	1462.3	5707.5	1727.1
3096.3	2672.9	3966.4	2272
6557.6	8368.9	2026.8	2634
3913.9	4130.1	7286.1	6194.1
6165.7	5429.1	4889.1	2893.8
8224.7	8589.4	13591.1	13208.1
3381.9	5003.7	8658.9	7151.6
6366	4642.3	6139.1	7214.5
7600.8	12364.4	18491.4	18199.5
6677.5	4787	4877	4928.6
5738.8	6448.5	7964.8	7985.7
3205.8	3815.1	8398.1	4843.1
4455.2	6493.9	7958.4	6974.9
9117	17801	21079.5	34199.9
5440.8	4718.1	11190.5	7528.3
3488.7	4290.7	8011.4	6887
1595.2	2638.2	3511.8	5244.5
6120.2	6703.7	9050.8	10333.1
5438.4	4416.2	7013.3	5810.8
2526.9	2535.6	4053.4	1446.8
6828.1	7442.2	11044.9	8998.5
4028	3754.8	4535.6	7755.3
2030.7	2900.8	3400.9	3589.6
5293.8	6804.2	13316.7	11734
3847	5432.6	6078	6367.9
13621.7	17168.4	27421.9	23304.9
3275.7	4356.9	5747.1	6626.5
3636.1	5235	6298	7582
6127.4	11138.4	10252.5	13925.3
5377.2	8186.2	11630.3	10637.8
7791.7	9536.2	10587.1	9711.6
2995.1	3940	5093.5	3883.1
4693.2	5204.7	7125.8	7284.7

4412	6351.8	10931.8	10660.1
5656.7	3964.9	5373.8	6281.4
5417.4	6370.6	8288	8582
4183.3	4505.5	7099.5	7733.8
13177.6	12879.7	10626.7	10549.1
4458.9	6764	8105.7	9119.7
4264.4	7310.2	10801.4	13262
3637.5	4620.4	5942.3	5586.2
7799.5	9571	13821.5	12098.7
16019.1	17954.6	20737.5	20957
12841.6	6681	5502	9499.7
7414.4	7114.8	6197.6	9308.2
2116	3082.2	3920.7	3124.2
10062.5	7775.2	13749.2	13308.6
3525.5	5280.1	7653.1	9277.6
4841.8	5649.6	7409.4	7883.9
9017.8	3823.7	4697.4	3530.8
6040.9	8562.5	7465.8	9632.1
5290.2	7111.1	12174.9	12309
1456.7	988.6	1458.9	1223.9
4864.9	9204.1	9185.2	10807.9
7014.7	8072.8	10204.7	10209.9
4556	5813.8	8673.6	8243.7
4595.9	7174.6	10585.2	25220.3
23619	440.6	4628.3	861.9
4629.2	5854.7	7714	7797.5
6794	5981.6	7527.7	7886
2217.7	6736.1	6462.2	14531.4
4489.3	4981.7	8263.3	6741.1
6811.6	10536.2	15345.4	16971.7
4718	4379.2	6480.8	6352.9
4345.4	6861.4	9184	11744
5103.8	11072.9	12353.9	20893.9

7243.4	10520.3	15413.6	11970.8
5766	6850.8	11288.3	9498.3
5669.8	7325.3	9508.2	9520.2
4534.7	6306.7	9790.3	11221.6
5192.1	4611.4	8265.5	8809.2
5825.9	6294.5	9644.4	8622.7
7380.2	5650.2	6258.8	7989.8
5838.4	5191	8170	6834.7
3133.8	4336.3	4706.4	3935.7
5274.3	6307.6	8228.4	8756
4441.2	5377.3	8090.1	7777.5
4285.1	5750.1	7907.1	8032.9
3741.2	7610.1	7626.9	5974.3
220.6	186.8	241.2	199.7
5630.7	4648.9	6270.7	6627.8
3332.5	5679.1	4917.7	5958.6
4575.5	5268.8	7106.1	6997.4
5200.7	6204.1	9620.5	9742
3134.1	4395.7	3067	3460.7
3379.7	3918.4	5813.9	5786.6
4358.8	5601.1	4442.6	4876.9
1122.3	7575.6	4462.2	15716.7
6209.5	9139.7	15636.3	10075.8
6633.1	3168.2	6426.2	3966.8
5021.1	4453	7286.5	8282.7
3339.8	2926.6	5269.3	2926.5
2801.3	3588.9	6200.1	4492.2
7925.6	8863.9	12006.7	11934.5
3081.2	3066.6	3564.6	3074
6521.5	7877.7	8828	8287.1
6369.9	5095.5	8843.7	7477.1
4657.6	5533.4	6045.1	5745.4
14727.2	20184.1	24031.4	26255.2

4959.3	3506.3	5145.1	3888.1
5026	4984.9	6330.7	3263.9
6395.6	6273.7	9422.2	10460.4
9445.3	12715.9	18131.1	16407.4
6595.6	6760.6	7053	6495
5629.3	6471	9210.4	8157.3
5324.5	6501	9037.7	9296.2
7497.4	11096.3	7560.3	22680.2
2968.4	1337.3	1936.3	2378.5
4275.9	5007.9	7015.8	6665.5
7769.8	3361	5015.9	4597.8
4132.9	5636.3	10333.9	10379.8
3877.9	4141.5	6852	6183.3
8720.4	13957.8	13158.3	17557.3
2871.3	3857.7	2255.7	2602
2539.7	3354	5239.9	5770.1
1152.3	1124.1	1341.2	1038.7
4230.1	5128.7	9106.8	8524.3
4222.2	5265	6353.8	5277.5
4228.6	4718	6224.1	5797.6
4774.5	5843.2	7953.3	8184.1
3611.6	9983.4	7980	6981.1
1316.8	1438	1927.7	1604.8
3423.6	4210.4	6395.6	6027.7
5927.8	7458.7	10450.1	10290.1
6150	3760.1	8645.8	7520.5
5724.6	7301.2	8829.5	12053.5
3975.7	6244.9	8664.4	9628
4598.7	8766.1	12912.5	13583.2
6140.7	8034.6	11744.8	9492.8
2376.5	2461.7	2374.3	2232.6
5000.9	5921.8	7871.4	8819.3
1705.3	2571.9	2840.6	2088.7

9483.2	10384.1	12585.5	10980.7
5149.5	7542.3	11588.2	10898.9
2683.3	2722	6297.2	3563
1568.5	1836.6	2162.4	2431.8
3450.6	5435.5	8629.9	8913.1
3158	4657.7	5795.9	7037.6
4858.5	7333.5	11022.1	11319.3
4950	3687.5	4555.5	3332.4
2753.9	2982.7	6075.3	4703.2
5078.2	5513.5	8114.1	6891.9
3808.2	5032.5	7270.2	6855.1
2309.1	8763.3	10761	11767.2
6442.4	8033.3	12137	9442.1
3957.5	5488.6	7926.8	8546.8
2272.8	2299.4	3886.4	2094.5
3860.3	5065.2	6932.5	7583.6
4284.4	6318	9089.4	9284.5
4320.7	5933	10262.3	8991
4660	8343.8	10549.3	6967.2
7928.3	15451.2	9310.5	11114.1
3172.3	4823	6923.6	7247.4
3945.1	4828.1	6878.7	6438.2
5889.8	6723.5	9421.1	8581.5
1979.4	3248.6	2515.1	2877.2
4751.2	5753.4	5371.6	4553.3
4538.7	6418.4	10511.3	10477.2
5828.7	6669.4	9736.6	9610
4977.2	7329.9	9873.3	8646.1
4646.5	7387.7	10805.9	10499.1
3976.5	6929	5843.5	5333.1
4296.7	6158.3	8511	8720.7
4470.3	3961.1	6616.4	6661.9
6553.2	2984.9	5743.4	3717.7

4612.2	5865.7	16660.3	5886.6
4476.7	4093.8	8055.2	5379.8
2493.2	2442.1	4256.9	7917
4969.4	2354.7	7375.2	5407.4
10096	13933.3	6471.1	8234.9
3506	4255.9	5985.4	5569.8
2851.1	3819.9	4767.6	4219.9
3267.3	4347.7	5611.2	5629.8
1693.2	3312.4	2702	4289.3
4184.4	4331.3	6094.7	6784.5
6025.7	7840.8	11540.8	9340.6
3332.7	5029.5	6066	4488.6
2834.1	3831.2	7130.7	7260.9
4007.8	5455.8	6247.3	8996.2
4498.8	5749.2	6885.1	7806.6
8170.5	5627.7	8167.7	6730.2
3465.3	5696.6	6221.7	6635.5
4198.6	3803.9	3001.3	4087.4
7479.2	9715.6	13749.7	11863.5
3749.9	4172.8	5771.6	5563.7
5412.2	5460.4	11616.9	6865.3
2462.7	2833.6	3708	1642.6
2393.9	2034.7	8453.7	2473.2
1844	1723.8	3320.9	2438.4
1827.7	2790.2	3626	3607.1
5031.9	5447.6	7149.1	6187.3
1590	1373.2	1947.8	2060.2
6995.5	6851.1	11099.1	8796.1
1536.2	1331.5	3583.9	1551.8
2194.5	1209.9	1532.8	1546.3
4301.6	6434.2	8239.1	7146
3718	4417.7	4867.6	4577.4
2877.3	3662.5	5228.4	5010.1

4978.4	4861.9	6746.4	5324.7
3226.7	4618	6900.8	6471
22186.4	5641.2	5048.6	5652
4819.9	6548	7535.1	6298.5
4559.9	5664.1	7182.2	6976.8
3220.5	3808	5867.9	6509.1
6853.8	6983.5	10427.9	8131.4
6214.5	8182.5	11036.2	12161.3
2987.2	4221.6	6258.7	8237.1
4420.2	6385.4	11138.4	11195.1
4962.1	6522.2	4939.7	9174.8
604	1165.2	1584.1	5835.1
4024.4	5021	7804.6	7517.9
3601.7	5687.7	8772.9	7781.6
5058.8	6941.2	8701.1	11778.7
2717.3	3327.2	3605.3	5271.5
3168.8	3729.9	6007.3	6399.3
3728.2	5192.1	6035	7919.7
4200.2	6834.7	6992.7	7018.5
4108.4	5504.6	7061.9	8053.1
14.2	88.7	123.9	19
4770.8	5062.6	6215.1	5854.6
3966.6	4248.7	6049.4	7356.7
4599.1	6881	8560.9	8013.4
3192.7	3867.7	4473.8	4960.8
1978.4	1698	3214.9	2296.2
4130.4	5892	4501.7	7525.8
3304.2	4009.2	6644.4	4565.9
2934.5	5498.3	4559	4551.1
5448	6471.6	8840.6	7713.3
3105.9	3578.7	5336.9	4756.3
5528.5	5907.8	8542.5	8006.1
3723	4579.6	7749.7	7297.5

3699.4	4950.8	7652.4	7118.7
3148	3785.7	5041.6	4040.7
3967.9	3283	3856.9	4955.8
14858.2	19626.1	27186.4	34528.6
5790.3	4283.4	7135.5	7299.4
3352.2	4126.7	3907.8	3267.3
4917.2	4499.7	3670.1	2474.3
3482.2	4561.7	5754.8	5079
6601	8185.1	11899.9	9781.3
4124.9	5025.8	7549.3	7716.4
3388.8	4200.5	7736.7	7687.1
4382.6	5395.1	8358.3	4832.2
5706.9	5224.2	6586.7	7539.3
3143.7	4155.9	5960.8	5951.6
5722.1	5863.1	8141.9	9132.1
167.4	776.7	346.4	1098.5
88.4	109.8	90.7	57.9
3915.5	5077	5873.8	7354.2
5397.7	5437.2	6487.8	6962.5
3133.4	3568.5	5022.6	5944.8
2498.9	3317.8	6545	4626.8
1124.2	7599.9	389.8	468.9
2777.7	4109.3	5778.3	5732.3
6135.1	5796	7436.8	4729
4787.8	6566	7623.7	11621
5056.2	8293.5	14499.6	9933.2
4226.6	4735.6	5991.9	7539.9
3026.5	3631.3	5170.5	4229.6
2328.2	3520.3	2636.8	5854.7
2227.6	3332.9	5076.3	5012
1844.1	2425.5	4994.5	3122.8
4489.4	4456.9	5738.1	6125.2
4092	6745.3	9901	7357.9

4033	4921.5	9383.4	5374
3107.1	2855	3103.8	2244.2
6343.8	7202.1	8454.2	8440
2215.2	2523.6	2619	3031.8
1909.8	1893.1	5356	2123.6
1962.3	707.2	956	816.2
2478.6	3687.3	5365.3	5225.7
4424.7	4249.5	6117.3	6528.8
2251.2	2732.1	3792.7	4203.2
2951.9	3694.6	5315.3	4429.6
2795.3	4320	5531	5844.2
5780.2	5254.6	3916.2	3965.2
2853.5	3918.7	4767.7	5358.7
3171.9	2718.9	2988.4	6617.1
3607.7	4689.8	5946	7590.3
3694.5	6342.9	9446.9	12278
3991.2	720.1	1161.5	1066.4
4284.6	5509.1	7414.4	7559.2
5641.9	7497.1	6847.9	7417.8
4401	5402.2	13062.5	9974.3
2506.2	3617.4	4414.5	4896.3
3205.2	4314.8	7315.4	7405.2
3304.6	4127.2	5867.9	5504.6
4598.7	5506.2	8301.5	8932.3
9281.2	11174.8	13846.4	14839.4
2188.2	9900.4	7469.2	2554.2
2958.4	3530.4	5100.8	4225
3744	3274.6	7283.3	4732.3
4236.4	5446	6663.9	6596.3
1963.1	4552.8	4832.8	13484.6
4088.6	4483.6	6286.1	6229.9
3123	3435.7	3039.9	4586.5
2745	3577	5101.3	5153.4

6256.5	7487.5	8156.2	7654.9
1336.3	12795.1	1271.4	4558
4231.5	5720.9	10098.2	9350
1983.1	2406.8	3345.2	3156.1
3013.4	4021.7	4439.3	8153.2
1170.6	2201.7	2631.4	3779.4
4010.8	3703.2	5359.3	4658
4719.2	6797.9	10303.7	10530
6761.7	4677.6	4126.8	2845
4856.8	5738.3	7293	8313
46998.3	370.6	1188.4	1102.5
2170.6	2325.1	3003.4	1727.2
3718.4	7039.2	7325.7	9694.9
2891.7	3293.3	4821.9	5436.1
3713.7	3425.9	5618.2	5207.6
3518	4284	6715.8	5235
2687.3	3798.5	5756.1	5352.9
5146.2	7072.7	4756.6	16425.9
4920.7	9949.2	16168.9	17252.7
3239.4	4185	5464.2	6658
3389.3	4239	6886.5	5833.4
5501.7	4372	5625.8	5594.6
3219.6	4579.9	7838.4	5868.5
2979	4612.8	6922.9	8416.5
2240.2	2334.3	3346.7	3566.2
3658	2288.7	4913.6	5052.2
4428.4	5893.5	6758	7873.7
3666.5	4558.3	6594	4793
4102.5	4493.5	4734.2	4344.6
3115.3	4459.3	5025.3	4826
2602.1	3784.2	5592	5149.1
4017.8	3881.5	4256.7	4698.8
2433.7	4593.6	5073.2	9053.4

2556.3	2386.3	2915.7	2950.9
5254.1	6776.8	9895.2	8756.6
2401.2	2546.7	2603.7	1730.4
2708.6	3094.6	3227.7	3438.8
3093	2956.7	3920.8	4204
3465.9	3175.1	2775.3	3336.2
4595	4922.7	7586.7	4624.8
2121.9	1433	1302.9	945.7
1822.5	2470.8	2866.1	3018.8
3714.6	4689.9	6416	6340.4
1739	1796.4	5191.6	3375.8
4350.4	4506.1	7319.4	7639.6
4682.2	4005.2	7052.1	6109.7
3756.7	5251.3	6644.4	7265
4252.9	7194.3	8588.7	12178.3
6494.1	3882.8	7369.1	4334.4
3438.5	7671.5	7860.4	12813.2
5349.4	6209.2	7797.8	8030.1
3596.2	4207.1	5118.8	5146.2
4355.9	3979.9	5873.2	4524.6
3221.7	4354.9	5816.6	5840.8
5387.7	6788.1	9366.6	8373.6
4977.1	5963.2	7995	4158.6
2466.6	2427.3	4487.5	3817.6
3758.2	6723.5	6061	4785.4
2350.2	3730.6	5317.8	7255.3
2364.3	3205.2	3991.3	4730.8
3798.1	3983.5	4324.5	3781.7
4347.1	6678.5	8584.8	10610.8
2780.2	2662.7	4803.6	5010
2774.8	2386.9	3965.1	3873
1908.8	4154.1	4821.5	6394.3
3086.1	3692.7	4544.9	5288

3513.4	4379.9	5076.6	5678.5
4313.2	6503.8	9885.2	8910.3
3101	2680.7	2777.7	2711.8
2657.7	3610.9	4144.8	5182.1
3647.6	4908.8	3744.7	2407.9
1370.3	2751.4	3796.5	10889.1
2737.5	1073.2	2311.3	1541.8
5388.1	8922.2	7396.5	15104.2
2574.5	2299.1	6275.5	2992.7
13117.5	17865.5	24503.6	31287.3
2566.1	3047.5	4254.3	4085.2
2273.5	2735.4	3349.7	3341.6
7890.2	1221.4	1546.2	1453.7
2875.6	3537	4629.7	4429.9
3130.8	3230.6	4491.3	4203.4
6664.1	7698.1	12871	11165.2
3035.7	3000.9	4735.2	4027.9
4402	5972.8	7259	6840.3
275.3	446.8	515.5	709.3
3136	3488	5398.6	4959.2
5113.8	4881.7	6648	6157.4
3138.7	3552	4660.8	3923.4
2762.5	3598.9	5274.7	5141.4
3226.8	3451.2	3391.7	3067.5
3397.5	2693.8	4613.4	1726.4
3646.3	4683.1	5690.3	6157.8
4029.2	4466	5382.6	4464
133.7	235.9	216.9	327.6
2952.5	4159	4563.8	6758.2
1083.6	1532.4	2609.9	2017.3
1986	2216.3	2590.7	2133.4
2159.7	2976	4261.7	5165.5
3191.9	2732.6	3586.8	3179.6

651.5	705.6	1279.3	1316
2369.5	2885.8	4017	4898.2
195.7	274.2	355.5	530.9
4319.5	5938.9	8064.4	7337.6
2063.1	2831.3	3962.8	4467.6
2252.8	2583.1	3697	3253.5
2293.2	4239.6	6194.7	6811.4
3283.2	3880.5	5855.1	5422.2
3176.7	3089.5	4498.2	4186.1
5111.7	2348.4	2432.3	3006.3
1728.9	3804.3	3658	4328.2
5477.6	7180.6	9685.2	9905.2
4741.3	6624	9152.4	10404.2
8121.6	6231.1	4194.3	14168.8
3156.3	3044.8	2656.7	1737.8
3010.2	6198.4	7001.2	7922.4
3038.8	2944.7	4211.5	3759.1
5967.8	5448.3	4362	3870.6
3854.4	4407.5	5483.3	6131.9
3589.6	3377.6	5419.1	4097.4
3822	4863.2	5748.8	5516.1
1973	2675.1	4058.3	4385.2
2756.1	3589.4	6583.1	5904.5
2234.6	3059.6	4279.9	3753
10034.9	6731.1	9311.7	7604.7
1965	2655.5	4079.7	4226.8
2243.3	1440.7	1642.6	1831.3
4997	6795.1	9203.9	8657.7
3167.1	2628.5	3638.7	3191.6
2681.2	3540.9	3870.8	4231.4
3087.5	3104.5	4033.8	7957.1
3609.7	4452.8	6870.1	5129.9
2183	3631.5	3541	6274.1

3289.6	4286.3	7404.7	5699.8
9830.6	14661.9	2119	3330.7
5525.6	7664.9	10923.6	9362.3
1732.7	10091.6	5281.8	15000.2
4047.9	4448.7	6348.6	5273.2
2140.9	3747.4	3218.1	2857.8
2077.6	2192.5	2594.6	2485.4
2793.6	3507.4	4613	5867.9
4422.4	5801	6990.2	7619
6892.5	3105.1	3947.3	4608.5
3602.4	5898.4	9689.8	9153.7
3528.2	3565.6	3259.9	2491.7
4813.1	4925.9	6304.3	5626.8
1591.1	2132.9	2060.1	3002.9
4511.2	5002.3	2867.3	7112.4
3567.9	2741.4	3883.2	3196
2255.9	2701.2	3812.7	3537.5
2405.4	2523	3244.3	2650.5
2782.3	3888.5	4268.4	4514.4
1188.7	1099	1158	1506.7
2087.5	2415.9	5413.2	4906
2706.2	3060.2	3813.8	5211.9
2523.5	3270.5	4373.5	5910.5
116.8	118.3	181.7	226.3
3336.1	4595.5	6604.3	6055.6
1804.5	2712.9	3400.4	3983.1
2425.6	4041.3	4014.8	11149.4
2588.4	3523.8	3144.7	4269.1
5198	3834.9	4172.8	3087.1
3394.9	3049	4719.9	4132
4614.1	3782.5	2640.1	1811.7
2804.7	4172.8	4808.6	5647.6
1864.7	2896.3	2354.1	2186.4

3553.2	4658.8	6520.5	5566.6
2478.2	3562.5	5350.5	5183.6
3341.7	3637.8	3493.1	2716.1
3184.7	1643.5	2838.4	1845.9
3122.4	3925.2	6135.2	6193.3
4872.7	5322.4	7148.6	7504.2
3343.8	4330.1	5391.5	5291.5
1149	1189	1254.5	939.8
3156.6	3783.5	5950.3	4583.4
2606.6	3775.8	4484.9	4457.5
4736.7	2218.1	3855.8	5615.7
4400.8	4246.9	2469.5	1856.9
1099.2	641.7	753.6	978.4
1559.6	2049.2	2576.8	2378.2
1314.7	1660.9	3833.7	3011.2
2612.3	3004.1	3820.6	3657.7
3111.7	3411.4	4642.5	4560.6
2896.6	2101	3158.4	3291
320.2	323.2	427.2	443.2
2452.3	3281.9	4318.3	4976.5
2830	3055.6	4928	4117.5
5484	910	2043.9	1043.6
3201.8	3661.5	6083.4	5651.6
2971.2	2842.4	4872.6	4981.2
933.8	947.8	847.7	1013.4
3650	5410.5	6862.4	7333
4258.6	5651.8	8350.2	6630.9
3543.9	2224.8	4956.3	3148.6
2855.2	3740.5	5010.8	5089.8
3022.3	1410.5	1894.1	1698.3
2559.1	2988.9	9647.9	7482.1
5993.3	5617.2	3487.8	4685.4
2009.5	2913.6	4142	3554.4

4775.1	5246.6	6948.1	7034.3
1968.7	1905.2	2597.7	1994.8
2650.6	3332.9	4741.2	4514.2
2722.8	2987.1	2358.1	1392.6
3003.9	2833.5	4649.4	3609.1
3030.3	1445.3	3267.3	1316.2
3221.4	2207.1	2174.4	2512
2224.6	2542.9	2948.2	3978.4
1999.4	2467.9	2966.9	4084.1
1460.1	6301.7	3199.4	5038.8
2679.5	3203.8	4245.2	4051.6
4401	4473.9	6454.1	7604.8
3700	3010.2	4748.6	4031.6
3185.3	4121.4	5498	5640.3
1338.4	948.3	863.6	659.6
3021.2	4320.7	5266.1	5680
2550.8	2755.4	6926.5	6338.2
4469.9	6805.3	11257.2	11205
1720.7	1650.6	1619.8	923.8
1814.6	2034.4	2634.5	2814.5
3697.5	3813.8	5068.1	4963.8
4663.5	5478.4	6680.2	7738.9
3066.7	2855.4	4157.2	3291
2193.1	3318.7	4719.6	4053.9
3486.1	3830.2	5126.8	4869.4
2579.4	1769.1	2920.9	2254.4
2209.3	3460.6	6254.5	5679.4
1203	80.9	164.7	147.7
2530.8	3473.8	5400.4	5204.9
3244.8	4167.4	5920.7	4836
2482.7	3177.6	4613.2	4876.5
1937.4	3722.3	1869	2826
2237.2	3039.7	3448.8	4999.5

2946.9	2366.4	2919.2	2839.4
2624.6	2118.3	3813.6	2790.3
1973.5	2706	2545.2	4482.2
5377.1	6228.2	8151.4	8125.9
2074.6	2663.1	3668.4	4127
2373.3	3362.4	4701.1	4495.4
2594.7	2483	2219	4130.2
2066.7	2889.4	6164.7	3632.4
2170.3	2569.1	3570.3	3645.4
13158.2	516.9	1090.9	1013.6
2798.1	2707.5	6459	4142.5
2724.8	2886.3	3949	3991.2
2400	3116.2	7260.7	3664.3
2707	3181.3	3464.6	3635
1645.4	1892.6	2140.4	2793.8
2634.3	2836.9	4104.3	3774.9
3307.8	4046.4	5311.8	5841.2
6053.1	6275.8	8312.3	8340.3
2855.3	3374.9	3692.6	6012.1
3772.9	6021	6776.2	7585.1
2469.6	3473.5	4139.1	4522.7
2652.9	2822.2	4729.9	3844.4
2598.1	7391.1	2751.8	1821.3
2154.3	2864.6	3966.5	3835.3
1729.4	2332.6	2900.4	2348.1
3549.4	4576.4	6859.3	6633.5
1882.4	2797.9	4799.8	4295.3
1403.4	2061.8	2796.7	2941.1
2089.6	1209.9	1582	1967.7
425.9	544	835	793.9
8274.3	2901.8	3468.4	3253.8
4597.5	4560.8	5798.8	5474.5
2215.4	4149.3	5047.3	3839.6

2413.7	5049.9	4878.5	5801.3
4097.7	5781.9	7618.2	9412.4
2286	3861.6	5619.8	5952.3
798	1014.1	1320.9	1481.7
1127.7	1828.1	3500.3	2284.6
2546.4	3815.8	5310.3	5430.8
1842.5	2417.4	3449.3	3461.2
2804.9	2591.7	8311.9	7118.3
9277.8	157.1	377.4	488.3
2033	1788.1	3710.2	2606
2083.7	2343.9	3215.7	4619.5
2208.4	3351.9	3943.1	4620
4752.3	5187.7	7345.6	9112.9
3302.3	5225.2	9712.6	10807.3
3037.4	3212.4	4762	4434
1961.2	3333.9	1853.7	1606.1
536.9	764.7	975.6	1107.5
2696	2956.8	4629	4481.9
2954.6	3804.4	4191.7	4931.1
3902.1	3702.1	5626.1	5887.1
5449.3	7485.8	10932.2	9184.7
2576.1	2960.6	3151.5	2924.7
2085.7	2859.5	3257.4	3527.4
1961.5	2275.2	2984.4	2906
3519.3	3119.3	5319.7	5563.9
3197.2	3260.7	4793.2	4376.4
1652.7	3129.8	2687.3	4100
1071.1	1097	1771.8	1606.8
3028.3	2526.5	4755.4	4596
577.7	1554.3	643.3	621.2
2135	1628.7	2391.2	1756.6
1775.3	2460.9	3420	3807.7
1734	2563.5	2188.3	2553

3729.4	4780.9	9485.3	5707.3
2893.8	3755	3270.4	7713.3
2488	2890.2	4972.3	3559
1840.7	1916.1	2431.4	2457.4
1251.3	1569.2	1632.7	1599.7
3643.3	3771.8	5292.3	4333.6
2806.7	2839.4	2032.7	1054.3
2186.4	2802.5	2699.6	4227.4
3671.2	4615.1	6564.6	5668.3
2780.5	2667.3	4342.4	3990
1857.1	2773.6	4504.7	3820.3
7260.5	991.6	1201.6	1524.3
2659	3486.6	4289.2	4487.5
6608.3	8449.7	12365.3	10758.9
2666.6	2876.6	6802.7	3807.5
2296.2	2506.4	3302.5	3363.5
1657.6	1801.8	3416	3758.9
1468.8	2333.1	2919.8	3349
2348.2	2938.5	4889.8	5138.6
2979.8	3396	4105	4946.5
3435	5395.5	5377.8	4995.6
2862.3	3366.1	7957	7271.8
2189.6	2651.6	3681.7	3838.7
24675.5	2944.1	3685	4443.4
841.6	1089.8	1510.5	1344.3
1907	1645.7	1844.6	2198.5
4512.9	6068.9	7569.3	7000
2271.6	4678.8	4527.5	3615.5
20477.2	2967.2	3915.2	4612.2
2072.6	2392.5	2390.5	1865.1
1745.1	2438.9	3553.5	3932
5924.8	4483.1	2820.4	1852
4871.5	5549	8679.1	5649.3

2539.5	3704.8	3867.8	3698.6
2486.1	2227.5	3783.3	3484.1
1056.9	984.3	1246	1039.4
1129.1	459.8	614.2	537.5
1109.1	1184.4	1785	1555.4
3686.4	4392.5	7244.1	6858.2
3247.6	3171.3	4317.8	4897.8
2228.4	3039.2	4492.5	4336.5
1984.6	2610.2	3645.7	3963
1971.1	2405.8	3375.1	3032.2
4199.7	2457.3	5905.7	3627.9
1377.2	3193	5367.8	4259.5
2698.7	3540.4	4480.5	4617.7
2414.3	2209.8	3705.1	2921.4
1759.4	1851.1	2767.9	2837.1
2683.6	3457.2	5399.1	5002.1
160.5	224.4	348.2	233.2
2372.8	3499.4	2283.7	1520.8
2956.3	3156.2	5411.2	4036.4
1752.1	1529.4	2437.6	2465.3
2769.3	3732.4	5352	3354.6
3292	3213.4	5037.1	4457.2
2736.8	3614.2	4984.7	6136.9
2627.9	3406.5	4415.1	4390.8
1617.9	1505	2344.1	2340.9
1789.3	1952.4	2017.4	1167.5
2675.7	2371.2	4300.5	1954.6
5041.2	6637	9622.4	8631.4
3712.2	3866.7	6071.7	4112.7
2683.8	2983.6	4909.4	5024.2
3492.1	4899	4995.2	6214.7
6618	8597.7	11622.1	13836.3

1218.8	997.2	1517.5	1329.5
3376.3	3988.3	5616.3	5259.1
2188.9	2155.3	3609.3	3220
2355.3	2696.8	3633.1	3375.8
4222.2	4863	6376.6	6174.8
2537.7	3083.6	4399.4	3254.7
1352.3	1840.2	2717.8	2551.6
2495.4	2631.2	3300.3	3153.2
2625.3	3305	4505	4030.4
2731	2961.7	3584.6	4555.5
2866.2	4119.5	6098.2	6079.4
3057.5	4329.6	5681.9	6037.6
1696.9	2165.7	2805.6	2857.8
2396.8	3504.8	5098.5	4752
4296.4	4245.8	5936.6	5811
3820.1	3076.9	5475.8	5976.6
2873	3201.4	5086	4920.2
2860.5	3638	3907	5596.4
3132.2	3373.1	5193.5	3915.1
2484.7	3042.5	4157	4099
2826.2	4745.9	6577.1	7046.8
2799.7	2712.4	4455.2	4288
6658.7	2128.1	2443.1	2028.8
3683.7	3368.5	4275.6	3483.1
1717.1	2723.3	3517	3096.9
1331.3	1279.9	1929.2	1591.6
2798.2	4312.5	6603.3	6125.8
785.6	986.9	1410.2	1308.7
1212.7	2379.3	3195.2	4395.2
4087.8	3589.2	4505.8	4744.5
2428.2	3008	4523.4	4353.3
1730.2	2264.9	1959.7	1855.6
4366.4	5673.3	5791.2	5003.4

3917.9	4697.5	6447.4	4575.8
2473.9	2593.6	2175.9	1801.1
2822.5	3431.5	4428.1	4169.9
2978.7	4873.2	7796.8	7779.4
2745.3	3246.3	3619.2	4736.5
2172.1	2250.6	1725.5	1205.3
3032.1	3429.8	4768.7	4959.2
2574.8	1745.2	2685.4	1665.6
3472.4	4368.1	6391.6	6495.1
2046.3	1069.9	1580	1624.8
458.6	591.9	866	703.1
3450	4458.1	6083.2	6113.6
3778.2	1648.4	2027.2	1790.9
703.4	581.2	794.2	1347
3124	3732	3227.8	3816
2261.1	2543.5	3814.3	4752.3
1758.9	1876.5	3594.6	2814.7
2017.8	3419.1	3354.3	3776.7
4274.5	3634.8	3040.1	4408.5
1597.4	2473.5	4217.7	3870.3
3118.6	3031.8	4499.8	4413.9
1860.4	3154.1	2783.6	3850.3
2300.9	3130.6	5709.1	5360
7563.6	9194	13475.9	11468.4
3374.3	3732.7	3753.4	3575.9
1969.5	2736.3	4098.3	3010.8
3109.8	5668.5	5464.7	13894.6
1692.3	2030.5	2006.6	2505.8
1448.1	1943.7	2673.8	2540.4
1718.4	2189.7	3686.3	3321
1570.5	2270.5	3834.6	1742.4
2472.5	2808.4	3556.5	3155.5
2245.5	2693.6	3971.9	3918.2

1353.8	1953	2572.4	2937.4
2642.9	3669.9	4044.3	3453.7
1060.6	1883.7	4288.3	3208.6
1921.4	1941.9	2244.2	1847.5
2428.1	3165	4981.4	1916
2062.5	3161.8	4974.5	4474.1
1896.9	1701.2	2898.2	1886.6
3354.3	2920.4	3663.2	4702.1
1245.4	1285.6	1906.2	2123.7
3812.6	5664.9	7023.2	7228.6
1519.1	2524.3	2959.3	3552.2
1494.9	1817.6	3464.9	2887.6
3446.8	3585.8	4421.8	5182.2
2595.7	3736.6	4712.9	3986.2
2894.7	3558	4842.5	4567.8
2065.5	649	6421	875.4
2286.9	2684.5	2708.8	2750.7
2113.9	3181.1	3712.4	3463.6
1839.8	2154.1	2796	2751.4
2006.1	2743.2	2965	4711.2
1026.7	2313.2	2686.3	6326.3
2758.6	3182.2	4395.9	4471
1707.9	2182.3	2989.6	3084
1443.4	613	1128.9	1871.8
2484.9	2607.1	3256.8	3246.8
629.1	738.8	901	1059.6
2300.4	2359.1	3488.5	3524
3662.6	3595.4	3227.6	5343.8
1716.1	1952.5	2800.7	2338
2208.5	2423.8	3418.6	3473.2
2723	3304.5	4709.6	4845.5
2837.1	3439.6	5445.3	4288.1
1764.5	2077	3220.7	2558.1

2133.3	2782.3	3063.1	2897
1858.5	2970.7	4146	3708.1
2251.6	3149.9	4103.6	3940.4
1769.3	1827.7	2918.6	2730.3
945.3	1120.6	1531.5	1299.7
3797.7	5836.3	9954.2	7810.7
2727.5	3460.2	2713	2226.7
2050.1	3478.3	4709.6	6928.4
2444.8	3592.1	4568	5205.8
2479.3	2711.9	1340.7	1139.7
1924.8	2453.3	2928	4406.1
1790.9	1691.6	2447.4	2858.1
2059.2	3329.4	3663.8	2818.1
2840.7	3588.8	3702.5	5318.7
2121.2	3135.1	5795.6	6062.4
1312.1	1509	1897.9	1769.4
3044.5	4044.4	5703.1	4791.8
2201.4	2658.1	3098.2	3500.3
1176.4	1565.9	2940.6	2194.4
2947.2	3344.6	4710.2	4573.5
2870.1	2747.1	3894.3	3525.5
1433.1	1838	3060	2579.2
2668.5	3643	4892.5	4335.5
1963.8	2320	3197.5	3466.5
1655.2	2329.4	3280.7	3808.7
1368.5	1294.7	3459.4	2429.3
2383.8	3282.6	3570.7	4408.5
2700.3	3108.4	3845.6	3911.2
1506.3	1958.5	2758.9	2975.3
1154.7	1271	1877.1	1540.6
1537.1	2563.4	4297.3	3993.3
5184.2	5687.6	8166.7	6951
2507.5	3165	4034	4444.6

2243.2	2433.1	2971.5	3159.1
5306.3	7342.1	9864.2	9035.9
1105.1	1513.9	2340.9	1977.1
1653.3	2046.7	2825	2646.8
1503.4	1479.7	1962.9	1739.2
1162.1	1930.1	2774.1	2877.8
1913.7	2537	2723.7	2747.3
2519.1	2744.9	3194.9	2811.7
1431.9	1380.3	1610	1359.7
2556.8	3274.1	4108.9	4108
4385.4	4291.8	6577.5	6799.6
2637.4	4019.6	4544.3	7227.9
967.6	2604.8	2346	2636.7
1986.2	2718.4	4359.3	4407
2046.7	3154	4015.3	4998.7
2192.5	2695	4249.6	3720.9
1597	2098.7	2892.4	2904.6
4818.7	5684.5	8118.6	7036.4
726.9	1240.4	2159.6	4701.8
2383.1	2823.9	2901.7	3764.3
1801.5	2395.4	3549	3494.3
16505.9	452.4	1572.8	930.6
1693.5	1683.4	2109.9	1187.6
2066.8	2248.9	2722.3	3171.6
1797	2346.4	2840.2	2312.5
1554.3	1822.3	2312.9	2581.3
1496.2	3184.4	1910.5	1901.5
8740.8	6289.4	5386.8	1903.2
6676.9	5202.5	5548.4	3177.7
1688	4889.3	5076.6	1779.7
1937	2617.2	2685.6	2787.5
2323.2	2751.9	3316.9	3746.1
5210.4	6400.8	9706.1	8023.7

2461.2	2796.3	3546.1	3340.4
1994	2441.4	2542.1	6977.9
1809.2	2189.3	2777.4	2589
1237.6	706.3	1101.8	630.7
2391.1	3890.5	4240.6	4833.4
1520.2	2134.2	3028.6	2319.8
1479.5	2001.7	2864.1	2864
3829.9	4717.5	6423	6372.1
1877.3	2804.6	3437.4	3505.9
1853.8	2498.9	2249.7	2969
2191	1760.2	2546.4	2113.4
1173.3	1744.6	2189.9	2077.6
1759.2	2189.1	3575.9	2801.3
8130.1	9608.8	12929	14734.6
612.2	623	798.9	763.8
1828.2	3083.3	3184	3171.1
4049.2	2488.9	2962.4	3514.5
3081.5	1630.1	2600.2	2261.7
3138.5	3583.5	5299.6	4974.6
1222.9	1901.1	2193.9	4189.8
2498	3140.9	7054.7	5601.1
2788.1	2659.4	2288.1	1955.3
3689	3592.4	4918.1	3359.4
2617.3	2986.9	3965.5	3743.7
2359.4	2949.9	3551.6	3252.5
1466.1	1514.8	1447.7	1239.4
1674.9	1598.4	3134.8	2729.2
1342	2287.2	1937	3261.8
1620.1	1964.6	4637.1	2647.6
2211	4144.7	6989.5	8184.1
1176.1	2397.7	3681.9	2010
1575.9	2164.5	3465.9	2910.8
1431.5	2538.6	1931.8	2068.1

3270.9	3179.1	5260.5	4746.7
1776.9	1848.8	2768.7	2725.9
6329.6	11175.6	11128.6	13885.9
1305	1729.9	1322.1	1012.3
1289.3	1277.5	4202.8	2368.4
2552.3	5564.9	2817.6	2847.5
1461	2016.9	2699.2	2308
1516.1	1954.6	3247.9	2808
2896.7	4178.4	5894.9	4760.6
2248.3	3198.9	5316.3	4459.1
3573.1	4925.8	6362.8	6028.9
1615.1	1814.1	2718	2816
1715	1925.7	2199.6	2599.3
935.3	986.9	1093.6	1316.3
1668.2	2240.5	3160.2	3197.2
1636.9	1159.7	1165.9	625.9
2336.5	2912.2	3823.6	4013.8
2827.9	5199.9	8091	9927
2086.6	3291.1	3973.8	3456.3
2013.4	2026.8	2688.7	2281.7
3822.3	2660.9	4486.7	4277.3
2432.6	3287.2	4381.7	3971.1
2156.2	2314.5	3317.5	3476.6
646.2	881.4	967.2	825.2
2746.3	2253.2	1231.9	545.8
2752.8	1458.9	1510.6	1526.1
1845.6	2153.7	3231.3	3355.3
2181.3	3106.5	5026.8	4265
969.7	1002.2	1571.1	1629.2
1719.4	1260.1	2543.1	1969.8
1385.6	1611.5	2091.8	1895.1
1181.4	1790.1	2605.3	2974.3
2982.3	3229	3888.5	4069.2

2448	4814.3	5808.7	9800.9
1385.7	2258.1	2745	2462.1
2783.1	4748.8	6164.7	6236.6
1337.3	1120.6	2273.1	1426.4
2797.5	2951.5	3851.4	3532
1860.6	2415.1	2993.2	3257.7
2727.6	2784.3	5453.1	6506.5
2927.5	1809.3	2930.8	1491.6
1475.1	6359.2	2531.9	1213.2
1098	1479.7	2173.1	2387.2
1409.2	1627.6	2620.5	3148.7
2949.7	3030.1	4784	4561.6
2675.2	3912.5	6026.4	5328
1659.6	1889.7	3061.2	2073.7
1984.7	2086.2	5737.4	3265.7
2171.6	2485.7	3494.3	3175.8
2037	2220.5	2990.4	3385
1396.3	1959.4	2989.3	2775.3
2852.7	3082.3	2817.9	2867.8
1182.2	1472.5	1951.4	2037.5
891.9	955.9	1916.6	1154.3
891.5	1182	1710.9	1642.2
656.7	893.4	1669.1	1787.4
2089.1	3539.9	4650.9	4856.4
1465.6	1455.3	1847.9	948.1
1514.2	2931.8	2552	5797.1
2763.3	1620	5386.8	5076.2
1794.2	2221.1	4262.4	3248.7
2337	1682.9	2704.7	1687.3
90.6	172.7	154.9	208.5
2330.2	2731.1	4285	3779.7
1741.4	2492	3496.3	3405.8
1641	2605.6	3960.5	4315.1

2123.1	2278.4	2300.1	2016.4
1776.5	2000.6	2502.4	2551.8
1725.8	2348.2	3226.2	2656.8
2200.3	2375.7	3347.3	3072.3
2722.7	3383.6	4642.9	3635.9
1869.7	2223.3	1911.5	2259
509.5	730.8	873.5	705.6
5294.6	6938.9	10492.6	8155.7
2902.7	3711.4	6181.6	5316.7
1780.6	2177.5	2693.8	2632.8
2221.5	2226.3	3397	3195.1
947.8	980.9	1556.2	1215.9
1456.8	1856.8	2195.2	2371
1612.7	1662.2	1612.7	1738
4185.6	6596.2	6964.8	6552.7
3656.2	4611.3	5210.6	5683.9
1418.6	1677.4	2192	1836.8
1536.9	2204.8	1921.3	2454.9
1559	1712.2	2637.6	1955.2
1635.7	2287.8	3371.7	2994.8
1214.3	2194.8	2898.3	3712.3
487.1	619.2	1937.2	534.1
1887.7	2493.7	3590	3467.6
1626.4	2137.4	2910.1	2922.7
3964.9	5859.9	8641.2	8395.1
3216.9	4837.6	3266.7	9302.6
1846.7	2296.6	2732.6	2625.1
1708.4	3456.1	1986.8	4375.2
1538	1984.7	3535.2	1175.6
1465.9	1894.7	2548.1	2529.4
2522	2350.2	3779.1	3604.1
897.6	1253.9	1958.8	1909.4
1391.1	1817.8	2882.3	3031.5

1827	2364.8	3612.9	2978.5
1379.8	1961.4	1931.3	2524
1109.3	1469.3	2855.1	2645
1770.5	1930.4	2387.1	2736.1
2499	1515.7	5604.7	2771.8
3007.1	4438.4	6981.9	7576.1
592.8	452.7	760.6	641
2393.3	3000.5	4140.9	3646.3
835.1	1460.7	1685.1	1021.7
1390.9	1855.3	3779.1	3944.5
1870	2540.8	3278.2	3153.5
2775.4	3693.2	4547	3909
1330.3	2055.2	2345.2	2304.2
1756.8	2076.3	3791.4	3653
992.4	3324.4	1563.5	6007.7
1203	991.8	1430	1557.5
3404.9	4023.9	6315.6	7115.3
1787.9	2006.7	2914.1	2632.9
1806.8	2603	3761.6	3973.7
2768.1	2728.7	4520.6	4240.2
3095.6	1934.6	3609.1	2496.9
1483	2034.9	4038.8	2801.3
2142.7	3128.6	5446	5015.8
1800.3	2553.7	4826.6	5550.4
1335.4	1261.2	2172.8	1155.5
1035.7	991	1124	710.3
2010.7	2524.9	4114.9	3185.3
2457.6	2924.5	4552.3	4003.6
1712	1480	2002.5	1821.7
2527.1	4209.8	1985.9	2753.7
2708.7	3499.3	3750	3854.7
1023.4	1829.5	2770.9	3136.7
1564.2	1806.8	4349.5	1834.7

3164.5	680.5	904.6	873.1
1047.2	605.1	1805.5	1919.9
1929.6	2583.5	3416.1	3332.6
3111.4	4141.4	6340.4	6184.8
1974.9	2418.1	3329.5	2803.3
1967.9	2693.9	3548.2	3692.4
1403.8	1808.3	2446.4	2288.6
1410.7	1789.2	2364.8	2199.9
2655	3323.5	3442.1	3782.3
2617.6	3958.8	4008	4447.4
5533.7	7095.1	11748.8	9561.7
2570.2	3242.5	5331.8	5661.6
3095.2	3537	3693.4	3692.9
2386.5	2693.9	3979.6	3416.9
1628.8	1650.7	2242.6	1982.7
1015.7	1053.1	1780.3	1550.4
1249.4	2412.2	2876.5	2411.5
2049.3	2319.3	5114.4	3880.6
1578.7	1892.3	2621.6	2372.8
1817	1907.2	6004.6	3145.3
1911.1	1886.4	2491.8	2764.1
2501.2	2548.8	4148.9	3751.9
2148.4	3178.5	4228.9	4548.9
2144.3	3541.3	2769.7	4570.5
1743.1	2213.4	4149	2439.7
1465.4	1755.3	2402.9	2305.4
1418.3	1789.2	1896.2	1850.5
2563.8	3578.8	4642.1	4477.9
5318.7	5003.6	5595.5	6988.6
1574.9	2465.1	2900.3	1085.9
1750.9	2238.9	2893.2	3208.2
1444.2	1914.8	1994.7	1884.4
1723.1	1831.8	2212.6	2462.4

1564.9	1545.3	1468	1029.3
142.6	40.3	85.8	77.1
2791	4476	5321.3	5978.4
1157	2297.8	3172.7	2834.4
2343.6	2394.8	2319.3	2633
1681.3	2721	3038.8	3058.2
1300.8	2453.7	4075.1	3285.3
2036.7	2183.5	3274.5	2739.8
1580.4	1816.4	2260.4	2390.2
819	994.1	2643.9	1249.5
2503.2	3182	4215.9	3952.5
967.2	1035.4	1002.4	1251.1
1490.1	1611.8	2058	2207.6
1726.9	2010.9	3002.1	3633.6
2190.5	1819.8	3139.8	2624.2
2253	1990.8	3288.3	2869.7
1806.5	2721.6	3259.1	3512.5
1417.7	1785.9	2887.2	2773.1
1102.2	1451.1	1362.6	869.6
1040.8	1608.8	2580.1	2560.6
2100	2329.8	3333.6	3018.2
1036.1	1687.7	2474.4	4831.9
1451	2156.3	2847.1	3522.4
5205.8	4901.6	6888.3	5307.6
2045.6	2391.3	3485.9	3272.5
2677.4	3712.8	4181.1	2882.1
1268.6	1629.4	1679.8	2830.9
2039.5	2742.3	4420.4	4566.2
1677.9	2360.1	3996.7	3625.7
3511.9	4683.5	6503.8	5648.1
2431.7	2844.6	3659.1	3455.8
1553.3	1683.3	2795.8	2522.4
860	540.6	838.2	730

432.6	699.9	790.3	997.6
3475.5	2264.6	3190	2865.9
1532	2121.8	2464.7	3227
1471.8	1442.5	1622.8	1734.3
12287.1	4376.3	1698.6	1875.6
1652.9	1873.8	2273.2	3189.8
1919.9	2431.6	3934	3422
2092.9	1783.2	2655.2	2402.1
3972.3	5492.8	7625.6	6476.3
2346.2	2166.5	2997.3	3151.5
1756.3	1515.9	2337.7	1956.7
81.4	68.5	80.1	84.3
863.2	954.4	1294.2	1249.6
2832.7	2897.7	3150.3	2882.9
1547.8	1614.1	4978	2437.4
1187.7	1419.1	1936.9	1919.3
1376.2	2307.7	3093.8	3910.1
1073.4	734.9	3137.2	1293.3
809.4	967.5	1229.6	1425.1
1494.7	1826.9	2554.4	2388.3
1338.8	1518.3	1904.2	1958.6
2311.4	3764.4	4092.3	3971.3
2170.9	3297.7	4671.9	5145.9
2752.9	2836.7	3179.2	3840.1
2449.1	3591.6	6351.7	4721.4
2378.3	3385.1	4220	3752.6
2885.1	2510.8	2937.9	4053.1
2038.4	2059.3	2388.1	2666.5
2112	3077.9	3756.6	4071.1
1298.8	1574.3	2398.6	2648.8
2891.8	3174.9	4423.3	4211.8
2407.6	2790	3132.3	3461
1893.4	2068.9	2008.6	3158

1611.9	1551.6	2468.1	2009.9
1239.1	1483.5	2049.6	2260.7
1848.4	1634.4	2706.3	2224.8
810.9	1020.9	1307.4	1528.7
1437.3	1945.1	2289	2604
382.5	416.2	526.8	500.5
1703.8	2582.5	3094.4	3055.6
1222.5	2299.4	2238.2	2345.3
2362.4	3183.4	4549.5	3633
2042.4	2195.8	3296.1	2799.6
1673.9	1603.1	1940.5	1030
1649.2	1228.9	1573	1566.4
1950.6	2157.9	2646.4	3171.2
1336.7	1897.2	2842.5	2611.2
1878.3	2978.5	3996.1	4459.5
1789.2	1809.1	1930.8	1369.4
1164	1166.1	1565.5	3390
1797	1653.3	2154.6	1215.4
1259.2	1354.5	1855	1840.4
481.9	531.3	746	988.8
646.8	925	1010.4	1722.1
1404.8	1705.2	3245.6	2127.4
2265.7	2348.1	3243.5	2967.5
2094.8	2483.2	4861.8	5546.9
2248	2863.6	3320.8	2822.8
4567.5	5238.8	6602.2	7411.5
1686.7	1880.7	2630.8	2250.1
1744	1791.3	2108.7	2119.9
1328.6	1330.5	1780.8	1973.1
1238.2	1508.5	3607.6	1566.5
347.4	247.6	387.3	392.6
2270.9	1964.1	2179.7	2069.5
2192.1	2832.9	5013.2	4289.7

1312.9	1288.5	1827.8	2567
2118.5	2925.9	2373.7	6185.3
1564.9	1758.3	2119.4	2773.8
3715.4	7140.4	2428.5	3128.7
2546.8	3286.8	4127.3	4388.6
2066.3	3265.7	2641.6	5439.4
2488.7	3217.8	4915.3	4386.8
801.2	900.6	1798.5	1488.8
1422	1931.3	1236.2	1784.4
934.4	1319.4	2267.4	1046.8
1502.6	1521.2	2213.8	2304.6
1522.8	1780.7	2866.3	2699
2266.9	2945.1	4067.1	3641.9
418.9	333.1	805.8	794.4
1323.3	2639.2	4149.4	4539.5
3260.9	3621.1	2521.9	1524.1
1849.1	1728.7	2971.1	2091.3
793.3	632.1	1188.7	1087.4
1368	1950.3	2984.4	3254.4
1738.1	1768.9	1488.7	1655.8
1212.1	1751.2	2364.9	2389.9
1046.2	1682.6	2501.4	2515.1
1228	1712.5	718	726.1
1636.2	1991.1	2692.3	2804.2
1636.9	1833.5	2782.3	1770.5
1043.4	1291.4	1853	1689.3
901.1	1113.4	1520.6	1657.1
1035.6	1345.4	1619.4	1801
3662.5	4708.5	6760.4	6039.4
1791.5	1830.8	2378	2550.2
1997	2292.9	3065.5	3274.6
1306.5	1691.8	1791.7	2579.4
1443.6	1634.7	2049.1	2189.2

1271.7	1618.7	3462.2	2980.3
3179.2	4312.3	6156.5	5225.1
1238.2	1172.1	1835.3	4414
3814.1	4814.3	6547.5	6076.4
1401.8	1044.4	2903.7	2499.4
1738.1	1760.4	2642.4	2160.5
1327.8	1705.7	2309.1	2284
2307.7	1798.1	2450.5	2440.7
1552.6	2536.7	3850.1	3914
528.7	435.7	577.8	494.1
1402.7	1682.3	1996.1	2230.7
1332.4	926.1	1026.2	1005.3
2788.3	2206.4	4625.8	3967
5995.6	4129.1	4565.5	4493.3
1091.1	820.7	1011	858.5
1699.7	2588.3	4235	3765.8
251.6	301.7	304.4	256.4
1934.3	2224.5	2390.1	1987.3
2338.9	1328.2	1479.4	3249.9
3526.6	4766.7	6755.3	5808.2
1101.9	1448	2139.3	2217
1717.6	2391.9	2823.3	2915.6
1071.6	1076.9	1481.5	1558.7
1653	2216	3380.6	2719.3
1026.5	1594	2014.2	1774.8
1517	1135.5	1534.1	2515
1894.9	2055.6	3608.7	2793.4
1552.2	2050.2	2828.1	3003
2161.6	2534.5	3013.5	3230.5
2410.7	2702.3	4384.7	3516.3
2242.7	2965.6	4450	3893.6
909.8	1268.9	1610.5	1856.2
2074.7	2861.8	3557.3	3321

3423.3	3385.5	4630.6	4556
977.1	1567.9	1251.9	1501.6
1512.4	1350.4	1825.9	2348
1154.8	1287.6	1697.9	1408.5
2010.9	3072.3	4071.6	3601.6
2758.2	2837.1	3802.9	4094.7
1471.2	1203.3	1457.8	1903.4
1287.3	1319.6	1556.9	1482.4
1048.3	1223.9	2238.1	2035.3
1572.9	1814.2	2380.9	2220.4
3618.8	1281.2	1428.8	1522.8
1259.3	752	2506.2	1474.9
2103.1	2368.8	3240.6	4017.1
2297.2	1223	1184.5	1142.7
2480	2676.8	3707.1	3636.8
950.8	801.1	1426.9	986.2
1864.9	1613.5	1885.7	2872.2
829	997.5	1755.1	2192.4
1578.7	1794.1	1823.9	1905.4
1091.2	1901.5	3452.2	5166.4
2092.5	2917.3	3750.8	4550.7
1284.8	1024.4	1028.4	1361.3
1249.2	1547.6	2130.2	1944.3
1775.7	1820.1	1243	1368.2
610.9	836	848.3	1500.8
1365.6	1479.3	2445.2	1970.3
1056.4	1544.6	1448.4	1346.4
1398.5	1334.4	2629.6	5538.3
1429.4	1774.3	2539.1	2328.4
1796.8	2473	3835.8	4115.7
1824.1	2278.9	3848.2	3859.3
1220.3	1399.7	2667.2	1987.6
2068	2579.8	4073.6	2821.6

169.3	144.8	164.2	172.2
1532.3	1891.4	2725.3	2913.4
1603.8	2453.3	3305.1	3108.9
1121.2	2670.1	2397	4865.7
3011.7	4244.8	6171	5067.3
1852.5	2039.8	2472.1	2123.2
321.7	351.8	356.6	377.9
182.7	322.2	340.4	281
2147.9	2240.8	3404.3	2941
760.5	713.8	703.7	426.9
2208.1	2688.4	3665.2	3560.5
1341	1587.7	2509.7	2459.3
1209.3	1456.5	2120.6	2095.5
2329.4	3103.8	3846.1	3823.6
933	1190.9	1599.9	1762.6
1116.1	1471.3	1821.4	1913.7
1205.2	1602.2	2478.1	2132.1
1452	2595.9	2517.9	2760.4
1668	2629.5	2206.4	1974
3049.3	395.6	1049.1	556.6
5347.9	10332.4	10288.1	14286.5
1368.9	1812.4	2577.4	2128.2
1450.1	2767.4	3822.6	3274.5
1662.7	2097.4	2390.9	1917
2805.3	3918.9	4542.6	4348.3
2264.4	3189.5	4819.6	5104.7
1127.4	1697.3	1813	2264.8
1082.8	1267.4	1705.4	1742.8
1380	1764.6	2626.4	2567
3210.8	3825.5	4377.1	8197.8
1108.6	1035.4	1684	1014.6
1388.8	1977.8	2263.8	2789
1324.4	1569.1	1926.2	1843.9

881.9	740	842.2	639.9
1135.3	1083.6	1872	1987.8
2871.2	3675.3	4814.2	4512.5
3566.3	1488.3	1683.5	1597.1
493.1	528.9	1012.2	840
262.1	326.7	735.5	468.3
2045.9	2701.7	3356.7	2515.1
1481.1	1619.2	2227.8	2481.7
754.5	890	1335.6	1001.6
919.5	1996.5	1655.6	2092.4
403.2	568.6	850.4	520.4
1455.1	1662.4	2081.1	2918.9
1215.5	1616	2352.4	2365.4
1569.9	1778.5	2518.2	2211.4
76.2	130.9	169	173.7
1019.6	1439.8	2257.9	2076.2
46.1	55.6	60.6	70.1
1530.2	1746.7	2656.2	2461.3
1906.1	1851.8	3490.3	2616.3
1676.4	1822.2	2609.4	2428.1
1122.1	1003.6	1454	1186.6
3450.7	1349	1536.9	1430.3
1094.9	1268.2	2641.2	2015.5
554.6	3005.5	1438.4	1659
1172.5	1892.9	3066.7	2732.2
891.2	1078.3	1241.1	1466.7
2847.9	4034.6	4736.1	5649.4
1380	1694.7	2524.4	2285.2
878.5	1117.3	1477.5	1456
1583.2	1905.3	2887.4	2469.7
1257.9	1594.3	1544.3	2591.4
4123.7	4256.8	5942.7	5953.4
2053.2	2332.7	2782.5	2370.1

1611.3	1924.5	3207	3232.6
3550.9	4918.4	7034.3	5871.5
415.8	585.6	692	727.8
1169.2	2100.6	3396.1	2505.5
1188.8	2333.3	2265.4	3505.6
1950.7	2226	2803.4	3914.9
1782.9	2289.5	3160	3219.7
1649.7	1662.3	2246.4	2035.6
2037.3	1891.5	2342.6	2059.2
876.7	1166.4	1423.1	1726.7
2856.2	2828.9	4594.6	4857
1230.1	1540.8	1943.2	1796.7
2922.8	764.1	1062.5	847.7
1302.9	1853	2164.9	2037.3
960.8	1200.2	1470.3	1611.5
566	12015.9	862.8	3494.7
1396.3	1721	2374.5	2227
1267.8	1694.8	2239.7	2144
871.4	1093.2	1737.4	1495.1
1576.9	2170.5	3233.9	2907.3
945.9	1198.7	1175.8	1425.7
1118.2	1174.7	1935.6	1073.4
1574.2	2079.8	2393	2377.3
4304.3	5871.4	7974.9	10303.3
4304.2	4175.2	6741.1	7490.9
708.3	709	1052	539.3
5727.9	250.8	676.1	760.5
2300.5	2505.2	3138.1	3005.5
635.3	517.6	779.1	1207.1
1246.3	1508.5	1763.2	2213.4
1605.9	2147.1	2744.7	2984.4
2035.1	2263.2	2948.2	3024.2
759.9	904.6	1378.1	1020.8

1341.3	1959.2	2610.8	2803
1919.4	2315.9	4150	3296
769.9	1054.5	1429.3	1355
1258.5	1669.9	3027.2	2385.9
1959.5	2025.3	2461	2657
1543	2163.9	3292.2	2927.2
4791.3	1599	2437	1250.5
1404.7	1552.3	2236.1	2134.4
5772.4	8579.8	12163.2	12707.1
1917.8	2353.3	3183.3	3375.1
1006.3	1104.4	1563.7	1484.1
1424.9	1844.6	2829.3	2306.3
1786.4	2167.8	3439.7	3574.8
584.2	976.7	1204.8	1827.1
2786.7	4942.9	4596.4	3746.9
1178.3	1156	1593.2	1778.5
1423.3	535	1746	2195
66.4	43.7	44.1	49.3
1183.1	1419.4	2090	1695.9
94.6	91.6	144.7	121.1
752.4	688.4	792.5	448.4
1208	1286.8	1987.3	1967.5
1193.4	1112.7	1482.9	1422.9
1921.3	3270.5	4652.3	3395.9
520.9	458.1	785.4	694.8
1268.9	1581.6	2246.6	2119.7
1145.7	1080.3	2013.3	1989.9
893.7	1589.1	2087.4	2559.2
1661.6	2361.2	2384.3	2940
1545.1	2004.2	2773.9	2157.8
876.3	1528.9	1081.8	1096.7
1799.7	2413.5	4624.1	3712.6
1542.4	1477.4	1930.8	1681.8

2148.2	2942.3	4013.8	4508.3
506.8	599.7	740	913.8
1051.3	1740.5	1773.2	2379
906.9	1353.5	1688.3	1744.7
1168.8	3314.5	2241.2	2528.3
1240.4	1830.8	2144.1	1921.2
1149.2	1448.7	2007.2	1927.3
1528.6	1895.8	2257.4	2223.6
772.7	1096	1314.2	1290.5
1581.1	1341.4	1953.2	1575.8
1259.7	1546.1	2395.7	3412
1841.9	2017.5	2630.7	2699.8
4264.8	3352.5	3981.9	5950.5
1633.4	1932	2917.2	2763.6
2268.2	3021.6	3388.7	3908.3
2245.7	2927.5	6973.8	8481.1
2287.6	2320.1	3731.6	3127.6
972.7	1328.3	2196.7	1935.2
1453.4	2078.6	3041.7	3121.7
675.2	623.7	764.1	941.6
524	624.3	855.7	750.7
1437.6	1401.8	2440.1	1874.4
1442.3	2150.6	2900.7	3237.7
782.9	706	1134.3	915.3
3552.7	3696.6	4862.1	4830.9
1204.1	1330	1924.8	2000.7
1588.9	1775	2228.7	2298
1992.5	2102.2	1214.2	2146.5
1671.6	2130	2747.6	3558.8
452.5	590.5	1292.9	939.4
1456.8	1366.9	2260	1891.2
1371.8	1608.3	2260.4	1922.1

318.3	493.7	366	510.1
740	655.9	724.5	850.1
1486	1643.1	2417.9	1897
1398.5	1678.5	1537.3	2308.9
2579.7	2898.5	3021.2	3986.3
709	878.9	1283.3	1437.2
968.3	928.7	689.2	2109.7
1185.2	1967.8	2203.5	2598
1269.6	1222.6	1388.1	1430.6
763.1	1273.5	1671.5	1798.4
1291.1	2877.6	2974.7	4657.3
1302.8	1655.7	2296.8	1731.5
1044.4	1575	2227.7	1904.4
2681.3	917	2035.5	1249.5
1964.1	2314.5	2778.2	2400.9
1343.6	1915.1	1942.2	2203.8
566.4	650.8	838.3	729.3
2345.3	2983.3	4897.5	4103.1
1422.1	1616.3	2477.9	2475.3
1425.7	1805.9	2583.2	1880.5
1067.8	1309.7	1747.6	1798.5
3212.3	4108.2	5671.8	4937.9
1858.1	1543.7	1999.4	2627.6
1167.4	1598.3	2122.8	2057.6
930.2	1092	1585	1377.6
1637.7	2102.3	2874.4	2865.9
1025.7	831	1161.8	1086.9
922.8	807.4	2510.6	1445.9
1234	1613.3	1916.4	2113.1
1171.3	1574.2	2446.5	3693.1
3357.3	4429.9	6061.7	5695.6
1326.9	1614	1980.2	2380.6
1024.1	1322	1842.8	2025.3

1396.8	1947.1	3844.7	2406.6
1626.3	2012.1	2663.2	2593.9
1939.5	2002.8	2789.5	3827.3
645.7	799	1168.5	1452.9
1257.4	2736.4	2972.2	3437.6
1379.7	1828.6	2297.6	2370.3
1172	1546.6	2546.7	2068.1
1205	1929	1721.4	3284.3
2017.4	1970.6	2593.3	2868.9
1084.1	635.6	746.7	669.2
2062.7	2684.5	3465	3626
585	783.3	701.3	725
2512.7	2578.6	4681.7	3392.6
1540	2282.3	3243.4	3091.3
1333.8	1864.8	2532.3	1774.3
1357.2	1752.2	2267.2	2235.5
1429.5	1638.6	2583.6	2150.7
1144.4	2113.3	2449.9	2198.2
476.2	416.4	425.5	407.7
1774	1579	2852.2	2228.9
1364.1	4472.5	2710.9	11825.9
1215.8	1118.8	1493.9	1730.6
1013.3	1334.2	1594.3	1678.5
809.4	838.2	1249.6	1015.9
830.1	1434	1817.9	1812.1
1633	2018.6	3190.2	2779.5
1208.9	1390.2	1940.4	2091.7
960.1	1129.8	2030.8	1535.6
4235.9	1161.6	10832.9	3873.4
565.2	619.9	1189.4	778.6
32.2	38.4	54.3	51.1
1042.5	924.9	1550.4	1565.9
1057.1	1209.9	1787.2	1557.7

854.4	1278.2	1794.6	1896.9
3222.2	4502	6643.6	5486
2459	6337.4	3979.4	3117.7
1217.6	1354	2374.8	2009
578.5	705.6	988.3	895.2
289.9	248	352.4	309.4
1725.2	2105.1	2950.9	2551.6
1316.8	1614.7	2283.3	2203.9
1143.9	1542.9	1788.1	2016.6
1932.5	2173.9	2752.1	2909.2
2556.5	2852	4777.9	3913.1
1433.6	1910.9	3035.9	2836.7
1442.3	2982.7	2548.4	3656.4
1883.2	1452.7	1898.3	1708.9
1599.9	2164.1	3073.6	2708.5
1141.5	1301.9	1524.6	1580.5
621.3	936.5	1581.4	1483.2
1827.4	3244.9	3571.9	4070.8
501.6	625.2	826.4	745.1
1638.2	1864.8	2418.8	2202.1
1350.4	1329.3	2600.5	1823
2618.7	2996.9	3379.4	2984.2
1493.9	1836.7	1686.9	1469.3
2966.9	1633.4	2131.7	1854.3
1078.6	1159.8	1473.7	1488.7
2011.8	2414.8	2527.3	2299.6
653.1	607.9	851.7	885.1
2851.7	3180.9	3843.8	5823.1
754.7	773.2	855.5	654.3
1031.3	1161.3	1542.3	1903.3
2383	2380.2	3598.9	3663.4
2709.9	3487.2	4748.7	3857.5
1452.4	1829.8	3474.2	3303.4

905.4	1336.3	1582.4	1732.9
731.3	1064.6	1942.3	2114.3
681.7	2150.7	1879.8	1581.6
356.2	283.9	464.6	467.2
1010.6	1623.2	1993.7	2400.3
1583.1	1790.4	2043.1	2512
1490.3	1747.6	2180.9	2514.8
903.3	1195.5	1853.9	1271.5
9920.8	1767.2	2369.7	1535.2
1382.6	1940.6	2178.5	2620.9
1621	1648.1	1880.4	2277.2
1467.6	1741.3	1957.4	2079.7
1256.4	1622.2	1964	3824.6
1900.1	1725.1	3262.7	3250.9
2440.4	3037.8	5319.8	5178.7
1759.5	2450.8	1825.6	1374.4
2007.9	2469.1	3893.2	3263.9
381.4	606.6	695.5	813.7
542.2	499.2	1134.3	608.4
1091.5	1437.2	2139.5	1733.2
801.5	1111.1	1165.2	1192.6
1606.2	2318.2	3190.3	2552.6
2205.9	1640.7	2759.1	2373.6
430.6	2763	1639.8	2100.8
1901.7	2698.3	3916.4	3807.4
1553.9	1911.9	3113.1	3120.4
2351.2	3344.4	4700.3	3883.9
1167.4	1563.9	2216.9	1924.7
2010.5	2563.9	3248.5	3030.7
1147.7	1668.8	1903.9	1289.3
1108.1	1607.1	2174.1	2346.8
1705.3	2440.7	3420.4	3415.6
1562.7	1725.8	1895.8	2033.4

1328.1	1475.2	2072.6	1826.5
2176.2	1402.7	1265.1	1787.7
1011	1675.8	1321.7	3148.9
1694.1	2114.1	2941.1	2637.7
1116.2	1385.1	2206.2	1743.9
1491.8	2039.6	2432.3	2490
847.8	946.1	1108.2	1085.7
1297.4	1262.5	1295.5	1422.6
1057.5	1472.6	1506.5	1561.2
1648.9	2057.5	2687.2	6231.6
1111.9	1482.4	1871.6	1749.4
972.8	1198.5	1851.9	1452.4
620.3	704.7	1412.1	757.6
910.7	1237.4	1348.6	1710.5
1154.6	1555.4	1620.4	2288.8
1411.9	1548	2323.1	2199.3
1982	2354.3	2538	3051.9
1152.8	1512.6	2054.5	1818.7
1570.7	734.2	978.7	820.7
761.8	901.2	1079.9	846.5
1498.4	1315.3	1226.2	598.3
1374.6	2080.6	1227.2	1134.8
209.5	273.5	348.1	366.8
970	1483.2	1785.6	1774.5
2111.6	2107.8	3401.9	2493.9
925.9	877.4	1203.2	1065.4
2076.9	2710.2	4156.3	3627.3
1276.2	1735.5	2593.3	2475.6
1620.9	1492.8	1777.2	1605
1708.1	2178.2	2856.2	2650.2
2358.5	2693.1	3415	3542.1
1388.3	1739.6	1678.5	1378.7
1308.5	1427.3	1518	1483.6

2444.7	959.4	1156.1	1359.6
2124	2472.1	3495	3143.6
752.6	1080.2	1370.6	1140.8
887.5	1901.3	1129.4	1448.2
374.5	378.1	446.7	596.5
1051.5	1038.8	1054.5	1078.8
874.9	1550.2	1342.2	2073.9
1052.6	1389.6	2958.4	1042.6
1001.8	1393.3	2023.5	1895.9
1358.5	1763	2049.3	2069.7
1034.7	1215	2096.2	1550.5
1577.8	1507.1	1736.3	1935
1227.6	1633.7	3869.8	1756.6
1323.8	1893.2	1772.6	1026.8
2376.2	902.5	920.3	1115.4
294.6	148	137.1	129.2
1040.5	1198.3	1677.9	1567.7
1253	1688.6	2307	2012.7
790	806.1	1105.5	1053.4
1284.8	2214.7	2217.7	2982.8
3749.1	5492.3	8054.3	7108.8
1346.1	1140.7	1636.9	1200.5
1387.4	1971.4	2379	2152.2
1451	1950.9	2890.5	2611.2
1159.6	1277.8	2311.7	1799.7
1426.1	1449.1	1828.9	1607
1279	1603.1	2205.4	2080.1
1490.8	1768.2	2680.2	2013.2
1651.6	1208.8	2984.4	1871.1
1383.8	1439.2	1958.5	1766.4
894.9	1532.5	1506.8	1659.1
1144.9	944.3	1166	1488.6
2588.1	3477.1	5009.7	4169.9

1758.3	1708.1	2811.8	2562.5
1310	1620.7	2420.8	2066.7
985	773.4	1751	1259.9
1645.5	1708.6	2165.9	1966.5
973	895.5	814.7	810.5
1239.7	1605	1939.4	1689.3
82.8	106.3	101.6	104
886	1197.1	1112.2	1668.5
1015.7	1272.2	1935.8	1502.3
1462	2296.6	3423.7	3066.8
1189.7	1688.1	2211.6	1998.5
529.3	624.5	731.6	960.9
3008.5	3306.1	4190.1	3463.4
1095.2	1632.1	2503.6	2738
943.5	1627.2	1337.7	1286.8
199.7	231.5	325.9	406
880.5	779.9	1633.3	1414.1
1356.9	1694	2533.2	2502
828.4	839.9	1239.3	1104.7
1680.9	2364.5	4152.5	4113.3
736.8	589.8	898.5	727.5
689.7	957.7	834.4	1138.4
1279.9	1043.6	1520.2	1442.8
1436.5	1478.3	2154.2	2174.4
639.3	603.1	772.6	692.9
949.2	1100.2	1482.7	1484.5
757.6	641.6	905.9	1501.2
1751.7	2249	3221.4	2831.6
1239	1277.1	3284.4	1836
2165.1	2216.8	4071.8	2228.5
1246.9	1424.4	1694.8	1543.1
1382.6	1883.1	1411.1	1061.2
1245	1822	2604.7	2467.2

1025.8	1415	1598.6	1531.4
3714.2	5363.2	5091.1	7369.5
1822.9	1622	1783.9	2189.9
1376.3	1945.4	2757.8	2401.4
830.5	1186.6	1632.6	1675.2
1446	2194.7	3241.4	3560.8
1274.6	1404.2	2120.8	1876.2
1500.7	1732.1	1738.2	1684.3
4041	3015.8	3048.4	3255.9
1556.1	2960	3445.1	6961.4
1185.4	2022.2	2398.1	4483.7
1739	1923.7	1822.5	2110.8
2158.6	2701.9	3330.4	3352.1
2585.5	1000.2	1200.1	1070.8
1033	1413.4	1617.4	1626.2
2151.8	491.6	886	625.9
903.3	1121.7	1657.7	1701.2
1424.5	1431.6	2216.5	1756.7
1453.4	1798.8	2440.7	2514
905.7	958.8	1336.6	1820.1
824.8	860.2	1168.9	1961.8
627.2	1037.4	852.5	1781
1051.5	1056.9	1447.1	1554.7
526.7	640.2	772.3	923.8
2139.2	4279.6	5597.4	6733.5
1193	1156.2	1792.1	1836.9
2689.2	3460.3	5227.9	4432
1524.4	1362.2	2261.8	1375.4
1474.2	2745.4	2847.9	2634.7
1102.7	1393.5	1724.7	1929
1341.6	2263.4	2931.7	3270.7
972	1640.4	2056.4	1991.7
1245.5	1420.9	2039.6	2100.3

1021.7	1427.9	1510.7	1853.6
1426.6	1584.9	1338.6	1217.7
976.2	1469.9	1710.5	2065.4
1107.1	856.5	1942.3	1799.8
1186.2	1718.2	1825.2	2111.4
1627.7	1994.8	3801.4	3187.3
1021.5	1059.6	1608.5	1603.5
1203.1	1495.3	1865.3	2297.2
1378.7	2032.2	2226.5	2738.4
798.3	1007.1	1874.9	1575.6
1065.8	1249.8	1796.5	1520.9
881.3	1075.2	1856.3	2036.6
3105.2	1152.6	546.5	1021.6
955.6	1009.3	1426	1322.2
2062.9	2169	3118.1	2615
2053.7	1979.7	2868.2	2283.9
1286.6	783.1	998	951.6
1382.9	1188.2	1981.1	1303.5
2610.2	3032.4	4337.3	4572
933.9	1272.8	1503.7	2441.1
1123.9	1494	2095.6	1868.3
1639.5	2040.7	3220.5	3070.4
1049.5	649.8	564.8	445.9
1158.8	1250.6	1468.2	1344.7
728.6	938.6	1220.3	1290.5
1269.4	1841.6	2371.5	3014.5
670.2	992	1240	1360.7
460.1	610.7	840.1	838.6
1976.3	2807.8	2130	3576.4
816.8	958.9	1260.4	1258.7
937.6	1154.8	1627.1	1631.1
734.9	990.2	1076.2	1288.1
804.9	743.1	1090	1219.7

976.3	936.1	2342.3	1592.9
455.3	614.8	877.4	886
1679.8	2064.5	2886.6	2878
1125.5	1183	1844.7	2537.8
1724	2802.3	4120.7	4241.6
350.2	98.9	64.8	70.3
477.3	394.9	511.2	474.4
1066.9	1563.9	1842.4	2080.1
680.9	910.5	1178.1	1249.2
1623.6	1749.6	4982.3	2475.3
1059.4	896	1690	1352.6
2181.4	2315.6	3107.1	3156.5
937.9	550.8	703.8	653.4
1345.4	1206	1690.5	1405.4
1210.9	1502	1835.4	3524.7
883.3	945.8	1229.6	1206.9
1054.4	1124.5	1375.8	1282.4
274	532.4	539	414.4
876.5	1023.4	1486.5	1551.7
1175.1	961.5	1581.1	1408.1
483.3	600.1	718.2	752.4
1043.8	1307.1	1628	1383.7
704.4	701	1209.4	966.4
1016.3	1125.5	1670.5	1726.6
2877.6	2431.1	3856.5	3450.5
1000.8	1002.5	1408.3	1064.5
797.8	922.3	1147.8	1222.3
1580.1	1350.8	2180	3248.5
1007.5	1412.5	2163.6	2202.5
1019.4	1236.1	1950.7	1897.5
584.1	507.4	682.5	557.6
1340	1486.1	1093.4	1168.4
1554.9	1465.6	1225.2	1552

409.8	399.8	670.4	476.1
982.1	1052.9	1447.1	1260.6
990.7	324.1	455.5	343.6
1247.4	1693.8	2166.2	2296.6
724.6	1109.8	1479	1610.9
1230.2	1705.8	2153.8	2072.9
1599.3	1669.3	1996.9	1834.9
1247.3	1426	1759.3	1793.1
940.3	1106.2	1495.9	1513.6
947.6	1213.4	1668.5	1389.5
931.3	1072.2	1360.7	1406.1
602.2	736	818.8	768.4
1852.4	1987.7	2782.8	2516.7
1172.2	1900.3	2750.8	3084.9
1180.9	1483.7	1726.8	1763.7
1500.9	1131.9	1435.1	1015.2
942.3	1142.3	1773.4	2099.7
552.4	1049.3	1238.9	1241.1
1056.8	1575.3	2250.6	2313.3
1807.3	1630.2	2589.6	2448.3
861.7	916.2	1376.7	1197.9
1482.2	1479.2	1469.5	1461.8
1924.1	3571.8	4985.6	4698.1
1510.7	1561.8	2410.9	2045.9
765.4	888.6	1021	1160
491.3	1441.8	1282	4979
1294.6	1620.2	2249.9	2190.6
825.8	1101.9	1556.2	1488.5
1214	1466.5	2224.5	3080.9
15.2	14.5	19.8	19.7
1031.9	1441.2	1824.2	1909.4
595.1	760.7	786.6	1252.9
327.3	245.8	403	413.2

1388.9	1674.6	1676.8	1516.8
1429.2	1544.8	1777.5	1095.8
612.5	650.1	1077.8	1076.8
1748.8	2096.4	3526.7	4628
873.3	1018.9	1315.1	1345.4
766.7	947.2	1163.6	1243.8
926.4	1072.2	1553.9	1291.9
908.5	1147.8	1676.2	1491.1
1232	1551.4	2106.1	1992
835	607.4	1576.3	884.1
931.3	971.6	1497.8	1711
1951.7	2780.1	1788.7	2286.7
1044.3	1310.7	1641	1816
1404.1	1255	1461.9	1495.1
799	1318.3	1488.5	2638.9
385.5	504.8	668.7	601.2
1445.7	1268.5	1486	1454.5
535.2	736.1	868.5	1105.3
970.7	1052.8	1350.2	1269.3
1329.2	1889.6	2070.4	3314.1
1005.2	1209.2	1660.8	2109.5
1393.5	597.3	689	600
1483.7	1765.1	2464.8	2338.4
932	1578.8	3446.4	1071.6
1109.9	1192.3	1326.1	1705.2
415	516.3	780.7	868.1
938.9	1708.1	2087.4	2588.6
1635.5	1654.4	1934.8	2066.8
1005	1139.8	1226.6	1621.8
1078.2	1373.5	1102.2	2134.7
853.2	1062	1081.9	1222.9
1007.3	1347.2	1856.7	1806.9
622.4	957.9	1207.9	1489.8

847.1	909.7	1051.4	1143.9
844.4	1080.3	1969.2	1967.8
984.6	1132.4	1983.5	1741.5
1203	1573.4	1484.4	1638.6
319.2	364	443.3	602.2
1990.9	2704.5	4845.7	3785.6
1083.6	1114.5	1452.6	1517.7
1096.7	1235.2	1675.7	1796.3
1095.2	1281.6	1727.8	1695.7
847.1	1144	1529.4	1285.6
795.1	1620.2	1292.8	3684.4
595	944.7	1034	1367.2
922.8	1291.8	1874.6	2130.1
942.7	755.4	1150.3	1473
1396	1386.7	2834.8	3444.4
661.2	840.3	708	992
1282.4	1454.3	2009.3	2064.9
868.7	807.4	1974.3	1492.8
905.1	1508	762.4	1032.5
1189.6	1573	3279.4	3483.6
1213.1	1073.6	1134.9	998.8
527.6	969.7	968.3	871.2
789.8	831.6	1544.9	1084.5
1385.3	1108.6	1786.7	1381.2
576.6	828.6	1032.7	1584.8
1761.8	2126.6	2871.7	2820.9
1392.6	1541.6	1760.9	2186
2253.1	4214.3	4906.2	4262.4
314.2	121.3	181	121.8
1048.5	1169.7	1774.5	1392.5
627.3	1031.1	1326.4	1203.7
491.2	640.8	662.2	949.8
2283.8	2893.3	4264.9	3732.7

1846.7	1629.4	1326.7	821.5
993.8	1218	1660.8	1727.3
999	1152.6	1521.2	1518.3
815.9	1117.3	1150.3	1189.4
1737.4	2474	2730.6	3802.1
500.3	392.6	375.5	339.9
1943.7	2195.3	3488.7	3413
745.2	1020.9	1842.3	1796.2
669	982.6	1535.6	1337.5
1346.4	1457.5	2280	1825.5
762.8	810.6	1167.2	1542.7
555.6	736.9	901.8	978
866.7	1715.7	1979.2	2433.5
6856.3	162.6	706.4	342.9
564	583.3	807	899.9
944.5	965	1381.6	1335.1
1578	1877.1	2609.3	2384.9
460.6	518.3	606.9	626.5
917.6	1662	1630.2	2808.1
832.1	1531.4	1088.2	2114
1064.4	1261.4	1964.4	1967
935.3	1698.4	1992.8	3084.4
1095.1	1370.4	1543	1209.3
1915.9	2400.2	2961.3	3378.7
1597.8	1937.4	2901.6	2464.7
1154.1	751	1320	1164.7
1476.3	2047.8	3165.2	3105.1
408	630.3	830.3	936.9
1387.5	1745.7	1962.5	1871.3
801.5	1014	1903.9	1956.9
925.2	1198.5	1586	1549.4
877.8	1220.7	1584.7	1588.3
411.4	716.5	1082.1	1141.3

1178	1451.1	2273	2309.9
1951.2	2924.9	4189.9	4556.5
1443	1248.8	2205.2	2204.3
2167.7	3491.3	5017.3	5619.8
1748.7	2202.6	2335.8	3219.9
988.5	476.8	645.3	633.8
1019.6	1318.8	1268.5	1526.5
1121.8	597.8	739.2	739.7
885	1197.8	1629.1	1624.7
1036.8	1296.3	2002.9	1890.1
180.4	181.6	261	216.8
1523	1190.5	1755.6	1682
1553.5	2440.4	3624.6	3880.4
744.2	1027	1477	1390.3
1014.7	1027.7	1973.5	1613.9
1236.6	630	976.6	476.9
829.2	1144.1	1550	1220.7
1088.4	1032.2	1937	1614.6
899.5	932.7	1519.2	1237.9
790.9	793.6	969.8	1033.9
1012.9	1371.2	1923.4	2017.7
3377.1	830.6	1446.1	864.3
1465.3	1300.4	2015.5	1717.5
2387	3004.2	3929.8	5880.9
1329	1292	2150.6	2266.5
1231.6	1608.7	2261.1	2526.7
839	790.7	1231.4	1044.6
330.2	307.4	463.5	373.4
908.3	966.4	1547.8	1060.1
1160.9	1298	1821.2	1560.4
1094	2160.3	2893.3	3409.6
973.9	1254.7	1899.6	2047.9
1091.8	1161.2	2169.9	1920.1

967.5	1009.7	1170.6	1435.9
645.2	1603.5	1592.2	1572
1062.7	1323.2	1835.5	1782.3
1312.9	1303.2	2614.1	2850.7
945.3	812.7	1398.7	974.4
693.1	985	1245.4	1279.3
783.7	1167.1	1767.2	1532
510.5	546.5	639	705.7
2228.3	3711.9	4359	4289.3
1064	1507.8	1990.9	2111.6
178.3	318.8	340	269.7
605.2	793.8	1278.5	1253.2
780.2	960	1457	1643.2
998.9	1126.5	1414.8	1764
421.8	548.9	473.7	569.7
941.3	1002.9	1148.1	1027.5
699.1	1027.2	1655.3	1446.4
1524	1818.7	2133.5	2139.6
1032.5	935.3	1333.7	1693.2
637.1	915.6	1465.5	1236.1
1029.1	1538.4	2286.5	2487.1
1026.4	920	2265.6	1283.1
763.9	1021	1648.9	1257.3
1034.4	931.9	2819.5	2759.9
1114	1591.7	2078.5	2374.7
368.7	486.4	655.6	572.5
811.7	1154.7	1379.1	1545.1
964	1234.3	1801.7	1436.7
892.8	999.5	1306.4	1371.6
83.1	121.8	122	148.4
670.1	970.3	1334.2	1430.1
1232.9	1059.4	1541.5	1456.3
778.8	1082.3	1219.5	1646.3

946.7	996.8	1392.9	1268.6
986	1293.5	1657.3	1514.4
469.1	605.4	1340	672.8
1235.1	1706.9	2459.5	2646.4
563.6	727.8	1027.2	1156.3
383	570.9	802.5	728.4
507.4	593.5	983.2	940.2
1068.5	1318.3	2030	1678.8
749.1	897.7	988.6	1631.5
982.4	1214.9	1736.6	1924.2
907.1	956.2	1134.5	1199.3
1349.4	1888.6	2789.1	2574.6
1228.7	1577.4	2223.7	2132.7
1319.4	1572.6	2333.1	2155
7332.5	847	1000.7	985.2
819	985.2	1988.4	1373.9
1353.6	2037.3	2186.8	2537.7
984.4	1117.4	1666.2	1293.4
1215.6	1393	2023.1	2010.3
1807.1	1686.3	1419.2	1872.3
1529.5	2036.1	2582.9	1983.3
1025.9	1236.2	1610.1	1836.4
1141.8	1269.6	1473.5	1307.5
780.1	1052.5	1756.7	1500.6
1146.2	1426.9	2060.8	1808.2
909.3	1590.5	3172.2	2228.9
1686.5	1983.4	2744.1	2495.1
3158.7	4612.4	6521	5780.6
978.6	814.7	1285.4	1467.1
1056.2	1201.8	1939.1	1650.1
1098.6	1211.8	1171.4	2079.9
660.9	908.3	1433.5	1197.5
1924.6	1647.4	2880.1	2148.9

1044.4	712.3	1113.5	875.9
2555.1	1049.3	1774.4	1960.1
1265.8	1229.5	1197.7	1055.8
692	904.8	1293.9	1202.8
320.9	334.1	446.5	486.4
1218.1	1550.2	2268.9	2785.9
530.8	1860.4	1174	1219
830.3	689.2	1415.3	738.9
418.6	534.5	854.9	827.8
92.4	88.5	176.9	142.1
736.8	948.5	1445.6	1373.3
336.3	422	583	655.4
796.3	1081.1	1753.4	2308.3
681.2	925.3	1268.8	1049.3
788.5	853.1	1111.4	1096.8
1014.9	1314.2	1530.3	2275.5
775.6	994.5	1479.5	1193
568.5	720.3	861.6	926.4
743.5	475.5	949.8	871
1262.8	891.9	1207.2	1230.4
1207.8	1452.2	2193.8	1661.1
977.2	1664.3	1781.6	2295.1
433.5	615.1	851.1	754.1
941	1418.6	1485.9	2096.1
1187.2	985.1	1857.1	1454.2
678.4	645.3	903.7	870.8
558.3	662	1191.8	2310.1
1409.8	1245.2	1449.3	1348.2
594.8	687.6	967.7	1053.3
818.2	1030.5	1301.6	1384.6
910.6	1322.7	1927.9	2013
823.9	908.4	1502.5	1658.8
765.1	799.4	1168.3	1724

473.8	365.8	713.5	510
829.7	905.1	1615.8	1524.7
1269.9	1462.3	2436.2	1999.8
418.7	239.9	252.6	370.4
1499.2	2332.1	3033.1	2945.8
745.9	914.9	1520.4	1302.8
916.5	1318.3	2065.1	1338.8
657.2	752.2	1060.4	1195
608.1	690.9	863.5	880.1
776	1245.8	1313.2	2272.8
731.5	1003.1	1621.1	1571.5
447.3	756.1	806.8	1629.6
884.3	1097.4	1589.4	1601.4
1785.4	2738.8	4272.2	3651.3
870.4	1053.2	1557.4	1458
3276.9	838.2	1206.8	1101.7
1395.6	2207.3	3204.9	3140.6
1400.5	1677.8	2625.6	2464.6
816	779.8	1455.1	1540
974.9	1502.6	1995.7	1438.4
985.6	1245.4	2191.7	1778.3
932.1	1386	3419.3	2481.7
1043.6	1126.3	1396.5	1341.1
1429.5	1404.4	1797	1427.9
817.7	888.3	1828	1195.2
768.3	755.9	1694.6	2295.2
2366.9	2307.1	3144.2	3020
2641.3	1179.3	1582.6	1282.5
1010.7	1656.8	2265.7	2185.8
902.9	1023.1	1280.7	1480.4
550.9	715.3	716	638.6
672.6	877.8	1295	1274.5
648.8	795.4	1039.8	1023

281.1	319.6	484.8	540.3
1141.5	1396.2	1866.1	1664.4
1348.9	1454.8	2332.2	3106.5
1695	2523.1	1571.5	1416.4
680.5	867.7	974.3	666.4
850.5	1102.9	1322.3	1586.7
577.6	891.4	1172.8	1148.7
1026.9	1407.8	2092.6	2237.5
274.3	394.4	647	460.1
1117.8	1707.5	2215.2	2319.1
1107.8	1382.8	2120.3	2042.6
688.3	872.5	836.1	870.8
479.5	711.9	711.6	746.7
1500.7	1498.5	1742.4	1722.7
1025.1	1448.9	2188.7	2033.6
826.4	1725.6	1738.2	1458.6
999.1	843.1	1244.6	864.8
282.1	324.4	375.1	370.5
162.5	145.4	256.2	238.9
272.3	21031.2	2403.4	4566.8
699	904.6	937.2	2612.9
880.4	1040.9	1299.6	1127.9
117.2	186.5	186.1	784
1029.4	881.3	1927.9	1524.2
1237.8	1212.7	1885.2	1450
1354.9	1646.9	2257	2109.6
603.4	1220.3	1649.5	1551.1
775.9	1041.8	1378.1	1231.4
997.5	897.5	1412.3	944.3
1956.6	962.9	980.5	3250.9
1854.8	1467.3	1888.9	1692.6
646.6	2306.8	1086.6	1085.9
445.1	573.9	746.5	912

2187.7	2842.9	4000.4	3528.6
788.3	1341.2	2216.3	1948.2
1526.5	1487.3	1901.2	2313
1297.5	1606.1	1966.1	2492.5
520.4	466.5	742.1	1132.2
1112.2	1196.1	2918.9	1579.6
856.3	1099	1157.3	1204
629.1	706.3	1050.8	985
933	1182.2	1569.4	1623.2
240.2	580	371.7	372.9
698	659	854.8	581.1
88.1	105.3	149.7	145.1
833.4	587.2	1300.2	925.9
763.9	940.9	1414.8	1887.6
1074.6	1349.1	1772.3	1832.9
773.4	800.7	1402.7	972.5
1860.9	2756.9	3606.2	3484.2
1209.9	2134.7	3858	2893.3
452.1	977.4	1288.5	1983.9
1935.8	2282.8	3290.2	2828
1455.3	1444.4	2129.5	1779.1
404.7	814.5	526.6	502.8
1766.2	2342.1	3434.7	2790.4
661.2	471.5	754.2	819.5
423.6	611.1	766.5	725.8
1199.7	1557.9	2160.6	1908.9
740.4	697.4	1607	1260.5
580	723.5	834.3	1049.6
769.5	1032.9	1615.4	1312.4
736.9	1006.4	1260.8	1179.9
452.2	567.5	737.5	598.5
2144.3	1095.7	848.8	2661.8
745.6	636.9	699.8	665.7

505.1	645.6	904.6	746.8
414.5	483.5	808	743.4
1358.6	2105.7	2226.8	2059.5
1029.2	989.4	1922.5	1932.7
3389.1	1798	1722.7	3423.7
1913.5	886.3	1247.1	860.4
1039	1087.1	1457.5	1496.1
615.5	849.6	1156	1316.6
310.2	563.4	537.3	1047.9
1828.5	1602.5	2533.4	2471.7
1084.8	1285.4	1814.5	1641.8
907.4	1199.7	1585	1815
1098.3	932.6	2106.6	1784.9
1041.7	1226.1	1923.7	2071.4
617.9	818.9	937.9	1033.4
1466.5	1719.1	1169.1	2427.9
767.9	773.8	1441.3	1042.6
793.9	1004.7	1747.2	1562.3
800.3	1543.8	1682.5	1590.3
543.9	764.1	1049.7	1212.6
495.6	484.5	563.2	591.2
810.4	1270.8	1676.9	1342.4
1224.6	819.6	1224.6	1090.5
758.7	808.3	912.7	983.6
766.5	1184.8	1445.6	1792.3
1053.9	1587.1	2422.2	2466.1
835.9	1190.7	1792.1	1650.3
729.8	1037.9	831.2	2431.6
1071.9	1221.2	1594.9	2385.3
1192.6	1477.7	2554.5	2073.2
1110.4	1056.9	1645.5	1943.2
700.9	671.3	1313	1227
1106.3	1327.4	2032.5	2294.9

1314.3	1724.7	1949.1	1990.7
1139.7	1396.9	1334.7	1380.7
942.7	1423	1948.6	1939.5
1106.3	2138	2580.7	1988.8
813	1181.7	1518.8	1351.7
126.3	1156.5	291.8	1485
684.5	672.4	1078.3	1002.6
1468.8	1211.1	1143.4	738.9
654.8	703.5	1001.2	958.8
891.2	1147.8	1022.7	1498.7
2158.9	2003.4	2958	2628.5
474.6	1173	949.1	855.1
1481.3	1868.8	2664.5	2318.9
790.8	1029.9	1203.2	1125.7
397.3	582.5	863.2	866.4
624.5	895.4	1325.1	620.9
426.1	533	757.1	674.1
812.9	780.7	1755.8	1807.6
642.4	826.2	1111.8	1239.4
1156.2	1447.6	1817.3	1794.3
1007.4	1388.6	1764.5	1381.1
1108.8	1270.5	1655.4	1497.2
594.8	736.2	1004.1	824
1111.6	1368.9	1787.3	1862.9
579.4	683.1	850.2	887.9
766.2	928.1	1432.9	1346
496.5	308.1	398.1	398.6
362.2	491.8	728.5	725.7
714.3	707.3	1593.3	1451.2
1175.7	1679	2847.5	3010.8
1910.4	2110.9	2850.4	3674.2
733.5	1015.3	1331	1193
287.4	341	627.7	607.3

657.9	784.2	930.2	933.6
885.7	1064.4	1476.3	1276.1
1607.3	2606.8	2214.9	2113.5
904	1229.3	1814.7	1684.6
395.9	567.9	547.2	688.7
619.3	945.6	1238.9	1197.9
834	1112	1733.2	1126.4
886.3	1165.8	1646.8	1547.1
294.1	340.6	363.8	757.6
727.7	1158.7	1880.1	1609.6
824.8	945.5	1353.6	1489.5
922.9	1203.3	1849.6	1441.9
619.2	766.2	1091.5	1104.2
1879.2	1839.3	2443.9	2193.1
302.7	565.2	657.1	1172.9
1720	2006.9	3370.5	2350.4
708.9	786.4	932.8	1110.4
635	963.1	816.7	802.7
1539.3	1901.1	2797.5	3082.1
1088.1	1657.4	1956	1615.4
615.6	921	1581.6	1454.5
1266.3	1582.6	2278.2	2554.5
1278.8	1954.5	2237.3	2961.6
248.6	213.5	385.4	368.3
974.6	1361.3	1898.8	2129.2
1041.6	1479.3	1398.6	1849.5
901.1	1141.5	1660.4	1451.6
1578.9	1990.6	2630.9	1957.9
572.9	1015.1	1194.7	955.1
552.9	842.4	1361.2	1291.6
708.5	1193.6	1914.3	1225
1010.9	1141.9	1608.8	1452.8
4259.4	701.3	823	812.8

908	1024	1843.7	1270.9
933.5	1526.7	1536.1	2138.2
387.1	449.6	587.9	527.7
886	1113.5	1331.8	2373.6
1207.8	1115.5	1802.8	1336.6
1404.4	1647.3	2533.8	2187.5
909.2	1071.5	1182.3	1646.9
319.9	14984.2	592.4	225.9
573.1	709.1	996.5	827.2
101.2	99.3	126.8	198.1
737.6	956.4	1308.1	1775.9
939.3	1044.1	2019.3	1249.6
858.7	1131	1255.7	1354.8
1029.4	1329.8	2301.8	1965.4
496.4	793.4	1186.4	1729.3
759.3	1168.7	1967.4	2058.3
1878.3	1568.6	1705.9	1747.4
719.6	847.8	1369.3	1368.3
1280.1	1532.1	2305.9	1621.9
525.5	602.4	906.5	917
532	584.1	529.3	780.8
716.7	843.9	1205.7	1080.5
864.5	1106.5	1764	1598.9
1444.8	1605.7	2328.8	2030.1
1156.1	1411.2	1986	1851.9
862.8	1153.6	1468.9	1789.5
515.7	702.8	923.5	758.5
430.9	439.2	894.4	596.7
1646.7	1646	1338.2	1429
639.8	626.3	1232	1179.1
799.8	921.2	1186.9	1088.4
771.8	1195.9	1160.6	1599.8
611.1	521.8	1409.9	746

171.5	201.9	413.6	228.1
805.6	940.7	2415.1	1394.6
693.7	1048.8	1314.8	1383.3
999.5	1133.3	1611.8	1816.7
787.5	499.3	552.9	556.2
402.5	679.9	753.6	789.2
686.9	873.3	1109.8	1174.7
1084	1360	1609	1467.4
1491.4	1479.5	2113	2018.5
304.3	421.4	519.6	611.9
885	1130.5	1294.2	1608.8
102.3	60	108.7	107.2
539.2	689.2	898.4	1015.6
328.2	509.3	534.2	597.5
705.5	872	1390.4	1220.2
665.7	1115.9	1407.5	1537.3
1047.8	1202.7	2096.2	1803
913.2	915.1	633	593.7
1077	1115.1	1575.5	1800.3
833.4	527	818.8	698.1
1019.8	1182.4	2062.1	1277.1
611.6	584.7	726.2	512.9
375.1	516.9	634.1	740
438.8	481.8	545.2	589.9
919.9	1063.6	1220.4	1369.2
4753.2	1760.6	577.2	259.9
264	351.5	444.9	459.9
1309.5	1683.2	2198.2	2016.6
496.6	943.2	602.2	724.2
633.6	696.8	851.6	1397
1175.5	1324.9	1752.7	1955.8
707	851.4	1351.7	1183.1
1146	1622.5	2152.3	2023

492.8	778.7	1002.6	967.8
1014.2	1608.9	3500.6	2508.9
800.1	1000.2	1181	1813.9
1151.3	1152.8	1594.4	1748.4
661.3	726	1021.3	1012.7
784.7	1096	1619.2	1530
931.5	1131.1	1255.2	1656.2
1138.5	1402.6	2187.7	2060.6
614.1	558.8	885.7	880.8
1178.4	1252.2	1227.3	600.9
1169.7	1576	2175	2143.8
1087.5	986	1631.2	1516.4
951.4	1337.4	1662.2	1756.6
1439.5	1236.8	865.9	2072.6
1991	1614.6	2625.8	2385.9
645.5	917.9	1122.5	1208.8
1175.6	1718.6	2624.3	2125.8
190.9	222.8	401.9	292.4
743.2	1208.4	1295.3	1262.2
537.4	583.1	788.5	666.9
436.6	491.5	559.7	744.4
1160.2	1443.1	1905.5	1733.4
485.1	593.6	696.7	737.2
930	1274.3	2475.8	1832.9
806.5	849.4	1143.2	1312.8
760.7	825.4	1270.3	2836.7
459.5	406.1	393.7	353.5
824.1	589.5	1011	1225
417	403.3	668.9	513.7
872	1001.7	1435.1	1260.1
909.1	902.4	1292.9	1470.5
703.2	730.4	1241.6	1039.7
1124	918.1	1397.3	1514.3

694.7	685.2	1137.7	847.6
955.5	1027.6	1935.8	1331.4
351	824.6	1315.4	1352.8
600.7	545	673.1	682.3
667.6	1094.8	1523.9	1506.3
708.6	822	956.3	1060
1309.8	1633.1	1825.8	2234.3
348.4	780.8	958.4	1090
1218	1421.8	1412.9	1816.2
785.6	1145.6	1668.6	1777.3
219	262.9	287.3	304.5
725.9	754.4	1422.4	867.6
975.6	499.5	560.3	410.5
937.7	1302.2	1798	1908.2
393.3	183.4	175.4	98
844.2	1382.4	1403.6	1957.9
723.9	914.8	1310.1	1278.9
554.6	555.6	766.6	605.8
933.6	1137.2	1601.3	1525.4
320.5	392.2	292	814.9
911.9	657.5	632.5	648.3
848.8	900.7	1162.5	1233.4
570.1	670.3	882.5	878.6
677.3	505	802.5	703.6
657.5	852	1373.1	876.8
912.7	738.6	1092.3	2335.7
1657.8	1242.2	1886.2	1665
1006.8	1415.1	1792.1	1875.2
586.5	488.7	476.8	462.1
664.3	819	1208.3	1055.6
643.8	859.5	1039.3	1184
467	490.6	846.7	641
863.9	1289.9	1242.5	1856.3

599.6	747.9	960.1	915.4
439.7	669.5	929.5	1078.2
825.3	1039.3	1496.2	1231.4
434.1	486	712.4	703.7
486.3	640.7	645.8	831.4
732.4	1116.3	1611.6	1266.3
471.8	767.5	1002.7	940.8
1613.7	654.1	796.5	972.1
454.1	513.9	603.9	749.8
1231.8	2133.7	1558.9	3795.1
1006.1	1225.5	1885.7	1670.3
621.8	634.3	1262.3	1121
320.6	598.8	949.8	924.3
1227.4	1263.1	1467.9	2817.7
675.2	1060.9	1356.7	1169.2
839.2	505.5	1142.5	1010.6
926.5	1152.6	1167.4	1410.4
1818.1	3126.7	2889.6	2508.2
701	1175.4	1462.1	1167.6
397.9	465.6	463.9	495.7
565.5	357.9	1207.1	432.9
722.2	705.9	1003.7	1106.4
1187.3	1570.1	2390.5	2067.5
846.1	710.4	1023.9	931.2
563.2	782.2	1942.3	313.3
1104.2	740.7	1027.4	426.4
859.7	1066	1269.1	1191.7
870.9	1021.1	1425.7	1532.5
963.4	1246.6	1539.9	1917.5
720.5	841.3	1360.1	1366.9
673.9	965.6	1021.5	1022.7
529	596.1	885.4	696.2
1093.7	1480.2	1792.1	1785.3

1064.9	1587.3	2304.2	2101.3
487.8	1236.4	758	606.9
1027.9	1172.9	1721.6	1495.9
669.6	700.6	947.3	869.8
773.7	944	1453.5	1134.8
1286.2	1496.2	1798.3	2103.2
400	466.6	560.3	578.7
784.1	906.7	1467.4	1232.9
881.4	1032.6	1867.1	1872
456.7	577.3	737.3	687.9
854.5	1473.4	1076.7	1363.4
1115.7	1296.6	1605.5	1406.8
586.3	604.3	743.3	760.3
474.1	641.2	1067.4	909.9
1819.9	2830	4043.1	3381.1
676.5	419.3	1415.8	1248.4
765.6	1136.9	1193.8	1047.2
627.3	522.8	788.2	696.6
900.8	1220.3	1936.4	2212.1
443.5	410.2	631.7	590.2
992.5	1208	1791.4	1405.1
752	809.7	1238.2	1064.1
654.8	843.3	1347.9	1226
318.7	582.5	267.6	212.4
1015.4	1261.3	1861.9	1502.4
450	1103.5	759.4	690.1
1149.3	1148.3	4285.3	1905.3
1546.5	1800.2	1739.7	1780.9
821.1	1234.9	1244.9	1462.3
1125.1	1079.3	1884.2	1583.2
537.8	784.5	972	1052.9
553.8	711.9	669.7	734.7

799	1118.9	1712.1	1240
342.5	620.7	647.3	600.4
672.7	562.8	548.1	809.1
1471.8	702.3	662	960
1185.1	1231.9	1642.6	1648
872.8	1400.5	1779.2	1723
472.4	574.6	802.2	861.8
547.1	412.8	469	435.5
663.4	751	846.8	891.6
1306.5	1814.9	2200.7	1659.5
1747.3	2081.1	2866.8	2650.8
1053.2	1181.5	1629	1564.4
530.2	569.1	858.3	749.8
336.7	497.4	446.4	1032.6
1023	1252	1536.3	1579.2
493	572.1	752.4	1761.5
1420.3	1236.2	1240.4	2046
666.8	604.4	966.8	743.1
454.4	590.9	868.1	873
427	520.7	865.5	983.1
741.3	763.4	1064.8	1126
1081.3	1046.5	1952.9	1416.5
578.2	763.3	1125.7	1215.4
995.6	1179.4	1463.6	1484.7
469.2	586.4	629	759.4
1377.2	3293.8	5597.2	7448.6
577.1	810.4	1067.3	1311.6
610.1	749.8	1065.2	969.5
119.7	214.5	318.3	276.4
398.5	663.5	681.3	699.4
588.6	795.8	1091.5	1255.3
621.1	983	1309.5	1565.4
423	525.6	526.5	680.1

393.2	359.4	682.9	563.3
582.8	1078.1	845.2	899.4
241	377.3	364.7	508.8
395.9	479.7	665.5	725.2
872.5	1259.6	1130.1	2028.2
512.3	604.8	791.4	927.8
1523.9	6714.7	1518.9	2048.6
1101.1	934.7	1214.6	1018.8
760	982.3	1256.9	1074.2
764.7	1065.3	2066.9	2016
1718.7	1728.6	2670.6	2430.8
773.3	960.3	1658.4	1072.2
375.7	246.1	385.4	413.9
491.4	449.7	514.7	390.2
1181.9	1133.2	1536.1	1555.9
725.7	1022.2	1603.1	1288.3
417	489.7	751.5	626.6
766	612.6	897.3	675.8
521.3	849.6	932.4	933.7
652.3	754.1	1029.4	986.4
692.3	1042.8	1115.1	1165.7
388.5	450.6	619	598.4
1671.4	1672.2	2486.7	2655.2
278.5	777.9	421.7	497.4
687.5	1047.8	1085.6	1532.7
611.6	872.8	1265.2	1162.4
708.6	928.5	1267.7	1197.4
636.5	660.6	1050.6	619.4
805.5	1072.6	1556.7	1667.2
278.2	402.4	607.6	617
432.4	299.3	302.8	414.8
3681.1	3688.4	5093.5	5137.2
3677.3	918	696.5	788.9

1541.1	1667	2176	2199.3
1040.5	1804	1994	1168.5
801.4	898.2	2000.5	1473.6
1167.5	1364.1	3854.4	3008.6
671.5	1080.7	1626.6	1523.9
449.3	522.8	620.3	2537.6
818	1211.3	1754.6	1585.1
679.2	785.3	878.9	769.3
1597.9	1477.5	1013.3	1299.1
513.2	748.4	1137.5	1159
308.3	568.4	587.4	727.2
917.5	925.4	1087.2	1002.3
682	848.4	984.3	1010.8
819.9	1136.1	1228.6	1601.3
720.2	664.5	2048.6	1289.5
454.4	938.1	691.4	1762.8
300.2	666.8	534.4	2054.9
631.2	700.4	858	811.3
889.4	947.8	1160.1	1076.9
631.5	1020.1	2659.5	1682.6
1316.3	1461.8	1870.2	2487.9
608.6	674	878.9	880.7
432.8	523.3	700.3	699.8
659.1	941.7	1293.8	1138.9
552.8	518.6	746.7	836
2137.8	646.9	492.7	463.2
661.3	976.6	1284.1	1448.6
254.1	314.7	469.3	403.9
782	989.5	1317.3	1307.4
496.8	913.1	1089.4	1054.8
581.6	716.4	1275.3	1128.3
641.4	739.2	1106.8	1017.1
2004.7	883.6	594.3	792.4

332.5	418	491	504.4
911.8	1494	1493.7	1892.4
607.8	331.5	418.7	454.5
936.2	947.1	1363.3	1586.2
547.6	763.8	864.1	1051
1329.9	2189.2	2839.4	2615.4
626.4	1125.1	1356.3	1445.4
514.9	683.9	782.4	683.4
761.8	960.5	1134.1	1242.8
574.9	888.1	1318.2	1260.4
491.4	526.3	679.3	729.5
1111.5	906.5	1088.4	465.5
2731.1	2528.2	1740.8	2434.9
554.8	706.8	956.6	1083.8
1055.2	1065	1526.1	1242.6
1308.6	1498	2199.4	2250.3
750	704.7	924.6	1020.4
752.7	1108.8	1210.9	1306.6
537.6	667.5	785.9	811.4
1541.9	335.8	479.2	360.9
534.6	557.7	632.2	1069.6
525.3	448.9	645.5	1087
673.5	590.3	913.1	808
333.9	445.2	620.1	571.5
529.2	1182	958.5	4193.2
66.7	43.4	37.3	34
1576.8	1188.2	1162.8	643.7
614.7	966.1	834.3	709.2
394.2	551.2	719	1211.8
679.9	881.3	1239.7	1068.6
345.1	452.6	598.5	660.9
421.8	376.2	431.8	486.6
702.5	666.5	1668.5	976.9

248.4	273.2	603.2	548.7
499.7	524.1	781.7	756.3
749	863	1131.3	1116.6
297.7	405.9	617.4	678
1261.6	1637.2	2375.5	2051.4
464.2	389.4	425.3	515.3
543.5	709.7	974.3	916.6
845.7	1029.6	1496.1	1275.3
264.6	297.2	449	399
1832	1954.7	1827.8	1850.5
337.3	31.4	42	50.6
783.4	1110.9	2081.1	1818.9
870.1	1089.9	1153.2	1400.1
2916.3	1876	2206.3	1788.4
451.6	882.3	579.4	760.9
1877.1	1432.2	731.2	778.7
940.9	1453.1	1811.6	1750.3
311.7	331.3	383	333.6
605	935	1146.5	1478.2
438.4	558.3	764.3	724.6
686.5	983.3	1584.3	1259.9
1012.6	1299.1	1686.5	1417.7
782.6	1223.7	1749	1417.6
780.7	973.1	1158.3	1320.8
712	1240.4	1599.4	1405.6
507.9	532.4	687.9	928.2
682.4	726.8	859.5	868
613.3	687.3	826.1	1107.4
545.7	788.8	881.6	1156.1
689.8	847.9	1079.4	1443.8
667.6	692	913.9	783.8
547.1	609.9	827.7	780
302.7	301.4	702.8	583.4

653.5	782.3	886.8	1077.9
225.6	133.6	123.2	61.5
733	1154.1	1285	1233
1460.6	1142	2753.1	1663.9
362.8	345.5	325.4	380.5
476.5	766.7	958.8	904.9
769.2	705.9	913.5	905.8
636.2	554.8	884	883.9
254.9	537.6	384.8	1354.9
420.1	548.8	679.1	673.7
336.2	340.7	485.3	385.4
335.8	392.8	666.5	691.3
29.4	76.5	52	67.8
566.9	838	855.2	887.5
560.6	748.8	1189.4	1514
535.8	873.3	885.3	946.7
895.8	690.4	619.1	387.9
510.5	722	1018.1	824.4
762.4	935.8	1117	1067.4
786	1123.9	1089.1	1089.3
1410.4	2121	2285.6	3115.7
855.5	535.4	634.5	656.9
529.1	662.2	936	999.8
649.9	607.8	708.7	608
454.7	502.2	784.6	759.1
571.2	981.3	1369.9	1142.2
502.9	525.6	654.2	708.4
2305	2882.5	4094.6	3572.9
222.6	328.2	336.5	436.7
772.2	761.2	888.3	892.8
1409.2	1399.8	2007.9	2073.1
940.6	1267.4	1594.7	1628.3
2053.1	1046.1	1514.3	1223.3

769.8	642.9	692.4	584.6
831.1	552	696.3	445.6
1265	1914.5	2238.1	2836.6
680.6	803.3	1159.9	1141.4
104.6	93.8	130.9	125.1
1699.3	238.3	330.2	362.6
1171.3	1299.8	2020.8	2164.5
424.5	862.2	904.3	838
671.9	888	1330.4	1207.3
240.3	340.8	376.7	374
657.6	666.9	1047.8	1195.1
989.7	1020.9	1615.5	1396.8
384.3	490.3	760.3	790.4
415	523.1	456.8	669.9
556.1	558.3	652.6	614.1
626.3	765.1	1557.3	940.7
82.1	132.9	153.6	158.3
689	724.3	1099.5	1004.6
1494.7	1363.8	1969.3	1889.8
310.2	543.4	704.3	653.1
440.8	524.7	758.1	675.6
436	896.6	1512.6	1508.5
390.1	492.4	639.1	625.6
293.1	334.4	391.3	365.1
347.1	566.7	1016.7	1120.8
620.8	631.8	395.5	227.6
526.6	683	918.9	921.5
559.9	758.6	1127.6	1260.3
544	599	960.2	822.7
332.3	237.6	292.5	1307.2
873.3	799.2	1667.2	1496.3
549.4	628.5	934.8	961.2
77.9	205	113.2	108.8

354.3	418.7	614.1	660.3
602.7	724.1	795.8	1054.5
166.7	187.6	228.7	263.4
426	434.5	546.2	587.2
9594.8	2511.2	1216.1	858.6
42.6	50.6	91.1	91.8
2429.6	1505.2	1947.7	1491.4
755.1	552.5	734.2	823.1
316.3	395.2	493.6	466.2
387.9	385.2	451	456.5
750.2	846.3	1682.4	1571.1
31	56	59.6	61.8
640.6	796	934.2	1036.2
438.3	502.1	746.5	542.4
970.5	1245.5	1861.8	1095.2
262.9	355	383	435.7
443.9	492.7	591.4	604.2
472.5	636.4	1165.9	471.1
673.6	828.2	1416.6	1313.9
625.6	904.6	1337.5	1313
1873	1005.8	833	440.2
499	600.2	782.9	784.1
763.7	843.8	1505.9	1355.2
1616	613.5	965.4	468.5
376.7	1057.9	934.4	1041.9
630.3	790.2	1253.9	1054.9
1191.2	1053.9	1988	1847.9
677.3	966.1	1338.7	1252.4
462.1	609.7	778.1	712.5
281.8	344.3	599.7	362.3
910.5	1119.3	1453.4	1476
670.1	549.8	1732.9	745.1
210.8	388.1	478	395.1

628.6	673	749.6	748.5
605.7	773.6	1027.2	1041.6
458.1	416.5	480.7	541.7
867.7	1295.6	1551.4	1740.2
249.9	465.6	666.4	433.9
798.6	1232.3	1815.2	1743
942.7	801.4	762.2	716.4
1563.2	1374	1455.2	1122.7
811.7	794.6	1069.8	995.4
730.9	769.4	1338.3	1239.1
459.9	631.4	589.8	1258.4
719.7	858.2	1054	1279.1
407.6	782	1020	1234.5
1445.6	1551.3	1202.6	1102.6
699.9	642	1336.8	973
935.1	1168	1427	1469
818.4	1186.8	1635.3	1449.3
1829.6	1276.5	1182.9	673.2
981.8	891.6	1097.3	1209
1049.1	1461.4	1780.7	2110.1
542.5	625.6	949.4	795.2
906.3	952.4	945.5	854.5
731.4	867.2	1162.5	1048.6
488.7	581.9	653.4	659.3
381	544.6	550.5	603
764.1	1134.8	1560.4	1125.7
889.3	1018.8	779.7	1024.5
1127.2	739.9	1055.1	653.1
556.3	602.6	769.5	688.5
799.7	1074.9	1241.8	1396.3
316.8	3371.9	812.1	1015.5
509.3	627.5	898.4	906.2
490.4	582	724.4	717.7

779.9	882	1363.5	1503.9
638.1	780.7	962	926.9
535.7	1178.6	1921.1	2105.6
392.5	304.9	447.8	468.4
895.8	433.5	893.6	528.7
594.7	665.2	1096.1	812.1
683.6	796	1011.6	810.5
787.9	1017.1	1366.8	1260.6
765.1	1006	1216.8	1189.5
476.4	219.9	410.2	261.4
448.2	650.9	838.2	836.9
1049.9	1505.9	1895.2	1899.8
382.3	464.8	1200.3	600.2
234.5	290.7	610.3	692.7
2256.1	422.3	688.2	667.6
522	667	839.4	732.1
504.3	641.4	931.5	858.9
462.1	257.9	638.8	398.3
297	401.2	469.5	565
337.6	586.3	599.6	1013.3
453.7	467.6	642.3	414.5
507.7	498.5	796.1	524.6
438.2	488.6	624.3	583.2
575.1	639	656.8	688.5
586.9	767	1012.2	703.2
403.2	847.1	540	811.8
269.3	338.6	392	375
598	700.2	1082.8	880.5
404.9	443.2	606.3	465.5
358.8	397.6	522.3	652.7
1169.3	1106.9	1716.3	1468
816.8	758.1	1155	985.1
2546.9	455.1	506.4	427.4

451.4	328.2	519.3	536.1
470.8	523.4	744	660
916.3	1172	1258.2	1471.2
504.7	714.5	946	897.2
407.8	487.6	636.5	575.8
1398.1	1672.2	1749.8	2141.5
704.1	811.4	1042.3	1111
634.3	756.6	1384.2	1329.8
223.1	516.3	1022.3	2403.6
412.6	420.3	497.2	580.1
455.8	329.6	445.2	474.9
231.5	656.3	303.7	634.5
2746	384.6	645.6	557.4
517.5	627.6	1052.4	1222.3
327.9	365.1	512.5	411.6
977.8	1405.6	1668.8	1892.2
711	975.4	2305.3	2127
507.9	1215	2240.5	2894
675.5	768.2	1389.5	1258.1
1538.7	1116.7	1019.5	1283.6
853.7	1215	1370.5	1862.1
804.6	826	1189.4	1073.2
1816.3	1991.8	2727.8	2532.6
437.5	560.7	795.9	819.7
624.7	824.8	1056.2	1202.7
514.3	640.3	775.7	743.8
290	356	510.8	575.7
517	759.1	998.7	1604.1
322.9	558.3	696.4	560.6
1624.8	1511.2	1797.9	1989.8
1823.8	2107.7	2163.4	1788.1
625.4	536.8	833.2	678.4
523.9	460	599.9	620.3

610.8	695.3	688.6	850.5
282	396.9	370	464.4
625.6	592.3	782.3	779.9
429.7	416.5	576.2	474.4
514.1	228.2	1758.7	1124.1
431.6	567.6	645.8	715
494.8	719.6	876.9	942.6
601.9	583	779.9	831.6
1831.1	1792.5	3285.5	3073.7
1053.8	1582.6	2082.5	2051.9
939.3	717.4	817.6	771
355.3	381.8	757	698.3
677.4	690.2	1044.3	898.5
73.9	113.9	153	133.3
748.8	945.9	1411.6	1209.8
400.7	486.6	692.5	665.7
644.7	876.4	478.9	844.7
1449.3	2233.8	3210.8	2529.8
1240	1233.7	2106.4	1920.8
278.7	353.5	386.5	452.5
664.3	159.7	1704.9	293.1
231.2	411.6	321.3	624.2
563.9	898.4	760.9	639.9
419	619.5	1037.2	1032.7
697.2	1068.8	1324.1	1322.3
1016.6	2437.5	1219.6	976.1
624.5	716.4	1016.8	1067.5
616.2	731.2	1262.6	993.1
583.1	927.3	1200.5	2027.2
489	613.1	726.3	835.4
436	661.5	808	828.2
380.7	442.6	503.2	770.5
473.8	596	653.8	1027.5

168.7	303.2	340.2	173.3
276.5	455.5	727.6	676.6
448.4	501.1	765.1	553.6
3244.3	4522	6829.7	5836.5
426.6	509	675.5	668.7
671.8	875.5	928.3	1422
503.2	544.8	1001.1	913.7
154.3	150.5	263.1	335.6
92.1	146.6	107.4	173
675.7	744.8	932	828.6
512.5	536.2	664.3	784.7
536.7	572.4	719.2	1118.2
928.9	850.9	1254.2	1217.2
588.1	565.8	820.3	785.6
698.9	1048.2	1573	1512.8
543.8	827.5	804.4	542.9
310.9	505.7	582.9	553.8
231.7	391.1	432.5	430.6
835.5	930.8	1380.3	1320.8
1099.4	1264.5	1890.7	1930.2
1472.7	225	349.6	194.4
421	411.2	1086.6	955.6
1747.1	2231.6	2856	2755
487.7	616.4	834.7	755.7
522.2	594.2	1050.6	1167.1
684.4	335.1	390.2	471.2
428.7	589.7	701.4	709.9
453.3	566.1	761.2	739
2190.7	623.5	577.9	653.8
1077.3	1890.5	2365	2892.7
664.8	823.7	1082.7	1070.5
547	687.5	802	589.2
847.4	855.4	1099.7	906.8

4837.2	810.5	1034.6	1195
267.2	373.9	430.3	473.2
419.8	400	523.6	355.1
353.2	422.6	659	613.4
606.3	697.3	945.5	724
659.4	812.4	1242	1095.3
382	429.6	555.8	465
585.2	570.7	960.7	1038.2
372.8	588.1	736.2	723.7
1389.9	1278.4	2058.7	1803
283.2	308.3	379.9	318.4
599.5	1024.3	1317.5	1456.6
809.1	908.8	1344.2	1292.8
604	780	1028.3	930.4
616	651.5	881.6	856.3
750.7	758.6	1468.5	1333.6
369.4	535	642.4	599.1
709.4	789.2	1270.2	1105.5
1086.1	1549	1785.5	1855.4
108.4	297.1	173.1	664.8
897.8	989.3	1274.6	1239.6
408.9	450.7	515.3	620
2862.6	702.4	737.4	693.7
1017.8	1135.7	1538.8	1458.1
237.7	251.5	379.3	311.1
659.8	725.6	1113.7	1062.2
520.5	748	912.3	942.7
547.9	1181.6	1586.4	1443.2
307.2	386.1	443.8	434.9
320.7	489	660.2	663.6
1711.1	932.2	726.4	522.9
285.5	355	619.1	447.8
158.1	202.6	409.4	278.6

281.2	356.2	394.6	398.5
23.7	21	21.2	36.5
583.3	656.3	804.2	1231.8
462.8	295.6	755.8	534.3
664.5	660.3	965.4	898.7
1562.8	1631.1	2162.5	2293.8
750	748.4	1218.4	1148.8
487	548.7	677.8	852.2
154.9	144.4	184.8	150.4
491.8	577.7	802.2	1021.6
478.2	355.5	389.9	400.2
753.2	887.4	1305.7	1248.5
641.4	626.8	869	768.2
376.5	501.6	682.6	562.3
568.4	580.3	752.3	700
561.4	630.3	597	1314.3
863.4	907.7	1266.8	1088.1
527.8	543.7	545.5	578.9
255	300.5	446.6	361
200.2	219.6	308.5	272.8
459.2	544.7	765.9	989.4
271	281	364	394.3
347.7	456.7	590.6	497.5
544.6	658.1	947.5	881.9
152.9	186.5	206.3	188.3
729.4	594.9	1751.4	892.7
573.6	452.8	549.6	379.9
553.2	752.2	1008.8	959.3
250.4	352.3	416	353.1
776	777.4	1308.5	804.5
250.4	326.2	344.1	429.9
746.8	713.1	1203.3	1002.2
220.5	293.6	404.7	435.5

435.4	586	781.7	850.9
949.4	968.6	1729.6	1118.4
285	428.1	496.7	581.2
483.4	738.4	905.4	878
367.2	428.5	569.7	553.3
770	735.3	669.5	918.4
857.2	827.9	1060	879.7
1230.7	1582.5	2191.9	2622.2
316.7	445.6	506.3	450
703	789.1	1046.7	848.1
643.2	1030.4	1100.7	1211.1
447.4	531.7	710.8	687.1
187.2	237.3	379.8	336.3
1134.5	1206.9	1383.2	1457
619.8	787	1299.9	1110.8
365.3	499.4	608.8	638.1
1009.7	1054.4	1519.3	1543.1
375.6	543.5	720.4	669
416.4	525	524.9	579
872.4	411.3	346.6	139.8
578	756.1	952.2	894.6
587.3	556.8	724.6	764.9
709.7	711.4	977.2	1107.3
598	686.5	880.7	1051.2
334.5	387.7	499.6	505.4
245.3	216.1	419.3	320.5
834.2	947.4	1339.5	1080.7
525.9	366.8	420.4	377.6
619.4	767.4	1068.5	944.3
365	429.5	566	546.4
444.2	696.4	834.4	1007
184.1	395.2	258.2	335.3
1361.6	1252.3	1253.4	876.2

405.4	537.6	708.4	583.2
462.6	478.9	639.2	773.9
699.5	732.5	960.7	884.4
204.3	240.2	272.3	268.3
650.6	1228.9	1741.8	1590
324.1	484.1	505.6	896.2
254	387.1	367.7	761
616.3	784.1	1129.9	1006.5
204.2	185.1	327.3	282.1
483.5	680	859	925.7
808.2	429.9	377.7	304.4
1098.9	754.9	1072.9	1098.3
832.7	404.9	529.4	548.6
245.9	370.8	382.2	657.5
501.9	636.6	894.2	996.2
428.1	590.8	646.5	695.4
415.6	604.3	823.2	879.2
253.6	498.1	849	426.5
1949.7	2021.9	2840.1	2619.2
469	573.8	746.2	673.5
409.1	483.2	1253.6	1082.3
425.4	449.3	809.7	749.8
621	1315	1742.7	2603.7
1035.5	830.9	1584.6	1060.9
597.4	641.9	1502.7	2386.9
845.2	850.7	1376.8	1218.8
417.9	558.5	744.9	668.5
628	706.4	883.5	984.6
229.8	351.3	435.7	485.6
351	612	781.4	788
898.8	756.6	1019.8	629.4
483.4	556.8	619.9	672.3
603.7	922.7	1060.1	1101

886.3	1105.5	1161.6	1352.5
459.9	379.2	539	564.5
460.8	488.9	697.8	651.9
311.6	350.6	606.9	528
710.6	853.3	1174.7	1405.2
380.2	585.8	771.3	700.2
14241.9		133.4	201.3
409.6	416.6	700.4	817.5
264.9	342.4	445.5	463.3
212.6	253.9	288.8	242.1
1280.7	765.8	941.5	815.3
540.1	617.1	787.6	706.8
357.7	658	1120.6	1173.4
364.8	396.5	683.7	671.2
483.5	671.7	834.4	1129
746.4	717.6	1038.7	866.7
291.5	149.7	213	236.4
611.6	592.3	747.1	429.5
498.8	640.4	1131.9	1122.5
784.8	1263.1	2152.4	2234.3
292.1	372	502.6	476.2
480.1	639.6	862.7	862.9
2198.4	1491	1092.3	662.2
204.2	250.8	307.3	304.8
179.7	232.1	520.4	447.6
264.3	423.8	484.2	459.7
2776.2	17.9	102.1	133.7
300.8	280.9	358.7	389
691.5	928.5	1208.5	981.9
589.8	674.7	1013.9	1087.3
679.7	668.2	1164.1	920.8
563.1	756.8	1183.3	852.6
555.7	654.4	778.7	738

270.2	356.5	385.3	428.6
334.8	482	623.3	686.1
429.6	746.9	934.4	455
417.1	668.3	752.1	600.2
672.6	779.7	603	808.7
355.5	431.3	559.3	537.1
318.1	269.2	492.1	363.3
467	608.9	630.2	633.2
307.7	521.2	549.3	563.1
424.1	559.5	695.7	718.6
392.6	585.5	853	832.5
572.3	741.4	1449.2	1428.5
166.4	219.6	289.4	297.6
763.3	637.6	972.1	652.1
590.1	601.6	762.7	745.8
250.5	548.8	812.8	942.2
692.4	584.7	1337.8	1081.7
430.2	697.6	955.6	873.7
508.3	719.5	651.9	1146.1
651.2	1083.5	1263.4	1295.8
305.3	335.7	466.1	419.6
366.7	438.3	551	560
949.9	1191.5	1078.2	1635
405.6	489.2	691.1	561.5
383.1	433.1	704.5	516.3
425.1	475.8	557.7	633.9
513.2	716.6	1021.2	1512.8
4390.1	468.2	541.1	749.8
574.8	963.8	1209.5	1210.7
308.6	358.5	400.5	437.1
305.7	292.5	605.9	470.6
511.8	551	744.1	663.1
440.9	647.5	824.6	699.1

265.5	391.3	421.2	475.2
760.6	901	1530.2	1768.2
502.2	990.6	849.8	744.7
812	1076.9	1200	1261
408.6	466.3	623	566.8
235.9	337.1	452.3	440.2
613.8	782.3	1246.2	1016.6
329.9	405.3	504.5	489
647.5	720.6	1056.1	1067.5
118.9	135.3	225.8	366
664.6	814.7	1090.8	1757.9
364	448.3	859.4	795.7
993.5	1035.8	1890.3	1358.1
301.4	339.5	469.4	388.2
367.8	604.5	621.4	898.4
470.9	33.7	106.3	76.4
534.3	816.9	1232.6	1113.7
500.1	602.3	827.9	752.4
513	493.6	743.3	684.8
439	530.5	801.9	696.4
683.4	806.9	1174.3	1226.7
823.3	1168.4	1283.7	1293.9
528.7	806.8	1225.8	1120.8
443.6	1676.2	394.5	1870.1
368.2	465	629.3	564.2
350.2	661.2	681.9	652.1
268.9	233.3	472.3	381.7
999.9	1229.9	828	1076.7
1848.3	1173.5	1694	1733.5
293.5	744	261.9	1482.5
407.9	558.6	608.4	808.8
944.9	826.2	619.2	724.7
1104	1437.9	1984.4	3080.7

232.1	421	438.4	391
186.8	358.5	386.2	331.7
167.2	239.7	286.7	251.4
352.1	440.7	458.1	387.2
452.4	597.6	878.8	711.6
1434.7	1616	2037.7	2385.9
316.8	346	457.6	464.6
456	828.7	737.6	1289.4
1089.8	995.8	1091.2	1051.4
779.6	816.6	1009.3	953.6
252.5	295.6	382.7	650.3
1046.3	1305	847.5	998.7
304	687.6	885.9	534.8
424	624.6	793.5	803.8
217.5	235.3	288.3	233.8
165.2	260.2	390.8	331.6
534.1	626.7	793.4	738.4
166.7	211	297.5	318.2
1146	1296.3	1683.2	1850.2
227	223.8	326.2	341.2
953.5	789.2	957	612.6
364.9	531	725.6	616.4
788.5	649.6	1265.8	676.1
27.6	48.8	66.4	60.1
472.1	526.5	899	858.7
443.2	561.9	793.8	712.8
298	392.6	519	506.7
475.8	403	111.3	165.8
247.8	352.2	418.3	412.1
147.4	202.8	232	198.3
406.1	802.4	768.5	775.1
271.6	306.8	506.9	378.9
648.6	734.6	1020	846.7

673.1	750.4	1043.3	1134.4
301.3	388.8	610.8	497.7
67.7	55.4	81.3	57.9
772.9	501.5	855.5	505.1
395.3	459.6	694.3	854.6
309.9	296.1	527.3	480.2
139.2	158.2	183.5	178.3
231.1	441	1192.2	710.8
850.4	408.5	740.3	491.9
510.1	615.6	524.9	573.6
859.3	875	1358.2	1157.1
499.6	559.9	752	513.3
984.5	1028.3	1792.8	2254.3
881.9	480	563.2	503.4
495.6	613.6	574.1	714.3
546.7	619.4	806.6	1194.9
146.7	175.5	246.3	321.2
380.1	383.8	573.1	508.2
402.7	620	991	984.9
837	739	1404.8	1169.1
294.3	247.7	360.7	308.1
279.4	1127.6	491.4	376.7
584.6	775	1320.2	1173.1
319.7	272.9	436.1	417.5
281.8	449.5	480.7	453.4
484.2	383.3	503.3	468.9
924	1329	1585	1713.5
157	146.2	217.3	248.8
770.4	661.9	665.8	410.5
425.2	478.3	577	566.7
401.9	473.6	536.1	688.1
352.9	358.1	490.9	443.4
575	760.6	1088.3	971.7

234.3	282.1	273.1	346.7
884.9	845	1227.3	1388.9
577.2	561.1	777	820
278.6	154.7	228.3	283.3
610.6	830.6	1041.6	1002.7
620.1	680.7	984.7	1050.1
283.9	370.2	465.4	559.8
1038.2	1087.3	1495.4	1901.6
540.6	628.1	1035.2	935
321	380.2	453.1	511.7
342.6	488	604.8	546.9
660.6	642.9	768.8	566.6
397.8	515.3	588.5	674.3
382.1	485.8	663.4	677.6
319.1	365.3	564.7	484.7
423.7	611.7	773.6	854.1
142.3	171.9	175.9	158.1
309.7	393.2	580.3	523.6
464	653.6	740.8	785.4
371.1	434.2	498.2	566.6
490.3	253	537.4	445.8
375.5	435.2	496.6	508
627.6	645.7	818.9	832.3
425.1	166.1	233.9	209.6
960	902.3	1501.7	1140.6
540.9	601.7	1021	1702
502.1	361.3	193.7	155.5
266.9	294.7	383.1	340.4
665.9	712.8	1061.9	911.3
352.4	574.1	606.4	640
581.9	521.1	515.2	622.5
782.5	990.5	1267.2	1173.7
300.8	412.6	602.6	538.5

575	733.6	1051.2	1016.6
293.5	433.7	745.2	646.6
378.4	515.4	718.9	722.7
544.5	700.2	1182.4	840.5
297.1	401.5	576.2	558.1
356.1	417.1	637.6	609
292.3	322.4	464	432
855.6	893.2	1256.8	1021.7
370.1	415.9	675.3	575.4
478.5	633.9	774.1	823.1
320.2	1091.4	1003.6	1279.5
549.3	419.9	535.2	673.2
780.8	1139.6	1742	1119.2
359.9	524.8	606.7	620.8
745.4	910.5	1125.7	1078.9
778.9	617.2	808.1	949.1
593.6	512.8	546.7	910.3
138.2	164.2	494.8	195.9
243.9	129.5	292.6	211.2
691.9	803.3	902	1152.2
588.1	582.4	944.1	970.3
49.2	30.9	50.3	35.2
557	121	140.4	201.5
1056.1	1055.5	1558.6	1372.8
192.5	231.5	289.4	278.2
642.3	301.7	305.9	291.8
689.9	920.3	1116.3	1311.4
3503.5	1651.3	2190.8	2075.5
85	245.9	81.4	147.5
961.6	1345.6	1944.6	1639.6
620.5	814.1	1042.4	957.2
318.5	396	545.1	548.7
434.6	733.8	943.3	981.8

419.4	467.9	554.3	643.1
125.6	187	279	267.1
680.1	817.3	1020.2	738.9
534.3	716.7	1075.5	908.5
596.5	744.4	802.8	921
553.4	863.5	997.2	1258.8
309.7	462.9	661.9	627.3
227.4	288	412.2	371.2
279.4	294.7	194	420.6
257.4	303	401.1	363.3
212.5	263.2	357.4	360.4
198.4	266.8	444.9	490.8
680	754.2	1212.1	944.8
422.9	546.3	783	704.3
385.7	423.5	492.5	487.1
288.9	338.2	438	399
478.8	534	521.7	549.7
367.2	247.5	1166.2	726.7
384.8	579.4	680.5	690.5
194.5	454.3	293.4	389.5
260	391.4	451.4	736.3
314.8	454.3	722.9	881.7
1061.3	1059.8	1534.6	1483.5
548.6	412.7	570.2	755.8
529.5	350.9	391.6	346.8
788.1	1010.5	1708.7	1461.9
228.8	308.1	441.1	476.8
509.7	881.4	1377.2	1451.4
368.2	550	727.8	716.6
684.5	703.9	857.7	923.2
468.5	764.2	1112	806.5
213.7	803.3	259.5	381.1

23.5	22.6	31.6	36.6
135.3	147.7	267.6	185.9
165.5	197.8	307.6	698.2
530.5	504.5	819	900.2
1202.3	861.9	1299.5	1132.6
402.1	587.6	737.1	779.9
577.7	765.3	1044.9	1172.5
688.1	741.7	550.8	578.4
290.1	335.5	369.9	453.2
354.2	449.8	687.6	658
380.9	534.3	606.7	670.9
191.7	151.5	213.7	193.3
173.1	314.3	297	451.5
538.3	619.9	1040.2	989.3
438.4	657.4	604.8	726
434.3	626.7	867.8	722.3
315.7	404	419.3	501.7
323.9	347.4	510	434.4
566	444.2	715.8	406.5
361.4	408.9	521.1	540.5
256.9	313.7	536.4	532.7
179.5	242.4	234.9	280.5
280.6	393	426.7	562.3
247.6	427.7	264.1	395.6
707.4	520.5	718.3	906.2
397.5	390.6	599	702.3
233.5	260.4	339.4	307.4
432.9	527.1	890.4	985.7
595.1	4256.2	1426.9	1963.2
570.3	772.9	1068.6	1219.8
435.4	567.5	854.7	756.4
917.3	1297.9	1741.2	1873.3
1004.2	678.1	722.7	500.4

280.4	359.5	578.7	448.8
268.9	304.9	433.2	436.1
632.4	858.2	1438.1	1294.4
404.5	1015.9	812.8	922.7
957.5	339.5	438.6	533.8
92.3	91.6	99.2	101.1
529.1	662.1	854.1	736.9
176.9	182.3	235.9	181.4
476.5	534.8	826.1	702.2
360.7	224.3	335	249.4
445.1	573	773.3	825.1
226.9	331.8	432.6	406.5
507.3	705.8	1211.6	1111.1
590.4	703.2	849.3	827.7
609.8	604.2	754.8	753.4
860.3	688.8	911.9	823.5
198.4	450.5	218.3	793.4
271.6	326.4	466	610.6
461.9	598.2	906.6	827.7
918	261.3	663.6	305.5
756.6	1054.7	1476.8	1331.5
747.1	852	900.2	877.2
401.5	616.9	855.1	785.7
397.8	726	842.2	718.4
334.6	333.5	745.7	778.5
578.9	1144.2	657.5	1626.4
446.9	592.4	783.1	799.2
229.3	135.7	288.3	141.5
286.1	306.5	626.7	631.2
257.9	267.6	374.6	249
193.4	230.6	282.1	303.6
434.5	661.3	795.6	803.2
999.1	882.3	1639.6	1398.9

390.2	452.1	539.5	602.1
1108.4	180.6	272.7	222.6
286.1	339.4	423.8	497.2
608.1	413.4	470.6	397.1
599.5	733.6	1326	981.8
583.9	596.8	695.9	702.4
153.9	114.8	147.9	166.1
606.3	513.4	429.5	540.1
386.2	394.8	741.6	918.5
164.1	191.2	566.9	234.1
288.9	239	585.6	439.5
304.8	345.7	527.9	405.6
636.5	793.6	1155.3	930.3
187.1	124	143.2	122
379.8	369.6	614.4	528.7
252.2	1066.1	385	515.3
532.4	463.3	634.4	640
280.8	209.2	503.3	541.1
232.8	249.6	337.7	297
388.5	747.5	852.3	919.3
314.5	403	405.6	384.2
351.2	415.6	460.6	516.7
539.8	676.3	739.7	796.1
182.2	247.8	680.5	605
216.2	254.4	353.2	329.4
198.4	252.8	225.9	249.8
297.3	230.1	481.7	472.7
494	522.2	805.8	517.1
349.9	428.3	685.6	630.8
140	133.6	147.9	134.6
224	311.3	362.6	441
236.2	254.3	440.4	299
688.2	973	1056.1	1456.2

754	1026.2	791.2	1083.4
371.2	356.5	493.3	431.4
810.2	667.9	876.5	942.2
225.7	275	363.3	320.9
737.1	687	439.5	1778.2
340.2	946	1225.6	1199.4
309.5	379.4	466.3	486.7
362.7	350.4	557.4	514.7
683	845.4	983.1	1042.7
1014.5	1300.2	1968.4	2030.2
406.8	414.8	555.7	529.3
351.5	429.9	587	620.2
115.6	128.3	195.6	123
285.6	213	318.4	255.8
981.1	1394.3	1801.8	1836.1
612	801.2	1058.1	688.6
628.3	359.7	582.8	963.8
168.6	186	322.9	372
304.2	294.6	446.5	392.4
240.6	320.6	389.1	311.3
357.6	441.9	598.8	512
341	204.6	279.3	266.6
187.9	54.2	70.6	55.9
475.5	387.6	451.4	422.5
292.6	332.3	481.4	398.1
453.9	634	924.9	931.4
407.3	550.8	703.3	740.9
299.3	327.4	443.3	547.7
342.9	393.5	542.6	449.7
1168.9	1482.1	1921.4	1811.4
331.9	237	201.7	104.7
160.3	144.5	200.1	224.8
176	224.9	342.1	301.4

351	353.1	485.7	553.7
590.4	540	255.9	367.9
181.6	280.9	224.7	210
603.4	743.5	1087.2	823.6
246.6	406	500.5	577.6
410.4	462.9	647.2	559
232.9	288.2	408.7	500.3
229.8	262.9	322.4	288.4
187.8	223.6	267.3	266.1
288.1	422.2	766.2	925.9
430.7	555.1	908.3	1024.6
386.8	378.4	316.9	266.4
7.9	6	16.2	8.7
791.7	895.7	1208.4	1249.8
92	109.6	140.4	132.8
280.7	290.8	313.7	343.7
668.8	609.1	1049.8	775.6
271.7	432.5	519.1	530.6
290.8	152.3	448.1	302.6
481.1	395.3	526.8	673.6
530	867.1	1273.5	1397.3
1222.1	1169.9	1554.6	1209.2
750.8	835.7	1115.2	975.3
446.7	581.1	730.5	784
467.8	498.9	653.7	653.8
328.4	374.2	482.4	448.5
96.4	169.4	103.6	179
316.5	414.3	625	855.2
468.3	499.3	891.6	680.4
722.6	919.7	1187.2	1082.3
246.5	211.1	268	270
849.3	836.1	1541.8	1466.4
776.9	811.6	1188.8	1283.3

404.6	639.5	917.8	915.9
539.9	442	541.2	460.5
437.7	515.7	847.3	673.2
584.4	743.7	1259.2	1167.4
408.1	436.6	573.7	528.4
511.1	488.7	853.2	708.8
170.7	234.1	244.2	243
601.5	1054.6	1034	901.8
553.3	661.1	902.9	837.4
419.4	489.6	639.7	563.2
307.7	416.9	396.1	528.6
307.8	415.3	612	497
1045.1	1221.6	1747.5	1690.2
244.7	317.5	433.8	446.4
185.7	245.7	493.6	400.6
633.3	621.6	730.3	1033.8
808.6	952.6	1301.4	1281.8
334.9	422.7	571.1	543.6
265.6	421	515.6	418
519.5	632	1055.3	911.8
158.8	259.8	339.7	353.1
648	662.6	918.4	904.3
192.6	187.1	288	229.9
122.2	142.3	151.4	92.2
275.1	389.4	541	495.5
99.7	73.5	103.8	82.1
278.1	272.8	546.7	339.7
639.1	597.2	1324.9	1298.7
335.3	386.4	566.6	580.3
607.9	902.8	1110.3	1331.3
562	737.2	1121.4	1146.7
660.5	527.4	754.2	351.2
646.7	869.3	1146.3	1179.5

618.4	711.8	733.3	461.9
894.4	975.5	899.8	853.1
399	473.1	588.7	624.2
1060.1	594.6	927.8	1528.9
174.1	170	373.3	239.3
560.4	788.4	961.5	983.2
249.9	269.2	484.7	387
231.2	264.4	837.7	646.5
302.4	400.7	517.7	461.2
376.4	452.3	728.2	552.5
295.9	406.8	445.2	545.1
315.6	396.6	648.3	584.8
581.1	651.6	1003.3	841.5
466.5	618.9	813.4	788.3
649.1	1464.2	724	2245.5
560	699.3	863.4	992.3
292.2	446.3	540	554.7
54.2	79.1	111.9	114.2
357.6	758.4	888.8	2730
249.9	297.6	389.6	429.5
349.6	468.3	613.7	552.4
696.5	1077.3	1342	1776.7
1307.7	248	281.9	214.5
182.5	232	329.2	279.8
601	844.3	1124.7	1124.6
316.3	433.8	612.9	488.4
695.9	882.2	1092.6	1042.6
1012.4	338.6	605.6	543.8
403.7	346.6	521.3	578.4
223	436.4	432	762
458.7	516.5	664.5	643.4
481.4	741.3	736.2	749.6
206.5	178.7	259.1	257.6

446	580.5	631.1	601
220.9	316.6	412.6	387.7
163.1	220.9	366.7	339.7
378.5	402.7	784.3	650.7
486.2	461.8	707	589.6
419.1	460.6	732.6	882.4
370.7	402.7	486.5	313.1
341.8	453.4	747	571.9
509.7	754.6	825.7	748
548.7	616.9	776.1	897.5
282.4	349.1	463.7	410.1
526	518.6	1188.5	764.1
155.3	150.1	264.6	206
186.1	225.7	286.8	255.2
296.3	391.2	547.8	503.6
309.8	445.9	872.2	683.3
295.6	393.3	517.7	465.3
525.9	712.8	1000	1097.5
403.6	478.8	750.8	675.1
133.4	175	226.4	217.8
1079.6	940.1	1079.3	1081.9
304.5	461.6	597.7	594.7
340.9	395.1	632.1	539.1
580.2	765.3	873.2	1190.8
87.6	82.6	143.8	123
225.1	259.9	389	312.7
170.5	195.4	332	358.2
405.3	492.6	596.1	532
286.7	315.4	471.7	459
279.7	631.8	1427.4	1447.7
278.9	345.1	361.9	341.3
315.6	330.3	433.6	467.5
965	816.7	1118.5	840.4

420.1	541.2	592.9	707
157.9	214.5	237.2	295.4
355.4	520.8	851.3	883.8
278.8	342.1	439.1	463.6
216.5	291.1	300.2	259.1
367.6	596.5	1064.6	844.8
198.3	180.1	291.4	230.5
305.3	177.4	286.9	185.9
335.6	425.4	580.8	557.8
361	434.1	434.1	573.5
432.5	690.4	1012.7	904.3
207.2	376	281.8	559.7
469.2	688.2	881	898.7
481.2	588	741.2	907.5
781.4	1293.1	1677.2	1635.3
495.3	265.4	207.4	275.2
151.6	185.5	477.9	227.8
254	396.7	434.9	614.6
488	513.1	1017.8	675.9
242.3	328.5	405	434.1
563.4	1294.3	1188.1	626.3
711.9	876.8	1120.8	1708.1
531.7	396.6	478.3	462.7
383.3	677.8	869.4	925.9
1000.3	464.2	769.2	453.2
368.6	557	572.3	583.6
226	362.9	520.5	567.4
108.2	173.4	232.1	206.6
429.2	600.8	798.8	800.8
540.7	687.9	1015.6	890.2
97.5	227.6	156.2	464.3
346.2	414.6	644	557.7
43.8	44.2	66.1	63.2

265	325.3	419	479.4
376.5	300.1	295.4	375.8
284.5	492	486.1	543.2
766.3	758.2	920.7	833.3
114.1	106.5	150.1	123.3
744.6	903	621.6	764.5
1534.6	1179.4	2443.5	2717.1
268.6	364	416.5	489
995.8	834.2	1507.9	656.3
231	326.2	392.3	402
291.4	398.3	577.8	470
407.6	436.1	504.2	595.4
382.5	556.3	817.9	833.9
399.8	487.2	660.9	575.4
483.9	489.9	675	584.5
171.5	236.7	249	180.7
570.9	185.7	181.6	242.8
276.2	242.1	242.5	230.9
337.9	259.2	372.4	347.5
847.7	1029.4	1314.5	1288
411.1	544.1	820.1	784
191.8	242.3	278.4	310.2
732.5	633.9	1225.6	1009.6
332.6	708.6	728.3	870.2
318.6	423.2	652.8	538.3
401.5	418.2	620.7	528.4
361.8	412.6	477.4	514.5
302	440.5	429.8	442
933.2	588.3	511.8	298.8
219.6	405.2	328.3	247.9
1743.5	163.8	208.8	251.1
614.8	628	734.1	701.7
821.2	271.6	324.4	281.3

587.7	660.6	652.1	564.5
208.5	262.4	258.8	260.5
247	581.3	360.7	556.3
308.2	448.8	594.8	348.6
168.2	199.4	305.2	285.3
265.7	195.9	453.1	340.3
1323.9	1071.1	729.2	432.3
240.3	240.5	223.4	219.5
563.5	553.2	574.2	516.6
57.3	68.5	208.7	207.9
529.1	266.7	244.7	191
208.6	153.9	243.2	205.9
584.7	385.7	580.4	565.1
162.2	254.5	301	325.5
255.5	301.8	505.7	472
231.8	311.9	482.2	775.3
376.9	481.7	730.9	608.5
479.9	583	673.6	757.7
340.4	396.6	549.1	521.1
199.6	345.7	436.5	466.6
265.3	295.9	405	475.9
394	469.1	665.4	548.9
188.7	228.8	327.2	469
317.1	373	487.6	534.3
124.6	285.2	353.5	408.5
351.1	419.4	398.8	789
354.1	414.1	428.8	392.9
114.2	138.3	182.3	183.7
242.4	503.2	389	1631
145.1	165.1	299.2	192.7
126	157.6	158.7	169.6
231.7	307.6	404.2	333.7
174	155.4	279	174.4

174.1	244.9	420.2	391.1
323.2	394.7	603.4	653.4
400.3	514.6	821.2	913.4
116.2	124.6	164	180.3
345.8	459.1	584.4	575.8
308.7	394.9	753.7	629.3
110.5	139.3	207.2	201.4
260	293.4	424.3	363.3
450	624	494	920.8
69.8	87.1	100.8	126.8
617.3	819.3	1097	1544.8
4277.7	414.5	519	708.3
545.2	604.1	860.1	843.6
639.6	756.1	1051.9	884.1
265.1	346	392	320
269.5	281.4	338.4	375.5
281.3	408.9	456.5	662.6
121.6	153.8	244.5	229
333.3	307.8	623.3	485.1
210.3	230.6	317.3	296.8
264.4	340.2	404.9	403.7
83.1	83.1	120.2	158.4
389.4	431.3	511.6	526.1
541	634.6	999.2	610.2
190.3	225.4	338.8	315.4
190.5	313.1	389.1	439.3
316.2	224.3	311.7	345.6
106.9	145.7	198.6	212.8
618.1	841.2	1169.4	1154.1
238.5	257.4	211.5	273
414.9	706.3	360.2	410.4
277.9	376.9	521.5	528.7
323.1	520.6	461.6	364

214	229.5	295.4	315.8
205.7	249.7	374.3	325.4
387.8	409.8	536.2	582.1
500.5	572.9	691.2	457.7
590.8	693.9	970.2	885.3
685.1	705	1046	887.3
304.4	195.1	624	452.5
1072.4	1236.9	1560.8	1712
415.8	328.6	607.9	569.2
486.7	754.8	939.2	861.3
220.9	262.6	337.6	427.9
184.2	158.1	125.6	114.8
400.8	428.7	810.6	781.9
304.5	329.8	416.6	387.6
428.1	612.7	727.7	745.5
231.9	276.9	436.2	385.9
269.3	423.7	489.8	511.2
422.3	487.3	600.6	633.9
143.5	134.8	165.8	171.6
216.7	342.7	347.8	435.1
171.7	193.6	330.4	269.4
401.8	307.9	562.7	341
242.8	259.6	443.7	572.5
231.4	238.8	471.5	334.8
56.2	66.1	113.9	85.3
167.9	248.6	346	295.4
1085.5	1365.8	1516.8	1519.9
398	462.8	493.2	422.7
338.9	592.6	837.7	817.4
490.5	438.7	582	537.2
324.8	343.2	465	445.8
184.1	525.5	854.7	578.2
216.5	271.4	376.7	333.8

74.9	103.1	106.3	173.6
290.3	310.4	442.5	440.8
116.8	143.6	215.5	198.5
309.5	304.5	282.7	176.4
364.7	373.3	616.2	502.3
103	142.6	169.4	204.4
216.4	433.1	509.7	1492.3
470.5	523.3	722.8	897.8
603.6	324.6	615.6	487.8
140.4	329.4	359.1	655.7
455	431.7	452.9	619.2
345.8	347.3	884.1	367.7
392.5	420.3	603	622.2
349.5	383.2	594.3	526.3
110.8	148.4	213	122.5
688.5	674.9	733.5	832.5
231.1	360.5	398.2	444.9
377.6	477.8	654.9	602.3
279.1	366.6	417.9	551
193.1	255.9	307.7	313.4
223.5	203.5	256.2	274.1
284.7	567.2	690.9	607.9
292.6	273.7	427.4	355.2
372.4	854	425.9	446.2
444.2	354.9	569.2	339.5
364.3	390.9	557.5	491.5
272.7	411.7	502.8	484
554.8	577.3	1003.5	909.9
1900.4	304	313.2	358.3
265.9	285.4	458.7	413.5
290.1	294	504.7	529.3
819.2	839.7	1172.8	1095.3
299.3	275.5	273.7	344.5

414.9	489.1	529.6	537.9
303.5	202	267.7	271.2
101.6	160.4	226.5	273.2
514.9	580.1	788.7	768.7
356.2	362.7	598	492.1
155.8	187.5	188.3	298.1
182	429.8	411.4	666.8
246.5	186.5	278.2	167.3
315.7	363.6	536.9	696
176	165.1	215.6	216.8
235.5	280.8	272.8	297.2
214.2	244.6	331.3	334.8
355.3	431.3	410	742.1
456.1	444.6	771	596.4
366.3	437	494.7	525.1
439.4	536.8	795.4	639
396.5	405.5	436.4	463.1
306.8	356.8	442	467.1
370.9	536.6	622.9	663.6
279.4	367.6	478.1	563.8
552.5	678	951.9	768.9
101.9	93.9	156.2	148.9
240.1	263.7	364.4	266.9
681.6	338	433.1	474.8
184.4	246.6	331.1	276.5
378.2	511.2	713.1	657.9
517.1	562	769.6	746.6
247.5	379	622.5	594.8
642.4	437.3	407.1	211.8
636.6	631.1	1047.2	992.9
477.8	237.4	364.7	375.1
224.2	213.7	359.6	259.7
256.7	287.7	418.2	480.9

519.7	630.3	673.6	910.2
164.7	185.7	213.1	187.8
184.4	208	243.9	263.9
188	234.1	360.4	411.3
284.5	231.9	206.8	158.2
415.1	2288.5	115.1	67.8
593.5	653.8	761.8	891.8
179.8	197.8	232.5	279
154.4	185.2	309.4	236.7
506.6	553.3	932.9	898.9
403.9	314.2	353.7	398.7
207.5	298.3	394.2	391
209.7	236.1	329.4	315
539.7	560.9	807.8	845.3
251.7	311.9	362.1	390.3
754.3	1230.9	2387.5	2809.1
47.3	60.6	108.7	81.1
158.4	224.5	287.2	276.8
613.4	375.5	320.5	125.4
130.8	209.6	270.8	285.3
722.6	1358.8	1759.9	1611.1
29.1	814.6	405.6	18.9
317.5	358.3	512.6	462.3
393.1	267.9	632.5	437.3
353	458.1	583.3	540.5
360.9	331.6	603.8	566
437.1	459.2	575.6	549.9
1872.8	301.7	300.2	576
232.1	329.6	377	457.3
892.5	948.5	1400.9	1303.1
275.9	814.6	636	1392.8
312.2	415.6	520.3	715.9

428.5	808.9	903.8	782.8
266.7	161.1	449.8	387.2
292.5	391.3	408.7	483.1
102.2	139.2	171.9	204.1
854.8	1037.5	1421.9	1431.2
1916.6	313.3	502	668.6
449.4	398.8	651.2	426.7
226.2	353.2	403.9	460.6
118.7	142.3	198.6	199.6
209.8	228.2	321.1	363.8
420.5	453.5	737.3	625.1
210.5	225.9	366.2	374.3
282.5	301.7	446.4	331.8
572.2	861.2	1213	1522.7
216.5	251.6	307.4	293.8
237.4	418.1	555	439.4
306.9	392.2	499.3	519.1
126.9	181.2	278	282.8
465.9	497.2	577.2	576.7
356.8	425.1	351.3	329.6
154.2	229.9	314.1	382.1
1247.7	1340.2	1590.2	2477.7
246	291.2	340.9	365.7
671	736.4	1039	873.8
401	451.6	857.1	692.1
615	778	798.8	658.5
59.8	124.4	159.4	104
486	459.5	758	491.9
580.1	776.3	996.2	1340.9
89.7	108.4	143.5	163.8
201.4	256.2	299.4	309.1
241.4	392.6	473.3	726.2
475.5	587.7	385.1	848.8

307.9	295.4	359.6	415.4
402.3	471.4	643.6	681.7
327.6	329.7	479.5	549.3
274	347.5	429.9	408.7
293.8	426.4	685.1	316.2
256.2	260.6	341.2	458.6
300.4	343.2	850.7	538.5
154.2	212.1	310.2	191.9
546	496.2	677.3	505.2
573.9	469.6	698.6	1025.9
267.8	337.8	356.8	196
209.9	352.1	361	344.5
325	360.2	553.4	494.3
120.3	148	194.7	179.6
60.9	67.9	87.8	82.7
541.4	502.6	725.4	605.3
463.2	425.5	601.1	268.8
159.4	271.9	231	399.2
1166.3	1241.4	1828.7	1570
490.3	556.8	784.8	666
273.4	362.3	431.8	651.4
602.1	523.4	748	594.9
600	538.6	649.3	769.9
180.3	372.9	676.2	534.7
328.9	378.1	433	421.4
366.2	366	453.9	517.5
239.4	219.3	233	206.5
598.2	754.9	944.5	1009.8
335.3	294.4	401	486.8
666.7	729.1	1085.8	1044.6
161.7	192.4	296.8	244.5
320.6	433.3	602.1	531.6
138.7	212.1	270	246.7

116.3	100.3	138.8	116.8
625.5	428.3	1164.7	1120.2
276.3	322.1	387.9	452.5
379.8	528.4	602.7	583.8
346	430.9	580.9	804.6
144.2	223.1	335.8	303.8
351.9	338.1	560.3	496.6
444.3	451.1	708.9	403.6
323.5	282.2	472.1	310.4
238.7	206.4	252.3	213.2
170.7	280.3	181.1	416.3
567.6	337.7	383.8	423.6
184.7	421.6	378.6	640.4
468.3	527.8	701.1	696.3
398.3	460.9	620.1	724.3
292.9	384.5	443.7	611.5
351.7	453.6	450.6	588.2
79.3	83.5	111.3	108.8
242.8	219.2	409	455
84.2	124.2	132.7	144.5
298.9	560	400.6	365
114.9	133.7	246.8	222.4
180.3	306.4	425.5	521.8
451.9	413.7	465.1	342.7
345.4	325.6	529.5	480.9
101	116.9	159.4	144
282.8	333.2	426.4	491.8
214.7	178.4	281.4	267.1
352.4	324.1	557.6	598
790.6	416.8	632	527.3
109.4	102	218.1	115.7
370.2	396.2	590.6	545.3
333.6	395.8	613.5	624.1

179.2	287.4	190.7	197.6
347.2	294.7	371.2	523.6
439.3	1033.5	811.9	427.3
207.9	258.9	354.7	355.9
905.9	538	791.7	641.2
189.8	288.6	457.7	583
119.4	147.5	180.5	154.5
228.4	279.3	376.6	408.6
259.8	341.2	430.5	448.2
1378.8	26.5	35.4	44.4
162.5	229.7	276.3	358.9
127.3	161.9	204.2	184
432.4	360.1	569.4	659.9
105.3	125.3	140.3	117.1
131.7	151.3	225.6	229.4
393.5	533	2117.4	1094.7
115.5	184.7	228.5	228.1
292.7	538.4	346.7	379.4
361.7	165.6	449.1	267.7
322.2	356.8	535.7	422.3
327.6	450.3	679.5	547.8
336.3	299.7	402.2	368.4
20.2	34.9	33.2	32.2
1004	1681.7	1317.8	2946.8
54.5	73.8	81.4	104.1
433.7	452.9	701.3	606.5
204.9	321.5	348.9	603.5
194.1	139.1	264.6	281.4
105.4	192.9	285.1	274.9
438.9	529.1	724.8	716.8
675.6	684.9	1606.4	672.3
262.1	140	257.1	142.7
205.8	233.2	199.5	282.1

332.1	368.7	552.4	459
295.9	396.8	490.6	425.7
204.7	174.5	357.9	310.7
119.9	152.5	242.9	197.1
555.8	760.3	603.9	1006.5
233.9	260.9	317.3	316.2
216.9	131.9	158.1	124.2
234.3	225.2	260.1	244.5
225.9	364.9	151.1	938
274.7	267.5	323.3	276.8
90.8	96.5	116	104.6
130.1	180.2	238	185.9
81.6	86.1	132.7	111.5
126.3	136.4	197.6	183
151	158.8	243.2	236
152.8	225.5	306.6	309.4
106.8	102	125.8	153.5
832.7	1016.9	1011.7	1212.1
239.2	291.8	291.9	231.7
176.8	445.5	308.1	706.7
128.7	127.3	156.7	158.1
229.7	243.8	411.2	312.5
188.2	231.3	420.9	381.7
257.9	367	378	406.4
1174.6	149.9	144.9	285.1
146.5	140.9	197.6	217.6
146.9	237.4	364.6	274.4
73.2	105.7	119.2	162.5
180.3	196.8	287.5	263.1
608.2	544.6	798.2	741.8
173.3	243.1	397.3	300.9
459.1	453.5	663.6	485
846.7	983.8	1771.2	1640.1

158.7	187	131.9	203.5
49.4	49.7	49.2	52.6
125.6	148.7	216.4	151.3
179.5	177.8	304.2	266.3
110.2	141.3	170.1	166
99.6	111.8	98.3	152.9
86.5	108.6	135.1	124.8
309.6	386.6	470.1	372.5
537.9	644.2	942.6	802
330.1	704	847.4	786.4
326.7	723.3	848.7	1000
643.2	503.6	544.4	881.4
185.1	192.6	287.9	238.6
721.5	831.2	1545.8	1368.7
258.7	226.1	223.8	223.7
85.7	135.8	171.1	156.5
642.4	679.6	765.1	718.5
323.2	516	847.4	1222.2
192	204.4	328	288.9
192	278.2	328.5	323.9
237.7	247.9	234.9	249.9
310.4	378.1	464.6	517.5
357.6	447.5	532.1	765.2
285.3	340.7	525.8	457.8
391.7	554.8	711.7	608
335	343.3	371.5	476
303.4	299.1	398.9	279.7
316.6	535.2	604	709
303.9	472.7	488.4	502.7
306.3	439.8	612.9	535.5
730.4	1025.3	1558.3	1431.2
392.3	272.5	732	1516.2
83.9	118.1	137.1	136.9

180.2	186	213.1	185.9
344.8	410.9	520.8	512.1
522	165.1	209	200.8
309.5	313.4	490.4	460.6
169.9	207	222.3	281.2
600.2	920.2	1384.9	1180
82	82	113.2	108.2
623.2	522	500.3	595.5
345.4	508.2	568.4	634.8
267.6	362.8	541.4	497.7
169	212.3	234.9	281.9
315.3	342.3	494.8	503.4
124.9	129.3	184.9	206.8
456.3	354.3	613.3	563
612.5	380.4	328.8	326.1
757.9	778.2	947.2	1034.9
290.7	301.1	559.9	357.2
536.8	799	1045.8	1106.4
347.1	338.1	391.6	301.9
246.3	270.5	426.5	478.9
873.8	978.8	1395.7	1460.8
66.2	64	114.9	101.8
102.6	101	185.8	139.3
183.8	226.2	281.2	227.9
335.5	382.5	549.9	464.4
540.5	568.6	730.4	725.5
104.4	90.5	129.6	96.2
409.7	510.4	674.8	752.7
189.7	200.2	254.8	366.1
158.2	108.7	169.1	154.8
280.5	377.1	500.2	516.3
195.2	287.6	417.7	364.8
169.2	210.7	438.1	395.6

179	164	240.2	164.2
460.4	576.8	859.6	1039
108.7	133.7	192.2	170.2
305	396	393.7	342.7
247.8	329.5	434.3	337.8
91.4	101.5	145.5	112.2
833.4	796.9	980.2	622.1
219.8	235.4	316.5	265.3
139.2	124.1	153.7	180.7
314.4	393.7	436.9	382.1
215.7	294.6	330.5	377.7
294.8	459.8	497.7	421.1
151.3	152.9	216.6	200.1
143.8	134.8	141	154.2
161.5	157.3	233.1	211.5
84.2	108.4	157.4	142.3
217.6	298.8	396.1	327.4
454.7	726	438.4	524.6
451.2	651.8	913.3	951.5
735.6	272.1	180.5	355
162.6	229.3	343.6	316.8
90.9	99.5	164.8	152.8
1360	1630.4	2059.9	2375.7
166.5	203.1	304.2	177.6
494.8	147.8	171.9	265.9
196	228.1	404	464.9
92.1	118.6	182.7	156.7
115.3	155.3	204.7	179.9
319.3	395.9	334.6	514.6
490.3	638.6	648.6	1049.5
256.8	385.8	498	600.8
682.2	945.1	968.1	933.5
83	123.5	113.7	116.3

36.8	44	53.7	85.3
346.1	413	367.1	754.5
257.9	391.4	518.8	431.7
123.8	236.8	367.8	384.6
460	534.7	831.9	847.9
164.6	224	335.6	238.1
197.3	275.9	325.9	411.8
350.7	219.4	760.3	1119.2
193.5	171.1	274.9	141.7
190.3	160	222	181.8
198.2	233.4	293.7	296.9
788.9	812.9	1455.5	1618.8
201.8	224.4	302	298.4
500	570.4	863.5	697.2
55.2	50.7	81.9	75.3
223.9	247.5	318	191.7
265.7	600.7	82.2	90.6
43.6	48.2	80.3	69.4
112.7	115.8	133.2	111.7
303.7	369.9	464.1	489.3
223.1	195.9	471.2	608
334.2	459.7	541.1	503.3
161.5	208.6	371	873.8
249.1	246.1	324.7	308.2
615.7	664.1	916.2	967.1
337.6	420.3	469.9	425.6
305.8	412.4	398.5	475.8
1020	164.9	250.4	207.7
237.3	309.6	420.6	408.9
42	47.7	46.8	50.1
149.8	462.3	192	178.8
336.5	264	443.6	398.1
335.5	408.1	957.7	482.7

61.9	82	115.6	125.4
509.7	500.1	994.1	733.6
259.5	280.8	373.6	329
510.7	516.8	894.8	717
112.9	109.1	152.1	179.9
99.4	182.8	188.5	226.1
82.4	112.6	165.4	149
431.6	390.6	530.4	519.5
312.4	372.3	519	517.1
166.2	208.1	253.2	258.6
405.6	410.9	401.1	214.1
455.9	342.8	495.5	497.1
157.8	189.1	277.9	311.2
290.2	320.6	494.4	448.1
393.8	620.5	864.6	842.9
170.6	190.7	262.1	309.9
328.8	420.2	515.9	398.5
81.7	107.4	163.1	162.6
390.1	477.2	531.6	553.8
669.2	1147.6	1299.5	1377.8
164.9	180.5	270.8	300.6
448.1	468.9	553.1	440
96.5	94.3	100.1	124.8
389.2	377.5	440.9	424.5
320.8	367.4	531.8	512.7
352.2	338.2	575.8	545.7
237.3	338.8	480.8	693
634.1	583.7	681.3	641.1
541.8	705.8	758.4	908.1
210.1	159.5	631.3	426.2
232.3	271.8	368.6	379.8
534.8	878.1	1003.4	841.9
227.1	310.9	397.3	428.7

472.6	473	857.6	852.4
91.5	85	240.6	90.1
106.8	117.8	147.6	137.2
411.3	363.4	522	485.8
433.2	529.4	1007.9	682
295.6	196.3	324.7	254.9
51.5	52.2	77	68.6
211.1	255.9	363.7	318
237.4	371.8	603.9	375.9
188.7	312	431.7	374.3
386	452.1	644.9	557.1
261.6	275.2	341.3	328.6
431.8	433.7	685.5	583.8
241.5	334.8	512.9	389.2
539.2	324.1	494	427.9
71.3	80.5	213.4	155.6
160.4	222.6	266.6	242
163.8	181.9	321	181
839.1	975.1	1240.9	1172
1752.7	255.5	328.4	279.1
417.6	355.4	436.5	382.9
369.2	372.3	602.1	473.5
441.3	616	833.7	733.9
192.1	258.4	364.3	281.6
177.4	100.1	244.8	130.5
327.2	432.6	644.8	588.9
359.4	505	534.7	601.5
592.2	742.9	1144.6	1015
554.9	1006.5	1335.2	1314.9
91.4	112.5	81.6	106.8
146.2	218.5	416.1	292
668.3	388.2	844.3	720.4
175.2	116.6	143.4	165.6

281.3	313.6	485.2	277
197.9	225.9	298.5	396.6
243.7	465.3	184.3	299
186.5	284.7	357.3	350.8
251	288.9	318.5	312
198.1	171.9	197.4	264.5
115.9	127.4	174.3	153.6
517.7	626	863.3	1357.5
196.7	267.1	377.5	367
65.2	84	85.7	83.1
196.8	247.2	285.3	257.7
194.9	199.2	260	281.6
67.9	101.6	136.3	149.5
179.2	252.8	325.5	289.9
315.9	392.3	598.9	515.9
247.5	210.4	320.2	255.3
298.4	367.8	376.1	401.4
187.6	182.7	191.2	210.8
60.7	22.6	18.2	12.9
319.9	466.9	975.8	689.5
32.6	47.6	46.1	50.6
154.3	159.9	230.9	167.3
108.6	101.6	149.2	130.5
168.5	194.8	310.5	261
301	383.9	459.6	501.8
118.9	130.8	147.3	148.5
251.4	269	405	452.9
209.1	126.9	198.3	180
733.9	877.8	1102.4	965.7
489.5	776.3	986.7	923.7
811.6	386.3	461.6	626.8
360.9	168.5	154.7	77.1
378.2	318	524.1	444.4

184.5	191.7	174.3	242.6
416	398.7	624.2	586
223.2	272.2	328.3	386.2
381.4	369.4	490.9	609.9
247.2	290.5	334.6	341
104.1	144	203.5	187.9
363.4	1702.9	468.9	677.3
256.9	406.3	728.8	599.8
28.6	29.9	35.9	43.3
178.9	224.1	351.9	375.1
338.7	392.1	559.9	505.6
156.7	175.5	251.1	249.9
472.9	515.4	693.6	600.8
140.8	161.7	169.2	225.1
311.4	467.7	557	523.5
261.2	289.1	376.6	372.2
386.5	483.4	484.1	396.9
212.1	197.7	402.5	301.3
198.6	173.3	307.7	264.1
135.9	152	252	230
274.7	334.8	489.7	405.2
1060.8	79.4	183.4	216.6
195.5	218.2	335.9	292.7
318.8	372.5	295.1	553.2
320.7	468.5	458.8	696.5
413.5	527.7	677	626.2
149.9	103.8	118.4	59.9
77	100.5	149.2	94.4
262.5	227.5	427	366.4
130.8	158.4	209.6	194.5
106.4	321.5	354	1892.2
178	253.1	364.7	436.1
124.7	194.4	317.4	333.4

120	161.1	199.1	204.4
314	329.7	568.2	483.8
325.4	331.3	497.7	409.4
193.5	267.1	325	303.8
502.9	776.7	1079.2	1000.8
156.2	157.7	175.8	253.6
187.3	267.4	286.6	358
56.1	71	105.9	84.4
180.2	250.7	380.2	254.8
124.4	119.4	149.3	169.4
70.9	73.7	134.6	166.4
147.2	174.6	226.1	250.9
259.5	276	401.2	410.6
257	275.3	302	272.9
233.3	239.4	172.6	118.7
149.1	127.7	180.5	180.6
262.1	190.3	351.9	264
187.3	207.5	244.3	184.4
185.3	188.1	295.5	212.9
616.2	850.2	1257.6	1239.3
272.6	271.2	443.2	378.2
127.4	170.1	200.7	243.3
123.4	140.8	208.2	153.3
188.1	200.2	220.3	242.5
168.8	227	253.8	295.8
80.5	111.3	165.5	207
264.1	283.3	329.3	285.9
327.3	358.7	429.9	474.5
172.9	253.5	270.7	272.6
284	402.2	638.2	740.7
416.4	380.5	598	480.2
118.5	135.3	202.1	159.6
60.8	56.9	119	81.5

321	381.4	363.8	631.2
312.6	345.4	392	714.4
115.1	111.9	127.7	157.2
277.7	299.4	459	454.4
294.5	252.1	459.7	465.7
159.5	167	341.9	290.5
245.6	330.4	385.5	388.5
236.4	227.7	318.4	306.2
128.4	184	236.7	202.6
323.4	382.9	578.6	584.6
289.3	282.3	455.4	509.1
62.3	64.3	75.2	99.2
242.6	347.6	492	445.2
175.2	248.3	400	537.3
198.5	213.7	329.4	364.5
171.4	172.4	277.1	196.5
146.8	473.4	91.2	66.4
818.8	975.6	1073.9	831
163.8	185.7	265.6	252.7
737.4	1388.8	1660.5	1845.8
143.4	141.1	214.5	220.7
350.5	593.3	784.3	628.7
111	149.6	221.6	167.4
140.5	172	197.3	216.8
247.6	279.7	372.4	264.4
162.2	155.7	497.1	392.3
176.3	177.9	240.9	251.7
160.9	166.8	253.7	220.4
193.6	280.9	348.4	390.6
118.6	160.5	193.8	181.9
213.6	335.1	547	448.2
110.5	130.7	88.1	113.1
74.8	118.2	156.3	120.1

95.6	126.8	163.3	165.8
404.4	276.4	223	457.9
256.5	373.8	491	503.1
152.2	622.7	328.6	999.5
897.3	329.3	489.5	376.4
319	494.3	533.1	476.6
260	279.2	330.7	356.1
182.1	213.9	303.6	232.1
718.3	730.6	900	923.3
145.1	193.3	240.5	273.6
508	550.9	806	805.1
343	416.2	827.9	713.2
214.4	192.7	295.8	308.4
323.2	737.3	1149.1	1152.3
249.3	328.3	363.2	352.9
210.4	178	274.9	239.2
216.9	211.1	246.6	280.4
94.5	131.2	126.1	135.1
198.3	218.9	297	345.4
90.4	94.7	143	145
133.2	216.1	258.6	326.2
629.5	691.2	1179.6	1054.3
502.2	336.5	611.4	465.8
113.4	97	172.3	85.3
270.8	481.6	606.4	536.7
228.9	265.5	409.8	294.5
31.8	48.1	43.9	28.3
352.7	356.7	606.6	331.7
145.1	143.3	197.3	211.1
189.3	210.8	274.8	259.5
333	428	540.9	504.9
307.8	321.3	456.8	424.2
186.7	138.7	185	223.1

235.6	286.9	299.6	446
105	109.9	168.1	149.8
151.1	157.4	219.7	212.6
87.5	93.4	81.5	111.6
252.5	396.3	726.1	336.4
75.5	96.8	147.4	118.3
111.3	126.4	151.4	151.6
225.6	190.9	212.9	415.4
175.2	337.2	279.7	331
150.5	145.7	126.3	137.6
252.5	259.4	361.1	432.8
221.8	302	194.6	332.6
134.6	186.8	277.8	256.7
242	279.1	370.4	368.6
120.6	116.1	143.4	178.5
125.6	169.8	266	218.1
222.8	232.8	337.5	288.7
252.5	230.5	295.5	308
210.2	333.3	1260.3	538
200.4	232.7	243.6	469
241.9	205.3	270	210.8
81.6	79.8	83.3	85.9
226.1	295.7	419.3	385.4
205.1	254	262.6	260.8
108.3	137.2	179.8	205.5
518.7	1046.8	826.1	1769
50.6	79.4	73.9	86.7
173.9	268.3	388.7	321.3
254.6	329.2	651.5	373
385.6	372.4	481.6	448.2
543.4	606.4	646.9	852.3
421	358.9	480.8	2572.4
529.1	761.8	1061.9	822.5

49.7	63.1	64.8	70.6
58.7	85.5	75.4	105.1
89.7	125.8	125.4	69.3
118.6	140.2	278.8	208.8
202.6	337.3	430.1	400.6
239.6	318.8	416.6	458.1
113.1	152.8	286.9	450.7
88	91.1	128.2	115.4
491.8	783.1	329.2	274.3
321.6	426	557.6	531.2
978.1	1133.7	1577.8	1676.2
208.3	254.5	353.6	379.2
203.1	277.6	352.8	380.9
97.3	72.2	73.6	73.8
238.6	243.2	324.4	602.7
95.9	123.1	176.2	156.7
493.4	464.4	945.8	558.7
193.1	155.7	174.7	180.6
171.1	168.2	213.1	274.4
148.6	182.6	200.5	277.5
246.6	298	429.9	408.8
784.6	605.5	791.3	646.4
416.8	481.3	687.2	621.7
85.1	120.5	240.9	254.3
163.6	220.5	282.5	265.7
145.2	240	351.1	323.1
347.5	419.6	566	606.5
22.4	38.1	36.6	57.6
113.4	737	167.3	288.4
92.3	94.8	159.1	164.8
112.8	183	181.8	251.4
153.7	164	288.2	299
83.7	104	145.1	138.6

243.3	176	366.3	148.7
219.2	275.9	401	368.4
593.6	443.1	643	420.3
229.6	269.3	341.4	344.9
568.6	831.6	1223.5	1122.4
240.4	313.1	483	462.6
111.2	169	248.4	200.3
310.6	403.2	521.4	551.9
268.7	248.2	511	335.9
157.9	188.7	207.9	233.3
316.2	346.5	380.6	449.7
785.1	640.6	963.5	1048.2
73.4	62.8	73.6	82.7
114	61.2	59.6	106.9
156.8	201	346.6	293.6
366.2	387.4	580.9	466.9
143.4	181.6	275.7	246.6
976.8	450	405.2	538
202.3	243.4	208.7	206.9
300.5	344.7	550.3	564.9
363.3	381.6	594.7	549.5
127.8	131.2	149.2	175.2
323.4	453	570	638.3
25.7	26.3	36.2	37.5
412.7	543.3	582.2	653.1
139.3	198.4	247.7	209.3
339.6	422.7	549.1	545
113.4	77.2	179.8	111.9
101.4	181.2	243.2	188.8
661	579.1	730.7	578.7
182.5	327.8	323	376
70.8	94.2	142.1	140.2
322.3	347.2	484.7	457.9

552.8	631.8	1168.8	918.9
477.1	606.6	944.7	730.2
305.9	446.7	752.3	980.5
48.6	25.9	34.8	43.6
140.2	227.7	289.6	266.8
19.4	33.6	27.8	48.9
69	84.2	113.2	99.3
1150.2	1229.8	1753.1	1492.1
567.3	886.5	1488.7	1527.5
133	143.9	186.1	106.8
402	414.6	748.2	539.4
285.3	407.2	423.1	482.3
252.4	274.6	395.9	399.8
135.7	105.4	197	190
215.6	253	380.5	325.7
234.3	266.8	345.8	290.8
28.3	35.1	37.3	33.7
320.8	428.7	933.4	646.7
172.8	164.7	203.5	208.3
305.3	276.2	383	415.9
321.7	380.7	404.9	408
1057.9	1089.7	588.2	850.5
457.9	322.5	309.7	217.1
136.3	179.2	337.7	357.7
585.7	657.7	291.6	397.3
630.2	677.6	1039.6	814.8
119	165.5	148	225.8
221.7	234.1	314.9	280.9
335.4	355.4	559.1	530.2
150.1	131.7	295	252.5
83.7	72.5	92.8	84.2
128.8	194.2	151.6	188.2
264.3	358.7	500.2	459.7

1063.1	26	41.2	38.8
106.8	107.8	180.6	182.8
109.8	143	179	197.5
251.9	315.9	393.9	413.4
44	56.2	59.2	64
146.7	154.7	200.4	210
359	315.9	357.6	479.6
171.7	285.4	326.4	336.3
232.3	49.3	60	73.9
110.5	171.1	194	185.4
101.8	275.6	108.8	61.8
262.7	308.1	369.3	391
108.3	131.3	117.3	159.1
194.6	261.5	407.8	583.4
628.2	1016.8	1408.5	1241.7
114.1	112.1	155.2	169.2
370.8	362.1	564.7	515.3
428.2	510.9	741.8	629.4
168.2	213.1	304.9	279.8
264.7	306.4	410.1	558.5
351.5	561.9	908.6	809.6
96.1	129.5	152.8	322.1
203.3	264.9	509.9	444.8
160.4	143.2	253	261.4
174.3	214.4	222	276.3
110.5	126.8	139.5	163.2
112.5	133.3	128.8	197.5
377.5	257.7	445.1	367.1
194.6	176.1	303.4	237.6
179.1	203.1	279.9	400.2
133	159.1	173.5	213.2
286.2	273.3	296.7	399.9

428.2	507.9	696.9	657.7
164.6	209.4	297.2	352.6
493.8	641.9	906.9	858.5
97.1	124.8	143.3	145.1
406.3	670.9	648.9	695.2
404.5	391.5	474.8	424.3
174.6	268.3	412	200.8
293.6	367.4	471.4	530.5
120.8	172.5	267.5	229.8
237	317.4	480.9	629.3
137.1	150	240.5	237.9
139.4	219.6	237.8	246.6
139.6	162.9	174.2	200
123.5	152.7	255.2	249.1
178.6	174.4	269.8	231.4
209.3	132.1	195.9	194.9
286.2	319.8	392	418.6
308.2	306.3	501.2	443.1
254.1	265.9	348.3	302.6
369.4	485.2	643.4	629.3
213.7	263.5	434.3	354.9
121.4	150.7	223.9	221.8
88.5	46.3	78.9	129.9
177.2	230.9	325.1	238.5
129	108.9	139.3	148.9
154.3	303.3	209.2	182.3
203.9	207.1	268.5	380.3
333.2	316.7	1036.7	856.8
82.1	110.4	254	230.6
81.8	109.9	121	124.9
22.7	31.7	55.9	35
123.9	154.3	168	208.1
345.9	509.2	629.7	568.4

47.3	82.9	84.9	82.3
249.7	247.6	250.1	70.1
217.5	277.3	396.1	392
203.9	259.2	189	262.7
41.1	76.8	63.4	79.2
196.4	222.3	298.3	330.6
51.6	59.4	87.4	80.7
28.9	27.3	45.1	42.5
183.6	211.8	372.1	344.2
213.6	294.4	263.9	416.5
285.9	328.4	330.4	698.4
351.9	484.6	580.5	529.5
368.5	398.1	597	407
190.5	263.4	384.3	332.1
20	20	30.1	28
162.4	231.9	302	373.9
162.8	147.9	149.7	127.1
147.9	154.9	199.5	194.4
195.8	213.8	343.1	488.9
56.9	158.6	102.5	102.4
108.8	114.4	161.8	127.8
254.2	287.7	171.1	144.9
277.9	273.1	368.9	361.8
336.6	376.3	464.5	443.4
149.4	205.6	282.6	276.6
247.1	229.5	339.3	269.5
75	117.7	177.6	171.7
201.5	222.8	289.1	290.7
157.3	253.7	495.8	576.6
102.1	50.8	210.6	67.6
14.9	15.2	16.1	14.4
92.9	90.4	162.8	105.4
187.4	235.7	280.5	387.5

233.9	239	347.5	275.2
166	214.3	375.9	259
372.6		10.4	9.2
166.4	239.1	309.1	284.8
89.3	91.1	122.3	111.4
238.4	200.8	265.4	244.6
202.1	267.1	467.3	253.1
138.7	194.6	236.1	289.1
71.7	108.6	163.5	158.7
155.5	128.6	159.1	121.2
153.8	133.1	214.7	222.2
99.4	93	139	110.6
148.5	134.4	158.2	169.9
180	237.1	376.3	573.5
99.6	102.6	186.3	172.6
273.1	255.6	400	303.1
719.5	394.1	375.2	358.4
91.5	72.2	113.2	115.8
96.3	134.4	113.8	188.1
218.6	223.6	346.2	340.3
165.4	145	118.8	101.8
176	286.1	440	514.6
21	31.9	21.6	59.5
342.2	397.8	425.8	412.6
286.6	236.4	419.3	309.9
296.2	417.3	498.8	624
451.2	165.2	579.7	628.5
214.1	214.9	326.2	317.6
247.7	342.5	430	425.2
175.4	191.7	175.9	235.1
162.4	223.1	158.9	222.1
77	133.7	252.5	257.8

291.3	329.1	464.6	457.7
273.8	497.7	486.9	578
45.2	44.9	50.5	61
109.4	149.6	164.7	295.6
221	277.7	358.4	354.4
127.5	139	155.7	209.2
69.5	138.7	172.1	173.1
32.4	41.5	34.1	44.6
233.1	403.4	485.7	431.9
415.8	419	509.7	721
51.7	57.9	75.2	75.5
228	258.3	390.6	335.3
120.7	189.4	164.7	963.7
48	44.7	47	44.6
119.3	145.1	190.7	174.9
210.2	324	333.7	397
347.7	336.3	542.3	535.7
35.7	40.6	62.4	55.4
38.3	95.5	61.7	68.6
179	155	279.9	256
166.7	204	248.9	285.4
230.6	516.5	424.7	342.1
386	538.8	671.9	631.2
176.6	304.9	404.9	354.5
93.3	127.1	177.8	222.8
210.6	237.7	349.2	356.3
127.3	165.6	218.8	245.9
163	193.2	224.6	192.6
55.9	74.8	82.5	79.4
316.8	360.4	525	500.9
61.7	68.2	131.7	67.4
380.7	401.2	567.3	556.1
153.7	195.8	304.3	256.2

215.6	189.7	257.4	280.3
151.7	176	245.3	235.3
262.5	312.1	426.2	537.6
177.8	133.1	212.8	202.2
36.2	36.5	41.4	51.5
175.7	198.2	213	208.4
60.9	72.4	77.5	82.6
132.5	66.9	290	287.4
135.4	310.3	186	267
179.9	160.4	242	264.1
12.7	16	22.6	24.5
117.7	147.8	168.5	194.1
28.4	37.7	37.4	37.4
262.3	355	490.4	372.3
175	135.9	346.4	274.3
206.2	192.2	256.9	242.4
150.5	177	216.9	194.1
110.3	60.5	181.1	77.8
70.3	96.6	79.7	88.7
119	121.8	144.2	175.1
317.3	400.6	601	529.2
377.5	402	247.1	413.8
97.7	114.8	126.3	133.3
618.8	1053.6	1164.9	1464.1
190.2	272.1	358.6	396.8
240.9	299.6	296.1	370.4
28.1	28.1	37.4	37.1
104.5	141.9	207.8	228.7
218	240.4	488.9	279.2
154.4	221.5	324.2	271.9
176.5	198.8	263.4	241.8
514	522	537.7	570
190.7	281.4	340.8	263

43	86.1	93.4	107.4
205.5	307	324.8	268.1
153.1	132.8	200.2	171.5
83.3	109.7	181.4	177
135.3	134.9	189.4	192.3
148.9	230.2	231.9	244.6
132.1	169.6	259.1	236.3
860	1083.8	1458	1233.8
48.9	51	84.1	67.5
280.7	292	520.7	439.3
325.9	409.5	400.8	508.1
312.2	255.5	329	379.6
309	350.7	480.4	583.9
70.5	114.8	100.1	126.8
64.7	86.1	114.9	93.5
93.3	150.3	122	121.3
204.4	290.2	429.6	526.9
148.2	160.4	361.6	1206.6
80	109.6	145.6	101.6
164.1	116.8	211.3	241
88	94.3	119.2	98
32.9	40.2	53	48.1
86.6	97.6	115.1	135.5
99.6	111.9	146.9	144.5
228.1	243.4	458.6	403.4
115.4	83.9	108.1	99
180.1	189.9	246.6	248.9
256.7	251.5	358.1	387.4
326	582.1	479.3	875.3
215.4	206	269.2	238.7
497.5	549.2	944.3	911.9
219.4	190.9	261.6	284.9
104.3	115.2	195.4	173.3

122.4	185.3	198.1	179.8
72.6	70.4	127	91.7
423.4	535	717.8	881.7
76.5	84.5	88.5	129
162.6	255.6	363.4	298.5
81.9	106.1	95.8	138.9
235.8	274	452.7	663.4
131.8	433.2	135.3	91.3
55.8	78.7	68.7	79.9
108.5	177.7	164.9	306.9
342.5	302.4	992.5	725.8
72.7	83.9	105	110.7
74.9	70.8	141.1	83.1
90.6	102.9	119.8	157.9
498.6	84.2	187.7	106.1
156.1	172.4	282.1	254.1
131	165.2	210.6	184.6
145.9	245.2	394.8	295.6
213.1	266.4	355.8	315.2
197.1	272.9	382.7	308.1
404.8	363.6	506.9	467.1
234	316.4	274.9	583.9
292.8	356.2	404.4	396.4
23.3	31.5	32.4	34.5
111	89	124.9	182.3
99.8	150.2	141.8	131.3
143.6	213.5	275.3	174.8
211.9	274.9	369.5	475
148	184.5	300.7	296.8
264.8	306.2	415.3	405.6
233.7	233.3	339.1	337.3
26.4	13.4	10.4	15
474.5	518.6	821.1	724.6

173.1	264.5	387.8	306.3
324.8	313.6	400.2	428.1
326.6	321.1	585	494.8
216.9	250.3	327.1	300.7
92	115.7	176.6	139
127.9	164	148.7	130.2
145.9	197.3	224.6	190.7
292.4	263.5	394.1	384.1
457.2	618.4	1286.8	1282
58	54.5	42.8	30.1
50	61.6	59.5	72.6
106.1	129	180.5	142.2
297.5	355.6	345.9	350.7
262	108.4	69.5	109.3
594	687.7	1064.4	967.6
226.3	406.1	458.7	398.6
345.6	352.5	500.1	547.3
465.5	414.1	743.8	314.5
69	83.3	128.8	103.1
259.5	313.7	416.6	379.7
29.6	29.2	42.7	30
61.5	72.2	123.5	106.5
193.9	313.6	404.2	373.6
121.4	132.5	312.7	145.3
133.8	231.6	234.3	287.3
356.6	307.2	325.3	500.5
135.1	129.7	177.1	195.3
59.2	37.2	145.7	54.2
149.1	171.3	231.6	242.7
156.4	105.9	301.9	416.3
117.5	180.1	184.9	231.3
177	289.9	337.1	311.1

252.6	259.9	314	343.2
175.5	187.6	272	254.5
126	120	155.7	106.4
280.9	143.2	194.6	146.2
140.2	95.6	132.9	161.6
143.2	146	241.5	207.8
47.9	56.5	67.6	84.8
112.1	259.3	180.8	141
117	135.1	175.8	193.2
179.5	248.7	319.1	322.5
275.5	209.9	298.3	284
181.5	243.6	367.1	384.7
267.7	292.1	479.6	440.5
134.4	138.4	206	193.4
323.2	206	406.4	368.2
241.6	312.6	407.1	381.5
194.4	227.4	363.8	292.1
179	201.4	393	309.8
68.2	561.5	69.7	100.1
650.6	361.5	455.6	251
224.3	67.7	232.1	139.8
364.7	337.3	556.8	583.6
72.6	107.8	141.1	115
200.6	224.9	338.6	225.5
173.1	196.9	283	205.2
132.1	149.3	217	192.1
266.8	275.4	481.6	363.1
374.5	346.9	606.8	669.6
415.3	581.6	856.5	970.3
255.5	322.4	401.6	415.9
189.1	295.7	277.1	260.6
195.5	261.7	225	298.3

218.7	272.2	351.2	356.1
187.4	241.1	355.6	309.5
275	426.6	558.8	594.8
147.9	181.2	269.1	285
213.8	415.2	603.5	466.5
62.7	53.7	58	41.1
29.8	41.9	51.6	70.2
309	315.8	476.6	476.5
88.7	88.1	98.1	87
243.7	326.1	534.5	431.1
604.7	808.3	774.7	1004.7
314.9	352.8	582.7	457.3
190.5	220.3	252.4	255.9
63.1	103	96.1	166.3
189.3	312	272.2	237.7
257.2	320.7	441.5	388.6
192.7	81.7	118.9	84.9
409.1	280.9	385.8	360
314	194.6	140.5	260.7
567	697.5	872.4	1926.3
87.2	116.7	193.9	214.4
121.6	164	196	196.3
110.7	115.1	89	87.7
92.5	95.9	170.7	185.5
37.6	18.2	27.5	19.9
53.8	44.4	75.4	70.7
700.3	258.1	327.4	330.7
70.5	86.2	119.5	103.3
117.4	154.2	243.8	191.6
158.8	207	290.9	249.8
38.7	36.7	34.7	41.3
67.6	65	77.5	129.8

172.1	115.3	123.3	84.4
214.3	141.4	157.4	144.8
61.4	141.1	185.5	218.8
24.1	57.3	59.6	92.6
155.7	214.1	289	336.6
120.5	145.3	235.7	229.4
77.4	79.3	87.4	78.5
191.6	238.4	281.2	361.9
147	138.7	195.4	220.8
58.9	80.6	106.4	96.5
100.6	165.6	231.4	167.4
100	123	210.9	205
95.9	114.5	133.4	123.8
174.1	202.7	282.4	200.4
108.1	173.7	112.8	121.5
189.3	400.1	338	268.2
323.1	411.7	679.2	712.6
45.4	32.1	147.3	148.1
52.9	56.8	80.8	68.8
175.1	181.2	257	219.2
275.5	253.9	407.7	404.6
149.4	177.1	191.2	281.5
99.4	140.1	159.9	159.1
693.2	605.9	860.6	811.5
188	156.3	273.4	87.2
90.6	113.8	164.7	181.4
65.8	91.4	153.1	524.3
417.4	275.5	312.9	274.8
161.4	190.5	230.2	268.5
486.7		37.2	39
107.8	122.1	123.1	151.3
62.2	96.4	121.9	93.5
108.7	92.2	158.2	145.6

31.2	183.2	54.1	39.4
31.1	38.2	48.3	52
151.2	171.7	227.2	186.1
34.6	32.9	47	36.5
206.2	175.8	271.4	157.8
125.5	130.7	194.6	184.4
247.7	274.9	341.8	294.4
177.5	181.3	237.4	164.7
144.7	143.6	189.9	176.3
23.2	29.2	38.3	30.1
355.5	411.8	509.9	618.3
147.5	156.3	533.5	338.8
126.4	220.3	233.9	243.8
82.7	73.2	90.9	118.2
176.5	183	279.2	244.6
436.2	258.4	274.6	393.7
244.4	313.3	358	358.4
149.7	109.7	225.3	98.4
84.7	119.9	117.9	89.7
121.8	118.5	130.1	146.4
101.9	75.4	147.4	115.4
131.2	137.2	87.6	86.2
197	189.2	199.5	266.6
247.5	310.5	385.3	494.6
173.5	185.1	198.5	232.2
38.7	83.7	88.6	90.8
198	172.5	172.6	72.1
143.1	20.7	46	32.8
114.2	144.3	198.4	208.7
36.5	42.8	52.9	46.9
59	80	85.9	111.4
400.7	440.9	566.4	471.8
262.5	335.1	570.1	512

172.9	192.3	272.6	232.8
45.5	61.2	61.6	92.7
99.6	174.1	214.2	222
32	32.9	34.4	38.8
206.8	252.3	273.2	297.4
412.3	353.6	550.1	495.1
40	72	48.4	86.3
56.9	76.2	71.5	54.6
103.9	151.3	145.9	220.4
226	201.7	298.2	363.4
92.2	96.8	130.1	142.6
136.5	168.2	287.5	258.3
41.4	49.8	57.7	56.6
132.8	146.5	160.3	171.7
239.1	325.8	470.1	457.5
36.9	38.2	53.8	56.8
202.1	270.1	274.6	341.7
135.7	126.4	107.9	105.4
2946.5	5533.8	3730.3	8724.6
60.5	50.5	108.2	61
175.7	267.7	332.5	338.1
69.5	78.8	84.8	112.1
59.6	87.3	83.3	88.6
22.4	23.1	23.2	16.6
136.5	43.8	147.9	172
43.3	46.6	50.9	57.4
50.7	48.9	71	77.5
21.4	22	19.7	31
172.4	219.3	271.8	271.2
207.5	335.2	358.9	476.9
31	27.9	34	46.1
56.6	83.3	118.2	170.8

290.7	293.9	523.2	456
54.3	64.5	89.5	104.7
99.7	109.1	133.8	129
122.2	164	276	214.5
538.2	640.5	997.2	849.8
138.6	110.9	141.3	126.9
13.1	15.9	18	19.7
177.9	178.5	251.6	213.7
331.9	255.9	390.7	323.3
87.8	65.7	89.3	60.4
313.3	507.9	463.5	491.9
318.9	348.5	610.9	474
93.9	32.4	68.1	119.7
164.8	144.5	184.3	175.7
62.3	49	61.7	68.9
111.6	257.8	175.4	269.6
109.5	82.6	92.1	85.2
58.4	134.3	163.5	106.4
63.5	76.8	119.2	114.6
159.5	186.2	234.8	225.7
85.5	74	162.4	132.4
86.6	125.1	139.8	207.3
81	86.7	125.4	106.2
176	201.6	136.5	137.5
120	127.9	190.3	126.7
98.5	85.3	187.2	202
66.3	68.7	95.9	102.1
249.8	454.6	459.7	554.8
174.6	132.9	125.8	135.1
370.3	138.1	104.5	159.1
23.6	34.3	50.5	41.8
97.7	83.7	181.6	153
59.9	103.2	79.3	73.4

132.8	284.6	376.9	217.4
51.1	44.3	65.8	48.3
170.9	206.1	268.8	209.4
109.7	199.1	213.8	210.2
72.9	84.9	113.6	90
85.2	46.4	64.4	59.6
201.1	742.7	303.7	314.6
34.3	23.1	39.2	64.7
215.1	134.8	226.8	161.4
192.7	165.2	182.9	188.5
96.1	101.4	119.1	137.2
24.3	29.6	30.1	46.7
42.5	70.3	97.8	78.7
169.8	124.5	256.8	148.6
521	109.2	110.4	56.2
76.9	77.9	98.9	115.5
94.3	123.5	165	148.9
378.6	382	482.8	480.2
136.3	224.2	241.7	348.9
192.3	280.2	425.6	354
211.6	465.8	465.1	750.7
46.8	53.5	78.7	76.2
110.2	152.3	222.2	227
53.1	46.9	79.6	87
92.5	181.9	246	265.6
107.2	93.9	185.8	116.9
248.1	278.9	416.7	406.1
158	138.6	201.2	147.1
311	326.2	380.8	349.2
101.7	115.1	198.9	151.4
68.2	49.7	66.1	75.8
62.8	70.3	103.5	105.6
68.4	108.7	161.3	131

149.7	129.4	178	169.4
62.3	151.7	215	198.6
169.1	172.2	298.3	225.3
165.9	356.7	242.4	212.5

147.1	146.9	208.6	213.8
149.9	159.5	178.2	197.7
72.1	136.6	269.1	221
330.6	415.9	626.7	572.5
203.2	242.9	327.9	280
264.6	269	770.8	223.5
43.1	28.9	38.2	27.3
318	408.6	508.1	578.5
147.6	142.6	276.7	183.8
71.5	100.4	127.8	78.1
238.2	269	452.7	401.4
117.9	157.7	272.1	202.6
208.8	56.3	106	42.9
68.4	83	115.3	110.8
16.6	26.9	35.4	33.6
149.6	166.5	239.1	201.1
60.3	76.6	91.4	100
61	66.5	122.2	109.8
43.9	70	69.4	54.2
97.8	128.9	172.3	144.8
84.2	162.9	200.2	154.8
97.3	204.2	111.2	311.8
47.7	41.5	95.7	38.4
97.7	117.6	188.6	165.7
206.2	250.1	320.1	255.3
44.9	53.2	72.1	71.2
441.3	51.1	94.3	54.1

116	168.1	219.5	232.1
451.2	277.6	623.3	564.5
58.6	72.2	73.7	78.8
219.3	238.4	368.6	312.1
162	193.5	278.3	148.4
38.3	37	45.9	31.4
189.4	213	358.8	273.5
65.4	139.5	149.9	175.5
92.2	86.7	138.1	162.8
65.1	56.3	95	77.2
157.9	162	220.4	231.1
276.2	179.1	280.3	216.1
35.5	51.6	68.4	91
233.5	310.1	661.6	317
394.2	293.2	757.9	610.5
39.9	58.5	67.6	62.1
29.9	38.8	44.9	56.4
105.6	158.8	182.3	119.2
42.5	28.1	42.2	35.2
55.3	66.3	123.6	96.7
27.9	36.1	29.8	49.8
62.2	93.3	83.5	250
43.4	45.7	56.7	72.5
146.2	100.8	258.7	122.5
202	143.9	204.3	223.4
85.5	89.8	145.3	142.1
112.2	224.7	197.3	272
110	141.8	155.4	118.5
82.9	123.9	163.2	136.1
712.8	482.3	744.6	592.4
61.7	60.5	58.5	61.3
19.7	44.2	38.1	38.6

164.6	227.7	335.7	272.4
167.2	84.1	121.9	176.7
195.9	255.6	316.6	234.8
361.2	369.2	449.5	599.8
58	91	116.6	83.5
140.7	190.4	289.6	242.7
132.6	219.7	294.1	418.7
328	610.7	406	230.5
48.7	85.2	77.2	140.8
190.2	388.4	249.4	530.6
61.1	55.7	62.1	55.7
85.1	127.2	223.1	183.1
304.3	487.8	472.3	1322.5
58	60.7	96.5	127.3
68	61.4	62.3	26.1
204.6	220.9	237.2	290.9
104.9	231	96.3	182.9
272.2	211.6	321.9	323.2
214.2	309	312.3	306.7
254.3	518.9	715.1	611.8
320.4	355.4	621.8	649.8
46.5	65.1	91.3	102.5
22.1	30.9	38	25.4
94	218	154	300
137.2	121.8	153.8	136.8
75.3	73	103.1	78.9
86	99.9	132.9	94.6
76.2	57.3	98	88.7
38.9	56.4	73.3	61.2
44.2	43.7	52.6	45.4
117.4	85.9	118.1	131.6
219.9	113	128.9	127
209.5	323.9	303.8	394.2

45.5	36	52.8	34
70.7	77.2	125.5	103.5
360	679.3	1061.9	1032
101.6	97.5	151.6	136.1
96.3	118.1	168.3	169.2
14	15.2	15.8	22.5
160.8	293.6	208.2	222.4
740	514.3	163	109.1
104	114.4	218.7	171.7
179.8	223.5	339.4	547.1
112.7	89.1	162	126.5
243	189	141.8	117.6
174.2	209.8	285.3	302.9
307	431	670.6	756.4
116.8	128	168	145.3
88.2	71	113.8	89.7
247.1	378.3	350.5	426.3
190.7	166.5	260.1	206
24	30.3	37.4	45
87.6	93.3	98.1	135.3
246	228.4	830.4	391.6
111.1	157.1	251.4	246.9
154	156.8	210.2	320.1
43.1	50.3	60.2	57.5
224.9	173.9	236.3	301.2
70.7	100.5	103.2	92.3
145.3	150.2	208.6	218.3
90.1	119.4	106.4	151.3
70.6	200.7	413.5	915.5
39.3	47.5	43.9	61
305	311	434.6	413.8
440.5	122.4	105.9	173.2

163.4	223.7	315.8	304.8
169	241.4	312.9	357.3
54.3	47.8	63.4	61.1
144.7	145.6	185	222
480.3	423.4	566.4	487
41.9	61.9	66.5	69.6
120.4	188.9	329.8	257
41.1	53.8	84.8	68.2
97.2	142.9	168.6	176.2
222.2	236.8	308	290.6
23.7	38.5	38.5	44.9
255.6	406.5	662.8	768.4
33.7	39.9	63.6	52.5
279.5	274.5	770.9	471.4
190.7	136.4	121.8	143.3
69.6	74.4	117.2	128.7
188.1	254.8	265	372.7
77.6	102.5	129.3	121.7
58.8	69.1	72.2	71.5
153.6	202.9	187.7	185.6
65.1	102.4	85.6	124.2
66.8	136.1	185.4	170.4
133	127.8	208.9	182.7
219.2	194	396.6	282.2
98.8	120.8	184.7	192.7
274.3	497.1	602.5	214.7
101	154.7	149.2	241.3
134.4	131.4	168	183.5
103	142.2	204.6	199.9
110.7	175.7	246.9	234.4
119.6	133.1	204.2	145.1

447.9	696.1	875.4	686.1
17.1	27	17.4	30.2
202.2	203.7	351.1	377.2
278.6	252.1	191.9	638.7
158.2	347.5	290	638.1
328.7	420.9	462.4	344.7
103.7	148.8	198.7	151.7
45.2	54.3	66.8	60.5
302.6	243.3	260.7	216.8
83.7	112	112.8	161.5
189.5	88.1	74.1	115.3
130.8	170.1	367.8	307.9
180.1	184.4	259.7	227.4
21.5	39.2	25.8	42.8
85.8	71.5	93.9	174.4
51.6	43.9	66.3	99.2
84	57.8	81.9	81.9
222.4	312.8	330.4	473.2
61	80.4	92.2	110.7
171.6	234.5	252.8	193.5
104.5	95.8	78.2	89.8
35.6	42.4	54.4	41.2
65.8	68.6	93.9	125.8
215.4	123	125.5	38.1
316.3	45.5	59.3	73.3
23.8	36.9	33.4	49.4
78.6	93.7	110.8	107
67.6	57.9	72.8	73.6
216.3	275	435.7	494.1
46.6	72.4	52	203.8
243.3	299.7	387	434.8
1098.4	1649.7	917.2	819.1
120.1	180	199.4	211.6

97.9	110.2	152.4	144.8
417.1	291	364.5	423.5
86	89.1	136.7	111.1
211.2	223.4	167.9	207.5
66.5	59.9	81.2	88.4
24.4	33	38.6	41.5
43.8	49.8	61	62.9
74.2	97.4	152.6	125.2
215.6	175.2	322	235
135.3	158.8	195.5	146.7
289.5	332.6	417.1	398.5
59.1	81.7	84.2	94.9
248.3	262.2	391.4	326.1
138.9	153.5	239.9	185
37.8	62.3	88.1	73.2
123.8	96	523.9	85.7
69.4	75.8	123.2	97.4
24.9	32	53.7	47.7
266	232.6	287.2	490.6
75.7	37.7	207.6	41.3
61	58	67.2	51.3
43.2	50.4	78.3	50.3
102.4	145.6	176.1	183.7
106.1	102	149.9	115.1
305.1	284.5	437.2	405.2
216.7	240.3	364.5	334.1
86.3	115.1	144.3	112.9
27.9	43	54.8	46.9
294.2	848.9	1306.5	1552
72	52.4	51.1	57.6
161.1	172.7	159	147

53.9	64.9	74.9	84
151.8	181.1	260.6	235
227.7	205.1	210	238.7
76.5	68.7	57.8	16.8
49.9	54.1	60.1	81.8
175.6	231.1	452.5	269
76.7	58.7	116.5	105.6
69.4	57.9	72.8	54.7
55.5	71.4	99.2	85.8
225.9	209.3	235.7	341.1
51	38.7	56.8	64.2
24.4	45.3	40.4	59.1
41.8	44.9	51.1	67.1
30	51	61.3	59.4
125.5	152.3	204.9	259.5
645	606	436.7	309.8
40.3	50.8	50	49.2
42.7	28	44.5	59.4
185.6	188.6	290.6	258.3
86.2	128.7	163.5	153
167.4	196.2	311.3	269.8
70.2	58.8	92.7	83.5
31.9	40.5	51.5	76.1
487.6	1341.5	687.2	671.9
307	94	136	53.1
32.5	36.7	39.3	33.2
123.2	187	315.3	257.7
136.4	129.4	213.9	211.5
96.4	124.1	122	140.9
87.7	107.3	133.9	108.4
167.7	212.8	253.8	273.4

149.9	173.9	286.9	239
69.5	84.3	149.9	104.2
56.8	44.9	87.5	99.9
49.9	41.3	68.1	58.5
82.7	86.9	122.7	123.7
53.5	43.6	157.2	205.8
144.3	181.6	241.9	225.1
232.1	6.7		6.4
93.6	121.4	147.4	150.1
281.2	499.6	709.9	848.5
1408.1	2106.7	1376.1	4239.6
222.7	261.1	356.4	329.5
44	42.6	49.3	75.3
191.4	241.2	277	201.4
28.2	46	106.7	87.3
46.4	52.2	80.2	64
134.9	247.6	410.1	534.8
65.6	69.6	68.9	63.8
136.2	133.6	197.2	193.5
72.3	88.3	121.7	106.5
92.1	88.2	112.7	91.4
147.2	84.8	129.1	128.8
24.9	34.3	44.4	42.3
35	44.7	46.2	61
157.2	208	251.7	300.8
153.9	195.4	271.9	230.9
9.8	12.4	16.2	46.9
57.7	50.2	78.3	84
32.9	46	64.2	68.1
84.9	95.5	102.5	79.5
177.2	220.9	417.8	212.2
92.5	58.4	89.1	176.9

39.4	50.7	56.2	56.6
177	232.2	356.2	339.3
386.1	481	783.9	597.1
43.5	55.1	71.4	69.9
56.6	65.1	69.5	99.9
80.1	84.2	87.3	99.7
29.1	54.2	34.2	70.5
63.6	76.9	172.1	113.9
82	145.7	187.4	128.3
120.8	157.1	225.7	217.8
96.9	95.7	153	140.8
95.8	108.7	136.1	103.4
47.8	64	72.2	83.7
113.4	126.6	144.7	161.7
41.6	60.2	74.8	91.6
61.1	62.4	121.5	143
99	99.8	159	158.4
59.4	43.7	92.1	68.3
67	65.9	100.2	91.9
112.4	68.3	103.9	92.9
155.2	107.4	165	150
70.5	89.1	93.2	155.3
41.3	48.8	47.2	57.4
108.4	101.3	126.7	102.5
105.3	79.8	85.3	99
97.9	67.9	128.2	186
112.8	115.3	154.8	157.1
188.7	235.1	300.2	357.5
108.4	64.5	107.5	133.6
49.8	43	45.6	63.3
620.6	462.5	625.7	588.6
127.6	139	168.9	192.3
50.5	73.2	75.8	74.4

104.1	140.2	159	182.7
352.6	350.1	477.8	461.9
73.8	90.7	122.6	130.6
161	171.6	366.7	306.7
95.3	80	102.8	104.5
156.3	365.3	96.7	72.2
205.3	181.3	229	221.2
90.4	122.5	192.4	174.8
117.5	130	181.6	198.1
89.3	83.4	176.2	102.7
64.8	90.9	109.2	76.3
65	67.3	87.4	86.4
84.7	64.2	93.6	93.1
79.4	43.4	53.5	69.2
272	307.1	406.1	386.5
361.8	121.3	159.4	69.8
53.5	42.3	64.8	65.8
27.5	33	36.9	42
88.4	97	129.2	190.2
103.5	226.7	141.6	139.1
43.7	58	65.3	74.3
257.9	80.3	122.2	108.1
69.9	94.1	104.6	117.9
90.8	120.3	175.6	167.6
294.7	469.7	679.5	511.4
86	91.8	116.2	94.1
59.7	71.7	82.4	95.3
90.4	78.4	70.9	109.6
76.9	60.7	102.8	86.5
43.1	69.3	67.1	94.5
69.5	93.3	107.9	131.9
64.3	40.2	103.4	82.2

111.8	37	61.6	81.2
127.4	154.9	270.1	216.5
101.1	131.4	200.4	202.9
86.2	80.5	166.1	96.3
444.4	500.6	642.5	737.8
48.4	56.1	80.5	95.9
101.9	101.9	214.5	153.6
79	114.2	178.5	250.6
40.1	61.5	78.5	73.4
96.5	95.5	78.4	152.1
34	129.2	46.1	66.8
55.1	107.3	117.3	151.3
85.4	93.6	116.2	114.1
101.8	142.3	151.6	216.8
103	125.8	195.8	220.4
23.3	26.1	31.6	38.8
252.7	237.5	313	289
82.6	96.4	122	137.5
370.7	366.9	504.8	464.4
68.5	33.9	91.1	97.5
68.5	58	88.4	68.8
98.7	110.6	159.7	109.9
30.5	47.8	43	42.5
107.6	143.9	79.9	125.5
77.1	100.9	132.3	100.4
221.1	266.9	378.7	319.5
123.6	152.1	236.3	173.8
47.7	72.1	143.9	90.7
81.9	73.8	128.6	153
224.3	296.6	410	420.2
189	218.4	317.7	424.4
32.2	46.6	113.2	44.5

18.3	19.1	47.1	24.9
141	116.7	216.4	88
189.2	178	221.7	216.1
76.7	96.2	117.9	146.9
139.3	172.6	248.6	202.4
76.4	97.7	197	135.5
104.9	119.1	185.6	163.5
184.6	172.5	179.2	209.3
63	64.3	94.4	72
73.1	65.2	109.5	91.2
123.9	181	147.4	123
128.8	168	264.8	156.8
166	186.7	264.2	258.5
80.4	117.6	140.1	141.8
156.4	178	95.8	115.1
41.3	34.4	46.7	56.4
161	166.8	172.9	178.9
151.7	61.1	67.3	39.4
165.6	214.5	300.2	341.4
78.2	89.8	158.6	135
132.3	201.3	289.4	263.5
73.2	97.7	109.7	125.2
130	400.7	290.6	248
330.8	194.9	235.8	177.7
35.7	27.7	35.2	49.7
436.8	515.6	680.2	631.3
327	286.4	387.5	427.7
79.3	621.7	102.1	82.5
97.6	105.8	144	153.5
343	214.5	549.2	409.1
90.7	89.9	96.4	93.2

21.3	37	34	49.6
42.5	54.5	62.6	137.3
59.8	70.9	151.5	122.7
72.4	75.7	102.6	89.1
76.1	71.5	99.6	86.3
31.5	31.8	43.8	35.3
86.3	90.3	129.5	184.1
38.8	45.9	52.9	48.3
105.6	115.8	154.1	164.8
59.5	87	89.8	74.5
223.4	248.7	226.9	300.4
95.2	185	140.7	210.6
98.9	98.2	122.3	119.6
54.3	72.1	76.9	94
175.2	234	289.8	234
68.2	115.2	96	86.6
131.2	153.1	190.9	214
126.7	111.5	136.4	122.9
449.8	638.4	733.2	849
105.2	122.7	166.5	162.2
44.4	50.5	60.9	58.4
120.5	103.7	64.1	80.5
72.3	90	116.5	92.3
92.1	128.4	165.9	158.2
59.3	59.7	95.8	90.7
263.3	323.5	443	477.2
183.5	207.2	260.4	261.1
470.6	487.6	472.1	662.6
26.1	38.4	39.5	44.4
77.6	92.7	95.9	103.5
73.2	103.6	109.3	101
62.4	94.4	80.5	122.7

100.3	108.6	179.5	174.5
71.3	102.8	139.9	116.9
224.6	241.3	299.7	383.3
227.9	269.5	387.3	296.7
167.5	71.7	96.3	107.5
69.5	95.7	112.2	150.4
85.9	113.4	135	127.3
65.4	80.3	100.8	89.4
170.8	178.3	233	240.6
52.7	82.7	94.8	136.8
195.8	221.2	380.6	348.2
57.5	79.6	97.7	114.1
71.4	76.4	106.5	105.2
148.4	161.1	277.2	210.7
129.6	237	196.7	254.4
42.9	41.9	56.4	37.1
33.4	39.4	66.8	66.4
47.3	32.9	67.9	62.7
66.6	70	91	81.8
147.1	161.7	189.1	180.3
31.4	36.7	32.6	44.2
1297.4			9.1
66.6	79.8	70.1	146.7
86	106	115.5	134.7
173	128.4	219	194.8
269	332.9	555.3	672
55.8	58	89.2	67.5
44.5	33.8	122.2	79.8

167.9	187.4	324.1	261.9
50.6	54.5	77	86.1
36.7	48	46	69.7
448.2	236.1	362.2	299.8
158	244.8	273.8	260.9
197.2	983.9	491.3	947.6
102.5	100.3	124.5	137.7
139.3	225.2	317.4	281
114.2	140.9	122.6	169.1
18	22.3	22.1	29.6
177.7	73.6	57	53.5
58	60.4	88.6	74.7
119.2	142.4	139	165
36.7	46.4	54.7	59.4
120.9	131.9	194.2	221.9
88.9	79.6	104.7	94.4
14.4	14.9	19.3	16.1
234	304	424.6	381.9
180.4	266.7	383.6	346.4
153.3	227.7	381.7	292.6
23.6	22.3	35.1	20.2
34.1	42.9	50	43.3
785.9		105	153.2
55.8	53.9	80.3	72.4
333.4	323.8	416.5	481.7
25	33.2	41	47.3
82.5	130.1	81.4	109.6
74.9	97.6	112	136.4
144.2	178.5	144.8	198.6
37.9	33.6	46.1	42.2
56.3	64.9	82.9	91.3

75	92.4	115.6	101.3
145.1	382.5	550.7	556.6
76.8	94.3	136.4	124.9
97	146	126.9	150.4
76.4	84.3	146.7	68
44.9	67.5	119.4	77.3
102.6	168.2	150.5	211.1
88.9	123.2	166.1	159.3
55.9	56.5	74.9	90
98.5	125.7	146.7	136.6
145.6	170.4	297.5	239
15.5	22.5	41.8	34.8
318.3	329.9	351.2	211.5
123.6	134.3	174.5	161.9
3509.4	6592.8	4476.4	10419.2
34.9	50.3	51.7	53.5
60.8	51	71.3	96.2
26.3	34	47.3	43.7
348	128.2	45.8	96.6
196.1	212.4	364.2	323.9
36.3	46	39	45
35.5	56.7	142.5	77.3
290.1	304.9	532.1	269.5
199.8	272.1	326.7	712.5
81.9	135.5	104.8	235.5
78.4	101.7	98.9	243
77.5	94.5	103.1	121.6
87.1	132.6	164	136.1
65.5	110.8	122.1	151.2
231.9	185.7	125	130.1

112.9	86.5	156.8	94.1
62.4	87.6	98.8	90.6
46.6	83.2	96.6	110.2
133.8	157.1	226.8	219.7
59.8	46.2	86.8	70.4
248.4	103.5	183.3	43.5
116.4	223.4	131.5	132.9
121.3	78.3	87	66
99	87	123.2	114.7
471.4	213.2	152	144.9
65.3	54.2	66.2	53.4
77.6	91.3	160.7	119.6
183.8	191.9	231.2	298.3
67.9	107.2	113.4	124.3
86.3	92.6	133.5	195.4
47.7	65.1	78	75.2
33.5	40.9	37.5	42.4
236.2	162.7	233.8	261.5
316.9	331.2	430.2	518.9
80.3	78.9	100.9	96.7
93.7	110.7	149.9	143.3
141.2	246.1	303.5	405.6
318.7	324.6	453.9	452.4
143.1	102.2	197.3	122.4
217.1	337.2	542.6	496.4
33.9	214.6	158.1	318.6
52.7	63.3	75.5	66.5
65.1	96.4	135.3	128.2
41.4	51.3	59.7	81.3
67.5	86.6	98.4	118.5
146.2	142.8	170.4	131.2

47.3	73.3	98.3	99
185.1	261.7	258.1	370.5
31.9	57.5	65.4	60.2
83.5	76.8	111.2	133.8
88	35.2	37.4	51.2
117.7	135.3	225.4	254.1
88.9	106	116.1	63.6
92.1	94.4	147.4	152.5
256.9	432	337.6	728.3
15	18.1	31	21.1
155.5	63.5	80.2	58.3
51.4	27	36.4	45.7
30.5	32.4	51.2	40.1
92.5	123.2	132.5	99.8
62.1	80.7	79.6	150.4
68.5	85.6	106.2	108.5
62.7	143.4	157	296.2
315.1	296.3	402.1	276.9
37.3	39.5	104	48.6
54.7	96.9	100.5	137.7
112.4	70.1	92.7	139.3
111.4	116	252.2	188.5
118.3	246.2	161.1	322.8
38.4	44.6	55	61.1
43.6	70.7	93.2	86.1
180	131.3	254	228.6
67.6	73.8	82.1	72.2
30.1	35.6	36.4	38.9
160.1	173.3	204.9	151.3

28.7	35	43.5	34.3
223.3	291.5	341.9	353.9
97	131.1	194.8	171.7
102.7	102.4	213.9	186.4
121.5	92.4	136	168.3
99.1	81.3	162.1	155.6
89.4	122.6	170.6	123.4
61.7	53.7	126.5	78.5
98.9	97.5	127.7	104
171.4	59.3	146.7	91.1
196.7	79.4	99.5	138.1
69.4	93.4	164.5	136.5
135	177.9	247.6	216.9
31.5	16.5	47.5	52.9
306.8	230.9	132.5	240.5
171.7	131.3	123	134.2
182.9	260.9	357.9	310.8
19.6	18.9	43.5	48.4
12.2	34.4	41	79.2
312	319.2	434.9	554.2
54.9	44.4	59.3	61.5
51.9	64.5	90.9	85.9
164.4	122.8	247.5	254.9
111.8	161.4	155.2	177.5
288.8	402.1	443.3	445.6
66.4	55.8	68.7	106.9
380.6	389.8	457.1	342.1
95.1	127.1	136.4	67.8
31.7	35.3	69.2	49.6
43.3	53.5	50.7	53
138.6	153.1	136.4	157.5
553.3	464.3	217.5	228.1
86.1	87.2	138.7	125.4

21.4	24.3	28.7	32.9
1489.7	182.5	190.2	229.9
84.1	82.4	101.2	110.1
61.2	67.5	65.7	81.3
101.7	71.8	191.3	71.4
106.5	132.9	175.6	377.8
107.4	182.9	179.2	156.4
51.6	105.7	78.8	63.1
203.7	283.8	465.7	393.2
133.8	73.5	103.8	63.1
33.2	54.7	34.8	52.1
34.9	41.4	30.3	52.2
57.7	66.6	91.7	56.4
210.7	218	213.4	201.8
51.7	49.3	51.1	308.3
35.1	57.6	76.6	71.4
150	155.6	225.5	189.1
144.4	101.7	168.1	137.1
189	181.6	184.7	217.7
72.1	26.4	30.7	38
102.2	142.9	183.6	137.2
99.7	85.2	119.8	118
135.7	139	150.2	135.4
44.5	46.8	63.7	62
101.7	59.5	112.4	152.8
71	80.1	120.8	101.8
127.2	81.7	141.2	125.2
31.4	31.7	38.5	43.1
216.4	242.2	323	282.2
89.2	105.6	106.4	135.5

71.7	118.3	115.5	170.7
81.4	102	148.6	135.6
28.4	46.3	49.9	69.2
66.6	60.7	71	53
27.5	34.1	37.6	61.4
266.4	137.6	179.9	195.5
107.3	145.1	198.5	179.1
236.9	225.4	291.6	279.4
116.6	157.5	147	160.2
47.5	82	114.4	85.9
171.4	239.7	361.9	365.8
65.3	79.5	94.1	109.7
112.1	142.6	226.8	214.4
112.3	40.7	66.8	52.4
47.9	64.7	66.4	81.8
236.9	291.8	417.5	344.8
215.1	129.3	229.1	177.9
110.7	108.1	149.2	142.4
109	128	200	158.5
118.8	111.2	184.2	137.6
40.1	70.3	95.2	130.4
55.8	71.4	83.5	101.3
45	13.9	96.8	52.1
100.5	94.8	179	143.6
82.2	9.3	21	17.6
82	72.8	112.5	107.5
103.4	118.7	192.3	139.9
50.4	91.3	108.5	135.2
75	82.6	154	148.1
28.4	41.1	94.5	34.9
76.5	95.6	126.8	157.9
92.9	131	149	168.7

93.5	87.4	107.6	111.8
25.4	68.8	70.8	45.4
128.9	199.3	299.2	237.8
124	152.3	238.5	214.8
127.5	119.9	227.8	170.9
66.7	76.9	65.2	80.5
80.1	188.5	227.8	193.8
98.6	114.4	175	183.2
133.7	123.4	165.8	102.4
120.8	151.8	221.8	213.9
52.9	56	77.4	61.5
266.9	199.2	300	245.2
169.5	189.7	200.9	165.6
28.7	30.6	29.3	27.3
188	90.5	128.1	144.6
54.3	68.2	140.5	102.8
99.5	135.2	220.8	165.5
227	218.7	262.8	205.7
34.4	53.6	85.3	83.6
125	173.8	250.4	197.2
102.7	97.4	147.2	135
20.1	25.8	51	38.2
74.1	95.2	113.8	105.1
92.3	98.2	124.6	132.3
34.8	45.8	77	65.9
111.8	74.1	115.8	111.2
138.8	156.3	239.8	188.9
29.2	60.7	79.2	67.8
134.9	223.5	303.3	529.5
41.4	43.6	52.8	46.9

71.6	80.2	110.5	90.1
126.8	137.8	162.3	164.7
138.4	156.5	203.2	225.3
66.4	87.1	128.7	104.9
18.2	18.1	31.8	26.9
107.9	112.7	198.7	167
42.2	50	55.5	53.9
13.5	16.1	21.5	14.3
66	113.1	146.5	132.3
198.5	237.7	351.9	386.3
28.7	42.7	47.1	40.4
327.7	212.6	172.9	95.8
38.8	41.5	75.1	51.8
67.8	60.1	68.9	73.6
50.3	41.6	64.3	99
490	930.2	1132.8	1470.9
19.2	23.4	22.2	29.4
165.2	172.5	296.5	215
69.2	31.5	27.9	35
100.9	532	54.4	52.3
18.7	26	42	27.2
28.7	25.8	28	38.7
84.4	91.5	113	127.2
185.1	223.6	390.4	237.7
94.6	104.1	106.7	112.1
469.4	526.8	861.9	809.2
38.9	30.5	49.3	39
170.2	130.4	129.6	142.5
139.1	175.2	214.3	164.3
29.2	48.2	48.7	53.7
163.6	213.9	289.2	284.4

32.9	38.1	55.6	62.5
93.9	89	170.3	143.6
7.7	7.9	7.2	10.3
237	230.2	313.8	294.6
31.2	62.2	45.7	65.6
50.5	41.6	52.6	28.9
185	155.6	218.2	220.6
93.5	116.2	125.9	174.6
72.5	79.3	130.8	113.7
87	96.1	135.3	150.5
202.3	221.7	290.4	194.6
85.1	76.5	168.5	147.5
67.3	75	109.5	67.7
78.8	72.6	80.3	69.8
38.6	67.2	55.2	60.8
62.3	61.2	70	66.7
44.5	51.8	59.8	55.6
95	75.9	100.9	104.5
176	109.8	136.6	100.5
108.5	275.6	158.2	197.8
181.8	206.8	273.9	293.1
21.3	45.3	29.9	59
172.5	255.1	361	333.1
22.1	66.5	105.3	100
58.8	67.9	83.2	68.3
167.2	233.4	287.1	264.2
208.8	236.1	388.7	340.6
270.5	263.3	278.1	339
81	89.4	163.8	116.6
278.2	291.3	317.7	277.7

19.4	22.5	36.6	27.1
87.8	59.2	90.7	70.5
41.7	75.4	72.9	75.1
26.7	37.4	49	41.4
81.3	105.4	135.5	99.8
31.5	40.1	33.9	40.9
114.2	105.7	112.7	155.9
151	185	210	200.9
45.9	78	68.5	126.4
57.7	94.7	100	134.5
30	39.1	69.1	59.3
90	68.6	114.4	75.6
115.4	116.3	178.4	97.2
60.8	88.2	88.7	188
192.8	74.6	136.2	80.7
56.7	56.9	94.8	79.3
46	75.5	59.7	97.6
49.1	38.9	87.3	29.6
41.9	43.3	49.8	64.1
40.5	57.4	68.6	60
57.1	53.5	62.7	59.1
57	78.8	154.5	131.1
111.2	121.6	184.1	168.3
41.9	45.9	45.8	38.8
131.9	140.9	164.5	123.7
132.4	158.7	186.7	263.4
126.7	196	293.6	247.3
120	119.8	196.3	344.3
352	194.6	216.6	212.9
338.3	395.8	539.1	491.7

66.1	55.7	71.2	101.2
34.8	44.9	53.6	46.4
46.7	59.6	51.1	87.7
30.6	27.1	51.5	40.4
239.6	328.8	536.5	490
92.5	107.6	110.1	166.3
56.5	302.4	40.8	58.2
91.2	155.5	212	213.3
79.4	130.4	212.3	147.6
137.3	212.2	158.2	169
36.5	48	60.2	73
92.2	92.9	116.5	155.9
213.3	246.5	613.4	416.4
133.3	128.6	165	141.2
58.6	52.2	65.5	98.9
56.9	96.6	79	85.1
264.3	430.9	619.5	501.8
32.7	50.2	58.7	56
30.2	30.2	24.9	35.2
95	125.5	149.1	178.3
43.6	92.1	68.5	60
27.2	50.5	66.8	60.9
76.2	103.5	125.7	102.7
67.9	99.3	128.8	78.8
88.8	93.3	109.2	98.2
85.5	87.9	106.6	82.9
56.4	106.6	101	93.4
262.6	257.6	350.5	284.9
82.9	140	163.3	140.5
122.1	131	159.3	155.1
81.4	124.2	132.2	115.4
196	179.7	235.6	202.3
120.1	199.5	321.1	340.5

19.1	18	50.8	39.7
231.3	221	245.7	317.6
202.5	224.6	194.7	156.5
30.2	44.9	39.2	80.6
57.8	62.7	108.9	74.8
106.3	157.7	198.8	167.4
77.2	95.4	128.2	140.9
55.9	74.8	77.7	124.6
58.8	71.3	72.1	69
94.2	82.1	117.2	73.7
87.2	114.3	143.4	117
167	193.3	261.8	182.6
15	22.6	20	32.4
215.4	275	470.3	364.2
11	10.9	16.9	17.8
17.6	28.7	17	35.3
331.8	620.4	1151.4	923.6
158.5	182.2	204.6	195.8
20.4	37.7	44.3	45.3
49.9	53.8	47.6	18.3
138.2	213.7	272.4	291.6
81.9	117.3	159.4	167.2
25.2	30.5	43.7	44.1
164.3	116.8	145.1	176.8
44	58.6	72.3	62.9
56.7	59.7	61	119.9
62	70.5	88.1	85.5
38.1	40	90.4	57.5

54.2	49.3	77.8	83.3
184.7	223.3	285.2	307.8
214.9	246.1	336.2	276.4
94.1	109.2	145.4	175.9
116.9	114.4	156.2	149.1
89.4	83.3	115.3	84.3
81.1	113.9	130.7	140.6
153.4	211.6	204.2	231.9
22.5	23.8	38.3	36.9
26.7	36.3	46.8	40.7
257.6	167.8	100.8	96.3
25	36.2	81.8	41.2
67.3	89.5	125.9	132.4
52.2	69.6	75.9	97.9
147.7	132.9	92.8	185.4
97.1	122.1	115.5	177.2
97.1	94.7	136	153.2
71.9	118.8	164.9	117.3
112.3	91.8	224.8	211.9
155.2	100	207	124.6
72.8	89	104.4	111.6
98.1	122.2	159	168.6
53	59.6	75.7	69.1
153.4	191.1	272.6	253.4
179.2	260.3	375.4	423.6
100.4	105.1	134.8	145.8
181.6	133.6	545	274.6
38.5	38	44.9	40.3
78.5	52.6	68	67.3
67.7	89	121	117.8
43.6	44.1	64.8	53.6
67.3	73.2	86.1	101.3

49.1	53.6	78.9	94.5
92	121	216.9	224.4
61	80	112.1	105.7
174.2	151.2	310.1	300.2
423.1	282.4	871.8	444.6
104.6	116	158.4	147.2
43.9	51.7	62.7	55.9
119.3	112.7	201.4	131.9
175.4	182.3	330.8	304.2
66.1	111.5	191.7	95.2
58.4	72.5	81.9	82
89.8	121.2	160.8	128.4
84.6	72.7	103.8	108.5
57.9	78.2	94.1	161.6
37.5	57.9	90	100.1
27.5	34.4	54	47.7
58.1	79.8	63.7	91.2
114.8	132.5	189.6	161.7
164.7	208.7	383.7	386.2
48.8	46.1	56.4	45.7
92.9	132.9	168.7	111
92	130.8	128.6	137.1
163.5	139.9	204.1	229.7
81	114.1	129.9	123.4
152.1	175.4	221	183
189.3	237.5	282.6	281.2
235.7	130.6	260.6	181.1
78.2	106.5	93.8	131.7
53.8	73.4	94.7	120.9

149.8	209.7	214.8	286.7
18.6	23.3	20.9	30.1
105.6	118.2	179.3	175.2
28.5	38.7	64.5	65.7
124	143.1	177.3	147.3
18.7	24.7	18.1	25.2
225.2	252.6	358.7	193.8
86.6	104.1	120.4	112.8
150.5	116.9	147.6	154.1
340.8	267.3	318.4	313.6
39.1	38.9	57.5	53.7
79.4	91.8	105.4	108.6
46.9	61.3	66.9	56
90.2	80.5	138.2	142.2
78	79.2	108.4	88.4
22.6	33.4	61.6	29.8
80.8	62	37.3	23.1
142	149.5	299.8	268.9
10.3	14.6	23.8	22.2

57.5	75.4	76.8	86.6
57.1	76.9	95.5	114.1
86	99.9	150	147.1
216.7	246.9	387.9	298
64.4	77.3	90.5	110.1
256.6	297.9	438.4	318.9
39.2	30.3	52.4	42.1
141	114.6	156.9	153
18.1	11.9	26.7	18
135.6	179.2	137	273.4
25.7	30.7	38.7	46.5
31.8	28.1	47.5	42.5

122.7	176.8	278.2	196.2
593.1	327.1	404.8	315.4
118	179.9	203.6	204.2
86.6	81	112.7	125.1
110.7	118.7	178.1	141.5
71.2	80.9	132.9	121.2
192.9	256.9	362.2	281.1
148	144	230.3	132.7
87.2	233.5	329	352.1
71.5	86.9	103.6	69.3
138.4	168.9	134.9	237.2
40	57.8	77.7	70.9
53.7	65.8	63.9	94.6
115.7	91.8	89.7	135.5
39.5	85.8	76.3	113.2
169.2	253	247.2	262.8
35.7	50.7	54.5	59.9
115.9	135.8	149.1	130.3
84.4	100.6	155.6	150.3
83.9	83.3	106.1	96.6
140	128.2	447	366.3
90.7	125.9	111.8	165.1
41.4	42.5	62.9	68.2
129.7	225.1	195.3	187.5
99.9	103.1	164	142.9
64.8	45.8	62.9	73.3
111.9	108	167.7	131.9
129.2	142.2	207.3	194.5
88.8	94	138.6	127.9
42.6	45.3	49.4	70.8

37.9	71	84.7	126.2
105	101.6	154.4	125.2
116.2	87.2	152.7	420.2
32.8	42.1	42.6	49.3
71.3	77	118.4	105.6
23.9	28.5	24.8	30.9
59.6	65	110.1	79
181	297.8	245	272.5
128.3	73.9	59.8	50.2
35.7	47.5	61.3	78.1
907	145	938.6	112.3
168.8	117.5	343.3	176.3
85	77.7	118.2	107.8
139	115.5	143.6	158.9
81.2	51.5	62.5	65.3
124.3	129.5	187.6	204.9
173	213.8	412	259.5
36	34.1	53.7	50.6
217.3	274.2	445.3	451.7
1485.4		28.2	13.5
110.9	97.5	95.3	114.9
78.6	80.7	111.1	117.1
139.4	93.2	110.8	157.5
212.7	311.1	387.2	478.2
92.7	102.6	114.6	106.9
21	29.4	30.2	35.8
24.9	42.1	47.5	53.7
41.3	7.4	11.8	8.2
76.3	130.5	103.4	221.5

149.5	116.1	165.7	138.5
55.2	60.4	75	59.6
78.5	75.5	139	169.4
325.5	300.8	262.1	150.8
64.8	90.4	84	145.6
121.1	140.5	198.9	198.5
98.7	194.7	224.5	192.1
891.8	956.3	1042	715.8
54.7	47.5	79.3	114.2
30.4	19.4	26.9	20.5
123	137.2	168.5	180.3
126.6	202.1	270.7	245.3
116.3	175.2	221.9	174.4
74.1	77.2	79.8	60.7
47.7	53	75.7	64.4
89.9	127.7	163.5	114.6
216.1	316.5	350.2	470
40.1	46.8	59.6	66.1
64.2	98.7	118	145.9
90.4	149.7	173.2	204.1
71	64.8	92	83.7
54.4	66.2	77.1	73.8
128.1	149.6	207.2	167.1
36.7	50.4	52.1	54.5
132	163.3	169.3	117.6
132.3	123.3	153.8	180
206.5	297.3	381.7	351.1
19.9	33.7	29.8	61.1
106.2	146.5	133.8	145.7
181.1	243.7	428.8	303.8
41.5	31.9	49.6	18.8

63.6	103	130.9	111.8
26.9	44.5	111	17.3
40.5	50.5	57.7	64.1
44.4	85.3	73.8	52.1
92.8	89.2	106.7	97.9
134.8	249.4	197.1	271.6
105.2	70.9	173.7	184.7
43.4	40.2	101.3	74.9
146.9	174	263.9	248.4
34.5	85.8	123.4	100.3
23.7	21.2	26.3	32.1
21.6	33.7	44.5	40.8
73.9	138.6	183.8	203.8
93.3	125.1	111.8	225.2
57.7	108.9	100.8	78.4
116.4	143	203.9	189.4
116	115.8	160.4	141.8
306.9	280.9	353.2	270.7
112.8	145.2	178.5	150.9
175.2	239	203.7	546
32.1	38.9	38.1	54.3
36.5	31.8	26	39.2
93.3	103.7	224.8	209.7
73.7	53.2	88.1	66
50.9	47	55.2	54.5
1146.3	557.4	439.3	463.5
75.1	57.8	88.9	82.4
159.4	168.8	260.6	210.7
116.6	126.3	177.4	179.8
46.1	26.4	83.7	72.2
18.1	30.4	29.1	39.2

79.2	103	151.2	130.3
95.1	91.4	149.1	106.5
39.8	32.7	75.2	55.5
199.9	196.3	139.3	222.8
102.6	103.3	142.3	131.4
72.1	116.8	118.5	150.7
60.9	93.1	100	110.8
15	17.4	18.2	13.7
50.7	70.4	72.3	109.4
42.1	47.8	67.4	62.4
37.3	45.1	75.7	65
85.9	135.3	150	150.3
105	126.9	177.3	189.1
148.1	188.1	380	253.3
41.2	35.5	58	70.5
100.6	115.4	170.8	147.9
105.1	148	113.3	209.9
57.6	60.9	73.6	90.4
112.2	54.2	139	68.2
148.6	264.6	475	329.6
362.5	442.6	647.7	609.7
92.1	61.9	84.6	56.8
382	122.4	178.8	175.3
119.9	156.5	225.4	177.1
116.9	130.2	214.2	184.8
224	60	45.9	92.9
57.2	63.5	86.2	123.5

34.6	40.3	35.7	34.8
55	60.2	92.3	64.3
162.1	175.3	144.6	82.4
16.6	19.5	23.2	16.2
100	122.8	132.8	182.6
66.3	120.8	204.6	168.4
136	129.4	195.3	166.9
95.9	105.7	119.3	133.1
74.7	78.4	112.9	108.1
106	131.8	179	143.4
45.4	62.6	86.3	91.4
154	85.5	102.5	123.4
35.3	47.6	58.7	74.7
95.8	108	181.2	142.5
33.9	48.1	60.9	71.3
755.5	863.5	1152.7	1054.5
162.7	205.2	267.8	131
216.8	301.2	392.5	435.2
82.1	122.1	163.6	123.6
75.1	74.6	85.5	94.7
20.9	42.4	47.7	54.1
748.5	676.4	691.1	441.2
39.1	49.8	78.2	68.8
95.1	82.5	111.6	124.7
20.6	24.9	23.5	24.9
58.7	81	106.4	115.5
52.8	56.4	74.6	89.6
305	516.9	531.7	644.3
94.4	95	137.3	105.7
9.2	23.1	95.8	40.6
158.5	216.4	319.4	313.4

54	38	48.4	27.5
271.7	236.9	339.6	315.9
122.6	100.2	173.7	197.5
64	76.8	81.5	89.3
49.3	54.7	117.8	60.8
79.3	152.1	133.1	171.3
91.4	98.4	135.8	126.9
38.6	54.8	63.1	58.8
127.4	110.6	167.9	126.4
39.6	43	53.9	33.9
151.4	157.6	186.1	228.8
470.1	481.3	759	892
40.4	48.1	62.6	57.4
69.5	87.1	71.9	88.1
95.9	91	161.2	120.6
71	102.9	122.6	70.3
21	19.3	74	24.4
65	73.6	97.6	76.4
80.4	98.8	224.2	118.7
115.5	209.7	357.8	304.1
52.3	56.1	71.1	67.6
129.1	208.8	305.7	272.9
111.2	99.8	203.3	145.1
23.4	44.6	45.9	53.6
162.6	125.7	88.4	114.9
36.4	46.3	65.4	62.2
53.2	30.2	87.8	57.4
85.3	87.7	80.8	104.2
192.6	233.8	400.5	344.2
98.9	68.3	85.6	85.2
520.7	833	1185.1	1160.3

159.6	136.7	198	198.9
142.9	214.7	198.1	148.2
19.1	17.5	29.6	104.1
159.7	195.5	226.4	227.8
56.1	90	113.2	117.6
23.8	14.3	18.7	18.3
95.1	119.6	144.5	175.2
142.8	183.3	292.9	309.3
13.7	8.7	11.8	9
56.1	85.7	109.2	104.1
78.6	101.9	128	116.6
27.7	26.8	28.4	41.4
328.4	612	1010.6	1005.5
208.1	84.3	158.5	81.8
30.3	45.8	53	45.2
30.3	46.4	44.5	52.1
53.1	64.6	74.6	78.9
56.8	45.3	78.7	76.2
48.9	48	68.3	40.1
142.3	92.7	133.9	98.2
78.6	101.4	118.7	123.7
148.1	139.1	126.4	145.1
89.4	92	181.2	124.1
48.9	112.8	133.7	184.6
57.7	58.7	110.4	100
87.9	88.6	139.5	121.8
60.7	79.2	88.6	68
120.8	89.4	77.4	68.8
81.6	106.9	142.3	141.8

106.1	103.6	111.2	105.4
60.4	65.4	85.8	83.8
74.8	38.1	64.2	54
108	66.7	92.5	82
231.4	261.6	403.9	365.9
128.3	141	243.6	312.6
37.1	43.3	32.8	29.8
351	342.3	642.6	505.2
213.4	327.9	412.9	542.1
107.6	163.1	115.8	335.3
277.3	403.2	450.5	406
38.6	39.6	54.7	48.8
165.4	94.6	176.8	85.9
28.6	49.9	26.3	35.8
320.2	327.7	625	540.6
17.8	22	34.3	37.6
142.7	147	276.1	282.3
437.7	553.5	493.7	534.3
269.3	195	458.6	274.9
84.8	128.7	132.6	117.5
66.6	80.9	137.9	158.8
206.6	296	445	430.1
85.5	155.3	201.3	202.9
154.1	159.2	223.6	236.3
113.4	115.6	171	160.8
100.6	59.8	66	104.7
203.7	299.4	648.4	427.9
162.4	154.2	190.8	159.3
457.8	456.9	581.4	209.5

62	77.6	93.5	106.1
187.4	133.6	159.9	236.1
98.1	130.3	92.1	114.6
152.3	214.9	372.1	320.8
117.4	113.2	205.9	158
149.2	103.4	169.1	113.8
196.9	232.7	374.5	441.7
109.1	121.2	183.6	207
40.7	48.9	47.6	25
87.3	269.9	372.4	370.2
231.8	238.5	336	313.6
159.1	255	328.6	326.7
158.7	67.6	112.5	102.5
74.6	85.2	121.1	139.7
131.3	182.7	271	220.7
123.4	198.4	186.1	143.2
89.9	270.4	332.9	410
221.2	348.1	526.7	411
46.7	50	82.3	62.8
95.8	119.3	211.7	152.2
82.9	108	148.3	135
147.3	228.7	312.3	363.4
