# Supplementary material for: Disrupted Tuzzerella abundance and impaired l-glutamine levels induce Treg accumulation in ovarian endometriosis: a comprehensive multi-omics analysis
Source: Metabolomics. 2024 Feb 29;20(2):32. doi: 10.1007/s11306-023-02072-0 (PMC10904428; doi:10.1007/s11306-023-02072-0)
Supplement: Supplementary file 8 — Supplementary file8 (DOCX 20 KB) [file 11306_2023_2072_MOESM8_ESM.docx]

**Table S1 16s rRNA sequencing sample information**

| Sample | RawReads | filtered | percentage of input passed filter | denoised | merged | percentage of input merged | non_chimeric | percentage of input non-chimeric | Pre_ASV_counts | Pre_Total_ASVs | Post_ASV_counts | Post_Total_ASVs |
| --- | --- | --- | --- | --- | --- | --- | --- | --- | --- | --- | --- | --- |
| C1 | 78333 | 76250 | 97.34 | 74724 | 67905 | 86.69 | 61821 | 78.92 | 681 | 16025 | 668 | 14718 |
| C10 | 79876 | 75466 | 94.48 | 74456 | 68601 | 85.88 | 62385 | 78.1 | 270 | 16025 | 262 | 14718 |
| C11 | 79573 | 74928 | 94.16 | 73575 | 68391 | 85.95 | 61629 | 77.45 | 236 | 16025 | 226 | 14718 |
| C12 | 81746 | 78822 | 96.42 | 77087 | 67242 | 82.26 | 59902 | 73.28 | 837 | 16025 | 821 | 14718 |
| C13 | 80733 | 77973 | 96.58 | 76396 | 69897 | 86.58 | 64765 | 80.22 | 410 | 16025 | 398 | 14718 |
| C14 | 80925 | 77941 | 96.31 | 77052 | 73673 | 91.04 | 71428 | 88.26 | 314 | 16025 | 308 | 14718 |
| C15 | 81962 | 78707 | 96.03 | 77608 | 72099 | 87.97 | 65659 | 80.11 | 263 | 16025 | 238 | 14718 |
| C16 | 80112 | 77823 | 97.14 | 76418 | 69710 | 87.02 | 61093 | 76.26 | 611 | 16025 | 596 | 14718 |
| C17 | 80482 | 78225 | 97.2 | 76697 | 69286 | 86.09 | 62023 | 77.06 | 742 | 16025 | 722 | 14718 |
| C18 | 79063 | 74378 | 94.07 | 73286 | 69226 | 87.56 | 63692 | 80.56 | 220 | 16025 | 217 | 14718 |
| C19 | 81117 | 78342 | 96.58 | 76741 | 68386 | 84.31 | 60683 | 74.81 | 785 | 16025 | 768 | 14718 |
| C2 | 80087 | 76336 | 95.32 | 75302 | 70723 | 88.31 | 66491 | 83.02 | 261 | 16025 | 258 | 14718 |
| C20 | 80409 | 77697 | 96.63 | 75939 | 68208 | 84.83 | 60993 | 75.85 | 698 | 16025 | 680 | 14718 |
| C21 | 81497 | 79000 | 96.94 | 77768 | 71453 | 87.68 | 65012 | 79.77 | 471 | 16025 | 460 | 14718 |
| C22 | 81338 | 78657 | 96.7 | 77831 | 74351 | 91.41 | 70057 | 86.13 | 245 | 16025 | 242 | 14718 |
| C3 | 78799 | 75706 | 96.07 | 74692 | 71259 | 90.43 | 65318 | 82.89 | 169 | 16025 | 168 | 14718 |
| C4 | 80533 | 76597 | 95.11 | 73205 | 53190 | 66.05 | 43820 | 54.41 | 1256 | 16025 | 1236 | 14718 |
| C5 | 80080 | 77605 | 96.91 | 76584 | 71799 | 89.66 | 65178 | 81.39 | 266 | 16025 | 260 | 14718 |
| C6 | 81792 | 77787 | 95.1 | 77129 | 74428 | 91 | 71116 | 86.95 | 149 | 16025 | 146 | 14718 |
| C7 | 80620 | 78030 | 96.79 | 77069 | 72475 | 89.9 | 68797 | 85.33 | 242 | 16025 | 235 | 14718 |
| C8 | 79076 | 76627 | 96.9 | 75327 | 68785 | 86.99 | 62234 | 78.7 | 665 | 16025 | 654 | 14718 |
| C9 | 81883 | 79844 | 97.51 | 78820 | 73602 | 89.89 | 67439 | 82.36 | 587 | 16025 | 569 | 14718 |
| O1 | 78188 | 74004 | 94.65 | 73066 | 68078 | 87.07 | 60394 | 77.24 | 262 | 16025 | 252 | 14718 |
| O10 | 79160 | 75884 | 95.86 | 74757 | 70503 | 89.06 | 66155 | 83.57 | 182 | 16025 | 176 | 14718 |
| O11 | 79537 | 76584 | 96.29 | 74881 | 62192 | 78.19 | 48768 | 61.31 | 1274 | 16025 | 1255 | 14718 |
| O12 | 80599 | 77884 | 96.63 | 76980 | 72960 | 90.52 | 67192 | 83.37 | 160 | 16025 | 159 | 14718 |
| O13 | 80529 | 77953 | 96.8 | 77199 | 74466 | 92.47 | 70765 | 87.88 | 216 | 16025 | 214 | 14718 |
| O14 | 81821 | 79251 | 96.86 | 78213 | 73464 | 89.79 | 67112 | 82.02 | 321 | 16025 | 309 | 14718 |
| O15 | 80210 | 77545 | 96.68 | 75992 | 69507 | 86.66 | 62924 | 78.45 | 614 | 16025 | 596 | 14718 |
| O16 | 78619 | 76442 | 97.23 | 75335 | 69480 | 88.38 | 62515 | 79.52 | 569 | 16025 | 557 | 14718 |
| O17 | 81110 | 77143 | 95.11 | 76010 | 71237 | 87.83 | 66281 | 81.72 | 302 | 16025 | 297 | 14718 |
| O18 | 79331 | 76543 | 96.49 | 75545 | 71796 | 90.5 | 67248 | 84.77 | 165 | 16025 | 158 | 14718 |
| O19 | 79260 | 76460 | 96.47 | 74969 | 66900 | 84.41 | 58360 | 73.63 | 904 | 16025 | 877 | 14718 |
| O2 | 78532 | 75574 | 96.23 | 74585 | 70584 | 89.88 | 66140 | 84.22 | 206 | 16025 | 200 | 14718 |
| O20 | 80321 | 77580 | 96.59 | 76523 | 72463 | 90.22 | 68857 | 85.73 | 209 | 16025 | 203 | 14718 |
| O21 | 78420 | 75931 | 96.83 | 74967 | 72220 | 92.09 | 71203 | 90.8 | 176 | 16025 | 172 | 14718 |
| O22 | 79761 | 77296 | 96.91 | 76173 | 71254 | 89.33 | 65178 | 81.72 | 359 | 16025 | 351 | 14718 |
| O23 | 81130 | 78879 | 97.23 | 77454 | 71199 | 87.76 | 64718 | 79.77 | 647 | 16025 | 631 | 14718 |
| O3 | 80902 | 78211 | 96.67 | 72525 | 51160 | 63.24 | 43034 | 53.19 | 939 | 16025 | 906 | 14718 |
| O4 | 79084 | 76032 | 96.14 | 74878 | 70166 | 88.72 | 64796 | 81.93 | 196 | 16025 | 189 | 14718 |
| O5 | 79597 | 76882 | 96.59 | 76189 | 72879 | 91.56 | 69629 | 87.48 | 201 | 16025 | 196 | 14718 |
| O6 | 80133 | 77668 | 96.92 | 76658 | 71561 | 89.3 | 67308 | 84 | 286 | 16025 | 277 | 14718 |
| O7 | 78417 | 76260 | 97.25 | 74779 | 67910 | 86.6 | 60129 | 76.68 | 802 | 16025 | 781 | 14718 |
| O8 | 78451 | 75589 | 96.35 | 74375 | 68216 | 86.95 | 60127 | 76.64 | 606 | 16025 | 598 | 14718 |
| O9 | 80228 | 75221 | 93.76 | 73374 | 63596 | 79.27 | 57131 | 71.21 | 719 | 16025 | 700 | 14718 |
| YX-1 | 80335 | 77667 | 96.68 | 76546 | 71567 | 89.09 | 67100 | 83.53 | 256 | 16025 |  |  |
| YX-2 | 78016 | 75560 | 96.85 | 74295 | 67198 | 86.13 | 60185 | 77.14 | 760 | 16025 |  |  |
| YX-3 | 78081 | 75702 | 96.95 | 74487 | 68040 | 87.14 | 61571 | 78.86 | 691 | 16025 |  |  |
