# Supplementary material for: Disrupted Tuzzerella abundance and impaired l-glutamine levels induce Treg accumulation in ovarian endometriosis: a comprehensive multi-omics analysis
Source: Metabolomics. 2024 Feb 29;20(2):32. doi: 10.1007/s11306-023-02072-0 (PMC10904428; doi:10.1007/s11306-023-02072-0)
Supplement: Supplementary file 9 — Supplementary file9 (DOCX 80 KB) [file 11306_2023_2072_MOESM9_ESM.docx]

Table S2 Wilcoxon rank sum tests

| Biomarker | Logarithm value | Groups | LDA_value | p-value |
| --- | --- | --- | --- | --- |
| k__Bacteria.p__Firmicutes.c__Clostridia.o__Clostridia_UCG_014.f__Clostridia_UCG_014 | 4.177544 |  |  | - |
| k__Bacteria.p__Dependentiae | 1.565043 |  |  | - |
| k__Bacteria.p__Bacteroidota.c__Bacteroidia.o__Bacteroidales.f__Paludibacteraceae | 1.583741 |  |  | - |
| k__Bacteria.p__Firmicutes.c__Clostridia.o__Lachnospirales.f__Lachnospiraceae.g__Dorea | 2.625535 |  |  | - |
| k__Bacteria.p__Acidobacteriota.c__Thermoanaerobaculia.o__Thermoanaerobaculales | 2.402628 |  |  | - |
| k__Bacteria.p__Proteobacteria.c__Alphaproteobacteria.o__Rhizobiales | 3.56374 |  |  | - |
| k__Bacteria.p__Bacteroidota.c__Bacteroidia.o__Chitinophagales.f__Chitinophagaceae.g__Filimonas | 1.213735 |  |  | - |
| k__Bacteria.p__Firmicutes.c__Clostridia.o__Peptococcales.f__Peptococcaceae | 2.671824 |  |  | - |
| k__Bacteria.p__Firmicutes.c__Bacilli.o__Erysipelotrichales.f__Erysipelotrichaceae.g__Turicibacter | 3.845584 |  |  | - |
| k__Bacteria.p__Actinobacteriota.c__Actinobacteria.o__Micrococcales.f__Micrococcaceae.g__Arthrobacter | 0.799633 |  |  | - |
| k__Bacteria.p__Acidobacteriota.c__Acidobacteriae.o__Acidobacteriales.f__Acidobacteriaceae__Subgroup_1_.g__Occallatibacter | 2.850896 |  |  | - |
| k__Bacteria.p__Proteobacteria.c__Alphaproteobacteria.o__Defluviicoccales.f__Defluviicoccaceae | 1.682376 |  |  | - |
| k__Bacteria.p__Firmicutes.c__Clostridia.o__Oscillospirales.f__Ruminococcaceae.g__Subdoligranulum | 1.601317 |  |  | - |
| k__Bacteria.p__Proteobacteria.c__Alphaproteobacteria.o__Rhizobiales.f__Devosiaceae.g__Devosia | 2.371618 |  |  | - |
| k__Bacteria.p__Bacteroidota.c__Kapabacteria.o__Kapabacteriales.f__Kapabacteriales.g__Kapabacteriales | 2.039643 |  |  | - |
| k__Bacteria.p__Firmicutes.c__Clostridia.o__Peptostreptococcales_Tissierellales.f__Peptostreptococcaceae.g__Romboutsia | 3.400896 |  |  | - |
| k__Bacteria.p__Firmicutes.c__Clostridia.o__Lachnospirales.f__Lachnospiraceae.g__Butyrivibrio | 2.476869 |  |  | - |
| k__Bacteria.p__MBNT15.c__MBNT15.o__MBNT15 | 0.861553 |  |  | - |
| k__Bacteria.p__Proteobacteria.c__Gammaproteobacteria.o__Xanthomonadales.f__Xanthomonadaceae.g__Stenotrophomonas | 1.942058 |  |  | - |
| k__Bacteria.p__Firmicutes.c__Limnochordia.o__Limnochordales.f__Limnochordaceae | 1.724432 |  |  | - |
| k__Bacteria.p__Proteobacteria.c__Alphaproteobacteria.o__Caulobacterales.f__Hyphomonadaceae.g__SWB02 | 0.799633 |  |  | - |
| k__Bacteria.p__Firmicutes.c__Bacilli.o__Acholeplasmatales | 2.706587 |  |  | - |
| k__Bacteria.p__Firmicutes.c__Clostridia.o__Oscillospirales.f__Ruminococcaceae.g___Eubacterium__siraeum_group | 3.328715 |  |  | - |
| k__Bacteria.p__Actinobacteriota.c__Actinobacteria.o__Micromonosporales.f__Micromonosporaceae | 3.524141 |  |  | - |
| k__Bacteria.p__Bacteroidota.c__Bacteroidia | 5.352274 |  |  | - |
| k__Bacteria.p__Firmicutes.c__Bacilli.o__Erysipelotrichales.f__Erysipelatoclostridiaceae.g__Coprobacillus | 2.997645 |  |  | - |
| k__Bacteria.p__Acidobacteriota | 4.174453 |  |  | - |
| k__Bacteria.p__Bacteroidota.c__Bacteroidia.o__Chitinophagales.f__Chitinophagaceae.g__Lacibacter | 1.348791 |  |  | - |
| k__Bacteria.p__Firmicutes.c__Clostridia.o__Christensenellales.f__Christensenellaceae.g__Christensenellaceae_R_7_group | 2.529438 |  |  | - |
| k__Bacteria.p__Firmicutes.c__Bacilli.o__Mycoplasmatales.f__Mycoplasmataceae.g__Mycoplasma | 2.742824 |  |  | - |
| k__Bacteria.p__Actinobacteriota.c__Actinobacteria.o__Propionibacteriales.f__Nocardioidaceae.g__Marmoricola | 0.720452 |  |  | - |
| k__Bacteria.p__Acidobacteriota.c__Subgroup_5.o__Subgroup_5 | 2.67495 |  |  | - |
| k__Bacteria.p__Firmicutes.c__Bacilli.o__Lactobacillales.f__Streptococcaceae | 4.074503 |  |  | - |
| k__Bacteria.p__Firmicutes.c__Bacilli.o__Lactobacillales.f__P5D1_392.g__P5D1_392 | 1.391543 |  |  | - |
| k__Bacteria.p__Elusimicrobiota.c__Elusimicrobia.o__MVP_88 | 2.53396 |  |  | - |
| k__Bacteria.p__Bacteroidota.c__Bacteroidia.o__Flavobacteriales.f__Flavobacteriaceae.g__Capnocytophaga | 2.942175 |  |  | - |
| k__Bacteria.p__Firmicutes.c__Bacilli.o__Lactobacillales.f__Carnobacteriaceae | 2.454446 |  |  | - |
| k__Bacteria.p__Bacteroidota.c__Bacteroidia.o__Bacteroidales | 5.339906 |  |  | - |
| k__Bacteria.p__Firmicutes.c__Bacilli.o__Paenibacillales | 3.817755 |  |  | - |
| k__Bacteria.p__Firmicutes.c__Clostridia.o__Lachnospirales.f__Lachnospiraceae.g__Lachnoanaerobaculum | 2.815438 |  |  | - |
| k__Bacteria.p__Firmicutes.c__Clostridia.o__Oscillospirales.f__Ruminococcaceae.g__Negativibacillus | 2.779624 |  |  | - |
| k__Bacteria.p__Bacteroidota.c__Bacteroidia.o__Bacteroidales.f__Rikenellaceae | 4.168206 |  |  | - |
| k__Bacteria.p__Proteobacteria.c__Alphaproteobacteria.o__Defluviicoccales.f__Defluviicoccaceae.g__Defluviicoccus | 1.682376 |  |  | - |
| k__Bacteria.p__Proteobacteria.c__Alphaproteobacteria.o__Rhizobiales.f__Rhizobiaceae.g__Phyllobacterium | 0.835553 |  |  | - |
| k__Bacteria.p__Firmicutes.c__Bacilli.o__Lactobacillales.f__Lactobacillaceae.g__Lactobacillus | 4.806666 |  |  | - |
| k__Bacteria.p__Firmicutes.c__Clostridia.o__Lachnospirales.f__Lachnospiraceae.g__Acetatifactor | 2.656879 |  |  | - |
| k__Bacteria.p__Bacteroidota.c__Bacteroidia.o__Bacteroidales.f__Muribaculaceae.g__Muribaculum | 2.262312 |  |  | - |
| k__Bacteria.p__Proteobacteria.c__Gammaproteobacteria.o__Burkholderiales.f__Nitrosomonadaceae.g__IS_44 | 1.905733 |  |  | - |
| k__Bacteria.p__Actinobacteriota.c__Actinobacteria.o__Pseudonocardiales.f__Pseudonocardiaceae | 2.458929 |  |  | - |
| k__Bacteria.p__Firmicutes.c__Clostridia.o__Oscillospirales.f__Oscillospiraceae.g__NK4A214_group | 2.844527 |  |  | - |
| k__Bacteria.p__Firmicutes.c__Clostridia.o__Clostridia.f__Hungateiclostridiaceae | 1.54264 |  |  | - |
| k__Bacteria.p__Actinobacteriota.c__Actinobacteria.o__Catenulisporales.f__Catenulisporaceae.g__Catenulispora | 2.503451 |  |  | - |
| k__Bacteria.p__Chloroflexi.c__Anaerolineae.o__Anaerolineae.f__Anaerolineae.g__Anaerolineae | 2.088972 |  |  | - |
| k__Bacteria.p__Actinobacteriota.c__Actinobacteria.o__Micromonosporales.f__Micromonosporaceae.g__Actinocatenispora | 1.960924 |  |  | - |
| k__Bacteria.p__Actinobacteriota.c__Actinobacteria.o__Pseudonocardiales.f__Pseudonocardiaceae.g__Amycolatopsis | 1.760628 |  |  | - |
| k__Bacteria.p__Verrucomicrobiota.c__Verrucomicrobiae.o__Pedosphaerales.f__Pedosphaeraceae | 2.601756 |  |  | - |
| k__Bacteria.p__Nitrospirota.c__Nitrospiria.o__Nitrospirales.f__Nitrospiraceae | 2.967335 |  |  | - |
| k__Bacteria.p__Verrucomicrobiota.c__Verrucomicrobiae.o__Verrucomicrobiales.f__Akkermansiaceae | 2.391624 | C | 2.232119 | 0.034381 |
| k__Bacteria.p__Bacteroidota.c__Bacteroidia.o__Chitinophagales.f__Chitinophagaceae.g__Edaphobaculum | 1.972673 |  |  | - |
| k__Bacteria.p__Actinobacteriota.c__Actinobacteria.o__Micromonosporales.f__Micromonosporaceae.g__Dactylosporangium | 0.924572 |  |  | - |
| k__Bacteria.p__Proteobacteria.c__Alphaproteobacteria.o__Rhizobiales.f__Rhizobiaceae.g__Pseudaminobacter | 1.771329 |  |  | - |
| k__Bacteria.p__Firmicutes.c__Clostridia.o__Clostridia_UCG_014.f__Clostridia_UCG_014.g__Clostridia_UCG_014 | 4.177544 |  |  | - |
| k__Bacteria.p__Actinobacteriota.c__Coriobacteriia.o__Coriobacteriales.f__Eggerthellaceae | 3.551183 |  |  | - |
| k__Bacteria.p__Proteobacteria.c__Gammaproteobacteria.o__Enterobacterales.f__Morganellaceae | 3.929166 |  |  | - |
| k__Bacteria.p__Bacteroidota.c__Bacteroidia.o__Sphingobacteriales.f__Lentimicrobiaceae.g__Lentimicrobium | 1.710802 |  |  | - |
| k__Bacteria.p__Campilobacterota.c__Campylobacteria | 4.241276 |  |  | - |
| k__Bacteria.p__Firmicutes.c__Clostridia.o__Oscillospirales.f___Eubacterium__coprostanoligenes_group.g___Eubacterium__coprostanoligenes_group | 3.821704 |  |  | - |
| k__Bacteria.p__Acidobacteriota.c__Holophagae.o__Subgroup_7.f__Subgroup_7 | 1.691859 |  |  | - |
| k__Bacteria.p__Bacteroidota.c__Bacteroidia.o__Sphingobacteriales.f__KD3_93 | 0.872054 |  |  | - |
| k__Bacteria.p__Firmicutes.c__Bacilli.o__RF39 | 2.404655 |  |  | - |
| k__Bacteria.p__Patescibacteria.c__Saccharimonadia.o__Saccharimonadales.f__Saccharimonadales.g__Saccharimonadales | 2.129247 |  |  | - |
| k__Bacteria.p__Proteobacteria.c__Alphaproteobacteria.o__Rhizobiales.f__Hyphomicrobiaceae | 1.703733 |  |  | - |
| k__Bacteria.p__Firmicutes.c__Bacilli.o__Lactobacillales.f__Carnobacteriaceae.g__Atopostipes | 0.820788 |  |  | - |
| k__Bacteria.p__Actinobacteriota.c__Actinobacteria.o__Micromonosporales | 3.524141 |  |  | - |
| k__Bacteria.p__Verrucomicrobiota | 2.858017 | C | 2.436423 | 0.044125 |
| k__Bacteria.p__Firmicutes.c__Bacilli.o__Lactobacillales.f__Lactobacillaceae | 4.806666 |  |  | - |
| k__Bacteria.p__Proteobacteria.c__Alphaproteobacteria.o__Paracaedibacterales.f__Paracaedibacteraceae | 2.526187 |  |  | - |
| k__Bacteria.p__Actinobacteriota.c__Actinobacteria.o__Micrococcales.f__Microbacteriaceae.g__Amnibacterium | 1.616794 |  |  | - |
| k__Bacteria.p__Actinobacteriota.c__Actinobacteria.o__Frankiales.f__Acidothermaceae | 2.808803 |  |  | - |
| k__Bacteria.p__Firmicutes.c__Bacilli.o__Staphylococcales.f__Gemellaceae.g__Gemella | 2.982478 |  |  | - |
| k__Bacteria.p__Firmicutes.c__Bacilli.o__Bacillales.f__Bacillaceae.g__Geobacillus | 1.280682 |  |  | - |
| k__Bacteria.p__Proteobacteria | 5.560698 |  |  | - |
| k__Bacteria.p__Firmicutes.c__Clostridia.o__Peptococcales.f__Peptococcaceae.g__Peptococcus | 1.748571 |  |  | - |
| k__Bacteria.p__MBNT15.c__MBNT15.o__MBNT15.f__MBNT15 | 0.861553 |  |  | - |
| k__Bacteria.p__Proteobacteria.c__Alphaproteobacteria.o__Acetobacterales.f__Acetobacteraceae.g__Rhodovastum | 1.350824 |  |  | - |
| k__Bacteria.p__Actinobacteriota.c__Actinobacteria.o__Frankiales.f__Geodermatophilaceae | 2.439748 |  |  | - |
| k__Bacteria.p__Firmicutes.c__Clostridia.o__Clostridiales.f__Clostridiaceae.g__Candidatus_Arthromitus | 2.913474 |  |  | - |
| k__Bacteria.p__Proteobacteria.c__Alphaproteobacteria.o__Rhizobiales.f__Xanthobacteraceae | 3.308007 |  |  | - |
| k__Bacteria.p__Actinobacteriota.c__Actinobacteria.o__Kineosporiales.f__Kineosporiaceae.g__Angustibacter | 1.430005 |  |  | - |
| k__Bacteria.p__Bacteroidota.c__Bacteroidia.o__Bacteroidales.f__Rikenellaceae.g__Rikenellaceae_RC9_gut_group | 3.245447 |  |  | - |
| k__Bacteria.p__Actinobacteriota.c__Actinobacteria.o__Propionibacteriales.f__Nocardioidaceae.g__Nocardioides | 2.597283 |  |  | - |
| k__Bacteria.p__Acidobacteriota.c__Holophagae.o__Holophagales.f__Holophagaceae.g__Holophaga | 2.736379 |  |  | - |
| k__Bacteria.p__Actinobacteriota.c__Acidimicrobiia.o__Acidimicrobiales | 1.373436 |  |  | - |
| k__Bacteria.p__Firmicutes.c__Bacilli.o__Erysipelotrichales.f__Erysipelatoclostridiaceae | 3.387771 |  |  | - |
| k__Bacteria.p__Proteobacteria.c__Gammaproteobacteria.o__Burkholderiales.f__Alcaligenaceae | 2.394382 |  |  | - |
| k__Bacteria.p__Bacteroidota.c__Bacteroidia.o__Cytophagales.f__Microscillaceae | 3.10618 |  |  | - |
| k__Bacteria.p__Elusimicrobiota.c__Lineage_IIa | 1.570129 |  |  | - |
| k__Bacteria.p__Acidobacteriota.c__Acidobacteriae.o__Solibacterales | 2.917332 |  |  | - |
| k__Bacteria.p__Latescibacterota.c__Latescibacteria.o__Latescibacterales.f__Latescibacteraceae | 1.644063 |  |  | - |
| k__Bacteria.p__Chloroflexi.c__Ktedonobacteria.o__Ktedonobacterales.f__JG30_KF_AS9 | 1.558491 |  |  | - |
| k__Bacteria.p__Proteobacteria.c__Alphaproteobacteria.o__Rhizobiales.f__Rhizobiaceae.g__Ensifer | 0.691442 |  |  | - |
| k__Bacteria.p__Actinobacteriota.c__Coriobacteriia.o__Coriobacteriales.f__Atopobiaceae.g__Atopobium | 2.656835 |  |  | - |
| k__Bacteria.p__Proteobacteria.c__Gammaproteobacteria.o__Pasteurellales | 4.370593 |  |  | - |
| k__Bacteria.p__Firmicutes.c__Clostridia.o__Christensenellales | 2.665072 |  |  | - |
| k__Bacteria.p__Myxococcota.c__Polyangia.o__Polyangiales.f__Polyangiaceae.g__Polyangium | 1.102866 |  |  | - |
| k__Bacteria.p__Proteobacteria.c__Gammaproteobacteria.o__Xanthomonadales.f__Rhodanobacteraceae.g__Frateuria | 2.387501 |  |  | - |
| k__Bacteria.p__Firmicutes.c__Bacilli.o__Alicyclobacillales.f__Alicyclobacillaceae | 2.380073 |  |  | - |
| k__Bacteria.p__Campilobacterota.c__Campylobacteria.o__Campylobacterales.f__Arcobacteraceae | 2.394875 |  |  | - |
| k__Bacteria.p__Proteobacteria.c__Alphaproteobacteria.o__Rhizobiales.f__Rhizobiales_Incertae_Sedis.g__Phreatobacter | 1.752676 |  |  | - |
| k__Bacteria.p__Firmicutes.c__Bacilli.o__Alicyclobacillales | 2.380073 |  |  | - |
| k__Bacteria.p__Acidobacteriota.c__Subgroup_22 | 1.339608 |  |  | - |
| k__Bacteria.p__Firmicutes.c__Bacilli.o__Bacillales.f__Bacillaceae.g__Bacillus | 3.196403 |  |  | - |
| k__Bacteria.p__Chloroflexi.c__Ktedonobacteria.o__Ktedonobacterales.f__JG30_KF_AS9.g__JG30_KF_AS9 | 1.558491 |  |  | - |
| k__Bacteria.p__Firmicutes.c__Bacilli.o__Mycoplasmatales.f__Mycoplasmataceae.g__Ureaplasma | 2.008988 |  |  | - |
| k__Bacteria.p__Firmicutes.c__Bacilli.o__Erysipelotrichales | 4.024997 |  |  | - |
| k__Bacteria.p__Acidobacteriota.c__Holophagae.o__Holophagales.f__Holophagaceae | 2.736379 |  |  | - |
| k__Bacteria.p__Proteobacteria.c__Gammaproteobacteria.o__Enterobacterales.f__Erwiniaceae | 1.252629 |  |  | - |
| k__Bacteria.p__Myxococcota.c__Polyangia.o__Polyangiales.f__Polyangiaceae.g__Aetherobacter | 1.780304 |  |  | - |
| k__Bacteria.p__Firmicutes.c__Clostridia.o__Monoglobales.f__Monoglobaceae.g__Monoglobus | 3.019813 |  |  | - |
| k__Bacteria.p__Proteobacteria.c__Gammaproteobacteria.o__Burkholderiales.f__Burkholderiaceae | 2.476458 |  |  | - |
| k__Bacteria.p__Proteobacteria.c__Alphaproteobacteria.o__Rhizobiales.f__Beijerinckiaceae | 2.283134 |  |  | - |
| k__Bacteria.p__Desulfobacterota.c__Desulfuromonadia.o__Geobacterales | 2.056841 |  |  | - |
| k__Bacteria.p__Firmicutes.c__Clostridia.o__Oscillospirales.f___Clostridium__methylpentosum_group | 1.089182 |  |  | - |
| k__Bacteria.p__Fusobacteriota.c__Fusobacteriia.o__Fusobacteriales | 4.171527 |  |  | - |
| k__Bacteria.p__Nitrospirota.c__Nitrospiria.o__Nitrospirales | 2.967335 |  |  | - |
| k__Bacteria.p__Actinobacteriota.c__Actinobacteria.o__Corynebacteriales.f__Mycobacteriaceae | 2.377942 |  |  | - |
| k__Bacteria.p__Proteobacteria.c__Alphaproteobacteria.o__Sphingomonadales | 4.828159 |  |  | - |
| k__Bacteria.p__Proteobacteria.c__Gammaproteobacteria.o__Xanthomonadales.f__Xanthomonadaceae.g__Pseudoxanthomonas | 2.368319 |  |  | - |
| k__Bacteria.p__Proteobacteria.c__Gammaproteobacteria.o__Gammaproteobacteria_Incertae_Sedis | 2.982249 |  |  | - |
| k__Bacteria.p__Proteobacteria.c__Gammaproteobacteria.o__Xanthomonadales.f__Rhodanobacteraceae.g__Dokdonella | 1.631392 |  |  | - |
| k__Bacteria.p__Firmicutes.c__Bacilli.o__Staphylococcales.f__Gemellaceae | 2.982478 |  |  | - |
| k__Bacteria.p__Proteobacteria.c__Gammaproteobacteria.o__Legionellales | 1.640638 |  |  | - |
| k__Bacteria.p__Acidobacteriota.c__Subgroup_5.o__Subgroup_5.f__Subgroup_5.g__Subgroup_5 | 2.67495 |  |  | - |
| k__Bacteria.p__Gemmatimonadota.c__S0134_terrestrial_group | 1.714819 |  |  | - |
| k__Bacteria.p__Acidobacteriota.c__Acidobacteriae.o__Acidobacteriales | 3.819034 |  |  | - |
| k__Bacteria.p__Firmicutes.c__Clostridia.o__Oscillospirales.f__Ruminococcaceae.g__Fournierella | 1.068349 |  |  | - |
| k__Bacteria.p__Bacteroidota.c__Bacteroidia.o__Chitinophagales.f__Chitinophagaceae.g__Niastella | 1.967141 |  |  | - |
| k__Bacteria.p__Patescibacteria.c__Saccharimonadia.o__Saccharimonadales.f__Saccharimonadaceae | 2.370368 |  |  | - |
| k__Bacteria.p__Patescibacteria.c__Saccharimonadia.o__Saccharimonadales.f__Saccharimonadales | 2.129247 |  |  | - |
| k__Bacteria.p__Bacteroidota.c__Bacteroidia.o__Bacteroidales.f__Prevotellaceae.g__Prevotellaceae_Ga6A1_group | 2.089879 |  |  | - |
| k__Bacteria.p__Acidobacteriota.c__Holophagae | 2.773908 |  |  | - |
| k__Bacteria.p__Proteobacteria.c__Gammaproteobacteria.o__CHAB_XI_27.f__CHAB_XI_27 | 1.467696 |  |  | - |
| k__Bacteria.p__Proteobacteria.c__Gammaproteobacteria.o__Enterobacterales.f__Yersiniaceae | 1.842238 |  |  | - |
| k__Bacteria.p__Bacteroidota.c__Bacteroidia.o__Bacteroidales.f__Bacteroidaceae | 4.554887 |  |  | - |
| k__Bacteria.p__Campilobacterota.c__Campylobacteria.o__Campylobacterales.f__Helicobacteraceae | 4.193765 |  |  | - |
| k__Bacteria.p__Firmicutes.c__Bacilli.o__RF39.f__RF39.g__RF39 | 2.404655 |  |  | - |
| k__Bacteria.p__Actinobacteriota.c__Acidimicrobiia.o__Microtrichales | 2.60417 |  |  | - |
| k__Bacteria.p__Firmicutes.c__Clostridia.o__Oscillospirales.f__Oscillospiraceae.g__Colidextribacter | 3.941067 |  |  | - |
| k__Bacteria.p__Patescibacteria.c__Gracilibacteria.o__Absconditabacteriales__SR1_.f__Absconditabacteriales__SR1_.g__Absconditabacteriales__SR1_ | 2.491286 |  |  | - |
| k__Bacteria.p__Proteobacteria.c__Gammaproteobacteria.o__Burkholderiales.f__Neisseriaceae.g__Eikenella | 2.402205 |  |  | - |
| k__Bacteria.p__Myxococcota.c__Myxococcia | 2.111731 |  |  | - |
| k__Bacteria.p__Acidobacteriota.c__Thermoanaerobaculia.o__Thermoanaerobaculales.f__Thermoanaerobaculaceae.g__Subgroup_10 | 2.402628 |  |  | - |
| k__Bacteria.p__Proteobacteria.c__Alphaproteobacteria.o__Rhizobiales.f__Beijerinckiaceae.g__Methylobacterium_Methylorubrum | 2.225355 |  |  | - |
| k__Bacteria.p__Firmicutes.c__Bacilli.o__Lactobacillales.f__P5D1_392 | 1.391543 |  |  | - |
| k__Bacteria.p__Firmicutes.c__Negativicutes.o__Veillonellales_Selenomonadales.f__Veillonellaceae.g__Dialister | 1.611203 |  |  | - |
| k__Bacteria.p__Acidobacteriota.c__Thermoanaerobaculia.o__Thermoanaerobaculales.f__Thermoanaerobaculaceae | 2.402628 |  |  | - |
| k__Bacteria.p__Actinobacteriota.c__Acidimicrobiia.o__Acidimicrobiales.f__Acidimicrobiaceae | 1.373436 |  |  | - |
| k__Bacteria.p__Bacteroidota.c__Bacteroidia.o__Chitinophagales.f__Chitinophagaceae.g__Terrimonas | 0.872054 |  |  | - |
| k__Bacteria.p__Bacteroidota.c__Bacteroidia.o__Bacteroidales.f__Tannerellaceae.g__Tannerellaceae | 1.466176 |  |  | - |
| k__Bacteria.p__Proteobacteria.c__Gammaproteobacteria.o__KF_JG30_C25.f__KF_JG30_C25 | 2.94646 |  |  | - |
| k__Bacteria.p__Firmicutes.c__Clostridia.o__Lachnospirales.f__Lachnospiraceae.g__Anaerostignum | 1.248347 |  |  | - |
| k__Bacteria.p__Proteobacteria.c__Gammaproteobacteria.o__JG36_TzT_191.f__JG36_TzT_191.g__JG36_TzT_191 | 0.460793 |  |  | - |
| k__Bacteria.p__Proteobacteria.c__Alphaproteobacteria.o__Rhodobacterales.f__Rhodobacteraceae.g__Rhodobacter | 2.525254 |  |  | - |
| k__Bacteria.p__SAR324_clade_Marine_group_B_.c__SAR324_clade_Marine_group_B_.o__SAR324_clade_Marine_group_B_.f__SAR324_clade_Marine_group_B_ | 1.124738 |  |  | - |
| k__Bacteria.p__Proteobacteria.c__Alphaproteobacteria.o__Rhizobiales.f__Beijerinckiaceae.g__Psychroglaciecola | 1.498735 |  |  | - |
| k__Bacteria.p__Chloroflexi.c__Anaerolineae | 2.088972 |  |  | - |
| k__Bacteria.p__Firmicutes.c__Clostridia.o__Lachnospirales.f__Lachnospiraceae.g__Sellimonas | 1.449886 |  |  | - |
| k__Bacteria.p__Bacteroidota.c__Bacteroidia.o__Sphingobacteriales.f__Sphingobacteriaceae.g__Pedobacter | 2.19042 |  |  | - |
| k__Bacteria.p__RCP2_54.c__RCP2_54 | 2.540402 |  |  | - |
| k__Bacteria.p__Myxococcota.c__Polyangia.o__Polyangiales.f__BIrii41 | 2.573832 |  |  | - |
| k__Bacteria.p__Nitrospirota.c__Nitrospiria | 2.967335 |  |  | - |
| k__Bacteria.p__Bacteroidota.c__Bacteroidia.o__Chitinophagales.f__Chitinophagaceae | 3.11107 |  |  | - |
| k__Bacteria.p__Actinobacteriota.c__Actinobacteria.o__Streptomycetales.f__Streptomycetaceae.g__Streptomyces | 3.054707 |  |  | - |
| k__Bacteria.p__Actinobacteriota.c__Actinobacteria.o__Kineosporiales.f__Kineosporiaceae | 1.430005 |  |  | - |
| k__Bacteria.p__Bacteroidota.c__Bacteroidia.o__Sphingobacteriales.f__AKYH767 | 2.295261 |  |  | - |
| k__Bacteria.p__Bdellovibrionota.c__Oligoflexia.o__0319_6G20.f__0319_6G20.g__0319_6G20 | 2.313704 |  |  | - |
| k__Bacteria.p__Proteobacteria.c__Gammaproteobacteria.o__Burkholderiales.f__Comamonadaceae.g__Delftia | 1.816932 |  |  | - |
| k__Bacteria.p__Proteobacteria.c__Gammaproteobacteria.o__Burkholderiales.f__Rhodocyclaceae | 1.436586 |  |  | - |
| k__Bacteria.p__Proteobacteria.c__Gammaproteobacteria.o__Burkholderiales.f__Sutterellaceae.g__Sutterella | 2.294233 |  |  | - |
| k__Bacteria.p__Patescibacteria.c__Saccharimonadia.o__Saccharimonadales | 2.964665 | C | 2.610632 | 0.033108 |
| k__Bacteria.p__Firmicutes.c__Bacilli.o__Erysipelotrichales.f__Erysipelotrichaceae.g__Dielma | 0.912705 |  |  | - |
| k__Bacteria.p__Proteobacteria.c__Gammaproteobacteria.o__CHAB_XI_27.f__CHAB_XI_27.g__CHAB_XI_27 | 1.467696 |  |  | - |
| k__Bacteria.p__Acidobacteriota.c__Subgroup_5 | 2.67495 |  |  | - |
| k__Bacteria.p__Fibrobacterota.c__Fibrobacteria.o__Fibrobacterales.f__Fibrobacteraceae | 2.674465 |  |  | - |
| k__Bacteria.p__Fibrobacterota.c__Fibrobacteria.o__Fibrobacterales.f__Fibrobacteraceae.g__Fibrobacter | 2.674465 |  |  | - |
| k__Bacteria.p__Myxococcota.c__bacteriap25.o__bacteriap25 | 2.572408 |  |  | - |
| k__Bacteria.p__Chloroflexi.c__Ktedonobacteria.o__Ktedonobacterales.f__Ktedonobacteraceae | 2.233237 |  |  | - |
| k__Bacteria.p__Patescibacteria.c__Parcubacteria.o__Candidatus_Jorgensenbacteria.f__Candidatus_Jorgensenbacteria.g__Candidatus_Jorgensenbacteria | 1.905844 |  |  | - |
| k__Bacteria.p__Myxococcota.c__Polyangia.o__Polyangiales.f__Sandaracinaceae | 2.583684 |  |  | - |
| k__Bacteria.p__Firmicutes.c__Clostridia.o__Clostridiales.f__Clostridiaceae | 4.143666 |  |  | - |
| k__Bacteria.p__Proteobacteria.c__Gammaproteobacteria.o__Burkholderiales.f__Nitrosomonadaceae.g__Ellin6067 | 2.944846 |  |  | - |
| k__Bacteria.p__Proteobacteria.c__Gammaproteobacteria.o__Pasteurellales.f__Pasteurellaceae | 4.370593 |  |  | - |
| k__Bacteria.p__Patescibacteria.c__Gracilibacteria | 2.491286 |  |  | - |
| k__Bacteria.p__Firmicutes.c__Bacilli.o__Mycoplasmatales.f__Mycoplasmataceae | 2.799039 |  |  | - |
| k__Bacteria.p__Firmicutes.c__Clostridia.o__Oscillospirales.f__Butyricicoccaceae.g__UCG_009 | 3.262092 |  |  | - |
| k__Bacteria.p__Firmicutes.c__Clostridia.o__Oscillospirales.f__Oscillospiraceae.g__Flavonifractor | 2.470133 |  |  | - |
| k__Bacteria.p__Proteobacteria.c__Alphaproteobacteria.o__Caulobacterales.f__Hyphomonadaceae.g__Hirschia | 1.773332 |  |  | - |
| k__Bacteria.p__Verrucomicrobiota.c__Chlamydiae.o__Chlamydiales | 1.875206 |  |  | - |
| k__Bacteria.p__Firmicutes.c__Negativicutes | 1.841575 |  |  | - |
| k__Bacteria.p__Elusimicrobiota | 2.59146 |  |  | - |
| k__Bacteria.p__Bdellovibrionota.c__Oligoflexia.o__Oligoflexales | 0.865093 |  |  | - |
| k__Bacteria.p__Firmicutes.c__Clostridia.o__Lachnospirales.f__Lachnospiraceae.g__Marvinbryantia | 2.737237 |  |  | - |
| k__Bacteria.p__Bdellovibrionota.c__Oligoflexia.o__0319_6G20 | 2.313704 |  |  | - |
| k__Bacteria.p__Firmicutes.c__Bacilli.o__Staphylococcales.f__Staphylococcaceae.g__Staphylococcus | 2.641893 |  |  | - |
| k__Bacteria.p__Proteobacteria.c__Alphaproteobacteria.o__Rhizobiales.f__Beijerinckiaceae.g__Microvirga | 1.779009 |  |  | - |
| k__Bacteria.p__Actinobacteriota.c__Coriobacteriia.o__Coriobacteriales.f__Eggerthellaceae.g__Adlercreutzia | 2.230035 |  |  | - |
| k__Bacteria.p__Proteobacteria.c__Alphaproteobacteria.o__Rhizobiales.f__Methyloligellaceae | 1.442695 |  |  | - |
| k__Bacteria.p__Fibrobacterota.c__Fibrobacteria.o__Fibrobacterales.f__Fibrobacterales.g__BBMC_4 | 0.865093 |  |  | - |
| k__Bacteria.p__Acidobacteriota.c__Acidobacteriae.o__Acidobacteriales.f__Koribacteraceae | 2.564413 |  |  | - |
| k__Bacteria.p__Acidobacteriota.c__Acidobacteriae.o__PAUC26f | 1.811791 |  |  | - |
| k__Bacteria.p__Firmicutes.c__Clostridia.o__Oscillospirales.f__Ruminococcaceae | 4.090733 |  |  | - |
| k__Bacteria.p__Actinobacteriota.c__Actinobacteria.o__Bifidobacteriales.f__Bifidobacteriaceae.g__Bifidobacterium | 2.884511 |  |  | - |
| k__Bacteria.p__Firmicutes.c__Clostridia.o__Oscillospirales.f__Ruminococcaceae.g__Anaerotruncus | 2.909493 |  |  | - |
| k__Bacteria.p__Firmicutes.c__Clostridia.o__Peptostreptococcales_Tissierellales.f__Anaerovoracaceae.g___Eubacterium__brachy_group | 1.620296 |  |  | - |
| k__Bacteria.p__Acidobacteriota.c__Acidobacteriae.o__Acidobacteriales.f__Acidobacteriaceae__Subgroup_1_.g__Acidipila | 2.611701 |  |  | - |
| k__Bacteria.p__Firmicutes.c__Clostridia.o__Lachnospirales.f__Lachnospiraceae.g__Blautia | 3.892868 |  |  | - |
| k__Bacteria.p__Firmicutes.c__Bacilli.o__Mycoplasmatales | 2.799039 |  |  | - |
| k__Bacteria.p__Firmicutes.c__Bacilli.o__Thermoactinomycetales.f__Thermoactinomycetaceae | 1.705432 |  |  | - |
| k__Bacteria.p__Desulfobacterota.c__Desulfovibrionia.o__Desulfovibrionales | 4.256902 |  |  | - |
| k__Bacteria.p__Bacteroidota.c__Kapabacteria.o__Kapabacteriales.f__Kapabacteriales | 2.039643 |  |  | - |
| k__Bacteria.p__Bacteroidota.c__Bacteroidia.o__Chitinophagales.f__Chitinophagaceae.g__Taibaiella | 1.404907 |  |  | - |
| k__Bacteria.p__Bacteroidota.c__Bacteroidia.o__Cytophagales.f__Hymenobacteraceae.g__Hymenobacter | 2.468411 | C | 2.266619 | 0.033705 |
| k__Bacteria.p__Latescibacterota.c__Latescibacteria.o__Latescibacterales.f__Latescibacteraceae.g__Latescibacteraceae | 1.644063 |  |  | - |
| k__Bacteria.p__Spirochaetota.c__Spirochaetia.o__Spirochaetales.f__Spirochaetaceae.g__Treponema | 2.827824 |  |  | - |
| k__Bacteria.p__Proteobacteria.c__Gammaproteobacteria.o__Xanthomonadales.f__Xanthomonadaceae.g__Lysobacter | 2.556527 |  |  | - |
| k__Bacteria.p__Deferribacterota.c__Deferribacteres.o__Deferribacterales.f__Deferribacteraceae.g__Mucispirillum | 2.70096 |  |  | - |
| k__Bacteria.p__Acidobacteriota.c__Subgroup_11 | 1.397506 |  |  | - |
| k__Bacteria.p__Proteobacteria.c__Alphaproteobacteria.o__Parvibaculales.f__Parvibaculaceae | 1.609603 |  |  | - |
| k__Bacteria.p__Bacteroidota.c__Bacteroidia.o__Sphingobacteriales | 2.919565 |  |  | - |
| k__Bacteria.p__Proteobacteria.c__Gammaproteobacteria.o__Burkholderiales.f__Oxalobacteraceae.g__Duganella | 1.721664 |  |  | - |
| k__Bacteria.p__Firmicutes.c__Bacilli.o__Lactobacillales.f__Carnobacteriaceae.g__Granulicatella | 2.444232 |  |  | - |
| k__Bacteria.p__Gemmatimonadota.c__Gemmatimonadetes.o__Gemmatimonadales | 3.90019 |  |  | - |
| k__Bacteria.p__Firmicutes.c__Bacilli.o__Staphylococcales.f__Staphylococcaceae | 2.641893 |  |  | - |
| k__Bacteria.p__Bacteroidota.c__Bacteroidia.o__Chitinophagales.f__Chitinophagaceae.g__Parasegetibacter | 1.749825 |  |  | - |
| k__Bacteria.p__Myxococcota.c__Polyangia.o__Polyangiales.f__Phaselicystidaceae | 2.620191 |  |  | - |
| k__Bacteria.p__Proteobacteria.c__Alphaproteobacteria.o__Azospirillales | 2.251293 |  |  | - |
| k__Bacteria.p__MBNT15 | 0.861553 |  |  | - |
| k__Bacteria.p__Proteobacteria.c__Alphaproteobacteria.o__Rickettsiales.f__Rickettsiaceae.g__Rickettsia | 1.651854 |  |  | - |
| k__Bacteria.p__Actinobacteriota.c__Actinobacteria.o__Streptosporangiales | 1.671626 |  |  | - |
| k__Bacteria.p__Proteobacteria.c__Gammaproteobacteria.o__Burkholderiales.f__Nitrosomonadaceae | 3.386021 |  |  | - |
| k__Bacteria.p__Firmicutes.c__Bacilli.o__Brevibacillales.f__Brevibacillaceae | 2.800757 |  |  | - |
| k__Bacteria.p__Proteobacteria.c__Gammaproteobacteria.o__Burkholderiales.f__Comamonadaceae.g__Aquabacterium | 1.624496 |  |  | - |
| k__Bacteria.p__Bacteroidota.c__Bacteroidia.o__Sphingobacteriales.f__Sphingobacteriaceae.g__Pseudopedobacter | 1.27515 |  |  | - |
| k__Bacteria.p__Firmicutes.c__Bacilli.o__Lactobacillales.f__Aerococcaceae | 2.383371 |  |  | - |
| k__Bacteria.p__Bacteroidota.c__Bacteroidia.o__Sphingobacteriales.f__Sphingobacteriaceae.g__Mucilaginibacter | 2.266017 |  |  | - |
| k__Bacteria.p__Proteobacteria.c__Gammaproteobacteria.o__Enterobacterales.f__Yersiniaceae.g__Serratia | 1.842238 |  |  | - |
| k__Bacteria.p__Proteobacteria.c__Gammaproteobacteria.o__Burkholderiales.f__Oxalobacteraceae.g__Undibacterium | 0.930046 |  |  | - |
| k__Bacteria.p__Proteobacteria.c__Gammaproteobacteria.o__Pseudomonadales.f__Moraxellaceae.g__Cavicella | 1.183143 |  |  | - |
| k__Bacteria.p__Firmicutes.c__Clostridia.o__Oscillospirales.f__Oscillospiraceae | 4.446879 |  |  | - |
| k__Bacteria.p__Proteobacteria.c__Alphaproteobacteria.o__Sphingomonadales.f__Sphingomonadaceae.g__Novosphingobium | 0.471526 |  |  | - |
| k__Bacteria.p__Proteobacteria.c__Gammaproteobacteria.o__KF_JG30_C25 | 2.94646 |  |  | - |
| k__Bacteria.p__Firmicutes.c__Clostridia.o__Lachnospirales.f__Defluviitaleaceae | 1.704693 |  |  | - |
| k__Bacteria.p__Proteobacteria.c__Gammaproteobacteria.o__Coxiellales | 1.430461 |  |  | - |
| k__Bacteria.p__Proteobacteria.c__Gammaproteobacteria.o__Burkholderiales.f__Oxalobacteraceae.g__Oxalicibacterium | 1.937685 |  |  | - |
| k__Bacteria.p__Proteobacteria.c__Gammaproteobacteria.o__JG36_TzT_191.f__JG36_TzT_191 | 0.460793 |  |  | - |
| k__Bacteria.p__Proteobacteria.c__Gammaproteobacteria.o__Enterobacterales.f__Enterobacteriaceae | 5.351713 |  |  | - |
| k__Bacteria.p__Bacteroidota.c__Bacteroidia.o__Bacteroidales.f__Porphyromonadaceae.g__Porphyromonas | 3.501228 |  |  | - |
| k__Bacteria.p__Proteobacteria.c__Gammaproteobacteria.o__Burkholderiales.f__Comamonadaceae.g__Variovorax | 1.234145 |  |  | - |
| k__Bacteria.p__Proteobacteria.c__Alphaproteobacteria.o__Rhodospirillales.f__Rhodospirillaceae | 2.496126 |  |  | - |
| k__Bacteria.p__Verrucomicrobiota.c__Verrucomicrobiae.o__Pedosphaerales | 2.601756 |  |  | - |
| k__Bacteria.p__Proteobacteria.c__Gammaproteobacteria.o__Burkholderiales.f__Alcaligenaceae.g__Alcaligenes | 2.373181 | O | 2.133991 | 0.047049 |
| k__Bacteria.p__SAR324_clade_Marine_group_B_.c__SAR324_clade_Marine_group_B_.o__SAR324_clade_Marine_group_B_ | 1.124738 |  |  | - |
| k__Bacteria.p__Myxococcota.c__Polyangia.o__Polyangiales | 3.098297 |  |  | - |
| k__Bacteria.p__Proteobacteria.c__Alphaproteobacteria.o__Rhizobiales.f__Amb_16S_1323 | 2.305054 |  |  | - |
| k__Bacteria.p__Bacteroidota.c__Bacteroidia.o__Sphingobacteriales.f__env_OPS_17.g__env_OPS_17 | 2.37614 |  |  | - |
| k__Bacteria.p__Actinobacteriota.c__Actinobacteria.o__Micrococcales.f__Intrasporangiaceae.g__Phycicoccus | 2.302518 |  |  | - |
| k__Bacteria.p__Firmicutes.c__Clostridia.o__Lachnospirales.f__Lachnospiraceae.g__ASF356 | 3.359108 |  |  | - |
| k__Bacteria.p__Gemmatimonadota | 3.949477 |  |  | - |
| k__Bacteria.p__Proteobacteria.c__Gammaproteobacteria.o__Acidiferrobacterales.f__Acidiferrobacteraceae.g__Sulfurifustis | 1.712442 |  |  | - |
| k__Bacteria.p__Actinobacteriota.c__Thermoleophilia.o__Solirubrobacterales.f__Solirubrobacteraceae.g__Solirubrobacter | 1.158687 |  |  | - |
| k__Bacteria.p__Desulfobacterota.c__Desulfovibrionia.o__Desulfovibrionales.f__Desulfovibrionaceae.g__Bilophila | 3.017898 |  |  | - |
| k__Bacteria.p__Firmicutes.c__Clostridia.o__Lachnospirales.f__Lachnospiraceae.g__Lachnospira | 0.624754 |  |  | - |
| k__Bacteria.p__Acidobacteriota.c__Acidobacteriae.o__Acidobacteriales.f__Acidobacteriaceae__Subgroup_1_.g__Edaphobacter | 1.914788 |  |  | - |
| k__Bacteria.p__Firmicutes.c__Clostridia.o__Peptostreptococcales_Tissierellales.f__Peptostreptococcaceae.g__Peptostreptococcus | 2.103597 |  |  | - |
| k__Bacteria.p__Actinobacteriota.c__Coriobacteriia.o__Coriobacteriales.f__Eggerthellaceae.g__Eggerthella | 1.828079 | O | 2.207171 | 0.022104 |
| k__Bacteria.p__Myxococcota.c__Polyangia.o__Polyangiales.f__Polyangiaceae | 2.324292 |  |  | - |
| k__Bacteria.p__Bacteroidota.c__Bacteroidia.o__Chitinophagales.f__Chitinophagaceae.g__Puia | 1.591488 |  |  | - |
| k__Bacteria.p__Patescibacteria.c__Saccharimonadia.o__Saccharimonadales.f__S32.g__TM7 | 2.512441 |  |  | - |
| k__Bacteria.p__Myxococcota.c__bacteriap25 | 2.572408 |  |  | - |
| k__Bacteria.p__Firmicutes.c__Clostridia.o__Lachnospirales.f__Lachnospiraceae.g__Roseburia | 3.921603 |  |  | - |
| k__Bacteria.p__Cyanobacteria.c__Cyanobacteriia | 1.828422 |  |  | - |
| k__Bacteria.p__Fusobacteriota | 4.171527 |  |  | - |
| k__Bacteria.p__Armatimonadota.c__Fimbriimonadia.o__Fimbriimonadales.f__Fimbriimonadaceae.g__Fimbriimonadaceae | 0.322512 |  |  | - |
| k__Bacteria.p__Proteobacteria.c__Gammaproteobacteria.o__CCD24.f__CCD24 | 1.826682 |  |  | - |
| k__Bacteria.p__Cyanobacteria | 2.413215 |  |  | - |
| k__Bacteria.p__Actinobacteriota.c__Coriobacteriia.o__Coriobacteriales.f__Coriobacteriaceae.g__Collinsella | 0.425226 |  |  | - |
| k__Bacteria.p__Proteobacteria.c__Alphaproteobacteria.o__Rhodobacterales.f__Rhodobacteraceae.g__Paracoccus | 1.222616 |  |  | - |
| k__Bacteria.p__Elusimicrobiota.c__Lineage_IIa.o__Lineage_IIa | 1.570129 |  |  | - |
| k__Bacteria.p__Acidobacteriota.c__Blastocatellia | 1.80122 |  |  | - |
| k__Bacteria.p__Firmicutes.c__Clostridia.o__Peptostreptococcales_Tissierellales.f__Peptostreptococcales_Tissierellales.g__Alkaliphilus | 1.300886 |  |  | - |
| k__Bacteria.p__Proteobacteria.c__Gammaproteobacteria.o__Gammaproteobacteria_Incertae_Sedis.f__Unknown_Family.g__Acidibacter | 2.955775 |  |  | - |
| k__Bacteria.p__Firmicutes.c__Bacilli.o__Brevibacillales | 2.800757 |  |  | - |
| k__Bacteria.p__Patescibacteria.c__Saccharimonadia.o__Saccharimonadales.f__S32 | 2.512441 |  |  | - |
| k__Bacteria.p__Myxococcota.c__Polyangia.o__Polyangiales.f__Polyangiaceae.g__Pajaroellobacter | 2.288091 |  |  | - |
| k__Bacteria.p__Bacteroidota.c__Bacteroidia.o__Sphingobacteriales.f__Lentimicrobiaceae | 1.710802 |  |  | - |
| k__Bacteria.p__Patescibacteria.c__Parcubacteria | 1.905844 |  |  | - |
| k__Bacteria.p__Actinobacteriota.c__Coriobacteriia.o__Coriobacteriales.f__Coriobacteriaceae | 0.425226 |  |  | - |
| k__Bacteria.p__Proteobacteria.c__Gammaproteobacteria.o__WD260.f__WD260 | 0.872054 |  |  | - |
| k__Bacteria.p__Proteobacteria.c__Gammaproteobacteria.o__Cellvibrionales.f__Cellvibrionaceae.g__Cellvibrio | 2.088493 |  |  | - |
| k__Bacteria.p__Proteobacteria.c__Alphaproteobacteria.o__Rhizobiales.f__D05_2.g__D05_2 | 2.3923 |  |  | - |
| k__Bacteria.p__Acidobacteriota.c__Acidobacteriae | 4.136067 |  |  | - |
| k__Bacteria.p__Proteobacteria.c__Alphaproteobacteria.o__Rhodobacterales.f__Rhodobacteraceae | 2.555381 | O | 2.286802 | 0.045759 |
| k__Bacteria.p__Firmicutes.c__Clostridia.o__Peptostreptococcales_Tissierellales.f__Peptostreptococcales_Tissierellales.g__Peptoniphilus | 1.365384 |  |  | - |
| k__Bacteria.p__Firmicutes.c__Clostridia.o__Oscillospirales.f__Ruminococcaceae.g__Paludicola | 1.572672 |  |  | - |
| k__Bacteria.p__Proteobacteria.c__Gammaproteobacteria.o__WD260.f__WD260.g__WD260 | 0.872054 |  |  | - |
| k__Bacteria.p__Proteobacteria.c__Gammaproteobacteria.o__Xanthomonadales.f__Rhodanobacteraceae | 3.644922 |  |  | - |
| k__Bacteria.p__MBNT15.c__MBNT15 | 0.861553 |  |  | - |
| k__Bacteria.p__Proteobacteria.c__Gammaproteobacteria.o__JG36_TzT_191 | 0.460793 |  |  | - |
| k__Bacteria.p__Proteobacteria.c__Alphaproteobacteria.o__Rhizobiales.f__D05_2 | 2.3923 |  |  | - |
| k__Bacteria.p__Acidobacteriota.c__Subgroup_22.o__Subgroup_22 | 1.339608 |  |  | - |
| k__Bacteria.p__Actinobacteriota.c__MB_A2_108.o__MB_A2_108 | 2.355094 |  |  | - |
| k__Bacteria.p__Proteobacteria.c__Alphaproteobacteria.o__Rhodospirillales | 2.680541 |  |  | - |
| k__Bacteria.p__Proteobacteria.c__Gammaproteobacteria.o__Gammaproteobacteria_Incertae_Sedis.f__Unknown_Family | 2.982249 |  |  | - |
| k__Bacteria.p__Actinobacteriota.c__Actinobacteria.o__Streptomycetales.f__Streptomycetaceae | 3.064692 |  |  | - |
| k__Bacteria.p__Actinobacteriota.c__Coriobacteriia.o__Coriobacteriales.f__Atopobiaceae.g__Olsenella | 2.121184 |  |  | - |
| k__Bacteria.p__Actinobacteriota.c__Actinobacteria.o__Kineosporiales | 1.430005 |  |  | - |
| k__Bacteria.p__Proteobacteria.c__Alphaproteobacteria.o__Defluviicoccales | 1.682376 |  |  | - |
| k__Bacteria.p__Firmicutes.c__Clostridia.o__Oscillospirales.f__Ruminococcaceae.g__UBA1819 | 3.115719 |  |  | - |
| k__Bacteria.p__Myxococcota.c__Myxococcia.o__Myxococcales.f__Anaeromyxobacteraceae.g__Anaeromyxobacter | 1.915474 |  |  | - |
| k__Bacteria.p__Acidobacteriota.c__Acidobacteriae.o__Subgroup_2 | 3.657284 |  |  | - |
| k__Bacteria.p__Proteobacteria.c__Gammaproteobacteria.o__Burkholderiales.f__Nitrosomonadaceae.g__Nitrosospira | 0.720452 |  |  | - |
| k__Bacteria.p__Bacteroidota.c__Bacteroidia.o__Sphingobacteriales.f__CWT_CU03_E12.g__CWT_CU03_E12 | 0.322512 |  |  | - |
| k__Bacteria.p__Chloroflexi.c__Ktedonobacteria.o__C0119.f__C0119.g__C0119 | 1.378622 |  |  | - |
| k__Bacteria.p__Proteobacteria.c__Gammaproteobacteria.o__Burkholderiales.f__SC_I_84 | 3.06635 |  |  | - |
| k__Bacteria.p__Acidobacteriota.c__Acidobacteriae.o__Subgroup_13.f__Subgroup_13.g__Subgroup_13 | 2.70973 |  |  | - |
| k__Bacteria.p__Proteobacteria.c__Alphaproteobacteria.o__Parvibaculales | 1.609603 |  |  | - |
| k__Bacteria.p__Actinobacteriota.c__Thermoleophilia.o__Solirubrobacterales.f__Solirubrobacteraceae.g__Conexibacter | 1.090513 |  |  | - |
| k__Bacteria.p__Gemmatimonadota.c__Gemmatimonadetes | 3.90019 |  |  | - |
| k__Bacteria.p__Proteobacteria.c__Alphaproteobacteria.o__Rickettsiales.f__Rickettsiaceae | 1.757546 |  |  | - |
| k__Bacteria.p__Acidobacteriota.c__Blastocatellia.o__11_24 | 1.530422 |  |  | - |
| k__Bacteria.p__Proteobacteria.c__Gammaproteobacteria.o__Burkholderiales.f__Nitrosomonadaceae.g__mle1_7 | 1.978863 |  |  | - |
| k__Bacteria.p__Proteobacteria.c__Alphaproteobacteria.o__Rhizobiales.f__Labraceae.g__Labrys | 1.483583 |  |  | - |
| k__Bacteria.p__Chloroflexi.c__Ktedonobacteria.o__C0119.f__C0119 | 1.378622 |  |  | - |
| k__Bacteria.p__Elusimicrobiota.c__Lineage_IIa.o__Lineage_IIa.f__Lineage_IIa | 1.570129 |  |  | - |
| k__Bacteria.p__Bacteroidota.c__Bacteroidia.o__Bacteroidales.f__Bacteroidaceae.g__Bacteroides | 4.554887 |  |  | - |
| k__Bacteria.p__Fusobacteriota.c__Fusobacteriia.o__Fusobacteriales.f__Fusobacteriaceae.g__Fusobacterium | 3.908271 |  |  | - |
| k__Bacteria.p__Proteobacteria.c__Gammaproteobacteria.o__Salinisphaerales.f__Solimonadaceae.g__Polycyclovorans | 1.904372 |  |  | - |
| k__Bacteria.p__Actinobacteriota.c__Actinobacteria.o__Corynebacteriales.f__Corynebacteriaceae.g__Lawsonella | 0.524509 |  |  | - |
| k__Bacteria.p__Acidobacteriota.c__Subgroup_5.o__Subgroup_5.f__Subgroup_5 | 2.67495 |  |  | - |
| k__Bacteria.p__Actinobacteriota.c__Actinobacteria.o__Streptomycetales.f__Streptomycetaceae.g__Kitasatospora | 1.421278 |  |  | - |
| k__Bacteria.p__Firmicutes.c__Clostridia.o__Lachnospirales.f__Lachnospiraceae.g___Eubacterium__hallii_group | 2.295835 |  |  | - |
| k__Bacteria.p__Bacteroidota.c__Bacteroidia.o__Flavobacteriales.f__Weeksellaceae | 2.749258 |  |  | - |
| k__Bacteria.p__Firmicutes.c__Clostridia.o__Peptostreptococcales_Tissierellales | 3.907632 |  |  | - |
| k__Bacteria.p__Firmicutes.c__Limnochordia.o__Limnochordales.f__Limnochordaceae.g__Limnochordaceae | 1.724432 |  |  | - |
| k__Bacteria.p__Actinobacteriota.c__Actinobacteria.o__Streptosporangiales.f__Thermomonosporaceae.g__Actinomadura | 1.56001 |  |  | - |
| k__Bacteria.p__Firmicutes.c__Clostridia.o__Oscillospirales | 4.708465 |  |  | - |
| k__Bacteria.p__Acidobacteriota.c__Holophagae.o__Subgroup_7 | 1.691859 |  |  | - |
| k__Bacteria.p__Firmicutes.c__Bacilli.o__Paenibacillales.f__Paenibacillaceae | 3.817755 |  |  | - |
| k__Bacteria.p__Gemmatimonadota.c__S0134_terrestrial_group.o__S0134_terrestrial_group.f__S0134_terrestrial_group | 1.714819 |  |  | - |
| k__Bacteria.p__Proteobacteria.c__Gammaproteobacteria.o__Burkholderiales.f__Comamonadaceae.g__Diaphorobacter | 1.398447 |  |  | - |
| k__Bacteria.p__Firmicutes.c__Clostridia.o__Lachnospirales.f__Lachnospiraceae.g__Agathobacter | 2.20401 |  |  | - |
| k__Bacteria.p__Proteobacteria.c__Gammaproteobacteria.o__PLTA13 | 1.500696 |  |  | - |
| k__Bacteria.p__Cyanobacteria.c__Sericytochromatia.o__Sericytochromatia.f__Sericytochromatia | 2.282365 |  |  | - |
| k__Bacteria.p__Fibrobacterota.c__Fibrobacteria.o__Fibrobacterales | 2.674465 |  |  | - |
| k__Bacteria.p__Proteobacteria.c__Alphaproteobacteria.o__Rhizobiales.f__Rhizobiales_Incertae_Sedis.g__Bauldia | 1.259493 |  |  | - |
| k__Bacteria.p__Firmicutes.c__Clostridia.o__Lachnospirales.f__Lachnospiraceae.g__A2 | 2.563435 |  |  | - |
| k__Bacteria.p__Proteobacteria.c__Gammaproteobacteria.o__Acidiferrobacterales | 1.712442 |  |  | - |
| k__Bacteria.p__Actinobacteriota.c__Actinobacteria.o__Micrococcales.f__Microbacteriaceae | 1.780255 |  |  | - |
| k__Bacteria.p__Elusimicrobiota.c__Lineage_IIb.o__Lineage_IIb.f__Lineage_IIb.g__Lineage_IIb | 1.88581 |  |  | - |
| k__Bacteria.p__Bacteroidota.c__Bacteroidia.o__Chitinophagales.f__Chitinophagaceae.g__Flavisolibacter | 2.595579 |  |  | - |
| k__Bacteria.p__Firmicutes.c__Clostridia.o__Lachnospirales.f__Lachnospiraceae.g__Lachnospiraceae_ND3007_group | 2.2711 | O | 2.292495 | 0.043012 |
| k__Bacteria.p__Actinobacteriota.c__Actinobacteria.o__Micromonosporales.f__Micromonosporaceae.g__Hamadaea | 2.004316 |  |  | - |
| k__Bacteria.p__SAR324_clade_Marine_group_B_.c__SAR324_clade_Marine_group_B_.o__SAR324_clade_Marine_group_B_.f__SAR324_clade_Marine_group_B_.g__SAR324_clade_Marine_group_B_ | 1.124738 |  |  | - |
| k__Bacteria.p__Proteobacteria.c__Gammaproteobacteria.o__Legionellales.f__Legionellaceae.g__Legionella | 1.640638 |  |  | - |
| k__Bacteria.p__Proteobacteria.c__Gammaproteobacteria.o__Burkholderiales.f__Methylophilaceae | 2.635157 |  |  | - |
| k__Bacteria.p__Proteobacteria.c__Alphaproteobacteria.o__Paracaedibacterales | 2.526187 |  |  | - |
| k__Bacteria.p__Proteobacteria.c__Alphaproteobacteria.o__Rhizobiales.f__Xanthobacteraceae.g__Afipia | 2.568155 |  |  | - |
| k__Bacteria.p__Actinobacteriota.c__Actinobacteria.o__Corynebacteriales.f__Nocardiaceae.g__Rhodococcus | 1.393062 |  |  | - |
| k__Bacteria.p__Firmicutes.c__Clostridia.o__Lachnospirales.f__Lachnospiraceae.g__GCA_900066575 | 3.116179 |  |  | - |
| k__Bacteria.p__Firmicutes.c__Clostridia.o__Oscillospirales.f__Ruminococcaceae.g__Ruminococcus | 3.097008 |  |  | - |
| k__Bacteria.p__Proteobacteria.c__Gammaproteobacteria.o__Enterobacterales.f__Enterobacteriaceae.g__Escherichia_Shigella | 5.303136 |  |  | - |
| k__Bacteria.p__Proteobacteria.c__Gammaproteobacteria.o__Burkholderiales.f__Comamonadaceae | 3.078163 |  |  | - |
| k__Bacteria.p__Verrucomicrobiota.c__Verrucomicrobiae.o__Verrucomicrobiales | 2.391624 | C | 2.200361 | 0.034381 |
| k__Bacteria.p__Actinobacteriota.c__Actinobacteria.o__Micrococcales.f__Micrococcaceae.g__Pseudarthrobacter | 2.72058 |  |  | - |
| k__Bacteria.p__Bdellovibrionota.c__Oligoflexia.o__0319_6G20.f__0319_6G20 | 2.313704 |  |  | - |
| k__Bacteria.p__Actinobacteriota.c__Actinobacteria.o__Micrococcales | 3.333663 |  |  | - |
| k__Bacteria.p__Firmicutes.c__Clostridia.o__Clostridia.f__Hungateiclostridiaceae.g__Ruminiclostridium | 1.54264 |  |  | - |
| k__Bacteria.p__Proteobacteria.c__Gammaproteobacteria.o__Cardiobacteriales | 2.166667 |  |  | - |
| k__Bacteria.p__Proteobacteria.c__Gammaproteobacteria.o__Coxiellales.f__Coxiellaceae.g__Coxiella | 1.430461 |  |  | - |
| k__Bacteria.p__Proteobacteria.c__Gammaproteobacteria.o__Ga0077536.f__Ga0077536 | 1.85477 |  |  | - |
| k__Bacteria.p__Firmicutes.c__Clostridia.o__Lachnospirales.f__Defluviitaleaceae.g__Defluviitaleaceae_UCG_011 | 1.704693 |  |  | - |
| k__Bacteria.p__Acidobacteriota.c__Blastocatellia.o__Blastocatellales.f__Blastocatellaceae | 1.467696 |  |  | - |
| k__Bacteria.p__Elusimicrobiota.c__Elusimicrobia.o__MVP_88.f__MVP_88.g__MVP_88 | 2.53396 |  |  | - |
| k__Bacteria.p__Gemmatimonadota.c__Longimicrobia.o__Longimicrobiales | 2.955762 |  |  | - |
| k__Bacteria.p__Actinobacteriota.c__Thermoleophilia.o__Solirubrobacterales.f__67_14.g__67_14 | 1.701832 |  |  | - |
| k__Bacteria.p__Firmicutes.c__Clostridia.o__Lachnospirales.f__Lachnospiraceae.g___Eubacterium__fissicatena_group | 2.042438 |  |  | - |
| k__Bacteria.p__Proteobacteria.c__Gammaproteobacteria.o__Enterobacterales.f__Enterobacteriaceae.g__Klebsiella | 2.882181 |  |  | - |
| k__Bacteria.p__Firmicutes.c__Clostridia.o__Peptostreptococcales_Tissierellales.f__Peptostreptococcales_Tissierellales | 2.007771 |  |  | - |
| k__Bacteria.p__Verrucomicrobiota.c__Chlamydiae | 1.875206 |  |  | - |
| k__Bacteria.p__Firmicutes.c__Clostridia.o__Lachnospirales.f__Lachnospiraceae.g__Anaerostipes | 2.686042 |  |  | - |
| k__Bacteria.p__Bacteroidota.c__Bacteroidia.o__Bacteroidales.f__Muribaculaceae | 5.07271 |  |  | - |
| k__Bacteria.p__Proteobacteria.c__Gammaproteobacteria.o__Burkholderiales.f__Hydrogenophilaceae.g__Thiobacillus | 2.027286 |  |  | - |
| k__Bacteria.p__Acidobacteriota.c__Holophagae.o__Holophagales | 2.736379 |  |  | - |
| k__Bacteria.p__Firmicutes.c__Clostridia.o__Lachnospirales.f__Lachnospiraceae.g___Eubacterium__eligens_group | 2.010916 |  |  | - |
| k__Bacteria.p__Proteobacteria.c__Gammaproteobacteria.o__Gammaproteobacteria_Incertae_Sedis.f__Unknown_Family.g__Unknown_Family | 1.754117 |  |  | - |
| k__Bacteria.p__Desulfobacterota | 4.256902 |  |  | - |
| k__Bacteria.p__Proteobacteria.c__Alphaproteobacteria.o__Caulobacterales.f__Caulobacteraceae.g__Phenylobacterium | 2.331239 |  |  | - |
| k__Bacteria.p__Proteobacteria.c__Gammaproteobacteria.o__Pasteurellales.f__Pasteurellaceae.g__Rodentibacter | 4.108031 |  |  | - |
| k__Bacteria.p__Bdellovibrionota.c__Oligoflexia | 2.313704 |  |  | - |
| k__Bacteria.p__Actinobacteriota.c__Thermoleophilia | 3.571209 |  |  | - |
| k__Bacteria.p__Armatimonadota.c__Fimbriimonadia | 0.322512 |  |  | - |
| k__Bacteria.p__Proteobacteria.c__Gammaproteobacteria.o__Burkholderiales.f__Oxalobacteraceae.g__Noviherbaspirillum | 1.835809 |  |  | - |
| k__Bacteria.p__Proteobacteria.c__Gammaproteobacteria.o__Burkholderiales.f__Comamonadaceae.g__Rhizobacter | 1.673043 |  |  | - |
| k__Bacteria.p__Bacteroidota.c__Bacteroidia.o__Chitinophagales.f__Chitinophagaceae.g__UTBCD1 | 1.38388 |  |  | - |
| k__Bacteria.p__Bacteroidota.c__Bacteroidia.o__Chitinophagales.f__Chitinophagaceae.g__Chitinophaga | 1.505886 |  |  | - |
| k__Bacteria.p__Firmicutes.c__Clostridia.o__Oscillospirales.f__UCG_010 | 3.242145 |  |  | - |
| k__Bacteria.p__Nitrospirota | 2.967335 |  |  | - |
| k__Bacteria.p__Firmicutes.c__Clostridia.o__Oscillospirales.f__UCG_010.g__UCG_010 | 3.242145 |  |  | - |
| k__Bacteria.p__Campilobacterota.c__Campylobacteria.o__Campylobacterales.f__Campylobacteraceae.g__Campylobacter | 3.288683 |  |  | - |
| k__Bacteria.p__Firmicutes.c__Clostridia.o__Lachnospirales.f__Lachnospiraceae.g__Coprococcus | 1.712551 |  |  | - |
| k__Bacteria.p__Firmicutes.c__Clostridia.o__Lachnospirales.f__Lachnospiraceae.g__Lachnospiraceae_AC2044_group | 2.32628 |  |  | - |
| k__Bacteria.p__Firmicutes.c__Limnochordia.o__Limnochordales | 1.724432 |  |  | - |
| k__Bacteria.p__Bacteroidota.c__Bacteroidia.o__Cytophagales.f__Cytophagaceae.g__Cytophaga | 2.628722 |  |  | - |
| k__Bacteria.p__Elusimicrobiota.c__Elusimicrobia.o__MVP_88.f__MVP_88 | 2.53396 |  |  | - |
| k__Bacteria.p__Fusobacteriota.c__Fusobacteriia | 4.171527 |  |  | - |
| k__Bacteria.p__Firmicutes.c__Clostridia.o__Lachnospirales | 5.096946 |  |  | - |
| k__Bacteria.p__Bacteroidota.c__Bacteroidia.o__Cytophagales | 3.242575 |  |  | - |
| k__Bacteria.p__Proteobacteria.c__Alphaproteobacteria.o__Acetobacterales | 2.728683 |  |  | - |
| k__Bacteria.p__Firmicutes.c__Bacilli.o__Erysipelotrichales.f__Erysipelotrichaceae | 3.97477 |  |  | - |
| k__Bacteria.p__Firmicutes.c__Clostridia.o__Oscillospirales.f__Oscillospiraceae.g__Oscillibacter | 3.369194 |  |  | - |
| k__Bacteria.p__Campilobacterota.c__Campylobacteria.o__Campylobacterales.f__Helicobacteraceae.g__Helicobacter | 4.193765 |  |  | - |
| k__Bacteria.p__Proteobacteria.c__Alphaproteobacteria.o__Rhizobiales.f__Rhizobiaceae | 2.767002 |  |  | - |
| k__Bacteria.p__Proteobacteria.c__Alphaproteobacteria.o__Rhizobiales.f__Xanthobacteraceae.g__Bradyrhizobium | 2.637736 |  |  | - |
| k__Bacteria.p__Bacteroidota.c__Bacteroidia.o__Cytophagales.f__Microscillaceae.g__OLB12 | 0.958463 |  |  | - |
| k__Bacteria.p__Firmicutes.c__Bacilli.o__Thermoactinomycetales | 1.705432 |  |  | - |
| k__Bacteria.p__Bacteroidota.c__Bacteroidia.o__Bacteroidales.f__Rikenellaceae.g__Alistipes | 4.085219 |  |  | - |
| k__Bacteria.p__Proteobacteria.c__Gammaproteobacteria.o__Enterobacterales.f__Enterobacteriaceae.g__Citrobacter | 3.190916 |  |  | - |
| k__Bacteria.p__Proteobacteria.c__Gammaproteobacteria.o__Burkholderiales.f__Neisseriaceae | 4.207285 |  |  | - |
| k__Bacteria.p__Firmicutes.c__Clostridia.o__Lachnospirales.f__Lachnospiraceae | 5.095259 |  |  | - |
| k__Bacteria.p__Proteobacteria.c__Alphaproteobacteria.o__Azospirillales.f__Inquilinaceae | 1.203048 |  |  | - |
| k__Bacteria.p__Bdellovibrionota.c__Bdellovibrionia.o__Bdellovibrionales.f__Bdellovibrionaceae.g__OM27_clade | 1.490055 |  |  | - |
| k__Bacteria.p__Proteobacteria.c__Gammaproteobacteria.o__Xanthomonadales.f__Rhodanobacteraceae.g__Rudaea | 2.257267 | C | 2.101711 | 0.034381 |
| k__Bacteria.p__Firmicutes.c__Bacilli.o__Erysipelotrichales.f__Erysipelotrichaceae.g__Faecalitalea | 0.731491 |  |  | - |
| k__Bacteria.p__Proteobacteria.c__Gammaproteobacteria.o__Burkholderiales.f__Oxalobacteraceae | 2.976848 |  |  | - |
| k__Bacteria.p__Actinobacteriota.c__Acidimicrobiia.o__Microtrichales.f__Iamiaceae.g__Iamia | 2.025786 |  |  | - |
| k__Bacteria.p__Actinobacteriota.c__Thermoleophilia.o__Solirubrobacterales.f__Solirubrobacteraceae | 1.842668 |  |  | - |
| k__Bacteria.p__Firmicutes.c__Bacilli.o__Erysipelotrichales.f__Erysipelatoclostridiaceae.g__Erysipelotrichaceae_UCG_003 | 1.667412 |  |  | - |
| k__Bacteria.p__Patescibacteria.c__Parcubacteria.o__Candidatus_Jorgensenbacteria.f__Candidatus_Jorgensenbacteria | 1.905844 |  |  | - |
| k__Bacteria.p__Actinobacteriota.c__Actinobacteria.o__Catenulisporales.f__Actinospicaceae.g__Actinospica | 2.437292 |  |  | - |
| k__Bacteria.p__Proteobacteria.c__Alphaproteobacteria.o__Rickettsiales | 2.46349 | C | 2.234009 | 0.034381 |
| k__Bacteria.p__Proteobacteria.c__Alphaproteobacteria.o__Rickettsiales.f__SM2D12.g__SM2D12 | 2.368306 |  |  | - |
| k__Bacteria.p__Proteobacteria.c__Gammaproteobacteria.o__Burkholderiales.f__Oxalobacteraceae.g___Aquaspirillum__arcticum_group | 1.966293 |  |  | - |
| k__Bacteria.p__Verrucomicrobiota.c__Chlamydiae.o__Chlamydiales.f__Parachlamydiaceae | 1.875206 |  |  | - |
| k__Bacteria.p__Firmicutes.c__Clostridia.o__Oscillospirales.f__Ruminococcaceae.g__Faecalibacterium | 2.512986 |  |  | - |
| k__Bacteria.p__Myxococcota.c__Polyangia | 3.269298 |  |  | - |
| k__Bacteria | 6 |  |  | - |
| k__Bacteria.p__Actinobacteriota.c__Acidimicrobiia.o__IMCC26256.f__IMCC26256.g__IMCC26256 | 2.845244 |  |  | - |
| k__Bacteria.p__Elusimicrobiota.c__Lineage_IIa.o__Lineage_IIa.f__Lineage_IIa.g__Lineage_IIa | 1.570129 |  |  | - |
| k__Bacteria.p__Actinobacteriota.c__Actinobacteria.o__Catenulisporales | 2.670748 |  |  | - |
| k__Bacteria.p__Proteobacteria.c__Gammaproteobacteria.o__Diplorickettsiales | 2.488212 |  |  | - |
| k__Bacteria.p__Firmicutes.c__Bacilli.o__Erysipelotrichales.f__Erysipelotrichaceae.g__Dubosiella | 2.18121 |  |  | - |
| k__Bacteria.p__Myxococcota.c__Polyangia.o__Polyangiales.f__Phaselicystidaceae.g__Phaselicystis | 2.620191 |  |  | - |
| k__Bacteria.p__Proteobacteria.c__Gammaproteobacteria.o__Pseudomonadales | 3.052172 |  |  | - |
| k__Bacteria.p__Bacteroidota | 5.352278 |  |  | - |
| k__Bacteria.p__Cyanobacteria.c__Sericytochromatia | 2.282365 |  |  | - |
| k__Bacteria.p__Actinobacteriota.c__MB_A2_108 | 2.355094 |  |  | - |
| k__Bacteria.p__Proteobacteria.c__Gammaproteobacteria.o__Pasteurellales.f__Pasteurellaceae.g__Actinobacillus | 2.636778 |  |  | - |
| k__Bacteria.p__Gemmatimonadota.c__BD2_11_terrestrial_group.o__BD2_11_terrestrial_group | 2.375087 |  |  | - |
| k__Bacteria.p__Proteobacteria.c__Alphaproteobacteria.o__Reyranellales.f__Reyranellaceae.g__Reyranella | 2.768592 |  |  | - |
| k__Bacteria.p__Bacteroidota.c__Bacteroidia.o__Bacteroidales.f__Rikenellaceae.g__Rikenella | 2.881786 |  |  | - |
| k__Bacteria.p__Firmicutes.c__Bacilli.o__RF39.f__RF39 | 2.404655 |  |  | - |
| k__Bacteria.p__Firmicutes.c__Clostridia.o__Lachnospirales.f__Lachnospiraceae.g__Oribacterium | 3.04471 |  |  | - |
| k__Bacteria.p__Firmicutes.c__Clostridia.o__Clostridia_vadinBB60_group.f__Clostridia_vadinBB60_group.g__Clostridia_vadinBB60_group | 2.998598 |  |  | - |
| k__Bacteria.p__Proteobacteria.c__Gammaproteobacteria.o__Pasteurellales.f__Pasteurellaceae.g__Aggregatibacter | 2.767992 |  |  | - |
| k__Bacteria.p__Proteobacteria.c__Gammaproteobacteria.o__Xanthomonadales.f__Rhodanobacteraceae.g__Rhodanobacteraceae | 2.074636 |  |  | - |
| k__Bacteria.p__Verrucomicrobiota.c__Chlamydiae.o__Chlamydiales.f__Parachlamydiaceae.g__Neochlamydia | 1.875206 |  |  | - |
| k__Bacteria.p__Actinobacteriota.c__Actinobacteria.o__Catenulisporales.f__Actinospicaceae | 2.437292 |  |  | - |
| k__Bacteria.p__Proteobacteria.c__Gammaproteobacteria.o__Enterobacterales.f__Enterobacteriaceae.g__Enterobacter | 2.658624 |  |  | - |
| k__Bacteria.p__Firmicutes.c__Clostridia.o__Lachnospirales.f__Lachnospiraceae.g__Lachnospiraceae_NK4A136_group | 4.69349 |  |  | - |
| k__Bacteria.p__Proteobacteria.c__Gammaproteobacteria.o__Xanthomonadales.f__Rhodanobacteraceae.g__Chujaibacter | 3.159273 |  |  | - |
| k__Bacteria.p__Proteobacteria.c__Alphaproteobacteria.o__Sphingomonadales.f__Sphingomonadaceae.g__Sphingobium | 1.549307 |  |  | - |
| k__Bacteria.p__Actinobacteriota.c__Actinobacteria.o__Propionibacteriales.f__Nocardioidaceae | 2.661582 |  |  | - |
| k__Bacteria.p__Gemmatimonadota.c__Gemmatimonadetes.o__Gemmatimonadales.f__Gemmatimonadaceae | 3.90019 |  |  | - |
| k__Bacteria.p__Bacteroidota.c__Bacteroidia.o__Chitinophagales.f__Chitinophagaceae.g__Chitinophagaceae | 1.42872 |  |  | - |
| k__Bacteria.p__Proteobacteria.c__Gammaproteobacteria.o__Burkholderiales.f__Burkholderiaceae.g__Burkholderia_Caballeronia_Paraburkholderia | 2.081963 |  |  | - |
| k__Bacteria.p__Actinobacteriota.c__Actinobacteria.o__Corynebacteriales.f__Mycobacteriaceae.g__Mycobacterium | 2.377942 |  |  | - |
| k__Bacteria.p__Proteobacteria.c__Alphaproteobacteria.o__Sphingomonadales.f__Sphingomonadaceae.g__Sphingomonas | 4.826944 |  |  | - |
| k__Bacteria.p__Gemmatimonadota.c__BD2_11_terrestrial_group | 2.375087 |  |  | - |
| k__Bacteria.p__Acidobacteriota.c__Acidobacteriae.o__Subgroup_13 | 2.70973 |  |  | - |
| k__Bacteria.p__Bacteroidota.c__Bacteroidia.o__Bacteroidales.f__Prevotellaceae.g__Prevotellaceae_NK3B31_group | 3.269496 |  |  | - |
| k__Bacteria.p__Elusimicrobiota.c__Elusimicrobia.o__Lineage_IV | 1.68491 |  |  | - |
| k__Bacteria.p__Actinobacteriota.c__Actinobacteria.o__Micrococcales.f__Micrococcaceae.g__Nesterenkonia | 1.042298 |  |  | - |
| k__Bacteria.p__Bacteroidota.c__Bacteroidia.o__Chitinophagales.f__Chitinophagaceae.g__Arachidicoccus | 1.634894 |  |  | - |
| k__Bacteria.p__Firmicutes.c__Clostridia.o__Lachnospirales.f__Lachnospiraceae.g__Tyzzerella | 2.139844 |  |  | - |
| k__Bacteria.p__Proteobacteria.c__Alphaproteobacteria.o__Reyranellales.f__Reyranellaceae | 2.828224 |  |  | - |
| k__Bacteria.p__Firmicutes.c__Limnochordia | 1.724432 |  |  | - |
| k__Bacteria.p__Acidobacteriota.c__Acidobacteriae.o__PAUC26f.f__PAUC26f.g__PAUC26f | 1.811791 |  |  | - |
| k__Bacteria.p__Firmicutes.c__Clostridia.o__Clostridia_UCG_014 | 4.177544 |  |  | - |
| k__Bacteria.p__Acidobacteriota.c__Subgroup_22.o__Subgroup_22.f__Subgroup_22 | 1.339608 |  |  | - |
| k__Bacteria.p__Actinobacteriota.c__Actinobacteria.o__0319_7L14.f__0319_7L14 | 1.54392 |  |  | - |
| k__Bacteria.p__Bacteroidota.c__Bacteroidia.o__Cytophagales.f__Hymenobacteraceae | 2.468411 |  |  | - |
| k__Bacteria.p__Elusimicrobiota.c__Lineage_IIb.o__Lineage_IIb.f__Lineage_IIb | 1.88581 |  |  | - |
| k__Bacteria.p__Actinobacteriota.c__Actinobacteria.o__Actinomycetales.f__Actinomycetaceae.g__F0332 | 1.777951 |  |  | - |
| k__Bacteria.p__Firmicutes.c__Clostridia.o__Lachnospirales.f__Lachnospiraceae.g__Stomatobaculum | 2.568806 |  |  | - |
| k__Bacteria.p__Proteobacteria.c__Alphaproteobacteria.o__Rhizobiales.f__Rhizobiales_Incertae_Sedis | 1.878631 |  |  | - |
| k__Bacteria.p__Spirochaetota.c__Spirochaetia.o__Spirochaetales.f__Spirochaetaceae | 2.827824 |  |  | - |
| k__Bacteria.p__Proteobacteria.c__Alphaproteobacteria.o__Sphingomonadales.f__Sphingomonadaceae.g__Sphingopyxis | 1.507171 |  |  | - |
| k__Bacteria.p__RCP2_54.c__RCP2_54.o__RCP2_54.f__RCP2_54.g__RCP2_54 | 2.540402 |  |  | - |
| k__Bacteria.p__Bdellovibrionota.c__Bdellovibrionia | 1.8076 |  |  | - |
| k__Bacteria.p__Proteobacteria.c__Gammaproteobacteria.o__Burkholderiales | 4.377793 |  |  | - |
| k__Bacteria.p__Firmicutes.c__Bacilli.o__Staphylococcales.f__Staphylococcaceae.g__Jeotgalicoccus | 0.557703 |  |  | - |
| k__Bacteria.p__Firmicutes.c__Clostridia.o__Peptostreptococcales_Tissierellales.f__Anaerovoracaceae.g__Anaerovorax | 1.829863 |  |  | - |
| k__Bacteria.p__Bacteroidota.c__Bacteroidia.o__Sphingobacteriales.f__NS11_12_marine_group | 2.118011 |  |  | - |
| k__Bacteria.p__Bacteroidota.c__Bacteroidia.o__Bacteroidales.f__Paludibacteraceae.g__F0058 | 1.583741 |  |  | - |
| k__Bacteria.p__Firmicutes.c__Clostridia.o__Lachnospirales.f__Lachnospiraceae.g__GCA_900066755 | 0.791002 |  |  | - |
| k__Bacteria.p__Proteobacteria.c__Gammaproteobacteria.o__Burkholderiales.f__Alcaligenaceae.g__Pusillimonas | 1.533146 |  |  | - |
| k__Bacteria.p__Firmicutes.c__Clostridia.o__Christensenellales.f__Christensenellaceae | 2.665072 |  |  | - |
| k__Bacteria.p__Acidobacteriota.c__Acidobacteriae.o__Solibacterales.f__Solibacteraceae.g__Candidatus_Solibacter | 2.917332 |  |  | - |
| k__Bacteria.p__Verrucomicrobiota.c__Verrucomicrobiae.o__Pedosphaerales.f__Pedosphaeraceae.g__Pedosphaeraceae | 1.541125 |  |  | - |
| k__Bacteria.p__Proteobacteria.c__Alphaproteobacteria.o__Elsterales | 3.119307 |  |  | - |
| k__Bacteria.p__Firmicutes.c__Clostridia.o__Lachnospirales.f__Lachnospiraceae.g___Eubacterium__xylanophilum_group | 3.69654 |  |  | - |
| k__Bacteria.p__Proteobacteria.c__Gammaproteobacteria.o__Oceanospirillales | 1.134599 |  |  | - |
| k__Bacteria.p__Proteobacteria.c__Alphaproteobacteria.o__Caulobacterales.f__Hyphomonadaceae | 1.817183 |  |  | - |
| k__Bacteria.p__Proteobacteria.c__Gammaproteobacteria.o__Enterobacterales.f__Enterobacteriaceae.g__Salmonella | 3.857863 |  |  | - |
| k__Bacteria.p__Actinobacteriota.c__Acidimicrobiia.o__IMCC26256 | 2.845244 |  |  | - |
| k__Bacteria.p__Actinobacteriota.c__Actinobacteria.o__Frankiales.f__Geodermatophilaceae.g__Geodermatophilus | 2.387448 |  |  | - |
| k__Bacteria.p__Firmicutes.c__Clostridia.o__Lachnospirales.f__Lachnospiraceae.g__Lachnospiraceae_UCG_010 | 2.505616 |  |  | - |
| k__Bacteria.p__Actinobacteriota.c__Acidimicrobiia.o__IMCC26256.f__IMCC26256 | 2.845244 |  |  | - |
| k__Bacteria.p__Bacteroidota.c__Bacteroidia.o__Flavobacteriales.f__Flavobacteriaceae.g__Flavobacterium | 2.946488 |  |  | - |
| k__Bacteria.p__Actinobacteriota.c__Thermoleophilia.o__Solirubrobacterales.f__67_14 | 1.701832 |  |  | - |
| k__Bacteria.p__Proteobacteria.c__Gammaproteobacteria.o__Burkholderiales.f__Chromobacteriaceae.g__Pseudogulbenkiania | 0.504077 |  |  | - |
| k__Bacteria.p__Proteobacteria.c__Gammaproteobacteria.o__PLTA13.f__PLTA13.g__PLTA13 | 1.500696 |  |  | - |
| k__Bacteria.p__Bacteroidota.c__Bacteroidia.o__Bacteroidales.f__Tannerellaceae.g__Parabacteroides | 3.519659 |  |  | - |
| k__Bacteria.p__Patescibacteria.c__Saccharimonadia | 2.964665 | C | 2.608334 | 0.033108 |
| k__Bacteria.p__Firmicutes.c__Clostridia.o__Oscillospirales.f__Ruminococcaceae.g__Candidatus_Soleaferrea | 1.32559 |  |  | - |
| k__Bacteria.p__Fusobacteriota.c__Fusobacteriia.o__Fusobacteriales.f__Leptotrichiaceae.g__Leptotrichia | 3.825281 |  |  | - |
| k__Bacteria.p__Bacteroidota.c__Bacteroidia.o__Bacteroidales.f__Marinifilaceae.g__Butyricimonas | 2.051684 |  |  | - |
| k__Bacteria.p__Cyanobacteria.c__Sericytochromatia.o__Sericytochromatia | 2.282365 |  |  | - |
| k__Bacteria.p__Actinobacteriota.c__Actinobacteria.o__Corynebacteriales.f__Corynebacteriaceae.g__Corynebacterium | 2.702242 |  |  | - |
| k__Bacteria.p__Actinobacteriota.c__Actinobacteria.o__Micrococcales.f__Microbacteriaceae.g__Galbitalea | 0.725926 |  |  | - |
| k__Bacteria.p__Proteobacteria.c__Gammaproteobacteria.o__Diplorickettsiales.f__Diplorickettsiaceae.g__Aquicella | 2.365365 |  |  | - |
| k__Bacteria.p__Proteobacteria.c__Alphaproteobacteria.o__Rhizobiales.f__Hyphomicrobiaceae.g__Hyphomicrobium | 1.703733 |  |  | - |
| k__Bacteria.p__Proteobacteria.c__Alphaproteobacteria.o__Rhizobiales.f__Rhizobiaceae.g__Allorhizobium_Neorhizobium_Pararhizobium_Rhizobium | 1.569153 |  |  | - |
| k__Bacteria.p__Proteobacteria.c__Gammaproteobacteria.o__EPR3968_O8a_Bc78.f__EPR3968_O8a_Bc78 | 1.606979 |  |  | - |
| k__Bacteria.p__Bacteroidota.c__Bacteroidia.o__Cytophagales.f__Hymenobacteraceae.g__Adhaeribacter | 1.377567 |  |  | - |
| k__Bacteria.p__Bacteroidota.c__Bacteroidia.o__Chitinophagales.f__Chitinophagaceae.g__Flavitalea | 1.484763 |  |  | - |
| k__Bacteria.p__Firmicutes.c__Clostridia.o__Clostridia_vadinBB60_group | 2.998598 |  |  | - |
| k__Bacteria.p__Firmicutes.c__Clostridia.o__Lachnospirales.f__Lachnospiraceae.g___Ruminococcus__torques_group | 2.317912 |  |  | - |
| k__Bacteria.p__Proteobacteria.c__Gammaproteobacteria.o__Burkholderiales.f__Hydrogenophilaceae | 2.027286 |  |  | - |
| k__Bacteria.p__Acidobacteriota.c__Blastocatellia.o__11_24.f__11_24 | 1.530422 |  |  | - |
| k__Bacteria.p__Actinobacteriota.c__Actinobacteria.o__Actinomycetales.f__Actinomycetaceae.g__Actinomyces | 3.390911 |  |  | - |
| k__Bacteria.p__Gemmatimonadota.c__S0134_terrestrial_group.o__S0134_terrestrial_group.f__S0134_terrestrial_group.g__S0134_terrestrial_group | 1.714819 |  |  | - |
| k__Bacteria.p__SAR324_clade_Marine_group_B_.c__SAR324_clade_Marine_group_B_ | 1.124738 |  |  | - |
| k__Bacteria.p__SAR324_clade_Marine_group_B_ | 1.124738 |  |  | - |
| k__Bacteria.p__Chloroflexi.c__Ktedonobacteria.o__Ktedonobacterales | 2.233237 |  |  | - |
| k__Bacteria.p__Bacteroidota.c__Bacteroidia.o__Chitinophagales.f__Chitinophagaceae.g__Niabella | 0.921326 |  |  | - |
| k__Bacteria.p__Proteobacteria.c__Gammaproteobacteria.o__Pseudomonadales.f__Moraxellaceae.g__Acinetobacter | 2.184034 |  |  | - |
| k__Bacteria.p__Bacteroidota.c__Bacteroidia.o__Sphingobacteriales.f__AKYH767.g__AKYH767 | 2.295261 |  |  | - |
| k__Bacteria.p__Firmicutes.c__Bacilli.o__Acholeplasmatales.f__Acholeplasmataceae.g__Anaeroplasma | 2.706587 |  |  | - |
| k__Bacteria.p__Actinobacteriota.c__Acidimicrobiia.o__Microtrichales.f__Ilumatobacteraceae | 1.768183 |  |  | - |
| k__Bacteria.p__Acidobacteriota.c__Subgroup_11.o__Subgroup_11.f__Subgroup_11.g__Subgroup_11 | 1.397506 |  |  | - |
| k__Bacteria.p__Firmicutes.c__Bacilli.o__Paenibacillales.f__Paenibacillaceae.g__Paenibacillus | 3.79508 |  |  | - |
| k__Bacteria.p__Bacteroidota.c__Bacteroidia.o__Flavobacteriales.f__Weeksellaceae.g__Bergeyella | 2.749258 |  |  | - |
| k__Bacteria.p__Proteobacteria.c__Alphaproteobacteria.o__Rhizobiales.f__Xanthobacteraceae.g__Rhodoplanes | 1.582714 |  |  | - |
| k__Bacteria.p__Proteobacteria.c__Gammaproteobacteria.o__Ga0077536 | 1.85477 |  |  | - |
| k__Bacteria.p__Proteobacteria.c__Gammaproteobacteria.o__CCD24.f__CCD24.g__CCD24 | 1.826682 |  |  | - |
| k__Bacteria.p__Proteobacteria.c__Alphaproteobacteria.o__Caulobacterales.f__Caulobacteraceae.g__Brevundimonas | 2.407316 |  |  | - |
| k__Bacteria.p__Actinobacteriota.c__Coriobacteriia.o__Coriobacteriales.f__Eggerthellaceae.g__DNF00809 | 0.85481 |  |  | - |
| k__Bacteria.p__Actinobacteriota.c__Actinobacteria.o__Propionibacteriales.f__Nocardioidaceae.g__Kribbella | 2.362504 |  |  | - |
| k__Bacteria.p__Elusimicrobiota.c__Lineage_IIb | 1.88581 |  |  | - |
| k__Bacteria.p__Firmicutes.c__Clostridia.o__Lachnospirales.f__Lachnospiraceae.g__Lachnospiraceae_NK4B4_group | 1.962938 |  |  | - |
| k__Bacteria.p__Myxococcota.c__Polyangia.o__Haliangiales | 2.948741 |  |  | - |
| k__Bacteria.p__Myxococcota.c__Myxococcia.o__Myxococcales.f__Myxococcaceae | 1.803713 |  |  | - |
| k__Bacteria.p__Actinobacteriota.c__Actinobacteria.o__Bifidobacteriales.f__Bifidobacteriaceae | 2.884511 |  |  | - |
| k__Bacteria.p__Proteobacteria.c__Gammaproteobacteria.o__Burkholderiales.f__Methylophilaceae.g__Methylotenera | 2.595321 |  |  | - |
| k__Bacteria.p__Proteobacteria.c__Gammaproteobacteria.o__Burkholderiales.f__Comamonadaceae.g__Ramlibacter | 2.249338 |  |  | - |
| k__Bacteria.p__Proteobacteria.c__Gammaproteobacteria.o__Burkholderiales.f__Burkholderiaceae.g__Lautropia | 2.289713 |  |  | - |
| k__Bacteria.p__Actinobacteriota.c__MB_A2_108.o__MB_A2_108.f__MB_A2_108.g__MB_A2_108 | 2.355094 |  |  | - |
| k__Bacteria.p__Firmicutes.c__Clostridia.o__Lachnospirales.f__Lachnospiraceae.g__Lachnospiraceae_FCS020_group | 2.90375 |  |  | - |
| k__Bacteria.p__Firmicutes.c__Clostridia.o__Clostridiales.f__Clostridiaceae.g__Clostridium_sensu_stricto_1 | 4.118861 |  |  | - |
| k__Bacteria.p__Actinobacteriota.c__Actinobacteria.o__Corynebacteriales.f__Corynebacteriaceae | 2.702242 |  |  | - |
| k__Bacteria.p__Bdellovibrionota.c__Bdellovibrionia.o__Bdellovibrionales.f__Bdellovibrionaceae.g__Bdellovibrio | 1.8076 |  |  | - |
| k__Bacteria.p__Firmicutes.c__Clostridia.o__Oscillospirales.f__Ruminococcaceae.g__Harryflintia | 2.476869 |  |  | - |
| k__Bacteria.p__Proteobacteria.c__Gammaproteobacteria.o__Burkholderiales.f__Sutterellaceae | 2.834436 |  |  | - |
| k__Bacteria.p__Proteobacteria.c__Alphaproteobacteria.o__Azospirillales.f__Inquilinaceae.g__Inquilinus | 1.203048 |  |  | - |
| k__Bacteria.p__Dependentiae.c__Babeliae | 1.565043 |  |  | - |
| k__Bacteria.p__Deferribacterota.c__Deferribacteres.o__Deferribacterales | 2.70096 |  |  | - |
| k__Bacteria.p__Acidobacteriota.c__Acidobacteriae.o__Solibacterales.f__Solibacteraceae | 2.917332 |  |  | - |
| k__Bacteria.p__Proteobacteria.c__Gammaproteobacteria.o__Diplorickettsiales.f__Diplorickettsiaceae | 2.488212 |  |  | - |
| k__Bacteria.p__Fusobacteriota.c__Fusobacteriia.o__Fusobacteriales.f__Leptotrichiaceae | 3.829122 |  |  | - |
| k__Bacteria.p__Firmicutes.c__Clostridia.o__Peptococcales | 2.671824 |  |  | - |
| k__Bacteria.p__Proteobacteria.c__Gammaproteobacteria.o__Burkholderiales.f__Burkholderiaceae.g__Ralstonia | 1.591734 |  |  | - |
| k__Bacteria.p__Firmicutes.c__Clostridia.o__Oscillospirales.f__Ruminococcaceae.g__Pygmaiobacter | 1.872211 |  |  | - |
| k__Bacteria.p__Actinobacteriota.c__Actinobacteria.o__Actinomycetales.f__Actinomycetaceae | 3.401372 |  |  | - |
| k__Bacteria.p__Dependentiae.c__Babeliae.o__Babeliales.f__Vermiphilaceae | 1.363779 |  |  | - |
| k__Bacteria.p__Firmicutes.c__Bacilli.o__Lactobacillales.f__Aerococcaceae.g__Abiotrophia | 2.383371 |  |  | - |
| k__Bacteria.p__Proteobacteria.c__Gammaproteobacteria.o__Acidiferrobacterales.f__Acidiferrobacteraceae | 1.712442 |  |  | - |
| k__Bacteria.p__Deferribacterota | 2.70096 |  |  | - |
| k__Bacteria.p__Proteobacteria.c__Gammaproteobacteria.o__Burkholderiales.f__Nitrosomonadaceae.g__MND1 | 3.163262 |  |  | - |
| k__Bacteria.p__Proteobacteria.c__Alphaproteobacteria.o__Caulobacterales.f__Caulobacteraceae | 3.349668 |  |  | - |
| k__Bacteria.p__Actinobacteriota.c__Thermoleophilia.o__Gaiellales.f__Gaiellaceae.g__Gaiella | 2.845348 |  |  | - |
| k__Bacteria.p__Bacteroidota.c__Bacteroidia.o__Sphingobacteriales.f__KD3_93.g__KD3_93 | 0.872054 |  |  | - |
| k__Bacteria.p__Acidobacteriota.c__Acidobacteriae.o__Acidobacteriales.f__Acidobacteriaceae__Subgroup_1_.g__Granulicella | 2.658841 |  |  | - |
| k__Bacteria.p__Acidobacteriota.c__Acidobacteriae.o__Subgroup_2.f__Subgroup_2 | 3.657284 |  |  | - |
| k__Bacteria.p__Bacteroidota.c__Bacteroidia.o__Sphingobacteriales.f__Sphingobacteriaceae | 2.752256 |  |  | - |
| k__Bacteria.p__Firmicutes.c__Clostridia.o__Oscillospirales.f___Clostridium__methylpentosum_group.g___Clostridium__methylpentosum_group | 1.089182 |  |  | - |
| k__Bacteria.p__Proteobacteria.c__Gammaproteobacteria.o__Burkholderiales.f__TRA3_20 | 2.832236 |  |  | - |
| k__Bacteria.p__Spirochaetota.c__Spirochaetia | 2.827824 |  |  | - |
| k__Bacteria.p__Verrucomicrobiota.c__Verrucomicrobiae.o__Verrucomicrobiales.f__Akkermansiaceae.g__Akkermansia | 2.391624 | C | 2.180154 | 0.034381 |
| k__Bacteria.p__MBNT15.c__MBNT15.o__MBNT15.f__MBNT15.g__MBNT15 | 0.861553 |  |  | - |
| k__Bacteria.p__Actinobacteriota.c__Actinobacteria.o__Micrococcales.f__Micrococcaceae | 3.319135 |  |  | - |
| k__Bacteria.p__Actinobacteriota.c__Actinobacteria | 4.109904 |  |  | - |
| k__Bacteria.p__Firmicutes.c__Negativicutes.o__Veillonellales_Selenomonadales.f__Veillonellaceae | 1.841575 |  |  | - |
| k__Bacteria.p__Actinobacteriota.c__Thermoleophilia.o__Gaiellales | 3.565581 |  |  | - |
| k__Bacteria.p__Firmicutes.c__Clostridia.o__Lachnospirales.f__Lachnospiraceae.g__Johnsonella | 1.620306 |  |  | - |
| k__Bacteria.p__Actinobacteriota.c__Actinobacteria.o__Frankiales.f__Acidothermaceae.g__Acidothermus | 2.808803 |  |  | - |
| k__Bacteria.p__Bacteroidota.c__Bacteroidia.o__Chitinophagales.f__Saprospiraceae | 1.300829 |  |  | - |
| k__Bacteria.p__Dependentiae.c__Babeliae.o__Babeliales | 1.565043 |  |  | - |
| k__Bacteria.p__Actinobacteriota.c__Actinobacteria.o__Propionibacteriales | 2.661582 |  |  | - |
| k__Bacteria.p__Actinobacteriota.c__Actinobacteria.o__Pseudonocardiales.f__Pseudonocardiaceae.g__Pseudonocardia | 2.324305 |  |  | - |
| k__Bacteria.p__Proteobacteria.c__Gammaproteobacteria.o__Burkholderiales.f__Comamonadaceae.g__Caenimonas | 1.511161 |  |  | - |
| k__Bacteria.p__Firmicutes.c__Clostridia.o__Lachnospirales.f__Lachnospiraceae.g___Eubacterium__oxidoreducens_group | 2.723841 |  |  | - |
| k__Bacteria.p__Bacteroidota.c__Bacteroidia.o__Cytophagales.f__Cytophagaceae | 2.628722 |  |  | - |
| k__Bacteria.p__Firmicutes.c__Bacilli.o__Lactobacillales.f__Enterococcaceae | 3.469769 |  |  | - |
| k__Bacteria.p__Proteobacteria.c__Gammaproteobacteria.o__Steroidobacterales.f__Steroidobacteraceae | 1.855086 |  |  | - |
| k__Bacteria.p__Bacteroidota.c__Bacteroidia.o__Bacteroidales.f__Marinifilaceae.g__Sanguibacteroides | 0.259493 |  |  | - |
| k__Bacteria.p__Verrucomicrobiota.c__Verrucomicrobiae.o__Pedosphaerales.f__Pedosphaeraceae.g__ADurb_Bin063_1 | 2.011637 |  |  | - |
| k__Bacteria.p__Myxococcota.c__bacteriap25.o__bacteriap25.f__bacteriap25.g__bacteriap25 | 2.572408 |  |  | - |
| k__Bacteria.p__Chloroflexi.c__Ktedonobacteria.o__C0119 | 1.378622 |  |  | - |
| k__Bacteria.p__Gemmatimonadota.c__Gemmatimonadetes.o__Gemmatimonadales.f__Gemmatimonadaceae.g__Gemmatimonas | 3.502531 |  |  | - |
| k__Bacteria.p__Firmicutes.c__Clostridia.o__Oscillospirales.f__Ruminococcaceae.g__Ruminococcaceae | 2.709058 |  |  | - |
| k__Bacteria.p__Chloroflexi.c__Ktedonobacteria | 2.246535 |  |  | - |
| k__Bacteria.p__Actinobacteriota.c__Actinobacteria.o__Pseudonocardiales | 2.458929 |  |  | - |
| k__Bacteria.p__Bacteroidota.c__Kapabacteria.o__Kapabacteriales | 2.039643 |  |  | - |
| k__Bacteria.p__Verrucomicrobiota.c__Verrucomicrobiae.o__Pedosphaerales.f__Pedosphaeraceae.g__Ellin516 | 1.799341 |  |  | - |
| k__Bacteria.p__Verrucomicrobiota.c__Verrucomicrobiae.o__Pedosphaerales.f__Pedosphaeraceae.g__Ellin517 | 2.522687 |  |  | - |
| k__Bacteria.p__Actinobacteriota.c__Coriobacteriia.o__Coriobacteriales | 3.603668 |  |  | - |
| k__Bacteria.p__Proteobacteria.c__Alphaproteobacteria.o__Caulobacterales | 3.362229 | O | 2.662928 | 0.034658 |
| k__Bacteria.p__Actinobacteriota.c__Actinobacteria.o__Micrococcales.f__Micrococcaceae.g__Garicola | 0.512387 |  |  | - |
| k__Bacteria.p__Firmicutes.c__Clostridia.o__Peptostreptococcales_Tissierellales.f__Peptostreptococcaceae | 3.799328 |  |  | - |
| k__Bacteria.p__Firmicutes.c__Clostridia.o__Eubacteriales | 1.964712 |  |  | - |
| k__Bacteria.p__Proteobacteria.c__Gammaproteobacteria.o__Enterobacterales.f__Yersiniaceae.g__Yersinia | 1.502934 |  |  | - |
| k__Bacteria.p__Proteobacteria.c__Alphaproteobacteria.o__Caulobacterales.f__Caulobacteraceae.g__Caulobacter | 2.080253 |  |  | - |
| k__Bacteria.p__Proteobacteria.c__Gammaproteobacteria.o__Burkholderiales.f__Oxalobacteraceae.g__Massilia | 2.879348 |  |  | - |
| k__Bacteria.p__Acidobacteriota.c__Acidobacteriae.o__Bryobacterales | 3.174221 |  |  | - |
| k__Bacteria.p__Spirochaetota | 2.827824 |  |  | - |
| k__Bacteria.p__Proteobacteria.c__Gammaproteobacteria.o__KF_JG30_C25.f__KF_JG30_C25.g__KF_JG30_C25 | 2.94646 |  |  | - |
| k__Bacteria.p__Elusimicrobiota.c__Elusimicrobia.o__Lineage_IV.f__Lineage_IV | 1.68491 |  |  | - |
| k__Bacteria.p__Proteobacteria.c__Alphaproteobacteria.o__Dongiales | 2.825556 |  |  | - |
| k__Bacteria.p__Acidobacteriota.c__Acidobacteriae.o__Subgroup_13.f__Subgroup_13 | 2.70973 |  |  | - |
| k__Bacteria.p__Proteobacteria.c__Alphaproteobacteria | 4.893986 |  |  | - |
| k__Bacteria.p__Firmicutes | 5.497968 |  |  | - |
| k__Bacteria.p__Firmicutes.c__Clostridia.o__Peptostreptococcales_Tissierellales.f__Peptostreptococcaceae.g__Clostridioides | 3.482279 |  |  | - |
| k__Bacteria.p__Bdellovibrionota.c__Bdellovibrionia.o__Bdellovibrionales | 1.8076 |  |  | - |
| k__Bacteria.p__Proteobacteria.c__Alphaproteobacteria.o__Rhizobiales.f__Labraceae | 1.483583 |  |  | - |
| k__Bacteria.p__Proteobacteria.c__Gammaproteobacteria.o__Legionellales.f__Legionellaceae | 1.640638 |  |  | - |
| k__Bacteria.p__Proteobacteria.c__Gammaproteobacteria.o__Burkholderiales.f__Alcaligenaceae.g__Achromobacter | 1.072406 |  |  | - |
| k__Bacteria.p__Proteobacteria.c__Gammaproteobacteria.o__CHAB_XI_27 | 1.467696 |  |  | - |
| k__Bacteria.p__Actinobacteriota.c__Actinobacteria.o__Propionibacteriales.f__Nocardioidaceae.g__Aeromicrobium | 1.743777 |  |  | - |
| k__Bacteria.p__Proteobacteria.c__Alphaproteobacteria.o__Acetobacterales.f__Acetobacteraceae | 2.728683 |  |  | - |
| k__Bacteria.p__Proteobacteria.c__Alphaproteobacteria.o__Rhizobiales.f__KF_JG30_B3 | 1.021482 |  |  | - |
| k__Bacteria.p__Firmicutes.c__Bacilli.o__Bacillales.f__Bacillaceae | 3.228548 |  |  | - |
| k__Bacteria.p__Myxococcota.c__Polyangia.o__Nannocystales.f__Nannocystaceae.g__Nannocystis | 1.449249 |  |  | - |
| k__Bacteria.p__Cyanobacteria.c__Cyanobacteriia.o__Cyanobacteriales | 1.746441 |  |  | - |
| k__Bacteria.p__Firmicutes.c__Clostridia.o__Oscillospirales.f__Oscillospiraceae.g__Intestinimonas | 3.306161 |  |  | - |
| k__Bacteria.p__Proteobacteria.c__Gammaproteobacteria.o__Pasteurellales.f__Pasteurellaceae.g__Haemophilus | 3.962946 |  |  | - |
| k__Bacteria.p__Proteobacteria.c__Gammaproteobacteria.o__Enterobacterales.f__Morganellaceae.g__Morganella | 3.782234 |  |  | - |
| k__Bacteria.p__Actinobacteriota.c__Actinobacteria.o__Pseudonocardiales.f__Pseudonocardiaceae.g__Actinophytocola | 1.442695 |  |  | - |
| k__Bacteria.p__Proteobacteria.c__Gammaproteobacteria.o__WD260 | 0.872054 |  |  | - |
| k__Bacteria.p__Firmicutes.c__Clostridia.o__Oscillospirales.f__Butyricicoccaceae.g__Butyricicoccus | 2.803461 |  |  | - |
| k__Bacteria.p__Firmicutes.c__Clostridia.o__Monoglobales.f__Monoglobaceae | 3.019813 |  |  | - |
| k__Bacteria.p__Proteobacteria.c__Gammaproteobacteria.o__Thiotrichales.f__Thiotrichaceae | 0.799633 |  |  | - |
| k__Bacteria.p__Firmicutes.c__Clostridia.o__Lachnospirales.f__Lachnospiraceae.g__Robinsoniella | 2.532371 |  |  | - |
| k__Bacteria.p__Firmicutes.c__Clostridia.o__Clostridiales | 4.143666 |  |  | - |
| k__Bacteria.p__Actinobacteriota.c__Actinobacteria.o__Micrococcales.f__Microbacteriaceae.g__Agromyces | 1.106137 |  |  | - |
| k__Bacteria.p__Firmicutes.c__Clostridia.o__Monoglobales | 3.019813 |  |  | - |
| k__Bacteria.p__Myxococcota | 3.372524 |  |  | - |
| k__Bacteria.p__Myxococcota.c__Myxococcia.o__Myxococcales.f__Anaeromyxobacteraceae | 1.915474 |  |  | - |
| k__Bacteria.p__Firmicutes.c__Clostridia.o__Oscillospirales.f__Oscillospiraceae.g__UCG_005 | 3.153438 |  |  | - |
| k__Bacteria.p__Proteobacteria.c__Gammaproteobacteria.o__Coxiellales.f__Coxiellaceae | 1.430461 |  |  | - |
| k__Bacteria.p__Acidobacteriota.c__Subgroup_11.o__Subgroup_11.f__Subgroup_11 | 1.397506 |  |  | - |
| k__Bacteria.p__Proteobacteria.c__Gammaproteobacteria.o__Oceanospirillales.f__Pseudohongiellaceae | 1.134599 |  |  | - |
| k__Bacteria.p__Proteobacteria.c__Gammaproteobacteria.o__Xanthomonadales | 3.695297 |  |  | - |
| k__Bacteria.p__Actinobacteriota.c__Actinobacteria.o__Micromonosporales.f__Micromonosporaceae.g__Actinoplanes | 2.782233 |  |  | - |
| k__Bacteria.p__Proteobacteria.c__Alphaproteobacteria.o__Rickettsiales.f__SM2D12 | 2.368306 |  |  | - |
| k__Bacteria.p__Bacteroidota.c__Bacteroidia.o__Sphingobacteriales.f__NS11_12_marine_group.g__NS11_12_marine_group | 2.118011 |  |  | - |
| k__Bacteria.p__Firmicutes.c__Bacilli.o__Lactobacillales.f__Streptococcaceae.g__Lactococcus | 1.753873 |  |  | - |
| k__Bacteria.p__Firmicutes.c__Clostridia.o__Peptostreptococcales_Tissierellales.f__Peptostreptococcales_Tissierellales.g__Parvimonas | 1.895483 |  |  | - |
| k__Bacteria.p__Myxococcota.c__Polyangia.o__Nannocystales | 1.449249 |  |  | - |
| k__Bacteria.p__Gemmatimonadota.c__BD2_11_terrestrial_group.o__BD2_11_terrestrial_group.f__BD2_11_terrestrial_group | 2.375087 |  |  | - |
| k__Bacteria.p__Proteobacteria.c__Gammaproteobacteria.o__Burkholderiales.f__Comamonadaceae.g__Rhodoferax | 1.702247 |  |  | - |
| k__Bacteria.p__Proteobacteria.c__Gammaproteobacteria.o__Enterobacterales.f__Morganellaceae.g__Proteus | 3.482817 |  |  | - |
| k__Bacteria.p__Proteobacteria.c__Gammaproteobacteria.o__Xanthomonadales.f__Rhodanobacteraceae.g__Ahniella | 0.623542 |  |  | - |
| k__Bacteria.p__Proteobacteria.c__Gammaproteobacteria.o__Xanthomonadales.f__Rhodanobacteraceae.g__Rhodanobacter | 2.840555 |  |  | - |
| k__Bacteria.p__Bacteroidota.c__Bacteroidia.o__Bacteroidales.f__Prevotellaceae.g__Prevotella | 4.328279 |  |  | - |
| k__Bacteria.p__Proteobacteria.c__Alphaproteobacteria.o__Parvibaculales.f__Parvibaculaceae.g__Parvibaculum | 1.609603 |  |  | - |
| k__Bacteria.p__Bacteroidota.c__Bacteroidia.o__Bacteroidales.f__Prevotellaceae.g__Alloprevotella | 4.058497 |  |  | - |
| k__Bacteria.p__Proteobacteria.c__Alphaproteobacteria.o__Paracaedibacterales.f__Paracaedibacteraceae.g__Candidatus_Paracaedibacter | 2.526187 |  |  | - |
| k__Bacteria.p__Actinobacteriota.c__Actinobacteria.o__Catenulisporales.f__Catenulisporaceae | 2.503451 |  |  | - |
| k__Bacteria.p__Firmicutes.c__Clostridia.o__Peptostreptococcales_Tissierellales.f__Anaerovoracaceae.g___Eubacterium__nodatum_group | 2.821434 |  |  | - |
| k__Bacteria.p__Desulfobacterota.c__Desulfuromonadia.o__Geobacterales.f__Geobacteraceae | 2.056841 |  |  | - |
| k__Bacteria.p__Actinobacteriota.c__Actinobacteria.o__Micromonosporales.f__Micromonosporaceae.g__Luedemannella | 2.184929 |  |  | - |
| k__Bacteria.p__Proteobacteria.c__Gammaproteobacteria.o__Cellvibrionales.f__Cellvibrionaceae | 2.088493 |  |  | - |
| k__Bacteria.p__Acidobacteriota.c__Subgroup_22.o__Subgroup_22.f__Subgroup_22.g__Subgroup_22 | 1.339608 |  |  | - |
| k__Bacteria.p__Myxococcota.c__Polyangia.o__Haliangiales.f__Haliangiaceae.g__Haliangium | 2.948741 |  |  | - |
| k__Bacteria.p__Bacteroidota.c__Bacteroidia.o__Sphingobacteriales.f__env_OPS_17 | 2.37614 |  |  | - |
| k__Bacteria.p__Firmicutes.c__Bacilli.o__Brevibacillales.f__Brevibacillaceae.g__Brevibacillus | 2.800757 |  |  | - |
| k__Bacteria.p__Firmicutes.c__Clostridia.o__Oscillospirales.f__Oscillospiraceae.g__Oscillospira | 1.367121 |  |  | - |
| k__Bacteria.p__Firmicutes.c__Clostridia.o__Peptostreptococcales_Tissierellales.f__Anaerovoracaceae.g__Family_XIII_AD3011_group | 2.658085 |  |  | - |
| k__Bacteria.p__Chloroflexi.c__Anaerolineae.o__Anaerolineae | 2.088972 |  |  | - |
| k__Bacteria.p__Desulfobacterota.c__Desulfovibrionia.o__Desulfovibrionales.f__Desulfovibrionaceae | 4.256245 |  |  | - |
| k__Bacteria.p__Actinobacteriota.c__Acidimicrobiia | 3.039907 |  |  | - |
| k__Bacteria.p__Actinobacteriota.c__Actinobacteria.o__Frankiales | 3.135574 |  |  | - |
| k__Bacteria.p__Proteobacteria.c__Alphaproteobacteria.o__Micropepsales.f__Micropepsaceae.g__Micropepsis | 1.827762 |  |  | - |
| k__Bacteria.p__Fibrobacterota.c__Fibrobacteria.o__Fibrobacterales.f__Fibrobacterales | 0.865093 |  |  | - |
| k__Bacteria.p__Proteobacteria.c__Gammaproteobacteria.o__PLTA13.f__PLTA13 | 1.500696 |  |  | - |
| k__Bacteria.p__Elusimicrobiota.c__Elusimicrobia.o__Lineage_IV.f__Lineage_IV.g__Lineage_IV | 1.68491 |  |  | - |
| k__Bacteria.p__Actinobacteriota.c__Actinobacteria.o__Bifidobacteriales | 2.884511 |  |  | - |
| k__Bacteria.p__Bacteroidota.c__Bacteroidia.o__Flavobacteriales | 3.34053 |  |  | - |
| k__Bacteria.p__Myxococcota.c__Polyangia.o__Nannocystales.f__Nannocystaceae | 1.449249 |  |  | - |
| k__Bacteria.p__Acidobacteriota.c__Subgroup_11.o__Subgroup_11 | 1.397506 |  |  | - |
| k__Bacteria.p__Proteobacteria.c__Gammaproteobacteria.o__Burkholderiales.f__Oxalobacteraceae.g__Collimonas | 1.745041 |  |  | - |
| k__Bacteria.p__Firmicutes.c__Bacilli.o__Lactobacillales | 4.885713 |  |  | - |
| k__Bacteria.p__Desulfobacterota.c__Desulfuromonadia | 2.056841 |  |  | - |
| k__Bacteria.p__Proteobacteria.c__Gammaproteobacteria.o__Salinisphaerales | 1.904372 |  |  | - |
| k__Bacteria.p__Proteobacteria.c__Gammaproteobacteria.o__Burkholderiales.f__Comamonadaceae.g__Polaromonas | 2.620059 |  |  | - |
| k__Bacteria.p__Proteobacteria.c__Gammaproteobacteria.o__Pseudomonadales.f__Moraxellaceae.g__Enhydrobacter | 1.068349 |  |  | - |
| k__Bacteria.p__Actinobacteriota.c__Actinobacteria.o__Micrococcales.f__Intrasporangiaceae | 2.39631 |  |  | - |
| k__Bacteria.p__Elusimicrobiota.c__Elusimicrobia | 2.59146 |  |  | - |
| k__Bacteria.p__Gemmatimonadota.c__Longimicrobia | 2.955762 |  |  | - |
| k__Bacteria.p__Patescibacteria.c__Saccharimonadia.o__Saccharimonadales.f__Saccharimonadaceae.g__Candidatus_Saccharimonas | 2.370368 |  |  | - |
| k__Bacteria.p__Gemmatimonadota.c__Longimicrobia.o__Longimicrobiales.f__Longimicrobiaceae.g__Longimicrobiaceae | 2.955762 |  |  | - |
| k__Bacteria.p__Acidobacteriota.c__Acidobacteriae.o__Acidobacteriales.f__Koribacteraceae.g__Candidatus_Koribacter | 2.564413 |  |  | - |
| k__Bacteria.p__Actinobacteriota.c__Actinobacteria.o__Corynebacteriales.f__Nocardiaceae | 1.393062 |  |  | - |
| k__Bacteria.p__Proteobacteria.c__Gammaproteobacteria.o__Burkholderiales.f__Burkholderiaceae.g__Robbsia | 0.629016 |  |  | - |
| k__Bacteria.p__Bacteroidota.c__Kapabacteria | 2.039643 |  |  | - |
| k__Bacteria.p__Proteobacteria.c__Gammaproteobacteria.o__Salinisphaerales.f__Solimonadaceae | 1.904372 |  |  | - |
| k__Bacteria.p__Firmicutes.c__Clostridia.o__Lachnospirales.f__Lachnospiraceae.g__Hungatella | 0.731491 |  |  | - |
| k__Bacteria.p__Firmicutes.c__Clostridia.o__Eubacteriales.f__Anaerofustaceae | 1.964712 |  |  | - |
| k__Bacteria.p__Proteobacteria.c__Gammaproteobacteria.o__Burkholderiales.f__Chromobacteriaceae | 0.504077 |  |  | - |
| k__Bacteria.p__Myxococcota.c__Polyangia.o__Polyangiales.f__BIrii41.g__BIrii41 | 2.573832 |  |  | - |
| k__Bacteria.p__Proteobacteria.c__Alphaproteobacteria.o__Rhodobacterales.f__Rhodobacteraceae.g__Rubellimicrobium | 0.86803 |  |  | - |
| k__Bacteria.p__Campilobacterota.c__Campylobacteria.o__Campylobacterales.f__Campylobacteraceae | 3.288683 |  |  | - |
| k__Bacteria.p__Patescibacteria | 3.090542 | C | 2.696871 | 0.02503 |
| k__Bacteria.p__Proteobacteria.c__Gammaproteobacteria.o__Burkholderiales.f__TRA3_20.g__TRA3_20 | 2.832236 |  |  | - |
| k__Bacteria.p__Gemmatimonadota.c__S0134_terrestrial_group.o__S0134_terrestrial_group | 1.714819 |  |  | - |
| k__Bacteria.p__Acidobacteriota.c__Acidobacteriae.o__Acidobacteriales.f__Acidobacteriaceae__Subgroup_1_ | 3.46647 |  |  | - |
| k__Bacteria.p__Actinobacteriota.c__Actinobacteria.o__Frankiales.f__Cryptosporangiaceae | 2.265274 |  |  | - |
| k__Bacteria.p__Proteobacteria.c__Alphaproteobacteria.o__Micropepsales | 3.32053 |  |  | - |
| k__Bacteria.p__Acidobacteriota.c__Blastocatellia.o__11_24.f__11_24.g__11_24 | 1.530422 |  |  | - |
| k__Bacteria.p__Acidobacteriota.c__Holophagae.o__Subgroup_7.f__Subgroup_7.g__Subgroup_7 | 1.691859 |  |  | - |
| k__Bacteria.p__Proteobacteria.c__Gammaproteobacteria.o__Burkholderiales.f__Comamonadaceae.g__Pelomonas | 2.529337 |  |  | - |
| k__Bacteria.p__Proteobacteria.c__Alphaproteobacteria.o__Rhodobacterales | 2.555381 | O | 2.292295 | 0.045759 |
| k__Bacteria.p__Firmicutes.c__Clostridia.o__Lachnospirales.f__Lachnospiraceae.g___Ruminococcus__gauvreauii_group | 2.585738 |  |  | - |
| k__Bacteria.p__Myxococcota.c__Polyangia.o__Haliangiales.f__Haliangiaceae | 2.948741 |  |  | - |
| k__Bacteria.p__Campilobacterota | 4.241276 |  |  | - |
| k__Bacteria.p__Proteobacteria.c__Gammaproteobacteria.o__Enterobacterales.f__Erwiniaceae.g__Pantoea | 1.252629 |  |  | - |
| k__Bacteria.p__Firmicutes.c__Clostridia.o__Oscillospirales.f__Ruminococcaceae.g__Incertae_Sedis | 3.392422 |  |  | - |
| k__Bacteria.p__Actinobacteriota.c__Actinobacteria.o__Streptosporangiales.f__Thermomonosporaceae | 1.671626 |  |  | - |
| k__Bacteria.p__Proteobacteria.c__Alphaproteobacteria.o__Sphingomonadales.f__Sphingomonadaceae.g__Ellin6055 | 2.41635 |  |  | - |
| k__Bacteria.p__Deferribacterota.c__Deferribacteres | 2.70096 |  |  | - |
| k__Bacteria.p__Firmicutes.c__Clostridia.o__Clostridia_vadinBB60_group.f__Clostridia_vadinBB60_group | 2.998598 |  |  | - |
| k__Bacteria.p__Proteobacteria.c__Alphaproteobacteria.o__Reyranellales | 2.828224 |  |  | - |
| k__Bacteria.p__Bacteroidota.c__Bacteroidia.o__Bacteroidales.f__Tannerellaceae | 3.598511 |  |  | - |
| k__Bacteria.p__Proteobacteria.c__Alphaproteobacteria.o__Acetobacterales.f__Acetobacteraceae.g__Acidicaldus | 1.478977 |  |  | - |
| k__Bacteria.p__Actinobacteriota.c__Thermoleophilia.o__Solirubrobacterales | 2.078964 |  |  | - |
| k__Bacteria.p__Myxococcota.c__Myxococcia.o__Myxococcales | 2.111731 |  |  | - |
| k__Bacteria.p__Actinobacteriota.c__Coriobacteriia | 3.603668 |  |  | - |
| k__Bacteria.p__Firmicutes.c__Clostridia.o__Clostridiales.f__Clostridiaceae.g__Clostridium_sensu_stricto_12 | 2.274651 |  |  | - |
| k__Bacteria.p__Firmicutes.c__Clostridia.o__Clostridia | 1.54264 |  |  | - |
| k__Bacteria.p__RCP2_54.c__RCP2_54.o__RCP2_54 | 2.540402 |  |  | - |
| k__Bacteria.p__Firmicutes.c__Clostridia.o__Lachnospirales.f__Lachnospiraceae.g__Catonella | 1.444215 |  |  | - |
| k__Bacteria.p__Firmicutes.c__Bacilli.o__Erysipelotrichales.f__Erysipelotrichaceae.g__Solobacterium | 2.388282 |  |  | - |
| k__Bacteria.p__Deferribacterota.c__Deferribacteres.o__Deferribacterales.f__Deferribacteraceae | 2.70096 |  |  | - |
| k__Bacteria.p__Bacteroidota.c__Bacteroidia.o__Sphingobacteriales.f__Sphingobacteriaceae.g__Sphingobacterium | 2.353589 |  |  | - |
| k__Bacteria.p__Actinobacteriota.c__Coriobacteriia.o__Coriobacteriales.f__Atopobiaceae | 2.76361 |  |  | - |
| k__Bacteria.p__Bacteroidota.c__Bacteroidia.o__Bacteroidales.f__Tannerellaceae.g__Tannerella | 2.682102 | O | 2.296216 | 0.018748 |
| k__Bacteria.p__Proteobacteria.c__Gammaproteobacteria.o__Burkholderiales.f__A21b.g__A21b | 2.523568 |  |  | - |
| k__Bacteria.p__Proteobacteria.c__Alphaproteobacteria.o__Rhizobiales.f__Rhizobiaceae.g__Mesorhizobium | 2.671901 |  |  | - |
| k__Bacteria.p__Firmicutes.c__Bacilli.o__Erysipelotrichales.f__Erysipelotrichaceae.g___Clostridium__innocuum_group | 3.143642 |  |  | - |
| k__Bacteria.p__Bacteroidota.c__Bacteroidia.o__Chitinophagales.f__Chitinophagaceae.g__Asinibacterium | 1.459386 |  |  | - |
| k__Bacteria.p__Firmicutes.c__Bacilli.o__Bacillales | 3.230455 |  |  | - |
| k__Bacteria.p__Bacteroidota.c__Bacteroidia.o__Cytophagales.f__Microscillaceae.g__Ohtaekwangia | 1.722617 |  |  | - |
| k__Bacteria.p__Firmicutes.c__Bacilli.o__Erysipelotrichales.f__Erysipelatoclostridiaceae.g__Erysipelatoclostridium | 3.155093 |  |  | - |
| k__Bacteria.p__Latescibacterota | 1.644063 |  |  | - |
| k__Bacteria.p__Firmicutes.c__Bacilli.o__Thermoactinomycetales.f__Thermoactinomycetaceae.g__Thermoactinomyces | 0.629016 |  |  | - |
| k__Bacteria.p__RCP2_54 | 2.540402 |  |  | - |
| k__Bacteria.p__Actinobacteriota.c__Actinobacteria.o__Corynebacteriales | 2.884938 |  |  | - |
| k__Bacteria.p__Proteobacteria.c__Gammaproteobacteria.o__Xanthomonadales.f__Rhodanobacteraceae.g__Dyella | 2.756894 |  |  | - |
| k__Bacteria.p__Firmicutes.c__Clostridia.o__Oscillospirales.f___Eubacterium__coprostanoligenes_group | 3.821704 |  |  | - |
| k__Bacteria.p__Firmicutes.c__Negativicutes.o__Veillonellales_Selenomonadales | 1.841575 |  |  | - |
| k__Bacteria.p__Proteobacteria.c__Alphaproteobacteria.o__Dongiales.f__Dongiaceae | 2.825556 |  |  | - |
| k__Bacteria.p__Actinobacteriota.c__Coriobacteriia.o__Coriobacteriales.f__Eggerthellaceae.g__Enterorhabdus | 3.501568 |  |  | - |
| k__Bacteria.p__Proteobacteria.c__Gammaproteobacteria.o__EPR3968_O8a_Bc78 | 1.606979 |  |  | - |
| k__Bacteria.p__Acidobacteriota.c__Blastocatellia.o__Blastocatellales | 1.467696 |  |  | - |
| k__Bacteria.p__Actinobacteriota.c__Thermoleophilia.o__Gaiellales.f__Gaiellaceae | 2.845348 |  |  | - |
| k__Bacteria.p__Firmicutes.c__Clostridia.o__Lachnospirales.f__Lachnospiraceae.g__Lachnospiraceae_UCG_006 | 3.043824 |  |  | - |
| k__Bacteria.p__Firmicutes.c__Clostridia.o__Lachnospirales.f__Lachnospiraceae.g__Lachnospiraceae_UCG_004 | 2.187439 |  |  | - |
| k__Bacteria.p__Actinobacteriota.c__Actinobacteria.o__Micrococcales.f__Microbacteriaceae.g__Humibacter | 0.322512 |  |  | - |
| k__Bacteria.p__Actinobacteriota.c__Actinobacteria.o__Frankiales.f__Geodermatophilaceae.g__Blastococcus | 1.494578 |  |  | - |
| k__Bacteria.p__Firmicutes.c__Clostridia.o__Lachnospirales.f__Lachnospiraceae.g__Lachnospiraceae_UCG_001 | 3.183033 |  |  | - |
| k__Bacteria.p__Bacteroidota.c__Bacteroidia.o__Bacteroidales.f__Prevotellaceae.g__Prevotellaceae_UCG_001 | 3.594566 |  |  | - |
| k__Bacteria.p__Bdellovibrionota | 2.431577 |  |  | - |
| k__Bacteria.p__Bacteroidota.c__Bacteroidia.o__Bacteroidales.f__Muribaculaceae.g__Muribaculaceae | 5.072115 |  |  | - |
| k__Bacteria.p__Proteobacteria.c__Gammaproteobacteria.o__Burkholderiales.f__Comamonadaceae.g__Acidovorax | 1.820396 |  |  | - |
| k__Bacteria.p__Armatimonadota.c__Fimbriimonadia.o__Fimbriimonadales | 0.322512 |  |  | - |
| k__Bacteria.p__Firmicutes.c__Clostridia.o__Lachnospirales.f__Lachnospiraceae.g__Lachnospiraceae_UCG_008 | 1.474477 |  |  | - |
| k__Bacteria.p__Actinobacteriota.c__Actinobacteria.o__0319_7L14 | 1.54392 |  |  | - |
| k__Bacteria.p__Proteobacteria.c__Gammaproteobacteria.o__Enterobacterales | 5.369072 |  |  | - |
| k__Bacteria.p__Firmicutes.c__Bacilli.o__Erysipelotrichales.f__Erysipelotrichaceae.g__Erysipelotrichaceae_UCG_006 | 1.553659 |  |  | - |
| k__Bacteria.p__Firmicutes.c__Bacilli | 4.983723 |  |  | - |
| k__Bacteria.p__Gemmatimonadota.c__Gemmatimonadetes.o__Gemmatimonadales.f__Gemmatimonadaceae.g__Roseisolibacter | 2.253392 |  |  | - |
| k__Bacteria.p__Bacteroidota.c__Bacteroidia.o__Bacteroidales.f__Marinifilaceae | 3.663065 |  |  | - |
| k__Bacteria.p__Firmicutes.c__Clostridia.o__Lachnospirales.f__Lachnospiraceae.g___Eubacterium__ventriosum_group | 2.822903 |  |  | - |
| k__Bacteria.p__Bacteroidota.c__Bacteroidia.o__Bacteroidales.f__Barnesiellaceae | 1.415477 |  |  | - |
| k__Bacteria.p__Gemmatimonadota.c__BD2_11_terrestrial_group.o__BD2_11_terrestrial_group.f__BD2_11_terrestrial_group.g__BD2_11_terrestrial_group | 2.375087 |  |  | - |
| k__Bacteria.p__Proteobacteria.c__Alphaproteobacteria.o__Rhizobiales.f__Amb_16S_1323.g__Amb_16S_1323 | 2.305054 |  |  | - |
| k__Bacteria.p__Proteobacteria.c__Gammaproteobacteria.o__Pseudomonadales.f__Pseudomonadaceae.g__Pseudomonas | 2.955673 |  |  | - |
| k__Bacteria.p__Chloroflexi | 2.262048 |  |  | - |
| k__Bacteria.p__Myxococcota.c__bacteriap25.o__bacteriap25.f__bacteriap25 | 2.572408 |  |  | - |
| k__Bacteria.p__Elusimicrobiota.c__Lineage_IIb.o__Lineage_IIb | 1.88581 |  |  | - |
| k__Bacteria.p__Gemmatimonadota.c__Longimicrobia.o__Longimicrobiales.f__Longimicrobiaceae.g__YC_ZSS_LKJ147 | 1.952523 |  |  | - |
| k__Bacteria.p__Proteobacteria.c__Alphaproteobacteria.o__Rhizobiales.f__Beijerinckiaceae.g__Roseiarcus | 0.725926 |  |  | - |
| k__Bacteria.p__Cyanobacteria.c__Sericytochromatia.o__Sericytochromatia.f__Sericytochromatia.g__Sericytochromatia | 2.282365 |  |  | - |
| k__Bacteria.p__Bacteroidota.c__Bacteroidia.o__Flavobacteriales.f__Flavobacteriaceae | 3.245367 |  |  | - |
| k__Bacteria.p__Firmicutes.c__Clostridia.o__Oscillospirales.f__Oscillospiraceae.g__UCG_007 | 0.981199 |  |  | - |
| k__Bacteria.p__Proteobacteria.c__Gammaproteobacteria.o__Burkholderiales.f__Sutterellaceae.g__Parasutterella | 2.826752 |  |  | - |
| k__Bacteria.p__Firmicutes.c__Clostridia.o__Oscillospirales.f__Oscillospiraceae.g__UCG_003 | 2.688315 |  |  | - |
| k__Bacteria.p__Firmicutes.c__Clostridia.o__Oscillospirales.f__Oscillospiraceae.g__UCG_002 | 2.765162 | O | 2.524839 | 0.041992 |
| k__Bacteria.p__Proteobacteria.c__Gammaproteobacteria.o__CCD24 | 1.826682 |  |  | - |
| k__Bacteria.p__Firmicutes.c__Clostridia.o__Peptostreptococcales_Tissierellales.f__Anaerovoracaceae | 3.22971 |  |  | - |
| k__Bacteria.p__Bacteroidota.c__Bacteroidia.o__Chitinophagales.f__Chitinophagaceae.g__Ferruginibacter | 1.561747 |  |  | - |
| k__Bacteria.p__Acidobacteriota.c__Thermoanaerobaculia | 2.402628 |  |  | - |
| k__Bacteria.p__Patescibacteria.c__Parcubacteria.o__Candidatus_Jorgensenbacteria | 1.905844 |  |  | - |
| k__Bacteria.p__Proteobacteria.c__Gammaproteobacteria.o__Cellvibrionales | 2.088493 |  |  | - |
| k__Bacteria.p__Firmicutes.c__Clostridia.o__Lachnospirales.f__Lachnospiraceae.g__Tuzzerella | 3.350067 | C | 2.852277 | 0.030727 |
| k__Bacteria.p__Proteobacteria.c__Gammaproteobacteria.o__Oceanospirillales.f__Pseudohongiellaceae.g__BIyi10 | 1.134599 |  |  | - |
| k__Bacteria.p__Proteobacteria.c__Gammaproteobacteria.o__Burkholderiales.f__A21b | 2.523568 |  |  | - |
| k__Bacteria.p__Proteobacteria.c__Gammaproteobacteria.o__Xanthomonadales.f__Rhodanobacteraceae.g__Mizugakiibacter | 3.123347 |  |  | - |
| k__Bacteria.p__Bdellovibrionota.c__Bdellovibrionia.o__Bdellovibrionales.f__Bdellovibrionaceae | 1.8076 |  |  | - |
| k__Bacteria.p__Firmicutes.c__Clostridia.o__Oscillospirales.f__Butyricicoccaceae | 3.458567 |  |  | - |
| k__Bacteria.p__Latescibacterota.c__Latescibacteria.o__Latescibacterales | 1.644063 |  |  | - |
| k__Bacteria.p__Proteobacteria.c__Alphaproteobacteria.o__Rhizobiales.f__Devosiaceae | 2.392701 |  |  | - |
| k__Bacteria.p__Firmicutes.c__Bacilli.o__Acholeplasmatales.f__Acholeplasmataceae | 2.706587 |  |  | - |
| k__Bacteria.p__Proteobacteria.c__Gammaproteobacteria.o__Burkholderiales.f__SC_I_84.g__SC_I_84 | 3.06635 |  |  | - |
| k__Bacteria.p__Proteobacteria.c__Gammaproteobacteria.o__Pseudomonadales.f__Moraxellaceae | 2.351552 |  |  | - |
| k__Bacteria.p__Firmicutes.c__Bacilli.o__Lactobacillales.f__Streptococcaceae.g__Streptococcus | 4.073575 |  |  | - |
| k__Bacteria.p__Fibrobacterota.c__Fibrobacteria | 2.674465 |  |  | - |
| k__Bacteria.p__Gemmatimonadota.c__Longimicrobia.o__Longimicrobiales.f__Longimicrobiaceae | 2.955762 |  |  | - |
| k__Bacteria.p__Firmicutes.c__Bacilli.o__Thermoactinomycetales.f__Thermoactinomycetaceae.g__Shimazuella | 1.705432 |  |  | - |
| k__Bacteria.p__Actinobacteriota.c__Actinobacteria.o__Micrococcales.f__Micrococcaceae.g__Rothia | 3.305731 |  |  | - |
| k__Bacteria.p__Proteobacteria.c__Gammaproteobacteria.o__Xanthomonadales.f__Xanthomonadaceae | 2.873564 |  |  | - |
| k__Bacteria.p__Proteobacteria.c__Gammaproteobacteria.o__Ga0077536.f__Ga0077536.g__Ga0077536 | 1.85477 |  |  | - |
| k__Bacteria.p__Actinobacteriota.c__Actinobacteria.o__Streptomycetales | 3.064692 |  |  | - |
| k__Bacteria.p__Actinobacteriota.c__Acidimicrobiia.o__Microtrichales.f__Iamiaceae | 2.025786 |  |  | - |
| k__Bacteria.p__Firmicutes.c__Negativicutes.o__Veillonellales_Selenomonadales.f__Veillonellaceae.g__Veillonella | 1.456114 |  |  | - |
| k__Bacteria.p__Nitrospirota.c__Nitrospiria.o__Nitrospirales.f__Nitrospiraceae.g__Nitrospira | 2.967335 |  |  | - |
| k__Bacteria.p__Firmicutes.c__Bacilli.o__Erysipelotrichales.f__Erysipelotrichaceae.g__Faecalibaculum | 2.72174 |  |  | - |
| k__Bacteria.p__Campilobacterota.c__Campylobacteria.o__Campylobacterales.f__Arcobacteraceae.g__Halarcobacter | 2.394875 |  |  | - |
| k__Bacteria.p__Proteobacteria.c__Gammaproteobacteria.o__Cardiobacteriales.f__Cardiobacteriaceae.g__Cardiobacterium | 2.166667 |  |  | - |
| k__Bacteria.p__Actinobacteriota.c__MB_A2_108.o__MB_A2_108.f__MB_A2_108 | 2.355094 |  |  | - |
| k__Bacteria.p__Actinobacteriota.c__Actinobacteria.o__Micromonosporales.f__Micromonosporaceae.g__Micromonospora | 2.440817 |  |  | - |
| k__Bacteria.p__Firmicutes.c__Clostridia | 5.335791 |  |  | - |
| k__Bacteria.p__Proteobacteria.c__Gammaproteobacteria.o__Burkholderiales.f__Burkholderiaceae.g__Limnobacter | 1.727158 |  |  | - |
| k__Bacteria.p__Bacteroidota.c__Bacteroidia.o__Bacteroidales.f__Prevotellaceae | 4.600144 |  |  | - |
| k__Bacteria.p__Firmicutes.c__Clostridia.o__Lachnospirales.f__Lachnospiraceae.g__Lachnoclostridium | 3.999117 |  |  | - |
| k__Bacteria.p__Bacteroidota.c__Bacteroidia.o__Bacteroidales.f__Porphyromonadaceae | 3.501228 |  |  | - |
| k__Bacteria.p__Armatimonadota | 0.322512 |  |  | - |
| k__Bacteria.p__Chloroflexi.c__Anaerolineae.o__Anaerolineae.f__Anaerolineae | 2.088972 |  |  | - |
| k__Bacteria.p__Spirochaetota.c__Spirochaetia.o__Spirochaetales | 2.827824 |  |  | - |
| k__Bacteria.p__Patescibacteria.c__Gracilibacteria.o__Absconditabacteriales__SR1_.f__Absconditabacteriales__SR1_ | 2.491286 |  |  | - |
| k__Bacteria.p__Acidobacteriota.c__Acidobacteriae.o__PAUC26f.f__PAUC26f | 1.811791 |  |  | - |
| k__Bacteria.p__Dependentiae.c__Babeliae.o__Babeliales.f__Vermiphilaceae.g__Vermiphilaceae | 1.363779 |  |  | - |
| k__Bacteria.p__Proteobacteria.c__Gammaproteobacteria | 5.459552 |  |  | - |
| k__Bacteria.p__Firmicutes.c__Bacilli.o__Staphylococcales | 2.984108 |  |  | - |
| k__Bacteria.p__Firmicutes.c__Clostridia.o__Oscillospirales.f__Ethanoligenenaceae | 2.534136 |  |  | - |
| k__Bacteria.p__Bacteroidota.c__Bacteroidia.o__Flavobacteriales.f__Weeksellaceae.g__Chryseobacterium | 1.620968 |  |  | - |
| k__Bacteria.p__Actinobacteriota.c__Actinobacteria.o__Frankiales.f__Frankiaceae | 2.556523 |  |  | - |
| k__Bacteria.p__Firmicutes.c__Bacilli.o__Erysipelotrichales.f__Erysipelotrichaceae.g__Allobaculum | 2.346134 |  |  | - |
| k__Bacteria.p__Armatimonadota.c__Fimbriimonadia.o__Fimbriimonadales.f__Fimbriimonadaceae | 0.322512 |  |  | - |
| k__Bacteria.p__Desulfobacterota.c__Desulfovibrionia.o__Desulfovibrionales.f__Desulfovibrionaceae.g__Desulfovibrio | 4.057162 |  |  | - |
| k__Bacteria.p__Proteobacteria.c__Alphaproteobacteria.o__Rhizobiales.f__Xanthobacteraceae.g__Pseudolabrys | 2.629605 |  |  | - |
| k__Bacteria.p__Proteobacteria.c__Alphaproteobacteria.o__Micropepsales.f__Micropepsaceae.g__Micropepsaceae | 1.823139 |  |  | - |
| k__Bacteria.p__Proteobacteria.c__Gammaproteobacteria.o__Pseudomonadales.f__Pseudomonadaceae | 2.955673 |  |  | - |
| k__Bacteria.p__Acidobacteriota.c__Acidobacteriae.o__Bryobacterales.f__Bryobacteraceae.g__Bryobacter | 3.174221 |  |  | - |
| k__Bacteria.p__Fusobacteriota.c__Fusobacteriia.o__Fusobacteriales.f__Fusobacteriaceae | 3.908271 |  |  | - |
| k__Bacteria.p__Bacteroidota.c__Bacteroidia.o__Cytophagales.f__Cytophagaceae.g__Sporocytophaga | 1.584639 |  |  | - |
| k__Bacteria.p__Bacteroidota.c__Bacteroidia.o__Bacteroidales.f__Marinifilaceae.g__Odoribacter | 3.65213 |  |  | - |
| k__Bacteria.p__Verrucomicrobiota.c__Verrucomicrobiae | 2.810307 |  |  | - |
| k__Bacteria.p__Proteobacteria.c__Alphaproteobacteria.o__Dongiales.f__Dongiaceae.g__Dongia | 2.825556 |  |  | - |
| k__Bacteria.p__Firmicutes.c__Clostridia.o__Peptostreptococcales_Tissierellales.f__Anaerovoracaceae.g__Family_XIII_UCG_001 | 1.817897 |  |  | - |
| k__Bacteria.p__Acidobacteriota.c__Acidobacteriae.o__Bryobacterales.f__Bryobacteraceae | 3.174221 |  |  | - |
| k__Bacteria.p__Proteobacteria.c__Gammaproteobacteria.o__Cardiobacteriales.f__Cardiobacteriaceae | 2.166667 |  |  | - |
| k__Bacteria.p__Actinobacteriota.c__Actinobacteria.o__Bifidobacteriales.f__Bifidobacteriaceae.g__Gardnerella | 2.052461 |  |  | - |
| k__Bacteria.p__Proteobacteria.c__Gammaproteobacteria.o__Thiotrichales | 0.799633 |  |  | - |
| k__Bacteria.p__Actinobacteriota.c__Actinobacteria.o__Actinomycetales | 3.401372 |  |  | - |
| k__Bacteria.p__Proteobacteria.c__Alphaproteobacteria.o__Sphingomonadales.f__Sphingomonadaceae | 4.828159 |  |  | - |
| k__Bacteria.p__Proteobacteria.c__Alphaproteobacteria.o__Micropepsales.f__Micropepsaceae | 3.32053 |  |  | - |
| k__Bacteria.p__Proteobacteria.c__Gammaproteobacteria.o__Steroidobacterales | 1.855086 |  |  | - |
| k__Bacteria.p__Proteobacteria.c__Alphaproteobacteria.o__Rhizobiales.f__KF_JG30_B3.g__KF_JG30_B3 | 1.021482 |  |  | - |
| k__Bacteria.p__Firmicutes.c__Bacilli.o__Lactobacillales.f__Enterococcaceae.g__Enterococcus | 3.469769 |  |  | - |
| k__Bacteria.p__Actinobacteriota.c__Coriobacteriia.o__Coriobacteriales.f__Atopobiaceae.g__Coriobacteriaceae_UCG_002 | 2.226329 |  |  | - |
| k__Bacteria.p__Campilobacterota.c__Campylobacteria.o__Campylobacterales | 4.241276 |  |  | - |
| k__Bacteria.p__Latescibacterota.c__Latescibacteria | 1.644063 |  |  | - |
| k__Bacteria.p__Proteobacteria.c__Gammaproteobacteria.o__EPR3968_O8a_Bc78.f__EPR3968_O8a_Bc78.g__EPR3968_O8a_Bc78 | 1.606979 |  |  | - |
| k__Bacteria.p__Firmicutes.c__Clostridia.o__Peptostreptococcales_Tissierellales.f__Anaerovoracaceae.g__Mogibacterium | 2.588466 | O | 2.349479 | 0.022104 |
| k__Bacteria.p__Bacteroidota.c__Bacteroidia.o__Chitinophagales | 3.203875 |  |  | - |
| k__Bacteria.p__Actinobacteriota | 4.331684 |  |  | - |
| k__Bacteria.p__Fibrobacterota | 2.674465 |  |  | - |
| k__Bacteria.p__Firmicutes.c__Clostridia.o__Eubacteriales.f__Anaerofustaceae.g__Anaerofustis | 1.964712 |  |  | - |
| k__Bacteria.p__Actinobacteriota.c__Actinobacteria.o__0319_7L14.f__0319_7L14.g__0319_7L14 | 1.54392 |  |  | - |
| k__Bacteria.p__Patescibacteria.c__Gracilibacteria.o__Absconditabacteriales__SR1_ | 2.491286 |  |  | - |
| k__Bacteria.p__Actinobacteriota.c__Actinobacteria.o__Micromonosporales.f__Micromonosporaceae.g__Phytomonospora | 1.682043 |  |  | - |
| k__Bacteria.p__RCP2_54.c__RCP2_54.o__RCP2_54.f__RCP2_54 | 2.540402 |  |  | - |
| k__Bacteria.p__Bacteroidota.c__Bacteroidia.o__Sphingobacteriales.f__CWT_CU03_E12 | 0.322512 |  |  | - |
| k__Bacteria.p__Firmicutes.c__Bacilli.o__Alicyclobacillales.f__Alicyclobacillaceae.g__Tumebacillus | 2.380073 |  |  | - |
| k__Bacteria.p__Desulfobacterota.c__Desulfovibrionia | 4.256902 |  |  | - |
| k__Bacteria.p__Firmicutes.c__Bacilli.o__Bacillales.f__Sporolactobacillaceae | 1.090826 |  |  | - |
| k__Bacteria.p__Proteobacteria.c__Gammaproteobacteria.o__Burkholderiales.f__Neisseriaceae.g__Neisseria | 4.20367 |  |  | - |
| k__Bacteria.p__Actinobacteriota.c__Actinobacteria.o__Frankiales.f__Frankiaceae.g__Jatrophihabitans | 2.556523 |  |  | - |
| k__Bacteria.p__Acidobacteriota.c__Acidobacteriae.o__Subgroup_2.f__Subgroup_2.g__Subgroup_2 | 3.657284 |  |  | - |
