# Supplementary material for: Disrupted Tuzzerella abundance and impaired l-glutamine levels induce Treg accumulation in ovarian endometriosis: a comprehensive multi-omics analysis
Source: Metabolomics. 2024 Feb 29;20(2):32. doi: 10.1007/s11306-023-02072-0 (PMC10904428; doi:10.1007/s11306-023-02072-0)
Supplement: Supplementary file 10 — Supplementary file10 (DOCX 15 KB) [file 11306_2023_2072_MOESM10_ESM.docx]

**Table S3 Quantification of microbial-related metabolites**

| IDs | C5 | C3 | O1 | O3 | O8 | O4 | O6 | O2 | C1 | O5 | O7 | C7 | C4 | C2 | C6 | C8 |
| --- | --- | --- | --- | --- | --- | --- | --- | --- | --- | --- | --- | --- | --- | --- | --- | --- |
| Glutaric acid | 3631.804 | 8054.993 | 3243.32 | 3687.08 | 3931.98 | 3607.057 | 4628.151 | 3864.544 | 6528.351 | 3488.336 | 4153.205 | 5661.925 | 7051.251 | 36554.99 | 11577.48 | 22550.83 |
| Succinic acid | 5643.592 | 6599.094 | 9920.442 | 34678.17 | 6340.937 | 6125.804 | 5263.112 | 5386.961 | 5945.679 | 4460.104 | 4968.96 | 23194.43 | 12726.77 | 66634.19 | 175101.2 | 43412.08 |
| Isovaleric acid | 82.46807 | 171.3586 | 209.3913 | 111.7293 | 75.88709 | 140.1761 | 158.7692 | 108.7102 | 151.6706 | 156.5156 | 176.6265 | 746.6006 | 148.6489 | 133.0862 | 139.9913 | 129.2087 |
| Glycoursodeoxycholic acid | 9.75E-07 | 3202.14 | 1555.653 | 177.5207 | 178.8664 | 1304.285 | 4635.976 | 600.6967 | 463.1621 | 836.6067 | 219.6494 | 1065.674 | 54.29396 | 2596.349 | 130.1538 | 9.75E-07 |
| Glycocholic acid | 4967.58 | 14160.64 | 9819.485 | 1108.032 | 1588.811 | 1435.241 | 3635.304 | 593.4576 | 985.132 | 1965.216 | 1698.002 | 923.1963 | 98.55161 | 3407.968 | 917.3367 | 852.0305 |
| Isocaproic acid | 309303.7 | 276740.6 | 322874.2 | 425748.1 | 559736 | 500052.8 | 471784.6 | 312071.6 | 425889 | 350365.3 | 322378.3 | 430570.6 | 200812.4 | 225010.5 | 352577.2 | 451581.1 |
