# Supplementary material for: Disrupted Tuzzerella abundance and impaired l-glutamine levels induce Treg accumulation in ovarian endometriosis: a comprehensive multi-omics analysis
Source: Metabolomics. 2024 Feb 29;20(2):32. doi: 10.1007/s11306-023-02072-0 (PMC10904428; doi:10.1007/s11306-023-02072-0)
Supplement: Supplementary file 11 — Supplementary file11 (DOCX 989 KB) [file 11306_2023_2072_MOESM11_ESM.docx]

Table S4 The correlation between microbiota and metabolites

| Microbiome | Metabolite | Correlation | Pvalue | AdjPvalue |
| --- | --- | --- | --- | --- |
| RF39 | 2-Deoxy-2,3-dehydro-n-acetyl-neuraminic acid | 0.938089 | 7.93E-08 | 0.001074 |
| RF39 | N-(1-Deoxy-1-fructosyl)tyrosine | 0.929012 | 2.02E-07 | 0.001074 |
| TRA3-20 | DG(17:2(9Z,12Z)/22:5(7Z,10Z,13Z,16Z,19Z)/0:0)[iso2] | 0.927678 | 2.29E-07 | 0.001074 |
| Anaerotruncus | Lucuminic acid | 0.919617 | 4.69E-07 | 0.001448 |
| Tuzzerella | Glutamylleucylarginine | 0.914002 | 7.41E-07 | 0.001448 |
| RF39 | 1H-Pyrrole-2,5-dione, 3-(1-methyl-1h-indol-3-yl)-4-(1-methyl-6-nitro-1h-indol-3-yl)- | 0.913689 | 7.60E-07 | 0.001448 |
| RF39 | BEFLOXATONE | 0.912228 | 8.51E-07 | 0.001448 |
| Tuzzerella | DG(18:0/20:4(8Z,11Z,14Z,17Z)/0:0) | 0.910269 | 9.88E-07 | 0.001448 |
| RF39 | 4'-O-Glucopyranosylsinapic acid | 0.909887 | 1.02E-06 | 0.001448 |
| Tuzzerella | PI(18:0/20:4(8Z,11Z,14Z,17Z)) | 0.906257 | 1.33E-06 | 0.001448 |
| RF39 | Neosaxitoxin | 0.905789 | 1.37E-06 | 0.001448 |
| Tuzzerella | Notoginsenoside H | 0.905615 | 1.39E-06 | 0.001448 |
| MND1 | Tyrosylglycine | 0.905578 | 1.39E-06 | 0.001448 |
| Christensenellaceae_R-7_group | Tyrosylglycine | 0.905116 | 1.44E-06 | 0.001448 |
| RF39 | N-(1-Deoxy-1-fructosyl)leucine | 0.90355 | 1.61E-06 | 0.001509 |
| Tuzzerella | Am-PE(16:0/18:0) | 0.889192 | 4.08E-06 | 0.003594 |
| Aquicella | Streptidine | 0.886712 | 4.74E-06 | 0.003923 |
| RF39 | 2-(2-Thienylmethylene)-1,6-dioxaspiro[4.4]non-3-ene | 0.882925 | 5.90E-06 | 0.004588 |
| RF39 | Xanthinol | 0.882077 | 6.19E-06 | 0.004588 |
| TRA3-20 | Asparaginyl-Proline | 0.876575 | 8.39E-06 | 0.005907 |
| Haliangium | Tyrosylglycine | 0.87506 | 9.10E-06 | 0.006102 |
| Tuzzerella | L-Glutamine | 0.873558 | 9.85E-06 | 0.006307 |
| Anaerostipes | 3-Methyl-3-butenyl apiosyl-(1->6)-glucoside | 0.872367 | 1.05E-05 | 0.006326 |
| Treponema | LysoPC(20:3(8Z,11Z,14Z)/0:0) | 0.871833 | 1.08E-05 | 0.006326 |
| RF39 | 2-C-methyl-D-erythritol-4-phosphate | 0.869637 | 1.21E-05 | 0.006632 |
| TRA3-20 | Prolyl-Asparagine | 0.869353 | 1.22E-05 | 0.006632 |
| TRA3-20 | Docosatrienoic acid | 0.865961 | 1.45E-05 | 0.00757 |
| RF39 | Cysteinyl-Aspartate | 0.86361 | 1.63E-05 | 0.008191 |
| Jatrophihabitans | 5'-Methylthioadenosine | 0.862724 | 1.70E-05 | 0.008255 |
| Rikenellaceae_RC9_gut_group | Phenylalanylproline | 0.859522 | 1.98E-05 | 0.009294 |
| Christensenellaceae_R-7_group | N2-gamma-Glutamylglutamine | 0.858241 | 2.10E-05 | 0.009329 |
| Tuzzerella | 25-Hydroxyvitamin D3-26,23-lactol | -0.85806 | 2.12E-05 | 0.009329 |
| Anaerotruncus | Dipotassium phosphate | 0.856749 | 2.25E-05 | 0.009501 |
| IMCC26256 | 3-Methyl-3-butenyl hexadecanoate | 0.856 | 2.33E-05 | 0.009501 |
| Muribaculum | 5'-Methylthioadenosine | 0.855492 | 2.39E-05 | 0.009501 |
| Haliangium | N2-gamma-Glutamylglutamine | 0.855108 | 2.43E-05 | 0.009501 |
| bacteriap25 | LysoPC(20:3(8Z,11Z,14Z)/0:0) | 0.848855 | 3.21E-05 | 0.012028 |
| Acidothermus | Selinexor | 0.84773 | 3.37E-05 | 0.012028 |
| bacteriap25 | Sulisobenzone | 0.847595 | 3.39E-05 | 0.012028 |
| Jatrophihabitans | 3-Hydroxyeicosanoylcarnitine | 0.847035 | 3.47E-05 | 0.012028 |
| RF39 | N-[(2R,3R,4S,6R)-4,6-Dihydroxy-6-methyl-2-[(1R,2R)-1,2,3-trihydroxypropyl]oxan-3-yl]acetamide | 0.846827 | 3.50E-05 | 0.012028 |
| Faecalibaculum | Selinexor | 0.845975 | 3.63E-05 | 0.012178 |
| Nitrospira | 5'-Methylthioadenosine | 0.840863 | 4.50E-05 | 0.014736 |
| MND1 | PE-NMe2(18:3(9Z,12Z,15Z)/18:1(11Z)) | 0.838667 | 4.92E-05 | 0.015754 |
| [Eubacterium]_oxidoreducens_group | Streptidine | 0.83799 | 5.06E-05 | 0.015832 |
| Flavonifractor | 3-Methyl-3-butenyl hexadecanoate | 0.836171 | 5.44E-05 | 0.016557 |
| Acidothermus | Phenylalanylproline | 0.835289 | 5.64E-05 | 0.016557 |
| Tuzzerella | 1-(10H-Phenothiazin-2-yl)ethanone | 0.834742 | 5.76E-05 | 0.016557 |
| Jatrophihabitans | Fucoxanthinol 3-(4Z,7Z,10Z,13Z,16Z,19Z-docosahexaenoate) 3'-palmitoleate | 0.834035 | 5.92E-05 | 0.016557 |
| TRA3-20 | Serylproline | 0.833921 | 5.95E-05 | 0.016557 |
| Nitrospira | Fucoxanthinol 3-(4Z,7Z,10Z,13Z,16Z,19Z-docosahexaenoate) 3'-palmitoleate | 0.832714 | 6.24E-05 | 0.016557 |
| Nitrospira | N-(1-Deoxy-1-fructosyl)alanine | 0.832256 | 6.35E-05 | 0.016557 |
| Anaerotruncus | [6-Hydroxy-8-methyl-3-[3,4,5-trihydroxy-6-(hydroxymethyl)oxan-2-yl]oxy-9,10-dioxatetracyclo[4.3.1.02,5.03,8]decan-4-yl]methyl benzoate | 0.832045 | 6.40E-05 | 0.016557 |
| TRA3-20 | Eicosadienoic acid | 0.831898 | 6.44E-05 | 0.016557 |
| TRA3-20 | 11,14,17-eicosatrienoic acid | 0.83179 | 6.47E-05 | 0.016557 |
| Tuzzerella | alpha-D-Galactopyranuronosyl-(1->4)-alpha-D-galactopyranuronosyl-(1->4)-D-galacturonic acid | 0.830737 | 6.74E-05 | 0.016937 |
| Bryobacter | Nopalinic acid | 0.827022 | 7.76E-05 | 0.01917 |
| Muribaculum | Inosine | 0.824076 | 8.66E-05 | 0.020528 |
| Nitrospira | 3-Hydroxyeicosanoylcarnitine | 0.823985 | 8.69E-05 | 0.020528 |
| Anaerotruncus | Sulisobenzone | 0.823694 | 8.79E-05 | 0.020528 |
| TM7 | (R)-3-Amino-2-fluoropropyl phosphenite | 0.823361 | 8.89E-05 | 0.020528 |
| MND1 | Phytosphingosine | 0.82286 | 9.06E-05 | 0.020535 |
| Tuzzerella | m7G(5')pppAm | 0.822111 | 9.31E-05 | 0.020535 |
| Granulicella | 3-Methyl-3-butenyl hexadecanoate | 0.822042 | 9.33E-05 | 0.020535 |
| MND1 | 5'-Hydroxytenoxicam | 0.82136 | 9.57E-05 | 0.020728 |
| TRA3-20 | 20-HETE-d6 | 0.820761 | 9.78E-05 | 0.020863 |
| Alcaligenes | Neryl 8-methyldecanoate | 0.818638 | 0.000106 | 0.022183 |
| Coprobacillus | 5'-Methylthioadenosine | 0.817285 | 0.000111 | 0.022936 |
| Tuzzerella | thiamphenicol | 0.81687 | 0.000112 | 0.022938 |
| Anaerostipes | 1H-Pyrrole-2,5-dione, 3-(1-methyl-1h-indol-3-yl)-4-(1-methyl-6-nitro-1h-indol-3-yl)- | 0.813643 | 0.000126 | 0.025319 |
| Anaerotruncus | N-gamma-L-Glutamyl-D-alanine | 0.812657 | 0.00013 | 0.02583 |
| Tuzzerella | Terizidone | 0.811547 | 0.000135 | 0.026463 |
| Solobacterium | 3-Methyl-3-butenyl hexadecanoate | 0.810591 | 0.00014 | 0.026968 |
| Rikenellaceae_RC9_gut_group | L-Carnitine | 0.81016 | 0.000142 | 0.026997 |
| Coprobacillus | N-(1-Deoxy-1-fructosyl)alanine | 0.809636 | 0.000144 | 0.027115 |
| TRA3-20 | Docosadienoate (22:2n6) | 0.809128 | 0.000147 | 0.027223 |
| RF39 | (R)-Propyl 2-amino-3-mercaptopropanoate | 0.808694 | 0.000149 | 0.027267 |
| Acidothermus | L-Carnitine | 0.808093 | 0.000152 | 0.027468 |
| MND1 | N2-gamma-Glutamylglutamine | 0.806927 | 0.000158 | 0.02768 |
| bacteriap25 | Lucuminic acid | 0.80688 | 0.000158 | 0.02768 |
| Rikenellaceae_RC9_gut_group | Selinexor | 0.806737 | 0.000159 | 0.02768 |
| Treponema | 3-Hydroxybutyrylcarnitine | 0.805517 | 0.000166 | 0.028475 |
| Subgroup_13 | Aspartyl-Alanine | 0.80472 | 0.00017 | 0.028635 |
| Subgroup_13 | 2,2,3,3,4,4,5,5,6,6,7,7,8,8,9,9-Hexadecafluorononanoic acid | 0.804618 | 0.000171 | 0.028635 |
| Jatrophihabitans | (13Z)-3-Hydroxyicos-13-enoylcarnitine | 0.804171 | 0.000173 | 0.028718 |
| Rikenellaceae_RC9_gut_group | Pentadecanoylcarnitine | 0.801812 | 0.000187 | 0.030659 |
| Rikenellaceae_RC9_gut_group | (S1)-Methoxy-3-heptanethiol | 0.800979 | 0.000192 | 0.031136 |
| TRA3-20 | Fructose-1,6-diphosphate | 0.800478 | 0.000196 | 0.031284 |
| Tuzzerella | Genistein 7-O-glucoside-6''-malonate | 0.799871 | 0.000199 | 0.031542 |
| Jatrophihabitans | Inosine | 0.79833 | 0.000209 | 0.032768 |
| Subgroup_13 | Creatine | 0.797249 | 0.000217 | 0.033444 |
| Acidothermus | (S1)-Methoxy-3-heptanethiol | 0.796994 | 0.000219 | 0.033444 |
| Tuzzerella | Allitridin | 0.79467 | 0.000235 | 0.035592 |
| Tuzzerella | Tercatain | 0.793459 | 0.000244 | 0.036525 |
| Subgroup_13 | N,N'-Diethylthiourea | 0.793154 | 0.000246 | 0.036525 |
| Anaerostipes | 2-Deoxy-2,3-dehydro-n-acetyl-neuraminic acid | 0.792392 | 0.000252 | 0.037006 |
| Tuzzerella | [6-Hydroxy-8-methyl-3-[3,4,5-trihydroxy-6-(hydroxymethyl)oxan-2-yl]oxy-9,10-dioxatetracyclo[4.3.1.02,5.03,8]decan-4-yl]methyl benzoate | 0.790495 | 0.000267 | 0.038748 |
| Muribaculum | 3-Hydroxyeicosanoylcarnitine | 0.790221 | 0.00027 | 0.038748 |
| TRA3-20 | Pentadeca-3,5,7-trienedioylcarnitine | 0.789423 | 0.000276 | 0.03899 |
| Coprobacillus | N-Acetylneuraminic acid | 0.789353 | 0.000277 | 0.03899 |
| Intestinimonas | Phytosphingosine | 0.788556 | 0.000284 | 0.039353 |
| Pseudolabrys | Fructose-1,6-diphosphate | 0.787742 | 0.000291 | 0.039353 |
| Tuzzerella | Pentadeca-3,5,7-trienedioylcarnitine | 0.787568 | 0.000292 | 0.039353 |
| Anaerotruncus | Benzyl gentiobioside | 0.78724 | 0.000295 | 0.039353 |
| Treponema | Sulisobenzone | 0.787164 | 0.000296 | 0.039353 |
| Acidothermus | Pentadecanoylcarnitine | 0.787082 | 0.000297 | 0.039353 |
| Tuzzerella | N2-gamma-Glutamylglutamine | 0.786802 | 0.000299 | 0.039353 |
| Anaerostipes | BEFLOXATONE | 0.78641 | 0.000303 | 0.03945 |
| Bryobacter | Dehydroascorbide(1-) | 0.784561 | 0.00032 | 0.040726 |
| Solobacterium | 3-Hydroxy-10'-apo-b,y-carotenal | 0.784331 | 0.000322 | 0.040726 |
| Anaerostipes | (R)-Propyl 2-amino-3-mercaptopropanoate | 0.783859 | 0.000326 | 0.040726 |
| Subgroup_2 | Tyrosylglycine | 0.783332 | 0.000332 | 0.040726 |
| Tuzzerella | PE(22:5(4Z,7Z,10Z,13Z,16Z)/PGD2) | -0.78326 | 0.000332 | 0.040726 |
| Tuzzerella | 2-[(4-{2-[(4-Cyclohexylbutyl)(cyclohexylcarbamoyl)amino]ethyl}phenyl)sulfanyl]-2-methylpropanoic acid | -0.78307 | 0.000334 | 0.040726 |
| Enterococcus | Nopalinic acid | 0.782933 | 0.000335 | 0.040726 |
| Rikenellaceae_RC9_gut_group | Aspartyl-Methionine | 0.782925 | 0.000336 | 0.040726 |
| Tuzzerella | Kuguacin E | -0.78176 | 0.000347 | 0.041625 |
| Muribaculum | Fucoxanthinol 3-(4Z,7Z,10Z,13Z,16Z,19Z-docosahexaenoate) 3'-palmitoleate | 0.781465 | 0.00035 | 0.041625 |
| Tuzzerella | 4-(Methylnitrosamino)-1-(3-pyridyl)-1-butanol glucuronide | 0.781305 | 0.000352 | 0.041625 |
| Achromobacter | Neryl 8-methyldecanoate | 0.780386 | 0.000361 | 0.041996 |
| Coprobacillus | Fucoxanthinol 3-(4Z,7Z,10Z,13Z,16Z,19Z-docosahexaenoate) 3'-palmitoleate | 0.780246 | 0.000363 | 0.041996 |
| Bryobacter | Valylserine | 0.780047 | 0.000365 | 0.041996 |
| Tuzzerella | 3-Ketosphingosine | -0.77986 | 0.000367 | 0.041996 |
| TRA3-20 | Erucic acid | 0.778967 | 0.000376 | 0.042448 |
| bacteriap25 | 3-Hydroxybutyrylcarnitine | 0.778682 | 0.00038 | 0.042448 |
| Treponema | LysoPI(16:0/0:0) | 0.778599 | 0.00038 | 0.042448 |
| Flavonifractor | 3-Hydroxy-10'-apo-b,y-carotenal | 0.778248 | 0.000384 | 0.042448 |
| Bryobacter | N6-Acetyl-L-lysine | 0.778084 | 0.000386 | 0.042448 |
| Muribaculum | N-(1-Deoxy-1-fructosyl)alanine | 0.777833 | 0.000389 | 0.042448 |
| TRA3-20 | 2',3'-Dideoxyuridine | 0.776772 | 0.000401 | 0.043417 |
| Anaerotruncus | Aspartyl-Alanine | 0.776091 | 0.000409 | 0.043755 |
| Bryobacter | Eleutherazine B; N,N'-((3,6-Dioxo-2,5-piperazinediyl)bis(trimethylene))bis(5-hydroxy-3-methyl-2-pentenamide) | 0.775962 | 0.00041 | 0.043755 |
| Nitrospira | N-Acetylneuraminic acid | 0.775256 | 0.000419 | 0.044303 |
| Muribaculum | (13Z)-3-Hydroxyicos-13-enoylcarnitine | 0.774358 | 0.000429 | 0.0451 |
| Tuzzerella | Camptothecin sodium | 0.773709 | 0.000437 | 0.04559 |
| Pseudolabrys | N-lactoyl-Methionine | 0.772824 | 0.000448 | 0.04639 |
| Tuzzerella | Diethylamino 2,5-dihydroxybenzenesulfonate | 0.772519 | 0.000452 | 0.046444 |
| Anaerostipes | Neosaxitoxin | 0.772248 | 0.000455 | 0.046457 |
| Bryobacter | Fructose-1,6-diphosphate | 0.771955 | 0.000459 | 0.046501 |
| Faecalibaculum | Azeloprazole | 0.771681 | 0.000463 | 0.046522 |
| Tuzzerella | PC(11:0/23:0) | 0.771115 | 0.00047 | 0.046663 |
| Tuzzerella | Fiacitabine | 0.77106 | 0.000471 | 0.046663 |
| Bryobacter | N-Lactoylphenylalanine | 0.769761 | 0.000488 | 0.047543 |
| Pseudolabrys | Asparaginyl-Proline | 0.769709 | 0.000489 | 0.047543 |
| TRA3-20 | 3-Methyl-3-butenyl hexadecanoate | 0.769625 | 0.00049 | 0.047543 |
| Pseudolabrys | N-Lactoylleucine | 0.769321 | 0.000494 | 0.047614 |
| Monoglobus | Fructose-1,6-diphosphate | 0.76873 | 0.000502 | 0.048062 |
| Treponema | 23S,25,26-Trihydroxyvitamin D3 | 0.768215 | 0.000509 | 0.048355 |
| Enterococcus | Prolyl-Asparagine | 0.768013 | 0.000512 | 0.048355 |
| Anaerostipes | 2-C-methyl-D-erythritol-4-phosphate | 0.767668 | 0.000517 | 0.048487 |
| Bryobacter | Valylhistidine | 0.767323 | 0.000521 | 0.04862 |
| MND1 | Tercatain | 0.766506 | 0.000533 | 0.049384 |
| Muribaculum | hydroxyhexadecenoylcarnitine | 0.765822 | 0.000543 | 0.049773 |
| TM7 | 2-Methylthioadenosine | 0.765731 | 0.000544 | 0.049773 |
| Coprobacillus | Inosine | 0.763672 | 0.000575 | 0.051377 |
| bacteriap25 | LysoPI(16:0/0:0) | 0.763516 | 0.000578 | 0.051377 |
| Jatrophihabitans | N-(1-Deoxy-1-fructosyl)alanine | 0.763489 | 0.000578 | 0.051377 |
| Pseudolabrys | Histidylisoleucine | 0.763417 | 0.000579 | 0.051377 |
| Anaerotruncus | Genistein 7-O-glucoside-6''-malonate | 0.76331 | 0.000581 | 0.051377 |
| TM7 | D-erythro-L-galacto-Nonulose | 0.763096 | 0.000584 | 0.051377 |
| Christensenellaceae_R-7_group | Phytosphingosine | 0.76289 | 0.000588 | 0.051377 |
| Anaerostipes | 4'-O-Glucopyranosylsinapic acid | 0.761866 | 0.000604 | 0.05222 |
| Achromobacter | Aspartyl-Gamma-glutamate | 0.761772 | 0.000605 | 0.05222 |
| Faecalibaculum | Creatine | 0.761581 | 0.000608 | 0.05222 |
| Tuzzerella | Beta-Citryl-L-glutamic acid | 0.761067 | 0.000617 | 0.052614 |
| Subgroup_13 | Azeloprazole | 0.760297 | 0.000629 | 0.053199 |
| [Eubacterium]_oxidoreducens_group | 3-Hydroxy-10'-apo-b,y-carotenal | 0.760095 | 0.000633 | 0.053199 |
| Tuzzerella | N-Acetyl-L-methionine | 0.759901 | 0.000636 | 0.053199 |
| Bryobacter | Prolylproline | 0.759566 | 0.000641 | 0.053199 |
| Alcaligenes | Dihydrozeatin O-beta-D-Glucoside | 0.759511 | 0.000642 | 0.053199 |
| Anaerotruncus | Aspartyl-Gamma-glutamate | 0.758878 | 0.000653 | 0.05324 |
| Acidothermus | Aspartyl-Methionine | 0.758744 | 0.000655 | 0.05324 |
| Gemella | 3-Methyl-3-butenyl hexadecanoate | 0.758739 | 0.000655 | 0.05324 |
| Anaerostipes | Cysteinyl-Aspartate | 0.758593 | 0.000658 | 0.05324 |
| Treponema | LysoPI(18:0/0:0) | 0.758307 | 0.000663 | 0.053332 |
| Faecalibaculum | N,N'-Diethylthiourea | 0.758018 | 0.000668 | 0.053429 |
| TM7 | scyllo-Inositol | 0.757557 | 0.000676 | 0.053555 |
| Anaerostipes | 2-(2-Thienylmethylene)-1,6-dioxaspiro[4.4]non-3-ene | 0.757386 | 0.000679 | 0.053555 |
| RF39 | N-(1-Deoxy-1-fructosyl)valine | 0.757277 | 0.000681 | 0.053555 |
| Anaerostipes | (R)-3-Amino-2-fluoropropyl phosphenite | 0.75621 | 0.0007 | 0.054747 |
| Gemella | 3-Hydroxy-10'-apo-b,y-carotenal | 0.755773 | 0.000708 | 0.055062 |
| Treponema | Methacholine | 0.755515 | 0.000713 | 0.055124 |
| Treponema | 6-Hydroxytricetin 5-rhamnoside | 0.7552 | 0.000718 | 0.05527 |
| Acidothermus | Azeloprazole | 0.754574 | 0.00073 | 0.055858 |
| TM7 | 3-Methyl-3-butenyl apiosyl-(1->6)-glucoside | 0.754093 | 0.000739 | 0.056004 |
| Muribaculum | N-Acetylneuraminic acid | 0.75405 | 0.00074 | 0.056004 |
| Achromobacter | 3-Methyl-3-butenyl hexadecanoate | 0.753806 | 0.000744 | 0.056052 |
| Tuzzerella | PC(TXB2/20:2(11Z,14Z)) | -0.75327 | 0.000755 | 0.056275 |
| TM7 | (R)-Propyl 2-amino-3-mercaptopropanoate | 0.753233 | 0.000755 | 0.056275 |
| Anaerostipes | Xanthinol | 0.752659 | 0.000767 | 0.056802 |
| Coprobacillus | 3-Hydroxyeicosanoylcarnitine | 0.752062 | 0.000778 | 0.057366 |
| IMCC26256 | 3-Hydroxy-10'-apo-b,y-carotenal | 0.751565 | 0.000788 | 0.057789 |
| Lachnospiraceae_UCG-006 | Neryl 8-methyldecanoate | 0.749707 | 0.000826 | 0.060241 |
| Bryobacter | PE(19:0/20:2(11Z,14Z)) | -0.74946 | 0.000831 | 0.060305 |
| Tuzzerella | Oleoyl Serotonin | -0.74916 | 0.000837 | 0.060414 |
| Tuzzerella | 16,17-Dihydro-16alpha,17-dihydroxygibberellin A4 17-glucoside | -0.74889 | 0.000843 | 0.060414 |
| Tuzzerella | 4-Bis(2-hydroxyethyl)amino-L-phenylalanine | 0.748637 | 0.000848 | 0.060414 |
| Pseudolabrys | N-Lactoylphenylalanine | 0.748569 | 0.00085 | 0.060414 |
| TRA3-20 | N-Lactoylleucine | 0.74791 | 0.000864 | 0.061104 |
| bacteriap25 | Camptothecin sodium | 0.746681 | 0.00089 | 0.061673 |
| Bryobacter | L-arginino-succinate | 0.746656 | 0.000891 | 0.061673 |
| Tuzzerella | Bis(2-propoxyethyl) 2,6-dimethyl-4-(3-nitrophenyl)-3,4-dihydropyridine-3,5-dicarboxylate | -0.74643 | 0.000896 | 0.061673 |
| Bryobacter | N-Lactoylleucine | 0.746366 | 0.000897 | 0.061673 |
| Enterococcus | Asparaginyl-Proline | 0.74631 | 0.000899 | 0.061673 |
| Subgroup_13 | Cysteine-glutathione disulfide | 0.746285 | 0.000899 | 0.061673 |
| Achromobacter | Dihydrozeatin O-beta-D-Glucoside | 0.74579 | 0.00091 | 0.061673 |
| Enterococcus | DG(17:2(9Z,12Z)/22:5(7Z,10Z,13Z,16Z,19Z)/0:0)[iso2] | 0.745749 | 0.000911 | 0.061673 |
| TRA3-20 | L-arginino-succinate | 0.745565 | 0.000915 | 0.061673 |
| Subgroup_2 | 4-(Methylnitrosamino)-1-(3-pyridyl)-1-butanol glucuronide | 0.745258 | 0.000922 | 0.061673 |
| Bryobacter | Fluazifop | 0.745245 | 0.000922 | 0.061673 |
| Enterococcus | Dehydroascorbide(1-) | 0.744954 | 0.000929 | 0.061673 |
| bacteriap25 | LysoPI(18:0/0:0) | 0.744808 | 0.000932 | 0.061673 |
| Pseudolabrys | L-arginino-succinate | 0.744428 | 0.000941 | 0.061673 |
| Muribaculum | 3-Hydroxy-11Z-octadecenoylcarnitine | 0.744419 | 0.000941 | 0.061673 |
| Tuzzerella | 2',3'-Dideoxyuridine | 0.744398 | 0.000942 | 0.061673 |
| Bryobacter | N-lactoyl-Methionine | 0.743941 | 0.000952 | 0.062077 |
| Enterococcus | Docosatrienoic acid | 0.74361 | 0.00096 | 0.062293 |
| TRA3-20 | N-lactoyl-Methionine | 0.743374 | 0.000966 | 0.062364 |
| RF39 | scyllo-Inositol | 0.743041 | 0.000974 | 0.062553 |
| RF39 | Glutamylleucylarginine | 0.742851 | 0.000978 | 0.062553 |
| Treponema | Ribonolactone | 0.742513 | 0.000986 | 0.062553 |
| Tuzzerella | 20-HETE-d6 | 0.742354 | 0.00099 | 0.062553 |
| Pseudolabrys | DG(17:2(9Z,12Z)/22:5(7Z,10Z,13Z,16Z,19Z)/0:0)[iso2] | 0.742173 | 0.000994 | 0.062553 |
| Tuzzerella | Resorufin | 0.742011 | 0.000998 | 0.062553 |
| Tuzzerella | Dolichyl b-D-glucosyl phosphate | -0.74191 | 0.001001 | 0.062553 |
| RF39 | (R)-3-Amino-2-fluoropropyl phosphenite | 0.741658 | 0.001007 | 0.062553 |
| Tuzzerella | D-Malic acid | 0.741484 | 0.001011 | 0.062553 |
| Tuzzerella | 4E,14Z-Sphingadiene | -0.7414 | 0.001013 | 0.062553 |
| bacteriap25 | NAD | 0.740729 | 0.00103 | 0.063295 |
| bacteriap25 | 6-Hydroxytricetin 5-rhamnoside | 0.739688 | 0.001056 | 0.064615 |
| Acetatifactor | Aspartyl-Alanine | 0.736965 | 0.001126 | 0.068396 |
| Tuzzerella | Phytosphingosine | 0.736841 | 0.00113 | 0.068396 |
| Nitrospira | (13Z)-3-Hydroxyicos-13-enoylcarnitine | 0.736586 | 0.001136 | 0.068396 |
| Subgroup_2 | N2-gamma-Glutamylglutamine | 0.736574 | 0.001137 | 0.068396 |
| Tuzzerella | Nipradilol | 0.735666 | 0.001161 | 0.069311 |
| Muribaculum | 11-Hydroxyoctadecanoylcarnitine | 0.735349 | 0.00117 | 0.069311 |
| bacteriap25 | 23S,25,26-Trihydroxyvitamin D3 | 0.735286 | 0.001172 | 0.069311 |
| Monoglobus | Serylproline | 0.735236 | 0.001173 | 0.069311 |
| Lachnospiraceae_UCG-006 | D-Mannose | 0.735114 | 0.001177 | 0.069311 |
| Tuzzerella | 11,14,17-eicosatrienoic acid | 0.734769 | 0.001186 | 0.069368 |
| Bryobacter | Dehydroascorbic acid | 0.734618 | 0.00119 | 0.069368 |
| Treponema | NAD | 0.734547 | 0.001192 | 0.069368 |
| bacteriap25 | Methacholine | 0.734193 | 0.001202 | 0.069657 |
| Jatrophihabitans | 11-Hydroxyoctadecanoylcarnitine | 0.733976 | 0.001208 | 0.069725 |
| Tuzzerella | PE(19:0/20:2(11Z,14Z)) | -0.73327 | 0.001228 | 0.070373 |
| Anaerostipes | N-[(2R,3R,4S,6R)-4,6-Dihydroxy-6-methyl-2-[(1R,2R)-1,2,3-trihydroxypropyl]oxan-3-yl]acetamide | 0.73323 | 0.00123 | 0.070373 |
| Treponema | Adomeglivant | 0.732816 | 0.001242 | 0.070767 |
| Alcaligenes | Aspartyl-Gamma-glutamate | 0.732413 | 0.001253 | 0.070942 |
| Treponema | Camptothecin sodium | 0.732363 | 0.001255 | 0.070942 |
| Christensenellaceae_R-7_group | 4'-O-Glucopyranosylsinapic acid | 0.731677 | 0.001275 | 0.07179 |
| Anaerostipes | N-(1-Deoxy-1-fructosyl)tyrosine | 0.731311 | 0.001286 | 0.072112 |
| TRA3-20 | N-Lactoylphenylalanine | 0.730755 | 0.001302 | 0.072755 |
| Tuzzerella | Fexaramine | -0.73008 | 0.001323 | 0.073343 |
| Anaerostipes | scyllo-Inositol | 0.730062 | 0.001323 | 0.073343 |
| Anaerotruncus | 3-Methyl-3-butenyl hexadecanoate | 0.729173 | 0.00135 | 0.074506 |
| Coprobacillus | Glucose-6-glutamate | 0.729033 | 0.001355 | 0.074506 |
| Bryobacter | PC(TXB2/20:2(11Z,14Z)) | -0.72871 | 0.001365 | 0.074506 |
| Anaerotruncus | 3-Hydroxy-10'-apo-b,y-carotenal | 0.728694 | 0.001365 | 0.074506 |
| Rikenellaceae_RC9_gut_group | trans-Hexadec-2-enoyl carnitine | 0.728504 | 0.001371 | 0.074541 |
| IMCC26256 | O-phosphonato-L-homoserine(2-) | 0.727265 | 0.001411 | 0.07638 |
| Treponema | Lucuminic acid | 0.72684 | 0.001424 | 0.076825 |
| Aquicella | D-Mannose | 0.7263 | 0.001442 | 0.077475 |
| Tuzzerella | (R)-Propyl 2-amino-3-mercaptopropanoate | 0.726065 | 0.001449 | 0.077591 |
| Anaerotruncus | Cysteine-glutathione disulfide | 0.725619 | 0.001464 | 0.077712 |
| TRA3-20 | 4-Bis(2-hydroxyethyl)amino-L-phenylalanine | 0.725607 | 0.001464 | 0.077712 |
| Anaerotruncus | O-phosphonato-L-homoserine(2-) | 0.725379 | 0.001472 | 0.077712 |
| TRA3-20 | Nopalinic acid | 0.725012 | 0.001484 | 0.077712 |
| Bryobacter | Histidylisoleucine | 0.724855 | 0.00149 | 0.077712 |
| Christensenellaceae_R-7_group | N-(1-Deoxy-1-fructosyl)leucine | 0.724839 | 0.00149 | 0.077712 |
| Faecalibaculum | Phenylalanylproline | 0.724716 | 0.001494 | 0.077712 |
| Tuzzerella | Tyrosylglycine | 0.724667 | 0.001496 | 0.077712 |
| Flavonifractor | Streptidine | 0.724256 | 0.00151 | 0.07795 |
| Coprobacillus | (13Z)-3-Hydroxyicos-13-enoylcarnitine | 0.724203 | 0.001512 | 0.07795 |
| Tuzzerella | PC(18:1(17Z)/18:1(17Z)) | 0.723854 | 0.001523 | 0.078276 |
| Acetatifactor | 3-Methyl-3-butenyl hexadecanoate | 0.723568 | 0.001533 | 0.078492 |
| bacteriap25 | Pevonedistat | 0.722833 | 0.001559 | 0.079503 |
| bacteriap25 | Ribonolactone | 0.72262 | 0.001566 | 0.079579 |
| Rikenellaceae_RC9_gut_group | Azeloprazole | 0.722439 | 0.001572 | 0.079579 |
| Flavonifractor | Genistein 7-O-glucoside-6''-malonate | 0.722204 | 0.001581 | 0.079579 |
| Tuzzerella | N-Acetyl-D-Glucosamine 6-Phosphate | 0.721928 | 0.00159 | 0.079579 |
| TM7 | 2-phospho-4-(cytidine 5'-diphospho)-2-C-methyl-D-erythritol | 0.721639 | 0.0016 | 0.079579 |
| Nitrospira | Glucose-6-glutamate | 0.721602 | 0.001602 | 0.079579 |
| Haliangium | Phytosphingosine | 0.721291 | 0.001613 | 0.079579 |
| Faecalibaculum | L-Carnitine | 0.721097 | 0.00162 | 0.079579 |
| Pseudolabrys | Fluazifop | 0.721053 | 0.001621 | 0.079579 |
| Anaerostipes | N-(1-Deoxy-1-fructosyl)leucine | 0.72091 | 0.001627 | 0.079579 |
| Acetatifactor | 2,2,3,3,4,4,5,5,6,6,7,7,8,8,9,9-Hexadecafluorononanoic acid | 0.720755 | 0.001632 | 0.079579 |
| Rikenellaceae_RC9_gut_group | Tetradecanoylcarnitine | 0.720625 | 0.001637 | 0.079579 |
| Pseudolabrys | Napelline | 0.720407 | 0.001645 | 0.079579 |
| Anaerostipes | D-erythro-L-galacto-Nonulose | 0.7203 | 0.001649 | 0.079579 |
| Tuzzerella | (S)-a-Amino-2,5-dihydro-5-oxo-4-isoxazolepropanoic acid N2-glucoside | 0.720251 | 0.00165 | 0.079579 |
| Treponema | 13-(3,4-Dimethyl-5-propylfuran-2-yl)tridecanoylcarnitine | 0.72025 | 0.00165 | 0.079579 |
| MND1 | 2-(2-Thienylmethylene)-1,6-dioxaspiro[4.4]non-3-ene | 0.720031 | 0.001658 | 0.079691 |
| Muribaculum | N-Acetyl-D-Glucosamine 6-Phosphate | 0.719749 | 0.001669 | 0.079915 |
| Tuzzerella | Pelargonidin 3,5-di-(6-acetylglucoside) | -0.71902 | 0.001696 | 0.080938 |
| Faecalibaculum | 2,2,3,3,4,4,5,5,6,6,7,7,8,8,9,9-Hexadecafluorononanoic acid | 0.718331 | 0.001722 | 0.081331 |
| Treponema | Doxepin | 0.718275 | 0.001724 | 0.081331 |
| Bryobacter | PC(20:5(5Z,8Z,11Z,14Z,17Z)/PGJ2) | -0.71825 | 0.001725 | 0.081331 |
| Lachnospiraceae_UCG-006 | Symmetric dimethylarginine | 0.718181 | 0.001727 | 0.081331 |
| Tuzzerella | PE-NMe2(18:3(9Z,12Z,15Z)/18:1(11Z)) | 0.717979 | 0.001735 | 0.081418 |
| Haliangium | 4'-O-Glucopyranosylsinapic acid | 0.717763 | 0.001743 | 0.081532 |
| Tuzzerella | 2-Deoxy-2,3-dehydro-n-acetyl-neuraminic acid | 0.717342 | 0.001759 | 0.081827 |
| Tuzzerella | Orotidine | 0.717199 | 0.001765 | 0.081827 |
| Subgroup_13 | PGP(18:3(9Z,12Z,15Z)/18:1(12Z)-O(9S,10R)) | 0.717143 | 0.001767 | 0.081827 |
| Christensenellaceae_R-7_group | 2-(2-Thienylmethylene)-1,6-dioxaspiro[4.4]non-3-ene | 0.715288 | 0.001839 | 0.084482 |
| Tuzzerella | Benzyl gentiobioside | 0.715247 | 0.001841 | 0.084482 |
| Tuzzerella | PC(P-18:0/20:4(5Z,8Z,11Z,14Z)-OH(16R)) | 0.71522 | 0.001842 | 0.084482 |
| Tuzzerella | 2-phospho-4-(cytidine 5'-diphospho)-2-C-methyl-D-erythritol | 0.714932 | 0.001854 | 0.084732 |
| bacteriap25 | Benzyl gentiobioside | 0.714614 | 0.001866 | 0.084831 |
| Tuzzerella | Sulisobenzone | 0.714393 | 0.001875 | 0.084831 |
| Bryobacter | Pro-Pro-Pro | 0.71434 | 0.001878 | 0.084831 |
| Tuzzerella | S-Adenosylhomocysteine | 0.71428 | 0.00188 | 0.084831 |
| MND1 | 4'-O-Glucopyranosylsinapic acid | 0.713572 | 0.001909 | 0.085295 |
| Subgroup_13 | Selinexor | 0.713516 | 0.001911 | 0.085295 |
| MND1 | 1H-Pyrrole-2,5-dione, 3-(1-methyl-1h-indol-3-yl)-4-(1-methyl-6-nitro-1h-indol-3-yl)- | 0.713359 | 0.001918 | 0.085295 |
| Tuzzerella | PC(20:4(5Z,8Z,11Z,14Z)-OH(16R)/2:0) | 0.713218 | 0.001923 | 0.085295 |
| Nitrospira | Inosine | 0.713138 | 0.001927 | 0.085295 |
| Treponema | Pevonedistat | 0.712902 | 0.001936 | 0.085295 |
| TM7 | PC(20:4(5Z,8Z,11Z,14Z)-OH(16R)/2:0) | 0.712714 | 0.001944 | 0.085295 |
| Anaerostipes | 2-Methylthioadenosine | 0.71264 | 0.001947 | 0.085295 |
| Enterococcus | PC(20:5(5Z,8Z,11Z,14Z,17Z)/PGJ2) | -0.7126 | 0.001949 | 0.085295 |
| Anaerotruncus | D-Malic acid | 0.711976 | 0.001975 | 0.085295 |
| Anaerotruncus | 2,2,3,3,4,4,5,5,6,6,7,7,8,8,9,9-Hexadecafluorononanoic acid | 0.711966 | 0.001976 | 0.085295 |
| Tuzzerella | N-(1-Deoxy-1-fructosyl)leucine | 0.711853 | 0.00198 | 0.085295 |
| TRA3-20 | N2-(3-Carboxy-2-hydroxy-1-oxopropyl)arginine | 0.711601 | 0.001991 | 0.085295 |
| Subgroup_13 | 3-Hydroxy-10'-apo-b,y-carotenal | 0.711576 | 0.001992 | 0.085295 |
| Christensenellaceae_R-7_group | N-(1-Deoxy-1-fructosyl)tyrosine | 0.711538 | 0.001994 | 0.085295 |
| Bryobacter | Glutaminylproline | 0.711445 | 0.001998 | 0.085295 |
| Acidothermus | trans-Hexadec-2-enoyl carnitine | 0.711261 | 0.002005 | 0.085295 |
| Tuzzerella | Pelargonidin 3-sophoroside | -0.71092 | 0.00202 | 0.085295 |
| Enterococcus | 11,14,17-eicosatrienoic acid | 0.710898 | 0.002021 | 0.085295 |
| Enterococcus | Docosadienoate (22:2n6) | 0.710886 | 0.002022 | 0.085295 |
| Tuzzerella | 12-Hydroxydodecanoic acid | -0.71088 | 0.002022 | 0.085295 |
| Bryobacter | 5-[1-Carboxy-2-(trimethylazaniumyl)ethoxy]-5-oxopentanoate | 0.710804 | 0.002025 | 0.085295 |
| Haliangium | N-(1-Deoxy-1-fructosyl)tyrosine | 0.710699 | 0.00203 | 0.085295 |
| RF39 | D-erythro-L-galacto-Nonulose | 0.71043 | 0.002041 | 0.085343 |
| IMCC26256 | Genistein 7-O-glucoside-6''-malonate | 0.710106 | 0.002055 | 0.085343 |
| bacteriap25 | Adomeglivant | 0.710069 | 0.002057 | 0.085343 |
| Anaerotruncus | Fiacitabine | 0.709919 | 0.002063 | 0.085343 |
| Treponema | Aminovaleric acid betaine | 0.709873 | 0.002065 | 0.085343 |
| Haliangium | 1H-Pyrrole-2,5-dione, 3-(1-methyl-1h-indol-3-yl)-4-(1-methyl-6-nitro-1h-indol-3-yl)- | 0.709835 | 0.002067 | 0.085343 |
| Tuzzerella | Thiazolidine-4-carboxylic acid | 0.70943 | 0.002085 | 0.085475 |
| Treponema | Tridecanoylcarnitine | 0.709314 | 0.00209 | 0.085475 |
| Haliangium | 2-(2-Thienylmethylene)-1,6-dioxaspiro[4.4]non-3-ene | 0.709163 | 0.002097 | 0.085475 |
| Pseudolabrys | Eleutherazine B; N,N'-((3,6-Dioxo-2,5-piperazinediyl)bis(trimethylene))bis(5-hydroxy-3-methyl-2-pentenamide) | 0.709149 | 0.002097 | 0.085475 |
| Enterococcus | Fructose-1,6-diphosphate | 0.709073 | 0.002101 | 0.085475 |
| Faecalibaculum | (S1)-Methoxy-3-heptanethiol | 0.707998 | 0.002149 | 0.087179 |
| Tuzzerella | Tuberonic acid glucoside | -0.70753 | 0.00217 | 0.087793 |
| Enterorhabdus | PIP(20:0/18:1(12Z)-2OH(9,10)) | -0.70692 | 0.002198 | 0.088654 |
| Tuzzerella | Fructose-1,6-diphosphate | 0.706353 | 0.002224 | 0.089461 |
| Tuzzerella | Cortisol | -0.70599 | 0.002241 | 0.089887 |
| IMCC26256 | Streptidine | 0.705176 | 0.002279 | 0.091159 |
| Jatrophihabitans | 3-Hydroxy-11Z-octadecenoylcarnitine | 0.704985 | 0.002288 | 0.091263 |
| Jatrophihabitans | N-Acetylneuraminic acid | 0.704841 | 0.002295 | 0.091276 |
| Anaerotruncus | Beta-Citryl-L-glutamic acid | 0.704456 | 0.002313 | 0.091444 |
| Tuzzerella | Pseudouridine | 0.704449 | 0.002314 | 0.091444 |
| Haliangium | N-(1-Deoxy-1-fructosyl)leucine | 0.704109 | 0.00233 | 0.091444 |
| Christensenellaceae_R-7_group | 1H-Pyrrole-2,5-dione, 3-(1-methyl-1h-indol-3-yl)-4-(1-methyl-6-nitro-1h-indol-3-yl)- | 0.704107 | 0.00233 | 0.091444 |
| Enterococcus | Dehydroascorbic acid | 0.704075 | 0.002332 | 0.091444 |
| Achromobacter | Symmetric dimethylarginine | 0.703919 | 0.002339 | 0.091485 |
| Tuzzerella | 8-Amino-7-oxononanoic acid | -0.70312 | 0.002378 | 0.092754 |
| RF39 | N2-gamma-Glutamylglutamine | 0.702647 | 0.002401 | 0.093376 |
| Jatrophihabitans | hydroxyhexadecenoylcarnitine | 0.702524 | 0.002408 | 0.093376 |
| Achromobacter | O-phosphonato-L-homoserine(2-) | 0.702099 | 0.002429 | 0.093935 |
| Tuzzerella | FAD | 0.701565 | 0.002455 | 0.09471 |
| Acidothermus | Tetradecanoylcarnitine | 0.701254 | 0.002471 | 0.095055 |
| Flavonifractor | O-phosphonato-L-homoserine(2-) | 0.700958 | 0.002486 | 0.09537 |
| Monoglobus | 4-(Methylnitrosamino)-1-(3-pyridyl)-1-butanol glucuronide | 0.70075 | 0.002497 | 0.095517 |
| Enterococcus | 20-HETE-d6 | 0.700361 | 0.002516 | 0.096016 |
| Bryobacter | Docosadienoate (22:2n6) | 0.699783 | 0.002546 | 0.09689 |
| Tuzzerella | Ethynodiol | -0.69905 | 0.002584 | 0.097737 |
| Muribaculum | 3'-Ketolactose | 0.699049 | 0.002585 | 0.097737 |
| Bryobacter | N-alpha-Acetyl-L-lysine | 0.698957 | 0.002589 | 0.097737 |
| Alcaligenes | 3-Methyl-3-butenyl hexadecanoate | 0.698063 | 0.002637 | 0.099254 |
| TRA3-20 | N-alpha-Acetyl-L-lysine | 0.697594 | 0.002662 | 0.099932 |
| bacteriap25 | PPM-18 | 0.697406 | 0.002672 | 0.100045 |
| TRA3-20 | Fluazifop | 0.69693 | 0.002698 | 0.100741 |
| bacteriap25 | Aminovaleric acid betaine | 0.69553 | 0.002774 | 0.103114 |
| Anaerotruncus | Tyrosyl-Alanine | 0.695503 | 0.002776 | 0.103114 |
| Pseudolabrys | Valylserine | 0.695374 | 0.002783 | 0.103114 |
| Acidothermus | a-L-Arabinofuranosyl-(1->3)-b-D-xylopyranosyl-(1->4)-D-xylose | 0.695224 | 0.002791 | 0.103152 |
| bacteriap25 | Avenin | 0.694173 | 0.002851 | 0.105061 |
| Alcaligenes | Symmetric dimethylarginine | 0.693748 | 0.002875 | 0.105629 |
| [Eubacterium]_oxidoreducens_group | Genistein 7-O-glucoside-6''-malonate | 0.693639 | 0.002881 | 0.105629 |
| Bryobacter | Nipradilol | 0.693363 | 0.002897 | 0.105934 |
| Enterococcus | 5beta-Cholane-3alpha,24-diol | 0.693223 | 0.002905 | 0.105952 |
| Muribaculum | 16-Methylheptadecanoylcarnitine | 0.693077 | 0.002913 | 0.105965 |
| Granulicella | Neryl 8-methyldecanoate | 0.692956 | 0.00292 | 0.105965 |
| Gemella | Genistein 7-O-glucoside-6''-malonate | 0.692616 | 0.00294 | 0.106053 |
| Bryobacter | Serylproline | 0.692552 | 0.002944 | 0.106053 |
| Anaerotruncus | PPM-18 | 0.692525 | 0.002945 | 0.106053 |
| TRA3-20 | N-lactoyl-Tyrosine | 0.692357 | 0.002955 | 0.106088 |
| Enterococcus | PE(19:0/20:2(11Z,14Z)) | -0.69225 | 0.002961 | 0.106088 |
| Tuzzerella | Phytal | -0.69202 | 0.002975 | 0.106264 |
| Bryobacter | N-lactoyl-Tyrosine | 0.69191 | 0.002981 | 0.106264 |
| Bryobacter | N-Acetyl-b-glucosaminylamine | 0.691693 | 0.002994 | 0.106286 |
| Tuzzerella | Dehydrocyclopeptine; (3E)-3-Benzylidene-4-methyl-3,4-dihydro-1H-1,4-benzodiazepine-2,5-dione | -0.69164 | 0.002997 | 0.106286 |
| Bryobacter | Phenylalanylproline | 0.691438 | 0.003009 | 0.106447 |
| Tuzzerella | Medicagol | 0.691226 | 0.003022 | 0.106625 |
| TRA3-20 | 5beta-Cholane-3alpha,24-diol | 0.690252 | 0.00308 | 0.107994 |
| Tuzzerella | Octadecanamide | -0.69025 | 0.003081 | 0.107994 |
| Tuzzerella | N6-Acetyl-L-lysine | 0.690195 | 0.003084 | 0.107994 |
| Monoglobus | Phytosphingosine | 0.690034 | 0.003093 | 0.108067 |
| Tuzzerella | Cannabidivarin | -0.68954 | 0.003124 | 0.108855 |
| bacteriap25 | Doxepin | 0.688831 | 0.003167 | 0.110091 |
| Monoglobus | Fluazifop | 0.68851 | 0.003187 | 0.110251 |
| Tuzzerella | 3-O-acetylecdysone 2-phosphate | -0.68843 | 0.003192 | 0.110251 |
| Rikenellaceae_RC9_gut_group | Maltotriose | 0.688378 | 0.003195 | 0.110251 |
| Bryobacter | DG(17:2(9Z,12Z)/22:5(7Z,10Z,13Z,16Z,19Z)/0:0)[iso2] | 0.688198 | 0.003206 | 0.110369 |
| bacteriap25 | Phenethylamine glucuronide | 0.687752 | 0.003234 | 0.111058 |
| Achromobacter | N-gamma-L-Glutamyl-D-alanine | 0.68758 | 0.003245 | 0.111159 |
| Tuzzerella | 6-Hydroxytricetin 5-rhamnoside | 0.687326 | 0.003261 | 0.11137 |
| Tuzzerella | 3-Hydroxy-10'-apo-b,y-carotenal | 0.687162 | 0.003271 | 0.11137 |
| Gemella | PGP(18:3(9Z,12Z,15Z)/18:1(12Z)-O(9S,10R)) | 0.687107 | 0.003275 | 0.11137 |
| TRA3-20 | (E,E)-3,7,11-Trimethyl-2,6,10-dodecatrienyl octanoate | 0.686974 | 0.003283 | 0.111387 |
| Enterococcus | Eicosadienoic acid | 0.686643 | 0.003304 | 0.111835 |
| Enterococcus | 2',3'-Dideoxyuridine | 0.686339 | 0.003324 | 0.112163 |
| Enterorhabdus | PE(22:4(7Z,10Z,13Z,16Z)/19:0) | -0.68624 | 0.00333 | 0.112163 |
| Enterococcus | N-Lactoylphenylalanine | 0.685826 | 0.003357 | 0.112475 |
| Monoglobus | N6-Acetyl-L-lysine | 0.685815 | 0.003358 | 0.112475 |
| bacteriap25 | 13-(3,4-Dimethyl-5-propylfuran-2-yl)tridecanoylcarnitine | 0.685728 | 0.003363 | 0.112475 |
| Bryobacter | Prolyl-Asparagine | 0.685304 | 0.003391 | 0.11313 |
| Tuzzerella | (卤)-(Z)-2-(5-Tetradecenyl)cyclobutanone | -0.68481 | 0.003423 | 0.113788 |
| MND1 | N-(1-Deoxy-1-fructosyl)leucine | 0.684589 | 0.003438 | 0.113788 |
| Tuzzerella | Uridine | 0.684571 | 0.003439 | 0.113788 |
| Tuzzerella | Cichorioside K | -0.68451 | 0.003443 | 0.113788 |
| Tuzzerella | Pantothenic acid | 0.684177 | 0.003465 | 0.11425 |
| Enterococcus | Withaferin A | -0.68385 | 0.003487 | 0.11427 |
| Treponema | Peruvianoside II | 0.683772 | 0.003492 | 0.11427 |
| Anaerotruncus | NAD | 0.683705 | 0.003497 | 0.11427 |
| Treponema | Ciadox | 0.683681 | 0.003498 | 0.11427 |
| Faecalibaculum | Aspartyl-Alanine | 0.683273 | 0.003526 | 0.1149 |
| Clostridioides | Phytosphingosine | 0.682998 | 0.003544 | 0.115237 |
| Solobacterium | Genistein 7-O-glucoside-6''-malonate | 0.682652 | 0.003568 | 0.115734 |
| Tuzzerella | 3-{[(1s)-2,2-Difluoro-1-Hydroxy-7-(Methylsulfonyl)-2,3-Dihydro-1h-Inden-4-Yl]oxy}-5-Fluorobenzonitrile | 0.682349 | 0.003588 | 0.116136 |
| Bryobacter | Asparaginyl-Proline | 0.681807 | 0.003625 | 0.117069 |
| Enterococcus | L-arginino-succinate | 0.681677 | 0.003634 | 0.117091 |
| Tuzzerella | LysoSM(d18:0) | -0.68153 | 0.003644 | 0.11714 |
| Subgroup_13 | 3-{[(1s)-2,2-Difluoro-1-Hydroxy-7-(Methylsulfonyl)-2,3-Dihydro-1h-Inden-4-Yl]oxy}-5-Fluorobenzonitrile | 0.681381 | 0.003655 | 0.11714 |
| Tuzzerella | Ethyl hexadecanoate | -0.68118 | 0.003669 | 0.11714 |
| Acidothermus | Maltotriose | 0.681058 | 0.003677 | 0.11714 |
| Pseudolabrys | N-lactoyl-Tyrosine | 0.681055 | 0.003678 | 0.11714 |
| Anaerostipes | N-(1-Deoxy-1-fructosyl)valine | 0.680869 | 0.00369 | 0.117287 |
| Rikenellaceae_RC9_gut_group | a-L-Arabinofuranosyl-(1->3)-b-D-xylopyranosyl-(1->4)-D-xylose | 0.680733 | 0.0037 | 0.117325 |
| Tuzzerella | Sodium nitrate (NaNO3) | -0.67979 | 0.003767 | 0.118995 |
| Muribaculum | Tephcalostan | 0.679747 | 0.00377 | 0.118995 |
| Tuzzerella | 2-Methylthioadenosine | 0.679429 | 0.003792 | 0.119376 |
| Tuzzerella | 2-Phenylethyl octanoate | -0.67934 | 0.003799 | 0.119376 |
| Tuzzerella | Xanthinol | 0.67876 | 0.00384 | 0.120079 |
| Aquicella | 3-Hydroxy-10'-apo-b,y-carotenal | 0.678759 | 0.00384 | 0.120079 |
| Enterorhabdus | Pseudouridine | 0.678591 | 0.003852 | 0.120079 |
| Enterococcus | Pentadeca-3,5,7-trienedioylcarnitine | 0.678371 | 0.003868 | 0.120079 |
| Tuzzerella | N-(1-Deoxy-1-fructosyl)threonine | 0.678352 | 0.00387 | 0.120079 |
| Nitrospira | N-Acetyl-D-Glucosamine 6-Phosphate | 0.678319 | 0.003872 | 0.120079 |
| Lachnospiraceae_UCG-006 | Dihydrozeatin O-beta-D-Glucoside | 0.677213 | 0.003953 | 0.122322 |
| RF39 | 2-Methylthioadenosine | 0.676666 | 0.003994 | 0.122459 |
| Anaerotruncus | Avenin | 0.676586 | 0.004 | 0.122459 |
| MND1 | N-(1-Deoxy-1-fructosyl)tyrosine | 0.676556 | 0.004002 | 0.122459 |
| bacteriap25 | [6-Hydroxy-8-methyl-3-[3,4,5-trihydroxy-6-(hydroxymethyl)oxan-2-yl]oxy-9,10-dioxatetracyclo[4.3.1.02,5.03,8]decan-4-yl]methyl benzoate | 0.676556 | 0.004002 | 0.122459 |
| Tuzzerella | N-(1-Deoxy-1-fructosyl)tyrosine | 0.676463 | 0.004009 | 0.122459 |
| Anaerotruncus | Azeloprazole | 0.67645 | 0.00401 | 0.122459 |
| Enterorhabdus | PS(22:5(7Z,10Z,13Z,16Z,19Z)/22:6(4Z,7Z,10Z,13Z,16Z,19Z)) | -0.67632 | 0.00402 | 0.122493 |
| Tuzzerella | 5beta-Cholane-3alpha,24-diol | 0.676096 | 0.004036 | 0.122613 |
| Muribaculum | Glucose-6-glutamate | 0.676034 | 0.004041 | 0.122613 |
| Achromobacter | PI(18:1(9Z)/0:0) | 0.675735 | 0.004063 | 0.122812 |
| Jatrophihabitans | N-Acetyl-D-Glucosamine 6-Phosphate | 0.675716 | 0.004065 | 0.122812 |
| Muribaculum | True blue | 0.675295 | 0.004097 | 0.123509 |
| Tuzzerella | Lucuminic acid | 0.675034 | 0.004117 | 0.123844 |
| Tuzzerella | 2-[4-(3-Hydroxypropyl)-2-methoxyphenoxy]-1,3-propanediol 1-xyloside | -0.67491 | 0.004126 | 0.123871 |
| Tuzzerella | 2-Cyclotetradecen-1-one | -0.67474 | 0.004139 | 0.123984 |
| Pseudolabrys | Serylproline | 0.674528 | 0.004155 | 0.124212 |
| Pseudolabrys | Prolyl-Asparagine | 0.674246 | 0.004177 | 0.124218 |
| Jatrophihabitans | 16-Methylheptadecanoylcarnitine | 0.674212 | 0.00418 | 0.124218 |
| bacteriap25 | D-Malic acid | 0.674002 | 0.004196 | 0.124218 |
| RF39 | 3-Methyl-3-butenyl apiosyl-(1->6)-glucoside | 0.67399 | 0.004197 | 0.124218 |
| Alcaligenes | PI(18:1(9Z)/0:0) | 0.673954 | 0.0042 | 0.124218 |
| Christensenellaceae_R-7_group | N-[(2R,3R,4S,6R)-4,6-Dihydroxy-6-methyl-2-[(1R,2R)-1,2,3-trihydroxypropyl]oxan-3-yl]acetamide | 0.67381 | 0.004211 | 0.124287 |
| Treponema | LysoPC(P-16:0/0:0) | 0.673649 | 0.004223 | 0.124374 |
| Tuzzerella | 16-hydroxy-6-hexadecenoic acid | -0.67355 | 0.004231 | 0.124374 |
| Intestinimonas | Tyrosylglycine | 0.673237 | 0.004256 | 0.124822 |
| Faecalibaculum | Cysteine-glutathione disulfide | 0.673057 | 0.00427 | 0.124976 |
| TRA3-20 | N6-Acetyl-L-lysine | 0.67279 | 0.004291 | 0.125331 |
| Monoglobus | Histidylisoleucine | 0.672578 | 0.004308 | 0.125559 |
| Enterococcus | N-Lactoylleucine | 0.672466 | 0.004316 | 0.125559 |
| Enterorhabdus | (S)-a-Amino-2,5-dihydro-5-oxo-4-isoxazolepropanoic acid N2-glucoside | 0.672234 | 0.004335 | 0.125832 |
| Subgroup_13 | Genistein 7-O-glucoside-6''-malonate | 0.671525 | 0.004391 | 0.127216 |
| Muribaculum | Cerulenin | 0.67125 | 0.004414 | 0.127596 |
| Tuzzerella | AB-MECA | -0.67077 | 0.004453 | 0.128461 |
| Coprobacillus | N-Acetyl-D-Glucosamine 6-Phosphate | 0.670105 | 0.004507 | 0.129755 |
| Bryobacter | D-erythro-L-galacto-Nonulose | 0.669658 | 0.004543 | 0.130547 |
| Faecalibaculum | Pentadecanoylcarnitine | 0.669202 | 0.004581 | 0.131294 |
| MND1 | Peperomin E | 0.669119 | 0.004588 | 0.131294 |
| Granulicella | O-phosphonato-L-homoserine(2-) | 0.668495 | 0.00464 | 0.132517 |
| Tuzzerella | 7-oxo-8-amino-nonanoic acid | -0.66797 | 0.004685 | 0.133522 |
| Nitrospira | 11-Hydroxyoctadecanoylcarnitine | 0.667162 | 0.004753 | 0.135198 |
| Tuzzerella | Lemborexant | -0.66666 | 0.004797 | 0.136152 |
| Bryobacter | Withaferin A | -0.66628 | 0.004829 | 0.136744 |
| Tuzzerella | 4'-O-Glucopyranosylsinapic acid | 0.666193 | 0.004837 | 0.136744 |
| Tuzzerella | N-[(2R,3R,4S,6R)-4,6-Dihydroxy-6-methyl-2-[(1R,2R)-1,2,3-trihydroxypropyl]oxan-3-yl]acetamide | 0.665962 | 0.004857 | 0.136803 |
| Muribaculum | 5'-Hydroxytenoxicam | 0.665946 | 0.004858 | 0.136803 |
| Tuzzerella | Neosaxitoxin | 0.665386 | 0.004907 | 0.137905 |
| Subgroup_13 | Dipotassium phosphate | 0.665216 | 0.004922 | 0.138051 |
| Monoglobus | DG(17:2(9Z,12Z)/22:5(7Z,10Z,13Z,16Z,19Z)/0:0)[iso2] | 0.664997 | 0.004942 | 0.138179 |
| Bryobacter | 9-Octadecenal | -0.66484 | 0.004955 | 0.138179 |
| Jatrophihabitans | 5'-Hydroxytenoxicam | 0.66483 | 0.004956 | 0.138179 |
| Tuzzerella | 4-Chloro-2-nitrobenzylalcohol | -0.66448 | 0.004987 | 0.138772 |
| RF39 | PE-NMe2(18:3(9Z,12Z,15Z)/18:1(11Z)) | 0.664343 | 0.005 | 0.138837 |
| Treponema | Benzyl gentiobioside | 0.663894 | 0.00504 | 0.139676 |
| Tuzzerella | 3-Methyl-3-butenyl apiosyl-(1->6)-glucoside | 0.66378 | 0.00505 | 0.139684 |
| MND1 | True blue | 0.663601 | 0.005066 | 0.139855 |
| [Eubacterium]_oxidoreducens_group | 3-Methyl-3-butenyl hexadecanoate | 0.663294 | 0.005094 | 0.140345 |
| Tuzzerella | Cysteinyl-Aspartate | 0.663179 | 0.005104 | 0.140358 |
| Monoglobus | Docosadienoate (22:2n6) | 0.662855 | 0.005134 | 0.140892 |
| Tuzzerella | Serylproline | 0.662468 | 0.005169 | 0.141585 |
| Alcaligenes | O-phosphonato-L-homoserine(2-) | 0.661471 | 0.005261 | 0.143823 |
| Treponema | L-2-Hydroxyglutaric acid | 0.66132 | 0.005275 | 0.143928 |
| Tuzzerella | Docosatrienoic acid | 0.660857 | 0.005318 | 0.144826 |
| Anaerotruncus | alpha-D-Galactopyranuronosyl-(1->4)-alpha-D-galactopyranuronosyl-(1->4)-D-galacturonic acid | 0.660579 | 0.005344 | 0.145256 |
| Enterococcus | 2,2,3,3,4,4,5,5,6,6,7,7,8,8,9,9-Hexadecafluorononanoic acid | 0.65975 | 0.005423 | 0.147103 |
| Muribaculum | Peperomin E | 0.659246 | 0.005471 | 0.148071 |
| Enterococcus | N-lactoyl-Methionine | 0.659156 | 0.005479 | 0.148071 |
| TRA3-20 | Valylserine | 0.658556 | 0.005537 | 0.149176 |
| Enterococcus | Serylproline | 0.658512 | 0.005542 | 0.149176 |
| TM7 | 2-C-methyl-D-erythritol-4-phosphate | 0.658187 | 0.005573 | 0.149738 |
| Flavonifractor | 2,2,3,3,4,4,5,5,6,6,7,7,8,8,9,9-Hexadecafluorononanoic acid | 0.658055 | 0.005586 | 0.149798 |
| Aquicella | O-phosphonato-L-homoserine(2-) | 0.657527 | 0.005637 | 0.150894 |
| Tuzzerella | 2-C-methyl-D-erythritol-4-phosphate | 0.657094 | 0.00568 | 0.151675 |
| Tuzzerella | N-(1-Deoxy-1-fructosyl)valine | 0.657012 | 0.005688 | 0.151675 |
| IMCC26256 | Docosatrienoic acid | 0.656583 | 0.005731 | 0.15252 |
| Tuzzerella | Hexadecenal | -0.65641 | 0.005748 | 0.15262 |
| bacteriap25 | U-75302 | 0.656157 | 0.005773 | 0.15262 |
| Tuzzerella | N-gamma-L-Glutamyl-D-alanine | 0.656121 | 0.005777 | 0.15262 |
| Bryobacter | PE(22:5(4Z,7Z,10Z,13Z,16Z)/PGD2) | -0.65596 | 0.005793 | 0.15262 |
| Solobacterium | 2,2,3,3,4,4,5,5,6,6,7,7,8,8,9,9-Hexadecafluorononanoic acid | 0.655923 | 0.005797 | 0.15262 |
| Bryobacter | Glycyl-D-proline | 0.655826 | 0.005806 | 0.15262 |
| Coprobacillus | 11-Hydroxyoctadecanoylcarnitine | 0.655786 | 0.00581 | 0.15262 |
| Enterorhabdus | 4Z,7-octadienoic acid | -0.65554 | 0.005835 | 0.15298 |
| Jatrophihabitans | True blue | 0.655375 | 0.005852 | 0.15298 |
| Acetatifactor | Neryl 8-methyldecanoate | 0.65517 | 0.005873 | 0.15298 |
| Treponema | Octadec-6-enoylcarnitine | 0.655059 | 0.005884 | 0.15298 |
| Tuzzerella | Phenethylamine glucuronide | 0.654962 | 0.005894 | 0.15298 |
| Tuzzerella | 6,10,14-Trimethyl-5,9,13-pentadecatrien-2-one | -0.65493 | 0.005897 | 0.15298 |
| Intestinimonas | Fluazifop | 0.654655 | 0.005925 | 0.15298 |
| Anaerotruncus | PI(18:1(9Z)/0:0) | 0.654576 | 0.005933 | 0.15298 |
| bacteriap25 | Peruvianoside II | 0.654373 | 0.005954 | 0.15298 |
| Anaerotruncus | 6-Hydroxytricetin 5-rhamnoside | 0.654287 | 0.005963 | 0.15298 |
| Anaerotruncus | LysoPI(18:0/0:0) | 0.65411 | 0.005981 | 0.15298 |
| bacteriap25 | Ciadox | 0.65398 | 0.005994 | 0.15298 |
| Enterococcus | N6-Acetyl-L-lysine | 0.653973 | 0.005995 | 0.15298 |
| Faecalibaculum | 3-{[(1s)-2,2-Difluoro-1-Hydroxy-7-(Methylsulfonyl)-2,3-Dihydro-1h-Inden-4-Yl]oxy}-5-Fluorobenzonitrile | 0.653923 | 0.006 | 0.15298 |
| TM7 | Cysteinyl-Aspartate | 0.653773 | 0.006016 | 0.15298 |
| Pseudolabrys | Docosadienoate (22:2n6) | 0.65366 | 0.006027 | 0.15298 |
| Aquicella | 3-Methyl-3-butenyl apiosyl-(1->6)-glucoside | 0.65365 | 0.006028 | 0.15298 |
| Acidothermus | Tephcalostan C | 0.653469 | 0.006047 | 0.15298 |
| Granulicella | Symmetric dimethylarginine | 0.653466 | 0.006047 | 0.15298 |
| Rikenellaceae_RC9_gut_group | 2-(Methylthiomethyl)-3-phenyl-2-propenal | 0.653435 | 0.006051 | 0.15298 |
| Muribaculum | Thiazolidine-4-carboxylic acid | 0.653419 | 0.006052 | 0.15298 |
| Anaerotruncus | Glycerol 3-phosphate | 0.653003 | 0.006096 | 0.153799 |
| Enterococcus | PC(TXB2/20:2(11Z,14Z)) | -0.65276 | 0.006121 | 0.153807 |
| Anaerotruncus | 4-Methylcatechol 1-glucuronide | 0.652588 | 0.006139 | 0.153807 |
| Treponema | Avenin | 0.652578 | 0.00614 | 0.153807 |
| TRA3-20 | PI(20:3(8Z,11Z,14Z)/0:0) | 0.652555 | 0.006143 | 0.153807 |
| TM7 | Nipradilol | 0.652479 | 0.006151 | 0.153807 |
| Treponema | PPM-18 | 0.651551 | 0.006249 | 0.155986 |
| Enterococcus | Fluazifop | 0.651429 | 0.006262 | 0.156034 |
| TM7 | BEFLOXATONE | 0.651248 | 0.006281 | 0.15624 |
| TM7 | Neosaxitoxin | 0.651029 | 0.006305 | 0.156546 |
| Pseudolabrys | 4-Bis(2-hydroxyethyl)amino-L-phenylalanine | 0.650892 | 0.006319 | 0.156566 |
| Treponema | Phenethylamine glucuronide | 0.650751 | 0.006334 | 0.156566 |
| Alcaligenes | PI(20:3(8Z,11Z,14Z)/0:0) | 0.650708 | 0.006339 | 0.156566 |
| Bryobacter | Dolichyl b-D-glucosyl phosphate | -0.65051 | 0.006361 | 0.156566 |
| Tuzzerella | AM2201 N-(4-hydroxypentyl) metabolite | -0.65051 | 0.006361 | 0.156566 |
| Enterorhabdus | 7-Hydroxy-R-acenocoumarol | 0.650381 | 0.006374 | 0.156573 |
| IMCC26256 | 2,2,3,3,4,4,5,5,6,6,7,7,8,8,9,9-Hexadecafluorononanoic acid | 0.650213 | 0.006393 | 0.156573 |
| bacteriap25 | Tridecanoylcarnitine | 0.650195 | 0.006395 | 0.156573 |
| Jatrophihabitans | L-2-Hydroxyglutaric acid | 0.649821 | 0.006435 | 0.157292 |
| Bryobacter | Amobarbital | 0.649241 | 0.006499 | 0.158092 |
| Lachnospiraceae_UCG-006 | Docosatrienoic acid | 0.649139 | 0.00651 | 0.158092 |
| Haliangium | PE-NMe2(18:3(9Z,12Z,15Z)/18:1(11Z)) | 0.64912 | 0.006512 | 0.158092 |
| Subgroup_2 | N-(1-Deoxy-1-fructosyl)leucine | 0.649113 | 0.006513 | 0.158092 |
| Acetatifactor | Creatine | 0.649005 | 0.006525 | 0.158107 |
| Enterorhabdus | 3-(3-(Pyridin-3-yl)-1,2,4-oxadiazol-5-yl)benzonitrile | 0.648053 | 0.00663 | 0.160299 |
| Treponema | PI(18:1(9Z)/0:0) | 0.64797 | 0.00664 | 0.160299 |
| TM7 | Xanthinol | 0.647796 | 0.006659 | 0.160299 |
| Intestinimonas | Histidylisoleucine | 0.647782 | 0.006661 | 0.160299 |
| Tuzzerella | PE(22:4(7Z,10Z,13Z,16Z)/19:0) | -0.64751 | 0.006691 | 0.160754 |
| Faecalibaculum | Aspartyl-Methionine | 0.646962 | 0.006753 | 0.161968 |
| Acetatifactor | N,N'-Diethylthiourea | 0.646594 | 0.006795 | 0.16243 |
| bacteriap25 | PI(18:1(9Z)/0:0) | 0.64646 | 0.00681 | 0.16243 |
| Lachnospiraceae_UCG-006 | (R)-3-Amino-2-fluoropropyl phosphenite | 0.646339 | 0.006824 | 0.16243 |
| Lachnospiraceae_UCG-006 | 3-Methyl-3-butenyl hexadecanoate | 0.646232 | 0.006836 | 0.16243 |
| Tuzzerella | [(2S,4R,5R,6R,14S,16R)-14-Hydroxy-7,11-dimethyl-6-(2-oxopyran-4-yl)-3-oxapentacyclo[8.8.0.02,4.02,7.011,16]octadecan-5-yl] acetate | -0.64606 | 0.006855 | 0.16243 |
| Achromobacter | N2-(3-Carboxy-2-hydroxy-1-oxopropyl)arginine | 0.646004 | 0.006862 | 0.16243 |
| Tuzzerella | Fluazifop | 0.646004 | 0.006862 | 0.16243 |
| Bryobacter | 1-Octadecene | -0.64598 | 0.006865 | 0.16243 |
| Enterococcus | 5-[1-Carboxy-2-(trimethylazaniumyl)ethoxy]-5-oxopentanoate | 0.645121 | 0.006964 | 0.164513 |
| Subgroup_13 | Fiacitabine | 0.644866 | 0.006994 | 0.164935 |
| Tuzzerella | Glucose-6-glutamate | 0.64445 | 0.007042 | 0.165254 |
| Acidothermus | 2-(Methylthiomethyl)-3-phenyl-2-propenal | 0.644303 | 0.00706 | 0.165254 |
| Jatrophihabitans | Cerulenin | 0.644296 | 0.007061 | 0.165254 |
| Anaerotruncus | N2-(3-Carboxy-2-hydroxy-1-oxopropyl)arginine | 0.644207 | 0.007071 | 0.165254 |
| Tuzzerella | Aspartyl-Gamma-glutamate | 0.644157 | 0.007077 | 0.165254 |
| Tuzzerella | PI(20:3(8Z,11Z,14Z)/0:0) | 0.644099 | 0.007084 | 0.165254 |
| Bryobacter | 4-Bis(2-hydroxyethyl)amino-L-phenylalanine | 0.643986 | 0.007097 | 0.165254 |
| TM7 | 2-Deoxy-2,3-dehydro-n-acetyl-neuraminic acid | 0.643915 | 0.007105 | 0.165254 |
| Solobacterium | N,N'-Diethylthiourea | 0.643851 | 0.007113 | 0.165254 |
| Subgroup_2 | 16-hydroxy-6-hexadecenoic acid | -0.64343 | 0.007163 | 0.165393 |
| IMCC26256 | Symmetric dimethylarginine | 0.643423 | 0.007164 | 0.165393 |
| Enterorhabdus | All trans decaprenyl diphosphate | -0.64341 | 0.007165 | 0.165393 |
| Muribaculum | L-2-Hydroxyglutaric acid | 0.643403 | 0.007166 | 0.165393 |
| Lachnospiraceae_UCG-006 | O-phosphonato-L-homoserine(2-) | 0.643174 | 0.007193 | 0.16575 |
| bacteriap25 | (2S)-3-[3-[(4-Carbamimidoylbenzoyl)amino]propanoylamino]-2-[(4-ethylphenyl)sulfonylamino]propanoic acid | 0.642907 | 0.007225 | 0.166214 |
| Subgroup_2 | 2-Cyclotetradecen-1-one | -0.64228 | 0.007301 | 0.167675 |
| Treponema | (2S)-3-[3-[(4-Carbamimidoylbenzoyl)amino]propanoylamino]-2-[(4-ethylphenyl)sulfonylamino]propanoic acid | 0.642146 | 0.007317 | 0.167769 |
| Solobacterium | Creatine | 0.641986 | 0.007336 | 0.16794 |
| Tuzzerella | Nopalinic acid | 0.641776 | 0.007361 | 0.168248 |
| TRA3-20 | Flumazenil | 0.641273 | 0.007423 | 0.16898 |
| Tuzzerella | Docosadienoate (22:2n6) | 0.6411 | 0.007444 | 0.16898 |
| [Eubacterium]_oxidoreducens_group | O-phosphonato-L-homoserine(2-) | 0.640948 | 0.007462 | 0.16898 |
| Rikenellaceae_RC9_gut_group | Heptadecanoyl carnitine | 0.640892 | 0.007469 | 0.16898 |
| Granulicella | Erucic acid | 0.640879 | 0.007471 | 0.16898 |
| MND1 | thiamphenicol | 0.640856 | 0.007474 | 0.16898 |
| Gemella | 2,2,3,3,4,4,5,5,6,6,7,7,8,8,9,9-Hexadecafluorononanoic acid | 0.640798 | 0.007481 | 0.16898 |
| Christensenellaceae_R-7_group | N-(1-Deoxy-1-fructosyl)valine | 0.640728 | 0.007489 | 0.16898 |
| Rikenellaceae_RC9_gut_group | 12-Hydroxy-12-octadecanoylcarnitine | 0.640505 | 0.007517 | 0.169246 |
| Anaerotruncus | Medicagol | 0.640437 | 0.007525 | 0.169246 |
| Anaerotruncus | PC(16:1(9Z)/17:1(9Z)) | -0.64026 | 0.007547 | 0.16947 |
| Aquicella | Genistein 7-O-glucoside-6''-malonate | 0.640141 | 0.007562 | 0.169531 |
| Anaerostipes | Glutamylleucylarginine | 0.640007 | 0.007579 | 0.169632 |
| Jatrophihabitans | Glucose-6-glutamate | 0.639714 | 0.007615 | 0.170015 |
| Treponema | U-75302 | 0.639675 | 0.00762 | 0.170015 |
| Enterococcus | alpha-D-Galactopyranuronosyl-(1->4)-alpha-D-galactopyranuronosyl-(1->4)-D-galacturonic acid | 0.639419 | 0.007652 | 0.17028 |
| Clostridioides | N6-Acetyl-L-lysine | 0.639387 | 0.007656 | 0.17028 |
| Pseudolabrys | (E,E)-3,7,11-Trimethyl-2,6,10-dodecatrienyl octanoate | 0.639141 | 0.007687 | 0.170647 |
| Lachnospiraceae_UCG-006 | 1-[3,4-Dihydroxy-5-(hydroxymethyl)-2-oxolanyl]-1,2,4-triazole-3-carboxamide | 0.639062 | 0.007697 | 0.170647 |
| Lachnospiraceae_UCG-006 | Erucic acid | 0.638865 | 0.007721 | 0.170839 |
| Coprobacillus | hydroxyhexadecenoylcarnitine | 0.638801 | 0.00773 | 0.170839 |
| Tuzzerella | D-erythro-L-galacto-Nonulose | 0.638696 | 0.007743 | 0.170861 |
| Subgroup_2 | N-(1-Deoxy-1-fructosyl)tyrosine | 0.638335 | 0.007789 | 0.171155 |
| Monoglobus | Asparaginyl-Proline | 0.638273 | 0.007796 | 0.171155 |
| Pseudolabrys | 2',3'-Dideoxyuridine | 0.638151 | 0.007812 | 0.171155 |
| Faecalibaculum | a-L-Arabinofuranosyl-(1->3)-b-D-xylopyranosyl-(1->4)-D-xylose | 0.638068 | 0.007822 | 0.171155 |
| TM7 | N-(1-Deoxy-1-fructosyl)threonine | 0.638061 | 0.007823 | 0.171155 |
| Enterorhabdus | Uridine | 0.637811 | 0.007855 | 0.171155 |
| Faecalibaculum | Streptidine | 0.637773 | 0.00786 | 0.171155 |
| Faecalibaculum | Fiacitabine | 0.63774 | 0.007864 | 0.171155 |
| Tuzzerella | Bacillamidin C | -0.63765 | 0.007875 | 0.171155 |
| Lachnospiraceae_UCG-006 | PC(20:4(5Z,8Z,11Z,14Z)-OH(16R)/2:0) | 0.637546 | 0.007889 | 0.171155 |
| Rikenellaceae_RC9_gut_group | Tephcalostan C | 0.637541 | 0.00789 | 0.171155 |
| Tuzzerella | D-Mannose | 0.637334 | 0.007916 | 0.171469 |
| Jatrophihabitans | Peperomin E | 0.637222 | 0.007931 | 0.171516 |
| Tuzzerella | LysoPI(18:0/0:0) | 0.636886 | 0.007974 | 0.172179 |
| bacteriap25 | L-2-Hydroxyglutaric acid | 0.636795 | 0.007986 | 0.172179 |
| Enterococcus | 4-Bis(2-hydroxyethyl)amino-L-phenylalanine | 0.636687 | 0.008 | 0.172203 |
| Coprobacillus | 3-Hydroxy-11Z-octadecenoylcarnitine | 0.636598 | 0.008011 | 0.172203 |
| Clostridioides | 25-Hydroxyvitamin D3-26,23-lactol | -0.63618 | 0.008066 | 0.172558 |
| Coprobacillus | Thiazolidine-4-carboxylic acid | 0.636137 | 0.008071 | 0.172558 |
| Pseudolabrys | Pro-Pro-Pro | 0.636058 | 0.008082 | 0.172558 |
| MND1 | N-[(2R,3R,4S,6R)-4,6-Dihydroxy-6-methyl-2-[(1R,2R)-1,2,3-trihydroxypropyl]oxan-3-yl]acetamide | 0.635887 | 0.008104 | 0.172558 |
| Tuzzerella | TG(8:0/8:0/a-13:0)[rac] | -0.63579 | 0.008117 | 0.172558 |
| Intestinimonas | Fructose-1,6-diphosphate | 0.635779 | 0.008118 | 0.172558 |
| Tuzzerella | Glycyl-D-proline | 0.635698 | 0.008129 | 0.172558 |
| Acidothermus | Heptadecanoyl carnitine | 0.635666 | 0.008133 | 0.172558 |
| Enterorhabdus | PE(18:0/19:1(9Z)) | -0.63563 | 0.008138 | 0.172558 |
| TM7 | Zidebactam | 0.63545 | 0.008162 | 0.172679 |
| TM7 | Streptidine | 0.635397 | 0.008168 | 0.172679 |
| Coprobacillus | Cerulenin | 0.635243 | 0.008189 | 0.172771 |
| TRA3-20 | Pro-Pro-Pro | 0.635054 | 0.008214 | 0.172771 |
| Acetatifactor | Genistein 7-O-glucoside-6''-malonate | 0.63498 | 0.008224 | 0.172771 |
| Solobacterium | PGP(18:3(9Z,12Z,15Z)/18:1(12Z)-O(9S,10R)) | 0.634925 | 0.008231 | 0.172771 |
| Treponema | Palmitoylcarnitine | 0.6349 | 0.008234 | 0.172771 |
| Intestinimonas | 4-(Methylnitrosamino)-1-(3-pyridyl)-1-butanol glucuronide | 0.634467 | 0.008292 | 0.173569 |
| Tuzzerella | Azeloprazole | 0.634429 | 0.008297 | 0.173569 |
| MND1 | Glutamylleucylarginine | 0.634319 | 0.008312 | 0.173617 |
| Solobacterium | 5beta-Cholane-3alpha,24-diol | 0.634138 | 0.008336 | 0.173867 |
| Acidothermus | Cysteine-glutathione disulfide | 0.63375 | 0.008388 | 0.174695 |
| Bryobacter | 11,14,17-eicosatrienoic acid | 0.633578 | 0.008411 | 0.174753 |
| bacteriap25 | Beta-Citryl-L-glutamic acid | 0.633545 | 0.008416 | 0.174753 |
| Bryobacter | 1-O-(2R-methoxy-hexadecyl)-sn-glycerol | -0.63329 | 0.00845 | 0.175201 |
| Anaerotruncus | PGP(18:3(9Z,12Z,15Z)/18:1(12Z)-O(9S,10R)) | 0.632541 | 0.008552 | 0.176648 |
| TM7 | D-Mannose | 0.632469 | 0.008562 | 0.176648 |
| Granulicella | Aspartyl-Gamma-glutamate | 0.632437 | 0.008566 | 0.176648 |
| TRA3-20 | Symmetric dimethylarginine | 0.632409 | 0.00857 | 0.176648 |
| Acetatifactor | 3-Hydroxy-10'-apo-b,y-carotenal | 0.632223 | 0.008595 | 0.176648 |
| Lachnospiraceae_UCG-006 | scyllo-Inositol | 0.632211 | 0.008597 | 0.176648 |
| Enterorhabdus | Phenethylamine glucuronide | 0.632137 | 0.008607 | 0.176648 |
| Intestinimonas | N6-Acetyl-L-lysine | 0.631888 | 0.008642 | 0.177095 |
| Flavonifractor | PGP(18:3(9Z,12Z,15Z)/18:1(12Z)-O(9S,10R)) | 0.631717 | 0.008665 | 0.177311 |
| Bryobacter | Eicosadienoic acid | 0.631629 | 0.008677 | 0.177311 |
| Tuzzerella | Dihydrozeatin O-beta-D-Glucoside | 0.631437 | 0.008704 | 0.177432 |
| MND1 | DG(LTE4/22:0/0:0) | 0.631404 | 0.008708 | 0.177432 |
| TRA3-20 | O-phosphonato-L-homoserine(2-) | 0.631273 | 0.008727 | 0.177544 |
| [Eubacterium]_oxidoreducens_group | N,N'-Diethylthiourea | 0.630471 | 0.008838 | 0.179532 |
| IMCC26256 | D-Mannose | 0.630391 | 0.00885 | 0.179532 |
| Tuzzerella | BEFLOXATONE | 0.630255 | 0.008869 | 0.179662 |
| Bryobacter | 2',3'-Dideoxyuridine | 0.629597 | 0.008962 | 0.181213 |
| TM7 | Dehydroascorbide(1-) | 0.629384 | 0.008992 | 0.181213 |
| Bryobacter | Docosatrienoic acid | 0.629355 | 0.008996 | 0.181213 |
| Christensenellaceae_R-7_group | PE-NMe2(18:3(9Z,12Z,15Z)/18:1(11Z)) | 0.629349 | 0.008997 | 0.181213 |
| Anaerotruncus | 2',3'-Dideoxyuridine | 0.629104 | 0.009032 | 0.181489 |
| Haliangium | N-[(2R,3R,4S,6R)-4,6-Dihydroxy-6-methyl-2-[(1R,2R)-1,2,3-trihydroxypropyl]oxan-3-yl]acetamide | 0.629037 | 0.009042 | 0.181489 |
| Tuzzerella | Ricinoleic acid | -0.62897 | 0.009051 | 0.181489 |
| Enterococcus | 3-Methyl-3-butenyl hexadecanoate | 0.628892 | 0.009062 | 0.181489 |
| Monoglobus | Napelline | 0.628771 | 0.009079 | 0.181523 |
| Tuzzerella | Quinagolida | -0.62865 | 0.009096 | 0.181523 |
| Tuzzerella | Glutaminylproline | 0.6285 | 0.009118 | 0.181523 |
| Rikenellaceae_RC9_gut_group | 14-Methylpentadecanoylcarnitine | 0.628471 | 0.009123 | 0.181523 |
| Acetatifactor | Dipotassium phosphate | 0.628431 | 0.009128 | 0.181523 |
| Rikenellaceae_RC9_gut_group | Valylhistidine | 0.62811 | 0.009175 | 0.182048 |
| Bryobacter | L-Homocystine | 0.627998 | 0.009191 | 0.182048 |
| Acidothermus | 12-Hydroxy-12-octadecanoylcarnitine | 0.627979 | 0.009194 | 0.182048 |
| Achromobacter | Lucuminic acid | 0.627735 | 0.009229 | 0.182256 |
| Enterococcus | 4-Chloro-L-phenylalanine | -0.62773 | 0.00923 | 0.182256 |
| TRA3-20 | Neryl 8-methyldecanoate | 0.627394 | 0.009279 | 0.18265 |
| Muribaculum | Tercatain | 0.627372 | 0.009282 | 0.18265 |
| Achromobacter | Erucic acid | 0.62731 | 0.009291 | 0.18265 |
| Jatrophihabitans | 3'-Ketolactose | 0.627235 | 0.009302 | 0.18265 |
| Jatrophihabitans | Thiazolidine-4-carboxylic acid | 0.626998 | 0.009336 | 0.182948 |
| [Eubacterium]_oxidoreducens_group | Creatine | 0.626945 | 0.009344 | 0.182948 |
| Tuzzerella | 2,2,3,3,4,4,5,5,6,6,7,7,8,8,9,9-Hexadecafluorononanoic acid | 0.626865 | 0.009356 | 0.182948 |
| RF39 | 1-(10H-Phenothiazin-2-yl)ethanone | 0.626447 | 0.009417 | 0.183801 |
| Gemella | Creatine | 0.626189 | 0.009456 | 0.183801 |
| Tuzzerella | N-alpha-Acetyl-L-lysine | 0.626164 | 0.009459 | 0.183801 |
| Flavonifractor | N,N'-Diethylthiourea | 0.626163 | 0.009459 | 0.183801 |
| Enterococcus | Genistein 7-O-glucoside-6''-malonate | 0.626126 | 0.009465 | 0.183801 |
| Achromobacter | PI(20:3(8Z,11Z,14Z)/0:0) | 0.625629 | 0.009539 | 0.184981 |
| bacteriap25 | LysoPC(P-16:0/0:0) | 0.625498 | 0.009558 | 0.185104 |
| Alcaligenes | N2-(3-Carboxy-2-hydroxy-1-oxopropyl)arginine | 0.625358 | 0.009579 | 0.185128 |
| Gemella | N,N'-Diethylthiourea | 0.625314 | 0.009586 | 0.185128 |
| Aquicella | Docosatrienoic acid | 0.625203 | 0.009602 | 0.185194 |
| Monoglobus | 4-Bis(2-hydroxyethyl)amino-L-phenylalanine | 0.625085 | 0.00962 | 0.185282 |
| Tuzzerella | PE(O-20:0/0:0) | -0.62456 | 0.009699 | 0.186556 |
| Flavonifractor | Creatine | 0.62432 | 0.009735 | 0.186992 |
| Enterococcus | Nipradilol | 0.624069 | 0.009773 | 0.187467 |
| MND1 | 4-(Methylnitrosamino)-1-(3-pyridyl)-1-butanol glucuronide | 0.623948 | 0.009792 | 0.187563 |
| Monoglobus | Valylserine | 0.6238 | 0.009814 | 0.187738 |
| Tuzzerella | Withaferin A | -0.62369 | 0.009832 | 0.187816 |
| Tuzzerella | 1-Octadecene | -0.62332 | 0.009887 | 0.188017 |
| Anaerotruncus | Camptothecin sodium | 0.623306 | 0.00989 | 0.188017 |
| Monoglobus | N-alpha-Acetyl-L-lysine | 0.623219 | 0.009903 | 0.188017 |
| Alcaligenes | Erucic acid | 0.623201 | 0.009906 | 0.188017 |
| Treponema | Maraviroc | 0.623177 | 0.00991 | 0.188017 |
| Achromobacter | Dipotassium phosphate | 0.623024 | 0.009933 | 0.188017 |
| Nitrospira | 3-Hydroxy-11Z-octadecenoylcarnitine | 0.622792 | 0.009969 | 0.188017 |
| Enterococcus | Iguratimod | -0.62276 | 0.009974 | 0.188017 |
| Bryobacter | scyllo-Inositol | 0.622724 | 0.009979 | 0.188017 |
| Tuzzerella | scyllo-Inositol | 0.622682 | 0.009986 | 0.188017 |
| Christensenellaceae_R-7_group | 2-Deoxy-2,3-dehydro-n-acetyl-neuraminic acid | 0.622445 | 0.010023 | 0.188017 |
| Clostridioides | (卤)-(Z)-2-(5-Tetradecenyl)cyclobutanone | -0.62242 | 0.010026 | 0.188017 |
| Anaerotruncus | LysoPI(16:0/0:0) | 0.622394 | 0.010031 | 0.188017 |
| Enterococcus | D-Mannose | 0.62223 | 0.010056 | 0.188017 |
| Treponema | Dimethylaminoparthenolide | 0.622122 | 0.010073 | 0.188017 |
| Anaerotruncus | L-Glutamine | 0.622086 | 0.010078 | 0.188017 |
| Alcaligenes | Terizidone | 0.622019 | 0.010089 | 0.188017 |
| Bryobacter | 20-HETE-d6 | 0.621973 | 0.010096 | 0.188017 |
| Alcaligenes | N-gamma-L-Glutamyl-D-alanine | 0.621973 | 0.010096 | 0.188017 |
| Lachnospiraceae_UCG-006 | S-[(3R,3As,6S,6aS)-3-nitrooxy-2,3,3a,5,6,6a-hexahydrofuro[3,2-b]furan-6-yl] ethanethioate | 0.621612 | 0.010152 | 0.188816 |
| Subgroup_2 | Cannabidivarin | -0.6214 | 0.010186 | 0.188941 |
| MND1 | 2-Deoxy-2,3-dehydro-n-acetyl-neuraminic acid | 0.621398 | 0.010186 | 0.188941 |
| Acidothermus | Creatine | 0.620949 | 0.010256 | 0.189785 |
| Enterococcus | [(2S,4R,5R,6R,14S,16R)-14-Hydroxy-7,11-dimethyl-6-(2-oxopyran-4-yl)-3-oxapentacyclo[8.8.0.02,4.02,7.011,16]octadecan-5-yl] acetate | -0.62085 | 0.010272 | 0.189785 |
| Christensenellaceae_R-7_group | 4-(Methylnitrosamino)-1-(3-pyridyl)-1-butanol glucuronide | 0.620782 | 0.010283 | 0.189785 |
| RF39 | Tyrosylglycine | 0.620666 | 0.010301 | 0.189785 |
| Tuzzerella | Mycobactins | -0.62061 | 0.01031 | 0.189785 |
| Lachnospiraceae_UCG-006 | 2-phospho-4-(cytidine 5'-diphospho)-2-C-methyl-D-erythritol | 0.620596 | 0.010312 | 0.189785 |
| Flavonifractor | Docosatrienoic acid | 0.620316 | 0.010357 | 0.190356 |
| Anaerotruncus | 3-Amino-3-methylbutanoic acid | 0.620051 | 0.010399 | 0.190881 |
| Monoglobus | N-lactoyl-Methionine | 0.619759 | 0.010446 | 0.191273 |
| Bryobacter | 2-hydroxy-9Z,12Z-Octadecadienoic acid | -0.61975 | 0.010447 | 0.191273 |
| IMCC26256 | N-gamma-L-Glutamyl-D-alanine | 0.619464 | 0.010493 | 0.191857 |
| Acetatifactor | alpha-D-Galactopyranuronosyl-(1->4)-alpha-D-galactopyranuronosyl-(1->4)-D-galacturonic acid | 0.619295 | 0.01052 | 0.192104 |
| Granulicella | Docosatrienoic acid | 0.619088 | 0.010553 | 0.192308 |
| Coprobacillus | True blue | 0.619056 | 0.010559 | 0.192308 |
| Treponema | D-Malic acid | 0.618878 | 0.010587 | 0.192583 |
| Tuzzerella | Dimethylaminoparthenolide | 0.618482 | 0.010652 | 0.193502 |
| Anaerotruncus | 1-Hydroxy-2-naphthoic acid | 0.618323 | 0.010677 | 0.193601 |
| RF39 | N-(1-Deoxy-1-fructosyl)threonine | 0.618279 | 0.010685 | 0.193601 |
| Tuzzerella | N-(N-(3-Amino-3-carboxypropyl)-3-amino-3-carboxypropyl)azetidine-2-carboxylic acid | 0.617949 | 0.010738 | 0.194328 |
| bacteriap25 | Octadec-6-enoylcarnitine | 0.617635 | 0.01079 | 0.194789 |
| Flavonifractor | N-gamma-L-Glutamyl-D-alanine | 0.617568 | 0.010801 | 0.194789 |
| Nitrospira | hydroxyhexadecenoylcarnitine | 0.617541 | 0.010805 | 0.194789 |
| Bryobacter | D-erythro-Sphingosine C-20 | -0.61735 | 0.010836 | 0.195097 |
| Nitrospira | Uridine | 0.617065 | 0.010884 | 0.195325 |
| Monoglobus | N-Lactoylleucine | 0.617056 | 0.010885 | 0.195325 |
| Alcaligenes | (E,E)-3,7,11-Trimethyl-2,6,10-dodecatrienyl octanoate | 0.617024 | 0.010891 | 0.195325 |
| bacteriap25 | 3-Amino-3-methylbutanoic acid | 0.616928 | 0.010907 | 0.195362 |
| Enterococcus | N-lactoyl-Tyrosine | 0.616695 | 0.010945 | 0.195548 |
| Subgroup_2 | Phytosphingosine | 0.616663 | 0.010951 | 0.195548 |
| Bryobacter | N-(2-Hydroxyethyl)-2-(1-isoquinolinylmethylene)hydrazinecarbothioamide | 0.616614 | 0.010959 | 0.195548 |
| Monoglobus | 11,14,17-eicosatrienoic acid | 0.616491 | 0.010979 | 0.195611 |
| Faecalibaculum | PGP(18:3(9Z,12Z,15Z)/18:1(12Z)-O(9S,10R)) | 0.616426 | 0.01099 | 0.195611 |
| Tuzzerella | PPM-18 | 0.616215 | 0.011025 | 0.195852 |
| MND1 | Notoginsenoside H | 0.616178 | 0.011031 | 0.195852 |
| Acidothermus | N,N'-Diethylthiourea | 0.615981 | 0.011064 | 0.196093 |
| Tuzzerella | N1-Methyl-2-pyridone-5-carboxamide | -0.61593 | 0.011073 | 0.196093 |
| Tuzzerella | 2-(2-Thienylmethylene)-1,6-dioxaspiro[4.4]non-3-ene | 0.615812 | 0.011093 | 0.196197 |
| Bryobacter | (R)-3-Amino-2-fluoropropyl phosphenite | 0.615687 | 0.011114 | 0.196322 |
| Tuzzerella | Oliceridine | -0.6153 | 0.011179 | 0.197231 |
| Tuzzerella | Carissanol | -0.61519 | 0.011198 | 0.197313 |
| Acidothermus | 14-Methylpentadecanoylcarnitine | 0.615105 | 0.011212 | 0.197314 |
| Jatrophihabitans | Uridine | 0.614747 | 0.011273 | 0.198135 |
| Tuzzerella | 1H-Pyrrole-2,5-dione, 3-(1-methyl-1h-indol-3-yl)-4-(1-methyl-6-nitro-1h-indol-3-yl)- | 0.614287 | 0.011351 | 0.199143 |
| Tuzzerella | Pro-Pro-Pro | 0.614244 | 0.011358 | 0.199143 |
| Tuzzerella | Phosphohydroxypyruvic acid | -0.61411 | 0.01138 | 0.199283 |
| Anaerotruncus | Ac-Ser-Asp-Lys-Pro-OH | 0.613725 | 0.011447 | 0.200036 |
| Bryobacter | Monooctyl phthalate | -0.6137 | 0.011452 | 0.200036 |
| Achromobacter | L-Glutamine | 0.613552 | 0.011477 | 0.20012 |
| Tuzzerella | Dehydroascorbide(1-) | 0.613506 | 0.011485 | 0.20012 |
| Treponema | [6-Hydroxy-8-methyl-3-[3,4,5-trihydroxy-6-(hydroxymethyl)oxan-2-yl]oxy-9,10-dioxatetracyclo[4.3.1.02,5.03,8]decan-4-yl]methyl benzoate | 0.613275 | 0.011525 | 0.200568 |
| Treponema | O-Linoleoylcarnitine | 0.613143 | 0.011548 | 0.200716 |
| Enterococcus | 5-Hydroxyindoxyl sulfate | -0.61298 | 0.011576 | 0.20075 |
| Enterococcus | Pro-Pro-Pro | 0.612936 | 0.011584 | 0.20075 |
| Tuzzerella | Avenin | 0.612819 | 0.011604 | 0.20075 |
| Bryobacter | Bis(2-propoxyethyl) 2,6-dimethyl-4-(3-nitrophenyl)-3,4-dihydropyridine-3,5-dicarboxylate | -0.61277 | 0.011612 | 0.20075 |
| Haliangium | 2-Deoxy-2,3-dehydro-n-acetyl-neuraminic acid | 0.612643 | 0.011635 | 0.20075 |
| Nitrospira | Thiazolidine-4-carboxylic acid | 0.612639 | 0.011635 | 0.20075 |
| IMCC26256 | 5beta-Cholane-3alpha,24-diol | 0.612429 | 0.011672 | 0.201138 |
| Muribaculum | Uridine | 0.612058 | 0.011737 | 0.201365 |
| Lachnospiraceae_UCG-006 | PI(20:3(8Z,11Z,14Z)/0:0) | 0.612024 | 0.011743 | 0.201365 |
| Lachnospiraceae_UCG-006 | Serylproline | 0.612011 | 0.011745 | 0.201365 |
| Achromobacter | Terizidone | 0.611855 | 0.011772 | 0.201365 |
| Enterorhabdus | Orotidine | 0.611709 | 0.011798 | 0.201365 |
| Acetatifactor | Fiacitabine | 0.611697 | 0.0118 | 0.201365 |
| Coprobacillus | Uridine | 0.611608 | 0.011816 | 0.201365 |
| Solobacterium | N-gamma-L-Glutamyl-D-alanine | 0.611464 | 0.011841 | 0.201365 |
| Acetatifactor | Azeloprazole | 0.611419 | 0.011849 | 0.201365 |
| Tuzzerella | PGP(18:3(9Z,12Z,15Z)/18:1(12Z)-O(9S,10R)) | 0.611407 | 0.011852 | 0.201365 |
| Bryobacter | Diethylamino 2,5-dihydroxybenzenesulfonate | 0.611298 | 0.011871 | 0.201365 |
| Lachnospiraceae_UCG-006 | L-Arginine | 0.61126 | 0.011877 | 0.201365 |
| Enterorhabdus | N-Acetyl-D-Glucosamine 6-Phosphate | 0.611234 | 0.011882 | 0.201365 |
| Bryobacter | 5'-Carboxy-gamma-chromanol | -0.61122 | 0.011885 | 0.201365 |
| Enterococcus | 1-Ethoxymethyl-5-fluorouracil | 0.610948 | 0.011933 | 0.201782 |
| TRA3-20 | Dehydroascorbide(1-) | 0.610901 | 0.011941 | 0.201782 |
| Bryobacter | Hydroxy-2-naphthalenylmethylphosphonic acid tris-acetoxymethyl ester | 0.610835 | 0.011953 | 0.201782 |
| Coprobacillus | 5'-Hydroxytenoxicam | 0.610634 | 0.011989 | 0.202026 |
| Bryobacter | alpha-D-Galactopyranuronosyl-(1->4)-alpha-D-galactopyranuronosyl-(1->4)-D-galacturonic acid | 0.610584 | 0.011998 | 0.202026 |
| Enterococcus | JP83 | -0.61044 | 0.012024 | 0.202026 |
| Enterorhabdus | benfluorex | 0.610233 | 0.01206 | 0.202026 |
| Acidothermus | Alpha-Lactose | 0.61018 | 0.01207 | 0.202026 |
| MND1 | N-(1-Deoxy-1-fructosyl)valine | 0.610157 | 0.012074 | 0.202026 |
| Tuzzerella | 3-Hydroxybutyrylcarnitine | 0.61013 | 0.012079 | 0.202026 |
| Bryobacter | LysoSM(d18:0) | -0.61008 | 0.012088 | 0.202026 |
| Enterococcus | L-Homocystine | 0.610032 | 0.012097 | 0.202026 |
| Acetatifactor | D-Mannose | 0.60976 | 0.012145 | 0.202371 |
| Aquicella | Symmetric dimethylarginine | 0.609687 | 0.012159 | 0.202371 |
| Anaerostipes | Streptidine | 0.609645 | 0.012166 | 0.202371 |
| Tuzzerella | benfluorex | 0.609598 | 0.012175 | 0.202371 |
| Bryobacter | [(2S,4R,5R,6R,14S,16R)-14-Hydroxy-7,11-dimethyl-6-(2-oxopyran-4-yl)-3-oxapentacyclo[8.8.0.02,4.02,7.011,16]octadecan-5-yl] acetate | -0.60932 | 0.012225 | 0.202971 |
| Enterococcus | Fiacitabine | 0.609207 | 0.012245 | 0.203011 |
| Enterococcus | Diethylamino 2,5-dihydroxybenzenesulfonate | 0.60902 | 0.01228 | 0.203011 |
| Solobacterium | O-phosphonato-L-homoserine(2-) | 0.608967 | 0.012289 | 0.203011 |
| [Eubacterium]_oxidoreducens_group | 2,2,3,3,4,4,5,5,6,6,7,7,8,8,9,9-Hexadecafluorononanoic acid | 0.60893 | 0.012296 | 0.203011 |
| Enterococcus | Streptidine | 0.608893 | 0.012302 | 0.203011 |
| Bryobacter | 5S-HETE di-endoperoxide | -0.60881 | 0.012318 | 0.203011 |
| [Eubacterium]_oxidoreducens_group | PGP(18:3(9Z,12Z,15Z)/18:1(12Z)-O(9S,10R)) | 0.60875 | 0.012329 | 0.203011 |
| Tuzzerella | Polypropylene glycol (m w 1,200-3,000) | -0.6084 | 0.012393 | 0.203837 |
| Anaerotruncus | Flumazenil | 0.608196 | 0.01243 | 0.204201 |
| MND1 | Fucoxanthinol 3-(4Z,7Z,10Z,13Z,16Z,19Z-docosahexaenoate) 3'-palmitoleate | 0.608098 | 0.012448 | 0.20426 |
| Faecalibaculum | 2-(Methylthiomethyl)-3-phenyl-2-propenal | 0.607976 | 0.01247 | 0.20439 |
| Pseudolabrys | Nopalinic acid | 0.607629 | 0.012534 | 0.205198 |
| Enterococcus | Prolylproline | 0.607312 | 0.012593 | 0.205664 |
| Treponema | Alpha-Lactose | 0.607132 | 0.012626 | 0.205664 |
| Nitrospira | Cerulenin | 0.607105 | 0.012631 | 0.205664 |
| Muribaculum | Lycoperdic acid | 0.607085 | 0.012635 | 0.205664 |
| Tuzzerella | LysoPC(20:3(8Z,11Z,14Z)/0:0) | 0.607033 | 0.012645 | 0.205664 |
| Flavonifractor | 5beta-Cholane-3alpha,24-diol | 0.606964 | 0.012658 | 0.205664 |
| TRA3-20 | PE(22:4(7Z,10Z,13Z,16Z)/19:0) | -0.6069 | 0.012669 | 0.205664 |
| Rikenellaceae_RC9_gut_group | 11-Hydroxyhexadecanoylcarnitine | 0.606846 | 0.01268 | 0.205664 |
| Treponema | Beta-Citryl-L-glutamic acid | 0.606622 | 0.012721 | 0.205965 |
| TM7 | 1-[3,4-Dihydroxy-5-(hydroxymethyl)-2-oxolanyl]-1,2,4-triazole-3-carboxamide | 0.60659 | 0.012727 | 0.205965 |
| Subgroup_13 | Streptidine | 0.606255 | 0.01279 | 0.206406 |
| Tuzzerella | 2-Methyl-2-[4-[3-[1-(4-methylbenzyl)-5-oxo-4,5-dihydro-1H-1,2,4-triazol-3-yl]propyl]phenoxy]propanoic acid | -0.60621 | 0.012799 | 0.206406 |
| Treponema | 3-Hydroxyoctadecanoylcarnitine | 0.60621 | 0.012799 | 0.206406 |
| Tuzzerella | N2-(3-Carboxy-2-hydroxy-1-oxopropyl)arginine | 0.605975 | 0.012843 | 0.206882 |
| Bryobacter | Phenylalanyl-Glycine | 0.605833 | 0.01287 | 0.207074 |
| Bryobacter | S-cucujolide III | -0.60575 | 0.012886 | 0.207101 |
| Faecalibaculum | Tephcalostan C | 0.605651 | 0.012904 | 0.207154 |
| Coprobacillus | Peperomin E | 0.605463 | 0.01294 | 0.20749 |
| bacteriap25 | N-gamma-L-Glutamyl-D-alanine | 0.605266 | 0.012977 | 0.207698 |
| Treponema | Hexadecadienylcarnitine | 0.605239 | 0.012982 | 0.207698 |
| Pseudolabrys | Eicosadienoic acid | 0.605111 | 0.013006 | 0.207851 |
| Coprobacillus | 16-Methylheptadecanoylcarnitine | 0.604896 | 0.013047 | 0.208269 |
| Monoglobus | 20-HETE-d6 | 0.604715 | 0.013082 | 0.208558 |
| Acetatifactor | Aspartyl-Gamma-glutamate | 0.604646 | 0.013095 | 0.208558 |
| Enterorhabdus | Polypropylene glycol (m w 1,200-3,000) | -0.60456 | 0.013112 | 0.208597 |
| Bryobacter | Berkeleylactone L | -0.60413 | 0.013194 | 0.209501 |
| Monoglobus | Tyrosylglycine | 0.604048 | 0.01321 | 0.209501 |
| Clostridioides | Kuguacin E | -0.60403 | 0.013214 | 0.209501 |
| Pseudolabrys | 11,14,17-eicosatrienoic acid | 0.603632 | 0.01329 | 0.210473 |
| Enterococcus | 7-oxo-8-amino-nonanoic acid | -0.60336 | 0.013343 | 0.210916 |
| TRA3-20 | 1-[3,4-Dihydroxy-5-(hydroxymethyl)-2-oxolanyl]-1,2,4-triazole-3-carboxamide | 0.603333 | 0.013348 | 0.210916 |
| TRA3-20 | D-Mannose | 0.603167 | 0.01338 | 0.211188 |
| Enterococcus | Glutaminylproline | 0.602907 | 0.013431 | 0.211363 |
| Enterococcus | Eleutherazine B; N,N'-((3,6-Dioxo-2,5-piperazinediyl)bis(trimethylene))bis(5-hydroxy-3-methyl-2-pentenamide) | 0.60288 | 0.013436 | 0.211363 |
| Tuzzerella | Skepinone-L | 0.602849 | 0.013442 | 0.211363 |
| Bryobacter | Oleic acid | -0.6028 | 0.013451 | 0.211363 |
| TRA3-20 | Tyrosyl-Alanine | 0.602609 | 0.013489 | 0.211719 |
| Subgroup_2 | 5'-Hydroxytenoxicam | 0.602341 | 0.013542 | 0.212307 |
| Nitrospira | Tercatain | 0.602106 | 0.013588 | 0.212794 |
| IMCC26256 | N,N'-Diethylthiourea | 0.601938 | 0.013621 | 0.213076 |
| Tuzzerella | 3,4-Dimethyl-5-propyl-2-furanpentadecanoic acid | -0.60177 | 0.013654 | 0.21335 |
| Tuzzerella | 7-Hydroxy-R-acenocoumarol | 0.601468 | 0.013714 | 0.213994 |
| Anaerotruncus | 1-p-Menthen-3-one | 0.60141 | 0.013725 | 0.213994 |
| Nitrospira | 16-Methylheptadecanoylcarnitine | 0.601085 | 0.01379 | 0.214765 |
| Tuzzerella | 13,14-dihydro-15-keto-PGD2-d4 | -0.60093 | 0.01382 | 0.214937 |
| Enterorhabdus | S-cucujolide III | -0.60088 | 0.013831 | 0.214937 |
| Gemella | N-gamma-L-Glutamyl-D-alanine | 0.60068 | 0.013871 | 0.215309 |
| Granulicella | D-Mannose | 0.600239 | 0.013959 | 0.216387 |
| Faecalibaculum | trans-Hexadec-2-enoyl carnitine | 0.600157 | 0.013975 | 0.216387 |
| IMCC26256 | Creatine | 0.600104 | 0.013986 | 0.216387 |
| Rikenellaceae_RC9_gut_group | Alpha-Lactose | 0.599901 | 0.014027 | 0.216622 |
| Lachnospiraceae_UCG-006 | L-Glutamine | 0.599876 | 0.014032 | 0.216622 |
| Bryobacter | 4-Chloro-L-phenylalanine | -0.59962 | 0.014084 | 0.217181 |
| Anaerotruncus | xi-2,3-Octadiene-5,7-diyn-1-ol | 0.59953 | 0.014102 | 0.217225 |
| bacteriap25 | Alpha-Lactose | 0.599452 | 0.014118 | 0.21723 |
| Enterorhabdus | (13Z)-3-Hydroxyicos-13-enoylcarnitine | 0.599247 | 0.014159 | 0.217256 |
| Monoglobus | (E,E)-3,7,11-Trimethyl-2,6,10-dodecatrienyl octanoate | 0.599241 | 0.014161 | 0.217256 |
| Tuzzerella | L-Arginine | 0.599153 | 0.014179 | 0.217256 |
| Nitrospira | m7G(5')pppAm | 0.599119 | 0.014185 | 0.217256 |
| Bryobacter | Pentadeca-3,5,7-trienedioylcarnitine | 0.599064 | 0.014197 | 0.217256 |
| Alcaligenes | Amobarbital | 0.598924 | 0.014225 | 0.217347 |
| Alcaligenes | L-Glutamine | 0.598884 | 0.014234 | 0.217347 |
| Tuzzerella | LysoPS(18:1(9Z)/0:0) | -0.59873 | 0.014264 | 0.217492 |
| Acidothermus | 3-{[(1s)-2,2-Difluoro-1-Hydroxy-7-(Methylsulfonyl)-2,3-Dihydro-1h-Inden-4-Yl]oxy}-5-Fluorobenzonitrile | 0.598466 | 0.014319 | 0.217492 |
| Enterococcus | N-alpha-Acetyl-L-lysine | 0.598423 | 0.014328 | 0.217492 |
| Acetatifactor | O-phosphonato-L-homoserine(2-) | 0.598406 | 0.014331 | 0.217492 |
| Enterorhabdus | Ricinoleic acid | -0.59839 | 0.014334 | 0.217492 |
| Anaerotruncus | 3-Hydroxybutyrylcarnitine | 0.598362 | 0.01434 | 0.217492 |
| Subgroup_13 | m7G(5')pppAm | 0.59826 | 0.014361 | 0.217492 |
| Coprobacillus | L-2-Hydroxyglutaric acid | 0.598234 | 0.014367 | 0.217492 |
| bacteriap25 | Palmitoylcarnitine | 0.598017 | 0.014411 | 0.217779 |
| Anaerotruncus | Symmetric dimethylarginine | 0.597992 | 0.014416 | 0.217779 |
| TM7 | 1H-Pyrrole-2,5-dione, 3-(1-methyl-1h-indol-3-yl)-4-(1-methyl-6-nitro-1h-indol-3-yl)- | 0.597501 | 0.014518 | 0.218688 |
| Jatrophihabitans | Tephcalostan | 0.597501 | 0.014518 | 0.218688 |
| IMCC26256 | 4-Methylcatechol 1-glucuronide | 0.597475 | 0.014523 | 0.218688 |
| IMCC26256 | PGP(18:3(9Z,12Z,15Z)/18:1(12Z)-O(9S,10R)) | 0.596968 | 0.014629 | 0.219858 |
| Bryobacter | Terbufos | 0.596951 | 0.014632 | 0.219858 |
| Bryobacter | PC(20:4(5Z,8Z,11Z,14Z)-OH(16R)/2:0) | 0.596314 | 0.014765 | 0.221623 |
| Tuzzerella | D-erythro-Sphingosine C-20 | -0.59612 | 0.014806 | 0.22199 |
| Monoglobus | Pentadeca-3,5,7-trienedioylcarnitine | 0.596048 | 0.014821 | 0.22199 |
| Nitrospira | Lycoperdic acid | 0.595851 | 0.014863 | 0.222097 |
| Rikenellaceae_RC9_gut_group | S-Adenosylhomocysteine | 0.595851 | 0.014863 | 0.222097 |
| Bryobacter | All trans decaprenyl diphosphate | -0.59579 | 0.014876 | 0.222097 |
| RF39 | L-Glutamine | 0.595163 | 0.015009 | 0.223173 |
| Monoglobus | 4'-O-Glucopyranosylsinapic acid | 0.595127 | 0.015016 | 0.223173 |
| Subgroup_13 | [6-Hydroxy-8-methyl-3-[3,4,5-trihydroxy-6-(hydroxymethyl)oxan-2-yl]oxy-9,10-dioxatetracyclo[4.3.1.02,5.03,8]decan-4-yl]methyl benzoate | 0.594864 | 0.015072 | 0.223173 |
| Lachnospiraceae_UCG-006 | Zidebactam | 0.594811 | 0.015084 | 0.223173 |
| Anaerostipes | N-(1-Deoxy-1-fructosyl)threonine | 0.594751 | 0.015097 | 0.223173 |
| Enterococcus | O-phosphonato-L-homoserine(2-) | 0.594705 | 0.015107 | 0.223173 |
| Monoglobus | L-arginino-succinate | 0.594691 | 0.015109 | 0.223173 |
| Monoglobus | N-Lactoylphenylalanine | 0.594668 | 0.015114 | 0.223173 |
| Enterorhabdus | Dimethylaminoparthenolide | 0.594645 | 0.015119 | 0.223173 |
| TRA3-20 | Histidylisoleucine | 0.59455 | 0.01514 | 0.223173 |
| Gemella | Streptidine | 0.594507 | 0.015149 | 0.223173 |
| Achromobacter | 1-p-Menthen-3-one | 0.594485 | 0.015153 | 0.223173 |
| Tuzzerella | N-Undecanoylglycine | -0.59446 | 0.015159 | 0.223173 |
| Pseudolabrys | 20-HETE-d6 | 0.594315 | 0.01519 | 0.223173 |
| Achromobacter | alpha-D-Galactopyranuronosyl-(1->4)-alpha-D-galactopyranuronosyl-(1->4)-D-galacturonic acid | 0.594309 | 0.015191 | 0.223173 |
| Tuzzerella | NAD | 0.594261 | 0.015202 | 0.223173 |
| Acidothermus | 9-Hydroxy-4-methoxypsoralen 9-glucoside | 0.594092 | 0.015238 | 0.223473 |
| Tuzzerella | Phenylalanyl-Glycine | 0.593957 | 0.015267 | 0.223625 |
| Enterococcus | Terbufos | 0.593897 | 0.01528 | 0.223625 |
| Lachnospiraceae_UCG-006 | Eicosadienoic acid | 0.593689 | 0.015325 | 0.22405 |
| bacteriap25 | Diethylamino 2,5-dihydroxybenzenesulfonate | 0.593455 | 0.015375 | 0.224393 |
| MND1 | Ethyl hexadecanoate | -0.59343 | 0.01538 | 0.224393 |
| Solobacterium | Streptidine | 0.593066 | 0.01546 | 0.225206 |
| Bryobacter | Alacepril | -0.593 | 0.015473 | 0.225206 |
| Acidothermus | Aspartyl-Alanine | 0.592956 | 0.015484 | 0.225206 |
| Tuzzerella | N-Acetylneuraminic acid | 0.592881 | 0.0155 | 0.22521 |
| Enterococcus | Valylserine | 0.592619 | 0.015557 | 0.225807 |
| Clostridioides | 6,10,14-Trimethyl-5,9,13-pentadecatrien-2-one | -0.59247 | 0.015591 | 0.225863 |
| Achromobacter | (E,E)-3,7,11-Trimethyl-2,6,10-dodecatrienyl octanoate | 0.592455 | 0.015593 | 0.225863 |
| Tuzzerella | PS(22:5(7Z,10Z,13Z,16Z,19Z)/22:6(4Z,7Z,10Z,13Z,16Z,19Z)) | -0.59209 | 0.015675 | 0.226806 |
| Achromobacter | Pentadeca-3,5,7-trienedioylcarnitine | 0.5919 | 0.015716 | 0.227071 |
| TRA3-20 | 4-Chloro-L-phenylalanine | -0.59186 | 0.015725 | 0.227071 |
| Enterorhabdus | 1-Octadecene | -0.59141 | 0.015824 | 0.228271 |
| bacteriap25 | Dipotassium phosphate | 0.591144 | 0.015883 | 0.228719 |
| Lachnospiraceae_UCG-006 | 11,14,17-eicosatrienoic acid | 0.591079 | 0.015897 | 0.228719 |
| Enterococcus | Hydroxy-2-naphthalenylmethylphosphonic acid tris-acetoxymethyl ester | 0.591048 | 0.015904 | 0.228719 |
| Aquicella | 1-[3,4-Dihydroxy-5-(hydroxymethyl)-2-oxolanyl]-1,2,4-triazole-3-carboxamide | 0.590943 | 0.015927 | 0.228819 |
| Acidothermus | Thidiazuron | 0.590806 | 0.015958 | 0.229024 |
| Monoglobus | benfluorex | 0.590591 | 0.016006 | 0.229481 |
| Bryobacter | 9-Hydroxy-4-methoxypsoralen 9-glucoside | 0.590334 | 0.016064 | 0.229717 |
| Subgroup_13 | Am-PE(16:0/18:0) | 0.590288 | 0.016074 | 0.229717 |
| bacteriap25 | Orotidine | 0.590206 | 0.016092 | 0.229717 |
| Tuzzerella | 5,5-Diphenyl-2-thiohydantoin | 0.590155 | 0.016104 | 0.229717 |
| Anaerotruncus | All trans decaprenyl diphosphate | -0.59015 | 0.016104 | 0.229717 |
| Clostridioides | Hexadecenal | -0.58981 | 0.016182 | 0.23059 |
| Tuzzerella | 1-Hydroxy-2-naphthoic acid | 0.5897 | 0.016206 | 0.230708 |
| Enterorhabdus | Dihydrozeatin O-beta-D-Glucoside | 0.589578 | 0.016234 | 0.230866 |
| MND1 | FAD | 0.589392 | 0.016276 | 0.230945 |
| Tuzzerella | N-Myristoyl Asparagine | -0.58933 | 0.016291 | 0.230945 |
| Tuzzerella | 9-Octadecenal | -0.58931 | 0.016294 | 0.230945 |
| Muribaculum | 3-Hydroxyoctadecanoylcarnitine | 0.589253 | 0.016307 | 0.230945 |
| Tuzzerella | 2-carboxy-L-threo-pentonate | -0.58915 | 0.01633 | 0.230945 |
| Monoglobus | Prolyl-Asparagine | 0.589084 | 0.016346 | 0.230945 |
| Acidothermus | 11-Hydroxyhexadecanoylcarnitine | 0.589047 | 0.016354 | 0.230945 |
| Granulicella | Dihydrozeatin O-beta-D-Glucoside | 0.588761 | 0.016419 | 0.231459 |
| TRA3-20 | Withaferin A | -0.58872 | 0.016429 | 0.231459 |
| Faecalibaculum | Tetradecanoylcarnitine | 0.588671 | 0.01644 | 0.231459 |
| Clostridioides | Octadecanamide | -0.58833 | 0.016518 | 0.232322 |
| Nitrospira | L-2-Hydroxyglutaric acid | 0.588193 | 0.016549 | 0.232534 |
| Acidothermus | S-Adenosylhomocysteine | 0.58797 | 0.016601 | 0.232964 |
| Flavonifractor | 4-Methylcatechol 1-glucuronide | 0.587916 | 0.016613 | 0.232964 |
| Bryobacter | Iguratimod | -0.5877 | 0.016664 | 0.233333 |
| Tuzzerella | Kaltostat | 0.587658 | 0.016673 | 0.233333 |
| IMCC26256 | Erucic acid | 0.58745 | 0.016721 | 0.233775 |
| Coprobacillus | Lycoperdic acid | 0.587173 | 0.016785 | 0.234256 |
| Nitrospira | 5'-Hydroxytenoxicam | 0.587125 | 0.016796 | 0.234256 |
| Tuzzerella | Niacinamide | 0.587086 | 0.016805 | 0.234256 |
| Granulicella | 3-Hydroxy-10'-apo-b,y-carotenal | 0.586669 | 0.016902 | 0.235335 |
| TRA3-20 | Dehydroascorbic acid | 0.58661 | 0.016916 | 0.235335 |
| Enterococcus | 8-Amino-7-oxononanoic acid | -0.58637 | 0.016973 | 0.235896 |
| Jatrophihabitans | Tercatain | 0.586204 | 0.017011 | 0.23619 |
| Bryobacter | Hexadecadienylcarnitine | 0.58612 | 0.017031 | 0.236229 |
| [Eubacterium]_oxidoreducens_group | D-Mannose | 0.586044 | 0.017048 | 0.236246 |
| Faecalibaculum | Maltotriose | 0.585715 | 0.017126 | 0.237083 |
| Enterorhabdus | Lemborexant | -0.58562 | 0.017148 | 0.237161 |
| Achromobacter | D-Mannose | 0.585549 | 0.017165 | 0.237161 |
| Flavonifractor | Symmetric dimethylarginine | 0.585363 | 0.017209 | 0.237535 |
| bacteriap25 | Maraviroc | 0.585245 | 0.017237 | 0.237689 |
| bacteriap25 | Pseudouridine | 0.585066 | 0.017279 | 0.237894 |
| TRA3-20 | PS(22:5(7Z,10Z,13Z,16Z,19Z)/22:6(4Z,7Z,10Z,13Z,16Z,19Z)) | -0.58501 | 0.017292 | 0.237894 |
| Bryobacter | 2-phospho-4-(cytidine 5'-diphospho)-2-C-methyl-D-erythritol | 0.584944 | 0.017308 | 0.237894 |
| Lachnospiraceae_UCG-006 | 20-HETE-d6 | 0.584794 | 0.017344 | 0.237894 |
| Tuzzerella | (R)-3-Amino-2-fluoropropyl phosphenite | 0.584725 | 0.017361 | 0.237894 |
| Intestinimonas | Glutaminylproline | 0.58467 | 0.017374 | 0.237894 |
| bacteriap25 | Tyrosyl-Alanine | 0.5846 | 0.01739 | 0.237894 |
| Lachnospiraceae_UCG-006 | D-erythro-L-galacto-Nonulose | 0.584581 | 0.017395 | 0.237894 |
| Granulicella | Eicosadienoic acid | 0.584542 | 0.017404 | 0.237894 |
| Achromobacter | Tyrosyl-Alanine | 0.584335 | 0.017454 | 0.238248 |
| Haliangium | 4-(Methylnitrosamino)-1-(3-pyridyl)-1-butanol glucuronide | 0.584223 | 0.01748 | 0.238248 |
| Clostridioides | Fructose-1,6-diphosphate | 0.584169 | 0.017493 | 0.238248 |
| Tuzzerella | 1-(O-alpha-D-glucopyranosyl)-(1,3S,25R)-hexacosanetriol | -0.58415 | 0.017498 | 0.238248 |
| Acetatifactor | S-[(3R,3As,6S,6aS)-3-nitrooxy-2,3,3a,5,6,6a-hexahydrofuro[3,2-b]furan-6-yl] ethanethioate | 0.583985 | 0.017537 | 0.238287 |
| Enterococcus | Erucic acid | 0.583921 | 0.017553 | 0.238287 |
| Alcaligenes | D-Mannose | 0.583883 | 0.017562 | 0.238287 |
| Treponema | 3-Amino-3-methylbutanoic acid | 0.583857 | 0.017568 | 0.238287 |
| Enterorhabdus | Camptothecin sodium | 0.583706 | 0.017605 | 0.238551 |
| Subgroup_13 | Niacinamide | 0.583591 | 0.017632 | 0.238695 |
| Lachnospiraceae_UCG-006 | (R)-Propyl 2-amino-3-mercaptopropanoate | 0.583519 | 0.01765 | 0.238703 |
| Anaerotruncus | DG(18:0/20:4(8Z,11Z,14Z,17Z)/0:0) | 0.583388 | 0.017681 | 0.238902 |
| Clostridioides | PI(20:4(5Z,8Z,11Z,14Z)/18:0) | -0.58322 | 0.017721 | 0.239204 |
| Tuzzerella | 1-p-Menthen-3-one | 0.582657 | 0.017859 | 0.240276 |
| Enterorhabdus | 3-Hydroxyeicosanoylcarnitine | 0.582552 | 0.017884 | 0.240276 |
| Anaerotruncus | (E)-2-(hexa-3,5-dien-1-yn-1-yl)-5-(prop-1-yn-1-yl)thiophene | 0.582508 | 0.017895 | 0.240276 |
| Enterorhabdus | 3'-Deoxythymidine | 0.582401 | 0.017921 | 0.240276 |
| Treponema | Orotidine | 0.582387 | 0.017924 | 0.240276 |
| Enterococcus | 3-Methyl-3-butenyl apiosyl-(1->6)-glucoside | 0.582384 | 0.017925 | 0.240276 |
| Monoglobus | Docosatrienoic acid | 0.582324 | 0.01794 | 0.240276 |
| Granulicella | Genistein 7-O-glucoside-6''-malonate | 0.582312 | 0.017943 | 0.240276 |
| Clostridioides | N-alpha-Acetyl-L-lysine | 0.58226 | 0.017955 | 0.240276 |
| Enterococcus | 3-Hydroxy-10'-apo-b,y-carotenal | 0.582197 | 0.017971 | 0.240276 |
| bacteriap25 | Hexadecadienylcarnitine | 0.582105 | 0.017993 | 0.240349 |
| Acidothermus | 2,2,3,3,4,4,5,5,6,6,7,7,8,8,9,9-Hexadecafluorononanoic acid | 0.581938 | 0.018034 | 0.240659 |
| Achromobacter | 20-HETE-d6 | 0.581871 | 0.018051 | 0.240659 |
| Achromobacter | 11,14,17-eicosatrienoic acid | 0.581736 | 0.018084 | 0.240865 |
| Achromobacter | [6-Hydroxy-8-methyl-3-[3,4,5-trihydroxy-6-(hydroxymethyl)oxan-2-yl]oxy-9,10-dioxatetracyclo[4.3.1.02,5.03,8]decan-4-yl]methyl benzoate | 0.581509 | 0.01814 | 0.240865 |
| Anaerotruncus | N,N'-Diethylthiourea | 0.581502 | 0.018141 | 0.240865 |
| Bryobacter | S-[(3R,3As,6S,6aS)-3-nitrooxy-2,3,3a,5,6,6a-hexahydrofuro[3,2-b]furan-6-yl] ethanethioate | 0.581398 | 0.018167 | 0.240865 |
| Tuzzerella | 5-[1-Carboxy-2-(trimethylazaniumyl)ethoxy]-5-oxopentanoate | 0.581292 | 0.018193 | 0.240865 |
| Faecalibaculum | Am-PE(16:0/18:0) | 0.581271 | 0.018199 | 0.240865 |
| Anaerotruncus | Ethynodiol | -0.58125 | 0.018205 | 0.240865 |
| Subgroup_13 | 3-Methyl-3-butenyl hexadecanoate | 0.581203 | 0.018215 | 0.240865 |
| Tuzzerella | L-Lysinamide | -0.58118 | 0.01822 | 0.240865 |
| Faecalibaculum | 3-Hydroxy-10'-apo-b,y-carotenal | 0.580952 | 0.018277 | 0.241244 |
| Pseudolabrys | N6-Acetyl-L-lysine | 0.58093 | 0.018283 | 0.241244 |
| Subgroup_13 | DG(18:0/20:4(8Z,11Z,14Z,17Z)/0:0) | 0.58051 | 0.018387 | 0.242393 |
| Rikenellaceae_RC9_gut_group | Cysteine-glutathione disulfide | 0.580436 | 0.018406 | 0.242409 |
| TRA3-20 | Nipradilol | 0.580321 | 0.018434 | 0.242559 |
| IMCC26256 | Aspartyl-Gamma-glutamate | 0.579788 | 0.018568 | 0.243694 |
| TRA3-20 | Iguratimod | -0.57977 | 0.018572 | 0.243694 |
| Enterococcus | N2-(3-Carboxy-2-hydroxy-1-oxopropyl)arginine | 0.579769 | 0.018573 | 0.243694 |
| Anaerotruncus | Terizidone | 0.579504 | 0.018639 | 0.244341 |
| Anaerostipes | 2-phospho-4-(cytidine 5'-diphospho)-2-C-methyl-D-erythritol | 0.579433 | 0.018657 | 0.244349 |
| MND1 | PI(18:0/20:4(8Z,11Z,14Z,17Z)) | 0.579199 | 0.018716 | 0.244895 |
| Rikenellaceae_RC9_gut_group | Hydroxy-2-naphthalenylmethylphosphonic acid tris-acetoxymethyl ester | 0.57895 | 0.018779 | 0.245488 |
| Monoglobus | 5'-Carboxy-gamma-chromanol | -0.57853 | 0.018886 | 0.246661 |
| Tuzzerella | 1-O-(2R-hydroxy-hexadecyl)-sn-glycerol | -0.57822 | 0.018965 | 0.247275 |
| Lachnospiraceae_UCG-006 | Pentadeca-3,5,7-trienedioylcarnitine | 0.578205 | 0.018968 | 0.247275 |
| Bryobacter | 1-Ethoxymethyl-5-fluorouracil | 0.578094 | 0.018997 | 0.247417 |
| Enterorhabdus | 1-O-(2R-methoxy-hexadecyl)-sn-glycerol | -0.578 | 0.01902 | 0.247491 |
| Anaerotruncus | Dihydrozeatin O-beta-D-Glucoside | 0.577857 | 0.019057 | 0.24763 |
| Anaerotruncus | LysoPC(20:3(8Z,11Z,14Z)/0:0) | 0.577727 | 0.019091 | 0.24763 |
| Flavonifractor | D-Mannose | 0.577712 | 0.019095 | 0.24763 |
| Gemella | Aspartyl-Alanine | 0.577686 | 0.019101 | 0.24763 |
| Flavonifractor | Dipotassium phosphate | 0.577558 | 0.019134 | 0.247678 |
| IMCC26256 | Dipotassium phosphate | 0.577523 | 0.019143 | 0.247678 |
| Tuzzerella | DG(17:2(9Z,12Z)/22:5(7Z,10Z,13Z,16Z,19Z)/0:0)[iso2] | 0.577466 | 0.019158 | 0.247678 |
| bacteriap25 | (E)-2-(hexa-3,5-dien-1-yn-1-yl)-5-(prop-1-yn-1-yl)thiophene | 0.577225 | 0.01922 | 0.248253 |
| Tuzzerella | Medica 16 | -0.57709 | 0.019256 | 0.248487 |
| bacteriap25 | O-Linoleoylcarnitine | 0.577013 | 0.019275 | 0.248506 |
| Nitrospira | True blue | 0.576458 | 0.019418 | 0.25013 |
| TRA3-20 | N-gamma-L-Glutamyl-D-alanine | 0.576378 | 0.019439 | 0.25017 |
| Achromobacter | Aspartyl-Alanine | 0.576215 | 0.019482 | 0.250433 |
| Tuzzerella | Serotinose | 0.576163 | 0.019495 | 0.250433 |
| Coprobacillus | Tercatain | 0.576057 | 0.019523 | 0.250556 |
| Subgroup_13 | L-Carnitine | 0.575643 | 0.019631 | 0.251718 |
| Rikenellaceae_RC9_gut_group | Aspartyl-Isoleucine | 0.575465 | 0.019678 | 0.252087 |
| MND1 | m7G(5')pppAm | 0.57518 | 0.019753 | 0.252817 |
| Alcaligenes | alpha-D-Galactopyranuronosyl-(1->4)-alpha-D-galactopyranuronosyl-(1->4)-D-galacturonic acid | 0.575098 | 0.019774 | 0.252862 |
| Pseudolabrys | Docosatrienoic acid | 0.575006 | 0.019799 | 0.252944 |
| Treponema | 7-Hydroxy-R-acenocoumarol | 0.574858 | 0.019838 | 0.253213 |
| Bryobacter | 2-Methylthioadenosine | 0.574635 | 0.019896 | 0.253358 |
| MND1 | Am-PE(16:0/18:0) | 0.574606 | 0.019904 | 0.253358 |
| Tuzzerella | Biliverdin | -0.57457 | 0.019914 | 0.253358 |
| bacteriap25 | 7-Hydroxy-R-acenocoumarol | 0.574513 | 0.019929 | 0.253358 |
| IMCC26256 | Aspartyl-Alanine | 0.574475 | 0.019939 | 0.253358 |
| Clostridioides | Dolichyl b-D-glucosyl phosphate | -0.57432 | 0.01998 | 0.25345 |
| Achromobacter | Benzyl gentiobioside | 0.574294 | 0.019987 | 0.25345 |
| Tuzzerella | 5'-Carboxy-gamma-chromanol | -0.57424 | 0.02 | 0.25345 |
| Bryobacter | (E,E)-3,7,11-Trimethyl-2,6,10-dodecatrienyl octanoate | 0.57412 | 0.020033 | 0.253533 |
| bacteriap25 | Dimethylaminoparthenolide | 0.57402 | 0.02006 | 0.253533 |
| Aquicella | N,N'-Diethylthiourea | 0.574017 | 0.020061 | 0.253533 |
| Bryobacter | 5-Hydroxyindoxyl sulfate | -0.57369 | 0.020148 | 0.254402 |
| Gemella | 5beta-Cholane-3alpha,24-diol | 0.573486 | 0.020203 | 0.25487 |
| Tuzzerella | 5'-Hydroxytenoxicam | 0.573402 | 0.020225 | 0.254925 |
| Alcaligenes | Pentadeca-3,5,7-trienedioylcarnitine | 0.573281 | 0.020258 | 0.255032 |
| Subgroup_13 | PI(18:0/20:4(8Z,11Z,14Z,17Z)) | 0.573195 | 0.020281 | 0.255032 |
| Tuzzerella | 5S-HETE di-endoperoxide | -0.57313 | 0.020297 | 0.255032 |
| Alcaligenes | 1-p-Menthen-3-one | 0.5731 | 0.020306 | 0.255032 |
| [Eubacterium]_oxidoreducens_group | Docosatrienoic acid | 0.573001 | 0.020333 | 0.255142 |
| Rikenellaceae_RC9_gut_group | 9-Hydroxy-4-methoxypsoralen 9-glucoside | 0.572771 | 0.020395 | 0.255554 |
| Achromobacter | Docosatrienoic acid | 0.572744 | 0.020402 | 0.255554 |
| Rikenellaceae_RC9_gut_group | Thidiazuron | 0.57254 | 0.020457 | 0.256017 |
| Bryobacter | 1-[3,4-Dihydroxy-5-(hydroxymethyl)-2-oxolanyl]-1,2,4-triazole-3-carboxamide | 0.572085 | 0.020581 | 0.257202 |
| Granulicella | N-gamma-L-Glutamyl-D-alanine | 0.57196 | 0.020615 | 0.257202 |
| Faecalibaculum | 9-Hydroxy-4-methoxypsoralen 9-glucoside | 0.571908 | 0.020629 | 0.257202 |
| Monoglobus | 2',3'-Dideoxyuridine | 0.571901 | 0.020631 | 0.257202 |
| Achromobacter | Flumazenil | 0.571792 | 0.020661 | 0.257202 |
| Tuzzerella | 3'-Deoxythymidine | 0.571788 | 0.020662 | 0.257202 |
| Clostridioides | Fluazifop | 0.571612 | 0.02071 | 0.257299 |
| Monoglobus | N-(1-Deoxy-1-fructosyl)tyrosine | 0.571591 | 0.020715 | 0.257299 |
| TM7 | Glutamylleucylarginine | 0.571528 | 0.020733 | 0.257299 |
| Anaerotruncus | Phenethylamine glucuronide | 0.5714 | 0.020768 | 0.257299 |
| Anaerotruncus | Creatine | 0.571398 | 0.020768 | 0.257299 |
| Clostridioides | PE(22:5(4Z,7Z,10Z,13Z,16Z)/PGD2) | -0.57136 | 0.020779 | 0.257299 |
| Anaerotruncus | U-75302 | 0.571235 | 0.020813 | 0.257392 |
| Faecalibaculum | L-Homocystine | 0.571197 | 0.020823 | 0.257392 |
| Bryobacter | Zidebactam | 0.571043 | 0.020866 | 0.257605 |
| Flavonifractor | Aspartyl-Alanine | 0.57089 | 0.020907 | 0.257605 |
| Clostridioides | Withaferin A | -0.57088 | 0.020911 | 0.257605 |
| MND1 | PC(18:1(17Z)/18:1(17Z)) | 0.570868 | 0.020914 | 0.257605 |
| Tuzzerella | 1-[3,4-Dihydroxy-5-(hydroxymethyl)-2-oxolanyl]-1,2,4-triazole-3-carboxamide | 0.570633 | 0.020978 | 0.257982 |
| Rikenellaceae_RC9_gut_group | L-Homocystine | 0.570603 | 0.020987 | 0.257982 |
| Enterococcus | Flumazenil | 0.570492 | 0.021017 | 0.257982 |
| bacteriap25 | 3-Hydroxyoctadecanoylcarnitine | 0.570445 | 0.02103 | 0.257982 |
| TRA3-20 | alpha-D-Galactopyranuronosyl-(1->4)-alpha-D-galactopyranuronosyl-(1->4)-D-galacturonic acid | 0.570312 | 0.021067 | 0.257982 |
| Christensenellaceae_R-7_group | 5'-Hydroxytenoxicam | 0.570299 | 0.021071 | 0.257982 |
| Achromobacter | Genistein 7-O-glucoside-6''-malonate | 0.570287 | 0.021074 | 0.257982 |
| Bryobacter | Thidiazuron | 0.570226 | 0.021091 | 0.257982 |
| Tuzzerella | PC(20:5(5Z,8Z,11Z,14Z,17Z)/PGJ2) | -0.57015 | 0.021113 | 0.258026 |
| Tuzzerella | DG(LTE4/22:0/0:0) | 0.569941 | 0.02117 | 0.258329 |
| Treponema | Pseudouridine | 0.569925 | 0.021174 | 0.258329 |
| IMCC26256 | Xanthosine | 0.569825 | 0.021202 | 0.258444 |
| Coprobacillus | 3'-Ketolactose | 0.569639 | 0.021254 | 0.258448 |
| Tuzzerella | 3-Amino-2-methoxynonadec-5-en-4-ol | -0.5696 | 0.021264 | 0.258448 |
| Clostridioides | Oleoyl Serotonin | -0.56959 | 0.021268 | 0.258448 |
| Tuzzerella | 2-(Methylthiomethyl)-3-phenyl-2-propenal | 0.569539 | 0.021282 | 0.258448 |
| Achromobacter | L-Arginine | 0.569495 | 0.021294 | 0.258448 |
| Lachnospiraceae_UCG-006 | Aspartyl-Gamma-glutamate | 0.569311 | 0.021346 | 0.258849 |
| Clostridioides | Notoginsenoside H | 0.569159 | 0.021388 | 0.258904 |
| Aquicella | Creatine | 0.569095 | 0.021406 | 0.258904 |
| TRA3-20 | Dolichyl b-D-glucosyl phosphate | -0.56898 | 0.021439 | 0.258904 |
| Alcaligenes | Zidebactam | 0.568962 | 0.021443 | 0.258904 |
| Christensenellaceae_R-7_group | BEFLOXATONE | 0.568948 | 0.021447 | 0.258904 |
| Muribaculum | Napelline | 0.568901 | 0.02146 | 0.258904 |
| Aquicella | 3-Methyl-3-butenyl hexadecanoate | 0.56876 | 0.0215 | 0.258975 |
| Intestinimonas | 4-Bis(2-hydroxyethyl)amino-L-phenylalanine | 0.568749 | 0.021503 | 0.258975 |
| Monoglobus | Monooctyl phthalate | -0.56861 | 0.021541 | 0.259094 |
| Tuzzerella | Monooctyl phthalate | -0.56858 | 0.02155 | 0.259094 |
| Achromobacter | 4-Methylcatechol 1-glucuronide | 0.568457 | 0.021585 | 0.259301 |
| Aquicella | scyllo-Inositol | 0.568215 | 0.021654 | 0.259891 |
| Intestinimonas | Docosadienoate (22:2n6) | 0.568097 | 0.021687 | 0.259891 |
| Enterorhabdus | L-2-Hydroxyglutaric acid | 0.568029 | 0.021706 | 0.259891 |
| Monoglobus | (R)-Propyl 2-amino-3-mercaptopropanoate | 0.568021 | 0.021708 | 0.259891 |
| Enterococcus | Glycerol 3-phosphate | 0.567877 | 0.021749 | 0.259925 |
| TRA3-20 | L-Arginine | 0.567724 | 0.021793 | 0.259925 |
| MND1 | Inosine | 0.56766 | 0.021811 | 0.259925 |
| Faecalibaculum | Hydroxy-2-naphthalenylmethylphosphonic acid tris-acetoxymethyl ester | 0.567638 | 0.021817 | 0.259925 |
| Faecalibaculum | S-Adenosylhomocysteine | 0.567633 | 0.021819 | 0.259925 |
| MND1 | Cysteinyl-Aspartate | 0.567621 | 0.021822 | 0.259925 |
| Haliangium | 5'-Hydroxytenoxicam | 0.567246 | 0.021929 | 0.259996 |
| Subgroup_2 | 2-Deoxy-2,3-dehydro-n-acetyl-neuraminic acid | 0.567215 | 0.021938 | 0.259996 |
| Acetatifactor | PGP(18:3(9Z,12Z,15Z)/18:1(12Z)-O(9S,10R)) | 0.567206 | 0.02194 | 0.259996 |
| Enterorhabdus | Tuberonic acid glucoside | -0.56707 | 0.021979 | 0.259996 |
| Monoglobus | 2-C-methyl-D-erythritol-4-phosphate | 0.567003 | 0.021998 | 0.259996 |
| Intestinimonas | Asparaginyl-Proline | 0.566911 | 0.022025 | 0.259996 |
| Clostridioides | Am-PE(16:0/18:0) | 0.566723 | 0.022079 | 0.259996 |
| Alcaligenes | 11,14,17-eicosatrienoic acid | 0.566712 | 0.022082 | 0.259996 |
| Lachnospiraceae_UCG-006 | N-alpha-Acetyl-L-lysine | 0.566656 | 0.022098 | 0.259996 |
| MND1 | 2-C-methyl-D-erythritol-4-phosphate | 0.566579 | 0.02212 | 0.259996 |
| Monoglobus | 2-Deoxy-2,3-dehydro-n-acetyl-neuraminic acid | 0.566521 | 0.022136 | 0.259996 |
| TRA3-20 | PE(22:5(4Z,7Z,10Z,13Z,16Z)/PGD2) | -0.56651 | 0.022139 | 0.259996 |
| Tuzzerella | (2R,4S)-2-Aminoformyl-6-fluoro-spiro[chroman-4,4'-imidazolidine]-2',5'-dione | -0.56651 | 0.02214 | 0.259996 |
| Enterorhabdus | Lycoperdic acid | 0.56647 | 0.022151 | 0.259996 |
| Clostridioides | PI(18:0/20:4(8Z,11Z,14Z,17Z)) | 0.566467 | 0.022152 | 0.259996 |
| Clostridioides | 2',3'-Dideoxyuridine | 0.566427 | 0.022164 | 0.259996 |
| Treponema | Maltotriose | 0.566414 | 0.022167 | 0.259996 |
| bacteriap25 | Medicagol | 0.566389 | 0.022174 | 0.259996 |
| Acidothermus | NAD | 0.566374 | 0.022179 | 0.259996 |
| Acetatifactor | 5beta-Cholane-3alpha,24-diol | 0.566248 | 0.022215 | 0.260205 |
| RF39 | 2-phospho-4-(cytidine 5'-diphospho)-2-C-methyl-D-erythritol | 0.566039 | 0.022275 | 0.260695 |
| Alcaligenes | 20-HETE-d6 | 0.565945 | 0.022303 | 0.260798 |
| Alcaligenes | L-Arginine | 0.565807 | 0.022342 | 0.260845 |
| Pseudolabrys | N-alpha-Acetyl-L-lysine | 0.565802 | 0.022344 | 0.260845 |
| Bryobacter | (R)-4A-(Ethoxymethyl)-1-(4-fluorophenyl)-6-((4-(trifluoromethyl)phenyl)sulfonyl)-4,4a,5,6,7,8-hexahydro-1H-pyrazolo[3,4-g]isoquinoline | -0.56572 | 0.022369 | 0.260924 |
| Bryobacter | Oleoyl Serotonin | -0.56565 | 0.022388 | 0.260929 |
| Tuzzerella | N,N'-Diethylthiourea | 0.565508 | 0.022429 | 0.261002 |
| Faecalibaculum | Thidiazuron | 0.565476 | 0.022439 | 0.261002 |
| Haliangium | N-(1-Deoxy-1-fructosyl)valine | 0.565398 | 0.022461 | 0.261002 |
| Faecalibaculum | Dipotassium phosphate | 0.565315 | 0.022485 | 0.261002 |
| Enterorhabdus | Ribonolactone | 0.56507 | 0.022557 | 0.261002 |
| Clostridioides | Phytal | -0.56506 | 0.02256 | 0.261002 |
| Monoglobus | Cysteinyl-Aspartate | 0.565007 | 0.022575 | 0.261002 |
| Intestinimonas | Serylproline | 0.564907 | 0.022604 | 0.261002 |
| Flavonifractor | Xanthosine | 0.564906 | 0.022605 | 0.261002 |
| Achromobacter | LysoPI(18:0/0:0) | 0.564834 | 0.022626 | 0.261002 |
| Pseudolabrys | benfluorex | 0.564816 | 0.022631 | 0.261002 |
| TRA3-20 | 1-O-Galloylglycerol | 0.564747 | 0.022651 | 0.261002 |
| Rikenellaceae_RC9_gut_group | NAD | 0.564722 | 0.022659 | 0.261002 |
| Subgroup_13 | (S1)-Methoxy-3-heptanethiol | 0.564705 | 0.022663 | 0.261002 |
| Tuzzerella | S-cucujolide III | -0.56464 | 0.022682 | 0.261002 |
| Achromobacter | xi-2,3-Octadiene-5,7-diyn-1-ol | 0.564611 | 0.022691 | 0.261002 |
| Monoglobus | Eicosadienoic acid | 0.564449 | 0.022738 | 0.261154 |
| Treponema | Aspartyl-Isoleucine | 0.564439 | 0.022741 | 0.261154 |
| MND1 | DG(18:0/20:4(8Z,11Z,14Z,17Z)/0:0) | 0.564188 | 0.022815 | 0.261789 |
| Rikenellaceae_RC9_gut_group | 3-hydroxyhexadecanoyl carnitine | 0.564112 | 0.022838 | 0.261799 |
| Tuzzerella | (2S)-1,1,1-Trifluoro-2-(4-pyrazol-1-ylphenyl)-3-[5-[[1-(trifluoromethyl)cyclopropyl]methyl]-1H-imidazol-2-yl]propan-2-ol | -0.56404 | 0.022859 | 0.261799 |
| Alcaligenes | Lucuminic acid | 0.563937 | 0.022889 | 0.261799 |
| Acidothermus | Hydroxy-2-naphthalenylmethylphosphonic acid tris-acetoxymethyl ester | 0.563933 | 0.02289 | 0.261799 |
| Alcaligenes | Dipotassium phosphate | 0.563695 | 0.022961 | 0.262199 |
| TM7 | Dehydroascorbic acid | 0.563688 | 0.022963 | 0.262199 |
| Acidothermus | L-Homocystine | 0.563584 | 0.022994 | 0.262339 |
| Enterorhabdus | Ethynodiol | -0.56309 | 0.023139 | 0.263781 |
| Haliangium | BEFLOXATONE | 0.563029 | 0.023158 | 0.263791 |
| Bryobacter | D-Mannose | 0.562763 | 0.023238 | 0.264482 |
| RF39 | FAD | 0.562621 | 0.02328 | 0.264749 |
| Anaerotruncus | Pentadeca-3,5,7-trienedioylcarnitine | 0.562527 | 0.023308 | 0.264854 |
| Aquicella | 4-Methylcatechol 1-glucuronide | 0.562385 | 0.02335 | 0.265122 |
| Tuzzerella | Streptidine | 0.562247 | 0.023392 | 0.265189 |
| Rikenellaceae_RC9_gut_group | LysoPC(P-16:0/0:0) | 0.56214 | 0.023424 | 0.265189 |
| Bryobacter | Glycerol 3-phosphate | 0.562119 | 0.02343 | 0.265189 |
| Achromobacter | Prolyl-Asparagine | 0.562053 | 0.02345 | 0.265189 |
| Subgroup_2 | 1-(10H-Phenothiazin-2-yl)ethanone | 0.562052 | 0.023451 | 0.265189 |
| Faecalibaculum | Niacinamide | 0.561986 | 0.02347 | 0.265201 |
| Anaerotruncus | Xanthosine | 0.56192 | 0.02349 | 0.265213 |
| Subgroup_13 | Notoginsenoside H | 0.561769 | 0.023536 | 0.265514 |
| Nitrospira | thiamphenicol | 0.561589 | 0.02359 | 0.265558 |
| Nitrospira | Peperomin E | 0.561572 | 0.023595 | 0.265558 |
| Subgroup_13 | PC(P-18:0/20:4(5Z,8Z,11Z,14Z)-OH(16R)) | 0.561568 | 0.023596 | 0.265558 |
| Bryobacter | 3'-Deoxythymidine | 0.561319 | 0.023672 | 0.266192 |
| Lachnospiraceae_UCG-006 | alpha-D-Galactopyranuronosyl-(1->4)-alpha-D-galactopyranuronosyl-(1->4)-D-galacturonic acid | 0.561182 | 0.023713 | 0.266227 |
| Tuzzerella | Tyrosyl-Alanine | 0.561132 | 0.023728 | 0.266227 |
| Acetatifactor | Symmetric dimethylarginine | 0.561122 | 0.023731 | 0.266227 |
| TRA3-20 | 5-Hydroxyindoxyl sulfate | -0.56074 | 0.023849 | 0.26724 |
| Bryobacter | 3-Methyl-3-butenyl apiosyl-(1->6)-glucoside | 0.560643 | 0.023877 | 0.26724 |
| Acetatifactor | JP83 | -0.56063 | 0.023879 | 0.26724 |
| [Eubacterium]_oxidoreducens_group | 4-Methylcatechol 1-glucuronide | 0.560575 | 0.023898 | 0.26724 |
| Anaerostipes | PC(20:4(5Z,8Z,11Z,14Z)-OH(16R)/2:0) | 0.560395 | 0.023953 | 0.267512 |
| Monoglobus | PE(22:4(7Z,10Z,13Z,16Z)/19:0) | -0.56034 | 0.023971 | 0.267512 |
| Solobacterium | Aspartyl-Alanine | 0.560281 | 0.023988 | 0.267512 |
| Enterorhabdus | PE(22:5(4Z,7Z,10Z,13Z,16Z)/PGD2) | -0.56025 | 0.023998 | 0.267512 |
| Enterorhabdus | Nipradilol | 0.560145 | 0.024029 | 0.267647 |
| Faecalibaculum | Beta-Citryl-L-glutamic acid | 0.559946 | 0.02409 | 0.268076 |
| Anaerotruncus | PI(18:0/20:4(8Z,11Z,14Z,17Z)) | 0.559895 | 0.024106 | 0.268076 |
| Intestinimonas | Eleutherazine B; N,N'-((3,6-Dioxo-2,5-piperazinediyl)bis(trimethylene))bis(5-hydroxy-3-methyl-2-pentenamide) | 0.559796 | 0.024136 | 0.268205 |
| Tuzzerella | Pevonedistat | 0.55972 | 0.02416 | 0.268251 |
| Alcaligenes | LysoPI(18:0/0:0) | 0.559422 | 0.024251 | 0.269058 |
| Acidothermus | Valylhistidine | 0.559343 | 0.024276 | 0.269116 |
| Pseudolabrys | Prolylproline | 0.559139 | 0.024339 | 0.269603 |
| Treponema | 3-hydroxyhexadecanoyl carnitine | 0.559025 | 0.024374 | 0.269727 |
| Faecalibaculum | [6-Hydroxy-8-methyl-3-[3,4,5-trihydroxy-6-(hydroxymethyl)oxan-2-yl]oxy-9,10-dioxatetracyclo[4.3.1.02,5.03,8]decan-4-yl]methyl benzoate | 0.558932 | 0.024403 | 0.269727 |
| Anaerostipes | PE-NMe2(18:3(9Z,12Z,15Z)/18:1(11Z)) | 0.558917 | 0.024407 | 0.269727 |
| Jatrophihabitans | Napelline | 0.558811 | 0.02444 | 0.26988 |
| Clostridioides | Bis(2-propoxyethyl) 2,6-dimethyl-4-(3-nitrophenyl)-3,4-dihydropyridine-3,5-dicarboxylate | -0.55874 | 0.024463 | 0.269918 |
| Clostridia_vadinBB60_group | Inosine | 0.558597 | 0.024507 | 0.270191 |
| TRA3-20 | Skepinone-L | 0.558189 | 0.024634 | 0.271186 |
| Acetatifactor | [6-Hydroxy-8-methyl-3-[3,4,5-trihydroxy-6-(hydroxymethyl)oxan-2-yl]oxy-9,10-dioxatetracyclo[4.3.1.02,5.03,8]decan-4-yl]methyl benzoate | 0.55813 | 0.024652 | 0.271186 |
| Enterococcus | 1-Hydroxy-2-naphthoic acid | 0.558122 | 0.024655 | 0.271186 |
| Christensenellaceae_R-7_group | Neosaxitoxin | 0.557974 | 0.024701 | 0.271232 |
| Subgroup_2 | 4'-O-Glucopyranosylsinapic acid | 0.557929 | 0.024715 | 0.271232 |
| Alcaligenes | Serylproline | 0.557877 | 0.024732 | 0.271232 |
| Jatrophihabitans | Lycoperdic acid | 0.557862 | 0.024736 | 0.271232 |
| Subgroup_13 | 2-(Methylthiomethyl)-3-phenyl-2-propenal | 0.557734 | 0.024776 | 0.271315 |
| Clostridia_vadinBB60_group | 5'-Methylthioadenosine | 0.557715 | 0.024782 | 0.271315 |
| Enterococcus | 1-[3,4-Dihydroxy-5-(hydroxymethyl)-2-oxolanyl]-1,2,4-triazole-3-carboxamide | 0.557526 | 0.024842 | 0.271578 |
| Clostridioides | DG(18:0/20:4(8Z,11Z,14Z,17Z)/0:0) | 0.557468 | 0.02486 | 0.271578 |
| Granulicella | 2,2,3,3,4,4,5,5,6,6,7,7,8,8,9,9-Hexadecafluorononanoic acid | 0.557454 | 0.024864 | 0.271578 |
| Lachnospiraceae_UCG-006 | N2-(3-Carboxy-2-hydroxy-1-oxopropyl)arginine | 0.557378 | 0.024888 | 0.271628 |
| Acidothermus | (2S)-3-[3-[(4-Carbamimidoylbenzoyl)amino]propanoylamino]-2-[(4-ethylphenyl)sulfonylamino]propanoic acid | 0.557069 | 0.024985 | 0.271942 |
| Tuzzerella | Creatine | 0.557066 | 0.024986 | 0.271942 |
| Tuzzerella | Lycoperdic acid | 0.557048 | 0.024992 | 0.271942 |
| Bryobacter | 5,5-Diphenyl-2-thiohydantoin | 0.556971 | 0.025017 | 0.271942 |
| Faecalibaculum | Alpha-Lactose | 0.556928 | 0.02503 | 0.271942 |
| Aquicella | (R)-Propyl 2-amino-3-mercaptopropanoate | 0.556874 | 0.025047 | 0.271942 |
| Subgroup_13 | Lucuminic acid | 0.556815 | 0.025066 | 0.271942 |
| MND1 | BEFLOXATONE | 0.556797 | 0.025071 | 0.271942 |
| Pseudolabrys | Pentadeca-3,5,7-trienedioylcarnitine | 0.556542 | 0.025152 | 0.272569 |
| TRA3-20 | S-cucujolide III | -0.55643 | 0.025186 | 0.272569 |
| Enterorhabdus | (E)-2-(hexa-3,5-dien-1-yn-1-yl)-5-(prop-1-yn-1-yl)thiophene | 0.556432 | 0.025187 | 0.272569 |
| Tuzzerella | Hexadecadienylcarnitine | 0.556008 | 0.025322 | 0.273676 |
| Granulicella | 5beta-Cholane-3alpha,24-diol | 0.555988 | 0.025328 | 0.273676 |
| Acetatifactor | Cysteine-glutathione disulfide | 0.555564 | 0.025464 | 0.274929 |
| RF39 | 1-[3,4-Dihydroxy-5-(hydroxymethyl)-2-oxolanyl]-1,2,4-triazole-3-carboxamide | 0.55524 | 0.025568 | 0.2757 |
| Treponema | Tyrosyl-Aspartate | 0.555219 | 0.025575 | 0.2757 |
| Bryobacter | PI(20:3(8Z,11Z,14Z)/0:0) | 0.555107 | 0.025611 | 0.275878 |
| Pseudolabrys | Glutaminylproline | 0.554938 | 0.025665 | 0.276066 |
| Enterorhabdus | Avenin | 0.554909 | 0.025675 | 0.276066 |
| Achromobacter | Amobarbital | 0.55487 | 0.025687 | 0.276066 |
| Faecalibaculum | Genistein 7-O-glucoside-6''-malonate | 0.55474 | 0.025729 | 0.276156 |
| Monoglobus | PI(20:3(8Z,11Z,14Z)/0:0) | 0.554722 | 0.025735 | 0.276156 |
| Achromobacter | Sulisobenzone | 0.554644 | 0.02576 | 0.276217 |
| Acetatifactor | Streptidine | 0.554579 | 0.025781 | 0.276233 |
| IMCC26256 | Eicosadienoic acid | 0.554494 | 0.025808 | 0.276315 |
| Bryobacter | JP83 | -0.55434 | 0.025859 | 0.276607 |
| Alcaligenes | Docosatrienoic acid | 0.55422 | 0.025897 | 0.276607 |
| Muribaculum | Octadec-6-enoylcarnitine | 0.554217 | 0.025898 | 0.276607 |
| Monoglobus | Xanthinol | 0.554168 | 0.025914 | 0.276607 |
| Bryobacter | N-(1-Deoxy-1-fructosyl)threonine | 0.553713 | 0.026062 | 0.277509 |
| Aquicella | (R)-3-Amino-2-fluoropropyl phosphenite | 0.553674 | 0.026075 | 0.277509 |
| TRA3-20 | PC(20:5(5Z,8Z,11Z,14Z,17Z)/PGJ2) | -0.55363 | 0.026089 | 0.277509 |
| Jatrophihabitans | 3-Hydroxyoctadecanoylcarnitine | 0.553559 | 0.026112 | 0.277509 |
| Gemella | Xanthosine | 0.553475 | 0.02614 | 0.277509 |
| Intestinimonas | Napelline | 0.553455 | 0.026146 | 0.277509 |
| Treponema | 14-Methylpentadecanoylcarnitine | 0.553396 | 0.026166 | 0.277509 |
| Alcaligenes | JP83 | -0.55338 | 0.02617 | 0.277509 |
| Acetatifactor | Erucic acid | 0.553364 | 0.026176 | 0.277509 |
| TRA3-20 | All trans decaprenyl diphosphate | -0.55294 | 0.026314 | 0.278757 |
| Bryobacter | (R)-Propyl 2-amino-3-mercaptopropanoate | 0.552672 | 0.026403 | 0.279349 |
| Intestinimonas | N-Lactoylleucine | 0.552654 | 0.026409 | 0.279349 |
| Coprobacillus | Tephcalostan | 0.552591 | 0.02643 | 0.279356 |
| TRA3-20 | 5-[1-Carboxy-2-(trimethylazaniumyl)ethoxy]-5-oxopentanoate | 0.552464 | 0.026472 | 0.27943 |
| bacteriap25 | N-(2-Hydroxyethyl)-2-(1-isoquinolinylmethylene)hydrazinecarbothioamide | 0.552447 | 0.026477 | 0.27943 |
| Christensenellaceae_R-7_group | Tercatain | 0.552381 | 0.026499 | 0.27943 |
| Clostridioides | N-(1-Deoxy-1-fructosyl)valine | 0.552314 | 0.026521 | 0.27943 |
| Solobacterium | Docosatrienoic acid | 0.552264 | 0.026537 | 0.27943 |
| Monoglobus | PE(18:0/19:1(9Z)) | -0.5521 | 0.026593 | 0.27943 |
| Enterorhabdus | Phytal | -0.55206 | 0.026604 | 0.27943 |
| Alcaligenes | Tyrosyl-Alanine | 0.552032 | 0.026614 | 0.27943 |
| Lachnospiraceae_UCG-006 | 2-Methylthioadenosine | 0.552029 | 0.026615 | 0.27943 |
| Enterococcus | Tyrosyl-Alanine | 0.551868 | 0.026669 | 0.279688 |
| Alcaligenes | PC(20:4(5Z,8Z,11Z,14Z)-OH(16R)/2:0) | 0.551835 | 0.02668 | 0.279688 |
| IMCC26256 | 11,14,17-eicosatrienoic acid | 0.551718 | 0.026719 | 0.27989 |
| Achromobacter | JP83 | -0.55155 | 0.026773 | 0.280253 |
| Rikenellaceae_RC9_gut_group | Palmitoylcarnitine | 0.551459 | 0.026805 | 0.280375 |
| Lachnospiraceae_UCG-006 | Glutamylleucylarginine | 0.551168 | 0.026902 | 0.281006 |
| Enterorhabdus | 4E,14Z-Sphingadiene | -0.55116 | 0.026905 | 0.281006 |
| Treponema | Glycyl-D-proline | 0.550884 | 0.026997 | 0.281553 |
| Acidothermus | Aspartyl-Isoleucine | 0.550832 | 0.027014 | 0.281553 |
| Anaerotruncus | Selinexor | 0.550822 | 0.027017 | 0.281553 |
| Faecalibaculum | Terbufos | 0.550495 | 0.027127 | 0.28249 |
| Anaerostipes | Diethylamino 2,5-dihydroxybenzenesulfonate | 0.55037 | 0.027169 | 0.282716 |
| Muribaculum | 3-(3-(Pyridin-3-yl)-1,2,4-oxadiazol-5-yl)benzonitrile | 0.54997 | 0.027304 | 0.28389 |
| Tuzzerella | Valylserine | 0.549821 | 0.027354 | 0.28389 |
| Bryobacter | N-Phenyl-p-phenylenediamine | -0.54978 | 0.027367 | 0.28389 |
| Anaerostipes | Nipradilol | 0.549748 | 0.027379 | 0.28389 |
| Muribaculum | Tyrosyl-Aspartate | 0.54968 | 0.027402 | 0.28389 |
| Enterococcus | Valylhistidine | 0.549664 | 0.027408 | 0.28389 |
| TRA3-20 | Napelline | 0.549618 | 0.027423 | 0.28389 |
| Treponema | N-(2-Hydroxyethyl)-2-(1-isoquinolinylmethylene)hydrazinecarbothioamide | 0.549351 | 0.027514 | 0.284205 |
| Lachnospiraceae_UCG-006 | Streptidine | 0.549351 | 0.027514 | 0.284205 |
| MND1 | 5'-Methylthioadenosine | 0.54935 | 0.027514 | 0.284205 |
| Tuzzerella | Dirithromycin | -0.54922 | 0.027559 | 0.284376 |
| Tuzzerella | 4Z,7-octadienoic acid | -0.54918 | 0.027571 | 0.284376 |
| Muribaculum | (S)-a-Amino-2,5-dihydro-5-oxo-4-isoxazolepropanoic acid N2-glucoside | 0.548957 | 0.027648 | 0.284659 |
| bacteriap25 | Glycyl-D-proline | 0.548889 | 0.027671 | 0.284659 |
| Enterococcus | PE(22:5(4Z,7Z,10Z,13Z,16Z)/PGD2) | -0.54886 | 0.027682 | 0.284659 |
| Acidothermus | Beta-Citryl-L-glutamic acid | 0.548838 | 0.027688 | 0.284659 |
| Aquicella | 2,2,3,3,4,4,5,5,6,6,7,7,8,8,9,9-Hexadecafluorononanoic acid | 0.548743 | 0.027721 | 0.284659 |
| Aquicella | PC(20:4(5Z,8Z,11Z,14Z)-OH(16R)/2:0) | 0.548712 | 0.027732 | 0.284659 |
| Granulicella | Aspartyl-Alanine | 0.548682 | 0.027742 | 0.284659 |
| Monoglobus | N-(1-Deoxy-1-fructosyl)leucine | 0.548581 | 0.027776 | 0.284659 |
| Tuzzerella | PC(16:1(9Z)/17:1(9Z)) | -0.54856 | 0.027785 | 0.284659 |
| IMCC26256 | Pentadeca-3,5,7-trienedioylcarnitine | 0.548439 | 0.027825 | 0.284659 |
| Rikenellaceae_RC9_gut_group | (2S)-3-[3-[(4-Carbamimidoylbenzoyl)amino]propanoylamino]-2-[(4-ethylphenyl)sulfonylamino]propanoic acid | 0.548394 | 0.02784 | 0.284659 |
| Alcaligenes | N-alpha-Acetyl-L-lysine | 0.548392 | 0.027841 | 0.284659 |
| MND1 | Neosaxitoxin | 0.548163 | 0.02792 | 0.285254 |
| Monoglobus | BEFLOXATONE | 0.547931 | 0.028 | 0.285846 |
| TM7 | N-Acetylneuraminic acid | 0.547839 | 0.028031 | 0.285846 |
| Pseudolabrys | PE(22:4(7Z,10Z,13Z,16Z)/19:0) | -0.54775 | 0.028062 | 0.285846 |
| Clostridioides | PE-NMe2(18:3(9Z,12Z,15Z)/18:1(11Z)) | 0.547712 | 0.028075 | 0.285846 |
| Bryobacter | Napelline | 0.5477 | 0.028079 | 0.285846 |
| Monoglobus | Neosaxitoxin | 0.547484 | 0.028154 | 0.286343 |
| Aquicella | Dehydroascorbide(1-) | 0.547396 | 0.028184 | 0.286343 |
| Anaerotruncus | Hexadecadienylcarnitine | 0.547363 | 0.028196 | 0.286343 |
| Rikenellaceae_RC9_gut_group | Maraviroc | 0.547324 | 0.028209 | 0.286343 |
| Tuzzerella | Alpha-Lactose | 0.547228 | 0.028242 | 0.286472 |
| Faecalibaculum | m7G(5')pppAm | 0.547086 | 0.028292 | 0.286766 |
| Tuzzerella | N-Acetyl-b-glucosaminylamine | 0.54695 | 0.028339 | 0.286897 |
| Enterorhabdus | 2-hydroxy-9Z,12Z-Octadecadienoic acid | -0.54689 | 0.028361 | 0.286897 |
| Alcaligenes | Flumazenil | 0.546873 | 0.028366 | 0.286897 |
| Solobacterium | Dipotassium phosphate | 0.546593 | 0.028463 | 0.287676 |
| Rikenellaceae_RC9_gut_group | Phenylalanyl-Glycine | 0.546393 | 0.028533 | 0.288175 |
| MND1 | PC(11:0/23:0) | 0.546258 | 0.02858 | 0.288445 |
| IMCC26256 | 20-HETE-d6 | 0.545912 | 0.028701 | 0.289401 |
| Acetatifactor | Hydroxy-2-naphthalenylmethylphosphonic acid tris-acetoxymethyl ester | 0.54587 | 0.028716 | 0.289401 |
| TRA3-20 | 9-Octadecenal | -0.54577 | 0.028751 | 0.289547 |
| Faecalibaculum | DG(18:0/20:4(8Z,11Z,14Z,17Z)/0:0) | 0.545522 | 0.028838 | 0.290189 |
| Tuzzerella | LysoPE(20:3(8Z,11Z,14Z)/0:0) | 0.545472 | 0.028856 | 0.290189 |
| Enterorhabdus | PC(20:4(5Z,8Z,11Z,14Z)-OH(16R)/2:0) | 0.545377 | 0.02889 | 0.290274 |
| Clostridioides | 4E,14Z-Sphingadiene | -0.54527 | 0.028927 | 0.290274 |
| Clostridioides | LysoSM(d18:0) | -0.54524 | 0.028938 | 0.290274 |
| Rikenellaceae_RC9_gut_group | Adomeglivant | 0.545196 | 0.028954 | 0.290274 |
| Tuzzerella | 1-O-(2R-methoxy-hexadecyl)-sn-glycerol | -0.54511 | 0.028985 | 0.290274 |
| Monoglobus | Dolichyl b-D-glucosyl phosphate | -0.5451 | 0.028988 | 0.290274 |
| Intestinimonas | N-Lactoylphenylalanine | 0.545017 | 0.029017 | 0.290347 |
| Lachnospiraceae_UCG-006 | DG(17:2(9Z,12Z)/22:5(7Z,10Z,13Z,16Z,19Z)/0:0)[iso2] | 0.544739 | 0.029115 | 0.290347 |
| Enterorhabdus | L-Lysinamide | -0.54466 | 0.029142 | 0.290347 |
| Pseudolabrys | Monooctyl phthalate | -0.54464 | 0.02915 | 0.290347 |
| Bryobacter | N-Undecanoylglycine | -0.54462 | 0.029159 | 0.290347 |
| Intestinimonas | Valylserine | 0.544609 | 0.029161 | 0.290347 |
| Achromobacter | Eicosadienoic acid | 0.544522 | 0.029192 | 0.290347 |
| Achromobacter | Serylproline | 0.544514 | 0.029195 | 0.290347 |
| TRA3-20 | 12-Hydroxydodecanoic acid | -0.54448 | 0.029207 | 0.290347 |
| Solobacterium | m7G(5')pppAm | 0.544461 | 0.029214 | 0.290347 |
| Faecalibaculum | Heptadecanoyl carnitine | 0.544389 | 0.02924 | 0.290347 |
| Bryobacter | a-L-Arabinofuranosyl-(1->3)-b-D-xylopyranosyl-(1->4)-D-xylose | 0.544362 | 0.029249 | 0.290347 |
| Achromobacter | DG(17:2(9Z,12Z)/22:5(7Z,10Z,13Z,16Z,19Z)/0:0)[iso2] | 0.544322 | 0.029264 | 0.290347 |
| Alcaligenes | PS(22:5(7Z,10Z,13Z,16Z,19Z)/22:6(4Z,7Z,10Z,13Z,16Z,19Z)) | -0.54376 | 0.029464 | 0.292037 |
| RF39 | PC(20:4(5Z,8Z,11Z,14Z)-OH(16R)/2:0) | 0.543729 | 0.029475 | 0.292037 |
| Alcaligenes | Benzyl gentiobioside | 0.543615 | 0.029516 | 0.292156 |
| Nitrospira | 3-Hydroxy-10'-apo-b,y-carotenal | 0.543579 | 0.029529 | 0.292156 |
| Enterococcus | Aspartyl-Alanine | 0.543353 | 0.02961 | 0.292753 |
| Alcaligenes | Prolyl-Asparagine | 0.543092 | 0.029704 | 0.293476 |
| Enterococcus | Ethynodiol | -0.54295 | 0.029755 | 0.293775 |
| TRA3-20 | Eleutherazine B; N,N'-((3,6-Dioxo-2,5-piperazinediyl)bis(trimethylene))bis(5-hydroxy-3-methyl-2-pentenamide) | 0.542844 | 0.029793 | 0.293943 |
| Tuzzerella | PI(18:1(9Z)/0:0) | 0.542743 | 0.02983 | 0.293943 |
| Christensenellaceae_R-7_group | 2-C-methyl-D-erythritol-4-phosphate | 0.542726 | 0.029836 | 0.293943 |
| Acetatifactor | Glycerol 3-phosphate | 0.542672 | 0.029856 | 0.293943 |
| Faecalibaculum | PI(18:0/20:4(8Z,11Z,14Z,17Z)) | 0.542592 | 0.029884 | 0.29402 |
| Aquicella | 20-HETE-d6 | 0.542394 | 0.029956 | 0.294522 |
| Achromobacter | PS(22:5(7Z,10Z,13Z,16Z,19Z)/22:6(4Z,7Z,10Z,13Z,16Z,19Z)) | -0.54216 | 0.030042 | 0.295076 |
| Acetatifactor | L-Glutamine | 0.542115 | 0.030058 | 0.295076 |
| Faecalibaculum | PE(19:0/20:2(11Z,14Z)) | -0.54207 | 0.030076 | 0.295076 |
| TM7 | Glucose-6-glutamate | 0.5419 | 0.030136 | 0.29529 |
| Bryobacter | 12-Hydroxydodecanoic acid | -0.54179 | 0.030175 | 0.29529 |
| Subgroup_13 | Ac-Ser-Asp-Lys-Pro-OH | 0.541765 | 0.030185 | 0.29529 |
| Clostridioides | Serylproline | 0.541743 | 0.030193 | 0.29529 |
| Bryobacter | Polypropylene glycol (m w 1,200-3,000) | -0.54163 | 0.030235 | 0.29529 |
| Aquicella | Glutamylleucylarginine | 0.541619 | 0.030238 | 0.29529 |
| Haliangium | Neosaxitoxin | 0.541603 | 0.030244 | 0.29529 |
| Enterorhabdus | Eleutherazine B; N,N'-((3,6-Dioxo-2,5-piperazinediyl)bis(trimethylene))bis(5-hydroxy-3-methyl-2-pentenamide) | 0.541503 | 0.030281 | 0.29544 |
| Clostridioides | 2-[(4-{2-[(4-Cyclohexylbutyl)(cyclohexylcarbamoyl)amino]ethyl}phenyl)sulfanyl]-2-methylpropanoic acid | -0.54137 | 0.03033 | 0.295554 |
| Bryobacter | Ricinoleic acid | -0.54136 | 0.030334 | 0.295554 |
| Rikenellaceae_RC9_gut_group | Beta-Citryl-L-glutamic acid | 0.541059 | 0.030443 | 0.296237 |
| Acidothermus | Adomeglivant | 0.541051 | 0.030446 | 0.296237 |
| Bryobacter | Erucic acid | 0.540938 | 0.030488 | 0.296351 |
| Aquicella | 11,14,17-eicosatrienoic acid | 0.540832 | 0.030527 | 0.296351 |
| bacteriap25 | Maltotriose | 0.540581 | 0.030619 | 0.296351 |
| Tuzzerella | Dipotassium phosphate | 0.540561 | 0.030626 | 0.296351 |
| Lachnospiraceae_UCG-006 | N-(1-Deoxy-1-fructosyl)threonine | 0.54054 | 0.030634 | 0.296351 |
| TM7 | 2-(2-Thienylmethylene)-1,6-dioxaspiro[4.4]non-3-ene | 0.540402 | 0.030685 | 0.296351 |
| Aquicella | Fiacitabine | 0.54032 | 0.030715 | 0.296351 |
| TM7 | 4'-O-Glucopyranosylsinapic acid | 0.540314 | 0.030718 | 0.296351 |
| Tuzzerella | 2-hydroxy-9Z,12Z-Octadecadienoic acid | -0.54028 | 0.03073 | 0.296351 |
| Alcaligenes | Eicosadienoic acid | 0.540256 | 0.030739 | 0.296351 |
| Faecalibaculum | PC(P-18:0/20:4(5Z,8Z,11Z,14Z)-OH(16R)) | 0.540246 | 0.030743 | 0.296351 |
| Pseudolabrys | S-cucujolide III | -0.54022 | 0.030752 | 0.296351 |
| MND1 | 4-Phenylbutanoylcarnitine | -0.54021 | 0.030755 | 0.296351 |
| Nitrospira | N,N'-Diethylthiourea | 0.54009 | 0.030801 | 0.296351 |
| Tuzzerella | LysoPE(20:4(5Z,8Z,11Z,14Z)/0:0) | -0.54004 | 0.030818 | 0.296351 |
| Monoglobus | N-lactoyl-Tyrosine | 0.540002 | 0.030833 | 0.296351 |
| Rikenellaceae_RC9_gut_group | Ciadox | 0.539987 | 0.030839 | 0.296351 |
| TRA3-20 | N-Undecanoylglycine | -0.53988 | 0.03088 | 0.296351 |
| Enterorhabdus | 9-Octadecenal | -0.53987 | 0.030881 | 0.296351 |
| TRA3-20 | 5,5-Diphenyl-2-thiohydantoin | 0.539834 | 0.030895 | 0.296351 |
| Anaerotruncus | m7G(5')pppAm | 0.539787 | 0.030913 | 0.296351 |
| Coprobacillus | PE-NMe2(18:3(9Z,12Z,15Z)/18:1(11Z)) | 0.539765 | 0.030921 | 0.296351 |
| Subgroup_2 | Medica 16 | -0.53964 | 0.030967 | 0.296472 |
| Rikenellaceae_RC9_gut_group | Aspartyl-Alanine | 0.539616 | 0.030976 | 0.296472 |
| Tuzzerella | Xanthosine | 0.539561 | 0.030997 | 0.296472 |
| Bryobacter | N-(1-Deoxy-1-fructosyl)valine | 0.539486 | 0.031025 | 0.296536 |
| Anaerotruncus | 5beta-Cholane-3alpha,24-diol | 0.539312 | 0.03109 | 0.296954 |
| Anaerotruncus | 5-[1-Carboxy-2-(trimethylazaniumyl)ethoxy]-5-oxopentanoate | 0.539233 | 0.031119 | 0.297035 |
| Anaerotruncus | 1-(10H-Phenothiazin-2-yl)ethanone | 0.539163 | 0.031145 | 0.297084 |
| Acidothermus | 3-hydroxyhexadecanoyl carnitine | 0.538931 | 0.031232 | 0.297511 |
| Enterorhabdus | N-(N-(3-Amino-3-carboxypropyl)-3-amino-3-carboxypropyl)azetidine-2-carboxylic acid | 0.538878 | 0.031252 | 0.297511 |
| TRA3-20 | 1-p-Menthen-3-one | 0.538814 | 0.031276 | 0.297511 |
| IMCC26256 | xi-2,3-Octadiene-5,7-diyn-1-ol | 0.538772 | 0.031292 | 0.297511 |
| Christensenellaceae_R-7_group | Cysteinyl-Aspartate | 0.538761 | 0.031296 | 0.297511 |
| TRA3-20 | Prolylproline | 0.538378 | 0.031439 | 0.298674 |
| Tuzzerella | Prolylproline | 0.538193 | 0.031509 | 0.29913 |
| Alcaligenes | 2-phospho-4-(cytidine 5'-diphospho)-2-C-methyl-D-erythritol | 0.538097 | 0.031545 | 0.29913 |
| Anaerotruncus | Pseudouridine | 0.538011 | 0.031578 | 0.29913 |
| Tuzzerella | 12,13-DHOME | -0.53801 | 0.031578 | 0.29913 |
| Solobacterium | Ac-Ser-Asp-Lys-Pro-OH | 0.537938 | 0.031606 | 0.29913 |
| Clostridioides | Cannabidivarin | -0.53791 | 0.031615 | 0.29913 |
| bacteriap25 | 2',3'-Dideoxyuridine | 0.537835 | 0.031644 | 0.299207 |
| Faecalibaculum | NAD | 0.537495 | 0.031773 | 0.29939 |
| Pseudolabrys | 5'-Carboxy-gamma-chromanol | -0.53749 | 0.031776 | 0.29939 |
| Enterorhabdus | 12-Hydroxydodecanoic acid | -0.53747 | 0.031784 | 0.29939 |
| TRA3-20 | Diethylamino 2,5-dihydroxybenzenesulfonate | 0.537409 | 0.031806 | 0.29939 |
| TRA3-20 | (R)-Propyl 2-amino-3-mercaptopropanoate | 0.537406 | 0.031807 | 0.29939 |
| RF39 | L-Arginine | 0.53737 | 0.03182 | 0.29939 |
| Acetatifactor | Amobarbital | 0.537352 | 0.031827 | 0.29939 |
| Treponema | Diethylamino 2,5-dihydroxybenzenesulfonate | 0.537335 | 0.031834 | 0.29939 |
| Treponema | (E)-2-(hexa-3,5-dien-1-yn-1-yl)-5-(prop-1-yn-1-yl)thiophene | 0.537242 | 0.031869 | 0.299519 |
| Gemella | O-phosphonato-L-homoserine(2-) | 0.537187 | 0.03189 | 0.299519 |
| Anaerotruncus | 20-HETE-d6 | 0.537029 | 0.03195 | 0.299716 |
| TRA3-20 | PE(19:0/20:2(11Z,14Z)) | -0.53702 | 0.031953 | 0.299716 |
| Pseudolabrys | 9-Octadecenal | -0.53695 | 0.031979 | 0.299756 |
| Anaerotruncus | JP83 | -0.53682 | 0.032029 | 0.29983 |
| Rikenellaceae_RC9_gut_group | 3-{[(1s)-2,2-Difluoro-1-Hydroxy-7-(Methylsulfonyl)-2,3-Dihydro-1h-Inden-4-Yl]oxy}-5-Fluorobenzonitrile | 0.53682 | 0.03203 | 0.29983 |
| TM7 | N-[(2R,3R,4S,6R)-4,6-Dihydroxy-6-methyl-2-[(1R,2R)-1,2,3-trihydroxypropyl]oxan-3-yl]acetamide | 0.536417 | 0.032184 | 0.300903 |
| Granulicella | 11,14,17-eicosatrienoic acid | 0.536355 | 0.032208 | 0.300903 |
| Pseudolabrys | Nipradilol | 0.536336 | 0.032215 | 0.300903 |
| Subgroup_2 | Neosaxitoxin | 0.536297 | 0.03223 | 0.300903 |
| Tuzzerella | 9-Hydroxy-4-methoxypsoralen 9-glucoside | 0.536172 | 0.032278 | 0.300909 |
| Anaerotruncus | Am-PE(16:0/18:0) | 0.536122 | 0.032297 | 0.300909 |
| Enterorhabdus | Glucose-6-glutamate | 0.53608 | 0.032313 | 0.300909 |
| TRA3-20 | Alacepril | -0.53607 | 0.032316 | 0.300909 |
| Enterorhabdus | 20-HETE-d6 | 0.535925 | 0.032373 | 0.301239 |
| Bryobacter | 8-Amino-7-oxononanoic acid | -0.53573 | 0.032447 | 0.30144 |
| Acidothermus | Palmitoylcarnitine | 0.535691 | 0.032463 | 0.30144 |
| Enterorhabdus | Oleic acid | -0.53553 | 0.032523 | 0.30144 |
| Acetatifactor | Docosatrienoic acid | 0.535502 | 0.032536 | 0.30144 |
| Tuzzerella | 3-hydroxyhexadecanoyl carnitine | 0.53543 | 0.032563 | 0.30144 |
| Lachnospiraceae_UCG-006 | Amobarbital | 0.535253 | 0.032632 | 0.30144 |
| Muribaculum | thiamphenicol | 0.535238 | 0.032638 | 0.30144 |
| Anaerotruncus | 16,17-Dihydro-16alpha,17-dihydroxygibberellin A4 17-glucoside | -0.53512 | 0.032682 | 0.30144 |
| Muribaculum | DG(LTE4/22:0/0:0) | 0.535047 | 0.032712 | 0.30144 |
| Alcaligenes | xi-2,3-Octadiene-5,7-diyn-1-ol | 0.535041 | 0.032714 | 0.30144 |
| bacteriap25 | Aspartyl-Isoleucine | 0.535039 | 0.032715 | 0.30144 |
| Jatrophihabitans | 3-(3-(Pyridin-3-yl)-1,2,4-oxadiazol-5-yl)benzonitrile | 0.53502 | 0.032722 | 0.30144 |
| TRA3-20 | (R)-4A-(Ethoxymethyl)-1-(4-fluorophenyl)-6-((4-(trifluoromethyl)phenyl)sulfonyl)-4,4a,5,6,7,8-hexahydro-1H-pyrazolo[3,4-g]isoquinoline | -0.53502 | 0.032722 | 0.30144 |
| Solobacterium | Xanthosine | 0.535017 | 0.032723 | 0.30144 |
| Clostridioides | [(2S,4R,5R,6R,14S,16R)-14-Hydroxy-7,11-dimethyl-6-(2-oxopyran-4-yl)-3-oxapentacyclo[8.8.0.02,4.02,7.011,16]octadecan-5-yl] acetate | -0.53499 | 0.032734 | 0.30144 |
| Enterococcus | N-Undecanoylglycine | -0.53498 | 0.032737 | 0.30144 |
| Anaerotruncus | 4-Bis(2-hydroxyethyl)amino-L-phenylalanine | 0.534811 | 0.032803 | 0.301854 |
| Christensenellaceae_R-7_group | 1-(10H-Phenothiazin-2-yl)ethanone | 0.534592 | 0.032888 | 0.302438 |
| Tuzzerella | Thidiazuron | 0.534418 | 0.032956 | 0.302686 |
| Granulicella | L-Arginine | 0.534411 | 0.032959 | 0.302686 |
| Tuzzerella | 2,5,7-trihydroxy-4'-methoxyisoflavanone | -0.53436 | 0.03298 | 0.302686 |
| Tuzzerella | Prolyl-Asparagine | 0.534177 | 0.03305 | 0.302943 |
| Gemella | Dipotassium phosphate | 0.534129 | 0.033069 | 0.302943 |
| Alcaligenes | [6-Hydroxy-8-methyl-3-[3,4,5-trihydroxy-6-(hydroxymethyl)oxan-2-yl]oxy-9,10-dioxatetracyclo[4.3.1.02,5.03,8]decan-4-yl]methyl benzoate | 0.534121 | 0.033072 | 0.302943 |
| Muribaculum | 11-Hydroxyhexadecanoylcarnitine | 0.533975 | 0.033129 | 0.302998 |
| Muribaculum | PE-NMe2(18:3(9Z,12Z,15Z)/18:1(11Z)) | 0.533942 | 0.033142 | 0.302998 |
| Bryobacter | 3-Ketosphingosine | -0.53386 | 0.033174 | 0.302998 |
| Anaerostipes | 1-[3,4-Dihydroxy-5-(hydroxymethyl)-2-oxolanyl]-1,2,4-triazole-3-carboxamide | 0.533802 | 0.033197 | 0.302998 |
| Tuzzerella | Micronomicin | -0.53374 | 0.03322 | 0.302998 |
| Enterococcus | 4E,14Z-Sphingadiene | -0.53366 | 0.033253 | 0.302998 |
| Intestinimonas | L-arginino-succinate | 0.533623 | 0.033267 | 0.302998 |
| Enterococcus | 3-Ketosphingosine | -0.53361 | 0.033271 | 0.302998 |
| Anaerotruncus | Notoginsenoside H | 0.533559 | 0.033292 | 0.302998 |
| Faecalibaculum | 12-Hydroxy-12-octadecanoylcarnitine | 0.533532 | 0.033303 | 0.302998 |
| Intestinimonas | N-lactoyl-Methionine | 0.533501 | 0.033315 | 0.302998 |
| Nitrospira | 3-(3-(Pyridin-3-yl)-1,2,4-oxadiazol-5-yl)benzonitrile | 0.533443 | 0.033338 | 0.303009 |
| Enterorhabdus | Pentadeca-3,5,7-trienedioylcarnitine | 0.533358 | 0.033371 | 0.303118 |
| Rikenellaceae_RC9_gut_group | Peruvianoside II | 0.533174 | 0.033444 | 0.303444 |
| Aquicella | Pentadeca-3,5,7-trienedioylcarnitine | 0.533117 | 0.033466 | 0.303444 |
| Enterorhabdus | Kuguacin E | -0.53308 | 0.033483 | 0.303444 |
| Acetatifactor | Am-PE(16:0/18:0) | 0.533049 | 0.033493 | 0.303444 |
| IMCC26256 | Fiacitabine | 0.532868 | 0.033564 | 0.303893 |
| Tuzzerella | Adenosine monophosphate | -0.53267 | 0.033642 | 0.304013 |
| Enterorhabdus | PPM-18 | 0.532661 | 0.033647 | 0.304013 |
| Intestinimonas | 4'-O-Glucopyranosylsinapic acid | 0.53266 | 0.033647 | 0.304013 |
| Nitrospira | Creatine | 0.532616 | 0.033664 | 0.304013 |
| Flavonifractor | Aspartyl-Gamma-glutamate | 0.53254 | 0.033694 | 0.304092 |
| Monoglobus | Eleutherazine B; N,N'-((3,6-Dioxo-2,5-piperazinediyl)bis(trimethylene))bis(5-hydroxy-3-methyl-2-pentenamide) | 0.532295 | 0.033792 | 0.304738 |
| Anaerotruncus | Niacinamide | 0.532251 | 0.033809 | 0.304738 |
| bacteriap25 | Cerulenin | 0.532157 | 0.033847 | 0.304879 |
| Enterococcus | D-erythro-L-galacto-Nonulose | 0.531989 | 0.033914 | 0.305011 |
| Faecalibaculum | Notoginsenoside H | 0.531927 | 0.033938 | 0.305011 |
| Aquicella | Flumazenil | 0.531916 | 0.033942 | 0.305011 |
| Achromobacter | D-Malic acid | 0.531902 | 0.033948 | 0.305011 |
| Acidothermus | Ciadox | 0.531348 | 0.034169 | 0.306278 |
| Bryobacter | PC(16:1(9Z)/17:1(9Z)) | -0.53135 | 0.03417 | 0.306278 |
| Monoglobus | N-[(2R,3R,4S,6R)-4,6-Dihydroxy-6-methyl-2-[(1R,2R)-1,2,3-trihydroxypropyl]oxan-3-yl]acetamide | 0.531317 | 0.034182 | 0.306278 |
| Subgroup_13 | 5beta-Cholane-3alpha,24-diol | 0.531276 | 0.034198 | 0.306278 |
| Tuzzerella | LysoPI(16:0/0:0) | 0.531257 | 0.034206 | 0.306278 |
| Alcaligenes | DG(17:2(9Z,12Z)/22:5(7Z,10Z,13Z,16Z,19Z)/0:0)[iso2] | 0.531223 | 0.034219 | 0.306278 |
| Clostridioides | PC(TXB2/20:2(11Z,14Z)) | -0.53117 | 0.034242 | 0.306287 |
| Faecalibaculum | PC(16:1(9Z)/17:1(9Z)) | -0.53095 | 0.034328 | 0.306685 |
| Tuzzerella | N-lactoyl-Tyrosine | 0.530947 | 0.03433 | 0.306685 |
| Enterococcus | Histidylisoleucine | 0.530646 | 0.034452 | 0.307525 |
| Enterococcus | scyllo-Inositol | 0.530606 | 0.034468 | 0.307525 |
| Alcaligenes | S-[(3R,3As,6S,6aS)-3-nitrooxy-2,3,3a,5,6,6a-hexahydrofuro[3,2-b]furan-6-yl] ethanethioate | 0.530511 | 0.034506 | 0.307557 |
| Bryobacter | 7-oxo-8-amino-nonanoic acid | -0.53035 | 0.034573 | 0.307557 |
| bacteriap25 | Tephcalostan C | 0.530328 | 0.03458 | 0.307557 |
| Anaerotruncus | Prolyl-Asparagine | 0.530308 | 0.034588 | 0.307557 |
| Tuzzerella | Fucoxanthinol 3-(4Z,7Z,10Z,13Z,16Z,19Z-docosahexaenoate) 3'-palmitoleate | 0.530226 | 0.034621 | 0.307557 |
| TRA3-20 | Bis(2-propoxyethyl) 2,6-dimethyl-4-(3-nitrophenyl)-3,4-dihydropyridine-3,5-dicarboxylate | -0.53015 | 0.034654 | 0.307557 |
| Acidothermus | Maraviroc | 0.530123 | 0.034663 | 0.307557 |
| Nitrospira | DG(LTE4/22:0/0:0) | 0.530026 | 0.034702 | 0.307557 |
| TRA3-20 | benfluorex | 0.529923 | 0.034744 | 0.307557 |
| Subgroup_2 | Xanthinol | 0.529897 | 0.034754 | 0.307557 |
| Bryobacter | Tyrosyl-Alanine | 0.52983 | 0.034782 | 0.307557 |
| Pseudolabrys | PI(20:3(8Z,11Z,14Z)/0:0) | 0.529797 | 0.034795 | 0.307557 |
| Aquicella | L-Glutamine | 0.529761 | 0.03481 | 0.307557 |
| Granulicella | Pentadeca-3,5,7-trienedioylcarnitine | 0.529745 | 0.034817 | 0.307557 |
| RF39 | D-Mannose | 0.52968 | 0.034843 | 0.307557 |
| Clostridioides | Glutaminylproline | 0.529661 | 0.034851 | 0.307557 |
| Acetatifactor | DG(18:0/20:4(8Z,11Z,14Z,17Z)/0:0) | 0.52966 | 0.034851 | 0.307557 |
| Anaerostipes | D-Mannose | 0.529626 | 0.034865 | 0.307557 |
| Aquicella | Glucose-6-glutamate | 0.529359 | 0.034974 | 0.308325 |
| Enterorhabdus | Thiazolidine-4-carboxylic acid | 0.529191 | 0.035042 | 0.30857 |
| Tuzzerella | Ribonolactone | 0.529183 | 0.035045 | 0.30857 |
| Subgroup_13 | Phenylalanylproline | 0.528937 | 0.035146 | 0.309173 |
| Clostridioides | Asparaginyl-Proline | 0.528869 | 0.035174 | 0.309173 |
| Anaerotruncus | 11,14,17-eicosatrienoic acid | 0.528823 | 0.035193 | 0.309173 |
| Subgroup_13 | S-Adenosylhomocysteine | 0.528801 | 0.035202 | 0.309173 |
| Acidothermus | Allitridin | 0.528712 | 0.035238 | 0.309299 |
| Clostridioides | N-[(2R,3R,4S,6R)-4,6-Dihydroxy-6-methyl-2-[(1R,2R)-1,2,3-trihydroxypropyl]oxan-3-yl]acetamide | 0.528414 | 0.03536 | 0.310073 |
| Tuzzerella | Glutaminylarginine | -0.52839 | 0.03537 | 0.310073 |
| Achromobacter | Glycerol 3-phosphate | 0.52827 | 0.03542 | 0.310144 |
| Treponema | Heptadecanoyl carnitine | 0.528177 | 0.035458 | 0.310144 |
| Flavonifractor | Erucic acid | 0.528153 | 0.035468 | 0.310144 |
| Jatrophihabitans | Octadec-6-enoylcarnitine | 0.528066 | 0.035504 | 0.310144 |
| Clostridioides | 4-Bis(2-hydroxyethyl)amino-L-phenylalanine | 0.527885 | 0.035579 | 0.310144 |
| Enterorhabdus | Napelline | 0.527881 | 0.03558 | 0.310144 |
| Intestinimonas | N-(1-Deoxy-1-fructosyl)valine | 0.527868 | 0.035585 | 0.310144 |
| TRA3-20 | Cortisol | -0.52777 | 0.035628 | 0.310144 |
| Intestinimonas | 1-(10H-Phenothiazin-2-yl)ethanone | 0.527624 | 0.035687 | 0.310144 |
| Rikenellaceae_RC9_gut_group | Allitridin | 0.527611 | 0.035692 | 0.310144 |
| Muribaculum | Phenylalanylaspartic acid | 0.527591 | 0.0357 | 0.310144 |
| IMCC26256 | L-Glutamine | 0.52756 | 0.035713 | 0.310144 |
| RF39 | Phytosphingosine | 0.527515 | 0.035732 | 0.310144 |
| Nitrospira | 3'-Ketolactose | 0.527497 | 0.035739 | 0.310144 |
| Acidothermus | Terbufos | 0.527491 | 0.035742 | 0.310144 |
| Tuzzerella | Tridecanoylcarnitine | 0.527444 | 0.035761 | 0.310144 |
| Enterorhabdus | 25-Hydroxyvitamin D3-26,23-lactol | -0.52744 | 0.035763 | 0.310144 |
| Enterorhabdus | Berkeleylactone L | -0.5274 | 0.035778 | 0.310144 |
| Acidothermus | Fiacitabine | 0.527358 | 0.035797 | 0.310144 |
| Granulicella | 20-HETE-d6 | 0.527034 | 0.035932 | 0.311119 |
| Achromobacter | N-alpha-Acetyl-L-lysine | 0.526918 | 0.03598 | 0.311279 |
| Flavonifractor | Fiacitabine | 0.526871 | 0.036 | 0.311279 |
| Bryobacter | Tephcalostan C | 0.52683 | 0.036016 | 0.311279 |
| Bryobacter | 1-O-Galloylglycerol | 0.526722 | 0.036062 | 0.311439 |
| Enterorhabdus | 11,14,17-eicosatrienoic acid | 0.52668 | 0.036079 | 0.311439 |
| Nitrospira | (S)-a-Amino-2,5-dihydro-5-oxo-4-isoxazolepropanoic acid N2-glucoside | 0.526465 | 0.036169 | 0.312024 |
| Achromobacter | PC(20:4(5Z,8Z,11Z,14Z)-OH(16R)/2:0) | 0.526358 | 0.036214 | 0.312217 |
| Acetatifactor | Dihydrozeatin O-beta-D-Glucoside | 0.526206 | 0.036277 | 0.312574 |
| Tuzzerella | Flumazenil | 0.526136 | 0.036307 | 0.312637 |
| [Eubacterium]_oxidoreducens_group | 5beta-Cholane-3alpha,24-diol | 0.525982 | 0.036371 | 0.312993 |
| Clostridioides | Docosatrienoic acid | 0.525924 | 0.036396 | 0.312993 |
| Subgroup_2 | BEFLOXATONE | 0.525879 | 0.036415 | 0.312993 |
| Treponema | Medicagol | 0.52578 | 0.036456 | 0.313157 |
| IMCC26256 | alpha-D-Galactopyranuronosyl-(1->4)-alpha-D-galactopyranuronosyl-(1->4)-D-galacturonic acid | 0.525624 | 0.036522 | 0.313231 |
| TRA3-20 | 5'-Carboxy-gamma-chromanol | -0.52562 | 0.036524 | 0.313231 |
| [Eubacterium]_oxidoreducens_group | Fiacitabine | 0.525602 | 0.036531 | 0.313231 |
| Enterococcus | Alacepril | -0.52538 | 0.036627 | 0.313714 |
| Clostridia_vadinBB60_group | Tephcalostan | 0.525362 | 0.036632 | 0.313714 |
| Enterococcus | PC(16:1(9Z)/17:1(9Z)) | -0.52524 | 0.036682 | 0.313949 |
| Coprobacillus | (S)-a-Amino-2,5-dihydro-5-oxo-4-isoxazolepropanoic acid N2-glucoside | 0.524976 | 0.036796 | 0.314039 |
| Tuzzerella | PC(16:0/16:0) | 0.524959 | 0.036803 | 0.314039 |
| Muribaculum | trans-Hexadec-2-enoyl carnitine | 0.524938 | 0.036812 | 0.314039 |
| Granulicella | Xanthosine | 0.524928 | 0.036816 | 0.314039 |
| Alcaligenes | 4-Methylcatechol 1-glucuronide | 0.524902 | 0.036827 | 0.314039 |
| Granulicella | L-Glutamine | 0.524901 | 0.036827 | 0.314039 |
| Subgroup_13 | Beta-Citryl-L-glutamic acid | 0.524851 | 0.036849 | 0.314039 |
| Christensenellaceae_R-7_group | Xanthinol | 0.524449 | 0.037019 | 0.315304 |
| Bryobacter | PIP(20:0/18:1(12Z)-2OH(9,10)) | -0.52429 | 0.037086 | 0.315679 |
| Tuzzerella | Aminovaleric acid betaine | 0.524223 | 0.037116 | 0.315745 |
| Acidothermus | Peruvianoside II | 0.524054 | 0.037188 | 0.316119 |
| Acetatifactor | PI(18:0/20:4(8Z,11Z,14Z,17Z)) | 0.524014 | 0.037205 | 0.316119 |
| Rikenellaceae_RC9_gut_group | Terbufos | 0.523871 | 0.037266 | 0.316161 |
| Pseudolabrys | PE(18:0/19:1(9Z)) | -0.52387 | 0.037267 | 0.316161 |
| Muribaculum | PC(11:0/23:0) | 0.523691 | 0.037343 | 0.316161 |
| Bryobacter | 5beta-Cholane-3alpha,24-diol | 0.523657 | 0.037358 | 0.316161 |
| Tuzzerella | L-arginino-succinate | 0.52365 | 0.037361 | 0.316161 |
| Acetatifactor | Eicosadienoic acid | 0.523603 | 0.037381 | 0.316161 |
| Enterorhabdus | Hexadecenal | -0.52355 | 0.037404 | 0.316161 |
| Tuzzerella | (2Z,4E,6Z)-Decatrienoylcarnitine | -0.52353 | 0.037412 | 0.316161 |
| TRA3-20 | [(2S,4R,5R,6R,14S,16R)-14-Hydroxy-7,11-dimethyl-6-(2-oxopyran-4-yl)-3-oxapentacyclo[8.8.0.02,4.02,7.011,16]octadecan-5-yl] acetate | -0.52353 | 0.037412 | 0.316161 |
| Enterorhabdus | D-erythro-Sphingosine C-20 | -0.52324 | 0.037537 | 0.316953 |
| Anaerotruncus | 3-Ketosphingosine | -0.52314 | 0.037578 | 0.316953 |
| Muribaculum | Ribonolactone | 0.523116 | 0.03759 | 0.316953 |
| Treponema | Tephcalostan C | 0.523103 | 0.037596 | 0.316953 |
| Alcaligenes | Aspartyl-Alanine | 0.522927 | 0.037672 | 0.317403 |
| Coprobacillus | DG(LTE4/22:0/0:0) | 0.522698 | 0.037771 | 0.318048 |
| bacteriap25 | Aspartyl-Gamma-glutamate | 0.522489 | 0.037861 | 0.318617 |
| Subgroup_13 | alpha-D-Galactopyranuronosyl-(1->4)-alpha-D-galactopyranuronosyl-(1->4)-D-galacturonic acid | 0.522403 | 0.037898 | 0.31874 |
| Aquicella | 2-phospho-4-(cytidine 5'-diphospho)-2-C-methyl-D-erythritol | 0.522303 | 0.037942 | 0.318916 |
| Tuzzerella | N-Lactoylphenylalanine | 0.522203 | 0.037985 | 0.319089 |
| TM7 | N-(1-Deoxy-1-fructosyl)tyrosine | 0.522026 | 0.038062 | 0.319427 |
| Anaerotruncus | Doxepin | 0.522006 | 0.038071 | 0.319427 |
| Clostridioides | 5beta-Cholane-3alpha,24-diol | 0.521876 | 0.038127 | 0.319631 |
| Achromobacter | Zidebactam | 0.521822 | 0.038151 | 0.319631 |
| TM7 | Diethylamino 2,5-dihydroxybenzenesulfonate | 0.521794 | 0.038163 | 0.319631 |
| Enterorhabdus | PE(O-20:0/0:0) | -0.52173 | 0.03819 | 0.319665 |
| Subgroup_2 | PE-NMe2(18:3(9Z,12Z,15Z)/18:1(11Z)) | 0.521622 | 0.038238 | 0.319875 |
| Pseudolabrys | Erucic acid | 0.52155 | 0.038269 | 0.319947 |
| Anaerotruncus | 7-Hydroxy-R-acenocoumarol | 0.521445 | 0.038315 | 0.320109 |
| bacteriap25 | 1-p-Menthen-3-one | 0.521386 | 0.038341 | 0.320109 |
| Tuzzerella | Berkeleylactone L | -0.52135 | 0.038357 | 0.320109 |
| Bryobacter | Ethynodiol | -0.52125 | 0.038399 | 0.32027 |
| TRA3-20 | L-Glutamine | 0.521123 | 0.038456 | 0.320558 |
| Solobacterium | DG(18:0/20:4(8Z,11Z,14Z,17Z)/0:0) | 0.520846 | 0.038578 | 0.321048 |
| Lachnospiraceae_UCG-006 | Xanthinol | 0.520831 | 0.038584 | 0.321048 |
| Lachnospiraceae_UCG-006 | JP83 | -0.52072 | 0.038633 | 0.321048 |
| Enterococcus | N,N'-Diethylthiourea | 0.52065 | 0.038664 | 0.321048 |
| Clostridia_vadinBB60_group | N-(1-Deoxy-1-fructosyl)alanine | 0.520588 | 0.038691 | 0.321048 |
| Enterorhabdus | Bacillamidin C | -0.52059 | 0.038692 | 0.321048 |
| MND1 | Tuberonic acid glucoside | -0.52058 | 0.038695 | 0.321048 |
| Jatrophihabitans | DG(LTE4/22:0/0:0) | 0.520568 | 0.0387 | 0.321048 |
| Rikenellaceae_RC9_gut_group | Phenylalanylaspartic acid | 0.520522 | 0.03872 | 0.321048 |
| Haliangium | 2-C-methyl-D-erythritol-4-phosphate | 0.520337 | 0.038802 | 0.321494 |
| Treponema | Tyrosyl-Alanine | 0.520297 | 0.038819 | 0.321494 |
| Monoglobus | N2-gamma-Glutamylglutamine | 0.520183 | 0.03887 | 0.32172 |
| Enterococcus | PC(20:4(5Z,8Z,11Z,14Z)-OH(16R)/2:0) | 0.520059 | 0.038925 | 0.321985 |
| Intestinimonas | PC(TXB2/20:2(11Z,14Z)) | -0.51988 | 0.039002 | 0.322156 |
| Coprobacillus | thiamphenicol | 0.519879 | 0.039004 | 0.322156 |
| TRA3-20 | LysoSM(d18:0) | -0.51986 | 0.039014 | 0.322156 |
| Clostridioides | PE(19:0/20:2(11Z,14Z)) | -0.5198 | 0.039037 | 0.322161 |
| bacteriap25 | 14-Methylpentadecanoylcarnitine | 0.519532 | 0.039158 | 0.322804 |
| Enterococcus | Creatine | 0.519525 | 0.039161 | 0.322804 |
| Tuzzerella | xi-2,3-Octadiene-5,7-diyn-1-ol | 0.519425 | 0.039206 | 0.322982 |
| Granulicella | Prolyl-Asparagine | 0.519213 | 0.0393 | 0.32352 |
| bacteriap25 | Tyrosyl-Aspartate | 0.519175 | 0.039317 | 0.32352 |
| Tuzzerella | 4-Phenylbutanoylcarnitine | -0.51906 | 0.03937 | 0.323767 |
| [Eubacterium]_oxidoreducens_group | Symmetric dimethylarginine | 0.518871 | 0.039452 | 0.324254 |
| IMCC26256 | L-Arginine | 0.518691 | 0.039533 | 0.324727 |
| Granulicella | 4-Methylcatechol 1-glucuronide | 0.518449 | 0.039641 | 0.325377 |
| Coprobacillus | Napelline | 0.518388 | 0.039669 | 0.325377 |
| Bryobacter | LysoPE(20:3(8Z,11Z,14Z)/0:0) | 0.518359 | 0.039681 | 0.325377 |
| IMCC26256 | Prolyl-Asparagine | 0.518226 | 0.039741 | 0.325505 |
| Granulicella | alpha-D-Galactopyranuronosyl-(1->4)-alpha-D-galactopyranuronosyl-(1->4)-D-galacturonic acid | 0.518222 | 0.039743 | 0.325505 |
| Monoglobus | PS(22:5(7Z,10Z,13Z,16Z,19Z)/22:6(4Z,7Z,10Z,13Z,16Z,19Z)) | -0.51805 | 0.039819 | 0.32588 |
| Monoglobus | 2-(2-Thienylmethylene)-1,6-dioxaspiro[4.4]non-3-ene | 0.517988 | 0.039848 | 0.32588 |
| Pseudolabrys | Oleic acid | -0.51797 | 0.039858 | 0.32588 |
| MND1 | AB-MECA | -0.51751 | 0.040066 | 0.327078 |
| bacteriap25 | 3-hydroxyhexadecanoyl carnitine | 0.517485 | 0.040075 | 0.327078 |
| Clostridioides | Docosadienoate (22:2n6) | 0.517463 | 0.040085 | 0.327078 |
| TRA3-20 | Genistein 7-O-glucoside-6''-malonate | 0.517396 | 0.040115 | 0.327078 |
| Bryobacter | N2-(3-Carboxy-2-hydroxy-1-oxopropyl)arginine | 0.51729 | 0.040163 | 0.327078 |
| Enterococcus | PI(18:0/20:4(8Z,11Z,14Z,17Z)) | 0.51726 | 0.040177 | 0.327078 |
| Aquicella | Dehydroascorbic acid | 0.517193 | 0.040207 | 0.327078 |
| Lachnospiraceae_UCG-006 | Flumazenil | 0.517166 | 0.040219 | 0.327078 |
| Tuzzerella | (2S)-3-[3-[(4-Carbamimidoylbenzoyl)amino]propanoylamino]-2-[(4-ethylphenyl)sulfonylamino]propanoic acid | 0.517144 | 0.040229 | 0.327078 |
| Achromobacter | 3-Hydroxy-10'-apo-b,y-carotenal | 0.517127 | 0.040237 | 0.327078 |
| Enterococcus | DG(18:0/20:4(8Z,11Z,14Z,17Z)/0:0) | 0.517014 | 0.040288 | 0.327272 |
| Lachnospiraceae_UCG-006 | Prolyl-Asparagine | 0.516962 | 0.040312 | 0.327272 |
| Gemella | m7G(5')pppAm | 0.516795 | 0.040388 | 0.327272 |
| Pseudolabrys | Dehydroascorbide(1-) | 0.516775 | 0.040397 | 0.327272 |
| Enterococcus | 9-Octadecenal | -0.51669 | 0.040436 | 0.327272 |
| Enterococcus | All trans decaprenyl diphosphate | -0.51668 | 0.040438 | 0.327272 |
| Rikenellaceae_RC9_gut_group | Creatine | 0.516667 | 0.040446 | 0.327272 |
| TRA3-20 | PIP(20:0/18:1(12Z)-2OH(9,10)) | -0.51664 | 0.040457 | 0.327272 |
| Anaerotruncus | 1-Ethoxymethyl-5-fluorouracil | 0.516614 | 0.04047 | 0.327272 |
| Tuzzerella | 4-Methylcatechol 1-glucuronide | 0.516536 | 0.040505 | 0.327279 |
| Jatrophihabitans | Ribonolactone | 0.51651 | 0.040518 | 0.327279 |
| Aquicella | alpha-D-Galactopyranuronosyl-(1->4)-alpha-D-galactopyranuronosyl-(1->4)-D-galacturonic acid | 0.516405 | 0.040565 | 0.327334 |
| Treponema | N-gamma-L-Glutamyl-D-alanine | 0.516393 | 0.040571 | 0.327334 |
| Enterococcus | Cortisol | -0.51627 | 0.040629 | 0.327579 |
| Nitrospira | Phenethylamine glucuronide | 0.516224 | 0.040648 | 0.327579 |
| Enterococcus | (卤)-(Z)-2-(5-Tetradecenyl)cyclobutanone | -0.51602 | 0.040741 | 0.327829 |
| Clostridia_vadinBB60_group | 3'-Ketolactose | 0.516005 | 0.040748 | 0.327829 |
| Intestinimonas | N-[(2R,3R,4S,6R)-4,6-Dihydroxy-6-methyl-2-[(1R,2R)-1,2,3-trihydroxypropyl]oxan-3-yl]acetamide | 0.515872 | 0.040809 | 0.327829 |
| Clostridioides | 4'-O-Glucopyranosylsinapic acid | 0.515854 | 0.040817 | 0.327829 |
| Enterorhabdus | Valylserine | 0.515837 | 0.040825 | 0.327829 |
| Anaerotruncus | 4E,14Z-Sphingadiene | -0.51576 | 0.040859 | 0.327829 |
| Faecalibaculum | PC(TXB2/20:2(11Z,14Z)) | -0.51571 | 0.040882 | 0.327829 |
| Monoglobus | 12-Hydroxydodecanoic acid | -0.51561 | 0.040928 | 0.327829 |
| Haliangium | 1-(10H-Phenothiazin-2-yl)ethanone | 0.515593 | 0.040937 | 0.327829 |
| Clostridioides | DG(17:2(9Z,12Z)/22:5(7Z,10Z,13Z,16Z,19Z)/0:0)[iso2] | 0.515589 | 0.040939 | 0.327829 |
| Faecalibaculum | 14-Methylpentadecanoylcarnitine | 0.515529 | 0.040966 | 0.327829 |
| Tuzzerella | O-phosphonato-L-homoserine(2-) | 0.515466 | 0.040995 | 0.327829 |
| IMCC26256 | DG(17:2(9Z,12Z)/22:5(7Z,10Z,13Z,16Z,19Z)/0:0)[iso2] | 0.515457 | 0.040999 | 0.327829 |
| Tuzzerella | 1-(O-alpha-D-glucopyranosyl)-(1,3R,25S,27R)-octacosanetetrol | -0.51539 | 0.041031 | 0.327829 |
| Acetatifactor | N-alpha-Acetyl-L-lysine | 0.515267 | 0.041087 | 0.327829 |
| Enterorhabdus | Cerulenin | 0.515162 | 0.041135 | 0.327829 |
| Acetatifactor | L-Homocystine | 0.51514 | 0.041145 | 0.327829 |
| TRA3-20 | Monooctyl phthalate | -0.51513 | 0.041151 | 0.327829 |
| Enterococcus | 12-Hydroxydodecanoic acid | -0.51512 | 0.041152 | 0.327829 |
| Enterococcus | Selinexor | 0.515116 | 0.041156 | 0.327829 |
| [Eubacterium]_oxidoreducens_group | Dipotassium phosphate | 0.515091 | 0.041168 | 0.327829 |
| Tuzzerella | Peperomin E | 0.514814 | 0.041296 | 0.32855 |
| Alcaligenes | PE(22:4(7Z,10Z,13Z,16Z)/19:0) | -0.51475 | 0.041323 | 0.32855 |
| bacteriap25 | Pro-Pro-Pro | 0.514744 | 0.041328 | 0.32855 |
| Tuzzerella | Glycerol 3-phosphate | 0.514677 | 0.041359 | 0.328611 |
| Granulicella | Dipotassium phosphate | 0.514595 | 0.041397 | 0.328727 |
| Lachnospiraceae_UCG-006 | Terizidone | 0.514286 | 0.04154 | 0.329248 |
| Anaerotruncus | D-Mannose | 0.514278 | 0.041544 | 0.329248 |
| TRA3-20 | 8-Amino-7-oxononanoic acid | -0.51427 | 0.041546 | 0.329248 |
| Subgroup_2 | N-[(2R,3R,4S,6R)-4,6-Dihydroxy-6-methyl-2-[(1R,2R)-1,2,3-trihydroxypropyl]oxan-3-yl]acetamide | 0.514226 | 0.041568 | 0.329248 |
| Alcaligenes | 5-[1-Carboxy-2-(trimethylazaniumyl)ethoxy]-5-oxopentanoate | 0.514163 | 0.041598 | 0.329248 |
| Subgroup_13 | Pentadecanoylcarnitine | 0.514044 | 0.041653 | 0.329248 |
| Haliangium | Cysteinyl-Aspartate | 0.514015 | 0.041667 | 0.329248 |
| Tuzzerella | (E)-2-(hexa-3,5-dien-1-yn-1-yl)-5-(prop-1-yn-1-yl)thiophene | 0.514003 | 0.041672 | 0.329248 |
| Flavonifractor | xi-2,3-Octadiene-5,7-diyn-1-ol | 0.514 | 0.041673 | 0.329248 |
| Enterorhabdus | 4-Bis(2-hydroxyethyl)amino-L-phenylalanine | 0.513892 | 0.041724 | 0.329307 |
| Jatrophihabitans | m7G(5')pppAm | 0.513883 | 0.041728 | 0.329307 |
| Anaerotruncus | Mycobactins | -0.5138 | 0.041768 | 0.329441 |
| Intestinimonas | N-alpha-Acetyl-L-lysine | 0.513536 | 0.04189 | 0.329501 |
| Coprobacillus | PC(11:0/23:0) | 0.513528 | 0.041894 | 0.329501 |
| Clostridia_vadinBB60_group | 3-{[(1s)-2,2-Difluoro-1-Hydroxy-7-(Methylsulfonyl)-2,3-Dihydro-1h-Inden-4-Yl]oxy}-5-Fluorobenzonitrile | 0.513519 | 0.041898 | 0.329501 |
| Monoglobus | PE(22:5(4Z,7Z,10Z,13Z,16Z)/PGD2) | -0.51351 | 0.041903 | 0.329501 |
| Treponema | 3'-Deoxythymidine | 0.513426 | 0.041941 | 0.329501 |
| Acetatifactor | 5-[1-Carboxy-2-(trimethylazaniumyl)ethoxy]-5-oxopentanoate | 0.513412 | 0.041948 | 0.329501 |
| Faecalibaculum | (2S)-3-[3-[(4-Carbamimidoylbenzoyl)amino]propanoylamino]-2-[(4-ethylphenyl)sulfonylamino]propanoic acid | 0.513362 | 0.041971 | 0.329501 |
| Achromobacter | Fiacitabine | 0.513267 | 0.042016 | 0.329501 |
| Acidothermus | LysoPC(P-16:0/0:0) | 0.513233 | 0.042031 | 0.329501 |
| Granulicella | xi-2,3-Octadiene-5,7-diyn-1-ol | 0.513222 | 0.042037 | 0.329501 |
| Solobacterium | PI(18:0/20:4(8Z,11Z,14Z,17Z)) | 0.513202 | 0.042046 | 0.329501 |
| IMCC26256 | Flumazenil | 0.51318 | 0.042056 | 0.329501 |
| Clostridioides | PE(O-20:0/0:0) | -0.51309 | 0.042097 | 0.329563 |
| Enterococcus | Dolichyl b-D-glucosyl phosphate | -0.51292 | 0.042178 | 0.329563 |
| Acetatifactor | Notoginsenoside H | 0.512902 | 0.042187 | 0.329563 |
| Treponema | Cerulenin | 0.512878 | 0.042198 | 0.329563 |
| MND1 | Xanthinol | 0.512859 | 0.042207 | 0.329563 |
| Solobacterium | PC(16:0/16:0) | 0.512832 | 0.04222 | 0.329563 |
| Clostridioides | Fexaramine | -0.51281 | 0.042228 | 0.329563 |
| Solobacterium | Aspartyl-Gamma-glutamate | 0.512702 | 0.042281 | 0.329753 |
| IMCC26256 | N2-(3-Carboxy-2-hydroxy-1-oxopropyl)arginine | 0.512662 | 0.042299 | 0.329753 |
| Tuzzerella | N-lactoyl-Methionine | 0.512426 | 0.042411 | 0.330389 |
| Subgroup_2 | 12,13-DHOME | -0.51239 | 0.042428 | 0.330389 |
| Enterorhabdus | (卤)-(Z)-2-(5-Tetradecenyl)cyclobutanone | -0.51231 | 0.042466 | 0.330389 |
| Bryobacter | Neryl 8-methyldecanoate | 0.51223 | 0.042503 | 0.330389 |
| Monoglobus | Pro-Pro-Pro | 0.512159 | 0.042537 | 0.330389 |
| MND1 | 2-[4-(3-Hydroxypropyl)-2-methoxyphenoxy]-1,3-propanediol 1-xyloside | -0.5121 | 0.042563 | 0.330389 |
| Acetatifactor | Withaferin A | -0.51207 | 0.042577 | 0.330389 |
| Achromobacter | All trans decaprenyl diphosphate | -0.51206 | 0.042584 | 0.330389 |
| Achromobacter | 2,2,3,3,4,4,5,5,6,6,7,7,8,8,9,9-Hexadecafluorononanoic acid | 0.512042 | 0.042592 | 0.330389 |
| Bryobacter | benfluorex | 0.511973 | 0.042625 | 0.330458 |
| Faecalibaculum | PC(20:5(5Z,8Z,11Z,14Z,17Z)/PGJ2) | -0.51167 | 0.042768 | 0.331388 |
| Clostridioides | 3-Ketosphingosine | -0.51157 | 0.042814 | 0.331558 |
| Lachnospiraceae_UCG-006 | 2-Deoxy-2,3-dehydro-n-acetyl-neuraminic acid | 0.51147 | 0.042863 | 0.33171 |
| Alcaligenes | Glycerol 3-phosphate | 0.511249 | 0.042969 | 0.33171 |
| Enterorhabdus | 2-phospho-4-(cytidine 5'-diphospho)-2-C-methyl-D-erythritol | 0.511227 | 0.042979 | 0.33171 |
| TRA3-20 | PC(20:4(5Z,8Z,11Z,14Z)-OH(16R)/2:0) | 0.511197 | 0.042993 | 0.33171 |
| Rikenellaceae_RC9_gut_group | N,N'-Diethylthiourea | 0.511194 | 0.042995 | 0.33171 |
| bacteriap25 | Cysteine-glutathione disulfide | 0.511174 | 0.043004 | 0.33171 |
| Flavonifractor | 11,14,17-eicosatrienoic acid | 0.511129 | 0.043026 | 0.33171 |
| Anaerotruncus | Glycyl-D-proline | 0.51112 | 0.04303 | 0.33171 |
| Lachnospiraceae_UCG-006 | Neosaxitoxin | 0.511087 | 0.043046 | 0.33171 |
| Solobacterium | Fiacitabine | 0.511039 | 0.043069 | 0.33171 |
| Tuzzerella | Zidebactam | 0.510756 | 0.043204 | 0.332568 |
| Anaerotruncus | PIP(20:0/18:1(12Z)-2OH(9,10)) | -0.51066 | 0.043248 | 0.332729 |
| Flavonifractor | Pentadeca-3,5,7-trienedioylcarnitine | 0.510552 | 0.043301 | 0.332955 |
| Enterorhabdus | 11-Hydroxyoctadecanoylcarnitine | 0.510333 | 0.043406 | 0.33358 |
| MND1 | AM2201 N-(4-hydroxypentyl) metabolite | -0.51023 | 0.043455 | 0.333638 |
| Bryobacter | Fiacitabine | 0.510217 | 0.043462 | 0.333638 |
| Clostridioides | 16-hydroxy-6-hexadecenoic acid | -0.51017 | 0.043485 | 0.333638 |
| bacteriap25 | N2-(3-Carboxy-2-hydroxy-1-oxopropyl)arginine | 0.510044 | 0.043545 | 0.333919 |
| Christensenellaceae_R-7_group | Glutamylleucylarginine | 0.50993 | 0.0436 | 0.334157 |
| Anaerotruncus | Methacholine | 0.509793 | 0.043666 | 0.334355 |
| Intestinimonas | 7-oxo-8-amino-nonanoic acid | -0.50978 | 0.043674 | 0.334355 |
| Enterorhabdus | 6,10,14-Trimethyl-5,9,13-pentadecatrien-2-one | -0.50971 | 0.043706 | 0.334421 |
| Enterococcus | Am-PE(16:0/18:0) | 0.509524 | 0.043796 | 0.334928 |
| TRA3-20 | PC(TXB2/20:2(11Z,14Z)) | -0.50945 | 0.043832 | 0.334928 |
| Acetatifactor | Lucuminic acid | 0.509425 | 0.043843 | 0.334928 |
| Muribaculum | Phenylalanyl-Glycine | 0.50937 | 0.043871 | 0.334953 |
| Bryobacter | PE(22:4(7Z,10Z,13Z,16Z)/19:0) | -0.50924 | 0.043934 | 0.335258 |
| Granulicella | DG(17:2(9Z,12Z)/22:5(7Z,10Z,13Z,16Z,19Z)/0:0)[iso2] | 0.509117 | 0.043993 | 0.335523 |
| MND1 | Cichorioside K | -0.50898 | 0.04406 | 0.335851 |
| Pseudolabrys | 2-hydroxy-9Z,12Z-Octadecadienoic acid | -0.50872 | 0.044184 | 0.336615 |
| Pseudolabrys | Diethylamino 2,5-dihydroxybenzenesulfonate | 0.508613 | 0.044238 | 0.336842 |
| Rikenellaceae_RC9_gut_group | N-(1-Deoxy-1-fructosyl)valine | 0.508486 | 0.044299 | 0.337129 |
| Alcaligenes | Skepinone-L | 0.50831 | 0.044385 | 0.337431 |
| Achromobacter | 5-[1-Carboxy-2-(trimethylazaniumyl)ethoxy]-5-oxopentanoate | 0.508307 | 0.044387 | 0.337431 |
| Achromobacter | 2-phospho-4-(cytidine 5'-diphospho)-2-C-methyl-D-erythritol | 0.508145 | 0.044466 | 0.337697 |
| Clostridioides | Histidylisoleucine | 0.508137 | 0.04447 | 0.337697 |
| Tuzzerella | (R)-4A-(Ethoxymethyl)-1-(4-fluorophenyl)-6-((4-(trifluoromethyl)phenyl)sulfonyl)-4,4a,5,6,7,8-hexahydro-1H-pyrazolo[3,4-g]isoquinoline | -0.5079 | 0.044585 | 0.33839 |
| bacteriap25 | 3'-Deoxythymidine | 0.507827 | 0.044621 | 0.338483 |
| TRA3-20 | 7-oxo-8-amino-nonanoic acid | -0.5077 | 0.044686 | 0.33879 |
| Intestinimonas | Prolyl-Asparagine | 0.507472 | 0.044795 | 0.339438 |
| Achromobacter | Xanthosine | 0.507316 | 0.044872 | 0.339663 |
| Aquicella | Mycobactins | -0.50724 | 0.044911 | 0.339663 |
| bacteriap25 | Thidiazuron | 0.507231 | 0.044914 | 0.339663 |
| Achromobacter | PE(22:4(7Z,10Z,13Z,16Z)/19:0) | -0.50721 | 0.044922 | 0.339663 |
| Tuzzerella | N-Lactoylleucine | 0.507166 | 0.044946 | 0.339663 |
| TM7 | Fructose-1,6-diphosphate | 0.507066 | 0.044995 | 0.339671 |
| Bryobacter | Selinexor | 0.507057 | 0.045 | 0.339671 |
| Clostridioides | Nopalinic acid | 0.507018 | 0.045019 | 0.339671 |
| Anaerotruncus | Aminovaleric acid betaine | 0.506916 | 0.04507 | 0.339868 |
| Enterorhabdus | Octadec-6-enoylcarnitine | 0.506798 | 0.045127 | 0.339986 |
| Alcaligenes | N-(1-Deoxy-1-fructosyl)threonine | 0.506786 | 0.045133 | 0.339986 |
| Faecalibaculum | Glycerol 3-phosphate | 0.506574 | 0.045238 | 0.340593 |
| Solobacterium | Notoginsenoside H | 0.506508 | 0.045271 | 0.340657 |
| Flavonifractor | 20-HETE-d6 | 0.506412 | 0.045319 | 0.340835 |
| Clostridioides | Bacillamidin C | -0.50629 | 0.045379 | 0.341104 |
| Gemella | Ac-Ser-Asp-Lys-Pro-OH | 0.506157 | 0.045445 | 0.341421 |
| Pseudolabrys | Alacepril | -0.50609 | 0.045478 | 0.341484 |
| Enterorhabdus | (E,E)-3,7,11-Trimethyl-2,6,10-dodecatrienyl octanoate | 0.506021 | 0.045513 | 0.341511 |
| Acetatifactor | Selinexor | 0.505986 | 0.04553 | 0.341511 |
| MND1 | Tephcalostan | 0.505889 | 0.045578 | 0.341534 |
| bacteriap25 | N-lactoyl-Tyrosine | 0.505883 | 0.045581 | 0.341534 |
| Tuzzerella | 3-Amino-3-methylbutanoic acid | 0.505768 | 0.045638 | 0.341779 |
| Aquicella | N2-(3-Carboxy-2-hydroxy-1-oxopropyl)arginine | 0.505716 | 0.045664 | 0.341791 |
| Bryobacter | 25-Hydroxyvitamin D3-26,23-lactol | -0.50567 | 0.04569 | 0.341798 |
| Subgroup_2 | 2-(2-Thienylmethylene)-1,6-dioxaspiro[4.4]non-3-ene | 0.505577 | 0.045734 | 0.341836 |
| Alcaligenes | Genistein 7-O-glucoside-6''-malonate | 0.505553 | 0.045746 | 0.341836 |
| Anaerostipes | Dehydroascorbide(1-) | 0.505509 | 0.045768 | 0.341836 |
| Enterococcus | 6,10,14-Trimethyl-5,9,13-pentadecatrien-2-one | -0.50536 | 0.045841 | 0.341909 |
| Enterorhabdus | 3-O-acetylecdysone 2-phosphate | -0.50534 | 0.045853 | 0.341909 |
| TM7 | N-(1-Deoxy-1-fructosyl)leucine | 0.505313 | 0.045866 | 0.341909 |
| Tuzzerella | Valylhistidine | 0.505295 | 0.045874 | 0.341909 |
| Tuzzerella | N-(1-Deoxy-1-fructosyl)alanine | 0.505225 | 0.04591 | 0.341991 |
| Enterococcus | Glycyl-D-proline | 0.504976 | 0.046034 | 0.34251 |
| Subgroup_2 | Peperomin E | 0.504967 | 0.046039 | 0.34251 |
| Achromobacter | Skepinone-L | 0.50494 | 0.046052 | 0.34251 |
| TRA3-20 | 4-Methylcatechol 1-glucuronide | 0.504813 | 0.046116 | 0.342805 |
| Enterococcus | Hexadecenal | -0.50433 | 0.046358 | 0.34406 |
| Flavonifractor | Eicosadienoic acid | 0.504325 | 0.046361 | 0.34406 |
| Jatrophihabitans | (S)-a-Amino-2,5-dihydro-5-oxo-4-isoxazolepropanoic acid N2-glucoside | 0.504299 | 0.046375 | 0.34406 |
| Aquicella | D-erythro-L-galacto-Nonulose | 0.504239 | 0.046405 | 0.34406 |
| Flavonifractor | DG(18:0/20:4(8Z,11Z,14Z,17Z)/0:0) | 0.504234 | 0.046407 | 0.34406 |
| Tuzzerella | True blue | 0.504086 | 0.046482 | 0.344315 |
| Monoglobus | PE-NMe2(18:3(9Z,12Z,15Z)/18:1(11Z)) | 0.504061 | 0.046495 | 0.344315 |
| Nitrospira | Notoginsenoside H | 0.504021 | 0.046515 | 0.344315 |
| Rikenellaceae_RC9_gut_group | N-Acetyl-b-glucosaminylamine | 0.503965 | 0.046543 | 0.344343 |
| Enterorhabdus | 3-Ketosphingosine | -0.50389 | 0.046579 | 0.344426 |
| Anaerotruncus | 4-Chloro-2-nitrobenzylalcohol | -0.50363 | 0.046712 | 0.345048 |
| Intestinimonas | DG(17:2(9Z,12Z)/22:5(7Z,10Z,13Z,16Z,19Z)/0:0)[iso2] | 0.503629 | 0.046713 | 0.345048 |
| Granulicella | Serylproline | 0.503544 | 0.046756 | 0.345048 |
| Haliangium | Xanthinol | 0.503535 | 0.046761 | 0.345048 |
| Granulicella | N2-(3-Carboxy-2-hydroxy-1-oxopropyl)arginine | 0.503225 | 0.046919 | 0.346028 |
| Enterorhabdus | Terizidone | 0.503025 | 0.04702 | 0.346445 |
| Enterococcus | Dipotassium phosphate | 0.503017 | 0.047024 | 0.346445 |
| Bryobacter | PC(11:0/23:0) | 0.502941 | 0.047063 | 0.346549 |
| Aquicella | PGP(18:3(9Z,12Z,15Z)/18:1(12Z)-O(9S,10R)) | 0.502883 | 0.047093 | 0.346587 |
| Tuzzerella | Tephcalostan C | 0.502816 | 0.047127 | 0.346657 |
| Bryobacter | 4Z,7-octadienoic acid | -0.50264 | 0.047216 | 0.346903 |
| Enterorhabdus | Histidylisoleucine | 0.502627 | 0.047224 | 0.346903 |
| Acetatifactor | 3-{[(1s)-2,2-Difluoro-1-Hydroxy-7-(Methylsulfonyl)-2,3-Dihydro-1h-Inden-4-Yl]oxy}-5-Fluorobenzonitrile | 0.502606 | 0.047234 | 0.346903 |
| Haliangium | Tercatain | 0.502389 | 0.047345 | 0.347534 |
| Subgroup_13 | Aspartyl-Methionine | 0.502319 | 0.047381 | 0.347617 |
| Nitrospira | PI(18:0/20:4(8Z,11Z,14Z,17Z)) | 0.502267 | 0.047407 | 0.34763 |
| Nitrospira | Tephcalostan | 0.502204 | 0.04744 | 0.347689 |
| Achromobacter | 1-Hydroxy-2-naphthoic acid | 0.502149 | 0.047468 | 0.347715 |
| Coprobacillus | m7G(5')pppAm | 0.502054 | 0.047517 | 0.347891 |
| Jatrophihabitans | thiamphenicol | 0.501996 | 0.047547 | 0.347926 |
| Clostridioides | Sodium nitrate (NaNO3) | -0.50193 | 0.047578 | 0.347976 |
| Anaerotruncus | Nopalinic acid | 0.501812 | 0.047641 | 0.348256 |
| TRA3-20 | 4E,14Z-Sphingadiene | -0.5015 | 0.047804 | 0.348414 |
| Anaerostipes | Fructose-1,6-diphosphate | 0.501488 | 0.047808 | 0.348414 |
| Subgroup_13 | Hydroxy-2-naphthalenylmethylphosphonic acid tris-acetoxymethyl ester | 0.501461 | 0.047822 | 0.348414 |
| bacteriap25 | a-L-Arabinofuranosyl-(1->3)-b-D-xylopyranosyl-(1->4)-D-xylose | 0.501439 | 0.047833 | 0.348414 |
| Subgroup_13 | PC(16:1(9Z)/17:1(9Z)) | -0.50142 | 0.047843 | 0.348414 |
| Clostridioides | Prolyl-Asparagine | 0.501415 | 0.047846 | 0.348414 |
| Enterorhabdus | Phenylalanyl-Glycine | 0.501355 | 0.047877 | 0.348414 |
| Muribaculum | O-Linoleoylcarnitine | 0.501341 | 0.047884 | 0.348414 |
| Aquicella | N-Acetylneuraminic acid | 0.501338 | 0.047886 | 0.348414 |
| Anaerotruncus | N-(2-Hydroxyethyl)-2-(1-isoquinolinylmethylene)hydrazinecarbothioamide | 0.501042 | 0.048039 | 0.349262 |
| Subgroup_2 | PI(20:4(5Z,8Z,11Z,14Z)/18:0) | -0.50102 | 0.048052 | 0.349262 |
| Coprobacillus | Phenethylamine glucuronide | 0.500836 | 0.048146 | 0.349763 |
| Acidothermus | Am-PE(16:0/18:0) | 0.50052 | 0.04831 | 0.350611 |
| Jatrophihabitans | Phenethylamine glucuronide | 0.500496 | 0.048322 | 0.350611 |
| Clostridioides | 11,14,17-eicosatrienoic acid | 0.500396 | 0.048374 | 0.350611 |
| Acidothermus | PE(19:0/20:2(11Z,14Z)) | -0.50039 | 0.048378 | 0.350611 |
| Clostridioides | Pentadeca-3,5,7-trienedioylcarnitine | 0.500371 | 0.048387 | 0.350611 |
| bacteriap25 | N-Acetyl-L-methionine | 0.500119 | 0.048518 | 0.351379 |
| Intestinimonas | 8-Amino-7-oxononanoic acid | -0.50007 | 0.048545 | 0.351379 |
| Acetatifactor | xi-2,3-Octadiene-5,7-diyn-1-ol | 0.49999 | 0.048585 | 0.351379 |
| Clostridioides | 2-(2-Thienylmethylene)-1,6-dioxaspiro[4.4]non-3-ene | 0.499922 | 0.048621 | 0.351379 |
| bacteriap25 | 9-Hydroxy-4-methoxypsoralen 9-glucoside | 0.499859 | 0.048654 | 0.351379 |
| Subgroup_2 | 1H-Pyrrole-2,5-dione, 3-(1-methyl-1h-indol-3-yl)-4-(1-methyl-6-nitro-1h-indol-3-yl)- | 0.499852 | 0.048658 | 0.351379 |
| IMCC26256 | DG(18:0/20:4(8Z,11Z,14Z,17Z)/0:0) | 0.499813 | 0.048678 | 0.351379 |
| Acidothermus | Phenylalanylaspartic acid | 0.499785 | 0.048692 | 0.351379 |
| Enterococcus | Notoginsenoside H | 0.499708 | 0.048733 | 0.351492 |
| TRA3-20 | N-Phenyl-p-phenylenediamine | -0.49961 | 0.048782 | 0.351667 |
| TM7 | N-(1-Deoxy-1-fructosyl)valine | 0.499446 | 0.04887 | 0.351689 |
| Enterococcus | D-Malic acid | 0.49941 | 0.048889 | 0.351689 |
| Acetatifactor | Xanthosine | 0.49939 | 0.0489 | 0.351689 |
| Tuzzerella | Methacholine | 0.49934 | 0.048925 | 0.351689 |
| Alcaligenes | D-Malic acid | 0.499238 | 0.048979 | 0.351689 |
| RF39 | N6-Acetyl-L-lysine | 0.499171 | 0.049014 | 0.351689 |
| Gemella | DG(18:0/20:4(8Z,11Z,14Z,17Z)/0:0) | 0.49916 | 0.04902 | 0.351689 |
| Bryobacter | 1-Hydroxy-2-naphthoic acid | 0.499158 | 0.049021 | 0.351689 |
| Nitrospira | Napelline | 0.499096 | 0.049054 | 0.351689 |
| Acetatifactor | Benzyl gentiobioside | 0.498954 | 0.049129 | 0.351689 |
| Treponema | a-L-Arabinofuranosyl-(1->3)-b-D-xylopyranosyl-(1->4)-D-xylose | 0.498944 | 0.049134 | 0.351689 |
| Bryobacter | Kaltostat | 0.498936 | 0.049138 | 0.351689 |
| Lachnospiraceae_UCG-006 | xi-2,3-Octadiene-5,7-diyn-1-ol | 0.49882 | 0.049199 | 0.351689 |
| Nitrospira | PC(11:0/23:0) | 0.498786 | 0.049217 | 0.351689 |
| Enterorhabdus | LysoSM(d18:0) | -0.49867 | 0.049277 | 0.351689 |
| TM7 | 1-O-(2R-hydroxy-hexadecyl)-sn-glycerol | -0.49865 | 0.049291 | 0.351689 |
| Enterorhabdus | O-phosphonato-L-homoserine(2-) | 0.498592 | 0.049319 | 0.351689 |
| Anaerotruncus | Pro-Pro-Pro | 0.498529 | 0.049352 | 0.351689 |
| Muribaculum | Tetradecanoylcarnitine | 0.498519 | 0.049358 | 0.351689 |
| Christensenellaceae_R-7_group | PC(18:1(17Z)/18:1(17Z)) | 0.498494 | 0.049371 | 0.351689 |
| Acetatifactor | 1-Ethoxymethyl-5-fluorouracil | 0.498491 | 0.049372 | 0.351689 |
| Enterococcus | 25-Hydroxyvitamin D3-26,23-lactol | -0.49844 | 0.049398 | 0.351689 |
| Bryobacter | Flumazenil | 0.498441 | 0.049399 | 0.351689 |
| bacteriap25 | Heptadecanoyl carnitine | 0.498367 | 0.049438 | 0.351689 |
| Faecalibaculum | Allitridin | 0.498341 | 0.049452 | 0.351689 |
| Intestinimonas | 2',3'-Dideoxyuridine | 0.498309 | 0.049469 | 0.351689 |
| Faecalibaculum | 3-Methyl-3-butenyl apiosyl-(1->6)-glucoside | 0.498301 | 0.049473 | 0.351689 |
| Treponema | 12-Hydroxy-12-octadecanoylcarnitine | 0.498242 | 0.049504 | 0.351689 |
| Muribaculum | Ethyl hexadecanoate | -0.49823 | 0.04951 | 0.351689 |
| Tuzzerella | 11-Hydroxyhexadecanoylcarnitine | 0.498081 | 0.04959 | 0.35178 |
| Intestinimonas | Pantothenic acid | 0.498075 | 0.049593 | 0.35178 |
| Alcaligenes | All trans decaprenyl diphosphate | -0.49807 | 0.049598 | 0.35178 |
| Enterorhabdus | PI(20:3(8Z,11Z,14Z)/0:0) | 0.497912 | 0.049679 | 0.352182 |
| Intestinimonas | PE(19:0/20:2(11Z,14Z)) | -0.49782 | 0.049729 | 0.352358 |
| Faecalibaculum | Benzyl gentiobioside | 0.497743 | 0.049769 | 0.35243 |
| MND1 | S-Adenosylhomocysteine | 0.497671 | 0.049807 | 0.35243 |
| Aquicella | 2-Methylthioadenosine | 0.49765 | 0.049819 | 0.35243 |
| Enterococcus | (R)-3-Amino-2-fluoropropyl phosphenite | 0.497611 | 0.049839 | 0.35243 |
| Tuzzerella | Aspartyl-Alanine | 0.497462 | 0.049918 | 0.352702 |
| Aquicella | L-Arginine | 0.497444 | 0.049928 | 0.352702 |
| [Eubacterium]_oxidoreducens_group | DG(18:0/20:4(8Z,11Z,14Z,17Z)/0:0) | 0.497367 | 0.049969 | 0.352815 |
| Rikenellaceae_RC9_gut_group | Amobarbital | 0.497271 | 0.05002 | 0.352999 |
| Treponema | Pro-Pro-Pro | 0.497208 | 0.050054 | 0.353059 |
| bacteriap25 | Resorufin | 0.49696 | 0.050187 | 0.353658 |
| Enterorhabdus | Ac-Ser-Asp-Lys-Pro-OH | 0.496909 | 0.050214 | 0.353658 |
| Monoglobus | Bis(2-propoxyethyl) 2,6-dimethyl-4-(3-nitrophenyl)-3,4-dihydropyridine-3,5-dicarboxylate | -0.49685 | 0.050245 | 0.353658 |
| Nitrospira | PE-NMe2(18:3(9Z,12Z,15Z)/18:1(11Z)) | 0.496848 | 0.050246 | 0.353658 |
| Tuzzerella | Heptadecanoyl carnitine | 0.496807 | 0.050268 | 0.353658 |
| Rikenellaceae_RC9_gut_group | 2,2,3,3,4,4,5,5,6,6,7,7,8,8,9,9-Hexadecafluorononanoic acid | 0.496743 | 0.050303 | 0.353658 |
| Faecalibaculum | alpha-D-Galactopyranuronosyl-(1->4)-alpha-D-galactopyranuronosyl-(1->4)-D-galacturonic acid | 0.496652 | 0.050351 | 0.353658 |
| Enterococcus | (R)-4A-(Ethoxymethyl)-1-(4-fluorophenyl)-6-((4-(trifluoromethyl)phenyl)sulfonyl)-4,4a,5,6,7,8-hexahydro-1H-pyrazolo[3,4-g]isoquinoline | -0.49663 | 0.050363 | 0.353658 |
| Clostridioides | AB-MECA | -0.49663 | 0.050365 | 0.353658 |
| Lachnospiraceae_UCG-006 | Genistein 7-O-glucoside-6''-malonate | 0.496285 | 0.050548 | 0.354772 |
| TRA3-20 | D-erythro-Sphingosine C-20 | -0.49612 | 0.050636 | 0.355058 |
| Faecalibaculum | 11-Hydroxyhexadecanoylcarnitine | 0.496079 | 0.050659 | 0.355058 |
| Tuzzerella | Symmetric dimethylarginine | 0.49594 | 0.050734 | 0.355058 |
| Subgroup_13 | Glycerol 3-phosphate | 0.49591 | 0.05075 | 0.355058 |
| Pseudolabrys | LysoPE(20:3(8Z,11Z,14Z)/0:0) | 0.495905 | 0.050753 | 0.355058 |
| Clostridia_vadinBB60_group | 3-(3-(Pyridin-3-yl)-1,2,4-oxadiazol-5-yl)benzonitrile | 0.495862 | 0.050776 | 0.355058 |
| Enterococcus | L-Glutamine | 0.49584 | 0.050788 | 0.355058 |
| Clostridioides | Phenylalanylproline | 0.495834 | 0.050791 | 0.355058 |
| bacteriap25 | Terizidone | 0.495707 | 0.05086 | 0.355363 |
| Tuzzerella | N-Lauroyl Glutamine | -0.49565 | 0.050892 | 0.355412 |
| MND1 | 3'-Ketolactose | 0.495582 | 0.050927 | 0.355479 |
| Flavonifractor | PI(18:0/20:4(8Z,11Z,14Z,17Z)) | 0.495488 | 0.050978 | 0.355659 |
| Subgroup_2 | L-Glutamine | 0.495302 | 0.051079 | 0.356096 |
| Monoglobus | Nopalinic acid | 0.495279 | 0.051091 | 0.356096 |
| Pseudolabrys | PC(20:5(5Z,8Z,11Z,14Z,17Z)/PGJ2) | -0.49514 | 0.051167 | 0.356203 |
| Pseudolabrys | Dolichyl b-D-glucosyl phosphate | -0.49508 | 0.051199 | 0.356203 |
| Enterococcus | 2-phospho-4-(cytidine 5'-diphospho)-2-C-methyl-D-erythritol | 0.495048 | 0.051216 | 0.356203 |
| Clostridioides | Eleutherazine B; N,N'-((3,6-Dioxo-2,5-piperazinediyl)bis(trimethylene))bis(5-hydroxy-3-methyl-2-pentenamide) | 0.495034 | 0.051224 | 0.356203 |
| [Eubacterium]_oxidoreducens_group | N-gamma-L-Glutamyl-D-alanine | 0.495017 | 0.051233 | 0.356203 |
| Nitrospira | DG(18:0/20:4(8Z,11Z,14Z,17Z)/0:0) | 0.494907 | 0.051293 | 0.356444 |
| Tuzzerella | PE(18:0/19:1(9Z)) | -0.49477 | 0.05137 | 0.356802 |
| Bryobacter | 2-C-methyl-D-erythritol-4-phosphate | 0.494675 | 0.051419 | 0.356968 |
| Lachnospiraceae_UCG-006 | 4-Methylcatechol 1-glucuronide | 0.494475 | 0.051528 | 0.357549 |
| Bryobacter | Benzyl gentiobioside | 0.494412 | 0.051563 | 0.35761 |
| Tuzzerella | Demethylzeylasteral | -0.49433 | 0.051606 | 0.357735 |
| Alcaligenes | Sulisobenzone | 0.494258 | 0.051647 | 0.357843 |
| Achromobacter | S-[(3R,3As,6S,6aS)-3-nitrooxy-2,3,3a,5,6,6a-hexahydrofuro[3,2-b]furan-6-yl] ethanethioate | 0.494169 | 0.051695 | 0.358001 |
| Acetatifactor | PC(16:1(9Z)/17:1(9Z)) | -0.49407 | 0.051751 | 0.358169 |
| Enterococcus | Bis(2-propoxyethyl) 2,6-dimethyl-4-(3-nitrophenyl)-3,4-dihydropyridine-3,5-dicarboxylate | -0.49402 | 0.051776 | 0.358169 |
| Bryobacter | Cysteinyl-Aspartate | 0.493976 | 0.051801 | 0.358169 |
| Clostridioides | L-Glutamine | 0.493939 | 0.051821 | 0.358169 |
| Anaerotruncus | [(2S,4R,5R,6R,14S,16R)-14-Hydroxy-7,11-dimethyl-6-(2-oxopyran-4-yl)-3-oxapentacyclo[8.8.0.02,4.02,7.011,16]octadecan-5-yl] acetate | -0.49389 | 0.051848 | 0.358179 |
| Muribaculum | m7G(5')pppAm | 0.493829 | 0.051881 | 0.358234 |
| Enterococcus | S-cucujolide III | -0.49362 | 0.051998 | 0.358864 |
| Treponema | 11-Hydroxyoctadecanoylcarnitine | 0.493496 | 0.052064 | 0.359145 |
| [Eubacterium]_oxidoreducens_group | 3-Methyl-3-butenyl apiosyl-(1->6)-glucoside | 0.493409 | 0.052112 | 0.359276 |
| Intestinimonas | 11,14,17-eicosatrienoic acid | 0.493368 | 0.052134 | 0.359276 |
| Subgroup_2 | L-Lysinamide | -0.49329 | 0.052177 | 0.359396 |
| Intestinimonas | 5beta-Cholane-3alpha,24-diol | 0.493173 | 0.052242 | 0.359578 |
| Tuzzerella | Americine | -0.49315 | 0.052255 | 0.359578 |
| Enterorhabdus | N-Phenyl-p-phenylenediamine | -0.49309 | 0.052286 | 0.359618 |
| Tuzzerella | Amobarbital | 0.493007 | 0.052334 | 0.359643 |
| Coprobacillus | 3-Hydroxyoctadecanoylcarnitine | 0.492993 | 0.052341 | 0.359643 |
| Clostridioides | Tyrosylglycine | 0.492894 | 0.052396 | 0.359843 |
| Intestinimonas | PE-NMe2(18:3(9Z,12Z,15Z)/18:1(11Z)) | 0.492707 | 0.052499 | 0.360054 |
| Haliangium | Glutamylleucylarginine | 0.492651 | 0.05253 | 0.360054 |
| Tuzzerella | (S)-Laudanosine | -0.49265 | 0.052531 | 0.360054 |
| Tuzzerella | Histidylisoleucine | 0.492613 | 0.052551 | 0.360054 |
| Acetatifactor | Terbufos | 0.492544 | 0.052589 | 0.360054 |
| Anaerotruncus | N-lactoyl-Tyrosine | 0.492527 | 0.052599 | 0.360054 |
| Lachnospiraceae_UCG-006 | Withaferin A | -0.49252 | 0.052605 | 0.360054 |
| Anaerotruncus | Adomeglivant | 0.492219 | 0.052769 | 0.360932 |
| Clostridioides | 4Z,7-octadienoic acid | -0.49219 | 0.052785 | 0.360932 |
| Treponema | 16-Methylheptadecanoylcarnitine | 0.4919 | 0.052947 | 0.361746 |
| Granulicella | Streptidine | 0.491885 | 0.052955 | 0.361746 |
| Pseudolabrys | 4Z,7-octadienoic acid | -0.49179 | 0.053005 | 0.361896 |
| IMCC26256 | PI(18:0/20:4(8Z,11Z,14Z,17Z)) | 0.491752 | 0.053029 | 0.361896 |
| Tuzzerella | PI(20:4(5Z,8Z,11Z,14Z)/18:0) | -0.49169 | 0.053062 | 0.361896 |
| Tuzzerella | Palmitoylcarnitine | 0.491661 | 0.05308 | 0.361896 |
| RF39 | N-Undecanoylglycine | -0.49154 | 0.053146 | 0.362165 |
| Treponema | Terizidone | 0.491497 | 0.053171 | 0.362165 |
| Subgroup_2 | 3,4-Dimethyl-5-propyl-2-furanpentadecanoic acid | -0.49143 | 0.053209 | 0.362248 |
| RF39 | Serylproline | 0.491295 | 0.053284 | 0.362586 |
| bacteriap25 | Glycerol 3-phosphate | 0.491169 | 0.053354 | 0.362887 |
| Anaerotruncus | 3-{[(1s)-2,2-Difluoro-1-Hydroxy-7-(Methylsulfonyl)-2,3-Dihydro-1h-Inden-4-Yl]oxy}-5-Fluorobenzonitrile | 0.491079 | 0.053405 | 0.363011 |
| Monoglobus | 9-Octadecenal | -0.49102 | 0.053439 | 0.363011 |
| Tuzzerella | indoprofen | -0.49093 | 0.053488 | 0.363011 |
| Rikenellaceae_RC9_gut_group | Tephcalostan | 0.490907 | 0.053501 | 0.363011 |
| Enterorhabdus | Dehydroascorbide(1-) | 0.490907 | 0.053502 | 0.363011 |
| Subgroup_13 | L-Homocystine | 0.49074 | 0.053595 | 0.363236 |
| [Eubacterium]_oxidoreducens_group | PI(18:0/20:4(8Z,11Z,14Z,17Z)) | 0.490696 | 0.05362 | 0.363236 |
| Lachnospiraceae_UCG-006 | N6-Acetyl-L-lysine | 0.490669 | 0.053635 | 0.363236 |
| Lachnospiraceae_UCG-006 | BEFLOXATONE | 0.490664 | 0.053638 | 0.363236 |
| Monoglobus | 1H-Pyrrole-2,5-dione, 3-(1-methyl-1h-indol-3-yl)-4-(1-methyl-6-nitro-1h-indol-3-yl)- | 0.490618 | 0.053664 | 0.363237 |
| Achromobacter | 5beta-Cholane-3alpha,24-diol | 0.490401 | 0.053786 | 0.36383 |
| Pseudolabrys | 3'-Deoxythymidine | 0.490349 | 0.053815 | 0.36383 |
| Intestinimonas | Valylhistidine | 0.490291 | 0.053847 | 0.36383 |
| Treponema | Thidiazuron | 0.490278 | 0.053855 | 0.36383 |
| Pseudolabrys | 1-Octadecene | -0.49016 | 0.053922 | 0.364106 |
| Flavonifractor | PC(16:0/16:0) | 0.490049 | 0.053984 | 0.364354 |
| TM7 | Resorufin | 0.489946 | 0.054042 | 0.364572 |
| Treponema | Pantothenic acid | 0.489791 | 0.05413 | 0.364768 |
| Enterococcus | D-erythro-Sphingosine C-20 | -0.48976 | 0.054146 | 0.364768 |
| Treponema | N-Acetyl-L-methionine | 0.489757 | 0.054149 | 0.364768 |
| TRA3-20 | 3-Hydroxy-10'-apo-b,y-carotenal | 0.489659 | 0.054205 | 0.364968 |
| Tuzzerella | Maraviroc | 0.48954 | 0.054272 | 0.365097 |
| Tuzzerella | 5'-Methylthioadenosine | 0.489533 | 0.054276 | 0.365097 |
| TRA3-20 | 3-Ketosphingosine | -0.48948 | 0.054308 | 0.365138 |
| Gemella | PI(18:0/20:4(8Z,11Z,14Z,17Z)) | 0.489421 | 0.054339 | 0.365177 |
| Lachnospiraceae_UCG-006 | Skepinone-L | 0.48933 | 0.054391 | 0.36535 |
| bacteriap25 | 1-Hydroxy-2-naphthoic acid | 0.489265 | 0.054428 | 0.365421 |
| Intestinimonas | N2-gamma-Glutamylglutamine | 0.489157 | 0.054489 | 0.365659 |
| Lachnospiraceae_UCG-006 | Cysteinyl-Aspartate | 0.489051 | 0.05455 | 0.36589 |
| Clostridioides | N-Lactoylphenylalanine | 0.488781 | 0.054703 | 0.36627 |
| Lachnospiraceae_UCG-006 | 2-C-methyl-D-erythritol-4-phosphate | 0.488773 | 0.054708 | 0.36627 |
| RF39 | Zidebactam | 0.488751 | 0.05472 | 0.36627 |
| Enterorhabdus | Docosadienoate (22:2n6) | 0.488694 | 0.054753 | 0.36627 |
| Clostridioides | Histamine | -0.48868 | 0.054764 | 0.36627 |
| Anaerotruncus | Docosatrienoic acid | 0.488617 | 0.054797 | 0.36627 |
| bacteriap25 | PC(16:1(9Z)/17:1(9Z)) | -0.48851 | 0.054858 | 0.36627 |
| Pseudolabrys | PS(22:5(7Z,10Z,13Z,16Z,19Z)/22:6(4Z,7Z,10Z,13Z,16Z,19Z)) | -0.48849 | 0.054867 | 0.36627 |
| Acetatifactor | [(2S,4R,5R,6R,14S,16R)-14-Hydroxy-7,11-dimethyl-6-(2-oxopyran-4-yl)-3-oxapentacyclo[8.8.0.02,4.02,7.011,16]octadecan-5-yl] acetate | -0.48846 | 0.054884 | 0.36627 |
| Clostridioides | Ethynodiol | -0.48845 | 0.05489 | 0.36627 |
| Muribaculum | 7-Hydroxy-R-acenocoumarol | 0.48845 | 0.054892 | 0.36627 |
| Muribaculum | 13-(3,4-Dimethyl-5-propylfuran-2-yl)tridecanoylcarnitine | 0.488298 | 0.054979 | 0.366675 |
| Flavonifractor | alpha-D-Galactopyranuronosyl-(1->4)-alpha-D-galactopyranuronosyl-(1->4)-D-galacturonic acid | 0.488218 | 0.055025 | 0.366754 |
| Pseudolabrys | N2-(3-Carboxy-2-hydroxy-1-oxopropyl)arginine | 0.488128 | 0.055076 | 0.366754 |
| Treponema | Resorufin | 0.488112 | 0.055086 | 0.366754 |
| Acidothermus | Niacinamide | 0.488095 | 0.055095 | 0.366754 |
| Anaerotruncus | Orotidine | 0.487998 | 0.055151 | 0.366953 |
| Clostridioides | alpha-D-Galactopyranuronosyl-(1->4)-alpha-D-galactopyranuronosyl-(1->4)-D-galacturonic acid | 0.487932 | 0.055189 | 0.367016 |
| Pseudolabrys | Berkeleylactone L | -0.48789 | 0.055213 | 0.367016 |
| Subgroup_2 | Kuguacin E | -0.48781 | 0.055256 | 0.367132 |
| Pseudolabrys | (R)-4A-(Ethoxymethyl)-1-(4-fluorophenyl)-6-((4-(trifluoromethyl)phenyl)sulfonyl)-4,4a,5,6,7,8-hexahydro-1H-pyrazolo[3,4-g]isoquinoline | -0.48759 | 0.055383 | 0.367802 |
| Enterorhabdus | N-Acetyl-L-methionine | 0.487513 | 0.05543 | 0.367937 |
| Pseudolabrys | 5S-HETE di-endoperoxide | -0.48741 | 0.055491 | 0.368029 |
| Solobacterium | 4-Methylcatechol 1-glucuronide | 0.487398 | 0.055496 | 0.368029 |
| Anaerotruncus | 25-Hydroxyvitamin D3-26,23-lactol | -0.48732 | 0.055538 | 0.368137 |
| TRA3-20 | 1-Octadecene | -0.48718 | 0.055624 | 0.368462 |
| Christensenellaceae_R-7_group | S-Adenosylhomocysteine | 0.487149 | 0.05564 | 0.368462 |
| Tuzzerella | 11-Hydroxyoctadecanoylcarnitine | 0.486861 | 0.055806 | 0.369324 |
| Flavonifractor | Notoginsenoside H | 0.486833 | 0.055822 | 0.369324 |
| Tuzzerella | MG(20:4(5Z,8Z,11Z,14Z)/0:0/0:0) | -0.48666 | 0.055919 | 0.369745 |
| Rikenellaceae_RC9_gut_group | Prolylproline | 0.486632 | 0.055938 | 0.369745 |
| Tuzzerella | N-(2-Hydroxyethyl)-2-(1-isoquinolinylmethylene)hydrazinecarbothioamide | 0.486481 | 0.056026 | 0.369861 |
| Acetatifactor | Terizidone | 0.486464 | 0.056036 | 0.369861 |
| Clostridia_vadinBB60_group | Fucoxanthinol 3-(4Z,7Z,10Z,13Z,16Z,19Z-docosahexaenoate) 3'-palmitoleate | 0.486439 | 0.056051 | 0.369861 |
| Enterorhabdus | 16-Methylheptadecanoylcarnitine | 0.486421 | 0.056061 | 0.369861 |
| Enterococcus | Octadecanamide | -0.48637 | 0.056092 | 0.369893 |
| bacteriap25 | All trans decaprenyl diphosphate | -0.48628 | 0.05614 | 0.370035 |
| Nitrospira | Orotidine | 0.486176 | 0.056203 | 0.370277 |
| Enterorhabdus | N-Acetylneuraminic acid | 0.486105 | 0.056245 | 0.370379 |
| [Eubacterium]_oxidoreducens_group | Mycobactins | -0.48605 | 0.056274 | 0.370401 |
| MND1 | 25-Hydroxyvitamin D3-26,23-lactol | -0.48598 | 0.056316 | 0.370501 |
| Pseudolabrys | N-Acetyl-b-glucosaminylamine | 0.485927 | 0.056348 | 0.370511 |
| Lachnospiraceae_UCG-006 | Docosadienoate (22:2n6) | 0.485889 | 0.05637 | 0.370511 |
| Tuzzerella | Eleutherazine B; N,N'-((3,6-Dioxo-2,5-piperazinediyl)bis(trimethylene))bis(5-hydroxy-3-methyl-2-pentenamide) | 0.485737 | 0.056459 | 0.370776 |
| Monoglobus | 1-(10H-Phenothiazin-2-yl)ethanone | 0.48573 | 0.056463 | 0.370776 |
| Acetatifactor | m7G(5')pppAm | 0.485639 | 0.056516 | 0.370827 |
| Nitrospira | Am-PE(16:0/18:0) | 0.485626 | 0.056524 | 0.370827 |
| Acidothermus | Phenylalanyl-Glycine | 0.485524 | 0.056583 | 0.370887 |
| Enterococcus | Phenylalanylproline | 0.485426 | 0.056641 | 0.370887 |
| Faecalibaculum | Dehydroascorbide(1-) | 0.485344 | 0.056688 | 0.370887 |
| Gemella | Notoginsenoside H | 0.485288 | 0.056722 | 0.370887 |
| Acidothermus | [6-Hydroxy-8-methyl-3-[3,4,5-trihydroxy-6-(hydroxymethyl)oxan-2-yl]oxy-9,10-dioxatetracyclo[4.3.1.02,5.03,8]decan-4-yl]methyl benzoate | 0.485145 | 0.056805 | 0.370887 |
| Monoglobus | Erucic acid | 0.485144 | 0.056806 | 0.370887 |
| Bryobacter | Alpha-Lactose | 0.485133 | 0.056812 | 0.370887 |
| Monoglobus | Oleoyl Serotonin | -0.48513 | 0.056816 | 0.370887 |
| Tuzzerella | Tyrosyl-Aspartate | 0.485112 | 0.056824 | 0.370887 |
| Tuzzerella | 1-O-(2R-methoxy-4Z-docosenyl)-sn-glycerol | -0.48511 | 0.056826 | 0.370887 |
| TRA3-20 | LysoPE(20:3(8Z,11Z,14Z)/0:0) | 0.485097 | 0.056833 | 0.370887 |
| Tuzzerella | DG(i-15:0/20:4(8Z,11Z,14Z,17Z)-2OH(5S,6R)/0:0) | -0.48506 | 0.056853 | 0.370887 |
| Tuzzerella | L-2-Hydroxyglutaric acid | 0.485026 | 0.056875 | 0.370887 |
| Enterococcus | Benzyl gentiobioside | 0.484934 | 0.056929 | 0.371064 |
| Tuzzerella | biliverdin-IX-alpha | -0.4848 | 0.057008 | 0.371407 |
| Tuzzerella | Peruvianoside II | 0.484717 | 0.057056 | 0.371498 |
| Bryobacter | N-Acetyl-L-methionine | 0.484647 | 0.057097 | 0.371498 |
| Intestinimonas | 5'-Carboxy-gamma-chromanol | -0.48463 | 0.057109 | 0.371498 |
| Flavonifractor | Flumazenil | 0.484597 | 0.057127 | 0.371498 |
| Bryobacter | 4E,14Z-Sphingadiene | -0.48443 | 0.057226 | 0.371967 |
| Clostridioides | N-Lactoylleucine | 0.484362 | 0.057265 | 0.372054 |
| Rikenellaceae_RC9_gut_group | Doxepin | 0.484181 | 0.057372 | 0.372575 |
| Enterorhabdus | 2',3'-Dideoxyuridine | 0.484136 | 0.057399 | 0.372577 |
| Anaerotruncus | Adenosine monophosphate | -0.484 | 0.05748 | 0.372931 |
| Subgroup_13 | Benzyl gentiobioside | 0.483889 | 0.057545 | 0.373082 |
| Rikenellaceae_RC9_gut_group | 6-Hydroxytricetin 5-rhamnoside | 0.483843 | 0.057572 | 0.373082 |
| MND1 | Thiazolidine-4-carboxylic acid | 0.483749 | 0.057628 | 0.373082 |
| Anaerotruncus | Streptidine | 0.483718 | 0.057646 | 0.373082 |
| Flavonifractor | L-Glutamine | 0.483708 | 0.057652 | 0.373082 |
| Acidothermus | PC(TXB2/20:2(11Z,14Z)) | -0.48369 | 0.057662 | 0.373082 |
| Clostridioides | 20-HETE-d6 | 0.483622 | 0.057703 | 0.373171 |
| TRA3-20 | Oleic acid | -0.48358 | 0.057729 | 0.373171 |
| Enterorhabdus | (R)-4A-(Ethoxymethyl)-1-(4-fluorophenyl)-6-((4-(trifluoromethyl)phenyl)sulfonyl)-4,4a,5,6,7,8-hexahydro-1H-pyrazolo[3,4-g]isoquinoline | -0.48351 | 0.057771 | 0.37327 |
| Intestinimonas | Pentadeca-3,5,7-trienedioylcarnitine | 0.483431 | 0.057816 | 0.373342 |
| Lachnospiraceae_UCG-006 | 5,5-Diphenyl-2-thiohydantoin | 0.483399 | 0.057835 | 0.373342 |
| Nitrospira | PC(P-18:0/20:4(5Z,8Z,11Z,14Z)-OH(16R)) | 0.483292 | 0.057899 | 0.373582 |
| Christensenellaceae_R-7_group | Notoginsenoside H | 0.48324 | 0.05793 | 0.373586 |
| Anaerotruncus | Ribonolactone | 0.48317 | 0.057972 | 0.373586 |
| Bryobacter | U-75302 | 0.483157 | 0.057979 | 0.373586 |
| [Eubacterium]_oxidoreducens_group | Notoginsenoside H | 0.482888 | 0.058139 | 0.373946 |
| IMCC26256 | Notoginsenoside H | 0.482865 | 0.058153 | 0.373946 |
| Enterococcus | Symmetric dimethylarginine | 0.482813 | 0.058184 | 0.373946 |
| Treponema | N-lactoyl-Tyrosine | 0.482797 | 0.058193 | 0.373946 |
| Enterococcus | 1-(10H-Phenothiazin-2-yl)ethanone | 0.482763 | 0.058214 | 0.373946 |
| Acetatifactor | Beta-Citryl-L-glutamic acid | 0.482763 | 0.058214 | 0.373946 |
| Enterorhabdus | 3-Amino-2-methoxynonadec-5-en-4-ol | -0.48275 | 0.058221 | 0.373946 |
| Enterorhabdus | Cortisol | -0.48263 | 0.058294 | 0.374101 |
| Rikenellaceae_RC9_gut_group | hydroxyhexadecenoylcarnitine | 0.482619 | 0.0583 | 0.374101 |
| Aquicella | DG(17:2(9Z,12Z)/22:5(7Z,10Z,13Z,16Z,19Z)/0:0)[iso2] | 0.482578 | 0.058325 | 0.374101 |
| TRA3-20 | Oleoyl Serotonin | -0.4825 | 0.058372 | 0.374162 |
| Clostridioides | Cortisol | -0.48247 | 0.058387 | 0.374162 |
| Flavonifractor | Prolyl-Asparagine | 0.482326 | 0.058475 | 0.374509 |
| TRA3-20 | Dihydrozeatin O-beta-D-Glucoside | 0.482294 | 0.058495 | 0.374509 |
| Tuzzerella | Prostaglandin B-1 | -0.48211 | 0.058605 | 0.374788 |
| Jatrophihabitans | Tyrosyl-Aspartate | 0.482056 | 0.058637 | 0.374788 |
| Pseudolabrys | D-erythro-Sphingosine C-20 | -0.48205 | 0.058638 | 0.374788 |
| Pseudolabrys | 4-Chloro-L-phenylalanine | -0.48204 | 0.058645 | 0.374788 |
| Treponema | Allitridin | 0.481794 | 0.058794 | 0.375437 |
| Bryobacter | Resorufin | 0.481785 | 0.058799 | 0.375437 |
| Clostridioides | Dirithromycin | -0.48169 | 0.058855 | 0.375619 |
| Nitrospira | Tuberonic acid glucoside | -0.48155 | 0.058942 | 0.375786 |
| Alcaligenes | Withaferin A | -0.48152 | 0.058956 | 0.375786 |
| Clostridioides | 1-(10H-Phenothiazin-2-yl)ethanone | 0.481431 | 0.059013 | 0.375786 |
| Anaerotruncus | Diethylamino 2,5-dihydroxybenzenesulfonate | 0.481394 | 0.059035 | 0.375786 |
| Lachnospiraceae_UCG-006 | N-Undecanoylglycine | -0.48139 | 0.059036 | 0.375786 |
| Tuzzerella | Adomeglivant | 0.481384 | 0.059041 | 0.375786 |
| Enterorhabdus | DG(17:2(9Z,12Z)/22:5(7Z,10Z,13Z,16Z,19Z)/0:0)[iso2] | 0.481334 | 0.059071 | 0.375808 |
| Tuzzerella | 1-O-Galloylglycerol | 0.481243 | 0.059126 | 0.375989 |
| Aquicella | DG(18:0/20:4(8Z,11Z,14Z,17Z)/0:0) | 0.481144 | 0.059186 | 0.376163 |
| Alcaligenes | 5,5-Diphenyl-2-thiohydantoin | 0.481109 | 0.059207 | 0.376163 |
| Enterorhabdus | Tyrosyl-Aspartate | 0.481061 | 0.059236 | 0.376177 |
| Clostridia_vadinBB60_group | Peperomin E | 0.481001 | 0.059272 | 0.376239 |
| Enterococcus | Bisazobiphenyl | 0.480873 | 0.05935 | 0.376559 |
| Clostridioides | Polypropylene glycol (m w 1,200-3,000) | -0.48065 | 0.059483 | 0.377237 |
| Alcaligenes | 1-Hydroxy-2-naphthoic acid | 0.480483 | 0.059587 | 0.377722 |
| Treponema | 9-Hydroxy-4-methoxypsoralen 9-glucoside | 0.480429 | 0.059619 | 0.377758 |
| Gemella | 4-Methylcatechol 1-glucuronide | 0.480365 | 0.059658 | 0.377832 |
| Tuzzerella | 7b-Hydroxy-3-oxo-5b-cholanoic acid | -0.48028 | 0.05971 | 0.377933 |
| Bryobacter | Kuguacin E | -0.48021 | 0.05975 | 0.377933 |
| Enterorhabdus | Monooctyl phthalate | -0.48021 | 0.059754 | 0.377933 |
| TRA3-20 | 4Z,7-octadienoic acid | -0.48014 | 0.059797 | 0.378037 |
| Achromobacter | Withaferin A | -0.47996 | 0.059904 | 0.378402 |
| Intestinimonas | Prolylproline | 0.479953 | 0.059909 | 0.378402 |
| Anaerostipes | Zidebactam | 0.479758 | 0.060027 | 0.378872 |
| Monoglobus | 4Z,7-octadienoic acid | -0.47974 | 0.060037 | 0.378872 |
| Intestinimonas | 20-HETE-d6 | 0.479614 | 0.060116 | 0.378978 |
| Subgroup_2 | 25-Hydroxyvitamin D3-26,23-lactol | -0.47958 | 0.060137 | 0.378978 |
| Tuzzerella | Dehydroascorbic acid | 0.479507 | 0.060181 | 0.378978 |
| Tuzzerella | 1-Ethoxymethyl-5-fluorouracil | 0.479499 | 0.060186 | 0.378978 |
| Muribaculum | Phenethylamine glucuronide | 0.479495 | 0.060189 | 0.378978 |
| Enterorhabdus | Resorufin | 0.479446 | 0.060218 | 0.378997 |
| Anaerotruncus | 4-Chloro-L-phenylalanine | -0.47925 | 0.060336 | 0.379358 |
| Clostridioides | Genistein 7-O-glucoside-6''-malonate | 0.479186 | 0.060377 | 0.379358 |
| Acetatifactor | N6-Acetyl-L-lysine | 0.479144 | 0.060403 | 0.379358 |
| Acetatifactor | N-gamma-L-Glutamyl-D-alanine | 0.479143 | 0.060404 | 0.379358 |
| Treponema | (13Z)-3-Hydroxyicos-13-enoylcarnitine | 0.479132 | 0.060411 | 0.379358 |
| Enterorhabdus | N-Lauroyl Glutamine | -0.47906 | 0.060453 | 0.379454 |
| Achromobacter | 1-(10H-Phenothiazin-2-yl)ethanone | 0.47898 | 0.060504 | 0.379506 |
| Tuzzerella | 1-O-(2R-methoxy-4Z-eicosenyl)-sn-glycerol | -0.47895 | 0.060525 | 0.379506 |
| Tuzzerella | Obacunone 17-O-beta-D-glucoside | -0.47892 | 0.060542 | 0.379506 |
| Achromobacter | 7-Hydroxy-R-acenocoumarol | 0.478717 | 0.060666 | 0.380112 |
| Clostridioides | Biliverdin | -0.47862 | 0.060728 | 0.380238 |
| Clostridioides | Prolylproline | 0.478597 | 0.06074 | 0.380238 |
| Treponema | Dipotassium phosphate | 0.478503 | 0.060797 | 0.380428 |
| TM7 | 9-Hydroxy-4-methoxypsoralen 9-glucoside | 0.478425 | 0.060845 | 0.380561 |
| Alcaligenes | Hexadecadienylcarnitine | 0.478366 | 0.060882 | 0.380619 |
| Enterococcus | N-gamma-L-Glutamyl-D-alanine | 0.478174 | 0.061 | 0.381192 |
| Enterorhabdus | 5'-Carboxy-gamma-chromanol | -0.47812 | 0.061031 | 0.381212 |
| Faecalibaculum | 3-Amino-3-methylbutanoic acid | 0.478058 | 0.061072 | 0.381301 |
| Aquicella | PI(18:0/20:4(8Z,11Z,14Z,17Z)) | 0.47793 | 0.061151 | 0.381625 |
| Tuzzerella | Ornithokinin | -0.47784 | 0.06121 | 0.381823 |
| Enterococcus | 1-O-Galloylglycerol | 0.477758 | 0.061258 | 0.381952 |
| Anaerotruncus | Withaferin A | -0.47745 | 0.06145 | 0.38298 |
| Acidothermus | N-(1-Deoxy-1-fructosyl)valine | 0.477338 | 0.061518 | 0.383179 |
| TRA3-20 | 1-Ethoxymethyl-5-fluorouracil | 0.477277 | 0.061556 | 0.383179 |
| Clostridioides | Quinagolida | -0.47724 | 0.061577 | 0.383179 |
| TRA3-20 | Glutaminylproline | 0.477222 | 0.061591 | 0.383179 |
| Subgroup_2 | True blue | 0.477168 | 0.061624 | 0.383217 |
| Tuzzerella | Aspartyl-Isoleucine | 0.47711 | 0.06166 | 0.383272 |
| TRA3-20 | JP83 | -0.47702 | 0.061718 | 0.383464 |
| Tuzzerella | 23S,25,26-Trihydroxyvitamin D3 | 0.476967 | 0.061749 | 0.383488 |
| bacteriap25 | 4-Bis(2-hydroxyethyl)amino-L-phenylalanine | 0.476843 | 0.061826 | 0.383737 |
| Clostridioides | L-arginino-succinate | 0.476773 | 0.06187 | 0.383737 |
| Acetatifactor | Nopalinic acid | 0.476753 | 0.061883 | 0.383737 |
| TM7 | PE(22:5(4Z,7Z,10Z,13Z,16Z)/PGD2) | -0.47673 | 0.061898 | 0.383737 |
| Alcaligenes | 7-Hydroxy-R-acenocoumarol | 0.476624 | 0.061963 | 0.383972 |
| Intestinimonas | Polypropylene glycol (m w 1,200-3,000) | -0.47651 | 0.062034 | 0.38413 |
| Clostridioides | Medica 16 | -0.47646 | 0.062063 | 0.38413 |
| Intestinimonas | Pro-Pro-Pro | 0.476452 | 0.062071 | 0.38413 |
| Clostridia_vadinBB60_group | True blue | 0.476303 | 0.062164 | 0.384334 |
| IMCC26256 | PC(16:0/16:0) | 0.47628 | 0.062179 | 0.384334 |
| Christensenellaceae_R-7_group | FAD | 0.476217 | 0.062218 | 0.384334 |
| Coprobacillus | Orotidine | 0.476196 | 0.062231 | 0.384334 |
| TM7 | 1-O-(2R-methoxy-hexadecyl)-sn-glycerol | -0.47618 | 0.06224 | 0.384334 |
| Acidothermus | PC(16:1(9Z)/17:1(9Z)) | -0.47607 | 0.062309 | 0.384591 |
| Gemella | PC(16:0/16:0) | 0.475987 | 0.062362 | 0.384609 |
| Flavonifractor | DG(17:2(9Z,12Z)/22:5(7Z,10Z,13Z,16Z,19Z)/0:0)[iso2] | 0.47598 | 0.062367 | 0.384609 |
| Coprobacillus | Ethyl hexadecanoate | -0.47586 | 0.06244 | 0.384893 |
| Enterococcus | 5,5-Diphenyl-2-thiohydantoin | 0.475697 | 0.062545 | 0.385078 |
| Enterococcus | Adenosine monophosphate | -0.47558 | 0.062619 | 0.385078 |
| Aquicella | Nipradilol | 0.47553 | 0.06265 | 0.385078 |
| Anaerotruncus | Pelargonidin 3,5-di-(6-acetylglucoside) | -0.47553 | 0.062652 | 0.385078 |
| Muribaculum | Aspartyl-Isoleucine | 0.475519 | 0.062657 | 0.385078 |
| Muribaculum | Orotidine | 0.475505 | 0.062665 | 0.385078 |
| RF39 | PE(22:5(4Z,7Z,10Z,13Z,16Z)/PGD2) | -0.4755 | 0.062671 | 0.385078 |
| Achromobacter | 1-Ethoxymethyl-5-fluorouracil | 0.475407 | 0.062727 | 0.385078 |
| Clostridia_vadinBB60_group | N-Acetylneuraminic acid | 0.475382 | 0.062743 | 0.385078 |
| [Eubacterium]_oxidoreducens_group | PC(16:0/16:0) | 0.47536 | 0.062757 | 0.385078 |
| Solobacterium | PC(P-18:0/20:4(5Z,8Z,11Z,14Z)-OH(16R)) | 0.475337 | 0.062771 | 0.385078 |
| Monoglobus | Neryl 8-methyldecanoate | 0.475262 | 0.062819 | 0.385203 |
| Monoglobus | LysoSM(d18:0) | -0.47514 | 0.062896 | 0.385343 |
| Bryobacter | Bisazobiphenyl | 0.475139 | 0.062896 | 0.385343 |
| Enterococcus | Amobarbital | 0.475086 | 0.06293 | 0.385379 |
| Nitrospira | Cortisol | -0.475 | 0.062986 | 0.385555 |
| TRA3-20 | Ethynodiol | -0.47494 | 0.06302 | 0.385594 |
| Enterococcus | (R)-Propyl 2-amino-3-mercaptopropanoate | 0.474848 | 0.06308 | 0.385605 |
| Subgroup_13 | Terbufos | 0.474822 | 0.063097 | 0.385605 |
| Clostridioides | 1H-Pyrrole-2,5-dione, 3-(1-methyl-1h-indol-3-yl)-4-(1-methyl-6-nitro-1h-indol-3-yl)- | 0.474811 | 0.063103 | 0.385605 |
| Anaerotruncus | Pantothenic acid | 0.474737 | 0.06315 | 0.385722 |
| Tuzzerella | Dodecanamide | -0.47468 | 0.063189 | 0.385722 |
| Granulicella | N-alpha-Acetyl-L-lysine | 0.474609 | 0.063232 | 0.385722 |
| Acetatifactor | Prolyl-Asparagine | 0.474608 | 0.063232 | 0.385722 |
| TM7 | Thidiazuron | 0.474422 | 0.06335 | 0.386276 |
| Achromobacter | 2',3'-Dideoxyuridine | 0.474198 | 0.063493 | 0.386659 |
| TRA3-20 | 2-phospho-4-(cytidine 5'-diphospho)-2-C-methyl-D-erythritol | 0.474192 | 0.063497 | 0.386659 |
| Muribaculum | 12-Hydroxy-12-octadecanoylcarnitine | 0.474172 | 0.063509 | 0.386659 |
| Bryobacter | (2S)-3-[3-[(4-Carbamimidoylbenzoyl)amino]propanoylamino]-2-[(4-ethylphenyl)sulfonylamino]propanoic acid | 0.474141 | 0.063529 | 0.386659 |
| Intestinimonas | Nopalinic acid | 0.474107 | 0.063551 | 0.386659 |
| Enterococcus | N-Phenyl-p-phenylenediamine | -0.47393 | 0.063664 | 0.38718 |
| Clostridioides | 9-Octadecenal | -0.47363 | 0.063855 | 0.388176 |
| Enterorhabdus | Dehydrocyclopeptine; (3E)-3-Benzylidene-4-methyl-3,4-dihydro-1H-1,4-benzodiazepine-2,5-dione | -0.47347 | 0.06396 | 0.388308 |
| Jatrophihabitans | Phenylalanylaspartic acid | 0.473434 | 0.06398 | 0.388308 |
| Tuzzerella | Alacepril | -0.47341 | 0.063998 | 0.388308 |
| Enterorhabdus | Octadecanamide | -0.47336 | 0.064029 | 0.388308 |
| Enterorhabdus | 3-Hydroxyoctadecanoylcarnitine | 0.473314 | 0.064057 | 0.388308 |
| Enterococcus | 1-Octadecene | -0.47328 | 0.064076 | 0.388308 |
| Anaerotruncus | LysoPC(P-16:0/0:0) | 0.473263 | 0.06409 | 0.388308 |
| Anaerotruncus | PC(P-18:0/20:4(5Z,8Z,11Z,14Z)-OH(16R)) | 0.473251 | 0.064097 | 0.388308 |
| Granulicella | Flumazenil | 0.473082 | 0.064205 | 0.388464 |
| Bryobacter | Sodium nitrate (NaNO3) | -0.47304 | 0.06423 | 0.388464 |
| MND1 | 3-{[(1s)-2,2-Difluoro-1-Hydroxy-7-(Methylsulfonyl)-2,3-Dihydro-1h-Inden-4-Yl]oxy}-5-Fluorobenzonitrile | 0.472999 | 0.064259 | 0.388464 |
| Tuzzerella | Lamivudine-monophosphate | -0.473 | 0.064259 | 0.388464 |
| Clostridia_vadinBB60_group | N,N'-Diethylthiourea | 0.472974 | 0.064275 | 0.388464 |
| Bryobacter | Cortisol | -0.47295 | 0.064289 | 0.388464 |
| Acetatifactor | S-Adenosylhomocysteine | 0.472875 | 0.064339 | 0.388511 |
| RF39 | PI(20:3(8Z,11Z,14Z)/0:0) | 0.472854 | 0.064352 | 0.388511 |
| Enterorhabdus | 3-Hydroxy-11Z-octadecenoylcarnitine | 0.472746 | 0.064421 | 0.388764 |
| Clostridioides | Valylserine | 0.472652 | 0.064482 | 0.388785 |
| Clostridioides | Phosphohydroxypyruvic acid | -0.47265 | 0.064484 | 0.388785 |
| Intestinimonas | 12-Hydroxydodecanoic acid | -0.47261 | 0.064507 | 0.388785 |
| Rikenellaceae_RC9_gut_group | 3'-Ketolactose | 0.472526 | 0.064563 | 0.388954 |
| Bryobacter | N-(N-(3-Amino-3-carboxypropyl)-3-amino-3-carboxypropyl)azetidine-2-carboxylic acid | 0.472464 | 0.064603 | 0.389027 |
| Aquicella | 5beta-Cholane-3alpha,24-diol | 0.47237 | 0.064663 | 0.389074 |
| Tuzzerella | Bisazobiphenyl | 0.47236 | 0.06467 | 0.389074 |
| Achromobacter | N-(1-Deoxy-1-fructosyl)threonine | 0.47224 | 0.064747 | 0.389074 |
| Alcaligenes | Fiacitabine | 0.472228 | 0.064755 | 0.389074 |
| TRA3-20 | D-erythro-L-galacto-Nonulose | 0.47222 | 0.06476 | 0.389074 |
| Intestinimonas | 2-(2-Thienylmethylene)-1,6-dioxaspiro[4.4]non-3-ene | 0.472168 | 0.064794 | 0.389074 |
| Tuzzerella | All trans decaprenyl diphosphate | -0.47215 | 0.064804 | 0.389074 |
| Monoglobus | Polypropylene glycol (m w 1,200-3,000) | -0.47192 | 0.064955 | 0.389337 |
| TRA3-20 | Berkeleylactone L | -0.47189 | 0.064976 | 0.389337 |
| Achromobacter | Beta-Citryl-L-glutamic acid | 0.471885 | 0.064977 | 0.389337 |
| Bryobacter | L-Glutamine | 0.471744 | 0.065068 | 0.389337 |
| Flavonifractor | N2-(3-Carboxy-2-hydroxy-1-oxopropyl)arginine | 0.471721 | 0.065083 | 0.389337 |
| Flavonifractor | Lucuminic acid | 0.471654 | 0.065126 | 0.389337 |
| [Eubacterium]_oxidoreducens_group | 20-HETE-d6 | 0.471648 | 0.06513 | 0.389337 |
| Haliangium | PC(18:1(17Z)/18:1(17Z)) | 0.4716 | 0.065161 | 0.389337 |
| bacteriap25 | 16-Methylheptadecanoylcarnitine | 0.471577 | 0.065176 | 0.389337 |
| Achromobacter | PIP(20:0/18:1(12Z)-2OH(9,10)) | -0.47151 | 0.065219 | 0.389337 |
| Pseudolabrys | N-Phenyl-p-phenylenediamine | -0.47149 | 0.065231 | 0.389337 |
| [Eubacterium]_oxidoreducens_group | 11,14,17-eicosatrienoic acid | 0.471466 | 0.065248 | 0.389337 |
| Aquicella | Diethylamino 2,5-dihydroxybenzenesulfonate | 0.47139 | 0.065297 | 0.389337 |
| Intestinimonas | N-(1-Deoxy-1-fructosyl)leucine | 0.471369 | 0.065311 | 0.389337 |
| Clostridia_vadinBB60_group | Creatine | 0.471335 | 0.065333 | 0.389337 |
| RF39 | Tuberonic acid glucoside | -0.47119 | 0.065426 | 0.389337 |
| Pseudolabrys | Valylhistidine | 0.471188 | 0.065428 | 0.389337 |
| bacteriap25 | 11-Hydroxyoctadecanoylcarnitine | 0.471105 | 0.065483 | 0.389337 |
| Alcaligenes | 1-Ethoxymethyl-5-fluorouracil | 0.471061 | 0.065511 | 0.389337 |
| Solobacterium | N-(1-Deoxy-1-fructosyl)alanine | 0.471035 | 0.065528 | 0.389337 |
| Coprobacillus | Octadec-6-enoylcarnitine | 0.471015 | 0.065541 | 0.389337 |
| [Eubacterium]_oxidoreducens_group | Flumazenil | 0.470911 | 0.065609 | 0.389337 |
| Monoglobus | 8-Amino-7-oxononanoic acid | -0.47089 | 0.06562 | 0.389337 |
| Achromobacter | Hexadecadienylcarnitine | 0.470826 | 0.065665 | 0.389337 |
| [Eubacterium]_oxidoreducens_group | Xanthosine | 0.470819 | 0.065669 | 0.389337 |
| bacteriap25 | 12-Hydroxy-12-octadecanoylcarnitine | 0.470813 | 0.065673 | 0.389337 |
| Monoglobus | Glutaminylproline | 0.470766 | 0.065704 | 0.389337 |
| Achromobacter | [(2S,4R,5R,6R,14S,16R)-14-Hydroxy-7,11-dimethyl-6-(2-oxopyran-4-yl)-3-oxapentacyclo[8.8.0.02,4.02,7.011,16]octadecan-5-yl] acetate | -0.47074 | 0.065718 | 0.389337 |
| [Eubacterium]_oxidoreducens_group | Pentadeca-3,5,7-trienedioylcarnitine | 0.470703 | 0.065745 | 0.389337 |
| Enterorhabdus | Bis(2-propoxyethyl) 2,6-dimethyl-4-(3-nitrophenyl)-3,4-dihydropyridine-3,5-dicarboxylate | -0.47069 | 0.065752 | 0.389337 |
| Bryobacter | Pantothenic acid | 0.470682 | 0.065758 | 0.389337 |
| Lachnospiraceae_UCG-006 | 1-O-Galloylglycerol | 0.470625 | 0.065796 | 0.389337 |
| Clostridioides | 3-Methyl-3-butenyl apiosyl-(1->6)-glucoside | 0.470615 | 0.065802 | 0.389337 |
| Achromobacter | 5,5-Diphenyl-2-thiohydantoin | 0.470561 | 0.065837 | 0.389337 |
| Acetatifactor | 4-Methylcatechol 1-glucuronide | 0.470529 | 0.065858 | 0.389337 |
| Aquicella | xi-2,3-Octadiene-5,7-diyn-1-ol | 0.470499 | 0.065878 | 0.389337 |
| Lachnospiraceae_UCG-006 | (E,E)-3,7,11-Trimethyl-2,6,10-dodecatrienyl octanoate | 0.470481 | 0.06589 | 0.389337 |
| Nitrospira | Ribonolactone | 0.470467 | 0.065899 | 0.389337 |
| Aquicella | Notoginsenoside H | 0.470242 | 0.066046 | 0.389708 |
| Anaerostipes | 1-(10H-Phenothiazin-2-yl)ethanone | 0.47024 | 0.066047 | 0.389708 |
| Jatrophihabitans | PE-NMe2(18:3(9Z,12Z,15Z)/18:1(11Z)) | 0.470208 | 0.066068 | 0.389708 |
| MND1 | 2-[(4-{2-[(4-Cyclohexylbutyl)(cyclohexylcarbamoyl)amino]ethyl}phenyl)sulfanyl]-2-methylpropanoic acid | -0.47019 | 0.066079 | 0.389708 |
| Pseudolabrys | PE(19:0/20:2(11Z,14Z)) | -0.47015 | 0.066103 | 0.389708 |
| Subgroup_2 | Dehydrocyclopeptine; (3E)-3-Benzylidene-4-methyl-3,4-dihydro-1H-1,4-benzodiazepine-2,5-dione | -0.47011 | 0.066132 | 0.389708 |
| Tuzzerella | a-L-Arabinofuranosyl-(1->3)-b-D-xylopyranosyl-(1->4)-D-xylose | 0.470041 | 0.066178 | 0.389708 |
| Bryobacter | 2-[(4-{2-[(4-Cyclohexylbutyl)(cyclohexylcarbamoyl)amino]ethyl}phenyl)sulfanyl]-2-methylpropanoic acid | -0.47003 | 0.066183 | 0.389708 |
| Monoglobus | D-erythro-Sphingosine C-20 | -0.46979 | 0.066343 | 0.390342 |
| Nitrospira | 7-Hydroxy-R-acenocoumarol | 0.46978 | 0.066349 | 0.390342 |
| Intestinimonas | N-lactoyl-Tyrosine | 0.469742 | 0.066374 | 0.390342 |
| Enterococcus | S-[(3R,3As,6S,6aS)-3-nitrooxy-2,3,3a,5,6,6a-hexahydrofuro[3,2-b]furan-6-yl] ethanethioate | 0.469661 | 0.066427 | 0.390492 |
| RF39 | 2-[4-(3-Hydroxypropyl)-2-methoxyphenoxy]-1,3-propanediol 1-xyloside | -0.4696 | 0.066466 | 0.390556 |
| Enterorhabdus | Dodecanamide | -0.46944 | 0.066576 | 0.390964 |
| Enterococcus | Ac-Ser-Asp-Lys-Pro-OH | 0.469412 | 0.066591 | 0.390964 |
| Enterorhabdus | Erucic acid | 0.469274 | 0.066682 | 0.391167 |
| Subgroup_2 | 4Z,7-octadienoic acid | -0.46926 | 0.066689 | 0.391167 |
| Clostridioides | 2-C-methyl-D-erythritol-4-phosphate | 0.469219 | 0.066718 | 0.391167 |
| Tuzzerella | U-75302 | 0.469191 | 0.066736 | 0.391167 |
| Alcaligenes | [(2S,4R,5R,6R,14S,16R)-14-Hydroxy-7,11-dimethyl-6-(2-oxopyran-4-yl)-3-oxapentacyclo[8.8.0.02,4.02,7.011,16]octadecan-5-yl] acetate | -0.46892 | 0.066915 | 0.391967 |
| Acidothermus | Tephcalostan | 0.4689 | 0.066929 | 0.391967 |
| Faecalibaculum | Lucuminic acid | 0.468764 | 0.067019 | 0.392143 |
| Muribaculum | Dimethylaminoparthenolide | 0.468761 | 0.067021 | 0.392143 |
| Bryobacter | Am-PE(16:0/18:0) | 0.468724 | 0.067045 | 0.392143 |
| Achromobacter | Medicagol | 0.468686 | 0.06707 | 0.392143 |
| Haliangium | Notoginsenoside H | 0.468637 | 0.067103 | 0.392172 |
| Tuzzerella | Cerulenin | 0.468462 | 0.067219 | 0.392685 |
| MND1 | N-Acetyl-D-Glucosamine 6-Phosphate | 0.468382 | 0.067272 | 0.392803 |
| Tuzzerella | Ciadox | 0.468348 | 0.067295 | 0.392803 |
| Nitrospira | 2-[4-(3-Hydroxypropyl)-2-methoxyphenoxy]-1,3-propanediol 1-xyloside | -0.46811 | 0.067451 | 0.39337 |
| Rikenellaceae_RC9_gut_group | Hexadecadienylcarnitine | 0.468078 | 0.067474 | 0.39337 |
| Enterococcus | PI(20:3(8Z,11Z,14Z)/0:0) | 0.468075 | 0.067476 | 0.39337 |
| Monoglobus | N-Undecanoylglycine | -0.46794 | 0.067564 | 0.39372 |
| TRA3-20 | 5S-HETE di-endoperoxide | -0.46782 | 0.067644 | 0.394025 |
| Coprobacillus | Ribonolactone | 0.467647 | 0.067761 | 0.394506 |
| Enterococcus | 5'-Carboxy-gamma-chromanol | -0.46761 | 0.067783 | 0.394506 |
| Anaerotruncus | DG(17:2(9Z,12Z)/22:5(7Z,10Z,13Z,16Z,19Z)/0:0)[iso2] | 0.467549 | 0.067827 | 0.3946 |
| Rikenellaceae_RC9_gut_group | Glutaminylproline | 0.467494 | 0.067863 | 0.39465 |
| Nitrospira | 3-Hydroxyoctadecanoylcarnitine | 0.467406 | 0.067922 | 0.394823 |
| IMCC26256 | Lucuminic acid | 0.467365 | 0.067949 | 0.394823 |
| Enterorhabdus | Skepinone-L | 0.467215 | 0.06805 | 0.395244 |
| Monoglobus | PC(TXB2/20:2(11Z,14Z)) | -0.46709 | 0.068132 | 0.3954 |
| Enterococcus | [6-Hydroxy-8-methyl-3-[3,4,5-trihydroxy-6-(hydroxymethyl)oxan-2-yl]oxy-9,10-dioxatetracyclo[4.3.1.02,5.03,8]decan-4-yl]methyl benzoate | 0.467091 | 0.068133 | 0.3954 |
| Flavonifractor | L-Arginine | 0.467043 | 0.068165 | 0.395422 |
| TM7 | S-[(3R,3As,6S,6aS)-3-nitrooxy-2,3,3a,5,6,6a-hexahydrofuro[3,2-b]furan-6-yl] ethanethioate | 0.466947 | 0.068229 | 0.395469 |
| Enterococcus | Phytal | -0.46692 | 0.068244 | 0.395469 |
| Intestinimonas | Withaferin A | -0.46691 | 0.068257 | 0.395469 |
| Flavonifractor | m7G(5')pppAm | 0.466781 | 0.06834 | 0.395774 |
| Enterococcus | Polypropylene glycol (m w 1,200-3,000) | -0.46662 | 0.068445 | 0.395774 |
| Bryobacter | Phosphohydroxypyruvic acid | -0.46661 | 0.068457 | 0.395774 |
| Treponema | 1-p-Menthen-3-one | 0.466569 | 0.068483 | 0.395774 |
| Achromobacter | LysoPI(16:0/0:0) | 0.466507 | 0.068524 | 0.395774 |
| Enterorhabdus | Pro-Pro-Pro | 0.466456 | 0.068559 | 0.395774 |
| Bryobacter | N-[(2R,3R,4S,6R)-4,6-Dihydroxy-6-methyl-2-[(1R,2R)-1,2,3-trihydroxypropyl]oxan-3-yl]acetamide | 0.466425 | 0.068579 | 0.395774 |
| MND1 | (2S)-1,1,1-Trifluoro-2-(4-pyrazol-1-ylphenyl)-3-[5-[[1-(trifluoromethyl)cyclopropyl]methyl]-1H-imidazol-2-yl]propan-2-ol | -0.46635 | 0.068631 | 0.395774 |
| Clostridia_vadinBB60_group | thiamphenicol | 0.46629 | 0.068671 | 0.395774 |
| Coprobacillus | 3-(3-(Pyridin-3-yl)-1,2,4-oxadiazol-5-yl)benzonitrile | 0.466256 | 0.068693 | 0.395774 |
| Granulicella | 1-[3,4-Dihydroxy-5-(hydroxymethyl)-2-oxolanyl]-1,2,4-triazole-3-carboxamide | 0.466211 | 0.068724 | 0.395774 |
| MND1 | (2R,4S)-2-Aminoformyl-6-fluoro-spiro[chroman-4,4'-imidazolidine]-2',5'-dione | -0.46621 | 0.068728 | 0.395774 |
| Enterococcus | Berkeleylactone L | -0.46618 | 0.068747 | 0.395774 |
| RF39 | Resorufin | 0.466149 | 0.068766 | 0.395774 |
| RF39 | Cichorioside K | -0.46615 | 0.068766 | 0.395774 |
| Nitrospira | 3-{[(1s)-2,2-Difluoro-1-Hydroxy-7-(Methylsulfonyl)-2,3-Dihydro-1h-Inden-4-Yl]oxy}-5-Fluorobenzonitrile | 0.46614 | 0.068772 | 0.395774 |
| IMCC26256 | Dihydrozeatin O-beta-D-Glucoside | 0.466117 | 0.068787 | 0.395774 |
| MND1 | Fexaramine | -0.46599 | 0.068874 | 0.396107 |
| IMCC26256 | 1-[3,4-Dihydroxy-5-(hydroxymethyl)-2-oxolanyl]-1,2,4-triazole-3-carboxamide | 0.46593 | 0.068913 | 0.396107 |
| Subgroup_2 | Cysteinyl-Aspartate | 0.465894 | 0.068937 | 0.396107 |
| Rikenellaceae_RC9_gut_group | N-(N-(3-Amino-3-carboxypropyl)-3-amino-3-carboxypropyl)azetidine-2-carboxylic acid | 0.465809 | 0.068995 | 0.396107 |
| Monoglobus | L-Arginine | 0.465804 | 0.068998 | 0.396107 |
| Pseudolabrys | PE(22:5(4Z,7Z,10Z,13Z,16Z)/PGD2) | -0.46578 | 0.069014 | 0.396107 |
| RF39 | Notoginsenoside H | 0.465663 | 0.069094 | 0.396154 |
| Tuzzerella | 14-Methylpentadecanoylcarnitine | 0.465596 | 0.069139 | 0.396154 |
| Enterococcus | Ricinoleic acid | -0.46559 | 0.06914 | 0.396154 |
| TM7 | N-Acetyl-L-methionine | 0.465569 | 0.069157 | 0.396154 |
| Acidothermus | Glycerol 3-phosphate | 0.465552 | 0.069169 | 0.396154 |
| Treponema | Phenylalanylaspartic acid | 0.465438 | 0.069246 | 0.396154 |
| Treponema | 2',3'-Dideoxyuridine | 0.465417 | 0.069261 | 0.396154 |
| Rikenellaceae_RC9_gut_group | Fiacitabine | 0.465354 | 0.069303 | 0.396154 |
| Clostridioides | PC(P-18:0/20:4(5Z,8Z,11Z,14Z)-OH(16R)) | 0.465338 | 0.069314 | 0.396154 |
| Bryobacter | Serotinose | 0.465323 | 0.069325 | 0.396154 |
| Enterococcus | PGP(18:3(9Z,12Z,15Z)/18:1(12Z)-O(9S,10R)) | 0.465312 | 0.069332 | 0.396154 |
| Faecalibaculum | Adomeglivant | 0.465251 | 0.069373 | 0.396197 |
| MND1 | Cortisol | -0.46522 | 0.069396 | 0.396197 |
| Lachnospiraceae_UCG-006 | 3-Hydroxy-10'-apo-b,y-carotenal | 0.46517 | 0.069428 | 0.396222 |
| Alcaligenes | 1-(10H-Phenothiazin-2-yl)ethanone | 0.464954 | 0.069575 | 0.396578 |
| Tuzzerella | Cysteine-glutathione disulfide | 0.464885 | 0.069622 | 0.396578 |
| Faecalibaculum | Valylhistidine | 0.464875 | 0.069628 | 0.396578 |
| Nitrospira | Cichorioside K | -0.46484 | 0.069655 | 0.396578 |
| Alcaligenes | 1-[3,4-Dihydroxy-5-(hydroxymethyl)-2-oxolanyl]-1,2,4-triazole-3-carboxamide | 0.46482 | 0.069666 | 0.396578 |
| Rikenellaceae_RC9_gut_group | PE(19:0/20:2(11Z,14Z)) | -0.46481 | 0.069674 | 0.396578 |
| Acetatifactor | 2-(Methylthiomethyl)-3-phenyl-2-propenal | 0.464738 | 0.069722 | 0.396578 |
| Clostridioides | Cysteinyl-Aspartate | 0.464723 | 0.069732 | 0.396578 |
| bacteriap25 | Allitridin | 0.464705 | 0.069744 | 0.396578 |
| Acidothermus | Glutaminylproline | 0.464384 | 0.069963 | 0.397528 |
| Subgroup_2 | Sulisobenzone | 0.464258 | 0.070049 | 0.397528 |
| Aquicella | N-(1-Deoxy-1-fructosyl)alanine | 0.464247 | 0.070057 | 0.397528 |
| Subgroup_13 | NAD | 0.464234 | 0.070065 | 0.397528 |
| Acidothermus | 6-Hydroxytricetin 5-rhamnoside | 0.464206 | 0.070085 | 0.397528 |
| Gemella | 3-{[(1s)-2,2-Difluoro-1-Hydroxy-7-(Methylsulfonyl)-2,3-Dihydro-1h-Inden-4-Yl]oxy}-5-Fluorobenzonitrile | 0.464172 | 0.070108 | 0.397528 |
| Lachnospiraceae_UCG-006 | PE(22:5(4Z,7Z,10Z,13Z,16Z)/PGD2) | -0.46417 | 0.070109 | 0.397528 |
| Anaerotruncus | (2S)-3-[3-[(4-Carbamimidoylbenzoyl)amino]propanoylamino]-2-[(4-ethylphenyl)sulfonylamino]propanoic acid | 0.464089 | 0.070165 | 0.397687 |
| Achromobacter | Camptothecin sodium | 0.46396 | 0.070253 | 0.397912 |
| Acidothermus | PGP(18:3(9Z,12Z,15Z)/18:1(12Z)-O(9S,10R)) | 0.463915 | 0.070284 | 0.397912 |
| Faecalibaculum | Dehydroascorbic acid | 0.463844 | 0.070333 | 0.397912 |
| Lachnospiraceae_UCG-006 | Nopalinic acid | 0.463837 | 0.070337 | 0.397912 |
| Granulicella | N,N'-Diethylthiourea | 0.463824 | 0.070346 | 0.397912 |
| Intestinimonas | N-Acetyl-b-glucosaminylamine | 0.463655 | 0.070463 | 0.398411 |
| Subgroup_13 | 3-Amino-3-methylbutanoic acid | 0.463556 | 0.07053 | 0.398626 |
| Clostridioides | N2-gamma-Glutamylglutamine | 0.463422 | 0.070622 | 0.398626 |
| Enterococcus | 4-Methylcatechol 1-glucuronide | 0.463414 | 0.070628 | 0.398626 |
| TRA3-20 | Zidebactam | 0.463407 | 0.070633 | 0.398626 |
| Granulicella | Creatine | 0.463393 | 0.070642 | 0.398626 |
| Gemella | Cysteine-glutathione disulfide | 0.463345 | 0.070675 | 0.398651 |
| Aquicella | Dipotassium phosphate | 0.463196 | 0.070778 | 0.398848 |
| Achromobacter | PC(16:1(9Z)/17:1(9Z)) | -0.46319 | 0.070779 | 0.398848 |
| Subgroup_2 | Glutamylleucylarginine | 0.463171 | 0.070795 | 0.398848 |
| Enterococcus | N-(2-Hydroxyethyl)-2-(1-isoquinolinylmethylene)hydrazinecarbothioamide | 0.46299 | 0.07092 | 0.399393 |
| Clostridioides | 3-Amino-2-methoxynonadec-5-en-4-ol | -0.46278 | 0.071062 | 0.400032 |
| Jatrophihabitans | Orotidine | 0.46274 | 0.071092 | 0.400042 |
| Clostridioides | m7G(5')pppAm | 0.462687 | 0.071129 | 0.400091 |
| Monoglobus | 5S-HETE di-endoperoxide | -0.46252 | 0.071243 | 0.400571 |
| Pseudolabrys | Dehydroascorbic acid | 0.46248 | 0.071272 | 0.400575 |
| MND1 | Lemborexant | -0.4624 | 0.071328 | 0.400585 |
| Alcaligenes | Xanthosine | 0.462391 | 0.071334 | 0.400585 |
| Tuzzerella | 3-Hydroxyeicosanoylcarnitine | 0.462354 | 0.071359 | 0.400585 |
| Pseudolabrys | PC(TXB2/20:2(11Z,14Z)) | -0.46225 | 0.071432 | 0.400767 |
| Treponema | Aspartyl-Gamma-glutamate | 0.462177 | 0.071482 | 0.400767 |
| Enterococcus | PIP(20:0/18:1(12Z)-2OH(9,10)) | -0.46215 | 0.071501 | 0.400767 |
| Anaerotruncus | PC(18:1(17Z)/18:1(17Z)) | 0.462143 | 0.071506 | 0.400767 |
| Subgroup_2 | 3-O-acetylecdysone 2-phosphate | -0.46201 | 0.0716 | 0.401032 |
| IMCC26256 | Neryl 8-methyldecanoate | 0.461993 | 0.07161 | 0.401032 |
| Granulicella | S-[(3R,3As,6S,6aS)-3-nitrooxy-2,3,3a,5,6,6a-hexahydrofuro[3,2-b]furan-6-yl] ethanethioate | 0.461779 | 0.071759 | 0.401543 |
| Enterorhabdus | Cichorioside K | -0.46178 | 0.07176 | 0.401543 |
| bacteriap25 | Pantothenic acid | 0.461685 | 0.071824 | 0.401543 |
| Alcaligenes | PIP(20:0/18:1(12Z)-2OH(9,10)) | -0.46164 | 0.071858 | 0.401543 |
| Acidothermus | hydroxyhexadecenoylcarnitine | 0.461589 | 0.071891 | 0.401543 |
| Anaerotruncus | N-Acetyl-L-methionine | 0.461566 | 0.071907 | 0.401543 |
| Jatrophihabitans | 7-Hydroxy-R-acenocoumarol | 0.4615 | 0.071953 | 0.401543 |
| Monoglobus | 2-hydroxy-9Z,12Z-Octadecadienoic acid | -0.4615 | 0.071954 | 0.401543 |
| Tuzzerella | Tephcalostan | 0.461493 | 0.071958 | 0.401543 |
| Acetatifactor | PC(P-18:0/20:4(5Z,8Z,11Z,14Z)-OH(16R)) | 0.461399 | 0.072023 | 0.401748 |
| Gemella | PC(P-18:0/20:4(5Z,8Z,11Z,14Z)-OH(16R)) | 0.461277 | 0.072108 | 0.402063 |
| Subgroup_2 | PE(18:0/19:1(9Z)) | -0.46118 | 0.072179 | 0.402105 |
| Acetatifactor | Pentadeca-3,5,7-trienedioylcarnitine | 0.461156 | 0.072192 | 0.402105 |
| Acetatifactor | 11,14,17-eicosatrienoic acid | 0.461138 | 0.072206 | 0.402105 |
| Subgroup_2 | 2-C-methyl-D-erythritol-4-phosphate | 0.4611 | 0.072232 | 0.402105 |
| Monoglobus | Cortisol | -0.46105 | 0.072269 | 0.402105 |
| Enterorhabdus | 16,17-Dihydro-16alpha,17-dihydroxygibberellin A4 17-glucoside | -0.46102 | 0.072287 | 0.402105 |
| TRA3-20 | scyllo-Inositol | 0.460915 | 0.072362 | 0.402278 |
| Clostridia_vadinBB60_group | Ac-Ser-Asp-Lys-Pro-OH | 0.460884 | 0.072383 | 0.402278 |
| Monoglobus | Skepinone-L | 0.460854 | 0.072404 | 0.402278 |
| Clostridioides | thiamphenicol | 0.460716 | 0.072501 | 0.40232 |
| Subgroup_2 | Camptothecin sodium | 0.460691 | 0.072518 | 0.40232 |
| Granulicella | JP83 | -0.46066 | 0.072539 | 0.40232 |
| Anaerotruncus | 23S,25,26-Trihydroxyvitamin D3 | 0.46065 | 0.072547 | 0.40232 |
| Achromobacter | Nopalinic acid | 0.460639 | 0.072554 | 0.40232 |
| Acetatifactor | 1-Hydroxy-2-naphthoic acid | 0.460512 | 0.072644 | 0.402513 |
| Intestinimonas | thiamphenicol | 0.460465 | 0.072677 | 0.402513 |
| Enterococcus | Kuguacin E | -0.46044 | 0.072695 | 0.402513 |
| MND1 | Carissanol | -0.46043 | 0.072703 | 0.402513 |
| Alcaligenes | Nopalinic acid | 0.460352 | 0.072756 | 0.402647 |
| Muribaculum | 3-hydroxyhexadecanoyl carnitine | 0.460296 | 0.072795 | 0.402705 |
| Tuzzerella | Selinexor | 0.460195 | 0.072866 | 0.402939 |
| Faecalibaculum | Aspartyl-Isoleucine | 0.46013 | 0.072912 | 0.402958 |
| Treponema | Serotinose | 0.460109 | 0.072927 | 0.402958 |
| Gemella | Lucuminic acid | 0.459955 | 0.073035 | 0.403398 |
| Anaerotruncus | Erucic acid | 0.459871 | 0.073094 | 0.403567 |
| Rikenellaceae_RC9_gut_group | Glycerol 3-phosphate | 0.45974 | 0.073187 | 0.403699 |
| Monoglobus | 5beta-Cholane-3alpha,24-diol | 0.45967 | 0.073236 | 0.403699 |
| Clostridia_vadinBB60_group | 2,2,3,3,4,4,5,5,6,6,7,7,8,8,9,9-Hexadecafluorononanoic acid | 0.459598 | 0.073287 | 0.403699 |
| Enterorhabdus | LysoPE(20:3(8Z,11Z,14Z)/0:0) | 0.459588 | 0.073294 | 0.403699 |
| Clostridioides | 4-(Methylnitrosamino)-1-(3-pyridyl)-1-butanol glucuronide | 0.459562 | 0.073313 | 0.403699 |
| Enterorhabdus | LysoPI(16:0/0:0) | 0.45956 | 0.073314 | 0.403699 |
| Lachnospiraceae_UCG-006 | 5beta-Cholane-3alpha,24-diol | 0.459543 | 0.073326 | 0.403699 |
| Monoglobus | S-cucujolide III | -0.45951 | 0.073348 | 0.403699 |
| Bryobacter | 2,2,3,3,4,4,5,5,6,6,7,7,8,8,9,9-Hexadecafluorononanoic acid | 0.45927 | 0.073519 | 0.404482 |
| Clostridioides | PC(11:0/23:0) | 0.4592 | 0.073569 | 0.404512 |
| Achromobacter | Ethynodiol | -0.45918 | 0.073582 | 0.404512 |
| Anaerotruncus | PI(20:3(8Z,11Z,14Z)/0:0) | 0.459033 | 0.073687 | 0.40473 |
| Enterococcus | 2-Methylthioadenosine | 0.459014 | 0.0737 | 0.40473 |
| Jatrophihabitans | 13-(3,4-Dimethyl-5-propylfuran-2-yl)tridecanoylcarnitine | 0.458983 | 0.073723 | 0.40473 |
| Tuzzerella | Histamine | -0.45896 | 0.073736 | 0.40473 |
| [Eubacterium]_oxidoreducens_group | Aspartyl-Alanine | 0.458902 | 0.07378 | 0.404812 |
| Intestinimonas | Monooctyl phthalate | -0.45867 | 0.073947 | 0.405418 |
| Intestinimonas | benfluorex | 0.458665 | 0.073948 | 0.405418 |
| Clostridioides | PC(18:1(17Z)/18:1(17Z)) | 0.458559 | 0.074024 | 0.405676 |
| Anaerotruncus | Iguratimod | -0.45841 | 0.074127 | 0.406083 |
| Enterorhabdus | N-Myristoyl Asparagine | -0.45832 | 0.074196 | 0.406303 |
| Solobacterium | Erucic acid | 0.458173 | 0.074299 | 0.406708 |
| Anaerotruncus | Pevonedistat | 0.457931 | 0.074471 | 0.407493 |
| Pseudolabrys | 1-O-(2R-methoxy-hexadecyl)-sn-glycerol | -0.45782 | 0.074549 | 0.407691 |
| Enterococcus | Azeloprazole | 0.457749 | 0.074601 | 0.407691 |
| Pseudolabrys | Iguratimod | -0.45775 | 0.074604 | 0.407691 |
| Alcaligenes | LysoPI(16:0/0:0) | 0.457719 | 0.074623 | 0.407691 |
| Granulicella | Fiacitabine | 0.457587 | 0.074717 | 0.408048 |
| Jatrophihabitans | 11-Hydroxyhexadecanoylcarnitine | 0.457486 | 0.07479 | 0.408283 |
| Pseudolabrys | N-(2-Hydroxyethyl)-2-(1-isoquinolinylmethylene)hydrazinecarbothioamide | 0.457346 | 0.07489 | 0.40852 |
| Tuzzerella | Maltotriose | 0.457345 | 0.074891 | 0.40852 |
| Anaerotruncus | L-Arginine | 0.457245 | 0.074962 | 0.408751 |
| Enterorhabdus | Docosatrienoic acid | 0.457114 | 0.075057 | 0.409108 |
| Monoglobus | N-(1-Deoxy-1-fructosyl)valine | 0.457057 | 0.075098 | 0.409171 |
| MND1 | PC(P-18:0/20:4(5Z,8Z,11Z,14Z)-OH(16R)) | 0.456966 | 0.075163 | 0.409187 |
| Tuzzerella | Eicosadienoic acid | 0.456931 | 0.075189 | 0.409187 |
| Pseudolabrys | Bis(2-propoxyethyl) 2,6-dimethyl-4-(3-nitrophenyl)-3,4-dihydropyridine-3,5-dicarboxylate | -0.45683 | 0.07526 | 0.409187 |
| Acidothermus | 3-Amino-3-methylbutanoic acid | 0.456819 | 0.075269 | 0.409187 |
| Treponema | 11-Hydroxyhexadecanoylcarnitine | 0.456801 | 0.075282 | 0.409187 |
| Enterorhabdus | Nopalinic acid | 0.456792 | 0.075288 | 0.409187 |
| bacteriap25 | (13Z)-3-Hydroxyicos-13-enoylcarnitine | 0.45677 | 0.075304 | 0.409187 |
| Lachnospiraceae_UCG-006 | [(2S,4R,5R,6R,14S,16R)-14-Hydroxy-7,11-dimethyl-6-(2-oxopyran-4-yl)-3-oxapentacyclo[8.8.0.02,4.02,7.011,16]octadecan-5-yl] acetate | -0.45672 | 0.075339 | 0.409196 |
| Subgroup_2 | Thiazolidine-4-carboxylic acid | 0.456671 | 0.075375 | 0.409196 |
| Enterococcus | N-Acetyl-b-glucosaminylamine | 0.456647 | 0.075393 | 0.409196 |
| Nitrospira | Fiacitabine | 0.456503 | 0.075497 | 0.409456 |
| Pseudolabrys | 12-Hydroxydodecanoic acid | -0.45647 | 0.075521 | 0.409456 |
| Clostridioides | Oliceridine | -0.45646 | 0.075528 | 0.409456 |
| [Eubacterium]_oxidoreducens_group | xi-2,3-Octadiene-5,7-diyn-1-ol | 0.456184 | 0.075727 | 0.410316 |
| Alcaligenes | N-Acetyl-L-methionine | 0.456107 | 0.075783 | 0.410316 |
| Tuzzerella | 16-Methylheptadecanoylcarnitine | 0.45603 | 0.075838 | 0.410316 |
| Alcaligenes | 2,2,3,3,4,4,5,5,6,6,7,7,8,8,9,9-Hexadecafluorononanoic acid | 0.456018 | 0.075847 | 0.410316 |
| Aquicella | Eicosadienoic acid | 0.456011 | 0.075852 | 0.410316 |
| TRA3-20 | 25-Hydroxyvitamin D3-26,23-lactol | -0.45594 | 0.075901 | 0.410316 |
| Subgroup_13 | trans-Hexadec-2-enoyl carnitine | 0.455928 | 0.075912 | 0.410316 |
| Monoglobus | 7-oxo-8-amino-nonanoic acid | -0.45592 | 0.07592 | 0.410316 |
| TRA3-20 | PE(18:0/19:1(9Z)) | -0.45585 | 0.075966 | 0.41041 |
| Enterococcus | Oleoyl Serotonin | -0.45573 | 0.076059 | 0.410754 |
| Alcaligenes | 12-Hydroxydodecanoic acid | -0.45565 | 0.076113 | 0.410758 |
| Clostridioides | Monooctyl phthalate | -0.45564 | 0.076121 | 0.410758 |
| Bryobacter | Phytosphingosine | 0.455549 | 0.076187 | 0.410758 |
| Subgroup_2 | (R)-Propyl 2-amino-3-mercaptopropanoate | 0.455527 | 0.076203 | 0.410758 |
| Flavonifractor | Mycobactins | -0.45552 | 0.076206 | 0.410758 |
| Treponema | PI(20:4(5Z,8Z,11Z,14Z)/18:0) | -0.45541 | 0.076289 | 0.410961 |
| Anaerotruncus | PS(22:5(7Z,10Z,13Z,16Z,19Z)/22:6(4Z,7Z,10Z,13Z,16Z,19Z)) | -0.45539 | 0.076302 | 0.410961 |
| Gemella | Fiacitabine | 0.455301 | 0.076367 | 0.411065 |
| Clostridioides | N-lactoyl-Methionine | 0.455281 | 0.076382 | 0.411065 |
| Enterococcus | (E,E)-3,7,11-Trimethyl-2,6,10-dodecatrienyl octanoate | 0.455241 | 0.076411 | 0.411065 |
| Muribaculum | N-(N-(3-Amino-3-carboxypropyl)-3-amino-3-carboxypropyl)azetidine-2-carboxylic acid | 0.455205 | 0.076438 | 0.411065 |
| Solobacterium | Am-PE(16:0/18:0) | 0.455075 | 0.076532 | 0.411414 |
| RF39 | 25-Hydroxyvitamin D3-26,23-lactol | -0.45496 | 0.076616 | 0.411711 |
| Enterorhabdus | Dolichyl b-D-glucosyl phosphate | -0.45481 | 0.076727 | 0.412149 |
| Enterococcus | thiamphenicol | 0.45475 | 0.076769 | 0.412218 |
| Tuzzerella | (4E,15E)-Bilirubin | -0.45463 | 0.076859 | 0.412543 |
| Enterococcus | PC(11:0/23:0) | 0.454436 | 0.076999 | 0.413135 |
| Enterococcus | 9-Hydroxy-4-methoxypsoralen 9-glucoside | 0.454335 | 0.077073 | 0.413268 |
| MND1 | 3-Methyl-3-butenyl apiosyl-(1->6)-glucoside | 0.454285 | 0.077109 | 0.413268 |
| Intestinimonas | Docosatrienoic acid | 0.454282 | 0.077112 | 0.413268 |
| Enterococcus | PE(22:4(7Z,10Z,13Z,16Z)/19:0) | -0.45401 | 0.077311 | 0.413839 |
| Anaerotruncus | Prolylproline | 0.453996 | 0.077321 | 0.413839 |
| RF39 | 4-(Methylnitrosamino)-1-(3-pyridyl)-1-butanol glucuronide | 0.453977 | 0.077335 | 0.413839 |
| Anaerotruncus | PC(16:0/16:0) | 0.453968 | 0.077342 | 0.413839 |
| Tuzzerella | Inosine | 0.45392 | 0.077377 | 0.413839 |
| Clostridia_vadinBB60_group | hydroxyhexadecenoylcarnitine | 0.453876 | 0.07741 | 0.413839 |
| Solobacterium | Pentadeca-3,5,7-trienedioylcarnitine | 0.453782 | 0.077478 | 0.413839 |
| Tuzzerella | 3-Methyl-3-butenyl hexadecanoate | 0.453779 | 0.077481 | 0.413839 |
| Monoglobus | (R)-4A-(Ethoxymethyl)-1-(4-fluorophenyl)-6-((4-(trifluoromethyl)phenyl)sulfonyl)-4,4a,5,6,7,8-hexahydro-1H-pyrazolo[3,4-g]isoquinoline | -0.45376 | 0.077496 | 0.413839 |
| Solobacterium | 3-{[(1s)-2,2-Difluoro-1-Hydroxy-7-(Methylsulfonyl)-2,3-Dihydro-1h-Inden-4-Yl]oxy}-5-Fluorobenzonitrile | 0.453737 | 0.077512 | 0.413839 |
| Bryobacter | Azeloprazole | 0.453667 | 0.077563 | 0.413956 |
| Rikenellaceae_RC9_gut_group | PC(TXB2/20:2(11Z,14Z)) | -0.45351 | 0.077679 | 0.414352 |
| IMCC26256 | Mycobactins | -0.45349 | 0.077697 | 0.414352 |
| Anaerotruncus | Glutamylleucylarginine | 0.453385 | 0.077771 | 0.414594 |
| Enterorhabdus | Glycyl-D-proline | 0.453296 | 0.077836 | 0.41478 |
| Tuzzerella | LysoPC(P-16:0/0:0) | 0.453257 | 0.077865 | 0.41478 |
| Tuzzerella | 13-(3,4-Dimethyl-5-propylfuran-2-yl)tridecanoylcarnitine | 0.453104 | 0.077979 | 0.415046 |
| Alcaligenes | PE(22:5(4Z,7Z,10Z,13Z,16Z)/PGD2) | -0.45306 | 0.07801 | 0.415046 |
| Anaerotruncus | Neryl 8-methyldecanoate | 0.453053 | 0.078016 | 0.415046 |
| Granulicella | PGP(18:3(9Z,12Z,15Z)/18:1(12Z)-O(9S,10R)) | 0.45303 | 0.078033 | 0.415046 |
| Anaerotruncus | S-Adenosylhomocysteine | 0.452932 | 0.078106 | 0.415275 |
| Alcaligenes | Glutamylleucylarginine | 0.452851 | 0.078166 | 0.415437 |
| Acetatifactor | 1-[3,4-Dihydroxy-5-(hydroxymethyl)-2-oxolanyl]-1,2,4-triazole-3-carboxamide | 0.452748 | 0.078241 | 0.41567 |
| RF39 | N-Acetyl-L-methionine | 0.452694 | 0.078282 | 0.41567 |
| Christensenellaceae_R-7_group | thiamphenicol | 0.452672 | 0.078298 | 0.41567 |
| Acidothermus | Benzyl gentiobioside | 0.452304 | 0.078571 | 0.416914 |
| Faecalibaculum | PC(11:0/23:0) | 0.452276 | 0.078591 | 0.416914 |
| Tuzzerella | Doxepin | 0.452131 | 0.078699 | 0.417318 |
| [Eubacterium]_oxidoreducens_group | alpha-D-Galactopyranuronosyl-(1->4)-alpha-D-galactopyranuronosyl-(1->4)-D-galacturonic acid | 0.452094 | 0.078727 | 0.417318 |
| TRA3-20 | N-(2-Hydroxyethyl)-2-(1-isoquinolinylmethylene)hydrazinecarbothioamide | 0.451882 | 0.078885 | 0.417916 |
| Gemella | Docosatrienoic acid | 0.451839 | 0.078917 | 0.417916 |
| Achromobacter | 12-Hydroxydodecanoic acid | -0.45182 | 0.078929 | 0.417916 |
| TRA3-20 | U-75302 | 0.451532 | 0.079146 | 0.418907 |
| Lachnospiraceae_UCG-006 | 5-[1-Carboxy-2-(trimethylazaniumyl)ethoxy]-5-oxopentanoate | 0.451472 | 0.079191 | 0.418989 |
| Clostridioides | 13,14-dihydro-15-keto-PGD2-d4 | -0.45137 | 0.079265 | 0.419189 |
| Alcaligenes | Resorufin | 0.451341 | 0.079288 | 0.419189 |
| Achromobacter | Glutamylleucylarginine | 0.451283 | 0.079332 | 0.419263 |
| Subgroup_13 | Xanthosine | 0.45122 | 0.079379 | 0.419356 |
| Acidothermus | PC(20:5(5Z,8Z,11Z,14Z,17Z)/PGJ2) | -0.451 | 0.079545 | 0.420021 |
| Achromobacter | Pelargonidin 3,5-di-(6-acetylglucoside) | -0.45097 | 0.079565 | 0.420021 |
| bacteriap25 | (S)-a-Amino-2,5-dihydro-5-oxo-4-isoxazolepropanoic acid N2-glucoside | 0.450809 | 0.079687 | 0.420509 |
| Pseudolabrys | Glycyl-D-proline | 0.450757 | 0.079726 | 0.420557 |
| Enterorhabdus | 13-(3,4-Dimethyl-5-propylfuran-2-yl)tridecanoylcarnitine | 0.450652 | 0.079805 | 0.420672 |
| TRA3-20 | Ricinoleic acid | -0.45063 | 0.079819 | 0.420672 |
| Rikenellaceae_RC9_gut_group | Tyrosyl-Aspartate | 0.450605 | 0.07984 | 0.420672 |
| Clostridioides | Pro-Pro-Pro | 0.450529 | 0.079898 | 0.420672 |
| Enterococcus | 5S-HETE di-endoperoxide | -0.45051 | 0.079909 | 0.420672 |
| MND1 | Uridine | 0.450489 | 0.079927 | 0.420672 |
| Rikenellaceae_RC9_gut_group | Am-PE(16:0/18:0) | 0.450371 | 0.080016 | 0.420983 |
| Enterococcus | PS(22:5(7Z,10Z,13Z,16Z,19Z)/22:6(4Z,7Z,10Z,13Z,16Z,19Z)) | -0.45024 | 0.080118 | 0.421209 |
| Treponema | N-Acetyl-b-glucosaminylamine | 0.450234 | 0.080119 | 0.421209 |
| Faecalibaculum | thiamphenicol | 0.450166 | 0.08017 | 0.421321 |
| TRA3-20 | xi-2,3-Octadiene-5,7-diyn-1-ol | 0.450028 | 0.080274 | 0.421709 |
| Rikenellaceae_RC9_gut_group | O-Linoleoylcarnitine | 0.449886 | 0.080381 | 0.422115 |
| Monoglobus | L-Glutamine | 0.449609 | 0.080591 | 0.422851 |
| Enterorhabdus | 2-[4-(3-Hydroxypropyl)-2-methoxyphenoxy]-1,3-propanediol 1-xyloside | -0.4496 | 0.080595 | 0.422851 |
| [Eubacterium]_oxidoreducens_group | Glucose-6-glutamate | 0.44955 | 0.080636 | 0.422851 |
| Anaerotruncus | OKOHA-PA | -0.44954 | 0.080642 | 0.422851 |
| Lachnospiraceae_UCG-006 | 1-(10H-Phenothiazin-2-yl)ethanone | 0.449391 | 0.080756 | 0.423292 |
| RF39 | Lemborexant | -0.44932 | 0.080808 | 0.423406 |
| [Eubacterium]_oxidoreducens_group | m7G(5')pppAm | 0.449209 | 0.080894 | 0.423699 |
| MND1 | 2-(Methylthiomethyl)-3-phenyl-2-propenal | 0.449109 | 0.08097 | 0.423942 |
| TRA3-20 | (R)-3-Amino-2-fluoropropyl phosphenite | 0.449058 | 0.081009 | 0.423985 |
| Acetatifactor | 20-HETE-d6 | 0.448903 | 0.081127 | 0.424445 |
| Acidothermus | Prolylproline | 0.448856 | 0.081162 | 0.424475 |
| Anaerotruncus | 5-Hydroxyindoxyl sulfate | -0.44881 | 0.081197 | 0.4245 |
| Acidothermus | 3'-Ketolactose | 0.448751 | 0.081242 | 0.424575 |
| TM7 | PI(20:3(8Z,11Z,14Z)/0:0) | 0.448652 | 0.081318 | 0.424813 |
| Acetatifactor | L-Arginine | 0.448612 | 0.081348 | 0.424814 |
| [Eubacterium]_oxidoreducens_group | L-Glutamine | 0.448487 | 0.081443 | 0.425153 |
| Achromobacter | 1-[3,4-Dihydroxy-5-(hydroxymethyl)-2-oxolanyl]-1,2,4-triazole-3-carboxamide | 0.448385 | 0.081521 | 0.4254 |
| Clostridioides | 8-Amino-7-oxononanoic acid | -0.44825 | 0.081627 | 0.425795 |
| Alcaligenes | 5beta-Cholane-3alpha,24-diol | 0.448135 | 0.081712 | 0.42608 |
| [Eubacterium]_oxidoreducens_group | N-(1-Deoxy-1-fructosyl)alanine | 0.44804 | 0.081784 | 0.426185 |
| RF39 | 8-Amino-7-oxononanoic acid | -0.44803 | 0.081792 | 0.426185 |
| Treponema | LysoPE(20:3(8Z,11Z,14Z)/0:0) | 0.447924 | 0.081873 | 0.42645 |
| Subgroup_2 | Tercatain | 0.447826 | 0.081948 | 0.426683 |
| Nitrospira | Ac-Ser-Asp-Lys-Pro-OH | 0.447746 | 0.082009 | 0.426842 |
| IMCC26256 | m7G(5')pppAm | 0.447659 | 0.082076 | 0.427003 |
| Clostridioides | 12-Hydroxydodecanoic acid | -0.4476 | 0.082123 | 0.427003 |
| Aquicella | Erucic acid | 0.447581 | 0.082136 | 0.427003 |
| Achromobacter | N-Acetyl-L-methionine | 0.447547 | 0.082161 | 0.427003 |
| Intestinimonas | PE(22:4(7Z,10Z,13Z,16Z)/19:0) | -0.44746 | 0.082227 | 0.427187 |
| RF39 | Dolichyl b-D-glucosyl phosphate | -0.44738 | 0.08229 | 0.427302 |
| bacteriap25 | LysoPE(20:3(8Z,11Z,14Z)/0:0) | 0.447354 | 0.08231 | 0.427302 |
| Enterococcus | 1-O-(2R-methoxy-hexadecyl)-sn-glycerol | -0.44726 | 0.082382 | 0.427448 |
| bacteriap25 | Azeloprazole | 0.447238 | 0.082399 | 0.427448 |
| Rikenellaceae_RC9_gut_group | PC(16:1(9Z)/17:1(9Z)) | -0.44712 | 0.082491 | 0.427769 |
| Alcaligenes | 1-O-Galloylglycerol | 0.447046 | 0.082547 | 0.4279 |
| Enterococcus | 3'-Deoxythymidine | 0.446966 | 0.082609 | 0.427913 |
| Acidothermus | Dipotassium phosphate | 0.446964 | 0.08261 | 0.427913 |
| MND1 | LysoPS(18:1(9Z)/0:0) | -0.44677 | 0.08276 | 0.428314 |
| Christensenellaceae_R-7_group | PI(18:0/20:4(8Z,11Z,14Z,17Z)) | 0.446736 | 0.082786 | 0.428314 |
| Monoglobus | D-erythro-L-galacto-Nonulose | 0.446701 | 0.082813 | 0.428314 |
| TRA3-20 | Glycyl-D-proline | 0.446701 | 0.082813 | 0.428314 |
| Intestinimonas | Cysteinyl-Aspartate | 0.446662 | 0.082843 | 0.428314 |
| Clostridioides | PE(18:0/19:1(9Z)) | -0.44663 | 0.08287 | 0.428314 |
| Intestinimonas | 2-C-methyl-D-erythritol-4-phosphate | 0.446527 | 0.082947 | 0.428554 |
| Enterorhabdus | 1-p-Menthen-3-one | 0.446472 | 0.08299 | 0.428617 |
| Anaerostipes | L-Glutamine | 0.446361 | 0.083075 | 0.428903 |
| Clostridioides | Valylhistidine | 0.446231 | 0.083176 | 0.429263 |
| Rikenellaceae_RC9_gut_group | 5-[1-Carboxy-2-(trimethylazaniumyl)ethoxy]-5-oxopentanoate | 0.446159 | 0.083231 | 0.429299 |
| Bryobacter | (卤)-(Z)-2-(5-Tetradecenyl)cyclobutanone | -0.44614 | 0.083244 | 0.429299 |
| Alcaligenes | benfluorex | 0.446013 | 0.083345 | 0.429663 |
| Enterorhabdus | Prostaglandin B-1 | -0.44593 | 0.083408 | 0.429835 |
| Anaerostipes | Resorufin | 0.44585 | 0.083471 | 0.429999 |
| Achromobacter | 4-Bis(2-hydroxyethyl)amino-L-phenylalanine | 0.445783 | 0.083523 | 0.430108 |
| Bryobacter | Terizidone | 0.445742 | 0.083555 | 0.430118 |
| Pseudolabrys | 5-Hydroxyindoxyl sulfate | -0.44555 | 0.083704 | 0.430727 |
| MND1 | Allitridin | 0.445485 | 0.083755 | 0.430831 |
| Lachnospiraceae_UCG-006 | Fiacitabine | 0.445142 | 0.084022 | 0.431687 |
| Clostridia_vadinBB60_group | 3-Hydroxyeicosanoylcarnitine | 0.445137 | 0.084025 | 0.431687 |
| Enterorhabdus | Neryl 8-methyldecanoate | 0.445135 | 0.084027 | 0.431687 |
| Acidothermus | m7G(5')pppAm | 0.445015 | 0.08412 | 0.431687 |
| Lachnospiraceae_UCG-006 | 2,2,3,3,4,4,5,5,6,6,7,7,8,8,9,9-Hexadecafluorononanoic acid | 0.445006 | 0.084127 | 0.431687 |
| Enterococcus | LysoSM(d18:0) | -0.44498 | 0.084149 | 0.431687 |
| [Eubacterium]_oxidoreducens_group | PC(P-18:0/20:4(5Z,8Z,11Z,14Z)-OH(16R)) | 0.444972 | 0.084154 | 0.431687 |
| Bryobacter | Maltotriose | 0.444956 | 0.084167 | 0.431687 |
| Subgroup_2 | 12-Hydroxydodecanoic acid | -0.44473 | 0.084339 | 0.431862 |
| Monoglobus | Prolylproline | 0.444708 | 0.08436 | 0.431862 |
| Acetatifactor | Tyrosyl-Alanine | 0.44469 | 0.084374 | 0.431862 |
| Alcaligenes | Beta-Citryl-L-glutamic acid | 0.444684 | 0.084379 | 0.431862 |
| Subgroup_13 | Tetradecanoylcarnitine | 0.444666 | 0.084393 | 0.431862 |
| Acetatifactor | All trans decaprenyl diphosphate | -0.44465 | 0.084403 | 0.431862 |
| IMCC26256 | Serylproline | 0.444638 | 0.084415 | 0.431862 |
| Pseudolabrys | U-75302 | 0.44457 | 0.084468 | 0.43195 |
| Treponema | Cysteine-glutathione disulfide | 0.444537 | 0.084494 | 0.43195 |
| Anaerotruncus | Pelargonidin 3-sophoroside | -0.44449 | 0.084532 | 0.43199 |
| Tuzzerella | Octadec-6-enoylcarnitine | 0.444301 | 0.084679 | 0.432521 |
| Aquicella | N-Undecanoylglycine | -0.44428 | 0.084698 | 0.432521 |
| bacteriap25 | Phenylalanylaspartic acid | 0.444146 | 0.0848 | 0.432705 |
| RF39 | PC(11:0/23:0) | 0.444043 | 0.084881 | 0.432705 |
| bacteriap25 | Serotinose | 0.444027 | 0.084894 | 0.432705 |
| Anaerotruncus | N-(N-(3-Amino-3-carboxypropyl)-3-amino-3-carboxypropyl)azetidine-2-carboxylic acid | 0.44399 | 0.084923 | 0.432705 |
| Bryobacter | Beta-Citryl-L-glutamic acid | 0.443925 | 0.084974 | 0.432705 |
| Aquicella | Prolyl-Asparagine | 0.443923 | 0.084975 | 0.432705 |
| bacteriap25 | Ethynodiol | -0.44389 | 0.085004 | 0.432705 |
| MND1 | 1-(10H-Phenothiazin-2-yl)ethanone | 0.44388 | 0.085009 | 0.432705 |
| Clostridioides | 5'-Carboxy-gamma-chromanol | -0.44388 | 0.08501 | 0.432705 |
| Subgroup_13 | O-phosphonato-L-homoserine(2-) | 0.443808 | 0.085066 | 0.432831 |
| RF39 | Cortisol | -0.44366 | 0.085179 | 0.433252 |
| Faecalibaculum | Ciadox | 0.443559 | 0.085262 | 0.43335 |
| Alcaligenes | 3-Hydroxy-10'-apo-b,y-carotenal | 0.443547 | 0.085272 | 0.43335 |
| Enterorhabdus | Oleoyl Serotonin | -0.44351 | 0.085304 | 0.43335 |
| Bryobacter | Prostaglandin B-1 | -0.44348 | 0.085322 | 0.43335 |
| Enterorhabdus | LysoPE(20:4(5Z,8Z,11Z,14Z)/0:0) | -0.44337 | 0.085409 | 0.433474 |
| Christensenellaceae_R-7_group | Am-PE(16:0/18:0) | 0.443369 | 0.085411 | 0.433474 |
| Coprobacillus | 2-[4-(3-Hydroxypropyl)-2-methoxyphenoxy]-1,3-propanediol 1-xyloside | -0.4433 | 0.085468 | 0.433474 |
| Rikenellaceae_RC9_gut_group | [6-Hydroxy-8-methyl-3-[3,4,5-trihydroxy-6-(hydroxymethyl)oxan-2-yl]oxy-9,10-dioxatetracyclo[4.3.1.02,5.03,8]decan-4-yl]methyl benzoate | 0.443296 | 0.085469 | 0.433474 |
| Faecalibaculum | 3-hydroxyhexadecanoyl carnitine | 0.443207 | 0.08554 | 0.433675 |
| Treponema | Kaltostat | 0.443138 | 0.085594 | 0.433794 |
| Alcaligenes | Pelargonidin 3,5-di-(6-acetylglucoside) | -0.44306 | 0.085657 | 0.433934 |
| Achromobacter | PE(22:5(4Z,7Z,10Z,13Z,16Z)/PGD2) | -0.44302 | 0.085683 | 0.433934 |
| Lachnospiraceae_UCG-006 | 1-p-Menthen-3-one | 0.442884 | 0.085795 | 0.434341 |
| Bryobacter | PS(22:5(7Z,10Z,13Z,16Z,19Z)/22:6(4Z,7Z,10Z,13Z,16Z,19Z)) | -0.44279 | 0.085866 | 0.434431 |
| TRA3-20 | 1-Hydroxy-2-naphthoic acid | 0.442784 | 0.085874 | 0.434431 |
| Achromobacter | 1-O-Galloylglycerol | 0.442741 | 0.085908 | 0.434447 |
| Anaerotruncus | PE(22:5(4Z,7Z,10Z,13Z,16Z)/PGD2) | -0.44267 | 0.085965 | 0.434491 |
| Acidothermus | Doxepin | 0.442652 | 0.085979 | 0.434491 |
| Jatrophihabitans | PC(11:0/23:0) | 0.442564 | 0.086049 | 0.434689 |
| Acetatifactor | Glutamylleucylarginine | 0.442497 | 0.086101 | 0.434721 |
| Intestinimonas | 2-Deoxy-2,3-dehydro-n-acetyl-neuraminic acid | 0.442454 | 0.086136 | 0.434721 |
| Bryobacter | Allitridin | 0.442439 | 0.086148 | 0.434721 |
| Clostridioides | PE(22:4(7Z,10Z,13Z,16Z)/19:0) | -0.4423 | 0.08626 | 0.434966 |
| Nitrospira | Niacinamide | 0.442274 | 0.086278 | 0.434966 |
| Tuzzerella | Oleic acid | -0.44226 | 0.086289 | 0.434966 |
| Rikenellaceae_RC9_gut_group | Kaltostat | 0.442136 | 0.086389 | 0.435313 |
| Enterococcus | Monooctyl phthalate | -0.44206 | 0.08645 | 0.435467 |
| Nitrospira | Octadec-6-enoylcarnitine | 0.441924 | 0.086557 | 0.435742 |
| Anaerotruncus | Resorufin | 0.441912 | 0.086567 | 0.435742 |
| Solobacterium | Lucuminic acid | 0.441612 | 0.086806 | 0.43679 |
| RF39 | PI(18:0/20:4(8Z,11Z,14Z,17Z)) | 0.441535 | 0.086867 | 0.436941 |
| Acetatifactor | Serylproline | 0.441372 | 0.086997 | 0.437288 |
| Solobacterium | 11,14,17-eicosatrienoic acid | 0.441371 | 0.086998 | 0.437288 |
| Jatrophihabitans | O-Linoleoylcarnitine | 0.44128 | 0.087071 | 0.437497 |
| Enterococcus | Oleic acid | -0.44121 | 0.08713 | 0.43751 |
| Enterorhabdus | 5S-HETE di-endoperoxide | -0.44114 | 0.087184 | 0.43751 |
| Alcaligenes | 2-Methylthioadenosine | 0.441119 | 0.0872 | 0.43751 |
| Treponema | N-(N-(3-Amino-3-carboxypropyl)-3-amino-3-carboxypropyl)azetidine-2-carboxylic acid | 0.441048 | 0.087256 | 0.43751 |
| Enterorhabdus | 5beta-Cholane-3alpha,24-diol | 0.441044 | 0.08726 | 0.43751 |
| Flavonifractor | PC(P-18:0/20:4(5Z,8Z,11Z,14Z)-OH(16R)) | 0.441042 | 0.087261 | 0.43751 |
| Bryobacter | Aspartyl-Alanine | 0.440993 | 0.087301 | 0.43751 |
| Coprobacillus | Tuberonic acid glucoside | -0.44094 | 0.087346 | 0.43751 |
| Acidothermus | PC(P-18:0/20:4(5Z,8Z,11Z,14Z)-OH(16R)) | 0.440928 | 0.087353 | 0.43751 |
| Treponema | 4-Bis(2-hydroxyethyl)amino-L-phenylalanine | 0.440861 | 0.087406 | 0.437623 |
| Granulicella | PI(20:3(8Z,11Z,14Z)/0:0) | 0.440715 | 0.087524 | 0.438054 |
| Muribaculum | AB-MECA | -0.44066 | 0.087571 | 0.438134 |
| Clostridioides | L-Arginine | 0.440455 | 0.087733 | 0.438788 |
| Nitrospira | 5beta-Cholane-3alpha,24-diol | 0.440351 | 0.087816 | 0.43905 |
| Pseudolabrys | 5-[1-Carboxy-2-(trimethylazaniumyl)ethoxy]-5-oxopentanoate | 0.440271 | 0.087881 | 0.439216 |
| TRA3-20 | Aspartyl-Gamma-glutamate | 0.440171 | 0.087961 | 0.439442 |
| Clostridioides | PC(20:5(5Z,8Z,11Z,14Z,17Z)/PGJ2) | -0.44014 | 0.087988 | 0.439442 |
| Monoglobus | PE(19:0/20:2(11Z,14Z)) | -0.44008 | 0.088035 | 0.43952 |
| RF39 | Tercatain | 0.440011 | 0.08809 | 0.439636 |
| Coprobacillus | Cichorioside K | -0.43992 | 0.088163 | 0.439763 |
| Faecalibaculum | Palmitoylcarnitine | 0.439895 | 0.088183 | 0.439763 |
| Achromobacter | Resorufin | 0.439825 | 0.08824 | 0.439763 |
| Anaerostipes | N-Undecanoylglycine | -0.43981 | 0.088252 | 0.439763 |
| Anaerotruncus | L-arginino-succinate | 0.439726 | 0.08832 | 0.439763 |
| Anaerostipes | PC(11:0/23:0) | 0.439722 | 0.088323 | 0.439763 |
| Clostridioides | (R)-Propyl 2-amino-3-mercaptopropanoate | 0.439672 | 0.088364 | 0.439763 |
| Solobacterium | Cysteine-glutathione disulfide | 0.43967 | 0.088365 | 0.439763 |
| Tuzzerella | L-Carnitine | 0.439587 | 0.088432 | 0.439942 |
| Nitrospira | Avenin | 0.439507 | 0.088497 | 0.440108 |
| Lachnospiraceae_UCG-006 | Cortisol | -0.43926 | 0.088696 | 0.440816 |
| Clostridioides | 3-Methyl-3-butenyl hexadecanoate | 0.439254 | 0.088702 | 0.440816 |
| Enterococcus | L-Arginine | 0.439193 | 0.088751 | 0.440832 |
| TRA3-20 | Resorufin | 0.439145 | 0.08879 | 0.440832 |
| Monoglobus | 5,5-Diphenyl-2-thiohydantoin | 0.439134 | 0.088799 | 0.440832 |
| Acetatifactor | 25-Hydroxyvitamin D3-26,23-lactol | -0.43906 | 0.08886 | 0.44092 |
| Granulicella | Docosadienoate (22:2n6) | 0.439035 | 0.088879 | 0.44092 |
| Bryobacter | LysoPC(P-16:0/0:0) | 0.4389 | 0.088989 | 0.441145 |
| Intestinimonas | N-(1-Deoxy-1-fructosyl)tyrosine | 0.438893 | 0.088994 | 0.441145 |
| Clostridioides | 3,4-Dimethyl-5-propyl-2-furanpentadecanoic acid | -0.43886 | 0.089019 | 0.441145 |
| Bryobacter | Dodecanamide | -0.43882 | 0.08905 | 0.441146 |
| Anaerotruncus | PC(20:4(5Z,8Z,11Z,14Z)-OH(16R)/2:0) | 0.438774 | 0.089091 | 0.441194 |
| Tuzzerella | 3'-Ketolactose | 0.438733 | 0.089125 | 0.4412 |
| Intestinimonas | Eicosadienoic acid | 0.438696 | 0.089155 | 0.4412 |
| Clostridia_vadinBB60_group | (2S)-1,1,1-Trifluoro-2-(4-pyrazol-1-ylphenyl)-3-[5-[[1-(trifluoromethyl)cyclopropyl]methyl]-1H-imidazol-2-yl]propan-2-ol | -0.43864 | 0.089197 | 0.441254 |
| IMCC26256 | Docosadienoate (22:2n6) | 0.438536 | 0.089285 | 0.441374 |
| Bryobacter | Hexadecenal | -0.43851 | 0.089308 | 0.441374 |
| Faecalibaculum | (E)-2-(hexa-3,5-dien-1-yn-1-yl)-5-(prop-1-yn-1-yl)thiophene | 0.438498 | 0.089316 | 0.441374 |
| Aquicella | Cortisol | -0.43845 | 0.089356 | 0.441396 |
| Solobacterium | Eicosadienoic acid | 0.438416 | 0.089383 | 0.441396 |
| TRA3-20 | (卤)-(Z)-2-(5-Tetradecenyl)cyclobutanone | -0.4383 | 0.08948 | 0.441638 |
| Acetatifactor | Iguratimod | -0.43825 | 0.089521 | 0.441638 |
| Monoglobus | N2-(3-Carboxy-2-hydroxy-1-oxopropyl)arginine | 0.43824 | 0.089526 | 0.441638 |
| Faecalibaculum | Peruvianoside II | 0.438183 | 0.089572 | 0.441676 |
| Alcaligenes | (R)-3-Amino-2-fluoropropyl phosphenite | 0.438154 | 0.089596 | 0.441676 |
| RF39 | Fructose-1,6-diphosphate | 0.438114 | 0.089629 | 0.441679 |
| Gemella | Aspartyl-Gamma-glutamate | 0.438076 | 0.08966 | 0.441679 |
| Bryobacter | PI(18:0/20:4(8Z,11Z,14Z,17Z)) | 0.437955 | 0.089759 | 0.442012 |
| Bryobacter | D-Malic acid | 0.437649 | 0.090009 | 0.443092 |
| Alcaligenes | PC(16:1(9Z)/17:1(9Z)) | -0.43757 | 0.090076 | 0.443264 |
| Bryobacter | PE(18:0/19:1(9Z)) | -0.43749 | 0.090141 | 0.443345 |
| Alcaligenes | Medicagol | 0.437471 | 0.090155 | 0.443345 |
| MND1 | Pelargonidin 3,5-di-(6-acetylglucoside) | -0.43727 | 0.090316 | 0.44391 |
| Clostridioides | D-erythro-Sphingosine C-20 | -0.43725 | 0.090337 | 0.44391 |
| Solobacterium | D-Mannose | 0.437195 | 0.090381 | 0.44391 |
| Pseudolabrys | LysoSM(d18:0) | -0.43718 | 0.090396 | 0.44391 |
| Anaerotruncus | (S)-a-Amino-2,5-dihydro-5-oxo-4-isoxazolepropanoic acid N2-glucoside | 0.437124 | 0.09044 | 0.443968 |
| RF39 | Streptidine | 0.436938 | 0.090593 | 0.444566 |
| Subgroup_13 | Heptadecanoyl carnitine | 0.43685 | 0.090665 | 0.444576 |
| Acetatifactor | PC(20:4(5Z,8Z,11Z,14Z)-OH(16R)/2:0) | 0.436848 | 0.090667 | 0.444576 |
| Solobacterium | 20-HETE-d6 | 0.43682 | 0.09069 | 0.444576 |
| Tuzzerella | (S1)-Methoxy-3-heptanethiol | 0.436773 | 0.090729 | 0.444612 |
| Christensenellaceae_R-7_group | DG(18:0/20:4(8Z,11Z,14Z,17Z)/0:0) | 0.436729 | 0.090765 | 0.444634 |
| Treponema | 1-Hydroxy-2-naphthoic acid | 0.436559 | 0.090905 | 0.444811 |
| Clostridioides | benfluorex | 0.436553 | 0.09091 | 0.444811 |
| Subgroup_13 | PC(18:1(17Z)/18:1(17Z)) | 0.43653 | 0.090929 | 0.444811 |
| RF39 | AM2201 N-(4-hydroxypentyl) metabolite | -0.43646 | 0.090983 | 0.444811 |
| Enterorhabdus | N-(1-Deoxy-1-fructosyl)alanine | 0.436431 | 0.091011 | 0.444811 |
| Tuzzerella | 3-Hydroxyoctadecanoylcarnitine | 0.436381 | 0.091052 | 0.444811 |
| Clostridia_vadinBB60_group | Cerulenin | 0.436367 | 0.091064 | 0.444811 |
| IMCC26256 | [6-Hydroxy-8-methyl-3-[3,4,5-trihydroxy-6-(hydroxymethyl)oxan-2-yl]oxy-9,10-dioxatetracyclo[4.3.1.02,5.03,8]decan-4-yl]methyl benzoate | 0.436316 | 0.091105 | 0.444811 |
| TRA3-20 | N-Acetyl-L-methionine | 0.436293 | 0.091124 | 0.444811 |
| Bryobacter | 3-O-acetylecdysone 2-phosphate | -0.43627 | 0.091144 | 0.444811 |
| MND1 | Kuguacin E | -0.43626 | 0.091149 | 0.444811 |
| Intestinimonas | 25-Hydroxyvitamin D3-26,23-lactol | -0.43612 | 0.091267 | 0.445237 |
| Alcaligenes | Ethynodiol | -0.43601 | 0.091361 | 0.445539 |
| Rikenellaceae_RC9_gut_group | Benzyl gentiobioside | 0.435964 | 0.091396 | 0.445557 |
| Clostridia_vadinBB60_group | (2R,4S)-2-Aminoformyl-6-fluoro-spiro[chroman-4,4'-imidazolidine]-2',5'-dione | -0.43588 | 0.091467 | 0.445747 |
| Pseudolabrys | Tyrosyl-Alanine | 0.435743 | 0.091579 | 0.445955 |
| Alcaligenes | Camptothecin sodium | 0.435652 | 0.091655 | 0.445955 |
| bacteriap25 | Kaltostat | 0.43563 | 0.091673 | 0.445955 |
| Subgroup_2 | 4-Chloro-2-nitrobenzylalcohol | -0.4356 | 0.0917 | 0.445955 |
| Clostridioides | N-Acetyl-b-glucosaminylamine | 0.435594 | 0.091704 | 0.445955 |
| bacteriap25 | PI(20:4(5Z,8Z,11Z,14Z)/18:0) | -0.43556 | 0.091728 | 0.445955 |
| Lachnospiraceae_UCG-006 | 1-Ethoxymethyl-5-fluorouracil | 0.43556 | 0.091731 | 0.445955 |
| Pseudolabrys | Polypropylene glycol (m w 1,200-3,000) | -0.43547 | 0.091808 | 0.446174 |
| Bryobacter | Octadecanamide | -0.43518 | 0.092048 | 0.447188 |
| Monoglobus | 1-Octadecene | -0.43508 | 0.092133 | 0.447443 |
| Pseudolabrys | All trans decaprenyl diphosphate | -0.435 | 0.092193 | 0.447583 |
| Rikenellaceae_RC9_gut_group | S-[(3R,3As,6S,6aS)-3-nitrooxy-2,3,3a,5,6,6a-hexahydrofuro[3,2-b]furan-6-yl] ethanethioate | 0.434834 | 0.092336 | 0.448059 |
| Aquicella | Serylproline | 0.434793 | 0.09237 | 0.448059 |
| bacteriap25 | L-arginino-succinate | 0.434754 | 0.092403 | 0.448059 |
| Bryobacter | Pevonedistat | 0.434734 | 0.092419 | 0.448059 |
| Intestinimonas | PS(22:5(7Z,10Z,13Z,16Z,19Z)/22:6(4Z,7Z,10Z,13Z,16Z,19Z)) | -0.43466 | 0.092483 | 0.448215 |
| Monoglobus | 25-Hydroxyvitamin D3-26,23-lactol | -0.43451 | 0.092605 | 0.448651 |
| Intestinimonas | Am-PE(16:0/18:0) | 0.434193 | 0.092871 | 0.449789 |
| Enterorhabdus | LysoPI(18:0/0:0) | 0.434014 | 0.093021 | 0.450344 |
| [Eubacterium]_oxidoreducens_group | N2-(3-Carboxy-2-hydroxy-1-oxopropyl)arginine | 0.433937 | 0.093086 | 0.450344 |
| Treponema | 16-hydroxy-6-hexadecenoic acid | -0.43388 | 0.093134 | 0.450344 |
| Muribaculum | 4-Phenylbutanoylcarnitine | -0.43385 | 0.093158 | 0.450344 |
| Granulicella | 1-p-Menthen-3-one | 0.433834 | 0.093172 | 0.450344 |
| Tuzzerella | N-Phenyl-p-phenylenediamine | -0.43382 | 0.093181 | 0.450344 |
| RF39 | Terizidone | 0.43374 | 0.093251 | 0.450344 |
| Treponema | benfluorex | 0.433732 | 0.093258 | 0.450344 |
| Acidothermus | O-Linoleoylcarnitine | 0.433713 | 0.093274 | 0.450344 |
| Anaerostipes | PE(22:5(4Z,7Z,10Z,13Z,16Z)/PGD2) | -0.43359 | 0.093375 | 0.450443 |
| Faecalibaculum | 5-[1-Carboxy-2-(trimethylazaniumyl)ethoxy]-5-oxopentanoate | 0.433569 | 0.093394 | 0.450443 |
| Clostridioides | 1-Octadecene | -0.43353 | 0.093431 | 0.450443 |
| bacteriap25 | Flumazenil | 0.433467 | 0.093481 | 0.450443 |
| Pseudolabrys | PIP(20:0/18:1(12Z)-2OH(9,10)) | -0.43341 | 0.093528 | 0.450443 |
| Anaerostipes | Dehydroascorbic acid | 0.433401 | 0.093536 | 0.450443 |
| Clostridioides | Eicosadienoic acid | 0.433366 | 0.093566 | 0.450443 |
| Anaerotruncus | Alpha-Lactose | 0.433363 | 0.093568 | 0.450443 |
| MND1 | Quinagolida | -0.43332 | 0.093602 | 0.450443 |
| Solobacterium | Niacinamide | 0.433308 | 0.093614 | 0.450443 |
| Intestinimonas | 1H-Pyrrole-2,5-dione, 3-(1-methyl-1h-indol-3-yl)-4-(1-methyl-6-nitro-1h-indol-3-yl)- | 0.433247 | 0.093666 | 0.450524 |
| Treponema | N2-(3-Carboxy-2-hydroxy-1-oxopropyl)arginine | 0.433188 | 0.093716 | 0.450524 |
| Treponema | All trans decaprenyl diphosphate | -0.43317 | 0.093727 | 0.450524 |
| Achromobacter | N-lactoyl-Tyrosine | 0.433128 | 0.093766 | 0.450555 |
| Subgroup_13 | thiamphenicol | 0.43302 | 0.093857 | 0.450655 |
| Monoglobus | N-Acetyl-b-glucosaminylamine | 0.432945 | 0.09392 | 0.450655 |
| RF39 | 1-O-(2R-hydroxy-hexadecyl)-sn-glycerol | -0.43294 | 0.093928 | 0.450655 |
| Enterorhabdus | 2-Phenylethyl octanoate | -0.43292 | 0.093943 | 0.450655 |
| Christensenellaceae_R-7_group | DG(LTE4/22:0/0:0) | 0.432914 | 0.093947 | 0.450655 |
| Enterorhabdus | Fructose-1,6-diphosphate | 0.4328 | 0.094043 | 0.450962 |
| Faecalibaculum | Maraviroc | 0.432697 | 0.09413 | 0.451228 |
| IMCC26256 | Cortisol | -0.4325 | 0.094301 | 0.451671 |
| Acetatifactor | Ethynodiol | -0.43249 | 0.094303 | 0.451671 |
| Anaerotruncus | 12-Hydroxydodecanoic acid | -0.43246 | 0.09433 | 0.451671 |
| Acidothermus | DG(18:0/20:4(8Z,11Z,14Z,17Z)/0:0) | 0.432436 | 0.094351 | 0.451671 |
| TRA3-20 | 6,10,14-Trimethyl-5,9,13-pentadecatrien-2-one | -0.43222 | 0.094531 | 0.452092 |
| Bryobacter | DG(18:0/20:4(8Z,11Z,14Z,17Z)/0:0) | 0.432187 | 0.094561 | 0.452092 |
| Acetatifactor | Pelargonidin 3,5-di-(6-acetylglucoside) | -0.43218 | 0.094567 | 0.452092 |
| Haliangium | DG(LTE4/22:0/0:0) | 0.432181 | 0.094567 | 0.452092 |
| bacteriap25 | 16,17-Dihydro-16alpha,17-dihydroxygibberellin A4 17-glucoside | -0.43194 | 0.094772 | 0.452792 |
| Faecalibaculum | N-(1-Deoxy-1-fructosyl)valine | 0.431917 | 0.094791 | 0.452792 |
| RF39 | thiamphenicol | 0.431895 | 0.09481 | 0.452792 |
| Anaerotruncus | Amobarbital | 0.431781 | 0.094907 | 0.452949 |
| Subgroup_2 | benfluorex | 0.43178 | 0.094907 | 0.452949 |
| Acidothermus | PI(18:0/20:4(8Z,11Z,14Z,17Z)) | 0.431709 | 0.094968 | 0.453084 |
| Coprobacillus | N,N'-Diethylthiourea | 0.43127 | 0.095341 | 0.454611 |
| Treponema | 3-Hydroxy-11Z-octadecenoylcarnitine | 0.43125 | 0.095359 | 0.454611 |
| Treponema | (S)-a-Amino-2,5-dihydro-5-oxo-4-isoxazolepropanoic acid N2-glucoside | 0.431187 | 0.095413 | 0.454611 |
| Monoglobus | Withaferin A | -0.43118 | 0.095417 | 0.454611 |
| RF39 | DG(18:0/20:4(8Z,11Z,14Z,17Z)/0:0) | 0.430994 | 0.095577 | 0.455221 |
| Tuzzerella | (13Z)-3-Hydroxyicos-13-enoylcarnitine | 0.430857 | 0.095694 | 0.455625 |
| Subgroup_13 | Histamine | -0.4308 | 0.095744 | 0.455709 |
| TM7 | N-Undecanoylglycine | -0.43075 | 0.09579 | 0.455771 |
| Clostridioides | LysoPS(18:1(9Z)/0:0) | -0.43065 | 0.095873 | 0.45601 |
| bacteriap25 | PIP(20:0/18:1(12Z)-2OH(9,10)) | -0.43057 | 0.095939 | 0.456174 |
| Enterococcus | Thidiazuron | 0.430531 | 0.095974 | 0.456184 |
| Alcaligenes | 2',3'-Dideoxyuridine | 0.430437 | 0.096054 | 0.456399 |
| Achromobacter | 4-Chloro-L-phenylalanine | -0.43037 | 0.096113 | 0.456399 |
| Anaerotruncus | Kuguacin E | -0.43036 | 0.096116 | 0.456399 |
| Intestinimonas | Dolichyl b-D-glucosyl phosphate | -0.43021 | 0.096248 | 0.456776 |
| Haliangium | PI(18:0/20:4(8Z,11Z,14Z,17Z)) | 0.430193 | 0.096264 | 0.456776 |
| Aquicella | 3-Ketosphingosine | -0.43016 | 0.096293 | 0.456776 |
| Monoglobus | Nipradilol | 0.429955 | 0.096469 | 0.457454 |
| Intestinimonas | (E,E)-3,7,11-Trimethyl-2,6,10-dodecatrienyl octanoate | 0.42986 | 0.09655 | 0.457688 |
| Achromobacter | 3-Ketosphingosine | -0.42978 | 0.096622 | 0.457872 |
| Pseudolabrys | Withaferin A | -0.42971 | 0.096676 | 0.457974 |
| Anaerotruncus | Glucose-6-glutamate | 0.429604 | 0.096771 | 0.458268 |
| Acidothermus | N-Acetyl-b-glucosaminylamine | 0.429537 | 0.096828 | 0.458389 |
| RF39 | 7-oxo-8-amino-nonanoic acid | -0.42944 | 0.096915 | 0.458565 |
| Anaerotruncus | Allitridin | 0.429418 | 0.096931 | 0.458565 |
| Enterorhabdus | N-lactoyl-Tyrosine | 0.429375 | 0.096969 | 0.45859 |
| Aquicella | PC(16:0/16:0) | 0.429028 | 0.097269 | 0.459597 |
| Aquicella | 7-oxo-8-amino-nonanoic acid | -0.42901 | 0.09728 | 0.459597 |
| Solobacterium | Symmetric dimethylarginine | 0.429015 | 0.09728 | 0.459597 |
| Enterorhabdus | N-lactoyl-Methionine | 0.428875 | 0.097401 | 0.459796 |
| Bryobacter | PE(O-20:0/0:0) | -0.42878 | 0.097486 | 0.459796 |
| Subgroup_13 | PC(16:0/16:0) | 0.428735 | 0.097522 | 0.459796 |
| Enterococcus | PE(O-20:0/0:0) | -0.42873 | 0.097525 | 0.459796 |
| Treponema | 2-Cyclotetradecen-1-one | -0.4287 | 0.097556 | 0.459796 |
| Flavonifractor | [6-Hydroxy-8-methyl-3-[3,4,5-trihydroxy-6-(hydroxymethyl)oxan-2-yl]oxy-9,10-dioxatetracyclo[4.3.1.02,5.03,8]decan-4-yl]methyl benzoate | 0.428618 | 0.097624 | 0.459796 |
| Bryobacter | N-Acetyl-D-Glucosamine 6-Phosphate | 0.428603 | 0.097636 | 0.459796 |
| Anaerotruncus | 5,5-Diphenyl-2-thiohydantoin | 0.428536 | 0.097694 | 0.459796 |
| Nitrospira | PC(16:0/16:0) | 0.428536 | 0.097695 | 0.459796 |
| Tuzzerella | OKOHA-PA | -0.42846 | 0.097763 | 0.459796 |
| Tuzzerella | 3-Hydroxy-11Z-octadecenoylcarnitine | 0.428377 | 0.097833 | 0.459796 |
| IMCC26256 | Am-PE(16:0/18:0) | 0.428364 | 0.097844 | 0.459796 |
| Treponema | Glycerol 3-phosphate | 0.428319 | 0.097883 | 0.459796 |
| Gemella | Am-PE(16:0/18:0) | 0.428283 | 0.097914 | 0.459796 |
| Lachnospiraceae_UCG-006 | Dolichyl b-D-glucosyl phosphate | -0.42826 | 0.097932 | 0.459796 |
| Acetatifactor | PI(18:1(9Z)/0:0) | 0.428261 | 0.097934 | 0.459796 |
| Monoglobus | Pantothenic acid | 0.428177 | 0.098007 | 0.459796 |
| Aquicella | Nopalinic acid | 0.42815 | 0.09803 | 0.459796 |
| Intestinimonas | Cortisol | -0.42814 | 0.098036 | 0.459796 |
| Acetatifactor | Flumazenil | 0.428055 | 0.098113 | 0.459796 |
| Bryobacter | Xanthinol | 0.428045 | 0.098122 | 0.459796 |
| Treponema | Thiazolidine-4-carboxylic acid | 0.42803 | 0.098135 | 0.459796 |
| Tuzzerella | JP83 | -0.42796 | 0.098197 | 0.459796 |
| RF39 | Oleoyl Serotonin | -0.42796 | 0.098199 | 0.459796 |
| Pseudolabrys | 1-O-Galloylglycerol | 0.427954 | 0.098201 | 0.459796 |
| Treponema | 4-(Methylnitrosamino)-1-(3-pyridyl)-1-butanol glucuronide | 0.427945 | 0.098208 | 0.459796 |
| Intestinimonas | Notoginsenoside H | 0.427888 | 0.098258 | 0.459796 |
| TRA3-20 | Kuguacin E | -0.42784 | 0.098296 | 0.459796 |
| [Eubacterium]_oxidoreducens_group | 1-[3,4-Dihydroxy-5-(hydroxymethyl)-2-oxolanyl]-1,2,4-triazole-3-carboxamide | 0.427825 | 0.098313 | 0.459796 |
| Intestinimonas | PE(18:0/19:1(9Z)) | -0.4278 | 0.098332 | 0.459796 |
| Enterococcus | Neryl 8-methyldecanoate | 0.427801 | 0.098334 | 0.459796 |
| Bryobacter | 6,10,14-Trimethyl-5,9,13-pentadecatrien-2-one | -0.42774 | 0.098384 | 0.459878 |
| TRA3-20 | N-(1-Deoxy-1-fructosyl)threonine | 0.427516 | 0.098583 | 0.46059 |
| Lachnospiraceae_UCG-006 | PI(18:1(9Z)/0:0) | 0.427471 | 0.098622 | 0.46059 |
| Tuzzerella | 12-Hydroxy-12-octadecanoylcarnitine | 0.427457 | 0.098635 | 0.46059 |
| Clostridia_vadinBB60_group | 16-Methylheptadecanoylcarnitine | 0.427408 | 0.098677 | 0.460635 |
| Treponema | PC(16:1(9Z)/17:1(9Z)) | -0.42736 | 0.098722 | 0.460693 |
| Anaerostipes | 1-O-(2R-hydroxy-hexadecyl)-sn-glycerol | -0.42722 | 0.09884 | 0.461088 |
| Subgroup_2 | Notoginsenoside H | 0.42713 | 0.09892 | 0.461311 |
| Subgroup_2 | N-(1-Deoxy-1-fructosyl)valine | 0.427072 | 0.098972 | 0.461398 |
| Monoglobus | Diethylamino 2,5-dihydroxybenzenesulfonate | 0.426971 | 0.09906 | 0.461466 |
| TRA3-20 | D-Malic acid | 0.426952 | 0.099076 | 0.461466 |
| Clostridioides | N-(1-Deoxy-1-fructosyl)leucine | 0.426943 | 0.099085 | 0.461466 |
| MND1 | (R)-Propyl 2-amino-3-mercaptopropanoate | 0.42672 | 0.09928 | 0.462108 |
| Granulicella | (E,E)-3,7,11-Trimethyl-2,6,10-dodecatrienyl octanoate | 0.426698 | 0.0993 | 0.462108 |
| Achromobacter | benfluorex | 0.426652 | 0.09934 | 0.462108 |
| Monoglobus | Alacepril | -0.42661 | 0.099378 | 0.462108 |
| Subgroup_2 | Terizidone | 0.426599 | 0.099386 | 0.462108 |
| Flavonifractor | Am-PE(16:0/18:0) | 0.426531 | 0.099446 | 0.462231 |
| Tuzzerella | Asparaginyl-Proline | 0.426408 | 0.099554 | 0.462583 |
| TRA3-20 | Xanthosine | 0.426333 | 0.09962 | 0.462735 |
| Faecalibaculum | Glutaminylproline | 0.426229 | 0.099712 | 0.462876 |
| Acetatifactor | 5-Hydroxyindoxyl sulfate | -0.42621 | 0.099729 | 0.462876 |
| Aquicella | 16,17-Dihydro-16alpha,17-dihydroxygibberellin A4 17-glucoside | -0.42614 | 0.099789 | 0.462876 |
| Gemella | Niacinamide | 0.426109 | 0.099818 | 0.462876 |
| Acidothermus | Kaltostat | 0.426096 | 0.099829 | 0.462876 |
| Flavonifractor | Ac-Ser-Asp-Lys-Pro-OH | 0.426054 | 0.099866 | 0.462876 |
| Alcaligenes | N-lactoyl-Tyrosine | 0.426038 | 0.09988 | 0.462876 |
| Monoglobus | LysoPE(20:3(8Z,11Z,14Z)/0:0) | 0.425861 | 0.100036 | 0.463289 |
| Faecalibaculum | Nopalinic acid | 0.425854 | 0.100042 | 0.463289 |
| Aquicella | Docosadienoate (22:2n6) | 0.425809 | 0.100082 | 0.463289 |
| RF39 | S-[(3R,3As,6S,6aS)-3-nitrooxy-2,3,3a,5,6,6a-hexahydrofuro[3,2-b]furan-6-yl] ethanethioate | 0.425787 | 0.100101 | 0.463289 |
| Bryobacter | L-Lysinamide | -0.42568 | 0.100194 | 0.463305 |
| Coprobacillus | Tyrosyl-Aspartate | 0.425669 | 0.100206 | 0.463305 |
| Achromobacter | 4E,14Z-Sphingadiene | -0.42566 | 0.100213 | 0.463305 |
| Acetatifactor | Niacinamide | 0.425635 | 0.100236 | 0.463305 |
| Enterococcus | xi-2,3-Octadiene-5,7-diyn-1-ol | 0.425552 | 0.100309 | 0.463337 |
| Acetatifactor | Docosadienoate (22:2n6) | 0.425525 | 0.100333 | 0.463337 |
| Bryobacter | Symmetric dimethylarginine | 0.425515 | 0.100342 | 0.463337 |
| [Eubacterium]_oxidoreducens_group | DG(17:2(9Z,12Z)/22:5(7Z,10Z,13Z,16Z,19Z)/0:0)[iso2] | 0.425314 | 0.10052 | 0.463776 |
| Enterorhabdus | Alacepril | -0.42531 | 0.100521 | 0.463776 |
| IMCC26256 | PC(P-18:0/20:4(5Z,8Z,11Z,14Z)-OH(16R)) | 0.425296 | 0.100536 | 0.463776 |
| TM7 | Dolichyl b-D-glucosyl phosphate | -0.42513 | 0.100684 | 0.464215 |
| Anaerotruncus | Asparaginyl-Proline | 0.425108 | 0.100702 | 0.464215 |
| bacteriap25 | N-(N-(3-Amino-3-carboxypropyl)-3-amino-3-carboxypropyl)azetidine-2-carboxylic acid | 0.425077 | 0.10073 | 0.464215 |
| Faecalibaculum | 6-Hydroxytricetin 5-rhamnoside | 0.424928 | 0.100862 | 0.464673 |
| bacteriap25 | Ac-Ser-Asp-Lys-Pro-OH | 0.424849 | 0.100932 | 0.464796 |
| RF39 | Skepinone-L | 0.424783 | 0.100991 | 0.464796 |
| bacteriap25 | Prolylproline | 0.424763 | 0.101009 | 0.464796 |
| Haliangium | FAD | 0.424749 | 0.101021 | 0.464796 |
| Bryobacter | Fexaramine | -0.42447 | 0.10127 | 0.465789 |
| Alcaligenes | N-(2-Hydroxyethyl)-2-(1-isoquinolinylmethylene)hydrazinecarbothioamide | 0.424152 | 0.101553 | 0.466936 |
| Anaerotruncus | Ciadox | 0.423964 | 0.101721 | 0.467202 |
| Achromobacter | Pelargonidin 3-sophoroside | -0.42382 | 0.101849 | 0.467202 |
| Enterorhabdus | 23S,25,26-Trihydroxyvitamin D3 | 0.423782 | 0.101883 | 0.467202 |
| Clostridioides | PS(22:5(7Z,10Z,13Z,16Z,19Z)/22:6(4Z,7Z,10Z,13Z,16Z,19Z)) | -0.42375 | 0.101915 | 0.467202 |
| Christensenellaceae_R-7_group | 2-(Methylthiomethyl)-3-phenyl-2-propenal | 0.423711 | 0.101947 | 0.467202 |
| Jatrophihabitans | trans-Hexadec-2-enoyl carnitine | 0.423678 | 0.101977 | 0.467202 |
| Tuzzerella | S-[(3R,3As,6S,6aS)-3-nitrooxy-2,3,3a,5,6,6a-hexahydrofuro[3,2-b]furan-6-yl] ethanethioate | 0.423592 | 0.102053 | 0.467202 |
| Pseudolabrys | Neryl 8-methyldecanoate | 0.423543 | 0.102097 | 0.467202 |
| Monoglobus | Oleic acid | -0.42354 | 0.102099 | 0.467202 |
| Acidothermus | Streptidine | 0.423539 | 0.1021 | 0.467202 |
| TM7 | 9-Octadecenal | -0.42353 | 0.102113 | 0.467202 |
| bacteriap25 | N-lactoyl-Methionine | 0.42351 | 0.102127 | 0.467202 |
| Tuzzerella | O-Linoleoylcarnitine | 0.423499 | 0.102137 | 0.467202 |
| bacteriap25 | Fluazifop | 0.423429 | 0.102199 | 0.467202 |
| RF39 | Kuguacin E | -0.42339 | 0.102238 | 0.467202 |
| bacteriap25 | N-Lactoylleucine | 0.423378 | 0.102245 | 0.467202 |
| Intestinimonas | (卤)-(Z)-2-(5-Tetradecenyl)cyclobutanone | -0.42333 | 0.102285 | 0.467202 |
| Enterococcus | N-(1-Deoxy-1-fructosyl)threonine | 0.423316 | 0.1023 | 0.467202 |
| Acetatifactor | 4-Chloro-L-phenylalanine | -0.42332 | 0.102301 | 0.467202 |
| Rikenellaceae_RC9_gut_group | Glycyl-D-proline | 0.423298 | 0.102316 | 0.467202 |
| Anaerotruncus | PE(22:4(7Z,10Z,13Z,16Z)/19:0) | -0.42329 | 0.102323 | 0.467202 |
| Enterococcus | Kaltostat | 0.42325 | 0.10236 | 0.467202 |
| Alcaligenes | Pantothenic acid | 0.423235 | 0.102374 | 0.467202 |
| MND1 | Cannabidivarin | -0.42319 | 0.102413 | 0.467232 |
| TRA3-20 | Glycerol 3-phosphate | 0.423137 | 0.102461 | 0.467284 |
| Anaerotruncus | Lycoperdic acid | 0.423104 | 0.102491 | 0.467284 |
| Anaerostipes | N-Acetyl-L-methionine | 0.422996 | 0.102588 | 0.467573 |
| MND1 | 3,4-Dimethyl-5-propyl-2-furanpentadecanoic acid | -0.42282 | 0.102744 | 0.468134 |
| Coprobacillus | Creatine | 0.422784 | 0.102778 | 0.468139 |
| Acidothermus | Notoginsenoside H | 0.422569 | 0.102972 | 0.468671 |
| bacteriap25 | N-Lactoylphenylalanine | 0.422564 | 0.102976 | 0.468671 |
| Haliangium | 3-Methyl-3-butenyl apiosyl-(1->6)-glucoside | 0.422512 | 0.103024 | 0.468671 |
| TRA3-20 | 2-hydroxy-9Z,12Z-Octadecadienoic acid | -0.42251 | 0.103028 | 0.468671 |
| Alcaligenes | Dolichyl b-D-glucosyl phosphate | -0.42244 | 0.10309 | 0.46877 |
| RF39 | alpha-D-Galactopyranuronosyl-(1->4)-alpha-D-galactopyranuronosyl-(1->4)-D-galacturonic acid | 0.422408 | 0.103117 | 0.46877 |
| Haliangium | thiamphenicol | 0.422335 | 0.103183 | 0.468782 |
| Tuzzerella | Pentadecanoylcarnitine | 0.422332 | 0.103186 | 0.468782 |
| Anaerotruncus | Peruvianoside II | 0.422152 | 0.103348 | 0.469092 |
| TRA3-20 | 2,2,3,3,4,4,5,5,6,6,7,7,8,8,9,9-Hexadecafluorononanoic acid | 0.422143 | 0.103356 | 0.469092 |
| Monoglobus | Berkeleylactone L | -0.42211 | 0.103384 | 0.469092 |
| Tuzzerella | Terbufos | 0.422083 | 0.103411 | 0.469092 |
| Muribaculum | AM2201 N-(4-hydroxypentyl) metabolite | -0.42206 | 0.103436 | 0.469092 |
| TRA3-20 | Bacillamidin C | -0.42203 | 0.103454 | 0.469092 |
| Muribaculum | 23S,25,26-Trihydroxyvitamin D3 | 0.42197 | 0.103513 | 0.469135 |
| Treponema | Prolylproline | 0.421951 | 0.10353 | 0.469135 |
| Nitrospira | Lemborexant | -0.4219 | 0.103576 | 0.469193 |
| bacteriap25 | 11-Hydroxyhexadecanoylcarnitine | 0.421821 | 0.103647 | 0.469364 |
| Treponema | Glutaminylproline | 0.421694 | 0.103763 | 0.469532 |
| Alcaligenes | 4-Bis(2-hydroxyethyl)amino-L-phenylalanine | 0.421585 | 0.103861 | 0.469532 |
| Coprobacillus | AB-MECA | -0.42158 | 0.103868 | 0.469532 |
| Bryobacter | 1-(10H-Phenothiazin-2-yl)ethanone | 0.421572 | 0.103873 | 0.469532 |
| Coprobacillus | Notoginsenoside H | 0.42156 | 0.103884 | 0.469532 |
| Intestinimonas | PC(20:5(5Z,8Z,11Z,14Z,17Z)/PGJ2) | -0.42151 | 0.103927 | 0.469532 |
| Clostridioides | 5,5-Diphenyl-2-thiohydantoin | 0.421512 | 0.103927 | 0.469532 |
| Clostridioides | Xanthosine | 0.421418 | 0.104013 | 0.469532 |
| Monoglobus | 1-[3,4-Dihydroxy-5-(hydroxymethyl)-2-oxolanyl]-1,2,4-triazole-3-carboxamide | 0.421405 | 0.104025 | 0.469532 |
| TM7 | Bis(2-propoxyethyl) 2,6-dimethyl-4-(3-nitrophenyl)-3,4-dihydropyridine-3,5-dicarboxylate | -0.4214 | 0.104032 | 0.469532 |
| bacteriap25 | Dihydrozeatin O-beta-D-Glucoside | 0.421333 | 0.10409 | 0.469532 |
| Aquicella | 8-Amino-7-oxononanoic acid | -0.42132 | 0.104099 | 0.469532 |
| Intestinimonas | Octadecanamide | -0.4213 | 0.104118 | 0.469532 |
| Muribaculum | Eleutherazine B; N,N'-((3,6-Dioxo-2,5-piperazinediyl)bis(trimethylene))bis(5-hydroxy-3-methyl-2-pentenamide) | 0.42112 | 0.104283 | 0.470126 |
| Enterorhabdus | PI(18:1(9Z)/0:0) | 0.42108 | 0.10432 | 0.470139 |
| Nitrospira | PPM-18 | 0.421013 | 0.104381 | 0.470264 |
| Achromobacter | 16,17-Dihydro-16alpha,17-dihydroxygibberellin A4 17-glucoside | -0.42095 | 0.104438 | 0.470371 |
| Coprobacillus | 7-Hydroxy-R-acenocoumarol | 0.42082 | 0.104556 | 0.470753 |
| Enterorhabdus | L-arginino-succinate | 0.420768 | 0.104604 | 0.470818 |
| Acidothermus | (E)-2-(hexa-3,5-dien-1-yn-1-yl)-5-(prop-1-yn-1-yl)thiophene | 0.420692 | 0.104672 | 0.470884 |
| Clostridioides | N-lactoyl-Tyrosine | 0.420648 | 0.104713 | 0.470884 |
| Subgroup_2 | 4-Phenylbutanoylcarnitine | -0.42062 | 0.104741 | 0.470884 |
| TRA3-20 | 3'-Deoxythymidine | 0.420605 | 0.104752 | 0.470884 |
| Haliangium | DG(18:0/20:4(8Z,11Z,14Z,17Z)/0:0) | 0.42054 | 0.104812 | 0.471001 |
| Intestinimonas | [(2S,4R,5R,6R,14S,16R)-14-Hydroxy-7,11-dimethyl-6-(2-oxopyran-4-yl)-3-oxapentacyclo[8.8.0.02,4.02,7.011,16]octadecan-5-yl] acetate | -0.42039 | 0.104945 | 0.471142 |
| Intestinimonas | PE(22:5(4Z,7Z,10Z,13Z,16Z)/PGD2) | -0.42037 | 0.104969 | 0.471142 |
| Solobacterium | Fucoxanthinol 3-(4Z,7Z,10Z,13Z,16Z,19Z-docosahexaenoate) 3'-palmitoleate | 0.42036 | 0.104976 | 0.471142 |
| Pseudolabrys | 5,5-Diphenyl-2-thiohydantoin | 0.420337 | 0.104996 | 0.471142 |
| Treponema | 4Z,7-octadienoic acid | -0.42032 | 0.10501 | 0.471142 |
| Solobacterium | xi-2,3-Octadiene-5,7-diyn-1-ol | 0.420195 | 0.105127 | 0.471398 |
| Enterococcus | Glucose-6-glutamate | 0.420161 | 0.105158 | 0.471398 |
| Lachnospiraceae_UCG-006 | N-gamma-L-Glutamyl-D-alanine | 0.420129 | 0.105187 | 0.471398 |
| Acidothermus | 5-[1-Carboxy-2-(trimethylazaniumyl)ethoxy]-5-oxopentanoate | 0.420113 | 0.105202 | 0.471398 |
| Enterorhabdus | PC(TXB2/20:2(11Z,14Z)) | -0.41989 | 0.105409 | 0.472015 |
| Rikenellaceae_RC9_gut_group | Tridecanoylcarnitine | 0.419864 | 0.10543 | 0.472015 |
| Lachnospiraceae_UCG-006 | N-(1-Deoxy-1-fructosyl)tyrosine | 0.419853 | 0.10544 | 0.472015 |
| Muribaculum | Am-PE(16:0/18:0) | 0.419777 | 0.105509 | 0.472135 |
| Alcaligenes | Pelargonidin 3-sophoroside | -0.41965 | 0.105624 | 0.472135 |
| RF39 | Bis(2-propoxyethyl) 2,6-dimethyl-4-(3-nitrophenyl)-3,4-dihydropyridine-3,5-dicarboxylate | -0.41956 | 0.105706 | 0.472135 |
| Clostridioides | N-Myristoyl Asparagine | -0.41956 | 0.105712 | 0.472135 |
| TRA3-20 | Polypropylene glycol (m w 1,200-3,000) | -0.41955 | 0.105713 | 0.472135 |
| Anaerotruncus | Palmitoylcarnitine | 0.419511 | 0.105753 | 0.472135 |
| Lachnospiraceae_UCG-006 | Xanthosine | 0.419507 | 0.105757 | 0.472135 |
| Achromobacter | Pantothenic acid | 0.419475 | 0.105786 | 0.472135 |
| Pseudolabrys | Skepinone-L | 0.419441 | 0.105818 | 0.472135 |
| TRA3-20 | 2-Methylthioadenosine | 0.419428 | 0.105829 | 0.472135 |
| Achromobacter | Iguratimod | -0.41942 | 0.105835 | 0.472135 |
| Acetatifactor | Cortisol | -0.41933 | 0.105918 | 0.472238 |
| Achromobacter | N-(2-Hydroxyethyl)-2-(1-isoquinolinylmethylene)hydrazinecarbothioamide | 0.419289 | 0.105957 | 0.472238 |
| TM7 | 5,5-Diphenyl-2-thiohydantoin | 0.419287 | 0.105959 | 0.472238 |
| bacteriap25 | Fiacitabine | 0.419195 | 0.106044 | 0.472415 |
| Subgroup_13 | PPM-18 | 0.419169 | 0.106067 | 0.472415 |
| Treponema | Fluazifop | 0.419112 | 0.10612 | 0.472415 |
| RF39 | 5,5-Diphenyl-2-thiohydantoin | 0.41907 | 0.106158 | 0.472415 |
| Enterococcus | Bacillamidin C | -0.41906 | 0.106167 | 0.472415 |
| bacteriap25 | N-(1-Deoxy-1-fructosyl)threonine | 0.418962 | 0.106258 | 0.472501 |
| RF39 | 5'-Carboxy-gamma-chromanol | -0.41894 | 0.106277 | 0.472501 |
| TRA3-20 | Fiacitabine | 0.418931 | 0.106287 | 0.472501 |
| Acetatifactor | scyllo-Inositol | 0.418825 | 0.106385 | 0.472787 |
| Enterorhabdus | N-alpha-Acetyl-L-lysine | 0.418746 | 0.106457 | 0.472961 |
| Anaerotruncus | N-Lactoylphenylalanine | 0.418628 | 0.106566 | 0.473067 |
| bacteriap25 | Glutaminylproline | 0.418605 | 0.106588 | 0.473067 |
| Bryobacter | Notoginsenoside H | 0.4186 | 0.106592 | 0.473067 |
| Achromobacter | 25-Hydroxyvitamin D3-26,23-lactol | -0.41857 | 0.106616 | 0.473067 |
| Treponema | Cannabidivarin | -0.41844 | 0.106741 | 0.473372 |
| Alcaligenes | Glycyl-D-proline | 0.418378 | 0.106797 | 0.473372 |
| Enterococcus | Hexadecadienylcarnitine | 0.418354 | 0.10682 | 0.473372 |
| Nitrospira | Ethyl hexadecanoate | -0.41834 | 0.106834 | 0.473372 |
| Anaerotruncus | 2-phospho-4-(cytidine 5'-diphospho)-2-C-methyl-D-erythritol | 0.418298 | 0.106871 | 0.473372 |
| Enterorhabdus | N1-Methyl-2-pyridone-5-carboxamide | -0.41826 | 0.106904 | 0.473372 |
| Enterorhabdus | Zidebactam | 0.418245 | 0.10692 | 0.473372 |
| Solobacterium | alpha-D-Galactopyranuronosyl-(1->4)-alpha-D-galactopyranuronosyl-(1->4)-D-galacturonic acid | 0.418199 | 0.106962 | 0.473411 |
| Rikenellaceae_RC9_gut_group | Niacinamide | 0.418026 | 0.107122 | 0.473817 |
| Enterococcus | N-Myristoyl Asparagine | -0.41802 | 0.107132 | 0.473817 |
| Enterococcus | 16,17-Dihydro-16alpha,17-dihydroxygibberellin A4 17-glucoside | -0.41799 | 0.107155 | 0.473817 |
| Clostridioides | 16,17-Dihydro-16alpha,17-dihydroxygibberellin A4 17-glucoside | -0.41794 | 0.107204 | 0.473885 |
| Coprobacillus | PI(18:0/20:4(8Z,11Z,14Z,17Z)) | 0.417864 | 0.107272 | 0.473913 |
| Faecalibaculum | 1-Ethoxymethyl-5-fluorouracil | 0.417842 | 0.107293 | 0.473913 |
| Acetatifactor | N2-(3-Carboxy-2-hydroxy-1-oxopropyl)arginine | 0.417823 | 0.107311 | 0.473913 |
| Enterorhabdus | Dehydroascorbic acid | 0.417688 | 0.107436 | 0.474087 |
| Christensenellaceae_R-7_group | Peperomin E | 0.417682 | 0.107442 | 0.474087 |
| RF39 | 2-[(4-{2-[(4-Cyclohexylbutyl)(cyclohexylcarbamoyl)amino]ethyl}phenyl)sulfanyl]-2-methylpropanoic acid | -0.41767 | 0.107452 | 0.474087 |
| Achromobacter | Docosadienoate (22:2n6) | 0.417593 | 0.107524 | 0.474258 |
| Aquicella | 1-O-Galloylglycerol | 0.417456 | 0.107651 | 0.47461 |
| Pseudolabrys | (R)-Propyl 2-amino-3-mercaptopropanoate | 0.417435 | 0.107671 | 0.47461 |
| Rikenellaceae_RC9_gut_group | Serotinose | 0.417393 | 0.10771 | 0.474631 |
| Intestinimonas | PI(18:0/20:4(8Z,11Z,14Z,17Z)) | 0.417343 | 0.107757 | 0.474689 |
| [Eubacterium]_oxidoreducens_group | Lucuminic acid | 0.417212 | 0.107879 | 0.475078 |
| IMCC26256 | 1-p-Menthen-3-one | 0.417122 | 0.107962 | 0.475122 |
| Anaerotruncus | Dodecanamide | -0.4171 | 0.107984 | 0.475122 |
| RF39 | 4-Phenylbutanoylcarnitine | -0.41709 | 0.10799 | 0.475122 |
| Achromobacter | Pseudouridine | 0.416855 | 0.108211 | 0.475894 |
| Subgroup_13 | Aspartyl-Gamma-glutamate | 0.416823 | 0.108242 | 0.475894 |
| Enterococcus | Zidebactam | 0.416796 | 0.108267 | 0.475894 |
| Jatrophihabitans | Dimethylaminoparthenolide | 0.416569 | 0.108479 | 0.476677 |
| TM7 | Nopalinic acid | 0.416477 | 0.108564 | 0.476813 |
| TRA3-20 | 1-O-(2R-methoxy-hexadecyl)-sn-glycerol | -0.41646 | 0.108578 | 0.476813 |
| Aquicella | N-(1-Deoxy-1-fructosyl)threonine | 0.416336 | 0.108697 | 0.477156 |
| RF39 | Pentadeca-3,5,7-trienedioylcarnitine | 0.416306 | 0.108725 | 0.477156 |
| Granulicella | PI(18:1(9Z)/0:0) | 0.416271 | 0.108757 | 0.477156 |
| Alcaligenes | 4-Chloro-L-phenylalanine | -0.41621 | 0.108815 | 0.477258 |
| Enterococcus | LysoPE(20:3(8Z,11Z,14Z)/0:0) | 0.416107 | 0.108911 | 0.477454 |
| Nitrospira | Pseudouridine | 0.41609 | 0.108927 | 0.477454 |
| Tuzzerella | (E,E)-3,7,11-Trimethyl-2,6,10-dodecatrienyl octanoate | 0.415842 | 0.109159 | 0.478254 |
| TRA3-20 | Sodium nitrate (NaNO3) | -0.41582 | 0.109177 | 0.478254 |
| bacteriap25 | Thiazolidine-4-carboxylic acid | 0.415763 | 0.109234 | 0.478351 |
| Subgroup_13 | PE(19:0/20:2(11Z,14Z)) | -0.41571 | 0.109286 | 0.478432 |
| Jatrophihabitans | Avenin | 0.415658 | 0.109332 | 0.478486 |
| Anaerotruncus | 13-(3,4-Dimethyl-5-propylfuran-2-yl)tridecanoylcarnitine | 0.415588 | 0.109397 | 0.478556 |
| Treponema | N-(1-Deoxy-1-fructosyl)threonine | 0.41555 | 0.109434 | 0.478556 |
| Solobacterium | PC(18:1(17Z)/18:1(17Z)) | 0.415532 | 0.10945 | 0.478556 |
| bacteriap25 | PI(20:3(8Z,11Z,14Z)/0:0) | 0.415324 | 0.109646 | 0.479265 |
| TRA3-20 | S-[(3R,3As,6S,6aS)-3-nitrooxy-2,3,3a,5,6,6a-hexahydrofuro[3,2-b]furan-6-yl] ethanethioate | 0.415177 | 0.109785 | 0.47972 |
| Enterorhabdus | PE(19:0/20:2(11Z,14Z)) | -0.41511 | 0.109844 | 0.479832 |
| Monoglobus | Kuguacin E | -0.41498 | 0.10997 | 0.480014 |
| Granulicella | Terizidone | 0.414965 | 0.109985 | 0.480014 |
| Granulicella | Tyrosyl-Alanine | 0.414951 | 0.109998 | 0.480014 |
| Aquicella | 1-(10H-Phenothiazin-2-yl)ethanone | 0.414925 | 0.110022 | 0.480014 |
| Faecalibaculum | Phenylalanylaspartic acid | 0.414805 | 0.110136 | 0.480362 |
| Solobacterium | Cortisol | -0.41464 | 0.110293 | 0.480786 |
| Monoglobus | Resorufin | 0.414629 | 0.110302 | 0.480786 |
| TRA3-20 | Hexadecenal | -0.41455 | 0.110376 | 0.480959 |
| Treponema | 2-hydroxy-9Z,12Z-Octadecadienoic acid | -0.41442 | 0.110499 | 0.481309 |
| Lachnospiraceae_UCG-006 | PS(22:5(7Z,10Z,13Z,16Z,19Z)/22:6(4Z,7Z,10Z,13Z,16Z,19Z)) | -0.41439 | 0.110524 | 0.481309 |
| [Eubacterium]_oxidoreducens_group | N-Acetylneuraminic acid | 0.414281 | 0.110631 | 0.481347 |
| Intestinimonas | Phytal | -0.41423 | 0.110683 | 0.481347 |
| Enterorhabdus | N-Lactoylleucine | 0.414225 | 0.110685 | 0.481347 |
| Alcaligenes | 3-Ketosphingosine | -0.41419 | 0.110719 | 0.481347 |
| Achromobacter | 6-Hydroxytricetin 5-rhamnoside | 0.414146 | 0.11076 | 0.481347 |
| Gemella | xi-2,3-Octadiene-5,7-diyn-1-ol | 0.414139 | 0.110766 | 0.481347 |
| Granulicella | DG(18:0/20:4(8Z,11Z,14Z,17Z)/0:0) | 0.414128 | 0.110776 | 0.481347 |
| Achromobacter | Asparaginyl-Proline | 0.414074 | 0.110827 | 0.481347 |
| Acidothermus | Tyrosyl-Aspartate | 0.41406 | 0.110841 | 0.481347 |
| Achromobacter | DG(18:0/20:4(8Z,11Z,14Z,17Z)/0:0) | 0.41402 | 0.110879 | 0.481365 |
| Jatrophihabitans | Ethyl hexadecanoate | -0.41386 | 0.111034 | 0.481887 |
| RF39 | N-alpha-Acetyl-L-lysine | 0.413802 | 0.111086 | 0.481967 |
| Alcaligenes | Docosadienoate (22:2n6) | 0.413765 | 0.111121 | 0.481972 |
| Achromobacter | U-75302 | 0.413696 | 0.111187 | 0.482105 |
| Alcaligenes | Bis(2-propoxyethyl) 2,6-dimethyl-4-(3-nitrophenyl)-3,4-dihydropyridine-3,5-dicarboxylate | -0.41363 | 0.111246 | 0.482108 |
| bacteriap25 | 4Z,7-octadienoic acid | -0.41362 | 0.111256 | 0.482108 |
| Tuzzerella | Erucic acid | 0.413404 | 0.111464 | 0.482864 |
| Intestinimonas | Hexadecenal | -0.41328 | 0.11158 | 0.483034 |
| Monoglobus | AB-MECA | -0.41327 | 0.111592 | 0.483034 |
| Enterococcus | Sodium nitrate (NaNO3) | -0.41323 | 0.111633 | 0.483034 |
| Faecalibaculum | Ac-Ser-Asp-Lys-Pro-OH | 0.413165 | 0.111692 | 0.483034 |
| [Eubacterium]_oxidoreducens_group | Am-PE(16:0/18:0) | 0.413127 | 0.111729 | 0.483034 |
| MND1 | L-Glutamine | 0.413098 | 0.111756 | 0.483034 |
| Solobacterium | Mycobactins | -0.41308 | 0.111772 | 0.483034 |
| RF39 | AB-MECA | -0.41308 | 0.111778 | 0.483034 |
| Tuzzerella | Ac-Ser-Asp-Lys-Pro-OH | 0.412992 | 0.111858 | 0.483229 |
| TM7 | PC(11:0/23:0) | 0.412956 | 0.111892 | 0.483231 |
| Achromobacter | Glycyl-D-proline | 0.412867 | 0.111977 | 0.483449 |
| [Eubacterium]_oxidoreducens_group | L-Arginine | 0.412692 | 0.112144 | 0.483913 |
| Nitrospira | Tyrosyl-Aspartate | 0.412651 | 0.112184 | 0.483913 |
| Clostridia_vadinBB60_group | Glucose-6-glutamate | 0.412647 | 0.112188 | 0.483913 |
| Aquicella | Fructose-1,6-diphosphate | 0.412592 | 0.11224 | 0.48393 |
| Enterorhabdus | alpha-D-Galactopyranuronosyl-(1->4)-alpha-D-galactopyranuronosyl-(1->4)-D-galacturonic acid | 0.412571 | 0.11226 | 0.48393 |
| Enterorhabdus | Dirithromycin | -0.41249 | 0.112342 | 0.484134 |
| Bryobacter | Bacillamidin C | -0.41244 | 0.112387 | 0.484178 |
| Flavonifractor | 2',3'-Dideoxyuridine | 0.412318 | 0.112503 | 0.484387 |
| Tuzzerella | Neryl 8-methyldecanoate | 0.412268 | 0.11255 | 0.484387 |
| Lachnospiraceae_UCG-006 | Dehydroascorbic acid | 0.412264 | 0.112555 | 0.484387 |
| IMCC26256 | 2',3'-Dideoxyuridine | 0.412245 | 0.112573 | 0.484387 |
| Anaerotruncus | Tephcalostan C | 0.412141 | 0.112672 | 0.484618 |
| [Eubacterium]_oxidoreducens_group | Prolyl-Asparagine | 0.412106 | 0.112705 | 0.484618 |
| Enterococcus | PC(P-18:0/20:4(5Z,8Z,11Z,14Z)-OH(16R)) | 0.412037 | 0.112772 | 0.484618 |
| Lachnospiraceae_UCG-006 | Resorufin | 0.412026 | 0.112783 | 0.484618 |
| TRA3-20 | N-Myristoyl Asparagine | -0.41201 | 0.112799 | 0.484618 |
| Granulicella | Withaferin A | -0.41189 | 0.112912 | 0.484753 |
| Granulicella | Lucuminic acid | 0.411881 | 0.112922 | 0.484753 |
| Monoglobus | scyllo-Inositol | 0.411813 | 0.112988 | 0.484753 |
| Clostridia_vadinBB60_group | PGP(18:3(9Z,12Z,15Z)/18:1(12Z)-O(9S,10R)) | 0.411785 | 0.113014 | 0.484753 |
| Enterococcus | 3-{[(1s)-2,2-Difluoro-1-Hydroxy-7-(Methylsulfonyl)-2,3-Dihydro-1h-Inden-4-Yl]oxy}-5-Fluorobenzonitrile | 0.411767 | 0.113032 | 0.484753 |
| Clostridioides | N-Lauroyl Glutamine | -0.41176 | 0.113037 | 0.484753 |
| Jatrophihabitans | Tuberonic acid glucoside | -0.4117 | 0.113097 | 0.484865 |
| TM7 | 1-Octadecene | -0.41165 | 0.113146 | 0.484925 |
| Clostridioides | 3-{[(1s)-2,2-Difluoro-1-Hydroxy-7-(Methylsulfonyl)-2,3-Dihydro-1h-Inden-4-Yl]oxy}-5-Fluorobenzonitrile | 0.411459 | 0.113329 | 0.485561 |
| Alcaligenes | Valylserine | 0.411364 | 0.113419 | 0.485687 |
| TRA3-20 | Octadecanamide | -0.41136 | 0.113427 | 0.485687 |
| Clostridioides | Dehydrocyclopeptine; (3E)-3-Benzylidene-4-methyl-3,4-dihydro-1H-1,4-benzodiazepine-2,5-dione | -0.41128 | 0.113501 | 0.485857 |
| Alcaligenes | D-erythro-L-galacto-Nonulose | 0.411163 | 0.113614 | 0.486066 |
| Gemella | PC(18:1(17Z)/18:1(17Z)) | 0.411157 | 0.113619 | 0.486066 |
| Bryobacter | Adenosine monophosphate | -0.41111 | 0.113665 | 0.486113 |
| Haliangium | Peperomin E | 0.411073 | 0.113701 | 0.486119 |
| Bryobacter | Glutamylleucylarginine | 0.410944 | 0.113826 | 0.486421 |
| Subgroup_2 | Pantothenic acid | 0.410889 | 0.113878 | 0.486421 |
| RF39 | Am-PE(16:0/18:0) | 0.410837 | 0.113929 | 0.486421 |
| bacteriap25 | 16-hydroxy-6-hexadecenoic acid | -0.41081 | 0.113957 | 0.486421 |
| Clostridioides | Diethylamino 2,5-dihydroxybenzenesulfonate | 0.410751 | 0.114012 | 0.486421 |
| Aquicella | D-Malic acid | 0.410745 | 0.114018 | 0.486421 |
| Anaerotruncus | Fluazifop | 0.410663 | 0.114097 | 0.486421 |
| Enterococcus | N-Acetyl-L-methionine | 0.410613 | 0.114145 | 0.486421 |
| Enterococcus | Beta-Citryl-L-glutamic acid | 0.410604 | 0.114154 | 0.486421 |
| Bryobacter | Phytal | -0.41059 | 0.114167 | 0.486421 |
| Rikenellaceae_RC9_gut_group | 3-Amino-3-methylbutanoic acid | 0.410584 | 0.114174 | 0.486421 |
| Enterorhabdus | LysoPC(20:3(8Z,11Z,14Z)/0:0) | 0.410571 | 0.114186 | 0.486421 |
| Tuzzerella | L-Homocystine | 0.410502 | 0.114253 | 0.486438 |
| Flavonifractor | Cortisol | -0.4105 | 0.114259 | 0.486438 |
| Anaerotruncus | Aspartyl-Isoleucine | 0.410287 | 0.114462 | 0.487155 |
| Anaerostipes | 9-Hydroxy-4-methoxypsoralen 9-glucoside | 0.410053 | 0.114689 | 0.487972 |
| Monoglobus | N-Acetyl-L-methionine | 0.409977 | 0.114763 | 0.48814 |
| Anaerotruncus | Skepinone-L | 0.409826 | 0.114909 | 0.488615 |
| Bryobacter | thiamphenicol | 0.409783 | 0.114951 | 0.488647 |
| Achromobacter | Dolichyl b-D-glucosyl phosphate | -0.40965 | 0.115085 | 0.488836 |
| TRA3-20 | 1-(10H-Phenothiazin-2-yl)ethanone | 0.409632 | 0.115098 | 0.488836 |
| Anaerotruncus | PI(20:4(5Z,8Z,11Z,14Z)/18:0) | -0.4096 | 0.115133 | 0.488836 |
| Enterorhabdus | U-75302 | 0.409595 | 0.115135 | 0.488836 |
| Christensenellaceae_R-7_group | m7G(5')pppAm | 0.409558 | 0.115171 | 0.488842 |
| Acetatifactor | 2-phospho-4-(cytidine 5'-diphospho)-2-C-methyl-D-erythritol | 0.409453 | 0.115273 | 0.489015 |
| Subgroup_2 | N-Acetyl-L-methionine | 0.409399 | 0.115325 | 0.489015 |
| Coprobacillus | Cortisol | -0.40931 | 0.115408 | 0.489015 |
| IMCC26256 | Tyrosyl-Alanine | 0.409281 | 0.115441 | 0.489015 |
| Aquicella | PC(P-18:0/20:4(5Z,8Z,11Z,14Z)-OH(16R)) | 0.409274 | 0.115448 | 0.489015 |
| [Eubacterium]_oxidoreducens_group | Eicosadienoic acid | 0.409226 | 0.115494 | 0.489015 |
| Anaerotruncus | Dehydrocyclopeptine; (3E)-3-Benzylidene-4-methyl-3,4-dihydro-1H-1,4-benzodiazepine-2,5-dione | -0.40922 | 0.115504 | 0.489015 |
| Acetatifactor | 1-(10H-Phenothiazin-2-yl)ethanone | 0.409131 | 0.115587 | 0.489015 |
| bacteriap25 | N-Acetyl-b-glucosaminylamine | 0.409114 | 0.115603 | 0.489015 |
| Enterorhabdus | N-Lactoylphenylalanine | 0.409096 | 0.115622 | 0.489015 |
| Acetatifactor | DG(17:2(9Z,12Z)/22:5(7Z,10Z,13Z,16Z,19Z)/0:0)[iso2] | 0.409075 | 0.115642 | 0.489015 |
| Acidothermus | Dehydroascorbide(1-) | 0.409044 | 0.115672 | 0.489015 |
| Pseudolabrys | Ricinoleic acid | -0.40903 | 0.115689 | 0.489015 |
| Acidothermus | N-(N-(3-Amino-3-carboxypropyl)-3-amino-3-carboxypropyl)azetidine-2-carboxylic acid | 0.409018 | 0.115698 | 0.489015 |
| Subgroup_13 | 1-Hydroxy-2-naphthoic acid | 0.408887 | 0.115826 | 0.489408 |
| Anaerotruncus | Bis(2-propoxyethyl) 2,6-dimethyl-4-(3-nitrophenyl)-3,4-dihydropyridine-3,5-dicarboxylate | -0.40882 | 0.11589 | 0.489445 |
| Clostridioides | Glutamylleucylarginine | 0.408806 | 0.115905 | 0.489445 |
| Anaerotruncus | N-Lactoylleucine | 0.408755 | 0.115955 | 0.489445 |
| Bryobacter | Genistein 7-O-glucoside-6''-malonate | 0.408736 | 0.115973 | 0.489445 |
| Coprobacillus | Am-PE(16:0/18:0) | 0.40854 | 0.116166 | 0.49011 |
| MND1 | 2-Cyclotetradecen-1-one | -0.40837 | 0.116332 | 0.490225 |
| Subgroup_13 | JP83 | -0.40836 | 0.116344 | 0.490225 |
| Intestinimonas | Oleoyl Serotonin | -0.40833 | 0.116373 | 0.490225 |
| Coprobacillus | PC(P-18:0/20:4(5Z,8Z,11Z,14Z)-OH(16R)) | 0.408298 | 0.116403 | 0.490225 |
| Clostridioides | Tercatain | 0.408297 | 0.116404 | 0.490225 |
| Enterorhabdus | Medicagol | 0.408297 | 0.116404 | 0.490225 |
| Lachnospiraceae_UCG-006 | N-Acetyl-L-methionine | 0.408264 | 0.116437 | 0.490225 |
| Alcaligenes | scyllo-Inositol | 0.408206 | 0.116494 | 0.490319 |
| Achromobacter | OKOHA-PA | -0.40806 | 0.116635 | 0.490682 |
| Nitrospira | Phenylalanylaspartic acid | 0.408047 | 0.11665 | 0.490682 |
| Flavonifractor | 1-[3,4-Dihydroxy-5-(hydroxymethyl)-2-oxolanyl]-1,2,4-triazole-3-carboxamide | 0.407937 | 0.116758 | 0.490851 |
| TRA3-20 | PE(O-20:0/0:0) | -0.40792 | 0.116771 | 0.490851 |
| Subgroup_2 | Resorufin | 0.407896 | 0.116798 | 0.490851 |
| Faecalibaculum | Prolylproline | 0.407864 | 0.116829 | 0.490851 |
| Monoglobus | N-(1-Deoxy-1-fructosyl)threonine | 0.407725 | 0.116966 | 0.491118 |
| Clostridioides | 2,2,3,3,4,4,5,5,6,6,7,7,8,8,9,9-Hexadecafluorononanoic acid | 0.407722 | 0.116969 | 0.491118 |
| Intestinimonas | DG(18:0/20:4(8Z,11Z,14Z,17Z)/0:0) | 0.407675 | 0.117016 | 0.491118 |
| Clostridia_vadinBB60_group | 3-Hydroxy-11Z-octadecenoylcarnitine | 0.407628 | 0.117062 | 0.491118 |
| Granulicella | PI(18:0/20:4(8Z,11Z,14Z,17Z)) | 0.40757 | 0.117119 | 0.491118 |
| Subgroup_2 | DG(18:0/20:4(8Z,11Z,14Z,17Z)/0:0) | 0.407563 | 0.117126 | 0.491118 |
| Enterococcus | Napelline | 0.407502 | 0.117186 | 0.491118 |
| Pseudolabrys | 5beta-Cholane-3alpha,24-diol | 0.407491 | 0.117197 | 0.491118 |
| IMCC26256 | Glutamylleucylarginine | 0.407477 | 0.117211 | 0.491118 |
| Enterococcus | U-75302 | 0.407446 | 0.117242 | 0.491118 |
| TM7 | 3-Ketosphingosine | -0.40732 | 0.117369 | 0.491378 |
| Anaerotruncus | 3'-Deoxythymidine | 0.407263 | 0.117422 | 0.491378 |
| TRA3-20 | Phosphohydroxypyruvic acid | -0.40725 | 0.117434 | 0.491378 |
| Enterococcus | Glutamylleucylarginine | 0.407196 | 0.117488 | 0.491378 |
| TRA3-20 | Amobarbital | 0.407188 | 0.117496 | 0.491378 |
| Acidothermus | Tridecanoylcarnitine | 0.407128 | 0.117556 | 0.491378 |
| Coprobacillus | DG(18:0/20:4(8Z,11Z,14Z,17Z)/0:0) | 0.407123 | 0.11756 | 0.491378 |
| Subgroup_13 | N-gamma-L-Glutamyl-D-alanine | 0.40705 | 0.117633 | 0.491378 |
| Aquicella | Am-PE(16:0/18:0) | 0.407024 | 0.117658 | 0.491378 |
| Intestinimonas | Kuguacin E | -0.40702 | 0.117667 | 0.491378 |
| Jatrophihabitans | 3-{[(1s)-2,2-Difluoro-1-Hydroxy-7-(Methylsulfonyl)-2,3-Dihydro-1h-Inden-4-Yl]oxy}-5-Fluorobenzonitrile | 0.406994 | 0.117688 | 0.491378 |
| Treponema | L-arginino-succinate | 0.406951 | 0.117731 | 0.491413 |
| Christensenellaceae_R-7_group | Cannabidivarin | -0.40687 | 0.117816 | 0.491621 |
| Anaerotruncus | LysoSM(d18:0) | -0.40673 | 0.117947 | 0.492023 |
| bacteriap25 | 1-(10H-Phenothiazin-2-yl)ethanone | 0.406689 | 0.11799 | 0.492056 |
| Bryobacter | 2-Phenylethyl octanoate | -0.40663 | 0.118045 | 0.492139 |
| Christensenellaceae_R-7_group | True blue | 0.406209 | 0.118466 | 0.493669 |
| Subgroup_2 | PI(18:0/20:4(8Z,11Z,14Z,17Z)) | 0.406166 | 0.11851 | 0.493669 |
| Lachnospiraceae_UCG-006 | 25-Hydroxyvitamin D3-26,23-lactol | -0.40616 | 0.118517 | 0.493669 |
| Subgroup_13 | Adenosine monophosphate | -0.40606 | 0.118614 | 0.493813 |
| Anaerotruncus | Maraviroc | 0.406051 | 0.118624 | 0.493813 |
| bacteriap25 | Prostaglandin B-1 | -0.40602 | 0.118657 | 0.493813 |
| TM7 | Docosatrienoic acid | 0.405923 | 0.118751 | 0.494059 |
| Alcaligenes | Iguratimod | -0.40576 | 0.118918 | 0.494609 |
| Enterorhabdus | Serylproline | 0.405666 | 0.119008 | 0.494834 |
| IMCC26256 | JP83 | -0.40553 | 0.11914 | 0.495146 |
| RF39 | Fexaramine | -0.40552 | 0.119153 | 0.495146 |
| TM7 | Skepinone-L | 0.405351 | 0.119322 | 0.495584 |
| Bryobacter | [6-Hydroxy-8-methyl-3-[3,4,5-trihydroxy-6-(hydroxymethyl)oxan-2-yl]oxy-9,10-dioxatetracyclo[4.3.1.02,5.03,8]decan-4-yl]methyl benzoate | 0.405343 | 0.11933 | 0.495584 |
| Pseudolabrys | Flumazenil | 0.405309 | 0.119364 | 0.495584 |
| Nitrospira | 25-Hydroxyvitamin D3-26,23-lactol | -0.40524 | 0.119431 | 0.495605 |
| Gemella | [6-Hydroxy-8-methyl-3-[3,4,5-trihydroxy-6-(hydroxymethyl)oxan-2-yl]oxy-9,10-dioxatetracyclo[4.3.1.02,5.03,8]decan-4-yl]methyl benzoate | 0.405193 | 0.11948 | 0.495605 |
| Lachnospiraceae_UCG-006 | Dipotassium phosphate | 0.405152 | 0.119521 | 0.495605 |
| TM7 | Symmetric dimethylarginine | 0.40515 | 0.119523 | 0.495605 |
| Faecalibaculum | LysoPC(P-16:0/0:0) | 0.405127 | 0.119545 | 0.495605 |
| Intestinimonas | Xanthinol | 0.404941 | 0.119732 | 0.496035 |
| Clostridioides | Dehydroascorbide(1-) | 0.404934 | 0.119739 | 0.496035 |
| Monoglobus | Ricinoleic acid | -0.40492 | 0.119754 | 0.496035 |
| [Eubacterium]_oxidoreducens_group | 2',3'-Dideoxyuridine | 0.404753 | 0.11992 | 0.496369 |
| Aquicella | Xanthosine | 0.404749 | 0.119924 | 0.496369 |
| Acidothermus | alpha-D-Galactopyranuronosyl-(1->4)-alpha-D-galactopyranuronosyl-(1->4)-D-galacturonic acid | 0.404732 | 0.119941 | 0.496369 |
| Clostridioides | Americine | -0.40468 | 0.119991 | 0.496432 |
| Subgroup_13 | D-Mannose | 0.404571 | 0.120102 | 0.496744 |
| Enterococcus | Resorufin | 0.404469 | 0.120205 | 0.49684 |
| Lachnospiraceae_UCG-006 | Bis(2-propoxyethyl) 2,6-dimethyl-4-(3-nitrophenyl)-3,4-dihydropyridine-3,5-dicarboxylate | -0.40443 | 0.120248 | 0.49684 |
| bacteriap25 | 4-Methylcatechol 1-glucuronide | 0.40441 | 0.120264 | 0.49684 |
| Coprobacillus | 13-(3,4-Dimethyl-5-propylfuran-2-yl)tridecanoylcarnitine | 0.404394 | 0.12028 | 0.49684 |
| TM7 | Pevonedistat | 0.404353 | 0.120321 | 0.49684 |
| Muribaculum | 3-{[(1s)-2,2-Difluoro-1-Hydroxy-7-(Methylsulfonyl)-2,3-Dihydro-1h-Inden-4-Yl]oxy}-5-Fluorobenzonitrile | 0.404321 | 0.120354 | 0.49684 |
| Jatrophihabitans | N,N'-Diethylthiourea | 0.404249 | 0.120426 | 0.49684 |
| Achromobacter | Bis(2-propoxyethyl) 2,6-dimethyl-4-(3-nitrophenyl)-3,4-dihydropyridine-3,5-dicarboxylate | -0.4042 | 0.120475 | 0.49684 |
| Subgroup_13 | 1-Ethoxymethyl-5-fluorouracil | 0.404183 | 0.120493 | 0.49684 |
| Alcaligenes | 25-Hydroxyvitamin D3-26,23-lactol | -0.40418 | 0.120496 | 0.49684 |
| Christensenellaceae_R-7_group | Ethyl hexadecanoate | -0.40416 | 0.120513 | 0.49684 |
| Anaerostipes | 2-[4-(3-Hydroxypropyl)-2-methoxyphenoxy]-1,3-propanediol 1-xyloside | -0.40411 | 0.120565 | 0.496907 |
| Bryobacter | Doxepin | 0.403939 | 0.120738 | 0.497476 |
| Anaerostipes | Thidiazuron | 0.40365 | 0.12103 | 0.498081 |
| Intestinimonas | Phenylalanylproline | 0.403641 | 0.121038 | 0.498081 |
| Treponema | N-lactoyl-Methionine | 0.403553 | 0.121127 | 0.498081 |
| Acidothermus | Hexadecadienylcarnitine | 0.403544 | 0.121136 | 0.498081 |
| Achromobacter | Cysteine-glutathione disulfide | 0.403473 | 0.121208 | 0.498081 |
| Muribaculum | Notoginsenoside H | 0.403447 | 0.121234 | 0.498081 |
| Anaerotruncus | L-Homocystine | 0.403367 | 0.121315 | 0.498081 |
| TRA3-20 | Streptidine | 0.403279 | 0.121404 | 0.498081 |
| Bryobacter | Dehydrocyclopeptine; (3E)-3-Benzylidene-4-methyl-3,4-dihydro-1H-1,4-benzodiazepine-2,5-dione | -0.40327 | 0.121413 | 0.498081 |
| Acetatifactor | OKOHA-PA | -0.40319 | 0.121495 | 0.498081 |
| Christensenellaceae_R-7_group | L-Glutamine | 0.403185 | 0.121499 | 0.498081 |
| Clostridioides | TG(8:0/8:0/a-13:0)[rac] | -0.40316 | 0.12152 | 0.498081 |
| Intestinimonas | 6,10,14-Trimethyl-5,9,13-pentadecatrien-2-one | -0.40308 | 0.121609 | 0.498081 |
| [Eubacterium]_oxidoreducens_group | Erucic acid | 0.403059 | 0.121626 | 0.498081 |
| Intestinimonas | N-Undecanoylglycine | -0.40305 | 0.121633 | 0.498081 |
| Achromobacter | 2-Methylthioadenosine | 0.403051 | 0.121635 | 0.498081 |
| TM7 | PE-NMe2(18:3(9Z,12Z,15Z)/18:1(11Z)) | 0.403003 | 0.121683 | 0.498081 |
| Enterorhabdus | Fiacitabine | 0.402998 | 0.121688 | 0.498081 |
| Acidothermus | Aminovaleric acid betaine | 0.402977 | 0.12171 | 0.498081 |
| Enterococcus | 2-hydroxy-9Z,12Z-Octadecadienoic acid | -0.40288 | 0.12181 | 0.498081 |
| Muribaculum | Serotinose | 0.402841 | 0.121848 | 0.498081 |
| Lachnospiraceae_UCG-006 | Glycerol 3-phosphate | 0.402839 | 0.121849 | 0.498081 |
| Subgroup_2 | Pelargonidin 3-sophoroside | -0.40283 | 0.121861 | 0.498081 |
| Anaerostipes | Cichorioside K | -0.40279 | 0.121902 | 0.498081 |
| Anaerotruncus | Heptadecanoyl carnitine | 0.40274 | 0.12195 | 0.498081 |
| Granulicella | [6-Hydroxy-8-methyl-3-[3,4,5-trihydroxy-6-(hydroxymethyl)oxan-2-yl]oxy-9,10-dioxatetracyclo[4.3.1.02,5.03,8]decan-4-yl]methyl benzoate | 0.402727 | 0.121963 | 0.498081 |
| [Eubacterium]_oxidoreducens_group | Dehydroascorbide(1-) | 0.402726 | 0.121964 | 0.498081 |
| Anaerotruncus | Eicosadienoic acid | 0.402724 | 0.121966 | 0.498081 |
| Coprobacillus | 3,4-Dimethyl-5-propyl-2-furanpentadecanoic acid | -0.40272 | 0.121969 | 0.498081 |
| Alcaligenes | 4E,14Z-Sphingadiene | -0.4027 | 0.121986 | 0.498081 |
| [Eubacterium]_oxidoreducens_group | Glutamylleucylarginine | 0.402664 | 0.122027 | 0.498081 |
| bacteriap25 | 5-[1-Carboxy-2-(trimethylazaniumyl)ethoxy]-5-oxopentanoate | 0.402644 | 0.122047 | 0.498081 |
| MND1 | PE(O-20:0/0:0) | -0.40264 | 0.122053 | 0.498081 |
| Treponema | Prostaglandin B-1 | -0.40257 | 0.122123 | 0.498172 |
| Enterorhabdus | Iguratimod | -0.40251 | 0.122184 | 0.498172 |
| Lachnospiraceae_UCG-006 | 12-Hydroxydodecanoic acid | -0.40248 | 0.122209 | 0.498172 |
| TRA3-20 | PI(18:0/20:4(8Z,11Z,14Z,17Z)) | 0.402428 | 0.122267 | 0.498172 |
| Enterococcus | Lucuminic acid | 0.402419 | 0.122276 | 0.498172 |
| Monoglobus | 1-O-Galloylglycerol | 0.402407 | 0.122287 | 0.498172 |
| Subgroup_2 | Polypropylene glycol (m w 1,200-3,000) | -0.40237 | 0.122328 | 0.498194 |
| Pseudolabrys | Oleoyl Serotonin | -0.40232 | 0.122375 | 0.498241 |
| Monoglobus | Bacillamidin C | -0.40224 | 0.122456 | 0.498243 |
| Nitrospira | 2-[(4-{2-[(4-Cyclohexylbutyl)(cyclohexylcarbamoyl)amino]ethyl}phenyl)sulfanyl]-2-methylpropanoic acid | -0.40223 | 0.12247 | 0.498243 |
| Muribaculum | N,N'-Diethylthiourea | 0.402216 | 0.122482 | 0.498243 |
| Enterorhabdus | Phenylalanylaspartic acid | 0.40214 | 0.12256 | 0.498416 |
| Flavonifractor | Docosadienoate (22:2n6) | 0.402011 | 0.122691 | 0.498762 |
| Nitrospira | Streptidine | 0.401956 | 0.122747 | 0.498762 |
| Anaerotruncus | PC(20:5(5Z,8Z,11Z,14Z,17Z)/PGJ2) | -0.40195 | 0.122751 | 0.498762 |
| Muribaculum | 2-[4-(3-Hydroxypropyl)-2-methoxyphenoxy]-1,3-propanediol 1-xyloside | -0.40189 | 0.122814 | 0.498874 |
| Coprobacillus | Phenylalanylaspartic acid | 0.401788 | 0.122918 | 0.499152 |
| Clostridioides | indoprofen | -0.40175 | 0.122959 | 0.499177 |
| Monoglobus | Valylhistidine | 0.401595 | 0.123115 | 0.499426 |
| Clostridioides | 3-Hydroxy-10'-apo-b,y-carotenal | 0.401588 | 0.123122 | 0.499426 |
| Granulicella | PC(20:4(5Z,8Z,11Z,14Z)-OH(16R)/2:0) | 0.401567 | 0.123143 | 0.499426 |
| Alcaligenes | Monooctyl phthalate | -0.40149 | 0.123225 | 0.499426 |
| RF39 | 11,14,17-eicosatrienoic acid | 0.401469 | 0.123243 | 0.499426 |
| Intestinimonas | Neosaxitoxin | 0.401434 | 0.123279 | 0.499426 |
| Aquicella | Withaferin A | -0.40143 | 0.123281 | 0.499426 |
| Subgroup_2 | N-gamma-L-Glutamyl-D-alanine | 0.401371 | 0.123344 | 0.499426 |
| RF39 | Ethyl hexadecanoate | -0.40136 | 0.12335 | 0.499426 |
| TM7 | N-(1-Deoxy-1-fructosyl)alanine | 0.40134 | 0.123375 | 0.499426 |
| Clostridioides | Napelline | 0.401192 | 0.123526 | 0.499892 |
| TRA3-20 | DG(18:0/20:4(8Z,11Z,14Z,17Z)/0:0) | 0.401038 | 0.123684 | 0.500299 |
| Bryobacter | Neosaxitoxin | 0.401025 | 0.123698 | 0.500299 |
| bacteriap25 | 3-Hydroxyeicosanoylcarnitine | 0.400944 | 0.12378 | 0.50049 |
| Treponema | 12,13-DHOME | -0.40088 | 0.123851 | 0.500555 |
| Subgroup_13 | (E)-2-(hexa-3,5-dien-1-yn-1-yl)-5-(prop-1-yn-1-yl)thiophene | 0.400859 | 0.123868 | 0.500555 |
| Treponema | N-Lactoylleucine | 0.400713 | 0.124017 | 0.500811 |
| Clostridia_vadinBB60_group | Niacinamide | 0.400696 | 0.124035 | 0.500811 |
| Solobacterium | [6-Hydroxy-8-methyl-3-[3,4,5-trihydroxy-6-(hydroxymethyl)oxan-2-yl]oxy-9,10-dioxatetracyclo[4.3.1.02,5.03,8]decan-4-yl]methyl benzoate | 0.400693 | 0.124038 | 0.500811 |
| Anaerotruncus | 1-O-Galloylglycerol | 0.400567 | 0.124167 | 0.500919 |
| Granulicella | Cortisol | -0.40056 | 0.124172 | 0.500919 |
| Acidothermus | PC(11:0/23:0) | 0.40054 | 0.124195 | 0.500919 |
| bacteriap25 | PS(22:5(7Z,10Z,13Z,16Z,19Z)/22:6(4Z,7Z,10Z,13Z,16Z,19Z)) | -0.40053 | 0.124207 | 0.500919 |
| Enterorhabdus | Glutaminylproline | 0.400471 | 0.124266 | 0.500974 |
| RF39 | 20-HETE-d6 | 0.400446 | 0.124292 | 0.500974 |
| Bryobacter | Aspartyl-Methionine | 0.400394 | 0.124344 | 0.500989 |
| Subgroup_13 | 12-Hydroxy-12-octadecanoylcarnitine | 0.400373 | 0.124367 | 0.500989 |
| Lachnospiraceae_UCG-006 | 3-Ketosphingosine | -0.4002 | 0.124544 | 0.501308 |
| Muribaculum | Avenin | 0.400186 | 0.124558 | 0.501308 |
| TRA3-20 | Glucose-6-glutamate | 0.400094 | 0.124654 | 0.501308 |
| Alcaligenes | 5'-Carboxy-gamma-chromanol | -0.40006 | 0.124686 | 0.501308 |
| MND1 | N6-Acetyl-L-lysine | 0.400046 | 0.124703 | 0.501308 |
| Christensenellaceae_R-7_group | 16-hydroxy-6-hexadecenoic acid | -0.40003 | 0.124716 | 0.501308 |
| MND1 | Oliceridine | -0.39993 | 0.124818 | 0.501308 |
| Anaerotruncus | PE(19:0/20:2(11Z,14Z)) | -0.39991 | 0.124841 | 0.501308 |
| Coprobacillus | 4-Phenylbutanoylcarnitine | -0.39991 | 0.124847 | 0.501308 |
| Clostridioides | AM2201 N-(4-hydroxypentyl) metabolite | -0.39981 | 0.124942 | 0.501308 |
| Bryobacter | 16,17-Dihydro-16alpha,17-dihydroxygibberellin A4 17-glucoside | -0.39966 | 0.125101 | 0.501308 |
| TM7 | 1-O-Galloylglycerol | 0.399647 | 0.125114 | 0.501308 |
| Muribaculum | PI(18:0/20:4(8Z,11Z,14Z,17Z)) | 0.399634 | 0.125128 | 0.501308 |
| Acetatifactor | Adenosine monophosphate | -0.39961 | 0.125155 | 0.501308 |
| Monoglobus | FAD | 0.399571 | 0.125193 | 0.501308 |
| bacteriap25 | 3-Hydroxy-11Z-octadecenoylcarnitine | 0.399563 | 0.125202 | 0.501308 |
| bacteriap25 | Aspartyl-Alanine | 0.399545 | 0.12522 | 0.501308 |
| Coprobacillus | 11-Hydroxyhexadecanoylcarnitine | 0.399492 | 0.125274 | 0.501308 |
| Anaerotruncus | Cerulenin | 0.399479 | 0.125288 | 0.501308 |
| Anaerostipes | FAD | 0.399372 | 0.125399 | 0.501308 |
| Acetatifactor | Hexadecadienylcarnitine | 0.39931 | 0.125463 | 0.501308 |
| Granulicella | Notoginsenoside H | 0.399272 | 0.125502 | 0.501308 |
| Alcaligenes | 16,17-Dihydro-16alpha,17-dihydroxygibberellin A4 17-glucoside | -0.39927 | 0.125504 | 0.501308 |
| Intestinimonas | Bis(2-propoxyethyl) 2,6-dimethyl-4-(3-nitrophenyl)-3,4-dihydropyridine-3,5-dicarboxylate | -0.39926 | 0.125513 | 0.501308 |
| Achromobacter | PI(18:0/20:4(8Z,11Z,14Z,17Z)) | 0.399238 | 0.125537 | 0.501308 |
| Anaerotruncus | (E,E)-3,7,11-Trimethyl-2,6,10-dodecatrienyl octanoate | 0.39922 | 0.125556 | 0.501308 |
| Clostridioides | 5S-HETE di-endoperoxide | -0.39921 | 0.125562 | 0.501308 |
| Treponema | PI(20:3(8Z,11Z,14Z)/0:0) | 0.399192 | 0.125585 | 0.501308 |
| Achromobacter | Pro-Pro-Pro | 0.399191 | 0.125586 | 0.501308 |
| Clostridioides | 3-O-acetylecdysone 2-phosphate | -0.39919 | 0.125589 | 0.501308 |
| Acidothermus | Amobarbital | 0.399152 | 0.125626 | 0.501308 |
| RF39 | Docosatrienoic acid | 0.399102 | 0.125678 | 0.501308 |
| TM7 | Alacepril | -0.39909 | 0.125686 | 0.501308 |
| Achromobacter | Azeloprazole | 0.399085 | 0.125695 | 0.501308 |
| bacteriap25 | benfluorex | 0.39906 | 0.125721 | 0.501308 |
| Intestinimonas | Phenylalanyl-Glycine | 0.399053 | 0.125729 | 0.501308 |
| TM7 | 20-HETE-d6 | 0.39902 | 0.125763 | 0.501308 |
| Faecalibaculum | Withaferin A | -0.39882 | 0.125969 | 0.50197 |
| Aquicella | 2',3'-Dideoxyuridine | 0.398753 | 0.126039 | 0.50197 |
| Muribaculum | Cichorioside K | -0.39872 | 0.126069 | 0.50197 |
| Treponema | N-Lactoylphenylalanine | 0.398722 | 0.126072 | 0.50197 |
| bacteriap25 | Lycoperdic acid | 0.398532 | 0.12627 | 0.502615 |
| Intestinimonas | D-erythro-Sphingosine C-20 | -0.39833 | 0.126475 | 0.503271 |
| Haliangium | Am-PE(16:0/18:0) | 0.398304 | 0.126506 | 0.503271 |
| Haliangium | True blue | 0.398153 | 0.126663 | 0.503755 |
| Clostridia_vadinBB60_group | 11-Hydroxyoctadecanoylcarnitine | 0.397958 | 0.126867 | 0.504421 |
| Faecalibaculum | D-Mannose | 0.397911 | 0.126916 | 0.504474 |
| Jatrophihabitans | Notoginsenoside H | 0.397813 | 0.127017 | 0.504735 |
| Clostridia_vadinBB60_group | (S)-a-Amino-2,5-dihydro-5-oxo-4-isoxazolepropanoic acid N2-glucoside | 0.397742 | 0.127092 | 0.504748 |
| bacteriap25 | PE(22:4(7Z,10Z,13Z,16Z)/19:0) | -0.39774 | 0.127092 | 0.504748 |
| Intestinimonas | alpha-D-Galactopyranuronosyl-(1->4)-alpha-D-galactopyranuronosyl-(1->4)-D-galacturonic acid | 0.397671 | 0.127166 | 0.504885 |
| Bryobacter | O-phosphonato-L-homoserine(2-) | 0.39764 | 0.127199 | 0.504885 |
| Subgroup_2 | 4-Bis(2-hydroxyethyl)amino-L-phenylalanine | 0.397522 | 0.127321 | 0.50491 |
| Aquicella | N-gamma-L-Glutamyl-D-alanine | 0.397457 | 0.127389 | 0.50491 |
| Bryobacter | (S)-a-Amino-2,5-dihydro-5-oxo-4-isoxazolepropanoic acid N2-glucoside | 0.397449 | 0.127398 | 0.50491 |
| bacteriap25 | 3-Ketosphingosine | -0.39738 | 0.12747 | 0.50491 |
| Acetatifactor | thiamphenicol | 0.397375 | 0.127475 | 0.50491 |
| Enterococcus | Dehydrocyclopeptine; (3E)-3-Benzylidene-4-methyl-3,4-dihydro-1H-1,4-benzodiazepine-2,5-dione | -0.39737 | 0.12748 | 0.50491 |
| Monoglobus | L-Lysinamide | -0.39737 | 0.12748 | 0.50491 |
| Treponema | Azeloprazole | 0.397282 | 0.127573 | 0.50491 |
| Nitrospira | 2,2,3,3,4,4,5,5,6,6,7,7,8,8,9,9-Hexadecafluorononanoic acid | 0.39727 | 0.127585 | 0.50491 |
| RF39 | Symmetric dimethylarginine | 0.397257 | 0.127598 | 0.50491 |
| Bryobacter | Pentadecanoylcarnitine | 0.397224 | 0.127633 | 0.50491 |
| Jatrophihabitans | Creatine | 0.397218 | 0.127639 | 0.50491 |
| MND1 | 3-O-acetylecdysone 2-phosphate | -0.39719 | 0.127671 | 0.50491 |
| TM7 | Ricinoleic acid | -0.39705 | 0.127817 | 0.504996 |
| Anaerotruncus | 2-Phenylethyl octanoate | -0.39705 | 0.127819 | 0.504996 |
| Nitrospira | 3,4-Dimethyl-5-propyl-2-furanpentadecanoic acid | -0.39704 | 0.127824 | 0.504996 |
| Achromobacter | 5-Hydroxyindoxyl sulfate | -0.39703 | 0.127836 | 0.504996 |
| Intestinimonas | BEFLOXATONE | 0.396974 | 0.127894 | 0.505058 |
| Acetatifactor | Medicagol | 0.396885 | 0.127988 | 0.505058 |
| Faecalibaculum | JP83 | -0.39688 | 0.127995 | 0.505058 |
| Clostridioides | Adenosine monophosphate | -0.39687 | 0.128001 | 0.505058 |
| Muribaculum | Pseudouridine | 0.396822 | 0.128054 | 0.505058 |
| Aquicella | S-[(3R,3As,6S,6aS)-3-nitrooxy-2,3,3a,5,6,6a-hexahydrofuro[3,2-b]furan-6-yl] ethanethioate | 0.39681 | 0.128067 | 0.505058 |
| TM7 | PE(19:0/20:2(11Z,14Z)) | -0.39669 | 0.128192 | 0.505408 |
| Jatrophihabitans | Tetradecanoylcarnitine | 0.396509 | 0.128383 | 0.506019 |
| TM7 | L-Glutamine | 0.396377 | 0.128521 | 0.506423 |
| Bryobacter | 2-Deoxy-2,3-dehydro-n-acetyl-neuraminic acid | 0.396341 | 0.128559 | 0.506431 |
| Alcaligenes | Pseudouridine | 0.396275 | 0.128629 | 0.506526 |
| Coprobacillus | Lemborexant | -0.39625 | 0.128655 | 0.506526 |
| Clostridia_vadinBB60_group | Kaltostat | 0.396133 | 0.128778 | 0.506869 |
| Aquicella | 2-C-methyl-D-erythritol-4-phosphate | 0.396095 | 0.128818 | 0.506885 |
| RF39 | 5'-Hydroxytenoxicam | 0.396061 | 0.128855 | 0.506886 |
| Subgroup_13 | Ethynodiol | -0.39598 | 0.128935 | 0.507062 |
| Clostridioides | biliverdin-IX-alpha | -0.39594 | 0.128981 | 0.5071 |
| Alcaligenes | N-(N-(3-Amino-3-carboxypropyl)-3-amino-3-carboxypropyl)azetidine-2-carboxylic acid | 0.395858 | 0.129069 | 0.507302 |
| Muribaculum | Aspartyl-Methionine | 0.395763 | 0.129168 | 0.507442 |
| Enterococcus | Dirithromycin | -0.39575 | 0.129187 | 0.507442 |
| Clostridioides | 7-oxo-8-amino-nonanoic acid | -0.39572 | 0.129212 | 0.507442 |
| Enterococcus | Terizidone | 0.395519 | 0.129426 | 0.50814 |
| Subgroup_2 | PE(22:5(4Z,7Z,10Z,13Z,16Z)/PGD2) | -0.39548 | 0.129463 | 0.508143 |
| Subgroup_13 | PC(20:5(5Z,8Z,11Z,14Z,17Z)/PGJ2) | -0.39536 | 0.129599 | 0.508177 |
| Aquicella | 4E,14Z-Sphingadiene | -0.39535 | 0.1296 | 0.508177 |
| [Eubacterium]_oxidoreducens_group | Cortisol | -0.39534 | 0.129618 | 0.508177 |
| Bryobacter | 3-Methyl-3-butenyl hexadecanoate | 0.39521 | 0.129753 | 0.508177 |
| Treponema | PE(22:4(7Z,10Z,13Z,16Z)/19:0) | -0.39516 | 0.129803 | 0.508177 |
| Muribaculum | Creatine | 0.395156 | 0.12981 | 0.508177 |
| Bryobacter | 4'-O-Glucopyranosylsinapic acid | 0.395141 | 0.129826 | 0.508177 |
| Acetatifactor | Pelargonidin 3-sophoroside | -0.39508 | 0.129895 | 0.508177 |
| MND1 | TG(8:0/8:0/a-13:0)[rac] | -0.39501 | 0.129963 | 0.508177 |
| Anaerostipes | 8-Amino-7-oxononanoic acid | -0.39501 | 0.129967 | 0.508177 |
| Faecalibaculum | Phenylalanyl-Glycine | 0.394998 | 0.129977 | 0.508177 |
| Faecalibaculum | [(2S,4R,5R,6R,14S,16R)-14-Hydroxy-7,11-dimethyl-6-(2-oxopyran-4-yl)-3-oxapentacyclo[8.8.0.02,4.02,7.011,16]octadecan-5-yl] acetate | -0.39492 | 0.130065 | 0.508177 |
| Achromobacter | Orotidine | 0.394912 | 0.130069 | 0.508177 |
| Acetatifactor | PC(16:0/16:0) | 0.394891 | 0.130091 | 0.508177 |
| Acidothermus | Serotinose | 0.394886 | 0.130096 | 0.508177 |
| Jatrophihabitans | 2-[4-(3-Hydroxypropyl)-2-methoxyphenoxy]-1,3-propanediol 1-xyloside | -0.39487 | 0.130113 | 0.508177 |
| Anaerotruncus | N-(1-Deoxy-1-fructosyl)threonine | 0.39487 | 0.130114 | 0.508177 |
| Alcaligenes | 1-O-(2R-hydroxy-hexadecyl)-sn-glycerol | -0.39486 | 0.130121 | 0.508177 |
| Aquicella | 2-Deoxy-2,3-dehydro-n-acetyl-neuraminic acid | 0.394828 | 0.130158 | 0.508179 |
| Alcaligenes | 2-Phenylethyl octanoate | -0.39478 | 0.130204 | 0.508219 |
| Treponema | 1-Octadecene | -0.39472 | 0.13027 | 0.508309 |
| Jatrophihabitans | Am-PE(16:0/18:0) | 0.394694 | 0.1303 | 0.508309 |
| Bryobacter | N-Myristoyl Asparagine | -0.39465 | 0.130344 | 0.508341 |
| Clostridioides | 12,13-DHOME | -0.39461 | 0.130385 | 0.508361 |
| IMCC26256 | Asparaginyl-Proline | 0.394555 | 0.130448 | 0.50838 |
| Anaerostipes | Ethyl hexadecanoate | -0.39451 | 0.130496 | 0.50838 |
| Haliangium | Tuberonic acid glucoside | -0.39451 | 0.130498 | 0.50838 |
| Clostridioides | 2-hydroxy-9Z,12Z-Octadecadienoic acid | -0.39445 | 0.130562 | 0.508488 |
| Acidothermus | Dehydroascorbic acid | 0.394298 | 0.13072 | 0.508634 |
| Bryobacter | 3-(3-(Pyridin-3-yl)-1,2,4-oxadiazol-5-yl)benzonitrile | 0.394287 | 0.130732 | 0.508634 |
| bacteriap25 | 1-Octadecene | -0.39425 | 0.130769 | 0.508634 |
| Rikenellaceae_RC9_gut_group | (E)-2-(hexa-3,5-dien-1-yn-1-yl)-5-(prop-1-yn-1-yl)thiophene | 0.39425 | 0.130772 | 0.508634 |
| Nitrospira | (2R,4S)-2-Aminoformyl-6-fluoro-spiro[chroman-4,4'-imidazolidine]-2',5'-dione | -0.39421 | 0.130812 | 0.508634 |
| Alcaligenes | 6-Hydroxytricetin 5-rhamnoside | 0.394208 | 0.130816 | 0.508634 |
| Muribaculum | Tuberonic acid glucoside | -0.39412 | 0.13091 | 0.508859 |
| Lachnospiraceae_UCG-006 | 1-O-(2R-hydroxy-hexadecyl)-sn-glycerol | -0.394 | 0.131035 | 0.509204 |
| MND1 | N1-Methyl-2-pyridone-5-carboxamide | -0.39392 | 0.131125 | 0.509344 |
| Subgroup_2 | Orotidine | 0.393876 | 0.131171 | 0.509344 |
| Anaerotruncus | Hydroxy-2-naphthalenylmethylphosphonic acid tris-acetoxymethyl ester | 0.393867 | 0.13118 | 0.509344 |
| bacteriap25 | Cannabidivarin | -0.39382 | 0.131226 | 0.509383 |
| Nitrospira | (2S)-1,1,1-Trifluoro-2-(4-pyrazol-1-ylphenyl)-3-[5-[[1-(trifluoromethyl)cyclopropyl]methyl]-1H-imidazol-2-yl]propan-2-ol | -0.3937 | 0.131359 | 0.509436 |
| Clostridioides | Ricinoleic acid | -0.3937 | 0.131361 | 0.509436 |
| Clostridia_vadinBB60_group | N-Acetyl-D-Glucosamine 6-Phosphate | 0.393687 | 0.131372 | 0.509436 |
| Monoglobus | PE(O-20:0/0:0) | -0.39365 | 0.131413 | 0.509436 |
| TM7 | D-erythro-Sphingosine C-20 | -0.39364 | 0.131421 | 0.509436 |
| Jatrophihabitans | PI(18:0/20:4(8Z,11Z,14Z,17Z)) | 0.393584 | 0.131483 | 0.509535 |
| bacteriap25 | Nipradilol | 0.393525 | 0.131546 | 0.50964 |
| Muribaculum | PC(P-18:0/20:4(5Z,8Z,11Z,14Z)-OH(16R)) | 0.393487 | 0.131586 | 0.509656 |
| Solobacterium | DG(17:2(9Z,12Z)/22:5(7Z,10Z,13Z,16Z,19Z)/0:0)[iso2] | 0.393354 | 0.131728 | 0.510064 |
| Coprobacillus | Nipradilol | 0.393149 | 0.131947 | 0.510551 |
| Alcaligenes | N-Undecanoylglycine | -0.39314 | 0.131959 | 0.510551 |
| Faecalibaculum | S-[(3R,3As,6S,6aS)-3-nitrooxy-2,3,3a,5,6,6a-hexahydrofuro[3,2-b]furan-6-yl] ethanethioate | 0.393135 | 0.131962 | 0.510551 |
| Aquicella | Skepinone-L | 0.393015 | 0.132091 | 0.510676 |
| Enterococcus | 3-O-acetylecdysone 2-phosphate | -0.39299 | 0.132119 | 0.510676 |
| Enterococcus | Phenylalanyl-Glycine | 0.392934 | 0.132177 | 0.510676 |
| Anaerostipes | N-Acetylneuraminic acid | 0.392934 | 0.132177 | 0.510676 |
| Clostridioides | 1-O-Galloylglycerol | 0.392908 | 0.132205 | 0.510676 |
| Monoglobus | N-Myristoyl Asparagine | -0.39289 | 0.132221 | 0.510676 |
| Monoglobus | Phosphohydroxypyruvic acid | -0.39286 | 0.132262 | 0.510676 |
| Muribaculum | 2-[(4-{2-[(4-Cyclohexylbutyl)(cyclohexylcarbamoyl)amino]ethyl}phenyl)sulfanyl]-2-methylpropanoic acid | -0.39283 | 0.132285 | 0.510676 |
| Jatrophihabitans | PC(P-18:0/20:4(5Z,8Z,11Z,14Z)-OH(16R)) | 0.392671 | 0.13246 | 0.510885 |
| Bryobacter | LysoPI(18:0/0:0) | 0.39266 | 0.132471 | 0.510885 |
| Anaerotruncus | Dolichyl b-D-glucosyl phosphate | -0.39266 | 0.132475 | 0.510885 |
| Anaerotruncus | Glutaminylproline | 0.392649 | 0.132484 | 0.510885 |
| Jatrophihabitans | PPM-18 | 0.392529 | 0.132612 | 0.510947 |
| Aquicella | PE(22:5(4Z,7Z,10Z,13Z,16Z)/PGD2) | -0.39252 | 0.132627 | 0.510947 |
| MND1 | 16-hydroxy-6-hexadecenoic acid | -0.3925 | 0.132639 | 0.510947 |
| Haliangium | m7G(5')pppAm | 0.392486 | 0.132659 | 0.510947 |
| Aquicella | Cichorioside K | -0.39246 | 0.132681 | 0.510947 |
| Anaerotruncus | LysoPE(20:3(8Z,11Z,14Z)/0:0) | 0.392334 | 0.132822 | 0.511128 |
| Lachnospiraceae_UCG-006 | PE(22:4(7Z,10Z,13Z,16Z)/19:0) | -0.39232 | 0.132837 | 0.511128 |
| Faecalibaculum | N-(1-Deoxy-1-fructosyl)alanine | 0.392302 | 0.132856 | 0.511128 |
| Haliangium | Ethyl hexadecanoate | -0.39226 | 0.132903 | 0.511128 |
| Anaerotruncus | 16-hydroxy-6-hexadecenoic acid | -0.39223 | 0.132932 | 0.511128 |
| Alcaligenes | OKOHA-PA | -0.39218 | 0.132991 | 0.511128 |
| Alcaligenes | (R)-Propyl 2-amino-3-mercaptopropanoate | 0.39216 | 0.13301 | 0.511128 |
| bacteriap25 | 4-Chloro-2-nitrobenzylalcohol | -0.39213 | 0.133036 | 0.511128 |
| Alcaligenes | Pro-Pro-Pro | 0.392117 | 0.133055 | 0.511128 |
| TM7 | 11,14,17-eicosatrienoic acid | 0.391873 | 0.133319 | 0.512002 |
| Bryobacter | Streptidine | 0.391739 | 0.133463 | 0.512307 |
| Monoglobus | alpha-D-Galactopyranuronosyl-(1->4)-alpha-D-galactopyranuronosyl-(1->4)-D-galacturonic acid | 0.391724 | 0.133479 | 0.512307 |
| bacteriap25 | O-phosphonato-L-homoserine(2-) | 0.391698 | 0.133508 | 0.512307 |
| Flavonifractor | Sulisobenzone | 0.391659 | 0.13355 | 0.512329 |
| Coprobacillus | 3-Hydroxy-10'-apo-b,y-carotenal | 0.39161 | 0.133603 | 0.512392 |
| Solobacterium | Prolyl-Asparagine | 0.391502 | 0.133719 | 0.512474 |
| Alcaligenes | U-75302 | 0.391498 | 0.133724 | 0.512474 |
| Subgroup_13 | 5-[1-Carboxy-2-(trimethylazaniumyl)ethoxy]-5-oxopentanoate | 0.391458 | 0.133767 | 0.512474 |
| Aquicella | 2-[4-(3-Hydroxypropyl)-2-methoxyphenoxy]-1,3-propanediol 1-xyloside | -0.39146 | 0.13377 | 0.512474 |
| bacteriap25 | L-Glutamine | 0.391322 | 0.133914 | 0.51284 |
| bacteriap25 | 2-Cyclotetradecen-1-one | -0.39122 | 0.13402 | 0.51284 |
| Achromobacter | Lycoperdic acid | 0.391217 | 0.134027 | 0.51284 |
| Clostridia_vadinBB60_group | Serotinose | 0.391204 | 0.134041 | 0.51284 |
| Lachnospiraceae_UCG-006 | 8-Amino-7-oxononanoic acid | -0.3912 | 0.134047 | 0.51284 |
| Jatrophihabitans | Cichorioside K | -0.39116 | 0.134092 | 0.512873 |
| Achromobacter | 2-Phenylethyl octanoate | -0.39112 | 0.134135 | 0.512897 |
| RF39 | N1-Methyl-2-pyridone-5-carboxamide | -0.39105 | 0.134207 | 0.513034 |
| Achromobacter | Avenin | 0.390999 | 0.134264 | 0.513067 |
| Enterorhabdus | hydroxyhexadecenoylcarnitine | 0.390942 | 0.134325 | 0.513067 |
| Intestinimonas | 9-Octadecenal | -0.39092 | 0.134346 | 0.513067 |
| Faecalibaculum | 1-Hydroxy-2-naphthoic acid | 0.390776 | 0.134505 | 0.513067 |
| MND1 | Pelargonidin 3-sophoroside | -0.39074 | 0.13454 | 0.513067 |
| Subgroup_13 | Avenin | 0.390725 | 0.134561 | 0.513067 |
| Achromobacter | PPM-18 | 0.390699 | 0.134589 | 0.513067 |
| Clostridioides | Allitridin | 0.39068 | 0.134609 | 0.513067 |
| Subgroup_2 | Am-PE(16:0/18:0) | 0.390615 | 0.13468 | 0.513067 |
| Intestinimonas | (R)-Propyl 2-amino-3-mercaptopropanoate | 0.390548 | 0.134752 | 0.513067 |
| Subgroup_13 | Medicagol | 0.390544 | 0.134757 | 0.513067 |
| Enterococcus | Xanthosine | 0.390542 | 0.13476 | 0.513067 |
| RF39 | 2-Cyclotetradecen-1-one | -0.39051 | 0.134795 | 0.513067 |
| Subgroup_13 | 11-Hydroxyhexadecanoylcarnitine | 0.390434 | 0.134877 | 0.513067 |
| Acidothermus | 3-Methyl-3-butenyl apiosyl-(1->6)-glucoside | 0.390367 | 0.134949 | 0.513067 |
| Rikenellaceae_RC9_gut_group | PC(20:5(5Z,8Z,11Z,14Z,17Z)/PGJ2) | -0.39036 | 0.134953 | 0.513067 |
| Faecalibaculum | Ethynodiol | -0.39035 | 0.134963 | 0.513067 |
| TM7 | Oleic acid | -0.39035 | 0.134969 | 0.513067 |
| Nitrospira | 11-Hydroxyhexadecanoylcarnitine | 0.390339 | 0.13498 | 0.513067 |
| Acetatifactor | 3-Ketosphingosine | -0.39033 | 0.134986 | 0.513067 |
| Bryobacter | (E)-2-(hexa-3,5-dien-1-yn-1-yl)-5-(prop-1-yn-1-yl)thiophene | 0.390332 | 0.134988 | 0.513067 |
| Anaerotruncus | Dimethylaminoparthenolide | 0.39025 | 0.135077 | 0.513067 |
| Subgroup_13 | 14-Methylpentadecanoylcarnitine | 0.390249 | 0.135078 | 0.513067 |
| Anaerotruncus | Cichorioside K | -0.39015 | 0.135182 | 0.513067 |
| Anaerotruncus | 3-hydroxyhexadecanoyl carnitine | 0.390118 | 0.135221 | 0.513067 |
| Clostridioides | Glycyl-D-proline | 0.390075 | 0.135267 | 0.513067 |
| Aquicella | 25-Hydroxyvitamin D3-26,23-lactol | -0.39005 | 0.135295 | 0.513067 |
| Clostridioides | scyllo-Inositol | 0.390004 | 0.135345 | 0.513067 |
| Nitrospira | Pelargonidin 3,5-di-(6-acetylglucoside) | -0.39 | 0.135353 | 0.513067 |
| Jatrophihabitans | Phenylalanyl-Glycine | 0.389959 | 0.135394 | 0.513067 |
| Treponema | PS(22:5(7Z,10Z,13Z,16Z,19Z)/22:6(4Z,7Z,10Z,13Z,16Z,19Z)) | -0.38996 | 0.135394 | 0.513067 |
| Achromobacter | Mycobactins | -0.38992 | 0.135431 | 0.513067 |
| Aquicella | Cysteinyl-Aspartate | 0.389884 | 0.135476 | 0.513067 |
| Lachnospiraceae_UCG-006 | Aspartyl-Alanine | 0.389816 | 0.13555 | 0.513067 |
| Anaerotruncus | 2-[(4-{2-[(4-Cyclohexylbutyl)(cyclohexylcarbamoyl)amino]ethyl}phenyl)sulfanyl]-2-methylpropanoic acid | -0.38981 | 0.135561 | 0.513067 |
| Treponema | L-Lysinamide | -0.3898 | 0.135568 | 0.513067 |
| Flavonifractor | N-(1-Deoxy-1-fructosyl)alanine | 0.389761 | 0.13561 | 0.513067 |
| Enterorhabdus | Pelargonidin 3-sophoroside | -0.38974 | 0.135634 | 0.513067 |
| bacteriap25 | Uridine | 0.389707 | 0.135669 | 0.513067 |
| TRA3-20 | Valylhistidine | 0.389702 | 0.135674 | 0.513067 |
| Anaerotruncus | 2-[4-(3-Hydroxypropyl)-2-methoxyphenoxy]-1,3-propanediol 1-xyloside | -0.38967 | 0.135712 | 0.513075 |
| Alcaligenes | Lycoperdic acid | 0.389533 | 0.135859 | 0.513411 |
| Faecalibaculum | Tercatain | 0.389469 | 0.135928 | 0.513411 |
| Acidothermus | thiamphenicol | 0.389429 | 0.135973 | 0.513411 |
| RF39 | Carissanol | -0.38942 | 0.135982 | 0.513411 |
| Enterorhabdus | N-Acetyl-b-glucosaminylamine | 0.389368 | 0.136039 | 0.513411 |
| Coprobacillus | (2S)-1,1,1-Trifluoro-2-(4-pyrazol-1-ylphenyl)-3-[5-[[1-(trifluoromethyl)cyclopropyl]methyl]-1H-imidazol-2-yl]propan-2-ol | -0.38935 | 0.136062 | 0.513411 |
| Clostridioides | Obacunone 17-O-beta-D-glucoside | -0.38934 | 0.136064 | 0.513411 |
| Treponema | PIP(20:0/18:1(12Z)-2OH(9,10)) | -0.38931 | 0.136099 | 0.513411 |
| Clostridioides | 2-Deoxy-2,3-dehydro-n-acetyl-neuraminic acid | 0.38911 | 0.136321 | 0.513411 |
| Anaerotruncus | 14-Methylpentadecanoylcarnitine | 0.389083 | 0.136351 | 0.513411 |
| Bryobacter | L-Arginine | 0.389079 | 0.136355 | 0.513411 |
| Anaerotruncus | Tridecanoylcarnitine | 0.389011 | 0.13643 | 0.513411 |
| Anaerotruncus | Maltotriose | 0.388993 | 0.13645 | 0.513411 |
| Achromobacter | N2-gamma-Glutamylglutamine | 0.38899 | 0.136453 | 0.513411 |
| Bryobacter | 2-Methyl-2-[4-[3-[1-(4-methylbenzyl)-5-oxo-4,5-dihydro-1H-1,2,4-triazol-3-yl]propyl]phenoxy]propanoic acid | -0.38898 | 0.136467 | 0.513411 |
| Coprobacillus | (2R,4S)-2-Aminoformyl-6-fluoro-spiro[chroman-4,4'-imidazolidine]-2',5'-dione | -0.38892 | 0.136529 | 0.513411 |
| RF39 | Monooctyl phthalate | -0.38891 | 0.136538 | 0.513411 |
| Alcaligenes | Asparaginyl-Proline | 0.388862 | 0.136594 | 0.513411 |
| Granulicella | PS(22:5(7Z,10Z,13Z,16Z,19Z)/22:6(4Z,7Z,10Z,13Z,16Z,19Z)) | -0.38884 | 0.136617 | 0.513411 |
| Nitrospira | PC(18:1(17Z)/18:1(17Z)) | 0.388838 | 0.13662 | 0.513411 |
| Anaerostipes | Dolichyl b-D-glucosyl phosphate | -0.38883 | 0.136624 | 0.513411 |
| Solobacterium | L-Glutamine | 0.38882 | 0.136639 | 0.513411 |
| TM7 | Serylproline | 0.388802 | 0.136659 | 0.513411 |
| Rikenellaceae_RC9_gut_group | Aminovaleric acid betaine | 0.388729 | 0.136739 | 0.513411 |
| Subgroup_13 | xi-2,3-Octadiene-5,7-diyn-1-ol | 0.388726 | 0.136743 | 0.513411 |
| Clostridioides | 5-[1-Carboxy-2-(trimethylazaniumyl)ethoxy]-5-oxopentanoate | 0.38872 | 0.136749 | 0.513411 |
| Muribaculum | Cortisol | -0.38862 | 0.136863 | 0.513703 |
| Jatrophihabitans | Aspartyl-Isoleucine | 0.388515 | 0.136974 | 0.51396 |
| Coprobacillus | 2-[(4-{2-[(4-Cyclohexylbutyl)(cyclohexylcarbamoyl)amino]ethyl}phenyl)sulfanyl]-2-methylpropanoic acid | -0.38848 | 0.137018 | 0.51396 |
| Aquicella | 4-Chloro-2-nitrobenzylalcohol | -0.38845 | 0.137041 | 0.51396 |
| Acetatifactor | PIP(20:0/18:1(12Z)-2OH(9,10)) | -0.3883 | 0.137211 | 0.51446 |
| MND1 | 7-oxo-8-amino-nonanoic acid | -0.38805 | 0.137488 | 0.514905 |
| Tuzzerella | Phenylalanylaspartic acid | 0.388041 | 0.137495 | 0.514905 |
| TRA3-20 | PC(16:1(9Z)/17:1(9Z)) | -0.38802 | 0.137514 | 0.514905 |
| Subgroup_2 | Fexaramine | -0.38801 | 0.137526 | 0.514905 |
| Treponema | Ethynodiol | -0.38801 | 0.137532 | 0.514905 |
| Coprobacillus | O-Linoleoylcarnitine | 0.387986 | 0.137557 | 0.514905 |
| Clostridioides | 2-carboxy-L-threo-pentonate | -0.38795 | 0.137593 | 0.514905 |
| Monoglobus | (R)-3-Amino-2-fluoropropyl phosphenite | 0.387926 | 0.137623 | 0.514905 |
| Jatrophihabitans | 23S,25,26-Trihydroxyvitamin D3 | 0.3877 | 0.137872 | 0.5157 |
| Coprobacillus | AM2201 N-(4-hydroxypentyl) metabolite | -0.38758 | 0.138002 | 0.515916 |
| [Eubacterium]_oxidoreducens_group | Sulisobenzone | 0.387581 | 0.138003 | 0.515916 |
| Granulicella | Glutamylleucylarginine | 0.387443 | 0.138155 | 0.516205 |
| Treponema | Uridine | 0.38742 | 0.138181 | 0.516205 |
| Nitrospira | 3-Ketosphingosine | -0.38741 | 0.13819 | 0.516205 |
| Muribaculum | DG(18:0/20:4(8Z,11Z,14Z,17Z)/0:0) | 0.387327 | 0.138284 | 0.516317 |
| bacteriap25 | alpha-D-Galactopyranuronosyl-(1->4)-alpha-D-galactopyranuronosyl-(1->4)-D-galacturonic acid | 0.387318 | 0.138293 | 0.516317 |
| Intestinimonas | 5-[1-Carboxy-2-(trimethylazaniumyl)ethoxy]-5-oxopentanoate | 0.38725 | 0.138369 | 0.516391 |
| Subgroup_13 | 4-Methylcatechol 1-glucuronide | 0.387234 | 0.138386 | 0.516391 |
| Pseudolabrys | N-Undecanoylglycine | -0.38703 | 0.138612 | 0.516822 |
| Clostridioides | 2-Cyclotetradecen-1-one | -0.38702 | 0.138628 | 0.516822 |
| Aquicella | m7G(5')pppAm | 0.386957 | 0.138693 | 0.516822 |
| Acetatifactor | 7-oxo-8-amino-nonanoic acid | -0.38692 | 0.138736 | 0.516822 |
| Achromobacter | N-(N-(3-Amino-3-carboxypropyl)-3-amino-3-carboxypropyl)azetidine-2-carboxylic acid | 0.386914 | 0.138741 | 0.516822 |
| Intestinimonas | PI(20:4(5Z,8Z,11Z,14Z)/18:0) | -0.3869 | 0.138753 | 0.516822 |
| Achromobacter | N-Undecanoylglycine | -0.3869 | 0.138759 | 0.516822 |
| Anaerotruncus | L-Carnitine | 0.386716 | 0.13896 | 0.517272 |
| MND1 | N-Myristoyl Asparagine | -0.38662 | 0.139065 | 0.517272 |
| Intestinimonas | Diethylamino 2,5-dihydroxybenzenesulfonate | 0.386582 | 0.139109 | 0.517272 |
| Enterococcus | 1-p-Menthen-3-one | 0.386581 | 0.139111 | 0.517272 |
| [Eubacterium]_oxidoreducens_group | [6-Hydroxy-8-methyl-3-[3,4,5-trihydroxy-6-(hydroxymethyl)oxan-2-yl]oxy-9,10-dioxatetracyclo[4.3.1.02,5.03,8]decan-4-yl]methyl benzoate | 0.386563 | 0.13913 | 0.517272 |
| TM7 | Berkeleylactone L | -0.38656 | 0.139139 | 0.517272 |
| Anaerotruncus | 3-Hydroxyeicosanoylcarnitine | 0.386525 | 0.139172 | 0.517272 |
| Aquicella | 5,5-Diphenyl-2-thiohydantoin | 0.386524 | 0.139174 | 0.517272 |
| Achromobacter | (R)-3-Amino-2-fluoropropyl phosphenite | 0.386489 | 0.139213 | 0.517275 |
| Clostridioides | Ethyl hexadecanoate | -0.3864 | 0.139313 | 0.517275 |
| Acetatifactor | N-(N-(3-Amino-3-carboxypropyl)-3-amino-3-carboxypropyl)azetidine-2-carboxylic acid | 0.386396 | 0.139316 | 0.517275 |
| Enterococcus | a-L-Arabinofuranosyl-(1->3)-b-D-xylopyranosyl-(1->4)-D-xylose | 0.386391 | 0.139321 | 0.517275 |
| Subgroup_2 | Allitridin | 0.386342 | 0.139376 | 0.51734 |
| IMCC26256 | Ac-Ser-Asp-Lys-Pro-OH | 0.38625 | 0.139478 | 0.517582 |
| Monoglobus | N-Phenyl-p-phenylenediamine | -0.38618 | 0.139551 | 0.517717 |
| Clostridia_vadinBB60_group | Fiacitabine | 0.38615 | 0.139589 | 0.517722 |
| Acetatifactor | Dehydroascorbic acid | 0.385998 | 0.139759 | 0.518032 |
| Alcaligenes | 9-Octadecenal | -0.38592 | 0.139843 | 0.518032 |
| Subgroup_13 | Withaferin A | -0.3859 | 0.139868 | 0.518032 |
| Clostridioides | N-(1-Deoxy-1-fructosyl)tyrosine | 0.385871 | 0.1399 | 0.518032 |
| Subgroup_2 | Dimethylaminoparthenolide | 0.385844 | 0.139931 | 0.518032 |
| Clostridia_vadinBB60_group | Carissanol | -0.38584 | 0.139937 | 0.518032 |
| Solobacterium | Glucose-6-glutamate | 0.385832 | 0.139944 | 0.518032 |
| Pseudolabrys | Zidebactam | 0.385811 | 0.139967 | 0.518032 |
| Nitrospira | Dihydrozeatin O-beta-D-Glucoside | 0.38577 | 0.140013 | 0.518065 |
| Lachnospiraceae_UCG-006 | DG(18:0/20:4(8Z,11Z,14Z,17Z)/0:0) | 0.385724 | 0.140064 | 0.518118 |
| Muribaculum | 3'-Deoxythymidine | 0.385589 | 0.140215 | 0.518459 |
| Aquicella | JP83 | -0.38558 | 0.14023 | 0.518459 |
| Subgroup_2 | Ethyl hexadecanoate | -0.38553 | 0.140285 | 0.518528 |
| Tuzzerella | Aspartyl-Methionine | 0.385443 | 0.140378 | 0.518735 |
| bacteriap25 | 2-hydroxy-9Z,12Z-Octadecadienoic acid | -0.38538 | 0.140443 | 0.51884 |
| Treponema | (S1)-Methoxy-3-heptanethiol | 0.385322 | 0.140513 | 0.518961 |
| Achromobacter | PGP(18:3(9Z,12Z,15Z)/18:1(12Z)-O(9S,10R)) | 0.38519 | 0.14066 | 0.519335 |
| Aquicella | Zidebactam | 0.385166 | 0.140688 | 0.519335 |
| Acetatifactor | 4E,14Z-Sphingadiene | -0.38506 | 0.140807 | 0.51937 |
| Clostridioides | N-Undecanoylglycine | -0.38503 | 0.140845 | 0.51937 |
| Anaerotruncus | Thidiazuron | 0.384901 | 0.140984 | 0.51937 |
| Jatrophihabitans | Pseudouridine | 0.384891 | 0.140996 | 0.51937 |
| Faecalibaculum | 25-Hydroxyvitamin D3-26,23-lactol | -0.38482 | 0.141074 | 0.51937 |
| Faecalibaculum | Kaltostat | 0.384802 | 0.141095 | 0.51937 |
| Flavonifractor | Tyrosyl-Alanine | 0.384782 | 0.141118 | 0.51937 |
| Anaerostipes | 7-oxo-8-amino-nonanoic acid | -0.38477 | 0.14113 | 0.51937 |
| Clostridioides | MG(20:4(5Z,8Z,11Z,14Z)/0:0/0:0) | -0.38476 | 0.141146 | 0.51937 |
| TRA3-20 | 2-[(4-{2-[(4-Cyclohexylbutyl)(cyclohexylcarbamoyl)amino]ethyl}phenyl)sulfanyl]-2-methylpropanoic acid | -0.38472 | 0.141189 | 0.51937 |
| Enterococcus | m7G(5')pppAm | 0.384709 | 0.141199 | 0.51937 |
| Acetatifactor | Prolylproline | 0.384682 | 0.14123 | 0.51937 |
| Acidothermus | Methacholine | 0.384552 | 0.141375 | 0.51937 |
| Anaerostipes | L-Arginine | 0.384547 | 0.141381 | 0.51937 |
| Acetatifactor | Kuguacin E | -0.38451 | 0.141423 | 0.51937 |
| Treponema | Dihydrozeatin O-beta-D-Glucoside | 0.38449 | 0.141446 | 0.51937 |
| bacteriap25 | Skepinone-L | 0.384477 | 0.14146 | 0.51937 |
| Enterococcus | Pelargonidin 3-sophoroside | -0.38448 | 0.141461 | 0.51937 |
| Lachnospiraceae_UCG-006 | Hydroxy-2-naphthalenylmethylphosphonic acid tris-acetoxymethyl ester | 0.384442 | 0.1415 | 0.51937 |
| Jatrophihabitans | 3-hydroxyhexadecanoyl carnitine | 0.384425 | 0.141518 | 0.51937 |
| Treponema | 1-(10H-Phenothiazin-2-yl)ethanone | 0.38441 | 0.141535 | 0.51937 |
| Anaerotruncus | N-lactoyl-Methionine | 0.384366 | 0.141585 | 0.51937 |
| Anaerotruncus | Sodium nitrate (NaNO3) | -0.38436 | 0.141588 | 0.51937 |
| Nitrospira | AB-MECA | -0.38431 | 0.141646 | 0.51937 |
| Granulicella | [(2S,4R,5R,6R,14S,16R)-14-Hydroxy-7,11-dimethyl-6-(2-oxopyran-4-yl)-3-oxapentacyclo[8.8.0.02,4.02,7.011,16]octadecan-5-yl] acetate | -0.38431 | 0.141648 | 0.51937 |
| Enterococcus | Pantothenic acid | 0.384302 | 0.141656 | 0.51937 |
| Anaerotruncus | Lamivudine-monophosphate | -0.38424 | 0.141725 | 0.519485 |
| Anaerostipes | PC(TXB2/20:2(11Z,14Z)) | -0.38418 | 0.141789 | 0.51954 |
| Lachnospiraceae_UCG-006 | PI(18:0/20:4(8Z,11Z,14Z,17Z)) | 0.384145 | 0.141834 | 0.51954 |
| Jatrophihabitans | DG(18:0/20:4(8Z,11Z,14Z,17Z)/0:0) | 0.384084 | 0.141902 | 0.51954 |
| Monoglobus | 2-[(4-{2-[(4-Cyclohexylbutyl)(cyclohexylcarbamoyl)amino]ethyl}phenyl)sulfanyl]-2-methylpropanoic acid | -0.38408 | 0.141912 | 0.51954 |
| Granulicella | Glycerol 3-phosphate | 0.384064 | 0.141924 | 0.51954 |
| Jatrophihabitans | Ac-Ser-Asp-Lys-Pro-OH | 0.383726 | 0.142305 | 0.520587 |
| TM7 | PC(TXB2/20:2(11Z,14Z)) | -0.38372 | 0.142316 | 0.520587 |
| TRA3-20 | Notoginsenoside H | 0.383672 | 0.142366 | 0.520587 |
| Subgroup_2 | 16,17-Dihydro-16alpha,17-dihydroxygibberellin A4 17-glucoside | -0.38367 | 0.14237 | 0.520587 |
| Subgroup_13 | PC(TXB2/20:2(11Z,14Z)) | -0.38365 | 0.142395 | 0.520587 |
| Subgroup_2 | PE(22:4(7Z,10Z,13Z,16Z)/19:0) | -0.38354 | 0.142516 | 0.520702 |
| Clostridioides | (2Z,4E,6Z)-Decatrienoylcarnitine | -0.3835 | 0.142561 | 0.520702 |
| bacteriap25 | 12,13-DHOME | -0.38349 | 0.14257 | 0.520702 |
| Intestinimonas | Histamine | -0.38341 | 0.142657 | 0.520702 |
| Treponema | 3-Hydroxyeicosanoylcarnitine | 0.383413 | 0.142658 | 0.520702 |
| Clostridia_vadinBB60_group | Pelargonidin 3,5-di-(6-acetylglucoside) | -0.3834 | 0.142677 | 0.520702 |
| Enterococcus | 3-Amino-2-methoxynonadec-5-en-4-ol | -0.38339 | 0.142685 | 0.520702 |
| bacteriap25 | Selinexor | 0.383302 | 0.142783 | 0.520924 |
| Tuzzerella | Tetradecanoylcarnitine | 0.383239 | 0.142855 | 0.52105 |
| Intestinimonas | Allitridin | 0.383176 | 0.142926 | 0.521176 |
| Anaerotruncus | 9-Hydroxy-4-methoxypsoralen 9-glucoside | 0.383143 | 0.142964 | 0.521177 |
| Alcaligenes | 1-Octadecene | -0.38309 | 0.143028 | 0.521275 |
| Pseudolabrys | 1-[3,4-Dihydroxy-5-(hydroxymethyl)-2-oxolanyl]-1,2,4-triazole-3-carboxamide | 0.383027 | 0.143095 | 0.521384 |
| TRA3-20 | Dodecanamide | -0.38287 | 0.143268 | 0.521881 |
| Gemella | Mycobactins | -0.3828 | 0.143354 | 0.522048 |
| TRA3-20 | N-Acetyl-b-glucosaminylamine | 0.382765 | 0.143392 | 0.522048 |
| Lachnospiraceae_UCG-006 | OKOHA-PA | -0.38272 | 0.143443 | 0.522048 |
| Faecalibaculum | Adenosine monophosphate | -0.38267 | 0.143496 | 0.522048 |
| Acidothermus | Glycyl-D-proline | 0.382656 | 0.143516 | 0.522048 |
| Rikenellaceae_RC9_gut_group | alpha-D-Galactopyranuronosyl-(1->4)-alpha-D-galactopyranuronosyl-(1->4)-D-galacturonic acid | 0.382611 | 0.143567 | 0.522048 |
| Coprobacillus | Pseudouridine | 0.382586 | 0.143594 | 0.522048 |
| Treponema | L-Carnitine | 0.382572 | 0.14361 | 0.522048 |
| Achromobacter | L-arginino-succinate | 0.382515 | 0.143675 | 0.522142 |
| MND1 | (2Z,4E,6Z)-Decatrienoylcarnitine | -0.38248 | 0.14371 | 0.522142 |
| Subgroup_2 | Bis(2-propoxyethyl) 2,6-dimethyl-4-(3-nitrophenyl)-3,4-dihydropyridine-3,5-dicarboxylate | -0.38233 | 0.143883 | 0.522555 |
| Monoglobus | [(2S,4R,5R,6R,14S,16R)-14-Hydroxy-7,11-dimethyl-6-(2-oxopyran-4-yl)-3-oxapentacyclo[8.8.0.02,4.02,7.011,16]octadecan-5-yl] acetate | -0.38232 | 0.143898 | 0.522555 |
| Nitrospira | 13-(3,4-Dimethyl-5-propylfuran-2-yl)tridecanoylcarnitine | 0.382187 | 0.144048 | 0.52264 |
| IMCC26256 | Sulisobenzone | 0.382132 | 0.14411 | 0.52264 |
| Enterococcus | 2-Phenylethyl octanoate | -0.38211 | 0.144133 | 0.52264 |
| Anaerotruncus | 1-O-(2R-hydroxy-hexadecyl)-sn-glycerol | -0.3821 | 0.144142 | 0.52264 |
| TM7 | Lycoperdic acid | 0.382101 | 0.144146 | 0.52264 |
| Anaerostipes | Glucose-6-glutamate | 0.382025 | 0.144232 | 0.52264 |
| Jatrophihabitans | 12-Hydroxy-12-octadecanoylcarnitine | 0.382023 | 0.144235 | 0.52264 |
| Enterorhabdus | O-Linoleoylcarnitine | 0.382006 | 0.144254 | 0.52264 |
| Alcaligenes | Alacepril | -0.382 | 0.144256 | 0.52264 |
| Subgroup_13 | 25-Hydroxyvitamin D3-26,23-lactol | -0.38182 | 0.14447 | 0.523136 |
| RF39 | Nipradilol | 0.381806 | 0.144481 | 0.523136 |
| Subgroup_2 | Phytal | -0.38179 | 0.144504 | 0.523136 |
| Anaerotruncus | a-L-Arabinofuranosyl-(1->3)-b-D-xylopyranosyl-(1->4)-D-xylose | 0.381714 | 0.144586 | 0.523297 |
| Clostridia_vadinBB60_group | (13Z)-3-Hydroxyicos-13-enoylcarnitine | 0.381671 | 0.144636 | 0.523342 |
| Intestinimonas | PE(O-20:0/0:0) | -0.38158 | 0.144741 | 0.523588 |
| Enterorhabdus | Beta-Citryl-L-glutamic acid | 0.381546 | 0.144778 | 0.523588 |
| Achromobacter | Notoginsenoside H | 0.381451 | 0.144886 | 0.523801 |
| Treponema | 16,17-Dihydro-16alpha,17-dihydroxygibberellin A4 17-glucoside | -0.38141 | 0.144939 | 0.523801 |
| Granulicella | Asparaginyl-Proline | 0.381376 | 0.144973 | 0.523801 |
| TM7 | Pentadeca-3,5,7-trienedioylcarnitine | 0.381359 | 0.144991 | 0.523801 |
| Alcaligenes | 5-Hydroxyindoxyl sulfate | -0.38131 | 0.145049 | 0.523801 |
| Granulicella | 1-Ethoxymethyl-5-fluorouracil | 0.381268 | 0.145095 | 0.523801 |
| Anaerotruncus | (S1)-Methoxy-3-heptanethiol | 0.381224 | 0.145146 | 0.523801 |
| Enterococcus | Dodecanamide | -0.3812 | 0.145178 | 0.523801 |
| Intestinimonas | L-Glutamine | 0.381138 | 0.145244 | 0.523801 |
| Clostridioides | BEFLOXATONE | 0.381132 | 0.145251 | 0.523801 |
| [Eubacterium]_oxidoreducens_group | Aspartyl-Gamma-glutamate | 0.38113 | 0.145253 | 0.523801 |
| Rikenellaceae_RC9_gut_group | Dipotassium phosphate | 0.38104 | 0.145357 | 0.523801 |
| Achromobacter | Dodecanamide | -0.38104 | 0.145362 | 0.523801 |
| bacteriap25 | 1-O-(2R-hydroxy-hexadecyl)-sn-glycerol | -0.38099 | 0.145417 | 0.523801 |
| Bryobacter | Peruvianoside II | 0.380929 | 0.145483 | 0.523801 |
| Subgroup_2 | 2-Phenylethyl octanoate | -0.38092 | 0.145498 | 0.523801 |
| Faecalibaculum | PPM-18 | 0.380903 | 0.145513 | 0.523801 |
| Pseudolabrys | D-erythro-L-galacto-Nonulose | 0.380893 | 0.145524 | 0.523801 |
| IMCC26256 | Withaferin A | -0.38088 | 0.145544 | 0.523801 |
| [Eubacterium]_oxidoreducens_group | Dehydroascorbic acid | 0.380757 | 0.14568 | 0.524158 |
| Enterorhabdus | L-Glutamine | 0.380655 | 0.145797 | 0.524169 |
| Subgroup_13 | 5-Hydroxyindoxyl sulfate | -0.38065 | 0.145803 | 0.524169 |
| Achromobacter | Valylserine | 0.380646 | 0.145807 | 0.524169 |
| Acidothermus | S-[(3R,3As,6S,6aS)-3-nitrooxy-2,3,3a,5,6,6a-hexahydrofuro[3,2-b]furan-6-yl] ethanethioate | 0.380624 | 0.145832 | 0.524169 |
| Flavonifractor | 1-p-Menthen-3-one | 0.380543 | 0.145925 | 0.52437 |
| Lachnospiraceae_UCG-006 | Oleoyl Serotonin | -0.38045 | 0.146027 | 0.524591 |
| TRA3-20 | Aspartyl-Alanine | 0.380372 | 0.146121 | 0.524591 |
| Acetatifactor | 8-Amino-7-oxononanoic acid | -0.38033 | 0.146165 | 0.524591 |
| Enterorhabdus | AB-MECA | -0.38031 | 0.146188 | 0.524591 |
| Alcaligenes | N6-Acetyl-L-lysine | 0.380292 | 0.146214 | 0.524591 |
| Subgroup_13 | Maltotriose | 0.380165 | 0.14636 | 0.524591 |
| Monoglobus | (卤)-(Z)-2-(5-Tetradecenyl)cyclobutanone | -0.38016 | 0.146362 | 0.524591 |
| Subgroup_13 | [(2S,4R,5R,6R,14S,16R)-14-Hydroxy-7,11-dimethyl-6-(2-oxopyran-4-yl)-3-oxapentacyclo[8.8.0.02,4.02,7.011,16]octadecan-5-yl] acetate | -0.38016 | 0.146367 | 0.524591 |
| Monoglobus | 2-Methylthioadenosine | 0.380157 | 0.146369 | 0.524591 |
| Achromobacter | NAD | 0.380104 | 0.14643 | 0.524591 |
| Monoglobus | N-Acetyl-D-Glucosamine 6-Phosphate | 0.380104 | 0.14643 | 0.524591 |
| Subgroup_13 | L-Glutamine | 0.380078 | 0.14646 | 0.524591 |
| [Eubacterium]_oxidoreducens_group | 7-oxo-8-amino-nonanoic acid | -0.38007 | 0.146471 | 0.524591 |
| Alcaligenes | (R)-4A-(Ethoxymethyl)-1-(4-fluorophenyl)-6-((4-(trifluoromethyl)phenyl)sulfonyl)-4,4a,5,6,7,8-hexahydro-1H-pyrazolo[3,4-g]isoquinoline | -0.37986 | 0.146709 | 0.525308 |
| Acetatifactor | (E,E)-3,7,11-Trimethyl-2,6,10-dodecatrienyl octanoate | 0.379819 | 0.146758 | 0.525351 |
| TRA3-20 | 16,17-Dihydro-16alpha,17-dihydroxygibberellin A4 17-glucoside | -0.37974 | 0.146853 | 0.525441 |
| Tuzzerella | Iguratimod | -0.37972 | 0.146872 | 0.525441 |
| Intestinimonas | PC(11:0/23:0) | 0.379676 | 0.146922 | 0.525441 |
| TM7 | N6-Acetyl-L-lysine | 0.379595 | 0.147015 | 0.525441 |
| Intestinimonas | FAD | 0.37959 | 0.147021 | 0.525441 |
| Intestinimonas | Bisazobiphenyl | 0.379571 | 0.147043 | 0.525441 |
| Christensenellaceae_R-7_group | Medica 16 | -0.37952 | 0.147107 | 0.525441 |
| IMCC26256 | OKOHA-PA | -0.37949 | 0.14714 | 0.525441 |
| Enterorhabdus | Eicosadienoic acid | 0.379465 | 0.147165 | 0.525441 |
| TRA3-20 | Adenosine monophosphate | -0.37945 | 0.147179 | 0.525441 |
| Lachnospiraceae_UCG-006 | 5'-Carboxy-gamma-chromanol | -0.37937 | 0.147277 | 0.525441 |
| Bryobacter | BEFLOXATONE | 0.379317 | 0.147336 | 0.525441 |
| Aquicella | PC(20:5(5Z,8Z,11Z,14Z,17Z)/PGJ2) | -0.3793 | 0.14736 | 0.525441 |
| Acetatifactor | PC(18:1(17Z)/18:1(17Z)) | 0.379279 | 0.14738 | 0.525441 |
| Subgroup_2 | LysoPE(20:4(5Z,8Z,11Z,14Z)/0:0) | -0.37926 | 0.147406 | 0.525441 |
| Bryobacter | AB-MECA | -0.37924 | 0.14742 | 0.525441 |
| Anaerotruncus | Serylproline | 0.379194 | 0.147479 | 0.525441 |
| Rikenellaceae_RC9_gut_group | Methacholine | 0.379167 | 0.14751 | 0.525441 |
| Flavonifractor | Asparaginyl-Proline | 0.379139 | 0.147542 | 0.525441 |
| Tuzzerella | Hydroxy-2-naphthalenylmethylphosphonic acid tris-acetoxymethyl ester | 0.379117 | 0.147567 | 0.525441 |
| Alcaligenes | Orotidine | 0.379111 | 0.147574 | 0.525441 |
| Enterorhabdus | PC(20:5(5Z,8Z,11Z,14Z,17Z)/PGJ2) | -0.37908 | 0.147609 | 0.525441 |
| Faecalibaculum | 4-Chloro-2-nitrobenzylalcohol | -0.37905 | 0.14765 | 0.525441 |
| MND1 | 8-Amino-7-oxononanoic acid | -0.37902 | 0.147682 | 0.525441 |
| Jatrophihabitans | Cortisol | -0.37899 | 0.147716 | 0.525441 |
| Acetatifactor | PE(22:5(4Z,7Z,10Z,13Z,16Z)/PGD2) | -0.37884 | 0.147885 | 0.525909 |
| Clostridioides | Xanthinol | 0.378691 | 0.148061 | 0.526401 |
| Jatrophihabitans | N-(N-(3-Amino-3-carboxypropyl)-3-amino-3-carboxypropyl)azetidine-2-carboxylic acid | 0.378645 | 0.148114 | 0.526457 |
| Enterococcus | (S)-a-Amino-2,5-dihydro-5-oxo-4-isoxazolepropanoic acid N2-glucoside | 0.378584 | 0.148185 | 0.526541 |
| Anaerotruncus | Tuberonic acid glucoside | -0.37854 | 0.14824 | 0.526541 |
| Clostridioides | S-Adenosylhomocysteine | 0.378528 | 0.14825 | 0.526541 |
| Clostridioides | D-Mannose | 0.378496 | 0.148287 | 0.526541 |
| Acidothermus | 3-Hydroxy-10'-apo-b,y-carotenal | 0.378385 | 0.148415 | 0.526767 |
| Lachnospiraceae_UCG-006 | Notoginsenoside H | 0.378277 | 0.14854 | 0.526767 |
| Subgroup_2 | Inosine | 0.378224 | 0.148603 | 0.526767 |
| Monoglobus | 4E,14Z-Sphingadiene | -0.3782 | 0.148635 | 0.526767 |
| Aquicella | OKOHA-PA | -0.37814 | 0.148701 | 0.526767 |
| Alcaligenes | Dodecanamide | -0.37813 | 0.148707 | 0.526767 |
| Bryobacter | Pelargonidin 3-sophoroside | -0.37812 | 0.148719 | 0.526767 |
| Lachnospiraceae_UCG-006 | Tyrosyl-Alanine | 0.378104 | 0.148741 | 0.526767 |
| Aquicella | Dihydrozeatin O-beta-D-Glucoside | 0.37809 | 0.148758 | 0.526767 |
| Enterococcus | Tephcalostan C | 0.378066 | 0.148786 | 0.526767 |
| Anaerotruncus | N-alpha-Acetyl-L-lysine | 0.377979 | 0.148887 | 0.526767 |
| Alcaligenes | N2-gamma-Glutamylglutamine | 0.37797 | 0.148898 | 0.526767 |
| Clostridioides | 1-[3,4-Dihydroxy-5-(hydroxymethyl)-2-oxolanyl]-1,2,4-triazole-3-carboxamide | 0.377943 | 0.148928 | 0.526767 |
| Enterorhabdus | Prolyl-Asparagine | 0.377939 | 0.148934 | 0.526767 |
| Rikenellaceae_RC9_gut_group | 1-Ethoxymethyl-5-fluorouracil | 0.377915 | 0.148962 | 0.526767 |
| TM7 | Oleoyl Serotonin | -0.3779 | 0.148977 | 0.526767 |
| Granulicella | PC(16:0/16:0) | 0.377893 | 0.148987 | 0.526767 |
| Intestinimonas | S-Adenosylhomocysteine | 0.377818 | 0.149075 | 0.526945 |
| IMCC26256 | 4E,14Z-Sphingadiene | -0.37769 | 0.149221 | 0.527157 |
| Subgroup_2 | 5'-Carboxy-gamma-chromanol | -0.37768 | 0.149229 | 0.527157 |
| Rikenellaceae_RC9_gut_group | Pantothenic acid | 0.37767 | 0.149247 | 0.527157 |
| Intestinimonas | 4E,14Z-Sphingadiene | -0.37759 | 0.149334 | 0.527333 |
| Flavonifractor | Serylproline | 0.377537 | 0.149402 | 0.527438 |
| Alcaligenes | Oleoyl Serotonin | -0.3774 | 0.149557 | 0.527856 |
| Haliangium | L-Glutamine | 0.377321 | 0.149654 | 0.527899 |
| Achromobacter | 1-O-(2R-hydroxy-hexadecyl)-sn-glycerol | -0.37732 | 0.149657 | 0.527899 |
| Monoglobus | AM2201 N-(4-hydroxypentyl) metabolite | -0.3773 | 0.149682 | 0.527899 |
| Flavonifractor | Dihydrozeatin O-beta-D-Glucoside | 0.377229 | 0.149761 | 0.528045 |
| Aquicella | PE(19:0/20:2(11Z,14Z)) | -0.37716 | 0.149847 | 0.528215 |
| Muribaculum | (2S)-1,1,1-Trifluoro-2-(4-pyrazol-1-ylphenyl)-3-[5-[[1-(trifluoromethyl)cyclopropyl]methyl]-1H-imidazol-2-yl]propan-2-ol | -0.3771 | 0.149915 | 0.528325 |
| IMCC26256 | 1-(10H-Phenothiazin-2-yl)ethanone | 0.376957 | 0.150078 | 0.528696 |
| Enterococcus | 3-(3-(Pyridin-3-yl)-1,2,4-oxadiazol-5-yl)benzonitrile | 0.376942 | 0.150096 | 0.528696 |
| Anaerotruncus | L-2-Hydroxyglutaric acid | 0.376886 | 0.150161 | 0.528795 |
| Muribaculum | Fexaramine | -0.3768 | 0.150262 | 0.528896 |
| Enterococcus | 2-[(4-{2-[(4-Cyclohexylbutyl)(cyclohexylcarbamoyl)amino]ethyl}phenyl)sulfanyl]-2-methylpropanoic acid | -0.37678 | 0.150288 | 0.528896 |
| Monoglobus | Octadecanamide | -0.37677 | 0.150303 | 0.528896 |
| Clostridioides | Neosaxitoxin | 0.376634 | 0.150457 | 0.529306 |
| Subgroup_2 | 4E,14Z-Sphingadiene | -0.37654 | 0.150569 | 0.529568 |
| IMCC26256 | N-(1-Deoxy-1-fructosyl)alanine | 0.376491 | 0.150624 | 0.529629 |
| bacteriap25 | Genistein 7-O-glucoside-6''-malonate | 0.376428 | 0.150698 | 0.529758 |
| Aquicella | PC(16:1(9Z)/17:1(9Z)) | -0.3763 | 0.150853 | 0.529956 |
| Jatrophihabitans | Niacinamide | 0.376281 | 0.150871 | 0.529956 |
| Intestinimonas | 5S-HETE di-endoperoxide | -0.37626 | 0.150893 | 0.529956 |
| Faecalibaculum | 5beta-Cholane-3alpha,24-diol | 0.376251 | 0.150905 | 0.529956 |
| Anaerostipes | 5,5-Diphenyl-2-thiohydantoin | 0.376194 | 0.150972 | 0.529986 |
| Achromobacter | Am-PE(16:0/18:0) | 0.376171 | 0.150999 | 0.529986 |
| Alcaligenes | L-arginino-succinate | 0.376148 | 0.151027 | 0.529986 |
| Subgroup_2 | Uridine | 0.376108 | 0.151073 | 0.529988 |
| Haliangium | 2-[4-(3-Hydroxypropyl)-2-methoxyphenoxy]-1,3-propanediol 1-xyloside | -0.37608 | 0.151102 | 0.529988 |
| Pseudolabrys | Hexadecadienylcarnitine | 0.375961 | 0.151246 | 0.530188 |
| Anaerostipes | Bis(2-propoxyethyl) 2,6-dimethyl-4-(3-nitrophenyl)-3,4-dihydropyridine-3,5-dicarboxylate | -0.37594 | 0.15127 | 0.530188 |
| MND1 | Octadecanamide | -0.37594 | 0.151273 | 0.530188 |
| Muribaculum | (2R,4S)-2-Aminoformyl-6-fluoro-spiro[chroman-4,4'-imidazolidine]-2',5'-dione | -0.37581 | 0.151422 | 0.530513 |
| Aquicella | PC(11:0/23:0) | 0.375783 | 0.151455 | 0.530513 |
| MND1 | Phytal | -0.37576 | 0.151478 | 0.530513 |
| Tuzzerella | hydroxyhexadecenoylcarnitine | 0.375663 | 0.151597 | 0.530797 |
| Acidothermus | Nopalinic acid | 0.375603 | 0.151667 | 0.530912 |
| Aquicella | [(2S,4R,5R,6R,14S,16R)-14-Hydroxy-7,11-dimethyl-6-(2-oxopyran-4-yl)-3-oxapentacyclo[8.8.0.02,4.02,7.011,16]octadecan-5-yl] acetate | -0.37553 | 0.151748 | 0.531045 |
| Nitrospira | N-(N-(3-Amino-3-carboxypropyl)-3-amino-3-carboxypropyl)azetidine-2-carboxylic acid | 0.375496 | 0.151794 | 0.531045 |
| Intestinimonas | Tercatain | 0.375475 | 0.151819 | 0.531045 |
| Monoglobus | 1-O-(2R-methoxy-hexadecyl)-sn-glycerol | -0.37537 | 0.151938 | 0.531324 |
| Acetatifactor | Ac-Ser-Asp-Lys-Pro-OH | 0.375328 | 0.151991 | 0.531324 |
| MND1 | Bacillamidin C | -0.37527 | 0.152055 | 0.531324 |
| Acetatifactor | N-Undecanoylglycine | -0.37526 | 0.152072 | 0.531324 |
| Acidothermus | Genistein 7-O-glucoside-6''-malonate | 0.375216 | 0.152124 | 0.531324 |
| Alcaligenes | Xanthinol | 0.375215 | 0.152125 | 0.531324 |
| Acidothermus | 1-Ethoxymethyl-5-fluorouracil | 0.375078 | 0.152287 | 0.531748 |
| Lachnospiraceae_UCG-006 | Mycobactins | -0.37504 | 0.152329 | 0.531748 |
| Intestinimonas | N-Myristoyl Asparagine | -0.37501 | 0.152363 | 0.531748 |
| Coprobacillus | Carissanol | -0.37498 | 0.152398 | 0.531748 |
| Subgroup_13 | 6-Hydroxytricetin 5-rhamnoside | 0.374949 | 0.152439 | 0.531762 |
| Enterococcus | Serotinose | 0.374831 | 0.152578 | 0.531997 |
| Tuzzerella | 4-Chloro-L-phenylalanine | -0.37483 | 0.152583 | 0.531997 |
| Intestinimonas | Hexadecadienylcarnitine | 0.374796 | 0.15262 | 0.531997 |
| Acidothermus | Tercatain | 0.374658 | 0.152783 | 0.532115 |
| Rikenellaceae_RC9_gut_group | Nopalinic acid | 0.374618 | 0.152831 | 0.532115 |
| Intestinimonas | Glycyl-D-proline | 0.374595 | 0.152858 | 0.532115 |
| Anaerotruncus | Dehydroascorbide(1-) | 0.374583 | 0.152871 | 0.532115 |
| Intestinimonas | 5-Hydroxyindoxyl sulfate | -0.37455 | 0.152913 | 0.532115 |
| Subgroup_13 | Iguratimod | -0.37454 | 0.152922 | 0.532115 |
| Anaerotruncus | Cannabidivarin | -0.37453 | 0.152938 | 0.532115 |
| Coprobacillus | Fiacitabine | 0.374505 | 0.152964 | 0.532115 |
| Faecalibaculum | hydroxyhexadecenoylcarnitine | 0.37448 | 0.152994 | 0.532115 |
| Aquicella | Aspartyl-Alanine | 0.374419 | 0.153066 | 0.532235 |
| Nitrospira | Carissanol | -0.37434 | 0.153158 | 0.532421 |
| Clostridia_vadinBB60_group | 2-carboxy-L-threo-pentonate | 0.374145 | 0.15339 | 0.533099 |
| Lachnospiraceae_UCG-006 | N-(1-Deoxy-1-fructosyl)leucine | 0.374102 | 0.153442 | 0.533147 |
| Haliangium | Cichorioside K | -0.37401 | 0.153551 | 0.533395 |
| Aquicella | (卤)-(Z)-2-(5-Tetradecenyl)cyclobutanone | -0.37397 | 0.153605 | 0.533448 |
| TM7 | 5S-HETE di-endoperoxide | -0.37385 | 0.153737 | 0.533651 |
| Enterococcus | 1-(O-alpha-D-glucopyranosyl)-(1,3S,25R)-hexacosanetriol | -0.37385 | 0.153744 | 0.533651 |
| Granulicella | Amobarbital | 0.37382 | 0.153777 | 0.533651 |
| Muribaculum | PPM-18 | 0.373764 | 0.153843 | 0.533671 |
| Clostridioides | (4E,15E)-Bilirubin | -0.37375 | 0.153858 | 0.533671 |
| Treponema | Monooctyl phthalate | -0.37344 | 0.154223 | 0.534743 |
| Anaerotruncus | LysoPE(20:4(5Z,8Z,11Z,14Z)/0:0) | -0.37343 | 0.154243 | 0.534743 |
| bacteriap25 | Zidebactam | 0.373381 | 0.154299 | 0.534806 |
| Nitrospira | Dimethylaminoparthenolide | 0.373274 | 0.154426 | 0.535065 |
| Aquicella | Aspartyl-Gamma-glutamate | 0.373211 | 0.154502 | 0.535065 |
| RF39 | 16,17-Dihydro-16alpha,17-dihydroxygibberellin A4 17-glucoside | -0.3732 | 0.154515 | 0.535065 |
| Coprobacillus | Phenylalanyl-Glycine | 0.373191 | 0.154526 | 0.535065 |
| Nitrospira | (卤)-(Z)-2-(5-Tetradecenyl)cyclobutanone | -0.3731 | 0.15463 | 0.535126 |
| RF39 | (2R,4S)-2-Aminoformyl-6-fluoro-spiro[chroman-4,4'-imidazolidine]-2',5'-dione | -0.37308 | 0.154662 | 0.535126 |
| Enterorhabdus | Aspartyl-Isoleucine | 0.373062 | 0.154679 | 0.535126 |
| Clostridia_vadinBB60_group | Phytal | -0.37305 | 0.154696 | 0.535126 |
| Lachnospiraceae_UCG-006 | 4E,14Z-Sphingadiene | -0.37298 | 0.154778 | 0.535148 |
| Subgroup_13 | Nopalinic acid | 0.372952 | 0.154811 | 0.535148 |
| Lachnospiraceae_UCG-006 | 16,17-Dihydro-16alpha,17-dihydroxygibberellin A4 17-glucoside | -0.37289 | 0.154887 | 0.535148 |
| TRA3-20 | Terbufos | 0.372854 | 0.154928 | 0.535148 |
| TM7 | S-cucujolide III | -0.37282 | 0.154965 | 0.535148 |
| Anaerotruncus | Phytal | -0.3728 | 0.154994 | 0.535148 |
| RF39 | (2S)-1,1,1-Trifluoro-2-(4-pyrazol-1-ylphenyl)-3-[5-[[1-(trifluoromethyl)cyclopropyl]methyl]-1H-imidazol-2-yl]propan-2-ol | -0.37266 | 0.155159 | 0.535148 |
| Faecalibaculum | 7-oxo-8-amino-nonanoic acid | -0.37264 | 0.155185 | 0.535148 |
| TRA3-20 | L-Homocystine | 0.372603 | 0.155228 | 0.535148 |
| Aquicella | 6,10,14-Trimethyl-5,9,13-pentadecatrien-2-one | -0.37256 | 0.155284 | 0.535148 |
| Acidothermus | Lucuminic acid | 0.372477 | 0.155378 | 0.535148 |
| Lachnospiraceae_UCG-006 | 7-oxo-8-amino-nonanoic acid | -0.3724 | 0.155474 | 0.535148 |
| Coprobacillus | Avenin | 0.372389 | 0.155484 | 0.535148 |
| Aquicella | Xanthinol | 0.372382 | 0.155492 | 0.535148 |
| Clostridioides | Pantothenic acid | 0.372348 | 0.155533 | 0.535148 |
| Treponema | Pentadecanoylcarnitine | 0.372324 | 0.155562 | 0.535148 |
| Anaerostipes | PE(19:0/20:2(11Z,14Z)) | -0.3723 | 0.155589 | 0.535148 |
| Clostridioides | Azeloprazole | 0.372297 | 0.155594 | 0.535148 |
| Intestinimonas | 3-Methyl-3-butenyl apiosyl-(1->6)-glucoside | 0.372297 | 0.155595 | 0.535148 |
| Achromobacter | Tuberonic acid glucoside | -0.37229 | 0.155599 | 0.535148 |
| Acetatifactor | D-Malic acid | 0.372279 | 0.155616 | 0.535148 |
| Coprobacillus | trans-Hexadec-2-enoyl carnitine | 0.372255 | 0.155645 | 0.535148 |
| Anaerotruncus | Oleoyl Serotonin | -0.3722 | 0.155706 | 0.535148 |
| Tuzzerella | 5-Hydroxyindoxyl sulfate | -0.3722 | 0.155709 | 0.535148 |
| Subgroup_13 | N-(1-Deoxy-1-fructosyl)alanine | 0.372195 | 0.155717 | 0.535148 |
| Achromobacter | Kuguacin E | -0.37219 | 0.155724 | 0.535148 |
| Enterorhabdus | Prolylproline | 0.372185 | 0.155729 | 0.535148 |
| Granulicella | N6-Acetyl-L-lysine | 0.372089 | 0.155844 | 0.535166 |
| IMCC26256 | Glucose-6-glutamate | 0.372057 | 0.155882 | 0.535166 |
| IMCC26256 | 7-oxo-8-amino-nonanoic acid | -0.37205 | 0.155886 | 0.535166 |
| Anaerotruncus | Nipradilol | 0.372051 | 0.155889 | 0.535166 |
| Intestinimonas | (R)-4A-(Ethoxymethyl)-1-(4-fluorophenyl)-6-((4-(trifluoromethyl)phenyl)sulfonyl)-4,4a,5,6,7,8-hexahydro-1H-pyrazolo[3,4-g]isoquinoline | -0.37202 | 0.155931 | 0.535166 |
| Alcaligenes | S-cucujolide III | -0.37194 | 0.156024 | 0.535166 |
| Anaerotruncus | Octadecanamide | -0.37191 | 0.156062 | 0.535166 |
| Jatrophihabitans | AB-MECA | -0.3719 | 0.156072 | 0.535166 |
| Monoglobus | Sodium nitrate (NaNO3) | -0.37189 | 0.156079 | 0.535166 |
| Bryobacter | Biliverdin | -0.37186 | 0.156114 | 0.535166 |
| Alcaligenes | DG(18:0/20:4(8Z,11Z,14Z,17Z)/0:0) | 0.371772 | 0.156224 | 0.535217 |
| bacteriap25 | S-cucujolide III | -0.37176 | 0.156234 | 0.535217 |
| Granulicella | Am-PE(16:0/18:0) | 0.371756 | 0.156243 | 0.535217 |
| Monoglobus | Glycyl-D-proline | 0.371707 | 0.156302 | 0.535291 |
| Haliangium | (R)-Propyl 2-amino-3-mercaptopropanoate | 0.371636 | 0.156388 | 0.535335 |
| Treponema | 1-O-(2R-hydroxy-hexadecyl)-sn-glycerol | -0.37159 | 0.156442 | 0.535335 |
| Subgroup_2 | Monooctyl phthalate | -0.37158 | 0.156461 | 0.535335 |
| Clostridioides | D-erythro-L-galacto-Nonulose | 0.371549 | 0.156492 | 0.535335 |
| Intestinimonas | Pelargonidin 3-sophoroside | -0.37149 | 0.156563 | 0.535335 |
| Lachnospiraceae_UCG-006 | N,N'-Diethylthiourea | 0.371475 | 0.156581 | 0.535335 |
| Intestinimonas | 2-hydroxy-9Z,12Z-Octadecadienoic acid | -0.37147 | 0.156582 | 0.535335 |
| Subgroup_2 | Mycobactins | -0.37137 | 0.15671 | 0.535646 |
| Anaerostipes | 2-[(4-{2-[(4-Cyclohexylbutyl)(cyclohexylcarbamoyl)amino]ethyl}phenyl)sulfanyl]-2-methylpropanoic acid | -0.37127 | 0.156827 | 0.535913 |
| Lachnospiraceae_UCG-006 | 9-Octadecenal | -0.37119 | 0.156925 | 0.536104 |
| Muribaculum | Carissanol | -0.37113 | 0.156992 | 0.536104 |
| TRA3-20 | Phytal | -0.37113 | 0.156997 | 0.536104 |
| Aquicella | [6-Hydroxy-8-methyl-3-[3,4,5-trihydroxy-6-(hydroxymethyl)oxan-2-yl]oxy-9,10-dioxatetracyclo[4.3.1.02,5.03,8]decan-4-yl]methyl benzoate | 0.371097 | 0.157036 | 0.536108 |
| Subgroup_2 | thiamphenicol | 0.371006 | 0.157146 | 0.536173 |
| Enterococcus | N-(1-Deoxy-1-fructosyl)alanine | 0.370983 | 0.157173 | 0.536173 |
| Subgroup_2 | LysoPC(20:3(8Z,11Z,14Z)/0:0) | 0.370956 | 0.157207 | 0.536173 |
| Aquicella | Neosaxitoxin | 0.370943 | 0.157222 | 0.536173 |
| Alcaligenes | N-Phenyl-p-phenylenediamine | -0.37092 | 0.157245 | 0.536173 |
| RF39 | Medica 16 | -0.37086 | 0.157319 | 0.536292 |
| Jatrophihabitans | 3-Hydroxy-10'-apo-b,y-carotenal | 0.370794 | 0.157402 | 0.536342 |
| MND1 | (卤)-(Z)-2-(5-Tetradecenyl)cyclobutanone | -0.37079 | 0.157409 | 0.536342 |
| Pseudolabrys | Resorufin | 0.370719 | 0.157492 | 0.536349 |
| MND1 | 13,14-dihydro-15-keto-PGD2-d4 | -0.37071 | 0.157501 | 0.536349 |
| Rikenellaceae_RC9_gut_group | m7G(5')pppAm | 0.370692 | 0.157526 | 0.536349 |
| TM7 | 16,17-Dihydro-16alpha,17-dihydroxygibberellin A4 17-glucoside | -0.37057 | 0.157674 | 0.536509 |
| TRA3-20 | Terizidone | 0.370562 | 0.157682 | 0.536509 |
| Flavonifractor | Niacinamide | 0.370528 | 0.157723 | 0.536509 |
| Intestinimonas | 3'-Deoxythymidine | 0.370527 | 0.157725 | 0.536509 |
| Enterorhabdus | N-(1-Deoxy-1-fructosyl)threonine | 0.370393 | 0.157886 | 0.536927 |
| Aquicella | BEFLOXATONE | 0.370362 | 0.157924 | 0.536927 |
| Lachnospiraceae_UCG-006 | Alacepril | -0.37006 | 0.15829 | 0.538039 |
| bacteriap25 | 12-Hydroxydodecanoic acid | -0.36996 | 0.158406 | 0.538305 |
| TM7 | alpha-D-Galactopyranuronosyl-(1->4)-alpha-D-galactopyranuronosyl-(1->4)-D-galacturonic acid | 0.369759 | 0.158655 | 0.53901 |
| Achromobacter | Oleoyl Serotonin | -0.36973 | 0.158691 | 0.53901 |
| Muribaculum | Niacinamide | 0.369698 | 0.158728 | 0.53901 |
| Christensenellaceae_R-7_group | 4-Phenylbutanoylcarnitine | -0.36959 | 0.15886 | 0.539188 |
| Coprobacillus | 3-{[(1s)-2,2-Difluoro-1-Hydroxy-7-(Methylsulfonyl)-2,3-Dihydro-1h-Inden-4-Yl]oxy}-5-Fluorobenzonitrile | 0.369544 | 0.158916 | 0.539188 |
| Pseudolabrys | [(2S,4R,5R,6R,14S,16R)-14-Hydroxy-7,11-dimethyl-6-(2-oxopyran-4-yl)-3-oxapentacyclo[8.8.0.02,4.02,7.011,16]octadecan-5-yl] acetate | -0.36952 | 0.158943 | 0.539188 |
| Intestinimonas | Bacillamidin C | -0.3695 | 0.158965 | 0.539188 |
| TRA3-20 | PI(18:1(9Z)/0:0) | 0.369498 | 0.158972 | 0.539188 |
| Monoglobus | Glutamylleucylarginine | 0.36943 | 0.159054 | 0.539336 |
| [Eubacterium]_oxidoreducens_group | Diethylamino 2,5-dihydroxybenzenesulfonate | 0.369394 | 0.159098 | 0.539356 |
| Anaerotruncus | O-Linoleoylcarnitine | 0.369351 | 0.159151 | 0.539403 |
| Lachnospiraceae_UCG-006 | Creatine | 0.369259 | 0.159262 | 0.539436 |
| Monoglobus | 5-[1-Carboxy-2-(trimethylazaniumyl)ethoxy]-5-oxopentanoate | 0.369232 | 0.159295 | 0.539436 |
| Clostridioides | DG(LTE4/22:0/0:0) | 0.369202 | 0.159331 | 0.539436 |
| Intestinimonas | 4-Chloro-L-phenylalanine | -0.36918 | 0.159355 | 0.539436 |
| Enterococcus | Niacinamide | 0.369167 | 0.159375 | 0.539436 |
| Subgroup_13 | a-L-Arabinofuranosyl-(1->3)-b-D-xylopyranosyl-(1->4)-D-xylose | 0.369127 | 0.159424 | 0.539436 |
| bacteriap25 | (S1)-Methoxy-3-heptanethiol | 0.369123 | 0.159428 | 0.539436 |
| IMCC26256 | N-alpha-Acetyl-L-lysine | 0.368998 | 0.15958 | 0.539599 |
| Subgroup_2 | 3-Hydroxybutyrylcarnitine | 0.368978 | 0.159605 | 0.539599 |
| Anaerotruncus | PC(TXB2/20:2(11Z,14Z)) | -0.36896 | 0.159629 | 0.539599 |
| Subgroup_2 | (S)-Laudanosine | -0.36895 | 0.159638 | 0.539599 |
| Rikenellaceae_RC9_gut_group | PC(P-18:0/20:4(5Z,8Z,11Z,14Z)-OH(16R)) | 0.3689 | 0.1597 | 0.539599 |
| Coprobacillus | Dimethylaminoparthenolide | 0.368895 | 0.159706 | 0.539599 |
| Achromobacter | Monooctyl phthalate | -0.36882 | 0.159797 | 0.539776 |
| Clostridioides | Dodecanamide | -0.36871 | 0.159931 | 0.540097 |
| IMCC26256 | 25-Hydroxyvitamin D3-26,23-lactol | -0.36868 | 0.159973 | 0.54011 |
| Bryobacter | Heptadecanoyl carnitine | 0.368592 | 0.160076 | 0.540329 |
| Gemella | alpha-D-Galactopyranuronosyl-(1->4)-alpha-D-galactopyranuronosyl-(1->4)-D-galacturonic acid | 0.368513 | 0.160172 | 0.540461 |
| Monoglobus | Glutaminylarginine | -0.36845 | 0.16025 | 0.540461 |
| TM7 | Dihydrozeatin O-beta-D-Glucoside | 0.36841 | 0.160299 | 0.540461 |
| Acetatifactor | Pantothenic acid | 0.368407 | 0.160302 | 0.540461 |
| Nitrospira | Fexaramine | -0.36837 | 0.160351 | 0.540461 |
| Haliangium | S-Adenosylhomocysteine | 0.368339 | 0.160385 | 0.540461 |
| Aquicella | PI(20:3(8Z,11Z,14Z)/0:0) | 0.36829 | 0.160445 | 0.540461 |
| bacteriap25 | L-Lysinamide | -0.36828 | 0.160454 | 0.540461 |
| bacteriap25 | L-Carnitine | 0.368255 | 0.160487 | 0.540461 |
| Rikenellaceae_RC9_gut_group | JP83 | -0.36825 | 0.160499 | 0.540461 |
| Solobacterium | N-Acetylneuraminic acid | 0.368192 | 0.160564 | 0.540501 |
| Bryobacter | Skepinone-L | 0.368173 | 0.160588 | 0.540501 |
| Clostridioides | Glutaminylarginine | -0.36808 | 0.160699 | 0.540747 |
| Lachnospiraceae_UCG-006 | 1-Hydroxy-2-naphthoic acid | 0.368011 | 0.160786 | 0.540831 |
| TRA3-20 | Hexadecadienylcarnitine | 0.367999 | 0.160801 | 0.540831 |
| Rikenellaceae_RC9_gut_group | PC(11:0/23:0) | 0.367949 | 0.160862 | 0.540866 |
| Alcaligenes | Hydroxy-2-naphthalenylmethylphosphonic acid tris-acetoxymethyl ester | 0.367928 | 0.160888 | 0.540866 |
| Flavonifractor | Glucose-6-glutamate | 0.367859 | 0.160973 | 0.541004 |
| Subgroup_2 | Diethylamino 2,5-dihydroxybenzenesulfonate | 0.367832 | 0.161006 | 0.541004 |
| Lachnospiraceae_UCG-006 | Kuguacin E | -0.36768 | 0.161194 | 0.541277 |
| Muribaculum | Quinagolida | -0.36768 | 0.161197 | 0.541277 |
| Bryobacter | Ciadox | 0.367671 | 0.161203 | 0.541277 |
| Anaerotruncus | (2R,4S)-2-Aminoformyl-6-fluoro-spiro[chroman-4,4'-imidazolidine]-2',5'-dione | -0.36743 | 0.161495 | 0.542068 |
| Achromobacter | 5'-Carboxy-gamma-chromanol | -0.36739 | 0.161546 | 0.542068 |
| RF39 | 16-hydroxy-6-hexadecenoic acid | -0.36737 | 0.161577 | 0.542068 |
| Enterorhabdus | MG(20:4(5Z,8Z,11Z,14Z)/0:0/0:0) | -0.36734 | 0.161615 | 0.542068 |
| Anaerotruncus | Kaltostat | 0.367322 | 0.161631 | 0.542068 |
| TM7 | 2-hydroxy-9Z,12Z-Octadecadienoic acid | -0.36724 | 0.161729 | 0.542193 |
| Anaerotruncus | 12-Hydroxy-12-octadecanoylcarnitine | 0.367188 | 0.161796 | 0.542193 |
| Faecalibaculum | Tephcalostan | 0.367171 | 0.161817 | 0.542193 |
| Achromobacter | scyllo-Inositol | 0.367114 | 0.161887 | 0.542193 |
| Treponema | 5-[1-Carboxy-2-(trimethylazaniumyl)ethoxy]-5-oxopentanoate | 0.367111 | 0.161891 | 0.542193 |
| Granulicella | 2-phospho-4-(cytidine 5'-diphospho)-2-C-methyl-D-erythritol | 0.367104 | 0.161899 | 0.542193 |
| Flavonifractor | Cysteine-glutathione disulfide | 0.367054 | 0.161961 | 0.54227 |
| Achromobacter | (R)-4A-(Ethoxymethyl)-1-(4-fluorophenyl)-6-((4-(trifluoromethyl)phenyl)sulfonyl)-4,4a,5,6,7,8-hexahydro-1H-pyrazolo[3,4-g]isoquinoline | -0.367 | 0.162024 | 0.542352 |
| MND1 | Histamine | -0.36695 | 0.162086 | 0.542433 |
| TM7 | Prostaglandin B-1 | -0.36685 | 0.162218 | 0.54244 |
| Gemella | Symmetric dimethylarginine | 0.366801 | 0.162272 | 0.54244 |
| Achromobacter | LysoPC(20:3(8Z,11Z,14Z)/0:0) | 0.366795 | 0.16228 | 0.54244 |
| Enterorhabdus | Fluazifop | 0.366784 | 0.162293 | 0.54244 |
| [Eubacterium]_oxidoreducens_group | Ac-Ser-Asp-Lys-Pro-OH | 0.366769 | 0.162312 | 0.54244 |
| Anaerotruncus | 2-carboxy-L-threo-pentonate | -0.36676 | 0.16232 | 0.54244 |
| bacteriap25 | 4E,14Z-Sphingadiene | -0.36667 | 0.162437 | 0.542703 |
| RF39 | 12-Hydroxydodecanoic acid | -0.36663 | 0.162483 | 0.542727 |
| Muribaculum | 3,4-Dimethyl-5-propyl-2-furanpentadecanoic acid | -0.36659 | 0.162536 | 0.542776 |
| Alcaligenes | Prolylproline | 0.366489 | 0.162657 | 0.542856 |
| Lachnospiraceae_UCG-006 | D-Malic acid | 0.36646 | 0.162693 | 0.542856 |
| Subgroup_2 | N-Acetyl-D-Glucosamine 6-Phosphate | 0.366449 | 0.162707 | 0.542856 |
| Flavonifractor | 3-{[(1s)-2,2-Difluoro-1-Hydroxy-7-(Methylsulfonyl)-2,3-Dihydro-1h-Inden-4-Yl]oxy}-5-Fluorobenzonitrile | 0.366443 | 0.162714 | 0.542856 |
| Faecalibaculum | 2-[(4-{2-[(4-Cyclohexylbutyl)(cyclohexylcarbamoyl)amino]ethyl}phenyl)sulfanyl]-2-methylpropanoic acid | -0.36625 | 0.162958 | 0.543377 |
| [Eubacterium]_oxidoreducens_group | 4-Chloro-2-nitrobenzylalcohol | -0.36621 | 0.163001 | 0.543377 |
| Anaerostipes | Tuberonic acid glucoside | -0.36621 | 0.163005 | 0.543377 |
| Faecalibaculum | Medicagol | 0.366163 | 0.163061 | 0.543377 |
| Solobacterium | Docosadienoate (22:2n6) | 0.366134 | 0.163096 | 0.543377 |
| Clostridioides | 2-Methyl-2-[4-[3-[1-(4-methylbenzyl)-5-oxo-4,5-dihydro-1H-1,2,4-triazol-3-yl]propyl]phenoxy]propanoic acid | -0.36613 | 0.163102 | 0.543377 |
| Intestinimonas | 2-Cyclotetradecen-1-one | -0.36603 | 0.163229 | 0.543672 |
| Anaerotruncus | (2S)-1,1,1-Trifluoro-2-(4-pyrazol-1-ylphenyl)-3-[5-[[1-(trifluoromethyl)cyclopropyl]methyl]-1H-imidazol-2-yl]propan-2-ol | -0.36575 | 0.163577 | 0.544585 |
| Subgroup_2 | 7b-Hydroxy-3-oxo-5b-cholanoic acid | -0.36574 | 0.16358 | 0.544585 |
| Enterorhabdus | 4-Chloro-L-phenylalanine | -0.3657 | 0.163637 | 0.544622 |
| Lachnospiraceae_UCG-006 | (R)-4A-(Ethoxymethyl)-1-(4-fluorophenyl)-6-((4-(trifluoromethyl)phenyl)sulfonyl)-4,4a,5,6,7,8-hexahydro-1H-pyrazolo[3,4-g]isoquinoline | -0.36564 | 0.163708 | 0.544622 |
| Pseudolabrys | 4-(Methylnitrosamino)-1-(3-pyridyl)-1-butanol glucuronide | 0.365602 | 0.163755 | 0.544622 |
| Rikenellaceae_RC9_gut_group | N6-Acetyl-L-lysine | 0.365547 | 0.163823 | 0.544622 |
| Coprobacillus | Fexaramine | -0.36554 | 0.163831 | 0.544622 |
| IMCC26256 | Glycerol 3-phosphate | 0.365537 | 0.163836 | 0.544622 |
| Clostridioides | Fiacitabine | 0.365509 | 0.16387 | 0.544622 |
| Anaerostipes | 4-Phenylbutanoylcarnitine | -0.36547 | 0.16392 | 0.544622 |
| Anaerostipes | 16,17-Dihydro-16alpha,17-dihydroxygibberellin A4 17-glucoside | -0.36543 | 0.163971 | 0.544622 |
| Rikenellaceae_RC9_gut_group | PI(18:0/20:4(8Z,11Z,14Z,17Z)) | 0.365422 | 0.163978 | 0.544622 |
| Bryobacter | Glucose-6-glutamate | 0.365324 | 0.1641 | 0.544631 |
| Granulicella | 1-(10H-Phenothiazin-2-yl)ethanone | 0.36531 | 0.164117 | 0.544631 |
| Rikenellaceae_RC9_gut_group | Dehydroascorbic acid | 0.365307 | 0.164121 | 0.544631 |
| Enterococcus | Mycobactins | -0.36527 | 0.164165 | 0.544631 |
| Monoglobus | Fexaramine | -0.36523 | 0.164211 | 0.544631 |
| Anaerostipes | 3,4-Dimethyl-5-propyl-2-furanpentadecanoic acid | -0.36523 | 0.164213 | 0.544631 |
| Anaerostipes | 2-Cyclotetradecen-1-one | -0.36511 | 0.164368 | 0.544863 |
| Solobacterium | 2',3'-Dideoxyuridine | 0.365091 | 0.164389 | 0.544863 |
| Solobacterium | thiamphenicol | 0.365083 | 0.164399 | 0.544863 |
| Treponema | Zidebactam | 0.365041 | 0.164451 | 0.544886 |
| Flavonifractor | Glutamylleucylarginine | 0.365015 | 0.164484 | 0.544886 |
| Acetatifactor | Valylhistidine | 0.364912 | 0.164612 | 0.545015 |
| RF39 | Glutaminylarginine | -0.36491 | 0.164615 | 0.545015 |
| Muribaculum | Fiacitabine | 0.36489 | 0.164639 | 0.545015 |
| Achromobacter | D-erythro-L-galacto-Nonulose | 0.36483 | 0.164714 | 0.545136 |
| [Eubacterium]_oxidoreducens_group | Docosadienoate (22:2n6) | 0.36477 | 0.164788 | 0.545253 |
| Rikenellaceae_RC9_gut_group | Tercatain | 0.364726 | 0.164843 | 0.545306 |
| MND1 | Oleoyl Serotonin | -0.36468 | 0.164905 | 0.545351 |
| Gemella | Sulisobenzone | 0.364638 | 0.164953 | 0.545351 |
| Rikenellaceae_RC9_gut_group | DG(18:0/20:4(8Z,11Z,14Z,17Z)/0:0) | 0.364614 | 0.164983 | 0.545351 |
| Anaerostipes | Lemborexant | -0.36458 | 0.16503 | 0.545351 |
| Bryobacter | Lamivudine-monophosphate | -0.36455 | 0.165067 | 0.545351 |
| Coprobacillus | Niacinamide | 0.364529 | 0.165089 | 0.545351 |
| Enterorhabdus | AM2201 N-(4-hydroxypentyl) metabolite | -0.36447 | 0.16516 | 0.54539 |
| Granulicella | 5-[1-Carboxy-2-(trimethylazaniumyl)ethoxy]-5-oxopentanoate | 0.364457 | 0.165178 | 0.54539 |
| Pseudolabrys | L-Lysinamide | -0.36443 | 0.165217 | 0.54539 |
| Muribaculum | Nipradilol | 0.364197 | 0.165503 | 0.546093 |
| IMCC26256 | PC(20:4(5Z,8Z,11Z,14Z)-OH(16R)/2:0) | 0.364193 | 0.165508 | 0.546093 |
| Achromobacter | Adenosine monophosphate | -0.36416 | 0.165547 | 0.546096 |
| Achromobacter | Alacepril | -0.36405 | 0.165687 | 0.546393 |
| Anaerostipes | AB-MECA | -0.36403 | 0.165715 | 0.546393 |
| Bryobacter | 2-Cyclotetradecen-1-one | -0.36396 | 0.165798 | 0.546401 |
| Muribaculum | 14-Methylpentadecanoylcarnitine | 0.363924 | 0.165845 | 0.546401 |
| Clostridioides | L-Lysinamide | -0.3639 | 0.165873 | 0.546401 |
| Clostridia_vadinBB60_group | Phenylalanylaspartic acid | 0.36386 | 0.165925 | 0.546401 |
| Anaerotruncus | Zidebactam | 0.36383 | 0.165961 | 0.546401 |
| Subgroup_13 | Tyrosyl-Alanine | 0.363824 | 0.16597 | 0.546401 |
| Achromobacter | LysoSM(d18:0) | -0.3638 | 0.165995 | 0.546401 |
| Anaerotruncus | Tyrosyl-Aspartate | 0.363754 | 0.166057 | 0.546401 |
| Anaerostipes | Notoginsenoside H | 0.363678 | 0.166153 | 0.546401 |
| Subgroup_2 | Fluazifop | 0.363646 | 0.166192 | 0.546401 |
| Christensenellaceae_R-7_group | 3-{[(1s)-2,2-Difluoro-1-Hydroxy-7-(Methylsulfonyl)-2,3-Dihydro-1h-Inden-4-Yl]oxy}-5-Fluorobenzonitrile | 0.363637 | 0.166204 | 0.546401 |
| Subgroup_2 | (2Z,4E,6Z)-Decatrienoylcarnitine | -0.36361 | 0.16624 | 0.546401 |
| Coprobacillus | Streptidine | 0.363579 | 0.166276 | 0.546401 |
| Alcaligenes | LysoSM(d18:0) | -0.36356 | 0.166297 | 0.546401 |
| Solobacterium | Dihydrozeatin O-beta-D-Glucoside | 0.363537 | 0.166328 | 0.546401 |
| Coprobacillus | 3-Ketosphingosine | -0.36353 | 0.166338 | 0.546401 |
| Acetatifactor | PI(20:3(8Z,11Z,14Z)/0:0) | 0.363479 | 0.166401 | 0.54648 |
| Muribaculum | Kaltostat | 0.363382 | 0.166522 | 0.546751 |
| TRA3-20 | Am-PE(16:0/18:0) | 0.363177 | 0.16678 | 0.547469 |
| Subgroup_2 | 2-[(4-{2-[(4-Cyclohexylbutyl)(cyclohexylcarbamoyl)amino]ethyl}phenyl)sulfanyl]-2-methylpropanoic acid | -0.36299 | 0.16701 | 0.547746 |
| Muribaculum | 25-Hydroxyvitamin D3-26,23-lactol | -0.36298 | 0.16703 | 0.547746 |
| MND1 | Glutaminylarginine | -0.36297 | 0.167038 | 0.547746 |
| Aquicella | 1-Ethoxymethyl-5-fluorouracil | 0.362903 | 0.167124 | 0.547746 |
| Achromobacter | 9-Octadecenal | -0.3629 | 0.167128 | 0.547746 |
| IMCC26256 | D-Malic acid | 0.362893 | 0.167136 | 0.547746 |
| Monoglobus | 6,10,14-Trimethyl-5,9,13-pentadecatrien-2-one | -0.36289 | 0.167137 | 0.547746 |
| Rikenellaceae_RC9_gut_group | Dehydroascorbide(1-) | 0.362792 | 0.167264 | 0.547885 |
| Enterococcus | 4Z,7-octadienoic acid | -0.36278 | 0.167281 | 0.547885 |
| Muribaculum | 2-Methyl-2-[4-[3-[1-(4-methylbenzyl)-5-oxo-4,5-dihydro-1H-1,2,4-triazol-3-yl]propyl]phenoxy]propanoic acid | -0.36277 | 0.167296 | 0.547885 |
| Clostridioides | 2,5,7-trihydroxy-4'-methoxyisoflavanone | -0.36271 | 0.167361 | 0.547955 |
| Anaerotruncus | N-(1-Deoxy-1-fructosyl)alanine | 0.362657 | 0.167434 | 0.547955 |
| Gemella | Azeloprazole | 0.362637 | 0.167458 | 0.547955 |
| [Eubacterium]_oxidoreducens_group | Niacinamide | 0.362626 | 0.167473 | 0.547955 |
| MND1 | 2-Methyl-2-[4-[3-[1-(4-methylbenzyl)-5-oxo-4,5-dihydro-1H-1,2,4-triazol-3-yl]propyl]phenoxy]propanoic acid | -0.36255 | 0.167571 | 0.54815 |
| Subgroup_2 | 2',3'-Dideoxyuridine | 0.362285 | 0.167903 | 0.548896 |
| [Eubacterium]_oxidoreducens_group | 16,17-Dihydro-16alpha,17-dihydroxygibberellin A4 17-glucoside | -0.36227 | 0.167922 | 0.548896 |
| RF39 | Dehydrocyclopeptine; (3E)-3-Benzylidene-4-methyl-3,4-dihydro-1H-1,4-benzodiazepine-2,5-dione | -0.36222 | 0.167983 | 0.548896 |
| Granulicella | Nopalinic acid | 0.362213 | 0.167992 | 0.548896 |
| Gemella | Histamine | -0.3622 | 0.168014 | 0.548896 |
| Anaerostipes | AM2201 N-(4-hydroxypentyl) metabolite | -0.36213 | 0.168103 | 0.548896 |
| Subgroup_2 | Dolichyl b-D-glucosyl phosphate | -0.36211 | 0.168117 | 0.548896 |
| Granulicella | Skepinone-L | 0.362089 | 0.168149 | 0.548896 |
| Enterococcus | (S)-Laudanosine | -0.36209 | 0.16815 | 0.548896 |
| Subgroup_2 | Pentadeca-3,5,7-trienedioylcarnitine | 0.362053 | 0.168194 | 0.548912 |
| Achromobacter | Prolylproline | 0.36198 | 0.168286 | 0.549086 |
| Granulicella | OKOHA-PA | -0.36184 | 0.168458 | 0.549473 |
| Aquicella | Ethynodiol | -0.36182 | 0.168483 | 0.549473 |
| Enterorhabdus | 1-O-(2R-hydroxy-hexadecyl)-sn-glycerol | -0.36177 | 0.168555 | 0.549539 |
| Intestinimonas | 2-Phenylethyl octanoate | -0.36175 | 0.168582 | 0.549539 |
| Alcaligenes | Kuguacin E | -0.36169 | 0.168653 | 0.549646 |
| Muribaculum | PC(20:4(5Z,8Z,11Z,14Z)-OH(16R)/2:0) | 0.361511 | 0.168879 | 0.550255 |
| Enterococcus | (E)-2-(hexa-3,5-dien-1-yn-1-yl)-5-(prop-1-yn-1-yl)thiophene | 0.361468 | 0.168935 | 0.550307 |
| RF39 | Cannabidivarin | -0.36141 | 0.169013 | 0.550434 |
| Acetatifactor | NAD | 0.3613 | 0.169147 | 0.550744 |
| Aquicella | Lucuminic acid | 0.361235 | 0.169229 | 0.550848 |
| Clostridioides | S-cucujolide III | -0.36121 | 0.169262 | 0.550848 |
| Enterococcus | N-Acetylneuraminic acid | 0.361182 | 0.169296 | 0.550848 |
| Flavonifractor | 4E,14Z-Sphingadiene | -0.36114 | 0.169344 | 0.550876 |
| Granulicella | N2-gamma-Glutamylglutamine | 0.361104 | 0.169396 | 0.550918 |
| Treponema | Berkeleylactone L | -0.36105 | 0.169468 | 0.550943 |
| Lachnospiraceae_UCG-006 | All trans decaprenyl diphosphate | -0.36097 | 0.169562 | 0.550943 |
| Faecalibaculum | Glutamylleucylarginine | 0.36097 | 0.169565 | 0.550943 |
| Alcaligenes | LysoPE(20:3(8Z,11Z,14Z)/0:0) | 0.360954 | 0.169586 | 0.550943 |
| Faecalibaculum | Doxepin | 0.360897 | 0.169658 | 0.550943 |
| Tuzzerella | trans-Hexadec-2-enoyl carnitine | 0.360892 | 0.169664 | 0.550943 |
| Achromobacter | 1-Octadecene | -0.36088 | 0.169677 | 0.550943 |
| Bryobacter | L-Carnitine | 0.36085 | 0.169717 | 0.550945 |
| Anaerotruncus | 8-Amino-7-oxononanoic acid | -0.3608 | 0.16978 | 0.550996 |
| Aquicella | Sulisobenzone | 0.360718 | 0.169886 | 0.550996 |
| Enterococcus | Micronomicin | -0.3607 | 0.169905 | 0.550996 |
| Subgroup_13 | Tephcalostan C | 0.360676 | 0.169939 | 0.550996 |
| Bryobacter | Dihydrozeatin O-beta-D-Glucoside | 0.360654 | 0.169966 | 0.550996 |
| Lachnospiraceae_UCG-006 | Glucose-6-glutamate | 0.360636 | 0.169989 | 0.550996 |
| Acetatifactor | Mycobactins | -0.36062 | 0.170007 | 0.550996 |
| RF39 | Dihydrozeatin O-beta-D-Glucoside | 0.360356 | 0.170346 | 0.551967 |
| Monoglobus | Hexadecenal | -0.36028 | 0.170446 | 0.552017 |
| Lachnospiraceae_UCG-006 | Ethynodiol | -0.36021 | 0.170532 | 0.552017 |
| Aquicella | Tuberonic acid glucoside | -0.36021 | 0.170537 | 0.552017 |
| Christensenellaceae_R-7_group | 25-Hydroxyvitamin D3-26,23-lactol | -0.3602 | 0.170547 | 0.552017 |
| Tuzzerella | PIP(20:0/18:1(12Z)-2OH(9,10)) | -0.36019 | 0.170557 | 0.552017 |
| Enterococcus | Sulisobenzone | 0.360152 | 0.170606 | 0.552048 |
| Granulicella | 1-Hydroxy-2-naphthoic acid | 0.360092 | 0.170682 | 0.552166 |
| Lachnospiraceae_UCG-006 | N2-gamma-Glutamylglutamine | 0.359959 | 0.170852 | 0.552447 |
| Pseudolabrys | N-Acetyl-L-methionine | 0.35995 | 0.170862 | 0.552447 |
| Alcaligenes | Azeloprazole | 0.359918 | 0.170903 | 0.552447 |
| bacteriap25 | 4-(Methylnitrosamino)-1-(3-pyridyl)-1-butanol glucuronide | 0.359901 | 0.170925 | 0.552447 |
| TRA3-20 | Benzyl gentiobioside | 0.359826 | 0.171022 | 0.552631 |
| Nitrospira | O-Linoleoylcarnitine | 0.359794 | 0.171061 | 0.552633 |
| Achromobacter | Hydroxy-2-naphthalenylmethylphosphonic acid tris-acetoxymethyl ester | 0.359708 | 0.171172 | 0.552679 |
| Bryobacter | NAD | 0.359699 | 0.171184 | 0.552679 |
| Alcaligenes | D-erythro-Sphingosine C-20 | -0.35963 | 0.171275 | 0.552679 |
| Flavonifractor | 7-oxo-8-amino-nonanoic acid | -0.35962 | 0.17128 | 0.552679 |
| Acetatifactor | (R)-3-Amino-2-fluoropropyl phosphenite | 0.359622 | 0.171282 | 0.552679 |
| TRA3-20 | 2-C-methyl-D-erythritol-4-phosphate | 0.359599 | 0.171311 | 0.552679 |
| Anaerotruncus | (卤)-(Z)-2-(5-Tetradecenyl)cyclobutanone | -0.35955 | 0.171374 | 0.552754 |
| Subgroup_13 | 7-oxo-8-amino-nonanoic acid | -0.3595 | 0.171436 | 0.552813 |
| IMCC26256 | 8-Amino-7-oxononanoic acid | -0.35944 | 0.171518 | 0.552813 |
| Anaerotruncus | 1-Octadecene | -0.35942 | 0.171545 | 0.552813 |
| RF39 | Bacillamidin C | -0.3594 | 0.171571 | 0.552813 |
| Intestinimonas | Iguratimod | -0.35938 | 0.171588 | 0.552813 |
| [Eubacterium]_oxidoreducens_group | D-Malic acid | 0.359275 | 0.171725 | 0.553016 |
| Granulicella | Iguratimod | -0.35924 | 0.171766 | 0.553016 |
| Achromobacter | S-cucujolide III | -0.35924 | 0.171769 | 0.553016 |
| Alcaligenes | PI(18:0/20:4(8Z,11Z,14Z,17Z)) | 0.359097 | 0.171953 | 0.553481 |
| Achromobacter | LysoPE(20:3(8Z,11Z,14Z)/0:0) | 0.359016 | 0.172057 | 0.553688 |
| TRA3-20 | Hydroxy-2-naphthalenylmethylphosphonic acid tris-acetoxymethyl ester | 0.358971 | 0.172114 | 0.553716 |
| Enterococcus | Dihydrozeatin O-beta-D-Glucoside | 0.358928 | 0.172169 | 0.553716 |
| Coprobacillus | Eleutherazine B; N,N'-((3,6-Dioxo-2,5-piperazinediyl)bis(trimethylene))bis(5-hydroxy-3-methyl-2-pentenamide) | 0.358918 | 0.172183 | 0.553716 |
| Achromobacter | (R)-Propyl 2-amino-3-mercaptopropanoate | 0.35883 | 0.172296 | 0.553799 |
| IMCC26256 | Ethynodiol | -0.35878 | 0.172363 | 0.553799 |
| Lachnospiraceae_UCG-006 | L-Homocystine | 0.358757 | 0.172389 | 0.553799 |
| RF39 | 2-Phenylethyl octanoate | -0.35875 | 0.172397 | 0.553799 |
| Subgroup_13 | All trans decaprenyl diphosphate | -0.35873 | 0.172424 | 0.553799 |
| Clostridia_vadinBB60_group | Tercatain | 0.358714 | 0.172445 | 0.553799 |
| Faecalibaculum | 3-O-acetylecdysone 2-phosphate | -0.35864 | 0.172541 | 0.553831 |
| Bryobacter | 2-carboxy-L-threo-pentonate | -0.35862 | 0.172569 | 0.553831 |
| Clostridioides | Hexadecadienylcarnitine | 0.358614 | 0.172573 | 0.553831 |
| bacteriap25 | PE(22:5(4Z,7Z,10Z,13Z,16Z)/PGD2) | -0.35856 | 0.172644 | 0.553872 |
| Solobacterium | 3-Hydroxyeicosanoylcarnitine | 0.358542 | 0.172665 | 0.553872 |
| bacteriap25 | Berkeleylactone L | -0.35848 | 0.172741 | 0.553992 |
| Lachnospiraceae_UCG-006 | Terbufos | 0.358436 | 0.172801 | 0.554007 |
| Enterorhabdus | 5-Hydroxyindoxyl sulfate | -0.35842 | 0.172825 | 0.554007 |
| TRA3-20 | Cysteinyl-Aspartate | 0.358312 | 0.17296 | 0.554315 |
| Coprobacillus | Quinagolida | -0.35827 | 0.173012 | 0.554356 |
| Lachnospiraceae_UCG-006 | FAD | 0.358202 | 0.173102 | 0.554428 |
| RF39 | 3,4-Dimethyl-5-propyl-2-furanpentadecanoic acid | -0.35819 | 0.173114 | 0.554428 |
| Pseudolabrys | Phenylalanyl-Glycine | 0.358155 | 0.173163 | 0.55446 |
| Pseudolabrys | 8-Amino-7-oxononanoic acid | -0.35812 | 0.173207 | 0.554476 |
| Treponema | Lycoperdic acid | 0.357934 | 0.173448 | 0.554877 |
| Nitrospira | 6,10,14-Trimethyl-5,9,13-pentadecatrien-2-one | -0.35793 | 0.173449 | 0.554877 |
| TM7 | 5'-Carboxy-gamma-chromanol | -0.35793 | 0.173454 | 0.554877 |
| Jatrophihabitans | Lemborexant | -0.3579 | 0.17349 | 0.554877 |
| Clostridioides | Demethylzeylasteral | -0.35785 | 0.17356 | 0.554975 |
| Lachnospiraceae_UCG-006 | Benzyl gentiobioside | 0.357807 | 0.17361 | 0.555009 |
| Christensenellaceae_R-7_group | 3-Methyl-3-butenyl apiosyl-(1->6)-glucoside | 0.357754 | 0.17368 | 0.555034 |
| Subgroup_13 | PC(11:0/23:0) | 0.35774 | 0.173697 | 0.555034 |
| Anaerostipes | Skepinone-L | 0.357708 | 0.173739 | 0.555042 |
| TRA3-20 | 3-Methyl-3-butenyl apiosyl-(1->6)-glucoside | 0.357641 | 0.173825 | 0.555192 |
| Intestinimonas | 4Z,7-octadienoic acid | -0.35754 | 0.173951 | 0.555299 |
| Haliangium | 3-(3-(Pyridin-3-yl)-1,2,4-oxadiazol-5-yl)benzonitrile | -0.35748 | 0.17403 | 0.555299 |
| TM7 | 8-Amino-7-oxononanoic acid | -0.35741 | 0.174129 | 0.555299 |
| Anaerostipes | N6-Acetyl-L-lysine | 0.357397 | 0.174141 | 0.555299 |
| Intestinimonas | LysoPE(20:3(8Z,11Z,14Z)/0:0) | 0.357372 | 0.174173 | 0.555299 |
| Clostridioides | 7b-Hydroxy-3-oxo-5b-cholanoic acid | -0.35737 | 0.174177 | 0.555299 |
| Achromobacter | N,N'-Diethylthiourea | 0.357332 | 0.174224 | 0.555299 |
| IMCC26256 | [(2S,4R,5R,6R,14S,16R)-14-Hydroxy-7,11-dimethyl-6-(2-oxopyran-4-yl)-3-oxapentacyclo[8.8.0.02,4.02,7.011,16]octadecan-5-yl] acetate | -0.35729 | 0.174275 | 0.555299 |
| Subgroup_13 | 4-Chloro-L-phenylalanine | -0.35728 | 0.174289 | 0.555299 |
| Coprobacillus | PC(20:4(5Z,8Z,11Z,14Z)-OH(16R)/2:0) | 0.35728 | 0.174291 | 0.555299 |
| [Eubacterium]_oxidoreducens_group | 8-Amino-7-oxononanoic acid | -0.35728 | 0.174293 | 0.555299 |
| Gemella | Pentadeca-3,5,7-trienedioylcarnitine | 0.357129 | 0.174487 | 0.555688 |
| Rikenellaceae_RC9_gut_group | Notoginsenoside H | 0.35709 | 0.174536 | 0.555688 |
| Intestinimonas | N-Acetyl-D-Glucosamine 6-Phosphate | 0.357082 | 0.174547 | 0.555688 |
| Subgroup_13 | Allitridin | 0.357062 | 0.174572 | 0.555688 |
| Acetatifactor | PE(19:0/20:2(11Z,14Z)) | -0.35696 | 0.174701 | 0.555972 |
| RF39 | Quinagolida | -0.35693 | 0.174749 | 0.555998 |
| [Eubacterium]_oxidoreducens_group | 4E,14Z-Sphingadiene | -0.35686 | 0.17483 | 0.55601 |
| Flavonifractor | OKOHA-PA | -0.35679 | 0.174926 | 0.55601 |
| Enterorhabdus | PC(11:0/23:0) | 0.356761 | 0.174963 | 0.55601 |
| Enterorhabdus | thiamphenicol | 0.356725 | 0.175009 | 0.55601 |
| Treponema | S-cucujolide III | -0.35672 | 0.175014 | 0.55601 |
| Rikenellaceae_RC9_gut_group | Eleutherazine B; N,N'-((3,6-Dioxo-2,5-piperazinediyl)bis(trimethylene))bis(5-hydroxy-3-methyl-2-pentenamide) | 0.356658 | 0.175096 | 0.55601 |
| Anaerostipes | 25-Hydroxyvitamin D3-26,23-lactol | -0.35665 | 0.1751 | 0.55601 |
| Achromobacter | N-Phenyl-p-phenylenediamine | -0.35664 | 0.175117 | 0.55601 |
| Clostridioides | PGP(18:3(9Z,12Z,15Z)/18:1(12Z)-O(9S,10R)) | 0.356602 | 0.175169 | 0.55601 |
| Acetatifactor | 5,5-Diphenyl-2-thiohydantoin | 0.356593 | 0.17518 | 0.55601 |
| Monoglobus | PC(20:5(5Z,8Z,11Z,14Z,17Z)/PGJ2) | -0.35659 | 0.175187 | 0.55601 |
| bacteriap25 | Bis(2-propoxyethyl) 2,6-dimethyl-4-(3-nitrophenyl)-3,4-dihydropyridine-3,5-dicarboxylate | -0.35651 | 0.175282 | 0.556134 |
| Anaerotruncus | 6,10,14-Trimethyl-5,9,13-pentadecatrien-2-one | -0.35644 | 0.175377 | 0.556134 |
| Enterorhabdus | Dipotassium phosphate | 0.356422 | 0.175402 | 0.556134 |
| Lachnospiraceae_UCG-006 | Pelargonidin 3-sophoroside | -0.35641 | 0.175414 | 0.556134 |
| Lachnospiraceae_UCG-006 | Monooctyl phthalate | -0.35637 | 0.175476 | 0.556134 |
| Subgroup_13 | Tercatain | 0.356341 | 0.175507 | 0.556134 |
| Faecalibaculum | 3-Ketosphingosine | -0.35633 | 0.175517 | 0.556134 |
| Treponema | Aspartyl-Methionine | 0.356314 | 0.175542 | 0.556134 |
| Coprobacillus | 25-Hydroxyvitamin D3-26,23-lactol | -0.35627 | 0.175598 | 0.556187 |
| TM7 | 2-[4-(3-Hydroxypropyl)-2-methoxyphenoxy]-1,3-propanediol 1-xyloside | -0.35615 | 0.175755 | 0.556558 |
| Enterococcus | 1-O-(2R-methoxy-4Z-docosenyl)-sn-glycerol | -0.35609 | 0.175828 | 0.556664 |
| Treponema | 12-Hydroxydodecanoic acid | -0.35589 | 0.176096 | 0.557298 |
| Nitrospira | PE(O-20:0/0:0) | -0.35588 | 0.176108 | 0.557298 |
| Anaerostipes | Oleoyl Serotonin | -0.35575 | 0.176282 | 0.557677 |
| IMCC26256 | PC(16:1(9Z)/17:1(9Z)) | -0.3557 | 0.176344 | 0.557677 |
| Lachnospiraceae_UCG-006 | Pelargonidin 3,5-di-(6-acetylglucoside) | -0.35569 | 0.176357 | 0.557677 |
| Rikenellaceae_RC9_gut_group | Withaferin A | -0.35564 | 0.17642 | 0.557677 |
| Enterorhabdus | N6-Acetyl-L-lysine | 0.355635 | 0.176425 | 0.557677 |
| Faecalibaculum | Avenin | 0.355404 | 0.176726 | 0.558234 |
| TRA3-20 | N-Acetyl-D-Glucosamine 6-Phosphate | 0.355402 | 0.17673 | 0.558234 |
| Enterorhabdus | (4E,15E)-Bilirubin | -0.35539 | 0.176748 | 0.558234 |
| Christensenellaceae_R-7_group | (R)-Propyl 2-amino-3-mercaptopropanoate | 0.355368 | 0.176774 | 0.558234 |
| Gemella | Erucic acid | 0.355348 | 0.1768 | 0.558234 |
| Bryobacter | Lycoperdic acid | 0.355299 | 0.176864 | 0.558268 |
| Muribaculum | Pelargonidin 3,5-di-(6-acetylglucoside) | -0.35528 | 0.17689 | 0.558268 |
| Alcaligenes | Tuberonic acid glucoside | -0.35514 | 0.177068 | 0.558599 |
| Acetatifactor | LysoSM(d18:0) | -0.3551 | 0.17713 | 0.558599 |
| Muribaculum | 3-Ketosphingosine | -0.35505 | 0.177191 | 0.558599 |
| MND1 | 2-Phenylethyl octanoate | -0.355 | 0.17726 | 0.558599 |
| Anaerotruncus | thiamphenicol | 0.354996 | 0.17726 | 0.558599 |
| Anaerotruncus | Phenylalanylaspartic acid | 0.354993 | 0.177264 | 0.558599 |
| Rikenellaceae_RC9_gut_group | N-(2-Hydroxyethyl)-2-(1-isoquinolinylmethylene)hydrazinecarbothioamide | 0.354986 | 0.177273 | 0.558599 |
| Monoglobus | thiamphenicol | 0.354901 | 0.177384 | 0.558722 |
| Haliangium | 4-Phenylbutanoylcarnitine | -0.3549 | 0.177391 | 0.558722 |
| Monoglobus | PC(11:0/23:0) | 0.354856 | 0.177443 | 0.558759 |
| Granulicella | All trans decaprenyl diphosphate | -0.35479 | 0.177533 | 0.558839 |
| Treponema | Valylhistidine | 0.354776 | 0.177547 | 0.558839 |
| Nitrospira | Phytal | -0.35472 | 0.177622 | 0.558951 |
| IMCC26256 | N-Undecanoylglycine | -0.35467 | 0.177684 | 0.558954 |
| Anaerotruncus | Terbufos | 0.354651 | 0.177711 | 0.558954 |
| Intestinimonas | Ricinoleic acid | -0.35463 | 0.177742 | 0.558954 |
| Muribaculum | Aminovaleric acid betaine | 0.35457 | 0.177818 | 0.559065 |
| TM7 | Cichorioside K | -0.35453 | 0.177867 | 0.559097 |
| Achromobacter | N6-Acetyl-L-lysine | 0.354429 | 0.178001 | 0.559393 |
| Tuzzerella | Phenylalanylproline | 0.354399 | 0.178041 | 0.559394 |
| IMCC26256 | 1-Hydroxy-2-naphthoic acid | 0.354319 | 0.178146 | 0.559542 |
| IMCC26256 | 1-Ethoxymethyl-5-fluorouracil | 0.354302 | 0.178168 | 0.559542 |
| Faecalibaculum | Aminovaleric acid betaine | 0.354221 | 0.178275 | 0.559752 |
| Subgroup_13 | 4-Chloro-2-nitrobenzylalcohol | -0.35415 | 0.178365 | 0.559752 |
| Granulicella | N-Undecanoylglycine | -0.35414 | 0.178385 | 0.559752 |
| IMCC26256 | 3-Ketosphingosine | -0.35413 | 0.178394 | 0.559752 |
| Rikenellaceae_RC9_gut_group | [(2S,4R,5R,6R,14S,16R)-14-Hydroxy-7,11-dimethyl-6-(2-oxopyran-4-yl)-3-oxapentacyclo[8.8.0.02,4.02,7.011,16]octadecan-5-yl] acetate | -0.35399 | 0.178581 | 0.560114 |
| Faecalibaculum | N-(N-(3-Amino-3-carboxypropyl)-3-amino-3-carboxypropyl)azetidine-2-carboxylic acid | 0.353958 | 0.17862 | 0.560114 |
| Granulicella | m7G(5')pppAm | 0.353928 | 0.178659 | 0.560114 |
| Lachnospiraceae_UCG-006 | Tuberonic acid glucoside | -0.35383 | 0.178792 | 0.560114 |
| Enterorhabdus | Inosine | 0.353794 | 0.178835 | 0.560114 |
| Aquicella | 1H-Pyrrole-2,5-dione, 3-(1-methyl-1h-indol-3-yl)-4-(1-methyl-6-nitro-1h-indol-3-yl)- | 0.353745 | 0.1789 | 0.560114 |
| Rikenellaceae_RC9_gut_group | thiamphenicol | 0.353742 | 0.178903 | 0.560114 |
| Lachnospiraceae_UCG-006 | 4'-O-Glucopyranosylsinapic acid | 0.3537 | 0.178959 | 0.560114 |
| Acetatifactor | 1-p-Menthen-3-one | 0.353688 | 0.178975 | 0.560114 |
| Granulicella | Mycobactins | -0.35367 | 0.178999 | 0.560114 |
| Muribaculum | Lemborexant | -0.35363 | 0.179054 | 0.560114 |
| Lachnospiraceae_UCG-006 | Dehydroascorbide(1-) | 0.353588 | 0.179106 | 0.560114 |
| Nitrospira | Phenylalanyl-Glycine | 0.353579 | 0.179119 | 0.560114 |
| Bryobacter | Quinagolida | -0.35358 | 0.179121 | 0.560114 |
| Bryobacter | 3-Hydroxy-10'-apo-b,y-carotenal | 0.353561 | 0.179141 | 0.560114 |
| Alcaligenes | Mycobactins | -0.35356 | 0.179146 | 0.560114 |
| Bryobacter | 12,13-DHOME | -0.35344 | 0.179303 | 0.560374 |
| Granulicella | 25-Hydroxyvitamin D3-26,23-lactol | -0.35343 | 0.179308 | 0.560374 |
| Nitrospira | Glutamylleucylarginine | 0.353365 | 0.1794 | 0.560525 |
| Subgroup_2 | Adenosine monophosphate | -0.35334 | 0.179437 | 0.560525 |
| Bryobacter | 1-p-Menthen-3-one | 0.353271 | 0.179523 | 0.560548 |
| Anaerostipes | Fexaramine | -0.35326 | 0.179537 | 0.560548 |
| IMCC26256 | S-[(3R,3As,6S,6aS)-3-nitrooxy-2,3,3a,5,6,6a-hexahydrofuro[3,2-b]furan-6-yl] ethanethioate | 0.353241 | 0.179563 | 0.560548 |
| Bryobacter | 2,5,7-trihydroxy-4'-methoxyisoflavanone | -0.35321 | 0.179603 | 0.560549 |
| TM7 | Withaferin A | -0.35305 | 0.179817 | 0.561082 |
| MND1 | Medica 16 | -0.35299 | 0.179891 | 0.561082 |
| RF39 | 1-O-Galloylglycerol | 0.35299 | 0.179894 | 0.561082 |
| Clostridioides | Nipradilol | 0.352935 | 0.179966 | 0.561168 |
| Clostridia_vadinBB60_group | m7G(5')pppAm | 0.352909 | 0.180001 | 0.561168 |
| Aquicella | Kuguacin E | -0.35286 | 0.180066 | 0.561236 |
| Achromobacter | Cortisol | -0.35283 | 0.180103 | 0.561236 |
| Solobacterium | 1-p-Menthen-3-one | 0.352784 | 0.180166 | 0.561311 |
| Enterorhabdus | N-(2-Hydroxyethyl)-2-(1-isoquinolinylmethylene)hydrazinecarbothioamide | 0.352747 | 0.180215 | 0.561336 |
| Enterorhabdus | Hexadecadienylcarnitine | 0.3527 | 0.180278 | 0.561336 |
| Pseudolabrys | 1-p-Menthen-3-one | 0.352678 | 0.180307 | 0.561336 |
| Subgroup_2 | LysoPS(18:1(9Z)/0:0) | -0.35264 | 0.180357 | 0.561336 |
| Enterococcus | N-(1-Deoxy-1-fructosyl)valine | 0.352618 | 0.180386 | 0.561336 |
| Lachnospiraceae_UCG-006 | LysoSM(d18:0) | -0.35259 | 0.180423 | 0.561336 |
| Enterococcus | PC(16:0/16:0) | 0.352526 | 0.180508 | 0.561336 |
| Lachnospiraceae_UCG-006 | [6-Hydroxy-8-methyl-3-[3,4,5-trihydroxy-6-(hydroxymethyl)oxan-2-yl]oxy-9,10-dioxatetracyclo[4.3.1.02,5.03,8]decan-4-yl]methyl benzoate | 0.352505 | 0.180535 | 0.561336 |
| Clostridia_vadinBB60_group | 7-Hydroxy-R-acenocoumarol | 0.352424 | 0.180643 | 0.561336 |
| Christensenellaceae_R-7_group | Tuberonic acid glucoside | -0.35242 | 0.180647 | 0.561336 |
| Coprobacillus | LysoPS(18:1(9Z)/0:0) | -0.35239 | 0.180682 | 0.561336 |
| Acidothermus | Eleutherazine B; N,N'-((3,6-Dioxo-2,5-piperazinediyl)bis(trimethylene))bis(5-hydroxy-3-methyl-2-pentenamide) | 0.352366 | 0.180719 | 0.561336 |
| TRA3-20 | L-Lysinamide | -0.35235 | 0.180741 | 0.561336 |
| TM7 | Amobarbital | 0.352347 | 0.180745 | 0.561336 |
| Lachnospiraceae_UCG-006 | 1H-Pyrrole-2,5-dione, 3-(1-methyl-1h-indol-3-yl)-4-(1-methyl-6-nitro-1h-indol-3-yl)- | 0.352296 | 0.180812 | 0.561336 |
| [Eubacterium]_oxidoreducens_group | 3-Ketosphingosine | -0.35227 | 0.18084 | 0.561336 |
| Intestinimonas | Alacepril | -0.35217 | 0.180977 | 0.561336 |
| RF39 | N-Myristoyl Asparagine | -0.35215 | 0.181002 | 0.561336 |
| Acidothermus | JP83 | -0.35215 | 0.181006 | 0.561336 |
| Enterococcus | 1-(O-alpha-D-glucopyranosyl)-(1,3R,25S,27R)-octacosanetetrol | -0.35209 | 0.181082 | 0.561336 |
| Treponema | Tetradecanoylcarnitine | 0.352089 | 0.181085 | 0.561336 |
| Alcaligenes | Berkeleylactone L | -0.35208 | 0.181095 | 0.561336 |
| Anaerostipes | Mycobactins | -0.35206 | 0.181119 | 0.561336 |
| Lachnospiraceae_UCG-006 | Am-PE(16:0/18:0) | 0.352039 | 0.181152 | 0.561336 |
| Enterorhabdus | Aminovaleric acid betaine | 0.351861 | 0.181389 | 0.561336 |
| Lachnospiraceae_UCG-006 | PC(16:1(9Z)/17:1(9Z)) | -0.35186 | 0.181396 | 0.561336 |
| Nitrospira | Kuguacin E | -0.35181 | 0.181459 | 0.561336 |
| IMCC26256 | 16,17-Dihydro-16alpha,17-dihydroxygibberellin A4 17-glucoside | -0.35179 | 0.18148 | 0.561336 |
| Enterococcus | N-Acetyl-D-Glucosamine 6-Phosphate | 0.35177 | 0.181509 | 0.561336 |
| Aquicella | 2-[(4-{2-[(4-Cyclohexylbutyl)(cyclohexylcarbamoyl)amino]ethyl}phenyl)sulfanyl]-2-methylpropanoic acid | -0.35175 | 0.181533 | 0.561336 |
| Enterorhabdus | 5-[1-Carboxy-2-(trimethylazaniumyl)ethoxy]-5-oxopentanoate | 0.351749 | 0.181537 | 0.561336 |
| Acetatifactor | 16,17-Dihydro-16alpha,17-dihydroxygibberellin A4 17-glucoside | -0.35172 | 0.181576 | 0.561336 |
| Enterorhabdus | (R)-Propyl 2-amino-3-mercaptopropanoate | 0.351697 | 0.181607 | 0.561336 |
| Monoglobus | 3'-Deoxythymidine | 0.351671 | 0.181641 | 0.561336 |
| Anaerotruncus | Prostaglandin B-1 | -0.35164 | 0.181675 | 0.561336 |
| [Eubacterium]_oxidoreducens_group | (卤)-(Z)-2-(5-Tetradecenyl)cyclobutanone | -0.35163 | 0.181689 | 0.561336 |
| Treponema | Skepinone-L | 0.351633 | 0.181691 | 0.561336 |
| IMCC26256 | Nopalinic acid | 0.351633 | 0.181691 | 0.561336 |
| Clostridia_vadinBB60_group | trans-Hexadec-2-enoyl carnitine | 0.351604 | 0.181729 | 0.561336 |
| Enterococcus | 1-O-(2R-methoxy-4Z-eicosenyl)-sn-glycerol | -0.35157 | 0.181776 | 0.561355 |
| Rikenellaceae_RC9_gut_group | PGP(18:3(9Z,12Z,15Z)/18:1(12Z)-O(9S,10R)) | 0.351456 | 0.181926 | 0.561698 |
| Bryobacter | S-Adenosylhomocysteine | 0.35142 | 0.181975 | 0.561723 |
| Alcaligenes | NAD | 0.351378 | 0.182029 | 0.561769 |
| Lachnospiraceae_UCG-006 | N-Acetylneuraminic acid | 0.351136 | 0.182352 | 0.562641 |
| Bryobacter | 2-(2-Thienylmethylene)-1,6-dioxaspiro[4.4]non-3-ene | 0.351081 | 0.182425 | 0.562745 |
| Enterorhabdus | DG(i-15:0/20:4(8Z,11Z,14Z,17Z)-2OH(5S,6R)/0:0) | -0.35104 | 0.182474 | 0.562771 |
| Bryobacter | 6-Hydroxytricetin 5-rhamnoside | 0.35098 | 0.18256 | 0.562808 |
| Gemella | 2',3'-Dideoxyuridine | 0.350972 | 0.182571 | 0.562808 |
| Bryobacter | 1-(O-alpha-D-glucopyranosyl)-(1,3S,25R)-hexacosanetriol | -0.35095 | 0.182606 | 0.562808 |
| Anaerostipes | PI(18:0/20:4(8Z,11Z,14Z,17Z)) | 0.350914 | 0.182647 | 0.562813 |
| TM7 | L-Arginine | 0.350769 | 0.182841 | 0.563286 |
| Intestinimonas | Micronomicin | -0.35073 | 0.182896 | 0.563332 |
| RF39 | PE(O-20:0/0:0) | -0.35061 | 0.183058 | 0.563478 |
| Clostridioides | (R)-4A-(Ethoxymethyl)-1-(4-fluorophenyl)-6-((4-(trifluoromethyl)phenyl)sulfonyl)-4,4a,5,6,7,8-hexahydro-1H-pyrazolo[3,4-g]isoquinoline | -0.35059 | 0.183075 | 0.563478 |
| bacteriap25 | xi-2,3-Octadiene-5,7-diyn-1-ol | 0.350591 | 0.183079 | 0.563478 |
| Achromobacter | Creatine | 0.350542 | 0.183145 | 0.563478 |
| Aquicella | Hexadecenal | -0.35054 | 0.183148 | 0.563478 |
| Muribaculum | 2-phospho-4-(cytidine 5'-diphospho)-2-C-methyl-D-erythritol | 0.350447 | 0.183271 | 0.563478 |
| Anaerotruncus | S-cucujolide III | -0.35043 | 0.183292 | 0.563478 |
| Granulicella | Pelargonidin 3,5-di-(6-acetylglucoside) | -0.35036 | 0.183385 | 0.563478 |
| Alcaligenes | Prostaglandin B-1 | -0.35034 | 0.18342 | 0.563478 |
| Gemella | L-Glutamine | 0.350317 | 0.183445 | 0.563478 |
| Anaerostipes | PI(20:3(8Z,11Z,14Z)/0:0) | 0.350277 | 0.183498 | 0.563478 |
| Solobacterium | Histamine | -0.35026 | 0.183527 | 0.563478 |
| Alcaligenes | L-Lysinamide | -0.35025 | 0.183529 | 0.563478 |
| Monoglobus | Phytal | -0.35025 | 0.183536 | 0.563478 |
| TRA3-20 | Dipotassium phosphate | 0.350239 | 0.183549 | 0.563478 |
| Treponema | (E,E)-3,7,11-Trimethyl-2,6,10-dodecatrienyl octanoate | 0.350197 | 0.183605 | 0.563478 |
| Anaerostipes | Cortisol | -0.35018 | 0.183624 | 0.563478 |
| TM7 | Flumazenil | 0.349874 | 0.184037 | 0.564202 |
| Alcaligenes | Dimethylaminoparthenolide | 0.349868 | 0.184045 | 0.564202 |
| Anaerotruncus | 4Z,7-octadienoic acid | -0.34977 | 0.18417 | 0.564202 |
| TRA3-20 | Dehydrocyclopeptine; (3E)-3-Benzylidene-4-methyl-3,4-dihydro-1H-1,4-benzodiazepine-2,5-dione | -0.34977 | 0.184181 | 0.564202 |
| Nitrospira | Pelargonidin 3-sophoroside | -0.34972 | 0.184239 | 0.564202 |
| Aquicella | Glycerol 3-phosphate | 0.349724 | 0.184239 | 0.564202 |
| Clostridioides | Phenylalanyl-Glycine | 0.349682 | 0.184295 | 0.564202 |
| Anaerostipes | 3-Ketosphingosine | -0.34968 | 0.184304 | 0.564202 |
| Monoglobus | Notoginsenoside H | 0.349675 | 0.184305 | 0.564202 |
| Flavonifractor | JP83 | -0.34965 | 0.184333 | 0.564202 |
| Solobacterium | Azeloprazole | 0.349641 | 0.184349 | 0.564202 |
| Coprobacillus | PPM-18 | 0.349586 | 0.184424 | 0.564202 |
| Clostridioides | Alacepril | -0.34957 | 0.184439 | 0.564202 |
| Acetatifactor | 2-Phenylethyl octanoate | -0.34956 | 0.184462 | 0.564202 |
| Monoglobus | 3-Ketosphingosine | -0.34954 | 0.184481 | 0.564202 |
| Christensenellaceae_R-7_group | Allitridin | 0.349529 | 0.184501 | 0.564202 |
| Subgroup_2 | PC(18:1(17Z)/18:1(17Z)) | 0.349452 | 0.184603 | 0.564392 |
| Enterorhabdus | 3-hydroxyhexadecanoyl carnitine | 0.349302 | 0.184805 | 0.564516 |
| Acidothermus | [(2S,4R,5R,6R,14S,16R)-14-Hydroxy-7,11-dimethyl-6-(2-oxopyran-4-yl)-3-oxapentacyclo[8.8.0.02,4.02,7.011,16]octadecan-5-yl] acetate | -0.34928 | 0.18483 | 0.564516 |
| Clostridia_vadinBB60_group | 5beta-Cholane-3alpha,24-diol | 0.34927 | 0.184848 | 0.564516 |
| Acidothermus | Withaferin A | -0.34925 | 0.184871 | 0.564516 |
| Bryobacter | 13,14-dihydro-15-keto-PGD2-d4 | -0.34925 | 0.184872 | 0.564516 |
| Subgroup_2 | Fructose-1,6-diphosphate | 0.349243 | 0.184884 | 0.564516 |
| Coprobacillus | (卤)-(Z)-2-(5-Tetradecenyl)cyclobutanone | -0.34904 | 0.185157 | 0.565189 |
| Nitrospira | Octadecanamide | -0.34901 | 0.185204 | 0.565189 |
| Acetatifactor | D-erythro-L-galacto-Nonulose | 0.348989 | 0.185225 | 0.565189 |
| Enterococcus | N-Lauroyl Glutamine | -0.34896 | 0.185265 | 0.565189 |
| Lachnospiraceae_UCG-006 | 2-Phenylethyl octanoate | -0.34889 | 0.185365 | 0.565318 |
| Nitrospira | Quinagolida | -0.34887 | 0.185388 | 0.565318 |
| Faecalibaculum | Glycyl-D-proline | 0.348773 | 0.185516 | 0.565574 |
| Achromobacter | N-Lactoylleucine | 0.348747 | 0.185552 | 0.565574 |
| Enterorhabdus | PI(20:4(5Z,8Z,11Z,14Z)/18:0) | -0.34865 | 0.185679 | 0.565642 |
| bacteriap25 | Monooctyl phthalate | -0.34864 | 0.185698 | 0.565642 |
| RF39 | LysoSM(d18:0) | -0.34863 | 0.185712 | 0.565642 |
| Intestinimonas | Pelargonidin 3,5-di-(6-acetylglucoside) | -0.34861 | 0.185735 | 0.565642 |
| Muribaculum | Biliverdin | -0.34856 | 0.185803 | 0.565725 |
| [Eubacterium]_oxidoreducens_group | 6,10,14-Trimethyl-5,9,13-pentadecatrien-2-one | -0.34849 | 0.185901 | 0.565887 |
| Faecalibaculum | N6-Acetyl-L-lysine | 0.348462 | 0.185936 | 0.565887 |
| Nitrospira | trans-Hexadec-2-enoyl carnitine | 0.348404 | 0.186014 | 0.566001 |
| Intestinimonas | LysoSM(d18:0) | -0.34834 | 0.186102 | 0.566148 |
| Clostridioides | Pelargonidin 3-sophoroside | -0.3483 | 0.186156 | 0.566171 |
| RF39 | D-erythro-Sphingosine C-20 | -0.34822 | 0.186256 | 0.566171 |
| Nitrospira | Hexadecenal | -0.34822 | 0.18626 | 0.566171 |
| bacteriap25 | N-Phenyl-p-phenylenediamine | -0.34819 | 0.1863 | 0.566171 |
| Subgroup_13 | Prolylproline | 0.348174 | 0.186324 | 0.566171 |
| Aquicella | 1-p-Menthen-3-one | 0.348138 | 0.186373 | 0.566171 |
| Granulicella | 1-O-Galloylglycerol | 0.348124 | 0.186391 | 0.566171 |
| Lachnospiraceae_UCG-006 | Valylserine | 0.348077 | 0.186455 | 0.566234 |
| Christensenellaceae_R-7_group | AM2201 N-(4-hydroxypentyl) metabolite | -0.34804 | 0.186505 | 0.566234 |
| Bryobacter | PI(20:4(5Z,8Z,11Z,14Z)/18:0) | -0.34802 | 0.186533 | 0.566234 |
| Anaerostipes | Serylproline | 0.347934 | 0.186649 | 0.566376 |
| Alcaligenes | LysoPC(20:3(8Z,11Z,14Z)/0:0) | 0.347909 | 0.186683 | 0.566376 |
| Bryobacter | (S1)-Methoxy-3-heptanethiol | 0.34787 | 0.186735 | 0.566376 |
| TRA3-20 | Camptothecin sodium | 0.347866 | 0.18674 | 0.566376 |
| Solobacterium | Flumazenil | 0.347772 | 0.186867 | 0.566621 |
| MND1 | Hexadecenal | -0.34775 | 0.186901 | 0.566621 |
| Acetatifactor | Dolichyl b-D-glucosyl phosphate | -0.34749 | 0.187255 | 0.567478 |
| Coprobacillus | Aspartyl-Isoleucine | 0.347479 | 0.187265 | 0.567478 |
| MND1 | Terizidone | 0.347427 | 0.187334 | 0.567567 |
| Bryobacter | FAD | 0.347378 | 0.187402 | 0.567606 |
| Enterococcus | 2-C-methyl-D-erythritol-4-phosphate | 0.347358 | 0.187428 | 0.567606 |
| RF39 | Docosadienoate (22:2n6) | 0.347292 | 0.187518 | 0.567756 |
| Muribaculum | LysoPS(18:1(9Z)/0:0) | -0.34725 | 0.187581 | 0.567804 |
| Clostridia_vadinBB60_group | Hexadecenal | -0.34722 | 0.187614 | 0.567804 |
| RF39 | Sodium nitrate (NaNO3) | -0.34717 | 0.187687 | 0.567898 |
| Jatrophihabitans | 4-Phenylbutanoylcarnitine | -0.34711 | 0.187767 | 0.567898 |
| Treponema | Amobarbital | 0.346999 | 0.187915 | 0.567898 |
| Achromobacter | N-Lactoylphenylalanine | 0.346968 | 0.187958 | 0.567898 |
| Subgroup_2 | Pseudouridine | 0.346925 | 0.188016 | 0.567898 |
| Pseudolabrys | N-(1-Deoxy-1-fructosyl)threonine | 0.346891 | 0.188062 | 0.567898 |
| Lachnospiraceae_UCG-006 | D-erythro-Sphingosine C-20 | -0.34683 | 0.188148 | 0.567898 |
| bacteriap25 | 5,5-Diphenyl-2-thiohydantoin | 0.346813 | 0.188168 | 0.567898 |
| Clostridia_vadinBB60_group | Orotidine | 0.346791 | 0.188199 | 0.567898 |
| Flavonifractor | D-Malic acid | 0.346789 | 0.188201 | 0.567898 |
| Enterococcus | Cysteinyl-Aspartate | 0.346784 | 0.188208 | 0.567898 |
| Anaerostipes | 1-O-Galloylglycerol | 0.34677 | 0.188227 | 0.567898 |
| Intestinimonas | Berkeleylactone L | -0.34675 | 0.188252 | 0.567898 |
| Subgroup_13 | Aspartyl-Isoleucine | 0.346735 | 0.188275 | 0.567898 |
| Granulicella | 4-Chloro-L-phenylalanine | -0.34665 | 0.188395 | 0.567898 |
| Anaerostipes | Carissanol | -0.34662 | 0.188424 | 0.567898 |
| Anaerotruncus | TG(8:0/8:0/a-13:0)[rac] | -0.3466 | 0.188463 | 0.567898 |
| Rikenellaceae_RC9_gut_group | 3-Hydroxy-11Z-octadecenoylcarnitine | 0.346596 | 0.188463 | 0.567898 |
| Anaerotruncus | Hexadecenal | -0.34655 | 0.188523 | 0.567898 |
| Intestinimonas | S-cucujolide III | -0.3465 | 0.188595 | 0.567898 |
| Granulicella | scyllo-Inositol | 0.346469 | 0.188636 | 0.567898 |
| Nitrospira | AM2201 N-(4-hydroxypentyl) metabolite | -0.34644 | 0.18867 | 0.567898 |
| Anaerotruncus | 3-O-acetylecdysone 2-phosphate | -0.34644 | 0.188678 | 0.567898 |
| Nitrospira | 4E,14Z-Sphingadiene | -0.34642 | 0.188698 | 0.567898 |
| Alcaligenes | Neosaxitoxin | 0.346345 | 0.188805 | 0.567898 |
| Aquicella | Bacillamidin C | -0.34631 | 0.18885 | 0.567898 |
| Bryobacter | Dirithromycin | -0.3463 | 0.188865 | 0.567898 |
| Haliangium | Lemborexant | -0.34628 | 0.188894 | 0.567898 |
| Coprobacillus | Tetradecanoylcarnitine | 0.346272 | 0.188904 | 0.567898 |
| Christensenellaceae_R-7_group | PC(11:0/23:0) | 0.346246 | 0.18894 | 0.567898 |
| Nitrospira | N-Myristoyl Asparagine | -0.34624 | 0.188951 | 0.567898 |
| IMCC26256 | Benzyl gentiobioside | 0.346171 | 0.189042 | 0.567898 |
| bacteriap25 | 9-Octadecenal | -0.34617 | 0.189044 | 0.567898 |
| Subgroup_13 | Mycobactins | -0.34617 | 0.189047 | 0.567898 |
| Flavonifractor | 1-(10H-Phenothiazin-2-yl)ethanone | 0.34616 | 0.189057 | 0.567898 |
| Enterorhabdus | Serotinose | 0.346102 | 0.189137 | 0.567994 |
| Monoglobus | Dehydroascorbide(1-) | 0.346078 | 0.18917 | 0.567994 |
| TM7 | Fiacitabine | 0.345943 | 0.189353 | 0.568422 |
| Gemella | Prolyl-Asparagine | 0.345887 | 0.18943 | 0.568481 |
| Clostridioides | Streptidine | 0.34587 | 0.189453 | 0.568481 |
| Anaerotruncus | Docosadienoate (22:2n6) | 0.3457 | 0.189685 | 0.568796 |
| IMCC26256 | Cysteine-glutathione disulfide | 0.345655 | 0.189746 | 0.568796 |
| Coprobacillus | Glutamylleucylarginine | 0.345638 | 0.18977 | 0.568796 |
| Enterorhabdus | Micronomicin | -0.3456 | 0.189826 | 0.568796 |
| TRA3-20 | Ac-Ser-Asp-Lys-Pro-OH | 0.345597 | 0.189826 | 0.568796 |
| Bryobacter | Dipotassium phosphate | 0.345584 | 0.189844 | 0.568796 |
| Faecalibaculum | 3'-Ketolactose | 0.34557 | 0.189863 | 0.568796 |
| Anaerotruncus | Serotinose | 0.345556 | 0.189882 | 0.568796 |
| Treponema | Nipradilol | 0.345525 | 0.189925 | 0.568801 |
| Enterococcus | Skepinone-L | 0.345496 | 0.189964 | 0.568801 |
| IMCC26256 | Adenosine monophosphate | -0.3454 | 0.1901 | 0.569088 |
| RF39 | 3-Ketosphingosine | -0.34535 | 0.190169 | 0.569174 |
| TM7 | Mycobactins | -0.34523 | 0.190327 | 0.569416 |
| Enterococcus | benfluorex | 0.34522 | 0.190341 | 0.569416 |
| Gemella | Eicosadienoic acid | 0.345198 | 0.190371 | 0.569416 |
| Christensenellaceae_R-7_group | AB-MECA | -0.34514 | 0.190458 | 0.569456 |
| Subgroup_13 | Glutaminylproline | 0.345087 | 0.190524 | 0.569456 |
| Faecalibaculum | PC(18:1(17Z)/18:1(17Z)) | 0.345082 | 0.19053 | 0.569456 |
| Intestinimonas | 3-{[(1s)-2,2-Difluoro-1-Hydroxy-7-(Methylsulfonyl)-2,3-Dihydro-1h-Inden-4-Yl]oxy}-5-Fluorobenzonitrile | 0.345071 | 0.190547 | 0.569456 |
| Intestinimonas | 3-O-acetylecdysone 2-phosphate | -0.345 | 0.190642 | 0.569585 |
| IMCC26256 | Niacinamide | 0.34496 | 0.190698 | 0.569585 |
| Gemella | 11,14,17-eicosatrienoic acid | 0.34495 | 0.190711 | 0.569585 |
| RF39 | DG(LTE4/22:0/0:0) | 0.344889 | 0.190796 | 0.569717 |
| Alcaligenes | L-Homocystine | 0.344812 | 0.190901 | 0.569849 |
| Enterococcus | OKOHA-PA | -0.3448 | 0.190923 | 0.569849 |
| Nitrospira | Genistein 7-O-glucoside-6''-malonate | 0.344768 | 0.190962 | 0.569849 |
| Clostridioides | [6-Hydroxy-8-methyl-3-[3,4,5-trihydroxy-6-(hydroxymethyl)oxan-2-yl]oxy-9,10-dioxatetracyclo[4.3.1.02,5.03,8]decan-4-yl]methyl benzoate | 0.344656 | 0.191115 | 0.570076 |
| Clostridioides | Oleic acid | -0.3446 | 0.191189 | 0.570076 |
| Subgroup_2 | N-(1-Deoxy-1-fructosyl)threonine | 0.344579 | 0.191221 | 0.570076 |
| Rikenellaceae_RC9_gut_group | Cannabidivarin | -0.34452 | 0.191306 | 0.570076 |
| Granulicella | Benzyl gentiobioside | 0.344513 | 0.191312 | 0.570076 |
| Anaerostipes | Phytosphingosine | 0.344475 | 0.191364 | 0.570076 |
| Muribaculum | Tridecanoylcarnitine | 0.344448 | 0.191401 | 0.570076 |
| Nitrospira | 3-O-acetylecdysone 2-phosphate | -0.34444 | 0.191407 | 0.570076 |
| Solobacterium | 4E,14Z-Sphingadiene | -0.34439 | 0.191485 | 0.570076 |
| Faecalibaculum | Amobarbital | 0.344383 | 0.191491 | 0.570076 |
| Anaerostipes | Pevonedistat | 0.344346 | 0.191542 | 0.570076 |
| Clostridioides | Berkeleylactone L | -0.34433 | 0.191559 | 0.570076 |
| TM7 | 2-[(4-{2-[(4-Cyclohexylbutyl)(cyclohexylcarbamoyl)amino]ethyl}phenyl)sulfanyl]-2-methylpropanoic acid | -0.34433 | 0.191564 | 0.570076 |
| Subgroup_13 | S-[(3R,3As,6S,6aS)-3-nitrooxy-2,3,3a,5,6,6a-hexahydrofuro[3,2-b]furan-6-yl] ethanethioate | 0.344279 | 0.191634 | 0.570164 |
| TRA3-20 | PC(11:0/23:0) | 0.344103 | 0.191876 | 0.570592 |
| Enterorhabdus | Methacholine | 0.344098 | 0.191882 | 0.570592 |
| TM7 | N-Phenyl-p-phenylenediamine | -0.34409 | 0.1919 | 0.570592 |
| TM7 | (R)-4A-(Ethoxymethyl)-1-(4-fluorophenyl)-6-((4-(trifluoromethyl)phenyl)sulfonyl)-4,4a,5,6,7,8-hexahydro-1H-pyrazolo[3,4-g]isoquinoline | -0.34405 | 0.191948 | 0.570615 |
| Subgroup_13 | Phenylalanylaspartic acid | 0.343948 | 0.192089 | 0.570615 |
| Treponema | 3-Ketosphingosine | -0.34392 | 0.192133 | 0.570615 |
| Subgroup_2 | 2-hydroxy-9Z,12Z-Octadecadienoic acid | -0.34391 | 0.192143 | 0.570615 |
| IMCC26256 | 3-{[(1s)-2,2-Difluoro-1-Hydroxy-7-(Methylsulfonyl)-2,3-Dihydro-1h-Inden-4-Yl]oxy}-5-Fluorobenzonitrile | 0.343892 | 0.192166 | 0.570615 |
| Aquicella | Resorufin | 0.343863 | 0.192207 | 0.570615 |
| Subgroup_2 | m7G(5')pppAm | 0.343856 | 0.192216 | 0.570615 |
| Flavonifractor | 25-Hydroxyvitamin D3-26,23-lactol | -0.34384 | 0.192242 | 0.570615 |
| Bryobacter | PC(P-18:0/20:4(5Z,8Z,11Z,14Z)-OH(16R)) | 0.343815 | 0.192272 | 0.570615 |
| Muribaculum | PE(18:0/19:1(9Z)) | -0.34372 | 0.192406 | 0.57082 |
| MND1 | 6,10,14-Trimethyl-5,9,13-pentadecatrien-2-one | -0.3437 | 0.192437 | 0.57082 |
| RF39 | Pelargonidin 3-sophoroside | -0.34366 | 0.192484 | 0.57082 |
| Anaerotruncus | 12,13-DHOME | -0.3436 | 0.192567 | 0.57082 |
| Acetatifactor | Glycyl-D-proline | 0.343592 | 0.192581 | 0.57082 |
| Intestinimonas | 1-Octadecene | -0.34359 | 0.192584 | 0.57082 |
| Monoglobus | Hexadecadienylcarnitine | 0.343536 | 0.192658 | 0.570864 |
| Clostridioides | (E,E)-3,7,11-Trimethyl-2,6,10-dodecatrienyl octanoate | 0.343501 | 0.192706 | 0.570864 |
| Faecalibaculum | Serotinose | 0.34349 | 0.192721 | 0.570864 |
| Subgroup_2 | PS(22:5(7Z,10Z,13Z,16Z,19Z)/22:6(4Z,7Z,10Z,13Z,16Z,19Z)) | -0.34345 | 0.192771 | 0.570888 |
| Acidothermus | PPM-18 | 0.343425 | 0.19281 | 0.570888 |
| Alcaligenes | 4Z,7-octadienoic acid | -0.34338 | 0.192877 | 0.570966 |
| Intestinimonas | PI(20:3(8Z,11Z,14Z)/0:0) | 0.343219 | 0.193095 | 0.571027 |
| Christensenellaceae_R-7_group | 2-[4-(3-Hydroxypropyl)-2-methoxyphenoxy]-1,3-propanediol 1-xyloside | -0.34321 | 0.193112 | 0.571027 |
| Alcaligenes | Am-PE(16:0/18:0) | 0.34319 | 0.193135 | 0.571027 |
| Christensenellaceae_R-7_group | Pantothenic acid | 0.343138 | 0.193207 | 0.571027 |
| Anaerotruncus | 2-(Methylthiomethyl)-3-phenyl-2-propenal | 0.343128 | 0.193221 | 0.571027 |
| Coprobacillus | 12-Hydroxy-12-octadecanoylcarnitine | 0.343105 | 0.193253 | 0.571027 |
| Monoglobus | PI(18:0/20:4(8Z,11Z,14Z,17Z)) | 0.3431 | 0.193259 | 0.571027 |
| Pseudolabrys | 7-oxo-8-amino-nonanoic acid | -0.34309 | 0.193279 | 0.571027 |
| Treponema | Flumazenil | 0.343069 | 0.193303 | 0.571027 |
| TM7 | Monooctyl phthalate | -0.34307 | 0.193307 | 0.571027 |
| Enterococcus | Aspartyl-Gamma-glutamate | 0.343024 | 0.193366 | 0.571027 |
| bacteriap25 | (E,E)-3,7,11-Trimethyl-2,6,10-dodecatrienyl octanoate | 0.34301 | 0.193384 | 0.571027 |
| Jatrophihabitans | (2S)-1,1,1-Trifluoro-2-(4-pyrazol-1-ylphenyl)-3-[5-[[1-(trifluoromethyl)cyclopropyl]methyl]-1H-imidazol-2-yl]propan-2-ol | -0.3429 | 0.193534 | 0.571029 |
| Pseudolabrys | 3-Methyl-3-butenyl hexadecanoate | 0.342866 | 0.193583 | 0.571029 |
| Subgroup_13 | 3-hydroxyhexadecanoyl carnitine | 0.342858 | 0.193595 | 0.571029 |
| Alcaligenes | Notoginsenoside H | 0.342755 | 0.193738 | 0.571029 |
| Jatrophihabitans | (2R,4S)-2-Aminoformyl-6-fluoro-spiro[chroman-4,4'-imidazolidine]-2',5'-dione | -0.34275 | 0.19375 | 0.571029 |
| Clostridioides | JP83 | -0.34274 | 0.19376 | 0.571029 |
| Anaerotruncus | Ricinoleic acid | -0.3427 | 0.193811 | 0.571029 |
| Intestinimonas | D-erythro-L-galacto-Nonulose | 0.3427 | 0.193814 | 0.571029 |
| Achromobacter | N-lactoyl-Methionine | 0.342663 | 0.193865 | 0.571029 |
| Haliangium | Cannabidivarin | -0.34265 | 0.19389 | 0.571029 |
| Alcaligenes | N-Lactoylleucine | 0.342622 | 0.193922 | 0.571029 |
| Aquicella | PC(TXB2/20:2(11Z,14Z)) | -0.34258 | 0.193974 | 0.571029 |
| Faecalibaculum | O-Linoleoylcarnitine | 0.342579 | 0.193982 | 0.571029 |
| Anaerostipes | (2S)-1,1,1-Trifluoro-2-(4-pyrazol-1-ylphenyl)-3-[5-[[1-(trifluoromethyl)cyclopropyl]methyl]-1H-imidazol-2-yl]propan-2-ol | -0.34256 | 0.194005 | 0.571029 |
| Alcaligenes | Cysteine-glutathione disulfide | 0.342432 | 0.194185 | 0.571029 |
| Pseudolabrys | Phytosphingosine | 0.342416 | 0.194207 | 0.571029 |
| Monoglobus | 2-Phenylethyl octanoate | -0.34241 | 0.194221 | 0.571029 |
| Anaerostipes | (2R,4S)-2-Aminoformyl-6-fluoro-spiro[chroman-4,4'-imidazolidine]-2',5'-dione | -0.34235 | 0.194301 | 0.571029 |
| Subgroup_13 | Docosatrienoic acid | 0.342349 | 0.194301 | 0.571029 |
| Muribaculum | (S1)-Methoxy-3-heptanethiol | 0.34231 | 0.194354 | 0.571029 |
| Anaerotruncus | 11-Hydroxyhexadecanoylcarnitine | 0.342277 | 0.194401 | 0.571029 |
| Granulicella | 4E,14Z-Sphingadiene | -0.34226 | 0.194423 | 0.571029 |
| Bryobacter | Maraviroc | 0.342246 | 0.194443 | 0.571029 |
| bacteriap25 | Dodecanamide | -0.34223 | 0.194472 | 0.571029 |
| Jatrophihabitans | 3,4-Dimethyl-5-propyl-2-furanpentadecanoic acid | -0.34222 | 0.194475 | 0.571029 |
| Alcaligenes | N-lactoyl-Methionine | 0.342188 | 0.194525 | 0.571029 |
| Enterorhabdus | 2,5,7-trihydroxy-4'-methoxyisoflavanone | -0.34214 | 0.194595 | 0.571029 |
| Monoglobus | 4-Chloro-L-phenylalanine | -0.34212 | 0.194613 | 0.571029 |
| [Eubacterium]_oxidoreducens_group | Cichorioside K | -0.34212 | 0.194622 | 0.571029 |
| Granulicella | PE(22:4(7Z,10Z,13Z,16Z)/19:0) | -0.34211 | 0.194627 | 0.571029 |
| Alcaligenes | N-Lactoylphenylalanine | 0.342086 | 0.194665 | 0.571029 |
| Intestinimonas | Dirithromycin | -0.34207 | 0.194694 | 0.571029 |
| Anaerostipes | DG(18:0/20:4(8Z,11Z,14Z,17Z)/0:0) | 0.342045 | 0.194724 | 0.571029 |
| Faecalibaculum | PC(16:0/16:0) | 0.341958 | 0.194844 | 0.571156 |
| Coprobacillus | 23S,25,26-Trihydroxyvitamin D3 | 0.341924 | 0.194891 | 0.571156 |
| Monoglobus | Terizidone | 0.341907 | 0.194915 | 0.571156 |
| Faecalibaculum | 8-Amino-7-oxononanoic acid | -0.34185 | 0.194995 | 0.571156 |
| Anaerotruncus | Octadec-6-enoylcarnitine | 0.341827 | 0.195025 | 0.571156 |
| Christensenellaceae_R-7_group | Cichorioside K | -0.34181 | 0.195051 | 0.571156 |
| TRA3-20 | 3-O-acetylecdysone 2-phosphate | -0.34179 | 0.195082 | 0.571156 |
| Lachnospiraceae_UCG-006 | 2-[4-(3-Hydroxypropyl)-2-methoxyphenoxy]-1,3-propanediol 1-xyloside | -0.34178 | 0.195091 | 0.571156 |
| Alcaligenes | Cortisol | -0.34165 | 0.195272 | 0.57151 |
| TRA3-20 | 4-(Methylnitrosamino)-1-(3-pyridyl)-1-butanol glucuronide | 0.341629 | 0.195302 | 0.57151 |
| Granulicella | 2',3'-Dideoxyuridine | 0.341606 | 0.195334 | 0.57151 |
| Bryobacter | Glutaminylarginine | -0.34148 | 0.195515 | 0.571778 |
| Intestinimonas | Nipradilol | 0.341473 | 0.195519 | 0.571778 |
| Alcaligenes | Avenin | 0.341453 | 0.195547 | 0.571778 |
| TRA3-20 | Glutamylleucylarginine | 0.341399 | 0.195622 | 0.571788 |
| Achromobacter | 2-[4-(3-Hydroxypropyl)-2-methoxyphenoxy]-1,3-propanediol 1-xyloside | -0.34138 | 0.195643 | 0.571788 |
| Clostridioides | Tyrosyl-Alanine | 0.341357 | 0.195681 | 0.571788 |
| MND1 | scyllo-Inositol | 0.341334 | 0.195713 | 0.571788 |
| Lachnospiraceae_UCG-006 | Nipradilol | 0.341265 | 0.195809 | 0.571851 |
| Enterorhabdus | 2-Methyl-2-[4-[3-[1-(4-methylbenzyl)-5-oxo-4,5-dihydro-1H-1,2,4-triazol-3-yl]propyl]phenoxy]propanoic acid | -0.34126 | 0.195817 | 0.571851 |
| [Eubacterium]_oxidoreducens_group | 1-(10H-Phenothiazin-2-yl)ethanone | 0.341196 | 0.195906 | 0.571851 |
| Rikenellaceae_RC9_gut_group | 16-hydroxy-6-hexadecenoic acid | -0.34119 | 0.195907 | 0.571851 |
| Treponema | 9-Octadecenal | -0.34117 | 0.195938 | 0.571851 |
| Granulicella | 5-Hydroxyindoxyl sulfate | -0.34105 | 0.196107 | 0.571956 |
| Subgroup_13 | Pelargonidin 3,5-di-(6-acetylglucoside) | -0.34097 | 0.196221 | 0.571956 |
| Subgroup_2 | AB-MECA | -0.34094 | 0.196268 | 0.571956 |
| Bryobacter | AM2201 N-(4-hydroxypentyl) metabolite | -0.34091 | 0.196303 | 0.571956 |
| IMCC26256 | N-Acetylneuraminic acid | 0.340866 | 0.196366 | 0.571956 |
| Flavonifractor | Neryl 8-methyldecanoate | 0.340847 | 0.196393 | 0.571956 |
| Granulicella | Ethynodiol | -0.34084 | 0.196405 | 0.571956 |
| Treponema | 5S-HETE di-endoperoxide | -0.34077 | 0.196504 | 0.571956 |
| Bryobacter | Adomeglivant | 0.340761 | 0.196513 | 0.571956 |
| Enterorhabdus | Glycerol 3-phosphate | 0.340744 | 0.196537 | 0.571956 |
| Gemella | D-Mannose | 0.340737 | 0.196547 | 0.571956 |
| Subgroup_2 | Lemborexant | -0.34073 | 0.196551 | 0.571956 |
| Monoglobus | 3-O-acetylecdysone 2-phosphate | -0.34073 | 0.196552 | 0.571956 |
| RF39 | Withaferin A | -0.3407 | 0.196601 | 0.571956 |
| Anaerostipes | Kuguacin E | -0.34067 | 0.196639 | 0.571956 |
| Acetatifactor | LysoPI(18:0/0:0) | 0.340611 | 0.196723 | 0.571956 |
| Jatrophihabitans | AM2201 N-(4-hydroxypentyl) metabolite | -0.34061 | 0.196727 | 0.571956 |
| Faecalibaculum | Kuguacin E | -0.34059 | 0.196747 | 0.571956 |
| Clostridioides | Dehydroascorbic acid | 0.340586 | 0.196758 | 0.571956 |
| Rikenellaceae_RC9_gut_group | 3-Hydroxybutyrylcarnitine | 0.340566 | 0.196786 | 0.571956 |
| Lachnospiraceae_UCG-006 | 2-(2-Thienylmethylene)-1,6-dioxaspiro[4.4]non-3-ene | 0.340479 | 0.196908 | 0.572191 |
| Anaerostipes | 1-O-(2R-methoxy-hexadecyl)-sn-glycerol | -0.34031 | 0.197137 | 0.57274 |
| bacteriap25 | 5S-HETE di-endoperoxide | -0.34012 | 0.19741 | 0.572773 |
| Treponema | Bis(2-propoxyethyl) 2,6-dimethyl-4-(3-nitrophenyl)-3,4-dihydropyridine-3,5-dicarboxylate | -0.34012 | 0.197414 | 0.572773 |
| Achromobacter | L-Homocystine | 0.340106 | 0.19743 | 0.572773 |
| Acetatifactor | 2-[(4-{2-[(4-Cyclohexylbutyl)(cyclohexylcarbamoyl)amino]ethyl}phenyl)sulfanyl]-2-methylpropanoic acid | -0.3401 | 0.197434 | 0.572773 |
| TM7 | 7-oxo-8-amino-nonanoic acid | -0.34005 | 0.197505 | 0.572773 |
| TM7 | Cortisol | -0.34004 | 0.197521 | 0.572773 |
| Haliangium | PIP(20:0/18:1(12Z)-2OH(9,10)) | 0.340033 | 0.197532 | 0.572773 |
| Clostridia_vadinBB60_group | 6,10,14-Trimethyl-5,9,13-pentadecatrien-2-one | -0.33999 | 0.197593 | 0.572773 |
| TM7 | N2-(3-Carboxy-2-hydroxy-1-oxopropyl)arginine | 0.339954 | 0.197644 | 0.572773 |
| Flavonifractor | 8-Amino-7-oxononanoic acid | -0.33995 | 0.197649 | 0.572773 |
| [Eubacterium]_oxidoreducens_group | 2-[4-(3-Hydroxypropyl)-2-methoxyphenoxy]-1,3-propanediol 1-xyloside | -0.33991 | 0.197706 | 0.572773 |
| Granulicella | 5,5-Diphenyl-2-thiohydantoin | 0.339895 | 0.197726 | 0.572773 |
| Muribaculum | 3-O-acetylecdysone 2-phosphate | -0.33986 | 0.197778 | 0.572773 |
| Subgroup_2 | D-Malic acid | 0.339759 | 0.197917 | 0.572773 |
| Lachnospiraceae_UCG-006 | Cichorioside K | -0.3397 | 0.197998 | 0.572773 |
| Muribaculum | FAD | 0.339676 | 0.198034 | 0.572773 |
| Anaerotruncus | 1-(O-alpha-D-glucopyranosyl)-(1,3S,25R)-hexacosanetriol | -0.33967 | 0.198043 | 0.572773 |
| Achromobacter | Dimethylaminoparthenolide | 0.339576 | 0.198174 | 0.572773 |
| Achromobacter | D-erythro-Sphingosine C-20 | -0.33957 | 0.198183 | 0.572773 |
| Enterorhabdus | Maraviroc | 0.339556 | 0.198203 | 0.572773 |
| Anaerostipes | S-[(3R,3As,6S,6aS)-3-nitrooxy-2,3,3a,5,6,6a-hexahydrofuro[3,2-b]furan-6-yl] ethanethioate | 0.339531 | 0.198237 | 0.572773 |
| Nitrospira | Mycobactins | -0.33952 | 0.198254 | 0.572773 |
| Achromobacter | Streptidine | 0.339431 | 0.198378 | 0.572773 |
| IMCC26256 | Skepinone-L | 0.339423 | 0.19839 | 0.572773 |
| Gemella | 20-HETE-d6 | 0.339419 | 0.198395 | 0.572773 |
| Monoglobus | N1-Methyl-2-pyridone-5-carboxamide | -0.33942 | 0.198396 | 0.572773 |
| Coprobacillus | 2-phospho-4-(cytidine 5'-diphospho)-2-C-methyl-D-erythritol | 0.339417 | 0.198398 | 0.572773 |
| Aquicella | Lemborexant | -0.33941 | 0.198411 | 0.572773 |
| Solobacterium | L-Arginine | 0.339403 | 0.198418 | 0.572773 |
| Coprobacillus | Biliverdin | -0.33935 | 0.19849 | 0.572773 |
| bacteriap25 | Pentadeca-3,5,7-trienedioylcarnitine | 0.339298 | 0.198565 | 0.572773 |
| Lachnospiraceae_UCG-006 | 1-Octadecene | -0.33929 | 0.198579 | 0.572773 |
| Anaerotruncus | N-Undecanoylglycine | -0.33924 | 0.198652 | 0.572773 |
| Faecalibaculum | Hexadecadienylcarnitine | 0.339236 | 0.198653 | 0.572773 |
| Aquicella | Benzyl gentiobioside | 0.339225 | 0.198669 | 0.572773 |
| Achromobacter | Fluazifop | 0.339213 | 0.198685 | 0.572773 |
| Solobacterium | N2-(3-Carboxy-2-hydroxy-1-oxopropyl)arginine | 0.339213 | 0.198686 | 0.572773 |
| Intestinimonas | Kaltostat | 0.339206 | 0.198696 | 0.572773 |
| Anaerostipes | alpha-D-Galactopyranuronosyl-(1->4)-alpha-D-galactopyranuronosyl-(1->4)-D-galacturonic acid | 0.339178 | 0.198735 | 0.572773 |
| Subgroup_13 | Dehydroascorbic acid | 0.339062 | 0.198898 | 0.573123 |
| Subgroup_2 | (R)-4A-(Ethoxymethyl)-1-(4-fluorophenyl)-6-((4-(trifluoromethyl)phenyl)sulfonyl)-4,4a,5,6,7,8-hexahydro-1H-pyrazolo[3,4-g]isoquinoline | -0.33899 | 0.198994 | 0.573283 |
| Flavonifractor | Ethynodiol | -0.33882 | 0.199235 | 0.573766 |
| TRA3-20 | Xanthinol | 0.338802 | 0.199265 | 0.573766 |
| Anaerotruncus | S-[(3R,3As,6S,6aS)-3-nitrooxy-2,3,3a,5,6,6a-hexahydrofuro[3,2-b]furan-6-yl] ethanethioate | 0.338764 | 0.199319 | 0.573766 |
| RF39 | S-Adenosylhomocysteine | 0.33867 | 0.199451 | 0.573766 |
| Achromobacter | Cichorioside K | -0.33865 | 0.199476 | 0.573766 |
| Anaerotruncus | Carissanol | -0.33864 | 0.199488 | 0.573766 |
| Enterorhabdus | Kaltostat | 0.33862 | 0.199521 | 0.573766 |
| Coprobacillus | N-(N-(3-Amino-3-carboxypropyl)-3-amino-3-carboxypropyl)azetidine-2-carboxylic acid | 0.338593 | 0.19956 | 0.573766 |
| Aquicella | N-Acetyl-L-methionine | 0.338589 | 0.199565 | 0.573766 |
| RF39 | Phosphohydroxypyruvic acid | -0.33857 | 0.199592 | 0.573766 |
| Jatrophihabitans | Pelargonidin 3,5-di-(6-acetylglucoside) | -0.33851 | 0.199674 | 0.573766 |
| Anaerotruncus | benfluorex | 0.338488 | 0.199708 | 0.573766 |
| bacteriap25 | Lamivudine-monophosphate | -0.33848 | 0.199725 | 0.573766 |
| Enterorhabdus | D-Malic acid | 0.338471 | 0.199732 | 0.573766 |
| Acidothermus | 1-Hydroxy-2-naphthoic acid | 0.338388 | 0.19985 | 0.573943 |
| RF39 | 9-Octadecenal | -0.33837 | 0.199875 | 0.573943 |
| Coprobacillus | PE(O-20:0/0:0) | -0.33834 | 0.199916 | 0.573943 |
| Flavonifractor | PC(16:1(9Z)/17:1(9Z)) | -0.33831 | 0.199957 | 0.573945 |
| Solobacterium | OKOHA-PA | -0.33825 | 0.200048 | 0.574086 |
| Flavonifractor | Adenosine monophosphate | -0.33822 | 0.200089 | 0.574086 |
| Treponema | Oleic acid | -0.33817 | 0.200154 | 0.574086 |
| Nitrospira | Oliceridine | -0.33816 | 0.20017 | 0.574086 |
| Enterococcus | LysoPE(20:4(5Z,8Z,11Z,14Z)/0:0) | -0.3381 | 0.200253 | 0.574187 |
| Monoglobus | 3-Methyl-3-butenyl apiosyl-(1->6)-glucoside | 0.338079 | 0.200286 | 0.574187 |
| Aquicella | Terbufos | 0.338051 | 0.200327 | 0.574187 |
| Treponema | L-Glutamine | 0.337977 | 0.200431 | 0.574328 |
| Muribaculum | Oliceridine | -0.33796 | 0.200458 | 0.574328 |
| Bryobacter | Palmitoylcarnitine | 0.337897 | 0.200544 | 0.574452 |
| Subgroup_2 | Tridecanoylcarnitine | 0.337801 | 0.200681 | 0.574452 |
| Jatrophihabitans | 2-[(4-{2-[(4-Cyclohexylbutyl)(cyclohexylcarbamoyl)amino]ethyl}phenyl)sulfanyl]-2-methylpropanoic acid | -0.33779 | 0.200698 | 0.574452 |
| Anaerostipes | Bacillamidin C | -0.33775 | 0.200747 | 0.574452 |
| Monoglobus | 2-Cyclotetradecen-1-one | -0.33775 | 0.200751 | 0.574452 |
| Pseudolabrys | PC(20:4(5Z,8Z,11Z,14Z)-OH(16R)/2:0) | 0.337732 | 0.200779 | 0.574452 |
| Alcaligenes | Oleic acid | -0.3377 | 0.200823 | 0.574452 |
| Lachnospiraceae_UCG-006 | N-Phenyl-p-phenylenediamine | -0.3377 | 0.200828 | 0.574452 |
| Bryobacter | Oliceridine | -0.33762 | 0.200933 | 0.57457 |
| Solobacterium | 25-Hydroxyvitamin D3-26,23-lactol | -0.33761 | 0.20095 | 0.57457 |
| Nitrospira | Docosatrienoic acid | 0.337548 | 0.20104 | 0.574709 |
| Alcaligenes | PPM-18 | 0.33746 | 0.201164 | 0.574946 |
| Enterococcus | Allitridin | 0.337392 | 0.201261 | 0.575064 |
| IMCC26256 | 1-O-Galloylglycerol | 0.337347 | 0.201324 | 0.575064 |
| Pseudolabrys | Amobarbital | 0.337339 | 0.201336 | 0.575064 |
| TM7 | PC(20:5(5Z,8Z,11Z,14Z,17Z)/PGJ2) | -0.33729 | 0.201408 | 0.575064 |
| bacteriap25 | Amobarbital | 0.337288 | 0.201409 | 0.575064 |
| Achromobacter | 4Z,7-octadienoic acid | -0.33726 | 0.201452 | 0.57507 |
| Alcaligenes | Adenosine monophosphate | -0.3372 | 0.201534 | 0.575183 |
| Treponema | 4-Chloro-2-nitrobenzylalcohol | -0.33717 | 0.201573 | 0.575183 |
| [Eubacterium]_oxidoreducens_group | OKOHA-PA | -0.33713 | 0.201631 | 0.575231 |
| Intestinimonas | Adenosine monophosphate | -0.33709 | 0.201691 | 0.575284 |
| Enterorhabdus | 5'-Methylthioadenosine | 0.336983 | 0.201842 | 0.575455 |
| Anaerostipes | Ricinoleic acid | -0.33698 | 0.201848 | 0.575455 |
| Anaerostipes | 20-HETE-d6 | 0.336961 | 0.201873 | 0.575455 |
| Clostridioides | 2-Phenylethyl octanoate | -0.33682 | 0.202072 | 0.575882 |
| Monoglobus | Zidebactam | 0.336799 | 0.202105 | 0.575882 |
| Bryobacter | N,N'-Diethylthiourea | 0.336585 | 0.202409 | 0.576633 |
| Acetatifactor | Asparaginyl-Proline | 0.336476 | 0.202564 | 0.576957 |
| Achromobacter | Lemborexant | -0.33642 | 0.202648 | 0.577081 |
| Aquicella | N-Myristoyl Asparagine | -0.33635 | 0.202741 | 0.577099 |
| Alcaligenes | 2-Deoxy-2,3-dehydro-n-acetyl-neuraminic acid | 0.33634 | 0.202758 | 0.577099 |
| Lachnospiraceae_UCG-006 | Lemborexant | -0.3363 | 0.202813 | 0.577099 |
| TRA3-20 | Bisazobiphenyl | 0.336214 | 0.202939 | 0.577099 |
| Acetatifactor | 3-(3-(Pyridin-3-yl)-1,2,4-oxadiazol-5-yl)benzonitrile | 0.336197 | 0.202962 | 0.577099 |
| Coprobacillus | Phytal | -0.33616 | 0.203008 | 0.577099 |
| Anaerotruncus | 3-Hydroxyoctadecanoylcarnitine | 0.336118 | 0.203075 | 0.577099 |
| Enterococcus | Medicagol | 0.336062 | 0.203156 | 0.577099 |
| Intestinimonas | Peperomin E | 0.33606 | 0.203158 | 0.577099 |
| Achromobacter | PC(16:0/16:0) | 0.336059 | 0.20316 | 0.577099 |
| Anaerostipes | thiamphenicol | 0.335935 | 0.203336 | 0.577099 |
| TM7 | FAD | 0.335925 | 0.203352 | 0.577099 |
| Solobacterium | Tuberonic acid glucoside | -0.3359 | 0.203386 | 0.577099 |
| Nitrospira | PC(20:4(5Z,8Z,11Z,14Z)-OH(16R)/2:0) | 0.335871 | 0.203429 | 0.577099 |
| Lachnospiraceae_UCG-006 | Iguratimod | -0.33586 | 0.203449 | 0.577099 |
| Acidothermus | 3-Hydroxybutyrylcarnitine | 0.335854 | 0.203453 | 0.577099 |
| Subgroup_2 | Pelargonidin 3,5-di-(6-acetylglucoside) | -0.33585 | 0.203456 | 0.577099 |
| Clostridioides | Aspartyl-Alanine | 0.335813 | 0.203511 | 0.577099 |
| Flavonifractor | PC(18:1(17Z)/18:1(17Z)) | 0.33581 | 0.203515 | 0.577099 |
| bacteriap25 | 1-Ethoxymethyl-5-fluorouracil | 0.335729 | 0.203632 | 0.577099 |
| Bryobacter | Creatine | 0.335708 | 0.203662 | 0.577099 |
| Faecalibaculum | 5'-Methylthioadenosine | 0.335703 | 0.203669 | 0.577099 |
| Aquicella | Dolichyl b-D-glucosyl phosphate | -0.33568 | 0.203706 | 0.577099 |
| Anaerostipes | 9-Octadecenal | -0.33566 | 0.203734 | 0.577099 |
| Enterococcus | (2S)-3-[3-[(4-Carbamimidoylbenzoyl)amino]propanoylamino]-2-[(4-ethylphenyl)sulfonylamino]propanoic acid | 0.335657 | 0.203734 | 0.577099 |
| Coprobacillus | 3-O-acetylecdysone 2-phosphate | -0.33558 | 0.203845 | 0.577099 |
| Flavonifractor | 16,17-Dihydro-16alpha,17-dihydroxygibberellin A4 17-glucoside | -0.33552 | 0.203927 | 0.577099 |
| Aquicella | thiamphenicol | 0.335477 | 0.203992 | 0.577099 |
| Anaerotruncus | N-Phenyl-p-phenylenediamine | -0.33548 | 0.203992 | 0.577099 |
| bacteriap25 | Nopalinic acid | 0.335446 | 0.204037 | 0.577099 |
| Acetatifactor | L-Carnitine | 0.335436 | 0.204051 | 0.577099 |
| Enterorhabdus | 1-Hydroxy-2-naphthoic acid | 0.335418 | 0.204076 | 0.577099 |
| Acetatifactor | 6-Hydroxytricetin 5-rhamnoside | 0.335412 | 0.204085 | 0.577099 |
| Aquicella | 3,4-Dimethyl-5-propyl-2-furanpentadecanoic acid | -0.33538 | 0.204128 | 0.577099 |
| TRA3-20 | PC(16:0/16:0) | 0.335376 | 0.204137 | 0.577099 |
| Aquicella | Octadecanamide | -0.33535 | 0.204177 | 0.577099 |
| Anaerotruncus | N-Acetylneuraminic acid | 0.335315 | 0.204224 | 0.577099 |
| Alcaligenes | Fluazifop | 0.335305 | 0.204238 | 0.577099 |
| Treponema | Phenylalanyl-Glycine | 0.335245 | 0.204326 | 0.577099 |
| bacteriap25 | Alacepril | -0.33524 | 0.204327 | 0.577099 |
| Haliangium | 16-hydroxy-6-hexadecenoic acid | -0.33524 | 0.204335 | 0.577099 |
| Clostridioides | N-gamma-L-Glutamyl-D-alanine | 0.335119 | 0.204505 | 0.577253 |
| IMCC26256 | PI(20:3(8Z,11Z,14Z)/0:0) | 0.335106 | 0.204525 | 0.577253 |
[truncated: 734,115 more chars]
