# Supplementary material for: Disrupted Tuzzerella abundance and impaired l-glutamine levels induce Treg accumulation in ovarian endometriosis: a comprehensive multi-omics analysis
Source: Metabolomics. 2024 Feb 29;20(2):32. doi: 10.1007/s11306-023-02072-0 (PMC10904428; doi:10.1007/s11306-023-02072-0)
Supplement: Supplementary file 2 — Supplementary file2 (PDF 46585 KB) [file 11306_2023_2072_MOESM2_ESM.pdf]

ID	m/z	Retention ti	Ion mode	Metabolites	HMDB	METLIN	InChIKey	Super Class	Class	Sub Class
10.63_540.3	540.33054	10.6260167	neg	LysoPC(0:0/	HMDB02402	40340	NEGQHKSY	Lipids and lip	Glycerophos	Glycerophos
1.08_612.15	613.158663	1.07531667	pos	Oxidized glut	HMDB00033	322837	YPZRWBKM	Organic acids	Carboxylic ac	Amino acids,
10.06_538.3	538.315184	10.0622	neg	LysoPC(16:1	HMDB00103	40288	LFUDDCMN	Lipids and lip	Glycerophos	Glycerophos
11.42_481.3	480.309265	11.42155	neg	LysoPE(0:0/1	HMDB00111	61992	KIHAGWUU	Lipids and lip	Glycerophos	Glycerophos
10.57_453.2	452.278194	10.5716833	neg	LysoPE(0:0/1	HMDB00114	62261	CKPBBEOJH	Lipids and lip	Glycerophos	Glycerophos
1.08_169.07	187.107606	1.07531667	pos	Pyridoxine	HMDB0000239		LXNHXLLTX	Organoheter	Pyridines and	Pyridoxines
1.07_148.03	147.029723	1.06741667	neg	L-2-Hydroxy	HMDB00006	4151	HWXBTNAV	Organic acids	Hydroxy acid	Short-chain h
10.34_564.3	564.330745	10.3358333	neg	LysoPC(0:0/	HMDB0061700		LSUXCWJO	Lipids and lip	Glycerophos	Glycerophos
10.84_566.3	566.346156	10.8434333	neg	LysoPC(0:0/	HMDB00617	40348	SULIDBRAX	Lipids and lip	Glycerophos	Glycerophos
0.67_154.06	154.062064	0.6745	neg	L-Histidine	HMDB00001	21	HNDVDQJC	Organic acids	Carboxylic ac	Amino acids,
0.81_173.00	173.008905	0.81161667	neg	Dehydroasco	HMDB00012	342	SBJKKFFYI	Organoheter	Lactones	Gamma buty
1.25_283.09	282.084172	1.2521	neg	Guanosine	HMDB00001	87	NYHBQMYG	Nucleosides,	Purine nucle	Unclassified
0.65_174.11	175.119016	0.6518	pos	L-Arginine	HMDB00005	5502	ODKSFYDX	Organic acids	Carboxylic ac	Amino acids,
10.86_521.3	522.355035	10.8590333	pos	LysoPC(18:1	HMDB00028	40298	YAMUFBLW	Lipids and lip	Glycerophos	Glycerophos
3.80_784.15	784.15039	3.80496667	neg	FAD	HMDB00012	6106	VWWQXMA	Nucleosides,	Flavin nucle	Unclassified
5.28_285.19	286.20095	5.28283333	pos	Octenoyl-L-c	HMDB0240723		SMMAHUIW	Organic acids	Keto acids a	Medium-chai
14.60_401.3	401.34102	14.60285	pos	7a-Hydroxy-	HMDB00019	6419	IOIZWEJGG	Lipids and lip	Steroids and	Cholestane s
9.11_581.24	581.240766	9.1134	neg	Biliverdin	HMDB00010	5938	QBUVFDKT	Organoheter	Tetrapyrroles	Bilirubins
1.07_168.02	167.020781	1.06741667	neg	Uric acid	HMDB00002	88	LEHOTFFK	Organoheter	Imidazopyri	Purines and
1.07_244.06	243.061981	1.06741667	neg	Uridine	HMDB00002	90	DRTQHJPV	Nucleosides,	Pyrimidine n	Unclassified
0.70_300.04	300.048717	0.70456667	neg	N-Acetyl-D-G	HMDB00010	5975	BRGMHAYQ	Organic oxy	Organooxyge	Carbohydrate
0.63_338.98	338.988546	0.62835	neg	Fructose-1,6	HMDB0244240		RNBGYGVW	Organic oxy	Organooxyge	Carbohydrate
1.08_219.07	218.066621	1.08208333	neg	O-Succinyl-L	HMDB0255868		GNISQJGXJ	Organic acids	Carboxylic ac	Amino acids,
1.08_123.05	123.055415	1.07531667	pos	Niacinamide	HMDB00014	1497	DFPAKSUC	Organoheter	Pyridines and	Pyridinecarb
0.70_147.05	148.060346	0.69861667	pos	L-Glutamic a	HMDB00001	19	WHUUTDBJ	Organic acids	Carboxylic ac	Amino acids,
11.56_568.3	568.361759	11.5642333	neg	2-Lysophosp	HMDB0258493		IHNKQIMGV	Lipids and lip	Glycerophos	Glycerophos
0.77_172.08	173.092083	0.7729	pos	Glycyl-D-prol	HMDB02528	4185	KZNQNBZM	Organic acids	Carboxylic ac	Amino acids,
1.32_103.03	103.039911	1.32371667	neg	3-Hydroxybu	HMDB00000	35598	WHBMMWS	Organic acids	Hydroxy acid	Beta hydroxy
0.72_185.99	184.985412	0.71928333	neg	3-Phosphogl	HMDB0000807		OSJPPGNT	Organic oxy	Organooxyge	Carbohydrate
0.80_244.06	243.062053	0.79596667	neg	Pseudouridin	HMDB00007	5734	PTJWIQPHV	Nucleosides,	Nucleoside a	Unclassified
1.07_151.02	151.025882	1.06741667	neg	Xanthine	HMDB00002	82	LRFVTYWO	Organoheter	Imidazopyri	Purines and
0.72_90.031	135.030131	0.71928333	neg	D-Lactic acid	HMDB00013	6150	JVTAAEKCZ	Organic acids	Hydroxy acid	Alpha hydrox

1.47_144.06	144.066464	1.46783333	neg	4-Acetamido	HMDB00036	44719	UZTFMUBK	Organic acid	Carboxylic a	Amino acids,
0.81_145.01	145.0141	0.81161667	neg	Oxoglutaric a	HMDB00002	5218	KPGXRSRH	Organic acid	Keto acids a	Gamma-keto
11.08_522.2	522.283537	11.07825	neg	1-Oleoylglyc	HMDB0061694		JZWNYZVV	Lipids and lip	Glycerophos	Glycerophos
11.76_594.3	594.377708	11.7562167	neg	LysoPC(20:1	HMDB00103	61701	YYSFWGQA	Lipids and lip	Glycerophos	Glycerophos
0.70_147.07	147.076461	0.69861667	pos	L-Glutamine	HMDB00006	5614	ZDXPYRJPN	Organic acid	Carboxylic a	Amino acids,
1.10_180.06	180.066397	1.09681667	neg	L-Tyrosine	HMDB00001	34	OUYCCCAS	Organic acid	Carboxylic a	Amino acids,
0.75_322.04	322.044538	0.74898333	neg	Cytidine mon	HMDB00000	3452	IERHLVCPS	Nucleosides,	Pyrimidine n	Pyrimidine ri
10.90_320.2	319.227578	10.8978167	neg	16(R)-HETE	HMDB00046	36321	JEKNPVYF	Lipids and lip	Fatty Acyls	Eicosanoids
6.23_405.19	405.191727	6.23498333	neg	19-Oic-deox	HMDB0012613		RUPOMAOZ	Lipids and lip	Steroids and	Hydroxyster
6.97_392.19	393.206889	6.96501667	pos	Dexamethas	HMDB00153	537	UREBDLICK	Lipids and lip	Steroids and	Hydroxyster
1.14_305.09	305.097839	1.13681667	pos	N-Acetylasp	HMDB0001067		OPVPGKGA	Organic acid	Carboxylic a	Amino acids,
3.95_293.11	293.114437	3.95231667	neg	gamma-Glut	HMDB00005	5573	XHHOHZPN	Organic acid	Carboxylic a	Amino acids,
1.35_131.09	132.10195	1.34996667	pos	L-Alloisoleuc	HMDB00005	315303	AGPKZVBTJ	Organic acid	Carboxylic a	Amino acids,
0.76_276.09	277.102883	0.75766667	pos	gamma-Glut	HMDB00117	62460	OWQDWQK	Organic acid	Carboxylic a	Amino acids,
1.06_189.12	189.123308	1.06053333	pos	N6-Acetyl-L-	HMDB00002	5216	DTERQYGM	Organic acid	Carboxylic a	Amino acids,
13.38_283.2	283.262691	13.38305	pos	Vaccenic aci	HMDB00032	3407	UWHZIFQP	Lipids and lip	Fatty Acyls	Fatty acids a
0.70_126.02	126.022105	0.69861667	pos	Taurine	HMDB00002	31	XOAAWQZA	Organic acid	Organic sulf	Organosulfo
0.70_258.11	258.109968	0.69861667	pos	Glycerophos	HMDB00000	370	SUHOQUVV	Lipids and lip	Glycerophos	Glycerophos
1.71_296.09	296.099843	1.7074	neg	1-Methylgua	HMDB00015	6324	UTAIYTHAJ	Nucleosides,	Purine nucle	Unclassified
10.57_527.3	528.308223	10.5732333	pos	LysoPE(0:0/2	HMDB00114	62283	KZXGPOLA	Lipids and lip	Glycerophos	Glycerophos
10.28_476.2	476.278176	10.2812167	neg	LysoPE(0:0/1	HMDB00114	62265	SVRBKLJID	Lipids and lip	Glycerophos	Glycerophos
1.44_275.07	275.075089	1.43671667	pos	Deoxyinosin	HMDB00000	3383	VGONTNSX	Nucleosides,	Purine nucle	Purine 2'-de
9.92_219.17	219.174117	9.92105	pos	Calamuseno	HMDB00382	92934	WJQUHMZH	Lipids and lip	Prenol lipids	Sesquiterpe
4.21_165.01	165.019148	4.20936667	neg	Phthalic acid	HMDB00021	6489	XNGIFLGAS	Benzenoids	Benzene and	Benzoic acid
10.79_480.3	480.308042	10.7875833	pos	LysoPE(18:1	HMDB00115	40778	PYVRVRFVL	Lipids and lip	Glycerophos	Glycerophos
0.62_112.08	112.087257	0.6215	pos	Histamine	HMDB00008	68	NTYJJOPFI	Organic nitro	Organonitro	Amines
0.65_187.16	188.175859	0.6518	pos	N1-Acetylsp	HMDB00012	3323	MQTAVJHIC	Organic acid	Carboximidic	Carboximidic
0.73_549.16	549.167335	0.7342	neg	Galactotrios	HMDB00299	86590	DBTMGCOV	Organic oxy	Organooxyg	Carbohydrat
0.69_132.03	132.030055	0.68961667	neg	L-Aspartic ac	HMDB00001	5206	CKLJMWTZI	Organic acid	Carboxylic a	Amino acids,
0.71_132.07	132.076786	0.71368333	pos	Creatine	HMDB00000	7	CVSVTCOR	Organic acid	Carboxylic a	Amino acids,
0.73_115.06	116.070858	0.72825	pos	L-Proline	HMDB00001	29	ONIBWKKT	Organic acid	Carboxylic a	Amino acids,
11.18_523.2	524.298055	11.1809167	pos	LysoPS(18:1	HMDB02406	40829	JZWNYZVV	Lipids and lip	Glycerophos	Glycerophos
2.70_218.10	218.103119	2.7013	neg	Pantothenic	HMDB00002	241	GHOKWGT	Organic oxy	Organooxyg	Alcohols and

1.08_385.128	385.128541	1.07531667	pos	S-Adenosylh	HMDB00009	296	ZJUKTBDSG	Organoheter	Lactones	Gamma buty
1.17_314.12	314.123397	1.1705	pos	Arginylthreor	HMDB0028719		XNSKSTRG	Organic acid	Carboxylic ac	Amino acids,
10.72_323.2	323.222417	10.7169	neg	Punicic acid	HMDB0030963		CUXYLFPM	Lipids and lip	Fatty Acyls	Lineolic acids
10.84_312.2	295.226327	10.8409	pos	octadecened	HMDB0304442		YDPSQMGC	Lipids and lip	Fatty Acyls	Fatty acids a
4.76_259.17	260.185437	4.75875	pos	Hexanoylcar	HMDB0000756		VVPRQWTY	Lipids and lip	Fatty Acyls	Fatty acid es
0.72_308.09	308.09862	0.71928333	neg	Glucose-6-gl	HMDB0252776		NQJPQZNY	Organic acid	Carboxylic ac	Amino acids,
10.03_279.1	279.196412	10.0257	neg	12-Hydroxyh	HMDB0244447		KUKJHGXX	Lipids and lip	Fatty Acyls	Fatty acids a
10.05_398.3	398.326101	10.0490667	pos	trans-Hexad	HMDB00063	58388	SOYNLLDA	Lipids and lip	Fatty Acyls	Fatty acid es
10.23_492.3	492.333034	10.2257833	neg	Arachidonoyl	HMDB00064	58409	RBFQHRAL	Lipids and lip	Fatty Acyls	Fatty acid es
3.29_231.14	232.154367	3.28915	pos	Isobutyryl-L-	HMDB00007	5704	LRCNOZRC	Lipids and lip	Fatty Acyls	Fatty acid es
10.91_281.2	281.247029	10.9125667	pos	Ricinoleic aci	HMDB0034297		WBHHMMIM	Lipids and lip	Fatty Acyls	Fatty acids a
3.70_280.12	280.129097	3.69811667	pos	Asparaginyl-I	HMDB00287	23675	OMSMPWHI	Organic acid	Carboxylic ac	Amino acids,
3.75_275.17	276.180646	3.75006667	pos	4-Hydroxyhe	HMDB0241665		JAXWMXMF	Lipids and lip	Fatty Acyls	Fatty acid es
1.49_219.07	219.077335	1.4863	neg	Tyr-Gly	HMDB0259337		HPYDSVWY	Organic acid	Carboxylic ac	Amino acids,
10.23_543.3	544.339404	10.2295	pos	LysoPC(0:0/2	HMDB00616	46690	IGJKYDBBI	Lipids and lip	Glycerophos	Glycerophos
0.70_203.15	203.150198	0.69861667	pos	Symmetric di	HMDB00033	44873	HVPFXCBJH	Organic acid	Carboxylic ac	Amino acids,
0.78_134.02	133.014086	0.7799	neg	D-Malic acid	HMDB00315	63096	BJEPYKJPY	Organic acid	Hydroxy acid	Beta hydroxy
1.56_283.06	283.068358	1.56021667	neg	Xanthosine	HMDB00002	3408	UBORTCND	Nucleosides,	Purine nucle	Unclassified
12.62_280.2	281.247141	12.6205	pos	Dihomolinole	HMDB00618	34800	GKJZMAHZ	Lipids and lip	Fatty Acyls	Lineolic acids
1.04_118.08	118.086502	1.0442	pos	Norvaline	HMDB00137	85259	SNDPXSYFE	Organic acid	Carboxylic ac	Amino acids,
1.07_135.03	135.031072	1.06741667	neg	Hypoxanthine	HMDB00001	83	FDGQSTZJE	Organoheter	Imidazopyrim	Purines and
3.14_311.12	312.13016	3.1446	pos	N2,N2-Dimet	HMDB00048	7086	RSPURTUNI	Nucleosides,	Purine nucle	Unclassified
1.25_335.06	335.060904	1.2521	neg	Glucose pyru	HMDB0252788		KFQNTCAR	Organic oxyg	Organooxyge	Carbohydrate
1.29_130.05	130.050794	1.2875	neg	N-Acetyl-L-al	HMDB00007	5733	KTHDTJVBE	Organic acid	Carboxylic ac	Amino acids,
9.61_484.32	484.328074	9.60636667	neg	(12Z,15Z)-10	HMDB0241545		PRXCKJGVE	Lipids and lip	Fatty Acyls	Fatty acid es
9.83_372.31	372.310222	9.83063333	pos	3-Methyltride	HMDB0240825		GYBMNPBD	Lipids and lip	Fatty Acyls	Fatty acid es
0.62_161.12	161.128535	0.6215	pos	Isoputreanine	HMDB00060	357148	JUBNBYBUF	Organic acid	Carboxylic ac	Amino acids,
10.21_423.3	424.341609	10.2120667	pos	O-Linoleoylca	HMDB0240780		MJLXQSQY	Lipids and lip	Fatty Acyls	Fatty acid es
0.69_140.01	140.011691	0.68961667	neg	O-Phosphoe	HMDB00002	5233	SUHOOTKU	Organic acid	Organic phos	Phosphate e
1.25_268.08	267.073348	1.2521	neg	Inosine	HMDB00001	84	UGQMRVRM	Nucleosides,	Purine nucle	Unclassified
10.73_219.1	219.174067	10.7334833	pos	(+)-4,11-Eud	HMDB00370	92039	KUFXJZXMV	Lipids and lip	Prenol lipids	Sesquiterper
10.79_478.2	478.293685	10.7894833	neg	LysoPE(0:0/1	HMDB00114	62263	CZYOFWNC	Lipids and lip	Glycerophos	Glycerophos
12.89_330.2	329.248402	12.8930167	neg	Docosapenta	HMDB00019	6412	AVKOENOB	Lipids and lip	Fatty Acyls	Fatty acids a

0.78_306.07	306.076359	0.7799	neg	Glutathione	HMDB00001	44	RWSXRVCM	Organic acid	Carboxylic a	Amino acids,
12.59_264.2	282.278568	12.5856	pos	(±)-(Z)-2-(5-T	HMDB00375	92402	ITFDRFJPW	Organic oxyg	Organooxyge	Carbonyl cor
0.71_114.06	114.066531	0.71368333	pos	Creatinine	HMDB00005	8	DDRJAANPI	Organic acid	Carboxylic a	Amino acids,
0.73_161.06	162.076176	0.72825	pos	Aminoadipic	HMDB00005	3271	OYIFNHCXN	Organic acid	Carboxylic a	Amino acids,
0.73_342.11	387.114489	0.7342	neg	Alpha-Lactos	HMDB00001	984806	GUBGYTABI	Organic oxyg	Organooxyge	Carbohydrate
10.43_444.3	444.333045	10.42685	neg	Palmitoylcarr	HMDB00002	5231	XOMRRQXK	Lipids and lip	Fatty Acyls	Fatty acid es
1.07_150.04	150.041805	1.06741667	neg	8-Hydroxyad	HMDB00005	5527	RGKBRPAA	Organoheter	Imidazopyrim	Purines and
9.34_460.26	460.268924	9.34135	pos	Cinobufagin	HMDB0250266		SCULJPGY	Lipids and lip	Steroids and	Steroid lacto
4.06_295.16	295.165492	4.05526667	pos	Tyrosyl-Isole	HMDB0029108		QJKMCQRF	Organic acid	Carboxylic a	Amino acids,
0.70_119.05	120.065683	0.69861667	pos	4-Amino-3-hy	HMDB00618	313521	YQGDEPYY	Organic acid	Carboxylic a	Amino acids,
3.31_246.12	247.1289	3.30741667	pos	Aspartyl-Leu	HMDB0028757		ZVDPYSVO	Organic acid	Carboxylic a	Amino acids,
7.43_404.26	404.265294	7.43236667	neg	3-hydroxydo	HMDB0061638		ULWDPUHF	Lipids and lip	Fatty Acyls	Fatty acid es
0.71_129.06	129.06597	0.71368333	pos	3-ureido-isob	HMDB0304156		PHENTZNAI	Organic acid	Organic carb	Ureas
1.07_129.04	128.035168	1.06741667	neg	Pyroglutamic	HMDB00002	314555	ODHCTXKN	Organic acid	Carboxylic a	Amino acids,
0.62_132.09	133.097269	0.6215	pos	Ornithine	HMDB00002	45121	AHLPHDHH	Organic acid	Carboxylic a	Amino acids,
1.07_174.01	173.008849	1.06741667	neg	Dehydroascc	HMDB0062706		JMMUPWYG	Organoheter	Dihydrofuran	Furanones
1.32_239.10	239.103185	1.31801667	pos	Tyrosylglycin	HMDB00291	23898	HPYDSVWY	Organic acid	Carboxylic a	Amino acids,
1.61_296.12	296.124247	1.61191667	pos	Tyrosyl-Aspa	HMDB00291	85992	ONWMQOR	Organic acid	Carboxylic a	Amino acids,
11.86_378.2	379.283727	11.8596167	pos	MG(0:0/20:4	HMDB00115	62332	JCKNHZDBF	Lipids and lip	Endocannabi	Unclassified
11.93_303.2	303.231299	11.9300167	pos	Crispane	HMDB00386	93349	NXPBEWQM	Lipids and lip	Prenol lipids	Monoterpenc
3.68_181.05	181.050407	3.67736667	neg	Hydroxypher	HMDB00007	5723	JVGVDSSU	Phenylpropa	Phenylpropa	Unclassified
9.77_460.32	460.328227	9.77068333	neg	3-hydroxyhe	HMDB0061642		XKAZIAFZA	Lipids and lip	Fatty Acyls	Fatty acid es
10.41_450.3	450.357244	10.41135	pos	23S,25,26-Tr	HMDB0060134		GDIBDBUYV	Lipids and lip	Steroids and	Vitamin D an
11.74_464.3	464.314211	11.7378333	neg	LysoPE(P-18	HMDB02405	46720	KACDZDUL	Lipids and lip	Glycerophos	Glycerophos
10.21_448.3	448.341155	10.2120667	pos	(8Z,11Z,13E,	HMDB0241584		NZRDWBUU	Lipids and lip	Fatty Acyls	Fatty acid es
2.04_207.07	207.077269	2.03931667	neg	Formyl-5-hyc	HMDB00129	63540	CKAXPTWY	Organic oxyg	Organooxyge	Carbonyl cor
4.61_342.23	342.238736	4.61381667	pos	Diprotin A	HMDB0251473		JNTMAZFVY	Organoheter	Pyrroles	Substituted p
0.74_243.08	244.092567	0.74288333	pos	Cytidine	HMDB00000	3376	UHDGCWIW	Nucleosides,	Pyrimidine nu	Unclassified
3.72_280.10	281.113178	3.71513333	pos	Phenylalanyl	HMDB00289	23739	HWMGTNO	Organic acid	Carboxylic a	Amino acids,
10.66_295.2	295.227588	10.66295	neg	alpha-Dimorp	HMDB00046	45662	NPDSHTNEI	Lipids and lip	Fatty Acyls	Lineolic acids
10.72_363.2	363.253976	10.7169	neg	16,16-Dimeth	HMDB0244726		RQOFITYRY	Lipids and lip	Fatty Acyls	Eicosanoids
4.96_204.06	204.066321	4.96071667	neg	Indolelactic a	HMDB0000671		XGILAAMKE	Organoheter	Indoles and	Indolyl carbo
1.78_131.03	131.03479	1.78133333	neg	Glutaric acid	HMDB00006	3254	JFCQEDHGI	Organic acid	Carboxylic a	Dicarboxylic

10.79_321.24	321.243082	10.7894833	neg	5a-Androstar	HMDB00058	57827	DJTOLSNIK.	Lipids and lip	Steroids and	Androstane s
12.36_309.24	309.241844	12.3586333	pos	2-[2-(4-Nonyl	HMDB02557	332346	BLXVTZPGE	Benzenoids	Phenol ether	Unclassified
0.81_174.04	174.040525	0.81161667	neg	4-Oxo-L-proli	HMDB0246561		HFXAFXVXF	Organic acid	Carboxylic ac	Amino acids,
1.07_334.10	333.093848	1.06741667	neg	(S)-a-Amino-	HMDB00294	86247	OURYTICKC	Organic oxyg	Organooxyge	Carbohydrate
9.78_467.30	468.307935	9.77705	pos	PC(0:0/14:0)		40339	IGZPHNNYP	Lipids and lip	Glycerophos	Glycerophos
0.71_309.10	310.113028	0.71368333	pos	N-Acetylneur	HMDB00002	5764	SQVRNKJH\	Organic oxyg	Organooxyge	Carbohydrate
10.50_397.3	397.309626	10.50225	pos	Diosgenin	HMDB0242671		WQLVFSAG	Lipids and lip	Prenol lipids	Triterpenoids
3.82_190.05	190.054185	3.82341667	neg	N-Acetyl-L-m	HMDB0011745		XUYPXLNMI	Organic acid	Carboxylic ac	Amino acids,
4.13_263.13	263.139228	4.12765	pos	Phenylalanyl	HMDB0011177		WEQJQNW)	Organic acid	Carboxylic ac	Amino acids,
10.01_452.2	452.276773	10.0126833	pos	LysoPE(0:0/1	HMDB00114	62262	MAFRHGZTI	Lipids and lip	Glycerophos	Glycerophos
8.14_354.24	377.229437	8.13956667	pos	8-iso-13,14-c	HMDB0006562		VKTIONYPM	Lipids and lip	Fatty Acyls	Eicosanoids
11.56_523.3	524.370674	11.5576	pos	LysoPC(0:0/	HMDB00111	40342	IQGPMZRCI	Lipids and lip	Glycerophos	Glycerophos
2.82_188.11	189.123443	2.82056667	pos	Glycyl-Isoleu	HMDB0028844		KGVHCTWY	Organic acid	Carboxylic ac	Amino acids,
3.97_252.08	252.08766	3.97085	neg	N-lactoyl-Tyr	HMDB0062177		XBOGARDIN	Organic acid	Carboxylic ac	Amino acids,
10.56_426.3	426.357221	10.5560833	pos	3-Hydroxyoct	HMDB0240769		PWZJXSPDI	Lipids and lip	Fatty Acyls	Fatty acid es
1.10_269.16	269.160562	1.1046	pos	Histidylisoleu	HMDB00288	23749	IDXZDKMBE	Organic acid	Carboxylic ac	Amino acids,
10.52_317.2	317.211941	10.5172833	neg	5S-hydroxy-6	HMDB00624	36346	FTAGQROY	Lipids and lip	Fatty Acyls	Eicosanoids
10.95_427.3	428.372921	10.9478667	pos	16-Methylhep	HMDB0240877		PTGBTBDV	Lipids and lip	Fatty Acyls	Fatty acid es
12.48_304.2	305.246014	12.4811	pos	Eicosatetrae	HMDB02517	356740	IQLUYYHUN	Lipids and lip	Fatty Acyls	Fatty acids a
2.11_165.07	164.071505	2.11061667	neg	L-Phenylalan	HMDB00001	28	COLNVLDH\	Organic acid	Carboxylic ac	Amino acids,
0.70_132.06	132.065763	0.69861667	pos	4-Amino-4-de	HMDB0246348		YECZZWUIA	Organic oxyg	Organooxyge	Carbohydrate
1.32_232.10	233.113179	1.31801667	pos	gamma-Amir	HMDB0252609		FUDYOSHJT	Organic acid	Carboxylic ac	Amino acids,
4.92_357.19	358.201102	4.91941667	pos	(S)-Laudanos	HMDB00302	1154	KGPAYJZAN	Organoheter	Isoquinolines	Benzylisoqui
9.79_512.29	512.299446	9.78913333	neg	LysoPC(14:0	HMDB00103	40278	VXUOFDJK\	Lipids and lip	Glycerophos	Glycerophos
1.23_117.01	117.019194	1.23491667	neg	Succinic acid	HMDB00002	114	KDYFGRWQ	Organic acid	Carboxylic ac	Dicarboxylic
1.76_296.12	296.124242	1.75533333	pos	Asparaginyl-	HMDB00287	85658	FYRVDDJMI	Organic acid	Carboxylic ac	Amino acids,
11.93_302.2	301.217054	11.9285167	neg	Retinyl ester	HMDB00035	6966	WWDMJSS\	Lipids and lip	Prenol lipids	Retinoids
3.96_246.13	269.126305	3.96453333	pos	Lenticin	HMDB0061115		AOHCBEAZ)	Organic acid	Carboxylic ac	Amino acids,
0.70_504.16	527.158219	0.69861667	pos	Maltotriose	HMDB00012	3585	FYGDTMLN	Organic oxyg	Organooxyge	Carbohydrate
10.84_343.2	343.227602	10.8434333	neg	(+/-)-13-HDoHE			SEVOKGDV	Lipids and lip	Fatty Acyls	Docosanoids
1.22_268.10	268.103882	1.2188	pos	Adenosine	HMDB00000	86	OIRDTQYFT	Nucleosides,	Purine nucle	Unclassified
0.79_118.08	118.086427	0.7877	pos	3-Amino-3-m	HMDB02458	449944	NFQAIWOM	Organic acid	Carboxylic ac	Amino acids,
10.16_364.3	364.320445	10.1580333	pos	Hexadecadie	HMDB0253126		HVWOQFIB\	Lipids and lip	Fatty Acyls	Fatty acids a

11.91_552.3(	552.367016	11.9099667	neg		PC(O-18:1(11Z)/0:0)		76582	HEEMBWYZ	Lipids and lip	Glycerophos	Glycerophos
9.94_302.30	302.304948	9.93923333	pos		(2R,3R)-2-Ar	HMDB0242634		OTKJDMGTI	Organic nitro	Organonitrog	Amines
10.15_335.2	335.222791	10.15385	neg		3-alpha-hydr	HMDB0245800		QGXBDMJG	Lipids and lip	Steroids and	Androstane s
10.59_570.2	570.282228	10.5914167	pos		PS(22:6(4Z,7Z,10Z,13Z,1		78840	FZUFCKYW	Lipids and lip	Glycerophos	Glycerophos
5.23_242.08	243.087475	5.22825	pos		Lumichrome	HMDB02541	64742	ZJTJUVIJVL	Organoheter	Pteridines an	Alloxazines a
8.35_243.16	243.160014	8.34551667	neg		1,11-Undeca	HMDB00023	6622	DXNCZXXFI	Lipids and lip	Fatty Acyls	Fatty acids a
9.72_335.22	335.22289	9.71598333	neg		8,20-DiHETE	HMDB0062687		AEHUQHOA	Lipids and lip	Fatty Acyls	Eicosanoids
6.23_361.20	361.200527	6.23411667	pos		(8S,9S,10R,1	HMDB0246907		OIGNJSKKL	Lipids and lip	Steroids and	Hydroxysterc
0.78_362.05	362.050704	0.7799	neg		Guanosine m	HMDB00013	6216	RQFCJASXJ	Nucleosides,	Purine nucle	Purine ribonu
11.10_550.3	550.351483	11.0963	neg		LysoPC(P-18	HMDB00104	46693	KJUNGQXF	Lipids and lip	Glycerophos	Glycerophos
4.03_194.04	194.045593	4.02623333	neg		N-acetyl-5-ar	HMDB00606	1146	GEFDRROB	Benzenoids	Benzene and	Benzoic acid
8.79_351.21	351.2139	8.7919	pos		(9R,10S,12Z	HMDB0040611		NIOKCFABU	Lipids and lip	Fatty Acyls	Lineolic acids
4.09_320.12	320.124375	4.09135	pos		Aspartyl-Tryp	HMDB0028764		ZARXTZFG(	Organic acids	Carboxylic ac	Amino acids,
0.72_179.07	160.061308	0.71928333	neg		Glucosamine	HMDB00015	63216	MSWZFWKM	Organic oxyg	Organooxyge	Carbohydrate
11.61_507.3	508.339489	11.61145	pos		LysoPE(20:1	HMDB00115	62297	JEAGLCKG/	Lipids and lip	Glycerophos	Glycerophos
4.54_131.07	131.071174	4.54091667	neg		2-Ethyl-2-Hy	HMDB00019	6411	LXVSANCQ	Lipids and lip	Fatty Acyls	Fatty acids a
8.89_334.21	333.207168	8.89388333	neg		Delta-12-Pro	HMDB00042	7034	TUXFWOHF	Lipids and lip	Fatty Acyls	Eicosanoids
4.17_159.06	159.066053	4.17231667	neg		Pimelic acid	HMDB00008	3280	WLJVNTCW	Lipids and lip	Fatty Acyls	Fatty acids a
0.67_131.04	131.046048	0.6745	neg		DL-Asparagi	HMDB0251512		DCXYFEDJ(	Organic acids	Carboxylic ac	Amino acids,
0.76_245.11	245.113114	0.75766667	pos		Nopalinic aci	HMDB00294	65631	UXZAXFPFS	Organic acids	Carboxylic ac	Amino acids,
4.63_209.04	209.04536	4.63258333	neg		Vanilpyruvic	HMDB00117	62448	YGQHQTMF	Benzenoids	Benzene and	Phenylpyruvi
4.83_166.06	165.055457	4.83273333	neg		L-3-Phenylla	HMDB00007	5745	VOXXWSYK	Phenylpropa	Phenylpropa	Unclassified
5.96_288.21	288.21658	5.95905	pos		6-Methylhept	HMDB0241043		JJGNMUWK	Lipids and lip	Fatty Acyls	Fatty acid es
0.61_204.13	204.134393	0.60603333	pos		Glycyl-Lysine	HMDB0028846		IKAIKUBBJH	Organic acids	Carboxylic ac	Amino acids,
10.59_325.2	325.23813	10.59005	neg		Avocadyne 2	HMDB00310	87372	LQFANUWE	Lipids and lip	Fatty Acyls	Fatty alcohol
0.83_147.02	147.029728	0.82703333	neg		Ribonolacton	HMDB00019	6380	CUOKHACJI	Organic oxyg	Organooxyge	Carbohydrate
0.71_275.11	276.118825	0.71368333	pos		N2-gamma-C	HMDB00117	429700	JBFYFLXEJI	Organic acids	Carboxylic ac	Amino acids,
1.60_258.08	303.083375	1.59701667	neg		Ribothymidin	HMDB00008	5843	DWRXFEIT\	Nucleosides,	Pyrimidine nu	Unclassified
5.22_187.09	187.09734	5.21915	neg		Azelaic acid	HMDB00007	5750	BDJRBEYX(	Lipids and lip	Fatty Acyls	Fatty acids a
5.88_201.11	201.113019	5.88396667	neg		Sebacic acid	HMDB00007	4240	CXMXRPHR	Lipids and lip	Fatty Acyls	Fatty acids a
8.25_342.26	342.263485	8.24955	pos		trans-2-Dode	HMDB0013326		JEOZLTJHD	Lipids and lip	Fatty Acyls	Fatty acid es
0.74_112.05	112.050889	0.74288333	pos		Cytosine	HMDB00006	283	OPTASPLR(	Organoheter	Diazines	Pyrimidines a
1.08_364.06	364.064866	1.07531667	pos		[(2R,5R)-5-(2	HMDB0257693		RQFCJASXJ	Nucleosides,	Purine nucle	Purine ribonu

0.75_259.02	259.022306	0.74898333	neg	beta-D-Fruct	HMDB0243977	HXRNACQB	Organic acid	Organic phos	Phosphate e
10.19_361.2	361.273113	10.1937667	pos	METENEPR	HMDB0254509	WMLGLMGS	Lipids and lip	Fatty Acyls	Fatty acids a
4.71_173.08	173.081649	4.70578333	neg	Suberic acid	HMDB00008 4243	TYFQFVWC	Lipids and lip	Fatty Acyls	Fatty acids a
2.93_383.10	384.115035	2.92891667	pos	N6-Succinyl	HMDB0255303	VKGZCEJT	Organic acid	Carboxylic a	Dicarboxylic
0.61_146.10	147.112864	0.60603333	pos	2,5-Diamino-	HMDB0248233	LNDPCYHW	Organic acid	Carboxylic a	Amino acids,
0.70_162.11	162.112393	0.69861667	pos	L-Carnitine	HMDB0000062	PHIQHXFUZ	Organic nitro	Organonitrog	Quaternary a
1.47_217.13	218.138751	1.47146667	pos	Propionylcar	HMDB00008 965	UFAHZIUFP	Lipids and lip	Fatty Acyls	Fatty acid es
4.92_284.18	284.185276	4.91941667	pos	Octa-3,6-die	HMDB0241718	XDXCICDB	Lipids and lip	Fatty Acyls	Fatty acid es
9.15_448.30	448.306855	9.14948333	neg	Deoxycholyl	HMDB0242414	WVULKSPC	Lipids and lip	Steroids and	Bile acids, al
4.92_212.09	212.091738	4.91941667	pos	Ferulic acid	HMDB00009 4156	KSEBMYQB	Phenylpropa	Cinnamic aci	Hydroxycinna
5.24_151.03	151.039858	5.23773333	neg	Penicillic aci	HMDB0256217	VOUGEZYP	Organic acid	Keto acids a	Medium-chai
7.41_352.22	351.217678	7.41378333	neg	8-isoprostagl	HMDB00058 36377	XEYBRNLF	Lipids and lip	Fatty Acyls	Eicosanoids
4.12_169.99	169.99159	4.11676667	neg	Lanthionine	HMDB00048 7085	XIVVIYYWX	Organic acid	Carboxylic a	Amino acids,
6.20_407.20	407.207093	6.19793333	neg	Cortisol	HMDB00000 272	JYGXADMD	Lipids and lip	Steroids and	Hydroxyster
0.70_159.07	159.076384	0.69861667	pos	N5-formyl-N	HMDB0304431	KORLZEIJS	Organic acid	Carboxylic a	Amino acids,
1.08_347.06	348.070042	1.07531667	pos	Vidarabine p	HMDB0245755	UDMBCSSL	Nucleosides,	Purine nucle	Purine ribon
12.38_509.2	509.287835	12.3811333	neg	1-(11Z-eicos	HMDB00623 82345	OAYFHKCH	Lipids and lip	Glycerophos	Glycerophos
0.70_309.12	309.12904	0.69861667	pos	2-Deoxy-2,3-	HMDB0245680	JINJZWSZQ	Organic acid	Carboxylic a	Carboxylic a
0.76_187.10	187.107707	0.75766667	pos	Valylserine	HMDB00291 23667	STTYIMSDI	Organic acid	Carboxylic a	Amino acids,
11.33_295.2	295.22758	11.3319667	neg	9,10-Epoxy-1	HMDB0302107	ITTPZDMHC	Lipids and lip	Fatty Acyls	Fatty acids a
9.25_269.12	269.128291	9.24935	pos	3-(1-Azatricy	HMDB0244618	JXENNHTVE	Organoheter	Quinolines a	Hydroquinoli
0.67_104.03	104.035181	0.6745	neg	2,3-Dihydrox	HMDB0245395	XLGVHAQD	Organic oxyg	Organooxyge	Carbohydrate
0.79_219.09	219.097401	0.7877	pos	N-gamma-L-	HMDB00363 91446	WQXXXVRA	Organic acid	Carboxylic a	Amino acids,
12.41_380.2	381.299456	12.4112833	pos	MG(20:3(11Z	HMDB00115 62357	IKBDZTUBA	Lipids and lip	Glycerolipids	Monoradylgly
4.23_263.10	263.103641	4.22771667	neg	Phenylacetyl	HMDB00063 58397	JFLIEFSWG	Organic acid	Carboxylic a	Amino acids,
4.47_257.16	258.169994	4.47151667	pos	2-Hexenoylc	HMDB0013161	JUAQYRSU	Lipids and lip	Fatty Acyls	Fatty acid es
4.30_243.17	243.171199	4.30156667	neg	Leucyl-Isole	HMDB0028932	AZLASBBH	Organic acid	Carboxylic a	Amino acids,
4.91_171.06	171.066118	4.90568333	neg	6-(2-Hydroxy	HMDB0061681	YXCHMHAN	Lipids and lip	Fatty Acyls	Fatty acids a
7.12_401.08	401.087689	7.11838333	neg	Dihydrohydr	HMDB00336 89456	ATDWDHGT	Phenylpropa	Sterigmatocy	Unclassified
0.71_127.05	127.050424	0.71368333	pos	Imidazoleace	HMDB00020 6445	PRJKNHOM	Organoheter	Azoles	Imidazoles
1.07_322.04	322.044551	1.06741667	neg	Cytidine 3'-m	HMDB02403 63414	UOOOPKAN	Organic oxyg	Organooxyge	Carbohydrate
1.08_230.18	230.186179	1.07531667	pos	N1,N8-Diace	HMDB0041947	BKCVMAZD	Organic acid	Carboxylic a	Carboxylic a
1.34_202.10	202.108288	1.34166667	neg	1-Methyl-4-p	HMDB02439 359004	RWPYOAUY	Organoheter	Piperidines	Unclassified

11.06_454.38	454.388448	11.0558	pos	12-Hydroxyic	HMDB0241569		SOEGPOBG	Lipids and lip	Fatty Acyls	Fatty acid es
2.11_107.049	107.049507	2.10798333	pos	Benzaldehyd	HMDB00061	58358	HUMNYLRZI	Benzenoids	Benzene anc	Benzoyl deriv
1.08_279.13	280.13891	1.07531667	pos	N-(1-Deoxy-1	HMDB00378	92652	OANCGDPJI	Organic acid	Carboxylic ac	Amino acids,
0.61_309.16	309.165596	0.60603333	pos	1-Deoxy-1-(N	HMDB0062186		ZAWLGBRD	Organic oxyg	Organooxyge	Carbohydrate
1.08_248.14	248.149049	1.07531667	pos	3-Hydroxybut	HMDB0013127		UEFRDQSM	Lipids and lip	Fatty Acyls	Fatty acid es
1.38_152.058	175.047885	1.3848	pos	N1-Methyl-2-	HMDB00041	7025	JLQSXXWT	Organoheter	Pyridines and	Pyridinecarbo
3.66_179.05	179.057228	3.65995	neg	Theophylline	HMDB00018	1458	ZFXYFBGIU	Organoheter	Imidazopyrim	Purines and
3.70_238.14	261.130697	3.69811667	pos	Pentaethylen	HMDB0256256		JLFNLZLINW	Organic oxyg	Organooxyge	Ethers
8.76_313.21	313.215605	8.75856667	pos	Metabolite M	HMDB0254496		MGYSTOQC	Lipids and lip	Steroids and	Hydroxystero
0.78_217.08	217.082648	0.7799	neg	Serylhydroxy	HMDB0029040		IISAHBYOIF	Organic acid	Carboxylic ac	Amino acids,
0.77_136.06	136.061781	0.7729	pos	FAPy-adenin	HMDB00048	7080	MVYUVUOS	Organoheter	Diazines	Pyrimidines a
1.07_306.07	306.076065	1.06741667	neg	Glutathionate	HMDB0062697		RWSXRVCM	Organic acid	Carboxylic ac	Amino acids,
6.03_353.23	353.231844	6.03235	pos	8-iso-PGF3a	HMDB0002132		SAKGBZWJ	Lipids and lip	Fatty Acyls	Eicosanoids
0.77_188.11	189.123336	0.7729	pos	N-alpha-Acet	HMDB00004	5435	VEYYWZRY	Organic acid	Carboxylic ac	Amino acids,
10.23_612.3	612.330772	10.2257833	neg	LysoPC(22:6	HMDB00104	40322	LSOWKZUL	Lipids and lip	Glycerophos	Glycerophos
10.37_488.3	488.359308	10.37205	neg	9-Hydroxyoct	HMDB0241520		PMIHTPDPP	Lipids and lip	Fatty Acyls	Fatty acid es
10.52_621.3	621.302743	10.5204167	pos	LysoPI(20:4(	HMDB0061690		LXUGKKVC	Lipids and lip	Glycerophos	Glycerophos
10.73_431.3	431.315083	10.7334833	pos	Kuguacin E	HMDB0302099		FGMDCYOF	Lipids and lip	Prenol lipids	Sesquiterper
4.23_178.05	178.05078	4.22771667	neg	Hippuric acid	HMDB00007	1301	QIAFMBKCN	Benzenoids	Benzene anc	Benzoic acid
0.64_108.06	126.102796	0.63663333	pos	2,6-Dimethyl	HMDB00352	318701	HJFZAYHYI	Organoheter	Diazines	Pyrazines
0.77_192.03	192.032432	0.7729	pos	2,4-Thiazolid	HMDB0244750		JCAKCGQZI	Organic acid	Carboxylic ac	Amino acids,
1.32_380.11	380.112028	1.31801667	pos	S-Lactoylglut	HMDB00010	5979	VDYDCVUW	Organic acid	Carboxylic ac	Amino acids,
3.80_158.08	158.082047	3.80496667	neg	N-Acetylvalin	HMDB0011757		IHYJTAOFM	Organic acid	Carboxylic ac	Amino acids,
11.18_417.3	417.335708	11.1809167	pos	1alpha,24-Di	HMDB0244150		BJYLYJCXY	Lipids and lip	Steroids and	Vitamin D an
12.11_510.3	510.391594	12.1088667	pos	LysoPC(O-18	HMDB00111	40388	XKBJVQHMI	Lipids and lip	Glycerophos	Glycerophos
0.78_119.03	119.034794	0.7799	neg	2,4-Dihydrox	HMDB00003	5349	UFYGCFHQ	Organic acid	Hydroxy acid	Short-chain h
1.07_346.05	346.055472	1.06741667	neg	Adenosine 2'	HMDB0011617		QDFHPFSB	Organic oxyg	Organooxyge	Carbohydrate
1.07_166.01	166.014217	1.06741667	neg	Quinolinic ac	HMDB00002	330	GJAWHXHK	Organoheter	Pyridines and	Pyridinecarbo
1.15_116.01	115.003518	1.14921667	neg	Maleic acid	HMDB00001	268686	VZCYOOQT	Organic acid	Carboxylic ac	Dicarboxylic
11.07_547.3	548.370632	11.0736	pos	LysoPC(20:2	HMDB00103	61702	YYQVCMMX	Lipids and lip	Glycerophos	Glycerophos
10.41_399.3	400.341659	10.41135	pos	14-Methylper	HMDB0240853		LEOBKKBBF	Lipids and lip	Fatty Acyls	Fatty acid es
10.91_302.2	303.231354	10.9125667	pos	Oxendolone	HMDB0255978		FCKLFGKAT	Lipids and lip	Steroids and	Estrane stero
11.54_352.2	375.25004	11.5398167	pos	MG(0:0/18:3	HMDB00115	62323	BXAXVVVVF	Lipids and lip	Fatty Acyls	Lineolic acids

9.34_285.13(	286.143419	9.34135	pos	Piperine	HMDB00293	43568	MXXWOMGl	Alkaloids anc	Unclassified	Unclassified
0.75_105.01	105.019122	0.74898333	neg	2-(Hydroxym	HMDB0257950		ZUCOIFUQE	Organic oxyg	Organooxyge	Ethers
1.36_161.04	161.045364	1.3586	neg	2-Hydroxyad	HMDB00003	5310	OTTXIFWBF	Lipids and lip	Fatty Acyls	Fatty acids a
4.78_349.18	349.184356	4.7769	pos	Foeniculosid	HMDB00415	95929	IQQCVQGVI	Lipids and lip	Prenol lipids	Terpene glyc
0.79_203.11	204.122979	0.7877	pos	L-Acetylcarni	HMDB0000201		RDHQFKQIC	Lipids and lip	Fatty Acyls	Fatty acid es
1.25_379.08	379.08705	1.2521	neg	Kaltostat	HMDB0253761		GKFPPCXIB	Organic oxyg	Organooxyge	Carbohydrate
10.84_293.2	293.212123	10.8434333	neg	13-Oxo-9,11-	HMDB0246143		JHXAZBBVC	Lipids and lip	Fatty Acyls	Lineolic acids
11.91_300.2	300.289278	11.9122333	pos	Palmitoyletha	HMDB00021	43210	HXYVTAGFY	Organic acids	Carboximidic	Carboximidic
6.66_215.12	215.12858	6.65806667	neg	Undecanedic	HMDB00008	5846	LWBHHRRT	Lipids and lip	Fatty Acyls	Fatty acids a
7.88_305.08	305.081868	7.88408333	neg	Moracin L	HMDB00412	95710	FQHZBTIVE	Organic acids	Organic carb	Organic carb
8.46_351.21	351.21771	8.45608333	neg	Sterebin C	HMDB00353	90639	ZFSCNCIWZ	Lipids and lip	Prenol lipids	Sesquiterper
10.19_501.2	502.292413	10.1937667	pos	LysoPE(20:4	HMDB00115	46715	ROPRRXYV	Lipids and lip	Glycerophos	Glycerophos
5.92_310.20	310.200436	5.92246667	pos	Octanoylcarr	HMDB0000791		CXTATJFJD	Lipids and lip	Fatty Acyls	Fatty acid es
0.66_225.06	225.06143	0.65943333	neg	D-Mannose	HMDB0000169		WQZGKKKJ	Organic oxyg	Organooxyge	Carbohydrate
1.95_281.08	281.089025	1.9471	neg	1-Methylinos	HMDB00027	3780	WJNGQIYE(	Nucleosides,	Purine nucle	Unclassified
12.34_256.2	256.26302	12.3412167	pos	Hexadecenal	HMDB00604	75364	KLJFYXOVG	Lipids and lip	Fatty Acyls	Fatty aldehyc
2.04_303.08	303.083267	2.03931667	neg	3-Methyluridi	HMDB0004813		UTQUILVPB	Nucleosides,	Pyrimidine nu	Unclassified
8.12_353.22	353.229641	8.1212	pos	13,14-Dihydr	HMDB00600	3817	VSRXYLYXI	Lipids and lip	Fatty Acyls	Eicosanoids
9.99_441.34	442.352305	9.99425	pos	3-Hydroxy-11	HMDB0013339		FMLYQPQCI	Lipids and lip	Fatty Acyls	Fatty acid es
0.74_174.10	175.107976	0.74288333	pos	N2-Acetylorn	HMDB00033	327320	JRLGPAXAC	Organic acids	Carboxylic ac	Amino acids,
1.35_293.14	294.154548	1.34996667	pos	N-(1-Deoxy-1	HMDB00378	92648	KGTRBDVO	Organic acids	Carboxylic ac	Amino acids,
11.06_297.2	320.255515	11.0558	pos	3-Ketosphing	HMDB0245914		VWTPJNGTI	Organic oxyg	Organooxyge	Carbonyl cor
9.95_263.16	263.165123	9.95315	neg	Patchoulenoi	HMDB00357	90999	JAWSHISYW	Lipids and lip	Prenol lipids	Sesquiterper
10.66_318.2	341.207365	10.6629	pos	15-Deoxypro	HMDB0244664		AWSUSADY	Lipids and lip	Fatty Acyls	Eicosanoids
9.50_239.12	239.128824	9.4972	neg	Cnidilide	HMDB03022	71445	UXDIXFDKS	Organoheter	Isobenzofura	Unclassified
12.02_271.2	271.22774	12.0185833	neg	2-Hydroxyhe	HMDB0031057		JGHSBPIZN	Lipids and lip	Fatty Acyls	Fatty acids a
7.15_314.23	314.232231	7.14843333	pos	(1(10)E,4a,5I	HMDB0038798		RTFVWCRR	Lipids and lip	Prenol lipids	Sesquiterper
0.78_323.02	323.028496	0.7799	neg	Uridine 5'-mc	HMDB0000288		DJJCXFVJD	Nucleosides,	Pyrimidine nu	Pyrimidine rik
1.44_129.06	129.066141	1.43671667	pos	Dihydrothymi	HMDB0000079		NBAKTGXDI	Organoheter	Diazines	Pyrimidines a
10.88_524.3	524.335557	10.8794667	neg	LysoPC(P-16	HMDB00104	40402	HTZINLFNXI	Lipids and lip	Glycerophos	Glycerophos
4.25_376.13	377.145977	4.25448333	pos	Riboflavin	HMDB00002	233	AUNGANRZ	Organoheter	Pteridines an	Alloxazines a
0.70_195.05	195.050739	0.70456667	neg	Gluconic acic	HMDB00006	345	RGHNJXZE(	Organic oxyg	Organooxyge	Carbohydrate
1.42_127.05	127.050466	1.41946667	pos	Thymine	HMDB00002	290	RWQNBRD(	Organoheter	Diazines	Pyrimidines a

11.08_321.24	321.243198	11.07825	neg	8-HETrE	HMDB00600	36269	SKIQVURLE	Lipids and lip	Fatty Acyls	Eicosanoids
2.33_309.10	309.109263	2.3315	neg	Tyrosyl-Gluta	HMDB00291	85996	PDSLRCZIN	Organic acids	Carboxylic ac	Amino acids,
3.99_137.02	137.024252	3.98933333	neg	3,4-Dihydroxy	HMDB00599	63101	IBGBGRVKF	Organic oxyg	Organooxyge	Carbonyl cor
4.91_236.09	236.092707	4.90568333	neg	N-Lactoylphe	HMDB0062175		IIRJJZHHNG	Organic acids	Carboxylic ac	Amino acids,
9.32_375.21	375.213851	9.32303333	pos	Thromboxan	HMDB00014	63351	DSNBHJFQC	Lipids and lip	Fatty Acyls	Eicosanoids
0.71_348.06	348.069409	0.71368333	pos	3'-AMP	HMDB00035	63401	LNQVTSRO	Nucleosides,	Ribonucleosi	Unclassified
0.76_112.04	112.040243	0.76461667	neg	Trans-3-hydr	HMDB0059659		BJBUEDPLE	Organic acids	Carboxylic ac	Amino acids,
0.76_193.04	194.048195	0.75766667	pos	S-(2-Carboxy	HMDB0246645		FBPINGSGH	Organic acids	Carboxylic ac	Amino acids,
11.93_354.2	355.282939	11.9300167	pos	Glyceryl mon	HMDB02421	4252	WECGLUPZ	Lipids and lip	Fatty Acyls	Lineolic acids
0.71_205.08	205.081967	0.71368333	pos	Aspartyl-Alar	HMDB00287	23696	DVUFTQLHI	Organic acids	Carboxylic ac	Amino acids,
1.88_246.12	247.128894	1.88096667	pos	Glutamylvalir	HMDB00288	23993	SITLTJHOQ	Organic acids	Carboxylic ac	Amino acids,
10.26_315.2	315.253837	10.2627833	neg	Pelargonic ac	HMDB00008	5810	FBUKVWPV	Lipids and lip	Fatty Acyls	Fatty acids a
10.90_526.3	526.351534	10.8978167	neg	1-O-Hexadec	HMDB02438	24070	VLBPIWYTP	Lipids and lip	Glycerophos	Glycerophos
8.34_386.28	386.289696	8.34093333	pos	hydroxytetra	HMDB0253296		FAHASWCZ	Lipids and lip	Fatty Acyls	Fatty acids a
0.77_152.05	152.055676	0.7729	pos	Guanine	HMDB00001	315	UYTPUPDQI	Organoheter	Imidazopyrim	Purines and
8.14_353.23	353.233321	8.14243333	neg	Prostaglandi	HMDB0302209		PXGPLTODI	Lipids and lip	Fatty Acyls	Eicosanoids
0.70_175.09	176.102932	0.69861667	pos	Citrulline	HMDB00009	16	RHGKLRLOI	Organic acids	Carboxylic ac	Amino acids,
1.07_146.02	191.019439	1.06741667	neg	Methoxymali	HMDB0302351		AHJCDQGR	Organic acids	Carboxylic ac	Dicarboxylic
6.25_239.09	239.092495	6.2534	neg	3-Hydroxyph	HMDB00416	96026	CMLIEOOX	Lipids and lip	Fatty Acyls	Fatty acids a
11.06_554.3	554.34601	11.0597833	neg	LysoPC(17:0	HMDB00121	24068	SRRQPVVY	Lipids and lip	Glycerophos	Glycerophos
4.71_192.06	192.066476	4.70578333	neg	2-Methylhipp	HMDB00117	24085	YOEBAVRJH	Benzenoids	Benzene anc	Benzoic acid
1.15_260.16	260.160531	1.1535	pos	Isoleucyl-Gai	HMDB00289	85826	PFUZINYUQ	Organic acids	Carboxylic ac	Amino acids,
1.97_296.09	296.099836	1.96566667	neg	2-Methylguar	HMDB00058	58332	SLEHRORO	Nucleosides,	Purine nucle	Unclassified
10.01_337.2	337.238518	10.00765	neg	6,7-dihydro-1	HMDB0012838		ZMZNVSWC	Lipids and lip	Fatty Acyls	Eicosanoids
2.61_246.12	247.128872	2.6067	pos	Aspartyl-Isol	HMDB0028756		BSWHERGF	Organic acids	Carboxylic ac	Amino acids,
4.76_424.06	423.061441	4.76071667	neg	Cefuroxime	HMDB00152	1628	JFPVXVDW	Organoheter	Lactams	Beta lactams
8.05_269.12	269.128215	8.04801667	pos	Nafazatrom	HMDB02554	315926	ISBUYSPRIJ	Benzenoids	Naphthalene	Unclassified
0.71_342.11	365.105205	0.71368333	pos	Melibiose	HMDB0000048		DLRVVLDZN	Organic oxyg	Organooxyge	Carbohydrate
2.46_219.13	219.133956	2.46275	pos	Ser-Leu	HMDB0258242		NFDYGNFE	Organic acids	Carboxylic ac	Amino acids,
0.70_102.05	102.055846	0.70456667	neg	1-Amino-4-hy	HMDB02475	693104	NHIYVTWY	Organic oxyg	Organooxyge	Carbonyl cor
0.74_249.10	249.107997	0.74288333	pos	gamma-Gluta	HMDB00291	86051	GWNXFCYU	Organic acids	Carboxylic ac	Amino acids,
10.01_262.1	263.200094	10.0126833	pos	Crithmumdio	HMDB0033957		LCHMNLCUI	Lipids and lip	Fatty Acyls	Fatty alcohol
3.77_259.12	259.129765	3.7682	neg	gamma-Gluta	HMDB00111	23750	MYFMARDIC	Organic acids	Carboxylic ac	Amino acids,

4.56_187.00(	187.006807	4.55865	neg	p-Cresol sulf	HMDB00116	62391	WGNAKZGU	Organic acid	Organic sulfu	Arylsulfates
0.74_141.06!	141.065802	0.74288333	pos	Oxiracetam	HMDB0255986		IHLAQQPQK	Organic acid	Carboxylic ac	Amino acids,
4.01_221.07:	220.064731	4.0077	neg	N-lactoyl-Met	HMDB0062182		UFMNJPDG	Organic acid	Carboxylic ac	Amino acids,
5.42_137.02	137.02421	5.42216667	neg	Salicylic acid	HMDB00018	616	YGSDEFSM	Benzenoids	Benzene and	Benzoic acid
9.76_299.28:	300.289306	9.7588	pos	Sphingosine	HMDB0000252		WWUZIQQU	Organic nitro	Organonitrog	Amines
10.28_252.1	253.179461	10.2840833	pos	3-Methyl-5-p	HMDB0112086		OVQQXXHY	Lipids and lip	Fatty Acyls	Fatty acids a
3.44_117.05!	117.055546	3.44081667	neg	2-Hydroxy-3-	HMDB00004	5396	NGEWQZID	Lipids and lip	Fatty Acyls	Fatty acids a
5.16_189.11:	189.113005	5.16371667	neg	(3R,5Z)-5-Oc	HMDB00303	86920	QTHIKVMVV	Lipids and lip	Fatty Acyls	Fatty alcohol
9.76_415.32!	416.336533	9.7588	pos	11-Hydroxyh	HMDB0241465		BXHAMSWF	Lipids and lip	Fatty Acyls	Fatty acid es
0.80_268.08(	291.069844	0.80285	pos	Fructose lact	HMDB0252491		ZMDXTLMY	Organic oxyg	Organooxyge	Carbohydrate
0.70_152.00:	152.002061	0.70456667	neg	3-Sulfinoalan	HMDB0000996		ADVPTQAUI	Organic acid	Carboxylic ac	Amino acids,
0.70_209.03(	209.030098	0.70456667	neg	Galactaric ac	HMDB00006	5612	DSLZVSRJT	Organic oxyg	Organooxyge	Carbohydrate
4.60_176.07(	176.070643	4.5955	pos	Phenylacetyl	HMDB00008	4237	UTYVDVLM	Organic acid	Carboxylic ac	Amino acids,
4.79_326.19:	326.193553	4.79411667	pos	5-Hydroxyoct	HMDB0241694		QGIAELIQC	Lipids and lip	Fatty Acyls	Fatty acid es
10.41_277.1	277.144296	10.4089833	neg	alpha-Amylci	HMDB00362	91354	AWNFWGNI	Benzenoids	Benzene and	Unclassified
6.78_370.23!	393.224245	6.7816	pos	19-Hydroxyp	HMDB0244832		QVVXWHIDI	Lipids and lip	Fatty Acyls	Eicosanoids
9.70_293.17!	293.175894	9.69793333	neg	(4E,7E,10E,1	HMDB0253125		IVTCJQZAG	Lipids and lip	Fatty Acyls	Fatty acids a
13.49_284.2!	284.294309	13.4891167	pos	Octadecanar	HMDB00341	34494	LYRFLYHAG	Organic acid	Carboximidic	Carboximidic
9.12_286.19:	287.200228	9.12051667	pos	Androstened	HMDB00000	2795	AEMFNILZO	Lipids and lip	Steroids and	Androstane s
0.78_346.05!	346.055563	0.7799	neg	Adenosine m	HMDB00000	5111	UDMBCSSL	Nucleosides,	Purine nucle	Purine ribonu
0.79_113.03	113.034879	0.7877	pos	Uracil	HMDB00003	258	ISAKRJDGN	Organoheter	Diazines	Pyrimidines a
10.38_444.3(	444.367356	10.3754333	pos	11-Hydroxyo	HMDB0241525		ZSRAEYNFJ	Lipids and lip	Fatty Acyls	Fatty acid es
10.55_470.3	470.348449	10.5532667	neg	Octadec-6-er	HMDB0241528		BUWRQWRI	Lipids and lip	Fatty Acyls	Fatty acid es
12.25_483.2	483.272516	12.2531167	neg	LysoPA(18:0	HMDB0007854		LAYXSTYJR	Lipids and lip	Glycerophos	Glycerophos
13.60_308.2	307.264092	13.59865	neg	Eicosadienoi	HMDB00050	62964	XSXIVVZCU	Lipids and lip	Fatty Acyls	Fatty acids a
2.15_141.01!	141.019124	2.1466	neg	Sumiki's acid	HMDB00024	6680	PCSKKIUUR	Organoheter	Furans	Furoic acid a
1.44_265.08!	265.085322	1.43671667	pos	Aspartyl-Met	HMDB00287	85673	DYDKXJWQ	Organic acid	Carboxylic ac	Amino acids,
10.14_359.2:	359.222877	10.1353167	neg	10S,17S-DiH	HMDB02442	45053	CRDZYJSQI	Lipids and lip	Fatty Acyls	Fatty acids a
11.00_506.3:	506.323508	11.0025333	pos	LysoPE(0:0/2	HMDB00114	62271	NHBRVTKHI	Lipids and lip	Glycerophos	Glycerophos
7.20_377.22!	377.229441	7.20335	pos	Prostaglandi	HMDB00011	6032	PXGPLTODI	Lipids and lip	Fatty Acyls	Eicosanoids
9.83_294.21!	295.226467	9.83063333	pos	17-Hydroxylir	HMDB00111	61976	PSQSSLVX(	Lipids and lip	Fatty Acyls	Lineolic acids
0.61_218.15(	218.150023	0.60603333	pos	Alanyllysine	HMDB00286	85608	QXRNAOYB	Organic acid	Carboxylic ac	Amino acids,
7.49_229.14	229.144296	7.48786667	neg	Dodecanedic	HMDB00006	5596	TVIDDXQYH	Lipids and lip	Fatty Acyls	Fatty acids a

9.97_311.22	311.222842	9.97076667	neg		(±)-(E)-13-Hy	HMDB00409	74711		CZGIUGHM.	Lipids and lip	Fatty Acyls	Lineolic acids
0.64_189.15	189.159848	0.63663333	pos		N6,N6,N6-Tr	HMDB00013	6161		MXNRLFUSI	Organic acid	Carboxylic ac	Amino acids,
0.73_133.11	134.11741	0.72825	pos		Diisopropanc	HMDB02513	44741		LVTYICIALW	Organic nitro	Organonitrog	Amines
10.81_437.2	438.297422	10.80565	pos		LysoPE(P-16	HMDB00111	46719		QYTPGOPLI	Lipids and lip	Glycerophos	Glycerophos
12.31_328.2	329.246928	12.3062167	pos		Docosahexa	HMDB00021	3457		MBMBGCFC	Lipids and lip	Fatty Acyls	Fatty acids a
8.29_355.24	355.248929	8.29023333	neg		13,14-Dihydr	HMDB0004239			LLQBSJQTC	Lipids and lip	Fatty Acyls	Eicosanoids
10.90_313.2	313.238417	10.8978167	neg		Octadecane	HMDB00007	5748		BNJOQKFEN	Lipids and lip	Fatty Acyls	Fatty acids a
4.78_318.18	318.180818	4.7769	pos		Tryptophyl-L	HMDB00290	85980		LYMVXFSTA	Organic acid	Carboxylic ac	Amino acids,
5.28_146.06	146.059991	5.28283333	pos		2-Methyl-2H-	HMDB0249616			MOFJSTXFU	Organoheter	Benzoxazine	Unclassified
10.14_309.2	309.207117	10.1353167	neg		(9Z,11R,12S	HMDB00335	89322		XHMGEKKH	Lipids and lip	Fatty Acyls	Fatty acids a
12.51_322.2	345.239393	12.51485	pos		12R-hydroxy	HMDB00622	36270		RFAKHEWA	Lipids and lip	Fatty Acyls	Eicosanoids
0.73_131.11	131.118061	0.72825	pos		N-Acetylputr	HMDB00020	3252		KLZGKIDSE	Organic acid	Carboximidic	Carboximidic
0.77_203.10	203.102662	0.7729	pos		N-Acetyl-b-gl	HMDB00011	6006		MCGXOCXF	Organic oxy	Organooxyge	Carbohydrate
8.90_449.31	472.30282	8.90103333	pos		Chenodeoxy	HMDB00006	43193		GHCZAUBVI	Lipids and lip	Steroids and	Bile acids, al
10.41_291.1	291.160134	10.4089833	neg		Isotrichoderm	HMDB0034738			OZXJPPYW	Lipids and lip	Prenol lipids	Sesquiterper
0.90_156.04	156.042153	0.89581667	pos		L-Valine	HMDB00008	5842		KZSNJWFQI	Organic acid	Carboxylic ac	Amino acids,
3.72_297.08	298.096785	3.71513333	pos		5'-Methylthio	HMDB00011	3425		WUUGFSXJ	Nucleosides,	5'-deoxyribor	5'-deoxy-5'-th
5.53_199.09	199.09733	5.53265	neg		2-Hydroxyde	HMDB00004	5413		LPIOYESQK	Organic acid	Hydroxy acid	Medium-chai
0.75_241.01	241.011973	0.74898333	neg		D-mannose 6	HMDB0304312			NBSCHQHZI	Organic oxy	Organooxyge	Carbohydrate
11.66_456.4	456.404209	11.6642167	pos		18-methylnor	HMDB0240909			QBGXQOUL	Lipids and lip	Fatty Acyls	Fatty acid es
0.74_229.08	230.095709	0.74288333	pos		Ergothionein	HMDB00030	53		SSISHJJTAX	Organic acid	Carboxylic ac	Amino acids,
0.75_209.06	209.066448	0.74898333	neg		Sedoheptulos	HMDB0003219			HAIWUXASL	Organic oxy	Organooxyge	Carbohydrate
1.09_213.12	213.123261	1.08996667	pos		Prolylproline	HMDB00111	23801		RWCOTTLH	Organic acid	Carboxylic ac	Amino acids,
1.72_253.11	253.118374	1.71971667	pos		Hypoglycin B	HMDB00294	66881		UYDZYCPIQ	Organic acid	Carboxylic ac	Amino acids,
12.64_331.2	331.262534	12.6383167	pos		Docosapenta	HMDB00065	194		YUFFSWGQ	Lipids and lip	Fatty Acyls	Fatty acids a
8.90_386.17	409.161653	8.90103333	pos		Porson	HMDB0030810			VHBRVHUP	Phenylpropa	Diarylheptan	Cyclic diarylh
9.54_377.22	377.229544	9.54118333	pos		5,6beta-Dihy	HMDB0246867			RJADQDXZY	Lipids and lip	Fatty Acyls	Eicosanoids
9.59_337.23	337.238603	9.58798333	neg		14,15-DiHET	HMDB0002265			SYAWGTIV	Lipids and lip	Fatty Acyls	Eicosanoids
0.63_145.09	145.098102	0.62835	neg		(3S)-3,6-Diar	HMDB00121	62789		QKEWQOJC	Organic acid	Carboxylic ac	Amino acids,
0.73_221.08	244.079107	0.72825	pos		Aldehydo-N-a	HMDB0062641			MBLBDJOUI	Organic oxy	Organooxyge	Carbohydrate
0.76_134.02	134.027052	0.75766667	pos		Thiazolidine-	HMDB0258979			DZLNHFMRI	Organic acid	Carboxylic ac	Amino acids,
1.79_297.10	297.108284	1.79108333	pos		Tyrosyl-Aspa	HMDB00291	85993		QZOSVNLXI	Organic acid	Carboxylic ac	Amino acids,
11.29_228.2	228.231898	11.2883167	pos		Tetradec-2-e	HMDB00325	75355		WHOZNOZY	Lipids and lip	Fatty Acyls	Fatty aldehy

4.47_234.17	235.18037	4.47151667	pos	Lidocaine	HMDB00144	995	NNJVILVZK	Benzenoids	Benzene an	Xylenes
8.30_321.24	321.241721	8.3042	pos	Sterebin E	HMDB0035379		RMAJUEUH	Lipids and lip	Prenol lipids	Diterpenoids
0.67_214.04	214.048603	0.6745	neg	Glycerylphos	HMDB00001	5151	JZNWSCPG	Lipids and lip	Glycerophos	Glycerophos
0.79_247.13	247.139918	0.7877	pos	Asparaginyl-	HMDB0028739		GADKFYNE	Organic acid	Carboxylic a	Amino acids,
1.45_260.14	260.149311	1.45418333	pos	3-Oxopentan	HMDB0241663		MMSQJKWG	Lipids and lip	Fatty Acyls	Fatty acid es
5.00_275.10	275.103887	4.99758333	neg	N-lactoyl-Try	HMDB0062178		AQHJZWISL	Organic acid	Carboxylic a	Amino acids,
1.04_243.08	244.092747	1.0442	pos	Cytarabine	HMDB00151	567	UHDGCWIW	Nucleosides,	Pyrimidine nu	Unclassified
1.34_311.15	292.140215	1.34166667	neg	Xanthinol	HMDB02599	320377	DSFGXPJYD	Organoheter	Imidazopyrim	Purines and
1.95_277.08	277.086211	1.9471	neg	gamma-Glut	HMDB00343	86047	RQNSKRXM	Organic acid	Carboxylic a	Amino acids,
10.45_289.2	289.215773	10.4474333	pos	(8S,9R,10S,	HMDB0260183		RAVRUVQH	Lipids and lip	Steroids and	Androstane s
9.15_257.17	257.175849	9.14948333	neg	4-(3-Hydroxy	HMDB00398	94354	QIHMBTNCJ	Lipids and lip	Prenol lipids	Sesquiterper
12.02_279.2	279.231372	12.0192167	pos	12,13-EpOM	HMDB00047	63352	CCPPLLJZD	Lipids and lip	Fatty Acyls	Fatty acids a
3.95_236.09	236.091893	3.94663333	pos	N-Acetylvanil	HMDB00117	62449	UKDKTHYZL	Organic acid	Carboxylic a	Amino acids,
6.79_367.15	367.158297	6.7873	neg	Dehydroepia	HMDB00010	4095	CZWCKYRV	Lipids and lip	Steroids and	Sulfated ster
7.68_375.21	375.213828	7.68096667	pos	15-Keto-pros	HMDB00042	7035	LOLJEILMP	Lipids and lip	Fatty Acyls	Eicosanoids
9.51_318.29	318.299896	9.50591667	pos	Phytosphing	HMDB00046	7066	AERBNCYC	Organic nitro	Organonitrog	Amines
10.17_524.2	524.278315	10.1716833	neg	LysoPE(0:0/	HMDB00114	62284	TWBVHOYV	Lipids and lip	Glycerophos	Glycerophos
10.21_526.3	526.314112	10.20805	neg	LysoPC(15:0	HMDB00103	40282	RJZVWDTY	Lipids and lip	Glycerophos	Glycerophos
10.39_241.1	241.180825	10.3905167	neg	4-(2,6,6-Trim	HMDB00361	91321	VSYLEWGI	Lipids and lip	Prenol lipids	Sesquiterper
10.83_616.3	616.361827	10.82535	neg	LysoPC(22:4	HMDB00104	61710	ZOJBSSVHF	Lipids and lip	Glycerophos	Glycerophos
11.55_275.2	275.201336	11.5461833	neg	Stearidonic a	HMDB00065	34838	JIWBIWFOS	Lipids and lip	Fatty Acyls	Lineolic acids
9.33_335.22	335.222939	9.3325	neg	10,11-dihydr	HMDB0012498		AHHXLFNP	Lipids and lip	Fatty Acyls	Eicosanoids
0.80_218.06	218.066744	0.79596667	neg	L-Proline, 1-a	HMDB0253913		BAPRUDZD	Organic acid	Carboxylic a	Amino acids,
7.19_353.23	353.233317	7.19245	neg	Sterebin G	HMDB00353	90676	VJXXTZUXT	Lipids and lip	Prenol lipids	Diterpenoids
7.30_181.08	181.085898	7.29506667	pos	5-(3E-Penter	HMDB00309	87335	KHWZQNG	Organoheter	Lactones	Gamma buty
7.90_377.22	377.229511	7.90143333	pos	Isoprostane	HMDB0243554		RZCPXIZGL	Lipids and lip	Fatty Acyls	Eicosanoids
0.70_245.04	245.042986	0.70456667	neg	Glycerophos	HMDB0240316		LLCSXHMJ	Organic acid	Organic phos	Phosphate e
0.74_187.05	187.057661	0.74288333	pos	1,5-Anhydros	HMDB00027	3775	MPCAJMNY	Organic oxy	Organooxyge	Carbohydrat
4.44_135.04	135.044229	4.435	pos	o-Phthalalde	HMDB0255866		ZWLUXSQA	Benzenoids	Benzene an	Benzoyl deri
4.91_267.05	267.051111	4.90568333	neg	p-Coumaroyl	HMDB0301727		RMPWEBYR	Phenylpropa	Cinnamic aci	Hydroxycinna
9.83_333.20	333.207193	9.82575	neg	Leukotriene	HMDB00050	36241	BISQPGCQ	Lipids and lip	Fatty Acyls	Eicosanoids
0.70_281.11	326.109086	0.70456667	neg	N-(1-Deoxy-1	HMDB00378	92651	JUBGLICOZ	Organic oxy	Organooxyge	Carbohydrat
10.16_440.2	440.276802	10.1580333	pos	LysoPE(0:0/1	HMDB00114	62260	GIUNBNKD	Lipids and lip	Glycerophos	Glycerophos

11.01_223.1	223.170103	11.0055	neg	Bornyl butyra	HMDB00382	92972	VIPNQHBVII	Lipids and lip	Prenol lipids	Monoterpeno
2.80_228.12	228.120564	2.80321667	pos	Panthenol	HMDB00042	3978	SNPLKNRPJ	Organic oxyg	Organooxyge	Alcohols and
3.59_204.08	203.082357	3.58845	neg	L-Tryptophar	HMDB00009	5879	QIVBCDIJIA	Organoheter	Indoles and	Indolyl carbo
9.34_359.21	359.218828	9.34135	pos	(+/-)-11,12-D	HMDB0242269		DNPZYIPMA	Lipids and lip	Fatty Acyls	Eicosanoids
9.46_414.28	414.286157	9.46038333	neg	Myristoleoylc	HMDB0240588		ABVVZYXTZ	Lipids and lip	Fatty Acyls	Fatty acid es
11.13_552.3	552.367198	11.1325833	neg	LysoPC(P-18	HMDB00131	40405	WBOMIOWF	Lipids and lip	Glycerophos	Glycerophos
3.70_180.10	180.101932	3.69811667	pos	alpha-Methyl	HMDB00292	86102	HYOWVAAE	Phenylpropa	Phenylpropa	Unclassified
0.62_146.16	146.165422	0.6215	pos	Spermidine	HMDB00012	254	ATHGHQPF	Organic nitro	Organonitrog	Amines
0.83_122.02	122.024594	0.82703333	neg	Isonicotinic a	HMDB00606	849	TWBYWOBI	Organoheter	Pyridines and	Pyridinecarb
0.84_226.01	226.011996	0.84308333	neg	L-Glutamic a	HMDB00012	6093	PJRXVIJAEF	Organic acid	Carboxylic a	Amino acids,
1.42_270.14	270.144842	1.41946667	pos	Tyrosyl-Alani	HMDB0029098		NLKUJNGE	Organic acid	Carboxylic a	Amino acids,
11.01_462.2	462.29898	11.0055	neg	LysoPE(P-18	HMDB0240599		URGVIWXY	Lipids and lip	Glycerophos	Glycerophos
3.97_138.05	138.055892	3.97085	neg	3-Methylcrot	HMDB00004	5447	PFWQSHXP	Organic acid	Carboxylic a	Amino acids,
4.19_243.17	243.171267	4.1908	neg	Isoleucyl-Isol	HMDB0028910		BCVIOZZGJ	Organic acid	Carboxylic a	Amino acids,
5.28_274.20	274.201065	5.28283333	pos	Heptanoylca	HMDB0013238		VDPCTFWU	Lipids and lip	Fatty Acyls	Fatty acid es
8.79_412.30	412.305154	8.7919	pos	3-Hydroxyhe	HMDB0013335		CPHHQDCI	Lipids and lip	Fatty Acyls	Fatty acid es
9.74_119.08	119.085601	9.74061667	pos	4-Methylstyr	HMDB0246518		JLBJTVDPS	Benzenoids	Benzene and	Styrenes
0.68_219.02	219.026511	0.68255	pos	scyllo-Inosito	HMDB0006088		CDAISMWE	Organic oxyg	Organooxyge	Alcohols and
10.52_590.3	590.346511	10.5172833	neg	LysoPC(20:3	HMDB00103	61704	BBNHCUBQ	Lipids and lip	Glycerophos	Glycerophos
6.49_225.11	225.113077	6.49351667	neg	Jasmolone	HMDB00300	86681	SVRKACAGI	Organic oxyg	Organooxyge	Alcohols and
0.61_130.08	130.086392	0.60603333	pos	Vigabatrin	HMDB00152	3024	PJDFLNIOA	Organic acid	Carboxylic a	Amino acids,
0.68_106.05	106.050121	0.68255	pos	L-Serine	HMDB00001	5203	MTCFGRXM	Organic acid	Carboxylic a	Amino acids,
0.72_168.99	168.990473	0.71928333	neg	Glyceraldehy	HMDB00011	149	LXJXRIRHZI	Organic oxyg	Organooxyge	Carbohydrate
0.74_154.02	154.026443	0.74288333	pos	5-(Hydroxym	HMDB0246731		HOBJEFOCI	Organoheter	Pyrrolidines	Pyrrolidones
1.20_230.04	230.045684	1.20368333	pos	(2R,2'S)-Isob	HMDB00304	86955	QSPWUNSF	Organic acid	Carboxylic a	Amino acids,
11.55_225.1	225.185742	11.5461833	neg	5Z-Tetradec	HMDB00005	5506	AFGUVBVUI	Lipids and lip	Fatty Acyls	Fatty acids a
2.48_189.12	189.123545	2.48101667	pos	Glycylleucine	HMDB00007	5727	DKEXFJVM	Organic acid	Carboxylic a	Amino acids,
2.87_233.14	233.149616	2.8749	pos	Threonylisole	HMDB00290	23987	LUMXICQAC	Organic acid	Carboxylic a	Amino acids,
3.80_413.14	413.142022	3.80401667	pos	Todatriol glu	HMDB00372	92213	OFPQILBGS	Lipids and lip	Fatty Acyls	Fatty acyl gly
4.13_195.08	195.087871	4.12765	pos	Caffeine	HMDB00018	1455	RYYVLZVUV	Organoheter	Imidazopyrim	Purines and
4.91_255.08	255.087371	4.90568333	neg	5,6-Dihydro-	HMDB00335	89329	MEZHTUKD	Organoheter	Pyrans	Pyranones a
7.57_377.22	377.229496	7.57063333	pos	11-Epi-PGF2	HMDB0011137		PXGPLTOD	Lipids and lip	Fatty Acyls	Eicosanoids
0.70_184.07	184.073332	0.69861667	pos	4-Trimethyla	HMDB0001161		JHPNVNIEX	Lipids and lip	Fatty Acyls	Fatty acids a

0.72_130.06	130.061972	0.71928333	neg	Beta-Guanidi	HMDB00132	44712	KMXXSJLYV	Organic nitro	Organonitrog	Guanidines
0.89_160.06	160.061329	0.8943	neg	beta-D-Gluc	HMDB00300	266	MSWZFWKN	Organic oxyg	Organooxyge	Carbohydrate
1.30_205.06	205.064143	1.30165	pos	Threonylcyst	HMDB00290	23771	CUTPSEKW	Organic acid	Carboxylic a	Amino acids,
10.35_337.2	337.238408	10.3534833	neg	7-[3-Hydroxy	HMDB02445	45575	DPOINJQW	Lipids and lip	Fatty Acyls	Eicosanoids
9.88_314.24	337.23445	9.88461667	pos	12,13-DHOM	HMDB0004705		CQSLTKIXA	Lipids and lip	Fatty Acyls	Fatty acids a
0.70_131.03	131.034049	0.69861667	pos	Glutaconic a	HMDB00006	5593	XVOUMQNX	Organic acid	Carboxylic a	Dicarboxylic
0.70_244.02	244.022444	0.70456667	neg	O-phosphona	HMDB00626	63612	FXDNYOAN	Organic acid	Carboxylic a	Amino acids,
1.14_367.12	367.122335	1.13681667	pos	Melibiitol	HMDB00067	3483	PYZZIILDSA	Lipids and lip	Fatty Acyls	Fatty acyl gly
4.61_172.09	172.097747	4.61413333	neg	N-Acetylisole	HMDB0061684		JDTWZSUN	Organic acid	Carboxylic a	Amino acids,
8.23_366.26	366.263411	8.23126667	pos	Alfadolone	HMDB0248250		XWYBFXIUI	Lipids and lip	Steroids and	Hydroxyster
3.72_326.20	326.207477	3.71513333	pos	L-Proline, 1-(	HMDB0253022		DPURXCQC	Organic acid	Carboxylic a	Amino acids,
3.82_231.17	231.170343	3.82191667	pos	Valylleucine	HMDB00291	23950	XCTHZFGS	Organic acid	Carboxylic a	Amino acids,
8.44_253.14	253.144406	8.43766667	neg	(5R,6S)-5,6-I	HMDB00349	90350	ZTJZJYUGO	Organoheter	Oxepanes	Unclassified
0.79_142.08	142.086268	0.7877	pos	(2R,3S)-3-Hy	HMDB0242121		DJMIFDZBC	Organic acid	Carboxylic a	Amino acids,
1.14_197.08	197.089669	1.13681667	pos	Formyllysine	HMDB0252452		LIEIRDDOGI	Organic acid	Carboxylic a	Amino acids,
1.20_260.19	260.196814	1.20368333	pos	Lysylleucine	HMDB00289	23967	ATIPDCIQT	Organic acid	Carboxylic a	Amino acids,
1.92_267.13	267.13402	1.9162	pos	Phenylalanyl	HMDB00290	23662	NYQBYASW	Organic acid	Carboxylic a	Amino acids,
4.58_359.12	359.124982	4.57715	neg	Cyclopropyl-r	HMDB0259618		DRAFVCKN	Organoheter	Azoles	Imidazoles
0.68_189.13	189.134608	0.68255	pos	Homo-L-argi	HMDB00006	5640	QUOGESRF	Organic acid	Carboxylic a	Amino acids,
0.70_176.05	176.056135	0.70456667	neg	cis-4-Hydrox	HMDB0240251		PMMYEEVY	Organic acid	Carboxylic a	Amino acids,
0.70_243.02	243.026517	0.69861667	pos	Galactose 1-	HMDB00006	455416	HXXFSFRB	Organic oxyg	Organooxyge	Carbohydrate
1.88_217.15	217.1547	1.88096667	pos	Valylvaline	HMDB00291	23988	KRNYOVHE	Organic acid	Carboxylic a	Amino acids,
10.32_299.2	299.236525	10.3206667	pos	Norbolethone	HMDB00060	412263	FTBJKONN	Lipids and lip	Steroids and	Pregnane ste
11.52_574.3	574.313146	11.5217833	pos	PG(22:6(4Z,7Z,10Z,13Z,1		80004	MGCOSBZS	Lipids and lip	Glycerophos	Glycerophos
2.46_298.12	298.128583	2.46275	pos	Phenethylam	HMDB00103	61646	OYNLGXUY	Organic oxyg	Organooxyge	Carbohydrate
4.42_271.08	271.084624	4.41721667	pos	Aminogluteth	HMDB00145	945	ROBVIMPU	Benzenoids	Benzene anc	Aniline and s
7.85_327.21	327.217595	7.84718333	neg	9-F1-phytopr	HMDB0304641		GKBLNJXQ	Lipids and lip	Fatty Acyls	Eicosanoids
11.98_251.2	251.201454	11.98215	neg	D6-Ambretto	HMDB00378	92617	HMWPDRY	Phenylpropa	Macrolides a	Unclassified
5.08_601.26	601.26557	5.08273333	pos	APC	HMDB00606	71230	BSVVZICJF	Organic oxyg	Organooxyge	Carbonyl cor
5.51_151.03	151.039862	5.51431667	neg	Furaneol ace	HMDB00329	88865	VPKIUOQJ	Organoheter	Dihydrofuran	Furanones
0.73_227.06	227.063684	0.72825	pos	L-beta-aspar	HMDB00111	62011	ARSVRKCV	Organic acid	Peptidomime	Hybrid peptic
0.76_178.01	178.017682	0.76461667	neg	S-Carboxym	HMDB00294	86253	GBFLZEXE	Organic acid	Carboxylic a	Amino acids,
0.96_145.04	145.049602	0.96066667	pos	(2S)-2-[(2S,3	HMDB0246074		WZYRMLAW	Organoheter	Oxolanes	Unclassified

1.08_215.01(	215.016088	1.07531667	pos	Citric acid	HMDB00000	124	KRKNYBCH;	Organic acid:	Carboxylic ac	Tricarboxylic
1.27_251.06:	251.063739	1.26745	pos	1-[(2R,4R,5F	HMDB0246409		RPQZTTQVI	Nucleosides,	Pyrimidine nu	Unclassified
5.76_262.10	262.107094	5.75788333	pos	Niazirin	HMDB00328	411218	OBJREHLZE	Organic oxyg	Organooxyge	Carbohydrate
5.89_455.09	455.09436	5.88578333	pos	Kaempferol 3	HMDB00405	95033	FFFIPDPCG	Phenylpropa	Flavonoids	Flavonoid gly
0.73_175.05	175.057614	0.72825	pos	Ribitol	HMDB0000508		HEBKCHPV(	Organic oxyg	Organooxyge	Carbohydrate
1.45_215.13	215.139188	1.45418333	pos	Prolyl-Valine	HMDB00290	23999	AWJGUZSY	Organic acid:	Carboxylic ac	Amino acids,
12.53_342.2!	342.299507	12.5329667	pos	N-Myristoyl L	HMDB0242054		GAYBJDZLM	Organic acid:	Carboxylic ac	Amino acids,
4.43_163.03!	163.03992	4.43021667	neg	cis-p-Couma	HMDB00306	87135	NGSWKAQJ	Phenylpropa	Cinnamic aci	Hydroxycinna
5.22_231.12:	231.123647	5.21915	neg	(1R,2R,4R)-1	HMDB00415	95928	PEWQMISW	Lipids and lip	Prenol lipids	Monoterpenc
5.39_377.08(	377.080291	5.38528333	neg	2-Hydroxyqu	HMDB02451	449258	XOQQVKDB	Organoheter	Quinolines a	Quinoline ca
9.18_301.21!	301.215686	9.17608333	pos	Ineketone	HMDB00366	67434	BWRPYSJNI	Lipids and lip	Prenol lipids	Diterpenoids
0.71_261.09!	262.103138	0.71368333	pos	Aspartyl-Gan	HMDB00287	85681	BEVYCXGD	Organic acid:	Carboxylic ac	Amino acids,
0.78_202.07	202.071881	0.7799	neg	N-Acetylman	HMDB00011	6024	OVRNDRQM	Organic oxyg	Organooxyge	Carbohydrate
1.28_219.13:	219.133769	1.28425	pos	Threoninyl-V	HMDB03047	85967	CKHWEVXP	Organic acid:	Carboxylic ac	Amino acids,
12.69_599.3:	599.320069	12.6870167	neg	LysoPI(18:0/	HMDB0240261		MXAFDFDAI	Lipids and lip	Glycerophos	Glycerophos
4.52_241.07	241.071923	4.52246667	neg	3-(3,4-Dihydr	HMDB0060735		GIIOASILGC	Phenylpropa	Phenylpropa	Unclassified
9.73_283.19	283.191393	9.73451667	neg	Guaidiol	HMDB00332	89095	RQHOQQOE	Lipids and lip	Prenol lipids	Sesquiterper
0.61_216.13!	216.1351	0.61033333	neg	beta-Alanyl-L	HMDB0060442		PLDCWKCP	Organic acid:	Peptidomime	Hybrid peptic
0.71_205.06	205.068161	0.71368333	pos	D-Iditol	HMDB0250766		FBPFZTCFM	Organic oxyg	Organooxyge	Carbohydrate
0.72_367.13!	367.135381	0.71928333	neg	N6-Galacturc	HMDB00331	88997	CFQJLIHML	Organic oxyg	Organooxyge	Carbohydrate
0.76_201.08	201.087822	0.76461667	neg	1-Aminocycl	HMDB00364	6029	PAJPWUMX	Organic acid:	Carboxylic ac	Amino acids,
1.38_133.05(	133.050435	1.37511667	neg	5-Deoxy-D-ri	HMDB0246776		WDRISBUVI	Organic oxyg	Organooxyge	Carbohydrate
10.91_318.2	341.208186	10.9125667	pos	4-Oxahexacy	HMDB0260287		ZHJDOAVXL	Organoheter	Oxepanes	Unclassified
10.98_467.3(	468.308047	10.9842333	pos	1-Heptadeca	HMDB00616	77696	RVNBVQKDI	Lipids and lip	Glycerophos	Glycerophos
11.54_501.3:	501.333071	11.5398167	pos	6-Deoxo-25-i	HMDB0301797		LCHQVKGT	Lipids and lip	Steroids and	Bile acids, al
13.88_295.2(	295.26396	13.87805	neg	10Z-Nonade	HMDB00136	35026	BBOWBNGL	Lipids and lip	Fatty Acyls	Fatty acids a
3.86_441.14(	442.147744	3.85685	pos	Folic acid	HMDB00001	246	OVBPIULPV	Benzenoids	Phenols	Methoxypher
4.60_251.10	251.102622	4.5955	pos	N-Acetylkynu	HMDB0240342		PPIMFXHQD	Organic oxyg	Organooxyge	Carbonyl cor
5.63_227.12	227.128719	5.6251	neg	3-Hydroxydo	HMDB00004	5402	FYVQCLGZI	Organic acid:	Hydroxy acid	Medium-chai
9.34_241.17!	241.179581	9.34135	pos	Tetradecane	HMDB00008	5833	HQHCYKULI	Lipids and lip	Fatty Acyls	Fatty acids a
9.78_499.26!	500.276621	9.77705	pos	LysoPE(20:5	HMDB00115	62303	KCWGBXXA	Lipids and lip	Glycerophos	Glycerophos
0.69_239.01(	239.016545	0.68961667	neg	L-Cystine	HMDB00001	5207	LEVWYRKD	Organic acid:	Carboxylic ac	Amino acids,
2.87_281.14!	281.149543	2.8749	pos	Valyltyrosine	HMDB00291	23812	VEYJKJORL	Organic acid:	Carboxylic ac	Amino acids,

3.68_199.03	198.032451	3.67736667	neg	4-Chloro-L-p	HMDB02446	44308	NIGWMJHC	Organic acid	Carboxylic ac	Amino acids,
4.83_748.50	749.515284	4.8302	pos	(2R,3S,4R,5	HMDB0242518		MQTOSJVF	Organic oxyg	Organooxyge	Carbohydrate
0.67_241.12	241.129596	0.66691667	pos	Homocarnos	HMDB00007	270	CCLQKVKJC	Organic acid	Peptidomime	Hybrid peptic
1.23_307.04	307.043684	1.23451667	pos	Arabinosylhy	HMDB0003040		UGQMRVRM	Nucleosides,	Purine nucle	Unclassified
10.26_293.2	293.212196	10.2627833	neg	9(S)-HOTrE	HMDB0247705		RIGGEAZDT	Lipids and lip	Fatty Acyls	Lineolic acids
10.81_247.1	247.170127	10.8069333	neg	Dimethylben	HMDB00322	88277	IRKDZQZMT	Benzenoids	Benzene and	Phenylpropa
10.93_266.2	266.211107	10.9300167	pos	2-Phenylethy	HMDB00377	351357	ASETYIALR	Lipids and lip	Fatty Acyls	Fatty acid es
12.20_303.2	303.231236	12.1975333	pos	2-Hydroxy-6-	HMDB00385	449737	VEPUCZUJL	Benzenoids	Benzene and	Benzoic acid
4.83_206.08	206.081984	4.83273333	neg	N-Acetyl-DL-	HMDB0255056		CBQJSKKF	Organic acid	Carboxylic ac	Amino acids,
4.99_316.21	316.211657	4.99255	pos	6-Oxononan	HMDB0241788		AHMPHMIC	Lipids and lip	Fatty Acyls	Fatty acid es
0.65_227.11	227.114218	0.6518	pos	beta-Alanyl-L	HMDB0256117		CQOVPNPJI	Organic acid	Peptidomime	Hybrid peptic
0.69_127.05	127.051046	0.68961667	neg	Alanylglycine	HMDB00068	23977	CXISPYVYM	Organic acid	Carboxylic ac	Amino acids,
0.73_157.03	157.036495	0.7342	neg	Allantoic acic	HMDB00012	343	NUCLJNSW	Organic acid	Carboxylic ac	Amino acids,
13.21_256.2	255.232791	13.21015	neg	Palmitic acid	HMDB00002	187	IPCSVZSSV	Lipids and lip	Fatty Acyls	Fatty acids a
3.56_294.14	294.14481	3.56223333	pos	Glutaminylph	HMDB0028804		VHLZDSUA	Organic acid	Carboxylic ac	Amino acids,
4.11_265.15	265.154667	4.10951667	pos	Valylphenyla	HMDB00291	24009	GJNDXQBA	Organic acid	Carboxylic ac	Amino acids,
4.34_297.12	297.126661	4.34396667	pos	Methionyl-Ph	HMDB0028980		HGCNKOLV	Organic acid	Carboxylic ac	Amino acids,
6.86_314.23	314.232157	6.85513333	pos	3-Decenoylc	HMDB0241067		YMJNFVUJ	Lipids and lip	Fatty Acyls	Fatty acid es
1.10_260.02	260.0232	1.09681667	neg	O-Sulfotyrosi	HMDB0155722		CIQHWLTY	Organic acid	Carboxylic ac	Amino acids,
10.92_317.2	317.211939	10.9157	neg	12-KETE	HMDB00136	35314	GURBRQGD	Lipids and lip	Fatty Acyls	Fatty acids a
2.53_246.14	246.144835	2.53481667	pos	Asparaginylis	HMDB00287	85649	MQLZLIYPF	Organic acid	Carboxylic ac	Amino acids,
2.70_149.02	149.024135	2.7013	neg	3, 5-Dihydro	HMDB0094709		IOVOJJDSF	Benzenoids	Phenols	Benzenediols
4.44_176.07	176.07077	4.435	pos	7-Amino-4-m	HMDB02472	65587	GLNDAGDH	Phenylpropa	Coumarins a	Unclassified
4.69_249.10	249.109706	4.68661667	pos	7-Epi-12-hyd	HMDB03037	36075	RZGFUGXQ	Lipids and lip	Fatty Acyls	Lineolic acids
6.31_341.17	341.173283	6.30893333	neg	Cortisone	HMDB00028	271	MFYSYFVPE	Lipids and lip	Steroids and	Hydroxysterc
9.24_267.11	267.113938	9.2416	neg	N'-Benzoyl-N	HMDB02552	343153	PQEZHJRK	Benzenoids	Benzene and	Benzoic acid
11.81_275.2	275.201352	11.8104333	neg	4,8,12,15-Oc	HMDB00326	88612	SIVLURJES	Lipids and lip	Fatty Acyls	Lineolic acids
3.58_223.10	223.107616	3.58011667	pos	Glycyl-Pheny	HMDB0028848		JBCLFWXM	Organic acid	Carboxylic ac	Amino acids,
4.36_251.13	251.138903	4.3621	pos	3-Hydroxyme	HMDB0060742		DWMWOUA	Organoheter	Indoles and	Indolines
5.07_373.14	373.140338	5.07146667	neg	(2S)-2-Aminc	HMDB0257556		FEFCOLGU	Organic acid	Carboxylic ac	Amino acids,
5.54_200.04	200.047148	5.53846667	pos	Tryptophol	HMDB00034	6932	MBBOMCVG	Organoheter	Indoles and	Indoles
6.81_417.19	417.191982	6.80563333	neg	Penicildione C			JRPKEULLJ	Lipids and lip	Steroids and	Steroids
8.99_343.27	344.279145	8.99243333	pos	Dodecanoylc	HMDB00022	6573	FUJLYHJRO	Lipids and lip	Fatty Acyls	Fatty acid es

0.76_127.08(	127.086596	0.75766667	pos		2-Aminophenol		491		CDAWCLOX	Unclassified	Unclassified	Unclassified
1.47_261.14	261.144611	1.47146667	pos		Leucyl-Glutaı	HMDB00289	85832		NFNVDJGXF	Organic acids	Carboxylic ac	Amino acids,
10.37_363.2	363.215279	10.37205	neg		18-Hydroxye	HMDB0257623			IVVBLUGHD	Lipids and lip	Fatty Acyls	Eicosanoids
10.66_277.1	277.180682	10.66295	neg		8-Acetoxy-4-	HMDB00309	87324		LDEHIZSFHI	Organic oxyg	Organooxyge	Carbonyl cor
2.28_102.05	102.055441	2.28218333	pos		4-Hydroxy-2-	HMDB00323	71806		VIHAEDVKX	Organoheter	Dihydrofuran	Furanones
7.62_498.28	498.289526	7.61696667	neg		Chenodeoxy	HMDB0242411			BHTRKEVKT	Lipids and lip	Steroids and	Bile acids, al
9.15_239.12	239.128818	9.14948333	neg		(2,2-Diethoxy	HMDB00416	327610		FYERTDTXC	Benzenoids	Benzene and	Unclassified
9.88_354.26	354.263472	9.88461667	pos		5(S),11(R)-D	HMDB00626	45981		GVBURXXH	Lipids and lip	Fatty Acyls	Eicosanoids
0.61_202.11	202.11957	0.61033333	neg		Lysylglycine	HMDB00289	23747		HGNRJCINZ	Organic acids	Carboxylic ac	Amino acids,
0.69_213.01	213.016907	0.68961667	neg		Polyribosylrib	HMDB0249913			VJDOAZKNE	Organic oxyg	Organooxyge	Carbohydrate
0.71_166.05	166.053179	0.71368333	pos		Ethiin	HMDB00370	92035		CSZTZFUEC	Organic acids	Carboxylic ac	Amino acids,
1.08_175.10	175.107884	1.07531667	pos		L-Theanine	HMDB00343	44433		DATAGRPVI	Organic acids	Carboxylic ac	Amino acids,
1.15_170.05	169.050454	1.14921667	neg		Herierin IV	HMDB00387	93410		QWSHNEPC	Organoheter	Pyrans	Pyranones ar
12.07_377.2	377.272578	12.0685667	neg		MG(20:4(8Z,	HMDB00115	62361		LWXLRBPW	Lipids and lip	Glycerolipids	Monoradylgly
2.26_282.14	282.142444	2.26408333	pos		Leucyl-Gamr	HMDB00289	85845		GSRPWCGE	Organic acids	Carboxylic ac	Amino acids,
4.07_334.14	334.140311	4.07303333	pos		Ac-Pro-Gly-F	HMDB0245745			GSBMVIVQF	Organic acids	Carboxylic ac	Amino acids,
4.12_263.14	263.140027	4.11676667	neg		Richter	HMDB0257218			DSSRNNGLI	Organic acids	Carboxylic ac	Amino acids,
4.56_279.16	279.169674	4.5592	pos		Leucylphenyl	HMDB00132	24020		KFKWRHQB	Organic acids	Carboxylic ac	Amino acids,
5.16_242.10	242.103268	5.15551667	pos		Sinapic acid	HMDB00326	45738		PCMORTLO	Phenylpropa	Cinnamic aci	Hydroxycinna
7.50_315.24	316.247864	7.49721667	pos		Decanoylcarı	HMDB00006	36669		LZOSYCMH	Lipids and lip	Fatty Acyls	Fatty acid es
0.79_167.11	167.11787	0.7877	pos		Venoterpine	HMDB00301	86772		IOIGOIPHPL	Organoheter	Pyridines and	Unclassified
1.15_199.07	199.07235	1.14921667	neg		1-(2-Hydroxy	HMDB0247200			ATKOZYBRE	Organoheter	Diazines	Pyrimidines a
10.14_386.3	386.326008	10.14065	pos		Pentadecanc	HMDB0062517			MDDWQHVI	Lipids and lip	Fatty Acyls	Fatty acid es
10.21_443.3	482.323775	10.2120667	pos		5-Hydroxyoct	HMDB0241522			UELBJSHFW	Lipids and lip	Fatty Acyls	Fatty acid es
4.88_168.10	168.101953	4.88355	pos		3-Hepteneoy	HMDB0094729			VCELCMWD	Organic acids	Carboxylic ac	Amino acids,
5.05_273.12	273.124606	5.05298333	neg		4-((6-Methox	HMDB02473	2082		KIMKJIXTIW	Organic acids	Carboxylic ac	Amino acids,
5.09_173.08	173.081647	5.08986667	neg		2,4-Dimethyl-	HMDB00398	94367		DMHLGGQH	Lipids and lip	Fatty Acyls	Fatty acids a
0.62_110.07	110.071598	0.6215	pos		3,4-Diaminop	HMDB02460	315851		OYTKINVCD	Organoheter	Pyridines and	Aminopyridin
0.67_239.11	239.114975	0.6745	neg		Azimexon	HMDB0248804			RPZOFMHR	Organic acids	Carboxylic ac	Amino acids,
0.72_118.99	118.998387	0.71928333	neg		Glyoxylic acid	HMDB00001	3213		HHLFWLYX	Organic acids	Carboxylic ac	Carboxylic ac
0.72_189.08	189.087715	0.71928333	neg		2-Amino-4-[(	HMDB00314	87663		BKHCIFAZU	Organic acids	Carboxylic ac	Amino acids,
0.72_229.04	229.047704	0.71928333	neg		4-Oxo-2-azet	HMDB0246560			YSPMLLKK	Organoheter	Lactams	Beta lactams
0.74_273.04	273.048175	0.74288333	pos		Serylglutami	HMDB00290	85931		LAFKUZYWI	Organic acids	Carboxylic ac	Amino acids,

10.38_319.2:	319.224017	10.3754333	pos	Prostaglandi	HMDB0302204		YBHMPNRD	Lipids and lip	Fatty Acyls	Eicosanoids
10.45_401.1:	401.122649	10.4453167	neg	sauchinone	HMDB0257508		GMTJIWUFF	Organoheter	Benzopyrans	1-benzopyra
10.73_260.2	260.200501	10.7334833	pos	Falcarinone	HMDB0033693		UQEBOJRX	Organic oxyg	Organooxyge	Carbonyl cor
4.76_307.13	307.13004	4.76071667	neg	Prolylphenyla	HMDB00111	23690	IWIANZLCJ\	Organic acid	Carboxylic a	Amino acids,
6.80_373.20	373.2008	6.79993333	pos	(Z)-7-[(1R,3F	HMDB0247474		CBOMORHD	Lipids and lip	Fatty Acyls	Eicosanoids
1.77_155.03	155.031499	1.77345	pos	Monoethyl m	HMDB00005	5558	HGINADPHJ	Organic acid	Carboxylic a	Dicarboxylic
0.61_325.16	325.160545	0.60603333	pos	Glutaminylar	HMDB00287	85705	OPINTGHFE	Organic acid	Carboxylic a	Amino acids,
0.67_283.06	283.069807	0.66691667	pos	Hydroxyproly	HMDB0028864		HXWNRESJ	Organic acid	Carboxylic a	Amino acids,
1.07_247.09	247.09323	1.06741667	neg	Serylproline	HMDB00290	23688	WBAXJMCU	Organic acid	Carboxylic a	Amino acids,
1.08_175.04	198.037246	1.07531667	pos	N-Acetyl-L-a	HMDB00008	5776	OTCCIMWX	Organic acid	Carboxylic a	Amino acids,
1.28_464.08	464.081344	1.28425	pos	Adenylsuccir	HMDB00005	3551	OFBHPPMP	Benzenoids	Benzene anc	Biphenyls an
1.61_154.08	154.083857	1.61191667	pos	L-Norleucine	HMDB00016	6334	LRQKBLKVF	Organic acid	Carboxylic a	Amino acids,
1.89_365.13	365.135238	1.89173333	neg	Semilepidino	HMDB0033108		ASPNXCICL	Organic oxyg	Organooxyge	Carbohydrate
2.26_284.08	284.088649	2.2575	neg	N4-Acetylcyti	HMDB00059	58335	NIDVTARKF	Nucleosides,	Pyrimidine nu	Unclassified
3.77_267.13	267.133664	3.76795	pos	N5-(4-Metho:	HMDB00335	89401	MLNAECTV	Organic acid	Carboxylic a	Amino acids,
3.93_194.11	194.115384	3.92891667	pos	Gabapentin	HMDB00050	2989	UGJMXCAK	Organic acid	Carboxylic a	Amino acids,
3.97_182.04	182.045585	3.97085	neg	4-Pyridoxic a	HMDB00000	239	HXACOUQI	Organoheter	Pyridines anc	Pyridinecarb
4.44_521.25	521.257511	4.435	pos	3b,6a-Dihydr	HMDB00415	95957	FCEJNLVNF	Lipids and lip	Fatty Acyls	Fatty acyl gly
8.22_352.22	351.217688	8.21615	neg	13,14-Dihydr	HMDB00027	2311	CUJMXIQZW	Lipids and lip	Fatty Acyls	Eicosanoids
8.60_311.22	311.222946	8.59883333	neg	9-Hydroxyno	HMDB0257844		VSOPTMREI	Lipids and lip	Fatty Acyls	Fatty alcohol
8.23_315.18	315.181141	8.23463333	neg	Menthol-gluc	HMDB0060004		JOHLBVHV\	Lipids and lip	Prenol lipids	Terpene glyc
0.70_152.99	152.995569	0.70456667	neg	beta-Glycero	HMDB00025	6708	DHCLVCXQI	Lipids and lip	Glycerophos	Glycerophos
0.70_229.01	229.011563	0.70456667	neg	1-deoxy-L-gly	HMDB03040	63860	OKYHYXLC	Organic acid	Organic phos	Phosphate e
0.71_180.08	180.086632	0.71368333	pos	(2R,3R,4S,5I	HMDB0242589		FZHXIRIBWI	Organic oxyg	Organooxyge	Carbohydrate
0.76_248.11	248.112737	0.75766667	pos	(3S,4R,5R)-1	HMDB0243864		MQDTXDVZI	Organoheter	Oxazinanes	Morpholines
1.28_132.06	132.06653	1.28425	pos	cis-Acetylacr	HMDB00604	66578	XGTKSWVC	Organic acid	Keto acids ar	Short-chain k
10.97_329.2	329.247355	10.9659	pos	Grifolin	HMDB00304	86985	PZHNKNRP	Lipids and lip	Prenol lipids	Sesquiterper
11.00_237.1	237.184566	11.0025333	pos	beta-Kessyl I	HMDB0036446		CSLBUZGPI	Lipids and lip	Prenol lipids	Sesquiterper
4.63_217.10	217.107904	4.63258333	neg	3-Hydroxysel	HMDB0000350		OQYZCCKC	Organic acid	Hydroxy acid	Medium-chai
0.62_331.14	331.147576	0.6215	pos	(2R)-2-Amin	HMDB0242138		BFSYFTQD	Organic acid	Carboxylic a	Amino acids,
0.70_190.07	190.07193	0.70456667	neg	L-cis-4-(Hydr	HMDB00294	86260	CISMGNUPI	Organic acid	Carboxylic a	Amino acids,
0.70_191.06	191.066161	0.69861667	pos	Aspartyl-Gly	HMDB0028753		JHFNSBBHK	Organic acid	Carboxylic a	Amino acids,
0.73_104.07	104.071664	0.72825	pos	Tromethamir	HMDB02402	43366	LENZDBCJC	Organic nitro	Organonitrog	Amines

0.74_150.05	173.04206	0.74288333	pos	D-Xylose	HMDB00000	314	SRBFZHDQ	Organic oxy	Organooxyge	Carbohydrate
0.80_385.06	385.069408	0.79596667	neg	5,6-Dioxo-3,7	HMDB0260428		QIPLCSYNR	Organic acid	Carboxylic ac	Amino acids,
1.10_148.04	148.047354	1.1046	pos	2-O-Methylcy	HMDB00043	58237	DHYLZDVD	Organoheter	Diazines	Pyrimidines a
1.14_190.04	213.037009	1.13681667	pos	2-Hydroxy-2-	HMDB0059927		FBBVDCTX	Organic acid	Keto acids ar	Medium-chai
10.08_333.2	333.207162	10.0807333	neg	Resolvin E3	HMDB0257164		WYCMUVN	Lipids and lip	Fatty Acyls	Eicosanoids
10.68_318.2	318.23989	10.6803667	pos	Ethynodiol	HMDB0252090		JYILPERKV	Lipids and lip	Steroids and	Estrane sterc
10.75_285.2	285.220794	10.7516833	pos	Norethandrol	HMDB0255730		ZDHCJEIGT	Lipids and lip	Steroids and	Pregnane ste
11.50_323.2	324.28919	11.5038	pos	Linoleoyl etha	HMDB00122	3718	KQXDGUVS	Organic nitro	Organonitrog	Amines
12.45_524.2	524.298981	12.4499667	neg	1-Stearoylgly	HMDB0061698		ZPDQFUYP	Lipids and lip	Glycerophos	Glycerophos
3.80_350.13	350.1356	3.80496667	neg	Threonyltrypt	HMDB00290	23729	KAFKKRJQ	Organic acid	Carboxylic ac	Amino acids,
4.04_186.07	186.076322	4.03688333	pos	3-Methoxysa	HMDB00597	96279	AUZQQIPZE	Benzenoids	Benzene and	Benzoic acid
5.59_245.13	245.139298	5.58803333	neg	Hexyl glucos	HMDB00316	87864	JVAZJLFFS	Lipids and lip	Fatty Acyls	Fatty acyl gly
7.56_421.22	421.220989	7.5615	neg	4-Hydroxy-D	HMDB0012777		IDXBOXWU	Lipids and lip	Fatty Acyls	Eicosanoids
9.74_197.15	197.153385	9.74061667	pos	Yuzu lactone	HMDB00329	88810	FZKPUQQW	Phenylpropa	Macrolides a	Unclassified
0.69_118.05	118.050829	0.68961667	neg	Aminoethoxy	HMDB0060587		GNRLUBOJI	Organic acid	Carboxylic ac	Amino acids,
0.73_258.03	258.038291	0.7342	neg	D-Glucosami	HMDB0250754		AEJSSXDYD	Organic oxy	Organooxyge	Carbohydrate
0.75_711.22	711.220113	0.74898333	neg	Stachyose	HMDB00035	6951	UQZIYBXSH	Organic oxy	Organooxyge	Carbohydrate
1.08_245.11	245.112974	1.07531667	pos	Pretyrosine	HMDB0304809		MIEILDYWG	Organic acid	Carboxylic ac	Amino acids,
1.89_331.07	331.070033	1.89173333	neg	2-Galloylgluc	HMDB00396	94182	DZAITDYEH	Benzenoids	Benzene and	Benzoic acid
10.34_405.2	405.227587	10.3358333	neg	Neuroprotect	HMDB00036	36435	CRDZYJSQ	Lipids and lip	Fatty Acyls	Fatty acids a
10.90_479.3	480.344282	10.8950333	pos	PC(O-16:1(11Z)/0:0)		76579	CSVZQGDL	Lipids and lip	Glycerophos	Glycerophos
4.58_381.10	381.106772	4.57715	neg	xi-2,3-Dihydr	HMDB00355	90795	ILGMGHZP	Organoheter	Indoles and	Indolyl carbo
4.72_203.11	202.108304	4.72411667	neg	N-Lactoylleu	HMDB0062176		BUMIGZVUJ	Organic acid	Carboxylic ac	Amino acids,
5.07_193.05	193.050354	5.07146667	neg	cis-Isoferulic	HMDB0259693		QURCVMIE	Phenylpropa	Cinnamic aci	Hydroxycinna
6.42_255.06	255.06266	6.41748333	pos	Chalconaring	HMDB00296	3402	YQHMWTPY	Phenylpropa	Linear 1,3-di	Chalcones ar
0.77_188.09	188.091759	0.7729	pos	3,4-Dihydrox	HMDB00003	5307	MTVWFVDW	Benzenoids	Phenols	Benzenediols
1.07_111.00	111.008585	1.06741667	neg	Gamma-delta	HMDB00132	319338	YHUFRVYV	Organic acid	Keto acids ar	Gamma-keto
1.63_129.05	129.05474	1.62985	pos	Solerol	HMDB00021	6526	QFCPIZSGD	Organoheter	Lactones	Unclassified
1.88_153.05	153.052204	1.88096667	pos	2H-Pyran-2-c	HMDB02448	44780	JYVXNLLUY	Organoheter	Lactones	Delta valerola
10.32_432.3	477.321947	10.31745	neg	4-cholesten-7	HMDB00623	57931	JZKUXZQO	Lipids and lip	Steroids and	Bile acids, al
10.73_804.5	804.57602	10.73455	neg	PE(18:0/19:1(9Z))		76866	AHZWSXIVF	Lipids and lip	Glycerophos	Glycerophos
4.13_138.09	138.091441	4.12765	pos	Lentialexin	HMDB00389	93603	AGWFAJXE	Lipids and lip	Fatty Acyls	Fatty alcohol
4.20_231.11	231.113221	4.19935	pos	Metomidate	HMDB02546	320926	FHFZEKYDS	Organoheter	Azoles	Imidazoles

8.23_317.21(	317.210337	8.23126667	pos		Prostaglandi	HMDB00600	3467		CMBOTAQM	Lipids and lip	Fatty Acyls	Eicosanoids
0.83_663.11	663.114098	0.82703333	neg		NAD	HMDB00009	101		BAWFJGJZ(	Nucleosides,	(5'->5')-dinuc	Unclassified
0.98_112.03	112.039682	0.97735	pos		1-Methylpyrr	HMDB0255336			ZIKUNELYR	Organoheter	Pyrroles	Substituted p
1.18_497.11	497.113971	1.18473333	neg		b-D-Glucuror	HMDB00397	94282		ZQXWNXNN	Organic oxyg	Organooxyge	Carbohydrate
10.98_353.2(	353.208877	10.9842333	pos		9,10-Epoxyo	HMDB00616	36010		ITTPZDMHC	Lipids and lip	Fatty Acyls	Fatty acids a
12.18_325.2	326.30483	12.17975	pos		Oleoylethanc	HMDB00020	24103		BOWVQLFM	Organic nitro	Organonitrog	Amines
3.61_231.17(	231.170117	3.6144	pos		Isoleucyl-Val	HMDB0028920			BCXBIONYY	Organic acid	Carboxylic ac	Amino acids,
4.16_184.03	167.034123	4.163	pos		Methyl gallat	HMDB02545	334339		FBSFWRHW	Benzenoids	Benzene anc	Benzoic acid
4.32_149.02	149.024187	4.32	neg		Vanillic acid	HMDB00004	5471		WKOLLVMJI	Benzenoids	Benzene anc	Benzoic acid
9.06_279.15	279.159224	9.05798333	neg		2-Octynoic a	HMDB02452	35231		BQDKCWCM	Lipids and lip	Fatty Acyls	Fatty acids a
0.61_234.14	234.144912	0.60603333	pos		Lysinoalanin	HMDB00294	86274		IMSOBGJSY	Organic acid	Carboxylic ac	Amino acids,
0.66_241.09	241.091819	0.65943333	neg		(S,S)-Nt-Hist	HMDB00402	94778		IZQGEZGZC	Organic acid	Carboxylic ac	Amino acids,
0.69_242.07	242.079528	0.68961667	neg		N-Acetylhisti	HMDB0032055			KBOJOGQF	Organic acid	Carboxylic ac	Amino acids,
0.73_426.02	426.022498	0.7342	neg		dGDP	HMDB00009	5906		CIKGWCTVF	Phenylpropa	Cinnamic aci	Hydroxycinna
10.77_528.3	528.309595	10.77115	neg		LysoPE(22:4	HMDB00115	62307		NJHKGJJIK(	Lipids and lip	Glycerophos	Glycerophos
12.36_254.2	253.217095	12.3628333	neg		Palmitoleic a	HMDB00032	188		SECPZKHBE	Lipids and lip	Fatty Acyls	Fatty acids a
12.74_451.3	451.306325	12.7441833	neg		[14]-Gingerol	HMDB0303091			GLKIRXHYC	Benzenoids	Phenols	Methoxypher
14.56_336.3	335.295427	14.5550833	neg		Docosadienc	HMDB00617	35068		HVGRZDAS	Lipids and lip	Fatty Acyls	Fatty acids a
3.66_118.06	118.065315	3.66498333	pos		Indole	HMDB00007	286		SIKJAQJRH	Organoheter	Indoles and	Indoles
4.91_261.03	261.035422	4.90568333	neg		N-acetyl-S-(	HMDB0060011			DJFUZUUKZ	Organic acid	Carboxylic ac	Amino acids,
5.70_151.03	151.039803	5.69895	neg		3-Methoxybe	HMDB00326	338731		XHQZJYCNI	Benzenoids	Benzene anc	Benzoic acid
0.72_161.04	161.045349	0.71928333	neg		3-Hydroxy-2-	HMDB0029169			JJSWRLIXM	Lipids and lip	Fatty Acyls	Fatty acids a
0.73_263.14(	263.146034	0.72825	pos		Streptidine	HMDB0258506			MSXMXWJP	Organic oxyg	Organooxyge	Alcohols and
1.03_239.07	239.077097	1.0341	neg		D-glycero-L-	HMDB00299	86612		HYWDXGG	Organic oxyg	Organooxyge	Carbohydrate
1.31_327.05	327.057287	1.30545	neg		Lumazine	HMDB02541	337520		UYEUUXMD	Organoheter	Pteridines an	Unclassified
10.18_351.2	351.213726	10.17625	pos		(5S)-hydrope	HMDB0062616			JIOJPWROV	Lipids and lip	Fatty Acyls	Eicosanoids
10.68_802.5	802.560777	10.6808167	neg		PE(20:2(11Z,14Z)/17:0)		77114		BYXDEQHY	Lipids and lip	Glycerophos	Glycerophos
12.26_449.2	449.290621	12.2643333	neg		MG(22:5(7Z,	HMDB00115	62368		IDSLCYURG	Lipids and lip	Glycerolipids	Monoradylgly
12.64_356.2	357.299326	12.6383167	pos		MG(18:1(9Z)/0:0/0:0)[rac]		4251		RZRNAYUH	Lipids and lip	Glycerolipids	Monoradylgly
13.21_781.5	804.549907	13.2068667	pos		PE-NMe2(15	HMDB0113934			MCAXWYRI	Lipids and lip	Glycerophos	Glycerophos
3.02_149.02	149.024167	3.0151	neg		2',4',6'-Trihyc	HMDB00296	337497		XLEYFDVV	Organic oxyg	Organooxyge	Carbonyl cor
7.39_512.26	512.267248	7.38693333	pos		N-[(3a,5b,7a	HMDB00024	57984		GLYPHOJM	Homogeneou	Homogeneou	Unclassified
8.09_395.18	395.189718	8.08708333	neg		Pregnenolon	HMDB0000774			DIJBBUIOW	Lipids and lip	Steroids and	Sulfated ster

9.61_415.32(	415.320562	9.61401667	pos		1alpha,23(S)	HMDB0062686		NHRGJVVEI	Lipids and lip	Steroids and	Vitamin D an
0.74_144.10	144.101935	0.74288333	pos		(R)-1-Methyl	HMDB0242107		BPSLZWSRI	Organic acid	Carboxylic ac	Amino acids,
0.83_132.06	132.065749	0.83391667	pos		3-Methylader	HMDB0011600		FSASIHFSF	Organoheter	Imidazopyrim	Purines and
1.17_232.12	232.129114	1.1705	pos		Asparaginyl-	HMDB00287	23904	KWBQPGIYI	Organic acid	Carboxylic ac	Amino acids,
10.10_315.2	315.253862	10.0986	neg		(9S,10S)-9,1	HMDB00596	45832	VACHUYIRE	Lipids and lip	Fatty Acyls	Fatty acids a
11.86_262.2	280.263031	11.8596167	pos		6,10,14-Trim	HMDB00344	89972	LTUMRKDL\	Lipids and lip	Prenol lipids	Diterpenoids
4.32_337.09	337.09275	4.32	neg		Ferulic acid 4	HMDB0301717		IEMIRSXOYI	Organic oxyg	Organooxyge	Carbohydrate
0.61_232.13	232.130161	0.61033333	neg		Lysylserine	HMDB00289	359296	YSZNURNV	Organic acid	Carboxylic ac	Amino acids,
0.70_293.09	293.098883	0.70456667	neg		3-(Carboxym	HMDB00335	89360	GAUBNQMY	Organic acid	Carboxylic ac	Amino acids,
0.91_282.15	282.155697	0.91118333	neg		Histidinyl-Lys	HMDB0253180		CZVQSYNVI	Organic acid	Carboxylic ac	Amino acids,
1.45_182.04	182.045617	1.44935	neg		Methyl nicoti	HMDB00298	86513	YNBADRVTZ	Organoheter	Pyridines and	Pyridinecarb
10.06_437.2	437.290601	10.0622	neg		3,7-Dihydrox	HMDB0246080		RUDATBOH	Lipids and lip	Steroids and	Bile acids, al
10.18_405.2	405.260855	10.17625	pos		13,14-Dihydr	HMDB0244515		TVHAZVBUY	Lipids and lip	Fatty Acyls	Eicosanoids
10.28_309.2	309.207073	10.2812167	neg		(11S,12S,13	HMDB0302825		XHMGEKKH	Lipids and lip	Fatty Acyls	Fatty acids a
4.22_334.14	334.140031	4.21765	pos		Glutamyltryp	HMDB00288	85744	LLEUXCDZF	Organic acid	Carboxylic ac	Amino acids,
5.22_241.07	241.073014	5.21915	neg		Hydroxytyros	HMDB0253298		FGJGLFPNI	Benzenoids	Phenols	Tyrosols and
9.06_293.17	293.175962	9.05798333	neg		[2-(Dimethox	HMDB00362	91367	QCHZKUPVI	Benzenoids	Benzene and	Unclassified
9.83_416.30	416.301868	9.82575	neg		Tetradecano	HMDB0005066		PSHXNVGS	Lipids and lip	Fatty Acyls	Fatty acid es
0.73_299.09	299.098132	0.7342	neg		D-Xylulose	HMDB0001644		LQXVFWRQ	Organic oxyg	Organooxyge	Carbohydrate
1.31_232.10	231.098428	1.30545	neg		Aspartyl-Vali	HMDB0028766		CPMKYMGG	Organic acid	Carboxylic ac	Amino acids,
10.14_267.1	267.19644	10.1353167	neg		beta-Eudesm	HMDB0249127		BOPIMTNSY	Lipids and lip	Prenol lipids	Sesquiterper
10.92_365.2	365.230681	10.9157	neg		Isocupressic	HMDB0253626		DOYKMKZYI	Lipids and lip	Prenol lipids	Diterpenoids
11.31_387.2	387.215378	11.3135833	neg		2-Hydroxy-6-	HMDB00310	87379	QUVGEKPN	Benzenoids	Benzene and	Benzoic acid
11.54_254.2	254.247408	11.5398167	pos		Palmitoleami	HMDB0256086		YRPQTVNC	Lipids and lip	Fatty Acyls	Fatty amides
3.70_275.11	275.10998	3.69811667	pos		Pantoyllactor	HMDB00412	95683	BFCZPWYLI	Lipids and lip	Fatty Acyls	Fatty acyl gly
5.40_255.11	255.114023	5.40378333	neg		Imazamethal	HMDB0034885		KFEFNHNXZ	Organic acid	Carboxylic ac	Amino acids,
5.48_399.11	399.117341	5.47755	neg		Allithiamine	HMDB0031758		WNCAVNGL	Organoheter	Diazines	Pyrimidines a
6.23_391.13	391.13748	6.23498333	neg		4-Hydroxy-6-	HMDB00406	95115	CVTPSMRX	Organic oxyg	Organooxyge	Carbonyl cor
6.38_191.10	191.103922	6.3809	pos		6-exo-Hydro	HMDB0182798		RBJVDSYEN	Lipids and lip	Prenol lipids	Monoterpeno
9.36_338.26	338.266074	9.35948333	pos		Mutilin	HMDB0254962		OBUUFWIMI	Lipids and lip	Prenol lipids	Diterpenoids
9.40_412.30	412.305437	9.39628333	pos		(9Z,12Z)-3-H	HMDB0240762		CPHHQDCI	Lipids and lip	Fatty Acyls	Fatty acid es
9.73_424.24	424.247196	9.73451667	neg		Sphingosine	HMDB00002	3891	DUYSYHSSI	Lipids and lip	Sphingolipids	Phosphosphi
0.61_246.14	246.14581	0.61033333	neg		Threonyllysir	HMDB0029066		YKRQRPFOI	Organic acid	Carboxylic ac	Amino acids,

0.79_148.09(	148.096716	0.7877	pos	Daunosamin	HMDB0250880		WPJRFCZK	Organic nitro	Organonitrog	Amines
10.14_359.2	359.218723	10.14065	pos	8S,15S-Dihy	HMDB0247570		NNPWRKSG	Lipids and lip	Fatty Acyls	Eicosanoids
10.43_317.2	317.210344	10.4295333	pos	7'-Carboxy-g	HMDB0012850		JFXDIXKMIN	Organoheter	Benzopyrans	1-benzopyra
10.95_344.2	344.257229	10.9478667	pos	3a-Hydroxyd	HMDB0060755		ZMLDTNLD	Lipids and lip	Steroids and	Estrane sterc
7.88_253.14	253.14443	7.88408333	neg	(5alpha,8bet	HMDB00346	90126	ULAJGYNC	Organoheter	Benzopyrans	Unclassified
0.73_221.08	266.087931	0.7342	neg	(2R,5S)-5-Ar	HMDB0255319		KFTDHDXFE	Organic oxyg	Organooxyge	Carbohydrate
0.82_276.14	276.144067	0.81883333	pos	3,4,5-Trihydr	HMDB0241652		CLDWQVMF	Lipids and lip	Fatty Acyls	Fatty acid es
10.16_468.3	468.367972	10.1580333	pos	3alpha,7alph	HMDB0062207		CNWPIIOQK	Lipids and lip	Steroids and	Bile acids, al
11.17_199.1	199.170059	11.1685	neg	Dodecanoic	HMDB00006	5611	POULHZVOI	Lipids and lip	Fatty Acyls	Fatty acids a
15.27_648.6	648.627974	15.2723833	pos	Cer(d18:1(4E)/24:0(3OH))			JUKHHDVP	Lipids and lip	Sphingolipids	Ceramides
4.06_245.16	246.170138	4.05526667	pos	Isovalerylcar	HMDB0000688		IGQBPDJNU	Lipids and lip	Fatty Acyls	Fatty acid es
5.11_245.01	245.012384	5.10843333	neg	4-Vinylpheno	HMDB0062775		IETVQHUKT	Organic acid	Organic sulfu	Arylsulfates
5.17_275.13(	275.1307	5.17355	pos	Chlorphenira	HMDB00019	1734	SOYKEARSI	Organoheter	Pyridines and	Pheniramines
6.59_387.20	387.204924	6.58565	neg	(Z)-5-((2R,3S	HMDB0245411		RJHNVFKNI	Lipids and lip	Fatty Acyls	Eicosanoids
7.59_347.18	347.182469	7.58896667	pos	12-Oxo-20-c	HMDB0012550		VLMCDTMN	Lipids and lip	Fatty Acyls	Fatty acids a
9.97_379.21(	379.210278	9.97076667	neg	Prostaglandi	HMDB0302205		PRFXRIUZN	Lipids and lip	Fatty Acyls	Eicosanoids
0.69_174.08	174.088073	0.68961667	neg	Carbamoyl (2	HMDB0257951		CFSBKLYCF	Organic acid	Carboxylic ac	Amino acids,
0.76_125.07	125.071142	0.75766667	pos	Ectoine	HMDB02406	43384	WQXNXVUD	Organic acid	Carboxylic ac	Amino acids,
0.80_344.13	344.133796	0.80285	pos	N-(1-Deoxy-1	HMDB00378	92653	QLJKHQIOD	Organic acid	Carboxylic ac	Amino acids,
1.07_288.12(	288.119992	1.06741667	neg	Ophthalmic a	HMDB00057	58300	JCMUOFQH	Organic acid	Carboxylic ac	Amino acids,
10.48_347.1	347.183724	10.48035	neg	Testololactor	HMDB0258855		CNIXJDVUM	Organoheter	Naphthopyra	Unclassified
10.75_301.2	301.217142	10.7528167	neg	Eicosatetrae	HMDB0244671		GQHZVWMI	Lipids and lip	Fatty Acyls	Eicosanoids
11.40_331.2(	331.262575	11.3962667	pos	Sclareol	HMDB0036827		XVULBTBTF	Lipids and lip	Prenol lipids	Diterpenoids
2.41_295.12	295.12893	2.40855	pos	L-gamma-Gl	HMDB00385	93191	IDPGQVFK	Organic acid	Carboxylic ac	Amino acids,
4.00_262.11	262.118956	4.00056667	pos	Tryptophyl-G	HMDB0029083		UYKREHOK	Organic acid	Carboxylic ac	Amino acids,
4.97_391.23(	391.230578	4.97433333	pos	(3S,5R,6S,7	HMDB00411	95598	MRPDHXXP	Lipids and lip	Fatty Acyls	Fatty acyl gly
5.08_207.09	207.099075	5.08273333	pos	1,3-Bis(hydr	HMDB0303357		SJGVIHKTO	Lipids and lip	Prenol lipids	Monoterpenc
6.03_369.22	369.228122	6.0318	neg	6-Keto-prost	HMDB00028	6782	KFGOFTHOI	Lipids and lip	Fatty Acyls	Eicosanoids
7.20_336.22	337.236608	7.20335	pos	Prostaglandi	HMDB00026	407	BGKHCLZF	Lipids and lip	Fatty Acyls	Eicosanoids
9.73_351.21	351.21777	9.73451667	neg	Prostaglandi	HMDB00013	3495	YIBNHAJFJ	Lipids and lip	Fatty Acyls	Eicosanoids
0.61_163.10	163.107814	0.60603333	pos	5-Hydroxylys	HMDB00004	5439	YSMODUON	Organic acid	Carboxylic ac	Amino acids,
0.70_291.12	291.129675	0.69861667	pos	N2-(3-Carbo	HMDB00394	93983	JRDIRZIEGS	Organic acid	Carboxylic ac	Amino acids,
0.77_160.13	160.133181	0.7729	pos	Aminovaleric	HMDB0240732		CDLVFVFTF	Lipids and lip	Fatty Acyls	Fatty acids a

0.79_136.03	137.045773	0.7877	pos	Erythronic ac	HMDB00006	5586	JPIJQSOTBS	Organic oxy	Organooxyge	Carbohydrate
0.79_278.06	278.064928	0.7877	pos	beta-D-ribos	HMDB0304540		PUEDDPCU	Organic oxy	Organooxyge	Carbohydrate
1.07_280.06	280.068544	1.06741667	neg	8-Oxoguanos	HMDB0247459		FPGSEBKFE	Nucleosides,	Purine nucle	Unclassified
1.08_148.04	148.043794	1.08208333	neg	L-Methionine	HMDB00006	5664	FFEARJCKV	Organic acid	Carboxylic a	Amino acids,
1.08_355.07	355.075697	1.08208333	neg	Glycyl-Cystei	HMDB0028838		MFBYPDKT	Organic acid	Carboxylic a	Amino acids,
1.28_103.03	103.039376	1.28425	pos	Acetoacetic	HMDB00000	276	WDJHALXB	Organic acid	Keto acids a	Short-chain
10.27_339.2	339.250675	10.2662	pos	(+)-15,16-Di	HMDB00310	74574	LSFLNLHTO	Lipids and lip	Fatty Acyls	Fatty acids a
11.51_257.2	257.212044	11.5124	neg	2-Tetradecar	HMDB00309	457097	POQLVOYR	Organic oxy	Organooxyge	Carbonyl cor
4.02_139.07	139.07553	4.01898333	pos	Tyrosol	HMDB00042	7044	YCCILVSKP	Benzenoids	Phenols	Tyrosols and
4.30_505.15	505.158792	4.30156667	neg	Methyl salicy	HMDB00331	89024	CTLIPTKQW	Organic oxy	Organooxyge	Carbohydrate
5.70_179.03	179.034878	5.69895	neg	(E)-3-(2,3-Di	HMDB0032057		SIUKXCMDY	Phenylpropa	Cinnamic aci	Hydroxycinna
7.63_377.29	377.298376	7.62578333	pos	12-Hydroxyd	HMDB0241208		FGDJUUIZT	Lipids and lip	Fatty Acyls	Fatty acid es
9.86_277.14	277.144374	9.86181667	neg	Collybial	HMDB00410	95471	HMBNCKSB	Organic oxy	Organooxyge	Carbonyl cor
0.70_210.13	210.133566	0.69861667	pos	2-[Bis-(2-hyd	HMDB02492	353689	OWMVSZAM	Organic nitro	Organonitrog	Amines
0.80_151.02	151.025952	0.79596667	neg	Glycolic acid	HMDB00001	3219	AEMRFAOFI	Organic acid	Hydroxy acid	Alpha hydrox
1.22_202.07	202.070938	1.2188	pos	3,4-Dihydrox	HMDB00018	696	RGHMISIYK	Benzenoids	Phenols	Benzenediols
1.32_275.04	275.047631	1.31801667	pos	Serylmethion	HMDB00290	23979	PBUXMVYW	Organic acid	Carboxylic a	Amino acids,
10.21_627.5	627.534015	10.2120667	pos	DG(18:0/20:	HMDB00071	58760	BRKLTVAHE	Lipids and lip	Glycerolipids	Diradylglycer
10.90_719.5	719.534702	10.8978167	neg	PE-Cer(d14:1(4E)/21:0)		103046	BPYVWEJRI	Lipids and lip	Sphingolipids	Phosphosphi
11.50_347.2	348.289142	11.5038	pos	Anandamide	HMDB00040	3721	LGEQQWM	Organic nitro	Organonitrog	Amines
3.64_172.99	172.991095	3.64241667	neg	Phenol sulph	HMDB0060015		CTYRPMDG	Organic acid	Organic sulfu	Arylsulfates
4.88_354.05	355.063144	4.88355	pos	Phenolsulfonphthalein		69460	BELBBZDIHI	Unclassified	Unclassified	Unclassified
5.68_409.22	409.222764	5.68048333	neg	Reichstein's	HMDB0245599		AWWCEQO	Lipids and lip	Steroids and	Hydroxysterc
8.60_291.19	291.192676	8.5985	pos	16-B1-phyto	HMDB03046	985566	WJSAFQZK	Lipids and lip	Fatty Acyls	Eicosanoids
9.81_357.20	357.202562	9.81245	pos	Resolvin E2	HMDB0257163		KPRHYAOS	Lipids and lip	Fatty Acyls	Eicosanoids
0.61_261.15	261.155713	0.60603333	pos	Lysylasparag	HMDB00289	23920	JPNRPAJIT	Organic acid	Carboxylic a	Amino acids,
0.70_233.07	233.077647	0.70456667	neg	H-Hyp-gly-O	HMDB0253018		WFDSWNX	Organic acid	Carboxylic a	Amino acids,
0.76_303.01	303.011856	0.76461667	neg	6-Phosphon	HMDB00011	6022	IJOJIVNDF	Organic oxy	Organooxyge	Carbohydrate
1.10_135.06	136.075834	1.1046	pos	2-Aminoacet	HMDB00326	327691	HEQOJEGT	Organic oxy	Organooxyge	Carbonyl cor
3.53_367.15	367.150476	3.52578333	pos	Tryptophan	HMDB0240296		CPXSBHKD	Organoheter	Indoles and	Indolyl carbo
3.60_146.06	146.060087	3.59728333	pos	6-Hydroxy-3,	HMDB0246939		HOSGXJW	Organoheter	Quinolines a	Quinolones a
4.61_193.04	193.049631	4.61381667	pos	5-Hydroxyfer	HMDB0035484		YFXWTVLD	Phenylpropa	Cinnamic aci	Hydroxycinna
4.63_318.18	318.181298	4.63161667	pos	Isoleucyl-Try	HMDB0028918		BVRPESWO	Organic acid	Carboxylic a	Amino acids,

7.10_528.26	528.263507	7.0999	neg		Glycochenod	HMDB00024	6702		DKXXSIJHW	Lipids and lip	Steroids and	Bile acids, al
7.39_347.18	347.182594	7.38693333	pos		Cibaric acid	HMDB00385	93247		LFTUCYCU	Lipids and lip	Fatty Acyls	Lineolic acids
8.23_352.22	375.213658	8.23126667	pos		(5Z)-(15S)-1	HMDB0012481			CUJMXIQZW	Lipids and lip	Fatty Acyls	Eicosanoids
0.70_110.02	110.027308	0.69861667	pos		Hypotaurine	HMDB00009	281		VVIUBCNYA	Organic acid	Sulfinic acids	Sulfinic acids
1.10_164.04	182.081152	1.1046	pos		2-Hydroxycin	HMDB00026	6727		PMOWTIHVI	Phenylpropa	Cinnamic aci	Hydroxycinna
13.24_333.2	333.27811	13.2417667	pos		3,4-Dimethyl	HMDB01120	922615		IGZNOKSSN	Lipids and lip	Fatty Acyls	Fatty acids a
4.34_302.15	302.150118	4.34396667	pos		Prolyl-Trypto	HMDB0029028			UEKYKRQIA	Organic acid	Carboxylic ac	Amino acids,
5.07_343.13	343.139483	5.07146667	neg		2-Octenedioi	HMDB00003	5330		BNTPVRGY	Lipids and lip	Fatty Acyls	Fatty acids a
7.47_464.30	464.30147	7.46943333	neg		Glycocholic a	HMDB00001	202		RFDAIACW	Lipids and lip	Steroids and	Bile acids, al
7.94_353.22	353.229336	7.93806667	pos		Sterebin B	HMDB0035338			BNOWPELK	Lipids and lip	Prenol lipids	Sesquiterper
9.52_201.14	201.149474	9.51548333	neg		Dihydrocitror	HMDB00378	92618		CBOBADCV	Organic oxyg	Organooxyge	Carbonyl cor
9.85_307.22	307.224063	9.84861667	pos		Avocadyne	HMDB00354	43517		OHLQBKZX	Lipids and lip	Fatty Acyls	Fatty alcohol
0.98_851.26	851.26354	0.97735	pos		Verbascose	HMDB0259784			FLUADVWH	Organic oxyg	Organooxyge	Carbohydrate
1.15_312.10	312.108981	1.14921667	neg		5'-O-beta-D-	HMDB0246884			MDLTWTOQ	Organoheter	Pyridines and	Pyridoxines
1.74_283.12	283.12892	1.73755	pos		Tyrosyl-Thre	HMDB00291	86007		MFEVVAXTE	Organic acid	Carboxylic ac	Amino acids,
10.32_199.1	200.200693	10.3206667	pos		Dodecanami	HMDB02515	36671		ILRSCQWRE	Lipids and lip	Fatty Acyls	Fatty amides
10.88_306.2	306.278726	10.8770833	pos		Candol B	HMDB0036727			TUJQVRFW	Lipids and lip	Prenol lipids	Diterpenoids
11.39_397.2	397.259161	11.3861833	neg		MG(0:0/18:3	HMDB00115	62324		ZCCLDKGW	Lipids and lip	Fatty Acyls	Lineolic acids
9.55_360.27	360.275577	9.55203333	neg		Dehydrophyt	HMDB0038057			CQKNELOTI	Organic nitro	Organonitrog	Amines
0.63_199.00	199.001091	0.62835	neg		D-Erythrose	HMDB00013	6158		NGHMDNPX	Organic oxyg	Organooxyge	Carbohydrate
0.70_315.08	315.080984	0.70456667	neg		1-(5-Hydroxy	HMDB0248150			MLVVTNIFH	Organoheter	Azoles	Imidazoles
0.79_252.10	252.108777	0.7877	pos		Muramic acic	HMDB0003254			MSFSPUZXI	Organic oxyg	Organooxyge	Carbohydrate
1.07_122.02	122.024549	1.06741667	neg		Nicotinic acic	HMDB00014	6272		PVNIIMVLH	Organoheter	Pyridines and	Pyridinecarb
10.77_496.3	496.364115	10.77115	neg		(11Z,14Z)-Ei	HMDB0240747			OLZWDVKT	Lipids and lip	Fatty Acyls	Fatty acid es
11.84_425.2	425.265586	11.8413833	pos		7b-Hydroxy-	HMDB00005	5526		UZRRNRRC	Lipids and lip	Steroids and	Bile acids, al
3.84_327.07	327.072226	3.84145	neg		bergenin	HMDB0249087			YWJXCIXBA	Benzenoids	Benzene and	Benzoic acid
8.67_377.23	377.233276	8.67263333	neg		21-Hydroxyp	HMDB00040	7002		MOIQRAOBI	Lipids and lip	Steroids and	Hydroxysterc
0.71_274.09	274.09182	0.71368333	pos		N-(1-Deoxy-1	HMDB00386	93320		BDMCSTCU	Organic oxyg	Organooxyge	Carbohydrate
0.76_155.00	155.009503	0.76461667	neg		Orotic acid	HMDB00002	318		PXQPEWDE	Organoheter	Diazines	Pyrimidines a
11.59_457.3	457.306523	11.5931667	pos		20a,22b-Dihy	HMDB0006763			ISBSSBGEY	Lipids and lip	Steroids and	Bile acids, al
11.82_304.2	304.263128	11.82365	pos		Arachidonoyl amine		36680		BNBSCAZC	Lipids and lip	Fatty Acyls	Fatty amides
13.10_299.2	299.258911	13.0968333	neg		7-Hydroxyoc	HMDB01121	35437		AROVHWXX	Lipids and lip	Fatty Acyls	Fatty acids a
7.23_355.24	355.248853	7.22931667	neg		Prostaglandi	HMDB00026	3503		DZUXGQBLI	Lipids and lip	Fatty Acyls	Eicosanoids

9.11_397.22	397.222165	9.1134	neg	20-hydroxyle	HMDB0245567		PTJFJXLGR	Lipids and lip	Fatty Acyls	Eicosanoids
9.74_426.26	426.26078	9.74061667	pos	LysoPE(0:0/1	HMDB00114	62258	DDUZNLUW	Lipids and lip	Glycerophos	Glycerophos
0.76_205.03	205.035095	0.76461667	neg	3-(Carboxym	HMDB0059932		CQOIYSASV	Organic acid	Carboxylic ac	Tricarboxylic
0.90_275.00	275.008632	0.89581667	pos	Glucuronic a	HMDB0252795		FHYMOZUIS	Organic oxyg	Organooxyge	Carbohydrate
1.11_211.10	211.108611	1.1137	neg	Hydroxyproly	HMDB00288	85782	OBSIPTVJS	Organic acid	Carboxylic ac	Amino acids,
1.49_259.12	259.129871	1.4863	neg	Valylproline	HMDB00291	23715	GIAZPLMM	Organic acid	Carboxylic ac	Amino acids,
11.22_498.2	498.282415	11.2158833	pos	Demethylzey	HMDB0250994		ZDZSFWLP	Benzenoids	Phenanthren	Unclassified
11.98_401.2	401.265624	11.9841667	pos	MG(20:4(5Z,	HMDB00115	62360	DCPCOKIYJ	Lipids and lip	Glycerolipids	Monoradylgly
0.79_342.13	342.139306	0.7877	pos	6-(alpha-D-G	HMDB0011668		HEPUIGACZ	Organic oxyg	Organooxyge	Carbohydrate
1.00_588.07	588.074959	0.99793333	neg	GDP-D-Rhar	HMDB00014	6283	LQEBEXMHI	Nucleosides,	Purine nucle	Purine nucle
11.16_472.3	472.312025	11.16325	pos	Vertilmicin	HMDB0242753		QVRLHIRFL	Organic oxyg	Organooxyge	Carbohydrate
5.70_407.20	407.207692	5.69895	neg	18-Hydroxyc	HMDB00003	5308	HFSXHZZD	Lipids and lip	Steroids and	Hydroxystero
7.20_255.11	255.112612	7.20335	pos	1-Amino-2-m	HMDB02438	73030	ZLCUIOWQ	Benzenoids	Anthracenes	Anthraquinon
9.87_518.32	518.323842	9.86676667	pos	LysoPC(18:3	HMDB00103	40304	WKQNRCYK	Lipids and lip	Glycerophos	Glycerophos
10.45_361.2	361.238617	10.4453167	neg	Pregnenolon	HMDB00002	24078	ORNBQBCI	Lipids and lip	Steroids and	Pregnane ste
12.22_271.2	271.227812	12.2214667	neg	Pentadecana	HMDB00310	346685	XGQJZNCF	Lipids and lip	Fatty Acyls	Fatty aldehy
4.00_180.10	180.102173	4.00056667	pos	Methyl l-phe	HMDB0245704		VSDUZFOS	Organic acid	Carboxylic ac	Amino acids,
4.29_362.15	362.151376	4.29085	pos	Levofloxacin	HMDB00019	58066	GSDSWSVV	Organoheter	Quinolines a	Quinoline ca
6.53_253.10	253.107871	6.53055	neg	(Z)-3-Oxo-2-	HMDB00301	86789	QAAHGFINE	Organic oxyg	Organooxyge	Carbonyl co
0.64_239.16	239.161722	0.63663333	pos	12-Hydroxyd	HMDB00020	6465	ZDHCZVWC	Organic acid	Hydroxy acid	Medium-chai
0.68_287.24	287.244019	0.68255	pos	N1,N12-Diac	HMDB00021	6525	NPDTUDWG	Organic acid	Carboximidic	Carboximidic
0.80_111.00	111.008621	0.79596667	neg	Pentenedioic	HMDB0256281		XVOUMQNX	Organic acid	Carboxylic ac	Dicarboxylic
0.82_123.04	123.044275	0.81883333	pos	4-Hydroxybe	HMDB00117	335656	RGHHSNMV	Organic oxyg	Organooxyge	Carbonyl co
10.46_502.2	502.29426	10.4632167	neg	LysoPE(20:3	HMDB00115	62301	RNWSKZCV	Lipids and lip	Glycerophos	Glycerophos
10.70_830.5	830.591662	10.6988167	neg	PE(19:0/20:2(11Z,14Z))		77018	FMNJZWZZ	Lipids and lip	Glycerophos	Glycerophos
3.82_144.06	144.065709	3.82191667	pos	N-methyl-L-g	HMDB00626	63297	XLBVNMSM	Organic acid	Carboxylic ac	Amino acids,
9.99_281.08	281.080629	9.99425	pos	Xanthoxyleti	HMDB00339	89626	JSJIIHRND	Phenylpropa	Coumarins a	Pyranocouma
0.71_263.10	302.063461	0.71368333	pos	Neplanocin A	HMDB0255538		XUGWUUD	Nucleosides,	Nucleoside a	Cyclopentyl
1.56_269.11	269.113296	1.55895	pos	Ser-Tyr	HMDB02582	85944	MALNXHYE	Organic acid	Carboxylic ac	Amino acids,
10.73_854.5	854.59159	10.73455	neg	PE(22:4(7Z,10Z,13Z,16Z)/		77354	HZHBKGHW	Lipids and lip	Glycerophos	Glycerophos
11.20_267.1	267.192814	11.198	pos	5-Hexyl-2-fur	HMDB0112088		HPFDAHYF	Lipids and lip	Fatty Acyls	Fatty acids a
13.21_829.5	830.565539	13.2068667	pos	PC(20:4(8Z,1	HMDB00084	59923	BFCSBEFT	Lipids and lip	Glycerophos	Glycerophos
14.94_508.4	508.435969	14.9361167	pos	Aragusteroketal		84262	YQWZCFGL	Lipids and lip	Steroids and	Sterols

5.28_223.09	223.095373	5.28283333	pos	2-Hydroxyca	HMDB0301821		OBFKTGKIC	Lipids and lip	Prenol lipids	Monoterpeno
5.59_223.09	223.097542	5.58803333	neg	Diplosporin	HMDB0030680		BFQQKLXDI	Organoheter	Pyrans	Pyranones a
7.17_330.24	353.229581	7.16671667	pos	9S,10S,11R-trihydroxy-12.		74943	JHGVFGJXF	Lipids and lip	Fatty Acyls	Octadecanoi
8.05_227.12	227.128659	8.05003333	neg	2-Propylacryl	HMDB0245232		HEBDGRTW	Lipids and lip	Fatty Acyls	Fatty acids a
9.74_460.26	460.268648	9.74061667	pos	[(2S,4R,5R,6	HMDB0247800		BNZAGRNEI	Lipids and lip	Steroids and	Steroid ester
0.65_245.23	245.233592	0.6518	pos	N1-Acetylspe	HMDB00011	3369	GUNURVWA	Organic acids	Carboxylic ac	Carboxylic ac
1.08_159.02	159.029465	1.08208333	neg	3-Oxoadipic	HMDB00003	5387	RTGHRDFW	Organic acids	Keto acids an	Medium-chai
1.67_188.11	189.123523	1.66533333	pos	Leucyl-Glycir	HMDB0028929		LESXFEZIF	Organic acids	Carboxylic ac	Amino acids,
15.08_419.3	419.315089	15.0786333	pos	Bis(3,5,5-trimethylhexyl) p		330325	GDJOUZYAI	Unclassified	Unclassified	Unclassified
3.97_186.05	186.0558	3.97085	neg	3-Indolehydr	HMDB0059765		HWRVAGPV	Organoheter	Indoles and	Indolyl carbo
9.94_421.31	422.326065	9.93923333	pos	Alpha-linolen	HMDB00063	58390	WJCRATGU	Lipids and lip	Fatty Acyls	Fatty acid es
0.70_118.06	118.061333	0.69861667	pos	Guanidoacet	HMDB00001	5163	BPMFZUMJ	Organic acids	Carboxylic ac	Amino acids,
0.70_381.07	381.079158	0.69861667	pos	Trehalose	HMDB00009	5913	HDTRYLNU	Organic oxyg	Organooxyge	Carbohydrate
0.73_231.13	231.134087	0.72825	pos	4-(1-Hydroxy	HMDB0260272		NRSDIKQZA	Organoheter	Pyridines and	Pyridoxines
0.73_234.10	234.108368	0.72825	pos	Asparaginyl-	HMDB00287	85656	VBKIFHUVG	Organic acids	Carboxylic ac	Amino acids,
1.03_116.07	116.071522	1.0341	neg	N-Methyl-a-a	HMDB00021	6508	DLAMVQGY	Organic acids	Carboxylic ac	Amino acids,
1.14_104.07	104.071071	1.13681667	pos	2-Butyne-1,4	HMDB02450	65776	DLDJFQGPI	Organic oxyg	Organooxyge	Alcohols and
13.65_310.3	310.309954	13.6501333	pos	3-Amino-2-m	HMDB0257752		PALMKPQNI	Organic oxyg	Organooxyge	Alcohols and
4.20_226.07	226.07134	4.19935	pos	Vanilloylglyci	HMDB00600	410680	LOODYTDRI	Benzenoids	Benzene and	Benzoic acid
4.20_262.11	262.118752	4.19935	pos	Glycyl-Trypto	HMDB0028852		AJHCSUXXE	Organic acids	Carboxylic ac	Amino acids,
9.54_583.25	583.254442	9.54118333	pos	21H-Biline-8,	HMDB0245624		RCNSAJSGI	Organoheter	Tetrapyrroles	Bilirubins
0.73_122.02	122.027269	0.72825	pos	L-Cysteine	HMDB00005	5556	XUJNEKJLA	Organic acids	Carboxylic ac	Amino acids,
10.19_885.5	885.550061	10.18955	neg	PI(20:4(5Z,8Z,11Z,14Z)/18:0)			CBYJKEBKE	Lipids and lip	Glycerophos	Glycerophos
11.33_293.2	293.212094	11.3319667	neg	12(13)-epoxy	HMDB0062288		TVHXKPMF	Lipids and lip	Fatty Acyls	Fatty acids a
2.26_246.14	246.1448	2.26408333	pos	Leucyl-Aspar	HMDB00289	85828	MLTRLIITQF	Organic acids	Carboxylic ac	Amino acids,
3.47_252.11	253.118506	3.47126667	pos	Serylphenyla	HMDB00290	23895	PPQRSMGD	Organic acids	Carboxylic ac	Amino acids,
5.29_257.13	257.139465	5.29295	neg	Daumone	HMDB0250877		KBTQMAFDI	Organic oxyg	Organooxyge	Carbohydrate
7.39_349.19	349.198113	7.38693333	pos	(Z)-5-[(2R,3S	HMDB0244345		CMBTXTLLE	Organic acids	Hydroxy acid	Medium-chai
7.42_397.19	397.195783	7.42368333	pos	Trospectomy	HMDB0259301		KHAUBYTY	Organoheter	Dioxanes	1,4-dioxanes
0.72_248.07	248.077421	0.71928333	neg	N-(1-Deoxy-1	HMDB00378	92650	KUVQLWYP	Organic acids	Carboxylic ac	Amino acids,
1.08_247.13	247.139941	1.07531667	pos	Prolyl-Aspara	HMDB0029012		JQOHKCDM	Organic acids	Carboxylic ac	Amino acids,
10.50_295.2	295.226125	10.50225	pos	13-HOTE	HMDB00102	36032	KLLGGGQN	Lipids and lip	Fatty Acyls	Lineolic acids
11.40_597.3	597.304453	11.40315	neg	PI(18:1(9Z)/(	HMDB0256522		UGDOFRYH	Lipids and lip	Glycerophos	Glycerophos

5.14_585.27	585.27067	5.13745	pos	Lumirubin	HMDB0254205		BYVDUQYJI	Organoheter	Tetrapyrroles	Unclassified
5.64_255.11	255.114098	5.64363333	neg	2-(p-Acetami	HMDB0247904		PBWKSBSB	Organoheter	Piperidines	Phenylpiperid
9.38_286.27	286.273688	9.37788333	pos	D-erythro-Sphingosine C-1		41557	RBEJCQPPI	Unclassified	Unclassified	Unclassified
1.08_342.11	342.119036	1.08208333	neg	De-O-methyl	HMDB00412	95628	SDYUFOBSI	Organic oxyg	Organooxyge	Carbohydrate
10.72_855.6	855.657363	10.7169	neg	(24R)-24,25-	HMDB0242655		BPEQZNMK	Lipids and lip	Steroids and	Vitamin D an
12.13_227.2	227.201407	12.12775	neg	Myristic acid	HMDB00008	196	TUNFSRHW	Lipids and lip	Fatty Acyls	Fatty acids a
12.48_808.5	808.58187	12.4811	pos	PC(18:1(17Z)/18:1(17Z))		39599	XHXDCKBFI	Lipids and lip	Glycerophos	Glycerophos
2.06_147.02	147.029689	2.0574	neg	1-Methylxant	HMDB00107	327993	MVOYJPOZI	Organoheter	Imidazopyrim	Purines and
4.67_363.10	363.10534	4.66926667	neg	Isohomovani	HMDB00003	5322	BWXLCOBS	Benzenoids	Phenols	Methoxypher
5.03_231.12	231.123659	5.03453333	neg	Isopentyl bet	HMDB00347	90176	PPOCTRFLI	Lipids and lip	Fatty Acyls	Fatty acyl gly
5.72_352.24	352.248102	5.72123333	pos	15d PGD2	HMDB0060046		QUGBPWLP	Lipids and lip	Fatty Acyls	Eicosanoids
7.04_151.07	151.075256	7.03826667	pos	2-(2-Furanyl)	HMDB00409	95374	JWRBBXQU	Organoheter	Heteroaroma	Unclassified
9.40_374.28	374.289586	9.39628333	pos	13,14-Dihydr	HMDB00026	36193	DPOINJQW	Lipids and lip	Fatty Acyls	Eicosanoids
9.67_396.31	396.310398	9.66891667	pos	(10Z)-12-Hyc	HMDB0241477		NNIGBOAA	Lipids and lip	Fatty Acyls	Fatty acid es
9.71_312.28	312.289187	9.70535	pos	13-Azaprosta	HMDB0244503		RZZNGDIYQ	Lipids and lip	Fatty Acyls	Fatty acids a
0.72_143.03	143.034765	0.71928333	neg	D-1-Deoxy-e	HMDB00299	86599	DTUGXZBP	Organic oxyg	Organooxyge	Carbohydrate
0.90_184.05	184.058074	0.89581667	pos	Acetylhomos	HMDB00294	3282	FCXZBWSIA	Organic acid	Carboxylic ac	Amino acids,
0.93_187.00	187.000218	0.92838333	pos	D-Xylono-1,5	HMDB00116	62415	XXBSUZSOI	Organoheter	Lactones	Delta valerola
11.31_335.1	335.198037	11.3062	pos	(+)-Norgestre	HMDB0242257		WWYNJERN	Lipids and lip	Steroids and	Estrane sterc
12.94_305.2	305.24498	12.9406833	pos	(Z)-13-Octad	HMDB00414	34954	BDLLSHRIFI	Lipids and lip	Fatty Acyls	Fatty acids a
12.94_307.2	307.254939	12.9357167	neg	20-HETE-d6		36334	NNDIXBJHN	Lipids and lip	Fatty Acyls	Eicosanoids
2.86_222.10	223.107665	2.85705	pos	Phenylalanyl	HMDB03047	85897	GLUBLISJV	Organic acid	Carboxylic ac	Amino acids,
4.38_225.14	225.148799	4.38046667	pos	Vomifoliol	HMDB03035	66125	KPQMCAKZI	Lipids and lip	Prenol lipids	Sesquiterper
4.61_199.09	199.097364	4.61413333	neg	delta-Valerol	HMDB02509	65736	OZJPLYNZG	Organoheter	Lactones	Delta valerola
6.23_329.17	329.175768	6.23498333	neg	Gibberellin A	HMDB00320	88115	SFGDEUSM	Lipids and lip	Prenol lipids	Diterpenoids
7.66_384.27	384.274028	7.6626	pos	Arbaprostil	HMDB0248565		XSGQFHNP	Lipids and lip	Fatty Acyls	Eicosanoids
9.40_361.23	361.234253	9.39628333	pos	8,9-DiHETrE	HMDB0002311		DCJBINATH	Lipids and lip	Fatty Acyls	Eicosanoids
0.79_192.08	192.086602	0.7877	pos	Shikimic acid	HMDB00030	338	JXOHGGNK	Organic oxyg	Organooxyge	Alcohols and
10.09_480.3	480.308359	10.0853667	pos	(12Z)-10-Hyc	HMDB0241535		YJDCDSFTL	Lipids and lip	Fatty Acyls	Fatty acid es
10.21_886.5	909.544697	10.2120667	pos	PI(18:0/20:4(	HMDB0009816		OJESNYFIP	Lipids and lip	Glycerophos	Glycerophos
10.38_522.2	522.282406	10.3754333	pos	LysoPS(18:2	HMDB02406	78838	HLISFLFEEI	Lipids and lip	Glycerophos	Glycerophos
14.71_309.2	309.278372	14.7074	pos	Ethyl linoleate		316156	FMMOOAYV	Unclassified	Unclassified	Unclassified
4.01_212.00	212.002169	4.0077	neg	Indoxyl sulfa	HMDB00006	253	BXFFHSIDQ	Organic acid	Organic sulfu	Arylsulfates

4.04_101.06	101.060621	4.04478333	neg		Tetrahydro-2	HMDB00311	87468		BSYVTEYKT	Organoheter	Tetrahydrofu	Unclassified
4.36_355.19	355.197724	4.3621	pos		Furylacryloyl	HMDB0247373			BZUABSYGI	Organic acid	Carboxylic a	Amino acids,
4.39_309.10	309.10703	4.39318333	neg		N(2)-phenyla	HMDB0062645			JFLIEFSWG	Organic acid	Carboxylic a	Amino acids,
5.65_310.20	310.200727	5.64811667	pos		(3Z,5E,7E)-D	HMDB0241124			QOXHDLWE	Lipids and lip	Fatty Acyls	Fatty acid es
7.42_316.20	317.210785	7.42368333	pos		Ovoideal E				QHBVEFZXL	Lipids and lip	Prenol lipids	Isoprenoids
9.35_430.29	430.29619	9.35091667	neg		Deoxycholic	HMDB00006	5604		WVULKSPC	Lipids and lip	Steroids and	Bile acids, al
9.96_329.14	329.149035	9.95751667	pos		(9Z,11E,13E	HMDB00310	87406		ZYOSGENS	Lipids and lip	Fatty Acyls	Lineolic acids
0.61_275.17	275.171314	0.60603333	pos		Lysyl-Gamma	HMDB00289	85867		MEFNTMKE	Organic acid	Carboxylic a	Amino acids,
1.67_310.13	310.139945	1.66533333	pos		Tyrosyl-alany	HMDB0253030			DLZKEQQW	Organic acid	Carboxylic a	Amino acids,
11.88_321.2	321.239684	11.8768833	pos		(12R)-12-Hy	HMDB0242322			WBHHMMIM	Lipids and lip	Fatty Acyls	Fatty acids a
2.32_311.12	311.123813	2.31811667	pos		gamma-Gluta	HMDB00117	62426		VVLXCWVS	Organic acid	Carboxylic a	Amino acids,
2.50_357.08	357.089046	2.49773333	neg		Pantetheine	HMDB00014	398		JDMUPRLR	Organic acid	Carboxylic a	Amino acids,
5.98_213.11	213.113032	5.97626667	neg		6-Hydroxy-2,	HMDB00346	90124		ILDAKDTXK	Lipids and lip	Prenol lipids	Monoterpen
9.60_361.23	361.23416	9.59576667	pos		5,8,11-Eicos	HMDB0244588			SYAWGTIV	Lipids and lip	Fatty Acyls	Eicosanoids
10.50_432.3	477.322008	10.4988	neg		7alpha,12alp	HMDB00624	57920		WOODKEC	Lipids and lip	Steroids and	Bile acids, al
10.84_478.3	478.388677	10.8409	pos		(3beta,5alph	HMDB00336	89434		OVIAMXWK	Lipids and lip	Steroids and	Ergostane st
11.26_571.2	571.288706	11.25965	neg		LysoPI(16:0/	HMDB0061695			UOXRPRZM	Lipids and lip	Glycerophos	Glycerophos
11.63_308.2	308.257934	11.6289333	pos		4-Androsten	HMDB00058	57835		BTTWKVFK	Lipids and lip	Steroids and	Androstane s
11.90_361.2	361.259763	11.89535	neg		MG(i-15:0/0:	HMDB0072878			CALSYPDG	Lipids and lip	Glycerolipids	Monoradylgly
4.06_384.19	384.199504	4.05526667	pos		5-Hydroxyde	HMDB0241133			FKPIGZRW	Lipids and lip	Fatty Acyls	Fatty acid es
4.90_155.07	155.070242	4.90175	pos		3-Isopropeny	HMDB00323	88387		PULOWZXU	Lipids and lip	Fatty Acyls	Fatty acids a
6.42_317.21	317.210723	6.41748333	pos		Prostaglandi	HMDB00027	3465		MYHXHCUN	Lipids and lip	Fatty Acyls	Eicosanoids
9.26_285.20	285.207078	9.25955	neg		16-Hydroxy-1	HMDB00412	95701		ZNMHENLY	Lipids and lip	Fatty Acyls	Fatty acids a
9.39_379.21	379.210173	9.3876	neg		12-Keto-leuk	HMDB00042	36254		SJVWVCVZ	Lipids and lip	Fatty Acyls	Eicosanoids
9.45_370.29	370.294822	9.45123333	pos		3-Hydroxytet	HMDB0061640			GFAZJTUX	Lipids and lip	Fatty Acyls	Fatty acid es
1.08_160.06	160.06126	1.08208333	neg		N-Acetylthre	HMDB00625	440051		PEDXUVCG	Organic acid	Carboxylic a	Amino acids,
1.17_328.04	328.045161	1.16698333	neg		Cyclic AMP	HMDB00000	5120		IVOMOUWH	Nucleosides,	Purine nucle	Cyclic purine
10.56_284.2	284.294424	10.5560833	pos		(2S,3R)-2-Ar	HMDB0242695			ZREXGWPJ	Organic oxy	Organooxyg	Alcohols and
10.68_414.3	414.357246	10.6803667	pos		Heptadecan	HMDB00062	58363		DWSFAVOT	Lipids and lip	Fatty Acyls	Fatty acid es
13.35_305.2	305.244778	13.3474333	pos		Octadec-5-e	HMDB0246833			AQWHMKSI	Lipids and lip	Fatty Acyls	Fatty acids a
4.39_241.11	241.119235	4.39318333	neg		Cyclo(L-prol	HMDB0240493			XLUAWXQC	Organic acid	Carboxylic a	Amino acids,
4.52_187.12	210.110073	4.52393333	pos		8-Amino-7-o	HMDB02406	331097		GUAHPAJO	Lipids and lip	Fatty Acyls	Fatty acids a
5.19_331.17	331.17236	5.19183333	pos		(3b,4b,5b)-4,	HMDB00389	93587		BMTDVVBL	Organic oxy	Organooxyg	Carbohydrat

6.80_391.20	391.208527	6.79993333	pos		Thromboxan	HMDB0005099			OYPPJMLK	Lipids and lip	Fatty Acyls	Eicosanoids
8.86_215.14	215.142913	8.86416667	pos		1(10),11-Ere	HMDB00354	90703		CEDQTRQW	Lipids and lip	Prenol lipids	Sesquiterper
1.07_176.05	176.055991	1.06741667	neg		5-Hydroxypr	HMDB0246811			KYBXNPIAS	Organic acid	Carboxylic ac	Amino acids,
1.20_197.12	197.128508	1.20368333	pos		1,2,3,4,5,6-H	HMDB0039655			BJPWZQDK	Organoheter	Pyridines and	Hydropyridin
1.34_242.10	242.100905	1.34166667	neg		5-[1-Carboxy	HMDB0247852			HYVXOSFJ	Organic acid	Carboxylic ac	Tricarboxylic
10.32_381.2	381.228169	10.31745	neg		9alpha-(3-Me	HMDB00391	93765		RJNJFKDVP	Lipids and lip	Prenol lipids	Sesquiterper
2.11_292.01	292.014736	2.11061667	neg		2-Hydroxyac	HMDB0062547			HRLQZTOIK	Organic acid	Organic sulfu	Arylsulfates
2.15_235.07	235.072024	2.1466	neg		2-Hydroxyfell	HMDB00606	2655		VTXNIAJKDI	Lipids and lip	Glycerolipids	Unclassified
2.64_260.16	260.160522	2.64221667	pos		Glutaminyllet	HMDB00288	806826		ARPVSMCN	Organic acid	Carboxylic ac	Amino acids,
7.70_393.22	393.224185	7.69936667	pos		Thromboxan	HMDB00032	422		XNRNNGPB	Lipids and lip	Fatty Acyls	Eicosanoids
9.92_265.18	265.180533	9.91656667	neg		Tetranorpros	HMDB0258926			VDSTWEBF	Lipids and lip	Fatty Acyls	Fatty acids a
11.66_360.2	360.256157	11.6642167	pos		Napelline		67134		AZAZKLKDE	Unclassified	Unclassified	Unclassified
12.59_304.2	304.260472	12.5856	pos		Oleamide	HMDB00021	4115		FATBGEAM	Lipids and lip	Fatty Acyls	Fatty amides
4.27_304.16	304.165735	4.27276667	pos		Tryptophyl-V	HMDB0029096			LWFWZRAN	Organic acid	Carboxylic ac	Amino acids,
5.43_176.07	176.070502	5.42866667	pos		Indoleacetic	HMDB00001	70		SEOVTRFCI	Organoheter	Indoles and	Indolyl carbo
7.26_584.26	585.270304	7.25836667	pos		(4E,15E)-Bili	HMDB0240584			BPYKTIZUT	Organoheter	Tetrapyrroles	Bilirubins
7.44_328.24	328.247825	7.44203333	pos		(4E)-Undec-	HMDB0241159			KTWZODJO	Lipids and lip	Fatty Acyls	Fatty acid es
7.51_367.21	367.212528	7.50616667	neg		19-hydroxypr	HMDB0062683			MTVPUJQVI	Lipids and lip	Fatty Acyls	Eicosanoids
0.62_347.14	347.142222	0.6215	pos		5-(Galactosy	HMDB0242167			OWGKYELX	Lipids and lip	Fatty Acyls	Fatty acyl gly
0.70_426.08	427.095012	0.69861667	pos		Cysteine-glu	HMDB0250707			GNTARDAH	Organic acid	Carboxylic ac	Amino acids,
1.62_103.03	103.039914	1.61501667	neg		isopropenylo	HMDB0253674			HNUKTDKIS	Organic oxyg	Organooxyge	Carbonyl cor
11.43_394.2	394.271192	11.4320667	pos		Docosahexa	HMDB00136	36744		GEEHOLRS	Organic nitro	Organonitrog	Amines
11.70_848.5	848.539583	11.7002333	pos		PS(19:1(9Z)/20:3(8Z,11Z,		78166		PHPYTPYV	Lipids and lip	Glycerophos	Glycerophos
5.59_284.12	284.121215	5.59335	pos		Dehydroevo	HMDB0250954			VXHNSVKJ	Organoheter	Diazanaphth	Benzodiazine
9.11_267.12	267.123719	9.1134	neg		Methyl 3-epi-	HMDB0303326			RRZQRPDF	Organic acid	Carboxylic ac	Carboxylic ac
1.08_321.06	320.062089	1.08208333	neg		Beta-Citryl-L-	HMDB0013220			GAQNUGIS	Organic acid	Carboxylic ac	Amino acids,
10.47_504.3	504.308151	10.46565	pos		LysoPE(0:0/	HMDB00114	62272		OQKGGFIW	Lipids and lip	Glycerophos	Glycerophos
10.61_267.1	267.196403	10.6077	neg		(-)-Valeranor	HMDB0036421			HDVXJTYH	Lipids and lip	Prenol lipids	Sesquiterper
5.59_301.11	301.119496	5.58803333	neg		Formylfusarc	HMDB00389	93578		ZSARLQOB	Organoheter	Benzopyrans	1-benzopyra
5.90_209.07	209.078177	5.90411667	pos		1-Methylcycl	HMDB0243937			PMUPSYZV	Organic acid	Carboxylic ac	Dicarboxylic
0.73_150.01	149.008918	0.7342	neg		Tartaric acid	HMDB00009	4197		FEWJPZIEW	Organic oxyg	Organooxyge	Carbohydrate
0.74_246.10	246.108307	0.74288333	pos		Creatine ribo	HMDB0240254			BGJFEKXAL	Organic oxyg	Organooxyge	Carbohydrate
0.79_251.15	251.150945	0.7877	pos		[(2S)-2-Amin	HMDB0257734			GYZQEUDX	Organic acid	Carboxylic ac	Amino acids,

0.83_180.06(	180.066429	0.82703333	neg	2-Acetyl-1,5,(	HMDB00351	90504	OISIGIMXBV	Organoheter	Azepines	Unclassified
1.07_310.09(	310.096341	1.06741667	neg	N-(1-Deoxy-1	HMDB00378	92649	KEGDHFMP'	Organic acid	Carboxylic ac	Amino acids,
1.10_219.13	219.133825	1.1046	pos	Carboxyethyl	HMDB0249659		UENLDOJJK	Organic acid	Carboxylic ac	Amino acids,
10.18_472.34	472.341715	10.17625	pos	(11Z,14Z,17Z	HMDB0241582		SRNPFTYGI	Lipids and lip	Fatty Acyls	Fatty acid es
10.32_474.35	474.357355	10.3206667	pos	Deoxycholylv	HMDB0242423		RVPFAKQTI	Unclassified	Unclassified	Unclassified
10.48_293.2	293.210418	10.4838833	pos	12(13)Ep-9-k	HMDB00136	35761	RCMABBHQ	Lipids and lip	Fatty Acyls	Fatty acids a
13.88_312.3	312.325591	13.8842333	pos	N,N-dimethyl-Safingol		53956	BYBHBTCYJ	Lipids and lip	Sphingolipids	Sphingoid ba
5.65_301.11	301.11827	5.64811667	pos	4'-Hydroxym	HMDB0246635		MTMBJMKM	Organoheter	Diazines	Pyrimidines a
5.89_202.12(	225.109342	5.88578333	pos	N-Lactoylisol	HMDB0062180		DYYQDDUC	Organic acid	Keto acids ar	Medium-chai
9.12_326.21	326.214673	9.12051667	pos	Farnesylcyst	HMDB00116	62388	SYSLNQMKI	Lipids and lip	Prenol lipids	Sesquiterper
9.41_263.16	263.16149	9.41486667	pos	3b-Hydroxy-6	HMDB00407	95220	OBOOZVIHE	Lipids and lip	Prenol lipids	Terpene lact
9.61_495.33	495.333041	9.60636667	neg	Coprocholic a	HMDB00006	5577	CNWPIIOQK	Lipids and lip	Steroids and	Bile acids, al
10.05_349.1	349.198099	10.0490667	pos	1-[2,3-Dimetl	HMDB0247708		BSIAHHIPM(	Lipids and lip	Fatty Acyls	Fatty alcohol
10.09_314.3(	314.304881	10.0853667	pos	13-Cyclohexyltridecanoic a		45891	JBTBXKNLIT	Lipids and lip	Fatty Acyls	Fatty Acids a
10.23_279.2	279.232666	10.2257833	neg	3-Methylene-	HMDB00371	92138	VBZQKPYXI	Organic oxyg	Organooxyge	Carbonyl cor
10.70_307.2	307.226251	10.6974667	pos	1,17-dihydroxy-androstan-3-one			MFLXKQKZI	Lipids and lip	Steroids and	Steroids
1.37_588.13	589.139716	1.36776667	pos	[(2R,5R)-5-(2	HMDB0260224		OEZOLYYGI	Nucleosides,	(5'->5')-dinuc	Unclassified
12.02_405.2	405.299548	12.0192167	pos	MG(22:5(4Z,	HMDB00115	62367	HDIQCISTZI	Lipids and lip	Glycerolipids	Monoradylgly
12.49_629.4	629.454798	12.4924667	neg	(2S,5S,6R)-2,6-cyclolycopene-1-methox			RRKGUCMF	Lipids and lip	Prenol lipids	Isoprenoids
3.62_229.15	229.155459	3.62421667	neg	Leucyl-Valine	HMDB00289	85844	MDSUKZSL(	Organic acid	Carboxylic ac	Amino acids,
9.74_135.08(	135.080402	9.74061667	pos	2,3,6-Trimetl	HMDB00320	88118	NHMDCMLC	Benzenoids	Benzene anc	Benzoyl deriv
9.84_474.26	474.26117	9.84338333	neg	LysoPE(0:0/1	HMDB00114	62267	JGWWMNVI	Lipids and lip	Glycerophos	Glycerophos
0.74_238.03	238.038045	0.74288333	pos	S-Cysteinosu	HMDB00294	86255	XPKKFTKCI	Organic acid	Carboxylic ac	Amino acids,
1.07_333.05	333.058287	1.06741667	neg	Orotidine	HMDB00007	5754	FKCRAVPPI	Nucleosides,	Pyrimidine nu	Unclassified
10.45_366.3	366.336129	10.4474333	pos	(E,E)-3,7,11-Trimethyl-2,6		97253	HHSVAJNBV	Lipids and lip	Fatty Acyls	Fatty esters
11.88_286.2	287.236401	11.8768833	pos	13-cis Retinc	HMDB00062	41507	FPIPGXGPP	Lipids and lip	Prenol lipids	Retinoids
13.85_334.2	333.279799	13.84655	neg	Docosatrienc	HMDB00028	195	WBBQTNCIS	Lipids and lip	Fatty Acyls	Fatty acids a
3.91_167.06	167.068027	3.91066667	pos	4-Hydroxycy(	HMDB00019	44761	HCFRWBBJI	Organic oxyg	Organooxyge	Alcohols and
4.26_376.13	421.136581	4.26475	neg	7,8-Dimethyl-	HMDB0247501		AUNGANRZ	Organoheter	Pteridines an	Alloxazines a
4.26_511.10	511.105835	4.26475	neg	Dihydromyric	HMDB0303822		QMGYCEQX	Phenylpropa	Flavonoids	Flavonoid gly
5.94_331.23	332.24272	5.94078333	pos	3-hydroxyde(	HMDB0061636		VCRSQDIR(	Lipids and lip	Fatty Acyls	Fatty acid es
8.55_255.16(	255.160135	8.5484	neg	Monomenthy	HMDB00361	91299	BLILOGGUT	Lipids and lip	Prenol lipids	Monoterpenc
9.90_359.21	359.218717	9.90308333	pos	5,15-DiHETE	HMDB00102	36301	UXGXCGPW	Lipids and lip	Fatty Acyls	Eicosanoids

1.10_296.07	296.079532	1.1046	pos	(S)-5'-Deoxy-	HMDB00336	89459	WXOJULRV	Nucleosides,	5'-deoxyribor	Unclassified
1.36_587.12	587.125825	1.3586	neg	Cytidylyl-(3',5	HMDB0245649		CTMZLDSMI	Nucleosides,	(3'->5')-dinuc	(3'->5')-dinuc
1.51_212.09	212.091923	1.50628333	pos	3-Hydroxy-al	HMDB0251525		CJCSPKMFH	Phenylpropa	Phenylpropa	Unclassified
1.58_181.03	181.036561	1.57865	neg	1-Methyluric	HMDB00030	2821	QFDRTQON	Organoheter	Imidazopyrim	Purines and
10.30_281.2	281.212043	10.2994667	neg	Zingiberol	HMDB0303118		GMZKBWZW	Organic oxyg	Organooxyge	Alcohols and
10.43_324.2	323.222522	10.42685	neg	Raspailyne A1			PWEKCZHK	Lipids and lip	Glycerolipids	Monoradylgly
13.21_725.5	725.555905	13.2068667	pos	SM(d18:0/16	HMDB0013464		CGVOQSQI	Lipids and lip	Sphingolipids	Phosphosphi
3.72_258.18	258.18129	3.71513333	pos	(2S)-2-Aminc	HMDB0255325		KVXSPXCU	Organic acids	Carboxylic ac	Amino acids,
6.51_514.28	514.284316	6.51206667	neg	Taurocholic a	HMDB00000	5104	WBWWGRH	Lipids and lip	Steroids and	Bile acids, al
9.30_379.24	379.249097	9.29605	neg	Enisoprost	HMDB0251811		CIBHDGPIO	Lipids and lip	Fatty Acyls	Fatty acid es
9.34_277.17	277.177337	9.34135	pos	Embelin	HMDB02517	43201	IRSFLDGTO	Organic oxyg	Organooxyge	Carbonyl cor
9.38_341.19	341.195641	9.37788333	pos	11-Dehydro-2	HMDB0244346		PJAAKFHM	Organic acids	Hydroxy acid	Medium-chai
9.47_302.30	302.304921	9.46953333	pos	Stearic acid	HMDB00008	189	QIQXTHQID	Lipids and lip	Fatty Acyls	Fatty acids a
9.51_334.21	357.203099	9.50591667	pos	5-Oxo-6-tran	HMDB0012824		MLZJFLKEK	Lipids and lip	Fatty Acyls	Eicosanoids
0.70_512.10	512.102149	0.69861667	pos	Citicoline	HMDB00014	6229	RZZPDXZPF	Organoheter	Pteridines an	Pterins and c
0.78_211.00	211.001256	0.7799	neg	Phosphoribos	HMDB0256491		DILVWDXO	Organic oxyg	Organooxyge	Carbohydrate
11.50_281.2	281.210775	11.5038	pos	8E-Heptadecenedioic acid		74925	VDTSYDDU	Lipids and lip	Fatty Acyls	Fatty Acids a
12.02_277.2	277.217019	12.0185833	neg	alpha-Linoler	HMDB00013	6208	DTOSIQBPF	Lipids and lip	Fatty Acyls	Lineolic acids
4.99_321.09	321.094226	4.99255	pos	4-coumaroyls	HMDB0304174		GVECSFFLZ	Phenylpropa	Cinnamic aci	Hydroxycinna
6.77_327.21	327.217715	6.76876667	neg	Auxin a	HMDB00384	93162	RKOUCPMG	Lipids and lip	Prenol lipids	Monoterpenc
7.88_399.21	399.211776	7.88303333	pos	11-Hydroxy-I	HMDB0062283		LFXPXGFIT	Lipids and lip	Fatty Acyls	Fatty alcohol
9.32_358.29	358.294701	9.32303333	pos	Tridecanoylc	HMDB0241308		OLAOWMDK	Lipids and lip	Fatty Acyls	Fatty acid es
0.64_333.05	333.05928	0.64391667	neg	Glycerophos	HMDB0011649		BMVUIWJCU	Lipids and lip	Glycerophos	Glycerophos
0.75_110.97	110.975562	0.74898333	neg	Methyl sulfat	HMDB02546	319749	JZMJDSHXV	Organic acids	Organic sulfu	Sulfuric acid
0.80_230.99	230.990134	0.80285	pos	Isocitric acid	HMDB00001	3328	ODBLHEXUI	Organic acids	Carboxylic ac	Tricarboxylic
1.52_149.02	149.024201	1.52331667	neg	p-Hydroxyma	HMDB00008	5785	YHXHKYRQ	Benzenoids	Phenols	1-hydroxy-2-
11.31_850.5	850.560218	11.3135833	neg	PE(22:1(13Z	HMDB0283842		CABMTENU	Unclassified	Unclassified	Unclassified
7.01_357.06	357.061686	7.00806667	neg	Succinyldisal	HMDB0251143		PREOBXYM	Organic acids	Carboxylic ac	Tetracarboxy
7.09_365.19	365.19317	7.09336667	pos	T2 Triol	HMDB00353	90690	DDAUKBBL	Lipids and lip	Prenol lipids	Sesquiterper
9.86_239.16	239.165189	9.86181667	neg	7,8-Dehydro-	HMDB00361	91333	WPGXAVCJ	Lipids and lip	Prenol lipids	Sesquiterper
0.70_113.00	113.000078	0.69861667	pos	Glycidol	HMDB02528	68689	CTKINSOIS	Organoheter	Epoxides	Unclassified
0.74_115.03	115.037134	0.74288333	pos	Glycerol	HMDB00001	105	PEDCQBHI	Organic oxyg	Organooxyge	Carbohydrate
0.79_250.12	250.12839	0.7877	pos	Voglibose	HMDB00155	85543	FZNCGRZW	Organic oxyg	Organooxyge	Alcohols and

1.17_322.10(	322.106695	1.1705	pos	Glycine, L-g-	HMDB0260312		GZFOMNDC	Organic acid:	Carboxylic ac	Amino acids,
1.29_179.04(	179.045958	1.2875	neg	2-Aminopurir	HMDB0245025		MWBWWFC	Organoheter	Imidazopyrim	Purines and
10.34_269.2	269.210769	10.3389333	pos	Hexadecane	HMDB00006	5642	QQHJDPRO	Lipids and lip	Fatty Acyls	Fatty acids a
10.75_335.2	335.222626	10.7528167	neg	10-hydroxy-1	HMDB0062479		DWNBPRRX	Lipids and lip	Fatty Acyls	Eicosanoids
10.77_610.3	610.370233	10.7697	pos	2-O-Glutaroy	HMDB0256378		CDZVJFRXJ	Lipids and lip	Glycerophos	Glycerophos
3.73_149.02	149.02419	3.73108333	neg	3,4-Dihydrox	HMDB00013	6170	CFFZDZCDL	Benzenoids	Phenols	Benzenediols
4.31_134.10	152.143511	4.30826667	pos	p-Mentha-1,3	HMDB00370	444756	XNMPFDIYA	Lipids and lip	Prenol lipids	Monoterpenc
4.52_224.05(	224.056296	4.52246667	neg	4-Acetamido	HMDB02463	2830	QCXJEYYX\	Benzenoids	Benzene anc	Benzoic acid
4.91_437.14	437.142776	4.90568333	neg	Cichorioside	HMDB0302057		SNIHMWOC	Organic oxyg	Organooxyge	Carbohydrate
5.47_167.07(	167.070425	5.46526667	pos	3-Methyltetra	HMDB02459	984830	LWMIDUUVI	Organoheter	Isobenzofura	Unclassified
6.62_269.04	269.045845	6.62148333	neg	3-(3,4-Dihydr	HMDB0245776		GSBNFGRT	Phenylpropa	Linear 1,3-dia	Chalcones ar
8.27_330.26	330.263488	8.26751667	pos	Undecanoylc	HMDB0013321		FPUYBGZE\	Lipids and lip	Fatty Acyls	Fatty acid es
0.70_312.09	312.093416	0.70456667	neg	Neuraminic a	HMDB0000830		CERZMXAJ\	Lipids and lip	Fatty Acyls	Fatty acid es
0.80_347.04	347.041857	0.79596667	neg	Phosphoribo	HMDB00014	3506	ABCOOORL	Nucleosides,	Imidazole rib	1-ribosyl-imic
1.20_277.04	277.047604	1.20271667	neg	3-Hydroxypic	HMDB00131	64950	BRARRAHG	Organoheter	Pyridines anc	Pyridinecarb
14.92_463.3	463.377987	14.9180833	pos	2-Deoxy-28-l	HMDB0301792		DYENWMU)	Lipids and lip	Steroids and	Stigmastane
4.04_140.07	140.071556	4.04478333	neg	Isovalerylgly	HMDB00006	5647	ZRQXMKMB	Organic acid:	Carboxylic ac	Amino acids,
4.12_201.07(	201.076675	4.11676667	neg	1,2-O-Isopro	HMDB0242729		BGGCXQKY	Organic oxyg	Organooxyge	Ethers
4.27_160.13	160.133346	4.27276667	pos	DL-2-Aminoc	HMDB00009	5923	AKVBCGQV	Organic acid:	Carboxylic ac	Amino acids,
3.95_265.15	265.154841	3.94663333	pos	Propacetamc	HMDB02568	330020	QTGAJCQTI	Organic acid:	Carboxylic ac	Amino acids,
4.85_217.10	217.107857	4.85055	neg	5-Hydroxyse	HMDB00291	86076	QDRVFLVAI	Organic acid:	Hydroxy acid	Medium-chai
9.06_221.15	221.154371	9.05798333	neg	Kobusone	HMDB0036790		UETZJEZFL	Organic oxyg	Organooxyge	Carbonyl cor
0.77_174.08	174.087343	0.7729	pos	4-Imidazolon	HMDB00010	319	HEXMLHKQ	Organoheter	Azoles	Imidazoles
1.17_187.06(	187.060712	1.16698333	neg	4,6-O-Ethylid	HMDB0246615		CYJNDOQN	Organoheter	Dioxanes	1,3-dioxanes
1.56_133.04	133.049668	1.55895	pos	Formyl 3-hyd	HMDB0257659		QMGUAHPN	Organic acid:	Hydroxy acid	Beta hydroxy
12.98_355.2	355.264021	12.9771167	neg	20-Dihydrody	HMDB0060729		YUDUQGLC	Phenylpropa	Phenylpropa	Unclassified
2.21_239.09	262.083862	2.21128333	pos	Carbazolepro	HMDB0249628		NHIYJOFCM	Organoheter	Indoles and	Carbazoles
2.41_310.12	310.128338	2.40855	pos	N-(1-Deoxy-1	HMDB00378	92654	FAVRCIXPI\	Organic acid:	Carboxylic ac	Amino acids,
6.95_389.19	389.197046	6.95326667	neg	11-Dehydroc	HMDB0004029		FUFLCEKSE	Lipids and lip	Steroids and	Hydroxysterc
9.07_277.17	277.178804	9.0656	pos	Tanacetol A	HMDB0035722		IULUGGNZL	Lipids and lip	Prenol lipids	Sesquiterper
9.25_297.26(	298.273753	9.24935	pos	3-ketosphingosine		43208	VWTPJNGTI	Lipids and lip	Sphingolipids	Sphingoid ba
1.07_261.07	261.072463	1.06741667	neg	L-beta-aspar	HMDB00111	62013	DBMVITQDC	Organic acid:	Peptidomime	Hybrid peptic
1.77_155.08	155.081492	1.77345	pos	m-Aminoben	HMDB00018	6374	XFDUHJPV(	Benzenoids	Benzene anc	Benzoic acid

10.70_309.2(	309.206976	10.6988167	neg	9-Oxo-11-(3-	HMDB02466	331165	RCMABBHQ	Lipids and lip	Fatty Acyls	Fatty acids a
13.21_797.5!	780.550699	13.2068667	pos	PC(20:4(5Z,8	HMDB0285914		ACPBRRYY)	Unclassified	Unclassified	Unclassified
3.99_211.05!	211.059837	3.98933333	neg	Vanillactic ac	HMDB00009	5868	SVYIZYRTO	Phenylpropa	Phenylpropa	Unclassified
4.63_183.06	183.062768	4.63161667	pos	Propylenegly	HMDB00416	95980	MLHOXUWV	Organic acid	Carboxylic ac	Dicarboxylic
7.03_227.12	227.128726	7.02585	neg	Traumatic ac	HMDB00009	5882	MAZWDMBC	Lipids and lip	Fatty Acyls	Fatty acids a
9.56_616.17	616.175986	9.55885	pos	Pelargonidin	HMDB0033679		MJCMQFQZ	Phenylpropa	Flavonoids	Flavonoid gly
9.73_215.16!	215.165004	9.73451667	neg	(±)-(E)-3-Met	HMDB00310	87385	JDCKKTBNC	Lipids and lip	Fatty Acyls	Fatty alcohol
9.92_295.19	295.191313	9.91656667	neg	10-Deoxymethynolide		40998	NZUJVBSYQ	Lipids and lip	Polyketides	Macrolides a
0.68_290.14!	290.145834	0.68255	pos	Aspartyl-Argi	HMDB00287	85662	PSZNHSNIG	Organic acid	Carboxylic ac	Amino acids,
11.81_741.5	722.513317	11.8104333	neg	PE(20:3(6,8,	HMDB0285269		GVNIYKUTY	Unclassified	Unclassified	Unclassified
13.49_298.3	298.309906	13.4891167	pos	Palmitoyl N-Isopropylamid		64930	TZYVUGCY	Unclassified	Unclassified	Unclassified
4.13_318.19	318.191405	4.12765	pos	3-methylhept	HMDB0241046		FFVIWPKOC	Lipids and lip	Fatty Acyls	Fatty acid es
6.84_290.26	290.268613	6.83671667	pos	16-Hydroxyh	HMDB00062	337737	UGAGPNKC	Lipids and lip	Fatty Acyls	Fatty acids a
0.62_168.07	168.076804	0.6215	pos	(S)-Spinacin	HMDB00298	86559	YCFJXOFFQ	Organic acid	Carboxylic ac	Amino acids,
10.41_569.3	570.355025	10.41135	pos	LysoPC(22:5	HMDB00104	61712	BIEOSECQF	Lipids and lip	Glycerophos	Glycerophos
4.67_208.06	208.061317	4.66926667	neg	3-Methyldiox	HMDB00041	7024	XCHBYBKNI	Organoheter	Indoles and	Indolines
5.27_141.09	141.091965	5.27448333	neg	Ethyl 2-meth	HMDB0037622		HOWBPXBY	Lipids and lip	Fatty Acyls	Fatty acid es
6.44_291.12	291.121371	6.4382	neg	Descyclopro	HMDB0251034		ZKJUXHDSL	Nucleosides,	Nucleoside a	Cyclopentyl r
6.98_319.15	319.153577	6.98325	pos	Zearalenone		40980	MBMQEIFVC	Lipids and lip	Polyketides	Macrolides a
7.74_221.08	221.081751	7.7395	neg	Dihydrodeox	HMDB00316	87847	JVUYBLROE	Organoheter	Azaphilones	Unclassified
8.47_500.27	500.277201	8.46991667	pos	PE(0:0/20:4;O)			UNKGTCKG	Lipids and lip	Glycerophos	Oxidized glyc
8.71_457.28	457.28313	8.7078	pos	1alpha-hydroxy-24-(dimethylphosphoryl			HRIMPHJRX	Lipids and lip	Steroids and	Secosteroids
8.75_531.25	531.254199	8.74946667	neg	Protegenin A			AMCJPHJAN	Lipids and lip	Fatty Acyls	Fatty Acids a
1.14_181.00	181.009751	1.13681667	pos	2-Maleylacet	HMDB0060348		SOXXPQLIZ	Organic acid	Keto acids ar	Medium-chai
10.48_254.2	255.231448	10.4838833	pos	Hypogeic aci	HMDB00021	45058	PJHOFUXB	Lipids and lip	Fatty Acyls	Fatty acids a
12.48_809.5	810.598133	12.4811	pos	PC(P-18:0/2(	HMDB0289543		AFUGAELDE	Unclassified	Unclassified	Unclassified
3.25_306.14	306.14483	3.2533	pos	Tryptophyl-T	HMDB00290	85986	YBRHKUNW	Organic acid	Carboxylic ac	Amino acids,
4.56_327.11	327.110535	4.5592	pos	hydroxymedi	HMDB0304581		IEPQZIWEK	Phenylpropa	Cinnamic aci	Hydroxycinna
7.46_465.30	466.315822	7.4605	pos	N-Choloylgly	HMDB02551	88551	RFDAIACWV	Lipids and lip	Steroids and	Bile acids, al
9.61_295.22	295.227706	9.60636667	neg	9,10-DHOME	HMDB0004704		XEBKSQSGI	Lipids and lip	Fatty Acyls	Fatty acids a
9.72_403.21	403.210387	9.71598333	neg	Cannabidioli	HMDB0249587		WVOLTBSC	Benzenoids	Benzene anc	Benzoic acid
9.73_508.32	508.327856	9.73451667	neg	(5Z,8Z)-10-[(	HMDB0241882		IPMJHHMW	Lipids and lip	Fatty Acyls	Eicosanoids
9.87_309.18	309.182337	9.86676667	pos	Cannabidiva	HMDB0249588		REOZWEGF	Lipids and lip	Prenol lipids	Monoterpenc

0.85_182.10	182.103397	0.84881667	pos	2-Deoxygala	HMDB00123	44704	PMMURAAU	Organic oxyg	Organooxyge	Carbohydrate
1.08_174.16	174.159995	1.07531667	pos	N-(3-(Dimeth	HMDB0246231		ADTJPOBHA	Organic acid	Carboxylic ac	Acrylic acids
3.77_331.08	332.089617	3.76795	pos	Ggstop	HMDB0252708		NTFPDEDRI	Organic acid	Carboxylic ac	Amino acids,
4.31_153.05	153.054773	4.30826667	pos	p-Hydroxyph	HMDB00000	130	XQXPVVBIM	Benzenoids	Phenols	1-hydroxy-2-
4.72_246.07	246.0748	4.72411667	neg	N-Phenylace	HMDB0059772		PTSRBZOZS	Organic acid	Carboxylic ac	Amino acids,
5.37_371.16	371.169616	5.37381667	pos	(1S,2S,4S)-1	HMDB00347	90208	IULDBYINDU	Organic oxyg	Organooxyge	Carbohydrate
7.27_391.21	391.212981	7.26623333	neg	21-Deoxycor	HMDB0004030		LCZBQMKVI	Lipids and lip	Steroids and	Pregnane ste
9.54_449.34	449.348028	9.54118333	pos	LysoSM(d18	HMDB00120	41562	GSEOJHIBP	Lipids and lip	Sphingolipids	Phosphosphi
0.78_301.04	301.042537	0.7799	neg	uric acid lact	HMDB0259719		XDJHJQPBG	Organoheter	Imidazopyrim	Purines and
0.91_357.10	357.105076	0.91118333	neg	Lactobionic a	HMDB0253945		JYTUSYBCF	Lipids and lip	Fatty Acyls	Fatty acyl gly
10.86_355.2	355.227511	10.8611	neg	14-HDoHE	HMDB0060044		CWSPVLXIS	Lipids and lip	Fatty Acyls	Lineolic acids
11.22_343.2	343.223724	11.2158833	pos	5-Oxoicosa-	HMDB0260375		ILNUTMKQM	Lipids and lip	Fatty Acyls	Fatty acids a
11.28_507.2	507.272406	11.2776833	neg	1-(11Z,14Z-e	HMDB0062303		PIWSAHYRI	Lipids and lip	Glycerophos	Glycerophos
13.38_790.5	790.56757	13.38305	pos	SM(d16:2(4E	HMDB0290344		NBBAYTVKZ	Unclassified	Unclassified	Unclassified
6.80_349.19	349.19806	6.79993333	pos	Gibberellin A	HMDB0303454		NJEWNTGS	Lipids and lip	Prenol lipids	Diterpenoids
8.45_362.32	362.325883	8.4515	pos	10,20-Dihydr	HMDB00319	88038	RIIQXYZWF	Lipids and lip	Fatty Acyls	Fatty acids a
8.79_375.21	375.21389	8.7919	pos	20-Hydroxy-l	HMDB00015	6287	PTJFJXLGR	Lipids and lip	Fatty Acyls	Eicosanoids
0.80_171.00	171.007646	0.79596667	neg	Glycerophos	HMDB02528	65927	AWUCVROL	Lipids and lip	Glycerophos	Glycerophos
4.60_151.03	151.039888	4.59571667	neg	Methyl furfur	HMDB00323	88423	PLNJUBIUG	Lipids and lip	Fatty Acyls	Fatty acid es
8.29_381.26	381.26476	8.29023333	neg	Pregnanetrio	HMDB0006070		SCPADBBIS	Lipids and lip	Steroids and	Pregnane ste
1.08_250.12	250.128358	1.07531667	pos	1-Deoxy-1-m	HMDB0243858		JPANQHVKL	Organoheter	Oxazinanes	Morpholines
1.17_367.05	367.053422	1.16698333	neg	5-Thymidylic	HMDB00012	6092	GYOZYWVX	Nucleosides,	Pyrimidine nu	Pyrimidine de
11.00_405.2	405.260958	11.0025333	pos	(11R,16S)-m	HMDB02423	45377	OJLOPKGSI	Lipids and lip	Fatty Acyls	Eicosanoids
13.05_328.3	328.320521	13.0475833	pos	Stearoyletha	HMDB00130	3720	OTGQIQQTI	Organic nitro	Organonitrog	Amines
3.60_144.08	144.080818	3.59728333	pos	Quinaldine	HMDB00420	96204	SMUQFGGV	Organoheter	Quinolines ai	Unclassified
4.24_105.03	105.033972	4.23603333	pos	1,3-Benzodic	HMDB02441	326436	FTNJQNQLE	Organoheter	Benzodioxole	Unclassified
6.03_317.21	317.210617	6.03235	pos	5,6-Ep-15S-HETE		36327	YNHSGCYE	Lipids and lip	Fatty Acyls	Eicosanoids
6.86_225.09	225.090863	6.85513333	pos	Equol	HMDB00022	44865	ADFCQWZH	Phenylpropa	Isoflavonoids	Isoflavans
8.21_201.08	201.088336	8.21301667	pos	5-Phenylvale	HMDB00020	6456	BYHDDXPK	Lipids and lip	Fatty Acyls	Fatty acids a
8.86_272.25	272.258175	8.86416667	pos	Hexadecaspl	HMDB02421	53900	BTUSGZZC	Organic nitro	Organonitrog	Amines
9.83_541.31	542.323643	9.83063333	pos	LysoPC(20:5	HMDB00103	40316	PDIGSOAO	Lipids and lip	Glycerophos	Glycerophos
11.30_367.2	367.246217	11.2956167	neg	12(S)-HETrE	HMDB0062747		YYIXZLMPK	Lipids and lip	Fatty Acyls	Eicosanoids
12.48_807.5	830.565186	12.4811	pos	PC(20:4(6E,8	HMDB0289744		AQVFPLNO	Unclassified	Unclassified	Unclassified

14.60_617.2(	617.259991	14.60285	pos	Obacunone 1	HMDB0302542		MXZVBPOY(	Lipids and lip	Prenol lipids	Triterpenoids
4.43_277.03:	277.032739	4.43021667	neg	Caffeoylmali(	HMDB0029318		PMKQSEYPI	Phenylpropa	Cinnamic aci	Hydroxycinna
5.05_317.11	317.114045	5.05298333	neg	Camonagrel	HMDB0249574		IRWWXLHSI	Benzenoids	Indanes	Unclassified
6.45_271.15:	271.15347	6.45406667	pos	ascr#13			OZXQOEFF'	Lipids and lip	Fatty Acyls	Fatty acyl gly
6.89_264.23:	264.231921	6.89188333	pos	Ginsenoyne .	HMDB00403	94885	AZYMFOSY:	Lipids and lip	Fatty Acyls	Fatty alcohol
7.33_365.19:	365.19301	7.3317	pos	Tetramethylc	HMDB0258916		GFTUVGXU'	Organic oxyç	Organooxyge	Carbohydrate
0.78_240.01:	240.018928	0.7799	neg	Glucosamine	HMDB00005	5571	MTDHILKWI	Organic oxyç	Organooxyge	Carbohydrate
0.79_217.11:	217.118044	0.7877	pos	Prolyl-Threor	HMDB00290	85920	GVUVRRPY'	Organic acid:	Carboxylic a(	Amino acids,
10.65_546.2:	546.282204	10.6454167	pos	PS(20:4(5Z,8Z,11Z,14Z)/0		46725	XHWSRRGL	Lipids and lip	Glycerophos	Glycerophos
11.13_642.3(	642.362047	11.1325833	neg	Am-LPE(18:0/0:0)			CSDPYUQTI	Lipids and lip	Glycerophos	Glycerophos
11.68_325.2:	325.23439	11.6823667	pos	MG(0:0/14:0/	HMDB00115	332664	TVIMZSOUÇ	Lipids and lip	Glycerolipids	Monoradylgly
14.42_367.3:	367.335593	14.4236667	pos	delta-24-Cho	HMDB0302890		AVSXSVCZ\	Lipids and lip	Steroids and	Cholestane s
2.14_236.07!	237.086903	2.14131667	pos	L-Formylkynı	HMDB00604	3373	BYHJHXPTÇ	Organic oxyç	Organooxyge	Carbonyl cor
5.50_341.06	341.06748	5.49605	neg	Phyllocoumarin		47286	JXVQELAUF	Lipids and lip	Polyketides	Flavonoids
0.80_369.00	369.007292	0.79596667	neg	Phosphoseri	HMDB00002	5270	BZQFBWGG	Organic acid:	Carboxylic a(	Amino acids,
2.46_105.03	105.033964	2.46275	pos	Tropolone	HMDB02592	324038	MDYOLVRU	Hydrocarbon	Tropones	Tropolones
8.03_181.12:	181.122149	8.0298	pos	Dihydroactini	HMDB00352	90552	IMKHDCBNF	Organoheter	Benzofurans	Unclassified
1.23_286.10:	286.103757	1.23451667	pos	(R)-3-hydrox	HMDB0062735		UEFRDQSM	Lipids and lip	Fatty Acyls	Fatty acid es
1.74_147.06(	147.066107	1.74428333	neg	Isovaleric aci	HMDB00007	109	GWYFCOCF	Lipids and lip	Fatty Acyls	Fatty acids a
10.73_430.3(	475.306195	10.73455	neg	25-Hydroxyvi	HMDB0060127		SFOPTSUKU	Lipids and lip	Steroids and	Vitamin D an
11.31_282.2!	283.262667	11.3062	pos	Oleic acid	HMDB00002	190	ZQPPMHVW	Lipids and lip	Fatty Acyls	Fatty acids a
12.48_755.5	756.551198	12.4811	pos	PC(P-16:0/1:	HMDB0289513		MTFXTJCEI(	Unclassified	Unclassified	Unclassified
3.66_227.10:	227.103354	3.65995	neg	Pyroglutamyl	HMDB00946	452554	DTSWLLBB(	Organic acid:	Carboxylic a(	Amino acids,
5.08_163.07!	163.075255	5.08273333	pos	4-Hydroxy-2,	HMDB00376	92445	COUDKMJL'	Organic oxyç	Organooxyge	Carbonyl cor
5.76_223.09!	223.095553	5.75788333	pos	(±)-Camphor	HMDB00344	89970	LSPHULWD'	Organic acid:	Carboxylic a(	Dicarboxylic
5.76_474.25:	474.251804	5.75788333	pos	Dibekacin	HMDB0251157		JJCQSGDBI	Organic oxyç	Organooxyge	Carbohydrate
7.98_202.15(	201.14943	7.97648333	neg	3-Hydroxyno	HMDB00324	88466	RTYFAKSW.	Lipids and lip	Fatty Acyls	Fatty alcohol
1.09_318.11!	318.119385	1.08996667	pos	4-Methylcate	HMDB0240458		PLHZIQGSG	Organic oxyç	Organooxyge	Carbohydrate
11.46_353.2:	353.211816	11.4580167	neg	Bendigole E			QZZDIOJZZI	Lipids and lip	Steroids and	Bile acids an
4.52_186.11:	186.113374	4.52246667	neg	7-oxo-8-amino-nonanoic acid			GUAHPAJO:	Lipids and lip	Fatty Acyls	Fatty Acids a
4.63_293.11	293.114382	4.63258333	neg	6-Hydroxyme	HMDB00040	7013	OMYMRCXC	Organoheter	Indoles and (	Hydroxyindol
6.12_190.08(	190.086185	6.12398333	pos	Indole-3-prop	HMDB00023	6602	GOLXRNDW	Organoheter	Indoles and (	Indolyl carbo
6.82_225.11:	225.113072	6.824	neg	2,2,7,7-Tetra	HMDB00300	86659	XBYJEPZKX	Organic oxyç	Organooxyge	Ethers

7.04_415.25	416.257941	7.03826667	pos		N-Demethyl	HMDB0255109			IBLXOBHAB	Lipids and lip	Steroids and	Oxosteroids
7.20_300.20	301.215729	7.20335	pos		all-trans-Reti	HMDB00018	2277		SHGAZHPC	Lipids and lip	Prenol lipids	Retinoids
7.28_239.09	239.092429	7.28471667	neg		2-(2-Methylp	HMDB0059943			NCMQNBRX	Benzenoids	Benzene and	Toluenes
9.10_251.12	251.127173	9.10213333	pos		5-Heptyltetra	HMDB00309	87338		FCCIDBCBP	Organoheter	Lactones	Gamma buty
9.46_244.08	243.082422	9.46038333	neg		(S)-Flurbiprofen		44881		SYTBZMRGI	Unclassified	Unclassified	Unclassified
10.15_447.2	447.275135	10.15385	neg		3-Methyl-5-p	HMDB0112084			QZGKHISFV	Lipids and lip	Fatty Acyls	Fatty acids a
10.56_363.2	363.250218	10.5560833	pos		Annoglabasi	HMDB0033398			ZKXLEDOC	Lipids and lip	Prenol lipids	Diterpenoids
11.91_490.3	490.330126	11.9099667	neg		LysoPE(0:0/2	HMDB00114	62269		QEQAJJJSR	Lipids and lip	Glycerophos	Glycerophos
12.94_324.2	307.262603	12.9406833	pos		15R-HEDE				ZTRWPEHM	Lipids and lip	Fatty Acyls	Eicosanoids
2.65_215.99	215.996926	2.64586667	neg		2-Aminopher	HMDB0304922			BUJVAHSU	Unclassified	Unclassified	Unclassified
3.07_279.13	279.134413	3.07325	pos		Tyrosyl-Proli	HMDB00291	86005		VNYDHJARL	Organic acid	Carboxylic a	Amino acids,
3.33_172.07	172.071765	3.32576667	pos		Metronidazol	HMDB00150	573		VAOCPAMS	Organoheter	Azoles	Imidazoles
3.96_337.11	337.113639	3.96453333	pos		3-[4-(3-Triflu	HMDB0249941			MAHBIUDTZ	Organic acid	Carboxylic a	Amino acids,
5.07_359.12	359.124937	5.07146667	neg		Isodityrosine	HMDB0302972			FWZXNPNH	Phenylpropa	Flavonoids	Flavonoid gly
5.36_418.06	418.067548	5.35556667	pos		2-Amino-5-[[	HMDB0257811			CAQPTAZC	Nucleosides,	Purine nucle	Purine 2'-dec
6.99_354.16	353.160454	6.98976667	neg		Propofol gluc	HMDB00609	2159		JZSJIASBM	Organic oxyg	Organooxyge	Carbohydrate
10.14_492.3	492.368005	10.14065	pos		(3beta,17alp	HMDB00351	90480		FGFZDTLKZ	Lipids and lip	Prenol lipids	Triterpenoids
11.00_349.2	349.234387	11.0025333	pos		Avocadyne 4	HMDB00310	87373		FHGZOCAZI	Lipids and lip	Fatty Acyls	Fatty alcohol
12.32_311.2	311.254982	12.3237167	pos		Glyceryl paln	HMDB0243951			KVYUBFKS	Lipids and lip	Glycerolipids	Monoradylgly
4.51_529.40	529.400218	4.50633333	pos		Ergosteryl-3beta-O-L-aspartate				DHFWBWIM	Lipids and lip	Steroids and	Sterols
7.83_173.11	173.118079	7.82873333	neg		3,5-Dimethyl	HMDB0061913			WIYNOPYN	Organic oxyg	Organooxyge	Alcohols and
8.16_399.21	399.211525	8.15795	pos		sinefungin	HMDB0258302			LMXOHSDX	Nucleosides,	5'-deoxyribor	Unclassified
8.76_435.30	435.300978	8.75856667	pos		cholesta-5,24(28),25-trien-		84008		ALRMOHDS	Lipids and lip	Steroids and	Sterols
9.12_384.28	384.289258	9.12051667	pos		Apo-12'-zeax	HMDB0302023			PAUIQDPAE	Lipids and lip	Prenol lipids	Diterpenoids
9.36_414.32	414.320999	9.35948333	pos		hydroxyhexa	HMDB0253270			ATGNGWON	Lipids and lip	Fatty Acyls	Fatty acids a
9.44_438.28	438.286078	9.44245	neg		(7Z,11Z,14Z)	HMDB0241497			OOYBPMDZ	Lipids and lip	Fatty Acyls	Fatty acid es
0.74_229.11	229.118194	0.74288333	pos		Valylglutamic	HMDB00291	23976		UPJONISHZ	Organic acid	Carboxylic a	Amino acids,
11.54_440.3	463.30252	11.5398167	pos		Acetolein	HMDB0303184			KXLSJQTXS	Lipids and lip	Glycerolipids	Triradylcglyc
14.71_366.2	366.299793	14.7074	pos		Carbocyclic t	HMDB0249633			ZIWNJZLXP	Lipids and lip	Fatty Acyls	Fatty acids a
14.92_392.3	392.315284	14.9180833	pos		3alpha-Hydroxy-5beta-cho		84524		FEGCPHIPS	Lipids and lip	Steroids and	Bile acids an
4.91_244.11	244.118811	4.90568333	neg		2-octenoylgly	HMDB0094801			HNXMRPDB	Organic acid	Carboxylic a	Amino acids,
6.07_269.10	269.102966	6.06866667	neg		Alongside	HMDB0248179			BKNQGBKA	Phenylpropa	Macrolides a	Unclassified
6.99_489.13	489.13534	6.98976667	neg		(2S)-1,1,1-Tr	HMDB0254787			UJINBEQCD	Organoheter	Azoles	Pyrazoles

8.51_223.13	223.133906	8.51168333	neg	13-Oxo-9,11-	HMDB00345	90028	XHDHWGKE	Lipids and lip	Fatty Acyls	Fatty acids a
1.07_225.06	225.061985	1.06741667	neg	1-[3,4-Dihydr	HMDB0243988		IWUCXVSUI	Nucleosides,	Triazole ribo	Unclassified
10.84_361.1	361.199537	10.8434333	neg	4-Hydroxyret	HMDB00062	2281	KGUMXGDK	Lipids and lip	Prenol lipids	Retinoids
4.21_187.06	187.060983	4.20936667	neg	ethyl-4-hydro	HMDB0303864		KXYCNEIOR	Organoheter	Dihydrofuran	Furanones
0.63_443.12	443.126297	0.62835	neg	L-Cystathioni	HMDB00000	39	ILRYLPWNY	Organic acid	Carboxylic ac	Amino acids,
7.20_265.21	265.215912	7.20335	pos	12-hydroxy-8E,10E-heptac		74572	INVCGLRLLI	Lipids and lip	Fatty Acyls	Fatty Acids a
0.72_104.01	207.014366	0.71928333	neg	Hydroxypyru	HMDB00013	482	HHDDCCUII	Organic acid	Hydroxy acid	Beta hydroxy
10.47_376.2	376.262976	10.46565	pos	C19 Sphingosine-1-phosp		83701	ZWOGUYCC	Lipids and lip	Sphingolipids	Sphingoid ba
4.94_373.21	373.212999	4.93775	pos	D-Tetrahydro	HMDB02507	84992	AEQDJSLRV	Alkaloids and	Protoberberi	Unclassified
8.76_357.27	357.274857	8.75856667	pos	3E,13Z,16Z-Docosatrienoic acid			DFTHKRVOI	Lipids and lip	Fatty Acyls	Docosanoids
8.97_356.27	356.278995	8.97426667	pos	Tridec-3-eno	HMDB0241309		ZMBYKPOU	Lipids and lip	Fatty Acyls	Fatty acid es
0.70_272.17	272.171474	0.69861667	pos	Valylhistidine	HMDB00291	23909	BNQVUHQW	Organic acid	Carboxylic ac	Amino acids,
11.30_323.2	323.258926	11.2956167	neg	11R-HEDE		36083	HDMIRVFIV	Lipids and lip	Fatty Acyls	Eicosanoids
11.47_267.2	267.267814	11.4676667	pos	9-Octadecen	HMDB00309	75373	ZENZJGDPV	Lipids and lip	Fatty Acyls	Fatty aldehy
2.11_148.05	166.086234	2.10798333	pos	trans-Cinnam	HMDB00009	5880	WBYWAXJH	Phenylpropa	Cinnamic aci	Cinnamic aci
3.29_246.14	246.144874	3.28915	pos	Asparaginyl-l	HMDB00287	23810	HXWUJJADI	Organic acid	Carboxylic ac	Amino acids,
3.45_417.24	417.246101	3.45331667	pos	Glutamylleuc	HMDB0252758		VSRCAOIHM	Organic acid	Carboxylic ac	Amino acids,
3.93_236.11	237.123453	3.92891667	pos	Alanylphenyl	HMDB0028694		OMNVYXHO	Organic acid	Carboxylic ac	Amino acids,
9.37_223.13	223.133897	9.36935	neg	Methyl jasmo	HMDB00365	3361	GEWDNTWI	Lipids and lip	Fatty Acyls	Lineolic acids
9.52_346.33	346.331092	9.52293333	pos	20-hydroxy-eicosanoic aci		74505	JLDIWYKSF	Lipids and lip	Fatty Acyls	Fatty Acids a
9.72_271.19	271.191474	9.71598333	neg	2,4,7,9-Tetra	HMDB0245484		LXOFYPKXC	Organic oxyg	Organooxyge	Carbonyl cor
9.87_191.17	191.179228	9.86676667	pos	5,8-tetradecadienal		75358	NGKCMQUF	Lipids and lip	Fatty Acyls	Fatty aldehy
0.67_284.09	284.098601	0.6745	neg	N-Gluconyl e	HMDB00322	88337	DTMUKVUZI	Organic nitro	Organonitrog	Amines
1.08_243.12	244.128931	1.07531667	pos	Glutaminylpr	HMDB00288	85719	NJMYZEJOF	Organic acid	Carboxylic ac	Amino acids,
1.70_162.07	162.076171	1.70151667	pos	3-Hexenedio	HMDB00003	5382	YHGNXQAF	Lipids and lip	Fatty Acyls	Fatty acids a
10.34_632.3	632.318238	10.3358333	neg	Retaspimycir	HMDB0257169		OAKGNIRU	Phenylpropa	Macrolactam	Unclassified
11.24_450.2	450.298874	11.2413667	neg	1-(2-methoxy-14-methyl-pentadecanyl)-			JCYSKRLDV	Lipids and lip	Glycerophos	Glycerophos
12.31_631.4	631.472813	12.3147833	neg	PA(O-16:0/16:1(9Z))		82154	BTZXRXSZM	Lipids and lip	Glycerophos	Glycerophos
3.56_195.11	195.113073	3.56223333	pos	1,2,3,4-Tetra	HMDB0244117		BWKMGYQJ	Organoheter	Tetrahydrois	Unclassified
4.10_155.07	155.071239	4.10018333	neg	8-Hydroxy-5,	HMDB00311	87408	LNDWPMYW	Organic acid	Hydroxy acid	Medium-chai
4.56_107.05	107.050098	4.55865	neg	Enol-3-Ethyl-	HMDB00312	87504	JHWFWLUA	Organic oxyg	Organooxyge	Carbonyl cor
9.22_237.14	237.14946	9.22325	neg	Edulan I	HMDB00349	340537	HUXGOQHT	Organoheter	Benzopyrans	Unclassified
9.87_135.11	135.116777	9.86676667	pos	(-)-Perillyl alcohol		41074	NDTYTMIUV	Lipids and lip	Prenol lipids	Isoprenoids

1.54_401.21	401.214643	1.54085	pos	Dihydrozeati	HMDB0302649		QRZHDHJU	Lipids and lip	Fatty Acyls	Fatty acyl gly
3.58_312.19	312.191683	3.58011667	pos	Val-pro-pro	HMDB0253034		DOFAQXCY	Organic acid	Carboxylic ac	Amino acids,
4.19_293.07	293.078014	4.1908	neg	Hydroxynalid	HMDB0060826		FRJDYPGZH	Organoheter	Diazanaphtha	Naphthyridin
4.40_516.31	516.314829	4.3987	pos	Val-Gly-Val-A	HMDB0253033		RLCSROTYI	Organic acid	Carboxylic ac	Amino acids,
5.20_286.09	286.094465	5.2006	neg	Tetraphyllin E	HMDB00299	86584	JRCWYCAE	Organic oxyg	Organooxyge	Carbohydrate
5.43_263.12	263.125127	5.42866667	pos	1,10-Epoxy-3	HMDB0303776		SCDDMRZF	Lipids and lip	Prenol lipids	Terpene lact
7.15_251.16	251.161619	7.14843333	pos	Menthone 1,2	HMDB00361	91295	ZBJCYZPAN	Lipids and lip	Prenol lipids	Monoterpeno
9.03_267.15	267.156267	9.02893333	pos	Dihydroarten	HMDB0061088		BJDCWCLM	Organic oxyg	Organooxyge	Carbohydrate
9.73_309.20	309.207177	9.73451667	neg	6-Methylging	HMDB0303099		HDNGHNOE	Benzenoids	Benzene and	Methoxybenz
0.68_104.10	104.107359	0.68255	pos	(E)-2-Penten	HMDB00316	46063	BTSIZIIPFN	Organic oxyg	Organooxyge	Alcohols and
0.88_360.15	360.149975	0.87973333	pos	Sucrose	HMDB00002	137	CZMRCDWA	Organic oxyg	Organooxyge	Carbohydrate
10.54_431.3	431.315117	10.5380833	pos	7alpha-Hydro	HMDB0012458		SATGKQGF	Lipids and lip	Steroids and	Bile acids, al
11.84_349.2	372.286575	11.8413833	pos	Dihomo-gam	HMDB0013625		ULQWKETU	Organic nitro	Organonitrog	Amines
4.92_473.19	473.192751	4.92405	neg	1-Carboxyeth	HMDB0240628		MSBSJGQKI	Organic acid	Carboxylic ac	Amino acids,
5.63_462.17	462.177136	5.6251	neg	Etodolac acy	HMDB0060916		XJZNMEMK	Organic oxyg	Organooxyge	Carbohydrate
6.73_154.99	154.990377	6.73181667	neg	m-Chloroben	HMDB00015	6311	LULAYUGMI	Benzenoids	Benzene and	Benzoic acid
6.98_399.13	399.138595	6.98325	pos	Carissanol	HMDB00300	86713	LHQJDCXE	Lignans, neo	Furanoid lign	Tetrahydrofu
8.29_405.26	405.260607	8.2859	pos	Lovastatin ac	HMDB0254177		QLJODMDS	Organic acid	Hydroxy acid	Medium-chai
8.81_377.22	377.229921	8.81001667	pos	Prostaglandi	HMDB00051	3499	CIMMACUR	Lipids and lip	Fatty Acyls	Eicosanoids
9.43_419.31	419.315164	9.43288333	pos	Oblatone H			HZNDUVVD	Lipids and lip	Polyketides	Aromatic poly
1.07_221.03	221.030008	1.06741667	neg	1,2,3-Propan	HMDB00311	334328	KQTIIICEAU	Organic acid	Carboxylic ac	Tricarboxylic
1.36_276.01	276.017454	1.3586	neg	2,5-Dioxopyr	HMDB0247382		FLCQLSRLQ	Organoheter	Pyrrolidines	Pyrrolidones
10.98_322.2	322.273655	10.9842333	pos	3-Dehydrosp	HMDB00014	3428	KBUNOSOG	Organic oxyg	Organooxyge	Carbonyl con
4.65_231.08	231.087256	4.65108333	neg	Glutarate ser	HMDB00122	35847	VBKPPDYGI	Lipids and lip	Fatty Acyls	Fatty acids a
9.52_333.20	333.207172	9.51548333	neg	bicyclo-PGE2	HMDB00600	3824	CGCZPIJMG	Lipids and lip	Fatty Acyls	Eicosanoids
1.19_299.07	300.08424	1.1884	pos	D-4'-Phosph	HMDB00010	3427	XHFVGHPG	Organic acid	Carboxylic ac	Amino acids,
1.54_258.11	258.119581	1.54085	pos	Tetrahydrone	HMDB00009	5890	XHIXPVCTD	Organoheter	Pteridines an	Pterins and d
10.06_256.1	256.192167	10.0622	neg	N-Lauroylgly	HMDB0013272		JWGGSJFIG	Organic acid	Carboxylic ac	Amino acids,
11.15_494.3	494.324884	11.1450667	pos	PE(19:1(9Z)/0:0)		77685	QXOXODCL	Lipids and lip	Glycerophos	Glycerophos
11.31_328.3	328.320484	11.3062	pos	D-erythro-Sphingosine C-2		45095	HTJSZHKGN	Unclassified	Unclassified	Unclassified
12.74_406.3	407.315062	12.7447333	pos	MG(0:0/22:4	HMDB00115	62337	DVYDCUGIJ	Lipids and lip	Glycerolipids	Monoradylgly
3.43_169.04	169.047053	3.43491667	pos	3-(Hydroxym	HMDB0255410		RAZMGTPZ	Lipids and lip	Fatty Acyls	Fatty acids a
4.39_203.00	203.001697	4.39318333	neg	O-methoxyca	HMDB0060013		AQTYXAPIH	Organic acid	Organic sulfu	Arylsulfates

7.22_320.234	321.24193	7.2217	pos	20-Hydroxye	HMDB00059	35316	NNDIXBJHN	Lipids and lip	Fatty Acyls	Fatty acids a
9.29_310.17	311.184864	9.28653333	pos	Botrydial		53517	SJFIYVCSGI	Lipids and lip	Prenol lipids	Isoprenoids
4.44_251.12	251.125449	4.435	pos	Kamahine C	HMDB00389	93546	NKEIHMMKF	Organic oxyg	Organooxyge	Ethers
4.44_265.14	265.141094	4.435	pos	Pisumic acid	HMDB00392	93831	GBHKILLDPI	Lipids and lip	Prenol lipids	Sesquiterper
6.20_456.24	456.240988	6.1974	pos	20-Hydroxy-l	HMDB00126	36255	BJRMBXPQ	Lipids and lip	Fatty Acyls	Eicosanoids
1.03_276.11	276.118952	1.02758333	pos	Imidazoleace	HMDB0002331		AHPWEWAS	Nucleosides,	Imidazole rib	Unclassified
11.00_398.2	421.232182	11.0025333	pos	Tris(2-butoxy	HMDB02592	70068	WTLBZVNB	Organic acid	Organic phos	Phosphate e
13.38_340.2	340.284109	13.38305	pos	Crassumol A			DDUWQTPS	Lipids and lip	Prenol lipids	Isoprenoids
3.72_290.15	290.159587	3.71513333	pos	3-Methylgluta	HMDB00005	5536	HFCPFJNSE	Lipids and lip	Fatty Acyls	Fatty acid es
5.79_251.16	251.161534	5.7943	pos	4,11,13,15-T	HMDB00361	91305	GAPZIAIPN	Lipids and lip	Prenol lipids	Terpene lact
9.67_375.21	375.213795	9.66891667	pos	15-Epi-lipoxi	HMDB0012587		IXAQOQZEC	Lipids and lip	Fatty Acyls	Eicosanoids
0.74_219.13	219.133845	0.74288333	pos	epsilon-(Carl	HMDB0251886		ALXLJPPLHI	Organic acid	Carboxylic ac	Amino acids,
3.60_187.06	205.097032	3.59728333	pos	Indoleacrylic	HMDB00007	5702	PLVPPLCLB	Organoheter	Indoles and	Indoles
4.27_290.19	290.196278	4.27276667	pos	5-Hydroxyhe	HMDB0241678		JGYMSEVET	Lipids and lip	Fatty Acyls	Fatty acid es
4.43_185.02	185.027522	4.43021667	neg	2,4-Dimethylbenzenesulfo		316804	CHZLVSBM	Unclassified	Unclassified	Unclassified
4.88_351.20	351.200166	4.88355	pos	(1S,2R,4R,8	HMDB00399	94519	KRTKKVIKR	Lipids and lip	Prenol lipids	Terpene glyc
1.85_232.08	232.08249	1.85476667	neg	5'-Deoxyade	HMDB00019	63895	XGYIMTFOT	Nucleosides,	5'-deoxyribor	Unclassified
12.24_425.2	425.290832	12.2397667	neg	MG(0:0/20:3	HMDB00115	62331	FHCHFKIVIF	Lipids and lip	Endocannabi	Unclassified
13.38_755.5	756.550984	13.38305	pos	PE-NMe2(15	HMDB0113927		XPHGGUYB	Lipids and lip	Glycerophos	Glycerophos
4.78_228.14	251.136267	4.7769	pos	Isoleucylproli	HMDB00111	23858	BBIXOODYV	Organic acid	Carboxylic ac	Amino acids,
6.18_292.22	292.226704	6.1791	pos	Exaprolol	HMDB0252141		ABXHHEZNI	Benzenoids	Phenol ether	Unclassified
7.17_294.21	295.226465	7.16671667	pos	Sterebin D	HMDB00368	91845	BQUAFPABI	Lipids and lip	Prenol lipids	Sesquiterper
8.12_386.28	386.289691	8.1212	pos	(12E)-10-Hy	HMDB0241362		LKICWTKOZ	Lipids and lip	Fatty Acyls	Fatty acid es
1.33_246.14	246.14475	1.33403333	pos	Isoleucyl-Asp	HMDB0028902		HZYHBDVR	Organic acid	Carboxylic ac	Amino acids,
1.56_280.12	280.12933	1.55895	pos	Phenylalanyl	HMDB00289	23916	BXNGIHFNN	Organic acid	Carboxylic ac	Amino acids,
12.02_323.2	323.222501	12.0185833	neg	Octadec-11-e	HMDB0255892		VENIIVIRET	Lipids and lip	Fatty Acyls	Fatty acids a
14.44_390.2	390.299789	14.4418	pos	Cervonoyl et	HMDB0013627		CXWASNUD	Lipids and lip	Fatty Acyls	Fatty acid es
8.69_311.18	311.186293	8.68903333	neg	Tetranor 12-I	HMDB00600	44857	KBOVKDIBC	Lipids and lip	Fatty Acyls	Fatty acids a
9.46_393.22	393.228354	9.46038333	neg	Ataprost	HMDB0248693		DKLGLHQHI	Lipids and lip	Fatty Acyls	Eicosanoids
9.88_466.35	466.352358	9.88461667	pos	12-Hydroxy-1	HMDB0013154		QAIWREXEZ	Lipids and lip	Fatty Acyls	Fatty acid es
1.20_280.09	280.091842	1.20368333	pos	5-Methylcytic	HMDB00009	5919	ZAYHVCMS	Nucleosides,	Pyrimidine nu	Unclassified
10.23_345.1	345.168124	10.2257833	neg	(2R,5R)-2-[6-	HMDB0255318		ARVGZMRD	Nucleosides,	Purine nucle	Unclassified
11.26_283.1	283.170076	11.25965	neg	9,17-hydroxy-androsta-1,4-dien-3-one			BDTCDYJFC	Lipids and lip	Steroids and	Steroids

3.56_302.19	302.196373	3.56223333	pos		4-Hydroxy-6-	HMDB0241704		VUSWVDXH	Lipids and lip	Fatty Acyls	Fatty acid es
3.65_363.08	364.096607	3.64815	pos		Cefadroxil	HMDB00152	3948	BOEGTKLJZ	Organoheter	Benzoxepine	Dibenzoxepi
3.73_156.06	156.066492	3.73108333	neg		Tiglylglycine	HMDB00009	4246	WRUSVQOK	Organic acids	Carboxylic ac	Amino acids,
3.80_306.11	306.119598	3.80401667	pos		pyroglutamyl	HMDB0256990		VTKWFWXC	Organic acids	Carboxylic ac	Amino acids,
8.69_294.18	295.188765	8.68985	pos		Gingerol	HMDB0005783		NLDDIKRKF	Benzenoids	Phenols	Methoxypher
11.07_345.2	345.239488	11.0736	pos		15(S)-Hydrox	HMDB00050	36268	IUKXMNDGT	Lipids and lip	Fatty Acyls	Eicosanoids
13.19_340.2	340.210556	13.19245	neg		Glycyllysylarg	HMDB0252886		CLNSYANKY	Organic acids	Carboxylic ac	Amino acids,
15.39_338.3	337.31126	15.3874667	neg		Erucic acid	HMDB00020	6470	DPUOLQHD	Lipids and lip	Fatty Acyls	Fatty acids a
2.04_103.03	103.03941	2.04021667	pos		Succinic acid	HMDB00012	6114	UIUJIQZEAC	Lipids and lip	Fatty Acyls	Fatty acids a
2.48_233.14	233.149525	2.48101667	pos		Isoleucyl-Thr	HMDB00289	85822	DRCKHKZYI	Organic acids	Carboxylic ac	Amino acids,
4.56_242.17	242.174886	4.5592	pos		Annuionone	HMDB00326	88627	DZVNWYFIA	Organoheter	Oxepanes	Unclassified
4.65_211.14	211.144117	4.64996667	pos		3-Hydroxy-3-	HMDB0245886		MOHYRCCD	Benzenoids	Benzene and	Phenylpropar
5.32_330.22	330.227129	5.3192	pos		5-Hydroxyde	HMDB0241076		HXIJIIXNWA	Lipids and lip	Fatty Acyls	Fatty acid es
7.30_240.09	223.096278	7.29506667	pos		3-Carboxy-4-	HMDB00611	45041	WMCQWXZI	Lipids and lip	Fatty Acyls	Fatty acids a
7.85_227.12	227.128683	7.84718333	neg		Dibutyl malat	HMDB00316	87872	PDSCSYLDF	Organic acids	Hydroxy acid	Beta hydroxy
9.92_463.30	463.306646	9.91656667	neg		(1R)-5-[2-[(1	HMDB0242365		DTXXSJZBS	Lipids and lip	Steroids and	Vitamin D an
0.86_339.09	339.091091	0.86366667	pos		(5R)-5-[(1S)-	HMDB0260164		LSMAUOJG	Lipids and lip	Fatty Acyls	Fatty acyl gly
10.84_454.2	454.292525	10.8409	pos		LysoPE(16:0	HMDB00115	40776	YVYMBNSK	Lipids and lip	Glycerophos	Glycerophos
11.35_311.1	311.168525	11.3501	neg		N-Undecylbe	HMDB00325	88521	UCDCOJNN	Benzenoids	Benzene and	Benzenesulf
11.86_325.2	325.216867	11.8613	neg		Anacardic ac	HMDB00338	89601	KAOMOVYH	Benzenoids	Benzene and	Benzoic acid
3.09_218.01	218.012555	3.08933333	neg		Sulfanilic aci	HMDB0258581		HVBSAKJJO	Benzenoids	Benzene and	Benzenesulf
3.90_284.16	284.161582	3.89685	neg		6-n-Octylami	HMDB0247103		PFSWASUQ	Organoheter	Diazines	Pyrimidines a
4.38_355.14	355.147758	4.38046667	pos		Indole-3-acet	HMDB0031182		XUACNUJF	Organoheter	Indoles and	Indolyl carbo
4.43_436.17	436.172469	4.43021667	neg		N-(1-Carboxy	HMDB0257086		WURJETPE	Organic acids	Carboxylic ac	Amino acids,
4.69_313.15	313.154565	4.68661667	pos		Phenylalanyl	HMDB0013302		GKZIWHRNI	Organic acids	Carboxylic ac	Amino acids,
5.10_314.14	314.141713	5.10093333	pos		Laurolitsine	HMDB0040974		KYVJVURXK	Alkaloids and	Aporphines	Unclassified
8.12_430.28	430.280862	8.12398333	neg		(5E)-8-Hydro	HMDB0241364		FDDDTNWQ	Lipids and lip	Fatty Acyls	Fatty acid es
9.54_472.13	473.143787	9.54118333	pos		Formononetin 7-O-(6''-acetylglucoside)			HZJBDSMA	Lipids and lip	Polyketides	Flavonoids
10.45_370.2	370.294646	10.4474333	pos		Persediene			IMNSNWGU	Lipids and lip	Fatty Acyls	Fatty alcohol
11.47_616.4	634.451641	11.4676667	pos		Nonoxynol-9	HMDB00156	43278	FBWNMEQM	Organic oxyg	Organooxyge	Ethers
14.74_115.0	115.050444	14.7422167	pos		N-Methylhyda	HMDB00036	329576	RHYBFKMFI	Organoheter	Azolines	Imidazolines
3.84_338.13	338.135019	3.83913333	pos		2-(3-carboxy-	HMDB00596	96237	CBQVLMCH	Organic acids	Carboxylic ac	Amino acids,
4.02_245.18	245.185952	4.01898333	pos		(2S)-6-Amino	HMDB0253148		DEAFEFSHU	Organic acids	Carboxylic ac	Amino acids,

8.05_198.18	198.185091	8.04801667	pos	12-amino-dodecanoic acid		35924	PBLZLIFKVF	Lipids and lip	Fatty Acyls	Fatty Acids a
0.74_253.07	276.068907	0.74288333	pos	Neopterin	HMDB0000845		BMQYVXCP.	Organoheter	Pteridines an	Pterins and d
0.76_142.99	142.998401	0.76461667	neg	4-Hydroxy-2-	HMDB00020	6471	WXSKVKPS	Organic acids	Keto acids ar	Gamma-keto
0.90_504.16	522.203026	0.89581667	pos	Levan	HMDB00035	6945	AIHDCSAXV	Organic oxyg	Organooxyge	Carbohydrate
1.34_361.10	361.106806	1.34166667	neg	3-O-Methylgl	HMDB00338	48361	ACDSUMGM	Phenylpropa	Isoflavonoids	Coumestans
10.28_340.3	340.320476	10.2840833	pos	Neryl 8-methyldecanoate			CGTSMPFIF	Lipids and lip	Fatty Acyls	Fatty esters
10.73_497.2	497.288426	10.73455	neg	Cucurbitacin S		41679	MBYLRWSU	Lipids and lip	Steroids and	Sterols
2.73_398.20	398.203522	2.73206667	pos	3-Methyl-3-b	HMDB00319	88066	DZEALCQV	Lipids and lip	Fatty Acyls	Fatty acyl gly
3.58_304.15	304.150423	3.58011667	pos	N5-Acetyl-N2	HMDB00394	93997	JAAJGIIASA	Organic acids	Carboxylic ac	Amino acids,
4.70_197.07	197.078286	4.70481667	pos	3-Methylpime	HMDB00597	3799	NXDCNRPLI	Lipids and lip	Fatty Acyls	Fatty acids a
4.72_158.11	158.117609	4.72301667	pos	5,5-Dimethyl-	HMDB0251369		BADXJIPKFI	Organic oxyg	Organooxyge	Carbonyl cor
5.29_369.15	369.155367	5.29295	neg	Tuberonic acid glucoside			JFDNMLUPL	Lipids and lip	Fatty Acyls	Fatty acyl gly
6.11_313.08	313.08892	6.10565	pos	Osmundalin	HMDB00313	87571	KZOPXYPPI	Organic oxyg	Organooxyge	Carbohydrate
6.76_358.25	358.258415	6.76326667	pos	(9E)-7-Hydro	HMDB0241224		CPNSTMOH	Lipids and lip	Fatty Acyls	Fatty acid es
8.91_432.29	432.296628	8.91221667	neg	2-Hydroxymy	HMDB0013166		WWDZVVW	Lipids and lip	Fatty Acyls	Fatty acid es
9.20_215.16	215.165003	9.20481667	neg	2-Hydroxydo	HMDB0245154		YDZIJQXINJ	Lipids and lip	Fatty Acyls	Fatty acids a
12.74_270.2	270.278704	12.7447333	pos	13-Heptadec	HMDB00138	85269	DZJZIJZUKA	Lipids and lip	Fatty Acyls	Fatty alcohol
3.38_290.17	290.171324	3.38003333	pos	Dhlnl	HMDB0246853		COYAOJMP	Organic acids	Carboxylic ac	Amino acids,
1.87_215.13	215.139781	1.87326667	neg	2-Amino-3-m	HMDB0246938		IHBAVXVTG	Organic acids	Carboxylic ac	Amino acids,
11.49_327.2	327.250061	11.4858667	pos	1-Acetoxy-2-	HMDB00310	87344	MSSXBCMV	Lipids and lip	Fatty Acyls	Fatty alcohol
12.09_492.3	492.344903	12.0909333	pos	LysoPE(20:0	HMDB00115	62296	HEQMDGOE	Lipids and lip	Glycerophos	Glycerophos
12.49_659.5	659.525162	12.4924667	neg	DG(i-18:0/0:	HMDB0299582		ACOCDXOV	Unclassified	Unclassified	Unclassified
13.07_385.3	385.345856	13.0659	pos	27-Hydroxycl	HMDB00021	6487	FYHRJWME	Lipids and lip	Steroids and	Bile acids, al
2.15_394.11	394.111712	2.1466	neg	BEFLOXATC	HMDB0248929		IALVDLPLCL	Benzenoids	Phenol ether	Unclassified
4.00_301.12	301.119998	4.00056667	pos	Pantetheine	HMDB00034	6928	ZNXZGRMV	Organic acids	Carboxylic ac	Amino acids,
4.10_232.11	232.118873	4.10018333	neg	4-[(2,4-Dihyd	HMDB0246316		SBBDHANT	Organic acids	Carboxylic ac	Amino acids,
4.26_255.08	255.088217	4.26475	neg	xi-5-Acetyltet	HMDB00380	92841	AHLDCEZSC	Organic oxyg	Organooxyge	Carbonyl cor
4.30_236.05	236.056305	4.30156667	neg	5-Phenyl-1,3	HMDB00604	71253	YPIQXPKZX	Benzenoids	Benzene anc	Unclassified
5.23_194.11	194.115005	5.22825	pos	1-Hydroxy-2,	HMDB0243896		KMEUSKGE	Organoheter	Piperidines	Piperidinones
5.57_187.06	187.060916	5.5696	neg	2-Methyliden	HMDB0257753		AOWRXQW	Lipids and lip	Fatty Acyls	Fatty acids a
7.67_313.11	313.119528	7.67051667	neg	(+)-Lysergic	HMDB0242256		ZAGRKAFMI	Alkaloids anc	Ergoline and	Lysergic acid
7.68_429.26	430.27357	7.68096667	pos	Mifepristone	HMDB00149	1303	VKHAHZOO	Lipids and lip	Steroids and	Oxosteroids
1.58_486.22	486.220619	1.57865	neg	Acetyl-Ser-As	HMDB0247929		HJDRXEQUI	Organic acids	Carboxylic ac	Amino acids,

10.99_621.3	621.303488	10.9877	neg	PI(20:3(8Z,11Z,14Z)/0:0)			LBUGPTQSI	Lipids and lip	Glycerophos	Glycerophos
14.60_299.1	299.138453	14.60285	pos	indoprofen	HMDB02534	313404	RJMIEHBSY	Phenylpropa	Phenylpropa	Unclassified
3.04_125.05	125.059946	3.03666667	pos	4-Hydroxybe	HMDB00117	4157	BVJSUAQZ	Benzenoids	Benzene anc	Benzyl alcoh
3.47_202.13	203.13936	3.47126667	pos	Alanylleucine	HMDB00286	44268	RDIKFPRVL	Organic acid	Carboxylic ac	Amino acids,
5.90_256.10	256.100008	5.90411667	pos	Rufinamide	HMDB02573	315231	POGQSBRI	Organic acid	Carboxylic ac	Carboxylic ac
0.75_226.08	226.083139	0.74898333	neg	Deoxycytidin	HMDB00000	3367	CKTSBUTU	Nucleosides,	Pyrimidine nu	Pyrimidine 2'
1.79_294.14	294.144984	1.79108333	pos	Phenylalanyl	HMDB00290	85907	XWHWNYM	Organic acid	Carboxylic ac	Amino acids,
14.53_436.3	436.341693	14.53075	pos	1alpha,25-dihydroxy-23-oxavitamin D3 /			BLWGJQZU	Lipids and lip	Steroids and	Secosteroids
3.99_455.09	455.097622	3.98933333	neg	Flavin monor	HMDB00015	6295	FVTCRASF	Nucleosides,	Flavin nuclec	Unclassified
4.89_399.12	399.12963	4.88748333	neg	Methyl helian	HMDB00407	95262	KMCLOJYU	Lipids and lip	Fatty Acyls	Fatty acyl gly
4.91_369.15	369.155401	4.90568333	neg	2-[4-(3-Hydro	HMDB00415	95935	OWQQMQR	Organic oxyg	Organooxyge	Carbohydrate
7.41_315.19	315.196364	7.41378333	neg	(ent-16betaC	HMDB00367	91786	QSJIZGQG	Phenylpropa	Stilbenes	Unclassified
7.99_263.16	263.161417	7.99311667	pos	(1R,7S,13S,1	HMDB0242339		KQNZDYYTI	Phenylpropa	Macrolides a	Unclassified
8.79_293.20	293.208281	8.7919	pos	Auxin b	HMDB00384	93163	MQETZQLZ	Lipids and lip	Prenol lipids	Monoterpeno
1.56_162.05	162.058418	1.55895	pos	S-Allyl-L-cysteine		71319	ZFAHNWW	Unclassified	Unclassified	Unclassified
10.48_271.2	271.227756	10.48035	neg	14S-hydroxy-hexadecanoi		35623	KBDZCYDP	Lipids and lip	Fatty Acyls	Fatty Acids a
11.42_983.6	983.608011	11.42155	neg	24(28)-Dehy	HMDB0302988		XTSYLJLNV	Lipids and lip	Steroids and	Bile acids, al
12.48_744.5	744.552598	12.4811	pos	PE(P-18:0/18	HMDB0285328		HLQPNCNJE	Unclassified	Unclassified	Unclassified
15.43_251.0	251.046504	15.4298167	pos	Diphenyl pho	HMDB02514	320267	ASMQGLCH	Organic acid	Organic phos	Phosphate es
5.02_567.17	567.176885	5.016	neg	Phloretin-2'-(	HMDB0301670		ZPVXZDCY	Phenylpropa	Flavonoids	Flavonoid gly
6.44_159.10	159.10244	6.4382	neg	(R)-2-Hydrox	HMDB00022	6582	JKRDADVRI	Lipids and lip	Fatty Acyls	Fatty acids a
8.43_254.15	277.140888	8.43301667	pos	Gallicynoic acid A			WSAOFJBM	Lipids and lip	Fatty Acyls	Fatty Acids a
8.76_341.27	341.276704	8.75856667	pos	CUDA		44975	HPTJABJPZ	Unclassified	Unclassified	Unclassified
9.23_349.19	349.198122	9.23096667	pos	12,20-Dioxo-	HMDB0060094		CPTWPKCL	Lipids and lip	Fatty Acyls	Eicosanoids
9.63_208.18	209.189771	9.63246667	pos	2-Cyclotetrac	HMDB00398	94428	RYRDADIPP	Organic oxyg	Organooxyge	Carbonyl cor
9.72_316.32	316.320588	9.72246667	pos	Tuberculostearic acid			BEOUGZFC	Lipids and lip	Fatty Acyls	Fatty Acids a
9.98_664.34	664.344993	9.97586667	pos	Aypgkf	HMDB0248753		VULJVZXXU	Organic acid	Carboxylic ac	Amino acids,
0.71_246.09	246.090496	0.71368333	pos	Asparaginyl-I	HMDB00287	23817	IQTUDDBAN	Organic acid	Carboxylic ac	Amino acids,
10.27_277.1	277.179311	10.2662	pos	Nordihydroca	HMDB00347	454234	BXBVPYSHE	Benzenoids	Phenols	Methoxypher
12.48_805.5	806.566399	12.4811	pos	PC(20:5(5Z,8	HMDB0289696		AAVYMDYU	Unclassified	Unclassified	Unclassified
14.74_395.3	395.3629	14.7422167	pos	N-Stearoyl Ly	HMDB0241946		VWYVABSA	Organic acid	Carboxylic ac	Amino acids,
4.50_276.12	276.127392	4.50406667	neg	4-hydroxy-2-i	HMDB0304176		XJUXNVLIAI	Organic acid	Carboxylic ac	Amino acids,
5.06_219.14	219.149039	5.06456667	pos	(E)-1-Cinnan	HMDB00388	93470	JSIGICUAXL	Phenylpropa	Cinnamic aci	Unclassified

8.10_410.28	410.289642	8.10283333	pos		20, 22-Dihyd	HMDB0060730		MWPLZPAH	Lipids and lip	Steroids and	Steroid lacto
8.90_316.20	317.210744	8.90103333	pos		19-Nordeoxy	HMDB0244837		YNCNEPHW	Lipids and lip	Steroids and	Hydroxyster
1.06_136.06	136.062074	1.06053333	pos		1H-Pyrazolo[	HMDB02448	345720	LHCPRYRLD	Organohetero	Pyrazolopyri	Pyrazolo[3,4
10.17_239.1	239.165081	10.1716833	neg		(¬±)-trans-Ne	HMDB0303821		JIFKIUVSUF	Lipids and lip	Prenol lipids	Monoterpen
10.61_335.1	335.183763	10.6077	neg		2-Methoxy-3-	HMDB0257874		SNZLFTJKIL	Benzenoids	Benzene and	Phenylpyruvi
10.92_481.2	481.256804	10.9157	neg		PG(16:1(9Z)/0:0)		80001	JSKJPUCKV	Lipids and lip	Glycerophos	Glycerophos
13.10_823.5	823.573179	13.1012833	pos		3-O-Sulfogal	HMDB0012317		NXJDPFSYC	Lipids and lip	Sphingolipids	Glycosphing
4.83_285.15	285.159537	4.8302	pos		Tropisetron		69620	ZNRGQMMC	Unclassified	Unclassified	Unclassified
7.17_329.23	329.233319	7.17388333	neg		9S,10S,13R-trihydroxyoctadec-11E-eno			NTVFQBIHL	Lipids and lip	Fatty Acyls	Octadecanoi
7.70_269.12	269.128276	7.69936667	pos		Cromakalim	HMDB0250542		TVZCRIROJ	Organohetero	Benzopyrans	1-benzopyra
10.08_379.2	379.248974	10.0807333	neg		(8R,9S,10S,1	HMDB0257629		QPLFSAZMH	Lipids and lip	Steroids and	Pregnane ste
10.41_333.1	333.135307	10.4089833	neg		Amifloxacin	HMDB00418	96152	RUXPNBWP	Phenylpropa	Macrolides a	Unclassified
11.07_361.2	361.234322	11.0736	pos		(3S,4S)-4-[(E	HMDB0251671		WOISDAHQ	Lipids and lip	Prenol lipids	Terpene lact
2.32_268.11	268.117984	2.31811667	pos		Samin	HMDB00389	93538	DRUQKRWF	Organohetero	Benzodioxole	Unclassified
3.60_317.04	317.048387	3.59728333	pos		3,4-Bis(carb	HMDB0258023		SZRYIORVL	Organic acids	Carboxylic ac	Tetracarboxy
9.25_265.17	265.177395	9.24935	pos		L-Menthyl (R	HMDB00323	88397	XSJPRWBZI	Lipids and lip	Prenol lipids	Monoterpen
9.38_285.13	285.132982	9.37788333	pos		karalicin			JATBUOZGJ	Lipids and lip	Fatty Acyls	Fatty alcohol
0.80_256.10	256.103614	0.80285	pos		3-deoxy-D-m	HMDB03041	63650	KYQCXUMV	Organic oxyg	Organooxyge	Carbohydrate
1.12_238.06	261.058289	1.11995	pos		2-Keto-3-Dec	HMDB0250768		NNLZBVFSC	Organic oxyg	Organooxyge	Carbohydrate
10.59_372.3	372.310254	10.5914167	pos		MG(0:0/18:2	HMDB00115	62322	IEPGNWMP	Lipids and lip	Fatty Acyls	Lineolic acids
11.88_482.4	482.419374	11.8768833	pos		2-Hydroxydo	HMDB0241600		QVDHNULO	Lipids and lip	Fatty Acyls	Fatty acid es
12.49_605.4	605.454904	12.4924667	neg		DG(17:2(9Z,12Z)/20:5(5Z,		4422	SSTJYHLUK	Lipids and lip	Glycerolipids	Diradylglycer
4.22_346.19	346.197538	4.21765	pos		N-Jasmonoyl	HMDB00293	72477	IBZYPBGPO	Organic acids	Carboxylic ac	Amino acids,
5.37_177.12	177.127299	5.37381667	pos		Cucujolide IV		97393	FAMIQKMUI	Lipids and lip	Fatty Acyls	Fatty esters
5.41_262.23	262.237408	5.41035	pos		omega-hydroxy myristic ac		35422	JOSXCARTD	Lipids and lip	Fatty Acyls	Fatty Acids a
0.63_239.03	239.039695	0.62835	neg		Pectin	HMDB00034	6916	AEMOLEFT	Organic oxyg	Organooxyge	Carbohydrate
1.25_357.10	357.105061	1.2521	neg		Serotinose	HMDB00298	86575	QYNRIDLOT	Organic oxyg	Organooxyge	Carbohydrate
10.68_291.2	291.231278	10.6803667	pos		3-Methyl-5-p	HMDB00310	87402	TUQVXFOS	Lipids and lip	Fatty Acyls	Fatty acids a
11.71_391.2	391.285378	11.7053667	neg		Dodecadien	HMDB0302621		HQSBWLQF	Lipids and lip	Fatty Acyls	Fatty acids a
12.83_321.2	321.239586	12.83355	pos		5-Hexyltetra	HMDB00311	831350	AQIVDANQK	Lipids and lip	Fatty Acyls	Eicosanoids
4.81_198.12	198.127676	4.81226667	pos		2',3-Dimethyl	HMDB02463	420435	HSQVHYAN	Benzenoids	Benzene and	Biphenyls an
5.46_145.08	145.086832	5.45918333	neg		omega-hydroxy enanthoic		35401	PNAJBOZY	Lipids and lip	Fatty Acyls	Fatty Acids a
9.37_482.31	482.312468	9.36935	neg		9-(5-Pentylfu	HMDB0241862		CPZLPCLBV	Lipids and lip	Fatty Acyls	Fatty acid es

0.70_432.17(	432.170942	0.69861667	pos	a-L-Arabinofu	HMDB00388	93494	FUDXMJHO.	Organic oxyg	Organooxyge	Carbohydrate
1.67_113.02:	113.023809	1.66533333	pos	Citraconic ac	HMDB00006	5607	HNEGQIOM'	Lipids and lip	Fatty Acyls	Fatty acids a
13.25_613.5	613.51986	13.2463167	neg	DG(17:1(9Z)/20:2(11Z,14Z		4385	FZXGUSKHI	Lipids and lip	Glycerolipids	Diradylglycer
4.11_190.10	190.107542	4.10951667	pos	N-Lactoylvali	HMDB0062181		IJKKTQLUX(	Organic acids	Carboxylic ac	Amino acids,
4.19_363.11	363.119917	4.1908	neg	3-iodo-hexadecanoic acid		35914	NCMJGOFM	Lipids and lip	Fatty Acyls	Fatty Acids a
4.76_135.04	135.045023	4.76071667	neg	2-Hydroxyac	HMDB02451	338652	ZWVHTXAY	Organic oxyg	Organooxyge	Carbonyl cor
6.79_413.21	413.218166	6.7873	neg	5(6)-Epoxy P	HMDB0012110		CBOBGOJU	Lipids and lip	Fatty Acyls	Eicosanoids
0.70_295.11:	295.113369	0.69861667	pos	Distichonic a	HMDB00387	93405	MQDGZZFO	Organic acids	Carboxylic ac	Amino acids,
1.67_243.13	243.134865	1.67041667	neg	Isoleucylhydr	HMDB0028908		IFZSZULHIW	Organic acids	Carboxylic ac	Amino acids,
11.19_165.0	165.040527	11.1870167	neg	S-Propyl 1-pr	HMDB00343	89901	XPRZAEWS	Organic acids	Thiosulfinic a	Unclassified
11.38_349.2	349.273043	11.3787167	pos	ent-16b,19-K	HMDB00368	91816	AELNFENJK	Lipids and lip	Prenol lipids	Diterpenoids
15.12_413.3	413.342681	15.11695	neg	6beta-Hydro:	HMDB0302854		GDTCMKSK	Lipids and lip	Steroids and	Ergostane st
3.59_349.01	349.012621	3.58845	neg	7-Chloroemodin		329619	AVSZOSVPT	Lipids and lip	Polyketides	Aromatic poly
3.62_200.00	200.002237	3.62421667	neg	2-Mercaptoni	HMDB0245193		WYKHFQKC	Organoheter	Pyridines and	Pyridinecarb
7.79_321.24	321.241849	7.7913	pos	(ent-2alpha,3	HMDB00386	93343	HFKTUZJPX	Lipids and lip	Prenol lipids	Diterpenoids
8.12_211.13:	211.133807	8.12398333	neg	Vulgarole	HMDB00350	90432	QRRSWTCV	Lipids and lip	Prenol lipids	Monoterpeno
11.06_243.1	243.196298	11.0597833	neg	Tridecanal	HMDB00309	75351	BGEHHAVM	Lipids and lip	Fatty Acyls	Fatty aldehy
14.92_335.2	335.294049	14.9180833	pos	10,13,16-Docosatrienoic a		835808	RILVNGKQI	Lipids and lip	Fatty Acyls	Docosanoids
15.18_253.1	253.179593	15.1842167	pos	L-Monoment	HMDB0303264		CTMTYSVT	Lipids and lip	Prenol lipids	Monoterpeno
4.74_156.13	156.138315	4.74081667	pos	9-amino-nonanoic acid		404334	VWPQCOZM	Lipids and lip	Fatty Acyls	Fatty Acids a
6.71_246.24	246.24247	6.70808333	pos	2,5-dimethyl-dodecanoic a		73635	ABQDDNHU	Lipids and lip	Fatty Acyls	Fatty Acids a
7.00_615.24	615.244053	7.00153333	pos	chrysoeriol 7	HMDB0304575		FITCDKVKQ	Lignans, neo	Furanoid lign	Unclassified
9.38_396.25	419.240202	9.37788333	pos	PGF2alpha-11-acetate			HQOFFMPY	Lipids and lip	Fatty Acyls	Eicosanoids
0.71_133.08	133.080015	0.71368333	pos	N,N'-Diethylthiourea		73103	FLVIGYVXZI	Unclassified	Unclassified	Unclassified
1.58_488.23	488.235525	1.57645	pos	Ac-Ser-Asp-L	HMDB00625	329432	HJDRXEQUI	Organic acids	Carboxylic ac	Amino acids,
12.00_478.3	478.329444	12.0014833	pos	1-Nonadecar	HMDB0243971		GEWUBFCX	Lipids and lip	Glycerophos	Glycerophos
14.05_319.2	319.262774	14.0456833	pos	3-Methyl-5-p	HMDB00310	87343	QDTBMEGP	Lipids and lip	Fatty Acyls	Fatty acids a
5.05_424.17	425.186822	5.04685	pos	Clindamycin		525	KDLRVYVG	Unclassified	Unclassified	Unclassified
1.40_177.04	177.040896	1.40198333	pos	Diglyceride	HMDB0251280		YNCKCWJJI	Organoheter	Dioxanes	1,4-dioxanes
1.49_220.08	221.092047	1.48828333	pos	5-Hydroxy-L-	HMDB0000472		LDCYZAJDB	Organoheter	Indoles and c	Tryptamines
10.38_292.1	292.118681	10.3754333	pos	(R)-Methystic	HMDB0033590		GTEXBOVB	Phenylpropa	Kavalactones	Unclassified
5.50_409.05	409.054884	5.49605	neg	Theaflavic ac	HMDB00406	95089	SDSXQESY(	Phenylpropa	Flavonoids	Flavans
6.68_411.23	411.238608	6.6766	neg	Tetrahydrocc	HMDB0000949		AODPIQQIL(	Lipids and lip	Steroids and	Bile acids, al

1.08_266.08	266.088097	1.07531667	pos	hydroxydeox	HMDB0253263		KERZOWOH	Organoheter	Imidazopyrim	Purines and
1.29_331.11	331.114898	1.2875	neg	Mytilin A	HMDB0039002		WXEQFJUH	Organic acid	Carboxylic ac	Amino acids,
1.93_296.10	279.101135	1.93406667	pos	4'-Hydroxy-R	HMDB0060885		ZPNYHQZR	Phenylpropa	Coumarins a	Hydroxycoun
10.68_342.3	342.336018	10.6803667	pos	3-Methyl-3-butenyl hexade		97325	ZYMJMSWG	Lipids and lip	Fatty Acyls	Fatty esters
14.78_156.0	156.076677	14.7777667	pos	4-Hydroxynic	HMDB0246459		UDTVJEZIO	Organoheter	Pyridines and	Pyridinecarb
3.96_277.03	277.031273	3.96453333	pos	Acefylline	HMDB02478	330005	HCYFGRCY	Organoheter	Imidazopyrim	Purines and
4.76_303.08	286.083915	4.75875	pos	5-Methyl-6-hydroxyluteolinidin			BPBQZNOR	Lipids and lip	Polyketides	Flavonoids
5.65_304.28	304.284177	5.64811667	pos	17-hydroxy-heptadecanoic		74500	JHRQMZPL	Lipids and lip	Fatty Acyls	Fatty Acids a
5.78_326.08	326.085155	5.77613333	pos	Midazolam	HMDB00148	1293	DDLIGBOFA	Organoheter	Benzodiazep	1,4-benzodia
7.20_399.21	399.211292	7.20335	pos	14-Hydroxy-E	HMDB0012580		BCQOUZGZ	Lipids and lip	Fatty Acyls	Eicosanoids
0.90_428.03	428.036919	0.89581667	pos	monophosph	HMDB0254870		KRHRWPPV	Organic oxyg	Organooxyge	Carbohydrate
1.10_121.06	121.065025	1.1046	pos	4-Methylben	HMDB00296	66526	FXLOVSHX	Benzenoids	Benzene and	Benzoyl deri
12.43_128.1	128.118164	12.4295667	pos	L-Lysinamide	HMDB0253898		HKXLAGBD	Organic acid	Carboxylic ac	Amino acids,
12.48_754.5	754.535425	12.4811	pos	PE-NMe2(15	HMDB0113855		HMTCQBST	Unclassified	Unclassified	Unclassified
4.52_472.25	495.241237	4.52393333	pos	(2R,4S)-4-Ca	HMDB0250552		UKTIJASCFI	Lipids and lip	Fatty Acyls	Fatty acid es
4.92_271.06	271.06751	4.92405	neg	2,3,4,5,6,7-H	HMDB0240292		KWMLJOLK	Organic oxyg	Organooxyge	Carbohydrate
8.74_508.26	508.26503	8.74186667	pos	Bis(2-propox	HMDB0260364		AQSVPVSO	Organoheter	Pyridines and	Hydropyridin
9.56_340.23	340.230119	9.55885	pos	S-Farnesyl-L-cysteine ME		43437	SIEHZFPZQ	Unclassified	Unclassified	Unclassified
1.08_331.09	331.090976	1.08208333	neg	Xylan	HMDB0301774		HEHIOFQJT	Organic oxyg	Organooxyge	Carbohydrate
1.58_262.16	262.164977	1.57645	pos	3-Hydroxyiso	HMDB0061189		IGLHHSKNB	Lipids and lip	Fatty Acyls	Fatty acid es
13.74_346.3	346.309923	13.74075	pos	Macamide B	HMDB00399	94496	MLGPKWUK	Lipids and lip	Fatty Acyls	Fatty amides
3.29_322.12	322.123613	3.28915	pos	Saxitoxin	HMDB00293	69686	RPQXVSUA	Phenylpropa	Saxitoxins, g	Unclassified
3.86_113.08	114.091591	3.85685	pos	Epsilon-capr	HMDB00627	44753	JBKVHLHDH	Organoheter	Lactams	Caprolactam
4.11_159.12	182.115381	4.10951667	pos	Pregabalin	HMDB00143	313704	AYXYPKUFH	Organic acid	Carboxylic ac	Amino acids,
4.15_251.01	251.017163	4.1538	neg	2-Hydroxy-4-	HMDB0060715		XMLFPUBZF	Benzenoids	Benzene and	Benzoic acid
5.27_244.98	244.985278	5.27448333	neg	Perfluoroisol	HMDB0256325		DAFIBNSJXI	Organic oxyg	Organooxyge	Ketene aceta
3.48_251.10	251.103605	3.47753333	neg	Phenylalanyl	HMDB00290	85902	ROHDXJUF	Organic acid	Carboxylic ac	Amino acids,
4.74_281.09	281.095045	4.74081667	pos	(5R,6R)-3-(2	HMDB0256886		MHSNTZYK	Organoheter	Lactams	Beta lactams
6.07_293.09	293.099305	6.06898333	pos	(2S,3R,4R,5	HMDB0243491		GKHCBYYBI	Organic oxyg	Organooxyge	Carbohydrate
6.92_371.24	371.243859	6.91643333	neg	8,8a-Deoxyoleandolide		40994	OLPAVFVRS	Lipids and lip	Polyketides	Macrolides a
6.93_466.19	466.196981	6.92858333	pos	Biotripyrrin-a	HMDB00033	6881	DXWHHYOV	Organoheter	Pyrroles	Substituted p
7.26_246.15	246.151914	7.25836667	pos	S-3-oxodeca	HMDB0059773		LGPPHMDEI	Lipids and lip	Fatty Acyls	Fatty acyl thi
7.51_448.30	448.306969	7.50616667	neg	Glycoursode	HMDB00007	5676	GHCZAUBVI	Lipids and lip	Steroids and	Bile acids, al

9.51_333.20	333.203151	9.50591667	pos		20-oxo-leuko	HMDB0012641		LVLQYGYNE	Lipids and lip	Fatty Acyls	Eicosanoids
10.79_389.2	389.230964	10.7894833	neg		4-HDoHE	HMDB0060049		IFRKCNPQV	Lipids and lip	Fatty Acyls	Fatty acids a
11.30_249.1	249.149409	11.2956167	neg		Quadrangolin	A; 2-[(1S,2S	985308	QTSQCCVL	Unclassified	Unclassified	Unclassified
12.48_833.5	856.580381	12.4811	pos		PC(22:6(4Z,7	HMDB0289612		AUXOYFPW	Unclassified	Unclassified	Unclassified
13.25_332.2	331.26409	13.2463167	neg		Docosa-2,4,6	HMDB0251563		FPRKGXIOS	Lipids and lip	Fatty Acyls	Fatty acids a
4.25_327.13	327.135173	4.24626667	neg		Tyrosyl-Pher	HMDB00291	23970	CGWAPUBC	Organic acids	Carboxylic ac	Amino acids,
6.95_310.16	310.163617	6.95326667	neg		Arginylhistidi	HMDB00287	23714	BNODVYXZ	Organic acids	Carboxylic ac	Amino acids,
7.28_195.10	195.102538	7.28471667	neg		4-Phenyl-2-b	HMDB00316	87808	GDWRKZLR	Benzenoids	Benzene and	Unclassified
7.98_251.09	251.092375	7.97648333	neg		2,3-Dihydro-6	HMDB00414	95809	ZCZUDOBLF	Organoheter	Benzopyrans	1-benzopyra
0.88_253.09	253.09273	0.87703333	neg		Dambonitol	HMDB0033942		MMCIFJWG	Organic oxyg	Organooxyge	Alcohols and
10.41_305.2	305.248369	10.4089833	neg		ent-16b-Kaur	HMDB0302662		XZESVXVYT	Lipids and lip	Prenol lipids	Diterpenoids
10.75_337.2	337.23834	10.7528167	neg		11-Deoxy-PG	HMDB0251584		DPNOTBLP	Lipids and lip	Fatty Acyls	Eicosanoids
14.08_497.2	497.278814	14.0819167	pos		Fexaramine	HMDB0252239		VLQTUNDJH	Benzenoids	Benzene and	Biphenyls an
3.97_128.01	128.017457	3.97085	neg		2-Acetyl-4,5-	HMDB00335	89371	FZOZFDAM	Organoheter	Azolines	Thiazolines
4.85_242.15	242.153717	4.84713333	pos		N,N-Didemet	HMDB0061165		BJVWYJOLT	Benzenoids	Benzene and	Diphenylmet
5.05_157.08	157.086772	5.05298333	neg		xi-3-Methyl-3	HMDB00343	89883	HKVFKEJBS	Organic oxyg	Organooxyge	Alcohols and
5.66_431.11	431.11919	5.66205	neg		1-O-Sinapoyl	HMDB00411	95597	XRKBRPFTF	Phenylpropa	Cinnamic aci	Hydroxycinna
7.59_375.15	375.154614	7.58896667	pos		Gravacridone	HMDB00293	86194	GJDBWKOC	Organoheter	Quinolines a	Benzoquinoli
7.77_185.11	185.118022	7.7731	neg		(E)-10-Hydro	HMDB00395	74538	RCXJFGCV	Organic acids	Hydroxy acid	Medium-chai
8.29_240.06	239.0596	8.29023333	neg		Propyl 1-(pro	HMDB00330	88963	NIBWKLISD	Organosulfu	Sulfoxides	Unclassified
8.75_845.59	845.599111	8.74946667	neg		All trans deca	HMDB00062	58382	UAYWNGPE	Lipids and lip	Prenol lipids	Isoprenoid p
1.12_118.12	118.122879	1.11995	pos		2-Diethylami	HMDB00339	89647	BFSVOASY	Organic nitro	Organonitrog	Amines
10.84_556.3	556.317567	10.8434333	neg		Fosteabine	HMDB0252470		YJTVZHOYE	Nucleosides,	Pyrimidine n	Pyrimidine ri
11.23_451.3	452.312857	11.2342333	pos		PC(P-14:0/0:0)		40398	AAFONNYQ	Lipids and lip	Glycerophos	Glycerophos
2.48_149.02	149.024168	2.47923333	neg		Homogentisi	HMDB00001	331	IGMNYECM	Benzenoids	Benzene and	Phenylacetic
3.71_178.08	178.087014	3.71251667	neg		3,4-Methylen	HMDB0041931		NGBBVGZW	Organoheter	Benzodioxole	Unclassified
4.88_339.20	362.190876	4.88355	pos		Bursopoietin	HMDB0249448		SHAVAUDE	Organic acids	Carboxylic ac	Amino acids,
7.17_195.13	195.137877	7.16671667	pos		Cucurbic acid	HMDB00293	86234	LYSGIJUGU	Lipids and lip	Fatty Acyls	Lineolic acids
7.24_446.12	469.110352	7.24003333	pos		Isokaempferide 7-rhamnoside			BIFYEBAZZ	Lipids and lip	Polyketides	Flavonoids
9.03_334.29	334.294585	9.02893333	pos		7,8-dihydroxy stearic acid		35465	AZZCJRLYI	Lipids and lip	Fatty Acyls	Octadecanoi
9.30_309.17	309.1708	9.29605	neg		dinor-12-oxo	PDA		SZVNKXCD	Lipids and lip	Fatty Acyls	Octadecanoi
9.38_462.32	462.320938	9.37788333	pos		(5Z)-7-[(1R,2	HMDB0241800		XPMPGBFE	Lipids and lip	Fatty Acyls	Eicosanoids
9.90_446.32	446.325986	9.90308333	pos		(10Z,12Z)-oc	HMDB0240822		HQMPRWW	Lipids and lip	Fatty Acyls	Fatty acid es

1.19_170.04:	170.04234	1.1884	pos	N-Acetylserir	HMDB00029	308	JJIHLJJYMX	Organic acid:	Carboxylic ac	Amino acids,
4.28_200.03	200.038505	4.28308333	neg	Penicillanic a	HMDB0256227		RBKMMJSQ	Organic acid:	Carboxylic ac	Amino acids,
4.44_279.16	279.169604	4.435	pos	Phenylalanyl	HMDB0253025		RFCVXVPW	Organic acid:	Carboxylic ac	Amino acids,
6.89_193.11	193.11982	6.89188333	pos	1-Cyclopropy	HMDB00369	91980	NTSNDVXM	Organic oxyg	Organooxyge	Alcohols and
8.53_288.28	288.28932	8.52503333	pos	2-Methylhexa	HMDB02452	4492	AXPAUZGVI	Lipids and lip	Fatty Acyls	Fatty acids a
8.75_496.28:	496.282561	8.74946667	neg	PC(18:4(9E,11E,13E,15E)		40306	WAONNIUK	Lipids and lip	Glycerophos	Glycerophos
0.76_176.12	176.128035	0.75766667	pos	Mycinonic acid I			SUTQROWY	Lipids and lip	Fatty Acyls	Fatty Acids a
1.08_251.06	251.069579	1.07531667	pos	Homolanthio	HMDB00020	6449	CMACTJDD	Organic acid:	Carboxylic ac	Amino acids,
10.12_325.1	326.174917	10.1222	pos	(2E,4E,8E)-F	HMDB00386	93304	XWQSYLYF	Organoheter	Benzodioxole	Unclassified
10.66_364.2	364.283887	10.6629	pos	1alpha,22-dihydroxy-23,24,25,26,27-pe			AEKVRHKK	Lipids and lip	Steroids and	Secosteroids
10.99_496.2	496.267938	10.9877	neg	LysoPS(16:0	HMDB02406	40830	XIVOBOJQP	Lipids and lip	Glycerophos	Glycerophos
11.11_269.2	269.212066	11.1143	neg	Ambrettolic acid		35483	MKIFOPBVD	Lipids and lip	Fatty Acyls	Fatty Acids a
14.36_591.3	591.39014	14.3632	neg	cholest-5,24-dien-3beta-yl		83949	QSPACSWM	Lipids and lip	Steroids and	Sterols
3.73_272.06	272.068401	3.73238333	pos	p-coumaroylt	HMDB0304447		ZUILKEWVV	Phenylpropa	Cinnamic aci	Hydroxycinna
3.95_347.16	347.168003	3.94663333	pos	Nepetaside	HMDB00381	92890	CUIDBUWF	Lipids and lip	Prenol lipids	Terpene glyc
4.58_454.03	455.037516	4.57725	pos	Cefazolin	HMDB00154	535	MLYYVTUW	Organoheter	Lactams	Beta lactams
7.60_183.10	183.102339	7.59848333	neg	alpha-Camph	HMDB0301822		VRCGDJCVI	Lipids and lip	Prenol lipids	Monoterpeno
9.52_359.22	359.223077	9.51548333	neg	5-cis-15(R)-Iloprost		45130	HIFJCPQKF	Unclassified	Unclassified	Unclassified
1.11_451.12	451.120101	1.1137	neg	anthralin	HMDB02484	1030	NUZWLKWV	Benzenoids	Anthracenes	Unclassified
10.54_311.1	311.125797	10.5380833	pos	Decursin	HMDB0250923		CUKSFECW	Phenylpropa	Coumarins a	Pyranocouma
11.23_406.3	406.35223	11.2342333	pos	Aminopentol	HMDB0248328		UWWVLQOI	Organic nitro	Organonitrog	Amines
13.40_585.4	585.486288	13.39515	neg	DG(15:0/20::	HMDB00070	58717	ARFQXCQTI	Lipids and lip	Glycerolipids	Diradylglycer
4.69_185.04	185.044553	4.68661667	pos	Succinylacet	HMDB02402	352772	OMFWHSRZ	Organic acid:	Keto acids ar	Medium-chai
6.03_427.18	427.186822	6.0318	neg	(2S,3As,6aS	HMDB0257995		DLLRWMXF	Organic acid:	Carboxylic ac	Amino acids,
9.28_593.13	593.130493	9.2775	neg	3''-O-Caffeoy	HMDB00373	92277	GKSHQQDC	Phenylpropa	Flavonoids	Flavonoid gly
9.38_383.21	383.218636	9.37788333	pos	Macrophorin	HMDB00304	86989	OOIYDCZXV	Organic oxyg	Organooxyge	Carbonyl cor
1.03_371.10	371.101259	1.02758333	pos	20R-Campto	HMDB0245602		VSJKWCGY	Alkaloids anc	Camptotheci	Unclassified
1.06_146.09	146.092318	1.06053333	pos	4-Guanidinol	HMDB00034	6938	TUHVEAJXII	Organic acid:	Carboxylic ac	Amino acids,
10.75_364.2	387.249975	10.7516833	pos	Clinprost	HMDB0250326		QIGRQPVO	Lipids and lip	Prenol lipids	Monoterpeno
11.54_484.3	507.328917	11.5398167	pos	(25S)-5alpha-cholestan-3		83929	WXGHOWLI	Lipids and lip	Steroids and	Sterols
12.46_321.2	321.239577	12.46405	pos	Dihydroxyeic	HMDB0251343		AVUQWNIW	Lipids and lip	Fatty Acyls	Fatty acids a
13.74_269.2	269.248431	13.7387167	neg	(S)-14-Methy	HMDB0031067		FXUKWLSZ	Lipids and lip	Fatty Acyls	Fatty acids a
2.02_145.05	146.060113	2.02305	pos	OXYQUINOLINE		44006	MCJGNVYP	Unclassified	Unclassified	Unclassified

4.22_190.08!	190.089881	4.21765	pos		S-Prenyl-L-cy	HMDB00122	62914		ULHWZNAS	Organic acid	Carboxylic a	Amino acids,
6.33_367.11	367.114107	6.32576667	pos		3-Hydroxy-4-	HMDB00393	93961		IILRAPUPZQ	Lipids and lip	Prenol lipids	Terpene glyc
9.07_332.31!	332.31548	9.0656	pos		19-hydroxy-nonadecanoic		74502		LSEAKSNIG	Lipids and lip	Fatty Acyls	Fatty Acids a
9.90_195.10	195.101548	9.90308333	pos		Butylparaber	HMDB00325	88536		QFOHBWFC	Benzenoids	Benzene and	Benzoic acid
9.90_256.26	256.263157	9.90308333	pos		Palmitic amic	HMDB00122	62905		HSEMFIZW	Lipids and lip	Fatty Acyls	Fatty amides
1.25_176.09	176.091698	1.251	pos		Glabrin A	HMDB0301777			VORPREYJN	Organic acid	Carboxylic a	Amino acids,
11.65_368.2!	368.258578	11.6461	pos		Palmitoyl Se	HMDB00136	64849		MZUNFYMZI	Lipids and lip	Fatty Acyls	Fatty amides
3.60_226.07	227.078961	3.59728333	pos		lysine phospl	HMDB0254265			UGPVGRMT	Organic acid	Carboxylic a	Amino acids,
3.91_344.18	344.182161	3.91066667	pos		Nipradilol	HMDB0255620			OMCPLEZZI	Organoheter	Benzopyrans	1-benzopyra
4.15_350.17	350.171691	4.14533333	pos		meta-O-Deal	HMDB0060831			FVJPPEWH	Benzenoids	Benzene and	Benzoic acid
4.27_461.24!	462.25645	4.27276667	pos		STROPHANTHIDIN SEMI		44254		GFJQQPYC	Unclassified	Unclassified	Unclassified
4.71_202.05	202.05076	4.70578333	neg		Methyl dioxin	HMDB00389	709353		BHWUWPH	Organoheter	Indoles and	Indolyl carbo
5.98_169.04!	169.049454	5.9774	pos		Ethanone, 1-(2,3,4-trihydr		337901		XIROXSOO	Unclassified	Unclassified	Unclassified
0.89_350.03	350.038942	0.8943	neg		Cytidine 2',3'	HMDB00116	62429		NMPZCCZX	Organic acid	Carboxylic a	Amino acids,
1.18_233.05	233.057853	1.18473333	neg		1-Ethoxymetl	HMDB0243872			OUAPULXY	Organoheter	Diazines	Pyrimidines a
11.58_278.0	278.067599	11.5827667	neg		Casimiroin	HMDB00333	68468		DPXXJCMM	Organoheter	Quinolines a	Quinolones a
2.19_100.07	100.076163	2.19353333	pos		2-Piperidinor	HMDB00117	340777		XUWHAWM	Organoheter	Piperidines	Piperidinone
2.87_237.12	237.123235	2.8749	pos		ALANYL-dl-PHENYLALAN		44266		OMNVYXHC	Unclassified	Unclassified	Unclassified
3.97_295.13!	295.139665	3.97085	neg		(2-Methoxye	HMDB00597	96276		JIXHYWCLU	Organic acid	Carboxylic a	Carboxylic a
4.23_131.07	131.071177	4.22771667	neg		(5R)-5-Hydro	HMDB00004	5398		YDCRNMJQ	Lipids and lip	Fatty Acyls	Fatty acids a
4.74_168.03	168.02999	4.74241667	neg		2-Methoxy-5-nitrophenol		316778		KXKCTSZY	Unclassified	Unclassified	Unclassified
4.81_271.17	272.185464	4.81226667	pos		Hept-5-enoyl	HMDB0241683			HHQHYPIBD	Lipids and lip	Fatty Acyls	Fatty acid es
8.97_392.27!	392.279677	8.97426667	pos		8-(5-Butylfur	HMDB0241857			IALAQKCSP	Lipids and lip	Fatty Acyls	Fatty acid es
9.81_312.22!	335.218843	9.81245	pos		9,14-DiHODE		35507		NYDHMIHPI	Lipids and lip	Fatty Acyls	Octadecanoi
1.32_171.02	171.027325	1.32371667	neg		Fluroxene	HMDB02524	337098		DLEGDLSLF	Organohalog	Organofluori	Unclassified
1.34_360.12	360.127681	1.34166667	neg		Neosaxitoxin	HMDB0029369			PPEKGEBBI	Phenylpropa	Saxitoxins, g	Unclassified
10.28_810.5	810.529147	10.2812167	neg		PE(20:4(8Z,1	HMDB00094	60817		DKEUOION	Lipids and lip	Glycerophos	Glycerophos
10.35_262.0!	262.090545	10.3534833	neg		N-(2-(Mercap	HMDB0257118			ABBSOQIXY	Organic acid	Carboxylic a	Amino acids,
4.31_242.09	265.081244	4.30826667	pos		beta-Lapach	HMDB02491	68281		QZPQTZZN	Organoheter	Naphthopyra	Naphthopyra
4.31_429.17	429.171255	4.30826667	pos		Crosatoside	HMDB0039253			BMZAMCOQ	Organic oxyg	Organooxyge	Carbohydrate
4.63_277.03	277.032787	4.63258333	neg		cis-Coutaric	HMDB00346	90083		INYJZRKTY	Phenylpropa	Cinnamic aci	Hydroxycinna
4.69_280.10!	281.113203	4.68661667	pos		Aspartylphen	HMDB00007	5674		YZQCXOFQ	Organic acid	Carboxylic a	Amino acids,
5.20_201.02	201.022302	5.2006	neg		3-ethylpheny	HMDB0062721			DMIBREBM	Organic acid	Organic sulfu	Arylsulfates

8.77_467.238	467.238652	8.77465	pos	Dolichyl b-D-	HMDB00010	5969	RHJMCOLW	Lipids and lip	Prenol lipids	Sesquiterper
10.16_330.33	330.336149	10.1580333	pos	Emulphor	HMDB0251781		KWVPFECT(	Organic oxyg	Organooxyge	Ethers
12.22_375.31	376.320368	12.2158833	pos	Adrenoyl etha	HMDB0013626		FMVHVRYF(	Organic nitro	Organonitrog	Amines
3.60_130.065	130.065268	3.59728333	pos	Isoquinoline	HMDB00342	318771	AWJUIBRHM	Organoheter	Isoquinolines	Unclassified
3.92_451.160	451.159998	3.9154	neg	4-Hydroxy-5-	HMDB0240455		QKRQBODP	Organic acids	Hydroxy acid	Medium-chai
4.20_252.123	252.123179	4.19935	pos	Aloesol	HMDB00357	90970	ZYCNQWOK	Organoheter	Benzopyrans	1-benzopyrar
4.74_144.102	144.10281	4.74241667	neg	7-Aminohept	HMDB0247233		XDOLZJYET	Lipids and lip	Fatty Acyls	Fatty acids a
4.74_188.092	188.092611	4.74241667	neg	3-hydroxyhex	HMDB0094720		AYNJYFWP(	Organic acids	Carboxylic ac	Amino acids,
5.23_281.135	281.135669	5.22825	pos	Dihydromara	HMDB00360	91229	OWJATCNS	Organoheter	Furofurans	Unclassified
9.94_265.179	265.179449	9.93923333	pos	12-Oxo-2,3-d	HMDB00320	88174	SZVNKXCDJ	Lipids and lip	Fatty Acyls	Eicosanoids
9.96_195.137	195.137843	9.95751667	pos	Neocnidolide	HMDB03022	71493	UPJFTVFLS	Organoheter	Isobenzofura	Unclassified
1.67_310.176	310.176186	1.66533333	pos	Pro-Pro-Pro	HMDB0256689		SBVPYBFMI	Organic acids	Carboxylic ac	Amino acids,
10.43_297.20	320.255558	10.4295333	pos	4E,14Z-Sphingadiene		53902	KWDXKYNW	Lipids and lip	Sphingolipids	Sphingoid ba
11.16_291.22	291.228943	11.16325	pos	1,1,1-Trifluor	HMDB02560	63017	MAHYXYTY	Organic oxyg	Organooxyge	Carbonyl cor
12.64_609.48	609.489194	12.6402	neg	DG(15:0/0:0/	HMDB0056002		HKYDBZXJJ	Lipids and lip	Glycerolipids	Diradylglycer
14.81_429.37	429.372312	14.8127	pos	13'-Hydroxy-a	HMDB0012559		URYLCCKXI	Lipids and lip	Prenol lipids	Quinone and
2.04_145.052	190.050754	2.03931667	neg	4-Hydroxyqu	HMDB02464	326574	PMZDQRJGI	Organoheter	Quinolines ar	Quinolones a
3.95_282.168	305.157254	3.94663333	pos	Hexaethylene	HMDB00618	332442	IIRDTKBZIN	Organic oxyg	Organooxyge	Ethers
4.24_328.223	328.223287	4.23603333	pos	DIPROTIN B		44661	NHXZRXLF(	Unclassified	Unclassified	Unclassified
6.97_397.259	397.258978	6.97153333	neg	Isoraspailyne A			JPVHGPAQJ	Lipids and lip	Glycerolipids	Monoradylgly
8.55_179.071	179.071166	8.5484	neg	10-hydroxy-(	HMDB0062773		YKLIFQPPZ	Lipids and lip	Fatty Acyls	Fatty acids a
9.39_311.222	311.222857	9.3876	neg	hydroxynone	HMDB0253280		STRVVRPNE	Organic oxyg	Organooxyge	Carbonyl cor
1.17_170.094	188.128105	1.1705	pos	1,4-Ipomeadi	HMDB00304	87001	AORCXYMS	Organoheter	Heteroaroma	Unclassified
1.35_363.121	363.121084	1.34996667	pos	AF Toxin II	HMDB0033504		PXZGUAVTZ	Lipids and lip	Fatty Acyls	Fatty acids a
1.45_210.044	210.044058	1.44935	neg	2-Hydroxy-L-	HMDB02469	814132	AQPJRTNUE	Organic acids	Carboxylic ac	Amino acids,
10.19_279.11	279.111095	10.1937667	pos	Dehydrocyclopeptine; (3E)		985221	FYVKHLSOI	Unclassified	Unclassified	Unclassified
10.91_263.23	263.236553	10.9125667	pos	Malvalic acid	HMDB03022	35954	HPSSZFFAY	Lipids and lip	Fatty Acyls	Fatty acids a
4.27_370.220	393.209863	4.27276667	pos	Octaethylene	HMDB00946	332404	GLZWNFNQ	Organic oxyg	Organooxyge	Ethers
4.50_259.021	259.021627	4.50406667	neg	Glucose 6-ph	HMDB0001401		NBSCHQHZI	Organic oxyg	Organooxyge	Carbohydrate
4.76_336.064	336.064544	4.75875	pos	isoxicam	HMDB02537	44108	YYUAYBYLJ	Organoheter	Benzothiazin	Unclassified
1.89_433.122	433.122707	1.89173333	neg	Sulfinpyrazon	HMDB0060943		PLGXGMUJ(	Organosulfur	Thioethers	Aryl thioether
10.57_498.25	498.256129	10.5732333	pos	Copanlisib	HMDB0250446		MWYDSXOC	Organoheter	Diazanaphtha	Benzodiazine
14.32_620.48	638.519128	14.3159167	pos	1-(O-alpha-D-glucopyranosyl)-(1,3R,25S			YTSUVFCO	Lipids and lip	Fatty Acyls	Fatty acyl gly

2.77_194.11	217.105497	2.76745	pos	Tetraethylen	HMDB0094708		UWHCKJMY	Organic oxyg	Organooxyge	Ethers
4.83_357.22	375.2614	4.8302	pos	N-(8-Amino-1	HMDB0247737		BVFMABDUI	Nucleosides,	Purine nucle	Unclassified
8.03_198.12	199.13289	8.0298	pos	7-hydroxy-2,4,7-trimethyl-2E,4E-octadie			AHNOKIIXV	Lipids and lip	Fatty Acyls	Fatty Acids a
0.91_313.05	313.051799	0.91118333	neg	L-Homocysti	HMDB0000676		ZTVZLYBCZ	Organic acid	Carboxylic ac	Amino acids,
10.25_286.3	286.309934	10.24785	pos	Spisulosine	HMDB0258433		YRYJJIXWW	Organic nitro	Organonitrog	Amines
10.72_322.2	323.221214	10.71525	pos	17-hydroxy-1-oxo-2,3-seco-androstan-3			UVMGMKYQ	Lipids and lip	Steroids and	Steroids
12.53_247.1	247.16618	12.5329667	pos	S-cucujolide III		97400	UNYFVMZKI	Lipids and lip	Fatty Acyls	Fatty esters
2.00_261.05	261.054907	2.0024	neg	Bisnorbiotin	HMDB00048	7083	QDFGCLSCI	Organic acid	Carboxylic ac	Amino acids,
3.73_159.09	159.091831	3.73238333	pos	4-Methylamir	HMDB0246502		LJQBMYDFV	Benzenoids	Benzene and	Unclassified
4.03_172.09	172.097778	4.02623333	neg	N-Acetylleuci	HMDB00117	313505	WXNXCEHX	Organic acid	Carboxylic ac	Amino acids,
4.51_261.03	261.036335	4.50633333	pos	D-Glucose 2-	HMDB0247860		GBXZONVF	Organic oxyg	Organooxyge	Carbohydrate
4.58_360.13	361.139394	4.57725	pos	Nitrendipine	HMDB00151	44354	PVHUJELLJ	Organoheter	Pyridines and	Hydropyridin
4.90_192.10	192.101871	4.90175	pos	L-Tyrosine e	HMDB0253924		SBBWEQLN	Organic acid	Carboxylic ac	Amino acids,
9.66_323.22	323.22258	9.66158333	neg	7Z-Octadecene-9-ynoic acid			BWSIKEZDJ	Lipids and lip	Fatty Acyls	Fatty Acids a
9.87_270.21	293.208382	9.86676667	pos	16-hydroxy-6-hexadeceno		35627	UEGSWNJQ	Lipids and lip	Fatty Acyls	Fatty Acids a
1.49_113.10	113.107764	1.48828333	pos	2-Imino-4-methylpiperidine		64866	GGDLOMFA	Unclassified	Unclassified	Unclassified
10.15_227.1	227.165157	10.15385	neg	9Z,11-Dodecadien-1-ol		46108	OIMIXJLPIJ	Lipids and lip	Fatty Acyls	Fatty alcohol
10.47_508.3	508.339115	10.46565	pos	PC(17:1(9Z)/0:0)		76568	LPMGFNAQ	Lipids and lip	Glycerophos	Glycerophos
4.00_358.19	358.197919	4.00056667	pos	1-[6-(2-Carb	HMDB0257669		HZLAWYIBL	Organic acid	Carboxylic ac	Amino acids,
4.01_215.00	215.00184	4.0077	neg	3- Acetylphe	HMDB0304930		CGFRVKXG	Organic oxyg	Organooxyge	Carbonyl cor
4.40_218.10	219.112635	4.3987	pos	N-Acetylsero	HMDB00012	366	MVAWJSID	Organoheter	Indoles and	Hydroxyindol
5.61_267.07	267.079328	5.61161667	pos	Tyrosyl-Cyste	HMDB00291	85994	WJKJJGXZ	Organic acid	Carboxylic ac	Amino acids,
6.22_223.09	223.097475	6.21651667	neg	4-(4-Methoxy	HMDB00335	317264	PCBSXBYC	Benzenoids	Phenol ether	Anisoles
6.67_297.08	297.089867	6.67121667	pos	Pelubiprofen	HMDB0256212		AUZUGWXL	Phenylpropa	Phenylpropa	Unclassified
8.32_343.29	343.294993	8.32255	pos	N-Myristoyl F	HMDB0242058		OFWGTRJQ	Organic acid	Carboxylic ac	Amino acids,
1.20_318.99	318.99722	1.20271667	neg	(2S,3S,4S,5	HMDB0258086		OQFRCXVR	Organic oxyg	Organooxyge	Carbohydrate
1.65_454.18	454.182307	1.64773333	pos	Fusarin C	HMDB0033337		FZFYFSUIO	Lipids and lip	Prenol lipids	Sesquiterper
10.77_235.1	235.168939	10.7697	pos	Petasalbin	HMDB00356	90907	ANKFPIBCT	Lipids and lip	Prenol lipids	Sesquiterper
11.30_318.2	317.211867	11.2956167	neg	5R-HEPE			FTAGQROY	Lipids and lip	Fatty Acyls	Eicosanoids
11.54_486.3	486.378416	11.5398167	pos	Herbasterol		83894	UWPWCNUI	Lipids and lip	Steroids and	Sterols
13.12_284.2	284.294325	13.1190833	pos	Safingol (L-threo-sphinga		53955	OTKJDMGT	Lipids and lip	Sphingolipids	Sphingoid ba
3.63_210.12	210.121009	3.6313	pos	(2S)-6-Amin	HMDB0253432		NNRMAICN	Organic acid	Carboxylic ac	Amino acids,
4.03_204.03	204.033332	4.02623333	neg	Citiolone	HMDB02503	313545	NRFJZTXWI	Organic acid	Carboxylic ac	Amino acids,

5.55_539.25(	539.250042	5.55113333	neg		Tetrahydroal(	HMDB00103	61672	CGIURIOFM	Lipids and lip	Steroids and	Steroidal glyc
6.95_243.18:	244.190429	6.94688333	pos		N-Undecano)	HMDB00132	331610	HEUQYIQQ(	Organic acid:	Carboxylic ac	Amino acids,
7.83_199.13:	199.133741	7.82873333	neg		p-Mentha-3-e	HMDB0302600		CQRYMYGK	Lipids and lip	Prenol lipids	Monoterpenc
0.73_120.10:	120.102081	0.72825	pos		Tetrahydro-2	HMDB00397	94348	ZFVWBUVYI	Organoheter	Tetrahydrofu	Unclassified
0.88_282.05!	281.051212	0.87703333	neg		N-(2-Hydroxy	HMDB0258458		BTLNRJMEC	Organic 1,3-(	Allyl-type 1,3	Organic nitro
1.10_119.04!	119.04935	1.1046	pos		M-toluic Acid	HMDB0062810		GPSDUZXP'	Unclassified	Unclassified	Unclassified
1.25_198.07:	198.073578	1.251	pos		Calystegine I	HMDB0038594		FXFBVZOJV	Alkaloids anc	Tropane alka	Unclassified
1.25_445.12(	445.120113	1.251	pos		Alacepril	HMDB0248095		FHHHOYXPI	Organic acid:	Carboxylic ac	Amino acids,
10.59_356.3!	356.38813	10.5914167	pos		Tricosanal	HMDB0259187		IALIDHPAWI	Lipids and lip	Fatty Acyls	Fatty aldehyc
10.98_440.3	440.372703	10.9842333	pos		(9Z)-Nonade	HMDB0241563		AIZSZHDPQ	Lipids and lip	Fatty Acyls	Fatty acid es
11.50_545.3!	545.359536	11.5038	pos		LysoPA(24:0	HMDB0114756		NNKULLNAF	Lipids and lip	Glycerophos	Glycerophos
12.48_734.5(	734.568215	12.4811	pos		PC(16:0/16:(	HMDB00005	5548	KILNVBDSW	Lipids and lip	Glycerophos	Glycerophos
14.72_122.0	122.071862	14.72495	pos		Pyridin-4-ylm	HMDB0257681		QXWSHDPE	Organoheter	Pyridines anc	Unclassified
3.58_584.28(	585.288024	3.58011667	pos		Ouabain	HMDB00152	1670	LPMXVESGI	Benzenoids	Naphthalene	Unclassified
4.28_209.00:	209.003898	4.28308333	neg		2,2,2-Trifluor	HMDB0244040		NLOLSXYRJ	Organohalog	Organofluori	Unclassified
4.54_441.18:	441.182063	4.5416	pos		4-O-Methylas	HMDB0246555		XUAAAHFMI	Benzenoids	Phenols	Methoxypher
8.49_357.26	357.26462	8.4932	neg		9,12-dimethoxy-13-hydrox		74816	NMFUHXXKI	Lipids and lip	Fatty Acyls	Fatty Acids a
0.90_229.01	229.011742	0.89581667	pos		3-Dehydroqu	HMDB00127	3327	WVMWZWG	Organic acid:	Hydroxy acid	Alpha hydrox
1.34_256.11(	256.116615	1.34166667	neg		Glutarylcarni	HMDB0013130		NXJAXUYO(	Lipids and lip	Fatty Acyls	Fatty acid es
1.49_144.10:	144.10288	1.4863	neg		2-Methylpipe	HMDB00324	88440	NNWUEBIE(	Organoheter	Piperidines	Unclassified
1.52_271.06	271.064788	1.52326667	pos		6-Sulfanilami	HMDB02471	423645	RLNLIVBLEZ	Benzenoids	Benzene anc	Benzenesulfc
10.30_349.1!	349.19952	10.2994667	neg		10-Acetoxy-8	HMDB00397	94287	AUQPBBOK	Lipids and lip	Fatty Acyls	Fatty alcohol
10.52_461.2!	462.297288	10.5204167	pos		AMC Arachidonoyl Amide		45116	IQPBUDMTX	Unclassified	Unclassified	Unclassified
11.08_389.2:	389.230857	11.07825	neg		10-HDoHE	HMDB0060037		DDCYKEYD'	Lipids and lip	Fatty Acyls	Fatty acids a
13.21_537.4!	537.488656	13.21015	neg		31-methyl-32-dotriacontalactone			BZXJIOXRIF	Lipids and lip	Fatty Acyls	Fatty esters
14.08_520.3!	520.354822	14.0819167	pos		2-[(4-{2-[(4-C	HMDB02529	45134	PKNYXWMT	Organosulfur	Thioethers	Aryl thioether
3.87_243.14	244.154603	3.87373333	pos		2-Ethylacrylo	HMDB02407	6417	AUFJTEGVQ	Lipids and lip	Fatty Acyls	Fatty acid es
5.85_171.10:	171.102471	5.84688333	neg		9-hydroxy-5Z-nonenoic ac		74606	WPQRIXDVI	Lipids and lip	Fatty Acyls	Fatty Acids a
5.85_277.13	300.120327	5.84918333	pos		3'-Deaminofu	HMDB00413	95737	GUILTLWLD	Organoheter	Benzopyrans	1-benzopyrar
8.88_197.15:	197.153361	8.88258333	pos		cis-3-Hexeny	HMDB00316	87868	WGKGNHCL	Lipids and lip	Fatty Acyls	Fatty acid es
8.90_432.23	432.237546	8.90103333	pos		Clausarinol	HMDB00414	95806	AMSRLBLJH	Phenylpropa	Coumarins a	Pyranocoum
8.94_349.19!	349.198019	8.93783333	pos		Carnosolic a(	HMDB0302928		XMKKZBYHI	Lipids and lip	Prenol lipids	Diterpenoids
0.79_279.13	280.138883	0.7877	pos		N-[(2R,3R,4S	HMDB0257998		GSXAFFVIB	Organoheter	Oxanes	Unclassified

1.40_174.14	174.148974	1.40198333	pos		(2S,4R,5S)-N	HMDB00299	86601	UQOFGTXD	Organic oxyg	Organooxyge	Carbohydrate
10.56_600.3	600.34976	10.5560833	pos		POV-PG			QLZLSMOBI	Lipids and lip	Glycerophos	Oxidized glyc
11.01_511.2	511.290903	11.0055	neg		Pregnanetrio	HMDB0247360		DUXHVCKAI	Lipids and lip	Steroids and	Steroidal glyc
12.64_635.5	635.525486	12.6402	neg		DG(18:0/0:0/	HMDB0295933		AFWZQXYZ	Unclassified	Unclassified	Unclassified
14.09_359.2	359.295431	14.0865333	neg		3,4-Dimethyl-	HMDB0112080		OUOHNCBM	Lipids and lip	Fatty Acyls	Fatty acids a
3.60_159.09	159.09173	3.59728333	pos		1,5-Naphthal	HMDB02442	73157	KQSABULTK	Benzenoids	Naphthalene	Unclassified
4.52_141.11	142.122702	4.52393333	pos		Allylisopropyl	HMDB0248168		HINLFAQOK	Lipids and lip	Fatty Acyls	Fatty amides
4.58_225.10	225.109687	4.57725	pos		2-Ethylsuberi	HMDB00597	96277	WUDDSDIH.	Lipids and lip	Fatty Acyls	Fatty acids a
4.69_183.02	183.029708	4.6877	neg		Sesamol	HMDB00338	68615	LUSZGTFNY	Organoheter	Benzodioxole	Unclassified
4.99_256.09	256.090056	4.99255	pos		Threonylaspa	HMDB00290	23892	UQTNIFUCM	Organic acids	Carboxylic ac	Amino acids,
5.05_328.23	351.224949	5.04685	pos		N-Lauroyl Gl	HMDB0242031		USMCNVFG	Organic acids	Carboxylic ac	Amino acids,
5.08_360.13	361.139007	5.08273333	pos		Eddha	HMDB0251699		PZZHMLOHI	Organic acids	Carboxylic ac	Amino acids,
8.94_367.27	368.27915	8.93783333	pos		(10Z,12E)-Te	HMDB0241375		KBLSLAYMF	Lipids and lip	Fatty Acyls	Fatty acid es
9.09_213.14	213.149477	9.09495	neg		12-hydroxy-10-dodecenoic		74546	MEUNNLKX	Lipids and lip	Fatty Acyls	Fatty Acids a
0.79_226.09	244.128958	0.7877	pos		3'-Deoxythyn	HMDB0246094		XKKCQTLDI	Nucleosides,	Pyrimidine nu	Pyrimidine 2'
10.01_302.1	325.175641	10.0126833	pos		11b-Hydroxy	HMDB00067	41861	WSCUHXPG	Organoheter	Oxazinanes	Morpholines
12.11_839.5	839.568529	12.1088667	pos		SM(d19:0/20	HMDB0290629		KFNYSZOB	Unclassified	Unclassified	Unclassified
14.35_576.4	594.493406	14.3515667	pos		1-(O-alpha-D-glucopyranosyl)-(1,3S,25			ZTDUIGVZK	Lipids and lip	Fatty Acyls	Fatty acyl gly
3.77_334.08	334.086719	3.76795	pos		Taxiphyllin	HMDB0030704		NVLTYOJHF	Organic oxyg	Organooxyge	Carbohydrate
4.12_324.06	324.067573	4.11676667	neg		Phospholipas	HMDB03029	403900	ZIIUUSVHCI	Benzenoids	Benzene and	Sulfanilides
4.32_1038.5	1038.58472	4.32	neg		Ornithokinin	HMDB00382	425918	ASWVOLDX	Organic acids	Carboxylic ac	Amino acids,
4.81_356.03	356.036692	4.81226667	pos		7,8-dihydron	HMDB0304239		PLSQMGZY	Organoheter	Pteridines an	Pterins and c
5.51_214.14	214.144711	5.51431667	neg		Hexaminolev	HMDB02531	426114	RYQOILLJDI	Organic acids	Carboxylic ac	Amino acids,
8.69_342.26	342.263479	8.68985	pos		Dodecenoylc	HMDB0251567		VFYGOZNDI	Lipids and lip	Fatty Acyls	Fatty acids a
9.21_209.15	209.153471	9.21266667	pos		4,5-Dihydrov	HMDB00406	95095	IHDJYDVWN	Lipids and lip	Prenol lipids	Sesquiterper
9.74_437.19	437.192882	9.74061667	pos		Armillaripin	HMDB00304	86949	BGKXQRPQ	Lipids and lip	Prenol lipids	Sesquiterper
1.61_184.09	184.096084	1.61191667	pos		Normetanepl	HMDB00008	66	YNYAYWLB.	Benzenoids	Phenols	Methoxypher
10.79_465.3	488.313286	10.7875833	pos		PE(O-18:1(9Z)/0:0)		46718	CDONWGC	Lipids and lip	Glycerophos	Glycerophos
11.07_594.3	594.375157	11.0736	pos		PA(8:0/18:1(	HMDB0266645		BAOKIHSKC	Unclassified	Unclassified	Unclassified
12.64_379.1	379.157953	12.6402	neg		Ecabet	HMDB00156	316325	IWCWQNVIU	Lipids and lip	Prenol lipids	Diterpenoids
13.21_728.5	728.519458	13.2068667	pos		PC(13:0/17:0)		39203	AZEAMUNCI	Lipids and lip	Glycerophos	Glycerophos
14.60_581.2	581.238795	14.60285	pos		biliverdin-IX-	HMDB0304275		QBUVFDKTZ	Organoheter	Tetrapyrroles	Bilirubins
2.11_276.08	276.084956	2.11061667	neg		Asparagine g	HMDB0248666		XGPQZYND	Organic acids	Carboxylic ac	Amino acids,

3.80_627.32[illegible]	627.328452	3.80401667	pos		Ergocristine	HMDB0251908		HEFIYUQVA	Alkaloids and	Ergoline and	Lysergic acid
4.04_170.08[illegible]	170.082105	4.04478333	neg		2-Ethyl-5-imi	HMDB00395	94142	QWYZDNYE	Organic nitro	Organonitrog	Imines
4.69_259.11[illegible]	259.111034	4.68661667	pos		2-(Ethylsulfin	HMDB00402	94814	OMOLDRXZ	Benzenoids	Benzene and	Phenyl methy
5.67_227.17[illegible]	227.17508	5.66635	pos		epsilon-(Hex	HMDB0251888		CBVKLEOG	Organic acids	Carboxylic ac	Amino acids,
7.42_287.14[illegible]	287.140181	7.42368333	pos		Phthalanilic a	HMDB02447	441229	NANJTVUHV	Benzenoids	Benzene and	Anilides
1.03_224.99[illegible]	224.999164	1.0341	neg		Fluoromethyl	HMDB02469	2424	VMCHRPIQI	Organohalog	Vinyl halides	Vinyl fluoride
1.04_217.06[illegible]	217.068295	1.0442	pos		D-Pinitol	HMDB0034219		DSCFFEYY	Organic oxyg	Organooxyge	Alcohols and
1.08_130.12[illegible]	130.122663	1.07531667	pos		2,5-Heptadie	HMDB0040339		ZKKRKBOEI	Lipids and lip	Fatty Acyls	Fatty alcohol
1.87_119.03[illegible]	119.034765	1.87326667	neg		L-Erythrulose	HMDB00062	58385	UQPHVQVX	Organic oxyg	Organooxyge	Carbohydrate
10.37_243.1[illegible]	243.196347	10.37205	neg		2-Hydroxymy	HMDB00022	6579	JYZJYKOZG	Lipids and lip	Fatty Acyls	Fatty acids a
13.58_357.2[illegible]	357.279768	13.5756833	neg		Tetracosape	HMDB0258880		KXVFBCSU	Lipids and lip	Fatty Acyls	Fatty acids a
3.36_204.03[illegible]	204.032647	3.36203333	pos		5-(Methoxyca	HMDB02467	408012	HYHZGBOW	Organohetero	Thiophenes	Thiophene ca
4.11_144.10[illegible]	144.102031	4.10951667	pos		beta-Hydroxy	HMDB0244982		DREONSBN	Organic acids	Carboxylic ac	Amino acids,
4.28_185.02[illegible]	185.027502	4.28308333	neg		3,4-Dimethylbenzenesulfo		319855	WYCOJIVD	Unclassified	Unclassified	Unclassified
4.67_105.03[illegible]	105.037263	4.66833333	pos		3-(Methylthio	HMDB00318	84977	CLUWOWR	Organic oxyg	Organooxyge	Carbonyl con
4.74_200.10[illegible]	223.093834	4.74081667	pos		alpha-Carbo	HMDB00309	87334	AVHURGYC	Organohetero	Lactones	Delta valerol
5.09_245.13[illegible]	245.139325	5.08986667	neg		3-Hydroxy-2,8-dimethyl-1,		102868	FHLZKMYU	Lipids and lip	Polyketides	Polyether an
6.51_265.06[illegible]	265.063783	6.50861667	pos		Difluorodeox	HMDB0251268		UTTULGRFE	Nucleosides,	Pyrimidine nu	Pyrimidine 2'
8.25_273.26[illegible]	274.273743	8.24955	pos		C16 Sphinganine		41556	ZKLREJQHF	Lipids and lip	Sphingolipids	Sphingoid ba
8.62_187.13[illegible]	187.133706	8.61736667	neg		10-Hydroxyd	HMDB02442	35414	YJCJVMMD	Organic acids	Hydroxy acid	Medium-chai
9.88_883.53[illegible]	883.535556	9.8803	neg		PI(20:3(6,8,1	HMDB0276647		CAZFJDCJB	Unclassified	Unclassified	Unclassified
0.61_355.07[illegible]	355.072119	0.61033333	neg		Cysteinylglyc	HMDB00000	5134	ZUKPVRWZ	Organic acids	Carboxylic ac	Amino acids,
0.69_287.00[illegible]	287.008208	0.68961667	neg		Uridine 2',3'-	HMDB00116	3438	HWDMHJDY	Nucleosides,	Pyrimidine nu	Cyclic pyrimi
0.72_367.10[illegible]	367.104925	0.71928333	neg		4'-O-Glucopy	HMDB0303764		KKLWTTVT	Organic oxyg	Organooxyge	Carbohydrate
0.89_269.08[illegible]	269.087825	0.8943	neg		D-erythro-L-g	HMDB00299	86613	USEZWDIV	Organic oxyg	Organooxyge	Carbohydrate
1.04_160.13[illegible]	160.133189	1.0442	pos		Methacholine	HMDB00156	316603	NZWOPGCL	Organic nitro	Organonitrog	Quaternary a
11.17_555.2[illegible]	555.272585	11.1685	neg		OKOHA-PA			TVFXQYCF	Lipids and lip	Glycerophos	Oxidized glyc
3.60_159.06[illegible]	160.075778	3.59728333	pos		1H-Indole, 1-acetyl-		338560	UUCUQJHY	Unclassified	Unclassified	Unclassified
3.87_245.99[illegible]	245.994962	3.87373333	pos		S-Nitroso-L-c	HMDB0257413		UOHAKHBE	Organic acids	Carboxylic ac	Amino acids,
4.52_152.10[illegible]	152.107011	4.52393333	pos		N-Methyltyra	HMDB00036	58167	AXVZFRBSC	Benzenoids	Benzene and	Phenethylam
4.83_225.10[illegible]	225.109503	4.8302	pos		6-Amino-9H-	HMDB00299	86603	QXAYJKFBN	Organohetero	Imidazopyrim	Purines and
5.11_394.07[illegible]	394.07107	5.10843333	neg		3-Dibenzofur	HMDB0245712		CRUVAUSV	Organohetero	Benzofurans	Dibenzofuran
6.76_144.13[illegible]	144.138226	6.76326667	pos		4-octenal		75311	ICPZCFJMC	Lipids and lip	Fatty Acyls	Fatty aldehyd

9.61_234.12	257.11446	9.61401667	pos		Dehydroorea	HMDB00360	91224		RBUVATMD	Organoheter	Naphthofura	Unclassified
9.83_228.20	227.201452	9.82575	neg		12-Methyltrid	HMDB00310	4286		YYVJAABUJ	Lipids and lip	Fatty Acyls	Fatty acids a
9.83_249.14	249.149463	9.82575	neg		Heliannuol A	HMDB0039644			FWVBSUZW	Organoheter	Oxocins	Unclassified
0.88_306.02	306.022325	0.87973333	pos		2-Amino-4-o	HMDB00014	6226		QBHQZPRD	Organoheter	Pteridines ar	Pterins and d
1.10_418.02	418.028609	1.09681667	neg		Glucoraphan	HMDB0038404			GMMLNKINI	Organic oxyg	Organooxyge	Carbohydrate
1.28_233.14	233.149371	1.28425	pos		Spermic acid	HMDB0013075			DGMOQCHI	Organic acid	Carboxylic ac	Amino acids,
1.76_161.98	161.986474	1.76276667	neg		Acesulfame	HMDB00335	319828		YGCFIWIQZ	Organic acid	Organic sulfu	Unclassified
10.59_291.1	291.1965	10.59005	neg		[7]-Paradol	HMDB00408	95270		CNKCFVAE	Benzenoids	Phenols	Methoxypher
12.49_583.4	583.473068	12.4924667	neg		DG(15:0/20:	HMDB00070	58718		ATYAOFUC	Lipids and lip	Glycerolipids	Diradylglycer
4.81_471.04	494.034502	4.81226667	pos		Cefmetazole	HMDB00144	3950		SNBUBQHD	Organic acid	Carboxylic ac	Amino acids,
7.52_173.11	173.118041	7.52448333	neg		(1R,2R,4R,5	HMDB0301947			OBMLZGDT	Organic oxyg	Organooxyge	Alcohols and
7.74_266.12	266.12059	7.7361	pos		Thiamine	HMDB00002	229		JZRWCGZR	Organoheter	Diazines	Pyrimidines a
8.96_172.16	172.169413	8.95591667	pos		cis-p-Menth-	HMDB0302611			IZXYHAXVIZ	Lipids and lip	Prenol lipids	Monoterpeno
9.83_266.10	266.103364	9.82575	neg		Hydrocotarni	HMDB00337	43460		XXANNZJIZ	Organoheter	Tetrahydrois	Unclassified
9.88_510.34	510.343571	9.8803	neg		(8S,9Z,11E,1	HMDB0241585			FFLOHEWY	Lipids and lip	Fatty Acyls	Fatty acid es
1.59_284.14	284.14696	1.59411667	pos		(2S,3R)-3-Hy	HMDB0241646			BFUHRGXPI	Lipids and lip	Fatty Acyls	Fatty acid es
10.59_313.2	313.234902	10.5914167	pos		(10E,12Z)-(9	HMDB00624	36019		JGUNZIWGI	Lipids and lip	Fatty Acyls	Lineolic acids
10.59_326.0	327.077463	10.5914167	pos		TRIPHENYL	HMDB02592	69897		XZZNDPSIH	Organic acid	Organic phos	Phosphate es
10.73_204.1	204.138091	10.7334833	pos		N,N,O-Tride	HMDB0060850			MJTYLDGVL	Benzenoids	Benzene and	Cyclohexylph
10.73_358.3	358.367402	10.7334833	pos		19-methyl-heneicosanoic a		73673		CDGJKEUOI	Lipids and lip	Fatty Acyls	Fatty Acids a
13.81_162.9	162.982134	13.8116333	neg		Pentafluorop	HMDB02563	337159		LRMSQVBR	Organohalog	Alkyl halides	Alkyl fluoride
15.10_99.03	100.039694	15.096	pos		3-Cyanoprop	HMDB0245845			BXYQHDXD	Lipids and lip	Fatty Acyls	Fatty acids a
7.13_570.30	570.30565	7.13003333	pos		ibho#26				HVABDZVIN	Lipids and lip	Fatty Acyls	Fatty acyl gly
7.17_237.18	237.184769	7.16671667	pos		Tuberonone	HMDB0039688			JNIVOKDEG	Lipids and lip	Prenol lipids	Sesquiterper
1.12_284.11	284.112613	1.11995	pos		Succinylcarn	HMDB0061717			HAEVNYBC	Lipids and lip	Fatty Acyls	Fatty acid es
1.68_467.18	467.188865	1.68358333	pos		dihydrophaseic acid 4-O-b		53369		UHHVHDDI	Lipids and lip	Prenol lipids	Isoprenoids
12.64_581.4	581.454854	12.6402	neg		DG(20:5(5Z,	HMDB00075	59049		JTSRFZCOF	Lipids and lip	Glycerolipids	Diradylglycer
3.89_286.17	286.176416	3.8922	pos		4-Bis(2-hydr	HMDB0247322			WHGUXSYE	Organic acid	Carboxylic ac	Amino acids,
4.80_159.10	159.102454	4.79645	neg		4-Hydroxyval	HMDB00139	2994		OASKNPCLI	Lipids and lip	Fatty Acyls	Fatty acids a
5.14_274.20	274.200982	5.13745	pos		5-Methylhexa	HMDB0241037			BINXESBTX	Lipids and lip	Fatty Acyls	Fatty acid es
5.59_206.15	229.141079	5.59335	pos		2-[2-(2-Butoxyethoxy)etho		268699		COBPKKZHI	Unclassified	Unclassified	Unclassified
7.04_384.27	384.27399	7.03826667	pos		O-(13-Carbo	HMDB0240776			AHBCTRVD	Lipids and lip	Fatty Acyls	Fatty acid es
9.07_244.26	244.263226	9.0656	pos		9-pentadecen-1-ol		36507		CMWLZVMF	Lipids and lip	Fatty Acyls	Fatty alcohol

10.50_280.2	303.192528	10.50225	pos	12-hydroxy-5,8,10-heptad		74573	KUKJHGXX	Lipids and lip	Fatty Acyls	Fatty Acids a
13.21_753.5	754.535322	13.2068667	pos	PE-NMe2(15	HMDB0113928		AWNOGRCE	Lipids and lip	Glycerophos	Glycerophos
2.25_250.09	250.093451	2.24641667	pos	3'-Azido-3'-d	HMDB0246088		HBOMLICN	Nucleosides,	Pyrimidine n	Pyrimidine 2'
4.33_364.22	364.226859	4.32651667	pos	5-Oxo-ETE-d7		36335	MEASLHGIL	Lipids and lip	Fatty Acyls	Eicosanoids
5.03_437.23	437.236582	5.02876667	pos	Ethyl cellulos	HMDB00333	89214	ZZSNKZQZ	Organic oxyg	Organooxyge	Carbohydrate
8.75_913.58	913.58741	8.74946667	neg	DGDG(16:0/18:3(9Z,12Z,15Z))			WVWINZZVI	Lipids and lip	Glycerolipids	Glycosyldirac
9.50_195.13	195.138793	9.4972	neg	9-keto lauric acid		35734	RGTQITVBY	Lipids and lip	Fatty Acyls	Fatty Acids a
0.74_261.18	261.180795	0.74288333	pos	Tiglylcarnitin	HMDB0002366		WURBQCVE	Lipids and lip	Fatty Acyls	Fatty acid es
0.77_212.07	230.113452	0.7729	pos	2',3'-Dideoxy	HMDB0245547		BTOTXLJHD	Nucleosides,	Pyrimidine n	Pyrimidine 2'
1.08_104.05	104.053212	1.07531667	pos	(±)-2-Methylt	HMDB00316	87859	DQMLFUME	Organohetero	Azolidines	Thiazolidines
1.51_123.09	123.091919	1.50628333	pos	2,5-Diaminotoluene		73089	OBCSAIDCZ	Unclassified	Unclassified	Unclassified
10.50_470.3	470.383602	10.50225	pos	(13Z)-3-Hydr	HMDB0241573		LPCNRUGE	Lipids and lip	Fatty Acyls	Fatty acid es
2.37_248.05	248.058769	2.37246667	pos	4-Allylpyroca	HMDB0304934		USWXDXYE	Organic acid	Organic sulfu	Arylsulfates
2.84_232.06	232.061434	2.8388	pos	3[N-Morpholi	HMDB0254880		DVLFYONB	Organohetero	Oxazinanes	Morpholines
3.72_272.16	272.160521	3.71513333	pos	Pyrraline	HMDB00331	89025	VTYFITADL	Organic acid	Carboxylic ac	Amino acids,
4.03_262.03	262.033168	4.02623333	neg	7-(Trifluorom	HMDB0247224		IJFPXNWBA	Organohetero	Indoles and	Indolines
4.44_313.08	313.085283	4.435	pos	4-(Dipropylsu	HMDB02452	461800	QVUYRWCZ	Benzenoids	Benzene and	Benzoic acid
4.87_318.12	341.110615	4.86546667	pos	fluvoxamino	HMDB00609	2951	KUIZEDQDE	Benzenoids	Benzene and	Trifluorometh
6.00_232.10	232.099972	5.99566667	pos	L-Proline, 1-(	HMDB0257407		QIAJJGXZCI	Organic acid	Carboxylic ac	Amino acids,
9.85_490.35	490.352436	9.84861667	pos	(11Z,14Z)-3-	HMDB0241577		SMJJEGJNW	Lipids and lip	Fatty Acyls	Fatty acid es
11.10_283.2	283.227623	11.0963	neg	MG(14:0/0:0	HMDB00115	62344	DCBSHORR	Lipids and lip	Glycerolipids	Monoradylgly
12.41_633.5	633.509359	12.4138167	neg	DG(20:3(6,8,	HMDB0299339		AGZZXXAH	Unclassified	Unclassified	Unclassified
12.94_633.4	633.486311	12.9357167	neg	DG(17:2(9Z,12Z)/22:5(7Z,		4529	FSAPHEIJIF	Lipids and lip	Glycerolipids	Diradylglycer
15.43_123.0	123.079778	15.4298167	pos	4Z,7-octadienoic acid		35096	DANMCTRT	Lipids and lip	Fatty Acyls	Fatty Acids a
3.66_362.08	362.082313	3.65995	neg	Seminaphtha	HMDB0244697		DYPYMMHZ	Organohetero	Benzopyrans	1-benzopyra
3.79_208.13	231.120429	3.78573333	pos	5S,6S-epoxy	HMDB0062236		ZNYRFEPB	Organic oxyg	Organooxyge	Ethers
3.98_251.04	251.047929	3.98231667	pos	(-)-Mycomycin			FYRXMBRL	Lipids and lip	Fatty Acyls	Fatty Acids a
8.08_195.10	195.101633	8.08456667	pos	2-ISOPRENYL-3-HYDRO		44640	COFJZDUA	Unclassified	Unclassified	Unclassified
1.13_253.09	253.092742	1.13148333	neg	Ethyl beta-D-	HMDB0303035		KQQFKZUG	Organic oxyg	Organooxyge	Carbohydrate
1.38_232.04	232.046039	1.37511667	neg	(1R,4R,5S,6	HMDB0242337		YASVRZWV	Organic acid	Carboxylic ac	Amino acids,
1.56_150.08	151.096587	1.55895	pos	TRIETHYLE	HMDB0259193		ZIBGPFATK	Organic oxyg	Organooxyge	Ethers
10.14_370.2	393.259872	10.14065	pos	MG(18:1(9Z)	HMDB0260492		FYCPUPBO	Unclassified	Unclassified	Unclassified
11.07_323.1	323.182451	11.0736	pos	Berkeleylactone F			OXTXYWGS	Lipids and lip	Fatty Acyls	Fatty esters

3.79_158.11	158.117732	3.78573333	pos	Statine	HMDB0258487		DFVFTMTW	Organic acid	Carboxylic ac	Amino acids,
4.12_126.00	126.001748	4.11676667	neg	Penem	HMDB0256222		HHXMXAQD	Organoheter	Lactams	Beta lactams
4.15_157.11	180.099664	4.14533333	pos	Tranexamic	HMDB0014447		GYDJEQRTZ	Organic acid	Carboxylic ac	Amino acids,
4.72_213.15	213.159701	4.72301667	pos	Brivaracetam	HMDB0249388		MSYKRHVO	Organic acid	Carboxylic ac	Amino acids,
8.74_287.28	288.28913	8.74186667	pos	C17 Sphinganine		41558	KFQUQCFJI	Lipids and lip	Sphingolipids	Sphingoid ba
9.00_200.17	245.175737	9.00285	neg	2-(L-Mentho	HMDB0303254		FVBGFZNFX	Lipids and lip	Prenol lipids	Monoterpenc
1.42_150.09	150.091423	1.41946667	pos	Phenylephrin	HMDB00021	699	SONNWYBII	Benzenoids	Phenols	1-hydroxy-4-
14.78_179.0	179.092617	14.7777667	pos	4-Acetylimida	HMDB00348	90276	WTOOGKIO	Organoheter	Imidazopyrid	Imidazo-[4,5-
4.81_328.04	328.041765	4.81226667	pos	N-Succinyl-2-	HMDB00122	62901	SDVXSCSN	Organic acid	Carboxylic ac	Amino acids,
5.64_134.06	134.060997	5.64363333	neg	Norfenefrine	HMDB02557	320581	LRCXRAABI	Benzenoids	Phenols	1-hydroxy-4-
0.89_217.01	217.011764	0.8943	neg	Glycerol 3-ph	HMDB00001	5161	AWUCVROL	Lipids and lip	Glycerophos	Glycerophos
1.45_181.04	181.047042	1.45418333	pos	Isopropylmal	HMDB00122	3279	NJMGRJLQI	Lipids and lip	Fatty Acyls	Fatty acids a
1.78_154.02	199.022118	1.78133333	neg	1,1,1-Trifluor	HMDB02462	336899	SHXHPUAKI	Organic oxyg	Organooxyge	Carbonyl cor
10.03_174.1	174.127661	10.0311	pos	n-diethyl-m-t	HMDB0255127		ZVRWNVUY	Benzenoids	Benzene anc	Phenylpropar
10.63_1035.	1035.66309	10.6260167	neg	GM4(d18:1/16:0)			PKLSFJOTJ	Lipids and lip	Sphingolipids	Acidic glycos
10.75_432.1	432.191252	10.7516833	pos	2-Methyl-2-[4	HMDB0254247		PNHFDVSKI	Benzenoids	Benzene anc	Phenoxyacet
12.62_487.1	487.137836	12.6234833	neg	Khelmarin D	HMDB00413	95795	SVNBKSLEK	Phenylpropa	Coumarins a	Pyranocoum
13.63_384.2	384.289309	13.63205	pos	Acetylleucyl-leucyl-norleuc		68977	FMYKJLXRF	Unclassified	Unclassified	Unclassified
2.17_149.02	149.024156	2.16503333	neg	3-Hydroxyma	HMDB00007	732	OLSDAJRAV	Benzenoids	Phenols	1-hydroxy-4-
2.32_276.18	276.180678	2.31811667	pos	3-Hydroxyhe	HMDB0013131		VYEONLJQF	Lipids and lip	Fatty Acyls	Fatty acid es
2.95_181.07	181.07206	2.9472	pos	Theobromine	HMDB00028	1456	YAPQBXQYI	Organoheter	Imidazopyrim	Purines and
4.02_180.14	180.147132	4.01898333	pos	(S)-carnitiniu	HMDB0062634		PHIQHXFUZ	Organic nitro	Organonitrog	Quaternary a
4.39_419.12	419.120944	4.39318333	neg	Swertiamarin	HMDB0258644		HEYZWPRK	Organic oxyg	Organooxyge	Carbohydrate
5.63_213.02	213.022394	5.6251	neg	4-Allylphenol	HMDB0170765		KFDHKOFCI	Organic acid	Organic sulfu	Arylsulfates
5.77_134.06	134.06098	5.773	neg	5-(2-Furanyl)	HMDB00400	94554	RSWIYWDW	Organoheter	Heteroaroma	Unclassified
7.86_177.12	177.127284	7.86466667	pos	Hexylresorcir	HMDB00325	4064	WFJIVOKAV	Benzenoids	Phenols	Benzenediols
0.76_294.02	294.028986	0.76461667	neg	S-[(3R,3As,6	HMDB0253932		BXZDULYKE	Organoheter	Furofurans	Unclassified
0.76_304.16	304.161248	0.75766667	pos	gamma-Gluta	HMDB00291	86035	AKAHWGGI	Organic acid	Carboxylic ac	Amino acids,
0.79_272.07	295.066097	0.7877	pos	Pyridine N-o	HMDB0061177		KITHDEVLY	Organic oxyg	Organooxyge	Carbohydrate
1.32_191.07	214.06856	1.31801667	pos	2-amino-2,3,7-trideoxy-D-l		63567	IFMHGOAD	Lipids and lip	Fatty Acyls	Fatty Acids a
4.69_139.03	139.039928	4.6877	neg	xi-2,3-Dihydr	HMDB00363	91523	JOWYHBUW	Organoheter	Pyrans	Pyranones ar
5.36_364.05	364.059426	5.35556667	pos	Avenanthram	HMDB00331	89067	FRNDILDQF	Benzenoids	Benzene anc	Benzoic acid
6.58_339.21	339.213635	6.58	pos	Phenylalanyl	HMDB00289	23741	OZILORBBP	Organic acid	Carboxylic ac	Amino acids,

6.74_241.17	241.177651	6.7449	pos	8,8-Dimetho	HMDB00402	318465	QCJVKUULZ	Organic oxyg	Organooxyge	Alcohols and
6.77_402.24	402.249667	6.76876667	neg	3-oxododeca	HMDB0241290		BSVXIHNW	Lipids and lip	Fatty Acyls	Fatty acid es
9.96_288.22	306.263498	9.95751667	pos	(S)-10,16-Dil	HMDB00377	35689	VJZBXAQG	Lipids and lip	Fatty Acyls	Fatty acids a
0.70_433.11	433.113006	0.70456667	neg	Lycoperdic a	HMDB00304	86958	ZQVZYZVR	Organic acids	Carboxylic ac	Amino acids,
10.81_482.2	482.261304	10.80565	pos	Linalool 3,6-c	HMDB00354	90771	CXCRZTAN	Organic oxyg	Organooxyge	Carbohydrate
13.40_381.1	381.1736	13.39515	neg	S-Japonin	HMDB0035802		HDHDUJDLH	Lipids and lip	Prenol lipids	Sesquiterpen
4.18_222.14	245.136137	4.1814	pos	Tetraglyme	HMDB0258894		ZUHZGEOKI	Organic oxyg	Organooxyge	Ethers
4.44_209.11	209.114808	4.435	pos	3R-hydroxy-4R-decanolide			HFYZTGBQI	Lipids and lip	Fatty Acyls	Fatty esters
5.34_137.10	137.107223	5.33735	pos	Tetramethylp	HMDB00365	316948	FINHMKGKII	Organoheter	Diazines	Pyrazines
7.87_221.11	221.118127	7.86566667	neg	Rhubafuran	HMDB00360	324773	GPMLJOOQ	Benzenoids	Benzene and	Unclassified
8.32_230.24	230.247566	8.32255	pos	3Z-Tetradecen-1-ol		46132	DQELOVNS	Lipids and lip	Fatty Acyls	Fatty alcohol
9.99_295.25	318.240004	9.99425	pos	(4E,8E,10E-d18:3)sphingo		53913	PZVYDNWE	Lipids and lip	Sphingolipids	Sphingoid ba
0.67_237.09	237.091233	0.6745	neg	15(R)-hydrop	HMDB0062295		JKMHFZQW	Organoheter	Diazinanes	Piperazines
0.78_226.99	226.996284	0.7799	neg	alpha-D-xylo	HMDB0304536		ILXHFXFPP	Organic oxyg	Organooxyge	Carbohydrate
1.49_157.07	157.07691	1.4863	neg	Serotonin	HMDB00002	74	QZAYGJVTT	Organoheter	Indoles and	Tryptamines
10.57_656.2	656.240674	10.5716833	neg	Lewis b	HMDB0254067		OXNGKCPR	Organic oxyg	Organooxyge	Carbohydrate
14.26_617.4	617.474604	14.2613833	pos	DG(i-15:0/20	HMDB0299081		CNLVDWFX	Unclassified	Unclassified	Unclassified
14.90_138.0	138.065984	14.9013667	pos	L-2-Amino-3-	HMDB00342	89814	PIGOPELHG	Organic acids	Carboxylic ac	Amino acids,
2.02_258.03	258.03815	2.02081667	neg	Aminofructos	HMDB00604	63735	KYORZMST	Organic oxyg	Organooxyge	Carbohydrate
4.61_465.23	465.232116	4.61381667	pos	glas#3			USHOVWZT	Lipids and lip	Fatty Acyls	Fatty acyl gly
6.05_265.14	265.140742	6.05065	pos	Hymenoxon	HMDB0253305		PYINVOHSC	Organoheter	Oxanes	Unclassified
8.43_349.19	349.198134	8.43301667	pos	(3b,6b,8a,12	HMDB00319	88075	CLQUNRBW	Organoheter	Naphthofura	Unclassified
11.31_299.2	299.258892	11.3135833	neg	11Z-heptadecen-1-ol		36509	BVPFCRZLY	Lipids and lip	Fatty Acyls	Fatty alcohol
11.43_280.1	280.136285	11.4320667	pos	2-Methyl-4'-(	HMDB0247316		LWRBVKNF	Organic oxyg	Organooxyge	Carbonyl cor
2.44_230.13	230.138924	2.4445	pos	3-Butenylcar	HMDB0241650		CJETZUCGL	Lipids and lip	Fatty Acyls	Fatty acid es
3.53_187.06	187.060899	3.53321667	neg	Erinapyrone	HMDB0041426		FXGUGSUN	Organoheter	Pyrans	Pyranones ar
3.59_497.14	497.141794	3.58845	neg	Nitrotryptoph	HMDB0255666		DAWPREIES	Organic acids	Carboxylic ac	Amino acids,
3.73_302.07	302.079031	3.73238333	pos	2,5,7-trihydro	HMDB0304042		IIQJLBKXW	Phenylpropa	Isoflavonoids	O-methylated
3.75_276.13	276.134824	3.75006667	pos	Tryptophyl-A	HMDB00290	85969	OHGNSVAC	Organic acids	Carboxylic ac	Amino acids,
5.63_342.25	365.240737	5.6299	pos	N-Myristoyl A	HMDB0242047		XCYGCBVZ	Organic acids	Carboxylic ac	Amino acids,
6.44_223.09	223.09749	6.4382	neg	3-hexynoic acid		74155	JHBKYZNFF	Lipids and lip	Fatty Acyls	Fatty Acids a
8.56_403.24	403.244726	8.56171667	pos	Telocinobufa	HMDB0258793		PBSOJKPTQ	Lipids and lip	Steroids and	Steroid lacto
8.71_683.25	683.254904	8.7071	neg	Veranisatin A	HMDB00406	95141	LXPKORXZ	Lipids and lip	Prenol lipids	Terpene lact

0.70_166.04	165.040137	0.70456667	neg	Ribonic acid	HMDB00008	3290	QXKAIJAYH	Organic oxyg	Organooxyge	Carbohydrate
1.29_371.12	371.120668	1.2875	neg	2-O-a-L-Fuc	HMDB00065	58476	BQEBASLZI	Lipids and lip	Fatty Acyls	Fatty acyl gly
1.51_304.15	304.150303	1.50628333	pos	N-(N-(3-Amir	HMDB0255025		KRGPXXHM	Organic acid	Carboxylic ac	Amino acids,
12.02_377.1	377.142291	12.0185833	neg	Pradofloxacir	HMDB0256725		LZLXHGFNC	Organoheter	Quinolines ar	Quinoline car
13.21_791.5	792.55081	13.2068667	pos	PE(20:4(8Z,1	HMDB00094	60825	AONMKBNB	Lipids and lip	Glycerophos	Glycerophos
3.77_326.17	326.171201	3.76795	pos	2-Carboxy-1-	HMDB00351	90500	DJTFHMOAI	Organic acid	Carboxylic ac	Amino acids,
3.91_316.18	316.187039	3.91066667	pos	Dimethylamir	HMDB0251394		UJNSFDHVI	Lipids and lip	Prenol lipids	Terpene lact
5.06_434.19	434.195128	5.06456667	pos	3b,8b-Dihydr	HMDB00365	91619	IYBZGGXFP	Lipids and lip	Prenol lipids	Terpene lact
5.85_159.10	159.102434	5.84688333	neg	8-Hydroxyoc	HMDB00619	35405	KDMSVYIHK	Organic acid	Hydroxy acid	Medium-chai
9.68_287.22	287.222919	9.68	neg	7(14)-Farnes	HMDB00409	95437	VBYQXYSS	Lipids and lip	Prenol lipids	Sesquiterper
1.36_446.19	491.193574	1.3586	neg	Estrone gluc	HMDB00044	7063	FJAZVHYPA	Lipids and lip	Steroids and	Steroidal glyc
1.83_311.12	311.123815	1.8272	pos	Glutamyltyro	HMDB00288	23927	YSWHPLCD	Organic acid	Carboxylic ac	Amino acids,
12.30_325.1	325.184051	12.3037667	neg	4-Dodecylbe	HMDB00599	324404	KWXICGTUI	Benzenoids	Benzene anc	Benzenesulf
12.71_446.3	469.31305	12.70945	pos	bhas#32			FHMZFRKIY	Lipids and lip	Fatty Acyls	Fatty acyl gly
12.98_296.2	296.294394	12.9755	pos	2-Pentadecyl	HMDB00336	89411	LSVQFJUSF	Organoheter	Heteroaroma	Unclassified
3.98_415.25	415.255677	3.98231667	pos	PC(9:0/0:0)		40334	VKTRCGYRI	Lipids and lip	Glycerophos	Glycerophos
4.36_176.08	199.076527	4.3621	pos	Ethyl 5-(metl	HMDB0303228		PAXAYUXLF	Lipids and lip	Fatty Acyls	Fatty acid es
5.28_270.10	270.105677	5.28283333	pos	Threoninyl-G	HMDB00290	85968	BTVBLYICT	Organic acid	Carboxylic ac	Amino acids,
9.68_440.30	440.30184	9.68	neg	9,12-Hexade	HMDB0013334		CMVGCHSJ	Lipids and lip	Fatty Acyls	Fatty acid es
0.72_163.02	163.024616	0.71928333	neg	Erythrono-1,4	HMDB00003	5338	SGMJBNSH	Organoheter	Lactones	Gamma buty
0.73_263.19	263.196292	0.72825	pos	Valerylcarniti	HMDB0013128		VSNFQQXVI	Lipids and lip	Fatty Acyls	Fatty acid es
0.90_293.02	293.025172	0.89581667	pos	Iobenguane	HMDB02535	321013	PDWUPXJE	Benzenoids	Benzene anc	Halobenzene
1.09_503.15	503.150535	1.08996667	pos	[6-Hydroxy-8	HMDB0257573		DNODDECD	Organic oxyg	Organooxyge	Carbohydrate
1.44_147.04	147.047463	1.43671667	pos	Dihydro-2-m	HMDB00402	94768	FCDCVDDF	Organosulfur	Thioacetals	Monothioace
10.14_407.2	407.276279	10.14065	pos	4-Hydroxydo	HMDB0241286		JGNYBJMU	Lipids and lip	Fatty Acyls	Fatty acid es
10.86_341.2	341.211852	10.8611	neg	Guggulsteror	HMDB00027	58133	WOVLRQSV	Lipids and lip	Steroids and	Androstane s
11.74_402.2	425.21398	11.7358167	pos	5S-HETE di-endoperoxide		74966	DOXGUXRZ	Lipids and lip	Fatty Acyls	Eicosanoids
11.93_676.3	699.358712	11.9300167	pos	Granadaene			PPFISAQUK	Lipids and lip	Fatty Acyls	Fatty amides
12.49_403.1	403.157789	12.4924667	neg	Sericetin		44599	BPGVKIFGC	Lipids and lip	Polyketides	Flavonoids
2.13_192.03	192.033355	2.1282	neg	2-Methylthiaz	HMDB0246657		FHTPNEYXN	Organic acid	Carboxylic ac	Amino acids,
4.72_316.07	316.074043	4.72301667	pos	Dehydroapor	HMDB00333	89199	YUWBTKIVD	Alkaloids anc	Aporphines	Unclassified
4.98_232.05	232.059073	4.97913333	neg	N-Carboxyac	HMDB00391	93703	IHAMOIOWI	Organic acid	Carboxylic ac	Amino acids,
5.00_197.08	197.081726	4.99758333	neg	Isoketocamp	HMDB0301824		WLXCRBOZ	Lipids and lip	Fatty Acyls	Fatty acids a

9.36_438.32	438.321102	9.35948333	pos	(11E)-Octade	HMDB0241538		ADGLXDYW	Lipids and lip	Fatty Acyls	Fatty acid es
0.80_175.04	198.037274	0.80285	pos	Alanine pyru	HMDB0248106		NVEYLYRW	Organic acid	Carboxylic ac	Amino acids,
1.18_255.04	255.039986	1.18473333	neg	cis,trans-5'-H	HMDB00138	85290	HHTOWVWI	Organoheter	Isoindoles ar	Isoindolines
10.41_390.3	390.336194	10.41135	pos	(+)-Dysideapalaunic acid		53743	VMXBEUNFI	Lipids and lip	Prenol lipids	Isoprenoids
11.54_396.2	419.276189	11.5398167	pos	MG(20:3(6,8	HMDB0260512		KHYYDOVN	Unclassified	Unclassified	Unclassified
11.61_242.2	242.247487	11.61145	pos	(+/-)-Dihydro	HMDB0032218		OOOOFOPL	Lipids and lip	Prenol lipids	Sesquiterper
11.95_362.3	385.291879	11.9481833	pos	Tetraethylen	HMDB02469	350763	WPMWEFX	Organic oxyg	Organooxyge	Ethers
12.04_500.2	523.254399	12.0372667	pos	Adavosertib	HMDB0254782		BKWJAKQV	Organoheter	Diazinanes	Piperazines
2.39_173.11	196.105713	2.39026667	pos	apo-[3-methy	HMDB00596	96232	DKPYGQKM	Organic acid	Carboximidic	Carboximidic
4.58_224.07	224.07404	4.57725	pos	Methyl (Z)-5-	HMDB00339	89654	OZOCRDZZ	Lipids and lip	Fatty Acyls	Fatty acid es
4.60_237.10	260.08934	4.5955	pos	2,3-dihydro-1,4-benzodiox		96365	WSCKMRCI	Unclassified	Unclassified	Unclassified
7.90_343.07	343.077356	7.90265	neg	Sitostanol-be	HMDB0304734		FPSPMXPR	Organic acid	Carboxylic ac	Amino acids,
8.53_633.25	633.25475	8.52503333	pos	Linalool (8-hy	HMDB03047	66514	FYIKIBQJAJ	Lipids and lip	Prenol lipids	Triterpenoids
8.60_163.12	163.122811	8.5985	pos	Nicotine	HMDB00019	1526	SNICXCGAK	Organoheter	Pyridines and	Pyrrolidinylpy
1.09_276.07	276.071124	1.08996667	pos	Dioxolane gu	HMDB0251437		DXNPMWVL	Organoheter	Imidazopyrim	Purines and
11.40_249.1	249.185779	11.40315	neg	Manaoic acid			NUYKZLPZC	Lipids and lip	Fatty Acyls	Fatty Acids a
11.50_572.3	595.380933	11.5038	pos	IC202A			BVRULQCEI	Lipids and lip	Fatty Acyls	Fatty amides
3.89_295.16	295.165327	3.8922	pos	Tyrosyl-Leuc	HMDB00291	24037	AUEJLPRZG	Organic acid	Carboxylic ac	Amino acids,
4.40_242.12	265.116482	4.3987	pos	3'-Hydroxyan	HMDB02460	426963	PUVZPWLD	Organoheter	Diazines	Pyrimidines a
4.45_393.12	393.129367	4.45335	pos	cyclic N-Acet	HMDB0060812		DHEUSGDH	Organic oxyg	Organooxyge	Carbohydrate
4.63_255.08	255.083955	4.63161667	pos	Bis(2-hydrox	HMDB02492	328740	QPKOBORK	Benzenoids	Benzene and	Benzoic acid
5.47_257.13	257.13563	5.46526667	pos	O2'-4a-cyclic	HMDB0013031		JZTDUNXDD	Organoheter	Pteridines an	Pterins and d
6.03_369.17	369.174171	6.0318	neg	4-Androsten-	HMDB0240429		KMTOEENO	Lipids and lip	Steroids and	Sulfated ster
7.23_423.23	423.236846	7.22931667	neg	(3R,5R)-7-[(1	HMDB0260155		HIZIJHNJVQ	Organic acid	Hydroxy acid	Medium-chai
8.60_267.16	267.160123	8.59883333	neg	(10betaH,11	HMDB00376	92448	YAQFZWZU	Lipids and lip	Prenol lipids	Sesquiterper
11.45_226.1	226.158689	11.4499667	pos	BRN 2646116		68710	APSXSFZAT	Lipids and lip	Fatty Acyls	Fatty amides
11.50_278.2	278.247508	11.5038	pos	3-Gonal	HMDB0245878		QVZYOPAD	Lipids and lip	Steroids and	Unclassified
11.82_494.3	517.370807	11.82365	pos	Heptaethyler	HMDB02515	347359	DWHIUNMO	Organic oxyg	Organooxyge	Ethers
12.48_359.1	359.166096	12.4811	pos	(2R,3S,4R,5	HMDB0242759		BJUAGLWL	Organic oxyg	Organooxyge	Carbohydrate
13.90_298.3	298.310012	13.9018833	pos	Tridemorph	HMDB00318	6168	YTOPFCCW	Organoheter	Oxazinanes	Morpholines
2.02_275.06	275.064955	2.02081667	neg	Flutamide	HMDB00146	553	MKXKFYHW	Benzenoids	Benzene and	Trifluorometh
4.29_171.09	171.099153	4.29085	pos	(2R,3R)-heptane-1,2,3-triol			HXYCHJFUE	Lipids and lip	Fatty Acyls	Fatty alcohol
4.36_575.31	575.310656	4.3621	pos	Cholylglutam	HMDB0242387		CNGHUSPO	Lipids and lip	Steroids and	Bile acids, al

4.40_156.10:	156.102064	4.3987	pos	6-Acetamidol	HMDB02470	313526	WDSCBUNM	Lipids and lip	Fatty Acyls	Fatty acids a
4.50_120.01:	120.012312	4.50406667	neg	Tramiprosate	HMDB02532	43475	SNKZJIOFVI	Organic acid:	Organic sulfo	Organosulfor
4.83_137.09(	137.09613	4.8302	pos	(R)-2-Phenyl	HMDB03039	446974	RNDNSYIPL	Benzenoids	Benzene and	Unclassified
5.76_157.13:	157.133454	5.75788333	pos	4,5-Diethyl-2	HMDB00378	92676	DIGAWDVO	Organoheter	Azoles	Oxazoles
6.03_199.13(	199.130339	6.03235	pos	1-Hexylglycerol		46621	MQVMITUCT	Lipids and lip	Glycerolipids	Monoradylgly
6.59_315.21	315.217564	6.58565	neg	(+)-12-hydroxy-9Z-hexade		35625	JPBAMKISC.	Lipids and lip	Fatty Acyls	Fatty Acids a
6.82_413.13(	413.13655	6.81833333	pos	Mammea B/E	HMDB00306	87117	JKOPHYFSC	Phenylpropa	Coumarins a	Pyranocouma
8.23_158.15:	158.15383	8.23126667	pos	2,6-Nonadier	HMDB0031262		AMXYRHBJZ	Lipids and lip	Fatty Acyls	Fatty alcohol
8.34_301.28	301.284524	8.34093333	pos	Prosafrinine		53951	FNYMOMSU	Lipids and lip	Sphingolipids	Sphingoid ba
8.75_936.57(	981.574415	8.74946667	neg	PI(20:5(5Z,8Z,11Z,14Z,17Z)/22:2(13Z,1			NGWSYZILF	Lipids and lip	Glycerophos	Glycerophos
1.08_247.04	247.044597	1.08208333	neg	2-(2-Nitroimi	HMDB0251709		ZMQGRZRW	Organic 1,3-(	Allyl-type 1,3	Organic nitro
1.49_175.14	175.144282	1.48828333	pos	methyl 1-met	HMDB0254560		LLEOWWWI	Organoheter	Piperidines	Piperidinecar
10.79_546.2	546.282037	10.7894833	neg	PS(20:3(8Z,11Z,14Z)/0:0)		78849	FTZJCZOBX	Lipids and lip	Glycerophos	Glycerophos
14.26_207.1:	207.123863	14.2613833	pos	N-Nitrosoana	HMDB0255199		ZJOFAFWT(	Organoheter	Pyridines and	Hydropyridin
14.99_445.3(	445.36726	14.9898167	pos	11alpha-ethyl-1alpha,25-dihydroxyvitam			BNJQRWCY	Lipids and lip	Steroids and	Secosteroids
4.13_326.19	349.183503	4.12765	pos	Heptaethyler	HMDB0061835		XPJRQAIZZ(	Organic oxyg	Organooxyge	Ethers
5.10_134.07	134.071246	5.10093333	pos	3-Methylpyrr	HMDB00331	89049	HSNADAQX	Organoheter	Pyrrolotriazin	Unclassified
5.12_462.19(	462.190332	5.11926667	pos	N-Oleoyl Gly	HMDB0241916		HPFXACZRF	Organoheter	Diazines	Pyrimidines a
5.54_201.17:	202.179998	5.53846667	pos	11-amino-undecanoic acid		35923	GUOSQNAU	Lipids and lip	Fatty Acyls	Fatty Acids a
7.87_357.09:	357.093175	7.86566667	neg	Tipepidine	HMDB02591	314749	YDDXVAXD	Organoheter	Azoles	Thiazoles
9.25_460.25	460.251203	9.24935	pos	Panax ginser	HMDB0302146		LTKLLKUVW	Organic acid:	Carboxylic ac	Amino acids,
0.59_192.97(	192.97662	0.58926667	pos	(S)-3-Sulfona	HMDB00601	69074	CQQGIWJSI	Organic acid:	Hydroxy acid	Alpha hydrox
0.69_168.04:	168.04284	0.68961667	neg	Phosphodime	HMDB00602	63348	BLHVJAAEH	Organic acid:	Organic phos	Phosphate e
1.14_203.11	226.104945	1.13681667	pos	Acetyl-L-carnitine		956	RDHQFKQI(	Lipids and lip	Fatty Acyls	Fatty esters
10.79_578.2	578.219341	10.7894833	neg	Elexacaftor/I	HMDB0304881		MVRHVFSO	Unclassified	Unclassified	Unclassified
14.42_356.3:	379.317749	14.4236667	pos	22-Hydroxyd	HMDB0302963		IBPVZXPSTI	Lipids and lip	Fatty Acyls	Fatty acids a
2.86_146.11	146.11761	2.85705	pos	N,N-dimethyl-L-Valine		44985	APGLTERDI	Unclassified	Unclassified	Unclassified
2.93_252.07:	252.072608	2.92891667	pos	4,5-Dihydro-1	HMDB02471	356468	CPXGGWXJ	Benzenoids	Benzene and	Trifluorometh
4.76_150.00	150.001644	4.76071667	neg	2(3H)-Benzo	HMDB02490	341971	YEDUAINPP	Organoheter	Benzothiazol	Unclassified
5.29_194.04	194.04559	5.29295	neg	1-3-(4,5-dihy	HMDB0302882		XUVJDFTVU	Organic acid:	Carboxylic ac	Amino acids,
5.41_193.11	193.11965	5.41035	pos	(+)-Angelicoi	HMDB0303084		HLVIHBJQD	Lipids and lip	Prenol lipids	Monoterpenc
5.50_319.05	319.058352	5.5019	pos	4-Sulfoxyphe	HMDB02465	424737	BTLKSOMRI	Organic acid:	Carboxylic ac	Amino acids,
8.99_319.18	319.187437	8.99243333	pos	3beta-Acetoxy-4-oplopano		53436	YNNDVHBD	Lipids and lip	Prenol lipids	Isoprenoids

9.72_464.33	464.336396	9.72246667	pos	(12E)-9-Hydr	HMDB0241534		BUGMCCNA	Lipids and lip	Fatty Acyls	Fatty acid es
1.08_206.04	206.048049	1.07531667	pos	o-Cresol sulf	HMDB0240655		CYGSXDXR	Organic acid	Organic sulf	Arylsulfates
11.22_394.2	417.263244	11.2158833	pos	11-Hydroxye	HMDB00125	85230	SSHLWLAPI	Lipids and lip	Endocannab	Unclassified
13.25_431.1	431.1891	13.2463167	neg	Androsta-1,4	HMDB0244564		QOEJEWOZ	Lipids and lip	Steroids and	Steroid ester
13.91_441.2	441.202901	13.90995	neg	Carbofuran	HMDB00317	44573	DUEPRVBV	Organoheter	Coumarans	Unclassified
14.34_385.3	385.311089	14.3415	neg	Somaliensene B			BGFULIZGC	Lipids and lip	Prenol lipids	Isoprenoids
2.11_344.13	344.134753	2.11061667	neg	N-((Tetrahyd	HMDB0252610		KZAVRHNK	Organic acid	Carboxylic a	Amino acids,
3.02_207.12	208.133295	3.01843333	pos	SALSOLIDINE		44388	HMYJLVDKF	Unclassified	Unclassified	Unclassified
3.61_524.15	524.152906	3.6144	pos	Metaflumizor	HMDB0254498		MIFOMMKA	Phenylpropa	Stilbenes	Unclassified
4.34_170.04	170.045723	4.33765	neg	pyramid	HMDB0256941		MMTVHLPX	Organoheter	Pyrans	Unclassified
4.61_647.36	647.364959	4.61381667	pos	Goshonoside	HMDB0038541		QIOMMMCQ	Lipids and lip	Prenol lipids	Terpene glyc
4.83_283.14	283.145159	4.83273333	neg	7-[4-(Dimeth	HMDB0260424		SOGYGBQC	Benzenoids	Benzene and	Phenylpropa
5.03_264.15	264.159261	5.02876667	pos	8-Epixanthati	HMDB0036473		RBRPTFMV	Lipids and lip	Prenol lipids	Terpene lact
5.30_174.18	174.185165	5.30103333	pos	p-Menthan-1	HMDB00370	92003	CMLYGGFIX	Lipids and lip	Prenol lipids	Monoterpen
5.36_516.29	516.293811	5.35556667	pos	(5Z)-7-[(1R,2	HMDB0241876		ARBYNSMIZ	Lipids and lip	Fatty Acyls	Eicosanoids
8.21_278.08	301.074968	8.21301667	pos	Triphenylphosphine oxide		64889	FIQMHBFVR	Unclassified	Unclassified	Unclassified
0.63_260.12	260.125068	0.62835	neg	Lysylaspartic	HMDB00289	23693	CIOWSLJGL	Organic acid	Carboxylic a	Amino acids,
0.64_205.15	205.154793	0.63663333	pos	1-Hydroxy-2,	HMDB0243895		CLKPVQZF	Organoheter	Pyrrolidines	Pyrrolidine c
1.63_174.13	175.144296	1.62985	pos	Ne,Ne dimet	HMDB0013287		XXEWFEBM	Organic acid	Carboxylic a	Amino acids,
10.39_305.2	306.24228	10.3935333	pos	Amafolone	HMDB0248273		QPRBHGIR	Lipids and lip	Steroids and	Androstane s
10.45_369.2	369.200763	10.4474333	pos	(1R,4E,6S,7	HMDB0242334		BCJMNZRQ	Phenylpropa	Macrolides a	Unclassified
10.59_372.1	372.13545	10.5914167	pos	Flumioxazin	HMDB00348	68790	FOUWCSDK	Organoheter	Benzoxazine	Benzoxazino
11.36_363.2	363.250016	11.36075	pos	PGE1 alcohol		36124	CMHCOGHT	Lipids and lip	Fatty Acyls	Eicosanoids
12.31_332.2	355.281368	12.3062167	pos	1-O-(2R-hydroxy-hexadecyl)-sn-glycero			TUBKELMMI	Lipids and lip	Glycerolipids	Monoradylgly
13.01_282.2	282.221233	13.0115667	pos	3,7-Dimethyl	HMDB0250571		NUXWXMZV	Lipids and lip	Prenol lipids	Retinoids
2.46_229.06	229.06962	2.46275	pos	trioxyethylen	HMDB0259257		HRDHVEJYL	Organic acid	Carboxylic a	Dicarboxylic
3.40_184.12	207.110574	3.39796667	pos	N-(3-acetami	HMDB0061384		OAUYENAPI	Organoheter	Pyrrolidines	N-alkylpyrroli
3.59_329.02	329.028557	3.58845	neg	2-O-(6-Phos	HMDB00121	62816	BOLXAGHG	Organic oxy	Organooxyge	Carbohydrate
3.72_274.16	274.164852	3.71513333	pos	(4E)-3-Hydro	HMDB0241670		JNWLVTSZ	Lipids and lip	Fatty Acyls	Fatty acid es
4.44_346.11	347.123952	4.435	pos	2,6-Dimethyl	HMDB0255502		OSUCQKNX	Organoheter	Pyridines and	Hydropyridin
4.47_220.06	220.063828	4.47151667	pos	N-Acetylalliin	HMDB0242184		CCCNUHFR	Organic acid	Carboxylic a	Amino acids,
4.72_474.05	474.058009	4.72411667	neg	Altropane	HMDB0248262		GTQLIPQFX	Alkaloids and	Tropane alka	Phenyltropa
5.26_361.21	361.219417	5.26461667	pos	Tributyl citrate		324874	ZFOZVQLO	Unclassified	Unclassified	Unclassified

6.20_229.16	252.156885	6.1974	pos		N-Decanoylg	HMDB0013267		WRRYZYAS	Organic acid	Carboxylic ac	Amino acids,
7.90_163.11	163.112669	7.90265	neg		xi-2-Hexyl-5-	HMDB00380	92782	AAUWBAWS	Organoheter	Dihydrofuran	Furanones
9.05_239.13	240.137984	9.04725	pos		Nornefopam	HMDB02557	1484	ZMOHJRCO	Organic nitro	Organonitrog	Amines
1.32_262.01	263.021165	1.31801667	pos		2-Hydroxy-4-	HMDB00416	96011	IFFSTUJRUZ	Organic oxyg	Organooxyge	Carbonyl cor
10.19_568.2	568.266068	10.18955	neg		4-Chloro-2-ni	HMDB0249542		HIYAVKIYRI	Organoheter	Benzoxazole	Unclassified
11.77_582.4	605.423136	11.77015	pos		Polidocanol	HMDB02566	69582	ONJQDTZCI	Organic oxyg	Organooxyge	Ethers
4.27_417.17	417.177353	4.27276667	pos		S-Adenosylm	HMDB00011	6064	MEFKEPWM	Nucleosides,	5'-deoxyribor	5'-deoxy-5'-th
4.70_552.31	552.31784	4.70481667	pos		Corchoroside	HMDB00338	89568	QBILRDAMJ	Lipids and lip	Steroids and	Steroid lactor
4.72_248.08	248.086819	4.72301667	pos		Streptozocin	HMDB00145	592	ZSJLQEPLLI	Organic oxyg	Organooxyge	Carbohydrate
4.94_182.19	182.190358	4.93775	pos		Dicyclohexyla	HMDB02512	70260	XBPCUCUW	Organic nitro	Organonitrog	Cyclohexylar
5.08_221.11	221.114592	5.08273333	pos		methyl-6S-hydroxy-2E,4E-decadienoate			YTLQMZKEI	Lipids and lip	Fatty Acyls	Fatty esters
5.48_301.16	301.161044	5.48356667	pos		1-Methyl-2-hy	HMDB0243923		MRYABSBQ	Organic oxyg	Organooxyge	Carbohydrate
0.65_174.13	175.143711	0.6518	pos		Ethyl lysine	HMDB0252051		PVPFJQSVZ	Organic acid	Carboxylic ac	Amino acids,
0.72_323.07	323.078739	0.71928333	neg		1-O-Caffeoyl	HMDB0036937		WQSDYZZE	Phenylpropa	Cinnamic aci	Hydroxycinna
1.02_298.94	298.943272	1.01586667	neg		Perfluorobuta	HMDB0246220		JGTNAGYH	Organohalog	Alkyl halides	Alkyl fluoride
1.04_195.07	195.074017	1.0442	pos		Glycylproline	HMDB00007	23672	KZNQNBZM	Organic acid	Carboxylic ac	Amino acids,
10.52_658.3	658.333874	10.5172833	neg		OHOOA-PS			AWBZXBXH	Lipids and lip	Glycerophos	Oxidized glyc
13.38_730.5	730.5372	13.38305	pos		PE(15:0/20:2	HMDB00089	60344	FXYFFYOQI	Lipids and lip	Glycerophos	Glycerophos
14.92_150.1	150.102438	14.9180833	pos		2-Methylbenz	HMDB02452	339584	LDZYRENCL	Organoheter	Benzimidazo	Unclassified
2.15_267.07	267.073372	2.1466	neg		Formycin b	HMDB0252449		MTCJZZBQN	Organic oxyg	Organooxyge	Carbohydrate
3.27_139.08	139.086689	3.27091667	pos		(2-Ethylcroto	HMDB0242468		QCUPYFTW	Organic acid	Organic carb	Ureas
3.79_178.13	178.134055	3.78573333	pos		Tolazoline	HMDB00149	2867	JIVZKJJQOZ	Benzenoids	Benzene anc	Unclassified
4.06_130.08	130.087113	4.06311667	neg		Aminocaproi	HMDB00019	3926	SLXKOJJOQ	Lipids and lip	Fatty Acyls	Fatty acids a
4.30_242.09	241.084594	4.30156667	neg		Thymidine	HMDB00002	3375	IQFYYKKMV	Nucleosides,	Pyrimidine nu	Pyrimidine 2'
5.98_235.02	235.021916	5.97626667	neg		4-(Trifluorom	HMDB02463	337338	SWKPKONE	Benzenoids	Benzene anc	Trifluorometh
8.23_180.13	180.135687	8.23126667	pos		Sec-Butylpro	HMDB0257395		ICMNKCCFF	Lipids and lip	Fatty Acyls	Fatty amides
8.93_357.26	357.264745	8.93068333	neg		18-oxo-nonadecanoic acid		74767	ZNFQHZMN	Lipids and lip	Fatty Acyls	Fatty Acids a
9.44_249.18	249.185842	9.44245	neg		7,10,13-hexadecatrienoic a		96765	KBGYPXOSI	Lipids and lip	Fatty Acyls	Fatty Acids a
9.96_145.12	145.122204	9.95751667	pos		4,4'-Oxydi-2-	HMDB0245040		AFSYRVDDZ	Organic oxyg	Organooxyge	Alcohols and
0.73_293.06	293.068622	0.7342	neg		3-(3-(Pyridin-	HMDB0247644		HGFXDSQLI	Organoheter	Azoles	Oxadiazoles
1.10_366.04	366.040578	1.1046	pos		3-{[(1s)-2,2-D	HMDB0256905		ONBSHRSJ	Organic oxyg	Organooxyge	Ethers
1.47_106.05	106.050313	1.47146667	pos		2-hydroxyacr	HMDB0062676		FEWFXBUN	Organic acid	Carboxylic ac	Carboxylic ac
12.41_114.1	114.102671	12.4112833	pos		N-Carbamoy	HMDB00334	6041	YANFYYGAI	Organic acid	Carboximidic	Carboximidic

14.90_183.08	183.087461	14.9013667	pos		Sorbitol	HMDB00002	143		FBPFZTCFM	Organic oxyg	Organooxyge	Carbohydrate
4.15_206.142	207.149362	4.14533333	pos		Oxotremorine	HMDB02560	44356		RSDOPYMF.	Organoheter	Pyrrolidines	N-alkylpyrroli
4.45_245.035	246.043168	4.45335	pos		4-Methoxyinc	HMDB0304935			IBLCRTNVH	Unclassified	Unclassified	Unclassified
4.51_566.298	566.298263	4.50633333	pos		15beta-hydroxycalactin				ZABRUXSLN	Lipids and lip	Steroids and	Sterols
6.73_255.062	255.062647	6.7265	pos		4',5,8-Trihydr	HMDB00318	52594		SMRHIFAZR	Phenylpropa	Flavonoids	Flavans
7.51_171.138	171.138912	7.50616667	neg		(1S,3R,4R)-p	HMDB00373	343371		IIZCEIWXLS	Lipids and lip	Prenol lipids	Monoterpeno
8.95_224.177	223.17024	8.94916667	neg		Alepric acid		34858		DTHSUAKYT	Lipids and lip	Fatty Acyls	Fatty Acids a
9.61_250.216	250.21619	9.61401667	pos		1-(2,6,6-Trim	HMDB00297	318611		FXCYGAGBI	Lipids and lip	Prenol lipids	Sesquiterper
1.03_228.085	228.085295	1.0341	neg		Malonylcarnit	HMDB0002095			ZGNBLKBZJ	Lipids and lip	Fatty Acyls	Fatty acid es
1.25_134.022	152.056665	1.251	pos		Pyrazolopyrir	HMDB02569	831145		DOTPSQVY(	Organoheter	Pyrazolopyrir	Unclassified
10.14_528.17	528.177639	10.1353167	neg		Nilotinib	HMDB00155	45263		HHZIURLSW	Benzenoids	Benzene anc	Anilides
11.50_387.24	387.249761	11.5038	pos		Dodeca-8,10	HMDB0241270			AMDSWHJA	Lipids and lip	Fatty Acyls	Fatty acid es
12.94_306.25	305.248376	12.9357167	neg		11,14,17-eicosatrienoic ac		73854		AHANXAKGI	Lipids and lip	Fatty Acyls	Fatty Acids a
3.47_345.216	368.205747	3.47126667	pos		2-[4-[4-(4-Ca	HMDB0252929			QGEGSJUS'	Organoheter	Diazinanes	Piperazines
3.80_351.143	352.150507	3.80401667	pos		benfluorex	HMDB02489	321112		CJAVTWRY(	Benzenoids	Benzene anc	Benzoic acid
4.91_278.070	323.074562	4.90568333	neg		3-(5,7-Dimethoxy-4-oxo-4H		985191		AKJIPZYHXY	Unclassified	Unclassified	Unclassified
9.74_186.185	186.185022	9.74061667	pos		(+/-)-trans- a	HMDB00322	88287		NYPOJSCNH	Lipids and lip	Prenol lipids	Monoterpeno
9.84_229.180	229.180702	9.84338333	neg		3R-hydroxy-tridecanoic ac		35679		CWSNHZHH	Lipids and lip	Fatty Acyls	Fatty Acids a
1.10_363.154	363.154774	1.1046	pos		N-(1-Deoxy-1	HMDB00378	92655		WACCHYXN	Organoheter	Indoles and (	Tryptamines
1.42_229.154	229.154654	1.41946667	pos		Pro-Ile	HMDB0304810			OCYROESY	Organic acid	Carboxylic ac	Amino acids,
10.41_452.27	452.276787	10.41135	pos		LysoPE(16:1	HMDB00115	62290		DSOWUEHX	Lipids and lip	Glycerophos	Glycerophos
10.91_342.22	343.22375	10.9125667	pos		14-oxo-DoHE	HMDB0062685			CTOFGDVN'	Lipids and lip	Fatty Acyls	Fatty acids a
11.43_341.30	341.304072	11.4320667	pos		20-oxo-heneicosanoic acic		74774		MAGPQMMJ	Lipids and lip	Fatty Acyls	Fatty Acids a
2.01_262.062	263.069563	2.00526667	pos		Furano[2'',3'':6,7]aurone		52416		YVBYFIDGV	Lipids and lip	Polyketides	Flavonoids
3.64_428.072	428.072546	3.64241667	neg		Pantoprazole	HMDB00050	1697		IQPSEEYGB	Organoheter	Benzimidazo	Sulfinylbenzir
3.75_236.128	236.128171	3.75006667	pos		Pandamarilac	HMDB00336	89412		AEEIFNXEU'	Organoheter	Dihydrofuran	Furanones
4.72_443.250	443.250035	4.72301667	pos		(S)-4-(2-(4-A	HMDB0247670			KGPGFQWE	Organoheter	Imidazopyrid	Imidazo-[4,5-
4.76_207.040	207.040879	4.76071667	neg		5H-Indazole-	HMDB0244329			JGVHSSOIV	Organoheter	Azolines	Pyrazolines
4.83_123.117	123.116973	4.8302	pos		(3Z,6Z)-3,6-N	HMDB00309	46088		PICGPEBVZ	Lipids and lip	Fatty Acyls	Fatty alcohol
6.93_495.367	518.356312	6.92858333	pos		PE(O-20:0/0:0)		77700		PKPGKYPSI	Lipids and lip	Glycerophos	Glycerophos
6.98_299.109	299.109807	6.98325	pos		Triethyl citrat	HMDB00342	89812		DOOTYTYQ	Organic acid	Carboxylic ac	Tricarboxylic
7.63_198.172	199.180204	7.62578333	pos		Cycluron		72851		DQZCVNGC	Unclassified	Unclassified	Unclassified
9.88_360.310	360.310383	9.88461667	pos		Medica 16	HMDB02544	62960		HYSMCRNF	Lipids and lip	Fatty Acyls	Fatty acids a

0.64_239.89(	238.891415	0.64391667	neg	Trimetaphos	HMDB00599	422356	AZSFNUJOC	Homogeneou	Non-metal o:	Non-metal pl
0.70_119.03	142.026428	0.69861667	pos	Benzoxazole	HMDB02490	336429	BCMCBBGG	Organoheter	Benzoxazole	Unclassified
1.07_129.03(	129.038501	1.06741667	neg	Ethyl 3-merc	HMDB00322	88317	FPBCNQQY	Lipids and lip	Fatty Acyls	Fatty acid es
1.07_633.12(	633.12644	1.06741667	neg	Petunidin	HMDB0003173		AFOLOMGW	Phenylpropa	Flavonoids	O-methylated
1.08_205.10(	205.100497	1.07531667	pos	BUTHIONINI	HMDB02494	66203	KJQFBVYM(	Organic acid:	Carboxylic ac	Amino acids,
1.36_202.07	202.071881	1.3586	neg	Avenic acid E	HMDB0038751		YVTYLIZWV	Organic acid:	Carboxylic ac	Amino acids,
11.22_278.1	323.171259	11.2230667	neg	(x)-2-Heptan	HMDB00350	90386	NZLSMMMV	Lipids and lip	Fatty Acyls	Fatty acyl gly
13.25_367.2	367.240136	13.2463167	neg	N-Lauroyl Tr	HMDB0242042		MTYLJFSVE	Organic acid:	Carboxylic ac	Amino acids,
2.12_187.09(	187.096866	2.12441667	pos	5-(2-Methylp	HMDB00309	87332	ODKYFEYT\	Organoheter	Lactones	Gamma buty
4.31_323.10(	323.106095	4.30826667	pos	Dansylsarco:	HMDB0250853		BRLJKBOXI\	Benzenoids	Naphthalene	Naphthalene
4.69_251.01(	251.015992	4.6877	neg	[(5-methyl-1,	HMDB0242147		XBGUTAISS	Organoheter	Dithiolanes	1,3-dithiolane
4.83_112.02	112.022947	4.83273333	neg	Butenyl isoth	HMDB0302343		XDDPFUCCI	Organosulfur	Isothiocyanat	Unclassified
5.39_432.25	432.252975	5.39208333	pos	Longichalasin A			KQJMIGIIKJI	Lipids and lip	Polyketides	Cytochalasin
6.42_231.00!	231.005732	6.41976667	neg	1-Fluoro-2,4-	HMDB0245460		LOTKRQAV(	Benzenoids	Benzene anc	Nitrobenzene
9.57_179.10	179.107575	9.56983333	neg	Butylbenzene	HMDB00598	72067	OCKPCBLVI	Benzenoids	Benzene anc	Unclassified
9.96_334.29	334.294731	9.95751667	pos	11,12-dihydroxy stearic ac		35470	BXMZIUSKY	Lipids and lip	Fatty Acyls	Octadecanoi
0.70_147.04!	147.049004	0.70456667	neg	(+/-)-3-Merca	HMDB0303256		GMXSGLCD	Organic acid:	Carboxylic ac	Carboxylic ac
0.73_161.00(	161.008937	0.7342	neg	1,4-Dioxane-	HMDB02442	337676	RKDVKSZUI	Organoheter	Dioxanes	1,4-dioxanes
1.08_282.11!	282.119449	1.07531667	pos	Lysylproline	HMDB00289	85861	AIXUQKMME	Organic acid:	Carboxylic ac	Amino acids,
10.26_361.1!	361.199722	10.2627833	neg	Isohumulone	HMDB00300	86672	QARXXMMQ	Lipids and lip	Prenol lipids	Monoterpenc
12.71_492.3(	492.388954	12.70945	pos	bhos#36			GQQORDYS	Lipids and lip	Fatty Acyls	Fatty acyl gly
13.28_361.2!	362.304709	13.2766833	pos	N-methyl N-(2-hydroxy-eth		36697	DZOXWGMU	Lipids and lip	Fatty Acyls	Fatty amides
4.06_364.18	364.187122	4.05526667	pos	Libenzapril	HMDB0254077		AXTCRUUIT	Organic acid:	Carboxylic ac	Amino acids,
4.65_250.11(	250.110714	4.64996667	pos	Camphorsulf	HMDB0243496		MIOPJNTWI	Lipids and lip	Prenol lipids	Monoterpenc
4.85_371.19	371.197305	4.84713333	pos	17a-Hydroxy	HMDB00003	5352	JERGUCIJO	Lipids and lip	Steroids and	Pregnane ste
4.98_144.01	144.012289	4.97913333	neg	3-Thiocyanat	HMDB00297	86465	IFVYHJRLW	Organosulfur	Allyl sulfur cc	Unclassified
5.20_350.08	350.08154	5.2006	neg	Fenoldopam	HMDB00149	4024	TVURRHSHI	Organoheter	Benzazepine	Unclassified
5.35_207.04(	207.040871	5.3483	neg	3-Nitrotyrosir	HMDB0001904		FBTSQILOG	Organic acid:	Carboxylic ac	Amino acids,
5.88_223.09	223.094926	5.88396667	neg	2,6-Diamino-	HMDB02469	316920	CSMCRCLIF	Organoheter	Imidazopyrim	Purines and
9.68_194.08	194.082009	9.68	neg	4-(Dimethyla	HMDB0246303		BGNGWHSE	Benzenoids	Benzene anc	Benzoyl deriv
1.05_390.03	390.032288	1.0516	neg	Glucotropae	HMDB0038419		QQGLQYQX	Organic oxyg	Organooxyge	Carbohydrate
1.10_172.16!	172.169604	1.1046	pos	(-)-trans-Ison	HMDB0303068		HHDDWVZS	Lipids and lip	Prenol lipids	Monoterpenc
1.31_215.05	215.053745	1.30545	neg	(2Z,4'Z)-2-(5-	HMDB00326	88601	SOSDBVUE!	Organic oxyg	Organooxyge	Ethers

1.32_222.98	222.989247	1.32371667	neg		2-Thiophene	HMDB00297	328775		CNUDBTRU	Organic oxy	Organooxyge	Carbonyl cor
1.35_262.18	285.178245	1.34996667	pos		1,4,10,13-Te	HMDB02442	458479		NLMDJJTUQ	Organic nitro	Organonitrog	Amines
1.99_210.14	210.148658	1.9877	pos		Butyl phenyla	HMDB00404	335606		LDOXTQYW	Benzenoids	Benzene anc	Unclassified
10.77_596.2	596.296794	10.77115	neg		1-Oleoyl-sn-g	HMDB0242160			UGDOFRYH	Lipids and lip	Glycerophos	Glycerophos
11.17_339.2	339.253735	11.1685	neg		Ethyl (E)-2-o	HMDB00374	92375		AISZSTYLO	Lipids and lip	Fatty Acyls	Fatty acid es
11.81_538.4	561.396852	11.80575	pos		Octaethylene	HMDB0246126			YYELLDKEC	Organic oxyg	Organooxyge	Ethers
2.11_331.16	331.164985	2.10798333	pos		N-cis-Feruloy	HMDB00363	91524		NPNNKDMS	Phenylpropa	Cinnamic aci	Hydroxycinna
3.64_247.04	247.044661	3.64241667	neg		Ethyl aconita	HMDB00402	405567		ANIDMCPGE	Organic acids	Carboxylic ac	Tricarboxylic
3.65_146.11	146.117578	3.64815	pos		[(3-Methylbut	HMDB0245644			FMZPWUNP	Organic acids	Carboxylic ac	Amino acids,
4.74_116.08	115.076218	4.74241667	neg		2,4,5-Trimeth	HMDB00402	94804		HLEXJAVJC	Organoheter	Dioxolanes	1,3-dioxolane
5.06_208.13	208.130658	5.06456667	pos		Dihydralazine	HMDB0251288			VQKLRVZQ	Organoheter	Diazanaphtha	Benzodiazine
5.53_226.10	226.108325	5.53265	neg		(R)-2-(4-(Ter	HMDB0247375			QVOPNRRQ	Organoheter	Oxazinanes	Morpholines
5.96_215.11	215.115368	5.95905	pos		Tocainide	HMDB00151	2864		BUJAGSGYI	Benzenoids	Benzene anc	Xylenes
6.03_437.21	437.215653	6.0318	neg		3-[[(2S)-2,4-I	HMDB02507	317442		GHOKWGTU	Organic acids	Carboxylic ac	Amino acids,
7.77_627.37	627.374949	7.7731	neg		Kahweol	HMDB00356	90878		JEKMKNDUI	Lipids and lip	Prenol lipids	Monoterpeno
1.70_342.16	342.166033	1.70151667	pos		PC(0:0/5:0)		40357		FUNVSMBQ	Lipids and lip	Glycerophos	Glycerophos
10.25_373.3	373.303401	10.24785	pos		Tridec-5-eno	HMDB0241310			BJGDRSVPY	Lipids and lip	Fatty Acyls	Fatty acid es
14.38_310.2	309.279757	14.3838667	neg		9Z-Octadecenyl acetate		46388		GYGAZRPD	Lipids and lip	Fatty Acyls	Fatty esters
14.65_312.3	312.325706	14.6543333	pos		Phytal	HMDB00356	90919		RAFZYSUIC	Lipids and lip	Prenol lipids	Diterpenoids
6.23_369.15	369.155395	6.23498333	neg		Blumealacto	HMDB00353	90657		ZZIWIHVMLI	Lipids and lip	Prenol lipids	Terpene lact
6.89_376.22	377.229431	6.89188333	pos		CRASSIN ACETATE		43734		WMGWWFH	Unclassified	Unclassified	Unclassified
8.22_378.23	423.236667	8.21615	neg		8-iso-16-cyclohexyl-tetran		45175		PIPQNTASW	Unclassified	Unclassified	Unclassified
9.46_226.19	225.185874	9.46038333	neg		Myristoleic ac	HMDB00020	6424		YWWVWXA	Lipids and lip	Fatty Acyls	Fatty acids a
9.52_287.22	287.222806	9.51548333	neg		3R-Methylheptanoic acid		45764		DVESMWJF	Lipids and lip	Fatty Acyls	Fatty Acids a
1.07_217.08	217.082614	1.06741667	neg		gamma-Gluta	HMDB00062	58376		WQXXXVRA	Organic acids	Carboxylic ac	Amino acids,
1.22_261.05	261.052861	1.21911667	neg		5-Fluorouridi	HMDB00603	71273		FHIDNBAQC	Nucleosides,	Pyrimidine nu	Unclassified
1.31_135.01	135.012018	1.30545	neg		2-Methylprop	HMDB0245236			XEEYSDHE	Organic acids	Organic sulfo	Organosulfo
11.20_428.2	428.285892	11.198	pos		Dynorphin B	HMDB0012936			VSLCIGXQL	Organic acids	Carboxylic ac	Amino acids,
14.78_508.5	508.544679	14.7777667	pos		2-Hexadecyl	HMDB0255386			XHLSSYAPL	Lipids and lip	Fatty Acyls	Fatty aldehy
3.53_391.19	391.190613	3.52578333	pos		Prostaglandi	HMDB00014	6221		BHMBVRSPI	Lipids and lip	Fatty Acyls	Eicosanoids
4.54_173.08	173.080945	4.5416	pos		3-Hydroxysu	HMDB00003	5314		ARJZZFJXSI	Organic acids	Hydroxy acid	Medium-chai
6.95_395.23	395.239894	6.94688333	pos		oscr#23				DIYWZICEC	Lipids and lip	Fatty Acyls	Fatty acyl gly
8.73_1004.5	1049.56237	8.73261667	neg		PIP(20:0/18:	HMDB0279506			LYZZEJQVW	Unclassified	Unclassified	Unclassified

8.80_211.13	211.133735	8.80223333	neg		Traumatin	HMDB00373	35783	INMKWUNQ	Lipids and lip	Fatty Acyls	Fatty acids a
9.40_361.19	361.198141	9.39628333	pos		Tryptophyl-A	HMDB00290	85970	LCPVBXOH	Organic acid	Carboxylic ac	Amino acids,
9.52_401.19	401.194493	9.51548333	neg		N-(3-hydroxy-pentanoyl)-homoserine lac			IZXCXPQW	Lipids and lip	Fatty Acyls	Fatty amides
0.61_252.08	270.119691	0.60603333	pos		Cndac	HMDB0250381		DCYBPMFX	Nucleosides,	Pyrimidine nu	Pyrimidine 2'
0.62_295.15	295.149964	0.6215	pos		Queuine	HMDB00014	6279	WYROLENT	Organoheter	Pyrrolopyrimi	Pyrrolo[2,3-d
0.73_256.05	256.059251	0.7342	neg		Avenin	HMDB02487	338418	XXRYFVCIM	Organic acid	Organic phos	Phosphate e
0.82_118.04	136.075766	0.81883333	pos		xi-2,3-Octadi	HMDB00405	95038	MFQBYQJL	Lipids and lip	Fatty Acyls	Fatty alcohol
1.99_397.17	397.171841	1.9877	pos		1-(3-Methylb	HMDB00399	94495	ZIEQNJFDW	Lipids and lip	Fatty Acyls	Fatty acyl gly
10.14_953.5	953.537882	10.1353167	neg		PI(5-iso PGF	HMDB0277840		CWZNGDHS	Unclassified	Unclassified	Unclassified
10.19_546.2	546.256446	10.1937667	pos		16,17-Dihydr	HMDB00406	95125	XKXOQMAK	Lipids and lip	Prenol lipids	Terpene glyc
12.33_421.2	421.22649	12.33275	neg		1,4-bis[(2-ethylhexyl)oxy]-		4003	HNSDLXPS	Lipids and lip	Fatty Acyls	Fatty esters
12.34_297.2	297.289576	12.3412167	pos		Linoleamide	HMDB00626	43435	SFIHQZFZM	Lipids and lip	Fatty Acyls	Fatty amides
2.43_249.12	249.126781	2.42631667	pos		Val-Met	HMDB0259742		YSGSDAIMS	Organic acid	Carboxylic ac	Amino acids,
4.69_451.21	451.218808	4.68661667	pos		Bedoradrine	HMDB0248928		OANCEOSL	Benzenoids	Tetralins	Unclassified
5.52_199.09	199.093761	5.52015	pos		6,8-dihydroxy-octanoic aci		74602	LJWTVHKB	Lipids and lip	Fatty Acyls	Fatty Acids a
9.08_297.16	297.166849	9.08396667	pos		3-Hydroxytet	HMDB00003	5383	CEDZIURHI	Lipids and lip	Fatty Acyls	Fatty acids a
0.62_136.07	136.073487	0.6215	pos		2-Acetylpyrr	HMDB00372	357571	FYCFJMPAS	Organoheter	Pyrrolidines	Unclassified
1.09_241.15	241.154504	1.08996667	pos		Cerulenin	HMDB0015168		GVEZIHKRY	Organoheter	Epoxides	Oxirane carb
1.15_324.13	325.143877	1.1535	pos		Glabranin		52653	DAWSYIQA	Lipids and lip	Polyketides	Flavonoids
1.33_409.24	410.253415	1.33403333	pos		Pentadeca-3	HMDB0241450		AEESIZVTD	Lipids and lip	Fatty Acyls	Fatty acid es
1.49_330.11	330.117089	1.4863	neg		N'-Hydroxyn	HMDB0033665		YBDAUZJLB	Phenylpropa	Saxitoxins, g	Unclassified
1.51_202.11	202.118832	1.50628333	pos		DL-Ornithino	HMDB00294	86275	BKYZUPUA	Organic acid	Carboxylic ac	Amino acids,
10.63_608.3	608.318229	10.6260167	neg		Boc-val-pro-	HMDB0249335		DFOSAUPK	Lipids and lip	Steroids and	Steroid ester
11.36_386.3	386.325917	11.36075	pos		Octadecyl fu	HMDB00380	345857	MHQJUHSH	Lipids and lip	Fatty Acyls	Fatty acid es
12.04_464.3	464.31446	12.0372667	pos		N-Docosahe	HMDB0242016		JEAQPBYM	Unclassified	Unclassified	Unclassified
14.42_400.3	423.34391	14.4236667	pos		1-O-(2R-methoxy-4Z-eicosenyl)-sn-glyc			QCTNOIZAG	Lipids and lip	Glycerolipids	Monoradylgly
2.02_368.14	368.145101	2.02305	pos		4-(Methylnitr	HMDB00603	73260	KNPUXTWH	Lipids and lip	Fatty Acyls	Fatty acyl gly
2.02_376.10	376.101617	2.02305	pos		6''-O-Acetylh	HMDB00363	91475	NIVXKMKLZ	Organic oxy	Organooxyge	Carbohydrate
3.24_157.08	157.083636	3.23546667	pos		Polypropylen	HMDB00324	88489	DUFKCOQIS	Organic oxy	Organooxyge	Alcohols and
3.66_351.20	351.200282	3.66498333	pos		trans-p-Ment	HMDB00347	90206	PHDIOUHO	Organic oxy	Organooxyge	Carbohydrate
4.32_321.09	321.09128	4.32	neg		Quinoline-4,	HMDB00602	63549	PYELIMVFII	Organoheter	Quinolines a	Quinolones a
4.36_229.10	229.104708	4.3621	pos		6-Carboxy-5,	HMDB0060410		QSIYONWV	Organoheter	Pteridines an	Pterins and d
4.63_243.12	243.120278	4.63161667	pos		Aciclovir	HMDB00149	789	MKUXAQIIE	Organoheter	Imidazopyrim	Purines and

4.81_314.14	314.14176	4.81226667	pos		Laurelliptine	HMDB00333	89201		HORZNQYQ	Alkaloids and	Aporphines	Unclassified
5.03_197.00	197.000832	5.03453333	neg		Benzodioxap	HMDB0249013			MDPMTBHL	Benzenoids	Unclassified	Unclassified
5.74_316.21	316.211563	5.73951667	pos		Tetranor-PGD1		74980		WWXNENYS	Lipids and lip	Fatty Acyls	Eicosanoids
5.94_310.22	333.214313	5.94078333	pos		Bacillamidin C				LCKMULXMU	Lipids and lip	Fatty Acyls	Fatty amides
5.96_143.10	143.10757	5.95783333	neg		Cycloheptyl	HMDB0250647			HXCKCCRK	Hydrocarbon	Saturated hy	Unclassified
7.19_489.20	489.208133	7.19245	neg		Gly-arg-gly-a	HMDB0252824			RGNVSYKV	Organic acids	Carboxylic ac	Amino acids,
1.14_191.10	191.104036	1.13681667	pos		(1r,3r)-1-Ami	HMDB0242328			YFYNOWXB	Organic acids	Carboxylic ac	Amino acids,
1.20_263.98	262.975806	1.20271667	neg		Perfluorovale	HMDB02563	346583		CXZGQIAOT	Organohalog	Alkyl halides	Alkyl fluoride
10.90_527.1	527.132749	10.8978167	neg		hypocrellin a	HMDB0253310			VANSZAOQ	Benzenoids	Perylenequin	Unclassified
11.20_324.2	324.216427	11.198	pos		5'-Carboxy-g	HMDB0012799			CNGKBUHO	Organoheter	Benzopyrans	1-benzopyran
12.48_706.5	706.588029	12.4811	pos		SM(d16:1/17	HMDB02406	83739		CGMZWBZS	Lipids and lip	Sphingolipids	Phosphosphi
13.33_522.5	522.596988	13.32975	pos		(21-Methyl-8Z-pentatriaco		97494		PXCAACLCF	Lipids and lip	Fatty Acyls	Hydrocarbon
2.39_179.03	179.034758	2.387	neg		Aspirin	HMDB00018	346		BSYNRYMU	Benzenoids	Benzene and	Benzoic acid
2.91_187.09	187.094071	2.9106	pos		Triethylene G	HMDB0062666			JLGLQAWT	Organic oxyg	Organooxyge	Ethers
2.92_450.08	450.087802	2.92268333	neg		4-Phenylbuty	HMDB00384	93113		PJZFULXME	Organic oxyg	Organooxyge	Carbohydrate
3.03_267.09	267.096119	3.03363333	neg		1-[5-(Thiophe	HMDB0249491			ALFGDCNSE	Organoheter	Indoles and d	Tryptamines
3.68_249.03	249.037816	3.67736667	neg		Diflunisal	HMDB00149	2029		HUPFGZXOI	Benzenoids	Benzene and	Biphenyls an
4.34_442.24	442.242214	4.34396667	pos		8beta-Angelo	HMDB00390	93619		LOQBOYSP	Lipids and lip	Prenol lipids	Terpene lact
4.69_215.16	238.152688	4.68661667	pos		Diethylcarba	HMDB00608	2005		KAJAFGMEF	Organoheter	Diazinanes	Piperazines
7.04_369.17	369.174011	7.04441667	neg		Tecastemizo	HMDB0255721			SFOVDSLXF	Organoheter	Benzimidazo	Unclassified
7.13_213.17	236.161898	7.13003333	pos		N-(9-Oxodecyl)acetamide		984916		JRJBWKJIC	Unclassified	Unclassified	Unclassified
9.03_213.14	213.145815	9.02893333	pos		2-Amino-4-hy	HMDB02471	6665		ARHXUJTYU	Organoheter	Pteridines an	Pterins and d
9.03_228.26	228.268449	9.02893333	pos		Pentadecylar	HMDB02438	34540		JPZYXGPCH	Organic nitro	Organonitrog	Amines
1.19_206.04	229.031859	1.1884	pos		Methylisocitri	HMDB00064	58419		HHKPKXCSI	Organic acids	Carboxylic ac	Tricarboxylic
1.20_240.99	240.993668	1.20271667	neg		5-Methylthior	HMDB00012	6143		CNSJRYUM	Organic oxyg	Organooxyge	Carbonyl cor
1.39_259.00	259.000575	1.39358333	neg		2-Mercapto-3	HMDB0246169			HTRUBLMPI	Organoheter	Thienopyrimi	Unclassified
10.75_827.5	826.560803	10.7528167	neg		PS(20:3(8Z,11Z,14Z)/19:0		78270		FOKQGHJZ	Lipids and lip	Glycerophos	Glycerophos
13.87_336.3	336.325579	13.8664333	pos		Anandamide (20:1, n-9)		3722		YDKRGMXLI	Lipids and lip	Fatty Acyls	Fatty amides
3.68_183.00	183.003329	3.67736667	neg		2,4-Dinitroph	HMDB02454	43994		UFBJCMHM	Benzenoids	Phenols	Nitrophenols
3.98_155.15	155.154408	3.98231667	pos		Pyrrole, 2-pe	HMDB0256996			SBEGSTRV	Organoheter	Pyrroles	Substituted p
4.69_119.05	119.053168	4.6877	neg		3-Mercapto-2	HMDB00323	88400		MRVKDGZL	Organosulfur	Thiols	Alkylthiols
4.81_354.02	354.022297	4.81485	neg		Succinylsulfa	HMDB02585	44631		SKVLYVHUL	Benzenoids	Benzene and	Benzenesulfo
5.39_179.06	179.063799	5.39208333	pos		2-Ethylsulfan	HMDB0244644			UGCOPUIBN	Organoheter	Benzimidazo	Unclassified

5.40_541.26!	541.265394	5.40378333	neg	Cortolone-3-(	HMDB00103	61643	OKNSFVSKI	Lipids and lip	Steroids and	Steroidal gly(
5.96_188.09	188.092564	5.95783333	neg	2-Hydroxypr(	HMDB0245163		WXFIFTYQC	Organic acid	Carboxylic ac	Carboxylic ac
8.29_223.02	223.028266	8.29023333	neg	2,3-Dihydrox	HMDB0240388		XMZBEAXQ	Lipids and lip	Fatty Acyls	Fatty acids a
8.97_222.08	245.078136	8.97426667	pos	2,3-Dihydro-3	HMDB00413	95782	YTGHSHJES	Organoheter	Benzopyrans	1-benzopyra
9.74_488.30	488.300076	9.74061667	pos	Withaferin A		67219	DBRXOUCR	Lipids and lip	Steroids and	Sterols
0.69_263.08	263.083337	0.68961667	neg	Perlolyrine	HMDB00303	67538	KFUCYPGCI	Alkaloids and	Harmala alka	Unclassified
0.75_223.02	268.022935	0.74898333	neg	2,2,4,4-Tetra	HMDB02457	460024	ZENKESXKV	Organoheter	Benzodioxan	Benzo-1,3-di
0.99_271.98	271.985148	0.99473333	pos	Aminobutane	HMDB0249923		XLIREHXMR	Organic acid	Organic phos	Bisphosphon
1.31_247.07	247.073523	1.30545	neg	5-Hydroxyinc	HMDB00041	58225	LFUCRBSG	Organic acid	Carboxylic ac	Amino acids,
1.47_160.10	160.108081	1.47146667	pos	delta-Guanid	HMDB02509	69638	UKUBCVAQ	Lipids and lip	Fatty Acyls	Fatty acids a
10.27_293.1	293.13552	10.2662	pos	(2S,3S,5R)-5	HMDB0257938		ZWYXPSXIV	Organoheter	Imidazopyrim	Purines and
10.70_243.1	243.135189	10.6974667	pos	6-Benzylamir	HMDB00392	68947	NWBJYWHL	Organoheter	Imidazopyrim	Purines and
12.21_293.1	293.179138	12.20735	neg	Sodium Tetra	HMDB00146	85386	URLJMZWT	Organic acid	Organic sulfu	Sulfuric acid
12.30_677.4	677.454654	12.3037667	neg	3b-Hydroxy-1	HMDB0249984		OKJRJJXKS	Lipids and lip	Steroids and	Androstane s
12.82_308.2	308.294304	12.8165833	pos	(3S,6E,10E)-	HMDB00350	90431	IQDXAJNQK	Lipids and lip	Prenol lipids	Diterpenoids
3.95_128.09	129.102489	3.94663333	pos	piperidine-1-	HMDB02565	345218	JSPCTNUQ	Organoheter	Piperidines	Piperidinecar
4.25_257.09	257.099609	4.25448333	pos	9-Carboxyme	HMDB0060805		MICNQLKUS	Organoheter	Imidazopyrim	Purines and
5.22_226.02	226.027526	5.21915	neg	Ethyl 4-nitro	HMDB0246217		BARJAAQSI	Benzenoids	Benzene and	Nitrobenzene
5.54_547.29	547.291698	5.53846667	pos	Macrolactin B			NDMBRILKN	Lipids and lip	Fatty Acyls	Fatty esters
6.16_371.04	371.046702	6.16076667	pos	5'-Hydroxyte	HMDB0014098		INPPRTDYE	Organoheter	Thienothiazir	Unclassified
6.20_228.16	228.160272	6.19793333	neg	Icaridin	HMDB0253340		QLHULAHO	Organoheter	Piperidines	Piperidinecar
6.92_409.22	409.223132	6.91643333	neg	4,5-Dihydroc	HMDB0246609		ACSFOIGNU	Lipids and lip	Steroids and	Hydroxysterc
7.25_491.11	491.119491	7.24778333	neg	Swertisin		49400	ABRULANJV	Lipids and lip	Polyketides	Flavonoids
7.61_229.08	229.080874	7.60728333	pos	Deoxyuridine	HMDB00000	5086	MXHRCPNR	Nucleosides,	Pyrimidine nu	Pyrimidine 2'
8.71_520.27	520.278851	8.7078	pos	LysoPE(18:0	HMDB00111	40775	BBYWOYAF	Lipids and lip	Glycerophos	Glycerophos
8.95_412.27	412.270663	8.94916667	neg	(2E,4E)-Tetra	HMDB0241377		DBJDCEJXE	Lipids and lip	Fatty Acyls	Fatty acid es
9.79_367.24	367.245099	9.79426667	pos	9,10,11-trihydroxy-12Z-octadecenoic ac			LPFKOMDPI	Lipids and lip	Fatty Acyls	Fatty alcohol
0.85_271.00	271.001046	0.84881667	pos	Thorium	HMDB0029215		ZSLUVFAKF	Homogeneou	Homogeneou	Unclassified
1.07_241.05	241.057402	1.06741667	neg	2-Carboxyara	HMDB0303760		XONDRGRA	Lipids and lip	Fatty Acyls	Fatty acids a
10.59_476.3	476.373125	10.5914167	pos	13-(3,4-Dime	HMDB0241838		QHFUBAWG	Lipids and lip	Fatty Acyls	Fatty acid es
11.33_250.1	250.14466	11.3319667	neg	2-Nitropheny	HMDB0245262		CXVOIIMJZF	Benzenoids	Benzene and	Nitrobenzene
11.40_347.2	347.243692	11.40315	neg	4-hydroxy pelargonic acid		35408	IKOSQCITE	Lipids and lip	Fatty Acyls	Fatty Acids a
11.54_442.3	442.352198	11.5398167	pos	isopropyl unc	HMDB0253677		XXUPXHKCI	Lipids and lip	Fatty Acyls	Eicosanoids

14.28_284.2	283.264023	14.2783333	neg	Ethyl hexade	HMDB00298	318358	XIRNKXNNC	Lipids and lip	Fatty Acyls	Fatty acid es
3.59_424.03	424.035111	3.58845	neg	[(2R,5R)-5-(2	HMDB0258063		BPZXYEUJB	Nucleosides,	Purine nucle	Purine ribonu
3.59_429.15	429.154067	3.58845	neg	Kanzonol N	HMDB00411	95533	ANRYVYXU	Phenylpropa	Isoflavonoids	O-methylated
3.82_326.02	326.029153	3.82341667	neg	Guanosine 2	HMDB00116	3487	UASRYODFI	Lipids and lip	Sphingolipids	Glycosphingo
4.03_279.02	279.023278	4.02623333	neg	2,2,2-Trifluor	HMDB0247856		RDHSJZAEI	Benzenoids	Benzene and	Nitrobenzene
4.44_209.01	209.018651	4.435	pos	1-Iodoheptar	HMDB02439	349466	LMHCYRULI	Organohalog	Organoiodide	Unclassified
4.45_200.03	200.038448	4.44846667	neg	5-Acetyl-2,4-	HMDB00414	318888	BLQOKWQU	Organoheter	Azoles	Thiazoles
4.47_263.09	263.094658	4.47151667	pos	Indoline, 1-(2	HMDB02493	430784	JZHBBTBIFN	Organoheter	Indoles and	Unclassified
4.51_274.13	274.136127	4.50633333	pos	N-ethyl-2-(4-	HMDB02551	419417	AWAAHDPC	Organoheter	Azoles	Oxazoles
4.54_328.09	328.094522	4.5416	pos	4-coumaroyl-	HMDB0304173		LVPGCTXCE	Phenylpropa	Cinnamic aci	Hydroxycinna
4.69_347.08	347.085902	4.6877	neg	7,7,8,8-Tetra	HMDB0258044		BLAGUIMGU	Organic oxyg	Organooxyge	Carbonyl cor
4.90_282.07	282.071051	4.90175	pos	1-(6-Aminop	HMDB0257910		XNKUEYOAI	Organoheter	Imidazopyrim	Purines and
4.91_239.05	239.053703	4.90568333	neg	Temozolomid	HMDB00149	85179	BPEGJWRS	Organoheter	Imidazotetra	Unclassified
5.16_130.15	130.159031	5.15551667	pos	3-Methyl-3-h	HMDB0061909		AAUHUDBD	Hydrocarbon	Unsaturated	Branched un
5.19_262.07	262.074025	5.19183333	pos	Mepanipyrim	HMDB02544	68688	CIFWZNRJI	Benzenoids	Benzene and	Aniline and s
5.25_267.15	267.156436	5.24643333	pos	3alpha,15-Di	HMDB00360	91217	INMDHOFDL	Organoheter	Furofurans	Unclassified
5.34_162.12	185.114737	5.33735	pos	1-O-Pentylgl	HMDB0243976		FQJXITFHAI	Lipids and lip	Glycerolipids	Monoradylgly
5.85_227.08	227.089873	5.84688333	neg	Dacarbazine	HMDB0014989		FDKXTQMXI	Organic acid	Carboxylic ac	Carboxylic ac
6.99_416.15	416.156129	6.98976667	neg	2-Methoxy-4-	HMDB0244645		HCHNINKPF	Organic oxyg	Organooxyge	Carbohydrate
9.15_382.27	382.275304	9.14948333	neg	N-benzyl-13-oxo-9E,11E-octadecadiena			BJLMUUKAI	Lipids and lip	Fatty Acyls	Fatty amides
1.20_310.04	309.041764	1.20271667	neg	Maximaisoflavone A		316121	HDEHMKSX	Lipids and lip	Polyketides	Flavonoids
1.23_340.09	679.187642	1.23491667	neg	3'-Ketolactos	HMDB00010	5955	HKKHTABTH	Organic oxyg	Organooxyge	Carbohydrate
1.31_283.04	283.041126	1.30545	neg	Sodium diac	HMDB0303659		BHZOKUMU	Organic acid	Carboxylic ac	Carboxylic ac
1.51_201.07	201.073362	1.50628333	pos	(2S,3R,4S,5F	HMDB0242656		HIPAIKSXHJ	Organic oxyg	Organooxyge	Carbohydrate
11.36_311.1	311.161557	11.36075	pos	Delgocitinib	HMDB0250969		LOWWYYZE	Organoheter	Pyrrolopyrimi	Pyrrolo[2,3-d
11.42_616.2	616.284211	11.42155	neg	Kinetensin 4-	HMDB0012986		NKRLKOMB	Organic acid	Carboxylic ac	Amino acids,
12.16_165.0	165.040525	12.1558667	neg	A,b-Dihydrox	HMDB00026	6723	DGADNPLB	Organic acid	Hydroxy acid	Beta hydroxy
12.48_794.6	794.602878	12.4811	pos	PC(18:3(9Z,1	HMDB00082	59654	CFHMUBPSI	Lipids and lip	Glycerophos	Glycerophos
12.57_435.8	435.80933	12.56865	pos	Iodoquinol	HMDB02535	44166	UXZFQZANI	Organoheter	Quinolines a	Haloquinoline
12.94_351.2	351.226382	12.9406833	pos	(R-(R*,S*))-3	HMDB02494	45416	ZIDQIOZJEJ	Organoheter	Azoles	Imidazoles
13.54_168.9	168.989014	13.5393333	neg	1,1,1,2,3,3,3-	HMDB0244037		YFMFNYKEU	Organohalog	Organofluori	Unclassified
14.26_374.3	397.328285	14.2613833	pos	1-O-(2R-hydroxy-nonadecyl)-sn-glycero			TYAPEMWQ	Lipids and lip	Glycerolipids	Monoradylgly
14.81_101.0	101.034921	14.8127	pos	N-carbamoyl	HMDB03044	337428	KZVRXPPUJ	Organic acid	Carboxylic ac	Amino acids,

15.43_202.1	202.108562	15.4298167	pos	Zeatin	HMDB00122	45500	UZKQTCBA	Organoheter	Imidazopyrim	Purines and
2.23_142.94	142.947997	2.22953333	pos	Sodium phos	HMDB0303430		AJPJDKMHJ	Mixed metal/	Alkali metal	Alkali metal
2.75_184.09	184.094495	2.74981667	pos	N-(2-Hydrox	HMDB0254990		AJNYQRXD	Organic acid	Carboxylic a	Amino acids,
3.96_289.12	289.122495	3.96453333	pos	1-Pyrenebut	HMDB02440	348134	QXYRRCOJ	Benzenoids	Pyrenes	Unclassified
3.99_305.04	305.042511	3.98933333	neg	Pyochelin	HMDB0256933		NYBZAGXT	Organic acid	Carboxylic a	Amino acids,
4.13_501.27	501.279807	4.12765	pos	Valyl-leucyl-	HMDB0250805		CAJXYXPLL	Organic acid	Carboxylic a	Amino acids,
4.31_286.05	287.063157	4.30826667	pos	Glycineamid	HMDB0002022		OBQMLSFO	Nucleosides,	Glycinamide	Unclassified
4.79_627.32	627.319957	4.79411667	pos	Niguldipine(+	HMDB0255603		MFGFAKST	Benzenoids	Benzene and	Diphenylmet
4.97_234.18	257.172294	4.97433333	pos	Triethylene g	HMDB0259194		RGICCULPC	Organic oxy	Organooxyge	Ethers
5.27_167.07	167.07128	5.27448333	neg	1-Methoxy-4-	HMDB00320	88162	CHLICZRVG	Benzenoids	Phenol ether	Anisoles
5.32_178.11	179.117739	5.3192	pos	Glycinexylidi	HMDB00606	1001	IXYVBZOSG	Benzenoids	Benzene and	Xylenes
5.35_240.04	240.04323	5.3483	neg	4-Nitropheny	HMDB0246536		YSHVIWJFW	Benzenoids	Benzene and	Nitrobenzene
5.44_213.07	213.074102	5.44071667	neg	(Methyl-triaz	HMDB0257597		SKOGEBGD	Organic acid	Carboxylic a	Carboxylic a
5.72_130.12	130.122674	5.72123333	pos	5-Methyl-1-hexen-3-one		453731	CMXNFXBF	Lipids and lip	Fatty Acyls	Oxygenated
6.51_401.23	402.242309	6.50861667	pos	[2-Amino-2-(	HMDB0252505		DQKSVFVIW	Benzenoids	Benzene and	Phenylbutyla
7.17_465.20	465.207833	7.17388333	neg	6alpha,9alph	HMDB00607	457737	BQEJAAIPK	Lipids and lip	Steroids and	Hydroxyster
8.07_193.08	193.086726	8.06858333	neg	Zingerone	HMDB00325	318121	OJYLAHXKV	Benzenoids	Phenols	Methoxyphe
8.41_367.11	367.114012	8.41473333	pos	tetranor-PGDM		45422	VNJBSPJILL	Lipids and lip	Fatty Acyls	Eicosanoids
8.60_244.26	244.263195	8.5985	pos	2-Pentadeca	HMDB00310	345615	CJPNOLIZC	Organic oxy	Organooxyge	Carbonyl co
8.76_905.57	905.571102	8.75856667	pos	Sarcinaxanthin monoglucoside			BRWOXKPP	Lipids and lip	Prenol lipids	Isoprenoids
9.34_414.20	437.193069	9.34135	pos	Armillarin	HMDB00393	93956	ISKWRTCZ	Organic oxy	Organooxyge	Carbohydrate
9.54_236.10	236.106255	9.54118333	pos	Cysteinyl-Pr	HMDB0028783		ZSRSLWKG	Organic acid	Carboxylic a	Amino acids,
9.90_285.20	285.20706	9.89818333	neg	octanoate	HMDB0304443		WWZKQHO	Lipids and lip	Fatty Acyls	Fatty acids a
0.65_186.91	186.912104	0.6518	pos	Sodium phos	HMDB0303418		RYFMWSXC	Mixed metal/	Alkali metal	Alkali metal
0.69_215.03	215.032602	0.68961667	neg	2-C-methyl-D	HMDB03040	330202	XMWHRVN	Organic acid	Organic phos	Phosphate e
0.70_139.02	277.032633	0.70456667	neg	(R)-3-Amino-	HMDB0248770		ZUHQHFXK	Organic oxy	Organic oxoa	Unclassified
0.73_144.04	145.049634	0.72825	pos	Methylglutar	HMDB0303007		AQYCMVICE	Lipids and lip	Fatty Acyls	Fatty acids a
0.73_185.02	185.021919	0.7342	neg	TRIMETHYL	HMDB0259237		WVLBCYQIT	Organic acid	Organic phos	Phosphate e
0.80_311.04	311.049457	0.79596667	neg	Allantoxanan	HMDB0247204		HJXJVORIL	Organic acid	Carboxylic a	Carboxylic a
0.86_146.94	146.94659	0.8592	neg	Sodium meta	HMDB0303299		AQMNWCRS	Mixed metal/	Alkali metal	Alkali metal
0.89_162.95	162.956811	0.8943	neg	Phosphite	HMDB0001443		OJMIONKXN	Homogeneou	Non-metal o	Non-metal p
0.90_135.00	135.002975	0.89581667	pos	Sodium lacta	HMDB03048	329181	NGSFWBMY	Organic acid	Carboxylic a	Carboxylic a
0.90_150.97	150.976811	0.89581667	pos	Phosphoric a	HMDB0257619		VFNOXQPP	Organic acid	Organic phos	Phosphate e

1.34_473.12	473.120184	1.34166667	neg	N-Benzoylas	HMDB00342	89804	DJLTZJGULI	Organic acid	Carboxylic ac	Amino acids,
1.43_201.08	201.087942	1.43078333	neg	N-(Hydroxym	HMDB02550	73150	CNCOEDDP	Organic acid	Carboxylic ac	Acrylic acids
1.49_287.06	287.064904	1.4863	neg	5-phosphonc	HMDB00596	63462	WLPXLNNU	Organic acid	Carboxylic ac	Amino acids,
1.62_215.00	215.009749	1.61501667	neg	2,4-Dinitro-1-	HMDB02454	339217	FFRBMBIXV	Benzenoids	Naphthalene	Nitronaphtha
1.86_173.11	173.117292	1.86321667	pos	5-Pentyl-1,4-	HMDB00599	92114	AYJJZBHSZ	Organoheter	Dioxanes	1,4-dioxanes
10.47_479.2	479.260379	10.46565	pos	Diflucortolon	HMDB0251261		HHJIUUAMY	Lipids and lip	Steroids and	Pregnane ste
11.07_184.9	184.985383	11.0736	pos	Phosphohyd	HMDB00010	484	LFLUCDOS	Organic oxyg	Organooxyge	Carbonyl cor
11.54_331.2	331.223785	11.5398167	pos	3-Hydroxyno	HMDB0241771		BOGFDYMN	Lipids and lip	Fatty Acyls	Fatty acid es
12.16_149.0	167.128953	12.1619333	pos	1-Benzylgua	HMDB0243835		ABSNGNUG	Benzenoids	Benzene and	Unclassified
13.17_563.4	563.428485	13.17195	pos	TG(8:0/8:0/a	HMDB0071246		HCDORBWZ	Lipids and lip	Glycerolipids	Triradylglyc
13.21_748.5	748.527858	13.21015	neg	PE(18:0/20:4	HMDB00090	40516	ANRKEHNW	Lipids and lip	Glycerophos	Glycerophos
15.33_311.2	311.295561	15.32745	neg	14S-Methyl-1-octadecene		97436	NAEZQVWA	Lipids and lip	Fatty Acyls	Hydrocarbon
2.04_267.03	267.033137	2.03931667	neg	Cyclonite	HMDB0253161		XTFIVUDBN	Organoheter	Triazinanes	1,3,5-triazina
2.13_504.00	504.008902	2.1282	neg	alpha,beta-M	HMDB0248246		CAWZRIXW	Nucleosides,	Purine nucle	Purine ribonu
2.43_207.08	207.087724	2.42631667	pos	Nitrobenzodi	HMDB0255641		VMGLACFX	Organoheter	Benzodiazep	Unclassified
2.48_225.08	225.087897	2.47923333	neg	DL-Propargy	HMDB02515	45299	DGYHPLMP	Organic acid	Carboxylic ac	Amino acids,
2.53_317.12	317.125546	2.5348	neg	Asparaginyl-	HMDB00287	24016	RGGVDKVX	Organic acid	Carboxylic ac	Amino acids,
3.25_226.97	226.974942	3.2533	pos	Trona	HMDB0303538		YVDUOWXD	Organic acid	Organic carb	Organic carb
3.58_462.22	462.226343	3.58011667	pos	Nvp-aew541	HMDB02558	45579	AECDBHGV	Organoheter	Pyrroles	Substituted p
4.36_191.03	191.034818	4.35616667	neg	2-Furancarb	HMDB00329	69936	HYBBIBNJH	Organic oxyg	Organooxyge	Carbonyl cor
4.41_194.98	194.988324	4.41166667	neg	Trifluoroacet	HMDB02592	332942	JVTSHOJDB	Organohalog	Organofluori	Unclassified
4.44_273.13	273.130913	4.435	pos	(S)-2-Methyl-	HMDB0303770		DMALNKYC	Lipids and lip	Fatty Acyls	Fatty acyl gly
4.58_321.04	321.040986	4.57715	neg	2-beta-d-Rib	HMDB0245037		KFYKWWBL	Organic oxyg	Organooxyge	Carbohydrate
4.67_193.07	216.06663	4.66833333	pos	S-Butylcystei	HMDB0302178		CBFHOPPJE	Organic acid	Carboxylic ac	Amino acids,
4.69_242.07	242.07476	4.68661667	pos	Serylasparag	HMDB00290	23733	LTFSLKWFM	Organic acid	Carboxylic ac	Amino acids,
4.70_180.10	180.10205	4.70481667	pos	3,6-octadien	HMDB0094793		ALNNZFFDA	Organic acid	Carboxylic ac	Amino acids,
4.88_204.10	204.105319	4.88355	pos	3-Cyclohexyl	HMDB02444	342879	PJWWRFAT	Organic nitro	Organonitrog	Cyclohexylar
5.05_271.11	271.115177	5.04685	pos	part#9			NZMXMOQN	Lipids and lip	Fatty Acyls	Fatty acyl gly
5.16_192.05	192.050858	5.15551667	pos	2,5-Dimethyl-	HMDB00402	94734	ZZXDUGZY	Organoheter	Heteroaroma	Unclassified
5.34_481.39	481.39979	5.33735	pos	(5Z,14Z,17Z)	HMDB0241599		XLTQIAYAM	Lipids and lip	Fatty Acyls	Fatty acid es
5.74_197.14	220.130616	5.73951667	pos	Estreptoquin	HMDB0258508		XRZWVSXE	Organic acid	Carboxylic ac	Amino acids,
5.77_246.03	246.038287	5.773	neg	m-Trifluoromethylhippuric		2682	ZDGGJQMS	Unclassified	Unclassified	Unclassified
5.94_170.03	170.036621	5.94078333	pos	Benzeneprop	HMDB00342	340659	ACRWYXSK	Benzenoids	Benzene and	Unclassified

5.94_195.06(	195.066063	5.93928333	neg	5-Methyl-2(3I	HMDB00296	86373	BGLUXFNV\	Organoheter	Dihydrofuran	Furanones
6.01_186.22	186.221575	6.01398333	pos	(E)-2-Dodece	HMDB0061819		ADOQBZAVI	Hydrocarbon	Unsaturated	Unsaturated
6.38_272.15(	272.15035	6.38276667	neg	N-octanoyl-homoserine lac		45309	JKEJEOJPJ\	Lipids and lip	Fatty Acyls	Fatty amides
6.44_178.09!	178.099178	6.43578333	pos	quinolizidine	HMDB0257048		LJPZHJUSIC	Organoheter	Quinolizines	Unclassified
8.72_547.27!	547.279876	8.71673333	neg	Hordatine A	HMDB0030461		FCINNGNG(	Phenylpropa	2-arylbenzofu	Unclassified
8.75_903.55!	903.558922	8.74946667	neg	PG(i-20:0/20	HMDB0271952		HFIMISQJTF	Unclassified	Unclassified	Unclassified
9.98_358.27	381.260758	9.97586667	pos	Siegesbeckin C			GGDBPLUAI	Lipids and lip	Fatty Acyls	Fatty esters
0.61_407.17	407.172712	0.61033333	neg	Idazoxan	HMDB02533	68730	HPMRFMKY	Organoheter	Benzodioxan	Benzo-1,4-di
0.76_231.01	231.011058	0.75766667	pos	2-carboxy-L-t	HMDB0304062		CQIRJDZGD	Lipids and lip	Fatty Acyls	Fatty acids a
1.10_200.12!	200.128224	1.1046	pos	Amyl 2-furoa	HMDB00294	343311	BGJZMKPSI	Organoheter	Furans	Furoic acid a
1.15_182.99(	182.990876	1.14921667	neg	Polymaleic a	HMDB03032	324496	VRVKOZSIJ	Organic acid	Carboxylic ac	Dicarboxylic
1.45_187.00(	187.000282	1.45418333	pos	1h,8h-Pyran(	HMDB0244906		XZFSVGMV!	Organoheter	Pyrans	Pyranones ar
1.47_308.10!	308.10799	1.47146667	pos	Aspergilloma	HMDB00294	86277	XFTWUNOV	Organic acid	Carboxylic ac	Amino acids,
10.04_253.2	253.217149	10.0440667	neg	Cadinene	HMDB00321	88249	FZZNNPQZI	Lipids and lip	Prenol lipids	Sesquiterper
10.45_477.2	477.246131	10.4453167	neg	Methylglutary	HMDB0254648		APURPPSD!	Organic acid	Keto acids ar	Medium-chai
10.81_589.2	589.248652	10.8069333	neg	N(6),O(2)-Dir	HMDB0255287		GRYSXUXXI	Nucleosides,	Purine nucle	Unclassified
11.25_217.1	217.179636	11.25255	pos	7-hydroxy-dodecanoic aci		74544	BNWKMHUF	Lipids and lip	Fatty Acyls	Fatty Acids a
11.40_276.1	276.160222	11.40315	neg	alpha-Pyrroli	HMDB02446	85222	YDIIDRWHP	Organic oxyg	Organooxyge	Carbonyl cor
11.63_435.2!	435.293519	11.6289333	pos	Polysorbate (	HMDB00371	92151	CRBBOOXG	Lipids and lip	Fatty Acyls	Fatty acid es
12.49_388.2	388.21028	12.4924667	neg	CP-642931		317565	FTEQMUXM	Unclassified	Unclassified	Unclassified
12.50_115.0	115.086668	12.4972833	pos	2-Hydroxype	HMDB0245162		AKXFVHOXI	Unclassified	Unclassified	Unclassified
13.15_334.3	334.309887	13.1542167	pos	n-arachidony	HMDB0255084		LJKYKFRQA	Organic nitro	Organonitrog	Amines
2.15_236.09	236.092615	2.1466	neg	(E)-5-(3,4,5,(	HMDB00335	89370	HKIPZBSQJI	Organoheter	Pyridines and	Hydropyridin
2.29_265.08	265.082885	2.29455	neg	N-(4-Aminob	HMDB0255007		GADGMZDH	Organic acid	Carboxylic ac	Amino acids,
2.62_179.06	179.067513	2.62451667	pos	N-Methyl-N-r	HMDB0247094		HTWGDSKV	Organoheter	Imidazopyrim	Purines and
2.87_289.08(	289.080532	2.86726667	neg	4-Amino-1-(6	HMDB0257570		HCUAWQYI!	Organoheter	Imidazopyrim	Purines and
3.49_176.95!	176.959527	3.48938333	pos	Terbium	HMDB03039	354264	GZCRRIHWI	Homogeneou	Homogeneou	Unclassified
3.68_438.18!	438.188376	3.68235	pos	Candoxatrila	HMDB0249582		ACZWIDANI	Organic acid	Carboxylic ac	Dicarboxylic
3.82_197.07!	197.078433	3.82191667	pos	Ethylene gly(	HMDB0252071		AOBIOSPN>	Organoheter	Epoxides	Unclassified
3.86_295.09	295.094236	3.85685	pos	1-Methoxy-1-	HMDB00317	87929	IXNRGYSRF	Benzenoids	Benzene and	Phenylpropa
3.95_282.14!	282.145066	3.94663333	pos	Lufironil	HMDB02541	415579	JUCNGMPT(	Organoheter	Pyridines and	Pyridinecarb
4.00_159.01(	159.016494	4.00056667	pos	2,6-Dihydrox	HMDB0245515		BFHKYHMCI	Organoheter	Pyridines and	3-pyridinecar
4.22_196.03	196.036991	4.21765	pos	N-Lactoyl eth	HMDB00323	88392	CKXSSEHW	Organic acid	Organic phos	Phosphate e:

4.34_227.08	227.089052	4.34396667	pos		2-Hydroxyglu	HMDB03039	688177	DYLHSDCN	Lipids and lip	Fatty Acyls	Fatty acid es
4.34_265.10	265.104639	4.34396667	pos		Diethylene gl	HMDB0251246		XFCMNSHQ	Organic acid	Carboxylic ac	Dicarboxylic
4.36_291.98	291.982962	4.3621	pos		2-Iodo-l-pher	HMDB0245177		BKXVGLPBX	Organic acid	Carboxylic ac	Amino acids,
4.45_373.11	373.116109	4.45335	pos		Veranisatin C	HMDB0031756		JTVRXANW	Lipids and lip	Prenol lipids	Terpene lacto
4.50_344.11	344.114905	4.50406667	neg		N-trans-p-Co	HMDB00328	88726	VATOSFCFN	Phenylpropa	Cinnamic aci	Hydroxycinna
4.61_238.00	236.999042	4.61413333	neg		Disodium hyd	HMDB0303751		CEYULKASI	Organic acid	Carboxylic ac	Tricarboxylic
4.67_546.32	569.314774	4.66833333	pos		Phytyl 2-O-methyl-dimethylarsinoylribos			DERBZIOJE	Lipids and lip	Prenol lipids	Isoprenoids
4.76_284.06	284.067701	4.76071667	neg		Theasaponin	HMDB0302079		FNBGTIABO	Organoheter	Diazanaphtha	Benzodiazine
4.78_203.14	203.143069	4.7769	pos		(Z)-6-Tetrade	HMDB00389	93576	AGXXOOILF	Lipids and lip	Fatty Acyls	Fatty alcohol
4.96_303.17	303.177521	4.95603333	pos		Arginyl-Gam	HMDB00287	85638	NSSFBLAW	Organic acid	Carboxylic ac	Amino acids,
5.03_400.96	400.963126	5.03453333	neg		1H-Perfluoro	HMDB0244895		KBHBUUBX	Organohalog	Organofluori	Unclassified
5.07_254.06	254.066994	5.07146667	neg		3-Carbamoyl	HMDB0060367		AYRIVCKWI	Benzenoids	Benzene anc	Unclassified
5.24_219.02	219.027405	5.23773333	neg		4-(Trifluorom	HMDB02463	337330	BEOBZEOP	Benzenoids	Benzene anc	Trifluorometh
5.68_143.10	143.107562	5.68048333	neg		(E)-4,4-Dime	HMDB0061894		BIDIHFPLDF	Hydrocarbon	Unsaturated	Unsaturated
5.96_167.03	167.034808	5.95783333	neg		3-Hydroxycy	HMDB0245898		IHXWECHP	Organic acid	Vinylogous a	Unclassified
5.96_444.22	444.221446	5.95783333	neg		Arginyl-glycy	HMDB0248574		MDNRBNZIC	Organic acid	Carboxylic ac	Amino acids,
5.98_213.01	213.013187	5.9774	pos		Trinitrosotrim	HMDB02592	425523	HFWOSHML	Organoheter	Triazinanes	1,3,5-triazina
6.03_199.15	222.14626	6.03235	pos		11-nitro-1-un	HMDB00626	353288	QSFROPSP	Organic 1,3-	Allyl-type 1,3	Organic nitro
6.82_318.14	318.145979	6.81833333	pos		Puquitinib	HMDB0256925		QUTFBURL	Organoheter	Imidazopyrim	Purines and
6.86_225.10	225.109356	6.85513333	pos		3-Methylazel	HMDB0059754		PLOUQIGE	Lipids and lip	Fatty Acyls	Fatty acids a
7.23_491.22	491.223793	7.22931667	neg		Ellipticine	HMDB02517	67470	CTSPAMFJE	Organoheter	Indoles and	Carbazoles
7.51_237.11	237.113258	7.50616667	neg		1-Ethoxy-2-m	HMDB00377	92606	AOSKXPFB	Benzenoids	Phenol ether	Anisoles
7.60_251.08	251.089923	7.59848333	neg		Amdoxovir	HMDB0248284		RLAHNGKR	Organoheter	Imidazopyrim	Purines and
9.37_293.21	293.212247	9.36935	neg		Isoraspailyne B			AAYSBNXVI	Lipids and lip	Glycerolipids	Monoradylgly
9.54_268.20	291.192673	9.54118333	pos		Berkeleylactone L			JVNFUDLQC	Lipids and lip	Fatty Acyls	Fatty esters
1.18_309.10	309.109054	1.18473333	neg		Phenylacetyl	HMDB0256432		IHFRBIZNQI	Organic acid	Carboxylic ac	Amino acids,
1.23_412.11	411.106668	1.23491667	neg		Peperomin E	HMDB0256298		HSEIBOLBS	Organoheter	Benzodioxol	Unclassified
1.25_110.03	110.035172	1.251	pos		1-Methyl-4-ni	HMDB0060659		HSCOLDQJ	Organoheter	Azoles	Imidazoles
1.25_357.06	357.068683	1.2521	neg		FIC	HMDB03033	44300	WTXBRZCV	Organoheter	Indoles and	Indolecarbox
1.40_297.08	297.082432	1.40198333	pos		Fluzinamide	HMDB0252423		YULWJRNIK	Benzenoids	Benzene anc	Trifluorometh
1.45_180.05	180.052566	1.44935	neg		Adenine	HMDB00000	85	GFFGJBXGE	Organoheter	Imidazopyrim	Purines and
1.52_244.98	244.984984	1.52331667	neg		Octafluorocy	HMDB00312	87562	BCCOBQSF	Organohalog	Organofluori	Unclassified
10.24_449.2	449.254474	10.2441667	neg		3alpha-Hydroxy-1,7-dioxo-		84589	IVUZTHWHI	Lipids and lip	Steroids and	Bile acids an

10.35_330.2(	330.20725	10.3534833	neg		Pentazocine	HMDB00147	1751	VOKSWYLN	Alkaloids anc	6,7-benzomc	2,6-dimethyl-
10.57_283.2:	283.22766	10.5716833	neg		3-Methylcycl	HMDB00341	327218	ALHUZKCOI	Organic oxyg	Organooxyge	Carbonyl cor
10.63_586.3(	586.308387	10.6275	pos		Gly-Pro-Gly-/	HMDB0252833		BRENMGSW	Organic acid:	Carboxylic ac	Amino acids,
10.75_209.1!	209.15332	10.7516833	pos		5,9:6,9-Diep	HMDB00385	93202	AKKNWGRL	Organoheter	Oxepanes	Unclassified
10.91_270.3:	270.315102	10.9125667	pos		1-Octadecene		97733	CCCMONHA	Lipids and lip	Fatty Acyls	Hydrocarbon
12.48_269.2:	269.225913	12.4811	pos		Abietinal	HMDB00347	330387	HOFSYSONI	Lipids and lip	Prenol lipids	Diterpenoids
12.57_494.5(	494.565489	12.56865	pos		Monocyclic botryococcane		53759	FQHXBUADI	Lipids and lip	Prenol lipids	Isoprenoids
13.96_202.1(	202.108508	13.9559833	pos		2-Aminodipy	HMDB00297	72966	BUXVIXBGM	Organoheter	Imidazopyrid	Unclassified
14.28_420.4(	420.404486	14.2796333	pos		1-O-(2R-hydroxy-heneicosanyl)-sn-glyc			ZWVPLFSZ:	Lipids and lip	Glycerolipids	Monoradylgly
2.02_319.09(	319.090831	2.02081667	neg		1,6-anhydro-	HMDB0304012		ZFEGYUMHI	Organoheter	Oxepanes	Unclassified
2.04_234.98!	234.989922	2.04021667	pos		Protocatechu	HMDB0124993		GSFKEOSQ	Organic acid:	Organic sulfu	Arylsulfates
2.30_281.14	281.147511	2.30001667	pos		Tyrosyl-Valin	HMDB0029118		OYOQKMOV	Organic acid:	Carboxylic ac	Amino acids,
2.87_149.09(	149.096156	2.8749	pos		2,4,6,8-decatetraenal		75339	ADCGETJXL	Lipids and lip	Fatty Acyls	Fatty aldehy(
3.18_169.08(	169.086117	3.18115	pos		4-Trimethyla	HMDB00013	6179	OITBLCDW›	Organic oxyg	Organooxyge	Carbonyl cor
3.20_161.09(	161.096154	3.1994	pos		3-Methyl-4-pl	HMDB00315	87772	BQJFBHBD(	Benzenoids	Benzene anc	Unclassified
3.56_226.19:	226.191445	3.56223333	pos		Esaprazole	HMDB02519	314167	RTFADALJK	Organic acid:	Carboxylic ac	Amino acids,
3.56_256.16!	256.165709	3.56223333	pos		Secobarbital	HMDB00145	43331	KQPKPCNLI	Organoheter	Diazines	Pyrimidines a
3.59_226.07:	271.069914	3.58845	neg		L-arogenate	HMDB0304400		MIEILDYWG	Organic acid:	Carboxylic ac	Amino acids,
3.73_291.09!	291.098666	3.73108333	neg		Methylpheno	HMDB00149	43243	ALARQZQTE	Organoheter	Diazines	Pyrimidines a
3.84_159.14!	159.149167	3.83913333	pos		Hygrine	HMDB03029	3268	ADKXZIOQK	Alkaloids anc	Unclassified	Unclassified
4.25_174.18!	174.185382	4.25448333	pos		4-Methylnonan-3-one		98151	BYSPXHLWI	Lipids and lip	Fatty Acyls	Oxygenated
4.32_331.15:	331.151069	4.32	neg		Spectinomyc	HMDB00150	2476	UNFWWIHT	Organoheter	Dioxanes	1,4-dioxanes
4.40_340.21!	340.219082	4.3987	pos		Neamine	HMDB0255495		SYJXFKPQI	Organic oxyg	Organooxyge	Carbohydrate
4.43_399.11	399.117302	4.43021667	neg		Benzo[alpha]	HMDB0249001		AXFBAIOSE	Benzenoids	Pyrenes	Unclassified
4.51_331.15:	331.151594	4.50633333	pos		B-Octylglucoside		24055	HEGSGKPQ	Unclassified	Unclassified	Unclassified
4.58_187.06(	187.060939	4.57715	neg		5-ethyl-4-hyd	HMDB0303906		QJYOEDXNI	Organoheter	Dihydrofuran	Furanones
4.67_257.16	257.16485	4.66833333	pos		(-)-Fumigacl	HMDB00302	86792	JUXRVSRUE	Organic acid:	Carboxylic ac	Amino acids,
4.76_162.99!	162.998517	4.76071667	neg		Trifluoroethyl	HMDB02592	336867	MIZLGWKEZ	Organohalog	Vinyl halides	Vinyl fluoride
4.76_226.99	226.994715	4.76071667	neg		urea citrate	HMDB0259715		DZIYQKGGZ	Organic acid:	Carboxylic ac	Tricarboxylic
4.76_385.15(	385.150401	4.76071667	neg		15-Deacetylr	HMDB00361	985297	CSYVMZME	Lipids and lip	Prenol lipids	Sesquiterper
5.05_197.15:	197.15354	5.04685	pos		Buibuilactone		97387	ADZMLQKJF	Lipids and lip	Fatty Acyls	Fatty esters
5.23_257.13!	257.135632	5.22825	pos		Dihydrobiopt	HMDB00000	5106	FEMXZDUTI	Organoheter	Pteridines ar	Pterins and c
5.29_245.04!	245.048903	5.29295	neg		2,2-Difluoro-I	HMDB0253829		VOXUFOPZ(	Organic nitro	Organonitro(	Amines

5.52_182.11	182.117481	5.52015	pos	3-Phenylprop	HMDB00344	89957	AMHRXSOV	Benzenoids	Benzene anc	Unclassified
6.45_247.09	247.093862	6.45406667	pos	Dhelwangin	HMDB00307	87144	AJFJTORMN	Organic oxyg	Organooxyge	Carbonyl cor
6.45_292.12	293.135522	6.45406667	pos	Histidylhistidi	HMDB00288	23880	SGCGMORC	Organic acid	Carboxylic ac	Amino acids,
6.49_227.12	227.128692	6.49351667	neg	Pyroterebic acid		4309	CQJHAULYL	Lipids and lip	Fatty Acyls	Fatty Acids a
6.59_589.16	589.162075	6.58565	neg	Piperolactam	HMDB00385	93250	PHKYZFGDI	Alkaloids anc	Aristolactams	Unclassified
6.68_217.09	217.097952	6.6766	neg	1,2,3,4-Tetra	HMDB00124	437874	CFTOTSJVC	Organoheter	Indoles and c	Pyridoindoles
7.63_200.04	200.047162	7.62578333	pos	3,4-Dihydro-4	HMDB00400	94589	BCWSVRKZ	Organoheter	Heteroaroma	Unclassified
7.71_199.09	199.097362	7.70598333	neg	3-Methylbuty	HMDB00021	6521	ALZLTHLQM	Organoheter	Lactones	Gamma buty
7.87_289.10	289.105765	7.86566667	neg	5'-N-Ethylcar	HMDB0255498		JADDQZYHC	Nucleosides,	Purine nucle	Unclassified
8.08_211.16	211.169056	8.08456667	pos	4-tridecynoic acid		74254	AOYWRTRIE	Lipids and lip	Fatty Acyls	Fatty Acids a
8.55_277.14	277.142338	8.5484	neg	4-Amino-5-hy	HMDB02473	3265	VUTBELPRE	Organoheter	Diazines	Pyrimidines a
8.75_474.30	474.30037	8.74946667	neg	1-(9Z-Nonad	HMDB0243799		QXOXODCL	Lipids and lip	Glycerophos	Glycerophos
8.84_267.19	267.196423	8.83935	neg	Ledol		67892	AYXPYQRX	Unclassified	Unclassified	Unclassified
8.97_177.05	177.054515	8.97426667	pos	10-Hydroxy-2	HMDB00310	87377	AJSNUBLNF	Lipids and lip	Fatty Acyls	Fatty acids a
9.12_221.11	221.114352	9.12051667	pos	(5-Phenyl-1,2,4-triazol-3-yl		70853	RUQGXFJIP	Unclassified	Unclassified	Unclassified
9.41_338.98	338.988354	9.40613333	neg	Dihydroxyace	HMDB00014	6262	GNGACRAT	Organic oxyg	Organooxyge	Carbohydrate
9.51_197.11	197.117057	9.50591667	pos	3-Methyliden	HMDB0302499		BYOAHAKIX	Lipids and lip	Fatty Acyls	Fatty acid es
9.84_279.16	279.160047	9.84338333	neg	(E,E)-7-hydroxy-6-methylh		72003	ARBHCEGTI	Lipids and lip	Fatty Acyls	Fatty alcohol
9.90_137.04	137.045745	9.90308333	pos	2-Hydroxypu	HMDB02451	456454	CRIZPXKIC	Organoheter	Imidazopyrim	Purines and
0.62_351.04	351.049919	0.6215	pos	Diacerein	HMDB02511	44094	TYNLGDBU	Benzenoids	Anthracenes	Anthracenec
0.68_169.03	170.042323	0.68255	pos	Monosodium	HMDB0303331		LPUQAYUQ	Organic acid	Carboxylic ac	Amino acids,
0.69_217.02	217.029433	0.68961667	neg	Cysteinyl-As	HMDB00287	85685	TULNGKSIL	Organic acid	Carboxylic ac	Amino acids,
0.74_191.11	192.12291	0.74288333	pos	N-(2,3-Dihyd	HMDB0254994		XSRKHYQA	Organic acid	Carboxylic ac	Amino acids,
0.83_445.07	445.076748	0.82703333	neg	6-Hydroxytricetin 5-rhamnoside			BVXAICGLD	Lipids and lip	Polyketides	Flavonoids
0.84_164.01	164.011827	0.84308333	neg	2-Amino-4-pl	HMDB0244992		DDOQBQRIE	Organic acid	Carboxylic ac	Amino acids,
0.89_208.98	208.983699	0.8943	neg	3,5-Dinitrosa	HMDB02460	339342	LWFUFLREC	Benzenoids	Phenols	Nitrophenols
0.91_178.00	178.008791	0.9113	pos	Sodium fuma	HMDB03036	439714	MSJMDZAO	Organic acid	Carboxylic ac	Dicarboxylic
1.17_191.07	174.076148	1.1705	pos	2-amino-3,7-	HMDB0304057		DYIXWHPDZ	Organic acid	Carboxylic ac	Amino acids,
1.19_203.08	203.081377	1.1884	pos	VASICINONE	HMDB0259770		SDIVYZXRQ	Organoheter	Diazanaphth	Benzodiazine
1.25_274.08	274.0845	1.2521	neg	Endalin	HMDB00418	96164	QBEIABZPR	Nucleosides,	Pyrimidine nu	Pyrimidine 2'
1.25_365.04	365.049515	1.2521	neg	N-Nitro-N-ph	HMDB0248976		LZGUHMNO	Benzenoids	Benzene anc	Phenylhydra
1.31_269.05	269.055599	1.30545	neg	9-(2-Phosph	HMDB02566	405829	XHXFQGAZ	Organoheter	Imidazopyrim	Purines and
1.36_281.97	281.973856	1.3586	neg	Iodohistamin	HMDB0253529		XOZBOZIQL	Organoheter	Azoles	Imidazoles

1.36_487.12!	487.125807	1.3586	neg	Caryatin gluc	HMDB00373	92283	GXSXKFFH(	Phenylpropa	Flavonoids	Flavonoid gly
1.56_353.07	353.074471	1.55895	pos	[(2R,3S,4R,5	HMDB0255441		JOUIQRNQJ	Nucleosides,	Pyridine nucl	Nicotinic acid
1.56_375.18	375.187607	1.55895	pos	16-dimethylarsinoyl-9Z-hexadecenoic ac			FZOQEVBD(	Lipids and lip	Fatty Acyls	Other Fatty A
1.65_351.13	351.131001	1.65196667	neg	(+/-)-(e)-4-Et	HMDB0242266		BSMGQVOY	Organoheter	Pyridines and	Pyridinecarb
10.23_312.1	312.134329	10.2295	pos	1-(4-Aminopl	HMDB0249842		MJKADKZSY	Organoheter	Benzodiazep	Unclassified
10.23_413.0	413.022088	10.2257833	neg	4-Nitro-3-(trif	HMDB0246527		ZEFMBAFM(	Benzenoids	Phenols	Nitrophenols
10.43_301.2	301.238283	10.42685	neg	4-hexyl-decanoic acid		73623	AZHXWUMC	Lipids and lip	Fatty Acyls	Fatty Acids a
10.54_363.2	363.213826	10.5380833	pos	2-beta-Ethox	HMDB0302962		WZOSFKXV	Lipids and lip	Prenol lipids	Sesquiterper
10.65_124.9	124.999782	10.6454167	pos	phosphonoa	HMDB02564	436116	YEMKIGUKN	Organic acid	Organic phos	Organic phos
10.70_221.1!	221.154403	10.6988167	neg	1-Methyl-2-n	HMDB00618	343758	BNXNQXKAI	Benzenoids	Benzene and	Toluenes
11.17_267.1!	267.157561	11.1685	neg	Sodium dode	HMDB0303651		BTURAGWY	Lipids and lip	Fatty Acyls	Fatty acids a
11.24_347.1	347.171344	11.2413667	neg	oscr#3			CYRZSEWK	Lipids and lip	Fatty Acyls	Fatty acyl gly
11.53_225.0	225.061411	11.52945	neg	alpha-D-Allof	HMDB0304814		AVVWPBAE	Organic oxyg	Organooxyge	Carbohydrate
12.30_283.2	283.242856	12.3037667	neg	18-fluoro-octadecanoic aci		34674	GYUYQQCJ	Lipids and lip	Fatty Acyls	Fatty Acids a
12.48_443.1!	443.150369	12.4754333	neg	Euchrenone b3		47832	AOFNFHUUI	Lipids and lip	Polyketides	Flavonoids
13.19_323.2	323.220097	13.19245	neg	Sodium palm	HMDB0303302		GGXKEBAC	Lipids and lip	Fatty Acyls	Fatty acids a
14.48_622.5	622.524387	14.47795	pos	1-(O-alpha-D-galactopyranosyl)-(1,3R,2			NFLSAIRBII	Lipids and lip	Fatty Acyls	Fatty acyl gly
2.09_445.11	445.119379	2.0924	neg	Benzo[d][2]b	HMDB0251445		CHEIXKXUY	Organoheter	Benzazepine	Unclassified
2.68_116.03!	116.035062	2.68286667	neg	(+)-threo-2-A	HMDB00293	86235	JBNUARFQ(	Organic acid	Carboxylic ac	Amino acids,
2.92_473.07	472.069626	2.92268333	neg	(2,5-Dihydro	HMDB0242489		AROXVHWE	Organoheter	Benzopyrans	1-benzopyra
3.29_244.13	244.130095	3.29291667	neg	Vinyl-L-NIO		62972	LMDRHVQX	Unclassified	Unclassified	Unclassified
3.51_213.03	213.037236	3.50783333	pos	2,4-dihydroxy-2-heptenedi		36007	APNIDHDQY	Lipids and lip	Fatty Acyls	Fatty Acids a
3.59_315.06	315.059981	3.58845	neg	2-Cyano-3-o:	HMDB0257847		NABQBYDEI	Benzenoids	Benzene and	Trifluorometh
3.87_215.08	215.089154	3.87373333	pos	(5S,6R)-Meth	HMDB0247393		RNMFWAFZ	Lipids and lip	Fatty Acyls	Fatty alcohol
4.06_401.15	401.150064	4.06311667	neg	Carbaryl	HMDB02496	1496	CVXBEEMK(	Benzenoids	Naphthalene	Unclassified
4.15_440.25	440.250973	4.14533333	pos	1-Octen-3-yl	HMDB00329	88859	VXNMHZVTI	Lipids and lip	Fatty Acyls	Fatty acyl gly
4.18_302.20	302.207524	4.1814	pos	1-(1-(Tert-Bu	HMDB02475	811391	PJUNYYOGI	Organoheter	Piperidines	Piperidinecar
4.27_249.18!	249.185127	4.27276667	pos	7-Hydroxy-10E-Hexadecen-8-ynoic acid			AGJDSYBSV	Lipids and lip	Fatty Acyls	Fatty Acids a
4.34_227.01	227.012946	4.33765	neg	1-(Difluorom	HMDB0243802		DFRYLFZTE	Benzenoids	Benzene and	Halobenzene
4.36_200.15	223.141754	4.3621	pos	Dimethyl sub	HMDB0251407		FRTGEIHSC	Organic acid	Carboximidic	Imidoesters
4.39_243.02	243.027719	4.39318333	neg	7-Methoxy-4-	HMDB0247269		HAZHUELNI	Phenylpropa	Coumarins a	Unclassified
4.45_213.14	236.137099	4.45335	pos	Propenyl-L-NIO		45344	JUSNBVJTF	Unclassified	Unclassified	Unclassified
4.47_325.05	325.056531	4.46708333	neg	D-Malic acid	HMDB0303755		QVPHNABU	Phenylpropa	Cinnamic aci	Hydroxycinna

4.54_269.12	269.128756	4.5416	pos		1-(2-Benzoyl	HMDB0260229			BCTHZXMP	Benzenoids	Benzene and	Benzophenon
4.61_356.18	356.183258	4.61381667	pos		PC(0:0/6:0)		40359		MYMVJSVDI	Lipids and lip	Glycerophos	Glycerophos
4.61_357.08	357.085905	4.61413333	neg		N1-(5-Phosp	HMDB00038	58181		ZMRGXEJK	Nucleosides,	Benzimidazo	Unclassified
4.65_279.08	279.089743	4.64996667	pos		3-[2-(6-Meth	HMDB0247757			CNNZLFXC	Organoheter	Pyridines and	Methylpyridin
4.65_295.12	295.128894	4.64996667	pos		Lidofenin	HMDB02540	314845		DJQJFMSH	Organic acid	Carboxylic ac	Amino acids,
4.69_230.08	230.082094	4.68661667	pos		2-(Fluoromet	HMDB0248213			NRNSHPCD	Phenylpropa	Phenylpropa	Unclassified
4.74_229.15	229.158653	4.74081667	pos		alpha-Amylci	HMDB00362	91355		CMJSVJIGL	Benzenoids	Benzene and	Unclassified
4.83_197.00	197.002462	4.83273333	neg		7-Aminobenz	HMDB0257745			TVTYWZAU	Organoheter	Benzothiazol	Unclassified
4.89_270.07	270.078067	4.88748333	neg		1-Hydroxy-1	HMDB00329	88882		NHYDBJFG	Organoheter	Quinolines an	Benzoquinoli
4.91_127.07	127.076296	4.90568333	neg		2-[(Tetrahydr	HMDB0252869			CTPDSKVQI	Organoheter	Tetrahydrofu	Unclassified
4.98_247.15	247.155008	4.97913333	neg		3S-hydroxy-undecanoic ac		35680		FARPMBPKI	Lipids and lip	Fatty Acyls	Fatty Acids a
5.01_271.15	271.151513	5.01075	pos		ascr#6.1				IIHKPFPJXR	Lipids and lip	Fatty Acyls	Fatty acyl gly
5.03_345.10	345.108965	5.03453333	neg		1-{2-[(3-Ethyl	HMDB0243809			DZJOJMJM	Organoheter	Pyridines and	Pyridinecarb
5.05_181.12	181.122247	5.04685	pos		trans-Carvyl	HMDB0302762			BPBPWBGJ	Lipids and lip	Prenol lipids	Monoterpeno
5.16_230.98	230.988306	5.16371667	neg		Hexafluorobe	HMDB0253133			ZQBFAOFF	Benzenoids	Benzene and	Halobenzene
5.33_189.01	189.016835	5.32985	neg		Sodium benz	HMDB0303720			WXMKPNIT	Benzenoids	Benzene and	Benzoic acid
5.35_257.00	257.005202	5.3483	neg		(2S)-3,4-Dio	HMDB0258122			GLLOVWGU	Organic acid	Carboxylic ac	Amino acids,
5.39_269.13	269.13569	5.39208333	pos		9H-Purine-9-	HMDB0251658			HNKGMGPC	Organoheter	Imidazopyrim	Purines and
5.40_291.14	291.146149	5.40378333	neg		Benzoyl L-ar	HMDB0249040			KAOMIKSY	Benzenoids	Benzene and	Benzoic acid
5.52_220.16	243.156435	5.52015	pos		Tetra-ethoxy	HMDB0258865			AIZCZOKUS	Organic oxyg	Organooxyge	Ortho esters
5.96_244.09	244.097555	5.95905	pos		Benzoic anh	HMDB0249022			CHIHQLCVL	Benzenoids	Benzene and	Benzyloxycar
6.03_505.20	505.203372	6.0318	neg		6-[(2R,4S,5R	HMDB0260369			DKGALSINF	Organic oxyg	Organooxyge	Carbohydrate
6.09_356.24	356.242755	6.0873	pos		(6E,10E)-3-	HMDB0241252			BIBUSSSIIO	Lipids and lip	Fatty Acyls	Fatty acid es
6.14_273.13	273.134368	6.14265	neg		9-hydroxy-traumatin				ADPDWAFV	Lipids and lip	Fatty Acyls	Fatty Acids a
6.42_245.07	245.079328	6.41976667	neg		N-(2-Pyrimidi	HMDB0252442			ANCSTOBS	Organic nitro	Organonitrog	N-arylamides
6.48_371.24	371.244232	6.47505	neg		2-Methylbuty	HMDB00402	94738		BAAJXLRV	Organic acid	Hydroxy acid	Beta hydroxy
6.77_355.24	355.248946	6.76876667	neg		METHOPRENE (S)		44012		NFGXHKAS	Unclassified	Unclassified	Unclassified
6.84_287.15	287.149982	6.8424	neg		Tetrahydrofu	HMDB00311	87469		AAQDYYFA	Organoheter	Tetrahydrofu	Unclassified
6.88_313.20	313.202345	6.8794	neg		Petroraspailyne B2				CRMYJHMH	Lipids and lip	Glycerolipids	Monoradylgly
6.99_411.11	411.119019	6.98976667	neg		6-iodo-5-hydroxy-eicosa-8		96922		FCRONHVV	Lipids and lip	Fatty Acyls	Eicosanoids
7.42_227.16	227.163933	7.42368333	pos		Dimethyl (3,3	HMDB0247330			AIUDWMLX	Lipids and lip	Fatty Acyls	Fatty acid es
7.45_327.21	327.217668	7.451	neg		12S-hydroxy-16-heptadec		35515		HBFCVDJQ	Lipids and lip	Fatty Acyls	Fatty Acids a
7.46_281.17	281.171954	7.4605	pos		2-methyl-tridecanedioic ac		74906		BNDKRNYI	Lipids and lip	Fatty Acyls	Fatty Acids a

7.56_371.24:	371.243943	7.5615	neg	Ceriporic acid A		45935	CJCOOGIIQ	Lipids and lip	Fatty Acyls	Fatty Acids a
7.83_159.10:	159.102444	7.82873333	neg	(±)-2-Methylb	HMDB00401	94726	BHVGMUDW	Organic oxyg	Organooxyge	Carbonyl cor
8.38_197.078	197.078073	8.37785	pos	Isoxanthopte	HMDB00007	44874	GLKCOBIIZK	Organoheter	Pteridines an	Pterins and c
9.17_295.19:	295.191441	9.168	neg	3E,8Z,11Z-Tetradecatrieny		46305	HWPJPNQE	Lipids and lip	Fatty Acyls	Fatty esters
9.55_297.207	297.207144	9.55203333	neg	3Z,9Z-hexadecadienoic ac		34935	AKJLYECXIE	Lipids and lip	Fatty Acyls	Fatty Acids a
9.63_351.21:	351.213822	9.63246667	pos	Arginyl-prolyl	HMDB0249363		YCYXHLZRU	Organic acids	Carboxylic ac	Amino acids,
9.68_251.20:	251.201498	9.68	neg	7,11-Dimethyl-3-methylene		97505	APAPGJJZP	Lipids and lip	Fatty Acyls	Hydrocarbon
9.77_307.19:	307.191333	9.77068333	neg	(2-Hydroxymethyl-cyclohe:		97379	AYMWCZFE	Lipids and lip	Fatty Acyls	Fatty esters
9.88_167.07(	167.070176	9.88461667	pos	Phosphorylch	HMDB00015	6326	YHHSONZF(	Organic nitro	Organonitrog	Quaternary a
9.95_452.998	452.99815	9.95315	neg	Nitroglycerin	HMDB00148	1578	SNIOPGDIG	Organic oxyg	Organic oxoa	Organic nitra
0.67_248.05(	271.040092	0.66691667	pos	Sodium gluc	HMDB0303297		FMYOMWC(	Organic oxyg	Organooxyge	Carbohydrate
0.70_148.05(	149.063716	0.69861667	pos	3-Mercapto-3	HMDB00361	91342	VTAPYUYITI	Organic acids	Carboxylic ac	Carboxylic ac
0.70_597.097	597.096974	0.70456667	neg	Phosphocrea	HMDB0256473		WORFGIDBI	Organic acids	Carboxylic ac	Amino acids,
0.76_150.997	150.997801	0.75766667	pos	Hexafluoroiso	HMDB00606	2423	BYEAHWXP	Organohalog	Halohydrins	Fluorohydrins
0.89_593.19:	593.193565	0.8943	neg	(S)-Annocher	HMDB00387	93378	XWZBLZPLF	Organoheter	Isoquinolines	Benzylisoquir
1.10_424.13(	424.136041	1.09681667	neg	Skepinone-L	HMDB0258326		HXMGCTFLI	Benzenoids	Dibenzocyclo	Unclassified
1.17_272.96(	272.960568	1.16698333	neg	Ribose-1-ars	HMDB00122	62913	RYJJOMQP/	Organic oxyg	Organooxyge	Carbohydrate
1.34_793.279	793.2799	1.34166667	neg	Radiprodil	HMDB0257096		GKGRZLGA(	Organoheter	Piperidines	Benzylpiperic
1.39_547.967	547.967575	1.39358333	neg	Difluorodeox	HMDB0251267		UWXMJKZO	Nucleosides,	Pyrimidine nu	Pyrimidine de
1.97_279.098	279.098488	1.96566667	neg	2-Methoxyida	HMDB0245206		HQGWKNG/	Organoheter	Benzodioxan	Benzo-1,4-di
10.23_656.3:	656.318391	10.2257833	neg	OKOOA-PS			JWSLONXE(	Lipids and lip	Glycerophos	Oxidized glyc
10.34_195.1:	195.138765	10.3358333	neg	Aleprylic acid		34856	GMYNCKRS	Lipids and lip	Fatty Acyls	Fatty Acids a
10.43_584.2!	584.251041	10.42685	neg	Nintedanib	HMDB02556	96478	CPMDPSXJE	Organoheter	Indoles and (	Indolecarbox
10.81_640.2	640.245694	10.8069333	neg	Taurocholic acid 3-sulfate		57982	LKTXOQJJF	Lipids and lip	Steroids and	Steroid conju
11.20_278.1!	301.140548	11.198	pos	Monooctyl ph	HMDB02548	328993	PKIYFBICNI(	Benzenoids	Benzene anc	Benzoic acid
11.49_304.2(	305.26814	11.4858667	pos	1-O-(2R-hydroxy-tetradecyl)-sn-glycerol			NWPUZHCK	Lipids and lip	Glycerolipids	Monoradylgly
11.64_418.2!	418.29616	11.6371833	neg	Arachidonoylmorpholine		36720	RUKZVIXSX	Lipids and lip	Fatty Acyls	Fatty amides
12.30_467.1!	467.150193	12.3037667	neg	Dichamanetin			WBZSDBHJ	Lipids and lip	Polyketides	Flavonoids
12.41_367.2	367.24334	12.4138167	neg	1,4-Dimethyl-	HMDB00364	41200	GXGJIOMUZ	Lipids and lip	Prenol lipids	Sesquiterper
12.64_418.2	418.280927	12.6402	neg	3-hydroxytrid	HMDB0061639		DOFFCMLM	Lipids and lip	Fatty Acyls	Fatty acid es
12.81_194.9!	194.988148	12.8107167	neg	Hexafluoropr	HMDB0253134		HCDGVLDPI	Organohalog	Vinyl halides	Vinyl fluoride
2.02_271.26:	271.263171	2.02305	pos	Pentadecyl acetate		341759	AAAIZLQILB	Lipids and lip	Fatty Acyls	Fatty esters
2.11_444.237	444.237841	2.10798333	pos	Kushenol T		52607	PMFICSJNA	Lipids and lip	Polyketides	Flavonoids

2.26_219.07	219.077303	2.2575	neg	1-Oxo-2,3-dil	HMDB0243983		ATXHUWAE	Benzenoids	Indanes	Indanones
2.28_267.09	267.097597	2.28218333	pos	Macromomy	HMDB0254293		HXKWEZFTI	Organoheter	Benzoxazine	Benzoxazino
2.68_281.14	281.149671	2.67816667	pos	4-(1,3-Dihydr	HMDB0244875		JBDLPHRPJ	Organoheter	Azolidines	Imidazolidine
2.87_135.04	135.044181	2.8749	pos	2-METHYL-3-HYDROXYE		44405	AFMHCDNE	Unclassified	Unclassified	Unclassified
2.87_135.11	135.116942	2.8749	pos	(S)-Phelland	HMDB00356	90877	AEVLWICM	Lipids and lip	Prenol lipids	Monoterpeno
3.22_167.03	167.034085	3.21716667	pos	Methyl 2,4,6-	HMDB00296	347537	AQDIJIAUY	Benzenoids	Benzene and	Benzoic acid
3.46_323.08	323.088344	3.45915	neg	4'-Nitropheny	HMDB0246183		OMRLTNCL	Organic oxyg	Organooxyge	Carbohydrate
3.80_323.12	323.128037	3.80496667	neg	3-(4-Methoxy	HMDB00326	68348	AXCXHFKZH	Phenylpropa	Cinnamaldeh	Unclassified
3.89_209.12	209.126106	3.8922	pos	N-(5-ACETAMIDOPENTY		985531	FQKKPLXFG	Unclassified	Unclassified	Unclassified
3.97_337.06	337.065372	3.97085	neg	2-hydroxyflut	HMDB00609	674	YPQLFJODE	Benzenoids	Benzene and	Trifluorometh
4.11_174.07	192.105418	4.10951667	pos	4,4'-Thiobis-2	HMDB00371	92123	CIBUHUTVS	Organic oxyg	Organooxyge	Carbonyl con
4.15_331.16	331.165455	4.14533333	pos	Enalaprilat	HMDB00418	2533	LZFZMUME	Organic acids	Carboxylic ac	Amino acids,
4.16_341.18	341.180722	4.163	pos	Valaciclovir	HMDB0014716		HDOVUKNU	Organic acids	Carboxylic ac	Amino acids,
4.18_223.09	223.094111	4.1814	pos	3,6-dioxo-decanoic acid		74717	AEVOBXGBI	Lipids and lip	Fatty Acyls	Fatty Acids a
4.31_272.06	272.068548	4.30826667	pos	1-(1,3-Benzo	HMDB0243831		BXBNADAPI	Organoheter	Piperidines	N-acylpiperid
4.69_253.10	253.10458	4.68661667	pos	6-Succinoam	HMDB0013204		PQBHLXAM	Organoheter	Imidazopyrim	Purines and
4.78_213.07	213.074067	4.77798333	neg	5-(3-Methyl-1	HMDB02549	628	MVBPAIHFZ	Organic acids	Carboxylic ac	Carboxylic ac
4.88_255.12	255.120072	4.88355	pos	2-Amino-6-(2	HMDB0257758		HKMZFFKYI	Organoheter	Pteridines an	Pterins and c
4.91_335.03	335.038203	4.90568333	neg	D-Sedohept	HMDB0001068		CBIDVWSRU	Organic oxyg	Organooxyge	Carbohydrate
5.12_317.19	317.193276	5.11926667	pos	Thiazinamiur	HMDB0240235		CDXCCYNIN	Organoheter	Benzothiazin	Phenothiazin
5.47_223.07	241.104285	5.46526667	pos	Succinylamir	HMDB0258547		SEXVKBABC	Organic acids	Carboxylic ac	Carboxylic ac
5.50_240.18	263.172866	5.5019	pos	Cropropamid	HMDB0250543		CYZWCBZIE	Organic acids	Carboxylic ac	Amino acids,
5.58_223.16	223.169062	5.57501667	pos	Isokobusone	HMDB00367	44491	BSFUDCIRZ	Organic oxyg	Organooxyge	Alcohols and
5.58_553.18	553.189001	5.57501667	pos	2-O-(beta-D-galactopyranosyl-(1->6)-be			WPSTUWW	Lipids and lip	Fatty Acyls	Fatty acyl gly
5.68_308.21	331.208637	5.68461667	pos	1,2,3-Tris(1-e	HMDB00371	92130	NSVOKCWM	Lipids and lip	Glycerolipids	Unclassified
5.81_403.08	403.088505	5.80983333	neg	Dihydrocaffei	HMDB00417	827479	AELQNMHO	Organic oxyg	Organooxyge	Carbohydrate
5.83_375.23	375.234959	5.83081667	pos	E-64		44981	LTLYEAJON	Unclassified	Unclassified	Unclassified
5.87_226.14	226.144681	5.8654	neg	N-Ethyl trans	HMDB0032273		ARSJQOAY	Lipids and lip	Fatty Acyls	Fatty amides
5.92_323.01	323.013405	5.92093333	neg	N-Nitrosothia	HMDB00401	94727	LGLMHMRN	Organic acids	Carboxylic ac	Amino acids,
6.29_439.21	439.213312	6.29061667	neg	cis-3-Hexeny	HMDB0061942		IEPWIPZLLI	Benzenoids	Benzene and	Benzoic acid
6.66_351.21	351.217605	6.65806667	neg	Methyl 8-hydroxy-11E,17-Octadecadien			FSRPQTYQ	Lipids and lip	Fatty Acyls	Fatty Acids a
6.81_170.98	170.985174	6.80563333	neg	5-Chlorosalic	HMDB02467	336569	NKBASRXW	Benzenoids	Benzene and	Benzoic acid
7.15_184.16	184.169511	7.14843333	pos	2-Heptylfurar	HMDB00361	348715	BHTUFJXTY	Organoheter	Heteroaroma	Unclassified

7.35_239.12	239.125022	7.35016667	pos	2'-Deoxysepi	HMDB00003	5378	MXQYRFPIG	Organoheter	Pteridines an	Pterins and d
7.39_415.15	415.152115	7.38693333	pos	12alpha-Hyd	HMDB00395	94116	CKRHSGZM	Lipids and lip	Prenol lipids	Terpene lacto
7.99_300.21	300.217912	7.99473333	neg	N-Lauroyl Th	HMDB0242041		BHPUZXHJE	Organic acids	Carboxylic ac	Amino acids,
8.14_301.11	301.119345	8.14243333	neg	Ancymidol	HMDB02483	72544	HUTDUHSN	Benzenoids	Phenol ether	Anisoles
8.19_502.29	502.292543	8.19463333	pos	LysoPA(22:5	HMDB0114753		BAJTXVWX	Lipids and lip	Glycerophos	Glycerophos
8.30_223.06	223.06342	8.3042	pos	Harmalan	HMDB00298	86538	CWOYLIJQL	Alkaloids and	Harmala alka	Unclassified
8.38_255.23	255.231471	8.37785	pos	(Z)-7-Dodecenyl butyrate		97243	ATOKUZXHI	Lipids and lip	Fatty Acyls	Fatty esters
8.45_452.16	475.157115	8.4515	pos	Cichorioside	HMDB0302056		PIBJAPSKIW	Organic oxyg	Organooxyge	Carbohydrate
8.88_199.13	199.132257	8.88258333	pos	N,N'-Bis(2-ar	HMDB02466	350169	UWMHHZFH	Organic nitro	Organonitrog	Amines
8.91_322.99	322.993643	8.91221667	neg	2-C-Methyl-D-erythritol 2,4		64016	SFRQRNJMI	Unclassified	Unclassified	Unclassified
8.95_295.10	295.108734	8.94916667	neg	Methaqualon	HMDB02402	66682	JEYCTXHKT	Organoheter	Diazanaphtha	Benzodiazine
8.95_303.00	303.007121	8.94916667	neg	Ethylene glyc	HMDB0252070		UQXKXGWC	Organic oxyg	Organic oxoa	Organic nitra
8.97_199.13	199.133757	8.96598333	neg	9-Decenal	HMDB00348	326816	AKMSQWLD	Organic oxyg	Organooxyge	Carbonyl cor
9.16_179.15	179.154085	9.15776667	pos	4-[(2S)-2-Am	HMDB0246317		ADMXVTGQ	Benzenoids	Benzene and	Phenethylam
9.29_295.15	295.151326	9.28653333	pos	Entecavir	HMDB00145	315023	QDGZDCVA	Nucleosides,	Nucleoside a	Unclassified
9.37_269.21	269.212136	9.36935	neg	7-Ethyl-4-tridecen-6-one		98252	AXQLANMO	Lipids and lip	Fatty Acyls	Oxygenated
9.46_379.05	379.057574	9.46038333	neg	(2s)-7-Tert-B	HMDB0260149		QGCKNIAM	Organoheter	Benzopyrans	1-benzopyran
9.60_433.25	433.256731	9.59576667	pos	N-Methylphe	HMDB0255190		LVCPRLFCF	Organic acids	Carboxylic ac	Amino acids,
9.74_298.14	299.148461	9.74061667	pos	Toxin T2 tetr	HMDB00361	91310	ZAXZBJSXS	Lipids and lip	Prenol lipids	Sesquiterper
9.85_405.26	405.260725	9.84861667	pos	urapidil	HMDB02597	44217	ICMGLRUYE	Organoheter	Diazinanes	Piperazines
9.88_269.21	269.210731	9.88461667	pos	N-(3-Aminop	HMDB0258032		NSLRHHMD	Organic nitro	Organonitrog	Amines
0.63_368.99	368.999161	0.62835	neg	Sedoheptulo	HMDB00602	156	OKHXOUGR	Organic oxyg	Organooxyge	Carbohydrate
0.64_141.93	180.894526	0.63663333	pos	Sodium sulfa	HMDB0303546		PMZURENO	Mixed metal/	Alkali metal c	Alkali metal s
0.70_110.98	110.985046	0.70456667	neg	Methylphosp	HMDB0061711		CAAULPUQI	Organic acids	Organic phos	Phosphate es
0.70_220.05	203.052598	0.69861667	pos	2-(2-Thienylr	HMDB00348	90249	LLZQYUSTI	Organic oxyg	Organooxyge	Ethers
0.73_121.07	121.071941	0.72825	pos	Carbamoylfo	HMDB0249609		ILTVGYDBX	Organic 1,3-c	Propargyl-typ	Unclassified
0.79_205.10	205.100441	0.7877	pos	(2R)-2-Amino	HMDB0242762		GFOGRACH	Organic acids	Carboxylic ac	Amino acids,
0.79_254.12	254.12453	0.7877	pos	Didanosine	HMDB00150	1998	BXZVVICBK	Nucleosides,	Purine nucle	Purine 2',3'-d
0.79_313.05	313.052152	0.7877	pos	N-(2-Hydroxy	HMDB0247709		HNBOCTKS	Organoheter	Isoquinolines	Unclassified
0.82_220.03	220.036943	0.81883333	pos	Benzoyl phos	HMDB00604	359001	SYLLWWIX	Benzenoids	Benzene and	Benzoic acid
0.83_400.00	400.002575	0.82703333	neg	thiamphenico	HMDB0258972		OTVAEFIXJI	Benzenoids	Benzene and	Benzenesulfo
0.85_260.97	260.972382	0.84881667	pos	Perflubutane	HMDB02509	336810	KAVGMUDT	Organohalog	Organofluori	Unclassified
0.91_336.97	336.970407	0.91118333	neg	Perflexane	HMDB02563	315350	ZJIJAJXFLBI	Organohalog	Organofluori	Unclassified

1.07_187.04	187.041676	1.06741667	neg		Bisazobipher	HMDB0249265		FBIBGSLAW	Benzenoids	Benzene anc	Unclassified
1.10_545.10	545.099977	1.09681667	neg		alpha-D-Gala	HMDB00397	94302	LCLHHZYHL	Organic oxyg	Organooxyge	Carbohydrate
1.20_552.18	551.17804	1.20271667	neg		(Z)-Resverat	HMDB00399	94453	YGQPMDDX	Phenylpropa	Stilbenes	Stilbene glyc
1.22_551.04	551.042921	1.21911667	neg		6-Phosphogl	HMDB00013	367	BIRSGZKFK	Organic oxyg	Organooxyge	Carbohydrate
1.27_348.07	348.070105	1.26745	pos		2'-Deoxyguai	HMDB0001044		LTFMZDNNI	Nucleosides,	Purine nucle	Purine deoxy
1.27_413.00	413.003844	1.26985	neg		Alginic acid	HMDB00299	86604	FHVDTGUD.	Organic oxyg	Organooxyge	Carbohydrate
1.31_461.09	461.090384	1.30545	neg		farnesyl triphosphate		53847	QIOOKVHMI	Lipids and lip	Prenol lipids	Isoprenoids
1.49_175.08	175.086629	1.48828333	pos		1-nitrosonapl	HMDB00621	453602	HXOPMUIUI	Benzenoids	Naphthalene	Unclassified
1.51_113.02	113.023793	1.50628333	pos		3-amino-prop	HMDB0062210		PCXDJQZLD	Organic oxyg	Organooxyge	Carbonyl cor
1.60_361.12	361.125216	1.59701667	neg		Egtazic acid	HMDB0251718		DEFVIWRAS	Organic acid	Carboxylic ac	Tetracarboxy
1.95_163.11	163.111818	1.95213333	pos		4-(4-Methylpl	HMDB00361	91332	BUIRDOGD(	Benzenoids	Benzene anc	Toluenes
10.25_449.3	449.317468	10.24785	pos		Calysterol		41799	CPOLWWXI	Lipids and lip	Steroids and	Sterols
10.28_628.3	628.362082	10.2812167	neg		PKOOA-PC			VZSPQCMFI	Lipids and lip	Glycerophos	Oxidized glyc
10.32_812.5	812.544593	10.31745	neg		PE-NMe2(18	HMDB0114129		ADVITIDKLX	Lipids and lip	Glycerophos	Glycerophos
10.45_344.2	367.244972	10.4474333	pos		1-O-Myristoy	HMDB0249917		DUCVQOXJ	Lipids and lip	Glycerolipids	Diradylglycer
10.59_402.3	402.393941	10.5914167	pos		3-hydroxy-tetracosanoic a		74591	DVDLWGAA	Lipids and lip	Fatty Acyls	Fatty Acids a
10.95_269.2	269.208676	10.9478667	pos		8,8-Diethoxy	HMDB00345	90022	XOJDKWNF	Organic oxyg	Organooxyge	Alcohols and
11.08_593.3	592.362026	11.07825	neg		PC(16:0/5:0(CHO))		45370	RKIDALSAC	Lipids and lip	Glycerophos	Oxidized glyc
11.50_128.1	128.118214	11.5038	pos		3-Propyl-1H-	HMDB0245977		QBNRQCHC	Organohetero	Azoles	Pyrazoles
11.62_349.2	349.274654	11.6192	neg		pregnan-21-ol		57868	DBMUNIJZU	Lipids and lip	Steroids and	Steroids
11.93_818.5	818.596671	11.9300167	pos		DG(LTE4/22	HMDB0296716		BIQUGRLMI	Unclassified	Unclassified	Unclassified
11.97_650.4	650.439031	11.9664167	pos		1-(2-methoxy-5Z,9Z-hexacosadienyl)-sn			GGQRNAKF	Lipids and lip	Glycerophos	Glycerophos
12.30_463.2	463.207712	12.3037667	neg		Acetamide, 2	HMDB0259996		RVMBDLSFI	Benzenoids	Phenol ether	Unclassified
12.65_401.2	401.290512	12.65015	neg		3-(2-Hepteny	HMDB00340	89668	AQZFORIM(	Lipids and lip	Glycerolipids	Diradylglycer
12.89_346.3	369.296978	12.8871833	pos		1-O-(2R-methoxy-hexadecyl)-sn-glycer			LMVRXHKE	Lipids and lip	Glycerolipids	Monoradylgly
13.05_107.9	107.967071	13.0475833	pos		Sodium nitra	HMDB0303521		VWDWKYIA	Mixed metal/	Alkali metal c	Alkali metal r
13.21_395.1	395.150292	13.21015	neg		HARMOL		43883	LBBJNGFCX	Unclassified	Unclassified	Unclassified
13.40_321.2	321.239614	13.4006833	pos		1-[(5-Methox	HMDB0260241		IIRCVWRUK	Organohetero	Indoles and c	Indolines
13.60_407.1	407.189281	13.59865	neg		N-(4-Fluorop	HMDB0244857		LECZXZOBE	Organohetero	Tetrahydrois	Unclassified
13.73_403.3	403.306215	13.7268833	neg		MG(i-18:0/0:	HMDB0072842		ASKIVFGGC	Lipids and lip	Glycerolipids	Monoradylgly
14.28_409.2	409.204826	14.2783333	neg		Famitinib	HMDB0252164		GKEYKDOLI	Organohetero	Pyrrolopyridi	Unclassified
14.42_446.4	446.419817	14.4236667	pos		1-O-(2R-methoxy-4Z-docosenyl)-sn-glyc			KDTIXNAEP	Lipids and lip	Glycerolipids	Monoradylgly
2.09_394.97	394.979262	2.0924	neg		Dihydroxyace	HMDB00117	62468	IWFZHMQFZ	Organic oxyg	Organooxyge	Carbonyl cor

2.24_249.08	294.084399	2.23906667	neg		2',3'-Didehyd	HMDB0245542			FZYYPNOH	Organoheter	Imidazopyrim	Purines and
2.34_177.06	177.063519	2.33636667	pos		Methylene bi	HMDB0254632			FIKFOOMAL	Organic acid	Carboxylic ac	Carboxylic ac
2.50_249.08	249.084544	2.49885	pos		Porphobilino	HMDB00002	337525		QSHWIQZF	Organic nitro	Organonitrog	Amines
2.52_202.14	202.143847	2.51663333	pos		(S)-Oleurope	HMDB00369	91982		BFYWJELX	Lipids and lip	Prenol lipids	Monoterpenc
2.98_218.07	218.078821	2.98328333	pos		alpha-Methyl	HMDB02482	69236		CAHPJJJZL	Phenylpropa	Phenylpropa	Unclassified
3.09_277.11	277.119176	3.08933333	neg		Ethyl L-trypt	HMDB0252047			DABYEOZX	Organic acid	Carboxylic ac	Amino acids,
3.11_137.09	137.096229	3.1093	pos		2-Ethyl-4-me	HMDB00389	93595		AVVVXUXM	Benzenoids	Phenols	Cresols
3.45_424.21	424.219398	3.45331667	pos		3-N-(2-Fluor	HMDB0245941			MNARAEXG	Organic oxy	Organooxyge	Carbonyl cor
3.49_366.20	366.206583	3.48938333	pos		6-O-Desmet	HMDB00139	85321		DJRBBQJRE	Organoheter	Piperidines	Benzylpiperic
3.73_244.08	262.118769	3.73238333	pos		Indolylacrylo	HMDB0006005			DUIFVCFSA	Organic acid	Carboxylic ac	Amino acids,
3.82_250.14	250.143982	3.82191667	pos		6-hydroxytetradeca-7,8-dien-10,12-diyn				AVFVLLCW	Lipids and lip	Fatty Acyls	Fatty Acids a
3.91_198.05	198.056244	3.91066667	pos		2-Amino-3-(2	HMDB0247060			AIDLBEYXQ	Organic acid	Carboxylic ac	Amino acids,
3.93_231.15	254.147675	3.92891667	pos		gamma-Amir	HMDB00019	6403		OCBQYJFU	Organic acid	Peptidomime	Hybrid peptic
3.93_567.31	567.31462	3.92891667	pos		Cholic acid g	HMDB00025	57962		RBLDVEUU	Lipids and lip	Steroids and	Steroidal gly
3.97_152.04	152.046333	3.97085	neg		1H-Imidazo[1	HMDB0244874			MFGQIJCM	Organoheter	Azoles	Imidazoles
4.14_380.05	380.055848	4.13528333	neg		2-Methyl-N-(	HMDB0257580			SPTMROWF	Organoheter	Benzothiazin	Unclassified
4.24_158.09	158.096587	4.23603333	pos		4,8-Dimethyl	HMDB0302402			DULGUAMZ	Organoheter	Quinolines ar	Unclassified
4.27_213.10	213.109726	4.27276667	pos		6-hydroxyme	HMDB03042	3335		CQQNNQTX	Organoheter	Pteridines ar	Pterins and c
4.32_130.99	130.992375	4.32	neg		Pentafluorop	HMDB0256268			COWKRCC	Organohalog	Halohydrins	Fluorohydrin
4.34_279.03	279.030624	4.33765	neg		Bowdichione		48294		GCAIEHBYL	Lipids and lip	Polyketides	Flavonoids
4.43_377.13	377.135382	4.43021667	neg		17-Iodohepta	HMDB0244775			UBQLJCFBC	Lipids and lip	Fatty Acyls	Fatty acids a
4.44_210.05	233.042296	4.435	pos		Curvulinic acid; (2-Acetyl-3		985091		JAHPPWGW	Unclassified	Unclassified	Unclassified
4.52_212.97	212.979062	4.52246667	neg		Heptafluorob	HMDB02531	336951		YPJUNDFVD	Organohalog	Alkyl halides	Alkyl fluoride
4.58_212.99	212.997868	4.57715	neg		Pentafluorob	HMDB02562	336888		WACNXHCZ	Benzenoids	Benzene anc	Halobenzene
4.65_253.06	253.069293	4.65108333	neg		2-Methyl-5-ni	HMDB02452	340911		FFYTTYVSD	Organoheter	Azoles	Imidazoles
4.69_329.12	329.124084	4.6877	neg		3-carboxy-4-methyl-5-(4-hydroxypentyl)				AVEHUFGE	Lipids and lip	Fatty Acyls	Fatty Acids a
4.69_349.13	349.139507	4.68661667	pos		2-iodopalmitaldehyde				HMBUZXLD	Lipids and lip	Fatty Acyls	Fatty aldehy
4.83_156.01	156.012352	4.83273333	neg		4-Mercaptop	HMDB0246488			FHTDDANQ	Organoheter	Pyridines anc	Hydropyridin
4.98_261.13	261.134235	4.97913333	neg		Homononactinic acid; (R)-		985176		HTCUURQJ	Unclassified	Unclassified	Unclassified
4.98_453.19	453.193056	4.97913333	neg		6-beta-hydro	HMDB0061022			RVBSTEHLL	Lipids and lip	Steroids and	Hydroxystero
5.00_200.12	200.129017	4.99758333	neg		2,2,6,6-Tetra	HMDB00311	87472		JWUXJYZV	Organoheter	Piperidines	Piperidinone
5.05_225.06	225.069608	5.05298333	neg		Cyclosarin	HMDB02506	984326		SNTRKUOV	Organic acid	Organic phos	Phosphonic a
5.21_531.24	531.248369	5.20995	pos		Calcitriol pho	HMDB0249549			PRJNGGUU	Lipids and lip	Steroids and	Vitamin D an

5.58_261.06	262.074092	5.57501667	pos	Diethylamino	HMDB0257905		SJXACOOQ	Benzenoids	Benzene anc	Benzenesulf
5.64_385.22	385.223255	5.64363333	neg	Berkeleylactone M			GUYSGRRZ	Lipids and lip	Fatty Acyls	Fatty esters
5.85_189.11	189.112921	5.84688333	neg	Hexyl acetat	HMDB00299	46183	AOGQPLXW	Organic acid	Carboxylic ac	Carboxylic ac
5.85_217.08	217.080981	5.84918333	pos	Glutamine la	HMDB0252811		OEHVJXBNI	Organic acid	Carboxylic ac	Amino acids,
6.03_432.22	432.223888	6.0318	neg	3-Hydroxydo	HMDB0241231		AANSXJATF	Lipids and lip	Fatty Acyls	Fatty acid es
6.11_195.13	195.137878	6.10565	pos	(S)-p-Mentha	HMDB00370	92001	BCTDJPZNM	Lipids and lip	Prenol lipids	Monoterpenc
6.14_255.11	255.112627	6.1424	pos	(S)-MHD	HMDB0060702		BMPDWHID	Organoheter	Benzazepine	Dibenzazepir
6.46_367.21	367.212569	6.45656667	neg	(4S,8R)-8,9-I	HMDB0039052		AOKPDATZI	Lipids and lip	Prenol lipids	Monoterpenc
6.60_195.10	195.102696	6.60338333	neg	2,6,6-Trimetl	HMDB00351	90499	AXVHXJQW	Organic oxyg	Organic oxid	Unclassified
7.08_227.16	227.163817	7.07501667	pos	norspermine	HMDB03044	349954	ZAXCZCOUI	Organic nitro	Organonitrog	Amines
7.13_221.11	221.114332	7.13003333	pos	N-[(2Z)-2-Me	HMDB0258951		KWKJDWTY	Organoheter	Imidazopyrim	Purines and
7.40_251.09	251.092351	7.39546667	neg	(2RS,5RS)-(	HMDB00319	88084	AWGIRTJPI	Benzenoids	Benzene anc	Styrenes
7.63_210.11	233.104707	7.62578333	pos	N-ethyl phen	HMDB0255130		BYFDOJZLC	Organoheter	Diazanaphth	Benzodiazine
7.85_149.02	149.024191	7.84718333	neg	Orsellinic acid		41017	AMKYESDO	Lipids and lip	Polyketides	Aromatic poly
7.94_255.12	255.121302	7.93951667	neg	Decarbamoy	HMDB0038319		VRRIYZJUSI	Phenylpropa	Saxitoxins, g	Unclassified
8.05_149.02	149.024247	8.05003333	neg	N-dimethylet	HMDB0304425		XNORYOIO	Organic acid	Organic phos	Phosphate e
8.45_583.25	583.254486	8.4515	pos	(3Z)-phytoch	HMDB0303975		DKMLMZVD	Organoheter	Tetrapyrroles	Bilirubins
8.62_367.24	367.245335	8.61701667	pos	Siegesbeckin A			CKRCOYVD	Lipids and lip	Fatty Acyls	Fatty esters
8.67_213.14	213.149457	8.67263333	neg	10-Undecen-	HMDB00335	89386	FJHNSGVMI	Organic oxyg	Organooxyge	Carbonyl cor
8.85_223.09	223.096345	8.8465	pos	Vanillin isob	HMDB00376	317361	BGKAKRUFI	Benzenoids	Phenol ester	Unclassified
8.90_254.24	254.247393	8.90103333	pos	5-Cyclohexadecen-1-one		317173	ABRIMXGLN	Unclassified	Unclassified	Unclassified
8.98_463.04	463.040031	8.98443333	neg	6-Cyano-7-ni	HMDB02503	69622	RPXVIAFEQ	Organoheter	Diazanaphth	Benzodiazine
9.07_262.14	262.143369	9.0656	pos	3-(1,1-Dimetl	HMDB00307	451758	BTXKAWICC	Phenylpropa	Coumarins a	Unclassified
9.21_307.18	307.187633	9.21266667	pos	6,7-Dimetho	HMDB02471	2460	APKHJGDG	Organoheter	Diazinanes	Piperazines
9.28_145.08	145.086833	9.2775	neg	1-Hexen-3-ol	HMDB00315	87722	BVOSSZSHI	Organic oxyg	Organooxyge	Alcohols and
9.40_402.32	402.320889	9.39628333	pos	5-Hydroxype	HMDB0241434		FBGNJEDM	Lipids and lip	Fatty Acyls	Fatty acid es
9.47_324.28	324.286837	9.46953333	pos	Enigmol		53958	CILLLUONW	Lipids and lip	Sphingolipids	Sphingoid ba
9.51_285.10	285.106959	9.50591667	pos	Phosphatidyl	HMDB0302468		GDQGIGPG	Lipids and lip	Glycerophos	Glycerophos
9.57_511.02	511.02048	9.56983333	neg	4-Methylumb	HMDB02465	348000	BCHIXGBGI	Phenylpropa	Coumarins a	Unclassified
9.61_364.01	409.011053	9.60636667	neg	3,3,4,4,5,5,6,	HMDB0260260		GRJRKPMIF	Organohalog	Alkyl halides	Alkyl fluoride
0.61_485.19	485.198826	0.61033333	neg	Glucosylgala	HMDB00005	5567	UTIRJVJBK	Lipids and lip	Fatty Acyls	Fatty acyl gly
0.72_132.04	113.024216	0.71928333	neg	2-C-Methyl-1	HMDB00298	86563	OHTGZAWF	Organoheter	Lactones	Gamma buty
0.72_455.09	455.095345	0.71928333	neg	L-2-Aminoetl	HMDB0031950		UQDJGEHQ	Organic acid	Carboxylic ac	Amino acids,

0.73_370.08	370.080949	0.7342	neg	(2R)-3-(4-Cy	HMDB0242725		JNGVJMBLX	Benzenoids	Benzene anc	Trifluorometh
0.75_429.02	429.027755	0.74898333	neg	1,3,5-Trinitro	HMDB0255343		IAKZRHCDC	Organic 1,3-(	Allyl-type 1,3	Organic nitro
1.08_567.20	567.203704	1.08208333	neg	Glypallichalcone		52235	CTBBBAQH	Lipids and lip	Polyketides	Flavonoids
1.11_282.09	282.098184	1.1137	neg	L-Glutamic a	HMDB0250759		BGGHCRNC	Organic acid	Carboxylic ac	Amino acids,
1.15_365.01	365.01004	1.14921667	neg	4-Fluoro-7-ni	HMDB0246422		PGZIDERTD	Organoheter	Benzoxadiaz	Unclassified
1.17_301.99	301.995728	1.1705	pos	Olpadronic a	HMDB02559	338709	UGEPSJNL(	Organic acid	Organic phos	Bisphosphon
1.27_447.03	447.03893	1.26985	neg	2-Naphthyl d	HMDB0245251		CQTZJAKSN	Benzenoids	Naphthalene	Unclassified
1.32_548.10	548.103832	1.32371667	neg	Uricase	HMDB0302660		VPXDHZML	Nucleosides,	(3'->5')-dinuc	(3'->5')-dinuc
1.34_351.15	351.151217	1.34166667	neg	Threonylglyc	HMDB00290	23781	BIYXEUAFG	Organic acid	Carboxylic ac	Amino acids,
1.34_419.13	419.138543	1.34166667	neg	D-altro-D-ma	HMDB0029952		BGWQRWR	Organic oxyg	Organooxyge	Carbohydrate
1.34_472.07	472.076193	1.34166667	neg	CDP-N-dime	HMDB0304286		GAEJDVMR	Nucleosides,	Pyrimidine nu	Pyrimidine rib
1.35_176.06	176.06575	1.34996667	pos	Guanidinosu	HMDB00031	10	VVHOUVWJ	Organic acid	Carboxylic ac	Amino acids,
1.54_181.13	181.133732	1.54085	pos	1-(2,3-Dihydr	HMDB00400	94566	DJAGDEJX(	Organoheter	Pyrrolizines	Unclassified
1.68_251.07	251.077523	1.68358333	pos	Allopurinol rit	HMDB00004	5468	KFQUAMTW	Nucleosides,	Pyrazolo[3,4-	Unclassified
1.69_308.16	308.161705	1.68893333	neg	Tyrosyl-Lysir	HMDB0029110		AOLHUMAV	Organic acid	Carboxylic ac	Amino acids,
1.86_187.00	187.0002	1.86321667	pos	2-Phosphogl	HMDB0000362		GXIURPTVH	Organic oxyg	Organooxyge	Carbohydrate
10.01_358.1	358.145919	10.0126833	pos	N-[4-(3-Amin	HMDB0257638		ACPCMBPQ	Benzenoids	Benzene anc	N-phenylurea
10.19_316.2	316.224207	10.1937667	pos	2'-hydroxy-N-(isobutyl)-tetradeca-2E,4E			OMYRBCZB	Lipids and lip	Fatty Acyls	Fatty amides
10.32_222.1	222.113343	10.31745	neg	1-(2,3-Dihydr	HMDB00400	94568	BJTRWJSV(	Organoheter	Pyrrolizines	Unclassified
10.48_255.1	255.196443	10.48035	neg	(Z)-8-Tetrade	HMDB00325	75357	ANJAOCICJ	Lipids and lip	Fatty Acyls	Fatty aldehyc
10.54_201.1	201.112888	10.5357333	neg	(E)-3-Hepten	HMDB00376	92460	AAYIYWGT(	Organic acid	Carboxylic ac	Carboxylic ac
10.55_598.3	598.336205	10.5532667	neg	PS(20:0/0:0)		78839	JRLGKCGUI	Lipids and lip	Glycerophos	Glycerophos
10.66_499.1	499.190605	10.66295	neg	Threonylmetl	HMDB00290	23684	APIDTRXFG	Organic acid	Carboxylic ac	Amino acids,
10.91_179.1	179.142856	10.9125667	pos	3-Methyl-1-pl	HMDB00317	87902	AEJRTNBCF	Benzenoids	Benzene anc	Unclassified
10.95_346.2	391.252095	10.9517167	neg	6-((Z)-pentadec-8-en-1-yl)		43889	YXHVCZZLV	Lipids and lip	Polyketides	Phenolic lipic
11.20_342.1	342.167102	11.198	pos	7-Hydroxyoct	HMDB0241695		CFTUMAOU	Lipids and lip	Fatty Acyls	Fatty acid es
11.29_278.2	278.247603	11.2883167	pos	Gonal	HMDB0252913		DIFYHTVBK	Lipids and lip	Steroids and	Unclassified
11.43_526.2	526.287697	11.4320667	pos	LysoPE(22:6	HMDB00115	62310	XEVRBOQZ	Lipids and lip	Glycerophos	Glycerophos
11.50_232.1	232.169255	11.5038	pos	Farfugin A			QUNSESJRI	Lipids and lip	Prenol lipids	Isoprenoids
11.81_507.2	507.271516	11.80575	pos	PG(18:3(9Z,12Z,15Z)/0:0)		80020	AZCURGXII	Lipids and lip	Glycerophos	Glycerophos
12.11_408.2	431.279248	12.1088667	pos	3a,6b,7b-Trit	HMDB0000415		DKPMWHFF	Lipids and lip	Steroids and	Bile acids, al
12.14_795.5	795.544851	12.1439667	pos	SM(d19:0/18	HMDB0290653		QNHSVEGU	Unclassified	Unclassified	Unclassified
12.24_442.2	442.280554	12.2397667	neg	PGE1-EA		45430	HLQFDRCT	Lipids and lip	Fatty Acyls	Eicosanoids

12.29_432.3	450.378331	12.2879833	pos	Polyglycerol	HMDB0303271		DUUKZBGYI	Lipids and lip	Glycerolipids	Diradylglycer
12.89_397.2	397.235682	12.8930167	neg	Alfentanil	HMDB00149	844	IDBPHNDTY	Benzenoids	Benzene anc	Anilides
13.35_396.2	396.217729	13.34985	neg	1-(3,4-Dietho	HMDB0243791		NCVXEYAKI	Organoheter	Isoquinolines	Benzylisoqui
13.75_365.3	365.342129	13.7497667	neg	5,9-Tricosadiene		97848	MNTTZIKPN	Lipids and lip	Fatty Acyls	Hydrocarbon
14.57_371.3	371.315163	14.5668	pos	FAHFA(2:0/2-O-20:0)			HFHSNILEJ	Lipids and lip	Fatty Acyls	Fatty esters
14.87_465.3	465.304094	14.8718	neg	Cholesterol s	HMDB00006	5625	BHYOQNUE	Lipids and lip	Steroids and	Cholestane s
2.04_223.07	223.071174	2.04021667	pos	4,6-Dihydrox	HMDB00335	327834	CQUUHDQR	Organoheter	Quinolines ar	Quinoline car
2.11_317.03	317.036351	2.11061667	neg	Disodium eth	HMDB0303556		ZGTMUACC	Organic acids	Carboxylic ac	Tetracarboxy
2.11_362.01	362.016776	2.11061667	neg	4-Amino-5-h	HMDB0301920		PCFPIDTUH	Organic oxyg	Organic oxoa	Organic pyro
2.13_218.04	218.045649	2.1282	neg	Quinoline-3-	HMDB02570	351252	DJXNJVFEF	Organoheter	Quinolines ar	Quinoline car
2.43_304.15	304.150381	2.42631667	pos	Fe(II)-nicotia	HMDB0304350		DWMVIOITG	Organic acids	Carboxylic ac	Amino acids,
2.53_253.11	253.118223	2.53481667	pos	5-Isoxazolea	HMDB0253597		AIXMJTYHQ	Benzenoids	Phenols	1-hydroxy-2-
3.33_320.12	320.124267	3.32576667	pos	indole-3-acet	HMDB0304379		YRKLGWOH	Organic acids	Carboxylic ac	Amino acids,
3.40_392.11	392.119723	3.4039	neg	Dide-O-meth	HMDB0041207		MRQYIJOEL	Organic oxyg	Organooxyge	Carbohydrate
3.45_380.19	380.195113	3.45331667	pos	Ile Pro Ile		17467	JNTMAZFVY	Unclassified	Unclassified	Unclassified
3.59_171.01	171.011938	3.58845	neg	5-Methyl-2-th	HMDB00324	317273	VAUMDUIUE	Organoheter	Thiophenes	2,5-disubstitu
3.59_332.12	332.125178	3.58845	neg	Gabazine	HMDB0252568		ACVGNKYJ	Organoheter	Diazines	Pyridazines a
3.68_282.07	282.076111	3.67736667	neg	9-[2-(Phosph	HMDB0258068		RZVRUUYW	Organoheter	Imidazopyrim	Purines and
3.71_301.08	301.080439	3.71251667	neg	Nicotinic acid	HMDB00068	58552	PUEDDPCU	Organic oxyg	Organooxyge	Carbohydrate
3.73_319.09	319.096735	3.73108333	neg	1,5-Dihydroxynaphthalene		328498	BOKGTLAJQ	Unclassified	Unclassified	Unclassified
3.79_196.91	196.910258	3.78651667	neg	3-iodo-2E-acrylic acid		96864	IBFDLVHJH	Lipids and lip	Fatty Acyls	Fatty Acids a
3.86_603.35	603.351471	3.85685	pos	Cholylarginin	HMDB0242375		VFJSXFXCQ	Lipids and lip	Steroids and	Bile acids, al
3.93_130.08	130.087095	3.93381667	neg	beta-alanine	HMDB03045	66871	ACZVSMNF	Unclassified	Unclassified	Unclassified
3.96_515.26	515.263249	3.96453333	pos	12-[Methyl-(4	HMDB0255480		SAAVELCIP	Lipids and lip	Fatty Acyls	Fatty acids a
3.97_287.10	287.107029	3.97085	neg	2,8-decadiene-4,6-diyn-1-a		75340	CPEFMWOE	Lipids and lip	Fatty Acyls	Fatty aldehy
4.04_362.17	362.172457	4.04478333	neg	Leucyl-Trypt	HMDB00289	23823	BQVUABVG	Organic acids	Carboxylic ac	Amino acids,
4.10_197.04	197.042803	4.10018333	neg	8-Azaguanin	HMDB0247422		LPXQRXLU	Organoheter	Triazolopyrim	Unclassified
4.11_446.20	446.203807	4.10951667	pos	P-Hydroxyhip	HMDB0256043		IHVRJTCVM	Organic acids	Carboxylic ac	Amino acids,
4.15_341.07	341.078466	4.14533333	pos	Diphenyl phtl	HMDB0251450		DWNAQMUI	Phenylpropa	Depsides anc	Unclassified
4.22_270.08	270.089339	4.21765	pos	1-(m-Methox	HMDB00388	93474	LAPTWLCIZ	Phenylpropa	Cinnamic aci	Hydroxycinna
4.22_272.08	272.086313	4.21765	pos	1-Iodononan	HMDB02439	323611	OGSJMFCW	Organohalog	Organoiodide	Unclassified
4.23_331.09	331.091293	4.22771667	neg	9-Methylxant	HMDB00597	409598	DHNIKYWY	Organoheter	Imidazopyrim	Purines and
4.23_399.07	399.078432	4.22771667	neg	Glucose lact	HMDB0252783		FWQWYTQ	Organic oxyg	Organooxyge	Carbohydrate

4.27_330.20:	330.203847	4.27276667	pos		Betaxolol	HMDB00143	1319	NWIUTZDMI	Benzenoids	Phenols	Tyrosols and
4.30_164.07:	164.071519	4.30156667	neg		Isoindoline	HMDB0253648		GWVMLCQV	Organoheter	Isoindoles ar	Isoindoles
4.31_317.19:	340.187682	4.30826667	pos		Tetrabenazin	HMDB00155	2800	MKJIEFSOB	Organoheter	Tetrahydrois	Unclassified
4.32_299.04:	299.043213	4.32	neg		thienodiazep	HMDB0258988		CCDMBTCS	Organoheter	Thienodiazep	Unclassified
4.43_254.01	299.015528	4.43021667	neg		Imidazole citr	HMDB0253417		VYKJZGXZK	Organic acid	Carboxylic ac	Tricarboxylic
4.44_437.18	438.187593	4.435	pos		(2S)-2-[[(2S)-	HMDB0257866		JHJGVTJGJ	Organic acid	Carboxylic ac	Amino acids,
4.50_619.39	619.396598	4.50406667	neg		PG(14:0/12:0)		79757	CHMQOCXZ	Lipids and lip	Glycerophos	Glycerophos
4.51_319.16	319.165236	4.50633333	pos		Genomorphir	HMDB0029382		AMAPEXTUI	Lipids and lip	Prenol lipids	Sesquiterper
4.61_196.13	196.130797	4.61381667	pos		4-Piperidinol,	HMDB0243489		CSGAUKGQ	Organoheter	Piperidines	Unclassified
4.65_373.11	373.116026	4.64996667	pos		3'-N'-Acetylfu	HMDB00374	92377	VXRAAUKU	Organoheter	Benzopyrans	1-benzopyrar
4.72_441.23	441.235368	4.72411667	neg		Neurogard	HMDB0247303		LBOJYSIDW	Benzenoids	Dibenzocyclo	Unclassified
4.76_250.10	250.108352	4.76071667	neg		1-(2,3-Dihydr	HMDB00400	94587	BDWPPPWF	Organoheter	Pyrrolizines	Unclassified
4.81_253.07	253.071576	4.81485	neg		furaneol (ket	HMDB0304357		INAXVXBDK	Organoheter	Dihydrofuran	Furanones
4.81_597.97	597.975566	4.81226667	pos		Perfluoro-15-	HMDB02563	459153	CAKZCCWL	Organoheter	Oxacyclic cor	Unclassified
4.83_301.03	301.03049	4.83273333	neg		Bis(2,5-dihyd	HMDB0260371		GCJMLPAIV	Organoheter	Pyrroles	Substituted p
4.91_495.10	495.101623	4.90568333	neg		Nitrefazole	HMDB02556	322308	NMTBSNPBI	Organoheter	Azoles	Imidazoles
5.06_410.21	410.210488	5.06456667	pos		Dovitinib	HMDB0251597		PIQCTGMSN	Organoheter	Diazinanes	Piperazines
5.16_232.08	255.073637	5.15551667	pos		Dazoxiben	HMDB0250886		XQGZSYKG	Benzenoids	Benzene anc	Benzoic acid
5.16_325.08	325.087959	5.16371667	neg		4-Hydroxy-L-	HMDB00022	327898	HBDWQSHE	Organic acid	Carboxylic ac	Amino acids,
5.22_277.06	277.066738	5.21915	neg		(2S)-5-Aminc	HMDB0257975		FBZQQZFTY	Organic acid	Carboxylic ac	Amino acids,
5.39_195.09	195.09893	5.39208333	pos		4(1H)-Pteridi	HMDB0247091		UDOGNMDL	Organoheter	Pteridines ar	Pterins and c
5.46_451.14	451.140562	5.45918333	neg		7-Methyl-2-(2	HMDB0247633		INGWEZCO	Organoheter	Diazanaphth	Naphthyridin
5.48_301.11	301.118285	5.48356667	pos		Avenanthran	HMDB00292	86153	INBHLTYBR	Phenylpropa	Cinnamic aci	Hydroxycinna
5.50_212.12	212.129054	5.49605	neg		4-Butyl-2-eth	HMDB00378	92694	FQGHKCJA	Organoheter	Azoles	Oxazoles
5.50_334.23	334.237229	5.5019	pos		Trideca-2,5,8	HMDB0241344		BSYCWHHL	Lipids and lip	Fatty Acyls	Fatty acid es
5.61_266.05	266.057763	5.61161667	pos		Koenoline	HMDB00334	89284	KNKVHLASD	Organoheter	Indoles and c	Carbazoles
5.66_331.12	331.129925	5.66205	neg		EPIISOPILO	HMDB0251850		OLLOSKHC	Organoheter	Azoles	Imidazoles
5.74_209.07	209.078146	5.73951667	pos		6-Formylpter	HMDB0247066		LLJAQDVNM	Organoheter	Pteridines ar	Pterins and c
6.00_357.18	357.187961	5.99566667	pos		2,6-Dimethyl-	HMDB00332	89085	BTUZVFUR	Lipids and lip	Fatty Acyls	Fatty acyl gly
6.03_573.19	573.190631	6.0318	neg		Uvarinol		52638	DNSBHCGT	Lipids and lip	Polyketides	Flavonoids
6.23_243.15	243.159892	6.23498333	neg		Ethyl 4-decer	HMDB00392	93811	AWNIQMQA	Lipids and lip	Fatty Acyls	Fatty acid es
6.23_422.18	422.182044	6.23498333	neg		Neopetasiter	HMDB00303	86892	RNNVXCSF	Organoheter	Azaspirodeca	Unclassified
6.29_517.09	517.098641	6.29061667	neg		(-)-Epigalloca	HMDB00402	94817	BMJHAAZDI	Phenylpropa	Flavonoids	Flavans

6.35_367.13	367.139696	6.34593333	neg	3,4-Methylen	HMDB0059730		CSNVGEOLI	Lipids and lip	Fatty Acyls	Fatty acids a
6.35_411.23	411.238644	6.34593333	neg	5alpha-Tetra	HMDB00005	5511	AODPIQQIL	Lipids and lip	Steroids and	Hydroxysterc
6.38_411.23	411.23506	6.3809	pos	5,6-Dihydrox	HMDB0012109		SAAOVBGAI	Lipids and lip	Fatty Acyls	Eicosanoids
6.51_173.08	173.081632	6.51206667	neg	2-methyl-2E-hexenoic acic		4627	AKOVMBAFZ	Lipids and lip	Fatty Acyls	Fatty Acids a
6.59_521.17	521.174323	6.58565	neg	1-(3-Methylb	HMDB0249950		KRFSMLDUZ	Benzenoids	Benzene anc	Benzoyl deriv
6.73_351.14	351.144806	6.73181667	neg	Naematolone	HMDB00357	91020	BBRNVDBL(	Lipids and lip	Prenol lipids	Sesquiterper
6.90_489.20	489.208104	6.8979	neg	Difluprednate	HMDB00156	316011	WYQPLTPS	Lipids and lip	Steroids and	Pregnane ste
6.92_540.36	540.365534	6.91643333	neg	N(4)-Octadec	HMDB0248147		HQHQCEKU	Nucleosides,	Pyrimidine nu	Unclassified
6.99_451.12	451.127847	6.98976667	neg	3-[[(2S)-3-(4-	HMDB0249872		MVRLTBIHU	Organic acids	Peptidomime	Hybrid peptic
7.16_267.11	267.113991	7.15531667	neg	SCHRADAN	HMDB02581	72766	SZKKRCSOS	Organic acids	Organic phos	Organic phos
7.31_265.14	265.140725	7.31328333	pos	(+-)-Carbovir	HMDB0242235		XSSYCIGJY	Nucleosides,	Nucleoside a	Unclassified
7.42_231.07	231.077966	7.42368333	pos	3(2H)-Isoflav	HMDB02461	323177	CNNBJLXLT	Phenylpropai	Isoflavonoids	Isoflav-3-ene
7.60_139.11	139.112644	7.59848333	neg	7:0(6Me,6Me)		317434	AAOISIQFPI	Lipids and lip	Fatty Acyls	Fatty Acids a
7.90_275.09	275.090157	7.90265	neg	5'-N-Methylca	HMDB0246883		PLYRYAHDI	Nucleosides,	Purine nucle	Unclassified
7.96_423.00	423.00781	7.95811667	neg	3,5-Dinitrobe	HMDB02460	334360	VYWYYJYR	Benzenoids	Benzene anc	Benzoic acid
7.96_447.02	447.027367	7.95811667	neg	3-[(1R,2R,3S	HMDB0255411		ANVLMUZOI	Organic acids	Hydroxy acid	Medium-chai
8.01_173.11	173.118083	8.01323333	neg	2-Methyl-4-h	HMDB00138	340182	AKRJXOYAL	Organic oxyg	Organooxyge	Carbonyl cor
8.18_451.22	451.229702	8.17628333	pos	L-Alanine, 3-	HMDB0259936		SRRJCHGG	Organic acids	Carboxylic ac	Amino acids,
8.60_409.25	409.256141	8.5985	pos	oscr#25			FOEYOFVRI	Lipids and lip	Fatty Acyls	Fatty acyl gly
9.21_293.20	293.208462	9.21266667	pos	9S-hydroxy-7E-hexadecenoic acid			ACEMCBZW	Lipids and lip	Fatty Acyls	Fatty Acids a
9.24_309.20	309.207186	9.2416	neg	(Z)-heptadec-14-en-11-ynoic acid			AQRFGVGE	Lipids and lip	Fatty Acyls	Fatty Acids a
9.74_165.07	165.075626	9.74061667	pos	L-rhamnofura	HMDB03044	63161	AFNUZVCFK	Organic oxyg	Organooxyge	Carbohydrate
9.87_266.12	266.123989	9.86676667	pos	Histidylglutar	HMDB00288	23820	CTCFZNBRZ	Organic acids	Carboxylic ac	Amino acids,
9.94_304.29	304.299503	9.93923333	pos	(8S,9S,10R,1	HMDB0260190		HOWBJCLEI	Lipids and lip	Steroids and	Pregnane ste
0.64_368.12	368.128558	0.63663333	pos	2,5-Dioxopyr	HMDB02444	406418	YBNMDCCM	Benzenoids	Pyrenes	Unclassified
0.65_467.10	467.102239	0.6518	pos	Balipodect	HMDB0258682		KVHRYLNQI	Organoheter	Azoles	Pyrazoles
0.67_203.02	203.020408	0.6745	neg	Dihydro-3(2H	HMDB00312	342247	DSXFPRKPI	Organoheter	Thiolanes	Unclassified
0.84_394.97	394.977289	0.84308333	neg	Perfluoro(me	HMDB02563	336803	QIROQPWS	Organohalog	Alkyl halides	Cyclohexyl h
0.96_222.98	222.983364	0.96215	neg	5-(Methylthio	HMDB00596	407063	HKEAOVFN	Organic acids	Organic phos	Phosphate e
0.98_102.01	102.019545	0.97965	neg	Methyl isocya	HMDB0254594		HAMGRBXT	Organic nitro	Organonitrog	Isocyanates
1.08_250.03	250.038781	1.08208333	neg	3-(2-fluorophenyl)-1H-pyra		417094	DAFWBGVQ	Unclassified	Unclassified	Unclassified
1.10_316.10	316.105014	1.09681667	neg	zaprinast	HMDB02599	44292	REZGGXND	Organoheter	Triazolopyrim	Unclassified
1.10_419.17	419.177118	1.1046	pos	Kuguacin B	HMDB0302097		IZWGYTYFD	Benzenoids	Phenol ether	Unclassified

1.13_387.11	387.114656	1.13148333	neg	D-Glucose, 6	HMDB0259486		AYRXSINWI	Lipids and lip	Fatty Acyls	Fatty acyl gly
1.14_411.15	411.150888	1.13681667	pos	1,3,7-Trimeth	HMDB0258028		GRDCWACK	Organic nitro	Organonitrog	Amines
1.20_202.03	202.032559	1.20368333	pos	2-Amino-3,4-	HMDB00304	86952	SODSBJHVK	Organic acids	Carboxylic ac	Amino acids,
1.25_206.98	206.98836	1.2521	neg	Hexafluoroac	HMDB02531	343580	QAMFBRUW	Organic oxyg	Organooxyge	Carbonyl cor
1.29_310.11	310.11436	1.2875	neg	D-1-[(3-Carb	HMDB00386	93321	HUEOABWG	Organic acids	Carboxylic ac	Amino acids,
1.51_345.17	345.177039	1.50628333	pos	15-dimethylarsinoyl pentadecanoic acid			RJOXDLOOI	Lipids and lip	Fatty Acyls	Other Fatty A
1.56_374.04	375.056572	1.55895	pos	Iodopindolol	HMDB0253531		LOMQONAZ	Organoheter	Indoles and	Indoles
1.73_340.15	340.151231	1.72581667	neg	Butralin	HMDB02494	72384	SPNQRCTZI	Benzenoids	Benzene and	Aniline and s
1.78_104.03	104.035176	1.78133333	neg	N-Methylform	HMDB00011	58023	ATHHXGZT	Organic acids	Carboximidic	Carboximidic
1.78_385.06	385.062984	1.78133333	neg	Fosfluconazo	HMDB02524	321527	GHJWNRRC	Benzenoids	Benzene and	Halobenzene
1.78_663.12	663.129757	1.78133333	neg	Fluorescein	HMDB00148	2702	GNBHRKFJI	Organoheter	Benzopyrans	1-benzopyra
10.19_636.2	636.253557	10.18955	neg	Mycobactins	HMDB0254968		HQYXQBFM	Organic acids	Peptidomime	Depsipeptide
10.21_323.1	323.159122	10.2120667	pos	p-hydroxyber	HMDB0256040		GFOOTRIUF	Benzenoids	Benzene and	Benzoic acid
10.27_296.2	335.197931	10.2662	pos	2-hydroxy-9Z,12Z-Octadecadienoic acid			AFDSETGKY	Lipids and lip	Fatty Acyls	Octadecanoi
10.28_242.2	242.283867	10.2840833	pos	1-Hexadecer	HMDB0243889		GQEZCXVZI	Hydrocarbon	Unsaturated	Unsaturated
10.32_665.0	665.044034	10.31745	neg	beta-nicotina	HMDB0304543		JOUIQRNQJ	Lipids and lip	Prenol lipids	Tetraterpeno
10.43_494.3	494.348868	10.42685	neg	Icosa-5,8,11-	HMDB0241579		DBCKADUS	Lipids and lip	Fatty Acyls	Fatty acid es
10.61_374.2	374.265851	10.6097333	pos	Aacocf3	HMDB0247736		PLWROONZ	Organic oxyg	Organooxyge	Carbonyl cor
10.63_693.2	693.296092	10.6260167	neg	3-(4-((3-Pher	HMDB02529	45154	DGENZVKC	Benzenoids	Benzene and	Phenylmethy
10.79_268.1	268.118959	10.7875833	pos	(2E,4E)-Hexa	HMDB0241673		JMCSSDQFI	Lipids and lip	Fatty Acyls	Fatty acid es
10.79_353.2	354.299785	10.7875833	pos	N-oleoyl alanine		75506	CHQZBIVWI	Lipids and lip	Fatty Acyls	Fatty amides
10.92_385.1	385.199415	10.9157	neg	Pexacerfont	HMDB0256359		LBWQSAZE	Organoheter	Pyridines and	Pyrazolylpyri
10.95_283.2	283.263994	10.9517167	neg	1-Heptadece	HMDB03023	97727	ADOBXTDBI	Hydrocarbon	Unsaturated	Unsaturated
11.86_440.2	440.264973	11.8613	neg	PGE2-EA		3827	GKKWUSPP	Lipids and lip	Fatty Acyls	Eicosanoids
12.09_383.2	383.203532	12.0909333	pos	Isopentenyla	HMDB00122	62882	XEHLLUQVS	Organic oxyg	Organooxyge	Carbohydrate
12.31_373.2	373.210806	12.3062167	pos	2-((5-(Diethyl	HMDB0244283		NWCVGIME	Organoheter	Triazolopyrim	Unclassified
12.41_375.2	375.274863	12.4138167	neg	MG(0:0/16:0/	HMDB00115	457149	BBNYCLARE	Lipids and lip	Glycerolipids	Monoradylgly
12.64_419.1	419.150572	12.6402	neg	4'-Hydroxy-4-(4-hydroxysty		47444	RDUYMQQG	Lipids and lip	Polyketides	Flavonoids
13.40_589.5	589.520063	13.39515	neg	dihydrosterc	HMDB0304336		PDXZQLDU	Lipids and lip	Fatty Acyls	Fatty acids a
13.92_324.2	324.20682	13.9195167	pos	Fenazaquin	HMDB02521	72502	DMYHGDXA	Organoheter	Diazanaphtha	Benzodiazine
2.00_283.03	283.036799	2.0024	neg	Olsalazine	HMDB0015380		QQBDLJCY	Organoheter	Azobenzenes	Unclassified
2.06_394.13	394.138069	2.0574	neg	Morniflumate	HMDB02548	330013	LDXSPUSKE	Benzenoids	Benzene and	Trifluorometh
3.00_130.08	130.087121	2.99661667	neg	1-Methylpyrr	HMDB0243946		AVFZOVWC	Organoheter	Pyrrolidines	N-alkylpyrroli

3.31_609.15	609.15752	3.31146667	neg	7-(Diethylphc	HMDB0257886		HDPYDWW(	Phenylpropa	Coumarins a	Unclassified
3.34_272.13	272.135346	3.34363333	pos	Dyphylline	HMDB00147	3524	KSCFJBIXM	Organoheter	Imidazopyrim	Purines and
3.54_387.20	387.204898	3.54413333	pos	(Z)-7-[(1R,4S	HMDB0248811		XBVVXCYNI	Lipids and lip	Fatty Acyls	Fatty acids a
3.56_426.23	426.23493	3.56223333	pos	ascr#4			HFJWZOCW	Lipids and lip	Fatty Acyls	Fatty acyl gly
3.71_184.98	184.985402	3.71251667	neg	Acetylphospl	HMDB00014	6278	LIPOUNRJVI	Organic acid	Organic phos	Phosphate e
3.71_328.09	328.09153	3.71251667	neg	Profluralin		72813	ITVQAKZNY	Unclassified	Unclassified	Unclassified
3.80_718.30	718.308186	3.80401667	pos	Z-Vdvad-fmk	HMDB02599	806432	ANTIWMNLI	Organic acid	Peptidomime	Hybrid peptic
3.89_132.04	132.044687	3.8922	pos	(S)-4-Hydrox	HMDB00603	63618	HOOOPXDS	Benzenoids	Phenols	1-hydroxy-2-
3.97_475.97	475.977409	3.97085	neg	Dihydroneop	HMDB00009	5917	DGGUVLXVI	Organoheter	Pteridines an	Pterins and c
4.02_457.24	457.247366	4.01898333	pos	Phytyl diphos	HMDB0011116		ITPLBNCCP	Lipids and lip	Prenol lipids	Diterpenoids
4.15_332.21	332.218078	4.14533333	pos	E-64c		44982	SCMSYZJDI	Unclassified	Unclassified	Unclassified
4.15_403.20	403.208513	4.14533333	pos	n6-phenyliso	HMDB0255302		KYOSRONG	Nucleosides,	Purine nucle	Unclassified
4.20_199.14	199.144389	4.19935	pos	3,4-Dimethox	HMDB00418	335489	ANOUKFYB(	Benzenoids	Benzene and	Methoxybenz
4.26_373.20	373.209323	4.26475	neg	(1S,2S,3S,4F	HMDB0242447		XRQDFNLIN	Organic acid	Carboxylic ac	Amino acids,
4.33_251.09	251.094676	4.32651667	pos	Atipamezole	HMDB02486	323230	HSWPZIDYA	Benzenoids	Indanes	Unclassified
4.33_340.08	340.080796	4.32651667	pos	7,7'-Dihydrox	HMDB00335	89369	GGNRJMYA	Phenylpropa	Coumarins a	Hydroxycoun
4.42_639.32	639.327715	4.41721667	pos	(2R)-2-[[(2R)	HMDB0260127		QHSRPPJQI	Organic acid	Carboxylic ac	Amino acids,
4.49_540.04	540.040386	4.4888	pos	Phosphoamii	HMDB0252977		UQABYHGX	Nucleosides,	Purine nucle	Purine ribonu
4.58_167.02	167.028271	4.57715	neg	Carbimazole	HMDB0014533		CFOYWRHI	Organoheter	Azoles	Imidazoles
4.58_207.02	207.023109	4.57715	neg	4-phenyl-1,2,3-Thiadiazole		96416	AWPNFXRM	Unclassified	Unclassified	Unclassified
4.76_386.15	409.146977	4.75875	pos	Methylsyringi	HMDB0039570		KDZYNPVXU	Organic oxyg	Organooxyge	Carbohydrate
5.01_291.12	291.125727	5.01075	pos	Delpazolid	HMDB0250974		QLUWQAFD	Benzenoids	Benzene and	Aniline and s
5.05_213.11	213.113037	5.05298333	neg	Trans-eldanolide		97376	ARNPQYFY(	Lipids and lip	Fatty Acyls	Fatty esters
5.09_151.02	151.025902	5.08986667	neg	6,8-Dihydrox	HMDB00011	418706	BYUOBSUZ	Organoheter	Imidazopyrim	Purines and
5.18_118.04	118.040921	5.18221667	neg	1H-Pyrazolo[	HMDB0244900		AMFYRKOU	Organoheter	Pyrazolopyric	Unclassified
5.22_309.06	309.06066	5.21915	neg	6-hydroxy-2-	HMDB0304226		WEZWWZKI	Organic oxyg	Organooxyge	Carbonyl cor
5.22_357.23	357.239415	5.21915	neg	4,7,13,16,21,	HMDB02456	458476	AUFVJZSDS	Organic nitro	Organonitrog	Amines
5.26_293.13	293.135708	5.26461667	pos	1-((2-Methyl-	HMDB0255803		WJSVJNDM	Organoheter	Imidazopyrim	Purines and
5.51_515.28	515.285995	5.51431667	neg	DG(2:0/0:0/F	HMDB0296917		JXYJWJWQ	Unclassified	Unclassified	Unclassified
6.09_413.20	413.200221	6.08708333	neg	(2-((3,5-Di-t-I	HMDB0242455		ADVNUIUAD	Benzenoids	Benzene and	Phenylpropa
6.12_312.13	312.135734	6.12398333	pos	4-Methylprop	HMDB0246516		INIIAIIRDKM	Benzenoids	Naphthalene	Unclassified
6.38_265.17	265.17734	6.3809	pos	Peramine	HMDB0256303		GQWWGRU	Organoheter	Pyrrolopyrazi	Unclassified
7.17_331.23	331.239424	7.17388333	neg	Edrophonium	HMDB00151	316537	VWLHWLSR	Benzenoids	Benzene and	Aniline and s

7.37_305.17	305.171947	7.36856667	pos		Tert-butyl 4-(	HMDB0245674			PODFBDRFI	Organoheter	Diazinanes	Piperazines
7.57_280.09	280.099796	7.57063333	pos		Carboxymeth	HMDB0303207			QMGYPNKI	Organic oxyg	Organooxyge	Carbohydrate
7.83_393.29	393.296106	7.82796667	pos		3,9-Dihydrox	HMDB0241293			JPGLPKYXC	Lipids and lip	Fatty Acyls	Fatty acid es
8.03_457.24	457.24443	8.03168333	neg		[6]-Gingerdio	HMDB00361	91280		IJSYHUFAW	Lipids and lip	Fatty Acyls	Fatty acyl gly
8.18_249.07	249.076635	8.1793	neg		Anofinic acid	HMDB00333	89159		AXICIBPYBC	Organoheter	Benzopyrans	1-benzopyra
8.41_323.18	323.18258	8.41473333	pos		Glycyl-l-histi	HMDB0252882			MVORZMQF	Organic acid	Carboxylic ac	Amino acids,
8.54_201.08	201.088361	8.5434	pos		triazolobenz	HMDB0259157			KYTREVLPF	Organoheter	Benzotriazol	Unclassified
8.54_255.06	255.062629	8.5434	pos		Tuckolide; Decarestrictine		985145		HWMMWMJ	Unclassified	Unclassified	Unclassified
8.56_272.22	272.221804	8.56171667	pos		(3beta,9beta	HMDB00360	91231		FLJVCTAWI	Organic oxyg	Organooxyge	Alcohols and
8.58_270.20	270.207435	8.58033333	neg		Xestoaminol B				PDPUNMLG	Lipids and lip	Sphingolipids	Sphingoid ba
8.97_353.00	353.004295	8.96598333	neg		(R)-Mevaloni	HMDB00019	6415		SIGQQUBJQ	Organic oxyg	Organic oxoa	Organic pyro
9.57_361.23	361.238847	9.56983333	neg		18-Fluoro-9,10-Epoxyoctadecanoic acid				ASQQIVAJP	Lipids and lip	Fatty Acyls	Fatty Acids a
9.59_405.22	405.227008	9.58798333	neg		N-(3-Methylb	HMDB02456	457762		HYVABZIGR	Organoheter	Imidazopyrim	Purines and
9.63_415.26	415.260446	9.63246667	pos		6alpha-Hydroxy-5alpha-ch		84330		AOZMFMMC	Lipids and lip	Steroids and	Bile acids an
9.65_241.17	241.178266	9.6509	pos		Neostigmine	HMDB0015472			ALWKGYPQ	Benzenoids	Benzene and	Phenoxy com
9.75_451.03	451.038603	9.75281667	neg		Methyl 3,5-di	HMDB0254567			POGCCFLN	Benzenoids	Benzene and	Benzoic acid
9.76_329.14	329.148945	9.7588	pos		(R)-Ruxolitini	HMDB0243698			HFNKQEVN	Organoheter	Pyrrolopyrimi	Pyrrolo[2,3-d
9.96_149.13	149.132372	9.95751667	pos		2,4-Dimethyl-	HMDB0061810			AADQFNAA	Lipids and lip	Prenol lipids	Monoterpenc
0.61_156.01	156.01424	0.60603333	pos		S-(-)-ureidog	HMDB0304474			NWZYYCVI	Organic acid	Carboxylic ac	Amino acids,
0.63_381.17	381.173135	0.62835	neg		4-Hydroxycit	HMDB00293	86233		WSFLFFUIE	Organic acid	Carboxylic ac	Amino acids,
0.64_324.82	324.822287	0.63663333	pos		3,3-diiodoacrylic acid		96867		PMILBWLQI	Lipids and lip	Fatty Acyls	Fatty Acids a
0.64_327.05	327.051549	0.63663333	pos		Lamivudine-r	HMDB0060641			WUMZLKDC	Nucleosides,	Nucleoside a	Unclassified
0.70_247.05	247.057058	0.70456667	neg		Penmacric a	HMDB00294	86267		QEFCFJFZZ	Organic acid	Carboxylic ac	Amino acids,
0.70_278.05	278.052404	0.70456667	neg		(2S)-2-[(2-Ca	HMDB0257920			XREHQPOY	Organic acid	Carboxylic ac	Amino acids,
0.70_539.13	539.138374	0.70456667	neg		Peruvianoside II				JUTUYETVY	Lipids and lip	Polyketides	Flavonoids
0.72_437.04	437.040898	0.71928333	neg		Creatinine ni	HMDB0250532			BVMRJQKB	Organic acid	Carboxylic ac	Amino acids,
0.73_254.10	277.089432	0.72825	pos		3-beta-D-gal	HMDB03041	3385		NHJUPBDC	Lipids and lip	Glycerolipids	Glycosylglyc
0.76_153.10	153.102191	0.75766667	pos		2-Isopropyl-6	HMDB0245733			AJPIUNPJB	Organoheter	Diazines	Pyrimidines a
0.79_217.09	218.102165	0.7877	pos		3-[(2-Oxoace	HMDB0241791			PMHVEZKB	Lipids and lip	Fatty Acyls	Fatty acid es
0.82_464.01	464.013951	0.81883333	pos		2,2,3,3,4,4,5,	HMDB0247702			RARQGXBO	Lipids and lip	Fatty Acyls	Fatty acids a
0.83_559.96	559.960669	0.82703333	neg		[(1r,2s,4s,5s)	HMDB0254929			NMVWLEUC	Organoheter	Imidazopyrim	Purines and
0.98_180.98	180.989882	0.97735	pos		Kojic acid	HMDB00329	44532		BEJNERDR	Organoheter	Pyrans	Pyranones a
1.07_295.09	295.09328	1.06741667	neg		1-iodo-2-met	HMDB00627	354398		RTWBFGUV	Organohalog	Organoiodide	Unclassified

1.08_488.09(	488.090455	1.08208333	neg	Selinexor	HMDB0258217		DEVSOMFA(	Organoheter	Azoles	Triazoles
1.23_469.06!	469.065206	1.23491667	neg	Phosphohisti	HMDB0256480		FDIKHVQUP	Organic acid	Carboxylic ac	Amino acids,
1.34_368.14:	368.141166	1.34166667	neg	DV 7751a	HMDB0251645		UGGPRXAH	Organoheter	Quinolines ar	Quinoline car
1.34_531.15:	531.152308	1.34166667	neg	Dihydrocitrin	HMDB0251297		VVVMDYGN	Benzenoids	Benzene anc	Benzoic acid
1.38_188.11!	188.11552	1.3848	pos	Immepip	HMDB02534	432115	MCNGUYXR	Organic nitro	Organonitrog	Amines
1.38_258.14!	258.145751	1.37511667	neg	Guanadrel	HMDB00143	321290	HPBNRIOWI	Organic oxyg	Organooxyge	Ethers
1.43_245.11	245.114209	1.43078333	neg	gamma-Gluta	HMDB00111	62020	AQAKHZVP(	Organic acid	Carboxylic ac	Amino acids,
1.47_383.07	383.070965	1.46783333	neg	2-(Trifluorom	HMDB02439	460456	TUXDRFWD	Benzenoids	Phenol ether	Anisoles
1.61_277.02	277.021495	1.61191667	pos	(2S)-2-[(3-Nit	HMDB0243594		CDBXKLYO.	Benzenoids	Benzene anc	Benzoic acid
1.62_281.02!	281.025409	1.61501667	neg	Isosorbide Di	HMDB00150	313407	MOYKHGMN	Organoheter	Furofurans	Isosorbides
1.65_410.16	410.167247	1.64773333	pos	Q	HMDB00596	62373	QQXQGKSP	Nucleosides,	Pyrrolopyrimi	Unclassified
1.76_135.01	135.012022	1.76276667	neg	Tetrahydroth	HMDB02445	653	AMXKVIWW	Organoheter	Thiolanes	Unclassified
1.93_293.08	293.081262	1.92873333	neg	N-FORMYLMETHIONYLA		44169	GBWVAAKK	Unclassified	Unclassified	Unclassified
10.07_611.2	611.249378	10.0669833	pos	Rhodin G7	HMDB0059949		VMMFVPSM	Organoheter	Tetrapyrroles	Chlorins
10.19_374.3(	374.362407	10.1937667	pos	13-hydroxy-docosanoic ac		74587	BYCZEMFW	Lipids and lip	Fatty Acyls	Fatty Acids a
10.25_527.2(	527.261678	10.24785	pos	Phorbol-12,1	HMDB0246702		BQJRUJTZS	Lipids and lip	Prenol lipids	Diterpenoids
10.35_313.2:	313.238414	10.3534833	neg	Isopropyl 7Z-tetradecenoa		97355	BYTFCQILG	Lipids and lip	Fatty Acyls	Fatty esters
10.36_384.3	384.310435	10.357	pos	AX 048		64840	FMFKQXKY	Unclassified	Unclassified	Unclassified
10.55_281.2	281.248287	10.5532667	neg	6Z,9Z-Heptadecadiene		97554	AGMLTHHZ.	Lipids and lip	Fatty Acyls	Hydrocarbon
10.63_1103.	1103.65047	10.6260167	neg	Lithocholic a	HMDB0254126		GIQXKAXWI	Lipids and lip	Steroids and	Steroidal gly
10.86_339.2	339.213718	10.8590333	pos	(2S)-2-(2-Be	HMDB0257707		MBLBNYZS(	Organic acid	Carboxylic ac	Amino acids,
10.90_523.1!	523.190044	10.8978167	neg	Methionylhyd	HMDB0028974		SQTUSSZU	Organic acid	Carboxylic ac	Amino acids,
11.16_337.1!	337.156693	11.16325	pos	Norethynodr	HMDB0255736		ICTXHFFSO	Lipids and lip	Steroids and	Oxosteroids
11.36_494.3(	494.360148	11.36075	pos	PE(P-20:0/0:0)		77701	GBHFCJJMZ	Lipids and lip	Glycerophos	Glycerophos
11.38_438.3	438.378345	11.3787167	pos	Cerotic acid(d3)			XMHIUKTWI	Lipids and lip	Fatty Acyls	Fatty Acids a
11.54_227.1	227.127466	11.5398167	pos	N-[2-[2-(2-An	HMDB0244609		GMXQUOKV	Organic acid	Carboxylic ac	Carboxylic ac
11.56_323.2!	323.257459	11.5576	pos	FAHFA(12:1(5Z)/3-O-8:0)			AVVAQCBN	Lipids and lip	Fatty Acyls	Fatty esters
12.07_114.1(	114.102672	12.0728	pos	Pyrrolidine-2	HMDB0257000		ALSCEGDXI	Organoheter	Pyrrolidines	Unclassified
12.20_306.2	306.278682	12.1975333	pos	Ethylestrenol	HMDB0252076		AOXRBFRF	Lipids and lip	Steroids and	Pregnane ste
12.38_435.2	435.210149	12.3811333	neg	ribostamycin	HMDB02572	71793	NSKGQURZ	Organic oxyg	Organooxyge	Carbohydrate
12.38_507.1!	507.194779	12.3811333	neg	5-Oxo-prolyl-	HMDB0250211		DGYWXYFL	Organic acid	Carboxylic ac	Amino acids,
12.59_468.2!	468.296064	12.5908167	neg	Methymycin		41001	HUKYPYXOI	Lipids and lip	Polyketides	Macrolides a
12.65_469.1(	469.166096	12.65015	neg	Calix[4]arene	HMDB0249557		YPNHVQZZI	Benzenoids	Phenols	1-hydroxy-4-

12.69_485.2(	485.267205	12.6870167	neg		Frovatriptan	HMDB00151	85456	XPSQPHWE	Organoheter	Indoles and (	Carbazoles
13.38_377.2:	377.236252	13.3769167	neg		NG-amino-L-Arginine		96429	NCHSYZVV\	Unclassified	Unclassified	Unclassified
13.52_349.1!	349.198339	13.5248833	pos		12,17;13,17-Diepoxy-16-hydroxy-9Z-oct			AXRADOFM	Lipids and lip	Fatty Acyls	Eicosanoids
13.58_465.3!	465.354344	13.5784833	pos		24,24-Difluoro-25-hydroxy-26,27-dimeth			NATOOCTZI	Lipids and lip	Steroids and	Secosteroids
13.60_421.3:	421.328287	13.5968667	pos		Axillarenic acid		74707	AKVZFALHM	Lipids and lip	Fatty Acyls	Fatty Acids a
13.75_383.2!	383.295417	13.7497667	neg		1alpha,25-dihydroxy-21-norvitamin D3 /			AUXHOSNBI	Lipids and lip	Steroids and	Secosteroids
14.22_313.0	313.078309	14.2220333	neg		3-Deoxy-D-g	HMDB0000425		FQHUAUMY	Organic oxyg	Organooxyge	Carbohydrate
14.24_312.3:	312.325596	14.2436833	pos		6Z,9Z-Eicosadien-11-ol		97010	HLRILOHBD	Lipids and lip	Fatty Acyls	Fatty alcohol
14.28_423.1!	423.181883	14.2783333	neg		Salicyaldehy(	HMDB0302513		AWSLIWUM	Benzenoids	Benzene anc	Phenylhydraz
2.02_191.05	191.057681	2.02305	pos		(4-Aminophe	HMDB0243614		OAOBMEMV	Benzenoids	Benzene anc	Aniline and s
2.02_343.05:	343.052182	2.02081667	neg		Phoxim	HMDB0256498		ATROHALU(	Benzenoids	Benzene anc	Unclassified
2.41_184.04(	184.04033	2.40855	pos		Allyl cysteine	HMDB0248164		AMPHKYRL!	Organic acid:	Carboxylic ac	Amino acids,
2.61_274.17(	274.176154	2.6067	pos		5-Hydroxylys	HMDB0246805		BJACJWXYI	Organic acid:	Carboxylic ac	Amino acids,
2.75_140.06!	140.068508	2.74981667	pos		3-Amino-2,2-	HMDB02458	446701	DGNHGRLD	Organic acid:	Carboxylic ac	Amino acids,
3.09_243.13:	243.133861	3.091	pos		Glutamylleuc	HMDB00288	44684	YBAFDPFAU	Organic acid:	Carboxylic ac	Amino acids,
3.20_139.07!	139.075481	3.1994	pos		1,2-Dimetho›	HMDB00321	316927	ABDKAPXRI	Benzenoids	Benzene anc	Methoxybenz
3.48_335.13(	335.13616	3.47753333	neg		Trimethoprim	HMDB00145	2956	IEDVJHCEM	Benzenoids	Phenol ether	Anisoles
3.60_408.17!	409.186969	3.59728333	pos		3-O-Methylni	HMDB0039084		MEJPFLKDA	Lipids and lip	Prenol lipids	Terpene lact(
3.70_284.20(	284.206508	3.69811667	pos		Pentadecylic acid(d3)			WQEPLUUG	Lipids and lip	Fatty Acyls	Fatty Acids a
3.73_293.11·	293.114311	3.73108333	neg		2-Oxomelato	HMDB0060721		HNEAVHQA(	Organoheter	Indoles and (	Indolines
3.73_466.22!	466.229805	3.73238333	pos		22-dimethylarsinoyl-(5Z,8Z, 11Z,14Z,17			GJSMHUXG	Lipids and lip	Fatty Acyls	Other Fatty A
3.80_685.30!	685.308717	3.80496667	neg		JWH 073 4-hydroxyindole		64912	DKIVMJXJKI	Unclassified	Unclassified	Unclassified
3.82_304.10·	304.104624	3.82341667	neg		Osmaronin	HMDB0032769		DAVUWBZD	Lipids and lip	Fatty Acyls	Fatty acyl gly
3.84_336.12(	336.120257	3.84145	neg		Seryltryptopl	HMDB00290	23978	LZLREEUGS	Organic acid:	Carboxylic ac	Amino acids,
3.84_446.20	446.207666	3.83913333	pos		Aspergamide A; 6b-hydro›		985087	QCZUWKYB	Unclassified	Unclassified	Unclassified
3.95_203.09:	203.09239	3.95231667	neg		trans-4-Hydr(	HMDB0000909		ALTAAUJNH	Organic oxyg	Organooxyge	Alcohols and
3.95_363.14!	363.14867	3.94663333	pos		Citalopram N	HMDB00606	1784	DIOGFDCEV	Benzenoids	Benzene anc	Phenylbutyla
3.97_155.04(	155.046063	3.97085	neg		3-Hydroxy-4-	HMDB0060741		DBDKLFOU\	Organoheter	Pyridines anc	Aminopyridin
3.98_165.13!	165.138775	3.98231667	pos		N,N,N',N'-Te	HMDB0244279		CJAOGUFA/	Organic nitro	Organonitro(	Amines
4.01_226.11!	226.119665	4.0077	neg		Azepexole	HMDB02487	320945	ZNXAJGZPL	Organoheter	Azepines	Unclassified
4.08_449.20:	449.20363	4.08158333	neg		Pisumionosic	HMDB0039947		PASRVRCW	Lipids and lip	Prenol lipids	Terpene glyc
4.13_152.99!	152.995067	4.12765	pos		Norfuraneol	HMDB00318	318101	DLVYTANE(	Organoheter	Dihydrofuran	Furanones
4.20_347.12·	347.124003	4.19935	pos		DHAP(10:0)	HMDB00116	62414	DWBYHKXQ	Organic oxyg	Organooxyge	Carbonyl cor

4.21_216.048	216.048676	4.20936667	neg	Carboxin	HMDB02496	68941	GYSSRZJIH	Benzenoids	Benzene and	Anilides
4.22_177.050	177.050038	4.21765	pos	L-3-Amino-2-	HMDB00386	93325	FNXJKVNOU	Organic acids	Carboxylic ac	Amino acids,
4.23_312.150	312.156584	4.22771667	neg	Carbuterol	HMDB0249665		KEMXXQOF	Benzenoids	Benzene and	N-phenylurea
4.24_378.160	378.166608	4.23603333	pos	Dityrosine	HMDB0006045		FHOZGLMQ	Lignans, neo	Unclassified	Unclassified
4.32_367.030	367.030712	4.32	neg	Polystyrene s	HMDB0015435		MAGFQRLK	Benzenoids	Benzene and	Benzenesulfo
4.33_454.242	477.231203	4.32651667	pos	Methyl cellulo	HMDB00299	86592	YLGXILFCIX	Organic oxyg	Organooxyge	Carbohydrate
4.43_231.027	231.027408	4.43021667	neg	Sodium (4-m	HMDB0303300		DCRRSNRL	Benzenoids	Benzene and	Benzoic acid
4.50_329.137	329.137066	4.50406667	neg	TETRAHYDROSAPPANO		44617	KXFFLWDYU	Unclassified	Unclassified	Unclassified
4.50_412.102	412.102536	4.50406667	neg	Ribosylzeatir	HMDB00348	90215	IRILMCCKFA	Nucleosides,	Purine nucle	Purine ribonu
4.51_229.040	229.040495	4.50633333	pos	2,3,4,5,6-Per	HMDB02454	353165	WPWWHXP	Benzenoids	Benzene and	Benzoic acid
4.52_232.092	232.092021	4.52393333	pos	(+/-)-(E)-Metl	HMDB0242268		HCUOEKSZ	Organic nitro	Organonitrog	Oximes
4.58_277.051	277.051073	4.57715	neg	4-Fluoro-3-pl	HMDB0246421		VLXNXMTVF	Benzenoids	Benzene and	Diphenylethe
4.63_229.051	229.051182	4.63258333	neg	5-(3-Aminopr	HMDB0258034		XBVUKAHA	Organoheter	Pyrans	Pyranones a
4.69_449.204	449.204306	4.6877	neg	1-dimethylarsinoyl-(2Z,5Z, 8Z,11Z,14Z,1			NSAFCTHJI	Lipids and lip	Fatty Acyls	Hydrocarbon
4.76_408.139	407.132031	4.76071667	neg	Wybutoxine	HMDB00391	93724	FRYWGCQT	Organic acids	Carboxylic ac	Amino acids,
4.88_332.242	332.242823	4.88355	pos	2S-hydroxy-3-(10Z-tetradecenoyloxy)-pı			LIMKXIDXBK	Lipids and lip	Fatty Acyls	Fatty esters
4.96_249.109	249.10953	4.95603333	pos	5'-Amino-5'-c	HMDB0246876		GVSGUDGN	Nucleosides,	5'-deoxyribor	Unclassified
5.03_425.238	448.227472	5.02876667	pos	Gly-pro-arg-p	HMDB02528	148571	WXPZDDCN	Organic acids	Carboxylic ac	Amino acids,
5.37_183.102	183.102315	5.36688333	neg	(R)-Cryptone	HMDB00359	91122	AANMVENR	Organic oxyg	Organooxyge	Carbonyl cor
5.45_476.330	476.336881	5.44698333	pos	Pubesenolide	HMDB00337	89500	FYYIHVSEG	Lipids and lip	Prenol lipids	Triterpenoids
5.46_383.153	383.153304	5.45918333	neg	16alpha-Hyd	HMDB0062544		ALBNSVAJD	Unclassified	Unclassified	Unclassified
5.59_420.158	420.158307	5.59335	pos	6-[[3-Fluoro-5	HMDB02533	426095	SLZBEPLWC	Organoheter	Quinolines ar	Quinolones a
5.66_117.019	117.01909	5.66205	neg	1,3-Dioxole	HMDB0244174		ABADUMLIA	Organoheter	Dioxoles	1,3-dioxoles
5.70_301.094	302.101751	5.70286667	pos	Citpressine I	HMDB00293	86187	OQMSMWR	Organoheter	Quinolines ar	Benzoquinoli
6.33_475.192	475.192592	6.32741667	neg	4'-Methoxych	HMDB00325	44091	KJHHAPASN	Phenylpropa	Linear 1,3-dia	Chalcones ar
6.34_189.163	189.163624	6.34418333	pos	4-(2,5,6,6-Te	HMDB0246948		BGKCUGPV	Lipids and lip	Prenol lipids	Sesquiterper
6.71_229.144	229.144236	6.71335	neg	(2S,6S,8S)-2,8-Dimethyl-1,7-dioxaspiro			AHOKGTBIV	Lipids and lip	Polyketides	Polyether ant
6.71_407.207	407.20753	6.71335	neg	Triptolide Analog		85120	ABKTWBMM	Unclassified	Unclassified	Unclassified
6.73_169.080	169.086788	6.73181667	neg	3,5,5-Trimeth	HMDB00402	458778	AVXVDNJFG	Organic oxyg	Organooxyge	Carbonyl cor
6.79_327.135	327.134952	6.7873	neg	3-(2-(Benzox	HMDB0244561		MHUVLRNBI	Organoheter	Benzoxazole	Unclassified
6.98_382.154	382.153951	6.98325	pos	Grepafloxaci	HMDB00145	320606	AIJTTZAVM	Organoheter	Quinolines ar	Quinoline car
6.98_450.120	450.120513	6.98325	pos	Magon	HMDB0246903		JSSROCUN	Benzenoids	Naphthalene	Naphthalene
7.13_320.294	320.294312	7.13003333	pos	Pregnan-20-c	HMDB0256754		RSRDWHPV	Lipids and lip	Steroids and	Pregnane ste

7.19_416.228	416.228941	7.19245	neg	Dodec-7-ene	HMDB0241228		FHUOXFYHI	Lipids and lip	Fatty Acyls	Fatty acid es
7.19_625.183	625.183165	7.19245	neg	Benorilate	HMDB02489	1261	FEJKLNWAC	Phenylpropa	Depsides and	Unclassified
7.27_605.238	605.238364	7.26623333	neg	Isomorellic a	HMDB00307	87160	COVMVPHA	Lipids and lip	Fatty Acyls	Fatty acid es
7.41_555.179	555.17975	7.41378333	neg	CAY10503		64659	DADMQAUV	Unclassified	Unclassified	Unclassified
7.59_421.303	421.303165	7.58896667	pos	N-Stearoyl A	HMDB0241938		VRILDFZVO	Organic acids	Carboxylic ac	Amino acids,
7.85_375.261	375.261289	7.8463	pos	N-goshuyoyl lysine			KWVQXGZF	Lipids and lip	Fatty Acyls	Fatty amides
7.88_340.247	340.247856	7.88303333	pos	Rapanone		68305	AMKNOBHC	Lipids and lip	Polyketides	Phenolic lipic
8.18_495.296	495.296397	8.1793	neg	polypodine B		57642	GMFLGNRC	Lipids and lip	Steroids and	Sterols
8.40_290.268	290.268442	8.39623333	pos	3-Hydroxyhe	HMDB00616	45828	CBWALJHXI	Lipids and lip	Fatty Acyls	Fatty acids a
8.53_631.241	631.240963	8.5302	neg	Darexaban g	HMDB0249922		IOUMBCGO	Organic oxyg	Organooxyge	Carbohydrate
8.60_227.165	227.165139	8.59883333	neg	trans- and cis	HMDB00325	88515	BPIALUCKE	Lipids and lip	Prenol lipids	Monoterpenc
8.88_311.146	311.146088	8.88258333	pos	N-(Dimethyla	HMDB0247659		GDQDKKCG	Organoheter	Imidazopyrim	Purines and
9.00_209.045	209.045385	9.00285	neg	Phenylpyruvi	HMDB00002	328	BTNMPGBKI	Benzenoids	Benzene and	Phenylpyruvi
9.50_375.103	375.103702	9.4972	neg	L-alpha-amin	HMDB0304407		SEPMYZAQI	Organic acids	Carboxylic ac	Amino acids,
9.56_464.336	464.336691	9.55885	pos	12-Ketoporric	HMDB0031079		AEDBOXCT	Lipids and lip	Prenol lipids	Triterpenoids
9.58_303.231	303.231264	9.57733333	pos	Yucalexin A1	HMDB00367	91795	AFZCVSRIU	Lipids and lip	Prenol lipids	Diterpenoids
9.77_533.006	533.006633	9.77068333	neg	Piridronic aci	HMDB0256596		NGMZSXZB	Organic acids	Organic phos	Bisphosphon
9.87_200.995	200.995502	9.86676667	pos	Fosfosal	HMDB02524	44294	FFKUDWZIC	Organic acids	Organic phos	Phosphate es
9.98_859.530	859.530654	9.97586667	pos	PI(12:0/22:1(11Z))			CLMYXZFLN	Lipids and lip	Glycerophos	Glycerophos
0.55_281.165	281.165137	0.55448333	pos	Girinimbine	HMDB00302	86827	GAEQWKVG	Organoheter	Indoles and c	Carbazoles
0.59_124.983	124.983422	0.59213333	neg	Peroxynitrite	HMDB00021	429944	CMFNMSML	Homogeneou	Non-metal ox	Non-metal pe
0.61_211.083	211.083473	0.61033333	neg	3-(Methylthio	HMDB00317	337720	CZUGFKJYC	Organosulfur	Thioethers	Dialkylthioeth
0.61_527.260	527.26595	0.61033333	neg	Alectinib	HMDB02481	413783	KDGFLJKFZ	Organoheter	Indoles and c	Carbazoles
0.63_267.057	267.057382	0.62835	neg	(S)-Ureidogly	HMDB00010	5935	NWZYYCVIC	Organic acids	Carboxylic ac	Amino acids,
0.63_333.035	333.034966	0.62835	neg	Urate radical	HMDB0060260		RKXSGLUFS	Organoheter	Imidazopyrim	Purines and
0.65_193.025	194.032797	0.6518	pos	Phosphocrea	HMDB00416	95994	HIPLEPXPN	Organic acids	Carboxylic ac	Amino acids,
0.65_317.115	317.11499	0.6518	pos	alhpa-tocopheronolactone		53844	AHKCSBSO	Lipids and lip	Prenol lipids	Quinones an
0.70_417.154	417.154702	0.70456667	neg	6-(4-methyl-2	HMDB0304223		GACVANPZ	Organoheter	Pyrans	Pyranones a
0.70_541.135	541.135232	0.70456667	neg	Ciadox	HMDB0250601		MBCZYLSVS	Organoheter	Diazanaphth	Benzodiazine
0.72_261.072	261.072486	0.71928333	neg	Aspartyl-Glut	HMDB00287	23953	CKAJHWFHI	Organic acids	Carboxylic ac	Amino acids,
0.72_315.093	315.093169	0.71928333	neg	6-(1,2,3,4-Te	HMDB0260352		JFEBLWKYN	Organic oxyg	Organooxyge	Carbohydrate
0.83_415.976	415.976771	0.82703333	neg	Fiacitabine	HMDB0252247		GIMSJJHKK	Nucleosides,	Pyrimidine nu	Pyrimidine 2'
1.02_188.068	187.060742	1.01586667	neg	Tetrahydro-2	HMDB0059767		CVYAVSIMB	Organic acids	Carboxylic ac	Dicarboxylic

1.05_203.06	203.067054	1.0516	neg		N-Nitrosobis(	HMDB0255200		AKRYBBWY	Organic nitro	Organonitrog	Organic nitro
1.10_460.23	460.232683	1.1046	pos		Lonchocarpol B		52771	HSPZUROD	Lipids and lip	Polyketides	Flavonoids
1.13_192.06	192.069833	1.13148333	neg		S-Methyl N,N	HMDB0257402		AQTFQQHF	Organosulfur	Thiocarbonyl	Thiocarbamic
1.14_233.14	233.149472	1.13681667	pos		6-hydroxyoct	HMDB0094767		AAMIEIRENI	Organic acids	Carboxylic ac	Amino acids,
1.14_303.16	303.166156	1.13681667	pos		Glycylprolylh	HMDB00021	6524	SZEOBSAZV	Organic acids	Carboxylic ac	Amino acids,
1.25_275.17	275.171038	1.251	pos		Val-Cit	HMDB0259738		AGGWFDNF	Organic acids	Carboxylic ac	Amino acids,
1.27_231.07	231.072278	1.26745	pos		4-hydroxylam	HMDB03041	316990	HTTDEAQRS	Benzenoids	Benzene and	Toluenes
1.27_386.01	386.014994	1.26745	pos		[(2S,3R,4R,5	HMDB0257860		ZWIADYZPC	Nucleosides,	Pyrimidine nu	Pyrimidine rib
1.32_215.01	215.017313	1.32371667	neg		Sodium algin	HMDB0303715		MSXHSNHN	Organic oxyg	Organooxyge	Carbohydrate
1.38_527.98	527.983128	1.37511667	neg		Arabinofuran	HMDB0248548		PCDQPRRS	Nucleosides,	Pyrimidine nu	Pyrimidine rib
1.50_627.13	627.132383	1.50483333	neg		7-Oxo-7h-Be	HMDB0258504		MYKOWOGZ	Benzenoids	Naphthalene	Naphthalene
1.59_114.96	114.961242	1.59411667	pos		Phosphoroth	HMDB0256494		RYYWUUFW	Homogeneou	Non-metal ox	Non-metal th
1.61_357.21	357.213379	1.61191667	pos		Xamoterol		69212	DXPOSRCH	Unclassified	Unclassified	Unclassified
1.65_408.15	408.152513	1.65196667	neg		(2S)-1-[(2S)-	HMDB0250908		ADDJKOLVC	Organic acids	Carboxylic ac	Amino acids,
1.68_133.06	133.064918	1.68358333	pos		o-Tolyl aceta	HMDB00320	337990	AMZORBZS	Benzenoids	Phenol esters	Unclassified
1.68_247.11	247.111241	1.68358333	pos		N-(3-oxo-hexanoyl)-homoserine thiolact			CBTLHSMHI	Lipids and lip	Fatty Acyls	Fatty amides
1.69_465.17	465.173775	1.68893333	neg		Trh-gly	HMDB0259129		CSVCLHYF	Organic acids	Carboxylic ac	Amino acids,
1.89_127.05	127.051182	1.89173333	neg		Dihydropyrida	HMDB02513	321650	BKWQKVJY	Organoheter	Diazines	Pyridazines a
10.01_337.2	320.257827	10.0126833	pos		N-(9Z-hexadecenoyl)-hom		64726	BDQHAQLG	Lipids and lip	Fatty Acyls	Fatty amides
10.36_437.3	437.311541	10.357	pos		Ile-Ile-Ile-Pro	HMDB03048	162431	OCEZPDBEI	Organic acids	Carboxylic ac	Amino acids,
10.52_662.2	662.276309	10.5172833	neg		Neoacrimarin	HMDB00403	94896	MHPPNTXKI	Organoheter	Quinolines an	Benzoquinoli
10.91_472.3	472.399307	10.9125667	pos		3-Hydroxyeic	HMDB0240767		ACXPNSWG	Lipids and lip	Fatty Acyls	Fatty acid es
10.97_284.2	284.223036	10.9697333	neg		Myristoylglyc	HMDB00132	427157	DYUGTPXLI	Organic acids	Carboxylic ac	Amino acids,
11.18_434.1	434.169237	11.1809167	pos		N-[3,3-Dimet	HMDB0260415		IOLRUMINDI	Organic acids	Carboxylic ac	Amino acids,
11.20_579.2	579.291807	11.198	pos		PKODiA-PA			FRSACBBYZ	Lipids and lip	Glycerophos	Oxidized glyc
11.42_548.2	548.296904	11.42155	neg		LysoPE(0:0/2	HMDB00114	62274	AKEOFNYGA	Lipids and lip	Glycerophos	Glycerophos
11.49_299.2	299.201349	11.494	neg		18-Nor-4(19)	HMDB00413	95776	ZPQHNIHJS	Lipids and lip	Prenol lipids	Diterpenoids
11.91_401.1	401.142264	11.9099667	neg		N-(6,8-Difluo	HMDB0245742		JTARFZSNU	Organoheter	Quinolines an	Haloquinoline
12.02_339.2	339.214901	12.0185833	neg		Kinetensin 1-	HMDB00129	20333	JXUGDUWB	Organic acids	Carboxylic ac	Amino acids,
12.11_365.1	365.193311	12.1088667	pos		5-Amino-1-[(	HMDB0252921		LECUGLHMI	Organic oxyg	Organooxyge	Carbohydrate
12.14_416.3	461.327213	12.1411	neg		3beta,4beta-dihydroxycholest-8(9)-en-2			AALOHBFIU	Lipids and lip	Steroids and	Sterols
12.34_389.1	389.192446	12.3449333	neg		DAz-2		64820	GQYUHTIXS	Unclassified	Unclassified	Unclassified
12.36_575.1	575.182519	12.3628333	neg		Iprobenfos	HMDB00317	330799	FCOAHACK	Benzenoids	Benzene and	Unclassified

12.45_417.1!	417.198166	12.44695	pos	AB-MECA	HMDB0242721		LDYMCRRF(	Nucleosides,	Purine nucle	Unclassified
12.55_510.3!	510.355011	12.5506167	pos	PC(O-16:0/1:0)		40057	BBASNTQPI	Lipids and lip	Glycerophos	Glycerophos
12.87_427.3(	427.306035	12.86515	neg	methyl 13-butylperoxy-9,1:		74462	GUWYTNFT	Lipids and lip	Fatty Acyls	Fatty Acids a
12.88_267.2:	267.232687	12.8780833	neg	Methyl 16-hy	HMDB0254562		AOTMRIXFF	Lipids and lip	Fatty Acyls	Fatty alcohol
12.92_363.1!	363.182084	12.9225667	pos	Tetraisopro	HMDB0257971		FEOZJNALV	Organic acid	Organic phos	Phosphate e
14.51_142.0(	142.061086	14.5128167	pos	4-Nitropyridir	HMDB0246547		FEXIEMAAK	Organic 1,3-(	Allyl-type 1,3	Organic nitro
2.09_623.17	623.171038	2.0924	neg	Quinacridone	HMDB00340	89686	NRCMAYZC	Organoheter	Quinolines ar	Benzoquinoli
3.34_360.20	360.201327	3.34363333	pos	Jasmolone g	HMDB00328	88786	NGPYRFLYI	Organic oxy	Organooxyge	Carbohydrate
3.50_140.03!	140.035141	3.49608333	neg	4-Hydroxypyridine		3232	GCNTZFIIOI	Unclassified	Unclassified	Unclassified
3.55_775.29!	775.298795	3.5518	neg	(R)-Heraclen	HMDB00390	93665	AEXJMQYVI	Phenylpropa	Coumarins a	Furanocoum
3.61_243.08	243.084937	3.6144	pos	(+)-gamma-H	HMDB0301838		UFBPWFOD	Organic acid	Carboxylic ac	Amino acids,
3.88_273.03:	273.033851	3.8786	neg	ensulizole		4009	UVCJGUGA(	Unclassified	Unclassified	Unclassified
3.93_458.29(	458.296583	3.92891667	pos	Netilmicin	HMDB0015090		ZBGPYVZLY	Organic oxy	Organooxyge	Carbohydrate
3.95_287.16	287.161161	3.94663333	pos	Endralazine	HMDB0251804		ALAXZYHFV	Benzenoids	Benzene anc	Benzoic acid
4.12_397.18!	397.188622	4.11676667	neg	Clidinium	HMDB0014909		HOOSGZJR(	Benzenoids	Benzene anc	Diphenylmetl
4.17_260.15(	260.150261	4.17231667	neg	N-Nonanoylg	HMDB0013279		JXDFEUKNI	Organic acid	Carboxylic ac	Amino acids,
4.20_348.23!	348.238213	4.19935	pos	oscr#17			FDWVWTCE	Lipids and lip	Fatty Acyls	Fatty acyl gly
4.31_519.26!	519.269565	4.30826667	pos	PG(17:1(9Z)/0:0)		80024	HSPWLAMZ	Lipids and lip	Glycerophos	Glycerophos
4.34_229.02	229.027843	4.34396667	pos	Stattic	HMDB02584	462936	ZRRGOUHIT	Organic 1,3-(	Allyl-type 1,3	Organic nitro
4.36_384.25(	384.250469	4.35616667	neg	Actinonin	HMDB0242670		XJLATMLVM	Organic acid	Carboxylic ac	Amino acids,
4.42_380.18:	380.182054	4.41721667	pos	PC(4:0/4:0)		39956	QIJYAMAPP	Lipids and lip	Glycerophos	Glycerophos
4.43_233.09:	233.093051	4.43021667	neg	5-Hydroxytry	HMDB02566	458025	HASNCBJLQ	Organoheter	Indoles and (	Pyridoindoles
4.54_601.19	601.197939	4.54091667	neg	Flutriafol	HMDB02524	72509	JWUCHKBS	Unclassified	Unclassified	Unclassified
4.58_468.10(	468.100518	4.57725	pos	Dinofenate	HMDB0251423		HEJVROKEI	Benzenoids	Benzene anc	Nitrobenzene
4.61_258.04	258.044007	4.61413333	neg	Metanephrin	HMDB0240719		WGKGYPCZ	Organic acid	Organic sulfu	Arylsulfates
4.61_586.32(	609.309407	4.61381667	pos	Presqualene diphosphate			ATZKAUGGI	Lipids and lip	Prenol lipids	Isoprenoids
4.72_270.09!	270.095887	4.72411667	neg	L-arginino-su	HMDB0304399		KRGUQRPU	Organic acid	Carboxylic ac	Amino acids,
4.72_284.07!	284.078802	4.72411667	neg	1,4-Bisdesox	HMDB0244200		HMAUMHGF	Organoheter	Diazanaphth	Benzodiazine
4.83_255.00	255.001625	4.83273333	neg	Thiazole-4-c	HMDB02589	330712	PQQRHWFF	Organoheter	Azoles	Thiazoles
4.87_247.08:	247.08221	4.86903333	neg	3-(1-Hydroxy	HMDB00330	88984	FUMUSIMA(	Lipids and lip	Fatty Acyls	Fatty acids a
4.92_525.03:	525.03297	4.92405	neg	4-Pyridoxic a	HMDB0246577		FTQNPJLEF	Organoheter	Pyridines anc	Pyridinecarb
5.10_500.20(	500.206089	5.10093333	pos	Neoaureothin			IZICQJAGBL	Lipids and lip	Polyketides	Polyether an
5.39_249.18	249.184654	5.39208333	pos	(6Z,10E)-hexadec-6,10-dien-8-ynoic aci			AJOUVFQEI	Lipids and lip	Fatty Acyls	Fatty Acids a

5.61_187.08	187.087446	5.60643333	neg	Pheneturide	HMDB02564	69458	AJOQSQHYI	Benzenoids	Benzene anc	Phenylpropar
5.72_236.09	236.096062	5.71736667	neg	3-(Cyclohexy	HMDB0247339		INEWUCPYE	Organic nitro	Organonitrog	Cyclohexylar
5.77_367.00	367.003224	5.773	neg	2-Thiouric ac	HMDB02453	435839	JDAXHCJXS	Organoheter	Imidazopyrim	Purines and
5.79_369.17	369.173954	5.7914	neg	Piperonyl sul	HMDB02586	72888	ATTZFSUZZ	Organoheter	Benzodioxole	Unclassified
5.87_168.10	168.102747	5.8654	neg	4-Dihydrocoll	HMDB0246411		MUSRXJZX\	Organoheter	Pyridines anc	Hydropyridine
6.27_159.98	159.986128	6.27191667	neg	thiol-maleimi	HMDB0259015		AUDYZXNUI	Organoheter	Pyrrolidines	Pyrrolidones
6.49_465.25	465.250783	6.49025	pos	Obelin	HMDB0255880		INULNSAIIZI	Organoheter	Quinolines ai	Benzoquinoli
6.64_176.00	351.008478	6.63946667	neg	3,4,5-Trifluorobenzoic acic		411138	VJMYKESYF	Unclassified	Unclassified	Unclassified
6.73_222.97	222.977862	6.73181667	neg	4-thiodimethylarsenobutanoic acid			LPOQZCAOI	Lipids and lip	Fatty Acyls	Other Fatty A
6.99_443.15	443.155944	6.98976667	neg	Methyl 3,4-di	HMDB00401	94662	OZKMIACEG	Lipids and lip	Prenol lipids	Terpene glyc
7.19_397.20	397.205495	7.19245	neg	2-(4-Hydroxy	HMDB0251106		CQGSORMF	Benzenoids	Benzene anc	Phenylpropar
7.21_179.07	179.071159	7.21093333	neg	7-Nonene-3,5	HMDB00345	90027	BPNAUFVE(	Lipids and lip	Fatty Acyls	Fatty alcohol
8.18_414.12	414.124884	8.17628333	pos	Glucose lacta	HMDB0252780		ADHQBUXW	Lipids and lip	Fatty Acyls	Fatty acyl gly
8.22_197.02	197.020071	8.21615	neg	2,4-Dinitroan	HMDB0245458		CVYZVNVP(	Benzenoids	Benzene anc	Nitrobenzene
8.51_381.26	381.26064	8.50668333	pos	9,13-dihydroxy-10-ethoxy-		74511	BHDZXVBB\	Lipids and lip	Fatty Acyls	Fatty Acids a
8.64_229.18	229.180657	8.63586667	neg	3Z-Dodecen-1-ol		46105	BDGQTWOH	Lipids and lip	Fatty Acyls	Fatty alcohol
8.76_115.05	115.054331	8.75856667	pos	3,4-octadien-5,7-diyn-1-ol			WHVOFFPT	Lipids and lip	Fatty Acyls	Fatty alcohol
8.95_229.00	229.009045	8.94916667	neg	Pancrelipase	HMDB03048	72500	XRHVZWWF	Organic acid	Carboxylic ac	Dicarboxylic
8.95_480.25	480.258207	8.94916667	neg	7,14-Dihydro	HMDB0241558		BEUSUPRM	Lipids and lip	Fatty Acyls	Fatty acid es
9.31_210.07	210.076989	9.3144	neg	4-Ethoxyben:	HMDB0246418		AZEIRPAUJ	Benzenoids	Benzene anc	Benzoic acid
9.40_631.27	631.275723	9.39628333	pos	13-Acetyl-9-Dihydrobaccat		73546	WPPPFZJNI	Unclassified	Unclassified	Unclassified
9.52_305.17	305.17182	9.52293333	pos	(6E,8S,10Z)-	HMDB0062366		NWVJXHCR	Lipids and lip	Fatty Acyls	Fatty acids a
9.59_239.16	239.165196	9.58798333	neg	beta-Ionol	HMDB00368	91834	CNOPDZWC	Lipids and lip	Prenol lipids	Sesquiterper
9.79_349.19	349.197863	9.79426667	pos	7-Hydroxyoc	HMDB0241715		HCIPFHRBB	Lipids and lip	Fatty Acyls	Fatty acid es
9.81_307.02	307.029882	9.81245	pos	zardaverine	HMDB0259989		HJMQDJPM(	Organoheter	Diazines	Pyridazines a
9.90_301.21	301.215732	9.90308333	pos	CAY10410		45631	AFBJLDUEE	Unclassified	Unclassified	Unclassified
0.61_213.09	213.098295	0.60603333	pos	Histidylglycin	HMDB00288	85791	LYCVKHSJG	Organic acid	Carboxylic ac	Amino acids,
0.65_318.12	318.129284	0.6518	pos	N-(1-Deoxy-1	HMDB00378	92647	UCRYORFH	Organic acid	Carboxylic ac	Amino acids,
0.67_377.10	377.106228	0.66691667	pos	(6S)-6,7-Dih	HMDB0248078		ZLHZLMOSF	Benzenoids	Benzene anc	Benzylethers
0.68_243.18	243.183315	0.68255	pos	2-N,2-N-Dibu	HMDB0257613		ZWAAKLAH	Organic nitro	Organonitrog	Amines
0.70_439.07	439.076076	0.70456667	neg	Thidiazuron		72579	HFCYZXMH	Unclassified	Unclassified	Unclassified
0.71_132.02	132.027142	0.71368333	pos	Trifluoroaceti	HMDB00141	85337	DTQVDTLA(	Organic acid	Carboxylic ac	Alpha-haloca
0.72_383.09	383.099549	0.71928333	neg	4-Hydroxy-6-	HMDB0246447		PTFCNBWC	Organoheter	Pyrans	Pyranones ar

0.73_198.12	198.123685	0.72825	pos		L-2-Amino-3-	HMDB00303	86943	CMUHFUGD	Organic acid	Carboxylic ac	Amino acids,
0.88_310.05	310.057043	0.87973333	pos		Incadronic ac	HMDB02534	412751	LWRDQHOZ	Organic acid	Organic phos	Bisphosphon
1.00_159.02	159.027404	0.99793333	neg		1-Benzothiep	HMDB0243832		NIGNBCLEM	Organoheter	Benzothiepin	Unclassified
1.08_229.15	229.154532	1.07531667	pos		Isoproterenol	HMDB00151	852	JWZZKOKVI	Benzenoids	Phenols	Benzenediols
1.10_105.01	105.019097	1.09681667	neg		1,2-Dioxetan	HMDB0251435		BVTJGGGYI	Organoheter	Dioxetanes	Unclassified
1.10_563.12	563.122905	1.09681667	neg		Phenytoin qu	HMDB0060868		AWCFFJFKI	Organoheter	Azolidines	Imidazolidine
1.15_134.08	134.081246	1.1535	pos		L-2-Amino-5-	HMDB00316	87840	CZWARROQ	Organic acid	Carboxylic ac	Amino acids,
1.15_306.16	306.165826	1.1535	pos		Syndesine	HMDB0039161		QWGIOMIEV	Organic acid	Carboxylic ac	Amino acids,
1.22_233.02	233.021443	1.21911667	neg		(2-Methylphe	HMDB0243569		HUJOGFUFU	Organic acid	Organic phos	Phosphate es
1.22_283.03	283.03461	1.21911667	neg		D-Erythro-im	HMDB00122	327694	HFYBTHCYF	Organic acid	Organic phos	Phosphate es
1.23_362.07	723.151767	1.23491667	neg		Tephcalostan		48315	KKHJPBHZH	Lipids and lip	Polyketides	Flavonoids
1.25_387.02	387.023205	1.2521	neg		2,3-Dihydro-5	HMDB02444	412258	BJAJDJDOD	Organoheter	Thienopyrimi	Unclassified
1.27_112.99	112.998999	1.26985	neg		Oxaluric acid	HMDB03030	63388	UWBHMRBF	Organic acid	Carboxylic ac	Amino acids,
1.32_125.04	125.046655	1.32371667	neg		5-Aminoimida	HMDB00031	629	DVNYTAVYE	Organoheter	Azoles	Imidazoles
1.45_289.08	289.089662	1.45418333	pos		Glucose 3-hy	HMDB0252771		HIBYJNNRF	Organic oxyg	Organooxyge	Carbohydrate
1.56_175.08	175.082461	1.55895	pos		5-Diazo-4-ox	HMDB0246779		FMHPSVZSI	Organic acid	Carboxylic ac	Amino acids,
1.67_133.06	133.060935	1.66533333	pos		(Methyl-ONN	HMDB02436	72978	BELPJCDYW	Organic 1,3-c	Allyl-type 1,3	Azoxy compo
1.73_280.05	280.059095	1.72581667	neg		alpha-Cepha	HMDB0302282		CFWRDBDJ	Lipids and lip	Glycerophos	Glycerophos
10.01_265.2	265.206656	10.0126833	pos		(-)-Quebrach	HMDB0030260		FDNDLNFGI	Alkaloids and	Quebracham	Unclassified
10.09_379.2	379.248013	10.0853667	pos		ent-13-F4t-NeuroP			BGXODHLJN	Lipids and lip	Fatty Acyls	Docosanoids
10.30_318.2	318.239832	10.3023333	pos		Tetrahydrobungeanool			GJDPGFHVI	Lipids and lip	Fatty Acyls	Fatty amides
10.34_241.2	241.226984	10.3389333	pos		Pellitorine			MAGQQZHF	Lipids and lip	Fatty Acyls	Fatty amides
10.34_494.3	494.383215	10.3389333	pos		Homodolicho	HMDB00340	89701	DXUFRIYNC	Lipids and lip	Steroids and	Stigmastanes
10.41_422.2	422.267773	10.4089833	neg		1-(2-methoxy-tetradecanyl)-sn-glycero-3			WWHPIBOG	Lipids and lip	Glycerophos	Glycerophos
10.43_434.3	434.304141	10.42685	neg		PC(O-14:0/0:0)		40381	MBRLTZVQ	Lipids and lip	Glycerophos	Glycerophos
10.86_336.3	336.310264	10.8590333	pos		1-O-(2R-hydroxy-pentadecyl)-sn-glycer			XNBDVGMH	Lipids and lip	Glycerolipids	Monoradylgly
10.95_277.1	277.179448	10.9478667	pos		Myrsinone	HMDB00406	95124	JPFXYNDBK	Lipids and lip	Prenol lipids	Quinone and
11.01_902.5	902.519154	11.0055	neg		PC(20:5(5Z,8	HMDB0287691		ANYFJCAZN	Unclassified	Unclassified	Unclassified
11.23_548.2	548.298699	11.2342333	pos		PG(20:5(5Z,8Z,11Z,14Z,1		80018	UBJZUYNJJ	Lipids and lip	Glycerophos	Glycerophos
11.36_246.1	246.196144	11.36075	pos		Indalpine	HMDB02534	342160	SADQVAVF	Organoheter	Indoles and d	Indoles
11.43_299.1	299.143679	11.4320667	pos		4,7,10,13,16,19-Docosahe		74278	OYADDSIRD	Lipids and lip	Fatty Acyls	Fatty Acids a
11.49_345.2	345.243274	11.494	neg		Allylestrenol	HMDB00155	41879	ATXHVCQZ	Lipids and lip	Steroids and	Estrane stero
11.56_636.3	636.349365	11.5642333	neg		Allodesmosir	HMDB00407	95178	IQEPQBCFQ	Organic acid	Carboxylic ac	Pentacarbox

11.56_640.29	640.292267	11.5642333	neg	Lipoxin C4		45970	CYCIJLWHN	Lipids and lip	Fatty Acyls	Eicosanoids
11.65_358.24	358.242105	11.6538333	neg	N-Myristoyl M	HMDB0242056		CLXADVHCS	Organic acids	Carboxylic ac	Amino acids,
11.65_373.25	373.259267	11.6538333	neg	MG(0:0/16:1(	HMDB00115	62318	CXUXMSAC	Lipids and lip	Glycerolipids	Monoradylgly
11.66_207.01	207.017139	11.6642167	pos	2,3-Diaminos	HMDB0013159		CGKSFNAG	Benzenoids	Benzene and	Benzoic acid
11.79_375.25	375.254081	11.7918667	neg	2-METHYL GRAMINE		44003	AJNMXMZCI	Unclassified	Unclassified	Unclassified
11.93_573.16	573.166799	11.9285167	neg	Clitocine	HMDB00337	89495	OHEMBWZZ	Organic oxyg	Organooxyge	Carbohydrate
12.09_408.22	408.229364	12.0909333	pos	N-Arachidonyl Maleimide		45064	GZNZRHSG	Unclassified	Unclassified	Unclassified
12.13_295.18	295.188875	12.12775	neg	1,2-Bis(2-(4,5	HMDB0245595		LWMFAFLIW	Organoheter	Imidolactams	Unclassified
12.38_864.45	882.488573	12.37545	pos	PGP(18:3(9Z	HMDB0273179		AFJWDCCU	Unclassified	Unclassified	Unclassified
12.48_697.44	697.442283	12.4754333	neg	PA(a-15:0/20	HMDB0266899		ARGKIJVPJ	Unclassified	Unclassified	Unclassified
12.62_405.17	405.178779	12.6234833	neg	(S)-Norfenflu	HMDB02437	2681	MLBHFBKZU	Benzenoids	Benzene and	Phenethylam
13.19_370.27	370.276857	13.1900333	pos	N-palmitoyl methionine		75491	FCVASHHB	Lipids and lip	Fatty Acyls	Fatty amides
13.33_271.27	271.274016	13.32975	pos	Obscuraminol C			PKCDBAJES	Lipids and lip	Sphingolipids	Sphingoid ba
13.38_395.21	395.214081	13.38305	pos	13,14-dihydro-15-keto-PG		36225	VSRXYLYXI	Lipids and lip	Fatty Acyls	Eicosanoids
13.62_429.32	429.321797	13.6219833	neg	(Z)-3-(1,2-Dil	HMDB0255948		FCSMZCCJF	Lipids and lip	Fatty Acyls	Fatty alcohol
14.06_425.21	425.214277	14.0639333	pos	Dibutyryl ade	HMDB0251178		YBPWNFMP	Nucleosides,	Purine nucle	Unclassified
14.17_169.10	169.108269	14.1715833	pos	chondroitin s	HMDB0062464		MOCKWYU	Benzenoids	Benzene and	N-phenylurea
14.23_342.29	342.299908	14.2258	pos	Cer(d18:1/2:0)		411	BLTCBVOJN	Lipids and lip	Sphingolipids	Ceramides
14.28_593.55	593.551315	14.2783333	neg	Heneicosanyl palmitoleate			PXPRWWK	Lipids and lip	Fatty Acyls	Fatty esters
14.38_377.26	377.266816	14.3838667	neg	Diethylamino	HMDB0251243		QLAJPGCD	Organic oxyg	Organooxyge	Carbohydrate
14.57_393.29	393.297334	14.5668	pos	FAHFA(10:0/3-O-12:0)			ACHKZGVG	Lipids and lip	Fatty Acyls	Fatty esters
15.27_126.06	126.066157	15.2723833	pos	Cyclocreatine	HMDB02506	44140	AMHZIUVRY	Organic acids	Carboxylic ac	Amino acids,
2.04_366.130	366.13043	2.03931667	neg	Carboquone	HMDB02496	316296	SHHKQEUP	Organic oxyg	Organooxyge	Carbonyl cor
2.11_249.049	249.049026	2.11061667	neg	5,5-Diphenyl-	HMDB0246851		AMDPNECW	Benzenoids	Benzene and	Diphenylmeth
2.25_203.084	203.08485	2.24641667	pos	ethyl-2,4-dimethyl-Thiazole		96503	BXOIIRQIGY	Unclassified	Unclassified	Unclassified
2.39_147.112	147.112929	2.39026667	pos	D-Lysine	HMDB00034	6919	KDXKERNSI	Organic acids	Carboxylic ac	Amino acids,
2.64_449.206	449.206483	2.64221667	pos	PA(0:0/16:0)		59309	LPQBQFLKE	Lipids and lip	Glycerophos	Glycerophos
2.71_170.081	170.081218	2.71386667	pos	Vanillin	HMDB00123	62927	MWOOGOJI	Benzenoids	Phenols	Methoxypher
2.72_168.066	168.066415	2.7196	neg	2-Propionylpy	HMDB00324	88492	AEJPPSRYX	Organic oxyg	Organooxyge	Carbonyl cor
2.75_258.169	258.169278	2.74981667	pos	Mycinonic acid III			BMTLPJXRT	Lipids and lip	Fatty Acyls	Fatty Acids a
2.91_406.200	406.200918	2.9106	pos	Xambioona		52543	FGJUXFVUC	Lipids and lip	Polyketides	Flavonoids
3.16_139.039	139.039066	3.16281667	pos	IRETOL		43632	ASTPOGDW	Unclassified	Unclassified	Unclassified
3.54_241.069	241.06916	3.54413333	pos	1-Methyl-2-ni	HMDB0244741		YKBZOVFAC	Organic nitro	Organonitrog	Guanidines

3.54_446.23	446.237468	3.54413333	pos		Hypochoeros	HMDB0303759		CPKWYFUG	Lipids and lip	Prenol lipids	Terpene lact
3.55_364.20	727.400057	3.5518	neg		Spirorenone	HMDB0258430		NZUIUYWHI	Lipids and lip	Steroids and	Steroid lacto
3.59_261.04	261.041061	3.58845	neg		Isobergapter	HMDB03028	43733	AJSPSRWW	Phenylpropa	Coumarins a	Furanocoum
3.59_383.04	383.047277	3.58845	neg		2,3-Diketo-L-	HMDB00065	58437	GJQWCDSA	Organic oxyg	Organooxyge	Carbohydrate
3.68_319.97	319.978726	3.67736667	neg		3-Iodo-alpha	HMDB0245909		KPOIUSXAP	Phenylpropa	Phenylpropa	Unclassified
3.72_134.09	134.096505	3.71513333	pos		Tranylcypromine		2895	AELCINSCM	Unclassified	Unclassified	Unclassified
3.72_365.18	365.181994	3.71513333	pos		N-[[2-(4-Amir	HMDB0257527		MBCJUIJWF	Organoheter	Imidazopyrid	Imidazo-[4,5-
3.73_259.07	259.07553	3.73108333	neg		Methyl bisnor	HMDB00048	7084	ITHUGYGPF	Organoheter	Thienoimidaz	Unclassified
3.79_601.33	601.334866	3.78573333	pos		3beta,15alpha,16alpha,24alpha-tetrahy			WTXVPPRU	Lipids and lip	Steroids and	Sterols
3.80_439.10	439.101868	3.80401667	pos		Riboflavin cy	HMDB0059614		CVZKYDYR	Organoheter	Pteridines an	Alloxazines a
3.93_510.29	510.293225	3.92891667	pos		2-(8-[3]-ladderane-octanyl		77699	MUJGIVXHP	Lipids and lip	Glycerophos	Glycerophos
3.96_228.02	228.024486	3.96453333	pos		2,3,4-Trinitro	HMDB02454	321528	FPKOPBFLF	Benzenoids	Benzene and	Nitrobenzene
3.96_407.22	407.228967	3.96453333	pos		Litchioside C			KZBDSSNNS	Lipids and lip	Fatty Acyls	Fatty acyl gly
4.17_336.07	336.07259	4.17231667	neg		2,5-diamino-(	HMDB03040	72671	ACIVVGBVC	Organic oxyg	Organooxyge	Carbohydrate
4.19_615.34	615.347012	4.1908	neg		Gestrinone	HMDB00027	3087	BJJXHLWLU	Lipids and lip	Steroids and	Estrane stero
4.21_233.00	233.006776	4.20936667	neg		Potassium gl	HMDB0303731		HLCFGWHY	Organic acids	Hydroxy acid	Medium-chai
4.21_385.10	385.101666	4.20936667	neg		3-Fluoro-8-m	HMDB0257668		QTKJKYVHY	Organoheter	Quinolines a	Quinolones a
4.24_379.19	379.197996	4.23603333	pos		6-[Ethyl-(3-is	HMDB0260356		JUCWZKDT	Organic nitro	Organonitrog	Amines
4.30_603.00	603.003998	4.30156667	neg		Ellagic acid	HMDB00028	3430	AFSDNFLWI	Phenylpropa	Tannins	Hydrolyzable
4.43_434.98	434.989923	4.43021667	neg		Edtmp	HMDB02446	343344	NFDRPXJGI	Organic acids	Organic phos	Organic phos
4.47_491.33	491.335809	4.47151667	pos		5beta-Cholestane-3alpha,		84777	BUVRTWKY	Lipids and lip	Steroids and	Bile acids an
4.58_222.05	222.059224	4.57715	neg		1-(2,2-Difluor	HMDB0257565		RUESXRXDI	Organoheter	Pyrrolidines	Pyrrolidine ca
4.69_513.28	513.284571	4.68661667	pos		pedilstatin		53688	ALKHEZOKT	Lipids and lip	Prenol lipids	Isoprenoids
4.71_195.06	195.063482	4.70578333	neg		2,6-Diaminop	HMDB02455	344623	MSSXOMSJI	Organoheter	Imidazopyrim	Purines and
4.72_394.19	394.196992	4.72301667	pos		Diacetylfusar	HMDB00389	93579	NJAMLUYVC	Organoheter	Benzopyrans	1-benzopyra
4.74_279.08	279.080649	4.74241667	neg		1,1,1,2,2-Per	HMDB02522	45480	ROPVXAWE	Benzenoids	Benzene and	Unclassified
4.88_213.15	213.159602	4.88355	pos		[3-(2-Aminop	HMDB0257571		LKRVDGINS	Organic oxyg	Organooxyge	Alcohols and
4.92_248.13	248.132391	4.92405	neg		VERNOLATE	HMDB02597	72839	OKUGPJPKI	Organosulfur	Thiocarbonyl	Thiocarbami
4.96_314.10	314.104567	4.96071667	neg		1,2-Disehydr	HMDB0302245		CCGUUHSJ	Organoheter	Isoquinolines	Benzylisoqui
5.06_130.06	130.065142	5.06456667	pos		Cinnamamid	HMDB0250263		APEJMQOB	Phenylpropa	Cinnamic aci	Cinnamic aci
5.11_371.12	371.124828	5.10843333	neg		Scriptaid		64766	JTDYUFSDZ	Unclassified	Unclassified	Unclassified
5.47_167.05	150.055015	5.46526667	pos		Isopyridoxal	HMDB00042	7046	GNKXHPULS	Organoheter	Pyridines and	Pyridine carb
5.58_214.03	214.032825	5.57501667	pos		Nitrilotriaceti	HMDB02556	70268	MGFYIUFZL	Organic acids	Carboxylic ac	Amino acids,

5.61_346.258	346.258313	5.61161667	pos	(11E,15Z)-9,10,13-Trihydr		985181	ADHVWICOI	Unclassified	Unclassified	Unclassified
5.94_413.200	413.20038	5.93928333	neg	3-(2-Phenyle	HMDB0257743		CNPMTFIIJC	Benzenoids	Benzene anc	Styrenes
6.20_256.097	256.097299	6.1974	pos	1-Hydroxy-3-	HMDB00293	86186	OCUBFJMZY	Organoheter	Quinolines ai	Benzoquinoli
6.53_213.014	213.014916	6.53055	neg	1,2-Dinitrobe	HMDB02440	70273	IZUKQUVSC	Benzenoids	Benzene anc	Nitrobenzene
6.79_435.145	435.145716	6.7873	neg	6-Butyryl-5-hydroxy-4-phe		48383	HTYHJXFUS	Lipids and lip	Polyketides	Flavonoids
6.80_419.109	419.109435	6.79993333	pos	3-O-Demethylamorphigeni		48029	ABUBWVZS	Lipids and lip	Polyketides	Flavonoids
6.86_432.240	432.240944	6.85513333	pos	undecylprodi	HMDB0259405		HIYSWASSI	Organoheter	Pyrroles	Substituted p
6.90_271.018	271.018098	6.8979	neg	Imidazotetra	HMDB0253425		TWVFRCAH	Organoheter	Tetrazines	Unclassified
7.20_450.193	450.19333	7.20335	pos	dihydrogeran	HMDB0304335		YJGANOFPA	Lipids and lip	Prenol lipids	Diterpenoids
7.24_397.305	397.305473	7.24003333	pos	Leukotriene I	HMDB00023	36243	DQLVVNIINU	Organic nitro	Organonitrog	Amines
7.98_341.061	341.061666	7.97648333	neg	Trifluridine	HMDB00145	315515	VSQQQLOS	Nucleosides,	Pyrimidine nu	Pyrimidine 2'
8.03_175.148	175.147972	8.0298	pos	4-(2,6,6-Trim	HMDB00325	88514	BGTBFNDXY	Organic oxyg	Organooxyge	Carbonyl cor
8.53_287.121	287.121741	8.5302	neg	N,N'-dinitros	HMDB00419	96186	WNSYEWGY	Organoheter	Diazinanes	Piperazines
8.60_300.991	300.991569	8.59883333	neg	Erythrityl Tet	HMDB00155	85532	SNFOERUNI	Organic oxyg	Organic oxoa	Organic nitra
8.65_447.346	447.346303	8.65385	pos	Certonardosterol C			AHDGWLLY	Lipids and lip	Steroids and	Sterols
8.71_336.249	336.249932	8.7078	pos	Pegvaliase	HMDB0256204		NPOCDVAO	Organic acids	Carboxylic ac	Amino acids,
9.00_154.990	154.990349	9.00285	neg	Phenyl dihyd	HMDB02564	65822	CMPQUABW	Organic acids	Organic phos	Phosphate es
9.05_271.205	271.205377	9.04725	pos	Dehydroandr	HMDB00059	455964	FMGSKLZLN	Lipids and lip	Steroids and	Androstane s
9.07_339.154	339.15396	9.0656	pos	Niazirinin	HMDB00328	88730	QSGQMXJN	Organic oxyg	Organooxyge	Carbohydrate
9.27_594.130	617.125805	9.26791667	pos	7-O-(4-Hydro	HMDB00404	94971	CAHLQXBTN	Phenylpropa	Flavonoids	Flavonoid gly
9.47_330.245	330.245533	9.46953333	pos	EPA (d5)		96379	JAZBEHYOT	Lipids and lip	Fatty Acyls	Fatty Acids a
9.71_360.346	360.346676	9.70535	pos	2-hydroxy-heneicosanoic acid			DHCJPXKW	Lipids and lip	Fatty Acyls	Fatty Acids a
0.59_382.181	382.181934	0.58926667	pos	n-acetylmura	HMDB0255071		BHFOXNINB	Organic acids	Carboxylic ac	Amino acids,
0.61_276.155	276.155463	0.60603333	pos	Glutamyllysir	HMDB00042	23795	BBBXWRGI	Organic acids	Carboxylic ac	Amino acids,
0.64_196.061	241.059572	0.64391667	neg	Alafosfalin	HMDB0248096		BHAYDBSY	Organic acids	Carboxylic ac	Amino acids,
0.67_161.056	161.056581	0.6745	neg	3-Amino-4-o	HMDB0258109		FVRHIJPPPS	Organic acids	Carboxylic ac	Amino acids,
0.69_199.936	199.93681	0.68961667	neg	4-Iodoaniline	HMDB02464	338129	VLVCDUSVT	Benzenoids	Benzene anc	Aniline and s
0.75_202.996	202.996567	0.74898333	neg	2-Hydroxy-5-	HMDB02451	458292	HNYMLXYAI	Benzenoids	Benzene anc	Benzoic acid
0.75_312.075	312.075837	0.74898333	neg	2-Methylthio	HMDB0245242		AJNDEAZTA	Nucleosides,	Purine nucle	Unclassified
0.79_283.091	284.098736	0.7877	pos	8-Hydroxy-2-	HMDB0247436		HCAJQHYU	Nucleosides,	Purine nucle	Purine 2'-dec
0.85_224.995	224.995619	0.84881667	pos	Angelicin	HMDB00339	67393	XDROKJSW	Phenylpropa	Coumarins a	Furanocoum
0.89_349.105	349.104956	0.8943	neg	Indole-3-ace	HMDB0038665		YRKLGWOH	Organic acids	Carboxylic ac	Amino acids,
0.93_274.926	274.926295	0.92753333	neg	Dimethylarsi	HMDB00116	44215	OGGXGZAM	Organometal	Organometal	Organoarser

0.98_201.15	201.159817	0.97735	pos	Methyprylon	HMDB00152	85468	SIDLZWOQU	Organoheter	Piperidines	Piperidinones
1.03_990.32	1013.31623	1.02758333	pos	Pgg-glucan	HMDB0256373		KUMGPIKDD	Organic oxyg	Organooxyge	Carbohydrate
1.07_655.10	655.108647	1.06741667	neg	Bryaquinone		48231	GRCODZNX	Lipids and lip	Polyketides	Flavonoids
1.10_282.00	282.005769	1.1046	pos	Thioxanthine	HMDB0060876		OAOIXPPCC	Organoheter	Imidazopyrim	Purines and
1.11_350.03	350.038653	1.1137	neg	Cifostodine	HMDB0250240		NMPZCCZX	Nucleosides,	Pyrimidine nu	Cyclic pyrimi
1.12_486.17	486.171724	1.11995	pos	Arginyl-glycy	HMDB0248575		DJXDNYKQ	Organic acids	Carboxylic ac	Amino acids,
1.14_111.05	111.053261	1.13681667	pos	L-alaninamid	HMDB0253863		HQMLIDZJX	Organic acids	Carboxylic ac	Amino acids,
1.25_352.94	352.94489	1.2521	neg	2'-Fluoro-5-ic	HMDB0245538		IPVFGAYTK	Nucleosides,	Pyrimidine nu	Pyrimidine 2'
1.34_436.24	436.240322	1.34166667	neg	3-O-Demethy	HMDB0245956		OWTJVZIKR	Organic oxyg	Organooxyge	Carbohydrate
1.45_571.13	571.131055	1.44935	neg	3'-Methoxyfu	HMDB00294	86308	DDSGTDZC	Phenylpropa	Phenylpropa	Unclassified
1.47_181.07	181.073465	1.47146667	pos	Diethyl cyan	HMDB0251238		ZWWWLCM	Organic acids	Organic phos	Phosphonic a
1.51_413.20	413.203219	1.50628333	pos	Deoxypyridin	HMDB0000569		ZAHDXEIQW	Organic acids	Carboxylic ac	Amino acids,
1.62_239.01	239.014885	1.61501667	neg	Methacrifos		331172	NTAHCMPO	Unclassified	Unclassified	Unclassified
1.62_297.05	297.056624	1.61501667	neg	threo-hydrox	HMDB0259046		DZYHJDLBF	Organic acids	Carboxylic ac	Amino acids,
1.80_224.98	224.983848	1.79976667	neg	Sodium n-alk	HMDB03032	337830	MZSDGDXX	Benzenoids	Benzene and	Benzenesulfo
1.82_159.04	159.040923	1.81826667	neg	3-Cyanoalan	HMDB0245844		BXRLWGXP	Organic acids	Carboxylic ac	Amino acids,
10.03_312.1	312.159015	10.0311	pos	(S)-Reticulin	HMDB00036	6967	BHLYRWXG	Organoheter	Isoquinolines	Benzylisoqui
10.09_430.3	430.351704	10.0853667	pos	11-Hydroxyh	HMDB0241514		PHOYWWU	Lipids and lip	Fatty Acyls	Fatty acid es
10.18_344.3	344.315345	10.17625	pos	2-oxophytanic acid		53584	CQJGVSCAI	Lipids and lip	Prenol lipids	Isoprenoids
10.21_357.3	357.347044	10.2120667	pos	AM3102		45552	IPVYNYWIR	Unclassified	Unclassified	Unclassified
10.41_533.0	533.023641	10.4089833	neg	N-acetylgluta	HMDB0304422		OJJHFKVRJ	Organic acids	Carboxylic ac	Amino acids,
10.56_506.3	506.360376	10.5560833	pos	Platelet-activ	HMDB00621	34488	HVAUUPRF	Lipids and lip	Glycerophos	Glycerophos
10.90_455.2	455.202672	10.8978167	neg	Acetyl-arginy	HMDB0247924		GFOJSWCC	Organic acids	Carboxylic ac	Amino acids,
11.02_508.2	508.277227	11.0205667	pos	Isodesmosin	HMDB00007	5707	RGXCTRIQ	Organic acids	Carboxylic ac	Tetracarboxy
11.20_428.7	428.787801	11.198	pos	3,5-Diiodosal	HMDB02460	335958	DHZVWQPH	Benzenoids	Benzene and	Benzoic acid
11.52_574.4	574.430203	11.5217833	pos	DG(8:0/PGF	HMDB0297255		HPERHSSF	Unclassified	Unclassified	Unclassified
11.54_345.2	345.2394	11.5398167	pos	(4E,6Z)-3-Hy	HMDB0241110		GZINPZNSG	Lipids and lip	Fatty Acyls	Fatty acid es
11.56_653.3	653.33983	11.5642333	neg	Azaperone	HMDB02487	3933	XTKDAFGW	Organic oxyg	Organooxyge	Carbonyl cor
11.98_347.2	347.255185	11.9841667	pos	8-Oxodecan	HMDB0241140		AALNTBZQF	Lipids and lip	Fatty Acyls	Fatty acid es
12.04_113.1	113.107488	12.0372667	pos	(S)-2-Amino-	HMDB0247199		FORGMRSG	Organic acids	Carboxylic ac	Amino acids,
12.05_268.2	268.263924	12.0546333	pos	iso (4E,15-methyl-d16:1) s		53905	LZKPPSAEI	Lipids and lip	Sphingolipids	Sphingoid ba
12.07_622.4	622.407323	12.0728	pos	PA(10:0/18:1	HMDB0262701		DEXKYTZV	Unclassified	Unclassified	Unclassified
12.30_395.2	395.219932	12.3037667	neg	1-pentadeca	HMDB00623	82356	RZDCKQAR	Lipids and lip	Glycerophos	Glycerophos

12.31_653.4!	653.454979	12.3147833	neg		FAHFA(22:6(	HMDB0112171	RPHQLMFTI	Lipids and lip	Fatty Acyls	Fatty acids a
12.36_557.4!	557.457613	12.3628333	neg		Chatenaytrie	HMDB0031910	BALJQQWV	Lipids and lip	Fatty Acyls	Fatty alcohol
13.21_355.1!	355.157975	13.21015	neg		Hydroxyzine	HMDB00146 4069	ZQDWXGKK	Benzenoids	Benzene anc	Diphenylmetl
13.58_353.2	353.242142	13.5784833	pos		Mutanamide		IFXDDVMQZ	Lipids and lip	Fatty Acyls	Fatty amides
2.02_352.11!	352.115002	2.02081667	neg		Pugnac	HMDB0256921	PBLNJFVQN	Organic oxyg	Organooxyge	Carbohydrate
2.13_581.17	581.172869	2.1282	neg		Avenalumin I	HMDB0038956	ASJBOIQMT	Organoheter	Benzoxazine	Unclassified
2.39_180.01!	180.015653	2.387	neg		Dihydroxyeth	HMDB0251344	PCBLZWZC	Organic nitro	Organonitrog	Amines
2.87_172.09	172.094399	2.8749	pos		Triethanolam	HMDB00325 43365	GSEJCLTVZ	Organic nitro	Organonitrog	Amines
3.57_406.12!	406.125681	3.57018333	neg		Pyriminobac-	HMDB00405 331671	USSIUIGPBI	Organic oxyg	Organooxyge	Ethers
3.59_263.03	263.038233	3.58845	neg		Diphenyl sulf	HMDB0251453	KZTYYGOKI	Benzenoids	Benzene anc	Benzenesulf
3.59_387.18	387.188463	3.58845	neg		1-Octanesulf	HMDB0243978	WLGDAKIJY	Organic acid	Organic sulfo	Organosulfor
3.59_723.21	723.213693	3.58845	neg		Linoside A		QRFPSEZA(	Lipids and lip	Polyketides	Flavonoids
3.68_292.05	292.059147	3.67736667	neg		Fluoroazomy	HMDB0259428	LPZSRGRD	Organic oxyg	Organooxyge	Carbohydrate
3.71_369.06	369.067793	3.71251667	neg		Glucose pyru	HMDB0252787	LAJCJBGHR	Organic oxyg	Organooxyge	Carbohydrate
3.73_310.13	310.139911	3.73238333	pos		(2S,4S)-Mon	HMDB00413 95722	RMLYXMMB	Organic acid	Carboxylic ac	Amino acids,
3.80_297.06	297.066138	3.80496667	neg		6-Methylthioi	HMDB0242752	ZDRFDHHAI	Nucleosides,	Purine nucle	Unclassified
3.86_314.20	314.207914	3.85685	pos		Marimastat	HMDB00149 433613	OCSMOTCN	Organic oxyg	Organooxyge	Alcohols and
3.91_473.22	473.227089	3.91066667	pos		Lexaptepid p	HMDB0251765	QJAGBAPUI	Organic acid	Carboxylic ac	Amino acids,
3.97_206.04	206.043217	3.97085	neg		2-keto-3-deo	HMDB0304074	FRIWJYNKZ	Organic acid	Keto acids ar	Medium-chai
3.98_390.20	413.193322	3.98231667	pos		Bakkenolide	HMDB0035206	AVAGQVZSI	Lipids and lip	Prenol lipids	Terpene lact
4.15_289.14	289.140514	4.1538	neg		Amino 6-oxol	HMDB0258069	ADDOFPNT	Organic oxyg	Organooxyge	Carbonyl cor
4.16_537.28	537.281614	4.163	pos		Enkephalinar	HMDB0243765	YZXGODHV	Organic acid	Carboxylic ac	Amino acids,
4.24_283.12	283.126303	4.23603333	pos		Isoleucyl-Glu	HMDB00289 85812	KTGFOCFY	Organic acid	Carboxylic ac	Amino acids,
4.29_451.23	434.232596	4.29085	pos		14alpha-Hyd	HMDB00409 95419	ROIDNUNHI	Organoheter	Naphthopyra	Unclassified
4.33_288.18	288.180499	4.32651667	pos		3-Oxoheptan	HMDB0241693	NLTOPJKYH	Lipids and lip	Fatty Acyls	Fatty acid es
4.38_332.15	332.152284	4.38046667	pos		Amitraz	HMDB02483 3928	QXAITBQSY	Benzenoids	Benzene anc	Xylenes
4.38_460.31	460.311964	4.38046667	pos		Gentamicin	HMDB00149 3041	CEAZRRDEI	Organic oxyg	Organooxyge	Carbohydrate
4.60_343.09	343.090937	4.59571667	neg		L-alpha-Amir	HMDB00343 89846	BDHFFHBFJ	Organic acid	Carboxylic ac	Amino acids,
4.61_303.97!	303.975333	4.61413333	neg		1-hydroxy-2-r	HMDB0304014	MDSIZRKJV	Lipids and lip	Prenol lipids	Isoprenoid pl
4.76_359.06	359.063595	4.75875	pos		N-(N-L-gamn	HMDB0257412	HYHSBSXUI	Organic acid	Carboxylic ac	Amino acids,
4.78_102.05!	102.055341	4.7769	pos		Serine methy	HMDB02457 317316	ANSUDRAT	Organic acid	Carboxylic ac	Amino acids,
4.91_211.09	211.097374	4.90568333	neg		Rotundifolon	HMDB00370 344659	AKASWINDI	Organoheter	Oxepanes	Unclassified
4.91_385.16	385.169037	4.90568333	neg		Olamufloxaci	HMDB0255928	LEILBPMISZ	Organoheter	Quinolines ar	Quinoline car

4.92_348.07(	348.070026	4.92405	neg	Hpmpa	HMDB0253216		FRPXSOOH'	Organoheter	Imidazopyrim	Purines and
4.94_302.19(	302.195995	4.93775	pos	4-Oxooctano	HMDB0241739		AHTAWKLQ	Lipids and lip	Fatty Acyls	Fatty acid es
5.03_286.09	286.094529	5.03453333	neg	Peldesine	HMDB02562	419846	DOHVAKFY/	Organoheter	Pyrrolopyrimi	Unclassified
5.23_332.24	332.242771	5.22825	pos	7-Hydroxyde	HMDB0241056		CUNUJTYZII	Lipids and lip	Fatty Acyls	Fatty acid es
5.26_180.98	180.988816	5.25613333	neg	Sevoflurane	HMDB00153	2422	DFEYYRMX(	Organic oxyg	Organooxyge	Ethers
5.63_383.13	383.134971	5.6251	neg	Acetyldeoxyr	HMDB00361	91293	ADFIQZBYN	Lipids and lip	Prenol lipids	Sesquiterper
5.74_342.08(	342.080081	5.73951667	pos	Ribavirin mor	HMDB0257208		SDWIOXKH'	Nucleosides,	Triazole ribor	Unclassified
5.75_242.04	223.028096	5.75441667	neg	[(2S,3S,4S,5	HMDB0252508		NLMDZXKC'	Organoheter	Oxanes	Unclassified
5.88_291.08	291.082559	5.88396667	neg	Aspartylhydr	HMDB00111	62009	WJDUWENE	Organic acid	Carboxylic ac	Amino acids,
5.92_419.26	419.261191	5.92246667	pos	Amperozide	HMDB02483	320939	NNAIYOXJN	Benzenoids	Benzene anc	Diphenylmetl
6.20_299.14(	299.146716	6.1974	pos	O(6)-Methyl-	HMDB0255876		BCKDNMPY	Nucleosides,	Purine nucle	Purine 2'-dec
6.20_424.19	424.19785	6.19793333	neg	O-Desmethyl	HMDB0060856		DSBGQRZO	Organic oxyg	Organooxyge	Carbohydrate
6.49_237.18	237.184655	6.49025	pos	2-Hydroxyac	HMDB0030916		ADNFCKCPI	Organic oxyg	Organooxyge	Carbonyl cor
6.49_293.10	293.103082	6.49351667	neg	Monocyclohe	HMDB02548	329031	PMDKYLLIO	Benzenoids	Benzene anc	Benzoic acid
6.60_221.07(	221.070061	6.59813333	pos	1,9-Pyrazolo	HMDB02444	45266	ACPOUJIDA	Benzenoids	Anthracenes	Unclassified
6.60_386.20(	409.195664	6.59813333	pos	Sufentanil	HMDB00148	2524	GGCSSNBK	Benzenoids	Benzene anc	Anilides
6.63_255.15(	255.156665	6.63455	pos	6-Deoxypenc	HMDB0247053		WJOWACPJ	Organoheter	Imidazopyrim	Purines and
6.75_470.23(	470.236789	6.75028333	neg	2-Azetidineca	HMDB0249315		MFTQITSPG	Organoheter	Lactams	Beta lactams
6.95_269.13(	269.136942	6.95326667	neg	Arginylaspara	HMDB00287	23959	JSLGXODUI	Organic acid	Carboxylic ac	Amino acids,
7.17_533.19(	533.196079	7.17388333	neg	Prinomide	HMDB02567	68722	KBQUAIAGF	Benzenoids	Benzene anc	Anilides
7.41_624.33	624.338935	7.41378333	neg	Glycochenodeoxycholic ac		57966	ABFZMYIIUF	Lipids and lip	Steroids and	Steroid conju
7.43_472.25	472.252646	7.43236667	neg	epothilone D		4987	XOZIUKBZL	Lipids and lip	Polyketides	Macrolides a
7.94_323.10	323.108714	7.93951667	neg	N-(2-Hydroxy	HMDB02446	336311	URDCARMU	Organic acid	Carboxylic ac	Amino acids,
7.97_319.08	319.08403	7.97481667	pos	Cyclopeptine		985251	KSQNKZMA	Unclassified	Unclassified	Unclassified
8.14_454.27	454.272788	8.13956667	pos	N-Eicosapen	HMDB0242074		DPADGAXV'	Unclassified	Unclassified	Unclassified
8.58_265.17	265.177405	8.58011667	pos	7-Oxotetradecanoic acid		45870	CDYSHNKYI	Lipids and lip	Fatty Acyls	Fatty Acids a
8.65_197.15	197.153453	8.65385	pos	Hexamethylp	HMDB02531	72970	GNOIPBMMI	Organic acid	Organic phos	Organic phos
8.76_932.59(	932.590419	8.75856667	pos	PS(6 keto-P(	HMDB0283009		FRKIGMJEM	Unclassified	Unclassified	Unclassified
8.78_412.96(	412.966299	8.784	neg	Perfluorooct	HMDB00595	96224	SNGREZUH	Unclassified	Unclassified	Unclassified
8.81_419.31	419.315214	8.81001667	pos	Dihomo-alpha-muricholic acid			AYIHMMDC(	Lipids and lip	Steroids and	Bile acids an
9.38_454.29	454.294655	9.37788333	pos	Paraminabeolide A			TXZGYZBGI	Lipids and lip	Steroids and	Sterols
9.46_311.07(	311.070192	9.46038333	neg	Flubendazol	HMDB0252324		CPEUVMUX	Benzenoids	Benzene anc	Benzophenor
9.69_278.20	278.208561	9.68703333	pos	Carisoprodol	HMDB00145	3947	OFZCIYFFP	Organic acid	Carboxylic ac	Amino acids,

9.69_611.24!	611.249225	9.68703333	pos	Cfp-aaf-pab	HMDB0249846		UZKWDJFXI	Organic acid:	Carboxylic ac	Amino acids,
9.73_377.02:	377.021817	9.73451667	neg	Casoxin D	HMDB0303768		INBRDFNDL	Organic oxyg	Organooxyge	Ethers
9.73_419.12	419.127611	9.73451667	neg	Phenanthren	HMDB02548	65920	RLZZZVKAU	Benzenoids	Phenanthren	Phenanthrols
0.50_159.06!	159.065268	0.50076667	pos	2-Isopropylmalic acid		339	BITYXLXUC:	Unclassified	Unclassified	Unclassified
0.61_152.97	152.97838	0.61033333	neg	Dihydroxy-(2	HMDB0258062		IBYZHOSML	Organic acid:	Organic thiop	Thiophospho
0.61_327.06!	327.065946	0.61033333	neg	6-Sulfatoxym	HMDB00418	454490	QQEILXDLZ	Organic acid:	Carboxylic ac	Carboxylic ac
0.62_282.06	305.057036	0.6215	pos	Ethyl (E)-3-di	HMDB02573	984250	MZULHRQPI	Organic acid:	Organic thiop	Thiophospho
0.66_180.93	180.930918	0.65943333	neg	Potassium pl	HMDB0303429		GNSKLFRGI	Mixed metal/	Alkali metal c	Alkali metal p
0.72_123.98	122.975631	0.71928333	neg	1,3,2-Dioxatl	HMDB0252075		ZPFAVCIQZ	Organic acid:	Organic sulfu	Sulfuric acid
0.72_176.01!	176.019767	0.71928333	neg	Iminoasparti	HMDB00011	6025	NMUOATVLI	Organic acid:	Carboxylic ac	Dicarboxylic
0.72_309.06:	309.063138	0.71928333	neg	N-Methyletha	HMDB00601	63347	HZDCAHRLI	Organic acid:	Organic phos	Phosphate e:
0.89_187.04	187.041757	0.8943	neg	1-Hydroxy-2-	HMDB02438	2389	SJJCQDRG/	Benzenoids	Naphthalene	Naphthalene
1.08_183.01!	183.015744	1.08208333	neg	5-Diazouracil	HMDB02467	345908	XFLJNCDBY	Organoheter	Diazines	Pyrimidines a
1.10_340.10	340.101556	1.1046	pos	cyclo-dopa 5	HMDB03043	64475	PXMFPPFHI	Organic oxyg	Organooxyge	Carbohydrate
1.20_377.02!	377.029287	1.20271667	neg	4-Aminopher	HMDB0249961		FXZCCINCK	Organic acid:	Organic phos	Phosphate e:
1.34_477.18	477.18033	1.34166667	neg	2,3-Bis(4-hyc	HMDB0244616		GHZHWDW/	Phenylpropa	Stilbenes	Unclassified
1.34_681.14:	681.143125	1.34166667	neg	2-Hydroxy-1,	HMDB0240438		PXAGSWBD	Organic oxyg	Organooxyge	Carbohydrate
1.34_735.24	735.247209	1.34166667	neg	Kwakhurin		47591	CFMMELZRI	Lipids and lip	Polyketides	Flavonoids
1.36_220.02	220.028262	1.3586	neg	S-(3-Oxo-3-c	HMDB0002135		MXVUEJUCI	Organic acid:	Carboxylic ac	Amino acids,
1.40_237.08	237.086782	1.40198333	pos	8-Methoxyky	HMDB00604	63556	BXZSKDOP(	Organoheter	Quinolines ar	Quinoline car
1.45_201.03	201.03701	1.45418333	pos	3-Keto-b-D-g	HMDB00013	6206	APIQNBNBII	Organic oxyg	Organooxyge	Carbohydrate
1.58_254.16	254.161168	1.57645	pos	Prolyl-Argini	HMDB0029011		HMNSRTLZ/	Organic acid:	Carboxylic ac	Amino acids,
1.61_326.16	326.164238	1.61191667	pos	Dipropyl-5-C	HMDB0251472		DPXOFRGR	Organoheter	Indoles and c	Indolecarbox
1.67_232.10	232.108091	1.66533333	pos	2-Benzyl-3-n	HMDB0255644		FZQZRBQK)	Organic 1,3-(	Allyl-type 1,3	Organic nitro
1.76_271.07	271.07484	1.75533333	pos	Deferitrin	HMDB0250934		OEUUFNIKL	Organic acid:	Carboxylic ac	Amino acids,
1.77_227.99!	227.995444	1.77345	pos	5-Hydroxy-2-	HMDB0304929		JNOMTUUJN	Unclassified	Unclassified	Unclassified
1.81_302.17	302.171072	1.80931667	pos	Siguazodan	HMDB0258290		NUHPODZZI	Organoheter	Diazines	Pyridazines a
1.89_367.14	367.14081	1.89173333	neg	Mesocarb	HMDB0254478		OWFUPROY	Benzenoids	Benzene anc	Phenethylam
1.93_734.82	734.828501	1.92873333	neg	[(2S,3R,5S,6	HMDB0257969		QQASWOAL	Organic oxyg	Organooxyge	Alcohols and
10.12_494.2!	494.252567	10.11685	neg	PS(16:1(9Z)/0:0)		78837	XSJRZJQMA	Lipids and lip	Glycerophos	Glycerophos
10.28_599.2	599.249148	10.2840833	pos	Bafetinib	HMDB0240206		ZGBAJMQH.	Benzenoids	Benzene anc	Anilides
10.34_616.3	616.344583	10.3389333	pos	PG-PG			ORHWXQW	Lipids and lip	Glycerophos	Oxidized glyc
10.41_450.2	450.262629	10.4089833	neg	PC(O-11:1(10E)/2:0)		40007	BDCLOBVQI	Lipids and lip	Glycerophos	Glycerophos

10.43_410.34	410.347053	10.4295333	pos	Lignoceric acid(d3)			QZZGJDVW	Lipids and lip	Fatty Acyls	Fatty Acids a
10.48_332.33	332.330729	10.4838833	pos	3Z,6Z,9Z,12Z,15Z-Tricosa		97592	WZOLXZVF	Lipids and lip	Fatty Acyls	Hydrocarbon
10.48_427.28	427.285015	10.48035	neg	(R)-3,4-Dihy	HMDB00363	91511	GJJVAFUKC	Lipids and lip	Prenol lipids	Diterpenoids
10.52_327.21	327.213723	10.5204167	pos	2,15,16-trihydroxy palmitic		35476	IJBFCIWUL	Lipids and lip	Fatty Acyls	Fatty Acids a
10.54_857.67	857.674765	10.5357333	neg	SM(d18:2(4E	HMDB02406	83781	DACOGJMB	Lipids and lip	Sphingolipids	Phosphosphi
10.57_572.33	572.33411	10.5732333	pos	PC(16:0/3:1(2E))		39409	ABBJFAPTM	Lipids and lip	Glycerophos	Glycerophos
10.63_392.35	392.351592	10.6275	pos	5-((Z)-nonadec-8-en-1-yl)resorcinol			ACEHMJPBI	Lipids and lip	Polyketides	Phenolic lipic
10.86_719.31	719.311376	10.8611	neg	Lepidine C	HMDB00327	88647	GTJSRUGIL	Benzenoids	Benzene anc	Diphenylethe
11.41_291.23	291.231318	11.4141	pos	16:4(2E,4E,8E,10E)(7Me[		73780	AHLLEVZDN	Lipids and lip	Fatty Acyls	Fatty Acids a
12.05_412.29	411.290377	12.0535	neg	(25R)-5alpha,8alpha-epidi		84264	ACIGRLRKX	Lipids and lip	Steroids and	Sterols
12.05_479.27	479.277702	12.0535	neg	PA(8:0/i-12:0	HMDB0115685		AFEICCKBA	Lipids and lip	Glycerophos	Glycerophos
12.21_420.31	420.311585	12.20735	neg	N-(1,1-dimethyl-2-hydroxy		36691	BJEGAFQVE	Lipids and lip	Fatty Acyls	Fatty amides
12.31_378.35	378.357089	12.3062167	pos	1-O-(2R-hydroxy-octadecyl)-sn-glycerol			WSTMDVJU	Lipids and lip	Glycerolipids	Monoradylgly
12.34_356.35	356.351672	12.3412167	pos	Docosenic ac	HMDB0302285		ATNNLHXCF	Lipids and lip	Fatty Acyls	Fatty acids a
12.62_325.21	325.210834	12.6205	pos	4-Phenylbuta	HMDB0241867		AEOYRSISB	Lipids and lip	Fatty Acyls	Fatty acid es
12.62_627.43	627.432762	12.6205	pos	1-(2-methoxy-16Z-tricosenyl)-sn-glycero			QBDJBTWF	Lipids and lip	Glycerophos	Glycerophos
12.84_317.24	317.248321	12.8434833	neg	(1S,4S,9R,10	HMDB0260107		AJMOAIFBR	Lipids and lip	Prenol lipids	Diterpenoids
12.94_445.16	445.166026	12.9357167	neg	Euchrenone b4		47813	HEWXGYZX	Lipids and lip	Polyketides	Flavonoids
13.05_467.37	467.373749	13.0525	neg	(5Z,9Z)-2-methoxy-hexacosadienoic aci			USCVSTUZL	Lipids and lip	Fatty Acyls	Fatty Acids a
13.13_293.24	293.248601	13.1264	neg	3,7-Diethyl-11-methyltride		97781	AMUXOEXE	Lipids and lip	Fatty Acyls	Hydrocarbon
13.31_417.22	417.223079	13.31045	neg	4-(Methylnitr	HMDB00418	73239	OGRXKBUC	Organoheter	Pyridines anc	Unclassified
13.38_327.22	327.226491	13.38305	pos	(2Z,4E,6Z)-D	HMDB0241123		AEIBVNCGR	Lipids and lip	Fatty Acyls	Fatty acid es
13.44_138.06	138.065995	13.4363167	pos	Benzofuraza	HMDB0249016		AWBOSXFR	Organoheter	Benzoxadiaz	Unclassified
14.09_297.24	297.243281	14.0865333	neg	1,16-Heptadecadien-3-one		98187	BJUAHDDP	Lipids and lip	Fatty Acyls	Oxygenated
2.04_265.036	265.036074	2.03931667	neg	Echinochrom	HMDB0251689		DNRFNICCF	Benzenoids	Naphthalene	Naphthoquin
2.11_259.022	259.022257	2.11061667	neg	1-Deoxy-D-xy	HMDB00012	6085	AJPADPZSR	Organic oxyg	Organooxyge	Carbohydrate
2.29_266.103	266.103252	2.29455	neg	Butylone	HMDB02494	85072	CGKQZIULZ	Benzenoids	Benzene anc	Butyropheno
2.39_147.076	147.0766	2.39026667	pos	1-Pyrroline-4	HMDB00022	6564	AOMLMYXP	Organoheter	Pyrrolines	Unclassified
2.71_264.081	264.081801	2.71386667	pos	Propan-2-yl 3	HMDB0247799		BZNDWPRG	Lipids and lip	Fatty Acyls	Fatty acid es
2.87_306.124	307.132119	2.8749	pos	(-)-Isolonchocarpin		52505	GJRSGESH	Lipids and lip	Polyketides	Flavonoids
3.25_327.160	327.160668	3.2533	pos	(+)-Galeon	HMDB00315	87743	QUSSPXNP	Phenylpropa	Diarylheptan	Cyclic diarylh
3.46_144.066	144.066406	3.45915	neg	1-Pyrrolidine	HMDB00345	90050	AGRIQBHIK	Organoheter	Pyrrolidines	Unclassified
3.47_405.198	405.198908	3.47126667	pos	Blasticidin S	HMDB0030452		CXNPLSGK	Organic acids	Carboxylic ac	Amino acids,

3.66_155.04	155.045047	3.66498333	pos		Acipimox	HMDB02479	314370	DJQOOSBJ	Organoheter	Diazines	Pyrazines
3.73_223.09	223.095413	3.73238333	pos		Hydralazine	HMDB0060606		AKFJXINWD	Organoheter	Diazanaphth	Benzodiazine
3.93_426.23	426.236111	3.93381667	neg		Valyl-prolyl-g	HMDB0259877		LFTRJWKKL	Organic acid	Carboxylic ac	Amino acids,
3.98_467.17	467.177631	3.98231667	pos		N-(3-(3-Hydr	HMDB0259626		WJYNYVNIF	Organic acid	Carboxylic ac	Amino acids,
4.06_336.13	336.139428	4.05526667	pos		Diethylenetri	HMDB0251248		AGMNQPKG	Organic acid	Carboxylic ac	Tetracarboxy
4.12_428.25	428.251184	4.11676667	neg		Sisomicin sul	HMDB0242632		URWAJWIAI	Unclassified	Unclassified	Unclassified
4.16_241.06	241.064138	4.163	pos		5-(4-Hydroxy	HMDB0247358		NKOHRVBB	Organoheter	Azolidines	Thiazolidines
4.21_267.04	267.04611	4.20936667	neg		Tert-Butylbic	HMDB02587	332182	VTBHBNXGI	Organic acid	Organic thiop	Thiophospho
4.24_641.84	641.846613	4.23603333	pos		Tyropanoic a	HMDB02593	320416	YMOXVLQZI	Phenylpropa	Phenylpropa	Unclassified
4.34_385.24	385.244881	4.34396667	pos		Bambuterol	HMDB00154	321358	ANZXOIAKU	Benzenoids	Benzene and	Phenoxy con
4.44_303.14	303.141167	4.435	pos		arabinofuran	HMDB0061067		JEPAHPFDU	Nucleosides,	Purine nucle	Unclassified
4.51_580.80	580.809811	4.50633333	pos		Ipodate	HMDB02535	72022	YQNFBOJPT	Phenylpropa	Phenylpropa	Unclassified
4.56_308.07	308.077738	4.55865	neg		Bolton-Hunte	HMDB0246176		KYRUKRFV	Benzenoids	Phenols	1-hydroxy-2-
4.61_207.01	207.012006	4.61413333	neg		2-Naphthaler	HMDB02554	71144	KVBGVZZKJ	Benzenoids	Naphthalene	Naphthalene
4.65_301.19	301.199489	4.64996667	pos		L-Citronellol	HMDB00298	86545	BGAILLKFXI	Lipids and lip	Prenol lipids	Terpene glyc
4.69_343.09	343.095766	4.68661667	pos		Flupirtine	HMDB02524	321005	JUUFBMOD	Benzenoids	Benzene and	Benzylamine
4.72_287.08	287.085987	4.72411667	neg		Fluconazole	HMDB00143	2708	RFHAOTPX	Benzenoids	Benzene and	Halobenzene
4.74_362.13	362.135261	4.74241667	neg		Hydroxyproly	HMDB00288	85780	PMCORMXC	Organic acid	Carboxylic ac	Amino acids,
4.74_383.16	383.168749	4.74081667	pos		PSN632408		64655	LHZWKWCE	Unclassified	Unclassified	Unclassified
4.83_115.00	115.008171	4.83273333	neg		(Diaminomet	HMDB0252972		FZPKSSRLA	Organic nitro	Organonitrog	Guanidines
4.92_223.13	223.130127	4.91941667	pos		9-(trans-4-Hy	HMDB0250028		DYLIWHYU	Organoheter	Imidazopyrim	Purines and
4.96_120.01	120.012314	4.96071667	neg		THIOACETA	HMDB02589	73014	YUKQRDCY	Organosulfur	Thioamides	Unclassified
4.96_408.02	408.02879	4.96071667	neg		Disodium gua	HMDB0304828		PVBRXXAAI	Organoheter	Dihydrofuran	Furanones
5.02_186.02	186.022755	5.016	neg		(6S)-5-Thia-1	HMDB0257892		FZEVMBJW	Organoheter	Lactams	Beta lactams
5.11_179.03	179.034866	5.10843333	neg		Phenylglyoxa	HMDB0061916		OJUGVDOD	Benzenoids	Benzene and	Phenylacetal
5.12_330.13	330.136571	5.11926667	pos		Perlapine	HMDB02563	322055	PWRPUAKX	Organoheter	Benzazepine	Unclassified
5.16_258.03	258.034749	5.15551667	pos		AS-605240		45073	SQWZFLMP	Unclassified	Unclassified	Unclassified
5.33_364.12	364.120154	5.32985	neg		Gravacridone	HMDB00385	93226	HPCOINMYI	Organoheter	Quinolines a	Benzoquinoli
5.57_551.17	551.174874	5.5696	neg		2-[(E)-2-(3-N	HMDB0244962		URIXDBULD	Organoheter	Quinolines a	Unclassified
5.63_339.03	339.034392	5.6251	neg		2-Galloyl-1,4	HMDB00372	92167	DTDWLEXE	Benzenoids	Benzene and	Benzoic acid
5.72_512.20	512.203556	5.71736667	neg		Benzyloxyca	HMDB0244824		MIFGOLAMI	Organoheter	Azaspirodeca	Unclassified
5.74_387.23	387.238952	5.7359	neg		1-(4-Fluorobenzyl)piperazi		406661	OOSZCNKV	Unclassified	Unclassified	Unclassified
5.83_725.35	725.355019	5.8283	neg		PHOOA-PI			BZDATZLAQ	Lipids and lip	Glycerophos	Oxidized glyc

6.00_505.95	505.953794	5.99566667	pos	3'-Deoxy-cyti	HMDB0246090		CHKFLBOLY	Organoheter	Diazines	Pyrimidines a
6.01_385.16	385.169107	6.01328333	neg	2-Phenylindo	HMDB02452	342087	KLLLJCACIF	Organoheter	Indoles and	Indoles
6.07_275.16	275.161492	6.06898333	pos	(+)-2-Sterpurene-6,12,15-t		53509	AWMMKZCE	Lipids and lip	Prenol lipids	Isoprenoids
6.23_393.15	393.15156	6.23411667	pos	Vernodalol		329070	FZALQGCQ	Unclassified	Unclassified	Unclassified
6.69_330.17	330.172943	6.68961667	pos	6-(4,5-Dihydr	HMDB02514	349362	DOETVZCF	Organoheter	Indoles and	Indoles
6.81_707.09	707.095906	6.80563333	neg	2'-Phosphopl	HMDB0245540		PHNOWZISI	Phenylpropa	Linear 1,3-di	Chalcones ar
6.91_334.23	334.237128	6.9103	pos	2-Methoxyes	HMDB00003	5333	AVCFXYYR	Lipids and lip	Fatty Acyls	Fatty acids a
6.99_177.12	177.126872	6.98976667	neg	Pentamethyl	HMDB02562	437113	XIFVTSIIYV	Organic nitro	Organonitrog	Amines
6.99_438.13	438.138009	6.98976667	neg	4-(2-Aminoet	HMDB0257978		GBGYSOHU	Organic acid	Carboxylic ac	Pentacarbox
7.17_233.10	465.192727	7.17388333	neg	Serylglutamir	HMDB00290	24026	UJTZHGHXJ	Organic acid	Carboxylic ac	Amino acids,
7.49_166.05	166.050708	7.48786667	neg	2-Acetylpyrid	HMDB00352	90588	AJKVQEKCL	Organic oxyg	Organooxyge	Carbonyl cor
7.68_493.28	493.282118	7.68096667	pos	Sarcoehrendin C			CUCRZDJH	Lipids and lip	Fatty Acyls	Eicosanoids
7.72_289.06	289.069244	7.72445	neg	2-Keto-glutar	HMDB00015	6318	COJBGNAU	Organic acid	Keto acids ar	Short-chain k
8.01_230.08	230.084196	8.01143333	pos	(E)-3-(2-(1H-	HMDB0244544		JDBSZVDIUI	Organoheter	Indoles and	Indoles
8.11_335.01	335.013662	8.10548333	neg	6-Thioxanthir	HMDB02471	449236	RJOXFJDOL	Organoheter	Imidazopyrim	Purines and
8.27_615.17	615.17903	8.26751667	pos	Delphinidin 3-sambubioside			TWYYVOVD	Lipids and lip	Polyketides	Flavonoids
8.64_456.33	456.331609	8.6355	pos	DG(20:3(6,8,	HMDB0297052		HVBXMHWF	Unclassified	Unclassified	Unclassified
8.80_130.05	130.050731	8.80223333	neg	METHACRYI	HMDB0254513		FQPSGWSL	Organic acid	Carboxylic ac	Carboxylic ac
9.56_518.20	518.201413	9.55885	pos	Ikarisoside A		50126	RPLMLWBO	Lipids and lip	Polyketides	Flavonoids
9.61_291.24	291.24263	9.61401667	pos	1-(1-(3-Meth	HMDB0243769		BQQSZHHK	Benzenoids	Phenol ether	Anisoles
9.98_688.34	688.345024	9.97586667	pos	Am-LPE(22:6(4Z,7Z,10Z,13Z,16Z,19Z)/			AKXNPTXZ	Lipids and lip	Glycerophos	Glycerophos
0.61_564.14	563.141136	0.61033333	neg	Isovitexin 2''-	HMDB0037569		ACRIYYKEW	Phenylpropa	Flavonoids	Flavonoid gly
0.62_268.16	268.163297	0.6215	pos	Valyllysine	HMDB00291	455651	JKHXYJKMN	Organic acid	Carboxylic ac	Amino acids,
0.62_345.14	345.148542	0.6215	pos	Corrin	HMDB0250457		PXOPDYTV	Organoheter	Tetrapyrroles	Unclassified
0.63_621.17	621.17412	0.62835	neg	Icilin		64735	RCEFMOGV	Unclassified	Unclassified	Unclassified
0.64_429.24	429.240408	0.63663333	pos	PA(19:3(10Z,13Z,16Z)/0:0)			BOUZMDJRI	Lipids and lip	Glycerophos	Glycerophos
0.64_616.78	616.78401	0.64391667	neg	Iophenoxic a	HMDB02535	314571	GOIQOQCN	Phenylpropa	Phenylpropa	Unclassified
0.67_295.04	295.049729	0.6745	neg	(R,R)-chrysanthemyl diph		53301	AORLUAKW	Lipids and lip	Prenol lipids	Isoprenoids
0.68_214.00	214.006162	0.68255	pos	TETRANITR	HMDB02589	73012	NYTOUQBR	Organic nitro	Organonitrog	Ortho amide
0.85_130.04	130.046067	0.84881667	pos	2,4-Difluoroa	HMDB0245451		CEPCPXLLF	Benzenoids	Benzene anc	Aniline and s
1.14_176.05	194.084699	1.13681667	pos	Methylthio 2-	HMDB00400	94544	VCLWCQBH	Organic acid	Thiocarboxyl	Thioesters
1.15_227.13	227.13909	1.1535	pos	Propoxur	HMDB02568	44243	ISRUGXGC	Benzenoids	Phenol ether	Unclassified
1.15_418.93	418.932815	1.14921667	neg	3-thiodimethylarsenopropanoic acid			ICLSKJLFAI	Lipids and lip	Fatty Acyls	Other Fatty A

1.19_230.09	230.095617	1.1884	pos	(1E,3E)-1-(4-	HMDB0242307		HKROEBDH	Benzenoids	Benzene anc	Halobenzene
1.23_389.12	389.125117	1.23491667	neg	True blue	HMDB0259305		MBFLUZME	Organoheter	Benzofurans	Unclassified
1.25_506.97	506.971888	1.2521	neg	Inosine triphc	HMDB00001	3589	HAEJPQIAT	Nucleosides,	Purine nucle	Purine ribonu
1.25_669.14	669.14925	1.2521	neg	Oxonantenin	HMDB00333	433309	NFDVJYPML	Alkaloids anc	Aporphines	Unclassified
1.29_215.02	215.028806	1.2875	neg	(4-Carbamim	HMDB0243615		PMHUSCHK	Benzenoids	Benzene anc	Unclassified
1.44_378.08	378.083138	1.43671667	pos	1,1-Diphenyl-	HMDB02440	344095	WCBPJVKVI	Benzenoids	Benzene anc	Nitrobenzene
1.45_287.07	287.076237	1.45418333	pos	Catechol glu	HMDB0240490		ICPYZFZFSI	Organic oxyg	Organooxyge	Carbohydrate
1.47_320.08	320.088046	1.46783333	neg	Metronidazol	HMDB02546	418225	CUUCCLJJC	Benzenoids	Benzene anc	Benzoic acid
1.49_371.14	371.144926	1.48828333	pos	Sambucus ni	HMDB00355	90783	BQIOAYLGM	Organic oxyg	Organooxyge	Carbohydrate
1.54_262.13	262.134806	1.54085	pos	Contragesta	HMDB0250440		GXCZZAKP	Organoheter	Azoles	Triazoles
1.59_432.19	432.1939	1.59411667	pos	Acidissiminol	HMDB00386	93279	FDTDKBQV	Lipids and lip	Prenol lipids	Monoterpenc
1.62_188.01	188.017462	1.61501667	neg	(E)-3-Tosyla	HMDB0248889		DOEWDSDE	Benzenoids	Benzene anc	Toluenes
10.03_337.2	337.213703	10.0311	pos	Cannabidiol	HMDB0249586		QHMBSVQN	Lipids and lip	Prenol lipids	Monoterpenc
10.04_463.0	463.020855	10.0440667	neg	Phosphovani	HMDB0256496		GSWZGTLW	Organic acid	Organic phos	Phosphate e
10.15_661.0	661.031174	10.15385	neg	GDP-mannu	HMDB0304366		DNBSDUDY	Nucleosides,	Purine nucle	Purine nucle
10.19_721.2	721.230995	10.18955	neg	1-[(4-Aminop	HMDB0260034		YIBDNDSKE	Organoheter	Azoles	Imidazoles
10.25_279.1	279.194699	10.24785	pos	12-KHT			BEHRKNVA	Lipids and lip	Fatty Acyls	Eicosanoids
10.27_283.2	283.226195	10.2662	pos	MG(14:1(9Z)	HMDB00115	62345	ARCRKLOZ	Lipids and lip	Glycerolipids	Monoradylgly
10.43_512.3	512.320309	10.42685	neg	Maraviroc	HMDB00155	322747	GSNHKUDZ	Alkaloids anc	Tropane alka	Unclassified
10.61_266.1	267.171506	10.6097333	pos	Potassium m	HMDB0303280		PYJBVGYZ	Lipids and lip	Fatty Acyls	Fatty acids a
10.63_658.2	658.238735	10.6275	pos	Citbismine A	HMDB00410	95519	FTTSBKFAN	Organoheter	Quinolines a	Benzoquinoli
10.70_556.3	556.302979	10.6974667	pos	PS(22:4(7Z,10Z,13Z,16Z)/		78841	JTHCCKGE	Lipids and lip	Glycerophos	Glycerophos
10.86_455.1	455.159233	10.8590333	pos	4-Ethyl-2-pro	HMDB02515	425586	OFYWYKMC	Benzenoids	Benzene anc	Biphenyls an
10.90_459.1	459.145598	10.8978167	neg	N-desalkyl de	HMDB0061021		MTEFFPTU	Organoheter	Diazinanes	Piperazines
10.95_277.1	277.144278	10.9517167	neg	10,11-Epidio	HMDB00410	95495	ALXPNLYW	Benzenoids	Tetralins	Unclassified
11.02_478.8	478.847416	11.0234	neg	3-(3,5-Diiodo	HMDB0059636		ZPJHINFPR	Phenylpropa	Phenylpropa	Unclassified
11.02_504.4	504.404344	11.0205667	pos	3,7-Dihydrox	HMDB00393	93940	AEUOCNAZ	Lipids and lip	Steroids and	Cucurbitacin
11.30_531.2	531.272673	11.2956167	neg	LysoPA(22:4	HMDB0114752		KSDVXYAH	Lipids and lip	Glycerophos	Glycerophos
11.31_337.1	337.166588	11.3062	pos	Norfloxacin	HMDB00151	1598	OGJPXUAP	Organic acid	Carboxylic a	Amino acids,
11.35_618.3	618.377429	11.3501	neg	OOV-PC			QIJHXUVSE	Lipids and lip	Glycerophos	Oxidized glyc
11.74_405.2	405.237753	11.7358167	pos	trimebutine	HMDB0259230		LORDFXWU	Benzenoids	Benzene anc	Benzoic acid
12.22_324.2	347.255081	12.2158833	pos	(Z)-15-Oxo-1	HMDB00297	86504	BMSUKKKA	Lipids and lip	Fatty Acyls	Fatty acids a
12.43_351.3	374.302325	12.4295667	pos	Anandamide (20:2, n-6)		36738	MWQCBVW	Lipids and lip	Fatty Acyls	Fatty amides

12.62_335.1	335.166148	12.6205	pos	Almotriptan	HMDB00150	43879	WKEMJKQC	Organoheter	Indoles and	Tryptamines
12.98_387.0	387.025709	12.9771167	neg	5-Fluorouridi	HMDB00603	71274	RNBMPPYR	Nucleosides,	Pyrimidine nu	Pyrimidine ri
13.08_821.0	821.081699	13.0830833	pos	m7G(5')pppA	HMDB0304410		UFVBPMPLS	Lipids and lip	Fatty Acyls	Fatty acyl thi
13.12_499.3	499.359848	13.1190833	pos	1-O-alpha-D-glucopyranosyl-1,2-eicosa			NNSQMUPT	Lipids and lip	Fatty Acyls	Fatty acyl gly
14.55_158.0	158.092517	14.5488333	pos	3-Methylthym	HMDB0245938		IIDONYMEG	Organoheter	Diazines	Pyrimidines a
14.74_361.3	361.311076	14.7365	neg	5beta-Cholane-3alpha,24-		84625	ACKIMFUPM	Lipids and lip	Steroids and	Bile acids an
2.11_180.06	180.066319	2.11061667	neg	2-Acetyl-6-m	HMDB00370	92048	FPQMUQPP	Organic oxyg	Organooxyge	Carbonyl cor
2.11_555.09	555.09466	2.11061667	neg	Garcinia bifla	HMDB0252640		SNFOQVRN	Phenylpropa	Flavonoids	Biflavonoids
2.18_490.19	490.191874	2.17555	pos	b-D-fructosyl	HMDB00292	86152	ASHAUBLEL	Phenylpropa	Cinnamic aci	Cinnamic aci
2.23_223.13	223.13147	2.22953333	pos	9-(Tetrahydr	HMDB0244787		UKHMZCMK	Organoheter	Imidazopyrim	Purines and
2.39_132.04	132.048673	2.387	neg	2-Amino-4-m	HMDB0258128		AMRLMYBW	Organosulfur	Thioethers	Dialkylthioeth
2.72_371.06	371.068214	2.7196	neg	Edetate diso	HMDB03017	324130	OVBJJZOQF	Organic acid	Carboxylic ac	Tetracarboxy
3.02_403.07	403.071006	3.0151	neg	Tyrphostin A	HMDB02593	45247	YZOFLYUA	Benzenoids	Phenols	Benzenetriol
3.35_457.08	457.087115	3.3483	neg	Flonicamid		72269	RLQJEEJIS	Unclassified	Unclassified	Unclassified
3.54_183.06	183.062695	3.54413333	pos	Dihydro-3-hydroxy-3-(1-hy		97373	AAMXRZJYS	Lipids and lip	Fatty Acyls	Fatty esters
3.59_158.08	158.082001	3.58845	neg	Methoxyisob	HMDB0254551		CEYVKTKJM	Organic nitro	Organonitrog	Organic isoc
3.66_204.04	204.049304	3.66498333	pos	8-Hydroxy-7-	HMDB00060	58348	VHPXSVXJE	Organoheter	Imidazopyrim	Purines and
3.68_283.01	283.01007	3.67736667	neg	S-(2-Furanyl	HMDB00377	92554	GFAOAYJTE	Organoheter	Heteroaroma	Unclassified
3.77_305.10	305.108402	3.76795	pos	Vicine	HMDB0034073		KGNGTSCI	Organic oxyg	Organooxyge	Carbohydrate
3.77_417.08	417.086852	3.7682	neg	Aza-anthrac	HMDB0248757		XYENXGAF	Organoheter	Isoquinolines	Isoquinolone
3.79_207.09	207.098995	3.78573333	pos	Pyrrolidino-[1	HMDB00324	88499	OJEOXDLL	Organoheter	Azacyclic co	Dithiazinanes
3.84_204.06	204.060669	3.83913333	pos	urea glutama	HMDB0259716		IPWDRBKG	Organic acid	Carboxylic ac	Amino acids,
3.87_243.99	243.997067	3.87373333	pos	Para-Iodoam	HMDB0256046		VZPKOWYC	Benzenoids	Benzene and	Phenethylam
3.87_382.16	382.161642	3.87373333	pos	Dec-5-enedi	HMDB0241083		GRNYXWC	Lipids and lip	Fatty Acyls	Fatty acid es
3.89_245.09	245.099911	3.8922	pos	Isopropyl bet	HMDB00327	88639	UOEFDXYU	Organic oxyg	Organooxyge	Carbohydrate
3.99_124.04	124.040224	3.98933333	neg	pyridinyl	HMDB0256969		FIULGFJIHJ	Organoheter	Pyridines an	Unclassified
4.02_352.20	352.202977	4.01898333	pos	1-Methyl-2-u	HMDB0243927		ZLIHBZFNM	Organoheter	Quinolines a	Quinolones a
4.04_195.00	195.007218	4.04478333	neg	1,2,3,4-Tetra	HMDB0244115		SOZFIIXUN	Benzenoids	Benzene and	Halobenzene
4.25_478.26	478.267642	4.25448333	pos	PE(O-16:0/0:0)		46716	OKXWJISJK	Lipids and lip	Glycerophos	Glycerophos
4.39_545.29	545.294339	4.39318333	neg	Desmethylcy	HMDB02510	425984	INPJSDMJY	Benzenoids	Dibenzocycl	Unclassified
4.40_698.37	698.372523	4.3987	pos	Fumonisin C4		53930	XRBVYXJC	Lipids and lip	Sphingolipids	Sphingoid ba
4.47_487.38	487.389665	4.47151667	pos	29:2(5Z,9Z)(22Me)		73786	CIDYATGW	Lipids and lip	Fatty Acyls	Fatty Acids a
4.47_528.24	528.245583	4.47151667	pos	Aeglin	HMDB00414	95813	HLXHWKRK	Lipids and lip	Fatty Acyls	Fatty acyl gly

4.50_755.37	755.371658	4.50406667	neg	Cyclosquam	HMDB0303371		XLTMDOFLU	Organic acid	Carboxylic ac	Amino acids,
4.65_462.19	462.191617	4.65108333	neg	4-Hydroxyno	HMDB0240578		ICRIFHIWW	Organic acid	Carboxylic ac	Amino acids,
4.65_464.28	464.284965	4.64996667	pos	Tert-butyl (2-	HMDB0248771		JRHUUZPSN	Benzenoids	Phenol ether	Unclassified
4.67_338.17	338.172143	4.66926667	neg	Phenylalanyl	HMDB00290	85900	FADYJNXDF	Organic acid	Carboxylic ac	Amino acids,
4.74_389.15	389.154229	4.74241667	neg	2-(3-pyridyl)-Benzimidazol		45439	BOUOQESV	Unclassified	Unclassified	Unclassified
4.78_116.07	116.071444	4.77798333	neg	4S-aminopentanoic acid		35941	ABSTXSZPC	Lipids and lip	Fatty Acyls	Fatty Acids a
4.90_311.06	311.069332	4.90175	pos	4-[5-(Pyridin-2-yl)-1H-pyra		331073	IBCXZJCWD	Unclassified	Unclassified	Unclassified
5.03_553.31	553.319156	5.02876667	pos	Arbekacin	HMDB00156	1113	MKKYBZZTJ	Organic oxyg	Organooxyge	Carbohydrate
5.05_339.09	339.09595	5.05298333	neg	1Propylthiou	HMDB0244907		HPJCTUJOH	Organoheter	Diazines	Pyrimidines a
5.31_709.30	709.305228	5.31133333	neg	JWH 018 N-(4,5-epoxyper		96601	WWVMNUM	Unclassified	Unclassified	Unclassified
5.33_320.03	320.030619	5.32985	neg	N5-Carboxya	HMDB00122	62902	JHLXDWGV	Organic oxyg	Organooxyge	Carbohydrate
5.35_325.02	325.021064	5.3483	neg	3-O-Galloyl-1	HMDB00372	92168	AKRXKEBXE	Benzenoids	Benzene and	Benzoic acid
5.75_196.02	196.024941	5.75441667	neg	4-Nitrobenza	HMDB0246531		BXRFQSNO	Benzenoids	Benzene and	Nitrobenzene
5.85_295.07	295.077288	5.84688333	neg	serine glutan	HMDB0258248		YFLQCMTW	Organic acid	Carboxylic ac	Amino acids,
5.87_297.14	297.147116	5.8654	neg	Olomoucine	HMDB00352	45229	GTVPOLSIJ	Organoheter	Imidazopyrim	Purines and
6.29_365.17	365.170164	6.28911667	pos	Naproxcinod	HMDB0255456		AKFJWRDC	Benzenoids	Naphthalene	Unclassified
6.34_373.21	373.219295	6.34418333	pos	Lubiprostone	HMDB0015180		WGFOBBZC	Lipids and lip	Fatty Acyls	Eicosanoids
6.38_323.14	323.149777	6.38276667	neg	Phenidone	HMDB02564	324081	CMCWWLV	Benzenoids	Benzene and	Unclassified
6.67_257.15	257.153003	6.67121667	pos	2-Butyl-5-[2-(	HMDB00409	95375	VFYKGOGE	Lipids and lip	Fatty Acyls	Fatty acids a
6.97_456.21	456.215156	6.96501667	pos	N-Eicosapen	HMDB0242067		AIQNGJJCE	Organic acid	Carboxylic ac	Amino acids,
7.06_231.15	231.158919	7.05653333	pos	Cladosporacid C			AOGGAUMD	Lipids and lip	Fatty Acyls	Fatty Acids a
7.11_325.15	325.152121	7.1117	pos	Emivirine	HMDB02517	431627	MLILORUFD	Organoheter	Diazines	Pyrimidines a
7.34_343.07	343.072102	7.3402	neg	[2,2'-Biquinol	HMDB02444	343174	AFYNADDZ	Organoheter	Quinolines a	Quinoline ca
7.36_437.16	437.161456	7.3586	neg	Calabaxanth	HMDB00306	87122	PLKQPRRVI	Organoheter	Benzopyrans	1-benzopyra
7.41_456.17	456.178581	7.4053	pos	Temsavir	HMDB0249319		QRPZBKAM	Organoheter	Pyridines and	Pyridyltriazol
7.59_227.12	227.125293	7.58896667	pos	(2xi,6xi)-7-M	HMDB00332	89084	GGDOZDCN	Lipids and lip	Fatty Acyls	Fatty alcohol
7.67_367.15	367.158443	7.67051667	neg	AB-Fubinaca	HMDB0247739		AKOOIMKX	Organic acid	Carboxylic ac	Amino acids,
7.90_253.08	253.080817	7.90143333	pos	(1R,2S,3R)-2	HMDB0029756		CQSIXFHVG	Organic oxyg	Organooxyge	Carbonyl cor
7.94_391.09	391.09643	7.93951667	neg	XANTHONE	HMDB02599	43571	JNELGWHK	Organoheter	Benzopyrans	1-benzopyra
8.29_315.00	315.007501	8.29023333	neg	Pentaerythrit	HMDB0256253		TZRXHJWU	Organic oxyg	Organic oxoa	Organic nitra
8.79_547.27	547.278424	8.7919	pos	SLF		64652	IIDSDBBDZ	Unclassified	Unclassified	Unclassified
8.92_267.12	267.121954	8.91945	pos	Veliparib	HMDB0259777		JNAHVYVR	Organoheter	Benzimidazo	Unclassified
9.06_351.13	351.134858	9.05798333	neg	Nb-p-Couma	HMDB00415	95902	CDMGLLBA	Phenylpropa	Cinnamic aci	Hydroxycinna

9.13_445.03	445.031365	9.13165	neg	FR	HMDB0303046		KLMCZVJOE	Homogeneou	Homogeneou	Unclassified
9.54_114.01	114.016674	9.54118333	pos	Trifluoroaceta	HMDB02453	336784	NRKYWOKH	Organic acids	Carboxylic ac	Carboxylic ac
0.59_102.01	102.013491	0.58926667	pos	3-S-methylm	HMDB0304155		CAOMCZAIA	Lipids and lip	Fatty Acyls	Fatty acids a
0.59_644.84	644.84792	0.59213333	neg	(4-Hydroxy-3	HMDB02556	462950	KPWFDZXS	Benzenoids	Phenols	Nitrophenols
0.61_302.09	325.088882	0.60603333	pos	Terizidone	HMDB0258834		ODKYYBOH	Benzenoids	Benzene and	Unclassified
0.63_485.03	485.035393	0.62835	neg	N-acetyl-2-carboxy Benzei		45011	ADVANFQV	Unclassified	Unclassified	Unclassified
0.67_262.06	262.068798	0.66691667	pos	2-Deoxy-D-a	HMDB0245100		MBPFNOMG	Organic acids	Organic phos	Phosphate es
0.67_520.16	543.14966	0.66691667	pos	6-Hydroxytricetin 6,7,3',5'-tetramethyl et			AVIYDUVKG	Lipids and lip	Polyketides	Flavonoids
0.72_339.07	339.073543	0.71928333	neg	S-(2-Furanyl	HMDB00377	92555	JNVPDFNCA	Organoheter	Heteroaroma	Unclassified
0.75_262.02	262.022962	0.74898333	neg	4-amino-2-m	HMDB0304169		PKYFHKIYH	Organoheter	Diazines	Pyrimidines a
0.75_393.08	393.084876	0.74898333	neg	2-Amino-5-pl	HMDB02473	69668	VOROEQBF	Organic acids	Carboxylic ac	Amino acids,
0.76_178.10	178.107297	0.75766667	pos	N-Methyl-1-d	HMDB00353	90659	AAKDPDFZN	Organoheter	Piperidines	Unclassified
0.91_152.01	152.01659	0.9113	pos	1,2-Benzisotl	HMDB00344	89917	DMSMPAJR	Organoheter	Benzothiazol	Unclassified
0.91_201.00	201.001867	0.91118333	neg	2-Amino-1,3,	HMDB0244972		QUKGLNCX	Organoheter	Azoles	Thiadiazoles
1.03_127.08	127.086378	1.02758333	pos	3-Hydroxy-2-Methylpyridin		103463	AQSRRZGQ	Unclassified	Unclassified	Unclassified
1.08_163.12	163.122878	1.07531667	pos	Rilmenidine	HMDB02572	68861	CQXADFVO	Organoheter	Azolines	Oxazolines
1.14_189.01	189.01747	1.13681667	pos	7-Methylhypo	HMDB0003162		CBQMKYHL	Organoheter	Imidazopyrim	Purines and
1.14_231.02	231.028054	1.13681667	pos	Enprofylline	HMDB00149	85437	SIQPXVQCL	Organoheter	Imidazopyrim	Purines and
1.15_214.05	214.05113	1.1535	pos	N-Isobutyryl-	HMDB0255162		BWBQXMAX	Organic acids	Carboxylic ac	Amino acids,
1.18_377.09	377.0916	1.18473333	neg	Thiotepa	HMDB00155	43348	FOCVUCIES	Organic acids	Organic thiop	Unclassified
1.25_511.14	511.142403	1.2521	neg	Hydrangenol		68205	DGKDFNDH	Unclassified	Unclassified	Unclassified
1.28_155.05	155.05072	1.28425	pos	3-Mercapto-2	HMDB00351	90503	FSAGSGCEI	Organosulfur	Thiols	Alkylthiols
1.32_148.00	148.007237	1.32371667	neg	4-Thiazolidin	HMDB0246586		NOLHRFLIX	Organoheter	Azolidines	Thiazolidines
1.34_172.06	172.061312	1.34166667	neg	Methylenecy	HMDB02546	460636	BCLPBTWF	Organic acids	Carboxylic ac	Amino acids,
1.34_260.04	260.045175	1.34166667	neg	Cidofovir	HMDB00145	66562	VWFCHDSQ	Organoheter	Diazines	Pyrimidines a
1.56_188.05	188.056231	1.56021667	neg	methoprolol	HMDB0254537		BQDKQPQP	Organic acids	Carboxylic ac	Amino acids,
1.56_272.91	272.91708	1.55895	pos	(alpha-D-ma	HMDB00622	340930	SYSZENVIJH	Benzenoids	Phenol ether	Anisoles
1.56_403.01	403.017249	1.56021667	neg	4-carboxy-2-hydroxy-cis,ci		45915	QWLUKZXO	Lipids and lip	Fatty Acyls	Fatty Acids a
1.65_242.09	242.095752	1.64773333	pos	(3As)-2,3,3a,	HMDB0257373		MNTIJYGEI	Organoheter	Thiadiazines	Benzothiadia
1.65_243.04	243.045198	1.64773333	pos	Chrycorin	HMDB00351	90485	FCVWHDAK	Organoheter	Oxanes	Unclassified
1.67_243.99	243.992023	1.67041667	neg	5-Mercapto-2	HMDB0245253		GANZODCW	Benzenoids	Benzene and	Benzoic acid
1.76_180.03	180.030146	1.76276667	neg	2-Benzoxazo	HMDB00329	323676	ASSKVPFEZ	Organoheter	Benzoxazole	Benzoxazolo
10.12_931.5	931.527299	10.1222	pos	Notoginseno	HMDB0032068		BIRZHRCAU	Lipids and lip	Prenol lipids	Triterpenoids

10.30_904.5!	904.589977	10.3023333	pos	Am-PE(16:0/18:0)			QLHGMDCR	Lipids and lip	Glycerophos|	Glycerophos|
10.47_494.3:	494.323636	10.46565	pos	PC(16:1(9E)/0:0)		40287	LFUDDCMNI	Lipids and lip	Glycerophos|	Glycerophos|
10.48_297.1	297.1462	10.4838833	pos	2-Polyprenyl-	HMDB0060354		CNUQZHQK	Lipids and lip	Prenol lipids	Quinone and
10.75_591.1	591.177485	10.7528167	neg	4-Carboxyne	HMDB0060759		WDXCMIDQ	Lipids and lip	Steroids and	Estrane sterc
10.84_276.1	299.161163	10.8409	pos	Sugeonyl acetate			FHGRIGPO(	Lipids and lip	Prenol lipids	Isoprenoids
10.86_634.3:	634.333764	10.8611	neg	Pentasine	HMDB0029803		OQTAHGSA	Organic acid:	Carboxylic ac	Pentacarbox
10.90_415.2	415.24558	10.8950333	pos	MG(0:0/20:5(	HMDB0260547		BFQHMGJPI	Unclassified	Unclassified	Unclassified
11.04_614.4(	637.393972	11.0380333	pos	Evasterioside D			NJUBTRJED	Lipids and lip	Steroids and	Steroid conju
11.13_257.1!	257.150764	11.12815	pos	N-benzyl-1-methyl-1H-pyra		96288	IGHOYJOUG	Unclassified	Unclassified	Unclassified
11.18_308.2	331.262592	11.1809167	pos	11,14-eicosadienoic acid		24088	XSXIVVZCU	Lipids and lip	Fatty Acyls	Fatty Acids a
11.41_283.2:	283.22397	11.4141	pos	3-Dodecylox	HMDB0244707		GBXRUYNQ	Lipids and lip	Glycerolipids	Monoradylgly
11.45_385.2!	385.291887	11.4499667	pos	l-lysyl-l-lysyl-l	HMDB0253899		WBSCNDJQ	Organic acid:	Carboxylic ac	Amino acids,
11.74_532.3(	532.301919	11.7378333	neg	POV-PE			NMWPZVLQ	Lipids and lip	Glycerophos|	Oxidized glyc
11.91_505.1	505.179514	11.9099667	neg	Devd-cho	HMDB0251076		ZMZRKOASI	Organic acid:	Peptidomime	Hybrid peptic
11.93_416.2(	416.264987	11.9285167	neg	3-Oxotrideca	HMDB0241348		PJXKBYRVX	Lipids and lip	Fatty Acyls	Fatty acid es
12.34_353.1	353.14212	12.3449333	neg	4-Amino-8-(2	HMDB0248777		KYDURMHF	Organoheter	Diazanaphth	Benzodiazine
12.36_299.1!	299.195241	12.3586333	pos	Diphthamide	HMDB0060473		FOOBQHKM	Organic acid:	Carboxylic ac	Amino acids,
13.21_587.5(	587.504701	13.21015	neg	Benzyl triacontanoate			GZQBDBZXI	Lipids and lip	Fatty Acyls	Fatty esters
13.85_401.2(	401.267024	13.84655	neg	N-Acetylamir	HMDB0059745		JJGNMSOJT	Organic acid:	Carboxylic ac	Amino acids,
14.27_593.4	593.478513	14.2674833	neg	Cohibin A	HMDB0031168		GFUJWCRM	Lipids and lip	Fatty Acyls	Fatty alcohol
14.72_619.4!	619.490434	14.72495	pos	TG(i-12:0/8:(	HMDB0094827		AGIVMWGN	Lipids and lip	Glycerolipids	Triradylcglyc
2.11_691.15	691.158213	2.11061667	neg	3-O-Methylflu	HMDB0245960		KDXNYSZN(	Organoheter	Benzopyrans	1-benzopyra
2.11_861.22!	861.229318	2.11061667	neg	Syringetin 3-rutinoside-7-glucoside			AKOYMFNXI	Lipids and lip	Polyketides	Flavonoids
2.32_148.00	148.003983	2.31811667	pos	Sulfonamido	HMDB0258596		LOHAMFKE(	Organic acid:	Organic sulfc	Organosulfor
2.33_248.02:	248.023421	2.3315	neg	Cystathionine	HMDB00020	6438	XJUQJVUY(	Organic acid:	Carboxylic ac	Amino acids,
2.53_131.07(	131.070463	2.53481667	pos	Glycerol 1-pr	HMDB00316	87830	AALUCPRYI	Lipids and lip	Glycerolipids	Monoradylgly
3.00_176.07	176.074058	3.0007	pos	2-n-Propylthi	HMDB0245245		QIJPWSGHI	Organic acid:	Carboxylic ac	Amino acids,
3.05_442.06(	442.066465	3.05498333	pos	CDP-N-meth	HMDB0304287		GWHZSYQF	Nucleosides,	Pyrimidine nu	Pyrimidine rit
3.47_308.08(	308.079961	3.47126667	pos	3,4-Dihydrox	HMDB0240447		CABZBRSE)	Unclassified	Unclassified	Unclassified
3.49_143.03	143.034078	3.48938333	pos	2-Methyl-4-o:	HMDB00394	94020	AVSMSTWC	Organic acid:	Keto acids ar	Gamma-keto
3.59_205.08	205.088493	3.58845	neg	4-Pentenoic	HMDB0246567		CYSFMOXXI	Lipids and lip	Fatty Acyls	Fatty acids a
3.70_460.21	460.21701	3.69811667	pos	Lusitanicosid	HMDB0034120		DAELTTGC(	Organic oxyg	Organooxyge	Carbohydrate
3.82_258.04	258.041606	3.82341667	neg	Resorufin	HMDB02571	340488	HSSLDCABI	Organoheter	Benzoxazine	Phenoxazine

3.86_481.24(	481.246124	3.85685	pos	Alnespirone	HMDB0248173		DLLULNTXJ	Organoheter	Azaspirodeca	Unclassified
4.04_749.12:	749.122616	4.03688333	pos	Scleroglucan	HMDB00299	86607	FEBUJFMRS	Organic oxyg	Organooxyge	Carbohydrate
4.09_458.25(	458.256663	4.09135	pos	10,11-Dihydr	HMDB0012501		FZJOTYMAF	Lipids and lip	Fatty Acyls	Eicosanoids
4.12_329.11:	329.111948	4.11676667	neg	N2-(2-Carbo:	HMDB00327	88693	VFYGIOOMI	Organic acid	Carboxylic ac	Amino acids,
4.30_304.08(	304.080269	4.30156667	neg	Tazarotenic a	HMDB00606	408666	IQIBKLWBV.	Organoheter	Thiochroman	Unclassified
4.33_627.33:	627.331419	4.32651667	pos	Tubocurarine	HMDB00153	320861	JFJZZMVDL	Organic oxyg	Organooxyge	Ethers
4.41_358.16:	358.162201	4.41166667	neg	Etoxazole	HMDB02521	72301	IXSZQYVWN	Benzenoids	Benzene anc	Phenylpropar
4.49_443.25:	443.253516	4.4888	pos	CPA(18:0)		75588	BAAJXXGE(	Lipids and lip	Glycerophos	Other Glycer
4.67_120.05(	120.056532	4.66926667	neg	(1E)-1-Pheny	HMDB0242314		FMTRXPVVZ	Benzenoids	Benzene anc	Unclassified
4.67_298.03(	298.030786	4.66926667	neg	Creatinine cit	HMDB0250529		UASUGYXG	Organic acid	Carboxylic ac	Amino acids,
4.70_630.34!	653.335146	4.70481667	pos	Remikiren	HMDB0014357		UXIGZRQVL	Organic acid	Carboxylic ac	Amino acids,
4.71_263.05:	263.051134	4.70578333	neg	Malonylglycir	HMDB0240730		UIDDUAFEZ	Unclassified	Unclassified	Unclassified
4.74_563.18:	563.181298	4.74241667	neg	Arabinopyrar	HMDB00296	86380	FVPQAMUW	Organic oxyg	Organooxyge	Carbohydrate
4.78_430.22:	430.222243	4.7769	pos	Sarpogrelate	HMDB02575	320633	FFYNAVGJS	Phenylpropa	Stilbenes	Unclassified
4.79_439.29:	439.291341	4.79411667	pos	CerP(d18:1/2:0)		103040	HWPZKJVGI	Lipids and lip	Sphingolipids	Ceramides
4.87_347.16'	347.167554	4.86546667	pos	Arginyl-glycy	HMDB0248572		IYMAXBFPH	Organic acid	Carboxylic ac	Amino acids,
4.92_350.05!	350.058288	4.91941667	pos	Citalopram p	HMDB00604	71261	JSYNRZJOD	Organoheter	Isocoumaran	Unclassified
5.11_328.12:	328.122465	5.10843333	neg	Reparixin		315241	KQDRVXQX	Unclassified	Unclassified	Unclassified
5.21_690.34!	690.345351	5.20995	pos	PS-PI			BOWZRUSZ	Lipids and lip	Glycerophos	Oxidized gly(
5.22_599.26!	599.268611	5.21915	neg	TMS		64732	JDBCWSHY	Unclassified	Unclassified	Unclassified
5.23_190.03:	190.033549	5.22825	pos	8-Hydroxygu	HMDB0002032		CLGFIVUFZI	Organoheter	Imidazopyrim	Purines and (
5.25_459.23!	459.238877	5.24643333	pos	Trillenogenin			BIKUIZPELK	Lipids and lip	Steroids and	Sterols
5.50_477.04:	477.041882	5.49605	neg	7-Amino-4-hy	HMDB0243485		KYARBIJYV(	Benzenoids	Naphthalene	Naphthalene
5.92_168.03(	168.030056	5.92093333	neg	2-Hydroxy-4-	HMDB00317	87887	FYWMPJHTI	Organic nitro	Organonitrog	Quinonimines
5.96_400.19!	400.195084	5.95783333	neg	L-902,688		45077	WPTLQOYL	Unclassified	Unclassified	Unclassified
6.09_481.18'	481.18781	6.08708333	neg	Artocarpin			AAXZLRPBJ	Lipids and lip	Polyketides	Flavonoids
6.20_352.24!	352.245695	6.1974	pos	4,8 Dimethyl	HMDB00062	58362	DDTDJDZHD	Lipids and lip	Fatty Acyls	Fatty acid es
6.64_346.95!	346.958218	6.63946667	neg	D-sedoheptu	HMDB0304323		OKHXOUGR	Organic oxyg	Organooxyge	Carbohydrate
6.69_328.15!	328.158717	6.69498333	neg	Dapivirine	HMDB02508	459491	ILAYIAGXTH	Benzenoids	Benzene anc	Benzonitriles
7.13_183.17	183.174197	7.13003333	pos	2-Nonanone	HMDB0303265		AEGTXRAM	Organic oxyg	Organooxyge	Ethers
7.17_365.20!	365.209791	7.17388333	neg	Iprovalicarb	HMDB0253572		NWUWYYSI	Organic acid	Carboxylic ac	Amino acids,
7.28_376.21(	376.216431	7.28471667	neg	N-Lauroyl Me	HMDB0242037		PFSGUIFKR	Organic acid	Carboxylic ac	Amino acids,
7.60_395.12:	395.121839	7.59848333	neg	Flecainide	HMDB00153	2701	DJBNUMBKI	Benzenoids	Benzene anc	Benzoic acid

7.92_399.22	399.220955	7.9211	neg	5alpha-Pregr	HMDB0240580		NOGKXXMA	Lipids and lip	Steroids and	Sulfated ster
8.36_269.13	269.130805	8.36401667	neg	Ciproxifan	HMDB02502	444884	ACQBHJXE	Organic oxyg	Organooxyge	Carbonyl cor
8.74_456.36	456.361145	8.74186667	pos	(+/-)N-(2-fluoro-ethyl)-2,16		75458	FNVGQXLOI	Lipids and lip	Fatty Acyls	Fatty amides
8.75_501.33	501.335587	8.74946667	neg	[3h]Thienylcy	HMDB0243761		PXXOGBVC	Organic nitro	Organonitrog	Cyclohexylar
8.90_414.29	414.299928	8.90103333	pos	1-hexadecyl-	HMDB00623	45224	XLVRFPVHC	Lipids and lip	Glycerophos	Glycerophos
8.91_193.08	193.0867	8.91221667	neg	3,4-Methylen	HMDB0059744		DHNSTMIM	Lipids and lip	Fatty Acyls	Fatty acids a
9.06_329.15	329.152298	9.05798333	neg	Imidocarb	HMDB02534	320697	SCEVFJUWI	Benzenoids	Benzene and	N-phenylurea
9.11_603.22	603.222855	9.1134	neg	Olmesartan r	HMDB02559	313637	UQGKUQLK	Benzenoids	Benzene and	Biphenyls an
9.19_549.10	549.104078	9.18648333	neg	BL II	HMDB00395	94124	BKYOIMKD	Organic acid	Carboxylic ac	Pentacarboxy
9.23_231.13	231.135382	9.23096667	pos	2-Amino-3,8-	HMDB00298	72975	DVCCCQNK	Organoheter	Diazanaphth	Benzodiazine
9.28_377.15	377.158139	9.2775	neg	Prenylcystein	HMDB0256764		PGSOFWOY	Organic acid	Carboxylic ac	Amino acids,
9.59_427.00	427.001489	9.58798333	neg	2-(6-Hydroxy	HMDB0255358		SNFKLBVOV	Organoheter	Diazines	Pyrimidines a
9.72_196.01	196.016527	9.72246667	pos	Phosphoric a	HMDB0246141		BQHATANC	Benzenoids	Benzene and	Benzonitriles
0.57_157.00	157.008383	0.57178333	pos	O,O,S-Trime	HMDB02558	432522	WTUNGUOZ	Organophos	Organothiopl	Unclassified
0.57_158.00	158.002965	0.57178333	pos	Benzothiazol	HMDB00329	88833	IOJUPLGTW	Organoheter	Benzothiazol	Unclassified
0.61_359.17	359.178659	0.61033333	neg	Pergolide	HMDB00153	1787	YEHCICAEU	Organoheter	Quinolines a	Indoloquinoli
0.62_573.24	573.244889	0.6215	pos	PA(2:0/PGE	HMDB0266575		LLCWLDRF	Unclassified	Unclassified	Unclassified
0.67_252.83	252.830229	0.6745	neg	Calcium diph	HMDB03034	984539	JUNWLZAG	Mixed metal/	Alkaline eartl	Alkaline eartl
0.67_357.24	357.249742	0.66691667	pos	1-dimethylarsinoyl-octadecane			FQKWIWFK	Lipids and lip	Fatty Acyls	Hydrocarbon
0.69_181.03	181.038309	0.68961667	neg	4-aminobenz	HMDB0304171		ALYNCZNDI	Benzenoids	Benzene and	Benzoic acid
0.70_273.07	273.074837	0.69861667	pos	Cyclo(his-pro	HMDB0250630		NAKUGCPA	Organic acid	Carboxylic ac	Amino acids,
0.70_380.08	379.082134	0.70456667	neg	Tephcalostan C		48317	CRYRNGYG	Lipids and lip	Polyketides	Flavonoids
0.72_279.05	279.052793	0.71928333	neg	2-Methoxybe	HMDB00325	353899	DSCJETUED	Benzenoids	Thiophenols	Unclassified
0.72_307.04	307.04781	0.71928333	neg	N-methyletha	HMDB0304429		OQZPTXREI	Organic acid	Organic phos	Phosphate e
0.75_370.10	369.098552	0.74898333	neg	Paulownin		416910	CAQZFLPW	Unclassified	Unclassified	Unclassified
0.80_200.05	200.056267	0.79596667	neg	methoxycate	HMDB0254544		KHTCLRGX	Benzenoids	Phenols	Methoxypher
0.88_240.04	240.049484	0.87703333	neg	1-(10H-Phen	HMDB02449	357941	JWGBOHJG	Organoheter	Benzothiazin	Phenothiazin
0.96_232.94	232.944138	0.96066667	pos	Sodium tartra	HMDB03036	330241	HELHAJAZN	Organic acid	Hydroxy acid	Beta hydroxy
1.07_395.09	395.095234	1.06741667	neg	(E)-2-(hexa-3,5-dien-1-yn-1-yl)-5-(prop-			BTXGXNRH	Lipids and lip	Fatty Acyls	Oxygenated
1.08_134.02	152.05633	1.07531667	pos	Malic acid	HMDB00001	45931	BJEPYKJPY	Organic acid	Hydroxy acid	Beta hydroxy
1.09_345.00	345.001415	1.08996667	pos	Minodronic a	HMDB02547	322136	VMMKGHQF	Organoheter	Imidazopyrid	Unclassified
1.18_324.10	324.100289	1.18473333	neg	phenyl [1-(N-succinylamino)pentyl]phos			FJQWWGC	Lipids and lip	Fatty Acyls	Fatty amides
1.20_179.00	179.002814	1.20271667	neg	Pentafluorop	HMDB0256267		BYCUWCJU	Benzenoids	Benzene and	Phenylhydra

1.20_241.00	241.002939	1.20271667	neg	(E)-C-HDMAPP		64798	YYYUHBYK	Unclassified	Unclassified	Unclassified
1.20_625.21	625.2128	1.20271667	neg	N-(4-Methylp	HMDB0255014		GPVHMPAA	Organoheter	Benzazepine	Unclassified
1.25_318.06	318.060402	1.2521	neg	N-Gluconyl e	HMDB00322	88338	JNXDTQSH	Organic acid	Organic phos	Phosphate e
1.32_228.98	228.985964	1.32371667	neg	2,6-Dithiopur	HMDB0245518		VQPMXSMU	Organoheter	Imidazopyrim	Purines and
1.34_559.18	559.180986	1.34166667	neg	Gravolenic a	HMDB0033884		DABSIZUNY	Phenylpropa	Cinnamic aci	Hydroxycinna
1.36_513.20	513.204101	1.3586	neg	Tolmetin	HMDB00146	2871	UPSPUYAD	Organic oxy	Organooxyge	Carbonyl cor
1.36_691.08	691.088716	1.3586	neg	3,3'-Bisjuglor	HMDB00339	89630	YSWLZVWS	Benzenoids	Naphthalene	Naphthoquin
1.52_367.19	367.197791	1.52326667	pos	(1S,2S,4R,8S	HMDB00399	94516	HKSOQIVAC	Lipids and lip	Prenol lipids	Terpene glyc
1.56_154.04	154.043499	1.55895	pos	Methioprim	HMDB0246995		XLVOWGDF	Organosulfur	Thioethers	Aryl thioether
1.62_213.03	213.037865	1.61501667	neg	fluorobenzoy	HMDB00609	447542	GYDFFFKC	Organic oxy	Organooxyge	Carbonyl cor
1.65_353.14	353.145713	1.64773333	pos	Cellulose, mi	HMDB00321	88252	PTHCMJGK	Organic oxy	Organooxyge	Carbohydrate
1.74_346.19	346.197383	1.73755	pos	(4E,6E)-Und	HMDB0241200		FCLCSNCQ	Lipids and lip	Fatty Acyls	Fatty acid es
1.93_215.04	215.048564	1.93406667	pos	7-Aminodeacetoxycephalc		329640	NVIAYEIXY	Unclassified	Unclassified	Unclassified
1.95_333.17	333.176919	1.95213333	pos	Isopropyl do	HMDB02533	45402	SFRALHFBK	Organic acid	Organic phos	Phosphonic a
10.03_551.1	551.146525	10.0257	neg	2-Propyl-4-p	HMDB02516	413112	RQGDXPDT	Benzenoids	Benzene and	Biphenyls an
10.15_403.2	403.210068	10.15385	neg	Strophanthidin		57775	ODJLBQGVI	Lipids and lip	Steroids and	Sterols
10.19_1021.	1021.62301	10.1937667	pos	PIM1(19:1(9Z)/18:1(9Z))			BAPVKEUOI	Lipids and lip	Glycerophos	Glycerophos
10.23_673.3	673.308091	10.2257833	neg	Oclacitinib	HMDB0255887		HJWLJNBZ	Organoheter	Pyrrolopyrimi	Pyrrolo[2,3-d
10.24_471.3	471.326367	10.2441667	neg	Reticulataxa	HMDB00368	91882	JNRFHJQRI	Lipids and lip	Prenol lipids	Triterpenoids
10.45_390.3	390.320929	10.4474333	pos	DG(10:0/0:0/	HMDB0092960		AMRVOUCE	Lipids and lip	Glycerolipids	Diradylglycer
10.50_433.1	433.127796	10.4988	neg	Calomelanol D-1		52298	AFAVBHVA	Lipids and lip	Polyketides	Flavonoids
10.52_499.3	499.304142	10.5172833	neg	Lactaronecat	HMDB00375	92395	ASKJTZIKS	Lipids and lip	Prenol lipids	Terpene lact
10.63_680.2	680.24813	10.6260167	neg	PA(LTE4/2:0	HMDB0266520		JQOXWAPV	Unclassified	Unclassified	Unclassified
10.79_502.3	502.388668	10.7875833	pos	(9Z,12Z,15Z,	HMDB0241635		AQYQJMYF	Lipids and lip	Fatty Acyls	Fatty acid es
10.86_310.1	310.154222	10.8590333	pos	Cinereain	HMDB00352	90533	CCHUDPAN	Organoheter	Diazines	Pyrimidines a
10.91_375.1	375.161133	10.9125667	pos	Sparfloxacin	HMDB00153	66707	DZZWHBIB	Organoheter	Quinolines a	Quinoline ca
11.20_352.2	352.24761	11.198	pos	8alpha-8-Hy	HMDB00358	91084	BDGVLOCE	Lipids and lip	Prenol lipids	Diterpenoids
11.36_574.3	574.386158	11.36075	pos	PC(18:1(9Z)/4:0)		39657	ANQYEWIK	Lipids and lip	Glycerophos	Glycerophos
11.86_218.1	218.105891	11.8596167	pos	4-N-[1-(Dime	HMDB0260286		QSTATYQR	Organic nitro	Organonitrog	Amines
11.96_557.2	557.288259	11.9644833	neg	Alcyopterosin H			QCCAHYCT	Lipids and lip	Prenol lipids	Isoprenoids
11.97_729.5	729.589576	11.9664167	pos	SM(d19:1/17:1)		83752	DELUIIJGTF	Lipids and lip	Sphingolipids	Phosphosphi
12.02_323.1	323.195173	12.0192167	pos	Perindoprilat	HMDB00605	1798	ODAIHABQ	Organic acid	Carboxylic a	Amino acids,
12.31_383.1	383.166212	12.3062167	pos	2-(acetylamir	HMDB00022	58114	MIDRXTGJG	Organic oxy	Organooxyge	Carbohydrate

12.71_536.37	536.371254	12.70945	pos	PC(O-16:0/3:1(2E))		40097	FZTHXYOLV	Lipids and lip	Glycerophos	Glycerophos
13.21_353.16	353.163638	13.21015	neg	n-octyl-beta-I	HMDB0255216		CGVLVOOF(	Organic oxyg	Organooxyge	Carbohydrate
13.25_685.51	685.517524	13.2463167	neg	Sitamaquine	HMDB0258315		RVAKDGYPI	Organoheter	Quinolines ar	Aminoquinoli
13.38_421.16	421.166137	13.3769167	neg	Kanzonol V	HMDB00297	86495	AKOSXIFOB	Phenylpropa	2-arylbenzofu	Unclassified
14.32_158.09	158.092615	14.3159167	pos	Argininic acid	HMDB00031	434779	BMFMQGXD	Organic acids	Carboxylic ac	Amino acids,
14.71_284.29	284.294442	14.7074	pos	2E-Octadecenal		46518	AHQSSWHC	Lipids and lip	Fatty Acyls	Fatty aldehyd
2.19_326.14	326.145799	2.19353333	pos	2-O-alpha-D-	HMDB00413	95745	CQLSQWAV	Organic oxyg	Organooxyge	Carbohydrate
2.23_628.25	628.257321	2.22953333	pos	Tri-N-acetylc	HMDB00066	58520	LRDDKCYRI	Organic oxyg	Organooxyge	Carbohydrate
2.39_149.11	149.117372	2.39026667	pos	Triethyl ortho	HMDB0259190		GKASDNZW	Organic oxyg	Organooxyge	Ortho esters
2.66_259.07	259.07459	2.66041667	pos	4'-Thiothymic	HMDB0246637		IVUDHNYLT	Nucleosides,	Nucleoside a	Thionucleosi
2.68_205.15	205.158771	2.67816667	pos	13-tetradecen-2,4-diyn-1-c		36473	CENJVOWT	Lipids and lip	Fatty Acyls	Fatty alcohol
2.71_158.03	158.035644	2.71386667	pos	Favipiravir	HMDB0252176		ZCGNOVWY	Organoheter	Diazines	Pyrazines
3.00_336.15	336.155347	3.0007	pos	N-(3-oxododecanoyl) hom		36731	PHSRRHGY	Lipids and lip	Fatty Acyls	Fatty amides
3.13_174.07	174.076905	3.1262	neg	2-Pyrrolidine	HMDB0029444		ADSALMJPJ	Organoheter	Pyrrolidines	Unclassified
3.36_297.18	297.180442	3.36203333	pos	Alcyopterosin G			QSUQUBXH	Lipids and lip	Prenol lipids	Isoprenoids
3.40_429.27	429.276593	3.39796667	pos	3S-hydroxy-4R-methyl-2S-(n-eicos-11?-			JFAPEZBOY	Lipids and lip	Polyketides	Annonaceae
3.51_148.05	148.051465	3.5146	neg	2,4-Diamino-	HMDB00603	357815	DFZSBQYO	Benzenoids	Benzene and	Nitrobenzene
3.54_392.17	392.170243	3.54413333	pos	Kievitol	HMDB0038112		AHXHRRAH	Phenylpropa	Isoflavonoids	Isoflavans
3.57_605.25	605.255324	3.57018333	neg	(2E)-4-Hydro	HMDB0241661		JBMXEKVFH	Lipids and lip	Fatty Acyls	Fatty acid es
3.66_583.10	583.104326	3.65995	neg	ENDECAPHYLLIN X		44079	JMPKPWBL(	Unclassified	Unclassified	Unclassified
3.68_281.99	281.993792	3.67736667	neg	Risedronate	HMDB00150	66852	IIDJRNMFW	Organic acids	Organic phos	Bisphosphon
3.68_558.03	557.026044	3.67736667	neg	Quercetin 3-glucuronide-3'-sulfate			BHFKPZSIVI	Lipids and lip	Polyketides	Flavonoids
3.70_220.09	221.103133	3.69811667	pos	2,4-Diamino-	HMDB00607	2461	ZCIPYVXKM	Organoheter	Diazanaphtha	Benzodiazine
3.73_136.05	136.051384	3.73108333	neg	D-Histidine	HMDB0250763		HNDVDQJCI	Organic acids	Carboxylic ac	Amino acids,
3.77_553.06	553.061848	3.7682	neg	trans-zeatin r	HMDB0304507		MXFPFNSSZ	Nucleosides,	Purine nucle	Purine ribonu
3.86_451.25	451.258887	3.85685	pos	Ivabradine	HMDB0253723		ACRHBAYQI	Organoheter	Benzazepine	Unclassified
3.95_519.05	519.058463	3.95231667	neg	1,3,5,8-Tetra	HMDB02510	68100	MPXAWSAB	Organoheter	Benzopyrans	1-benzopyrar
4.00_699.10	699.103465	4.00056667	pos	Diadenosine	HMDB00013	58036	FHISWBUCE	Nucleosides,	(3'->5')-dinuc	(3'->5')-dinuc
4.04_494.12	494.123475	4.04478333	neg	Dioxinoacrim	HMDB00407	95234	AEXHLHGCI	Organoheter	Quinolines ar	Benzoquinoli
4.06_298.22	298.222665	4.05526667	pos	Palmitic acid(d3)		96756	IPCSVZSSV	Lipids and lip	Fatty Acyls	Fatty Acids a
4.07_410.11	410.112045	4.07303333	pos	Isradipine	HMDB00144	872	HMJIYCCIJY	Organoheter	Benzoxadiaz	Unclassified
4.16_281.07	281.074544	4.163	pos	Mefenacet	HMDB02544	331279	XIGAUIHYSI	Organoheter	Benzothiazol	Unclassified
4.25_252.19	252.195849	4.25448333	pos	(1alpha,4beta	HMDB00364	91583	ITIGZFMSPA	Lipids and lip	Prenol lipids	Sesquiterper

4.36_465.19	465.199012	4.35616667	neg	Sibrafiban	HMDB0258287		WBNUCLPU	Benzenoids	Benzene and	Benzoic acid
4.36_553.79	553.797061	4.3621	pos	Iopanoic acid	HMDB02535	43213	OIRFJRBSR	Phenylpropa	Phenylpropa	Unclassified
4.38_618.34	618.340239	4.38046667	pos	OHHdiA-PE			HBDOLRKW	Lipids and lip	Glycerophos	Oxidized glyc
4.45_315.11	315.11077	4.45335	pos	(-)-Blebbistatin		45431	LZAXPYOBK	Unclassified	Unclassified	Unclassified
4.52_283.07	283.073885	4.52393333	pos	4'-Hydroxyflu	HMDB00609	2924	GTSMMBJBI	Benzenoids	Benzene and	Biphenyls an
4.54_757.29	757.298368	4.54091667	neg	Desmethyl E	HMDB0244811		KOQIAZNBA	Organoheter	Diazanaphtha	Benzodiazine
4.56_405.20	405.206063	4.55865	neg	OR-1855	HMDB0060860		GDMRFHZLI	Benzenoids	Benzene and	Aniline and s
4.58_286.08	286.084056	4.57725	pos	Tiflorex	HMDB0259085		HNONSDNC	Benzenoids	Benzene and	Phenethylam
4.65_411.01	411.012824	4.65108333	neg	dIDP	HMDB00035	58159	BKUSIKGSP	Nucleosides,	Purine nucle	Purine deoxy
4.76_535.03	535.038707	4.76071667	neg	UDP-L-arabi	HMDB00123	62922	DQQDLYVH	Nucleosides,	Pyrimidine nu	Pyrimidine rib
4.83_207.02	207.023041	4.83273333	neg	Isoquinolines	HMDB0253686		GZZCYMXZ	Organoheter	Isoquinolines	Unclassified
4.91_193.12	193.121719	4.90568333	neg	Sodium capr	HMDB0303292		FIWQZURF	Lipids and lip	Fatty Acyls	Fatty acids a
4.99_315.13	315.136836	4.99255	pos	Tritiozine	HMDB02592	314329	MVOUIYUW	Benzenoids	Phenol ether	Anisoles
5.01_544.30	567.29851	5.01075	pos	Gracilosulfate B			VIBNKBPPU	Lipids and lip	Steroids and	Steroid conju
5.03_459.14	459.149552	5.03453333	neg	Galactitol, 1,	HMDB0246021		MMAXAFOT	Organic acids	Carboxylic ac	Dicarboxylic
5.06_321.13	321.130379	5.06456667	pos	Triciribine		45227	HOGVTUZU	Unclassified	Unclassified	Unclassified
5.22_377.04	377.047209	5.21915	neg	Edta oxalate	HMDB0251707		BROHKIGYE	Organic acids	Carboxylic ac	Hexacarboxy
5.22_491.04	491.049212	5.21915	neg	[3-Methoxy-2	HMDB0255982		GSOXMQLW	Phenylpropa	Stilbenes	Unclassified
5.26_286.11	286.115086	5.25613333	neg	Tetrahydrobi	HMDB00000	5753	FNKQXYHW	Organoheter	Pteridines an	Pterins and d
5.30_393.15	393.151564	5.30103333	pos	Lemborexant	HMDB0254004		MUGXRYIU	Organic nitro	Organonitrog	N-arylamides
5.35_242.04	242.040371	5.3483	neg	Aminoparath	HMDB00015	6284	XIZOTXGJX	Organic acids	Organic thiop	Thiophospho
5.55_607.23	607.237333	5.55113333	neg	Lificiguat	HMDB02599	433815	OQQVFCKU	Organoheter	Benzopyrazo	Indazoles
5.56_399.11	399.110144	5.5568	pos	Celecoxib	HMDB00050	44362	RZEKVGVHI	Organoheter	Azoles	Pyrazoles
5.81_525.27	525.270147	5.80983333	neg	steviol-19-O-beta-D-glucoside			OQPOFZJZF	Lipids and lip	Prenol lipids	Isoprenoids
6.07_292.09	337.090417	6.06866667	neg	Edetic Acid	HMDB00151	4007	KCXVZYZYF	Organic acids	Carboxylic ac	Tetracarboxy
6.11_337.14	337.142158	6.1056	neg	6-Hydroxydo	HMDB00015	6307	DIVDFFZHC	Benzenoids	Phenols	Benzenediols
6.29_194.11	194.114911	6.28911667	pos	6-Methylpteri	HMDB0247090		MFNAKLLYS	Organoheter	Pteridines an	Unclassified
6.69_396.14	396.14604	6.69498333	neg	RCS-4 N-(5-carboxypentyl		96592	CJXFWFGL	Unclassified	Unclassified	Unclassified
6.73_209.08	209.085168	6.73181667	neg	2-Ethyl-1-he	HMDB00333	319761	MHGOKSLT	Organic acids	Organic sulfu	Sulfuric acid
6.80_329.14	329.149115	6.79993333	pos	(S)-Nandiger	HMDB00333	89206	CFUKKPQR	Alkaloids and	Aporphines	Unclassified
7.42_626.35	626.352406	7.42368333	pos	Benzonatate	HMDB0015006		MAFMQEKG	Benzenoids	Benzene and	Benzoic acid
7.54_465.24	465.248858	7.54296667	neg	Dimethyl 3-m	HMDB00320	88183	DSALQCDY	Organic acids	Keto acids ar	Beta-keto ac
7.87_374.08	374.083003	7.86566667	neg	2-hydroxylam	HMDB0304073		FDHKZWGR	Benzenoids	Benzene and	Toluenes

7.98_297.04(	297.046834	7.97648333	neg	Tretazicar	HMDB02591	454041	WOCXQMCI	Benzenoids	Benzene anc	Aniline and s
8.47_548.29!	548.295353	8.46991667	pos	1-(2-methoxy-6Z-heptadecenyl)-sn-glyc			VRCJKDPTI	Lipids and lip	Glycerophos	Glycerophos
8.56_271.18	271.18787	8.56171667	pos	alpha-Butyl-c	HMDB00321	88248	PXWCUJRV	Organic oxyg	Organooxyge	Ethers
8.69_593.25	593.257947	8.68985	pos	2-O-(beta-D-galactopyranosyl-(1->6)-be			MBOSFCFY(	Lipids and lip	Fatty Acyls	Fatty acyl gly
8.71_335.18	335.182113	8.7078	pos	Arteether	HMDB0242731		NLYNIRQVN	Lipids and lip	Prenol lipids	Sesquiterper
8.71_605.23!	605.239154	8.7071	neg	5-Pyrimidine(	HMDB0243486		GPASXNHO	Organoheter	Diazines	Pyrimidines a
8.94_225.11!	226.122378	8.93783333	pos	6-Acryloyl-2-(	HMDB0247971		HMWAJFNE	Benzenoids	Naphthalene	Unclassified
9.28_445.29(	445.295978	9.2775	neg	DHEA ENANTHATE		84949	HHENOUDB	Unclassified	Unclassified	Unclassified
9.46_301.04	301.041053	9.46038333	neg	5-((p-Hydrox	HMDB0253558		ALZDMJPU(	Organoheter	Diazines	Pyrimidines a
9.48_507.02!	507.029883	9.47878333	neg	Shikimic acid	HMDB03017	3384	QYOJSKGC	Organic acid	Organic phos	Phosphate e
9.81_383.21!	383.219085	9.81245	pos	1-tetradecan	HMDB00623	40945	FAZBDRGX(	Lipids and lip	Glycerophos	Glycerophos
9.83_317.13!	317.139207	9.82575	neg	(S)-Verimol F	HMDB00365	91638	NHUSVJTYI	Benzenoids	Benzene anc	Diphenylmetl
0.54_136.12(	136.120252	0.53628333	pos	Betaine	HMDB00000	96231	KWIUHFFTV	Organic acid	Carboxylic ac	Amino acids,
0.61_313.05(	313.050409	0.61033333	neg	Malaoxon	HMDB00606	1060	WSORODG\	Lipids and lip	Fatty Acyls	Fatty acid es
0.61_343.04	343.043245	0.61033333	neg	Polyribophos	HMDB0256690		OLXOHDAJ	Organic oxyg	Organooxyge	Carbohydrate
0.61_427.16!	427.165852	0.61033333	neg	3-Hydroxy-2-	HMDB0257515		BIAVGWDGI	Organoheter	Quinolines ai	Phenylquinol
0.63_281.07	281.073009	0.62835	neg	Flunoxaprofe	HMDB0252342		UFVXLFRB\	Organoheter	Azoles	Oxazoles
0.64_293.05	293.053853	0.64391667	neg	Pyridoxamine	HMDB00015	236	ZMJGSOSN	Organoheter	Pyridines anc	Pyridoxamine
0.70_307.07	307.073377	0.70456667	neg	Diethylphosp	HMDB00122	62856	UCQFCFPE(	Organic acid	Organic phos	Phosphate e
0.70_335.06	335.06775	0.70456667	neg	Tetraethyl py	HMDB02588	72768	IDCBOTIENI	Organic oxyg	Organic oxoa	Organic pyro
0.89_258.96(	258.960318	0.8943	neg	5-Nitrothiazo	HMDB02468	351834	VVVCJCRUF	Organoheter	Azoles	Thiazoles
0.99_243.02(	243.026781	0.99473333	pos	Beta-D-Fruct	HMDB0003971		BGWGXPAF	Organic oxyg	Organooxyge	Carbohydrate
1.08_139.05	139.052213	1.07531667	pos	Indene	HMDB0253451		YBYIRNPNP	Benzenoids	Indenes and	Unclassified
1.08_146.02(	146.026923	1.07531667	pos	THIOPHENE	HMDB02590	337894	QERYCTSH	Organoheter	Thiophenes	Thiophene ca
1.11_417.16	417.162779	1.1137	neg	2-Hydroxyimi	HMDB00606	53258	FKCUOSGT(	Organoheter	Benzazepine	Dibenzazepir
1.14_250.06	233.058998	1.13681667	pos	(4S)-4-Aminc	HMDB0257667		ANDRTDSFI	Organic acid	Carboxylic ac	Amino acids,
1.18_272.06	272.068863	1.18473333	neg	Dexelvucitab	HMDB0251085		HSBKFSPNI	Nucleosides,	Nucleoside a	Unclassified
1.25_365.04	365.042187	1.2521	neg	betanidin qui	HMDB0304274		MCTHLMSFI	Alkaloids anc	Betalains	Unclassified
1.34_172.09	172.097712	1.34166667	neg	Cyclohexane	HMDB02506	342774	PNZXMIKHJ	Organic acid	Carboxylic ac	Carboxylic ac
1.35_133.10	133.10493	1.34996667	pos	2-Heptanethi	HMDB00323	88347	DAZNOIJJVI	Organosulfur	Thiols	Alkylthiols
1.36_350.09	350.098936	1.3586	neg	Entacapone	HMDB00122	315007	JRURYQJSL	Phenylpropai	Cinnamic aci	Hydroxycinna
1.36_623.10	623.101302	1.3586	neg	3,5,7,8-Tetra	HMDB02599	45564	KLGQSVMIF	Organoheter	Pyranopyridii	Unclassified
1.45_169.09	169.097289	1.45418333	pos	2'-Hydroxyac	HMDB0061919		ADVGKWPZ	Benzenoids	Benzene anc	Anilides

1.49_277.14	277.141831	1.48828333	pos		Palitantin, (+)-		425698		MPOXQBRZ	Unclassified	Unclassified	Unclassified
1.56_284.16	284.160438	1.55895	pos		Threonylphe	HMDB00290	85961		IQHUITKNH	Organic acid	Carboxylic a	Amino acids,
1.56_395.08	395.081876	1.56021667	neg		N-(6-Aminop	HMDB0256368			ZESFDAKNY	Benzenoids	Benzene and	Biphenyls an
1.58_403.14	403.146984	1.57865	neg		Pyrene	HMDB00420	69974		BBEAQIROQ	Benzenoids	Pyrenes	Unclassified
1.63_308.04	308.044633	1.63346667	neg		Epinephrine	HMDB00018	58061		AELFRHHZC	Organic acid	Organic sulfu	Arylsulfates
1.76_137.10	137.107432	1.75533333	pos		N,N-Dimethy	HMDB02476	66134		BZORFPDS	Organic nitro	Organonitrog	Amines
1.93_278.03	278.033765	1.92873333	neg		Dopamine 4-	HMDB00041	58219		DEKNNWJX	Organic acid	Organic sulfu	Arylsulfates
10.23_605.3	605.321342	10.2257833	neg		Tribenzylamine n-oxide		24081		KIAARAUZZ	Unclassified	Unclassified	Unclassified
10.32_189.1	189.103282	10.3206667	pos		Fluorodopam	HMDB0252375			WODALLKZ	Benzenoids	Phenols	Benzenediols
10.43_265.1	265.14779	10.42685	neg		6,7-Dihydro-	HMDB00334	89304		XLWXLZKP	Benzenoids	Phenols	1-hydroxy-2-
10.65_675.4	675.450136	10.6454167	pos		H-Ser-Leu-Il	HMDB0253029			CNOKQOBE	Organic acid	Carboxylic a	Amino acids,
10.77_233.1	233.154421	10.77115	neg		1,10-Epoxyg	HMDB00354	90699		BRHJBHVGI	Lipids and lip	Prenol lipids	Sesquiterpe
10.79_440.3	440.372248	10.7875833	pos		11-HydroxyN	HMDB0241561			LZIQXDUPO	Lipids and lip	Fatty Acyls	Fatty acid es
10.79_631.2	631.259045	10.7894833	neg		Disperse Yel	HMDB02514	352464		VGKYEIFFS	Organohetero	Azobenzenes	Unclassified
10.84_583.3	583.336347	10.8434333	neg		Thioperamid	HMDB02590	45306		QKDDJDBF	Organohetero	Piperidines	Unclassified
11.00_572.2	572.298255	11.0025333	pos		4-(2,2,6,6-Te	HMDB0252920			MNYFRPDQ	Lignans, neo	Lignan lacto	Unclassified
11.29_341.2	341.207718	11.2883167	pos		(2E,8E)-10-h	HMDB0241109			GQJKEPWE	Lipids and lip	Fatty Acyls	Fatty acid es
11.30_926.5	926.52014	11.2956167	neg		PS(22:5(7Z,1	HMDB0116791			LIHIQTWQK	Lipids and lip	Glycerophos	Glycerophos
11.43_311.1	311.153504	11.4320667	pos		Bifonazole	HMDB00155	43556		OCAPBUJL	Benzenoids	Benzene and	Diphenylmet
11.58_332.1	332.176754	11.5754333	pos		Butriptyline	HMDB0249471			ALELTFCQZ	Benzenoids	Dibenzocycl	Unclassified
11.81_468.3	468.345731	11.80575	pos		PA(P-20:0/0:0)		82361		SPWZKHQA	Lipids and lip	Glycerophos	Glycerophos
11.91_441.1	441.134817	11.9099667	neg		OSU03012		96406		YULUCECV	Unclassified	Unclassified	Unclassified
11.97_632.3	655.332424	11.9664167	pos		16,16-dimethyl Prostaglan		45607		YDCXQLUD	Unclassified	Unclassified	Unclassified
12.09_587.3	587.348242	12.0909333	pos		Valnemulin	HMDB0259752			LLYYNOVS	Lipids and lip	Prenol lipids	Diterpenoids
12.20_823.5	823.572629	12.1975333	pos		SM(d19:0/20	HMDB0290657			OZEIXOVBZ	Unclassified	Unclassified	Unclassified
12.49_413.2	413.225519	12.4924667	neg		4-Fluorobuty	HMDB0255010			QZFMCYUB	Organohetero	Piperidines	Fentanyls
12.62_465.1	465.128548	12.6205	pos		Methacycline	HMDB0015066			IWFHNYCD	Phenylpropa	Tetracyclines	Unclassified
14.67_165.0	165.076974	14.67175	pos		Galactitol	HMDB00001	5148		FBPFZTCF	Organic oxyg	Organooxyge	Carbohydrate
2.11_555.02	555.021479	2.11061667	neg		5-Amino-1-[c	HMDB0260380			KLZLEXIXV	Organic acid	Carboxylic a	Amino acids,
2.15_393.02	393.029275	2.1466	neg		Dehydro-L-(+)-ascorbic ac		45119		MUYNUOXI	Unclassified	Unclassified	Unclassified
2.48_398.15	398.155483	2.48101667	pos		1,2,4-Triazol	HMDB0254773			GOIFCXRIF	Organohetero	Azoles	Triazoles
2.80_359.19	359.192348	2.80321667	pos		15-deoxy-delta-12,14-PGJ2-d4				VHRUMKCA	Lipids and lip	Fatty Acyls	Eicosanoids
3.13_262.16	262.164895	3.12683333	pos		Polyethylene	HMDB00324	88485		AZUZXOSW	Organic acid	Keto acids a	Medium-chai

3.31_295.17	295.178652	3.30741667	pos	Pilsicainide	HMDB02565	315614	BCQTVJKBT	Organic acid	Carboxylic a	Amino acids,
3.49_242.16	242.162037	3.48938333	pos	QUINPIROL	HMDB0257056		FTSUPYGM	Organic nitro	Organonitrog	Amines
3.59_514.13	514.131716	3.58845	neg	Bucladesine	HMDB0249421		CJGYSWNG	Nucleosides,	Purine nucle	Cyclic purine
3.64_430.06	430.069244	3.64241667	neg	2-Fluorobutyl	HMDB0252390		CLNODLMQ	Organoheter	Piperidines	Phenylpiperid
3.66_378.09	378.095871	3.65995	neg	Flurtamone	HMDB02524	331456	NYRMIJKDB	Benzenoids	Benzene and	Trifluoromet
3.68_419.05	419.054492	3.67736667	neg	Iguratimod	HMDB02533	322835	ANMATWQY	Organoheter	Benzopyrans	1-benzopyra
3.73_336.07	336.075937	3.73108333	neg	Orciprenaline	HMDB0060609		IJWHPUWE	Organic acid	Organic sulfu	Arylsulfates
3.80_406.16	406.164552	3.80401667	pos	3,4-Dehydro	HMDB0247344		GHALECSG	Organoheter	Quinolines a	Quinolones a
3.84_376.20	376.203725	3.83913333	pos	(E)-1-[(2R)-2	HMDB0252279		OPLOPFHU	Organoheter	Azoles	Pyrazoles
3.86_517.29	517.299117	3.85685	pos	dasc#1			ROTVIEONM	Lipids and lip	Fatty Acyls	Fatty acyl gly
3.89_276.17	315.134245	3.8922	pos	[6]-Shogaol		68362	OQWKEEOH	Unclassified	Unclassified	Unclassified
3.91_227.05	227.052683	3.91066667	pos	3,5,6-Trihydr	HMDB0041031		OPIUUJWC	Organic oxyg	Organooxyge	Carbonyl con
3.97_263.99	263.992345	3.97085	neg	4-Nitropheny	HMDB00602	6144	XZKIHKMTE	Organic acid	Organic phos	Phosphate e
3.98_522.27	522.272484	3.98231667	pos	Morphiceptin	HMDB00057	58312	LSQXZIURE	Organic acid	Carboxylic a	Amino acids,
4.00_452.26	452.261715	4.00056667	pos	(2s)-2-[[(2s)-	HMDB0250026		NXHZAKRR	Organic acid	Carboxylic a	Amino acids,
4.06_252.12	252.123271	4.05526667	pos	Coixinden B	HMDB00409	71942	BIUULCNW	Benzenoids	Indenes and	Unclassified
4.06_590.25	590.25796	4.05526667	pos	5alpha-Etho	HMDB00296	86386	GCRPDPKX	Lipids and lip	Steroids and	Physalins an
4.10_154.06	154.063422	4.10018333	neg	4-hydroxy-5-	HMDB0304179		DBYQGTLF	Organoheter	Dihydrofuran	Furanones
4.24_859.42	859.423069	4.23603333	pos	CDP-DG(i-12	HMDB0116157		DOQQEGQF	Lipids and lip	Glycerophos	CDP-glycero
4.29_548.28	548.286073	4.29085	pos	Myotoxin A	HMDB0254743		XHEPXCWC	Lipids and lip	Prenol lipids	Sesquiterpe
4.42_235.09	235.090391	4.41721667	pos	Thiolanilide		96349	SGMHGVVT	Unclassified	Unclassified	Unclassified
4.58_296.09	296.093625	4.57715	neg	Citpressine I	HMDB00293	86188	CHCYJKZB	Organoheter	Quinolines a	Benzoquinoli
4.61_161.06	161.063731	4.61413333	neg	1-Heptanesu	HMDB0243886		AKRQHOW	Organic acid	Organic sulfo	Organosulfo
4.63_316.14	316.149299	4.63161667	pos	7-Methylgua	HMDB00011	6008	OGHAROSJ	Nucleosides,	Purine nucle	Unclassified
4.71_231.05	231.051099	4.70578333	neg	Erinapyrone	HMDB0041026		ZXCJKDVK	Organoheter	Pyrans	Pyranones a
4.74_440.15	440.151231	4.74241667	neg	N-(2,5-Dimet	HMDB0250816		DCRZYADK	Benzenoids	Benzene and	Diphenylethe
4.80_487.24	487.240867	4.79645	neg	TRIPHENYL	HMDB02592	337847	AAAQKTZKL	Benzenoids	Triphenyl co	Unclassified
4.81_292.12	292.121101	4.81226667	pos	ANABASAMINE		44025	TZRDBHMK	Unclassified	Unclassified	Unclassified
4.83_1091.4	1091.44416	4.83273333	neg	(2S)-2-[(4-M	HMDB0260138		YLFFZEQHD	Organic acid	Carboxylic a	Amino acids,
4.91_229.02	229.024591	4.90568333	neg	Furylfuramid	HMDB0252550		LYAHJFZLD	Organoheter	Furans	Nitrofurans
5.08_624.03	624.039427	5.08273333	pos	2-phospho-4	HMDB03040	330211	HTJXTKBIU	Nucleosides,	Pyrimidine n	Pyrimidine ri
5.30_318.22	318.227032	5.30103333	pos	Tetranor-PGF1alpha		74978	NPXVWXIFJ	Lipids and lip	Fatty Acyls	Eicosanoids
5.54_359.10	359.106748	5.53846667	pos	3-Hydroxym	HMDB0033927		QPIOQLJXM	Organic acid	Carboxylic a	Amino acids,

5.59_193.08:	193.083158	5.59335	pos	Berteroin	HMDB00315	87781	HBVIMVJTU	Organosulfur	Isothiocyana	Unclassified
5.70_261.12(	261.126229	5.70286667	pos	N-(3-oxo-heptanoyl)-homoserine thiolac			NPRILBGCC	Lipids and lip	Fatty Acyls	Fatty amides
5.74_472.23	472.237003	5.7359	neg	15-HETE-T		75530	HTGCVHGW	Lipids and lip	Fatty Acyls	Fatty amides
6.59_443.15	443.158045	6.58565	neg	Camostat	HMDB0249575		XASIMHXSU	Phenylpropa	Depsides an	Unclassified
6.60_567.15	567.152395	6.59813333	pos	Estriol 3-sulfate 16-glucuronide			ATNWFRGU	Lipids and lip	Steroids and	Steroid conju
6.73_239.96	239.968084	6.73181667	neg	5-IAI		96598	BIHPYCDDP	Unclassified	Unclassified	Unclassified
6.99_413.11(	413.116197	6.98976667	neg	TRI-P-CRES	HMDB02591	328849	BOSMZFBH	Organic acid	Organic phos	Phosphate e
7.21_761.15	761.158348	7.21093333	neg	Netoglitazon	HMDB0255551		PKWDZWY	Benzenoids	Naphthalene	Unclassified
7.36_285.01(	285.016613	7.3586	neg	Ethyl 2-amino-4-(trifluorom		324983	XJRPTMOR	Unclassified	Unclassified	Unclassified
7.41_691.15	691.155142	7.41378333	neg	Hydroxydehy	HMDB02461	1538	ARDQNVJJI	Organoheter	Pyridines an	Phenylpyridi
7.59_245.18	245.18947	7.58896667	pos	Ginsenoyne	HMDB0040374		AOXSLJSDF	Lipids and lip	Fatty Acyls	Fatty alcohol
8.76_868.58	869.594316	8.75856667	pos	PG(i-22:0/20	HMDB0272149		GVOXUSYC	Unclassified	Unclassified	Unclassified
8.88_599.24	599.249296	8.88258333	pos	Ptilosaponoside B			KTFHNNJQ	Lipids and lip	Steroids and	Steroid conju
9.07_360.12	360.126958	9.0656	pos	4-O-alpha-D-	HMDB00382	92956	IWKGAFMT	Organic oxy	Organooxyge	Carbohydrat
9.43_447.34(	447.346364	9.43288333	pos	1alpha,25-dihydroxy-24a,24b-dihomo-2			AXZXYLZES	Lipids and lip	Steroids and	Secosteroids
9.53_603.22	603.222799	9.53388333	neg	3'-Hydroxy-3	HMDB00416	96016	DLSNLGWO	Phenylpropa	Stilbenes	Unclassified
9.99_575.05	575.052841	9.98928333	neg	5-(3',5'-Dihyc	HMDB0240474		CVGUPVHD	Organic acid	Organic sulfu	Arylsulfates
0.59_133.01(	133.016606	0.58926667	pos	Methylpyrazi	HMDB00331	318704	CAWHJQAV	Organoheter	Diazines	Pyrazines
0.61_279.07	279.077631	0.61033333	neg	4,4'-methanc	HMDB0061037		JNJWXPZH	Benzenoids	Benzene anc	Diphenylmet
0.63_517.14	517.147889	0.62835	neg	5-(1H-Indol-3	HMDB0255500		TXUWMXQF	Organic acid	Carboxylic ac	Amino acids,
0.70_226.03	226.038898	0.70456667	neg	Acamprosate	HMDB00147	753	AFCGFAGU	Organic acid	Organic sulfo	Organosulfor
0.72_152.98(	152.986056	0.71928333	neg	VINYLSULF(	HMDB02598	316770	NLVXSWCK	Organic acid	Organic sulfo	Organosulfor
0.73_202.10(	202.100793	0.72825	pos	Isoflurophate	HMDB00148	43220	MUCZHBLJL	Organic acid	Organic phos	Phosphate e
0.74_160.03	160.034525	0.74288333	pos	Alloxan	HMDB00028	252	HIMXGTXN	Organoheter	Diazines	Pyrimidines a
0.75_324.04	369.042922	0.74898333	neg	2-Amino-3-[[(	HMDB0257578		IUBBHHOZV	Organic acid	Carboxylic ac	Amino acids,
0.75_326.06	326.064724	0.74898333	neg	Flufenamic a	HMDB02523	43461	LPEPZBJOK	Benzenoids	Benzene anc	Benzoic acid
0.77_274.13	274.139496	0.7729	pos	Methyl-3H-th	HMDB0254620		IPDRTIBDO	Nucleosides,	Pyrimidine n	Pyrimidine 2'
0.82_471.12	471.127825	0.81883333	pos	Benzyl gentic	HMDB00415	95899	LLEMMFPEL	Organic oxy	Organooxyge	Carbohydrat
0.89_346.12	346.127005	0.8943	neg	Gum arabic	HMDB03048	450130	UOENJXXS	Benzenoids	Benzene anc	Benzoic acid
0.90_383.11	383.115866	0.89581667	pos	Invert sugar	HMDB0303239		PJVXUVWG	Organic oxy	Organooxyge	Carbohydrat
0.91_222.07	261.036989	0.9113	pos	Ethyl glucuro	HMDB00103	61648	IWJBVMJWS	Organic oxy	Organooxyge	Carbohydrat
0.93_149.00	149.007414	0.92838333	pos	Dihydroxyfun	HMDB00020	6460	KAPRQAPA	Organic acid	Keto acids a	Short-chain k
1.00_344.95	344.957549	0.99793333	neg	Perfluorohep	HMDB0256322		ZWBAMYVP	Organohalog	Alkyl halides	Alkyl fluoride

1.04_108.04	108.04477	1.0442	pos		2-Propenami	HMDB0251122			CHDKQNHK	Organic acid	Carboxylic ac	Carboxylic ac
1.04_396.23	396.23795	1.0442	pos		(2E)-2-[(4,5-I	HMDB0251668			AALSSIXXBI	Lipids and lip	Prenol lipids	Quinone and
1.06_259.17	259.170834	1.06053333	pos		O-1821		44953		GKVOVXWE	Unclassified	Unclassified	Unclassified
1.07_434.08	434.087156	1.06741667	neg		Delphinidin 3	HMDB0301665			WIEYMFHX	Phenylpropa	Flavonoids	Flavonoid gly
1.10_495.13	495.13693	1.09681667	neg		2-(5'-methylt	HMDB0304052			OKIIWFXZW	Organic acid	Hydroxy acid	Medium-chai
1.13_325.06	325.065193	1.13148333	neg		1-Benzenesu	HMDB0245613			HWWVPVU	Organoheter	Azolidines	Imidazolidine
1.14_359.01	359.017406	1.13681667	pos		(2S)-4,5,7-Tr	HMDB0255326			AWMNMVKF	Phenylpropa	Flavonoids	Hydroxyflavo
1.20_391.07	391.072812	1.20271667	neg		Thioglucose	HMDB0259013			ABXYOVCS	Organic oxyg	Organooxyge	Carbohydrate
1.31_253.08	253.080574	1.30545	neg		Ganciclovir	HMDB0015139			IRSCQMHQ	Organoheter	Imidazopyrim	Purines and
1.59_408.10	408.105786	1.59411667	pos		Niazicinin	HMDB00319	88060		RHLFBIFJRZ	Organic oxyg	Organooxyge	Carbohydrate
1.74_376.17	376.171603	1.73755	pos		2-Phenylaminoadenosine		5981		SCNILGOVB	Unclassified	Unclassified	Unclassified
1.85_452.09	452.094501	1.84545	pos		trans-zeatin	HMDB0304508			IRILMCCKF	Nucleosides,	Purine nucle	Purine ribonu
10.39_464.2	464.278089	10.3905167	neg		LysoPC(14:1	HMDB00103	40280		JXGLGTFMA	Lipids and lip	Glycerophos	Glycerophos
10.43_516.2	516.2632	10.42685	neg		valeryl 4-hyd	HMDB0259747			JXPAKNRYZ	Organic acid	Carboxylic ac	Amino acids,
10.56_642.3	642.359958	10.5560833	pos		OG-PG				RDNYSWRF	Lipids and lip	Glycerophos	Oxidized glyc
10.77_628.2	628.234882	10.77115	neg		ITX-5061 fre	HMDB0260339			UUROSJLZN	Benzenoids	Naphthalene	Unclassified
11.00_370.3	370.331072	11.0025333	pos		Pantheric Acid A				KKPWDPXW	Lipids and lip	Fatty Acyls	Fatty Acids a
11.02_878.5	878.53744	11.0205667	pos		PS(18:0/TXB	HMDB0281356			BAVKJNLFY	Unclassified	Unclassified	Unclassified
11.93_655.4	655.494	11.9285167	neg		Stanozolol	HMDB00031	2487		LKAJKIOFIW	Lipids and lip	Steroids and	Estrane sterc
12.51_651.4	651.43262	12.51485	pos		OON-PE				ZYICSGMYF	Lipids and lip	Glycerophos	Oxidized glyc
12.53_576.3	576.331876	12.5329667	pos		Denticulaflavonol		50412		VLQNALFJV	Lipids and lip	Polyketides	Flavonoids
12.64_685.5	685.540824	12.6402	neg		DG(20:0/0:0/	HMDB0296377			AAUYDFBY	Unclassified	Unclassified	Unclassified
12.82_391.3	391.341273	12.8165833	pos		Oleoylcholine	HMDB0240596			PVXGXBXE	Organic nitro	Organonitrog	Quaternary a
13.05_537.4	537.415826	13.0525	neg		16-oxo-palmi	HMDB0304028			NKVUEIHJD	Lipids and lip	Fatty Acyls	Fatty acids a
13.60_343.2	343.240443	13.59865	neg		16b-Hydroxy	HMDB0003166			IZGBPAAEP	Lipids and lip	Steroids and	Estrane sterc
14.58_584.2	584.262298	14.5847667	pos		Americine	HMDB0034441			SBDJWBSJF	Organic acid	Carboxylic ac	Amino acids,
2.02_150.09	150.091457	2.02305	pos		2-Methylcum	HMDB0059847			GBGPVUAO	Organoheter	Benzofurans	Unclassified
2.04_472.24	472.24009	2.04021667	pos		3-O-(alpha-L-arabinopyranosyl-(1->6)-b				MLIAQAVEA	Lipids and lip	Fatty Acyls	Eicosanoids
2.11_284.03	284.030179	2.11061667	neg		(2R)-1-(4-Iod	HMDB02426	321029		ISEHJSHTIV	Benzenoids	Benzene anc	Phenethylam
2.11_565.05	565.050051	2.11061667	neg		Uridine diphc	HMDB00003	5291		HSCJRCZFD	Nucleosides,	Pyrimidine nu	Pyrimidine nu
2.11_623.08	623.082159	2.11061667	neg		1-Sulfooxym	HMDB02440	417493		JEHJDOIFM	Benzenoids	Pyrenes	Unclassified
2.71_315.06	315.063615	2.71386667	pos		Elephantopinolide M				DIOKUCNW	Lipids and lip	Prenol lipids	Isoprenoids
2.75_278.03	278.033014	2.74981667	pos		3,5-Dihydrox	HMDB0240449			KTPBHOMU	Phenylpropa	Cinnamic aci	Hydroxycinna

3.40_304.182	304.182134	3.39796667	pos	Aptiganel	HMDB02485	320991	BFNCJMUR	Benzenoids	Naphthalene	Unclassified
3.49_392.148	392.148903	3.48938333	pos	Epiroprim	HMDB0251860		NMARPFMJ	Organoheter	Pyrroles	Substituted p
3.49_395.220	395.220069	3.48938333	pos	Norgestomet	HMDB0255739		IWSXBCZCF	Lipids and lip	Steroids and	Pregnane ste
3.60_521.283	521.283073	3.59728333	pos	PG(17:0/0:0)		80021	WVMGNTLV	Lipids and lip	Glycerophos	Glycerophos
3.66_201.030	202.043818	3.66498333	pos	Thiabendazo	HMDB00148	2823	WJCNZQLZ	Organoheter	Benzimidazo	Unclassified
3.68_385.969	385.969052	3.67736667	neg	5'-Hydroxylor	HMDB00139	85327	NQOMZUZL	Organic acids	Carboxylic ac	Amino acids,
3.77_362.192	362.192612	3.76795	pos	Bursin	HMDB0249447		DIBAKBGYJ	Organic acids	Carboxylic ac	Amino acids,
3.96_447.251	447.251456	3.96453333	pos	Cuminone J			GLPOORBZI	Lipids and lip	Polyketides	Aromatic poly
4.04_292.041	292.041486	4.04478333	neg	Emtricitabine	HMDB00150	69459	XQSPYNMV	Nucleosides,	Nucleoside a	3'-thia pyrimi
4.15_401.238	401.238879	4.14533333	pos	L-Proline, 1-(	HMDB0249520		ZEZGJKSEB	Organic acids	Carboxylic ac	Amino acids,
4.16_237.072	237.072902	4.163	pos	Curvulalide			XVHODQZC	Lipids and lip	Fatty Acyls	Fatty esters
4.21_453.089	453.089273	4.20936667	neg	N-ethyl-7H-p	HMDB0255132		RMQGEAPU	Organoheter	Imidazopyrim	Purines and
4.23_538.995	538.995915	4.22771667	neg	Phosphatidyl	HMDB0252943		VQSJAWPF	Lipids and lip	Glycerophos	Glycerophos
4.27_416.285	416.285851	4.27276667	pos	N-(R)-[2-(Hy	HMDB0258720		LMIQCBIEA	Organic acids	Carboxylic ac	Amino acids,
4.33_360.192	360.192633	4.32651667	pos	(S)-N-Benzyl	HMDB0253741		ICULFJDHZ	Organic acids	Carboxylic ac	Amino acids,
4.45_375.077	375.077758	4.45335	pos	Chlorophacir	HMDB02501	72320	UDHXJZHVN	Benzenoids	Benzene anc	Diphenylmetl
4.50_397.124	397.124267	4.50406667	neg	2-(4-Methyl-5	HMDB00324	88448	KXYXTLJGF	Organoheter	Azoles	Thiazoles
4.56_232.985	232.985568	4.5592	pos	(E)-3-methyl-6-(pent-3-en-1-yn-1-yl)-1,2			VZBIMBFEZ	Lipids and lip	Fatty Acyls	Oxygenated
4.63_210.983	210.983263	4.63258333	neg	Pentafluorob	HMDB02562	339162	YZERDTRE	Benzenoids	Benzene anc	Benzoic acid
4.67_218.062	218.062281	4.66833333	pos	Glucosaminic acid		3334	UFYKDFXCZ	Unclassified	Unclassified	Unclassified
4.70_495.278	495.277967	4.70481667	pos	glas#18			QBDRWZBY	Lipids and lip	Fatty Acyls	Fatty acyl gly
4.76_188.997	188.997786	4.76071667	neg	6-Sulfanyl-1H	HMDB0255801		HCFLFBOW	Organoheter	Diazines	Pyrimidines a
4.87_347.203	347.203901	4.86546667	pos	Hydroxyethyl	HMDB0240229		PDSVTRQO	Organoheter	Benzothiazin	Phenothiazin
4.87_839.327	839.327233	4.86903333	neg	Amtolmetin g	HMDB0248367		CWJNMKKM	Organic acids	Carboxylic ac	Amino acids,
4.96_382.092	382.092001	4.96071667	neg	Dda-tetracyc	HMDB0250895		XCCHQGIGI	Phenylpropa	Tetracyclines	Unclassified
5.01_150.127	150.127725	5.01075	pos	4-Phenylbuty	HMDB02465	418493	AGNFWIZBE	Benzenoids	Benzene anc	Phenylbutyla
5.05_432.181	432.180987	5.05298333	neg	Pcera-1	HMDB0256170		WSWXSQN	Benzenoids	Benzene anc	Phenylpropa
5.26_245.060	245.066903	5.25613333	neg	(Z)-3-(1-Form	HMDB00330	88983	IVZLDVMNN	Lipids and lip	Fatty Acyls	Fatty acids a
5.35_249.022	249.022603	5.3483	neg	Bisphenol S	HMDB02407	69883	VPWNQTHU	Benzenoids	Benzene anc	Benzenesulfo
5.85_311.061	311.061083	5.84918333	pos	n-methyl-2-(4	HMDB0255169		OKPLJXUHF	Organoheter	Benzothiazol	Unclassified
5.87_307.077	307.077323	5.8654	neg	1-Phenyl-2,4	HMDB00329	88847	ZKQQJUBG	Benzenoids	Benzene anc	Benzoyl deriv
6.20_297.090	297.089966	6.1974	pos	N-(2-Cyclohe	HMDB02557	63036	KTDZCOWX	Benzenoids	Benzene anc	Nitrobenzene
6.38_383.151	383.151521	6.3809	pos	2-Methoxyestradiol-17beta 3-sulfate			HKVMUVAM	Lipids and lip	Steroids and	Steroid conju

6.44_170.987	170.987381	6.4382	neg		3-Hydroxy-4-	HMDB0061120			UHYGMFPQ	Organic acid	Organic sulfu	Arylsulfates
6.90_369.035	369.035316	6.8979	neg		[(2S,3R,4R,5	HMDB0247871			DJJCXFVJD	Nucleosides,	Pyrimidine nu	Pyrimidine rib
7.17_392.228	392.228893	7.17388333	neg		3,7-Dihydrox	HMDB0241145			CFJJLQJGW	Lipids and lip	Fatty Acyls	Fatty acid es
7.28_467.152	467.152355	7.2767	pos		Pyridinoline	HMDB00008	5814		LCYXYLLJXI	Organic acid	Carboxylic ac	Amino acids,
9.06_236.105	236.10532	9.05798333	neg		AL 34662		64719		WBYHTZYH	Unclassified	Unclassified	Unclassified
9.06_429.150	429.150546	9.05798333	neg		N-Methacrylo	HMDB0255166			BCKKKQIAP	Organic acid	Carboxylic ac	Amino acids,
9.32_255.195	255.19519	9.32303333	pos		Canavalmine	HMDB00394	94003		RZOHQCYU	Organic nitro	Organonitrog	Amines
9.54_773.492	773.492693	9.54118333	pos		PG(20:4(5Z,8	HMDB0270649			ACQGHOCP	Unclassified	Unclassified	Unclassified
0.57_119.049	119.049394	0.57178333	pos		1,4-Benzodic	HMDB0244196			BNBQRQQY	Organoheter	Benzodioxan	Benzo-1,4-di
0.59_215.102	215.102584	0.58926667	pos		N-Hydroxy-L-	HMDB0038750			CNIUEVQJA	Organic acid	Carboxylic ac	Amino acids,
0.64_218.904	218.904901	0.64391667	neg		5-Iodouracil	HMDB02468	340908		KSNXJLQDQ	Organoheter	Diazines	Pyrimidines a
0.64_365.034	365.034246	0.64391667	neg		8-Hydroxythi	HMDB0060804			TWLCFFLQI	Organoheter	Imidazopyrim	Purines and
0.64_377.066	377.066664	0.64391667	neg		Quisqualic acid		66893		ASNFTDCKZ	Unclassified	Unclassified	Unclassified
0.70_163.115	163.115673	0.69861667	pos		(S1)-Methoxy	HMDB00323	88407		HRYCNFLXC	Organic oxyg	Organooxyge	Ethers
0.70_272.067	253.049986	0.70456667	neg		Butein		45732		AYMYWHCQ	Lipids and lip	Polyketides	Flavonoids
0.72_295.083	295.083761	0.71928333	neg		Olprinone	HMDB02559	321093		JPAWFIIYTJ	Organoheter	Pyridines and	Bipyridines a
0.72_353.089	353.089411	0.71928333	neg		2-Ethoxy-6(5	HMDB0247563			MPTIYRLFO	Organoheter	Benzopyrans	1-benzopyrar
0.73_228.010	228.009997	0.7342	neg		Acephate	HMDB02479	44455		YASYVMFA	Organophos	Organothiopl	Unclassified
0.76_150.022	150.022848	0.76461667	neg		2-Amino-3-su	HMDB0244781			BOTPEONK	Organosulfur	Thiols	Alkylthiols
0.79_431.140	431.140751	0.7877	pos		Sabiporide	HMDB0257436			SRRHGTUD	Organoheter	Diazinanes	Piperazines
0.80_381.030	381.030005	0.79596667	neg		2,3-dioxo-L-g	HMDB0304040			GJQWCDSA	Organic oxyg	Organooxyge	Carbohydrate
0.83_131.047	131.047053	0.83391667	pos		4-Methoxyph	HMDB02464	458428		GPKUICFDW	Benzenoids	Phenol ether	Anisoles
0.99_135.116	135.116922	0.99473333	pos		2-Ethyl-p-xyle	HMDB00598	344283		AXIUBBVSO	Benzenoids	Benzene and	Xylenes
1.08_374.119	374.119455	1.07531667	pos		Acarviosin	HMDB0247888			KFHKERRGI	Organic oxyg	Organooxyge	Carbohydrate
1.10_370.076	370.076518	1.09681667	neg		Dihyroxy-1H-	HMDB0059997			IAHCDPVIVI	Organic oxyg	Organooxyge	Carbohydrate
1.11_361.075	360.06777	1.1137	neg		SPARSOMY	HMDB0258407			XKLZIVIOZD	Organoheter	Diazines	Pyrimidines a
1.12_113.096	113.096423	1.11995	pos		Propyl 2-met	HMDB00362	349198		AZFUASHXS	Organic acid	Carboxylic ac	Carboxylic ac
1.15_203.150	203.150358	1.1535	pos		Arginine, ethy	HMDB0248577			AKGWUHIOI	Organic acid	Carboxylic ac	Amino acids,
1.20_407.009	407.009065	1.20271667	neg		ascorbic acid	HMDB0248653			RALJLUURQ	Organic acid	Carboxylic ac	Tetracarboxy
1.20_436.173	436.173492	1.20271667	neg		PS(6:0/6:0)		40805		MIQYPPGTN	Lipids and lip	Glycerophos	Glycerophos
1.22_283.057	283.057467	1.2188	pos		Tensyuic acid F				DFYGVGBRI	Lipids and lip	Fatty Acyls	Fatty Acids a
1.23_523.120	523.120551	1.23491667	neg		Lanceolatin B		985115		NQNOTBRH	Unclassified	Unclassified	Unclassified
1.25_303.050	303.049974	1.2521	neg		Alternariol	HMDB00308	347565		CEBXXEKPI	Phenylpropai	Coumarins a	Unclassified

1.31_405.03	405.030326	1.30545	neg		(2R,3S)-2-m	HMDB0303970			HHKPKXCSI	Organic acid	Carboxylic ac	Tricarboxylic
1.36_419.02	419.027364	1.3586	neg		TIFLAMIZOL	HMDB02590	336678		XUOAKFNM	Organoheter	Azoles	Imidazoles
1.36_449.10	449.104915	1.3586	neg		6-Aminometh	HMDB0248281			LBSXFXZKP	Organoheter	Thiadiazines	Benzothiadia
1.36_489.19	489.19802	1.3586	neg		Cynaroside A	HMDB00405	95023		MVSPYXAIG	Lipids and lip	Prenol lipids	Terpene lact
1.40_382.07	382.07377	1.40198333	pos		Triglochinin	HMDB0030934			LABCALMTG	Organic oxyg	Organooxyge	Carbohydrate
1.41_549.08	549.087862	1.4122	neg		3-Methylellag	HMDB00370	92058		BQACUXSQ	Phenylpropa	Tannins	Hydrolyzable
10.18_570.2	570.256368	10.17625	pos		Prupaside	HMDB00389	93562		GLRJVMYT	Lignans, neo	Lignan glyco	Unclassified
10.48_643.2	643.288966	10.48035	neg		PI(22:6(4Z,7Z,10Z,13Z,16Z,19Z)/0:0)				CAPHXUZAI	Lipids and lip	Glycerophos	Glycerophos
10.63_318.1	318.161063	10.6275	pos		2-[5-[(3,5-Dir	HMDB0247477			CVCLJVVBH	Organoheter	Pyrroles	Substituted p
10.66_331.2	331.204186	10.66295	neg		Thalassotalamide A				MRBDSURQ	Lipids and lip	Fatty Acyls	Fatty amides
10.81_536.2	536.208467	10.8069333	neg		5-O-[(3S)-1-E	HMDB0260407			XHEATHCD	Organoheter	Pyridines and	Hydropyridin
10.88_592.3	592.323431	10.8794667	neg		OG-PE				HNCJTFUKD	Lipids and lip	Glycerophos	Oxidized glyc
11.01_525.2	525.294471	11.0055	neg		Alcyopterosin B				UJHFESNVII	Lipids and lip	Prenol lipids	Isoprenoids
11.07_361.1	361.166745	11.0736	pos		Acetylfentany	HMDB0246153			FYIUUQUPC	Benzenoids	Benzene and	Anilides
11.20_850.6	850.607975	11.198	pos		PE(24:1(15Z	HMDB00097	61162		FAEYIXWOZ	Lipids and lip	Glycerophos	Glycerophos
11.93_359.1	359.15105	11.9300167	pos		Antofloxacin	HMDB0248493			MHFCVNDH	Organoheter	Quinolines a	Quinoline ca
12.30_531.1	531.194929	12.3037667	neg		3-Deazaader	HMDB0245849			DBZQFUNL	Nucleosides,	Imidazole[4,5	Unclassified
12.57_297.2	320.255603	12.56865	pos		2S-amino-octadeca-4E,6E		53959		BXFSVOSKI	Lipids and lip	Sphingolipids	Sphingoid ba
12.62_393.1	393.198117	12.6205	pos		AM2201 N-(4-hydroxypent		96633		HTFNNXALS	Unclassified	Unclassified	Unclassified
12.83_425.2	425.287074	12.83355	pos		ascr#28				HSLYPVWJ	Lipids and lip	Fatty Acyls	Fatty acyl gly
12.89_635.5	635.50446	12.8930167	neg		Alloepipregn	HMDB00014	57867		AURFZBICLI	Lipids and lip	Steroids and	Pregnane ste
14.27_383.1	383.189297	14.2674833	neg		Schisanheno	HMDB0258169			FYSHYFPJB	Phenylpropa	Tannins	Hydrolyzable
14.28_353.3	353.302127	14.2796333	pos		Phytantriol	HMDB0256517			CGIHFIDUL	Lipids and lip	Prenol lipids	Diterpenoids
14.37_368.3	368.315486	14.3693667	pos		N-(octadecanoyl)-homose		45493		HGMDJDYX	Lipids and lip	Fatty Acyls	Fatty amides
2.11_593.14	593.146536	2.11061667	neg		Kuwanon Z	HMDB0033297			HEAKAXOV	Phenylpropa	Flavonoids	Flavans
2.34_347.17	347.179034	2.33636667	pos		(8R,9S,10S,1	HMDB0260176			LNSIGBRLIF	Lipids and lip	Steroids and	Androstane s
2.53_385.11	385.113118	2.5348	neg		6-Hydroxyme	HMDB0247079			XGWIBNWD	Organoheter	Pteridines an	Pterins and d
3.02_223.06	223.063675	3.01843333	pos		N-Phenyl-p-p	HMDB0246356			ATGUVEKS	Benzenoids	Benzene and	Aniline and s
3.27_572.26	572.269558	3.27091667	pos		Ssioriside	HMDB00389	93545		UTPBCUCEI	Lignans, neo	Lignan glyco	Unclassified
3.33_588.14	588.145384	3.32576667	pos		Pelargonidin 3-sambubioside				NKUOSFBSI	Lipids and lip	Polyketides	Flavonoids
3.40_488.25	488.254356	3.39796667	pos		N-Eicosapen	HMDB0242077			PXVMEXBCI	Unclassified	Unclassified	Unclassified
3.49_220.02	220.027646	3.48938333	pos		Benzoic acid	HMDB0240374			HGKLFYLYV	Benzenoids	Benzene and	Benzoic acid
3.62_522.13	522.138086	3.62421667	neg		Malvidin 3-rh	HMDB0303639			IBKIPCPWN	Phenylpropa	Kavalactones	Unclassified

3.66_138.02	138.022182	3.66498333	pos	3-Mercaptop	HMDB00013	6195	OJOLFAIGO	Organic acid	Keto acids a	Alpha-keto a
3.71_363.11	363.114971	3.71251667	neg	Tebupirimfos	HMDB02587	72470	AWYOMXWI	Organic acid	Organic thio	Thiophospho
3.73_388.20	388.200907	3.73238333	pos	Caroverine	HMDB0249687		MSPRUJDU	Organoheter	Diazanaphtha	Benzodiazine
3.77_533.23	533.236389	3.7682	neg	4-amino-MX	HMDB0061009		PWFQZSWK	Benzenoids	Benzene and	Aniline and s
3.82_204.08	204.086865	3.82191667	pos	5-Amino-4-cyclohexylisoxa		339610	CQTRVZZZ	Unclassified	Unclassified	Unclassified
3.99_340.11	340.118171	3.98933333	neg	PC(2:0/2:0)		39733	GAZIIOYXD	Lipids and lip	Glycerophos	Glycerophos
4.04_548.28	548.286729	4.03688333	pos	Ergosine	HMDB0251913		NESVMZOP	Alkaloids and	Ergoline and	Lysergic acid
4.13_521.28	521.280009	4.12765	pos	Dolichyl diph	HMDB00015	53862	MXGLYEVG	Lipids and lip	Prenol lipids	Polyprenols
4.14_659.25	659.253531	4.13528333	neg	Flumezapine	HMDB0252339		JBHUBOISL	Organoheter	Benzodiazep	Unclassified
4.20_740.36	740.363099	4.19935	pos	Leucomycin	HMDB0254035		XYJOGTQL	Organic oxy	Organooxyge	Carbohydrate
4.22_499.27	499.279081	4.21765	pos	PS(15:1(9Z)/0:0)		78842	OOFVILSDF	Lipids and lip	Glycerophos	Glycerophos
4.25_502.26	502.262719	4.25448333	pos	Micronomicin	HMDB0254707		DNYGXMIC	Organic oxy	Organooxyge	Carbohydrate
4.25_609.25	609.252597	4.24626667	neg	Zaleplon	HMDB00150	85038	HUNXMJYC	Organoheter	Diazines	Pyrimidines a
4.33_299.10	299.105864	4.32651667	pos	Asn Asn Gly His		195985	LDSFSKFAT	Unclassified	Unclassified	Unclassified
4.39_696.35	696.357974	4.39318333	neg	Naloxegol	HMDB0255436		XNKCCCKF	Benzenoids	Phenanthren	Unclassified
4.45_652.36	652.36109	4.45335	pos	Janthitrem G	HMDB00305	87044	PGYSJEQV	Organic acid	Peptidomime	Depsipeptide
4.58_295.12	589.232629	4.57715	neg	Indobufen	HMDB02534	329985	AYDXAULL	Organoheter	Isoindoles ar	Isoindolines
4.60_615.31	615.317037	4.5955	pos	4-[[(1R)-2-[[(	HMDB0244531		FVQSSYMR	Organoheter	Indoles and	Tryptamines
4.67_691.25	691.258104	4.66926667	neg	Dihydrorhoda	HMDB02513	64881	FNEZBBILN	Organoheter	Benzopyrans	1-benzopyra
4.72_375.11	375.119752	4.72411667	neg	N-Acetylsero	HMDB0060833		DRKQFNYK	Organic oxy	Organooxyge	Carbohydrate
4.76_379.07	379.071681	4.76071667	neg	4-Nitroquinol	HMDB02465	24073	YHQDZJICG	Organoheter	Quinolines a	Nitroquinolin
4.81_276.01	276.016601	4.81226667	pos	Caffeic acid	HMDB0304903		VWQNTRNA	Phenylpropa	Cinnamic aci	Hydroxycinna
4.83_588.40	588.409183	4.8302	pos	DG(8:0/6 ket	HMDB0297119		CPSDCXICH	Unclassified	Unclassified	Unclassified
4.87_415.29	415.291436	4.86546667	pos	Phytosphingosine 1-phosp		53901	AYGOSKUL	Lipids and lip	Sphingolipids	Sphingoid ba
4.92_159.04	159.043986	4.91941667	pos	1,4-Naphtho	HMDB02442	65798	FRASJONU	Benzenoids	Naphthalene	Naphthoquin
4.92_167.03	167.030278	4.91941667	pos	trans-2-Hexe	HMDB00133	45913	HSBSUGYT	Lipids and lip	Fatty Acyls	Fatty acids a
5.03_266.98	266.985185	5.03453333	neg	Acetazolami	HMDB00149	782	BZKPWHYZ	Organoheter	Azoles	Thiadiazoles
5.11_515.21	515.217815	5.10843333	neg	6-Nitroquipa	HMDB0247098		GGDBEAVV	Organoheter	Quinolines a	Nitroquinolin
5.22_677.34	677.340955	5.21915	neg	7-[(4-Fluorop	HMDB0247864		NXPLYKRKI	Benzenoids	Benzene and	Halobenzene
5.27_466.16	466.166921	5.27448333	neg	Pitavastatin	HMDB00419	320829	VGYFMXBA	Organoheter	Quinolines a	Phenylquinol
5.54_218.12	218.120755	5.53846667	pos	Propionic aci	HMDB0249401		NEHWDLUE	Lipids and lip	Fatty Acyls	Fatty acids a
6.11_240.17	263.161488	6.10565	pos	(1S,2S)-3-oxo-2-pentyl-cy		74961	MQWNXLZV	Lipids and lip	Fatty Acyls	Octadecanoi
6.99_557.12	557.122624	6.98976667	neg	(2R,4S)-2-Ar	HMDB0242567		WAAPEIZFC	Organoheter	Azolidines	Imidazolidine

7.04_195.03!	195.039084	7.03826667	pos	Hydantoin-5-	HMDB00012	6084	VWFWNXQ/	Organoheter	Azolidines	Imidazolidine
7.28_319.03:	319.032774	7.28471667	neg	Thiacremonc	HMDB00359	91104	JYMIRUWYS	Organic oxyg	Organooxyge	Carbonyl cor
7.92_206.06(	206.066648	7.91976667	pos	(Z)-But-1-ene	HMDB00603	327647	BJYPZFUW\	Organic acid:	Carboxylic ac	Tricarboxylic
8.05_478.19(	478.196872	8.04801667	pos	Proversilin D			DJUJOUYGE	Lipids and lip	Prenol lipids	Isoprenoids
8.30_263.05!	263.055859	8.3042	pos	METHYL 7-DESHYDROX`		43947	DQWQITFU\	Unclassified	Unclassified	Unclassified
8.62_279.00(	279.006204	8.61736667	neg	Riluzole	HMDB00148	2321	FTALBRSUT	Organoheter	Benzothiazol	Unclassified
8.64_298.08!	298.084989	8.63586667	neg	5,4'-Dimethyl-6-hydroxyluteolinidin			PAGRNMMU	Lipids and lip	Polyketides	Flavonoids
8.77_469.26:	469.263329	8.76651667	neg	Camicinal	HMDB0249573		RZKDEGZIF	Benzenoids	Benzene anc	Phenylacetar
8.78_553.23(	553.236399	8.784	neg	D-Pantethine	HMDB00038	316134	DJWYOLJPS	Organic acid:	Carboxylic ac	Amino acids,
8.79_500.27(	500.276512	8.7919	pos	PA(22:6(4Z,7Z,10Z,13Z,1(		82338	UWHSPTWE	Lipids and lip	Glycerophos|	Glycerophos|
9.06_633.11:	633.113376	9.05798333	neg	Nilutamide	HMDB00148	44648	XWXYUMMI	Organoheter	Azolidines	Imidazolidine
9.39_458.23!	458.23988	9.3876	neg	Apstatin	HMDB0248539		YVUUZAPYL	Organic acid:	Peptidomime	Hybrid peptic
9.61_552.31!	552.315668	9.60636667	neg	Chenodeoxy	HMDB0242402		TZJSDNVVG	Lipids and lip	Steroids and	Bile acids, al
9.97_686.33	686.33143	9.97076667	neg	PC(22:6(4Z,7	HMDB0288887		FHCMBRRT	Unclassified	Unclassified	Unclassified
0.61_495.15:	495.153722	0.61033333	neg	(R)-Byakang	HMDB00390	93675	CVNASKZT(	Phenylpropa	Coumarins a	Furanocoum:
0.70_218.02!	435.043829	0.70456667	neg	2-Methoxy-4-	HMDB0304925		LILXPJXZNJ	Organic acid:	Organic sulfu	Arylsulfates
0.71_249.07	249.071515	0.71368333	pos	Aspartyl-Asp	HMDB00287	85664	FRYULLIZUI	Organic acid:	Carboxylic ac	Amino acids,
0.72_278.08	278.08769	0.71928333	neg	Nelarabine	HMDB00154	316204	IXOXBSCIX:	Nucleosides,	Purine nucle	Unclassified
0.90_216.12	216.1219	0.89581667	pos	Alvameline	HMDB02482	409886	RNMOMKCF	Organoheter	Pyridines anc	Hydropyridin
0.91_299.05!	299.055474	0.91118333	neg	Phenylglyoxy	HMDB00015	326632	FAQJJMHZN	Benzenoids	Benzene anc	Benzoyl deriv
0.93_251.16(	251.166471	0.92838333	pos	Antibiotic JBIR 27; 6-Hydr(		985361	VXPARNCTI	Unclassified	Unclassified	Unclassified
1.17_282.03	305.023537	1.1705	pos	3-[[(2R)-2-Ac	HMDB0247775		ZLCOWUKV	Organic acid:	Carboxylic ac	Amino acids,
1.20_414.19	414.191652	1.20271667	neg	PC(7:0/0:0)		40330	RGIOGDXW	Lipids and lip	Glycerophos|	Glycerophos|
1.22_404.13!	404.135795	1.21911667	neg	Oxodipine	HMDB0255993		MSOAVHHA	Organoheter	Benzodioxole	Unclassified
1.22_675.08!	675.0895	1.21911667	neg	S-sulfanylglu	HMDB0304477		PRJBNAOAE	Organic acid:	Carboxylic ac	Amino acids,
1.30_281.02!	281.025567	1.30165	pos	Glucaron	HMDB0247896		ZOZKYEHVI	Organic acid:	Carboxylic ac	Tetracarboxy
1.31_337.04:	337.043192	1.30545	neg	Imidazoleace	HMDB00060	58346	RDQUQBHP	Nucleosides,	Purine nucle	Unclassified
1.32_343.14!	343.148358	1.32371667	neg	VORTIOXET	HMDB0259875		YQNWZWMI	Organoheter	Diazinanes	Piperazines
1.34_725.29:	725.292204	1.34166667	neg	CAY10581		45721	WKHJQIQD(	Unclassified	Unclassified	Unclassified
1.36_610.11(	609.108689	1.3586	neg	Quercetin 3-(	HMDB0301686		DKHSNOLJF	Phenylpropa	Flavonoids	Flavonoid gly
1.38_319.11:	319.113445	1.3848	pos	4-Nitropheny	HMDB0245678		IFBHRQDFS	Organic oxyg	Organooxyge	Carbohydrate
1.39_286.03(	286.03672	1.39358333	neg	Coumarin-3-(	HMDB02446	430874	NTXGVUJAL	Phenylpropa	Coumarins a	Unclassified
1.39_371.03!	371.035382	1.39358333	neg	Calcium prop	HMDB03036	72191	BCZXFFBUY	Organic acid:	Carboxylic ac	Carboxylic ac

1.43_300.10	300.106454	1.43078333	neg		4-Hydroxy-5-	HMDB0260283			AHIWRJZVL	Benzenoids	Phenol ether	Unclassified
1.52_116.01	116.017395	1.52331667	neg		N-Methylcyst	HMDB0255186			DVOOXRTY	Organic acid	Carboxylic ac	Amino acids,
1.54_245.03	245.038952	1.54175	neg		1-[(2R,4S,5S	HMDB0257970			DGVFFRFC	Organoheter	Diazines	Pyrimidines a
1.54_399.19	399.19973	1.54175	neg		cis-4-Decene	HMDB00006	74921		CXGDCGIPE	Lipids and lip	Fatty Acyls	Fatty acids a
1.62_271.08	271.079979	1.61501667	neg		Arbutin	HMDB00299	43989		BJRNKVDFD	Organic oxyg	Organooxyge	Carbohydrate
1.68_214.11	214.118766	1.68358333	pos		5-Aminosalic	HMDB0246754			OCIBQVQZF	Organic acid	Carboxylic ac	Amino acids,
1.70_370.05	370.053483	1.70151667	pos		3-Phosphoac	HMDB0245974			AGQFLEFSF	Nucleosides,	Purine nucle	Unclassified
1.90_411.11	411.113988	1.89901667	pos		Deacetyldiltia	HMDB00028	2271		NZHUXMZTS	Organoheter	Benzothiazep	Unclassified
1.95_349.07	349.076479	1.9471	neg		Lactate carbo	HMDB0253943			CWIQNOUW	Organic acid	Carboxylic ac	Tetracarboxy
10.19_1027.5	1027.57546	10.1937667	pos		PI(22:2(13Z,1	HMDB0277719			HNQRXDUH	Unclassified	Unclassified	Unclassified
10.21_560.3	560.320459	10.20805	neg		ibha#28				DVKTXYSVN	Lipids and lip	Fatty Acyls	Fatty acyl gly
10.30_417.1	417.187153	10.2994667	neg		Proflavine	HMDB00152	68897		WDVSHHCD	Organoheter	Quinolines a	Benzoquinoli
10.32_391.2	391.245296	10.3206667	pos		gamma-Eud	HMDB00379	92751		QKXSRJAH	Lipids and lip	Prenol lipids	Sesquiterpen
10.63_1175.	1175.57988	10.6260167	neg		IV2Fuc-nLc4	HMDB0062482			NEEVJWBFI	Lipids and lip	Sphingolipids	Glycosphing
10.63_557.3	557.320532	10.6260167	neg		Doxepin		2463		ODQWQRR	Unclassified	Unclassified	Unclassified
10.63_748.2	748.235686	10.6260167	neg		Antibiotic CC	HMDB0248478			UOWVMDUE	Organoheter	Indoles and	Pyrroloindole
10.73_415.2	415.260849	10.7334833	pos		3-Hydroxy-1	HMDB00390	93625		HQPQTQQU	Lipids and lip	Prenol lipids	Sesterterpen
10.84_322.2	322.236712	10.8409	pos		4-Hydroxytestosterone		57832		BQOIJSIMM	Lipids and lip	Steroids and	Steroids
10.90_321.2	321.234297	10.8978167	neg		1-Phenylpipe	HMDB02439	349111		LLSKXGRDU	Organoheter	Piperidines	Phenylpiperi
11.07_207.0	207.017343	11.0736	pos		2-Pyrroloylgl	HMDB0059778			GCBXCOXV	Organic acid	Carboxylic ac	Amino acids,
11.40_317.1	317.173115	11.40315	neg		Methyl 5-(hy	HMDB0061678			BSILTUUPJE	Organoheter	Pyrrolidines	Pyrrolidine ca
11.98_469.2	469.253499	11.9841667	pos		4-Hydroxyval	HMDB00138	85284		ICSQZMPILL	Organic acid	Carboxylic ac	Amino acids,
12.48_742.5	742.571456	12.4811	pos		PE(O-20:0/15:0)		77544		CKGVEHXG	Lipids and lip	Glycerophos	Glycerophos
12.83_661.5	661.541432	12.8323333	neg		1-(9,14-dimethyl-pentadec		75577		HGKBESRA	Lipids and lip	Glycerolipids	Diradylglycer
12.89_365.2	365.224772	12.8930167	neg		(-)-Pyrifolidin	HMDB00335	89335		BTDUGGGJ	Alkaloids anc	Aspidosperm	Unclassified
14.58_656.2	656.270626	14.5847667	pos		Hexyl heptan	HMDB00323	88365		JMHXWPSJ	Lipids and lip	Fatty Acyls	Fatty acid es
2.02_479.02	479.027102	2.02081667	neg		Kaempferol 3-sulfate-7-alpha-arabinopy				WQCLONBE	Lipids and lip	Polyketides	Flavonoids
2.09_166.01	166.016686	2.09156667	pos		DL-Homocys	HMDB0251520			VBOQYPQE	Organic acid	Carboxylic ac	Amino acids,
2.11_300.04	300.046361	2.11061667	neg		N-Acetyl-alpha-D-glucosar		384		FZLJPEPAY	Unclassified	Unclassified	Unclassified
2.11_759.14	759.1456	2.11061667	neg		4-Methoxybe	HMDB00347	90180		LILHPDXFNI	Organic oxyg	Organooxyge	Carbohydrate
2.39_247.02	247.022298	2.387	neg		Triflusal	HMDB0259212			RMWVZGDJ	Benzenoids	Benzene anc	Benzoic acid
2.52_177.09	177.091404	2.51663333	pos		3-(4-Methoxy	HMDB00371	92113		BNBBFUJNM	Phenylpropa	Cinnamaldeh	Unclassified
2.59_348.16	348.169763	2.58846667	pos		(9Z,11E,14Z)	HMDB0303985			UKAYSZOB	Lipids and lip	Fatty Acyls	Lineolic acids

2.61_292.17	292.176075	2.6067	pos	Ormetoprim		315759	KEEYRKYKL	Unclassified	Unclassified	Unclassified
2.83_323.08	323.083572	2.83016667	neg	9-dimethylarsinoyl-nonanoic acid			KCRYGWRE	Lipids and lip	Fatty Acyls	Other Fatty A
2.91_275.10	275.106045	2.9106	pos	2-(Ethylsulfo	HMDB00402	94813	IOPTXXRNX	Benzenoids	Benzene and	Phenyl methy
2.92_540.05	540.057305	2.92268333	neg	ADP Ribose	HMDB0248024		PWJFNRJRI	Nucleosides,	Purine nucle	Purine ribonu
3.34_464.25	464.250405	3.34363333	pos	(3S)-4-[[(1S)	HMDB0257534		PLSJXKVBQ	Organic acid	Carboxylic a	Amino acids,
3.59_655.22	655.226306	3.58845	neg	Triphyllin A			KIOGDCNEF	Lipids and lip	Polyketides	Flavonoids
3.60_204.08	204.089568	3.59728333	pos	1-(4-Methyl-1	HMDB0257832		ABAWEANE	Organic nitro	Organonitrog	Amines
3.68_317.02	317.025009	3.67736667	neg	Succinimidyl	HMDB02585	422364	ZJIFDEVVTF	Organoheter	Pyrrolidines	Pyrrolidones
3.77_366.08	366.08915	3.7682	neg	2-Amino-4-o	HMDB00121	62809	XWORXFNM	Organoheter	Pteridines an	Pterins and d
3.92_369.17	369.177814	3.9154	neg	L-Prolinamid	HMDB0253911		YWZAPMXA	Organic acid	Carboxylic a	Amino acids,
3.92_432.13	432.136499	3.9154	neg	Tegoprazan	HMDB0258778		CLIQCDHNF	Organoheter	Benzopyrans	1-benzopyra
3.93_525.89	525.899351	3.92891667	pos	3,3'-Diiodoth	HMDB0005869		CPCJBZABT	Organic acid	Carboxylic a	Amino acids,
3.93_737.34	737.347102	3.92891667	pos	OHHdiA-PI			GJKJHBUGZ	Lipids and lip	Glycerophos	Oxidized glyc
4.02_529.27	529.275243	4.01898333	pos	(5alpha,6bet	HMDB00331	89071	AYSGOPOJ	Lipids and lip	Steroids and	Steroid lacto
4.04_731.83	731.833177	4.04478333	neg	Hexacosane	HMDB00618	340424	HMSWAIKSI	Hydrocarbon	Saturated hy	Alkanes
4.06_461.01	461.012982	4.05526667	pos	Hexanitrohex	HMDB0253147		NDYLCHGX	Organoheter	Diazinanes	Piperazines
4.11_622.32	622.323322	4.10951667	pos	Scilliroside			QDGKTFOP	Lipids and lip	Steroids and	Sterols
4.14_681.23	681.235266	4.13528333	neg	3-Amino-N-[(	HMDB02598	411786	MDNWGCQ	Organoheter	Thienopyridi	Unclassified
4.27_538.27	538.276043	4.27276667	pos	Purothionin A	HMDB00390	93607	PVEVXUMVI	Benzenoids	Benzene and	Benzoic acid
4.34_432.13	432.138068	4.34396667	pos	Ezetimibe	HMDB00151	43875	OLNTVTPD	Organoheter	Lactams	Beta lactams
4.34_929.44	929.446789	4.33765	neg	Luminespib	HMDB0254202		NDAZATDQI	Benzenoids	Benzene and	Cumenes
4.36_292.06	292.064768	4.3621	pos	Glucose asp	HMDB0252772		RZTRGWJVI	Organic acid	Carboxylic a	Amino acids,
4.36_401.24	401.240431	4.35616667	neg	Merodesmos	HMDB00304	86951	ZUXGPPMN	Organic acid	Carboxylic a	Amino acids,
4.39_514.29	514.299455	4.39318333	neg	Tumonoic Acid G		65398	NHPBYMFQ	Unclassified	Unclassified	Unclassified
4.43_299.09	299.089951	4.43021667	neg	Butanoic aci	HMDB0245609		UGIGKLCVA	Organic acid	Organic phos	Phosphate e
4.51_352.12	353.13362	4.50633333	pos	Cotinine gluc	HMDB00010	5943	XWZCZWKL	Nucleosides,	Pyrimidine n	Pyrimidine ri
4.63_470.02	470.025057	4.63258333	neg	hydroxytyros	HMDB0253299		JLMHVOFX	Organic acid	Peptidomime	Depsipeptide
4.71_675.34	675.344585	4.70578333	neg	sufac#1			JZVLQNZGN	Lipids and lip	Fatty Acyls	Fatty Acids a
4.72_359.13	359.134772	4.72301667	pos	7,8-Dimetho	HMDB0249844		BABUXLQH	Organoheter	Benzodiazep	Unclassified
4.74_473.27	473.273203	4.74081667	pos	10-Hydroxy-	HMDB0060120		IRADJQUIA	Lipids and lip	Saccharolipi	Unclassified
4.83_180.01	180.012235	4.83273333	neg	2-Phenylethy	HMDB0256545		ZZUZYEMRI	Benzenoids	Benzene and	Unclassified
4.91_313.04	313.045849	4.90568333	neg	5'-Phosphori	HMDB0001308		VDXLUNDM	Nucleosides,	Glycinamide	Unclassified
4.91_432.15	432.15094	4.90568333	neg	Benzyladeni	HMDB0302642		KRUUBWXA	Organic oxyg	Organooxyge	Carbohydrate

4.92_380.15	380.157482	4.92405	neg	Hippuryl-L-ar	HMDB0253174		GFLCPYUSF	Organic acid	Carboxylic ac	Amino acids,
4.94_195.06	195.066061	4.9421	neg	4-Hydroxy-3-	HMDB0059824		LXBHHIZIQ	Organic oxyg	Organooxyge	Carbonyl cor
4.96_509.29	509.293285	4.95603333	pos	glas#20			VJWJXLJAP	Lipids and lip	Fatty Acyls	Fatty acyl gly
5.00_219.06	219.063673	4.99758333	neg	N7-(2-Carbal	HMDB02553	825115	JYHHZNJOF	Organoheter	Imidazopyrim	Purines and
5.23_182.03	182.03658	5.22825	pos	1-Benzazepi	HMDB0244865		DQFQCHIDF	Organoheter	Benzazepine	Unclassified
5.30_388.19	388.196279	5.30103333	pos	Terazosin	HMDB00152	2721	VCKUSRYTF	Organoheter	Diazinanes	Piperazines
5.98_320.21	343.208854	5.9774	pos	1,3,7-Trimetl	HMDB0255344		LZDCTQSVD	Organoheter	Imidazopyrim	Purines and
6.03_474.25	474.251571	6.03235	pos	(2R)-5-(Diam	HMDB0242607		KUWBXRGF	Benzenoids	Benzene anc	Diphenylmetl
6.09_399.17	399.178433	6.08708333	neg	1-dodecanoy	HMDB00623	82334	STTKJLVEX	Lipids and lip	Glycerophos	Glycerophos
6.23_222.14	222.146205	6.23411667	pos	Triethylenem	HMDB02591	66702	IUCJMVBFZ	Organic nitro	Organonitrog	Amines
6.23_395.16	395.162762	6.23498333	neg	Mocetinostat	HMDB0254820		HRNLUBSXI	Benzenoids	Benzene anc	Anilides
6.40_413.02	413.024744	6.40135	neg	Orotidylic aci	HMDB0000218		KYOBSHFO	Nucleosides,	Pyrimidine nu	Pyrimidine rib
6.60_516.15	516.155513	6.59813333	pos	5-O-Benzyl 3	HMDB0248917		GQCIGLRU	Phenylpropa	Cinnamic aci	Cinnamic aci
6.97_426.14	426.145173	6.97153333	neg	2-Methylthio-	HMDB0301782		VZQXUWKZ	Nucleosides,	Purine nucle	Unclassified
6.99_465.13	465.13765	6.98976667	neg	1-Hydroxy-2-	HMDB0244631		CVFXMLXL	Organic acid	Carboxylic ac	Amino acids,
6.99_533.12	533.125545	6.98976667	neg	Sulfisoxazole	HMDB00144	43339	NHUHCSRW	Benzenoids	Benzene anc	Benzenesulf
7.38_454.15	454.151548	7.37703333	neg	Acetylcoryno	HMDB0247932		PUHCFWFO	Alkaloids anc	Benzophenai	Hexahydrobe
8.23_371.01	371.01449	8.23463333	neg	8-Nitro-cGMI	HMDB0247452		XRKODJJSF	Nucleosides,	Purine nucle	Cyclic purine
8.75_503.31	503.316499	8.74946667	neg	Didesipramin	HMDB02512	451737	XJWJQDZW	Organoheter	Benzazepine	Dibenzazepir
9.10_259.12	259.127677	9.10213333	pos	Methocarban	HMDB00145	43251	GNXFOGHN	Benzenoids	Phenol ether	Anisoles
9.44_506.27	506.2734	9.44245	neg	DELTALINE		44191	DTTPWCNK	Unclassified	Unclassified	Unclassified
9.47_357.04	357.048025	9.46953333	pos	Atreleuton	HMDB0248714		MMSNEKOT	Benzenoids	Benzene anc	Halobenzene
9.61_307.22	307.22426	9.61401667	pos	Dihydro-2,4,6	HMDB00322	88271	RQGPQWU	Organoheter	Azacyclic cor	Dithiazinanes
0.59_190.97	190.979374	0.58926667	pos	Mercaptopuri	HMDB00151	503	GLVAUDGF	Organoheter	Imidazopyrim	Purines and
0.59_246.14	246.144784	0.58926667	pos	Tetraacetylet	HMDB00405	95058	BGRWYDH	Organic acid	Carboxylic ac	Carboxylic ac
0.61_182.09	182.097404	0.60603333	pos	2-Fluoromethcathinone		96735	DCMOUMKI	Unclassified	Unclassified	Unclassified
0.73_258.05	258.056168	0.7342	neg	PPM-18		64815	BIVQBWSIG	Unclassified	Unclassified	Unclassified
0.73_270.03	270.038478	0.7342	neg	2-[(6-Aminop	HMDB0244961		WGADUGL	Organoheter	Imidazopyrim	Purines and
0.73_363.07	363.074013	0.7342	neg	6-[Ethoxy(me	HMDB0257850		GESBUOGN	Benzenoids	Phenols	1-hydroxy-4-
0.74_371.11	371.112268	0.74288333	pos	1-p-Menthen	HMDB0302750		DIJWSVXPJ	Lipids and lip	Prenol lipids	Monoterpen
0.75_337.05	337.058873	0.74898333	neg	S-methylaza	HMDB0061068		CAGKKYGO	Organoheter	Imidazopyrim	Purines and
0.76_158.02	158.027072	0.75766667	pos	Benzenesulf	HMDB02489	334230	KHBQMWC	Benzenoids	Benzene anc	Benzenesulf
0.76_235.03	235.039254	0.76461667	neg	Aspartyl-Cys	HMDB00287	85665	FKBFDTRIL	Organic acid	Carboxylic ac	Amino acids,

0.82_441.13	441.134752	0.81883333	pos		Oleoside dim	HMDB00313	87604	KYVUMEGN	Lipids and lip	Prenol lipids	Terpene glyc
0.91_551.01	551.011703	0.91118333	neg		Phosphoami	HMDB0248355		PVKSNHVPL	Nucleosides,	Purine nucle	Purine ribonu
0.99_313.01	313.011413	0.99473333	pos		Alloathyriol	HMDB00387	93370	CLZONBOP	Organoheter	Benzopyrans	1-benzopyra
1.11_297.09	342.09573	1.1137	neg		Teroxirone	HMDB0258835		OUPZKGBU	Organoheter	Triazines	Triazinones
1.17_243.00	243.008443	1.16698333	neg		(2R,3R,4S,5	HMDB0242595		CMLYNMGU	Organic acid	Organic phos	Phosphate e
1.20_259.03	259.031292	1.20368333	pos		Dehydroxyzy	HMDB00139	85324	CKVDCQYU	Organoheter	Benzothioph	1-benzothiop
1.20_365.11	365.113597	1.20271667	neg		Camptotheci	HMDB0259530		SARNOWGF	Alkaloids and	Camptotheci	Unclassified
1.29_529.07	529.076901	1.2875	neg		Dotmp	HMDB0247657		RCXMQNID	Organic acid	Organic phos	Organic phos
1.32_215.07	215.072757	1.32371667	neg		EUPARIN		43728	OPUFDNZT	Unclassified	Unclassified	Unclassified
1.36_395.16	395.167807	1.3586	neg		Guaifenesin	HMDB00049	4057	HSRJKNPTN	Benzenoids	Phenol ether	Anisoles
1.36_630.17	630.173491	1.3586	neg		2-(4-(Bis(car	HMDB0253463		AMHAQOBU	Organic acid	Carboxylic ac	Pentacarbox
1.44_227.10	227.100594	1.43671667	pos		3,4-Dihydrox	HMDB00399	94490	ARJIGPTXX	Organoheter	Pyrrolidines	N-alkylpyrroli
1.54_611.13	611.137028	1.54175	neg		(-)-Epigalloca	HMDB00383	44010	XMOCLSLC	Phenylpropa	Flavonoids	Flavans
1.56_229.05	228.048886	1.56021667	neg		2-Methoxy-1	HMDB02452	442492	DLYKFPHPE	Organoheter	Benzothiazin	Phenothiazin
1.60_293.05	293.054803	1.59701667	neg		5-Hydroxy-2-	HMDB0246804		JSXNJGKW	Benzenoids	Benzene and	Trifluorometh
1.61_216.13	216.134521	1.61191667	pos		Metharbital	HMDB00146	85385	FWJKNZON	Organoheter	Diazines	Pyrimidines a
10.05_275.1	275.126666	10.0490667	pos		3alpha-Hydr	HMDB00360	91225	QWJVXAZU	Organoheter	Naphthofura	Unclassified
10.10_665.3	665.324189	10.1038333	pos		PA(20:4(6E,8	HMDB0262690		FWCYLFSJH	Unclassified	Unclassified	Unclassified
10.41_366.3	366.321153	10.41135	pos		Prostaglandin F1a-d9		96410	DZUXGQBLI	Unclassified	Unclassified	Unclassified
10.50_742.5	742.539243	10.4988	neg		PC(16:1(9Z)/17:1(9Z))		75761	AEPBKRNQ	Lipids and lip	Glycerophos	Glycerophos
10.54_365.1	365.140115	10.5357333	neg		Sodium decy	HMDB0303294		RLJSXMVTL	Benzenoids	Benzene and	Benzenesulf
10.55_640.3	640.346879	10.5532667	neg		PS-PC			GEUVQGBC	Lipids and lip	Glycerophos	Oxidized glyc
10.66_361.1	361.166834	10.6629	pos		Finerenone	HMDB0252261		BTBHLEZXC	Organoheter	Diazanaphth	Naphthyridin
10.77_509.3	548.297601	10.7697	pos		Cholylthreon	HMDB0242443		WRLSMXLT	Unclassified	Unclassified	Unclassified
10.79_403.2	403.243147	10.7875833	pos		Bisnorcholic	HMDB00020	43151	BBSJMECO	Lipids and lip	Steroids and	Bile acids, al
10.79_602.2	602.214638	10.7875833	pos		(E)-3-Hydrox	HMDB0244742		LLJQVSGW	Phenylpropa	Stilbenes	Stilbene glyc
10.98_581.3	581.364805	10.9842333	pos		3-O-alpha-L-rhamnopyranosyl-3-hydrox			ACCQJSCH	Lipids and lip	Fatty Acyls	Fatty acyl gly
11.20_303.1	303.146683	11.198	pos		Tetracaine	HMDB02588	2807	GKCBAIGFK	Benzenoids	Benzene and	Benzoic acid
11.56_1046.	1047.73335	11.5576	pos		GM4(d18:1/20:0)			ZHNKBYJDA	Lipids and lip	Sphingolipids	Acidic glycos
11.56_585.3	585.352001	11.5642333	neg		10-Hydroxyamitriptyline		952	GHWBJXOK	Unclassified	Unclassified	Unclassified
11.64_716.5	716.523173	11.6371833	neg		PE(19:1(9Z)/15:0)		77032	BAXWXJKC	Lipids and lip	Glycerophos	Glycerophos
11.74_281.1	281.140966	11.7358167	pos		codeinone	HMDB0304293		FFGRXXWI	Alkaloids and	Morphinans	Unclassified
12.02_413.1	413.192015	12.0185833	neg		4-(Methylnitr	HMDB00116	62378	FLAQQSHRI	Organic oxyg	Organooxyge	Carbonyl cor

12.49_387.27	387.27654	12.4924667	neg	N-(1-Adamar	HMDB02445	417254	QYYHPAUO	Organic acids	Organic carb	Ureas
12.49_401.16	401.163554	12.4924667	neg	Bay-41-8543	HMDB02488	461606	AQYFUZRYE	Organoheter	Pyrazolopyric	Unclassified
12.60_881.61	881.615563	12.6029833	pos	PC(MonoMe	HMDB0061448		FKBKOIXSZ	Lipids and lip	Glycerophos	Glycerophos
12.62_609.50	609.508668	12.6234833	neg	FAHFA(18:1(	HMDB0112173		APKQIGMSV	Lipids and lip	Fatty Acyls	Fatty acids a
12.62_814.57	814.570283	12.6205	pos	SM(d18:2(4E	HMDB0290569		KCDPHYMV	Unclassified	Unclassified	Unclassified
12.64_649.44	649.442187	12.6402	neg	PA(O-16:0/14:1(9Z))		82151	MAMOADSF	Lipids and lip	Glycerophos	Glycerophos
12.94_361.18	361.181821	12.9406833	pos	9-Fluorocortis	HMDB0247594		BHDHELFRE	Lipids and lip	Steroids and	Hydroxysterc
13.55_843.55	843.550242	13.5489167	neg	SM(d18:1/20	HMDB0290524		DHRIQIMLX	Unclassified	Unclassified	Unclassified
14.17_357.27	357.278348	14.1715833	pos	(1S)-1-hydroxy-23,24-dide		57900	DLAROWCG	Lipids and lip	Steroids and	Secosteroids
2.01_316.178	316.178367	2.00526667	pos	N-Desisopro	HMDB0255116		LOOAPMDV	Organic acids	Carboxylic ac	Amino acids,
2.32_237.109	237.10973	2.31811667	pos	2-Carboxy-5,	HMDB00309	87330	FEFSWWMY	Organoheter	Lactones	Gamma buty
2.46_364.101	364.101504	2.46066667	neg	4-Hydroxyvo	HMDB0060894		ICGJRJXWK	Benzenoids	Benzene and	Phenylpropa
2.55_243.044	243.044265	2.55331667	neg	(5R,6S)-3-(A	HMDB0248479		RXCNILVLO	Organic acids	Carboxylic ac	Amino acids,
2.72_439.055	439.055699	2.7196	neg	4-Methyl-2-(pyridin-3-yl)-1,		453164	CJLQNROYE	Unclassified	Unclassified	Unclassified
2.77_185.042	185.042506	2.77471667	neg	Diphenyl sulf	HMDB0251452		LTYMSROW	Organosulfur	Thioethers	Aryl thioether
2.84_318.160	318.165954	2.8388	pos	Esmolol	HMDB00143	2576	AQNDDEOP	Benzenoids	Phenol ether	Unclassified
3.27_412.120	412.120005	3.27433333	neg	Riodipine	HMDB02572	314170	AZVFIZVKG	Organoheter	Pyridines and	Hydropyridine
3.33_281.165	281.165235	3.32576667	pos	Carazolol	HMDB0249603		BQXQGZPY	Organoheter	Indoles and c	Carbazoles
3.38_345.194	345.194532	3.38003333	pos	BUTACAINE		44369	HQFWVSGE	Unclassified	Unclassified	Unclassified
3.40_177.127	177.127587	3.39796667	pos	N-butyryl-L-Homoserine la		96403	VFFNZZXXT	Unclassified	Unclassified	Unclassified
3.47_271.170	271.176533	3.47126667	pos	N-hexanoyl histidine			ORIZVAWR(	Lipids and lip	Fatty Acyls	Fatty amides
3.47_329.200	329.200638	3.47126667	pos	Roxindole	HMDB0257319		HGEYJZMM	Organoheter	Indoles and c	Hydroxyindol
3.54_365.211	365.210966	3.54413333	pos	Gancaonin R	HMDB00375	421749	QFAPONVN	Phenylpropa	Stilbenes	Unclassified
3.56_435.215	435.215	3.56223333	pos	3beta-formyloxyresibufogenin			ANZIXHORK	Lipids and lip	Steroids and	Sterols
3.62_353.048	353.048128	3.62421667	neg	Sulforaphane	HMDB00057	58318	SUVMJBTUF	Organosulfur	Sulfoxides	Unclassified
3.66_272.050	272.050736	3.65995	neg	Acetamide, N	HMDB02421	2555	CYLYVXPHA	Benzenoids	Benzene and	Benzenesulfc
3.82_146.063	146.063495	3.82191667	pos	2,5-Dimethyl-	HMDB00401	94731	DBBHCZMXI	Organoheter	Heteroaroma	Unclassified
3.82_275.032	275.031957	3.82341667	neg	Wasalexin A	HMDB00347	90189	JJQPWCPYI	Organoheter	Indoles and c	Unclassified
3.82_451.235	451.235919	3.82191667	pos	Belotecan	HMDB0248935		LNHWXBUN	Alkaloids and	Camptotheci	Unclassified
3.89_309.174	309.174574	3.8922	pos	Calcium octa	HMDB03036	327506	NDWWLJQH	Lipids and lip	Fatty Acyls	Fatty acids a
3.96_374.207	374.207024	3.96453333	pos	Sphingosine	HMDB00600	83702	IQHNJQKWE	Organic acids	Organic phos	Phosphate es
4.06_476.083	476.083348	4.05526667	pos	7-Methylguar	HMDB00596	96235	SBASPRREC	Nucleosides,	Purine nucle	Purine ribonu
4.07_366.204	366.204833	4.07303333	pos	PC(8:0/0:0)		40332	ZVPMBHRQ	Lipids and lip	Glycerophos	Glycerophos

4.10_273.12	273.127957	4.10018333	neg		Tenax GC	HMDB0258813		TWADJGWU	Benzenoids	Benzene anc	Terphenyls
4.12_273.07	273.070388	4.11676667	neg		Antigastrin	HMDB0248486		FOQYDURH	Organoheter	Pyridines anc	Unclassified
4.13_701.33	701.336607	4.12765	pos		Posaconazol	HMDB00153	85484	RAGOYPUP	Organoheter	Diazinanes	Piperazines
4.19_402.13	402.134065	4.1908	neg		3-[Bis(4-meth	HMDB0258731		KUEYYIJXBI	Benzenoids	Benzene anc	Diphenylmetl
4.25_301.09	301.090309	4.24626667	neg		2-Hydroxyad	HMDB00004	5392	DRAVOWXC	Organoheter	Imidazopyrim	Purines and
4.27_705.38	705.388243	4.27276667	pos		PA(16:1(9Z)/	HMDB01148	82067	BFIPEVORC	Lipids and lip	Glycerophos	Glycerophos
4.30_865.44	865.442745	4.30156667	neg		Hericenone E	HMDB0036548		ZJTHOPGQZ	Organoheter	Isoindoles ar	Isoindolines
4.34_517.19	517.197266	4.33765	neg		Doxepin N-o	HMDB0061139		NLZMYODD	Organoheter	Benzoxepine	Dibenzoxepir
4.47_1030.4	1030.41305	4.47151667	pos		1-(2,4-Dinitro	HMDB0247153		KWDJWQW	Unclassified	Unclassified	Unclassified
4.47_226.07	226.071142	4.47151667	pos		1-[3,4-Dihydr	HMDB0243807		CBOKZNLSF	Organic oxyg	Organooxyge	Carbohydrate
4.54_243.13	243.134829	4.54091667	neg		4-Amino-3-hy	HMDB0241810		AOCBVTCN	Lipids and lip	Fatty Acyls	Fatty acid es
4.54_339.07	339.07831	4.54091667	neg		Cyanthoate	HMDB02506	72733	TWDJIKFUV	Organophos	Organothiopl	Unclassified
4.54_353.11	353.117708	4.54091667	neg		Penilloic acid	HMDB0256237		LRWFMQCG	Organic acids	Carboxylic ac	Amino acids,
4.54_390.07	390.075974	4.54091667	neg		Thiamine mo	HMDB00026	320846	HZSAJDVW	Organoheter	Diazines	Pyrimidines a
4.58_233.06	233.06131	4.57715	neg		7-Hydroxy-2-	HMDB00339	47524	BBCDTCKKF	Phenylpropa	Isoflavonoids	Isoflav-2-ene
4.71_717.29	717.299571	4.70578333	neg		Officinoterpenoside A1			NCVFPCPIH	Lipids and lip	Prenol lipids	Isoprenoids
4.78_601.30	601.300833	4.7769	pos		Pinometostat	HMDB0256563		LXFOLMYKS	Nucleosides,	5'-deoxyribor	Unclassified
4.91_372.06	372.067426	4.90568333	neg		Fluazifop	HMDB02523	331255	YUVKUEAF	Benzenoids	Benzene anc	2-phenoxypr
4.92_495.17	495.175012	4.92405	neg		Nb-trans-Fer	HMDB00327	88688	RZBNXMNIC	Organoheter	Indoles and	Tryptamines
4.96_403.22	426.214613	4.95603333	pos		metergoline	HMDB0254511		WZHJKEUHI	Organoheter	Quinolines ar	Indoloquinoli
4.96_617.22	617.227516	4.95603333	pos		Lactodifucote	HMDB00065	58473	BRHHWBDL	Lipids and lip	Fatty Acyls	Fatty acyl gly
5.13_881.37	881.379823	5.12693333	neg		Bacmecillinar	HMDB0248840		XHKFBADIZ	Organic acids	Carboxylic ac	Amino acids,
5.21_232.04	232.043438	5.20995	pos		Bis-A-tda	HMDB0249237		WMAHFAYL	Organic nitro	Organonitrog	Amines
5.81_471.07	471.076089	5.80983333	neg		Diflunisal glu	HMDB0251263		HDNSISKAK	Organic oxyg	Organooxyge	Carbohydrate
6.48_171.01	171.019805	6.47505	neg		Nitroxoline	HMDB00154	314644	RJIWZDNTC	Organoheter	Quinolines ar	Nitroquinolin
6.59_371.18	371.189426	6.58565	neg		4-Dodecan-6	HMDB02464	984301	GHNRTXCR	Benzenoids	Benzene anc	Benzenesulfo
6.59_511.14	511.145639	6.58565	neg		Chlormidazol	HMDB02501	314151	WNAQOLSM	Organoheter	Benzimidazo	Unclassified
6.99_355.16	355.166016	6.98976667	neg		O-Desmethyl	HMDB0247073		VJFMSYZSF	Alkaloids anc	Cinchona alk	Unclassified
7.04_437.16	437.161361	7.04441667	neg		Asparaginyl-S	HMDB00287	85655	SONUFGRS	Organic acids	Carboxylic ac	Amino acids,
7.28_297.05	297.050954	7.28471667	neg		Nigakinone	HMDB0030228		XPMYKCPEI	Alkaloids anc	Indolonaphth	Unclassified
7.41_409.17	409.175974	7.41378333	neg		Quinaprilat	HMDB00420	2217	FLSLEGPOV	Organic acids	Carboxylic ac	Amino acids,
7.42_460.19	460.191639	7.42368333	pos		Methyltetrahy	HMDB0254666		OSSQVWUX	Organoheter	Pteridines ar	Pterins and c
8.71_537.25	537.251429	8.7071	neg		Declopramid	HMDB0250921		YEYAKZXEE	Benzenoids	Benzene anc	Benzoic acid

8.89_584.28	584.281793	8.89388333	neg	Amikacin	HMDB00146	943	LKCWBDHB	Organic oxyg	Organooxyge	Carbohydrate
8.98_241.07	241.072164	8.98443333	neg	phlorisobutyr	HMDB03044	66633	BNEBXEZRE	Organic oxyg	Organooxyge	Carbonyl cor
9.45_462.34	462.342098	9.45123333	pos	[(3S,4S,5S,6	HMDB0260228		LUXUAZKG	Lipids and lip	Fatty Acyls	Fatty acyl gly
9.59_660.16	660.168106	9.58798333	neg	Pelargonidin 3,5-di-(6-acetylglucoside)			MKERTHRU	Lipids and lip	Polyketides	Flavonoids
0.61_165.02	183.061772	0.60603333	pos	8-Oxoguanin	HMDB00418	96151	UBKVUFQG	Organoheter	Imidazopyrim	Purines and
0.61_356.92	356.923781	0.61033333	neg	IODODIFLU	HMDB0253524		SSYOLLIRA	Benzenoids	Benzene anc	Biphenyls an
0.62_427.15	427.151741	0.6215	pos	Fragransin D	HMDB00335	89350	AGYZMBXY	Lignans, neo	Furanoid lign	Tetrahydrofu
0.64_375.13	375.138022	0.63663333	pos	2-Methoxyac	HMDB0240215		GDCCQRYS	Organic oxyg	Organooxyge	Carbohydrate
0.69_325.12	325.125015	0.68961667	neg	(R)-Propyl 2-	HMDB0243697		AZDDIYKMQ	Organic acid	Carboxylic ac	Amino acids,
0.78_224.97	224.979205	0.7799	neg	Thiophene, 2	HMDB0259028		KFYLNCXBN	Organoheter	Heteroaroma	Unclassified
0.78_245.12	245.125309	0.7799	neg	(2S)-2-(Carb	HMDB0260137		BEUMZLYCE	Organic acid	Carboxylic ac	Amino acids,
0.88_173.02	173.025979	0.87703333	neg	8-Hydroxy-7-	HMDB00306	447904	KIGCGZUA	Phenylpropa	Coumarins a	Hydroxycoun
0.89_314.08	295.066925	0.8943	neg	Sulfaphenaz	HMDB00156	4101	QWCJHSGM	Organoheter	Azoles	Pyrazoles
0.93_177.12	177.127354	0.92838333	pos	4-Methyl-1-pl	HMDB00315	350957	DTYGTEGD	Benzenoids	Benzene anc	Unclassified
1.03_341.12	341.125787	1.0341	neg	Deoxyloganic	HMDB0037028		DSXFHNSG	Lipids and lip	Prenol lipids	Terpene glyc
1.08_274.08	274.087294	1.07531667	pos	Preparation	HMDB0256765		FCZCIXQGZ	Organic acid	Organic thiop	Thiophospho
1.08_281.03	281.035786	1.08208333	neg	Tazobactam	HMDB00155	581	LPQZKKCYT	Organic acid	Carboxylic ac	Amino acids,
1.12_429.11	429.112935	1.11995	pos	Gonyautoxin	HMDB00335	89323	CETRDCWB	Phenylpropa	Saxitoxins, g	Unclassified
1.22_340.05	340.056438	1.2188	pos	Sulfotep	HMDB02586	72469	XIUROWKZ	Organic acid	Organic thiop	Thiophospho
1.25_537.16	537.168788	1.251	pos	Conivaptan	HMDB00150	451915	IKENVDNFQ	Benzenoids	Benzene anc	Anilides
1.27_430.08	430.088009	1.26745	pos	PharmaGSID_47330		317802	PCZLQMGF	Unclassified	Unclassified	Unclassified
1.29_393.10	393.103129	1.2875	neg	Creatinol-O-Phosphate		73577	FOIPWTMK	Unclassified	Unclassified	Unclassified
1.34_199.07	199.077788	1.34166667	neg	Propranolol g	HMDB0256835		BYNNMWG	Benzenoids	Naphthalene	Unclassified
1.34_446.19	446.195712	1.34166667	neg	Risdiplam	HMDB0304853		ASKZRYGF	Organoheter	Diazinanes	Piperazines
1.36_497.08	497.081759	1.3586	neg	Lirodenine	HMDB03022	317647	LOQRLFDL	Benzenoids	Benzene anc	Benzonitriles
1.38_456.20	456.207767	1.3848	pos	Turofexorate	HMDB0247151		INASOKQDN	Organoheter	Indoles and	Indoles
1.41_317.04	317.044775	1.4122	neg	7-Aminocephalosporanic a		64538	HSHGZXNA	Unclassified	Unclassified	Unclassified
1.47_208.14	208.141106	1.47146667	pos	N(omega)-Hy	HMDB00042	58229	FQWRAVYM	Organic acid	Carboxylic ac	Amino acids,
1.49_439.06	439.067787	1.4863	neg	8-Methoxy-6,	HMDB00398	94368	GDJFGAQX	Phenylpropa	Coumarins a	Unclassified
1.70_206.08	206.084958	1.70151667	pos	N-Propionylm	HMDB0094704		RBAAEQRIT	Organic acid	Carboxylic ac	Amino acids,
1.87_267.09	267.096174	1.87326667	neg	Seryltyrosine	HMDB00290	23778	MALNXHYE	Organic acid	Carboxylic ac	Amino acids,
10.03_619.1	619.133776	10.0257	neg	Mono-trans-p	HMDB0029783		WZHTXSWU	Phenylpropa	Cinnamic aci	Hydroxycinna
10.18_278.1	278.111077	10.17625	pos	Dezinamide	HMDB0251101		LIAILJLSVR	Benzenoids	Benzene anc	Trifluorometh

10.38_414.3	414.335512	10.3754333	pos	Type III cyanolipid 20:0 ester			LQHZOOCH	Lipids and lip	Fatty Acyls	Fatty esters
10.52_769.5	769.502501	10.5172833	neg	PA(18:0/20:4	HMDB01148	3884	AXJKOPKP	Lipids and lip	Glycerophos	Glycerophos
10.81_409.2	432.268562	10.80565	pos	N-Linoleoyl	HMDB0241976		KUOWDMB	Organic acid	Carboxylic a	Amino acids,
10.81_841.5	841.548856	10.80565	pos	SM(d18:0/P	HMDB0290467		ATLZIKJCA	Unclassified	Unclassified	Unclassified
10.86_506.3	506.324955	10.8611	neg	LysoPE(0:0/	HMDB00114	62270	DGAUETYR	Lipids and lip	Glycerophos	Glycerophos
11.22_497.4	480.404413	11.2158833	pos	(16Z)-14-Hy	HMDB0241603		FPDJEXDJC	Lipids and lip	Fatty Acyls	Fatty acid es
11.26_530.3	530.325067	11.25965	neg	PC(19:3(10Z,13Z,16Z)/0:0		40269	YWILQZMM	Lipids and lip	Glycerophos	Glycerophos
11.31_838.5	838.594316	11.3135833	neg	PC(20:3(8Z,	HMDB00084	59870	CCNAQOMJ	Lipids and lip	Glycerophos	Glycerophos
11.35_379.1	379.155877	11.3501	neg	4-hydroxy-2-	HMDB0304175		XLGVEZUM	Organic acid	Carboxylic a	Amino acids,
11.39_626.3	626.367072	11.3861833	neg	PS(22:0/0:0)		78852	HRFWJAJQ	Lipids and lip	Glycerophos	Glycerophos
11.42_283.2	283.262311	11.42155	neg	Sar chelate	HMDB02574	347975	NVOVSXGZ	Organic nitro	Organonitro	Amines
11.70_600.4	600.402272	11.7002333	pos	PC(22:1(11Z)/0:0)		76576	DNYZTHZO	Lipids and lip	Glycerophos	Glycerophos
11.91_509.1	509.122023	11.9099667	neg	Mahuannin D			BHGCRZW	Lipids and lip	Polyketides	Flavonoids
12.02_345.2	345.204364	12.0185833	neg	8,8a-Diepisw	HMDB0247470		FXUAIOOA	Organohete	Indolizidines	Unclassified
12.36_579.4	579.438833	12.3628333	neg	Stearidonic Acid methyl es		44892	BIRKCHKC	Unclassified	Unclassified	Unclassified
12.64_762.5	762.505502	12.6383167	pos	PE(18:4(6Z,	HMDB00092	60606	AJSCMAKD	Lipids and lip	Glycerophos	Glycerophos
12.84_561.3	561.357692	12.8434833	neg	PA(14:1(9Z)/12:0)		81273	FCBXHNCH	Lipids and lip	Glycerophos	Glycerophos
13.40_379.1	379.179116	13.39515	neg	Bisantrene	HMDB0249263		NJSMWLQO	Benzenoids	Anthracenes	Unclassified
13.60_509.3	509.380981	13.5968667	pos	Fasciculol C	HMDB00358	91069	YRXIDKUVB	Lipids and lip	Prenol lipids	Triterpenoids
2.04_333.02	333.023612	2.03931667	neg	(5R,6S)-3-(2-	HMDB0247681		LVCPLOQIO	Organic acid	Carboxylic a	Amino acids,
2.06_462.12	462.125834	2.0574	neg	Temafloxaci	HMDB0258796		QKDHBVNJ	Benzenoids	Anthracenes	Unclassified
2.11_477.07	477.07835	2.11061667	neg	7-methylsulf	HMDB0304241		LQZALQLZC	Organic oxy	Organooxyg	Carbohydrate
2.42_317.05	317.050082	2.42373333	neg	Fukiic acid	HMDB00294	86306	PHFSBARL	Phenylpropa	Phenylpropa	Unclassified
3.46_176.03	176.038443	3.45915	neg	N-Formyl-L-	HMDB0001015		PYUSHNKN	Organic acid	Carboxylic a	Amino acids,
3.46_456.17	456.175358	3.45915	neg	Tfa-VAL-TYR-VAL-OH		44312	SAVSLMGB	Unclassified	Unclassified	Unclassified
3.47_436.21	436.218011	3.47126667	pos	2-[4-(3-Hydr	HMDB00403	94867	LRRKTNFBE	Organic oxy	Organooxyg	Carbohydrate
3.51_202.05	202.051071	3.50783333	pos	Cyclamic aci	HMDB0031340		HCAJEUSO	Organic acid	Sulfamic aci	Cyclamates
3.54_158.09	158.096584	3.54413333	pos	5-Phenyl-1,3	HMDB00329	71875	ZZGANZXIT	Benzenoids	Benzene an	Unclassified
3.62_402.12	402.122247	3.62421667	neg	Desmethylte	HMDB0304920		IJPRFXKJW	Organic acid	Organic sulf	Arylsulfates
3.66_581.10	581.107286	3.65995	neg	5'-Methoxybi	HMDB00381	92859	RQNXAFVR	Phenylpropa	Flavonoids	Biflavonoids
3.97_378.03	378.033793	3.97085	neg	cefetamet	HMDB0249761		MQLRYUCJ	Organohete	Lactams	Beta lactams
3.99_356.03	356.039803	3.98933333	neg	Aristolochic	HMDB0248599		MEEXETVZ	Benzenoids	Phenanthren	Aristolochic
4.10_632.22	632.224822	4.10018333	neg	N-Acetyl-D-g	HMDB00014	6251	OZGJPDIBI	Organic oxy	Organooxyg	Carbohydrate

4.11_285.05:	285.053262	4.10951667	pos		PRENYLETIN		43675	AWEFUQDN	Unclassified	Unclassified	Unclassified
4.12_189.09(	189.095962	4.11676667	neg		3-Mercaptoh	HMDB00323	88399	JWESHEXX:	Organic acid:	Carboxylic ac	Carboxylic ac
4.13_499.26	499.26727	4.12765	pos		PG(8:0/8:0)		40862	BQEXNLVNI	Lipids and lip	Glycerophos	Glycerophos
4.28_253.01!	253.015236	4.28308333	neg		quinalizarin	HMDB02570	328448	VBHKTXLEJ	Benzenoids	Anthracenes	Anthraquinor
4.28_545.21!	545.219036	4.28308333	neg		Hhcfu	HMDB0247349		QGVFMTLBI	Organoheter	Diazines	Pyrimidines a
4.29_406.23!	406.239626	4.29085	pos		7alpha-Thion	HMDB0246238		FWRDLPQB	Lipids and lip	Steroids and	Steroid lacto
4.30_394.16:	394.161707	4.30156667	neg		16, 17-dihydr	HMDB0304025		OXEMPUBM	Lipids and lip	Prenol lipids	Diterpenoids
4.30_468.03(	467.028752	4.30156667	neg		Primisulfuron	HMDB0256773		ZTYVMAQSI	Organic nitro	Organonitrog	Sulfonylureas
4.31_326.21	326.217355	4.30826667	pos		(R)-1-O-b-D-	HMDB00293	86223	PDTNYXYW	Lipids and lip	Fatty Acyls	Fatty acyl gly
4.36_315.21!	338.205052	4.3621	pos		Val-Val-Val	HMDB0094676		LSLXWOCIII	Organic acid:	Carboxylic ac	Amino acids,
4.40_544.29!	544.298211	4.3987	pos		2-O-(beta-D-galactopyranosyl-(1->6)-be			LMSCLROPI	Lipids and lip	Fatty Acyls	Fatty acyl gly
4.43_467.10	467.104701	4.43021667	neg		2-(4'-methyltl	HMDB0304051		MHYITDUVX	Organic acid:	Hydroxy acid	Medium-chai
4.45_214.06:	214.063085	4.45335	pos		4-(Dimethylamino)cinnama		85013	RUKJCCIJLI	Unclassified	Unclassified	Unclassified
4.54_244.07:	244.072034	4.54091667	neg		Olaquindox	HMDB0255930		TURHTASYU	Organoheter	Diazanaphth	Benzodiazine
4.56_254.99	254.994737	4.55865	neg		4-Methylumb	HMDB02404	4083	FUYLLJCBC	Phenylpropa	Coumarins a	Unclassified
4.58_276.12(	276.12649	4.57725	pos		Cybutryne		68695	HDHLIWCXI	Unclassified	Unclassified	Unclassified
4.76_262.19(	262.190757	4.75875	pos		Verruculotoxin		68439	CUANCTHYI	Unclassified	Unclassified	Unclassified
4.83_369.01!	369.01826	4.83273333	neg		2-hydroxy-3-I	HMDB0304069		MYHSVHWC	Organic oxyg	Organooxyge	Carbohydrate
4.91_232.10:	232.10123	4.90568333	neg		Molinate		68828	DEDOPGXG	Unclassified	Unclassified	Unclassified
4.92_250.14	250.146963	4.91941667	pos		DIHYDROFLAVOPEREIR		84953	DAGPUIPHA	Unclassified	Unclassified	Unclassified
4.94_640.99:	640.991288	4.93775	pos		ADP ribose 1	HMDB0304258		NPSPRYXP(	Nucleosides,	Purine nucle	Purine nucle
5.03_332.97!	332.975795	5.03453333	neg		Menadiol dis	HMDB00327	88652	JYJGXBHAX	Benzenoids	Naphthalene	Unclassified
5.18_529.23:	529.23381	5.18221667	neg		Nicotredole	HMDB0255590		ZDAZUJBAS	Organoheter	Indoles and (	Indoles
5.29_392.14	437.142701	5.29295	neg		Marmesin rha	HMDB00395	94123	HOSJGHRJ!	Phenylpropa	Coumarins a	Furanocoum
5.39_237.07(	237.07671	5.38528333	neg		Dehydrozing	HMDB0032591		AFWKBSMF	Phenylpropa	Cinnamic aci	Hydroxycinna
5.59_271.15:	271.151317	5.59335	pos		Penciclovir	HMDB00144	2651	JNTOCHDNI	Organoheter	Imidazopyrim	Purines and
6.22_642.30	642.304807	6.2158	pos		Glycylalanylp	HMDB00332	89108	WSOXREPF	Organic acid:	Carboxylic ac	Amino acids,
6.60_213.08:	213.083914	6.59813333	pos		(2R,6S)-2,6-I	HMDB02425	352	GMKMEZVLI	Organic acid:	Carboxylic ac	Amino acids,
6.81_425.11	425.11738	6.80563333	neg		Bmn-673 8R	HMDB0244463		HWGQMRY(	Organoheter	Quinolines a	Phenylquinol
7.17_215.08	215.087416	7.17388333	neg		1-Methylpyre	HMDB0243945		KBSPJIWZD	Benzenoids	Pyrenes	Unclassified
7.55_229.11!	229.119932	7.55221667	pos		alpha-Methyl	HMDB00363	91534	ANJRGSHRI	Benzenoids	Benzene anc	Phenylpropa
8.76_458.37(	458.376771	8.75856667	pos		Oleoyl Serotonin		62948	LCQKHZYXI	Lipids and lip	Fatty Acyls	Fatty amides
8.81_542.26:	542.261294	8.81001667	pos		(3,4,5-trihydroxy-6-((4-(2,6		985446	CBGLETFGT	Unclassified	Unclassified	Unclassified

9.68_508.28	508.289054	9.68	neg	Colforsin dar	HMDB0250404		RSOZZQTUI	Lipids and lip	Prenol lipids	Triterpenoids
9.68_609.23	609.235626	9.68	neg	VU 0357121		433737	AHCYOTLTL	Unclassified	Unclassified	Unclassified
9.79_282.13	282.130382	9.79426667	pos	Northiaden	HMDB0255753		FTELXJYVT	Organoheter	Benzothiepin	Dibenzothiep
9.90_499.02	499.021083	9.89818333	neg	Hydroxymeth	HMDB0060000		DCXMBZPFI	Benzenoids	Phenols	Methoxypher
0.57_117.05	117.054883	0.57178333	pos	N-Methylpyri	HMDB0240545		PQBAWAQII	Organoheter	Pyridines anc	Methylpyridir
0.61_536.22	536.226441	0.61033333	neg	Ethyl 4-[[2-[[(	HMDB0248639		BVABUBJIQ	Benzenoids	Benzene anc	Benzoic acid
0.62_249.14	249.144753	0.6215	pos	Isovalerylglut	HMDB00007	5694	HCVIJONZM	Organic acid	Carboxylic ac	Amino acids,
0.70_244.16	244.165495	0.69861667	pos	Amobarbital	HMDB00154	496	VIROVYVQC	Organoheter	Diazines	Pyrimidines a
0.73_241.08	241.085036	0.72825	pos	(R)-6-(3-Metl	HMDB0243665		USAKGOYB	Organoheter	Diazinanes	Piperazines
0.74_212.95	212.953361	0.74288333	pos	Phenyl phos	HMDB0256424		NAKDJXIEB	Benzenoids	Benzene anc	Unclassified
0.78_341.03	341.030519	0.7799	neg	Medicagol	HMDB00338	448700	URMVEUAW	Phenylpropa	Isoflavonoids	Coumestans
0.79_407.23	407.238292	0.7877	pos	Adenosine, 8	HMDB0258096		FZFITDOIQ	Nucleosides,	Purine nucle	Unclassified
0.91_425.06	425.065614	0.91118333	neg	Hydroxy-2-na	HMDB0260374		HMQXNCRP	Benzenoids	Naphthalene	Unclassified
1.01_160.07	160.073761	1.0117	pos	2-(Dimethyla	HMDB0245103		AUABZJZJX	Organic nitro	Organonitrog	Amines
1.07_264.04	263.039674	1.06741667	neg	tri-glyceride	HMDB0259133		JQPUZFQX	Organic acid	Carboxylic ac	Tricarboxylic
1.10_400.10	399.095906	1.09681667	neg	Sinapic acid	HMDB0240383		SVMPWPW	Organic oxyg	Organooxyge	Carbohydrate
1.10_417.02	417.022517	1.1046	pos	3,4,5-trihydro	HMDB0129424		VLWJVRAC	Organic oxyg	Organooxyge	Carbohydrate
1.10_577.03	577.036732	1.09681667	neg	Xct790	HMDB0259925		HQFNFOOG	Phenylpropa	Cinnamic aci	Cinnamic aci
1.10_598.14	598.147557	1.1046	pos	Neoacrimarir	HMDB00331	89066	XRRKNHPP	Organoheter	Quinolines ai	Benzoquinoli
1.15_227.01	227.011336	1.1535	pos	2-Methoxy-1,4-naphthoqui		345630	OBGBGHKY	Unclassified	Unclassified	Unclassified
1.20_180.99	180.999861	1.20271667	neg	S-2-Propenyl	HMDB00327	88672	BBZQGJLFM	Organic acid	Thiosulfinic a	Unclassified
1.20_216.00	216.009487	1.20368333	pos	Cycloalliin	HMDB00294	86254	JYMHODZX	Phenylpropa	Flavonoids	Flavonoid gly
1.22_442.11	442.119149	1.2188	pos	Dolutegravir	HMDB0251580		RHWKPHLQ	Organoheter	Pyridines anc	Pyridinecarb
1.36_183.02	183.027152	1.3586	neg	Dibenzothiop	HMDB02511	319029	IYYZUPMFV	Organoheter	Benzothiophe	Dibenzothiop
1.36_266.06	266.062141	1.3586	neg	Flunoxaprofe	HMDB0252341		ARPYQKTVF	Organoheter	Azoles	Oxazoles
1.36_547.32	547.329389	1.3586	neg	Esculentic ac	HMDB0034639		CLXOLTFMI	Lipids and lip	Prenol lipids	Triterpenoids
1.43_285.05	285.052829	1.43078333	neg	Doqualast	HMDB02515	346075	HMZDSBSM	Organoheter	Pyridopyrimic	Unclassified
1.52_258.16	258.163192	1.52326667	pos	Cyclobenzap	HMDB00150	316488	JURKNVYFZ	Benzenoids	Dibenzocyclo	Unclassified
1.62_230.02	230.028012	1.61501667	neg	1-Naphthyl is	HMDB0243961		JBDOSUUXI	Benzenoids	Naphthalene	Unclassified
1.79_331.19	331.197864	1.79108333	pos	Oxybuprocai	HMDB00150	321283	CMHHMUW	Benzenoids	Benzene anc	Benzoic acid
1.89_401.11	401.111982	1.89173333	neg	N-(2,4-Dinitro	HMDB0254997		ODQMCTXU	Benzenoids	Benzene anc	Aniline and s
10.06_606.3	606.302834	10.0622	neg	OOB-PS			ANNYJABKE	Lipids and lip	Glycerophos	Oxidized glyc
10.23_302.1	302.132222	10.2295	pos	2,4-Diamino-	HMDB0303378		IYLIKMWNA	Organoheter	Diazines	Pyrimidines a

10.50_554.28	554.287668	10.50225	pos		Vignatic acid	HMDB00335	89402	OEJLUFSEW	Organic acids	Carboxylic ac	Amino acids,
10.57_616.32	616.325252	10.5716833	neg		PC(2:0/PGE1	HMDB0288894		CTVXUMCEI	Unclassified	Unclassified	Unclassified
10.73_285.14	285.147299	10.7334833	pos		9-Nonylpurin	HMDB0260401		IEXOEQIFRI	Organoheter	Imidazopyrim	Purines and
10.81_576.20	576.201199	10.8069333	neg		3-[[2-(2-Benz	HMDB0257429		LROWLFSPI	Organic acids	Carboxylic ac	Amino acids,
10.90_570.31	570.314028	10.8950333	pos		Glu-Ile-Leu-A	HMDB0252757		VPKCCODFI	Organic acids	Peptidomime	Hybrid peptic
10.92_433.21	433.218261	10.9157	neg		Tafluprost	HMDB00157	44903	WSNODXPE	Lipids and lip	Fatty Acyls	Eicosanoids
11.29_658.50	658.508837	11.2883167	pos		(2S,3R,4E,8E)-3-Hydroxy-2-[methyl(ste			RIMXVCHDY	Lipids and lip	Sphingolipids	Other Sphing
11.82_449.37	449.371428	11.82365	pos		OMDM-2		64738	ICDMLAQPC	Unclassified	Unclassified	Unclassified
11.84_532.34	532.340515	11.84315	neg		1-linoleoyl-sn-glycero-3-phosphocholine			FKSLBYVOV	Lipids and lip	Glycerophos	Glycerophos
12.31_385.16	385.166164	12.3062167	pos		Mesulergine	HMDB0254486		JLVHTNZNK	Organoheter	Quinolines ar	Indoloquinoli
12.43_429.17	429.178181	12.4319167	neg		FK-409		62986	MZAGXDHQ	Unclassified	Unclassified	Unclassified
12.48_652.44	675.432647	12.4811	pos		1-Cyclohepty	HMDB0259956		LZQSLXDZJ	Benzenoids	Benzene and	N-phenylurea
12.48_892.57	892.579135	12.4811	pos		PE-NMe2(22	HMDB0114519		MMAGQVVE	Lipids and lip	Glycerophos	Glycerophos
12.58_555.12	555.125	12.5775167	neg		Cefluprenam	HMDB0249765		XAKKNLNAJ	Organoheter	Lactams	Beta lactams
12.62_337.19	337.191649	12.6234833	neg		Dimethinden	HMDB00156	85587	MVMQESMQ	Benzenoids	Indenes and	Unclassified
12.94_436.20	436.204282	12.9406833	pos		3-Pyridineme	HMDB0248052		MEKASOQE	Organoheter	Benzimidazo	Unclassified
13.58_716.52	716.521728	13.5784833	pos		PA(20:2(11Z	HMDB01155	81729	BIYPJVJCTN	Lipids and lip	Glycerophos	Glycerophos
13.76_821.59	821.590341	13.7582	pos		PI(O-16:0/19:0)			HBZWWUBL	Lipids and lip	Glycerophos	Glycerophos
2.00_374.08	374.086976	2.0024	neg		(Z)-N-Feruloy	HMDB00385	93245	JXFZHMCSC	Phenylpropa	Cinnamic aci	Hydroxycinna
2.02_309.01	309.016706	2.02305	pos		4,6,4'-Trihyd	HMDB01375	52446	ACGXIFQKQ	Lipids and lip	Polyketides	Flavonoids
2.64_346.19	346.197234	2.64221667	pos		N-3-oxo-tetradec-7(Z)-eno		45495	MGGGOCSK	Unclassified	Unclassified	Unclassified
2.82_190.12	190.126812	2.82056667	pos		S-Ethyl dipro	HMDB02518	68824	GUVLYNGU	Organosulfur	Thiocarbonyl	Thiocarbamic
3.44_456.99	456.992839	3.44081667	neg		Dimethoate	HMDB02513	44564	MCWXGJITA	Organic acids	Organic dithi	Dithiophosph
3.53_313.12	313.121972	3.52578333	pos		Tyrosyl-Meth	HMDB0029111		KYPMKDGK	Organic acids	Carboxylic ac	Amino acids,
3.54_729.41	729.4142	3.54413333	pos		PA(PGJ2/18:	HMDB0264337		CBXKTAHTE	Unclassified	Unclassified	Unclassified
3.60_371.16	389.203166	3.59728333	pos		Lenperone	HMDB02540	314089	WCIBOXFOI	Organic oxyg	Organooxyge	Carbonyl cor
3.68_202.03	202.034158	3.67736667	neg		4-(2-Aminoet	HMDB0246285		MGSKVZWG	Benzenoids	Benzene and	Phenethylam
3.72_258.05	258.052813	3.71513333	pos		Paraoxon	HMDB00130	44800	WYMSBXTX	Benzenoids	Benzene and	Nitrobenzene
3.77_194.13	194.139688	3.76795	pos		1-[Ethyl-(6-hy	HMDB0243808		OWMCFGLD	Organic nitro	Organonitrog	Amines
3.82_583.30	583.302241	3.82191667	pos		PG(22:4(7Z,10Z,13Z,16Z)		80005	GTUVDMUY	Lipids and lip	Glycerophos	Glycerophos
3.89_426.13	426.13711	3.8922	pos		Fasidotril	HMDB0252173		KKBIUAUSZ	Organic acids	Carboxylic ac	Amino acids,
3.96_279.00	279.006544	3.96453333	pos		Quinizarin	HMDB0257042		GUEIZVNYD	Benzenoids	Anthracenes	Anthraquinor
4.04_358.14	358.148458	4.04478333	neg		Octa-2,6-dier	HMDB0241730		FSIXZKJXBH	Lipids and lip	Fatty Acyls	Fatty acid es

4.06_229.028	229.02863	4.06311667	neg	Asulam	HMDB02486	72170	VGPYEHKO	Benzenoids	Benzene and	Benzenesulfo
4.06_256.073	256.07365	4.05526667	pos	Glutethimide	HMDB00155	66661	JMBQKKAJII	Organoheter	Piperidines	Phenylpiperid
4.07_432.094	432.093995	4.07303333	pos	Rangbatti	HMDB02571	352186	RHAXKFFKC	Benzenoids	Anthracenes	Anthracenec
4.07_451.219	451.219255	4.07303333	pos	Lomerizine	HMDB02541	320839	JQSAYKKFZ	Benzenoids	Benzene and	Diphenylmeth
4.09_427.227	427.227097	4.09135	pos	Teneligliptin	HMDB0258815		WGRQANOI	Organic acids	Carboxylic ac	Amino acids,
4.09_484.243	485.250926	4.09135	pos	DIHYDROGEDUNIN		43658	ROWFSYNH	Unclassified	Unclassified	Unclassified
4.10_341.115	341.115138	4.10018333	neg	Phenylalanyl	HMDB00290	85901	PYOHODCE	Organic acids	Carboxylic ac	Amino acids,
4.33_201.138	201.138822	4.32651667	pos	tetrahydrozol	HMDB02589	319327	BYJAVTDNI	Benzenoids	Tetralins	Unclassified
4.33_423.258	423.258848	4.32651667	pos	Lisinopril	HMDB00019	1009	RLAWWYSC	Organic acids	Carboxylic ac	Amino acids,
4.34_211.130	211.130809	4.34396667	pos	7-hydroxy-decanoic acid		74533	BUAGALXJU	Lipids and lip	Fatty Acyls	Fatty Acids a
4.47_244.198	244.198253	4.47151667	pos	2-[(Z)-[(4Z)-4	HMDB0244416		HXWLLFLQU	Organic nitro	Organonitrog	Guanidines
4.54_345.143	345.143143	4.54091667	neg	Laninamivir	HMDB0253972		QNRRHYPP	Organic acids	Carboxylic ac	Carboxylic ac
4.60_528.292	551.28162	4.5955	pos	MGMG(18:4;O/0:0)			AABZWWBX	Lipids and lip	Glycerolipids	Glycosylmon
4.76_215.013	215.013302	4.76071667	neg	Diethylthioph	HMDB00014	6256	PKUWKAXT	Organic acids	Organic thiop	Thiophospho
4.76_357.066	357.066816	4.75875	pos	Pazufloxacin	HMDB0256145		XAGMUUZP	Organoheter	Quinolines ar	Quinoline car
4.83_263.990	263.990858	4.8302	pos	Nitro 2-acety	HMDB0257748		MABOJVBZC	Benzenoids	Benzene and	Benzoic acid
4.92_333.052	333.052727	4.91941667	pos	7-Acetoxy-2-	HMDB00293	47526	DPIAJERHFI	Phenylpropa	Isoflavonoids	Isoflav-2-ene
4.96_269.005	269.005413	4.96071667	neg	3-deoxy-D-ar	HMDB0304124		PJWIPEXIFF	Organic oxyg	Organooxyge	Carbohydrate
4.99_255.083	255.083676	4.99255	pos	Glycerol 1-pr	HMDB00316	87831	LVONJPHZH	Lipids and lip	Glycerolipids	Triradylcglyc
5.09_498.190	498.190735	5.08986667	neg	Benzyloxycar	HMDB0259975		SUUHZYLYA	Organic acids	Peptidomime	Hybrid peptic
5.12_779.401	779.401101	5.11926667	pos	Vinorelbine	HMDB0014505		GBABOYUK	Alkaloids and	Vinca alkaloi	Unclassified
5.22_269.150	269.150551	5.21915	neg	Isamoxole	HMDB0253591		VMBNXRJYI	Organoheter	Azoles	Oxazoles
5.22_452.149	452.149713	5.21915	neg	CAY10650		96446	HTOJZPHW	Unclassified	Unclassified	Unclassified
5.27_398.179	398.179589	5.27448333	neg	(5E,7E)-Und	HMDB0241202		BFROTNJJY	Lipids and lip	Fatty Acyls	Fatty acid es
5.47_338.129	361.118705	5.46526667	pos	Ro 90-7501		409038	PAGZCEHLF	Unclassified	Unclassified	Unclassified
5.87_346.038	346.038752	5.8654	neg	Fludarabine	HMDB00152	675	GIUYCYHIAI	Nucleosides,	Purine nucle	Purine ribonu
5.89_453.097	453.097417	5.88578333	pos	Lignans	HMDB00314	87691	YJGVMLPVU	Lignans, neo	Lignan lactor	Podophylloto
6.53_294.053	294.053887	6.53055	neg	Sulfapyridine	HMDB00150	2552	GECHUMIMI	Benzenoids	Benzene and	Benzenesulfo
6.59_497.177	497.177022	6.58565	neg	Daniquidone		330027	SRIOCKJKF	Unclassified	Unclassified	Unclassified
6.62_564.295	564.295063	6.61625	pos	Pikromycin		41002	UZQBOFAU	Lipids and lip	Polyketides	Macrolides a
6.73_290.965	290.965143	6.73181667	neg	4-Mercapto-5	HMDB00398	94376	PQKQGARV	Organic acids	Vinylogous th	Unclassified
6.93_347.230	347.230155	6.92858333	pos	Tralkoxydim		72540	DQFPEYAR	Unclassified	Unclassified	Unclassified
6.97_505.185	505.185583	6.97153333	neg	Tovophyllin E	HMDB00305	87058	NKTLGMPG	Lipids and lip	Prenol lipids	Sesquiterper

6.98_484.11	484.116267	6.98325	pos		KBT 1585 hy	HMDB0247783		ZKUKMWMS	Organoheter	Lactams	Beta lactams
6.99_473.10	473.109548	6.98976667	neg		Tambulin 3,5-diacetate		51585	AMJOOTWK	Lipids and lip	Polyketides	Flavonoids
7.19_613.35	613.359157	7.19245	neg		Cuscutic resinoside A			GITOCQGO	Lipids and lip	Saccharolipi	Other acyl su
8.14_169.15	169.158523	8.13956667	pos		3,7-Dimethyl-6-octenyl for		97300	AGARWNGU	Lipids and lip	Fatty Acyls	Fatty esters
8.72_1117.5	1117.54896	8.71673333	neg		Benzamide, I	HMDB0258400		DNDVBYOV	Benzenoids	Benzene and	Benzoic acid
9.00_309.17	309.170698	9.00285	neg		all-trans-7-hydroxyhexadeca-2,4,8,10-te			CMIWOGJR	Lipids and lip	Fatty Acyls	Fatty Acids a
9.26_363.00	363.008391	9.25955	neg		Chelidamic acid		424137	XTLJJHGQA	Unclassified	Unclassified	Unclassified
9.32_215.18	215.18791	9.32303333	pos		Hept-2-en-1-	HMDB0303232		ZCECLCUTN	Lipids and lip	Fatty Acyls	Fatty acids a
9.44_586.34	586.346053	9.44245	neg		Poststatin	HMDB0256711		UNPBSZUD	Organic acid	Carboxylic ac	Amino acids,
9.63_430.34	430.345859	9.63246667	pos		7-dehydrocholesterol-d7		103336	UCTLRSWJ	Lipids and lip	Steroids and	Sterols
9.85_373.25	373.255639	9.84861667	pos		TXB1		36262	JSDWWNLT	Lipids and lip	Fatty Acyls	Eicosanoids
0.61_379.17	379.177829	0.61033333	neg		(1S)-1-(3-Me	HMDB0250376		WGUILOMY	Organoheter	Azoles	Triazoles
0.62_495.13	495.139401	0.6215	pos		11-keto Fluprostenol		45702	MDMHJAKZ	Unclassified	Unclassified	Unclassified
0.64_180.92	180.921304	0.64391667	neg		Potassium hy	HMDB03034	318976	CHKVPARO	Mixed metal/	Alkali metal c	Alkali metal s
0.67_410.96	410.967063	0.6745	neg		Pyrogallol-2-	HMDB0060018		XADBYTWT	Organic acid	Organic sulfu	Arylsulfates
0.69_292.07	292.071596	0.68961667	neg		Etamicastat	HMDB0251986		CWWWTTY	Organoheter	Benzopyrans	1-benzopyran
0.70_296.06	277.050561	0.70456667	neg		Gyrocyanin	HMDB00341	89728	DIFAFZFNM	Benzenoids	Phenols	1-hydroxy-2-
0.86_354.81	354.81834	0.8592	neg		Iodate	HMDB0001061		VNFOOPCK	Homogeneou	Other non-m	Unclassified
1.03_329.98	329.989578	1.0341	neg		AS-604850		45220	SRLVNYDX	Unclassified	Unclassified	Unclassified
1.04_334.12	334.124277	1.0442	pos		Domoic acid		69666	VZFRNCSO	Unclassified	Unclassified	Unclassified
1.07_254.98	254.980403	1.06741667	neg		Ascorbic acid	HMDB0240641		FBFZPSQPK	Organoheter	Dihydrofuran	Furanones
1.22_310.04	311.05617	1.2188	pos		S-(N,N-Dieth	HMDB0259477		DNPJGGSX	Organic acid	Carboxylic ac	Amino acids,
1.25_356.12	356.121093	1.2521	neg		1,7-Dimethyl	HMDB00019	58069	AISVAXNAL	Nucleosides,	Purine nucle	Unclassified
1.27_693.11	693.111021	1.26985	neg		5'-Hydroxypi	HMDB0014096		CCKOORAN	Organoheter	Benzothiazin	Unclassified
1.31_295.04	295.046857	1.30545	neg		Diacetyl ben	HMDB0257835		HCGVMMG	Organic acid	Carboxylic ac	Tetracarboxy
1.35_268.14	268.14033	1.34996667	pos		3-Deaza-2'-deoxyadenosi		96553	KMQPIRJQF	Unclassified	Unclassified	Unclassified
1.52_171.08	171.080609	1.52326667	pos		(E)-dodeca-2-en-4,6,8-triyne-1,1-diol			CMSDNYVA	Lipids and lip	Fatty Acyls	Fatty alcohol
1.59_365.14	365.149057	1.59411667	pos		1,2-Dimetho	HMDB00410	95496	FYDZPRHX	Organoheter	Quinolines a	Benzoquinoli
1.63_242.01	242.011011	1.62985	pos		2,8-Quinoline	HMDB0240658		ZLYUIVZAQ	Organoheter	Quinolines a	Quinolones a
1.77_211.07	211.075487	1.77345	pos		5,6-Dihydro-	HMDB00342	68015	DKKJNZYH	Phenylpropa	Kavalactone	Unclassified
1.83_389.14	389.14927	1.8272	pos		S-(N,N-Dieth	HMDB0257384		WZXBYDBY	Organic acid	Carboxylic ac	Amino acids,
1.89_450.11	450.112938	1.89173333	neg		Cabotegravir	HMDB0249523		WCWSTNLS	Organoheter	Pyridines and	Pyridinecarb
10.01_378.1	378.156859	10.0126833	pos		1',3'-Dimethy	HMDB0254789		JRDUBBHIP	Organoheter	Azaspirodeca	Unclassified

10.03_347.18	347.183878	10.0257	neg	(8S,9R,10R,1	HMDB0258073		AFBIOJNOX	Lipids and lip	Steroids and	Androstane s
10.17_439.35	439.353831	10.1716833	neg	alpha-Cyper	HMDB0035026		APWLDLGO	Lipids and lip	Prenol lipids	Sesquiterper
10.19_503.28	503.286496	10.18955	neg	1-(1-Hydroxy	HMDB0243771		ATLHCOBC	Organoheter	Pyrrolidines	Pyrrolidones
10.73_750.54	750.544086	10.73455	neg	PE(22:4(7Z,1	HMDB00096	60998	CBTPUGMC	Lipids and lip	Glycerophos	Glycerophos
10.75_527.13	527.132934	10.7528167	neg	8-dimethylarsinoyl-octanoic acid			OGWMGQJE	Lipids and lip	Fatty Acyls	Other Fatty A
10.88_596.26	596.266549	10.8794667	neg	TOG10			SKKVPWLM	Lipids and lip	Fatty Acyls	Eicosanoids
10.93_394.24	394.241564	10.9300167	pos	N-arachidonoyl taurine		44906	YUNYSWCR	Lipids and lip	Fatty Acyls	Fatty amides
11.01_291.15	291.157789	11.0055	neg	Acetylvalerer	HMDB00356	90950	VBBXZFLAY	Lipids and lip	Prenol lipids	Sesquiterper
11.08_660.34	660.349523	11.07825	neg	THYMOPEN	HMDB0259057		PSWFFKRA	Organic acid	Carboxylic ac	Amino acids,
11.13_624.29	624.296994	11.1325833	neg	S-(9-deoxy-d	HMDB0013057		FRARAXHW	Organic acid	Carboxylic ac	Amino acids,
11.42_633.27	633.274897	11.42155	neg	Sialorphin	HMDB0258277		RHFWFWR	Organic acid	Carboxylic ac	Amino acids,
11.50_542.32	542.323648	11.5038	pos	PC(0:0/20:4;O)			GLFHTTFIW	Lipids and lip	Glycerophos	Oxidized glyc
11.58_502.35	502.350472	11.5754333	pos	10-(3,4-Dime	HMDB0241827		DUIRYWVEI	Lipids and lip	Fatty Acyls	Fatty acid es
11.60_386.23	386.236873	11.6011	neg	N-linoleoyl taurine		75520	YIWODQVLE	Lipids and lip	Fatty Acyls	Fatty amides
11.81_584.47	584.472438	11.80575	pos	DG(15:0/0:0/15:0) (d5)		4685	HZEBMMLX	Lipids and lip	Glycerolipids	Diradylglycer
12.46_434.18	434.188512	12.46405	pos	Quinagolida	HMDB0257030		GDFGTRDC	Organoheter	Quinolines ar	Benzoquinoli
12.48_326.22	349.21072	12.4811	pos	Anordiol	HMDB0248450		HUUUMTTW	Lipids and lip	Steroids and	Androstane s
12.48_361.16	361.166524	12.4811	pos	sufac#2			LOHIJOXSS	Lipids and lip	Fatty Acyls	Fatty Acids a
12.50_936.68	936.680948	12.4972833	pos	PC(20:2(11Z	HMDB00083	59801	AXKQBIMXE	Lipids and lip	Glycerophos	Glycerophos
13.21_239.05	239.059533	13.21015	neg	6(5H)-Phena	HMDB0247116		RZFVLEJOH	Organoheter	Quinolines ar	Benzoquinoli
13.40_485.28	485.282378	13.39515	neg	N-(2'-(4-benzenesulfonam		36721	PCSYNZSW	Lipids and lip	Fatty Acyls	Fatty amides
13.73_390.26	390.268142	13.7268833	neg	N-Stearoyltau	HMDB02405	44909	LMIJIHJZVU	Lipids and lip	Fatty Acyls	Fatty amides
14.16_489.27	489.277372	14.1603833	neg	Bisnortilidin	HMDB0249273		BTKAMSWF	Organic acid	Carboxylic ac	Amino acids,
14.17_324.32	324.325732	14.1715833	pos	trans-11S,12S-Epoxy-6Z,9		98315	AZNAATMZI	Lipids and lip	Fatty Acyls	Oxygenated
2.09_168.08	168.085701	2.09156667	pos	D-Apiose	HMDB0029941		ASNHGEVA	Organic oxyg	Organooxyge	Carbohydrate
2.11_472.05	472.057271	2.11061667	neg	Monepantel	HMDB0254843		WTERNLDO	Benzenoids	Benzene anc	Benzoic acid
2.53_280.17	303.166279	2.53481667	pos	Mefexamide		321124	HUNIPYLVU	Unclassified	Unclassified	Unclassified
2.68_207.13	207.136775	2.67816667	pos	Undecylenic	HMDB00337	2980	FRPZMMHW	Lipids and lip	Fatty Acyls	Fatty acids a
2.93_428.07	428.079039	2.92891667	pos	Taisho	HMDB0258681		FQXBJYBYA	Organic acid	Carboxylic ac	Amino acids,
3.29_258.18	258.181184	3.28915	pos	Pirbuterol	HMDB00154	316502	VQDBNKDJI	Organoheter	Pyridines anc	Hydroxypyrid
3.33_462.20	462.202308	3.32576667	pos	LysoPE(14:1	HMDB00115	62288	ADNZOMZM	Lipids and lip	Glycerophos	Glycerophos
3.40_373.20	373.208751	3.39796667	pos	pre-putrebactin			MMTPETIUE	Lipids and lip	Fatty Acyls	Fatty amides
3.72_338.09	338.09059	3.71513333	pos	Niraxostat	HMDB02556	450986	AETHRPHB	Organoheter	Azoles	Pyrazoles

3.73_401.18!	401.185742	3.73238333	pos	Meropenem	HMDB00148	1142	DMJNNHOO	Organoheter	Lactams	Beta lactams
3.80_738.27!	738.275367	3.80496667	neg	Lomitapide	HMDB02541	444438	MBBCVAKA.	Benzenoids	Fluorenes	Unclassified
3.84_443.12	443.124131	3.84145	neg	CBHA		44839	OYKBQNOP	Unclassified	Unclassified	Unclassified
3.86_348.21	348.213933	3.85685	pos	18-Nitrooctac	HMDB0257646		AVXZEYRIV.	Lipids and lip	Fatty Acyls	Lineolic acids
3.86_462.11	462.114426	3.86	neg	Peonidin-3-g	HMDB0013689		ZZWPMFRO	Phenylpropa	Flavonoids	Flavonoid gly
3.95_458.22	459.235858	3.94663333	pos	1,3-DIDEACETYL-7-DEA(		43704	BBSPXZBAZ	Unclassified	Unclassified	Unclassified
3.97_320.07!	320.075024	3.97085	neg	lavendustin c	HMDB02539	413808	LULATDWLI	Benzenoids	Benzene anc	Phenylmethy
3.98_407.90	407.903551	3.98231667	pos	5-phospho-al	HMDB0304220		PQGCEDQV	Organic oxyg	Organooxyge	Carbohydrate
4.03_971.58!	971.58956	4.02623333	neg	Hydrocortisone cypionate		41876	DLVOSEUFI	Lipids and lip	Steroids and	Steroids
4.11_429.23!	429.235128	4.10951667	pos	PE(6:0/6:0)		40502	PELYUHWU	Lipids and lip	Glycerophos	Glycerophos
4.15_358.09	358.097197	4.14533333	pos	benfluralin	HMDB02489	44585	SMDHCQAY	Benzenoids	Benzene anc	Aniline and s
4.20_426.13	426.130984	4.19935	pos	(4-{2-[(4-Carl	HMDB0257276		ZCCMELPMI	Organic oxyg	Organooxyge	Carbonyl cor
4.21_431.23	431.233249	4.20936667	neg	p-[(R)-3-[Bis-	HMDB0247637		OPPQEWZC	Benzenoids	Benzene anc	Benzoic acid
4.28_528.22	528.228731	4.28308333	neg	(2R)-2-Acety	HMDB0260128		WBANUFIDI	Organic acid	Peptidomime	Hybrid peptic
4.37_353.09	353.098145	4.37481667	neg	(2S)-2-[Aceta	HMDB0255324		IOBAVJSNP	Organic acid	Carboxylic ac	Amino acids,
4.42_394.23	394.23967	4.41721667	pos	Fluocortolone	HMDB0252351		GAKMQHDJ	Lipids and lip	Steroids and	Hydroxysterc
4.45_441.29	441.296751	4.45335	pos	(3E,9E,12E)-	HMDB0241504		KPEYYOAQI	Lipids and lip	Fatty Acyls	Fatty acid es
4.51_407.22!	407.2254	4.50633333	pos	Kiwiionoside	HMDB00386	93346	SGUOENJPI	Lipids and lip	Prenol lipids	Terpene glyc
4.69_415.07	415.073688	4.6877	neg	3-Sulfopropy	HMDB0245985		KFNGWPXY	Organic acid	Organic sulfc	Organosulfor
4.74_211.01	211.017109	4.74081667	pos	1-Naphthoic acid		2759	LNETULKM	Unclassified	Unclassified	Unclassified
4.78_363.19	363.190906	4.7769	pos	(2-(2',6'-Dime	HMDB0242457		GYSZUJHY	Benzenoids	Benzene anc	Methoxybenz
4.79_248.03	271.028662	4.79411667	pos	Fludioxonil	HMDB02523	72268	MUJOIMFVI	Organoheter	Benzodioxole	Unclassified
4.81_241.16!	241.165853	4.81226667	pos	L-Gizzerosin	HMDB00391	93755	LFNFNJYYT	Organic acid	Carboxylic ac	Amino acids,
4.81_329.18	329.18666	4.81226667	pos	Labetalol	HMDB00147	912	SGUAFYQXI	Benzenoids	Benzene anc	Benzoic acid
4.87_271.07	271.078738	4.86546667	pos	2-(2-Phenyle	HMDB0258520		XJNHONCW	Benzenoids	Benzene anc	Styrenes
5.03_125.01	125.016146	5.03453333	neg	Ethchlorvyno	HMDB0014335		ZEHYJZXQE	Organic oxyg	Organooxyge	Carbonyl cor
5.21_503.27	503.276167	5.20995	pos	OOB-PA			LRGUKMWI	Lipids and lip	Glycerophos	Oxidized glyc
5.31_387.18	387.184749	5.31133333	neg	Quassin		41276	IOSXSVZRT	Lipids and lip	Prenol lipids	Isoprenoids
5.34_298.14	298.14681	5.33735	pos	Oxycodone	HMDB00146	43292	BRUQQQPB	Benzenoids	Phenanthren	Unclassified
5.37_671.30	671.308366	5.36688333	neg	Stelleracin E			JEJIRRUCW	Lipids and lip	Prenol lipids	Isoprenoids
5.44_213.05!	213.055521	5.44071667	neg	(2S,3S,4S)-5	HMDB00389	93524	CBKQFMQQ	Lipids and lip	Fatty Acyls	Fatty alcohol
5.45_277.10	277.100151	5.44698333	pos	Benzo[ghi]pe	HMDB02490	69963	GYFAGKUZ	Benzenoids	Pyrenes	Benzopyrene
5.48_530.96	530.964287	5.48356667	pos	2-[(3-Iodoph	HMDB0255366		INXCCTQPV	Benzenoids	Benzene anc	Halobenzene

6.03_437.16	437.16145	6.0318	neg	Anguvetin		52273	AJLNIEILVU	Lipids and lip	Polyketides	Flavonoids
6.07_359.07	359.072358	6.06866667	neg	VAS2870	HMDB0259768		HZSOKHVV	Organosulfur	Thioethers	Aryl thioether
6.55_397.20	397.204786	6.54903333	neg	2-(2-(4-(2-Flu	HMDB0259698		FLZZFWBNY	Organoheter	Azoles	Pyrazoles
6.80_417.11	417.112603	6.79993333	pos	Aloesone 7-(	HMDB0035734		AXUMVWMF	Organic oxyg	Organooxyge	Carbohydrate
7.17_229.06	229.066678	7.17388333	neg	1-Hydroxy-6-	HMDB00603	72105	BVDKRJLKV	Benzenoids	Pyrenes	Unclassified
7.24_215.08	215.081378	7.24003333	pos	3H-Phenoxa	HMDB02462	447045	UOMHBFAJ	Organoheter	Benzoxazine	Phenoxazine
7.28_346.05	346.05176	7.28471667	neg	N-Acetylman	HMDB0062500		BRGMHAYQ	Organic oxyg	Organooxyge	Carbohydrate
7.37_447.29	447.292391	7.36856667	pos	Vitamin D3 s	HMDB0259848		CAVKNZYPF	Lipids and lip	Steroids and	Vitamin D an
8.76_638.44	638.448684	8.75856667	pos	Butyl 4'-O-hexadecanoyl-neohesperidos			GSHHCUYN	Lipids and lip	Saccharolipic	Other acyl su
9.26_481.28	481.281038	9.25955	neg	DG(20:3(5Z,	HMDB0296856		ABDCTJHQ	Unclassified	Unclassified	Unclassified
9.37_409.03	409.030223	9.36935	neg	benzylidene	HMDB0249079		SGIZECXZF	Organoheter	Azolidines	Thiazolidines
9.44_391.02	391.020262	9.44245	neg	Calcium asc	HMDB0304830		BLORRZQT	Organoheter	Dihydrofuran	Furanones
9.46_482.27	482.273542	9.46038333	neg	Leukotriene D4-d5		96385	YEESKJGW	Unclassified	Unclassified	Unclassified
9.87_417.28	417.281663	9.86676667	pos	Agaric acid		315818	HZLCGUXU	Unclassified	Unclassified	Unclassified
9.96_303.06	303.062591	9.95751667	pos	3-(3-Hydroxy	HMDB0246020		JBWCTTBD	Benzenoids	Naphthalene	Naphthols ar
9.99_170.09	170.096243	9.99425	pos	Acenaphthyle	HMDB0247900		HXGDTGSA	Benzenoids	Acenaphthyle	Unclassified
0.61_158.97	158.97833	0.61033333	neg	Allitridin	HMDB00311	313764	UBAXRAHS	Organosulfur	Organic trisu	Unclassified
0.70_301.06	301.065061	0.70456667	neg	2-(4-(Methyla	HMDB0244929		ZQAQXZBS	Organoheter	Benzothiazol	Unclassified
0.71_270.06	270.06406	0.71368333	pos	TRIAZIQUO	HMDB02591	73219	PXSOHRWN	Organic oxyg	Organooxyge	Carbonyl cor
0.76_335.11	335.118937	0.75766667	pos	Triflupromazine hydrochlo		313351	FTNWXGFY	Unclassified	Unclassified	Unclassified
0.84_264.07	264.073519	0.84308333	neg	Adenosine di	HMDB0248007		ILMNSCQOS	Organoheter	Imidazopyrim	Purines and
1.08_193.06	193.068084	1.07531667	pos	2-(Methylthio	HMDB00371	92134	QJDHQEQD	Phenylpropa	Cinnamaldeh	Unclassified
1.15_238.07	238.074328	1.1535	pos	3-(3'-methylt	HMDB0304106		SQXVIIOPM	Organic acid	Hydroxy acid	Medium-chai
1.18_281.01	281.019384	1.18473333	neg	Cinnavalinin	HMDB00040	323767	FSBKJYLVD	Organoheter	Benzoxazine	Phenoxazine
1.20_453.07	453.075542	1.20271667	neg	Susalimod	HMDB02586	431446	WJLQPSZX	Benzenoids	Benzene anc	Benzenesulf
1.20_475.05	475.057246	1.20271667	neg	Nitrofurantoin		1576	NXFQHRVN	Unclassified	Unclassified	Unclassified
1.22_263.19	263.196446	1.2188	pos	isovaleryl-L-carnitine			IGQBPDJNU	Lipids and lip	Fatty Acyls	Fatty esters
1.27_398.13	398.131333	1.26985	neg	Difloxacin	HMDB02512	320705	NOCJXYPHI	Organoheter	Quinolines a	Phenylquinol
1.27_471.03	471.035612	1.26985	neg	Sulfacetamid	HMDB00147	2527	SKIVFJLND	Benzenoids	Benzene anc	Benzenesulf
1.28_376.04	376.047758	1.28425	pos	6-Methylthio	HMDB00604	71266	QJRQZGPAI	Nucleosides,	Purine nucle	Purine ribonu
1.31_324.06	324.067553	1.30545	neg	Coenzyme B	HMDB0304798		JBJSVEVEE	Organic acid	Carboxylic ac	Amino acids,
1.32_302.09	302.09492	1.31801667	pos	Taurolidine	HMDB02587	447634	AJKIRUJIDF	Organoheter	Thiadiazinan	Unclassified
1.34_640.20	640.202835	1.34166667	neg	2-Pyrrolino-d	HMDB0245311		NOPNWHSN	Phenylpropa	Anthracycline	Unclassified

1.35_329.14:	329.142117	1.34996667	pos		Azatadine	HMDB00148	1184	SEBMTIQKR	Organoheter	Benzocycloh	Unclassified
1.35_338.11	338.118431	1.34996667	pos		N'-Hydroxysa	HMDB00336	89461	AGUPJSLCN	Phenylpropa	Saxitoxins, g	Unclassified
1.36_283.05:	283.052252	1.3586	neg		Ataluren	HMDB02486	316025	OOUGLTULI	Organoheter	Azoles	Oxadiazoles
1.36_561.06	561.069683	1.3586	neg		Epitheaflavic	HMDB00406	95090	RQTWDINIJ	Phenylpropa	Flavonoids	Flavans
1.38_327.09	327.099546	1.37511667	neg		bendazac	HMDB02489	320649	BYFMCKSPI	Organoheter	Benzopyrazo	Indazoles
1.43_245.06:	245.063925	1.43078333	neg		Hydrazinecai	HMDB0247878		VEXRMMJO	Organic nitro	Organonitrog	Hydrazines a
1.49_491.02	491.027435	1.4863	neg		(5'-(but-3-en-1-yn-1-yl)-[2,2'-bithiophen]-			OWMCNWK	Lipids and lip	Fatty Acyls	Oxygenated
10.17_499.0:	499.039663	10.1716833	neg		(S)-Spirobras	HMDB00359	91160	FUHQSEOSI	Organoheter	Indoles and c	Indolines
10.75_430.1	430.176931	10.7528167	neg		Bifeprunox	HMDB0249198		CYGODHVA	Benzenoids	Benzene anc	Biphenyls an
10.79_614.2	614.268584	10.7894833	neg		Golvatinib	HMDB0252909		UQRCJCNVI	Organic oxyg	Organooxyge	Ethers
10.90_419.1	419.153146	10.8978167	neg		Pomiferin	HMDB02566	44075	GHCZYXUO	Phenylpropa	Isoflavonoids	Isoflavans
10.93_394.3	394.367361	10.9300167	pos		22:3(5Z,9Z,19Z)(13Me,17I		102890	LHDXYOPB.	Lipids and lip	Prenol lipids	Isoprenoids
11.02_615.2	615.26412	11.0234	neg		2-((2-(Dimeth	HMDB0245102		HYOLQGVN	Organoheter	Quinolines ai	Phenylquinol
11.41_429.3	429.317964	11.4141	pos		(24R)-24-fluoro-1alpha,25-dihydroxyvita			NYQPKLHIA	Lipids and lip	Steroids and	Secosteroids
11.41_771.4	771.485405	11.4141	pos		Notoginsenoside R2		90661	FNIRVWPHI	Unclassified	Unclassified	Unclassified
11.42_543.3	543.305027	11.42155	neg		DMABA-d10 NHS ester		96493	FNGFZOAQ	Unclassified	Unclassified	Unclassified
11.49_281.2	281.247094	11.4858667	pos		11Z,14Z-octadecadienoic		34963	ACYNXSLK	Lipids and lip	Fatty Acyls	Fatty Acids a
11.70_283.1	283.144025	11.7002333	pos		Eduline	HMDB00405	95079	GFUAPSNF	Organoheter	Quinolines ai	Phenylquinol
11.93_369.2	369.204397	11.9285167	neg		N-pentanoyl-homoserine lactone			BFIMURGDI	Lipids and lip	Fatty Acyls	Fatty amides
12.10_609.3	609.35761	12.1011333	neg		PA(10:0/20:3	HMDB0262641		BMOMTJKTI	Unclassified	Unclassified	Unclassified
12.14_434.3	434.304056	12.1439667	pos		Oleoyl 3-carbacyclic Phos		44869	IJSTZKWSIL	Unclassified	Unclassified	Unclassified
12.64_344.2	344.261177	12.6383167	pos		U-75302		43455	JNBOAUIJLI	Unclassified	Unclassified	Unclassified
12.78_612.5	612.503521	12.78065	pos		DG(16:0/0:0/16:0) (d5)		4686	GFAZGHRE.	Lipids and lip	Glycerolipids	Diradylglycer
12.94_390.2	390.226056	12.9357167	neg		Pentadeca-3	HMDB0241456		DNBDXDFD	Lipids and lip	Fatty Acyls	Fatty acid es
13.19_369.1	369.19837	13.1900333	pos		Oliceridine	HMDB0255950		DMNOVGJW	Organic oxyg	Organooxyge	Ethers
13.28_670.5	670.545281	13.2766833	pos		DG(20:4(5Z,8Z,11Z,14Z)/(		4691	BVPIMSGTE	Lipids and lip	Glycerolipids	Diradylglycer
13.49_904.4	904.47502	13.4891167	pos		PGP(18:3(9Z	HMDB0273189		BYNKDCSIN	Unclassified	Unclassified	Unclassified
2.11_487.10	487.107309	2.11061667	neg		1-O-Galloylgl	HMDB00391	93771	PDEQYQCQ	Benzenoids	Benzene anc	Benzoic acid
2.18_530.08	530.086831	2.18363333	neg		I-SAP		45634	SZNMERGTI	Unclassified	Unclassified	Unclassified
2.19_367.16	367.161052	2.19353333	pos		gamma-Gluta	HMDB0252616		GJOHGZXBI	Organic acids	Carboxylic ac	Amino acids,
2.25_208.10	208.100269	2.24641667	pos		Ethyl 4-(acet	HMDB00322	88307	AKUUBUQN	Lipids and lip	Fatty Acyls	Fatty acid es
2.61_267.09	267.096124	2.60876667	neg		2-p-Tolyl-5,6,7,8-tetrahydr		69104	IMUKUMUN	Unclassified	Unclassified	Unclassified
2.85_425.05	425.055467	2.84868333	neg		Taurine lacta	HMDB0258739		OBWFMYYM	Organic acids	Organic sulfo	Organosulfor

3.42_260.14!	260.149356	3.41641667	pos		Celgosivir	HMDB0249792		HTJGLYIJVS	Organoheter	Indolizidines	Unclassified
3.53_500.73	500.738665	3.52578333	pos		2,3,5-Triiodol	HMDB0245436		ZMZGFLUUZ	Benzenoids	Benzene anc	Benzoic acid
3.59_356.04	356.047487	3.58845	neg		2-Methylprop	HMDB0038421		SKLKAEFXB	Organic oxyg	Organooxyge	Carbohydrate
3.64_471.97	471.978863	3.64241667	neg		Deoxyadenos	HMDB00015	6303	SUYVUBYJA	Nucleosides,	Purine nucle	Purine deoxy
3.72_395.15	395.156045	3.71513333	pos		3-Methylthiof	HMDB0061718		SRARDYUH	Benzenoids	Benzene anc	Anilides
3.86_681.28	681.285061	3.86	neg		Mycotoxin T	HMDB00328	88782	UNNZZQOFI	Organic oxyg	Organooxyge	Carbohydrate
3.91_442.19	442.199206	3.91066667	pos		Chitobiose	HMDB00035	6953	HSRZMOSQ	Organic oxyg	Organooxyge	Carbohydrate
3.91_649.34	649.346526	3.91066667	pos		PC(2:0/20:5(	HMDB0288872		JEVKBGCAI	Unclassified	Unclassified	Unclassified
4.01_378.16	378.164862	4.0077	neg		Merafloxacin	HMDB0254459		BAYYCLWC	Organoheter	Quinolines a	Quinoline ca
4.06_288.16	288.166737	4.05526667	pos		Morinidazole	HMDB0254886		GAZGHCHC	Organoheter	Azoles	Imidazoles
4.18_267.14	267.143083	4.1814	pos		Ethyl N2-ace	HMDB0252056		VAPHLLYHD	Organic acid	Carboxylic ac	Amino acids,
4.25_857.37	857.379225	4.24626667	neg		2-(2-(4-Phen	HMDB0254187		DFFOSEJNF	Benzenoids	Benzene anc	Diphenylethe
4.36_223.18	223.180509	4.3621	pos		3,4-Dimethylethcathinone		96622	AOXOTNQJI	Unclassified	Unclassified	Unclassified
4.36_530.30	553.29622	4.3621	pos		L-Oleandrosyl-oleandolide		40997	WBLQSDZL	Lipids and lip	Polyketides	Macrolides a
4.40_348.23	348.230434	4.3987	pos		C17 Sphingosine-1-phosp		102933	VITGNIYTXH	Lipids and lip	Sphingolipids	Sphingoid ba
4.50_331.02	331.022635	4.50406667	neg		3-Hydroxyph	HMDB0304917		JNBVGGPM	Unclassified	Unclassified	Unclassified
4.52_343.11	343.116039	4.52246667	neg		LysoPA(8:0/(	HMDB0114740		VOEQFNGS	Lipids and lip	Glycerophos	Glycerophos
4.54_374.22	374.228375	4.5416	pos		N-3-oxo-hexadec-11(Z)-er		45315	UKVJHGAET	Unclassified	Unclassified	Unclassified
4.56_386.04	386.048519	4.55865	neg		N-(4-Acetyl-2	HMDB02444	407489	DIIYLGZNZ	Benzenoids	Benzene anc	Diphenylethe
4.61_285.00	285.005549	4.61381667	pos		Homovanillic	HMDB0011719		IACOAKYXF	Organic acid	Organic sulfu	Arylsulfates
4.70_402.17	402.177291	4.70481667	pos		Dacinostat	HMDB0250821		BWDQBBCL	Organoheter	Indoles and	Tryptamines
4.71_315.06	315.064168	4.70578333	neg		2-S-cysteinyl	HMDB00627	461445	IAGPESOLV	Organic acid	Carboxylic ac	Amino acids,
4.72_777.42	777.428957	4.72301667	pos		PG(i-13:0/18	HMDB0271207		DGISMXCDK	Unclassified	Unclassified	Unclassified
4.74_307.17	307.173653	4.74081667	pos		(2S)-N-[(2S)-	HMDB0254713		NOOJLZTTV	Organic acid	Carboxylic ac	Amino acids,
4.91_413.04	413.044284	4.90568333	neg		Bis-(4-methy	HMDB0246897		FEGSOIMPQ	Phenylpropa	Coumarins a	Unclassified
5.10_648.30	648.306824	5.10093333	pos		Nonioside B	HMDB0303582		CTKLGODDI	Organic oxyg	Organooxyge	Carbohydrate
5.10_701.49	701.493331	5.10093333	pos		DG(20:4(7E,	HMDB0299429		APLPHKPAZ	Unclassified	Unclassified	Unclassified
5.24_225.07	225.074133	5.23773333	neg		Homocystam	HMDB0253204		RSNVLCAXJ	Organosulfur	Organic disul	Dialkyldisulfi
5.26_483.27	483.2785	5.26461667	pos		Glycyl-histidy	HMDB0252877		YVHCULPW	Organic acid	Carboxylic ac	Amino acids,
5.81_389.21	389.214038	5.81251667	pos		Fluorogeston	HMDB0252379		JKQQZJHNU	Lipids and lip	Steroids and	Pregnane ste
6.18_170.00	170.001226	6.17958333	neg		4-Amino-2-cl	HMDB02463	345947	MBDUKNCP	Benzenoids	Benzene anc	Benzoic acid
6.60_499.16	499.165044	6.59813333	pos		Talampicillin	HMDB0258686		SOROUYSP	Organoheter	Benzodiazep	Unclassified
7.04_623.31	623.316461	7.03826667	pos		LysoPI(0:0/1	HMDB0061704		AGVRWBBF	Lipids and lip	Glycerophos	Glycerophos

7.20_462.20	462.206884	7.20335	pos		Difenacoum	HMDB02512	331118	FVQITOLOY	Benzenoids	Naphthalene	Phenylnaphtl
7.40_353.11	353.112885	7.39546667	neg		C.I. Solvent F	HMDB0037521		GJUABKCEX	Benzenoids	Naphthalene	Naphthols ar
7.94_272.11	272.111754	7.93951667	neg		N-Despropyl-	HMDB0060841		RDMWWGLI	Benzenoids	Tetralins	Unclassified
8.19_389.20	389.208263	8.19463333	pos		Tetrahydrode	HMDB00059	57990	UPTAPIKFK	Lipids and lip	Steroids and	Hydroxysterc
8.20_418.99	418.995996	8.1977	neg		Dimethyl 2,3-	HMDB0245398		VVOFTOVXI	Lipids and lip	Fatty Acyls	Fatty acid es
9.06_356.17	356.171349	9.05798333	neg		Noberastine	HMDB0255678		VNIOQSAWI	Organoheter	Imidazopyrid	Unclassified
9.46_187.05	187.05634	9.46038333	neg		Diphenylcycl	HMDB02514	315346	HCIBTBXNL	Benzenoids	Benzene anc	Unclassified
9.84_387.00	387.006794	9.84338333	neg		S-Acetyl dihy	HMDB00357	90974	PXKKBEIPD	Lipids and lip	Fatty Acyls	Fatty acids a
9.99_518.25	518.250325	9.98928333	neg		LysoPE(0:0/1	HMDB00114	62268	SYKRNJVTC	Lipids and lip	Glycerophos	Glycerophos
0.61_504.11	504.11639	0.61033333	neg		Deoxyribonu	HMDB0247632		AWBASQCA	Organic oxyg	Organooxyge	Carbohydrate
0.67_209.08	209.080984	0.6745	neg		Chlamydospordiol; 6-(3-Hy		985207	BSXCHPRM	Unclassified	Unclassified	Unclassified
0.70_219.04	219.044826	0.70456667	neg		Cystathionine	HMDB00023	6666	JNUGGJHCI	Organic acids	Carboxylic ac	Amino acids,
0.70_488.16	488.162044	0.70456667	neg		Pevonedistat	HMDB0256358		MPUQHZXIX	Benzenoids	Indanes	Unclassified
0.73_318.09	318.094094	0.7342	neg		5,9:7,10a-Dir	HMDB0246673		CFMYXEVW	Organoheter	Diazanaphth	Benzodiazine
0.78_217.01	217.015873	0.7799	neg		Tiazotic acid		350649	OJUNWHNR	Unclassified	Unclassified	Unclassified
0.78_289.01	289.017708	0.7799	neg		Sulisobenzor	HMDB02586	43340	CXVGEDCS	Benzenoids	Benzene anc	Benzophenor
0.79_243.10	243.100748	0.7877	pos		2-Hydroxy-3-	HMDB0254881		NUFBIAUZA	Organoheter	Oxazinanes	Morpholines
0.79_385.12	385.128561	0.7877	pos		Marbofloxaci	HMDB02543	315508	BPFYOAJND	Organoheter	Quinolines ar	Quinoline car
0.80_250.10	250.104586	0.80285	pos		N-(3-oxo-heptanoyl)-homoserine lactone			WMGXONS	Lipids and lip	Fatty Acyls	Fatty amides
0.81_204.03	204.033255	0.81161667	neg		(R)-2-Amino-	HMDB0246154		JAKVEOCMI	Organic acids	Carboxylic ac	Amino acids,
0.83_374.21	374.214314	0.83391667	pos		1-[4-[2-[2-(4-	HMDB0250008		CYCXZGUVI	Benzenoids	Phenol ether	Unclassified
0.88_379.07	379.072537	0.87973333	pos		1-(2-Hydroxy	HMDB02557	433314	YLFMCMWK	Organoheter	Benzimidazo	Phenylbenzir
1.10_557.06	557.069638	1.1046	pos		Genistein 7-O-glucoside-6		64302	FRAUJUKW	Lipids and lip	Polyketides	Flavonoids
1.11_412.03	412.038881	1.1137	neg		Desacetylcef	HMDB00418	605	FHYWAOQC	Organoheter	Lactams	Beta lactams
1.17_243.15	243.158991	1.1705	pos		2-Carboxy-4-	HMDB00309	87331	FMXVFWDI	Organoheter	Lactones	Gamma buty
1.18_262.98	262.985047	1.18473333	neg		(E)-4-(6-((formyloxy)methyl)-1,2-dithiin-3			GMPFMYDX	Lipids and lip	Fatty Acyls	Oxygenated
1.19_366.99	366.995977	1.1884	pos		Fluorodifen		68807	HHMCAJWV	Unclassified	Unclassified	Unclassified
1.22_373.06	373.066378	1.21911667	neg		Valeant	HMDB0259745		RNEACARJK	Organic acids	Carboxylic ac	Amino acids,
1.22_404.11	427.10833	1.2188	pos		1,2-Diphenyl-	HMDB0248909		AUMHDRMJ	Organoheter	Azoles	Pyrazoles
1.25_847.19	847.19922	1.2521	neg		Azimsulfuron	HMDB00331	72244	MAHPNPYY	Organic nitro	Organonitrog	Sulfonylureas
1.35_482.21	482.218654	1.34996667	pos		Pumilaisoflavone A		47779	KUMFNGIFC	Lipids and lip	Polyketides	Flavonoids
1.36_555.11	555.113341	1.3586	neg		Salvianolic ac	HMDB0302946		AZOCLKLJIK	Lignans, neo	Unclassified	Unclassified
1.36_708.19	708.190623	1.3586	neg		Pelargonidin 3-sambubioside-5-glucosic			NNEATHLRL	Lipids and lip	Polyketides	Flavonoids

1.56_381.03	381.035643	1.56021667	neg		Nicousamide	HMDB0255592			DOLJMUVM	Benzenoids	Benzene and	Anilides
1.62_217.05	217.050207	1.61501667	neg		4E-Undecene-6,8,10-triyn		74344		FENUSKPO	Lipids and lip	Fatty Acyls	Fatty Acids a
1.89_491.08	491.081427	1.89173333	neg		Baicalin	HMDB0041832			IKIIZLYTISP	Phenylpropa	Flavonoids	Flavonoid gly
10.30_481.0	481.010204	10.2994667	neg		3-Mercaptola	HMDB00065	58438		MAFDYIDM	Organic acid	Carboxylic ac	Amino acids,
10.34_786.5	786.53002	10.3358333	neg		PC(20:3(8Z,1	HMDB0285627			ACOLOYZT	Unclassified	Unclassified	Unclassified
10.79_371.2	371.239547	10.7875833	pos		Dodecyl glucoside		34493		PYIDGJJWB	Unclassified	Unclassified	Unclassified
10.97_566.2	566.218988	10.9697333	neg		CAY10573		45150		ZXWVCCFK	Unclassified	Unclassified	Unclassified
11.44_309.1	309.174078	11.4395167	neg		8-O-Methylol	HMDB00345	90043		QZJMBSSOI	Organoheter	Isoquinolines	Benzylisoqui
11.65_413.2	413.280023	11.6461	pos		Desacetyllev	HMDB0251028			YPYQDMBJ	Organoheter	Quinolines ar	Benzoquinoli
11.72_846.5	846.5479	11.71745	pos		PG(18:1(9Z)/	HMDB0269007			ADSYTFBY	Unclassified	Unclassified	Unclassified
12.59_563.5	563.550274	12.5856	pos		Dirithromycin		322781		WLOHNSSY	Unclassified	Unclassified	Unclassified
12.60_405.2	405.283053	12.6029833	pos		mepitiostane	HMDB0254455			IVDYZAAPO	Lipids and lip	Steroids and	Estrane sterc
12.62_655.4	655.488144	12.6205	pos		DG(20:4(5Z,8	HMDB0299166			ABRFGHSE	Unclassified	Unclassified	Unclassified
13.25_399.2	399.251322	13.2463167	neg		(6Ar,10aR)-1	HMDB0257598			QHGPTMAB	Organoheter	Benzopyrans	1-benzopyrar
13.99_190.1	190.108462	13.9923667	pos		Miglitol	HMDB00146	43885		IBAQFPQHF	Organoheter	Piperidines	Unclassified
2.02_505.13	505.13188	2.02081667	neg		6''-Acetylliqui	HMDB00413	95787		HKUBLIRXX	Phenylpropa	Flavonoids	Flavonoid gly
2.11_487.03	487.034166	2.11061667	neg		Cefditoren	HMDB0015199			KMIPKYQIO	Organoheter	Lactams	Beta lactams
2.18_216.14	216.142362	2.17555	pos		Cycloate	HMDB02506	72550		DFCAFRGA	Organosulfur	Thiocarbonyl	Thiocarbamic
3.51_561.53	561.539944	3.5146	neg		Laurocapram	HMDB0253992			AXTGDCSM	Organoheter	Lactams	Caprolactam
3.59_341.06	341.062239	3.58845	neg		Phenylmetha	HMDB02564	349925		ABOYDMHG	Benzenoids	Benzene and	Unclassified
3.59_769.09	769.091704	3.58845	neg		Mavacoxib	HMDB02543	439863		TTZNQDOU	Organoheter	Azoles	Pyrazoles
3.68_296.03	296.030168	3.67736667	neg		Metronidazol	HMDB0254689			GFFOAZOY	Organoheter	Azoles	Imidazoles
3.82_597.97	597.9793	3.82341667	neg		2-Mesatp	HMDB0245197			XNOBOKJV	Nucleosides,	Purine nucle	Purine ribonu
3.84_465.10	465.105845	3.84145	neg		5,7,4'-Trihydroxy-3,6,8,3',5		51774		DOUSAHOS	Lipids and lip	Polyketides	Flavonoids
3.87_585.04	585.047913	3.87373333	pos		dTDP-alpha-	HMDB0304344			YSYKRGRSI	Nucleosides,	Pyrimidine nu	Pyrimidine nu
3.93_418.23	418.239356	3.92891667	pos		Paxilline	HMDB00303	69702		ACNHBCIZL	Organoheter	Naphthopyra	Unclassified
3.96_366.16	366.165935	3.96453333	pos		Cilomilast	HMDB02502	315749		CFBUZOUX	Benzenoids	Phenol ether	Anisoles
3.97_123.03	123.032454	3.97085	neg		Glycoluril	HMDB0252872			VPVSTMAP	Organoheter	Azolidines	Imidazolidine
4.04_422.22	422.228068	4.03688333	pos		Phenylalanyl	HMDB0256435			KIJDUGBFU	Organic acid	Carboxylic ac	Amino acids,
4.06_192.01	192.014929	4.05526667	pos		s-Allyl cystei	HMDB0257393			CPUZZTUW	Organic acid	Carboxylic ac	Amino acids,
4.32_1060.5	1060.56663	4.32	neg		CDP-DG(18:	HMDB0293142			ARPPVCQO	Unclassified	Unclassified	Unclassified
4.49_540.29	540.291812	4.4888	pos		ethyl ((3,4,5-	HMDB0252023			UAOCLDQA	Organic oxyg	Organooxyge	Carbohydrate
4.51_866.43	866.437866	4.50633333	pos		PS(18:4(6Z,9	HMDB01125	78119		FRTSBNHS	Lipids and lip	Glycerophos	Glycerophos

4.72_259.00	259.009591	4.72411667	neg	4-Hydroxy-5-	HMDB00335	89358	GIVVLEIYPV	Organic acid	Vinylogous th	Unclassified
4.74_632.98	632.980195	4.74241667	neg	Phosmet	HMDB02564	72529	LMNZTLDVJ	Organoheter	Isoindoles ar	Isoindolines
4.76_467.02	467.025482	4.76071667	neg	Arctinol A	HMDB0302304		APCVAJQBZ	Organoheter	Bi- and oligo	Unclassified
4.83_211.03	211.034133	4.8302	pos	2-(Sec-butyl	HMDB0244601		BPBPYQWM	Organoheter	Azoles	Imidazoles
4.91_454.13	454.133188	4.90568333	neg	Dhurrin 6'-gl	HMDB00297	86483	FPYKJQSRV	Organic oxy	Organooxyge	Carbohydrate
4.91_460.13	505.130457	4.90568333	neg	Apremilast	HMDB0248531		IMOZEMNVL	Organoheter	Isoindoles ar	Isoindolines
5.24_239.08	239.085244	5.23773333	neg	Methiuron		72828	MMCJEAKIN	Unclassified	Unclassified	Unclassified
5.26_400.19	400.191192	5.25613333	neg	JWH 122 3-methylnaphthy		96704	AWAISCZQF	Unclassified	Unclassified	Unclassified
5.59_329.19	329.190187	5.58803333	neg	TTNPB		43445	FOIVPCKZD	Unclassified	Unclassified	Unclassified
5.98_1085.4	1085.4938	5.9774	pos	Melongoside	HMDB00409	95392	AENUWGNJ	Lipids and lip	Fatty Acyls	Fatty alcohol
6.03_371.23	371.234135	6.0318	neg	QUINACAIN	HMDB0257029		OPNPUTUBI	Organoheter	Quinolines a	4-quinolinem
6.59_386.20	385.199535	6.58565	neg	17-phenyl-18	HMDB00626	36138	FOBVMYJQ	Lipids and lip	Fatty Acyls	Eicosanoids
6.79_452.13	452.136282	6.7873	neg	gamma-L-Gl	HMDB00386	93329	OGZNUDJBI	Organic acid	Carboxylic a	Amino acids,
8.16_197.18	197.18984	8.15795	pos	Octyl 2-meth	HMDB00362	91365	BCOJVEMH	Lipids and lip	Fatty Acyls	Fatty alcohol
8.43_263.16	263.161409	8.43301667	pos	[(1R,5R)-5-(	HMDB0243753		SFLSLPRBW	Organoheter	Imidazopyrim	Purines and
8.71_591.26	591.269422	8.7071	neg	Cryptotanshi	HMDB00352	90535	GVKKJJOM	Lipids and lip	Prenol lipids	Diterpenoids
8.90_356.19	357.203254	8.90103333	pos	vamorolone	HMDB0259760		ZYTXTXAM	Lipids and lip	Steroids and	Hydroxyster
9.46_213.07	213.071938	9.46038333	neg	1-Methoxypy	HMDB00603	72103	CPPFVJLHD	Benzenoids	Pyrenes	Unclassified
9.99_529.06	529.066309	9.98928333	neg	(2-Carbamoy	HMDB0242465		NDCUAPJVL	Organic acid	Carboxylic a	Amino acids,
0.62_419.03	419.037551	0.6215	pos	C.I. Food Re	HMDB0033383		DZCOAQKTI	Benzenoids	Naphthalene	Naphthalene
0.63_349.06	349.060441	0.62835	neg	4-(2,6-Dioxo-	HMDB0256891		UYDRRQPG	Organoheter	Imidazopyrim	Purines and
0.64_222.05	245.048439	0.63663333	pos	3-Fluoro-5-[(	HMDB0245870		CYNKRSTZ	Benzenoids	Benzene and	Benzonitriles
0.64_264.08	287.07031	0.63663333	pos	Phenyl 1-hyd	HMDB0256421		QHDYIMWK	Benzenoids	Naphthalene	Naphthalene
0.67_173.88	196.87801	0.66691667	pos	Dipotassium	HMDB0303424		ZPWVASYFI	Mixed metal/	Alkali metal	Alkali metal
0.68_353.05	353.051724	0.68255	pos	8-Azido-cycli	HMDB0247425		KTMLKBFM	Nucleosides,	Purine nucle	Cyclic purine
0.75_392.07	373.05568	0.74898333	neg	5,7,3',4',5'-Pentahydroxy-3		51769	STOZTZBHY	Lipids and lip	Polyketides	Flavonoids
0.77_350.18	350.189436	0.7729	pos	Dansylamido	HMDB0254853		MDDJCYLRI	Benzenoids	Naphthalene	Naphthalene
0.80_368.07	368.076587	0.79596667	neg	7-Hydroxy-R	HMDB0060883		JTCREUDLE	Phenylpropa	Coumarins a	Hydroxycoun
0.82_336.03	336.035728	0.81883333	pos	6-(4-Fluorop	HMDB0258331		YOELZIQOL	Organoheter	Azoles	Imidazoles
0.82_469.13	469.129969	0.81883333	pos	Lucuminic ac	HMDB00350	90367	DUZCTLMSI	Organic oxy	Organooxyge	Carbohydrate
0.84_213.01	213.012729	0.84308333	neg	Rutalexin	HMDB0302037		NJGJFPPBM	Organoheter	Indoles and	Indoles
1.03_523.13	523.13098	1.02758333	pos	Cyanidin 3-O	HMDB00292	86121	VXFDROQY	Phenylpropa	Flavonoids	Flavonoid gly
1.07_357.05	357.054948	1.06741667	neg	Calmagite	HMDB02495	347535	VBRNLOQC	Benzenoids	Naphthalene	Naphthalene

1.07_369.09	369.098351	1.06741667	neg	Diphenylphos	HMDB0251460		GPAYUJZHT	Benzenoids	Benzene anc	Unclassified
1.07_411.09	411.090393	1.06741667	neg	Furocoumari	HMDB00296	86367	XRLPSAYLY	Organic oxyg	Organooxyge	Carbohydrate
1.08_270.14	270.144621	1.07531667	pos	Alanyl-Tyrosi	HMDB03047	85614	ALZVPLKYD	Organic acid	Carboxylic ac	Amino acids,
1.11_553.09	553.094634	1.1137	neg	4-Nitropheny	HMDB0246544		PCEUKRKD	Benzenoids	Benzene anc	Nitrobenzene
1.14_252.14	252.144801	1.13681667	pos	ascr#11			VQZVOFZW	Lipids and lip	Fatty Acyls	Fatty acyl gly
1.18_320.05	320.051718	1.18473333	neg	Nifuroxazide	HMDB0255597		YCWSUKQG	Benzenoids	Benzene anc	Benzoic acid
1.20_121.08	121.087454	1.20271667	neg	Metenamine	HMDB00295	43250	VKYKSIONX	Organoheter	Triazinanes	1,3,5-triazina
1.20_527.09	527.092776	1.20271667	neg	Furagin	HMDB0252536		DECBQELQ	Organoheter	Azolidines	Imidazolidine
1.25_352.08	352.089312	1.2521	neg	(S)-Succinylc	HMDB00011	6058	RJCJWONC	Lipids and lip	Fatty Acyls	Fatty acids a
1.30_257.06	257.069526	1.30165	pos	Abrine		44665	CZCIKBSVH	Unclassified	Unclassified	Unclassified
1.30_392.11	392.112	1.30165	pos	Neodiospyrin	HMDB00295	86326	LZAXNDGRI	Benzenoids	Naphthalene	Naphthoquin
1.34_429.09	429.093986	1.34166667	neg	Temocaprilat	HMDB00617	69022	KZVWEOXA	Organic acid	Carboxylic ac	Amino acids,
1.38_295.07	295.07364	1.3848	pos	3-Octylthio-1	HMDB0247568		WMQHRXUI	Organic oxyg	Organooxyge	Carbonyl cor
1.61_241.02	241.026934	1.61191667	pos	3,4',5-Bipher	HMDB00327	88643	HSZOQEGJI	Benzenoids	Benzene anc	Biphenyls an
1.62_519.08	519.083621	1.61501667	neg	Tiazofurin		320962	FVRDYQYE	Unclassified	Unclassified	Unclassified
1.65_314.17	314.170939	1.64773333	pos	Levobunolol	HMDB00153	934	IXHBTMCLR	Benzenoids	Tetralins	Unclassified
1.74_270.16	270.168803	1.73755	pos	(3Z,5E)-Hept	HMDB0241692		CDRGTXOC	Lipids and lip	Fatty Acyls	Fatty acid es
1.86_291.13	291.137339	1.86321667	pos	N-Ethyl-N-(2-	HMDB0255133		ZTQGWROH	Benzenoids	Benzene anc	Toluenes
10.43_628.4	651.427964	10.4295333	pos	2-(9R-(15Z-docosenoyloxy		97367	UVAQPIXHY	Lipids and lip	Fatty Acyls	Fatty esters
10.57_588.2	588.253244	10.5716833	neg	Glycocholena	HMDB0242178		OTUHTIDAA	Lipids and lip	Steroids and	Bile acids, al
10.63_296.1	296.145905	10.6275	pos	Pizotifen	HMDB02566	2036	FIADGNVRK	Organoheter	Cycloheptath	Unclassified
10.63_610.2	610.26607	10.6260167	neg	7-O-acetyl-19,20-epoxycytochalasin C			SYQABGFB	Lipids and lip	Polyketides	Cytochalasin
10.75_624.3	624.351739	10.7528167	neg	POB-PC			BHSGUPRVI	Lipids and lip	Glycerophos	Oxidized glyc
11.24_518.2	518.286169	11.2413667	neg	[(3R,4S)-1,1-	HMDB0258345		KQPGDBVG	Lipids and lip	Fatty Acyls	Fatty amides
11.93_206.1	206.105467	11.9300167	pos	N-(2,5-Dihyd	HMDB00407	95233	BYQQTWRC	Benzenoids	Phenols	Benzenediols
11.93_445.2	445.253423	11.9300167	pos	Monoketoch	HMDB0254862		IYFKRBTYSI	Lipids and lip	Steroids and	Bile acids, al
12.13_327.1	327.126787	12.12775	neg	Lepidine D	HMDB00327	88646	KDBJPLXCL	Benzenoids	Benzene anc	Diphenylethe
12.14_398.2	398.275156	12.1439667	pos	Linusic acid			HCPSZQRU	Lipids and lip	Fatty Acyls	Fatty Acids a
12.78_779.0	779.067277	12.78065	pos	Syzyginin B	HMDB0030237		MAXKGPWN	Phenylpropa	Tannins	Hydrolyzable
13.21_805.5	806.564824	13.2068667	pos	LacCer(d18:	HMDB0004866		KNWHKVBH	Unclassified	Unclassified	Unclassified
13.21_896.5	896.561329	13.2068667	pos	PS(18:1(12Z	HMDB0283139		AFPVOHMJ	Unclassified	Unclassified	Unclassified
2.53_281.14	304.132511	2.53481667	pos	N-Nornucifer	HMDB0255212		QQKAHDMN	Alkaloids anc	Aporphines	Unclassified
2.59_217.15	217.158656	2.58846667	pos	10-Hydroxy-isolaurene		53403	AGMIVVAYC	Lipids and lip	Prenol lipids	Isoprenoids

2.79_119.064	119.064296	2.78548333	pos	TRIMETHYL	HMDB0259244		JAEZSIYNW	Organosulfur	Thioureas	Unclassified
3.38_321.189	321.189947	3.38003333	pos	bhos#10			DGLIIKDKPY	Lipids and lip	Fatty Acyls	Fatty acyl gly
3.49_295.187	295.187391	3.48938333	pos	Pentadecanedioic acid		35977	BTZVDPWK	Lipids and lip	Fatty Acyls	Fatty Acids a
3.59_791.201	791.201142	3.58845	neg	PtdIns-(3,5)-P2 (1,2-diocta		45083	QXHVLVSUI	Unclassified	Unclassified	Unclassified
3.61_393.970	393.97005	3.6144	pos	3-Iodothyrona	HMDB0060524		XIINYOJWN	Benzenoids	Benzene and	Diphenylethe
3.64_496.060	496.060308	3.64241667	neg	Cefozopran	HMDB0249772		QDUIJCOKQ	Organoheter	Lactams	Beta lactams
3.71_295.127	295.127273	3.71251667	neg	Methdilazine	HMDB00150	43285	HTMIBDQKF	Organoheter	Benzothiazin	Phenothiazin
3.75_662.332	662.332213	3.75006667	pos	Apicidin		96431	JWOGUUIO	Unclassified	Unclassified	Unclassified
3.79_283.167	283.166969	3.78573333	pos	(R)-3-Hydrox	HMDB00107	35675	ATRNZOYKS	Lipids and lip	Fatty Acyls	Fatty acids a
3.82_396.140	396.140658	3.82191667	pos	(2R)-3-Cyclo	HMDB0242661		NEQSWPCD	Benzenoids	Benzene and	Phenylacetar
3.86_300.192	300.192525	3.85685	pos	2-N,N-Di-n-p	HMDB0257124		FPAHQTSJK	Benzenoids	Indanes	Unclassified
3.95_315.096	315.096332	3.95231667	neg	Diphenylhyda	HMDB02514	1857	UESCARMR	Benzenoids	Benzene and	Diphenylmeth
3.95_353.162	353.162367	3.95231667	neg	Derenofylline	HMDB0258338		RBZNJGHIK	Organoheter	Pyrrolopyrimi	Pyrrolo[2,3-d
4.12_246.051	246.051325	4.11676667	neg	Isoniazid alpl	HMDB0060663		CCBSJXHUN	Organoheter	Pyridines and	Pyridinecarbo
4.31_604.993	604.993549	4.30826667	pos	Adenosine te	HMDB00013	6191	WWMWAMF	Nucleosides,	Purine nucle	Purine ribonu
4.38_286.118	309.107425	4.38046667	pos	CAY10616		45520	UQIWTPQG	Unclassified	Unclassified	Unclassified
4.38_680.377	703.366594	4.38046667	pos	Tenuifolin	HMDB0258821		DBJLNNAUD	Lipids and lip	Prenol lipids	Terpene glyc
4.39_377.094	377.094105	4.39318333	neg	Phosphine ox	HMDB0254193		PODOEQVN	Benzenoids	Benzene and	Benzoic acid
4.54_326.079	326.079869	4.54091667	neg	Ofurace		331151	OWDLFBLN	Unclassified	Unclassified	Unclassified
4.67_187.040	187.040649	4.66926667	neg	methyl 10-hydroxy-8,9-epoxy-2Z-decen			DHUOSWGS	Lipids and lip	Fatty Acyls	Fatty Acids a
4.67_377.214	377.214726	4.66833333	pos	(6RS)-22-hydroxy-23,24,25,26,27-penta			ZMPGFJMAS	Lipids and lip	Steroids and	Secosteroids
4.70_613.309	613.30961	4.70481667	pos	OHOOA-PA			OABJQCLPC	Lipids and lip	Glycerophos	Oxidized glyc
4.72_260.066	260.066933	4.72411667	neg	Naftazone	HMDB0255426		AGSIRJFXA	Benzenoids	Naphthalene	Naphthols ar
4.74_937.100	937.100352	4.74241667	neg	Chebulinic acid		68180	YGVHOSGN	Unclassified	Unclassified	Unclassified
4.78_552.165	552.165209	4.77798333	neg	Umbralisib	HMDB0304848		IUVCFHHAE	Phenylpropa	Isoflavonoids	Isoflav-2-ene
4.79_325.154	325.154212	4.79411667	pos	Carbocystein	HMDB0249634		ZXFWVUHY	Organic acid	Carboxylic ac	Amino acids,
4.81_736.380	736.380371	4.81226667	pos	Progesterone 3-biotin		64895	YDTYUCKID	Unclassified	Unclassified	Unclassified
4.83_457.233	457.233059	4.8302	pos	LysoPA(0:0/1	HMDB0007852		PALVOIADD	Lipids and lip	Glycerophos	Glycerophos
4.90_367.050	367.050159	4.90175	pos	Glucose citra	HMDB0252775		DDUFRMAO	Organic acid	Carboxylic ac	Tricarboxylic
4.99_422.128	422.128123	4.99255	pos	Methyl (1-(4-	HMDB0254816		FRFFLYJQP	Organic acid	Carboxylic ac	Amino acids,
5.19_518.112	518.112367	5.19183333	pos	Mdz-glucuror	HMDB0247486		ICIUMXQTL	Organoheter	Benzodiazep	1,4-benzodia
5.51_291.007	291.007352	5.51431667	neg	O-Ethyl S,S-	HMDB00317	70059	AWZOLILCC	Benzenoids	Benzene and	Unclassified
5.68_333.213	333.213937	5.68461667	pos	1-dimethylarsinoyl-pentadecane			JUBXTBKUC	Lipids and lip	Fatty Acyls	Hydrocarbon

5.78_203.12	203.121212	5.77613333	pos	isoleucyl-thia	HMDB0253651		ARNUPLMO.	Organoheter	Azolidines	Thiazolidines
5.79_281.12	281.12817	5.7943	pos	3-Hydroxy-2-naphthanilide	328643		JFGQHAHJ	Unclassified	Unclassified	Unclassified
6.11_463.28	463.287319	6.10565	pos	(6S)-1alpha,25-dihydroxyvitamin D3 6,1			BFYSIYCQJ	Lipids and lip	Steroids and	Secosteroids
7.27_673.22	673.225818	7.26623333	neg	indole-3-acet	HMDB0304391		DKHLXGMLI	Organoheter	Indoles and	Indolyl carbo
7.90_326.17	326.178031	7.90143333	pos	BENALAXYL	HMDB02489	68696	CJPQIRJHIZ	Organic acid	Carboxylic ac	Amino acids,
8.07_268.18	268.187984	8.06628333	pos	(3Z)-2-Propy	HMDB0241903		DKKYRZFDI	Lipids and lip	Fatty Acyls	Fatty acid es
8.75_507.29	507.292975	8.74946667	neg	Lucidenic aci	HMDB00359	91159	DPRVTGUH	Phenylpropa	Flavonoids	O-methylated
8.91_516.29	516.294002	8.91221667	neg	3-[(1R,4S,5S	HMDB0241815		FGVGZPKAI	Lipids and lip	Fatty Acyls	Eicosanoids
9.46_423.04	423.046629	9.46038333	neg	Zonisamide	HMDB00150	313884	UBQNRHZM	Organoheter	Benzisoxazol	Unclassified
9.61_517.31	517.314763	9.60636667	neg	HECOGENIN ACETATE		43700	CVKZWRTY	Unclassified	Unclassified	Unclassified
9.73_449.17	449.173729	9.73451667	neg	C-Quens	HMDB0249505		QMBJSIBW	Lipids and lip	Steroids and	Pregnane ste
9.79_580.28	580.287075	9.78913333	neg	POB-PS			IMHWZIBEX	Lipids and lip	Glycerophos	Oxidized glyc
9.83_338.30	338.302506	9.83063333	pos	N-Hexadecyl-L-hydroxypro		34538	FDSLJAYOP	Unclassified	Unclassified	Unclassified
0.61_393.15	393.15721	0.61033333	neg	Eperezolid	HMDB0251836		SIMWTRCFI	Organoheter	Diazinanes	Piperazines
0.62_169.09	192.080009	0.6215	pos	Diphenylamir	HMDB00325	68774	DMBHHRLKI	Benzenoids	Benzene and	Aniline and s
0.63_295.15	295.150889	0.62835	neg	17-Ethynyl-1	HMDB0244769		IFDRGXJMF	Lipids and lip	Steroids and	Estrane sterc
0.64_372.04	395.039113	0.63663333	pos	NORSTICTIC ACID		43603	IEVVSJFLBY	Unclassified	Unclassified	Unclassified
0.67_311.02	311.023812	0.6745	neg	resorcinol sulfoxide		4087	YNHHSHCJI	Unclassified	Unclassified	Unclassified
0.69_133.03	133.033388	0.68961667	neg	1-Pentanesu	HMDB0243986		RJQRCOMH	Organic acid	Organic sulfo	Organosulfor
0.73_235.06	235.062797	0.7342	neg	2'-Fluoro-2',3	HMDB0245537		SLWSQPYU	Nucleosides,	Purine nucle	Purine 2',3'-d
0.74_277.21	277.211952	0.74288333	pos	4-Methylpent	HMDB0241032		GCKJJXWIY	Lipids and lip	Fatty Acyls	Fatty acid es
0.78_230.01	230.016097	0.7799	neg	3-Amino-4-su	HMDB0257610		GHOHRLXV	Organic acid	Organic sulfo	Organosulfor
0.85_243.00	243.006008	0.84881667	pos	Angelical	HMDB0302137		JZYCZVLVW	Phenylpropa	Coumarins a	Unclassified
1.03_252.04	252.046522	1.0341	neg	Sulfamethoxa	HMDB00151	2546	JLKIGFTWX	Benzenoids	Benzene and	Benzenesulfo
1.08_374.20	374.203095	1.07531667	pos	Biriperone	HMDB0249235		YCNCIZWA	Organoheter	Indoles and	Pyridoindoles
1.10_534.14	534.14623	1.09681667	neg	Hydroxy Des	HMDB0253253		JXXCMZWR	Organoheter	Diazines	Pyrimidines a
1.22_265.07	265.077941	1.21911667	neg	6-Formylindolo [3,2-B] carl		43424	ZUDXFBWD	Unclassified	Unclassified	Unclassified
1.22_697.20	697.207045	1.21911667	neg	Ampicillin	HMDB00145	536	AVKUERGKI	Organoheter	Lactams	Beta lactams
1.31_201.05	201.057345	1.30545	neg	TUBAIC ACID		44615	CMXGHLJK	Unclassified	Unclassified	Unclassified
1.56_613.15	613.151579	1.55895	pos	Cassiaoccidentalin C			CIQAFMGH	Lipids and lip	Polyketides	Flavonoids
1.65_351.16	351.164406	1.64773333	pos	(2S)-2-Butan	HMDB00412	95673	DANFNCOJI	Organic oxyg	Organooxyge	Carbohydrate
1.67_172.95	172.956493	1.66533333	pos	1,2-Ethanedi	HMDB0244098		AFAXGSQY	Organic acid	Organic sulfo	Organosulfor
10.03_483.1	483.158876	10.0257	neg	(4S,7S,12Br)	HMDB0254372		RIWRWPXU	Organic acid	Carboxylic ac	Amino acids,

10.04_611.0:	611.03464	10.0440667	neg		(-)-Mintlactor	HMDB0302763		NRBDZRMC	Benzenoids	Benzene anc	Benzenesulfo
10.46_625.3(	625.308588	10.4632167	neg		Hordatine B	HMDB0030459		LRLXAXGC(	Phenylpropa	2-arylbenzofu	Unclassified
10.63_1052.(	1052.65259	10.6260167	neg		Surfactin A	HMDB0258633		PBEDWPIW.	Organic acid:	Peptidomime	Depsipeptide
10.63_584.3:	584.321146	10.6260167	neg		PS(19:0/0:0)		78855	QZTQWDNE	Lipids and lip	Glycerophos	Glycerophos
10.92_377.1	377.18632	10.9157	neg		3-(2-Propylpe	HMDB02457	433416	HUMCKIRUE	Organoheter	Azolidines	Imidazolidine
11.01_308.1	308.147778	11.0055	neg		Metixene	HMDB00144	319349	MJFJKKXQD	Organoheter	Benzothiopyr	1-benzothiop
11.51_358.2!	358.251615	11.5124	neg		Eptastigmine	HMDB0251892		RRGMXBQN	Organoheter	Indoles and (	Pyrroloindole
12.03_327.1	327.180351	12.0349667	neg		Kynuramine	HMDB00122	43923	QLPVTIQQF	Organic oxyg	Organooxyge	Carbonyl cor
12.29_819.6	819.613187	12.2879833	pos		PG(22:0/17:1(9Z))		79533	AEKHDJYUS	Lipids and lip	Glycerophos	Glycerophos
12.48_511.1:	511.137505	12.4754333	neg		Hydrangeic a	HMDB0253236		UWXXIBUTk	Phenylpropa	Stilbenes	Unclassified
13.07_190.1(	190.108409	13.0659	pos		1-(2-Hydroxy	HMDB0243780		IBAQFPQHF	Organoheter	Piperidines	Unclassified
13.26_491.3	491.37204	13.2592333	pos		Barringtogen	HMDB00345	326048	AYDKOFQQ	Lipids and lip	Prenol lipids	Triterpenoids
14.28_368.2	368.241465	14.2783333	neg		3-Hydroxytric	HMDB0241326		DPBKTUXYk	Lipids and lip	Fatty Acyls	Fatty acid es
2.11_419.04(	419.046944	2.11061667	neg		Etoricoxib 1'-	HMDB00609	806932	KMLFAHIIJS	Organoheter	Pyridines anc	Bipyridines a
2.41_128.01!	128.019494	2.40855	pos		S-Methyl met	HMDB00327	88666	RRGUMJYE(	Organic acid:	Thiosulfinic a	Unclassified
3.42_336.15!	336.155772	3.41641667	pos		Nona-4,6-die	HMDB0241759		BLCJDVPTD	Lipids and lip	Fatty Acyls	Fatty acid es
3.43_394.21!	394.21982	3.43491667	pos		MeTRH	HMDB0254684		DJLHRAHTT	Organic acid:	Carboxylic ac	Amino acids,
3.46_483.21	483.218434	3.45915	neg		Amcinonide	HMDB00144	313604	ILKJAFIWWI	Lipids and lip	Steroids and	Pregnane ste
3.51_211.02	211.02214	3.5146	neg		Thioxanthen-	HMDB02590	337565	YRHRIQCW(	Organoheter	Thiochromen	Unclassified
3.58_350.98	350.98722	3.58011667	pos		N-Succinimic	HMDB0258411		JWDFQMWE	Organoheter	Pyridines anc	Unclassified
3.60_300.19	300.191723	3.59728333	pos		Terbucarb		72871	PNRAZZZISI	Unclassified	Unclassified	Unclassified
3.60_674.32	674.327332	3.59728333	pos		PC(6 keto-P(	HMDB0288831		ITAKUNZFE.	Unclassified	Unclassified	Unclassified
3.68_448.16	448.161661	3.67736667	neg		O-Ethyl-4-[(2	HMDB0031949		GTQXJGMY.	Organic oxyg	Organooxyge	Carbohydrate
3.73_368.05	368.051671	3.73108333	neg		Cytosine aral	HMDB0243501		IERHLVCPS	Nucleosides,	Pyrimidine nu	Pyrimidine rit
3.77_466.04!	466.049618	3.7682	neg		Glucoerucin	HMDB0038403		GKUMMDFL	Organic oxyg	Organooxyge	Carbohydrate
3.88_785.33!	785.339281	3.8786	neg		PKODiA-PI			GXYNPAFQ'	Lipids and lip	Glycerophos	Oxidized glyc
3.89_333.14	333.148076	3.8922	pos		Dienestrol di	HMDB0251232		YWLLGDVB	Phenylpropa	Stilbenes	Unclassified
3.89_352.22	352.221361	3.8922	pos		Ile-Val-Val	HMDB0304801		YHFPHRUW	Organic acid:	Carboxylic ac	Amino acids,
3.91_501.25	501.253573	3.91066667	pos		Geranylfarne	HMDB00122	53738	JMVSBFJBM	Lipids and lip	Prenol lipids	Polyprenols
3.93_759.37(	759.370281	3.93381667	neg		3-Methyl-2-pl	HMDB0257514		MQNYRKWJ	Organoheter	Quinolines ai	Phenylquinol
3.97_456.05(	456.050101	3.97085	neg		Carboxycele(	HMDB0060571		WTHNOVFE	Organoheter	Azoles	Pyrazoles
4.11_341.11	341.116954	4.10951667	pos		Diphacinone	HMDB02514	72476	JYGLAHSAI:	Benzenoids	Benzene anc	Diphenylmetl
4.12_375.13	375.138602	4.11676667	neg		Ketanserin	HMDB02537	889	FPCCSQOG	Organic oxyg	Organooxyge	Carbonyl cor

4.15_510.24	510.242935	4.14533333	pos		4-[2-[2-[3-Ac	HMDB0246932			BSOQXXWZ	Organic acid	Carboxylic ac	Amino acids,
4.15_725.31	725.319043	4.1538	neg		PKHdiA-PI				TTZZKCXTZ	Lipids and lip	Glycerophos	Oxidized glyc
4.31_297.01	297.018563	4.30826667	pos		Disulfoton	HMDB02514	44559		DOFZAZXD	Organic acid	Organic dithi	Dithiophosph
4.31_414.98	414.98669	4.30826667	pos		5-Iodotubercidin		45255		WHSIXKUP	Unclassified	Unclassified	Unclassified
4.33_342.07	342.077751	4.32651667	pos		N-(p-Toluene	HMDB0244558			CGRCVIZBN	Organic acid	Carboxylic ac	Amino acids,
4.45_370.24	370.243666	4.45335	pos		Carrageenan	HMDB0303208			HKKMJRVJH	Organic acid	Carboxylic ac	Amino acids,
4.50_372.17	372.177571	4.50406667	neg		(1-(5-Fluorop	HMDB02422	422173		IGBHZHCGV	Organoheter	Indoles and	Naphthoylind
4.51_265.20	265.203539	4.50633333	pos		Y-27632 Dih	HMDB0259944			IYOZTVGME	Organoheter	Pyridines an	Hydropyridin
4.72_610.03	610.03312	4.72411667	neg		3,4-Difluoro-	HMDB0257282			FIMYFEGKM	Benzenoids	Benzene and	Benzoic acid
4.72_641.37	641.374171	4.72301667	pos		PHODiA-PE				GHAGXTIPF	Lipids and lip	Glycerophos	Oxidized glyc
4.74_232.01	232.014912	4.74081667	pos		quinoxaline-	HMDB0257054			MXQPKTHZI	Organoheter	Diazanaphth	Benzodiazine
4.78_539.30	539.303992	4.7769	pos		Ulimorelin	HMDB0259396			WGYPAJVJI	Organic acid	Carboxylic ac	Amino acids,
4.79_394.18	394.180793	4.79411667	pos		Fluvastatin	HMDB0015227			FJLGEFLZQ	Organoheter	Pyrroles	Substituted p
4.96_272.05	272.054022	4.96071667	neg		Tolcapone	HMDB00144	43355		MIQPIUSUK	Benzenoids	Benzene and	Benzophenon
5.00_411.07	411.078523	4.99758333	neg		4-Amino-5-m	HMDB0250828			DIJCILWNO	Organoheter	Benzopyrans	1-benzopyra
5.08_600.33	600.33339	5.08273333	pos		Penitrem E	HMDB0256241			LTCFBVUSII	Organoheter	Naphthopyra	Unclassified
5.18_303.13	303.138301	5.18221667	neg		Derricin		51815		HMGRVRIPI	Lipids and lip	Polyketides	Flavonoids
5.19_304.14	327.134184	5.19183333	pos		Cedpht	HMDB0251608			SINKVNIAFS	Phenylpropa	Phenylpropa	Unclassified
5.56_227.21	227.211529	5.5568	pos		Labienoxime	HMDB00360	328459		MEJYWDUB	Organic nitro	Organonitrog	Oximes
5.85_363.06	363.064979	5.84688333	neg		Belinostat	HMDB0248932			NCNRHFGM	Phenylpropa	Cinnamic aci	Unclassified
6.05_481.24	481.244237	6.05016667	neg		11-beta-hydroxyandrosterone-3-glucuro				BRPLOVMH	Lipids and lip	Steroids and	Steroid conju
6.60_389.21	389.218982	6.59813333	pos		Nemonoxaci	HMDB0255512			AVPQPGFL	Organoheter	Quinolines a	Quinoline ca
6.99_761.08	761.085205	6.98976667	neg		Desacetycef	HMDB0037269			IOFHZPVEQ	Hydrocarbon	Saturated hy	Alkanes
7.19_355.23	355.235417	7.18508333	pos		(Z)-7-[(1S,4R	HMDB0247875			PEZZZRQW	Lipids and lip	Fatty Acyls	Fatty acids a
7.90_292.08	292.080299	7.90265	neg		Zierin	HMDB0301769			KCVXNPDA	Organic oxy	Organooxyg	Carbohydrate
8.21_408.12	408.129515	8.21301667	pos		5,6,3'-Trihydroxy-3,7,8,4'-t		51749		AHABJNGV	Lipids and lip	Polyketides	Flavonoids
8.42_307.01	307.018527	8.41935	neg		2-Hydroxyet	HMDB0245158			KYNFOMQI	Organosulfu	Organic disu	Dialkyldisulfi
8.71_751.24	751.242513	8.7071	neg		Leonoside A	HMDB0040342			ZPJGTPAAE	Organic oxy	Organooxyg	Carbohydrate
8.79_493.25	493.259873	8.7919	pos		Ezlopitant	HMDB0252150			XPNMCDYO	Benzenoids	Benzene and	Diphenylmet
9.08_334.23	334.234827	9.08396667	pos		N-tetradecanoyl-homoseri		64716		ZQAYHOXX	Lipids and lip	Fatty Acyls	Fatty amides
9.13_476.18	476.182681	9.13165	neg		Lafutidine	HMDB02402	314993		KMZQAVXS	Organic oxy	Organooxyg	Ethers
9.53_439.00	439.002122	9.53388333	neg		Methylpyrog	HMDB0240694			ARLAWMCE	Organic acid	Organic sulf	Arylsulfates
0.63_375.13	375.138397	0.62835	neg		Oxomemazi	HMDB0240230			QTQPVLDZ	Organoheter	Benzothiazin	Phenothiazin

0.66_174.10	174.10748	0.65943333	neg	4-(Dimethyla	HMDB0246305		UFYJLJINU(	Organosulfur	Isothioureas	Unclassified
0.70_358.16	358.164094	0.69861667	pos	1-Hydroxy-3,	HMDB00412	95669	ADQOAYRZ	Organoheter	Benzopyrans	1-benzopyra
0.74_361.16	361.164653	0.74288333	pos	(-)-jasmonoyl	HMDB0303944		IBZYPBGPO	Organic acid	Carboxylic ac	Amino acids,
0.75_447.13	447.13552	0.74898333	neg	Setipiprant	HMDB0258261		IHAXLPDVO	Benzenoids	Naphthalene	Naphthalene
0.76_471.09	471.095837	0.75766667	pos	(Z)-Resverat	HMDB00370	92052	AUBJHOOEI	Phenylpropa	Stilbenes	Stilbene glyc
0.77_341.04	341.047094	0.7729	pos	Cambendazo	HMDB02495	316234	QZWHWHN(	Organoheter	Benzimidazo	Unclassified
1.02_112.00	112.003946	1.01586667	neg	2-Nitrofuran	HMDB02452	70043	FUBFWTUFI	Organoheter	Furans	Nitrofurans
1.12_297.05	297.057278	1.11995	pos	N-Acetyldjen	HMDB00294	86258	KXLLUVVNY	Organic acid	Carboxylic ac	Amino acids,
1.15_270.06	309.026858	1.1535	pos	4,4'-dinitrostilbene		53255	CLVIIRIMEIE	Lipids and lip	Polyketides	Aromatic poly
1.22_453.17	453.171041	1.2188	pos	N-Palmitoyl A	HMDB0241920		DENIQPHQL	Lipids and lip	Steroids and	Androstane s
1.22_699.17	699.174478	1.21911667	neg	Penicillin V	HMDB00145	1739	BPLBGHOL)	Organic acid	Carboxylic ac	Amino acids,
1.37_258.00	258.005808	1.36776667	pos	3-Hydroxyhip	HMDB0304909		ZYEMKIHYS	Benzenoids	Benzene and	Benzoic acid
1.45_639.11	639.118585	1.44935	neg	Quercetin 3-glucosyl-(1->2)-glucuronide			FVVVGDCT	Lipids and lip	Polyketides	Flavonoids
1.67_383.16	383.161999	1.66533333	pos	(E,E)-Boviqu	HMDB00307	87169	ADRVEZVEK	Lipids and lip	Prenol lipids	Diterpenoids
1.89_428.13	428.130754	1.89173333	neg	Fluazifop-but	HMDB02523	68784	VAIZTNZGP	Benzenoids	Benzene and	2-phenoxypro
1.90_369.15	369.155491	1.89901667	pos	hbas#9			GOULJBFAJ	Lipids and lip	Fatty Acyls	Fatty acyl gly
10.01_838.5	838.594836	10.00765	neg	PE(24:1(15Z	HMDB0284636		AYCPXRZVI	Unclassified	Unclassified	Unclassified
10.24_741.2	741.295461	10.2441667	neg	JWH 018 N-pentanoic acid		64911	BYIVGHIYNI	Unclassified	Unclassified	Unclassified
10.43_648.2	648.295428	10.42685	neg	MCTR1			NWYKIASJH	Lipids and lip	Fatty Acyls	Docosanoids
10.73_380.3	380.349287	10.7334833	pos	C-6 Ceramide		63012	NPRJSFWNI	Unclassified	Unclassified	Unclassified
10.83_411.2	411.215064	10.82535	neg	Umbelliprenin	HMDB00305	87086	GNMUGVNE	Lipids and lip	Prenol lipids	Terpene lact
11.43_538.2	538.244434	11.4320667	pos	L-Alaninamid	HMDB0258719		AWNBSWDI	Organic acid	Carboxylic ac	Amino acids,
12.36_518.3	518.341859	12.3586333	pos	Tuftsin	HMDB0005770		IESDGNYHX	Organic acid	Carboxylic ac	Amino acids,
12.48_358.2	359.218555	12.4811	pos	Ferutinin	HMDB0252237		CYSHNJQM	Lipids and lip	Prenol lipids	Sesquiterpe
12.48_744.5	744.587281	12.4811	pos	PC(11:0/23:0)		39162	ARPFEYKJA	Lipids and lip	Glycerophos	Glycerophos
12.49_303.2	303.218106	12.4924667	neg	3S-hydroxy-pentadecanoic		35673	ATMSEJBAE	Lipids and lip	Fatty Acyls	Fatty Acids a
12.62_432.1	432.197481	12.6234833	neg	(4-Ethylnaph	HMDB02596	85079	NSCXPXDW	Organoheter	Indoles and	Naphthoylind
13.29_1185.	1185.84559	13.29445	pos	Fucoxanthinol 3-(4Z,7Z,10Z,13Z,16Z,19			GSYOIRYMI	Lipids and lip	Prenol lipids	Isoprenoids
13.38_380.2	380.208812	13.38305	pos	Atevirdine	HMDB02486	423196	UCPOMLWZ	Organoheter	Diazinanes	Piperazines
14.90_139.0	139.073904	14.9013667	pos	Isocaproic ac	HMDB00006	4191	FGKJLKRYE	Lipids and lip	Fatty Acyls	Fatty acids a
2.16_440.21	440.21361	2.15823333	pos	Cilazapril	HMDB00154	1748	HHHKFGXW	Organic acid	Carboxylic ac	Amino acids,
2.46_226.09	226.097801	2.46275	pos	Imidazo(1,2-a	HMDB0254914		BWZMTZXT	Organoheter	Imidazopyraz	Unclassified
3.34_517.24	517.243694	3.34363333	pos	2beta-hydroxymelianthusigenin			CKMXQHHD	Lipids and lip	Steroids and	Sterols

3.56_269.17	269.174702	3.56223333	pos	3,11,12-Trihy	HMDB00381	92895	DQOOWVDI	Lipids and lip	Prenol lipids	Sesquiterper
3.59_397.01	397.015849	3.58845	neg	Pyrrole-2,3,5	HMDB0256995		UGSAVTSXI	Organic acid	Carboxylic ac	Tricarboxylic
3.66_202.13	202.137058	3.66498333	pos	1-Azidosulfa	HMDB0255333		DWSCKFBU	Organic salts	Organic azid	Unclassified
3.75_1046.5	1046.53995	3.74966667	neg	Angiotensin	HMDB02484	981569	CHCOFDZS	Organic acid	Carboxylic ac	Amino acids,
3.75_613.33	613.331058	3.75006667	pos	(2R)-2-[[(1R,	HMDB0254924		ZTENZJJCF	Organic acid	Carboxylic ac	Amino acids,
3.75_693.31	693.319723	3.75006667	pos	POV-PI			VVEZLCRC	Lipids and lip	Glycerophos	Oxidized glyc
3.80_411.12	411.127006	3.80496667	neg	Methylpicraq	HMDB00383	93067	YCIAHAYEV	Organic oxyg	Organooxyge	Carbohydrate
3.91_973.51	973.515275	3.91066667	pos	PGP(i-21:0/F	HMDB0275750		BLBRYHIDW	Unclassified	Unclassified	Unclassified
3.95_412.22	412.223757	3.94663333	pos	BRUCINE		43921	RRKTZKIUP	Unclassified	Unclassified	Unclassified
3.98_195.08	195.080754	3.98231667	pos	anthracen-9-	HMDB0248459		AUKRYONW	Benzenoids	Anthracenes	Unclassified
3.99_309.11	309.112269	3.98933333	neg	Cinnamyl cin	HMDB00338	89559	NQBWNECT	Phenylpropa	Cinnamic aci	Cinnamic aci
4.21_366.16	366.164515	4.20936667	neg	Gabexate	HMDB02525	321409	YKGYIDJEE	Benzenoids	Benzene anc	Benzoic acid
4.26_1001.5	1001.50332	4.26475	neg	PIP(18:1(9Z)	HMDB0279093		AFNZZWWF	Unclassified	Unclassified	Unclassified
4.30_387.01	387.01486	4.30156667	neg	Ranelic acid	HMDB02571	320325	DJSXNILVA	Organic acid	Carboxylic ac	Amino acids,
4.41_388.17	388.172551	4.41166667	neg	MAM2201 N-(4-hydroxype		85212	KKTSWDJP	Unclassified	Unclassified	Unclassified
4.43_210.98	210.98335	4.43021667	neg	Hexafluoropr	HMDB0253135		PGFXOWRD	Organoheter	Epoxides	Unclassified
4.45_403.20	403.208424	4.45335	pos	Isopropylphe	HMDB0253681		FOQCCKILB	Nucleosides,	Purine nucle	Unclassified
4.69_578.31	561.30671	4.68661667	pos	Strebloside			BGGIZHKHJ	Lipids and lip	Steroids and	Sterols
4.83_343.08	343.08396	4.83273333	neg	Kuhlmannin		44662	ABUBCBFU	Lipids and lip	Polyketides	Flavonoids
4.91_374.07	374.07265	4.90568333	neg	N-(2-Hydroxy	HMDB0240542		PRQFWBNV	Organic oxyg	Organooxyge	Carbohydrate
4.97_415.22	415.2235	4.97433333	pos	CPA(16:0)		75590	WLYRJURL	Lipids and lip	Glycerophos	Other Glycer
5.03_198.99	198.998003	5.03453333	neg	3,5-Dinitroca	HMDB02460	44188	VDCDWNDT	Benzenoids	Phenols	Nitrophenols
5.25_351.15	351.154569	5.24643333	pos	Guaiaretic acid		985426	OPAORDVB	Unclassified	Unclassified	Unclassified
5.39_527.30	527.30441	5.39208333	pos	Dipyridamole	HMDB00151	2341	IZEKFCXSFI	Organic nitro	Organonitrog	Amines
5.45_317.19	317.193162	5.44698333	pos	3,7-Dimethyl	HMDB00347	90195	ZCDKJIRLF	Lipids and lip	Fatty Acyls	Fatty acyl gly
5.64_305.02	305.02294	5.64363333	neg	4'-Hydroxy N	HMDB0246630		XYHFQSKA	Benzenoids	Benzene anc	Diphenylethe
6.22_507.31	507.313774	6.2158	pos	16alpha,17alpha-epoxy-sinusta-5-en-3b			MBEVKYVYI	Lipids and lip	Steroids and	Steroid conju
6.38_257.15	257.153097	6.3809	pos	4,4'-Methyler	HMDB02466	351026	AZZWZMUX	Benzenoids	Benzene anc	Diphenylmetl
6.98_238.09	238.090928	6.98325	pos	N-(1-Deoxy-1	HMDB00378	92656	UKKRCTOBI	Organic oxyg	Organooxyge	Carbohydrate
8.75_881.57	881.576456	8.74946667	neg	Sulfated Dihydromenaquir		102915	YLFBUPJPG	Lipids and lip	Prenol lipids	Quinones an
9.93_447.00	447.007924	9.93468333	neg	3-Amino-5-(3	HMDB02575	45240	BMUACLAD	Organoheter	Bi- and oligo	Unclassified
9.95_307.17	307.172306	9.95315	neg	7,7-Difluoro-8Z-dodecenyl		46260	QXRHOBW	Lipids and lip	Fatty Acyls	Fatty esters
0.61_210.11	210.117361	0.60603333	pos	Ametryn	HMDB02482	72478	RQVYBGPQ	Organoheter	Triazines	1,3,5-triazine

0.61_298.06:	298.062983	0.61033333	neg	6-Thioguano:	HMDB0247107		OTDJAMXE!	Nucleosides,	Purine nucle	Unclassified
0.61_553.18(	553.186844	0.61033333	neg	Letermovir	HMDB0254025		FWYSMLBE'	Organoheter	Diazinanes	Piperazines
0.62_266.05!	289.044176	0.6215	pos	4-Methoxybr	HMDB00407	95255	JVKOHVNW	Organoheter	Indoles and (	Indoles
0.73_372.09(	372.09688	0.7342	neg	Zidebactam	HMDB0260005		YCZPXRQPI	Organic acid:	Carboxylic ac	Amino acids,
0.99_238.16!	238.16554	0.99473333	pos	Lidamidine	HMDB02540	323215	RRHJHSBDJ	Benzenoids	Benzene anc	N-phenylurea
1.07_286.05!	286.05511	1.06741667	neg	Morin-5'-sulf(	HMDB0254884		IEYFQXRAJI	Benzenoids	Benzene anc	Anilides
1.08_390.11!	390.115273	1.08208333	neg	3-(3-Amino-3	HMDB0242130		YXNIEZJFC(	Nucleosides,	Pyrimidine nu	Unclassified
1.09_397.09(	397.09654	1.08996667	pos	Succinylamir	HMDB02402	837240	XNKLTOHYI	Nucleosides,	Imidazole rib	1-ribosyl-imic
1.13_401.05(	401.050371	1.13148333	neg	BISSALICYL	FUMARATE	43539	DMCJLRGW	Unclassified	Unclassified	Unclassified
1.15_239.01(	239.016675	1.14921667	neg	Pyrrolidin-1-y	HMDB0257676		NHZGRUILD	Organoheter	Pyrrolidines	Unclassified
1.22_325.01(	325.016045	1.21911667	neg	Endothion	HMDB02518	72741	YCAGGFXSI	Organoheter	Pyrans	Pyranones a
1.25_420.97	420.974543	1.251	pos	Olsalazine-O	HMDB0060600		BAHPNDUIX	Organic acid:	Organic sulfu	Arylsulfates
1.31_315.06:	315.061411	1.30545	neg	9H-Purin-6-ol,	9-(p-nitrobe	418719	AWOOIVCG	Unclassified	Unclassified	Unclassified
1.34_404.15:	404.153654	1.34166667	neg	Neolinustatin		66915	WOSYVGNE	Unclassified	Unclassified	Unclassified
1.34_545.16	545.16765	1.34166667	neg	S)-2-(5(((1,2-	HMDB0244576		BRVFNEZM'	Organic acid:	Carboxylic ac	Amino acids,
1.36_487.05:	487.052788	1.3586	neg	Dimethylellag	HMDB0251398		KHTUHWMI	Phenylpropa	Tannins	Hydrolyzable
1.38_274.00:	274.002225	1.37511667	neg	3- Hydroxyin(	HMDB0304928		HLYCAHON(	Organoheter	Indoles and (	Indolines
1.49_347.10	347.10724	1.4863	neg	Dansylprolin(	HMDB0250852		ZHJIWURD(	Benzenoids	Naphthalene	Naphthalene
1.49_423.04(	423.040008	1.4863	neg	Mikanin 3-O-sulfate		51296	OABRFHRM	Lipids and lip	Polyketides	Flavonoids
1.51_278.16(	278.160402	1.50628333	pos	Glycerol tripr	HMDB00328	325642	YZWRNSAR	Lipids and lip	Glycerolipids	Triradylcglyc(
10.21_536.3:	536.320461	10.20805	neg	Cholylglutam	HMDB0242384		CBUKBSJQ\	Unclassified	Unclassified	Unclassified
10.28_576.2(	576.203606	10.2812167	neg	Acrimarine J	HMDB00403	94898	ZWXOIPZAT	Organoheter	Quinolines ar	Benzoquinoli
10.41_749.5:	748.527408	10.4089833	neg	PE(22:5(4Z,7	HMDB00096	61031	DWRNCYXS	Lipids and lip	Glycerophos	Glycerophos
10.54_898.5	898.579338	10.5357333	neg	PC(TXB2/20	HMDB0287181		ANPYNPWN	Unclassified	Unclassified	Unclassified
10.57_275.1:	275.122525	10.5732333	pos	1,5,5,8-Tetra	HMDB0036468		RNAZTWUZ	Organoheter	Thiiranes	Unclassified
10.90_591.1	591.177727	10.8978167	neg	rhamnetin 3-	HMDB0304579		MWIKBJCXY	Phenylpropa	Cinnamic aci	Hydroxycinna
11.58_682.3:	682.326966	11.5754333	pos	Murnac-tripe	HMDB0254953		NMAKGCME	Organic acid:	Carboxylic ac	Amino acids,
12.05_809.0	809.082194	12.0546333	pos	Tercatain	HMDB0039263		NALYUPYCI	Phenylpropa	Tannins	Hydrolyzable
12.30_535.1:	535.137838	12.3037667	neg	7-HYDROXY-2'-METHOX		43943	AHLHZSCEN	Unclassified	Unclassified	Unclassified
12.60_837.5	837.588915	12.6029833	pos	SM(d18:0/22	HMDB0013467		DRKCMBAZI	Lipids and lip	Sphingolipids	Phosphosphi
12.64_415.2(	415.207658	12.6402	neg	11-(Dansylar	HMDB02508	44899	CEPGVMDM	Benzenoids	Naphthalene	Naphthalene
12.66_684.9(	684.960872	12.65635	pos	Adenosine 5'	HMDB00596	96250	WYJWVZZC	Nucleosides,	Purine nucle	Purine ribonu
2.12_465.09	465.098125	2.12441667	pos	Vericiguat	HMDB0304849		QZFHIXARH	Organoheter	Pyrazolopyric	Unclassified

2.83_255.09	255.096112	2.83016667	neg	2-(2-Aminop	HMDB0255353		UPSXYNJD	Organic acid	Carboxylic a	Amino acids,
3.33_418.20	418.202503	3.32576667	pos	CAY10585		45359	BJRPPNOJY	Unclassified	Unclassified	Unclassified
3.40_405.24	405.240405	3.39796667	pos	PA(17:1(9Z)/0:0)		3887	PIVUMJCNA	Lipids and lip	Glycerophos	Glycerophos
3.43_503.22	503.227665	3.43491667	pos	Isocyclocalar	HMDB00352	90537	BPAWUWIF	Lipids and lip	Steroids and	Steroid lacto
3.43_575.25	575.255674	3.43491667	pos	<i>ortho</i>-	HMDB0061015		CZBPKFICA	Organoheter	Pyrroles	Substituted p
3.53_190.07	190.071425	3.52578333	pos	Glutarylglycir	HMDB00005	5569	NBLKTRLPZ	Organic acid	Carboxylic a	Amino acids,
3.54_404.22	404.223307	3.54413333	pos	(-)-AS 115		45507	BTYYXSHZ	Unclassified	Unclassified	Unclassified
3.59_487.11	487.113032	3.58845	neg	S-(4-Nitrobenzyl)glutathior		4098	OAWORKDF	Unclassified	Unclassified	Unclassified
3.61_388.15	388.158921	3.60671667	neg	Paroxetine	HMDB00148	1710	AHOUBRCZ	Organoheter	Piperidines	Phenylpiperi
3.65_708.10	708.106841	3.64815	pos	Chymosin pr	HMDB00321	66338	SERBHKJM	Organic acid	Peptidomime	Depsipeptide
3.71_431.10	431.10221	3.71251667	neg	6-Aminopeni	HMDB00606	314018	NGHVIOIJC	Organic acid	Carboxylic a	Amino acids,
3.75_485.07	485.074183	3.74966667	neg	(6-Iodo-2-me	HMDB02447	437656	JHOTYHDSI	Organoheter	Indoles and	Benzoylindol
3.87_354.16	355.171006	3.87373333	pos	Valganciclovi	HMDB00155	320490	WPVFJKSG	Organic acid	Carboxylic a	Amino acids,
3.88_352.14	352.149167	3.8786	neg	Hippuryl-L-ly	HMDB0253017		LRCZLURYH	Organic acid	Carboxylic a	Amino acids,
3.89_438.22	438.224535	3.8922	pos	PS(13:0/0:0)		46724	JHFVXCSIK	Lipids and lip	Glycerophos	Glycerophos
3.95_452.24	452.24593	3.94663333	pos	p-MPPF	HMDB0244701		YJZYDPRM	Organoheter	Diazinanes	Piperazines
3.96_357.21	357.219883	3.96453333	pos	Edivoxetine	HMDB0251701		CPBHSHYQ	Benzenoids	Phenol ether	Anisoles
3.99_651.29	651.292553	3.98933333	neg	Preglabridin		984968	HJYNNFAP	Unclassified	Unclassified	Unclassified
4.00_284.19	284.197137	4.00056667	pos	CC(=O)Nccc	HMDB0257739		AUVGDBMR	Organoheter	Pyridines an	Methylpyridir
4.04_426.13	426.135942	4.04478333	neg	Amoxicilloyl	HMDB0248351		BNIOMQSG	Organic acid	Carboxylic a	Amino acids,
4.11_416.22	417.236665	4.10951667	pos	Ramipril	HMDB00143	2243	HDACQVRG	Organic acid	Carboxylic a	Amino acids,
4.13_581.31	581.313249	4.12765	pos	LysoPI(18:1(	HMDB0061693		UGDOFRYH	Lipids and lip	Glycerophos	Glycerophos
4.15_703.21	703.217117	4.1538	neg	Penicilloic ac	HMDB0060617		HCYWNSXL	Organic acid	Carboxylic a	Amino acids,
4.25_730.36	730.368581	4.25448333	pos	Pinokalant	HMDB02565	320887	PYWYBTRA	Organoheter	Isoquinolines	Benzylisoqui
4.25_879.36	879.361001	4.24626667	neg	Dorsmanin H		52894	AZJHCBAWI	Lipids and lip	Polyketides	Flavonoids
4.26_489.12	489.124059	4.26475	neg	6-Azauridine	HMDB0247037		WYXSYVWA	Organic oxyg	Organooxyge	Carbohydrate
4.29_463.24	463.245645	4.29085	pos	3alpha,6alpha,7beta,12alp		84433	COCMFMBN	Lipids and lip	Steroids and	Bile acids an
4.31_354.00	372.040699	4.30826667	pos	uric acid citra	HMDB0259718		DEVMSNHD	Organoheter	Imidazopyrim	Purines and
4.45_425.24	425.248763	4.45335	pos	Dexanabinol		405153	SSQJFGME	Unclassified	Unclassified	Unclassified
4.52_383.23	384.245018	4.52393333	pos	N-(1-Adamar	HMDB02445	982248	UCMFSGVIE	Organoheter	Benzopyrazo	Indazoles
4.54_737.33	737.330599	4.5416	pos	HEUDELOTTIN C		43978	BYWKXGOE	Unclassified	Unclassified	Unclassified
4.65_328.12	328.121211	4.64996667	pos	N-(4-Hydrox	HMDB00395	94071	LEEDEKWK	Organic acid	Carboxylic a	Amino acids,
4.70_315.17	338.162158	4.70481667	pos	Dihydroethidi	HMDB0251312		XYJODUBP	Organoheter	Quinolines a	Phenylquinol

4.74_576.12(	576.126287	4.74241667	neg		3-Caffeoylpe	HMDB00380	92850	KYTVPWHU	Phenylpropa	Flavonoids	Flavonoid gly
4.90_600.30:	600.303905	4.90175	pos		Antiaroside H			PQWNIWYZ	Lipids and lip	Steroids and	Sterols
5.05_566.32:	589.311489	5.04685	pos		25-Cinnamoy	HMDB00413	95772	SXPHINZZV	Lipids and lip	Prenol lipids	Sesterterpen
5.08_459.24!	459.249886	5.08273333	pos		[(1R)-1-(hydroxymethyl)-2-phosphonoox			BDCFJMBXZ	Lipids and lip	Glycerophos	Glycerophos
5.21_514.26	514.26726	5.20995	pos		Benzyl N-[(2!	HMDB0258106		GXZZQHQQ	Organic acid	Carboxylic ac	Amino acids,
5.53_282.13:	282.132225	5.53265	neg		Hept-4-enedi	HMDB0241688		JLIHWXBJCI	Lipids and lip	Fatty Acyls	Fatty acid es
5.57_529.19:	529.19262	5.5696	neg		Plantacyanin	HMDB0303586		VFUDMQLBI	Organohetero	Indoles and c	Carbazoles
5.77_418.14	418.144243	5.773	neg		Pazopanib	HMDB02561	321971	CUIHSIWYW	Organic nitro	Organonitrog	Amines
5.81_234.15	234.157474	5.81251667	pos		N-a-Acetyl-L-	HMDB00046	58241	SNEIUMQYF	Organic acid	Carboxylic ac	Amino acids,
6.20_296.14	296.14779	6.19793333	neg		Rotigotine hydrochloride		413692	CEXBONHIC	Unclassified	Unclassified	Unclassified
6.53_296.05(	296.050784	6.53055	neg		Oxfendazole	HMDB00318	43289	BEZZFPOZA	Organohetero	Benzimidazo	Unclassified
6.92_557.35!	557.35549	6.91643333	neg		Moxisylyte	HMDB0254909		VRYMTAVO:	Lipids and lip	Prenol lipids	Monoterpeno
6.99_547.09	547.094002	6.98976667	neg		4-Hydroxy-5-	HMDB0059981		ZCVMPBGBI	Benzenoids	Benzene and	Unclassified
7.17_438.20(	438.206925	7.16671667	pos		n-Formyl-me	HMDB0257949		BYNDVMFG	Organic acid	Carboxylic ac	Amino acids,
7.19_479.17!	479.179239	7.19245	neg		vincosamide; (3?,15?,16?)		985212	LBRPLJCNR	Unclassified	Unclassified	Unclassified
7.41_623.16	623.167398	7.41378333	neg		isorhamnetin	HMDB0304580		PSYRONNZ	Phenylpropa	Cinnamic aci	Hydroxycinna
8.16_536.16!	536.16503	8.15795	pos		(R)-4A-(Etho	HMDB0250459		SLKURXRZH	Organohetero	Azoles	Pyrazoles
8.75_501.30(	501.300462	8.74946667	neg		2,6-Di-tert-butyl-4-nitrophe		268917	FCGKUUOT	Unclassified	Unclassified	Unclassified
8.94_370.28!	370.285456	8.93783333	pos		Isopropamide	HMDB0015562		JTPUMZTWI	Benzenoids	Benzene and	Diphenylmet
9.06_295.18:	295.181836	9.05798333	neg		Palonosetror	HMDB00145	85372	CPZBLNMU(	Organohetero	Isoquinolines	Isoquinolone
9.09_281.13(	281.136907	9.09495	neg		Artemisin		532	BLUAFEHZU	Lipids and lip	Prenol lipids	Isoprenoids
9.13_498.16	498.164579	9.13165	neg		Fluphenazine	HMDB02524	85049	UFPPOFUTI	Organohetero	Benzothiazin	Phenothiazin
9.53_437.24(	437.240495	9.53388333	neg		Ritalinic acid	HMDB00420	1231	INGSNVSER	Organic nitro	Organonitrog	Amines
9.86_509.02	509.024747	9.86181667	neg		Phosfolan		72762	ILBONRFSL	Unclassified	Unclassified	Unclassified
0.59_231.13!	231.139614	0.58926667	pos		Dehydrocostus lactone		67654	NETSQGRTI	Unclassified	Unclassified	Unclassified
0.61_174.06!	174.068805	0.60603333	pos		(R)-(+)-beta-Methylphenet		426011	AXORVIZLP	Unclassified	Unclassified	Unclassified
0.61_389.03	389.037269	0.61033333	neg		4-iodo-SAHA		64756	BYVHZKAHE	Unclassified	Unclassified	Unclassified
0.70_385.15:	386.158943	0.69861667	pos		Papaverrubir	HMDB00301	86769	JEUAVYPZV	Alkaloids and	Rhoeadine a	Unclassified
0.74_229.08	229.087849	0.74288333	pos		Trioxsalen	HMDB00155	43364	FMHHVULE	Phenylpropa	Coumarins a	Furanocouma
0.76_341.10:	341.101037	0.75766667	pos		Phenylalanyl	HMDB00289	23660	OHUXOEXB	Organic acid	Carboxylic ac	Amino acids,
0.77_301.15(	301.15007	0.7729	pos		Imidazolone .	HMDB0253421		HZDLKNOGI	Organic acid	Carboxylic ac	Amino acids,
0.77_405.20	405.208875	0.7729	pos		(Z)-7-[(1S,4R	HMDB0260212		JEUSDRLWI	Lipids and lip	Fatty Acyls	Fatty acids a
0.78_314.10:	314.101257	0.7799	neg		4-Hydroxy Tr	HMDB02464	2909	QNJVMSAS	Organohetero	Pteridines an	Unclassified

0.79_384.08	384.084364	0.7877	pos	Aminomethyl	HMDB0248324		UPNIAQIADI	Organoheter	Benzopyrans	1-benzopyrai
0.82_447.20	447.205848	0.81883333	pos	Azeloprazole	HMDB0248793		DWDKHTXM	Organoheter	Benzimidazo	Sulfinylbenzi
0.89_244.04	244.043742	0.8943	neg	Phenols	HMDB0302434		WJFKNYWR	Organoheter	Benzothiazin	Phenothiazin
1.03_339.05	320.033954	1.0341	neg	Cefacetrile	HMDB00154	85512	RRYMAQUW	Organoheter	Lactams	Beta lactams
1.09_320.12	320.125696	1.08996667	pos	3-{4-[2-Hydro	HMDB0250054		ILSCWPMG	Phenylpropa	Phenylpropa	Unclassified
1.27_737.13	737.137272	1.26985	neg	Lansoprazole	HMDB00050	919	MJIHNNLFO	Organoheter	Benzimidazo	Sulfinylbenzi
1.34_493.08	493.082738	1.34166667	neg	Curcumin sul	HMDB0240492		NEJVQQBB	Phenylpropa	Diarylheptan	Linear diaryl
1.42_382.10	365.105496	1.41946667	pos	Enterodiol su	HMDB0240504		QFWYXPNN	Lignans, neo	Dibenzylbuta	Dibenzylbuta
10.21_368.1	413.155689	10.20805	neg	N-Desmethyl	HMDB00139	85306	HHTDENYTS	Organoheter	Indoles and	Indoles
10.55_538.3	538.335973	10.5532667	neg	2-thio-PAF		63005	YPPZOKNKE	Unclassified	Unclassified	Unclassified
10.79_536.2	536.251862	10.7894833	neg	Enkephalin L	HMDB00010	24069	URLZCHNOI	Organic acid	Carboxylic ac	Amino acids,
10.98_394.3	394.328788	10.9842333	pos	7a-Hydroxy-5	HMDB00024	6700	JVMCMMXF	Lipids and lip	Steroids and	Bile acids, al
12.43_515.3	515.348819	12.4295667	pos	Lobophysterol D			FQRVYQKVI	Lipids and lip	Steroids and	Sterols
12.64_376.1	376.19261	12.6383167	pos	(-)-Tylophorir	HMDB0242221		SSEUDFYBE	Benzenoids	Phenanthren	Phenanthroir
12.94_325.2	325.270839	12.9406833	pos	11(S)-HEDE		45655	HDMIRVFIV	Unclassified	Unclassified	Unclassified
13.70_444.3	444.305796	13.7043333	pos	Dynorphin A	HMDB0012932		BHSYMWWI	Organic acid	Carboxylic ac	Amino acids,
13.92_567.3	567.380491	13.9195167	pos	ELV-N34			RELOZBYG	Lipids and lip	Fatty Acyls	Fatty Acids a
2.11_165.07	165.074661	2.11061667	neg	3-(4-Methyl-3	HMDB00381	92919	UAGFAMQT	Organoheter	Heteroaroma	Unclassified
3.31_402.96	402.961832	3.30741667	pos	D-myo-Inositol-1,2,6-triphc		45088	GKDKOMAJ	Unclassified	Unclassified	Unclassified
3.59_684.19	684.19141	3.58845	neg	Malvidin 3-rh	HMDB0303640		GNUHZLJAF	Phenylpropa	Flavonoids	Flavonoid gly
3.68_181.00	181.005934	3.67736667	neg	2-(4-Chloropl	HMDB02449	69646	DKHJWWRY	Benzenoids	Benzene and	2-phenoxypro
3.68_725.05	725.054131	3.67736667	neg	2-Hydroxyeth	HMDB0301921		RFCSADBE	Organic oxyg	Organooxyge	Carbohydrate
3.72_805.33	805.339077	3.71513333	pos	Antibiotic 20	HMDB0255695		KGTDRFCX	Lipids and lip	Prenol lipids	Triterpenoids
3.73_390.18	390.187695	3.73238333	pos	Dodeca-4,6,8	HMDB0241284		DGPCVQPN	Lipids and lip	Fatty Acyls	Fatty acid es
3.75_358.15	358.154942	3.75006667	pos	Afn-1252	HMDB0260335		QXTWSUQC	Organoheter	Diazanaphtha	Naphthyridin
3.82_192.04	192.049947	3.82341667	neg	5-Hydroxy-4-	HMDB0246802		FRTKOPTW	Organic acid	Carboxylic ac	Amino acids,
3.89_695.09	695.09843	3.8922	pos	Quercetagetin 4'-methyl ether 7-(6-(E)-c			VOLQPULS	Lipids and lip	Polyketides	Flavonoids
3.93_650.31	650.317081	3.92891667	pos	16alpha-Acetoxyasclepin			WDMVIHZX	Lipids and lip	Steroids and	Sterols
3.99_819.39	819.391906	3.98933333	neg	4,4'-(Isopropylidenebis(4,1		325497	KMKWGXGS	Unclassified	Unclassified	Unclassified
4.06_472.29	472.290597	4.05526667	pos	18-acetoxy-PGF2alpha-11-acetate			RPLPBOQM	Lipids and lip	Fatty Acyls	Eicosanoids
4.12_709.33	709.335725	4.11676667	neg	Perazine sulf	HMDB02563	450812	QGGFCTLXI	Organoheter	Benzothiazin	Phenothiazin
4.13_516.30	516.309332	4.12765	pos	LysoPC(18:4	HMDB00103	61699	RVSJNDKZL	Lipids and lip	Glycerophos	Glycerophos
4.20_433.20	433.205269	4.19935	pos	S-Furanopet	HMDB0036131		DZIJJEZRPI	Lipids and lip	Prenol lipids	Sesquiterper

4.28_596.21(	596.216286	4.28308333	neg	4-Hydroxytar	HMDB0061124		ZGQCEPMG	Phenylpropa	Stilbenes	Unclassified
4.31_339.20!	339.209856	4.30826667	pos	18-fluoro-9E-octadecenoic		35912	XZCKFYWU	Lipids and lip	Fatty Acyls	Fatty Acids a
4.36_434.21	434.218134	4.3621	pos	Armillaric aci	HMDB00370	92019	AOCKXACX(	Lipids and lip	Prenol lipids	Sesquiterper
4.36_563.31:	564.31969	4.3621	pos	Dihydroergo	HMDB0251308		SEALOBQTI	Alkaloids and	Ergoline and	Lysergic acid
4.38_445.24:	445.243192	4.38046667	pos	xi-Linalool 3-	HMDB00304	86963	SZTBSKBBY	Lipids and lip	Fatty Acyls	Fatty acyl gly
4.43_571.30	571.301499	4.43021667	neg	Abacavir	HMDB00151	315018	MCGSCOLB	Nucleosides,	Nucleoside a	Cyclopentyl r
4.45_313.09(	313.096328	4.44846667	neg	Benzoic acid, p-(9-acridiny		351644	FNNMCOQM	Unclassified	Unclassified	Unclassified
4.54_475.05:	475.053573	4.54091667	neg	8-Hydroxytricetin 7-glucuronide			GEISOXGZD	Lipids and lip	Polyketides	Flavonoids
4.63_436.28:	436.28308	4.63161667	pos	CPA(18:1(11	HMDB00070	58640	AXUULXHJK	Lipids and lip	Glycerophos	Glycerophos
4.63_515.27(	515.270627	4.63258333	neg	Propoxate	HMDB02568	330067	LKGPZAQFN	Organoheter	Azoles	Imidazoles
4.67_310.10	310.108553	4.66833333	pos	Methoprotryne		72846	DDUIUBPJP	Unclassified	Unclassified	Unclassified
4.76_208.04	208.044173	4.76071667	neg	2-Ethylbenzo	HMDB00400	94575	CVULGJIRG	Organoheter	Benzothiazol	Unclassified
4.81_291.00	291.001649	4.81485	neg	Isopentenyl p	HMDB00013	6181	NUHSROFQ	Lipids and lip	Prenol lipids	Isoprenoid pl
4.91_389.05	389.057789	4.90568333	neg	3,4,9,10-Per	HMDB0247483		KJOLVZJFM	Benzenoids	Phenanthren	Unclassified
4.91_427.11:	427.113785	4.90568333	neg	Nesbuvir	HMDB0255547		WTDWVLJJ.	Phenylpropa	2-arylbenzof	Unclassified
4.91_617.00(	617.00653	4.90568333	neg	Refametinib	HMDB0257134		RDSACQWT	Benzenoids	Benzene and	Sulfanilides
4.96_460.20	460.208481	4.95603333	pos	Hydrocortiso	HMDB0253247		BGSOJVFOI	Lipids and lip	Steroids and	Pregnane ste
5.16_240.14!	240.149269	5.15551667	pos	3'-Methyl-4-d	HMDB0246103		LVTFSVIRYI	Organoheter	Azobenzenes	Unclassified
5.22_425.22	425.227217	5.21915	neg	Octhilinone		72525	JPMIIZHYYV	Unclassified	Unclassified	Unclassified
5.32_505.29	505.291521	5.3192	pos	icas#19			CQSBSCBIG	Lipids and lip	Fatty Acyls	Fatty acyl gly
5.94_184.11!	184.115135	5.94078333	pos	4-Butyl-5-pro	HMDB00400	94608	AHPGPUJQI	Organoheter	Azoles	Thiazoles
6.60_431.17	431.177597	6.59813333	pos	(-)-alpha-Nar	HMDB00334	43280	AKNNEGZIB	Alkaloids and	Phthalide iso	Unclassified
6.65_350.18	350.184915	6.65295	pos	(9S,10E,12Z	HMDB0303982		JGUNZIWGI	Lipids and lip	Fatty Acyls	Lineolic acids
6.98_409.09:	409.093942	6.98325	pos	(3-(2-(4-(2-(T	HMDB0249906		AEDMQUAP	Benzenoids	Benzene and	Trifluorometh
7.60_387.06	387.06469	7.59848333	neg	(E)-3-[1-(Ber	HMDB0249930		NBHWQUHE	Phenylpropa	Cinnamic aci	Unclassified
7.64_419.03:	419.032482	7.63533333	neg	Methyl 1-(2-p	HMDB00330	88968	GAWXJHIAH	Organosulfu	Sulfoxides	Unclassified
8.76_879.54!	879.549025	8.75856667	pos	PE(22:5(4Z,7	HMDB0284156		AWNFSGUX	Unclassified	Unclassified	Unclassified
9.10_671.46!	671.46983	9.10213333	pos	19-Hexanoyloxymytiloxanthin			UFJHVUSMI	Lipids and lip	Prenol lipids	Isoprenoids
9.41_359.01:	359.013424	9.40613333	neg	trans-Propen	HMDB0303136		CKVNYTRY(	Organosulfu	Organic trisu	Unclassified
9.57_377.00	377.004542	9.56983333	neg	1-[[Difluoro-(:	HMDB0257902		BLAVPXRZC	Benzenoids	Benzene and	Halobenzene
0.61_148.11(	148.116039	0.60603333	pos	3-Methylcycl	HMDB00315	87746	PWHHTYJQ	Organosulfu	Thiols	Alkylthiols
0.61_413.15(	413.150498	0.61033333	neg	Diltiazem	HMDB00144	2268	HSUGRBWC	Organoheter	Benzothiaze	Unclassified
0.70_594.22:	594.223219	0.69861667	pos	Adomeglivan	HMDB0248020		FASLTMSUI	Benzenoids	Benzene and	Biphenyls an

0.74_391.06	409.094891	0.74288333	pos		Glucoconring	HMDB0038418		DYAQCRHE	Organic oxyg	Organooxyge	Carbohydrate
0.79_361.07	361.079072	0.7877	pos		7-Methylguar	HMDB00596	96234	AOKQNZVJ.	Organic acid	Carboxylic ac	Dicarboxylic
1.00_317.12	317.12543	0.99793333	neg		Tryptophyl-A	HMDB00290	85971	GRQCSEWE	Organic acid	Carboxylic ac	Amino acids,
1.07_318.07	318.076292	1.06741667	neg		Quinophthalone		984893	IZMJMCDDV	Unclassified	Unclassified	Unclassified
1.20_287.22	287.221588	1.20368333	pos		Isooctanedio	HMDB0061936		AOINFOWS.	Lipids and lip	Fatty Acyls	Fatty acid es
1.23_205.01	205.015444	1.23491667	neg		Dehydrochor	HMDB00363	91459	LKNPFOZIO	Benzenoids	Benzene anc	Phenoxyacet
1.25_263.04	263.044305	1.2521	neg		3-Methyl sulf	HMDB00596	343032	FAYFWMOS	Organoheter	Dihydrothiop	Unclassified
1.25_625.12	625.123767	1.2521	neg		Triazophos	HMDB02591	72443	AMFGTOFW	Organoheter	Azoles	Triazoles
1.31_275.04	275.04964	1.30545	neg		6-Methoxy-3-	HMDB00386	93293	FGXYYCWD	Organoheter	Indoles and c	Indoles
1.32_283.12	283.127645	1.32371667	neg		N-Desmethyl	HMDB02510	2944	NCLWFOLZ	Organoheter	Benzothiazin	Phenothiazin
1.32_347.10	347.109452	1.32371667	neg		indole-3-acet	HMDB0304380		DVJIJAYHBZ	Organic acid	Carboxylic ac	Amino acids,
1.36_274.02	274.02234	1.3586	neg		Meticrane	HMDB0254673		FNQQBFNIY	Organoheter	Thiochroman	Unclassified
1.38_328.14	328.142188	1.3848	pos		Dimethylphys	HMDB0243517		IUHMWLMD.	Organoheter	Indoles and c	Pyrroloindole
1.42_306.18	306.181122	1.41946667	pos		Imazamethal	HMDB00349	69069	FFCCBBNQI	Benzenoids	Benzene anc	Benzoic acid
1.44_305.08	305.080133	1.43671667	pos		Nevirapine	HMDB00143	43275	NQDJXKOV.	Organic nitro	Organonitrog	Amines
1.45_462.10	462.102308	1.45418333	pos		Dhghab	HMDB0243628		XIOUDSNTN	Phenylpropa	Coumarins a	Furanocoum
10.70_485.3	485.342162	10.6988167	neg		Triangulyne G			OURGQUM	Lipids and lip	Fatty Acyls	Fatty alcohol
10.73_895.5	894.547894	10.73455	neg		PS(20:4(6Z,8	HMDB0283121		AOVHKFVHI	Unclassified	Unclassified	Unclassified
10.97_534.2	534.281187	10.9697333	neg		PS-PE			HXZAJHUMI	Lipids and lip	Glycerophos	Oxidized glyc
11.13_620.3	620.35444	11.1325833	neg		OS-PC			YTDJKPAW	Lipids and lip	Glycerophos	Oxidized glyc
11.22_404.2	404.296675	11.2158833	pos		Spergualin	HMDB0258417		GDVNLLJNA	Organic acid	Carboxylic ac	Amino acids,
12.48_361.1	361.191583	12.4754333	neg		Benzetimide	HMDB02489	314266	LQQIVYSCP	Organoheter	Piperidines	Benzylpiperid
13.17_343.2	343.285166	13.17195	pos		3-Acetoxystearic acid			FCQLMPOG	Lipids and lip	Fatty Acyls	Fatty Acids a
13.92_716.5	716.521829	13.9195167	pos		PE(19:1(9Z)/15:1(9Z))		77033	BERWUBMU	Lipids and lip	Glycerophos	Glycerophos
2.09_353.13	353.138868	2.0924	neg		2',4'-Dihydroxy-3'-(1,1-dim		51814	ATYPSCXFV	Lipids and lip	Polyketides	Flavonoids
2.13_258.06	303.059614	2.1282	neg		4-(5-Fluoro-1	HMDB0245710		IFWLHIIUGS	Organoheter	Benzothiazol	Unclassified
2.13_435.94	435.947682	2.1282	neg		(6-Aminopuri	HMDB0258010		OBQGMEIRS	Organoheter	Imidazopyrim	Purines and
3.80_248.09	248.095434	3.80401667	pos		Thymol Sulfa	HMDB0062720		NODSEPOU	Organic acid	Organic sulfu	Arylsulfates
3.82_297.14	297.143109	3.82341667	neg		AG-879		96449	XRZYELWZI	Unclassified	Unclassified	Unclassified
3.89_555.27	556.280439	3.8922	pos		Butirosina	HMDB0248282		XEQLFNPSY	Organic oxyg	Organooxyge	Carbohydrate
3.98_445.23	445.235332	3.98231667	pos		5-methoxy-3-(2R-acetoxy-pentadecyl)-1			CVZNKLNAH	Lipids and lip	Polyketides	Phenolic lipic
4.11_506.95	506.959543	4.10951667	pos		[[(2S,5S)-5-(2	HMDB02581	3579	PGAVKCOV	Nucleosides,	Pyrimidine nu	Pyrimidine rib
4.43_458.15	458.154524	4.43021667	neg		Pavinetant	HMDB0256143		QYTBBBAHI	Organoheter	Quinolines ai	Phenylquinol

4.45_334.22	334.22251	4.45335	pos	2,3,4,5-tetranor-thromboxane B1			AXWSHCHV	Lipids and lip	Fatty Acyls	Fatty alcohol
4.54_517.22	517.223594	4.5416	pos	Ustiloxin D	HMDB0041054		GDXLZSYAC	Organic acid	Carboxylic ac	Amino acids,
4.70_327.17	327.171122	4.70481667	pos	Koenigine	HMDB00302	86798	CZZZOTXCA	Organoheter	Indoles and c	Carbazoles
4.81_423.17	423.17119	4.81226667	pos	Riociguat	HMDB0257236		WXXSNCNJ	Organoheter	Pyrazolopyric	Unclassified
4.87_501.25	501.259357	4.86546667	pos	5-deoxykaladasterone			HHSAPBUK	Lipids and lip	Steroids and	Sterols
4.88_247.09	247.099453	4.88355	pos	Benzamide, I	HMDB0256659		WECKJOND	Benzenoids	Benzene anc	Benzoic acid
5.03_446.25	446.251631	5.02876667	pos	PG(12:0/0:0)		79999	IACXMECMZ	Lipids and lip	Glycerophos	Glycerophos
5.88_359.06	359.069861	5.88396667	neg	Valdecoxib	HMDB00050	43886	LNPDTQAFD	Benzenoids	Benzene anc	Benzenesulfo
6.48_359.21	359.210698	6.47505	neg	2-Undecyl-4(	HMDB0032995		PVFSIEJKJI	Organoheter	Quinolines ar	Quinolones a
6.99_389.13	389.137295	6.98976667	neg	Melatonin glu	HMDB0060830		GXNGLFUTE	Nucleosides,	Nucleoside a	1-pyranosylin
7.22_262.02	262.023827	7.2217	pos	4-(Phosphon	HMDB0246313		LPMRCCND	Organic acid	Carboxylic ac	Amino acids,
7.41_387.19	387.194291	7.41378333	neg	Lincomycin	HMDB0015564		OJMMVQQU	Organic acid	Carboxylic ac	Amino acids,
7.57_454.13	454.135984	7.57063333	pos	Imidazo(5,1-I	HMDB0259507		NXNPDXUSI	Organoheter	Azoles	Pyrazoles
8.35_329.00	329.003949	8.34551667	neg	Pyrroloquinol	HMDB00136	63125	MMXZSJMA	Organoheter	Quinolines ar	Pyrroloquinol
8.72_971.54	971.545697	8.71673333	neg	PI(20:5(5Z,8Z	HMDB0277130		BTRCCWKK	Unclassified	Unclassified	Unclassified
9.74_495.15	495.151281	9.74061667	pos	Picrosid II, H	HMDB0246243		AKNILCMFR	Phenylpropa	Tannins	Hydrolyzable
9.74_515.13	515.135882	9.74061667	pos	2-Methyl-1-hy	HMDB00068	58571	SSYCSHKTI	Organoheter	Diazines	Pyrimidines a
0.59_192.09	192.094442	0.58926667	pos	Talipexole	HMDB02586	314723	DHSSDEDRI	Organoheter	Azepines	Unclassified
0.59_273.12	273.122913	0.58926667	pos	Methyl 6-met	HMDB00386	93300	ANFDUFMLI	Organoheter	Indoles and c	Carbazoles
0.62_240.02	240.029191	0.6215	pos	2-Thenoyltrifl	HMDB02453	336589	TXBBUSUX	Organic oxyg	Organooxyge	Carbonyl cor
0.64_223.05	223.052764	0.64391667	neg	2-Amino-5-nitrobenzopher		1570	PZPZDEIASI	Unclassified	Unclassified	Unclassified
0.64_468.98	468.982886	0.63663333	pos	Deoxyuridine	HMDB00011	6069	AHCYMLUZI	Nucleosides,	Pyrimidine nu	Pyrimidine de
0.67_315.01	315.017649	0.66691667	pos	4H-1,3,2-Ber	HMDB0249713		LKQSEFCGI	Organic acid	Organic phos	Phosphate es
0.72_439.08	439.08512	0.71928333	neg	9-Hydroxy-4-	HMDB00390	93653	BPZBSASYS	Phenylpropa	Coumarins a	Coumarin gly
0.80_286.09	286.096672	0.80285	pos	Flumazenil	HMDB00153	2724	OFBIFZUFA	Organoheter	Benzodiazep	1,4-benzodia
0.81_196.99	196.995876	0.81161667	neg	1H,1'H-2,2'-D	HMDB0245174		UIOKLEZTPI	Organoheter	Azoles	Imidazoles
0.81_328.10	328.107038	0.81161667	neg	KINETIN RIBOSIDE		43508	CAGLGYNQ	Unclassified	Unclassified	Unclassified
0.91_254.00	254.007389	0.9113	pos	HPAA sulfate	HMDB0240381		JCVGTUVGI	Organic acid	Organic sulfu	Arylsulfates
0.93_269.02	269.025549	0.92753333	neg	Terbufos	HMDB02588	72471	XLNZEKHUL	Organic acid	Organic dithi	Dithiophosph
0.98_273.14	273.143069	0.97735	pos	Madam	HMDB0249942		UABIXNSHH	Organosulfur	Thioethers	Aryl thioether
1.08_314.11	297.107969	1.07531667	pos	7,8-Dihydrop	HMDB00014	6228	WBFYVDCH	Organoheter	Pteridines ar	Pterins and c
1.20_421.04	421.047492	1.20368333	pos	Resorcinolnaphthalein		64793	FTUOFHGO	Unclassified	Unclassified	Unclassified
1.22_105.04	105.048795	1.2188	pos	N,N'-DIMETH	HMDB0255274		VLCDUOXHI	Organosulfur	Thioureas	Unclassified

1.23_435.10	435.103402	1.23451667	pos		Methyl (3x,4E	HMDB00408	95350		CDSGJPSZ\	Lipids and lip	Fatty Acyls	Fatty acyl gly
1.34_667.25	667.259944	1.34166667	neg		Isolimonic ac	HMDB00393	93942		KEITVHVZEI	Lipids and lip	Saccharolipic	Unclassified
1.45_573.14	573.145647	1.45418333	pos		Balanol; azepinostatin		985341		XYUFCXJZF	Unclassified	Unclassified	Unclassified
1.56_450.98	450.980668	1.56021667	neg		2,2'-Dithenyl	HMDB00329	88881		PVTVLOME(	Organoheter	Heteroaroma	Unclassified
1.58_409.09	409.097573	1.57865	neg		Bms-707035	HMDB0249320			VNIWZCGZI	Organoheter	Diazines	Pyrimidines a
1.61_285.24	285.242566	1.61191667	pos		16-oxo-heptadecanoic aci		74745		APFVJHNDS	Lipids and lip	Fatty Acyls	Fatty Acids a
1.78_230.95	230.959581	1.78133333	neg		3,5-dihydroxy	HMDB0126639			DMNOQQUL	Benzenoids	Benzene and	Benzoic acid
10.07_604.3	604.323131	10.0669833	pos		3-O-acetylecdysone 2-pho		57633		MYOZIWALI	Lipids and lip	Steroids and	Sterols
10.19_502.2	502.262718	10.1937667	pos		4-[(E)-2-(7-H	HMDB0254074			JEIWQRITH	Unclassified	Unclassified	Unclassified
10.41_822.5	822.577742	10.41135	pos		PC(P-20:0/17:2(9Z,12Z))		76545		CMXVZLDXI	Lipids and lip	Glycerophos	Glycerophos
10.43_907.5	907.529785	10.4295333	pos		Tilmicosin	HMDB0259095			JTSDBFGMI	Organic oxyg	Organooxyge	Carbohydrate
10.72_706.2	706.264209	10.7169	neg		PBD dimer	HMDB0256148			OMRPLUKQ	Organoheter	Benzodiazep	1,4-benzodia
10.90_355.2	355.204206	10.8978167	neg		(R,E)-S-2-acetamido-13-(r		65470		SKFVXBGH	Unclassified	Unclassified	Unclassified
11.77_321.2	321.219734	11.7739	neg		Bis(2-ethylhe	HMDB0249242			SEGLCEQV	Organic acid	Organic phos	Phosphate e
12.02_356.2	356.205297	12.0185833	neg		Farnesyl Thiosalicylic Acid		44840		GZTMFRUG	Unclassified	Unclassified	Unclassified
12.14_445.2	445.294838	12.1439667	pos		(23S,25R)-25-hydroxyvitar		42060		DBDDUVJJI	Lipids and lip	Steroids and	Secosteroids
12.25_415.2	415.261898	12.2531167	neg		9-Octadecen	HMDB0245757			PUPWWTU	Lipids and lip	Fatty Acyls	Fatty acid es
12.29_480.1	480.197694	12.29065	neg		2-(4-(2-Carb	HMDB0249866			PAOANWZG	Nucleosides,	Purine nucle	Unclassified
12.48_307.2	307.253511	12.4811	pos		Linoleic acid(d4)		96528		OYHQOLUK	Lipids and lip	Fatty Acyls	Fatty Acids a
13.11_337.2	337.205136	13.11485	neg		Lisuride	HMDB00147	1010		BKRGVLQU	Organoheter	Quinolines a	Indoloquinoli
14.62_283.1	283.107443	14.6196833	pos		O-(N-Phthali	HMDB0255867			GWPXUNTV	Organic oxyg	Organooxyge	Carbonyl cor
2.79_301.19	301.190991	2.78548333	pos		Phosphonic acid, tetradec		350022		BVQJQTMS	Unclassified	Unclassified	Unclassified
3.36_331.18	331.182211	3.36203333	pos		Acrivastine	HMDB0240231			PWACSDKD	Benzenoids	Benzene and	Styrenes
3.88_763.35	763.356345	3.8786	neg		Elaterinide	HMDB00358	91097		QKEJRKXVL	Lipids and lip	Steroids and	Steroidal glyc
3.90_424.12	424.121954	3.89685	neg		((4-(3-(4-Fluc	HMDB0242230			JLRFZRFZF	Benzenoids	Benzene and	Diphenylmetl
3.99_673.27	673.2743	3.98933333	neg		Linezolid	HMDB00147	315181		TYZROVQL	Organoheter	Oxazinanes	Morpholines
4.00_554.28	554.288269	4.00056667	pos		RCTR2				RJHKUGKYI	Lipids and lip	Fatty Acyls	Docosanoids
4.02_484.27	484.273049	4.01898333	pos		Verdamicin	HMDB0259789			XUSXOPRD	Organic oxyg	Organooxyge	Carbohydrate
4.02_574.29	574.293966	4.01898333	pos		Pexmetinib	HMDB0256362			LNMRSSIMC	Organoheter	Azoles	Pyrazoles
4.06_460.12	460.121338	4.05526667	pos		(-)-Catechin	HMDB00292	416949		LSHVYAFM	Phenylpropa	Linear 1,3-di	Chalcones a
4.07_285.20	285.204904	4.07303333	pos		Arginyllysine	HMDB00287	23816		JQFZHHSQ	Organic acid	Carboxylic a	Amino acids,
4.07_308.18	308.184425	4.07303333	pos		5-(4-Methylpl	HMDB0257964			NOEZOTVSI	Organoheter	Pyrazolopyri	Unclassified
4.09_538.26	556.297478	4.09135	pos		Prolyl-phenyl	HMDB0259666			FMZSPKHRI	Organic acid	Carboxylic a	Amino acids,

4.15_339.16!	339.165881	4.1538	neg	TIADENOL	HMDB0259066		WRCITXQN)	Organosulfur	Thioethers	Dialkylthioeth
4.28_881.41	881.414546	4.28308333	neg	PS(12:0/0:0)		78835	OBMNLLRGI	Lipids and lip	Glycerophos	Glycerophos
4.31_446.22	485.191298	4.30826667	pos	Acetylstropha	HMDB0247937		JLZAERUVC	Lipids and lip	Steroids and	Steroid lacto
4.49_721.39	721.397061	4.4888	pos	Cyclopassiflo	HMDB00383	93096	ZNQCKSPN	Lipids and lip	Prenol lipids	Terpene glyc
4.50_687.38	687.384019	4.50406667	neg	Formebolone	HMDB00046	41845	AMVODTGM	Lipids and lip	Steroids and	Androstane s
4.63_377.25	377.25251	4.63161667	pos	bhas#22			ARJGFPSUJ	Lipids and lip	Fatty Acyls	Fatty acyl gly
4.72_889.33	889.331816	4.72411667	neg	tetrahydrofola	HMDB03044	714	MSTNYGQP	Organic oxyg	Organooxyge	Carbonyl cor
4.72_900.32	900.323023	4.72411667	neg	BETA-AMAN	HMDB0249096		IEQCUEXVA	Organic acids	Carboxylic ac	Amino acids,
4.81_256.00	256.004718	4.81226667	pos	Tyramine-O-s	HMDB00064	58408	DYDUXGMD	Organic acids	Organic sulfu	Arylsulfates
4.92_360.20	360.207928	4.91941667	pos	5-Fluoro-adb	HMDB0247023		PWEKNGSN	Organic acids	Carboxylic ac	Amino acids,
5.14_481.26	481.263122	5.13745	pos	Citronellyl be	HMDB00328	88759	UPVBTYUC(	Lipids and lip	Prenol lipids	Terpene glyc
5.35_376.01	376.018157	5.3483	neg	Cefdinir	HMDB00146	313994	RTXOFQZKI	Organoheter	Lactams	Beta lactams
5.36_737.03	737.036896	5.35556667	pos	Phosphoribos	HMDB00036	3690	RKNHJBVBF	Nucleosides,	Purine nucle	Purine ribonu
5.98_1074.5	1074.5026	5.9774	pos	Hypertrehalo	HMDB0253308		NHXHBXCO	Organic Poly	Polypeptides	Unclassified
6.47_288.16	288.162388	6.47233333	pos	Convolamine		319087	AEFPCFUCI	Unclassified	Unclassified	Unclassified
6.99_797.29	797.298278	6.98976667	neg	UTILIN		43671	HPSILLNWM	Unclassified	Unclassified	Unclassified
7.28_309.08	309.084934	7.28471667	neg	7-Hydroxy-6-	HMDB0004256		COXMGTTX	Organoheter	Pteridines an	Unclassified
7.28_324.07	324.070056	7.28471667	neg	HHPAA gluc	HMDB0240379		FGIQLOOXJ	Organic oxyg	Organooxyge	Carbohydrate
7.98_319.07	319.079691	7.97648333	neg	(Z)-4-Methox	HMDB00303	86928	BKJYMZRGI	Phenylpropa	Stilbenes	Unclassified
8.25_347.01	347.013772	8.25326667	neg	9-(6-Sulfanyl	HMDB0257704		JQOXUQIJW	Organoheter	Imidazopyrim	Purines and
8.74_915.59	915.59937	8.74186667	pos	PI(22:4(7Z,10Z,13Z,16Z)/18:0)			CRHVNDIIVI	Lipids and lip	Glycerophos	Glycerophos
9.37_377.20	377.200461	9.36935	neg	Isopetasosid	HMDB00296	86382	AVZCSGMW	Lipids and lip	Prenol lipids	Terpene glyc
9.74_416.20	439.198974	9.74061667	pos	JP83		45144	JMLPLSJWS	Unclassified	Unclassified	Unclassified
9.74_442.17	442.17077	9.74061667	pos	Mandelonitril	HMDB00328	88725	QSPJUYWA	Organic oxyg	Organooxyge	Carbohydrate
0.63_199.06	199.069801	0.62835	neg	Methyldiphenylphosphine		430862	UJNZOIKQA	Unclassified	Unclassified	Unclassified
0.69_352.12	352.121566	0.68961667	neg	Parfumine		67836	AHNUBWYC	Unclassified	Unclassified	Unclassified
0.70_204.15	204.153526	0.69861667	pos	S-(2-(N,N-Di	HMDB02446	427264	LZIAMMQBH	Organosulfur	Isothioureas	Unclassified
0.70_257.05	256.049755	0.70456667	neg	4-(N,N-Dime	HMDB02445	417927	VPDDJYGQ.	Organoheter	Benzoxadiaz	Unclassified
0.70_489.16	490.176444	0.69861667	pos	(2S)-3-[3-[(4-	HMDB0258340		YHPJWEBFI	Benzenoids	Benzene and	Benzenesulfo
0.73_349.05	349.058256	0.7342	neg	2-(3,4-Dihydr	HMDB0243555		BYVWXQRJ	Phenylpropa	Flavonoids	Hydroxyflavo
0.79_386.11	386.112373	0.7877	pos	Cetefloxacin	HMDB0249830		LTDOHCZUI	Organoheter	Quinolines ar	Phenylquinol
0.86_299.03	299.037555	0.8592	neg	Djenkolic aci	HMDB0251505		JMQMNWIBI	Organic acids	Carboxylic ac	Amino acids,
1.03_236.13	236.13546	1.0341	neg	Dimethametryn		72343	IKYICRRUVI	Unclassified	Unclassified	Unclassified

1.17_291.01(	291.016789	1.16698333	neg	3-[3-(Sulfoox	HMDB0094710		IQWLPDPK\	Organic acid:	Organic sulfu	Arylsulfates
1.20_284.04!	284.044958	1.20271667	neg	S-(6-Purinyl)(	HMDB0244484		XGNASSAY(	Organic acid:	Carboxylic ac	Amino acids,
1.22_119.03:	119.031802	1.21911667	neg	2-Ethylbenze	HMDB00413	349852	ABROBCBII\	Benzenoids	Thiophenols	Unclassified
1.25_553.03!	553.038003	1.2521	neg	Ceftriaxone	HMDB0015343		VAAUVRVF(	Organic oxyg	Organooxyge	Carbohydrate
1.27_488.02(	488.026056	1.26985	neg	8-oxo-dGDP	HMDB00596	96251	LJMLTZSNW	Nucleosides,	Purine nucle	Purine deoxy
1.49_434.26:	457.251816	1.48828333	pos	Erinacine C	HMDB0040389		DMPGFSQM	Organoheter	Quinolines ar	Benzoquinoli
1.69_683.16(	683.166247	1.68893333	neg	Malvidin 3-(6	HMDB0301903		HXQOVGDX	Phenylpropa	Flavonoids	Flavonoid gly
10.28_870.5	870.547897	10.2812167	neg	PE(PGJ2/20:	HMDB0261851		APOJPGQG	Unclassified	Unclassified	Unclassified
10.59_949.6:	949.623585	10.5914167	pos	di-trans,poly-cis-undecapr(		53855	NTXGVHCC:	Lipids and lip	Prenol lipids	Polyprenols
10.86_478.2:	478.235242	10.8590333	pos	1-(2-methoxy-6Z-tetradecenyl)-sn-glyce			AKCHPDUNI	Lipids and lip	Glycerophos	Glycerophos
10.86_644.4!	644.492853	10.8590333	pos	adenosylhopane		53872	YCIUCIBXUZ	Lipids and lip	Prenol lipids	Hopanoids
11.15_500.3(	500.360643	11.1450667	pos	7-[(1R,2R,3R	HMDB0241874		FRBMLOLIV	Lipids and lip	Fatty Acyls	Eicosanoids
11.30_821.5(	821.566811	11.2956167	neg	Tecoflex	HMDB0258770		DSUFPYCIL:	Organic acid:	Carboxylic ac	Amino acids,
11.36_441.2(	441.263589	11.36075	pos	Lucidenic aci	HMDB0037611		INIPQDKLX(	Lipids and lip	Prenol lipids	Sesquiterper
11.89_522.3!	522.356072	11.8942667	pos	PC(18:1(6Z)/	HMDB0256159		ADOCFGYW	Lipids and lip	Glycerophos	Glycerophos
11.93_441.2!	441.297074	11.9300167	pos	26,26,26-trifluoro-25-hydrc		41965	QZGLVEKNI	Lipids and lip	Steroids and	Secosteroids
12.48_772.5:	772.522816	12.4811	pos	PE(17:0/18:0)		77447	AJNYYCRN\	Lipids and lip	Glycerophos	Glycerophos
13.05_459.3:	459.310457	13.0475833	pos	(-)-Asbestinine 2		53662	CCQWAVHY	Lipids and lip	Prenol lipids	Isoprenoids
14.53_209.1(	209.103119	14.53075	pos	2'',6''-O-Diac	HMDB0302015		BQADMILW'	Organoheter	Pteridines ar	Unclassified
2.11_310.00:	310.001842	2.11061667	neg	Flurochlorido	HMDB02523	68841	OQZCSNDV	Organoheter	Pyrrolidines	Phenylpyrroli
2.20_384.08:	384.083155	2.20205	neg	Pelargonidin	HMDB0301663		BGOQMKHC	Phenylpropa	Flavonoids	Flavonoid gly
2.53_322.08	322.084671	2.53481667	pos	2-[Methyl(3-p	HMDB00328	88765	APLMLPAZZ	Benzenoids	Benzene anc	Benzoic acid
2.57_510.25	510.254327	2.57026667	pos	L-Alaninamid	HMDB0259949		KQCBPHUO	Organic acid:	Carboxylic ac	Amino acids,
3.33_306.08!	306.089693	3.32576667	pos	Apomorphine	HMDB00148	1097	VMWNQDU\	Alkaloids anc	Aporphines	Unclassified
3.40_379.22:	379.222229	3.39796667	pos	Arachidonyl Trifluoromethy		63007	PLWROONZ	Unclassified	Unclassified	Unclassified
3.53_545.95	545.95397	3.52578333	pos	dGTP	HMDB00014	6248	HAAZLUGHY	Nucleosides,	Purine nucle	Purine deoxy
3.64_614.98	614.984726	3.64241667	neg	Urolithin a 3-	HMDB0240567		WMPNAWQ'	Phenylpropa	Coumarins a	Unclassified
3.69_312.11	312.117721	3.69436667	neg	10H-Pyrido(3	HMDB0250728		PLFSKOIHTI	Organic nitro	Organonitrog	Amines
3.75_344.10	344.107375	3.74966667	neg	Biotinyl-p-am	HMDB0249231		BAMKNWRZ	Organoheter	Biotin and de	Unclassified
3.79_354.16(	354.166205	3.78573333	pos	1,1'-(1,8-Dio:	HMDB0259675		GFTIUQBZN	Organoheter	Pyrrolidines	N-acylpyrrolic
3.84_445.13!	445.138971	3.83913333	pos	Albanin D	HMDB00388	93498	KDUZVQDA\	Phenylpropa	Flavonoids	Flavones
3.86_269.15!	269.159775	3.85685	pos	L-Leucine-p-i	HMDB0246659		AXZJHDNQI	Organic acid:	Carboxylic ac	Amino acids,
4.04_228.07!	228.07953	4.03688333	pos	Benzenesulf(	HMDB0247357		CBGXSOAP:	Benzenoids	Benzene anc	Benzenesulfo

4.04_434.22	434.226473	4.03688333	pos	PC(O-8:0/2:0)		40155	GNSZJQDO	Lipids and lip	Glycerophos	Glycerophos
4.10_626.21	626.211905	4.10018333	neg	Ustiloxin B	HMDB0041373		BISPUFPES	Lipids and lip	Prenol lipids	Diterpenoids
4.15_545.34	546.349129	4.14533333	pos	Cholylhistidir	HMDB0242389		GOBIDZCU	Lipids and lip	Steroids and	Bile acids, alc
4.16_643.30	643.306001	4.163	pos	[(S)-1-Carbo:	HMDB0246226		SDNYTAYIC	Organic acids	Carboxylic ac	Amino acids,
4.18_674.34	674.344683	4.1814	pos	PHOHA-PC			JZEIFJXAJN	Lipids and lip	Glycerophos	Oxidized glyc
4.20_529.28	529.280858	4.19935	pos	Sapacitabine	HMDB0257481		LBGFKUUH	Nucleosides,	Pyrimidine nu	Pyrimidine 2'
4.22_457.25	457.256898	4.21765	pos	PG(14:0/0:0)		80000	LUTDZDAPS	Lipids and lip	Glycerophos	Glycerophos
4.34_449.23	449.232854	4.34396667	pos	1-tauro-dinor-PGE2			LDYNTZRUZ	Lipids and lip	Fatty Acyls	Fatty amides
4.47_975.36	975.361363	4.46708333	neg	SERICETIN DIACETATE		43609	IIKLIRVROM	Unclassified	Unclassified	Unclassified
4.89_256.04	256.043889	4.88748333	neg	Benzo[d][2,1	HMDB0258137		RCZNTCRH	Benzenoids	Unclassified	Unclassified
4.90_413.15	413.150725	4.90175	pos	CP-465394		317535	PTMPFOHR	Unclassified	Unclassified	Unclassified
4.90_512.28	512.281619	4.90175	pos	Flumethason	HMDB00148	322955	JWRMHDSI	Lipids and lip	Steroids and	Pregnane ste
4.92_641.32	641.323976	4.91941667	pos	dolichyl beta-D-glucosyl pl		53860	AGLXCEGQ	Lipids and lip	Prenol lipids	Polyprenols
5.12_608.30	608.304301	5.11926667	pos	MeOSuc-Ala	HMDB0247307		VLVGCNNW	Organic acids	Carboxylic ac	Amino acids,
5.19_449.26	472.256354	5.19183333	pos	6-(3-(Pyrrolid	HMDB0251669		YMYJXFUP	Organoheter	Azoles	Oxazoles
5.34_646.32	646.321883	5.33735	pos	Aconitinum	HMDB0247962		XFSBVAOIA	Lipids and lip	Prenol lipids	Diterpenoids
5.36_241.05	242.05751	5.35556667	pos	(S)-2-Acetam	HMDB0243712		HLORMWAK	Organic acids	Carboxylic ac	Amino acids,
5.58_604.33	604.334861	5.57501667	pos	Cholylcitrullir	HMDB0242383		RTDQSSMY	Unclassified	Unclassified	Unclassified
7.26_272.08	295.070255	7.25836667	pos	beta-naphtho	HMDB0249144		OUGIDAPQ	Phenylpropa	Flavonoids	Flavones
8.51_333.20	333.203144	8.50668333	pos	Alizapride	HMDB00154	85516	KSEYRUGY	Organoheter	Benzotriazole	Unclassified
8.71_937.58	937.581945	8.7078	pos	PI(20:3(8Z,11Z,14Z)/22:4(7Z,10Z,13Z,1			FDCPTJFIJV	Lipids and lip	Glycerophos	Glycerophos
8.90_390.31	390.311482	8.90103333	pos	Finasteride	HMDB00019	2698	DBEPLOCGI	Lipids and lip	Steroids and	Androstane s
9.99_439.01	439.019291	9.98928333	neg	2,2'-Dithiodip	HMDB02453	452491	HAXFWIACA	Organoheter	Pyridines and	Unclassified
0.59_156.96	156.962747	0.59213333	neg	Magnesium (	HMDB03029	338241	UHNWOJJP	Organic acids	Carboxylic ac	Dicarboxylic
0.61_314.10	314.103996	0.60603333	pos	4-(Methylsulf	HMDB0241872		ZIQIIRWBM	Lipids and lip	Fatty Acyls	Fatty acid es
0.62_220.93	220.934654	0.6215	pos	Succimer	HMDB00147	984292	ACTRVOBW	Lipids and lip	Fatty Acyls	Fatty acids a
0.67_216.88	216.880685	0.6745	neg	Dicalcium ort	HMDB0303416		JHLNERQLK	Mixed metal/	Alkaline eartl	Alkaline eartl
0.68_354.99	354.994623	0.68255	pos	9-(6-Methylsı	HMDB0260403		VMKUMCCF	Organoheter	Imidazopyrim	Purines and
0.73_345.09	345.0966	0.7342	neg	7,2'-Dihydroxy-8,4'-dimeth		48314	BEHFIWGOI	Lipids and lip	Polyketides	Flavonoids
0.76_297.07	297.070245	0.76461667	neg	Febuxostat	HMDB02521	321691	BQSJTQLCZ	Organoheter	Azoles	Thiazoles
0.80_312.93	312.939642	0.79596667	neg	2,2'-Dithiobis	HMDB0244457		AFZSMODL	Organoheter	Benzothiazol	Unclassified
0.89_304.00	304.008033	0.8943	neg	AS-252424		45160	OYYVWND	Unclassified	Unclassified	Unclassified
0.93_230.06	230.064333	0.92753333	neg	TG003		96422	BGVLELSCI	Unclassified	Unclassified	Unclassified

0.99_291.16	291.169866	0.99473333	pos	Norgalanthar	HMDB0255738		AIXQQSTVC	Alkaloids and	Amaryllidace	Galanthamin
1.14_455.09	455.090258	1.13681667	pos	Naphthofluorescein		45440	IXQIUDNVF	Unclassified	Unclassified	Unclassified
1.15_267.16	267.160014	1.1535	pos	Epinastine	HMDB00148	320775	WHWZLSFA	Organoheter	Benzazepine	Dibenzazepi
1.15_339.03	339.030689	1.14921667	neg	4-[(2,4-Dihyd	HMDB00378	92643	MHKGJUOE	Organoheter	Azobenzenes	Unclassified
1.23_301.10	301.107581	1.23491667	neg	2'-Methoxy-4'-hydroxydihy		52247	CSEOFFVA	Lipids and lip	Polyketides	Flavonoids
1.23_429.06	429.063605	1.23491667	neg	Sulfacarbami	HMDB02585	314968	WVAKABMN	Benzenoids	Benzene and	Benzenesulf
1.23_536.18	536.184501	1.23451667	pos	Bopta	HMDB0249345		OEIYJWYTU	Organic acid	Carboxylic ac	Pentacarbox
1.23_598.14	598.149599	1.23491667	neg	Adtepp	HMDB0248031		UNZWIPPZC	Organic acid	Carboxylic ac	Amino acids,
1.27_447.07	447.073896	1.26985	neg	Psoberan	HMDB0302635		IAMQACMB	Phenylpropa	Coumarins a	Furanocoum
1.34_333.05	333.051406	1.34166667	neg	s-Nitroglutat	HMDB0257410		YCXQDCDF	Organic acid	Carboxylic ac	Amino acids,
1.34_392.09	392.091382	1.34166667	neg	(4R)-4-Hydro	HMDB0257914		ZMMDIASCS	Organoheter	Benzothiazol	Unclassified
1.34_644.26	644.269947	1.34166667	neg	OXONITINE		43770	SVQZFPSK	Unclassified	Unclassified	Unclassified
1.42_469.10	469.105811	1.41946667	pos	Pinakryptol Yellow		326399	ZXQHSPWB	Unclassified	Unclassified	Unclassified
1.44_255.13	255.139316	1.43671667	pos	3-Mercaptoh	HMDB00377	92584	KVXKOWZL	Lipids and lip	Fatty Acyls	Fatty acid es
1.54_480.25	480.256781	1.54085	pos	Aceroside VII; 1,7-Bis(4-hy		985454	MAUAGULX	Unclassified	Unclassified	Unclassified
1.91_367.05	367.050329	1.91026667	neg	GN25		96472	DIIRLGMLD	Unclassified	Unclassified	Unclassified
10.19_626.2	626.224534	10.18955	neg	Pirarubicin	HMDB0256586		KMSKQZKK	Phenylpropa	Anthracycline	Unclassified
11.15_283.1	283.152598	11.1450667	pos	N6-Methyl-2'	HMDB0255300		DYSDOYRQ	Nucleosides,	Purine nucle	Purine 2'-dec
12.48_1029.	1029.79543	12.4811	pos	TG(22:5(4Z,7	HMDB0055083		CMOULRWL	Lipids and lip	Glycerolipids	Triradylglyc
13.22_786.5	786.537139	13.2235667	pos	SM(d16:1/20	HMDB0290285		FJHJAHQSF	Unclassified	Unclassified	Unclassified
14.28_402.1	402.163182	14.2783333	neg	4-Hydroxytor	HMDB0246469		OIUCUUXSM	Phenylpropa	Stilbenes	Unclassified
2.02_420.99	420.995007	2.02081667	neg	Ceftezole	HMDB0249781		DZMVCVMF	Organoheter	Lactams	Beta lactams
2.02_488.98	488.982318	2.02081667	neg	6-Hydroxyluteolin 6,3'-dim		49683	QFXPVCDO	Lipids and lip	Polyketides	Flavonoids
2.68_441.20	441.203679	2.67816667	pos	Gamabufotalin		71464	FMTLOAVO	Lipids and lip	Steroids and	Sterols
3.45_336.20	336.2082	3.45331667	pos	Robalzotan	HMDB0257283		MQTUXRKN	Organoheter	Benzopyrans	1-benzopyra
3.47_264.16	264.16889	3.47126667	pos	Octopine		3377	IMXSCCDU	Unclassified	Unclassified	Unclassified
3.49_486.24	486.24938	3.48938333	pos	Exiguaflavanone E		52966	BDVHKISNF	Lipids and lip	Polyketides	Flavonoids
3.72_838.90	838.907544	3.71513333	pos	Iohexol	HMDB00154	832	NTHXOOBQ	Benzenoids	Benzene and	Anilides
3.86_1034.5	1034.55931	3.86	neg	Deforolimus	HMDB0242756		BUROJSBIW	Phenylpropa	Macrolide lac	Unclassified
3.92_364.14	364.149658	3.9154	neg	Propericiazin	HMDB00155	313618	LUALIOATIC	Organoheter	Benzothiazin	Phenothiazin
3.92_971.50	971.501346	3.9154	neg	PGP(22:6(4Z	HMDB0116476		ABHMVWXF	Lipids and lip	Glycerophos	Glycerophos
3.95_330.23	330.238316	3.94663333	pos	Oseltamivir	HMDB00143	66811	VSZGPKBB	Organic acid	Carboxylic ac	Amino acids,
3.96_336.20	336.205948	3.96453333	pos	18-hydroxyol	HMDB0304033		LQUHZVLTT	Lipids and lip	Fatty Acyls	Fatty acids a

4.01_289.05!	288.052404	4.0077	neg	GW 610		45012	ZRLSVQBGI	Unclassified	Unclassified	Unclassified
4.06_509.23	509.237132	4.05526667	pos	Gibberellin A	HMDB00386	93272	BQKJRWQL	Lipids and lip	Prenol lipids	Terpene glyc
4.09_824.36(	824.366213	4.09135	pos	Senktide	HMDB0258234		HMHYXLVEI	Organic acid	Carboxylic ac	Amino acids,
4.15_285.17(	285.170578	4.14533333	pos	LYSERGAMI	HMDB0254261		GENAHGKE	Alkaloids and	Ergoline and	Lysergic acid
4.22_506.22:	506.223518	4.21765	pos	Tobramycin	HMDB00148	2863	NLVFBUXFD	Organic oxyg	Organooxyge	Carbohydrate
4.78_689.35	689.351805	4.7769	pos	3-O-(alpha-L-rhamnopyranosyl-(1-2)-alp			FCBUKWW(	Lipids and lip	Fatty Acyls	Fatty acyl gly
5.03_225.07	225.074534	5.03453333	neg	2-Phenyl-3,4	HMDB0245290		NRYGFMAO	Organoheter	Thiochroman	Thioflavans
5.14_624.32!	624.325831	5.13745	pos	PC(20:4(5Z,8	HMDB0288817		AVRKLRJEB	Unclassified	Unclassified	Unclassified
5.72_205.02!	205.029097	5.71736667	neg	1-Hydroxyan	HMDB02438	65876	BTLXPCBPY	Benzenoids	Anthracenes	Anthraquinor
6.99_479.10(	479.106513	6.98976667	neg	Bentazone	HMDB02489	44591	ZOMSMJKL(	Benzenoids	Unclassified	Unclassified
6.99_829.07:	829.07342	6.98976667	neg	Granatin A	HMDB00392	93860	BEAQEKRA	Phenylpropa	Tannins	Hydrolyzable
7.19_438.21(	438.210485	7.19245	neg	Pivmecillinan	HMDB00155	321318	NPGNOVNW	Organic acid	Carboxylic ac	Amino acids,
7.41_487.19	487.192505	7.41378333	neg	Estriol succir	HMDB0251979		VBRVDDFOI	Lipids and lip	Steroids and	Steroid ester
7.96_516.25(	516.25084	7.95645	pos	N2-Maltulosy	HMDB00415	95925	ZAZHPBXBN	Lipids and lip	Saccharolipic	Unclassified
8.38_353.02:	353.023792	8.38236667	neg	3-butenylgluc	HMDB0304115		PLYQBXHVY	Organic oxyg	Organooxyge	Carbohydrate
9.93_377.25	377.254557	9.93468333	neg	9,10,13-Trihy	HMDB00309	87299	CDWBHGX(	Lipids and lip	Fatty Acyls	Fatty acids a
0.59_267.15(	267.150388	0.58926667	pos	Tropicamide	HMDB00149	43367	BGDKAVGW	Benzenoids	Benzene and	Phenylacetar
0.64_667.64	712.646951	0.64391667	neg	Cer(d18:0/24:0(2OH))		103012	DGORBCXE	Lipids and lip	Sphingolipids	Ceramides
0.75_380.00:	380.003282	0.74898333	neg	5'-Carboxy m	HMDB00607	416775	MTQQFYLG	Organoheter	Benzothiazin	Unclassified
0.82_406.13	406.134006	0.81883333	pos	Ixoside			AYRQUPRK	Lipids and lip	Prenol lipids	Isoprenoids
0.90_205.98	205.987224	0.89581667	pos	Saccharin	HMDB00297	43328	CVHZOJJKT	Organoheter	Benzothiazol	Unclassified
1.00_287.00	287.001959	0.99793333	neg	2-Anthraquin	HMDB0245031		MMNWSHJJ	Benzenoids	Anthracenes	Anthraquinor
1.01_319.17	319.178821	1.0117	pos	Phygrine	HMDB00397	94305	UBFAEXSSI	Alkaloids and	Unclassified	Unclassified
1.07_357.03	357.031248	1.06741667	neg	Doxantrazole	HMDB0251600		VIDCTSLIEZ	Organoheter	Benzothiopy	1-benzothiop
1.10_415.06:	396.045181	1.09681667	neg	Floridin	HMDB03036	44138	CZTQZXZIAI	Organoheter	Lactams	Beta lactams
1.22_382.11!	405.10908	1.2188	pos	Amppd	HMDB0248363		XYIPYISRNJ	Organic acid	Organic phos	Phosphate e
1.22_577.12	577.122455	1.21911667	neg	6,7,3',4'-Tetrahydroxyaurone 6-(4'',6''-di			ADZWGGD	Lipids and lip	Polyketides	Flavonoids
1.23_701.17(	701.169998	1.23491667	neg	Hexaacetylpyracanthoside			NJOSVEIEK	Lipids and lip	Polyketides	Flavonoids
1.29_250.00(	250.000232	1.2875	neg	3,4-Dihydro-!	HMDB02537	818666	DHUAGGSH	Organoheter	Benzothiophe	1-benzothiop
1.40_145.02!	145.029405	1.40198333	pos	Diethyl sulfor	HMDB0251241		MBDUIEKYV	Organosulfur	Sulfonyls	Sulfones
1.42_320.18!	320.185861	1.41946667	pos	Enterodiol	HMDB00050	319378	DWONJCND	Lignans, neo	Dibenzylbuta	Dibenzylbuta
1.43_354.08(	354.086511	1.43078333	neg	Deferasirox	HMDB00155	321511	BOFQWVMA	Organoheter	Azoles	Triazoles
1.54_160.07!	160.079088	1.54085	pos	2,5-Dimethyl-	HMDB00401	340053	DWAOFMNI	Organosulfur	Thioethers	Aryl thioether

10.01_337.1	337.155306	10.00765	neg		Picrinine	HMDB0256537		BDXYPHKG	Organoheter	Indoles and	Pyridoindoles
10.19_653.2	653.243625	10.18955	neg		Eruberin C			MQPDBDMS	Lipids and lip	Polyketides	Flavonoids
10.45_498.3	498.399334	10.4474333	pos		N-Nervonoyl	HMDB0242096		UBYPIVPBR	Organic acid	Carboxylic a	Amino acids,
10.81_494.2	494.241721	10.8069333	neg		2-Amino-N-[	HMDB0247859		HPZJMUBD	Organic acid	Carboxylic a	Amino acids,
10.93_570.3	570.352275	10.9300167	pos		PC(O-18:2(9Z,12Z)/2:0)			ICPPVEDR	Lipids and lip	Glycerophos	Glycerophos
11.58_488.3	488.334941	11.5754333	pos		LysoSM(d18	HMDB0006482		JLVSPVFPB	Lipids and lip	Sphingolipids	Phosphosphi
12.25_658.4	658.418003	12.25135	pos		PE(P-16:0/12:0)		77585	XVUVOVJN	Lipids and lip	Glycerophos	Glycerophos
12.30_329.2	329.239225	12.3037667	neg		1-Cyclohexyl	HMDB0254934		IGBRRSIHE	Phenylpropa	Stilbenes	Unclassified
12.76_598.4	621.399266	12.763	pos		DG(6 keto-P	HMDB0294464		BQRVNLCF	Unclassified	Unclassified	Unclassified
13.10_895.6	895.632058	13.1012833	pos		PC(MonoMe	HMDB0061442		AVVMCGZS	Lipids and lip	Glycerophos	Glycerophos
13.40_319.2	319.222188	13.39515	neg		Prostaglandin A2-d4		96522	MYHXHCUN	Unclassified	Unclassified	Unclassified
13.60_392.2	392.241675	13.59865	neg		CAY10590		45481	ANTPELWB	Unclassified	Unclassified	Unclassified
14.24_192.1	192.124257	14.2436833	pos		Minoxidil	HMDB0014494		ZFMITUMM	Organic nitro	Organonitrog	Amines
14.39_549.4	549.415436	14.3875333	pos		DG(20:4(5Z,	HMDB0294414		ABZISRGTW	Unclassified	Unclassified	Unclassified
2.08_262.06	262.069491	2.07525	neg		Propetamphos		72453	BZNDWPRG	Unclassified	Unclassified	Unclassified
2.11_276.04	276.048651	2.11061667	neg		2-Phthalimidoglutaric acid		2816	FEFFSKLJN	Unclassified	Unclassified	Unclassified
3.42_508.27	508.271208	3.41641667	pos		8-desoxy-19,20-epoxycytochalasin C			ABSZCAQH	Lipids and lip	Polyketides	Cytochalasin
3.45_420.22	420.221772	3.45331667	pos		Methyl 7-epi-	HMDB00317	87926	AJWIFVNLX	Lipids and lip	Fatty Acyls	Fatty acyl gly
3.51_345.21	345.213553	3.50783333	pos		PC(O-5:0/0:0)[R]		40394	FBCWZRYN	Lipids and lip	Glycerophos	Glycerophos
3.54_458.23	458.236229	3.54413333	pos		(1S-Endo)-5-	HMDB0242368		SUGVYNSR	Organoheter	Azoles	Pyrazoles
3.57_324.11	324.117478	3.57018333	neg		Rabeprazole	HMDB0244427		BSXAHDOW	Organoheter	Benzimidazo	Unclassified
3.59_480.98	480.988711	3.58845	neg		PD 0325901		45435	SUDAHWBC	Unclassified	Unclassified	Unclassified
3.61_524.13	524.134376	3.60671667	neg		Monohydrox	HMDB0254860		DVDAQKZL	Benzenoids	Phenol ester	Unclassified
3.68_421.05	421.051447	3.67736667	neg		5-Hydroxyin	HMDB0304936		YCHNMDFA	Unclassified	Unclassified	Unclassified
3.69_214.02	214.02733	3.69436667	neg		Tyrphostin A	HMDB02480	408702	DUQADSPE	Benzenoids	Phenols	Nitrophenols
3.82_403.09	403.097784	3.82341667	neg		Glyceryl lact	HMDB00322	88343	FADKMVWC	Organoheter	Azoles	Pyrazoles
4.04_520.26	520.266642	4.03688333	pos		PS(18:3(9Z,12Z,15Z)/0:0)		78856	JHARNNCE	Lipids and lip	Glycerophos	Glycerophos
4.10_1074.0	1074.03983	4.10018333	neg		2-eicosyl-3-hydroxy-28,29-epoxy-30-me			BIUFDHMW	Lipids and lip	Fatty Acyls	Fatty Acids a
4.15_516.25	499.252483	4.14533333	pos		Eriojaposide	HMDB0038029		AFWVBXLX	Lipids and lip	Fatty Acyls	Fatty acyl gly
4.33_471.25	471.259054	4.32651667	pos		alpha-Ionol	HMDB00387	93385	MITNBWXS	Lipids and lip	Fatty Acyls	Fatty acyl gly
4.38_448.21	448.210926	4.38046667	pos		15-isoLG hydroxylactam-ethanolamine			HDLBUPJK	Lipids and lip	Fatty Acyls	Eicosanoids
4.49_501.94	501.944307	4.4888	pos		Iodothyronin	HMDB0253535		ATBCREBG	Organic acid	Carboxylic a	Amino acids,
5.10_645.31	645.318646	5.10093333	pos		OA-PA			IHHQFWLU	Lipids and lip	Glycerophos	Oxidized gly

5.34_698.40(	721.389633	5.33735	pos	PI(12:0/12:0)			VMJRELYAN	Lipids and lip	Glycerophos	Glycerophos
6.77_450.20(	450.206902	6.76876667	neg	trequinsin	HMDB02591	321085	MCMSJVMU	Organoheter	Pyridopyrimi	Unclassified
6.89_862.46	862.46768	6.89188333	pos	PA(20:4(8Z,1	HMDB0265273		DXUQAUXO	Unclassified	Unclassified	Unclassified
6.95_446.13	446.138117	6.95326667	neg	Bacampicillin	HMDB00155	319852	PFOLLRNAD	Organoheter	Lactams	Beta lactams
7.26_316.24	316.242767	7.25836667	pos	Perhexiline	HMDB00152	44340	CYXKNKQEI	Organoheter	Piperidines	Unclassified
7.43_238.99	238.994747	7.43236667	neg	Ethoxzolamic	HMDB00144	85362	OUZWUKM(	Organoheter	Benzothiazol	Unclassified
9.37_527.30	527.301549	9.36935	neg	Neocucurbitacin E			HEOGQIJKU	Lipids and lip	Steroids and	Sterols
9.54_377.25	400.247286	9.54118333	pos	15-HETE-Gly		75524	AFWJZEQU	Lipids and lip	Fatty Acyls	Fatty amides
9.88_433.02	433.029386	9.8803	neg	Potassium cy	HMDB0303276		PALPTWOX	Organic acid	Sulfamic acic	Cyclamates
0.59_224.13	224.131764	0.58926667	pos	Prometryn	HMDB02568	44589	AAEVYOVX(	Organoheter	Triazines	1,3,5-triazine
0.70_400.11	383.115805	0.69861667	pos	1H-Pyrrole-2	HMDB02547	413590	OVSKGTON	Organoheter	Indoles and (	N-alkylindole
0.73_451.01(	451.016923	0.7342	neg	Bis(2-furanyl	HMDB00299	349711	CBJPZHSWI	Organoheter	Heteroaroma	Unclassified
0.76_396.05	396.053765	0.75766667	pos	grayanotoxin	HMDB0242624		GMLBVLXDI	Unclassified	Unclassified	Unclassified
0.89_276.07	276.077176	0.8943	neg	Indirubin-3'-n	HMDB02534	45531	FQCPPVRJF	Organoheter	Indoles and (	Hydroxyindol
1.08_435.04(	435.040146	1.08208333	neg	2-(2-Hydroxy	HMDB0304908		YSUPYJPCF	Organic acid	Organic sulfu	Arylsulfates
1.08_542.05	542.057561	1.07531667	pos	Cefminox	HMDB0249766		JSDXOWVA	Organoheter	Lactams	Beta lactams
1.09_446.15	446.151319	1.08996667	pos	[4,5-Bis(4-me	HMDB0252483		UBHKJRYGI	Organoheter	Azoles	Thiazoles
1.17_303.00	303.008917	1.16698333	neg	Sulprofos	HMDB02586	72767	JXHJNEJVU	Benzenoids	Benzene and	Phenoxy com
1.18_429.12(	429.125954	1.18473333	neg	Pamicogrel	HMDB02561	322213	ISCHOARKJ	Organic acid	Carboxylic a(	Amino acids,
1.20_386.98	386.985115	1.20271667	neg	trans-Resver	HMDB00417	96133	AUJFEUYHE	Phenylpropa	Stilbenes	Unclassified
1.34_328.11(	328.116708	1.34166667	neg	EVOXINE		44387	FGANMDNH	Unclassified	Unclassified	Unclassified
1.34_589.31	589.317148	1.34166667	neg	Prorepensin		51912	AMMAXRCD	Lipids and lip	Polyketides	Flavonoids
1.51_215.15	215.155323	1.50628333	pos	Siduron		72242	JXVIIQLNUF	Unclassified	Unclassified	Unclassified
1.63_329.19	329.197469	1.62985	pos	3-(((4,7-Dime	HMDB02445	422298	VDZJXIOFIS	Organoheter	Benzoxazole	Unclassified
10.63_306.1	306.151335	10.6275	pos	7-[(2-Benzyl-	HMDB0244471		KJVKOEVEF	Lipids and lip	Fatty Acyls	Fatty acids a
10.63_592.2	592.292954	10.6260167	neg	Dihydroergo(	HMDB0251309		DEQITUUQF	Alkaloids anc	Ergoline and	Lysergic acid
10.64_563.0	563.031694	10.6444833	neg	UDP-alpha-D	HMDB0304527		HSCJRCZFD	Nucleosides,	Pyrimidine nu	Pyrimidine nu
10.86_289.1	289.141797	10.8590333	pos	indole-3-acet	HMDB0304386		STGVBTJKC	Organic acid	Carboxylic a(	Amino acids,
10.90_181.0	181.01429	10.8950333	pos	3-(but-3-en-1-yn-1-yl)-6-methyl-1,2-dithi			SEXDKVHSI	Lipids and lip	Fatty Acyls	Oxygenated
11.11_526.3	549.34251	11.1098667	pos	Hemiasterlin	HMDB0253082		KQODQNJL	Organic acid	Carboxylic a(	Amino acids,
11.15_339.1	339.157332	11.1450667	pos	(2R,3R,4E)-2	HMDB0241865		OUASMDLFI	Lipids and lip	Fatty Acyls	Fatty acid es
11.25_373.2	373.237166	11.25255	pos	Mevastatin		44654	AJLFOPYRI	Unclassified	Unclassified	Unclassified
11.56_638.2	638.29746	11.5642333	neg	Protorifamycin I			DWSNNJAN	Lipids and lip	Polyketides	Ansamycins

11.58_320.1(	320.16705	11.5754333	pos	Olopatadine	HMDB00149	66742	JBIMVDZLSI	Organoheter	Benzoxepine	Dibenzoxepir
12.83_554.3!	577.37345	12.83355	pos	DG(PGD1/8:	HMDB0297252		AQRZFENHJ	Unclassified	Unclassified	Unclassified
2.02_470.22!	470.225317	2.02081667	neg	Propanamide	HMDB0254792		XIYPJXKEM	Phenylpropa	Stilbenes	Unclassified
2.11_683.26(	683.266496	2.11061667	neg	Tame	HMDB0258705		FKMJXALNH	Organic acid	Carboxylic ac	Amino acids,
3.38_214.08!	214.089845	3.38003333	pos	2-(4-Methyl-5	HMDB00324	88442	GDRZNYCK!	Organoheter	Azoles	Thiazoles
3.56_373.14	373.142925	3.56223333	pos	3-Pyridazinar	HMDB0259582		UVUYWJWY	Organoheter	Piperidines	Benzylpiperic
3.58_358.17!	358.179382	3.58011667	pos	6-Acetyloxy-3	HMDB0247032		DNRCGFUT	Organoheter	Benzopyrans	1-benzopyrar
3.62_492.09	492.091691	3.62421667	neg	cyanidin 5-O-	HMDB0304305		GMRSKDNJ	Phenylpropa	Flavonoids	Flavonoid gly
3.66_475.25	475.252385	3.66498333	pos	Eleutherazine B; N,N'-((3,6		985542	CNQCBOHT	Unclassified	Unclassified	Unclassified
3.68_564.98	564.984702	3.67736667	neg	5-(3-Hydroxy	HMDB02467	406400	PTDJNLYHC	Organic oxyg	Organooxyge	Carbonyl cor
3.73_551.06!	551.065045	3.73238333	pos	UDP-L-rham	HMDB00123	62924	DRDCJEIZVI	Nucleosides,	Pyrimidine nu	Pyrimidine nu
3.75_1035.5	1035.54906	3.74966667	neg	PI(22:4(10Z,1	HMDB0277966		DDKKRJZQE	Unclassified	Unclassified	Unclassified
3.75_372.21	372.218906	3.75006667	pos	N3-Cyclopro	HMDB0250047		AVXQPEKZI	Organoheter	Diazanaphth	Benzodiazine
3.80_479.11	479.114397	3.80496667	neg	Sivelestat	HMDB02583	320535	BTGNGJJLZ	Benzenoids	Benzene and	Benzoic acid
3.84_279.18	302.171425	3.83913333	pos	Vanillyl octar	HMDB0302471		CMUQEPIYT	Benzenoids	Phenols	Methoxypher
3.84_422.21!	422.219207	3.83913333	pos	Ozanimod	HMDB0256027		XRVDGNKR	Organoheter	Azoles	Oxadiazoles
3.86_774.34	774.34753	3.86	neg	Jubanine B	HMDB00302	86797	FJSKSYVNU	Organic acid	Carboxylic ac	Amino acids,
3.91_958.50!	958.509468	3.91066667	pos	PGP(TXB2/1	HMDB0272954		FMNAEAMV	Unclassified	Unclassified	Unclassified
3.98_501.27(	501.270316	3.98231667	pos	Caffeoylferul	HMDB00334	89297	YUUGXIVVD	Phenylpropa	Cinnamic aci	Hydroxycinna
3.98_559.55(	559.555998	3.98231667	pos	4,8,16-Trimethyltetratriaco		97818	DYIKXRQRJ	Lipids and lip	Fatty Acyls	Hydrocarbon
3.99_753.39	753.394436	3.98933333	neg	Dioncophyllir	HMDB0251430		MXIZZLBQR	Organoheter	Isoquinolines	Naphthylisoq
3.99_841.37	841.373763	3.98933333	neg	FIPI		45560	LHABRXRGI	Unclassified	Unclassified	Unclassified
4.04_367.23!	367.235461	4.03688333	pos	N6-lauroyl lysine			GYDYJUYZE	Lipids and lip	Fatty Acyls	Fatty amides
4.06_421.21	421.214631	4.05526667	pos	(3S,5R,6S)-5	HMDB03039	53818	CAXVJDRXJ	Lipids and lip	Prenol lipids	Diterpenoids
4.08_964.44(	964.440816	4.08158333	neg	PGP(LTE4/1	HMDB0272431		GMIVCVSIN	Unclassified	Unclassified	Unclassified
4.09_431.02(	431.026403	4.09135	pos	6-Benzyl-1-(t	HMDB0249929		DZMYZUPN!	Organoheter	Diazines	Pyrimidines a
4.11_506.26	506.264577	4.10951667	pos	1-(2-methoxy-5Z-hexadecenyl)-sn-glyce			GIYHJKMZJI	Lipids and lip	Glycerophos	Glycerophos
4.12_1029.5!	1029.59587	4.11676667	neg	PI(22:3(10Z,1	HMDB0277837		DKXIVVRPT	Unclassified	Unclassified	Unclassified
4.33_600.31	600.313415	4.32651667	pos	PI(17:2(9Z,12Z)/0:0)			CXHDGZFL\	Lipids and lip	Glycerophos	Glycerophos
4.36_1153.5!	1153.59221	4.35616667	neg	PIM2(18:2(9Z,12Z)/16:2(9Z,12Z))			XWZYGZRT	Lipids and lip	Glycerophos	Glycerophos
4.50_125.02	125.024186	4.50406667	neg	3,6-Dimethyl-	HMDB0246077		JJTUDXZGH	Organoheter	Dioxanes	1,4-dioxanes
4.65_244.17	245.185763	4.64996667	pos	N-(6-aminohexanoyl)-6-an		45748	IWENLYKHS	Lipids and lip	Fatty Acyls	Other Fatty A
4.70_1012.9!	1012.98009	4.70481667	pos	Guanosine h	HMDB00015	58056	QESDWYZA	Nucleosides,	(5'->5')-dinuc	Unclassified

4.74_452.03!	452.035136	4.74081667	pos	4-Methylsulfc	HMDB0301922		ONOWOZM(	Organic oxyg	Organooxyge	Carbohydrate
4.83_268.13	268.137667	4.83273333	neg	2-(4-Methyl-5	HMDB00324	437328	OGJATLSJIN	Organoheter	Azoles	Thiazoles
5.14_622.34(	622.346292	5.13745	pos	1-(2-methoxy-heneicosanyl)-sn-glycero-			PREPKQNG	Lipids and lip	Glycerophos	Glycerophos
5.15_256.02(	256.020429	5.14531667	neg	Lorcaserin su	HMDB0061153		YZUNJIOOIH	Organoheter	Benzazepine	Unclassified
5.45_514.97	514.971655	5.44698333	pos	2'-Deoxyinos	HMDB00035	438284	UFJPAQSLH	Nucleosides,	Purine nucle	Purine deoxy
5.52_449.27	449.271762	5.52015	pos	Umbelliferyl Arachidonate		63019	SFTGFGOB(	Unclassified	Unclassified	Unclassified
5.96_334.24	334.248489	5.95905	pos	Dodecylphosphocholine		34530	QBHFVMDLI	Unclassified	Unclassified	Unclassified
6.11_375.15(	375.156	6.1056	neg	Z-PP-CHO	HMDB0259973		ORZXYSPO	Organic acids	Carboxylic ac	Amino acids,
6.23_473.17	473.179164	6.23498333	neg	RHODOMYRTOXIN		44391	DEUFHKWB	Unclassified	Unclassified	Unclassified
6.33_406.16	406.168734	6.32576667	pos	Dihydrozeatir	HMDB0012213		DRPMMLWY	Organic oxyg	Organooxyge	Carbohydrate
6.59_647.12(	647.120519	6.58565	neg	Grevilline A	HMDB0029535		PZEIKFYZAU	Organoheter	Pyrans	Pyranones ar
7.19_693.17(	693.17051	7.19245	neg	Syringetin 3-(6'''-acetylglucosyl)(1->3)-g			BHRTWWYZ	Lipids and lip	Polyketides	Flavonoids
8.69_527.31(	527.316607	8.68985	pos	3-O-alpha-L-rhamnopyranosyl-3-hydrox			PPMPLIBYTI	Lipids and lip	Fatty Acyls	Fatty acyl gly
8.71_955.20	955.204937	8.7071	neg	(R)-carnitinyl-CoA betaine			BBRISSLDTU	Lipids and lip	Fatty Acyls	Fatty esters
9.74_277.09	277.095414	9.74061667	pos	Cloethocarb	HMDB02503	72711	PITWUHDDI	Benzenoids	Benzene anc	Phenyl methy
9.78_881.51	881.513635	9.77705	pos	PI(18:3(9Z,12Z,15Z)/18:1(9Z))			CIBWCGFXI	Lipids and lip	Glycerophos	Glycerophos
0.50_115.11	115.111975	0.50076667	pos							
0.50_115.96	115.967948	0.50076667	pos							
0.50_117.01	117.019089	0.50026667	neg							
0.50_126.95	126.955653	0.50076667	pos							
0.50_167.03	167.034071	0.50076667	pos							
0.50_207.05(	207.050724	0.50026667	neg							
0.50_248.90	248.901668	0.50076667	pos							
0.50_452.84	452.849845	0.50026667	neg							
0.52_136.94(	136.946134	0.51898333	pos							
0.52_142.99(	142.995954	0.5187	neg							
0.52_176.95	176.959429	0.51898333	pos							
0.52_178.09	161.096096	0.51898333	pos							
0.52_200.96	200.965049	0.5187	neg							
0.52_304.89	304.894019	0.51898333	pos							
0.52_445.12(	445.120101	0.51898333	pos							
0.54_114.97	114.971695	0.53628333	pos							
0.54_125.96	125.964348	0.53628333	pos							

0.54_131.92[illegible]	131.929887	0.53628333	pos
0.54_133.01[illegible]	133.014057	0.53716667	neg
0.54_140.98[illegible]	140.980387	0.53716667	neg
0.54_171.17[illegible]	171.174312	0.53628333	pos
0.54_172.92[illegible]	172.929328	0.53716667	neg
0.54_190.88[illegible]	190.888102	0.53716667	neg
0.54_223.13[illegible]	223.131677	0.53628333	pos
0.54_253.91[illegible]	236.906914	0.53628333	pos
0.54_384.86[illegible]	384.862184	0.53716667	neg
0.54_469.83[illegible]	469.83891	0.53716667	neg
0.54_531.86[illegible]	531.867877	0.53716667	neg
0.54_588.82[illegible]	588.82394	0.53716667	neg
0.54_598.85[illegible]	598.85335	0.53716667	neg
0.54_666.84[illegible]	666.840858	0.53716667	neg
0.55_140.04[illegible]	140.042497	0.55448333	pos
0.55_170.03[illegible]	170.036812	0.55448333	pos
0.55_172.95[illegible]	172.956436	0.55448333	pos
0.55_193.12[illegible]	193.12193	0.55448333	pos
0.56_141.01[illegible]	141.019143	0.55561667	neg
0.56_210.98[illegible]	210.983372	0.55561667	neg
0.56_615.84[illegible]	615.841877	0.55561667	neg
0.57_113.96[illegible]	113.963991	0.57178333	pos
0.57_114.95[illegible]	114.956556	0.57178333	pos
0.57_114.96[illegible]	114.967246	0.57178333	pos
0.57_118.94[illegible]	118.942894	0.57178333	pos
0.57_129.00[illegible]	130.00821	0.57178333	pos
0.57_139.96[illegible]	139.963362	0.57178333	pos
0.57_145.95[illegible]	145.954602	0.57178333	pos
0.57_147.93[illegible]	147.939098	0.57393333	neg
0.57_152.04[illegible]	152.04631	0.57393333	neg
0.57_153.95[illegible]	153.955966	0.57178333	pos
0.57_158.96[illegible]	141.958682	0.57178333	pos
0.57_161.96[illegible]	161.969627	0.57178333	pos

0.57_170.961	170.961512	0.57178333	pos
0.57_180.989	180.989871	0.57178333	pos
0.57_181.122	181.122435	0.57178333	pos
0.57_182.985	182.985247	0.57178333	pos
0.57_188.445	188.445922	0.57178333	pos
0.57_188.932	188.932883	0.57393333	neg
0.57_193.623	193.623685	0.57178333	pos
0.57_208.943	208.94386	0.57178333	pos
0.57_231.120	231.120052	0.57178333	pos
0.57_281.663	281.663362	0.57178333	pos
0.57_411.881	411.880956	0.57393333	neg
0.57_479.868	479.868132	0.57393333	neg
0.57_488.886	488.886839	0.57393333	neg
0.57_490.884	490.883994	0.57393333	neg
0.57_515.906	515.906308	0.57393333	neg
0.57_547.855	547.855392	0.57393333	neg
0.57_566.903	566.903013	0.57393333	neg
0.57_583.893	583.893545	0.57393333	neg
0.57_635.897	634.890609	0.57393333	neg
0.57_703.885	702.878113	0.57393333	neg
0.57_712.835	712.835353	0.57393333	neg
0.57_770.865	770.865375	0.57393333	neg
0.59_101.950	101.950365	0.58926667	pos
0.59_116.972	116.972186	0.58926667	pos
0.59_117.990	117.99075	0.58926667	pos
0.59_121.012	122.019524	0.58926667	pos
0.59_121.993	121.993759	0.58926667	pos
0.59_124.024	124.024581	0.58926667	pos
0.59_132.003	132.003672	0.58926667	pos
0.59_136.909	136.909596	0.59213333	neg
0.59_140.961	140.961756	0.58926667	pos
0.59_141.006	141.006543	0.58926667	pos
0.59_142.976	142.976452	0.58926667	pos

0.59_143.03	143.039678	0.58926667	pos
0.59_143.95	143.95909	0.58926667	pos
0.59_144.00	145.009632	0.58926667	pos
0.59_145.03	145.035376	0.58926667	pos
0.59_148.94	148.949511	0.59213333	neg
0.59_149.95	149.952849	0.58926667	pos
0.59_149.99	194.989498	0.59213333	neg
0.59_150.04	150.047569	0.58926667	pos
0.59_150.94	150.946718	0.59213333	neg
0.59_150.98	150.98265	0.59213333	neg
0.59_151.94	151.949915	0.58926667	pos
0.59_153.99	153.990511	0.58926667	pos
0.59_164.92	164.927027	0.59213333	neg
0.59_168.02	168.025498	0.58926667	pos
0.59_173.03	173.030168	0.58926667	pos
0.59_173.11	173.11808	0.59213333	neg
0.59_180.89	180.899441	0.59213333	neg
0.59_182.89	182.896289	0.59213333	neg
0.59_188.11	188.111353	0.58926667	pos
0.59_191.94	191.946067	0.59213333	neg
0.59_192.59	192.595782	0.58926667	pos
0.59_195.62	195.621133	0.58926667	pos
0.59_196.12	196.122811	0.58926667	pos
0.59_197.99	178.977593	0.59213333	neg
0.59_200.91	200.919338	0.59213333	neg
0.59_202.62	202.62897	0.58926667	pos
0.59_203.13	203.130209	0.58926667	pos
0.59_206.97	206.972432	0.59213333	neg
0.59_209.62	209.624116	0.58926667	pos
0.59_210.13	210.134262	0.58926667	pos
0.59_210.95	210.950656	0.58926667	pos
0.59_215.99	215.993504	0.58926667	pos
0.59_216.93	216.936881	0.59213333	neg

0.59_217.142	217.142069	0.58926667	pos
0.59_221.930	221.930745	0.58926667	pos
0.59_221.95	244.946796	0.58926667	pos
0.59_222.98	222.98461	0.59213333	neg
0.59_224.63	224.633631	0.58926667	pos
0.59_224.95	224.950259	0.59213333	neg
0.59_232.92	232.923197	0.58926667	pos
0.59_235.97	235.973996	0.58926667	pos
0.59_239.13	239.137065	0.58926667	pos
0.59_245.95	245.95377	0.59213333	neg
0.59_250.95	250.949988	0.58926667	pos
0.59_258.14	258.144828	0.58926667	pos
0.59_258.98	258.989854	0.58926667	pos
0.59_259.65	259.650342	0.58926667	pos
0.59_259.93	259.933521	0.59213333	neg
0.59_260.14	260.147939	0.58926667	pos
0.59_262.09	262.098395	0.58926667	pos
0.59_265.93	265.930136	0.58926667	pos
0.59_272.62	272.620934	0.58926667	pos
0.59_272.94	255.939024	0.58926667	pos
0.59_282.00	282.005619	0.58926667	pos
0.59_285.93	284.924517	0.59213333	neg
0.59_288.65	288.653017	0.58926667	pos
0.59_294.14	294.142875	0.58926667	pos
0.59_294.64	294.644309	0.58926667	pos
0.59_294.87	294.879744	0.59213333	neg
0.59_294.95	294.953199	0.59213333	neg
0.59_296.96	296.965393	0.58926667	pos
0.59_299.90	344.901899	0.59213333	neg
0.59_300.90	300.90261	0.59213333	neg
0.59_301.91	301.914867	0.59213333	neg
0.59_304.90	304.909595	0.59213333	neg
0.59_311.94	311.943496	0.59213333	neg

0.59_321.903	321.903264	0.59213333	neg
0.59_327.921	327.92095	0.59213333	neg
0.59_336.941	336.941708	0.59213333	neg
0.59_345.674	345.674492	0.58926667	pos
0.59_353.919	352.91194	0.59213333	neg
0.59_354.908	354.908947	0.59213333	neg
0.59_360.925	360.92518	0.59213333	neg
0.59_362.940	362.940949	0.59213333	neg
0.59_388.875	388.875414	0.59213333	neg
0.59_398.904	398.90461	0.59213333	neg
0.59_408.933	408.933705	0.59213333	neg
0.59_421.900	420.899236	0.59213333	neg
0.59_430.928	430.928094	0.59213333	neg
0.59_440.884	440.884203	0.59213333	neg
0.59_457.874	457.874207	0.59213333	neg
0.59_476.921	476.921062	0.59213333	neg
0.59_493.911	493.911566	0.59213333	neg
0.59_499.923	498.915771	0.59213333	neg
0.59_508.872	508.87196	0.59213333	neg
0.59_525.861	525.861785	0.59213333	neg
0.59_562.904	562.904217	0.59213333	neg
0.59_576.859	576.859476	0.59213333	neg
0.59_593.849	593.849627	0.59213333	neg
0.59_608.877	608.877687	0.59213333	neg
0.59_624.856	624.856349	0.59213333	neg
0.59_628.873	628.873304	0.59213333	neg
0.59_631.913	612.896104	0.59213333	neg
0.59_661.836	661.83691	0.59213333	neg
0.59_675.869	675.869334	0.59213333	neg
0.59_690.843	690.843061	0.59213333	neg
0.61_100.024	100.024847	0.60603333	pos
0.61_102.970	102.97071	0.60603333	pos
0.61_105.003	105.003712	0.60603333	pos

0.61_108.479	108.479286	0.60603333	pos
0.61_108.988	108.988533	0.61033333	neg
0.61_109.479	109.479367	0.60603333	pos
0.61_109.579	109.579063	0.60603333	pos
0.61_109.979	109.979223	0.60603333	pos
0.61_111.012	111.012256	0.60603333	pos
0.61_115.578	115.578901	0.60603333	pos
0.61_115.989	115.989358	0.61033333	neg
0.61_116.035	116.035109	0.61033333	neg
0.61_116.986	116.98613	0.61033333	neg
0.61_117.484	117.48441	0.60603333	pos
0.61_117.576	117.576344	0.60603333	pos
0.61_117.983	117.983871	0.60603333	pos
0.61_118.484	118.484463	0.60603333	pos
0.61_118.984	118.98418	0.60603333	pos
0.61_120.981	120.981129	0.60603333	pos
0.61_124.043	124.043898	0.60603333	pos
0.61_124.120	163.083947	0.60603333	pos
0.61_124.584	124.5841	0.60603333	pos
0.61_125.982	126.989824	0.60603333	pos
0.61_126.594	126.594868	0.60603333	pos
0.61_127.012	110.009062	0.60603333	pos
0.61_130.491	130.491903	0.60603333	pos
0.61_130.983	130.98344	0.61033333	neg
0.61_132.998	132.998274	0.60603333	pos
0.61_134.100	134.100566	0.60603333	pos
0.61_138.107	138.107564	0.60603333	pos
0.61_139.497	139.497212	0.60603333	pos
0.61_140.043	123.040609	0.60603333	pos
0.61_142.086	142.086283	0.60603333	pos
0.61_142.589	142.589645	0.60603333	pos
0.61_142.989	125.986497	0.60603333	pos
0.61_144.042	145.049768	0.60603333	pos

0.61_144.10	144.102077	0.60603333	pos
0.61_146.03	146.030107	0.60603333	pos
0.61_146.08	146.087212	0.60603333	pos
0.61_146.96	146.961376	0.61033333	neg
0.61_149.59	149.597391	0.60603333	pos
0.61_151.03	151.03529	0.60603333	pos
0.61_152.03	170.066282	0.60603333	pos
0.61_152.90	152.906883	0.60603333	pos
0.61_154.90	154.907514	0.60603333	pos
0.61_154.97	154.97406	0.61033333	neg
0.61_157.00	158.012308	0.60603333	pos
0.61_159.03	142.035055	0.60603333	pos
0.61_160.98	160.982339	0.61033333	neg
0.61_161.09	161.092852	0.61033333	neg
0.61_163.58	163.58562	0.60603333	pos
0.61_167.01	167.012892	0.60603333	pos
0.61_168.44	168.44454	0.60603333	pos
0.61_173.62	173.62619	0.60603333	pos
0.61_174.95	174.955859	0.61033333	neg
0.61_175.96	175.968455	0.61033333	neg
0.61_176.96	176.960228	0.61033333	neg
0.61_178.94	178.948798	0.61033333	neg
0.61_179.90	179.902872	0.60603333	pos
0.61_181.60	181.605384	0.60603333	pos
0.61_181.90	181.903048	0.60603333	pos
0.61_182.05	182.057403	0.60603333	pos
0.61_182.90	182.902443	0.60603333	pos
0.61_182.96	182.96218	0.60603333	pos
0.61_188.61	188.613187	0.60603333	pos
0.61_198.94	198.940053	0.60603333	pos
0.61_214.91	214.917575	0.60603333	pos
0.61_216.63	216.632005	0.60603333	pos
0.61_217.97	200.972497	0.60603333	pos

0.61_219.13	219.134013	0.60603333	pos
0.61_222.92	223.928918	0.60603333	pos
0.61_226.96	226.965837	0.61033333	neg
0.61_228.96	228.969702	0.61033333	neg
0.61_230.07	230.075515	0.61033333	neg
0.61_231.91	276.913676	0.61033333	neg
0.61_232.60	255.596208	0.60603333	pos
0.61_233.95	216.950728	0.60603333	pos
0.61_233.96	233.966637	0.60603333	pos
0.61_240.99	241.999612	0.60603333	pos
0.61_242.94	242.943367	0.61033333	neg
0.61_244.61	244.612095	0.60603333	pos
0.61_244.96	244.96363	0.61033333	neg
0.61_245.06	245.062626	0.61033333	neg
0.61_247.15	230.148225	0.60603333	pos
0.61_247.89	230.88998	0.60603333	pos
0.61_248.92	271.91646	0.60603333	pos
0.61_249.04	249.049681	0.61033333	neg
0.61_251.16	251.160169	0.60603333	pos
0.61_255.09	255.094935	0.60603333	pos
0.61_256.96	239.966613	0.60603333	pos
0.61_259.07	259.078354	0.61033333	neg
0.61_259.14	259.140953	0.61033333	neg
0.61_262.13	262.139796	0.60603333	pos
0.61_262.93	262.930012	0.61033333	neg
0.61_265.01	265.015559	0.60603333	pos
0.61_268.04	268.042511	0.61033333	neg
0.61_272.89	272.89875	0.61033333	neg
0.61_272.95	272.958959	0.61033333	neg
0.61_273.13	274.139768	0.60603333	pos
0.61_274.14	274.140975	0.61033333	neg
0.61_283.56	283.563104	0.60603333	pos
0.61_285.95	286.95841	0.60603333	pos

0.61_287.084	287.084753	0.61033333	neg
0.61_288.073	271.070028	0.60603333	pos
0.61_288.936	288.936335	0.61033333	neg
0.61_289.949	289.948954	0.61033333	neg
0.61_291.028	291.028563	0.61033333	neg
0.61_300.065	301.073197	0.60603333	pos
0.61_300.117	300.117582	0.61033333	neg
0.61_301.019	301.019599	0.61033333	neg
0.61_305.926	305.926345	0.61033333	neg
0.61_306.634	306.634111	0.60603333	pos
0.61_307.151	307.151089	0.61033333	neg
0.61_308.218	308.217982	0.60603333	pos
0.61_308.631	308.631842	0.60603333	pos
0.61_309.974	309.97436	0.60603333	pos
0.61_310.930	310.930834	0.61033333	neg
0.61_312.951	312.951341	0.61033333	neg
0.61_314.605	314.605708	0.60603333	pos
0.61_317.037	317.037028	0.61033333	neg
0.61_323.145	323.145792	0.61033333	neg
0.61_325.590	325.590005	0.60603333	pos
0.61_333.077	333.077775	0.60603333	pos
0.61_333.579	333.579449	0.60603333	pos
0.61_334.010	334.010566	0.61033333	neg
0.61_336.027	336.027133	0.61033333	neg
0.61_340.885	340.885794	0.61033333	neg
0.61_340.946	340.946136	0.61033333	neg
0.61_342.139	342.139528	0.60603333	pos
0.61_350.055	395.053244	0.61033333	neg
0.61_359.016	359.016463	0.61033333	neg
0.61_373.058	373.058761	0.61033333	neg
0.61_387.184	387.18494	0.61033333	neg
0.61_391.133	391.13303	0.61033333	neg
0.61_404.014	404.014651	0.61033333	neg

0.61_411.030	411.030331	0.61033333	neg
0.61_422.110	422.110731	0.61033333	neg
0.61_424.911	424.911258	0.61033333	neg
0.61_426.931	426.93168	0.61033333	neg
0.61_436.126	436.126363	0.61033333	neg
0.61_441.046	441.046125	0.61033333	neg
0.61_449.025	449.025362	0.61033333	neg
0.61_454.863	454.863857	0.61033333	neg
0.61_456.924	456.924626	0.61033333	neg
0.61_457.024	457.024057	0.61033333	neg
0.61_463.041	463.040992	0.61033333	neg
0.61_471.904	471.904838	0.61033333	neg
0.61_472.001	472.001888	0.61033333	neg
0.61_473.158	473.158913	0.61033333	neg
0.61_487.213	487.213585	0.60603333	pos
0.61_492.898	492.898736	0.61033333	neg
0.61_507.272	507.27258	0.61033333	neg
0.61_509.033	509.033608	0.61033333	neg
0.61_511.130	511.130198	0.61033333	neg
0.61_525.011	525.011044	0.61033333	neg
0.61_532.035	531.028482	0.61033333	neg
0.61_540.986	540.986043	0.61033333	neg
0.61_560.885	560.88572	0.61033333	neg
0.61_566.624	566.624551	0.61033333	neg
0.61_580.125	579.117792	0.61033333	neg
0.61_592.998	592.998605	0.61033333	neg
0.61_625.186	625.186362	0.61033333	neg
0.61_627.200	627.200508	0.60603333	pos
0.61_632.135	631.128525	0.61033333	neg
0.61_645.009	645.009239	0.61033333	neg
0.61_660.986	660.986231	0.61033333	neg
0.61_693.098	693.098184	0.61033333	neg
0.61_693.174	693.174422	0.61033333	neg

0.61_878.74	878.742435	0.61033333	neg
0.61_959.76	959.76883	0.61033333	neg
0.62_113.09	113.0906	0.6215	pos
0.62_123.59	123.594488	0.6215	pos
0.62_131.57	131.573644	0.6215	pos
0.62_134.06	134.069011	0.6215	pos
0.62_152.94	152.94718	0.6215	pos
0.62_156.07	156.076846	0.6215	pos
0.62_157.08	157.080235	0.6215	pos
0.62_159.14	159.149341	0.6215	pos
0.62_168.08	169.094825	0.6215	pos
0.62_173.60	173.608108	0.6215	pos
0.62_178.12	178.120564	0.6215	pos
0.62_186.63	186.633959	0.6215	pos
0.62_193.97	193.973972	0.6215	pos
0.62_201.07	201.07209	0.6215	pos
0.62_204.95	204.957079	0.6215	pos
0.62_208.07	231.065282	0.6215	pos
0.62_214.08	215.08771	0.6215	pos
0.62_224.05	224.051611	0.6215	pos
0.62_226.89	226.891361	0.6215	pos
0.62_237.15	237.157732	0.6215	pos
0.62_243.10	243.109392	0.6215	pos
0.62_243.93	266.927619	0.6215	pos
0.62_249.95	272.944157	0.6215	pos
0.62_250.88	250.889875	0.6215	pos
0.62_275.20	275.207786	0.6215	pos
0.62_278.90	278.900844	0.6215	pos
0.62_287.05	287.057507	0.6215	pos
0.62_291.97	291.976484	0.6215	pos
0.62_292.19	315.187814	0.6215	pos
0.62_295.94	318.937353	0.6215	pos
0.62_297.18	298.187413	0.6215	pos

0.62_317.942	340.931869	0.6215	pos
0.62_336.174	359.16405	0.6215	pos
0.62_350.055	373.04448	0.6215	pos
0.62_383.162	383.162518	0.6215	pos
0.62_418.042	441.032013	0.6215	pos
0.62_429.124	429.12459	0.6215	pos
0.62_450.155	473.144525	0.6215	pos
0.62_508.188	509.196073	0.6215	pos
0.62_509.019	509.019833	0.6215	pos
0.62_527.252	527.252128	0.6215	pos
0.62_540.137	563.126598	0.6215	pos
0.62_577.006	577.006679	0.6215	pos
0.62_608.124	631.113754	0.6215	pos
0.63_146.101	146.101719	0.62835	neg
0.63_213.085	213.085544	0.62835	neg
0.63_232.922	232.922185	0.62835	neg
0.63_248.900	248.899963	0.62835	neg
0.63_270.107	270.107103	0.62835	neg
0.63_300.909	300.90953	0.62835	neg
0.63_306.866	306.866281	0.62835	neg
0.63_313.185	313.185813	0.62835	neg
0.63_330.841	330.841423	0.62835	neg
0.63_360.970	360.970756	0.62835	neg
0.63_363.981	363.981088	0.62835	neg
0.63_378.917	378.917431	0.62835	neg
0.63_380.015	380.015317	0.62835	neg
0.63_396.847	396.847566	0.62835	neg
0.63_401.900	446.904973	0.62835	neg
0.63_401.997	401.997344	0.62835	neg
0.63_409.179	409.17947	0.62835	neg
0.63_417.048	417.04803	0.62835	neg
0.63_423.004	423.004426	0.62835	neg
0.63_428.807	428.80753	0.62835	neg

0.63_428.958	428.958242	0.62835	neg
0.63_434.029	433.021765	0.62835	neg
0.63_444.784	444.784483	0.62835	neg
0.63_449.160	449.160626	0.62835	neg
0.63_451.949	496.947593	0.62835	neg
0.63_459.119	459.118998	0.62835	neg
0.63_477.166	477.166882	0.62835	neg
0.63_480.809	480.809483	0.62835	neg
0.63_486.765	486.765559	0.62835	neg
0.63_502.010	501.009531	0.62835	neg
0.63_512.920	512.920041	0.62835	neg
0.63_514.892	514.892024	0.62835	neg
0.63_519.934	518.927629	0.62835	neg
0.63_529.059	529.058983	0.62835	neg
0.63_545.154	545.154531	0.62835	neg
0.63_562.977	562.977911	0.62835	neg
0.63_582.879	582.879179	0.62835	neg
0.63_599.016	599.016063	0.62835	neg
0.63_609.694	609.694148	0.62835	neg
0.63_621.010	621.010086	0.62835	neg
0.63_632.757	632.757907	0.62835	neg
0.63_637.991	636.984178	0.62835	neg
0.63_650.689	650.689926	0.62835	neg
0.63_650.866	650.866267	0.62835	neg
0.63_667.003	667.003339	0.62835	neg
0.63_702.641	702.641487	0.62835	neg
0.63_734.828	734.828463	0.62835	neg
0.64_113.988	113.988734	0.64391667	neg
0.64_118.941	118.941948	0.64391667	neg
0.64_128.959	128.959466	0.64391667	neg
0.64_135.974	158.964127	0.63663333	pos
0.64_151.073	151.073056	0.63663333	pos
0.64_154.071	177.061049	0.63663333	pos

0.64_164.947	164.947328	0.64391667	neg
0.64_171.053	171.053091	0.63663333	pos
0.64_172.116	172.116571	0.63663333	pos
0.64_177.051	178.058725	0.63663333	pos
0.64_180.972	180.972909	0.64391667	neg
0.64_181.976	181.976222	0.64391667	neg
0.64_184.061	207.050735	0.63663333	pos
0.64_187.936	186.929236	0.64391667	neg
0.64_188.925	188.925075	0.64391667	neg
0.64_196.946	196.9468	0.64391667	neg
0.64_202.997	202.997104	0.63663333	pos
0.64_203.910	202.903295	0.64391667	neg
0.64_203.962	226.951525	0.63663333	pos
0.64_209.918	248.88187	0.63663333	pos
0.64_216.909	216.909171	0.64391667	neg
0.64_217.047	217.047209	0.63663333	pos
0.64_222.049	222.049488	0.64391667	neg
0.64_236.075	259.064194	0.63663333	pos
0.64_240.887	240.887064	0.64391667	neg
0.64_242.864	242.864603	0.63663333	pos
0.64_248.960	248.960315	0.64391667	neg
0.64_249.963	249.963696	0.64391667	neg
0.64_250.964	250.964533	0.64391667	neg
0.64_255.872	254.865339	0.64391667	neg
0.64_255.933	254.92642	0.64391667	neg
0.64_256.861	256.861857	0.64391667	neg
0.64_260.873	260.873082	0.64391667	neg
0.64_264.934	264.934285	0.64391667	neg
0.64_268.028	268.028008	0.63663333	pos
0.64_270.890	270.890932	0.64391667	neg
0.64_271.949	310.912886	0.63663333	pos
0.64_275.986	275.986539	0.64391667	neg
0.64_277.854	276.847056	0.64391667	neg

0.64_278.84	278.843862	0.64391667	neg
0.64_282.01	327.009859	0.64391667	neg
0.64_283.86	306.851476	0.63663333	pos
0.64_290.03	290.037099	0.64391667	neg
0.64_293.82	292.819669	0.64391667	neg
0.64_299.83	322.825752	0.63663333	pos
0.64_303.99	305.000921	0.63663333	pos
0.64_307.92	346.888373	0.63663333	pos
0.64_309.07	309.07934	0.64391667	neg
0.64_310.01	311.025997	0.63663333	pos
0.64_313.03	313.03594	0.63663333	pos
0.64_314.87	314.876967	0.64391667	neg
0.64_319.98	319.989608	0.63663333	pos
0.64_328.86	328.860404	0.64391667	neg
0.64_330.14	353.12956	0.63663333	pos
0.64_332.84	332.843527	0.63663333	pos
0.64_332.92	332.921953	0.64391667	neg
0.64_336.85	336.858756	0.64391667	neg
0.64_337.82	674.651595	0.64391667	neg
0.64_338.85	338.854336	0.64391667	neg
0.64_338.87	338.87819	0.64391667	neg
0.64_338.88	338.887262	0.64391667	neg
0.64_339.93	378.900415	0.63663333	pos
0.64_340.96	358.999517	0.63663333	pos
0.64_343.02	343.02557	0.63663333	pos
0.64_344.83	344.834189	0.64391667	neg
0.64_345.89	368.882688	0.63663333	pos
0.64_346.83	346.830992	0.64391667	neg
0.64_348.89	348.895856	0.64391667	neg
0.64_349.99	394.997173	0.64391667	neg
0.64_352.83	352.832676	0.64391667	neg
0.64_355.05	355.05782	0.63663333	pos
0.64_358.84	358.840852	0.64391667	neg

0.64_360.808	360.808504	0.64391667	neg
0.64_360.836	360.836484	0.64391667	neg
0.64_364.181	365.188338	0.63663333	pos
0.64_367.823	390.812838	0.63663333	pos
0.64_368.036	368.036414	0.64391667	neg
0.64_371.031	371.031694	0.63663333	pos
0.64_374.839	374.839291	0.63663333	pos
0.64_381.829	380.822542	0.64391667	neg
0.64_385.998	387.005918	0.63663333	pos
0.64_390.788	390.788917	0.64391667	neg
0.64_391.908	390.901002	0.64391667	neg
0.64_393.040	393.040708	0.64391667	neg
0.64_394.874	394.874467	0.63663333	pos
0.64_397.803	396.79644	0.64391667	neg
0.64_398.792	398.79223	0.64391667	neg
0.64_400.830	400.830806	0.63663333	pos
0.64_400.909	400.909239	0.64391667	neg
0.64_400.953	400.953699	0.64391667	neg
0.64_400.995	400.995706	0.63663333	pos
0.64_401.905	424.894637	0.63663333	pos
0.64_402.804	402.804217	0.64391667	neg
0.64_404.002	404.001999	0.63663333	pos
0.64_406.874	406.874695	0.64391667	neg
0.64_408.919	408.919356	0.63663333	pos
0.64_412.822	412.821984	0.64391667	neg
0.64_415.774	414.766873	0.64391667	neg
0.64_418.778	418.778445	0.64391667	neg
0.64_419.785	442.774705	0.63663333	pos
0.64_423.898	446.887878	0.63663333	pos
0.64_429.968	430.97527	0.63663333	pos
0.64_430.914	430.913992	0.63663333	pos
0.64_434.752	434.752403	0.64391667	neg
0.64_435.811	458.800437	0.63663333	pos

0.64_435.86[illegible]	436.871074	0.63663333	pos
0.64_437.79[illegible]	437.793918	0.64391667	neg
0.64_442.82[illegible]	442.826614	0.63663333	pos
0.64_443.12[illegible]	443.125712	0.63663333	pos
0.64_448.78[illegible]	448.785223	0.64391667	neg
0.64_450.72[illegible]	450.726171	0.64391667	neg
0.64_455.15[illegible]	455.157962	0.63663333	pos
0.64_455.77[illegible]	500.772129	0.64391667	neg
0.64_456.01[illegible]	479.000598	0.63663333	pos
0.64_456.80[illegible]	456.807919	0.64391667	neg
0.64_463.02[illegible]	463.026546	0.63663333	pos
0.64_464.83[illegible]	464.835393	0.64391667	neg
0.64_468.76[illegible]	467.762397	0.64391667	neg
0.64_468.89[illegible]	468.896617	0.64391667	neg
0.64_471.79[illegible]	470.792092	0.64391667	neg
0.64_475.74[illegible]	475.749553	0.64391667	neg
0.64_476.90[illegible]	476.906858	0.63663333	pos
0.64_477.75[illegible]	477.753562	0.63663333	pos
0.64_486.74[illegible]	486.74095	0.64391667	neg
0.64_494.76[illegible]	494.764107	0.64391667	neg
0.64_497.76[illegible]	497.768172	0.64391667	neg
0.64_498.90[illegible]	498.901585	0.63663333	pos
0.64_502.74[illegible]	502.740213	0.64391667	neg
0.64_502.75[illegible]	503.759743	0.63663333	pos
0.64_504.85[illegible]	504.856041	0.63663333	pos
0.64_506.73[illegible]	506.738504	0.63663333	pos
0.64_507.86[illegible]	546.823508	0.63663333	pos
0.64_508.02[illegible]	531.014107	0.63663333	pos
0.64_508.75[illegible]	508.759229	0.64391667	neg
0.64_511.11[illegible]	511.113264	0.63663333	pos
0.64_512.73[illegible]	512.734438	0.64391667	neg
0.64_513.98[illegible]	536.970982	0.63663333	pos
0.64_516.74[illegible]	516.745954	0.64391667	neg

0.64_523.76	522.753491	0.64391667	neg
0.64_524.72	542.76244	0.63663333	pos
0.64_525.72	548.71869	0.63663333	pos
0.64_525.74	524.733222	0.64391667	neg
0.64_527.73	527.737	0.64391667	neg
0.64_531.73	532.744733	0.63663333	pos
0.64_532.71	532.719911	0.64391667	neg
0.64_532.82	532.822863	0.64391667	neg
0.64_538.72	538.728435	0.64391667	neg
0.64_538.77	538.779143	0.64391667	neg
0.64_540.72	540.725023	0.64391667	neg
0.64_542.84	542.847801	0.64391667	neg
0.64_544.73	544.735356	0.64391667	neg
0.64_546.71	546.714307	0.64391667	neg
0.64_546.75	546.751089	0.64391667	neg
0.64_548.79	548.797116	0.64391667	neg
0.64_551.69	551.697479	0.63663333	pos
0.64_553.73	534.716662	0.64391667	neg
0.64_556.69	556.698852	0.64391667	neg
0.64_557.74	557.743216	0.64391667	neg
0.64_561.71	560.709701	0.64391667	neg
0.64_562.72	562.725414	0.64391667	neg
0.64_565.72	565.729479	0.64391667	neg
0.64_565.82	588.81752	0.63663333	pos
0.64_566.88	566.888728	0.63663333	pos
0.64_568.73	568.733478	0.64391667	neg
0.64_573.71	573.716592	0.64391667	neg
0.64_574.72	574.727538	0.63663333	pos
0.64_575.84	614.811065	0.63663333	pos
0.64_579.72	579.725221	0.64391667	neg
0.64_584.70	584.707417	0.64391667	neg
0.64_587.71	587.712447	0.64391667	neg
0.64_589.84	572.845691	0.63663333	pos

0.64_592.694	592.694212	0.64391667	neg
0.64_593.710	616.70572	0.63663333	pos
0.64_595.699	595.699923	0.64391667	neg
0.64_595.735	595.735253	0.64391667	neg
0.64_598.703	598.703633	0.64391667	neg
0.64_599.673	622.6626	0.63663333	pos
0.64_599.724	600.731994	0.63663333	pos
0.64_606.767	606.767312	0.64391667	neg
0.64_614.713	614.713521	0.64391667	neg
0.64_621.853	620.846297	0.64391667	neg
0.64_625.704	625.704134	0.64391667	neg
0.64_636.695	636.695873	0.64391667	neg
0.64_641.759	622.741211	0.64391667	neg
0.64_644.672	644.672896	0.64391667	neg
0.64_652.669	652.669839	0.64391667	neg
0.64_653.676	653.676868	0.63663333	pos
0.64_654.666	654.666228	0.64391667	neg
0.64_658.678	658.67817	0.64391667	neg
0.64_674.687	674.687724	0.64391667	neg
0.64_681.667	680.65984	0.64391667	neg
0.64_684.771	684.771451	0.64391667	neg
0.64_685.820	708.815562	0.63663333	pos
0.64_690.728	690.728776	0.64391667	neg
0.64_696.634	696.634278	0.64391667	neg
0.64_701.856	702.86341	0.63663333	pos
0.64_705.655	705.655078	0.64391667	neg
0.64_711.640	756.644725	0.64391667	neg
0.64_717.648	698.630765	0.64391667	neg
0.64_717.829	718.837196	0.63663333	pos
0.64_730.829	730.829086	0.64391667	neg
0.64_734.662	734.662307	0.64391667	neg
0.64_736.707	736.707191	0.63663333	pos
0.64_736.786	736.786001	0.64391667	neg

0.64_750.63!	750.635741	0.64391667	neg
0.64_766.85:	766.853397	0.64391667	neg
0.64_772.61!	772.619929	0.64391667	neg
0.64_791.81:	772.794885	0.64391667	neg
0.65_124.92:	124.922908	0.6518	pos
0.65_134.01!	134.018954	0.6518	pos
0.65_141.94(	142.948123	0.6518	pos
0.65_157.90!	196.868601	0.6518	pos
0.65_167.92:	190.912034	0.6518	pos
0.65_175.11!	176.122295	0.6518	pos
0.65_196.09:	197.101015	0.6518	pos
0.65_214.84(	214.84075	0.6518	pos
0.65_235.91(	258.89943	0.6518	pos
0.65_239.91	278.880913	0.6518	pos
0.65_247.18	247.184579	0.6518	pos
0.65_247.88	248.891356	0.6518	pos
0.65_252.97(	275.959783	0.6518	pos
0.65_255.06:	255.06318	0.6518	pos
0.65_255.97!	255.978045	0.6518	pos
0.65_262.88	262.887919	0.6518	pos
0.65_264.85!	264.855942	0.6518	pos
0.65_264.86!	264.865269	0.6518	pos
0.65_284.13	284.137209	0.6518	pos
0.65_284.91(	284.910192	0.6518	pos
0.65_293.82!	316.818042	0.6518	pos
0.65_293.88!	316.878655	0.6518	pos
0.65_299.89	300.904765	0.6518	pos
0.65_303.89	326.88693	0.6518	pos
0.65_320.95	343.947095	0.6518	pos
0.65_320.97	320.974918	0.6518	pos
0.65_345.88:	368.871987	0.6518	pos
0.65_348.82	348.826995	0.6518	pos
0.65_352.89	352.897613	0.6518	pos

0.65_384.86	384.865947	0.6518	pos
0.65_386.84	386.842923	0.6518	pos
0.65_388.96	388.962095	0.6518	pos
0.65_436.76	436.766595	0.6518	pos
0.65_452.84	452.849273	0.6518	pos
0.65_454.85	454.852258	0.6518	pos
0.65_520.83	520.836969	0.6518	pos
0.65_539.81	562.801774	0.6518	pos
0.65_578.69	578.699329	0.6518	pos
0.65_602.67	602.679618	0.6518	pos
0.65_83.961	122.924793	0.6518	pos
0.66_102.95	102.956709	0.65943333	neg
0.66_104.95	104.953692	0.65943333	neg
0.66_110.07	110.072285	0.65943333	neg
0.66_164.95	164.956812	0.65943333	neg
0.66_172.02	172.024174	0.65943333	neg
0.66_173.10	173.104143	0.65943333	neg
0.66_187.94	232.944334	0.65943333	neg
0.66_203.92	248.918519	0.65943333	neg
0.66_206.96	206.967492	0.65943333	neg
0.66_207.99	207.999134	0.65943333	neg
0.66_212.02	212.021554	0.65943333	neg
0.66_217.93	216.928114	0.65943333	neg
0.66_228.90	228.902794	0.65943333	neg
0.66_230.89	230.899864	0.65943333	neg
0.66_232.90	232.902211	0.65943333	neg
0.66_244.88	244.888038	0.65943333	neg
0.66_244.89	244.8975	0.65943333	neg
0.66_259.96	259.961109	0.65943333	neg
0.66_271.90	270.900365	0.65943333	neg
0.66_286.86	286.862142	0.65943333	neg
0.66_296.89	296.890339	0.65943333	neg
0.66_312.88	312.885129	0.65943333	neg

0.66_322.893	322.893303	0.65943333	neg
0.66_330.855	330.855071	0.65943333	neg
0.66_341.191	341.191835	0.65943333	neg
0.66_380.852	380.852185	0.65943333	neg
0.66_388.814	388.814912	0.65943333	neg
0.66_389.864	434.86249	0.65943333	neg
0.66_396.826	396.826083	0.65943333	neg
0.66_404.788	404.788885	0.65943333	neg
0.67_100.076	100.076196	0.66691667	pos
0.67_108.901	108.901746	0.6745	neg
0.67_110.898	110.898775	0.6745	neg
0.67_112.896	112.896043	0.66691667	pos
0.67_114.893	114.893169	0.66691667	pos
0.67_119.958	158.922075	0.66691667	pos
0.67_120.003	120.003475	0.66691667	pos
0.67_127.024	150.013844	0.66691667	pos
0.67_152.883	152.883521	0.6745	neg
0.67_154.035	177.024659	0.66691667	pos
0.67_154.928	154.928051	0.6745	neg
0.67_155.065	155.065432	0.6745	neg
0.67_156.925	156.925036	0.6745	neg
0.67_161.993	161.993817	0.6745	neg
0.67_163.001	164.008287	0.66691667	pos
0.67_163.990	163.9908	0.6745	neg
0.67_168.051	191.04032	0.66691667	pos
0.67_170.892	170.89248	0.6745	neg
0.67_170.901	170.901942	0.6745	neg
0.67_171.145	189.179099	0.66691667	pos
0.67_172.899	172.898967	0.6745	neg
0.67_183.060	206.055291	0.66691667	pos
0.67_184.983	207.972301	0.66691667	pos
0.67_186.038	186.038168	0.6745	neg
0.67_189.004	189.004618	0.6745	neg

0.67_195.93	234.893944	0.66691667	pos
0.67_195.93	194.927218	0.6745	neg
0.67_197.93	196.922847	0.6745	neg
0.67_197.93	215.964642	0.66691667	pos
0.67_197.97	197.970175	0.6745	neg
0.67_198.87	198.876122	0.66691667	pos
0.67_199.96	199.96717	0.6745	neg
0.67_206.89	206.894	0.66691667	pos
0.67_209.90	232.896754	0.66691667	pos
0.67_210.84	210.842187	0.6745	neg
0.67_212.83	212.839353	0.6745	neg
0.67_212.88	212.886748	0.6745	neg
0.67_213.94	213.944489	0.6745	neg
0.67_214.88	214.883832	0.6745	neg
0.67_215.85	238.841966	0.66691667	pos
0.67_216.92	216.922854	0.66691667	pos
0.67_222.98	222.988154	0.66691667	pos
0.67_225.03	225.034639	0.66691667	pos
0.67_225.94	225.944096	0.66691667	pos
0.67_226.81	226.816011	0.6745	neg
0.67_227.03	228.037348	0.66691667	pos
0.67_228.06	228.064191	0.6745	neg
0.67_230.81	230.810311	0.6745	neg
0.67_234.86	234.868563	0.6745	neg
0.67_234.94	217.938355	0.66691667	pos
0.67_236.86	236.865719	0.6745	neg
0.67_239.92	239.92016	0.66691667	pos
0.67_240.85	240.859854	0.66691667	pos
0.67_241.88	259.922845	0.66691667	pos
0.67_242.13	260.171588	0.66691667	pos
0.67_242.79	242.790132	0.6745	neg
0.67_243.86	266.851027	0.66691667	pos
0.67_244.01	244.01127	0.66691667	pos

0.67_250.83	250.833117	0.6745	neg
0.67_250.86	250.86804	0.66691667	pos
0.67_250.93	251.941215	0.66691667	pos
0.67_257.10	280.092044	0.66691667	pos
0.67_258.86	258.865378	0.66691667	pos
0.67_263.92	263.921499	0.6745	neg
0.67_264.84	264.844641	0.66691667	pos
0.67_265.93	265.930906	0.66691667	pos
0.67_266.80	266.80383	0.6745	neg
0.67_267.91	267.915224	0.66691667	pos
0.67_268.80	268.800801	0.6745	neg
0.67_270.79	270.797996	0.6745	neg
0.67_272.79	272.794708	0.66691667	pos
0.67_272.79	272.795121	0.6745	neg
0.67_273.03	274.042841	0.66691667	pos
0.67_278.82	278.829731	0.6745	neg
0.67_282.77	282.777715	0.6745	neg
0.67_284.77	284.774865	0.6745	neg
0.67_286.77	286.771924	0.6745	neg
0.67_286.81	286.819348	0.6745	neg
0.67_288.76	288.769162	0.6745	neg
0.67_290.87	290.87613	0.66691667	pos
0.67_291.86	272.842602	0.6745	neg
0.67_294.85	294.854941	0.66691667	pos
0.67_296.82	296.821167	0.66691667	pos
0.67_296.85	296.859094	0.6745	neg
0.67_297.04	297.04669	0.6745	neg
0.67_298.84	298.84543	0.6745	neg
0.67_298.94	321.934638	0.66691667	pos
0.67_310.78	310.789045	0.6745	neg
0.67_314.18	337.171422	0.66691667	pos
0.67_315.88	315.88479	0.6745	neg
0.67_326.75	326.759513	0.6745	neg

0.67_328.06	351.057427	0.66691667	pos
0.67_329.83	329.831123	0.6745	neg
0.67_330.80	330.801151	0.6745	neg
0.67_333.91	333.918274	0.66691667	pos
0.67_334.12	334.125665	0.6745	neg
0.67_336.83	336.837356	0.66691667	pos
0.67_342.73	342.733667	0.6745	neg
0.67_344.77	344.777941	0.6745	neg
0.67_346.77	346.775098	0.6745	neg
0.67_348.22	349.230939	0.66691667	pos
0.67_348.77	348.772185	0.6745	neg
0.67_353.00	353.008353	0.6745	neg
0.67_355.00	355.005482	0.6745	neg
0.67_380.78	380.783681	0.66691667	pos
0.67_387.78	387.789809	0.6745	neg
0.67_406.73	406.730774	0.6745	neg
0.67_429.77	429.774243	0.6745	neg
0.67_431.21	432.222015	0.66691667	pos
0.67_510.79	510.790509	0.66691667	pos
0.67_589.67	589.672459	0.6745	neg
0.68_132.05	133.060816	0.68255	pos
0.68_148.00	148.003983	0.68255	pos
0.68_155.01	178.008667	0.68255	pos
0.68_158.09	158.092511	0.68255	pos
0.68_162.97	185.959782	0.68255	pos
0.68_164.02	164.029327	0.68255	pos
0.68_167.07	167.07365	0.68255	pos
0.68_168.05	169.058353	0.68255	pos
0.68_169.98	169.985815	0.68255	pos
0.68_171.98	171.981572	0.68255	pos
0.68_179.98	179.982268	0.68255	pos
0.68_185.03	185.032326	0.68255	pos
0.68_194.10	194.102424	0.68255	pos

0.68_206.10:	206.10222	0.68255	pos
0.68_220.92!	243.918377	0.68255	pos
0.68_224.11:	224.112759	0.68255	pos
0.68_227.94	227.944335	0.68255	pos
0.68_229.98(	229.980108	0.68255	pos
0.68_238.09!	239.105935	0.68255	pos
0.68_245.18(	245.180332	0.68255	pos
0.68_256.82(	256.820595	0.68255	pos
0.68_264.91(	303.874	0.68255	pos
0.68_278.88	301.876991	0.68255	pos
0.68_278.98	278.98712	0.68255	pos
0.68_281.01(	299.043784	0.68255	pos
0.68_288.01!	289.022912	0.68255	pos
0.68_288.76!	288.768669	0.68255	pos
0.68_288.99:	311.98285	0.68255	pos
0.68_290.08	290.084213	0.68255	pos
0.68_302.90(	285.902965	0.68255	pos
0.68_305.91!	305.918211	0.68255	pos
0.68_316.98:	316.982259	0.68255	pos
0.68_317.19:	317.193056	0.68255	pos
0.68_338.01:	339.020638	0.68255	pos
0.68_360.07	360.074587	0.68255	pos
0.68_375.99(	414.95998	0.68255	pos
0.68_390.91!	390.915394	0.68255	pos
0.68_399.14	399.144466	0.68255	pos
0.69_124.03	124.037924	0.68961667	neg
0.69_131.08:	131.08242	0.68961667	neg
0.69_136.91	136.917431	0.68961667	neg
0.69_145.06	145.061727	0.68961667	neg
0.69_150.88(	150.886431	0.68961667	neg
0.69_154.88(	154.880581	0.68961667	neg
0.69_158.84(	158.84649	0.68961667	neg
0.69_160.84:	160.84184	0.68961667	neg

0.69_162.83[illegible]	162.838912	0.68961667	neg
0.69_164.83[illegible]	164.835855	0.68961667	neg
0.69_174.04[illegible]	174.045341	0.68961667	neg
0.69_176.04[illegible]	176.042522	0.68961667	neg
0.69_181.96[illegible]	181.965838	0.68961667	neg
0.69_183.96[illegible]	183.962701	0.68961667	neg
0.69_192.99[illegible]	237.988541	0.68961667	neg
0.69_197.93[illegible]	197.939619	0.68961667	neg
0.69_204.00[illegible]	204.004167	0.68961667	neg
0.69_206.00[illegible]	206.001297	0.68961667	neg
0.69_208.84[illegible]	208.845023	0.68961667	neg
0.69_209.00[illegible]	189.988617	0.68961667	neg
0.69_211.97[illegible]	211.970664	0.68961667	neg
0.69_214.97[illegible]	259.970337	0.68961667	neg
0.69_225.98[illegible]	225.98611	0.68961667	neg
0.69_227.94[illegible]	227.944444	0.68961667	neg
0.69_239.92[illegible]	239.924453	0.68961667	neg
0.69_241.96[illegible]	241.960221	0.68961667	neg
0.69_255.89[illegible]	255.898514	0.68961667	neg
0.69_272.01[illegible]	271.003857	0.68961667	neg
0.69_279.02[illegible]	279.026574	0.68961667	neg
0.69_283.94[illegible]	283.944921	0.68961667	neg
0.69_286.97[illegible]	286.977835	0.68961667	neg
0.69_289.11[illegible]	289.115252	0.68961667	neg
0.69_294.06[illegible]	294.069256	0.68961667	neg
0.69_296.00[illegible]	296.007975	0.68961667	neg
0.69_297.88[illegible]	297.883036	0.68961667	neg
0.69_299.88[illegible]	299.880127	0.68961667	neg
0.69_303.92[illegible]	303.9212	0.68961667	neg
0.69_307.11[illegible]	307.114589	0.68961667	neg
0.69_313.85[illegible]	313.857201	0.68961667	neg
0.69_315.85[illegible]	315.854	0.68961667	neg
0.69_316.98[illegible]	316.980836	0.68961667	neg

0.69_317.929	317.929183	0.68961667	neg
0.69_328.962	328.962301	0.68961667	neg
0.69_330.959	330.959107	0.68961667	neg
0.69_351.000	351.000908	0.68961667	neg
0.69_355.841	355.841879	0.68961667	neg
0.69_357.839	357.839022	0.68961667	neg
0.69_358.007	358.00761	0.68961667	neg
0.69_359.883	359.883262	0.68961667	neg
0.69_362.106	362.106064	0.68961667	neg
0.69_371.815	371.81554	0.68961667	neg
0.69_374.939	374.939757	0.68961667	neg
0.69_384.087	384.087788	0.68961667	neg
0.69_386.921	386.921165	0.68961667	neg
0.69_388.918	388.918314	0.68961667	neg
0.69_401.859	401.859621	0.68961667	neg
0.69_431.771	431.771465	0.68961667	neg
0.69_471.758	471.758155	0.68961667	neg
0.69_473.755	473.755905	0.68961667	neg
0.70_104.141	104.141942	0.69861667	pos
0.70_105.104	105.104317	0.69861667	pos
0.70_105.113	105.113355	0.69861667	pos
0.70_124.991	124.991136	0.70456667	neg
0.70_125.010	125.010459	0.70456667	neg
0.70_125.014	124.007209	0.70456667	neg
0.70_127.025	127.025332	0.69861667	pos
0.70_128.017	128.0179	0.69861667	pos
0.70_132.946	132.946121	0.70456667	neg
0.70_133.037	134.044766	0.69861667	pos
0.70_134.943	134.943193	0.70456667	neg
0.70_147.052	146.045671	0.70456667	neg
0.70_148.055	148.055537	0.69861667	pos
0.70_149.999	149.999667	0.69861667	pos
0.70_172.049	217.047383	0.70456667	neg

0.70_185.06	166.043397	0.70456667	neg
0.70_188.01	188.014417	0.69861667	pos
0.70_201.05	201.053798	0.69861667	pos
0.70_203.06	203.067171	0.70456667	neg
0.70_215.06	215.069667	0.69861667	pos
0.70_215.09	196.074179	0.70456667	neg
0.70_216.06	216.063053	0.69861667	pos
0.70_219.07	218.066548	0.70456667	neg
0.70_221.97	221.974796	0.70456667	neg
0.70_222.99	221.983814	0.70456667	neg
0.70_223.97	223.970768	0.70456667	neg
0.70_234.09	234.097062	0.69861667	pos
0.70_241.03	241.030998	0.69861667	pos
0.70_241.10	241.1017	0.69861667	pos
0.70_243.95	243.956694	0.70456667	neg
0.70_243.96	243.965831	0.70456667	neg
0.70_246.02	246.025323	0.70456667	neg
0.70_247.04	247.040162	0.70456667	neg
0.70_251.01	251.01749	0.70456667	neg
0.70_251.03	251.036362	0.69861667	pos
0.70_252.05	252.054474	0.70456667	neg
0.70_257.81	257.811042	0.70456667	neg
0.70_258.98	258.982007	0.70456667	neg
0.70_259.93	259.930439	0.70456667	neg
0.70_259.93	259.93979	0.70456667	neg
0.70_260.97	260.979464	0.70456667	neg
0.70_265.21	265.211909	0.69861667	pos
0.70_266.00	266.004435	0.70456667	neg
0.70_267.04	267.040764	0.69861667	pos
0.70_267.09	268.102396	0.69861667	pos
0.70_267.95	267.952985	0.69861667	pos
0.70_272.01	295.000267	0.69861667	pos
0.70_274.10	274.104471	0.70456667	neg

0.70_275.053	275.053916	0.70456667	neg
0.70_281.978	281.978383	0.70456667	neg
0.70_287.051	287.051529	0.70456667	neg
0.70_287.067	288.074548	0.69861667	pos
0.70_287.986	287.986578	0.70456667	neg
0.70_294.049	317.038694	0.69861667	pos
0.70_298.023	298.023756	0.70456667	neg
0.70_301.071	601.136265	0.70456667	neg
0.70_305.078	286.060131	0.70456667	neg
0.70_310.000	310.000208	0.70456667	neg
0.70_312.060	293.042385	0.70456667	neg
0.70_316.054	316.054829	0.69861667	pos
0.70_320.074	320.074657	0.70456667	neg
0.70_327.059	350.049155	0.69861667	pos
0.70_328.034	309.01629	0.70456667	neg
0.70_330.998	330.998112	0.70456667	neg
0.70_338.069	339.077186	0.69861667	pos
0.70_341.942	341.942694	0.70456667	neg
0.70_343.994	343.994697	0.70456667	neg
0.70_346.998	346.998667	0.70456667	neg
0.70_349.098	372.087913	0.69861667	pos
0.70_353.099	398.097536	0.70456667	neg
0.70_354.063	354.063298	0.70456667	neg
0.70_354.071	354.071598	0.70456667	neg
0.70_363.113	364.121021	0.69861667	pos
0.70_368.981	368.981153	0.70456667	neg
0.70_376.100	376.100818	0.70456667	neg
0.70_377.085	377.085285	0.70456667	neg
0.70_381.109	381.109941	0.70456667	neg
0.70_384.036	384.036889	0.70456667	neg
0.70_390.962	390.962682	0.70456667	neg
0.70_397.025	398.032959	0.69861667	pos
0.70_407.020	406.018976	0.70456667	neg

0.70_407.07	408.084162	0.69861667	pos
0.70_414.00	414.007092	0.69861667	pos
0.70_414.03	414.036964	0.69861667	pos
0.70_419.06	420.071487	0.69861667	pos
0.70_429.14	474.14612	0.70456667	neg
0.70_435.12	435.127581	0.69861667	pos
0.70_441.99	441.996731	0.69861667	pos
0.70_448.16	448.165761	0.69861667	pos
0.70_452.91	452.915412	0.69861667	pos
0.70_456.08	456.084299	0.69861667	pos
0.70_457.97	457.97065	0.69861667	pos
0.70_462.14	462.146409	0.69861667	pos
0.70_462.18	462.181413	0.69861667	pos
0.70_472.01	472.013368	0.70456667	neg
0.70_476.16	476.160666	0.69861667	pos
0.70_505.08	506.093893	0.69861667	pos
0.70_522.20	522.202787	0.69861667	pos
0.70_523.10	523.104077	0.69861667	pos
0.70_524.08	524.081836	0.70456667	neg
0.70_586.08	586.082548	0.70456667	neg
0.70_599.09	599.094113	0.70456667	neg
0.70_624.23	624.234081	0.69861667	pos
0.70_628.17	628.176284	0.70456667	neg
0.70_630.19	630.190068	0.69861667	pos
0.70_636.19	636.199117	0.70456667	neg
0.70_638.21	638.21295	0.69861667	pos
0.70_650.21	650.214733	0.70456667	neg
0.70_651.16	652.172544	0.69861667	pos
0.70_652.22	652.228803	0.69861667	pos
0.70_668.14	668.146538	0.69861667	pos
0.70_686.13	686.135088	0.70456667	neg
0.70_701.19	701.191317	0.70456667	neg
0.70_706.21	689.210409	0.69861667	pos

0.70_763.188	763.188453	0.70456667	neg
0.70_790.229	790.229159	0.70456667	neg
0.71_160.096	160.096861	0.71368333	pos
0.71_168.989	168.98965	0.71368333	pos
0.71_214.051	214.051152	0.71368333	pos
0.71_219.047	219.047383	0.71368333	pos
0.71_221.042	221.042048	0.71368333	pos
0.71_221.185	221.185825	0.71368333	pos
0.71_234.133	234.133526	0.71368333	pos
0.71_235.092	235.092277	0.71368333	pos
0.71_254.069	254.069042	0.71368333	pos
0.71_267.011	290.000707	0.71368333	pos
0.71_278.040	261.036913	0.71368333	pos
0.71_279.191	279.191238	0.71368333	pos
0.71_283.072	284.079306	0.71368333	pos
0.71_300.021	323.011042	0.71368333	pos
0.71_300.935	318.969713	0.71368333	pos
0.71_324.045	324.045526	0.71368333	pos
0.71_427.168	428.175889	0.71368333	pos
0.71_456.102	479.091461	0.71368333	pos
0.71_457.166	457.166913	0.71368333	pos
0.71_467.130	490.119963	0.71368333	pos
0.71_484.142	484.142049	0.71368333	pos
0.72_129.019	129.019175	0.71928333	neg
0.72_174.839	174.839282	0.71928333	neg
0.72_174.988	174.98813	0.71928333	neg
0.72_189.034	189.034408	0.71928333	neg
0.72_193.815	193.815262	0.71928333	neg
0.72_194.936	194.936667	0.71928333	neg
0.72_195.810	195.810692	0.71928333	neg
0.72_195.953	194.946178	0.71928333	neg
0.72_195.991	195.991885	0.71928333	neg
0.72_197.807	197.807698	0.71928333	neg

0.72_199.804	199.804775	0.71928333	neg
0.72_201.801	201.801867	0.71928333	neg
0.72_203.798	203.798846	0.71928333	neg
0.72_204.998	204.998708	0.71928333	neg
0.72_213.852	213.852776	0.71928333	neg
0.72_232.892	232.892207	0.71928333	neg
0.72_242.051	242.051636	0.71928333	neg
0.72_254.033	254.033808	0.71928333	neg
0.72_258.064	258.064664	0.71928333	neg
0.72_265.995	265.995177	0.71928333	neg
0.72_272.000	272.000184	0.71928333	neg
0.72_273.873	273.873651	0.71928333	neg
0.72_277.023	277.023391	0.71928333	neg
0.72_281.914	281.914106	0.71928333	neg
0.72_283.910	283.910191	0.71928333	neg
0.72_303.951	303.951105	0.71928333	neg
0.72_314.018	314.01863	0.71928333	neg
0.72_314.905	314.90517	0.71928333	neg
0.72_316.014	316.014229	0.71928333	neg
0.72_316.034	316.03419	0.71928333	neg
0.72_322.082	322.082568	0.71928333	neg
0.72_325.093	325.093838	0.71928333	neg
0.72_331.982	331.982032	0.71928333	neg
0.72_335.042	335.042735	0.71928333	neg
0.72_344.075	344.075052	0.71928333	neg
0.72_346.072	346.072021	0.71928333	neg
0.72_348.963	347.955901	0.71928333	neg
0.72_366.108	366.108731	0.71928333	neg
0.72_376.068	376.06866	0.71928333	neg
0.72_378.020	359.002462	0.71928333	neg
0.72_378.072	378.072604	0.71928333	neg
0.72_396.114	396.114564	0.71928333	neg
0.72_397.115	397.115499	0.71928333	neg

0.72_398.08[illegible]	379.069169	0.71928333	neg
0.72_404.10[illegible]	404.104302	0.71928333	neg
0.72_406.06[illegible]	406.066994	0.71928333	neg
0.72_406.07[illegible]	406.075296	0.71928333	neg
0.72_413.03[illegible]	413.032978	0.71928333	neg
0.72_419.09[illegible]	419.098903	0.71928333	neg
0.72_420.09[illegible]	420.095213	0.71928333	neg
0.72_467.13[illegible]	466.123193	0.71928333	neg
0.72_477.14[illegible]	477.146151	0.71928333	neg
0.72_496.14[illegible]	496.147794	0.71928333	neg
0.72_497.04[illegible]	497.044578	0.71928333	neg
0.72_559.04[illegible]	559.044445	0.71928333	neg
0.72_564.09[illegible]	564.09976	0.71928333	neg
0.73_114.05[illegible]	114.05583	0.7342	neg
0.73_116.03[illegible]	116.030476	0.72825	pos
0.73_116.11[illegible]	116.111863	0.72825	pos
0.73_117.07[illegible]	117.074025	0.72825	pos
0.73_139.98[illegible]	139.98435	0.7342	neg
0.73_142.02[illegible]	187.018989	0.7342	neg
0.73_146.11[illegible]	146.117573	0.72825	pos
0.73_154.91[illegible]	154.918646	0.7342	neg
0.73_156.91[illegible]	156.915592	0.7342	neg
0.73_173.09[illegible]	173.092834	0.7342	neg
0.73_185.05[illegible]	185.056471	0.7342	neg
0.73_187.00[illegible]	187.000175	0.72825	pos
0.73_192.98[illegible]	192.988494	0.7342	neg
0.73_207.97[illegible]	208.982121	0.72825	pos
0.73_212.87[illegible]	212.877322	0.7342	neg
0.73_214.87[illegible]	214.874365	0.7342	neg
0.73_216.99[illegible]	216.993145	0.7342	neg
0.73_217.92[illegible]	216.918369	0.7342	neg
0.73_218.05[illegible]	199.037589	0.7342	neg
0.73_224.93[illegible]	224.933612	0.7342	neg

0.73_233.00(	233.006941	0.7342	neg
0.73_233.06:	233.063088	0.72825	pos
0.73_236.02:	236.023228	0.7342	neg
0.73_236.07	236.077247	0.7342	neg
0.73_249.15!	249.155557	0.72825	pos
0.73_251.06:	232.045995	0.7342	neg
0.73_261.01:	261.011107	0.72825	pos
0.73_262.06(	263.073458	0.72825	pos
0.73_268.07:	268.0719	0.72825	pos
0.73_274.88	274.886951	0.7342	neg
0.73_283.07	283.078157	0.7342	neg
0.73_283.96(	282.952982	0.7342	neg
0.73_291.05:	291.052494	0.7342	neg
0.73_291.05:	290.04619	0.7342	neg
0.73_296.07:	296.073338	0.7342	neg
0.73_302.00(	302.006622	0.7342	neg
0.73_302.10(	302.100784	0.7342	neg
0.73_304.93!	304.935186	0.7342	neg
0.73_308.90(	308.906501	0.7342	neg
0.73_310.07	310.077757	0.7342	neg
0.73_318.05(	318.050564	0.7342	neg
0.73_332.03	332.038934	0.7342	neg
0.73_337.09	337.094158	0.7342	neg
0.73_344.93(	344.936931	0.7342	neg
0.73_356.11!	356.119721	0.7342	neg
0.73_363.90(	363.906457	0.7342	neg
0.73_365.90:	365.901516	0.7342	neg
0.73_369.15(	369.150045	0.72825	pos
0.73_379.87!	379.879834	0.7342	neg
0.73_385.88	385.887816	0.7342	neg
0.73_387.05	370.051354	0.72825	pos
0.73_394.22:	394.222254	0.72825	pos
0.73_410.09	410.098287	0.7342	neg

0.73_425.17(	425.176057	0.72825	pos
0.73_428.05'	428.057048	0.7342	neg
0.73_485.064	485.064166	0.72825	pos
0.73_659.093	659.093625	0.7342	neg
0.73_721.08(	721.086314	0.7342	neg
0.74_145.10!	145.10525	0.74288333	pos
0.74_163.11(	186.105944	0.74288333	pos
0.74_177.54(	177.546868	0.74288333	pos
0.74_186.06(	186.060155	0.74288333	pos
0.74_204.14(	204.140148	0.74288333	pos
0.74_235.06!	258.058453	0.74288333	pos
0.74_247.20:	247.201464	0.74288333	pos
0.74_257.13'	275.171166	0.74288333	pos
0.74_260.12:	260.123833	0.74288333	pos
0.74_274.05:	274.053076	0.74288333	pos
0.74_320.10{	320.108641	0.74288333	pos
0.74_370.05(	371.05749	0.74288333	pos
0.74_375.16:	375.161999	0.74288333	pos
0.74_430.03!	431.042258	0.74288333	pos
0.74_646.10:	647.110912	0.74288333	pos
0.75_125.00(	125.000752	0.74898333	neg
0.75_125.01'	125.017696	0.74898333	neg
0.75_125.87:	125.873007	0.74898333	neg
0.75_132.01:	132.012388	0.74898333	neg
0.75_134.00{	134.00816	0.74898333	neg
0.75_162.97'	162.977806	0.74898333	neg
0.75_164.974	164.974729	0.74898333	neg
0.75_181.94'	181.947042	0.74898333	neg
0.75_188.95(	188.956867	0.74898333	neg
0.75_190.01'	190.017694	0.74898333	neg
0.75_191.96'	191.96795	0.74898333	neg
0.75_192.03:	192.03338	0.74898333	neg
0.75_199.00:	199.001109	0.74898333	neg

0.75_205.839	205.839455	0.74898333	neg
0.75_206.059	205.052228	0.74898333	neg
0.75_207.836	207.836556	0.74898333	neg
0.75_216.003	214.996465	0.74898333	neg
0.75_224.053	223.045734	0.74898333	neg
0.75_224.968	224.968524	0.74898333	neg
0.75_228.081	228.081034	0.74898333	neg
0.75_229.884	229.883961	0.74898333	neg
0.75_229.989	229.989247	0.74898333	neg
0.75_240.084	285.082607	0.74898333	neg
0.75_249.992	249.992129	0.74898333	neg
0.75_252.015	252.015818	0.74898333	neg
0.75_256.959	256.95971	0.74898333	neg
0.75_262.060	262.060739	0.74898333	neg
0.75_274.868	274.868361	0.74898333	neg
0.75_275.088	275.088536	0.74898333	neg
0.75_281.960	326.964376	0.74898333	neg
0.75_289.037	270.020076	0.74898333	neg
0.75_290.088	290.088116	0.74898333	neg
0.75_304.982	304.98203	0.74898333	neg
0.75_330.079	329.072616	0.74898333	neg
0.75_334.095	334.095259	0.74898333	neg
0.75_337.077	337.077701	0.74898333	neg
0.75_358.091	358.091492	0.74898333	neg
0.75_388.055	388.055911	0.74898333	neg
0.75_420.011	420.011082	0.74898333	neg
0.75_424.015	424.015753	0.74898333	neg
0.75_441.993	441.993363	0.74898333	neg
0.76_118.941	118.941896	0.76461667	neg
0.76_135.030	135.030344	0.75766667	pos
0.76_136.019	119.016232	0.75766667	pos
0.76_145.922	126.904903	0.76461667	neg
0.76_160.962	160.962155	0.76461667	neg

0.76_163.030	162.022847	0.76461667	neg
0.76_165.07	166.084785	0.75766667	pos
0.76_176.09	176.091778	0.75766667	pos
0.76_179.84	179.841061	0.76461667	neg
0.76_184.86	184.863371	0.76461667	neg
0.76_186.86	186.86035	0.76461667	neg
0.76_187.10	187.1086	0.76461667	neg
0.76_189.97	189.970899	0.76461667	neg
0.76_196.04	196.043968	0.75766667	pos
0.76_199.62	199.62365	0.75766667	pos
0.76_200.83	200.837525	0.76461667	neg
0.76_201.15	201.159684	0.75766667	pos
0.76_202.95	202.951228	0.75766667	pos
0.76_206.04	229.031829	0.75766667	pos
0.76_209.83	209.833654	0.76461667	neg
0.76_211.88	211.882492	0.76461667	neg
0.76_212.10	212.104578	0.75766667	pos
0.76_223.03	223.033986	0.75766667	pos
0.76_224.05	247.042344	0.75766667	pos
0.76_227.07	228.079971	0.75766667	pos
0.76_230.99	230.997077	0.76461667	neg
0.76_231.88	231.881129	0.76461667	neg
0.76_238.01	238.012296	0.75766667	pos
0.76_243.00	243.008492	0.76461667	neg
0.76_245.00	245.006738	0.76461667	neg
0.76_251.00	252.011506	0.75766667	pos
0.76_251.01	251.013693	0.75766667	pos
0.76_251.08	252.09536	0.75766667	pos
0.76_261.99	261.995913	0.75766667	pos
0.76_271.15	271.152532	0.75766667	pos
0.76_291.09	314.084298	0.75766667	pos
0.76_304.19	305.206742	0.75766667	pos
0.76_306.12	306.129317	0.75766667	pos

0.76_306.93;	306.93207	0.76461667	neg
0.76_306.968	306.968437	0.76461667	neg
0.76_308.03	308.037584	0.75766667	pos
0.76_311.94;	311.942618	0.76461667	neg
0.76_318.73	318.737144	0.76461667	neg
0.76_319.93	318.931856	0.76461667	neg
0.76_321.01	321.013317	0.76461667	neg
0.76_321.06	321.066602	0.75766667	pos
0.76_323.05	324.058833	0.75766667	pos
0.76_334.12	334.12424	0.75766667	pos
0.76_347.99	347.995204	0.76461667	neg
0.76_348.99	348.990418	0.75766667	pos
0.76_350.95	350.958095	0.76461667	neg
0.76_357.91	357.917051	0.76461667	neg
0.76_361.07	361.073766	0.75766667	pos
0.76_361.18	361.182438	0.75766667	pos
0.76_366.99	366.991663	0.75766667	pos
0.76_374.20	374.203281	0.75766667	pos
0.76_374.81	374.814537	0.76461667	neg
0.76_375.99	375.991169	0.76461667	neg
0.76_378.97	359.958732	0.76461667	neg
0.76_390.03	390.033769	0.76461667	neg
0.76_391.99	391.996615	0.76461667	neg
0.76_469.02	469.021098	0.76461667	neg
0.76_631.07	631.074279	0.76461667	neg
0.77_114.05	114.055302	0.7729	pos
0.77_130.12	130.122645	0.7729	pos
0.77_139.04	157.080235	0.7729	pos
0.77_146.09	146.092356	0.7729	pos
0.77_163.03	146.027049	0.7729	pos
0.77_187.12	188.128064	0.7729	pos
0.77_190.01	190.012488	0.7729	pos
0.77_252.99	254.007213	0.7729	pos

0.77_263.15	286.139553	0.7729	pos
0.77_278.57	278.572278	0.7729	pos
0.77_282.11	282.118703	0.7729	pos
0.77_285.91	285.917148	0.7729	pos
0.77_295.03	318.025165	0.7729	pos
0.77_304.15	304.150047	0.7729	pos
0.77_326.06	327.06769	0.7729	pos
0.77_342.15	343.161011	0.7729	pos
0.78_116.07	116.071532	0.7799	neg
0.78_124.12	124.121727	0.7799	neg
0.78_136.02	136.027982	0.7799	neg
0.78_150.04	150.041887	0.7799	neg
0.78_159.01	159.010355	0.7799	neg
0.78_161.00	161.006383	0.7799	neg
0.78_168.02	167.020837	0.7799	neg
0.78_171.07	171.0773	0.7799	neg
0.78_190.97	190.972703	0.7799	neg
0.78_228.99	228.993219	0.7799	neg
0.78_235.00	235.008267	0.7799	neg
0.78_252.96	252.963543	0.7799	neg
0.78_254.01	254.015317	0.7799	neg
0.78_261.04	261.042211	0.7799	neg
0.78_264.98	264.988306	0.7799	neg
0.78_266.03	311.037776	0.7799	neg
0.78_277.02	322.021129	0.7799	neg
0.78_278.12	278.124412	0.7799	neg
0.78_279.98	279.987589	0.7799	neg
0.78_282.90	282.908334	0.7799	neg
0.78_286.17	286.174723	0.7799	neg
0.78_294.03	275.021329	0.7799	neg
0.78_294.93	294.933129	0.7799	neg
0.78_294.98	294.983327	0.7799	neg
0.78_302.93	302.936902	0.7799	neg

0.78_304.991	304.991421	0.7799	neg
0.78_328.058	328.05838	0.7799	neg
0.78_340.896	340.89656	0.7799	neg
0.78_358.046	358.046007	0.7799	neg
0.78_370.974	370.974501	0.7799	neg
0.78_411.090	411.090841	0.7799	neg
0.78_460.017	460.017609	0.7799	neg
0.78_611.144	611.144614	0.7799	neg
0.78_633.126	633.126573	0.7799	neg
0.78_651.097	651.097515	0.7799	neg
0.78_709.113	709.113019	0.7799	neg
0.79_110.035	110.035183	0.7877	pos
0.79_125.130	125.130513	0.7877	pos
0.79_126.090	126.090831	0.7877	pos
0.79_126.136	126.136576	0.7877	pos
0.79_127.139	127.139775	0.7877	pos
0.79_155.070	155.070204	0.7877	pos
0.79_156.003	178.99272	0.7877	pos
0.79_162.050	162.050106	0.7877	pos
0.79_169.035	169.035577	0.7877	pos
0.79_170.100	170.100302	0.7877	pos
0.79_172.133	172.133145	0.7877	pos
0.79_176.005	176.004989	0.7877	pos
0.79_176.037	176.037534	0.7877	pos
0.79_184.133	184.133188	0.7877	pos
0.79_189.100	190.10733	0.7877	pos
0.79_191.048	191.048396	0.7877	pos
0.79_193.068	193.067993	0.7877	pos
0.79_218.090	241.079229	0.7877	pos
0.79_222.037	245.026741	0.7877	pos
0.79_229.154	229.154513	0.7877	pos
0.79_230.124	269.087915	0.7877	pos
0.79_230.186	230.186183	0.7877	pos

0.79_244.06	267.058555	0.7877	pos
0.79_248.14	248.149077	0.7877	pos
0.79_251.04	251.046613	0.7877	pos
0.79_251.06	251.069489	0.7877	pos
0.79_258.17	258.178591	0.7877	pos
0.79_262.17	262.174793	0.7877	pos
0.79_287.18	288.189061	0.7877	pos
0.79_290.13	290.134372	0.7877	pos
0.79_307.08	307.08308	0.7877	pos
0.79_307.08	308.090777	0.7877	pos
0.79_307.58	307.584586	0.7877	pos
0.79_312.11	312.110755	0.7877	pos
0.79_347.02	347.024707	0.7877	pos
0.79_363.05	364.064994	0.7877	pos
0.79_369.08	392.073055	0.7877	pos
0.79_396.23	396.237827	0.7877	pos
0.79_424.10	424.101539	0.7877	pos
0.79_460.20	460.20208	0.7877	pos
0.79_612.15	613.158886	0.7877	pos
0.79_621.24	622.254823	0.7877	pos
0.80_104.05	104.053424	0.80285	pos
0.80_111.01	111.019845	0.79596667	neg
0.80_132.96	132.964261	0.79596667	neg
0.80_138.03	176.999768	0.80285	pos
0.80_145.97	144.9672	0.79596667	neg
0.80_153.04	153.040667	0.80285	pos
0.80_154.99	154.995946	0.79596667	neg
0.80_167.95	212.954665	0.79596667	neg
0.80_174.01	347.023457	0.79596667	neg
0.80_176.05	176.056033	0.79596667	neg
0.80_181.00	181.00958	0.80285	pos
0.80_191.01	191.019551	0.79596667	neg
0.80_192.96	192.969605	0.79596667	neg

0.80_214.008	215.01612	0.80285	pos
0.80_214.033	214.033064	0.79596667	neg
0.80_215.958	214.951651	0.79596667	neg
0.80_220.019	220.019181	0.80285	pos
0.80_222.983	222.983374	0.79596667	neg
0.80_234.980	234.980183	0.79596667	neg
0.80_237.009	217.991549	0.79596667	neg
0.80_241.154	241.154498	0.80285	pos
0.80_244.991	244.991293	0.79596667	neg
0.80_247.093	247.093361	0.79596667	neg
0.80_248.931	248.931395	0.79596667	neg
0.80_248.978	248.978221	0.79596667	neg
0.80_250.948	250.948268	0.79596667	neg
0.80_250.975	250.975399	0.79596667	neg
0.80_260.977	260.977351	0.79596667	neg
0.80_270.913	270.913514	0.79596667	neg
0.80_271.057	271.057106	0.79596667	neg
0.80_271.990	252.972927	0.79596667	neg
0.80_272.910	272.910583	0.79596667	neg
0.80_272.957	272.957489	0.79596667	neg
0.80_278.938	278.93828	0.79596667	neg
0.80_279.038	279.038798	0.79596667	neg
0.80_279.907	279.907736	0.79596667	neg
0.80_280.010	280.010816	0.79596667	neg
0.80_280.942	280.94213	0.79596667	neg
0.80_281.035	281.035895	0.79596667	neg
0.80_284.020	284.020412	0.80285	pos
0.80_286.925	286.925287	0.79596667	neg
0.80_295.030	313.063837	0.80285	pos
0.80_301.020	301.02092	0.79596667	neg
0.80_325.017	325.017107	0.80285	pos
0.80_330.917	330.917394	0.79596667	neg
0.80_332.924	332.924557	0.79596667	neg

0.80_332.951	332.95129	0.79596667	neg
0.80_342.068	342.068514	0.79596667	neg
0.80_350.09(	349.088719	0.79596667	neg
0.80_358.979	358.979356	0.79596667	neg
0.80_379.037	379.037139	0.79596667	neg
0.80_379.099	379.099302	0.79596667	neg
0.80_387.034	387.034384	0.79596667	neg
0.80_388.88(	388.886355	0.79596667	neg
0.80_404.044	404.044288	0.79596667	neg
0.80_409.027	409.027068	0.79596667	neg
0.80_427.10(	427.10692	0.80285	pos
0.80_430.132	475.13039	0.79596667	neg
0.81_135.991	270.975654	0.81161667	neg
0.81_178.897	178.89753	0.81161667	neg
0.81_207.999	207.999207	0.81161667	neg
0.81_230.967	230.967589	0.81161667	neg
0.81_238.025	238.025403	0.81161667	neg
0.81_239.03(	239.03623	0.81161667	neg
0.81_248.053	248.053883	0.81161667	neg
0.81_267.993	267.993006	0.81161667	neg
0.81_293.99(	293.990219	0.81161667	neg
0.81_300.01(	300.016065	0.81161667	neg
0.81_306.057	306.057885	0.81161667	neg
0.81_357.974	357.974926	0.81161667	neg
0.81_365.033	410.03152	0.81161667	neg
0.81_410.00(	410.006838	0.81161667	neg
0.81_534.071	534.071066	0.81161667	neg
0.81_543.044	543.044113	0.81161667	neg
0.82_132.024	150.058308	0.81883333	pos
0.82_164.047	182.081207	0.81883333	pos
0.82_165.05(	166.057853	0.81883333	pos
0.82_172.977	172.977045	0.81883333	pos
0.82_185.042	185.042075	0.81883333	pos

0.82_194.02	194.022349	0.81883333	pos
0.82_203.05	204.063195	0.81883333	pos
0.82_206.04	206.048166	0.81883333	pos
0.82_277.03	277.031909	0.81883333	pos
0.82_318.11	318.119499	0.81883333	pos
0.82_321.06	344.058695	0.81883333	pos
0.82_382.01	382.014498	0.81883333	pos
0.82_417.02	417.022281	0.81883333	pos
0.82_432.12	450.161539	0.81883333	pos
0.82_439.15	439.1557	0.81883333	pos
0.82_457.10	480.097353	0.81883333	pos
0.82_463.09	463.095799	0.81883333	pos
0.82_468.06	491.055403	0.81883333	pos
0.82_468.18	468.182247	0.81883333	pos
0.82_479.98	479.981628	0.81883333	pos
0.82_485.98	485.987079	0.81883333	pos
0.82_503.15	503.15077	0.81883333	pos
0.82_658.11	681.10164	0.81883333	pos
0.82_664.12	665.12816	0.81883333	pos
0.82_785.07	785.077101	0.81883333	pos
0.83_110.02	110.024623	0.82703333	neg
0.83_129.03	129.038524	0.82703333	neg
0.83_129.04	128.035212	0.82703333	neg
0.83_132.05	132.054336	0.83391667	pos
0.83_142.93	142.938604	0.83391667	pos
0.83_151.02	152.031799	0.83391667	pos
0.83_166.94	166.94307	0.82703333	neg
0.83_175.02	175.022616	0.83391667	pos
0.83_201.11	201.112197	0.83391667	pos
0.83_204.15	204.153521	0.83391667	pos
0.83_209.01	209.014915	0.83391667	pos
0.83_214.02	214.021447	0.82703333	neg
0.83_231.04	231.047509	0.83391667	pos

0.83_239.898	257.932315	0.83391667	pos
0.83_244.165	244.165486	0.83391667	pos
0.83_259.092	259.092372	0.83391667	pos
0.83_271.981	271.981622	0.83391667	pos
0.83_280.026	280.026227	0.82703333	neg
0.83_280.067	281.074296	0.83391667	pos
0.83_299.028	299.028379	0.83391667	pos
0.83_302.048	303.056179	0.83391667	pos
0.83_321.069	320.062151	0.82703333	neg
0.83_335.060	335.060181	0.82703333	neg
0.83_343.051	342.043958	0.82703333	neg
0.83_358.018	358.018023	0.82703333	neg
0.83_360.130	360.130099	0.82703333	neg
0.83_365.033	366.040693	0.83391667	pos
0.83_366.056	366.056843	0.82703333	neg
0.83_372.104	372.104891	0.82703333	neg
0.83_375.996	375.99613	0.83391667	pos
0.83_401.999	401.999395	0.82703333	neg
0.83_418.029	418.029746	0.82703333	neg
0.83_418.083	418.083026	0.83391667	pos
0.83_419.043	419.043432	0.82703333	neg
0.83_421.984	421.984753	0.82703333	neg
0.83_438.982	483.980882	0.82703333	neg
0.83_440.012	440.01219	0.82703333	neg
0.83_440.021	440.021351	0.82703333	neg
0.83_455.993	455.993285	0.82703333	neg
0.83_462.001	462.001486	0.82703333	neg
0.83_467.058	467.058661	0.82703333	neg
0.83_476.065	476.065815	0.82703333	neg
0.83_477.973	477.973666	0.82703333	neg
0.83_489.040	489.040633	0.82703333	neg
0.83_489.169	470.151256	0.82703333	neg
0.83_495.083	495.083305	0.83391667	pos

0.83_506.05	506.054787	0.82703333	neg
0.83_512.08	493.068801	0.82703333	neg
0.83_513.03	513.037603	0.83391667	pos
0.83_537.97	537.978576	0.82703333	neg
0.83_541.94	541.943471	0.82703333	neg
0.83_565.02	565.027371	0.82703333	neg
0.83_581.94	581.94234	0.82703333	neg
0.83_597.91	597.916076	0.82703333	neg
0.83_603.92	603.924439	0.82703333	neg
0.83_608.99	608.999477	0.82703333	neg
0.83_783.07	783.070896	0.82703333	neg
0.84_128.00	128.00268	0.84308333	neg
0.84_137.02	137.024181	0.84308333	neg
0.84_159.02	159.029743	0.84308333	neg
0.84_171.98	171.980889	0.84308333	neg
0.84_185.99	185.993711	0.84308333	neg
0.84_187.99	187.990745	0.84308333	neg
0.84_196.02	196.02249	0.84308333	neg
0.84_238.85	238.853297	0.84308333	neg
0.84_238.90	238.900203	0.84308333	neg
0.84_245.94	245.949835	0.84308333	neg
0.84_247.98	247.984575	0.84308333	neg
0.84_247.99	247.993812	0.84308333	neg
0.84_253.98	253.981334	0.84308333	neg
0.84_264.01	264.010174	0.84308333	neg
0.84_275.03	275.031985	0.84308333	neg
0.84_279.05	279.059418	0.84308333	neg
0.84_280.06	280.068651	0.84308333	neg
0.84_288.87	288.87695	0.84308333	neg
0.84_290.87	290.87393	0.84308333	neg
0.84_294.01	587.016335	0.84308333	neg
0.84_298.06	298.069559	0.84308333	neg
0.84_298.84	298.846988	0.84308333	neg

0.84_303.908	303.908092	0.84308333	neg
0.84_305.943	305.943602	0.84308333	neg
0.84_305.952	305.952267	0.84308333	neg
0.84_327.926	327.926525	0.84308333	neg
0.84_336.858	336.858889	0.84308333	neg
0.84_337.017	337.017899	0.84308333	neg
0.84_339.990	339.990552	0.84308333	neg
0.84_353.095	334.07793	0.84308333	neg
0.84_367.933	367.933838	0.84308333	neg
0.84_383.907	383.907843	0.84308333	neg
0.84_385.886	385.886922	0.84308333	neg
0.84_389.915	389.915692	0.84308333	neg
0.84_390.950	390.950542	0.84308333	neg
0.84_456.807	456.807845	0.84308333	neg
0.85_132.065	132.065787	0.84881667	pos
0.85_133.514	133.514824	0.84881667	pos
0.85_144.934	144.934309	0.84881667	pos
0.85_149.999	149.999807	0.84881667	pos
0.85_176.116	176.116283	0.84881667	pos
0.85_188.035	188.03515	0.84881667	pos
0.85_210.133	210.133581	0.84881667	pos
0.85_216.966	216.966801	0.84881667	pos
0.85_241.894	264.883611	0.84881667	pos
0.85_262.887	262.887854	0.84881667	pos
0.85_282.034	305.023365	0.84881667	pos
0.85_300.084	300.084462	0.84881667	pos
0.85_354.077	354.077069	0.84881667	pos
0.86_121.943	121.943908	0.8592	neg
0.86_128.901	128.901412	0.8592	neg
0.86_135.044	135.044103	0.86366667	pos
0.86_150.112	150.112376	0.86366667	pos
0.86_157.781	157.781248	0.86366667	pos
0.86_180.904	180.903989	0.86366667	pos

0.86_181.158	181.158778	0.86366667	pos
0.86_185.032	185.032214	0.86366667	pos
0.86_186.855	186.855908	0.8592	neg
0.86_205.056	205.056722	0.86366667	pos
0.86_231.031	231.031409	0.8592	neg
0.86_283.078	283.078237	0.8592	neg
0.86_296.091	277.073511	0.8592	neg
0.86_296.859	296.859375	0.8592	neg
0.86_304.090	327.07962	0.86366667	pos
0.86_304.090	303.083319	0.8592	neg
0.86_318.831	318.8319	0.8592	neg
0.86_323.897	323.897308	0.8592	neg
0.86_325.065	325.065144	0.8592	neg
0.86_332.904	332.904326	0.8592	neg
0.86_383.024	383.023954	0.8592	neg
0.86_401.050	401.050499	0.8592	neg
0.86_423.032	423.03228	0.8592	neg
0.88_148.943	148.943487	0.87703333	neg
0.88_150.014	150.013971	0.87973333	pos
0.88_164.930	164.930029	0.87973333	pos
0.88_198.097	198.097615	0.87973333	pos
0.88_199.017	199.017177	0.87703333	neg
0.88_213.888	213.888743	0.87703333	neg
0.88_215.886	215.886077	0.87703333	neg
0.88_239.161	239.161668	0.87973333	pos
0.88_247.044	247.044844	0.87703333	neg
0.88_262.900	262.900098	0.87703333	neg
0.88_266.894	266.894877	0.87703333	neg
0.88_287.986	287.986252	0.87703333	neg
0.88_298.007	298.007516	0.87703333	neg
0.88_315.066	315.06615	0.87973333	pos
0.88_322.004	323.01125	0.87973333	pos
0.88_344.897	344.897174	0.87703333	neg

0.88_360.00[illegible]	360.007275	0.87703333	neg
0.89_114.02[illegible]	114.024261	0.8943	neg
0.89_134.03[illegible]	115.02066	0.8943	neg
0.89_135.02[illegible]	135.027	0.8943	neg
0.89_146.98[illegible]	146.982918	0.8943	neg
0.89_148.97[illegible]	148.97989	0.8943	neg
0.89_187.00[illegible]	187.001331	0.8943	neg
0.89_188.05[illegible]	188.055836	0.8943	neg
0.89_201.03[illegible]	201.037873	0.8943	neg
0.89_204.94[illegible]	204.941283	0.8943	neg
0.89_208.97[illegible]	208.973648	0.8943	neg
0.89_224.95[illegible]	224.956981	0.8943	neg
0.89_225.95[illegible]	206.938508	0.8943	neg
0.89_229.97[illegible]	229.97199	0.8943	neg
0.89_231.91[illegible]	231.914791	0.8943	neg
0.89_236.02[illegible]	236.020848	0.8943	neg
0.89_240.91[illegible]	240.910956	0.8943	neg
0.89_256.02[illegible]	256.022895	0.8943	neg
0.89_258.05[illegible]	258.059374	0.8943	neg
0.89_266.94[illegible]	266.941899	0.8943	neg
0.89_268.92[illegible]	268.929488	0.8943	neg
0.89_268.93[illegible]	268.938771	0.8943	neg
0.89_278.01[illegible]	258.996575	0.8943	neg
0.89_278.98[illegible]	278.988418	0.8943	neg
0.89_283.92[illegible]	328.922956	0.8943	neg
0.89_285.02[illegible]	285.022672	0.8943	neg
0.89_285.95[illegible]	266.932457	0.8943	neg
0.89_289.92[illegible]	288.914627	0.8943	neg
0.89_289.99[illegible]	289.99734	0.8943	neg
0.89_304.88[illegible]	304.88825	0.8943	neg
0.89_310.11[illegible]	310.114368	0.8943	neg
0.89_314.97[illegible]	314.970583	0.8943	neg
0.89_316.95[illegible]	316.954993	0.8943	neg

0.89_318.952	318.952169	0.8943	neg
0.89_320.828	320.82867	0.8943	neg
0.89_320.988	320.988099	0.8943	neg
0.89_320.996	320.996375	0.8943	neg
0.89_328.941	328.941825	0.8943	neg
0.89_329.025	329.02559	0.8943	neg
0.89_352.062	352.06246	0.8943	neg
0.89_358.943	358.943214	0.8943	neg
0.89_367.887	348.869971	0.8943	neg
0.89_372.886	372.886744	0.8943	neg
0.89_378.955	378.955699	0.8943	neg
0.89_384.942	384.942874	0.8943	neg
0.89_386.984	386.984159	0.8943	neg
0.89_431.140	431.140492	0.8943	neg
0.89_454.058	454.057992	0.8943	neg
0.90_171.981	171.981685	0.89581667	pos
0.90_172.045	172.045531	0.89581667	pos
0.90_185.990	185.99018	0.89581667	pos
0.90_199.131	199.131676	0.89581667	pos
0.90_204.022	227.011341	0.89581667	pos
0.90_220.967	220.967811	0.89581667	pos
0.90_230.002	268.965299	0.89581667	pos
0.90_233.982	233.982148	0.89581667	pos
0.90_242.935	242.935204	0.89581667	pos
0.90_247.016	247.016634	0.89581667	pos
0.90_262.897	262.897402	0.89581667	pos
0.90_262.990	262.990543	0.89581667	pos
0.90_274.972	274.972285	0.89581667	pos
0.90_276.934	276.934207	0.89581667	pos
0.90_277.971	277.971945	0.89581667	pos
0.90_329.943	329.9433	0.89581667	pos
0.90_333.968	334.975503	0.89581667	pos
0.90_368.985	368.985604	0.89581667	pos

0.90_650.94(	650.946038	0.89581667	pos
0.91_172.95(	172.95645	0.9113	pos
0.91_180.50(	180.505954	0.9113	pos
0.91_189.97!	189.975544	0.91118333	neg
0.91_190.96:	190.963734	0.9113	pos
0.91_202.98	202.987483	0.9113	pos
0.91_214.89	214.896988	0.91118333	neg
0.91_216.89	216.894014	0.91118333	neg
0.91_221.02:	441.039036	0.91118333	neg
0.91_232.97!	232.975297	0.91118333	neg
0.91_246.03	246.031028	0.91118333	neg
0.91_250.94!	233.945764	0.9113	pos
0.91_252.02!	252.025365	0.91118333	neg
0.91_255.92	255.927679	0.9113	pos
0.91_262.15	262.157486	0.9113	pos
0.91_263.87!	263.875119	0.91118333	neg
0.91_268.01	267.009797	0.91118333	neg
0.91_268.95	268.95216	0.91118333	neg
0.91_270.91:	270.913416	0.91118333	neg
0.91_270.96(	270.966764	0.9113	pos
0.91_274.95!	274.955039	0.91118333	neg
0.91_274.97(	274.970651	0.91118333	neg
0.91_277.17!	277.179823	0.9113	pos
0.91_290.91	290.911435	0.91118333	neg
0.91_292.08(	292.08002	0.91118333	neg
0.91_296.01!	296.015194	0.91118333	neg
0.91_296.92	296.927381	0.91118333	neg
0.91_296.99!	296.999082	0.91118333	neg
0.91_303.03(	348.034901	0.91118333	neg
0.91_308.03(	308.036454	0.91118333	neg
0.91_315.00:	315.003847	0.9113	pos
0.91_330.88!	330.887955	0.91118333	neg
0.91_330.95	331.958668	0.9113	pos

0.91_332.961	332.961091	0.91118333	neg
0.91_342.968	342.968934	0.91118333	neg
0.91_343.944	343.944702	0.91118333	neg
0.91_343.985	388.983862	0.91118333	neg
0.91_349.910	349.910906	0.91118333	neg
0.91_350.914	350.914796	0.91118333	neg
0.91_352.924	352.924846	0.91118333	neg
0.91_359.030	359.030055	0.9113	pos
0.91_364.039	364.03899	0.91118333	neg
0.91_371.035	371.035354	0.91118333	neg
0.91_373.006	373.006901	0.91118333	neg
0.91_377.952	377.952334	0.91118333	neg
0.91_381.039	381.039136	0.91118333	neg
0.91_390.028	371.010286	0.91118333	neg
0.91_392.055	392.055193	0.91118333	neg
0.91_398.041	379.023457	0.91118333	neg
0.91_411.932	411.932315	0.91118333	neg
0.91_416.022	416.022045	0.91118333	neg
0.91_433.010	433.010548	0.91118333	neg
0.91_449.983	430.965644	0.91118333	neg
0.91_460.048	460.04837	0.91118333	neg
0.91_483.024	483.023954	0.91118333	neg
0.91_493.052	493.052854	0.91118333	neg
0.91_498.997	498.99784	0.91118333	neg
0.91_509.026	509.02662	0.91118333	neg
0.91_537.079	537.078986	0.91118333	neg
0.91_648.931	648.931571	0.91118333	neg
0.93_135.030	135.030406	0.92838333	pos
0.93_155.874	155.874059	0.92753333	neg
0.93_157.871	157.871918	0.92753333	neg
0.93_169.989	169.989184	0.92753333	neg
0.93_170.032	170.032759	0.92838333	pos
0.93_178.920	178.920205	0.92753333	neg

0.93_195.00[illegible]	213.037034	0.92838333	pos
0.93_216.04[illegible]	216.048825	0.92753333	neg
0.93_230.95[illegible]	230.955549	0.92753333	neg
0.93_230.98[illegible]	230.982625	0.92838333	pos
0.93_238.91[illegible]	238.918594	0.92753333	neg
0.93_239.46[illegible]	239.469411	0.92838333	pos
0.93_239.86[illegible]	239.866689	0.92753333	neg
0.93_248.05[illegible]	248.054033	0.92753333	neg
0.93_251.66[illegible]	251.668215	0.92838333	pos
0.93_286.01[illegible]	286.015719	0.92753333	neg
0.93_289.88[illegible]	289.889886	0.92753333	neg
0.93_298.86[illegible]	298.869615	0.92753333	neg
0.93_318.98[illegible]	318.981181	0.92753333	neg
0.93_328.98[illegible]	328.981016	0.92753333	neg
0.93_344.99[illegible]	344.994841	0.92753333	neg
0.93_346.00[illegible]	326.984083	0.92753333	neg
0.93_346.96[illegible]	346.968145	0.92753333	neg
0.93_350.98[illegible]	350.987058	0.92838333	pos
0.93_358.70[illegible]	358.700413	0.92838333	pos
0.93_397.01[illegible]	397.012908	0.92753333	neg
0.93_410.95[illegible]	410.956673	0.92753333	neg
0.93_449.02[illegible]	449.026658	0.92753333	neg
0.93_458.01[illegible]	438.997631	0.92753333	neg
0.93_561.03[illegible]	561.039928	0.92753333	neg
0.94_123.01[illegible]	123.019906	0.944	neg
0.94_159.04[illegible]	159.046796	0.944	neg
0.94_274.87[illegible]	274.877703	0.944	neg
0.94_275.01[illegible]	275.017551	0.944	neg
0.94_276.96[illegible]	276.961213	0.944	neg
0.94_284.07[illegible]	265.056449	0.944	neg
0.94_284.99[illegible]	284.999437	0.944	neg
0.94_337.01[illegible]	337.013042	0.944	neg
0.94_356.90[illegible]	356.901907	0.944	neg

0.94_465.00(	465.000205	0.944	neg
0.94_517.01:	517.013714	0.944	neg
0.94_576.91(	576.910868	0.944	neg
0.95_111.05!	111.055565	0.94518333	pos
0.95_131.50:	131.500982	0.94518333	pos
0.95_148.93:	131.929924	0.94518333	pos
0.95_168.04!	168.04911	0.94518333	pos
0.95_196.00!	196.009768	0.94518333	pos
0.95_199.98[illegible]	199.987783	0.94518333	pos
0.95_200.06!	200.068468	0.94518333	pos
0.95_208.06:	208.063864	0.94518333	pos
0.95_212.94!	212.945763	0.94518333	pos
0.95_230.94(	230.946204	0.94518333	pos
0.95_244.63:	244.632394	0.94518333	pos
0.95_329.52!	329.525767	0.94518333	pos
0.96_131.07(	131.070473	0.96066667	pos
0.96_138.51:	138.511904	0.96066667	pos
0.96_172.98!	172.985607	0.96215	neg
0.96_188.03(	187.022917	0.96215	neg
0.96_214.97(	214.970316	0.96215	neg
0.96_218.98:	218.982113	0.96215	neg
0.96_219.08:	219.082946	0.96066667	pos
0.96_222.65!	222.655704	0.96066667	pos
0.96_234.94[illegible]	234.947124	0.96066667	pos
0.96_234.95(	234.956526	0.96066667	pos
0.96_272.92!	272.92912	0.96215	neg
0.96_276.05:	276.052265	0.96215	neg
0.96_276.97(	276.970517	0.96215	neg
0.96_280.05:	280.05157	0.96066667	pos
0.96_294.15[illegible]	294.154606	0.96066667	pos
0.96_302.84[illegible]	302.846976	0.96215	neg
0.96_340.91(	340.916294	0.96215	neg
0.96_358.85(	358.850443	0.96215	neg

0.96_392.10!	392.10576	0.96066667	pos
0.96_394.97	394.971357	0.96215	neg
0.98_110.00!	110.009099	0.97735	pos
0.98_128.95(	128.95089	0.97735	pos
0.98_133.92	133.926954	0.97735	pos
0.98_147.11	147.117165	0.97735	pos
0.98_161.05!	161.059741	0.97735	pos
0.98_169.96!	169.965531	0.97735	pos
0.98_172.86	172.867709	0.97965	neg
0.98_174.95:	174.953485	0.97735	pos
0.98_181.12	181.122392	0.97735	pos
0.98_183.87(	183.876872	0.97965	neg
0.98_183.96!	164.947332	0.97965	neg
0.98_184.09	184.094429	0.97735	pos
0.98_186.95(	186.956279	0.97735	pos
0.98_187.13:	187.13287	0.97735	pos
0.98_193.93(	193.930128	0.97735	pos
0.98_213.04!	213.049072	0.97965	neg
0.98_222.91!	222.915542	0.97965	neg
0.98_230.89!	230.899863	0.97965	neg
0.98_231.02:	231.023133	0.97965	neg
0.98_231.89	231.894661	0.97965	neg
0.98_235.20!	235.205672	0.97735	pos
0.98_238.90(	238.900305	0.97965	neg
0.98_239.89	478.789775	0.97965	neg
0.98_240.13	240.134447	0.97735	pos
0.98_241.88	241.882445	0.97965	neg
0.98_244.88	244.888058	0.97965	neg
0.98_270.97	270.977651	0.97735	pos
0.98_280.17	280.178716	0.97735	pos
0.98_296.89(	296.890303	0.97965	neg
0.98_301.97!	282.957886	0.97965	neg
0.98_327.00!	327.009908	0.97965	neg

0.98_342.91	342.913423	0.97965	neg
0.98_352.98	352.986849	0.97965	neg
0.98_405.00	405.000351	0.97965	neg
0.98_827.26	827.267779	0.97965	neg
0.99_112.95	112.956263	0.99473333	pos
0.99_113.02	113.023683	0.99473333	pos
0.99_140.95	140.951308	0.99473333	pos
0.99_144.93	144.934344	0.99473333	pos
0.99_154.05	154.058706	0.99473333	pos
0.99_161.96	161.961028	0.99473333	pos
0.99_164.07	164.078829	0.99473333	pos
0.99_176.78	176.788412	0.99473333	pos
0.99_203.22	203.223091	0.99473333	pos
0.99_206.65	206.653717	0.99473333	pos
0.99_226.97	226.975018	0.99473333	pos
0.99_241.98	241.980701	0.99473333	pos
0.99_264.67	264.678849	0.99473333	pos
0.99_278.66	279.677183	0.99473333	pos
0.99_290.66	290.668138	0.99473333	pos
1.00_128.95	128.959471	0.99793333	neg
1.00_134.86	134.865074	0.99793333	neg
1.00_136.90	136.909685	0.99793333	neg
1.00_158.89	158.892923	0.99793333	neg
1.00_159.06	159.066026	0.99793333	neg
1.00_164.95	164.956793	0.99793333	neg
1.00_170.87	170.870728	0.99793333	neg
1.00_173.11	173.118128	0.99793333	neg
1.00_180.89	180.899535	0.99793333	neg
1.00_183.90	228.902719	0.99793333	neg
1.00_185.89	185.892561	0.99793333	neg
1.00_196.88	196.884752	0.99793333	neg
1.00_196.89	196.89409	0.99793333	neg
1.00_207.99	207.999018	0.99793333	neg

1.00_214.03[illegible]	214.033226	0.99793333	neg
1.00_235.03[illegible]	235.039369	0.99793333	neg
1.00_244.89[illegible]	244.897323	0.99793333	neg
1.00_246.88[illegible]	246.885069	0.99793333	neg
1.00_306.88[illegible]	306.887176	0.99793333	neg
1.00_308.96[illegible]	308.960836	0.99793333	neg
1.00_312.87[illegible]	312.876143	0.99793333	neg
1.00_341.10[illegible]	341.108776	0.99793333	neg
1.00_350.94[illegible]	350.945331	0.99793333	neg
1.00_367.90[illegible]	367.905886	0.99793333	neg
1.00_376.94[illegible]	376.948454	0.99793333	neg
1.00_418.93[illegible]	418.932915	0.99793333	neg
1.00_434.91[illegible]	434.917756	0.99793333	neg
1.00_462.95[illegible]	462.959072	0.99793333	neg
1.01_129.95[illegible]	129.958789	1.0117	pos
1.01_148.11[illegible]	148.118457	1.0117	pos
1.01_151.56[illegible]	151.560465	1.0117	pos
1.01_158.10[illegible]	158.105116	1.0117	pos
1.01_167.03[illegible]	167.034132	1.0117	pos
1.01_172.07[illegible]	173.081072	1.0117	pos
1.01_172.12[illegible]	172.120643	1.0117	pos
1.01_182.98[illegible]	182.985207	1.0117	pos
1.01_193.92[illegible]	193.920501	1.0117	pos
1.01_202.95[illegible]	202.951229	1.0117	pos
1.01_206.15[illegible]	206.151981	1.0117	pos
1.01_242.56[illegible]	242.561985	1.0117	pos
1.01_263.14[illegible]	263.148417	1.0117	pos
1.01_280.99[illegible]	280.99549	1.0117	pos
1.01_308.18[illegible]	308.187772	1.0117	pos
1.01_308.68[illegible]	308.689635	1.0117	pos
1.02_1025.2[illegible]	1025.29758	1.01586667	neg
1.02_103.92[illegible]	103.9201	1.01586667	neg
1.02_136.86[illegible]	136.863031	1.01586667	neg

1.02_147.02	293.049372	1.01586667	neg
1.02_152.89	152.894873	1.01586667	neg
1.02_166.99	166.995995	1.01586667	neg
1.02_172.96	172.962109	1.01586667	neg
1.02_182.99	182.990899	1.01586667	neg
1.02_186.98	186.986069	1.01586667	neg
1.02_207.91	207.918497	1.01586667	neg
1.02_226.98	226.980948	1.01586667	neg
1.02_227.01	227.017224	1.01586667	neg
1.02_232.94	232.94425	1.01586667	neg
1.02_233.91	233.910385	1.01586667	neg
1.02_241.98	241.989353	1.01586667	neg
1.02_249.99	249.992633	1.01586667	neg
1.02_250.97	250.978545	1.01586667	neg
1.02_254.92	254.926349	1.01586667	neg
1.02_256.93	256.936667	1.01586667	neg
1.02_260.87	260.873331	1.01586667	neg
1.02_268.98	268.989154	1.01586667	neg
1.02_272.96	272.960484	1.01586667	neg
1.02_284.95	284.955037	1.01586667	neg
1.02_288.93	288.934762	1.01586667	neg
1.02_290.97	290.971242	1.01586667	neg
1.02_295.00	295.004535	1.01586667	neg
1.02_298.88	298.887357	1.01586667	neg
1.02_304.04	304.047614	1.01586667	neg
1.02_314.89	314.895381	1.01586667	neg
1.02_324.92	324.923901	1.01586667	neg
1.02_329.86	329.86574	1.01586667	neg
1.02_339.03	339.030744	1.01586667	neg
1.02_352.94	352.942332	1.01586667	neg
1.02_359.84	358.840813	1.01586667	neg
1.02_360.97	360.974029	1.01586667	neg
1.02_372.03	372.035067	1.01586667	neg

1.02_380.86[illegible]	380.86311	1.01586667	neg
1.03_115.11[illegible]	115.112055	1.02758333	pos
1.03_120.04[illegible]	120.04459	1.02758333	pos
1.03_124.12[illegible]	124.121708	1.0341	neg
1.03_126.13[illegible]	126.136624	1.02758333	pos
1.03_127.13[illegible]	127.139797	1.02758333	pos
1.03_129.01[illegible]	129.016916	1.0341	neg
1.03_139.03[illegible]	139.039011	1.02758333	pos
1.03_142.96[illegible]	142.961909	1.02758333	pos
1.03_145.08[illegible]	145.086844	1.0341	neg
1.03_149.09[illegible]	149.096165	1.02758333	pos
1.03_152.97[illegible]	152.978061	1.0341	neg
1.03_157.01[illegible]	157.011627	1.0341	neg
1.03_158.96[illegible]	141.958649	1.02758333	pos
1.03_174.03[illegible]	174.029982	1.0341	neg
1.03_181.93[illegible]	181.937537	1.0341	neg
1.03_184.05[illegible]	184.058912	1.0341	neg
1.03_189.07[illegible]	189.073314	1.02758333	pos
1.03_192.10[illegible]	192.10921	1.0341	neg
1.03_199.98[illegible]	199.981197	1.0341	neg
1.03_205.91[illegible]	205.915285	1.0341	neg
1.03_209.09[illegible]	209.099307	1.0341	neg
1.03_211.93[illegible]	211.931023	1.02758333	pos
1.03_213.00[illegible]	211.999815	1.0341	neg
1.03_214.98[illegible]	428.961513	1.0341	neg
1.03_223.06[illegible]	223.063629	1.02758333	pos
1.03_226.06[illegible]	226.061194	1.02758333	pos
1.03_234.95[illegible]	234.95303	1.0341	neg
1.03_235.92[illegible]	235.925998	1.0341	neg
1.03_242.06[illegible]	242.063487	1.02758333	pos
1.03_260.09[illegible]	260.096623	1.0341	neg
1.03_262.00[illegible]	262.001859	1.0341	neg
1.03_270.05[illegible]	270.052279	1.0341	neg

1.03_281.487	281.487414	1.02758333	pos
1.03_291.19(	274.187286	1.02758333	pos
1.03_293.95:	293.951516	1.0341	neg
1.03_296.13!	296.135038	1.0341	neg
1.03_304.18!	304.18537	1.0341	neg
1.03_309.87(	309.870029	1.0341	neg
1.03_309.96	309.967509	1.0341	neg
1.03_309.97(	309.976724	1.0341	neg
1.03_312.00	292.986847	1.0341	neg
1.03_328.08	328.083984	1.0341	neg
1.03_332.10!	332.109908	1.0341	neg
1.03_354.00:	354.002973	1.0341	neg
1.03_380.82:	380.82253	1.0341	neg
1.03_388.02:	388.021595	1.0341	neg
1.03_429.02	429.027625	1.0341	neg
1.03_456.00!	456.009376	1.0341	neg
1.03_464.05!	464.058941	1.0341	neg
1.03_472.98	472.987588	1.0341	neg
1.03_496.95:	496.952295	1.0341	neg
1.03_523.63:	523.632564	1.02758333	pos
1.03_990.32!	1035.32672	1.0341	neg
1.04_107.95(	107.950808	1.0442	pos
1.04_112.05(	112.05088	1.0442	pos
1.04_1152.3	1175.36822	1.0442	pos
1.04_118.04	118.044787	1.0442	pos
1.04_120.09:	120.093075	1.0442	pos
1.04_125.13(	125.130606	1.0442	pos
1.04_130.60:	130.602188	1.0442	pos
1.04_135.94!	135.945393	1.0442	pos
1.04_147.11:	170.100362	1.0442	pos
1.04_161.96!	161.969707	1.0442	pos
1.04_162.05(	162.050152	1.0442	pos
1.04_181.10	181.107709	1.0442	pos

1.04_208.628	208.628807	1.0442	pos
1.04_271.263	271.263095	1.0442	pos
1.04_309.161	309.160984	1.0442	pos
1.04_393.720	393.72004	1.0442	pos
1.04_431.042	431.042357	1.0442	pos
1.04_446.120	446.120422	1.0442	pos
1.04_486.171	509.16049	1.0442	pos
1.04_604.157	604.156956	1.0442	pos
1.04_604.658	604.658336	1.0442	pos
1.05_187.108	187.108486	1.0516	neg
1.05_200.965	200.965237	1.0516	neg
1.05_228.915	228.91568	1.0516	neg
1.05_280.989	280.989311	1.0516	neg
1.05_305.003	305.003657	1.0516	neg
1.05_331.027	331.027355	1.0516	neg
1.05_377.992	377.992758	1.0516	neg
1.05_397.977	397.976957	1.0516	neg
1.05_444.023	444.023137	1.0516	neg
1.06_124.039	124.039485	1.06053333	pos
1.06_151.049	174.038587	1.06053333	pos
1.06_173.092	173.092084	1.06053333	pos
1.06_181.460	181.460184	1.06053333	pos
1.06_181.794	181.794692	1.06053333	pos
1.06_190.963	190.963675	1.06053333	pos
1.06_191.463	191.463616	1.06053333	pos
1.06_203.102	203.102649	1.06053333	pos
1.06_203.115	204.122962	1.06053333	pos
1.06_211.105	211.105232	1.06053333	pos
1.06_229.453	229.453797	1.06053333	pos
1.06_230.113	230.113463	1.06053333	pos
1.06_254.124	254.124519	1.06053333	pos
1.06_271.686	271.686523	1.06053333	pos
1.06_323.051	324.05897	1.06053333	pos

1.06_341.04[illegible]	341.04717	1.06053333	pos
1.06_362.08[illegible]	362.085769	1.06053333	pos
1.06_664.11[illegible]	664.1155	1.06053333	pos
1.07_111.01[illegible]	111.019838	1.06741667	neg
1.07_124.01[illegible]	124.014921	1.06741667	neg
1.07_176.00[illegible]	176.002063	1.06741667	neg
1.07_188.09[illegible]	188.092519	1.06741667	neg
1.07_208.02[illegible]	208.028303	1.06741667	neg
1.07_223.05[illegible]	204.033168	1.06741667	neg
1.07_245.12[illegible]	245.125196	1.06741667	neg
1.07_264.07[illegible]	264.073603	1.06741667	neg
1.07_265.01[illegible]	265.018896	1.06741667	neg
1.07_270.97[illegible]	270.975424	1.06741667	neg
1.07_278.12[illegible]	278.124288	1.06741667	neg
1.07_279.05[illegible]	279.059235	1.06741667	neg
1.07_286.17[illegible]	286.174645	1.06741667	neg
1.07_286.97[illegible]	286.970429	1.06741667	neg
1.07_294.02[illegible]	294.028856	1.06741667	neg
1.07_311.03[illegible]	311.037682	1.06741667	neg
1.07_311.04[illegible]	311.049327	1.06741667	neg
1.07_317.53[illegible]	634.069474	1.06741667	neg
1.07_319.05[illegible]	319.054423	1.06741667	neg
1.07_323.02[illegible]	323.028155	1.06741667	neg
1.07_328.03[illegible]	328.034171	1.06741667	neg
1.07_328.05[illegible]	328.058319	1.06741667	neg
1.07_334.05[illegible]	315.032335	1.06741667	neg
1.07_341.03[illegible]	341.029995	1.06741667	neg
1.07_342.06[illegible]	342.068331	1.06741667	neg
1.07_373.11[illegible]	372.10455	1.06741667	neg
1.07_391.09[illegible]	391.099233	1.06741667	neg
1.07_393.12[illegible]	392.113097	1.06741667	neg
1.07_398.06[illegible]	398.068949	1.06741667	neg
1.07_403.19[illegible]	403.194345	1.06741667	neg

1.07_434.05[illegible]	434.058061	1.06741667	neg
1.07_436.09[illegible]	417.078155	1.06741667	neg
1.07_449.11[illegible]	448.102928	1.06741667	neg
1.07_458.97[illegible]	458.97309	1.06741667	neg
1.07_498.08[illegible]	498.088974	1.06741667	neg
1.07_514.08[illegible]	514.08385	1.06741667	neg
1.07_522.19[illegible]	522.193695	1.06741667	neg
1.07_523.17[illegible]	523.177777	1.06741667	neg
1.07_530.24[illegible]	530.24373	1.06741667	neg
1.07_551.13[illegible]	551.137136	1.06741667	neg
1.07_591.13[illegible]	590.125007	1.06741667	neg
1.07_611.14[illegible]	611.144346	1.06741667	neg
1.07_628.06[illegible]	628.069205	1.06741667	neg
1.08_102.05[illegible]	102.055363	1.07531667	pos
1.08_108.02[illegible]	108.020125	1.08208333	neg
1.08_110.02[illegible]	110.02458	1.08208333	neg
1.08_110.03[illegible]	110.035165	1.07531667	pos
1.08_113.03[illegible]	113.034841	1.07531667	pos
1.08_114.03[illegible]	114.038153	1.07531667	pos
1.08_124.05[illegible]	124.058713	1.07531667	pos
1.08_129.04[illegible]	130.049998	1.07531667	pos
1.08_132.02[illegible]	150.058298	1.07531667	pos
1.08_136.03[illegible]	137.045765	1.07531667	pos
1.08_136.99[illegible]	136.9937	1.07531667	pos
1.08_138.04[illegible]	138.04274	1.07531667	pos
1.08_141.10[illegible]	141.102004	1.07531667	pos
1.08_148.09[illegible]	148.096777	1.07531667	pos
1.08_152.03[illegible]	153.040628	1.07531667	pos
1.08_166.07[illegible]	166.07223	1.07531667	pos
1.08_167.11[illegible]	167.117895	1.07531667	pos
1.08_168.02[illegible]	169.035586	1.07531667	pos
1.08_172.04[illegible]	172.040151	1.07531667	pos
1.08_172.13[illegible]	172.133111	1.07531667	pos

1.08_175.00	175.001682	1.07531667	pos
1.08_175.05	175.053469	1.07531667	pos
1.08_177.54	177.546899	1.07531667	pos
1.08_187.99	187.990714	1.08208333	neg
1.08_189.10	190.107339	1.07531667	pos
1.08_191.04	191.048441	1.07531667	pos
1.08_193.56	193.569822	1.07531667	pos
1.08_199.62	199.623519	1.07531667	pos
1.08_213.01	213.012584	1.08208333	neg
1.08_213.62	213.626391	1.07531667	pos
1.08_214.63	214.634201	1.07531667	pos
1.08_217.11	217.118112	1.07531667	pos
1.08_219.09	219.097432	1.07531667	pos
1.08_220.02	220.028152	1.08208333	neg
1.08_220.14	203.139083	1.07531667	pos
1.08_222.64	222.644242	1.07531667	pos
1.08_225.98	224.979408	1.08208333	neg
1.08_231.09	231.097359	1.07531667	pos
1.08_236.00	236.003578	1.08208333	neg
1.08_236.97	236.970406	1.07531667	pos
1.08_244.06	245.076698	1.07531667	pos
1.08_247.98	247.984423	1.08208333	neg
1.08_247.99	247.993832	1.08208333	neg
1.08_251.99	251.998422	1.08208333	neg
1.08_258.08	259.093312	1.07531667	pos
1.08_258.17	258.178612	1.07531667	pos
1.08_263.15	286.139447	1.07531667	pos
1.08_271.00	271.000796	1.07531667	pos
1.08_272.07	295.066013	1.07531667	pos
1.08_272.65	272.657819	1.07531667	pos
1.08_274.13	274.139459	1.07531667	pos
1.08_275.00	275.008239	1.07531667	pos
1.08_280.04	279.038801	1.08208333	neg

1.08_282.10[illegible]	282.100425	1.07531667	pos
1.08_285.66[illegible]	285.665729	1.07531667	pos
1.08_286.09[illegible]	286.096571	1.07531667	pos
1.08_287.18[illegible]	288.189009	1.07531667	pos
1.08_289.12[illegible]	312.110787	1.07531667	pos
1.08_300.20[illegible]	300.202737	1.07531667	pos
1.08_307.06[illegible]	306.057807	1.08208333	neg
1.08_307.58[illegible]	307.584519	1.07531667	pos
1.08_308.09[illegible]	308.090739	1.07531667	pos
1.08_312.03[illegible]	330.072676	1.07531667	pos
1.08_314.07[illegible]	359.072762	1.08208333	neg
1.08_316.14[illegible]	316.149574	1.07531667	pos
1.08_322.98[illegible]	322.988097	1.08208333	neg
1.08_324.98[illegible]	324.986264	1.08208333	neg
1.08_327.06[illegible]	327.067088	1.07531667	pos
1.08_332.96[illegible]	332.961091	1.08208333	neg
1.08_336.03[illegible]	336.035533	1.07531667	pos
1.08_338.18[illegible]	338.181815	1.07531667	pos
1.08_340.08[illegible]	340.082839	1.08208333	neg
1.08_343.16[illegible]	343.160777	1.07531667	pos
1.08_349.16[illegible]	349.161307	1.07531667	pos
1.08_359.05[illegible]	359.058653	1.08208333	neg
1.08_359.98[illegible]	359.982374	1.08208333	neg
1.08_362.05[illegible]	362.050646	1.08208333	neg
1.08_368.07[illegible]	368.076535	1.08208333	neg
1.08_371.11[illegible]	371.112148	1.07531667	pos
1.08_380.10[illegible]	381.114955	1.07531667	pos
1.08_386.00[illegible]	386.005082	1.08208333	neg
1.08_386.11[illegible]	386.112837	1.07531667	pos
1.08_387.17[illegible]	405.20888	1.07531667	pos
1.08_404.13[illegible]	404.130695	1.08208333	neg
1.08_404.17[illegible]	404.178438	1.08208333	neg
1.08_420.01[illegible]	420.010663	1.08208333	neg

1.08_447.219	447.219366	1.07531667	pos
1.08_457.107	458.115009	1.07531667	pos
1.08_457.974	457.974108	1.08208333	neg
1.08_489.146	489.146123	1.07531667	pos
1.08_493.068	493.068368	1.08208333	neg
1.08_510.072	510.071991	1.08208333	neg
1.08_606.120	606.120145	1.08208333	neg
1.08_651.096	651.096944	1.08208333	neg
1.08_653.110	653.110826	1.07531667	pos
1.08_663.113	663.113788	1.08208333	neg
1.08_668.132	667.12508	1.08208333	neg
1.08_708.085	707.077738	1.08208333	neg
1.09_152.070	152.0705	1.08996667	pos
1.09_219.079	219.079648	1.08996667	pos
1.09_257.069	257.069336	1.08996667	pos
1.09_288.118	288.118818	1.08996667	pos
1.09_310.536	310.536091	1.08996667	pos
1.09_311.039	311.03969	1.08996667	pos
1.09_321.069	322.076604	1.08996667	pos
1.09_343.126	344.133737	1.08996667	pos
1.09_406.192	406.192936	1.08996667	pos
1.09_463.095	463.095201	1.08996667	pos
1.09_471.127	471.127677	1.08996667	pos
1.09_620.064	620.064748	1.08996667	pos
1.09_665.128	665.128047	1.08996667	pos
1.09_697.064	697.064735	1.08996667	pos
1.10_107.049	107.049464	1.1046	pos
1.10_123.044	123.0444	1.1046	pos
1.10_132.065	132.065703	1.1046	pos
1.10_163.039	163.039881	1.09681667	neg
1.10_171.007	171.007592	1.09681667	neg
1.10_181.069	181.069706	1.09681667	neg
1.10_184.617	184.617777	1.1046	pos

1.10_188.05[illegible]	188.056071	1.09681667	neg
1.10_188.50[illegible]	188.501501	1.1046	pos
1.10_192.98[illegible]	192.989485	1.09681667	neg
1.10_196.06[illegible]	196.061148	1.09681667	neg
1.10_203.05[illegible]	204.063125	1.1046	pos
1.10_206.04[illegible]	206.049045	1.09681667	neg
1.10_207.05[illegible]	208.063724	1.1046	pos
1.10_209.01[illegible]	209.014833	1.1046	pos
1.10_209.51[illegible]	209.516689	1.1046	pos
1.10_217.11[illegible]	217.118988	1.09681667	neg
1.10_220.03[illegible]	220.036653	1.1046	pos
1.10_238.02[illegible]	238.025105	1.09681667	neg
1.10_240.02[illegible]	240.022195	1.09681667	neg
1.10_284.16[illegible]	284.160301	1.1046	pos
1.10_293.08[illegible]	292.080205	1.09681667	neg
1.10_294.06[illegible]	294.065244	1.09681667	neg
1.10_316.14[illegible]	361.140636	1.09681667	neg
1.10_320.04[illegible]	320.04648	1.09681667	neg
1.10_325.53[illegible]	325.536766	1.09681667	neg
1.10_332.85[illegible]	332.859905	1.09681667	neg
1.10_342.08[illegible]	341.079272	1.09681667	neg
1.10_343.05[illegible]	342.043836	1.09681667	neg
1.10_349.03[illegible]	349.036181	1.1046	pos
1.10_358.70[illegible]	358.700349	1.1046	pos
1.10_359.02[illegible]	358.01802	1.09681667	neg
1.10_365.03[illegible]	410.031568	1.09681667	neg
1.10_368.97[illegible]	368.978651	1.09681667	neg
1.10_375.99[illegible]	375.995991	1.1046	pos
1.10_383.12[illegible]	383.12253	1.09681667	neg
1.10_385.12[illegible]	385.128124	1.09681667	neg
1.10_386.04[illegible]	386.046937	1.1046	pos
1.10_400.00[illegible]	400.002538	1.09681667	neg
1.10_405.03[illegible]	405.039688	1.09681667	neg

1.10_417.07:	417.073616	1.1046	pos
1.10_421.98	421.984867	1.09681667	neg
1.10_423.11	404.093744	1.09681667	neg
1.10_427.02	427.021608	1.09681667	neg
1.10_438.97	483.976351	1.09681667	neg
1.10_440.01	440.01183	1.09681667	neg
1.10_450.16	450.161868	1.1046	pos
1.10_454.02	454.021617	1.09681667	neg
1.10_454.05	454.057712	1.09681667	neg
1.10_460.09	441.080932	1.09681667	neg
1.10_461.99	461.993974	1.09681667	neg
1.10_462.00	462.004057	1.09681667	neg
1.10_463.92	463.928237	1.09681667	neg
1.10_467.05	467.058559	1.09681667	neg
1.10_476.03	476.039317	1.09681667	neg
1.10_504.04	504.047381	1.09681667	neg
1.10_522.08	567.081479	1.09681667	neg
1.10_525.05	525.053807	1.09681667	neg
1.10_542.14	541.142435	1.09681667	neg
1.10_544.23	544.237273	1.09681667	neg
1.10_545.24	546.251632	1.1046	pos
1.10_555.05	555.054907	1.09681667	neg
1.10_557.13	557.137555	1.09681667	neg
1.10_565.10	565.105519	1.09681667	neg
1.10_586.17	586.17831	1.09681667	neg
1.10_588.20	588.203046	1.1046	pos
1.10_596.13	596.13365	1.09681667	neg
1.10_607.14	607.149734	1.09681667	neg
1.10_679.09	679.09208	1.09681667	neg
1.10_698.19	698.191958	1.09681667	neg
1.10_748.15	748.15909	1.09681667	neg
1.10_751.04	751.041566	1.09681667	neg
1.10_770.13	770.138087	1.09681667	neg

1.11_159.04	159.046782	1.1137	neg
1.11_173.02	173.026001	1.1137	neg
1.11_195.03	195.032908	1.1137	neg
1.11_203.05	248.053817	1.1137	neg
1.11_216.91	216.918479	1.1137	neg
1.11_221.04	441.091062	1.1137	neg
1.11_271.04	316.041358	1.1137	neg
1.11_292.04	292.043925	1.1137	neg
1.11_295.98	295.983837	1.1137	neg
1.11_300.02	300.02502	1.1137	neg
1.11_301.02	300.01623	1.1137	neg
1.11_313.01	313.015554	1.1137	neg
1.11_325.03	306.012828	1.1137	neg
1.11_328.86	328.860797	1.1137	neg
1.11_352.03	352.035653	1.1137	neg
1.11_357.98	357.983588	1.1137	neg
1.11_374.00	374.000035	1.1137	neg
1.11_379.95	379.956428	1.1137	neg
1.11_384.02	384.029488	1.1137	neg
1.11_423.06	423.063238	1.1137	neg
1.11_428.05	428.054882	1.1137	neg
1.11_432.01	432.013084	1.1137	neg
1.11_434.01	434.010896	1.1137	neg
1.11_452.01	452.016488	1.1137	neg
1.11_472.08	472.080903	1.1137	neg
1.11_489.57	489.571195	1.1137	neg
1.11_496.04	496.042798	1.1137	neg
1.11_528.12	528.121164	1.1137	neg
1.11_588.10	588.109362	1.1137	neg
1.11_646.13	646.134674	1.1137	neg
1.12_156.04	156.042176	1.11995	pos
1.12_163.07	163.075376	1.11995	pos
1.12_172.97	172.977163	1.11995	pos

1.12_192.59	192.597299	1.11995	pos
1.12_197.50	197.507103	1.11995	pos
1.12_203.22	203.223036	1.11995	pos
1.12_206.63	206.631266	1.11995	pos
1.12_228.63	228.631831	1.11995	pos
1.12_290.50	290.50812	1.11995	pos
1.12_290.94	290.949296	1.11995	pos
1.12_314.17	314.170902	1.11995	pos
1.12_324.01	324.01238	1.11995	pos
1.12_353.14	353.145554	1.11995	pos
1.12_354.12	354.129345	1.11995	pos
1.12_403.20	404.213863	1.11995	pos
1.13_129.01	129.016835	1.13148333	neg
1.13_149.98	149.983282	1.13148333	neg
1.13_171.02	171.027276	1.13148333	neg
1.13_174.00	174.001781	1.13148333	neg
1.13_185.89	185.892495	1.13148333	neg
1.13_188.02	169.011635	1.13148333	neg
1.13_188.94	188.948926	1.13148333	neg
1.13_191.04	191.044327	1.13148333	neg
1.13_203.01	203.018471	1.13148333	neg
1.13_213.03	213.037866	1.13148333	neg
1.13_215.05	215.05342	1.13148333	neg
1.13_220.97	220.973401	1.13148333	neg
1.13_226.00	226.002494	1.13148333	neg
1.13_236.99	236.999328	1.13148333	neg
1.13_264.01	264.010133	1.13148333	neg
1.13_266.93	266.932464	1.13148333	neg
1.13_271.04	252.025303	1.13148333	neg
1.13_271.90	271.907115	1.13148333	neg
1.13_276.97	276.970601	1.13148333	neg
1.13_287.00	287.001026	1.13148333	neg
1.13_288.91	288.914529	1.13148333	neg

1.13_299.037	299.037438	1.13148333	neg
1.13_300.963	300.963068	1.13148333	neg
1.13_304.090	303.083332	1.13148333	neg
1.13_320.996	320.996738	1.13148333	neg
1.13_328.922	328.922805	1.13148333	neg
1.13_331.997	331.997588	1.13148333	neg
1.13_332.859	332.859821	1.13148333	neg
1.13_340.916	340.916513	1.13148333	neg
1.13_342.969	342.969156	1.13148333	neg
1.13_347.978	347.978912	1.13148333	neg
1.13_354.003	354.003208	1.13148333	neg
1.13_372.886	372.886784	1.13148333	neg
1.13_383.024	383.023953	1.13148333	neg
1.13_393.052	393.052782	1.13148333	neg
1.13_400.847	400.847165	1.13148333	neg
1.13_410.956	410.956207	1.13148333	neg
1.13_415.035	415.03497	1.13148333	neg
1.13_423.032	423.032567	1.13148333	neg
1.13_449.026	449.026645	1.13148333	neg
1.13_473.102	473.102045	1.13148333	neg
1.13_483.953	483.953919	1.13148333	neg
1.14_105.520	105.520224	1.13681667	pos
1.14_116.972	116.972189	1.13681667	pos
1.14_136.006	136.006447	1.13681667	pos
1.14_149.018	149.018817	1.13681667	pos
1.14_155.974	155.974143	1.13681667	pos
1.14_156.101	156.101945	1.13681667	pos
1.14_161.096	161.096135	1.13681667	pos
1.14_164.017	164.017264	1.13681667	pos
1.14_184.094	184.094281	1.13681667	pos
1.14_197.984	197.984843	1.13681667	pos
1.14_198.096	198.096408	1.13681667	pos
1.14_200.000	200.000424	1.13681667	pos

1.14_200.52	200.525588	1.13681667	pos
1.14_202.62	202.628596	1.13681667	pos
1.14_208.09	231.083914	1.13681667	pos
1.14_242.86	242.863247	1.13681667	pos
1.14_245.12	245.127979	1.13681667	pos
1.14_247.98	270.977797	1.13681667	pos
1.14_281.07	281.074378	1.13681667	pos
1.14_326.07	327.079694	1.13681667	pos
1.14_401.09	401.091991	1.13681667	pos
1.14_491.58	491.585811	1.13681667	pos
1.15_107.95	107.950816	1.1535	pos
1.15_112.95	112.956255	1.1535	pos
1.15_116.03	116.034493	1.1535	pos
1.15_118.04	118.040938	1.14921667	neg
1.15_120.04	120.04466	1.1535	pos
1.15_121.94	121.943836	1.14921667	neg
1.15_125.01	125.010553	1.14921667	neg
1.15_131.93	131.930143	1.1535	pos
1.15_133.03	133.031239	1.14921667	neg
1.15_135.04	135.044123	1.1535	pos
1.15_144.95	144.95922	1.14921667	neg
1.15_145.95	145.954512	1.1535	pos
1.15_146.99	292.986842	1.14921667	neg
1.15_148.11	148.113976	1.1535	pos
1.15_158.98	158.98271	1.14921667	neg
1.15_161.05	161.059695	1.1535	pos
1.15_161.59	161.597379	1.1535	pos
1.15_164.00	164.008363	1.1535	pos
1.15_172.86	172.867654	1.14921667	neg
1.15_172.96	172.962078	1.14921667	neg
1.15_179.03	179.034133	1.14921667	neg
1.15_180.99	180.990083	1.1535	pos
1.15_181.94	181.946984	1.14921667	neg

1.15_186.001	186.001814	1.14921667	neg
1.15_187.132	187.132941	1.1535	pos
1.15_196.178	196.178312	1.1535	pos
1.15_197.889	197.889513	1.14921667	neg
1.15_199.096	199.096634	1.1535	pos
1.15_199.981	199.981142	1.14921667	neg
1.15_200.068	200.068504	1.1535	pos
1.15_202.951	202.951195	1.1535	pos
1.15_205.050	205.0506	1.1535	pos
1.15_205.980	250.978472	1.14921667	neg
1.15_211.999	211.999793	1.14921667	neg
1.15_214.014	214.014498	1.1535	pos
1.15_214.033	214.033184	1.14921667	neg
1.15_214.970	214.970291	1.14921667	neg
1.15_222.043	222.043077	1.1535	pos
1.15_223.652	223.652284	1.1535	pos
1.15_226.981	226.980999	1.14921667	neg
1.15_227.017	227.017237	1.14921667	neg
1.15_228.915	228.91567	1.14921667	neg
1.15_232.944	232.944055	1.1535	pos
1.15_232.944	232.944234	1.14921667	neg
1.15_234.952	234.952938	1.14921667	neg
1.15_234.962	234.962086	1.14921667	neg
1.15_238.918	238.918592	1.14921667	neg
1.15_241.980	241.980604	1.1535	pos
1.15_241.989	241.989399	1.14921667	neg
1.15_244.153	244.153739	1.1535	pos
1.15_244.888	244.887951	1.14921667	neg
1.15_244.944	244.944758	1.14921667	neg
1.15_247.166	247.166487	1.1535	pos
1.15_251.652	251.65276	1.1535	pos
1.15_252.990	252.990691	1.1535	pos
1.15_256.936	256.936533	1.14921667	neg

1.15_261.877	261.877045	1.14921667	neg
1.15_262.000	262.000322	1.14921667	neg
1.15_266.027	266.027926	1.14921667	neg
1.15_266.658	266.657962	1.1535	pos
1.15_267.968	267.968695	1.14921667	neg
1.15_268.989	268.989177	1.14921667	neg
1.15_272.013	271.006203	1.14921667	neg
1.15_274.018	274.01818	1.14921667	neg
1.15_280.995	280.995416	1.1535	pos
1.15_282.957	282.957909	1.14921667	neg
1.15_284.018	284.018627	1.14921667	neg
1.15_284.975	284.97573	1.14921667	neg
1.15_288.120	288.120098	1.14921667	neg
1.15_288.934	288.93476	1.14921667	neg
1.15_289.019	289.018968	1.14921667	neg
1.15_295.004	295.004572	1.14921667	neg
1.15_296.124	296.124	1.1535	pos
1.15_307.965	352.963418	1.14921667	neg
1.15_309.976	309.976821	1.14921667	neg
1.15_314.894	314.894737	1.14921667	neg
1.15_317.994	362.992397	1.14921667	neg
1.15_320.986	320.98633	1.14921667	neg
1.15_324.923	324.923679	1.14921667	neg
1.15_330.919	330.919183	1.14921667	neg
1.15_339.065	339.065082	1.1535	pos
1.15_350.903	350.903387	1.14921667	neg
1.15_355.069	355.069923	1.1535	pos
1.15_360.974	360.974171	1.14921667	neg
1.15_361.012	361.012906	1.14921667	neg
1.15_365.105	365.105357	1.1535	pos
1.15_369.963	350.945218	1.14921667	neg
1.15_379.037	379.037066	1.14921667	neg
1.15_381.859	381.859481	1.1535	pos

1.15_384.97	384.973984	1.14921667	neg
1.15_386.04	386.04719	1.14921667	neg
1.15_402.19	402.198039	1.1535	pos
1.15_405.00	405.000071	1.14921667	neg
1.15_428.96	428.961432	1.14921667	neg
1.15_472.98	472.987625	1.14921667	neg
1.15_480.03	480.036813	1.14921667	neg
1.15_496.94	496.949343	1.14921667	neg
1.15_527.15	527.158335	1.1535	pos
1.17_112.00	112.003902	1.16698333	neg
1.17_113.02	113.023705	1.1705	pos
1.17_115.11	115.111963	1.1705	pos
1.17_140.95	140.951282	1.1705	pos
1.17_141.03	141.030428	1.16698333	neg
1.17_161.96	161.969761	1.1705	pos
1.17_173.11	173.11809	1.16698333	neg
1.17_180.89	180.899482	1.16698333	neg
1.17_192.02	192.024394	1.1705	pos
1.17_202.10	202.107416	1.1705	pos
1.17_203.03	203.034601	1.16698333	neg
1.17_204.05	204.056257	1.1705	pos
1.17_206.03	206.038442	1.16698333	neg
1.17_210.93	193.930085	1.1705	pos
1.17_231.63	231.631558	1.1705	pos
1.17_235.92	235.925984	1.16698333	neg
1.17_258.66	258.664813	1.1705	pos
1.17_259.03	259.037129	1.16698333	neg
1.17_261.63	261.631533	1.1705	pos
1.17_262.13	262.133543	1.1705	pos
1.17_262.93	262.931611	1.16698333	neg
1.17_279.03	279.03649	1.1705	pos
1.17_281.02	281.026963	1.16698333	neg
1.17_295.06	295.066855	1.16698333	neg

1.17_304.15(	304.150158	1.1705	pos
1.17_312.99	312.998684	1.16698333	neg
1.17_316.99!	316.995502	1.16698333	neg
1.17_327.16(	327.166251	1.1705	pos
1.17_330.05!	330.059833	1.1705	pos
1.17_339.56(	339.566713	1.1705	pos
1.17_340.94	340.947724	1.16698333	neg
1.17_344.01	344.014401	1.16698333	neg
1.17_352.94	352.944913	1.16698333	neg
1.17_371.05(	371.0507	1.16698333	neg
1.17_377.96	377.963998	1.16698333	neg
1.17_396.97	396.971228	1.16698333	neg
1.17_408.93!	408.935246	1.16698333	neg
1.17_545.13	545.137737	1.16698333	neg
1.17_572.11!	572.115031	1.16698333	neg
1.17_574.12!	574.129424	1.1705	pos
1.18_211.01	211.01358	1.18473333	neg
1.18_213.01	213.017501	1.18473333	neg
1.18_219.97	264.97164	1.18473333	neg
1.18_264.99!	264.99545	1.18473333	neg
1.18_268.97	268.972264	1.18473333	neg
1.18_284.95	284.957432	1.18473333	neg
1.18_298.01	279.000991	1.18473333	neg
1.18_298.06!	298.0695	1.18473333	neg
1.18_298.95	298.952311	1.18473333	neg
1.18_346.98	346.988478	1.18473333	neg
1.18_353.00	353.008579	1.18473333	neg
1.18_382.92	382.924864	1.18473333	neg
1.18_391.01!	391.01503	1.18473333	neg
1.18_403.07(	403.070569	1.18473333	neg
1.18_404.90(	404.906696	1.18473333	neg
1.18_421.05!	421.055597	1.18473333	neg
1.18_450.97	450.973114	1.18473333	neg

1.18_486.078	486.078551	1.18473333	neg
1.18_595.14	595.14175	1.18473333	neg
1.19_110.52	110.52547	1.1884	pos
1.19_169.98	169.985874	1.1884	pos
1.19_172.95	172.956387	1.1884	pos
1.19_181.12	181.125469	1.1884	pos
1.19_199.13	199.131871	1.1884	pos
1.19_210.11	210.109969	1.1884	pos
1.19_224.63	224.632993	1.1884	pos
1.19_245.02	245.026871	1.1884	pos
1.19_248.04	249.053864	1.1884	pos
1.19_258.16	258.163108	1.1884	pos
1.19_288.03	288.039679	1.1884	pos
1.19_311.12	311.123658	1.1884	pos
1.19_326.16	326.164016	1.1884	pos
1.19_348.17	348.176263	1.1884	pos
1.19_375.22	375.223667	1.1884	pos
1.19_376.17	376.17133	1.1884	pos
1.19_395.03	395.030393	1.1884	pos
1.19_420.31	420.318858	1.1884	pos
1.20_121.07	121.074371	1.20271667	neg
1.20_122.03	122.033364	1.20368333	pos
1.20_144.03	287.059849	1.20271667	neg
1.20_201.11	201.112343	1.20368333	pos
1.20_202.98	202.981841	1.20271667	neg
1.20_220.96	220.968979	1.20271667	neg
1.20_221.09	221.091945	1.20368333	pos
1.20_247.03	247.03931	1.20271667	neg
1.20_268.04	268.040755	1.20271667	neg
1.20_272.93	272.930709	1.20271667	neg
1.20_285.96	286.972256	1.20368333	pos
1.20_288.06	575.127034	1.20271667	neg
1.20_288.95	288.956524	1.20271667	neg

1.20_294.91:	294.912645	1.20271667	neg
1.20_323.03(	323.036231	1.20271667	neg
1.20_334.16(	334.160687	1.20368333	pos
1.20_337.95:	337.95394	1.20368333	pos
1.20_345.01{	345.018254	1.20271667	neg
1.20_347.95:	347.952939	1.20271667	neg
1.20_350.05!	350.055463	1.20271667	neg
1.20_357.04:	357.04145	1.20368333	pos
1.20_360.94:	360.943159	1.20271667	neg
1.20_366.99!	366.999898	1.20271667	neg
1.20_372.03	372.037437	1.20271667	neg
1.20_383.99:	428.990616	1.20271667	neg
1.20_394.01!	394.019415	1.20271667	neg
1.20_403.97	403.974647	1.20271667	neg
1.20_430.13:	431.140756	1.20368333	pos
1.20_431.09:	431.093294	1.20271667	neg
1.20_439.95:	440.96119	1.20368333	pos
1.20_442.10:	442.103279	1.20271667	neg
1.20_458.15!	458.155388	1.20271667	neg
1.20_463.03(	463.03086	1.20271667	neg
1.20_465.04!	465.045457	1.20368333	pos
1.20_468.07	467.070198	1.20271667	neg
1.20_475.13:	475.131508	1.20271667	neg
1.20_492.12:	492.121797	1.20271667	neg
1.20_508.05!	508.059558	1.20271667	neg
1.20_511.03:	511.033471	1.20271667	neg
1.20_528.02(	573.024805	1.20271667	neg
1.20_573.15!	573.159806	1.20271667	neg
1.20_597.10!	597.109309	1.20271667	neg
1.22_104.04:	104.042503	1.2188	pos
1.22_117.01!	117.01581	1.21911667	neg
1.22_121.00:	121.000984	1.21911667	neg
1.22_121.04!	120.038117	1.21911667	neg

1.22_122.05[illegible]	121.04422	1.21911667	neg
1.22_132.05[illegible]	132.052191	1.2188	pos
1.22_136.06[illegible]	136.061826	1.2188	pos
1.22_189.00[illegible]	189.004729	1.2188	pos
1.22_193.98[illegible]	194.988851	1.2188	pos
1.22_207.02[illegible]	206.021716	1.21911667	neg
1.22_210.94[illegible]	210.940161	1.21911667	neg
1.22_211.99[illegible]	211.991503	1.2188	pos
1.22_219.02[illegible]	219.020931	1.21911667	neg
1.22_222.98[illegible]	222.983657	1.2188	pos
1.22_235.00[illegible]	235.007406	1.2188	pos
1.22_235.00[illegible]	218.004755	1.2188	pos
1.22_239.98[illegible]	239.987409	1.21911667	neg
1.22_250.11[illegible]	289.074413	1.2188	pos
1.22_255.00[illegible]	255.003487	1.21911667	neg
1.22_263.95[illegible]	263.951156	1.21911667	neg
1.22_264.07[illegible]	264.071759	1.21911667	neg
1.22_281.05[illegible]	281.051715	1.21911667	neg
1.22_295.01[illegible]	295.019538	1.21911667	neg
1.22_298.02[illegible]	299.029822	1.2188	pos
1.22_305.01[illegible]	305.016485	1.21911667	neg
1.22_308.07[illegible]	308.072562	1.2188	pos
1.22_315.02[illegible]	316.032494	1.2188	pos
1.22_320.09[illegible]	320.099183	1.2188	pos
1.22_325.97[illegible]	325.97144	1.21911667	neg
1.22_376.95[illegible]	376.955721	1.21911667	neg
1.22_384.08[illegible]	384.082297	1.21911667	neg
1.22_385.02[illegible]	385.028098	1.21911667	neg
1.22_399.03[illegible]	399.036699	1.21911667	neg
1.22_405.08[illegible]	405.0863	1.21911667	neg
1.22_409.11[illegible]	409.111483	1.21911667	neg
1.22_410.25[illegible]	410.253492	1.2188	pos
1.22_417.02[illegible]	417.027462	1.21911667	neg

1.22_419.06(	420.073617	1.2188	pos
1.22_421.01(	421.019198	1.21911667	neg
1.22_425.08(	425.086316	1.21911667	neg
1.22_438.15(	461.148957	1.2188	pos
1.22_449.08:	449.083288	1.21911667	neg
1.22_489.05:	489.052437	1.21911667	neg
1.22_513.22!	513.228056	1.2188	pos
1.22_516.07:	516.071217	1.21911667	neg
1.22_538.05:	538.053075	1.21911667	neg
1.22_551.11!	551.115784	1.21911667	neg
1.22_553.14!	553.145535	1.21911667	neg
1.22_555.14(	555.140371	1.21911667	neg
1.22_570.15:	570.151315	1.21911667	neg
1.22_574.00(	574.000479	1.21911667	neg
1.22_587.07	587.077414	1.21911667	neg
1.22_613.09!	613.099696	1.21911667	neg
1.22_696.14!	741.143211	1.21911667	neg
1.22_697.07:	697.071393	1.21911667	neg
1.22_721.15(	721.1565	1.21911667	neg
1.23_118.02!	118.02536	1.23491667	neg
1.23_132.15:	132.151279	1.23451667	pos
1.23_153.05!	153.059899	1.23451667	pos
1.23_162.99	162.997672	1.23451667	pos
1.23_169.05!	169.058375	1.23451667	pos
1.23_174.97	174.977619	1.23491667	neg
1.23_185.00(	185.006404	1.23491667	neg
1.23_189.03:	377.055003	1.23491667	neg
1.23_201.99(	201.996649	1.23491667	neg
1.23_210.00	210.007247	1.23491667	neg
1.23_213.97!	213.979563	1.23451667	pos
1.23_229.03:	229.032649	1.23491667	neg
1.23_236.96!	236.968427	1.23491667	neg
1.23_274.12(	274.120768	1.23491667	neg

1.23_274.954	274.954928	1.23451667	pos
1.23_283.091	284.098777	1.23451667	pos
1.23_313.071	313.071345	1.23491667	neg
1.23_345.079	345.079875	1.23491667	neg
1.23_364.090	364.090426	1.23491667	neg
1.23_376.136	376.13596	1.23491667	neg
1.23_381.119	426.118054	1.23491667	neg
1.23_388.118	388.118885	1.23491667	neg
1.23_388.140	388.140911	1.23491667	neg
1.23_400.110	400.110608	1.23491667	neg
1.23_400.182	400.182418	1.23451667	pos
1.23_441.033	441.03387	1.23451667	pos
1.23_442.034	442.034895	1.23491667	neg
1.23_443.044	443.044305	1.23491667	neg
1.23_445.016	445.016528	1.23491667	neg
1.23_445.058	445.058835	1.23451667	pos
1.23_479.094	479.094329	1.23491667	neg
1.23_496.084	496.084585	1.23491667	neg
1.23_531.055	531.055731	1.23491667	neg
1.23_547.089	547.089483	1.23491667	neg
1.23_562.060	607.064317	1.23491667	neg
1.23_609.060	609.059966	1.23491667	neg
1.23_672.216	672.216939	1.23491667	neg
1.23_698.140	743.138803	1.23491667	neg
1.25_119.035	119.035382	1.251	pos
1.25_167.033	167.033853	1.251	pos
1.25_170.000	215.004822	1.2521	neg
1.25_175.107	175.107798	1.251	pos
1.25_192.913	193.920544	1.251	pos
1.25_218.066	218.066685	1.2521	neg
1.25_219.074	242.063361	1.251	pos
1.25_253.054	253.054196	1.251	pos
1.25_267.061	267.061661	1.2521	neg

1.25_268.08	291.069766	1.251	pos
1.25_274.97	274.976223	1.2521	neg
1.25_305.04	305.046974	1.2521	neg
1.25_312.09	312.094785	1.2521	neg
1.25_325.03	325.032382	1.2521	neg
1.25_327.02	327.029179	1.2521	neg
1.25_330.06	330.06904	1.2521	neg
1.25_336.96	336.95995	1.2521	neg
1.25_350.07	350.071615	1.2521	neg
1.25_352.05	352.050937	1.2521	neg
1.25_356.94	356.943949	1.2521	neg
1.25_358.98	358.983866	1.251	pos
1.25_364.98	409.980169	1.2521	neg
1.25_372.11	372.115637	1.2521	neg
1.25_379.05	379.050555	1.2521	neg
1.25_395.01	395.015843	1.2521	neg
1.25_409.00	409.004795	1.2521	neg
1.25_450.16	450.161748	1.251	pos
1.25_488.13	488.13708	1.2521	neg
1.25_520.09	501.076529	1.2521	neg
1.25_527.14	572.147514	1.2521	neg
1.25_536.16	559.150558	1.251	pos
1.25_545.10	545.102507	1.2521	neg
1.25_550.16	550.165126	1.2521	neg
1.25_551.17	574.161273	1.251	pos
1.25_564.12	564.128043	1.251	pos
1.25_569.06	569.064095	1.2521	neg
1.25_593.11	593.113345	1.2521	neg
1.25_598.13	581.132416	1.251	pos
1.25_615.09	615.095246	1.2521	neg
1.25_640.13	640.135828	1.2521	neg
1.25_642.11	642.113827	1.2521	neg
1.25_674.12	655.10698	1.2521	neg

1.25_677.08	677.088472	1.2521	neg
1.25_803.23	803.234972	1.2521	neg
1.25_825.21	825.217345	1.2521	neg
1.27_114.02	114.024229	1.26985	neg
1.27_129.06	129.066216	1.26745	pos
1.27_133.03	133.031183	1.26985	neg
1.27_174.00	174.001739	1.26985	neg
1.27_195.99	195.993288	1.26745	pos
1.27_213.04	213.049068	1.26985	neg
1.27_222.64	222.644377	1.26745	pos
1.27_228.07	273.072536	1.26985	neg
1.27_259.04	259.042211	1.26745	pos
1.27_276.15	276.155129	1.26745	pos
1.27_289.09	334.097462	1.26985	neg
1.27_314.05	314.055227	1.26745	pos
1.27_314.14	314.145621	1.26745	pos
1.27_333.17	333.176608	1.26745	pos
1.27_362.15	362.155514	1.26745	pos
1.27_364.05	364.053992	1.26985	neg
1.27_369.01	369.01271	1.26745	pos
1.27_374.12	374.120392	1.26985	neg
1.27_403.04	403.048248	1.26985	neg
1.27_411.01	411.019014	1.26745	pos
1.27_414.97	414.975976	1.26985	neg
1.27_418.05	418.059284	1.26985	neg
1.27_420.03	420.038521	1.26985	neg
1.27_420.11	420.11367	1.26985	neg
1.27_435.99	480.991121	1.26985	neg
1.27_455.01	455.009991	1.26985	neg
1.27_461.00	461.007075	1.26985	neg
1.27_476.99	476.992304	1.26985	neg
1.27_486.04	486.046897	1.26985	neg
1.27_488.10	488.101081	1.26985	neg

1.27_502.973	502.973319	1.26985	neg
1.27_515.061	515.061928	1.26985	neg
1.27_528.954	528.954136	1.26985	neg
1.27_539.022	539.022762	1.26985	neg
1.27_544.979	544.979372	1.26985	neg
1.27_583.049	583.049771	1.26985	neg
1.27_607.010	607.010824	1.26985	neg
1.27_627.064	627.064545	1.26745	pos
1.27_674.998	674.998146	1.26985	neg
1.27_761.098	761.098035	1.26985	neg
1.28_114.055	114.055198	1.28425	pos
1.28_118.942	118.942854	1.28425	pos
1.28_122.027	122.027204	1.28425	pos
1.28_131.930	131.930595	1.28425	pos
1.28_149.096	149.096097	1.28425	pos
1.28_153.040	154.047452	1.28425	pos
1.28_161.059	161.059726	1.28425	pos
1.28_161.969	161.969672	1.28425	pos
1.28_169.085	169.085941	1.28425	pos
1.28_179.067	179.067544	1.28425	pos
1.28_184.094	184.094383	1.28425	pos
1.28_193.083	193.083348	1.28425	pos
1.28_226.104	226.104857	1.28425	pos
1.28_227.011	227.011339	1.28425	pos
1.28_231.139	231.139401	1.28425	pos
1.28_231.990	231.990743	1.28425	pos
1.28_254.090	237.086718	1.28425	pos
1.28_280.995	280.995356	1.28425	pos
1.28_304.180	304.186468	1.28425	pos
1.28_381.039	381.039137	1.28425	pos
1.28_400.010	400.010358	1.28425	pos
1.28_414.198	414.197993	1.28425	pos
1.28_435.208	435.208329	1.28425	pos

1.28_527.158	527.158529	1.28425	pos
1.29_148.979	148.979867	1.2875	neg
1.29_158.015	158.014986	1.2875	neg
1.29_158.892	158.892897	1.2875	neg
1.29_166.943	166.94311	1.2875	neg
1.29_188.009	188.009575	1.2875	neg
1.29_188.948	188.948918	1.2875	neg
1.29_196.884	196.884728	1.2875	neg
1.29_198.038	198.038207	1.2875	neg
1.29_219.013	219.013506	1.2875	neg
1.29_226.000	224.999122	1.2875	neg
1.29_230.955	230.955544	1.2875	neg
1.29_241.989	241.989467	1.2875	neg
1.29_242.064	242.064561	1.2875	neg
1.29_250.009	250.009503	1.2875	neg
1.29_256.936	256.936575	1.2875	neg
1.29_266.025	266.025873	1.2875	neg
1.29_296.066	296.066203	1.2875	neg
1.29_317.961	298.943213	1.2875	neg
1.29_324.924	324.923984	1.2875	neg
1.29_334.013	334.01339	1.2875	neg
1.29_337.013	337.013121	1.2875	neg
1.29_360.974	360.974127	1.2875	neg
1.29_366.930	366.930431	1.2875	neg
1.29_378.039	378.039467	1.2875	neg
1.29_402.000	402.000469	1.2875	neg
1.29_428.961	428.961324	1.2875	neg
1.29_462.066	462.066825	1.2875	neg
1.29_482.963	482.963137	1.2875	neg
1.29_496.949	496.949351	1.2875	neg
1.29_510.964	510.964422	1.2875	neg
1.30_135.945	135.94546	1.30165	pos
1.30_137.096	137.096212	1.30165	pos

1.30_140.95	140.951269	1.30165	pos
1.30_178.09	179.106908	1.30165	pos
1.30_186.95	186.956349	1.30165	pos
1.30_193.93	193.930122	1.30165	pos
1.30_226.96	226.961199	1.30165	pos
1.30_229.11	229.114586	1.30165	pos
1.30_257.57	257.57113	1.30165	pos
1.30_288.98	288.987838	1.30165	pos
1.30_290.50	290.507759	1.30165	pos
1.30_302.05	302.058546	1.30165	pos
1.30_303.14	303.146495	1.30165	pos
1.30_343.03	343.035027	1.30165	pos
1.30_348.99	348.990779	1.30165	pos
1.30_365.10	365.105423	1.30165	pos
1.30_369.66	369.661729	1.30165	pos
1.30_389.20	389.203036	1.30165	pos
1.31_118.94	118.941936	1.30545	neg
1.31_182.99	182.990846	1.30545	neg
1.31_187.00	187.001359	1.30545	neg
1.31_202.96	202.964672	1.30545	neg
1.31_206.96	206.967346	1.30545	neg
1.31_208.98	208.983421	1.30545	neg
1.31_219.95	219.954814	1.30545	neg
1.31_240.88	240.887195	1.30545	neg
1.31_245.94	245.946642	1.30545	neg
1.31_256.08	256.080304	1.30545	neg
1.31_260.87	260.873304	1.30545	neg
1.31_270.95	270.95229	1.30545	neg
1.31_282.95	282.957785	1.30545	neg
1.31_283.06	283.068356	1.30545	neg
1.31_309.87	309.870109	1.30545	neg
1.31_316.10	316.101178	1.30545	neg
1.31_338.93	338.939526	1.30545	neg

1.31_374.98	374.989726	1.30545	neg
1.31_381.92	381.921853	1.30545	neg
1.31_392.05	392.055341	1.30545	neg
1.31_406.92	406.927052	1.30545	neg
1.31_409.07	409.069972	1.30545	neg
1.31_432.94	432.94805	1.30545	neg
1.31_442.97	442.977136	1.30545	neg
1.31_501.01	501.018903	1.30545	neg
1.31_578.95	578.952297	1.30545	neg
1.32_126.02	149.01873	1.31801667	pos
1.32_156.13	156.138326	1.31801667	pos
1.32_157.99	157.990056	1.31801667	pos
1.32_160.99	160.998466	1.32371667	neg
1.32_162.99	162.995607	1.32371667	neg
1.32_171.02	171.020794	1.32371667	neg
1.32_175.04	175.041669	1.32371667	neg
1.32_176.90	176.900463	1.32371667	neg
1.32_176.98	176.984477	1.31801667	pos
1.32_188.01	188.017439	1.32371667	neg
1.32_190.07	190.071851	1.32371667	neg
1.32_200.00	200.000471	1.31801667	pos
1.32_201.63	201.639201	1.31801667	pos
1.32_202.98	202.983845	1.31801667	pos
1.32_203.97	203.971519	1.31801667	pos
1.32_204.97	204.979389	1.31801667	pos
1.32_214.07	214.076696	1.32371667	neg
1.32_222.99	222.998469	1.32371667	neg
1.32_229.06	229.069101	1.32371667	neg
1.32_239.01	239.014811	1.32371667	neg
1.32_241.02	241.026732	1.31801667	pos
1.32_244.99	244.997981	1.31801667	pos
1.32_245.97	290.977126	1.32371667	neg
1.32_246.95	247.961326	1.31801667	pos

1.32_253.118	253.118043	1.31801667	pos
1.32_256.004	256.00493	1.32371667	neg
1.32_258.005	258.005176	1.32371667	neg
1.32_258.059	258.059403	1.32371667	neg
1.32_260.078	305.076506	1.32371667	neg
1.32_262.013	262.013243	1.31801667	pos
1.32_283.103	283.103263	1.32371667	neg
1.32_286.944	286.944882	1.32371667	neg
1.32_296.973	296.973333	1.32371667	neg
1.32_297.056	297.056578	1.32371667	neg
1.32_298.970	298.970397	1.32371667	neg
1.32_304.047	304.047569	1.31801667	pos
1.32_307.002	307.002308	1.32371667	neg
1.32_312.049	312.049695	1.31801667	pos
1.32_312.959	312.958951	1.32371667	neg
1.32_323.992	323.992328	1.32371667	neg
1.32_351.028	351.02855	1.32371667	neg
1.32_355.015	355.015433	1.32371667	neg
1.32_364.960	364.960776	1.32371667	neg
1.32_380.946	380.945987	1.32371667	neg
1.32_382.145	382.145848	1.32371667	neg
1.32_389.187	389.187654	1.32371667	neg
1.32_391.980	391.980243	1.32371667	neg
1.32_399.023	399.023251	1.32371667	neg
1.32_410.031	410.031533	1.32371667	neg
1.32_424.029	424.02912	1.32371667	neg
1.32_433.031	433.031349	1.32371667	neg
1.32_450.050	450.050054	1.32371667	neg
1.32_450.133	450.133284	1.32371667	neg
1.32_455.171	455.171066	1.31801667	pos
1.32_528.065	528.065657	1.32371667	neg
1.32_550.117	550.117817	1.31801667	pos
1.33_452.158	470.192168	1.33403333	pos

1.34_119.034	119.034766	1.34166667	neg
1.34_130.038	130.038617	1.34166667	neg
1.34_131.090	131.090476	1.34166667	neg
1.34_131.094	130.087133	1.34166667	neg
1.34_145.086	145.086798	1.34166667	neg
1.34_146.997	292.986806	1.34166667	neg
1.34_154.918	154.918623	1.34166667	neg
1.34_173.104	173.1041	1.34166667	neg
1.34_188.045	188.045695	1.34166667	neg
1.34_188.056	188.056187	1.34166667	neg
1.34_190.042	190.042852	1.34166667	neg
1.34_198.074	198.074531	1.34166667	neg
1.34_207.998	207.998923	1.34166667	neg
1.34_215.064	215.064777	1.34166667	neg
1.34_215.994	215.994447	1.34166667	neg
1.34_244.118	244.118801	1.34166667	neg
1.34_244.129	244.129787	1.34166667	neg
1.34_265.988	265.98879	1.34166667	neg
1.34_283.163	283.163806	1.34166667	neg
1.34_290.071	290.071124	1.34166667	neg
1.34_301.083	301.083907	1.34166667	neg
1.34_310.088	310.088364	1.34166667	neg
1.34_317.020	317.02059	1.34166667	neg
1.34_324.103	324.103912	1.34166667	neg
1.34_329.158	374.15673	1.34166667	neg
1.34_340.006	340.005994	1.34166667	neg
1.34_355.135	355.135779	1.34166667	neg
1.34_378.075	378.075803	1.34166667	neg
1.34_386.021	386.021139	1.34166667	neg
1.34_395.177	395.177067	1.34166667	neg
1.34_402.109	402.109705	1.34166667	neg
1.34_404.088	404.088726	1.34166667	neg
1.34_404.125	404.125115	1.34166667	neg

1.34_409.19	409.19322	1.34166667	neg
1.34_418.14	418.14083	1.34166667	neg
1.34_419.06	419.065421	1.34166667	neg
1.34_428.11	428.114616	1.34166667	neg
1.34_435.05	435.050108	1.34166667	neg
1.34_437.58	437.582285	1.34166667	neg
1.34_460.07	460.07866	1.34166667	neg
1.34_463.16	463.165107	1.34166667	neg
1.34_476.09	476.092836	1.34166667	neg
1.34_477.09	477.096896	1.34166667	neg
1.34_503.03	503.037339	1.34166667	neg
1.34_504.22	504.228037	1.34166667	neg
1.34_514.18	514.183211	1.34166667	neg
1.34_540.06	540.063657	1.34166667	neg
1.34_562.18	562.18635	1.34166667	neg
1.34_572.21	572.215265	1.34166667	neg
1.34_574.28	574.287458	1.34166667	neg
1.34_576.28	576.282728	1.34166667	neg
1.34_582.17	582.170929	1.34166667	neg
1.34_613.15	613.155144	1.34166667	neg
1.34_650.15	650.158467	1.34166667	neg
1.34_698.24	698.244577	1.34166667	neg
1.34_810.38	810.381011	1.34166667	neg
1.34_878.36	878.368637	1.34166667	neg
1.34_888.32	888.322435	1.34166667	neg
1.35_132.15	132.151262	1.34996667	pos
1.35_134.10	134.108302	1.34996667	pos
1.35_232.02	232.026589	1.34996667	pos
1.35_339.09	339.097465	1.34996667	pos
1.35_348.20	348.201375	1.34996667	pos
1.35_492.17	492.173871	1.34996667	pos
1.36_121.94	121.943829	1.3586	neg
1.36_143.03	143.034795	1.3586	neg

1.36_154.92	154.928096	1.3586	neg
1.36_159.02	159.029765	1.3586	neg
1.36_173.08	173.081659	1.3586	neg
1.36_173.96	173.965253	1.3586	neg
1.36_181.94	181.947005	1.3586	neg
1.36_244.88	244.888008	1.3586	neg
1.36_251.01	251.014816	1.3586	neg
1.36_256.03	256.033393	1.3586	neg
1.36_267.98	267.985581	1.3586	neg
1.36_285.16	285.169218	1.3586	neg
1.36_293.00	293.007718	1.3586	neg
1.36_316.04	316.04122	1.3586	neg
1.36_317.09	317.098752	1.3586	neg
1.36_318.02	318.024006	1.3586	neg
1.36_319.03	319.035765	1.3586	neg
1.36_324.02	324.020787	1.3586	neg
1.36_326.01	326.017641	1.3586	neg
1.36_328.86	328.860851	1.3586	neg
1.36_333.97	333.976248	1.3586	neg
1.36_339.08	339.080669	1.3586	neg
1.36_349.96	349.961401	1.3586	neg
1.36_350.02	350.023474	1.3586	neg
1.36_351.07	351.077752	1.3586	neg
1.36_353.06	334.049704	1.3586	neg
1.36_361.10	406.104377	1.3586	neg
1.36_378.12	359.110943	1.3586	neg
1.36_386.98	386.987287	1.3586	neg
1.36_392.00	392.008336	1.3586	neg
1.36_402.03	402.036902	1.3586	neg
1.36_407.99	407.993342	1.3586	neg
1.36_417.94	417.948593	1.3586	neg
1.36_427.05	427.052597	1.3586	neg
1.36_436.12	436.128923	1.3586	neg

1.36_446.063	446.063229	1.3586	neg
1.36_462.240	462.240309	1.3586	neg
1.36_470.024	470.024108	1.3586	neg
1.36_474.092	474.092107	1.3586	neg
1.36_475.980	475.980691	1.3586	neg
1.36_504.117	504.117252	1.3586	neg
1.36_545.084	545.084574	1.3586	neg
1.36_565.069	565.069124	1.3586	neg
1.36_572.104	572.104013	1.3586	neg
1.36_613.072	613.072446	1.3586	neg
1.36_616.245	616.245045	1.3586	neg
1.36_718.145	718.145842	1.3586	neg
1.36_776.178	776.178826	1.3586	neg
1.36_861.268	861.268117	1.3586	neg
1.37_132.136	132.1364	1.36776667	pos
1.37_145.049	145.04944	1.36776667	pos
1.37_184.034	185.042014	1.36776667	pos
1.37_254.008	254.008514	1.36776667	pos
1.37_261.907	261.907912	1.36776667	pos
1.37_397.129	397.129538	1.36776667	pos
1.37_618.260	618.259964	1.36776667	pos
1.38_124.040	124.040219	1.37511667	neg
1.38_134.053	134.053847	1.37511667	neg
1.38_147.066	147.066091	1.37511667	neg
1.38_152.046	152.046364	1.37511667	neg
1.38_166.058	166.058	1.3848	pos
1.38_172.941	172.941006	1.37511667	neg
1.38_186.929	186.929272	1.37511667	neg
1.38_188.617	188.617313	1.3848	pos
1.38_204.615	204.61569	1.3848	pos
1.38_222.043	222.043123	1.3848	pos
1.38_244.053	244.053196	1.3848	pos
1.38_260.160	260.160403	1.3848	pos

1.38_267.482	267.482827	1.3848	pos
1.38_288.068	288.068819	1.3848	pos
1.38_300.972	300.972916	1.37511667	neg
1.38_312.040	312.040674	1.3848	pos
1.38_327.807	327.807873	1.3848	pos
1.38_344.004	344.004928	1.37511667	neg
1.38_377.150	377.150857	1.37511667	neg
1.38_411.992	411.992565	1.37511667	neg
1.38_440.965	440.965521	1.3848	pos
1.38_441.216	441.216551	1.3848	pos
1.38_454.193	454.193297	1.37511667	neg
1.38_459.995	459.995606	1.37511667	neg
1.38_479.979	479.979893	1.37511667	neg
1.38_487.015	487.015289	1.37511667	neg
1.38_538.011	538.01186	1.37511667	neg
1.38_587.618	587.618514	1.3848	pos
1.38_610.598	610.598245	1.37511667	neg
1.38_633.056	633.056727	1.37511667	neg
1.38_701.044	701.044174	1.37511667	neg
1.38_759.076	759.075997	1.37511667	neg
1.39_130.033	130.033109	1.39358333	neg
1.39_161.026	161.026228	1.39358333	neg
1.39_176.018	176.01871	1.39358333	neg
1.39_188.925	188.925188	1.39358333	neg
1.39_214.033	214.033164	1.39358333	neg
1.39_231.981	231.981514	1.39358333	neg
1.39_242.010	242.010476	1.39358333	neg
1.39_259.982	259.982142	1.39358333	neg
1.39_286.000	286.000396	1.39358333	neg
1.39_367.956	367.956279	1.39358333	neg
1.39_377.985	377.98521	1.39358333	neg
1.39_438.074	438.074358	1.39358333	neg
1.39_445.972	445.972806	1.39358333	neg

1.39_469.95	469.950968	1.39358333	neg
1.39_496.10	496.102695	1.39358333	neg
1.39_605.99	605.999614	1.39358333	neg
1.39_615.95	615.954942	1.39358333	neg
1.39_673.98	673.986924	1.39358333	neg
1.39_683.94	683.942623	1.39358333	neg
1.40_112.02	112.021877	1.40198333	pos
1.40_112.95	112.956314	1.40198333	pos
1.40_138.05	138.054772	1.40198333	pos
1.40_144.10	144.10185	1.40198333	pos
1.40_175.03	176.03765	1.40198333	pos
1.40_178.03	178.03342	1.40198333	pos
1.40_213.12	213.123505	1.40198333	pos
1.40_220.00	220.001491	1.40198333	pos
1.40_222.16	222.168713	1.40198333	pos
1.40_242.86	242.863274	1.40198333	pos
1.40_243.15	243.158296	1.40198333	pos
1.40_266.16	266.160323	1.40198333	pos
1.40_267.09	268.104004	1.40198333	pos
1.40_280.09	280.092029	1.40198333	pos
1.40_280.99	280.995558	1.40198333	pos
1.40_290.84	290.842404	1.40198333	pos
1.40_295.66	295.660866	1.40198333	pos
1.40_305.93	305.930344	1.40198333	pos
1.40_322.07	345.062294	1.40198333	pos
1.40_348.17	348.176549	1.40198333	pos
1.41_125.03	125.035473	1.4122	neg
1.41_146.94	146.946334	1.4122	neg
1.41_152.05	197.056604	1.4122	neg
1.41_193.02	193.022887	1.4122	neg
1.41_244.89	244.897424	1.4122	neg
1.41_312.93	312.932879	1.4122	neg
1.42_114.03	114.03804	1.41946667	pos

1.42_119.089	119.089878	1.41946667	pos
1.42_153.065	153.065924	1.41946667	pos
1.42_177.054	177.054912	1.41946667	pos
1.42_184.094	184.094445	1.41946667	pos
1.42_216.632	216.63195	1.41946667	pos
1.42_228.057	251.046418	1.41946667	pos
1.42_247.129	247.128967	1.41946667	pos
1.42_251.136	251.136551	1.41946667	pos
1.42_257.112	257.112834	1.41946667	pos
1.42_272.110	272.110487	1.41946667	pos
1.42_306.431	306.431875	1.41946667	pos
1.42_361.171	361.171867	1.41946667	pos
1.42_407.906	407.905977	1.41946667	pos
1.42_440.088	440.088896	1.41946667	pos
1.43_145.061	145.061692	1.43078333	neg
1.43_153.055	153.055613	1.43078333	neg
1.43_188.948	188.948944	1.43078333	neg
1.43_232.118	232.118903	1.43078333	neg
1.43_232.944	232.944241	1.43078333	neg
1.43_251.078	251.078517	1.43078333	neg
1.43_263.070	263.070707	1.43078333	neg
1.43_303.065	303.065545	1.43078333	neg
1.43_309.977	309.977214	1.43078333	neg
1.43_319.066	319.066004	1.43078333	neg
1.43_361.977	361.977883	1.43078333	neg
1.43_387.053	387.053713	1.43078333	neg
1.43_426.560	426.560723	1.43078333	neg
1.43_510.964	510.964438	1.43078333	neg
1.44_105.520	105.52033	1.43671667	pos
1.44_133.085	133.085835	1.43671667	pos
1.44_173.085	173.085789	1.43671667	pos
1.44_176.128	176.128186	1.43671667	pos
1.44_190.107	190.107525	1.43671667	pos

1.44_206.048	206.048311	1.43671667	pos
1.44_212.173	212.173353	1.43671667	pos
1.44_219.621	219.621067	1.43671667	pos
1.44_226.974	226.974946	1.43671667	pos
1.44_234.133	234.133638	1.43671667	pos
1.44_270.017	270.017363	1.43671667	pos
1.44_319.156	319.156034	1.43671667	pos
1.44_385.717	385.717238	1.43671667	pos
1.44_434.561	434.561549	1.43671667	pos
1.44_451.602	451.602389	1.43671667	pos
1.45_117.074	117.074251	1.45418333	pos
1.45_145.122	145.122359	1.45418333	pos
1.45_147.044	147.044209	1.45418333	pos
1.45_163.075	163.075444	1.45418333	pos
1.45_163.965	163.96505	1.45418333	pos
1.45_169.086	169.086053	1.45418333	pos
1.45_182.067	182.067504	1.45418333	pos
1.45_200.631	200.631533	1.45418333	pos
1.45_213.049	213.049197	1.44935	neg
1.45_225.086	225.08616	1.45418333	pos
1.45_229.044	229.044067	1.44935	neg
1.45_244.645	244.645052	1.45418333	pos
1.45_265.092	265.092945	1.44935	neg
1.45_287.578	287.578092	1.45418333	pos
1.45_287.663	287.663274	1.45418333	pos
1.45_314.036	314.035955	1.45418333	pos
1.45_314.537	314.537609	1.45418333	pos
1.45_324.923	324.923799	1.44935	neg
1.45_345.195	345.195099	1.45418333	pos
1.45_345.696	345.696797	1.45418333	pos
1.45_362.155	362.155666	1.45418333	pos
1.45_373.066	373.066186	1.44935	neg
1.45_428.961	428.961407	1.44935	neg

1.45_707.10	707.105434	1.44935	neg
1.47_103.99	142.961924	1.47146667	pos
1.47_126.02	126.022178	1.47146667	pos
1.47_135.04	135.044115	1.47146667	pos
1.47_135.06	135.067954	1.47146667	pos
1.47_145.07	146.081195	1.47146667	pos
1.47_146.99	169.985985	1.47146667	pos
1.47_150.09	150.099553	1.47146667	pos
1.47_169.05	169.050493	1.46783333	neg
1.47_173.11	196.105714	1.47146667	pos
1.47_184.07	184.073302	1.47146667	pos
1.47_188.09	188.092667	1.46783333	neg
1.47_193.93	193.930109	1.47146667	pos
1.47_197.10	197.109036	1.47146667	pos
1.47_202.95	202.951255	1.47146667	pos
1.47_207.63	207.639314	1.47146667	pos
1.47_212.05	212.053797	1.46783333	neg
1.47_232.02	232.026345	1.46783333	neg
1.47_234.95	234.956653	1.47146667	pos
1.47_245.13	268.126811	1.47146667	pos
1.47_246.88	246.88517	1.46783333	neg
1.47_256.08	256.08024	1.46783333	neg
1.47_263.13	263.137041	1.47146667	pos
1.47_270.08	271.092527	1.47146667	pos
1.47_270.08	315.083603	1.46783333	neg
1.47_283.65	283.650299	1.47146667	pos
1.47_305.05	305.054636	1.46783333	neg
1.47_309.18	309.188952	1.47146667	pos
1.47_332.07	332.073597	1.46783333	neg
1.47_335.51	335.513669	1.47146667	pos
1.47_444.55	444.55291	1.47146667	pos
1.47_456.06	456.06332	1.46783333	neg
1.47_603.10	603.101686	1.46783333	neg

1.49_135.11(	135.116937	1.48828333	pos
1.49_141.01‡	141.01797	1.4863	neg
1.49_145.99(	145.990047	1.48828333	pos
1.49_167.03⁄	167.034801	1.4863	neg
1.49_170.87(	170.870632	1.4863	neg
1.49_171.98(	171.980822	1.4863	neg
1.49_197.11	197.11771	1.48828333	pos
1.49_211.99!	211.999843	1.4863	neg
1.49_232.90‡	232.908229	1.48828333	pos
1.49_262.12!	262.129538	1.4863	neg
1.49_263.13(	264.144176	1.48828333	pos
1.49_290.17	290.171113	1.48828333	pos
1.49_296.04	591.087266	1.4863	neg
1.49_308.68(	308.68692	1.48828333	pos
1.49_310.11:	310.113095	1.48828333	pos
1.49_318.87	318.871383	1.4863	neg
1.49_354.12!	354.129809	1.48828333	pos
1.49_355.05:	355.052418	1.4863	neg
1.49_398.10⁄	398.104397	1.4863	neg
1.49_438.56(	438.566338	1.4863	neg
1.49_446.56:	446.563839	1.4863	neg
1.49_457.58!	457.585641	1.4863	neg
1.49_466.09	466.091761	1.4863	neg
1.49_483.08:	483.082242	1.4863	neg
1.49_496.94!	496.949438	1.4863	neg
1.49_534.07!	534.079521	1.4863	neg
1.49_602.06	602.067035	1.4863	neg
1.49_602.60	602.601049	1.4863	neg
1.49_670.05⁄	670.054706	1.4863	neg
1.50_141.03(	141.030444	1.50483333	neg
1.50_183.04(	183.040864	1.50483333	neg
1.50_247.03!	247.039376	1.50483333	neg
1.50_271.90	271.907157	1.50483333	neg

1.50_298.90(	298.906678	1.50483333	neg
1.50_312.87(	312.87608	1.50483333	neg
1.50_337.00{	337.008768	1.50483333	neg
1.50_404.99(	404.996213	1.50483333	neg
1.51_129.01{	129.018444	1.50628333	pos
1.51_163.06{	163.06849	1.50628333	pos
1.51_171.58	171.58184	1.50628333	pos
1.51_202.62(	202.620498	1.50628333	pos
1.51_211.93	211.931089	1.50628333	pos
1.51_248.04(	249.054009	1.50628333	pos
1.51_265.05(	265.055994	1.50628333	pos
1.51_266.16(	266.169781	1.50628333	pos
1.51_278.66	278.66235	1.50628333	pos
1.51_281.16	281.165569	1.50628333	pos
1.51_281.66	281.667574	1.50628333	pos
1.51_314.08(	314.089407	1.50628333	pos
1.51_315.07(	315.076864	1.50628333	pos
1.51_316.18(	316.186736	1.50628333	pos
1.51_332.18	332.181579	1.50628333	pos
1.51_459.60(	459.60022	1.50628333	pos
1.52_107.02	107.024899	1.52331667	neg
1.52_122.01(	122.019571	1.52326667	pos
1.52_129.95{	129.958823	1.52326667	pos
1.52_148.11	148.114109	1.52326667	pos
1.52_157.06	157.061657	1.52331667	neg
1.52_160.00	160.007241	1.52331667	neg
1.52_187.10{	187.108599	1.52331667	neg
1.52_189.12	189.123495	1.52326667	pos
1.52_216.62(	216.626433	1.52326667	pos
1.52_219.63(	219.6392	1.52326667	pos
1.52_220.06	220.063904	1.52326667	pos
1.52_227.99	227.994754	1.52331667	neg
1.52_264.87	264.872272	1.52331667	neg

1.52_274.157	274.1579	1.52326667	pos
1.52_274.176	274.176183	1.52326667	pos
1.52_288.504	288.504469	1.52326667	pos
1.52_299.390	299.390101	1.52326667	pos
1.52_302.135	302.135828	1.52331667	neg
1.52_308.134	308.134011	1.52326667	pos
1.52_339.894	339.894567	1.52331667	neg
1.52_380.863	380.863165	1.52331667	neg
1.52_400.846	400.846746	1.52331667	neg
1.54_135.945	135.945487	1.54085	pos
1.54_156.966	156.966161	1.54085	pos
1.54_158.586	158.586513	1.54085	pos
1.54_161.464	161.464049	1.54085	pos
1.54_162.022	162.021982	1.54085	pos
1.54_187.057	187.057739	1.54085	pos
1.54_210.983	210.983416	1.54175	neg
1.54_221.654	221.654901	1.54085	pos
1.54_228.031	228.03118	1.54175	neg
1.54_231.084	231.084042	1.54085	pos
1.54_239.647	222.644475	1.54085	pos
1.54_253.092	253.092835	1.54175	neg
1.54_318.166	318.166049	1.54085	pos
1.54_343.073	343.073488	1.54175	neg
1.54_469.590	469.590946	1.54175	neg
1.54_471.605	471.605862	1.54085	pos
1.56_138.055	138.055872	1.56021667	neg
1.56_142.050	142.050716	1.56021667	neg
1.56_160.061	160.061365	1.56021667	neg
1.56_161.068	184.058072	1.55895	pos
1.56_173.078	173.078611	1.55895	pos
1.56_173.116	173.116544	1.55895	pos
1.56_174.953	174.953475	1.55895	pos
1.56_178.120	196.154487	1.55895	pos

1.56_214.64	214.647091	1.55895	pos
1.56_237.63	237.637259	1.55895	pos
1.56_238.13	238.139087	1.55895	pos
1.56_284.07	285.083032	1.55895	pos
1.56_289.01	289.013071	1.55895	pos
1.56_296.03	296.036357	1.56021667	neg
1.56_334.03	334.038895	1.55895	pos
1.56_341.02	341.026879	1.56021667	neg
1.56_343.02	343.023812	1.56021667	neg
1.56_346.05	329.046911	1.55895	pos
1.56_351.05	351.057111	1.56021667	neg
1.56_354.67	354.674724	1.55895	pos
1.56_362.15	362.155782	1.55895	pos
1.56_368.04	368.045919	1.56021667	neg
1.56_371.19	371.192533	1.55895	pos
1.56_373.17	373.172839	1.56021667	neg
1.56_382.99	382.993245	1.56021667	neg
1.56_409.01	409.014579	1.56021667	neg
1.56_419.04	419.044176	1.56021667	neg
1.56_436.03	436.033693	1.56021667	neg
1.56_442.03	487.030906	1.56021667	neg
1.56_442.71	443.722774	1.55895	pos
1.56_443.21	444.224087	1.55895	pos
1.56_448.02	448.020413	1.55895	pos
1.56_476.69	476.695789	1.55895	pos
1.56_487.23	487.238855	1.55895	pos
1.56_487.74	487.740253	1.55895	pos
1.56_510.02	555.018561	1.56021667	neg
1.56_518.96	518.968404	1.56021667	neg
1.56_520.21	520.211765	1.55895	pos
1.56_623.00	623.006078	1.56021667	neg
1.58_114.97	114.971657	1.57645	pos
1.58_145.99	145.990053	1.57645	pos

1.58_169.08(	169.086168	1.57645	pos
1.58_179.63(	179.630252	1.57645	pos
1.58_211.07!	211.07548	1.57645	pos
1.58_213.12(	213.128882	1.57645	pos
1.58_271.58(	271.580905	1.57645	pos
1.58_280.66:	280.661375	1.57645	pos
1.58_289.08(	307.122249	1.57645	pos
1.58_361.17:	361.171917	1.57645	pos
1.58_363.55:	363.553645	1.57645	pos
1.58_447.72!	447.725191	1.57645	pos
1.58_477.08!	477.085304	1.57865	neg
1.58_508.20:	508.202319	1.57865	neg
1.58_576.19(	576.19013	1.57865	neg
1.59_105.52(	105.52033	1.59411667	pos
1.59_127.05(	127.050446	1.59411667	pos
1.59_146.96:	146.961392	1.59411667	pos
1.59_169.96!	169.965513	1.59411667	pos
1.59_187.13:	187.132938	1.59411667	pos
1.59_199.98(	199.987975	1.59411667	pos
1.59_258.08!	281.074463	1.59411667	pos
1.59_310.05!	310.059192	1.59411667	pos
1.59_334.16:	334.161227	1.59411667	pos
1.60_325.06!	325.06529	1.59701667	neg
1.60_393.05:	393.052688	1.59701667	neg
1.60_406.09(	406.090804	1.59701667	neg
1.60_438.07·	438.074396	1.59701667	neg
1.60_588.11(	588.109968	1.59701667	neg
1.60_611.09!	610.091733	1.59701667	neg
1.61_115.11:	115.111949	1.61191667	pos
1.61_131.93(	131.929978	1.61191667	pos
1.61_149.01!	149.019055	1.61191667	pos
1.61_172.09'	172.097302	1.61191667	pos
1.61_200.00(	200.00058	1.61191667	pos

1.61_204.97[illegible]	204.979504	1.61191667	pos
1.61_208.63[illegible]	208.634705	1.61191667	pos
1.61_216.63[illegible]	216.636305	1.61191667	pos
1.61_226.96[illegible]	226.961023	1.61191667	pos
1.61_237.64[illegible]	237.649865	1.61191667	pos
1.61_246.99[illegible]	246.990037	1.61191667	pos
1.61_273.16[illegible]	273.16772	1.61191667	pos
1.61_284.99[illegible]	284.993438	1.61191667	pos
1.61_288.98[illegible]	288.987928	1.61191667	pos
1.61_303.14[illegible]	303.146339	1.61191667	pos
1.61_363.88[illegible]	363.887916	1.61191667	pos
1.61_432.52[illegible]	432.528044	1.61191667	pos
1.61_440.08[illegible]	440.089194	1.61191667	pos
1.61_499.11[illegible]	499.116674	1.61191667	pos
1.61_590.12[illegible]	590.124544	1.61191667	pos
1.62_130.04[illegible]	129.036386	1.61501667	neg
1.62_146.05[illegible]	146.053859	1.61501667	neg
1.62_160.99[illegible]	160.998459	1.61501667	neg
1.62_171.02[illegible]	171.027344	1.61501667	neg
1.62_177.99[illegible]	222.989245	1.61501667	neg
1.62_187.00[illegible]	187.001371	1.61501667	neg
1.62_201.07[illegible]	201.076694	1.61501667	neg
1.62_202.96[illegible]	202.964705	1.61501667	neg
1.62_203.00[illegible]	203.009091	1.61501667	neg
1.62_214.07[illegible]	214.076699	1.61501667	neg
1.62_215.01[illegible]	215.017151	1.61501667	neg
1.62_215.07[illegible]	215.073084	1.61501667	neg
1.62_222.99[illegible]	222.998404	1.61501667	neg
1.62_228.98[illegible]	228.985904	1.61501667	neg
1.62_229.06[illegible]	229.06909	1.61501667	neg
1.62_244.97[illegible]	244.975438	1.61501667	neg
1.62_245.94[illegible]	245.946689	1.61501667	neg
1.62_256.00[illegible]	256.005077	1.61501667	neg

1.62_271.95	270.9523	1.61501667	neg
1.62_283.04	283.041354	1.61501667	neg
1.62_294.10	294.109779	1.61501667	neg
1.62_296.97	296.9734	1.61501667	neg
1.62_307.00	307.002382	1.61501667	neg
1.62_312.95	312.959435	1.61501667	neg
1.62_323.99	323.992458	1.61501667	neg
1.62_338.93	338.939513	1.61501667	neg
1.62_349.01	349.012725	1.61501667	neg
1.62_355.01	355.015236	1.61501667	neg
1.62_364.96	364.960799	1.61501667	neg
1.62_365.04	365.04399	1.61501667	neg
1.62_372.97	372.974225	1.61501667	neg
1.62_374.98	374.989735	1.61501667	neg
1.62_376.07	376.079561	1.61501667	neg
1.62_380.94	380.947545	1.61501667	neg
1.62_391.98	391.980065	1.61501667	neg
1.62_406.92	406.92713	1.61501667	neg
1.62_407.05	407.054813	1.61501667	neg
1.62_417.00	417.000386	1.61501667	neg
1.62_432.94	432.948225	1.61501667	neg
1.62_433.03	433.031354	1.61501667	neg
1.62_442.97	442.977214	1.61501667	neg
1.62_446.14	446.141632	1.61501667	neg
1.62_448.93	448.935577	1.61501667	neg
1.62_459.96	459.967545	1.61501667	neg
1.62_474.91	474.914457	1.61501667	neg
1.62_484.98	484.987694	1.61501667	neg
1.62_497.10	497.101878	1.61501667	neg
1.62_501.01	501.019192	1.61501667	neg
1.62_510.96	510.964275	1.61501667	neg
1.62_527.95	527.954885	1.61501667	neg
1.62_541.06	541.065609	1.61501667	neg

1.62_552.97[illegible]	552.975174	1.61501667	neg
1.62_569.00[illegible]	569.006186	1.61501667	neg
1.62_578.95[illegible]	578.952167	1.61501667	neg
1.62_610.88[illegible]	610.889481	1.61501667	neg
1.62_646.93[illegible]	646.939767	1.61501667	neg
1.62_714.92[illegible]	714.927297	1.61501667	neg
1.63_121.50[illegible]	121.509397	1.62985	pos
1.63_132.07[illegible]	132.077165	1.62985	pos
1.63_167.03[illegible]	167.034157	1.62985	pos
1.63_168.03[illegible]	169.047043	1.62985	pos
1.63_191.01[illegible]	191.017189	1.62985	pos
1.63_201.00[illegible]	201.008309	1.62985	pos
1.63_266.16[illegible]	266.160588	1.62985	pos
1.63_273.41[illegible]	273.418497	1.62985	pos
1.63_298.97[illegible]	298.970531	1.63346667	neg
1.63_306.08[illegible]	306.089656	1.62985	pos
1.63_359.19[illegible]	359.19265	1.62985	pos
1.63_496.57[illegible]	496.579117	1.63346667	neg
1.63_500.93[illegible]	500.935552	1.63346667	neg
1.63_572.11[illegible]	572.115067	1.63346667	neg
1.63_640.10[illegible]	640.102382	1.63346667	neg
1.65_114.95[illegible]	114.956577	1.64773333	pos
1.65_158.09[illegible]	158.096444	1.64773333	pos
1.65_161.05[illegible]	161.059799	1.64773333	pos
1.65_219.13[illegible]	219.135135	1.64773333	pos
1.65_232.63[illegible]	232.634509	1.64773333	pos
1.65_250.66[illegible]	250.663313	1.64773333	pos
1.65_290.13[illegible]	290.134865	1.64773333	pos
1.65_314.52[illegible]	314.527988	1.64773333	pos
1.65_328.69[illegible]	328.6957	1.64773333	pos
1.65_452.16[illegible]	452.167523	1.65196667	neg
1.65_573.12[illegible]	574.129765	1.64773333	pos
1.67_103.92[illegible]	103.920088	1.67041667	neg

1.67_162.02	162.02283	1.67041667	neg
1.67_168.07	151.075338	1.66533333	pos
1.67_187.10	187.108603	1.67041667	neg
1.67_195.12	195.122944	1.66533333	pos
1.67_201.00	181.991584	1.67041667	neg
1.67_245.14	245.149646	1.66533333	pos
1.67_252.15	252.15983	1.66533333	pos
1.67_286.17	286.176176	1.66533333	pos
1.67_300.89	300.895358	1.66533333	pos
1.67_310.85	310.85064	1.66533333	pos
1.67_311.97	311.979652	1.67041667	neg
1.67_332.86	332.860104	1.67041667	neg
1.67_358.15	358.157796	1.66533333	pos
1.67_358.65	358.659361	1.66533333	pos
1.67_383.66	383.663569	1.66533333	pos
1.67_449.58	449.587962	1.67041667	neg
1.67_475.87	475.870303	1.67041667	neg
1.67_543.85	543.857105	1.67041667	neg
1.68_226.97	226.974991	1.68358333	pos
1.68_284.86	284.869759	1.68358333	pos
1.68_305.64	305.645286	1.68358333	pos
1.68_352.13	352.134248	1.68358333	pos
1.68_451.60	451.602824	1.68358333	pos
1.69_182.89	182.896406	1.68893333	neg
1.69_335.06	335.06106	1.68893333	neg
1.69_437.87	437.87261	1.68893333	neg
1.69_458.57	458.579215	1.68893333	neg
1.69_468.83	468.834123	1.68893333	neg
1.70_166.07	166.072426	1.70151667	pos
1.70_175.61	175.613099	1.70151667	pos
1.70_176.11	176.115137	1.70151667	pos
1.70_297.10	320.096625	1.70151667	pos
1.70_386.52	386.5257	1.70151667	pos

1.70_460.09	460.092137	1.70151667	pos
1.71_162.98	162.982239	1.7074	neg
1.71_176.90	176.900497	1.7074	neg
1.71_364.08	364.087683	1.7074	neg
1.71_458.07	458.077485	1.7074	neg
1.72_145.95	145.954437	1.71971667	pos
1.72_157.97	157.974052	1.71971667	pos
1.72_193.04	194.048366	1.71971667	pos
1.72_243.19	266.183829	1.71971667	pos
1.72_334.87	334.871629	1.71971667	pos
1.73_166.94	166.943112	1.72581667	neg
1.73_194.02	194.029109	1.72581667	neg
1.73_251.10	251.103656	1.72581667	neg
1.73_260.02	260.020792	1.72581667	neg
1.73_319.09	319.091026	1.72581667	neg
1.73_328.00	328.008354	1.72581667	neg
1.73_536.82	536.822556	1.72581667	neg
1.74_113.96	113.964013	1.73755	pos
1.74_114.95	114.956588	1.73755	pos
1.74_142.95	142.955793	1.73755	pos
1.74_155.07	155.070458	1.73755	pos
1.74_158.10	181.094787	1.73755	pos
1.74_179.06	179.067549	1.73755	pos
1.74_194.10	194.103751	1.73755	pos
1.74_202.99	202.999503	1.74428333	neg
1.74_243.64	243.649888	1.73755	pos
1.74_462.20	462.20241	1.73755	pos
1.74_472.83	472.833416	1.74428333	neg
1.76_114.52	114.52533	1.75533333	pos
1.76_129.95	129.958783	1.75533333	pos
1.76_135.94	135.94549	1.75533333	pos
1.76_179.03	179.034776	1.76276667	neg
1.76_180.89	180.899505	1.76276667	neg

1.76_182.985	182.985265	1.75533333	pos
1.76_257.077	257.077881	1.76276667	neg
1.76_278.962	278.962924	1.76276667	neg
1.76_279.671	279.671464	1.75533333	pos
1.76_281.074	281.074182	1.75533333	pos
1.76_290.971	290.971052	1.76276667	neg
1.76_294.109	294.109643	1.76276667	neg
1.76_354.149	354.149436	1.75533333	pos
1.76_434.890	434.889973	1.75533333	pos
1.76_516.837	516.837852	1.76276667	neg
1.77_133.974	133.974816	1.77345	pos
1.77_176.000	177.013532	1.77345	pos
1.77_176.972	176.972054	1.77345	pos
1.77_187.132	187.132895	1.77345	pos
1.77_229.652	229.652376	1.77345	pos
1.77_276.896	276.896908	1.77345	pos
1.77_284.811	284.81191	1.77345	pos
1.78_156.996	156.996295	1.78133333	neg
1.78_181.036	181.036553	1.78133333	neg
1.78_188.993	188.993425	1.78133333	neg
1.78_210.975	210.975415	1.78133333	neg
1.78_216.012	216.012424	1.78133333	neg
1.78_221.004	221.004128	1.78133333	neg
1.78_237.994	237.994443	1.78133333	neg
1.78_248.887	248.887045	1.78133333	neg
1.78_288.991	288.991742	1.78133333	neg
1.78_295.093	295.093422	1.78133333	neg
1.78_298.947	298.947201	1.78133333	neg
1.78_305.981	305.981931	1.78133333	neg
1.78_317.075	317.0753	1.78133333	neg
1.78_356.979	356.979302	1.78133333	neg
1.78_366.934	366.934296	1.78133333	neg
1.78_424.966	424.966947	1.78133333	neg

1.78_434.92:	434.921698	1.78133333	neg
1.78_492.95	492.954107	1.78133333	neg
1.78_588.82	588.824667	1.78133333	neg
1.78_595.14	595.14211	1.78133333	neg
1.79_131.96	131.967172	1.79108333	pos
1.79_149.94	149.940444	1.79108333	pos
1.79_202.97	202.974951	1.79108333	pos
1.79_252.63	252.638904	1.79108333	pos
1.79_299.08	299.081873	1.79108333	pos
1.79_299.58	299.583621	1.79108333	pos
1.79_326.16	326.164071	1.79108333	pos
1.79_334.16	334.161041	1.79108333	pos
1.80_300.15	300.156433	1.79976667	neg
1.80_443.83	443.830442	1.79976667	neg
1.80_461.59	461.593736	1.79976667	neg
1.80_604.81	604.810385	1.79976667	neg
1.81_107.95	107.950873	1.80931667	pos
1.81_141.12	141.127582	1.80931667	pos
1.81_172.97	172.977289	1.80931667	pos
1.81_173.08	173.08108	1.80931667	pos
1.81_181.10	181.102896	1.80931667	pos
1.81_448.11	448.11751	1.80931667	pos
1.82_309.87	309.870118	1.81826667	neg
1.82_369.88	369.885098	1.81826667	neg
1.82_384.86	384.862203	1.81826667	neg
1.82_400.90	400.909197	1.81826667	neg
1.82_452.84	452.849714	1.81826667	neg
1.82_540.82	540.821181	1.81826667	neg
1.82_662.84	662.842128	1.81826667	neg
1.82_802.81	802.815941	1.81826667	neg
1.83_143.14	143.143153	1.8272	pos
1.83_163.07	163.075466	1.8272	pos
1.83_183.61	183.610494	1.8272	pos

1.83_208.982	208.982232	1.8272	pos
1.83_215.902	215.902509	1.8272	pos
1.83_226.974	226.974887	1.8272	pos
1.83_262.887	262.887899	1.8272	pos
1.83_318.886	318.886444	1.8272	pos
1.84_136.909	136.909623	1.83668333	neg
1.84_146.938	146.938538	1.83668333	neg
1.84_157.050	157.050393	1.83668333	neg
1.84_190.928	190.928298	1.83668333	neg
1.84_207.918	207.918436	1.83668333	neg
1.84_308.863	308.86391	1.83668333	neg
1.84_316.874	316.874535	1.83668333	neg
1.84_450.079	450.079825	1.83668333	neg
1.84_683.830	683.83046	1.83668333	neg
1.85_112.956	112.956368	1.84545	pos
1.85_115.039	115.039178	1.84545	pos
1.85_144.030	144.030015	1.85476667	neg
1.85_145.990	145.990026	1.84545	pos
1.85_151.061	151.061551	1.84545	pos
1.85_257.652	257.652693	1.84545	pos
1.85_258.005	258.005132	1.85476667	neg
1.85_272.135	272.135237	1.84545	pos
1.85_287.888	287.888851	1.84545	pos
1.85_318.166	318.165989	1.84545	pos
1.85_365.887	365.887949	1.85476667	neg
1.85_386.683	386.683046	1.84545	pos
1.85_387.184	387.184782	1.84545	pos
1.85_440.869	440.869308	1.84545	pos
1.85_463.608	463.608079	1.84545	pos
1.85_536.884	536.884005	1.85476667	neg
1.85_615.843	615.843238	1.85476667	neg
1.86_159.969	159.969214	1.86321667	pos
1.86_175.952	176.959444	1.86321667	pos

1.86_193.13	193.131565	1.86321667	pos
1.86_193.63	193.633476	1.86321667	pos
1.86_236.90	236.906956	1.86321667	pos
1.86_305.08	305.085683	1.86321667	pos
1.86_357.21	357.213487	1.86321667	pos
1.87_125.06	125.060635	1.87326667	neg
1.87_151.05	151.051086	1.87326667	neg
1.87_152.04	152.046334	1.87326667	neg
1.87_153.05	153.055596	1.87326667	neg
1.87_157.06	157.061614	1.87326667	neg
1.87_178.97	178.977553	1.87326667	neg
1.87_235.92	235.92599	1.87326667	neg
1.87_245.11	245.114167	1.87326667	neg
1.87_258.91	258.915706	1.87326667	neg
1.87_275.90	275.905905	1.87326667	neg
1.87_335.08	335.083686	1.87326667	neg
1.87_371.90	371.900764	1.87326667	neg
1.87_411.88	411.880905	1.87326667	neg
1.87_462.87	462.878303	1.87326667	neg
1.87_501.86	501.863084	1.87326667	neg
1.87_520.83	520.836777	1.87326667	neg
1.87_597.85	597.857191	1.87326667	neg
1.88_139.96	139.963368	1.88096667	pos
1.88_141.95	141.95867	1.88096667	pos
1.88_142.96	142.966508	1.88096667	pos
1.88_146.11	146.117638	1.88096667	pos
1.88_177.99	177.992337	1.88096667	pos
1.88_180.98	180.989919	1.88096667	pos
1.88_195.12	195.123294	1.88096667	pos
1.88_195.62	195.625189	1.88096667	pos
1.88_244.99	244.998175	1.88096667	pos
1.88_333.08	333.08469	1.88096667	pos
1.89_155.03	155.034812	1.89173333	neg

1.89_423.09	423.093996	1.89173333	neg
1.89_456.11	501.110475	1.89173333	neg
1.89_518.10	518.100455	1.89173333	neg
1.89_524.09	569.097734	1.89173333	neg
1.89_608.80	608.809016	1.89173333	neg
1.89_637.08	637.085426	1.89173333	neg
1.89_705.07	705.072933	1.89173333	neg
1.90_136.95	136.95334	1.89901667	pos
1.90_150.11	150.11261	1.89901667	pos
1.90_153.05	153.054542	1.89901667	pos
1.90_154.96	154.966986	1.89901667	pos
1.90_157.97	157.974035	1.89901667	pos
1.90_168.04	168.045449	1.89901667	pos
1.90_169.08	169.086055	1.89901667	pos
1.90_201.00	201.008353	1.89901667	pos
1.90_203.10	203.106959	1.89901667	pos
1.90_211.03	211.038305	1.89901667	pos
1.90_221.64	221.642291	1.89901667	pos
1.90_223.56	223.565435	1.89901667	pos
1.90_233.62	216.626401	1.89901667	pos
1.90_300.89	300.895486	1.89901667	pos
1.90_304.89	304.894265	1.89901667	pos
1.90_319.88	319.881321	1.89901667	pos
1.90_366.14	367.150095	1.89901667	pos
1.91_179.93	179.936095	1.91026667	neg
1.91_299.06	299.062874	1.91026667	neg
1.91_326.90	326.903269	1.91026667	neg
1.92_129.95	129.958752	1.9162	pos
1.92_230.62	230.629418	1.9162	pos
1.92_299.06	299.06169	1.9162	pos
1.93_102.94	102.948659	1.92873333	neg
1.93_195.09	195.099674	1.93406667	pos
1.93_210.98	210.983391	1.92873333	neg

1.93_215.139	215.139031	1.93406667	pos
1.93_231.044	231.044493	1.92873333	neg
1.93_283.103	283.103615	1.93406667	pos
1.93_329.674	329.674053	1.93406667	pos
1.93_394.890	394.89053	1.92873333	neg
1.93_470.704	470.704247	1.93406667	pos
1.93_479.868	479.868347	1.92873333	neg
1.93_530.865	530.8657	1.92873333	neg
1.93_547.856	547.856074	1.92873333	neg
1.93_598.853	598.853439	1.92873333	neg
1.93_666.840	666.840893	1.92873333	neg
1.95_105.033	105.033935	1.95213333	pos
1.95_172.977	172.977159	1.95213333	pos
1.95_199.975	199.975414	1.95213333	pos
1.95_233.618	233.618461	1.95213333	pos
1.95_256.920	256.920452	1.9471	neg
1.95_298.114	298.114611	1.95213333	pos
1.95_330.166	330.166204	1.95213333	pos
1.95_343.893	343.893456	1.9471	neg
1.95_372.881	372.881587	1.95213333	pos
1.95_386.859	386.859008	1.9471	neg
1.95_471.205	471.205738	1.95213333	pos
1.95_505.860	505.860079	1.9471	neg
1.97_122.019	122.019554	1.97033333	pos
1.97_123.117	123.117054	1.97033333	pos
1.97_142.966	142.966539	1.97033333	pos
1.97_163.928	163.928661	1.96566667	neg
1.97_166.072	166.072428	1.97033333	pos
1.97_187.111	187.111718	1.97033333	pos
1.97_191.104	191.104089	1.97033333	pos
1.97_213.124	213.124319	1.96566667	neg
1.97_281.113	281.11319	1.97033333	pos
1.97_297.082	297.08258	1.97033333	pos

1.97_304.66	304.661139	1.97033333	pos
1.97_314.47	314.472764	1.97033333	pos
1.97_345.18	345.182479	1.97033333	pos
1.97_355.07	355.069994	1.97033333	pos
1.97_364.08	364.087553	1.96566667	neg
1.97_390.19	390.197592	1.97033333	pos
1.97_395.15	395.156702	1.96566667	neg
1.97_417.06	417.064142	1.96566667	neg
1.97_432.07	432.074695	1.96566667	neg
1.97_568.77	568.772739	1.96566667	neg
1.98_177.02	177.022416	1.98383333	neg
1.98_351.02	351.024235	1.98383333	neg
1.98_656.81	656.812176	1.98383333	neg
1.99_140.95	140.951409	1.9877	pos
1.99_143.03	143.033934	1.9877	pos
1.99_149.09	149.096148	1.9877	pos
10.01_314.2	314.26853	10.0126833	pos
10.01_446.0	445.011658	10.00765	neg
10.01_450.2	450.262677	10.00765	neg
10.01_474.2	474.258814	10.0126833	pos
10.01_553.0	553.013506	10.00765	neg
10.01_554.3	554.330901	10.00765	neg
10.01_556.0	601.031236	10.00765	neg
10.01_629.0	629.025034	10.00765	neg
10.03_1049.	1049.32431	10.0311	pos
10.03_119.0	119.013733	10.0311	pos
10.03_180.9	180.989666	10.0311	pos
10.03_281.2	281.202866	10.0257	neg
10.03_325.2	325.201481	10.0257	neg
10.03_400.3	400.332264	10.0311	pos
10.03_415.1	415.171496	10.0257	neg
10.03_431.0	431.013719	10.0257	neg
10.03_447.0	447.062684	10.0257	neg

10.03_486.0:	531.026425	10.0257	neg
10.03_517.0:	517.030087	10.0257	neg
10.03_535.3(	535.300493	10.0311	pos
10.03_559.0:	559.022125	10.0257	neg
10.03_633.0:	633.037899	10.0257	neg
10.03_909.5!	909.550345	10.0257	neg
10.04_398.2(	398.265823	10.0440667	neg
10.04_442.3:	442.31747	10.0440667	neg
10.04_510.3(	510.304724	10.0440667	neg
10.05_186.9!	186.980819	10.0490667	pos
10.05_274.6:	274.624956	10.0490667	pos
10.05_287.0(	287.067657	10.0490667	pos
10.05_351.1!	351.193101	10.0490667	pos
10.05_422.2(	422.263079	10.0490667	pos
10.05_482.3:	482.323943	10.0490667	pos
10.05_493.3:	494.323847	10.0490667	pos
10.06_124.0(	124.007128	10.0622	neg
10.06_192.9:	192.92853	10.0622	neg
10.06_526.3:	526.314367	10.0622	neg
10.06_674.2!	674.289951	10.0622	neg
10.07_1048.!	1048.57179	10.0669833	pos
10.07_274.0!	274.090999	10.0669833	pos
10.07_282.9(	282.909946	10.0669833	pos
10.08_1021.!	1021.52566	10.0807333	neg
10.08_225.0(	225.061398	10.0807333	neg
10.08_459.0:	459.026583	10.0807333	neg
10.08_477.0:	477.017822	10.0807333	neg
10.08_483.0:	483.02658	10.0807333	neg
10.08_549.0:	549.018201	10.0807333	neg
10.08_585.0:	585.022006	10.0807333	neg
10.08_675.0:	675.047759	10.0807333	neg
10.09_1048.!	1048.82289	10.0853667	pos
10.09_1049.(	1049.07362	10.0853667	pos

10.09_207.0	207.017365	10.0853667	pos
10.10_131.5	131.533487	10.1038333	pos
10.10_282.2	282.278819	10.1038333	pos
10.10_328.9	328.903637	10.0986	neg
10.10_338.2	356.279024	10.1038333	pos
10.10_382.9	382.995156	10.0986	neg
10.10_383.2	383.241743	10.0986	neg
10.10_471.0	471.026054	10.0986	neg
10.10_491.0	491.014273	10.0986	neg
10.10_511.0	511.02047	10.0986	neg
10.10_513.0	513.016504	10.0986	neg
10.12_266.1	311.18398	10.11685	neg
10.12_299.2	299.257603	10.1222	pos
10.12_340.2	363.21359	10.1222	pos
10.12_372.9	372.990352	10.11685	neg
10.12_484.0	529.01089	10.11685	neg
10.12_546.8	546.835363	10.11685	neg
10.12_838.5	838.594071	10.11685	neg
10.14_183.1	183.175099	10.1353167	neg
10.14_331.1	331.189123	10.1353167	neg
10.14_383.2	383.218447	10.14065	pos
10.14_471.7	471.746974	10.14065	pos
10.14_503.0	503.033492	10.1353167	neg
10.14_583.2	583.254592	10.14065	pos
10.14_619.0	619.021453	10.1353167	neg
10.15_253.0	253.082876	10.15385	neg
10.15_285.2	285.207056	10.15385	neg
10.15_415.3	415.317817	10.15385	neg
10.15_416.9	416.997641	10.15385	neg
10.15_438.2	438.262351	10.15385	neg
10.15_476.2	476.278161	10.15385	neg
10.15_512.3	512.358994	10.15385	neg
10.15_546.0	545.039045	10.15385	neg

10.15_581.0[illegible]	581.027194	10.15385	neg
10.15_612.2[illegible]	612.253838	10.15385	neg
10.15_816.5[illegible]	861.549997	10.15385	neg
10.15_837.5[illegible]	837.545965	10.15385	neg
10.15_911.5[illegible]	911.566341	10.15385	neg
10.16_197.1[illegible]	197.189847	10.1580333	pos
10.16_199.1[illegible]	199.18023	10.1580333	pos
10.16_266.6[illegible]	266.609297	10.1580333	pos
10.16_352.3[illegible]	352.318124	10.1580333	pos
10.16_370.3[illegible]	393.307993	10.1580333	pos
10.16_477.2[illegible]	478.29244	10.1580333	pos
10.17_432.3[illegible]	432.307672	10.1716833	neg
10.17_456.3[illegible]	456.344401	10.1716833	neg
10.17_584.2[illegible]	584.237955	10.1716833	neg
10.17_587.0[illegible]	587.016192	10.1716833	neg
10.17_592.2[illegible]	592.265753	10.1716833	neg
10.17_624.2[illegible]	624.203489	10.1716833	neg
10.17_660.2[illegible]	660.256439	10.1716833	neg
10.17_728.2[illegible]	728.242466	10.1716833	neg
10.18_1049.[illegible]	1049.57277	10.17625	pos
10.18_270.6[illegible]	270.623078	10.17625	pos
10.18_277.6[illegible]	277.611665	10.17625	pos
10.18_290.6[illegible]	290.609197	10.17625	pos
10.18_291.1[illegible]	291.110865	10.17625	pos
10.18_293.2[illegible]	293.208401	10.17625	pos
10.18_394.3[illegible]	395.362779	10.17625	pos
10.18_440.4[illegible]	440.42042	10.17625	pos
10.18_525.2[illegible]	526.292714	10.17625	pos
10.18_541.7[illegible]	541.754009	10.17625	pos
10.19_1002.[illegible]	1003.5771	10.1937667	pos
10.19_1019.[illegible]	1019.61041	10.18955	neg
10.19_1023.[illegible]	1023.54088	10.18955	neg
10.19_1026.[illegible]	1026.56649	10.18955	neg

10.19_116.9	116.967513	10.1937667	pos
10.19_197.0	197.095844	10.1937667	pos
10.19_269.6	269.604008	10.1937667	pos
10.19_278.6	278.609307	10.1937667	pos
10.19_501.2	500.278388	10.18955	neg
10.19_505.3	505.300932	10.1937667	pos
10.19_529.2	529.252529	10.1937667	pos
10.19_553.0	553.026299	10.18955	neg
10.19_556.2	556.211727	10.1937667	pos
10.19_558.2	558.211438	10.1937667	pos
10.19_558.2	558.236714	10.18955	neg
10.19_563.2	563.273822	10.18955	neg
10.19_585.2	585.256155	10.18955	neg
10.19_600.2	600.203663	10.18955	neg
10.19_614.2	614.243149	10.1937667	pos
10.19_623.0	623.034839	10.18955	neg
10.19_624.1	624.199675	10.1937667	pos
10.19_629.8	629.892513	10.1937667	pos
10.19_640.1	640.196122	10.18955	neg
10.19_704.2	704.241006	10.18955	neg
10.19_708.1	708.183487	10.18955	neg
10.19_772.2	772.228319	10.18955	neg
10.19_796.2	796.228355	10.18955	neg
10.19_840.2	840.215605	10.18955	neg
10.19_908.2	908.203497	10.18955	neg
10.19_976.1	976.189916	10.18955	neg
10.21_1043.	1043.61066	10.20805	neg
10.21_1045.	1045.62309	10.2120667	pos
10.21_287.6	287.632637	10.2120667	pos
10.21_288.1	288.134326	10.2120667	pos
10.21_311.6	311.632685	10.2120667	pos
10.21_318.2	318.239857	10.2120667	pos
10.21_339.0	339.007746	10.20805	neg

10.21_423.3:	468.332934	10.20805	neg
10.21_450.3	450.347669	10.2120667	pos
10.21_470.3:	470.324376	10.2120667	pos
10.21_471.2	471.245422	10.2120667	pos
10.21_481.1	481.14317	10.20805	neg
10.21_519.3:	520.339371	10.2120667	pos
10.21_554.3(	554.301612	10.20805	neg
10.21_554.3:	554.320562	10.2120667	pos
10.21_564.3:	564.330725	10.20805	neg
10.21_567.3:	568.339963	10.2120667	pos
10.21_581.3:	581.320765	10.20805	neg
10.21_610.3(	610.308621	10.2120667	pos
10.21_632.3	632.318314	10.20805	neg
10.21_662.4	662.441932	10.2120667	pos
10.21_700.3(	700.3058	10.20805	neg
10.21_768.2!	768.292926	10.20805	neg
10.21_828.5!	828.550293	10.2120667	pos
10.21_836.2	836.280346	10.20805	neg
10.23_222.0	222.076925	10.2257833	neg
10.23_250.1:	250.129015	10.2295	pos
10.23_277.1	277.180651	10.2257833	neg
10.23_297.2(	320.255589	10.2295	pos
10.23_299.6:	299.63271	10.2295	pos
10.23_300.1:	300.134337	10.2295	pos
10.23_365.2(	365.269604	10.2257833	neg
10.23_436.2	436.282037	10.2295	pos
10.23_489.0:	489.037074	10.2257833	neg
10.23_495.0	495.045174	10.2257833	neg
10.23_547.3	547.348114	10.2295	pos
10.23_549.1:	549.130656	10.2257833	neg
10.23_566.8:	566.823439	10.2295	pos
10.23_578.3(	578.302089	10.2257833	neg
10.23_588.3:	588.33088	10.2257833	neg

10.23_594.3:	594.333788	10.2295	pos
10.23_604.3(	604.308075	10.2257833	neg
10.23_625.0·	625.049359	10.2257833	neg
10.23_634.3(	634.308575	10.2295	pos
10.23_646.2!	646.289633	10.2257833	neg
10.23_663.4!	686.441232	10.2295	pos
10.23_673.4:	673.436175	10.2295	pos
10.23_673.9:	673.937155	10.2295	pos
10.23_674.9·	674.945096	10.2295	pos
10.23_680.3:	680.318054	10.2257833	neg
10.23_724.3(	724.305856	10.2257833	neg
10.23_746.5:	746.512752	10.2257833	neg
10.23_792.2!	792.293357	10.2257833	neg
10.24_434.2(	434.267564	10.2441667	neg
10.24_473.0(	473.004804	10.2441667	neg
10.24_483.0·	483.044498	10.2441667	neg
10.24_515.0:	515.031149	10.2441667	neg
10.24_860.2!	860.280877	10.2441667	neg
10.25_261.6·	261.644022	10.24785	pos
10.25_408.3:	391.313724	10.24785	pos
10.26_275.1(	275.165118	10.2627833	neg
10.26_314.0!	313.078454	10.2627833	neg
10.26_383.2·	383.241812	10.2627833	neg
10.26_396.8!	396.8909	10.2627833	neg
10.26_413.0(	413.004745	10.2627833	neg
10.26_549.0:	549.03639	10.2627833	neg
10.27_277.2:	277.215399	10.2662	pos
10.27_293.2:	316.211522	10.2662	pos
10.27_298.2!	321.23963	10.2662	pos
10.27_314.0!	297.082075	10.2662	pos
10.27_369.3!	369.351126	10.2662	pos
10.27_583.3(	584.370626	10.2662	pos
10.27_594.4(	617.458916	10.2662	pos

10.28_266.6[illegible]	266.609358	10.2840833	pos
10.28_477.2[illegible]	478.292435	10.2840833	pos
10.28_521.0[illegible]	521.023618	10.2812167	neg
10.28_612.2[illegible]	612.253549	10.2812167	neg
10.28_629.0[illegible]	629.024224	10.2812167	neg
10.30_115.9[illegible]	115.964195	10.3023333	pos
10.30_117.9[illegible]	117.959631	10.3023333	pos
10.30_186.9[illegible]	186.980806	10.3023333	pos
10.30_282.2[illegible]	265.215777	10.3023333	pos
10.30_400.2[illegible]	400.265121	10.3023333	pos
10.30_411.7[illegible]	411.739845	10.3023333	pos
10.30_489.0[illegible]	489.017922	10.2994667	neg
10.30_544.2[illegible]	544.267586	10.2994667	neg
10.30_599.0[illegible]	599.031121	10.2994667	neg
10.32_124.9[illegible]	124.964453	10.3206667	pos
10.32_182.9[illegible]	182.921181	10.31745	neg
10.32_295.2[illegible]	295.226343	10.3206667	pos
10.32_351.2[illegible]	351.252399	10.3206667	pos
10.32_356.3[illegible]	356.351773	10.3206667	pos
10.32_397.1[illegible]	397.138415	10.31745	neg
10.32_413.2[illegible]	413.254391	10.31745	neg
10.32_414.3[illegible]	397.30968	10.3206667	pos
10.32_420.3[illegible]	420.30893	10.3206667	pos
10.32_499.3[illegible]	499.304259	10.31745	neg
10.32_615.3[illegible]	615.334759	10.31745	neg
10.34_165.9[illegible]	165.990736	10.3389333	pos
10.34_243.9[illegible]	243.941317	10.3389333	pos
10.34_285.2[illegible]	285.206981	10.3358333	neg
10.34_287.6[illegible]	287.63268	10.3389333	pos
10.34_288.1[illegible]	288.134431	10.3389333	pos
10.34_311.1[illegible]	311.183738	10.3358333	neg
10.34_339.2[illegible]	339.253783	10.3358333	neg
10.34_344.3[illegible]	344.315435	10.3389333	pos

10.34_412.34	412.341541	10.3389333	pos
10.34_519.33	520.339493	10.3389333	pos
10.34_554.30	554.301996	10.3358333	neg
10.34_581.32	581.3211	10.3358333	neg
10.34_610.30	610.308547	10.3389333	pos
10.34_700.30	700.305751	10.3358333	neg
10.34_768.29	768.292875	10.3358333	neg
10.34_777.96	777.964532	10.3389333	pos
10.34_836.28	836.280495	10.3358333	neg
10.35_493.86	493.866368	10.3534833	neg
10.35_730.25	730.256085	10.3534833	neg
10.36_172.95	172.956197	10.357	pos
10.36_298.63	298.635081	10.357	pos
10.36_301.21	301.215715	10.357	pos
10.36_361.23	361.234357	10.357	pos
10.36_385.23	385.234457	10.357	pos
10.37_207.10	207.102328	10.37205	neg
10.37_295.22	295.227667	10.37205	neg
10.37_318.90	318.904239	10.37205	neg
10.37_429.88	474.87903	10.37205	neg
10.37_431.20	431.202758	10.37205	neg
10.37_431.87	476.875926	10.37205	neg
10.37_433.01	433.011144	10.37205	neg
10.37_457.02	457.029912	10.37205	neg
10.37_472.88	472.88199	10.37205	neg
10.37_483.04	483.044941	10.37205	neg
10.37_489.87	489.872058	10.37205	neg
10.37_491.86	491.869453	10.37205	neg
10.37_526.29	526.293859	10.37205	neg
10.37_556.34	556.346476	10.37205	neg
10.37_594.28	594.281521	10.37205	neg
10.37_614.82	614.822767	10.37205	neg
10.37_663.27	663.273845	10.37205	neg

10.38_109.52	109.521847	10.3754333	pos
10.38_283.93	283.933776	10.3754333	pos
10.38_291.61	291.616995	10.3754333	pos
10.38_299.21	299.219696	10.3754333	pos
10.38_322.29	322.2948	10.3754333	pos
10.38_468.31	491.299761	10.3754333	pos
10.38_510.83	510.836287	10.3754333	pos
10.38_511.33	511.337748	10.3754333	pos
10.38_513.81	513.815441	10.3754333	pos
10.38_527.30	528.308185	10.3754333	pos
10.38_532.84	532.849195	10.3754333	pos
10.39_142.99	142.991021	10.3935333	pos
10.39_156.99	156.990518	10.3935333	pos
10.39_207.01	207.017267	10.3935333	pos
10.39_299.25	299.258883	10.3905167	neg
10.39_418.26	418.264094	10.3935333	pos
10.39_446.29	469.287231	10.3935333	pos
10.39_466.80	466.809422	10.3935333	pos
10.39_467.31	467.31148	10.3935333	pos
10.39_488.82	488.822681	10.3935333	pos
10.39_489.32	489.324258	10.3935333	pos
10.39_490.32	513.313878	10.3935333	pos
10.39_613.02	613.02958	10.3905167	neg
10.41_144.10	144.102806	10.4089833	neg
10.41_312.64	312.640608	10.41135	pos
10.41_320.25	343.245009	10.41135	pos
10.41_351.00	351.008149	10.4089833	neg
10.41_362.25	362.253184	10.41135	pos
10.41_379.24	379.248132	10.41135	pos
10.41_381.73	381.736383	10.41135	pos
10.41_389.77	389.779125	10.41135	pos
10.41_398.29	398.292365	10.41135	pos
10.41_398.32	398.327383	10.4089833	neg

10.41_400.34	400.343117	10.4089833	neg
10.41_402.27	425.261023	10.41135	pos
10.41_402.34	402.347575	10.41135	pos
10.41_402.77	425.76301	10.41135	pos
10.41_422.78	422.783147	10.41135	pos
10.41_423.28	423.28475	10.41135	pos
10.41_424.28	447.274003	10.41135	pos
10.41_424.78	447.775892	10.41135	pos
10.41_444.79	444.796285	10.41135	pos
10.41_445.29	445.298406	10.41135	pos
10.41_446.33	446.339354	10.4089833	neg
10.41_458.81	458.811807	10.41135	pos
10.41_466.29	466.292543	10.41135	pos
10.41_588.25	588.253325	10.4089833	neg
10.41_614.34	614.346626	10.4089833	neg
10.41_846.54	846.546346	10.4089833	neg
10.43_141.98	141.98323	10.4295333	pos
10.43_188.09	188.092537	10.42685	neg
10.43_223.98	223.988082	10.4295333	pos
10.43_255.23	255.232796	10.42685	neg
10.43_313.27	313.273163	10.4295333	pos
10.43_315.19	315.1963	10.42685	neg
10.43_315.23	315.231503	10.4295333	pos
10.43_343.33	343.331522	10.4295333	pos
10.43_346.21	391.210181	10.42685	neg
10.43_364.28	403.24529	10.4295333	pos
10.43_378.75	378.756871	10.4295333	pos
10.43_381.23	381.234571	10.4295333	pos
10.43_408.30	431.297444	10.4295333	pos
10.43_452.33	470.368246	10.4295333	pos
10.43_454.37	454.373312	10.4295333	pos
10.43_498.90	498.900826	10.4295333	pos
10.43_580.30	580.307594	10.42685	neg

10.43_584.4[illegible]	607.402052	10.4295333	pos
10.43_672.4[illegible]	695.454379	10.4295333	pos
10.43_716.2[illegible]	716.28335	10.42685	neg
10.43_716.4[illegible]	739.480323	10.4295333	pos
10.43_775.5[illegible]	775.597051	10.42685	neg
10.45_307.2[illegible]	307.226196	10.4474333	pos
10.45_324.2[illegible]	347.218756	10.4474333	pos
10.45_326.2[illegible]	327.244605	10.4474333	pos
10.45_399.7[illegible]	399.763505	10.4474333	pos
10.45_436.2[illegible]	436.283337	10.4453167	neg
10.45_496.3[illegible]	514.394429	10.4474333	pos
10.45_540.3[illegible]	563.375854	10.4474333	pos
10.45_542.4[illegible]	542.425805	10.4474333	pos
10.45_586.4[illegible]	586.451408	10.4474333	pos
10.45_597.2[illegible]	597.29865	10.4453167	neg
10.45_630.4[illegible]	630.476443	10.4474333	pos
10.46_305.2[illegible]	305.212023	10.4632167	neg
10.46_480.3[illegible]	480.309925	10.4632167	neg
10.46_531.3[illegible]	531.305224	10.4632167	neg
10.46_538.3[illegible]	538.314985	10.4632167	neg
10.46_543.3[illegible]	543.33875	10.4632167	neg
10.46_552.3[illegible]	552.330851	10.4632167	neg
10.46_614.2[illegible]	614.265977	10.4632167	neg
10.46_638.2[illegible]	638.268514	10.4632167	neg
10.46_680.2[illegible]	680.248202	10.4632167	neg
10.46_745.2[illegible]	745.296636	10.4632167	neg
10.47_223.1[illegible]	223.168928	10.46565	pos
10.47_279.6[illegible]	279.61696	10.46565	pos
10.47_361.2[illegible]	361.273204	10.46565	pos
10.47_466.3[illegible]	466.328703	10.46565	pos
10.47_650.9[illegible]	650.94486	10.46565	pos
10.48_184.0[illegible]	184.07314	10.4838833	pos
10.48_297.6[illegible]	297.647899	10.4838833	pos

10.48_339.2	339.215058	10.48035	neg
10.48_347.1	347.182465	10.4838833	pos
10.48_407.2	407.202691	10.48035	neg
10.48_534.2	534.295146	10.4838833	pos
10.48_586.3	586.308824	10.4838833	pos
10.48_610.3	610.323833	10.48035	neg
10.48_618.2	618.246277	10.4838833	pos
10.48_619.2	619.288846	10.48035	neg
10.48_639.4	662.441669	10.4838833	pos
10.48_649.4	649.435796	10.4838833	pos
10.48_687.2	687.276393	10.48035	neg
10.48_755.2	755.263828	10.48035	neg
10.48_812.2	812.280833	10.48035	neg
10.50_131.5	131.533474	10.50225	pos
10.50_297.1	297.152773	10.4988	neg
10.50_311.2	311.222737	10.4988	neg
10.50_333.2	333.205204	10.4988	neg
10.50_358.2	358.294505	10.50225	pos
10.50_431.0	431.013505	10.4988	neg
10.50_479.0	479.032645	10.4988	neg
10.50_552.2	552.27327	10.4988	neg
10.50_632.4	632.417408	10.50225	pos
10.50_636.3	636.324281	10.50225	pos
10.50_792.5	792.585562	10.50225	pos
10.50_938.5	938.535507	10.4988	neg
10.52_292.1	292.102213	10.5204167	pos
10.52_300.6	300.640523	10.5204167	pos
10.52_338.1	338.114458	10.5204167	pos
10.52_338.6	338.616245	10.5204167	pos
10.52_385.1	385.19957	10.5172833	neg
10.52_388.8	388.878167	10.5172833	neg
10.52_460.2	460.283406	10.5172833	neg
10.52_479.3	480.344334	10.5204167	pos

10.52_517.0	517.028258	10.5172833	neg
10.52_524.3	524.335589	10.5172833	neg
10.52_545.3	546.355022	10.5204167	pos
10.52_607.3	607.336723	10.5172833	neg
10.52_614.8	614.822782	10.5172833	neg
10.52_642.2	643.284676	10.5204167	pos
10.52_726.3	726.321585	10.5172833	neg
10.52_774.5	774.535679	10.5172833	neg
10.52_922.5	922.578583	10.5172833	neg
10.52_962.5	962.535178	10.5172833	neg
10.54_225.0	225.061368	10.5357333	neg
10.54_292.6	292.60684	10.5380833	pos
10.54_353.2	353.208445	10.5380833	pos
10.54_358.0	358.040519	10.5357333	neg
10.54_528.2	528.272851	10.5357333	neg
10.54_529.2	530.287693	10.5380833	pos
10.54_596.2	596.260153	10.5357333	neg
10.55_327.2	327.253909	10.5532667	neg
10.55_339.0	339.0078	10.5532667	neg
10.55_473.0	473.005001	10.5532667	neg
10.55_550.3	550.351571	10.5532667	neg
10.55_566.2	566.24993	10.5532667	neg
10.55_606.3	606.32358	10.5532667	neg
10.55_860.2	860.202426	10.5532667	neg
10.56_160.9	160.983444	10.5560833	pos
10.56_252.2	253.215722	10.5560833	pos
10.56_269.2	269.209039	10.5560833	pos
10.56_457.3	457.301011	10.5560833	pos
10.57_113.1	113.107472	10.5732333	pos
10.57_203.9	203.94563	10.5716833	neg
10.57_275.6	275.624344	10.5732333	pos
10.57_282.9	282.909905	10.5732333	pos
10.57_291.6	291.617066	10.5732333	pos

10.57_296.63	296.636106	10.5732333	pos
10.57_313.27	313.272954	10.5732333	pos
10.57_337.19	337.199683	10.5716833	neg
10.57_453.28	454.292299	10.5732333	pos
10.57_455.28	455.286705	10.5716833	neg
10.57_504.34	504.343672	10.5732333	pos
10.57_510.23	510.236659	10.5716833	neg
10.57_515.27	515.273796	10.5716833	neg
10.57_520.26	520.265916	10.5716833	neg
10.57_522.28	522.282208	10.5732333	pos
10.57_537.25	537.255819	10.5716833	neg
10.57_552.20	552.203554	10.5716833	neg
10.57_566.24	566.243407	10.5732333	pos
10.57_592.19	592.196155	10.5716833	neg
10.57_660.18	660.183653	10.5716833	neg
10.57_716.52	716.52333	10.5716833	neg
10.57_724.22	724.228047	10.5716833	neg
10.57_728.17	728.171282	10.5716833	neg
10.57_792.21	792.215356	10.5716833	neg
10.57_906.56	907.575572	10.5732333	pos
10.59_239.06	239.067101	10.59005	neg
10.59_326.24	327.252308	10.5914167	pos
10.59_349.23	349.234406	10.5914167	pos
10.59_361.01	361.010637	10.59005	neg
10.59_398.26	421.259114	10.5914167	pos
10.59_399.27	399.274344	10.5914167	pos
10.59_436.28	436.281202	10.5914167	pos
10.61_229.91	229.915718	10.6077	neg
10.61_312.22	312.229389	10.6097333	pos
10.61_336.31	336.310301	10.6097333	pos
10.61_434.26	434.267378	10.6077	neg
10.63_100.51	100.510266	10.6275	pos
10.63_105.11	105.110574	10.6275	pos

10.63_1081.(	1081.63958	10.6275	pos
10.63_1126.(	1171.63777	10.6260167	neg
10.63_275.63	275.632494	10.6275	pos
10.63_295.14	295.148126	10.6275	pos
10.63_295.64	295.648077	10.6275	pos
10.63_296.64	296.647521	10.6275	pos
10.63_306.65	306.653	10.6275	pos
10.63_316.65	316.659102	10.6275	pos
10.63_317.65	317.659443	10.6275	pos
10.63_478.32	478.328718	10.6275	pos
10.63_480.30	480.309387	10.6260167	neg
10.63_495.33	496.339165	10.6275	pos
10.63_499.34	499.347674	10.6275	pos
10.63_523.29	523.298939	10.6275	pos
10.63_523.80	523.800631	10.6275	pos
10.63_530.30	530.301571	10.6260167	neg
10.63_543.33	543.339046	10.6260167	neg
10.63_575.27	576.279124	10.6275	pos
10.63_592.26	592.264117	10.6275	pos
10.63_595.25	618.245997	10.6275	pos
10.63_598.28	598.289316	10.6260167	neg
10.63_603.29	603.294766	10.6275	pos
10.63_612.26	612.260737	10.6260167	neg
10.63_616.45	655.417263	10.6275	pos
10.63_621.93	621.938068	10.6275	pos
10.63_625.30	625.308105	10.6260167	neg
10.63_627.45	650.442584	10.6275	pos
10.63_627.95	650.944952	10.6275	pos
10.63_637.93	637.936679	10.6275	pos
10.63_640.25	640.25492	10.6260167	neg
10.63_649.93	649.937748	10.6275	pos
10.63_654.29	654.29536	10.6275	pos
10.63_654.91	654.914955	10.6275	pos

10.63_661.43	661.435916	10.6275	pos
10.63_663.95	663.951921	10.6275	pos
10.63_664.45	664.455152	10.6275	pos
10.63_666.27	666.277075	10.6260167	neg
10.63_676.30	676.305398	10.6260167	neg
10.63_734.26	734.264131	10.6260167	neg
10.63_744.29	744.293258	10.6260167	neg
10.63_752.17	752.17794	10.6260167	neg
10.63_761.28	761.283003	10.6260167	neg
10.63_765.98	765.986591	10.6275	pos
10.63_766.48	766.488776	10.6275	pos
10.63_799.97	799.979718	10.6275	pos
10.63_800.48	800.482014	10.6275	pos
10.63_812.28	812.280702	10.6260167	neg
10.63_816.22	816.223376	10.6260167	neg
10.63_880.26	880.267845	10.6260167	neg
10.63_948.25	948.255626	10.6260167	neg
10.63_990.66	991.670631	10.6275	pos
10.64_498.93	498.930069	10.6444833	neg
10.64_714.50	714.508148	10.6444833	neg
10.64_973.62	973.62651	10.6444833	neg
10.65_184.07	184.07303	10.6454167	pos
10.65_341.31	341.315749	10.6454167	pos
10.65_374.17	374.177039	10.6454167	pos
10.65_518.82	518.822818	10.6454167	pos
10.65_643.90	661.937568	10.6454167	pos
10.65_649.43	649.435787	10.6454167	pos
10.65_675.95	675.952686	10.6454167	pos
10.65_909.10	909.100687	10.6454167	pos
10.65_974.63	975.639121	10.6454167	pos
10.66_278.22	279.231372	10.6629	pos
10.66_297.23	297.234292	10.66295	neg
10.66_327.33	327.336536	10.6629	pos

10.66_339.2	339.253759	10.66295	neg
10.66_339.2	339.288732	10.6629	pos
10.66_363.2	363.21517	10.66295	neg
10.66_380.2	380.205356	10.66295	neg
10.66_394.1	394.188221	10.6629	pos
10.66_395.1	395.152853	10.66295	neg
10.66_431.2	431.202295	10.66295	neg
10.66_435.1	435.145262	10.66295	neg
10.66_567.1	567.177536	10.66295	neg
10.66_568.2	568.264177	10.6629	pos
10.66_744.5	744.554103	10.66295	neg
10.68_307.2	307.227722	10.6808167	neg
10.68_359.3	342.299766	10.6803667	pos
10.70_266.2	266.211052	10.6974667	pos
10.70_275.1	275.168612	10.6988167	neg
10.70_356.2	356.279196	10.6974667	pos
10.70_375.2	375.215315	10.6988167	neg
10.70_407.2	407.248872	10.6974667	pos
10.70_407.2	408.29236	10.6974667	pos
10.70_452.3	452.372941	10.6974667	pos
10.70_505.2	505.275955	10.6974667	pos
10.70_569.3	569.354319	10.6988167	neg
10.70_612.3	612.324285	10.6974667	pos
10.70_688.2	688.280046	10.6988167	neg
10.72_132.5	132.53115	10.71525	pos
10.72_270.2	271.226337	10.71525	pos
10.72_289.6	289.640614	10.71525	pos
10.72_290.1	290.142544	10.71525	pos
10.72_325.2	325.229013	10.7169	neg
10.72_420.7	420.760743	10.71525	pos
10.72_472.7	472.74743	10.71525	pos
10.72_481.3	481.353373	10.7169	neg
10.72_525.3	525.363058	10.71525	pos

10.72_563.3	586.360819	10.71525	pos
10.72_583.8	583.882436	10.71525	pos
10.72_636.3	636.33979	10.7169	neg
10.72_702.3	702.32135	10.7169	neg
10.72_770.3	770.308505	10.7169	neg
10.73_163.1	163.111558	10.7334833	pos
10.73_257.2	257.212114	10.73455	neg
10.73_284.1	284.147259	10.7334833	pos
10.73_335.1	335.197891	10.7334833	pos
10.73_418.7	418.747458	10.7334833	pos
10.73_539.8	539.856787	10.7334833	pos
10.73_540.3	540.358847	10.7334833	pos
10.73_541.3	564.347274	10.7334833	pos
10.73_541.8	564.84953	10.7334833	pos
10.73_561.8	561.869786	10.7334833	pos
10.73_562.3	562.371059	10.7334833	pos
10.73_779.5	778.560227	10.73455	neg
10.73_807.5	807.592839	10.73455	neg
10.73_875.5	875.555291	10.7334833	pos
10.73_904.5	904.58868	10.7334833	pos
10.75_197.1	197.190781	10.7528167	neg
10.75_293.1	293.126618	10.7516833	pos
10.75_377.1	377.161656	10.7516833	pos
10.75_377.1	377.186206	10.7528167	neg
10.75_393.2	393.260128	10.7516833	pos
10.75_393.7	393.739924	10.7516833	pos
10.75_453.3	453.322113	10.7528167	neg
10.75_468.3	468.308915	10.7528167	neg
10.75_497.8	520.823063	10.7516833	pos
10.75_506.8	506.85277	10.7516833	pos
10.75_509.3	509.33037	10.7516833	pos
10.75_515.3	515.366255	10.7516833	pos
10.75_517.8	517.843813	10.7516833	pos

10.75_519.34	542.334949	10.7516833	pos
10.75_519.84	542.836104	10.7516833	pos
10.75_528.86	528.865883	10.7516833	pos
10.75_574.28	574.287405	10.7516833	pos
10.77_116.92	116.921778	10.77115	neg
10.77_281.21	281.21194	10.77115	neg
10.77_292.62	292.624808	10.7697	pos
10.77_300.28	300.28928	10.7697	pos
10.77_344.25	344.257846	10.7697	pos
10.77_453.30	476.294909	10.7697	pos
10.77_453.80	476.796727	10.7697	pos
10.77_475.31	498.30811	10.7697	pos
10.77_475.82	498.809759	10.7697	pos
10.77_484.83	484.83959	10.7697	pos
10.77_487.31	487.317112	10.7697	pos
10.77_495.83	495.830526	10.7697	pos
10.77_497.02	497.023164	10.77115	neg
10.77_529.31	530.323753	10.7697	pos
10.77_624.19	624.197286	10.7697	pos
10.77_664.28	664.284459	10.77115	neg
10.77_732.27	732.272107	10.77115	neg
10.77_750.24	750.243209	10.77115	neg
10.77_824.53	824.537537	10.7697	pos
10.77_833.61	833.610222	10.77115	neg
10.79_267.61	267.617197	10.7875833	pos
10.79_289.13	289.13088	10.7875833	pos
10.79_338.28	339.288915	10.7875833	pos
10.79_344.23	327.231405	10.7875833	pos
10.79_369.23	369.239663	10.7875833	pos
10.79_431.29	449.326774	10.7875833	pos
10.79_431.79	454.783678	10.7875833	pos
10.79_440.81	440.813263	10.7875833	pos
10.79_441.31	441.315805	10.7875833	pos

10.79_443.29	443.290852	10.7875833	pos
10.79_451.80	451.804095	10.7875833	pos
10.79_452.30	452.30586	10.7875833	pos
10.79_462.29	462.296956	10.7875833	pos
10.79_462.82	462.826341	10.7875833	pos
10.79_463.34	463.340978	10.7875833	pos
10.79_465.30	465.304312	10.7875833	pos
10.79_465.81	465.819419	10.7875833	pos
10.79_473.81	473.817319	10.7875833	pos
10.79_474.31	474.319277	10.7875833	pos
10.79_480.29	480.299091	10.7894833	neg
10.79_486.29	486.298819	10.7894833	neg
10.79_487.83	487.832729	10.7875833	pos
10.79_524.27	524.272414	10.7875833	pos
10.79_563.27	563.271221	10.7894833	neg
10.79_592.25	592.259886	10.7875833	pos
10.79_618.21	618.21149	10.7894833	neg
10.79_682.25	682.255774	10.7894833	neg
10.79_686.19	686.198971	10.7894833	neg
10.79_818.23	818.231216	10.7894833	neg
10.79_862.58	885.57126	10.7875833	pos
10.79_915.58	915.584238	10.7894833	neg
10.79_958.60	959.608748	10.7875833	pos
10.81_160.98	160.983447	10.80565	pos
10.81_249.18	249.184536	10.80565	pos
10.81_266.12	266.127433	10.80565	pos
10.81_267.12	267.12519	10.80565	pos
10.81_267.62	267.626582	10.80565	pos
10.81_287.63	287.638146	10.80565	pos
10.81_368.35	368.351755	10.80565	pos
10.81_387.76	410.75752	10.80565	pos
10.81_394.32	394.328803	10.80565	pos
10.81_409.77	432.763984	10.80565	pos

10.81_418.8[illegible]	418.800117	10.80565	pos
10.81_421.2[illegible]	421.277398	10.80565	pos
10.81_429.7[illegible]	429.791167	10.80565	pos
10.81_436.2[illegible]	436.283086	10.8069333	neg
10.81_443.8[illegible]	443.806259	10.80565	pos
10.81_447.2[illegible]	430.292801	10.80565	pos
10.81_504.2[illegible]	504.27076	10.8069333	neg
10.81_521.2[illegible]	521.260593	10.8069333	neg
10.81_534.2[illegible]	534.214234	10.8069333	neg
10.81_572.2[illegible]	572.258207	10.8069333	neg
10.81_644.1[illegible]	644.188448	10.8069333	neg
10.81_657.2[illegible]	657.236318	10.8069333	neg
10.81_708.2[illegible]	708.23348	10.8069333	neg
10.81_712.1[illegible]	712.175557	10.8069333	neg
10.81_776.2[illegible]	776.220531	10.8069333	neg
10.81_865.5[illegible]	865.527818	10.80565	pos
10.81_874.5[illegible]	875.587216	10.80565	pos
10.81_916.5[illegible]	917.597545	10.80565	pos
10.82_365.2[illegible]	388.242701	10.82305	pos
10.82_365.7[illegible]	388.744487	10.82305	pos
10.82_377.2[illegible]	377.251867	10.82305	pos
10.82_385.7[illegible]	385.76476	10.82305	pos
10.82_386.2[illegible]	386.266297	10.82305	pos
10.82_387.2[illegible]	410.255576	10.82305	pos
10.82_399.2[illegible]	399.264424	10.82305	pos
10.82_407.7[illegible]	407.777926	10.82305	pos
10.82_438.2[illegible]	438.272854	10.82305	pos
10.82_507.2[illegible]	507.285879	10.82305	pos
10.82_550.2[illegible]	550.248852	10.82305	pos
10.82_571.3[illegible]	572.370606	10.82305	pos
10.82_582.1[illegible]	582.186258	10.82305	pos
10.82_730.5[illegible]	748.535439	10.82305	pos
10.82_958.6[illegible]	959.644775	10.82305	pos

10.83_499.02	499.020838	10.82535	neg
10.83_957.63	957.631503	10.82535	neg
10.84_1087.(	1087.69564	10.8434333	neg
10.84_193.08	193.086631	10.8434333	neg
10.84_277.21	277.213798	10.8409	pos
10.84_317.2(	317.208246	10.8409	pos
10.84_340.28	340.284153	10.8409	pos
10.84_558.31	558.314678	10.8434333	neg
10.84_624.3(	624.304637	10.8434333	neg
10.84_638.27	638.276656	10.8434333	neg
10.84_651.32	651.32381	10.8434333	neg
10.84_666.52	684.561104	10.8409	pos
10.84_686.48	709.46998	10.8409	pos
10.84_878.59	878.590942	10.8434333	neg
10.86_1042.(	1043.70121	10.8590333	pos
10.86_113.1(	113.107472	10.8590333	pos
10.86_288.64	288.640108	10.8590333	pos
10.86_291.25	291.252541	10.8590333	pos
10.86_525.3(	525.363111	10.8590333	pos
10.86_598.42	621.417469	10.8590333	pos
10.86_612.32	612.323973	10.8590333	pos
10.86_642.45	665.443662	10.8590333	pos
10.86_651.94	634.946147	10.8590333	pos
10.86_661.2(	644.262629	10.8590333	pos
10.86_663.45	663.450499	10.8590333	pos
10.86_674.44	674.443171	10.8590333	pos
10.86_688.51	688.519179	10.8590333	pos
10.86_702.32	702.321181	10.8611	neg
10.86_706.2(	706.264033	10.8611	neg
10.86_770.3(	770.308435	10.8611	neg
10.86_774.25	774.251401	10.8611	neg
10.86_838.29	838.295992	10.8611	neg
10.88_225.0(	225.061277	10.8794667	neg

10.88_260.2(	260.200375	10.8770833	pos
10.88_359.8(	359.86272	10.8794667	neg
10.88_510.3	533.365404	10.8770833	pos
10.88_514.3(	514.306795	10.8794667	neg
10.88_554.4(	577.391529	10.8770833	pos
10.88_600.4(	600.467027	10.8770833	pos
10.88_660.3	660.310711	10.8794667	neg
10.88_664.2!	664.253402	10.8794667	neg
10.88_728.2!	728.298419	10.8794667	neg
10.90_200.0	200.047032	10.8950333	pos
10.90_209.0	209.012792	10.8950333	pos
10.90_288.6!	288.650095	10.8950333	pos
10.90_334.2	357.260651	10.8950333	pos
10.90_378.2!	401.28687	10.8950333	pos
10.90_380.3	380.336867	10.8950333	pos
10.90_387.2	387.215177	10.8978167	neg
10.90_403.2!	403.292928	10.8950333	pos
10.90_404.2(	404.205171	10.8978167	neg
10.90_422.3	445.312907	10.8950333	pos
10.90_424.3(	424.362814	10.8950333	pos
10.90_466.3!	489.33928	10.8950333	pos
10.90_468.3	468.389062	10.8950333	pos
10.90_512.4	512.415285	10.8950333	pos
10.90_556.4	556.441293	10.8950333	pos
10.90_594.3	594.3394	10.8978167	neg
10.90_642.4	642.445432	10.8950333	pos
10.90_662.3	662.326133	10.8978167	neg
10.91_1100.	1100.783	10.9125667	pos
10.91_1101.	1124.78276	10.9125667	pos
10.91_177.0!	177.054401	10.9125667	pos
10.91_289.1!	289.155959	10.9125667	pos
10.91_364.2	364.28397	10.9125667	pos
10.91_377.1(	377.161577	10.9125667	pos

10.91_481.3!	482.360329	10.9125667	pos
10.91_534.4	534.472499	10.9125667	pos
10.92_297.2	297.243227	10.9157	neg
10.93_241.7	241.704965	10.9300167	pos
10.93_243.1	243.135204	10.9300167	pos
10.93_349.2	350.268519	10.9300167	pos
10.93_433.2	433.265092	10.9300167	pos
10.93_546.8	546.835041	10.9332333	neg
10.93_550.3	550.376872	10.9300167	pos
10.93_593.3	593.365303	10.9332333	neg
10.93_618.3	618.368739	10.9332333	neg
10.93_796.5	796.524086	10.9300167	pos
10.93_904.5	904.587672	10.9300167	pos
10.95_158.9	158.940781	10.9517167	neg
10.95_229.9	229.915685	10.9517167	neg
10.95_247.8	247.884352	10.9478667	pos
10.95_363.2	363.250097	10.9478667	pos
10.95_413.2	413.230781	10.9517167	neg
10.95_472.3	472.364119	10.9517167	neg
10.97_241.2	241.203255	10.9659	pos
10.97_286.2	286.23726	10.9659	pos
10.97_466.2	466.293619	10.9697333	neg
10.97_525.2	525.205581	10.9697333	neg
10.97_559.5	559.516862	10.9659	pos
10.98_181.0	181.014274	10.9842333	pos
10.98_456.3	479.297294	10.9842333	pos
10.98_595.2	595.284478	10.9842333	pos
11.00_280.1	280.132979	11.0025333	pos
11.00_281.1	281.133251	11.0025333	pos
11.00_318.2	318.239888	11.0025333	pos
11.00_489.2	489.281831	11.0025333	pos
11.00_529.3	529.30934	11.0025333	pos
11.01_235.1	235.170116	11.0055	neg

11.01_359.14	359.145037	11.0055	neg
11.01_504.30	504.309621	11.0055	neg
11.01_520.25	520.256692	11.0055	neg
11.01_530.28	530.286325	11.0055	neg
11.01_562.22	562.224144	11.0055	neg
11.01_666.26	666.261434	11.0055	neg
11.01_670.20	670.204514	11.0055	neg
11.02_225.06	225.061351	11.0234	neg
11.02_259.61	259.619633	11.0205667	pos
11.02_260.12	260.121294	11.0205667	pos
11.02_444.30	444.307978	11.0205667	pos
11.02_463.30	464.313134	11.0205667	pos
11.02_598.27	598.275078	11.0234	neg
11.02_602.21	602.216849	11.0234	neg
11.02_656.92	656.929491	11.0205667	pos
11.02_659.40	659.407371	11.0205667	pos
11.02_734.24	734.248777	11.0234	neg
11.02_820.52	820.523395	11.0205667	pos
11.02_824.53	824.537881	11.0205667	pos
11.04_319.00	319.002014	11.04175	neg
11.04_634.91	634.91662	11.0380333	pos
11.04_635.41	635.418136	11.0380333	pos
11.04_637.89	637.896115	11.0380333	pos
11.04_790.56	790.567138	11.0380333	pos
11.06_165.99	165.990763	11.0558	pos
11.06_351.00	351.008304	11.0597833	neg
11.06_544.26	544.265719	11.0597833	neg
11.06_592.39	615.381618	11.0558	pos
11.06_612.90	612.903587	11.0558	pos
11.06_613.40	613.405272	11.0558	pos
11.06_615.88	615.883006	11.0558	pos
11.06_622.33	622.332711	11.0597833	neg
11.06_626.27	626.276741	11.0597833	neg

11.06_690.32	690.320782	11.0597833	neg
11.07_111.52	111.522045	11.0736	pos
11.07_131.53	131.533463	11.0736	pos
11.07_139.98	139.987754	11.0736	pos
11.07_160.98	160.983525	11.0736	pos
11.07_171.95	171.95938	11.0736	pos
11.07_186.98	186.980805	11.0736	pos
11.07_198.01	198.017106	11.0736	pos
11.07_207.97	226.01196	11.0736	pos
11.07_228.00	228.007348	11.0736	pos
11.07_256.91	256.915589	11.0736	pos
11.07_258.91	258.911084	11.0736	pos
11.07_281.17	281.172028	11.0736	pos
11.07_285.24	285.242033	11.0736	pos
11.07_301.64	301.648201	11.0736	pos
11.07_303.15	303.153514	11.0736	pos
11.07_509.34	510.355151	11.0736	pos
11.07_570.37	593.367984	11.0736	pos
11.07_570.88	593.869809	11.0736	pos
11.07_582.37	582.377426	11.0736	pos
11.07_590.89	590.890708	11.0736	pos
11.07_645.30	645.299995	11.0736	pos
11.08_457.21	457.218806	11.07825	neg
11.08_461.16	461.161219	11.07825	neg
11.08_614.82	614.822385	11.07825	neg
11.08_664.29	664.292754	11.07825	neg
11.08_728.33	728.337242	11.07825	neg
11.09_506.36	506.360357	11.0915333	pos
11.09_548.86	571.856951	11.0915333	pos
11.09_557.88	557.886747	11.0915333	pos
11.09_560.36	560.364736	11.0915333	pos
11.09_566.40	566.400106	11.0915333	pos
11.09_568.87	568.877531	11.0915333	pos

11.09_569.37	569.379389	11.0915333	pos
11.09_616.35	616.359111	11.0915333	pos
11.11_155.14	155.142864	11.1098667	pos
11.11_162.93	162.939334	11.1143	neg
11.11_280.26	280.263027	11.1098667	pos
11.11_280.96	280.962882	11.1143	neg
11.11_337.88	337.880944	11.1143	neg
11.11_343.33	343.331382	11.1098667	pos
11.11_526.85	549.844151	11.1098667	pos
11.11_529.34	529.345557	11.1098667	pos
11.11_535.87	535.873818	11.1098667	pos
11.11_538.35	538.351064	11.1098667	pos
11.11_546.86	546.864608	11.1098667	pos
11.11_775.59	775.596497	11.1143	neg
11.11_785.64	785.649356	11.1098667	pos
11.13_282.15	282.15253	11.12815	pos
11.13_504.34	527.329227	11.12815	pos
11.13_504.84	527.83092	11.12815	pos
11.13_513.86	513.86055	11.12815	pos
11.13_516.33	516.338603	11.12815	pos
11.13_524.85	524.85157	11.12815	pos
11.13_525.35	525.353416	11.12815	pos
11.13_538.86	538.867812	11.12815	pos
11.13_542.33	542.338134	11.1325833	neg
11.13_644.37	644.375822	11.12815	pos
11.13_688.34	688.341972	11.1325833	neg
11.15_118.04	118.040676	11.1509667	neg
11.15_143.95	143.958874	11.1450667	pos
11.15_171.95	171.959357	11.1450667	pos
11.15_184.07	184.073123	11.1450667	pos
11.15_223.98	223.988087	11.1450667	pos
11.15_226.01	226.011962	11.1450667	pos
11.15_281.65	281.650956	11.1450667	pos

11.15_299.0!	299.095332	11.1509667	neg
11.15_339.0(	339.007968	11.1509667	neg
11.15_482.8:	505.817945	11.1450667	pos
11.15_491.8	491.84761	11.1450667	pos
11.15_492.3(	492.309583	11.1509667	neg
11.15_502.8:	502.838432	11.1450667	pos
11.15_507.3(	508.37588	11.1450667	pos
11.15_514.3	514.37678	11.1450667	pos
11.15_516.8!	516.854487	11.1450667	pos
11.15_900.5(	900.565975	11.1450667	pos
11.16_360.2	383.20348	11.16325	pos
11.16_406.2	406.279477	11.16325	pos
11.16_460.3	483.303119	11.16325	pos
11.16_460.8	483.804604	11.16325	pos
11.16_469.8:	469.834302	11.16325	pos
11.16_480.8:	480.825245	11.16325	pos
11.16_494.8	494.840774	11.16325	pos
11.17_142.9:	142.924747	11.1685	neg
11.17_182.9:	182.921064	11.1685	neg
11.17_203.9	203.94568	11.1685	neg
11.17_204.9	204.94204	11.1685	neg
11.17_225.0(	225.061329	11.1685	neg
11.17_284.1	284.147736	11.1685	neg
11.17_319.0(	319.00197	11.1685	neg
11.17_403.1:	403.132384	11.1685	neg
11.17_415.2:	415.231411	11.1685	neg
11.17_461.3:	461.327271	11.1685	neg
11.18_388.1(	411.093242	11.1809167	pos
11.18_416.1	434.206919	11.1809167	pos
11.18_438.3(	461.289731	11.1809167	pos
11.18_438.8(	461.791415	11.1809167	pos
11.18_447.8:	447.821082	11.1809167	pos
11.18_450.2!	450.298813	11.1809167	pos

11.18_458.8	458.812113	11.1809167	pos
11.18_459.3	459.313969	11.1809167	pos
11.18_470.3	470.34963	11.1809167	pos
11.18_472.8	472.827497	11.1809167	pos
11.18_876.6	899.589769	11.1809167	pos
11.19_388.1	433.10245	11.1870167	neg
11.20_208.0	208.069064	11.198	pos
11.20_208.5	208.570745	11.198	pos
11.20_416.2	439.276667	11.198	pos
11.20_416.7	439.778372	11.198	pos
11.20_425.8	425.808058	11.198	pos
11.20_426.3	426.309807	11.198	pos
11.20_436.7	436.798996	11.198	pos
11.20_437.3	437.300695	11.198	pos
11.20_448.3	448.336841	11.198	pos
11.20_450.8	450.81423	11.198	pos
11.20_502.2	502.292219	11.198	pos
11.21_226.9	226.994501	11.20545	neg
11.21_342.2	342.264649	11.20545	neg
11.21_427.8	427.849895	11.20545	neg
11.22_343.8	342.872423	11.2230667	neg
11.22_366.2	366.299847	11.2158833	pos
11.22_394.7	417.765189	11.2158833	pos
11.22_403.7	403.794896	11.2158833	pos
11.22_406.7	406.774091	11.2158833	pos
11.22_414.7	414.786099	11.2158833	pos
11.22_415.2	415.287728	11.2158833	pos
11.22_428.8	428.800881	11.2158833	pos
11.22_498.9	498.900969	11.2158833	pos
11.22_811.5	811.538348	11.2158833	pos
11.23_145.1	145.122197	11.2342333	pos
11.23_297.2	297.241969	11.2342333	pos
11.23_341.3	341.304339	11.2342333	pos

11.23_360.2[illegible]	378.321179	11.2342333	pos
11.23_362.2[illegible]	380.327076	11.2342333	pos
11.23_372.2[illegible]	395.250349	11.2342333	pos
11.23_372.7[illegible]	395.752184	11.2342333	pos
11.23_384.2[illegible]	384.259458	11.2342333	pos
11.23_384.7[illegible]	384.761124	11.2342333	pos
11.23_392.7[illegible]	392.772707	11.2342333	pos
11.23_408.3[illegible]	408.358308	11.2342333	pos
11.23_480.3[illegible]	480.308128	11.2342333	pos
11.23_489.3[illegible]	490.328801	11.2342333	pos
11.23_744.5[illegible]	767.512924	11.2342333	pos
11.23_785.6[illegible]	785.650193	11.2342333	pos
11.24_301.0[illegible]	301.011383	11.2413667	neg
11.24_488.3[illegible]	488.31445	11.2413667	neg
11.25_184.0[illegible]	184.073096	11.25255	pos
11.25_289.2[illegible]	289.237003	11.25255	pos
11.25_316.2[illegible]	316.230299	11.25255	pos
11.25_373.7[illegible]	373.739114	11.25255	pos
11.25_388.8[illegible]	388.850798	11.25255	pos
11.25_536.3[illegible]	536.334547	11.25255	pos
11.25_700.5[illegible]	718.534428	11.25255	pos
11.26_337.8[illegible]	337.88098	11.25965	neg
11.26_359.8[illegible]	359.862833	11.25965	neg
11.26_489.3[illegible]	489.358083	11.25965	neg
11.26_495.8[illegible]	495.837852	11.25965	neg
11.26_573.2[illegible]	573.299838	11.25965	neg
11.27_200.0[illegible]	200.047071	11.2702	pos
11.27_271.2[illegible]	271.26282	11.2702	pos
11.27_656.4[illegible]	679.459505	11.2702	pos
11.28_176.8[illegible]	176.883895	11.2776833	neg
11.28_252.9[illegible]	252.903195	11.2776833	neg
11.28_275.8[illegible]	320.890573	11.2776833	neg
11.29_207.0[illegible]	207.017325	11.2883167	pos

11.29_256.9	256.915591	11.2883167	pos
11.29_360.2	360.256146	11.2883167	pos
11.29_436.3	436.341605	11.2883167	pos
11.29_614.3	614.366073	11.2883167	pos
11.30_317.1	317.136746	11.2956167	neg
11.31_160.9	160.983516	11.3062	pos
11.31_172.9	172.956258	11.3062	pos
11.31_258.9	258.911138	11.3062	pos
11.31_544.3	544.338125	11.3062	pos
11.31_568.4	591.407247	11.3062	pos
11.31_612.4	635.433295	11.3062	pos
11.31_614.4	614.482718	11.3062	pos
11.31_872.5	872.535543	11.3062	pos
11.32_478.3	478.328747	11.3239833	pos
11.32_482.8	482.812299	11.3239833	pos
11.32_524.3	547.381193	11.3239833	pos
11.32_570.4	570.45675	11.3239833	pos
11.33_410.8	410.860007	11.3319667	neg
11.34_440.2	463.276831	11.3422833	pos
11.34_460.7	460.798939	11.3422833	pos
11.34_463.7	463.778459	11.3422833	pos
11.34_480.3	503.354909	11.3422833	pos
11.34_526.4	526.430789	11.3422833	pos
11.35_252.1	252.150906	11.3501	neg
11.35_570.3	570.367903	11.3501	neg
11.35_636.3	636.349413	11.3501	neg
11.35_640.2	640.292219	11.3501	neg
11.36_358.2	358.294518	11.36075	pos
11.36_436.3	459.328673	11.36075	pos
11.36_438.7	438.785933	11.36075	pos
11.36_441.7	441.765273	11.36075	pos
11.36_502.2	502.292242	11.36075	pos
11.38_341.2	341.265893	11.3787167	pos

11.38_367.24	367.245022	11.3787167	pos
11.38_392.31	415.302435	11.3787167	pos
11.38_417.30	417.308451	11.3787167	pos
11.38_685.95	685.950471	11.3787167	pos
11.38_688.42	688.428739	11.3787167	pos
11.38_792.52	815.511578	11.3787167	pos
11.38_859.53	859.533775	11.3787167	pos
11.39_366.27	347.258903	11.3861833	neg
11.39_415.24	415.246513	11.3861833	neg
11.40_288.63	288.639866	11.3962667	pos
11.40_295.16	295.164167	11.3962667	pos
11.40_353.26	353.266395	11.3962667	pos
11.40_385.16	385.16085	11.40315	neg
11.40_579.26	579.269338	11.3962667	pos
11.40_628.38	628.380968	11.3962667	pos
11.40_643.42	666.415589	11.3962667	pos
11.40_662.26	662.261752	11.3962667	pos
11.40_663.93	663.93753	11.3962667	pos
11.40_666.91	666.91734	11.3962667	pos
11.41_256.91	256.915574	11.4141	pos
11.41_309.65	309.651314	11.4141	pos
11.41_621.41	644.402696	11.4141	pos
11.41_621.91	644.904096	11.4141	pos
11.41_641.92	641.924155	11.4141	pos
11.41_642.42	642.426513	11.4141	pos
11.41_784.65	784.650008	11.4141	pos
11.42_416.28	416.280627	11.42155	neg
11.42_483.31	483.317887	11.42155	neg
11.42_538.26	538.268135	11.42155	neg
11.42_565.28	565.287055	11.42155	neg
11.42_578.24	578.240346	11.42155	neg
11.42_580.23	580.234625	11.42155	neg
11.42_606.25	606.255829	11.42155	neg

11.42_620.2:	620.227136	11.42155	neg
11.42_684.2	684.271719	11.42155	neg
11.42_688.2:	688.214547	11.42155	neg
11.42_752.2!	752.259233	11.42155	neg
11.42_756.2(	756.202306	11.42155	neg
11.42_820.2	820.246485	11.42155	neg
11.43_1184.8	1184.88918	11.4320667	pos
11.43_289.1:	289.138085	11.4320667	pos
11.43_289.6:	289.63978	11.4320667	pos
11.43_310.6!	310.651545	11.4320667	pos
11.43_348.2	371.276045	11.4320667	pos
11.43_350.2!	350.297499	11.4320667	pos
11.43_373.2!	373.292731	11.4320667	pos
11.43_394.3!	394.35228	11.4320667	pos
11.43_481.3:	482.323635	11.4320667	pos
11.43_485.3:	485.332082	11.4320667	pos
11.43_509.2	509.284125	11.4320667	pos
11.43_509.7	509.784879	11.4320667	pos
11.43_594.2	594.275017	11.4320667	pos
11.43_599.3!	622.388778	11.4320667	pos
11.43_599.9(	622.890477	11.4320667	pos
11.43_603.2:	626.21249	11.4320667	pos
11.43_619.9:	619.910777	11.4320667	pos
11.43_620.4:	620.412657	11.4320667	pos
11.43_666.2(	666.204626	11.4320667	pos
11.43_704.4	722.504602	11.4320667	pos
11.43_962.6:	963.63933	11.4320667	pos
11.44_701.2(	701.261591	11.4395167	neg
11.44_888.2:	888.233602	11.4395167	neg
11.45_577.8	600.877519	11.4499667	pos
11.45_589.3	589.38537	11.4499667	pos
11.45_597.8!	597.898249	11.4499667	pos
11.45_598.4(	598.400107	11.4499667	pos

11.45_660.4	683.433123	11.4499667	pos
11.46_214.8	214.859156	11.4580167	neg
11.46_254.8	254.851648	11.4580167	neg
11.46_284.8	284.840968	11.4580167	neg
11.47_141.1	141.127223	11.4676667	pos
11.47_555.8	578.864953	11.4676667	pos
11.47_577.3	600.376224	11.4676667	pos
11.47_662.4	662.482576	11.4676667	pos
11.47_913.6	913.605804	11.4676667	pos
11.48_332.8	332.84402	11.4761	neg
11.48_427.8	427.849899	11.4761	neg
11.48_794.5	794.54557	11.4761	neg
11.49_299.2	299.257607	11.4858667	pos
11.49_350.3	350.325995	11.4858667	pos
11.49_368.2	368.280271	11.494	neg
11.49_385.2	385.2708	11.494	neg
11.49_392.2	392.280511	11.494	neg
11.49_409.2	409.270533	11.494	neg
11.49_414.8	414.802929	11.494	neg
11.49_555.3	578.363342	11.4858667	pos
11.49_564.8	564.894853	11.4858667	pos
11.49_567.3	567.372433	11.4858667	pos
11.49_575.8	575.885361	11.4858667	pos
11.49_576.3	576.387479	11.4858667	pos
11.50_137.0	137.096009	11.5038	pos
11.50_528.3	551.354928	11.5038	pos
11.50_533.3	556.350162	11.5038	pos
11.50_533.8	556.851543	11.5038	pos
11.50_542.8	542.881647	11.5038	pos
11.50_553.8	553.872207	11.5038	pos
11.50_554.3	554.374414	11.5038	pos
11.50_618.4	618.456297	11.5038	pos
11.51_162.9	162.939315	11.5124	neg

11.52_173.0	173.078008	11.5217833	pos
11.52_367.2	367.244903	11.5217833	pos
11.52_511.3	534.33698	11.5217833	pos
11.52_511.8	534.839013	11.5217833	pos
11.52_520.8	520.868572	11.5217833	pos
11.52_531.8	531.859532	11.5217833	pos
11.52_532.3	532.360931	11.5217833	pos
11.52_543.3	543.397323	11.5217833	pos
11.52_545.8	545.875786	11.5217833	pos
11.54_184.0	184.073155	11.5398167	pos
11.54_398.3	398.325968	11.5398167	pos
11.54_489.3	512.324341	11.5398167	pos
11.54_489.8	512.825583	11.5398167	pos
11.54_498.8	498.855034	11.5398167	pos
11.54_509.8	509.846453	11.5398167	pos
11.54_510.3	510.348053	11.5398167	pos
11.54_521.3	521.384174	11.5398167	pos
11.54_523.8	523.862032	11.5398167	pos
11.54_530.4	530.403984	11.5398167	pos
11.55_280.9	280.96286	11.5461833	neg
11.56_1091.	1091.72555	11.5642333	neg
11.56_1159.	1159.71336	11.5642333	neg
11.56_200.0	200.047048	11.5576	pos
11.56_310.1	310.161511	11.5576	pos
11.56_310.6	310.663137	11.5576	pos
11.56_320.6	320.668867	11.5576	pos
11.56_331.6	331.675042	11.5576	pos
11.56_467.3	490.310633	11.5576	pos
11.56_508.3	508.338511	11.5642333	neg
11.56_527.3	527.379078	11.5576	pos
11.56_546.8	546.853842	11.5576	pos
11.56_552.8	552.832603	11.5576	pos
11.56_558.3	558.332999	11.5642333	neg

11.56_614.33	614.339712	11.5576	pos
11.56_626.32	626.320395	11.5642333	neg
11.56_651.95	651.953514	11.5576	pos
11.56_663.28	646.277557	11.5576	pos
11.56_663.95	663.952628	11.5576	pos
11.56_664.45	664.45826	11.5576	pos
11.56_664.96	664.960519	11.5576	pos
11.56_668.28	668.287524	11.5642333	neg
11.56_677.96	677.968561	11.5576	pos
11.56_688.45	688.457532	11.5576	pos
11.56_689.96	689.967793	11.5576	pos
11.56_694.30	694.308595	11.5642333	neg
11.56_704.33	704.336827	11.5642333	neg
11.56_708.27	708.279859	11.5642333	neg
11.56_721.32	721.32731	11.5642333	neg
11.56_772.32	772.324112	11.5642333	neg
11.56_776.26	776.267038	11.5642333	neg
11.56_840.31	840.311667	11.5642333	neg
11.56_844.25	844.254883	11.5642333	neg
11.56_908.29	908.298968	11.5642333	neg
11.56_934.64	952.676375	11.5576	pos
11.58_105.11	105.110584	11.5754333	pos
11.58_214.85	214.859073	11.5827667	neg
11.58_229.91	229.915695	11.5827667	neg
11.58_467.82	490.81256	11.5754333	pos
11.58_476.84	476.842451	11.5754333	pos
11.58_487.83	487.833186	11.5754333	pos
11.58_499.37	499.37086	11.5754333	pos
11.58_501.84	501.848814	11.5754333	pos
11.58_631.33	631.330299	11.5754333	pos
11.58_675.45	675.451468	11.5754333	pos
11.58_675.95	675.952658	11.5754333	pos
11.58_689.46	689.466485	11.5754333	pos

11.59_445.3(	468.297695	11.5931667	pos
11.59_445.8	468.799461	11.5931667	pos
11.59_454.8	454.828789	11.5931667	pos
11.59_455.3	455.331423	11.5931667	pos
11.59_457.8(	457.808758	11.5931667	pos
11.59_465.8	465.820112	11.5931667	pos
11.59_476.2	476.284639	11.5931667	pos
11.59_477.3	477.357425	11.5931667	pos
11.59_479.8	479.835325	11.5931667	pos
11.59_480.3	480.336959	11.5931667	pos
11.59_502.2	502.292426	11.5931667	pos
11.59_890.6	908.649935	11.5931667	pos
11.60_506.3	506.324771	11.6011	neg
11.60_574.3	574.312195	11.6011	neg
11.62_396.7(	396.763751	11.6192	neg
11.63_359.2	382.274455	11.6289333	pos
11.63_423.2	446.284417	11.6289333	pos
11.63_423.7	446.786074	11.6289333	pos
11.63_432.8	432.815804	11.6289333	pos
11.63_433.3	433.317844	11.6289333	pos
11.63_443.8(	443.806783	11.6289333	pos
11.63_444.3(	444.30852	11.6289333	pos
11.63_455.3	455.344262	11.6289333	pos
11.63_457.8	457.822177	11.6289333	pos
11.63_846.5	869.578435	11.6289333	pos
11.63_875.5	875.552618	11.6289333	pos
11.63_906.4	906.488414	11.6289333	pos
11.65_322.2	322.246714	11.6538333	neg
11.65_373.2	396.286802	11.6461	pos
11.65_392.8	392.821047	11.6538333	neg
11.65_401.2	424.271306	11.6461	pos
11.65_401.7	424.773205	11.6461	pos
11.65_410.8(	410.802791	11.6461	pos

11.65_411.30	411.304567	11.6461	pos
11.65_413.78	413.781711	11.6461	pos
11.65_421.79	421.793631	11.6461	pos
11.65_422.29	422.295427	11.6461	pos
11.65_435.80	435.809317	11.6461	pos
11.65_460.30	460.300166	11.6461	pos
11.65_802.50	825.551427	11.6461	pos
11.66_223.98	223.988002	11.6642167	pos
11.66_379.20	402.258633	11.6642167	pos
11.66_391.20	391.267749	11.6642167	pos
11.66_391.70	391.768931	11.6642167	pos
11.66_399.78	399.780511	11.6642167	pos
11.66_400.28	400.282189	11.6642167	pos
11.66_478.32	478.328458	11.6642167	pos
11.66_546.28	546.280864	11.6642167	pos
11.66_758.53	776.571159	11.6642167	pos
11.67_271.22	271.227743	11.6722833	neg
11.67_482.79	482.790265	11.6722833	neg
11.68_591.53	592.540526	11.6823667	pos
11.68_621.39	621.397413	11.6823667	pos
11.68_850.55	850.551413	11.6823667	pos
11.69_636.53	636.532232	11.6908	neg
11.69_653.52	653.522156	11.6908	neg
11.70_115.08	115.086719	11.7002333	pos
11.70_714.51	715.519403	11.7002333	pos
11.71_495.83	495.837755	11.7053667	neg
11.72_340.28	340.284172	11.71745	pos
11.72_437.24	437.246011	11.7200833	neg
11.72_447.27	447.275021	11.7200833	neg
11.72_619.43	619.438486	11.71745	pos
11.72_670.48	693.475205	11.71745	pos
11.72_716.55	716.550303	11.71745	pos
11.74_131.53	131.533443	11.7358167	pos

11.74_184.07	184.072999	11.7358167	pos
11.74_230.89	230.894083	11.7358167	pos
11.74_251.20	251.201449	11.7378333	neg
11.74_281.64	281.642348	11.7358167	pos
11.74_302.65	302.654339	11.7358167	pos
11.74_337.02	337.029123	11.7378333	neg
11.74_342.26	342.26328	11.7358167	pos
11.74_385.27	385.273425	11.7358167	pos
11.74_403.28	403.283745	11.7358167	pos
11.74_438.28	438.286559	11.7358167	pos
11.74_448.29	448.289997	11.7358167	pos
11.74_465.32	466.328472	11.7358167	pos
11.74_510.29	510.292627	11.7358167	pos
11.74_527.31	527.310111	11.7378333	neg
11.74_564.23	564.239646	11.7378333	neg
11.74_570.45	570.456633	11.7358167	pos
11.74_600.28	600.289565	11.7378333	neg
11.74_604.23	604.232428	11.7378333	neg
11.74_668.27	668.276874	11.7378333	neg
11.74_672.22	672.220031	11.7378333	neg
11.74_736.26	736.264509	11.7378333	neg
11.75_438.78	438.788613	11.7528	pos
11.75_443.29	443.298556	11.7528	pos
11.75_549.37	550.386622	11.7528	pos
11.75_575.41	575.411859	11.7528	pos
11.75_626.46	649.449265	11.7528	pos
11.75_672.52	672.524465	11.7528	pos
11.77_124.00	124.007055	11.7739	neg
11.77_319.00	319.001933	11.7739	neg
11.77_526.43	526.430724	11.77015	pos
11.77_628.49	628.49838	11.77015	pos
11.79_389.26	389.265658	11.7882667	pos
11.79_415.25	433.293032	11.7882667	pos

11.79_457.2	457.277893	11.7882667	pos
11.79_614.4	614.482839	11.7882667	pos
11.79_670.9	670.945057	11.7882667	pos
11.79_671.4	671.447361	11.7882667	pos
11.79_673.4	673.423412	11.7882667	pos
11.79_673.9	673.924889	11.7882667	pos
11.81_156.1	156.138198	11.80575	pos
11.81_173.0	173.077966	11.80575	pos
11.81_410.2	410.236785	11.8104333	neg
11.81_540.4	540.446625	11.80575	pos
11.81_574.8	574.897525	11.80575	pos
11.81_628.4	651.410198	11.80575	pos
11.81_628.9	651.911829	11.80575	pos
11.81_640.4	640.418834	11.80575	pos
11.81_648.9	648.932338	11.80575	pos
11.82_353.3	376.318015	11.82365	pos
11.82_356.2	356.27879	11.82365	pos
11.82_555.8	555.862245	11.82365	pos
11.82_606.4	624.441549	11.82365	pos
11.82_615.9	615.928441	11.82365	pos
11.83_359.8	359.862817	11.8283	neg
11.84_167.1	167.142874	11.8413833	pos
11.84_174.8	174.886785	11.84315	neg
11.84_394.2	394.295811	11.84315	neg
11.84_450.3	473.344278	11.8413833	pos
11.84_496.4	496.420235	11.8413833	pos
11.84_506.3	506.364812	11.8413833	pos
11.84_606.9	629.898593	11.8413833	pos
11.84_618.4	618.405645	11.8413833	pos
11.84_626.9	626.919341	11.8413833	pos
11.84_627.4	627.421299	11.8413833	pos
11.84_629.3	629.397052	11.8413833	pos
11.86_184.0	184.073103	11.8596167	pos

11.86_188.10	188.107914	11.8596167	pos
11.86_237.61	237.618363	11.8596167	pos
11.86_282.26	282.269532	11.8596167	pos
11.86_302.24	302.244893	11.8596167	pos
11.86_342.32	381.289902	11.8596167	pos
11.86_413.24	413.246113	11.8613	neg
11.86_423.27	423.275149	11.8613	neg
11.86_425.28	425.281194	11.8613	neg
11.86_427.84	427.849869	11.8613	neg
11.86_482.40	482.405495	11.8596167	pos
11.86_487.35	487.359622	11.8596167	pos
11.86_584.39	607.383702	11.8596167	pos
11.86_584.89	607.885759	11.8596167	pos
11.86_593.91	593.915147	11.8596167	pos
11.86_596.39	596.392757	11.8596167	pos
11.86_596.89	596.893112	11.8596167	pos
11.86_604.90	604.906169	11.8596167	pos
11.88_230.89	230.894029	11.8768833	pos
11.88_298.88	298.881308	11.8768833	pos
11.88_510.35	533.346681	11.8768833	pos
11.89_160.98	160.983483	11.8942667	pos
11.89_223.98	223.987993	11.8942667	pos
11.89_562.38	585.371003	11.8942667	pos
11.89_562.88	585.87261	11.8942667	pos
11.89_574.38	574.379998	11.8942667	pos
11.89_582.89	582.893108	11.8942667	pos
11.89_583.39	583.395116	11.8942667	pos
11.89_596.90	596.909328	11.8942667	pos
11.90_124.00	124.007056	11.89535	neg
11.90_337.88	337.880915	11.89535	neg
11.91_173.07	173.077974	11.9122333	pos
11.91_344.28	344.28032	11.9099667	neg
11.91_357.29	357.290505	11.9122333	pos

11.91_491.3	492.344943	11.9122333	pos
11.91_508.3	508.375806	11.9122333	pos
11.91_540.3	563.357804	11.9122333	pos
11.91_540.8	563.859484	11.9122333	pos
11.91_549.8	549.889213	11.9122333	pos
11.91_560.8	560.880309	11.9122333	pos
11.91_721.5	721.506169	11.9122333	pos
11.93_205.1	205.105201	11.9300167	pos
11.93_225.6	225.618431	11.9300167	pos
11.93_226.6	226.618545	11.9300167	pos
11.93_247.1	247.131862	11.9300167	pos
11.93_274.2	297.239858	11.9300167	pos
11.93_303.2	303.223815	11.9285167	neg
11.93_318.2	341.265727	11.9300167	pos
11.93_320.3	320.315406	11.9300167	pos
11.93_347.1	347.195162	11.9300167	pos
11.93_386.1	386.194675	11.9285167	neg
11.93_389.2	389.246277	11.9285167	neg
11.93_399.2	399.274865	11.9285167	neg
11.93_401.2	401.280861	11.9285167	neg
11.93_406.3	429.31808	11.9300167	pos
11.93_437.1	437.19183	11.9285167	neg
11.93_452.3	452.394025	11.9300167	pos
11.93_464.3	464.373097	11.9300167	pos
11.93_561.3	561.381315	11.9300167	pos
11.93_574.8	574.896875	11.9300167	pos
11.95_350.3	350.325733	11.9481833	pos
11.95_353.2	353.200208	11.9464	neg
11.95_364.3	364.341493	11.9481833	pos
11.95_408.3	408.367797	11.9481833	pos
11.95_511.3	511.334376	11.9481833	pos
11.95_511.8	511.836092	11.9481833	pos
11.95_518.3	541.344877	11.9481833	pos

11.95_518.8	541.846521	11.9481833	pos
11.95_527.8	527.876453	11.9481833	pos
11.95_530.3	530.354028	11.9481833	pos
11.95_530.8	530.855668	11.9481833	pos
11.95_538.8	538.867196	11.9481833	pos
11.95_539.3	539.369118	11.9481833	pos
11.95_550.4	550.405486	11.9481833	pos
11.95_552.8	552.884142	11.9481833	pos
11.95_738.5	738.508201	11.9464	neg
11.96_118.0	118.040616	11.9644833	neg
11.96_324.8	324.833547	11.9644833	neg
11.97_322.2	322.27259	11.9664167	pos
11.97_496.8	519.83361	11.9664167	pos
11.98_311.9	311.928585	11.9841667	pos
11.98_496.3	519.331875	11.9841667	pos
11.98_505.8	505.863227	11.9841667	pos
11.98_516.8	516.854181	11.9841667	pos
11.98_517.3	517.355739	11.9841667	pos
11.98_528.3	528.392361	11.9841667	pos
11.98_530.8	530.870284	11.9841667	pos
11.98_588.3	611.306241	11.9841667	pos
11.98_654.4	672.524785	11.9841667	pos
12.00_256.2	256.263051	12.0014833	pos
12.00_319.1	319.188669	12.00025	neg
12.00_439.2	439.261858	12.00025	neg
12.00_449.2	449.290618	12.00025	neg
12.00_474.3	497.31865	12.0014833	pos
12.00_474.8	497.820376	12.0014833	pos
12.00_483.8	483.84993	12.0014833	pos
12.00_486.3	486.327728	12.0014833	pos
12.00_489.8	489.822769	12.0014833	pos
12.00_494.8	494.840949	12.0014833	pos
12.00_495.3	495.342663	12.0014833	pos

12.00_506.8	506.880502	12.0014833	pos
12.00_508.8	508.856906	12.0014833	pos
12.00_509.3	509.359129	12.0014833	pos
12.00_544.2	567.280296	12.0014833	pos
12.00_948.6	971.647327	12.0014833	pos
12.02_335.1	335.150958	12.0192167	pos
12.02_347.2	347.258871	12.0185833	neg
12.02_362.1	362.19475	12.0185833	neg
12.02_391.1	391.182677	12.0192167	pos
12.02_481.1	481.179379	12.0185833	neg
12.02_485.1	485.122141	12.0185833	neg
12.02_506.3	506.378989	12.0192167	pos
12.02_876.5	876.56391	12.0192167	pos
12.04_386.2	386.271849	12.0372667	pos
12.04_452.3	475.305517	12.0372667	pos
12.04_452.8	475.807266	12.0372667	pos
12.04_461.8	461.836748	12.0372667	pos
12.04_462.3	462.339163	12.0372667	pos
12.04_472.8	472.827831	12.0372667	pos
12.04_473.3	473.329716	12.0372667	pos
12.04_484.3	484.36561	12.0372667	pos
12.04_486.8	486.84328	12.0372667	pos
12.04_487.3	487.345661	12.0372667	pos
12.04_545.3	545.365974	12.0372667	pos
12.04_546.3	546.331936	12.0372667	pos
12.04_904.6	922.665893	12.0372667	pos
12.05_173.0	173.07799	12.0546333	pos
12.05_230.8	230.894061	12.0546333	pos
12.05_413.3	413.304407	12.0546333	pos
12.05_452.3	475.386599	12.0546333	pos
12.05_497.3	497.395882	12.0535	neg
12.05_502.3	502.305331	12.0546333	pos
12.05_514.3	514.385758	12.0535	neg

12.05_610.4	633.453996	12.0546333	pos
12.05_701.5	701.558044	12.0546333	pos
12.07_307.2	307.226209	12.0728	pos
12.07_427.8	427.849909	12.0685667	neg
12.07_430.3	453.292356	12.0728	pos
12.07_430.8	453.794034	12.0728	pos
12.07_439.8	439.82362	12.0728	pos
12.07_440.3	440.325657	12.0728	pos
12.07_442.3	442.301353	12.0728	pos
12.07_444.3	467.308141	12.0728	pos
12.07_444.8	467.809712	12.0728	pos
12.07_450.8	450.814635	12.0728	pos
12.07_451.3	451.316335	12.0728	pos
12.07_464.8	464.830143	12.0728	pos
12.07_860.6	883.59513	12.0728	pos
12.07_866.5	867.543309	12.0728	pos
12.09_205.1	205.105264	12.0909333	pos
12.09_216.9	216.922487	12.0909333	pos
12.09_251.0	251.046315	12.0909333	pos
12.09_252.8	252.875976	12.0909333	pos
12.09_320.8	320.863344	12.0909333	pos
12.09_337.8	337.853403	12.0909333	pos
12.09_373.2	374.302409	12.0909333	pos
12.09_377.2	377.265867	12.0909333	pos
12.09_389.2	389.246213	12.0852167	neg
12.09_401.2	401.280617	12.0852167	neg
12.09_445.2	445.253335	12.0909333	pos
12.09_532.3	532.374842	12.0909333	pos
12.09_554.3	554.382484	12.0852167	neg
12.09_597.4	597.456706	12.0909333	pos
12.11_184.0	184.073035	12.1088667	pos
12.11_408.7	431.780875	12.1088667	pos
12.11_417.8	417.8105	12.1088667	pos

12.11_420.2	420.288277	12.1088667	pos
12.11_420.7	420.790312	12.1088667	pos
12.11_428.8	428.801622	12.1088667	pos
12.11_429.3	429.303682	12.1088667	pos
12.11_442.8	442.816982	12.1088667	pos
12.11_537.5	537.53477	12.1088667	pos
12.11_862.6	862.636059	12.1088667	pos
12.13_371.3	371.31514	12.1262833	pos
12.13_387.2	387.27511	12.12775	neg
12.13_393.2	393.297957	12.1262833	pos
12.13_398.7	398.776943	12.1262833	pos
12.14_239.0	239.059526	12.1411	neg
12.14_386.2	409.266207	12.1439667	pos
12.14_395.7	395.797601	12.1439667	pos
12.14_406.7	406.788355	12.1439667	pos
12.14_409.7	409.76782	12.1439667	pos
12.14_445.7	445.796619	12.1439667	pos
12.14_462.1	463.129966	12.1439667	pos
12.14_485.1	485.112091	12.1439667	pos
12.14_507.1	507.121018	12.1411	neg
12.14_508.1	508.188112	12.1439667	pos
12.14_785.0	785.083095	12.1439667	pos
12.14_819.0	819.066748	12.1439667	pos
12.14_824.5	824.540738	12.1439667	pos
12.16_115.0	115.086714	12.1619333	pos
12.16_184.8	184.888737	12.1619333	pos
12.16_223.0	223.028038	12.1558667	neg
12.16_230.8	230.894104	12.1619333	pos
12.16_284.8	284.840969	12.1558667	neg
12.16_298.8	298.881397	12.1619333	pos
12.16_323.2	323.259133	12.1558667	neg
12.16_388.8	388.850625	12.1619333	pos
12.16_566.4	589.42791	12.1619333	pos

12.16_603.9	603.91826	12.1619333	pos
12.16_882.5	865.527852	12.1619333	pos
12.17_313.2	313.238444	12.1744833	neg
12.18_173.0	173.077929	12.17975	pos
12.18_342.2	342.299827	12.17975	pos
12.18_581.9	581.905057	12.17975	pos
12.18_584.3	584.381211	12.17975	pos
12.18_699.9	699.966172	12.17975	pos
12.18_728.5	751.519135	12.17975	pos
12.18_775.5	775.599038	12.17975	pos
12.19_370.2	370.295945	12.1895	neg
12.20_294.2	294.27869	12.1975333	pos
12.20_383.2	383.276342	12.1975333	pos
12.20_423.2	423.281691	12.1975333	pos
12.20_684.5	707.491055	12.1975333	pos
12.22_420.8	420.803794	12.2158833	pos
12.22_423.7	423.783404	12.2158833	pos
12.22_433.0	433.011433	12.2214667	neg
12.22_657.4	680.430829	12.2158833	pos
12.22_657.9	680.932501	12.2158833	pos
12.22_677.9	677.953052	12.2158833	pos
12.22_730.5	730.566228	12.2158833	pos
12.23_328.3	328.32045	12.2338167	pos
12.23_559.8	559.892014	12.2338167	pos
12.23_582.5	582.592762	12.2338167	pos
12.23_640.4	663.464688	12.2338167	pos
12.24_254.8	254.851653	12.2397667	neg
12.24_359.8	359.86276	12.2397667	neg
12.24_453.1	453.178704	12.2397667	neg
12.25_380.2	381.299436	12.25135	pos
12.25_526.8	526.888115	12.25135	pos
12.25_562.3	562.367917	12.25135	pos
12.25_562.8	562.869819	12.25135	pos

12.25_635.9[illegible]	658.91938	12.25135	pos
12.25_655.9[illegible]	655.939985	12.25135	pos
12.25_656.4[illegible]	656.441923	12.25135	pos
12.26_297.2[illegible]	297.243286	12.2643333	neg
12.27_405.2[illegible]	405.299551	12.26975	pos
12.27_493.3[illegible]	493.335274	12.26975	pos
12.27_540.3[illegible]	540.355284	12.26975	pos
12.27_540.4[illegible]	540.446583	12.26975	pos
12.27_540.8[illegible]	540.856777	12.26975	pos
12.27_546.4[illegible]	546.405146	12.26975	pos
12.27_596.4[illegible]	619.438271	12.26975	pos
12.27_613.4[illegible]	636.404418	12.26975	pos
12.27_613.9[illegible]	636.90647	12.26975	pos
12.27_625.4[illegible]	625.413449	12.26975	pos
12.27_633.9[illegible]	633.92689	12.26975	pos
12.27_642.5[illegible]	642.513883	12.26975	pos
12.29_163.1[illegible]	163.122773	12.2879833	pos
12.29_452.3[illegible]	452.384379	12.2879833	pos
12.29_478.4[illegible]	478.409709	12.2879833	pos
12.29_490.8[illegible]	490.85783	12.2879833	pos
12.29_499.4[illegible]	482.404942	12.2879833	pos
12.29_552.4[illegible]	575.412324	12.2879833	pos
12.29_598.4[illegible]	598.487893	12.2879833	pos
12.29_599.1[illegible]	599.182521	12.29065	neg
12.29_657.5[illegible]	657.509035	12.29065	neg
12.30_327.0[illegible]	327.040809	12.3037667	neg
12.30_328.2[illegible]	327.232699	12.3037667	neg
12.30_363.2[illegible]	363.209412	12.3037667	neg
12.30_385.1[illegible]	385.191164	12.3037667	neg
12.30_397.2[illegible]	397.226274	12.3037667	neg
12.30_412.2[illegible]	412.210231	12.3037667	neg
12.30_427.1[illegible]	427.15776	12.3037667	neg
12.31_376.3[illegible]	399.307495	12.3062167	pos

12.31_422.38	422.383311	12.3062167	pos
12.31_441.19	441.198522	12.3062167	pos
12.31_513.12	513.128901	12.3062167	pos
12.31_591.40	614.391576	12.3062167	pos
12.31_591.90	614.893179	12.3062167	pos
12.31_600.92	600.922862	12.3062167	pos
12.31_603.40	603.400923	12.3062167	pos
12.31_611.91	611.913804	12.3062167	pos
12.31_612.41	612.415416	12.3062167	pos
12.31_700.44	701.451456	12.3062167	pos
12.32_375.21	375.217247	12.3237167	pos
12.32_508.39	531.385949	12.3237167	pos
12.32_529.36	529.364163	12.3237167	pos
12.32_554.46	554.461894	12.3237167	pos
12.32_676.44	699.432774	12.3237167	pos
12.34_258.26	258.269606	12.3412167	pos
12.34_333.27	333.27733	12.3412167	pos
12.34_338.19	338.194533	12.3449333	neg
12.34_387.02	387.025775	12.3449333	neg
12.34_515.86	515.864853	12.3412167	pos
12.34_518.84	518.843842	12.3412167	pos
12.34_569.38	592.378418	12.3412167	pos
12.34_569.89	592.880257	12.3412167	pos
12.34_578.90	578.909747	12.3412167	pos
12.34_581.38	581.38762	12.3412167	pos
12.34_589.90	589.900851	12.3412167	pos
12.34_590.40	590.402667	12.3412167	pos
12.34_819.60	819.603575	12.3412167	pos
12.36_255.23	255.231384	12.3586333	pos
12.36_321.20	321.204458	12.3628333	neg
12.36_324.22	347.21864	12.3586333	pos
12.36_346.27	346.273527	12.3586333	pos
12.36_396.76	396.763803	12.3628333	neg

12.36_397.2[illegible]	397.259371	12.3628333	neg
12.36_400.3[illegible]	400.341627	12.3586333	pos
12.36_547.3[illegible]	570.365621	12.3586333	pos
12.36_547.8[illegible]	570.867209	12.3586333	pos
12.36_567.8[illegible]	567.887934	12.3586333	pos
12.36_568.3[illegible]	568.389674	12.3586333	pos
12.38_507.3[illegible]	507.351169	12.37545	pos
12.38_524.1[illegible]	524.185587	12.3811333	neg
12.38_556.8[illegible]	556.896878	12.37545	pos
12.38_559.3[illegible]	559.374734	12.37545	pos
12.38_579.1[illegible]	579.125204	12.3811333	neg
12.38_581.9[illegible]	581.904296	12.37545	pos
12.38_873.5[illegible]	874.57954	12.37545	pos
12.39_405.2[illegible]	405.299455	12.3931167	pos
12.39_485.1[illegible]	485.186006	12.3931167	pos
12.39_487.3[illegible]	487.359917	12.3931167	pos
12.39_496.4[illegible]	496.420364	12.3931167	pos
12.39_510.4[illegible]	510.436013	12.3931167	pos
12.39_525.3[illegible]	548.352561	12.3931167	pos
12.39_525.8[illegible]	548.85428	12.3931167	pos
12.39_534.8[illegible]	534.883942	12.3931167	pos
12.39_535.3[illegible]	535.386757	12.3931167	pos
12.39_537.3[illegible]	537.361421	12.3931167	pos
12.39_545.8[illegible]	545.874868	12.3931167	pos
12.39_546.3[illegible]	546.376229	12.3931167	pos
12.39_557.4[illegible]	557.413388	12.3931167	pos
12.41_234.1[illegible]	234.131643	12.4112833	pos
12.41_234.6[illegible]	234.633337	12.4112833	pos
12.41_327.2[illegible]	327.250766	12.4112833	pos
12.41_330.2[illegible]	313.273112	12.4112833	pos
12.41_365.2[illegible]	365.245843	12.4138167	neg
12.41_465.3[illegible]	465.378096	12.4112833	pos
12.41_496.3[illegible]	496.327968	12.4112833	pos

12.41_559.89	559.891212	12.4112833	pos
12.43_342.29	342.299681	12.4295667	pos
12.43_463.32	463.324967	12.4295667	pos
12.43_485.33	485.337859	12.4295667	pos
12.43_493.85	493.851095	12.4295667	pos
12.43_494.35	494.352983	12.4295667	pos
12.43_503.35	526.339464	12.4295667	pos
12.43_503.85	526.841295	12.4295667	pos
12.43_512.87	512.870851	12.4295667	pos
12.43_523.86	523.861933	12.4295667	pos
12.43_537.87	537.878365	12.4295667	pos
12.43_691.49	691.495911	12.4295667	pos
12.45_249.18	249.182444	12.44695	pos
12.46_1106.7	1106.77071	12.46405	pos
12.46_439.20	439.207369	12.4626	neg
12.46_451.32	474.315673	12.46405	pos
12.46_481.33	504.326217	12.46405	pos
12.46_481.83	504.828034	12.46405	pos
12.46_490.85	490.85774	12.46405	pos
12.46_493.33	493.335236	12.46405	pos
12.46_501.84	501.848622	12.46405	pos
12.46_502.35	502.350405	12.46405	pos
12.46_513.38	513.386792	12.46405	pos
12.46_516.36	516.366586	12.46405	pos
12.48_1068.7	1051.77728	12.4811	pos
12.48_1107.7	1108.78511	12.4811	pos
12.48_1134.7	1134.79941	12.4811	pos
12.48_303.35	303.351527	12.4754333	neg
12.48_303.40	303.403983	12.4754333	neg
12.48_304.23	303.232613	12.4754333	neg
12.48_305.23	305.238997	12.4754333	neg
12.48_341.23	341.238863	12.4754333	neg
12.48_346.27	346.273616	12.4811	pos

12.48_371.2	371.219931	12.4754333	neg
12.48_373.2	373.226049	12.4754333	neg
12.48_383.2	383.203455	12.4811	pos
12.48_420.3	443.333684	12.4811	pos
12.48_456.1	456.197822	12.4754333	neg
12.48_466.4	466.409666	12.4811	pos
12.48_489.1	489.128648	12.4811	pos
12.48_522.5	522.59696	12.4811	pos
12.48_686.5	686.540263	12.4811	pos
12.48_720.5	720.587299	12.4811	pos
12.48_729.5	768.551616	12.4811	pos
12.48_740.5	740.554407	12.4811	pos
12.48_743.5	726.558627	12.4811	pos
12.48_748.5	748.580967	12.4811	pos
12.48_802.5	802.534464	12.4811	pos
12.48_812.5	812.544342	12.4811	pos
12.48_846.5	846.546747	12.4811	pos
12.48_859.5	859.599608	12.4811	pos
12.48_870.5	870.545509	12.4811	pos
12.48_902.6	941.636721	12.4811	pos
12.48_962.6	985.662757	12.4811	pos
12.49_116.9	116.922093	12.4924667	neg
12.49_303.0	303.061384	12.4924667	neg
12.49_339.2	339.208976	12.4924667	neg
12.49_399.2	399.220615	12.4924667	neg
12.49_633.4	633.490727	12.4924667	neg
12.50_459.3	482.312613	12.4972833	pos
12.50_468.8	468.844573	12.4972833	pos
12.51_111.0	111.091877	12.51485	pos
12.51_184.0	184.073042	12.51485	pos
12.51_459.8	482.814936	12.51485	pos
12.51_479.8	479.835558	12.51485	pos
12.51_480.3	480.33723	12.51485	pos

12.53_437.3[illegible]	460.299988	12.5329667	pos
12.53_437.8[illegible]	460.801604	12.5329667	pos
12.53_446.8[illegible]	446.831272	12.5329667	pos
12.53_449.3[illegible]	449.308957	12.5329667	pos
12.53_455.3[illegible]	455.344792	12.5329667	pos
12.53_457.8[illegible]	457.822273	12.5329667	pos
12.53_458.3[illegible]	458.324009	12.5329667	pos
12.53_471.8[illegible]	471.837924	12.5329667	pos
12.53_472.3[illegible]	472.339598	12.5329667	pos
12.53_874.6[illegible]	897.609039	12.5329667	pos
12.55_176.8[illegible]	176.883897	12.5465	neg
12.55_388.8[illegible]	388.878202	12.5465	neg
12.55_474.8[illegible]	474.817432	12.5506167	pos
12.55_508.3[illegible]	508.340216	12.5465	neg
12.57_406.3[illegible]	407.314932	12.56865	pos
12.57_415.2[illegible]	438.286815	12.56865	pos
12.57_415.7[illegible]	438.788471	12.56865	pos
12.57_424.8[illegible]	424.818223	12.56865	pos
12.57_427.2[illegible]	427.295902	12.56865	pos
12.57_436.3[illegible]	436.311123	12.56865	pos
12.57_449.8[illegible]	449.824702	12.56865	pos
12.57_830.5[illegible]	853.583379	12.56865	pos
12.58_297.2[illegible]	297.24315	12.5775167	neg
12.58_351.0[illegible]	351.008355	12.5775167	neg
12.58_453.3[illegible]	453.312617	12.5775167	neg
12.59_284.2[illegible]	284.285033	12.5856	pos
12.59_392.8[illegible]	392.82116	12.5908167	neg
12.59_429.3[illegible]	447.345979	12.5856	pos
12.59_441.2[illegible]	441.277083	12.5908167	neg
12.59_451.3[illegible]	451.306251	12.5908167	neg
12.59_624.4[illegible]	647.469499	12.5856	pos
12.60_393.2[illegible]	416.273847	12.6029833	pos
12.60_393.7[illegible]	416.775526	12.6029833	pos

12.60_461.18	461.185463	12.6029833	pos
12.61_483.19	483.194891	12.6119	neg
12.62_113.10	113.107499	12.6205	pos
12.62_337.16	337.166731	12.6205	pos
12.62_365.24	365.245747	12.6234833	neg
12.62_367.24	367.245006	12.6205	pos
12.62_375.22	375.220864	12.6234833	neg
12.64_1083.7	1084.78507	12.6383167	pos
12.64_1110.7	1110.79947	12.6383167	pos
12.64_280.23	279.232672	12.6402	neg
12.64_281.23	281.239246	12.6402	neg
12.64_297.24	297.241461	12.6383167	pos
12.64_315.20	315.209408	12.6402	neg
12.64_317.20	317.206025	12.6402	neg
12.64_330.25	329.248371	12.6402	neg
12.64_331.25	331.254887	12.6402	neg
12.64_347.22	347.219998	12.6402	neg
12.64_364.21	364.210407	12.6402	neg
12.64_365.22	365.224569	12.6402	neg
12.64_375.22	375.226365	12.6383167	pos
12.64_377.16	377.163501	12.6402	neg
12.64_391.26	391.262005	12.6402	neg
12.64_397.23	397.23567	12.6402	neg
12.64_410.18	410.188677	12.6383167	pos
12.64_414.22	414.22585	12.6402	neg
12.64_429.17	429.173394	12.6402	neg
12.64_566.89	566.899713	12.6383167	pos
12.64_631.47	631.470507	12.6402	neg
12.64_833.64	833.641465	12.6383167	pos
12.64_876.58	876.589541	12.6383167	pos
12.65_176.99	176.997633	12.65015	neg
12.65_403.29	403.296583	12.65015	neg
12.65_465.22	465.222949	12.65015	neg

12.66_441.31	441.317075	12.65635	pos
12.66_569.87	569.877725	12.65635	pos
12.66_664.44	687.438586	12.65635	pos
12.66_664.95	687.940749	12.65635	pos
12.66_685.46	685.461671	12.65635	pos
12.66_857.59	896.560945	12.65635	pos
12.67_167.12	167.128941	12.6741667	pos
12.67_243.13	243.135389	12.6741667	pos
12.67_569.37	569.375934	12.6741667	pos
12.67_698.51	716.550524	12.6741667	pos
12.67_849.54	850.552364	12.6741667	pos
12.69_252.90	252.903183	12.6870167	neg
12.69_274.88	274.885225	12.6870167	neg
12.69_430.28	430.28965	12.69225	pos
12.69_430.79	430.791262	12.69225	pos
12.69_534.35	534.356419	12.6870167	neg
12.69_642.43	665.425726	12.69225	pos
12.69_662.94	662.947707	12.69225	pos
12.69_663.44	663.449242	12.69225	pos
12.70_320.89	320.890527	12.7015667	neg
12.70_324.83	324.833542	12.7015667	neg
12.70_392.82	392.821115	12.7015667	neg
12.71_247.88	247.884254	12.70945	pos
12.71_299.25	299.258927	12.71275	neg
12.71_410.86	410.86006	12.71275	neg
12.71_542.40	542.408643	12.70945	pos
12.71_544.88	544.886518	12.70945	pos
12.71_580.45	603.443562	12.70945	pos
12.71_642.93	665.927631	12.70945	pos
12.72_254.85	254.851639	12.7228167	neg
12.73_130.15	130.159011	12.72735	pos
12.73_620.42	643.412417	12.72735	pos
12.73_620.92	643.914327	12.72735	pos

12.73_640.9[illegible]	640.934748	12.72735	pos
12.73_654.4[illegible]	677.479992	12.72735	pos
12.74_130.9[illegible]	130.992252	12.7441833	neg
12.74_173.0[illegible]	173.078035	12.7447333	pos
12.74_184.8[illegible]	184.888753	12.7447333	pos
12.74_190.1[illegible]	190.108444	12.7447333	pos
12.74_252.8[illegible]	252.876009	12.7447333	pos
12.74_641.4[illegible]	641.436339	12.7447333	pos
12.74_872.5[illegible]	872.561995	12.7441833	neg
12.76_207.0[illegible]	207.098889	12.763	pos
12.76_284.8[illegible]	284.840916	12.7625833	neg
12.76_523.3[illegible]	523.374616	12.763	pos
12.76_598.9[illegible]	621.901094	12.763	pos
12.76_607.9[illegible]	607.930729	12.763	pos
12.76_610.4[illegible]	610.408504	12.763	pos
12.76_610.4[illegible]	633.454079	12.763	pos
12.76_618.9[illegible]	618.92161	12.763	pos
12.76_619.4[illegible]	619.423252	12.763	pos
12.76_656.5[illegible]	656.52964	12.763	pos
12.76_762.5[illegible]	762.505512	12.763	pos
12.78_376.3[illegible]	376.318011	12.78065	pos
12.80_363.2[illegible]	363.20696	12.7995333	neg
12.80_522.8[illegible]	522.872809	12.7984	pos
12.80_566.4[illegible]	589.428384	12.7984	pos
12.80_576.3[illegible]	599.386275	12.7984	pos
12.80_576.8[illegible]	599.888168	12.7984	pos
12.80_585.9[illegible]	585.917867	12.7984	pos
12.80_588.3[illegible]	588.395342	12.7984	pos
12.80_596.9[illegible]	596.908706	12.7984	pos
12.80_597.4[illegible]	597.410544	12.7984	pos
12.81_214.8[illegible]	214.859089	12.8107167	neg
12.82_502.3[illegible]	525.35011	12.8165833	pos
12.82_514.3[illegible]	514.360677	12.8165833	pos

12.83_391.2(	391.261943	12.8323333	neg
12.83_536.4:	575.397078	12.83355	pos
12.83_554.8!	577.875157	12.83355	pos
12.83_563.9(	563.904845	12.83355	pos
12.83_566.3!	566.382746	12.83355	pos
12.83_574.8!	574.895717	12.83355	pos
12.84_269.2:	269.211994	12.8434833	neg
12.85_128.0!	128.081822	12.8511333	pos
12.85_452.3!	452.393852	12.8511333	pos
12.85_457.3⁄	457.349332	12.8511333	pos
12.85_503.8:	503.838387	12.8511333	pos
12.85_559.4:	559.417706	12.8511333	pos
12.87_390.3:	413.323106	12.8694	pos
12.87_392.3	392.372785	12.8694	pos
12.87_436.3!	436.399137	12.8694	pos
12.87_500.8!	500.859349	12.8694	pos
12.87_503.3:	503.336845	12.8694	pos
12.87_532.3	555.360331	12.8694	pos
12.87_532.8	555.862059	12.8694	pos
12.87_541.8!	541.891733	12.8694	pos
12.87_544.3(	544.369485	12.8694	pos
12.87_552.8!	552.882866	12.8694	pos
12.87_553.3!	553.384756	12.8694	pos
12.89_113.1(	113.107523	12.8871833	pos
12.89_241.2(	241.203282	12.8871833	pos
12.89_331.2!	331.255053	12.8930167	neg
12.89_331.2(	331.262634	12.8871833	pos
12.89_375.2:	375.226508	12.8871833	pos
12.89_414.2:	414.226	12.8930167	neg
12.89_492.3⁄	492.345879	12.8871833	pos
12.89_510.3!	533.347389	12.8871833	pos
12.91_130.1!	130.158949	12.9052833	pos
12.91_254.8!	254.851669	12.90795	neg

12.91_510.8(	533.849186	12.9052833	pos
12.91_520.38	520.380057	12.9052833	pos
12.91_522.35	522.356491	12.9052833	pos
12.91_522.4:	545.40241	12.9052833	pos
12.91_530.8(	530.869783	12.9052833	pos
12.91_531.37	531.371203	12.9052833	pos
12.91_568.47	568.477825	12.9052833	pos
12.92_184.07	184.073046	12.9225667	pos
12.92_519.87	519.878837	12.9225667	pos
12.94_302.82	302.824301	12.9406833	pos
12.94_317.24	317.24827	12.9357167	neg
12.94_341.22	341.224815	12.9357167	neg
12.94_348.28	348.289103	12.9406833	pos
12.94_373.23	373.235684	12.9357167	neg
12.94_401.23	401.236293	12.9357167	neg
12.94_405.17	405.173504	12.9357167	neg
12.94_419.21	419.213956	12.9406833	pos
12.94_441.22	441.223169	12.9357167	neg
12.94_488.34	511.334398	12.9406833	pos
12.94_497.8(	497.865602	12.9406833	pos
12.94_500.34	500.343402	12.9406833	pos
12.94_520.39	520.395498	12.9406833	pos
12.94_720.58	720.587181	12.9406833	pos
12.94_976.68	999.678715	12.9406833	pos
12.95_363.2(	363.206939	12.9541167	neg
12.96_169.1(	169.108227	12.9578	pos
12.96_467.85	467.854899	12.9578	pos
12.96_488.84	511.836075	12.9578	pos
12.96_508.85	508.856685	12.9578	pos
12.96_509.35	509.358418	12.9578	pos
12.96_767.0(	767.066808	12.9578	pos
12.98_395.3(	395.362896	12.9755	pos
12.98_397.29	398.304905	12.9755	pos

12.98_442.3[illegible]	481.323854	12.9755	pos
12.98_466.3[illegible]	489.321184	12.9755	pos
12.98_475.8[illegible]	475.852596	12.9755	pos
12.98_481.8[illegible]	481.82537	12.9755	pos
12.98_487.3[illegible]	487.345147	12.9755	pos
12.98_498.3[illegible]	498.381256	12.9755	pos
12.98_610.4[illegible]	628.498772	12.9755	pos
12.98_742.0[illegible]	742.060548	12.9755	pos
12.98_932.6[illegible]	955.652554	12.9755	pos
12.99_343.2[illegible]	343.285284	12.9938	pos
12.99_466.8[illegible]	489.823016	12.9938	pos
12.99_478.3[illegible]	478.330154	12.9938	pos
12.99_486.8[illegible]	486.843538	12.9938	pos
12.99_754.0[illegible]	754.059874	12.9938	pos
13.01_163.1[illegible]	163.12277	13.0115667	pos
13.01_444.3[illegible]	467.308063	13.0115667	pos
13.01_456.3[illegible]	456.317137	13.0115667	pos
13.01_476.3[illegible]	476.367999	13.0115667	pos
13.01_478.3[illegible]	501.375739	13.0115667	pos
13.01_478.8[illegible]	478.846002	13.0115667	pos
13.01_479.3[illegible]	479.347872	13.0115667	pos
13.01_492.4[illegible]	515.391546	13.0115667	pos
13.01_566.4[illegible]	589.427625	13.0115667	pos
13.01_700.5[illegible]	700.555569	13.0115667	pos
13.03_372.3[illegible]	372.311586	13.0341167	neg
13.03_444.8[illegible]	467.809866	13.0296833	pos
13.03_453.8[illegible]	453.839464	13.0296833	pos
13.03_464.8[illegible]	464.830368	13.0296833	pos
13.03_465.3[illegible]	465.332117	13.0296833	pos
13.03_524.4[illegible]	524.451725	13.0296833	pos
13.03_888.6[illegible]	911.626465	13.0296833	pos
13.05_130.1[illegible]	130.158913	13.0475833	pos
13.05_166.1[illegible]	167.128915	13.0475833	pos

13.05_226.9!	226.994464	13.0525	neg
13.05_422.3(	445.29487	13.0475833	pos
13.05_468.3!	491.370551	13.0475833	pos
13.05_746.5:	746.513203	13.0525	neg
13.05_765.0!	765.052357	13.0475833	pos
13.06_427.3(	427.306056	13.0641833	neg
13.07_150.1(	150.102436	13.0659	pos
13.07_154.1:	154.122524	13.0659	pos
13.07_169.1(	169.108244	13.0659	pos
13.07_307.0(	307.002146	13.0725833	neg
13.07_337.0:	337.029012	13.0725833	neg
13.07_422.8(	445.796648	13.0659	pos
13.07_431.8:	431.826278	13.0659	pos
13.07_434.3(	434.303895	13.0659	pos
13.07_442.8:	442.817164	13.0659	pos
13.07_443.3:	443.319374	13.0659	pos
13.07_456.8:	456.832717	13.0659	pos
13.07_844.6:	862.644569	13.0659	pos
13.08_420.8(	420.804047	13.0830833	pos
13.08_421.3(	421.305902	13.0830833	pos
13.08_448.3:	448.319558	13.0830833	pos
13.08_573.9(	573.90735	13.0830833	pos
13.08_576.3!	576.383632	13.0830833	pos
13.08_713.9!	713.981733	13.0830833	pos
13.08_726.0!	726.057628	13.0830833	pos
13.10_140.1(	140.106764	13.1012833	pos
13.10_173.0'	173.078029	13.1012833	pos
13.10_400.2!	423.281736	13.1012833	pos
13.10_408.3(	408.367665	13.1012833	pos
13.10_423.7!	423.78348	13.1012833	pos
13.10_436.3!	436.398943	13.1012833	pos
13.10_452.3!	452.393986	13.1012833	pos
13.10_819.6:	819.619353	13.1012833	pos

13.12_388.28	388.287455	13.1190833	pos
13.12_437.29	437.297499	13.1190833	pos
13.12_551.89	551.894375	13.1190833	pos
13.12_554.37	554.370757	13.1190833	pos
13.12_691.96	691.968622	13.1190833	pos
13.12_692.47	692.471526	13.1190833	pos
13.12_846.65	846.650019	13.1190833	pos
13.13_365.34	365.342122	13.1264	neg
13.14_113.10	113.107527	13.1368167	pos
13.14_434.81	434.819533	13.1368167	pos
13.14_552.39	552.396015	13.1368167	pos
13.14_638.49	661.485296	13.1368167	pos
13.14_774.59	774.594136	13.1368167	pos
13.15_339.00	339.008205	13.1490333	neg
13.15_649.44	672.433452	13.1542167	pos
13.15_658.96	658.96446	13.1542167	pos
13.15_669.95	669.955697	13.1542167	pos
13.15_670.45	670.457296	13.1542167	pos
13.15_851.60	851.605309	13.1542167	pos
13.17_1172.8	1172.83851	13.17195	pos
13.17_270.27	270.278732	13.17195	pos
13.19_1185.3	1185.34252	13.1900333	pos
13.19_301.21	301.210861	13.1900333	pos
13.19_307.28	308.294215	13.1900333	pos
13.19_533.45	533.454673	13.19245	neg
13.19_647.94	647.942475	13.1900333	pos
13.19_648.44	648.444935	13.1900333	pos
13.19_712.53	730.565025	13.1900333	pos
13.19_748.52	748.526293	13.1900333	pos
13.19_764.52	764.524897	13.19245	neg
13.19_833.64	833.642861	13.1900333	pos
13.21_115.96	115.961177	13.21015	neg
13.21_136.86	136.862851	13.21015	neg

13.21_193.08	193.086575	13.21015	neg
13.21_536.14	559.130838	13.2068667	pos
13.21_559.47	559.470458	13.21015	neg
13.21_581.52	581.526183	13.21015	neg
13.21_582.20	582.206664	13.2068667	pos
13.21_627.43	650.420021	13.2068667	pos
13.21_764.52	764.521646	13.2068667	pos
13.21_770.50	770.508757	13.2068667	pos
13.21_813.56	814.568648	13.2068667	pos
13.21_816.54	816.548481	13.2068667	pos
13.21_831.57	832.580496	13.2068667	pos
13.21_859.60	859.599996	13.2068667	pos
13.21_876.55	876.552525	13.2068667	pos
13.22_370.30	370.309572	13.2235667	pos
13.22_605.41	628.407348	13.2235667	pos
13.22_686.54	686.540549	13.2235667	pos
13.22_708.49	691.495894	13.2235667	pos
13.22_857.58	840.584519	13.2235667	pos
13.23_106.04	106.040827	13.2280333	neg
13.24_354.25	377.242108	13.2417667	pos
13.24_617.41	617.417399	13.2417667	pos
13.24_625.92	625.929376	13.2417667	pos
13.24_626.43	626.431216	13.2417667	pos
13.25_333.27	333.27055	13.2463167	neg
13.25_416.24	416.241541	13.2463167	neg
13.26_214.85	214.859105	13.26345	neg
13.26_530.38	530.382288	13.2592333	pos
13.26_592.92	592.925611	13.2592333	pos
13.26_595.40	595.40289	13.2592333	pos
13.26_624.48	647.469735	13.2592333	pos
13.26_802.62	802.624117	13.2592333	pos
13.28_1173.3	1173.3439	13.2766833	pos
13.28_529.88	529.88094	13.2766833	pos

13.28_532.3!	532.356665	13.2766833	pos
13.28_583.4(	606.394202	13.2766833	pos
13.28_583.9(	606.895966	13.2766833	pos
13.28_594.4(	617.458887	13.2766833	pos
13.28_603.9:	603.916535	13.2766833	pos
13.28_604.4:	604.418924	13.2766833	pos
13.29_1160.:	1160.33668	13.29445	pos
13.29_561.8!	584.883083	13.29445	pos
13.29_615.4!	615.457882	13.29445	pos
13.29_771.5(	754.566528	13.29445	pos
13.29_772.0‑	772.076002	13.29445	pos
13.31_494.5(	494.565611	13.3124333	pos
13.31_561.3!	584.3812	13.3124333	pos
13.31_570.9:	570.91248	13.3124333	pos
13.31_573.3!	573.390326	13.3124333	pos
13.31_581.9(	581.903551	13.3124333	pos
13.31_582.4(	582.405288	13.3124333	pos
13.31_796.0‑	819.067002	13.3124333	pos
13.32_387.0:	387.025676	13.3215333	neg
13.33_130.1!	130.158924	13.32975	pos
13.33_173.0‑	173.078026	13.32975	pos
13.33_383.2(	383.203567	13.32975	pos
13.33_489.1!	489.153426	13.3314333	neg
13.33_593.4‹	593.442886	13.32975	pos
13.35_125.0‑	125.070754	13.3474333	pos
13.35_504.4(	522.436189	13.3474333	pos
13.35_539.3‑	562.368054	13.3474333	pos
13.35_539.8i	562.870041	13.3474333	pos
13.35_548.8!	548.899921	13.3474333	pos
13.35_551.3‑	551.377534	13.3474333	pos
13.35_559.8!	559.890571	13.3474333	pos
13.35_560.3!	560.392808	13.3474333	pos
13.35_564.5!	565.566066	13.3474333	pos

13.36_337.0[illegible]	337.028848	13.3580667	neg
13.37_265.2[illegible]	265.252233	13.3655667	pos
13.37_342.2[illegible]	342.299812	13.3655667	pos
13.37_580.4[illegible]	603.44381	13.3655667	pos
13.38_1034.[illegible]	1057.72018	13.38305	pos
13.38_115.9[illegible]	115.961277	13.3769167	neg
13.38_145.9[illegible]	145.926512	13.3769167	neg
13.38_517.8[illegible]	540.857131	13.38305	pos
13.38_526.8[illegible]	526.886627	13.38305	pos
13.38_529.3[illegible]	529.364543	13.38305	pos
13.38_536.5[illegible]	559.516794	13.38305	pos
13.38_537.8[illegible]	537.877558	13.38305	pos
13.38_587.5[illegible]	587.547939	13.38305	pos
13.38_598.5[illegible]	598.516501	13.3769167	neg
13.38_757.5[illegible]	796.522519	13.38305	pos
13.38_789.5[illegible]	772.523155	13.38305	pos
13.38_814.5[illegible]	814.568691	13.38305	pos
13.38_833.5[illegible]	816.585502	13.38305	pos
13.40_100.9[illegible]	100.933396	13.39515	neg
13.40_116.9[illegible]	116.922489	13.39515	neg
13.40_130.9[illegible]	130.928728	13.39515	neg
13.40_132.9[illegible]	132.899959	13.39515	neg
13.40_132.9[illegible]	132.923219	13.39515	neg
13.40_134.8[illegible]	134.894447	13.39515	neg
13.40_136.8[illegible]	136.891359	13.39515	neg
13.40_145.9[illegible]	145.923367	13.39515	neg
13.40_257.9[illegible]	257.93879	13.39515	neg
13.40_282.2[illegible]	281.248358	13.39515	neg
13.40_283.2[illegible]	283.254881	13.39515	neg
13.40_317.2[illegible]	317.225012	13.39515	neg
13.40_349.2[illegible]	349.235722	13.39515	neg
13.40_366.2[illegible]	366.225766	13.39515	neg
13.40_517.3[illegible]	540.355259	13.4006833	pos

13.40_538.37	538.379365	13.4006833	pos
13.40_608.47	631.464292	13.4006833	pos
13.41_393.27	393.275885	13.41285	neg
13.42_183.08	183.087424	13.4184833	pos
13.42_504.87	504.873577	13.4184833	pos
13.42_507.35	507.351152	13.4184833	pos
13.42_510.84	510.84655	13.4184833	pos
13.44_495.35	518.342116	13.4363167	pos
13.44_495.85	518.844016	13.4363167	pos
13.44_510.34	510.344794	13.4363167	pos
13.44_515.86	515.864586	13.4363167	pos
13.44_516.36	516.366416	13.4363167	pos
13.44_610.62	610.623788	13.4363167	pos
13.44_763.55	763.553574	13.4363167	pos
13.45_251.04	251.046375	13.4534833	pos
13.45_482.86	482.860436	13.4534833	pos
13.45_485.33	485.338178	13.4534833	pos
13.45_488.33	488.331844	13.4534833	pos
13.45_822.08	822.089595	13.4534833	pos
13.47_464.35	464.356935	13.4718167	pos
13.47_473.33	496.32861	13.4718167	pos
13.47_473.84	496.830771	13.4718167	pos
13.47_493.85	493.85135	13.4718167	pos
13.47_494.35	494.353077	13.4718167	pos
13.47_505.89	505.891658	13.4718167	pos
13.47_507.86	507.867528	13.4718167	pos
13.47_508.36	508.369127	13.4718167	pos
13.47_573.43	573.433386	13.4718167	pos
13.47_582.49	582.493344	13.4718167	pos
13.47_946.67	964.712918	13.4718167	pos
13.49_286.30	286.300943	13.4891167	pos
13.49_472.34	472.340001	13.4891167	pos
13.49_485.85	485.85383	13.4891167	pos

13.49_486.3!	486.356052	13.4891167	pos
13.49_536.4:	559.41772	13.4891167	pos
13.50_393.2	393.277167	13.5029667	neg
13.50_403.3(	403.306179	13.5029667	neg
13.51_120.1(	120.102032	13.5068	pos
13.51_149.0!	167.128924	13.5068	pos
13.51_208.1!	208.15555	13.5068	pos
13.51_321.2	321.210275	13.5114167	neg
13.51_451.3:	469.360475	13.5068	pos
13.51_451.8:	474.817625	13.5068	pos
13.51_463.3:	463.324994	13.5068	pos
13.51_471.8:	471.838258	13.5068	pos
13.51_568.4	568.477751	13.5068	pos
13.51_902.6!	920.686636	13.5068	pos
13.52_321.2:	321.239647	13.5248833	pos
13.52_460.8	460.847175	13.5248833	pos
13.53_333.0	333.017578	13.53095	neg
13.54_438.8:	438.834272	13.5426	pos
13.54_443.3:	466.318222	13.5426	pos
13.54_449.8:	449.825047	13.5426	pos
13.54_450.3:	450.326786	13.5426	pos
13.54_463.8	463.840663	13.5426	pos
13.54_696.5:	719.527586	13.5426	pos
13.54_858.6:	876.660196	13.5426	pos
13.54_904.6!	904.692416	13.5426	pos
13.56_429.3	447.346801	13.5609833	pos
13.56_721.4!	721.491102	13.5609833	pos
13.57_194.9	194.988141	13.5673833	neg
13.58_366.3(	366.300072	13.5784833	pos
13.58_421.2:	421.230145	13.5784833	pos
13.58_430.2	430.289741	13.5784833	pos
13.58_698.9	698.976691	13.5784833	pos
13.58_814.6(	837.591007	13.5784833	pos

13.60_130.1!	130.158905	13.5968667	pos
13.60_309.2	309.270699	13.59865	neg
13.60_375.2!	375.251338	13.59865	neg
13.60_455.3	455.327382	13.5968667	pos
13.60_615.5	615.53586	13.59865	neg
13.61_151.1	151.122784	13.6149667	pos
13.63_173.0	173.078189	13.63205	pos
13.63_656.4!	679.441107	13.63205	pos
13.63_676.9	676.963459	13.63205	pos
13.63_860.6	860.665759	13.63205	pos
13.65_116.9!	116.956385	13.6549667	neg
13.65_634.4	652.472734	13.6501333	pos
13.65_677.4	677.464927	13.6501333	pos
13.67_115.0	115.086737	13.6681833	pos
13.67_654.9!	654.950387	13.6681833	pos
13.67_655.4!	655.451948	13.6681833	pos
13.67_744.5	744.583563	13.6681833	pos
13.67_880.4	880.475099	13.6681833	pos
13.69_612.4	635.415024	13.6865833	pos
13.70_152.9	152.996669	13.6964333	neg
13.70_599.5	599.500734	13.6964333	neg
13.70_612.9	630.961551	13.7043333	pos
13.70_627.5!	627.551127	13.6964333	neg
13.70_632.9	632.937423	13.7043333	pos
13.70_633.4	633.438795	13.7043333	pos
13.72_670.5	670.545732	13.72275	pos
13.72_675.5	675.500945	13.72275	pos
13.72_700.5!	700.556007	13.72275	pos
13.73_393.2	393.277627	13.7268833	neg
13.74_705.5	705.511565	13.74075	pos
13.75_212.9	212.978925	13.7497667	neg
13.76_130.1!	130.158914	13.7582	pos
13.76_506.4	529.407466	13.7582	pos

13.76_590.4	608.446758	13.7582	pos
13.76_590.9	608.948629	13.7582	pos
13.76_595.4	595.477531	13.7582	pos
13.76_610.9	610.924296	13.7582	pos
13.76_611.4	611.426007	13.7582	pos
13.78_169.1	169.108266	13.77595	pos
13.78_251.0	251.046451	13.77595	pos
13.78_568.3	586.433763	13.77595	pos
13.78_589.4	589.41297	13.77595	pos
13.79_113.1	113.10763	13.7936333	pos
13.79_568.9	586.935513	13.7936333	pos
13.79_577.9	577.920218	13.7936333	pos
13.79_588.9	588.911414	13.7936333	pos
13.80_609.5	609.557222	13.8015667	neg
13.81_256.2	256.263103	13.8118	pos
13.81_283.2	284.294385	13.8118	pos
13.83_173.0	173.078354	13.83005	pos
13.83_566.8	566.898424	13.83005	pos
13.84_469.0	469.028291	13.8377667	neg
13.85_371.3	371.315161	13.8483833	pos
13.85_546.3	564.420747	13.8483833	pos
13.85_567.3	567.399824	13.8483833	pos
13.85_578.4	578.436756	13.8483833	pos
13.85_638.4	661.485305	13.8483833	pos
13.87_524.3	542.407693	13.8664333	pos
13.87_524.8	542.909369	13.8664333	pos
13.87_533.8	533.894573	13.8664333	pos
13.87_544.8	544.88554	13.8664333	pos
13.87_545.3	545.386627	13.8664333	pos
13.88_556.4	556.424425	13.8842333	pos
13.90_473.2	473.282247	13.9001833	neg
13.91_194.9	194.988199	13.90995	neg
13.92_209.0	209.003803	13.92265	neg

13.92_502.3(	520.394622	13.9195167	pos
13.92_502.8(	520.896455	13.9195167	pos
13.94_514.8'	514.87543	13.9377667	pos
13.94_515.3'	515.376871	13.9377667	pos
13.94_522.8'	522.872415	13.9377667	pos
13.94_523.3'	523.374334	13.9377667	pos
13.96_142.1:	142.122562	13.9559833	pos
13.96_480.3	498.381601	13.9559833	pos
13.96_480.8	503.838675	13.9559833	pos
13.96_500.8!	500.859224	13.9559833	pos
13.96_501.3(	501.360877	13.9559833	pos
13.97_129.9:	129.936083	13.97315	neg
13.97_155.1	155.142844	13.9744167	pos
13.97_512.3!	512.397901	13.9744167	pos
13.99_157.9!	157.954809	13.99205	neg
13.99_173.0'	173.078389	13.9923667	pos
13.99_458.3:	476.36867	13.9923667	pos
13.99_467.8!	467.855147	13.9923667	pos
13.99_478.8	478.846085	13.9923667	pos
13.99_479.3	479.347958	13.9923667	pos
13.99_490.3!	490.383753	13.9923667	pos
13.99_492.8(	492.861884	13.9923667	pos
13.99_594.4(	617.458931	13.9923667	pos
13.99_640.5:	640.534685	13.9923667	pos
14.01_458.8:	476.870237	14.0101167	pos
14.01_772.6:	772.610757	14.0101167	pos
14.03_436.3:	459.310569	14.02765	pos
14.03_495.3:	495.339388	14.02765	pos
14.05_456.8:	456.83306	14.0456833	pos
14.05_457.3:	457.335108	14.0456833	pos
14.05_872.6	890.676533	14.0456833	pos
14.07_194.9!	194.988141	14.06525	neg
14.08_128.1:	128.118133	14.0819167	pos

14.08_158.0	158.092511	14.0819167	pos
14.08_828.6	846.649917	14.0819167	pos
14.09_280.9	280.962873	14.0865333	neg
14.14_464.4	464.430796	14.1357167	pos
14.14_599.4	599.448508	14.1357167	pos
14.14_802.6	802.624121	14.1357167	pos
14.15_142.0	142.061181	14.1537833	pos
14.15_179.1	179.129036	14.1537833	pos
14.15_226.9	226.994454	14.1513833	neg
14.15_638.6	638.655049	14.1537833	pos
14.15_661.9	661.958394	14.1537833	pos
14.16_180.8	180.842013	14.1603833	neg
14.16_209.0	209.003778	14.1603833	neg
14.17_140.1	140.117903	14.1715833	pos
14.17_206.1	206.103532	14.1715833	pos
14.17_229.1	229.14326	14.1715833	pos
14.19_639.9	639.945207	14.1897833	pos
14.19_728.5	728.588388	14.1897833	pos
14.21_116.9	116.956638	14.2110167	neg
14.21_610.1	628.193981	14.2076333	pos
14.21_656.2	656.225293	14.2076333	pos
14.23_166.0	166.097341	14.2258	pos
14.23_550.4	568.477771	14.2258	pos
14.23_615.4	615.455259	14.2258	pos
14.24_128.1	128.118144	14.2436833	pos
14.24_167.1	167.128902	14.2436833	pos
14.24_506.4	529.407149	14.2436833	pos
14.24_742.5	742.570289	14.2436833	pos
14.26_133.0	133.013998	14.2565833	neg
14.26_146.9	146.927405	14.2565833	neg
14.26_321.2	321.239604	14.2613833	pos
14.26_329.2	329.242225	14.2613833	pos
14.26_345.2	345.281357	14.2565833	neg

14.26_418.3(	441.354526	14.2613833	pos
14.26_462.3!	485.380822	14.2613833	pos
14.26_464.4:	464.430885	14.2613833	pos
14.26_508.4!	508.457116	14.2613833	pos
14.26_828.5!	828.550466	14.2613833	pos
14.27_100.9:	100.933401	14.2674833	neg
14.27_115.9:	115.920695	14.2674833	neg
14.27_144.9:	144.923198	14.2674833	neg
14.27_319.2‹	319.240576	14.2674833	neg
14.27_381.1!	381.19474	14.2674833	neg
14.28_130.9:	130.92875	14.2783333	neg
14.28_155.8(	155.865383	14.2783333	neg
14.28_160.9:	160.935255	14.2783333	neg
14.28_171.9!	171.980772	14.2783333	neg
14.28_379.2!	379.251942	14.2783333	neg
14.28_480.5:	480.513623	14.2796333	pos
14.29_153.8(	153.868311	14.2884333	neg
14.29_229.9(	229.900763	14.2884333	neg
14.30_664.5:	682.545343	14.2978	pos
14.32_310.3:	310.310003	14.3159167	pos
14.33_553.3!	571.428843	14.3338167	pos
14.33_573.9(	573.906189	14.3338167	pos
14.33_574.4(	574.40812	14.3338167	pos
14.33_696.5:	714.571815	14.3338167	pos
14.34_115.8!	115.880268	14.3415	neg
14.37_311.2!	311.286382	14.37455	neg
14.37_549.9:	549.917421	14.3693667	pos
14.37_551.8!	551.893096	14.3693667	pos
14.38_124.0(	124.007025	14.3838667	neg
14.39_355.2!	355.257998	14.3875333	pos
14.39_488.4(	511.396474	14.3875333	pos
14.39_532.4:	550.46726	14.3875333	pos
14.39_578.4!	578.498357	14.3875333	pos

14.41_171.1(	171.101614	14.4055833	pos
14.41_279.0!	279.093069	14.4055833	pos
14.41_444.3!	467.370178	14.4055833	pos
14.41_490.4	490.446117	14.4055833	pos
14.41_529.8!	529.880138	14.4055833	pos
14.42_402.3!	402.393601	14.4236667	pos
14.42_527.4(	527.402882	14.4236667	pos
14.44_652.5:	670.545471	14.4418	pos
14.46_350.2!	333.278316	14.4597833	pos
14.46_507.8(	507.867207	14.4597833	pos
14.46_623.6(	662.571128	14.4597833	pos
14.48_260.8(	260.860283	14.4766667	neg
14.48_372.2!	372.292672	14.47795	pos
14.53_134.0	134.071091	14.53075	pos
14.53_195.1(	195.101485	14.53075	pos
14.55_136.0!	136.086866	14.5488333	pos
14.55_359.2!	359.293731	14.5488333	pos
14.55_511.3!	511.396508	14.5488333	pos
14.55_648.6:	648.624983	14.5488333	pos
14.57_167.1:	167.128899	14.5668	pos
14.57_619.4!	619.490614	14.5668	pos
14.58_100.0!	100.050847	14.5847667	pos
14.58_583.2!	583.25376	14.5847667	pos
14.60_584.2(	585.269255	14.60285	pos
14.60_600.2!	600.256285	14.60285	pos
14.64_568.4:	568.42688	14.63725	pos
14.67_142.0!	142.097336	14.67175	pos
14.67_518.6:	518.630272	14.6654333	neg
14.71_124.1:	124.132159	14.7074	pos
14.72_228.1!	228.195564	14.72495	pos
14.72_338.3:	338.32485	14.72495	pos
14.72_411.3!	411.394145	14.72495	pos
14.74_320.3(	338.341288	14.7422167	pos

14.74_340.34	340.348017	14.7422167	pos
14.74_353.32	354.336208	14.7422167	pos
14.74_675.67	675.67528	14.7422167	pos
14.74_708.51	708.510538	14.7422167	pos
14.76_202.10	202.108546	14.7596667	pos
14.79_129.06	129.065567	14.7946833	pos
14.79_142.06	142.06115	14.7946833	pos
14.79_906.25	906.256843	14.7946833	pos
14.80_199.85	199.850707	14.80345	neg
14.80_214.85	214.859207	14.80345	neg
14.80_216.85	216.853439	14.80345	neg
14.81_100.05	100.050914	14.8127	pos
14.81_102.05	102.055321	14.8127	pos
14.81_169.10	169.108338	14.8127	pos
14.82_100.92	100.925792	14.8209333	neg
14.82_116.92	116.928228	14.8209333	neg
14.82_314.78	314.784528	14.8209333	neg
14.82_316.77	316.77873	14.8209333	neg
14.82_414.70	414.709749	14.8209333	neg
14.82_416.70	416.704152	14.8209333	neg
14.82_516.62	516.629348	14.8209333	neg
14.83_741.19	741.193756	14.8305333	pos
14.85_728.58	728.587164	14.84795	pos
14.87_667.17	667.175166	14.8652833	pos
14.90_166.08	149.082074	14.9013667	pos
14.90_181.10	181.108215	14.9013667	pos
14.97_593.59	593.597276	14.97215	pos
15.01_126.06	126.066208	15.0078167	pos
15.01_312.32	312.325692	15.0078167	pos
15.06_627.53	627.532185	15.0609333	neg
15.10_116.95	116.956909	15.0963667	neg
15.11_100.06	100.063507	15.1136333	pos
15.13_593.15	593.15681	15.1315833	pos

15.15_174.0:	174.015403	15.1477333	neg
15.18_447.3‹	447.346452	15.1842167	pos
15.24_190.1(	190.108477	15.23725	pos
15.26_403.2:	403.232317	15.25515	pos
15.29_176.1:	176.118226	15.2891167	pos
15.29_508.1(	508.109515	15.2907	neg
15.29_557.4!	557.457528	15.2907	neg
15.29_663.4!	663.452574	15.2891167	pos
15.29_709.6:	709.61019	15.2907	neg
15.31_620.4!	620.45364	15.31075	neg
15.38_165.1:	165.126813	15.3778833	pos
15.40_445.3(	445.366859	15.3950333	pos
15.41_654.5'	654.579093	15.4076333	neg
15.45_142.0(	142.061264	15.4477	pos
2.00_195.98(	195.986097	2.0024	neg
2.00_227.06{	227.068879	2.0024	neg
2.00_378.09'	378.097623	2.0024	neg
2.00_388.87'	388.877806	2.0024	neg
2.00_410.86(	410.8609	2.0024	neg
2.00_442.07‹	442.074446	2.0024	neg
2.00_692.94‹	692.944755	2.0024	neg
2.00_750.97(	750.976735	2.0024	neg
2.01_104.04!	104.049861	2.00526667	pos
2.01_112.95(	112.956325	2.00526667	pos
2.01_116.97:	116.972174	2.00526667	pos
2.01_201.00{	201.008293	2.00526667	pos
2.01_209.15:	209.153356	2.00526667	pos
2.01_223.98{	223.988301	2.00526667	pos
2.01_290.17(	290.17095	2.00526667	pos
2.01_315.67(	315.676497	2.00526667	pos
2.01_545.58{	545.588463	2.00526667	pos
2.02_120.04‹	120.044915	2.02305	pos
2.02_133.08!	133.085947	2.02305	pos

2.02_136.07	137.079077	2.02305	pos
2.02_146.06	146.060976	2.02081667	neg
2.02_159.89	159.89367	2.02081667	neg
2.02_164.07	164.073946	2.02305	pos
2.02_179.06	179.067694	2.02305	pos
2.02_191.05	209.092021	2.02305	pos
2.02_230.06	231.073906	2.02305	pos
2.02_279.67	279.675708	2.02305	pos
2.02_321.04	321.043226	2.02305	pos
2.02_354.12	354.129198	2.02305	pos
2.02_357.71	357.710746	2.02305	pos
2.02_360.04	360.042496	2.02081667	neg
2.02_388.17	388.172603	2.02081667	neg
2.02_389.03	389.030502	2.02305	pos
2.02_390.08	390.082098	2.02081667	neg
2.02_390.18	390.186919	2.02305	pos
2.02_391.08	392.096467	2.02305	pos
2.02_401.01	401.010518	2.02081667	neg
2.02_411.03	411.039671	2.02081667	neg
2.02_416.99	416.996422	2.02081667	neg
2.02_428.02	428.029645	2.02081667	neg
2.02_437.14	437.144087	2.02081667	neg
2.02_468.99	468.998272	2.02081667	neg
2.02_496.01	496.017415	2.02081667	neg
2.02_536.98	536.986122	2.02081667	neg
2.02_545.25	545.254629	2.02305	pos
2.02_547.01	547.014735	2.02081667	neg
2.02_556.96	556.969649	2.02081667	neg
2.02_573.11	573.119017	2.02081667	neg
2.02_615.00	615.002023	2.02081667	neg
2.02_624.95	624.957311	2.02081667	neg
2.02_682.98	682.989565	2.02081667	neg
2.04_112.98	112.98215	2.03931667	neg

2.04_114.02	114.024191	2.03931667	neg
2.04_133.05	133.050448	2.03931667	neg
2.04_144.10	144.101815	2.04021667	pos
2.04_156.04	157.047235	2.04021667	pos
2.04_181.08	181.083242	2.04021667	pos
2.04_201.03	201.037723	2.03931667	neg
2.04_269.02	269.025474	2.03931667	neg
2.04_292.05	292.055112	2.03931667	neg
2.04_327.02	327.026902	2.03931667	neg
2.04_329.18	329.18167	2.04021667	pos
2.04_337.01	337.012992	2.03931667	neg
2.04_405.00	405.00024	2.03931667	neg
2.04_566.76	566.765183	2.04021667	pos
2.04_666.08	666.088675	2.03931667	neg
2.04_734.07	734.075636	2.03931667	neg
2.06_530.11	530.113219	2.0574	neg
2.06_530.95	530.953678	2.0574	neg
2.06_598.10	598.100694	2.0574	neg
2.06_649.93	649.939161	2.0574	neg
2.07_182.08	182.080996	2.07461667	pos
2.07_255.15	255.157639	2.07461667	pos
2.07_320.16	320.163682	2.07461667	pos
2.08_299.97	299.973066	2.07525	neg
2.08_331.95	331.957969	2.07525	neg
2.08_510.96	510.969537	2.07525	neg
2.08_513.96	513.963849	2.07525	neg
2.09_120.03	120.038284	2.09156667	pos
2.09_166.08	166.08052	2.09156667	pos
2.09_249.03	497.062796	2.0924	neg
2.09_306.96	306.962206	2.0924	neg
2.09_326.99	326.991921	2.0924	neg
2.09_367.96	367.960447	2.0924	neg
2.09_377.98	377.989172	2.0924	neg

2.09_412.062	412.062177	2.0924	neg
2.09_425.992	425.992616	2.0924	neg
2.09_436.021	436.021166	2.0924	neg
2.09_445.976	445.976641	2.0924	neg
2.09_629.039	629.038967	2.0924	neg
2.09_681.040	681.040784	2.0924	neg
2.09_691.069	691.069562	2.0924	neg
2.09_827.044	827.044691	2.0924	neg
2.09_837.000	837.000114	2.0924	neg
2.09_895.031	895.031526	2.0924	neg
2.11_102.046	102.046807	2.10798333	pos
2.11_102.047	120.080944	2.10798333	pos
2.11_119.073	119.073427	2.10798333	pos
2.11_120.123	120.123596	2.10798333	pos
2.11_121.084	121.08411	2.10798333	pos
2.11_147.045	147.044953	2.11061667	neg
2.11_165.078	148.075611	2.10798333	pos
2.11_166.038	166.038117	2.10798333	pos
2.11_166.078	166.078056	2.11061667	neg
2.11_187.060	188.068106	2.10798333	pos
2.11_202.033	202.033034	2.11061667	neg
2.11_222.030	222.030092	2.11061667	neg
2.11_224.027	224.027156	2.11061667	neg
2.11_232.058	232.058841	2.11061667	neg
2.11_260.054	260.054105	2.11061667	neg
2.11_265.003	266.010834	2.10798333	pos
2.11_284.021	284.021028	2.11061667	neg
2.11_290.017	290.017664	2.11061667	neg
2.11_304.116	304.116478	2.11061667	neg
2.11_307.010	352.008776	2.11061667	neg
2.11_308.006	308.006233	2.11061667	neg
2.11_310.001	308.994019	2.11061667	neg
2.11_330.028	330.028755	2.11061667	neg

2.11_344.03	344.036119	2.11061667	neg
2.11_344.07	344.072331	2.11061667	neg
2.11_351.13	351.132389	2.11061667	neg
2.11_352.01	352.0171	2.11061667	neg
2.11_352.98	352.983669	2.11061667	neg
2.11_358.00	358.005068	2.11061667	neg
2.11_367.02	366.018097	2.11061667	neg
2.11_368.03	368.0337	2.11061667	neg
2.11_373.99	373.989979	2.11061667	neg
2.11_384.07	407.065913	2.10798333	pos
2.11_385.00	769.012585	2.11061667	neg
2.11_385.02	385.02394	2.11061667	neg
2.11_397.11	397.110707	2.10798333	pos
2.11_409.09	409.091058	2.11061667	neg
2.11_419.12	419.119959	2.11061667	neg
2.11_420.97	420.971701	2.11061667	neg
2.11_421.12	421.125721	2.11061667	neg
2.11_428.06	428.067137	2.11061667	neg
2.11_429.07	429.075108	2.11061667	neg
2.11_436.10	436.109652	2.11061667	neg
2.11_441.97	441.977128	2.11061667	neg
2.11_446.06	446.065153	2.11061667	neg
2.11_474.07	474.072838	2.11061667	neg
2.11_493.06	493.064002	2.11061667	neg
2.11_504.09	504.097742	2.11061667	neg
2.11_514.05	514.052489	2.11061667	neg
2.11_538.19	538.193327	2.11061667	neg
2.11_540.04	540.044801	2.11061667	neg
2.11_545.06	545.065924	2.11061667	neg
2.11_548.14	548.148615	2.11061667	neg
2.11_550.16	550.163139	2.10798333	pos
2.11_561.05	561.051005	2.11061667	neg
2.11_562.05	562.055895	2.11061667	neg

2.11_564.19	564.192944	2.11061667	neg
2.11_571.13	594.126533	2.10798333	pos
2.11_572.08	572.08517	2.11061667	neg
2.11_582.04	582.040424	2.11061667	neg
2.11_584.17	584.171305	2.10798333	pos
2.11_606.18	606.181037	2.11061667	neg
2.11_613.05	613.053467	2.11061667	neg
2.11_616.13	616.135997	2.11061667	neg
2.11_633.03	633.037577	2.11061667	neg
2.11_640.07	640.072804	2.11061667	neg
2.11_664.13	664.139709	2.11061667	neg
2.11_674.16	674.168214	2.11061667	neg
2.11_684.12	684.123599	2.11061667	neg
2.11_701.02	701.025119	2.11061667	neg
2.11_708.05	708.059697	2.11061667	neg
2.11_725.25	725.25444	2.11061667	neg
2.11_732.12	732.127184	2.11061667	neg
2.11_735.20	735.209521	2.11061667	neg
2.11_742.15	742.1559	2.11061667	neg
2.11_752.11	752.111179	2.11061667	neg
2.11_781.18	781.186307	2.10798333	pos
2.11_793.24	793.241686	2.11061667	neg
2.11_803.19	803.197636	2.11061667	neg
2.11_810.14	810.143471	2.11061667	neg
2.11_820.09	820.098911	2.11061667	neg
2.11_871.18	871.184238	2.11061667	neg
2.11_878.13	878.130466	2.11061667	neg
2.11_888.08	888.086031	2.11061667	neg
2.11_904.98	904.987481	2.11061667	neg
2.11_929.21	929.216656	2.11061667	neg
2.11_946.11	946.117675	2.11061667	neg
2.11_980.30	980.303407	2.11061667	neg
2.11_997.20	997.203716	2.11061667	neg

2.12_168.092	168.092206	2.12441667	pos
2.13_131.003	131.003065	2.1282	neg
2.13_158.045	158.045643	2.1282	neg
2.13_185.042	185.042529	2.1282	neg
2.13_307.961	307.961527	2.1282	neg
2.13_309.961	309.96186	2.1282	neg
2.13_334.957	334.957057	2.1282	neg
2.13_360.002	360.002018	2.1282	neg
2.13_434.005	434.005591	2.1282	neg
2.13_453.011	453.011481	2.1282	neg
2.13_462.966	462.966939	2.1282	neg
2.13_759.056	759.056921	2.1282	neg
2.14_138.091	138.091449	2.14131667	pos
2.14_195.610	195.610249	2.14131667	pos
2.14_218.138	218.138538	2.14131667	pos
2.14_255.157	255.157885	2.14131667	pos
2.14_278.037	278.037412	2.14131667	pos
2.14_327.131	328.138935	2.14131667	pos
2.14_346.024	346.024685	2.14131667	pos
2.14_372.101	372.101327	2.14131667	pos
2.15_174.055	174.0558	2.1466	neg
2.15_209.006	209.006532	2.1466	neg
2.15_260.020	260.020782	2.1466	neg
2.15_325.041	325.041534	2.1466	neg
2.15_326.124	326.1244	2.1466	neg
2.15_328.008	328.008266	2.1466	neg
2.15_493.979	493.979894	2.1466	neg
2.15_503.935	503.93566	2.1466	neg
2.15_509.964	509.964824	2.1466	neg
2.15_513.185	513.185104	2.1466	neg
2.15_520.998	520.998775	2.1466	neg
2.15_561.967	561.967366	2.1466	neg
2.15_571.996	571.995989	2.1466	neg

2.15_577.95	577.952968	2.1466	neg
2.15_581.95	581.951518	2.1466	neg
2.15_588.98	588.986653	2.1466	neg
2.15_629.95	629.954907	2.1466	neg
2.15_639.98	639.983528	2.1466	neg
2.15_656.97	656.974282	2.1466	neg
2.15_707.97	707.970979	2.1466	neg
2.15_717.92	717.926844	2.1466	neg
2.15_775.95	775.958457	2.1466	neg
2.15_785.91	785.914424	2.1466	neg
2.16_167.01	167.01222	2.15823333	pos
2.16_193.04	194.048206	2.15823333	pos
2.16_212.12	212.120962	2.15823333	pos
2.17_276.99	276.994025	2.16503333	neg
2.17_462.09	462.099533	2.16503333	neg
2.18_105.52	105.520257	2.17555	pos
2.18_112.95	112.95632	2.17555	pos
2.18_142.94	142.948103	2.17555	pos
2.18_153.04	153.040933	2.17555	pos
2.18_161.96	161.969924	2.17555	pos
2.18_178.97	178.977532	2.18363333	neg
2.18_180.98	180.989914	2.17555	pos
2.18_199.97	199.975389	2.17555	pos
2.18_273.16	273.165684	2.17555	pos
2.18_330.14	330.144309	2.17555	pos
2.18_652.24	652.244272	2.17555	pos
2.19_143.14	143.143162	2.19353333	pos
2.19_169.09	169.096845	2.19353333	pos
2.19_171.97	154.966967	2.19353333	pos
2.19_184.09	184.094392	2.19353333	pos
2.19_272.16	272.160398	2.19353333	pos
2.20_232.94	232.944147	2.20205	neg
2.20_370.13	370.136639	2.20205	neg

2.21_155.07	155.070408	2.21128333	pos
2.21_169.96	169.965475	2.21128333	pos
2.21_190.07	190.071276	2.21128333	pos
2.21_221.09	221.09208	2.21128333	pos
2.21_273.16	273.167408	2.21128333	pos
2.21_352.12	352.124459	2.21128333	pos
2.22_182.89	182.896328	2.22055	neg
2.22_197.88	197.889508	2.22055	neg
2.23_142.02	142.026245	2.22953333	pos
2.23_143.03	143.033936	2.22953333	pos
2.23_145.99	145.990011	2.22953333	pos
2.23_167.03	167.03407	2.22953333	pos
2.23_236.64	236.64192	2.22953333	pos
2.23_238.10	238.107372	2.22953333	pos
2.23_264.64	264.642372	2.22953333	pos
2.23_287.88	287.888781	2.22953333	pos
2.23_491.70	491.706617	2.22953333	pos
2.23_509.74	509.744205	2.22953333	pos
2.24_136.90	136.909611	2.23906667	neg
2.24_180.89	180.89947	2.23906667	neg
2.24_226.93	226.937121	2.23906667	neg
2.24_258.91	258.915654	2.23906667	neg
2.24_312.87	312.875975	2.23906667	neg
2.24_437.87	437.872508	2.23906667	neg
2.24_608.80	608.80886	2.23906667	neg
2.25_172.95	172.956463	2.24641667	pos
2.25_188.03	188.035159	2.24641667	pos
2.25_249.19	249.196117	2.24641667	pos
2.25_266.63	266.63988	2.24641667	pos
2.25_314.63	314.632312	2.24641667	pos
2.25_376.82	376.820996	2.24641667	pos
2.25_457.72	480.715361	2.24641667	pos
2.26_103.92	103.920074	2.2575	neg

2.26_144.943	144.943122	2.2575	neg
2.26_146.938	146.938511	2.2575	neg
2.26_170.870	170.870579	2.2575	neg
2.26_173.117	173.117209	2.26408333	pos
2.26_186.956	186.956261	2.26408333	pos
2.26_190.928	190.928265	2.2575	neg
2.26_231.621	231.621132	2.26408333	pos
2.26_251.155	251.155498	2.26408333	pos
2.26_316.684	316.684392	2.26408333	pos
2.26_318.871	318.871021	2.2575	neg
2.26_360.974	360.974085	2.2575	neg
2.26_365.541	365.541702	2.26408333	pos
2.28_102.948	102.94866	2.27598333	neg
2.28_107.950	107.950861	2.28218333	pos
2.28_221.642	221.642304	2.28218333	pos
2.28_364.873	364.87323	2.28218333	pos
2.28_365.207	365.207442	2.28218333	pos
2.29_180.033	180.033055	2.29455	neg
2.29_469.839	469.839181	2.29455	neg
2.29_663.846	663.84667	2.29455	neg
2.30_129.958	129.958744	2.30001667	pos
2.30_161.059	161.059796	2.30001667	pos
2.30_220.108	220.108218	2.30001667	pos
2.30_281.649	281.649363	2.30001667	pos
2.30_303.166	303.166298	2.30001667	pos
2.31_331.091	331.091057	2.31311667	neg
2.32_111.055	111.055657	2.31811667	pos
2.32_119.943	119.943421	2.31811667	pos
2.32_126.136	126.136671	2.31811667	pos
2.32_159.027	159.027849	2.31811667	pos
2.32_177.091	177.091308	2.31811667	pos
2.32_427.224	427.224475	2.31811667	pos
2.32_427.726	427.726045	2.31811667	pos

2.33_272.02[illegible]	272.020961	2.3315	neg
2.33_605.81[illegible]	605.814634	2.3315	neg
2.34_135.94[illegible]	135.945469	2.33636667	pos
2.34_188.01[illegible]	206.048276	2.33636667	pos
2.34_252.63[illegible]	252.63699	2.33636667	pos
2.34_318.88[illegible]	318.886351	2.33636667	pos
2.34_345.67[illegible]	346.677394	2.33636667	pos
2.34_358.69[illegible]	358.694867	2.33636667	pos
2.34_380.15[illegible]	380.151791	2.33636667	pos
2.35_140.96[illegible]	140.966653	2.35463333	pos
2.35_142.95[illegible]	142.955768	2.35463333	pos
2.35_146.94[illegible]	146.946305	2.34998333	neg
2.35_179.14[illegible]	179.143126	2.35463333	pos
2.35_191.10[illegible]	191.104071	2.35463333	pos
2.35_207.91[illegible]	207.918415	2.34998333	neg
2.35_264.16[illegible]	264.16864	2.35463333	pos
2.35_369.88[illegible]	369.885039	2.34998333	neg
2.35_379.65[illegible]	379.650268	2.35463333	pos
2.35_384.86[illegible]	384.862193	2.34998333	neg
2.35_452.84[illegible]	452.849728	2.34998333	neg
2.37_125.06[illegible]	125.060608	2.36853333	neg
2.37_163.92[illegible]	163.928628	2.36853333	neg
2.37_167.06[illegible]	167.068037	2.37246667	pos
2.37_210.98[illegible]	210.983387	2.36853333	neg
2.37_333.86[illegible]	333.864519	2.36853333	neg
2.37_440.86[illegible]	440.869253	2.37246667	pos
2.37_446.90[illegible]	446.905736	2.36853333	neg
2.37_472.83[illegible]	472.833334	2.36853333	neg
2.37_501.86[illegible]	501.86288	2.36853333	neg
2.37_615.84[illegible]	615.843078	2.36853333	neg
2.39_129.06[illegible]	129.066001	2.39026667	pos
2.39_131.07[illegible]	131.070469	2.39026667	pos
2.39_133.10[illegible]	133.10523	2.39026667	pos

2.39_134.04	134.044919	2.39026667	pos
2.39_137.04	137.045939	2.39026667	pos
2.39_140.06	140.068494	2.39026667	pos
2.39_142.96	142.966525	2.39026667	pos
2.39_146.99	148.003969	2.39026667	pos
2.39_147.11	147.1169	2.39026667	pos
2.39_150.05	150.058251	2.39026667	pos
2.39_153.04	153.040949	2.39026667	pos
2.39_167.03	167.03408	2.39026667	pos
2.39_175.11	175.119017	2.39026667	pos
2.39_179.93	179.936065	2.387	neg
2.39_182.08	182.081572	2.39026667	pos
2.39_186.95	186.956249	2.39026667	pos
2.39_200.04	200.047333	2.39026667	pos
2.39_204.12	204.123149	2.39026667	pos
2.39_299.06	299.061721	2.39026667	pos
2.39_316.87	316.874525	2.387	neg
2.39_326.90	326.903201	2.387	neg
2.39_394.89	394.890454	2.387	neg
2.39_462.87	462.87821	2.387	neg
2.39_530.86	530.865601	2.387	neg
2.39_598.85	598.853352	2.387	neg
2.39_662.84	662.84205	2.387	neg
2.41_187.06	187.06092	2.40555	neg
2.41_192.03	192.032574	2.40855	pos
2.41_221.65	221.654816	2.40855	pos
2.41_236.90	236.906926	2.40855	pos
2.41_260.16	260.160508	2.40855	pos
2.41_276.89	276.896812	2.40855	pos
2.41_492.91	492.91093	2.40855	pos
2.41_505.86	505.860012	2.40555	neg
2.41_569.84	569.849814	2.40555	neg
2.42_249.06	249.06265	2.42373333	neg

2.42_278.114	278.11451	2.42373333	neg
2.42_386.859	386.858989	2.42373333	neg
2.42_401.852	401.852	2.42373333	neg
2.42_411.880	411.880814	2.42373333	neg
2.43_114.956	114.956559	2.42631667	pos
2.43_151.075	151.075335	2.42631667	pos
2.43_173.984	173.984894	2.42631667	pos
2.43_187.111	187.111734	2.42631667	pos
2.43_203.106	203.10688	2.42631667	pos
2.43_237.157	237.157647	2.42631667	pos
2.43_250.070	273.059472	2.42631667	pos
2.43_280.129	280.129279	2.42631667	pos
2.44_113.960	113.960695	2.4445	pos
2.44_181.083	181.083813	2.4445	pos
2.44_215.655	215.65499	2.4445	pos
2.44_248.901	248.901397	2.4445	pos
2.44_537.827	537.827063	2.44226667	neg
2.46_115.968	115.967981	2.46275	pos
2.46_131.929	131.929918	2.46275	pos
2.46_296.113	296.113782	2.46066667	neg
2.46_318.166	318.16637	2.46275	pos
2.46_318.668	318.668349	2.46275	pos
2.46_605.814	605.81452	2.46066667	neg
2.48_137.096	137.096235	2.48101667	pos
2.48_167.055	167.055548	2.48101667	pos
2.48_176.959	176.959451	2.48101667	pos
2.48_199.987	199.987907	2.48101667	pos
2.48_209.078	209.078402	2.48101667	pos
2.48_217.119	217.119123	2.47923333	neg
2.48_227.102	227.102508	2.48101667	pos
2.48_289.655	289.655849	2.48101667	pos
2.48_666.840	666.840795	2.47923333	neg
2.48_734.828	734.828513	2.47923333	neg

2.50_140.951	140.951399	2.49885	pos
2.50_163.075	163.075447	2.49885	pos
2.50_188.091	188.091815	2.49885	pos
2.50_209.153	209.153655	2.49885	pos
2.50_234.911	234.911619	2.49885	pos
2.50_251.000	251.000231	2.49885	pos
2.50_288.191	288.191745	2.49885	pos
2.50_289.827	289.827601	2.49885	pos
2.50_547.855	547.855933	2.49773333	neg
2.52_135.945	135.945474	2.51663333	pos
2.52_136.953	136.95332	2.51663333	pos
2.52_145.990	145.990001	2.51663333	pos
2.52_163.111	163.111799	2.51663333	pos
2.52_208.647	208.647124	2.51663333	pos
2.52_276.955	276.955121	2.51621667	neg
2.52_304.894	304.894265	2.51663333	pos
2.52_343.893	343.89341	2.51621667	neg
2.52_403.716	403.716527	2.51663333	pos
2.53_107.950	107.950871	2.53481667	pos
2.53_107.967	107.967272	2.53481667	pos
2.53_137.046	137.045951	2.53481667	pos
2.53_186.956	186.956176	2.53481667	pos
2.53_223.131	223.131611	2.53481667	pos
2.53_226.974	226.974876	2.53481667	pos
2.53_257.174	257.173988	2.53481667	pos
2.53_275.905	275.905848	2.5348	neg
2.53_479.868	479.86824	2.5348	neg
2.53_656.812	656.812331	2.5348	neg
2.55_171.174	171.174269	2.5526	pos
2.55_173.080	173.080837	2.5526	pos
2.55_275.100	275.100102	2.5526	pos
2.55_372.881	372.881704	2.5526	pos
2.55_641.147	641.147324	2.55331667	neg

2.57_140.06	140.068504	2.57026667	pos
2.57_297.08	297.082477	2.57026667	pos
2.57_436.70	436.709256	2.57026667	pos
2.57_510.75	510.755886	2.57026667	pos
2.57_520.83	520.836751	2.5718	neg
2.59_145.08	145.086792	2.59035	neg
2.59_212.11	212.115539	2.58846667	pos
2.59_501.86	501.863004	2.59035	neg
2.61_142.93	142.938599	2.6067	pos
2.61_245.11	245.114142	2.60876667	neg
2.61_433.87	433.875331	2.60876667	neg
2.61_443.83	443.830476	2.60876667	neg
2.62_161.05	161.059803	2.62451667	pos
2.62_245.64	245.647281	2.62451667	pos
2.64_119.04	119.049366	2.64221667	pos
2.64_173.98	173.98501	2.64221667	pos
2.64_262.15	262.153411	2.64221667	pos
2.64_405.17	405.174806	2.64221667	pos
2.65_133.01	133.014078	2.64586667	neg
2.65_276.95	276.955146	2.64586667	neg
2.66_105.03	105.033975	2.66041667	pos
2.66_107.96	107.967229	2.66041667	pos
2.66_142.96	142.966606	2.66041667	pos
2.66_186.95	186.956256	2.66041667	pos
2.66_248.88	248.887012	2.66431667	neg
2.68_116.97	116.972156	2.67816667	pos
2.68_136.95	136.953348	2.67816667	pos
2.68_201.11	201.112471	2.67816667	pos
2.68_203.10	203.106806	2.67816667	pos
2.68_374.15	374.156802	2.68286667	neg
2.68_376.17	376.171274	2.67816667	pos
2.68_441.70	441.705164	2.67816667	pos
2.70_229.03	229.032657	2.7013	neg

2.70_279.66[illegible]	279.665869	2.69571667	pos
2.70_286.09[illegible]	286.090749	2.7013	neg
2.70_303.08[illegible]	303.080831	2.7013	neg
2.70_344.04[illegible]	344.049215	2.7013	neg
2.70_377.69[illegible]	377.690248	2.69571667	pos
2.70_378.19[illegible]	378.191744	2.69571667	pos
2.70_412.03[illegible]	412.036898	2.7013	neg
2.70_626.02[illegible]	626.027866	2.7013	neg
2.71_133.08[illegible]	133.086007	2.71386667	pos
2.71_158.53[illegible]	158.537381	2.71386667	pos
2.71_185.11[illegible]	185.117358	2.71386667	pos
2.71_219.11[illegible]	220.117943	2.71386667	pos
2.72_276.06[illegible]	276.061761	2.7196	neg
2.72_354.07[illegible]	354.078165	2.7196	neg
2.72_422.06[illegible]	422.065725	2.7196	neg
2.72_490.05[illegible]	490.053049	2.7196	neg
2.72_558.04[illegible]	558.040471	2.7196	neg
2.73_115.11[illegible]	115.111938	2.73206667	pos
2.73_128.14[illegible]	128.143556	2.73206667	pos
2.74_396.18[illegible]	396.188511	2.73811667	neg
2.74_588.82[illegible]	588.82465	2.73811667	neg
2.75_137.04[illegible]	137.045865	2.74981667	pos
2.75_169.08[illegible]	169.086014	2.74981667	pos
2.75_185.11[illegible]	185.117396	2.74981667	pos
2.75_212.14[illegible]	212.14932	2.74981667	pos
2.75_231.08[illegible]	231.083841	2.74981667	pos
2.76_168.03[illegible]	168.030066	2.75626667	neg
2.76_802.81[illegible]	802.815988	2.75626667	neg
2.77_129.95[illegible]	129.958736	2.76745	pos
2.77_138.07[illegible]	138.0715	2.76745	pos
2.77_171.13[illegible]	171.137986	2.76745	pos
2.77_182.06[illegible]	182.061739	2.77471667	neg
2.77_187.06[illegible]	187.060916	2.77471667	neg

2.77_222.14(	240.180507	2.76745	pos
2.77_225.06:	225.061426	2.77471667	neg
2.77_276.01i	276.018173	2.77471667	neg
2.77_287.88i	287.888788	2.76745	pos
2.77_521.92:	521.921576	2.76745	pos
2.79_134.06(	134.060284	2.78548333	pos
2.79_157.08:	157.08371	2.78548333	pos
2.79_173.98-	173.984923	2.78548333	pos
2.79_231.12!	231.128955	2.78548333	pos
2.79_231.15!	231.159145	2.78548333	pos
2.79_300.68!	300.68948	2.78548333	pos
2.79_683.83(	683.830439	2.7932	neg
2.80_112.95(	112.956336	2.80321667	pos
2.80_167.03-	167.034067	2.80321667	pos
2.80_408.20'	408.207094	2.80321667	pos
2.80_408.54:	408.5414	2.80321667	pos
2.81_118.04(	118.040892	2.81173333	neg
2.81_200.96!	200.965015	2.81173333	neg
2.81_204.12:	204.123796	2.81173333	neg
2.82_129.95:	129.951636	2.82056667	pos
2.82_135.94!	135.945445	2.82056667	pos
2.82_143.11'	143.117861	2.82056667	pos
2.82_176.95!	176.959431	2.82056667	pos
2.83_187.10i	187.108522	2.83016667	neg
2.83_201.12-	201.124264	2.83016667	neg
2.84_126.05!	126.055157	2.8388	pos
2.84_131.07(	131.070464	2.8388	pos
2.84_201.07:	201.073186	2.8388	pos
2.84_206.13i	206.138719	2.8388	pos
2.84_233.08'	233.087198	2.8388	pos
2.84_274.16-	274.164854	2.8388	pos
2.85_597.85'	597.857242	2.84868333	neg
2.86_209.07i	209.078365	2.85705	pos

2.87_181.12:	181.122454	2.8749	pos
2.87_221.09:	221.092936	2.86726667	neg
2.87_247.11:	247.111295	2.8749	pos
2.87_248.90:	248.901328	2.8749	pos
2.87_281.16!	281.164995	2.8749	pos
2.87_357.06:	357.068009	2.86726667	neg
2.87_391.71:	391.711276	2.8749	pos
2.87_464.68·	464.684839	2.8749	pos
2.89_105.04!	105.045141	2.89255	pos
2.89_109.06!	109.065197	2.89255	pos
2.89_322.67:	322.671668	2.89255	pos
2.89_344.69	344.69748	2.89255	pos
2.89_453.69:	453.693923	2.89255	pos
2.91_113.96(	113.960698	2.9106	pos
2.91_180.13!	180.13595	2.9106	pos
2.91_194.09:	195.099705	2.9106	pos
2.91_222.16:	222.168666	2.9106	pos
2.91_406.70:	406.70254	2.9106	pos
2.92_382.10(	382.10046	2.92268333	neg
2.92_467.07	467.077814	2.92268333	neg
2.92_505.86(	505.859996	2.92268333	neg
2.92_608.04·	608.044703	2.92268333	neg
2.92_676.03:	676.031903	2.92268333	neg
2.93_129.95:	129.951616	2.92891667	pos
2.93_287.88:	287.888723	2.92891667	pos
2.94_133.01·	133.014072	2.9412	neg
2.94_318.87:	318.871079	2.9412	neg
2.94_410.04:	410.041361	2.9412	neg
2.94_676.79!	676.795906	2.9412	neg
2.94_744.01!	744.019911	2.9412	neg
2.94_812.00	812.007167	2.9412	neg
2.96_297.90(	297.900419	2.95975	neg
2.97_179.06	179.067673	2.965	pos

2.97_467.10:	467.101989	2.965	pos
2.98_186.95(	186.956208	2.98328333	pos
2.98_197.078	197.078192	2.98328333	pos
2.98_197.15:	197.153821	2.98328333	pos
2.98_332.58!	332.589488	2.98328333	pos
2.98_443.83(	443.830407	2.97823333	neg
2.98_448.11	448.117675	2.98328333	pos
2.98_486.90:	486.90329	2.97823333	neg
2.98_662.15	662.157587	2.97823333	neg
2.98_664.17	664.171781	2.98328333	pos
3.00_131.07(	131.070449	3.0007	pos
3.00_173.98	173.984851	3.0007	pos
3.00_226.97	226.974873	3.0007	pos
3.00_294.86!	294.865468	3.0007	pos
3.00_297.08:	297.082485	3.0007	pos
3.00_347.19:	347.192673	3.0007	pos
3.00_401.85:	401.852058	2.99661667	neg
3.02_140.98(	140.980397	3.0151	neg
3.02_233.91:	233.91296	3.01843333	pos
3.02_246.12	247.12884	3.01843333	pos
3.02_271.26:	271.26317	3.01843333	pos
3.02_428.96	428.96128	3.0151	neg
3.03_246.12	245.114134	3.03363333	neg
3.03_335.08:	335.083781	3.03363333	neg
3.04_123.11	123.11706	3.03666667	pos
3.04_134.08	134.081265	3.03666667	pos
3.04_151.07:	151.072894	3.03666667	pos
3.04_163.11	163.111777	3.03666667	pos
3.04_171.17	171.174312	3.03666667	pos
3.04_440.86!	440.869258	3.03666667	pos
3.05_107.95(	107.950854	3.05498333	pos
3.05_126.13(	126.136674	3.05498333	pos
3.05_137.04!	137.045949	3.05498333	pos

3.05_142.94	142.948012	3.05498333	pos
3.05_184.09	184.094346	3.05498333	pos
3.05_333.15	333.155706	3.05498333	pos
3.05_440.05	440.052564	3.05223333	neg
3.05_444.06	444.063411	3.05498333	pos
3.05_446.90	446.905783	3.05223333	neg
3.05_636.82	636.828019	3.05223333	neg
3.07_135.94	135.945498	3.07325	pos
3.07_145.98	145.989945	3.07325	pos
3.07_203.12	203.127896	3.07325	pos
3.07_215.16	215.164309	3.07325	pos
3.07_304.15	304.150232	3.07325	pos
3.09_134.96	134.966574	3.091	pos
3.09_192.03	192.032556	3.091	pos
3.09_208.64	208.647125	3.091	pos
3.09_259.12	259.129725	3.08933333	neg
3.09_304.90	304.903299	3.08933333	neg
3.11_239.16	239.161731	3.1093	pos
3.11_355.07	355.069969	3.1093	pos
3.11_372.88	372.881633	3.1093	pos
3.13_243.94	243.941646	3.12683333	pos
3.13_244.88	244.887918	3.1262	neg
3.13_313.88	313.880526	3.1262	neg
3.13_361.17	361.171697	3.12683333	pos
3.14_105.03	105.03394	3.1446	pos
3.14_235.92	235.925954	3.14476667	neg
3.14_253.11	253.118287	3.1446	pos
3.14_267.16	267.168268	3.1446	pos
3.14_267.67	267.670126	3.1446	pos
3.14_310.11	310.115634	3.14476667	neg
3.14_468.89	468.896353	3.14476667	neg
3.16_173.08	173.080966	3.16281667	pos
3.16_173.08	173.085707	3.16281667	pos

3.16_197.07[illegible]	197.078209	3.16281667	pos
3.16_347.86[illegible]	347.86147	3.16281667	pos
3.18_365.88[illegible]	365.887891	3.18185	neg
3.18_371.90[illegible]	371.900702	3.18185	neg
3.18_389.11[illegible]	389.111753	3.18115	pos
3.20_171.17[illegible]	171.174318	3.1994	pos
3.20_173.98[illegible]	173.984872	3.1994	pos
3.20_221.11[illegible]	221.115703	3.1994	pos
3.20_255.06[illegible]	255.062873	3.1994	pos
3.20_480.26[illegible]	480.267348	3.1994	pos
3.22_130.08[illegible]	130.087083	3.21883333	neg
3.22_136.04[illegible]	136.047653	3.21716667	pos
3.22_272.91[illegible]	272.917	3.21716667	pos
3.22_273.16[illegible]	273.167594	3.21716667	pos
3.22_294.15[illegible]	294.155202	3.21716667	pos
3.22_299.06[illegible]	299.061738	3.21716667	pos
3.22_363.55[illegible]	363.55349	3.21716667	pos
3.22_363.88[illegible]	363.887763	3.21716667	pos
3.24_314.08[illegible]	314.087035	3.23546667	pos
3.24_314.58[illegible]	314.588795	3.23546667	pos
3.24_386.55[illegible]	386.555668	3.23546667	pos
3.24_387.22[illegible]	387.224265	3.23546667	pos
3.24_461.47[illegible]	461.47648	3.23546667	pos
3.25_142.94[illegible]	142.948027	3.2533	pos
3.25_145.12[illegible]	145.122359	3.2533	pos
3.25_147.11[illegible]	147.116841	3.2533	pos
3.25_167.06[illegible]	167.068089	3.2533	pos
3.25_167.07[illegible]	167.070346	3.2533	pos
3.25_194.11[illegible]	194.117925	3.2533	pos
3.25_327.66[illegible]	327.662336	3.2533	pos
3.25_356.66[illegible]	356.667396	3.2533	pos
3.25_375.45[illegible]	375.458321	3.2533	pos
3.25_413.70[illegible]	413.700769	3.2533	pos

3.25_414.20	414.202444	3.2533	pos
3.26_141.03	141.0304	3.25576667	neg
3.27_144.10	144.102802	3.27433333	neg
3.27_147.02	147.029705	3.27433333	neg
3.27_156.04	156.045254	3.27091667	pos
3.27_186.95	186.956273	3.27091667	pos
3.27_206.04	206.049001	3.27433333	neg
3.27_210.98	210.983372	3.27433333	neg
3.27_276.14	276.145022	3.27433333	neg
3.27_344.13	344.132513	3.27433333	neg
3.27_447.09	447.097769	3.27091667	pos
3.27_447.34	447.346866	3.27091667	pos
3.27_480.10	480.107448	3.27433333	neg
3.27_548.09	548.095092	3.27433333	neg
3.27_616.08	616.082245	3.27433333	neg
3.27_684.07	684.070006	3.27433333	neg
3.27_762.68	762.689499	3.27091667	pos
3.29_127.00	127.001203	3.29291667	neg
3.29_154.96	154.966976	3.28915	pos
3.29_169.08	169.086029	3.28915	pos
3.29_188.09	188.092574	3.29291667	neg
3.29_258.68	258.683148	3.28915	pos
3.29_270.11	270.109981	3.28915	pos
3.29_278.97	278.970698	3.29291667	neg
3.29_287.24	287.244108	3.28915	pos
3.29_295.67	295.679097	3.28915	pos
3.29_329.20	329.203954	3.28915	pos
3.29_329.45	329.454866	3.28915	pos
3.29_329.70	329.70555	3.28915	pos
3.29_346.96	346.963352	3.28915	pos
3.29_347.46	347.46481	3.28915	pos
3.29_355.55	355.553806	3.28915	pos
3.29_426.57	426.570313	3.28915	pos

3.29_554.89(	554.890274	3.29291667	neg
3.31_124.09	124.097126	3.30741667	pos
3.31_153.05	153.054603	3.30741667	pos
3.31_188.17	188.175794	3.30741667	pos
3.31_199.09	199.097265	3.31146667	neg
3.31_215.00	215.009618	3.31146667	neg
3.31_246.12	245.114142	3.31146667	neg
3.31_267.09	267.096072	3.31146667	neg
3.31_368.72	368.721333	3.30741667	pos
3.31_368.97	368.972052	3.30741667	pos
3.31_369.47	369.473429	3.30741667	pos
3.31_407.47	407.477627	3.30741667	pos
3.31_407.72	407.728357	3.30741667	pos
3.31_432.52	432.52164	3.30741667	pos
3.31_432.85	432.855805	3.30741667	pos
3.31_433.19	433.190196	3.30741667	pos
3.31_454.23	454.230907	3.31146667	neg
3.31_473.24	456.245478	3.30741667	pos
3.31_542.96	542.96809	3.30741667	pos
3.31_543.30	543.30204	3.30741667	pos
3.31_546.62	546.629018	3.30741667	pos
3.31_586.13	586.130546	3.31146667	neg
3.31_654.11	654.117978	3.31146667	neg
3.31_753.33	753.334477	3.30741667	pos
3.33_143.11	143.117893	3.32576667	pos
3.33_187.09	187.094087	3.32576667	pos
3.33_221.64	221.642369	3.32576667	pos
3.33_286.51	286.511813	3.32576667	pos
3.33_306.59	306.59144	3.32576667	pos
3.33_314.91	314.911411	3.32576667	pos
3.33_315.42	338.412687	3.32576667	pos
3.33_316.18	316.18633	3.32576667	pos
3.33_327.68	327.6885	3.32576667	pos

3.33_327.93!	327.939305	3.32576667	pos
3.33_328.18!	328.189925	3.32576667	pos
3.33_338.66:	338.66348	3.32576667	pos
3.33_360.87(	360.870002	3.32576667	pos
3.33_389.23!	389.239746	3.32576667	pos
3.33_393.55!	393.559589	3.32576667	pos
3.33_396.87{	396.878799	3.32576667	pos
3.33_409.94(	409.946698	3.32576667	pos
3.33_418.53(	418.536568	3.32576667	pos
3.33_418.70	418.703954	3.32576667	pos
3.33_436.91(	436.91674	3.32576667	pos
3.33_437.25	437.251143	3.32576667	pos
3.33_450.88	450.881326	3.32576667	pos
3.33_572.51{	572.518729	3.32576667	pos
3.33_572.76{	572.768597	3.32576667	pos
3.33_573.01{	573.018806	3.32576667	pos
3.33_649.95!	649.955622	3.32576667	pos
3.33_763.02(	763.020626	3.32576667	pos
3.34_226.01	226.014632	3.34363333	pos
3.34_289.48:	289.48384	3.34363333	pos
3.34_291.50!	291.505819	3.34363333	pos
3.34_301.19	301.191021	3.34363333	pos
3.34_344.18	344.181948	3.34363333	pos
3.34_360.53!	360.535709	3.34363333	pos
3.34_375.72:	375.723209	3.34363333	pos
3.34_375.97	375.973952	3.34363333	pos
3.34_376.22	376.224452	3.34363333	pos
3.34_376.86:	376.863469	3.34363333	pos
3.34_434.22	434.224929	3.34363333	pos
3.34_500.92(	500.92676	3.34363333	pos
3.34_501.26	501.261229	3.34363333	pos
3.34_501.59!	501.595386	3.34363333	pos
3.34_606.88(	606.880835	3.34363333	pos

3.34_758.09	758.099069	3.34363333	pos
3.34_758.34	758.347735	3.34363333	pos
3.34_758.59	758.598816	3.34363333	pos
3.34_758.84	758.849301	3.34363333	pos
3.35_152.04	152.046299	3.3483	neg
3.35_158.02	158.027919	3.3483	neg
3.35_199.02	199.022114	3.3483	neg
3.36_129.95	129.958825	3.36203333	pos
3.36_195.09	195.099735	3.36203333	pos
3.36_223.09	223.096331	3.36203333	pos
3.36_224.63	224.631512	3.36203333	pos
3.36_295.52	295.523857	3.36203333	pos
3.36_295.85	295.858339	3.36203333	pos
3.36_297.43	297.431228	3.36203333	pos
3.36_300.68	300.689563	3.36203333	pos
3.36_306.43	306.434594	3.36203333	pos
3.36_306.68	306.685149	3.36203333	pos
3.36_312.19	312.192047	3.36203333	pos
3.36_323.19	323.191229	3.36203333	pos
3.36_350.18	350.18407	3.36203333	pos
3.36_584.59	584.597817	3.36203333	pos
3.36_584.93	584.931524	3.36203333	pos
3.36_647.54	647.544758	3.36203333	pos
3.36_661.50	644.503916	3.36203333	pos
3.36_804.87	804.877141	3.36203333	pos
3.37_116.03	116.035054	3.36678333	neg
3.37_276.95	276.955127	3.36678333	neg
3.37_465.08	465.084562	3.36678333	neg
3.38_130.05	130.055829	3.38003333	pos
3.38_186.95	186.956478	3.38003333	pos
3.38_231.17	231.170362	3.38003333	pos
3.38_251.66	251.665534	3.38003333	pos
3.38_261.13	261.131081	3.38003333	pos

3.38_269.16[illegible]	269.169467	3.38003333	pos
3.38_270.16[illegible]	270.168901	3.38003333	pos
3.38_270.67[illegible]	270.670606	3.38003333	pos
3.38_276.15[illegible]	276.155784	3.38003333	pos
3.38_320.85[illegible]	320.855465	3.38003333	pos
3.38_321.52[illegible]	321.524221	3.38003333	pos
3.38_322.69[illegible]	322.690444	3.38003333	pos
3.38_338.43[illegible]	338.433419	3.38003333	pos
3.38_345.52[illegible]	345.528719	3.38003333	pos
3.38_359.85[illegible]	359.85692	3.38003333	pos
3.38_372.91[illegible]	395.904818	3.38003333	pos
3.38_373.25[illegible]	396.239239	3.38003333	pos
3.38_388.20[illegible]	388.201371	3.38003333	pos
3.38_396.57[illegible]	396.573465	3.38003333	pos
3.38_415.22[illegible]	415.222539	3.38003333	pos
3.38_415.55[illegible]	415.557235	3.38003333	pos
3.38_415.89[illegible]	415.891184	3.38003333	pos
3.38_428.97[illegible]	428.973908	3.38003333	pos
3.38_441.88[illegible]	441.881886	3.38003333	pos
3.38_442.21[illegible]	442.21597	3.38003333	pos
3.38_442.54[illegible]	442.549851	3.38003333	pos
3.38_480.77[illegible]	480.779921	3.38003333	pos
3.38_481.28[illegible]	481.281342	3.38003333	pos
3.38_489.00[illegible]	489.004663	3.38003333	pos
3.38_524.60[illegible]	524.607134	3.38003333	pos
3.38_524.94[illegible]	524.940878	3.38003333	pos
3.38_561.26[illegible]	561.263618	3.38003333	pos
3.38_644.10[illegible]	644.104238	3.38003333	pos
3.38_648.04[illegible]	648.044475	3.38003333	pos
3.38_679.81[illegible]	679.810702	3.38003333	pos
3.38_804.62[illegible]	804.627568	3.38003333	pos
3.39_152.11[illegible]	152.116558	3.38528333	neg
3.39_288.15[illegible]	288.156528	3.38528333	neg

3.40_172.97	172.977391	3.39796667	pos
3.40_302.68	302.687105	3.39796667	pos
3.40_304.43	304.432754	3.39796667	pos
3.40_312.69	312.695224	3.39796667	pos
3.40_314.93	314.93972	3.39796667	pos
3.40_315.19	315.190454	3.39796667	pos
3.40_315.44	315.441224	3.39796667	pos
3.40_361.45	361.455004	3.39796667	pos
3.40_361.70	361.704976	3.39796667	pos
3.40_363.69	363.695685	3.39796667	pos
3.40_363.94	363.946535	3.39796667	pos
3.40_367.86	367.86926	3.39796667	pos
3.40_370.87	370.875369	3.39796667	pos
3.40_372.70	372.707707	3.39796667	pos
3.40_372.95	372.958576	3.39796667	pos
3.40_373.86	373.860377	3.39796667	pos
3.40_382.22	382.221462	3.39796667	pos
3.40_419.58	419.584	3.39796667	pos
3.40_419.91	419.918327	3.39796667	pos
3.40_452.69	452.694023	3.39796667	pos
3.40_464.74	464.740391	3.39796667	pos
3.40_496.60	496.608168	3.39796667	pos
3.40_496.94	496.94245	3.39796667	pos
3.40_498.74	498.743469	3.39796667	pos
3.40_643.70	643.704092	3.39796667	pos
3.40_643.90	643.90393	3.39796667	pos
3.40_670.32	670.329363	3.4039	neg
3.40_672.34	672.344262	3.39796667	pos
3.40_720.32	720.322292	3.39796667	pos
3.40_720.65	720.656626	3.39796667	pos
3.40_726.07	726.075006	3.39796667	pos
3.42_105.03	105.033995	3.41641667	pos
3.42_132.06	132.06577	3.41641667	pos

3.42_133.10	133.10534	3.41641667	pos
3.42_235.01	235.019772	3.42245	neg
3.42_260.48	260.481577	3.41641667	pos
3.42_260.81	260.816009	3.41641667	pos
3.42_290.42	290.426746	3.41641667	pos
3.42_318.17	318.176471	3.41641667	pos
3.42_330.19	330.190784	3.41641667	pos
3.42_330.52	330.525239	3.41641667	pos
3.42_330.85	330.859654	3.41641667	pos
3.42_336.69	336.697992	3.41641667	pos
3.42_336.94	336.94855	3.41641667	pos
3.42_337.44	337.4499	3.41641667	pos
3.42_358.20	358.20512	3.41641667	pos
3.42_367.95	367.95437	3.41641667	pos
3.42_383.87	383.872272	3.41641667	pos
3.42_384.20	384.207446	3.41641667	pos
3.42_387.54	370.540461	3.41641667	pos
3.42_389.21	390.21875	3.41641667	pos
3.42_390.46	390.467128	3.41641667	pos
3.42_449.26	449.26322	3.41641667	pos
3.42_464.90	464.907376	3.41641667	pos
3.42_465.56	465.560742	3.41641667	pos
3.42_465.59	448.594939	3.41641667	pos
3.42_465.89	465.895222	3.41641667	pos
3.42_465.91	466.925349	3.41641667	pos
3.42_465.93	448.929057	3.41641667	pos
3.42_467.25	467.259806	3.41641667	pos
3.42_467.59	467.594074	3.41641667	pos
3.42_481.91	481.914391	3.41641667	pos
3.42_489.93	489.936148	3.41641667	pos
3.42_499.93	499.935768	3.41641667	pos
3.42_500.27	500.269966	3.41641667	pos
3.42_500.60	500.605421	3.41641667	pos

3.42_569.61[illegible]	569.611616	3.41641667	pos
3.42_569.94[illegible]	569.946144	3.41641667	pos
3.42_570.28[illegible]	570.280163	3.41641667	pos
3.42_804.37[illegible]	804.377343	3.41641667	pos
3.43_234.94[illegible]	234.947101	3.43491667	pos
3.43_272.16[illegible]	272.160834	3.43491667	pos
3.43_288.82[illegible]	288.820431	3.43491667	pos
3.43_304.84[illegible]	304.843463	3.43491667	pos
3.43_319.94[illegible]	319.94639	3.43491667	pos
3.43_320.19[illegible]	320.195811	3.43491667	pos
3.43_320.44[illegible]	320.447774	3.43491667	pos
3.43_321.94[illegible]	321.941198	3.43491667	pos
3.43_332.83[illegible]	332.833194	3.43491667	pos
3.43_333.18[illegible]	356.173287	3.43491667	pos
3.43_334.45[illegible]	334.452114	3.43491667	pos
3.43_343.70[illegible]	343.70016	3.43491667	pos
3.43_343.95[illegible]	343.950877	3.43491667	pos
3.43_344.67[illegible]	344.674613	3.43491667	pos
3.43_345.17[illegible]	345.176377	3.43491667	pos
3.43_356.70[illegible]	379.693709	3.43491667	pos
3.43_362.15[illegible]	362.156199	3.43491667	pos
3.43_394.55[illegible]	394.554096	3.43491667	pos
3.43_396.99[illegible]	396.992655	3.43491667	pos
3.43_397.49[illegible]	397.494199	3.43491667	pos
3.43_397.74[illegible]	397.744989	3.43491667	pos
3.43_408.87[illegible]	409.883561	3.43491667	pos
3.43_409.72[illegible]	409.722441	3.43491667	pos
3.43_417.21[illegible]	417.213081	3.43491667	pos
3.43_417.54[illegible]	417.54687	3.43491667	pos
3.43_418.90[illegible]	418.907441	3.43491667	pos
3.43_419.54[illegible]	419.548742	3.43491667	pos
3.43_430.69[illegible]	430.699312	3.43491667	pos
3.43_431.53[illegible]	431.532494	3.43491667	pos

3.43_437.91	437.914767	3.43491667	pos
3.43_457.24	457.240556	3.43491667	pos
3.43_481.57	481.579871	3.43491667	pos
3.43_485.75	485.751809	3.43491667	pos
3.43_495.97	495.972033	3.43491667	pos
3.43_496.47	496.473913	3.43491667	pos
3.43_497.21	497.210756	3.43491667	pos
3.43_503.56	503.561756	3.43491667	pos
3.43_547.75	547.750606	3.43491667	pos
3.44_133.01	133.014047	3.44081667	neg
3.44_143.05	143.051874	3.44081667	neg
3.44_168.03	167.032303	3.44081667	neg
3.44_185.04	185.042748	3.44081667	neg
3.44_202.03	202.033104	3.44081667	neg
3.44_253.03	253.030426	3.44081667	neg
3.44_322.02	321.01777	3.44081667	neg
3.44_389.00	389.005556	3.44081667	neg
3.45_163.03	163.034826	3.45331667	pos
3.45_176.07	176.074249	3.45331667	pos
3.45_228.65	228.650472	3.45331667	pos
3.45_233.14	233.149867	3.45331667	pos
3.45_234.82	234.820095	3.45331667	pos
3.45_237.65	237.649988	3.45331667	pos
3.45_237.66	237.668145	3.45331667	pos
3.45_311.92	311.929745	3.45331667	pos
3.45_314.69	314.69396	3.45331667	pos
3.45_331.50	331.506741	3.45331667	pos
3.45_331.84	331.841119	3.45331667	pos
3.45_338.53	338.537971	3.45331667	pos
3.45_340.14	340.148878	3.45331667	pos
3.45_341.84	341.842112	3.45331667	pos
3.45_355.67	355.671754	3.45331667	pos
3.45_356.43	356.439152	3.45331667	pos

3.45_377.528	377.528862	3.45331667	pos
3.45_390.538	390.538318	3.45331667	pos
3.45_395.888	395.888085	3.45331667	pos
3.45_396.222	396.222011	3.45331667	pos
3.45_403.541	421.575451	3.45331667	pos
3.45_403.870	421.910217	3.45331667	pos
3.45_420.472	420.472775	3.45331667	pos
3.45_449.254	472.244106	3.45331667	pos
3.45_479.548	479.548553	3.45331667	pos
3.45_482.248	482.248293	3.45331667	pos
3.45_482.582	482.582398	3.45331667	pos
3.45_488.734	488.734419	3.45331667	pos
3.45_521.707	521.707211	3.45331667	pos
3.45_555.700	555.700679	3.45331667	pos
3.45_578.025	578.025143	3.45331667	pos
3.45_599.637	599.637155	3.45331667	pos
3.45_672.839	672.839212	3.45331667	pos
3.46_145.061	145.061663	3.45915	neg
3.46_149.027	149.02739	3.45915	neg
3.46_155.034	155.034795	3.45915	neg
3.46_272.021	272.021004	3.45915	neg
3.46_278.970	278.970799	3.45915	neg
3.46_340.008	340.008212	3.45915	neg
3.46_382.550	382.550416	3.45915	neg
3.46_415.231	415.231038	3.45915	neg
3.46_535.585	535.585937	3.45915	neg
3.47_200.602	200.602892	3.47126667	pos
3.47_206.048	206.048395	3.47126667	pos
3.47_234.485	234.485605	3.47126667	pos
3.47_253.142	253.142257	3.47126667	pos
3.47_259.171	259.171171	3.47126667	pos
3.47_259.672	259.672934	3.47126667	pos
3.47_262.499	262.499039	3.47126667	pos

3.47_264.670	264.670616	3.47126667	pos
3.47_274.164	274.164145	3.47126667	pos
3.47_311.527	311.527727	3.47126667	pos
3.47_327.078	327.078429	3.47126667	pos
3.47_332.168	332.168789	3.47126667	pos
3.47_344.534	344.534493	3.47126667	pos
3.47_344.868	344.868861	3.47126667	pos
3.47_345.718	368.707478	3.47126667	pos
3.47_350.714	350.714437	3.47126667	pos
3.47_357.466	357.466117	3.47126667	pos
3.47_378.545	378.545312	3.47126667	pos
3.47_378.879	378.879748	3.47126667	pos
3.47_384.510	384.510191	3.47126667	pos
3.47_389.872	389.872382	3.47126667	pos
3.47_405.548	405.548191	3.47126667	pos
3.47_405.714	405.714903	3.47126667	pos
3.47_419.875	419.875865	3.47126667	pos
3.47_436.229	436.229337	3.47126667	pos
3.47_439.568	439.568728	3.47126667	pos
3.47_505.734	505.734179	3.47126667	pos
3.47_512.560	530.59995	3.47126667	pos
3.47_551.779	551.778989	3.47126667	pos
3.47_630.806	630.806652	3.47126667	pos
3.48_125.060	125.060603	3.47753333	neg
3.48_195.052	195.051995	3.47753333	neg
3.49_151.073	151.073002	3.48938333	pos
3.49_201.073	201.073269	3.48938333	pos
3.49_228.663	228.662959	3.48938333	pos
3.49_245.185	245.18572	3.48938333	pos
3.49_263.496	263.496307	3.48938333	pos
3.49_263.830	263.830668	3.48938333	pos
3.49_266.647	266.64776	3.48938333	pos
3.49_270.593	270.593511	3.48938333	pos

3.49_286.17[illegible]	286.176338	3.48938333	pos
3.49_296.50[illegible]	296.503482	3.48938333	pos
3.49_296.83[illegible]	296.837641	3.48938333	pos
3.49_298.67[illegible]	298.679675	3.48938333	pos
3.49_312.15[illegible]	312.155622	3.48938333	pos
3.49_312.65[illegible]	312.657469	3.48938333	pos
3.49_319.53[illegible]	319.537442	3.48938333	pos
3.49_334.69[illegible]	334.69034	3.48938333	pos
3.49_337.15[illegible]	337.151029	3.48938333	pos
3.49_350.46[illegible]	350.463742	3.48938333	pos
3.49_362.21[illegible]	362.216917	3.48938333	pos
3.49_366.70[illegible]	366.708303	3.48938333	pos
3.49_373.69[illegible]	373.697619	3.48938333	pos
3.49_374.44[illegible]	374.449307	3.48938333	pos
3.49_386.56[illegible]	369.557639	3.48938333	pos
3.49_393.73[illegible]	432.702252	3.48938333	pos
3.49_394.17[illegible]	394.172608	3.48938333	pos
3.49_394.24[illegible]	433.203996	3.48938333	pos
3.49_406.46[illegible]	406.463853	3.48938333	pos
3.49_406.73[illegible]	406.732367	3.48938333	pos
3.49_406.98[illegible]	406.983375	3.48938333	pos
3.49_408.20[illegible]	409.217206	3.48938333	pos
3.49_408.88[illegible]	408.883016	3.48938333	pos
3.49_423.18[illegible]	423.187731	3.48938333	pos
3.49_429.22[illegible]	429.227256	3.48938333	pos
3.49_439.54[illegible]	439.546584	3.48938333	pos
3.49_444.57[illegible]	444.574254	3.48938333	pos
3.49_453.57[illegible]	453.579547	3.48938333	pos
3.49_479.88[illegible]	479.88054	3.48938333	pos
3.49_480.21[illegible]	480.215029	3.48938333	pos
3.49_498.59[illegible]	498.595718	3.48938333	pos
3.49_498.92[illegible]	498.9295	3.48938333	pos
3.49_505.00[illegible]	505.006598	3.48938333	pos

3.49_505.23[illegible]	505.233378	3.48938333	pos
3.49_505.50[illegible]	505.507418	3.48938333	pos
3.49_542.25[illegible]	542.252283	3.48938333	pos
3.49_548.75[illegible]	548.749982	3.48938333	pos
3.50_194.98[illegible]	194.988566	3.49608333	neg
3.51_217.01[illegible]	217.012867	3.5146	neg
3.51_231.99[illegible]	231.991904	3.5146	neg
3.51_270.12[illegible]	270.120815	3.5146	neg
3.51_272.13[illegible]	272.135431	3.50783333	pos
3.51_278.11[illegible]	278.11459	3.5146	neg
3.51_292.17[illegible]	292.176072	3.50783333	pos
3.51_292.67[illegible]	292.67932	3.50783333	pos
3.51_343.17[illegible]	344.181759	3.50783333	pos
3.51_345.94[illegible]	345.942926	3.50783333	pos
3.51_346.44[illegible]	346.444251	3.50783333	pos
3.51_351.19[illegible]	351.195351	3.50783333	pos
3.51_351.51[illegible]	351.51767	3.50783333	pos
3.51_351.69[illegible]	351.698613	3.50783333	pos
3.51_351.85[illegible]	351.852025	3.50783333	pos
3.51_354.44[illegible]	354.442815	3.50783333	pos
3.51_360.21[illegible]	360.213429	3.50783333	pos
3.51_362.17[illegible]	362.174796	3.50783333	pos
3.51_367.46[illegible]	367.462393	3.50783333	pos
3.51_373.54[illegible]	373.54526	3.50783333	pos
3.51_378.21[illegible]	378.212188	3.50783333	pos
3.51_378.71[illegible]	378.71297	3.50783333	pos
3.51_391.95[illegible]	391.957744	3.50783333	pos
3.51_392.45[illegible]	392.45906	3.50783333	pos
3.51_402.18[illegible]	402.188429	3.50783333	pos
3.51_402.68[illegible]	402.689865	3.50783333	pos
3.51_419.70[illegible]	419.701295	3.50783333	pos
3.51_430.70[illegible]	430.704655	3.50783333	pos
3.51_433.56[illegible]	433.562621	3.50783333	pos

3.51_433.89[illegible]	433.897274	3.50783333	pos
3.51_434.23[illegible]	434.231231	3.50783333	pos
3.51_453.24[illegible]	453.240543	3.50783333	pos
3.51_460.92[illegible]	460.92155	3.50783333	pos
3.51_461.58[illegible]	461.589743	3.50783333	pos
3.51_468.24[illegible]	468.245882	3.50783333	pos
3.51_475.60[illegible]	475.600803	3.50783333	pos
3.51_491.98[illegible]	491.981992	3.50783333	pos
3.51_498.24[illegible]	521.238801	3.50783333	pos
3.51_509.23[illegible]	492.232927	3.50783333	pos
3.51_520.73[illegible]	520.739434	3.50783333	pos
3.51_520.98[illegible]	520.989594	3.50783333	pos
3.51_521.48[illegible]	521.489805	3.50783333	pos
3.51_524.75[illegible]	524.759002	3.50783333	pos
3.51_525.93[illegible]	525.93125	3.50783333	pos
3.51_530.76[illegible]	530.767703	3.50783333	pos
3.51_531.26[illegible]	531.268894	3.50783333	pos
3.51_540.75[illegible]	540.75261	3.50783333	pos
3.51_547.44[illegible]	547.441669	3.50783333	pos
3.51_547.60[illegible]	547.609126	3.50783333	pos
3.51_556.77[illegible]	556.77134	3.50783333	pos
3.51_563.55[illegible]	563.555321	3.50783333	pos
3.51_564.05[illegible]	564.056774	3.50783333	pos
3.51_693.98[illegible]	693.982964	3.50783333	pos
3.51_694.31[illegible]	694.316284	3.50783333	pos
3.51_694.64[illegible]	694.648323	3.50783333	pos
3.53_150.09[illegible]	150.099687	3.52578333	pos
3.53_179.06[illegible]	179.067683	3.52578333	pos
3.53_196.12[illegible]	196.120916	3.52578333	pos
3.53_209.07[illegible]	209.0785	3.52578333	pos
3.53_235.65[illegible]	235.658079	3.52578333	pos
3.53_279.70[illegible]	279.702604	3.52578333	pos
3.53_280.12[illegible]	280.12945	3.52578333	pos

3.53_280.20	280.204319	3.52578333	pos
3.53_282.18	282.180159	3.52578333	pos
3.53_282.51	282.514604	3.52578333	pos
3.53_297.16	297.168499	3.52578333	pos
3.53_297.67	297.670134	3.52578333	pos
3.53_300.17	300.179577	3.52578333	pos
3.53_303.68	303.684156	3.52578333	pos
3.53_325.52	325.522032	3.52578333	pos
3.53_327.84	327.846973	3.52578333	pos
3.53_349.20	349.201395	3.52578333	pos
3.53_349.53	349.536024	3.52578333	pos
3.53_356.53	356.532987	3.52578333	pos
3.53_365.13	365.13528	3.53321667	neg
3.53_375.68	375.680421	3.52578333	pos
3.53_391.69	391.691904	3.52578333	pos
3.53_394.71	394.717282	3.52578333	pos
3.53_404.19	404.191253	3.52578333	pos
3.53_409.71	409.717064	3.52578333	pos
3.53_409.96	409.967751	3.52578333	pos
3.53_420.88	420.885813	3.52578333	pos
3.53_433.12	433.122539	3.53321667	neg
3.53_443.58	443.580188	3.52578333	pos
3.53_444.24	444.246608	3.52578333	pos
3.53_449.17	449.179107	3.53321667	neg
3.53_481.74	481.746476	3.52578333	pos
3.53_513.54	513.543465	3.52578333	pos
3.53_513.79	513.794353	3.52578333	pos
3.53_514.29	514.294861	3.52578333	pos
3.53_540.80	540.80685	3.52578333	pos
3.53_546.28	546.288119	3.52578333	pos
3.53_581.97	581.976644	3.52578333	pos
3.53_582.31	582.310623	3.52578333	pos
3.53_817.36	817.369476	3.53321667	neg

3.54_231.159	231.159307	3.54413333	pos
3.54_236.633	236.633169	3.54413333	pos
3.54_246.497	246.497657	3.54413333	pos
3.54_271.507	271.507312	3.54413333	pos
3.54_284.827	284.827896	3.54413333	pos
3.54_295.170	334.139705	3.54413333	pos
3.54_319.666	319.666818	3.54413333	pos
3.54_345.144	345.144628	3.54413333	pos
3.54_347.192	347.192949	3.54413333	pos
3.54_354.195	354.195145	3.54413333	pos
3.54_364.969	364.968984	3.54413333	pos
3.54_365.180	365.180731	3.54413333	pos
3.54_365.712	365.712488	3.54413333	pos
3.54_380.209	380.209583	3.54413333	pos
3.54_386.703	386.703427	3.54413333	pos
3.54_404.557	404.557402	3.54413333	pos
3.54_423.898	423.898726	3.54413333	pos
3.54_426.708	426.708839	3.54413333	pos
3.54_427.210	427.210293	3.54413333	pos
3.54_427.544	427.544067	3.54413333	pos
3.54_446.739	446.739147	3.54413333	pos
3.54_447.476	447.476494	3.54413333	pos
3.54_451.194	451.194005	3.54413333	pos
3.54_457.733	457.732952	3.54413333	pos
3.54_457.909	457.909212	3.54413333	pos
3.54_464.725	482.759436	3.54413333	pos
3.54_472.683	472.683696	3.54413333	pos
3.54_478.769	478.769866	3.54413333	pos
3.54_479.271	479.27128	3.54413333	pos
3.54_509.757	509.756964	3.54413333	pos
3.54_510.730	528.770568	3.54413333	pos
3.54_546.502	546.502112	3.54413333	pos
3.54_546.750	546.749984	3.54413333	pos

3.54_548.94[illegible]	548.947395	3.54413333	pos
3.54_563.00[illegible]	546.001111	3.54413333	pos
3.54_563.25[illegible]	546.252082	3.54413333	pos
3.54_576.27[illegible]	576.271191	3.54413333	pos
3.54_588.78[illegible]	588.787785	3.54413333	pos
3.54_727.66[illegible]	727.66494	3.54413333	pos
3.54_727.99[illegible]	727.999894	3.54413333	pos
3.54_751.39[illegible]	751.395798	3.54413333	pos
3.55_155.07[illegible]	155.071179	3.5518	neg
3.55_178.97[illegible]	178.977558	3.5518	neg
3.55_424.22[illegible]	424.220255	3.5518	neg
3.55_471.67[illegible]	942.34524	3.5518	neg
3.55_482.66[illegible]	964.32832	3.5518	neg
3.55_749.38[illegible]	749.381657	3.5518	neg
3.55_775.80[illegible]	775.800362	3.5518	neg
3.56_208.00[illegible]	208.009682	3.56223333	pos
3.56_290.48[illegible]	290.48388	3.56223333	pos
3.56_343.19[illegible]	343.19795	3.56223333	pos
3.56_343.53[illegible]	343.532802	3.56223333	pos
3.56_368.95[illegible]	368.959294	3.56223333	pos
3.56_387.53[illegible]	387.535214	3.56223333	pos
3.56_420.73[illegible]	420.737878	3.56223333	pos
3.56_424.69[illegible]	424.698654	3.56223333	pos
3.56_425.20[illegible]	425.200781	3.56223333	pos
3.56_472.75[illegible]	472.756429	3.56223333	pos
3.56_490.26[illegible]	473.257915	3.56223333	pos
3.56_499.64[illegible]	499.643323	3.56223333	pos
3.56_525.27[illegible]	525.272085	3.56223333	pos
3.56_540.23[illegible]	540.236473	3.56223333	pos
3.56_557.92[illegible]	557.929191	3.56223333	pos
3.56_584.60[illegible]	602.640415	3.56223333	pos
3.56_602.30[illegible]	602.306227	3.56223333	pos
3.56_602.97[illegible]	602.974336	3.56223333	pos

3.57_107.02[illegible]	107.024874	3.57018333	neg
3.57_302.13[illegible]	302.135587	3.57018333	neg
3.57_346.16[illegible]	346.161629	3.57018333	neg
3.57_363.11[illegible]	363.115141	3.57018333	neg
3.57_463.14[illegible]	463.14748	3.57018333	neg
3.57_583.27[illegible]	583.273377	3.57018333	neg
3.58_157.07[illegible]	157.076325	3.58011667	pos
3.58_175.08[illegible]	175.086633	3.58011667	pos
3.58_221.64[illegible]	221.642242	3.58011667	pos
3.58_293.14[illegible]	293.14752	3.58011667	pos
3.58_309.69[illegible]	309.694715	3.58011667	pos
3.58_320.10[illegible]	320.10695	3.58011667	pos
3.58_327.52[illegible]	327.522046	3.58011667	pos
3.58_334.70[illegible]	334.702547	3.58011667	pos
3.58_335.87[illegible]	335.878367	3.58011667	pos
3.58_336.21[illegible]	336.212927	3.58011667	pos
3.58_336.54[illegible]	336.546928	3.58011667	pos
3.58_347.16[illegible]	348.176682	3.58011667	pos
3.58_358.67[illegible]	358.679592	3.58011667	pos
3.58_373.72[illegible]	373.723829	3.58011667	pos
3.58_374.22[illegible]	374.225548	3.58011667	pos
3.58_391.22[illegible]	391.227523	3.58011667	pos
3.58_391.56[illegible]	391.561867	3.58011667	pos
3.58_391.89[illegible]	391.896047	3.58011667	pos
3.58_418.71[illegible]	418.711323	3.58011667	pos
3.58_422.72[illegible]	422.727461	3.58011667	pos
3.58_422.97[illegible]	422.97821	3.58011667	pos
3.58_423.22[illegible]	423.228314	3.58011667	pos
3.58_442.18[illegible]	442.184514	3.58011667	pos
3.58_462.54[illegible]	462.548604	3.58011667	pos
3.58_465.73[illegible]	465.737173	3.58011667	pos
3.58_515.74[illegible]	515.740662	3.58011667	pos
3.58_525.77[illegible]	525.773611	3.58011667	pos

3.58_529.289	529.2894	3.58011667	pos
3.58_530.29!	531.302256	3.58011667	pos
3.58_531.55:	531.553017	3.58011667	pos
3.58_543.95:	543.95343	3.58011667	pos
3.58_544.28	544.288566	3.58011667	pos
3.58_580.269	580.269838	3.58011667	pos
3.58_586.33	586.337808	3.58011667	pos
3.58_586.839	586.839324	3.58011667	pos
3.58_686.20!	686.205616	3.58011667	pos
3.59_116.05(	116.050395	3.58845	neg
3.59_122.03!	122.035646	3.58845	neg
3.59_123.04!	123.044951	3.58845	neg
3.59_204.99:	204.992608	3.58845	neg
3.59_221.09:	221.092858	3.58845	neg
3.59_258.00!	258.005089	3.58845	neg
3.59_273.07!	273.075363	3.58845	neg
3.59_288.06(	288.06009	3.58845	neg
3.59_289.08(	289.080463	3.58845	neg
3.59_294.05	339.057145	3.58845	neg
3.59_315.09(	315.096091	3.58845	neg
3.59_323.03	323.031886	3.58845	neg
3.59_331.02!	331.025605	3.58845	neg
3.59_346.01	346.018641	3.58845	neg
3.59_362.04(	407.044742	3.58845	neg
3.59_400.11:	400.112332	3.58845	neg
3.59_405.029	405.02903	3.58845	neg
3.59_414.00!	414.005859	3.58845	neg
3.59_417.00(	417.000161	3.58845	neg
3.59_465.00:	465.003175	3.58845	neg
3.59_473.01(	473.016405	3.58845	neg
3.59_475.03:	475.032055	3.58845	neg
3.59_481.99:	481.993237	3.58845	neg
3.59_484.98	484.987521	3.58845	neg

3.59_492.022	492.022559	3.58845	neg
3.59_532.990	532.990856	3.58845	neg
3.59_543.019	543.019608	3.58845	neg
3.59_549.981	549.981007	3.58845	neg
3.59_552.975	552.975273	3.58845	neg
3.59_555.100	555.100347	3.58845	neg
3.59_560.010	560.01	3.58845	neg
3.59_565.129	565.129235	3.58845	neg
3.59_582.119	582.119358	3.58845	neg
3.59_600.978	600.978572	3.58845	neg
3.59_611.007	611.007159	3.58845	neg
3.59_620.962	620.96257	3.58845	neg
3.59_623.090	623.090517	3.58845	neg
3.59_627.997	627.99758	3.58845	neg
3.59_633.116	633.116727	3.58845	neg
3.59_650.107	650.107123	3.58845	neg
3.59_668.966	668.966172	3.58845	neg
3.59_678.994	678.994595	3.58845	neg
3.59_688.950	688.950305	3.58845	neg
3.59_691.075	691.07523	3.58845	neg
3.59_695.985	695.984959	3.58845	neg
3.59_701.104	701.104287	3.58845	neg
3.59_718.094	718.094	3.58845	neg
3.59_746.982	746.982361	3.58845	neg
3.59_756.937	756.936958	3.58845	neg
3.59_814.969	814.969887	3.58845	neg
3.59_824.925	824.925745	3.58845	neg
3.59_837.079	837.079098	3.58845	neg
3.59_859.188	859.188353	3.58845	neg
3.59_882.957	882.957621	3.58845	neg
3.59_905.066	905.066543	3.58845	neg
3.60_115.054	115.054544	3.59728333	pos
3.60_138.055	138.054964	3.59728333	pos

3.60_143.039	143.039584	3.59728333	pos
3.60_149.084	132.080861	3.59728333	pos
3.60_150.525	150.52508	3.59728333	pos
3.60_189.069	207.103464	3.59728333	pos
3.60_221.091	221.091862	3.59728333	pos
3.60_236.647	236.647347	3.59728333	pos
3.60_258.169	258.169675	3.59728333	pos
3.60_295.523	295.523594	3.59728333	pos
3.60_303.495	303.495177	3.59728333	pos
3.60_305.021	305.021838	3.59728333	pos
3.60_318.174	318.17461	3.59728333	pos
3.60_333.517	333.517428	3.59728333	pos
3.60_334.038	334.038403	3.59728333	pos
3.60_347.930	347.930193	3.59728333	pos
3.60_370.864	370.864432	3.59728333	pos
3.60_384.715	384.715677	3.59728333	pos
3.60_385.035	385.035795	3.59728333	pos
3.60_452.143	475.132637	3.59728333	pos
3.60_465.235	465.235722	3.59728333	pos
3.60_476.758	476.758525	3.59728333	pos
3.60_477.259	477.259534	3.59728333	pos
3.60_480.839	480.839753	3.59728333	pos
3.60_485.238	485.23806	3.59728333	pos
3.60_543.120	543.12035	3.59728333	pos
3.60_594.562	594.562768	3.59728333	pos
3.60_594.813	594.813062	3.59728333	pos
3.60_600.547	600.547537	3.59728333	pos
3.60_601.299	601.299684	3.59728333	pos
3.60_601.815	601.815086	3.59728333	pos
3.60_602.065	602.065673	3.59728333	pos
3.60_606.295	606.295229	3.59728333	pos
3.60_606.545	606.545558	3.59728333	pos
3.60_606.796	606.796157	3.59728333	pos

3.60_607.047	607.046988	3.59728333	pos
3.61_115.020	115.020561	3.60671667	neg
3.61_199.058	199.058274	3.60671667	neg
3.61_223.998	223.998584	3.6144	pos
3.61_229.104	229.104489	3.6144	pos
3.61_275.173	276.180467	3.6144	pos
3.61_288.045	575.084457	3.60671667	neg
3.61_294.171	294.171182	3.6144	pos
3.61_308.186	308.186052	3.6144	pos
3.61_320.171	320.171324	3.60671667	neg
3.61_324.687	307.684398	3.6144	pos
3.61_358.197	358.197108	3.6144	pos
3.61_362.204	362.20455	3.6144	pos
3.61_366.150	366.149973	3.6144	pos
3.61_394.220	394.220906	3.6144	pos
3.61_394.471	394.471546	3.6144	pos
3.61_400.559	400.559194	3.6144	pos
3.61_414.557	414.557751	3.6144	pos
3.61_415.978	415.978871	3.6144	pos
3.61_431.160	431.160123	3.60671667	neg
3.61_432.885	432.885337	3.6144	pos
3.61_497.861	497.861338	3.6144	pos
3.61_498.462	498.46284	3.6144	pos
3.61_508.782	508.782058	3.6144	pos
3.61_524.958	524.95803	3.6144	pos
3.61_525.626	525.626095	3.6144	pos
3.61_588.566	588.56641	3.6144	pos
3.61_604.291	622.325056	3.6144	pos
3.61_622.074	622.07431	3.6144	pos
3.61_622.575	622.575467	3.6144	pos
3.61_622.826	622.826518	3.6144	pos
3.62_1139.09	1139.09505	3.62421667	neg
3.62_126.001	126.001623	3.62421667	neg

3.62_156.012	156.012283	3.62421667	neg
3.62_169.991	169.991466	3.62421667	neg
3.62_236.999	236.999101	3.62421667	neg
3.62_240.978	240.978695	3.62421667	neg
3.62_257.969	257.968954	3.62421667	neg
3.62_267.989	267.989688	3.62421667	neg
3.62_335.977	335.977173	3.62421667	neg
3.62_424.104	424.104445	3.62421667	neg
3.62_539.966	539.966213	3.62421667	neg
3.62_560.079	560.079203	3.62421667	neg
3.62_564.047	564.047774	3.62421667	neg
3.62_586.157	586.157439	3.62421667	neg
3.62_605.957	605.957333	3.62421667	neg
3.62_615.912	615.912533	3.62421667	neg
3.62_622.947	622.947819	3.62421667	neg
3.62_628.066	628.06675	3.62421667	neg
3.62_696.053	696.053908	3.62421667	neg
3.62_892.955	892.955046	3.62421667	neg
3.63_181.072	181.071996	3.6313	pos
3.63_350.702	350.702775	3.6313	pos
3.63_411.227	411.227752	3.6313	pos
3.63_447.988	447.988532	3.6313	pos
3.63_448.239	448.239283	3.6313	pos
3.63_448.489	448.489935	3.6313	pos
3.63_448.741	448.741079	3.6313	pos
3.63_498.061	498.061718	3.6313	pos
3.63_523.674	523.674081	3.6313	pos
3.63_544.557	544.557842	3.6313	pos
3.63_560.809	543.805855	3.6313	pos
3.63_562.316	562.316912	3.6313	pos
3.63_569.067	569.067732	3.6313	pos
3.63_571.559	571.55926	3.6313	pos
3.63_572.766	572.766906	3.6313	pos

3.63_578.56	561.564939	3.6313	pos
3.63_579.06	562.06638	3.6313	pos
3.63_579.32	579.32443	3.6313	pos
3.63_587.81	570.807266	3.6313	pos
3.63_588.06	571.057889	3.6313	pos
3.63_596.98	596.98244	3.6313	pos
3.63_597.31	597.316619	3.6313	pos
3.63_597.65	597.651113	3.6313	pos
3.63_654.33	654.339262	3.6313	pos
3.63_654.84	654.840441	3.6313	pos
3.63_725.07	725.072131	3.6313	pos
3.63_760.73	760.739863	3.6313	pos
3.63_761.07	761.074402	3.6313	pos
3.63_761.40	761.408142	3.6313	pos
3.63_785.08	785.087248	3.6313	pos
3.63_829.43	829.430579	3.6313	pos
3.64_1099.5	1099.59452	3.64241667	neg
3.64_130.05	130.050733	3.64241667	neg
3.64_174.98	174.98713	3.64241667	neg
3.64_235.00	235.003251	3.64241667	neg
3.64_346.96	391.966158	3.64241667	neg
3.64_368.91	368.918032	3.64241667	neg
3.64_401.99	401.994746	3.64241667	neg
3.64_403.99	403.991528	3.64241667	neg
3.64_411.95	411.950199	3.64241667	neg
3.64_414.95	459.953554	3.64241667	neg
3.64_418.98	418.985136	3.64241667	neg
3.64_469.98	469.982063	3.64241667	neg
3.64_475.93	475.938786	3.64241667	neg
3.64_479.93	479.937491	3.64241667	neg
3.64_481.93	481.935265	3.64241667	neg
3.64_537.96	537.969657	3.64241667	neg
3.64_547.92	547.925304	3.64241667	neg

3.64_623.01	623.016912	3.64241667	neg
3.64_642.00	642.00417	3.64241667	neg
3.64_666.07	666.076557	3.64241667	neg
3.64_668.07	668.07335	3.64241667	neg
3.64_691.00	691.004351	3.64241667	neg
3.64_693.00	693.001585	3.64241667	neg
3.64_700.95	700.959937	3.64241667	neg
3.64_758.99	758.992006	3.64241667	neg
3.64_826.97	826.979584	3.64241667	neg
3.64_914.02	914.02351	3.64241667	neg
3.65_340.68	340.684149	3.64815	pos
3.65_361.09	362.09977	3.64815	pos
3.65_395.40	395.407092	3.64815	pos
3.65_406.06	406.063673	3.64815	pos
3.65_514.76	514.766471	3.64815	pos
3.65_517.25	517.256784	3.64815	pos
3.65_566.68	566.687363	3.64815	pos
3.65_566.88	566.888096	3.64815	pos
3.65_567.08	567.088297	3.64815	pos
3.65_568.31	551.30971	3.64815	pos
3.65_568.56	551.559005	3.64815	pos
3.65_568.81	551.809592	3.64815	pos
3.65_654.58	654.589814	3.64815	pos
3.65_665.30	665.304667	3.64815	pos
3.65_705.69	705.69747	3.64815	pos
3.65_708.35	708.357736	3.64815	pos
3.65_708.60	708.608335	3.64815	pos
3.65_708.85	708.858966	3.64815	pos
3.65_735.07	735.075307	3.64815	pos
3.65_735.40	735.409688	3.64815	pos
3.65_943.80	943.805733	3.64815	pos
3.65_944.47	944.47482	3.64815	pos
3.66_102.04	141.010098	3.66498333	pos

3.66_117.05	117.057509	3.66498333	pos
3.66_119.07	119.07313	3.66498333	pos
3.66_136.00	154.041743	3.66498333	pos
3.66_138.00	156.038699	3.66498333	pos
3.66_143.00	143.007187	3.66498333	pos
3.66_182.01	200.047313	3.66498333	pos
3.66_197.94	197.941834	3.65995	neg
3.66_199.95	199.955061	3.66498333	pos
3.66_200.13	200.138798	3.66498333	pos
3.66_201.95	201.950251	3.66498333	pos
3.66_221.02	222.028989	3.66498333	pos
3.66_246.00	246.008017	3.66498333	pos
3.66_270.05	270.053604	3.65995	neg
3.66_274.03	274.038943	3.65995	neg
3.66_299.97	299.971674	3.66498333	pos
3.66_323.97	323.978507	3.65995	neg
3.66_325.97	325.975584	3.65995	neg
3.66_328.18	328.187591	3.65995	neg
3.66_330.20	330.2021	3.66498333	pos
3.66_334.00	334.007453	3.65995	neg
3.66_336.00	336.004463	3.65995	neg
3.66_340.96	340.968507	3.65995	neg
3.66_343.96	343.962694	3.65995	neg
3.66_360.08	360.085394	3.65995	neg
3.66_398.07	421.068715	3.66498333	pos
3.66_400.07	423.065497	3.66498333	pos
3.66_403.07	403.075701	3.65995	neg
3.66_445.06	445.062949	3.65995	neg
3.66_475.50	475.502826	3.66498333	pos
3.66_478.19	478.198572	3.66498333	pos
3.66_506.02	506.028939	3.65995	neg
3.66_532.77	532.778998	3.66498333	pos
3.66_567.98	566.982191	3.65995	neg

3.66_632.97[illegible]	632.97244	3.65995	neg
3.66_634.96[illegible]	634.969684	3.65995	neg
3.66_640.00[illegible]	640.00729	3.65995	neg
3.66_649.09[illegible]	649.094795	3.65995	neg
3.66_651.09[illegible]	651.091868	3.65995	neg
3.66_687.04[illegible]	688.050464	3.66498333	pos
3.66_695.02[illegible]	695.029208	3.65995	neg
3.66_697.02[illegible]	697.026932	3.65995	neg
3.66_700.03[illegible]	700.031123	3.65995	neg
3.66_717.08[illegible]	717.082333	3.65995	neg
3.66_786.00[illegible]	786.00644	3.65995	neg
3.66_802.12[illegible]	802.128554	3.65995	neg
3.66_819.11[illegible]	819.110524	3.65995	neg
3.66_844.03[illegible]	844.038526	3.65995	neg
3.66_846.03[illegible]	846.035968	3.65995	neg
3.66_997.07[illegible]	997.072843	3.65995	neg
3.66_999.07[illegible]	999.070609	3.65995	neg
3.68_1084.1[illegible]	1084.11729	3.67736667	neg
3.68_1105.1[illegible]	1086.11532	3.67736667	neg
3.68_1152.1[illegible]	1152.10366	3.67736667	neg
3.68_163.06[illegible]	162.055804	3.67736667	neg
3.68_198.09[illegible]	198.095061	3.67736667	neg
3.68_200.02[illegible]	200.029345	3.67736667	neg
3.68_210.99[illegible]	255.991213	3.67736667	neg
3.68_234.00[illegible]	234.009106	3.67736667	neg
3.68_236.00[illegible]	236.006139	3.67736667	neg
3.68_240.00[illegible]	285.007118	3.67736667	neg
3.68_257.98[illegible]	257.98832	3.67736667	neg
3.68_266.02[illegible]	266.019976	3.67736667	neg
3.68_268.01[illegible]	268.016988	3.67736667	neg
3.68_283.99[illegible]	283.991206	3.67736667	neg
3.68_304.05[illegible]	304.056777	3.67736667	neg
3.68_310.00[illegible]	310.009828	3.67736667	neg

3.68_310.04[illegible]	310.046222	3.67736667	neg
3.68_312.00[illegible]	312.006642	3.67736667	neg
3.68_317.98[illegible]	317.981937	3.67736667	neg
3.68_331.06[illegible]	331.060385	3.67736667	neg
3.68_349.98[illegible]	349.981158	3.67736667	neg
3.68_350.99[illegible]	350.997508	3.67736667	neg
3.68_352.99[illegible]	352.994478	3.67736667	neg
3.68_397.01[illegible]	377.997151	3.67736667	neg
3.68_399.06[illegible]	399.069347	3.67736667	neg
3.68_435.23[illegible]	435.239183	3.68235	pos
3.68_465.03[illegible]	465.032562	3.68235	pos
3.68_470.05[illegible]	470.059276	3.67736667	neg
3.68_477.01[illegible]	477.013075	3.67736667	neg
3.68_487.04[illegible]	487.041858	3.67736667	neg
3.68_489.03[illegible]	489.038663	3.67736667	neg
3.68_493.76[illegible]	493.767198	3.68235	pos
3.68_496.99[illegible]	496.997372	3.67736667	neg
3.68_500.00[illegible]	545.000495	3.67736667	neg
3.68_504.03[illegible]	504.032185	3.67736667	neg
3.68_507.26[illegible]	507.268167	3.68235	pos
3.68_507.51[illegible]	507.51524	3.68235	pos
3.68_531.03[illegible]	531.03138	3.67736667	neg
3.68_541.00[illegible]	541.000791	3.67736667	neg
3.68_541.99[illegible]	541.994119	3.67736667	neg
3.68_546.99[illegible]	546.997457	3.67736667	neg
3.68_555.02[illegible]	555.029192	3.67736667	neg
3.68_564.54[illegible]	564.54268	3.68235	pos
3.68_565.04[illegible]	565.041701	3.68235	pos
3.68_572.01[illegible]	572.019697	3.67736667	neg
3.68_574.01[illegible]	574.016463	3.67736667	neg
3.68_580.01[illegible]	625.013843	3.67736667	neg
3.68_640.07[illegible]	640.076412	3.67736667	neg
3.68_642.07[illegible]	642.073349	3.67736667	neg

3.68_652.029	652.029047	3.67736667	neg
3.68_708.063	708.0639	3.67736667	neg
3.68_710.061	710.061091	3.67736667	neg
3.68_718.019	718.019574	3.67736667	neg
3.68_721.023	720.016423	3.67736667	neg
3.68_727.051	727.051388	3.67736667	neg
3.68_776.051	776.051852	3.67736667	neg
3.68_778.048	778.048838	3.67736667	neg
3.68_786.733	786.733134	3.68235	pos
3.68_788.003	788.003702	3.67736667	neg
3.68_821.108	821.108127	3.67736667	neg
3.68_848.065	848.064982	3.67736667	neg
3.68_850.061	850.061521	3.67736667	neg
3.68_861.098	861.09849	3.67736667	neg
3.68_863.095	863.095583	3.67736667	neg
3.68_929.086	929.086024	3.67736667	neg
3.68_931.083	931.083672	3.67736667	neg
3.69_151.039	151.039828	3.69436667	neg
3.69_227.139	227.13995	3.69436667	neg
3.69_266.008	266.008387	3.69436667	neg
3.69_278.114	278.114498	3.69436667	neg
3.69_284.161	284.161476	3.69436667	neg
3.69_387.966	387.965993	3.69436667	neg
3.69_393.177	393.177799	3.69436667	neg
3.69_393.962	393.962712	3.69436667	neg
3.69_407.951	407.951046	3.69436667	neg
3.69_475.938	475.938403	3.69436667	neg
3.69_486.972	486.972418	3.69436667	neg
3.69_543.926	543.926469	3.69436667	neg
3.70_203.142	203.142389	3.69811667	pos
3.70_228.147	229.154534	3.69811667	pos
3.70_277.104	277.104546	3.69811667	pos
3.70_286.175	286.175864	3.69811667	pos

3.70_311.998	311.998281	3.69811667	pos
3.70_369.213	369.213237	3.69811667	pos
3.70_379.985	379.985935	3.69811667	pos
3.70_401.247	401.247922	3.69811667	pos
3.70_435.484	435.484878	3.69811667	pos
3.70_500.513	500.513618	3.69811667	pos
3.70_500.764	500.764299	3.69811667	pos
3.70_509.606	509.606802	3.69811667	pos
3.70_509.941	509.94134	3.69811667	pos
3.70_555.588	555.588404	3.69811667	pos
3.70_561.535	561.535596	3.69811667	pos
3.70_590.054	590.054564	3.69811667	pos
3.70_590.303	590.303176	3.69811667	pos
3.70_593.299	593.29916	3.69811667	pos
3.70_593.799	593.799242	3.69811667	pos
3.70_618.561	618.56134	3.69811667	pos
3.70_618.811	618.811721	3.69811667	pos
3.70_619.062	619.06233	3.69811667	pos
3.70_622.065	622.065597	3.69811667	pos
3.70_640.842	640.842729	3.69811667	pos
3.70_824.412	824.412131	3.69811667	pos
3.71_251.113	251.113466	3.71251667	neg
3.71_280.105	279.098453	3.71251667	neg
3.71_342.087	342.087452	3.71251667	neg
3.71_394.137	394.137694	3.71251667	neg
3.71_461.950	461.950524	3.71251667	neg
3.71_527.940	527.940725	3.71251667	neg
3.71_554.959	554.959777	3.71251667	neg
3.71_616.915	616.914951	3.71251667	neg
3.71_673.944	673.94466	3.71251667	neg
3.71_683.900	683.900265	3.71251667	neg
3.71_732.288	732.28833	3.71251667	neg
3.71_741.932	741.932634	3.71251667	neg

3.71_754.26	754.269913	3.71251667	neg
3.71_776.25	776.251982	3.71251667	neg
3.71_809.92	809.920109	3.71251667	neg
3.71_836.89	836.894304	3.71251667	neg
3.71_837.39	837.394818	3.71251667	neg
3.71_844.23	844.239188	3.71251667	neg
3.71_859.37	859.375374	3.71251667	neg
3.72_180.07	163.075447	3.71513333	pos
3.72_180.99	180.990001	3.71513333	pos
3.72_227.65	227.654669	3.71513333	pos
3.72_235.67	235.670564	3.71513333	pos
3.72_258.68	258.683075	3.71513333	pos
3.72_260.08	261.09057	3.71513333	pos
3.72_272.26	272.266645	3.71513333	pos
3.72_272.66	272.662249	3.71513333	pos
3.72_276.16	276.163073	3.71513333	pos
3.72_322.14	322.147202	3.71513333	pos
3.72_332.18	332.181504	3.71513333	pos
3.72_341.71	341.71034	3.71513333	pos
3.72_366.69	366.698298	3.71513333	pos
3.72_377.08	395.116271	3.71513333	pos
3.72_394.61	394.614521	3.71513333	pos
3.72_399.72	399.727092	3.71513333	pos
3.72_401.71	401.719136	3.71513333	pos
3.72_432.88	432.88509	3.71513333	pos
3.72_437.23	420.228658	3.71513333	pos
3.72_455.56	455.569186	3.71513333	pos
3.72_473.25	473.258217	3.71513333	pos
3.72_473.74	474.754418	3.71513333	pos
3.72_476.99	476.996577	3.71513333	pos
3.72_478.24	478.249678	3.71513333	pos
3.72_485.74	485.745243	3.71513333	pos
3.72_497.23	497.237674	3.71513333	pos

3.72_557.02[illegible]	558.036617	3.71513333	pos
3.72_558.28[illegible]	558.286516	3.71513333	pos
3.72_558.53[illegible]	558.537948	3.71513333	pos
3.72_559.60[illegible]	559.608077	3.71513333	pos
3.72_575.26[illegible]	575.263656	3.71513333	pos
3.72_581.27[illegible]	581.270466	3.71513333	pos
3.72_581.52[illegible]	581.520778	3.71513333	pos
3.72_581.77[illegible]	581.771284	3.71513333	pos
3.72_585.57[illegible]	568.573173	3.71513333	pos
3.72_585.82[illegible]	568.824072	3.71513333	pos
3.72_594.04[illegible]	594.049485	3.71513333	pos
3.72_608.06[illegible]	608.061244	3.71513333	pos
3.72_650.82[illegible]	650.825437	3.71513333	pos
3.72_651.07[illegible]	651.072982	3.71513333	pos
3.72_676.08[illegible]	676.084099	3.71513333	pos
3.72_676.33[illegible]	676.334567	3.71513333	pos
3.72_676.58[illegible]	676.58538	3.71513333	pos
3.72_676.83[illegible]	676.836308	3.71513333	pos
3.72_733.29[illegible]	734.30217	3.71513333	pos
3.72_744.04[illegible]	744.046738	3.71513333	pos
3.72_758.09[illegible]	758.095292	3.71513333	pos
3.72_839.40[illegible]	839.408308	3.71513333	pos
3.72_901.10[illegible]	901.109092	3.71513333	pos
3.72_901.44[illegible]	901.444248	3.71513333	pos
3.72_901.77[illegible]	901.777994	3.71513333	pos
3.72_908.43[illegible]	908.436817	3.71513333	pos
3.73_1075.0[illegible]	1075.04742	3.73108333	neg
3.73_143.04[illegible]	143.04604	3.73108333	neg
3.73_184.61[illegible]	184.618436	3.73238333	pos
3.73_209.12[illegible]	209.127231	3.73238333	pos
3.73_209.46[illegible]	209.461698	3.73238333	pos
3.73_241.65[illegible]	241.657963	3.73238333	pos
3.73_247.54[illegible]	247.544565	3.73238333	pos

3.73_260.10	260.103924	3.73108333	neg
3.73_295.12	295.129129	3.73238333	pos
3.73_296.13	296.135102	3.73238333	pos
3.73_300.06	300.064312	3.73108333	neg
3.73_302.06	302.061246	3.73108333	neg
3.73_304.07	304.076117	3.73238333	pos
3.73_308.65	308.658034	3.73238333	pos
3.73_309.16	309.160015	3.73238333	pos
3.73_311.84	311.841076	3.73238333	pos
3.73_312.17	312.175432	3.73238333	pos
3.73_312.50	312.509458	3.73238333	pos
3.73_313.18	313.187078	3.73238333	pos
3.73_316.15	316.151338	3.73108333	neg
3.73_318.16	318.1659	3.73238333	pos
3.73_335.85	335.856593	3.73238333	pos
3.73_338.04	338.0414	3.73108333	neg
3.73_340.12	340.126029	3.73108333	neg
3.73_353.21	353.218501	3.73238333	pos
3.73_353.46	353.468225	3.73238333	pos
3.73_366.21	366.21455	3.73108333	neg
3.73_368.22	368.229467	3.73238333	pos
3.73_370.53	370.536792	3.73238333	pos
3.73_372.10	372.101572	3.73238333	pos
3.73_385.22	385.226492	3.73238333	pos
3.73_387.48	387.484956	3.73238333	pos
3.73_387.98	387.986462	3.73238333	pos
3.73_389.54	389.543904	3.73238333	pos
3.73_389.87	389.878492	3.73238333	pos
3.73_391.20	391.207549	3.73238333	pos
3.73_396.17	396.176766	3.73238333	pos
3.73_419.56	419.560775	3.73238333	pos
3.73_430.88	430.888814	3.73238333	pos
3.73_434.98	434.988	3.73238333	pos

3.73_436.039	436.039175	3.73108333	neg
3.73_454.890	472.924453	3.73238333	pos
3.73_464.214	464.21479	3.73108333	neg
3.73_466.250	489.239725	3.73238333	pos
3.73_466.687	467.69519	3.73238333	pos
3.73_535.967	574.930109	3.73238333	pos
3.73_547.621	547.621806	3.73238333	pos
3.73_550.814	550.814298	3.73238333	pos
3.73_582.804	583.81212	3.73238333	pos
3.73_598.817	598.817206	3.73238333	pos
3.73_622.301	640.33537	3.73238333	pos
3.73_624.317	624.317868	3.73238333	pos
3.73_624.652	624.652175	3.73238333	pos
3.73_640.000	640.000628	3.73238333	pos
3.73_640.668	640.668413	3.73238333	pos
3.73_705.611	705.611126	3.73238333	pos
3.73_706.113	706.113201	3.73238333	pos
3.73_718.042	718.042615	3.73238333	pos
3.73_718.376	718.376676	3.73238333	pos
3.73_725.369	725.369333	3.73238333	pos
3.73_734.082	734.082306	3.73238333	pos
3.73_734.416	734.416597	3.73238333	pos
3.73_735.714	718.710881	3.73238333	pos
3.73_742.705	743.712543	3.73238333	pos
3.73_775.024	775.024684	3.73238333	pos
3.73_877.907	877.907213	3.73108333	neg
3.73_932.368	932.36832	3.73108333	neg
3.73_934.382	934.382155	3.73238333	pos
3.75_1020.54	1020.54287	3.74966667	neg
3.75_1036.05	1036.05003	3.74966667	neg
3.75_1047.04	1047.04086	3.74966667	neg
3.75_107.024	107.024897	3.74966667	neg
3.75_132.074	132.074548	3.74966667	neg

3.75_155.034	155.03484	3.74966667	neg
3.75_196.010	196.010518	3.74966667	neg
3.75_198.006	198.006505	3.74966667	neg
3.75_211.133	211.133352	3.75006667	pos
3.75_250.645	250.645176	3.75006667	pos
3.75_263.998	263.998093	3.74966667	neg
3.75_275.173	320.171318	3.74966667	neg
3.75_282.146	282.146611	3.75006667	pos
3.75_300.707	300.707937	3.75006667	pos
3.75_306.603	306.603946	3.75006667	pos
3.75_314.692	314.692769	3.75006667	pos
3.75_329.681	329.681686	3.75006667	pos
3.75_335.522	335.522448	3.75006667	pos
3.75_336.689	336.689621	3.75006667	pos
3.75_344.854	344.854167	3.75006667	pos
3.75_349.099	349.099221	3.74966667	neg
3.75_349.189	349.189329	3.75006667	pos
3.75_350.722	333.718726	3.75006667	pos
3.75_358.656	358.656348	3.75006667	pos
3.75_366.150	366.150259	3.75006667	pos
3.75_372.720	372.720214	3.75006667	pos
3.75_383.714	383.714208	3.75006667	pos
3.75_386.563	386.562979	3.75006667	pos
3.75_388.159	388.159226	3.74966667	neg
3.75_393.214	393.214335	3.75006667	pos
3.75_414.481	414.480967	3.75006667	pos
3.75_414.731	414.731914	3.75006667	pos
3.75_437.570	437.57039	3.75006667	pos
3.75_437.904	437.904502	3.75006667	pos
3.75_445.253	445.253835	3.75006667	pos
3.75_448.571	448.571423	3.75006667	pos
3.75_448.906	448.905962	3.75006667	pos
3.75_456.146	456.146486	3.74966667	neg

3.75_466.58[illegible]	466.58806	3.75006667	pos
3.75_473.23[illegible]	473.238941	3.75006667	pos
3.75_475.91[illegible]	475.916491	3.75006667	pos
3.75_476.25[illegible]	476.250733	3.75006667	pos
3.75_476.58[illegible]	476.585088	3.75006667	pos
3.75_480.77[illegible]	480.777615	3.75006667	pos
3.75_498.90[illegible]	498.9073	3.75006667	pos
3.75_516.58[illegible]	534.621452	3.75006667	pos
3.75_522.78[illegible]	522.783405	3.75006667	pos
3.75_524.13[illegible]	524.13383	3.74966667	neg
3.75_534.95[illegible]	534.956314	3.75006667	pos
3.75_552.64[illegible]	552.640278	3.75006667	pos
3.75_556.30[illegible]	556.308954	3.75006667	pos
3.75_606.53[illegible]	606.532548	3.75006667	pos
3.75_606.78[illegible]	606.782915	3.75006667	pos
3.75_607.03[illegible]	607.033734	3.75006667	pos
3.75_607.28[illegible]	607.284015	3.75006667	pos
3.75_614.29[illegible]	614.294466	3.75006667	pos
3.75_614.62[illegible]	614.62872	3.75006667	pos
3.75_650.32[illegible]	650.324938	3.75006667	pos
3.75_662.66[illegible]	662.665038	3.75006667	pos
3.75_662.99[illegible]	662.998076	3.75006667	pos
3.75_682.04[illegible]	682.040838	3.75006667	pos
3.75_682.37[illegible]	682.375025	3.75006667	pos
3.75_682.70[illegible]	682.709522	3.75006667	pos
3.75_689.37[illegible]	689.37116	3.75006667	pos
3.75_692.04[illegible]	692.046147	3.75006667	pos
3.75_692.37[illegible]	692.378557	3.75006667	pos
3.75_692.71[illegible]	692.712819	3.75006667	pos
3.75_698.36[illegible]	699.371703	3.75006667	pos
3.75_699.70[illegible]	699.705624	3.75006667	pos
3.75_700.04[illegible]	700.0401	3.75006667	pos
3.75_707.03[illegible]	707.033184	3.75006667	pos

3.75_711.35!	711.355817	3.74966667	neg
3.75_713.37(	713.370461	3.75006667	pos
3.75_713.87:	713.872259	3.75006667	pos
3.75_722.85:	722.851106	3.74966667	neg
3.75_739.87!	739.878107	3.75006667	pos
3.75_740.38(	740.380178	3.75006667	pos
3.75_808.70!	808.708112	3.75006667	pos
3.77_1004.0(	1004.00578	3.7682	neg
3.77_1004.5(	1004.50776	3.7682	neg
3.77_176.81	176.817107	3.7682	neg
3.77_176.95!	176.959511	3.76795	pos
3.77_243.65!	243.655215	3.76795	pos
3.77_260.13	261.14447	3.76795	pos
3.77_273.65!	273.655551	3.76795	pos
3.77_274.15	274.157684	3.76795	pos
3.77_281.11:	281.111805	3.7682	neg
3.77_281.16!	304.157998	3.76795	pos
3.77_282.50(	282.506032	3.76795	pos
3.77_286.69:	286.692206	3.76795	pos
3.77_301.16!	301.169947	3.76795	pos
3.77_310.53:	310.533481	3.76795	pos
3.77_327.91(	327.910398	3.76795	pos
3.77_328.16(	328.160894	3.76795	pos
3.77_330.07!	330.074993	3.7682	neg
3.77_332.07:	332.072029	3.7682	neg
3.77_347.70:	347.703488	3.76795	pos
3.77_360.97‹	360.974231	3.7682	neg
3.77_374.53!	374.539309	3.76795	pos
3.77_375.55:	414.516057	3.76795	pos
3.77_386.85!	386.859341	3.7682	neg
3.77_392.11!	392.119801	3.7682	neg
3.77_395.89!	395.899799	3.76795	pos
3.77_400.05!	400.058986	3.7682	neg

3.77_412.89[illegible]	412.894277	3.76795	pos
3.77_413.22[illegible]	414.23377	3.76795	pos
3.77_414.98[illegible]	414.983201	3.76795	pos
3.77_424.21[illegible]	424.215598	3.76795	pos
3.77_426.55[illegible]	426.558725	3.76795	pos
3.77_429.46[illegible]	429.468605	3.76795	pos
3.77_429.71[illegible]	429.719679	3.76795	pos
3.77_437.76[illegible]	437.763342	3.76795	pos
3.77_439.74[illegible]	440.753821	3.76795	pos
3.77_440.24[illegible]	441.255403	3.76795	pos
3.77_452.69[illegible]	452.690178	3.76795	pos
3.77_464.56[illegible]	464.569579	3.76795	pos
3.77_464.90[illegible]	464.903754	3.76795	pos
3.77_487.23[illegible]	505.27001	3.76795	pos
3.77_498.01[illegible]	498.018507	3.76795	pos
3.77_498.26[illegible]	498.269289	3.76795	pos
3.77_498.77[illegible]	498.770326	3.76795	pos
3.77_504.93[illegible]	504.935612	3.76795	pos
3.77_506.75[illegible]	506.756794	3.76795	pos
3.77_508.98[illegible]	547.952831	3.76795	pos
3.77_513.23[illegible]	513.231547	3.76795	pos
3.77_513.56[illegible]	513.565194	3.76795	pos
3.77_526.53[illegible]	526.530198	3.76795	pos
3.77_526.77[illegible]	526.779811	3.76795	pos
3.77_533.66[illegible]	533.663646	3.76795	pos
3.77_533.86[illegible]	533.864015	3.76795	pos
3.77_534.03[illegible]	534.037829	3.7682	neg
3.77_534.26[illegible]	534.264818	3.76795	pos
3.77_534.46[illegible]	534.465564	3.76795	pos
3.77_535.25[illegible]	535.251766	3.76795	pos
3.77_579.29[illegible]	579.294286	3.76795	pos
3.77_579.54[illegible]	579.544941	3.76795	pos
3.77_611.31[illegible]	611.315778	3.7682	neg

3.77_663.68[illegible]	663.688775	3.76795	pos
3.77_664.02[illegible]	664.022876	3.76795	pos
3.77_664.35[illegible]	664.357177	3.76795	pos
3.77_666.82[illegible]	666.827474	3.76795	pos
3.77_667.07[illegible]	667.077125	3.76795	pos
3.77_667.32[illegible]	667.327055	3.76795	pos
3.77_689.30[illegible]	672.306018	3.76795	pos
3.77_689.81[illegible]	672.80852	3.76795	pos
3.77_691.30[illegible]	691.305809	3.7682	neg
3.77_888.76[illegible]	888.766362	3.76795	pos
3.77_889.09[illegible]	889.099794	3.76795	pos
3.77_896.42[illegible]	896.427135	3.76795	pos
3.77_993.01[illegible]	993.014995	3.7682	neg
3.79_188.03[illegible]	188.037736	3.78651667	neg
3.79_202.95[illegible]	202.951205	3.78573333	pos
3.79_207.91[illegible]	207.918435	3.78651667	neg
3.79_215.65[illegible]	215.655057	3.78573333	pos
3.79_216.15[illegible]	216.15689	3.78573333	pos
3.79_221.16[illegible]	221.169822	3.78573333	pos
3.79_254.19[illegible]	254.196346	3.78573333	pos
3.79_276.95[illegible]	276.955199	3.78651667	neg
3.79_282.83[illegible]	282.832819	3.78573333	pos
3.79_285.68[illegible]	285.684592	3.78573333	pos
3.79_295.16[illegible]	295.165459	3.78573333	pos
3.79_312.69[illegible]	312.695378	3.78573333	pos
3.79_324.15[illegible]	324.155811	3.78573333	pos
3.79_325.84[illegible]	325.846975	3.78573333	pos
3.79_326.67[illegible]	326.672842	3.78573333	pos
3.79_329.69[illegible]	329.692079	3.78573333	pos
3.79_330.19[illegible]	330.193611	3.78573333	pos
3.79_343.19[illegible]	343.198009	3.78573333	pos
3.79_389.87[illegible]	389.872151	3.78573333	pos
3.79_390.54[illegible]	390.540617	3.78573333	pos

3.79_412.559	412.559699	3.78573333	pos
3.79_426.893	426.893073	3.78573333	pos
3.79_427.227	427.226966	3.78573333	pos
3.79_428.287	428.28759	3.78651667	neg
3.79_435.763	435.763858	3.78573333	pos
3.79_437.261	437.261296	3.78573333	pos
3.79_442.558	442.558481	3.78573333	pos
3.79_442.892	442.892662	3.78573333	pos
3.79_455.998	455.998054	3.78573333	pos
3.79_470.759	470.759412	3.78573333	pos
3.79_487.256	487.256805	3.78573333	pos
3.79_487.507	487.507457	3.78573333	pos
3.79_488.008	488.008291	3.78573333	pos
3.79_507.007	507.007614	3.78573333	pos
3.79_523.277	523.277752	3.78573333	pos
3.79_526.934	526.934579	3.78573333	pos
3.79_527.269	527.269664	3.78573333	pos
3.79_527.603	527.603153	3.78573333	pos
3.79_534.064	534.064213	3.78573333	pos
3.79_586.284	586.284849	3.78573333	pos
3.79_607.661	607.661906	3.78573333	pos
3.79_643.783	661.817567	3.78573333	pos
3.79_649.673	649.673065	3.78573333	pos
3.79_650.006	650.006363	3.78573333	pos
3.79_744.694	744.694599	3.78573333	pos
3.79_745.030	745.030122	3.78573333	pos
3.80_145.053	146.060287	3.80401667	pos
3.80_145.061	145.061713	3.80496667	neg
3.80_197.154	197.15404	3.80401667	pos
3.80_217.012	217.012724	3.80496667	neg
3.80_232.136	232.136777	3.80401667	pos
3.80_346.684	346.684712	3.80401667	pos
3.80_348.070	348.070731	3.80401667	pos

3.80_355.175	373.209219	3.80401667	pos
3.80_355.928	355.927957	3.80401667	pos
3.80_356.429	356.429034	3.80401667	pos
3.80_359.657	359.657803	3.80401667	pos
3.80_371.315	371.315825	3.80401667	pos
3.80_372.117	372.117689	3.80496667	neg
3.80_374.975	374.975511	3.80401667	pos
3.80_381.691	399.725171	3.80401667	pos
3.80_386.617	386.617417	3.80401667	pos
3.80_402.229	402.229269	3.80401667	pos
3.80_403.887	403.887681	3.80401667	pos
3.80_405.193	405.193852	3.80401667	pos
3.80_410.156	410.156888	3.80496667	neg
3.80_411.163	412.171114	3.80401667	pos
3.80_416.215	416.215146	3.80496667	neg
3.80_417.223	418.230436	3.80401667	pos
3.80_417.867	417.867439	3.80401667	pos
3.80_418.250	418.25083	3.80401667	pos
3.80_420.546	420.545959	3.80401667	pos
3.80_421.047	421.047393	3.80401667	pos
3.80_428.922	428.922698	3.80401667	pos
3.80_434.530	417.533134	3.80401667	pos
3.80_434.874	434.874152	3.80401667	pos
3.80_435.209	435.209254	3.80401667	pos
3.80_440.105	440.105141	3.80496667	neg
3.80_441.498	441.498567	3.80401667	pos
3.80_442.000	442.000272	3.80401667	pos
3.80_458.552	458.552002	3.80401667	pos
3.80_458.886	458.886026	3.80401667	pos
3.80_463.555	463.555899	3.80401667	pos
3.80_474.569	474.569267	3.80401667	pos
3.80_478.144	478.14429	3.80496667	neg
3.80_499.699	499.699096	3.80401667	pos

3.80_521.762	521.762771	3.80401667	pos
3.80_529.791	529.791005	3.80401667	pos
3.80_530.292	530.292308	3.80401667	pos
3.80_548.284	548.284736	3.80401667	pos
3.80_550.254	550.254275	3.80401667	pos
3.80_555.917	555.917594	3.80401667	pos
3.80_583.636	583.636321	3.80401667	pos
3.80_612.938	612.938439	3.80401667	pos
3.80_613.272	613.272865	3.80401667	pos
3.80_613.606	613.606563	3.80401667	pos
3.80_622.812	622.812785	3.80401667	pos
3.80_623.318	623.318705	3.80401667	pos
3.80_627.829	627.829842	3.80401667	pos
3.80_630.810	630.810745	3.80401667	pos
3.80_631.061	631.061177	3.80401667	pos
3.80_651.808	651.808245	3.80401667	pos
3.80_661.316	661.31647	3.80401667	pos
3.80_670.679	670.679394	3.80401667	pos
3.80_716.293	716.29349	3.80496667	neg
3.80_746.285	746.285102	3.80496667	neg
3.80_786.164	786.164721	3.80401667	pos
3.80_806.132	806.132331	3.80496667	neg
3.80_874.120	874.120015	3.80496667	neg
3.82_104.053	104.053367	3.82191667	pos
3.82_148.043	148.043578	3.82341667	neg
3.82_148.051	148.051488	3.82341667	neg
3.82_167.046	167.046018	3.82341667	neg
3.82_167.061	150.058474	3.82191667	pos
3.82_178.110	179.1181	3.82191667	pos
3.82_191.061	214.051056	3.82191667	pos
3.82_194.065	194.064988	3.82191667	pos
3.82_229.155	229.15553	3.82341667	neg
3.82_236.032	236.032931	3.82191667	pos

3.82_250.00!	250.009884	3.82341667	neg
3.82_252.40(	252.406804	3.82191667	pos
3.82_255.04:	255.0424	3.82191667	pos
3.82_264.16	264.168707	3.82191667	pos
3.82_279.68(	279.686361	3.82191667	pos
3.82_287.01!	287.015026	3.82191667	pos
3.82_290.67	290.677324	3.82191667	pos
3.82_294.52	294.527835	3.82191667	pos
3.82_298.07:	321.063061	3.82191667	pos
3.82_342.17	342.17181	3.82191667	pos
3.82_343.01!	343.019136	3.82341667	neg
3.82_359.45	359.454629	3.82191667	pos
3.82_359.70!	359.705338	3.82191667	pos
3.82_359.95(	359.956211	3.82191667	pos
3.82_361.67	361.677508	3.82191667	pos
3.82_378.73	378.73468	3.82191667	pos
3.82_394.01(	394.016514	3.82341667	neg
3.82_401.54!	419.579026	3.82191667	pos
3.82_410.22	393.223827	3.82191667	pos
3.82_420.25	459.220169	3.82191667	pos
3.82_438.68:	438.683279	3.82191667	pos
3.82_438.93	477.894975	3.82191667	pos
3.82_439.25(	439.250377	3.82191667	pos
3.82_449.24:	449.243907	3.82191667	pos
3.82_449.74!	449.745497	3.82191667	pos
3.82_454.57	477.560854	3.82191667	pos
3.82_461.24!	461.249123	3.82191667	pos
3.82_461.58:	461.583781	3.82191667	pos
3.82_462.00	462.004217	3.82341667	neg
3.82_479.58	479.588508	3.82191667	pos
3.82_493.02:	493.023677	3.82191667	pos
3.82_505.26	505.264034	3.82191667	pos
3.82_505.59	505.598934	3.82191667	pos

3.82_511.268	511.268525	3.82191667	pos
3.82_528.520	528.520422	3.82191667	pos
3.82_529.991	529.991494	3.82341667	neg
3.82_582.967	582.967725	3.82191667	pos
3.82_591.306	591.306022	3.82191667	pos
3.82_595.983	595.983696	3.82191667	pos
3.82_670.344	670.344231	3.82191667	pos
3.82_760.257	760.257201	3.82341667	neg
3.82_785.055	785.055433	3.82191667	pos
3.82_785.390	785.390063	3.82191667	pos
3.82_808.146	808.146868	3.82191667	pos
3.84_172.956	172.956557	3.83913333	pos
3.84_177.099	177.099752	3.83913333	pos
3.84_190.928	190.928295	3.84145	neg
3.84_197.042	197.04271	3.84145	neg
3.84_244.657	244.657857	3.83913333	pos
3.84_258.915	258.915716	3.84145	neg
3.84_281.149	281.149758	3.83913333	pos
3.84_295.511	295.511933	3.83913333	pos
3.84_296.890	296.890196	3.84145	neg
3.84_302.179	302.179047	3.83913333	pos
3.84_306.180	329.175819	3.83913333	pos
3.84_320.693	320.692963	3.83913333	pos
3.84_322.703	322.70336	3.83913333	pos
3.84_326.903	326.903274	3.84145	neg
3.84_328.674	328.674225	3.83913333	pos
3.84_359.229	359.229563	3.83913333	pos
3.84_359.731	359.731007	3.83913333	pos
3.84_411.880	411.880905	3.84145	neg
3.84_421.722	421.722462	3.83913333	pos
3.84_422.239	445.228458	3.83913333	pos
3.84_422.384	422.384724	3.83913333	pos
3.84_445.706	445.706327	3.83913333	pos

3.84_452.85(	452.850053	3.84145	neg
3.84_454.72ɛ	454.728206	3.83913333	pos
3.84_458.584	458.584095	3.83913333	pos
3.84_458.91ɛ	458.91841	3.83913333	pos
3.84_462.87ɛ	462.878311	3.84145	neg
3.84_474.21	492.245508	3.83913333	pos
3.84_530.86!	530.865753	3.84145	neg
3.84_627.65ɛ	627.65832	3.83913333	pos
3.84_701.01	701.014481	3.83913333	pos
3.86_1013.0	1013.01379	3.85685	pos
3.86_103.92(	103.920083	3.86	neg
3.86_107.96	107.967238	3.85685	pos
3.86_115.094	115.094846	3.85685	pos
3.86_125.06(	125.060626	3.86	neg
3.86_152.99!	152.995059	3.85685	pos
3.86_161.05!	161.059915	3.85685	pos
3.86_178.12(	201.1099	3.85685	pos
3.86_178.97	178.977587	3.86	neg
3.86_181.13ɛ	181.138722	3.85685	pos
3.86_220.56!	440.132384	3.86	neg
3.86_221.57	221.577327	3.85685	pos
3.86_224.18!	224.18572	3.85685	pos
3.86_226.16ɛ	227.175418	3.85685	pos
3.86_245.66ɛ	268.657822	3.85685	pos
3.86_269.64	269.647351	3.85685	pos
3.86_292.13(	292.130422	3.86	neg
3.86_292.17:	292.173006	3.85685	pos
3.86_293.68	293.687471	3.85685	pos
3.86_294.14!	294.14506	3.85685	pos
3.86_321.95:	321.95314	3.85685	pos
3.86_335.20(	335.20611	3.85685	pos
3.86_350.98	350.987164	3.85685	pos
3.86_352.17!	370.209271	3.85685	pos

3.86_363.62	363.625773	3.85685	pos
3.86_370.54	370.543322	3.85685	pos
3.86_375.22	375.224557	3.85685	pos
3.86_380.14	380.146386	3.86	neg
3.86_406.69	406.6935	3.85685	pos
3.86_416.89	416.898803	3.85685	pos
3.86_419.76	419.761471	3.85685	pos
3.86_424.89	424.896383	3.85685	pos
3.86_474.25	474.25645	3.85685	pos
3.86_489.96	489.965578	3.85685	pos
3.86_500.76	500.765147	3.85685	pos
3.86_515.28	515.28343	3.86	neg
3.86_580.81	580.816529	3.85685	pos
3.86_624.26	624.263206	3.86	neg
3.86_683.29	683.299934	3.85685	pos
3.86_691.38	691.385081	3.85685	pos
3.86_691.71	691.719227	3.85685	pos
3.86_699.04	699.046647	3.85685	pos
3.86_774.84	774.84909	3.86	neg
3.87_199.98	199.988472	3.87373333	pos
3.87_285.68	285.684653	3.87373333	pos
3.87_286.95	286.958398	3.87373333	pos
3.87_337.70	337.708423	3.87373333	pos
3.87_350.17	350.171687	3.87373333	pos
3.87_354.66	355.672681	3.87373333	pos
3.87_357.71	357.711553	3.87373333	pos
3.87_391.96	391.966043	3.87373333	pos
3.87_397.18	415.218945	3.87373333	pos
3.87_398.45	398.451017	3.87373333	pos
3.87_409.72	409.725727	3.87373333	pos
3.87_412.24	412.243971	3.87373333	pos
3.87_429.71	429.719912	3.87373333	pos
3.87_470.72	470.723228	3.87373333	pos

3.87_474.288	474.288595	3.87373333	pos
3.87_490.300	490.300201	3.87373333	pos
3.87_498.257	498.257853	3.87373333	pos
3.87_510.583	510.58383	3.87373333	pos
3.87_510.918	510.918116	3.87373333	pos
3.87_511.252	511.252318	3.87373333	pos
3.87_536.285	536.285597	3.87373333	pos
3.87_545.631	545.631248	3.87373333	pos
3.87_552.787	552.787641	3.87373333	pos
3.87_558.104	558.104317	3.87373333	pos
3.87_558.271	558.271134	3.87373333	pos
3.87_562.560	585.549382	3.87373333	pos
3.87_563.610	563.610361	3.87373333	pos
3.87_563.944	563.944382	3.87373333	pos
3.87_585.298	585.298847	3.87373333	pos
3.87_588.525	588.525078	3.87373333	pos
3.87_588.776	588.775958	3.87373333	pos
3.87_589.026	589.026035	3.87373333	pos
3.87_589.276	589.276498	3.87373333	pos
3.87_591.277	591.277201	3.87373333	pos
3.87_632.822	632.822812	3.87373333	pos
3.87_761.709	784.698194	3.87373333	pos
3.87_762.042	785.031876	3.87373333	pos
3.87_765.371	765.371872	3.87373333	pos
3.87_765.872	765.872792	3.87373333	pos
3.87_862.425	862.425814	3.87373333	pos
3.88_136.909	136.909629	3.8786	neg
3.88_174.019	174.019413	3.8786	neg
3.88_241.982	241.982304	3.8786	neg
3.88_243.980	243.980042	3.8786	neg
3.88_309.969	309.96976	3.8786	neg
3.88_311.967	311.967533	3.8786	neg
3.88_313.119	313.119545	3.8786	neg

3.88_373.209	373.209311	3.8786	neg
3.88_433.875	433.875404	3.8786	neg
3.88_446.905	446.905908	3.8786	neg
3.88_547.856	547.855953	3.8786	neg
3.88_598.853	598.853464	3.8786	neg
3.88_666.841	666.840973	3.8786	neg
3.88_763.858	763.858196	3.8786	neg
3.89_217.155	217.155116	3.8922	pos
3.89_222.650	222.650308	3.8922	pos
3.89_236.907	236.907249	3.8922	pos
3.89_239.137	239.137008	3.8922	pos
3.89_273.168	273.168169	3.8922	pos
3.89_273.670	273.670627	3.8922	pos
3.89_286.678	286.678241	3.8922	pos
3.89_293.700	293.700013	3.8922	pos
3.89_304.894	304.894447	3.8922	pos
3.89_309.676	309.676086	3.8922	pos
3.89_327.522	327.522442	3.8922	pos
3.89_327.857	327.857075	3.8922	pos
3.89_337.954	337.954444	3.8922	pos
3.89_363.660	363.6605	3.8922	pos
3.89_390.900	390.899969	3.8922	pos
3.89_393.722	393.722323	3.8922	pos
3.89_396.177	396.177308	3.8922	pos
3.89_396.984	396.984916	3.8922	pos
3.89_409.474	409.474876	3.8922	pos
3.89_420.922	420.922701	3.8922	pos
3.89_421.257	421.257038	3.8922	pos
3.89_432.624	432.623951	3.8922	pos
3.89_437.890	437.890119	3.8922	pos
3.89_442.212	442.212371	3.8922	pos
3.89_442.714	442.714186	3.8922	pos
3.89_444.599	444.59901	3.8922	pos

3.89_473.72[illegible]	473.728951	3.8922	pos
3.89_513.64[illegible]	513.644792	3.8922	pos
3.89_513.97[illegible]	513.979071	3.8922	pos
3.89_518.77[illegible]	518.77071	3.8922	pos
3.89_540.52[illegible]	540.528557	3.8922	pos
3.89_540.77[illegible]	540.779324	3.8922	pos
3.89_558.43[illegible]	558.438154	3.8922	pos
3.89_597.30[illegible]	597.30604	3.8922	pos
3.89_597.64[illegible]	597.640399	3.8922	pos
3.89_597.97[illegible]	597.974811	3.8922	pos
3.89_695.59[illegible]	695.598309	3.8922	pos
3.89_713.35[illegible]	713.35816	3.8922	pos
3.90_232.94[illegible]	232.944217	3.89685	neg
3.90_235.92[illegible]	235.926017	3.89685	neg
3.90_325.90[illegible]	325.906068	3.89685	neg
3.90_328.15[illegible]	328.151383	3.89685	neg
3.90_365.88[illegible]	365.887975	3.89685	neg
3.90_371.90[illegible]	371.900847	3.89685	neg
3.90_384.86[illegible]	384.862326	3.89685	neg
3.90_442.27[illegible]	442.278404	3.89685	neg
3.90_479.86[illegible]	479.868304	3.89685	neg
3.90_501.86[illegible]	501.86305	3.89685	neg
3.91_167.08[illegible]	167.081848	3.91066667	pos
3.91_184.06[illegible]	184.060566	3.91066667	pos
3.91_213.07[illegible]	213.073488	3.91066667	pos
3.91_313.93[illegible]	313.93099	3.91066667	pos
3.91_314.43[illegible]	314.432507	3.91066667	pos
3.91_330.16[illegible]	330.166302	3.91066667	pos
3.91_330.20[illegible]	330.202735	3.91066667	pos
3.91_334.45[illegible]	334.457102	3.91066667	pos
3.91_339.44[illegible]	339.443825	3.91066667	pos
3.91_339.69[illegible]	339.694409	3.91066667	pos
3.91_352.94[illegible]	352.946644	3.91066667	pos

3.91_361.19	361.197743	3.91066667	pos
3.91_377.70	377.700296	3.91066667	pos
3.91_395.24	395.239989	3.91066667	pos
3.91_395.74	395.741766	3.91066667	pos
3.91_396.87	396.872397	3.91066667	pos
3.91_422.55	422.552193	3.91066667	pos
3.91_440.18	440.189216	3.91066667	pos
3.91_441.24	441.247635	3.91066667	pos
3.91_441.58	441.581853	3.91066667	pos
3.91_451.92	451.921712	3.91066667	pos
3.91_470.26	470.260603	3.91066667	pos
3.91_491.93	491.935209	3.91066667	pos
3.91_496.43	496.431911	3.91066667	pos
3.91_504.50	504.502226	3.91066667	pos
3.91_531.53	531.531046	3.91066667	pos
3.91_550.29	550.299813	3.91066667	pos
3.91_556.48	556.480628	3.91066667	pos
3.91_615.32	615.325217	3.91066667	pos
3.91_621.64	639.678431	3.91066667	pos
3.91_621.97	640.011309	3.91066667	pos
3.91_622.29	605.293632	3.91066667	pos
3.91_628.97	629.980474	3.91066667	pos
3.91_630.31	630.313282	3.91066667	pos
3.91_637.83	637.838294	3.91066667	pos
3.91_649.68	649.680978	3.91066667	pos
3.91_650.01	650.015202	3.91066667	pos
3.91_656.34	639.343175	3.91066667	pos
3.91_656.67	656.673829	3.91066667	pos
3.91_657.00	657.008288	3.91066667	pos
3.91_695.34	695.34818	3.91066667	pos
3.91_974.01	974.016967	3.91066667	pos
3.92_189.07	189.076605	3.9154	neg
3.92_276.15	276.156335	3.9154	neg

3.92_293.14	293.146618	3.9154	neg
3.92_303.06	303.065424	3.9154	neg
3.92_314.17	314.172368	3.9154	neg
3.92_342.16	342.166937	3.9154	neg
3.92_382.15	382.159644	3.9154	neg
3.92_396.13	396.138804	3.9154	neg
3.92_437.87	437.872563	3.9154	neg
3.92_576.24	576.242997	3.9154	neg
3.92_735.33	735.332493	3.9154	neg
3.92_803.85	803.85303	3.9154	neg
3.92_972.00	972.002342	3.9154	neg
3.93_111.00	111.008587	3.93381667	neg
3.93_201.07	201.073486	3.92891667	pos
3.93_234.07	234.077039	3.93381667	neg
3.93_241.15	241.154751	3.92891667	pos
3.93_275.90	275.905935	3.93381667	neg
3.93_288.18	288.180845	3.92891667	pos
3.93_305.70	305.700013	3.92891667	pos
3.93_313.69	313.697636	3.92891667	pos
3.93_349.19	349.198023	3.92891667	pos
3.93_349.69	349.698848	3.92891667	pos
3.93_353.19	371.225039	3.92891667	pos
3.93_374.54	374.548838	3.92891667	pos
3.93_377.54	377.549181	3.92891667	pos
3.93_377.88	377.883545	3.92891667	pos
3.93_381.16	381.161015	3.92891667	pos
3.93_391.72	391.724216	3.92891667	pos
3.93_415.48	415.48683	3.92891667	pos
3.93_418.57	418.573222	3.92891667	pos
3.93_422.98	422.982832	3.92891667	pos
3.93_423.48	423.484117	3.92891667	pos
3.93_463.90	463.90945	3.92891667	pos
3.93_466.72	466.724045	3.92891667	pos

3.93_478.27[illegible]	478.27007	3.92891667	pos
3.93_486.29[illegible]	486.292956	3.92891667	pos
3.93_507.92[illegible]	507.925921	3.92891667	pos
3.93_508.97[illegible]	508.972755	3.92891667	pos
3.93_509.64[illegible]	509.641131	3.92891667	pos
3.93_520.24[illegible]	520.24091	3.92891667	pos
3.93_525.56[illegible]	525.565026	3.92891667	pos
3.93_526.23[illegible]	526.233308	3.92891667	pos
3.93_527.04[illegible]	527.039976	3.92891667	pos
3.93_527.79[illegible]	527.792307	3.92891667	pos
3.93_528.29[illegible]	529.299598	3.92891667	pos
3.93_530.78[illegible]	530.780402	3.92891667	pos
3.93_564.05[illegible]	564.052252	3.92891667	pos
3.93_594.76[illegible]	594.769524	3.92891667	pos
3.93_595.23[illegible]	595.236337	3.92891667	pos
3.93_626.29[illegible]	626.293569	3.92891667	pos
3.93_695.84[illegible]	695.84839	3.92891667	pos
3.93_702.71[illegible]	702.718357	3.92891667	pos
3.93_758.86[illegible]	758.869109	3.93381667	neg
3.93_759.87[illegible]	760.883761	3.92891667	pos
3.93_760.37[illegible]	761.385276	3.92891667	pos
3.93_769.85[illegible]	769.859936	3.93381667	neg
3.93_769.89[illegible]	769.898228	3.92891667	pos
3.93_770.36[illegible]	770.361421	3.93381667	neg
3.93_770.86[illegible]	771.874666	3.92891667	pos
3.93_771.36[illegible]	772.376092	3.92891667	pos
3.93_773.94[illegible]	773.946717	3.92891667	pos
3.93_779.85[illegible]	779.858981	3.92891667	pos
3.93_786.83[illegible]	787.842961	3.92891667	pos
3.93_787.33[illegible]	788.344093	3.92891667	pos
3.95_222.98[illegible]	222.983571	3.95231667	neg
3.95_257.00[illegible]	257.005363	3.95231667	neg
3.95_272.06[illegible]	272.068663	3.94663333	pos

3.95_282.64(	282.646706	3.94663333	pos
3.95_295.12(	295.129164	3.94663333	pos
3.95_297.14	297.14479	3.94663333	pos
3.95_310.19(	328.233195	3.94663333	pos
3.95_316.69(	316.690159	3.94663333	pos
3.95_346.66(	346.666452	3.94663333	pos
3.95_357.86(	357.866241	3.94663333	pos
3.95_358.53	358.534776	3.94663333	pos
3.95_383.08	383.083724	3.95231667	neg
3.95_392.95(	392.959717	3.95231667	neg
3.95_411.72	411.722102	3.94663333	pos
3.95_430.22(	430.229706	3.94663333	pos
3.95_431.25	431.251024	3.94663333	pos
3.95_435.91	418.907992	3.94663333	pos
3.95_445.25	428.250667	3.94663333	pos
3.95_451.07	451.071243	3.95231667	neg
3.95_472.79	472.792934	3.94663333	pos
3.95_473.29	473.29471	3.94663333	pos
3.95_531.97	532.986012	3.94663333	pos
3.95_532.31	533.320035	3.94663333	pos
3.95_532.65	532.651489	3.94663333	pos
3.95_547.64(	547.640642	3.94663333	pos
3.95_547.76(	547.760084	3.94663333	pos
3.95_548.26	548.261367	3.94663333	pos
3.95_557.93	557.938186	3.94663333	pos
3.95_571.60	571.602942	3.94663333	pos
3.95_748.37	748.3731	3.94663333	pos
3.95_748.87	748.87465	3.94663333	pos
3.95_808.38	808.384394	3.94663333	pos
3.96_151.07	151.075424	3.96453333	pos
3.96_158.09(	158.09663	3.96453333	pos
3.96_158.11	158.117736	3.96453333	pos
3.96_174.02	174.022185	3.96453333	pos

3.96_181.08[illegible]	181.083494	3.96453333	pos
3.96_190.01[illegible]	190.017102	3.96453333	pos
3.96_287.66[illegible]	287.662775	3.96453333	pos
3.96_296.14[illegible]	319.136592	3.96453333	pos
3.96_301.67[illegible]	301.678969	3.96453333	pos
3.96_308.68[illegible]	308.686848	3.96453333	pos
3.96_309.18[illegible]	309.188657	3.96453333	pos
3.96_312.40[illegible]	312.409065	3.96453333	pos
3.96_318.17[illegible]	318.173775	3.96453333	pos
3.96_335.70[illegible]	335.704031	3.96453333	pos
3.96_341.11[illegible]	341.118498	3.96453333	pos
3.96_343.19[illegible]	343.19801	3.96453333	pos
3.96_347.21[illegible]	347.215006	3.96453333	pos
3.96_347.54[illegible]	347.5497	3.96453333	pos
3.96_347.88[illegible]	347.884115	3.96453333	pos
3.96_349.86[illegible]	349.867384	3.96453333	pos
3.96_374.70[illegible]	374.708576	3.96453333	pos
3.96_382.87[illegible]	382.878039	3.96453333	pos
3.96_383.54[illegible]	383.546395	3.96453333	pos
3.96_387.22[illegible]	387.224187	3.96453333	pos
3.96_400.21[illegible]	383.212773	3.96453333	pos
3.96_401.47[illegible]	401.470345	3.96453333	pos
3.96_401.72[illegible]	401.720459	3.96453333	pos
3.96_401.97[illegible]	401.971549	3.96453333	pos
3.96_402.54[illegible]	402.543996	3.96453333	pos
3.96_432.88[illegible]	432.885746	3.96453333	pos
3.96_447.34[illegible]	447.347518	3.96453333	pos
3.96_455.75[illegible]	455.754064	3.96453333	pos
3.96_469.92[illegible]	469.92552	3.96453333	pos
3.96_470.59[illegible]	470.594438	3.96453333	pos
3.96_480.99[illegible]	499.030641	3.96453333	pos
3.96_481.25[illegible]	464.253006	3.96453333	pos
3.96_494.02[illegible]	494.02743	3.96453333	pos

3.96_494.278	494.277969	3.96453333	pos
3.96_494.529	494.529232	3.96453333	pos
3.96_517.674	517.674722	3.96453333	pos
3.96_526.545	526.545313	3.96453333	pos
3.96_534.624	534.624498	3.96453333	pos
3.96_534.958	534.958778	3.96453333	pos
3.96_541.753	541.753191	3.96453333	pos
3.96_579.296	579.296672	3.96453333	pos
3.96_582.777	582.777291	3.96453333	pos
3.96_583.277	583.2779	3.96453333	pos
3.96_645.833	646.841011	3.96453333	pos
3.96_646.335	664.369313	3.96453333	pos
3.96_646.590	646.59041	3.96453333	pos
3.96_647.091	647.091283	3.96453333	pos
3.96_677.320	677.320777	3.96453333	pos
3.96_677.652	677.652088	3.96453333	pos
3.97_142.037	123.019828	3.97085	neg
3.97_142.066	142.066005	3.97085	neg
3.97_176.997	176.99778	3.97085	neg
3.97_196.004	196.004767	3.97085	neg
3.97_205.035	250.033204	3.97085	neg
3.97_209.984	209.98403	3.97085	neg
3.97_246.130	245.129493	3.97085	neg
3.97_261.986	261.986288	3.97085	neg
3.97_273.022	272.015402	3.97085	neg
3.97_307.982	307.982394	3.97085	neg
3.97_310.046	310.046441	3.97085	neg
3.97_331.979	331.97978	3.97085	neg
3.97_341.009	340.002392	3.97085	neg
3.97_375.969	375.969663	3.97085	neg
3.97_388.062	388.062789	3.97085	neg
3.97_405.052	405.052732	3.97085	neg
3.97_407.990	407.990009	3.97085	neg

3.97_443.95	443.95702	3.97085	neg
3.97_511.94	511.944399	3.97085	neg
3.97_524.03	524.037471	3.97085	neg
3.97_543.96	543.965078	3.97085	neg
3.97_579.93	579.932231	3.97085	neg
3.97_592.02	592.025057	3.97085	neg
3.97_611.95	611.952758	3.97085	neg
3.98_190.07	190.071509	3.98231667	pos
3.98_194.99	217.986046	3.98231667	pos
3.98_208.09	208.097184	3.98231667	pos
3.98_226.16	249.157382	3.98231667	pos
3.98_245.17	245.17493	3.98231667	pos
3.98_253.09	254.102468	3.98231667	pos
3.98_253.50	253.509629	3.98231667	pos
3.98_273.13	273.13122	3.98231667	pos
3.98_294.13	294.133919	3.98231667	pos
3.98_298.06	298.066522	3.98231667	pos
3.98_303.16	303.168651	3.98231667	pos
3.98_322.20	345.19468	3.98231667	pos
3.98_333.17	333.179135	3.98231667	pos
3.98_342.68	342.687547	3.98231667	pos
3.98_344.69	344.693081	3.98231667	pos
3.98_353.96	353.961234	3.98231667	pos
3.98_378.20	378.208333	3.98231667	pos
3.98_378.70	378.709851	3.98231667	pos
3.98_392.24	392.240106	3.98231667	pos
3.98_445.73	445.737114	3.98231667	pos
3.98_448.18	448.189533	3.98231667	pos
3.98_450.76	450.761829	3.98231667	pos
3.98_453.84	453.846879	3.98231667	pos
3.98_454.18	454.181221	3.98231667	pos
3.98_455.57	456.581468	3.98231667	pos
3.98_470.06	470.069432	3.98231667	pos

3.98_476.01!	459.011938	3.98231667	pos
3.98_490.59!	490.598424	3.98231667	pos
3.98_498.52!	498.52935	3.98231667	pos
3.98_498.78(	498.780011	3.98231667	pos
3.98_500.77(	500.770485	3.98231667	pos
3.98_515.22!	533.263563	3.98231667	pos
3.98_515.73	516.744924	3.98231667	pos
3.98_517.47	517.474049	3.98231667	pos
3.98_545.80(	545.806273	3.98231667	pos
3.98_546.30	546.307496	3.98231667	pos
3.98_564.59!	587.584977	3.98231667	pos
3.98_587.33	587.334621	3.98231667	pos
3.98_587.78	588.790854	3.98231667	pos
3.98_588.08(	588.086669	3.98231667	pos
3.98_612.01	612.013939	3.98231667	pos
3.98_618.33	618.333805	3.98231667	pos
3.98_645.80	645.801708	3.98231667	pos
3.98_646.30	646.30302	3.98231667	pos
3.98_651.79!	652.805932	3.98231667	pos
3.98_652.30(	653.30753	3.98231667	pos
3.98_662.78!	663.796893	3.98231667	pos
3.98_664.29!	664.298626	3.98231667	pos
3.98_664.70	664.703589	3.98231667	pos
3.98_665.03!	665.038025	3.98231667	pos
3.98_671.69	671.697081	3.98231667	pos
3.98_819.89	820.904898	3.98231667	pos
3.98_820.39!	821.406566	3.98231667	pos
3.98_831.89!	831.89563	3.98231667	pos
3.98_832.39	832.397145	3.98231667	pos
3.99_144.01	144.01228	3.98933333	neg
3.99_166.03	166.033115	3.98933333	neg
3.99_222.06(	222.060506	3.98933333	neg
3.99_239.99	239.994784	3.98933333	neg

3.99_277.12	277.122664	3.98933333	neg
3.99_278.02	278.023451	3.98933333	neg
3.99_280.02	280.020528	3.98933333	neg
3.99_369.88	369.885017	3.98933333	neg
3.99_424.02	424.027361	3.98933333	neg
3.99_434.13	434.130339	3.98933333	neg
3.99_492.01	492.014802	3.98933333	neg
3.99_507.74	507.74171	3.98933333	neg
3.99_508.24	508.243119	3.98933333	neg
3.99_560.00	560.00194	3.98933333	neg
3.99_650.79	650.791331	3.98933333	neg
3.99_661.78	661.782398	3.98933333	neg
3.99_662.28	662.283999	3.98933333	neg
3.99_672.77	672.773238	3.98933333	neg
3.99_683.76	683.764049	3.98933333	neg
3.99_684.26	684.26562	3.98933333	neg
3.99_818.89	818.890477	3.98933333	neg
3.99_829.88	829.881054	3.98933333	neg
3.99_830.38	830.382965	3.98933333	neg
3.99_840.87	840.871706	3.98933333	neg
4.00_1029.5	1029.5588	4.00056667	pos
4.00_1029.7	1029.72493	4.00056667	pos
4.00_1030.0	1030.05914	4.00056667	pos
4.00_1030.2	1030.22553	4.00056667	pos
4.00_1030.3	1030.39241	4.00056667	pos
4.00_1037.7	1037.74944	4.00056667	pos
4.00_121.08	121.084411	4.00056667	pos
4.00_173.11	173.117407	4.00056667	pos
4.00_179.14	179.143246	4.00056667	pos
4.00_221.07	244.061692	4.00056667	pos
4.00_225.10	225.109829	4.00056667	pos
4.00_228.13	228.134429	4.00056667	pos
4.00_236.05	236.058977	4.00056667	pos

4.00_266.04	266.043557	4.00056667	pos
4.00_284.69	284.698846	4.00056667	pos
4.00_327.68	327.682344	4.00056667	pos
4.00_329.19	329.199857	4.00056667	pos
4.00_346.68	346.684681	4.00056667	pos
4.00_380.96	380.966852	4.00056667	pos
4.00_383.89	383.896616	4.00056667	pos
4.00_384.56	384.565093	4.00056667	pos
4.00_431.21	431.214633	4.00056667	pos
4.00_436.14	436.145555	4.00056667	pos
4.00_442.59	442.590091	4.00056667	pos
4.00_444.75	444.753055	4.00056667	pos
4.00_452.59	452.596137	4.00056667	pos
4.00_478.91	478.913147	4.00056667	pos
4.00_496.26	496.263861	4.00056667	pos
4.00_507.62	507.620039	4.00056667	pos
4.00_507.95	507.954127	4.00056667	pos
4.00_508.28	508.288009	4.00056667	pos
4.00_509.75	509.757138	4.00056667	pos
4.00_515.61	515.615736	4.00056667	pos
4.00_515.94	515.9497	4.00056667	pos
4.00_516.28	516.285034	4.00056667	pos
4.00_520.74	520.74842	4.00056667	pos
4.00_524.78	524.780326	4.00056667	pos
4.00_640.60	640.60305	4.00056667	pos
4.00_666.84	666.842698	4.00056667	pos
4.00_667.09	667.093783	4.00056667	pos
4.00_667.34	667.344132	4.00056667	pos
4.00_694.01	694.015185	4.00056667	pos
4.00_694.34	694.349473	4.00056667	pos
4.00_694.68	694.683886	4.00056667	pos
4.00_699.35	699.353921	4.00056667	pos
4.00_699.60	699.604567	4.00056667	pos

4.00_699.85!	699.855573	4.00056667	pos
4.00_830.00(	830.000826	4.00056667	pos
4.00_830.20(	830.200559	4.00056667	pos
4.00_830.40:	830.401007	4.00056667	pos
4.01_1034.4!	1034.49512	4.0077	neg
4.01_1042.1!	1042.15631	4.0077	neg
4.01_1042.4!	1042.4897	4.0077	neg
4.01_178.13:	178.132373	4.0077	neg
4.01_246.12(	246.119985	4.0077	neg
4.01_256.98!	256.985036	4.0077	neg
4.01_279.98!	279.989545	4.0077	neg
4.01_356.18:	356.182797	4.0077	neg
4.01_371.15(	371.156908	4.0077	neg
4.01_429.19!	429.198903	4.0077	neg
4.01_492.06	492.067388	4.0077	neg
4.02_1029.3!	1029.39212	4.01898333	pos
4.02_1029.8!	1029.89228	4.01898333	pos
4.02_1036.5(	1036.50923	4.01898333	pos
4.02_1036.8	1036.84283	4.01898333	pos
4.02_1037.1	1037.1777	4.01898333	pos
4.02_1037.5(	1037.50436	4.01898333	pos
4.02_1043.8:	1043.8359	4.01898333	pos
4.02_1044.5(	1044.50435	4.01898333	pos
4.02_142.93!	142.938624	4.01898333	pos
4.02_150.05!	150.058411	4.01898333	pos
4.02_173.08:	173.081157	4.01898333	pos
4.02_173.10!	196.09468	4.01898333	pos
4.02_187.14	187.144338	4.01898333	pos
4.02_199.13:	222.121602	4.01898333	pos
4.02_224.49!	224.498867	4.01898333	pos
4.02_224.83:	224.83336	4.01898333	pos
4.02_225.16	225.167775	4.01898333	pos
4.02_227.17!	227.175527	4.01898333	pos

4.02_306.60[illegible]	306.604064	4.01898333	pos
4.02_325.54[illegible]	325.540898	4.01898333	pos
4.02_325.87[illegible]	325.875137	4.01898333	pos
4.02_329.71[illegible]	329.710663	4.01898333	pos
4.02_336.18[illegible]	337.195216	4.01898333	pos
4.02_336.24[illegible]	336.244798	4.01898333	pos
4.02_336.74[illegible]	336.746474	4.01898333	pos
4.02_337.52[illegible]	337.528884	4.01898333	pos
4.02_337.86[illegible]	337.861631	4.01898333	pos
4.02_363.20[illegible]	363.204875	4.01898333	pos
4.02_363.45[illegible]	363.455706	4.01898333	pos
4.02_380.87[illegible]	380.872899	4.01898333	pos
4.02_381.03[illegible]	381.039783	4.01898333	pos
4.02_386.87[illegible]	386.872253	4.01898333	pos
4.02_387.54[illegible]	387.540328	4.01898333	pos
4.02_432.53[illegible]	432.532056	4.01898333	pos
4.02_432.86[illegible]	432.866595	4.01898333	pos
4.02_433.19[illegible]	433.199597	4.01898333	pos
4.02_435.89[illegible]	435.895709	4.01898333	pos
4.02_436.06[illegible]	436.062757	4.01898333	pos
4.02_444.25[illegible]	444.251644	4.01898333	pos
4.02_450.93[illegible]	450.930319	4.01898333	pos
4.02_456.64[illegible]	456.645473	4.01898333	pos
4.02_456.84[illegible]	456.846016	4.01898333	pos
4.02_476.85[illegible]	476.85535	4.01898333	pos
4.02_477.05[illegible]	477.055756	4.01898333	pos
4.02_478.74[illegible]	478.746275	4.01898333	pos
4.02_479.08[illegible]	479.080199	4.01898333	pos
4.02_483.60[illegible]	501.635134	4.01898333	pos
4.02_487.30[illegible]	487.306621	4.01898333	pos
4.02_487.80[illegible]	487.808095	4.01898333	pos
4.02_501.96[illegible]	501.969497	4.01898333	pos
4.02_504.92[illegible]	504.920235	4.01898333	pos

4.02_522.873	522.873272	4.01898333	pos
4.02_523.073	523.073765	4.01898333	pos
4.02_538.109	538.109221	4.01898333	pos
4.02_555.964	555.964267	4.01898333	pos
4.02_556.861	574.895056	4.01898333	pos
4.02_560.269	560.269009	4.01898333	pos
4.02_560.519	560.519171	4.01898333	pos
4.02_570.555	570.555138	4.01898333	pos
4.02_574.494	574.494051	4.01898333	pos
4.02_574.694	574.694555	4.01898333	pos
4.02_579.313	579.313407	4.01898333	pos
4.02_582.554	605.543335	4.01898333	pos
4.02_596.067	596.067127	4.01898333	pos
4.02_605.793	605.793908	4.01898333	pos
4.02_606.045	606.045077	4.01898333	pos
4.02_626.317	626.31778	4.01898333	pos
4.02_638.299	638.299087	4.01898333	pos
4.02_638.633	638.632989	4.01898333	pos
4.02_651.989	651.989737	4.01898333	pos
4.02_653.338	653.338161	4.01898333	pos
4.02_663.670	663.670415	4.01898333	pos
4.02_664.004	664.004296	4.01898333	pos
4.02_696.325	696.325377	4.01898333	pos
4.02_717.614	717.614108	4.01898333	pos
4.02_717.865	717.865471	4.01898333	pos
4.02_718.115	718.115465	4.01898333	pos
4.02_724.863	724.863937	4.01898333	pos
4.02_741.665	741.665415	4.01898333	pos
4.02_741.999	741.999877	4.01898333	pos
4.02_758.772	758.772764	4.01898333	pos
4.02_758.972	758.972752	4.01898333	pos
4.02_759.172	759.172916	4.01898333	pos
4.02_759.373	759.373057	4.01898333	pos

4.02_762.054	762.054354	4.01898333	pos
4.02_762.722	762.722825	4.01898333	pos
4.02_769.382	769.382106	4.01898333	pos
4.02_777.634	777.634209	4.01898333	pos
4.02_777.884	777.884486	4.01898333	pos
4.02_778.135	778.134966	4.01898333	pos
4.02_778.385	778.385741	4.01898333	pos
4.02_791.583	791.583685	4.01898333	pos
4.02_792.985	792.98533	4.01898333	pos
4.02_793.185	793.185119	4.01898333	pos
4.02_793.384	793.384917	4.01898333	pos
4.02_793.786	793.786054	4.01898333	pos
4.02_925.724	948.71396	4.01898333	pos
4.02_947.963	947.963862	4.01898333	pos
4.02_948.213	948.213674	4.01898333	pos
4.02_948.463	948.463425	4.01898333	pos
4.02_953.459	953.458959	4.01898333	pos
4.02_953.708	953.70853	4.01898333	pos
4.02_953.959	953.959049	4.01898333	pos
4.02_954.209	954.209308	4.01898333	pos
4.02_954.459	954.45914	4.01898333	pos
4.02_959.453	959.453678	4.01898333	pos
4.02_959.705	959.705112	4.01898333	pos
4.02_961.693	961.692957	4.01898333	pos
4.02_965.198	965.19867	4.01898333	pos
4.02_991.229	991.22933	4.01898333	pos
4.02_991.479	991.479758	4.01898333	pos
4.02_991.728	991.728558	4.01898333	pos
4.03_162.982	162.982215	4.02623333	neg
4.03_171.007	171.007263	4.02623333	neg
4.03_224.999	224.999028	4.02623333	neg
4.03_238.069	238.069701	4.02623333	neg
4.03_240.085	240.085295	4.02623333	neg

4.03_789.84	789.847635	4.02623333	neg
4.04_1022.5	1022.51362	4.04478333	neg
4.04_179.93	179.936088	4.04478333	neg
4.04_180.89	180.899506	4.04478333	neg
4.04_195.09	195.099026	4.03688333	pos
4.04_202.07	202.071951	4.04478333	neg
4.04_206.04	206.048383	4.03688333	pos
4.04_237.65	237.650003	4.03688333	pos
4.04_248.03	248.038862	4.03688333	pos
4.04_254.05	254.058937	4.04478333	neg
4.04_278.97	278.970854	4.04478333	neg
4.04_281.14	281.149915	4.03688333	pos
4.04_288.19	288.191962	4.03688333	pos
4.04_289.65	289.659849	4.03688333	pos
4.04_290.16	290.160987	4.04478333	neg
4.04_290.16	290.161204	4.03688333	pos
4.04_306.94	306.947594	4.03688333	pos
4.04_320.16	320.160896	4.03688333	pos
4.04_324.19	324.192849	4.04478333	neg
4.04_324.19	325.206605	4.03688333	pos
4.04_327.52	327.522674	4.03688333	pos
4.04_327.85	327.857074	4.03688333	pos
4.04_366.90	366.901392	4.03688333	pos
4.04_372.88	372.881925	4.03688333	pos
4.04_376.20	376.203136	4.03688333	pos
4.04_389.51	389.517683	4.03688333	pos
4.04_389.85	389.85208	4.03688333	pos
4.04_390.18	390.185611	4.03688333	pos
4.04_421.72	421.725938	4.03688333	pos
4.04_450.59	450.598465	4.03688333	pos
4.04_452.73	452.730345	4.03688333	pos
4.04_453.23	453.232008	4.03688333	pos
4.04_458.10	458.106278	4.04478333	neg

4.04_490.26	473.263368	4.03688333	pos
4.04_490.78	490.780586	4.03688333	pos
4.04_494.77	494.775525	4.03688333	pos
4.04_494.92	494.923267	4.03688333	pos
4.04_495.09	495.090308	4.03688333	pos
4.04_495.42	495.424358	4.03688333	pos
4.04_495.75	495.758556	4.03688333	pos
4.04_501.31	502.322832	4.03688333	pos
4.04_512.26	512.260552	4.03688333	pos
4.04_519.76	519.765412	4.03688333	pos
4.04_544.78	545.78734	4.03688333	pos
4.04_553.78	553.784877	4.03688333	pos
4.04_554.03	554.035445	4.03688333	pos
4.04_554.53	554.536507	4.03688333	pos
4.04_554.94	593.906689	4.03688333	pos
4.04_562.11	562.110748	4.04478333	neg
4.04_565.06	565.061865	4.03688333	pos
4.04_588.24	588.242296	4.04478333	neg
4.04_593.70	593.706173	4.03688333	pos
4.04_594.10	594.1072	4.03688333	pos
4.04_648.68	648.686569	4.03688333	pos
4.04_653.58	653.58993	4.03688333	pos
4.04_683.02	683.020506	4.03688333	pos
4.04_683.35	683.354872	4.03688333	pos
4.04_706.37	745.339669	4.03688333	pos
4.04_712.32	712.328869	4.03688333	pos
4.04_712.52	712.529131	4.03688333	pos
4.04_732.33	732.33377	4.04478333	neg
4.04_733.84	733.847159	4.03688333	pos
4.04_734.34	734.348742	4.03688333	pos
4.04_736.16	736.164177	4.03688333	pos
4.04_738.37	738.377182	4.03688333	pos
4.04_741.88	741.88051	4.03688333	pos

4.04_742.13	742.131042	4.03688333	pos
4.04_742.38	742.381939	4.03688333	pos
4.04_742.82	742.823168	4.04478333	neg
4.04_744.83	744.83806	4.03688333	pos
4.04_749.62	749.624606	4.03688333	pos
4.04_749.87	749.875132	4.03688333	pos
4.04_753.81	753.814781	4.04478333	neg
4.04_766.37	749.37356	4.03688333	pos
4.04_943.95	943.957837	4.03688333	pos
4.04_970.51	970.510816	4.04478333	neg
4.04_971.51	972.524934	4.03688333	pos
4.04_973.02	973.026905	4.03688333	pos
4.04_998.82	998.828713	4.03688333	pos
4.06_188.99	188.9977	4.06311667	neg
4.06_209.07	209.078567	4.05526667	pos
4.06_217.09	217.097872	4.05526667	pos
4.06_229.10	229.104753	4.05526667	pos
4.06_252.15	275.14644	4.05526667	pos
4.06_284.83	284.836264	4.05526667	pos
4.06_301.17	301.176336	4.05526667	pos
4.06_323.84	323.840111	4.05526667	pos
4.06_331.67	331.679295	4.05526667	pos
4.06_332.18	332.181047	4.05526667	pos
4.06_350.98	350.98729	4.05526667	pos
4.06_351.70	351.700701	4.05526667	pos
4.06_368.69	368.698046	4.05526667	pos
4.06_382.22	382.220654	4.05526667	pos
4.06_391.94	391.947029	4.05526667	pos
4.06_392.19	392.197555	4.05526667	pos
4.06_392.44	392.448346	4.05526667	pos
4.06_405.21	405.21674	4.05526667	pos
4.06_406.16	406.161907	4.06311667	neg
4.06_456.76	456.767375	4.05526667	pos

4.06_461.26	461.263388	4.05526667	pos
4.06_461.51	461.514396	4.05526667	pos
4.06_461.76	461.76287	4.05526667	pos
4.06_475.91	475.916186	4.05526667	pos
4.06_476.25	476.250345	4.05526667	pos
4.06_476.41	476.417285	4.05526667	pos
4.06_476.58	476.58433	4.05526667	pos
4.06_477.92	477.928703	4.05526667	pos
4.06_491.78	491.78165	4.05526667	pos
4.06_499.96	499.963299	4.05526667	pos
4.06_504.75	504.754726	4.05526667	pos
4.06_522.59	522.595714	4.05526667	pos
4.06_523.27	523.274787	4.05526667	pos
4.06_540.11	540.119949	4.05526667	pos
4.06_540.45	540.453679	4.05526667	pos
4.06_543.27	543.278632	4.06311667	neg
4.06_543.61	543.616329	4.05526667	pos
4.06_543.95	543.950426	4.05526667	pos
4.06_545.29	545.294106	4.05526667	pos
4.06_551.80	551.801548	4.05526667	pos
4.06_559.80	559.80296	4.05526667	pos
4.06_560.05	560.053457	4.05526667	pos
4.06_570.89	570.897566	4.05526667	pos
4.06_571.09	571.09835	4.05526667	pos
4.06_571.29	571.299262	4.05526667	pos
4.06_571.49	571.498766	4.05526667	pos
4.06_594.30	594.307812	4.05526667	pos
4.06_594.50	594.507799	4.05526667	pos
4.06_614.68	614.68236	4.05526667	pos
4.06_655.03	655.037744	4.05526667	pos
4.06_655.37	655.372093	4.05526667	pos
4.06_668.60	668.601998	4.05526667	pos
4.06_668.85	668.853013	4.05526667	pos

4.06_687.32(	687.320704	4.05526667	pos
4.06_713.36(	713.369606	4.05526667	pos
4.06_713.62(	713.620618	4.05526667	pos
4.06_713.87:	713.871345	4.05526667	pos
4.06_714.12:	714.121543	4.05526667	pos
4.06_735.76:	735.763799	4.05526667	pos
4.06_735.96	735.964066	4.05526667	pos
4.06_736.36	736.365472	4.05526667	pos
4.06_788.89	788.892428	4.05526667	pos
4.06_794.32	794.325536	4.06311667	neg
4.06_794.82	794.827978	4.06311667	neg
4.06_796.33	796.339174	4.05526667	pos
4.06_812.90	812.905205	4.06311667	neg
4.06_861.36	861.367292	4.06311667	neg
4.06_863.38:	863.381281	4.05526667	pos
4.06_919.70:	919.703173	4.05526667	pos
4.06_919.95:	919.953547	4.05526667	pos
4.06_920.20:	920.203478	4.05526667	pos
4.06_920.45:	920.453925	4.05526667	pos
4.06_988.83(	988.836576	4.05526667	pos
4.06_989.17:	989.172705	4.05526667	pos
4.07_107.96	107.967281	4.07303333	pos
4.07_126.13(	126.136778	4.07303333	pos
4.07_171.10:	171.10177	4.07303333	pos
4.07_197.07	197.078533	4.07303333	pos
4.07_285.70(	285.706654	4.07303333	pos
4.07_296.51	296.515447	4.07303333	pos
4.07_343.23	343.234399	4.07303333	pos
4.07_343.73(	343.736011	4.07303333	pos
4.07_355.18(	373.214157	4.07303333	pos
4.07_366.53	366.539475	4.07303333	pos
4.07_366.87	366.874151	4.07303333	pos
4.07_373.71	373.715815	4.07303333	pos

4.07_380.208	380.208933	4.07303333	pos
4.07_387.730	387.730005	4.07303333	pos
4.07_392.732	392.732246	4.07303333	pos
4.07_393.234	393.234272	4.07303333	pos
4.07_437.864	437.864744	4.07303333	pos
4.07_441.986	441.986036	4.07303333	pos
4.07_442.236	442.236593	4.07303333	pos
4.07_442.487	442.487178	4.07303333	pos
4.07_491.530	491.530668	4.07303333	pos
4.07_492.032	492.032187	4.07303333	pos
4.07_511.758	511.758501	4.07303333	pos
4.07_512.008	512.008844	4.07303333	pos
4.07_519.000	519.000622	4.07303333	pos
4.07_536.776	536.776137	4.07303333	pos
4.07_539.628	539.628466	4.07303333	pos
4.07_539.961	539.961932	4.07303333	pos
4.07_566.324	589.314006	4.07303333	pos
4.07_587.313	587.313009	4.07303333	pos
4.07_587.980	587.980374	4.07303333	pos
4.07_683.715	683.715513	4.07303333	pos
4.08_140.980	140.980424	4.08158333	neg
4.08_211.024	211.02479	4.08158333	neg
4.08_296.002	296.002173	4.08158333	neg
4.08_318.109	318.109392	4.08158333	neg
4.08_340.091	340.091408	4.08158333	neg
4.08_408.097	408.096998	4.08158333	neg
4.08_588.216	588.216315	4.08158333	neg
4.08_743.406	743.40641	4.08158333	neg
4.08_953.449	953.449874	4.08158333	neg
4.08_953.951	953.951583	4.08158333	neg
4.08_964.942	964.942426	4.08158333	neg
4.09_222.662	222.662859	4.09135	pos
4.09_296.214	296.21458	4.09135	pos

4.09_302.19[illegible]	302.196589	4.09135	pos
4.09_333.70[illegible]	333.700687	4.09135	pos
4.09_334.20[illegible]	334.202148	4.09135	pos
4.09_340.08[illegible]	340.080952	4.09135	pos
4.09_342.07[illegible]	342.078009	4.09135	pos
4.09_353.71[illegible]	353.716399	4.09135	pos
4.09_354.21[illegible]	354.217822	4.09135	pos
4.09_382.19[illegible]	382.197517	4.09135	pos
4.09_385.24[illegible]	385.244671	4.09135	pos
4.09_402.52[illegible]	402.525716	4.09135	pos
4.09_409.72[illegible]	409.724231	4.09135	pos
4.09_427.56[illegible]	427.561189	4.09135	pos
4.09_427.89[illegible]	427.895274	4.09135	pos
4.09_431.22[illegible]	431.227008	4.09135	pos
4.09_431.42[illegible]	431.427347	4.09135	pos
4.09_431.62[illegible]	431.627814	4.09135	pos
4.09_457.59[illegible]	457.596034	4.09135	pos
4.09_458.75[illegible]	458.75809	4.09135	pos
4.09_466.88[illegible]	484.916525	4.09135	pos
4.09_467.56[illegible]	467.56579	4.09135	pos
4.09_478.23[illegible]	478.236421	4.09135	pos
4.09_478.48[illegible]	478.48717	4.09135	pos
4.09_478.73[illegible]	478.737575	4.09135	pos
4.09_478.98[illegible]	478.988189	4.09135	pos
4.09_485.94[illegible]	485.944543	4.09135	pos
4.09_503.92[illegible]	503.923673	4.09135	pos
4.09_511.50[illegible]	511.507389	4.09135	pos
4.09_528.61[illegible]	528.613275	4.09135	pos
4.09_538.53[illegible]	538.531289	4.09135	pos
4.09_538.78[illegible]	538.781822	4.09135	pos
4.09_539.03[illegible]	539.032547	4.09135	pos
4.09_545.47[illegible]	545.47922	4.09135	pos
4.09_548.80[illegible]	548.805004	4.09135	pos

4.09_556.63:	556.63181	4.09135	pos
4.09_556.96!	556.965928	4.09135	pos
4.09_587.64(	587.646237	4.09135	pos
4.09_605.23{	606.245782	4.09135	pos
4.09_605.81:	605.811544	4.09135	pos
4.09_622.31<	605.311344	4.09135	pos
4.09_637.31:	637.312333	4.09135	pos
4.09_637.64(	637.646526	4.09135	pos
4.09_637.98(	637.980634	4.09135	pos
4.09_681.34(	681.346298	4.09135	pos
4.09_681.59(	681.596614	4.09135	pos
4.09_681.84<	681.844687	4.09135	pos
4.09_682.00{	682.008302	4.09135	pos
4.09_717.70!	717.705467	4.09135	pos
4.09_718.03!	718.039615	4.09135	pos
4.09_718.37:	718.371881	4.09135	pos
4.09_834.44<	834.444332	4.09135	pos
4.10_107.03(	107.036151	4.10018333	neg
4.10_1074.5<	1074.54128	4.10018333	neg
4.10_1085.0:	1085.02966	4.10018333	neg
4.10_130.05{	130.058851	4.10018333	neg
4.10_314.03!	314.039177	4.10018333	neg
4.10_338.06(	338.066243	4.10018333	neg
4.10_339.10:	339.101812	4.10018333	neg
4.10_342.16(	342.166915	4.10018333	neg
4.10_399.17!	399.175101	4.10018333	neg
4.10_604.22!	604.229799	4.10018333	neg
4.10_638.32:	638.321316	4.10018333	neg
4.10_664.37{	664.378721	4.10018333	neg
4.10_822.35:	822.351844	4.10018333	neg
4.10_831.92'	831.927648	4.10018333	neg
4.10_832.42{	832.428902	4.10018333	neg
4.11_130.06!	130.065385	4.10951667	pos

4.11_131.162	131.162567	4.10951667	pos
4.11_136.088	137.096186	4.10951667	pos
4.11_145.105	145.105341	4.10951667	pos
4.11_163.543	163.543609	4.10951667	pos
4.11_170.999	172.006404	4.10951667	pos
4.11_197.128	197.128656	4.10951667	pos
4.11_203.128	203.127986	4.10951667	pos
4.11_211.082	212.089511	4.10951667	pos
4.11_215.970	215.970386	4.10951667	pos
4.11_243.120	243.120514	4.10951667	pos
4.11_256.115	256.115457	4.10951667	pos
4.11_266.952	266.952227	4.10951667	pos
4.11_284.125	284.125573	4.10951667	pos
4.11_307.697	307.697345	4.10951667	pos
4.11_335.204	335.204349	4.10951667	pos
4.11_335.955	335.954996	4.10951667	pos
4.11_345.686	345.686344	4.10951667	pos
4.11_360.966	360.966099	4.10951667	pos
4.11_381.250	381.250088	4.10951667	pos
4.11_383.229	383.229164	4.10951667	pos
4.11_394.875	394.875831	4.10951667	pos
4.11_402.745	402.745355	4.10951667	pos
4.11_408.220	408.220131	4.10951667	pos
4.11_413.726	413.726894	4.10951667	pos
4.11_413.883	413.882978	4.10951667	pos
4.11_416.735	416.735899	4.10951667	pos
4.11_416.986	416.98661	4.10951667	pos
4.11_427.630	427.630664	4.10951667	pos
4.11_430.266	430.266414	4.10951667	pos
4.11_430.727	430.727798	4.10951667	pos
4.11_447.269	447.269605	4.10951667	pos
4.11_447.840	447.840122	4.10951667	pos
4.11_448.241	448.24135	4.10951667	pos

4.11_474.23	474.235032	4.10951667	pos
4.11_474.56	474.569402	4.10951667	pos
4.11_477.61	477.614297	4.10951667	pos
4.11_481.28	481.287252	4.10951667	pos
4.11_491.93	491.932165	4.10951667	pos
4.11_493.85	493.858351	4.10951667	pos
4.11_504.59	504.591726	4.10951667	pos
4.11_505.93	505.931951	4.10951667	pos
4.11_506.62	506.625018	4.10951667	pos
4.11_510.27	510.275672	4.10951667	pos
4.11_520.78	520.781831	4.10951667	pos
4.11_521.03	521.032272	4.10951667	pos
4.11_521.61	521.615084	4.10951667	pos
4.11_534.53	534.537147	4.10951667	pos
4.11_535.03	535.03826	4.10951667	pos
4.11_536.63	536.630733	4.10951667	pos
4.11_536.96	536.965052	4.10951667	pos
4.11_546.54	546.546936	4.10951667	pos
4.11_559.54	559.548453	4.10951667	pos
4.11_617.07	617.070655	4.10951667	pos
4.11_617.32	617.32113	4.10951667	pos
4.11_617.57	617.57173	4.10951667	pos
4.11_617.82	617.822032	4.10951667	pos
4.11_648.78	648.789692	4.10951667	pos
4.11_695.82	696.835838	4.10951667	pos
4.11_696.33	697.338226	4.10951667	pos
4.11_707.82	707.826326	4.10951667	pos
4.11_708.32	708.327875	4.10951667	pos
4.12_144.10	144.102843	4.11676667	neg
4.12_171.98	171.986887	4.11676667	neg
4.12_189.09	188.092649	4.11676667	neg
4.12_227.95	227.950258	4.11676667	neg
4.12_237.97	237.979101	4.11676667	neg

4.12_246.17	246.170986	4.11676667	neg
4.12_254.96	254.969202	4.11676667	neg
4.12_254.98	254.989474	4.11676667	neg
4.12_256.08	256.080242	4.11676667	neg
4.12_305.96	305.966607	4.11676667	neg
4.12_341.05	341.057666	4.11676667	neg
4.12_373.95	373.953989	4.11676667	neg
4.12_381.21	381.214544	4.11676667	neg
4.12_392.05	392.05519	4.11676667	neg
4.12_409.04	409.04545	4.11676667	neg
4.12_441.94	441.941365	4.11676667	neg
4.12_444.18	444.188865	4.11676667	neg
4.12_460.04	460.042683	4.11676667	neg
4.12_477.03	477.032634	4.11676667	neg
4.12_509.92	509.928705	4.11676667	neg
4.12_528.03	528.030007	4.11676667	neg
4.12_577.91	577.916371	4.11676667	neg
4.12_596.01	596.01791	4.11676667	neg
4.12_630.09	630.098142	4.11676667	neg
4.12_664.00	664.005363	4.11676667	neg
4.12_694.82	694.821413	4.11676667	neg
4.13_130.15	130.159238	4.12765	pos
4.13_190.14	190.143888	4.12765	pos
4.13_196.09	196.091118	4.12765	pos
4.13_236.90	236.906954	4.12765	pos
4.13_254.64	254.641986	4.12765	pos
4.13_271.26	271.263186	4.12765	pos
4.13_291.98	291.983035	4.12765	pos
4.13_349.95	349.954343	4.12765	pos
4.13_351.18	351.188538	4.12765	pos
4.13_354.22	372.259422	4.12765	pos
4.13_360.74	360.742262	4.12765	pos
4.13_361.21	361.21739	4.12765	pos

4.13_389.73[illegible]	389.732385	4.12765	pos
4.13_399.20[illegible]	399.206461	4.12765	pos
4.13_399.54[illegible]	399.541045	4.12765	pos
4.13_414.74[illegible]	414.740297	4.12765	pos
4.13_481.56[illegible]	520.530633	4.12765	pos
4.13_496.01[illegible]	496.017726	4.12765	pos
4.13_501.61[illegible]	501.611853	4.12765	pos
4.13_501.94[illegible]	501.945143	4.12765	pos
4.13_510.02[illegible]	510.025327	4.12765	pos
4.13_516.81[illegible]	516.810674	4.12765	pos
4.13_521.53[illegible]	521.530694	4.12765	pos
4.13_542.29[illegible]	542.291822	4.12765	pos
4.13_542.54[illegible]	542.544067	4.12765	pos
4.13_575.30[illegible]	575.308371	4.12765	pos
4.13_583.85[illegible]	606.840956	4.12765	pos
4.13_591.76[illegible]	591.768136	4.12765	pos
4.13_592.26[illegible]	592.269617	4.12765	pos
4.13_607.34[illegible]	607.342451	4.12765	pos
4.13_694.86[illegible]	694.867334	4.12765	pos
4.13_695.37[illegible]	695.370542	4.12765	pos
4.13_701.83[illegible]	701.839703	4.12765	pos
4.13_714.85[illegible]	714.859644	4.12765	pos
4.13_715.36[illegible]	715.361187	4.12765	pos
4.13_727.33[illegible]	727.333433	4.12765	pos
4.13_727.83[illegible]	727.834765	4.12765	pos
4.13_734.37[illegible]	734.378664	4.12765	pos
4.13_734.71[illegible]	734.713769	4.12765	pos
4.14_287.01[illegible]	287.015747	4.13528333	neg
4.14_289.96[illegible]	289.968511	4.13528333	neg
4.14_357.95[illegible]	357.955858	4.13528333	neg
4.14_369.21[illegible]	369.214302	4.13528333	neg
4.14_438.23[illegible]	438.235824	4.13528333	neg
4.14_507.06[illegible]	507.06841	4.13528333	neg

4.14_736.31(	736.310243	4.13528333	neg
4.15_1156.6‹	1156.64843	4.1538	neg
4.15_1157.1‹	1157.14945	4.1538	neg
4.15_183.02!	183.029766	4.1538	neg
4.15_191.62(	191.626208	4.14533333	pos
4.15_192.12!	192.127828	4.14533333	pos
4.15_260.02:	260.023278	4.1538	neg
4.15_261.12‹	261.124291	4.1538	neg
4.15_268.00!	268.007524	4.1538	neg
4.15_284.66{	284.668894	4.14533333	pos
4.15_328.22:	328.223124	4.14533333	pos
4.15_340.57(	340.57686	4.14533333	pos
4.15_357.56(	357.560306	4.14533333	pos
4.15_357.71:	357.713148	4.14533333	pos
4.15_370.86{	370.868463	4.14533333	pos
4.15_371.22!	371.229182	4.14533333	pos
4.15_386.23(	386.236848	4.14533333	pos
4.15_386.57(	386.570787	4.14533333	pos
4.15_386.90‹	386.904895	4.14533333	pos
4.15_394.26(	394.259954	4.14533333	pos
4.15_396.22‹	435.187726	4.14533333	pos
4.15_400.58:	400.583832	4.14533333	pos
4.15_400.73!	400.735158	4.14533333	pos
4.15_400.91{	400.918189	4.14533333	pos
4.15_403.71(	403.710233	4.14533333	pos
4.15_404.54{	404.54801	4.14533333	pos
4.15_404.88:	404.882291	4.14533333	pos
4.15_410.73!	410.735616	4.14533333	pos
4.15_431.71:	431.712103	4.14533333	pos
4.15_451.27(	451.276338	4.14533333	pos
4.15_457.78!	457.787452	4.14533333	pos
4.15_465.75:	465.75307	4.14533333	pos
4.15_497.27‹	497.274894	4.14533333	pos

4.15_499.75	499.753401	4.14533333	pos
4.15_502.59	502.598599	4.14533333	pos
4.15_519.77	519.778752	4.14533333	pos
4.15_533.29	533.293577	4.14533333	pos
4.15_566.02	604.986458	4.14533333	pos
4.15_574.60	597.594678	4.14533333	pos
4.15_582.96	582.969428	4.14533333	pos
4.15_597.84	597.845451	4.14533333	pos
4.15_598.09	598.095919	4.14533333	pos
4.15_629.99	629.998054	4.14533333	pos
4.15_656.33	656.336781	4.14533333	pos
4.15_660.26	661.268051	4.14533333	pos
4.15_661.87	661.870736	4.1538	neg
4.15_725.19	725.199244	4.1538	neg
4.15_796.79	796.790806	4.14533333	pos
4.15_910.43	910.436066	4.1538	neg
4.15_911.95	911.950034	4.14533333	pos
4.15_912.45	912.450005	4.14533333	pos
4.16_138.02	156.057592	4.163	pos
4.16_156.03	156.039007	4.163	pos
4.16_261.64	261.640964	4.163	pos
4.16_281.57	281.576334	4.163	pos
4.16_343.53	343.538453	4.163	pos
4.16_343.87	343.872824	4.163	pos
4.16_359.21	359.213473	4.163	pos
4.16_362.15	363.162847	4.163	pos
4.16_375.71	358.713518	4.163	pos
4.16_396.47	396.471684	4.163	pos
4.16_396.97	396.973171	4.163	pos
4.16_407.88	407.887905	4.163	pos
4.16_442.25	443.260359	4.163	pos
4.16_442.92	442.92643	4.163	pos
4.16_443.59	443.594687	4.163	pos

4.16_496.601	496.601741	4.163	pos
4.16_507.936	507.936295	4.163	pos
4.16_520.029	520.029504	4.163	pos
4.16_527.958	527.958604	4.163	pos
4.16_528.627	528.627697	4.163	pos
4.16_566.832	566.832809	4.163	pos
4.16_579.835	579.835265	4.163	pos
4.16_580.086	580.086351	4.163	pos
4.16_580.336	580.336761	4.163	pos
4.16_580.587	580.58767	4.163	pos
4.16_582.634	582.634899	4.163	pos
4.16_590.033	590.033237	4.163	pos
4.16_606.786	589.783097	4.163	pos
4.16_631.308	632.316179	4.163	pos
4.16_653.677	653.676975	4.163	pos
4.16_654.011	654.011217	4.163	pos
4.16_663.885	663.885544	4.163	pos
4.16_664.386	664.386828	4.163	pos
4.16_665.323	665.323593	4.163	pos
4.16_674.875	674.875883	4.163	pos
4.16_698.332	699.339746	4.163	pos
4.16_703.366	703.366763	4.163	pos
4.16_772.777	772.777718	4.163	pos
4.16_773.111	773.111636	4.163	pos
4.16_773.445	773.445668	4.163	pos
4.16_780.104	780.104299	4.163	pos
4.16_780.772	780.772528	4.163	pos
4.16_786.040	786.040399	4.163	pos
4.16_786.375	786.375165	4.163	pos
4.16_786.706	786.706331	4.163	pos
4.17_141.996	141.996636	4.17231667	neg
4.17_181.047	181.047753	4.17231667	neg
4.17_286.177	286.177199	4.17231667	neg

4.17_316.187	316.187692	4.17231667	neg
4.17_319.004	319.00438	4.17231667	neg
4.17_386.133	386.133702	4.17231667	neg
4.17_446.906	446.906	4.17231667	neg
4.17_615.843	615.843124	4.17231667	neg
4.18_206.048	206.0484	4.1814	pos
4.18_225.148	225.148858	4.1814	pos
4.18_268.212	268.211994	4.1814	pos
4.18_288.192	288.192013	4.1814	pos
4.18_293.184	293.184	4.1814	pos
4.18_304.212	304.212028	4.1814	pos
4.18_318.202	318.202535	4.1814	pos
4.18_320.160	320.160926	4.1814	pos
4.18_326.531	326.530978	4.1814	pos
4.18_345.695	345.694989	4.1814	pos
4.18_351.700	351.70049	4.1814	pos
4.18_356.685	356.685399	4.1814	pos
4.18_357.187	357.187116	4.1814	pos
4.18_370.958	370.958817	4.1814	pos
4.18_371.209	371.209595	4.1814	pos
4.18_371.460	371.460307	4.1814	pos
4.18_388.221	388.221439	4.1814	pos
4.18_415.771	415.771478	4.1814	pos
4.18_437.987	437.987802	4.1814	pos
4.18_447.598	447.598294	4.1814	pos
4.18_447.932	447.932395	4.1814	pos
4.18_455.250	455.250724	4.1814	pos
4.18_465.268	465.268162	4.1814	pos
4.18_494.275	494.275779	4.1814	pos
4.18_494.610	494.610364	4.1814	pos
4.18_510.606	510.606386	4.1814	pos
4.18_524.607	524.607656	4.1814	pos
4.18_536.946	536.946846	4.1814	pos

4.18_537.61	537.615294	4.1814	pos
4.18_576.62	576.624729	4.1814	pos
4.18_576.95	576.958186	4.1814	pos
4.18_587.25	588.266801	4.1814	pos
4.18_588.76	588.7683	4.1814	pos
4.18_599.25	599.257915	4.1814	pos
4.18_599.75	599.759411	4.1814	pos
4.18_619.01	619.018023	4.1814	pos
4.18_619.35	619.352115	4.1814	pos
4.18_624.06	624.065204	4.1814	pos
4.18_624.56	624.567002	4.1814	pos
4.18_699.00	699.005793	4.1814	pos
4.18_699.67	699.673931	4.1814	pos
4.19_106.04	106.040905	4.1908	neg
4.19_124.00	124.007187	4.1908	neg
4.19_249.03	249.035289	4.1908	neg
4.19_318.14	318.145794	4.1908	neg
4.19_586.25	586.251656	4.1908	neg
4.20_225.07	225.073334	4.19935	pos
4.20_229.10	229.10484	4.19935	pos
4.20_231.17	231.170529	4.19935	pos
4.20_347.17	347.171759	4.19935	pos
4.20_378.75	378.7528	4.19935	pos
4.20_386.10	387.116667	4.19935	pos
4.20_396.21	397.226796	4.19935	pos
4.20_397.72	397.728256	4.19935	pos
4.20_449.23	449.239624	4.19935	pos
4.20_460.25	460.250362	4.19935	pos
4.20_488.22	488.221989	4.19935	pos
4.20_488.47	488.472772	4.19935	pos
4.20_488.72	488.72269	4.19935	pos
4.20_585.98	585.980023	4.19935	pos
4.20_586.64	586.648195	4.19935	pos

4.20_614.315	614.315211	4.19935	pos
4.20_640.592	640.592875	4.19935	pos
4.20_657.019	657.01982	4.19935	pos
4.20_667.306	667.30604	4.19935	pos
4.20_740.864	740.864521	4.19935	pos
4.20_775.070	775.070195	4.19935	pos
4.20_789.363	789.363489	4.19935	pos
4.20_795.397	795.397264	4.19935	pos
4.20_795.647	795.647719	4.19935	pos
4.21_180.066	180.066468	4.20936667	neg
4.21_188.074	188.074825	4.20936667	neg
4.21_189.029	189.029802	4.20936667	neg
4.21_260.096	260.096229	4.20936667	neg
4.21_260.103	260.103641	4.20936667	neg
4.21_284.036	284.0363	4.20936667	neg
4.21_300.994	300.994231	4.20936667	neg
4.21_332.125	332.125228	4.20936667	neg
4.21_335.033	335.033605	4.20936667	neg
4.21_344.182	344.182663	4.20936667	neg
4.21_354.107	354.107153	4.20936667	neg
4.21_403.020	403.020811	4.20936667	neg
4.21_439.219	439.219833	4.20936667	neg
4.21_471.008	471.008452	4.20936667	neg
4.22_167.068	167.068169	4.21765	pos
4.22_179.067	179.067722	4.21765	pos
4.22_193.083	193.083648	4.21765	pos
4.22_225.109	225.109821	4.21765	pos
4.22_236.120	259.115385	4.21765	pos
4.22_252.157	275.146762	4.21765	pos
4.22_294.210	294.210196	4.21765	pos
4.22_313.164	314.171468	4.21765	pos
4.22_315.543	315.543076	4.21765	pos
4.22_332.218	332.218089	4.21765	pos

4.22_334.947	334.947343	4.21765	pos
4.22_371.883	371.88346	4.21765	pos
4.22_432.241	433.24835	4.21765	pos
4.22_455.562	455.56216	4.21765	pos
4.22_493.039	493.03983	4.21765	pos
4.22_498.944	498.944826	4.21765	pos
4.22_505.722	505.722428	4.21765	pos
4.22_513.611	513.611498	4.21765	pos
4.22_513.946	513.945993	4.21765	pos
4.22_531.283	514.280182	4.21765	pos
4.22_542.948	565.938106	4.21765	pos
4.22_554.598	554.59825	4.21765	pos
4.22_578.640	578.640432	4.21765	pos
4.22_616.986	616.98644	4.21765	pos
4.22_619.621	619.621081	4.21765	pos
4.22_619.955	619.955471	4.21765	pos
4.22_730.870	730.870367	4.21765	pos
4.22_779.051	779.051683	4.21765	pos
4.22_779.385	779.385293	4.21765	pos
4.22_783.879	783.879264	4.21765	pos
4.22_784.380	784.38002	4.21765	pos
4.23_199.058	199.058447	4.22771667	neg
4.23_201.113	201.11305	4.22771667	neg
4.23_246.038	246.038344	4.22771667	neg
4.23_259.129	259.129772	4.22771667	neg
4.23_269.100	269.100644	4.22771667	neg
4.23_314.026	314.026018	4.22771667	neg
4.23_326.208	326.208497	4.22771667	neg
4.23_376.151	376.151684	4.22771667	neg
4.23_382.013	382.013336	4.22771667	neg
4.23_385.150	385.15044	4.22771667	neg
4.23_503.707	503.707524	4.22771667	neg
4.23_667.784	667.784876	4.22771667	neg

4.23_668.28	668.286351	4.22771667	neg
4.23_678.77	678.775265	4.22771667	neg
4.23_889.40	889.402951	4.22771667	neg
4.23_889.90	889.902857	4.22771667	neg
4.23_900.39	900.394452	4.22771667	neg
4.23_900.89	900.895847	4.22771667	neg
4.24_235.67	235.670757	4.23603333	pos
4.24_267.12	267.120366	4.23603333	pos
4.24_287.10	287.10046	4.23603333	pos
4.24_295.69	295.691222	4.23603333	pos
4.24_296.18	319.173018	4.23603333	pos
4.24_296.19	296.192812	4.23603333	pos
4.24_306.68	306.682082	4.23603333	pos
4.24_307.18	307.183513	4.23603333	pos
4.24_317.67	317.673128	4.23603333	pos
4.24_318.17	318.174757	4.23603333	pos
4.24_360.21	360.213261	4.23603333	pos
4.24_397.52	397.523568	4.23603333	pos
4.24_397.85	397.857583	4.23603333	pos
4.24_430.24	430.247838	4.23603333	pos
4.24_430.74	430.749788	4.23603333	pos
4.24_433.94	433.941189	4.23603333	pos
4.24_438.18	438.184886	4.23603333	pos
4.24_448.22	448.222979	4.23603333	pos
4.24_456.75	456.756152	4.23603333	pos
4.24_460.27	460.275895	4.23603333	pos
4.24_495.25	495.256418	4.23603333	pos
4.24_535.28	535.287354	4.23603333	pos
4.24_535.62	535.621103	4.23603333	pos
4.24_543.30	543.305895	4.23603333	pos
4.24_543.55	543.556527	4.23603333	pos
4.24_565.60	565.603769	4.23603333	pos
4.24_566.27	566.271894	4.23603333	pos

4.24_583.57	583.576675	4.23603333	pos
4.24_583.91	583.910768	4.23603333	pos
4.24_590.37	590.375353	4.23603333	pos
4.24_594.61	594.61409	4.23603333	pos
4.24_594.94	594.948657	4.23603333	pos
4.24_595.28	595.282317	4.23603333	pos
4.24_608.31	609.321257	4.23603333	pos
4.24_616.83	616.830352	4.23603333	pos
4.24_623.06	623.063739	4.23603333	pos
4.24_623.31	623.311183	4.23603333	pos
4.24_668.34	691.330031	4.23603333	pos
4.24_680.78	680.789444	4.23603333	pos
4.24_731.20	731.203548	4.23603333	pos
4.24_846.89	847.900886	4.23603333	pos
4.24_847.39	848.402381	4.23603333	pos
4.24_858.89	858.891663	4.23603333	pos
4.25_156.13	156.138474	4.25448333	pos
4.25_216.12	216.123924	4.24626667	neg
4.25_217.15	217.155808	4.25448333	pos
4.25_220.06	220.064018	4.25448333	pos
4.25_227.08	227.08895	4.25448333	pos
4.25_239.13	239.136865	4.25448333	pos
4.25_245.17	245.174893	4.25448333	pos
4.25_252.69	252.697579	4.25448333	pos
4.25_328.14	329.14984	4.25448333	pos
4.25_417.23	417.234185	4.25448333	pos
4.25_424.73	424.731588	4.25448333	pos
4.25_424.98	424.982303	4.25448333	pos
4.25_456.99	456.992197	4.25448333	pos
4.25_457.49	457.493636	4.25448333	pos
4.25_469.50	469.500215	4.25448333	pos
4.25_470.00	470.001442	4.25448333	pos
4.25_470.25	470.251796	4.25448333	pos

4.25_478.76	478.769645	4.25448333	pos
4.25_498.25	498.257912	4.25448333	pos
4.25_502.51	502.513405	4.25448333	pos
4.25_502.76	502.764045	4.25448333	pos
4.25_503.01	503.01442	4.25448333	pos
4.25_511.27	511.275904	4.25448333	pos
4.25_540.53	540.533903	4.25448333	pos
4.25_547.52	547.527568	4.25448333	pos
4.25_608.98	608.987067	4.25448333	pos
4.25_609.65	609.655386	4.25448333	pos
4.25_661.57	661.572636	4.25448333	pos
4.25_669.34	669.347505	4.25448333	pos
4.25_687.01	670.015864	4.25448333	pos
4.25_693.83	693.83277	4.25448333	pos
4.25_694.08	694.083197	4.25448333	pos
4.25_694.33	694.334295	4.25448333	pos
4.25_694.58	694.585302	4.25448333	pos
4.25_729.70	729.700428	4.25448333	pos
4.25_730.03	730.03496	4.25448333	pos
4.25_734.70	734.703428	4.25448333	pos
4.25_735.03	735.03681	4.25448333	pos
4.25_845.88	845.886579	4.24626667	neg
4.25_846.38	846.388244	4.24626667	neg
4.25_856.87	856.877852	4.24626667	neg
4.25_867.86	867.868587	4.24626667	neg
4.25_868.37	868.370286	4.24626667	neg
4.25_878.85	878.859537	4.24626667	neg
4.25_889.85	889.85138	4.24626667	neg
4.25_890.35	890.35481	4.24626667	neg
4.26_1002.0	1002.00447	4.26475	neg
4.26_233.10	233.102948	4.26475	neg
4.26_438.12	438.127851	4.26475	neg
4.27_114.12	114.128021	4.27276667	pos

4.27_132.04	132.047928	4.27276667	pos
4.27_185.11	185.117282	4.27276667	pos
4.27_191.02	191.029205	4.27276667	pos
4.27_195.03	218.028188	4.27276667	pos
4.27_201.04	202.05334	4.27276667	pos
4.27_241.10	241.10466	4.27276667	pos
4.27_258.59	258.591361	4.27276667	pos
4.27_319.67	319.67931	4.27276667	pos
4.27_344.70	344.705341	4.27276667	pos
4.27_346.51	346.510439	4.27276667	pos
4.27_359.10	359.100637	4.27276667	pos
4.27_375.22	375.2243	4.27276667	pos
4.27_376.56	376.561355	4.27276667	pos
4.27_376.89	376.895712	4.27276667	pos
4.27_377.22	377.22961	4.27276667	pos
4.27_393.45	393.451599	4.27276667	pos
4.27_393.70	393.702597	4.27276667	pos
4.27_393.95	393.952865	4.27276667	pos
4.27_482.75	482.755936	4.27276667	pos
4.27_492.29	492.29284	4.27276667	pos
4.27_513.30	513.309605	4.27276667	pos
4.27_518.29	518.296945	4.27276667	pos
4.27_518.79	518.797813	4.27276667	pos
4.27_523.93	523.932306	4.27276667	pos
4.27_528.79	528.792225	4.27276667	pos
4.27_588.95	588.954457	4.27276667	pos
4.27_589.28	589.288046	4.27276667	pos
4.27_589.62	589.622708	4.27276667	pos
4.27_598.57	598.571041	4.27276667	pos
4.27_598.81	598.819832	4.27276667	pos
4.27_630.31	630.310926	4.27276667	pos
4.27_630.64	630.645076	4.27276667	pos
4.27_634.33	634.330719	4.27276667	pos

4.27_669.681	669.681878	4.27276667	pos
4.27_887.442	887.44269	4.27276667	pos
4.27_938.774	938.774442	4.27276667	pos
4.28_153.017	153.017559	4.28308333	neg
4.28_156.048	156.04874	4.28308333	neg
4.28_268.026	268.026109	4.28308333	neg
4.28_330.167	330.167031	4.28308333	neg
4.28_357.085	357.085859	4.28308333	neg
4.28_430.230	430.230523	4.28308333	neg
4.28_460.241	460.241513	4.28308333	neg
4.28_664.203	664.203697	4.28308333	neg
4.28_732.191	732.191669	4.28308333	neg
4.28_880.913	880.913155	4.28308333	neg
4.29_152.995	152.994977	4.29085	pos
4.29_181.083	181.083479	4.29085	pos
4.29_199.988	199.988015	4.29085	pos
4.29_247.111	247.111281	4.29085	pos
4.29_276.163	276.163175	4.29085	pos
4.29_276.665	276.664952	4.29085	pos
4.29_301.095	301.095143	4.29085	pos
4.29_315.110	315.110741	4.29085	pos
4.29_320.205	321.21276	4.29085	pos
4.29_320.710	320.710879	4.29085	pos
4.29_328.094	328.094619	4.29085	pos
4.29_383.708	383.708733	4.29085	pos
4.29_429.238	429.238281	4.29085	pos
4.29_460.016	460.016643	4.29085	pos
4.29_462.743	462.743337	4.29085	pos
4.29_466.605	466.605287	4.29085	pos
4.29_470.226	470.226599	4.29085	pos
4.29_470.560	470.560798	4.29085	pos
4.29_470.894	470.894493	4.29085	pos
4.29_473.734	473.734694	4.29085	pos

4.29_506.26	506.261667	4.29085	pos
4.29_506.50	506.507463	4.29085	pos
4.29_506.75	506.758267	4.29085	pos
4.29_507.00	507.008481	4.29085	pos
4.29_507.25	507.259371	4.29085	pos
4.29_519.10	519.102562	4.29085	pos
4.29_521.02	521.022835	4.29085	pos
4.29_521.27	521.273223	4.29085	pos
4.29_548.62	548.619988	4.29085	pos
4.29_575.57	575.576357	4.29085	pos
4.29_581.31	581.313563	4.29085	pos
4.29_581.81	581.814452	4.29085	pos
4.29_595.06	595.066574	4.29085	pos
4.29_600.83	623.819495	4.29085	pos
4.29_632.80	632.804295	4.29085	pos
4.29_641.01	641.018298	4.29085	pos
4.29_675.00	675.007043	4.29085	pos
4.29_675.34	675.34073	4.29085	pos
4.29_730.07	730.079299	4.29085	pos
4.29_730.41	730.413469	4.29085	pos
4.29_730.74	730.747917	4.29085	pos
4.30_213.96	213.963659	4.30156667	neg
4.30_271.02	271.021094	4.30156667	neg
4.30_309.07	309.072077	4.30156667	neg
4.30_311.15	311.159001	4.30156667	neg
4.30_326.06	326.062132	4.30156667	neg
4.30_353.06	353.061783	4.30156667	neg
4.30_377.05	377.059453	4.30156667	neg
4.30_383.19	383.192411	4.30156667	neg
4.30_394.04	394.049273	4.30156667	neg
4.30_400.04	445.046939	4.30156667	neg
4.30_417.15	416.149901	4.30156667	neg
4.30_421.04	421.04916	4.30156667	neg

4.30_435.01	435.018001	4.30156667	neg
4.30_455.00	455.002187	4.30156667	neg
4.30_457.00	457.000405	4.30156667	neg
4.30_462.03	462.037208	4.30156667	neg
4.30_476.98	476.983985	4.30156667	neg
4.30_484.01	484.019122	4.30156667	neg
4.30_535.01	535.016255	4.30156667	neg
4.30_544.97	544.971272	4.30156667	neg
4.30_573.14	573.14588	4.30156667	neg
4.30_619.99	619.993854	4.30156667	neg
4.30_670.99	670.991423	4.30156667	neg
4.30_922.46	922.465004	4.30156667	neg
4.31_1044.7	1044.71352	4.30826667	pos
4.31_169.52	169.526278	4.30826667	pos
4.31_206.04	206.048283	4.30826667	pos
4.31_245.18	245.185822	4.30826667	pos
4.31_304.21	304.212009	4.30826667	pos
4.31_316.69	339.686181	4.30826667	pos
4.31_328.69	328.695181	4.30826667	pos
4.31_329.19	329.196859	4.30826667	pos
4.31_338.04	338.045027	4.30826667	pos
4.31_339.54	339.5442	4.30826667	pos
4.31_354.04	355.050581	4.30826667	pos
4.31_356.40	356.407217	4.30826667	pos
4.31_358.22	358.221401	4.30826667	pos
4.31_364.72	364.728523	4.30826667	pos
4.31_384.19	407.189157	4.30826667	pos
4.31_393.15	393.151859	4.30826667	pos
4.31_407.74	407.743043	4.30826667	pos
4.31_408.24	408.244424	4.30826667	pos
4.31_414.48	414.485614	4.30826667	pos
4.31_414.73	414.736378	4.30826667	pos
4.31_416.73	416.738688	4.30826667	pos

4.31_419.90!	419.909631	4.30826667	pos
4.31_428.98(	428.986445	4.30826667	pos
4.31_445.00	445.007148	4.30826667	pos
4.31_445.25	445.257362	4.30826667	pos
4.31_445.50	445.50793	4.30826667	pos
4.31_445.75!	445.758237	4.30826667	pos
4.31_451.73	434.734169	4.30826667	pos
4.31_491.60!	491.605243	4.30826667	pos
4.31_499.93	499.931324	4.30826667	pos
4.31_506.16(	507.173613	4.30826667	pos
4.31_508.98(	508.980948	4.30826667	pos
4.31_509.31!	509.315324	4.30826667	pos
4.31_509.64!	509.649568	4.30826667	pos
4.31_528.29!	528.298257	4.30826667	pos
4.31_539.77(	539.775996	4.30826667	pos
4.31_592.63!	592.638428	4.30826667	pos
4.31_592.97	592.9727	4.30826667	pos
4.31_604.65!	604.658425	4.30826667	pos
4.31_605.32	605.327616	4.30826667	pos
4.31_634.40	634.401406	4.30826667	pos
4.31_675.67!	675.674992	4.30826667	pos
4.31_721.39	704.394213	4.30826667	pos
4.32_552.00(	552.006715	4.32	neg
4.32_738.97!	738.978894	4.32	neg
4.33_247.65	247.652325	4.32651667	pos
4.33_262.20	262.201428	4.32651667	pos
4.33_353.21!	353.219792	4.32651667	pos
4.33_353.72	353.721448	4.32651667	pos
4.33_365.51(	365.510629	4.32651667	pos
4.33_415.68	415.681012	4.32651667	pos
4.33_423.76(	423.760023	4.32651667	pos
4.33_453.72(	471.760457	4.32651667	pos
4.33_470.50	471.509611	4.32651667	pos

4.33_471.00	471.008109	4.32651667	pos
4.33_482.26	482.265955	4.32651667	pos
4.33_485.51	485.514172	4.32651667	pos
4.33_519.43	519.436424	4.32651667	pos
4.33_519.79	520.803631	4.32651667	pos
4.33_520.29	521.304873	4.32651667	pos
4.33_530.78	531.794591	4.32651667	pos
4.33_531.28	532.295991	4.32651667	pos
4.33_554.94	554.949297	4.32651667	pos
4.33_564.76	564.767288	4.32651667	pos
4.33_565.26	565.268287	4.32651667	pos
4.33_626.83	626.83068	4.32651667	pos
4.33_628.00	628.009158	4.32651667	pos
4.33_777.39	777.391446	4.32651667	pos
4.34_150.99	150.998769	4.33765	neg
4.34_197.07	197.078296	4.34396667	pos
4.34_244.02	225.007478	4.33765	neg
4.34_248.99	248.99852	4.33765	neg
4.34_257.04	257.048903	4.33765	neg
4.34_281.65	281.652919	4.34396667	pos
4.34_300.13	300.135297	4.33765	neg
4.34_338.06	338.066205	4.33765	neg
4.34_408.14	408.141109	4.33765	neg
4.34_433.83	433.833552	4.34396667	pos
4.34_437.58	437.587443	4.34396667	pos
4.34_437.92	437.921695	4.34396667	pos
4.34_438.25	438.25578	4.34396667	pos
4.34_442.57	442.576551	4.34396667	pos
4.34_448.73	448.731357	4.34396667	pos
4.34_467.21	467.214183	4.34396667	pos
4.34_515.95	515.954327	4.34396667	pos
4.34_524.00	542.040077	4.34396667	pos
4.34_541.28	542.290693	4.34396667	pos

4.34_542.541	542.541519	4.34396667	pos
4.34_551.448	551.448356	4.34396667	pos
4.34_599.912	599.912475	4.34396667	pos
4.34_633.389	656.378905	4.34396667	pos
4.34_654.869	655.876655	4.34396667	pos
4.34_662.859	662.859718	4.34396667	pos
4.34_665.860	666.868115	4.34396667	pos
4.34_672.862	672.862537	4.34396667	pos
4.34_721.041	721.041556	4.34396667	pos
4.34_740.724	740.724663	4.34396667	pos
4.34_741.059	741.058978	4.34396667	pos
4.34_741.392	741.392154	4.34396667	pos
4.34_745.713	745.713416	4.34396667	pos
4.34_746.047	746.047524	4.34396667	pos
4.34_746.382	746.382149	4.34396667	pos
4.36_202.951	202.951243	4.3621	pos
4.36_249.139	249.139864	4.3621	pos
4.36_259.129	259.129825	4.35616667	neg
4.36_280.612	280.612331	4.3621	pos
4.36_287.146	287.14661	4.3621	pos
4.36_304.894	304.894366	4.3621	pos
4.36_339.252	362.241493	4.3621	pos
4.36_374.221	374.221493	4.35616667	neg
4.36_375.421	375.420985	4.3621	pos
4.36_424.977	424.977545	4.3621	pos
4.36_425.478	425.478916	4.3621	pos
4.36_434.034	434.034177	4.3621	pos
4.36_445.014	463.047808	4.3621	pos
4.36_462.847	462.847188	4.3621	pos
4.36_468.250	468.250388	4.3621	pos
4.36_494.274	494.274217	4.3621	pos
4.36_500.094	500.094869	4.3621	pos
4.36_500.429	500.429007	4.3621	pos

4.36_504.154	504.154723	4.35616667	neg
4.36_506.169	506.169643	4.3621	pos
4.36_528.331	551.320452	4.3621	pos
4.36_530.894	530.894346	4.3621	pos
4.36_540.832	540.832592	4.3621	pos
4.36_541.333	541.33362	4.3621	pos
4.36_548.802	548.802044	4.3621	pos
4.36_549.051	549.050989	4.3621	pos
4.36_551.615	551.615308	4.3621	pos
4.36_553.045	553.045447	4.3621	pos
4.36_553.546	553.546266	4.3621	pos
4.36_556.070	579.059251	4.3621	pos
4.36_563.814	564.821295	4.3621	pos
4.36_575.812	575.812118	4.3621	pos
4.36_578.555	578.555935	4.3621	pos
4.36_578.807	578.807356	4.3621	pos
4.36_585.140	585.13996	4.3621	pos
4.36_598.682	598.682338	4.3621	pos
4.36_599.016	599.016764	4.3621	pos
4.36_629.983	629.983671	4.3621	pos
4.36_663.364	663.36425	4.3621	pos
4.36_696.312	696.31244	4.3621	pos
4.36_737.051	737.05171	4.3621	pos
4.36_743.341	743.341806	4.3621	pos
4.36_743.676	743.675994	4.3621	pos
4.36_749.889	749.889665	4.3621	pos
4.36_829.726	829.726323	4.3621	pos
4.36_830.060	830.060859	4.3621	pos
4.37_1125.61	1125.61628	4.37481667	neg
4.37_1147.59	1147.59882	4.37481667	neg
4.37_124.007	124.007163	4.37481667	neg
4.37_175.039	175.039838	4.37481667	neg
4.37_285.110	285.110823	4.37481667	neg

4.37_289.96[illegible]	289.968521	4.37481667	neg
4.37_294.06[illegible]	294.065199	4.37481667	neg
4.37_358.95[illegible]	358.95876	4.37481667	neg
4.37_663.84[illegible]	663.846901	4.37481667	neg
4.38_192.13[illegible]	215.125554	4.38046667	pos
4.38_240.18[illegible]	240.180628	4.38046667	pos
4.38_243.94[illegible]	243.941743	4.38046667	pos
4.38_266.17[illegible]	289.162214	4.38046667	pos
4.38_296.07[illegible]	296.079826	4.38046667	pos
4.38_331.65[illegible]	331.650342	4.38046667	pos
4.38_338.71[illegible]	339.717343	4.38046667	pos
4.38_349.68[illegible]	349.689642	4.38046667	pos
4.38_350.70[illegible]	350.708201	4.38046667	pos
4.38_351.21[illegible]	351.210025	4.38046667	pos
4.38_358.24[illegible]	358.243746	4.38046667	pos
4.38_363.19[illegible]	363.199085	4.38046667	pos
4.38_375.67[illegible]	375.671511	4.38046667	pos
4.38_388.21[illegible]	388.215777	4.38046667	pos
4.38_399.71[illegible]	399.715398	4.38046667	pos
4.38_399.96[illegible]	399.966191	4.38046667	pos
4.38_400.24[illegible]	400.240326	4.38046667	pos
4.38_400.57[illegible]	400.574492	4.38046667	pos
4.38_414.24[illegible]	437.236105	4.38046667	pos
4.38_441.24[illegible]	441.240283	4.38046667	pos
4.38_442.58[illegible]	425.580366	4.38046667	pos
4.38_448.54[illegible]	448.545322	4.38046667	pos
4.38_448.87[illegible]	448.879557	4.38046667	pos
4.38_495.94[illegible]	495.946296	4.38046667	pos
4.38_500.26[illegible]	500.261631	4.38046667	pos
4.38_500.76[illegible]	500.762779	4.38046667	pos
4.38_500.93[illegible]	500.930155	4.38046667	pos
4.38_515.94[illegible]	515.94119	4.38046667	pos
4.38_516.27[illegible]	516.275304	4.38046667	pos

4.38_532.61	532.617223	4.38046667	pos
4.38_538.62	538.622024	4.38046667	pos
4.38_566.63	566.635263	4.38046667	pos
4.38_591.34	614.337286	4.38046667	pos
4.38_599.33	600.338842	4.38046667	pos
4.38_606.79	606.797339	4.38046667	pos
4.38_610.82	610.828653	4.38046667	pos
4.38_613.83	613.835879	4.38046667	pos
4.38_614.08	614.086654	4.38046667	pos
4.38_616.84	599.837969	4.38046667	pos
4.38_618.08	618.085772	4.38046667	pos
4.38_634.32	634.320676	4.38046667	pos
4.38_634.65	634.655707	4.38046667	pos
4.38_634.98	634.989004	4.38046667	pos
4.38_637.86	637.866836	4.38046667	pos
4.38_638.36	638.368659	4.38046667	pos
4.38_661.53	661.536561	4.38046667	pos
4.38_678.42	678.427413	4.38046667	pos
4.38_681.88	681.884278	4.38046667	pos
4.38_702.69	702.699404	4.38046667	pos
4.38_703.03	703.033402	4.38046667	pos
4.38_809.67	809.676718	4.38046667	pos
4.38_840.46	863.450571	4.38046667	pos
4.38_962.78	962.789056	4.38046667	pos
4.39_222.98	222.983416	4.39318333	neg
4.39_445.08	445.081855	4.39318333	neg
4.39_486.90	486.903347	4.39318333	neg
4.39_582.28	582.287219	4.39318333	neg
4.39_915.43	915.436012	4.39318333	neg
4.39_915.93	915.9372	4.39318333	neg
4.40_143.03	143.039715	4.3987	pos
4.40_276.05	276.055359	4.3987	pos
4.40_279.19	279.191893	4.3987	pos

4.40_279.69[illegible]	279.694022	4.3987	pos
4.40_294.20[illegible]	312.238152	4.3987	pos
4.40_298.20[illegible]	298.200033	4.3987	pos
4.40_319.17[illegible]	319.172861	4.3987	pos
4.40_348.73[illegible]	348.73235	4.3987	pos
4.40_375.13[illegible]	375.13006	4.3987	pos
4.40_419.71[illegible]	419.719053	4.3987	pos
4.40_420.22[illegible]	420.220646	4.3987	pos
4.40_459.22[illegible]	459.228852	4.3987	pos
4.40_459.47[illegible]	459.479483	4.3987	pos
4.40_459.73[illegible]	459.730289	4.3987	pos
4.40_459.98[illegible]	459.980909	4.3987	pos
4.40_500.59[illegible]	500.596124	4.3987	pos
4.40_525.26[illegible]	525.261618	4.3987	pos
4.40_525.51[illegible]	525.511893	4.3987	pos
4.40_547.30[illegible]	547.309384	4.3987	pos
4.40_553.29[illegible]	571.327547	4.3987	pos
4.40_571.82[illegible]	571.829079	4.3987	pos
4.40_572.81[illegible]	595.80681	4.3987	pos
4.40_584.97[illegible]	584.972611	4.3987	pos
4.40_600.11[illegible]	600.112752	4.3987	pos
4.40_611.96[illegible]	611.969101	4.3987	pos
4.40_612.30[illegible]	612.303195	4.3987	pos
4.40_652.68[illegible]	652.687227	4.3987	pos
4.40_653.02[illegible]	653.021838	4.3987	pos
4.40_863.19[illegible]	863.199781	4.3987	pos
4.40_863.70[illegible]	863.701253	4.3987	pos
4.40_863.95[illegible]	863.951983	4.3987	pos
4.41_293.11[illegible]	293.114459	4.41166667	neg
4.41_361.10[illegible]	361.10213	4.41166667	neg
4.41_427.21[illegible]	427.218397	4.41166667	neg
4.41_492.06[illegible]	492.067431	4.41166667	neg
4.41_544.20[illegible]	544.203228	4.41166667	neg

4.42_247.54	247.544601	4.41721667	pos
4.42_273.08	273.081591	4.41721667	pos
4.42_295.12	295.129024	4.41721667	pos
4.42_394.74	394.741196	4.41721667	pos
4.42_415.73	415.734915	4.41721667	pos
4.42_428.22	451.215601	4.41721667	pos
4.42_466.26	466.261485	4.41721667	pos
4.42_473.19	473.197414	4.41721667	pos
4.42_494.08	494.082471	4.41721667	pos
4.42_513.93	513.939367	4.41721667	pos
4.42_532.79	532.789956	4.41721667	pos
4.42_546.03	546.03392	4.41721667	pos
4.42_546.53	546.535183	4.41721667	pos
4.42_561.31	584.307381	4.41721667	pos
4.42_572.81	572.814752	4.41721667	pos
4.42_573.31	573.316589	4.41721667	pos
4.42_579.67	579.67536	4.41721667	pos
4.42_583.80	583.806029	4.41721667	pos
4.42_606.84	606.845734	4.41721667	pos
4.42_609.31	609.317494	4.41721667	pos
4.42_627.09	627.092261	4.41721667	pos
4.42_638.99	638.992833	4.41721667	pos
4.42_713.03	713.034634	4.41721667	pos
4.42_713.89	713.893315	4.41721667	pos
4.43_170.05	151.03987	4.43021667	neg
4.43_201.01	201.017935	4.43021667	neg
4.43_248.01	248.017534	4.43021667	neg
4.43_273.14	273.145613	4.43021667	neg
4.43_367.00	367.002449	4.43021667	neg
4.43_369.88	369.885095	4.43021667	neg
4.43_436.22	436.220101	4.43021667	neg
4.43_526.14	526.142055	4.43021667	neg
4.43_570.79	570.79978	4.43021667	neg

4.44_322.720	322.720581	4.435	pos
4.44_323.106	323.106111	4.435	pos
4.44_340.576	340.576712	4.435	pos
4.44_402.229	402.229438	4.435	pos
4.44_402.731	402.731239	4.435	pos
4.44_424.747	424.747715	4.435	pos
4.44_539.958	539.958718	4.435	pos
4.44_540.627	540.627339	4.435	pos
4.44_546.785	546.785402	4.435	pos
4.44_556.953	556.953216	4.435	pos
4.44_557.287	557.287923	4.435	pos
4.44_626.591	626.591095	4.435	pos
4.44_628.081	628.081916	4.435	pos
4.44_658.851	658.851577	4.435	pos
4.44_783.361	783.361191	4.435	pos
4.44_794.351	794.351861	4.435	pos
4.44_794.848	794.848172	4.435	pos
4.44_795.348	795.348013	4.435	pos
4.44_851.949	851.949312	4.435	pos
4.44_905.870	905.870563	4.435	pos
4.44_906.038	906.038128	4.435	pos
4.45_1003.13	1003.13739	4.45335	pos
4.45_118.040	118.04093	4.44846667	neg
4.45_204.099	185.081655	4.44846667	neg
4.45_219.027	219.027305	4.44846667	neg
4.45_231.993	231.993588	4.44846667	neg
4.45_233.990	233.990644	4.44846667	neg
4.45_234.008	234.008489	4.45335	pos
4.45_236.005	236.005338	4.45335	pos
4.45_271.263	271.26326	4.45335	pos
4.45_321.091	321.091358	4.44846667	neg
4.45_332.218	332.218072	4.45335	pos
4.45_357.871	357.871147	4.45335	pos

4.45_360.72:	361.730298	4.45335	pos
4.45_361.22·	362.232084	4.45335	pos
4.45_370.74!	370.7454	4.45335	pos
4.45_372.72	372.72126	4.45335	pos
4.45_373.06:	373.062956	4.44846667	neg
4.45_373.22:	373.22311	4.45335	pos
4.45_391.11·	391.114749	4.44846667	neg
4.45_403.70!	403.70986	4.45335	pos
4.45_468.22!	468.228206	4.45335	pos
4.45_507.77!	507.775246	4.45335	pos
4.45_508.02(	508.026116	4.45335	pos
4.45_508.27(	508.276796	4.45335	pos
4.45_508.52	508.527556	4.45335	pos
4.45_531.77	531.777761	4.45335	pos
4.45_540.88(	540.88068	4.45335	pos
4.45_556.32:	556.322134	4.45335	pos
4.45_556.82:	556.823511	4.45335	pos
4.45_623.33(	623.336503	4.45335	pos
4.45_647.37:	647.373913	4.45335	pos
4.45_647.70!	647.708153	4.45335	pos
4.45_652.02(	652.026875	4.45335	pos
4.45_722.45:	722.453514	4.45335	pos
4.45_781.34	781.347229	4.44846667	neg
4.45_781.84:	781.842502	4.44846667	neg
4.45_782.34:	782.34249	4.44846667	neg
4.45_782.85(	783.857855	4.45335	pos
4.45_784.35	784.357513	4.45335	pos
4.45_792.33!	792.338109	4.44846667	neg
4.45_792.83:	792.832334	4.44846667	neg
4.45_793.33:	793.333303	4.44846667	neg
4.45_803.82:	803.823664	4.44846667	neg
4.45_814.32(	814.320224	4.44846667	neg
4.45_814.81!	814.815632	4.44846667	neg

4.45_995.47(	995.476223	4.45335	pos
4.45_995.81(	995.810349	4.45335	pos
4.47_1019.92	1019.9235	4.47151667	pos
4.47_158.082	158.082057	4.46708333	neg
4.47_167.05!	167.055585	4.47151667	pos
4.47_223.094	223.094014	4.47151667	pos
4.47_226.06!	226.069515	4.46708333	neg
4.47_237.18(	237.186252	4.47151667	pos
4.47_244.69!	244.69994	4.47151667	pos
4.47_299.98	299.981129	4.46708333	neg
4.47_301.188	302.19622	4.47151667	pos
4.47_346.187	346.186964	4.46708333	neg
4.47_393.044	393.044079	4.46708333	neg
4.47_436.75:	436.75358	4.47151667	pos
4.47_461.03	461.031337	4.46708333	neg
4.47_491.767	491.767685	4.47151667	pos
4.47_535.314	535.314657	4.47151667	pos
4.47_587.637	570.634179	4.47151667	pos
4.47_589.31!	589.319763	4.47151667	pos
4.47_599.97(	599.970529	4.47151667	pos
4.47_606.07	589.067768	4.47151667	pos
4.47_655.238	655.238419	4.47151667	pos
4.47_711.358	711.358356	4.47151667	pos
4.47_758.86(	758.866569	4.46708333	neg
4.47_840.75!	840.759474	4.47151667	pos
4.47_841.094	841.094016	4.47151667	pos
4.47_841.428	841.428167	4.47151667	pos
4.47_954.38!	955.393099	4.47151667	pos
4.47_954.887	955.894292	4.47151667	pos
4.47_966.384	966.383998	4.47151667	pos
4.47_966.88!	966.885567	4.47151667	pos
4.47_972.397	953.379176	4.46708333	neg
4.47_972.898	953.880548	4.46708333	neg

4.47_975.862	975.862471	4.46708333	neg
4.47_983.387	964.370033	4.46708333	neg
4.47_983.889	964.872108	4.46708333	neg
4.49_148.025	148.025924	4.48555	neg
4.49_218.049	218.04903	4.48555	neg
4.49_432.763	432.763309	4.4888	pos
4.49_433.264	433.264726	4.4888	pos
4.49_457.257	457.257143	4.4888	pos
4.49_458.273	481.262706	4.4888	pos
4.49_463.217	463.2177	4.48555	neg
4.49_472.769	472.769853	4.4888	pos
4.49_481.601	481.601703	4.4888	pos
4.49_481.935	481.935629	4.4888	pos
4.49_492.102	492.102115	4.4888	pos
4.49_514.019	514.019756	4.4888	pos
4.49_514.521	514.521164	4.4888	pos
4.49_514.771	514.771488	4.4888	pos
4.49_514.893	514.893353	4.48555	neg
4.49_518.506	518.506456	4.4888	pos
4.49_518.757	518.757229	4.4888	pos
4.49_519.007	519.007713	4.4888	pos
4.49_535.809	535.809604	4.4888	pos
4.49_539.789	539.789777	4.4888	pos
4.49_540.542	540.54269	4.4888	pos
4.49_570.467	570.467044	4.4888	pos
4.49_570.801	570.801213	4.4888	pos
4.49_636.711	636.711273	4.4888	pos
4.49_685.357	685.357692	4.4888	pos
4.49_685.691	685.691638	4.4888	pos
4.49_696.017	696.01752	4.4888	pos
4.49_702.020	685.023074	4.4888	pos
4.49_710.852	710.8528	4.4888	pos
4.49_719.384	719.384242	4.4888	pos

4.49_756.601	756.601163	4.4888	pos
4.49_756.852	756.852196	4.4888	pos
4.49_819.382	819.38212	4.4888	pos
4.49_967.885	967.885253	4.48555	neg
4.50_122.032	122.032719	4.50406667	neg
4.50_168.989	168.989078	4.50406667	neg
4.50_326.031	326.031485	4.50406667	neg
4.50_327.009	327.009378	4.50406667	neg
4.50_382.013	382.013783	4.50406667	neg
4.50_480.089	480.089935	4.50406667	neg
4.50_609.367	609.367729	4.50406667	neg
4.50_816.866	816.866718	4.50406667	neg
4.50_827.858	827.857962	4.50406667	neg
4.51_183.091	183.09189	4.50633333	pos
4.51_265.704	265.704901	4.50633333	pos
4.51_277.134	260.131609	4.50633333	pos
4.51_302.207	302.207139	4.50633333	pos
4.51_399.220	399.220889	4.50633333	pos
4.51_399.722	399.72227	4.50633333	pos
4.51_442.756	442.7563	4.50633333	pos
4.51_443.007	443.007001	4.50633333	pos
4.51_487.252	487.252517	4.50633333	pos
4.51_536.247	536.247836	4.50633333	pos
4.51_536.582	536.582069	4.50633333	pos
4.51_554.458	554.458535	4.50633333	pos
4.51_570.968	570.968449	4.50633333	pos
4.51_577.289	577.289156	4.50633333	pos
4.51_577.790	577.790734	4.50633333	pos
4.51_770.732	770.73288	4.50633333	pos
4.51_779.917	818.880546	4.50633333	pos
4.51_866.939	866.939211	4.50633333	pos
4.52_125.097	125.096977	4.52246667	neg
4.52_209.102	254.100946	4.52246667	neg

4.52_230.99(	230.99675	4.52246667	neg
4.52_233.18!	233.185826	4.52393333	pos
4.52_234.08!	234.088299	4.52246667	neg
4.52_244.17!	244.178395	4.52393333	pos
4.52_246.03!	246.038269	4.52246667	neg
4.52_292.04:	292.043893	4.52246667	neg
4.52_314.02(	314.026025	4.52246667	neg
4.52_322.08!	322.088222	4.52246667	neg
4.52_346.23:	346.233205	4.52393333	pos
4.52_358.19!	358.198521	4.52246667	neg
4.52_360.21:	360.212791	4.52393333	pos
4.52_382.73!	383.743144	4.52393333	pos
4.52_392.25(	392.256555	4.52393333	pos
4.52_392.75!	392.758173	4.52393333	pos
4.52_394.73<	394.734187	4.52393333	pos
4.52_395.23(	395.236042	4.52393333	pos
4.52_420.85(	420.856918	4.52393333	pos
4.52_458.06:	458.063393	4.52246667	neg
4.52_471.24<	471.244393	4.52246667	neg
4.52_486.91!	486.918916	4.52393333	pos
4.52_526.05(	526.050806	4.52246667	neg
4.52_555.27!	555.27969	4.52393333	pos
4.52_583.56	583.567307	4.52393333	pos
4.52_626.64!	626.648613	4.52393333	pos
4.52_626.98:	626.982921	4.52393333	pos
4.52_632.81<	632.814744	4.52393333	pos
4.52_662.02!	662.025886	4.52246667	neg
4.52_668.99	687.031031	4.52393333	pos
4.52_687.36<	687.364884	4.52393333	pos
4.52_687.69!	687.698897	4.52393333	pos
4.52_730.01:	730.013034	4.52246667	neg
4.52_833.96(	833.96005	4.52393333	pos
4.52_988.89	988.897578	4.52246667	neg

4.54_177.049	177.049834	4.5416	pos
4.54_199.058	199.058415	4.54091667	neg
4.54_216.048	216.048641	4.54091667	neg
4.54_216.123	216.123829	4.54091667	neg
4.54_232.059	232.059117	4.54091667	neg
4.54_265.074	265.074031	4.5416	pos
4.54_267.046	267.045964	4.54091667	neg
4.54_271.091	271.091014	4.54091667	neg
4.54_285.037	285.036954	4.54091667	neg
4.54_310.199	333.188267	4.5416	pos
4.54_335.033	335.033724	4.54091667	neg
4.54_350.124	350.124324	4.54091667	neg
4.54_387.055	388.063072	4.5416	pos
4.54_403.020	403.020905	4.54091667	neg
4.54_418.223	419.230437	4.5416	pos
4.54_418.729	418.729291	4.5416	pos
4.54_466.763	466.763663	4.5416	pos
4.54_480.584	480.584636	4.5416	pos
4.54_502.870	502.87007	4.5416	pos
4.54_506.453	506.453772	4.5416	pos
4.54_506.654	506.654235	4.5416	pos
4.54_519.538	502.535615	4.5416	pos
4.54_538.625	539.632567	4.5416	pos
4.54_542.294	565.283467	4.5416	pos
4.54_580.631	580.631503	4.5416	pos
4.54_594.038	594.038323	4.54091667	neg
4.54_637.310	655.344276	4.5416	pos
4.54_690.318	735.317057	4.54091667	neg
4.54_724.325	724.32568	4.54091667	neg
4.54_724.827	724.827319	4.54091667	neg
4.54_725.045	725.04581	4.5416	pos
4.54_725.332	726.339565	4.5416	pos
4.54_725.833	726.84107	4.5416	pos

4.54_734.380	734.380482	4.5416	pos
4.54_735.818	735.817972	4.54091667	neg
4.54_737.832	737.832151	4.5416	pos
4.54_746.307	746.307503	4.54091667	neg
4.54_746.809	746.809077	4.54091667	neg
4.54_757.800	757.800221	4.54091667	neg
4.54_924.980	924.980611	4.5416	pos
4.56_167.118	167.118024	4.5592	pos
4.56_212.118	212.118272	4.5592	pos
4.56_271.984	271.984679	4.55865	neg
4.56_277.155	277.155589	4.55865	neg
4.56_278.970	278.970895	4.55865	neg
4.56_322.981	322.981906	4.55865	neg
4.56_361.131	361.13098	4.55865	neg
4.56_390.969	390.969582	4.55865	neg
4.56_441.968	441.968244	4.55865	neg
4.56_449.274	449.274334	4.5592	pos
4.56_451.503	451.503123	4.5592	pos
4.56_458.956	458.956906	4.55865	neg
4.56_466.589	466.589682	4.5592	pos
4.56_474.941	474.941752	4.5592	pos
4.56_502.774	502.774721	4.5592	pos
4.56_525.769	543.803768	4.5592	pos
4.56_527.016	527.016168	4.5592	pos
4.56_539.298	539.29872	4.5592	pos
4.56_543.303	543.303128	4.5592	pos
4.56_585.069	585.069034	4.55865	neg
4.56_608.252	608.252103	4.55865	neg
4.56_655.009	655.009641	4.5592	pos
4.56_655.677	655.677887	4.5592	pos
4.56_711.908	711.908894	4.5592	pos
4.56_712.410	712.410496	4.5592	pos
4.56_713.685	713.685242	4.5592	pos

4.56_714.02(	714.020021	4.5592	pos
4.56_714.35	714.354095	4.5592	pos
4.56_718.85(	718.85686	4.55865	neg
4.58_124.00	124.007156	4.57715	neg
4.58_142.03	142.032991	4.57715	neg
4.58_168.01	168.012297	4.57715	neg
4.58_187.13	187.132925	4.57725	pos
4.58_195.11	195.112981	4.57725	pos
4.58_236.16	259.151661	4.57725	pos
4.58_247.11	247.118663	4.57715	neg
4.58_253.01(	253.010312	4.57715	neg
4.58_274.11	274.111904	4.57715	neg
4.58_275.10	275.103844	4.57715	neg
4.58_284.07	284.078607	4.57715	neg
4.58_294.12	277.118317	4.57725	pos
4.58_294.61	294.614156	4.57715	neg
4.58_302.20	302.207345	4.57725	pos
4.58_362.05	362.052659	4.57715	neg
4.58_365.00	365.00492	4.57715	neg
4.58_373.06	373.062983	4.57715	neg
4.58_404.09	403.088596	4.57715	neg
4.58_404.74	405.756608	4.57725	pos
4.58_405.25(	428.239909	4.57725	pos
4.58_414.77	414.771597	4.57725	pos
4.58_416.74	416.747689	4.57725	pos
4.58_417.24	417.248992	4.57725	pos
4.58_426.95	426.958492	4.57715	neg
4.58_459.74	459.749729	4.57725	pos
4.58_464.01	464.018128	4.57725	pos
4.58_464.26	464.269055	4.57725	pos
4.58_464.52(	464.520054	4.57725	pos
4.58_472.08	517.081771	4.57715	neg
4.58_476.01	521.009978	4.57715	neg

4.58_496.98(	496.986807	4.57715	neg
4.58_502.29(	525.288615	4.57725	pos
4.58_516.01(	516.018644	4.57715	neg
4.58_538.00(	538.000872	4.57715	neg
4.58_543.99(	542.992276	4.57715	neg
4.58_549.98(	549.983268	4.57725	pos
4.58_604.81(	604.81183	4.57725	pos
4.58_675.63(	675.631237	4.57725	pos
4.58_675.96(	675.965854	4.57725	pos
4.58_676.29(	676.299513	4.57725	pos
4.58_701.35(	702.358845	4.57725	pos
4.60_166.12(	166.122759	4.5955	pos
4.60_249.08(	249.08769	4.59571667	neg
4.60_267.15(	267.156607	4.5955	pos
4.60_279.12(	279.120695	4.5955	pos
4.60_294.06(	294.065153	4.59571667	neg
4.60_296.07(	296.079751	4.5955	pos
4.60_359.22(	359.229053	4.5955	pos
4.60_375.07(	375.07763	4.5955	pos
4.60_440.87(	440.87033	4.5955	pos
4.60_446.90(	446.905957	4.59571667	neg
4.60_451.25(	451.252086	4.5955	pos
4.60_470.76(	470.760838	4.5955	pos
4.60_484.24(	484.241528	4.5955	pos
4.60_484.49(	484.491792	4.5955	pos
4.60_484.74(	484.742285	4.5955	pos
4.60_488.26(	471.262484	4.5955	pos
4.60_495.60(	495.601901	4.5955	pos
4.60_510.79(	510.790328	4.5955	pos
4.60_517.10(	517.105575	4.5955	pos
4.60_598.80(	598.809814	4.5955	pos
4.60_599.30(	599.307129	4.5955	pos
4.60_605.31(	605.313024	4.5955	pos

4.60_614.81!	614.815396	4.5955	pos
4.60_627.57(	627.57665	4.5955	pos
4.60_645.65:	645.653834	4.5955	pos
4.60_655.88	655.887997	4.5955	pos
4.60_656.38!	656.389325	4.5955	pos
4.60_741.75!	741.75585	4.5955	pos
4.60_759.06	759.068179	4.5955	pos
4.60_863.40	863.40805	4.5955	pos
4.60_863.60!	863.60935	4.5955	pos
4.60_888.03!	888.03905	4.5955	pos
4.60_930.38	930.381617	4.5955	pos
4.61_172.14	195.138088	4.61381667	pos
4.61_188.00	188.002865	4.61381667	pos
4.61_189.99!	189.999894	4.61381667	pos
4.61_209.05!	209.055752	4.61381667	pos
4.61_231.99:	231.993593	4.61413333	neg
4.61_233.99(	233.990636	4.61413333	neg
4.61_234.00	234.008361	4.61381667	pos
4.61_236.00!	236.00537	4.61381667	pos
4.61_238.00	238.002401	4.61381667	pos
4.61_238.24!	238.249206	4.61381667	pos
4.61_250.14	273.130845	4.61381667	pos
4.61_259.05	282.040567	4.61381667	pos
4.61_274.99!	274.999735	4.61413333	neg
4.61_283.00	283.007644	4.61381667	pos
4.61_291.94!	291.949514	4.61413333	neg
4.61_299.98	299.98111	4.61413333	neg
4.61_301.97	301.978164	4.61413333	neg
4.61_326.03	326.031584	4.61413333	neg
4.61_340.22	340.224029	4.61413333	neg
4.61_345.02(	345.020298	4.61413333	neg
4.61_359.10(	359.100366	4.61381667	pos
4.61_360.97	360.97429	4.61413333	neg

4.61_365.13!	365.135242	4.61413333	neg
4.61_367.96!	367.968527	4.61413333	neg
4.61_369.96!	369.965516	4.61413333	neg
4.61_381.06:	404.052385	4.61381667	pos
4.61_392.14(	392.146511	4.61413333	neg
4.61_393.15:	416.14253	4.61381667	pos
4.61_413.00	413.007814	4.61413333	neg
4.61_422.55(	422.550788	4.61381667	pos
4.61_422.88	422.884494	4.61381667	pos
4.61_480.99!	480.995291	4.61413333	neg
4.61_483.25	506.243415	4.61381667	pos
4.61_486.52	486.527463	4.61381667	pos
4.61_502.77!	503.786315	4.61381667	pos
4.61_503.03(	486.026802	4.61381667	pos
4.61_540.29(	540.289965	4.61381667	pos
4.61_546.98!	546.985752	4.61381667	pos
4.61_548.98:	548.982407	4.61413333	neg
4.61_551.78	551.784148	4.61381667	pos
4.61_552.81(	552.810421	4.61381667	pos
4.61_563.59!	563.599819	4.61381667	pos
4.61_646.95	646.954559	4.61381667	pos
4.61_647.69!	647.699595	4.61381667	pos
4.61_692.87(	692.870456	4.61381667	pos
4.61_725.37!	725.375112	4.61381667	pos
4.61_725.70!	725.709185	4.61381667	pos
4.61_726.04:	726.042829	4.61381667	pos
4.61_736.38(	736.386433	4.61381667	pos
4.61_742.09(	742.090265	4.61381667	pos
4.61_781.40:	781.403319	4.61381667	pos
4.61_781.73	781.73708	4.61381667	pos
4.61_782.07	782.071572	4.61381667	pos
4.61_787.72:	788.730175	4.61381667	pos
4.61_806.43!	829.428226	4.61381667	pos

4.61_815.92[illegible]	815.92164	4.61413333	neg
4.61_816.42[illegible]	816.423129	4.61413333	neg
4.61_816.92[illegible]	817.935446	4.61381667	pos
4.61_817.42[illegible]	818.437103	4.61381667	pos
4.61_826.91[illegible]	826.912517	4.61413333	neg
4.61_827.41[illegible]	827.413851	4.61413333	neg
4.61_828.92[illegible]	828.925836	4.61381667	pos
4.61_904.88[illegible]	904.886989	4.61381667	pos
4.61_905.38[illegible]	905.388485	4.61381667	pos
4.61_913.86[illegible]	913.864452	4.61413333	neg
4.61_914.36[illegible]	914.366079	4.61413333	neg
4.61_915.87[illegible]	915.8782	4.61381667	pos
4.61_916.37[illegible]	916.37921	4.61381667	pos
4.61_921.89[illegible]	902.873767	4.61413333	neg
4.61_922.39[illegible]	903.374434	4.61413333	neg
4.61_924.85[illegible]	924.856036	4.61413333	neg
4.61_930.21[illegible]	930.213978	4.61381667	pos
4.61_930.54[illegible]	930.548237	4.61381667	pos
4.61_930.71[illegible]	930.715325	4.61381667	pos
4.61_930.88[illegible]	930.882506	4.61381667	pos
4.63_1054.1[illegible]	1054.11986	4.63258333	neg
4.63_1064.1[illegible]	1064.12874	4.63161667	pos
4.63_173.11[illegible]	173.117283	4.63161667	pos
4.63_196.09[illegible]	196.094421	4.63161667	pos
4.63_223.01[illegible]	223.019662	4.63258333	neg
4.63_240.08[illegible]	240.08525	4.63258333	neg
4.63_241.10[illegible]	241.104576	4.63161667	pos
4.63_246.04[illegible]	246.04406	4.63258333	neg
4.63_254.17[illegible]	254.172687	4.63161667	pos
4.63_255.17[illegible]	255.175479	4.63161667	pos
4.63_277.06[illegible]	277.067999	4.63161667	pos
4.63_294.02[illegible]	294.023167	4.63258333	neg
4.63_316.65[illegible]	316.650811	4.63161667	pos

4.63_361.10[illegible]	361.101853	4.63258333	neg
4.63_361.21[illegible]	361.215595	4.63161667	pos
4.63_377.23[illegible]	377.236675	4.63161667	pos
4.63_377.73[illegible]	377.738593	4.63161667	pos
4.63_380.05[illegible]	380.055791	4.63258333	neg
4.63_426.76[illegible]	427.769843	4.63161667	pos
4.63_427.26[illegible]	428.271598	4.63161667	pos
4.63_436.78[illegible]	436.784761	4.63161667	pos
4.63_438.76[illegible]	438.760768	4.63161667	pos
4.63_439.26[illegible]	439.262386	4.63161667	pos
4.63_448.04[illegible]	448.04337	4.63258333	neg
4.63_464.20[illegible]	464.206643	4.63161667	pos
4.63_489.53[illegible]	489.530705	4.63161667	pos
4.63_503.53[illegible]	503.535386	4.63161667	pos
4.63_516.27[illegible]	539.267929	4.63161667	pos
4.63_555.29[illegible]	555.29603	4.63161667	pos
4.63_562.98[illegible]	585.96967	4.63161667	pos
4.63_563.93[illegible]	563.933875	4.63161667	pos
4.63_592.04[illegible]	592.041995	4.63258333	neg
4.63_616.65[illegible]	616.650906	4.63161667	pos
4.63_622.82[illegible]	622.822908	4.63161667	pos
4.63_671.04[illegible]	671.043713	4.63161667	pos
4.63_671.71[illegible]	671.711547	4.63161667	pos
4.63_794.72[illegible]	794.724513	4.63258333	neg
4.63_969.03[illegible]	969.031747	4.63258333	neg
4.65_195.09[illegible]	195.099033	4.64996667	pos
4.65_239.10[illegible]	257.135901	4.64996667	pos
4.65_285.09[illegible]	285.095581	4.65108333	neg
4.65_343.02[illegible]	343.025145	4.65108333	neg
4.65_377.05[illegible]	377.057804	4.65108333	neg
4.65_464.78[illegible]	464.786645	4.64996667	pos
4.65_474.10[illegible]	474.109877	4.64996667	pos
4.65_479.00[illegible]	479.000541	4.65108333	neg

4.65_523.30[illegible]	523.307295	4.64996667	pos
4.65_523.64[illegible]	523.641504	4.64996667	pos
4.65_543.27[illegible]	543.27826	4.64996667	pos
4.65_648.30[illegible]	648.307939	4.64996667	pos
4.65_648.97[illegible]	648.975799	4.64996667	pos
4.65_678.84[illegible]	678.84539	4.64996667	pos
4.65_731.37[illegible]	731.378913	4.64996667	pos
4.65_735.73[illegible]	735.737484	4.64996667	pos
4.65_735.88[illegible]	735.880865	4.64996667	pos
4.65_736.13[illegible]	736.131242	4.64996667	pos
4.65_756.74[illegible]	756.740663	4.64996667	pos
4.67_150.05[illegible]	150.055197	4.66833333	pos
4.67_192.06[illegible]	192.065555	4.66833333	pos
4.67_202.05[illegible]	202.051068	4.66833333	pos
4.67_209.07[illegible]	209.078375	4.66833333	pos
4.67_211.09[illegible]	211.093957	4.66833333	pos
4.67_276.04[illegible]	276.048786	4.66926667	neg
4.67_296.15[illegible]	296.151797	4.66833333	pos
4.67_296.65[illegible]	296.653405	4.66833333	pos
4.67_358.71[illegible]	358.713089	4.66833333	pos
4.67_359.21[illegible]	359.214654	4.66833333	pos
4.67_366.01[illegible]	366.018276	4.66926667	neg
4.67_420.76[illegible]	420.768959	4.66833333	pos
4.67_421.27[illegible]	421.270401	4.66833333	pos
4.67_560.97[illegible]	560.970656	4.66833333	pos
4.67_618.68[illegible]	618.682899	4.66833333	pos
4.67_938.39[illegible]	938.391986	4.66926667	neg
4.67_938.89[illegible]	938.893375	4.66926667	neg
4.69_143.11[illegible]	143.117908	4.68661667	pos
4.69_268.00[illegible]	268.007411	4.6877	neg
4.69_279.09[illegible]	279.09848	4.6877	neg
4.69_307.11[illegible]	307.115099	4.68661667	pos
4.69_319.00[illegible]	319.004156	4.6877	neg

4.69_334.168	334.168106	4.68661667	pos
4.69_334.997	334.997559	4.6877	neg
4.69_347.124	347.124707	4.6877	neg
4.69_386.992	386.992199	4.6877	neg
4.69_389.048	389.048755	4.6877	neg
4.69_449.782	449.782938	4.68661667	pos
4.69_450.284	450.284537	4.68661667	pos
4.69_454.979	454.979456	4.6877	neg
4.69_456.755	456.755778	4.68661667	pos
4.69_458.296	458.296344	4.68661667	pos
4.69_458.797	458.797867	4.68661667	pos
4.69_460.773	460.773773	4.68661667	pos
4.69_461.275	461.275316	4.68661667	pos
4.69_482.231	482.231579	4.68661667	pos
4.69_513.618	513.618649	4.68661667	pos
4.69_549.294	549.29472	4.68661667	pos
4.69_566.655	566.655445	4.68661667	pos
4.69_600.791	600.791222	4.68661667	pos
4.69_634.844	634.844196	4.68661667	pos
4.69_642.660	642.659989	4.68661667	pos
4.69_769.422	769.422224	4.68661667	pos
4.69_769.923	769.923589	4.68661667	pos
4.70_1012.47	1012.47932	4.70481667	pos
4.70_1012.73	1012.73012	4.70481667	pos
4.70_1013.48	1013.4812	4.70481667	pos
4.70_1018.72	1018.7265	4.70481667	pos
4.70_1018.97	1018.97706	4.70481667	pos
4.70_167.012	167.012885	4.70481667	pos
4.70_194.082	194.082083	4.70481667	pos
4.70_229.008	229.008324	4.70481667	pos
4.70_327.673	327.672978	4.70481667	pos
4.70_335.684	335.684504	4.70481667	pos
4.70_338.664	338.664016	4.70481667	pos

4.70_345.09	345.091255	4.70481667	pos
4.70_420.72	420.726765	4.70481667	pos
4.70_518.95	518.956759	4.70481667	pos
4.70_544.29	544.297533	4.70481667	pos
4.70_552.65	552.651911	4.70481667	pos
4.70_552.98	552.986302	4.70481667	pos
4.70_648.02	648.027787	4.70481667	pos
4.70_718.36	718.362446	4.70481667	pos
4.70_956.94	956.94377	4.70481667	pos
4.70_995.19	1013.23074	4.70481667	pos
4.71_230.98	230.988338	4.70578333	neg
4.71_260.05	260.05394	4.70578333	neg
4.71_321.09	321.094651	4.70578333	neg
4.71_331.03	331.038341	4.70578333	neg
4.71_332.15	332.153864	4.70578333	neg
4.71_399.02	399.025828	4.70578333	neg
4.71_418.14	418.144193	4.70578333	neg
4.72_1017.1	1017.18759	4.72301667	pos
4.72_169.04	169.049743	4.72301667	pos
4.72_170.08	170.082075	4.72411667	neg
4.72_170.55	170.551333	4.72301667	pos
4.72_201.01	201.017955	4.72411667	neg
4.72_203.11	204.123049	4.72301667	pos
4.72_207.05	207.050774	4.72411667	neg
4.72_211.16	211.16918	4.72301667	pos
4.72_224.99	224.99905	4.72411667	neg
4.72_246.57	246.576506	4.72411667	neg
4.72_259.51	259.511412	4.72411667	neg
4.72_280.18	303.177632	4.72301667	pos
4.72_299.06	299.068523	4.72301667	pos
4.72_328.05	328.054408	4.72411667	neg
4.72_333.06	333.064146	4.72301667	pos
4.72_338.08	338.08339	4.72411667	neg

4.72_355.07	355.073866	4.72411667	neg
4.72_406.07	406.070672	4.72411667	neg
4.72_451.20	451.202905	4.72301667	pos
4.72_492.14	492.14468	4.72411667	neg
4.72_509.22	509.223074	4.72411667	neg
4.72_509.95	509.954787	4.72301667	pos
4.72_518.62	518.622347	4.72301667	pos
4.72_542.04	542.045589	4.72411667	neg
4.72_560.30	583.293577	4.72301667	pos
4.72_575.99	575.996735	4.72301667	pos
4.72_576.33	576.330791	4.72301667	pos
4.72_591.82	591.829571	4.72301667	pos
4.72_602.82	602.8221	4.72301667	pos
4.72_637.68	637.688592	4.72301667	pos
4.72_641.87	641.875484	4.72301667	pos
4.72_678.02	678.020509	4.72411667	neg
4.72_746.00	746.008454	4.72411667	neg
4.72_777.93	777.930478	4.72301667	pos
4.72_862.11	862.113784	4.72301667	pos
4.72_889.83	889.832445	4.72411667	neg
4.72_890.83	891.846091	4.72301667	pos
4.72_891.09	891.094813	4.72301667	pos
4.72_900.82	900.824285	4.72411667	neg
4.72_902.33	902.335587	4.72301667	pos
4.72_902.83	902.837401	4.72301667	pos
4.74_179.04	179.044976	4.74081667	pos
4.74_183.06	183.064048	4.74241667	neg
4.74_204.02	204.027384	4.74241667	neg
4.74_215.18	260.186545	4.74241667	neg
4.74_221.01	221.01787	4.74241667	neg
4.74_240.00	240.000974	4.74081667	pos
4.74_241.01	241.010432	4.74241667	neg
4.74_252.15	252.156893	4.74081667	pos

4.74_252.528	252.528189	4.74081667	pos
4.74_256.080	256.080218	4.74241667	neg
4.74_259.178	304.1765	4.74241667	neg
4.74_271.262	271.262832	4.74081667	pos
4.74_275.072	274.065475	4.74241667	neg
4.74_292.074	292.07473	4.74081667	pos
4.74_295.175	318.164649	4.74081667	pos
4.74_295.677	318.666409	4.74081667	pos
4.74_301.161	301.161168	4.74081667	pos
4.74_310.199	333.188331	4.74081667	pos
4.74_314.085	314.085949	4.74241667	neg
4.74_315.687	315.687199	4.74081667	pos
4.74_327.165	372.163857	4.74241667	neg
4.74_365.728	365.728569	4.74081667	pos
4.74_366.229	366.229912	4.74081667	pos
4.74_381.062	364.05945	4.74081667	pos
4.74_382.073	382.073183	4.74241667	neg
4.74_386.041	386.041277	4.74081667	pos
4.74_396.041	396.041792	4.74241667	neg
4.74_419.979	419.979192	4.74081667	pos
4.74_427.206	427.205997	4.74241667	neg
4.74_462.275	463.282663	4.74081667	pos
4.74_462.781	462.781176	4.74081667	pos
4.74_470.788	471.795708	4.74081667	pos
4.74_473.607	473.607575	4.74081667	pos
4.74_473.941	473.941852	4.74081667	pos
4.74_477.987	477.987232	4.74081667	pos
4.74_478.994	478.994704	4.74081667	pos
4.74_482.786	482.786669	4.74081667	pos
4.74_483.288	483.288495	4.74081667	pos
4.74_508.138	508.138671	4.74241667	neg
4.74_524.619	524.619276	4.74081667	pos
4.74_524.953	524.953717	4.74081667	pos

4.74_525.129	525.129031	4.74241667	neg
4.74_543.010	543.01027	4.74241667	neg
4.74_564.992	564.992368	4.74241667	neg
4.74_590.350	613.339945	4.74081667	pos
4.74_595.331	595.331119	4.74081667	pos
4.74_595.665	595.665831	4.74081667	pos
4.74_644.113	644.113898	4.74241667	neg
4.74_742.375	742.375512	4.74081667	pos
4.74_869.112	869.112657	4.74241667	neg
4.74_870.119	871.126312	4.74081667	pos
4.76_118.040	118.04089	4.76071667	neg
4.76_137.047	120.044586	4.75875	pos
4.76_145.086	145.086856	4.76071667	neg
4.76_153.996	153.996651	4.76071667	neg
4.76_167.028	167.028251	4.76071667	neg
4.76_179.034	179.034859	4.76071667	neg
4.76_289.075	289.075153	4.76071667	neg
4.76_337.073	318.055188	4.76071667	neg
4.76_350.154	350.154584	4.75875	pos
4.76_357.061	357.061662	4.76071667	neg
4.76_377.034	377.034823	4.76071667	neg
4.76_381.063	362.045214	4.76071667	neg
4.76_384.027	384.027244	4.76071667	neg
4.76_417.978	417.978811	4.75875	pos
4.76_424.068	447.057844	4.75875	pos
4.76_446.050	491.048944	4.76071667	neg
4.76_452.014	452.014595	4.76071667	neg
4.76_459.005	459.005266	4.75875	pos
4.76_469.039	469.039854	4.75875	pos
4.76_481.020	481.020101	4.76071667	neg
4.76_483.017	483.01697	4.76071667	neg
4.76_486.057	486.057143	4.76071667	neg
4.76_504.009	503.002253	4.76071667	neg

4.76_508.03	508.038872	4.76071667	neg
4.76_514.03	513.030628	4.76071667	neg
4.76_531.02	576.026527	4.76071667	neg
4.76_537.02	537.027314	4.75875	pos
4.76_554.01	554.017509	4.75875	pos
4.76_571.99	570.989515	4.76071667	neg
4.76_582.02	581.018412	4.76071667	neg
4.76_590.97	590.974656	4.76071667	neg
4.76_599.01	598.009017	4.76071667	neg
4.76_605.01	605.01447	4.75875	pos
4.76_612.29	612.292074	4.75875	pos
4.76_619.34	619.344851	4.75875	pos
4.76_631.34	631.345317	4.75875	pos
4.76_638.19	621.193264	4.75875	pos
4.76_638.97	638.976891	4.76071667	neg
4.76_650.01	649.005882	4.76071667	neg
4.76_665.99	665.99628	4.76071667	neg
4.76_706.96	706.964414	4.76071667	neg
4.76_716.99	716.993641	4.76071667	neg
4.76_733.98	733.984369	4.76071667	neg
4.76_762.99	762.998785	4.76071667	neg
4.76_784.98	784.980811	4.76071667	neg
4.76_828.40	828.400155	4.76071667	neg
4.76_852.96	852.968234	4.76071667	neg
4.76_920.95	920.956168	4.76071667	neg
4.76_987.47	987.476489	4.75875	pos
4.76_987.72	987.727204	4.75875	pos
4.76_987.97	987.976051	4.75875	pos
4.76_988.22	988.227762	4.75875	pos
4.76_988.94	988.942686	4.76071667	neg
4.78_166.03	166.035506	4.7769	pos
4.78_170.03	170.036698	4.7769	pos
4.78_172.01	152.996754	4.77798333	neg

4.78_198.032	198.032411	4.77798333	neg
4.78_244.029	225.007463	4.77798333	neg
4.78_270.017	270.017403	4.77798333	neg
4.78_303.204	348.202714	4.77798333	neg
4.78_337.180	360.175214	4.7769	pos
4.78_338.005	338.005065	4.77798333	neg
4.78_338.173	338.173933	4.77798333	neg
4.78_346.706	346.706596	4.7769	pos
4.78_349.686	349.686075	4.7769	pos
4.78_357.697	357.697533	4.7769	pos
4.78_358.199	358.19925	4.7769	pos
4.78_360.676	360.676907	4.7769	pos
4.78_416.190	416.19032	4.77798333	neg
4.78_421.240	421.240852	4.7769	pos
4.78_435.220	435.220208	4.7769	pos
4.78_473.772	473.772281	4.7769	pos
4.78_484.177	484.177518	4.77798333	neg
4.78_502.322	502.322311	4.7769	pos
4.78_505.301	505.301749	4.7769	pos
4.78_530.622	530.622761	4.7769	pos
4.78_530.957	530.957042	4.7769	pos
4.78_531.289	531.289924	4.7769	pos
4.78_556.620	557.633546	4.7769	pos
4.78_591.972	591.972353	4.7769	pos
4.78_592.306	592.306652	4.7769	pos
4.78_620.152	620.152452	4.77798333	neg
4.78_674.371	697.361016	4.7769	pos
4.78_724.845	724.845647	4.7769	pos
4.78_725.011	725.011871	4.7769	pos
4.78_725.179	725.179515	4.7769	pos
4.78_755.856	755.856609	4.7769	pos
4.78_756.023	756.023332	4.7769	pos
4.78_756.524	756.524732	4.7769	pos

4.78_775.76	775.765826	4.7769	pos
4.78_869.61	869.613549	4.7769	pos
4.78_869.81	869.813633	4.7769	pos
4.78_870.01	870.013339	4.7769	pos
4.78_907.02	907.027217	4.7769	pos
4.79_1040.9	1040.99544	4.79411667	pos
4.79_1073.0	1073.01033	4.79411667	pos
4.79_143.14	143.143053	4.79411667	pos
4.79_171.12	194.115166	4.79411667	pos
4.79_212.12	212.125617	4.79411667	pos
4.79_217.19	217.190861	4.79411667	pos
4.79_236.16	259.151475	4.79411667	pos
4.79_246.04	269.031519	4.79411667	pos
4.79_265.15	265.151938	4.79411667	pos
4.79_304.21	304.211619	4.79411667	pos
4.79_306.21	306.217247	4.79411667	pos
4.79_325.65	325.656057	4.79411667	pos
4.79_488.24	489.255861	4.79411667	pos
4.79_493.80	493.809049	4.79411667	pos
4.79_598.64	598.644085	4.79411667	pos
4.79_602.62	602.620453	4.79411667	pos
4.79_676.37	715.342074	4.79411667	pos
4.79_715.17	715.175411	4.79411667	pos
4.79_715.50	715.509506	4.79411667	pos
4.79_715.67	715.676423	4.79411667	pos
4.79_746.35	746.353577	4.79411667	pos
4.79_746.52	746.520335	4.79411667	pos
4.79_762.70	762.707646	4.79411667	pos
4.79_765.35	765.351908	4.79411667	pos
4.79_765.60	765.601764	4.79411667	pos
4.79_765.85	765.85189	4.79411667	pos
4.79_832.79	832.798182	4.79411667	pos
4.79_858.20	858.208914	4.79411667	pos

4.79_858.40(	858.408689	4.79411667	pos
4.79_858.61(	858.610154	4.79411667	pos
4.79_891.80(	891.800683	4.79411667	pos
4.79_892.46(	892.469376	4.79411667	pos
4.79_895.62(	895.622923	4.79411667	pos
4.80_1185.1(	1185.1177	4.79645	neg
4.80_191.01(	191.013286	4.79645	neg
4.80_266.01(	266.017787	4.79645	neg
4.80_266.51(	266.519419	4.79645	neg
4.80_300.01(	300.011458	4.79645	neg
4.80_310.05(	355.052628	4.79645	neg
4.80_615.10(	615.102668	4.79645	neg
4.81_116.01(	116.011511	4.81485	neg
4.81_127.01(	127.014741	4.81485	neg
4.81_182.99(	182.995535	4.81485	neg
4.81_196.12(	196.120526	4.81226667	pos
4.81_205.04(	205.044509	4.81226667	pos
4.81_223.01(	223.014765	4.81485	neg
4.81_255.50(	255.503162	4.81226667	pos
4.81_263.48(	263.489335	4.81226667	pos
4.81_264.50(	264.508441	4.81226667	pos
4.81_268.02(	268.027541	4.81226667	pos
4.81_272.49(	272.494608	4.81226667	pos
4.81_281.02(	281.024802	4.81485	neg
4.81_317.18(	340.177794	4.81226667	pos
4.81_317.69(	340.67951	4.81226667	pos
4.81_326.70(	326.709178	4.81226667	pos
4.81_329.68(	329.688514	4.81226667	pos
4.81_337.70(	337.700032	4.81226667	pos
4.81_338.20(	338.201982	4.81226667	pos
4.81_415.75(	415.756566	4.81226667	pos
4.81_471.04(	941.083533	4.81485	neg
4.81_481.97(	481.97581	4.81485	neg

4.81_493.02	492.020049	4.81485	neg
4.81_504.99	504.992886	4.81485	neg
4.81_506.01	506.014293	4.81485	neg
4.81_515.00	514.001712	4.81485	neg
4.81_516.01	516.016553	4.81226667	pos
4.81_516.79	516.795817	4.81226667	pos
4.81_525.97	525.971821	4.81226667	pos
4.81_527.01	510.009254	4.81226667	pos
4.81_527.99	527.996504	4.81485	neg
4.81_529.99	529.993261	4.81485	neg
4.81_531.99	531.990157	4.81226667	pos
4.81_533.03	533.033688	4.81485	neg
4.81_555.01	555.015224	4.81485	neg
4.81_556.96	556.964121	4.81226667	pos
4.81_557.29	557.299314	4.81226667	pos
4.81_566.31	566.31065	4.81226667	pos
4.81_566.56	566.563202	4.81226667	pos
4.81_578.00	576.997705	4.81485	neg
4.81_584.00	584.003824	4.81226667	pos
4.81_591.94	591.944889	4.81485	neg
4.81_595.98	595.984422	4.81485	neg
4.81_615.31	598.310003	4.81226667	pos
4.81_620.33	620.333733	4.81485	neg
4.81_620.83	620.835437	4.81485	neg
4.81_621.34	622.347617	4.81226667	pos
4.81_622.84	622.849618	4.81226667	pos
4.81_633.84	633.840602	4.81226667	pos
4.81_634.37	657.366475	4.81226667	pos
4.81_644.98	644.985277	4.81485	neg
4.81_650.98	695.982399	4.81485	neg
4.81_676.63	694.667099	4.81226667	pos
4.81_679.37	679.375452	4.81485	neg
4.81_685.95	685.953907	4.81485	neg

4.81_693.83	693.832032	4.81226667	pos
4.81_693.99	693.999436	4.81226667	pos
4.81_694.16	694.166298	4.81226667	pos
4.81_694.50	694.500251	4.81226667	pos
4.81_700.86	700.861678	4.81226667	pos
4.81_712.97	712.973113	4.81485	neg
4.81_736.88	736.881345	4.81226667	pos
4.81_763.03	763.033288	4.81226667	pos
4.81_774.05	792.085057	4.81226667	pos
4.81_780.96	780.960458	4.81485	neg
4.81_799.08	799.084304	4.81226667	pos
4.81_799.41	799.417702	4.81226667	pos
4.81_799.75	799.752477	4.81226667	pos
4.81_831.95	831.957793	4.81485	neg
4.81_832.99	832.998598	4.81226667	pos
4.81_834.94	834.942117	4.81226667	pos
4.81_835.44	835.44314	4.81226667	pos
4.83_1090.9	1090.94246	4.83273333	neg
4.83_1093.4	1093.45746	4.8302	pos
4.83_1103.9	1103.94613	4.8302	pos
4.83_1104.4	1104.44772	4.8302	pos
4.83_119.05	119.050035	4.83273333	neg
4.83_126.98	126.98715	4.83273333	neg
4.83_165.01	165.012641	4.83273333	neg
4.83_180.00	180.007289	4.83273333	neg
4.83_193.04	193.04965	4.8302	pos
4.83_209.00	209.003042	4.83273333	neg
4.83_219.00	199.985765	4.83273333	neg
4.83_233.04	233.042933	4.83273333	neg
4.83_243.12	243.120131	4.8302	pos
4.83_250.03	250.033132	4.83273333	neg
4.83_253.00	253.005838	4.83273333	neg
4.83_261.01	284.002583	4.8302	pos

4.83_274.069	274.069501	4.83273333	neg
4.83_278.00	278.006168	4.83273333	neg
4.83_279.02	279.021563	4.8302	pos
4.83_281.97	281.977061	4.83273333	neg
4.83_284.50	284.504067	4.8302	pos
4.83_292.94	292.945543	4.83273333	neg
4.83_310.03	310.032617	4.83273333	neg
4.83_318.02	318.020607	4.83273333	neg
4.83_341.01	321.995986	4.83273333	neg
4.83_343.97	343.978073	4.83273333	neg
4.83_345.02	345.020211	4.83273333	neg
4.83_347.00	347.008892	4.8302	pos
4.83_376.00	376.00389	4.83273333	neg
4.83_418.14	418.143993	4.83273333	neg
4.83_426.97	426.976145	4.83273333	neg
4.83_446.90	446.905858	4.83273333	neg
4.83_453.99	453.9957	4.83273333	neg
4.83_515.82	515.821906	4.8302	pos
4.83_516.29	516.294161	4.8302	pos
4.83_516.54	516.54486	4.8302	pos
4.83_520.29	520.294763	4.8302	pos
4.83_526.81	526.813174	4.8302	pos
4.83_527.31	527.31559	4.8302	pos
4.83_548.77	566.813523	4.8302	pos
4.83_552.98	551.976125	4.83273333	neg
4.83_568.99	549.979479	4.83273333	neg
4.83_582.99	627.994948	4.83273333	neg
4.83_600.99	600.994207	4.8302	pos
4.83_617.96	617.967044	4.83273333	neg
4.83_619.96	619.962845	4.83273333	neg
4.83_640.96	640.968037	4.83273333	neg
4.83_651.99	651.990795	4.8302	pos
4.83_660.28	660.287427	4.83273333	neg

4.83_661.80	661.79996	4.8302	pos
4.83_662.30	662.301743	4.8302	pos
4.83_672.79	672.791299	4.8302	pos
4.83_673.29	673.292723	4.8302	pos
4.83_682.68	682.687578	4.8302	pos
4.83_688.39	688.390214	4.8302	pos
4.83_718.97	763.969887	4.83273333	neg
4.83_899.94	899.945273	4.83273333	neg
4.83_963.06	963.06569	4.83273333	neg
4.83_967.93	967.932421	4.83273333	neg
4.85_149.04	149.044855	4.84713333	pos
4.85_161.07	161.071872	4.85055	neg
4.85_191.11	191.115482	4.84713333	pos
4.85_233.00	233.003912	4.85055	neg
4.85_242.98	242.989606	4.85055	neg
4.85_254.06	254.066971	4.85055	neg
4.85_272.13	272.131396	4.84713333	pos
4.85_359.19	382.188348	4.84713333	pos
4.85_359.70	382.689994	4.84713333	pos
4.85_368.71	368.719707	4.84713333	pos
4.85_369.22	369.221344	4.84713333	pos
4.85_371.69	371.699122	4.84713333	pos
4.85_379.71	379.710664	4.84713333	pos
4.85_499.28	499.283394	4.84713333	pos
4.85_536.78	536.782497	4.84713333	pos
4.85_583.32	583.329105	4.84713333	pos
4.85_661.75	661.754994	4.85055	neg
4.85_718.39	741.387611	4.84713333	pos
4.85_720.40	720.408258	4.84713333	pos
4.85_729.64	729.644835	4.84713333	pos
4.85_763.39	763.397313	4.85055	neg
4.87_120.04	120.04527	4.86903333	neg
4.87_142.03	142.033011	4.86903333	neg

4.87_149.024	149.024194	4.86903333	neg
4.87_164.047	147.044056	4.86546667	pos
4.87_195.122	195.12259	4.86546667	pos
4.87_211.133	211.13306	4.86546667	pos
4.87_220.096	220.096733	4.86546667	pos
4.87_225.017	225.017633	4.86903333	neg
4.87_232.022	232.022612	4.86903333	neg
4.87_239.014	239.014721	4.86903333	neg
4.87_249.012	249.012833	4.86903333	neg
4.87_316.157	316.157489	4.86546667	pos
4.87_317.114	317.114053	4.86903333	neg
4.87_344.689	344.689819	4.86546667	pos
4.87_347.669	347.669138	4.86546667	pos
4.87_385.101	385.101596	4.86903333	neg
4.87_465.267	465.267071	4.86546667	pos
4.87_498.781	498.781655	4.86546667	pos
4.87_501.761	501.760965	4.86546667	pos
4.87_536.281	536.280965	4.86546667	pos
4.87_548.826	548.826228	4.86546667	pos
4.87_574.031	574.031383	4.86903333	neg
4.87_576.045	576.045886	4.86546667	pos
4.87_596.013	596.01302	4.86903333	neg
4.87_729.897	729.89745	4.86546667	pos
4.87_838.826	838.826369	4.86903333	neg
4.87_839.831	840.838895	4.86546667	pos
4.87_849.816	849.816802	4.86903333	neg
4.87_850.318	850.318107	4.86903333	neg
4.87_860.810	860.810786	4.86903333	neg
4.87_951.967	951.967633	4.86546667	pos
4.88_183.137	183.137943	4.88355	pos
4.88_234.115	234.115776	4.88355	pos
4.88_247.051	270.040523	4.88355	pos
4.88_330.073	330.07368	4.88355	pos

4.88_339.703	362.692606	4.88355	pos
4.88_348.722	348.722326	4.88355	pos
4.88_349.223	349.223752	4.88355	pos
4.88_351.701	351.701639	4.88355	pos
4.88_359.713	359.713193	4.88355	pos
4.88_360.214	360.214911	4.88355	pos
4.88_476.768	476.768647	4.88355	pos
4.88_479.246	479.246246	4.88355	pos
4.88_479.748	479.748047	4.88355	pos
4.88_487.623	487.623348	4.88355	pos
4.88_629.266	629.266561	4.88355	pos
4.88_668.343	668.343366	4.88355	pos
4.88_678.402	701.391879	4.88355	pos
4.88_942.393	942.393832	4.88355	pos
4.88_942.895	942.895604	4.88355	pos
4.89_202.090	202.090571	4.88748333	neg
4.89_218.082	218.082084	4.88748333	neg
4.89_246.044	246.044022	4.88748333	neg
4.89_286.069	286.069664	4.88748333	neg
4.89_311.016	311.016135	4.88748333	neg
4.89_315.027	315.027528	4.88748333	neg
4.89_328.059	328.05938	4.88748333	neg
4.89_353.048	353.048843	4.88748333	neg
4.89_376.038	421.036314	4.88748333	neg
4.89_411.007	411.007678	4.88748333	neg
4.89_413.004	413.004446	4.88748333	neg
4.89_438.026	438.026142	4.88748333	neg
4.89_444.025	443.018094	4.88748333	neg
4.89_460.009	460.008956	4.88748333	neg
4.89_940.380	940.380654	4.88748333	neg
4.89_940.881	940.881593	4.88748333	neg
4.89_951.371	951.371583	4.88748333	neg
4.89_951.872	951.872906	4.88748333	neg

4.89_962.36[illegible]	962.362427	4.88748333	neg
4.89_962.86[illegible]	962.863684	4.88748333	neg
4.90_1006.2[illegible]	1006.22921	4.90175	pos
4.90_1006.4[illegible]	1006.48058	4.90175	pos
4.90_1006.7[illegible]	1006.73145	4.90175	pos
4.90_237.10[illegible]	238.107296	4.90175	pos
4.90_245.12[illegible]	246.133427	4.90175	pos
4.90_278.07[illegible]	279.083915	4.90175	pos
4.90_334.03[illegible]	334.037053	4.90175	pos
4.90_381.21[illegible]	404.201595	4.90175	pos
4.90_381.71[illegible]	404.703231	4.90175	pos
4.90_390.73[illegible]	390.732806	4.90175	pos
4.90_392.14[illegible]	393.151773	4.90175	pos
4.90_393.21[illegible]	393.210426	4.90175	pos
4.90_393.71[illegible]	393.712271	4.90175	pos
4.90_401.72[illegible]	401.7239	4.90175	pos
4.90_402.22[illegible]	402.225499	4.90175	pos
4.90_406.23[illegible]	406.231395	4.90175	pos
4.90_406.73[illegible]	406.733118	4.90175	pos
4.90_415.73[illegible]	415.739226	4.90175	pos
4.90_438.19[illegible]	438.195214	4.90175	pos
4.90_500.28[illegible]	523.272645	4.90175	pos
4.90_500.78[illegible]	523.77424	4.90175	pos
4.90_512.78[illegible]	512.783125	4.90175	pos
4.90_520.79[illegible]	520.795281	4.90175	pos
4.90_521.77[illegible]	521.770899	4.90175	pos
4.90_521.93[illegible]	521.936922	4.90175	pos
4.90_522.27[illegible]	522.270233	4.90175	pos
4.90_522.43[illegible]	522.439227	4.90175	pos
4.90_600.10[illegible]	600.103508	4.90175	pos
4.90_600.50[illegible]	600.504687	4.90175	pos
4.90_626.12[illegible]	626.123392	4.90175	pos
4.90_643.32[illegible]	626.323846	4.90175	pos

4.90_749.87	749.877759	4.90175	pos
4.90_750.12	750.127683	4.90175	pos
4.90_750.62	750.62852	4.90175	pos
4.90_750.73	750.731742	4.90175	pos
4.90_767.38	750.377024	4.90175	pos
4.90_782.40	782.404544	4.90175	pos
4.90_804.98	804.984876	4.90175	pos
4.90_805.18	805.185621	4.90175	pos
4.90_805.38	805.385923	4.90175	pos
4.90_805.58	805.586787	4.90175	pos
4.91_112.98	112.982155	4.90568333	neg
4.91_238.09	238.097695	4.90568333	neg
4.91_257.00	257.005318	4.90568333	neg
4.91_257.09	257.092375	4.90568333	neg
4.91_278.02	278.025683	4.90568333	neg
4.91_293.02	293.025391	4.90568333	neg
4.91_294.05	294.051538	4.90568333	neg
4.91_299.08	299.088356	4.90568333	neg
4.91_304.08	304.080259	4.90568333	neg
4.91_321.07	321.07029	4.90568333	neg
4.91_329.02	329.023016	4.90568333	neg
4.91_340.06	340.064768	4.90568333	neg
4.91_345.05	345.056619	4.90568333	neg
4.91_346.01	346.013251	4.90568333	neg
4.91_347.01	347.017046	4.90568333	neg
4.91_370.04	351.028565	4.90568333	neg
4.91_371.16	371.160906	4.90568333	neg
4.91_397.01	397.010339	4.90568333	neg
4.91_405.13	405.13176	4.90568333	neg
4.91_430.02	430.02492	4.90568333	neg
4.91_440.05	440.05506	4.90568333	neg
4.91_457.04	457.04546	4.90568333	neg
4.91_464.99	464.997722	4.90568333	neg

4.91_481.03[illegible]	481.031858	4.90568333	neg
4.91_508.04[illegible]	508.042221	4.90568333	neg
4.91_522.12[illegible]	522.120477	4.90568333	neg
4.91_532.98[illegible]	532.986229	4.90568333	neg
4.91_549.01[illegible]	549.019166	4.90568333	neg
4.91_573.11[illegible]	573.117862	4.90568333	neg
4.91_576.02[illegible]	576.029759	4.90568333	neg
4.91_585.25[illegible]	585.252812	4.90568333	neg
4.91_590.10[illegible]	590.108301	4.90568333	neg
4.91_641.10[illegible]	641.105383	4.90568333	neg
4.91_644.01[illegible]	644.017596	4.90568333	neg
4.91_664.09[illegible]	663.087421	4.90568333	neg
4.91_695.81[illegible]	695.811041	4.90568333	neg
4.91_696.31[illegible]	696.311873	4.90568333	neg
4.91_712.00[illegible]	712.005165	4.90568333	neg
4.91_777.08[illegible]	777.080382	4.90568333	neg
4.91_807.42[illegible]	807.42344	4.90568333	neg
4.92_195.13[illegible]	195.137898	4.91941667	pos
4.92_224.06[illegible]	224.061671	4.92405	neg
4.92_224.56[illegible]	224.563349	4.92405	neg
4.92_241.10[illegible]	241.104431	4.91941667	pos
4.92_285.00[illegible]	285.00031	4.92405	neg
4.92_292.04[illegible]	292.049329	4.92405	neg
4.92_299.16[illegible]	299.161071	4.92405	neg
4.92_300.16[illegible]	323.157478	4.91941667	pos
4.92_336.16[illegible]	336.166301	4.91941667	pos
4.92_343.09[illegible]	343.095859	4.91941667	pos
4.92_344.18[illegible]	344.185531	4.91941667	pos
4.92_366.70[illegible]	366.702694	4.91941667	pos
4.92_388.19[illegible]	388.195551	4.91941667	pos
4.92_498.01[illegible]	498.013282	4.92405	neg
4.92_521.60[illegible]	521.603788	4.91941667	pos
4.92_566.08[illegible]	566.082349	4.91941667	pos

4.92_566.28	566.284918	4.91941667	pos
4.92_593.02	593.020925	4.92405	neg
4.92_600.70	600.704879	4.91941667	pos
4.92_625.72	625.722355	4.91941667	pos
4.92_625.92	625.92288	4.91941667	pos
4.92_640.65	640.656775	4.91941667	pos
4.92_696.77	696.771871	4.92405	neg
4.92_698.78	698.784957	4.91941667	pos
4.92_707.85	707.852209	4.91941667	pos
4.92_846.21	846.213234	4.91941667	pos
4.94_1042.9	1042.95082	4.93775	pos
4.94_118.04	118.040926	4.9421	neg
4.94_130.15	130.159095	4.93775	pos
4.94_215.12	215.128593	4.9421	neg
4.94_226.15	226.158883	4.93775	pos
4.94_258.05	258.055244	4.9421	neg
4.94_258.55	258.557075	4.9421	neg
4.94_268.05	251.046739	4.93775	pos
4.94_278.17	301.161427	4.93775	pos
4.94_305.15	305.15273	4.93775	pos
4.94_361.21	384.204026	4.93775	pos
4.94_361.71	384.705728	4.93775	pos
4.94_369.18	369.180394	4.93775	pos
4.94_369.68	369.682123	4.93775	pos
4.94_370.73	370.73543	4.93775	pos
4.94_371.23	371.237134	4.93775	pos
4.94_373.71	373.714746	4.93775	pos
4.94_381.72	381.726317	4.93775	pos
4.94_382.22	382.228338	4.93775	pos
4.94_388.69	388.69378	4.93775	pos
4.94_555.53	555.53175	4.93775	pos
4.94_695.63	695.637685	4.93775	pos
4.94_734.18	734.189744	4.93775	pos

4.94_772.337	772.337593	4.9421	neg
4.94_794.319	794.319693	4.9421	neg
4.96_195.099	195.099197	4.95603333	pos
4.96_206.081	206.081188	4.95603333	pos
4.96_247.124	248.131333	4.95603333	pos
4.96_250.045	250.04499	4.95603333	pos
4.96_267.120	267.120188	4.95603333	pos
4.96_275.162	275.162025	4.95603333	pos
4.96_276.126	276.126255	4.95603333	pos
4.96_293.160	293.160708	4.95603333	pos
4.96_340.041	340.041364	4.96071667	neg
4.96_403.232	403.232399	4.95603333	pos
4.96_415.725	415.725419	4.95603333	pos
4.96_424.238	424.238649	4.95603333	pos
4.96_522.290	545.285688	4.95603333	pos
4.96_522.798	545.787373	4.95603333	pos
4.96_534.294	534.294707	4.95603333	pos
4.96_534.796	534.796008	4.95603333	pos
4.96_542.808	542.808029	4.95603333	pos
4.96_543.309	543.30963	4.95603333	pos
4.96_555.782	555.782105	4.95603333	pos
4.96_593.058	593.058725	4.95603333	pos
4.96_655.380	655.380829	4.95603333	pos
4.96_734.356	734.356359	4.95603333	pos
4.96_734.690	734.689991	4.95603333	pos
4.96_809.850	809.850486	4.96071667	neg
4.97_193.083	193.083355	4.97433333	pos
4.97_202.044	202.044273	4.97433333	pos
4.97_236.077	236.077208	4.97433333	pos
4.97_245.128	245.12851	4.97433333	pos
4.97_285.130	285.130507	4.97433333	pos
4.97_299.111	299.111516	4.97433333	pos
4.97_304.894	304.89407	4.97433333	pos

4.97_403.72[illegible]	426.716362	4.97433333	pos
4.97_412.74[illegible]	412.745933	4.97433333	pos
4.97_423.73[illegible]	423.736895	4.97433333	pos
4.97_437.75[illegible]	437.752229	4.97433333	pos
4.97_660.48[illegible]	660.485719	4.97433333	pos
4.97_703.17[illegible]	703.175843	4.97433333	pos
4.97_703.34[illegible]	703.342203	4.97433333	pos
4.97_806.43[illegible]	824.467489	4.97433333	pos
4.97_808.79[illegible]	808.796917	4.97433333	pos
4.97_808.99[illegible]	808.996995	4.97433333	pos
4.97_809.92[illegible]	809.922723	4.97433333	pos
4.97_810.42[illegible]	810.42309	4.97433333	pos
4.97_824.13[illegible]	824.132711	4.97433333	pos
4.97_824.80[illegible]	824.802139	4.97433333	pos
4.98_211.99[illegible]	211.999708	4.97913333	neg
4.98_357.23[illegible]	357.23958	4.97913333	neg
4.99_170.03[illegible]	170.036651	4.99255	pos
4.99_200.14[illegible]	201.148458	4.99255	pos
4.99_201.13[illegible]	224.125584	4.99255	pos
4.99_249.16[illegible]	249.169444	4.99255	pos
4.99_269.13[illegible]	269.135884	4.99255	pos
4.99_298.22[illegible]	298.222161	4.99255	pos
4.99_388.71[illegible]	388.715806	4.99255	pos
4.99_391.19[illegible]	391.193233	4.99255	pos
4.99_448.15[illegible]	448.151662	4.99255	pos
4.99_544.81[illegible]	567.800365	4.99255	pos
4.99_564.82[illegible]	564.820884	4.99255	pos
4.99_703.50[illegible]	703.509267	4.99255	pos
4.99_843.80[illegible]	843.809664	4.99255	pos
4.99_844.01[illegible]	844.010408	4.99255	pos
5.00_201.11[illegible]	201.113021	4.99758333	neg
5.00_283.02[illegible]	283.021007	4.99758333	neg
5.00_343.09[illegible]	343.091258	4.99758333	neg

5.00_424.154	424.154858	4.99758333	neg
5.00_479.066	479.065953	4.99758333	neg
5.01_181.083	181.083595	5.01075	pos
5.01_255.120	255.120128	5.01075	pos
5.01_276.110	277.118027	5.01075	pos
5.01_383.227	406.216997	5.01075	pos
5.01_383.729	406.718818	5.01075	pos
5.01_392.748	392.748261	5.01075	pos
5.01_395.226	395.226093	5.01075	pos
5.01_395.727	395.727848	5.01075	pos
5.01_403.739	403.739432	5.01075	pos
5.01_553.830	553.830008	5.01075	pos
5.01_556.307	556.307663	5.01075	pos
5.01_556.809	556.809127	5.01075	pos
5.01_565.322	565.322448	5.01075	pos
5.01_680.671	680.671124	5.01075	pos
5.02_176.817	176.817085	5.016	neg
5.02_230.012	230.012617	5.016	neg
5.02_253.010	253.01024	5.016	neg
5.02_297.000	297.000339	5.016	neg
5.02_454.689	908.372381	5.016	neg
5.03_127.013	127.013305	5.03453333	neg
5.03_213.012	213.012172	5.03453333	neg
5.03_242.979	242.979263	5.02876667	pos
5.03_261.115	261.11515	5.02876667	pos
5.03_264.988	264.988393	5.03453333	neg
5.03_281.978	281.97857	5.03453333	neg
5.03_373.234	373.234259	5.03453333	neg
5.03_390.224	390.224694	5.03453333	neg
5.03_424.179	469.17797	5.03453333	neg
5.03_425.740	448.729249	5.02876667	pos
5.03_426.170	471.174397	5.03453333	neg
5.03_434.758	434.758894	5.02876667	pos

5.03_437.73	437.738417	5.02876667	pos
5.03_445.74	445.749833	5.02876667	pos
5.03_712.04	712.048574	5.02876667	pos
5.03_712.38	712.383405	5.02876667	pos
5.03_712.71	712.716489	5.02876667	pos
5.03_718.87	718.875579	5.02876667	pos
5.05_126.12	126.126548	5.04685	pos
5.05_225.57	225.571228	5.05298333	neg
5.05_231.00	231.008384	5.05298333	neg
5.05_237.16	237.169305	5.04685	pos
5.05_250.17	273.166968	5.04685	pos
5.05_255.02	255.02608	5.05298333	neg
5.05_275.17	275.171995	5.04685	pos
5.05_287.14	287.146173	5.04685	pos
5.05_296.24	296.242827	5.04685	pos
5.05_318.12	341.110505	5.04685	pos
5.05_426.17	427.183428	5.04685	pos
5.05_566.82	589.813323	5.04685	pos
5.05_586.83	586.833647	5.04685	pos
5.05_587.33	587.334927	5.04685	pos
5.05_617.73	617.737463	5.05298333	neg
5.05_618.23	618.237974	5.05298333	neg
5.05_624.18	624.182677	5.04685	pos
5.05_660.62	660.628937	5.04685	pos
5.05_728.21	728.211938	5.04685	pos
5.06_319.05	319.058833	5.06456667	pos
5.06_374.14	375.154677	5.06456667	pos
5.06_405.24	428.229974	5.06456667	pos
5.06_405.74	428.731768	5.06456667	pos
5.06_414.76	414.7614	5.06456667	pos
5.06_417.23	417.239129	5.06456667	pos
5.06_417.74	417.740866	5.06456667	pos
5.06_425.75	425.752434	5.06456667	pos

5.06_661.058	661.058477	5.06456667	pos
5.06_728.54(	728.546233	5.06456667	pos
5.06_795.07:	795.072794	5.06456667	pos
5.06_802.06!	802.065812	5.06456667	pos
5.06_802.39!	802.399728	5.06456667	pos
5.06_802.73	802.734003	5.06456667	pos
5.06_822.07	822.071803	5.06456667	pos
5.06_959.47!	959.479939	5.06456667	pos
5.06_969.968	969.968733	5.06456667	pos
5.07_243.08	243.084673	5.07146667	neg
5.07_295.07	295.077215	5.07146667	neg
5.07_381.10(	381.106592	5.07146667	neg
5.07_383.15:	383.153286	5.07146667	neg
5.07_395.12:	395.122192	5.07146667	neg
5.07_404.09!	449.094055	5.07146667	neg
5.07_472.08:	471.076085	5.07146667	neg
5.07_486.90:	486.903312	5.07146667	neg
5.07_539.06:	539.063197	5.07146667	neg
5.07_607.05	607.051001	5.07146667	neg
5.07_956.96!	956.965566	5.07146667	neg
5.07_957.46(	957.466749	5.07146667	neg
5.08_216.13!	239.125031	5.08273333	pos
5.08_240.08(	240.08639	5.08273333	pos
5.08_417.22:	435.257236	5.08273333	pos
5.08_447.25	470.240709	5.08273333	pos
5.08_447.75:	470.742328	5.08273333	pos
5.08_457.27:	457.273769	5.08273333	pos
5.08_467.76:	467.762989	5.08273333	pos
5.08_468.10(	468.099962	5.08273333	pos
5.08_468.26	468.264655	5.08273333	pos
5.08_546.41	546.411823	5.08273333	pos
5.08_624.46!	624.469261	5.08273333	pos
5.08_624.61:	624.612283	5.08273333	pos

5.08_633.969	633.969552	5.08273333	pos
5.08_718.103	718.103231	5.08273333	pos
5.08_727.877	727.877035	5.08273333	pos
5.08_728.713	728.7134	5.08273333	pos
5.08_833.080	833.080686	5.08273333	pos
5.08_833.415	833.41536	5.08273333	pos
5.08_878.345	878.345679	5.08273333	pos
5.08_878.847	878.847075	5.08273333	pos
5.08_889.336	889.33654	5.08273333	pos
5.08_950.451	950.451247	5.08273333	pos
5.09_241.107	241.107567	5.08986667	neg
5.09_259.000	259.00089	5.08986667	neg
5.09_278.970	278.970847	5.08986667	neg
5.09_317.006	317.006015	5.08986667	neg
5.09_351.032	351.032897	5.08986667	neg
5.09_699.805	699.805871	5.08986667	neg
5.09_700.307	700.307376	5.08986667	neg
5.09_876.333	876.333365	5.08986667	neg
5.09_876.832	876.832393	5.08986667	neg
5.09_887.826	887.826554	5.08986667	neg
5.10_179.067	179.067727	5.10093333	pos
5.10_211.169	211.169153	5.10093333	pos
5.10_212.091	212.091674	5.10093333	pos
5.10_213.159	213.159496	5.10093333	pos
5.10_265.104	265.104267	5.10093333	pos
5.10_269.135	269.135782	5.10093333	pos
5.10_322.198	345.188003	5.10093333	pos
5.10_347.203	347.203686	5.10093333	pos
5.10_486.231	486.23137	5.10093333	pos
5.10_486.482	486.482161	5.10093333	pos
5.10_486.732	486.732488	5.10093333	pos
5.10_486.982	486.982749	5.10093333	pos
5.10_532.747	532.747472	5.10093333	pos

5.10_533.24	533.24871	5.10093333	pos
5.10_588.33	611.324484	5.10093333	pos
5.10_588.83	611.826201	5.10093333	pos
5.10_597.85	597.855854	5.10093333	pos
5.10_598.35	598.355818	5.10093333	pos
5.10_608.84	608.846748	5.10093333	pos
5.10_609.34	609.34857	5.10093333	pos
5.10_647.97	647.972407	5.10093333	pos
5.10_648.64	648.640475	5.10093333	pos
5.11_180.03	180.030091	5.10843333	neg
5.11_193.07	238.07733	5.10843333	neg
5.11_238.57	238.579032	5.10843333	neg
5.11_272.07	272.0711	5.10843333	neg
5.11_272.57	272.572868	5.10843333	neg
5.11_416.05	416.053602	5.10843333	neg
5.11_710.79	710.797085	5.10843333	neg
5.11_711.29	711.298702	5.10843333	neg
5.11_969.44	969.441488	5.10843333	neg
5.12_206.15	229.141002	5.11926667	pos
5.12_209.07	209.078087	5.11926667	pos
5.12_252.21	252.216734	5.11926667	pos
5.12_275.16	275.161901	5.11926667	pos
5.12_396.08	396.085738	5.11926667	pos
5.12_418.06	418.067727	5.11926667	pos
5.12_532.99	532.998097	5.11926667	pos
5.12_546.53	546.536955	5.11926667	pos
5.12_608.63	608.638864	5.11926667	pos
5.12_700.81	701.819198	5.11926667	pos
5.12_701.31	702.321564	5.11926667	pos
5.12_712.81	712.810903	5.11926667	pos
5.12_779.73	779.735123	5.11926667	pos
5.12_780.06	780.069692	5.11926667	pos
5.12_976.72	976.7292	5.11926667	pos

5.13_253.01(	253.010322	5.12693333	neg
5.14_173.08!	173.085533	5.13745	pos
5.14_216.09!	239.088796	5.13745	pos
5.14_255.12(	255.120095	5.13745	pos
5.14_415.17	415.174158	5.13745	pos
5.14_427.25	450.243161	5.13745	pos
5.14_427.75!	450.74489	5.13745	pos
5.14_428.26	428.261034	5.13745	pos
5.14_429.88	429.887996	5.13745	pos
5.14_430.22	430.222499	5.13745	pos
5.14_436.77	436.774516	5.13745	pos
5.14_439.25	439.252271	5.13745	pos
5.14_439.75	439.753903	5.13745	pos
5.14_447.76!	447.765673	5.13745	pos
5.14_448.26	448.267513	5.13745	pos
5.14_469.76(	492.755617	5.13745	pos
5.14_546.66	546.662427	5.13745	pos
5.14_610.34	633.337848	5.13745	pos
5.14_622.84	622.847706	5.13745	pos
5.14_630.85!	630.859805	5.13745	pos
5.14_631.36	631.36142	5.13745	pos
5.14_667.83	667.832996	5.13745	pos
5.14_710.34!	728.379283	5.13745	pos
5.14_728.04	728.044838	5.13745	pos
5.14_882.89	882.891398	5.13745	pos
5.14_939.98	939.983512	5.13745	pos
5.14_940.48	940.484879	5.13745	pos
5.15_124.00	124.007136	5.14531667	neg
5.15_273.01(	273.016751	5.14531667	neg
5.15_324.00	324.007775	5.14531667	neg
5.16_1083.2(	1083.26618	5.15551667	pos
5.16_171.98(	171.980836	5.16371667	neg
5.16_173.08(	173.080747	5.15551667	pos

5.16_224.06	224.061649	5.16371667	neg
5.16_224.56	224.563338	5.16371667	neg
5.16_243.12	243.119986	5.15551667	pos
5.16_257.10	257.100628	5.16371667	neg
5.16_258.05	258.055229	5.16371667	neg
5.16_260.03	260.031696	5.15551667	pos
5.16_283.06	283.067413	5.16371667	neg
5.16_315.02	315.027387	5.16371667	neg
5.16_385.15	385.152929	5.15551667	pos
5.16_393.07	393.075789	5.16371667	neg
5.16_414.67	414.672447	5.15551667	pos
5.16_430.55	430.556761	5.15551667	pos
5.16_469.26	492.253683	5.15551667	pos
5.16_478.78	478.785212	5.15551667	pos
5.16_489.77	489.776249	5.15551667	pos
5.16_490.27	490.277872	5.15551667	pos
5.16_610.85	633.839521	5.15551667	pos
5.16_619.86	619.868747	5.15551667	pos
5.17_1083.5	1083.51612	5.17355	pos
5.17_1089.0	1089.01163	5.17355	pos
5.17_1089.2	1089.26043	5.17355	pos
5.17_151.07	151.07533	5.17355	pos
5.18_249.03	249.03175	5.18221667	neg
5.18_344.03	344.03642	5.18221667	neg
5.19_1083.7	1083.76576	5.19183333	pos
5.19_1084.0	1084.01614	5.19183333	pos
5.19_1084.2	1084.26609	5.19183333	pos
5.19_1089.5	1089.51154	5.19183333	pos
5.19_1089.7	1089.76142	5.19183333	pos
5.19_180.05	180.050997	5.19183333	pos
5.19_415.21	415.212494	5.19183333	pos
5.19_442.91	442.91175	5.19183333	pos
5.19_443.24	443.246141	5.19183333	pos

5.19_444.563	444.563509	5.19183333	pos
5.19_444.897	444.897946	5.19183333	pos
5.19_449.768	472.758005	5.19183333	pos
5.19_467.226	467.226202	5.19183333	pos
5.19_467.560	467.560605	5.19183333	pos
5.19_469.778	469.778813	5.19183333	pos
5.19_470.280	470.28027	5.19183333	pos
5.19_478.270	479.283234	5.19183333	pos
5.19_632.361	655.350956	5.19183333	pos
5.19_632.863	655.852581	5.19183333	pos
5.19_644.359	644.359617	5.19183333	pos
5.19_644.860	644.860615	5.19183333	pos
5.19_652.873	652.872958	5.19183333	pos
5.19_653.374	653.374551	5.19183333	pos
5.20_222.983	222.983521	5.2006	neg
5.20_229.144	229.144303	5.2006	neg
5.20_462.058	462.058946	5.2006	neg
5.20_677.842	677.842212	5.2006	neg
5.21_137.987	156.020964	5.20995	pos
5.21_149.044	149.044781	5.20995	pos
5.21_458.787	458.787507	5.20995	pos
5.21_503.777	503.777569	5.20995	pos
5.21_511.789	511.78947	5.20995	pos
5.21_512.291	512.291072	5.20995	pos
5.21_514.768	514.768898	5.20995	pos
5.21_531.379	531.379098	5.20995	pos
5.21_679.855	679.855738	5.20995	pos
5.21_690.847	690.846965	5.20995	pos
5.22_209.079	209.079243	5.21915	neg
5.22_216.123	216.123872	5.21915	neg
5.22_228.024	228.024559	5.21915	neg
5.22_294.015	294.01526	5.21915	neg
5.22_317.114	317.114039	5.21915	neg

5.22_318.05	318.055201	5.21915	neg
5.22_345.05	345.05418	5.21915	neg
5.22_362.00	362.002457	5.21915	neg
5.22_362.04	362.045042	5.21915	neg
5.22_514.03	559.036598	5.21915	neg
5.22_581.01	581.018631	5.21915	neg
5.22_599.76	599.769835	5.21915	neg
5.22_663.32	663.328307	5.21915	neg
5.23_1187.5	1187.54048	5.22825	pos
5.23_153.05	153.054667	5.22825	pos
5.23_158.01	158.018041	5.22825	pos
5.23_210.08	211.093828	5.22825	pos
5.23_228.04	228.041963	5.22825	pos
5.23_284.05	284.056725	5.22825	pos
5.23_336.06	336.06464	5.22825	pos
5.23_358.24	341.243171	5.22825	pos
5.23_364.05	364.059428	5.22825	pos
5.23_366.22	389.214204	5.22825	pos
5.23_442.10	442.102634	5.22825	pos
5.23_446.05	447.057817	5.22825	pos
5.23_552.28	552.288437	5.22825	pos
5.24_133.01	133.014044	5.23773333	neg
5.24_239.58	239.586991	5.23773333	neg
5.24_250.99	250.994628	5.23773333	neg
5.24_294.05	294.057272	5.23773333	neg
5.24_413.04	413.041345	5.23773333	neg
5.24_481.02	481.02909	5.23773333	neg
5.24_514.89	514.893258	5.23773333	neg
5.25_157.02	157.024358	5.24643333	pos
5.25_216.14	216.141597	5.24643333	pos
5.25_269.13	269.135917	5.24643333	pos
5.25_318.12	341.110423	5.24643333	pos
5.25_342.26	342.26329	5.24643333	pos

5.25_457.587	457.587139	5.24643333	pos
5.25_457.921	457.921525	5.24643333	pos
5.25_458.256	458.255966	5.24643333	pos
5.25_459.573	459.573375	5.24643333	pos
5.25_459.907	459.907444	5.24643333	pos
5.25_628.785	629.792705	5.24643333	pos
5.25_654.374	677.363421	5.24643333	pos
5.25_663.895	663.895316	5.24643333	pos
5.25_666.372	666.372484	5.24643333	pos
5.25_674.885	674.885822	5.24643333	pos
5.25_675.387	675.387388	5.24643333	pos
5.25_677.865	677.865398	5.24643333	pos
5.25_784.973	784.973001	5.24643333	pos
5.26_142.938	142.938645	5.26461667	pos
5.26_222.075	222.075866	5.26461667	pos
5.26_381.019	381.019043	5.25613333	neg
5.26_471.280	494.269404	5.26461667	pos
5.26_480.800	480.800574	5.26461667	pos
5.26_491.791	491.791884	5.26461667	pos
5.26_492.293	492.293652	5.26461667	pos
5.26_494.771	494.771307	5.26461667	pos
5.26_533.802	533.802579	5.26461667	pos
5.26_536.280	536.280059	5.26461667	pos
5.26_536.781	536.781707	5.26461667	pos
5.26_541.265	541.265391	5.25613333	neg
5.26_547.855	547.855933	5.25613333	neg
5.26_627.779	627.778965	5.25613333	neg
5.26_630.294	630.294338	5.26461667	pos
5.26_638.770	638.76997	5.25613333	neg
5.26_782.903	782.903712	5.26461667	pos
5.27_144.045	144.045315	5.27448333	neg
5.27_144.102	144.102805	5.27448333	neg
5.27_187.061	187.060975	5.27448333	neg

5.27_188.09:	188.092583	5.27448333	neg
5.27_306.05!	306.059393	5.27448333	neg
5.27_330.19:	330.192183	5.27448333	neg
5.27_343.14:	343.140983	5.27448333	neg
5.27_377.22:	377.221333	5.27448333	neg
5.27_387.25(	387.250183	5.27448333	neg
5.27_404.24(	404.240143	5.27448333	neg
5.27_415.17(	415.169961	5.27448333	neg
5.27_534.15⁄	534.15455	5.27448333	neg
5.27_602.14:	602.142148	5.27448333	neg
5.28_193.08:	193.083196	5.28283333	pos
5.28_217.20⁄	218.21129	5.28283333	pos
5.28_288.20(	288.206696	5.28283333	pos
5.28_342.25:	365.24074	5.28283333	pos
5.28_376.17(	376.170065	5.28283333	pos
5.28_424.18	424.187261	5.28283333	pos
5.28_470.61(	470.610776	5.28283333	pos
5.28_472.26:	472.262714	5.28283333	pos
5.28_472.59	472.597085	5.28283333	pos
5.28_474.24!	474.248998	5.28283333	pos
5.28_697.40(	697.400556	5.28283333	pos
5.28_699.37	699.377137	5.28283333	pos
5.28_699.87!	699.878953	5.28283333	pos
5.28_727.66⁄	727.664123	5.28283333	pos
5.29_212.12!	212.129062	5.29295	neg
5.29_328.04:	328.041572	5.29295	neg
5.29_371.16(	371.160916	5.29295	neg
5.29_427.11:	427.113691	5.29295	neg
5.29_432.15(	432.150626	5.29295	neg
5.29_454.13:	454.133212	5.29295	neg
5.29_460.13:	505.130502	5.29295	neg
5.29_573.11!	573.118023	5.29295	neg
5.29_641.10!	641.105241	5.29295	neg

5.29_709.09[illegible]	709.092877	5.29295	neg
5.30_157.13[illegible]	157.133414	5.30103333	pos
5.30_195.13[illegible]	195.137981	5.30103333	pos
5.30_229.17[illegible]	252.167735	5.30103333	pos
5.30_320.09[illegible]	320.092238	5.30103333	pos
5.30_415.13[illegible]	415.133576	5.30103333	pos
5.30_472.93[illegible]	472.931414	5.30103333	pos
5.30_473.91[illegible]	473.914417	5.30103333	pos
5.30_607.30[illegible]	607.30005	5.30103333	pos
5.30_607.55[illegible]	607.550693	5.30103333	pos
5.30_607.80[illegible]	607.801293	5.30103333	pos
5.30_695.88[illegible]	695.881527	5.30103333	pos
5.30_696.38[illegible]	696.38315	5.30103333	pos
5.30_696.89[illegible]	696.89905	5.30103333	pos
5.30_706.87[illegible]	706.871628	5.30103333	pos
5.30_787.09[illegible]	787.092728	5.30103333	pos
5.31_169.05[illegible]	169.05046	5.31133333	neg
5.31_178.97[illegible]	178.977571	5.31133333	neg
5.31_192.98[illegible]	192.988923	5.31133333	neg
5.31_243.12[illegible]	243.12354	5.31133333	neg
5.31_246.94[illegible]	246.949882	5.31133333	neg
5.32_196.07[illegible]	197.078176	5.3192	pos
5.32_267.12[illegible]	267.120042	5.3192	pos
5.32_292.92[illegible]	292.928846	5.3192	pos
5.32_513.80[illegible]	513.804863	5.3192	pos
5.32_514.30[illegible]	514.306658	5.3192	pos
5.32_516.78[illegible]	516.784454	5.3192	pos
5.32_706.36[illegible]	706.367137	5.3192	pos
5.33_206.00[illegible]	206.006825	5.32985	neg
5.33_208.01[illegible]	207.004293	5.32985	neg
5.33_252.04[illegible]	252.04314	5.32985	neg
5.33_254.04[illegible]	254.040305	5.32985	neg
5.33_289.07[illegible]	289.07511	5.32985	neg

5.33_296.13	296.132469	5.32985	neg
5.33_672.85	672.85939	5.32985	neg
5.34_177.08	200.071392	5.33735	pos
5.34_181.15	181.158605	5.33735	pos
5.34_183.13	183.137977	5.33735	pos
5.34_208.19	208.190631	5.33735	pos
5.34_241.20	241.203318	5.33735	pos
5.34_241.70	241.705004	5.33735	pos
5.34_254.05	254.057559	5.33735	pos
5.34_486.93	486.938091	5.33735	pos
5.34_487.27	487.272504	5.33735	pos
5.34_487.60	487.606784	5.33735	pos
5.34_488.92	488.924295	5.33735	pos
5.34_489.25	489.258347	5.33735	pos
5.34_494.30	494.300243	5.33735	pos
5.34_558.29	558.292741	5.33735	pos
5.34_647.07	647.074702	5.33735	pos
5.34_718.91	718.911444	5.33735	pos
5.34_719.41	719.413272	5.33735	pos
5.34_721.89	721.89132	5.33735	pos
5.34_861.42	861.426256	5.33735	pos
5.34_861.76	861.761082	5.33735	pos
5.35_274.99	274.996021	5.3483	neg
5.35_290.01	289.011641	5.3483	neg
5.35_297.00	297.000165	5.3483	neg
5.35_298.00	298.001912	5.3483	neg
5.35_298.99	298.996017	5.3483	neg
5.35_308.03	308.030779	5.3483	neg
5.35_310.02	310.028142	5.3483	neg
5.35_350.08	350.081575	5.3483	neg
5.35_357.06	357.062782	5.3483	neg
5.35_394.07	394.071049	5.3483	neg
5.35_444.00	444.005654	5.3483	neg

5.35_462.058	462.058891	5.3483	neg
5.35_554.890	554.890447	5.3483	neg
5.36_154.041	154.04173	5.35556667	pos
5.36_173.080	173.0804	5.35556667	pos
5.36_206.115	206.115092	5.35556667	pos
5.36_244.054	244.054569	5.35556667	pos
5.36_286.021	286.021396	5.35556667	pos
5.36_355.080	355.079986	5.35556667	pos
5.36_410.251	433.24056	5.35556667	pos
5.36_465.600	488.589902	5.35556667	pos
5.36_646.573	646.573198	5.35556667	pos
5.36_646.823	646.823817	5.35556667	pos
5.37_251.185	251.184985	5.37381667	pos
5.37_290.141	290.141739	5.37381667	pos
5.37_360.974	360.974288	5.36688333	neg
5.39_173.118	173.118033	5.38528333	neg
5.39_186.998	186.998392	5.38528333	neg
5.39_219.096	219.096457	5.39208333	pos
5.39_241.105	241.105588	5.38528333	neg
5.39_255.120	255.120003	5.39208333	pos
5.39_309.093	309.093268	5.38528333	neg
5.39_365.126	365.126068	5.39208333	pos
5.39_381.019	381.019027	5.38528333	neg
5.39_429.120	429.120626	5.39208333	pos
5.39_445.067	445.067834	5.38528333	neg
5.39_480.270	503.265393	5.39208333	pos
5.39_495.938	495.938337	5.39208333	pos
5.39_499.961	499.961562	5.39208333	pos
5.39_501.613	501.613406	5.39208333	pos
5.39_501.947	501.94792	5.39208333	pos
5.39_502.281	502.281843	5.39208333	pos
5.39_503.599	503.599808	5.39208333	pos
5.39_503.934	503.933988	5.39208333	pos

5.39_515.30	538.296057	5.39208333	pos
5.39_515.80	538.797625	5.39208333	pos
5.39_525.92	525.927816	5.39208333	pos
5.39_527.80	527.806411	5.39208333	pos
5.39_535.81	535.817979	5.39208333	pos
5.39_536.31	536.319911	5.39208333	pos
5.39_673.32	673.322283	5.39208333	pos
5.39_743.40	743.402678	5.39208333	pos
5.39_743.90	743.904208	5.39208333	pos
5.39_757.93	796.902385	5.39208333	pos
5.40_221.00	221.003883	5.40378333	neg
5.40_225.11	225.113076	5.40378333	neg
5.40_609.25	609.252986	5.40378333	neg
5.41_405.24	405.245605	5.41035	pos
5.41_624.18	624.18201	5.41035	pos
5.41_645.31	645.313173	5.41035	pos
5.41_645.56	645.563309	5.41035	pos
5.41_645.81	645.813761	5.41035	pos
5.41_646.06	646.064164	5.41035	pos
5.41_710.87	710.87807	5.41035	pos
5.41_711.37	711.379044	5.41035	pos
5.42_250.10	250.108417	5.42216667	neg
5.42_324.99	324.992886	5.42216667	neg
5.42_446.90	446.90592	5.42216667	neg
5.43_250.14	250.14158	5.42866667	pos
5.43_293.16	293.160456	5.42866667	pos
5.43_409.21	409.219389	5.42866667	pos
5.43_495.28	518.275092	5.42866667	pos
5.43_765.41	765.415526	5.42866667	pos
5.44_1150.5	1150.58438	5.44071667	neg
5.44_224.99	224.99931	5.44071667	neg
5.44_276.95	276.955199	5.44071667	neg
5.44_337.12	337.122884	5.44071667	neg

5.44_413.20	413.199962	5.44071667	neg
5.44_765.82	765.827345	5.44071667	neg
5.45_1152.0	1152.09564	5.44698333	pos
5.45_1152.5	1152.59699	5.44698333	pos
5.45_285.13	285.130665	5.44698333	pos
5.45_494.95	517.940888	5.44698333	pos
5.45_510.61	510.613931	5.44698333	pos
5.45_514.63	514.637141	5.44698333	pos
5.45_516.62	516.623537	5.44698333	pos
5.45_540.60	540.603142	5.44698333	pos
5.45_547.78	547.781016	5.44698333	pos
5.45_557.83	557.831068	5.44698333	pos
5.45_558.33	558.332678	5.44698333	pos
5.45_560.81	560.810394	5.44698333	pos
5.45_660.91	660.915177	5.44698333	pos
5.45_768.39	768.399223	5.44698333	pos
5.45_768.73	768.733024	5.44698333	pos
5.46_1150.0	1150.08239	5.45918333	neg
5.46_182.98	182.988209	5.45918333	neg
5.46_230.98	230.988319	5.45918333	neg
5.46_303.02	303.027224	5.45918333	neg
5.46_317.11	317.114094	5.45918333	neg
5.47_160.07	160.078893	5.46526667	pos
5.47_205.13	205.131011	5.46526667	pos
5.47_319.12	319.128611	5.46526667	pos
5.47_537.31	560.30889	5.46526667	pos
5.47_548.28	548.281928	5.46526667	pos
5.48_209.00	209.003823	5.47755	neg
5.48_227.08	227.089862	5.47755	neg
5.48_276.99	276.991489	5.47755	neg
5.48_287.03	287.03228	5.47755	neg
5.48_301.01	301.011589	5.47755	neg
5.48_331.12	331.129848	5.47755	neg

5.48_332.13(	355.125993	5.48356667	pos
5.48_464.58:	464.582593	5.48356667	pos
5.48_529.31:	529.312541	5.48356667	pos
5.48_529.64	529.647144	5.48356667	pos
5.48_531.29	531.298753	5.48356667	pos
5.48_545.02	545.029862	5.47755	neg
5.50_273.08(	273.080239	5.49605	neg
5.50_310.04	310.048831	5.49605	neg
5.50_358.05	358.057778	5.49605	neg
5.50_509.96	532.950676	5.5019	pos
5.50_532.61(	532.61625	5.5019	pos
5.50_533.28	533.2848	5.5019	pos
5.50_882.77	882.771039	5.5019	pos
5.50_889.76	889.764857	5.5019	pos
5.50_890.09	890.098331	5.5019	pos
5.52_215.15:	216.159351	5.52015	pos
5.52_286.15:	286.152188	5.52015	pos
5.52_394.22(	394.225984	5.52015	pos
5.52_395.87	395.877649	5.52015	pos
5.52_559.33:	582.321556	5.52015	pos
5.52_579.84	579.84433	5.52015	pos
5.53_202.00	202.008858	5.53265	neg
5.53_313.11	313.119488	5.53265	neg
5.53_381.10(	381.106327	5.53265	neg
5.53_403.08	403.08832	5.53265	neg
5.53_850.81	850.819252	5.53265	neg
5.53_918.80	918.805505	5.53265	neg
5.54_173.08(	173.080534	5.53846667	pos
5.54_197.15:	197.153527	5.53846667	pos
5.54_202.04	202.044211	5.53846667	pos
5.54_224.12	224.125498	5.53846667	pos
5.54_314.12(	315.133613	5.53846667	pos
5.54_545.63	545.639799	5.53846667	pos

5.54_545.97	545.974264	5.53846667	pos
5.54_547.62	547.625829	5.53846667	pos
5.54_547.96	547.960335	5.53846667	pos
5.55_177.05	177.055498	5.55113333	neg
5.55_200.01	200.011822	5.55113333	neg
5.55_218.98	218.985699	5.55113333	neg
5.55_220.10	220.10109	5.55113333	neg
5.55_283.11	283.118458	5.55113333	neg
5.55_332.21	332.219013	5.55113333	neg
5.55_376.13	376.133749	5.55113333	neg
5.56_287.21	310.20897	5.5568	pos
5.57_253.01	253.010207	5.5696	neg
5.57_260.05	260.059619	5.5696	neg
5.57_262.97	262.975788	5.5696	neg
5.57_286.99	286.996001	5.5696	neg
5.57_328.04	328.047176	5.5696	neg
5.57_348.99	348.99027	5.5696	neg
5.57_387.23	387.239549	5.5696	neg
5.57_554.89	554.890439	5.5696	neg
5.58_376.62	376.624227	5.57501667	pos
5.58_408.90	408.900998	5.57501667	pos
5.58_409.23	409.235019	5.57501667	pos
5.58_410.55	410.552793	5.57501667	pos
5.58_410.88	410.88733	5.57501667	pos
5.58_562.63	562.635189	5.57501667	pos
5.58_601.85	601.856848	5.57501667	pos
5.59_150.00	150.001616	5.58803333	neg
5.59_228.99	228.99386	5.58803333	neg
5.59_252.21	252.216665	5.59335	pos
5.59_306.03	306.037767	5.59335	pos
5.59_407.20	407.207574	5.58803333	neg
5.59_418.14	418.144184	5.58803333	neg
5.59_464.02	464.022002	5.58803333	neg

5.59_560.64	560.649419	5.59335	pos
5.59_560.98	560.983576	5.59335	pos
5.59_561.96	561.966725	5.59335	pos
5.61_222.97	222.978091	5.60643333	neg
5.61_361.22	361.219969	5.61161667	pos
5.61_395.03	395.034576	5.60643333	neg
5.61_446.90	446.905845	5.60643333	neg
5.63_142.93	142.938604	5.6299	pos
5.63_143.03	143.039553	5.6299	pos
5.63_193.11	193.119493	5.6299	pos
5.63_208.12	208.127149	5.6299	pos
5.63_387.24	387.249678	5.6251	neg
5.63_404.24	404.240207	5.6251	neg
5.63_423.91	423.910798	5.6299	pos
5.63_576.64	576.64192	5.6299	pos
5.63_679.20	679.209266	5.6299	pos
5.63_789.76	789.763617	5.6299	pos
5.63_790.09	790.098521	5.6299	pos
5.64_229.14	229.144285	5.64363333	neg
5.64_246.03	246.038336	5.64363333	neg
5.64_248.09	248.096237	5.64363333	neg
5.64_371.24	371.243957	5.64363333	neg
5.65_250.11	250.110484	5.64811667	pos
5.65_300.16	300.162524	5.64811667	pos
5.65_332.13	355.126181	5.64811667	pos
5.65_417.90	417.901201	5.64811667	pos
5.65_421.92	421.924581	5.64811667	pos
5.65_423.57	423.576513	5.64811667	pos
5.65_425.22	425.22775	5.64811667	pos
5.65_425.56	425.562568	5.64811667	pos
5.65_493.29	493.297965	5.64811667	pos
5.65_575.32	575.324592	5.64811667	pos
5.65_623.87	623.870033	5.64811667	pos

5.65_626.347	626.347392	5.64811667	pos
5.66_314.025	314.025888	5.66205	neg
5.66_315.027	315.02735	5.66205	neg
5.67_252.133	235.130258	5.66635	pos
5.67_345.187	345.18794	5.66635	pos
5.67_660.915	660.915046	5.66635	pos
5.67_954.154	937.1516	5.66635	pos
5.67_954.489	937.485763	5.66635	pos
5.68_170.987	170.987385	5.68048333	neg
5.68_196.096	196.096823	5.68461667	pos
5.68_293.135	293.135802	5.68461667	pos
5.68_413.664	413.664578	5.68461667	pos
5.68_704.365	704.365283	5.68461667	pos
5.68_868.239	868.239604	5.68461667	pos
5.68_868.406	868.406697	5.68461667	pos
5.68_936.817	936.817283	5.68461667	pos
5.68_977.828	977.828217	5.68461667	pos
5.68_984.487	984.48729	5.68461667	pos
5.68_984.821	984.821096	5.68461667	pos
5.68_985.154	985.154907	5.68461667	pos
5.70_180.030	180.030051	5.69895	neg
5.70_188.997	188.997733	5.69895	neg
5.70_243.159	243.159893	5.69895	neg
5.70_317.114	317.114027	5.69895	neg
5.70_416.914	439.903554	5.70286667	pos
5.70_417.248	440.238059	5.70286667	pos
5.70_438.251	438.251922	5.70286667	pos
5.70_440.572	440.572317	5.70286667	pos
5.70_461.067	461.067313	5.69895	neg
5.70_591.317	591.317072	5.70286667	pos
5.70_646.384	646.384544	5.70286667	pos
5.70_648.361	648.361116	5.70286667	pos
5.72_197.153	197.153522	5.72123333	pos

5.72_200.04[illegible]	200.047129	5.72123333	pos
5.72_213.11[illegible]	213.113019	5.71736667	neg
5.72_346.95[illegible]	346.95833	5.71736667	neg
5.74_156.94[illegible]	156.948408	5.73951667	pos
5.74_267.15[illegible]	267.156372	5.73951667	pos
5.74_344.07[illegible]	344.077076	5.73951667	pos
5.74_344.94[illegible]	344.942571	5.7359	neg
5.75_209.00[illegible]	209.003786	5.75441667	neg
5.75_260.09[illegible]	260.092562	5.75441667	neg
5.75_554.89[illegible]	554.89056	5.75441667	neg
5.76_195.09[illegible]	195.098928	5.75788333	pos
5.76_342.26[illegible]	342.263534	5.75788333	pos
5.76_420.15[illegible]	420.158438	5.75788333	pos
5.76_431.58[illegible]	454.579076	5.75788333	pos
5.76_431.92[illegible]	454.913587	5.75788333	pos
5.76_439.92[illegible]	439.924426	5.75788333	pos
5.76_447.25[illegible]	447.251839	5.75788333	pos
5.76_451.27[illegible]	451.27553	5.75788333	pos
5.76_451.60[illegible]	451.60994	5.75788333	pos
5.76_452.92[illegible]	452.927391	5.75788333	pos
5.76_453.26[illegible]	453.261685	5.75788333	pos
5.76_453.59[illegible]	453.596071	5.75788333	pos
5.76_667.89[illegible]	667.895737	5.75788333	pos
5.77_222.98[illegible]	222.983499	5.773	neg
5.77_299.01[illegible]	299.015895	5.773	neg
5.77_360.97[illegible]	360.974215	5.773	neg
5.77_486.90[illegible]	486.903247	5.773	neg
5.78_142.93[illegible]	142.938603	5.77613333	pos
5.78_328.08[illegible]	328.082165	5.77613333	pos
5.78_445.26[illegible]	446.268595	5.77613333	pos
5.78_660.77[illegible]	660.772036	5.77613333	pos
5.79_158.01[illegible]	158.018045	5.7943	pos
5.79_159.14[illegible]	159.149092	5.7943	pos

5.79_171.98	171.980816	5.7914	neg
5.79_283.02	283.021009	5.7914	neg
5.79_314.02	314.025967	5.7914	neg
5.79_409.21	409.219023	5.7943	pos
5.81_170.14	171.149015	5.81251667	pos
5.81_171.14	172.152328	5.81251667	pos
5.81_225.09	225.090888	5.81251667	pos
5.81_313.11	313.119516	5.80983333	neg
5.81_314.12	315.133498	5.81251667	pos
5.81_321.01	321.017666	5.80983333	neg
5.81_381.10	381.106551	5.80983333	neg
5.81_454.59	454.599511	5.81251667	pos
5.81_454.93	454.934156	5.81251667	pos
5.81_460.27	460.27522	5.81251667	pos
5.81_460.60	460.609759	5.81251667	pos
5.81_465.95	465.950886	5.81251667	pos
5.81_466.28	466.285467	5.81251667	pos
5.81_467.60	467.602843	5.81251667	pos
5.81_467.93	467.937301	5.81251667	pos
5.81_469.25	469.254419	5.81251667	pos
5.81_539.06	539.063606	5.80983333	neg
5.81_558.37	558.376096	5.80983333	neg
5.81_655.81	655.811078	5.80983333	neg
5.81_657.82	657.824081	5.81251667	pos
5.81_692.38	692.386393	5.81251667	pos
5.83_187.08	187.087421	5.8283	neg
5.83_196.14	196.141196	5.83081667	pos
5.83_220.16	243.156433	5.83081667	pos
5.83_239.08	239.089848	5.8283	neg
5.83_267.07	267.07944	5.83081667	pos
5.83_467.10	467.10175	5.83081667	pos
5.83_726.36	727.368107	5.83081667	pos
5.85_244.08	244.080124	5.84688333	neg

5.85_257.10(	257.100544	5.84688333	neg
5.85_363.17:	363.172147	5.84918333	pos
5.85_385.22:	385.223185	5.84688333	neg
5.85_431.05:	431.05245	5.84688333	neg
5.87_141.12	141.127307	5.86745	pos
5.87_205.08:	205.081251	5.86745	pos
5.87_227.16	227.163956	5.86745	pos
5.87_250.14:	250.141839	5.86745	pos
5.87_271.00(	271.000827	5.8654	neg
5.87_354.23	354.235099	5.86745	pos
5.87_365.13	365.134326	5.8654	neg
5.87_372.21	372.213992	5.8654	neg
5.87_474.95:	474.95104	5.86745	pos
5.87_961.11:	962.120502	5.86745	pos
5.87_961.44	962.455308	5.86745	pos
5.87_961.78:	962.789326	5.86745	pos
5.87_968.44(	969.448179	5.86745	pos
5.87_968.77	969.78224	5.86745	pos
5.87_969.10	970.116654	5.86745	pos
5.87_976.10:	977.109528	5.86745	pos
5.87_976.43(	977.444177	5.86745	pos
5.87_976.77	976.774855	5.86745	pos
5.88_245.00:	245.001574	5.88396667	neg
5.88_303.02	303.027106	5.88396667	neg
5.88_307.00:	307.002223	5.88396667	neg
5.88_339.22	339.228666	5.88396667	neg
5.88_346.95	346.95831	5.88396667	neg
5.88_451.08:	451.083277	5.88396667	neg
5.88_636.82	636.8277	5.88396667	neg
5.88_809.85(	809.850526	5.88396667	neg
5.89_191.10:	191.103863	5.88578333	pos
5.89_269.13	269.135609	5.88578333	pos
5.89_298.15	321.143581	5.88578333	pos

5.89_460.94	483.929994	5.88578333	pos
5.89_461.27	484.264369	5.88578333	pos
5.89_467.62	467.623488	5.88578333	pos
5.89_469.60	469.609707	5.88578333	pos
5.89_479.61	480.626417	5.88578333	pos
5.89_481.29	481.295119	5.88578333	pos
5.89_482.27	482.278297	5.88578333	pos
5.89_482.61	482.612633	5.88578333	pos
5.89_484.59	484.598807	5.88578333	pos
5.90_118.04	118.040903	5.90251667	neg
5.90_149.13	149.132397	5.90411667	pos
5.90_210.99	210.99957	5.90251667	neg
5.90_229.91	229.91581	5.90251667	neg
5.90_249.89	249.897988	5.90411667	pos
5.92_188.11	188.111146	5.92093333	neg
5.92_236.07	236.078269	5.92093333	neg
5.92_345.01	345.018702	5.92093333	neg
5.92_564.78	564.783682	5.92246667	pos
5.92_565.28	565.284874	5.92246667	pos
5.92_575.77	575.774467	5.92246667	pos
5.92_576.27	576.276031	5.92246667	pos
5.92_660.62	660.628892	5.92246667	pos
5.92_853.77	853.778825	5.92246667	pos
5.92_996.82	996.823747	5.92093333	neg
5.94_195.13	195.137884	5.94078333	pos
5.94_257.00	257.00554	5.93928333	neg
5.94_285.01	285.016616	5.93928333	neg
5.94_319.00	319.002074	5.93928333	neg
5.94_325.04	325.041544	5.93928333	neg
5.94_355.22	355.223726	5.93928333	neg
5.94_376.23	376.233942	5.93928333	neg
5.94_475.61	498.605351	5.94078333	pos
5.94_482.29	482.298621	5.94078333	pos

5.94_483.95(	483.950388	5.94078333	pos
5.94_484.28!	484.285121	5.94078333	pos
5.94_494.29	495.301873	5.94078333	pos
5.94_495.63(	495.636185	5.94078333	pos
5.94_495.97(	495.970568	5.94078333	pos
5.94_496.95:	496.953622	5.94078333	pos
5.94_497.62:	497.622289	5.94078333	pos
5.94_498.93!	498.939704	5.94078333	pos
5.94_663.84(	663.846712	5.93928333	neg
5.94_679.06(	679.065998	5.94078333	pos
5.96_290.22:	290.222499	5.95905	pos
5.96_306.24(	329.229429	5.95905	pos
5.96_332.20	332.207794	5.95783333	neg
5.96_349.01:	349.012675	5.95783333	neg
5.96_512.20!	512.208784	5.95783333	neg
5.96_928.83!	928.835741	5.95783333	neg
5.98_1075.0(	1075.00413	5.9774	pos
5.98_1085.9!	1085.99519	5.9774	pos
5.98_143.02!	124.007162	5.97626667	neg
5.98_156.94!	156.948411	5.9774	pos
5.98_248.09(	248.096139	5.97626667	neg
5.98_252.01:	252.012424	5.97626667	neg
5.98_308.99!	308.99798	5.97626667	neg
5.99_317.00(	317.005998	5.99473333	neg
6.00_213.10!	213.109331	5.99566667	pos
6.00_304.89	304.894149	5.99566667	pos
6.00_378.13!	378.138471	5.99566667	pos
6.00_490.29	513.280933	5.99566667	pos
6.00_490.62(	513.615359	5.99566667	pos
6.00_497.30!	497.308395	5.99566667	pos
6.00_498.62!	498.625738	5.99566667	pos
6.00_504.30	504.301794	5.99566667	pos
6.00_508.96!	509.977218	5.99566667	pos

6.00_510.31	510.311659	5.99566667	pos
6.00_511.62	511.629262	5.99566667	pos
6.00_511.96	511.96379	5.99566667	pos
6.01_225.10	225.109331	6.01398333	pos
6.01_225.61	225.611557	6.01398333	pos
6.01_226.99	226.994556	6.01328333	neg
6.01_284.03	284.032871	6.01328333	neg
6.01_654.86	654.869437	6.01328333	neg
6.03_223.01	223.019631	6.0318	neg
6.03_271.02	271.021327	6.0318	neg
6.03_276.95	276.955177	6.0318	neg
6.03_329.11	329.114208	6.0318	neg
6.03_331.02	331.022031	6.0318	neg
6.03_385.22	385.223058	6.0318	neg
6.03_392.21	393.224356	6.03235	pos
6.03_405.20	405.204789	6.0318	neg
6.03_454.20	454.206016	6.0318	neg
6.03_522.19	522.193593	6.0318	neg
6.03_528.26	528.263626	6.0318	neg
6.03_641.17	641.177867	6.0318	neg
6.03_709.16	709.165975	6.0318	neg
6.05_294.98	294.98207	6.05016667	neg
6.05_504.96	527.956304	6.05065	pos
6.05_505.30	528.290828	6.05065	pos
6.05_513.30	513.301279	6.05065	pos
6.05_518.97	518.977145	6.05065	pos
6.05_519.64	519.645877	6.05065	pos
6.05_520.62	520.628987	6.05065	pos
6.05_526.30	526.304257	6.05065	pos
6.05_526.63	526.639002	6.05065	pos
6.05_526.97	526.973299	6.05065	pos
6.07_173.00	173.002722	6.06866667	neg
6.07_200.01	200.011811	6.06866667	neg

6.07_225.061	225.061422	6.06866667	neg
6.07_262.164	262.164665	6.06898333	pos
6.07_287.182	287.182591	6.06898333	pos
6.07_319.038	319.03836	6.06866667	neg
6.07_346.958	346.958345	6.06866667	neg
6.07_350.986	350.986639	6.06898333	pos
6.09_324.992	324.992743	6.08708333	neg
6.09_514.893	514.89336	6.08708333	neg
6.09_528.311	528.311092	6.0873	pos
6.09_535.304	535.304802	6.0873	pos
6.09_835.636	835.636386	6.0873	pos
6.09_836.138	836.13801	6.0873	pos
6.11_187.060	187.060936	6.1056	neg
6.11_269.139	269.139554	6.1056	neg
6.11_333.017	333.017848	6.1056	neg
6.11_498.901	498.901115	6.10565	pos
6.11_519.642	542.631686	6.10565	pos
6.11_533.652	533.652622	6.10565	pos
6.11_539.328	539.327968	6.10565	pos
6.11_540.980	540.979964	6.10565	pos
6.11_541.314	541.31457	6.10565	pos
6.11_541.648	541.648734	6.10565	pos
6.11_542.960	542.966106	6.10565	pos
6.12_149.024	149.024176	6.12411667	neg
6.12_156.948	156.948382	6.12398333	pos
6.12_197.078	197.078014	6.12398333	pos
6.12_226.965	226.965804	6.12411667	neg
6.12_279.614	279.614071	6.12411667	neg
6.12_280.117	280.117614	6.12411667	neg
6.12_299.025	299.025944	6.12411667	neg
6.12_304.894	304.894061	6.12398333	pos
6.12_431.016	431.016314	6.12411667	neg
6.12_660.915	660.915114	6.12398333	pos

6.14_173.08	173.081631	6.14265	neg
6.14_271.10	271.109086	6.14265	neg
6.14_345.15	345.155282	6.14265	neg
6.14_346.16	369.151714	6.1424	pos
6.14_534.31	557.306971	6.1424	pos
6.14_534.65	557.641477	6.1424	pos
6.14_542.98	542.986236	6.1424	pos
6.14_548.32	548.327773	6.1424	pos
6.14_552.99	554.003298	6.1424	pos
6.14_555.65	555.655256	6.1424	pos
6.14_555.98	555.989824	6.1424	pos
6.14_556.32	556.324071	6.1424	pos
6.16_173.11	173.118024	6.1611	neg
6.16_200.04	200.047108	6.16076667	pos
6.16_213.11	213.113026	6.1611	neg
6.16_241.01	241.010373	6.1611	neg
6.16_241.10	241.10561	6.1611	neg
6.16_309.09	309.093224	6.1611	neg
6.16_312.21	312.216564	6.16076667	pos
6.16_335.03	335.033368	6.1611	neg
6.16_423.72	423.727258	6.16076667	pos
6.18_331.20	331.208761	6.1791	pos
6.18_352.89	352.897104	6.1791	pos
6.18_421.18	421.186916	6.17958333	neg
6.18_475.19	475.1949	6.17958333	neg
6.20_211.16	211.169075	6.1974	pos
6.20_313.11	313.119453	6.19793333	neg
6.20_314.12	315.133501	6.1974	pos
6.20_329.19	329.193065	6.1974	pos
6.20_335.10	335.101289	6.19793333	neg
6.20_363.21	363.216179	6.1974	pos
6.20_374.25	374.254537	6.19793333	neg
6.20_403.08	403.088397	6.19793333	neg

6.20_434.984	434.984749	6.1974	pos
6.20_454.226	454.226867	6.19793333	neg
6.20_471.076	471.076355	6.19793333	neg
6.20_473.072	473.072874	6.19793333	neg
6.20_484.053	484.053579	6.19793333	neg
6.20_508.050	508.050141	6.1974	pos
6.20_548.993	571.982185	6.1974	pos
6.20_549.327	572.316644	6.1974	pos
6.20_563.337	563.337938	6.1974	pos
6.20_564.654	564.654913	6.1974	pos
6.20_564.989	564.989819	6.1974	pos
6.20_568.678	568.678566	6.1974	pos
6.20_569.013	569.01319	6.1974	pos
6.20_570.330	570.330594	6.1974	pos
6.20_570.665	570.664954	6.1974	pos
6.20_570.999	570.999259	6.1974	pos
6.20_826.854	826.854309	6.19793333	neg
6.22_102.034	102.034181	6.2158	pos
6.22_162.055	162.055868	6.21651667	neg
6.22_163.075	163.075199	6.2158	pos
6.22_269.189	269.18964	6.2158	pos
6.22_278.970	278.97088	6.21651667	neg
6.22_284.015	284.015231	6.21651667	neg
6.22_286.957	286.957835	6.2158	pos
6.22_554.890	554.890588	6.21651667	neg
6.23_203.143	203.143	6.23411667	pos
6.23_224.999	224.999475	6.23498333	neg
6.23_236.993	236.993692	6.23498333	neg
6.23_303.007	303.007342	6.23498333	neg
6.25_171.980	171.980834	6.2534	neg
6.25_200.129	200.128963	6.2534	neg
6.25_227.163	227.163908	6.2524	pos
6.25_371.243	371.243879	6.2534	neg

6.25_415.179	415.17981	6.2534	neg
6.25_444.50	444.501579	6.2524	pos
6.25_446.24	446.241616	6.2524	pos
6.25_564.67	564.675331	6.2524	pos
6.25_582.34	583.35395	6.2524	pos
6.25_584.02	584.022652	6.2524	pos
6.25_585.00	585.005845	6.2524	pos
6.25_585.34	585.340416	6.2524	pos
6.25_586.99	586.991667	6.2524	pos
6.27_1065.5	1065.53124	6.27078333	pos
6.27_195.12	195.12259	6.27078333	pos
6.27_498.90	498.901073	6.27078333	pos
6.27_677.49	677.493772	6.27078333	pos
6.29_1058.2	1058.20141	6.28911667	pos
6.29_118.04	118.040917	6.29061667	neg
6.29_248.16	248.167645	6.28911667	pos
6.29_365.24	365.240702	6.28911667	pos
6.29_456.99	456.997806	6.28911667	pos
6.29_536.36	536.366823	6.28911667	pos
6.29_558.37	558.376186	6.29061667	neg
6.29_599.68	599.680782	6.28911667	pos
6.31_230.09	230.095713	6.3075	pos
6.31_232.10	232.099974	6.3075	pos
6.31_243.00	243.005812	6.30893333	neg
6.31_337.02	337.029259	6.30893333	neg
6.31_343.18	343.187728	6.3075	pos
6.31_456.74	456.747037	6.3075	pos
6.31_578.34	601.332931	6.3075	pos
6.33_182.98	182.987604	6.32741667	neg
6.33_221.00	221.00393	6.32741667	neg
6.33_385.22	385.223018	6.32741667	neg
6.33_386.23	409.219231	6.32576667	pos
6.33_405.66	405.667221	6.32576667	pos

6.33_414.94(	414.94615	6.32741667	neg
6.34_207.13!	207.135178	6.34418333	pos
6.34_213.10!	213.109513	6.34418333	pos
6.34_298.14(	298.146789	6.34418333	pos
6.34_316.14:	317.149234	6.34418333	pos
6.34_466.51·	466.514713	6.34418333	pos
6.34_467.75:	467.75336	6.34418333	pos
6.35_213.11:	213.113009	6.34593333	neg
6.35_276.95!	276.955161	6.34593333	neg
6.35_289.01:	289.011914	6.34593333	neg
6.35_297.12·	297.1245	6.34593333	neg
6.35_345.01!	345.018082	6.34593333	neg
6.35_360.97·	360.97425	6.34593333	neg
6.35_648.85:	648.853492	6.34593333	neg
6.36_222.99!	222.999604	6.36436667	neg
6.36_335.03:	335.033321	6.36436667	neg
6.36_385.15:	385.152871	6.36253333	pos
6.38_173.08(	173.080239	6.3809	pos
6.38_216.92:	216.922614	6.3809	pos
6.38_243.15	243.157257	6.3809	pos
6.38_253.01(	253.010292	6.38276667	neg
6.38_268.03!	268.038069	6.38276667	neg
6.38_270.10!	270.105618	6.3809	pos
6.38_672.85!	672.859129	6.38276667	neg
6.40_197.18!	197.189859	6.39921667	pos
6.40_205.00!	205.008908	6.40135	neg
6.40_243.15!	243.159909	6.40135	neg
6.40_274.16·	274.164612	6.39921667	pos
6.42_169.08(	169.086779	6.41976667	neg
6.42_209.00:	209.00383	6.41976667	neg
6.42_215.18!	238.1775	6.41748333	pos
6.42_229.91!	229.915852	6.41976667	neg
6.42_285.66:	285.663589	6.41748333	pos

6.42_326.988	326.988545	6.41976667	neg
6.42_369.228	369.228268	6.41976667	neg
6.42_393.224	393.224329	6.41748333	pos
6.42_437.215	437.21571	6.41976667	neg
6.42_505.203	505.203411	6.41976667	neg
6.42_573.190	573.190651	6.41976667	neg
6.42_677.493	677.493571	6.41748333	pos
6.44_179.107	179.107532	6.4382	neg
6.44_227.089	227.089821	6.4382	neg
6.44_269.139	269.139346	6.4382	neg
6.44_284.999	284.999735	6.4382	neg
6.44_285.167	285.167119	6.43578333	pos
6.44_313.202	313.202241	6.4382	neg
6.44_490.017	490.017401	6.43578333	pos
6.45_256.214	279.20358	6.45406667	pos
6.46_262.992	262.992654	6.45656667	neg
6.47_324.240	347.2301	6.47233333	pos
6.47_326.247	349.236369	6.47233333	pos
6.47_501.023	501.023737	6.47233333	pos
6.48_252.073	252.0732	6.47505	neg
6.48_324.240	369.239087	6.47505	neg
6.48_386.229	386.22946	6.47505	neg
6.49_151.039	151.039856	6.49351667	neg
6.49_194.066	194.06666	6.49025	pos
6.49_294.010	339.008365	6.49351667	neg
6.49_366.261	389.250507	6.49025	pos
6.49_417.245	417.245289	6.49025	pos
6.49_850.819	850.819479	6.49351667	neg
6.49_996.823	996.82359	6.49351667	neg
6.51_118.040	118.040911	6.51206667	neg
6.51_176.997	176.997744	6.51206667	neg
6.51_247.000	247.000814	6.51206667	neg
6.51_360.974	360.974243	6.51206667	neg

6.51_498.90	498.90098	6.50861667	pos
6.51_558.37	558.376114	6.51206667	neg
6.53_102.03	102.034165	6.52693333	pos
6.53_128.95	128.950783	6.52693333	pos
6.53_173.08	173.080553	6.52693333	pos
6.53_211.16	211.169009	6.52693333	pos
6.53_228.99	228.990177	6.53055	neg
6.53_295.06	296.068021	6.52693333	pos
6.53_362.04	362.041417	6.53055	neg
6.53_365.02	365.023965	6.53055	neg
6.53_378.13	378.138513	6.52693333	pos
6.53_536.36	536.366563	6.52693333	pos
6.53_877.83	877.837585	6.53055	neg
6.55_213.15	213.159462	6.54505	pos
6.55_240.99	240.990569	6.54903333	neg
6.55_261.01	261.016225	6.54903333	neg
6.55_295.15	295.151257	6.54505	pos
6.55_758.30	758.301638	6.54505	pos
6.56_414.33	414.336034	6.56313333	pos
6.57_271.15	271.155029	6.56738333	neg
6.57_331.02	331.021937	6.56738333	neg
6.57_355.01	355.019797	6.56738333	neg
6.58_246.11	246.115445	6.58	pos
6.58_710.60	710.609138	6.58	pos
6.59_112.98	112.982149	6.58565	neg
6.59_187.06	187.060879	6.58565	neg
6.59_229.91	229.915717	6.58565	neg
6.59_297.90	297.903027	6.58565	neg
6.59_336.99	336.99298	6.58565	neg
6.59_385.03	385.030054	6.58565	neg
6.59_385.44	385.445637	6.58565	neg
6.59_445.15	445.154722	6.58565	neg
6.59_453.18	453.187009	6.58565	neg

6.59_470.177	470.176992	6.58565	neg
6.59_538.164	538.164814	6.58565	neg
6.59_565.163	565.163738	6.58565	neg
6.59_579.133	579.133151	6.58565	neg
6.59_606.152	606.152579	6.58565	neg
6.59_657.149	657.149496	6.58565	neg
6.59_674.139	674.13979	6.58565	neg
6.59_715.108	715.108307	6.58565	neg
6.59_725.137	725.137277	6.58565	neg
6.59_742.127	742.127065	6.58565	neg
6.59_783.095	783.095037	6.58565	neg
6.59_793.124	793.124232	6.58565	neg
6.59_793.388	793.388043	6.58565	neg
6.59_810.114	810.114578	6.58565	neg
6.59_861.111	861.111738	6.58565	neg
6.59_861.375	861.3754	6.58565	neg
6.59_878.101	878.101596	6.58565	neg
6.59_929.099	929.099114	6.58565	neg
6.60_213.585	213.585622	6.59813333	pos
6.60_341.004	341.004025	6.60338333	neg
6.60_386.229	409.218591	6.59813333	pos
6.60_412.203	412.203386	6.59813333	pos
6.60_432.271	432.2715	6.59813333	pos
6.60_441.133	441.133009	6.59813333	pos
6.60_464.154	447.151445	6.59813333	pos
6.60_635.139	635.13946	6.59813333	pos
6.60_710.362	710.362365	6.59813333	pos
6.60_839.365	839.365653	6.59813333	pos
6.62_200.047	200.047179	6.61625	pos
6.62_223.019	223.019526	6.62148333	neg
6.62_241.010	241.010357	6.62148333	neg
6.62_298.200	298.200785	6.61625	pos
6.62_316.141	299.138517	6.61625	pos

6.62_316.14[illegible]	297.124426	6.62148333	neg
6.62_324.99[illegible]	324.992546	6.62148333	neg
6.62_489.13[illegible]	489.135909	6.61625	pos
6.62_672.85[illegible]	672.859288	6.62148333	neg
6.62_710.85[illegible]	710.859259	6.61625	pos
6.63_710.50[illegible]	710.506141	6.63455	pos
6.64_200.12[illegible]	200.128944	6.63946667	neg
6.64_224.99[illegible]	224.999224	6.63946667	neg
6.64_237.11[illegible]	237.110637	6.63946667	neg
6.64_381.01[illegible]	381.018934	6.63946667	neg
6.65_312.21[illegible]	312.216558	6.65295	pos
6.66_149.02[illegible]	149.024136	6.65806667	neg
6.66_300.03[illegible]	299.026059	6.65806667	neg
6.66_319.03[illegible]	319.038372	6.65806667	neg
6.66_353.02[illegible]	353.024033	6.65806667	neg
6.66_740.84[illegible]	740.846713	6.65806667	neg
6.67_257.65[illegible]	257.654598	6.67121667	pos
6.67_314.12[illegible]	315.133411	6.67121667	pos
6.67_325.24[illegible]	325.248083	6.67121667	pos
6.67_350.68[illegible]	350.686615	6.67121667	pos
6.67_677.49[illegible]	677.493762	6.67121667	pos
6.68_208.06[illegible]	208.061276	6.6766	neg
6.68_313.11[illegible]	313.119461	6.6766	neg
6.68_335.10[illegible]	335.101226	6.6766	neg
6.68_403.08[illegible]	403.088264	6.6766	neg
6.69_193.11[illegible]	193.119476	6.68961667	pos
6.69_213.10[illegible]	213.109502	6.68961667	pos
6.69_225.06[illegible]	225.061405	6.69498333	neg
6.69_227.16[illegible]	227.163858	6.68961667	pos
6.69_255.02[illegible]	255.025984	6.69498333	neg
6.69_257.02[illegible]	257.022622	6.69498333	neg
6.69_298.94[illegible]	298.942859	6.69498333	neg
6.69_376.21[illegible]	376.216287	6.69498333	neg

6.69_430.91	430.913357	6.68961667	pos
6.69_477.30	477.303116	6.68961667	pos
6.69_654.86	654.869494	6.69498333	neg
6.71_194.04	194.048089	6.70808333	pos
6.71_263.01	263.012793	6.71335	neg
6.71_280.01	279.006995	6.71335	neg
6.71_367.00	367.003424	6.71335	neg
6.71_426.94	426.940043	6.71335	neg
6.73_156.94	156.948334	6.7265	pos
6.73_156.98	156.987334	6.73181667	neg
6.73_179.97	358.952517	6.73181667	neg
6.73_239.08	239.089824	6.73181667	neg
6.73_494.92	494.927702	6.73181667	neg
6.73_498.90	498.901121	6.7265	pos
6.73_562.91	562.914768	6.73181667	neg
6.73_660.77	660.772151	6.7265	pos
6.74_267.15	267.156359	6.7449	pos
6.74_429.08	429.087951	6.7449	pos
6.74_593.30	593.308934	6.7449	pos
6.74_710.50	710.506225	6.7449	pos
6.75_356.99	356.999502	6.75028333	neg
6.75_360.97	360.97435	6.75028333	neg
6.75_368.99	368.999174	6.75028333	neg
6.75_421.22	421.220908	6.75028333	neg
6.75_809.85	809.850633	6.75028333	neg
6.76_175.14	175.147993	6.76326667	pos
6.76_377.22	377.229306	6.76326667	pos
6.77_159.10	159.102408	6.76876667	neg
6.77_372.02	371.014534	6.76876667	neg
6.77_383.01	383.014914	6.76876667	neg
6.77_663.84	663.846751	6.76876667	neg
6.78_128.95	128.950825	6.7816	pos
6.79_369.22	369.228186	6.7873	neg

6.79_437.21!	437.21572	6.7873	neg
6.79_505.20:	505.203471	6.7873	neg
6.79_571.12(	571.120697	6.7873	neg
6.79_639.10{	639.108035	6.7873	neg
6.80_229.17!	229.179517	6.79993333	pos
6.80_295.15:	295.151325	6.79993333	pos
6.80_363.17	363.177618	6.79993333	pos
6.80_411.13	411.134718	6.79993333	pos
6.80_454.16:	454.163183	6.79993333	pos
6.81_171.98(	171.980811	6.80563333	neg
6.81_241.14	241.144446	6.80563333	neg
6.81_276.95!	276.95515	6.80563333	neg
6.81_301.01:	301.011794	6.80563333	neg
6.81_319.03{	319.038451	6.80563333	neg
6.81_340.18{	385.187017	6.80563333	neg
6.81_375.15	375.157805	6.80563333	neg
6.81_399.01(	399.009981	6.80563333	neg
6.81_402.17(	402.176633	6.80563333	neg
6.81_503.13:	503.133447	6.80563333	neg
6.82_170.11	170.117412	6.81833333	pos
6.82_223.09(	223.095982	6.81833333	pos
6.82_223.09	223.09744	6.824	neg
6.82_272.08:	295.070254	6.81833333	pos
6.82_336.99:	336.993091	6.824	neg
6.82_646.48:	646.48291	6.81833333	pos
6.82_733.08	733.084004	6.81833333	pos
6.82_785.41(	785.410204	6.81833333	pos
6.82_785.74	785.744505	6.81833333	pos
6.84_155.14:	155.142884	6.83671667	pos
6.84_289.01:	289.01106	6.8424	neg
6.84_317.00!	317.005916	6.8424	neg
6.84_430.22(	430.226819	6.8424	neg
6.84_554.89(	554.890535	6.8424	neg

6.84_786.07	786.078543	6.83671667	pos
6.84_826.85	826.853778	6.8424	neg
6.86_241.17	241.179511	6.85513333	pos
6.86_241.99	241.996142	6.86086667	neg
6.86_244.13	244.136259	6.85513333	pos
6.86_244.88	244.887926	6.86086667	neg
6.86_283.00	283.000767	6.86086667	neg
6.87_142.93	142.938609	6.8735	pos
6.87_212.01	212.019654	6.8735	pos
6.87_243.94	243.94138	6.8735	pos
6.88_173.11	173.118028	6.8794	neg
6.88_241.10	241.105573	6.8794	neg
6.88_326.98	326.988623	6.8794	neg
6.88_758.86	758.867036	6.8794	neg
6.89_128.95	128.950794	6.89188333	pos
6.89_265.17	265.177256	6.89188333	pos
6.89_301.19	301.198085	6.89188333	pos
6.89_372.27	372.273968	6.89188333	pos
6.89_524.24	524.242622	6.89188333	pos
6.89_677.78	677.780201	6.89188333	pos
6.89_862.80	862.802109	6.89188333	pos
6.90_149.02	149.02416	6.8979	neg
6.90_280.62	280.621845	6.8979	neg
6.90_281.12	281.123529	6.8979	neg
6.90_346.95	346.958387	6.8979	neg
6.90_368.02	413.025221	6.8979	neg
6.90_421.22	421.220883	6.8979	neg
6.91_195.12	195.122552	6.9103	pos
6.91_593.05	593.058349	6.9103	pos
6.91_776.75	776.755399	6.9103	pos
6.91_777.42	777.423746	6.9103	pos
6.91_863.13	863.13614	6.9103	pos
6.92_150.00	150.001655	6.91643333	neg

6.92_207.91	207.91835	6.91643333	neg
6.92_229.91	229.915806	6.91643333	neg
6.92_297.90	297.903319	6.91643333	neg
6.92_355.24	355.248784	6.91643333	neg
6.92_356.99	356.999259	6.91643333	neg
6.93_118.04	118.04091	6.93491667	neg
6.93_202.05	202.050735	6.93491667	neg
6.93_215.15	216.159288	6.92858333	pos
6.93_217.08	217.080906	6.92858333	pos
6.93_379.24	379.244983	6.92858333	pos
6.93_399.01	399.010716	6.93491667	neg
6.93_677.35	677.350488	6.92858333	pos
6.93_753.07	753.076286	6.92858333	pos
6.93_753.41	753.410838	6.92858333	pos
6.93_777.08	777.089737	6.92858333	pos
6.93_993.50	993.503894	6.92858333	pos
6.95_242.17	242.176131	6.95326667	neg
6.95_268.17	268.177663	6.94688333	pos
6.95_273.01	273.016632	6.95326667	neg
6.95_288.15	288.154152	6.94688333	pos
6.95_327.15	327.15325	6.95326667	neg
6.95_376.21	376.216433	6.95326667	neg
6.95_378.15	378.150738	6.95326667	neg
6.95_398.26	421.255503	6.94688333	pos
6.95_514.12	514.12558	6.95326667	neg
6.95_592.93	592.933209	6.94688333	pos
6.95_790.07	790.074132	6.94688333	pos
6.97_198.18	198.185097	6.96501667	pos
6.97_207.17	207.174135	6.96501667	pos
6.97_313.11	313.119394	6.97153333	neg
6.97_314.12	315.133441	6.96501667	pos
6.97_324.99	324.992517	6.97153333	neg
6.97_336.10	335.101229	6.97153333	neg

6.97_373.01	373.010418	6.97153333	neg
6.97_403.08	403.088146	6.97153333	neg
6.97_437.19	437.19799	6.97153333	neg
6.97_454.18	454.188284	6.97153333	neg
6.97_471.07	471.076068	6.97153333	neg
6.98_197.18	197.189822	6.98325	pos
6.98_217.57	217.57771	6.98325	pos
6.98_226.58	226.582927	6.98325	pos
6.98_238.59	238.592659	6.98325	pos
6.98_249.10	249.109229	6.98325	pos
6.98_354.16	377.156674	6.98325	pos
6.98_378.15	379.162022	6.98325	pos
6.98_467.12	467.126067	6.98325	pos
6.98_535.11	535.113686	6.98325	pos
6.98_569.09	569.093791	6.98325	pos
6.98_660.77	660.772045	6.98325	pos
6.98_710.21	710.219943	6.98325	pos
6.98_739.27	739.274761	6.98325	pos
6.98_752.29	775.28774	6.98325	pos
6.99_112.98	112.982156	6.98976667	neg
6.99_421.14	421.148026	6.98976667	neg
6.99_424.15	424.156387	6.98976667	neg
6.99_506.12	506.125232	6.98976667	neg
6.99_563.07	563.079069	6.98976667	neg
6.99_574.11	574.113057	6.98976667	neg
6.99_615.08	615.081891	6.98976667	neg
6.99_625.11	625.110351	6.98976667	neg
6.99_642.10	642.100825	6.98976667	neg
6.99_683.06	683.06875	6.98976667	neg
6.99_693.09	693.097754	6.98976667	neg
6.99_710.08	710.088004	6.98976667	neg
6.99_729.31	729.310547	6.98976667	neg
6.99_751.05	751.056189	6.98976667	neg

6.99_778.07	778.075766	6.98976667	neg
6.99_865.28	865.285595	6.98976667	neg
6.99_897.06	897.060622	6.98976667	neg
7.00_457.09	457.097223	7.00153333	pos
7.00_459.09	459.094078	7.00153333	pos
7.01_130.05	130.05071	7.00806667	neg
7.01_209.08	209.081739	7.00806667	neg
7.01_379.04	379.042015	7.00806667	neg
7.02_274.18	274.183134	7.01995	pos
7.02_340.19	340.193693	7.01995	pos
7.02_646.48	646.483272	7.01995	pos
7.02_719.87	719.876872	7.01995	pos
7.03_397.03	397.030098	7.02585	neg
7.04_141.06	141.065769	7.03826667	pos
7.04_207.10	207.1024	7.04441667	neg
7.04_241.17	241.179567	7.03826667	pos
7.04_242.18	265.177193	7.03826667	pos
7.04_248.90	248.902298	7.04441667	neg
7.04_262.13	245.128246	7.03826667	pos
7.04_335.03	335.0332	7.04441667	neg
7.04_391.20	391.208861	7.03826667	pos
7.04_505.14	505.149026	7.04441667	neg
7.04_565.79	565.798529	7.03826667	pos
7.04_593.18	593.182446	7.03826667	pos
7.06_213.10	213.109522	7.05653333	pos
7.06_244.90	244.908083	7.06308333	neg
7.06_246.90	246.905199	7.06308333	neg
7.06_259.00	259.0008	7.06308333	neg
7.06_271.00	271.000914	7.06308333	neg
7.06_446.90	446.905844	7.06308333	neg
7.06_554.89	554.890619	7.06308333	neg
7.06_826.85	826.854271	7.06308333	neg
7.08_142.93	142.938598	7.07501667	pos

7.08_171.98(	171.980814	7.0814	neg
7.08_279.00	279.007092	7.0814	neg
7.08_319.19	319.198713	7.07501667	pos
7.08_360.97	360.974298	7.0814	neg
7.08_677.49	677.493777	7.07501667	pos
7.08_677.63	677.636549	7.07501667	pos
7.08_710.07	710.076748	7.07501667	pos
7.08_758.86	758.867163	7.0814	neg
7.09_197.15	197.153448	7.09336667	pos
7.09_246.11	246.115498	7.09336667	pos
7.10_244.88	244.887895	7.0999	neg
7.10_265.01	265.01016	7.0999	neg
7.10_276.99	276.991548	7.0999	neg
7.10_381.01	381.019055	7.0999	neg
7.11_286.18	309.178081	7.1117	pos
7.11_677.35	677.350239	7.1117	pos
7.12_205.00	205.008815	7.11838333	neg
7.12_222.99	222.999653	7.11838333	neg
7.12_224.99	224.999164	7.11838333	neg
7.12_260.99	260.996605	7.11838333	neg
7.12_261.01	261.016264	7.11838333	neg
7.12_469.07	469.07517	7.11838333	neg
7.13_498.90	498.901084	7.13003333	pos
7.13_660.77	660.772215	7.13003333	pos
7.13_754.39	754.3962	7.13003333	pos
7.14_188.95	188.951327	7.1368	neg
7.14_190.94	190.94837	7.1368	neg
7.14_255.02	255.02598	7.1368	neg
7.14_256.93	256.938963	7.1368	neg
7.14_258.93	258.935887	7.1368	neg
7.14_403.02	403.020856	7.1368	neg
7.14_413.02	413.025678	7.1368	neg
7.15_163.07	163.075191	7.14843333	pos

7.15_231.18[illegible]	254.172369	7.14843333	pos
7.15_274.18[illegible]	274.183048	7.14843333	pos
7.15_709.64[illegible]	709.647388	7.14843333	pos
7.15_710.36[illegible]	710.363051	7.14843333	pos
7.16_387.02[illegible]	387.026217	7.15531667	neg
7.17_255.12[illegible]	255.121241	7.17388333	neg
7.17_327.06[illegible]	327.064816	7.17388333	neg
7.17_375.21[illegible]	375.211427	7.16671667	pos
7.17_397.22[illegible]	397.22026	7.17388333	neg
7.17_414.21[illegible]	414.210993	7.17388333	neg
7.17_601.18[illegible]	601.183772	7.17388333	neg
7.17_646.62[illegible]	646.626245	7.16671667	pos
7.17_669.17[illegible]	669.171334	7.17388333	neg
7.17_710.50[illegible]	710.50669	7.16671667	pos
7.17_790.24[illegible]	790.241236	7.16671667	pos
7.19_103.92[illegible]	103.920064	7.19245	neg
7.19_187.13[illegible]	187.133699	7.19245	neg
7.19_227.16[illegible]	227.163884	7.18508333	pos
7.19_358.99[illegible]	358.994949	7.19245	neg
7.19_378.29[illegible]	401.286857	7.18508333	pos
7.19_389.00[illegible]	389.005841	7.19245	neg
7.19_389.20[illegible]	389.209818	7.19245	neg
7.19_392.27[illegible]	393.284332	7.18508333	pos
7.19_411.19[illegible]	411.191762	7.19245	neg
7.19_421.22[illegible]	421.220912	7.19245	neg
7.19_466.19[illegible]	466.196942	7.18508333	pos
7.19_506.19[illegible]	506.197803	7.19245	neg
7.19_557.19[illegible]	557.195437	7.19245	neg
7.19_709.93[illegible]	709.933664	7.18508333	pos
7.20_1030.1[illegible]	1030.16863	7.20335	pos
7.20_1030.5[illegible]	1030.50321	7.20335	pos
7.20_200.04[illegible]	200.047239	7.20335	pos
7.20_281.17[illegible]	281.17215	7.20335	pos

7.20_372.274	372.274194	7.20335	pos
7.20_660.628	660.628887	7.20335	pos
7.21_246.981	246.980976	7.21093333	neg
7.21_247.058	247.058717	7.21093333	neg
7.21_253.010	253.010216	7.21093333	neg
7.21_264.048	264.048858	7.21093333	neg
7.21_315.027	315.027502	7.21093333	neg
7.21_323.108	323.108635	7.21093333	neg
7.21_367.003	367.003264	7.21093333	neg
7.22_1030.83	1030.83757	7.2217	pos
7.22_1031.17	1031.17173	7.2217	pos
7.22_1037.82	1037.82957	7.2217	pos
7.22_1038.16	1038.1643	7.2217	pos
7.22_1038.49	1038.49777	7.2217	pos
7.22_259.205	259.205204	7.2217	pos
7.22_295.151	295.151228	7.2217	pos
7.23_218.988	218.988348	7.22931667	neg
7.23_238.994	238.994735	7.22931667	neg
7.23_273.016	273.016687	7.22931667	neg
7.23_292.038	292.038146	7.22931667	neg
7.24_173.080	173.080396	7.24003333	pos
7.24_292.634	292.634671	7.24003333	pos
7.24_293.136	293.136175	7.24003333	pos
7.24_356.255	379.245101	7.24003333	pos
7.24_593.308	593.308854	7.24003333	pos
7.25_367.019	367.019808	7.24778333	neg
7.25_413.302	413.302028	7.24778333	neg
7.25_740.847	740.846993	7.24778333	neg
7.26_190.150	213.145996	7.25836667	pos
7.26_299.679	299.679441	7.25836667	pos
7.26_318.146	318.146023	7.25836667	pos
7.26_709.790	709.790552	7.25836667	pos
7.27_187.060	187.060921	7.26623333	neg

7.27_229.14	229.144243	7.26623333	neg
7.27_301.01	301.011764	7.26623333	neg
7.27_321.01	321.017657	7.26623333	neg
7.27_399.01	399.010558	7.26623333	neg
7.27_443.15	443.155936	7.26623333	neg
7.27_583.25	583.256359	7.26623333	neg
7.27_624.33	624.339085	7.26623333	neg
7.28_207.13	207.135116	7.2767	pos
7.28_241.09	241.097374	7.28471667	neg
7.28_262.08	307.079869	7.28471667	neg
7.28_276.95	276.955184	7.28471667	neg
7.28_329.06	329.061827	7.28471667	neg
7.28_385.01	385.011026	7.28471667	neg
7.28_397.04	397.049004	7.28471667	neg
7.28_465.03	465.036459	7.28471667	neg
7.28_482.02	482.027359	7.28471667	neg
7.28_528.26	528.263433	7.28471667	neg
7.28_533.02	533.024573	7.28471667	neg
7.28_601.01	601.012162	7.28471667	neg
7.28_646.76	646.769756	7.2767	pos
7.30_201.07	201.07282	7.29506667	pos
7.30_255.00	255.005977	7.3031	neg
7.30_265.17	265.177265	7.29506667	pos
7.30_276.99	276.991472	7.3031	neg
7.30_284.06	285.070495	7.29506667	pos
7.30_430.20	453.199079	7.29506667	pos
7.30_677.49	677.493799	7.29506667	pos
7.30_710.50	710.506546	7.29506667	pos
7.31_227.16	227.163891	7.31328333	pos
7.32_259.00	259.000781	7.32175	neg
7.32_283.00	283.000836	7.32175	neg
7.32_307.00	307.002091	7.32175	neg
7.32_367.21	367.212316	7.32175	neg

7.32_401.04[illegible]	401.04183	7.32175	neg
7.32_554.89[illegible]	554.890551	7.32175	neg
7.33_323.18[illegible]	323.182503	7.3317	pos
7.33_498.90[illegible]	498.901	7.3317	pos
7.34_287.03[illegible]	287.032272	7.3402	neg
7.34_511.29[illegible]	511.29101	7.3402	neg
7.35_128.95[illegible]	128.950802	7.35016667	pos
7.35_142.93[illegible]	142.938573	7.35016667	pos
7.35_601.26[illegible]	601.264978	7.35016667	pos
7.35_660.77[illegible]	660.772049	7.35016667	pos
7.36_317.00[illegible]	317.005478	7.3586	neg
7.36_369.17[illegible]	369.174006	7.3586	neg
7.36_413.02[illegible]	413.025476	7.3586	neg
7.36_573.13[illegible]	573.13635	7.3586	neg
7.36_641.12[illegible]	641.123794	7.3586	neg
7.37_213.10[illegible]	213.109473	7.36856667	pos
7.37_255.23[illegible]	255.231447	7.36856667	pos
7.37_646.62[illegible]	646.626813	7.36856667	pos
7.38_140.98[illegible]	140.980424	7.37703333	neg
7.38_205.08[illegible]	205.086734	7.37703333	neg
7.38_255.63[illegible]	510.252825	7.37703333	neg
7.38_300.99[illegible]	300.991672	7.37703333	neg
7.38_319.03[illegible]	319.038553	7.37703333	neg
7.38_324.99[illegible]	324.992658	7.37703333	neg
7.38_405.01[illegible]	405.016648	7.37703333	neg
7.38_505.14[illegible]	505.149239	7.37703333	neg
7.39_156.94[illegible]	156.94836	7.38693333	pos
7.40_225.06[illegible]	225.06144	7.39546667	neg
7.40_285.12[illegible]	285.126013	7.39546667	neg
7.40_288.61[illegible]	288.616527	7.39546667	neg
7.40_391.00[illegible]	391.00133	7.39546667	neg
7.40_397.03[illegible]	397.030055	7.39546667	neg
7.40_397.25[illegible]	397.259034	7.39546667	neg

7.40_443.15!	443.155912	7.39546667	neg
7.41_193.08:	193.083131	7.4053	pos
7.41_260.99(	260.996492	7.41378333	neg
7.41_264.99:	264.99151	7.41378333	neg
7.41_269.13	269.137004	7.41378333	neg
7.41_327.13	327.134885	7.41378333	neg
7.41_346.95	346.95847	7.41378333	neg
7.41_353.22	353.224619	7.41378333	neg
7.41_373.24!	373.245693	7.4053	pos
7.41_381.01	381.018859	7.41378333	neg
7.41_401.00!	401.00548	7.41378333	neg
7.41_414.21:	414.213257	7.41378333	neg
7.41_419.20!	419.205108	7.41378333	neg
7.41_436.19!	436.195215	7.41378333	neg
7.41_504.18:	504.18323	7.41378333	neg
7.41_566.04!	566.049272	7.4053	pos
7.42_352.22:	335.22004	7.42368333	pos
7.42_359.26(	360.273978	7.42368333	pos
7.42_362.27!	362.279787	7.42368333	pos
7.42_677.49:	677.493705	7.42368333	pos
7.43_218.98	218.988393	7.43236667	neg
7.43_240.99(	240.990379	7.43236667	neg
7.43_255.00(	255.00604	7.43236667	neg
7.43_349.01:	349.012482	7.43236667	neg
7.43_413.30:	413.302175	7.43236667	neg
7.43_415.02:	415.021242	7.43236667	neg
7.44_272.08:	295.070177	7.44203333	pos
7.44_646.48:	646.482396	7.44203333	pos
7.45_309.17(	309.170791	7.451	neg
7.45_325.01:	325.012444	7.451	neg
7.45_335.01:	335.013717	7.451	neg
7.45_486.90:	486.903285	7.451	neg
7.45_540.24(	540.24035	7.451	neg

7.47_200.01	200.011842	7.46943333	neg
7.47_224.99	224.999266	7.46943333	neg
7.47_399.22	399.220982	7.46943333	neg
7.47_514.89	514.893362	7.46943333	neg
7.47_554.89	554.890742	7.46943333	neg
7.47_654.86	654.869479	7.46943333	neg
7.47_996.82	996.823739	7.46943333	neg
7.49_149.02	149.024143	7.48786667	neg
7.49_251.12	251.126786	7.48786667	neg
7.49_403.02	403.020833	7.48786667	neg
7.50_265.17	265.177171	7.49721667	pos
7.50_318.25	318.253922	7.49721667	pos
7.50_391.20	391.20856	7.49721667	pos
7.50_498.90	498.901017	7.49721667	pos
7.51_672.85	672.85962	7.50616667	neg
7.52_261.10	261.109367	7.5156	pos
7.52_273.01	273.016691	7.52448333	neg
7.53_190.15	213.145795	7.53386667	pos
7.53_319.08	319.08397	7.53386667	pos
7.53_646.62	646.626238	7.53386667	pos
7.53_710.50	710.506531	7.53386667	pos
7.54_202.99	202.993369	7.54296667	neg
7.54_229.91	229.915829	7.54296667	neg
7.54_360.97	360.974317	7.54296667	neg
7.54_399.02	399.025975	7.54296667	neg
7.54_850.81	850.819485	7.54296667	neg
7.55_173.08	173.080283	7.55221667	pos
7.56_197.02	197.020024	7.5615	neg
7.56_241.14	241.144461	7.5615	neg
7.57_502.29	502.292409	7.57063333	pos
7.58_118.04	118.040926	7.58008333	neg
7.58_356.99	356.999249	7.58008333	neg
7.58_758.86	758.867261	7.58008333	neg

7.59_430.91[illegible]	430.913326	7.58896667	pos
7.60_253.14[illegible]	253.144359	7.59848333	neg
7.60_311.01[illegible]	311.013476	7.59848333	neg
7.60_319.07[illegible]	319.077231	7.59848333	neg
7.60_373.14[illegible]	373.140406	7.59848333	neg
7.61_154.96[illegible]	154.966815	7.60728333	pos
7.61_216.92[illegible]	216.922587	7.60728333	pos
7.62_222.99[illegible]	222.999654	7.61696667	neg
7.62_263.93[illegible]	263.934903	7.61696667	neg
7.62_296.99[illegible]	296.997724	7.61696667	neg
7.62_412.31[illegible]	457.310929	7.61696667	neg
7.62_421.01[illegible]	421.012332	7.61696667	neg
7.62_479.29[illegible]	479.292591	7.61696667	neg
7.63_102.03[illegible]	102.034177	7.62578333	pos
7.63_200.18[illegible]	200.183606	7.62578333	pos
7.64_265.17[illegible]	265.177295	7.64415	pos
7.64_321.14[illegible]	321.147079	7.63533333	neg
7.64_660.91[illegible]	660.91523	7.64415	pos
7.65_238.99[illegible]	238.994739	7.65203333	neg
7.65_255.08[illegible]	255.084895	7.65203333	neg
7.65_349.01[illegible]	349.012476	7.65203333	neg
7.66_315.13[illegible]	315.133281	7.6626	pos
7.66_498.90[illegible]	498.901073	7.6626	pos
7.67_367.01[illegible]	367.019917	7.67051667	neg
7.67_409.27[illegible]	409.270962	7.67051667	neg
7.68_217.17[illegible]	217.179587	7.68096667	pos
7.68_387.26[illegible]	387.26132	7.68096667	pos
7.69_339.00[illegible]	339.008236	7.68903333	neg
7.69_389.00[illegible]	389.005795	7.68903333	neg
7.69_433.01[illegible]	433.011742	7.68903333	neg
7.69_465.24[illegible]	465.248988	7.68903333	neg
7.70_145.03[illegible]	145.035149	7.69936667	pos
7.70_255.21[illegible]	255.210268	7.69936667	pos

7.70_377.22[illegible]	377.229509	7.69936667	pos
7.70_415.68[illegible]	415.680269	7.69936667	pos
7.70_416.18[illegible]	416.182073	7.69936667	pos
7.71_267.11[illegible]	267.114019	7.70598333	neg
7.71_300.99[illegible]	300.991641	7.70598333	neg
7.71_486.90[illegible]	486.903283	7.70598333	neg
7.71_809.85[illegible]	809.850703	7.70598333	neg
7.72_195.91[illegible]	195.912647	7.72445	neg
7.72_255.00[illegible]	255.00598	7.72445	neg
7.72_343.02[illegible]	343.020171	7.72445	neg
7.72_360.97[illegible]	360.974459	7.72445	neg
7.72_417.01[illegible]	417.016903	7.72445	neg
7.72_762.34[illegible]	762.346609	7.72445	neg
7.74_265.11[illegible]	265.119772	7.7361	pos
7.74_276.19[illegible]	276.198969	7.7361	pos
7.74_400.28[illegible]	400.285636	7.7361	pos
7.74_554.89[illegible]	554.890653	7.7395	neg
7.75_279.19[illegible]	279.192255	7.7545	pos
7.77_158.96[illegible]	158.961173	7.77291667	pos
7.77_181.98[illegible]	181.988117	7.77291667	pos
7.77_187.06[illegible]	187.060929	7.7731	neg
7.77_265.17[illegible]	265.177249	7.77291667	pos
7.77_355.24[illegible]	355.248949	7.7731	neg
7.77_356.25[illegible]	379.245046	7.77291667	pos
7.77_423.23[illegible]	423.236733	7.7731	neg
7.77_502.29[illegible]	502.292471	7.77291667	pos
7.77_710.50[illegible]	710.506362	7.77291667	pos
7.77_767.36[illegible]	767.361338	7.7731	neg
7.79_158.09[illegible]	158.096343	7.7913	pos
7.79_269.21[illegible]	269.210738	7.7913	pos
7.79_278.98[illegible]	278.98721	7.79163333	neg
7.79_323.18[illegible]	323.182559	7.7913	pos
7.81_421.01[illegible]	421.01211	7.81013333	neg

7.81_996.82	996.823756	7.81013333	neg
7.83_283.15	283.152741	7.82873333	neg
7.83_305.17	305.171854	7.82796667	pos
7.83_324.99	324.992805	7.82873333	neg
7.83_343.01	343.019743	7.82873333	neg
7.83_410.29	409.289781	7.82873333	neg
7.83_660.77	660.772026	7.82796667	pos
7.85_297.12	297.124679	7.84718333	neg
7.85_303.00	303.007259	7.84718333	neg
7.85_319.03	319.038397	7.84718333	neg
7.85_710.36	710.363085	7.8463	pos
7.86_252.10	252.105047	7.86466667	pos
7.86_255.23	255.231519	7.86466667	pos
7.86_267.09	267.096424	7.86466667	pos
7.87_200.01	200.011818	7.86566667	neg
7.87_256.98	256.985367	7.86566667	neg
7.87_259.00	259.000795	7.86566667	neg
7.87_347.06	347.063782	7.86566667	neg
7.87_399.02	399.026691	7.86566667	neg
7.87_425.08	425.080669	7.86566667	neg
7.87_493.06	493.067349	7.86566667	neg
7.87_561.05	561.055913	7.86566667	neg
7.87_629.04	629.044237	7.86566667	neg
7.88_225.06	225.061446	7.88408333	neg
7.88_281.17	281.171967	7.88303333	pos
7.90_207.10	207.102424	7.90265	neg
7.90_245.17	245.174463	7.90143333	pos
7.90_287.01	287.012267	7.90265	neg
7.90_300.19	300.198772	7.90143333	pos
7.90_372.27	372.273967	7.90143333	pos
7.90_411.06	411.064842	7.90265	neg
7.90_421.22	421.220985	7.90265	neg
7.90_429.03	429.036078	7.90265	neg

7.90_433.04[illegible]	433.047082	7.90265	neg
7.90_489.20[illegible]	489.208414	7.90265	neg
7.92_102.03[illegible]	102.03421	7.91976667	pos
7.92_195.10[illegible]	195.101044	7.91976667	pos
7.92_224.99[illegible]	224.999269	7.9211	neg
7.92_502.29[illegible]	502.292468	7.91976667	pos
7.94_187.13[illegible]	187.13372	7.93951667	neg
7.94_229.91[illegible]	229.915834	7.93951667	neg
7.94_233.11[illegible]	233.112164	7.93806667	pos
7.94_268.16[illegible]	268.159953	7.93806667	pos
7.94_291.00[illegible]	291.0073	7.93951667	neg
7.94_319.00[illegible]	319.002227	7.93951667	neg
7.96_349.01[illegible]	349.012348	7.95811667	neg
7.96_409.04[illegible]	409.04922	7.95811667	neg
7.96_477.03[illegible]	477.036653	7.95811667	neg
7.97_710.36[illegible]	710.363007	7.97481667	pos
7.98_118.04[illegible]	118.040916	7.97648333	neg
7.98_229.00[illegible]	229.009032	7.97648333	neg
7.98_248.07[illegible]	248.079939	7.97648333	neg
7.98_300.99[illegible]	300.99164	7.97648333	neg
7.98_374.98[illegible]	374.98939	7.97648333	neg
7.99_173.07[illegible]	173.079207	7.99311667	pos
7.99_197.18[illegible]	197.189839	7.99311667	pos
7.99_214.99[illegible]	214.993449	7.99473333	neg
7.99_385.01[illegible]	385.01102	7.99473333	neg
7.99_416.18[illegible]	416.182053	7.99311667	pos
7.99_437.02[illegible]	437.023019	7.99473333	neg
7.99_461.04[illegible]	461.042492	7.99473333	neg
8.01_199.18[illegible]	199.18008	8.01143333	pos
8.01_227.16[illegible]	227.163915	8.01143333	pos
8.01_240.99[illegible]	240.990424	8.01323333	neg
8.01_301.01[illegible]	301.011799	8.01323333	neg
8.01_367.01[illegible]	367.019651	8.01323333	neg

8.01_389.00	389.005737	8.01323333	neg
8.01_415.68	415.68025	8.01143333	pos
8.01_451.00	451.003407	8.01323333	neg
8.03_252.18	252.18362	8.0298	pos
8.03_267.11	267.113961	8.03168333	neg
8.03_421.01	421.01217	8.03168333	neg
8.05_369.17	369.17406	8.05003333	neg
8.05_418.99	418.995888	8.05003333	neg
8.07_137.09	137.09601	8.06628333	pos
8.07_145.08	145.086802	8.06858333	neg
8.07_227.18	250.177445	8.06628333	pos
8.07_269.02	269.021582	8.06858333	neg
8.07_273.25	273.25321	8.06628333	pos
8.07_275.01	275.012263	8.06858333	neg
8.07_290.19	290.197349	8.06858333	neg
8.09_261.07	261.074362	8.08708333	neg
8.09_272.99	272.99659	8.08708333	neg
8.09_296.99	296.997674	8.08708333	neg
8.09_311.01	311.013303	8.08708333	neg
8.10_173.07	173.079357	8.10283333	pos
8.10_241.17	241.178963	8.10283333	pos
8.11_247.00	246.999953	8.10548333	neg
8.11_397.22	397.221066	8.10548333	neg
8.12_283.00	283.000877	8.12398333	neg
8.12_346.95	346.958399	8.12398333	neg
8.12_446.90	446.905912	8.12398333	neg
8.14_170.03	170.03658	8.13956667	pos
8.14_170.15	170.15378	8.13956667	pos
8.14_241.20	241.203222	8.13956667	pos
8.14_368.99	368.999141	8.14243333	neg
8.14_421.22	421.220982	8.14243333	neg
8.14_489.20	489.208279	8.14243333	neg
8.14_557.19	557.195589	8.14243333	neg

8.14_599.25[illegible]	599.251435	8.14243333	neg
8.14_600.25[illegible]	601.265183	8.13956667	pos
8.16_183.17[illegible]	183.174217	8.15795	pos
8.16_199.18[illegible]	199.180075	8.15795	pos
8.16_244.88[illegible]	244.887982	8.16083333	neg
8.16_374.28[illegible]	374.289641	8.15795	pos
8.16_379.24[illegible]	379.24491	8.15795	pos
8.18_102.03[illegible]	102.03413	8.17628333	pos
8.18_173.08[illegible]	173.081658	8.1793	neg
8.18_268.15[illegible]	268.159866	8.17628333	pos
8.18_323.18[illegible]	323.18258	8.17628333	pos
8.18_353.02[illegible]	353.023912	8.1793	neg
8.18_375.00[illegible]	375.009097	8.1793	neg
8.18_473.23[illegible]	473.238965	8.1793	neg
8.18_710.21[illegible]	710.219772	8.17628333	pos
8.19_179.14[illegible]	179.142888	8.19463333	pos
8.19_660.77[illegible]	660.772042	8.19463333	pos
8.20_173.11[illegible]	173.118043	8.1977	neg
8.20_269.13[illegible]	269.136968	8.1977	neg
8.20_337.02[illegible]	337.029267	8.1977	neg
8.20_465.01[illegible]	465.018047	8.1977	neg
8.22_733.36[illegible]	733.367526	8.21615	neg
8.23_223.98[illegible]	223.988053	8.23126667	pos
8.23_319.00[illegible]	319.002233	8.23463333	neg
8.23_343.01[illegible]	343.019491	8.23463333	neg
8.23_419.20[illegible]	419.205179	8.23463333	neg
8.23_487.19[illegible]	487.19263	8.23463333	neg
8.25_158.96[illegible]	158.961103	8.24955	pos
8.25_256.98[illegible]	256.985388	8.25326667	neg
8.25_258.98[illegible]	258.980884	8.25326667	neg
8.25_307.18[illegible]	307.187654	8.24955	pos
8.25_417.01[illegible]	417.01748	8.25326667	neg
8.25_581.23[illegible]	581.238843	8.24955	pos

8.25_710.36[illegible]	710.362823	8.24955	pos
8.27_142.93[illegible]	142.938592	8.26751667	pos
8.27_281.17[illegible]	281.1719	8.26751667	pos
8.27_349.01[illegible]	349.012292	8.27171667	neg
8.27_505.33[illegible]	505.334297	8.26751667	pos
8.29_267.15[illegible]	267.156235	8.2859	pos
8.29_386.17[illegible]	409.16158	8.2859	pos
8.30_183.13[illegible]	183.13779	8.3042	pos
8.30_223.09[illegible]	223.096065	8.3042	pos
8.30_259.12[illegible]	259.128106	8.3042	pos
8.30_265.17[illegible]	265.17733	8.3042	pos
8.30_269.21[illegible]	269.21068	8.3042	pos
8.30_478.19[illegible]	478.197063	8.3042	pos
8.31_256.63[illegible]	512.268572	8.3086	neg
8.31_283.15[illegible]	283.152675	8.3086	neg
8.31_440.99[illegible]	440.998438	8.3086	neg
8.31_534.25[illegible]	534.250817	8.3086	neg
8.32_241.21[illegible]	241.21581	8.32255	pos
8.32_374.28[illegible]	374.289446	8.32255	pos
8.32_710.21[illegible]	710.219871	8.32255	pos
8.33_368.99[illegible]	368.999112	8.32698333	neg
8.33_387.28[illegible]	387.286621	8.32698333	neg
8.33_431.01[illegible]	431.016016	8.32698333	neg
8.34_102.03[illegible]	102.034142	8.34093333	pos
8.35_255.16[illegible]	255.160101	8.34551667	neg
8.35_264.10[illegible]	264.106412	8.34551667	neg
8.35_265.14[illegible]	265.143265	8.34551667	neg
8.35_272.99[illegible]	272.996617	8.34551667	neg
8.35_333.01[illegible]	333.017825	8.34551667	neg
8.35_399.00[illegible]	399.00963	8.34551667	neg
8.36_215.16[illegible]	215.164982	8.36401667	neg
8.36_266.12[illegible]	266.120662	8.35931667	pos
8.36_317.29[illegible]	318.299919	8.35931667	pos

8.36_430.28[illegible]	430.280927	8.36401667	neg
8.36_455.01[illegible]	455.014143	8.36401667	neg
8.36_473.03[illegible]	473.039569	8.35931667	pos
8.36_483.00[illegible]	483.009025	8.36401667	neg
8.38_241.20[illegible]	241.203373	8.37785	pos
8.38_295.18[illegible]	295.187683	8.37785	pos
8.38_877.83[illegible]	877.83745	8.38236667	neg
8.40_156.94[illegible]	156.948372	8.39623333	pos
8.40_227.16[illegible]	227.16382	8.39623333	pos
8.40_241.17[illegible]	241.179556	8.39623333	pos
8.40_305.00[illegible]	305.002969	8.4009	neg
8.40_349.01[illegible]	349.012134	8.4009	neg
8.41_142.93[illegible]	142.938565	8.41473333	pos
8.42_399.02[illegible]	399.02576	8.41935	neg
8.44_177.09[illegible]	177.091873	8.43766667	neg
8.44_304.96[illegible]	304.968109	8.43766667	neg
8.44_321.13[illegible]	321.131845	8.43766667	neg
8.44_485.02[illegible]	485.024058	8.43766667	neg
8.46_353.00[illegible]	353.004362	8.45608333	neg
8.46_463.02[illegible]	463.022614	8.45608333	neg
8.47_213.10[illegible]	213.109624	8.46991667	pos
8.47_251.04[illegible]	251.046467	8.46991667	pos
8.47_710.21[illegible]	710.219803	8.46991667	pos
8.49_216.92[illegible]	216.922501	8.48831667	pos
8.49_225.06[illegible]	225.061409	8.4932	neg
8.49_433.04[illegible]	433.047167	8.4932	neg
8.49_500.27[illegible]	500.278331	8.4932	neg
8.49_733.36[illegible]	733.367993	8.4932	neg
8.51_156.94[illegible]	156.948349	8.50668333	pos
8.51_223.98[illegible]	223.988149	8.50668333	pos
8.51_224.14[illegible]	225.148289	8.50668333	pos
8.51_245.17[illegible]	245.174434	8.50668333	pos
8.51_334.20[illegible]	334.206679	8.50668333	pos

8.51_350.98[illegible]	350.986619	8.50668333	pos
8.51_365.19[illegible]	365.193081	8.50668333	pos
8.51_409.29[illegible]	454.289094	8.51168333	neg
8.51_421.01[illegible]	421.011815	8.51168333	neg
8.51_483.04[illegible]	483.044061	8.51168333	neg
8.53_249.10[illegible]	249.109455	8.52503333	pos
8.53_269.21[illegible]	269.210768	8.52503333	pos
8.53_307.18[illegible]	307.187688	8.52503333	pos
8.53_310.27[illegible]	310.271105	8.52503333	pos
8.53_369.03[illegible]	369.035299	8.5302	neg
8.53_386.03[illegible]	367.01981	8.5302	neg
8.53_473.00[illegible]	473.005556	8.5302	neg
8.53_478.19[illegible]	478.19695	8.52503333	pos
8.53_511.03[illegible]	511.038447	8.5302	neg
8.53_554.89[illegible]	554.890895	8.5302	neg
8.55_256.98[illegible]	256.985355	8.5484	neg
8.55_276.99[illegible]	276.99149	8.5484	neg
8.55_379.24[illegible]	379.248957	8.5484	neg
8.55_446.90[illegible]	446.906005	8.5484	neg
8.56_271.26[illegible]	271.262792	8.56171667	pos
8.56_272.25[illegible]	272.258332	8.56171667	pos
8.56_498.90[illegible]	498.90084	8.56171667	pos
8.57_300.02[illegible]	281.002999	8.5669	neg
8.58_159.10[illegible]	159.10245	8.58033333	neg
8.58_173.11[illegible]	173.118	8.58033333	neg
8.58_223.09[illegible]	223.09583	8.58011667	pos
8.58_267.11[illegible]	267.119903	8.58011667	pos
8.58_270.21[illegible]	293.208447	8.58011667	pos
8.58_281.17[illegible]	281.172086	8.58011667	pos
8.58_297.16[illegible]	297.166888	8.58011667	pos
8.58_320.99[illegible]	320.997825	8.58033333	neg
8.58_335.01[illegible]	335.013566	8.58033333	neg
8.58_356.99[illegible]	356.999073	8.58033333	neg

8.58_389.00	389.005442	8.58033333	neg
8.58_399.02	399.026016	8.58033333	neg
8.58_403.02	403.020404	8.58033333	neg
8.60_102.03	102.034209	8.5985	pos
8.60_142.93	142.938527	8.5985	pos
8.60_225.09	225.090893	8.5985	pos
8.60_302.21	302.214364	8.5985	pos
8.60_360.97	360.974414	8.59883333	neg
8.60_361.01	361.010657	8.59883333	neg
8.60_418.21	418.213698	8.5985	pos
8.62_200.04	200.047128	8.61701667	pos
8.62_224.01	224.011802	8.61736667	neg
8.62_255.12	255.121299	8.61736667	neg
8.62_353.02	353.02364	8.61736667	neg
8.62_519.02	519.026954	8.61736667	neg
8.62_529.03	529.030593	8.61736667	neg
8.64_158.98	158.985938	8.6355	pos
8.64_199.09	199.097345	8.63586667	neg
8.64_276.95	276.955212	8.63586667	neg
8.64_314.98	314.988589	8.63586667	neg
8.64_347.01	347.013237	8.63586667	neg
8.64_435.02	435.026555	8.63586667	neg
8.64_515.01	515.01577	8.63586667	neg
8.65_269.21	269.210883	8.65385	pos
8.65_307.22	307.224263	8.65385	pos
8.67_172.94	172.941001	8.67263333	neg
8.67_243.19	243.196403	8.67263333	neg
8.67_316.28	316.284164	8.67195	pos
8.67_336.99	336.99302	8.67263333	neg
8.67_346.99	346.994547	8.67263333	neg
8.67_398.01	379.001835	8.67263333	neg
8.67_488.02	488.023696	8.67263333	neg
8.67_542.26	542.261828	8.67195	pos

8.69_1091.1	1091.17825	8.68903333	neg
8.69_241.07	241.072116	8.68903333	neg
8.69_333.16	333.168255	8.68903333	neg
8.69_352.21	352.217134	8.68985	pos
8.69_373.04	373.044535	8.68903333	neg
8.69_419.31	419.315154	8.68985	pos
8.69_469.02	469.029217	8.68903333	neg
8.69_498.03	497.024525	8.68903333	neg
8.69_501.03	501.035502	8.68903333	neg
8.69_597.23	597.234999	8.68903333	neg
8.69_621.22	621.223606	8.68903333	neg
8.69_809.20	809.200416	8.68903333	neg
8.69_904.20	904.206716	8.68903333	neg
8.71_1023.1	1023.19241	8.7071	neg
8.71_1066.5	1066.54974	8.7071	neg
8.71_267.16	267.162778	8.7078	pos
8.71_267.66	267.664944	8.7078	pos
8.71_613.25	613.252287	8.7071	neg
8.71_614.25	614.259713	8.7071	neg
8.71_632.25	632.257604	8.7071	neg
8.71_673.22	673.225921	8.7071	neg
8.71_682.24	682.246578	8.7071	neg
8.71_700.24	700.245174	8.7071	neg
8.71_768.23	768.232171	8.7071	neg
8.71_819.23	819.230074	8.7071	neg
8.71_887.21	887.217783	8.7071	neg
8.72_1185.5	1185.53525	8.71673333	neg
8.72_457.62	457.626742	8.71673333	neg
8.72_495.26	495.265149	8.71673333	neg
8.72_550.28	550.289248	8.71673333	neg
8.72_563.25	563.253361	8.71673333	neg
8.72_564.27	564.27043	8.71673333	neg
8.72_615.26	615.26724	8.71673333	neg

8.73_274.97	274.975746	8.73261667	neg
8.73_479.29	479.292196	8.73261667	neg
8.73_487.32	487.321253	8.73261667	neg
8.73_502.28	503.288674	8.72548333	pos
8.73_870.09	870.095719	8.72548333	pos
8.74_238.66	238.661024	8.74186667	pos
8.74_246.64	246.649874	8.74186667	pos
8.74_247.15	247.151602	8.74186667	pos
8.74_401.81	440.780371	8.74186667	pos
8.74_432.79	432.794218	8.74186667	pos
8.75_411.30	456.304535	8.74946667	neg
8.75_412.31	457.310548	8.74946667	neg
8.75_413.31	413.311328	8.74946667	neg
8.75_446.27	446.274755	8.74946667	neg
8.75_447.28	447.281968	8.74946667	neg
8.75_457.53	457.531767	8.74946667	neg
8.75_460.31	460.319412	8.74946667	neg
8.75_473.29	473.293437	8.74946667	neg
8.75_509.27	509.271331	8.74946667	neg
8.75_523.28	523.281839	8.74946667	neg
8.75_841.62	822.611586	8.74946667	neg
8.75_859.59	859.593774	8.74946667	neg
8.75_863.61	844.592871	8.74946667	neg
8.75_930.57	930.577638	8.74946667	neg
8.76_128.06	128.061999	8.75856667	pos
8.76_375.28	358.281326	8.75856667	pos
8.76_376.29	377.298036	8.75856667	pos
8.76_377.27	377.278172	8.75856667	pos
8.76_378.29	379.304368	8.75856667	pos
8.76_401.31	440.278139	8.75856667	pos
8.76_411.30	434.294809	8.75856667	pos
8.76_412.31	430.34585	8.75856667	pos
8.76_414.31	437.307268	8.75856667	pos

8.76_432.29	432.292521	8.75856667	pos
8.76_472.35	472.356581	8.75856667	pos
8.76_476.32	476.327763	8.75856667	pos
8.76_486.37	486.371938	8.75856667	pos
8.76_510.26	510.265554	8.75856667	pos
8.76_607.47	646.434704	8.75856667	pos
8.76_638.95	638.950462	8.75856667	pos
8.76_646.93	646.936089	8.75856667	pos
8.76_823.61	824.624867	8.75856667	pos
8.76_824.62	825.631028	8.75856667	pos
8.76_845.10	845.106063	8.75856667	pos
8.77_173.08	173.080488	8.77465	pos
8.77_375.28	375.285293	8.77465	pos
8.77_410.29	455.297392	8.76651667	neg
8.77_411.03	411.031004	8.76651667	neg
8.77_411.57	411.574018	8.76651667	neg
8.77_479.29	478.285946	8.76651667	neg
8.77_482.30	482.301824	8.76651667	neg
8.77_551.29	551.292055	8.77465	pos
8.77_621.19	621.192258	8.77465	pos
8.77_912.58	912.580277	8.76651667	neg
8.78_546.27	546.273212	8.784	neg
8.79_244.26	244.263141	8.7919	pos
8.79_371.31	371.315366	8.7919	pos
8.79_576.22	594.261241	8.7919	pos
8.79_621.44	621.443223	8.7919	pos
8.80_1024.1	1024.1957	8.80223333	neg
8.80_1091.1	1091.17764	8.80223333	neg
8.80_349.01	349.011907	8.80223333	neg
8.80_421.22	421.220775	8.80223333	neg
8.80_431.03	431.03337	8.80223333	neg
8.80_451.03	451.038576	8.80223333	neg
8.80_516.27	516.273327	8.80223333	neg

8.80_525.05	525.055096	8.80223333	neg
8.80_622.22	622.227964	8.80223333	neg
8.80_741.21	741.213727	8.80223333	neg
8.80_769.23	769.235925	8.80223333	neg
8.80_836.21	836.219568	8.80223333	neg
8.80_904.20	904.206718	8.80223333	neg
8.81_231.15	231.158817	8.81001667	pos
8.81_247.13	247.130206	8.81001667	pos
8.81_268.15	268.159813	8.81001667	pos
8.81_290.29	290.295544	8.81001667	pos
8.81_332.31	332.315067	8.81001667	pos
8.81_515.24	515.242129	8.81001667	pos
8.81_522.28	522.284972	8.81001667	pos
8.81_828.42	828.421554	8.81001667	pos
8.82_463.02	463.022255	8.82083333	neg
8.83_200.04	200.047096	8.82821667	pos
8.83_414.31	414.311984	8.82821667	pos
8.83_478.19	478.196674	8.82821667	pos
8.84_365.02	365.023646	8.83935	neg
8.84_476.18	476.182781	8.83935	neg
8.85_225.09	225.090844	8.8465	pos
8.85_259.19	259.190069	8.8465	pos
8.85_311.22	311.220048	8.8465	pos
8.85_828.25	828.254474	8.8465	pos
8.86_102.03	102.034195	8.86416667	pos
8.86_119.94	119.946749	8.85703333	neg
8.86_167.01	167.012678	8.86416667	pos
8.86_177.12	177.127243	8.86416667	pos
8.86_243.94	243.941371	8.86416667	pos
8.86_365.00	365.004407	8.85703333	neg
8.86_828.58	828.588428	8.86416667	pos
8.88_187.06	187.060936	8.87543333	neg
8.88_227.16	227.16388	8.88258333	pos

8.88_307.01[illegible]	307.018419	8.87543333	neg
8.88_524.27[illegible]	524.274643	8.88258333	pos
8.89_319.00[illegible]	319.00223	8.89388333	neg
8.89_401.19[illegible]	401.194386	8.89388333	neg
8.90_241.17[illegible]	241.179541	8.90103333	pos
8.90_255.06[illegible]	255.062594	8.90103333	pos
8.90_363.17[illegible]	363.171418	8.90103333	pos
8.90_365.19[illegible]	365.192881	8.90103333	pos
8.90_388.30[illegible]	388.305283	8.90103333	pos
8.90_410.28[illegible]	410.287362	8.90103333	pos
8.90_425.13[illegible]	425.135543	8.90103333	pos
8.90_536.16[illegible]	536.165058	8.90103333	pos
8.91_241.57[illegible]	241.573792	8.91221667	neg
8.91_243.19[illegible]	243.19639	8.91221667	neg
8.91_276.95[illegible]	276.955191	8.91221667	neg
8.91_448.30[illegible]	448.306785	8.91221667	neg
8.91_451.00[illegible]	451.003426	8.91221667	neg
8.91_507.02[illegible]	507.029657	8.91221667	neg
8.91_928.83[illegible]	928.834961	8.91221667	neg
8.92_137.09[illegible]	137.09596	8.91945	pos
8.92_173.08[illegible]	173.080546	8.91945	pos
8.92_183.17[illegible]	183.174213	8.91945	pos
8.93_283.00[illegible]	283.000847	8.93068333	neg
8.93_332.99[illegible]	332.998029	8.93068333	neg
8.93_445.03[illegible]	445.031237	8.93068333	neg
8.95_301.99[illegible]	346.994704	8.94916667	neg
8.95_356.99[illegible]	356.999094	8.94916667	neg
8.95_393.01[illegible]	393.017454	8.94916667	neg
8.95_413.02[illegible]	413.024769	8.94916667	neg
8.96_295.18[illegible]	295.18773	8.95591667	pos
8.97_102.03[illegible]	102.034169	8.97426667	pos
8.97_145.08[illegible]	145.086818	8.96598333	neg
8.97_271.26[illegible]	271.262812	8.97426667	pos

8.97_279.19[illegible]	279.192426	8.97426667	pos
8.97_378.03[illegible]	377.023509	8.96598333	neg
8.97_379.00[illegible]	379.001269	8.96598333	neg
8.97_809.85[illegible]	809.850979	8.96598333	neg
8.98_159.10[illegible]	159.102443	8.98443333	neg
8.98_320.99[illegible]	320.99782	8.98443333	neg
8.98_389.00[illegible]	389.005328	8.98443333	neg
8.98_400.90[illegible]	400.909171	8.98443333	neg
8.98_439.02[illegible]	439.02213	8.98443333	neg
8.99_221.11[illegible]	221.114523	8.99243333	pos
8.99_287.28[illegible]	288.289309	8.99243333	pos
8.99_290.29[illegible]	290.295758	8.99243333	pos
8.99_346.28[illegible]	346.28534	8.99243333	pos
9.00_144.10[illegible]	144.102859	9.00285	neg
9.00_188.09[illegible]	188.092616	9.00285	neg
9.00_267.11[illegible]	267.113956	9.00285	neg
9.00_305.00[illegible]	305.002957	9.00285	neg
9.00_331.00[illegible]	331.002564	9.00285	neg
9.00_333.01[illegible]	333.017826	9.00285	neg
9.00_377.15[illegible]	377.157949	9.00285	neg
9.00_388.27[illegible]	388.270589	9.00285	neg
9.00_456.25[illegible]	456.258138	9.00285	neg
9.00_463.02[illegible]	463.022797	9.00285	neg
9.00_524.24[illegible]	524.245505	9.00285	neg
9.00_536.88[illegible]	536.884184	9.00285	neg
9.01_107.96[illegible]	107.967085	9.01088333	pos
9.01_197.18[illegible]	197.189877	9.01088333	pos
9.01_199.18[illegible]	199.180173	9.01088333	pos
9.01_216.92[illegible]	216.92258	9.01088333	pos
9.01_269.12[illegible]	269.12828	9.01088333	pos
9.01_484.04[illegible]	484.044044	9.01088333	pos
9.02_281.13[illegible]	281.129981	9.02115	neg
9.02_337.02[illegible]	337.029221	9.02115	neg

9.02_425.302	425.302392	9.02115	neg
9.02_431.033	431.033153	9.02115	neg
9.02_442.292	442.292256	9.02115	neg
9.03_156.948	156.948341	9.02893333	pos
9.03_380.303	403.292662	9.02893333	pos
9.04_173.118	173.118034	9.03956667	neg
9.04_338.988	338.988321	9.03956667	neg
9.04_381.264	381.264918	9.03956667	neg
9.05_241.215	241.215784	9.04725	pos
9.05_405.260	405.260688	9.04725	pos
9.06_247.000	246.999952	9.05798333	neg
9.06_253.144	253.144412	9.05798333	neg
9.06_356.023	356.023202	9.05798333	neg
9.06_361.163	361.163373	9.05798333	neg
9.06_378.153	378.15329	9.05798333	neg
9.06_385.029	385.029523	9.05798333	neg
9.06_429.035	429.035945	9.05798333	neg
9.06_446.140	446.140873	9.05798333	neg
9.06_470.032	470.032185	9.05798333	neg
9.06_475.022	475.022178	9.05798333	neg
9.06_497.138	497.138394	9.05798333	neg
9.06_565.125	565.125635	9.05798333	neg
9.07_193.174	193.174714	9.0656	pos
9.08_249.183	249.183466	9.08396667	pos
9.08_330.899	330.898993	9.07648333	neg
9.08_335.013	335.013593	9.07648333	neg
9.08_358.995	358.995217	9.07648333	neg
9.09_349.124	349.124398	9.09495	neg
9.09_369.035	369.035283	9.09495	neg
9.09_441.905	486.903304	9.09495	neg
9.10_200.047	200.047144	9.10213333	pos
9.11_290.113	335.111214	9.1134	neg
9.11_324.200	324.200176	9.1134	neg

9.11_357.09:	357.093176	9.1134	neg
9.11_425.08(	425.080719	9.1134	neg
9.11_473.00!	473.005602	9.1134	neg
9.11_671.21(	671.210272	9.1134	neg
9.12_137.04!	137.045746	9.12051667	pos
9.12_291.12(	291.12008	9.12051667	pos
9.12_477.18!	478.196949	9.12051667	pos
9.12_582.24	583.254677	9.12051667	pos
9.12_583.75(	583.756289	9.12051667	pos
9.13_319.01{	319.018398	9.13165	neg
9.13_355.24!	355.249017	9.13165	neg
9.14_365.19:	365.193089	9.1391	pos
9.15_199.09	199.097381	9.14948333	neg
9.15_225.06	225.061468	9.14948333	neg
9.15_351.00{	351.008224	9.14948333	neg
9.15_368.99!	368.999084	9.14948333	neg
9.15_385.01(	385.010547	9.14948333	neg
9.15_516.29	516.293968	9.14948333	neg
9.15_928.83!	928.835451	9.14948333	neg
9.16_449.31:	450.320898	9.15776667	pos
9.17_341.00	341.003973	9.168	neg
9.17_361.01(	361.010421	9.168	neg
9.17_401.02:	401.023343	9.168	neg
9.18_179.10(	179.106714	9.17608333	pos
9.18_255.06:	255.062655	9.17608333	pos
9.18_285.27{	285.278309	9.17608333	pos
9.18_319.18	319.18762	9.17608333	pos
9.18_437.19:	437.193362	9.17608333	pos
9.19_187.13:	187.133692	9.18648333	neg
9.19_283.15:	283.152661	9.18648333	neg
9.19_297.16{	297.168244	9.18648333	neg
9.19_303.00	303.007116	9.18648333	neg
9.19_319.00:	319.002099	9.18648333	neg

9.19_329.00[illegible]	329.003434	9.18648333	neg
9.19_331.00[illegible]	331.001606	9.18648333	neg
9.19_381.03[illegible]	381.03526	9.18648333	neg
9.19_397.02[illegible]	397.029908	9.18648333	neg
9.19_403.02[illegible]	403.020432	9.18648333	neg
9.19_405.26[illegible]	405.260769	9.19446667	pos
9.20_264.99[illegible]	264.990556	9.20481667	neg
9.20_311.01[illegible]	311.012566	9.20481667	neg
9.20_326.01[illegible]	325.009111	9.20481667	neg
9.20_384.02[illegible]	383.014591	9.20481667	neg
9.20_517.01[illegible]	517.014505	9.20481667	neg
9.21_271.22[illegible]	271.226507	9.21266667	pos
9.22_343.01[illegible]	343.01884	9.22325	neg
9.22_435.02[illegible]	435.026629	9.22325	neg
9.22_463.02[illegible]	463.022466	9.22325	neg
9.23_249.18[illegible]	249.182855	9.23096667	pos
9.24_357.01[illegible]	357.015652	9.2416	neg
9.24_425.30[illegible]	425.30236	9.2416	neg
9.24_442.29[illegible]	442.292278	9.2416	neg
9.24_535.02[illegible]	535.022758	9.2416	neg
9.25_380.30[illegible]	403.292738	9.24935	pos
9.25_525.30[illegible]	526.313071	9.24935	pos
9.25_631.27[illegible]	631.275643	9.24935	pos
9.26_401.04[illegible]	401.041272	9.25955	neg
9.28_118.04[illegible]	118.040908	9.2775	neg
9.28_239.16[illegible]	239.165236	9.2775	neg
9.28_287.01[illegible]	287.012092	9.2775	neg
9.28_321.01[illegible]	321.017237	9.2775	neg
9.28_367.01[illegible]	367.019635	9.2775	neg
9.28_379.00[illegible]	379.001343	9.2775	neg
9.28_445.14[illegible]	445.145586	9.2775	neg
9.29_241.20[illegible]	241.203255	9.28653333	pos
9.29_266.12[illegible]	266.123965	9.28653333	pos

9.29_526.29[illegible]	526.29329	9.28653333	pos
9.29_577.36[illegible]	577.363504	9.28653333	pos
9.30_377.02[illegible]	377.023335	9.29605	neg
9.30_513.13[illegible]	513.132874	9.29605	neg
9.31_411.00[illegible]	411.008134	9.3144	neg
9.31_431.26[illegible]	431.265003	9.3144	neg
9.33_173.08[illegible]	173.081656	9.3325	neg
9.33_340.03[illegible]	385.029637	9.3325	neg
9.33_393.01[illegible]	393.01713	9.3325	neg
9.33_397.22[illegible]	397.222671	9.3325	neg
9.33_413.04[illegible]	413.041206	9.3325	neg
9.33_459.20[illegible]	459.20265	9.3325	neg
9.33_545.02[illegible]	545.02947	9.3325	neg
9.34_416.32[illegible]	416.326579	9.34135	pos
9.34_487.05[illegible]	487.05501	9.34135	pos
9.35_297.20[illegible]	297.207153	9.35091667	neg
9.35_312.99[illegible]	312.991813	9.35091667	neg
9.35_322.99[illegible]	322.99361	9.35091667	neg
9.35_417.03[illegible]	417.036287	9.35091667	neg
9.35_584.04[illegible]	584.043261	9.35091667	neg
9.36_331.00[illegible]	331.001785	9.35948333	pos
9.36_409.25[illegible]	409.256424	9.35948333	pos
9.37_319.00[illegible]	319.002204	9.36935	neg
9.37_333.01[illegible]	333.017692	9.36935	neg
9.37_353.00[illegible]	353.004252	9.36935	neg
9.37_353.02[illegible]	353.023571	9.36935	neg
9.37_360.27[illegible]	360.275648	9.36935	neg
9.37_433.04[illegible]	433.047519	9.36935	neg
9.37_550.29[illegible]	550.299921	9.36935	neg
9.38_374.06[illegible]	374.062552	9.37788333	pos
9.39_225.06[illegible]	225.061466	9.3876	neg
9.39_337.00[illegible]	337.009329	9.3876	neg
9.39_396.25[illegible]	395.243878	9.3876	neg

9.39_440.998	440.998936	9.3876	neg
9.39_594.287	594.287535	9.3876	neg
9.40_131.533	131.5335	9.39628333	pos
9.40_312.229	295.226499	9.39628333	pos
9.40_343.285	343.285308	9.39628333	pos
9.41_351.217	351.217775	9.40613333	neg
9.41_364.091	365.098321	9.41486667	pos
9.41_401.005	401.005485	9.40613333	neg
9.41_413.024	413.024836	9.40613333	neg
9.41_419.205	419.205378	9.40613333	neg
9.41_453.018	453.017969	9.40613333	neg
9.41_527.035	527.035045	9.40613333	neg
9.42_211.133	211.133724	9.42405	neg
9.42_311.012	311.012824	9.42405	neg
9.42_329.003	329.003281	9.42405	neg
9.42_363.084	363.084364	9.42405	neg
9.42_370.994	370.994774	9.42405	neg
9.42_382.997	382.9971	9.42405	neg
9.42_387.026	387.025959	9.42405	neg
9.42_429.036	429.036208	9.42405	neg
9.42_489.018	489.018118	9.42405	neg
9.44_343.018	343.01868	9.44245	neg
9.44_411.025	411.02592	9.44245	neg
9.44_415.021	415.021117	9.44245	neg
9.44_421.011	421.011637	9.44245	neg
9.45_227.163	227.163836	9.45123333	pos
9.45_255.062	255.062637	9.45123333	pos
9.45_301.215	301.215685	9.45123333	pos
9.45_372.301	372.301075	9.45123333	pos
9.45_393.287	394.29462	9.45123333	pos
9.45_396.300	396.300932	9.45123333	pos
9.46_188.092	188.092597	9.46038333	neg
9.46_199.092	199.09269	9.46038333	neg

9.46_317.00	317.002712	9.46038333	neg
9.46_328.06	328.060178	9.46038333	neg
9.46_339.00	339.007843	9.46038333	neg
9.46_396.04	396.047486	9.46038333	neg
9.46_447.04	447.044676	9.46038333	neg
9.46_515.03	515.032462	9.46038333	neg
9.46_550.26	550.26139	9.46038333	neg
9.46_583.02	583.024402	9.46038333	neg
9.46_651.00	651.007634	9.46038333	neg
9.46_718.99	718.994997	9.46038333	neg
9.46_786.98	786.983021	9.46038333	neg
9.47_221.11	221.114351	9.46953333	pos
9.47_289.06	289.060721	9.46953333	pos
9.47_381.06	381.069105	9.46953333	pos
9.48_316.01	296.996933	9.47878333	neg
9.48_383.03	383.031281	9.47878333	neg
9.48_399.02	399.025514	9.47878333	neg
9.48_413.00	413.005806	9.47878333	neg
9.48_437.29	437.290553	9.47878333	neg
9.48_443.09	443.090738	9.47878333	neg
9.48_477.32	477.322038	9.47878333	neg
9.49_433.33	433.330792	9.4876	pos
9.50_307.11	307.11635	9.4972	neg
9.50_324.10	324.106344	9.4972	neg
9.50_345.01	345.015476	9.4972	neg
9.50_373.01	373.010371	9.4972	neg
9.50_545.02	545.027036	9.4972	neg
9.50_547.05	547.058598	9.4972	neg
9.51_209.15	209.153499	9.50591667	pos
9.52_118.04	118.040859	9.51548333	neg
9.52_295.19	295.191285	9.51548333	neg
9.52_296.19	319.187445	9.52293333	pos
9.52_301.11	302.120533	9.52293333	pos

9.52_309.20	309.207218	9.51548333	neg
9.52_321.01	321.017071	9.51548333	neg
9.52_380.02	425.023741	9.51548333	neg
9.52_394.99	394.995155	9.51548333	neg
9.52_438.05	419.032273	9.51548333	neg
9.52_631.27	631.275225	9.52293333	pos
9.53_185.11	185.118042	9.53388333	neg
9.53_287.01	287.012041	9.53388333	neg
9.53_343.00	343.000023	9.53388333	neg
9.53_362.29	362.291379	9.53388333	neg
9.53_375.25	420.250538	9.53388333	neg
9.53_382.02	427.020275	9.53388333	neg
9.53_410.22	410.221619	9.53388333	neg
9.53_422.25	422.256936	9.53388333	neg
9.53_457.01	457.011351	9.53388333	neg
9.53_477.03	477.037881	9.53388333	neg
9.53_519.02	519.026421	9.53388333	neg
9.53_571.03	571.038041	9.53388333	neg
9.53_581.24	581.240737	9.53388333	neg
9.54_236.60	236.607606	9.54118333	pos
9.54_257.61	257.619379	9.54118333	pos
9.54_375.25	376.258982	9.54118333	pos
9.54_398.24	398.240869	9.54118333	pos
9.54_421.31	421.316697	9.54118333	pos
9.54_423.32	423.323228	9.54118333	pos
9.54_466.22	466.228241	9.54118333	pos
9.54_495.12	495.125683	9.54118333	pos
9.54_583.75	583.756061	9.54118333	pos
9.55_398.01	397.010519	9.55203333	neg
9.55_403.02	403.020399	9.55203333	neg
9.56_308.12	308.125338	9.55885	pos
9.56_315.27	338.265951	9.55885	pos
9.57_405.01	405.0166	9.56983333	neg

9.58_196.01(	196.016561	9.57733333	pos
9.58_228.19!	228.195567	9.57733333	pos
9.58_300.24	283.241575	9.57733333	pos
9.58_323.25	323.257535	9.57733333	pos
9.58_381.26(	381.260668	9.57733333	pos
9.59_311.01	311.012483	9.58798333	neg
9.59_333.01	333.017454	9.58798333	neg
9.59_375.00(	375.006866	9.58798333	neg
9.59_457.02!	457.02991	9.58798333	neg
9.59_495.02	495.028298	9.58798333	neg
9.59_502.04	501.035158	9.58798333	neg
9.60_143.98(	143.980811	9.59576667	pos
9.60_173.08(	173.08051	9.59576667	pos
9.60_295.22(	295.22644	9.59576667	pos
9.60_299.25	299.25761	9.59576667	pos
9.60_356.27!	356.279007	9.59576667	pos
9.60_369.22	369.224507	9.59576667	pos
9.60_440.33(	440.336619	9.59576667	pos
9.60_462.31	462.318319	9.59576667	pos
9.61_137.04!	137.045728	9.61401667	pos
9.61_169.15!	169.159597	9.60636667	neg
9.61_225.14	225.148335	9.61401667	pos
9.61_266.12	266.121932	9.61401667	pos
9.61_279.19	279.192296	9.61401667	pos
9.61_385.02!	385.029436	9.60636667	neg
9.61_389.02	389.022068	9.60636667	neg
9.61_428.99	428.998639	9.60636667	neg
9.61_461.04	461.043019	9.60636667	neg
9.61_468.30	468.308097	9.61401667	pos
9.61_473.04	473.042958	9.60636667	neg
9.61_498.04(	543.044907	9.60636667	neg
9.61_512.29!	512.299337	9.60636667	neg
9.61_525.05	525.05427	9.60636667	neg

9.61_545.04[illegible]	545.04292	9.60636667	neg
9.62_248.58[illegible]	248.581565	9.62486667	neg
9.62_336.99[illegible]	336.993222	9.62486667	neg
9.62_339.21[illegible]	339.217451	9.62486667	neg
9.62_392.99[illegible]	392.998852	9.62486667	neg
9.62_504.04[illegible]	485.024044	9.62486667	neg
9.62_513.01[illegible]	513.018143	9.62486667	neg
9.63_114.52[illegible]	114.525233	9.63246667	pos
9.63_131.53[illegible]	131.533484	9.63246667	pos
9.63_135.11[illegible]	135.116759	9.63246667	pos
9.63_142.99[illegible]	142.991033	9.63246667	pos
9.63_337.21[illegible]	337.213619	9.63246667	pos
9.64_225.06[illegible]	225.06144	9.64313333	neg
9.64_331.00[illegible]	331.000524	9.64313333	neg
9.64_391.02[illegible]	391.02022	9.64313333	neg
9.64_395.01[illegible]	395.013962	9.64313333	neg
9.64_431.03[illegible]	431.032296	9.64313333	neg
9.64_431.31[illegible]	431.316441	9.64313333	neg
9.64_447.02[illegible]	447.027133	9.64313333	neg
9.64_451.00[illegible]	451.002747	9.64313333	neg
9.65_255.23[illegible]	255.231472	9.6509	pos
9.65_480.21[illegible]	480.212602	9.6509	pos
9.66_248.07[illegible]	248.079917	9.66158333	neg
9.66_382.99[illegible]	382.995138	9.66158333	neg
9.66_444.03[illegible]	489.036736	9.66158333	neg
9.66_463.03[illegible]	463.039669	9.66158333	neg
9.66_465.01[illegible]	465.01799	9.66158333	neg
9.67_149.09[illegible]	149.095974	9.66891667	pos
9.67_304.89[illegible]	304.893859	9.66891667	pos
9.67_314.26[illegible]	314.268404	9.66891667	pos
9.67_398.31[illegible]	398.316711	9.66891667	pos
9.67_447.34[illegible]	447.346189	9.66891667	pos
9.68_303.00[illegible]	303.007138	9.68	neg

9.68_355.000	355.000129	9.68	neg
9.68_358.994	358.994901	9.68	neg
9.68_530.03[illegible]	529.030379	9.68	neg
9.68_593.04[illegible]	593.042813	9.68	neg
9.69_174.95[illegible]	174.953627	9.68703333	pos
9.69_200.04[illegible]	200.047071	9.68703333	pos
9.69_295.18[illegible]	295.188748	9.68703333	pos
9.70_237.14[illegible]	237.146992	9.69793333	neg
9.70_345.03[illegible]	345.031038	9.69793333	neg
9.70_433.01[illegible]	433.011554	9.69793333	neg
9.70_449.02[illegible]	449.023455	9.69793333	neg
9.71_241.20[illegible]	241.203195	9.70535	pos
9.71_251.04[illegible]	251.046434	9.70535	pos
9.71_323.24[illegible]	346.23468	9.70535	pos
9.71_360.22[illegible]	360.222388	9.70535	pos
9.72_116.96[illegible]	116.967507	9.72246667	pos
9.72_245.17[illegible]	245.17439	9.72246667	pos
9.72_318.32[illegible]	318.326938	9.72246667	pos
9.72_417.01[illegible]	417.016959	9.71598333	neg
9.73_283.15[illegible]	283.152618	9.73451667	neg
9.73_351.14[illegible]	351.140147	9.73451667	neg
9.73_430.02[illegible]	475.020904	9.73451667	neg
9.73_459.20[illegible]	459.202643	9.73451667	neg
9.73_471.00[illegible]	471.008682	9.73451667	neg
9.73_476.19[illegible]	476.192735	9.73451667	neg
9.73_479.03[illegible]	479.033429	9.73451667	neg
9.73_516.02[illegible]	515.015856	9.73451667	neg
9.73_580.05[illegible]	561.036214	9.73451667	neg
9.74_100.51[illegible]	100.510277	9.74061667	pos
9.74_160.98[illegible]	160.983482	9.74061667	pos
9.74_235.06[illegible]	235.068656	9.74061667	pos
9.74_235.57[illegible]	235.570285	9.74061667	pos
9.74_247.59[illegible]	247.595752	9.74061667	pos

9.74_255.58	255.581814	9.74061667	pos
9.74_256.08	256.083454	9.74061667	pos
9.74_414.20	453.166797	9.74061667	pos
9.74_463.27	463.277889	9.74061667	pos
9.74_486.26	486.264875	9.74061667	pos
9.74_503.31	503.310572	9.74061667	pos
9.74_505.18	505.180344	9.74061667	pos
9.74_522.17	522.170553	9.74061667	pos
9.74_713.48	736.474497	9.74061667	pos
9.74_828.40	851.396267	9.74061667	pos
9.75_329.00	329.003106	9.75281667	neg
9.75_409.02	409.029351	9.75281667	neg
9.75_547.05	547.057889	9.75281667	neg
9.75_591.03	591.033591	9.75281667	neg
9.76_272.29	272.294399	9.7588	pos
9.76_279.23	279.231334	9.7588	pos
9.76_302.29	302.295862	9.7588	pos
9.76_327.25	327.25237	9.7588	pos
9.76_345.26	345.262944	9.7588	pos
9.76_418.34	418.342652	9.7588	pos
9.77_271.22	271.227812	9.77068333	neg
9.77_382.02	381.016229	9.77068333	neg
9.77_391.00	391.001328	9.77068333	neg
9.77_401.02	401.021878	9.77068333	neg
9.77_527.03	527.036061	9.77068333	neg
9.77_537.01	537.018537	9.77068333	neg
9.77_596.30	596.30296	9.77068333	neg
9.78_109.02	109.022614	9.77705	pos
9.78_199.18	199.180198	9.77705	pos
9.78_322.27	322.271139	9.77705	pos
9.78_384.31	384.310379	9.77705	pos
9.79_282.63	282.631994	9.79426667	pos
9.79_284.29	284.294319	9.79426667	pos

9.79_297.168	297.168126	9.78913333	neg
9.79_307.187	307.187598	9.79426667	pos
9.79_311.184	311.184388	9.78913333	neg
9.79_329.229	329.22957	9.79426667	pos
9.79_363.254	363.254245	9.78913333	neg
9.79_411.025	411.025477	9.78913333	neg
9.79_427.001	427.00152	9.78913333	neg
9.79_437.022	437.022872	9.78913333	neg
9.79_465.054	465.053961	9.78913333	neg
9.79_498.262	498.262866	9.78913333	neg
9.79_545.029	545.029614	9.78913333	neg
9.79_566.250	566.250186	9.78913333	neg
9.79_584.047	583.039799	9.78913333	neg
9.79_595.367	595.36735	9.79426667	pos
9.79_597.019	597.019197	9.78913333	neg
9.81_197.189	197.189841	9.81245	pos
9.81_277.215	277.215944	9.81245	pos
9.81_358.294	358.294528	9.81245	pos
9.81_392.998	392.997973	9.80731667	neg
9.81_481.029	481.02959	9.80731667	neg
9.81_495.045	495.045904	9.80731667	neg
9.81_568.053	549.035716	9.80731667	neg
9.83_119.957	119.957183	9.83063333	pos
9.83_144.102	144.102847	9.82575	neg
9.83_188.092	188.092578	9.82575	neg
9.83_334.091	334.090972	9.82575	neg
9.83_346.993	346.993888	9.82575	neg
9.83_374.316	374.316473	9.83063333	pos
9.83_379.000	379.000574	9.82575	neg
9.83_415.001	415.0014	9.82575	neg
9.83_484.289	484.289249	9.82575	neg
9.83_503.034	503.034654	9.82575	neg
9.83_552.276	552.27672	9.82575	neg

9.83_586.31[illegible]	586.315457	9.82575	neg
9.83_625.05[illegible]	625.050875	9.82575	neg
9.84_262.91[illegible]	262.911674	9.84338333	neg
9.84_313.23[illegible]	313.238523	9.84338333	neg
9.84_362.01[illegible]	407.013069	9.84338333	neg
9.84_423.00[illegible]	423.007881	9.84338333	neg
9.84_573.03[illegible]	573.036464	9.84338333	neg
9.85_227.16[illegible]	227.163927	9.84861667	pos
9.85_247.09[illegible]	247.099309	9.84861667	pos
9.86_209.04[illegible]	209.045358	9.86181667	neg
9.86_307.15[illegible]	307.152641	9.86181667	neg
9.86_375.14[illegible]	375.14028	9.86181667	neg
9.86_382.99[illegible]	382.995299	9.86181667	neg
9.86_435.00[illegible]	435.007865	9.86181667	neg
9.86_562.31[illegible]	562.315202	9.86181667	neg
9.87_172.95[illegible]	172.956196	9.86676667	pos
9.87_316.28[illegible]	316.284515	9.86676667	pos
9.87_360.34[illegible]	360.346744	9.86676667	pos
9.88_271.26[illegible]	271.262756	9.88461667	pos
9.88_339.24[illegible]	339.240712	9.88461667	pos
9.88_412.32[illegible]	412.326219	9.88461667	pos
9.88_451.00[illegible]	451.001905	9.8803	neg
9.88_469.02[illegible]	469.029119	9.8803	neg
9.88_473.04[illegible]	473.042803	9.8803	neg
9.88_539.03[illegible]	539.032602	9.8803	neg
9.88_542.03[illegible]	541.030682	9.8803	neg
9.88_577.03[illegible]	577.033635	9.8803	neg
9.88_599.03[illegible]	599.033094	9.8803	neg
9.90_409.01[illegible]	409.010997	9.89818333	neg
9.90_433.25[illegible]	433.257502	9.90308333	pos
9.90_438.31[illegible]	461.30775	9.90308333	pos
9.90_468.03[illegible]	513.035493	9.89818333	neg
9.90_479.01[illegible]	479.014306	9.89818333	neg

9.90_495.008	495.008393	9.89818333	neg
9.90_507.027	507.027688	9.89818333	neg
9.90_565.012	565.012846	9.89818333	neg
9.92_142.986	142.986492	9.92105	pos
9.92_163.075	163.075172	9.92105	pos
9.92_341.233	341.233081	9.91656667	neg
9.92_365.004	365.004072	9.91656667	neg
9.92_381.227	381.227553	9.91656667	neg
9.92_413.023	413.023139	9.91656667	neg
9.92_419.013	419.013398	9.91656667	neg
9.92_465.037	465.037026	9.91656667	neg
9.92_467.014	467.014215	9.91656667	neg
9.92_474.031	455.013994	9.91656667	neg
9.92_482.345	500.378791	9.92105	pos
9.92_493.029	493.029783	9.91656667	neg
9.92_527.015	527.015348	9.91656667	neg
9.92_531.046	531.046565	9.91656667	neg
9.92_545.027	545.027077	9.91656667	neg
9.92_559.039	559.039197	9.91656667	neg
9.92_589.031	589.031644	9.91656667	neg
9.93_403.210	403.210184	9.93468333	neg
9.93_483.318	483.318817	9.93468333	neg
9.93_506.038	487.020374	9.93468333	neg
9.93_534.048	533.041252	9.93468333	neg
9.93_535.023	535.023224	9.93468333	neg
9.93_557.026	557.026923	9.93468333	neg
9.93_597.056	597.056606	9.93468333	neg
9.93_615.047	615.047217	9.93468333	neg
9.93_951.524	951.524656	9.93468333	neg
9.94_227.200	227.200245	9.93923333	pos
9.94_249.182	249.182237	9.93923333	pos
9.94_304.893	304.893884	9.93923333	pos
9.94_348.250	348.250472	9.93923333	pos

9.94_365.22	365.229436	9.93923333	pos
9.94_424.33	424.332216	9.93923333	pos
9.94_456.16	456.168534	9.93923333	pos
9.94_851.39	851.395991	9.93923333	pos
9.95_330.89	330.899064	9.95315	neg
9.95_331.15	331.152861	9.95315	neg
9.95_416.99	416.998172	9.95315	neg
9.95_461.02	461.023648	9.95315	neg
9.95_466.31	466.317325	9.95315	neg
9.95_543.02	543.027311	9.95315	neg
9.95_556.32	556.325349	9.95315	neg
9.96_110.52	110.521954	9.95751667	pos
9.96_152.99	152.994794	9.95751667	pos
9.96_154.99	154.990086	9.95751667	pos
9.96_217.17	217.179527	9.95751667	pos
9.96_308.26	308.269407	9.95751667	pos
9.96_357.20	357.201693	9.95751667	pos
9.96_419.31	419.315104	9.95751667	pos
9.96_485.33	485.333087	9.95751667	pos
9.97_394.99	394.994856	9.97076667	neg
9.97_441.01	441.017461	9.97076667	neg
9.97_447.19	447.1978	9.97076667	neg
9.97_570.05	551.032814	9.97076667	neg
9.97_662.33	662.331477	9.97076667	neg
9.98_160.98	160.983508	9.97586667	pos
9.98_244.22	244.226864	9.97586667	pos
9.98_358.29	358.294509	9.97586667	pos
9.98_480.30	480.308349	9.97586667	pos
9.99_173.08	173.080592	9.99425	pos
9.99_174.95	174.953597	9.99425	pos
9.99_297.24	297.243293	9.98928333	neg
9.99_307.16	307.16664	9.99425	pos
9.99_340.24	340.249142	9.98928333	neg

9.99_363.25	363.254223	9.98928333	neg
9.99_408.25	408.252013	9.98928333	neg
9.99_410.26	410.265416	9.99425	pos
9.99_486.34	486.3438	9.98928333	neg
9.99_521.00	521.005355	9.98928333	neg
9.99_643.04	643.041349	9.98928333	neg
9.99_657.05	657.057439	9.98928333	neg

level	Score	Fragmentati	Adducts	Formula	Mass Error (	QC1-1	QC1-2	QC1-3	C1	C2
***	79.5	99.5	M+FA-H	C24H50NO7	-0.307843	3047257.04	2902463.83	2774154.95	1420253.52	6676798.76
***	78.1	99.9	M+H, M+Na	C20H32N6O	-0.9413232	263746.689	252697.437	215396.136	621066.733	227655.416
***	77.7	98.5	M+FA-H	C24H48NO7	0.28686522	68616.7783	71838.5226	71040.1495	53894.9118	186871.019
***	77.5	94.4	M-H, 2M-H	C23H48NO7	-0.6191144	1566540.73	1663467.44	1569433.63	534511.522	2353173.87
***	77.4	93.9	M-H, 2M-H	C21H44NO7	-0.1523651	434533.781	447557.604	426527.737	162083.8	740932.954
***	76.9	96.6	M+H, M+NH4	C8H11NO3	-0.6649479	33590.1902	37415.2919	34943.0431	11104.6999	45923.8971
***	76.5	86.3	M-H2O-H, M	C5H8O5	-1.1729027	45915.5549	44774.8205	44114.9564	31082.8107	189473.831
***	76.5	98.5	M+FA-H	C26H50NO7	0.10201568	199115.156	202367.058	209303.313	71115.5367	595442.065
***	76.5	98.7	M+FA-H	C26H52NO7	-0.3586132	777406.133	785996.093	758607.804	404129.391	1764763.56
***	76.1	93.5	M-H	C6H9N3O2	-0.8805609	121256.511	123525.313	108279.097	135301.957	109755.332
***	76.1	89.2	M-H	C6H6O6	-1.4751056	82199.1597	80740.2953	73088.5699	133780.631	109597.61
***	75.9	88.1	M-H, M+FA-H	C10H13N5O	-0.7777331	59096.5588	59818.9536	63257.7748	44785.3421	84220.036
***	75.8	91.3	M+K, M+H	C6H14N4O2	0.36401485	1547105.68	1569627.8	1597514.21	2247126.03	1827468.78
***	75.4	94.7	M+H, M+Na,	C26H52NO7	-0.7312756	3363477.03	3358335.98	3413857.61	2275539.01	8027473.63
***	75.4	95.7	M-H	C27H33N9O	0.67772395	92435.545	98422.568	96147.0329	99705.0151	113933.368
***	75.4	91.2	M+H, M+Na	C15H27NO4	-1.1748669	152585.818	156488.514	154207.622	30132.7538	918833.234
***	75.2	88	M+H	C27H44O2	-0.967855	23862.3863	23582.787	23253.2624	4797.8346	6433.0466
***	75.2	94.6	M-H	C33H34N4O	0.35727107	28363.0503	28536.6231	28560.8309	29959.144	6974.2459
***	75	96	M-H, 2M-H	C5H4N4O3	-1.6829915	489807.118	500806.866	470590.439	501609.618	357115.51
***	75	92.4	M-H, 2M-H, M	C9H12N2O6	-1.1410673	577732.197	600689.565	580754.046	665412.004	958608.266
***	74.9	83.4	M-H	C8H16NO9P	-0.9095576	118481.554	124619.837	120076.446	127159.474	190789.466
***	74.6	95.1	M-H	C6H14O12P	-0.6681255	34977.5052	35045.7513	31830.7173	66112.515	75986.9733
***	74.5	83.8	M-H2O-H, M	C8H13NO6	-1.7774816	13071.2785	14619.3096	13573.4948	22304.5012	70959.0953
***	74.4	84.3	M+H	C6H6N2O	1.02926735	1661514.35	1759925.1	1756230.97	2864264.74	2373007.89
***	74.3	93.4	M+H-H2O, M	C5H9NO4	-0.5979499	2346043.07	2431394.36	2474552.6	5379708.89	3729092.19
***	74.2	92.3	M+FA-H	C26H54NO7	-0.4462794	1177085.86	1151821.16	1135251.82	591369.393	2203699.66
***	74.1	73.2	M+H, M+H-H	C7H12N2O3	0.08135819	115497.347	111277.702	113963.714	86430.0042	326289.702
***	74.1	93	M-H	C4H8O3	-1.508943	22057.782	23813.3954	20939.8797	49181.1997	39960.4228
***	74	84.1	M-H2O-H, M	C3H7O7P	-1.3481312	283657.048	277638.581	285753.936	387763.996	315917.526
***	74	93.1	M-H, M+FA-H	C9H12N2O6	-0.8480448	251950.795	247621.623	238336.12	339344.475	564839.406
***	74	97.3	M-H	C5H4N4O2	-1.7530511	673248.452	733924.68	696908.515	828139.905	780726.616
***	73.9	78.8	M+FA-H, 2M	C3H6O3	2.60120646	518684.168	516252.435	518515.623	600284.451	487051.064

***	73.9	89.4	M-H	C6H11NO3	-1.050479	1632.94185	1749.31869	1759.14135	4427.99253	2122.29977
***	73.6	96.7	M-H	C5H6O5	-1.0087316	8582.92848	6337.19224	5584.38636	7951.90023	3889.9119
***	73.6	93.4	M-H	C24H46NO9	-0.3920116	317512.893	347108.007	316828.783	227972.724	250182.579
***	73.6	92.4	M+FA-H	C28H56NO7	0.11865334	19876.848	20421.1077	19314.1301	7669.19866	53702.6497
***	73.4	94.4	M+H	C5H10N2O3	0.2922425	706356.229	712284.645	725205.492	1122390.57	1094653.16
***	73.4	93.8	M-H	C9H11NO3	-1.2135365	160869.035	178499.205	180109.803	198334.049	181317.595
***	73.3	90.3	M-H	C9H14N3O8	-0.1135468	52736.2383	56794.1685	49962.7047	40520.0518	23631.6617
***	73.3	80.8	M-H, 2M-H	C20H32O3	-0.9075192	414942.462	424555.876	416324.554	417232.318	186389.685
***	73.3	76	M+FA-H	C21H28O5	-0.415212	10578.067	10919.5927	11019.0251	8430.7445	41184.4154
***	73.3	81.9	M+H, M+Na	C22H29FO5	-0.7374751	8854.47315	8810.23895	9283.95363	33212.2161	9.7531E-07
***	73.2	86.4	M+H	C11H16N2O	-0.3395443	49903.5216	46949.1494	45942.8416	99202.9325	33136.4953
***	73.1	70.1	M-H	C14H18N2O	0.48254389	9249.02625	9848.40286	9423.70281	27360.8223	13371.0991
***	73	76.9	M+H, M+Na	C6H13NO2	0.34399092	9864593.79	9599653.77	9224101.07	13809914.1	11757027.6
***	72.9	68.3	M+H, M+Na,	C10H16N2O	-0.522344	204378.327	197713.604	211248.879	483686.9	138385.128
***	72.9	80.9	M+H	C8H16N2O3	-0.32069	20690.8831	22279.5661	25031.8482	24486.8683	20050.2242
***	72.9	80.8	M+H	C18H34O2	-1.6509679	23596.9564	25755.0328	25076.0913	46146.7589	23120.7225
***	72.8	88.3	M+H	C2H7NO3S	1.31293705	306369.037	309626.943	319369.729	236690.074	194528.141
***	72.8	76.9	M+H	C8H20NO6P	-0.5160808	635240.386	630483.328	660629.12	84991.0497	1308214.9
***	72.8	86.8	M-H	C11H15N5O	-0.6704424	3653.90476	3821.60101	3491.93305	5474.54559	6606.16586
***	72.6	89.7	M+H, M+Na	C27H46NO7	-0.4614787	111093.407	105572.437	102833.315	186561.679	39302.9103
***	72.5	88.2	M-H	C23H44NO7	-0.1814471	59355.5253	55771.8331	54843.4164	22717.6489	87184.7138
***	72.4	81.6	M+Na	C10H12N4O	0.05372302	8534.17104	8967.10977	9756.60571	25836.275	5591.86782
***	72.4	68.8	M+H	C15H22O	-1.0304522	65246.1357	79629.6896	67934.4987	102512.744	61878.7803
***	72.3	66.2	M-H	C8H6O4	-1.1108572	21322.5615	21511.7665	21441.5204	21222.3696	22922.8622
***	72.2	88.8	M+H	C23H46NO7	-0.8846434	1467677.49	1448566.64	1493471.88	955865.296	2040861.84
***	72.1	84.5	M+H	C5H9N3	2.99878649	75189.3543	73899.2753	73010.2059	2456.65947	52806.2304
***	72.1	85.8	M+Na, M+K,	C9H21N3O	0.64171006	266863.93	222477.734	253878.283	31099.7099	1237742.59
***	72	85.1	M+FA-H	C18H32O16	0.19294989	88413.4644	94026.2822	91454.4116	9.7531E-07	555247.599
***	71.9	86.4	M-H	C4H7NO4	-1.3218459	270513.615	268688.741	272625.906	304917.682	187089.456
***	71.9	94.3	M+H	C4H9N3O2	0.24974519	2945073.91	2968080.55	2992861.78	8334219	2830090.49
***	71.9	97.7	M+H, M+Na	C5H9NO2	2.20053595	2465070.28	2500390.63	2514240.73	4888578.16	3105273.25
***	71.9	70.4	M+H, M+Na	C24H46NO9	-0.4580163	458504.917	475467.963	474631.093	398082.811	323479.469
***	71.9	97.8	M-H	C9H17NO5	-1.2666113	79533.2689	82550.8961	80392.7452	80470.8927	165597.73

***	71.7	84.2	M+H	C14H20N6O	-0.8437088	204086.202	193134.204	200140.406	282535.488	377926.888
***	71.5	68.5	M+K	C10H21N5O	3.21686266	12514.0779	11057.4496	11555.3206	21789.5754	32271.3025
***	71.5	66.3	M+FA-H	C18H30O2	-1.3140744	4402.78361	3869.49135	5006.49525	230.326476	728.541423
***	71.4	63	M+H-H2O, M	C18H32O4	-1.4233455	66677.3615	66043.8667	65720.9199	40816.2001	46838.8543
***	71.4	85.4	M+H, M+K, M	C13H25NO4	-0.764236	469169.501	473329.623	459460.147	66512.8147	2453726.2
***	71.3	73.9	M-H	C11H19NO9	-0.2746321	512415.378	498806.189	520549.595	987045.14	957392.832
***	71.3	64.9	M-H	C17H28O3	-0.5581841	71865.4814	73863.5569	72699.2946	7983.12348	229234.098
***	71.3	66.6	M+H	C23H43NO4	-0.9667988	451036.266	448110.379	450689.365	74052.7921	1323461.4
***	71.2	64.7	M+FA-H	C27H45NO4	-0.0623398	7799.16591	7058.96752	6842.16093	645.702859	33527.4954
***	71.2	57.4	M+H, M+Na	C11H21NO4	0.13961467	803738.565	794875.179	809541.367	327819.758	3181926.59
***	71.1	76.3	M+H-H2O	C18H34O3	-1.6008137	39952.9191	40787.4135	40253.1501	50793.6506	28969.0901
***	71.1	69.6	M+H	C13H17N3O	-0.3063137	3273.10819	3185.75069	3011.8292	2002.06333	11123.5709
***	71.1	61.7	M+H, M+Na	C13H25NO5	0.35220288	85869.2444	83343.6349	85805.4213	18604.7271	514582.095
***	71	65.5	M-H2O-H	C11H14N2O	-0.7590285	7755.0615	8081.54685	8037.86374	5522.76307	24138.3948
***	70.7	76.9	M+H, M+Na,	C28H50NO7	-0.6670107	2264798.87	2275807.39	2293029.96	1319339.01	3026214.75
***	70.6	78.4	M+H	C8H18N4O2	-0.2702724	199922.653	195328.142	212068.315	498259.281	379430.494
***	70.5	79.5	M-H2O-H, M	C4H6O5	-1.1997369	1294260.53	1329093.85	1294237.54	1503145.78	2263804.03
***	70.5	90.3	M-H	C10H12N4O	-0.1752337	74375.9568	76448.9607	75503.5076	170291.653	97661.3517
***	70.5	63.6	M+H-H2O, M	C18H32O2	-1.3060363	53717.2675	63449.0844	59206.0845	115832.347	57566.5689
***	70.3	84.8	M+H	C5H11NO2	2.11048925	591065.359	609855.013	575012.883	901538.747	737841.717
***	70.3	78.8	M-H	C5H4N4O	-1.1969329	332804.495	359873.378	340529.809	441328.758	310599.923
***	70.3	91.7	M+H, M+Na	C12H17N5O	-0.2736165	11858.4962	12273.5024	12438.2246	35231.3285	15505.1059
***	70.2	78.5	M-H2O-H	C12H18O12	-3.0517108	218970.93	225353.274	247204.923	140198.097	269893.212
***	70.2	84.7	M-H	C5H9NO3	-1.3196751	30872.1605	31914.4366	29534.8066	33023.0615	45060.118
***	70.2	53.8	M+FA-H	C25H45NO5	0.22213842	7883.92663	7804.40067	8011.69674	516.132195	46855.3311
***	70.2	71.7	M+H	C21H41NO4	-1.6518451	535497.813	551683.615	560305.705	85497.9401	1606339.29
***	70.1	69.3	M+H	C7H16N2O2	0.50408748	14825.9059	15291.6049	14888.9362	14879.7155	56231.0949
***	70.1	56.3	M+H, M+Na	C25H45NO4	-1.2443518	786649.353	790309.156	793509.84	120106.368	3266243.69
***	70	58.7	M-H	C2H8NO4P	-0.900153	48400.872	49658.5825	48588.7096	70597.8062	43422.2195
***	70	76.5	M-H, 2M-H	C10H12N4O	-0.541499	1118930.72	1141634.26	1159140.53	694905.768	1732062.7
***	70	77.8	M+H	C15H22O	-1.2591519	28295.3153	29412.4591	26727.9565	37403.1963	23686.7239
***	70	94.7	M-H	C23H46NO7	-0.4753292	510666.482	510751.761	507388.235	248657.374	718639.649
***	69.9	93.2	M-H, M+FA-I	C22H34O2	-0.6109928	353781.59	365154.184	340471.411	524686.866	192752.116

***	69.8	94.4	M-H	C10H17N3O	-0.5556947	35167.9497	30614.5944	29446.8862	11257.3817	27877.0107
***	69.8	63.5	M+H, M+NH4	C18H32O	-2.169051	4289684.77	4292248.67	4246906.67	497642.004	2443659.23
***	69.7	77.1	M+H	C4H7N3O	3.03478081	82288.1383	84996.8806	87139.6824	171159.779	117585.059
***	69.6	57.4	M+H, M+Na,	C6H11NO4	0.56985116	134033.306	141309.31	137261.32	130733.55	655827.631
***	69.6	66	M-H, M+FA-H	C12H22O11	0.21766184	66888.8158	66787.8225	66270.0823	10087.2327	274185.949
***	69.5	86.2	M+FA-H	C23H45NO4	-0.0413852	66426.3542	65910.4778	61536.3222	13796.8474	273392.736
***	69.4	60.4	M-H	C5H5N5O	-2.1734996	1444.9448	1302.84967	2017.15141	283.852801	351.291398
***	69.4	53.2	M+NH4	C26H34O6	-0.9960842	6263.1517	6097.48051	7494.02689	3461.80623	4174.8779
***	69.3	55	M+H	C15H22N2O	0.87844335	2768.70467	2646.89948	2495.86909	827.755579	4267.52396
***	69.2	59.7	M+H, M+Na,	C4H9NO3	1.37611801	219453.773	226968.745	230054.493	422788.332	268481.052
***	69.1	74.2	M+H, M+Na	C10H18N2O	0.21273179	19084.2032	18543.5291	18237.7646	34993.9013	13282.2542
***	69.1	73.3	M+FA-H	C19H37NO5	-0.2286409	6636.74706	6380.61109	6454.18762	555.286557	36231.082
***	68.9	50.4	M+H-H2O	C5H10N2O3	0.79401846	19110.2548	18474.9681	19008.2604	24940.0731	26026.2332
***	68.9	74.2	M-H, M+FA-H	C5H7NO3	-1.1510408	434875.328	439935.864	414894.805	650190.784	277309.621
***	68.7	85.5	M+H-H2O, M	C5H12N2O2	0.87246798	44985.8647	43472.6897	43281.9044	37873.576	106772.095
***	68.7	47.7	M-H, 2M-H, M	C6H6O6	-1.7951483	82752.8152	75436.8399	68111.932	140126.39	166853.763
***	68.7	59.5	M+H	C11H14N2O	2.31686105	69053.1601	67694.3969	69971.0883	26557.7246	145667.118
***	68.7	59.5	M+H	C13H17N3O	0.50889669	3966.29003	3320.45089	3752.41883	1535.0974	6353.86473
***	68.7	59.5	M+H-H2O, M	C23H38O4	-1.477119	185134.984	191133.619	145739.058	35510.143	8537.0972
***	68.7	50.2	M+H-H2O	C20H32O3	-1.7414	20200.3242	17327.9925	16685.8827	27207.4773	27349.9771
***	68.7	77.2	M-H	C9H10O4	-1.2397965	23713.4713	24576.5181	23066.1269	25594.1281	9359.46799
***	68.7	85.8	M+FA-H	C23H45NO5	0.60243279	23972.5448	24325.6452	24274.8059	6756.62832	92916.9681
***	68.6	51.9	M+NH4	C27H44O4	-1.2516649	263779.498	261518.921	263271.737	56485.4697	1421566.1
***	68.6	71.6	M-H	C23H48NO6	-0.9400318	340211.841	336678.858	335829.222	74527.2047	703295.017
***	68.5	55.8	M+H-H2O	C27H47NO5	-2.1068218	365603.956	362405.366	368171.484	90744.7533	1297495.73
***	68.5	61.1	M-H	C10H12N2O	-1.18664	25939.5501	26192.0879	26691.4199	24137.7718	72985.3715
***	68.4	61.5	M+H	C17H31N3O	0.00857825	6740.72459	6770.23788	6480.33665	3936.28697	8516.04065
***	68.3	80.8	M+H, M+Na,	C9H13N3O5	-0.9464909	78863.9983	79864.7261	76690.3817	37320.2626	159121.157
***	68.3	50.6	M+H, M+Na	C13H16N2O	-0.0709423	33375.3203	32376.6397	30848.078	29683.4634	98573.3028
***	68.2	75.2	M-H	C18H32O3	-0.9469171	119268.489	117441.931	121487.846	47211.3031	127836.146
***	68.2	53.7	M-H2O-H	C22H38O5	-0.2814791	7613.34742	7832.11603	7656.96279	3145.27695	698.355332
***	68.2	70.7	M-H	C11H11NO3	-1.4405068	4879.71013	5158.40986	4962.34794	8831.42567	1946.61841
***	68.1	84.6	M-H	C5H8O4	-1.4561608	8444.78056	8573.89701	8291.30528	6528.35096	36554.9879

***	68.1	54.6	M+FA-H	C19H32O	-1.5787029	20050.3231	20144.2117	20554.0898	8788.10394	6803.42969
***	68.1	50.4	M+H	C19H32O3	-1.8735736	75341.1403	70520.6919	73975.8973	2637.15225	1003.67171
***	68	43.9	M+FA-H	C5H7NO3	-2.0972326	122956.212	128962.605	119843.652	168080.424	39827.5925
***	68	67	M-H, M+FA-I	C12H18N2O	-0.3176813	1849358.49	2069614.61	1885567.85	2178202.97	3440749.37
***	68	74.4	M+H, M+Na	C22H46NO7	-1.1358899	69026.8252	68301.08	66004.6781	53341.3625	237472.52
***	67.9	80.3	M+H-H2O, M	C11H19NO9	-0.7415596	530543.071	534329.562	528127.796	825595.897	676504.495
***	67.9	54.9	M+H-H2O	C27H42O3	-1.1604654	2907.23084	2737.48447	2689.82396	2038.6441	1.76581927
***	67.9	96	M-H	C7H13NO3S	-0.7977101	42638.1879	44314.5718	43586.2447	42889.6518	85174.1246
***	67.9	43.5	M+H	C14H18N2O	0.79788806	61025.2809	63439.228	63782.7672	28661.4969	106744.265
***	67.8	74.5	M+H	C21H42NO7	-0.8699554	18817.9244	17648.4275	19056.3299	15030.1806	24090.9847
***	67.8	89.8	M+H-H2O, M	C20H34O5	-1.1521206	67151.8745	64966.3747	66048.6798	15116.1824	368260.796
***	67.7	70.4	M+H, M+Na,	C26H54NO7	-0.7496176	6411022.66	6435884.87	6710156.08	4614398.1	12222280.7
***	67.7	88.6	M+H, M+Na	C8H16N2O3	0.39357766	33468.5006	34161.0513	34545.6646	9924.9991	29903.8788
***	67.7	50.8	M-H	C12H15NO5	-0.3404432	27159.5438	27732.9101	27694.205	31526.9985	74055.3184
***	67.6	51.7	M+H-H2O	C25H49NO5	-1.2726316	1710270.4	1723524.8	1731909.26	351414.675	6329468.5
***	67.4	43.9	M+H	C12H20N4O	-0.9519912	23852.5598	24312.5918	24735.0004	4380.43727	49341.4427
***	67.4	42.8	M-H	C20H30O3	-0.8718263	12556.137	11905.7901	11632.0252	10860.8928	8060.60414
***	67.3	69	M+H, M+Na	C25H49NO4	-1.2047043	2173555.41	2177148.98	2217361.65	1325660.66	6538228.84
***	67.3	51.2	M+H-H2O, M	C20H32O2	-4.906492	765122.916	721225.522	722513.759	1123701.6	803920.702
***	67.3	97.7	M-H, 2M-H	C9H11NO2	-1.1968397	372288.455	393012.196	349389.11	411015.47	530140.169
***	67.2	49.3	M+H-H2O	C5H11NO4	1.6300733	13120.0892	13446.6358	11933.4892	35655.6234	8382.60225
***	67.2	44.2	M+H, M+Na	C9H16N2O5	-0.0819288	39918.8598	41787.1584	41140.5802	12785.7821	114321.629
***	67.2	95.3	M+H, M+Na	C21H27NO4	-0.5123685	79306.433	76997.9673	77548.4161	828.909614	679.659268
***	67.2	98	M+FA-H	C22H46NO7	0.11452928	24298.8619	24701.2311	24175.5987	14369.9426	87479.5556
***	67.1	73.5	M-H	C4H6O4	-1.1751972	24492.8313	26104.3096	25186.1849	5945.67886	66634.1905
***	67.1	63.5	M+H	C13H17N3O	0.49090464	3790.08123	3632.29926	3949.56815	2355.09834	13186.1853
***	67.1	59.6	M-H, M+FA-I	C20H30O2	-0.8271829	282230.718	260778.125	263071.943	244677.205	398965.449
***	67.1	39	M+H, M+K, I	C14H18N2O	1.04335385	162792.004	161781.885	163351.743	187944.369	101175.56
***	67	78.1	M+Na, M+K	C18H32O16	-0.0725488	880069	848266.641	906421.759	28790.6787	3721832.19
***	67	50	M-H	C22H32O3	-0.7743108	49118.5074	50220.083	50208.5308	21565.564	21126.7825
***	66.8	50.2	M+H	C10H13N5O	-0.5543555	157961.358	156614.347	155111.106	114864.484	110900.507
***	66.7	45.6	M+H	C5H11NO2	1.47124886	2137871.57	2123727.33	2212068.69	2955823.07	4227670.02
***	66.7	46.1	M+H-H2O	C23H43NO3	-1.4699241	14564.2362	14543.6414	14402.5791	6353.34224	47700.264

***	66.7	86.1	M+FA-H	C26H54NO6	-0.1215621	7161.8301	6830.85876	5549.97886	2091.08353	25430.3954
***	66.7	43.8	M+H	C18H39NO2	-1.3528809	31074.752	30101.9674	30486.7608	15517.1625	20375.5806
***	66.6	51	M+FA-H	C19H30O2	0.02620139	48480.3832	50427.4676	52114.1398	13096.1228	20828.6275
***	66.6	60.7	M+H	C28H44NO9	-0.7332094	91471.4445	87001.0537	86758.0906	107840.185	55088.8117
***	66.6	52.4	M+H, M+Na	C12H10N4O	-0.7318383	10637.5593	10810.316	9926.70962	15554.9764	12782.4189
***	66.6	43.4	M-H	C13H24O4	-0.6933403	3175.0008	3577.46268	3202.35167	2592.91112	2897.54594
***	66.6	38.7	M-H	C20H32O4	0.31857502	15417.8124	15769.6687	16421.8381	3342.81788	11280.3571
***	66.5	58.1	M+H	C21H28O5	-1.174338	4943.35448	5049.98546	5208.15552	4977.27899	17907.0665
***	66.4	60.7	M-H	C10H14N5O	-0.0519066	40520.6756	39522.3646	38063.1848	1923.78748	29579.8281
***	66.4	72.3	M+FA-H	C26H52NO6	0.10877018	11114.8428	11766.3889	10966.5488	2232.52332	37228.6945
***	66.4	47.6	M-H	C9H9NO4	-1.4771437	1989.317	1689.86165	1672.84248	919.923277	12913.1434
***	66.4	41.5	M+Na	C18H32O5	-0.8994605	16138.2591	14961.2	15223.2136	21130.307	12089.6237
***	66.3	91.8	M+H	C15H17N3O	0.87188122	5131.0006	5332.62677	5143.21206	2533.43156	15878.268
***	66.2	61.3	M-H2O-H, M	C6H13NO5	-1.2476518	9168.91059	10587.9124	10547.2866	13741.1143	65931.7627
***	66.1	87.9	M+H, M+Na	C25H50NO7	-0.5453098	78368.7897	74807.0202	77568.2234	23340.0154	120713.008
***	66.1	49.5	M-H	C6H12O3	-1.4679523	8564.77586	8675.54952	8378.544	12949.4224	14357.3884
***	66.1	50.5	M-H2O-H, M	C20H30O4	0.10347542	7166.95014	7808.5708	7268.10322	216.19886	16556.031
***	66	82	M-H	C7H12O4	-1.4304522	8599.14248	9095.19699	8608.77861	8006.47611	7731.75539
***	65.9	54.4	M-H	C4H8N2O3	-1.2720464	23408.4279	25162.6073	25115.6589	28029.0145	21660.8911
***	65.9	39	M+H-H2O	C10H18N2O	-0.3216067	16591.1242	15328.8376	16522.5444	23506.9049	30380.452
***	65.9	73.3	M-H	C10H10O5	-0.889489	89101.9853	91191.8523	90104.8973	89140.2955	80319.7852
***	65.9	87.4	M-H2O-H, M	C9H10O3	-1.569594	21331.9646	21108.7302	21608.7403	16566.1764	2035.41636
***	65.9	38.4	M+H	C15H29NO4	-1.2353108	96610.3226	97799.6814	96734.5388	6003.47682	286882.062
***	65.8	49.2	M+H	C8H17N3O3	0.61833725	11790.6302	12492.7758	12966.4488	5301.58891	26608.529
***	65.8	59.7	M-H	C19H34O4	-0.9298897	2884.22355	3384.45684	3244.04925	230.985032	931.575411
***	65.7	46.8	M-H	C5H8O5	-1.1431151	24115.9564	25447.9756	24242.9861	17262.0986	85600.255
***	65.6	54.3	M+H, M+Na	C10H17N3O	-0.6778543	54497.5873	57531.9227	53525.6697	118678.239	89714.0375
***	65.6	63.9	M-H, M+FA-I	C10H14N2O	-0.0547792	6857.09808	7881.84915	7313.45543	20979.4129	10348.7599
***	65.6	82.3	M-H	C9H16O4	-1.2895667	29579.8283	31085.8547	29192.2147	26177.4454	28907.4495
***	65.6	87.2	M-H	C10H18O4	-1.0548369	6625.04197	6608.73065	6504.82283	4699.95928	6185.9882
***	65.6	48.4	M+H	C19H35NO4	-1.1722963	38583.8398	38354.579	38849.33	3910.35764	75748.2704
***	65.5	56.4	M+H	C4H5N3O	3.1546406	16914.5993	17073.2152	18317.8254	7758.31484	26462.9631
***	65.5	76.7	M+H	C10H14N5O	-1.128103	89411.3968	78291.3334	86498.1908	2988.81398	89947.0391

***	65.4	42.2	M-H	C6H13O9P	-0.5223337	347699.629	358469.973	352047.986	355846.405	223029.865
***	65.4	64.6	M+H-H2O	C23H38O4	-1.6083404	15084.1725	16715.0569	16261.8332	9881.93992	7647.07953
***	65.4	82.3	M-H	C8H14O4	-1.6270392	16756.481	17358.4078	16377.852	14866.8759	15804.905
***	65.3	55.5	M+H, M+Na	C14H17N5O	0.12033684	234276.675	236268.402	241460.779	119093.249	182912.206
***	65.2	52.8	M+H-H2O, M	C6H14N2O2	0.40886537	1070140.72	1047070.94	1038150.36	1497012.18	1254624.75
***	65.2	60	M+H	C7H15NO3	-0.4767545	2023465.19	2004620.4	2142638.48	1742016.23	6358936.83
***	65.2	87.7	M+H, M+Na,	C10H19NO4	0.30635555	868111.548	888418.789	885893.959	122851.04	957580.093
***	65.2	56.5	M+H	C15H25NO4	-1.2656866	2210.80686	2533.69742	2410.80694	432.51011	17959.5694
***	65.2	37.9	M-H	C26H43NO5	0.01699678	2312.23162	2201.19258	2233.69462	1203.56728	10748.7414
***	65.1	35.5	M+NH4	C10H10O4	0.01836348	2182.57558	2260.42124	2319.3227	3041.58551	1343.48432
***	65.1	45.3	M-H2O-H	C8H10O4	-1.2338254	1240.26519	1213.82848	1248.922	960.02874	745.986067
***	65.1	84.2	M-H2O-H, M	C20H32O5	-0.055532	50501.703	51481.7296	51499.2064	3348.93678	97808.9357
***	64.8	41.8	M-H2O-H	C6H7NO4S	-0.7794829	8777.8426	9059.50025	9009.03386	17371.1877	3809.48635
***	64.8	67.9	M+FA-H	C21H30O5	-1.1981338	11362.3995	11139.9999	10736.4156	256.787631	13730.8574
***	64.7	39.3	M+H-H2O	C6H12N2O4	-0.1987953	22089.0113	20502.0878	24001.5532	26202.3381	22228.397
***	64.7	59.1	M+H, M+Na	C10H14N5O	-0.9178983	398611.337	401168.187	387017.123	77088.9491	454553.119
***	64.7	36.4	M+FA-H	C23H45O7P	-1.4177301	77425.3743	77277.8782	77420.8876	46649.788	43142.726
***	64.6	61.5	M+NH4	C11H17NO8	-0.6944471	64854.1108	65524.2958	67058.6769	6020.78094	103686.823
***	64.6	57.1	M+H-H2O	C8H16N2O4	-0.0565441	44988.2176	45748.8476	49768.8764	22016.0343	91924.8414
***	64.6	38.6	M-H2O-H	C18H34O4	-0.9166161	41012.2156	40395.5335	41446.4398	34787.9375	33362.5704
***	64.6	33.2	M+H	C16H16N2O	-0.6096957	5213.15955	4759.76614	5102.68457	7124.21066	1996.27941
***	64.5	46.3	M-H	C3H7NO3	-1.2915194	14470.5469	15083.3597	12592.4208	21707.6724	15210.7418
***	64.5	38.6	M+H	C8H14N2O5	-0.6724949	31668.7221	31391.9446	29887.9522	72074.9745	62540.3751
***	64.5	60.6	M+H, M+Na	C23H40O4	-1.2623998	8261.08131	8031.66557	6020.30995	9111.32915	1932.43342
***	64.5	66.9	M-H	C13H16N2O	-0.3387277	3019.16109	2856.42782	2822.41183	4882.65302	1892.68506
***	64.5	76.3	M+H, M+Na	C13H23NO4	0.03630689	18503.4036	19159.8882	18942.8058	4044.99549	121874.811
***	64.4	38.1	M-H	C12H24N2O	-0.8907296	944.928109	916.539278	928.581061	180.872248	949.733194
***	64.4	35.5	M-H2O-H	C8H14O5	-0.8639416	30924.0988	32177.568	31708.7892	35379.1757	28252.2094
***	64.4	35.5	M+FA-H	C19H16O7	-0.3267422	13022.6811	14578.6953	14631.403	14814.5543	13218.1133
***	64.3	40.2	M+H	C5H6N2O2	1.7492753	2904.4362	3325.2559	3031.16428	9.7531E-07	9.7531E-07
***	64.3	76.5	M-H	C9H14N3O8	-0.0726601	19655.3557	20001.1741	19161.8716	3484.78988	6783.16977
**	64.3	29.5	M+H	C11H23N3O	-0.5440115	149749.192	177573.893	177376.227	10264.6359	1292930.28
***	64.3	39.4	M+FA-H	C8H15NO2	-1.2304218	6524.69824	6498.87519	6506.27934	864.048192	15906.4878

***	64.3	58.1	M+H-H2O	C27H53NO5	-1.3519394	310610.732	318980.074	309668.709	211297.512	705653.204
***	64.3	73.3	M+H	C7H6O	3.44676819	33970.0349	35896.0394	38186.7774	51909.5899	39693.4024
***	64.2	50.6	M+H-H2O, M	C11H21NO7	-0.6034379	212575.585	218884.865	233266.08	7416.21563	216616.368
***	64.1	39.5	M+H	C12H24N2O	-0.1033538	57294.8999	61463.341	57323.8974	13432.6076	64133.2441
***	64.1	64.4	M+H	C11H21NO5	-0.8111651	645705.1	704095.003	725903.878	593271.857	2449002.05
***	64.1	59.7	M+Na, M+K	C7H8N2O2	0.57235789	15839.6218	17003.2742	16539.1389	17873.7421	945.420615
***	64.1	67.1	M-H	C7H8N4O2	-1.2271197	3290.40825	3621.05448	3435.16334	6105.56932	892.173995
***	64.1	38.4	M+H, M+Na,	C10H22O6	-0.6799703	565195.932	540239.872	510156.084	393298.897	1140118.36
***	64.1	58.6	M+H-H2O	C21H30O3	-1.8223299	13595.101	15882.3811	15890.3666	21856.97	12994.0367
***	64	33.2	M-H	C8H14N2O5	-1.5931227	9699.74176	9749.79854	9824.77904	17550.4868	22156.1983
***	63.9	51.6	M+H-H2O	C5H7N5O	0.05898598	4174.10627	3659.21477	4350.31781	3685.03889	4529.14693
***	63.9	70	M-H	C10H17N3O	-1.5136202	49674.3582	53158.1763	48371.7006	10733.4806	36081.4611
***	63.9	49.4	M+H	C20H32O5	-1.1556186	36023.7155	36830.8626	36898.1875	2308.86503	22093.9263
***	63.8	33.9	M+H, M+K, M	C8H16N2O3	-0.172801	49244.6418	50161.3064	52358.1645	59983.2581	60819.2288
***	63.8	97.6	M+FA-H	C30H50NO7	0.13978947	65297.585	59860.5081	61173.1134	30795.9931	132778.945
***	63.8	38.6	M+FA-H	C25H49NO5	0.07173181	8053.96017	8091.23806	7503.63571	4429.42153	33370.4912
***	63.8	94.1	M+H	C29H49O12I	-1.1232771	102830.211	94588.8533	93538.0436	107701.315	128329.303
***	63.8	38.2	M+H	C27H42O4	-1.1684705	65195.0525	64642.4752	64561.8821	26732.9405	16425.538
***	63.8	82.7	M-H	C9H9NO3	-1.0450302	2031.00587	1892.12142	1740.68055	2618.9148	997.074953
**	63.7	29.1	M+H, M+NH4	C6H8N2	2.0587807	14945.3495	15976.2619	15941.1029	14169.2645	16610.828
***	63.7	39.9	M+H	C6H9NO4S	-0.3819102	147026.126	140474.092	139278.03	392722.28	138275.379
***	63.7	47.2	M+H	C13H21N3O	-0.4848009	4220.97102	3641.34524	4954.5274	3745.89912	749.742083
***	63.7	64.5	M-H	C7H13NO3	-1.379108	1488.96826	1384.22484	1296.70604	1701.58383	1878.43865
***	63.6	54.5	M+H	C27H44O3	-1.4739575	8375.27599	8131.67209	8254.43694	5349.37841	1076.34704
***	63.6	32	M+H	C26H56NO6	-0.4078865	75052.7964	72531.8328	76008.7732	36437.832	120153.628
**	63.5	24.3	M-H	C4H8O4	-1.5648909	4053.43278	3904.29659	4413.89961	3463.82418	4647.6721
***	63.5	52.9	M-H	C10H14N5O	-0.967155	139665.95	148154.819	138847.765	21645.3827	154724.544
***	63.4	47.2	M-H	C7H5NO4	-2.1785201	11715.2549	12654.0453	11670.2195	5682.4507	64326.2895
***	63.4	55.2	M-H, 2M-H	C4H4O4	-1.4161927	26168.4584	25896.4776	28776.2411	38506.2227	54318.4258
***	63.4	70.6	M+H, M+Na	C28H54NO7	-0.7931919	274214.292	264736.212	256249.297	147723.447	628322.932
***	63.3	36.8	M+H, M+K, M	C23H45NO4	-1.1929014	3538806.27	3581917.83	3619866.85	944164.431	13114873.6
***	63.3	51.6	M+H-H2O, M	C20H30O2	-1.6641724	267152.378	271866.237	266449.44	412379.641	109773.477
**	63.3	27.2	M+Na, M+K,	C21H36O4	-1.5347786	155934.3	148084.282	151018.55	29913.7928	83994.5654

***	63.3	75.7	M+H, M+Na	C17H19NO3	-1.2303328	24820.2971	24947.3929	25516.9416	49231.7242	905.901315
***	63.2	49.3	M-H	C3H6O4	-1.9845153	52723.3637	60019.0188	58702.3966	54262.303	97047.5244
***	63.2	44.9	M-H	C6H10O5	-1.1304627	11193.5883	10475.8313	11611.2448	10645.6816	47469.9303
***	63.2	33.2	M+H	C16H28O8	-3.8447906	2912.13414	4192.50313	3592.47227	2379.44812	6119.27978
***	63.1	62.1	M+H-H2O, M	C9H17NO4	-0.2722304	3409962.62	3518850.66	3430162.03	1940198	4153741.73
***	63.1	60.3	M-H2O-H	C14H22O13	-2.8875957	30505.5671	31386.4433	32274.3773	25621.0974	76455.2045
**	63.1	19.8	M-H	C18H30O3	-0.32479	33958.2259	33581.0433	34567.3829	21220.0479	20677.915
***	63.1	81.3	M+H	C18H37NO2	-1.4281797	20398.6787	19371.6462	19551.2014	34895.2589	10182.2
***	63.1	81.2	M-H	C11H20O4	-1.3991023	2760.95618	2796.0395	2965.63426	2383.43163	2344.96573
**	63.1	24.3	M-H2O-H	C19H16O5	-0.1979112	11594.9639	9148.98983	9902.33126	12364.4811	8528.71701
**	63.1	20.9	M-H	C20H32O5	0.03601896	6979.05773	6387.04517	6676.11398	209.507212	9698.12649
***	63	87.7	M+H, M+K, M	C25H44NO7	-0.8034438	4633143.8	4640085.22	4714175.45	3879865.44	2577116.62
***	63	58.6	M+Na	C15H29NO4	5.42092155	20528.2733	21869.4687	20956.1066	14876.2034	62180.9145
***	62.9	61.6	M+FA-H	C6H12O6	-0.8967158	187915.106	188519.125	163228.1	310083.018	191750.191
***	62.9	34.5	M-H	C11H14N4O	-0.4172395	3499.14613	3554.45356	3758.97214	1805.42158	1726.68389
**	62.9	24	M+NH4	C16H30O	-1.9757709	675480.138	630016.231	691424.392	161148.401	366068.433
***	62.9	40.4	M+FA-H	C10H14N2O	-0.4731392	2922.11516	3056.40708	3172.38431	5553.04207	3006.62647
**	62.9	27.8	M+H	C20H32O5	-7.4091268	15636.2778	14118.7533	14241.5744	14429.6459	14454.0698
***	62.9	37.7	M+H, M+Na	C25H47NO5	-0.8945506	183924.567	181899.583	183077.744	52255.1789	659464.109
**	62.8	29.5	M+H, M+Na	C7H14N2O3	1.47517865	10148.2436	9910.935	10989.7028	12469.6056	3511.48114
***	62.8	62.4	M+H, M+Na,	C12H23NO7	-0.6170473	711132.202	689720.628	685591.355	27196.6163	1117672.74
***	62.8	31	M+H, M+Na	C18H35NO2	-1.6331059	49561.7682	49322.5622	48208.2824	37760.8672	24673.0166
**	62.8	16.6	M+FA-H	C15H22O	-0.6655977	70290.545	69133.7101	73752.3035	65228.6102	64679.1669
**	62.7	24.5	M+Na, M+K	C20H30O3	-4.2434719	12649.538	13554.2193	12791.5145	7712.45177	4410.16105
***	62.7	40.4	M+FA-H	C12H18O2	-0.3035191	46269.5349	50090.9568	49237.2683	48526.1562	38983.0246
***	62.6	76.6	M-H	C16H32O3	-0.472346	46387.0477	45231.192	43081.9179	86120.4438	14756.4694
***	62.6	33.2	M+NH4	C17H28O4	-1.1936719	4252.25222	4153.62244	4469.93722	209.219684	21345.3016
***	62.5	35.8	M-H	C9H13N2O9	-0.2895402	40604.8404	35916.2715	39626.8094	30410.5692	56200.5698
***	62.5	36.5	M+H	C5H8N2O2	2.24106581	6252.84159	6237.74821	6416.67313	13489.0372	3260.07075
***	62.5	97.4	M+FA-H	C24H50NO6	-0.4617158	140657.519	140886.94	132431.716	34112.1218	448264.905
***	62.5	74.6	M+H, M+Na	C17H20N4O	1.10572026	26631.6735	26428.3137	27590.9095	41221.5038	24046.0977
***	62.4	45.3	M-H	C6H12O7	-1.4634872	190687.492	166843.496	194350.505	235810.92	218045.697
***	62.4	42.4	M+H	C5H6N2O2	2.08118967	2905.85798	3042.07699	3006.89747	17698.6356	1672.43637

***	62.4	71.6	M-H	C20H34O3	-0.9950747	68260.2601	69362.0175	67601.9099	52320.7009	41267.2412
***	62.4	32.2	M-H	C14H18N2O	0.16991774	5359.96923	5222.75443	5300.97832	12992.2431	6543.16671
**	62.4	29.7	M-H	C7H6O3	-1.1992124	1678.90751	1758.78601	1590.62148	1651.06604	1291.50968
***	62.4	84.8	M-H	C12H15NO4	-0.523724	76498.097	78314.9708	75984.0321	84265.1702	190941.08
**	62.4	22.1	M+Na	C20H32O5	-0.9752005	19650.5181	18866.184	19901.7069	4638.05844	12588.9458
***	62.3	64.6	M+H	C10H14N5O	-2.7422004	146725.741	139870.365	142506.321	317856.635	260195.82
**	62.3	21.7	M-H2O-H	C5H9NO3	-1.2119347	4107.28127	3562.77851	3216.57662	14409.6194	7127.24119
***	62.3	38.2	M+H, M+K, M	C6H11NO4S	0.20745818	304417.468	300976.872	313697.065	222607.59	667842.017
***	62.3	87.8	M+H-H2O, M	C21H38O4	-3.8031777	64107.0491	56487.0019	44755.8692	22994.9719	10468.6577
**	62.2	23.6	M+H	C7H12N2O5	0.33964038	38509.4894	37263.214	36461.5526	83943.4952	63050.1356
***	62.2	42.6	M+H, M+Na	C10H18N2O	0.18842689	39467.0397	39352.8962	41795.4413	185647.485	58950.8989
***	62.2	73.5	2M-H	C9H18O2	-0.7776163	6231.19917	6577.5585	5947.27351	4595.17705	2759.69513
***	62.2	97.9	M+FA-H	C24H52NO6	0.22107606	63339.6338	66353.7408	66093.7175	20887.865	131041.784
***	62.2	56.7	M+H	C21H39NO5	-1.0482924	23250.8843	24070.4463	23273.2772	3592.83046	60592.7449
***	62.1	72.2	M+H	C5H5N5O	-6.6864017	89819.0075	88757.5149	88399.504	79837.2163	61621.077
***	62.1	55	M-H	C20H34O5	-0.0746873	59643.5454	59152.7626	60403.3641	6206.11695	225173.553
***	62	40.8	M+H, M+Na,	C6H13N3O3	-0.2023807	85063.2896	81861.0213	80381.9955	99273.9587	59532.048
***	62	57.7	M-H, M+FA-I	C5H6O5	-1.9696759	187206.452	181491.324	159045.654	32788.2892	59123.3658
**	62	22.3	M+FA-H	C11H14O3	-0.0132723	5740.5945	6719.77779	6742.88767	6278.14769	6455.43613
***	61.9	98.4	M+FA-H	C25H52NO7	-0.6537065	59116.7935	57846.5883	55362.8566	35575.3408	103908.345
***	61.9	39.4	M-H	C10H11NO3	-0.7274663	1127.0373	1237.83338	1144.73084	766.559339	626.004755
**	61.8	20.5	M+H	C11H21N3O	0.18614509	4529.03531	4805.91253	4672.31844	2846.58924	3583.4525
***	61.8	81.4	M-H	C11H15N5O	-0.6943584	5567.75263	5621.9917	6222.07584	9467.08285	8476.3441
***	61.8	36.2	M-H	C20H34O4	0.25157867	4373.88847	5114.84022	4279.60469	1793.75426	2890.34553
***	61.8	92.3	M+H, M+Na	C10H18N2O	0.09677806	14566.2658	14734.3838	15060.002	5324.63774	69728.0032
***	61.8	92.9	M-H, 2M-H	C16H16N4O	-0.3947031	486170.484	521692.559	518026.76	2845.42609	5335.91664
***	61.8	38.4	M+H	C16H16N2O	-0.8932564	5182.07205	5240.0987	5313.4863	8288.65298	2986.45664
**	61.7	22.5	M+H-H2O, M	C12H22O11	-0.6628228	737130.048	719751.938	736895.77	40233.4581	1434739.91
***	61.7	57.6	M+H	C9H18N2O4	0.10250775	7529.37529	7298.0746	7434.38755	3071.3928	6119.14422
**	61.6	12.6	M-H	C4H9NO2	-2.0018469	14560.0688	14517.7262	15282.2284	34929.1517	24706.0685
**	61.6	17.5	M+H	C9H16N2O6	-0.4663411	17693.8903	16490.7158	16328.5666	57173.2022	16615.759
***	61.6	43.1	M+H-H2O, M	C17H26O2	-1.7646897	79326.3569	80674.9017	76746.4306	20105.0185	243327.412
***	61.6	81.8	M-H	C11H20N2O	-0.6922005	24403.9084	25584.7845	24252.5884	74523.5053	33745.0204

***	61.6	85.8	M-H	C7H8O4S	-1.309296	14336.3717	14328.4019	14294.9327	5234.1368	2067.06928
**	61.5	11.7	M+H-H2O	C6H10N2O3	-0.3267806	19222.3085	19055.6222	19031.0102	14987.8188	8610.74732
***	61.5	34.1	M-H2O-H, M	C8H15NO4S	-0.7744933	19430.8725	20145.0596	20283.7826	19359.2524	50855.1125
***	61.5	83.5	M-H	C7H6O3	-1.5028048	3196.33386	3191.68648	3217.27904	3102.41222	2798.77132
***	61.5	87	M+H-H2O, M	C18H37NO2	-1.3363255	358736.101	357364.223	365715.067	311664.708	325328.641
***	61.4	43.5	M+H, M+Na	C15H24O3	-1.4290649	53805.021	56192.6035	57145.6668	76829.27	41469.5106
***	61.4	75.4	M-H	C5H10O3	-1.4589125	16286.1438	16812.5374	16441.2251	22806.7967	24061.5375
**	61.4	30	M+FA-H	C8H16O2	-1.5788591	5371.49265	5163.32888	5425.254	4495.1679	5260.55903
***	61.4	47.6	M+H, M+Na	C23H45NO5	-1.2449454	288518.983	291833.215	301059.311	126929.632	1054859.52
**	61.3	14.1	M+Na, M+K	C9H16O9	4.44226854	151219.234	164340.377	152940.375	153505.841	215004.104
***	61.2	37	M-H	C3H7NO4S	-1.5743161	15472.6087	15364.6027	15113.8586	30622.3466	4594.45074
***	61.2	33.2	M-H	C6H10O8	-0.9169841	33406.4881	30993.6738	35835.1851	25292.4723	5122.70572
**	61.2	8.78	M+H-H2O	C10H11NO3	0.19883397	628.491958	662.56913	628.150262	112.279442	405.332731
***	61.2	65.2	M+Na	C15H29NO5	-0.794646	7319.65301	7770.31961	7580.27689	598.215548	40072.8886
**	61.1	16.1	M+FA-H	C15H20O2	-1.0180623	3786.7642	4546.16833	3939.01111	3673.82431	4036.78976
***	61.1	65.7	M+H-H2O, M	C20H34O6	-1.3885054	63684.9409	62538.6304	65624.1376	12834.1403	119256.661
**	61.1	19.5	M+FA-H	C16H24O2	0.24634051	5386.05147	5520.18022	5565.78753	4731.18337	3899.62751
**	61	24.7	M+H	C18H37NO	-1.70269	204947.166	205476.279	213625.821	41229.4926	88323.9666
***	61	46.2	M+H, M+NH4	C19H26O2	-1.1488947	5102.20797	5373.63275	5440.52315	2885.41069	1867.34663
**	60.9	24.7	M-H	C10H14N5O	-0.7062049	29909.1995	28283.1722	31751.7647	11492.6522	15162.1408
***	60.9	69.3	M+H	C4H4N2O2	2.90574203	96017.7886	93905.0954	97069.561	210631.013	155884.391
***	60.9	30.2	M+H	C25H49NO5	-2.2415335	177204.416	178617.922	183031.801	141294.141	606304.091
***	60.9	34.2	M+FA-H	C25H47NO4	-0.6184902	24774.3353	24445.8295	21309.6432	2697.66616	96629.4994
***	60.9	47.8	M+FA-H	C21H43O7P	-0.7456328	82732.6175	80898.3333	79901.2714	9332.03416	53356.4346
***	60.9	67.8	M-H, M+FA-H	C20H36O2	-0.5244126	402616.277	418987.62	440067.024	603595.373	312160.544
***	60.9	33.2	M-H	C6H6O4	-1.4690167	6152.03183	4996.58167	4797.9122	3975.92332	4354.84965
***	60.8	50.7	M+H	C9H16N2O5	0.20143713	14719.9162	14490.6472	15648.6439	5273.22153	52367.2574
**	60.8	20.6	M-H	C22H32O4	0.26178085	5431.96194	6294.19157	6313.5237	229.977358	486.190921
***	60.8	42	M+H	C25H48NO7	-1.2031279	74969.798	72424.775	73245.69	42217.5575	91130.1229
***	60.8	83.3	M+Na	C20H34O5	-1.1409464	68857.4176	67342.909	69703.6439	5363.28918	296589.685
***	60.8	59.2	M+H, M+Na	C18H30O3	-1.0324292	20721.036	22753.4766	22650.4123	18874.7185	11674.1554
**	60.7	19.2	M+H	C9H19N3O3	0.48290447	7974.91271	7945.96885	7804.94853	2071.63217	8631.69348
***	60.7	74.3	M-H	C12H22O4	-1.0307574	3114.179	3587.14156	3393.16569	2414.25447	4252.84385

***	60.7	43.1	M-H	C18H32O4	0.18851707	44105.1723	46773.5351	45735.1421	12461.3279	15316.6597
**	60.6	21.7	M+H	C9H20N2O2	0.49854956	52440.0867	52346.9092	51078.8854	48478.5755	32366.4405
**	60.6	6.93	M+H, M+K	C6H15NO2	-1.0872264	68780.3791	69834.4343	71563.285	262447.164	30736.098
***	60.6	96	M+H, M+K, M	C21H44NO6	-1.0957093	2123843.43	2160215.88	2204264.94	817420.268	4519705.36
***	60.6	93.4	M+H-H2O, M	C22H32O2	-1.7617257	119881.386	86295.8978	118107.962	118627.395	106477.939
***	60.6	58.2	M-H	C20H36O5	-0.1940028	18298.3003	18467.3818	17875.3259	2919.41363	11052.3238
***	60.5	65.7	M-H	C18H34O4	-0.0517869	7877.70555	8362.68323	8145.40817	7030.5365	5251.75259
***	60.5	39.6	M+H	C17H23N3O	-1.2610851	1421.38894	1340.04776	1753.45996	116.016016	4345.24332
***	60.5	37.4	M+H-H2O	C9H9NO2	-0.3036915	1742.15128	1644.73515	1678.56166	1807.13359	994.884832
**	60.4	23.8	M-H	C18H30O4	-0.0515068	4623.80774	5603.85804	5357.71997	5775.09143	5428.26676
**	60.4	28	M+H, M+Na	C20H34O3	-1.9315274	7568.93968	6604.47248	6707.95788	1632.92779	1303.25808
***	60.3	36.5	M+H	C6H14N2O	1.31503275	22465.6894	22702.6464	22002.6247	13309.8324	8910.37017
***	60.3	47	M+H-H2O	C8H16N2O5	0.13107212	17010.4386	17250.019	17452.3521	4734.13392	43882.697
***	60.3	92	M+H, M+Na,	C26H43NO5	-1.166976	22148.6123	22951.7923	22556.398	12886.7013	51087.1573
***	60.2	30.6	M-H	C17H24O4	-0.1664386	10549.7772	10980.4174	10603.1299	9892.25705	10458.433
**	60.1	5.11	M+K	C5H11NO2	0.14325432	7311.14979	7141.416	7286.29406	12496.5873	50003.6168
***	60.1	32.3	M+H, M+Na	C11H15N5O	-0.1755415	18547.7314	18919.1673	19312.1754	48439.2148	18067.4158
***	60.1	38.4	M-H2O-H	C10H18O5	-1.160004	8722.94033	9129.38953	8548.41492	9181.86183	10073.0309
**	60	10.3	M-H2O-H	C6H13O9P	0.36735495	39311.26	39098.3565	38236.0675	55869.4974	10955.8261
***	60	36.6	M+H	C27H53NO4	-1.1565502	131732.122	129529.755	131565.024	336949.836	152217.911
***	59.9	36.4	M+H, M+K, M	C9H15N3O2	-0.2861073	157131.839	157245.274	161308.395	112365.258	141868.18
**	59.9	13.3	M-H	C7H14O7	-1.0879908	8774.50785	10285.4541	10275.6072	6533.77863	8784.22234
***	59.9	32.5	M+H	C10H16N2O	-0.5065716	213618.986	232786.422	228702.331	180615.564	521688.399
**	59.9	12.4	M+H-H2O	C12H18N2O	0.33698568	11193.7737	11347.8586	11422.9298	4265.5022	17784.4475
***	59.9	92	M+H	C22H34O2	-1.8862036	28742.5716	36184.3386	29052.782	57576.5207	23739.3036
**	59.9	19.8	M+H, M+Na,	C22H26O6	-1.3119887	113346.029	105349.678	107337.69	247.253317	12754.0473
**	59.9	4.72	M+Na	C20H34O5	-0.8498151	7623.02654	7242.84671	7296.7646	1975.33428	3212.19818
***	59.9	30.6	M-H	C20H34O4	0.50154553	4452.34692	4379.38155	4670.03197	853.454581	1577.2232
**	59.8	12.5	M-H	C6H14N2O2	-1.0234187	23746.742	27708.5557	20111.7758	36210.2161	42311.4686
***	59.8	31.9	M+H, M+Na,	C8H15NO6	-0.228765	260391.299	257675.127	277246.313	762043.722	824365.29
**	59.8	23.4	M+H	C4H7NO2S	0.19265358	434759.054	445095.5	467398.973	468228.933	1105471.38
***	59.8	59.6	M+H	C13H16N2O	0.57890858	16363.7163	16664.4065	16467.3658	4709.06856	66703.5515
**	59.8	17.9	M+NH4	C14H26O	-1.392229	24288.4215	24282.8325	23674.6328	8082.40894	10718.9986

***	59.8	61.7	M+H, M+Na	C14H22N2O	-0.5131775	277506.881	281438.485	291169.184	749192.81	681747.794
***	59.8	51.2	M+H-H2O	C20H34O4	-2.0713135	5190.69526	4840.14309	4939.81537	4164.90133	2628.40456
**	59.7	20.8	M-H	C5H14NO6P	0.02550161	19737.5487	20104.0165	20047.8731	4633.24908	45820.436
**	59.7	13.5	M+NH4	C9H15N3O4	-0.7138912	83023.9926	81901.6426	84157.2367	147300.776	108577.835
***	59.7	44.9	M+H	C12H21NO5	0.23767916	3025.5979	2877.24809	3065.14589	1372.24545	5611.22733
***	59.7	62.1	M-H	C14H16N2O	0.56815932	5486.08287	5763.10693	5936.72501	10330.2679	5037.11591
***	59.6	36.1	M+H, M+K, M	C9H13N3O5	-0.2061881	56392.0849	63115.1662	55612.6794	27704.5704	98603.8621
**	59.6	17.2	M-H2O-H, M	C13H21N5O	-4.1729534	69120.7249	69326.7351	67070.9611	4444.07351	130723.894
***	59.6	84.1	M-H	C10H18N2O	-0.5588575	2717.52046	3074.90372	2752.41438	7333.12818	4690.52379
***	59.6	54.2	M+H	C19H28O2	-1.5042183	1464.32089	1331.36689	1369.82841	9.7531E-07	923.018981
***	59.6	32	M+FA-H	C13H24O2	0.0751395	3536.52544	3673.97867	3314.37805	2448.11163	4683.27552
***	59.5	39	M+H-H2O	C18H32O3	-1.635286	17449.2007	17505.0104	16897.5702	31571.0593	10543.1209
**	59.5	15	M+H-H2O	C12H15NO5	0.6268996	795.217198	991.065587	771.215349	614.628999	1437.01001
**	59.5	16.5	M-H	C19H28O5S	-0.4669366	134579.959	136645.439	133603.034	174673.165	101293.949
***	59.5	38.3	M+Na	C20H32O5	-1.0405714	2921.387	3237.03493	3216.43556	706.649789	8012.99437
***	59.5	72	M+H	C18H39NO3	-1.1804707	20348.9601	19992.5982	19545.6195	12426.8515	20055.1889
***	59.4	91.1	M-H	C27H44NO7	0.09865393	404620.446	392675.166	413707.036	276493.797	299430.873
***	59.4	89.3	M+FA-H	C23H48NO7	-1.9322856	16823.067	16334.3044	15275.7235	7831.47632	39619.6088
**	59.4	25.7	M+FA-H	C13H24O	-0.4771276	79137.9331	82083.0875	79693.3426	77332.5543	65799.9256
***	59.4	93.9	M+FA-H	C30H54NO7	-0.2899674	31026.3878	33004.9396	29884.0439	25259.4561	30479.1494
**	59.4	24.1	M-H	C18H28O2	-1.1485029	2554.79672	2515.53436	2469.76521	4117.86272	1323.91527
***	59.4	37.9	M-H	C20H32O4	0.46242887	16564.0968	16665.4042	16878.8978	4176.40035	11321.2048
**	59.3	0	M+FA-H	C7H11NO4	-1.5429695	465.422129	643.304256	736.14351	253.694972	471.734462
***	59.3	50	M-H	C20H34O5	-0.0865786	82960.9489	84610.2652	79844.2416	3286.09536	421893.489
***	59.3	58.7	M+H-H2O	C10H14O4	-0.1139287	7683.98563	8033.05172	8038.01013	10656.9804	10146.0345
**	59.3	16.1	M+Na	C20H34O5	-0.9425328	15858.473	16004.3217	17300.2712	2125.42008	30179.0183
**	59.2	9.95	M-H	C6H15O8P	-0.7790805	51054.4136	51883.0605	55872.9668	34425.2409	90955.1797
**	59.2	10.9	M+Na	C6H12O5	-0.200755	7941.63041	6352.74002	8655.95315	8139.17114	3655.90561
**	59.2	0	M+H	C8H6O2	1.29057603	2104.58847	2362.30792	2202.75195	2963.76715	1750.25954
***	59.2	34.7	M+FA-H	C11H10O5	0.38055172	3502.53905	3805.28819	3506.92113	3572.81778	3280.17097
**	59.2	21	M-H	C20H30O4	0.17854529	3210.87401	3043.99568	3066.21015	520.680146	1586.40616
***	59.1	30.6	M-H, M+FA-H	C10H19NO8	-0.6528613	61181.7935	57776.8195	58706.0303	37738.992	158234.946
**	59.1	0	M+H	C20H42NO7	-0.8276761	2295.32931	2223.21534	2075.93125	508.751604	2543.47009

**	59.1	9.89	M-H	C14H24O2	-1.1185974	8551.40905	9126.48303	9563.61439	9498.7441	12271.5817
**	59.1	3.69	M+Na	C9H19NO4	-0.3176979	7073.34289	6638.17973	6886.54686	13935.4185	7114.0277
***	59.1	88.2	M-H, 2M-H, I	C11H12N2O	-1.1956619	183697.727	190579.708	191936.669	308428.267	91283.0255
***	59.1	37.3	M+Na	C20H32O4	-1.3457653	28989.1647	28206.3087	30227.5637	15152.8848	21123.8757
**	59.1	19.5	M+FA-H	C21H39NO4	0.12409742	5715.56388	6614.83524	6104.45702	9.7531E-07	16622.393
***	59	83.8	M+FA-H	C26H54NO6	0.23579027	54387.1185	56697.8963	53488.2505	16431.6703	125534.705
***	59	84.4	M+H	C10H13NO2	0.14846874	11790.4608	10963.9702	12142.9935	16404.5	10102.0913
***	58.9	37.5	M+H	C7H19N3	1.70796321	15195.4626	14184.7585	15588.4262	31118.5833	64693.4553
**	58.9	6.18	M-H	C6H5NO2	-1.2834493	2289.66572	2515.05667	2473.91739	965.131972	12019.2121
**	58.9	29.9	M-H	C5H10NO7P	-0.9512383	7610.74156	7492.95956	7846.64044	26690.066	18180.4073
**	58.9	5.34	M+NH4	C12H16N2O	0.03575119	33567.4557	34398.2558	35608.901	64487.0167	84823.5441
***	58.9	86.5	M-H	C23H46NO6	-0.0387061	344614.735	354997.355	332921.262	63786.281	573796.124
**	58.9	22.5	M-H2O-H	C7H11NO3	-1.0182928	15595.3135	16791.5616	16178.3991	14986.9862	12412.6579
**	58.9	0	M-H	C12H24N2O	-0.6127161	458.368357	443.755798	544.587914	111.418489	338.409233
***	58.9	74.6	M+H	C14H27NO4	-0.8054988	2697.59158	2848.44903	2748.12196	755.584053	12713.8661
**	58.9	8.72	M+H	C23H41NO5	-1.4496601	96479.1292	107279	95347.3564	18740.7265	312178.483
***	58.9	63.2	M+H	C9H10	0.6304579	5872.52826	6313.21693	6937.21765	6886.86253	5353.06669
**	58.8	0	M+K	C6H12O6	-0.195383	329220.911	298021.072	348671.999	314713.451	348545.998
***	58.8	80.9	M+FA-H	C28H52NO7	0.30808613	131264.083	124877.611	119701.967	69411.067	459218.789
**	58.8	0	M+FA-H	C11H16O2	-0.8631262	639.315815	580.345821	675.577543	1166.83879	1535.81906
***	58.7	35.8	M+H	C6H11NO2	1.06451595	460262.068	462428.563	446619.506	682336.525	551522.575
**	58.7	24.2	M+H	C3H7NO3	2.39411318	31664.3751	32236.1268	35438.5633	56162.1971	40935.8541
**	58.7	0	M-H	C3H7O6P	-1.6187817	7020.41163	6975.32178	7234.36152	6457.29011	5092.64229
**	58.7	0	M+K	C5H9NO2	-0.3755167	1991.91808	2696.65223	2055.69154	6185.91947	3176.41435
**	58.7	0	M+Na	C7H13NO4S	-0.3190754	3930.05132	4497.78402	3573.48416	4716.19968	6695.87238
**	58.7	0.217	M-H	C14H26O2	-1.1550056	2376.73431	2356.6061	2080.18649	2903.09352	1649.10765
***	58.7	76.4	M+H	C8H16N2O3	0.93520684	2792.36703	2846.35421	3087.65702	1622.82907	2246.66029
***	58.7	63.6	M+H	C10H20N2O	0.1382965	6262.23761	6106.25719	6064.15307	2466.54105	4621.12588
**	58.7	0	M+Na	C17H26O10	0.52330793	10452.1181	9860.99463	10606.1166	27127.7734	15505.1749
***	58.7	44.1	M+H	C8H10N4O2	1.1308172	23514.4411	24611.0309	24003.1952	67559.6008	517.228419
***	58.7	49.8	2M-H	C6H8O3	-0.1598698	28512.063	29662.7788	29401.4746	51738.2383	35385.6873
**	58.7	27.7	M+Na	C20H34O5	-0.9851633	4436.82621	4690.03638	4826.72203	1049.80677	15270.651
***	58.6	33.9	M+K	C7H15NO2	-0.7247027	766966.314	757577.577	830321.035	736304.719	539871.858

**	58.6	0	M-H	C4H9N3O2	-1.7439348	5331.2409	4652.42861	5031.72864	17498.8224	3763.55941
**	58.6	0	M-H2O-H	C6H13NO5	-1.1285675	2870.65387	2824.60737	3101.76376	4638.35338	9823.95602
**	58.6	10.6	M+H-H2O	C7H14N2O4	0.01473996	5622.8776	6068.56417	5900.16522	13702.585	1969.21575
***	58.6	72.7	M-H2O-H	C20H36O5	-0.0697714	25022.8941	26448.0382	26849.6594	6776.48942	7824.36714
**	58.6	27.9	M+Na, M+K,	C18H34O4	-1.5283508	458139.912	465518.807	465832.91	937501.424	374804.778
**	58.5	0	M+H	C5H6O4	1.26190902	1654.4008	2518.7058	1690.16911	4866.40106	3871.54688
**	58.5	15.6	M+FA-H	C4H10NO6P	-1.6693815	35285.9965	38272.3377	35062.8517	109876.944	73557.4017
**	58.5	17.8	M+Na	C12H24O11	3.64098097	4389.85242	4590.8504	5767.34719	11037.0585	5827.11855
**	58.5	0	M-H	C8H15NO3	-0.9791788	511.606233	600.389812	608.171732	783.468477	818.053232
**	58.5	8.03	M+NH4	C21H32O4	-1.3619191	3077.79039	3180.40296	2827.84134	9.7531E-07	3749.77643
**	58.4	0	M+H	C16H27N3O	0.13729858	2881.34812	2756.3929	3148.73354	1362.99796	8263.52185
***	58.4	65.5	M+H	C11H22N2O	0.10635533	13846.5067	13603.9156	13371.7336	4480.01247	9848.02332
***	58.4	36.4	M+FA-H	C13H20O2	-0.6084633	3609.51121	3666.80591	3778.96376	3135.43642	3188.6527
**	58.3	0	M+H-H2O	C7H13NO3	0.07968119	1892.42336	2120.17221	2365.63619	646.165386	3372.6774
**	58.3	0	M+Na	C7H14N2O3	0.03318522	1523.00434	1487.94553	1511.44235	637.111096	855.253008
**	58.3	0	M+H	C12H25N3O	-0.2082924	11201.4064	10945.0631	10632.8976	6036.90533	16284.2736
***	58.3	85.4	M+H	C13H18N2O	0.324311	4842.65285	4365.0253	4601.67641	1743.21206	7580.58799
***	58.3	44.7	M+FA-H	C17H18N2O	0.38834442	24890.2973	24788.9559	26101.5583	29561.6871	15449.7692
**	58.2	22.1	M+H	C7H16N4O2	0.02859193	28847.0256	27887.5427	29571.9891	37151.605	27283.9555
**	58.2	0	M+FA-H	C5H9NO3	-2.3718124	2814.96333	1633.12037	2809.99635	2649.78946	877.853958
**	58.2	0	M+H-H2O	C6H13O9P	0.3323855	2830.01036	2311.85327	2345.0926	321.561462	261.980122
***	58.2	75.2	M+H	C10H20N2O	0.14585944	9116.60776	9060.25748	9331.55945	6897.76269	10935.8947
**	58.2	0	M+H-H2O	C21H32O2	-1.3189342	2848.099	1764.86966	2312.46374	266.409878	9.7531E-07
**	58.2	0	M+NH4	C28H45O9P	-1.4363374	33698.4288	30363.6796	32051.272	60192.6877	15392.3909
*	58.2	95.4	M+H	C14H19NO6	0.23372951	25485.845	25365.2541	24838.2765	38628.6581	65127.8109
**	58.2	0	M+K	C13H16N2O	1.24232397	1309.7516	1341.07732	1361.724	2198.43582	482.087723
**	58.2	0	M-H	C18H32O5	-0.3122022	1856.1894	2090.07159	2426.33506	797.230407	754.633796
**	58.1	0.943	M-H	C16H28O2	-0.792112	5555.85146	5241.39744	4971.92635	21037.6741	1439.91157
**	58.1	24.1	M+H-H2O	C33H38N4O	-0.1712723	11034.9843	10317.6404	9967.96784	10906.14	323.200161
**	58.1	0	M-H2O-H	C8H10O4	-1.2083677	561.762062	578.834437	546.289068	480.205846	542.28858
**	58	0	M+Na	C7H12N2O5	-0.7744027	8405.04185	8653.2279	6930.9603	14430.712	7229.03167
**	58	6.02	M-H	C5H9NO4S	-1.5072986	5091.50695	4942.55811	5207.84534	464.49506	6309.94967
**	58	0	M+H-H2O	C6H10O5	0.40999336	1133.62074	1180.04507	1114.0678	891.969649	1770.43368

**	58	5.82	M+Na	C6H8O7	-0.7053505	24061.6208	21349.2958	21773.1543	3715.85986	1016.20324
**	58	0	M+Na	C9H12N2O5	-0.450556	1929.69048	1670.35063	1804.32715	9613.77236	1007.66814
**	58	0	M+H-H2O	C14H17NO5	-1.0400839	435.500568	598.667766	567.58126	454.42001	524.820842
**	58	0	M+Na	C21H20O10	-1.1734336	512.87741	789.299567	585.212584	9.7531E-07	9.7531E-07
**	57.9	0	M+Na	C5H12O5	-0.5280363	4888.43369	5072.94209	5682.4021	7349.89421	9415.84291
***	57.9	45.2	M+H	C10H18N2O	0.79096046	3065.51254	3540.41217	3381.36741	2787.87645	4240.53675
**	57.9	0	M+H	C20H39NO3	-2.2367121	2564.88466	2267.52925	2303.04704	5808.93988	3106.7707
**	57.9	8.21	M-H	C9H8O3	-0.8972439	8138.55809	8154.60557	7926.29819	4259.36197	2234.37197
**	57.9	0	M+FA-H	C10H18O3	-0.8054179	835.457929	830.703451	778.675236	875.736504	684.989424
**	57.9	0	2M-H	C10H7NO3	6.2985218	1052.43721	1079.77119	977.95672	1006.13676	680.973519
**	57.9	16.6	M+H-H2O	C20H30O3	-1.6359133	2913.98796	3212.39007	3374.48512	1655.21306	1539.23504
**	57.8	0	M+H, M+K	C9H15N3O6	-0.8574046	21970.4116	16700.0189	17873.8889	50154.8633	49939.5313
**	57.8	0	M-H2O-H	C8H15NO6	-0.9721307	333.024941	324.275526	272.335022	685.493911	9.7531E-07
**	57.8	0	M+H	C9H18N2O4	-0.7520166	1918.96575	1659.60734	1784.53195	981.146601	1310.44178
*	57.8	95.4	M-H	C27H53O12I	-0.1966929	234152.152	276030.296	272347.069	222101.6	684573.151
**	57.8	0	M+FA-H	C10H12O4	0.82368974	212.002996	174.345876	175.04945	197.563723	149.009032
**	57.8	0	M+FA-H	C15H26O2	-0.3795957	2687.20769	2721.84994	2473.81284	2349.85199	3328.64035
**	57.7	0	M-H	C9H19N3O3	-1.2217545	663.082211	655.817492	801.93602	9.7531E-07	626.456926
**	57.7	0	M+Na	C6H14O6	-0.5384724	14654.2548	15918.5216	15878.1948	2694.28895	16840.2864
**	57.7	0	M+FA-H	C12H22N2O	-1.3573758	5262.93701	3190.19989	3742.33252	22319.5407	50052.8687
**	57.7	0	2M-H	C4H7NO2	-1.2780042	187.716037	180.21881	180.334099	9.7531E-07	1068.48497
**	57.7	13	M-H	C5H10O4	-1.4742899	1404.0746	1147.68814	1655.74859	1386.76013	897.13578
**	57.7	10.2	M+H-H2O, M	C20H30O3	-1.6637507	15579.7244	17038.1971	18128.3457	8661.47699	8851.49316
***	57.7	88.3	M+H, M+Na	C22H46NO7	-0.8960693	75176.8762	73324.5076	74704.5329	46310.566	111749.909
**	57.7	19.2	M+K	C29H50O4	-2.1570011	15653.0357	12668.2264	13862.3574	812.04958	18775.6955
**	57.7	14.6	M-H	C19H36O2	-0.993247	10551.8209	10198.7882	8973.23312	14120.1162	6136.84414
**	57.7	0	M+H, M+Na	C19H19N7O	1.78272566	3080.00166	3592.77151	2345.72928	9.7531E-07	308.693239
**	57.7	0	M+H	C12H14N2O	-0.045382	280.486714	263.507889	204.71242	62.1629041	63.3836865
**	57.7	0	M-H2O-H	C12H22O5	-0.6637185	14615.763	15485.476	14854.8779	14361.7681	12490.1755
**	57.7	0	M+H-H2O	C14H26O4	-0.9305729	6772.09369	7054.73959	7502.86813	9202.44113	6019.95058
***	57.7	75.9	M+H, M+Na	C25H42NO7	-1.0910432	37943.8234	38700.8969	39273.5595	21604.924	41107.1147
**	57.6	13.7	M-H	C6H12N2O4	-0.1135833	15799.024	14425.891	16763.464	7636.48055	27944.3087
**	57.6	0	M+H	C14H20N2O	-0.1437774	2127.76771	2365.98157	2325.26048	748.005232	3282.57386

***	57.6	84.5	M-H, 2M-H, I	C9H10ClNO2	-1.4007881	1354384.21	1403366.66	1280474.69	1315724.39	1261275.51
*	57.6	89.9	M+H, M+Na	C38H72N2O	-0.692533	37567.7013	37993.8207	33030.6543	197.355612	250.628481
**	57.5	0	M+H	C10H16N4O	0.32884641	1083.61966	1109.90099	1519.41402	9.7531E-07	9.7531E-07
**	57.5	9.51	M+K	C10H12N4O	-0.9103959	245811.23	250802.309	260517.023	329332.631	303877.65
***	57.5	46.9	M-H	C18H30O3	-0.0745624	14490.1298	14511.7293	14864.6843	5511.08687	8969.45033
**	57.5	0	M-H	C16H24O2	-0.9126529	4016.59914	4272.18328	4676.66677	4082.80642	4341.5435
**	57.5	0	M+NH4	C16H24O2	-1.4032446	13069.1156	12425.8165	12853.2737	13872.8769	3711.19003
**	57.5	0	M+H-H2O	C20H32O3	-1.9391012	2056.81622	2016.32954	2046.28692	1191.8212	1674.83067
**	57.5	0	M-H	C11H13NO3	-1.364894	733.234659	694.871979	720.964189	1385.4762	681.71484
**	57.5	13.2	M+H	C16H29NO5	-0.6117293	570.306911	654.067902	668.784651	6656.92803	776.601199
**	57.4	0	M+H	C9H14N4O3	1.55416424	2654.90326	3682.15843	3179.25072	981.444358	2598.09011
**	57.4	0.954	M-H2O-H	C5H10N2O3	-1.7459217	3425.74151	3241.49029	3629.05028	6604.92327	4634.21388
**	57.4	0	M-H2O-H	C4H8N4O4	-1.2429739	385.225028	513.524719	361.623387	359.186938	9.7531E-07
**	57.4	4.61	M-H, 2M-H, I	C16H32O2	-0.633607	326982.319	336182.58	339092.777	358210.206	289280.21
**	57.4	0	M+H	C14H19N3O	-0.0757815	1243.27357	832.544258	1185.7445	551.951589	1737.10537
***	57.4	66.5	M+H	C14H20N2O	-0.0086539	7480.45614	7265.07251	7857.8387	4714.94975	7365.15805
**	57.4	0	M+H	C14H20N2O	-0.2679299	689.641686	853.715003	837.394883	389.264438	545.388869
**	57.4	22.6	M+H	C17H31NO4	-1.3669169	15909.5702	16459.2294	16520.294	2871.89481	28171.357
***	57.3	65.6	M-H	C9H11NO6S	-0.8886856	7704.64864	8905.07954	8523.60028	11974.6138	9536.81113
***	57.3	37.7	M-H	C20H30O3	-0.8775534	11550.7712	12486.9869	12167.0682	4194.17511	7114.42565
**	57.3	0	M+H	C10H19N3O	0.01054481	3054.21793	2890.61055	2915.14152	1033.77907	4731.91278
**	57.3	0	M-H2O-H	C8H8O4	-1.6819705	1249.2052	1281.4433	1131.277	926.664834	948.251833
**	57.3	10.7	M+H	C10H9NO2	0.94130216	409.492738	338.71158	408.191859	56.400142	434.555132
**	57.3	0	M+Na	C12H18O4	-0.1029583	2498.30968	2804.69191	2624.46265	4043.76099	2032.17631
**	57.3	10.2	M-H2O-H	C21H28O5	-7.0794273	2012.09017	1594.77675	1848.22618	4802.467	537.896379
***	57.3	35.6	M-H	C16H16N2O	0.13588326	15310.4319	12514.7376	19159.521	17687.2217	5415.91912
**	57.2	0	M-H	C18H28O2	-1.0927264	1325.55173	1440.05731	1554.116	1633.80997	758.085162
***	57.2	85.2	M+H	C11H14N2O	-0.4605801	10965.5168	10645.8872	10694.6352	4834.98937	11779.7602
**	57.2	0	M+H	C13H18N2O	-0.4614264	194.102065	119.054124	184.607678	203.696311	245.390471
**	57.2	0	M+FA-H	C18H20N2O	-0.5252056	3232.14633	3167.43715	3302.52126	5689.20755	694.294888
**	57.2	0	M+K	C10H11NO	-0.4573447	3600.8342	3785.64026	4865.34948	5775.0169	3209.08966
*	57.2	89.8	M+FA-H	C22H28O5	0.28257886	3378.40528	3131.94507	3013.27942	9.7531E-07	60.390662
*	57.2	90.6	M+H, M+Na	C19H37NO4	-1.1356334	221006.466	211487.935	207986.944	16260.8167	594840.074

**	57.1	0	M+NH4	C6H7NO	0.059748	1223.28402	1106.40569	1565.18617	926.595044	504.736692
***	57.1	39.2	M+H	C11H20N2O	0.43414344	7383.50431	7403.34318	7459.37226	5412.82786	7225.05569
**	57.1	0	M+FA-H	C20H30O3	-7.6022261	3085.43974	3779.22654	3219.46536	1135.44672	924.665366
**	57.1	0	M-H	C17H26O3	-0.8504525	1158.86587	1139.23383	1184.88775	754.230804	633.853872
**	57.1	0	M+NH4	C4H4O2	5.78524199	2158.74483	2395.25274	2220.78615	2774.71574	1855.4128
**	57.1	0	M-H	C26H45NO6	0.08653539	3373.35215	3140.89426	3391.59828	756.768561	6357.61268
**	57.1	0	M+FA-H	C12H18O2	-0.3353762	2789.64187	2838.38497	2727.09906	3040.6836	2442.98918
**	57.1	0	M+NH4	C20H32O4	-1.2279496	664.998428	719.316059	686.786882	113.145245	240.446999
**	57	0	M-H	C8H17N3O3	-0.7138693	712.250758	707.000222	877.526532	117.432443	1766.75774
**	57	0	M-H2O-H	C5H13O8P	-0.2423667	6510.00569	6809.12855	7055.18405	5786.91536	7770.8909
**	57	0	M+H	C5H11NO3S	-0.3760751	16278.6581	16512.0332	21118.5221	17040.604	25763.7458
**	57	0	M+H	C7H14N2O3	0.94947896	5520.57895	4936.08767	5450.01924	366.629058	2474.67628
**	57	0	M-H2O-H, M	C8H10O4	-1.0488904	431.517555	451.706658	394.355355	573.799904	136.681372
**	57	0	M-H	C23H38O4	7.52087624	89362.3534	86739.2585	83086.9472	82288.6393	73210.5873
**	57	0.163	M+Na	C11H21N3O	0.06593397	2039.42004	2075.63303	2583.09949	46.9223171	2457.69099
**	57	0	M+Na	C14H21N3O	9.54278268	572.473394	637.361559	559.300908	258.774259	169.00649
**	57	0	M+FA-H	C13H18N2O	-0.4100394	576.524425	604.390981	647.026413	133.326387	547.125916
***	57	50.8	M+H	C15H22N2O	-2.3181571	9189.11618	8976.35074	10557.3913	2947.75043	8308.56744
**	57	0	M+NH4	C11H12O5	4.32426469	443.982768	391.893745	542.113526	526.802285	73.4270947
*	57	89.7	M+H, M+Na	C17H33NO4	-1.1772102	51122.7351	51548.0939	50471.0698	1842.51459	87354.5669
**	56.9	0	M+NH4	C9H11NO	-0.1289972	3310.82597	3406.40179	3597.93456	6771.92034	510.431573
**	56.9	0	M-H	C8H12N2O4	-0.4003611	194.176735	130.990147	148.663528	412.467636	170.796364
***	56.9	59.3	M+H	C22H43NO4	-1.2376006	44465.0629	42832.2144	42946.3365	10365.7002	132296.24
***	56.9	40.1	M+H-H2O, M	C25H49NO5	-1.0304926	63084.2738	65749.9	65125.9604	36559.0794	200246.916
**	56.9	0	M+H-H2O	C9H15NO3	0.25649664	154.695685	163.561694	159.467181	189.065332	165.253049
**	56.9	0	M-H	C15H18N2O	0.51074377	495.425	613.775397	589.916078	387.919428	248.881207
**	56.9	0	M+FA-H	C7H12O2	-2.2299213	2903.15547	3118.48653	3149.61277	3299.95802	2895.85875
**	56.8	17.3	M+H	C5H7N3	2.97500518	34668.8482	39676.4132	37181.915	36444.2572	28426.7698
**	56.8	0	M+FA-H	C9H14N4O	0.05943419	746.899569	1016.59325	1229.47816	9.7531E-07	387.616945
**	56.8	0	M+FA-H	C2H2O3	-2.8370475	3178.7237	2900.16868	3104.7696	2076.87704	1086.77831
**	56.8	0	M-H	C7H14N2O4	-1.9202034	1041.55024	828.747224	1103.79388	3832.70692	1504.22575
**	56.8	0	2M-H	C4H5NO3	4.75663843	1732.28912	2091.69717	2009.29138	9680.61197	3358.44815
**	56.8	0	M+K	C8H14N2O6	-0.7243248	2620.86424	1965.46396	1598.88858	11505.7467	8737.45238

**	56.8	0	M+H-H2O	C20H32O4	-8.1913038	559579.777	559948.409	555962.099	777226.942	409690.852
**	56.8	0	M+FA-H	C20H20O6	-4.3299601	2198.31858	1860.06477	2119.54607	1686.99584	1281.97631
***	56.8	64	M+NH4	C17H22O	-1.6096943	23343.7261	23569.9694	23474.9107	31809.1687	18561.9933
**	56.8	0	M+FA-H	C14H18N2O	0.36122096	140.289818	149.972615	172.612987	17.6199203	273.914545
**	56.8	0	M+Na	C20H30O5	6.44006773	2553.20226	2951.02444	2513.11987	9.7531E-07	172.674517
**	56.7	0	M+Na	C5H8O4	0.14670307	318.230795	364.337665	370.990549	408.332051	1934.98026
**	56.6	0	M+Na	C11H22N6O	3.5454461	54808.6694	55659.1906	55046.3092	78193.9698	36991.2594
**	56.6	22	M+K	C10H16N2O	2.9785678	33289.6889	30937.3129	35745.2039	4384.8356	7307.3694
**	56.6	28.8	M+FA-H	C8H14N2O4	-1.633558	14569.8179	13251.1856	12275.7264	23124.4802	18881.4679
**	56.6	0	M+H, M+Na	C6H9NO5	-0.2689331	17700.3922	19962.2043	23600.4212	29264.9113	433.897582
**	56.6	0	M+H	C14H18N5O	0.05249933	723.785703	1135.29762	894.101479	9.7531E-07	286.588077
**	56.6	0	M+Na	C6H13NO2	0.05530199	621.459942	693.780944	625.985974	706.62515	591.48689
***	56.6	47.9	M-H	C17H22N2O	-0.5083709	49360.1814	49474.4809	48805.3242	50595.5117	48310.3426
**	56.6	0	M-H	C11H15N3O	-0.5591564	442.282825	426.685508	413.698851	979.163199	570.543585
**	56.6	0	M+H	C13H18N2O	-1.0113689	944.600635	760.121783	682.468295	1473.07697	885.667539
**	56.6	0	M+Na	C9H17NO2	1.37138909	447.810421	550.646759	478.695656	480.057461	292.537833
*	56.6	88	M-H	C8H9NO4	-1.6213393	4532.50013	4660.38432	4423.98821	4090.96238	3490.55442
**	56.6	0	M+H	C24H40O12	-3.3493095	9944.93381	10122.9493	9003.91676	7213.84247	21083.6317
***	56.6	32	M-H2O-H, M	C20H32O5	-0.0268701	13277.3668	13963.9081	12780.0275	333.157169	56731.4654
**	56.6	0	2M-H	C9H16O2	0.52118413	1711.01707	1008.33755	1262.86702	550.497983	758.172564
**	56.5	0	M-H	C16H28O6	-0.5404066	924.863365	958.171828	1083.77162	689.423569	916.138267
**	56.4	0	M-H2O-H	C3H9O6P	-1.5368611	1027.60269	1053.35345	1672.99357	1115.81448	690.609802
**	56.4	0	M+FA-H	C4H9O6P	-1.7106862	27259.0567	29633.2564	28954.6023	21800.1132	24948.9038
**	56.4	0	M+H	C6H13NO5	-0.0969739	5669.41177	5507.60493	4775.93091	9.7531E-07	9.7531E-07
**	56.4	0	M+H-H2O	C10H19NO7	-0.4796329	3888.13467	4163.82965	6203.83471	3232.49289	5191.916
**	56.4	0	M+NH4	C5H6O3	8.86048876	8629.66104	8543.23228	8020.48461	9720.30655	8981.43265
**	56.4	27.8	M+H	C22H32O2	-0.4613405	13013.8423	11703.1579	11783.1349	10387.5357	4056.96759
**	56.4	0	M+H	C15H24O2	-1.4419191	4516.60856	3639.59552	3652.69053	6988.39061	3360.73036
**	56.4	13.3	M-H	C10H18O5	-1.1129804	1562.77059	1600.01257	1562.39605	1312.5099	2150.84415
**	56.3	0	M+Na	C12H24N2O	0.01246153	4463.37161	3822.73395	4139.70325	9.7531E-07	4394.65384
**	56.3	0	M+FA-H	C6H11NO3	-1.1423217	4853.50516	4620.07841	4497.0873	6464.64146	2845.78911
**	56.3	0	M+H	C6H10N2O5	-0.4587726	6512.23675	6167.16451	4730.00875	6712.20784	5599.83965
**	56.3	0	M+H-H2O	C4H11NO3	8.74575623	13781.2679	13407.0398	15041.9154	33262.8152	14408.5396

**	56.3	29	M+Na, M+K	C5H10O5	0.10842948	27842.9388	24644.6355	28102.2266	17734.0918	19617.1425
**	56.3	0	2M-H	C9H7NO4	4.32258737	25611.3919	23532.0031	21372.9995	10821.5356	21384.9069
**	56.3	0	M+Na	C5H7N3O	-6.226595	7689.7882	6970.10066	6773.94442	12404.1845	9281.53822
**	56.3	0	M+H, M+Na	C7H10O6	0.26354462	7996.9944	8001.91069	8131.71313	5131.88481	2320.33139
**	56.3	20.3	M-H	C20H30O4	0.08808749	2177.26495	2339.29082	2390.51509	1044.04759	1079.12164
**	56.3	0	M+NH4	C20H28O2	-9.5472106	26082.7729	26903.7742	25820.6597	16891.337	7431.77092
***	56.3	32.1	M+H-H2O	C20H30O2	-1.6462957	7273.00458	7850.52811	8262.14959	5681.89509	4402.78336
***	56.3	72.8	M+H, M+Na	C20H37NO2	-1.5964975	32256.4361	32584.1554	31888.0937	55769.7277	12233.0908
***	56.3	63.4	M-H	C24H48NO9	-0.7823317	39673.328	52296.0959	48748.5062	13030.9481	37445.8328
***	56.3	51.4	M+FA-H	C15H19N3O	-0.5196557	5126.90074	5302.3749	5080.342	2436.14453	14904.9527
**	56.3	0	M+NH4	C8H8O4	1.41186263	1252.53723	1327.84531	1244.67868	2030.35229	423.958145
**	56.3	0	M-H2O-H	C12H24O6	-0.5653864	1104.24751	1222.54898	1007.52575	923.563252	1385.9641
**	56.3	0	M+FA-H	C22H32O5	-5.8162075	2621.81869	2897.67385	3067.73407	240.204956	15070.0668
**	56.3	0	M+H	C12H20O2	-1.1270519	2674.05065	2870.29785	2934.36749	3463.96754	2208.42062
**	56.2	0	M-H	C4H9NO3	-1.157922	15992.0155	16204.8252	17557.5023	40727.7141	23372.4621
**	56.2	0	M-H	C6H14NO8P	-0.5240902	2262.97798	1925.35856	1710.1296	1657.69483	12565.0549
**	56.2	0	M+FA-H	C24H42O21	0.07702462	6892.48692	7287.311	8539.7653	9.7531E-07	128971.295
**	56.2	0	M+NH4	C10H13NO5	-0.9856823	18798.433	19925.7459	19092.5035	13476.314	32651.1457
**	56.2	0	M-H	C13H16O10	8.92161424	466.446306	518.034872	461.473127	287.631593	469.323196
**	56.2	20.4	M+FA-H	C22H32O4	-1.8761749	12082.7671	10118.012	11738.0609	9251.11794	7896.92874
***	56.2	42.3	M+H, M+Na,	C24H50NO6	-1.1876396	617520.197	610738.165	636268.99	205103.035	2025044.95
**	56.2	0	2M-H	C10H9NO3	-6.380722	7513.93001	7469.19872	8308.25917	9424.08357	4847.69667
***	56.2	48.3	M-H, 2M-H, M	C9H17NO4	-0.8741616	102140.98	103853.556	103098.627	114745.381	253810.735
**	56.2	0	M-H	C10H10O4	-1.4319433	1487.12125	1455.2894	1639.71175	1547.44492	1327.43814
**	56.2	0	M+H-H2O	C15H12O5	-9.2806822	14267.1888	14340.8515	14395.3167	18769.0904	10938.1197
**	56.1	0	M+NH4	C8H10O4	0.14367444	526.581705	692.864936	778.499773	9.7531E-07	303.419858
**	56.1	6.46	M-H2O-H	C5H6O4	-1.4005721	3840.53175	3757.06209	3613.06056	1251.57437	1717.09883
**	56.1	0	M+H-H2O	C6H10O4	0.81527483	1756.14593	1907.04739	1861.43255	2675.39327	1863.42656
**	56.1	0	M+Na	C6H10O3	-0.0846247	529.998514	450.751385	476.14589	558.333744	529.864416
**	56.1	12	M-H, M+FA-H	C27H44O4	-0.4986242	4426.39947	4290.05619	3987.5566	9.7531E-07	168.927341
*	56.1	87.9	M+FA-H	C42H82NO8	0.0157833	6744970.67	7036097.28	4459915.42	6866277.52	4832562.12
**	56.1	0	M+NH4	C8H8O	0.84156384	110.116463	128.959401	141.161574	95.4587099	61.6639926
**	56.1	0	M+H	C13H14N2O	1.81120286	277.458667	322.567937	310.107386	499.174252	468.237899

**	56.1	0	M+H-H2O	C20H30O4	-2.3458038	597.483537	667.969734	730.805894	9.7531E-07	2796.72065
**	56	0	M-H	C21H28N7O	6.66740353	13775.14	15066.0455	13952.0998	88255.3531	350394.05
**	56	0	M+H-H2O	C5H7NO3	2.92324613	753.957636	568.0277	609.910402	639.917095	604.302602
**	56	0	M-H2O-H	C18H28O17	-1.621802	5142.07214	5373.4967	6426.22868	132.145538	17330.4509
**	56	0	M+K	C18H34O4	0.02884407	3583.27225	2761.41197	2671.86878	8994.33949	3551.04487
***	56	61.6	M+H, M+Na	C20H39NO2	-1.6152514	28790.8868	24572.9598	27017.2917	61638.3169	15145.6864
***	56	63.6	M+H	C11H22N2O	-0.8794683	6862.58801	7195.6032	6210.25324	3650.20276	6418.62611
**	56	0	M+H-H2O, M	C8H8O5	1.29428013	1644.46633	1559.24192	1300.59815	1840.80531	863.280628
**	56	0	M-H2O-H	C8H8O4	-1.3723293	862.103084	828.976339	771.516752	712.748067	674.590267
**	56	0	2M-H	C8H12O2	-3.4227652	2279.84665	2029.62009	2360.07033	1733.23213	2408.22395
**	55.9	0	M+H	C9H19N3O4	0.34239049	5794.1377	5589.29934	5688.96778	1919.21217	9567.39328
**	55.9	0	M-H	C9H14N4O4	-9.9514205	12863.0928	13999.6389	9285.36846	56588.8872	45861.164
**	55.9	0	M+FA-H	C8H11N3O3	6.51293379	6799.38643	7939.70779	7240.21789	1282.29991	15873.7851
**	55.9	0	M-H	C10H15N5O	0.84235583	48768.0771	50360.3853	48734.2884	47482.0942	45441.7788
***	55.9	83.1	M-H	C27H48NO7	0.06007889	301979.729	320497.542	294816.726	297678.61	191465.88
***	55.9	49	M-H, M+FA-H	C16H30O2	-0.8194775	54318.065	48515.9514	48182.4449	112359.231	25253.4
**	55.9	0	M+FA-H	C25H42O4	-0.4625258	13030.0369	14269.7436	12414.8035	2523.93348	292.467516
**	55.9	0	M-H, M+FA-H	C22H40O2	-0.3793529	105940.589	74929.2239	121383.996	127898.232	81057.3476
***	55.9	78.4	M+H	C8H7N	1.61944604	79058.0449	76906.0574	72433.8588	114394.763	59605.1204
**	55.9	0	M-H	C9H14N2O3	-7.1971182	5980.14834	5753.0503	5813.59834	7446.4445	5189.41024
**	55.9	0	M-H	C8H8O3	-1.7400821	387.556722	384.38102	348.244545	376.603845	337.016787
***	55.8	46.5	M-H	C6H10O5	-1.2242091	12151.0185	12320.5331	12455.6798	3586.30801	22128.0561
**	55.8	5.64	M+H	C8H18N6O4	-0.7450326	28899.8313	30731.6889	29857.1863	197892.498	22226.4593
**	55.8	0	M-H	C8H16O8	-0.601764	1080.15205	725.159081	845.471752	682.918158	2112.88937
**	55.8	0	2M-H	C6H4N4O2	-6.9714851	189.1589	305.285352	225.345323	38.8442038	958.385999
**	55.8	0	M+H	C20H30O5	-8.2087642	67046.0415	67729.3913	68634.08	89327.6951	49828.1776
*	55.8	90.5	M+FA-H	C42H80NO8	0.55336717	3148042.36	2195180.01	3474446.75	2005336.21	3036622.49
**	55.8	0	M+FA-H	C25H40O4	-0.5966037	20656.946	15632.0317	16445.2893	9310.68457	712.087426
*	55.8	86.7	M+H-H2O, M	C21H40O4	-1.7125544	24121.5023	25611.2745	27702.3974	19162.5479	7076.86461
*	55.8	92.9	M+Na, M+K	C44H80NO8	-1.8790341	13864538.8	8746870.18	11320486.6	15373519.6	1992829.9
**	55.8	0	M-H2O-H	C8H8O4	-1.4941896	496.58042	514.734817	411.979396	432.007809	369.209286
**	55.8	0	M+H-H2O	C26H43NO8	-0.7601768	2259.2555	2187.02703	2051.73098	2765.90745	2737.1797
**	55.8	4.03	M-H	C21H32O5S	-0.128392	4519.50902	4021.96269	4412.00361	1759.55467	5786.03895

**	55.8	0	M+H-H2O	C27H44O4	-0.2540975	2029.57472	1737.98047	1942.02596	726.755753	1250.84261
***	55.7	31.4	M+H	C7H13NO2	0.21215391	225834.505	227429.318	236238.381	196723.891	321381.777
**	55.7	0	M+H-H2O	C6H7N5	-7.434918	4576.36745	4830.42462	5772.19421	12466.4197	2770.4824
**	55.7	0	M+H	C9H17N3O4	-0.2972926	778.823349	586.954938	741.744802	348.15672	782.213069
**	55.7	15.5	M-H	C18H36O4	-0.6979997	3602.8258	4135.48161	3452.06557	2091.2267	1640.99349
***	55.7	47.6	M+H, M+NH4	C18H30O	-1.7558665	792151.165	761974.455	774172.13	59736.9602	386713.801
**	55.7	0	M-H2O-H	C16H20O9	-0.3952279	412.60492	314.681471	239.806734	364.986458	568.645638
**	55.6	0	M-H	C9H19N3O4	-0.5079669	601.628752	582.533669	605.299828	108.194332	909.66289
**	55.6	0	2M-H	C5H9NO4	-0.5320743	3430.70183	3068.16298	2158.84903	10852.4012	2919.98088
**	55.6	0	M-H	C12H21N5O	-5.1777002	1511.50128	1859.63787	1568.69545	5419.73423	7932.59174
**	55.6	0	M+FA-H	C7H7NO2	-1.9280232	977.306959	945.187896	968.499635	712.904765	287.308278
**	55.6	0	M+FA-H	C24H40O4	-0.6677657	963.738652	876.12605	935.25052	626.568053	657.871959
**	55.6	0	M+Na	C22H38O5	-0.7597259	5865.96892	5731.97334	6227.81205	1056.57161	3699.70442
**	55.6	0	M-H	C18H30O4	-0.1927235	9833.28626	10741.4728	12550.4202	5540.90461	5903.37723
***	55.6	47.4	M+H	C16H19N3O	0.85095967	6231.09978	6027.50769	6252.33047	20170.2604	1987.00007
**	55.6	0	M+FA-H	C10H12O4	6.38592299	466.049631	403.052683	366.145425	415.7277	470.796485
***	55.6	45.6	M+FA-H	C16H24O2	0.52151012	118450.163	121040.906	117592.805	55484.6024	116125.558
***	55.6	61.5	M+FA-H	C21H41NO4	0.28769062	15706.03	14027.7912	14437.7615	893.770889	48679.3088
**	55.5	0	2M-H	C5H10O5	-0.7951545	3041.60609	3901.63933	3242.48181	2952.68481	17238.7311
***	55.5	31.8	M-H2O-H, M	C9H16N2O5	-0.9377634	4772.44149	5063.92093	4743.90528	1619.80248	16515.4209
**	55.5	0	M+FA-H	C15H26O	-0.5779935	756.599694	857.865313	826.4114	459.709304	1528.74186
**	55.5	0	M+FA-H	C20H32O3	-8.3285474	8423.21095	8026.95558	7635.43731	4344.77971	3287.85399
**	55.5	0	M+FA-H	C22H30O3	-6.7786316	1976.84987	2400.13858	1768.37739	1516.87015	1348.14107
***	55.5	43.5	M+H	C16H31NO	-1.7084433	8132.23837	8722.78572	8965.75608	828.25436	3394.55387
**	55.5	0	M+H-H2O	C12H20O8	-8.7267154	10192.1548	9408.19942	8183.72771	3178.68556	4336.35771
**	55.5	0	M-H2O-H	C15H18N2O	0.44381015	1279.5814	1401.94776	1522.91691	992.972421	855.836951
**	55.5	0	M+FA-H	C15H22N4O	2.03271214	839.634841	676.944738	730.032578	450.82445	412.573374
**	55.5	0	2M-H	C10H12O4	-6.0221867	830.723478	976.515288	1076.56497	513.160341	571.534515
**	55.5	28.1	M+Na	C10H16O2	-1.954215	24403.9925	23800.8922	25332.0539	30638.2595	19169.8385
**	55.5	0	M+NH4	C20H32O3	-9.0457685	3631.83499	3377.56396	3781.34111	1923.21963	1632.55534
**	55.5	0	M+H	C23H41NO5	-0.7612131	614.520869	801.254271	790.05842	83.9573792	1860.23014
**	55.5	0	M+FA-H	C18H38NO5	0.6143813	1405.13023	1550.80348	1751.37564	501.486507	3464.71829
**	55.4	0	M-H	C10H21N3O	-0.4835612	363.845585	233.186017	347.988705	9.7531E-07	433.624487

**	55.4	0	M+H	C6H13NO3	-0.7066285	8285.78897	7300.96197	7786.47585	14470.3325	5893.52026
**	55.4	7.33	M+Na	C20H32O4	-1.6571253	53690.1205	54517.5146	55080.3854	19734.6439	20636.0242
**	55.4	0	M+H-H2O	C20H30O4	-2.3263595	2110.74096	2043.94312	1846.45972	2546.37325	3092.53299
**	55.4	0	M+NH4	C22H30O2	-3.6086371	1910.85273	2368.52575	1808.7234	1623.3079	532.193048
***	55.4	32.1	M+FA-H	C13H20O2	-0.4931355	1083.25967	1040.88855	1145.63491	1354.75119	3265.39169
**	55.3	0	M-H, M+FA-I	C8H15NO6	-0.9439927	7931.78385	7781.0004	8860.24458	14832.964	32863.2063
**	55.3	0	M+H-H2O	C12H23NO7	-0.3305445	1536.50623	2101.15093	2205.34758	9.7531E-07	16276.8442
**	55.3	12.6	M+NH4	C27H46O5	-0.839905	43913.2878	41586.4305	43174.2823	15869.445	178674.958
**	55.3	7.29	M-H	C12H24O2	-1.4727583	722.720628	711.318147	833.283628	955.861694	543.386979
*	55.3	87.1	M+H-H2O	C42H83NO4	-1.4237485	95771.2644	84476.9742	68032.6268	117427.694	56143.0472
***	55.3	64.2	M+H, M+Na	C12H23NO4	0.62377872	341316.167	360107.05	357029.766	216137.565	365613.857
**	55.3	0	M+FA-H	C8H8O4S	-0.7415678	367.693083	408.329043	297.489623	9.7531E-07	9.7531E-07
*	55.3	87.7	M+H	C16H19ClN2	-0.9234224	3070.8003	3195.91029	3362.54628	147.128074	125.27336
**	55.3	0	M+FA-H	C18H30O6	7.25555308	23857.5043	22554.348	24094.5965	57690.1167	9659.06836
**	55.3	0	M+H-H2O	C20H28O6	-7.7753687	2381.45876	2781.82722	2625.51257	3056.1556	2039.3301
**	55.3	0	M+FA-H	C20H30O4	-6.98501	7863.61239	8306.53936	8678.94429	993.350622	1427.23265
**	55.2	0	M-H	C6H13N3O3	-1.9550508	6418.41641	6472.72771	7366.89973	9698.87556	4269.81778
**	55.2	0	M+H-H2O	C6H10N2O2	1.42773389	1044.37552	893.075912	1253.54255	9.7531E-07	179.62938
**	55.2	0	M+H	C15H21NO8	-0.5748612	20753.9672	20550.5162	18666.2092	9.7531E-07	28003.1685
**	55.2	0	M-H	C11H19N3O	-0.4059667	1772.33649	1966.89786	1746.38141	2872.81867	5567.89712
**	55.2	0	M+FA-H	C19H26O3	-8.8458556	694.65387	806.876307	912.541868	9.7531E-07	3830.49814
**	55.2	0	M-H2O-H	C20H32O3	-0.5058928	586.579515	698.237957	821.687199	76.0333826	190.095432
**	55.2	0	M+Na	C20H36O2	5.91639014	9663.96004	8804.2067	8544.07536	9411.76166	1949.58097
**	55.2	19.2	M+H	C14H18N2O	0.27718331	5796.85253	5682.03151	5788.84243	2979.39801	4651.33556
**	55.2	0	M+H	C13H15N3O	1.29470122	683.522483	770.005335	724.821597	375.169281	631.194551
**	55.2	0	M+H	C19H34O8	-5.2949946	7596.83619	7113.91591	7557.21961	6179.98432	3036.72502
**	55.2	4.09	M+Na	C10H16O3	-0.4892729	78386.0474	78587.2535	77648.4181	114044.197	59372.7785
***	55.2	45.6	M-H	C20H34O6	-0.3797744	56204.2487	56582.3536	56104.815	2624.595	61285.8352
*	55.2	84.5	M+H, M+NH4	C20H32O4	-2.1658001	53840.2147	53811.445	55250.9639	7861.4304	226544.272
**	55.2	1.18	M-H	C20H32O5	0.20411032	3344.37807	3681.68871	3906.03059	348.046674	1985.31039
**	55.1	0	M+H	C6H14N2O3	0.58608697	10309.2201	9505.55139	9919.13518	17766.6816	12044.0832
**	55.1	0	M+H	C10H18N4O	-0.8115374	44856.9167	36836.9028	39232.5823	89184.3384	104501.046
**	55.1	7.65	M+H	C8H17NO2	-0.1493534	188217.118	193643.16	211882.055	162442.102	633814.011

**	55.1	0	M+H, M+K, M	C4H8O5	9.7280479	2454417.72	2491236.61	2461407	4897685.18	2704748.64
**	55.1	0	M+Na	C11H13NO6	5.56746007	2927.67363	4790.78575	4006.29706	8588.96316	1704.30813
***	55.1	35.8	M-H2O-H	C10H13N5O	-0.6615491	33413.3368	36734.6207	30878.0316	49027.86	24391.4011
**	55.1	0	M-H	C5H11NO2S	0.14017168	8433.54336	10154.5565	9463.03532	7430.47983	9401.67579
**	55.1	0	2M-H	C5H10N2O3	1.53640269	16823.3408	22330.2912	16673.9114	14345.5858	20346.1162
**	55.1	0	M+H	C4H6O3	3.97102219	113.058092	107.108023	127.707024	102.855396	135.841783
**	55.1	0	M+Na	C18H36O4	0.3007944	16419.7913	15300.1401	17147.4255	17671.2934	6793.78724
**	55.1	0	M+FA-H	C14H28O	-0.8203129	507.32709	714.222362	690.804874	1056.96777	201.724606
**	55.1	0	M+H	C8H10O2	1.26144074	14214.8811	15626.7701	15925.1976	19694.5635	11379.0865
**	55.1	0	M+FA-H	C20H28O12	5.46036145	869.038121	998.279891	954.932814	8145.55475	128.106443
**	55.1	0	M-H	C9H8O4	-0.5801733	434.177317	391.059758	375.551387	400.201574	348.305956
**	55.1	0	M+NH4	C19H37NO5	-7.2996708	4767.91654	4784.97654	5177.50478	6996.73617	3483.18389
***	55.1	34.6	M+FA-H	C15H20O2	-0.6821566	12015.5948	12316.7555	12166.616	12090.2733	10662.4752
**	55	0	M+H	C8H19NO5	-0.1563834	3710.77437	2710.58405	2892.63482	2022.95352	9.7531E-07
**	55	0	2M-H	C2H4O3	7.50023821	135888.396	141064.186	127197.666	208914.329	194880.229
**	55	0	M+NH4	C8H8O5	-0.3287226	2081.87174	2129.35825	2259.4672	1716.64039	3703.58174
**	55	0	M+K	C8H16N2O4	5.91061479	1947.13976	1777.47495	1695.22347	14682.8405	2976.86855
*	55	79.9	M+H-H2O	C41H72O5	-1.0433819	892024.31	907606.367	736182.918	1219017.39	728374.947
*	55	79.1	M+FA-H	C37H75N2O	0.33317227	450414.453	251634.397	427880.251	382753.079	327352.2
***	55	50.3	M+H, M+Na	C22H37NO2	-1.6237147	20973.3343	20484.0259	18998.4528	56253.3843	10628.6283
*	55	83.4	M-H	C6H6O4S	-1.7742109	34323.6903	36011.3135	33907.0276	95242.3749	54859.5757
*	55	81.8	M+H, M+Na	C19H14O5S	-0.9247139	33840.3531	33009.2824	33487.4603	11329.9288	4573.71515
**	55	0	M+FA-H	C21H32O5	-1.1341653	236.842747	286.910709	366.19595	9.7531E-07	9.7531E-07
**	55	0	M+H-H2O	C18H28O4	-9.0678377	35337.5291	34321.7073	32881.4313	47754.6048	27103.4787
**	55	0	M+Na	C20H30O4	-3.1968514	3070.09664	3132.1821	2945.53742	1139.8072	1429.25525
**	54.9	0	M+H	C10H20N4O	-0.070694	8759.76155	8311.30226	8014.28857	3617.83706	13343.57
**	54.9	0	M+FA-H	C7H12N2O4	-1.395617	7297.46576	6366.28135	7480.40323	9707.77192	12053.3483
**	54.9	0	M+FA-H	C6H11O9P	-1.6082009	2542.36823	2908.13678	2280.32136	6947.71133	2976.55779
*	54.9	78.3	M+H-H2O, M	C8H9NO	1.06264737	415576.511	422575.172	412124.986	687241.369	545326.396
***	54.9	61.7	M+H	C17H22N2O	1.36137081	19174.5129	18420.2815	18000.4436	1716.28566	7982.36503
***	54.9	42.5	M+H-H2O	C9H9NO2	0.2868265	16376.5856	16075.7416	16755.3579	37108.2023	7815.64059
**	54.9	0	M+H-H2O	C10H10O5	0.45493229	205.280306	157.982679	253.436646	31.066855	152.478993
**	54.9	0	M+H	C17H23N3O	0.25239402	1113.39172	1180.36574	1140.36319	224.780374	1802.86369

***	54.9	55.9	M-H	C26H43NO8	-0.2920458	4003.23217	3914.98445	4127.88221	2776.93841	2845.31169
**	54.9	0	M+Na	C18H28O5	-0.9286078	3733.13326	4236.45196	4273.42493	5724.53228	3080.62423
***	54.9	35.6	M+NH4, M+N	C20H32O5	-1.5228155	23778.9154	25035.0385	24469.4294	2295.75315	71886.4754
**	54.8	0	M+H	C2H7NO2S	2.58334537	4093.55067	4069.66257	4663.41517	15729.5261	9202.93932
*	54.8	74.6	M+H, M+NH4	C9H8O3	-0.1047612	5603877.77	5962540.53	5660732.94	9499237.18	7315464.59
***	54.8	31.9	M+H-H2O	C22H38O3	-1.9885793	17851.541	19173.8798	20109.2834	39822.1673	10630.7712
**	54.8	0	M+H	C16H19N3O	0.66500351	2810.80884	2958.43591	2357.35402	937.367179	10434.5718
**	54.8	0	2M-H	C8H12O4	-1.0417694	1806.69877	1745.60562	1665.65281	950.280008	2093.90635
***	54.8	52.5	M-H	C26H43NO6	-0.6258347	2868.2758	3076.74775	3502.58346	985.132039	3407.96799
**	54.8	0	M+H	C20H32O5	-8.2753875	12438.4762	13125.2911	13031.216	12622.0112	9305.06579
**	54.8	0	M+FA-H	C10H20O	-0.9213311	3119.08916	3540.59892	3105.70413	2371.83763	1276.67693
**	54.8	0	M+Na	C17H32O3	-1.0658473	2190.42684	2429.19849	1885.22939	2698.17167	1451.16441
**	54.7	0	M+Na	C30H52O26	-0.4375807	7571.81762	6924.90459	7409.29964	9.7531E-07	44836.3544
**	54.7	0	M-H2O-H	C14H21NO8	0.31917169	1040.33315	1066.29006	1200.19805	1797.13423	3331.98911
**	54.7	0	M+H	C13H18N2O	0.2551558	1360.73757	1520.05474	1398.1179	438.539934	1192.7149
**	54.7	21.3	M+H, M+Na	C12H25NO	-0.9953198	175876.708	169902.049	172939.078	142463.745	60298.444
**	54.7	13.5	M+NH4	C20H32O	-1.4397003	1887.29609	1973.59066	1912.6275	556.715009	1410.64963
**	54.7	0	M+FA-H	C21H36O4	-1.1383683	4097.77929	3718.32331	4052.46219	726.612108	9.7531E-07
**	54.7	0	M+FA-H	C18H37NO3	0.09692771	1247.68099	1145.39471	1058.25196	359.432398	387.2316
**	54.6	0	M-H	C4H9O7P	-1.1087034	124.359883	120.18574	107.106934	295.640587	776.264546
**	54.6	0	2M-H	C4H6N4O3	0.88611053	4817.19382	4505.13368	4215.71111	33433.638	6142.89996
**	54.6	0	M+H	C9H17NO7	3.97791067	38998.1563	40811.8588	37381.9933	32317.4669	14103.4263
**	54.6	0	M-H	C6H5NO2	-1.6534909	2233.30297	2524.6382	2338.48485	1023.01965	13982.2686
**	54.6	0	M+FA-H	C27H49NO4	-0.5468113	1750.0455	1150.23341	1840.98791	9.7531E-07	9385.20382
**	54.6	0	M+Na	C25H38O4	-1.6015403	16989.1164	15951.3951	16107.8905	7085.32708	3763.32697
***	54.6	34.8	M-H	C14H16O9	0.21553535	858.567449	849.475209	784.471266	370.101083	865.506747
**	54.6	0	M+FA-H	C21H32O3	-0.2148586	364.897012	278.764709	465.221045	9.7531E-07	45.0194523
**	54.5	0	M+Na	C9H17NO7	8.35141378	26462.2147	24607.8969	31867.1512	51697.1184	29582.8905
**	54.5	0	M-H	C5H4N2O4	-2.0978097	1695.27894	1333.30681	1477.0225	312.962245	473.353489
**	54.5	5.83	M+K	C27H46O3	-3.1806128	15093.5629	14741.4621	17679.3133	951.496121	19849.1035
*	54.5	79.4	M+H	C20H33NO	-1.1963399	3174.92562	2846.19653	3342.15804	1385.59605	9.7531E-07
***	54.5	33.5	M-H	C18H36O3	-0.8586926	13246.1304	13128.4436	12799.9585	19083.3204	4615.19272
*	54.5	77.2	M-H	C20H36O5	-0.4065355	9883.41733	10405.3437	9192.88472	1137.64295	61760.0209

**	54.5	0	M+FA-H	C20H32O5	-2.8732594	1479.69866	1497.94822	1125.17275	588.519608	448.084191
**	54.5	0	M+H	C19H40NO7	-1.7296681	2827.67643	2731.55856	2461.65736	1503.14275	3476.86242
**	54.4	0	M-H	C7H10O7	-1.364587	9418.64121	8384.27566	8985.59892	11331.8231	4220.15295
**	54.4	0	M+H	C6H10O10S	6.89098171	16179.227	16548.7846	16283.2764	33500.1637	24640.1985
**	54.4	0	M-H2O-H	C10H18N2O	-0.8925992	844.598381	742.293715	905.416174	181.665822	1854.23343
**	54.4	0	M+FA-H	C10H18N2O	-0.3471985	561.36408	537.235692	700.143177	381.213216	610.693978
**	54.4	0	M+NH4	C29H36O6	-5.4121489	11832.1029	12442.5781	11131.2023	1751.93349	2215.71707
**	54.4	2.55	M+Na	C23H38O4	-1.6017394	33459.1931	27414.1178	31751.4749	10091.7017	3290.03637
**	54.3	0	M+H	C12H23NO1	-0.487671	42789.9443	35374.1628	32848.6535	41111.7863	10095.177
**	54.3	0	M-H	C16H25N5O	-0.0046267	551.039267	601.388304	699.01429	984.763935	9.7531E-07
*	54.3	78.7	M+H-H2O	C22H43N5O	-1.9125187	14147.4528	14199.9459	16065.376	9.7531E-07	22587.4121
**	54.3	0	M+FA-H	C21H30O5	0.45513614	819.908045	480.463623	617.096605	856.846358	2397.58843
**	54.3	26.2	M+NH4	C15H11NO2	-0.8085094	1759.38887	1682.97034	1721.42448	1713.89566	1343.17649
***	54.3	37.9	M+H	C26H48NO7	-0.5301757	21914.337	21034.3696	22057.2258	17426.8187	55354.7265
**	54.2	0	M+FA-H	C21H32O2	0.5814412	4989.52114	5354.94761	5149.62008	1032.17712	1372.66872
**	54.2	0	M+FA-H	C15H30O	-0.250195	597.180126	725.982136	792.951407	989.718757	321.736116
***	54.2	30.8	M+H	C10H13NO2	1.495188	3672.32144	3894.25255	3993.92758	2792.26212	5360.03583
*	54.2	77.1	M+H	C18H20FN3(	0.87390487	1674.87796	1691.41016	1833.48793	9.7531E-07	9.7531E-07
**	54.2	0	M+FA-H	C12H16O3	-1.3257302	672.655177	657.931884	569.269258	955.6334	739.214273
*	54.1	73.6	M+Na	C12H24O3	-0.2011728	110078.662	139149.787	134745.1	151825.31	78479.561
***	54.1	50.5	M+H	C14H30N4O	-0.4662252	221000.845	225608.489	228595.999	9.7531E-07	2529265.51
**	54.1	20.6	M-H2O-H	C5H6O4	-1.1299564	10421.254	9768.49685	10339.6136	5204.30261	2682.13818
*	54.1	75.3	M+H	C7H6O2	1.79393952	32650.3255	30866.4913	32261.6417	52990.1394	43080.1394
***	54.1	70.1	M-H	C25H46NO7	0.68986305	73750.1738	74564.4775	72399.7921	59236.6397	119370.813
*	54.1	85.7	M+FA-H	C44H84NO8	0.00526385	3096405.69	2385580.22	2165966.46	2671371.24	2439727.77
*	54.1	76	M+H-H2O	C6H11NO4	1.17578779	6324.80238	6749.00191	6840.09404	8154.10057	8924.96694
**	54.1	0	M+Na	C15H14O4	8.52124639	589.889101	464.642484	475.421664	9.7531E-07	817.265813
**	54	0	M+H, M+K	C11H13N5O	-5.8403013	9231.53717	7483.09873	8041.33504	9.7531E-07	6035.2934
**	54	0	M+H	C12H16N2O	0.36418949	2930.44505	2881.57664	2851.23397	1093.98152	2581.87213
*	54	86.3	M+FA-H	C46H84NO8	-0.0844187	2880989.09	2712382.72	2259419.93	2327270.42	1736815.16
**	54	0	M+H	C16H26O3	-9.9837936	481.183341	472.320766	509.141678	108.333671	329.086762
*	54	89.6	M+H, M+Na,	C48H80NO8	-4.692452	1474231.53	1181277.39	981356.096	4632798.77	2231696.54
*	54	75.5	M+NH4	C31H54O4	-0.1368596	8638.14797	10767.8573	7677.67874	9.7531E-07	2910.75574

**	54	0	M+Na	C10H16O4	6.46152869	2232.58662	2374.31751	2226.79479	2925.73389	1764.0666
**	54	0	M-H	C12H16O4	-0.1794266	466.138598	492.82587	412.59635	75.1560202	245.466085
*	54	74.2	M+NH4, M+N	C18H34O5	-0.8002948	50780.146	52053.1107	53489.6472	16466.8674	22990.1328
**	54	0	2M-H	C6H10O2	-0.9822569	1061.81071	1088.32791	990.008855	1100.19596	913.635597
*	54	73.1	M+NH4	C26H34O6	-1.6201274	993607.67	1013844.46	1041268.09	1153056.72	943051.419
**	53.9	0	M+H	C12H28N4O	0.01578178	934.959775	635.336151	847.338775	9.7531E-07	43757.2913
**	53.9	0	M-H	C6H8O5	-2.6962162	168.696526	221.52514	224.38769	388.073386	40.2759946
**	53.9	12.8	M+H, M+Na	C8H16N2O3	0.8211781	13923.4786	14379.9985	14318.8245	6894.39524	11379.2893
*	53.9	76.9	M+H	C26H42O4	-1.1885879	442189.887	435890.909	428732.384	538537.787	348342.629
***	53.9	33.7	M-H2O-H	C11H11NO3	-1.2291013	3741.70684	3581.33918	3601.13667	2832.47916	2388.30529
***	53.9	35.7	M+H, M+Na	C25H43NO4	-0.9976076	127517.686	131428.05	128666.093	12602.3461	374391.194
**	53.8	0	M+H	C3H7N3O2	1.9690035	14149.6933	13884.2937	14977.0792	9058.66583	3848.35409
**	53.8	10.7	M+K	C12H22O11	-0.6181577	313086.969	298891.362	318143.648	142642.999	1188015.36
**	53.8	0	M+NH4	C10H15NO4	0.71803405	3064.36716	3309.22416	3334.82949	3831.35907	2088.63842
**	53.8	0	M+H	C8H15N3O5	-0.3370888	2672.4492	2036.24553	1534.22142	9.7531E-07	9.7531E-07
**	53.8	4.58	M-H	C5H11NO2	-1.541244	4096.4487	4211.79903	4874.72858	4890.50417	5511.2121
**	53.8	0	M+NH4	C4H6O2	5.41711537	397.721009	600.254482	389.490218	591.13006	259.588729
*	53.8	74.8	M+H-H2O	C20H41NO2	-1.4886622	95405.1228	96720.6685	98755.6452	20886.7589	35269.3018
**	53.8	0	M+H	C10H11NO5	1.51368573	413.629115	398.414222	322.676585	406.88691	296.077943
***	53.8	40.7	M+H	C13H15N3O	0.51392957	2002.51599	1776.23028	1829.70055	3976.30066	1167.22955
***	53.8	49.5	M+H	C33H34N4O	-1.150083	123374.463	128859.128	126167.95	391993.992	38808.0494
**	53.7	0	M+H	C3H7NO2S	2.01014659	3116.79258	3127.34834	3555.31998	5122.02797	14972.2516
*	53.7	76.7	M-H	C47H83O13I	0.23481905	5687166.75	6708782.24	6532022.04	6024631.3	4699772.65
**	53.7	0	M-H	C18H30O3	-0.4211941	1551.52484	1500.97283	1571.45097	1280.17772	567.444115
**	53.7	0	M+H	C10H19N3O	-0.1320293	840.029298	630.546629	748.26813	502.532308	1274.01726
***	53.7	73.1	M+H, M+Na	C12H16N2O	0.88340101	9164.67487	9241.1721	8851.8364	5640.74894	5719.73814
**	53.7	0	M-H2O-H	C13H24O6	0.06462893	1080.06097	1115.84747	876.571401	1661.14467	966.655283
**	53.7	0	M+Na	C18H30O5	-1.324821	18763.4293	18684.0202	18828.5878	24022.7382	15295.2214
*	53.7	74.5	M+Na	C17H30N2O	3.36968114	3736.39858	3944.46994	4005.2155	105.647563	7197.16881
**	53.6	0	M-H2O-H	C9H17NO8	-0.5769857	6619.50155	3916.6089	7210.64568	8696.96496	2605.42637
***	53.6	49.8	M+NH4	C9H15N3O4	-0.611533	76409.969	85912.5905	63824.237	139430.978	103795.141
**	53.6	0	M+H	C18H30O3	-2.1953644	4106.99529	4734.9508	4577.29809	5316.87238	3116.33475
*	53.6	75.6	M-H	C27H51O12I	-0.1408769	83966.19	85185.2108	79618.1619	88783.1198	177312.952

*	53.6	74.8	M+H	C33H36N4O	-0.1556934	1556.79176	1466.88496	1432.55584	4098.02946	93.4172764
**	53.6	25.2	M-H2O-H	C15H18N2O	0.71877906	844.96661	895.152446	746.960078	328.475071	399.604977
*	53.6	73	M+H	C17H35NO2	-1.2887491	17289.1475	17258.5976	18505.1486	16437.7175	20815.3078
**	53.5	0	M-H2O-H	C15H23NO9	-1.119775	10231.7993	10306.9394	10217.1322	9.7531E-07	10735.6643
**	53.5	0	2M-H	C28H44O3	7.64468762	52745.4381	59757.1139	42468.7387	57257.6905	7449.57939
**	53.5	28.9	M-H	C14H28O2	-1.0793873	3084.75396	2994.89423	2768.40245	3967.28848	1785.57313
*	53.5	81.3	M+Na	C44H84NO8	-1.0255397	20911558.3	19208305.3	18949877.5	26120295.1	20611559.9
**	53.5	0	M-H2O-H	C6H6N4O2	-9.3064397	309.17088	318.560166	329.096505	295.671547	238.967072
**	53.5	0	2M-H	C9H10O4	-8.7913255	436.601157	501.742605	481.111848	132.251295	415.970031
**	53.5	0	M-H2O-H	C11H22O6	-0.5530537	1448.11456	1547.78695	1619.42341	1565.57136	1407.01245
*	53.5	70.8	M+NH4	C20H30O4	-0.3970223	1314.30903	1400.42152	1146.14502	670.77851	274.01749
**	53.5	0	M+H	C9H10O2	-0.6681909	1253.83697	1258.39515	1265.08155	2472.80627	1034.82436
**	53.5	0	M+NH4	C20H36O5	-1.4427025	1317.42515	1520.43644	1809.44911	1974.59426	1267.93359
***	53.5	38.3	M+H-H2O	C23H43NO5	-1.0569907	134825.013	138777.364	143807.402	7817.78301	505566.06
**	53.5	0	M+H	C19H37NO2	-1.6676115	3213.14349	3403.48652	3281.1025	1055.51455	10370.982
**	53.4	25.9	M-H2O-H	C6H10O5	-1.3399073	9266.44706	8208.60251	8209.32127	3117.4301	7372.92175
**	53.4	0	M+Na	C6H11NO4	0.28411223	3450.58276	2994.07541	3008.78048	7361.58093	5669.74449
**	53.4	0	M+K	C5H8O5	-0.7638249	5222.40273	5275.04253	5080.12529	13200.6154	8165.51061
**	53.4	0	M+Na	C21H28O2	-0.3661362	1441.66201	1446.75777	887.717404	3016.64588	1384.39748
**	53.4	0	M+Na	C18H34O2	-0.4271934	1240.24592	1602.56058	1229.47892	4600.59819	1984.83662
*	53.4	82.2	M-H2O-H	C20H26D6O	-0.0779598	37073.7673	33651.0458	32241.2396	55493.6434	44781.9841
*	53.4	69.1	M+H, M+Na	C11H14N2O	-0.2418752	89885.2408	92055.0785	92026.0415	18101.0481	224354.639
**	53.4	0	M+H	C13H20O3	1.24237386	1363.12399	1381.61789	1397.64931	1870.31292	981.920486
**	53.4	0	2M-H	C5H8O2	-1.0927416	15458.089	16294.3139	15065.2373	14706.4469	14524.3716
**	53.4	0	M-H2O-H	C20H28O5	-0.1858467	296.400679	345.002705	291.36948	186.835218	1779.4604
**	53.4	7.2	M+NH4	C21H34O5	-1.1506552	11467.8347	11201.4732	11811.438	1652.57509	33722.188
**	53.4	0	M+Na	C20H34O4	-2.0016883	1141.95777	1370.35793	1192.81489	446.431796	266.29417
**	53.3	0	M+NH4	C7H10O5	-0.2692978	8486.92572	8056.12491	6335.37721	10249.8332	2392.135
**	53.3	0	M+K	C25H47NO5	-0.5054341	13038.6914	11242.6876	8780.96587	9840.14742	6491.16338
*	53.3	72	M+H, M+Na	C47H83O13I	-1.8641355	3699760.39	3592621.79	2988866.09	4901432.73	2816622.46
**	53.3	0	M+H	C24H44NO9	-0.4587064	2091.78266	1616.74882	2010.47614	2791.95232	3677.32468
*	53.3	74.2	M+H	C20H36O2	-1.4101824	3401.26889	2967.4256	2540.61204	12121.1113	2345.49603
***	53.3	62.2	M-H	C8H7NO4S	-0.6256188	3271.21051	3201.71226	3288.13937	3345.83615	4752.56143

**	53.3	4.23	M-H	C5H10O2	-1.7853404	231.599092	266.083419	255.185275	149.15493	238.543208
**	53.3	0	M+NH4	C16H23N3O	0.37808725	523.739004	623.544116	492.336133	9.7531E-07	9.7531E-07
**	53.3	0	M+FA-H	C13H16N2O	-8.2520545	2559.64283	2660.24925	2725.58038	4100.257	1480.9459
***	53.3	61	M+H	C17H27NO4	-1.8033241	9086.8438	9677.69268	9495.2457	7461.40352	25497.2087
*	53.3	69	M+H-H2O, M	C20H28O3	-1.0641945	13730.1442	13418.7416	13929.3174	2459.0358	23893.4701
**	53.3	0	M-H2O-H	C26H43NO5	-0.2059529	1841.23684	1725.34795	1490.61437	722.976721	1551.17833
**	53.3	0	M+K	C18H26O3	-7.9855745	3572.83762	3901.10914	3284.59929	7005.17261	1585.02566
**	53.2	0	M+H	C11H22N4O	-0.2481236	2228.29437	2938.48424	2770.05465	9.09124139	5279.23915
**	53.2	0	M+H	C14H19N3O	0.63919919	1281.14454	1246.13892	1148.28115	392.514532	1207.00729
**	53.2	0	M+Na	C18H34O3	-1.1134456	3216.41909	2939.85709	3409.01987	4242.29095	2110.92568
***	53.2	40.2	M+H	C14H18N2O	0.16185756	25954.2526	25279.2723	25736.1944	83756.7529	30853.8274
**	53.2	17.6	M-H	C11H23N2O	-0.1012167	2226.13646	1994.15005	1767.32849	53.2365258	2571.55609
**	53.2	16.6	M+FA-H	C10H16O2	-1.1923201	2482.83485	2294.92953	2353.6617	2864.05145	2449.2262
**	53.2	0	M+Na	C20H34O4	-2.2764658	1864.25634	1473.44317	1841.74947	1218.90919	803.11283
**	53.1	6.91	M-H, M+FA-H	C27H44O4	-0.3575817	8385.93408	8746.89171	8833.68245	4632.24587	1104.97259
**	53.1	4.95	M+NH4	C29H48O4	-0.8870198	76602.7194	74228.7864	77240.5607	35183.8253	348759.858
***	53.1	79.3	M-H	C25H49O12I	-0.3167267	109348.609	114369.636	110036.68	83749.8736	329309.994
*	53.1	71.1	M+NH4	C19H30O2	-1.6259345	3908.54098	3812.92398	4033.21123	9.7531E-07	9.7531E-07
**	53.1	0	M+FA-H	C18H36O4	0.63348295	513.790552	514.863703	722.396106	292.058037	21.5069733
**	53.1	0	M+Na	C17H31NO7	0.63874383	402.396092	228.475605	391.934477	207.453802	271.045105
**	53.1	0	M+H-H2O	C8H12O4	-0.1682926	1804.00509	1812.83505	1837.78359	2932.6504	1401.87032
*	53.1	71	M+H-H2O	C20H30O4	-1.1913825	3628.73366	3543.96764	3391.41185	817.084802	17148.8781
**	53.1	0	M-H	C16H30O4	-0.1920118	1324.43495	1189.50082	1293.10203	1032.24771	794.016935
**	53.1	0	M+FA-H	C20H30O4	-7.2981887	1024.18648	939.43543	1017.62384	9.7531E-07	252.955085
***	53.1	30.8	M+H-H2O	C21H41NO5	-0.9385622	259034.546	268517.517	272108.231	16938.6947	617260.404
**	53	16.6	M-H	C6H11NO4	-1.6870028	8984.42665	9534.86286	8311.73124	13080.0061	7638.49985
**	53	13.1	M-H	C10H12N5O	-0.2516723	2687.76727	3105.07711	2770.93664	1185.8517	5642.90111
**	53	0	M+H	C18H37NO	-1.295438	9537.37871	9415.67291	9520.50951	2893.87652	771.213633
***	53	36.7	M+H	C24H47NO4	-1.3054308	74648.8246	75314.057	76100.7476	42057.4692	243164.529
**	53	0	M+Na	C18H34O2	-1.1429129	1715.36932	1842.6903	2145.95448	4011.9814	1743.56808
**	53	6.83	M+FA-H	C10H16N2O	-0.7423257	11444.8617	11714.5532	11078.9559	16755.6492	10010.4836
*	53	67	M+H-H2O, M	C9H17NO3	0.04586811	69195.13	72837.8235	72359.393	30486.3787	68676.0856
**	53	0	M+H	C16H26O7	-8.3894354	5140.94403	5379.89372	6235.11521	11385.9002	2006.94429

**	53	21.1	M+Na	C20H32O6	-1.5808369	5769.1188	6015.66465	6266.1627	1000.7823	3142.18197
**	53	0	M+H-H2O	C15H20O2	-0.5560001	394.114148	292.894811	330.380941	429.214258	220.949973
**	52.9	0	M+FA-H	C5H9NO3	-3.4731359	1763.84427	1752.16463	1379.12848	2849.39482	4749.87088
**	52.9	14.8	M+NH4	C10H13NO2	0.29828111	758.921654	821.726282	898.707448	720.493342	9.7531E-07
**	52.9	0	M-H2O-H	C11H19NO6	-9.5387149	22086.8631	21229.4141	19949.8334	31098.4717	50878.8689
**	52.9	0.0436	M+FA-H	C20H32O4	-0.2791792	3343.76828	2902.69921	3328.12555	715.76614	827.6026
**	52.9	0	M+FA-H	C8H9NO6S	5.97343757	5456.94365	5619.71727	5229.50436	5792.34172	5764.48182
***	52.9	53.2	M-H2O-H	C11H14N2O	-1.5989601	2024.15385	2229.40406	2163.48961	3205.12346	710.229788
**	52.9	0	M+H	C11H21N3O	0.15347402	2207.08248	2184.5377	1675.68416	977.142171	2342.5246
**	52.9	0	M+Na	C20H34O6	-1.5526813	3024.13545	3131.83571	2930.87971	573.935898	6434.84167
**	52.9	0	M-H2O-H	C16H28O4	-1.3544643	2268.49854	2166.02163	2197.51857	2430.97721	3508.19495
*	52.8	78.2	M+H	C22H33NO3	7.89643552	16146.2905	13540.0925	14285.9293	4011.70892	8980.40694
**	52.8	7.72	M+Na	C18H35NO	-2.1817298	1232.06814	1350.00898	1911.06518	9.7531E-07	1158.66485
**	52.8	0	M+H	C16H21N3O	0.55078611	1079.56528	1159.8994	1270.29717	109.512047	3752.3606
**	52.8	13.8	M+H	C10H9NO2	-0.5884368	1177.66442	1107.28837	1106.37166	2214.62454	804.686138
*	52.8	66.7	M+H, M+Na	C33H36N4O	-0.7830294	64811.8935	61589.856	59690.1113	57593.3864	3972.299
**	52.8	0	M+H	C18H33NO4	-1.2519415	2418.22994	2147.45915	2202.07018	1042.36116	5212.63374
***	52.8	44.5	M-H	C20H32O6	-0.2293802	2448.02898	2790.86873	2477.38593	9.7531E-07	9800.23517
**	52.7	0	M+Na	C12H24N2O	-0.816921	2025.88154	1714.36536	1217.60983	3593.6841	593.994958
**	52.7	0	M+H, M+Na,	C13H22N4O	-0.3994652	122842.505	118652.481	116982.122	348277.642	347842.632
***	52.7	38.3	M+FA-H	C3H6O	-2.6524036	27476.7156	27493.2826	25386.3312	63731.9701	32146.6225
**	52.7	3.47	M+Na	C24H37NO2	-1.2347681	4249.63386	3288.75128	4246.24051	6415.14888	926.141352
*	52.7	81.6	M+Na	C45H80NO1	-1.9651781	559016.452	542218.705	476786.921	415582.46	439396.838
**	52.7	0	M+H-H2O	C19H15N3O	9.932313	56409.0146	57141.042	56748.0707	11674.258	5921.49751
**	52.7	25.5	M+FA-H	C13H18O3	-0.3508049	14374.2231	14217.0796	13854.4576	18448.7219	14371.4309
*	52.6	65.2	M-H, 2M-H, I	C11H15NO1	-0.7170601	1599775.01	1673531.53	1650718.31	2604673.03	4971564.38
***	52.6	61.7	M+H	C25H46NO7	-0.6253349	164428.065	156528.867	158046.283	180788.486	250816.657
**	52.6	5.85	M+FA-H	C15H26O	-0.7438499	15179.5674	15668.9404	14738.7004	18290.6061	17712.7209
**	52.6	0	M-H2O-H	C16H20N2O	0.36011974	630.245397	628.558486	576.859849	708.116144	207.811404
**	52.6	0	M+Na	C9H14O4	-1.3564594	1076.3334	1082.42699	1045.41302	1303.91499	796.014685
**	52.5	0	M-H2O-H, M	C4H6O6	-1.6198418	1521.59487	1171.83236	1573.8726	1373.63761	1150.56508
**	52.5	0	M+H-H2O	C9H17N3O6	-0.5319852	1831.92936	2433.73148	2701.90312	2012.72673	9.7531E-07
**	52.5	0	M+H-H2O	C12H20N4O	2.5831173	11430.0087	14309.2918	14163.0013	15304.8297	12440.0114

**	52.5	11.6	M-H2O-H	C9H13NO4	-0.943218	32823.4515	30774.914	29613.3291	40659.6129	51327.9693
**	52.5	0	M-H	C11H21NO7	-0.8204215	5912.45623	8156.79937	7483.677	1172.35297	8508.94774
**	52.5	0	M+H	C9H18N2O4	-0.4996551	12398.6072	12262.4708	11935.7528	1737.27277	7497.12095
*	52.5	69.5	M+Na	C27H47NO4	4.41892418	75795.2562	76454.449	78207.6994	4062.15638	202152.517
*	52.5	69.6	M+H-H2O	C29H49NO5	-0.8769158	43377.6931	44311.4811	44107.9659	3200.98902	161864.295
**	52.5	0	M+H-H2O	C18H30O4	-2.2679945	4927.30804	5263.24887	5586.6978	5159.94145	2810.60931
*	52.5	68.7	M+H-H2O	C20H43NO2	-1.517946	7099.47162	7207.0877	8180.21795	1168.0177	1691.5565
**	52.5	0	M+Na	C14H18N2O	8.60159471	281.66925	372.860852	504.771919	9.7531E-07	9.7531E-07
**	52.5	0	M+H, M+Na	C10H18O4	-1.9183562	2250.63164	2735.66985	2477.27574	2936.88268	2097.23925
**	52.5	16.1	M+H	C18H31NO2	-0.4739544	6963.69667	6696.71869	7206.61651	7036.62823	6125.00135
**	52.5	0	M+H-H2O	C16H24O4	-9.5699123	20288.7537	21391.1293	21342.1185	30091.2096	17820.167
*	52.5	68.4	M+FA-H	C27H46O5	0.69592492	3058.08277	2863.29547	2848.64502	9.7531E-07	9.7531E-07
**	52.4	0	M+Na	C18H30O5	-1.3659219	4897.0868	4866.77653	4742.36638	5158.3674	3662.64724
*	52.4	67.5	M+NH4	C19H36O2	-1.6042857	17743.1307	18173.4345	17321.4208	46041.511	19229.7795
**	52.4	3.96	2M-H	C9H16O	-1.0261548	3223.06666	2390.94422	2201.99488	152.064607	16096.5851
*	52.4	68	M+H	C19H30O3	-1.6997224	1481.00217	1768.37273	1656.12081	1359.3985	544.272656
*	52.3	70.6	M+H, M+Na	C19H25N8O	-0.8754272	8762.60641	8359.84109	9310.01212	9.7531E-07	6134.50186
***	52.3	37.3	M+H	C25H40O4	-0.9608231	11600.0602	10454.6747	10646.595	5103.00987	949.887438
*	52.3	71.3	M+FA-H	C41H60O2	-4.6812722	294225.25	276158.066	226397.505	252597.422	311463.303
**	52.3	0	M-H	C11H22N2O	-1.3358513	572.130817	600.667545	766.609991	237.41925	530.528757
*	52.3	67.4	M+H	C9H10O	-0.2958785	6783.03035	7275.55835	8243.83393	8457.07524	6024.00154
**	52.3	0	M-H	C23H42NO7	-3.0349742	1531.59273	1313.04437	2022.20889	1050.9362	4046.99385
**	52.2	0	M+H	C7H11NO6S	0.25368114	9609.71562	11272.5079	10567.671	10829.9285	29095.1519
*	52.2	67.6	M+FA-H	C10H12N2O	2.49406046	33633.0782	35663.6327	32468.1575	35935.2664	67059.4486
*	52.2	66.8	M+NH4	C23H40O2	-1.5132788	59966.5161	59973.0354	59253.0636	44827.4127	127686.751
*	52.2	69.2	M+H-H2O, M	C20H30O	-1.888719	5742.23124	5721.4661	3861.33598	124.431173	160.173391
**	52.2	0	M-H, M+FA-H	C22H38O2	-0.3137105	150964.032	161047.924	140472.405	281844.933	117150.598
**	52.2	0	M+Na	C7H12O3	1.12447553	4200.86851	4650.04147	4692.56405	6181.13167	3419.35594
**	52.2	0	M-H, M+FA-H	C17H20N4O	0.24918669	11768.5618	11976.6073	11442.5205	12400.0559	11723.8236
**	52.2	0	M+FA-H	C21H22O12	-7.4969237	798.458204	1160.47474	983.434615	1420.45773	1204.12437
***	52.2	51	M+H, M+Na	C17H33NO5	-1.2964786	73138.6444	72984.9396	74631.4041	6614.6626	414266.998
**	52.2	21.6	M-H	C14H24O4	-0.1880183	2898.22317	2884.04675	3033.70431	2873.94303	5855.9552
***	52.2	32.7	M+Na	C20H32O4	-1.674499	10193.2315	12636.5488	11686.7263	4735.4628	4948.15016

**	52.1	0	M+H-H2O	C11H15N5O	-5.2859364	1106.54176	920.685034	1279.42123	1996.72478	3249.87646
*	52.1	66.7	M-H	C19H25N8O	0.24892769	8777.68374	7884.1237	8047.04841	1090.78814	6247.03088
**	52.1	0	M+H	C10H13NO4	0.89349106	1736.5648	1886.98904	1802.60362	3076.46635	1899.19381
**	52.1	0	M-H	C6H6N4O3	-0.8362101	243.10595	223.16502	236.636219	371.091891	21.5359388
***	52.1	48.1	M+FA-H	C16H28O	-0.7407294	18579.2028	19757.5001	19317.5214	3575.08042	65239.6557
*	52.1	65.2	M-H, M+FA-I	C19H32O4	-0.8063879	27100.1421	29347.3795	28426.826	2738.66098	24491.6677
*	52.1	66.5	M+Na	C39H79N2O	-1.2674423	3248643.76	3113492.43	3044029.25	8370157.26	4760894.3
**	52.1	0	M+H	C12H23N3O	0.27865203	3145.08065	3591.61264	3109.31023	1798.53601	2342.63348
**	52.1	0	M-H	C26H45NO7	-0.1569773	695.414707	997.25952	1103.9082	9.7531E-07	883.088138
**	52.1	0	M-H	C22H36O5	0.26048363	1652.71914	1545.46264	1578.62569	9.7531E-07	730.845092
**	52.1	0	M+H-H2O	C17H26O4	-8.4432316	9198.74318	9031.07699	8819.64429	13655.9981	7944.74309
*	52.1	66	M+H	C18H28O6	-0.6588685	3615.88868	3296.81597	3160.44537	2204.68134	1496.96288
**	52.1	25.6	M+NH4	C18H36O2	-1.5284753	38450.9925	39798.0917	41096.4916	20776.1508	27190.8645
**	52.1	6.46	M+H-H2O, M	C20H30O4	-1.5876678	15019.1767	15225.4269	15252.0578	2249.00631	66263.2465
**	52	0	M+Na	C14H27N4O	-4.5551638	24549.0459	25616.0208	21970.0763	9.7531E-07	23974.6504
**	52	0	M-H2O-H	C5H11O8P	-0.2474136	1702.8819	1665.22118	2412.40384	1111.31848	684.388003
*	52	64.7	M+H-H2O	C17H30O4	-1.1611792	2607.47858	2824.3552	2220.08345	9.7531E-07	9.7531E-07
***	52	48.9	M-H	C18H30O2	-1.0227931	125374.958	112738.334	109089.518	187032.493	68900.1737
**	52	0	M+H	C16H16O7	-8.2887927	9077.37633	9544.94647	10119.2249	14622.0823	8495.33719
**	52	0	M-H	C18H32O5	0.05363491	1994.07621	1904.84927	1842.33348	128.956596	939.678419
**	52	0	M+Na	C22H32O5	-6.4294511	1595.4605	1691.28097	1804.9692	300.756412	2438.30303
***	52	59.8	M+H	C20H39NO4	-1.3546815	32357.0453	32905.3716	34422.6814	10755.2079	159427.558
**	51.9	0	M-H	C9H19O11P	0.17541046	13575.4748	15399.5634	12648.1259	11241.9962	37067.9807
*	51.9	68.3	M-H	CH4O4S	-1.7081778	3228.26312	2952.4644	3434.54707	865.399839	925.715386
**	51.9	0	M+K	C6H8O7	-0.1376215	14625.4801	12785.3449	11818.5942	10049.5938	1908.63575
**	51.9	0	M-H2O-H	C8H8O4	-1.2887784	407.371703	330.057084	430.454197	357.569473	349.355642
*	51.9	72.9	M-H2O-H	C47H84NO1	-0.1608858	876558.656	644718.55	1107180.29	411670.832	139337.356
***	51.9	30.4	M-H	C18H14O8	0.2660901	5470.78063	5243.39106	5308.47579	4573.82824	4346.83762
**	51.9	0	M+H-H2O	C20H30O7	-7.0504742	2044.66256	1964.82244	2010.04175	2692.46333	1409.95225
**	51.9	16.4	M+FA-H	C13H22O	-0.4078826	8696.06518	9152.7921	9244.81777	10776.2839	11459.3356
**	51.8	0	M+K	C3H6O2	1.89646514	738.193407	972.457092	947.212243	9.7531E-07	9.7531E-07
**	51.8	0	M+Na	C3H8O3	6.18672542	1630.56905	1975.58268	2624.42987	4631.4071	2373.74925
**	51.8	0	M+H-H2O	C10H21NO7	-0.4624573	12355.9315	14045.3954	15270.4691	15323.0754	29128.4774

**	51.8	0	M+H	C11H19N3O	-0.1178569	519.920389	806.707161	500.012759	1445.29753	2174.42638
**	51.8	0	M+FA-H	C5H4N5	8.09311136	217.795618	278.761797	302.223407	112.472319	41.3061779
**	51.8	0	M+H-H2O	C16H30O4	-1.2290194	9030.14348	8388.08808	8692.13311	12238.8661	5647.6925
**	51.8	0	M-H	C20H32O4	-0.4666755	2768.55265	2730.59023	2653.8085	1477.80756	2474.65196
**	51.8	0	M+H	C29H56NO1	-2.0139258	2433.7006	2009.97644	2067.17887	1248.15293	381.705904
**	51.8	0	M-H2O-H	C8H8O4	-1.3551791	628.478351	626.458441	647.536966	537.560519	480.037327
*	51.8	61.1	M+H, M+NH4	C10H14	1.00398129	12700.8564	12876.6173	13566.6003	2855.4421	1633.37207
**	51.8	29.5	M+FA-H	C9H9NO3	-0.8356119	4224.98466	4057.27438	4331.93326	3539.69645	3744.33407
**	51.8	0	M-H	C21H26O10	-5.8084215	24553.1067	25733.0086	24781.5687	9034.43405	8772.01586
**	51.8	0	M+H	C9H10O3	0.93215804	761.248718	829.066929	774.896927	1099.4012	691.21908
**	51.8	0	M-H2O-H	C15H12O6	1.03598777	353.200418	323.837817	363.654825	272.452821	275.518634
**	51.8	0	M+H	C18H35NO4	-1.2062825	1170.32838	1181.86587	1105.49252	101.575546	2306.11404
**	51.7	0	M+FA-H	C9H17NO8	-0.7629394	9029.72143	9301.40932	9215.8819	1556.59793	2045.16915
**	51.7	0	M-H2O-H	C10H15N4O	5.55426831	8883.83961	9825.00319	8798.93815	7832.31317	1278.30252
**	51.7	0	2M-H	C6H5NO3	3.57552538	990.26202	577.609298	1050.618	1754.79741	2469.25012
*	51.7	68.4	M+H	C29H50O4	-0.4314653	14434.7978	15325.214	13240.7315	3569.28375	8249.33812
**	51.7	0	M-H2O-H	C7H13NO3	-0.9217673	133.692971	96.5853071	122.107209	207.193075	36.7263427
**	51.7	0	M-H2O-H	C9H16O6	-0.7820888	2331.28359	2397.73721	2301.05942	2279.26862	2047.17341
*	51.7	61.4	M+H	C8H17NO2	0.88335699	2989.97224	2965.01842	2857.80464	3837.24506	4921.99046
**	51.6	0	M+H	C14H20N2O	0.6522294	2366.87574	2624.92836	2722.06781	2502.0104	2577.28154
**	51.6	0	M-H	C10H18O5	-1.330762	949.675833	1006.46008	1073.97555	1018.7677	926.641351
**	51.6	0	M-H	C14H22O2	-1.497194	1509.84883	1577.21498	1572.74194	730.786768	1416.21365
***	51.5	30.4	M+NH4	C6H8N2O3	0.15995069	14687.2081	14306.2268	14717.2137	3867.02201	231.037473
**	51.5	0	M-H2O-H	C8H14O6	-2.3515535	397.992161	411.984292	392.46666	230.35711	96.1963096
**	51.5	0	M+H	C5H8O4	1.00517104	546.6063	606.251871	492.00654	1269.17694	568.759319
**	51.5	26.9	M+FA-H	C23H34	-0.7501023	27022.3259	26200.9611	25176.8911	15711.802	21679.89
*	51.5	59.3	M+H, M+Na	C15H13NO2	0.05197654	10086.1722	9935.16215	9845.36138	11581.5593	38046.8571
**	51.5	0	M+H-H2O	C15H21NO7	-0.5369014	646.452318	839.723305	652.530559	9.7531E-07	866.359803
**	51.5	0	M+FA-H	C21H28O4	0.24372452	369.705824	463.87342	435.624842	9.7531E-07	2586.56356
***	51.5	45.7	M+H-H2O	C17H26O4	-3.4557669	12044.4401	11959.2084	12103.1851	11748.767	10798.0033
*	51.5	62.1	M+H, M+Na,	C18H35NO2	-1.0202048	76212.1987	72480.4746	77503.0568	156683.523	59620.3166
**	51.4	0	M-H	C9H14N2O7	-1.3791768	2810.90951	2416.22643	2484.4005	3895.37096	7012.33882
**	51.4	0	M+NH4	C7H7NO2	-0.0889102	389.498249	441.606202	438.480678	381.553159	225.049255

**	51.4	0	M-H	C18H30O4	-0.505935	931.377208	757.476604	978.089349	542.397054	461.926004
*	51.4	83.5	M+H-H2O, M	C44H80NO9	-3.8650314	6014106.01	7940398.94	5775709.66	6901299.04	3833291.81
**	51.4	0	M-H	C10H12O5	-6.4116794	506.150965	521.846241	525.19884	1354.43418	404.261804
**	51.4	0	M+Na	C7H12O4	-0.0744908	1571.23159	1544.04432	1578.79945	1587.21265	6531.68103
*	51.4	62.9	M-H	C12H20O4	-0.6881839	5468.24353	5540.86501	5206.29303	5809.45743	6647.07268
*	51.4	64.3	M+Na	C28H33O14	-0.4480344	268851.628	262960.033	263480.219	163240.203	5341.39121
**	51.4	5.85	M+FA-H	C11H22O	-1.5498131	14510.053	14986.7179	14228.4641	16961.4209	18144.4019
*	51.4	63.2	M-H	C17H28O4	-0.5736361	833.626566	834.128677	892.047823	376.537716	945.781725
**	51.3	0	M+H	C10H19N5O	-0.2109009	2291.23017	2077.44482	2414.33378	9.7531E-07	13337.2744
*	51.3	64.6	M-H2O-H, M	C41H76NO8	0.40832334	1391945.24	1096674.81	1918011.91	1046839.8	327108.714
*	51.3	61.3	M+H	C19H39NO	-1.8018987	1185.62366	2065.66326	1973.28283	1763.93596	435.031237
**	51.3	0	M+H	C15H27NO6	0.91818336	785.295682	935.770507	955.27546	666.261735	2494.73493
*	51.3	62.1	M+NH4	C16H32O3	-1.3143581	3273.39937	3499.80276	3458.45486	1706.74608	3294.3502
**	51.2	0	M+H	C7H9N3O2	0.30400091	2944.86763	2868.92146	3810.70571	5813.28319	872.54559
***	51.2	57.2	M+H, M+Na	C30H52NO7	-0.686453	76586.7405	73312.5658	76332.7612	82375.8846	120228.2
*	51.2	58.6	M+FA-H	C9H9NO2	-1.3133967	4785.95101	4808.76099	4876.80001	4254.69314	3216.21786
**	51.2	0	M-H	C8H14O2	-0.9705072	1017.63501	1068.67932	1023.75633	491.814664	4206.84623
*	51.2	59.2	M+FA-H	C11H14N6O	1.05045652	1430.99983	1623.90292	1607.90179	211.217906	426.794615
*	51.2	61.4	M+H	C18H22O5	-1.3307261	2428.62191	2325.43778	2431.01981	1907.72446	548.063567
**	51.2	16.1	M-H	C12H14O4	-0.8192006	5676.12567	5830.64039	6116.93593	7690.92322	5190.20084
*	51.2	62.7	M+H-H2O	C25H44NO8	0.0690459	520.501748	547.119922	558.259821	9.7531E-07	215.020991
*	51.2	68	M+Na	C26H43O3P	-2.468743	42774.1063	43780.3205	46970.8363	65524.2241	36508.3455
*	51.2	60.5	2M-H	C18H18O2	0.21849822	33320.335	39635.4937	27925.6273	19932.6004	17249.0235
**	51.1	0	M+Na	C6H6O5	-6.2855185	4548.94297	6184.10468	5381.64325	10508.7778	3092.70134
*	51.1	60.8	M+H-H2O, M	C16H30O2	-1.6070725	77431.0112	77128.6318	77710.3719	120405.684	65102.3114
*	51.1	65.5	M+H, M+Na,	C46H84NO8	-3.2102823	42094239.6	47430301.5	38013873.5	50780279.5	26575535.6
**	51.1	0	M+H	C15H19N3O	-0.0087393	2318.63897	2238.17691	2269.37914	496.794711	5316.28753
**	51.1	0	M+H	C15H18O8	9.47776952	108.211889	132.24926	136.579531	222.816921	9.7531E-07
**	51.1	0	M+H, M+Na	C26H43NO6	-1.0590874	3255.79734	3268.18427	3506.52432	1455.93314	5259.39664
**	51.1	0.465	M-H2O-H	C18H34O4	-0.5164856	976.639626	1058.48879	966.945049	183.733866	6391.28765
**	51.1	0	M+FA-H	C22H30O4	-6.2136001	1232.0116	1067.06851	1336.47816	217.268679	768.830117
*	51.1	60.7	M+FA-H	C27H45NO5	-0.2603109	2419.77458	2267.68245	1886.9491	9.7531E-07	10430.8334
**	51.1	1.4	M+Na	C19H26O2	-0.5735014	5623.80345	6452.58091	6065.23693	37921.5207	6676.70941

**	51	0	M+NH4	C6H12O5	6.69479561	3287.01037	3101.48532	4535.63126	413.826562	9.7531E-07
**	51	0	M+NH4	C8H16N2O	-0.6000466	921.527141	1392.65729	968.706027	9.7531E-07	9.7531E-07
*	51	59.8	M+H, M+Na,	C13H18NO7	0.76130937	53094.3727	49977.2639	50705.1937	78784.309	80184.722
**	51	0	M+H	C8H8O3	1.0029422	1390.04309	1410.43424	1509.36851	2015.71705	1163.67333
**	51	0	M-H2O-H	C13H15NO5	-8.9841896	2772.58584	3077.50972	2699.15362	1914.51691	2179.46436
**	51	0	M+Na	C16H28O8	5.68076822	1408.50072	1707.98039	1571.79865	629.98332	567.891697
**	51	0	M+FA-H	C21H30O4	1.06370424	518.16381	534.850374	633.629142	9.7531E-07	347.609767
*	51	63.4	M+H-H2O	C23H51N2O	-4.8088833	91667.3501	89767.3298	99526.0757	124146.793	87316.54
*	50.9	59.7	M+FA-H	C8H8N4O6	-0.1932996	99383.2034	106314.69	101887.181	132803.252	113886.827
**	50.9	0	M-H	C12H22O12	3.42302033	1978.05839	1649.52701	2317.62659	2011.66244	6071.09873
*	50.9	60.9	M-H	C23H32O3	-1.0028137	2510.02834	2665.66503	2670.2738	9.7531E-07	120.836204
**	50.9	0.603	M+Na	C20H32O3	-2.0031179	24458.9198	24540.3821	23355.3996	19164.7527	12136.8236
**	50.9	24.8	M+FA-H	C23H43O7P	-0.9468263	74031.8291	75114.43	70663.462	60175.3895	107575.247
*	50.9	68.4	M+NH4	C41H77N2O	-3.7849657	730545.868	526167.349	733242.5	1030756.84	446087.898
**	50.9	0	M+H	C20H28O5	-8.3006526	7990.81273	7649.088	7223.32236	10737.4166	5895.25215
*	50.9	59.9	M+NH4	C20H40O4	-1.7499615	5071.89824	4827.26184	4716.7005	849.68571	1301.85686
**	50.9	5	M+Na	C20H32O5	-0.8643383	3790.87328	3334.37998	3304.61713	499.741917	1565.52513
**	50.8	0	M-H	C3H9O6P	7.25331064	8236.34894	7876.05738	9034.27953	15449.0718	5341.2135
**	50.8	0	M-H	C8H8O3	-1.1788088	338.741111	370.943248	344.058064	328.474886	288.598185
***	50.8	45.1	M+FA-H	C21H36O3	0.33380451	3110.37854	2712.32116	2618.83449	360.747391	8821.61861
**	50.7	0	M+H	C10H19NO6	-0.6250305	17205.8235	18938.1939	16552.0149	27261.1776	29164.1706
**	50.7	0	M+FA-H	C10H15N2O	-4.2948093	1886.13471	1794.41349	1808.39251	3607.36979	4428.72909
**	50.7	0	M+Na	C22H38O5	-0.4887242	2147.12197	1769.31948	2692.05976	1233.8583	998.530537
**	50.7	0	M+H	C20H41NO2	-1.4825603	6871.1855	6795.58609	7119.08899	18149.7376	6213.04262
*	50.7	58.4	M+H	C10H9N	0.29840878	7661.80001	8231.50236	7742.93853	16640.3037	3533.24404
**	50.7	0	M+H-H2O	C7H6O2	3.94013147	572.4755	540.369493	563.051075	872.212125	384.830977
*	50.7	60.2	M+H-H2O	C20H30O4	-1.5094488	1323.72175	1379.37762	1483.92459	52.9195815	1094.32963
**	50.7	0	M+H-H2O	C15H14O3	-0.5913291	4043.23335	4216.20167	4079.63254	5192.48031	3114.67991
**	50.7	0	M+Na	C11H14O2	-1.4834368	2465.531	2877.65456	2804.26058	4096.04463	2123.69685
*	50.7	58.5	M+H	C16H33NO2	-0.8515515	13747.4069	13181.2317	13536.0906	19552.7165	8055.99397
*	50.7	61.7	M+H, M+Na	C28H48NO7	-0.8744703	23176.9943	22841.8676	21943.4934	10442.4908	56325.852
**	50.6	0	M+FA-H	C20H34O3	-8.6278317	6414.73811	7282.29258	6397.23173	6795.15601	5202.76985
*	50.6	69.2	M+H-H2O, M	C46H82NO8	-2.2778854	5424792.46	4181450.53	4321036.39	5213143.74	3827785.98

*	50.6	59.9	M+H-H2O	C32H42O13	1.16346096	28409.0618	28041.0319	27816.8893	9721.73077	610.23172
**	50.6	0	M-H2O-H	C13H12O8	-8.9086952	704.676611	596.925438	670.770339	597.335497	598.635433
***	50.6	36.1	M+FA-H	C15H16N2O	-0.9196365	3339.93845	3625.62737	3190.58684	3130.78921	2060.05967
*	50.6	58.5	M+H-H2O	C14H24O6	-1.8395959	2748.56773	2671.21617	2890.86232	2546.46755	1818.85618
*	50.6	57.2	M+NH4	C17H26O	-1.0985677	15852.15	15393.1155	15949.7592	15721.7268	11236.9763
**	50.6	0	M+H-H2O	C20H30O7	-7.4698997	5612.02657	5492.90342	6271.3799	7642.68316	4430.01619
**	50.5	0	M-H2O-H	C6H13NO8S	2.2460144	656.324822	789.574602	869.342317	369.201847	152.505466
**	50.5	0	M+H	C9H16N2O4	-1.1095895	12184.1749	14981.7308	14807.8973	4222.23575	26804.7888
***	50.5	44.7	M+H	C26H44NO9	-0.8080202	110108.348	107617.684	112886.868	84650.4424	35892.8572
*	50.5	60.2	M-H	C29H58NO1	-0.527197	12538.0549	13736.9971	11024.9966	98.8292306	26890.694
**	50.5	0.278	M+Na	C17H34O4	-1.7878079	2201.20359	2638.92179	2620.82603	501.68165	463.857086
**	50.5	0	M+H-H2O	C27H44O	-0.8699267	5598.74738	6704.01757	6209.861	3651.65671	2753.85704
*	50.5	57.2	M+H, M+Na	C11H12N2O	-0.3419513	21015.7313	21710.9566	22709.6014	43319.3525	4422.58833
*	50.5	61.4	M-H	C18H14O7	2.34805227	10376.74	10958.2089	10243.474	4802.65352	2980.36929
**	50.4	0	2M-H	C3H8NO6P	-8.8600452	1620.94128	2175.77573	2577.84721	3351.33761	1202.68398
**	50.4	0	M+H-H2O	C7H6O2	3.87630207	1040.18913	1049.09578	1201.61769	1398.87414	891.11463
*	50.4	55.8	M+H	C11H16O2	-0.8731475	9801.22686	10118.8696	10541.6712	10212.3138	6888.22159
**	50.3	0	M+K	C11H21NO5	-5.5609288	3563.69991	3688.48052	4119.18297	4403.93362	5318.47713
**	50.3	0	M+FA-H	C5H10O2	-1.7231167	148.308706	145.21825	113.174644	151.67062	133.086235
***	50.3	30.5	M-H, M+FA-I	C27H42O4	-0.7376002	39359.3042	41282.4848	39802.2351	10664.1399	10213.1746
*	50.3	56.9	M+H, M+K	C18H34O2	-1.7339628	187894.206	185610.153	185036.911	271465.086	150204.087
*	50.3	69.8	M+H, M+Na	C42H78NO8	-3.4198183	4557268.03	4941340.55	4341676.72	4281334.84	1962024.01
*	50.3	60.7	M-H	C10H16N2O	-1.6514708	1123.7601	1065.03724	1481.0533	3661.44585	1349.19836
**	50.3	0	M+H-H2O	C10H12O3	-0.5599507	3479.49776	3602.12774	3833.88336	4391.42765	2667.45822
**	50.3	0	M+Na	C10H16O4	7.36412438	21842.1905	22707.3842	21538.3492	28901.7319	17462.0049
*	50.3	61.6	M+Na	C18H37N5O	-3.6122207	2187.34867	2280.74977	1775.8078	9.7531E-07	11054.9035
**	50.3	5.39	M-H2O-H, M	C11H22O3	-0.9288519	81937.5683	84194.0761	77984.4503	79230.1744	67683.684
*	50.2	57.7	M+NH4	C13H16O8	3.47207422	830502.285	989231.93	994452.797	1889168.2	1486624.29
*	50.2	56	M-H2O-H	C23H32O4	-1.0809311	62258.6452	64592.4745	59759.1758	955.23501	5910.98085
*	50.2	54.2	M-H	C9H17NO3	-1.0329539	32679.4625	32883.593	31946.0784	9380.21127	35227.0785
**	50.2	21.5	M+FA-H	C13H16N2O	0.34984451	815.372939	1035.01664	911.780918	118.494506	3435.48126
**	50.2	0	M+H	C11H11NO2	-0.3723871	438.380289	555.730337	478.134189	732.6889	258.988483
**	50.2	0	M+FA-H	C11H16O2	-0.8925431	408.254978	441.238058	389.442533	516.320713	302.193283

*	50.2	57.3	M+H, M+Na,	C28H33NO2	-1.1181489	58260.7166	59168.7315	58105.5558	18.4257503	154186.855
*	50.2	57	M+H-H2O, M	C20H28O2	-1.591175	7986.07095	7453.76177	7948.5157	423.860454	36443.2809
**	50.2	28	M+FA-H	C11H14O3	-0.3500307	45933.6128	46928.8031	45757.4404	46718.3019	67906.4057
**	50.2	0	M+Na	C12H20O4	7.86044109	10648.9887	10630.5002	10907.2847	17538.9418	9994.10839
*	50.2	56.6	M-H, 2M-H, M	C15H13FO2	-1.0619575	1589.67007	1720.15883	1571.19254	6254.46556	9.7531E-07
**	50.1	0	2M-H	C13H20O3	-0.1728119	2051.365	1935.17941	2088.45896	788.281928	2048.61401
**	50.1	0	M+H	C22H34O4	-7.6399135	1970.99133	1337.59245	1959.51047	759.929646	516.500135
*	50.1	58.1	M-H2O-H	C25H52NO7	-0.3383485	7050.78457	7651.57474	6413.42022	454.678346	8924.67773
*	50.1	56.5	M+H-H2O, M	C20H36O3	-1.7082053	54355.2794	52109.609	53307.6347	171497.583	75812.7109
*	50.1	56.6	M-H	C7H7NO5S	-1.340343	5407.03003	5859.15366	5637.21143	45.2474924	54400.412
*	50.1	55.4	M+H	C14H18N2O	1.72503722	31787.1209	31220.9217	31714.4588	13246.6947	63091.6854
*	50.1	55.6	M+H	C6H9N3O3	0.56725315	7910.91129	7897.91097	7829.12891	1096.18878	692.29274
*	50.1	56.1	M+Na	C15H17F3N2	0.65560382	1646.83592	1801.43893	1897.4733	2803.32967	815.190608
*	50.1	54.7	M-H	C18H20N2O	0.21372584	36583.3165	35846.386	37803.6178	41316.6364	18411.5722
*	50.1	58	M+K	C13H21N3O	-1.4385448	2754.19314	3295.74154	2871.59551	9.7531E-07	9.7531E-07
*	50.1	54.2	M-H, 2M-H	C18H26O7	-0.3466764	692985.487	719889.634	704614.749	332179.632	144657.943
**	50	5.04	M+NH4	C29H46O5	-0.7284748	16274.1065	18801.0304	18084.1998	4041.13564	108972.048
**	50	0	M+Na	C19H34O4	-1.665206	1751.19302	1767.4779	1843.13289	559.975106	231.159512
**	50	0	M+H-H2O	C19H36O4	-9.412772	9436.3014	9038.79768	8973.79043	16481.6606	8642.77131
*	50	51.8	M+NH4	C32H49NO4	0.45678376	593527.678	562851.537	609707.005	2088451.61	582335.132
**	50	0	M+FA-H	C8H16O	-1.8624144	538.4367	494.238226	544.370083	492.206462	433.197935
*	50	58.2	M+NH4	C15H23N7O	4.28194126	2847.17561	2504.283	2719.84364	341.631809	14728.9169
*	50	59.9	M+K	C28H44O	-3.5226257	7846755.44	7911264.14	7940197.19	10500672.5	6188072.22
*	50	54.8	M+NH4	C25H34O2	-1.2220374	6022.29401	5484.93054	6158.51408	9.7531E-07	7045.175
**	50	10.5	M+H	C23H43NO5	-0.9700853	76388.8089	74952.5959	78157.7791	16539.6099	265400.3
***	50	45.3	M+FA-H	C23H39NO4	-0.0842298	2751.06855	3464.24107	3120.30144	9.7531E-07	8294.00338
**	49.9	16.2	M+H-H2O	C10H18N2O	-0.3615784	49751.6801	48679.6144	51077.2169	146083.137	67767.0634
*	49.9	54.7	M+NH4, M+N	C25H44O6	-1.1112892	142333.289	143488.691	143834.686	47768.6551	76723.0635
*	49.9	57.2	M+NH4	C22H36O3	-1.3711313	8461.71603	7311.22919	6223.58379	29749.1654	5354.81494
*	49.9	56.4	M+NH4	C24H38O3	-1.7012117	3787.54517	2582.25431	2601.55697	36029.331	3703.77629
*	49.9	53.8	M+FA-H	C10H17NO3	-1.1809534	238.334893	303.888828	248.440273	74.765189	3718.96138
*	49.9	52.3	M+FA-H	C12H16O4	-0.4281163	3658.26455	3963.13337	3577.74446	5087.74851	7217.50087
*	49.9	55.5	M+FA-H	C20H18F6N4	-3.0256781	103405.185	109408.746	105177.126	48303.6294	20279.6766

**	49.9	18.2	M-H	C13H20O3	-0.2789043	7249.39339	7021.40796	7888.37295	7562.62011	7314.17833
**	49.8	27.9	M-H2O-H	C8H12N4O5	-3.863202	99916.4413	103184.047	103419.099	135346.489	97101.0247
**	49.8	0	M+FA-H	C20H28O3	-7.9384049	2462.20673	2790.03016	2727.86533	1143.35944	1432.29289
**	49.8	0	M+FA-H	C7H10O3	-1.5061279	1117.74954	1098.91304	1162.25258	1082.73482	991.105691
**	49.7	0	2M-H	C7H14N2O4	-2.8877881	1066.26037	927.895382	699.741014	123.127388	1205.50179
*	49.7	52.5	M+H-H2O	C17H30O3	-1.0452937	1474.81763	1637.05828	1633.88531	437.613252	5780.01034
**	49.6	15.3	M-H, 2M-H	C3H4O4	-1.3205021	51893.3933	52408.8171	50717.6015	26888.6798	18142.6136
*	49.6	65.8	M+H-H2O	C19H40NO5	4.71536693	55432.7299	52930.1102	53020.0602	67203.8224	40696.0677
**	49.6	0	M+NH4	C21H25NO4	2.29633841	3607.67001	2894.28987	3299.42245	11216.6174	3173.72972
*	49.6	75	M+Na	C22H38O2	-4.6197672	40965.2893	38823.6629	38972.693	58503.2071	32294.6316
*	49.6	55.6	M+H	C20H37NO4	-1.5204222	7431.58941	7505.31329	7019.19651	3929.57096	31595.3216
**	49.5	0	M+NH4	C11H18N4O	-0.9526459	13900.4117	10260.9589	13494.6088	859.610845	45868.2598
*	49.5	55.8	M-H	C20H36O3	-0.7476628	6482.2065	6550.95626	6860.92467	3367.42142	10154.0043
*	49.5	53.3	M+H	C18H34O	-1.6086147	83020.1225	79125.414	82819.1047	132277.257	75235.909
*	49.5	48	M+H-H2O, M	C9H8O2	-0.1443136	10082661.4	9991247.85	10411183.7	15985870.3	14045188.3
*	49.5	51.3	M+H	C10H19N3O	0.16775027	3608.84147	3632.64658	3601.01123	760.053567	13624.5501
*	49.5	54.7	M+H	C17H32N6O	1.18213885	40237.2517	42068.2964	43033.5049	67775.0038	71582.605
**	49.5	11.5	M+H, M+Na	C12H16N2O	0.35522129	5794.27276	5537.65215	5468.68365	3170.25182	5738.97856
**	49.5	0	M-H	C13H20O3	-0.3177873	559.512244	602.267854	632.769299	593.782378	499.008341
*	49.5	53.8	M+NH4	C20H40O3	-1.4580208	6515.00081	6300.76827	7178.8831	1766.08874	2838.55696
**	49.5	0	M+FA-H	C14H26O2	-0.0376499	1457.53103	1370.61066	1478.85343	797.689269	1024.64589
*	49.5	51	M+H-H2O	C14H24O	-0.9579359	1772.79749	1924.51895	1744.96738	15447.1394	2601.85939
**	49.4	0	M+FA-H	C8H17NO7	-0.4340306	10729.6351	11256.5586	9080.9478	32958.8304	30994.8557
**	49.4	0	M+H, M+NH4	C10H17N3O	-1.03608	13586.4583	15495.5802	17223.018	15363.299	41241.2933
**	49.4	0	M+NH4	C6H8O4	0.59905966	172.353347	162.840743	138.511227	160.807283	370.854317
**	49.4	0.314	M+FA-H	C31H45N3O	-1.0726362	32751.5391	35687.4305	35264.7201	11489.701	99071.1645
*	49.4	51.7	M-H2O-H	C22H48NO7	-0.2657643	41134.4106	43742.0585	40966.5036	9234.04692	64464.8458
*	49.4	56.6	M-H	C35H69O7P	3.15944514	20635.1273	25973.2205	21839.6031	17818.5243	22435.2956
**	49.4	0	M+NH4	C10H11NO2	1.51652228	1172.65165	1055.02946	1376.37263	2030.50868	1535.22542
**	49.4	0	M-H	C8H12O3	-0.8263044	730.189403	719.478868	675.368956	867.711923	728.778486
**	49.4	0	M-H2O-H	C7H10O2	-1.1147726	39.452793	39.4777676	32.5098844	17.8477676	17.2232546
**	49.4	0	M+FA-H	C13H20O	-0.8215017	1066.33852	980.901687	1013.01695	855.330599	1106.11793
*	49.4	50.5	M+H-H2O	C10H16O	-0.3246849	2657.71105	3050.73151	3127.35144	6892.34361	2248.92834

*	49.3	54.2	M+NH4	C16H25N5O	0.87202263	17734.4739	18163.6806	18657.4226	28465.7942	40683.2268
*	49.3	60.8	M+H	C15H25N3O	-0.3190138	4114.69623	3793.04987	5356.21996	1319.24167	21561.0628
*	49.3	48.2	M+FA-H	C12H12N2O	0.42033429	8060.07724	7838.31786	9722.29147	9932.52468	2738.09722
*	49.3	53.1	M+NH4	C22H38N6O	1.61698136	16412.4055	14426.3855	15775.6763	49186.0676	16260.7868
*	49.3	56.7	M-H	C12H17NO7	4.31901612	1984.15837	1841.86627	2974.39031	3224.91434	456.393294
**	49.3	0	M+H-H2O	C15H20O5	-9.4901051	11300.7092	11455.2209	11304.7234	16128.6186	9141.22288
**	49.3	0	M+Na	C13H24O3	-0.6416567	6490.37262	6486.53423	6094.01986	8211.55735	5468.35149
**	49.3	0	M+H-H2O	C15H24O5	-9.918275	3708.1336	3830.27062	3514.20713	4590.28117	2543.30157
**	49.3	0	M-H	C18H30O4	0.14083995	384.091413	424.448365	481.79538	302.539808	245.015547
*	49.2	51.2	M+NH4	C5H10O	4.28324412	4874615.57	4718590.86	5091299.42	8116283.83	5835055.38
**	49.2	0	M+NH4	C12H22O11	-0.1800792	6220.98853	7172.18523	6871.60761	9.7531E-07	13080.3637
*	49.2	53.1	M+H	C27H42O4	-1.0900392	4289.48289	3504.81787	3665.70122	425.978731	59.1374404
*	49.2	56.7	M+H, M+Na	C22H39NO2	-2.07725	11804.7236	9721.26659	11215.4708	43336.5327	7352.16951
**	49.2	0	2M-H	C12H15NO4	-0.3971823	440.540819	335.069804	430.107414	368.749672	3830.83826
**	49.2	0	M-H	C23H29NO9	0.39096193	260.016278	305.044812	405.025847	912.944753	9.7531E-07
*	49.2	74.1	M-H	C7H5ClO2	-0.9877892	21499.6226	21739.2132	22312.9831	21532.9911	18590.4448
**	49.2	0	M+Na	C20H24O7	-7.519226	68128.9803	68245.69	71703.9597	44900.7244	13343.8624
**	49.2	0	M+H-H2O	C24H38O6	-6.9720557	2254.27903	2658.03592	2552.42014	552.822523	7333.76019
**	49.2	5.56	M+Na	C20H34O5	0.21568193	6528.86405	6030.81238	6495.2374	3652.99012	2701.5052
*	49.2	53.2	M+H	C26H42O4	-1.0093723	52395.8512	56294.0132	57981.3248	67284.5649	43264.9909
**	49.1	0	M+FA-H	C6H8O6	-1.6081516	3237.03659	3769.36522	4046.34525	4529.24726	2933.12276
*	49.1	56.2	M+FA-H	C8H9NO5S	-3.8603713	55768.6212	46193.8005	44933.9513	49689.0015	45481.2937
**	49.1	0	M+Na	C18H37NO2	6.70027221	1034.01485	682.773491	849.035907	702.095771	9.7531E-07
**	49.1	25	2M-H	C5H8O3	-0.6702748	1243.14494	1268.70807	1020.03165	282.751311	184.339871
***	49.1	41.3	M-H	C20H30O4	0.11810776	9203.29071	9213.57332	9075.83902	252.610966	46218.1042
*	49	51.3	M+H, M+Na	C9H18NO8P	-0.1320654	30761.1138	32307.6704	33119.2426	96003.2379	12916.1564
*	49	49.3	M+H	C9H15N5O4	-0.3854771	826.676657	797.576275	691.808337	73.1779941	9.7531E-07
**	49	0	M-H	C14H27NO3	1.35820242	491.173833	523.337519	519.571535	554.945864	434.497597
*	49	60.9	M+H	C24H48NO7	1.55756799	14454.3416	15529.5766	16787.7519	1811.09179	20815.5771
*	49	51.4	M+H	C20H41NO2	-1.5960736	123415.989	117200.772	120555.661	186885.493	100986.593
***	49	49.1	M+H, M+Na	C25H42O4	-1.2899851	3809.13196	2936.46565	3290.38031	2749.87159	187.082084
**	49	0	M+Na	C6H10O4	-0.5227658	908.361917	1095.19413	954.711421	1224.9736	1072.70717
**	49	0	M-H	C7H8O5S	-1.3279423	178.515555	258.342365	209.117837	12.5597553	43.0298614

*	49	50.6	M+H-H2O, M	C20H32O3	-1.5327056	4042.98048	4366.65148	4242.69374	1255.95038	24862.861
*	49	50.3	M+H, M+Na	C17H26O5	-1.4078057	61754.6505	59607.6537	62150.5564	84124.8275	51530.8485
**	48.9	0	M+H-H2O	C14H20O5	-8.7153741	11623.3639	12362.9354	11931.7121	16258.7789	10304.4325
**	48.9	0	M+H-H2O	C15H22O5	-8.3008026	1882.04132	2028.69935	2123.65196	2840.56284	1641.02419
*	48.9	55.9	M+H	C23H37NO6	-0.9817019	1603.41494	1691.78352	1775.99948	81.5724256	12360.2902
**	48.8	0	M+NH4	C10H14N2O	-0.2329363	1530.22257	1601.65459	1515.37457	2716.95638	4097.74129
*	48.8	47.6	M+H, M+Na	C18H39O7P	-0.9520125	99225.2462	101899.39	105390.682	154020.785	80417.5752
*	48.8	51.7	M+NH4	C20H34O3	-1.5882465	14227.7836	11977.2376	13092.1237	32855.6034	10057.2234
*	48.8	51.7	M+H	C13H23NO6	-0.7855369	1449.34692	1830.24814	1492.65942	218.683358	998.929579
**	48.8	0	M+H-H2O	C15H24O4	-9.832609	21014.9163	20329.6266	20879.7997	31106.9369	15949.1082
**	48.8	0	M+Na	C20H32O5	-1.134269	4028.62405	4649.9534	4340.73459	957.976714	2022.44786
*	48.7	48.8	M+H	C9H18N2O4	-0.4066691	28621.8521	27527.7741	27985.7515	70469.1215	41151.7275
*	48.7	45.3	M+H, M+NH4	C11H9NO2	-0.6535932	2045332.63	2092662.7	2016624.7	4492829.47	929351.819
*	48.7	48.1	M+H	C14H27NO5	0.2725596	1392.70495	1760.59567	1718.88516	496.152753	8517.79605
*	48.7	49.2	M-H	C8H10O3S	-1.4330705	1774.24498	1911.5243	1789.47906	58.1908297	6172.05593
**	48.7	0	M+H	C16H30O8	-3.3657665	3091.31834	3132.04057	3885.25001	12636.351	3378.10966
**	48.6	0	M-H2O-H	C10H13N5O	-6.0054327	474.895442	385.108255	376.32453	188.980679	4348.53611
**	48.6	0.0771	M+FA-H	C23H40O4	-0.0805277	132494.614	102264.519	109491.679	44159.9573	22234.5628
*	48.6	61.6	M+H-H2O, M	C42H78NO8	-3.7020366	4108083.52	3677327.27	3292626.73	3929821.33	1478962.82
*	48.6	49.9	M+H-H2O, M	C11H20N2O	-1.5163446	2408.0012	2552.3928	2394.00114	2104.58932	1907.00225
*	48.6	48.4	M+H	C18H29NO2	-1.378152	907.551847	943.422036	1010.2258	122.717886	302.076711
**	48.6	17.2	M+H-H2O, M	C18H30O3	-1.0417763	8549.90724	8527.94485	9237.48555	5054.78846	5849.54971
*	48.6	48.7	M+H	C21H39NO5	-1.0610446	29727.8091	28374.7948	29759.677	2166.55341	109519.878
**	48.5	0	M+H	C10H19N3O	-0.335769	1971.62035	1959.38885	1921.53542	9.7531E-07	8780.09531
**	48.5	0	M+H	C13H17N3O	0.52934691	2244.56511	2110.08754	1988.69865	558.774814	2767.15402
**	48.5	0	M+FA-H	C18H30O2	-1.0124209	4666.78732	4155.20773	3740.71955	5769.34555	2372.16005
*	48.5	54.6	M+NH4	C24H36O3	-1.2925045	4746.0338	4272.62158	5024.81492	22804.3202	5385.32538
***	48.5	48.1	M+FA-H	C16H26O3	-0.3942973	4316.56386	3808.51531	4314.67092	2149.49679	2776.75762
**	48.5	0	M+FA-H	C21H32O4	0.26431418	2553.97693	2311.33159	2951.75907	1908.9222	2108.55643
*	48.5	49.6	M+Na	C25H49NO5	4.65358027	56207.6754	57657.7403	59434.4623	14345.5022	253432.592
**	48.4	11.1	M+Na	C10H15N3O	5.64109005	15292.3944	15541.1528	17033.3625	2073.91914	24538.8572
*	48.4	47.4	M-H2O-H	C16H24N6O	0.17032393	4456.69693	4299.70826	4580.76142	1914.3288	2549.48263
*	48.4	47.3	M-H2O-H	C19H26O3	-0.9177363	2974.61082	3054.58476	2603.53639	9.7531E-07	286.07315

**	48.4	13.1	M+H	C15H27NO5	0.57742911	4487.27495	4722.50397	4773.46628	3899.60689	36275.9286
*	48.4	54.9	M+H, M+Na,	C16H17N3O	1.20943567	154990.077	158085.946	149015.036	6457.94222	134534.149
**	48.4	0	M-H	C7H11NO3	-0.7964661	980.726217	880.140896	952.858983	820.133952	860.96949
*	48.4	46.8	M+H-H2O	C13H17N5O	-0.2559746	819.329911	687.695888	760.418726	250.75149	215.135
**	48.4	12.5	M+H-H2O, M	C17H26O4	-5.5076999	9455.86348	9230.44526	8862.06479	11393.858	7634.53829
**	48.3	0	M+Na	C20H34O3	-1.6384382	4080.18386	3652.59016	2847.01775	4268.8188	1430.43895
*	48.3	49.8	M-H2O-H	C14H29N7O	0.82115681	18403.3073	20446.6197	18116.4896	20770.5592	21625.4488
**	48.3	0.123	M-H, M+FA-H	C22H42O2	0.16469005	165235.18	125010.634	99731.0598	160825.97	101455.174
*	48.3	48	M+H	C4H6O3	4.30941403	967.840668	1049.33751	938.917749	269.943484	309.535264
***	48.3	30.7	M+H	C10H20N2O	-0.2507253	2876.98986	2991.04301	2602.45176	752.18777	2257.97105
**	48.3	0	M+NH4	C13H20O3	-0.8223282	348.037918	288.768339	354.69223	544.780967	158.039028
**	48.3	14.4	M+NH4	C11H15NO2	0.0672009	2194.47106	2086.26861	2151.77981	1899.36633	1744.49723
***	48.3	32.1	M+H	C17H31NO5	-1.1259861	4988.12262	5070.47216	4916.85726	895.921034	32139.8725
*	48.3	45	M+H-H2O, M	C12H16O5	-0.864735	42528.4137	41825.9918	44223.1363	59810.4589	56975.369
**	48.3	0	M-H2O-H	C12H22O5	-0.8108421	754.168172	810.432914	747.093676	676.208491	575.274953
**	48.3	0	M+FA-H	C26H42O4	0.31770079	1773.3192	1779.74832	1555.87085	1234.74125	1266.88356
**	48.2	0	M+H	C12H18O11	-3.2439248	10030.6845	9495.44996	8935.04659	8369.76254	7117.92864
**	48.2	0	M+H	C21H44NO7	-0.6424512	2819.64828	2686.96567	2542.13546	1168.64507	4031.93777
*	48.2	44.8	M-H	C17H28O3S	-0.3663809	272534.799	283165.62	276485.497	259767.769	208829.558
**	48.2	3.29	M-H2O-H	C22H32O3	-1.2687371	3017.99088	2844.79618	3021.05255	1861.83841	1555.67366
*	48.2	51.4	M+FA-H	C6H7NO3S	-1.8004471	461.028045	529.708552	525.638808	9.7531E-07	9.7531E-07
*	48.2	44.3	M+FA-H	C12H21N3O	0.00811871	1660.36073	1639.66034	1354.92232	2222.78904	3628.52371
**	48.2	0	M+NH4	C16H19NO7	-6.584875	494.911513	690.055191	390.867156	500.678142	386.791776
**	48.2	0	M+FA-H	C19H25N3O	-0.1766696	5901.02335	6081.06341	7064.23835	9206.50786	8904.02111
**	48.2	0	M+H	C18H20N2O	-0.3319212	3039.75552	3085.31779	3241.98134	2013.33892	2588.20678
**	48.2	0	M+H	C18H19NO4	9.67158184	2212.72399	1998.80694	2042.37409	2537.31951	757.653772
*	48.2	45.7	M+FA-H	C21H39NO5	-0.426185	2323.55695	2332.42614	1941.80799	9.7531E-07	11139.173
*	48.2	46.1	M+H, M+NH4	C24H24O10	-0.9241342	34513.2334	36160.4752	36951.6158	78222.0601	12636.4616
*	48.1	46.7	M+NH4	C21H36O4	-1.530756	14881.9161	14171.7482	15215.7017	1357.61217	11004.4751
*	48.1	47.3	M+NH4, M+N	C33H60O10	-1.3504353	93540.2328	95745.7813	94522.2132	33266.0136	216867.048
*	48.1	44.9	M+H	C4H6N2O2	2.10690239	251575.242	251902.762	252820.954	319496.485	188534.179
*	48.1	42.5	M+K	C13H23N4O	-0.2310395	9427.82771	9485.92562	9475.11642	19808.1631	12206.6246
**	48.1	19.8	M+H	C12H24N2O	-0.069611	1094.75636	1321.0651	1071.76736	312.612155	694.85976

*	48.1	45.7	M+H-H2O	C12H25NO2	-0.6944252	4812.3573	4957.99032	5041.94734	5303.50905	2971.9142
**	48	0	M+Na, M+K	C9H11N5O4	-5.6019752	7138.72757	5428.91476	7741.6538	5386.67228	2648.92588
**	48	29.5	M-H2O-H	C5H6O6	-1.2067335	28005.5651	26706.263	24579.1038	40416.0107	14535.6578
**	48	0	M+H-H2O, M	C18H32O16	0.32813361	7869.00761	6774.97932	6470.87433	9.7531E-07	26829.6783
*	48	53.9	M-H2O-H	C22H20O6	-3.5292762	52161.2414	42222.7972	39795.4492	46240.7751	49879.6831
*	48	45.4	M+NH4	C21H38O2	-1.643379	23161.5854	22128.7277	22468.6828	27681.4311	31952.2126
*	48	54.5	M-H	C30H42O6	-4.8898904	6907.62595	7130.2734	6640.55543	1433.34954	1068.23164
*	48	48.7	M+NH4	C16H28O10	3.81127933	16526.5607	16399.3343	15703.3764	23766.862	7811.5629
*	48	46.3	M+H	C12H21N3O	0.36549329	10673.1519	10795.3926	11352.1049	20605.3979	10655.2283
**	48	0	M+Na	C8H14O4	-0.8227787	1735.58811	1820.60501	2035.13916	2517.38726	1736.97705
**	48	11.9	M+NH4	C8H12O2	0.38189568	7794.68248	7382.4004	7490.08956	12707.6253	18045.8433
*	48	44.2	M-H2O-H	C18H28O9	-0.3203903	52212.0546	54610.4555	54068.3809	21338.0326	18541.21
*	48	44.8	M+Na	C12H18O8	-1.6126198	535.42378	569.87776	616.109286	2424.33494	305.074591
*	48	45.3	M+H	C19H35NO5	-1.0779424	22620.974	22646.8665	22229.1183	2431.41428	96416.81
***	48	43	M+FA-H	C21H41NO5	-0.1239691	9126.23743	9203.16406	9082.37879	1114.68703	39767.5113
**	48	4.38	M-H	C12H24O3	-1.2262616	48431.6397	50547.582	45731.5404	48265.1236	39198.8026
**	47.9	4.79	M+NH4	C17H32O	-1.7342165	53706.2964	53163.8882	54075.9323	40085.885	25998.6255
*	47.9	44.6	M+H-H2O	C12H25N3O	0.8996652	4018.77475	4277.95484	4298.34849	1200.67718	7513.00941
**	47.8	0	M+FA-H	C9H18N2O	-1.9673768	370.481643	469.292767	403.536034	202.389571	634.256138
**	47.8	0	M+H	C19H34O4	-8.9644906	53313.993	52297.029	54854.079	36931.2002	84813.1786
**	47.8	0	M+H-H2O	C25H52NO7	0.10088021	2429.32091	2432.84835	1488.50415	9.7531E-07	1966.71184
*	47.8	48.6	M-H	C41H72O6	-0.6832904	18148.0292	16434.065	14344.6576	4345.87218	511.878738
**	47.8	0	M+H-H2O	C27H46O2	-1.5812095	3696.39811	4196.29821	4846.2614	1953.88305	562.160081
*	47.8	41.8	M+FA-H	C15H18F3N(	-0.568898	22672.995	24151.1193	24313.9256	295.987015	32100.2753
**	47.8	0	M+Na	C11H22N2O	2.69438908	834.129286	692.048801	742.354698	79.9991184	703.131184
**	47.8	0	M-H	C10H19NO5	-0.7430903	125.610364	81.7705586	79.0085726	9.7531E-07	994.203406
**	47.8	0	2M-H	C6H8O3	3.14431509	881.101698	825.754326	778.069347	847.250508	831.13645
*	47.8	42.1	M+FA-H	C10H9NO3	-0.7376345	848.166354	745.506087	857.707934	387.195703	154.856376
**	47.8	0	M+Na	C9H17NO2	-0.844592	1879.94643	1916.39001	1872.32435	1209.7192	1796.43394
**	47.8	0	M+FA-H	C7H10O3	-1.9792136	2977.21778	3163.83558	2892.186	3048.58491	2578.84622
*	47.8	43.9	M+FA-H	C16H16N2O	0.5498575	1195.62701	1381.20361	1573.97888	485.100547	124.16412
*	47.8	45.7	M+H, M+Na	C29H35NO2	-1.1314872	40884.7678	41942.9802	43522.2101	85.6915926	73290.1261
*	47.7	44.6	M-H	C20H33N5O	0.13982395	8658.9129	9403.8927	9036.00651	19222.827	21569.6338

*	47.7	47.4	M-H	C29H51O12I	-1.6856076	64706.9774	66754.024	63821.8369	69772.2766	172572.358
*	47.7	44.7	M+NH4	C17H15NO3	-2.0116368	26492.4339	26363.5223	27721.24	13581.8943	1339.71122
**	47.7	0	M+H	C7H8O2	1.93908996	23348.6741	25631.1921	25794.8118	31327.7269	20279.3986
*	47.7	48.9	M+H, M+Na	C9H18N2O3	1.68843141	4962.06309	5188.1327	5292.08203	2748.04441	4383.51841
**	47.7	0	M+NH4	C10H8F2N4(	-1.826256	886.226642	792.108858	858.329993	646.688375	555.645428
**	47.6	0	M-H	C9H13N3O4	-0.838105	851.746433	539.580231	522.331947	3002.14859	4179.92566
**	47.6	0	M+H	C14H19N3O	0.51581966	3069.86748	3028.70108	3074.73535	1144.56923	4356.92686
*	47.6	45.7	M+NH4	C26H42O4	-1.0578345	34871.6852	36785.2253	31169.5688	47390.7282	24540.096
**	47.6	0	M-H	C17H21N4O	0.62232052	4453.35835	5135.93032	5236.44949	5055.04684	5003.13204
**	47.6	0	M+FA-H	C17H22O8	-0.1133898	728.401326	742.674355	884.654102	261.207118	158.584004
**	47.6	27.2	M-H2O-H	C18H28O9	-0.2333356	86995.353	88225.4232	82983.4079	31893.8945	29329.1884
**	47.6	0	M-H2O-H	C20H30O4	-0.609938	465.188379	575.789107	610.947275	9.7531E-07	1112.48142
**	47.6	0	M+H-H2O	C16H24O4	-9.8281545	42875.1325	41096.934	42483.1843	59541.3653	33604.7416
**	47.6	0	M+H-H2O	C18H30O4	-9.1563059	7869.71777	7588.76729	7537.97626	10359.0777	5765.59411
*	47.5	41.8	M+H	C6H11NO2S	0.56949049	3442.35333	3532.59159	3497.17994	4377.25829	7050.17889
*	47.5	41.8	M-H	C16H32O3	-0.4122913	84317.3109	87231.0604	83426.189	90566.1082	65940.2545
*	47.5	54.9	2M-H	C28H44O7	-2.1530693	8663.68584	9327.67227	9548.657	9.7531E-07	25264.5283
*	47.5	50.9	M+H-H2O	C41H80NO9	-1.5536072	1176335.19	676794.065	1020155.15	1165515.45	377739.493
*	47.5	44.7	M+H	C12H11O4P	-1.069428	117539.842	112002.403	110357.492	148207.209	98328.7882
**	47.5	0	M-H	C26H32O14	8.72130151	335.989487	337.907432	508.764684	9.7531E-07	9.7531E-07
*	47.5	42.6	M-H	C8H16O3	-1.4246747	2853.24298	2835.80328	2752.2238	2959.38361	3058.27752
*	47.5	41.5	M+H, M+Na	C14H22O4	-0.5598029	6557.648	7566.18406	6962.00302	7960.35356	5611.31707
*	47.5	50.5	M+H	C19H36N2O	-9.3029145	33113.1445	37871.1235	38505.6716	55775.5707	32013.2427
**	47.5	0	M+H	C20H28O5	-8.1237712	18903.6908	19760.7007	20460.3606	27038.2371	16061.7734
*	47.5	41.1	M+H-H2O, M	C14H24O	-1.0593763	29245.2169	27828.2459	28803.9257	106983.421	31881.9444
*	47.5	41.8	M+NH4	C19H38O2	-1.4020237	728949.279	746603.068	754953.281	501716.78	821635.792
*	47.5	46.4	M+H-H2O	C34H47N7O	-0.483979	27811.6561	29064.3323	27982.8995	673.679439	13742.7361
*	47.4	42.3	M+H-H2O	C9H17N3O4	-0.7318158	235224.847	228709.772	226582.614	173018.646	233016.059
**	47.4	0	M+H-H2O	C17H26O4	-1.7324474	4482.41454	4113.55161	4947.57626	6555.40982	3642.6397
*	47.4	53.3	M+H, M+Na,	C46H80NO8	-3.7641452	13777319.7	12285105.6	14900062.4	11022719.8	6140891.12
*	47.4	45.4	M+H-H2O	C24H48N2O	-0.7392002	31003.9722	29502.2894	28827.4515	18554.0814	12027.6153
*	47.4	41.5	M-H	C12H23NO4	-0.3990911	3883.39996	3871.61651	3757.49943	1168.0871	5467.89315
**	47.4	0	M+NH4	C13H15NO	-0.7475032	478.459732	512.100396	398.001149	2295.02036	230.232211

*	47.4	43.4	M+NH4	C23H36O5	-1.1663878	5869.83267	5816.20227	5875.83472	75.2861105	23409.5965
***	47.4	31.9	M+H-H2O, M	C20H28O3	-1.1942195	6854.80526	7049.70969	6831.65024	977.287994	12064.3274
***	47.3	36	M+H	C5H5N5	2.23895781	7920.0664	7076.66744	7922.57585	3825.26153	12592.4313
**	47.3	0	M+FA-H	C13H22O	-0.9627024	830.450302	680.825871	678.570144	1778.6983	542.42222
**	47.3	0	M+FA-H	C18H26O3	-9.0777245	1996.85438	1490.66691	1839.53346	2740.10108	2522.10663
**	47.3	0	M-H	C22H43O9P	-0.8072397	4398.34235	3039.57212	3333.3024	1136.23748	252.003168
*	47.3	49.4	M+NH4	C42H79NO1	2.44559161	73399.9244	64388.2081	71176.2493	15560.9807	106184.852
*	47.3	40.4	M+H	C17H20N2O	-0.7639886	37027.2442	37856.128	37081.7543	158692.619	5.2880613
*	47.3	40	M-H	C18H34O5	-0.0859144	40606.1762	42661.1728	40870.71	5791.00985	14086.6682
*	47.3	41.3	M+H-H2O	C16H18N2O	-0.6233869	2784.45099	3117.77501	3091.93912	4337.53518	1888.05302
**	47.2	0	M+FA-H	C21H34O3	-0.0706957	634.454706	683.340199	373.14293	9.7531E-07	173.360113
*	47.2	49.5	M-H	C16H19FN4(	-4.5944654	7993.22107	8350.7203	8153.84531	7689.22535	6636.51143
**	47.2	0	M+Na	C20H34O4	-1.7988025	2649.61664	2249.07009	1997.56535	904.494808	1036.15134
*	47.2	40	M+NH4	C13H14O5	0.13822488	5330.83303	5251.78552	4836.61867	3878.34125	1682.47904
*	47.2	42.2	M+Na	C10H14O10	1.59732287	7753.49041	7911.71086	6624.23391	12932.3801	3352.76848
**	47.2	0	M+Na	C14H26O3	-0.0824021	6488.73704	6991.9411	7459.3613	9738.81121	6219.81573
*	47.2	52.5	M+H	C14H20O6	-0.9959885	656.446817	573.817631	637.977561	561.089426	270.690558
**	47.1	0	M+NH4	C8H14O8	3.86759514	6271.82369	6227.44576	3920.61436	5615.68507	4218.67397
*	47.1	40.6	M+Na, M+K	C8H14O8	0.84546024	31649.7863	30394.7574	29342.3943	37348.5884	2427.17957
**	47.1	1.7	M+NH4	C21H38O4	-1.6411851	2352.78102	2743.44777	2755.26494	540.394044	1016.98048
*	47.1	46.2	M+H-H2O	C29H57NO5	-2.0249784	56694.9572	54075.7095	58920.4789	157988.89	41525.2475
*	47.1	53.6	M-H2O-H	C40H64O5	-4.2121261	24459.5127	22495.3665	20513.5187	23756.0635	20666.381
*	47.1	46.7	M+Na	C18H29NO4	-4.1496021	8304.22641	8125.6289	7751.36133	1997.85392	31754.1776
*	47.1	40	M+H-H2O	C12H18O2	-0.4748343	3342.6432	3228.66745	3456.01738	3200.4309	1822.89089
*	47.1	40.7	M+NH4	C14H28O3	-1.0744722	5646.87089	5610.68092	5774.13175	3311.91648	5313.35516
**	47	0	M+FA-H	C6H10O7	-5.9816876	1255.33996	1415.11778	1293.09084	1358.83503	1082.47095
*	47	41.9	M+FA-H	C11H20O10	3.8813973	21556.2174	21347.8755	20398.3987	14598.0547	53392.1393
*	47	41.3	M+H-H2O	C19H32O3	-1.8779573	4835.02764	5135.18335	5118.30157	2069.033	5010.22196
**	47	0	2M-H	C12H20O2	-0.0146899	918.171073	823.697191	890.096745	2017.80602	220.905434
**	47	0	M+Na	C18H34O3	-1.4390229	1047.04911	984.954414	1060.0373	2777.81501	1443.68003
*	47	39.5	M+H	C14H15N	-0.2549826	14273.852	13890.5641	14195.0215	4844.77516	4254.93284
*	47	39	M-H	C7H14O3	-1.2748001	3176.10538	3281.00823	3348.37088	2942.84431	3274.94021
*	47	39.2	M+FA-H	C25H43NO5	0.32287621	3410.62464	3034.6598	3352.73877	97.3479229	12614.3964

**	46.9	0	M+NH4	C15H26O13	-0.5429692	29307.983	35026.107	32775.9186	9.7531E-07	113523.349
**	46.9	0	M+H-H2O	C5H6O4	3.75625963	371.526706	397.766794	393.716099	383.934688	392.222106
*	46.9	41.7	M-H2O-H	C40H72O5	-0.433932	16683.8432	14066.516	15536.191	26434.2061	5033.62171
*	46.9	39.2	M+H	C8H15NO4	0.83362945	16269.7398	17378.8082	17334.4389	32607.723	37203.1959
*	46.9	39.8	M-H2O-H	C16H31IO2	2.30126077	5077.79822	5360.30596	5582.14105	5563.08211	4835.777
*	46.9	41.4	M-H	C8H8O2	-0.9545857	1539.47849	1585.05561	1576.69844	1503.55791	1464.09874
**	46.9	0	M+FA-H	C20H32O6	0.20080941	527.402209	615.707255	770.055977	9.7531E-07	50.4291437
**	46.8	0	M+H	C10H18N2O	-0.7586386	12406.0668	15133.6381	11787.3871	18065.671	10597.2714
*	46.8	40.1	M-H	C11H20N2O	-0.6800322	3171.17264	3032.1939	3156.83612	2835.80999	7579.23863
*	46.8	44.4	M-H	C6H14OS2	-4.8393419	14016.913	13027.9555	16499.6259	7859.8127	5722.72918
**	46.8	0	M+H	C22H36O3	-1.9485724	2497.07497	2164.2868	3066.13599	2530.48643	3404.53939
**	46.8	0	M-H	C28H46O2	0.42686202	5152.14237	4728.66621	3279.06914	5240.28206	3971.70158
*	46.8	60.6	M+FA-H	C15H9ClO5	1.86638405	10317.5931	7970.83442	6976.42636	12759.7344	4357.30968
*	46.8	38.8	M+FA-H	C6H5NO2S	-0.4199135	858.005785	1020.70625	1051.80722	1032.11554	367.953002
**	46.8	0	M+H	C20H32O3	-1.7877138	571.159881	725.849225	535.353705	374.638929	3365.22306
**	46.8	0	M-H	C12H20O3	-0.7579991	2173.68446	2142.95576	2152.14785	2280.76048	2915.6646
**	46.7	0	M+FA-H	C13H26O	-1.3657754	1814.64249	1657.89996	1803.40516	3146.7237	829.425402
*	46.7	40.6	M+H	C22H38O2	-1.2188527	3092.39666	2279.55659	2867.12454	34448.5964	5313.30648
*	46.7	50.3	M+H-H2O	C15H26O4	-0.8434059	96757.706	95501.071	94537.998	131520.14	80803.1974
*	46.7	37.3	M+H-H2O	C9H19NO2	0.14031186	554.415328	591.352604	477.018096	324.022277	102.241158
*	46.7	37.5	M+NH4	C14H28O2	-1.2539041	20053.469	20120.2745	20231.9065	12810.0124	28647.041
*	46.7	38	M+H	C32H38O12	0.7320387	1638.64285	1235.56435	1329.69204	363.44865	59.6994572
*	46.7	38.1	M+H, M+NH4	C22H36O6	-0.522578	11841.6726	11000.8049	13125.9092	5239.57352	4824.033
*	46.6	49.3	M+H	C5H12N2S	4.68530374	128714.315	126688.261	129802.463	363612.732	129105.95
*	46.6	38.3	M+H	C20H33N5O	0.86377933	60164.4352	57856.881	63093.4282	170630.15	131362.538
**	46.6	0	M+H-H2O	C24H50NO7	0.49069184	20016.65	23647.2096	16875.8971	29844.2675	19863.6904
**	46.6	0	M+H-H2O	C21H36O3	-1.1376832	4326.03284	3607.41156	4610.42338	2363.01151	4631.18625
*	46.6	64.3	M+H, M+K, M	C18H33ClN2	-0.7677764	166561.541	166917.137	167811.964	284103.526	50947.5586
**	46.5	0	M+H	C6H8O6	8.699255	8659.9048	8967.12004	7831.58615	10783.9084	11274.8552
*	46.5	36.6	M+H, M+Na,	C11H12N2O	-0.0986343	58543.683	59892.5506	59873.7411	59609.6369	166228.774
**	46.5	0	M+NH4	C15H14O5	2.66977249	917.119435	1111.16747	661.923051	1972.18498	96.620641
*	46.5	49.2	M-H2O-H	C21H16O10	-3.7871583	3950.71923	4663.40958	3843.73324	1975.41101	1315.7313
**	46.5	0	M+FA-H	C21H34O5	-0.5980661	1853.50816	1623.44099	1784.12549	676.189406	2151.88329

**	46.4	0	M+H-H2O	C10H13N5O	-1.0024413	32355.4652	34390.6858	31568.1008	86235.6749	21701.9334
**	46.4	0	M-H	C13H20N2O	0.62961422	700.648338	500.989288	653.777012	4466.06991	625.189379
*	46.4	42.9	M+H-H2O, M	C18H16O4	-1.4704602	21880.9981	20783.5483	21718.5021	69305.1901	35579.1994
*	46.4	38	M+NH4	C21H40O2	-1.9671938	50704.8323	50152.3657	50938.2671	232061.569	55929.0784
*	46.4	38.2	M+NH4	C6H6N2O2	-0.5521901	223530.83	237844.133	255692.664	306342.11	174899.158
**	46.4	0	M+K	C9H10N4O4	-8.7790928	753.046863	722.04874	775.610695	1557.42395	632.471452
*	46.4	49.5	M+H-H2O, M	C16H15O6+	1.12166087	6128.9791	6124.84213	5835.79564	5997.04631	2006.58752
*	46.4	36.3	M+NH4	C17H34O3	-1.5486317	1842.3592	2399.3441	2185.27633	48.0345563	582.719952
*	46.4	60.4	M+H	C18H13ClFN	-0.9976069	18210.0518	17631.5276	17324.4424	34014.8768	32066.9614
**	46.4	0	M+Na	C22H32O5	-7.7166974	6720.70113	6674.64486	6875.83774	1643.54481	26245.1667
**	46.3	0	M+K	C11H20NO1	3.72504219	2519.16657	2792.42141	2534.34893	3840.49398	2841.66514
*	46.3	38.2	M+H	C8H8O	1.94508059	13801.6468	15314.842	17480.1421	25853.9629	16009.4381
*	46.3	36	M+H-H2O	C6H15N3O	-0.4151451	26935.1312	24185.9532	28565.5786	63167.9526	31813.7793
*	46.3	48.6	M+Na	C40H78NO8	-0.410472	1004973.97	668974.436	720657.828	1186061.86	650361.284
*	46.3	40.5	M+NH4, M+N	C21H36N4O	-2.7479889	17885.7044	17544.1792	19369.0538	1821.6841	6122.86662
*	46.3	39.2	M+FA-H	C7H14O8	1.94399248	2844.75567	2484.71228	2693.16647	4921.25822	5268.18776
*	46.3	37.1	M+NH4	C25H34N2O	-0.6356735	56419.0043	59080.8954	58739.1738	89678.7469	48543.4769
*	46.3	37.2	M+H	C19H33NO2	-1.0542477	10582.3603	11068.3821	10574.6502	4615.85681	14296.858
**	46.2	0	2M-H	C5H10O6	8.35995212	12491.6139	11649.3079	11840.596	19755.0665	9465.5064
**	46.2	20	M+H	C12H23NO5	0.29592186	56383.9149	55375.9901	57999.0566	67564.2027	54894.2409
*	46.2	36.7	M+H	C23H39NO	-1.5013546	7980.19893	8088.51513	8400.30032	9.7531E-07	192.92987
*	46.2	35.1	M+Na	C10H17N7O	0.63438544	31376.0553	28268.4087	30301.8597	19129.5109	39385.9436
*	46.2	39.3	M+H, M+Na	C6H11NO	2.21896605	289152.209	313997.239	320281.707	96329.0947	49607.0253
*	46.2	34	M+H, M+Na	C8H17NO2	1.45787893	7386.86904	7612.43123	7860.1162	9578.33617	5682.56878
*	46.2	32.5	M+FA-H	C8H5F3O3	-0.5742609	4368.95906	4528.33874	4843.92829	2787.38461	2338.8051
*	46.2	34.3	M+FA-H	C4F8	-0.7525241	13207.4405	13129.6069	13610.2479	12638.5747	11746.2665
**	46.1	0	M-H	C12H16N2O	-0.497857	910.065933	903.595873	1081.17699	231.647381	654.950274
*	46.1	36.5	M+H-H2O	C13H18N2O	-1.3233306	4253.88399	4239.11321	4045.36622	2339.81385	8142.44874
*	46.1	33.9	M+Na	C13H18O6	-0.9402549	1615.30971	2064.31496	1859.2925	3396.40312	4063.15399
*	46.1	36.2	M-H	C20H36O6	-0.1429155	8152.18584	8032.59431	8048.65341	490.731226	13500.1964
**	46.1	0	M+H	C25H27N3O	-0.6036107	2707.25369	2222.21202	2456.46362	8824.85239	2546.26875
*	46.1	36.4	M+H	C12H23NO2	-1.272772	7957.77795	8252.989	8303.96551	8135.95361	16158.5944
**	46.1	0	M-H	C26H43NO5	0.27122689	1247.09507	931.021805	1059.27392	463.162082	2596.34928

**	46.1	0	M+H-H2O	C20H30O5	-8.2377367	163618.398	166492.444	169129.043	218730.671	127676.823
**	46	0	M+FA-H	C22H32O3	-6.9248468	3108.17658	2819.12056	2736.27954	1504.41661	250.188408
*	46	46.4	M-H	C15H22O3	-0.8341881	11267.6234	11609.5881	11808.2596	11524.1795	11411.4722
*	46	61.4	M+H-H2O, M	C48H84NO8	-2.7526323	3059275.99	2800766.79	2984815.97	3600758.9	2306908.54
**	46	8.26	M-H, M+FA-H	C22H36O2	-0.492355	945086.287	958209.839	912796.36	1243849.79	487011.592
**	46	0	M-H	C18H20N2O	0.43219745	489.449264	453.286406	617.501577	45.0965032	1129.67765
*	46	34.2	M-H	C12H21N7O	0.98428906	10984.6976	11681.5146	11031.8144	6906.49111	11274.3021
**	46	0	M+FA-H	C10H14O	-0.868649	763.630719	986.909821	833.957506	925.539741	1296.92777
*	46	33	M+FA-H	C12H14O3	-0.5914475	11667.3056	11846.0201	12112.1354	11612.1284	10305.4182
**	45.9	0	M+FA-H	C8H16O6	-0.7735368	1209.33669	1007.05723	1166.36561	832.918144	1025.31163
**	45.9	0	M-H	C20H34O2	-0.7677265	633.177169	477.339083	578.946777	9.7531E-07	6085.42409
**	45.9	0	M-H	C20H34O4	-0.2742321	1538.44693	1791.63402	1677.24815	1736.97838	1576.20846
*	45.9	41.6	M+H	C32H36N2O	-2.1274489	2080.74183	2134.52596	2019.11675	21350.2629	11491.2349
*	45.9	40.9	M-H	C5H7NOS	-0.7894304	11537.351	12272.8252	12531.9105	12511.0754	7780.74655
*	45.9	32.7	M+H	C16H19NO	-0.929247	20862.3577	21202.0122	20740.0893	1173.48893	2439.55246
*	45.9	33.4	M+FA-H	C7H12O	-2.1908268	51272.5076	52415.6103	50582.0454	49917.4426	44919.8929
**	45.9	0	M+FA-H	C17H22O10	-0.8026212	693.711821	608.231361	785.46845	1715.66723	1094.51916
*	45.9	34.3	M+NH4	C19H19NO6	-1.2558299	6414.48146	6336.98036	6732.97685	10544.4362	2868.35193
*	45.9	34.7	M-H	C10H18O3	-1.5914575	60604.5214	63894.8747	60865.6538	62091.135	54646.8562
*	45.9	45.7	M-H, M+FA-H	C9H20OS3	-3.1303931	56656.1898	63017.2799	66283.6231	33836.1209	28394.0722
*	45.9	31.5	M-H2O-H	C50H90O7P	0.89523528	4466625.62	4295764.46	4737164.63	4343420.24	3755708.05
*	45.8	35.2	M+H	C6H15NO	2.03749146	2913.09029	3077.41048	2891.29124	3486.79603	2243.75453
*	45.8	60.6	M-H2O-H	C27H50N3O	3.22565229	10777.8581	11983.3873	11007.8277	6082.47619	25115.6129
*	45.8	34.4	M+H, M+Na	C22H46NO6	-1.5387357	125465.575	121497.471	119084.173	37200.126	199777.619
**	45.8	0	M-H2O-H	C8H8O4	-1.4872085	521.200991	517.963718	459.138027	482.493126	406.963814
**	45.8	0	M-H	C10H13NO2	-1.8893867	296.89581	365.186533	438.859335	407.034634	282.745422
*	45.8	41.6	M+H, M+NH4	C14H25N7O	-0.6840503	12353.6967	11713.0138	13075.6724	52205.477	11504.5338
*	45.8	32	M+H-H2O	C12H20O3	-0.3733299	4777.68008	5031.58429	5020.15966	5125.26988	3161.68034
*	45.8	32.8	M+H, M+Na,	C22H22O10	-0.3708674	20535.068	23442.2607	23950.0057	37591.0654	22284.9401
*	45.8	35.4	M+NH4	C18H36O4	-1.8970717	4047.60835	4360.17066	4093.72153	2527.34848	4109.31506
*	45.8	33.3	M+FA-H	C16H24O3	0.19857629	25665.9269	25744.124	26251.9905	23980.5371	21650.1752
*	45.8	36	M+H-H2O	C27H45NO6	-0.9646722	3412.03015	3278.09684	3674.87146	9.7531E-07	12358.2413
*	45.8	38.6	M+Na	C25H45NO4	4.50437352	16120.4737	15915.7455	15005.708	950.436712	52508.9456

*	45.7	32.7	M+Na	C5H9NO4	-0.2602926	5812.41498	5313.70562	4643.37266	7067.32454	3548.80683
*	45.7	37.1	M-H	C8H11NO3S	-0.9087557	1022.01909	1136.75681	1160.29699	668.500369	981.57141
**	45.7	0	M+H	C15H22N2O	-2.5697819	152.243493	95.1231155	150.821003	9.7531E-07	94.6597071
**	45.7	0	M+Na	C10H18O2	-0.4705836	2411.01618	2495.12938	2608.28092	3418.46023	1887.84634
*	45.7	33	M+NH4	C17H34O2	-1.4277207	264184.31	261616.189	266160.93	173879.638	332710.588
*	45.7	33.2	M-H2O-H	C26H46NO7	-1.5269486	426534.272	432057.93	529060.622	472551.075	417229.93
*	45.6	31.7	M+NH4	C8H14O3	-0.5339802	67066.6378	69164.3604	72080.7686	68855.7977	159573.272
*	45.6	43.2	M+H-H2O	C8H16N2O6	-0.1477117	14679.2144	17391.4467	15911.2736	14774.7573	20218.4458
*	45.6	31.4	M+H, M+Na	C20H23NO3	-0.470277	8497.97163	9407.90524	8742.15688	12509.1849	2535.8347
*	45.6	35.4	M+NH4	C22H34O3	-2.1169997	2282.82972	2161.88888	2174.7088	595.094531	9.7531E-07
**	45.6	0	M-H	C22H44NO9	-0.3103206	10470.8364	12165.2701	10695.7126	1095.0828	1729.77827
*	45.6	33.4	M-H	C16H30O3	-0.564996	84384.745	86633.9391	84071.3722	76188.4914	54692.0421
*	45.6	41.5	M+FA-H	C33H54O6	-0.1865993	5628.10849	5132.74614	3842.2717	19482.5524	2409.87083
*	45.6	42.9	M+H	C15H11O5-	1.75551416	8246.20105	9290.35607	8563.98355	4374.36474	14052.8424
**	45.6	0	M+H	C16H26O8	-5.8968519	1854.7846	1600.54908	1802.1027	1584.63282	2701.52136
*	45.6	35.4	M+H, M+Na,	C14H14N8O	0.49683859	60856.753	59883.2688	62544.0899	2.77804058	3.64203018
*	45.6	32.7	M-H	C10H16O3	-1.7875282	4295.49408	4454.26021	4407.75097	1916.48769	2389.00693
*	45.6	34.4	M-H	C22H32O4	0.815989	5370.93996	5416.58862	5650.98956	122.707551	658.105429
*	45.5	32	2M-H	C14H10O3	3.07231241	22457.8193	20488.6972	22571.4114	14082.1164	61256.9611
**	45.5	0	M+H-H2O	C19H20O5	-6.0585385	3411.03336	3407.9292	2837.75814	5313.13233	2153.69698
*	45.5	32.5	M+H	C22H47NO5	-1.1604318	91642.6358	88803.5558	91579.3885	1735.95543	306.496936
*	45.5	43.5	M-H2O-H	C38H68O5	-4.2113959	23047.345	21992.9101	17662.3847	30347.2987	12781.9154
*	45.5	31.2	M+H-H2O	C8H10O6	0.51292235	2746.47229	1873.08162	2081.19806	972.562429	1702.17718
**	45.5	0	M-H2O-H	C23H30N2O	-1.4294714	560.009517	533.864741	496.443385	9.7531E-07	350.126414
*	45.5	34.8	M-H	C30H26O13	0.7217064	15942.093	18526.7965	24591.1005	23535.2211	13308.0378
**	45.5	0	M+Na	C22H32O4	-1.7893623	2157.89114	2307.8285	2004.53619	853.852158	550.467814
*	45.4	37.4	M+Na	C20H16N2O	2.9619739	104301.713	96305.797	109509.379	156442.085	100557.132
**	45.4	0	M+H	C5H11N3O2	-0.5875944	1106.13943	1120.34521	1598.7885	9.7531E-07	66.5686723
***	45.4	31.9	M+H-H2O, M	C22H36O4	-1.6630235	14226.0952	11908.4313	14430.9179	3262.33295	1650.5265
*	45.4	32.1	M+NH4, M+N	C27H48O7	-0.6354405	124871.106	126674.441	130981.659	56661.8049	113690.643
**	45.4	0	M+H-H2O	C20H34O4	-8.4091334	12580.5879	11634.3353	12588.1796	25503.6466	13217.4346
*	45.4	31.8	M-H	C17H34O2	-0.6401609	14626.7348	14859.9719	14979.4367	17708.8248	7679.35375
*	45.4	32	M+H, M+NH4	C9H7NO	0.49981678	12737.9719	11993.0106	13480.122	14981.8004	32794.9883

*	45.4	33.8	M+H	C8H15NO2S	1.34890799	5397.95297	5257.70115	5173.70348	7864.77024	8781.23314
**	45.4	0	M+K	C16H24O7	-3.8210255	777.806145	1280.48916	808.006971	1313.47935	2364.78758
*	45.4	32.5	M+NH4	C19H38O3	-1.4007957	14280.313	13793.1125	13634.5607	10026.1294	15754.8519
*	45.4	29.5	M+H	C11H14O3	-0.1196576	8373.76315	10308.1235	8637.59861	13376.2346	8278.15768
*	45.4	31.5	M+H	C16H33NO	-1.3102605	12615.3726	13353.2403	13422.093	5558.86784	2053.39944
*	45.3	33.8	M+H	C7H13NO4	-0.2071244	29318.6709	30123.5031	28707.7435	261802.83	53355.6778
**	45.3	0	M+K	C19H39NO3	7.36619999	2292.38594	2290.13937	2870.422	18580.0004	6686.98933
*	45.3	31.3	M+H, M+Na	C6H15N2O5	-0.7685548	146219.37	150173.155	137675.67	296250.511	61718.9153
*	45.3	30.6	M+NH4	C15H22N2O	1.68465884	13058.8965	12259.1701	14502.0055	11677.0598	30896.3669
**	45.3	0	M+NH4	C15H19F3N:	9.29926716	563.670877	582.697914	680.303147	241.105201	520.719548
*	45.3	39	M+H, M+Na	C24H35N3O	-7.3969851	42787.4036	40938.2764	43819.1623	6557.3288	4308.72159
**	45.3	0	M-H2O-H	C11H11NO4	-0.9360934	157.98059	133.021121	136.237038	218.520742	9.7531E-07
*	45.3	28.8	M+H	C8H8O4	-0.4800527	4324.38904	4050.61102	5367.45215	3596.1013	3235.99519
**	45.2	0	M+FA-H	C9H12N3O7	-1.7925804	656.502168	806.986812	745.976662	1943.63629	1391.81943
*	45.2	27.5	M+FA-H	C7H9FN2O3	-0.3725452	56097.4933	57462.9884	58471.5088	78694.7381	90411.6638
*	45.2	33.5	M+FA-H	C12H11NO4	2.52522305	2959.90321	2761.5422	2941.26199	4218.21765	2287.87905
*	45.2	35	M+H	C5H9NO	4.77327191	10424.0802	10466.1092	10934.5298	13772.179	20107.0369
*	45.2	42.7	M+H	C12H16N2O	-0.5667717	7015.15335	7361.00095	7036.61132	3439.96625	9216.3753
**	45.2	0	2M-H	C6H12O4	-0.5939748	1202.83533	1102.79506	1150.19903	93.8934812	585.766284
**	45.2	14.8	M-H	C6H12O3	-1.4468941	5683.90502	5704.05042	5536.96737	6537.66077	14456.9664
*	45.2	35.8	M-H	C7H7NO4	-1.4268092	1074.60336	991.397051	989.25787	335.934644	287.305675
*	45.2	29.3	M+H, M+Na	C14H25NO4	-0.6295709	9326.25516	9140.89385	8956.39317	5613.07044	41364.5045
*	45.2	37.4	M+H-H2O	C23H39NO5	0.34589223	7206.38744	7160.98865	7401.48526	6070.73807	6266.3141
*	45.2	33.5	M+NH4, M+N	C18H32O4	-1.3996951	87844.9891	91794.5989	89078.716	70924.4298	45504.0833
*	45.1	30.7	M+FA-H	C4H5F3O	-1.013777	82530.9087	82958.663	80974.734	164546.298	119099.572
*	45.1	29.6	M+FA-H	C10H17N7O	1.14781653	23990.0089	25052.2041	27047.5121	303.921431	44690.236
*	45.1	38.2	M+FA-H	C43H76NO8	0.11684492	567486.537	573165.483	739339.99	573348.766	475772.973
*	45.1	35.6	M-H2O-H	C14H19NO3	-0.6329974	3300.40609	2864.58885	3306.62617	2813.96865	3162.46401
*	45.1	43.2	M+H, M+Na,	C15H14O3	-9.3797974	183141.993	184388.809	191024.894	677716.995	50667.6208
**	45.1	0	M+H-H2O	C20H30O11	-9.5675745	2017.12273	2062.95368	2020.07994	9.7531E-07	567.400221
**	45.1	0	M-H2O-H	C13H12O8	-8.7460802	26305.5674	26413.9169	25532.3901	24806.242	22709.6475
*	45.1	33.9	M+H, M+Na	C13H16N2O	0.01616228	6797.06503	7264.09281	7472.4222	8423.31228	27283.7466
*	45.1	35.7	M-H	C8H10O4S	-1.9876637	537.783792	543.229409	475.794962	100.663824	87.3375593

*	45.1	35.1	M+H	C21H39O9P	-3.8483194	34089.2592	36493.0015	40257.3147	62304.1688	37717.6602
*	45	30.3	M+NH4	C20H40O2	-1.622692	78251.6021	77324.9175	78625.9205	32603.5999	36286.8799
*	45	37.9	M+H, M+Na	C24H41NO2	-1.7004782	10436.4814	8674.15477	10229.6602	21237.2999	3338.3221
*	45	29.3	M+H	C9H7N	1.10115245	9888.33898	9771.29354	9599.70623	19918.8571	5373.43729
**	45	0	2M-H	C11H14O5	-2.1508609	215.710705	183.9083	147.94128	1025.4296	225.364391
*	45	32.6	M+NH4	C13H14O4	0.61708615	1557.31117	1803.32141	1636.94553	2003.06839	1468.52685
*	45	29.3	M-H	C7H15NO2	-1.3257077	640.220784	662.987881	694.333118	34.8351128	4504.00964
**	45	0.172	M-H	C8H15NO4	-1.1670835	1092.05269	1045.97342	994.58379	108.665468	7049.64502
**	45	0	M+H	C15H20O5	-9.5703289	6888.56349	6582.78303	7007.7863	9164.6551	5328.01176
*	45	29.6	M+H	C16H24O3	-1.4084637	15339.1252	16145.4221	14871.1035	22019.0087	12306.854
*	45	29.3	M+H	C12H18O2	-0.5814729	4624.11394	4980.96862	4915.48638	4781.94036	2815.39074
*	44.9	28.6	M+H	C15H23N3O	0.17304397	144226.272	145512.558	148048.581	151040.449	376555.575
*	44.9	29.7	M+H-H2O, M	C18H35NO2	-1.4861298	31778.2431	30940.7612	31806.3421	13925.6893	11864.7967
**	44.9	0	M+H-H2O	C17H31F3O	-1.5230465	5786.68716	4946.5323	5487.76967	8470.25908	4634.72098
*	44.9	40.3	M-H2O-H	C40H68O5	0.572144	16492.1322	13307.6756	11442.7951	13628.3751	7526.2805
*	44.9	30.3	M+H-H2O	C29H50O3	-0.8850989	106140.404	162518.34	165954.012	200005.524	165350.218
*	44.9	27.5	M-H, M+FA-I	C9H7NO	-1.4632701	18304.9193	19066.5493	20104.7606	17631.2117	52624.7243
*	44.9	26.1	M+Na, M+K,	C12H26O7	0.63864354	428440.285	446871.702	437489.085	347040.736	900271.185
*	44.9	31.5	M+H	C16H29N3O	0.62449433	4291.30075	4449.79752	4321.42146	2006.3277	14042.5327
*	44.9	32.6	M+FA-H	C21H36O4	-1.6603525	1738.28613	1902.85469	1875.93971	9.7531E-07	250.242793
*	44.9	29.7	M-H	C10H12O3	-1.118033	472.287112	470.22502	533.324791	327.987705	259.615412
**	44.9	19.9	2M-H	C9H16O2	0.23646249	8433.01342	8073.3839	7450.71183	4211.71879	3596.35919
*	44.8	26.5	M+NH4, M+N	C9H14O3	-0.0883192	2844.82286	2991.83313	2576.11744	1685.1697	951.749492
*	44.8	40.7	M+K	C17H24O6	1.9656817	14565.7175	10884.4705	11736.5114	16722.5191	13064.4942
*	44.8	26.8	M+FA-H	C5H11NO3S	-0.6625431	1256.28967	1110.27774	1146.55562	18.6596457	9.7531E-07
*	44.8	46.8	M+H	C17H14N2O	-6.1470495	21837.372	23652.4646	21820.3424	23682.9216	12225.8626
**	44.8	0	M+H-H2O	C18H32O2	-1.3878145	1354.75645	1520.79015	1158.04228	706.248959	712.559873
*	44.8	27.5	M+NH4, M+N	C16H34O9	0.97096587	151692.792	148226.67	157567.094	177694.157	304899.524
*	44.8	31.2	M-H	C6H13O9P	-3.134957	865.604805	900.665047	880.317065	920.97234	934.443358
*	44.8	30.2	M+H	C14H13N3O	-0.965896	30660.16	30078.8673	29490.7065	9.7531E-07	9.7531E-07
*	44.7	35.2	M+FA-H	C23H20N2O	-0.1147642	22151.0891	23054.4072	22045.7548	23156.0114	22328.912
*	44.7	34	M+NH4	C23H28N8O	-2.1830018	13038.5531	13988.2941	13888.5952	7432.97024	24393.8513
*	44.7	46.5	M+NH4, M+N	C34H68O9	-1.6613238	24731.0475	26236.5092	23441.6311	1341.73545	3127.99105

*	44.7	33.6	M+Na, M+K,	C8H18O5	4.39368097	825804.794	847835.361	836930	765771.222	1368783.37
*	44.7	37.2	M+NH4, M+N	C17H31N3O	3.36935043	93922.9913	96013.3883	94266.6847	2196.49777	7395.08646
*	44.7	27.3	M+H, M+Na	C11H18O3	0.09660826	8510.1115	8365.00305	8599.78981	8064.13915	5618.51536
**	44.6	0	M+FA-H	C8H16N2O4	-5.7934644	107081.133	115665.497	113398.325	186351.885	203712.781
**	44.6	0	M+H	C18H39NO	-1.778133	9398.10962	10759.2845	10803.7029	5160.80178	4156.81787
*	44.6	36.4	M+H, M+Na	C19H30O4	-1.4657098	4575.65578	4437.1235	4905.04772	1864.71276	800.5304
*	44.6	32.4	M+Na	C14H24O2	-2.9900854	190793.722	192616.214	205566.979	305860.446	174227.417
*	44.6	29.2	M+FA-H	C8H12N2O3	-0.7380799	2925.21285	2861.73218	2941.0818	7756.01469	3537.24427
**	44.6	0	M+H-H2O	C10H12N2O	0.88950106	265.831056	234.096687	364.045346	437.143743	241.967109
*	44.6	28	M-H	C8H15NO3	-0.8002542	1459.35931	1441.84634	1486.72372	505.604289	1092.38533
*	44.6	29.2	M+H	C6H13O9P	-2.5367557	664.670546	631.633398	624.699147	1547.28078	1075.75924
*	44.6	27.6	M+H, M+Na	C18H20N2O	-0.0516883	39065.3694	39908.8599	40700.4386	73436.3591	22460.8156
*	44.6	27.1	M+H-H2O	C11H15NO3	-0.1633378	2698.90875	2551.34687	2809.41131	4285.10241	6442.16202
*	44.6	29.7	M+FA-H	C18H30O2	-0.7297386	242.722894	445.078597	405.808028	9.7531E-07	128.985113
*	44.6	27.3	M+H-H2O, M	C16H30O3	-1.2323917	31944.8539	34361.4262	33853.9002	159418.641	31512.2204
*	44.5	34.2	M+H	C6H12N2	3.91482966	2975.90362	2994.02627	3031.76574	1000.36253	516.407097
*	44.5	25.7	M+FA-H	C12H22O	-0.6099434	73259.4756	75175.3492	71578.4666	68671.5038	55430.8422
**	44.5	0	M+H	C25H50NO7	-1.28407	9582.52128	9933.08215	10439.9672	8791.90608	24101.8818
*	44.5	29.7	M+NH4	C16H24N2O	1.93029598	9577.31209	9305.26043	9344.59548	5761.27222	24190.034
*	44.5	31.3	M-H	C8H8O5S	-0.5902847	179.118247	215.204478	160.034165	3.94027992	8.53185523
**	44.5	0	M+H, M+Na	C12H14N2O	-0.7763229	1918.3403	1905.02548	2040.83275	2221.96908	1308.89955
*	44.5	31.7	M+H-H2O	C12H16N2O	-1.6254962	3228.24584	3084.1984	3083.26935	5592.6973	6766.64076
*	44.5	26.1	M+FA-H	C11H14O2	-0.6052477	1431.97786	1478.6926	1366.94102	583.243454	749.271179
*	44.5	34.8	M+K	C16H18O3	4.31817281	2697.92587	2984.7206	2945.03894	4911.88209	4053.60466
*	44.5	29.1	M+NH4	C19H35NO3	-1.6191583	3997.32205	3864.27157	4013.5098	8271.07449	4944.4367
*	44.4	28.2	M+FA-H	C6H10O10S	-1.6437545	26351.8269	22336.5048	24126.9614	25313.1824	16046.8581
*	44.4	33.1	M+Na	C23H29NO7	-3.0507999	7153.0718	6859.54753	7385.89282	5899.51897	2609.56396
*	44.4	35	M+H	C15H22O2	-1.3560431	96479.9669	95503.2962	97361.8602	121969.63	76533.6131
*	44.4	30	M-H, M+FA-I	C20H30O3	-1.1051446	9154.45024	9290.16939	8525.3197	6121.89353	6600.31958
*	44.4	28.5	M+NH4	C27H48O6	-1.0652088	22403.0061	22284.9738	20862.8566	8577.65736	14243.6651
*	44.4	26.9	M+H-H2O	C18H39NO2	-1.5458023	20707.7698	20585.6963	19328.4621	4856.9748	3785.99034
*	44.4	28.8	M+Na	C8H17N3O2	-1.5430511	808.679423	856.421389	684.568128	123.721771	938.203722
*	44.4	32.3	M+FA-H	C6H9NO2S	-1.7032388	804.96528	865.672574	943.116255	1344.97081	654.945822

**	44.4	0	M-H	C27H40O11	0.47522781	4938.15711	5884.53643	5697.96347	5072.57317	3168.42036
*	44.4	26.3	M+H, M+Na,	C13H25NO3	-1.1985373	117906.346	118076.29	121338.451	101683.339	109834.802
*	44.4	25.8	M+FA-H	C10H18O	-1.4750283	44148.6061	45714.6729	44391.4641	43607.3909	37043.3244
*	44.3	29.1	M+NH4	C5H10O2	1.7249988	8455.86316	7466.55681	8194.21662	33741.6367	3689.96887
*	44.3	26.9	M-H2O-H, M	C8H9F3N4O	3.18797821	49216.5175	47268.4083	47642.6685	66664.5427	29649.6856
*	44.3	24.9	M+H-H2O	C8H8O2	1.53472555	63702.9457	64792.3917	65277.906	105988.978	86608.0164
*	44.3	25.5	M+Na	C7H13NO4	-0.5766493	2258.89896	1939.59685	2363.55708	21732.5856	2997.69752
*	44.3	39.8	M+K	C20H26N2O	1.75182835	734048.851	724194.311	745462.128	943812.758	596201.054
*	44.3	28.8	M+NH4	C23H46O	-1.659743	27945.2416	27716.5586	28778.7346	130.3722	172.653327
**	44.3	0	M+H	C26H49NO4	-1.6680966	7316.92071	5589.64078	5772.51306	3752.94478	14285.9863
*	44.3	52.5	M+Na	C27H55O7P	3.39718994	9945.50853	10595.8583	11091.0529	9.7531E-07	13875.8605
*	44.3	28.5	M+H	C40H80NO8	-1.6579819	30793469.6	33963765.1	20487175.1	37105540.1	16274762.6
*	44.3	32.6	M+H	C6H7N3	4.85855011	115719.598	97973.9619	97145.8982	127233.11	79070.2908
*	44.3	32	M+H, M+Na	C29H44O12	-4.3289895	60575.6914	63028.5587	69086.3275	75760.7346	62588.8295
*	44.3	29	M+FA-H	C4H2F6	-2.2841733	25158.5499	25247.486	25731.783	23090.9904	23145.9065
*	44.3	48.6	M+Na	C24H31ClO4	4.19717454	1619.7139	1354.1479	1468.44474	2165.80294	95.1717374
*	44.3	28.8	M-H	C20H38O5	-0.0781623	2223.27692	1983.91089	2201.59024	78.3830298	1349.04522
**	44.2	0	M+K	C7H10O6	4.44978781	3748.4462	3765.16688	3511.28982	7347.74359	4830.42834
**	44.2	0	M-H2O-H	C12H21NO6	-8.8379511	30233.0374	27252.8944	24582.6012	58212.8799	40293.3446
*	44.2	23.5	M+FA-H	C6H13N	-1.2334027	236.128939	222.592147	294.441017	9.7531E-07	325.265304
*	44.2	35.9	M+H-H2O	C13H12N4O	-0.0701361	225.408361	206.741795	223.024158	9.7531E-07	408.79287
**	44.2	0	M+FA-H	C19H28O3	-8.3101652	4722.41916	4756.3862	5034.11924	608.821314	15890.7366
*	44.2	38	M+H, M+Na	C30H39NO3	-6.4662466	87813.3253	89283.6791	87805.9105	16443.2023	130905.803
**	44.2	0	M+FA-H	C22H32O3	-7.2355095	12074.7991	11752.777	11558.8316	8726.1389	6267.45454
*	44.2	25	M+FA-H	C33H64O2	-0.361699	18819.8587	15837.3027	18548.656	22031.7641	12668.8565
*	44.2	33.3	M+NH4	C29H46N2O	-3.8187119	24180.3117	19839.5171	21001.1642	90139.8004	74058.4067
**	44.2	19.3	M+H, M+Na	C12H21NO4	1.10249222	11960.8779	12150.9114	12805.0602	8842.78335	12596.7651
*	44.2	25.4	M-H	C9H16O3	-1.1461465	60866.0715	62813.2315	61138.1334	59574.0774	52758.5849
*	44.2	27.2	M+Na, M+K,	C15H19NO4	-1.0871481	2623.59314	3240.33746	2728.09662	55.2620396	1399.07704
**	44.2	0	M+H	C12H20O2	-1.2522882	2744.65656	2984.77722	3006.27119	3604.11143	2189.32311
*	44.2	26.7	M+NH4	C24H30O6	-1.2516196	16898.282	19741.7636	18029.5779	9.7531E-07	1394.96968
**	44.2	0	M+H	C20H28O5	-8.41837	8070.10295	7238.55515	7757.7013	10219.8449	6159.62363
**	44.1	0	M+H, M+Na,	C11H21NO7	-0.7012893	206440.064	205323.487	205389.15	7912.52132	210141.719

*	44.1	26.5	M+H	C9H19NO2	0.68512334	1005.76003	1037.77049	973.631505	2262.93747	3603.78496
*	44.1	29.7	M+NH4	C27H51O11I	-1.656323	17976.7372	16163.4799	16411.8264	129.221219	49282.0502
*	44.1	28.5	M-H	C27H44O9	-0.6900765	5503.36839	5889.32886	5600.71912	9.7531E-07	16721.1055
*	44.1	24.4	M-H	C39H72O6	-0.199914	56390.7694	38816.9385	43005.1421	16481.8958	4864.32383
**	44.1	0.0241	M-H2O-H	C24H42O3	-0.3245943	84428.1904	81702.164	76268.0372	49967.4315	58739.9637
*	44.1	23.7	M+H	C10H10N2	0.35206979	12547.7989	13563.8616	13629.8114	30611.4414	6033.63815
*	44.1	23.6	M+H-H2O, M	C8H15NO	0.43835351	4222.24381	4244.9015	4198.27551	2084.83237	4030.34163
*	44.1	24.7	M+Na	C10H18O4	-0.2125113	20057.7881	20101.5674	20318.0864	29343.9982	17484.9265
**	44.1	17.6	M+FA-H	C7H6O3	-1.3700371	10846.7334	10446.0427	10691.4463	3453.81218	7739.9295
*	44.1	28.3	M+Na	C8H15N3O5	-1.4378529	11165.3248	10962.1291	10848.665	3202.98294	2155.23197
*	44.1	26.9	M+H, M+Na	C17H32N2O	-1.4600724	11814.6951	12647.4904	13129.9583	1103.16451	7639.45465
*	44.1	25.9	M+H, M+Na	C18H20N2O	-1.1264608	58990.5276	57549.6103	57774.1172	86864.033	25259.5694
*	44.1	25.7	M+H, M+Na	C21H37NO4	-1.0484091	205737.134	190133.228	190609.093	6876.63978	399291.959
*	44.1	24.7	M-H	C12H22O3	-0.6612696	64834.9124	65642.9419	63216.2066	60913.1134	57375.1768
*	44	35.2	M+H, M+NH4	C10H14N2O	-0.9942531	22348.6293	21919.4062	17794.6356	14672.4472	47494.7213
*	44	38.6	M+H, M+Na	C19H26O3	-5.8729839	16276.725	16939.8272	15775.2843	10607.4808	42335.7631
*	44	26.9	M+K	C44H85N2O	1.26980256	94941.2022	104169.351	107759.54	14881.3409	111844.85
*	44	26.9	M+NH4, M+N	C32H64O8	-0.9343644	70190.3791	71982.3633	69391.9845	871.431719	2012.1201
**	44	0	M+Na	C14H17NO7	-9.6535616	13406.9028	11999.696	13033.0722	20402.6326	20312.6281
*	44	32.4	M-H2O-H	C16H16F3N	0.08527431	7962.39991	7999.1908	7918.30853	10319.0906	19293.0372
*	44	28.7	M-H	C48H77N15	-0.6797013	16931.2534	17448.0084	13242.5811	3059.45515	9.7531E-07
*	44	27.4	M+Na	C9H12N5O7	0.1104336	33631.7599	34811.1998	33255.7464	73239.1486	57570.3649
*	44	24.7	M-H	C11H21NO3	-0.7264931	1842.43074	2052.36558	1888.43804	974.954439	1712.34031
**	44	22.4	M+H	C19H35NO4	-1.190894	7620.72282	6573.53132	7151.23	595.903575	36769.202
*	44	23.2	M+H-H2O	C13H22O3	-0.6001923	9581.73162	10104.96	10767.2605	15930.1562	8722.03301
**	44	15.4	M+Na	C24H30O6	-1.3940135	2077515.3	2035374.27	2202173.61	1957648.13	1701066.5
*	43.9	28.2	M+H	C9H13NO3	-4.0205052	3022.86454	3072.7151	3196.3787	3969.9658	10177.7114
*	43.9	39	M+Na, M+K	C23H48NO6	4.59892487	19063.0435	18411.984	19445.1088	5208.12194	40637.7824
*	43.9	36.7	M+NH4	C29H53O9P	-2.4084918	25985.3806	24814.7372	25282.536	15946.7493	12323.4742
*	43.9	28.6	M-H	C20H28O5S	-1.3563209	117405.144	148663.748	110236.02	141008.754	110255.947
*	43.9	29.4	M+Na	C38H76NO8	-0.8759116	519690.687	411270.236	295140.581	1475291.38	223736.914
*	43.9	35.6	M+H	C33H32N4O	-1.1483366	17936.0136	19851.8412	19047.6471	6326.21174	9.7531E-07
*	43.9	27.5	M-H	C9H15N3O7	4.44817038	4630.21049	4214.30672	5021.01243	6007.32957	11138.2575

*	43.9	36.2	M+NH4	C35H39N5O	-0.8085577	16680.9597	15186.4054	16554.9678	1471.62581	15304.987
*	43.9	24.1	M+FA-H	C7H11NO	-1.2964869	379.234382	368.728939	383.764503	96.3792023	448.009244
*	43.9	26.6	M+NH4	C11H15NO3	-0.2297243	238.729967	365.86614	331.492261	7916.17937	111.844305
*	43.9	26.4	M+H-H2O	C12H24N2O	-1.3280016	348.382426	323.516923	356.898116	9.7531E-07	135.462072
**	43.9	0	M+NH4	C16H15NO3	4.31742451	3798.64906	3585.44845	3830.34173	5124.80047	2622.65805
*	43.8	20.3	M+FA-H	C4H2F6O	-0.1283135	43167.7264	38855.4159	45190.8943	50204.7735	48977.1341
*	43.8	22.4	M+Na	C7H14O6	0.18495802	11329.3376	12050.973	10961.0735	4040.5446	34341.7039
*	43.8	20.9	M+NH4	C7H12O	0.19903579	2251.95132	2171.44531	2278.58436	9.7531E-07	4060.09417
*	43.8	22.9	M-H	C4H8O4	-1.8071838	2066.14722	1710.99105	1287.42792	1825.60889	1709.01431
*	43.8	23.7	M-H	C14H28O3	-0.9046923	77564.2586	81783.139	75647.7061	77249.9136	63572.0887
*	43.8	23.9	M-H	C24H38O2	-0.3804854	76763.0485	78830.1266	79136.5469	108218.147	43666.1487
*	43.8	25.3	M+NH4	C7H6O4S	0.76131463	4702.75782	5249.6482	4858.50772	2535.51469	347.92276
*	43.8	26.9	M+H-H2O	C7H15NO3	0.78033369	4305.62895	4478.66579	4634.613	8417.67569	9044.92492
*	43.8	24.2	M-H	C8H10O3S	-1.5421495	1890.80496	1978.54601	2255.1518	45.6739301	6107.0097
*	43.8	28.4	M+H	C4H8OS	3.85395523	208.422516	198.44862	203.173665	9.7531E-07	115.682459
*	43.8	25.8	M+H, M+Na	C10H16O4	-1.2296508	7913.70149	8190.81124	7650.71271	9214.87304	10859.6108
*	43.8	23	M+FA-H	C11H20O3	-0.6110947	2307.40686	2177.67673	2323.63684	1213.23488	3355.62134
*	43.8	24.6	M+H	C9H10F2N2(	2.7596885	1342.44494	1664.52441	1541.72883	2157.95045	1663.43292
*	43.8	23.1	M+H, M+Na	C16H35NO2	-1.1429181	181149.853	181702.465	182044.147	85521.4769	136609.594
*	43.8	24.2	M-H	C10H20O3	-1.3904831	2694.54994	2844.51563	2709.19647	2257.99947	2280.43877
*	43.8	36.3	M-H2O-H	C47H83O14I	1.49879668	714079.506	621078.459	663663.894	610910.011	689830.144
**	43.7	0	2M-H	C5H10N2O3	-8.5123085	1542.26796	1359.23822	1087.56227	1310.76528	3427.53048
**	43.7	3.1	M-H2O-H	C9H11N2O8	2.44241157	11670.4051	9660.47781	10311.3471	20634.1187	7254.67565
*	43.7	33	M-H2O-H	C17H22O10	3.80510401	35582.6586	36539.956	35302.0328	1187.42951	61391.8541
*	43.7	21.6	M-H	C9H18O9	0.07150316	221986.771	219114.511	216953.403	94619.9894	388595.266
*	43.7	21.2	M+H	C8H17NO2	-0.1038737	52650.6203	54283.7699	57475.4008	42252.0404	249699.852
*	43.7	25.4	M-H2O-H	C28H47O10I	-0.449344	83050.1672	84065.8726	80197.5857	32563.0988	45021.3399
*	43.7	23	M+H-H2O, M	C10H9NO	0.5489744	4430.73327	5017.27879	4056.53363	8981.57855	2309.45714
*	43.7	27	M+K	C5H9N3O4S	2.06419859	17234.1584	18141.8085	18318.3681	25120.7844	12943.1659
*	43.7	21.2	M+H	C9H13NO	0.13678378	620.762438	698.971235	859.480743	341.341682	753.328251
*	43.7	23.3	M+NH4	C8H9N5O2	0.25483146	28394.158	28877.6939	28149.1675	41955.756	22800.2057
*	43.7	25.8	M+FA-H	C16H16NO6	3.84754713	2671.5624	3321.03742	3537.16142	9.7531E-07	30.6935433
*	43.7	20.4	M+NH4	C8H14O	-0.511905	4042.21792	3975.57032	3927.04971	6180.13136	3735.38635

*	43.7	23.7	M+H, M+Na	C14H18O3	-1.5184175	16490.8054	17092.7205	16854.5647	20103.7399	11385.6821
*	43.7	23.2	M-H, M+FA-I	C14H28O2	-0.8830381	4552.56089	4897.03504	4627.08176	2027.9291	11401.3371
**	43.7	0	M-H	C15H22O3	-0.6190762	756.543884	742.229849	683.043795	673.027862	483.5674
*	43.6	29.4	M+K	C9H9N5O5	-4.4973591	10818.1176	9375.49777	9986.24239	58537.1109	22935.2537
*	43.6	41.8	M-H2O-H	C12H23NO1	-4.4814865	87679.9109	99274.1956	87346.2623	18756.1574	12273.4615
**	43.6	0	M+H	C10H20N2O	-0.915055	3105.28396	3561.33239	3614.54298	1461.86935	3117.09547
*	43.6	26.3	M-H	C4H5NO4S	-1.0934953	1149.73909	1369.05959	1231.47916	80.0728861	88.3293977
**	43.6	0	M-H	C18H28O3	-0.2350266	1454.19065	1567.38042	1349.84411	973.241518	864.720924
*	43.6	26.1	M-H2O-H	C38H66O5	-0.1921054	39802.7387	37263.7361	30373.7763	37878.7284	38965.5616
*	43.6	24.5	M+H, M+NH4	C15H17N7O	-0.1034052	493130.243	500814.302	488792.33	1137780.57	923169.519
*	43.6	23.6	M-H	C9H18O3	-1.5883064	2928.98829	3231.21104	3137.16297	3135.1674	2810.69395
*	43.6	37.4	M+H	C12H17N4O	3.79784577	1740.55206	1585.30189	1546.28928	1398.69208	612.969847
*	43.6	20.7	M+NH4	C10H18O	-1.1557238	20205.9181	19458.5263	19404.7019	27151.0856	18270.8225
*	43.6	19.7	M+FA-H	C12H15NO3	-0.1458978	7857.14713	8304.24177	8393.17249	8172.98032	6919.16675
*	43.6	23.7	M+FA-H	C27H47NO5	-0.1189976	3393.38219	3803.65658	3977.57823	285.262349	21904.4372
**	43.5	0	M+Na	C12H23NO5	0.44687866	4473.15627	4072.96238	4587.06151	6036.19241	4016.50126
**	43.5	0	M+H	C18H32O4	-7.7961077	9646.42408	9706.22801	10113.3591	8424.46317	10131.8538
**	43.5	0	M+H, M+Na	C18H15O4P	-1.867009	12706.1782	13213.1526	14138.0501	23887.0443	7929.22867
*	43.5	21.7	M+H-H2O	C13H19NO2	-0.9030029	223192.912	219064.177	218038.502	300769.425	177001.478
*	43.5	23.2	M+NH4	C22H44O2	-1.6284463	212663.498	215959.753	220665.398	83974.7616	63506.7606
*	43.5	21.9	M-H	C3HF5O2	-1.583684	17148.2161	17602.7887	16653.4211	15695.8068	14071.0318
*	43.5	22.5	M+H, M+NH4	C4H5NO2	3.93148995	139581.074	132270.286	122494.414	181257.553	130060.719
*	43.5	23.7	M+Na	C30H45NO8	3.49260383	5184.10685	4978.43701	5724.90658	9.7531E-07	140.108243
**	43.5	0	M+H	C15H24O2	-0.583023	1320.95861	1622.87282	1671.75178	1820.21932	1213.96861
**	43.4	0	M+Na	C11H19NO6	8.25199307	3174.5519	2992.63493	2962.77068	1306.911	9.7531E-07
*	43.4	23.5	M+Na	C21H32O10	0.2185249	8580.99937	8555.66033	9771.66056	4831.66354	37430.5365
*	43.4	32	M-H2O-H	C38H64O5	-4.4628221	34826.8894	27644.421	28494.4527	30726.289	23897.7949
*	43.4	23.7	M+NH4	C13H20N2O	1.05539903	14232.8001	13977.9733	14909.1801	25501.871	36231.4904
*	43.4	20.9	M-H	C8H16O3	-1.3393224	41909.0673	43579.4257	40127.1107	40113.4334	43342.2042
**	43.4	17.1	M+H	C14H27NO4	-1.1081313	2685.37468	3096.66443	2999.52022	1575.8892	19551.4717
*	43.4	18.8	M+H, M+NH4	C10H22O4	0.23708363	41426.521	41508.7214	42041.339	80583.0026	32607.271
*	43.4	22.2	M+H-H2O	C21H39NO6	-1.1460266	2303.79871	2241.40286	2328.38191	9.7531E-07	20977.5452
*	43.4	20.7	M+NH4	C15H30O	-1.1721201	22594.205	22677.9003	22582.6002	14301.8612	25438.2075

*	43.3	23	M+H, M+Na,	C17H28O3	-1.9183081	17593.4314	18812.2133	18693.4242	5692.59311	61621.5251
*	43.3	38.8	M+H, M+Na	C42H76NO8	-3.7284296	941493.079	727317.662	594662.17	829622.388	233917.766
*	43.3	20.4	M+H-H2O	C10H13N5O	-0.0562893	32192.1704	32929.1596	32399.2274	64011.6722	67548.7391
*	43.3	31.2	M+K	C20H23D7O	0.82596199	8583.90713	8382.12181	8999.44151	4821.5691	1499.44975
*	43.3	33.8	M+H-H2O	C20H38O11	-3.3931932	3989.78611	3296.54247	3840.00054	2398.75571	7811.63597
*	43.3	20.8	M-H	C49H86O15	-2.170517	755231.535	722413.976	794880.093	761899.655	628252.443
*	43.3	21.6	M-H2O-H	C12H22O3	-1.2138442	3414.22828	3583.38108	3398.32761	3623.1105	2988.93643
**	43.2	0	M+NH4	C12H21NO4	-0.3647335	8037.67377	6385.49565	8787.7392	1077.59743	15295.9735
*	43.2	24.7	M+H-H2O, M	C9H12N2O4	-0.3772873	34621.5965	34073.5605	33806.2598	71676.4905	81866.5446
*	43.2	25	M+H	C4H9NS	3.54357074	26799.4433	27618.6885	31132.8379	40175.2999	27898.7357
*	43.2	19.9	M+H	C7H10N2	2.00282945	51895.8778	52340.3203	51986.6689	33011.7595	46275.896
*	43.2	21.7	M+H	C27H51NO5	-0.8489119	48217.6967	44857.4778	47216.9071	31261.9257	175732.406
*	43.2	27.1	M+NH4	C9H10O5S	0.21464702	2676.07641	2492.36457	2555.91113	9.7531E-07	9.7531E-07
*	43.2	20.6	M+Na	C7H15NO4S	0.16331759	2583.39933	2652.35967	2815.47873	0.07774181	219.926985
**	43.2	0	M+NH4	C12H18N2O	0.15013026	4112.51121	3576.22618	3390.94894	2867.04708	5660.88072
*	43.2	21.8	M+FA-H	C9H6F3NO2	-0.4514258	638.802445	608.297639	559.70788	421.057081	3910.33918
*	43.2	23.5	M+H-H2O	C13H18N2O	0.04332478	3602.46687	3866.65286	3732.38506	6264.77243	8476.72658
*	43.2	25.4	M+H, M+Na	C14H17F3N	7.12829386	5269.90022	6191.60015	6253.40917	8347.43313	3320.39222
*	43.2	25.8	M+H	C10H17NO3	-0.9488233	1459.81829	1338.07541	1546.01349	1177.15552	1495.80041
*	43.2	23.9	M+Na	C27H49NO5	4.58342769	11736.393	11452.5744	12665.6131	553.654683	54395.489
**	43.1	0	M-H2O-H	C17H34O4	-0.8123618	975.794131	854.937483	886.096004	795.087608	473.348522
*	43.1	22.4	M-H	C39H70O6	-0.9521423	54357.0355	51564.821	51885.4409	50952.4028	16128.664
*	43.1	31	M-H2O-H	C42H68O5	-3.867551	15759.9153	12086.8184	11733.3959	34020.2751	22658.1929
*	43.1	23.6	M+H-H2O	C8H12O2	-4.733062	226970.984	260968.729	280506.554	348573.299	163843.468
*	43.1	28	M-H2O-H	C24H15NO4	0.12184522	29742.4693	32652.6187	31334.6533	964.323822	32600.9578
*	43.1	17.7	M+H, M+Na	C9H20O5	0.64782051	164385.419	158206.994	161627.348	341027.526	113500.965
*	43.1	26.9	M+K	C14H12O2	4.9119944	4689.26221	4622.99422	5031.85296	9573.45583	3654.56409
*	43.1	18	M+H	C11H14O3	0.31840954	7087.65995	6542.21582	6282.59205	8157.03588	4833.19009
*	43	18.2	M+FA-H	C8H16O6	-0.716084	1193.89211	1465.93398	1319.75918	1905.05086	1544.56587
*	43	21.3	M+FA-H	C7H9NO5	-1.2646736	813.548558	825.365148	919.046434	83.0317323	1386.78696
*	43	18.4	M+H, M+NH4	C6H14O4	0.6778682	16905.6571	16982.8602	16450.0691	15591.1387	32270.2129
*	43	33.1	M+H-H2O, M	C21H38O5	-3.4381173	14339.5843	12607.2794	13783.2651	2991.10711	1797.30472
*	43	20.6	M+Na	C16H28O5	-1.476696	1004.55824	1468.67043	1188.37869	24569.789	1031.11153

*	43	20	M+H-H2O	C8H17NO3	1.00774003	1922.51976	1999.1997	2209.56691	1871.75261	2004.16102
*	43	20.9	M-H	C5H5NOS	-1.2634141	6386.79502	6845.00089	6822.60107	11618.0798	3594.87879
*	43	19.1	M+H, M+Na	C8H15NO2	1.04695752	4787.5486	4894.05707	4645.27602	4080.67742	3605.67418
*	43	22.8	M+H	C11H20N2O	-0.2515267	836.234052	772.311582	865.266782	500.153944	499.346428
*	43	20.8	M+H, M+Na	C17H37NO2	-2.0054733	687510.612	684165.862	679490.842	489878.759	779498.836
*	43	18.9	M-H, M+FA-I	C12H24O2	-0.4774745	2197.46772	1992.51257	2115.39639	995.414501	4340.41426
**	42.9	0	M+H-H2O	C9H13NO2	0.49149975	238.161089	171.146294	287.603444	320.656737	185.136141
*	42.9	18	M+NH4	C8H7N3O	-0.7457021	304912.1	312045.417	312804.002	412252.272	230918.746
*	42.9	24.9	M+K	C11H15NO8	-4.0099355	16084.9735	17406.9048	17127.7991	36282.237	28318.6351
*	42.9	18.8	M-H2O-H	C8H11NO2	-0.9175192	494.382873	657.492891	623.99939	516.07411	408.728132
**	42.8	9.32	M+FA-H	C3H9O6P	-0.6592421	23673.3089	26422.7832	26045.1253	62404.8678	84700.4984
*	42.8	19.8	M+Na	C7H10O4	-0.5515985	4302.21589	4159.62421	4416.76506	1355.38688	4044.29749
*	42.8	17.2	M+FA-H, 2M	C5H5F3O2	-1.61401	3605.58319	3590.83601	3383.52143	3007.17853	21323.8441
*	42.8	18.2	M+H-H2O	C12H17NO	-0.3419929	2728.187	2806.24879	2670.44947	5988.5646	4617.99252
*	42.8	23.8	M+FA-H	C51H94N2O	4.59881731	133803.241	119997.447	110587.118	23672.1913	781426.625
*	42.8	24.7	M+Na	C23H27N3O	4.58199059	65545.266	69276.8951	70898.9156	105989.252	6857.89842
*	42.8	25.8	M-H	C28H24O8	-4.1080786	24265.8786	25340.4377	18248.6167	26692.6648	22110.8141
*	42.8	31.9	M+H	C20H37N3O	9.46008979	3355.75686	3183.85703	3340.54476	1444.23851	1819.92751
**	42.8	0	M-H2O-H	C8H8O4	-1.5567054	543.781466	557.466198	494.473922	479.234972	454.73595
*	42.8	42.9	M+H	C13H25NO5	0.46765379	18768.5396	18820.4951	20299.7574	19899.2885	40210.8619
**	42.8	0	M+H	C7H8N4O2	0.32378303	1798.29563	1961.9041	1896.23609	3475.74342	9.7531E-07
*	42.8	18.1	M+NH4	C7H16NO3+	1.77837487	864.182111	1098.21757	1001.13047	295.466765	344.536204
**	42.8	0	M+FA-H	C16H22O10	3.86005081	571.924841	571.647516	613.846708	578.186942	824.499946
*	42.8	21.3	M-H	C9H10O4S	-1.4452791	2600.88042	2442.867	2615.39376	2487.84786	275.87526
*	42.8	18.9	M-H	C8H9NO	-1.1627648	428.489967	442.861793	430.714114	444.955291	262.021649
**	42.8	0	M+H-H2O	C12H18O2	-0.5542855	3105.88806	3586.54439	3653.89185	4150.38004	2472.66419
*	42.7	20.3	M+FA-H	C8H11NO6S	0.30197744	54031.0223	51581.3796	50210.1028	69459.1238	73066.3035
*	42.7	22.6	M+H	C11H21N5O	-0.9802195	7837.50527	5893.07056	7871.04744	2779.50991	14425.1259
*	42.7	18.1	M+Na, M+K,	C11H14NO7	-0.5524961	307333.885	311906.933	304563.544	840097.955	333906.703
*	42.7	20.2	M+H, M+Na	C7H13NO5	-0.1736055	9758.24555	9048.47202	9732.59294	27617.1384	26745.6261
*	42.7	21.1	M-H2O-H	C7H10O4	-0.8866736	639.869352	553.84042	675.816772	410.750251	484.341507
*	42.7	24.1	M+K	C18H15NO5	3.83037565	1890.71185	2184.44438	2042.47089	9.7531E-07	9.7531E-07
*	42.7	18.8	M+NH4	C15H23N5O	-0.8716562	1221.45733	1617.48584	1512.93829	467.972747	770.062437

*	42.7	17.4	M+Na	C12H26O3	1.08034855	7050.36587	9744.69138	10028.2499	3991.43779	3358.56225
*	42.7	17.7	M+FA-H	C19H35NO5	-0.1652004	1863.42973	1763.38799	1896.51524	9.7531E-07	9340.08334
*	42.7	17.7	M+NH4, M+K	C16H32O4	-1.34194	110248.582	111880.114	110579.949	16472.243	9113.02526
**	42.6	0	2M-H	C8H11NO6	6.92884239	64244.8598	67209.489	55498.27	113594.885	212889.394
*	42.6	23.1	M+NH4	C21H36O11	3.69817724	20773.6244	23326.3027	24432.0025	11156.4155	38692.0452
*	42.6	22.2	M+FA-H	C19H28O3S	-1.5429414	186510.158	257123.94	203169.004	319242.482	152138.923
*	42.6	15.8	M+H, M+Na	C10H22O5	0.86442612	181960.02	187860.736	186614.033	418953.02	156373.892
*	42.6	17.1	M+Na	C10H18O3	-0.0364778	21384.1818	21581.5362	21741.2468	31670.2393	16521.9148
*	42.6	15.6	M+H	C8H12N2	-0.7489304	778.891807	740.97862	816.882879	260.165255	32.6400945
*	42.6	15.7	M+FA-H	C12H16O	-1.0867151	7022.74879	7642.19984	8128.31177	7080.01565	6240.34534
*	42.6	16.8	M+NH4	C14H28O	-1.2974991	7651.87103	7621.27637	7845.78375	6075.35334	5198.87184
*	42.6	20.1	M+H-H2O, M	C18H33NO2	-1.1721947	17067.6687	17141.3956	17173.7368	7662.09208	6176.24946
*	42.5	20	M-H	C8H18N2O4	-0.9184812	174883.188	180278.891	190155.822	226.07862	9.7531E-07
*	42.5	14.8	M-H	C5H9O8P-2	0.24799243	206864.398	201039.254	185890.215	176902.266	32046.9697
**	42.5	0	M-H2O-H	C10H12N2O	-1.2042798	219.860005	255.592147	221.083415	153.710614	823.005228
*	42.5	18	M-H2O-H	C26H45NO1	-0.0940179	12811.6722	10820.9588	11812.6731	3574.09486	17119.3171
*	42.5	27	M+H-H2O	C38H66O7	-4.6688236	4812.9888	8686.25806	6717.33458	11448.3578	5346.36478
*	42.5	17.8	M+H-H2O	C6H9N3O2	-1.3165981	54021.5074	56780.8875	58674.2158	70943.4006	39912.6993
*	42.5	17.9	M-H	C6H14NO8P	-1.0666073	3588.54068	3717.08252	3858.65786	3494.11535	9941.39177
*	42.5	19.3	M+H	C21H36O11	-1.9877439	1487.90647	2022.41194	1444.44019	1764.49234	415.850338
**	42.5	0	M+H-H2O	C15H22O5	-9.5469173	48663.3029	49997.0679	49315.2827	67585.9643	39620.8569
**	42.5	0	M+Na	C18H30O5	-1.2590593	10888.7319	11247.0895	11418.9368	12902.2384	8552.26871
*	42.4	15.8	M+FA-H	C17H34O	-1.0878745	58566.535	61898.1378	58020.4098	57422.8016	44425.7763
*	42.4	23.9	M+H	C15H21NO2	-1.0450181	198.291575	193.27311	168.573114	271.692546	78.0462917
*	42.4	17.8	M+H	C11H19NO4	1.04493333	3117.12978	3004.87611	2933.91358	511.173364	16035.246
**	42.4	0	M+FA-H	C7H10O3	-2.0993774	1827.3621	1750.27338	1607.05566	1527.47955	1551.81854
*	42.4	24.1	2M-H	C11H11N3O	-1.6884063	18691.8673	19661.0676	19208.0077	48311.0663	5976.30291
*	42.4	21.4	M+H	C16H13O6-	1.79913623	10102.2108	10409.6909	9075.93872	5390.39561	865.653525
**	42.4	0	M+H	C14H17N3O	2.02168246	1301.6961	1159.63365	1197.89263	451.429567	3254.34934
*	42.4	20.6	M+H-H2O, M	C18H34N2O	-0.9978043	18848.8845	18681.13	18671.1475	4277.36213	23671.4968
*	42.4	15.5	2M-H	C6H8O2	-0.4115988	775.099072	849.729457	864.740729	1369.52063	290.929209
**	42.4	0	M+H	C24H34O5	-7.8919985	1494.16813	1590.40347	1420.07732	908.199792	912.494913
*	42.4	25.3	2M-H	C16H22O8	-1.102908	289562.853	249780.2	281297.278	263796.847	237496.623

*	42.3	16.8	M-H2O-H, M	C5H10O6	-1.9554493	130995.486	122813.457	135999.923	120428.956	66372.7655
**	42.3	0	M+FA-H	C12H22O10	3.58272606	1202.51628	898.393771	1480.4188	1478.01042	4054.18506
*	42.3	15.2	M+H	C12H21N3O	-0.0293495	12663.2086	12309.519	13572.8018	11639.8663	31802.3551
*	42.3	17.4	M-H2O-H	C21H21FN4(	0.91666828	8698.71106	7016.42585	8161.23073	9191.88473	3299.04584
*	42.3	30.4	M+H-H2O, M	C45H78NO8	-3.7536438	469270.306	387201.328	410377.644	689189.841	199723.032
*	42.3	23.5	M+NH4	C15H20N2O	0.49765304	2144.03728	2409.67982	1740.92758	1570.1609	371.557793
*	42.3	22	M+Na	C17H27NO3	-4.3493387	11070.9433	10999.9343	12030.7264	2727.20409	42977.6785
*	42.3	43	M+NH4	C20H29ClO7	2.69574373	2359.35218	2506.96662	2412.50304	982.496169	1042.65335
*	42.3	15.3	M-H	C8H16O3	-1.4600318	7262.37059	7939.94695	7535.80443	9217.92859	18253.5546
**	42.3	0	M+FA-H	C15H30O2	0.56260976	607.251593	824.994058	589.100158	915.67852	436.881565
*	42.2	21.4	M-H, M+FA-I	C24H30O8	2.92156378	62402.1468	62120.4483	75208.5727	51538.5205	43917.6976
**	42.2	0	M+H	C14H18N2O	0.16785238	1814.98851	1736.35151	1632.1097	1048.27484	1390.63937
*	42.2	20.2	M-H	C18H30O3S	-0.7308287	303592.35	302213.808	290645.13	294069.551	234198.152
*	42.2	16.1	M+Na, M+K,	C24H46O7	-1.1740568	833128.664	835849.728	855059.781	1288141.61	738194.357
*	42.2	16.8	M+NH4	C19H34O	-1.4276169	2369.54881	1949.20747	2186.06581	9.7531E-07	365.546659
*	42.2	19.5	M+NH4	C17H36NO7	-2.7374619	836.986824	1037.60141	919.026962	9.7531E-07	227.198807
*	42.2	19	M+H, M+Na	C8H16O2S	1.16862619	1605.407	1804.40012	1706.14773	625.615362	657.253189
*	42.2	18.8	M+Na	C9H17N3O5	-1.4743586	29288.4504	29641.7723	29334.3965	5312.72538	3159.44895
*	42.2	15.8	M+FA-H	C23H41NO4	0.19780236	2719.54867	3213.54016	3177.35298	9.7531E-07	15361.4583
*	42.1	15.3	M+FA-H	C4H6O4	-1.6584863	21198.041	19980.569	20504.2642	6590.37246	8532.23541
**	42.1	0	M+NH4	C12H23NO4	-0.9862797	2872.55563	2854.01575	4472.85209	4796.7113	17665.2107
*	42.1	16.7	M+NH4	C8H10IN3	-2.1791971	1153.04951	1184.94101	1213.87956	345.745406	1.74534648
*	42.1	19.4	M+Na	C23H28O11	-3.8466835	1070898.03	1109750.17	1173726.18	2485325.7	3284760.86
*	42.1	18.3	M+H	C6H10O2S	0.24561429	2501.2668	2492.37584	2945.19742	5624.4522	10332.2571
*	42.1	17.2	M+NH4	C19H35NO7	2.82749512	2972.48142	2152.70813	3731.15135	9.7531E-07	9.7531E-07
**	42.1	0	M-H	C22H30O3	-1.0699187	1077.82059	1170.83236	1083.17486	9.7531E-07	126.755582
*	42.1	15.1	M+Na, M+K,	C20H34O8	-1.5141328	540594.961	521900.782	547529.462	861424.348	495978.492
*	42.1	19.2	M+NH4, M+N	C39H52N2O	-4.2505477	3466.47943	2714.86065	3235.38632	9.7531E-07	1232.38941
*	42.1	20	M-H	C25H24O5	6.65927219	182760.277	224437.13	158946.319	190678.916	217970.984
*	42.1	14.8	M+FA-H	C5H9NO2S	-1.6842252	10575.8718	10899.9385	10780.5447	9630.58663	28084.2617
*	42.1	24.7	M+K	C18H15NO2	2.18719254	1861.19277	2434.81939	2009.34629	4574.92884	4880.35167
**	42.1	0	M-H2O-H	C12H13NO5	-9.7912742	878.216968	931.225449	943.088145	384.391113	561.365895
**	42.1	16	M-H2O-H	C10H16O5	-0.9575934	1130.18686	1181.41916	1258.8106	1560.82242	1301.16836

*	42.1	14.3	M+H-H2O	C25H45NO6	-0.6549458	45006.5118	44014.6232	46571.3281	8411.70105	157804.484
**	42	3.31	M+H, M+Na,	C6H9NO5	-0.1063166	50240.2589	51759.1599	47669.3668	104786.911	11311.5022
*	42	24.5	M-H2O-H	C13H10N2O	-4.1758435	17398.0797	17149.8086	17499.769	24799.0846	23028.3562
*	42	18.5	M+NH4	C25H40O2	-1.2415874	13706.6789	13504.0637	13806.9798	27582.6624	25992.9661
*	42	16	M+NH4, M+N	C23H40O5	-1.5293603	182606.311	184482.781	192313.403	44235.6466	69878.3708
**	42	0	M+NH4	C15H28O	-1.5785754	1007.08947	758.873387	1017.22438	559.209643	508.522564
*	42	14.4	M+NH4, M+N	C20H42O5	-1.5632914	278426.219	276871.497	278186.974	97467.6534	283294.193
*	42	15.5	M+NH4, M+N	C27H32N8O	0.71225232	6388.6333	3610.03661	5602.95131	2645.55367	4026.27525
*	42	19.1	M+H, M+Na	C7H15N3O2	0.37750822	4188.85541	4411.49283	4355.02696	7.70046931	4335.99395
*	42	12.9	M+NH4	C11H10O2S	0.31039027	3787.89213	3493.34354	3650.1415	6901.00997	4599.71059
*	42	12.2	M+H, M+K, M	C12H15NO4	0.04851185	45514.5294	45577.5615	45368.2165	218.270716	28930.2142
*	42	18.4	M+FA-H	C12H14N2O	-3.1806097	2541.80057	2451.63037	2701.90341	747.618455	678.904384
*	42	13.8	M+H-H2O	C32H42O14	0.89499072	21375.5983	19464.7448	20599.0167	2376.4966	10459.6852
*	42	13.2	M+H	C10H14N2	-1.0080585	532.986864	752.014555	776.456765	580.211784	348.698203
**	41.9	0	M+Na	C9H11N5O4	3.16069583	4625.63038	2635.21848	4549.64677	7527.17257	11042.7464
*	41.9	13.4	M-H	C16H26O2	-0.897193	20217.9902	19216.4367	19767.9989	29310.6154	14731.724
*	41.9	16.7	M+NH4, M+N	C27H52N6O	3.43232311	113861.095	107738.778	111197.426	38607.4565	193122.68
**	41.9	0	M+H	C15H22N2O	0.31643914	708.844409	789.377088	700.819757	610.898	995.546051
*	41.9	16	M+H, M+Na	C11H18N2O	2.49498365	20013.1072	20586.7755	20458.3548	46335.693	16830.2679
*	41.9	14.7	M+H	C18H20N2O	0.31954874	1326.72643	1154.34044	864.826199	26953.8482	9.7531E-07
*	41.9	24.4	M+H	C12H14O6	-9.2861436	1866.21809	1884.84856	1946.92725	544.973349	301.794246
*	41.9	12.3	M+NH4	C9H13N5O3	-0.1450488	10970.3055	10628.4027	10560.5444	13969.9503	9662.0484
*	41.9	17.2	M-H	C19H30O5S	0.14069193	7058.26979	7373.99451	7270.67702	7131.03469	9809.11396
*	41.9	23.7	M-H	C23H36O7	-4.6687638	2421.82482	2898.04542	2237.9628	164.091267	15630.3698
**	41.9	0	M+FA-H	C14H22O2	-0.2688441	2097.57413	1950.48045	2022.34926	1950.71784	1839.21116
*	41.8	16.3	M+H-H2O	C16H21NO	-1.3865867	8202.57658	8809.86425	8931.51198	9079.96514	7829.63798
**	41.8	0	M+NH4	C18H28O	-1.2780654	7061.41685	5671.18278	5786.42364	108.259813	1592.80776
*	41.8	12.7	M+NH4, M+N	C26H54O8	-0.5701607	331954.627	337435.437	330788.984	92600.0143	436442.877
*	41.8	16.7	M+NH4	C12H23NO1	0.21854928	28522.8458	30027.8396	26500.977	51534.6733	35518.8384
*	41.8	13.1	M+H	C19H39NO	-1.4435053	8885.11184	9271.66308	10124.7006	495.578634	762.720687
*	41.8	10.9	M-H	C11H11F3N2	0.19593276	46442.9868	48270.3295	47646.8639	44035.3097	121405.417
*	41.8	12.6	M+Na	C7H16O3	-0.0782571	31253.6552	33421.0328	38285.1042	33475.167	21656.0099
*	41.8	27.1	M+K	C29H48N2O	2.50929866	4052.23561	3350.23604	4618.75025	720.655922	1178.70544

**	41.8	5.01	M+H-H2O	C8H15NO3	0.91528857	302.276058	365.660149	323.935988	281.555079	152.91888
*	41.8	18.2	M-H2O-H	C3H9NO3S	-1.1547329	204.589241	207.890601	207.726423	60.2815538	298.03743
**	41.8	0	M+H	C9H12O	0.28609768	899.56537	1039.81154	1022.59366	1298.69389	759.822352
*	41.8	18.7	M+NH4	C8H13NO	-0.6166341	326.473229	262.019108	236.464337	20.6957897	342.084716
*	41.8	13.9	M+Na	C9H20O3	-0.71916	10454.0041	10165.0782	10854.0345	14363.2676	8252.95465
*	41.8	11.6	M+FA-H	C16H30O3	-0.4962027	763.768241	1022.42736	767.602435	9.7531E-07	300.204224
*	41.8	13.7	M+K	C21H26O6	1.21261015	14863.9185	15876.1598	15409.7924	27072.9473	10473.2877
*	41.8	15.6	M+NH4	C9H16O	-0.7912184	65781.4409	65608.0866	66671.227	84150.1514	53659.0378
*	41.8	15.6	M+NH4	C17H33NO2	-1.5201897	1026.91794	1014.98926	1196.89881	4647.75937	3332.5881
*	41.8	16	M-H, M+FA-H	C51H85O13I	3.66475417	335973.318	302280.245	329321.398	325439.193	264543.983
*	41.7	10.6	M-H2O-H	C8H9F3N4O	-0.8914415	25336.2009	34084.9409	27589.9072	34432.4635	19314.1174
**	41.7	0	M+NH4	C8H15NO2	1.13101388	1533.36745	1691.31455	1549.20547	3891.54877	1618.29404
*	41.7	16.8	M-H	C26H46NO9	-3.1165947	129046.86	128458.433	122228.39	89225.3044	184113.145
*	41.7	13.2	M+NH4	C10H11N3O	-0.9233344	331437.654	337489.73	342477.737	462294.921	275744.994
*	41.7	19.2	M+H	C29H48O3	-0.8146291	35634.0362	62872.0522	58396.6673	72647.0044	50429.7599
*	41.7	11.9	M+Na, M+K,	C14H30O8	0.65803699	269014.578	278316.158	288610.972	249839.269	584631.844
*	41.7	16.9	M+H	C7H7N3	-0.2104331	1233.14794	1306.20889	1373.84911	257.218646	1423.54075
*	41.7	16.5	M+NH4	C20H21FN6(	1.71215692	1090.92379	1344.52611	1230.12462	543.777912	2816.00429
*	41.7	15.2	M+H, M+Na	C11H23NO2	-0.7843922	767.599852	771.861126	883.914447	308.757414	417.095528
*	41.7	21.6	M+FA-H	C10H16N8S:	3.3246122	13689.9438	14097.0961	14403.3587	14529.4146	12532.0816
*	41.7	14.9	M+H	C18H33N7O	-0.4796769	2600.70569	1934.96675	2362.45527	3.76144817	548.15077
**	41.6	0	M+Na	C3H6O6S	-6.5256898	1880.57756	1666.355	1709.06089	657.833913	1021.72653
*	41.6	14.3	M-H	C4H12NO4P	-1.646619	16473.8363	17817.6854	17393.6947	20615.3296	14695.4455
*	41.6	11.2	M+Na, M+K	C9H17NO4	-0.1666248	4198.92558	3980.15871	3639.12999	3408.13451	1639.01482
*	41.6	19.9	M-H2O-H	C26H34F3N7	4.47673319	36818.7012	36214.7253	37703.2545	15859.8088	55788.3726
*	41.6	15.2	M+NH4, M+N	C22H44O3	-1.450259	23462.391	26798.4533	25528.2521	9.7531E-07	9.7531E-07
*	41.6	42.7	M+H	C7H15NO2	0.3793022	2435.13634	2680.04425	2602.84115	2770.29489	2272.65913
*	41.6	14.5	M+Na	C10H10F3N:	3.07984758	6333.18372	7170.66899	7057.77955	3139.13663	5172.38964
*	41.6	32.7	M-H	C7H5NOS	-1.7486326	3851.12609	3889.03354	3765.21211	3207.04906	3115.02303
**	41.6	0	M-H	C9H9NO4	-1.4921486	645.870724	622.027937	642.779509	588.750109	484.696011
**	41.6	0	M+Na	C10H18O2	-1.4747652	2560.27135	2528.97757	2371.63909	3224.87657	1875.35473
*	41.6	17.7	M+NH4	C11H11NO7	-3.641984	7490.69369	7605.79824	8500.33224	5288.57003	2219.70074
*	41.6	14.2	M+Na	C17H28O4	-1.8339177	1986.05162	2090.29842	2044.70424	820.97196	729.371273

*	41.6	14.2	M+Na	C25H47NO5	3.96845512	41228.4206	37836.8295	40592.5285	8344.80354	139410.592
*	41.5	13.8	M+NH4	C7H8O4S	-0.5656858	58543.8789	69631.4403	62248.101	174325.026	122213.354
**	41.5	3.77	M+NH4, M+N	C23H38O5	5.32292971	61423.793	59042.1671	57298.0534	14851.9971	80079.7463
*	41.5	15.8	M-H2O-H	C24H31FO7	3.46484943	36745.754	49903.606	40217.7944	63649.0642	23709.4137
*	41.5	17	2M-H	C12H15NO3	-0.4733065	11771.8707	14113.8609	12205.1167	6338.23238	5772.74603
*	41.5	10.3	M+FA-H	C25H40	-0.3392253	47503.51	41726.5655	31655.2549	47288.5435	34438.9981
*	41.5	19.6	M-H2O-H	C16H21N5O	-4.6106644	2278.16891	2202.41982	1877.30435	770.579726	4764.50608
*	41.5	10.6	M+H, M+Na	C12H17NO2	0.43458253	11457.4475	11280.0887	11901.2539	532.680272	35231.204
*	41.5	33.4	M+NH4	C24H16F6N4	2.63753941	8173.5165	10029.162	9094.2373	9.7531E-07	43575.426
*	41.5	11.4	M+FA-H	C6H7NO2	-1.2679794	3257.57775	3115.24375	3160.42181	2858.97602	2911.57882
*	41.5	25.1	M+H	C32H54O13	1.91940615	4838.45844	6390.13634	6416.10172	346.311416	370.315896
*	41.5	11	M-H2O-H	C17H22N2O	-0.1414512	2309.99134	2181.78822	1984.83333	6372.04102	9.7531E-07
*	41.5	17.1	M+NH4	C15H18O3	-0.6453993	982.906706	1116.54735	1044.49433	12986.3564	2005.89481
*	41.5	10.6	M+NH4	C10H20O	-0.488072	1310.99037	1446.07893	1369.183	719.430904	1538.83541
*	41.5	14.6	M+Na	C27H43NO7	1.29278675	14253.6917	14508.6268	12101.6401	3039.55683	9940.69417
*	41.5	13	M+H, M+Na	C18H15OP	-1.0954784	5880.04156	6110.16595	6138.51222	8770.51995	4861.96317
**	41.4	0	M-H	C10H19N3O	-0.4854068	544.861416	493.382945	559.959965	151.106294	2530.0113
*	41.4	11.9	M+NH4	C9H17NO3	0.66273144	23004.6957	22882.846	22870.5075	14223.3858	39013.5517
*	41.4	11.6	M+H, M+Na	C8H18N2O2	1.10142871	5511.67089	5636.6732	6158.84129	16443.1882	6832.79849
*	41.4	11.2	M+H, M+Na	C19H31NO2	-1.5578252	1944.1916	1594.47636	1359.33271	294.77288	9.7531E-07
*	41.4	15	M+NH4	C18H25NO6	-3.5587322	1154.7273	1269.23748	1175.54894	145.043923	226.982035
*	41.4	12.2	M+NH4	C19H15FN2(	0.11129048	5885.64331	6795.68202	6699.9881	13932.7845	2667.82549
*	41.4	12.3	M+Na	C20H36O4	-1.6582904	8420.7384	8572.62904	8609.8708	2697.74961	602.381721
*	41.4	10.9	M+Na, M+K,	C19H40O4	-1.5414749	131152.912	124920.801	137938.224	196150.404	109382.162
*	41.4	11.6	M+H-H2O	C20H29NO	-1.3159175	890.38592	1055.3543	1099.02116	278.164661	2022.22317
*	41.4	15.5	M+H-H2O	C10H14O7	-4.243941	2726.33101	2805.03549	2584.40561	495.55201	26.9758029
*	41.4	12.1	M+H, M+Na	C9H16N2O2	0.95412043	28509.8434	28445.1743	28091.7143	35936.7349	29041.0739
*	41.4	13.2	M-H2O-H	C9H17O12P	1.82469764	8613.08175	9076.22515	8504.93623	12804.4695	4302.47357
*	41.4	10.3	M+H	C13H23NO5	-0.1734602	3595.24292	3511.28179	3949.70047	874.718686	30161.9165
*	41.4	17	M+H, M+Na	C17H18N2O	0.54773086	8280.77686	8972.42211	8821.42971	11951.5853	7648.92129
*	41.4	14	M+H	C8H13NO4S	0.10378205	3269.30606	3685.51742	3691.57905	4487.68039	5918.3482
*	41.4	14.9	M+FA-H	C18H21FIN(	-0.6978472	10098.8081	9698.55731	9333.49406	12174.9905	20415.7851
*	41.4	21.5	M+H	C18H32O7	-7.3932341	11146.8735	11152.108	11454.6028	19980.8798	9556.6732

*	41.4	12	M+H-H2O, M	C12H23NO3	-0.5619547	10192.0283	10246.19	10115.4935	8895.49028	8714.5745
*	41.4	11.2	M-H2O-H	C11H18O2	-0.9326986	1553.96188	1353.12922	1487.95994	469.795151	401.119421
*	41.4	16	M+H, M+Na	C16H17NO	-1.2812825	2329.82878	2708.12684	2540.1447	1638.14348	1031.03833
*	41.3	17.7	M+H, M+Na	C9H10O7S	-3.1888606	5174.49226	5905.38986	5436.68956	35297.0987	12763.037
*	41.3	12.7	M+FA-H	C29H37N3O	-0.7091312	414734.847	431809.735	399845.166	279697.751	255860.189
*	41.3	10.7	M+NH4, M+N	C30H62O10	-0.6577167	374409.577	383289.084	381129.167	77112.5935	514681.669
*	41.3	18.3	M+NH4	C15H23N6O	-3.8502679	2093.79623	1749.20515	1861.65033	1589.48395	2703.89359
*	41.3	28.6	M+NH4	C29H42O9	2.11860332	4747.85557	4339.46471	4790.71634	222.663312	169.64597
*	41.3	14.2	M+H-H2O	C8H15N3O7	-3.3683005	10218.8574	10810.436	10904.4677	19642.7458	24072.8843
*	41.3	9.56	M+H	C12H23N	0.17390541	2427.88763	2334.06978	2334.73692	3601.43161	1834.08764
*	41.3	14.9	M+Na	C11H18O3	-1.1258095	110004.282	111045.072	111234.243	158295.693	85680.6573
*	41.3	12.9	M+NH4	C11H17N5O	-2.9527628	4591.28678	4523.11957	4700.0756	9576.19296	2148.66855
*	41.2	14.7	M+H, M+Na	C8H18N2O2	-2.2603436	14376.911	13737.7906	13441.9446	5958.02062	11687.8963
*	41.2	19.2	M-H2O-H	C15H18O9	4.37886392	191176.115	187550.401	182561.098	43060.8174	96132.2825
*	41.2	22.9	M-H	C4HF9O3S	0.93116329	36662.4876	40962.3245	38158.9957	7128.36985	3093.09081
**	41.2	0	M+Na	C7H12N2O3	0.02651607	1085.96159	991.915081	1419.88975	523.728192	3274.74658
*	41.2	18.5	M-H2O-H	C32H56NO1	-3.3921885	13894.0535	14506.4827	13160.4752	6630.30846	53493.7802
*	41.2	22.1	M+H	C40H76NO8	-1.2768568	557495.597	817479.157	578678.188	134485.749	62353.428
*	41.2	15.4	M+NH4	C8H8N2	-1.0282365	4536.5712	4922.66899	5350.17474	7463.5902	3846.2757
*	41.2	12.6	M-H	C10H12N4O	-0.4501192	2216.77346	2787.06625	2559.02289	8042.95321	3269.46677
*	41.2	15	M+H-H2O	C7H12N2O2	0.64039795	1029.82422	1143.17363	1136.58069	2214.72226	194.131134
*	41.2	14.4	M+NH4	C10H12N2	1.12819146	14262.8559	13123.7268	14192.468	28416.2736	6122.1899
*	41.2	12.1	M-H	C6H13NO2	-1.8213592	581.59449	527.868457	594.077304	530.201276	581.752587
*	41.2	16.7	M-H, 2M-H, I	C10H14N2O	6.60250174	195585.53	196698.985	194052.441	499266.044	58895.6274
*	41.2	13	M+FA-H	C8H5F3O2	-2.3721419	1447.77007	1343.38874	1355.77023	897.590267	1222.33659
*	41.2	13.5	M+Na	C9H19NO	-1.259624	1273.50231	1909.89263	1268.68929	1915.86256	1383.01077
*	41.2	11.4	M+FA-H	C19H36O3	0.31203288	607.537221	568.693767	490.715731	53.9288119	9.7531E-07
*	41.2	11.3	M-H	C16H26O2	-0.6459922	1338.14553	1365.34265	1581.1653	9.7531E-07	3358.85508
*	41.2	12.1	M+H-H2O	C8H18O3	-0.6309839	3105.01711	3183.20754	3451.89081	1464.9881	933.586282
*	41.1	10.2	M+FA-H	C14H8N4O	2.45179161	75602.0501	76958.3278	77048.861	55889.0357	67937.0656
*	41.1	21.9	M+H-H2O	C17H12F3N(	-0.1236718	30790.3696	29807.8311	32581.5215	53438.7708	33747.8137
**	41.1	0	M+NH4	C3H4O3	5.03474064	269.413536	203.950278	221.509059	406.070935	302.317291
*	41.1	9.96	M+H-H2O	C5H13N3O	0.74253692	1086.64516	713.807637	1027.32179	1445.16848	635.080203

*	41.1	18.6	M+H	C6H14O6	6.29682925	18842.6671	22296.4129	18840.8552	27134.8488	16031.8513
*	41.1	8.51	M+H, M+Na,	C12H18N2O	0.83583818	41754.6669	43380.4353	42881.9945	272871.933	51226.2748
*	41.1	14.4	M+H, M+Na	C9H11NO5S	0.39936907	635.268313	558.311705	630.330854	9.7531E-07	9.7531E-07
*	41.1	26.4	M+NH4	C29H40O10	4.17624961	8231.63251	6206.10379	7864.36472	23361.4593	8491.27067
**	41.1	0	M+H-H2O	C15H12O5	-9.3310922	8645.67478	8748.70156	8278.96587	11185.4085	6490.23353
**	41.1	0	M-H	C10H20O2	-0.8211225	286.947781	332.350079	322.023347	156.56402	496.162842
*	41.1	8.94	M-H, M+FA-I	C14H24O2	-0.5085033	2790.26147	2827.32997	2850.48069	876.118211	5682.29777
*	41.1	10.1	M+NH4	C16H24O	-1.5099101	4222.8763	4365.92129	4468.01253	15817.4845	4848.31898
**	41	0	M-H2O-H	C10H17NO6	-9.917411	3979.63569	3560.02512	4596.32886	8777.14737	5658.47054
*	41	6.8	M+H, M+NH4	C5H2N4O	-0.1549321	64021.2009	62645.4609	65490.2415	63105.5081	72628.095
*	41	15.2	M-H	C28H22F3N	2.1202224	3601.26967	4531.58778	3676.26982	240.734628	978.548381
*	41	14.8	M+NH4	C19H31NO6	2.16048568	7160.12667	6706.10648	7873.44904	8664.06211	3683.79923
*	41	8.48	M-H, 2M-H, I	C20H34O2	-0.7443702	1968809.24	1785988.54	1734721.35	2990118.36	2311238.29
*	41	10.7	M+Na, M+K	C18H27N5O	0.14899877	9406.75654	9585.22583	9165.67642	3519.07975	5488.97313
*	41	15.6	M+H, M+Na	C19H20F3N	-3.9391867	22737.6884	22839.3617	23237.1576	16313.4909	51302.9429
*	41	20.5	M-H, M+FA-I	C14H14O6	-9.6333099	9415.64077	9778.88239	9377.9671	17225.5668	11282.0788
*	41	10	M+NH4	C11H20O	-1.3025021	3494.12399	3279.39623	3600.01637	6018.68413	3062.25164
*	41	8.99	M-H	C13H26O3	-0.938576	81403.6624	85799.2672	78121.0337	79128.6326	65169.8642
*	40.9	10.4	M+H-H2O	C18H24N2O	-0.7608492	63723.8269	74060.4676	66298.8935	99793.9116	64792.0832
*	40.9	13	M+H	C11H20N2O	-0.0654865	11011.9304	11534.623	11418.0076	15767.6298	18987.9455
*	40.9	11.6	M+H	C21H42NO7	-0.8396914	7624.06255	9073.15112	7912.5936	816.748287	24755.9093
**	40.9	0	M+H, M+Na	C22H30O3	-8.8286742	46385.9545	47568.8842	53721.7711	71441.55	17352.444
*	40.9	28.9	M+H	C21H40O3	-2.7917738	4537.0915	4754.94041	4410.85079	2431.57125	6471.83874
*	40.9	17.3	M+H, M+Na	C17H10O3	-2.7003817	23544.7552	23480.748	23898.8477	81608.347	25350.1389
*	40.9	16.5	M+FA-H	C16H15F2N	-2.0627952	30049.8388	31023.5617	29619.962	651.985185	31744.5331
*	40.9	8.39	M+H	C13H17NO3	0.2156457	675.006486	901.26458	783.041217	102.898291	122.936043
*	40.9	13.7	M+NH4	C21H27N7O	-3.1241212	1378.2559	1374.5327	1433.37077	9.7531E-07	9.7531E-07
*	40.9	16.8	M-H2O-H	C9H10N2O5	-1.1122655	14622.4071	15677.8192	14950.9668	9.7531E-07	9.7531E-07
*	40.9	11.7	M+H-H2O	C9H16O	1.0462876	4469.49396	4839.54123	5122.88983	6833.06284	3535.42791
*	40.9	10.3	M+H-H2O, M	C25H54NO6	-3.6017305	21202.3171	19534.7942	20238.2687	3999.33508	22557.1743
*	40.9	8.56	M+Na	C12H20O7	-1.1471823	24501.7374	23781.8381	25163.477	35181.7976	22148.5489
*	40.9	16.5	M+H, M+Na	C11H22N2O	-1.4438923	12447.671	12884.3559	12694.3228	18126.3417	11558.4698
*	40.9	9.58	M+NH4	C20H38O4	-1.3216806	16181.4419	18918.8294	17362.5526	107079.716	27033.0503

*	40.8	7.56	M-H, 2M-H, M	H3O9P3	-1.2525061	420973.747	416191.939	415563.606	7613.27662	6526.82354
*	40.8	10.9	M+NH4, M+N	C7H5NO	0.78800877	85923.2202	84484.4556	92479.1174	158164.394	54470.6963
*	40.8	15.7	M-H2O-H	C6H12O2S	3.65879202	15730.0997	16277.5322	15907.033	21054.9984	10401.8849
*	40.8	15.3	2M-H	C16H13O7+	2.30426964	27705.1753	34883.1592	23710.7847	57685.2052	20377.9284
*	40.8	10	M+H-H2O	C8H18N2O3	-0.1268334	20701.3233	22985.0169	24633.7751	79251.5476	15648.7863
**	40.8	0	M-H2O-H	C8H15NO6	-0.9727432	552.435617	488.852374	440.978382	404.970618	57.5981261
*	40.8	5.82	M-H, M+FA-H	C13H26O6	0.42430244	10608.0684	9548.65822	9094.24335	21149.2045	5791.21162
*	40.8	26.6	M-H2O-H	C23H34N2O	2.67618429	12672.8882	14225.7182	12214.2051	21380.6083	5319.98699
*	40.8	16.5	M+H	C9H14O4	2.0440359	1259.69866	1470.32114	1162.14543	445.897089	713.985301
*	40.8	11.3	M+H	C15H18N2O	0.28160078	4651.59001	4645.83195	5066.24428	13430.5494	7272.70008
*	40.8	16.2	M+FA-H	C6H10N2O2	-2.8168819	19880.8491	19738.2882	20458.8848	8973.40915	13570.2543
*	40.8	17.4	M-H	C5H7NS	2.6830426	2496.4588	2106.50095	2518.85556	3217.53729	4102.28665
*	40.8	10.6	M+H	C28H33NO3	-0.8001459	5527.87702	6092.58673	5320.75314	19.914797	14760.0422
*	40.8	9.49	M+FA-H	C6H3FN2O4	-0.8390988	7731.11288	7582.27461	7657.30727	7845.27046	6507.18403
*	40.8	6.78	M+FA-H	C10H14	-1.3281706	8733.95794	8158.15019	10566.9546	11341.9252	11121.4634
*	40.8	8.63	M+NH4	C18H36O4	-1.4350647	15577.0419	16496.4493	17081.6193	192.53569	230.048943
*	40.7	15.3	M-H	C6H12O2S	3.24039887	61870.7425	60189.6814	60347.8049	134873.626	92284.4755
*	40.7	8.89	M+FA-H	C4H4O4	-1.9324039	12133.8848	10796.0868	11400.6841	3807.59762	1704.96497
**	40.7	0	M+K	C11H21N3O	-8.2281755	39313.6123	43345.2074	42225.4054	130173.508	39998.8491
**	40.7	0	M-H	C21H30O5	-6.4216414	1824.99874	1597.28076	1596.49012	126.828842	557.992961
*	40.7	8.9	M+NH4	C26H50O7	-1.1077984	40945.1309	39564.1301	38125.8563	67342.9606	40631.5068
*	40.7	9.69	M+H, M+Na	C23H39NO2	-1.7913267	957.433857	910.765551	1472.01031	1010.26191	410.082315
*	40.7	15.2	M+H	C18H25N3O	1.16900816	6124.50782	7275.62126	6697.04809	610.706231	4281.99081
*	40.7	14.7	M+NH4	C10H16O4S	-0.1807537	561.294634	530.816282	536.383268	492.984119	204.979695
*	40.7	22	M+K	C21H32O3	-3.0037495	4547.96497	4146.19293	3809.02147	2989.95316	8562.24818
*	40.7	9.74	M+FA-H	C4H5NS	-1.8578881	1314.69854	1381.06869	1225.75887	135.671595	3452.40236
*	40.7	36.4	M+FA-H	C16H16ClNO	4.80450087	1664.00067	1874.23139	1823.97119	9.7531E-07	9.7531E-07
*	40.7	8.46	M-H2O-H	C9H10N2O5	-1.1483086	654.518285	689.159881	714.926297	9.7531E-07	9.7531E-07
*	40.7	6.73	M-H	C8H12N6O2	0.12676957	1637.09599	1775.33311	1670.88214	1093.11926	1674.04348
*	40.7	6.57	M+FA-H	C9H11NO	-1.7277378	77099.3747	83553.0086	85195.4741	79442.821	71776.2108
*	40.6	19.5	M-H2O-H	C14H19NO9	0.01392	1002.16279	976.461268	1177.61724	3.33117392	438.009181
*	40.6	6.53	M+NH4	C10H18O	0.08732327	1365.14329	1503.5433	1343.83613	243.763155	153.124274
*	40.6	15.8	M-H2O-H	C13H14O2S	0.57994431	23672.5957	23554.1841	21708.2423	58230.7124	62262.1466

*	40.6	8.8	2M-H	C5H4OS	-0.2151475	11003.234	12122.5497	10009.0545	5199.83232	2039.22415
*	40.6	10.5	M+H, M+Na	C12H26N2O	-0.8891305	30225.1792	25168.2079	23216.167	25075.8067	30586.7949
*	40.6	8.55	M+NH4	C12H16O2	-1.0267271	1544.38397	1695.03097	1612.61129	872.829594	9813.44254
*	40.6	8.54	M-H	C27H50O12I	0.13687081	29914.5842	30194.3501	29054.847	29969.0868	9056.69953
**	40.6	0	2M-H	C10H18O2	-1.0231129	1821.83192	1700.47103	1900.16182	1567.71912	931.332337
*	40.6	7.29	M+Na, M+K,	C28H58O9	-0.8402429	368033.515	379362.645	374575.782	87417.2512	514548.324
*	40.6	14.8	M+NH4	C18H19NO4	-0.7930326	13560.7681	10958.1946	10741.8781	22303.7579	28160.4309
**	40.6	0	M+FA-H	C8H10O6	-6.3368848	140.766569	146.251213	180.278809	492.965808	9.7531E-07
*	40.6	9.41	M+H	C7H15NO2	0.15775797	4192.6615	4364.45058	3847.82107	2046.41315	5796.38769
*	40.6	7.39	M-H, M+FA-I	C6H12O2	-2.0255965	968.725837	1035.53257	996.713438	106.874229	5966.01937
*	40.6	7.47	M+NH4	C8H10N6	0.72460421	3109.81816	3067.10041	3463.10064	2613.40103	1755.56601
**	40.6	0	M-H2O-H	C11H19NO5	-0.6387243	211.251637	190.144897	238.488829	82.2972499	205.485942
*	40.6	6.54	M+Na	C11H16N2O	-0.600425	582.056063	560.956315	565.501934	38.875095	263.285943
*	40.6	16.9	2M-H	C9H17NO5	3.6150064	15932.3144	15758.7036	16531.2651	532.586551	16419.0341
**	40.6	0	2M-H	C20H26O3	9.28730593	2417.8964	1958.85071	2194.7193	4490.85735	1772.00875
*	40.5	11.4	M+H	C13H28NO7	-4.6375798	15807.0568	14808.892	16174.9555	10950.6111	29577.0712
**	40.5	0	M+NH4	C20H37NO4	-7.5516154	2264.11939	2489.30962	1999.01167	3034.28288	1576.46414
*	40.5	6.19	M-H, 2M-H, I	C20H38O2	-0.4723269	348324.194	346370.223	317575.023	384066.734	278734.518
*	40.5	8.59	M+NH4	C20H38O	-1.3101106	30744.4229	30669.4813	30899.8283	13729.5282	12130.4302
*	40.5	6.84	M+FA-H	C17H24O6	-0.2985175	4187.34394	4714.91563	4753.29685	2207.06786	2550.04295
*	40.5	16.1	M+H, M+Na	C22H32O5	-7.4953546	7504.71261	6754.56383	6903.63382	155.879076	49322.0401
*	40.5	20.2	M-H, M+FA-I	C22H34O5	-5.7097775	7526.6974	7374.57549	7590.78056	391.542086	2911.49713
*	40.5	6.53	M-H, M+FA-I	C14H26O2	-0.5723649	3053.48914	3533.09363	3224.22222	796.271254	6302.21734
*	40.5	5.51	2M-H	C8H16O2	0.0810219	1560.35281	1697.26693	1492.04216	1831.90724	906.183067
**	40.4	0	M-H	C8H14N2O5	-1.7488322	13498.7124	12944.7746	12808.6019	22195.7477	37975.6912
*	40.4	4.82	M-H	C9H11FN2O	0.08916586	26623.6822	29596.7293	28068.7325	6510.3837	68652.5216
*	40.4	8.87	M-H	C4H8O3S	-0.8860407	2301.64403	2305.79412	2303.62142	1360.26264	2888.94117
*	40.4	13.3	M+H-H2O	C20H39N5O	-1.9166058	13513.2989	14417.6471	13745.1211	2394.55467	19150.392
*	40.4	6.27	M+NH4	C34H66O	-1.0460697	7811.56819	6074.49632	4519.7254	16562.3911	9525.35932
*	40.4	24.9	M+K	C20H32O5	7.04457938	6325.34154	6391.31471	6727.02764	509.839977	330.957355
**	40.4	0	M+H-H2O	C8H14O5	0.57914623	5433.17838	5795.34594	5325.58933	7088.22048	4288.86685
*	40.4	8.27	M+Na	C20H36O6	-1.3844057	4669.83095	5388.04627	5651.10699	1016.84635	7316.62355
*	40.4	12.9	M-H2O-H, M	C47H90O18I	3.90949194	139138.036	126612.041	146128.018	138032.891	114522.026

**	40.4	0	M-H	C12H20O3	-1.09898	675.270145	734.767187	599.984211	515.279379	659.548645
*	40.4	5.59	M+H	C17H24N6O	-0.343985	1069.09397	987.06031	749.474174	239.197078	78.1519232
*	40.4	13.4	2M-H	C9H15NO4	3.86294481	1119.29331	1486.43727	860.840313	9.7531E-07	9216.73048
**	40.3	0	M+NH4, M+K	C10H12N4O	0.0434046	3700.15206	3988.59625	3190.78082	2264.79719	7930.45138
**	40.3	0	M+NH4	C12H15N5O	-4.8761915	4763.04873	4112.85553	3868.95904	9.7531E-07	13534.8962
*	40.3	4.06	M+FA-H	C6H14NO5P	0.42035627	181068.07	176011.051	175560.18	528343.002	898141.422
*	40.3	4.11	M+H, M+NH4	C8H6O	0.64083722	99151.1082	97803.2105	99584.2818	169190.185	142620.139
*	40.3	10.2	M+H	C16H28O11	3.54212913	1931.4041	1722.30646	2090.9788	9.7531E-07	9.7531E-07
*	40.3	10.3	M-H2O-H	C50H85O16I	-1.8504532	643729.067	788401.736	757088.547	717501.028	510323.199
*	40.3	12.8	M+NH4	C25H36O12	3.68094061	56540.0805	54671.1136	58676.1838	38738.3435	21912.7386
*	40.3	7.61	M-H	C20H38O7S	-0.1366057	31559.4797	30787.4185	30353.9635	8146.76435	3015.40645
**	40.3	0	M+NH4	C18H33NO	-1.6624621	5771.3273	5046.02988	6041.89442	218.235921	2994.20049
**	40.3	0	M+H	C10H20N2O	0.16553044	3121.2095	2954.77279	3570.90361	1019.14059	2200.97673
*	40.3	13.3	M+Na	C24H32N2O	-3.5846689	6166.81802	6284.14576	6814.7216	773.972351	1104.13812
*	40.3	7.73	M+Na	C8H16O4	-1.8079403	2869.18816	3091.15358	2909.67792	4564.05155	2363.37368
*	40.3	6.8	M+Na	C14H26O5	-1.4439468	3005.35281	2494.60567	2960.45344	16802.5562	2889.11941
**	40.2	0	M+Na	C6H11NO	1.78685059	2208.18833	2177.52611	2318.80032	1764.40399	1202.46191
*	40.2	6.59	M+NH4	C12H17NO3	-0.740863	94864.049	94783.1559	98885.3947	112653.801	237969.716
*	40.2	14.3	M+H, M+Na	C20H20O4	1.36339735	1611.56836	1604.49901	1665.13142	80.907086	1062.18448
*	40.2	10.1	M+Na, M+K,	C22H35NO6	-0.7301471	80582.8601	67549.5458	62190.6339	115028.849	103058.089
*	40.2	5.24	M-H	C10H17N7O	1.00838569	4937.62961	5416.45804	5559.17508	2237.35722	11333.8743
*	40.2	12.5	M+H-H2O	C8H17N3O4	0.97817947	2804.02293	2770.43693	2671.23783	882.526413	6597.46497
*	40.2	13.1	M-H2O-H	C31H45N7O	-3.1505857	452439.255	434064.814	402880.442	226479.403	767910.799
*	40.2	8.29	M+NH4	C22H40O4	-1.5419776	2524.87744	2925.79611	2593.50167	920.894831	405.807765
*	40.2	25.8	M+Na	C28H43NO3	2.14059527	16313.8355	15959.4426	14252.2737	9.7531E-07	20301.6515
*	40.2	6.95	M+NH4, M+N	C24H48O4	-1.4252388	95487.8783	104172.487	103029.697	9.7531E-07	9.7531E-07
*	40.2	9.72	M+H-H2O	C16H23N3O	-0.326639	10622.2245	9193.38689	9328.90131	10383.1139	29220.6511
*	40.2	14.7	M+Na	C16H19NO8	3.76653706	10315.8039	9549.10172	9114.92649	3021.56575	11888.7843
*	40.2	8.13	M+Na	C6H14O3	0.90384255	38790.2643	39873.103	39551.7949	54824.4602	34037.5599
*	40.2	22.1	M+H	C16H30O8	-3.0341782	3289.43972	3995.06667	3825.30279	9.7531E-07	2471.87535
**	40.2	0	2M-H	C9H7NO2	9.93345488	1112.25867	833.711343	873.437888	1953.83425	1713.75451
*	40.2	5.25	M+NH4	C7H9N5O3	1.62822061	7141.47528	7210.76856	6680.62107	13881.1106	5641.8581
*	40.2	4.77	M+NH4	C8H11N5O3	1.16911957	6856.40298	7241.20716	7195.66595	13943.0762	5526.59634

**	40.2	0	M+H	C18H19NO4	9.82169801	3227.07873	2873.85827	2868.67287	703.723694	278.162782
*	40.2	3.56	M+FA-H	C7H5O2P	-0.5691582	7824.80491	8042.97037	7762.43839	7155.9209	3066.69616
*	40.2	6.62	M+NH4	C16H26O5	-0.9604088	976.825959	966.075639	1050.17605	8379.10111	1974.18183
*	40.2	6.49	M+H, M+Na	C17H30N2O	-1.7737002	12344.1387	12738.5942	12949.4295	4652.43029	11224.4179
*	40.2	5.56	M+FA-H	C7H14	-1.8700001	478.732327	539.684086	459.566107	155.691188	1204.52734
*	40.2	9.14	M-H	C17H30N8O	3.74202018	11313.0264	10522.4008	9759.59251	223.049202	52516.6511
**	40.1	0	M+NH4	C7H11NO4	8.10535333	4895.03172	5435.34293	4756.13126	5778.79456	3418.05256
*	40.1	8.63	M-H, 2M-H	C5HF9O2	-0.7605453	44314.9085	51261.5998	45406.8188	10870.9184	3870.70278
**	40.1	0	M-H2O-H	C30H26O10	-3.6738832	2420.57613	1566.23869	2011.31037	4727.9235	9.7531E-07
*	40.1	7.87	M+NH4	C18H26O4	-1.6591794	204029.167	165630.856	172773.47	271988.378	148147.967
*	40.1	32	M+NH4	C38H77N2O	3.30884169	561680.354	427670.106	543180.557	685558.351	504460.2
*	40.1	3.37	M+NH4	C36H72	-0.4745169	25719.2508	20325.9959	23570.1415	66582.6011	40607.7828
*	40.1	6.49	M-H	C9H8O4	-1.24719	3960.05618	4017.09069	3960.83599	2552.86616	1237.68054
*	40.1	2.71	M+Na	C7H16O4	-0.0510934	55482.7995	55740.315	55623.8771	91120.0283	42254.3369
*	40.1	19.6	M-H	C17H25NO9	-4.4217926	24549.6512	24958.5236	24986.3962	8640.13663	20387.2289
*	40.1	14	M-H2O-H	C16H18N2O	-0.0837166	3464.88322	3635.1661	3180.47777	554.359451	12968.5254
*	40.1	13	M-H	C13H8F2O3	3.7675077	6260.26829	6278.02247	6668.28222	7100.73663	2234.33171
*	40.1	20.6	M+NH4	C22H32O8	-3.1341915	6869.69377	5778.00215	6645.42198	4085.70753	8371.16359
*	40.1	6.22	M+H, M+Na	C10H21N3O	0.41844491	1188.21532	1342.49221	1206.79647	539.155541	452.883235
*	40.1	6.03	M+FA-H	C19H21FN4	2.41522021	21766.8039	22257.209	20952.3109	14577.0905	7111.16274
*	40.1	5.15	M+H, M+Na	C12H23NO2	-0.9461821	35460.5507	35789.5708	36585.6691	37381.2656	23367.8967
*	40.1	4.62	M+NH4	C8H13N5O	-0.1039817	18750.2381	18599.7244	19495.7436	20783.2839	13736.1568
*	40.1	3.25	M+H	C15H33N	-0.5624949	1876.01235	1913.19126	1749.40102	602.463904	500.133125
*	40	3.35	M+Na, M+K,	C7H10O7	-0.0699259	22847.5172	23047.1806	23235.3172	30262.1278	5582.51461
*	40	10.6	M-H2O-H	C6H13O7PS	-1.7357463	127697.409	142145.111	124418.745	21292.317	11241.1581
*	40	9.2	M-H	C12H8N2OS	0.17881735	6640.24024	6988.28882	7746.53046	7658.17273	12588.789
*	40	10.4	M-H, M+FA-H	C45H82NO1	0.53789658	3131640.87	2958719.46	3146882.85	4087893.95	3583225.9
*	40	5.99	M+H-H2O	C22H43NO2	-1.4487497	74921.199	72368.3689	84140.3208	26668.3448	23037.7635
*	40	13.8	M-H	C6H4N2O5	-7.6934648	4833.83086	5646.54295	5122.45387	4952.54058	5117.12772
*	40	5.78	M+NH4	C9H15N	0.96978649	7544.79825	7635.17239	7893.77641	5749.27233	3389.4117
*	40	11.4	M-H	C5H12OS	-3.67946	11992.0254	12121.4405	12317.2062	12144.3283	10243.4986
*	40	5.77	M-H	C13H13N3O	-0.2523224	38219.1759	38772.1915	40499.0773	63651.1335	78841.1309
*	40	7.58	M+H	C9H10N2S	0.29762016	2952.28344	2923.00112	2723.53459	4846.71596	2666.13464

*	40	6.3	M-H	C27H42O11	-0.077076	4224.22709	4936.38476	4762.48455	3935.83722	4284.4998
*	40	3.38	M+FA-H	C7H13NO2	-1.8674842	288.328279	211.658901	243.827419	9.7531E-07	980.674082
*	40	7.03	M+FA-H	C6H10O4S	0.46940045	3782.72219	4594.65168	5194.04583	909.13621	767.948931
*	40	4.89	M+H, M+Na	C12H14O4	-1.3204048	15325.2343	14180.2329	13654.1483	19396.3889	10092.9022
*	40	3.45	M+NH4	C28H38O6	-1.2513909	49095.6317	51631.6251	51541.7721	55267.9486	51903.8436
*	39.9	17.9	M-H	C16H12N2O	2.78572119	6466.20479	6006.65655	6336.40646	17064.8322	8117.38313
*	39.9	13.5	M+FA-H, 2M	C8H5F4NO2	-4.0765798	5687.80133	4976.85917	5550.53364	4769.72189	5577.56778
*	39.9	3.19	M+K	C4H13NO6P	0.77000607	995.959314	894.669937	889.762097	210.142467	9.7531E-07
*	39.9	11.4	M-H	C12H12N2O	4.40549146	3669.067	3535.78223	3511.50732	9821.9667	5373.67949
*	39.9	3.84	M+H	C6H13N3O2	0.17826615	562.601265	714.045199	721.440852	201.706777	29.6943901
*	39.9	3.27	M+NH4	C12H13N5O	-0.5256852	1379.98908	1382.49997	1281.37007	8672.59807	1020.89402
*	39.9	2.55	M+NH4	C12H11N5	-0.363429	15128.2654	17011.5829	16504.6196	21322.4625	6237.12418
*	39.9	8.83	M-H	C14H30O4S	-0.223679	50290.4861	48718.1709	46482.3626	42277.2543	32271.2305
*	39.9	17.1	2M-H	C21H29N3O	-0.2865388	9087.45455	11752.9984	8771.32772	5446.00625	6571.15655
**	39.9	0	M+NH4	C20H34O	-1.6771661	1859.67301	1918.535	1600.66453	273.663602	196.842011
*	39.9	7.3	M+H, M+Na	C6H12N2O	1.94446589	9370.21404	10017.5828	10032.2793	10580.6717	8005.32864
*	39.9	4.49	M+NH4	C8H9N5O4	1.38017103	3137.96537	3323.08889	3486.77541	4109.63264	2357.29298
*	39.9	2.06	M-H2O-H	C9H12NO5P	0.23722443	4127.72386	4318.73781	4484.16925	2892.50316	1862.84325
*	39.9	16.2	M+H-H2O	C30H44O10	2.72648499	3019.68793	3270.76404	3844.40366	1554.08192	4648.31532
*	39.9	18.5	M+NH4	C13H11N3O	-3.2193192	23258.5211	23784.9043	22780.3098	4198.47318	44335.7163
*	39.9	4.06	M-H	C12H23NO3	-1.0706509	3217.9597	3232.67803	3314.23508	1843.25048	2822.57901
**	39.9	0	M+FA-H	C21H32O5	-0.1246871	2247.35308	2174.38895	2110.92462	1123.2785	4465.83314
*	39.9	2.98	M+FA-H	C22H22O10	-0.019874	9597.74001	11925.6733	13287.0068	13304.6192	10249.9917
*	39.9	6.43	M+H	C9H12N2O5	-4.4886753	973.928354	1014.01826	1031.14767	121.158225	259.115528
*	39.9	5.53	M+K	C23H48NO7	-2.3830757	131674.67	129167.046	136494.912	186637.337	108460.432
*	39.9	3.96	M+FA-H	C21H37NO4	0.54827885	4574.67605	4624.67724	4431.4019	9.7531E-07	10530.9018
*	39.9	4.81	M+Na	C19H36O5	-1.1506573	87965.6115	95176.4129	94299.5881	129545.463	74151.2227
*	39.8	0	M+K	Th	-0.6968051	5426.38162	6213.20366	5870.18373	15101.4005	3700.35864
**	39.8	0	M+FA-H	C6H12O7	4.57146211	11668.2315	12661.3959	10986.2933	11642.6483	9696.40589
*	39.8	5.79	M+H-H2O	C29H51NO5	-0.6294365	81162.6137	77514.8755	81303.8606	14248.1938	386223.623
*	39.8	2.67	M-H	C14H21NO3	-0.8259221	227123.089	232655.856	229241.755	229810.252	207996.246
*	39.8	1.83	2M-H	C9H18O3	-0.6329842	964.005797	879.925185	885.108139	121.309108	9.7531E-07
*	39.8	4.24	M+NH4	C25H44O5	-1.1835945	36338.5341	39590.5478	39117.3565	9713.46219	19317.2687

*	39.8	2.58	M-H, 2M-H, I	C18H36O2	-0.8134412	1633018.92	1676787.1	928622.484	1132348.61	868771.729
*	39.8	11.9	M+FA-H	C10H14N5O	4.62340597	9324.84937	10598.0385	11076.7137	14777.9139	5456.16156
*	39.8	7.29	M+FA-H	C22H24O6	-3.7085572	24540.0623	25811.4233	24863.2819	68079.3248	7679.48629
*	39.8	5.3	M-H2O-H	C10H12N5O	-1.2765286	9757.4025	9799.79542	9329.63852	9488.05802	19094.7278
*	39.8	5.4	M+FA-H	C8H5F3N2O	-0.6496039	302.837652	324.332448	359.505431	59.1785074	4039.27925
*	39.8	0.16	M+H-H2O	C7H15I	0.40108917	1085.38371	1050.73727	979.081358	790.007594	93.3236704
*	39.8	10.6	M+FA-H	C7H9NOS	-1.5491469	576.603123	612.573431	651.28372	281.88131	252.892733
*	39.8	1.84	M+K	C15H16N2	0.67459526	385.38043	293.210402	422.60859	194.482868	613.082767
*	39.8	15.2	M+NH4	C15H13FN2(	4.33545449	1516.75851	1556.9341	1202.34178	9.7531E-07	106.240852
*	39.8	16.9	M+H	C18H15O6-	1.16789397	2266.72766	2052.38479	2227.0553	131.158588	9.7531E-07
*	39.8	0	M-H2O-H	C12H14N8O	0.30837657	761.107916	601.474952	770.29493	538.925566	3768.07664
*	39.8	0.775	M+Na	C10H9N7O2	0.22293395	7370.29935	8037.20341	7767.60056	12698.2634	17000.3921
*	39.8	0.955	M+FA-H	C6H6N6O2	1.4246451	6565.11263	6435.69825	6609.61014	7675.04238	5535.44171
*	39.8	0	M+NH4	C8H16	0.04260081	1333.82688	1530.61696	1461.59102	1855.3186	998.443527
*	39.8	8.83	M+K	C14H13N3	-0.3613942	1096.75392	964.085209	875.097242	548.936191	4620.08013
**	39.8	0	M+H	C15H22O4	-9.9548468	12342.5229	12381.6501	13175.9245	20852.4512	10559.161
*	39.8	3.44	M+H, M+Na,	C8H18O3	-0.4793101	135424.496	145148.794	153284.468	175125.669	108940.633
*	39.8	1.03	M+FA-H	C6H10N6O	0.33711477	3440.33672	3643.23937	3544.96884	4894.77835	11677.2113
*	39.8	2.82	M+FA-H	C17H25NO8	-0.2450168	11298.0342	13702.2241	14841.2758	6800.03271	2608.02476
*	39.8	6.45	M-H	C25H37NO2	0.39484789	3141.85987	2683.803	2929.45234	9.7531E-07	4558.73998
*	39.7	14.2	M-H, 2M-H	C17H10O6	4.20169717	92899.1944	87660.2794	100998.433	122210.847	65430.0241
**	39.7	0	M+FA-H, 2M	C12H20O11	-9.1222609	19628.458	26385.1474	17607.0698	6145.71926	60923.5793
*	39.7	0	2M-H	C4H7NaO4	-0.0117501	3701.77381	3821.89863	3740.19735	11154.1552	10858.383
*	39.7	2.48	M+Na	C7H14O5	0.10142931	2268.37811	2416.06371	2170.46861	21176.6871	3822.05028
*	39.7	0	M+H	C16H18N6O	0.23129285	944.065918	1107.01456	1462.96598	963.98061	1702.47841
*	39.7	5.02	M+FA-H	C26H37N9O	-1.1753908	87991.2101	86631.0973	83503.4069	27219.0186	114858.875
*	39.7	0	M+FA-H	C4H8O4	0.52478087	1041.04535	745.683793	1033.05941	663.847641	487.425123
*	39.7	35.6	M+H-H2O	C46H86NO8	-3.621392	491380.633	302194.415	310699.292	604388.604	311258.916
*	39.7	0	M+K	C9H5I2NO	0.28155835	20271.2005	19436.844	19534.965	598.319485	39770.8629
*	39.7	6.76	M+H-H2O	C19H32N2O	-3.9436827	32798.0251	36294.6291	34123.2187	221650.676	93189.7673
*	39.7	3.34	M-H	C3HF7	-2.0995729	9364.61904	10427.9967	10784.9616	9652.11352	7718.14465
*	39.7	6.3	M+H, M+Na,	C22H46O4	-1.4571231	14521.7424	15180.0877	15924.7511	4216.70592	3104.69259
*	39.7	4.1	M+H-H2O	C3H6N2O3	3.1078825	155931.594	157808.672	156283.476	217864.757	120204.525

*	39.7	7.02	M+H-H2O	C10H13N5O	-0.7309161	7966.25036	8117.99172	7461.34535	9041.76371	5061.63079
*	39.7	0	M+Na	H2NaO4P	-0.5255225	793.160115	748.524993	612.051968	1794.22742	968.161025
*	39.7	0	M+Na	C7H15NO3	0.50553563	511.689237	575.872541	622.581643	473.178516	2837.92416
*	39.7	8.07	M+H	C20H16O2	0.65361314	15835.9941	15446.9748	15679.4475	30138.547	29509.7146
*	39.7	15.4	M-H2O-H	C14H16N2O	0.36419679	1240.59878	1537.30528	1523.22076	1343.50991	5064.73776
*	39.7	7.92	M+Na	C23H38N6O	0.45617343	3134.24316	3508.38505	4197.83633	320.489753	123.481654
*	39.7	3.33	M+H, M+Na	C7H15N2O8	-2.5225814	26332.9743	26479.8249	27779.6169	102704.202	6972.95807
*	39.7	17.4	M+NH4	C36H39N3O	3.68476806	4355.49048	4756.52017	5264.08243	2121.04138	3454.12389
*	39.7	0.825	M+H, M+Na	C12H26O4	-0.1542104	3004.68902	2948.37879	3018.50369	4388.69167	1271.93015
*	39.7	0	M+FA-H	C8H10O	-0.7226201	178.682053	173.76492	112.821038	99.85687	96.7863994
*	39.7	6.63	M+H, M+Na	C10H14N2O	-0.8475351	6293.5001	6471.8493	6376.9688	53.5350251	1907.30085
*	39.7	1.06	M-H2O-H	C10H14NO5	0.43124399	14111.5472	13509.701	13537.6215	13847.6834	11602.991
*	39.7	1.54	M+FA-H	C5H8N6O	-0.3562641	965.57727	1036.36735	976.73662	779.140353	1048.51959
*	39.7	0	M+NH4	C7H12O	0.2984924	537.564356	588.51478	530.308923	721.342255	538.50712
*	39.7	11	M+H, M+Na	C20H36NO5	4.79099616	25931.2897	25611.6816	25220.213	2343.97556	83467.7039
*	39.7	10.1	M-H	C25H32F2O	-3.4010473	6667.45138	6556.11006	5755.0747	245.333929	3678.98298
*	39.7	2.63	M-H	C11H14O3	-1.50233	45227.8555	41238.4229	53056.9618	48824.7546	38386.9719
*	39.7	6.25	M+K	C16H24O7	-4.1104608	1768.89087	1882.28243	1924.81345	2916.62109	3532.0359
*	39.7	2.52	M+NH4	C15H30O	-1.3081293	10746.6241	11241.2062	10711.2978	6572.89773	10020.6142
*	39.7	20.4	M+K	C56H82O7	2.18003311	365312.367	401029.417	404929.146	520917.385	431966.419
*	39.7	3.55	M+Na, M+K,	C24H30O6	-0.943418	40586.8761	38220.2334	39221.1918	17129.945	18979.0909
*	39.7	7.29	M+NH4	C8H14N2O3	-0.3831588	4048.23117	3869.46255	3421.1978	5763.51937	3742.4915
*	39.7	0	2M-H	C8H15O2-	-0.2559275	2822.33692	2865.01286	2694.48116	2324.82722	1883.61343
*	39.6	0	M+Na	Na3O4P	0.94886068	2107.17313	1316.5692	1566.89364	7600.13456	3078.72242
*	39.6	0.316	M-H	C5H13O7P	-0.0510884	3303313.09	3237991.48	3418909.03	1701039.33	3368270.15
*	39.6	0.99	M+FA-H, 2M	C3H7FNO2F	0.79968315	88867.263	87943.5642	89755.4272	35084.4536	202135.446
*	39.6	0	M+H, M+H-H	C6H8O4-2	0.63723145	8997.88402	8603.53065	8432.808	1050.93436	10808.0579
*	39.6	0.337	M+FA-H	C3H9O4P	-0.9253313	10704.9862	10797.0624	11290.2765	13978.1508	22398.9884
*	39.6	0	2M-H	C4H4N4O3	0.17232471	8120.35084	6286.65249	5979.26528	17205.8697	11872.6137
*	39.6	0	M+FA-H	NaO3P	1.1009052	1452.88931	1515.08405	1639.70908	2240.34315	835.829927
*	39.6	0	2M-H	H3O3P	0.77233749	3213.54185	3511.74276	3354.27009	8194.47129	7500.48362
*	39.6	0	M+Na	C3H5NaO3	1.03644722	128055.266	127067.997	135464.653	236952.305	130788.191
*	39.6	0	M+Na	CH5O5P	1.01687338	10622.5434	10341.956	10545.0551	30042.1339	22287.2449

*	39.6	0	2M-H	C11H11NO5	0.03340796	2225.12129	1859.22783	1896.04217	3130.60087	3819.02498
*	39.6	0	2M-H	C4H7NO2	-0.6853396	394.952243	420.008643	403.3046	205.610679	1061.19202
*	39.6	2.96	M+FA-H	C6H15N2O6	-0.2942246	3815.4447	4206.96316	3980.61219	2789.1829	12841.9833
*	39.6	0	M-H2O-H	C10H6N2O5	-0.3458597	816.365762	593.810509	780.32183	1136.4242	998.77049
*	39.6	0	M+H	C9H16O3	0.41270461	3469.59969	3645.94139	3365.53265	3687.4833	2349.88106
*	39.6	2.64	M+H	C27H36F2O!	0.04631055	22513.9981	22469.3915	22515.5486	24636.0762	50089.7284
*	39.6	5.78	M+H	C3H5O7P	4.4442192	18719.1612	20087.7308	19161.7659	30638.2062	18631.1729
*	39.6	4.67	M+NH4	C16H27NO5	3.30901046	14575.06	14392.0965	14253.0882	4529.10315	7993.51686
*	39.6	0	M+H, M+NH4	C8H11N3	-1.1387304	2254.58573	3241.22765	2616.28912	5244.93419	3432.58264
*	39.6	9.01	M+Na	C32H60O6	0.50737296	3037.79981	2827.99078	2891.03359	4061.8998	2316.73563
*	39.6	16.1	M-H2O-H	C43H78NO8	-1.049539	142540.266	202567.361	142991.913	182224.405	27688.1603
*	39.6	0	M+FA-H	C19H38	0.0276846	75110.6551	59955.0679	44293.0597	61782.4452	51464.2203
*	39.6	0	M+FA-H	C3H6N6O6	0.23405104	722.333775	749.828055	854.284869	685.737721	2348.75012
*	39.6	0	M-H	C11H18N5O	-0.5996414	79256.815	81059.7088	80327.936	84751.8994	66337.8434
*	39.6	2.65	M+NH4	C9H7N3O2	0.3799629	3148.66273	3060.1381	3091.13005	171.599747	9.7531E-07
*	39.6	0	2M-H	C5H7NO2	-0.8122335	1142.26161	1102.57217	1347.42302	1395.87364	1256.31598
**	39.6	0	M-H	C15H18N4O	0.05521739	393.46033	336.613764	388.337703	9.7531E-07	989.121518
*	39.6	0	M+H	C2H5Na3O8	-0.3744097	4765.21365	4369.17892	4664.69685	6215.60074	3661.92042
*	39.6	7.06	M+Na	C27H29N5O	-0.2003553	6022.24166	6190.40662	5213.31229	3077.8891	2679.58965
*	39.6	0	2M-H	C5H4O2	-0.8556725	198.439148	182.330529	148.11677	98.5582276	118.131509
*	39.6	0	2M-H	C2HF3O	-1.5197374	17329.5363	16985.7533	16973.154	16057.9775	15177.5088
*	39.6	0	M+Na	C11H22O6	0.21717413	3327.5652	3436.77227	3592.20859	7842.26	2787.25586
*	39.6	4.77	M+FA-H	C9H12N2O6	4.26134788	14025.1836	13788.9705	14696.4933	319.6393	422.255219
*	39.6	5.7	M+Na, M+K	C7H15NO3S	0.7485883	8642.99589	8832.24276	8833.06519	141.835155	4715.35794
*	39.6	3.2	M+Na	C7H13N3O5	0.08497113	537.708063	455.344903	562.960578	395.075415	426.538359
*	39.6	0	M+H-H2O	C10H15NO3	0.73297553	261.650055	286.884728	249.20338	22.3008091	36.3202897
*	39.6	1.84	M+H-H2O	C9H19NO3S	0.19196738	7372.87217	7883.59714	7329.14879	3104.47781	11417.1521
*	39.6	1.12	M+Na	C11H20O6	-0.1287275	1436.26489	1402.50918	1404.07272	1416.82871	803.590571
*	39.6	11.4	M+NH4	C7H10OS2	-1.5744864	331.678513	374.938385	394.422168	57.6620328	67.0635471
*	39.6	1.89	M+NH4	C28H49NO4	-0.4207809	15326.2738	15387.8669	14518.0072	26399.1932	13502.143
*	39.6	4.2	M+H, M+Na	C11H19NO2	-0.9314402	4516.33861	4569.66553	4316.34777	1466.3994	1941.39659
*	39.6	0.0168	M-H	C10H8F3NO	-0.2600379	935.906289	1125.07491	1023.70051	943.01687	687.620973
*	39.6	0	M+K	C9H9N	-0.2789591	5167.49389	4927.95004	4808.51869	6445.30428	3984.41343

*	39.6	0	2M-H	C5H6O2	-1.1203021	175.904382	132.051619	127.877553	38.5502083	23.430719
*	39.6	0	M+NH4	C12H24	-0.3053569	164.427836	201.812279	139.441893	9.7531E-07	70.3373009
*	39.6	0	M+FA-H	C12H21NO3	0.01505283	214.907084	141.536049	172.536864	30.897559	2046.79927
*	39.6	8.59	M+K	C9H17N	-0.571077	2582.50159	2596.67718	2875.71532	4431.67607	2370.23521
*	39.6	4.34	M-H2O-H	C28H38N8O	2.1209986	1410599.3	1261367.71	1414396.96	1349935.34	1163193.85
*	39.6	14.3	M+FA-H	C46H83O12I	-1.7418389	96493.3751	93274.8934	105623.797	92101.5254	84397.5738
*	39.6	5.77	M+NH4, M+N	C20H38O5	-1.0793292	1365.95104	1909.65283	1626.28377	19319.139	4197.53236
*	39.5	0	2M-H	C11H12N2O	0.5719866	15373.7029	19422.7776	16720.6236	2891.99256	2436.69431
*	39.5	5.85	M+Na	C6H8O8-2	-0.3857216	9712.81686	5989.58182	8823.67836	3106.96691	9.7531E-07
*	39.5	0	M+NH4	C10H14O3	0.57350595	1433.4428	1766.51115	1422.94583	1583.45291	1881.03127
*	39.5	0	M+FA-H	C4H3NaO4	-1.6644456	13156.9559	13625.6955	14969.4809	18306.1598	17689.5206
*	39.5	0	M+Na	C8H4O4	0.62897619	745.810852	792.209959	817.119237	1925.49992	1107.43305
*	39.5	4.81	M+H	C10H17N3O	-2.7713676	13071.8823	12804.7628	13961.8369	4051.48307	10114.3247
*	39.5	0	M+FA-H	C15H28	-0.7435156	1147.8732	1457.68942	1214.29535	9.7531E-07	5416.38602
*	39.5	0	M+FA-H	C20H36N2O	1.76306585	4737.21186	4899.09166	5254.66139	4879.47975	13255.3093
*	39.5	0	2M-H	C12H17N5O	-0.3041295	3675.3437	3918.23899	3984.10547	775.27675	6091.30228
*	39.5	0	M+H	C12H24O3	-0.8572626	1509.81439	1699.91246	1943.42003	1114.46663	748.075378
*	39.5	3.08	M+FA-H	C15H21NO	-1.2772701	5056.11793	5020.08651	5161.16725	5024.99531	4121.52108
*	39.5	16.4	M+H	C22H42O8	-3.9738908	19158.8145	19229.8564	19017.6468	1543.20937	24320.972
*	39.5	0	M+FA-H	C17H25N7O	0.05297997	146229.22	166695.323	145915.085	166886.977	172042.395
*	39.5	0	M+NH4	C5H7NO	0.8108207	412.071624	559.050558	325.638938	791.6132	355.449256
*	39.5	5.99	M+H	C22H39NO	-1.6642133	11611.035	12223.0318	13749.5989	3105.8632	3234.11993
*	39.5	0	M+FA-H	C11H13NO2	-1.1353335	3819.7271	4004.98526	4032.80964	272.085863	6041.53562
*	39.5	0	M-H	C12H14N2O	-0.4135679	967.507917	745.013001	674.613405	879.700037	2539.231
*	39.5	0	M+H	C6H6N6O	-0.4064051	1796.55959	2131.22823	1969.80124	2550.77866	1566.06489
*	39.5	1.62	M+FA-H	C9H8N8O	0.91225606	5076.55054	4945.50892	4994.28535	423.925428	14295.1603
*	39.5	0	M+NH4	Tb	2.25498361	1069.2258	1021.30812	919.442468	1335.26503	736.722516
*	39.5	16.5	M+K	C20H33NO7	-1.2137774	42893.4877	34388.6799	40304.5586	34055.4978	87148.0714
*	39.5	0	M+Na	C8H14O4	0.01956536	1395.91776	1555.381	1534.31346	2088.80795	1295.85446
*	39.5	0	M+K	C13H20O5	0.01822998	1020.95263	771.245708	917.264491	9.7531E-07	91.8323301
*	39.5	12.2	M+H	C13H19N3O	0.83128819	3625.51599	4079.21897	3911.03206	7956.00446	6378.65918
*	39.5	0	M+Na	C6H4N2O2	-0.029175	376.588027	420.33947	504.148954	640.457242	945.784469
*	39.5	0	M+H-H2O	C5H12NO6P	0.2597822	131.62231	137.882306	144.89101	42.0405014	73.0202285

*	39.5	0	M+Na	C9H16O5	0.28348502	1626.22947	1621.85995	1734.17335	2423.4659	1412.0112
*	39.5	0	M+Na	C12H18O5	-0.0221644	1217.3466	1268.10855	962.363394	1012.3052	1305.89642
*	39.5	0	M+H	C9H10INO2	0.20357064	1966.07081	2031.61593	2047.412	2856.41998	1507.25693
**	39.5	0	M+H	C16H20O10	8.56217937	715.35868	465.591055	544.741669	1386.64038	102.530748
**	39.5	0	M+FA-H	C17H17NO4	3.15512302	2116.98161	2162.70329	2031.41415	373.597003	3073.4264
*	39.5	0	M-H2O-H, M	C6H8Na2O7	-0.93563	8681.75371	9102.03666	8795.2783	8260.42264	9414.72447
*	39.5	5.38	M+NH4, M+N	C28H55AsO	-1.8171135	43001.4784	44282.1549	44723.9501	181546.259	59697.0834
*	39.5	0	M-H	C14H11N3O	0.07493385	4138.17915	4979.46209	4374.57194	382.212247	465.757697
*	39.5	0	M+H-H2O	C14H20O2	0.12250412	687.663108	822.382545	924.501266	1004.89531	508.780261
*	39.5	0	M+H	C11H22N6O	-0.0289425	12065.8642	11606.2709	11855.7421	24568.8727	5030.39085
*	39.5	0	M-H2O-H	C8HF17	0.68363513	999.982583	1028.4496	1037.58367	1026.83406	342.505826
*	39.5	0	M+FA-H	C10H11NO4	-0.0812458	453.58834	496.380563	619.479387	387.215842	500.901421
*	39.5	0	M+FA-H	C8H5F3O	-0.272962	278.784163	263.398157	211.289986	9.7531E-07	20.7559458
*	39.5	0	M+FA-H	C7H14	-1.951583	200.702139	229.687793	240.757193	154.011093	144.657377
*	39.5	0	2M-H	C4H4O2	-1.0353346	893.670468	823.332698	833.914609	453.26791	644.695493
*	39.5	0.0639	M-H	C17H31N7O	0.50741496	3680.71808	3826.29891	3725.45366	9.7531E-07	26186.3467
*	39.5	0	M+K	C3H6N6O3	-0.6277005	280.118449	374.354107	239.323047	99.5532338	230.46938
*	39.5	0	M+H, M+Na	C11H21NO2	-0.9544065	2001.9807	2009.76778	1876.51503	751.901845	1141.76235
*	39.5	0	M+H	C17H15N7	-0.600991	407.599514	475.314641	579.941281	359.819616	178.62943
**	39.5	0	M+Na	C10H18O4	-1.8510471	3123.70677	3431.65319	3397.15619	4794.33505	2216.71487
*	39.5	0	2M-H	C17H14N2	-0.6648769	933.230099	1034.04978	916.610516	9.7531E-07	8301.18937
*	39.5	0	M+FA-H	C12H16O2	0.13029411	367.894323	314.835518	275.854063	325.664368	120.975829
*	39.5	0	M-H	C9H12N6O3	0.44102453	1099.76167	1520.80019	1492.47454	638.437188	730.841547
*	39.5	0	M-H	C18H30O3	0.0957616	630.73747	708.9281	812.310979	56.2336336	1770.20656
*	39.5	2.03	M+Na, M+K	C16H28O3	-1.4618938	192427.177	187063.127	188569.712	287930.479	152059.393
*	39.4	0	M+FA-H	C13H16N2O	-0.5894	1238.45147	940.931011	1319.43978	614.228908	2117.86315
*	39.4	12	M-H, 2M-H	C22H20O8	-4.5461907	130607.786	138000.882	121688.9	93890.8605	181401.366
*	39.4	5.27	M+H-H2O	C4H5N3O2	2.23480025	1318.14698	1174.30077	746.923455	884.589192	1653.79707
*	39.4	0	2M-H	C9H6FNO2	-1.5462059	8160.58013	7816.29136	8504.69093	4154.75206	12177.2786
*	39.4	0	M+Na	C12H13F3N2	1.09036352	1399.69999	1643.00736	1874.41626	2230.2994	1281.12001
*	39.4	0	M+FA-H	C5H5N5	-0.9797989	236.945383	206.651398	268.513486	254.149274	169.136774
*	39.4	0	M+FA-H	C4F8	-2.2244243	724.900406	769.589374	849.768791	735.004672	2039.7942
*	39.4	0.398	M+FA-H	C24H36O5	-0.0073835	7019.25221	6402.87987	6136.63814	4735.47886	4507.72839

*	39.4	0	M+FA-H	C19H27NO	-0.7610203	606.204757	433.506757	468.952311	322.296048	114.730586
*	39.4	0	M+FA-H	C16H30O	-0.8730056	426.539433	482.808182	385.965005	225.012693	1545.64021
*	39.4	6.68	M+H-H2O	C27H41N9O	-2.0212991	96868.7388	98462.3945	100239.407	72366.8735	179779.272
*	39.4	0	M+H	C13H20O2	-1.3763995	1671.83054	1938.4623	2005.53963	2469.08509	1575.92207
*	39.4	2.33	M+NH4	C18H36	-1.6816938	34140.9073	33442.6288	36110.02	53249.5478	24419.6749
**	39.4	0	M+H-H2O	C20H30O	-1.6215278	3898.42429	3608.67565	3071.01764	5081.01634	3330.36663
*	39.4	4.25	M+NH4	C34H68	-0.9195851	31007.4212	26539.6768	28715.7725	55555.2362	25847.4776
*	39.4	0	M+NH4	C10H8N4	-1.1589057	2747.6278	1843.85981	2418.98753	2567.88103	1421.6725
*	39.4	1.9	M+NH4	C24H50O4	-0.6210962	4085.15111	2880.31685	3939.35214	9.7531E-07	9.7531E-07
*	39.4	0	M+FA-H	C11H16NO7	-0.1793388	325.288499	266.714145	335.558834	349.430055	1918.54197
*	39.4	10.2	M+H	C7H6O7S	-3.323393	2217.4233	1659.68113	2380.66408	46.3626647	9.7531E-07
*	39.4	8.03	M+H	C14H20N2O	-7.3981422	5126.24164	4725.7297	4625.29842	10312.2088	1577.01875
*	39.4	0	M+H	C10H12O	0.43843655	31376.4217	34122.4858	39563.2194	33474.4575	20363.4953
*	39.4	0	M+K	C7H16NO+	-1.7673423	1756.18272	1822.38187	1839.65443	2195.79739	1391.05856
*	39.4	0	M+H	C11H12O	0.39180773	53874.249	58429.6351	62545.0672	63427.8356	40317.2609
*	39.4	2.15	M+H	C12H23N3O	0.24860216	214.101102	231.833308	208.71783	2.66028991	1.72754404
*	39.4	0	M+NH4	C12H18N2O	0.59311303	234.037938	264.732128	184.803729	1052.57235	1345.22293
*	39.4	0.647	M-H, M+FA-I	C10H12NO5	0.72522103	159311.829	162148.767	161532.094	253540.177	83713.4251
*	39.4	0	M+FA-H	C13H14N2O	0.08471624	1122.61753	1137.13138	1276.72826	1518.02404	631.803621
*	39.4	0	M+NH4	C8H15NO	-0.1622008	1078.80703	1216.84579	1424.19758	401.234876	128.249723
*	39.4	0	M+NH4	C10H20O	0.90223014	304.067683	261.411474	339.969433	1009.09499	40.8088228
*	39.4	0	M-H	C14H24N2O	-0.0164033	892.839493	929.296994	879.216763	511.581872	777.517385
*	39.4	0	M+NH4	C12H26N4O	0.06791553	1077.19998	839.11807	951.972261	419.642094	207.418295
*	39.4	0	2M-H	C16H8	-1.5550443	1652.99606	1712.72113	2109.05849	1420.88734	1684.3534
*	39.4	0	M+K	C14H28O6	-0.5232748	2461.99299	2626.23097	2667.18425	7129.56094	1219.83102
**	39.4	0	M+FA-H	C7H10O3	-1.8136462	1823.3007	1786.1528	1672.89777	1684.0219	1501.70047
*	39.4	0	M+H	C16H20N2O	0.04165905	563.864497	602.230128	624.034342	624.116087	665.238426
*	39.4	0	2M-H	C2HF3	-1.6809046	3784.40194	3897.54786	3655.48542	3698.06781	3440.92699
*	39.4	3.56	M-H2O-H	C7H6N2O8	0.57430277	48397.3794	49195.3963	49447.5045	46686.5041	43949.973
*	39.4	0.791	M+FA-H	C17H24O7	-0.013274	3740.99409	3741.86977	4346.94473	2421.18478	642.488506
*	39.4	0	M+H	C12H20O2	-0.3391683	1999.2757	1838.33591	1895.79938	2746.99652	1664.39104
*	39.4	0	M+NH4	C9H13N5O3	-0.1356145	6132.97076	5524.50729	5437.26082	9650.10479	4234.24937
*	39.4	0	M-H2O-H	C8H10F2N4(	-1.011731	779.630857	831.144925	809.280046	105.209396	1802.21251

*	39.4	0	M+NH4	C10H12O2	-0.4534178	234.366685	275.976892	285.458563	132.954031	272.947029
*	39.4	0	M+Na	C12H16O4	-0.9719695	1030.95678	1121.3529	988.085861	2597.10212	286.209254
*	39.4	0	M+H, M+Na	C12H16N6O	-0.4882451	9417.70463	9638.24901	9755.35241	5063.22998	4357.0066
*	39.4	0	2M-H	C6H10O2	-0.8372533	2557.47699	2851.12143	2616.35724	2489.29919	2364.70993
*	39.4	0	2M-H	C17H13NO4	0.73863835	49612.9527	49549.1029	49697.6717	115263.222	19673.9561
*	39.4	0	M+FA-H	C11H12N2	-1.7400102	266.94301	315.61866	389.528861	393.064617	374.032534
*	39.4	0	M+K	C10H11NO	-0.3721374	1394.21077	1491.28357	1479.02801	1831.58499	1090.3748
*	39.4	0	2M-H	C5H8O2	-1.1005825	992.565997	924.005827	957.479597	1050.21518	801.295937
*	39.4	1.12	M-H2O-H	C12H16N6O	0.98265141	15751.6223	16425.7717	16646.9741	16307.2537	13762.5503
*	39.4	0	M+H	C13H22O2	-0.9548626	4750.74604	4395.48696	4304.40236	4947.16771	3411.13881
*	39.4	0	2M-H	C6H9N3O	1.76476718	1020.91174	1220.36242	1131.98848	1128.27024	1895.45971
*	39.4	1.14	M-H2O-H	C24H48NO7	2.78007027	451309.513	518617.14	612217.617	537863.639	463213.875
*	39.4	0	M+FA-H	C15H26O	-0.653885	865.300384	898.64595	875.4464	40.0872599	3391.45279
*	39.4	0	M+H	C10H8O3	-0.6010235	1773.85978	2055.31734	2061.66908	2478.03349	1622.59684
*	39.4	0	M+NH4	C9H9N5O	-0.9037547	846.083764	1099.70655	1133.78967	990.636526	710.743897
*	39.4	0	2M-H	C3H7O6P	-1.2308879	7921.64582	7610.31018	8034.92004	8175.16595	6902.98587
*	39.4	0	M+H	C11H16O3	-0.8332292	1415.46929	1352.10975	1520.95084	1471.73998	1092.78695
*	39.4	0	2M-H	C8H12O2	-0.4834384	1695.51342	1903.86041	2148.54932	1857.00852	2035.62687
*	39.4	0	M+H	C5H4N4O	-0.31303	1001.56133	1043.80663	1149.05025	1828.04473	1239.55099
*	39.3	0	M+H-H2O	C19H12O8	-0.0272353	1713.17785	1052.70988	1111.37709	4105.79211	4621.74297
*	39.3	0	M+Na, M+K,	C5H8NNaO4	-0.3292585	284845.517	277308.69	299937.27	615734.297	179078.065
*	39.3	3.3	M-H2O-H	C7H12N2O5	2.46548369	1206843.84	1173100.08	1227769.6	623328.347	1222832.94
*	39.3	3.27	M+H-H2O, M	C8H17NO4	-0.650686	38528.2274	36174.5946	38281.1549	48555.6075	81196.4711
*	39.3	8.24	M-H2O-H	C21H20O12	-1.910763	8542.13216	8518.11264	7456.55215	22053.8045	89656.8124
*	39.3	0	M-H2O-H	C4H10NO5P	0.04858971	2685.34493	2682.39907	2576.4883	4662.21695	1621.30088
*	39.3	8.03	M-H2O-H	C7H4N2O7	-1.3633607	74400.0087	76459.4902	75731.088	189988.74	151452.809
*	39.3	0	M+NH4	C4H2Na2O4	0.73876994	1350.47352	1183.18135	1053.64328	1919.54	493.216645
*	39.3	0	M+H-H2O, M	C7H13NO5	0.33175373	1382.63423	1029.66167	1477.87888	1834.31222	2158.9545
*	39.3	0	M+H	C11H10N2O	-0.6286541	359.646532	301.247012	297.813642	245.405195	655.678147
*	39.3	0	M+FA-H	C9H12FN3O	0.11884108	3223.14698	2983.7339	2659.28026	3720.63337	5435.42502
*	39.3	0	2M-H	C6H5N3O4	2.12996071	4533.94636	4993.86755	4852.03384	8196.91336	20277.3987
*	39.3	0	M-H2O-H	C8H13N6O4	-0.5202918	1219.99856	1037.3613	935.438075	2928.48006	1972.30702
*	39.3	0	M+FA-H	C5H8IN3	-2.7118252	3356.1542	2240.58993	2512.55594	560.502797	148.171279

*	39.3	12.6	M-H2O-H	C24H26O12	2.41494939	27590.6018	24056.119	27536.3384	30882.1908	25923.5731
*	39.3	0	M+NH4	C11H14NO9	0.08218805	5498.02691	5022.13093	5125.6921	3645.33034	15183.9379
*	39.3	3.34	M+H	C18H35AsO:	0.30606461	10760.7831	10109.2803	10868.0331	2529.22456	51945.7352
*	39.3	0	M+FA-H	C14H18N4O	-0.0227314	444.884137	569.609904	566.08057	121.370468	939.250579
*	39.3	0	M+H	C17H17N3O	0.19757427	292.956036	332.787281	339.210284	159.756804	967.769881
*	39.3	0	2M-H	C7H4F3NO3	1.71231495	4203.45601	3154.01818	3766.73362	2979.37	3393.11392
*	39.3	0	M+FA-H	C16H32O2	-0.5849083	2408.64575	2628.88444	2804.92838	721.034378	12464.8064
*	39.3	3.06	M+Na	C19H32O5	-1.0848276	811.565353	1108.54345	862.430722	131.049204	429.749097
*	39.3	0	M+H	C2H5O4P	-0.3240355	789.947426	780.234156	757.719739	889.849536	1716.60083
*	39.3	0	M+FA-H	C13H20	-1.7084223	36728.9568	32913.7017	42372.2362	46023.9328	50209.2685
*	39.3	0	M+FA-H	C12H23NaO	-0.9708467	934.742288	733.124599	1034.67722	1325.97914	346.914608
*	39.3	0	M+FA-H	C15H26O6	0.67178567	1736.07187	1378.12738	1480.56306	187.255604	2405.56825
*	39.3	0	M+FA-H	C6H12O6	-1.0015677	2621.49503	1500.44488	2224.4439	2258.56841	2380.69762
*	39.3	4.7	M-H2O-H	C18H35FO2	-4.6706218	10889.1119	10738.4547	9666.21486	7672.88587	7240.53568
*	39.3	0	M-H2O-H	C27H26O7	0.77325678	108954.67	128648.01	92731.8329	121281.3	119068.332
*	39.3	0	M+FA-H	C16H31NaO	-1.0089718	36142.0297	39244.1379	34689.7299	38485.7545	35124.7916
*	39.3	5.03	M+NH4	C34H68O8	-1.4185229	15777.9861	11955.1683	12681.6926	16.8400164	9.7531E-07
*	39.3	0	2M-H	C14H9NO2	-0.0033122	6138.46321	5813.33555	5468.16415	4897.32021	3450.6169
*	39.3	0	M-H2O-H	C4H9NO4	-1.8847295	431.540345	400.739749	456.015931	439.605535	402.229136
*	39.3	15.1	M-H, M+FA-I	C25H15NO9	4.69586682	12365.1098	12414.0404	13248.4866	3736.64645	10091.1119
*	39.3	0	M+FA-H	C9H17N3O2	-0.9251456	416.514668	372.359744	407.616993	25.0638677	1637.10424
*	39.3	0	M+Na	C7H10O6	1.46042977	823.587498	1000.32263	858.933367	556.261819	4544.16307
*	39.3	0	M+FA-H	C12H9F3N2(	0.6159555	2256.09094	2529.1736	2584.75194	3868.10383	1440.93853
*	39.3	2.29	M+Na	C8H16O5	0.82918329	4122.38334	3978.93329	4134.08133	7804.20475	2840.95128
*	39.3	0	2M-H	C12H11NO2	-1.5344673	618.612397	756.364989	547.335426	553.673972	1723.92383
*	39.3	14.3	M+NH4	C19H34O10	4.61942985	4486.60179	4750.53445	5265.58405	9717.28681	12258.5091
*	39.3	0	M+NH4	C14H24N2O	0.31925689	1390.17313	1286.99765	1440.50589	440.045412	3261.41427
*	39.3	0	M+H-H2O	C16H26O3	0.82756436	1480.45716	1667.52366	1508.30312	1904.26379	1146.98605
*	39.3	6.68	M+FA-H	C7H3F5	-4.1086482	957.534519	885.79581	994.749727	3168.46268	414.87383
*	39.3	0.974	M+H, M+Na	C10H20N2O	0.27616819	3492.70365	3580.00494	3530.6364	431.729793	2844.47501
*	39.3	0	M-H	C11H7F3O3	1.09426426	128.479881	218.335581	148.131032	158.907793	184.611008
*	39.3	2.16	M+H, M+Na	C10H19N3O	0.71279577	914.990799	917.186459	996.96551	44.3624762	344.971988
*	39.3	0	M+FA-H	C13H12O7	0.09147606	3220.75291	3157.62923	3630.17583	3356.00781	2718.92947

*	39.3	0	M+H	C16H16N2O	1.12493761	1124.98116	1260.94034	1078.84209	3457.33756	1077.58158
*	39.3	0	M+H	C14H30NO7	-0.0199058	358.255224	253.256806	439.276289	9.7531E-07	4642.37854
*	39.3	0	M-H	C14H19N2O	0.54222039	327.314326	224.639808	394.140171	193.611614	35.7101479
*	39.3	0	M+K	C15H16N2O	1.34160223	593.271387	698.704087	694.097699	785.109759	27.101248
*	39.3	0	M+H	C14H18N2O	0.15494547	592.077699	487.987341	510.316266	211.001802	2073.20221
*	39.3	0	M+H	C10H12FNO	-0.9557204	142.571729	117.252462	105.476126	96.4267836	537.004913
*	39.3	0	M+H-H2O	C16H22O2	-0.1582047	510.293142	589.175952	319.588234	46.4221342	9.7531E-07
*	39.3	3.1	M-H	C7H6N2O3S	-0.8803814	1985.65428	1734.29622	1836.86853	2452.91659	2758.86615
*	39.3	3.13	M+FA-H	C14H11NO2	3.93516863	630.664788	644.379365	488.622704	138.278615	1083.46504
*	39.3	0.816	M-H2O-H	C7H14O3	-1.0733449	1937.31821	2054.36139	1932.35439	2345.92432	1837.56532
*	39.3	0	M+FA-H	C11H22O3	-0.4433017	303.359251	296.232815	182.754622	175.439615	170.954557
*	39.3	2.1	M+Na	C12H24O5	-0.3301526	7106.04537	7291.65165	7262.79495	9950.93677	6222.23252
*	39.3	0	M+FA-H	C16H16N2O	-0.8170541	1017.95264	839.826158	947.558318	1252.10099	423.107174
*	39.3	0	M+H	C11H16O2	-0.3260234	35342.1157	36685.146	36652.7936	48416.05	27801.6072
*	39.3	0	M+FA-H	C6F6	-1.6974687	1180.5866	1273.1468	1050.32166	985.804324	1014.8531
*	39.3	0	M+FA-H	C7H5NaO2	-0.6389571	582.648219	528.389031	511.743069	622.745688	369.075733
*	39.3	0	2M-H	C4H3NO4	0.24407417	5350.10932	5305.06758	5486.6895	4977.44745	4764.69413
*	39.3	0	M+NH4	C10H13N5O	0.10067175	8645.47139	8423.17889	8790.48844	11588.54	7757.6112
*	39.3	0	M-H	C14H20N4O	-0.3950849	536.20711	564.55575	550.59042	318.68086	359.140729
*	39.3	0.18	M+H, M+Na	C11H24O4	-1.1110767	2374.07527	2295.81469	2457.75358	18541.5897	2888.44414
*	39.3	3.83	M+NH4	C14H10O3	3.2520161	1628.9625	1575.41514	1518.66136	258.129011	4651.91616
*	39.3	8.91	2M-H	C11H15N3O	-3.6817391	7749.49271	7679.78465	7546.17675	9.7531E-07	5983.15763
*	39.3	2.92	M+H	C19H33NO5	-1.1101454	2310.6343	2068.82326	2279.4481	56.9798836	9979.49544
*	39.3	0	M+FA-H	C12H20O4	0.02785508	1681.95951	1605.69159	1460.23253	516.894824	2213.51007
*	39.3	0	2M-H	C5H5N3O	0.32828622	493.285547	524.27669	544.02652	908.286053	80.3468169
*	39.3	0	2M-H	C10H18O3	0.85921154	1897.10689	2074.39401	2205.40986	107.459656	6814.64022
*	39.3	0	M+FA-H	C19H34O3	-0.1655557	307.893728	463.22624	429.417422	9.7531E-07	3115.42177
*	39.3	0	2M-H	C7H12O3	-0.1055329	1029.26209	998.184978	941.784274	1279.47944	819.331286
*	39.3	0	M+FA-H	C16H28O3	1.11022804	329.505334	258.835924	329.618408	9.7531E-07	239.355514
*	39.3	2.15	M-H2O-H	C20H31IO2	-0.0416636	10280.2567	11705.4273	10578.5468	5419.26728	1853.26568
*	39.3	0	M+H-H2O	C13H24O4	-0.9754024	1437.55723	1528.04796	1548.38307	1989.84488	1258.21272
*	39.3	0	M+FA-H	C17H30O3	-0.1043839	433.458925	583.646469	625.91431	307.169533	208.750804
*	39.3	0	M+Na	C14H26O4	-1.4571311	7749.0868	8167.49316	8343.62567	10938.121	6787.60618

*	39.3	0	M+FA-H	C19H34O4	0.09298659	299.040014	372.402375	368.104534	9.7531E-07	574.287932
*	39.3	0	M+FA-H	C7H14O	-1.959215	650.47045	699.78845	626.392004	698.152018	551.560888
*	39.3	0	M+NH4	C6H5N5O2	-0.4271009	978.556185	992.175162	919.925298	1066.16812	770.579874
*	39.3	0	M+FA-H	C16H26O2	-0.1691463	1253.93478	1145.12358	1105.88041	9.7531E-07	129.664238
*	39.3	0	M+FA-H	C16H28O2	0.04314949	576.564976	730.808107	484.870611	9.7531E-07	163.146022
*	39.3	0	M+H-H2O	C16H28N6O	-0.2540711	78603.8065	79522.1113	85542.1803	101907.853	63424.5364
*	39.3	0	M+FA-H	C15H26	-0.7549943	532.914039	645.905999	485.432449	9.7531E-07	3260.62341
*	39.3	0	2M-H	C9H14O2	-0.4856784	562.94797	586.488832	682.56689	495.256373	462.258512
*	39.3	0	M+H-H2O	C5H15NO4P	-2.2033886	1064.48062	1112.30412	973.057923	1299.74743	1078.09729
*	39.3	0	2M-H	C3H5N3O9	0.59126296	5418.85258	5204.15844	4370.9581	5413.19662	4762.81711
*	39.2	0	M+Na, M+K	C7H13NaO8	0.24205091	19366.4551	20868.695	19684.0986	57227.8055	19246.1895
*	39.2	9.09	M+H-H2O, M	C6H12O2S	4.31494359	143447.435	150314.272	153242.538	340101.148	236646.492
*	39.2	0	2M-H	C7H14N3O8	0.91671343	26472.2884	22457.8878	22092.0465	2555.75867	59325.9728
*	39.2	0	M+H-H2O	C3H2F6O	0.62449411	1544.24936	2030.86541	1991.32311	3046.58798	1119.79053
*	39.2	0	2M-H	C17H15NO4	1.05346505	14503.2489	15570.7762	14432.8607	9.7531E-07	176309.301
*	39.2	5.6	M-H	C24H21F2N(	-1.2861977	86639.8827	93450.0475	102350.725	134438.092	185185.671
*	39.2	1.08	M-H	C5H11AsO8	3.12353523	4126.22383	4040.93375	4298.79128	5212.87938	2770.24098
*	39.2	0	2M-H	C21H20FN3(	-0.4940367	5554.18531	4524.46568	4456.99323	7073.73511	3933.23829
*	39.2	0	M+FA-H	C9H14F2N3(	-2.7462083	5829.03931	5381.73724	4790.17488	5355.99386	3864.20828
*	39.2	0	M+FA-H	C12H14N2O	-0.6723975	943.951096	849.127625	1129.78973	417.820852	978.660479
*	39.2	5.54	M-H2O-H	C32H54NO1	-3.1546416	103515.228	98096.1132	98707.2077	46610.0692	149247.057
*	39.2	2.12	M-H	C12H20O2	-1.4692144	533.122171	511.351335	587.663685	568.543777	188.678126
*	39.2	0	M+FA-H	C31H33N5O	-0.7714964	3770.08907	3177.19683	3391.76917	157.728781	17740.3732
*	39.2	15.4	M+FA-H	C26H45NO1	-1.6761209	20641.5666	18868.1519	16735.6399	5478.47938	33552.2442
*	39.2	6.47	M+Na, M+K	C16H22O4	-1.7321515	900121.225	802034.714	791202.839	1217163.75	646271.037
*	39.2	3.06	M+H, M+NH4	C17H36O4	-1.6294195	7809.05736	7893.39887	7772.89783	4549.27934	14694.2973
*	39.2	0	M+FA-H	C24H39NO2	-0.3265836	1660.73337	1312.37092	1060.88975	1603.09835	169.872479
*	39.2	0	M-H	C29H24O6	0.38544075	28293.1476	33723.6637	24803.6208	23942.5437	23178.8787
*	39.2	0	2M-H	C14H16	0.58588703	7172.97108	7514.70397	6837.98614	8241.78137	2432.59849
*	39.2	0	M+FA-H	C20H39NO5	-0.2656677	2204.85564	2395.86632	2265.07925	793.057783	56.9760286
*	39.2	0	M+FA-H	C3F6	-3.1642529	1298.37321	1182.72295	1508.68093	1323.52723	1090.72589
*	39.2	0	M+H	C17H34O2	0.0525783	74548.6919	70336.0826	75499.2718	98204.6899	55112.2317
*	39.2	10.3	M+NH4	C25H30O6	-0.5227695	24497.5736	22501.8141	20117.24	31445.2666	29548.9765

*	39.2	0	M+FA-H	C10H10N2O	-1.2198488	1836.45131	1863.46844	1840.64392	1766.60716	699.026141
*	39.2	0	M+NH4	C12H11NO5	0.19848413	3334.24432	3296.01213	3099.05026	4766.2636	8327.247
*	39.2	0	M+H	C14H20N2O	0.31293423	1525.11383	1535.67973	1578.23115	262.112952	1898.7995
*	39.2	0	M+H-H2O	C8H8O3	0.82431994	2068.80901	2256.78919	2645.36198	2952.31757	1781.52404
*	39.2	0	M+H-H2O	C10H16O	0.75696431	24430.0861	25973.5228	29601.178	28091.4032	17221.9283
*	39.2	0	M+H-H2O	C8H8O5	1.08682906	9057.22507	8944.51316	8911.18681	10605.4875	6386.40457
*	39.2	0	M-H2O-H	C14H18N2O	-0.3826359	149.546047	169.919508	165.49826	116.523612	387.443044
*	39.2	0	2M-H	C10H10O2	-2.6100706	257.932062	358.413238	328.318885	33.6119754	36.5513957
*	39.2	0	M+Na	C9H18N2O2	0.31137665	563.41008	535.793861	649.32398	294.595873	241.132107
*	39.2	0	M+FA-H	C11H11F3N:	0.26664049	788.994603	966.574419	1059.91338	1024.88794	3150.99739
*	39.2	2.09	M+H, M+NH4	C8H14O2S	0.81560945	3841.37442	4037.84437	3891.89343	8454.0975	14661.1156
*	39.2	0	M+H-H2O	C18H24N2O	0.63610669	1223.68743	1140.13974	1179.08704	785.672517	332.721928
*	39.2	0	M+NH4	C13H19N6O	0.36752247	1320.96571	1346.07139	1375.78093	9.7531E-07	432.336297
*	39.2	0	M+Na	C10H16O4	0.15879555	10790.5628	11225.0902	11101.1548	14948.8737	9258.81165
*	39.2	0	M+K	C13H15NO3	0.84406057	720.554128	890.629536	759.417609	737.275764	122.235807
*	39.2	0	M+NH4	C9H9N5O3	0.91541848	4553.35822	4585.25203	4715.58736	6118.04606	3380.60883
*	39.2	0	M+FA-H	C5H8N6O	-0.5611625	12708.6271	12885.8747	12466.0074	12772.4318	10867.9118
*	39.2	0	M+NH4	C9H11N5O3	0.24233226	2513.43663	2214.5277	2593.26358	4121.54041	2098.39339
*	39.2	0	M+FA-H	C7H15O10P	-0.9763692	1326.50932	1599.7621	1593.48479	1517.71854	1276.65527
*	39.2	12.3	M+NH4	C18H23N2S	4.19858425	12961.8724	13221.3323	12704.0636	21766.2799	6601.8036
*	39.2	0	M+NH4, M+N	C8H9N5O3	-0.3564648	3151.7013	3454.08722	3268.96495	4161.44793	2915.45595
*	39.2	0	M+H, M+Na	C13H24N2O	-0.5527539	1287.39773	1886.57328	1518.18274	2.10550261	1717.92905
*	39.2	0	M+H	C14H22O2	-0.8728644	6251.43114	6441.14664	6304.20912	7699.96883	3846.87879
*	39.2	0	M+K	C21H38O14	-0.608118	3401.94812	3469.97055	3291.10471	2985.30914	381.014863
*	39.2	0.904	M+H, M+NH4	C15H32O6	-1.5313405	339624.854	337420.301	342826.698	326933.196	541326.9
*	39.2	0	M+FA-H	C15H18O10	0.85239074	2531.05588	2855.2264	2780.38563	3171.55571	1263.39825
*	39.2	0	M+NH4	C15H27N5O	-0.2400093	6464.95265	6622.66217	6863.71381	4445.20255	4130.54424
*	39.2	0	M+FA-H	C11H19NO	-1.0264289	329.07392	303.207967	364.229528	235.738333	448.045714
*	39.2	0.00519	2M-H	C4H6N2O3S	2.64113348	78736.3996	79289.9115	81309.0722	80711.3269	71940.2131
*	39.2	0	2M-H	C13H16O3	1.58861353	154.171107	179.845425	231.520699	884.761766	110.052525
*	39.2	0	M+FA-H	C19H30O3	-0.3019444	274.87532	252.061403	168.112122	9.7531E-07	2045.57961
*	39.2	24.5	M-H	C7H5ClO3	-1.5775452	838.008785	1029.66258	956.043195	507.159619	637.518234
*	39.2	0	M+NH4	C11H18O	-0.4816671	205.272666	248.944778	214.125941	387.699412	286.109706

*	39.2	0	M+NH4	C9H11N5O2	-0.3535657	1093.22189	1110.30591	1156.6672	2053.6338	878.749923
*	39.2	0.0382	M+K	C21H28O6	0.97839445	8210.85063	9192.44848	8227.92686	7950.16582	2902.50618
*	39.2	0	M-H	C16H31NO4	-0.39945	659.039085	833.1493	819.733139	1518.26335	1531.48771
*	39.2	0	M+FA-H	C15H16N2O	-0.1404023	760.292595	685.254281	830.139778	462.447969	0.2178541
*	39.2	0	M+NH4	C25H41O7P	-0.5627672	16590.4074	11691.1965	13345.5595	26634.9495	10329.5448
*	39.2	7.24	M+K	C12H12N2	1.158709	16337.6835	19037.2716	20422.95	15927.356	9280.64128
*	39.2	0	M+H	C16H30O2	-1.5168094	2005.25146	2000.82616	2116.47844	2545.80852	1680.53777
*	39.2	0	M+H, M+Na	C22H28O10	-0.7810362	24987.3844	26814.1615	26559.045	53913.2039	8562.56772
*	39.2	0	M+K	C7H20N4	1.89005933	2490.14031	2470.64491	2275.40719	2992.61572	1899.93441
*	39.2	0	M+FA-H	C5H12O9P2	-0.7725208	2703.66357	2436.32797	2909.59918	2403.24173	2505.79246
*	39.2	0	M+FA-H	C16H14N2O	-0.3288539	2592.747	2432.70785	2959.67132	1976.54828	4887.93712
*	39.2	0	2M-H	C2H4N2O6	1.72966408	4154.01595	4241.63901	4315.25681	4707.06357	4236.76877
*	39.2	0	M+FA-H	C10H18O	-1.3690587	17741.1036	18162.6864	17216.9424	16905.2204	13880.4947
*	39.2	0	M+H	C11H18N2	-1.0667138	344.866416	400.069387	407.927533	73.6401639	9.7531E-07
*	39.2	0	M+NH4	C12H15N5O	0.0415488	2202.15132	2146.39998	2315.03121	7837.32204	1716.84725
*	39.2	0	M+FA-H	C15H28O	-0.3663584	1977.26076	2004.43571	1815.69387	1060.55092	4539.44712
*	39.2	25.3	M+FA-H	C15H14ClF3	3.035368	15504.795	16083.4662	15456.3764	41674.6566	9.7531E-07
*	39.2	11.6	M+H	C21H32N6O	2.20117635	2865.56449	2824.50714	4291.30019	4843.86528	3389.89061
*	39.2	1.01	M+H-H2O, M	C15H22O6	-1.5206473	29834.1701	27340.932	30535.9751	24479.4525	22423.9177
*	39.2	0	M+NH4	C20H29N5O	-0.3633346	2480.87585	2645.07942	2423.32538	637.699579	443.775121
*	39.2	0	M+K	C12H30N4	2.28242639	27721.7518	27903.2101	27664.5755	38943.733	21956.9552
*	39.1	0	M-H	C7H16O13P	-0.476991	2588.50295	2717.64104	2181.1705	3321.47234	7085.37671
*	39.1	0	M+Na, M+K,	Na2O4S	0.70667213	674534.106	656893.572	679946.077	9451.48841	2055.3951
*	39.1	0	M-H	CH5O4P	-1.9905037	2351.19902	2590.98441	2938.44383	1917.58044	1980.42252
*	39.1	7.21	M+H-H2O, M	C12H12O2S	0.3910576	1446645.32	1304910.84	1516326.41	830717.92	1002359.28
*	39.1	0	M+NH4	C2H5N3O2	-0.5933042	1078.82784	1059.69421	1562.33519	2041.62333	9.7531E-07
*	39.1	0	M+H-H2O	C8H18N2O3	-0.3781865	54418.8505	53049.5487	54009.1402	154060.59	31783.4855
*	39.1	0	M+NH4	C10H12N4O	-1.0002745	156319.136	172024.714	166312.632	251010.985	181915.894
*	39.1	2.07	M+K	C13H14N4O	0.58930863	26274.3662	25395.4992	22615.1808	16432.9767	73342.0204
*	39.1	0	M+NH4	C7H7O5P	0.03628515	7349.44008	8593.54684	7205.47309	15657.6183	8957.82647
*	39.1	45.3	M+FA-H	C12H15Cl2N	-1.2022602	15881.2174	17081.5576	16552.2969	63602.8968	45346.1507
*	39.1	0	M+Na	C4F10	-3.6577802	3967.48066	3198.87822	3518.83302	7530.69071	855.739146
*	39.1	0	M-H	C6F14	0.11460367	2481.77694	2453.19351	2756.23087	7238.55572	8801.44617

*	39.1	8.96	M-H2O-H	C12H6N4	1.31247013	679903.099	744022.559	627020.724	674724.775	808351.538
*	39.1	0	M-H	C18H26O19	0.77799231	69304.1387	61540.0554	64994.8409	111529.153	104264.275
*	39.1	8.65	M-H2O-H, M	C26H32O13	1.85636263	25648.2956	28659.9767	27633.2835	1952.0226	99151.45
*	39.1	0	2M-H	C6H13O10P	1.68634601	6945.28221	8933.96019	7525.47774	3716.84366	1580.06128
**	39.1	0	M+H	C10H14N5O	-0.7376337	1924.17799	1676.58471	2276.45268	9.7531E-07	3049.41262
*	39.1	0	M-H	C12H16O12I	-1.3984564	9699.01551	8248.39067	8950.31401	3814.3947	8581.78561
*	39.1	0	M-H	C15H29O10I	0.6565899	3532.31426	3480.14158	4798.96494	6266.79698	10411.6558
*	39.1	0	M+NH4	C10H7NO	0.25150498	1507.98396	1561.68339	1712.11179	2165.00248	4458.89773
*	39.1	0	M+K	C3H8NO+	0.61960805	795.767492	791.675552	831.192319	833.338997	789.736542
*	39.1	0	M-H2O-H	C14H24N2O	-0.0993769	385.571494	312.173623	299.954788	5593.36042	1126.04018
*	39.1	0	M+H	C11H14O	0.4709208	16206.7305	16906.3374	18847.083	20485.9465	12028.3559
*	39.1	3.1	M+K	C29H46O	-1.3561982	24379.79	25255.6195	24871.6141	32669.9036	13141.7259
*	39.1	0	M-H2O-H	C32H58NO1	0.13752131	2738.58094	2316.09473	1797.74285	1034.09152	8707.50494
*	39.1	0	M+FA-H	C43H78NO8	-0.1498521	436293.443	625771.061	746179.312	673022.333	562098.409
*	39.1	1.39	M+H, M+Na	C19H36O5	-1.518438	112493.191	117162.939	115936.365	173016.745	74299.3315
*	39.1	0	M+NH4	C24H48O3	-0.5982168	3763.50442	3966.71602	4744.51395	159.220795	9.7531E-07
*	39.1	0	M+Na	C14H30O3	-0.1592554	20205.9983	20176.3474	19231.8871	24842.6642	14097.4384
*	39.1	0	M-H, M+FA-I	C29H56NO9	0.05640837	48331.204	48446.6377	43899.5302	20775.7975	111990.759
*	39.1	0	M+NH4	C6H10N2	-0.0880438	863.535151	733.93298	863.599805	1855.08666	1288.5157
*	39.1	0	M+FA-H	C21H36O	-0.54063	735.995553	644.063285	584.068125	656.615076	121.08004
*	39.1	20.7	M+H-H2O	C48H85NO8	0.44203621	725558.604	775959.27	710701.791	582408.621	371275.998
*	39.1	0	M+H	C33H64NO9	-0.1761568	10231.7106	12430.679	11998.4674	6077.88845	5010.37435
*	39.1	0.00777	M+FA-H	C22H30N2O	-2.0979077	56463.533	63467.315	48442.7448	45716.4569	44873.1041
*	39.1	0	M+FA-H	C21H40O4	-0.9844834	245259.75	231753.806	239843.406	104799.722	33369.8956
*	39.1	1.37	M+NH4, M+I	C20H42O4	-1.5945627	140670.021	145577.623	146681.818	287369.877	147666.751
*	39.1	0	M+Na	NNaO3	3.0971662	11792.1337	12419.8728	12651.5295	26459.1008	16933.7514
*	39.1	0	2M-H	C12H10N2O	-2.6697965	845.135682	1260.86651	895.971031	690.651865	2004.19998
*	39.1	0	M+NH4	C16H25N5O	-0.4032346	6637.14188	6423.56649	5884.62618	14991.8596	8181.04222
*	39.1	0	M+FA-H	C22H23FN4	1.11271295	20358.409	22562.9782	25903.0922	30599.239	17742.1259
*	39.1	0	M+FA-H	C21H42O4	-0.8302928	22293.881	21385.7149	19473.4571	13757.9048	6581.80315
*	39.1	0	M-H	C23H27FN4(	0.72659071	842.061232	1065.88319	1274.04684	1565.85179	1005.52291
*	39.1	0.85	M+NH4	C26H52O4	-1.3281359	45386.0193	43249.5231	43182.0452	9.7531E-07	9.7531E-07
*	39.1	0	2M-H	C4H7O7P	1.66656164	4909.52804	4396.9678	4063.21298	4608.40787	4006.84025

*	39.1	0.224	M-H, M+FA-I	C10H11N5O	0.02956702	3842.47933	4093.78527	3707.49433	5431.92434	9846.89492
*	39.1	0	M+Na	C7H10N2O2	0.45632373	752.104438	701.864258	610.781505	1357.99883	422.810646
*	39.1	0	M+Na	C10H14N2O	-0.1498594	1369.55536	1313.33192	1213.19978	1871.68621	1237.42715
*	39.1	20.6	M+NH4	C10H16O3	0.42120841	4997.86514	5016.79288	5402.80546	3666.71722	2451.52918
*	39.1	0	M+Na	C10H13NO3	0.29022257	983.85848	947.910547	784.802593	613.91573	1373.17661
*	39.1	0	M+FA-H	C13H16N2O	-0.8810531	2190.78515	2165.75257	2185.34254	870.687266	5457.32097
*	39.1	0	M+H	C9H12O	1.00959006	14013.1792	15500.4623	16502.6183	18269.5295	11568.2699
*	39.1	0	M+H-H2O	C25H29F2N3	-0.2196934	2865.66065	3252.15101	2922.65677	6662.10378	3752.49466
*	39.1	9.93	M+H	C23H27NO3	0.58181036	4893.5175	4895.56982	5154.94231	1137.06013	2990.40661
*	39.1	0	M+H, M+NH4	C13H12N2O	0.61916976	6127.50336	6404.82834	6460.37352	802.890939	19578.6498
*	39.1	0	M+NH4	C14H16O3	0.91507879	549.448788	552.462656	624.289163	9.7531E-07	211.603378
*	39.1	0	M+H-H2O	C9H10FNO4	0.67873488	1424.38277	1390.27426	1270.48429	691.743557	1263.64018
*	39.1	0.629	M+H-H2O, M	C10H21N3O	0.70334455	4366.73664	4168.60792	4347.84305	4395.74747	3343.63825
*	39.1	8.6	M+H-H2O	C30H48O11	-3.002618	2842.6437	2751.27209	2895.00171	16.4779826	9.7531E-07
*	39.1	0	M+FA-H	C5H5N3	-2.0257399	381.566218	345.553136	349.625153	389.417104	360.096767
*	39.1	0	M+FA-H	C14H13N3O	0.16067528	198.356216	251.566017	238.714967	9.7531E-07	0.032321
*	39.1	0	M+H	C11H11N	1.0293257	988.27766	1270.21546	1156.27323	1410.66321	793.456659
*	39.1	0	M+NH4	C7H9N5O2	1.41330698	5458.31329	5591.31581	6144.90828	7686.49623	4507.71767
*	39.1	0	M-H2O-H	C3H3F5O	-1.2619086	6948.32021	7089.62013	7165.69346	6848.51831	6118.56862
*	39.1	6.86	M-H2O-H	C16H10O6	2.43889962	116.6604	94.3870032	76.3432185	64.8972395	577.826241
*	39.1	0	M-H2O-H	C17H33IO2	1.75432046	5347.76527	6069.38684	6329.91367	5487.76391	5900.61742
*	39.1	0	M+H-H2O, M	C10H10O5	1.20042049	364.785368	478.402789	451.936344	92.9591338	141.112371
*	39.1	0	M-H	C4HF7O2	-0.6484455	3496.7861	3439.99049	3531.17874	3017.40303	3051.77617
*	39.1	0	M+FA-H	C6HF5	-1.0472594	3423.4783	3369.76957	3450.83817	3116.79755	2947.94331
*	39.1	0	2M-H	C4H5N3O2	0.85185735	821.464777	838.533658	733.248034	311.11602	293.459077
*	39.1	0	M+FA-H	C14H20O6	-0.3766168	508.27539	518.168126	429.841114	52.6967251	2259.97509
*	39.1	0	M+H-H2O	C16H31IO	2.27006093	1107.40297	1045.22815	1159.41019	2062.57113	180.867315
*	39.1	0	M+FA-H	C5H5NS	-1.0888156	3225.16	3381.76475	3330.53722	5464.8121	6851.60429
*	39.1	0	M+FA-H	C11H20O4	-0.5883557	1774.65229	1886.43841	1830.62869	1870.1736	1379.70588
*	39.1	0	M+FA-H	C22H29FO6	0.08900566	792.384858	643.555188	760.696242	2101.3356	9.7531E-07
*	39.1	0	M+FA-H	C9H17NO	-1.2925205	625.370239	689.989986	673.533487	273.203359	537.285168
*	39.1	0	M+FA-H	C7H14FO2P	-0.7743576	1294.02724	1410.97481	1317.92387	725.194962	438.86485
*	39.1	0	M+Na	C27H41O7P	0.31042942	1644.16681	1680.16077	1657.36671	913.027851	8661.74272

*	39.1	1.47	M+H, M+Na	C10H15NO5	-1.0637136	40345.2027	39916.2186	40081.9535	37886.7894	95260.5297
*	39.1	0	M-H	C20H34O7	0.20241143	316.250576	364.258923	374.324295	9.7531E-07	297.242867
*	39.1	0	M+FA-H	C8H16O2	-2.1607019	603.871316	641.227186	584.62121	320.612928	451.84113
*	39.1	3.07	M+H-H2O	C8H14N2O6	-3.9153439	2248.54591	2430.29668	2356.67974	2075.3246	1964.37377
*	39.1	0	M+FA-H	C19H33NO7	-0.0434224	714.810223	884.861824	829.619781	9.7531E-07	542.99637
*	39.1	0	M+H	C12H18O2	-0.4016285	2941.38908	3151.04798	2985.46222	3868.52808	2365.06443
*	39.1	0	M+H	C15H14N2O	-0.6975005	586.039274	727.295625	605.096304	1050.85834	555.427682
*	39.1	0	2M-H	C10H16O3	-0.1185591	1474.94782	1383.79778	1279.70266	9.7531E-07	1275.46078
*	39.1	0	M+FA-H	C10H14O	0.18973805	1245.39413	1255.9213	1468.41822	1230.41002	1082.55857
*	39.1	0	M+K	C9H24N4	2.98619578	2306.1761	2368.26842	2353.33378	3349.81943	1868.94375
*	39.1	0	M+H	C9H12N6O	-0.9257335	1245.59065	1425.07471	1105.64896	1480.42183	1195.6722
*	39.1	0	M+FA-H	C12H14O3	-0.7070715	2640.75696	2887.20856	2757.93172	2653.42481	2182.0368
*	39.1	0	M+NH4, M+N	C14H14N2	-1.0089865	3645.02873	4341.52864	4090.83773	7223.39604	2960.76708
*	39.1	0	M-H2O-H	C8H8O4	-1.3456246	471.562392	494.911153	497.128782	482.217093	421.774222
*	39.1	0.227	M-H	C9H16N6O3	0.74251487	5449.23023	5389.2689	5206.69517	6921.95275	12549.2842
*	39.1	0	M-H2O-H	C4H11NO4P	-2.8668829	462.229837	455.696313	501.053175	567.648879	415.31427
*	39.1	0	M+H	C33H34N4O	-1.0747027	6447.91782	6499.85078	6293.78547	12029.5784	1329.56773
*	39.1	0	M+Na	C19H36O5	-0.4643714	710.493125	1029.30096	787.991534	346.518196	391.172045
*	39.1	0	M+FA-H	C11H20O	-0.9603979	26533.8404	28112.908	27228.8249	26161.9448	23543.147
*	39.1	0	M+H	C12H14O4	-0.6317989	1839.93606	1880.70589	2038.65743	2208.85598	1311.56938
*	39.1	0	M+NH4	C16H28O	-1.8978394	2370.23264	2285.0504	2297.53495	3319.2174	1246.45962
*	39.1	0	2M-H	C9H4N4O4	1.7193938	1746.43564	1152.41961	1649.05449	1062.44378	1398.83387
*	39.1	0	M+NH4	C15H16O3	-1.6433767	1177.97708	1448.80067	1200.81023	807.209398	1329.20021
*	39.1	0	M+NH4	C14H19N5O	-0.2344761	25403.0638	24548.5729	24919.4484	34786.8598	19261.0649
*	39.1	0	M+FA-H	C6H12O	-1.8476812	463.298491	571.81102	532.28261	548.715875	416.111898
*	39.1	1.13	M+H	C22H43NO5	-1.2725635	2770.59708	2883.58464	2567.97822	1006.16124	8765.68023
*	39.1	0	M+Na	C18H39NO2	-1.5372047	2466.09714	2050.99133	2642.20058	1078.45945	2273.44582
*	39.1	0	M+Na	C8H23O7P	-1.5312973	1170.5418	967.543678	1258.64457	1685.74543	791.450028
*	39.1	0	2M-H	C10H9O6P	0.7958948	2726.39314	1975.33159	1990.8678	2171.93372	3264.64049
*	39.1	0	M-H2O-H, M	C8H5F13O	-1.1843632	12194.9938	13432.7401	13337.4912	11162.8854	10763.376
*	39	0	M-H	C18H34N2O	0.02737274	11909.4431	11898.5437	12818.6208	13647.6034	5927.11711
**	39	20.1	M-H2O-H, M	C5H8O4	-1.5233856	21393.091	23077.3547	22187.0912	17670.4588	19276.3373
*	39	0	2M-H	C5H13N2O6	0.82441938	4168.36428	2739.75274	4752.6237	17945.17	10979.4982

*	39	0	M-H2O-H	C19H14F3N:	0.16502908	4781.69153	4268.88977	6063.58179	3068.8011	11416.8609
*	39	0	2M-H	C6H5N3O6	-1.4843742	8879.10609	5756.59898	7355.11476	2716.36852	9.7531E-07
*	39	0	2M-H	C17H16O4	2.22231958	1532.12634	1457.3184	1301.30273	650.408186	38100.2282
*	39	0	M+FA-H	C12H15NO4	-0.5366376	288.185701	271.272037	247.724148	318.848757	9.7531E-07
*	39	0	2M-H	C6H2FN3O3	3.49254712	702.110504	581.239775	639.667534	1723.48621	3753.45474
*	39	0	M+K	C5H15NO7P	0.74004897	1273.4926	1504.18952	1465.25247	1713.75239	330.501479
*	39	0	2M-H	C10H9O4P	-3.3138837	1335.97939	1154.03504	1845.79052	1165.16226	941.997306
*	39	0	M-H2O-H	C18H26N5O	0.5040632	7191.53151	7010.83686	7582.01809	850.054306	23409.1343
*	39	3.81	2M-H	C6H12N2O4	-2.6119674	29887.2934	28123.3453	26352.3611	33180.3383	39438.2287
*	39	6.1	2M-H	C7H14O7	-4.9644389	29113.828	28364.9357	28426.2723	35584.2626	34196.3692
*	39	0	M-H	C13H23N4O	-0.8171277	5389.92447	4479.99379	4982.84226	6584.30875	6823.33243
*	39	2.41	M+H	C5H9N3O4	-4.7541018	142170.065	132936.957	125129.158	220808.476	134987.613
*	39	0	M+NH4	C10H13NO	1.17753462	156.414527	159.139545	141.870811	91.8632977	26.1031434
*	39	0	M+H-H2O	C10H12N4O	0.1548761	1717.02392	1958.94905	1758.36678	3539.4878	98.4293846
*	39	0	M-H	C15H23N3O	0.40609042	2590.44356	2632.52454	2580.3937	1846.98893	7748.97696
*	39	0	M+H	C3H7O7P	-0.083651	547.899636	631.149578	555.776954	1337.00317	810.194562
*	39	0	M+H-H2O	C21H18FN5(	-0.8834267	2231.87471	2523.76526	1977.2355	127.765739	10845.0481
*	39	0	M+Na	C18H31NO2	-1.6799962	4633.63299	4498.59532	5072.71988	2973.69011	2226.17429
*	39	0	M+FA-H	C11H15NO	-1.2625391	1222.87569	1189.20019	1030.94795	79.4904199	2230.04388
*	39	0	M+FA-H	C14H26O	-0.5962041	43679.4685	44990.1259	44458.3671	41804.9778	33349.5674
*	39	0	M+FA-H	C9H16O2	-2.2056814	332.910689	250.543119	273.67954	243.204297	117.165899
*	39	1.67	M+FA-H	C26H52NO9	0.05975933	8091.10622	8656.66199	8078.08538	335.786709	26332.8789
*	39	4.63	2M-H	C9H18N2O4	0.84928246	3201.63336	2980.36098	2393.93442	908.547305	1640.72995
*	39	0	M+H	C12H18O	-1.0405361	1709.64189	1762.71782	1683.55535	1422.72504	1096.08733
*	39	12.4	M-H, M+FA-I	C22H34O3	8.9447653	102623.201	95817.699	92965.4932	81829.8841	67588.5604
*	39	0	M+K	C15H29NO5	-2.0752387	6572.65246	6408.90095	5450.23533	8636.30065	4526.95928
*	39	0	M+NH4	C18H28O	-0.9135107	2843.3886	2711.87183	2800.99753	113.510751	1199.09792
*	39	14.3	M+H	C27H44NO7	-9.7455949	62949.8453	61505.392	61709.4008	44922.166	87925.6579
*	39	0	M+NH4	C15H18O	-1.5666723	3543.38758	3786.30509	3427.83999	5152.30285	2953.35363
*	39	0	M+H	C24H43O9P	-0.4550674	6218.0096	5257.9666	5464.42611	2710.09244	1770.82194
*	39	17.3	M+NH4, M+N	C24H40O5	6.00666675	61619.1617	53872.4348	58372.9113	2397.87332	82599.6795
*	39	15.6	M+K	C42H81N2O	4.69653988	133465.154	166403.246	144165.82	144892.638	188894.531
*	39	0	M+FA-H	C22H39NO5	-1.1875033	929.974746	950.660429	1483.90261	399.629149	9.7531E-07

*	39	0.481	M+NH4, M+N	C24H48O6	-1.3511473	163800.111	158833.186	166846.448	1315.38539	1839.80276
*	39	0	M-H2O-H	C21H32N6O	-0.1586123	21897.2263	24924.9004	20320.2736	32943.9383	12654.345
*	39	0	M-H	C24H31NO4	-0.7639013	1020.97901	1018.29226	1012.86341	268.33916	9.7531E-07
*	39	0	M+FA-H	C23H44	-1.1701503	37844.2883	34027.5961	21468.0091	34667.8851	30054.9643
*	39	3.63	M+H	C22H42O4	-1.1425241	221819.341	219092.785	206476.351	272927.166	179752.939
*	39	7.39	M-H	C27H46O4S	-0.6664105	241828.301	214190.73	191333.759	97216.8744	187641.35
*	39	0	M+NH4	C10H7NO4	-0.7770656	736.605208	488.651818	724.3556	817.562055	2588.62958
*	39	0	M-H2O-H	C10H14N2N	-1.0780714	21265.0891	24333.2217	23035.6946	26311.2734	27443.5113
*	39	0	M+FA-H	C6H13N3O8	2.47849267	2141.62602	1768.68401	2162.98754	1995.3071	1756.7853
*	39	0	M+FA-H	C10H7NO2	-1.3442177	155.790707	191.705877	130.034833	270.655114	21.0005273
*	39	0	M+H	C12H21N3O	0.22789991	1835.62515	1980.5049	1917.73211	572.624905	8132.89273
*	39	0	M+NH4	C12H13NO4	-0.2551015	826.630342	765.981609	672.515264	117.30879	1082.24651
*	39	0	M+NH4	C15H14N2O	0.56185064	3831.69732	3898.32061	3962.77025	1805.50559	8563.18878
*	39	0	M+FA-H	C14H21NO9	-0.3196715	3327.92666	3315.10466	2872.1119	3153.49384	2780.70755
*	39	0	M+K	C17H31N3O	1.46131551	1396.08842	1206.41579	1121.89112	505.812068	534.625611
*	39	1.73	M+FA-H	C6H6OS	-1.5905519	2628.07443	2597.35934	2610.66145	1856.48249	2000.89506
*	39	0	M+FA-H	C15H17N3O	-0.0557688	2052.81281	1871.66286	1797.93198	431.883557	4509.29593
*	39	0	M+FA-H	C9H12N5OP	-0.1612102	3048.63895	2404.03112	2747.46827	2701.33019	3263.16291
*	39	0	M+FA-H	C11H14NO6	0.48446953	2205.00483	2348.87856	2282.88697	1186.71014	6479.38222
*	39	0	2M-H	C10H8O2	-2.6486347	357.833035	397.614566	417.135541	274.181517	630.124538
*	39	0	M-H	C3H3IO2	-1.2282079	4271.76365	4372.64422	4313.40585	4394.55718	3548.16729
*	39	0	M+K	C30H52N4O	-0.6591039	7573.01074	7359.95007	7769.67358	5679.35665	23728.1783
*	39	0	M-H	C6H13NO2	-1.9610015	362.015227	315.391954	366.152708	363.038013	403.450243
*	39	0	M+K	C25H40N4O	0.46335759	3149.26614	3435.60012	2933.95507	2681.96485	1697.53678
*	39	0	2M-H	C10H8O	-2.5132441	1610.48225	1522.6169	1627.51284	2059.20937	4316.29548
*	39	0	M+FA-H	C17H23N3O	0.98430368	694.864572	631.569097	617.784745	9.7531E-07	334.010039
*	39	1.29	M+FA-H	C4H4N6O	-0.3831898	2416.44037	2637.21328	2418.60356	1848.75124	2474.61276
*	39	0	M+H	C21H27N5O	0.89077709	13464.8981	14552.2298	13310.5114	26714.2411	16699.1137
*	39	0	M+Na	C20H14O4	0.1145246	51.7230291	66.1925259	79.5956219	1201.62248	158.225623
*	39	0	M+K	C14H17NO2	1.09161017	1660.70024	1662.39731	1684.3253	392.794397	66.1457618
*	39	0	M+NH4	C9H19I	-2.6014166	523.825943	489.109905	477.647482	126.722134	33.0271123
*	39	0	2M-H	C6H6N4O2	1.25923775	982.328358	921.701732	982.27144	1831.44894	475.395095
*	39	0	M+FA-H	C12H18O12	1.13950433	442.876232	456.783903	362.653938	902.577495	273.265584

*	39	0	M+Na	C18H29NO3	-0.3816006	2278.43959	1842.09082	2167.7898	4459.17317	1942.39096
*	39	0	M+FA-H	C8H9N	-1.5344266	260.702323	227.738247	244.354863	96.0780733	139.114107
*	39	0	M+H, M+Na	C19H27NO3	-1.9947779	1222.39664	799.924115	874.110042	112.257239	357.161742
*	39	0	2M-H	C7H6N2S	0.50469391	3252.34355	3177.27309	3082.81289	7982.26296	670.956973
*	39	2.59	M-H, M+FA-H	C9H6N2O7	-0.6888408	9628.5206	9671.63753	10236.2611	7568.36031	8461.54403
*	39	0	M+H, M+Na	C20H27N3O	1.14725272	8756.71488	9157.97623	8423.94062	17674.8213	12827.5653
*	39	0	M-H2O-H	C32H63O10I	-2.2650165	24213.2138	22982.7184	24291.3032	56627.5369	22736.6658
*	39	0	M+NH4	C17H19NO4	0.00874311	412.97186	384.219355	476.645607	29.6432772	761.723282
*	39	0	M+Na	C9H19NO2	-0.0139067	1198.36083	1298.09511	1087.08891	1434.95469	977.620017
*	39	0	M+K	C17H22N2O	-0.011996	712.617584	1254.22663	1128.19588	1361.29469	0.61083649
*	39	1.13	2M-H	C16H15N	3.94676959	3209.85648	3035.94494	2872.07887	202.252906	62.692034
*	39	0	M+FA-H	C12H15NO2	-0.6302322	194.634062	192.265226	236.957777	683.984695	890.817091
*	39	0	2M-H	C6H7O3-	-0.731645	789.726442	837.299033	826.236544	606.342709	730.338882
*	39	0	M+NH4	C10F20O5	-1.5469054	5530.16762	6113.83577	7485.34873	6627.20574	5501.76143
*	39	4.03	M+FA-H	C9H8N2O7	-3.372764	6211.10841	5557.74613	6271.84809	4674.93105	1965.36446
*	39	0	2M-H	C10H8N4O4	-0.4226807	702.367244	710.148357	662.457645	163.193287	9.7531E-07
*	39	0	M+NH4	C21H21FN6	1.46588393	863.137333	859.309729	771.207006	9.7531E-07	767.641016
*	39	1.49	M+H, M+Na	C12H12N2O	-1.6197567	636.715744	787.637092	788.364131	41.4060269	467.881718
*	39	0	2M-H	C5H9NO5	-2.7887965	1591.38746	1656.20993	1662.95295	1478.84345	1576.91985
*	39	2.41	M+FA-H	C8H12N2O6	-4.3126077	3696.68899	3827.03816	3830.11399	3043.82608	3604.77709
*	39	0	M+NH4	C7H7N5O	0.24981245	7281.42462	7262.27093	7029.12844	10154.8151	5928.30944
*	39	0	2M-H	C13H10N2O	-1.3640696	674.106989	873.933758	514.15448	466.620534	91.8811781
*	39	0	M+NH4	C16H13NO4	0.00539596	947.752064	959.602699	998.776327	873.144813	296.330186
*	39	0	M+FA-H	C10H17NO	-0.9727831	174.980619	193.584768	160.497847	46.2661898	194.814226
*	39	0	M+H-H2O	C20H33NO4	-1.2555683	551.381619	652.372843	676.168796	9.7531E-07	4784.11454
*	39	0	M+K	C14H13NO2	-0.1032058	179.907135	199.633457	157.088276	9.7531E-07	9.7531E-07
*	39	0	M+FA-H	C16H18N2O	-0.0714149	486.484708	678.907224	542.561737	357.790829	276.854813
*	39	0	M+NH4	C7H5N5O2	-0.0185804	1894.68221	1832.18131	1784.92198	2481.47372	1469.72776
*	39	0	M+Na	C16H30O7	-1.2354849	288.68167	390.964596	314.904473	151.524549	39.4178535
*	39	14.3	M-H	C36H30O7	-2.1701022	3808.40603	3658.23444	3803.48361	9.7531E-07	2679.58099
*	39	0	M+FA-H	C12H22O2	-1.4655932	917.79022	651.142642	865.312985	890.814153	739.652086
*	39	0	M-H	C21H29NO8	0.00888532	5974.51501	6347.78747	6851.94628	4493.20815	25879.8608
*	39	0	M+FA-H	C23H20O11	-0.2614818	4862.54283	4800.07755	5444.31894	5896.36088	4710.08235

*	39	0	2M-H	C9H12O4	-0.3958245	964.383053	790.268423	705.326464	287.681064	312.347928
*	39	0	M+FA-H	C21H34O5	-0.4996636	761.069132	778.651269	732.329472	470.735448	1493.63181
*	39	0	M+Na	C20H36O7	-0.6802204	788.307837	638.591812	851.17104	317.097404	5065.96763
*	39	0	M+FA-H	C7H12O2	-2.3486185	1898.9	1969.93315	1904.26361	2173.61356	1815.8239
*	39	0	2M-H	C16H11N3O	2.25119057	89575.774	90765.3565	93217.6277	210693.667	36943.5975
*	39	0	M+FA-H	C17H22O5	-0.3945834	808.772861	812.649747	788.389404	415.353592	9.7531E-07
*	39	8.89	M-H2O-H	C27H34F2O	-2.5873669	1038.44449	714.668201	833.175682	9.7531E-07	7564.64249
*	39	0	M+FA-H	C27H49N3O	0.22138099	2983.0073	3807.07082	3935.87299	152.135117	4084.88344
*	39	0	M+FA-H	C19H23N2O	0.66647888	625.504892	1095.84234	778.129986	105.916981	9.7531E-07
*	39	0	M-H2O-H	C8H24N4O3	-1.8575861	635.716387	727.04602	636.180949	947.861002	645.278885
*	39	0	M+NH4	C11H13N5O	-0.1022186	13076.3265	12972.773	13656.0604	18021.1963	10847.7669
*	39	0	M+Na	C15H12O	-0.3340331	523.078275	449.145429	492.815217	706.538816	294.572218
*	39	0	M-H2O-H	C9H18O2	-1.2292247	314.451472	409.771932	317.668978	91.0200851	153.026183
*	39	0	M-H2O-H	C11H14N6O	1.17444568	8655.48503	7815.09749	7956.13155	3641.18675	2809.26259
*	39	0	2M-H	C7H4N2O6	2.8647091	77089.0417	75088.6017	77052.5809	73286.4268	65106.953
*	39	0	2M-H	C6H8O9	2.18746984	71803.6128	74783.4237	73794.3504	75203.5746	68506.4458
*	39	0	M+FA-H	C8H16O	-1.8363063	482.865774	565.497057	526.276867	692.567431	405.904496
*	39	0	M+NH4	C20H27N5O	-0.592841	5785.04569	6405.2132	5850.77237	6954.61309	5615.61108
*	39	0	M+Na	C21H38O6	0.21125128	243.964468	432.953174	306.6771	9.7531E-07	9.7531E-07
*	39	0	M+Na	C16H30O3	-0.9370534	35965.7711	36605.7661	37287.3236	54865.496	31192.764
*	39	0	M+FA-H	C17H28O2	0.20163657	576.345847	461.020753	519.683751	174.933404	244.894408
*	39	0	M+H	C6H12O5	-0.7524321	1914.55717	1696.48546	1793.36475	1484.56207	1211.95094
*	39	0	M+H-H2O	C11H17N5O	-2.74445	638.429159	846.59161	493.759732	1560.75079	139.278513
*	39	0.926	M+NH4	C21H34	-1.3062841	10583.5711	10994.2974	10673.64	10535.9797	3575.12729
*	38.9	0	M+H-H2O	C24H19NO4	1.13753893	2048.51028	1206.62946	1632.26227	4332.36258	1465.31225
*	38.9	0	M+K	C23H17FN6	-1.4497396	2865.04846	3864.79899	3371.75377	7319.71375	2486.46634
*	38.9	0	2M-H	C4H6OS	-0.9171557	10071.0114	10495.1496	9018.92275	16036.893	10556.7611
*	38.9	0	M+FA-H	C7F14	4.11629966	2290.45716	2525.4943	1944.37806	5648.86646	32.2641124
*	38.9	0	M-H2O-H	C6H11O6PS	-0.7883044	2254.14407	1999.42745	1922.55378	2206.76438	2973.45724
*	38.9	0	M+FA-H	C2H3NO	-2.1271664	96.1752083	143.443554	126.473441	126.809089	89.1085098
*	38.9	0	M+FA-H	C10H6FN2O	-3.6710619	4693.96889	3797.09874	3628.22727	4977.24258	3525.91171
*	38.9	0	M+FA-H	C13H13N5O	-0.4172836	5342.54451	5073.0467	5833.79645	6744.6859	4535.37503
*	38.9	0	M+H	C25H23FN2	1.36487608	10923.375	11698.729	8501.21253	9.7531E-07	9554.51837

*	38.9	0	M+FA-H	C12H22O11	0.70689016	1435.04641	1555.48919	1865.80807	9.7531E-07	5454.9955
*	38.9	0	M+Na	C16H20N8O	2.35990785	989.54671	827.570021	589.567496	9.7531E-07	7084.34147
*	38.9	0	M+Na	C5H9NO6	1.96220331	3208.77866	2419.70497	2675.30863	3780.96327	1590.30725
*	38.9	0	M-H	C5H2F6O2	-1.2626429	1993.016	2093.58602	1986.89102	237.406923	3340.88631
*	38.9	0	M+FA-H	C10H19NO7	0.01903286	1277.54377	1305.04081	994.01455	841.987385	4173.8297
*	38.9	0	M+H-H2O	C17H35AsO:	0.30455667	1532.13977	1792.50961	1354.42605	507.716562	2527.64053
*	38.9	0	M+H, M+Na	C14H19IN2C	0.45326747	8750.46553	8499.75257	8663.27931	6751.2955	24660.4822
*	38.9	0	M+FA-H	C14H21N3O	-0.6032853	812.868404	824.044473	998.760128	228.745781	2075.40382
*	38.9	0	M+FA-H	C2H5NO	-2.3751787	91.4281785	74.237443	95.9581703	133.08635	112.833805
*	38.9	0	M-H	C13H13F2N(	-0.3522205	713.990699	582.383345	716.627939	48.4723376	3360.32208
*	38.9	0	2M-H	C20H12O5	0.130557	607.386328	777.782548	883.174228	9.7531E-07	1005.53781
*	38.9	0.543	M+FA-H	C27H37N5O	2.21895273	137306.157	140084.318	136425.289	77717.982	97716.5565
*	38.9	2.19	M+NH4	C16H19NO5	-3.36405	1187.56753	1324.26037	1385.86623	757.601586	877.223804
*	38.9	0	M+NH4, M+I	C18H32O3	-1.2559875	56847.1925	56993.8317	57143.9022	103333.464	49490.1029
*	38.9	0	M+NH4	C16H32	-1.6038784	12410.5252	13040.7785	13674.975	17873.5862	8312.64592
*	38.9	0	2M-H	C11H12NO9	2.06435259	7705.5106	10230.7764	9780.22594	8624.14247	7895.24224
*	38.9	0	M+FA-H	C27H47NO4	0.3470002	3385.39835	3808.02444	3500.93858	264.331293	30673.3128
*	38.9	0.0508	M+NH4	C21H31F3O	-1.8950738	3313.28455	2944.13025	2995.07424	415.78883	2823.13273
*	38.9	0	2M-H	C22H21NO3	-1.3224908	13220.9786	13127.5661	13737.8264	7051.97824	20990.2875
*	38.9	0	M+H-H2O	C13H19NO6	3.54303077	3565.57814	3696.52555	3496.22968	2452.72927	6752.63442
*	38.9	0	M+H, M+Na	C21H39NO3	-1.375275	1673.0473	1963.39376	1346.20614	390.770879	544.509816
*	38.9	0	M+FA-H	C18H24N6O	0.15613375	852.394677	927.865512	1182.28374	407.113781	753.878846
*	38.9	0	M+FA-H	C17H34	-1.0923726	5160.55565	5046.25205	4788.85496	3615.81817	11628.5053
*	38.9	0	M+FA-H	C22H37NO5	-1.0189011	7496.3884	9580.79829	8843.72706	868.283232	0.10274445
*	38.9	0	M+NH4	C16H23N5O	-0.5825609	5298.61007	3698.24592	4344.27456	6976.0395	4078.66391
*	38.9	0.275	M+K	C17H30N6O	-1.3823582	86625.2752	69375.8201	84196.4231	119909.125	81027.4446
*	38.9	1.31	M+FA-H	C19H38O4	-1.0569599	256491.121	233996.678	231107.77	226093.905	70380.5896
*	38.9	0	M+FA-H	C24H22O4	1.49676514	14889.4914	13618.062	11614.2284	14567.5854	10858.1324
*	38.9	0	2M-H	C19H35O2-	-0.1207583	3137.57631	1872.1264	2671.6166	4761.229	1728.94584
*	38.9	0	M+NH4	C20H22N2O	-0.716319	107.305857	99.196391	82.2898899	92.0204212	55.0678165
*	38.9	8.08	M-H2O-H	C14H10N2O	2.4976648	1547.95128	1581.36145	1454.81613	3977.001	1602.39472
*	38.9	0	M-H	C19H20F3N:	-0.8372686	3693.57165	3787.65582	3613.91508	3768.0091	16125.8107
*	38.9	0	M+FA-H	C5H11N	-2.7125211	677.817875	575.066155	697.361676	661.221868	1058.97105

*	38.9	0	2M-H	C15H16NO4	2.30665574	462.464208	534.006814	441.695465	9.7531E-07	9.7531E-07
*	38.9	0	M+NH4	C10H14N4O	0.0617929	201.638523	181.382862	259.636763	4.88195381	26.4931392
*	38.9	0	M+K	C20H32N2O	1.28409861	532.921212	524.384399	543.387144	149.899644	163.287889
*	38.9	1.61	M+NH4	C18H32O10	3.81495625	7860.05052	6350.37409	7596.18641	9834.18401	22307.3971
*	38.9	0	M+FA-H	C2H5O5P	-1.8651065	622.715196	533.762555	514.426068	856.832911	928.493477
*	38.9	0	M-H2O-H	C14H16F3N:	0.23108462	519.364393	455.29479	510.679138	43.6691152	1622.54855
*	38.9	0	M+Na	C32H46FN5(	1.69677722	32872.9877	32225.8331	32958.1103	67846.3892	25971.0129
*	38.9	0	M+H-H2O	C8H7NO2	1.98900228	639.128397	536.747853	618.046753	1403.72907	974.218378
*	38.9	0	M-H2O-H	C9H16N5O1	-1.0005956	3490.91279	3988.45852	3841.06735	3383.07907	2354.32459
*	38.9	0	M+H	C20H42O7P	-1.0679449	5565.08308	5793.1032	6224.52076	5098.49889	9068.30276
*	38.9	0	M+NH4	C15H26N2O	0.25669577	972.872544	951.30505	823.139932	116.164749	2531.53058
*	38.9	11.1	M+NH4	C19H23N5O	-0.8220954	3093.05388	3440.6795	3070.70027	1237.90913	3445.33717
*	38.9	0	M+NH4	C10H15NO2	1.57497451	211.151571	207.455879	231.226474	9.7531E-07	186.683769
*	38.9	0	M+FA-H	C15H28N4O	0.19833184	819.841666	871.868461	825.882572	72.8597814	60.6744422
*	38.9	0	M+K	C14H16N2	0.7962316	229.193642	234.404934	315.600808	779.392477	70.4091601
*	38.9	6.8	M+NH4	C18H10O6	-2.3824588	1435.45955	1731.8349	1654.32579	1252.42912	144.153247
*	38.9	16.4	M+H-H2O	C36H44N6O	-1.8745864	3410.58202	2230.34521	2774.34223	4117.50219	1652.75733
*	38.9	5.4	M+NH4	C10H17N6O	-0.160683	7561.58278	5304.63157	8022.26843	1615.02563	561.460336
*	38.9	0	M-H2O-H	C7H10N2O2	-1.0021539	1206.80201	1154.44907	1150.9877	274.061995	231.511803
*	38.9	0	M+FA-H	C8H6N2S	-1.6248196	419.632902	434.320798	481.50072	14.9545686	17.9654535
*	38.9	0	M+H-H2O, M	C18H26O9	0.19125402	3960.78981	3228.52953	2998.39933	3544.09217	559.71015
*	38.9	0	M+H-H2O	C14H17FN4(	1.77524981	622.203734	667.560605	408.519815	374.606016	88.2120748
*	38.9	0	M+FA-H	C10H16O2	-1.164999	14181.4743	14625.6702	13573.7415	13436.3172	11871.5177
*	38.9	0	M-H	C5H4N4O2	-1.6264825	313.49639	264.474106	307.391544	307.113467	104.719585
*	38.9	0	M-H	C6H5N3	-1.2610351	559.163578	588.531625	491.167091	521.13096	486.396562
*	38.9	0	2M-H	C7H7O4-	-3.0013778	573.855536	515.64013	355.274395	610.160064	666.824293
*	38.9	0	M-H2O-H	C18H36N2O	-0.2157707	4129.00489	3746.08818	4284.02049	676.877105	5841.45951
*	38.9	0	M+NH4	C12H13N5O	0.15863851	2914.68413	2958.93469	2260.1391	3869.877	2241.16952
*	38.9	0	M+FA-H	C25H42O8	-0.3755632	310.408197	257.575574	192.704372	515.15286	9.7531E-07
*	38.9	2.36	M+FA-H	C20H32O4S	-0.3053535	3796.78527	3750.20881	3391.45373	2492.10612	2998.57078
*	38.9	0	M+K	C17H23NO2	-1.1104631	537.184962	709.806395	601.453959	425.02165	288.25719
*	38.9	0	M+NH4	C12H17N5O	0.82578332	10208.6496	9474.43267	10105.491	13650.7697	8505.28575
*	38.9	0	2M-H	C10H16NO+	0.9702543	363.614667	581.633171	517.600714	9.7531E-07	120.92569

*	38.9	0	M+H	C14H20N6O	-0.3387065	5178.71768	5727.44828	5706.26671	7384.14765	4348.54624
*	38.9	0	M+NH4	C8H15NaO8	-1.8736381	843.965222	760.267054	826.500899	119.678433	172.49893
*	38.9	0	M+NH4	C19H37NO6	0.5133211	4773.59556	4916.62924	4490.86374	4411.6608	3184.52351
*	38.9	0	M-H	C23H38O9	0.27058298	426.013782	307.349875	304.622326	9.7531E-07	326.67266
*	38.9	0	M+FA-H	C12H12O3	-1.0388627	2810.40226	3001.52219	2819.61163	2305.20539	1882.70976
*	38.9	0	M+H-H2O	C14H24N6O	-0.1040381	5867.73019	6223.29963	6065.03353	7627.74108	4668.62068
*	38.9	0	M+NH4	C9H5N5	0.21836163	2727.71433	2679.1328	2766.61213	3234.71692	1489.77597
*	38.9	0	M+K	C10H16O5	-1.401886	15230.3865	15295.8232	14719.542	20735.5966	12101.6799
*	38.9	0	M+NH4	C15H26O3	-0.8515496	2581.58784	2817.60606	2772.46418	2427.17333	2303.79068
*	38.9	0	M+FA-H	C14H27NO	-0.1434561	1589.66011	1545.46906	1626.02122	851.968219	1546.248
*	38.9	0	M+FA-H	C6H14O10P	-0.4151301	9718.71288	9500.52872	10392.3128	9957.09552	9010.18028
*	38.9	0	M+FA-H	C18H33FO3	-2.3059019	1548.71066	1556.12213	1665.76069	129.79523	213.039993
*	38.9	0	2M-H	C10H13N5	0.22943347	2442.58793	2009.87493	2476.61151	1796.9794	1887.26514
*	38.9	0	M+K	C24H40O3	-1.2154055	4342.60346	4617.09486	3108.7	16226.489	3328.44557
*	38.9	0	M+NH4	C12H19N2O	-0.9529201	2102.79936	1902.58208	1868.64202	2948.0074	1510.47046
*	38.9	0	2M-H	C8H6N2O6	1.56433013	66404.9716	67038.439	64859.8837	61349.4799	58377.7195
*	38.9	0	M+Na	C17H18N6	1.40393424	1586.06741	1814.81593	2229.32787	3778.16435	795.035057
*	38.9	0	M+H	C11H16	-0.7078382	655.149235	673.102631	598.566126	835.914629	426.055588
*	38.8	0	M+Na	C3H5N2O4-	0.66115924	3237.22846	3910.01583	3451.52081	5005.29559	6457.93508
*	38.8	0	2M-H	C6H13N3O4	-2.0935116	4329.69938	4594.34459	4255.64963	6354.88453	4152.04571
*	38.8	0	M+H	C3H2I2O2	1.80716531	27453.3079	27693.6573	30050.7151	7037.25968	3247.87468
*	38.8	4.32	M+NH4	C8H12N3O6	-2.328724	24908.2221	28992.654	25805.1041	90844.8648	28636.6768
*	38.8	0	M+FA-H	C7H10N2O5	-0.5740849	5604.61585	4604.72703	5488.78387	7922.75052	13392.9364
*	38.8	7.43	M+FA-H	C8H11NO7	2.78468358	22739.7353	20136.0526	20617.7611	18888.5322	5434.67468
*	38.8	9.04	M+FA-H	C23H26O12	-4.5642391	461820.754	460958.011	459202.66	16808.3964	2151330.08
*	38.8	0	2M-H	C4H5N5O6	0.4769225	13137.925	10546.4723	9725.5172	339.304098	17866.6285
*	38.8	0.799	M+Na, M+K	C9H18O8	0.17272148	44520.0413	38111.1925	41056.642	36053.7143	181724.533
*	38.8	0	M+H	C8H12N2O	-0.3173651	2622.21511	1851.66181	2493.83984	3097.66911	9.7531E-07
*	38.8	0	M+H-H2O, M	C9H15NO5	-0.6173116	2555.14005	3135.17402	2215.94914	9.7531E-07	671.598167
*	38.8	0	M+NH4	C9H2F16O2	0.43615432	84223.8844	84716.6604	80400.5009	55578.3382	26684.694
*	38.8	0	M-H	C13H18IN5C	0.73440432	49101.9383	50772.7277	50279.5167	1972.98558	1588.04512
*	38.8	0	M+K	C6H6O4	0.81457428	3279.78236	3380.67545	2106.51057	3709.24875	2398.21482
*	38.8	0	M-H	C12H25I	1.54411914	3209.45507	3346.58441	3342.38342	513.339848	6724.46632

*	38.8	0	M+FA-H	C17H11F6N'	-1.5248185	46455.3379	51435.4984	49342.3199	72543.1555	102190.415
*	38.8	0	2M-H	C6H10N3O5	1.84788348	9444.86633	10316.95	10961.4777	9761.65646	11134.2893
*	38.8	0	M-H2O-H	C20H22FN3(	-1.1039442	967.371359	886.920334	659.350796	1220.78993	1923.9182
*	38.8	0	2M-H	C13H14O6	2.83410081	2821.33847	2131.81735	2066.23069	3904.35643	5048.43507
*	38.8	6.49	M+Na	C9H15N3	-1.8073876	5014.01262	5383.34529	5579.77351	10699.4033	4043.44125
*	38.8	0	M+FA-H	C10H19N3O	-0.8393723	255.315333	214.272905	193.455493	9.7531E-07	902.688158
*	38.8	0	M-H	C10H18N2O	-0.3514682	393.499874	466.360928	507.789871	598.181643	418.034811
*	38.8	0	2M-H	C8H7F3O2	-3.6113408	1193.67183	1333.03042	1200.12699	1610.42258	2111.55908
*	38.8	0	M+K	C10H10N2O	-2.6644604	752.800514	657.883936	818.592793	1771.73688	3551.05005
*	38.8	0.0919	M+FA-H	C6H8N2O8	-3.6394951	3543.61557	3264.49346	3461.08958	538.01928	406.045967
*	38.8	0	M+H	C17H23N5O	0.54464796	6668.49104	6380.20371	6922.157	2237.24884	19152.3058
*	38.8	0	M-H	C4H8O3S	-0.856701	206.524547	222.233868	184.251821	299.782326	248.845881
*	38.8	0	M+FA-H	C9H16N2O4	-0.0760839	583.686835	453.879526	742.244719	1433.411	825.85434
*	38.8	0	M+H	C34H34N4O	-1.0617938	3885.86747	3829.7508	3887.77986	4942.73954	351.714405
*	38.8	0	M+NH4	C22H44O3	-1.3017064	9378.58182	11370.7957	10233.1211	1764.80681	2227.6672
*	38.8	0	M+Na	C28H40O8	0.27609802	3294.56597	2709.73433	2498.03412	3879.64367	4856.32469
*	38.8	0	M+FA-H	C17H32O2	-0.069883	6392.73186	6466.77177	6537.75771	5273.57281	4231.18716
*	38.8	0	M+H	C22H41NO4	-1.0447151	940.874386	794.411867	917.612208	168.925587	146.46363
*	38.8	0	M+FA-H	C17H32	-1.3428098	4444.08825	4601.04585	4560.67277	810.934924	17773.7071
*	38.8	12.4	2M-H	C30H48O9	-1.7355556	36050.3391	35887.0381	30499.5848	5303.61028	157486.441
*	38.8	0	M+NH4	C15H23N5O	-0.6126304	7956.7047	8492.60437	8791.01062	13586.7088	5004.61512
*	38.8	6.36	2M-H	C10H18N2O	-0.2593405	9396.81413	10879.9151	10230.4309	14222.2005	3127.72547
*	38.8	0	M+K	C20H26O2	0.85301533	1104.19842	700.013609	750.48617	2348.68048	433.682333
*	38.8	0	M+H	C25H52NO6	-0.7160061	4692.90362	4562.08277	5007.09699	1592.1004	15569.6472
*	38.8	0.0939	M+K	C26H49D3O	-0.9358258	6396.17325	5683.15595	6748.24694	9.7531E-07	10390.9548
*	38.8	0	M+K	C8H20N4O	3.1725032	1802.5259	2219.49148	2012.24868	706.851466	1122.76254
*	38.8	0	M+H-H2O	C20H36O4	-1.7986968	2486.32474	2605.513	3086.57731	3429.99661	1095.52215
*	38.8	0	M+NH4	C5H8N2	1.02715675	598.10571	827.254681	544.759502	674.878523	545.987395
*	38.8	0	M+NH4	C20H32O	-1.593215	1047.08602	775.799023	748.136219	477.778333	9.7531E-07
*	38.8	0	M-H2O-H	C17H34N4O	1.09311097	49488.9958	47396.5719	48268.2552	46978.0277	38146.4737
*	38.8	1.06	M+FA-H	C19H26N8O	-2.0649415	39915.5453	49919.4736	32883.4254	43095.0157	41843.5454
*	38.8	0	M-H	C25H43NO7	-1.3040452	1178.53462	1570.03234	1254.58396	9.7531E-07	9.7531E-07
*	38.8	0	M+FA-H	C28H24O4	1.02283334	2944.59984	2890.40799	1848.96314	3658.83484	2036.1491

*	38.8	0	2M-H	C14H17N3O	0.32208236	1786.95379	1549.79341	1817.01321	1469.99006	2328.3319
*	38.8	0	2M-H	C6H15N5O2	-4.2852175	4884.72039	4693.71711	4979.86068	6066.84779	3247.03778
*	38.8	0	M+Na	C18H30O5	-0.6299316	373.512809	437.496834	545.554443	675.855495	832.141108
*	38.8	0	M+H	C29H46F2O:	1.0347439	2427.43885	2496.51016	2880.05063	892.054681	1388.67473
*	38.8	0	M+Na	C24H46O4	-1.3634968	1797.11898	1499.8126	1762.69481	9.7531E-07	121.873417
*	38.8	0	M-H2O-H	C26H42O3	-0.3419465	4875.67726	4654.75666	4476.8777	1014.59209	4882.08591
*	38.8	0	M+FA-H	C9H16O9	2.51307034	1615.79304	1529.4268	1095.60946	811.538127	782.093925
*	38.8	0	M+NH4	C20H38O	-1.6825176	5220.96111	6629.71757	6673.23678	3045.56593	2213.42772
*	38.8	0	2M-H	C13H12N2O	-1.8076574	11319.981	12844.2551	7245.06597	8515.83308	8594.07068
*	38.8	0	M+NH4	C6H8NO3P	-1.8770677	845.10634	858.488752	629.430023	568.793684	1870.38391
*	38.8	1.32	M+FA-H	C12H15N2O	-0.4052719	29525.2634	31007.5822	30364.5693	28537.1483	71948.9423
*	38.8	0	M+Na	C6H11NO2S	0.37167837	1832.79856	1896.37386	2079.9724	608.839017	2037.21095
*	38.8	0	M+H-H2O	C12H25N3O	0.07190008	416.147394	523.411427	364.268997	9.7531E-07	1255.56591
*	38.8	3.37	M+Na	C5H11NO2	2.63787732	772.648444	835.247533	946.442027	812.836221	3944.21264
**	38.8	0	M+H-H2O	C11H20N2O	-0.2791135	1588.30201	1534.21654	1692.79248	1214.79293	1357.67364
*	38.8	0	M+H	C8H10O2	0.90339938	2954.57632	3322.39798	3413.37733	3987.96627	2603.13823
*	38.8	0	M+FA-H	C14H18N4O	0.23067902	564.214208	543.390309	638.217885	45.863946	2439.24222
*	38.8	1.4	M+H, M+Na	C21H28O8	3.1222982	30502.753	29801.9084	27008.2033	96588.0456	7645.6111
*	38.8	0.658	M+K	C15H27D3O	-0.2474633	30114.6963	25342.8328	23934.7101	18870.7878	62434.9052
*	38.8	0	M+FA-H	C13H16N2O	0.06291805	492.771889	512.181532	530.23594	207.495099	357.508656
*	38.8	0	M+NH4	C24H37AsO:	0.25116985	3874.01422	4175.53227	4160.30752	275.821044	210.190907
*	38.8	0	2M-H	C23H21NO2	2.23738861	131.956004	163.548946	221.871086	9.7531E-07	9.7531E-07
*	38.8	0	M+FA-H	C11H17NO6	3.21680461	525.032286	499.161947	547.861296	98.5173664	49.4730474
*	38.8	0	M+FA-H	C14H17N3O	0.50856195	1623.24773	1721.06633	1974.4666	2589.56496	2531.37642
*	38.8	0	M+H-H2O	C26H29N3O	0.50290756	557.697385	853.324204	695.542895	119.264945	80.9831833
*	38.8	0	M+FA-H	C8H14O3	-0.675658	449.449168	436.777405	433.22964	327.61413	410.529704
*	38.8	0	M+Na	C20H21FN2(	2.18470068	466.256761	438.308201	469.476386	158.496162	389.325824
*	38.8	0	M+FA-H	C5H6N2O	-1.3855258	305.426039	248.907159	262.313056	307.029741	227.125625
*	38.8	0	M+H	C10H16N2	0.91441195	138.261932	138.03623	164.486496	9.7531E-07	34.9877077
*	38.8	0	M+FA-H	C9H15N3O	-0.2736062	224.383861	166.606456	205.919383	166.492023	160.208825
*	38.8	0	M+FA-H	C19H32O9	1.96520656	579.197698	824.29826	657.761688	230.019281	1121.21255
*	38.8	0	M+K	C5H6O3	1.88737218	1070.67584	1026.80339	1186.13899	1407.8076	735.727006
*	38.8	0	M+Na	C13H25O7P	3.06130316	3430.91913	3470.92996	3056.51171	4673.29333	2683.30799

*	38.8	4.14	M-H2O-H	C12H13NO2	-0.7771046	1457.90886	1414.42717	1516.41174	1584.10537	6123.34057
*	38.8	0	M+H	C5H8N2O5	-3.1813608	993.298433	999.179258	974.00882	2146.29881	4231.96113
*	38.8	0	M+FA-H	C13H21N3O	0.33554664	568.52965	525.51003	504.272201	343.307639	29.7906056
*	38.8	0	M+NH4	C18H20N2O	1.79273781	3537.82273	3646.74894	3279.2768	7117.77694	5060.98736
*	38.8	0	2M-H	C8H8O3S	-2.2876207	2115.41453	2071.05149	1917.50563	6037.72536	323.131406
*	38.8	0	M+H, M+Na	C20H38O11	1.25445458	6279.04008	5663.27735	5692.36396	5601.10659	11136.6076
*	38.8	0	M-H	C10H9NaO5	-0.3606035	6037.42888	6064.74322	6076.39112	2978.61604	1738.33787
*	38.8	10.5	M-H	C19H22O5	-7.2132979	4870.3673	5268.15113	4862.19702	10937.2077	2357.37779
*	38.8	0	M-H2O-H	C15H22N5O	-0.5161719	666.365164	676.012466	826.737965	113.771324	1430.66273
*	38.8	0	M+NH4	C7H2F5NO	4.80968623	137.177077	151.614807	91.9226627	9.7531E-07	160.743107
*	38.8	0	M+H	C8H13N3O5	-3.3591502	2934.44289	3149.30072	2919.74343	1411.64087	3053.21709
*	38.8	3.76	M+FA-H	C13H9FO3	-3.0270325	2330.67216	2265.09742	2388.85259	250.861553	288.809177
*	38.8	0	M-H	C9H13NO4P	1.03633661	2635.41821	2472.79561	2705.50094	2720.83498	2136.61093
*	38.8	0	M+FA-H	C23H37AsO	0.16203251	2072.79174	2062.53194	2406.08583	214.523968	266.512094
*	38.8	0	M-H, M+FA-H	C16H20N6O	-0.0958172	702.730419	502.44463	685.658497	489.909644	94.5902655
*	38.8	0	M+NH4	C17H30O5	-1.0389347	1810.14209	1838.49947	1632.72279	1336.42568	6858.18442
*	38.8	0	M+H-H2O	C10H14N6O	0.30028462	8200.64245	7961.35987	7762.62636	11783.8054	6333.00331
*	38.8	6.33	M+H, M+Na,	C18H31N7O	-0.9772207	16563.2757	16559.5429	15276.2361	13399.0447	31546.1487
*	38.8	0	M+FA-H	C9H14O	-2.5548809	904.180135	937.211452	893.1861	661.588448	764.758435
*	38.8	0	M+NH4	C28H42O5	-0.3686886	1380.69687	1433.91144	1091.51636	5894.36637	341.088253
*	38.8	2.02	M-H	C19H28O6S	-0.2053017	5759.59012	6186.98662	6186.03526	4136.78707	1216.99475
*	38.8	0	M+Na	C23H24FNO	0.37647298	3749.8284	3487.31235	3224.98286	2629.39601	1882.99861
*	38.8	0	M+FA-H	C3H4O2	-3.3607656	1125.5094	1340.62184	1222.27308	1256.87417	1130.1292
*	38.8	0	M+H, M+NH4	C16H15NO5	-1.8190335	834.322171	1160.3478	1042.50902	483.30484	397.741095
*	38.8	0	2M-H	C16H14O2	2.32826926	443.839593	331.571096	457.037169	9.7531E-07	4412.68759
*	38.8	0	M+H-H2O	C14H22O	-0.7409751	297.159635	221.688553	332.247747	253.650782	667.040391
*	38.8	0	M+FA-H	C11H20O2	-1.6120875	931.841998	1165.34081	1020.99886	1096.77811	841.211476
*	38.8	0	M+FA-H	C21H30O5	0.00982415	902.916552	912.536426	948.37947	515.888873	3311.58037
*	38.8	0	M+FA-H	C8H12O	-1.8498418	7910.08905	8305.72142	7862.83427	7960.75648	6856.62864
*	38.8	0	M+FA-H	C17H18N2O	-0.2799853	1795.48341	1847.14456	1383.30433	2301.57349	462.541486
*	38.8	0	M+Na	C19H22FN3O	0.58592383	3271.16475	3495.02382	3532.95425	12550.6906	4024.48558
*	38.8	3.81	M+K	C25H21N3O	-2.2780195	5810.51395	5985.49462	5439.44472	3324.29854	1004.90799
*	38.8	0	M+NH4	C21H34O	-1.5840972	3225.53215	3352.90519	3138.10888	1497.03455	9.7531E-07

*	38.8	0	M+FA-H	C19H33NO6	-0.1332847	1049.28629	1168.20823	1191.71971	9.7531E-07	7810.18436
*	38.8	0	2M-H	C17H15NO5	0.63292811	3228.7309	3617.30222	3208.86552	9.7531E-07	17210.766
*	38.8	0	M+FA-H	C33H36O8	-1.529395	2524.96575	2456.4405	2698.44215	1803.03836	9.7531E-07
*	38.8	0	2M-H	C18H14O3	-2.8080542	3443.55021	3699.87443	4036.59321	9.7531E-07	6945.12426
*	38.8	0	M+Na	C22H42N2O	-1.2885243	1097.00279	1739.70115	1287.97515	9.7531E-07	815.442189
*	38.8	0	M+Na	C20H36N2O	-1.4890163	1435.48277	1396.93214	1448.2245	261.003891	1220.88408
*	38.8	0	M+NH4	C19H30O4	-1.1769128	610.775874	682.853942	808.767311	9.7531E-07	3743.00844
*	38.8	0	M-H	C27H44O8	0.11010254	1915.12732	2017.94268	1882.26642	312.606954	7533.80205
*	38.8	0	M+NH4	C16H32O3	-1.941898	409.262939	513.232761	553.24337	602.823933	4729.73394
*	38.8	0.429	M-H2O-H	C33H38N4O	0.01701793	4458.87725	3728.95838	3783.21235	9.7531E-07	1365.88189
*	38.8	0	M+FA-H	C12H22O	-0.7098787	6096.99305	7050.74076	6239.1558	6326.24277	6309.04488
*	38.8	0	M+H	C12H18N6O	-0.4574478	2374.20228	2286.0095	1990.95996	12968.4129	1867.21726
*	38.8	0	M+FA-H	C9H8O3	-0.9865357	2878.38238	2850.22199	2991.05888	2916.42058	2408.7237
*	38.8	0	2M-H	C7H10NO5-	-2.1701337	3161.05615	3708.0306	3723.57339	3390.84781	2763.50725
*	38.8	0	M+NH4	C27H42O5	-0.8049366	2851.66791	2959.58405	3571.08959	9.7531E-07	16577.1096
*	38.8	0	M+H	C20H30O2	-1.9612763	607.512425	870.596413	613.79565	607.283498	431.189393
*	38.8	0	2M-H	C7H11NO6P	2.97548356	9860.68919	10549.5801	11475.2489	9965.69571	9761.74674
*	38.8	0	M+H-H2O	C7H7O6P	3.51379769	682.824339	598.698409	760.551758	1132.88421	756.400439
*	38.8	0	M+Na	C43H81O13I	-0.055164	171447.951	193565.172	183624.586	174950.689	140863.905
*	38.7	0	M+NH4	C18H17NO	1.13005954	4482.09688	4402.12634	2992.0304	4158.37726	2501.54589
*	38.7	0	2M-H	HNO3	-4.6623211	532.971677	413.333581	554.046318	295.834265	197.483059
*	38.7	0	2M-H	C4H10OS	1.30843579	621.41518	877.247617	738.431179	121.191045	662.534661
*	38.7	0	M+FA-H	C30H34N4O	-0.8891277	1237.03867	1344.0128	1535.54287	599.631811	607.077366
*	38.7	0	2M-H	C3H6N2O4	-3.1876513	1060.35475	836.589218	999.72551	1252.30351	3618.23921
*	38.7	0	2M-H	C5H3N4O3	3.63043372	1941.96693	2090.08909	2132.541	522.017315	176.620222
*	38.7	0	M+K, M+H	C4H8N3O4P	1.1458435	14109.2301	14653.4265	15077.2285	38961.9941	38096.3077
*	38.7	0	M+K	C16H22O4	0.08242725	41058.4695	42155.4045	39203.99	97775.0141	68325.2624
*	38.7	0	2M-H	C11H13O4-	-1.8870933	29994.3189	27917.4401	30196.673	20.6694106	9.7531E-07
*	38.7	0	2M-H	C12H9N5O3	2.63816167	137875.26	137611.608	149703.807	3825.0324	700369.397
*	38.7	0	M-H	C9H14N2O7	-1.291999	12272.052	11859.2251	11437.8798	21932.767	27671.8477
*	38.7	0	M+FA-H	C9H18O9	-0.4300546	19812.3546	16612.9493	18061.5695	925.530819	19830.6336
*	38.7	0	M+FA-H	C9H11FIN3C	1.98331094	3404.68433	3529.70409	3795.76704	24308.08	20099.7521
*	38.7	0	M-H2O-H, M	C8H12O5	-2.4194083	668.50981	543.804753	389.276342	753.154023	202.17688

*	38.7	0	M+FA-H	C6H10N2O3	-1.8423517	651.499253	649.54207	676.322762	1472.50205	1286.05821
*	38.7	0	M+NH4	C25H30O7	-0.6686874	6619.50023	10166.6674	10187.2993	13202.8334	8753.77713
*	38.7	0	M+FA-H	C6H13NOS	-1.0543118	156.51482	137.859611	150.034332	1116.00179	89.3860101
*	38.7	0	M+NH4	C10H17NO4	-0.5192417	2051.98215	2210.40046	2218.6357	531.96166	1449.94852
*	38.7	0	M+NH4	C12H19N3O	-0.491541	3757.50899	6170.28348	5591.99165	555.958818	21914.5791
*	38.7	0	M+H	C11H22N4O	-1.2543416	2296.08765	2061.70216	1940.46047	2598.19031	12194.5784
*	38.7	0	M+NH4	C7H7N3O5	-0.5515422	2428.97342	1767.93995	1929.09363	202.501781	9.7531E-07
*	38.7	0	M+H-H2O	C9H15N3O1	0.24929634	4856.73757	4016.04168	5372.67997	3673.84509	4290.21699
*	38.7	0	M-H	C6H9NaO7	-0.036419	1231.281	931.836163	1124.12544	2686.97528	1379.71616
*	38.7	0	M+FA-H	C9H16N3O1	0.85571216	1743.74535	1754.99559	1739.52799	2572.42253	1155.03546
*	38.7	0	2M-H	C19H10N2O	2.18882477	574.194823	593.532927	549.31319	9.7531E-07	123.920705
*	38.7	0	M+H	H3O3PS	-0.7514397	821.741654	759.999938	848.272454	1009.68979	779.035303
*	38.7	0	M+NH4	C16H25N3O	0.39221844	1618.02514	1857.06386	1769.27986	9046.73773	409.948077
*	38.7	0	M+FA-H	C16H21N5O	0.11470996	1143.81163	1261.95228	968.492142	275.523835	3279.40553
*	38.7	0	M+H-H2O	C9H10O2	0.84231303	7406.25626	8667.14962	9443.32541	9570.30056	6581.75929
*	38.7	0	M+NH4	C10H15NO3	0.65850953	820.864245	798.559625	785.815265	548.327515	878.109241
*	38.7	0	M+FA-H	C18H24N6O	-0.3806419	1719.8899	1528.79093	1849.00061	778.217076	7845.57163
*	38.7	0	M+FA-H	C4H6N2	-1.4474308	224.82764	196.659311	210.405517	217.070783	191.707491
*	38.7	0.0629	M+H-H2O, M	C20H35NO3	-1.717021	1679.10847	1685.91212	2571.13171	1045.0655	1146.06012
*	38.7	0	M+H-H2O	C23H42N4O	-1.5218844	1885.67291	1579.62164	1947.21583	3580.94887	3752.16232
*	38.7	0	M-H2O-H	C40H43NO9	0.54054976	4598.7049	5454.83941	5126.6959	4012.35018	21515.7644
*	38.7	0	M+H	C27H53NO5	-0.7284469	15689.0856	15881.215	16278.0309	28249.3971	46192.4498
*	38.7	0	M-H	C16H31NO3	-0.2849279	3073.63843	2753.32526	2948.45364	4256.62941	2280.02034
*	38.7	0	M+K	C19H29N3O	1.12135814	8653.21683	6000.54469	6260.88918	6463.79147	3255.18464
*	38.7	0	M+H	C27H47O11I	-1.8478084	17831.7045	16278.8303	12515.4753	25013.0844	10840.7595
*	38.7	2.13	M+FA-H	C25H46NO7	-4.9431997	222890.074	228797.479	220500.543	76728.5624	310691.435
*	38.7	0.0655	M+FA-H	C19H26	-1.1985738	818.079328	520.240745	553.199747	68.9321563	189.33984
*	38.7	0	M+FA-H	C19H18F2N4	-2.2643404	6201.41848	7217.95284	6497.49648	5239.50368	8222.75538
*	38.7	0	M-H2O-H	C15H30N6O	-0.3114916	5964.7978	5523.08475	5021.68639	11703.447	1163.13905
*	38.7	0	M+NH4	C16H21N5O	0.37842101	1970.73136	1366.71739	1788.48965	2938.65775	1532.8672
*	38.7	0	M-H, M+FA-I	C27H44O3	-0.0833543	2648.23155	2568.96116	2005.74225	87.1045166	89.9008637
*	38.7	0	2M-H	C9H13N3O2	-4.690992	5268.24247	4152.26826	4318.6871	12287.5295	1871.08883
*	38.7	0	2M-H	C13H21O3P	-0.0156045	10617.4194	13576.3551	7481.53806	11122.9503	9668.11522

*	38.7	0	M+NH4	C18H21N7O	-2.9097572	39811.5818	37836.8781	38849.7378	82628.6106	54124.8236
*	38.7	0	M+H	C25H52NO7	-0.7943261	8215.49792	7190.15396	7811.74443	1781.81685	35096.32
*	38.7	0	M+FA-H	C23H42O4	-1.2488872	12552.0897	12509.8643	11340.5283	2220.76532	911.704228
*	38.7	0	M-H2O-H	C17H34O3	-0.9314707	7648.40888	7777.46865	6784.41445	15415.3067	4285.14608
*	38.7	0	M+NH4	C12H29NO6	3.61474838	1666.27364	1901.53535	1714.7986	5950.90774	2774.16629
*	38.7	0	M+NH4	C5H4N2O2	-0.1347557	1412.88024	1382.5452	1474.15508	1264.64664	998.35516
*	38.7	0	2M-H	C20H12N2O	-2.308316	1441.14803	1540.9456	1326.48342	2364.50291	2772.68993
*	38.7	9.87	M+NH4	C17H26O7	-1.0286768	6323.00964	6344.32731	6542.63063	4686.45526	3584.08622
*	38.7	0	M+FA-H	C5H5NO	-1.8458716	365.240383	331.139451	383.970884	193.9028	211.654556
*	38.7	0	2M-H	C21H24O7	2.1447718	649.196403	406.945109	458.623451	9.7531E-07	3396.57293
*	38.7	0	M+K	C7H16N4O3	-2.260129	759.046164	966.990859	971.840622	1271.9761	178.132324
*	38.7	0	M-H	C13H10N2O	-0.312456	248.931831	288.595102	336.716507	586.244338	238.279402
*	38.7	0	M+H-H2O	C21H41N5O	-1.5301969	5658.37234	5969.42539	5055.3582	29.1515464	1047.76194
*	38.7	0	M+NH4	C14H15N5O	-1.2061833	1297.74126	1318.62922	1309.54258	1361.01532	1551.82533
*	38.7	0	M+FA-H	C22H26NO3	-2.4135909	285.364374	289.181215	271.584097	154.662007	934.278592
*	38.7	0	M+FA-H	C11H21NO3	-0.3992078	441.573101	510.10503	512.88445	114.225082	472.80453
*	38.7	0	M+NH4	C17H30O6	0.45164822	1273.58478	1335.46514	1324.72141	459.00454	4547.5148
*	38.7	0	M+Na	C23H45O9P	0.45287849	3653.1347	3535.95547	3778.7434	2992.14402	6937.21819
*	38.7	0	M+NH4	C8H5NO4S	0.42270861	250.56059	251.14629	245.219006	2163.88847	9.7531E-07
*	38.7	0	M-H	C19H35N3O	0.19227951	693.901341	625.737073	610.413058	117.337021	1348.47885
*	38.7	0	M+H-H2O	C16H32NO8	-3.0500468	855.801261	851.542101	827.241023	560.428233	2542.02266
*	38.7	0	M+FA-H	C11H12N2O	-0.6131325	537.791745	679.670014	618.497429	793.082455	1540.9088
*	38.7	0	2M-H	C16H13F2N:	-0.2018382	1036.31289	946.345457	1277.85017	2863.67737	579.272507
*	38.7	0	M+NH4	C17H14N4O	1.74400344	941.784511	972.433299	992.481277	1602.23302	791.681579
*	38.7	0	M-H2O-H	C10H15NO6	-0.5789628	2941.85731	2920.75558	2969.15647	2056.57244	3592.56014
*	38.7	0.113	M+NH4, M+N	C30H52O7P	2.31790254	26618.8014	31104.4186	28236.206	22878.7852	52759.8805
*	38.7	0.319	M-H2O-H	C10H17N4O	-3.7412323	39232.1039	40496.0804	38990.8383	45397.6019	93480.82
*	38.7	0	M+FA-H	C12H9N5O	-0.4631845	327.699647	465.618192	464.426216	781.996603	108.244539
*	38.7	0	2M-H	C4H4N2OS	0.13356481	856.097896	654.938479	760.142837	1516.18323	1841.84274
*	38.7	0	M+FA-H	C9H14O5	-0.5756412	439.38631	427.301186	365.286422	986.692532	359.081691
*	38.7	0	2M-H	C8H10NO7P	2.41324125	848.590028	1093.6656	1314.81343	1303.99132	3134.87966
*	38.7	6.48	M+Na	C28H31NO6	3.62571288	2645.92448	2045.48226	1722.49126	6881.59471	2687.18559
*	38.7	0	M+H	C16H24O2	-1.0161261	1626.88842	1619.77799	1427.19311	1960.61629	1079.22287

*	38.7	0	M-H2O-H	C11H14N2O	-1.1648314	485.162399	474.885357	451.170266	574.262309	1073.90997
*	38.7	1.65	M-H	C9H19NO4S	-0.5948417	2995.05767	3016.78144	3092.63169	1964.52182	3348.4299
*	38.7	0	2M-H	C5H4N4O2S	-1.3366786	1328.36651	1421.63145	1274.33762	1641.90804	1148.95881
*	38.7	0	M+FA-H	C18H28O3S	-0.508763	1291.99869	1055.16437	1104.54976	702.474898	503.041597
*	38.7	0	M+FA-H	C8H13N	-2.0750782	285.508204	320.642501	464.028924	476.119489	226.125179
*	38.7	0	M-H2O-H	C8H5NO2S	-0.7293989	826.537654	821.092272	922.113757	986.798696	876.571448
*	38.7	0	M+H	C26H32N4O	2.47977714	2444.15444	2235.21552	2055.33945	9.7531E-07	7530.67222
*	38.7	0	M+FA-H, 2M	C7H3F3O2	-3.6177415	71500.3088	71217.5163	71926.2075	72313.0483	64442.9489
*	38.7	0	M-H	C6H13AsO2	-0.3746849	16746.5402	16813.5901	16884.6915	16530.6706	14451.1258
*	38.7	0	M+FA-H	C19H26O9	0.14812322	2984.92838	2810.95919	3009.06199	1178.85723	280.19382
*	38.7	0	M+FA-H	C20H32O3S	0.21547039	14278.1767	15750.1674	12888.0214	10831.4731	17814.6356
*	38.7	0	M+FA-H	C9H10O	-1.5578599	2278.25744	2606.3514	2413.38427	2087.09653	1878.24464
*	38.7	0	M+H	C14H23NO1	1.61709334	290.664831	349.552738	409.652593	286.849966	9.7531E-07
*	38.7	0	M-H	C7H6N2O5	-1.6372599	6452.4893	6891.00335	6530.35761	6622.01014	5645.83025
*	38.7	0	M+Na	C20H38O5	-1.409347	1349.47072	1764.4373	1737.29701	224.21052	1032.05385
*	38.7	0	M+FA-H	C12H24O	-1.4198315	9424.76993	8984.92773	8896.25785	8302.34138	7074.42566
*	38.7	3.96	M+H-H2O	C9H8O	0.79295294	4076.19559	3960.67702	4292.54503	5120.49429	3024.86295
*	38.7	0	M-H	C8H8Na2O5	-1.6966631	896.881332	819.705706	762.989968	927.215641	731.362133
*	38.7	4.32	M-H	C25H39NO8	-4.3289345	4790.60381	4069.3518	3742.8043	9.7531E-07	10962.6742
*	38.7	0	M+FA-H	C9H11NO2	-1.164011	934.815925	811.064292	960.043139	804.227862	666.794653
*	38.7	0	M+H	C33H42O12	1.30111974	2782.1557	3841.57664	3304.60494	7587.57551	310.491474
*	38.7	1.82	M+Na	C16H26O4	-1.8061604	11015.2652	10934.2691	10743.3645	11038.8375	12006.5971
*	38.7	0	M+FA-H	C13H22O	-0.3733415	664.936367	750.538243	682.411317	607.531532	1186.95316
*	38.7	0	M+NH4	C15H25NO7	2.82528665	4010.95803	4379.57588	3704.23658	5342.11685	2914.4808
*	38.7	0	M+K	C12H10F2N2	2.89264838	1182.87155	1105.7105	862.395292	3163.92986	9.7531E-07
*	38.7	0	M+H-H2O	C20H30O3	-1.4902827	1566.45864	1658.23108	1880.63138	439.791252	580.304533
*	38.6	0	M+H	C8H12N4O3	0.36854547	5820.96967	6425.74811	5869.53394	3077.0797	9580.57448
*	38.6	0	M+H	C12H19N3O	-0.9228924	16991.3499	10726.0051	16373.268	5.04273905	3696.69273
*	38.6	0	M+NH4	C14H12F3N3	-1.3997683	3218.39767	1765.00556	2918.14693	9.7531E-07	9.7531E-07
*	38.6	0	M+Na	C14H24N2	0.66256309	307548.276	316004.067	303130.127	766094.351	249079.867
*	38.6	0	2M-H	C9H8N4OS	-0.9344634	315114.28	303451.618	301974.452	5851.07033	298692.99
*	38.6	0	M+NH4	C2HF3O2	3.97070547	2286.16301	2549.927	2145.45471	8768.37767	1532.33003
*	38.6	0	2M-H	C9H8N2O3	-0.4129284	47379.1473	47826.7546	46873.5346	14506.2203	30347.2081

*	38.6	0	M+NH4	C9H12N2O2	-0.0998379	7687.80434	7082.82017	8042.15895	9.7531E-07	6674.8078
*	38.6	0	M+Na	C8H19NO6P	-3.2681467	5760.70264	5143.97756	5452.69365	535.541374	9390.05348
*	38.6	0	M-H	C10H8S	0.05432639	369.043781	270.809913	354.029265	175.731228	31.8341274
*	38.6	0	M+NH4	C11H17NO3	-0.6497845	59081.6472	65287.0963	61235.2209	89603.5436	95271.8885
*	38.6	0	M+FA-H	C2H4O2	-3.9267698	283.05234	210.885068	263.212141	169.981673	277.508574
*	38.6	0	2M-H	C15H10N2O	3.66498011	4078.02474	4314.2952	5260.05159	6373.82627	1790.19747
*	38.6	0	M+H	C5H11NO3	0.57352925	56.0074768	35.9207916	40.2922552	51.6828232	9.7531E-07
*	38.6	0	M+H	C12H23N3O	-0.4450766	1138.06669	964.675842	1201.52453	9.7531E-07	5830.76315
*	38.6	0.79	M+FA-H	C7H9O4P	-3.2200844	11560.4922	11662.902	12986.8782	13826.6488	9684.62546
*	38.6	0	M+FA-H	C6H11N2O6	3.92645364	4209.69917	4346.80409	4255.53237	450.902763	7823.64175
*	38.6	0	M+FA-H, 2M	C21H14O6	1.3357333	17689.8421	20705.4913	15217.6581	5926.8502	44462.9145
*	38.6	0.218	2M-H	C8H6N2O2S	1.24913518	46157.9963	51646.0632	47329.0538	7794.31132	11915.3577
*	38.6	0	M-H2O-H	C3H4N2O4	-2.0172996	308.829665	371.65947	300.556203	193.090675	209.378003
*	38.6	0	M-H	C4H6N4O	-1.8234028	591.226864	622.148867	554.811213	878.632239	291.974108
*	38.6	0	M+Na	C10H18O8	1.02805145	310.020246	325.003281	419.938577	474.667747	4630.32075
*	38.6	0	M+NH4	C5H7N3O3	-0.6707098	1524.52416	1439.40424	1481.07186	1452.85494	2672.30967
*	38.6	0	M+H	C4H8N2O3	1.26292297	1027.26508	1118.74134	1062.88494	1689.46183	1435.35381
*	38.6	0	M-H2O-H	C9H18NO8P	-0.2251002	1164.78589	1174.72302	1305.84556	1823.46943	1071.3405
*	38.6	0	M+H-H2O	C19H26N2	1.22241784	1096.4012	1165.1906	1044.99806	1475.73647	3194.31366
*	38.6	0	M+H	C22H34O5	0.29590002	3133.36901	3442.91444	3330.93989	3623.50326	2012.1712
*	38.6	0	M+Na	C18H33NO2	-1.7532026	3761.57801	3512.48582	3086.1944	3734.21163	2430.0662
*	38.6	0	M+NH4	C14H25NO	-2.0441168	798.28522	752.813152	606.318258	599.928851	192.037102
*	38.6	0	M+NH4	C29H48O5	-1.6475954	7305.98574	7991.58903	7565.86999	1654.51465	50583.388
*	38.6	0	M-H2O-H	C20H44NO7	0.17033842	2382.72834	2912.00527	2910.48868	750.357104	7303.76813
*	38.6	0	M-H2O-H	C22H48NO6	0.12661138	1038.14993	1220.52983	1341.98706	9.7531E-07	6627.70655
*	38.6	0.281	M+NH4	C18H38O4	-1.795887	5632.95276	4866.15598	4593.30068	3238.08457	5310.38565
**	38.6	0	M+H-H2O	C17H26O4	-1.2668353	5728.64149	5107.45064	5358.64702	5584.36916	4900.18573
*	38.6	0	M+FA-H	C48H76NO1	0.31099085	188285.426	145370.106	128873.159	175322.884	162855.538
*	38.6	0	M+NH4	C26H43O9P	0.76072503	2328.92426	2722.73699	2131.79457	12983.5644	8328.57293
*	38.6	0	M+NH4	C15H20N2	-1.4455727	205.372692	162.319241	190.001633	9.7531E-07	1.57263957
*	38.6	0	M+H-H2O	C22H20O2	2.01556728	1687.97469	1681.54885	1429.83499	683.68298	2545.35589
*	38.6	0	M+FA-H	C21H32O	-0.8147115	2097.02512	2053.62414	2088.91544	1393.3385	880.70417
*	38.6	0	M-H2O-H	C30H51N6O	0.82217511	159589.734	158366.472	156466.105	82630.3393	282065.631

*	38.6	0	M-H	C30H47N3O	2.07081461	129672.805	137299.346	120148.134	54141.4955	218778.706
*	38.6	0	M-H	C19H37NO3	-0.0923191	1487.64766	908.39186	953.300833	63.1637526	713.934161
*	38.6	0	M+FA-H	C19H36O4	-0.9013327	14656.7462	13465.6847	12833.6661	9344.89408	705.914061
*	38.6	0	M+K	C7H8N2O3	2.90891439	2983.44971	2268.53052	2451.18926	3757.61188	2188.73169
*	38.6	0	2M-H	C12H16N2	-3.5606965	166.04861	198.870046	164.922836	1330.32532	9.7531E-07
*	38.6	0	2M-H	C9H13N5O6	1.58303599	2205.54742	2403.35327	1695.25334	1733.1924	3328.14578
*	38.6	0	M+K	C24H35NO2	-1.5531846	5122.07516	4546.24129	4539.04519	10740.6015	5118.98954
*	38.6	0	M+FA-H	C12H22N6	0.31107183	1502.27098	1118.35491	1063.04967	1909.54341	816.035371
*	38.6	0	M+H, M+NH4	C42H74O14I	-0.7325576	127648.611	184926.576	165448.028	396393.969	170833.106
*	38.6	4.87	M-H	C38H67O9P	-3.8819718	46196.8449	52072.2125	32299.0864	42302.889	51278.5395
*	38.6	0	2M-H	C10H12F3N	4.15409337	3272.12086	3234.72131	3155.84483	4001.35491	3267.4274
*	38.6	0	M+H-H2O	C21H41NO3	-1.471712	10608.1639	10678.0771	12323.8191	37019.5209	80024.0933
*	38.6	0	M+NH4	C16H31NO	-1.4764898	5064.6268	5503.30972	5623.89006	9814.98702	5671.36229
*	38.6	0.576	M+K	C20H28D4O	2.36295061	6754.44298	6732.98761	6448.74529	19086.4006	11148.735
*	38.6	0	M+FA-H	C23H44O4	-0.9521816	1077.03789	1280.5782	1278.38733	157.154032	9.7531E-07
*	38.6	0	M+NH4	C18H25N5O	-0.0782698	2117.16467	2316.68856	3087.00557	6760.49384	2986.45304
*	38.6	0	M+NH4	C7H9N3O	-0.7832427	860.255662	920.747959	1176.04604	1655.87318	827.346966
*	38.6	0	M+H	C20H39NO3	-1.0616989	4827.61766	4810.80215	5178.44783	7982.6168	5923.3357
*	38.6	0	M+FA-H	C37H72O2	-0.218366	11638.1201	12827.3978	8491.01111	12002.8423	6752.98492
*	38.6	0.0153	M-H	C18H38N2O	2.92167536	5615.76679	7918.87079	7736.40148	12907.459	9601.61151
*	38.6	0	M+Na	C22H42O4	-0.5299159	1529.86164	1125.3214	998.36878	2170.28743	1446.3702
*	38.6	0	M+H-H2O	C5H9N3O2	-0.2163582	1175.73019	1189.42606	1252.61171	1491.74072	733.838664
*	38.6	0	M+FA-H	C15H19N3O	-0.757088	2568.8034	2917.69757	2382.40086	2031.65053	7635.61448
*	38.6	5.01	M-H2O-H	C15H12N2O	-0.6213066	77380.4113	87797.0554	87028.4763	98769.6037	137335.412
*	38.6	0	M+NH4	C8H11NO2S	-0.1333873	2449.17276	2517.26834	2262.22275	802.188899	5671.36643
*	38.6	0	M+H	C6H14N2O2	0.85495534	3519.80852	3443.58027	3680.83108	5444.79958	5103.92426
*	38.6	0	M+K	C19H39O7P	-0.0370812	3820.60681	4325.47734	3244.80899	1839.19252	10815.6098
*	38.6	24.1	M+NH4	C8H8O3	0.3198818	4567.0631	4864.94594	4753.07142	4008.70894	4614.7175
*	38.6	0	M+FA-H	C7H9NO	-1.6382376	179.151869	172.995987	158.099261	16.1917665	243.520545
*	38.6	0	M+NH4	C13H20O4	-2.9441999	4041.09103	4332.98012	4487.90946	3271.39882	4812.95114
*	38.6	0	M+NH4	C25H24O4	-0.944871	10746.2445	10966.2217	11040.6195	4484.23484	2794.18406
*	38.6	0	M+H-H2O	C7H8O4	0.61228081	4621.73434	4628.78975	4369.52681	6562.71946	4239.68306
*	38.6	0	M+K	C7H14N4O3	-2.9099121	335.587399	442.510372	282.296562	616.51656	296.308824

*	38.6	0	M+NH4	C21H32O9	-2.3111746	3389.90733	4345.09143	4206.69074	137.08007	2333.22229
*	38.6	0	M-H2O-H, 2M	C24H28O3	-0.4892241	8939.80946	10621.767	9571.67687	731.952115	10118.5926
*	38.6	0	M+FA-H	C12H8O4	2.77431111	9468.86068	9815.45784	9665.02541	15837.5676	4796.07585
*	38.6	0	2M-H	C6H8O7	1.42834252	2544.97065	2932.27166	3085.21356	4547.21383	1410.96656
*	38.6	0	M-H	C10H12INO3	-0.5867242	4023.02728	5187.56845	2963.5318	2371.52011	1971.3691
*	38.6	0	M+H	C9H11N	0.59411288	807.900703	1002.49315	1046.35197	1684.01528	684.324324
*	38.6	0	M+Na	C16H22N8O	3.26245859	5481.73646	5271.47312	6032.44965	893.723187	21406.8567
*	38.6	0	M+FA-H	C9H14N2O2	-1.2683646	562.85976	637.9709	592.038129	519.692237	152.272397
*	38.6	0	M+Na	C32H50O9	0.28073307	1753.05026	1758.07021	1704.85288	13414.3132	3351.73394
*	38.6	0	M+H	C17H19N4O	1.23525725	10418.2892	9929.20769	11402.1026	16217.5006	10660.1835
*	38.6	6.07	M+Na	C25H46NO6	-4.6582766	3989.56394	4805.7689	3972.10125	3914.5613	759.108053
*	38.6	0	M+H	C7H5N3O6	-2.7553808	3164.76623	3101.87802	3343.75631	4340.66901	2090.20012
*	38.6	0	M+H	C19H34O9	3.46626784	1626.88607	2031.78063	1718.60522	2289.79988	1899.02563
*	38.6	0	M-H2O-H	C9H18N5O8	3.18723121	844.741082	846.072388	955.260165	910.99432	261.524992
*	38.6	0	2M-H	C21H24O2	-1.5769993	549.490999	705.275937	649.880814	189.089294	1070.59744
*	38.6	0	M-H	C6H11KO7	-0.5655341	5194.37692	5302.68709	5172.96611	5262.07654	5877.55695
*	38.6	0	2M-H	C10H8FNO2	2.9229796	1659.89965	1553.50026	1829.07822	1842.21469	1389.91761
*	38.6	0	M+Na	C21H28N2O	-3.4184059	790.371444	437.581386	666.976477	116.545919	1862.76185
*	38.6	0	2M-H	C14H6O8	-2.0854097	4432.98729	3651.01961	3579.37384	11780.2645	1066.18732
*	38.6	0	M-H	C6H20N2O1	1.21211843	929.775489	1040.74092	1365.90768	427.42521	9.7531E-07
*	38.6	0	M+Na	C27H48O6	3.20078086	1870.91815	1758.38448	1936.59441	5794.96384	12725.179
*	38.6	0	M-H	C8H11F2NO	3.97160652	319.862702	307.692859	341.975433	373.103176	474.471973
*	38.6	0	M+H	C30H40O7	-0.2137492	4822.24869	4108.93701	3641.06315	2813.89763	14768.7475
*	38.6	0.866	M+FA-H	C5H6N6	-0.7670267	2019.5179	2000.61033	1863.36125	1488.50883	1940.83552
*	38.6	0	M+NH4	C19H24N2O	-0.7180138	1511.94367	1417.53283	1380.72065	247.311794	3444.92184
*	38.6	0	M-H	C13H13F5O	-2.6074182	507.557344	559.218372	582.780064	99.0386935	918.441914
*	38.6	0	M+NH4	C11H17NO2	-0.7793402	696.554227	588.907148	679.86688	577.151744	434.592442
*	38.6	0	M+FA-H	C10H21NOS	-0.9692878	247.889698	220.104693	289.681981	66.6009971	50.9533157
*	38.6	0	M+FA-H	C16H15NO3	4.34934865	711.147208	813.466306	848.002142	350.759304	894.951098
*	38.6	0	M+H-H2O	C9H9NO	0.10961436	1456.37079	1691.99436	1472.14154	1576.83994	1220.49301
*	38.6	0	M+FA-H	C18H18N2O	-0.0969938	489.773357	468.76478	468.695331	498.308176	105.402657
*	38.6	0	M+H-H2O, M	C8H9NO3	0.3596021	784.232018	898.027873	740.657209	1013.52716	2394.93776
*	38.6	0	M+Na	C6H9NO6	3.23285786	315.888238	239.770815	293.383935	664.759045	35.8356347

*	38.6	0	M+NH4	C18H32O5	-1.4828074	181.701208	293.661965	218.145129	9.7531E-07	3695.4004
*	38.6	0	2M-H	C15H13N	-4.6885771	332.687359	310.746386	260.902534	228.730836	252.416216
*	38.6	5.66	M+H	C15H13NO3	1.87789047	1649.68666	1884.45586	1803.37052	1271.45502	817.442475
*	38.6	0	M+FA-H	C6H4N2O4	-2.3448366	1105.91788	1217.20118	989.142962	1265.89602	527.948345
*	38.6	0.981	M+FA-H	C24H22O5	2.02338946	22048.5624	22266.7788	22253.9423	28430.0592	16675.8247
*	38.6	0	M+Na	C22H20O7	-1.7379011	463.737768	439.505564	579.09086	94.4237304	9.7531E-07
*	38.6	0.0488	M+K	C25H35N3O	-0.577826	2421.10865	2630.48141	2548.18132	1158.80879	4396.28338
*	38.6	0	2M-H	C3N6O	-4.9379494	698.509615	827.023158	741.805707	593.882812	548.720297
*	38.6	0	M+H	C20H35O7P	0.56131498	1041.11991	928.02183	1003.40195	9.7531E-07	5255.36499
*	38.6	0	M+NH4	C22H37NO4	-1.612777	1598.34461	1307.22168	1500.61499	111.602036	2102.36871
*	38.6	0	M+FA-H	C10H11F3N:	4.9227797	3286.91497	2991.11957	3720.90901	3186.39127	2662.15817
*	38.6	0	M+H-H2O	C13H20O	-0.8085934	2117.53486	2092.89323	2065.81962	2467.96947	1720.81828
*	38.6	0	2M-H	C4H8N4O2	-1.5056123	3751.62591	3799.68805	3609.84387	3591.2031	3499.89154
*	38.6	0	M-H	C4H6N4O12	2.06485632	5284.0076	6073.65313	5456.12458	5186.90734	4816.97746
*	38.6	0	M+H-H2O	C28H48O5	-1.2561715	3784.30021	4745.86245	4544.15755	5618.58489	3620.42763
*	38.6	0	M+NH4	C15H30N2O	1.99441368	1076.52244	998.156144	786.551192	255.104593	430.349435
*	38.6	0	M-H2O-H	C6H7O4P	-0.0312133	13219.8511	13267.4528	13056.7145	13509.899	11483.3322
*	38.6	0	M+H-H2O	C19H28O2	-0.9205293	2046.97126	1973.96978	2007.48581	1431.287	1032.44116
*	38.6	0	M+NH4	C16H19NO6	-3.4351101	4743.7518	5403.94118	4669.97423	2876.14449	5075.29326
*	38.6	0	M+H, M+Na	C30H26O13	-1.2735188	14040.5423	17256.1703	21289.1925	29762.4302	17655.545
*	38.6	0	M+Na	C20H25D5O	1.13424227	4407.48435	4137.3853	4096.19787	6926.55137	4057.44538
*	38.6	0	M+NH4	C21H42O3	-1.5912506	10547.5607	11611.9122	12584.3258	10893.3464	7462.03219
*	38.5	0	M+NH4	C14H24N2O	-0.1967155	2294.49472	2548.34528	2326.66401	5152.93304	2116.40908
*	38.5	0	M+H	C11H21N3O	0.23944333	6428.8022	7372.61654	7290.9922	10313.5897	8729.47897
*	38.5	0	M+FA-H, 2M	C5H13N2O4	0.3851307	446.702124	635.267061	547.688026	2833.65179	3454.30302
*	38.5	0	M+FA-H	C4H8N2O2	-1.7137789	2713.03862	2260.87733	2846.59457	632.723835	2995.74767
*	38.5	0	M-H2O-H	C6H6IN	0.70357587	6020.0803	3880.55646	6749.37995	9764.21373	8487.88687
*	38.5	0	M-H2O-H	C8H5F3O4	1.86654464	3373.98633	3030.90223	3948.99934	3815.32207	7054.6041
*	38.5	0	M-H	C11H15N5O	-4.3501265	5911.27134	6499.18466	6450.72731	4027.69328	34392.8683
*	38.5	0	M+H, M+Na	C10H13N5O	-0.7364866	62431.2364	63950.5587	65216.5099	112866.927	91574.4397
*	38.5	0	M+K	C11H6O3	4.12028477	1612.15764	2620.82632	1873.54035	5611.15074	1948.22468
*	38.5	0	M+FA-H	C15H16N2O	2.73499468	1582.56716	873.509719	1489.49534	69.9779804	89.5600832
*	38.5	0	2M-H	C2H7AsO2	4.23903549	1712.74615	1916.43974	1935.25453	2719.39475	1117.86464

*	38.5	0	M+NH4	C10H17NO2	0.34346716	459.522384	473.87604	482.248717	9.7531E-07	4667.58702
*	38.5	0	M+H, M+Na	C36H62O31	-0.4962813	37189.3494	41829.0668	39304.2219	1298.82991	201990.38
*	38.5	0	2M-H	C17H12O7	-1.0397315	3630.9994	6238.98966	5720.25607	10947.1518	9.7531E-07
*	38.5	0	M+NH4	C5H5N4O5P	0.44255666	11698.9751	14421.6997	12074.8006	19410.8692	8089.2949
*	38.5	0	M+FA-H	C9H12N3O7	-2.7427472	5639.09261	5281.4717	6269.49939	7264.72969	3280.09343
*	38.5	0	M+K	C16H29N7O	1.80032908	25381.1056	23617.3599	26434.8661	57924.5025	20119.7837
*	38.5	1.47	M+Na	C3H8N2O	4.29068055	1379.63898	1442.44003	1249.14798	752.400341	2150.29992
*	38.5	0	M-H2O-H	C9H10FIN2C	2.37518337	2584.25571	2475.37413	3075.95523	1282.07631	441.677454
*	38.5	0	M+FA-H	C16H33N5O	-2.464672	6162.94401	5294.64562	4816.76447	7456.19199	10235.1304
*	38.5	0	2M-H	C12H14O8	1.04290512	9263.4025	10351.3359	11270.668	392.130561	9101.04926
*	38.5	0	M+NH4	C5H10NO3P	-1.166338	175.518016	246.417356	201.909042	47.1970668	114.247589
*	38.5	0	M+H	C18H28N4O	0.34830603	5229.36621	5586.43195	5741.74526	18812.6629	3495.70614
*	38.5	0	M-H	C7H13O5PS	0.12706592	12028.0958	12636.7819	11291.7629	28747.8356	13682.036
*	38.5	0.186	2M-H	C4H7NO5	-3.1693607	1286.46805	1789.70829	1239.97474	10624.9278	2420.9632
*	38.5	0	M+FA-H	C6H5NaO3S	-0.3564723	462.291832	619.27085	651.246412	55.2908583	22.1679762
*	38.5	0	M+FA-H	C4H6N2O2	-1.8134821	438.883574	455.851872	493.514315	422.493236	352.325752
*	38.5	0	M+H-H2O	C19H23NO4	-1.2312226	385.018821	479.805722	630.79009	808.53234	9.7531E-07
*	38.5	0	M+H	C24H47NO5	-2.3196342	2286.9818	2799.02577	3081.5186	2339.5981	9500.92089
*	38.5	0	M+NH4	C20H38O3	-1.7651394	5479.36629	6481.99637	6527.87236	7693.8083	5067.20315
*	38.5	0	M+NH4	C21H41NO2	-1.5071277	210.497801	346.010322	374.957516	623.134689	1976.12732
*	38.5	0	2M-H	C7H10NO8P	3.95338396	16401.534	14891.5778	16191.5278	15224.6899	13773.8253
*	38.5	0	M+H-H2O	C26H54NO7	-0.2396634	12504.2129	11452.4	11809.4653	5124.99692	17698.3955
*	38.5	1.38	M-H2O-H	C17H30N8O	3.90817276	26111.7739	26131.9216	24287.284	26075.0723	7951.52809
*	38.5	0	M+H-H2O	C24H39N5O	1.24053335	13015.1862	12310.7338	13860.8914	4125.3962	22525.0206
*	38.5	0	M+K	C7H4I2O3	-0.8907043	4606.21372	4720.30622	4012.27404	9.7531E-07	8548.63016
*	38.5	1.32	M+NH4	C31H56O8	-2.0503281	8065.59835	8057.49462	9754.0175	2827.68515	12739.546
*	38.5	0	M+NH4	C17H29NO5	3.05930343	1587.03896	929.52465	1392.55654	692.355598	9.7531E-07
*	38.5	0	2M-H	C19H22FN3(	-3.4764181	9869.88218	11501.6988	12900.5372	5935.76929	21109.0666
*	38.5	0	M+NH4	C17H31NO5	3.45054455	750.743144	709.979983	644.952139	367.552902	86.8544133
*	38.5	0	M+H-H2O	C6H14N2O	1.25130585	1045.5033	1514.42734	1385.54523	2314.26224	1438.83695
*	38.5	0	M+H-H2O	C17H35NO2	1.51884116	1160.88462	1600.80392	1471.02878	60.1783938	419.535409
*	38.5	0	M+NH4	C31H57O9P	-0.8648072	13620.6169	12399.0893	12915.6167	4436.79996	2413.22384
*	38.5	0	M-H	C18H37O7P	-1.215444	204708.2	210734.04	181247.268	154773.951	147010.089

*	38.5	11.7	M-H	C44H62O4	-3.9036982	11032.1708	14415.0838	11345.1562	9294.69144	12755.9951
*	38.5	0	M+FA-H	C35H60O2	0.15365342	1465.30253	1567.98924	2203.45485	4763.60087	760.539678
*	38.5	19.6	M-H2O-H	C21H27ClN2	-0.7734505	91373.7277	124206.481	98429.7137	118972.613	99756.8761
*	38.5	0	M+H-H2O	C19H34N2O	-3.6243092	2352.65669	2223.55698	2270.69219	9044.52211	7175.46925
*	38.5	0	M-H	C15H19N3O	-0.0610289	468.630874	368.535078	521.839737	385.28064	3003.50973
*	38.5	0	2M-H	C18H13NO3	1.8187243	920.909909	755.565828	1029.02493	9.7531E-07	1594.29524
*	38.5	7.5	M-H	C5H11NO2S	-1.0585202	812.276392	830.121936	901.465195	9.7531E-07	1040.99539
*	38.5	0	M+Na	C6H15NO3	-0.0982696	233.178714	261.749127	154.936415	603.875181	204.975337
*	38.5	0	M+FA-H	C17H19N3O	0.25813807	415.292484	482.363168	484.414805	573.036021	378.935113
*	38.5	0	M+FA-H	C12H10O2S	-0.553049	3011.76926	3242.7941	2848.72548	4940.50741	1419.3508
*	38.5	0	2M-H	C8H18O3S	1.05162443	581.437901	581.189537	594.16116	9.7531E-07	1423.42345
*	38.5	0	M+FA-H	C32H38O16	-0.7302104	1949.30166	2199.556	1885.82265	8069.44666	177.692346
*	38.5	0	M+FA-H	C8H10FN3O	2.00542687	1913.04945	1700.38142	1621.09425	1551.68421	1367.32764
*	38.5	0	M+FA-H	C11H16O11	1.01384303	893.903223	1327.04103	956.196533	561.648983	3332.38882
*	38.5	0	M+NH4	C14H16N2O	0.55957795	979.43221	860.386416	841.341983	243.786355	3354.71668
*	38.5	0	M-H	C11H14N4O	-0.5403899	820.040329	1068.93298	840.604815	2394.17609	738.189445
*	38.5	0	M+H-H2O	C15H29N3O	1.45279904	1143.25384	1149.52637	1048.48463	269.042461	3149.75146
*	38.5	0	M+H	C18H37N2O	2.60505838	1056.41826	976.515589	887.234085	9.7531E-07	38.318433
*	38.5	0	M+FA-H	C6H9O5-	0.09922729	1170.04953	1227.01294	1605.43851	1595.30199	1029.44066
*	38.5	0	M+NH4, M+N	C22H30O6	-0.3515532	301.341718	494.89638	426.493529	18.8945294	193.921731
*	38.5	0	2M-H	C6H11NO3	0.01256585	143.453012	100.323669	128.370247	113.386026	369.872704
*	38.5	0	M+H-H2O	C28H38N6O	-0.6871674	2311.74286	2140.2532	2977.35845	2916.86249	2999.55336
*	38.5	0	M+Na	C11H20N2O	-0.5366898	639.792615	544.598927	545.540603	639.601859	1318.38837
*	38.5	7.59	M+H, M+Na,	C27H33NO5	0.02380788	14425.7522	13941.6015	14546.0197	2568.37335	3671.23432
*	38.5	0	M+H	C14H25NO5	-0.1745597	140.276909	209.013065	134.015243	2755.46156	461.514495
*	38.5	0	M+K	C19H23N3	-0.244865	808.566122	904.462002	839.264483	530.776229	893.552113
*	38.5	0	M+H-H2O	C21H43N5O	-2.0879383	2630.58039	2434.98755	2530.49087	5213.5052	5159.00929
*	38.5	0	2M-H	C6H8N2O4	4.06981373	529.811121	572.863804	551.417969	649.44237	226.330749
*	38.5	0	M+FA-H	C5H9O8P2-3	-0.5228906	227.865567	243.139752	182.502583	304.162081	257.145363
*	38.5	0	M+Na	C10H16N4O	1.20276963	6708.72204	7025.02807	6209.05879	17317.568	7018.81149
*	38.5	0	M+H-H2O	C4H9NO3	3.24621025	847.294229	988.36838	952.939132	1412.22964	805.26528
*	38.5	0	M+FA-H	C10H14O2	-1.2538119	822.136151	1005.3633	837.767333	1190.59808	1097.06717
*	38.5	0	M-H	C20H23FN4	2.31561304	3175.23099	3589.65423	3154.21265	2344.00759	1485.98502

*	38.5	0	M+FA-H	C9H14N5O5	-4.7247867	375.107471	507.223174	387.005454	529.785076	1303.16331
*	38.5	0	M+H	C15H27NO5	-0.6772941	460.126152	398.555756	497.216607	372.813166	1733.56395
*	38.5	0	M+FA-H	C12H11N5O	-0.1411118	1487.61105	1511.75132	2117.26215	2530.00539	281.07229
*	38.5	0	M+H	C17H33NO5	-1.1434234	686.119275	630.923096	590.184097	9.7531E-07	4223.35302
*	38.5	0	M-H2O-H	C4H3F7O	-2.7740968	5897.89858	6093.81246	6238.7365	6002.13338	5587.79178
*	38.5	0	M+FA-H	C17H22O7	0.63487166	1313.95882	1674.86324	1566.13435	167.557554	240.817353
*	38.5	0	M+NH4	C8H13N4O8	-2.605585	913.328235	919.381951	924.696027	2916.94779	1257.52019
*	38.5	0	M-H2O-H, M	C7H14O7S	-0.3595391	1723.81767	2281.82969	2658.35048	212.982463	158.057868
*	38.5	0	M+FA-H	C9H14N2O6	-3.3734111	1035.64675	1051.08973	1082.35865	752.598712	1039.84354
*	38.5	0	M+NH4	C23H29F2N3	-1.255626	2366.4559	2609.47152	2709.21691	927.812656	2746.65015
*	38.5	0	M+NH4	C11H15N5O	1.73076638	713.360043	812.277997	887.605648	1185.02793	853.119089
*	38.5	0	M-H	C21H31NO8	0.37536856	2267.86144	2568.68423	2141.09915	9.7531E-07	656.61161
*	38.5	0	M+H	C15H24O2	-1.0635939	1730.06654	1576.70104	1726.37241	1588.97089	1309.32482
*	38.5	0	M+FA-H	C14H16O4	0.08298029	681.5272	675.459231	799.057974	756.142883	684.984674
*	38.5	0	M+H	C14H8N2O	-3.9915642	3697.87292	3439.76817	3706.21894	13490.6504	1229.5477
*	38.5	14.9	M+Na, M+K,	C22H30N2O	9.43591981	1320452.22	1318427.9	1317768.75	4298742.46	510060.856
*	38.5	0	M+NH4	C10H15N5O	1.11471796	3105.39729	3125.84739	3370.02447	4252.83053	2606.82855
*	38.5	0	M+FA-H	C18H31N7O	-0.1906792	596.785598	555.258364	554.178646	9.7531E-07	4795.51244
*	38.5	0	M-H2O-H	C10H20N6O	0.62297538	2035.63121	2078.00868	1724.61163	1654.3008	1525.05737
*	38.5	0	2M-H	C15H13N3O	3.37348583	2727.19503	3047.31093	2791.76728	182.433403	1372.68228
*	38.5	0	M-H	C32H51NO1	0.00023133	2333.84552	2686.98358	2732.78407	133.863285	11312.5383
*	38.5	6.72	M-H2O-H	C27H41NO5	-0.1169112	3314.89293	3491.25524	3100.54354	99.6954097	19355.3737
*	38.5	0.0721	M+FA-H	C10H18N2O	-3.1995242	2825.96592	2581.3656	2618.88383	3336.3481	6092.22471
*	38.5	0	M+K	C17H16N2O	-1.0933386	1299.57419	1231.87571	1316.55894	1483.67328	9.7531E-07
*	38.5	0	M+K	C26H41NO3	2.37299034	2410.80532	2455.05974	2135.67312	296.590638	4026.93616
*	38.5	0	M+Na	C14H26O3	-0.0432915	2819.23739	2623.31366	2856.22142	3729.78242	2246.55088
*	38.5	0	M+NH4	C6H18N3OP	4.90465826	1924.60383	1887.93838	1970.07855	2338.80311	1506.14769
*	38.5	2.35	M+H	C48H86NO1	4.88369487	388316.584	457129.781	452263.808	582906.311	365727.276
*	38.5	0	M-H	C8HF15O2	-0.3078822	6069.85806	6967.41821	7521.46871	16917.5311	1851.97342
*	38.5	0	M+H-H2O	C26H44O5	-0.8526658	52377.2617	53765.4424	55619.647	62166.1691	39580.1287
*	38.5	0	M+NH4	C28H36O4	-1.214776	1149.88988	1424.99053	1064.27667	477.072499	865.08824
*	38.5	0	M-H	C16H11FN3(	-3.1288066	27101.2959	29813.6854	28181.1001	76929.7262	9.7531E-07
*	38.5	0	M+NH4	C12H24N2O	4.33654155	3266.31905	3288.10755	3192.56535	3355.33074	1941.95882

*	38.5	0	M+Na	C32H36N4O	2.727683	11140.7101	10405.0457	13311.2781	19688.584	6427.63072
*	38.5	0	M-H	C9H7F13O	0.42652837	5094.25888	5341.62811	5944.80791	5208.53974	4543.85922
*	38.5	0	2M-H	C14H10O2	-3.0258312	847.803773	782.413945	664.886239	1086.09316	1073.28182
*	38.4	0	M+H-H2O	C7H12O5	0.47062137	1911.4306	1987.46229	1807.94431	2213.49647	1558.09488
*	38.4	0.495	M-H2O-H	C3H9O4PS	1.76938036	15165.767	15930.3915	15109.7453	8167.73946	10994.4051
*	38.4	0	M-H	C13H16N2O	0.960543	30715.4083	30789.3853	30676.553	28468.4547	45416.3098
*	38.4	0	M+Na, M+K	C10H19O5P	-4.4892663	12778.9117	11919.2287	11145.4861	24020.1305	18143.8978
*	38.4	0	M+FA-H	H2KO4P	-0.457712	2529.4316	2284.43991	1996.42365	9492.04128	15558.0897
*	38.4	0	M-H, M+FA-I	C2H4O4S	-0.9864204	6468.28656	6091.68988	7344.28069	2379.12513	2625.6284
*	38.4	0	M+FA-H	C4H5NO4	-2.2438424	2194.31833	1367.76519	2284.20401	9.7531E-07	9.7531E-07
*	38.4	0	2M-H	C3H10NO4P	2.98438647	13835.8889	12548.4849	13506.2984	12337.441	2081.55017
*	38.4	7.88	M-H	C11H8O3	8.98344136	607039.373	637891.125	634619.683	703591.135	882596.257
*	38.4	0	M+FA-H	C4H2N4O2	-1.6993894	149.134987	120.303941	147.51527	238.835919	118.619855
**	38.4	0	M+H-H2O	C15H19NO9	-3.184761	6412.69023	7368.71767	6698.75227	13815.2463	1816.61044
*	38.4	0	2M-H	C6H8NO4P	-4.2985034	8064.26395	8351.23368	10328.4159	11031.594	3752.35268
*	38.4	0	2M-H	C15H13NO2	-3.453013	3205.16698	2347.29144	2092.93555	7056.03864	4152.20866
*	38.4	0	2M-H	C14H15NO9	1.5231891	1829.82633	1212.41949	1788.64232	3527.48557	1579.68058
*	38.4	0	2M-H	C21H20O6	3.40713676	6821.11124	4527.93643	3962.12577	6078.38097	4570.98783
*	38.4	0	M-H	C7H11NO5S	-1.1549318	3554.43302	3666.82817	3749.75664	1142.85148	1312.33011
*	38.4	0	M+NH4	C11H9NO4	-0.9185991	895.80227	1076.07394	929.247001	1129.62522	1085.17356
*	38.4	0	M+Na	C6H10O6	0.28852004	1539.42933	1620.74033	1618.02665	1876.19753	467.605549
*	38.4	0	M+H-H2O	C11H21N5O	0.06151357	3277.14236	3679.02324	3750.78826	3501.17587	4281.71627
*	38.4	0	M+K	C17H25N3O	4.58790142	410.94571	628.322717	686.00076	1086.73237	4397.20112
*	38.4	0	M+NH4	C12H10N2O	0.1753825	504.212604	325.786224	344.042969	147.734763	258.914406
*	38.4	0	M+NH4	C11H11NO4	0.53657293	1353.25893	1190.3953	1139.52149	637.355149	1870.52361
*	38.4	3.64	M+H-H2O	C8H7NO6S	-2.758665	6034.0155	6067.10406	6211.21222	6694.25156	34536.823
*	38.4	2.17	M+NH4	C14H16N6O	-4.6209318	12946.6246	13819.5816	13382.6481	11679.1929	93611.4475
*	38.4	0	M+FA-H	C18H18N4O	-1.1458578	1234.86417	1072.56079	1220.98685	1115.55413	1915.06745
*	38.4	0	M+FA-H	C6H17O24P	4.33538268	39494.1251	34304.0376	35321.8782	38203.0316	24272.7487
*	38.4	0	M-H	C22H42NO9	0.25138141	740.347006	488.861007	544.489302	710.919358	145.077778
*	38.4	0	M+Na	C30H31F3N8	4.5725239	2240.10046	3068.86914	2285.72424	1823.06463	15.018221
*	38.4	0	M+NH4	C27H51O12I	-1.7648274	5033.95114	5418.36287	5650.85963	9.7531E-07	14970.1694
*	38.4	0	M-H	C21H42NO7	0.03577647	4130.12692	4160.58122	3677.91575	381.090866	12109.243

*	38.4	0	M+K	C24H45D3O	-0.9859997	2644.68861	2632.6164	3040.94315	261.133857	3968.64791
*	38.4	0	M+NH4	C23H38	-1.4253882	1649.28488	2004.31983	2183.79795	711.955476	515.09047
*	38.4	0	M+FA-H	C26H38O2	-0.9632067	398.310165	399.315307	585.568825	116.561493	363.914838
*	38.4	0	M+Na	C16H32O5	-1.5497564	3670.80099	3859.23942	4005.4019	624.153215	3441.55821
*	38.4	0	M+FA-H	C47H93N2O	-0.6932331	453027.07	494782.149	483926.369	588558.253	442362.939
*	38.4	0	M+Na	C27H52NO8	3.33967977	8599.9407	9020.18776	6835.31173	2972.08862	7372.76729
*	38.4	0	M+NH4	C25H42O2	-1.909022	8376.16227	8541.45995	9083.75809	15874.4408	17336.5621
*	38.4	0	2M-H	C21H20N4O	1.9445604	2052.98241	2356.61293	1518.91399	916.722868	5037.4165
*	38.4	0	M+H	C19H30O2	-1.8548926	1225.55032	1197.34331	1029.14893	1322.38658	853.243093
*	38.4	0	M-H, M+FA-H	C27H40O3	-0.2221577	5455.45893	5059.08071	5422.2671	806.476777	10.3656933
*	38.4	0	M-H	C23H45O8P	-0.4714791	295.96906	287.919445	455.547684	9.7531E-07	9.7531E-07
*	38.4	0	M+FA-H	C24H41NO2	-0.9263276	2905.50178	2988.36549	2509.48276	4363.33116	275.276306
*	38.4	0	M+NH4	C21H44O4	-1.9330041	20660.2507	17578.3146	19865.8294	33366.3103	19104.7451
*	38.4	0	M+NH4	C22H42O2	-1.8754185	6250.00113	6965.07607	10279.37	16698.1058	9596.18677
*	38.4	0	M+NH4	C17H25NO4	-4.3933972	83981.622	72414.4782	84284.3146	212805.37	118890.964
*	38.4	0	M+NH4	C30H60NO9	-2.6787344	6405.72023	7108.18772	7799.35194	29355.9371	15015.2471
*	38.4	0	M+FA-H	C20H32	-1.0374193	752.572245	980.258581	728.346054	1479.86919	552.551347
*	38.4	0	M-H2O-H	C27H28O7	0.78396611	10528.2113	11440.742	6298.49798	20209.7599	16578.894
*	38.4	0	M+FA-H	C27H50O3	-1.0636122	2256.40076	2677.14465	2189.22729	3254.91782	80.7674135
*	38.4	0	M+FA-H	C18H32	-0.0112155	1075.69791	899.3935	1210.57627	3524.11349	764.999546
*	38.4	0	2M-H	C10H15N3O	-5.9723505	9420.62695	11585.4668	7341.46677	9037.80596	10649.146
*	38.4	0.0425	M+NH4	C17H27NO4	-4.3429409	25989.8306	22600.9637	31237.2458	100236.886	41506.1038
*	38.4	0	M+NH4	C6H4N2O	-1.6064181	5206.87599	4785.30982	5780.80555	7655.49041	3583.80489
*	38.4	0	M+FA-H	C17H32O	-0.9419538	2231.32704	2309.18285	2533.90505	1486.31567	1021.48203
*	38.4	1.18	M-H	C12H10O7	2.62100819	2655.21833	2709.61385	2625.67554	2423.77111	6861.96614
*	38.4	0	M+FA-H	C5H11O7P	-0.8649816	1323.00293	1486.22783	1571.04425	937.477367	1470.29328
*	38.4	0	M+FA-H	C12H15NO3	-0.652598	630.387618	557.586529	480.448515	290.240652	208.411484
*	38.4	0	M+NH4	C5H7NO3	1.40725259	4245.94069	3729.2375	3595.55033	7932.37716	6626.2254
*	38.4	0	M+H-H2O	C10H20NO4	0.08411984	9655.8627	9788.6205	9764.07733	12128.613	18716.4087
*	38.4	3.4	M+H, M+Na	C20H18O3	-2.4549123	5681.86952	6237.83014	6417.81997	283.04373	175.680498
*	38.4	13.8	M+H	C20H22O4	4.85186436	4501.48712	4574.9229	4163.52062	590.806401	544.664003
*	38.4	0	M+FA-H	C5H9NO	-2.1266332	862.876344	776.789783	771.201982	576.065445	822.850709
*	38.4	0	M+H-H2O	C17H26N8O	-0.9938287	329.616554	374.561531	480.497088	962.248854	1139.50191

*	38.4	0	M+H	C6H6N2O3	-0.4668404	161365.345	152642.571	142926.752	208482.388	117861.685
*	38.4	0	M+Na	C11H12N4	-0.0179366	1547.18016	1254.98146	1721.71042	2025.14496	1079.87787
*	38.4	0	M-H	C19H33N5O	0.71172684	323.106315	235.202805	403.539539	9.7531E-07	1693.58777
*	38.4	0	M+Na	C23H28N2O	-2.7938589	670.283394	717.566994	775.39216	9.7531E-07	85.7147802
*	38.4	0	M+H	C12H21N3O	-2.1274578	2689.39281	2833.59864	2755.96609	1772.00671	2656.73247
*	38.4	0	M-H2O-H	C19H37N5O	-0.610888	258.751549	297.426787	166.597534	9.7531E-07	9.7531E-07
*	38.4	0	M+NH4	C10H9NO3S	-0.0063003	736.16288	787.360518	879.242043	1274.71269	2835.93758
*	38.4	0.26	M+FA-H	C8H15O3PS	-0.2021945	2231.78799	2069.77993	2199.93733	2859.29323	7937.65857
*	38.4	0	M+H	C15H18I3NC	-4.2931076	1387.13219	1165.82929	997.351937	889.000855	152.741166
*	38.4	0	M+NH4	C18H29N3O	0.91031494	3440.71315	3285.97975	2957.25376	3668.08061	6436.3707
*	38.4	0	M+NH4	C10H15N5O	0.08012392	3055.67677	3532.94869	3345.21525	8094.51071	1704.0974
*	38.4	0	M+H-H2O	C12H13I3N2	3.30771968	748.021391	498.283417	531.906938	285.266749	9.7531E-07
*	38.4	0	M+FA-H	C13H13NO5	0.61907898	207.407878	157.144659	197.945231	356.960818	95.7109148
*	38.4	0.414	M-H	C10H8O3S	-0.6380756	3598.00637	3662.09718	3552.14609	4000.75061	3439.96056
*	38.4	0	M+H-H2O	C16H30O6	-4.591865	520.727205	524.951751	298.839479	537.011998	815.706275
*	38.4	0	M+K	C15H17FN4	-3.1092835	485.114274	386.398132	596.710644	569.58044	624.865395
*	38.4	0	M-H2O-H	C13H12F2N	-0.7748704	5350.72275	6125.03262	6049.88023	6184.25986	15912.3036
*	38.4	0	M+FA-H	C16H19N3O	-1.5704197	512.629272	577.973745	492.708969	49.2784989	3924.82465
*	38.4	0	M+Na	C18H24N4O	-0.6303604	753.204502	893.64695	661.078961	9.7531E-07	137.211301
*	38.4	2.59	M-H	C2H4N4S	-1.8953327	9532.3643	8628.82094	8078.83703	12780.4408	13067.2764
*	38.4	0	M+NH4	C9H11N5O	-0.2855249	57172.398	57688.3076	57855.9761	81787.9508	44223.1506
*	38.4	0	M+FA-H	C2H5NS	-2.1241685	187.402322	214.556814	165.590962	112.003879	264.573629
*	38.4	0	M-H	C10H14N5N	-3.596874	834.598515	990.63349	773.149135	1651.8197	351.682346
*	38.4	0	M+FA-H	C6H7NOS	-2.0040178	110.462557	104.672782	79.2132301	113.591654	335.285381
*	38.4	0	M+FA-H	C8H6O2	-0.8697791	619.619331	633.054624	619.134067	526.726178	479.857004
*	38.4	0	M+K	C19H21N3	-0.4631916	172.104102	261.833483	244.862858	11.8567186	9.7531E-07
*	38.4	8.03	M+H	C12H7N3O2	6.12900224	10981.8	12011.1847	11788.9367	20090.195	10325.2885
*	38.4	0	M-H2O-H	C21H21NO6	2.89074632	736.094778	633.461569	954.908854	53.2646202	407.501421
*	38.4	0	2M-H	C17H12N2O	4.3381734	1402.43919	1458.56117	1228.27798	848.567146	9.7531E-07
*	38.4	0	M-H2O-H	C14H14O11	-3.8488469	481.71556	441.359734	470.138124	9.7531E-07	57.6631564
*	38.4	0	M+FA-H	C22H30FN3	-3.0517945	1329.38119	1118.55101	1418.69659	1569.79196	407.897977
*	38.4	0	2M-H	C11H15FN2	6.11692647	489.63665	378.088931	290.601723	166.685184	2471.64393
*	38.4	0	M-H	C33H59O15I	4.31964985	1189.32495	1666.9391	1291.16619	298.704113	119.874481

*	38.4	0	M+K	C9H16N3O1	2.22290406	710.496861	700.425848	1019.83237	2.11947231	156.716433
*	38.4	0	2M-H	C14H11N	-4.9587715	738.567247	698.851785	773.800856	352.526087	199.445423
*	38.4	0	M+Na	C15H24O3	-1.0834357	2376.93808	2486.84134	2432.20849	3232.65429	1728.76885
*	38.4	5.46	M+H	C20H24O8	-7.2266672	3010.65476	2943.58994	2955.96044	1934.58867	1574.90189
*	38.4	5.04	M+H	C20H19N5	4.92496391	2663.25731	2663.82422	2542.12525	602.718772	209.018422
*	38.4	0	2M-H	C15H15O8P	3.21244813	1652.50897	1523.96588	1208.2759	2023.84686	895.982114
*	38.4	0	M+NH4	C20H28O3	-1.7136591	2262.1151	2146.82828	2169.91497	4479.97133	905.89697
*	38.4	0	M-H2O-H	C8H16N6	5.45014223	2814.90092	2735.48861	2644.44882	1218.52424	481.832725
*	38.4	0.225	M+FA-H	C14H23N3O	3.71990534	24681.5284	28613.0841	29007.5226	12425.9471	4886.78331
*	38.4	0	M-H2O-H, 2M	C8H15N3O5	-5.0154221	1842.53153	1787.59573	1669.26297	1211.06315	938.89921
*	38.4	0	M+FA-H	C7H7NO	-2.1350326	1722.72632	1786.55247	1730.44203	1823.71287	1556.78817
*	38.4	0	M+H-H2O	C27H42O9	4.94454294	3065.64234	3417.49847	3043.63093	9.7531E-07	5704.51743
*	38.4	0	2M-H	C5H7NO4	5.18694347	1743.62533	2017.88396	1914.40129	2575.5033	1965.71342
*	38.4	0	M+H	C11H8FN5	2.38320633	559.523821	563.790248	538.780117	243.170668	306.7185
*	38.4	0	2M-H	C5H4N4OS	-0.6709164	7915.88797	8452.35435	8209.22961	8286.3816	6930.09085
*	38.4	0	M+NH4	C26H29O16	-0.5946756	11498.3989	10656.3985	10632.6379	8912.01666	8078.60503
*	38.4	0	M+NH4	C25H42O6	-0.8113088	1351.12705	1457.79626	1681.51223	672.530788	4796.29094
*	38.4	0	M+FA-H	C4H7NO	-2.7739041	257.212815	288.891398	237.476995	280.927555	223.236415
*	38.4	0	M+NH4	C26H28O10	-1.3194858	886.059232	677.623734	1000.50856	2408.98027	110.760888
*	38.4	0	M+NH4	C18H27NO	-1.6823344	452.267725	541.441742	496.474249	3199.20839	695.480238
*	38.4	0	M+H	C33H54NO1	-0.8946441	4615.00168	4816.65702	3135.2968	9.7531E-07	2155.64319
*	38.3	0	M-H, M+FA-H	C26H28O14	0.8985414	8102.7838	7889.26424	6970.98695	5595.69007	7589.93692
*	38.3	0	M+Na	C11H23N3O	0.54881113	7647.49068	4831.64367	5908.22489	8648.79058	8688.05858
*	38.3	0	M+K	C19H22N4	3.0595084	1312.94422	1364.87564	1458.20838	2408.43015	2356.10739
*	38.3	0	2M-H	C16H13N3O	0.29643515	1926.13425	1276.74821	1151.19215	1657.82178	284.922774
*	38.3	0	M+H-H2O	C22H39O7P	0.79894124	3190.0957	2091.23892	3728.89304	8491.58282	5355.98319
*	38.3	0	M+FA-H	C11H11I3O3	2.74343804	5286.29437	5224.03636	7072.50101	2979.99805	904.387105
*	38.3	0.25	M-H2O-H	C10H20O7P	-2.7243309	26348.807	27888.6342	28114.9346	313.57895	9.7531E-07
*	38.3	0	M+NH4	CN4O8	3.69435354	4746.2061	4414.15288	4947.68685	5857.60676	1973.8804
*	38.3	0	M+H	C6H5F2N	-1.6631908	175.104869	119.950188	119.25738	40.1766264	9.7531E-07
*	38.3	0	M+H, M+NH4	C7H12O3S	0.89789345	2342.66561	2195.4342	2795.10746	15428.4531	867.139984
*	38.3	0	M+NH4	C11H15NO3	0.34172223	767.871049	773.3121	743.577904	749.303077	1616.93638
*	38.3	0	2M-H	C5H11AsO2	2.25470204	747.0399	807.893507	970.413315	915.932599	165.866448

*	38.3	0	M+Na	C12H14FNO	2.19233868	754.853953	976.86065	899.780089	123.346119	374.261285
*	38.3	0	M+FA-H	C20H16N4O	-1.1950318	182951.214	212427.649	164991.904	121789.359	248058.529
*	38.3	0	M-H	C10H15N4O	-1.1740513	2855.03104	3497.06911	2006.83367	9.7531E-07	9.7531E-07
*	38.3	0	2M-H	C19H13NO5	-3.3110363	1240.63953	1517.03701	1579.5405	847.719467	9324.67019
*	38.3	0	M-H	C8H9FN2O2	-3.6769762	17500.6314	17470.3538	17238.3782	15728.9422	22112.907
*	38.3	0	M+H-H2O	C18H13N5O	-0.3970295	936.880211	1195.70328	1427.27669	529.353109	2032.56017
*	38.3	1.7	M+H	C12H14O8	0.32533183	22877.9076	24082.5086	24174.7149	2099.39197	22378.3337
*	38.3	0	M+FA-H	C13H13N3O	-2.773155	430.60052	378.823688	450.920994	77.3070307	897.927849
*	38.3	0	M+NH4	C16H19NO8	0.09727657	1482.30533	1221.07973	1764.89487	9.7531E-07	6123.28039
*	38.3	0	M+H-H2O	C17H17N3O	3.33883399	1203.05962	975.734954	822.5484	9.7531E-07	5934.54663
*	38.3	0	M+K	C25H31NO3	0.88496233	3688.24685	2759.7348	4034.40594	9.7531E-07	16749.5514
*	38.3	0	M-H2O-H	C10H9NO2S	-0.4658507	11379.0457	12863.5526	12234.4448	39191.6264	20947.9246
**	38.3	0	M+Na	C21H30O2	-0.3114728	15088.0083	15942.875	15329.1495	33113.1765	12494.0364
*	38.3	0	2M-H	C8H9O6P	1.68666762	9749.70704	9743.19349	9481.46524	9982.82338	8132.82824
*	38.3	0	M+FA-H	C16H20N5O	0.0459162	8850.53072	11884.1204	12038.9382	9332.27278	10627.7238
*	38.3	0	2M-H	C19H15N5O	4.55903003	4796.43124	4033.78448	6335.70038	2057.67353	1817.68203
*	38.3	0	M+H	C17H26O3	-2.7743867	3286.53378	3368.86696	3311.96337	1398.26552	6519.34164
**	38.3	0	M+H-H2O	C17H32O4	-1.9198683	1448.47182	1369.76054	1300.49636	1081.29791	4351.9814
*	38.3	0	M-H	C29H41F2N5	-0.6462187	36055.2794	35081.9864	33911.2387	5625.49835	148258.645
*	38.3	0	M+H, M+Na	C14H27KO2	-2.1882436	18081.7162	18735.6776	17150.3734	17452.2529	12796.0355
*	38.3	0	M+NH4	C35H32N2O	-1.2268462	14443.4931	13561.5674	13030.5645	10374.2094	10658.6907
*	38.3	0	M+H-H2O	C28H48NO9	-0.7011685	2588.64962	2943.81894	2448.91906	2037.45552	690.300142
*	38.3	0	M+K	C23H24N6O	0.00113397	1032.10876	754.367996	978.75836	2068.58245	9.7531E-07
*	38.3	0	M+FA-H	C19H22N6O	-0.0355926	6469.05582	7976.80847	8168.61738	10403.0872	1929.2791
*	38.3	0	M+FA-H	C15H20O2	-1.0992315	1490.26875	1505.61573	1508.24524	1281.82315	1393.85465
*	38.3	0	M+FA-H	C9H8I2O4	-4.5894924	175460.135	189229.432	180975.642	187832.86	127214.945
*	38.3	0	M+NH4	C31H50O4	-0.8044814	13601.8872	12725.6041	14125.762	6243.73262	48559.6772
*	38.3	0	M+FA-H	C25H43O7P	-0.3497181	25599.4056	25665.4697	26168.155	29843.093	12936.5559
*	38.3	0	M+NH4	C16H18FN3O	-1.4315104	2405.73754	1891.04135	3347.38552	1211.98497	1495.52335
*	38.3	0	M-H	C31H58NO9	-0.344337	1834.53872	2950.3651	2331.06739	2417.31071	8522.19001
*	38.3	0	M+NH4	C22H29NO5	-1.6673742	1787.93467	1543.1992	1452.85708	1620.10223	1294.55838
*	38.3	0	M+H-H2O, M	C20H36O3	-1.8042775	1225.64888	1022.49525	1233.40876	829.237732	437.100336
*	38.3	0	M+H, M+Na	C22H41NO2	-1.7809902	2220.59171	2454.52712	1981.29241	8231.69647	1729.22157

*	38.3	0	M+H	C17H24N3O	-0.155411	3218.52688	3235.41482	2350.04885	7011.34708	2936.87843
*	38.3	0	M+FA-H	C9H12FN2O	3.10270357	2593.2855	3735.04553	2506.74422	2249.17572	2098.46212
*	38.3	0	M+Na	C22H29N10(	-0.0269316	44402.9955	59509.6197	48056	88818.5906	47633.5014
*	38.3	0	M+Na	C26H52O7	-1.4205594	2507.82962	2256.39429	2081.6184	9.7531E-07	1429.92372
*	38.3	0	M+NH4	C6H8N2O2	0.8145661	2698.35755	2943.65288	2318.42313	4181.26129	2366.69117
*	38.3	0	M-H	C24H42O2	-0.3526922	64212.7265	43789.1468	50949.482	85958.3477	40106.415
*	38.3	0	M+FA-H	C8H9NO	-2.2020862	1505.61949	1438.7376	1455.22544	1770.24597	2072.74607
*	38.3	5.78	M-H2O-H	C30H22O12	2.39415696	66394.5595	67259.5689	69151.9772	83295.712	82053.9026
*	38.3	0	M+NH4	C21H28O12	-0.0597144	5609.05606	4005.88617	4533.83524	9.7531E-07	30430.7292
*	38.3	0	M+NH4	C9H11N5O	6.26292754	1310.66647	1240.91294	1293.96836	1597.13293	999.243296
*	38.3	0	M-H	C5H11NOS	-1.3919452	811.841179	720.063118	741.278081	52.941162	792.557036
*	38.3	0	M-H	C10H18N2N	-0.5195032	1691.71479	1741.45556	2108.93198	2059.30174	4706.34041
*	38.3	0	2M-H	C10H6N2O3	6.43056128	1022.4613	712.310531	1120.65366	9.7531E-07	4450.20826
*	38.3	0	2M-H	C9H6F3N3O	3.92670791	72.4266474	99.0123677	83.3471527	9.7531E-07	9.7531E-07
*	38.3	0	M+Na	C7H12O4	-0.5281581	1727.91787	1975.05349	1661.60627	3150.61521	2130.40998
*	38.3	0	M+FA-H	C6H11NO	-2.3497718	225.658834	223.070426	330.712116	138.197311	218.517167
*	38.3	0.0787	M+Na	C6H7N5O2	0.601091	22893.5334	22989.7286	24205.1454	30389.163	18612.6811
*	38.3	0	2M-H	C6H6O2S	-1.249435	194312.697	211843.876	202195.377	188764.625	209010.07
*	38.3	0	M+H	C10H16N4O	-2.5414541	1691.21776	1745.65638	1495.31439	8164.44473	2214.99799
*	38.3	0	2M-H	C13H7NO2	-2.9392849	2835.96519	2934.96768	2726.98286	9100.0187	3354.9503
*	38.3	0	M+NH4	C8H15NS2	3.05510029	3393.36638	3358.66578	3641.67989	4545.57473	2561.71153
*	38.3	0	M+H	C6H9N3O5	-4.0776722	525.730842	515.692141	570.489393	62.4618468	141.045835
*	38.3	0	M+H-H2O	C9H12IN	-4.1826839	18141.1737	19343.397	19957.3346	26445.2053	13702.7294
*	38.3	0	M+K	C17H29NO6	-2.9254872	1861.91898	2100.81675	2015.57897	618.287051	3518.33854
*	38.3	0	M+Na	C9H18O6	1.58533053	1355.87616	1474.87323	1356.38512	1993.82376	731.052878
*	38.3	0	M+FA-H	C5H5N	-2.2527077	151.676488	144.164044	148.833351	170.839508	115.760991
*	38.3	0	M+K	C21H31NO	-2.3799338	1157.64873	1233.90525	1627.74909	508.498052	1954.55968
*	38.3	0	M+FA-H	C6H2F4	-1.6497027	251.446285	213.748833	233.889564	274.747437	209.516989
*	38.3	0	M+K	C21H46NO6	-4.0767299	3773.77924	4097.63241	3565.39271	1584.66684	5239.03713
*	38.3	0	2M-H	C20H19N	-3.4483062	650.498919	557.658501	726.9467	9.7531E-07	276.422571
*	38.3	0	M+Na	C33H57NO1	0.4605109	4510.36796	4728.21917	4273.81647	4235.16154	635.02146
*	38.3	0	M+K	C30H56O2	-3.3989524	3719.87133	3945.76322	3572.12132	16279.0216	3724.0033
*	38.3	0	M+NH4	C25H34O11	3.22557872	1600.61195	1122.35639	1858.15242	186.393882	624.198926

*	38.3	0	M-H2O-H	C36H54N8O	-2.2018982	1835.90375	1865.94513	2022.24438	5274.92986	2174.88642
*	38.3	0	M-H	C19H33N3O	0.12323444	1086.40178	1097.90523	1370.74723	98.3200406	2505.29891
*	38.3	12.2	M+NH4	C23H34N4O	-3.9909523	3723.00543	4572.60723	3387.09634	170.356363	91.0006135
*	38.3	0	M+FA-H	C15H23N3O	-0.0042533	891.213404	814.625751	878.33325	1044.00813	350.742054
*	38.3	0	2M-H	C12H9N3	5.66699976	774.148519	983.349062	891.697777	9.7531E-07	6269.87658
*	38.3	0	M-H	C5H11NO2	-2.2058881	186.737412	190.127871	250.529434	209.801777	232.753806
*	38.3	0	M+K	C17H12N4	-0.0828046	1556.40722	1460.27746	1476.03666	2477.84178	2047.10658
*	38.3	0	M+H	C22H44N6O	-0.0224752	1224.65403	672.683014	995.35492	1272.5039	174.540567
*	38.3	0	2M-H	C7H10N2OS	1.34714229	393.933522	662.706222	491.490705	442.735488	237.466415
*	38.3	0	2M-H	C24H21NO2	-2.7504449	495.445133	559.568406	522.809967	9.7531E-07	9.7531E-07
*	38.3	0	M-H2O-H	C9H14N3O9	4.99805848	554.147849	567.330473	559.31752	53.1587049	88.1191025
*	38.3	0	M-H2O-H	C13H12O11	2.74480354	481.721918	596.414334	780.84816	599.041789	436.229583
*	38.3	0	M+FA-H	C7H5NO3	-1.355093	432.282412	470.502216	373.891707	651.844064	357.45459
*	38.3	0.398	M+FA-H	C8H14N2O7	-4.0606433	1607.21095	1895.47245	1609.08768	2226.30921	5795.83203
*	38.3	0	M-H	C15H18N6O	0.61323519	3496.82502	3369.66619	3496.08343	7743.4775	1421.15006
*	38.3	0	M+NH4	C18H21NO6	-1.5802303	1089.89757	1154.50783	1015.39011	4152.20761	708.631312
*	38.3	0.205	M+H-H2O	C20H32F2O5	2.05590069	2302.0348	2309.57604	1996.61993	9.7531E-07	326.334051
*	38.3	0	2M-H	C9H10N2O	-4.8521863	489.616486	609.005451	444.513593	9.7531E-07	494.750543
*	38.3	9.1	M+H-H2O	C17H22O3	-2.2021808	1785.26103	1991.96038	1840.77424	1430.86308	10398.1369
*	38.3	0	M+K	C24H35NO5	1.13771274	1252.58537	1810.34446	1695.20667	5634.57759	86.6569874
*	38.3	0	M+H	C12H22O4	-0.7235152	740.459304	825.143955	1011.95311	932.438543	519.539149
*	38.3	0	M+Na	C17H22N2O	-0.4696205	392.7989	382.388467	309.196589	78.1137105	45.9986834
*	38.3	0	M-H	C20H12N2O	-0.954495	1060.56298	941.236327	1019.52452	1095.49206	852.492401
*	38.3	0.389	M+FA-H	C24H24O5	2.24299102	16229.0968	16018.3693	16641.0861	10777.7071	5643.53606
*	38.3	0	M+H-H2O	C24H23N7O	1.51593383	3500.28688	3471.01881	3023.70722	3036.74306	1131.45077
*	38.3	0	M+Na	C10H20O4	-0.4235611	3286.82803	3260.12562	3479.83074	3604.65524	2538.0291
*	38.3	0	M-H	C20H21FN4(	2.34893093	2181.14582	2280.91252	2544.26441	2252.86608	2028.75943
*	38.3	0	M+Na	C9H14N2O5	5.75930315	828.454334	721.597066	902.370176	323.37149	120.300003
*	38.3	0	2M-H	C13H8O2	-2.9396248	1610.67508	1662.24315	1453.13396	2191.07617	3350.71558
*	38.3	0	M-H	C5H8N4O12	2.86503617	2920.09645	3604.8227	3119.87165	2989.39016	2627.56282
*	38.3	0	M+Na	C30H40N2O	1.07992483	5508.72517	7485.89409	5340.28281	6513.14958	4877.97843
*	38.3	0	M+Na	C13H16N4O	1.32008367	851.161621	821.982044	856.282998	799.012509	325.642502
*	38.3	0	M+FA-H	C19H18N2O	-0.5654988	1412.46642	1228.20609	1054.8963	906.079434	1013.82086

*	38.3	0	2M-H	Fr	-1.860632	6794.3972	5908.89151	6201.37184	6611.13189	5304.47548
*	38.3	0	M+H	C2H2F3NO	4.85935908	1072.53728	1181.64169	1002.53892	1563.81583	917.457712
*	38.2	0.366	M+H-H2O	C4H7O2S-	0.87015445	8421.85337	8905.25774	8916.28363	4345.06563	3773.02789
*	38.2	0	2M-H	C8H6INO5	-4.5581012	5040.44985	3093.43168	3348.11728	4043.06166	4964.49036
*	38.2	0	M+Na, M+K	C14H14N4O	-6.1026686	10446.4581	11057.3296	9066.14173	14540.113	29471.0818
*	38.2	0	2M-H	C9H9NO5S	4.90291861	6169.41484	5895.81176	3990.31593	23664.5292	17606.1302
*	38.2	0	M+NH4	C6H13O8P	0.68858205	3038.15162	2294.30396	2929.98222	597.664421	13498.5158
*	38.2	0	M+Na, M+K	C25H28O12	4.54219973	3490.50546	3354.96507	3554.86976	76.9949808	16543.7049
*	38.2	0	2M-H	C8H10O2S	1.52279829	14542.3681	14794.0628	13213.6812	10820.9578	1821.00651
*	38.2	0	M+FA-H	C6H8N3O4P	-2.2277252	2770.57489	2236.79497	2749.15889	3121.23245	9.7531E-07
*	38.2	0	2M-H	C5H12NO5P	3.89262343	2326.6639	1730.43065	2269.52258	882.539164	420.521217
*	38.2	0	M+H	C7H15NO4	-0.4916919	3336.01354	2115.75679	2991.30973	13480.383	9.7531E-07
*	38.2	0	M+H	C7H5NOS	0.85385064	549.923238	602.165418	540.992839	582.272202	457.457769
*	38.2	0	2M-H	C2H3N3S	-1.943606	9946.47222	9852.5563	9481.95411	14507.5809	11491.3184
*	38.2	0	M+NH4	C6H7NO	-1.9415828	173.760243	178.694134	131.022393	9.7531E-07	171.925878
*	38.2	0	M+H-H2O	C10H16N2O	-0.5383964	4252.10181	6134.09301	7238.21071	7083.66841	1440.59119
*	38.2	0	M+K	C6H6N4O	1.00447365	438.476908	494.612219	418.032731	920.782884	192.606055
*	38.2	0	M+K	C8H8N4O2	0.88976601	632.763698	496.67062	675.855089	1097.76712	1402.588
*	38.2	0	M+Na	C7H13NO3S	1.54340518	894.744821	554.535004	705.578736	1370.6258	3158.25141
*	38.2	0.422	2M-H	C6H12N3PS	2.55546799	12665.9122	12595.3598	12771.6084	19161.9991	15723.0546
*	38.2	0	2M-H	C15H12O4	5.00169305	1189.95987	857.107856	1308.61912	392.183734	7413.35758
*	38.2	0	M+Na	C6H12OS	4.64456932	2063.51474	2484.44073	2262.09182	3023.34533	2505.84986
*	38.2	0	M+FA-H	C3H5NOS	-1.4615842	1885.35582	1867.56973	1929.50962	1539.65397	1825.45847
*	38.2	0	M+FA-H	C6H9NO2	-1.7279673	235.436912	251.45863	262.639649	500.934368	265.176179
*	38.2	0	M-H2O-H	C8H14N3O6	3.56131196	1222.35601	1123.92944	1062.83636	1081.04156	1017.09057
*	38.2	0	M+FA-H	C6H9NO3	-1.5021782	288.538645	351.316962	273.656006	280.237046	114.017
*	38.2	0	M+K	C7H7IO	-1.0298184	13555.3196	14165.2752	14005.1438	4135.29875	879.403379
*	38.2	0	2M-H	C7H6O7	4.50654188	2122.7183	2382.77013	2012.64379	1452.01761	255.515456
*	38.2	0	M+NH4	C10H12N2O	-0.0973648	518.382176	622.841602	507.526324	9.7531E-07	9.7531E-07
*	38.2	0	M+Na	C12H12O2S	0.80103043	1642.56896	1562.98441	1933.7431	2588.20552	10539.3935
*	38.2	0	M+FA-H	C7H5NO4S	-0.5459191	1316.19782	1325.45696	1509.76419	1873.77887	3922.521
*	38.2	0	M+FA-H	C7H5NO2	-0.6282454	610.674324	475.360452	412.875501	422.923389	419.341778
*	38.2	0	M+H-H2O	C47H80O19	1.27265746	280787.437	275022.433	232185.951	368403.902	196694.892

*	38.2	0	M+Na	C45H88NO1	1.61950061	307259.159	321154.244	278059.434	398043.289	259554.37
*	38.2	0	M+H	C24H48NO7	-0.9732143	3177.5781	2168.24222	3372.37324	443.986688	10265.5617
*	38.2	0	M+Na	C17H22O3	0.30888535	8837.53517	8173.35432	8124.64058	8767.08211	12381.1763
*	38.2	0	2M-H	C15H12N4O	4.86408698	1135.05225	977.427558	660.461342	9.7531E-07	508.073532
*	38.2	0	M+NH4, M+N	C17H24O3	-2.1793075	3658.15858	3763.48023	4107.31881	6912.95084	2335.02918
*	38.2	0	M-H2O-H	C30H49N6O	0.89990799	115864.73	119503.92	113924.185	59729.8641	240075.168
*	38.2	0	M+Na	C23H36O5	0.21628057	111.382908	76.3395764	126.928906	109.5753	39.117443
*	38.2	0	M+NH4, M+N	C33H58O10	2.85434915	7466.66045	8094.65642	6201.9826	1142.82674	7322.3985
*	38.2	0	M+NH4	C13H13N5	-0.6570269	1384.82475	1256.79777	1310.65821	1408.82056	823.458722
*	38.2	1.31	M+NH4, M+N	C20H36O2	5.97179706	7138.54277	5789.76422	6351.62828	2856.52644	625.605515
*	38.2	0	M+Na	C15H32O3	-1.5189127	2219.60738	2224.06097	2306.87158	2753.5072	1586.18916
*	38.2	0	M+H-H2O	C18H38N6O	-0.6912047	4742.11167	5226.30961	4952.203	4108.95655	3415.25431
*	38.2	0.666	M-H2O-H	C26H50NO9	-4.6411236	45614.9328	42594.3803	44118.0214	13290.0767	91048.0329
*	38.2	0	M+FA-H	C18H28N4O	1.66803476	5917.95751	5604.03033	5300.70231	4752.88386	8416.8091
*	38.2	0	M+FA-H	C20H37NO5	-1.0476139	1248.68145	1229.65125	1564.7459	86.3857386	76.9827206
*	38.2	0	M-H	C19H19FN4	0.54376533	4309.59596	5499.1964	3995.89856	11132.6574	1782.29605
*	38.2	0	M+H	C13H24N5O	0.16714764	886.347002	1160.74042	1186.82652	6393.89217	564.390869
*	38.2	0	M+FA-H	C37H66O2	0.40019299	6180.28521	4419.86993	7289.86525	9106.04681	1438.10093
*	38.2	0	2M-H	C10H19NO3	3.26529335	2068.74336	2696.63752	1786.60011	4448.70965	2000.06326
*	38.2	0	M+FA-H	C35H64O4	-0.2734984	5792.32774	6191.82065	4513.62051	3225.57053	2119.71506
*	38.2	0	M+Na	C36H68O6	-0.6314968	9248.72996	7068.46621	7501.85172	4529.23263	4036.06754
*	38.2	0	2M-H	C21H14O5	-3.9834884	3912.91345	5666.00482	5842.54558	7185.80085	8471.24422
*	38.2	0	M+FA-H	C35H44O22	-1.6026696	10209.0309	9811.58038	8859.14393	16710.395	24007.018
*	38.2	0	M+Na	C2H7NO3S	0.78549249	386.426278	457.48479	421.284992	1719.09435	730.002786
*	38.2	0	M+FA-H	C7H9NO4S	-0.0524293	2744.12165	3011.90486	2977.38716	1643.11561	3576.40059
*	38.2	0	M+H-H2O	C6H12O4	1.29682216	755.25144	813.257128	796.404889	1028.3886	674.617879
*	38.2	0	M+H	C7H13NO2S	0.46592223	1665.13204	1800.29875	1566.4459	2130.76387	2951.78258
*	38.2	0	M+H-H2O	C12H21N4O	3.37194559	5289.48199	4684.30912	4329.68941	1825.95907	1490.62411
*	38.2	0	M+NH4	C11H14O7S	0.38553181	1727.1851	1686.372	1638.59591	1576.91068	569.113703
*	38.2	0	M+H-H2O	C6H8O5	1.20337427	348.006569	398.308587	406.193367	435.543107	201.575393
*	38.2	0	M+FA-H	C8H13FO2	2.0739208	742.159654	902.436305	868.491715	1302.24121	351.745823
*	38.2	0	M+NH4	C21H30O10	-1.6123537	1936.49617	2123.55881	1416.89831	460.159907	2441.8921
*	38.2	2.14	M+FA-H	C12H7NO3	3.80441377	21018.2424	21699.5428	21514.0327	20396.3856	43168.1171

*	38.2	0	M+K	C26H38N2O	-0.4342377	1629.90365	1195.99806	1072.47929	4168.71624	715.024819
*	38.2	0	M+K	C24H40O20I	0.90303959	6550.82354	6447.06053	5799.66181	41493.5877	2731.19899
*	38.2	9.89	M+H	C23H39NO6	-0.9246923	3663.20192	4261.83335	4037.67481	2608.50699	12736.199
*	38.2	0	M-H2O-H	C12H20N4O	4.81299432	2326.08507	2486.92226	2934.35888	395.588107	4672.9404
*	38.2	0	M-H2O-H	C19H17NO2	0.34135528	553.441451	688.620377	888.829794	1851.78593	144.688741
*	38.2	0	M+NH4	C37H41N2O	1.85668277	4849.28816	5024.20143	3935.06045	708.576588	2707.71427
*	38.2	0	M-H	C21H23F2N(	-0.5777136	249.463583	230.384883	294.825253	375.071458	237.949394
*	38.2	4.77	M+Na	C21H41O6P	0.52161892	6722.12885	6249.17159	7458.29624	1583.04594	7758.64047
*	38.2	0	M-H	C6H7N3	-1.5631874	724.357461	751.719423	716.425824	723.322792	686.272478
*	38.2	0	M-H	C10H9N3O8	-3.0145235	1112.66615	1008.85443	980.76356	1030.54254	666.522118
*	38.2	0.00543	M+NH4, M+I	C33H50N4O	1.29958262	25571.4925	25513.4535	24228.6807	17266.1923	52241.8251
*	38.2	0	M+FA-H	C7H10N2O6	-4.3788139	1396.47425	1356.33699	1492.10127	1243.8094	1408.26792
*	38.2	0	2M-H	C10H18O9	-2.8171215	143.513122	179.007485	218.772537	121.540702	1758.20297
*	38.2	0	M+H	C24H31NO6	-0.3983578	1154.97252	1075.13638	952.612088	9.7531E-07	131.470038
*	38.2	0	M+NH4	C20H40NO6	-4.2951189	710.839695	950.373375	836.202497	1655.07472	1664.60304
*	38.2	0	M+H	C12H22N6O	0.56350611	1931.64568	1908.05669	1322.63828	771.488013	576.130304
*	38.2	0	M+K	C18H14FNO	-2.0608365	1348.99549	1647.78698	1489.89447	2696.38779	3418.76388
*	38.2	0	M+FA-H	C14H21NO3	0.16714858	38.9904212	54.2447632	58.0160713	78.5920439	9.7531E-07
*	38.2	0	M+NH4	C29H53O15I	-1.014544	4952.17122	7014.6678	5375.98886	7400.25594	2999.71276
*	38.2	0	2M-H	C18H20O4	5.94526846	919.933066	1027.63995	1154.98592	461.812839	257.573897
*	38.2	0	M+Na	C5H5N5O2	0.02397567	227.224567	219.717215	195.452859	347.603821	241.45078
*	38.2	15.5	M+H-H2O	C26H36O8	2.40872746	6476.88653	6529.32571	6547.37172	3573.76209	14494.1171
*	38.2	0	2M-H	C10H9NO4S	-2.7176602	2937.27038	3465.81653	2535.18311	1274.33846	976.881191
*	38.2	0	M+FA-H	C6H5NO2	-1.4237376	1082.82231	811.575954	686.200099	805.833579	631.397724
*	38.2	0	M-H2O-H	C21H27F2N!	-0.8502567	2537.94344	2448.03957	2107.88962	9.7531E-07	8554.69312
*	38.2	0	M+FA-H	C26H28O6	2.33490047	267.550121	357.220002	233.675759	66.976327	94.20775
*	38.2	0	M+Na	C18H35NO4	-0.4090097	3322.72363	3810.47222	3659.56964	2402.75382	2607.28488
*	38.2	0	M-H2O-H	C7H12O13P	2.0373149	1675.92225	1669.0596	1778.01951	1593.07979	1563.20606
*	38.2	0	M-H	C20H19N5	5.91751361	694.622615	498.324706	725.748628	171.881615	225.053552
*	38.2	0	M+H-H2O	C12H24O2	-0.7233539	986.895789	940.887165	997.417424	1304.2473	863.675893
*	38.2	0	M+FA-H	C18H28N2O	4.9812496	305.987414	308.888904	287.118584	9.7531E-07	47.2936687
*	38.2	0	M+FA-H	C17H33NO3	0.34295164	975.424706	1118.54329	888.334375	246.537222	192.15138
*	38.2	0	M-H2O-H	C17H20F6N2	4.51015708	264.894969	277.750835	234.027775	611.784839	113.188348

*	38.2	1.44	M-H	C21H36O5S	-0.2835074	5117.28051	4878.51802	5181.0355	1446.81811	19524.9245
*	38.2	0	M-H	C16H18N2O	4.64000474	990.107717	866.741189	1067.75012	1037.62329	816.183614
*	38.2	0	M+Na	C28H48FNO	-0.1599408	17540.4711	15191.4183	13992.3355	21264.3313	11752.4583
*	38.2	0	2M-H	C15H25NS	2.63132946	18244.3717	18738.0789	23180.7321	21347.7842	21576.7386
*	38.2	0	M+NH4	C19H41O6P	5.11443639	2158.12899	1993.82339	2113.24941	942.231588	5098.65977
*	38.2	0	M-H2O-H	C11H16O4	-1.4970372	4111.3395	3813.6855	5148.52482	4586.63921	3915.87093
*	38.2	0	M-H2O-H	C19H20N6O	0.80365313	858.425065	780.343215	834.747297	224.810624	842.515232
*	38.2	0	M+FA-H	C29H30N6O	3.52805218	7310.0961	6116.06812	6488.89118	6080.9847	1398.23516
*	38.2	0	M-H	C28H22O12	0.41436261	4904.05389	4422.64069	5613.31072	4583.97993	3381.54868
*	38.2	0	M+NH4	C11H11N5	0.52346793	1698.77361	1561.90804	1678.41435	2830.98156	1389.40732
*	38.2	0	2M-H	C8H15NO2S	1.89456303	6110.6793	6318.01044	6225.5661	5975.21768	5421.29517
*	38.2	0	2M-H	C7H6N2O4S	-2.0791991	1130.23387	1658.86993	1818.92635	1490.04963	1242.52249
*	38.2	0	M+H-H2O	C8H8NO4P	3.38551283	643.853155	674.364109	456.712103	824.165515	373.660096
*	38.1	0	M+H	C3H9O3PS	0.6698036	995.351521	1126.04101	1002.55658	248.041651	135.616889
*	38.1	4.56	M+Na	C7H5NS	-3.8947902	12890.3377	11674.3956	13223.7427	7096.6705	4495.8846
*	38.1	0	M+FA-H	C19H26N2S	-3.8641932	5689.1654	5162.83903	6698.23845	3388.69984	7823.80942
*	38.1	0	M+Na	C25H43O11I	2.48944847	6901.22295	7283.71411	8934.46055	6534.49065	3097.51072
*	38.1	0	M-H	Ca2O7P2	1.56869277	13126.7437	10604.2288	11413.0495	9.7531E-07	9.7531E-07
*	38.1	0	M+H-H2O	C20H43AsO	0.11547463	3178.92214	2199.57068	1928.83261	1697.0547	760.006576
*	38.1	0	M+FA-H	C7H6NO2-	1.85983856	5500.62794	4125.93233	4093.50077	7938.05053	4392.04708
*	38.1	0	M+K	C11H14N4O	0.01238011	36742.6829	43813.4652	41935.7978	99601.4576	59532.5504
*	38.1	0	M-H, M+FA-H	C21H16O7	-0.5065814	236913.664	226667.286	228533.767	99178.7771	789789.988
*	38.1	0	2M-H	C7H8OS	3.20534376	69738.242	73130.6787	66601.0713	66169.3144	11717.0728
*	38.1	0	2M-H	C3H9NO4P-	4.05111146	10358.5045	9730.36956	10162.178	2061.97892	9.7531E-07
*	38.1	0	M-H, M+FA-H	C20H18O7	1.55512252	2218.06734	1716.38254	1878.78378	14932.6234	14174.9053
*	38.1	0	M+FA-H	C7H9NO3	-1.1560162	538.432022	385.515125	580.373703	976.319546	1439.14227
*	38.1	4.61	M-H	C14H11NOS	2.59400616	28541.8603	31770.5569	30120.2385	63287.7344	76022.0065
*	38.1	0	M+K	C4H4Na2O6	3.36889283	1369.65079	1020.85448	1097.19209	4659.62483	1312.51402
*	38.1	0	2M-H	C13H10S	4.71581377	198889.44	217912.921	206822.026	266363.991	417479.203
**	38.1	0	M+H, M+NH4	C4H6O5	7.32144588	135710.432	134968.446	141265.675	102803.785	84459.7624
*	38.1	0	M+Na	C9H12N2O7	0.68271646	30690.2247	29577.1275	31444.8328	39543.7535	29199.1875
*	38.1	0	M-H2O-H	C15H22NO6	-1.0034069	856.719563	1244.681	1023.36773	1236.15083	1600.67187
*	38.1	0	M-H2O-H	C6H3F5N2	-4.9680955	22971.0792	22949.8434	24013.2278	26869.673	19665.0258

*	38.1	0	M-H2O-H	C6H14O7P2	-2.6749884	12241.2221	12954.6683	12333.451	27508.5467	20900.3629
*	38.1	0	2M-H	C21H15NO2	-0.7677333	985.58016	1173.80464	634.546206	9.7531E-07	10647.9369
*	38.1	0.491	M-H	C8H18NO10	2.65011814	4841.52283	4278.55393	4293.77161	4420.39707	6451.8348
*	38.1	4.04	M+FA-H	C5H4N4S2	0.12691997	1241.95889	1318.89339	1181.67418	2969.90179	1394.23398
*	38.1	0	2M-H	C14H16O6	-1.9883434	5507.64644	5931.31685	6708.18541	5516.11609	5181.17899
*	38.1	0	2M-H	C15H15NO3	1.92610944	955.493979	1106.32367	1269.28765	9.7531E-07	2878.33869
*	38.1	0	2M-H	C20H10O6	0.74621279	3976.97197	4268.17096	4895.89172	5114.31373	1918.72207
*	38.1	0	M+H	C16H30O9	4.18376011	5126.98493	4961.0504	5348.2277	4498.5607	13291.8967
*	38.1	0	M+H-H2O	C6H9N3OS	0.90063789	1617.91523	1497.67964	1490.82429	3913.22313	1469.55348
*	38.1	0.392	M-H	C10H8F2O3	4.62859328	7035.86285	7037.46482	6950.19297	1439.34833	1084.23631
*	38.1	0	M+H-H2O	C14H26O11	4.02355287	3683.98798	3924.93942	4470.92454	1891.72541	7507.61815
*	38.1	0	M+Na	C18H29NO4	-4.629927	1375.78241	1596.69118	1409.45295	104.85704	5299.43572
*	38.1	0	M+H	C8H10N2O3	0.3490843	611.751514	491.783325	495.028828	1123.8092	9.7531E-07
*	38.1	0	M+K	C15H32FO2	4.64252248	1508.90154	1533.56207	1660.55514	737.113488	940.335083
*	38.1	0	M+FA-H	C23H19F5N	-1.2706701	2204.03484	2202.20984	2888.10133	140.344647	9162.74938
*	38.1	6.39	M-H	C23H32O6	-6.2935683	9040.75642	8778.85232	8481.05552	808.686447	2624.48284
*	38.1	0	M+H-H2O	C52H95O18	0.67414047	10049.7214	8873.25605	9835.3578	1788.52656	23387.199
*	38.1	0	2M-H	C15H23N5O	1.29887918	2837.58648	3500.42745	4222.67311	1468.50004	6488.96152
*	38.1	0	M-H	C33H44O2	-1.0311636	28632.089	27512.493	26084.975	25463.5195	18888.6336
*	38.1	0	M+NH4	C21H40O5	-1.2656538	5662.62386	5117.98464	4182.10989	5080.25615	2247.79096
*	38.1	0	M+FA-H	C24H20O5	-3.8135399	2204.07851	2967.66684	2100.70794	2288.55392	1528.19846
*	38.1	0	2M-H	C15H22O3	-4.7377386	1070.33289	655.12019	931.110703	552.820044	9.7531E-07
*	38.1	4.9	M+FA-H	C28H46NO1	-4.710165	54286.2101	50619.0267	51010.0661	26394.8153	73096.1167
*	38.1	0	M+H	C31H51NO4	-0.832069	4637.82849	3550.35815	5034.50797	9.7531E-07	26118.1174
*	38.1	0	M+H-H2O	C18H21N3O	-2.3884575	1074.81227	1298.56018	1107.95269	326.771894	2740.74163
*	38.1	0	M+H-H2O	C19H22F2N	-4.0180726	2469.73941	2616.15307	2563.02814	6109.2316	9.7531E-07
*	38.1	0	M+NH4	C20H30O4	-1.8685108	4062.40967	4909.22129	3548.75891	6273.48441	3134.94781
*	38.1	0	M+H-H2O	C30H58NO8	-0.9436941	11315.4011	11555.5547	12844.1304	15049.9832	36992.128
*	38.1	0	M+K	C10H17N3	2.70791978	3479.60869	3065.32773	3622.93345	1052.64892	731.247642
*	38.1	0	2M-H	C15H21NO4	2.5412459	19317.4869	17982.1673	17333.4361	24017.5762	6359.95858
*	38.1	3.9	M+H	C41H81N2O	-1.2696886	810655.119	480741.695	556434.855	377789.84	450155.449
*	38.1	0.0477	M+H-H2O	C17H28N2O	-3.9993849	6737.40284	5926.62537	6759.38397	16736.5213	3990.53941
*	38.1	0	M+NH4	C14H23NO1	0.52304549	7086.54753	6550.91103	5938.23907	8420.11219	6183.07033

*	38.1	0	M+H	C27H54NO7	0.3512118	13386.5814	16709.8824	15306.9662	2578.82621	38141.0288
*	38.1	0	M+FA-H	C14H28O5S	-1.0066901	1892.27226	3093.98883	2590.14229	2798.03383	2627.85694
*	38.1	0	2M-H	C21H33N3O	0.10959661	885.792302	1513.95675	991.419662	2952.37795	9.7531E-07
*	38.1	0	M+FA-H	C24H24O4	1.26346512	6143.97023	8033.15037	5366.83082	9565.87815	7138.14872
*	38.1	0	M+H-H2O	C6H13N3O3	1.21322141	1519.12209	1226.67626	1791.87719	2051.23638	2091.25145
*	38.1	0	M+NH4	C18H34O	-1.31001	8554.72894	9076.52346	9741.96985	11746.2047	5014.67151
*	38.1	0	M+H	C12H23NO9	3.81650693	16354.5322	16923.8373	17189.7856	25008.9227	15822.0906
*	38.1	0	M+H	C24H41N3O	2.17172184	2351.49319	2397.66733	1945.33928	1328.67462	9.7531E-07
*	38.1	0	M+H	C7H16O3	1.02239523	414.21605	420.016319	464.830522	421.033605	251.684786
*	38.1	0	M+H	C10H14N2O	-0.443231	1813.9869	1932.82301	1665.95566	2209.25881	4217.07243
*	38.1	0	M+H	C14H20O	0.3879502	2119.52319	2503.47102	2185.29508	2440.20511	1878.87017
*	38.1	0	M+H	C5H4FN3O2	-2.4662235	3828.04313	3878.6616	4228.1424	5262.29862	8227.40675
*	38.1	0	M+K	C16H27NO4	-6.1209972	6112.92443	6443.77353	6165.05987	16826.3808	19116.3358
*	38.1	0	M+FA-H	C6H11NO2	-2.1415914	343.134779	356.702635	341.712897	1158.65971	637.062315
*	38.1	4.65	M+NH4	C15H21NO4	-1.5809209	4157.80813	4939.51035	4181.35527	5985.76736	7437.45771
*	38.1	0	M+K	C25H42O3	0.10112784	518.350142	372.263725	545.643392	132.608169	613.031255
*	38.1	0	M-H2O-H	C7H9N3O2	-1.0212181	1315.24247	1402.4968	1437.71071	1454.70112	1080.0996
*	38.1	0	M+NH4	C20H22O7	-0.3613856	810.402333	548.059382	768.224472	57.2475872	863.507822
*	38.1	0	2M-H	C13H21NO7	-1.6553114	1858.0409	1632.93496	1719.1674	1274.32154	2703.02619
*	38.1	0	M-H	C18H24N4O	5.20860983	7359.51346	5959.39507	5748.8247	74.4542965	8092.95032
*	38.1	0	M-H	C7H11NO7P	-0.0219273	3563.49057	3266.41881	3847.04199	4125.26025	3897.61892
*	38.1	0	M-H2O-H, M	C21H18O16	3.16226755	73677.3286	73412.7471	72302.7736	67711.5283	72724.2445
*	38.1	0	M+H, M+Na	C10H12N4O	-0.7692155	2068.92524	3081.62305	2310.11079	657.121885	139.65703
*	38.1	0	M-H2O-H	C6H9N3O2	-1.6237721	776.70201	710.472195	736.27161	701.796836	741.449816
*	38.1	0	M+FA-H	C15H20N5O	0.37534567	840.342253	727.012173	801.962592	3816.0416	1024.75795
*	38.1	0	M+H-H2O	C27H36N2O	-0.5264434	1288.66161	1105.35125	1315.655	223.943291	453.250721
*	38.1	0	2M-H	C13H8O6	3.00570066	426.381212	567.409017	715.331319	2723.19287	981.796678
*	38.1	0	M+Na	C20H26N10(	-2.0105932	4438.50782	4273.79697	4486.35174	19309.4095	4108.92134
*	38.1	9.14	M-H2O-H	C29H23NO8	-2.0480071	3038.96264	2566.43634	2808.32331	1095.5805	2849.4023
*	38.1	0	M+K	C16H29D3O	1.723698	2422.79962	2261.61361	2730.50556	6695.98827	1036.2278
*	38.1	0	M+K	C19H21N3O	2.06439327	1662.93408	1750.67897	1834.70874	9.7531E-07	1940.54055
*	38.1	0	M+H-H2O	C16H14N2O	0.78436929	285.098347	431.779404	444.438143	724.701098	375.940094
*	38.1	4.02	M+NH4	C15H22O2	0.18739607	1780.46879	1763.67035	1881.68457	3950.26971	866.451838

*	38.1	0	M+FA-H	C20H28N4O	-0.180318	198.430506	202.478587	224.080029	9.7531E-07	9.7531E-07
*	38.1	0	M+H-H2O	C11H12I3NC	0.22111173	2892.80115	3514.3375	2668.08994	903.768485	2251.43575
*	38.1	8.05	M+H-H2O	C30H54NO1	0.12492173	6127.18896	6196.91397	7457.78183	2212.90721	3427.63422
*	38.1	0	M+Na	C18H16N2O	1.2714101	2362.25714	2341.86401	2468.04339	3819.81605	479.291341
*	38.1	0	M+Na	C15H13FO3	-0.8009379	698.983093	714.269989	796.10892	203.929594	613.138464
*	38.1	0	2M-H	C21H21N3O	-1.0129012	1823.42232	1389.77635	1722.1986	286.748817	9.7531E-07
*	38.1	0	2M-H	C11H13N3O	3.97713311	214.549563	188.945921	185.276707	9.7531E-07	24.3516235
*	38.1	0	M+Na	C12H16F3NS	-2.7367822	2701.39205	3004.38882	2846.04847	4363.73033	1678.13567
*	38.1	1.1	M-H	C10H14N4O	3.84684076	1423.52776	1551.17038	1739.60159	14863.4149	9.7531E-07
*	38.1	0	M-H	C14H22N2O	2.84949762	3028.64763	3159.27292	2835.79528	81.4488744	406.627153
*	38.1	2.36	M-H	C9H8N2O2S	-1.5935315	395.828018	366.935819	578.558085	785.839154	1037.21472
*	38.1	0	M-H	C10H19NaO	3.71813786	251.431919	347.424314	209.575738	34.4321364	55.8128798
*	38.1	0	M+NH4	C14H19NO4	-1.5756658	1819.89124	1872.77205	1564.85819	1543.48565	1259.1432
*	38.1	13.9	M+Na, M+K,	C28H48O8S	4.22540312	20264.304	21662.3589	21072.3728	11805.1536	48038.552
*	38.1	0	2M-H	C10H14O6	-2.7121965	363.682125	569.462864	586.186922	906.987736	139.755895
*	38.1	0	M+H	C13H16N6O	-0.6257241	2827.29728	2952.50161	3165.5915	2726.32209	3442.37312
*	38.1	0	M-H	C12H14N2O	-0.4974462	1195.78443	1166.43182	1502.038	1465.23818	1902.92901
*	38.1	0	M-H	C18H22O12I	-4.3919555	3169.4004	3153.27729	3287.99986	9.7531E-07	9.7531E-07
*	38.1	0	M+FA-H	C9H15N5O3	-2.5136795	158.683716	201.786407	113.021327	61.1309588	248.626453
*	38.1	0.435	M+H-H2O	C22H20F2N4	-1.4132224	25229.4738	27154.2585	25993.438	14005.4926	9255.44635
*	38.1	0	M-H2O-H	C10H16NO3	-2.4466168	4425.20799	4427.78935	4398.41574	4430.18989	3673.42568
*	38.1	0	2M-H	C19H16N2O	3.70637441	1738.08465	1360.02936	1412.87783	1322.69213	520.756378
*	38.1	0	M+NH4	C17H14F3N3	1.1437568	232.229299	162.079004	208.891211	9.7531E-07	9.7531E-07
*	38.1	0	M+FA-H	C26H40O8	-0.7786425	1415.54614	1309.63034	1645.52418	620.335436	544.798684
*	38.1	0	M-H, M+FA-I	C10H16N2O	5.30313943	1071.73342	1060.48662	1123.57689	1537.35475	2387.81907
*	38.1	0	2M-H	C8H11NO3	4.87224818	2121.98626	2105.42622	1895.19574	2890.9557	1580.73776
*	38.1	0	M+NH4	C7H8N6	0.23347451	862.940504	603.029073	870.013606	277.950889	997.981055
*	38.1	0	M+FA-H	C21H21NO4	2.21950918	180.702178	201.877165	248.289841	9.7531E-07	9.7531E-07
*	38.1	0	M-H	C8H18O4S	-0.6459449	279.315251	350.951134	358.010629	21.3373391	9.7531E-07
*	38.1	0	M+NH4	C18H17NO4	-1.5075966	1748.77126	2209.47815	1986.43447	3784.79094	522.581453
*	38.1	0	M+Na	C30H53NO1	2.19362206	1656.03338	2069.60291	1597.73429	107.570138	10695.998
*	38.1	0	M+FA-H	C24H36O6	-1.2709268	901.701773	606.149763	647.379461	626.949313	287.103109
*	38.1	0	M-H	C13H17N3O	-2.9704555	1020.50403	1034.79271	1070.77209	1134.10356	888.88328

*	38.1	0	M+FA-H	C9H8N4O5	-3.323882	1125.1363	1215.17046	1713.21598	108.675762	3.51882991
*	38.1	0	M+Na	C24H48NO9	-1.0203199	212.450089	262.233166	342.600506	2523.46727	711.169908
*	38.1	0	M+Na	C13H28O4	-0.4434483	3025.38421	3545.74765	3444.00997	4544.47211	2582.13831
*	38.1	0	M+K	C25H46O13	1.7090405	9168.05169	13356.8891	14774.6641	19979.5966	8724.72132
*	38.1	0	M+Na	C17H28O5	-2.5040605	5218.89695	6441.23378	6237.9026	4310.39752	3295.97486
*	38.1	14.6	2M-H	C15H17N3O	4.3193521	49555.1025	48833.7906	49756.2735	54621.9338	41881.9861
*	38.1	3.15	M+H, M+Na	C15H15NO	-1.1648162	10105.0093	10477.1045	10449.2601	5765.38285	4521.1444
*	38.1	0	M+FA-H	C26H40O3	0.07547999	930.783233	1137.42522	1266.32726	9.7531E-07	279.174567
*	38.1	0	M-H	C13H10N4O	3.2043787	394.54859	529.500309	454.220146	3173.00836	107.089204
*	38.1	0	2M-H	C7H11O8P	-2.2598224	8101.70247	9515.64741	8797.93614	7705.71386	8226.47865
*	38.1	0	M+H	C17H35O7P	-0.6046371	4389.09938	4569.52525	3994.81101	5743.71584	2470.3368
*	38.1	0	M+FA-H	C17H20O3	-0.8837041	1896.1215	1979.33276	1932.59863	1626.03504	2548.81032
*	38	0	M+NH4	C5H12NO2+	-3.1912161	71.5806068	78.5498695	44.0890389	102.003469	50.1560912
*	38	0	M-H	C10H19O7P	-3.9006332	1699.02259	1921.56812	1860.5008	916.466397	6693.17613
*	38	0	M-H2O-H	C10H19O12I	-0.9006544	5978.11363	6017.16632	5739.23953	5049.07605	6768.17881
*	38	0	M+FA-H	C25H22N2O	-1.253736	8615.08774	9908.44421	9922.70484	5730.25782	9167.43141
*	38	0	M-H	C16H11FN2(	-0.6028981	21704.5835	20281.0652	17499.2115	40174.1885	30364.1983
*	38	0	M+FA-H	C8H13N2O5	-2.2485022	6200.54583	6918.20516	6792.25372	634.938262	161.787763
*	38	0	2M-H	C4H11O4P	5.39643483	9073.13514	9948.67873	10649.0566	14500.7998	16765.2531
*	38	0	M+FA-H	C8H20O7P2	3.86345606	18130.8105	19068.072	16939.0849	9.7531E-07	921.608411
*	38	0	2M-H	C3H2N2O2S	0.75969623	1053.55295	1011.06528	1159.8049	2121.66837	520.639691
*	38	0	M+H-H2O	C6H13O9P	1.34909254	1261.64708	1553.916	1249.76886	9.7531E-07	2109.83656
*	38	0	M+Na	C9H8	3.37912393	3471.0405	1998.27696	2894.34071	4467.43569	3165.48982
*	38	0	M+NH4	C5H4O2S	-0.8032458	7570.47306	9842.85948	9412.74729	19226.8673	4845.484
*	38	0	2M-H	C14H11NO	4.61034555	1030.55749	909.049327	1226.31404	9.7531E-07	1653.54422
*	38	0	M+H-H2O, M	C8H14N2O5	-0.2235233	9002.80046	8527.48741	8907.47302	26081.2609	12099.8557
*	38	0	M+FA-H	C9H10FN3O	0.17854141	2423.60713	2796.1813	2219.40835	4763.19821	2715.20533
*	38	4.07	M-H2O-H	C18H12N2O	1.7268026	53364.9479	63337.6904	51774.861	27239.9757	34342.1913
*	38	0	M+FA-H	C7H13NO	-1.6106248	241.200358	251.111784	271.663583	14.6241297	428.387352
*	38	5.73	M+H	C7H16S	2.89250166	663376.631	642280.608	619267.721	941223.666	796839.718
*	38	0	M+FA-H	C14H15N3O	-1.4345871	2610.4718	3126.00841	3395.72134	188.602245	5708.33542
*	38	0	2M-H	C14H11F3N2	-0.4150113	10132.0791	10583.2399	10876.6785	11961.9183	7608.47932
*	38	0	M+NH4	C8H9NO2	0.8913496	491.105917	420.678488	514.073667	839.059332	43.4820933

*	38	0	M+Na	C14H22O4	3.15202414	929.258625	1190.44416	1276.57267	1544.63227	568.967764
*	38	0	M+NH4	C13H18N2O	-0.1676581	2895.62129	2904.76247	2804.65468	954.508205	11619.8223
*	38	0	M+FA-H	C18H14N4O	-0.2105266	423.988043	328.290411	390.019	1917.87577	1337.48493
*	38	0	2M-H	C16H10	-5.5435546	1461.38901	1467.90061	1561.99874	4723.95768	2530.2372
*	38	0	M+FA-H	C9H13NO6S	0.27358322	3384.00932	3053.18571	3520.0061	2105.7835	6345.65935
*	38	0	M+H	C8H12N2	0.78736497	1349.89599	1326.02121	1448.50557	1268.60763	585.63259
*	38	0	M+FA-H	C8H11NO5S	-0.991595	665.003746	586.142671	718.529484	0.52746452	9.7531E-07
*	38	0	2M-H	C21H21NO	6.58045185	13731.0381	15321.0175	19168.9459	6259.13451	29224.9943
*	38	0	M+NH4	C8H10FNO2	-0.5854225	475.002578	439.593013	325.388318	545.17402	99.9151198
*	38	0	M-H2O-H	C18H22NO2	2.03177413	88613.5372	89421.1547	91216.4745	81622.9272	77431.8962
*	38	0	M+NH4	C29H55N9O	-1.5963024	8713.162	9172.22094	8599.50883	4170.7608	9100.35495
*	38	1.29	M-H	C15H22O2	-1.2056699	90915.3006	90743.938	92308.2605	90302.0656	80448.401
**	38	0	M+H-H2O	C26H51NO5	-2.596195	308.085509	165.4293	267.840581	9.7531E-07	6006.49447
*	38	0	2M-H	C19H16N4O	2.37082381	1328.38421	1384.52652	1836.31968	483.325655	3207.65333
*	38	0	2M-H	C15H24N4S	-1.218103	15229.6248	16751.2435	17787.1619	9432.28391	44825.662
*	38	0	M+NH4	C30H38N2O	2.91013857	18843.5674	18993.7194	22250.6173	38352.9045	13070.311
*	38	0	M+NH4	C17H25NO5	1.91822528	2333.37285	1835.33802	1913.72483	1737.74951	1470.83142
*	38	0	M+FA-H	C50H76NO1	1.42172018	191835.668	163585.134	161350.428	133918.022	90169.3802
*	38	0	M+H	C22H18N2	-2.4874117	3833.92364	3315.90701	4185.92172	2621.41891	6293.1801
*	38	0	M+K	C21H27N	-2.570588	1350.66637	1360.70702	834.470314	1077.01613	3664.77537
*	38	0	M+NH4	C23H47O6P	1.95242419	11405.472	11139.8221	10313.3532	3626.87734	12166.0358
*	38	0	M-H2O-H	C26H19F3N	3.39496227	3489.52097	3553.33855	3355.32737	3403.48583	5343.53091
*	38	0	M+NH4, M+N	C37H48N2O	-4.6623032	6167.4371	6100.5518	6923.72181	9.7531E-07	2942.50519
*	38	0	M+Na	C31H52N2O	-1.191469	2010.47313	1787.57806	2423.55738	9.7531E-07	9.7531E-07
*	38	0	M+K	C44H85N2O	0.03915978	29646.899	27785.6493	29657.7507	17096.8091	44839.2535
*	38	0	M+FA-H	C23H29FN2	2.51003663	3415.42475	3373.71913	2503.49786	4768.32435	3421.28306
*	38	0	M+Na	C22H22N2O	3.87197772	3327.449	1968.69505	2299.08747	11358.0715	7396.33083
*	38	0	M+H-H2O	C6H14O6	6.72101016	1525.98832	1831.09525	1751.51272	1791.40298	1007.26798
*	38	0	M-H	C16H16N2O	-1.5924306	3016.69791	2233.2821	2251.19025	2209.42058	2260.2946
*	38	0	M+FA-H	C12H12O12	-5.1824813	275.567163	202.322615	275.998863	544.093855	9.7531E-07
*	38	0	M+H	C19H17F2N	4.88920735	317.657732	485.220125	398.382657	107.640074	2718.21981
*	38	0	M+K	C20H24D4O	0.74411945	5635.30055	6268.34869	5546.90322	242.509123	36757.8833
*	38	0	M+NH4	C12H20O5	-0.0164096	7119.60308	6561.05472	7252.21151	8563.34938	4829.11297

*	38	0	M+Na	C17H24N2O	2.08507304	2119.70923	1477.2078	2215.11264	1060.775	6985.76395
*	38	0	M+Na	C13H21N3	-3.3379298	411.847101	546.170457	697.237676	1684.94474	1072.05751
*	38	0	M+FA-H	C18H24N5O	-5.8327217	2472.8313	3161.6056	3679.9347	7741.08239	684.371405
*	38	0	M-H	C18H23FIN(	1.77630136	8924.34148	8720.12428	8139.67128	9.7531E-07	9052.94717
*	38	0	M+FA-H	C18H14F3N(	0.01441218	3476.33318	3196.81053	3012.13482	686.805468	4176.72236
*	38	0.473	M+FA-H	C17H14N2O	-2.5874493	382548.995	393601.544	342251.563	366427.449	370236.096
*	38	0	M+FA-H	C11H17NO6	0.26162041	1563.56445	1378.0826	1514.66558	4.46515174	2119.25742
*	38	0	M+K	C20H25N5O	1.5489889	335.811765	347.292908	348.782172	10641.0903	241.637833
*	38	0	M+H	C23H25N3O	4.72151336	349.032754	329.064599	282.601358	470.952683	74.4087278
*	38	0	M+H-H2O	C26H46O11	-3.0072704	3486.03784	4424.16885	3920.83697	359.4588	9.7531E-07
*	38	0	M+NH4, M+I	C17H24O3	-5.2768738	5582.73305	6057.30806	5922.57726	9133.44448	3903.39846
*	38	0	M+Na	C8H12O6	0.36272293	335.527086	362.701134	326.476707	66.4747057	34.3590501
*	38	0	M+FA-H	C6H6NO6P	3.96656705	4272.99082	4695.93471	4791.29044	4664.89438	2847.48537
*	38	0	M+H	C28H35N5O	2.66388174	4899.43004	4358.92341	6592.63548	5091.03099	4807.64585
*	38	0	M+NH4	C20H30N6O	0.28037312	2291.35925	2560.63125	2527.57649	1614.83465	1424.93512
*	38	0	M+NH4	C13H14O4	1.01044465	1270.9725	1463.15524	1285.62056	1809.13	1376.3901
*	38	0	M+NH4	C30H36O11	-2.8446674	2120.8333	1919.03439	2293.33557	152.10461	342.704808
*	38	0	M-H	C8H11O3-	-0.7771001	265.348538	305.354692	280.527552	756.979063	358.046013
*	38	0	M+NH4	C36H65N3O	0.17986562	629.73743	726.114758	1047.6926	262.186606	9.7531E-07
*	38	0	M+NH4	C29H38O9	1.25443058	1667.85685	1137.57166	1574.27981	876.808436	1647.42183
*	38	0	M+NH4	C12H11NOS	1.98114456	578.714378	649.139976	556.828929	1006.86816	583.181459
*	38	0	M-H2O-H	C17H17NO5	2.51829555	484.319458	374.905744	334.611205	263.062929	791.050302
*	38	0	M-H2O-H	C7H16O3S	-2.4598318	3345.472	3391.40617	3432.51681	3357.03815	2910.35075
*	38	0	M+NH4	C11H16N5O	1.10495936	3377.60939	2571.43857	2846.83789	2964.99309	5027.91759
*	38	0	M+FA-H	C8H10O5	0.39275536	285.199213	254.637532	276.369698	206.09008	254.164654
*	38	0	M+FA-H	C23H22FNO	-0.6544257	4900.74309	5015.40063	5346.74349	430.547044	29416.1578
*	38	0	2M-H	C19H16	-4.6232614	983.140585	983.589676	1184.86154	709.133438	438.554746
*	38	0	M+K	C16H19N3	0.17739386	1938.41757	1843.05952	1969.95229	1220.95446	1328.77994
*	38	0	2M-H	C25H34N6O	-0.6143777	5958.4258	5399.5216	4748.59656	9.7531E-07	9.7531E-07
*	38	0	M-H2O-H	C11H8N2O5	-3.5849735	175.93177	193.030897	209.498394	291.11446	163.071591
*	38	0	M+Na	C14H26N3O	4.49239869	16761.3992	14542.0256	14129.4302	11183.9546	31940.6796
*	38	0	M+NH4	C16H28O5	-1.5563424	781.501391	730.730572	754.252637	74.8318406	3878.81687
*	38	0	M+Na	C12H20N2O	1.92460271	1089.66296	1083.59401	1117.54915	663.881928	1489.51471

*	38	0	M+NH4	C7H13NS2	2.23633263	4472.27589	4529.71569	4434.49074	7514.76245	3445.39136
*	38	0	M+NH4	C11H17NO3	-2.1003799	219.487013	200.417064	214.511772	148.701792	151.13466
*	38	0	M+FA-H	C22H37NO5	-1.0393993	645.154756	503.462656	525.174644	9.7531E-07	4727.85329
*	38	2.49	M+FA-H	C20H22N4O	2.06583334	7229.50808	7548.69261	7739.98114	19090.5681	3085.19695
*	38	8.56	M+Na	C24H32O12	3.17421559	15332.2023	15751.7527	15543.505	73920.9902	4850.02168
*	38	0	M-H2O-H	C9H10IN	0.49467243	3854.1515	4600.64401	4724.86067	4691.90673	4378.16987
*	38	0	M+FA-H	C21H21O4P	0.67367946	2970.11086	3332.25042	2999.281	1399.45607	618.358227
*	38	0	2M-H	C21H16FNO	-1.7853507	883.506127	592.492428	890.405638	9.7531E-07	7563.9427
*	38	0	M+FA-H	C7H7F3N2O	1.57130381	1764.17359	1937.93958	2067.59601	1709.58353	1543.83839
*	38	0	2M-H	C16H14N2O	3.20319692	678.214705	631.153638	923.990425	9.7531E-07	2103.76008
*	38	0	M+H-H2O	C17H26O2	-1.9920117	6858.60085	7190.51228	7204.68595	8477.10401	5042.81814
*	38	0	M+H, M+Na	C48H85O11	4.70771645	412587.553	443230.711	478453.242	609030.919	334990.646
*	38	0	M+Na	C27H44O11	-0.6209764	2478.75039	3124.23193	2710.50035	4061.51245	344.620573
*	38	0	M+Na	C13H23NO9	1.35274267	195.492712	215.076265	157.419984	9.7531E-07	9.7531E-07
*	38	0	M+H	C28H46O4	-1.169841	6631.72953	7383.54525	7408.30248	8579.36503	5785.44196
*	38	0	2M-H	C17H18O5	-1.2773144	1781.40688	1429.0819	1690.97396	3563.94897	469.25388
*	38	0	2M-H	C11H12O7S	-1.0943038	23133.7442	24260.277	25926.4991	23539.2263	23275.3122
*	37.9	0	M+K	C5H6N2	3.71594563	129.774911	205.69228	133.000945	305.315421	148.412185
*	37.9	0	M+FA-H	C15H10N2O	0.49145342	1856.77709	2119.96615	2154.71317	771.456055	1100.2774
*	37.9	0	2M-H	C13H13N3O	-1.3516561	2877.11086	3330.88664	2506.67876	3720.03498	1993.3342
*	37.9	0	M+FA-H	C5H11NO4S	-1.0152777	10310.0547	9422.06469	10327.0612	6688.42967	2129.73638
*	37.9	0	M+FA-H	C2H4O3S	-2.4287521	2966.19573	1821.26784	2800.19739	7952.0181	720.800342
*	37.9	0	M+NH4	C6H14FO3P	2.75937785	1927.2165	1346.79984	1872.21635	9.7531E-07	1456.34086
*	37.9	0	M+NH4	C4H2N2O4	-5.3324017	1067.52014	719.975805	957.206906	2663.62572	49.7602272
*	37.9	0	M-H2O-H, M	C10H16N2O	-0.7997272	21862.1504	19918.7904	20041.5448	8833.13914	15142.6726
*	37.9	0	M+FA-H	C14H10F3N	0.56313283	6213.5302	4745.64139	4407.48403	16458.541	13157.6019
*	37.9	0	M+NH4	C11H16N2O	-0.9817832	3936.40508	4812.39322	2672.69911	2711.65297	9635.44766
*	37.9	0	M+K	C19H28O11	3.48371058	45194.5326	39110.2335	38913.3515	92846.6724	178233.164
*	37.9	0	M+FA-H	C15H20NNa	-0.6653781	2268.35952	2221.39674	2396.57508	9.7531E-07	11316.2126
*	37.9	0	M+Na	C12H24O12	-0.3630567	7220.31052	5987.80511	5992.89126	1380.04718	5435.83691
*	37.9	0	M+H-H2O, M	C8H14O7	-0.5503355	2389.72047	2362.14725	2830.18129	2033.14399	4702.59346
*	37.9	0	M+H	C4H4O6	-4.3924267	433.38886	278.362471	255.750648	444.738493	515.095815
*	37.9	0	M-H2O-H	C7HF13O2	-4.1375891	1212.83808	1412.16063	1658.54775	6255.9784	3829.98492

*	37.9	0	M+H-H2O	C6H7NO2	3.03343939	209.032233	252.034612	180.402984	139.082879	133.526919
*	37.9	0	M+NH4	C21H30O6	-0.3031923	4115.40596	4577.38678	3141.69695	4682.21864	4583.8578
*	37.9	0	M+H	C17H22O2	6.11198676	768.465513	416.334755	634.779106	352.975511	824.349061
*	37.9	0	M-H	C20H19O11-	3.89790205	5833.02304	4842.84396	3912.46429	7082.2244	9424.29506
*	37.9	0	2M-H	C10H16O5S	1.04146101	17437.2949	18640.0744	16762.4795	20942.6646	50084.0924
*	37.9	0	M-H2O-H	C17H16N2O	-0.1275312	19275.1443	21527.8042	20655.1464	38464.5417	13847.0009
*	37.9	0	M+K	C15H12O8	3.22003173	4639.59219	4635.61339	4539.30407	9516.11079	2262.57552
*	37.9	0	2M-H	C6H12O5S	-2.5523739	1554.49221	1906.81027	1934.95975	3760.99959	1095.97714
*	37.9	0	M-H	C9H12N5O4	-4.2444188	1480.45063	1534.31705	1356.51555	70.1647902	5335.59162
*	37.9	0	M+K	C17H23NO8	0.70708431	3096.60707	3060.95731	2973.35473	890.42925	2656.07463
*	37.9	0	M+NH4	C16H18N6O	-3.2813732	7783.60298	7820.68257	8722.09098	3015.65488	24223.0517
*	37.9	0	M+Na	C15H20N5O	0.77037955	1826.19532	1739.51784	1537.93946	9.7531E-07	9.7531E-07
*	37.9	0	M-H	C22H44NO7	-0.3745919	689.096166	649.439649	464.490959	138.928093	1405.80702
*	37.9	0	M-H2O-H	C29H37N5O	2.93684704	1477.91764	1612.60767	2261.51375	124.423671	14994.6154
*	37.9	0	M+NH4	C29H53O12I	-2.1317487	6360.12289	6103.6047	4223.57559	9.7531E-07	15021.966
*	37.9	0	M+FA-H	C30H37N3O	2.49951993	9216.00152	9655.9309	10357.9038	10131.6256	1511.39254
*	37.9	0	M+NH4	C22H40O3	-1.4154962	2021.6862	1695.08208	2071.49201	2361.71799	1821.10074
*	37.9	0	M+H	C44H80NO1	-1.6861905	132384.378	141646.206	124108.175	70660.015	45704.9298
*	37.9	0	2M-H	C21H32N2O	-2.5140051	2426.36181	1953.36762	2579.09394	9.7531E-07	9.7531E-07
*	37.9	0	M+NH4	C32H60NO9	-2.8014132	1423.77953	2006.14223	1782.35529	6098.89876	3466.94521
*	37.9	0	M+NH4	C35H42O6	-0.159108	625.839308	738.025002	651.67905	1201.28578	835.771946
*	37.9	0	M-H2O-H	C43H76O7	-0.623975	9520.79682	7412.58234	12345.1555	2994.48889	1674.9553
*	37.9	0	M+Na	C23H46NO2	-2.1770553	3820.18118	4938.22274	3813.9578	4944.9556	2970.49041
*	37.9	0	2M-H	C16H29O3-	-0.4408711	1421.45785	1166.0588	1232.63837	1024.61056	9.7531E-07
*	37.9	0	M-H	C21H32N2O	3.89642012	914.685213	1292.12681	1405.56122	1953.66728	832.769547
*	37.9	22.5	M+K	C31H39N5O	-1.9531039	124669.49	132149.128	131147.022	81222.9097	12059.0162
*	37.9	0	M+NH4	C9H8O	0.88168856	141.679419	137.109413	176.95234	108.229098	69.7800352
*	37.9	0	M+NH4	C19H34O12	2.72578195	13623.9157	13241.2082	11890.6029	46272.8069	36564.0546
*	37.9	0	M-H2O-H	C12H18IN	-1.24554	2230.65797	1708.03814	1754.71151	3931.193	4843.66469
*	37.9	0.313	M-H	C15H24N2O	4.07480388	27898.5039	22986.3076	24413.3194	27523.3572	26970.9659
*	37.9	0	2M-H	C17H12O4S	-2.9228932	43366.0505	43853.7515	44693.1719	51613.1556	43360.8155
*	37.9	0	M+K	C15H16O5	2.47492222	4968.72125	4392.47057	4001.64479	5586.32116	9425.41379
*	37.9	0	M+NH4	C9H8O7S	0.44341156	1316.97899	1245.56405	1434.91037	2129.7471	617.182035

*	37.9	0	M+H	C20H21N3	4.31895586	732.829887	1054.37315	973.35967	373.662601	1120.82872
*	37.9	0	M+K	C19H23N5O	1.61333615	517.685388	533.589878	328.315237	6252.10345	1751.6591
*	37.9	0	M+Na	C23H32O4	2.1181433	1600.3994	1570.03626	1239.59222	1641.26237	1375.2759
*	37.9	0	M+Na	C23H47O9P	-3.8469098	1581.07217	2054.6005	1286.21883	3951.9271	1742.09978
*	37.9	0	M+H, M+Na	C10H7N3S	2.35373494	6311862.34	6466020.89	6347865.99	8676892.65	4986055.04
*	37.9	34.1	M-H	C13H10ClN3	3.32894826	6804.76058	8745.27261	6808.75445	4301.27861	3854.56718
*	37.9	0	M+Na	C14H25N7O	4.4318305	2056.09369	1858.66153	1627.92605	513.064194	5286.8785
*	37.9	0	M+Na	C27H36O4	2.06515887	2039.28374	1517.79651	2728.18761	578.410039	1505.03489
*	37.9	0	M+FA-H	C8H10FN3O	2.40009403	181.046136	116.670632	124.043419	33.2996415	9.7531E-07
*	37.9	0	M+NH4	C18H29N3O	-1.5182917	680.016694	795.008928	1040.76237	102.870013	1607.58132
*	37.9	1.01	M+Na	C10H14O5	-2.0657147	1548.97013	1467.50654	1401.70249	615.034766	7505.46647
*	37.9	0	2M-H	C7H9N5O2S	2.55011453	927.177234	658.582737	718.990095	707.716891	627.68048
*	37.9	0	M+FA-H	C9H21O17P	-2.9297356	897.908955	705.310727	621.795275	1218.4793	2933.08532
*	37.9	0	M+H	C19H37N5O	-2.1552802	4046.6466	4241.29808	4462.05475	5243.25665	10684.3661
*	37.9	0	M+H	C19H25N3O	2.36595377	2577.9205	2477.62794	2660.41462	2366.08131	7318.21575
*	37.9	16.4	M+H	C23H15ClO3	-1.3104839	9279.93357	9289.04026	8709.91773	21069.4105	9556.58598
*	37.9	0	2M-H	C9H13NO2S	-4.662087	1053.52853	1043.63471	1127.49876	2458.70421	367.239092
*	37.9	4.42	M+K	C10H10S2	0.09285199	1084.40887	1346.29971	1151.84122	729.21069	98.7405526
*	37.9	0	M-H	C7HF5O2	4.09971432	729.078121	948.707443	778.680006	877.795915	752.983812
*	37.9	0	M+Na	C6H13NO6	-6.2898873	273.555726	261.874663	315.817609	9.7531E-07	113.734565
*	37.9	0	M+H	C23H42O11	-4.0898433	879.114709	1167.00658	854.387972	515.26951	563.68787
*	37.9	0	M+FA-H	C4H4N2O2S	1.62912623	9947.13462	9871.21074	9986.2767	9702.29894	8733.30794
*	37.9	3.58	M+NH4	C19H25N2O	3.99670366	13756.0287	13971.5298	13491.7093	36987.1025	6448.83554
*	37.9	0	2M-H	C24H24N2O	-3.0155631	4098.96815	3345.49359	3614.51549	9.7531E-07	9.7531E-07
*	37.9	0	M-H2O-H	C20H19NO8	-3.0530976	260.058551	286.97301	241.086601	134.123419	377.603334
*	37.9	0	M+H	C10H15N	-0.0044361	312.1636	323.308977	355.258265	315.38827	204.060894
*	37.9	0	M+FA-H	C18H30NO6	4.41702422	748.930694	631.873317	832.096834	210.203784	218.382227
*	37.9	0	M+FA-H	C9H12O5	1.13239533	1031.00752	991.508482	1057.89544	780.801809	1120.99642
*	37.9	0	M-H	C12H10O4S	-0.4013123	278.625793	326.856981	433.606519	416.737511	297.054346
*	37.9	0	M+K	C15H16N2O	-1.5037006	1133.53186	1477.67514	1221.60846	2149.06714	553.786982
*	37.9	0	2M-H	C11H6O	2.82172888	609.83265	616.586727	956.019672	656.286458	637.442806
*	37.9	0	M+H-H2O	C13H18N2O	-1.2356368	1506.10112	1568.10721	1608.02717	2297.13993	2276.09542
*	37.9	0	M+H	C19H26O6S	-2.0006024	350.998043	347.450459	419.47478	9.7531E-07	9.7531E-07

*	37.9	0	M-H2O-H	C5H6N2O4S	2.07705977	1677.00439	1807.4614	1787.60389	1865.08095	1631.93477
*	37.9	0	M+FA-H	C9H13N2O9	3.84657457	52186.4625	52919.3874	51640.9054	52858.6467	47585.0673
*	37.9	0	M+FA-H	C17H33NO6	-0.281931	451.57606	483.522574	389.150422	9.7531E-07	421.038012
*	37.9	0	M+K	C18H28N4O	-3.5432361	817.548422	785.94029	843.616147	2268.27373	9.7531E-07
*	37.9	0	M+FA-H	C10H13N3O	6.5682122	2719.88171	2795.88026	2731.69954	1226.26551	2973.31227
*	37.9	0.0276	2M-H	C9H13NO5	-2.1457859	16135.0396	16219.825	16260.4111	7000.16931	15060.9302
*	37.9	0	M+K	C11H28N4	2.93885347	1807.21818	1691.30357	1885.76986	2847.36798	1473.77444
*	37.9	0	M+H	C41H73O11I	-4.7036309	45157.1384	46467.5943	32855.8604	45155.9088	34382.838
*	37.8	0	M+H-H2O	C8H8O2	1.86041043	292.623843	319.863735	285.061767	285.504489	168.671256
*	37.8	0	M+NH4	C9H11NO4	-0.2486	670.458635	1064.08635	922.158454	176.676385	9.7531E-07
*	37.8	0	M-H2O-H	C4H3IN2O2	-4.9726286	89834.9659	88078.6941	91152.5146	9.7531E-07	9.7531E-07
*	37.8	0	2M-H	C5H5N5OS	-3.9316014	2499.43624	1845.48055	1670.88036	19964.3777	20938.6501
*	37.8	0	2M-H	C5H7N3O5	-8.463577	2354.71862	2024.474	1510.24735	12041.3108	7047.75096
*	37.8	0.467	M+H	C8H18OS	3.45560138	207260.914	204894.769	213383.511	158660.602	616469.442
*	37.8	0	M-H2O-H, M	C15H12O5	-2.3742905	27415.4368	23685.0214	28802.3478	56669.7047	42307.5158
*	37.8	0	M+FA-H	C14H10N4O	0.38680712	7714.68356	6738.93293	6510.30704	21291.0952	7334.79287
*	37.8	0	M+FA-H	C16H12N4O	0.87049011	48632.5516	44762.5948	44860.2006	12036.6644	19828.3075
*	37.8	0	M+FA-H	C4H10NO3P	-0.5847603	2415.60895	2341.864	2819.58273	3103.65529	6889.62491
*	37.8	0	M+FA-H	C3H7NOS	-1.8086713	506.029336	510.807831	361.92572	484.033841	166.565856
*	37.8	0	M+Na	C18H19F3N(	-1.5379933	24105.4943	20361.1054	17495.8826	9.7531E-07	72339.5543
*	37.8	0	2M-H	C6H7O7-	-2.8098239	376.688783	416.276356	414.821747	662.323295	9.7531E-07
*	37.8	0	M+Na	C7H8O	2.93608496	517.980012	584.360943	510.730685	1160.20089	610.005358
*	37.8	0	M+H	C10H14	0.71107043	4820.53799	4691.06284	5437.21984	4460.69429	3319.53222
*	37.8	0	M+K	C14H25NO8	-5.1321325	22391.5576	22618.7662	20422.4943	36569.549	25870.2929
*	37.8	0	M+FA-H	C14H15NO8	-4.4650969	3637.03652	1983.35293	3178.28156	2924.38377	566.75547
*	37.8	8.79	M-H, M+FA-I	C13H19N3O	-4.3375304	26618.5569	25182.5508	30042.1514	31939.9471	20406.5724
*	37.8	0	M+H-H2O	C7H14O2	2.54476894	4810.25678	4779.59983	4635.10498	5724.48007	3510.78031
*	37.8	0	M+H	C8H18N4O2	0.52076196	5354.28866	4869.33885	5142.35765	9958.72379	7192.44921
*	37.8	0	M+FA-H	C12H10O13	-3.5298425	24397.0462	26466.3606	26721.4198	9880.42927	3960.49858
*	37.8	0	M-H2O-H	C18H34NO1	-1.5369924	4119.47837	3741.2456	3244.08846	9.7531E-07	5439.43401
*	37.8	0	M+K	C11H16O6	-1.5533631	12393.8827	14217.5004	10955.3392	18004.4998	7342.61897
*	37.8	0	2M-H	C17H10O3	3.50858399	5595.41818	4961.10848	5485.2618	5618.49436	10883.8482
*	37.8	0.424	M+FA-H	C14H10O5	-4.0778754	37698.3842	40501.5706	40074.0259	22842.6666	49682.0234

*	37.8	0	2M-H	C7H7O7-3	-1.8548753	672.875088	750.301905	525.643196	1617.56605	226.963102
*	37.8	0	M-H	C17H10F6N2	-4.9451777	9036.16947	9248.78332	10588.3044	13476.0251	9795.36673
*	37.8	0	2M-H	C9H11N3O2	-4.8954392	2200.00004	2152.09855	2099.38844	3388.50624	7297.27253
*	37.8	0	M+FA-H	C21H32O10	0.60734903	2930.67068	2333.20273	3423.16697	2763.96773	1701.97701
*	37.8	0	M+Na	C14H17NO1	-1.9408204	4399.18855	5068.82511	5392.00755	13155.0133	6163.65412
*	37.8	0.749	M+FA-H	C23H20O13	-1.4514811	6113.44758	5748.67281	6461.39076	504.559182	6293.69861
*	37.8	0	M+NH4	C27H36O12	3.37905213	11148.9549	9792.96604	10213.7534	9413.40703	5674.49505
*	37.8	0	M-H	C31H49O12I	0.12226305	11459.288	11651.1903	11005.2845	2844.40577	43013.8731
*	37.8	0	M+H	C20H19N3O	3.07136364	3524.54777	3350.17133	3111.66835	2215.82102	6386.32061
*	37.8	0	M-H	C19H28N2O	4.42420637	240.966866	319.233532	268.247635	9.7531E-07	330.661432
*	37.8	0	M+FA-H	C27H29N3O	9.42346885	51759.4167	53824.3491	57073.4272	13752.878	113624.487
*	37.8	3.41	M-H	C28H52NO1	-3.6680154	18996.8621	20321.0865	17197.4706	3154.1465	59905.651
*	37.8	0	2M-H	C15H21NO3	-4.8261714	2289.05213	2283.17862	2385.83443	143.955607	4262.13742
*	37.8	0	M+K	C21H26N2O	-2.874266	9690.69564	11937.9006	8258.32347	22346.4008	12900.1881
*	37.8	0	M+K	C47H90NO7	-0.830933	13461.678	12161.6763	9331.89842	9.7531E-07	21334.3426
*	37.8	0	M+H-H2O	C18H21FN4	-0.9177388	1489.58971	1703.76819	1279.96222	2641.12342	2786.63898
*	37.8	0	2M-H	C11H14N4O	-1.5122617	22961.6237	23604.0802	19085.7767	17868.1978	17342.643
*	37.8	0	M+H-H2O, M	C18H35NO2	-1.3339773	2721.85089	3729.05758	3848.07943	9.7531E-07	2456.7702
*	37.8	0	M+NH4	C24H22FNO	2.22453377	20029.8895	17645.2545	19563.1498	56235.4895	28564.1332
*	37.8	0	M+Na	C22H42O6	-0.7104013	3930.11756	4253.7483	4168.43077	8471.21103	4091.86675
*	37.8	0	2M-H	C21H34O2	-0.0381614	3311.27932	2648.47024	3359.24765	9170.14196	963.266395
*	37.8	0	M-H2O-H	C23H30O6	7.20806399	73929.2501	67707.6858	56330.1203	59737.5394	38371.8918
*	37.8	0	M+Na	C20H42O3	-1.4803957	642.630701	1021.47181	707.20184	183.629343	257.819306
*	37.8	0	M+H	C22H41NO3	-1.1822717	1358.21176	1230.08976	1962.20783	1849.45159	1002.85838
*	37.8	0	M-H	C34H26O10	2.04637106	22256.4979	26878.9862	34231.9215	25496.4544	25217.0397
*	37.8	0	M+K	C19H29FO2	2.32803903	2128.07974	2027.38826	1693.34464	1165.84159	10132.5793
*	37.8	0	2M-H	C7H7N5O2	1.15278666	46.3309257	51.4915533	56.2298737	9.7531E-07	402.95853
*	37.8	0	M+K	C12H12N2	2.54229046	75680.9973	77715.4564	80333.8204	99609.519	58856.0931
*	37.8	0	M+NH4	C27H38O12	-1.0716179	5173.32984	5090.34831	4015.01103	3893.65377	11275.3277
*	37.8	0	M+Na	C26H29O14-	0.76491996	3415.96144	2997.11938	3203.69802	9.7531E-07	142.80655
*	37.8	0	M+K	C29H39NO3	-3.9968604	857.348192	601.630417	686.391965	766.007876	2689.2564
*	37.8	0	M+NH4	C7H6O5S	1.11913833	301.509456	305.523477	368.229874	9.7531E-07	20.1574603
*	37.8	0	M+FA-H	C23H25O11-	0.41286002	2627.69577	2903.64297	2342.7597	9.7531E-07	11939.4801

*	37.8	0	M+NH4	C3H4O3S	2.01165295	4909.75718	5242.21144	4563.91643	6372.91658	4040.20268
*	37.8	0	M+FA-H	C13H23N2O	0.21377929	2303.83845	2259.56691	2412.73397	600.138641	7373.60102
*	37.8	0	M+Na	C22H27N3O	3.72145659	2161.13197	1783.22827	1488.43536	446.175395	666.934847
*	37.8	0	2M-H	C12H17N3O	-0.274043	587.672463	385.993165	583.936619	9.7531E-07	191.620072
*	37.8	0	M+Na	C9H13N2O2	-0.3204279	671.937968	863.762621	767.842549	976.838125	770.251775
*	37.8	0	M-H	C12H24NO8	4.37863374	212.109264	347.536394	322.404362	72.2327745	319.743474
*	37.8	0	M+H	C30H37N5O	-0.0297155	973.400253	1379.26033	1007.72116	2115.88006	757.904045
*	37.8	0	M+H	C25H46O7P	1.64342958	4002.20724	4157.46067	3359.39296	9.7531E-07	6518.72224
*	37.8	0	2M-H	C17H19FN4!	-3.15702	9805.63036	9318.5127	8829.30977	12911.2528	5545.03101
*	37.8	0	M+K	C35H59NO1	1.85410468	1796.94072	1536.99406	1593.07696	3522.82894	1664.84533
*	37.8	0	M+NH4	C21H40NO9	2.46722134	1805.48559	1854.59719	1421.61897	1281.37243	1033.11272
*	37.8	4.69	M+K	C20H41N5O	-2.2390401	21719.3102	21776.0579	23390.8939	15874.0245	5477.40173
*	37.8	0	2M-H	C17H15N5O	7.46106335	484.204885	665.960066	431.113358	577.380094	9.7531E-07
*	37.8	0	M+H-H2O	C13H20N2O	-0.4446658	329.848565	313.658969	467.17812	442.807256	119.441551
*	37.8	0	M+FA-H	C34H53NO1	-3.2090779	1199.93163	1324.44036	1527.63316	598.354401	9.7531E-07
*	37.8	0	M+Na	C39H51NO6	0.3671843	6767.54621	5176.25166	4969.39066	2974.41674	692.432769
*	37.8	0	M-H, 2M-H	C18H17NO3	-3.0188009	1110.09045	1066.39375	1168.26082	706.486403	208.202666
*	37.8	0	M+H	C35H42N4O	-1.0976482	2059.69417	2895.39445	2723.22151	1062.50062	3246.03344
*	37.8	0	2M-H	C21H18N2O	2.73790231	300.916016	205.057456	236.838432	9.7531E-07	9.7531E-07
*	37.8	0	M-H2O-H	C18H22N2O	-0.0583387	504.071084	538.775061	553.921113	538.846854	239.32595
*	37.8	0	2M-H	C9H6N2O3	8.61180574	3143.27967	3862.49977	3751.20125	16.0998001	118.280922
*	37.8	0	M+NH4	C9H6O7S-2	-2.5137428	514.781287	738.373881	686.183951	1580.22774	1293.40888
*	37.8	0	M+NH4	C31H54O9	-2.4993211	1017.35695	600.087219	671.058174	9.7531E-07	674.700704
*	37.8	0	M+NH4	C18H40NO6	-4.3154833	380.553528	314.581738	285.497754	9.7531E-07	9.7531E-07
*	37.8	0	M+H	C10H6O2	-0.440442	349.937988	389.306405	331.848356	714.873487	790.634317
*	37.8	0	M+Na	C6H8O4	-8.3396278	900.548222	925.861332	1082.99475	1585.14504	2513.91438
*	37.8	0	M+FA-H	C4H6N4O3S	-5.1794412	1636.32134	1613.54869	1495.92857	1212.36858	1041.43468
*	37.8	0	2M-H	C13H14N4O	3.37020116	635.175091	880.709236	674.34248	86.6583955	9.7531E-07
*	37.8	0	2M-H	C20H22FN3(	-1.6946195	902.401359	1122.84281	1211.57348	951.25606	385.371533
*	37.8	0	M+FA-H	C25H24FNO	-0.5182425	1619.04946	1928.93577	1746.42921	179.094396	13287.7678
*	37.8	0	M+H	C10H19NO2	-0.7869197	198.905371	363.121992	297.540919	208.093183	836.49319
*	37.8	0	M+H, M+Na	C14H24O3	-1.1557195	5638.81037	6165.96642	6000.99546	8319.32716	5141.16479
*	37.8	0.0213	2M-H	C12H10FN3(	-2.0911855	63777.5146	66503.8558	64139.4959	28719.2542	12178.7789

*	37.8	0	M+Na	C6H8N2O4	8.46348343	207.873521	180.527962	155.21148	746.45718	155.971051
*	37.8	0	2M-H	C6H8O3S	3.81422289	621.59118	542.096132	537.058504	479.801176	892.734958
*	37.8	0	M+NH4	C7H8O6	3.90480324	630.908947	771.468962	658.939131	1084.63856	1036.6637
*	37.8	0	M+K	C26H33NO5	-4.9164056	6318.73693	6297.08434	5835.30909	7214.8574	9.7531E-07
*	37.8	0	M+H	C13H10O6	3.22304447	1253.43965	1674.2123	1601.71952	856.09231	398.766514
*	37.8	0	M+FA-H	C8H5F3N2O	2.27496957	1146.48569	1100.22909	1153.19169	1187.22337	1082.20749
*	37.8	0	M-H2O-H	C17H17O6+	0.99861826	676.464808	551.789594	648.33387	440.77109	385.988891
*	37.8	14.2	M+FA-H	C25H33FN4(	3.03181135	253890.56	242913.75	270278.82	260854.179	219611.243
*	37.8	25.6	M-H	C22H42N4O	-1.3193659	13290.1332	15384.8064	14123.3432	12134.5121	10800.3385
*	37.8	0	M+NH4	C25H39O7P	-1.3551044	569.954446	892.275985	596.486168	9.7531E-07	9.7531E-07
*	37.8	0	2M-H	C12H10F3N:	-6.3527828	2924.74638	1915.31351	2552.98134	882.040824	2106.84892
*	37.8	0	M-H	C23H33N5O	-2.2049289	1288.38322	902.480389	1239.81613	328.377903	412.188654
*	37.8	0.0719	M+FA-H	C28H45NO7	-4.213233	2963.69459	2605.46842	2339.34028	9.7531E-07	18571.756
*	37.8	0	M+FA-H	C32H52NO1	0.53594326	2588.38609	2546.61774	2300.88068	9.7531E-07	1229.89678
*	37.7	0	M-H	C23H28O12	5.88874002	6948.81611	6807.31694	6334.50612	4648.16918	5344.53483
*	37.7	0	M-H, 2M-H	C8H10O5S	3.01869917	23564.6124	21979.5713	22865.0868	5130.33793	26123.7363
*	37.7	0	M+H	C8H12N2O7	-0.8533037	5772.9267	7568.60849	6124.68731	8941.28627	9345.7579
*	37.7	0	M-H2O-H	C11H15N5O	-6.0146261	8980.76055	9633.47046	9440.85196	20960.0594	28982.1211
*	37.7	0	M+Na	C9H15N5	-0.3411767	1202.7774	1029.85625	860.19364	1325.63361	2636.86342
*	37.7	0	2M-H	C8H6O3	-2.125432	1259.76558	1743.76757	1712.95417	5336.11429	4622.63078
*	37.7	12.5	M+H	C15H22O3	9.19599344	9652.64576	10054.0748	9782.58066	8188.1846	18560.6004
*	37.7	0	M+H, M+Na	C8H14N2O5	-0.3453447	20351.0762	18299.9279	21918.6953	52514.8543	29071.6621
*	37.7	0	M+FA-H	C15H32NO7	4.90199215	3126.64468	3124.05551	2496.12058	9.7531E-07	4437.2181
*	37.7	0.185	M+FA-H	C19H21NO6	1.96220095	28206.72	33311.8197	28045.4949	25574.5681	37954.2264
*	37.7	0	2M-H	C10H16N3O	0.99340508	5854.65034	6743.46793	5154.19611	2709.05763	1990.53096
*	37.7	0	M+Na	C10H10O8	-4.7298789	2420.73399	3010.28836	1989.0801	8398.84291	3041.38274
*	37.7	0	M-H	C10H15N2O	-3.1003392	1377.88966	1561.47141	1192.30594	3599.58189	1612.43987
*	37.7	0	M+FA-H	C18H22N2S	-0.7199085	226.029379	284.276319	260.772481	679.468663	1911.10311
*	37.7	0	2M-H	C22H21NO4	7.38628834	7336.38192	5918.79429	5587.29214	8111.79681	7896.51263
*	37.7	0	M-H, M+FA-I	C26H26O17	-1.6943706	2351.64446	1626.53513	2063.05156	726.039522	723.166983
*	37.7	0	M+NH4	C12H15NO8	-0.4872014	3196.98356	2336.65237	3154.60284	4099.88693	1281.83756
*	37.7	0	M-H	C14H9NO6	3.51670453	1736.69537	1369.31646	1697.28304	2134.30236	6901.62243
*	37.7	0	2M-H	C6H10CaO4	4.46005453	236.484931	201.46097	204.452289	199.490397	773.954231

*	37.7	0	M-H2O-H	C17H21NO3	0.25222691	188.064682	218.267058	202.924576	190.937266	335.0406
*	37.7	0	M-H2O-H	C4H9NO2S	-1.2091997	209.411329	208.309877	206.566594	182.886183	634.697279
*	37.7	0	M-H2O-H	C9H10F2N2(	3.84532686	249.193215	259.869732	329.646009	468.262521	1620.79906
*	37.7	0	2M-H	C10H16O4	-6.7764116	1658.16004	2036.24363	1954.80715	2247.1652	3979.84063
*	37.7	6.18	M-H	C12H16O7	-8.6257752	582.15071	617.017701	655.872151	169.556395	6.02969414
*	37.7	0	M+NH4	C9H12N2O3	0.75479676	1117.60257	1100.12149	1240.20043	9.7531E-07	3234.48794
*	37.7	0	M+Na	C10H14N5O	3.39427408	289.251135	249.833572	222.959557	439.16274	695.680685
*	37.7	0	M+K	C20H24N2O	0.17813973	4874.63688	4849.8291	4193.19028	6801.64306	4426.82997
*	37.7	0	M-H2O-H	C13H20O12	-3.1408998	1054.90448	1072.11636	1254.56081	590.634605	545.032245
*	37.7	0	M+Na	C51H89O17I	2.4899534	22984.038	20615.1475	23358.453	7324.64037	5475.47843
*	37.7	0	M-H	C31H47NO8	-4.3321334	2297.96363	2561.37895	1959.24065	9.7531E-07	13212.244
*	37.7	0	2M-H	C13H11N3	9.17076388	1354.49454	1802.03195	1584.35752	107.409424	6807.78257
*	37.7	10.7	M+Na	C21H36O5	-0.5398268	7655.12576	8102.96038	8190.10088	778.235145	9.7531E-07
*	37.7	0	M-H	C52H92N2O	-1.3486473	1700.33594	2796.2656	2563.34068	9.7531E-07	15300.0806
*	37.7	0	2M-H	C19H21NO	5.69456119	87585.8445	100578.023	105489.288	48769.3416	225729.686
*	37.7	0	M+FA-H	C37H33N7O	-2.2438213	24078.6018	22450.818	22093.4019	10974.3922	25666.8438
*	37.7	0	M+Na	C27H36O2	0.24877096	165843.987	162790.751	110570.887	473425.846	161165.842
*	37.7	0.495	M+NH4	C19H28O3	-3.1494844	8367.16204	7668.83588	7473.44474	14494.1355	5321.2782
*	37.7	0	2M-H	C11H15N	2.09253832	5207.59459	5255.7799	5352.80632	7603.35001	1938.54957
*	37.7	3.58	M+K	C7H8N2O3	4.12515135	7581.36884	7644.49603	6553.76409	13871.4369	6285.34538
*	37.7	0	2M-H	C7H13NO3	4.10205315	4376.73338	3337.68432	3797.26135	6206.32309	2324.88164
*	37.7	0	M+NH4	C24H29N5O	-5.0548296	9091.25481	5480.47713	7992.17898	511.091299	9.7531E-07
*	37.7	0	M+Na	C40H82NO7	-0.9102624	198468.516	271671.933	237761.351	63876.6988	61540.3467
*	37.7	0	M+FA-H	C40H72O4	0.27288114	3638.8828	3484.43514	5502.05231	3108.32982	284.592737
*	37.7	0	M-H2O-H	C23H32N2O	3.43702325	998.946703	1166.08278	773.211253	2453.06769	474.820391
*	37.7	0.183	M+NH4	C35H34N4O	-1.3529956	8502.63488	6072.71303	7256.62817	2197.35848	9.7531E-07
*	37.7	6.88	M-H2O-H	C20H18O13!	-3.7525276	15105.0356	16531.3002	15455.8622	14100.1551	31124.1294
*	37.7	0	M+H-H2O	C4H9NO5S	-0.92451	9262.30805	9746.65509	10611.7131	16863.9346	16381.0407
*	37.7	0.767	M-H	C8H16NO9P	-8.7352439	253318.889	257934.847	254300.574	285727.016	300934.273
*	37.7	0	2M-H	C14H20O10!	-3.3667131	3867.22469	4170.39951	5456.41951	6575.29634	7955.93657
*	37.7	0	M-H	C10H7F3O4	-0.2796868	1443.68334	1553.66712	1611.51791	901.079621	127.684696
*	37.7	0	M+H	C11H12O2	2.25961724	4026.0237	4731.79047	4814.08241	4752.64831	3118.54944
*	37.7	0	M+K	C18H29O4-	0.06580498	1225.61466	1469.6909	1496.68714	874.033199	4177.93388

*	37.7	0	M+NH4	C14H18N4O	-2.6480149	7870.48629	8219.77504	8179.73107	6517.21541	13721.6531
*	37.7	0	M+FA-H	C11H23AsO:	-3.4055712	1386.53547	1227.49923	1418.59855	195.715271	1115.57305
*	37.7	0	M+NH4	C11H15NO4	0.15943129	1673.50525	1886.0682	1724.38458	3988.98417	2846.93638
*	37.7	0	M-H2O-H	C15H23N5O	6.21046896	8159.77945	8816.63247	8327.16972	2580.73973	6543.78928
*	37.7	0	M+H	C22H33N5O	0.09570842	1055.15079	1337.26661	1270.23085	1369.86008	3321.18993
*	37.7	0	M-H	C30H40O16	2.96763423	2240.7737	2776.57761	2404.2482	10392.2572	224.262942
*	37.7	0	M+K	C9H15N3	-1.1383621	996.169962	1074.20888	712.621033	2922.44385	341.572783
*	37.7	0	2M-H	C5H5NO5	-3.9577629	1564.28151	1521.09096	1330.53776	1181.06429	496.872419
*	37.7	0	M+FA-H	C9H15N5O8	-3.4727357	510.708203	474.309425	621.030833	2118.68426	654.745351
*	37.7	0	M+FA-H	C15H24N4O	-0.4436342	589.578042	652.157751	419.809881	535.449815	58.7345848
*	37.7	0	M+FA-H	C20H19F2N:	-2.9744087	232.797501	260.556811	271.500448	228.006267	1088.58616
*	37.7	0	M+H	C15H13I2NC	-2.531887	6353.20323	5673.73959	5352.42241	2893.13763	1263.84546
*	37.7	0	M+H-H2O	C34H59O16I	-4.8809874	5779.5215	4950.32019	5082.3552	6.96446557	276.142271
*	37.7	0	M+Na	C28H42O8	-3.8440383	1360.06836	954.729835	1111.42856	3422.11247	1292.66615
*	37.7	0	2M-H	C26H54	-6.3448042	6299.99944	7039.56951	6890.03606	6884.56837	1589.72239
*	37.7	0	M+Na	C6H6N12O1	2.16409796	4317.27349	3532.24573	3475.86739	2251.0734	6900.63742
*	37.7	0	M+NH4	C32H44O11	1.87619826	4077.41326	4503.80429	4688.93631	2283.65648	1738.13573
*	37.7	0	2M-H	C18H19N3O	4.31973298	2477.57254	3005.68377	2898.83905	4278.70262	1557.22013
*	37.7	0	M+NH4	C26H36N2O	0.26316545	1819.22089	2006.89376	2546.09806	588.020635	573.782828
*	37.7	0	M+Na	C24H21F2N(	-0.2507672	515.727698	635.225162	628.148069	593.215846	2687.4241
*	37.7	0	2M-H	C26H31N3O	1.42262375	271.699172	200.461826	281.388462	9.7531E-07	9.7531E-07
*	37.7	0	M+H-H2O	C10H15NO1	-4.9818523	314.402473	312.054251	322.144355	9.7531E-07	9.7531E-07
*	37.7	0	M-H	C18H34N4O	-0.3170601	1306.4051	1043.24097	1324.42727	315.435787	1869.63969
*	37.7	0	M+FA-H	C25H43NO7	-5.7544277	3719.98214	4017.28365	4214.43685	8449.33894	3963.81893
*	37.7	0	M+FA-H	C9H19O6P	-0.6975366	360.636719	349.7746	399.554726	973.343567	172.687296
*	37.7	0	M+H, M+Na	C16H20N2O	-2.0098714	4961.23591	4640.78865	4363.78144	13725.2572	2280.68015
*	37.7	0	M+FA-H	C13H15NO1	1.0446397	1027.94023	1319.13575	897.881894	100.743555	9.7531E-07
*	37.7	0	2M-H	C15H30O6S	-1.1212281	951.327809	741.729255	1122.91249	359.545038	1994.05544
*	37.7	0	M+NH4	C17H15N3O	-0.6563592	544.367683	551.754593	693.951866	999.535807	1277.58423
*	37.7	4.46	M+H-H2O	C24H42O10	-2.6653804	25018.5404	23565.7986	22680.2395	33350.9761	25051.7541
*	37.7	0	M-H	C8H7NO2S	-1.3142508	988.47067	796.214401	963.998195	1209.34802	1485.74065
*	37.7	0	M-H	C8H15N2O9	5.12153521	251.25474	315.259293	341.693906	561.036937	348.928981
*	37.7	0	M+FA-H	C18H21N5O	-3.9562176	1215.79001	990.722872	1495.66296	337.767255	529.856481

*	37.7	0	M+FA-H	C15H21N5O	-0.2245837	225.93917	237.610079	321.597066	995.021285	898.930142
**	37.7	0	M+FA-H	C9H10O2	-1.4736491	165.813947	163.015523	122.767573	778.122799	117.940012
*	37.7	0	M+H	C24H44O11	-4.6303564	1734.91369	1222.06195	1499.4713	1455.629	394.266668
*	37.7	0	M-H2O-H	C8H10N6O3	0.31993926	245.663548	230.326099	239.791485	388.639474	313.657376
*	37.7	0	M+K	C10H9N	-0.5435411	281.892728	335.509452	224.139562	213.753227	163.122487
*	37.7	0	M+H	C19H25N5O	-4.2655631	4020.26413	4597.07486	4072.17587	2152.41683	1547.2016
*	37.7	0	M+H, M+Na	C17H28N4O	-4.9745368	1856.8187	1705.76809	1719.97785	667.656731	368.761562
*	37.7	0	M+H	C27H31N5O	3.39104627	793.477995	1101.04432	1196.84559	173.283801	5931.90257
*	37.7	0	M+FA-H	C15H31O7P	-1.4399033	451.43507	488.313594	400.733158	1287.81145	130.838641
*	37.7	0	M+NH4	C9H12N6	0.17229202	2427.4318	2915.03485	2851.7284	3262.27768	2330.68798
*	37.7	0	M-H	C23H20N6O	0.45215137	300.741792	391.340767	349.842981	321.052079	1790.30518
*	37.7	0	M+FA-H	C10H13N2O	2.29580222	2886.8597	2710.50953	2782.02161	3145.77884	2811.92163
[truncated: 497,282 more chars]
