# Supplementary material for: Disrupted Tuzzerella abundance and impaired l-glutamine levels induce Treg accumulation in ovarian endometriosis: a comprehensive multi-omics analysis
Source: Metabolomics. 2024 Feb 29;20(2):32. doi: 10.1007/s11306-023-02072-0 (PMC10904428; doi:10.1007/s11306-023-02072-0)
Supplement: Supplementary file 3 — Supplementary file3 (PDF 122057 KB) [file 11306_2023_2072_MOESM3_ESM.pdf]

Annotated S	Modificatior	Qvality PEP	Qvality q-va	# Protein	# Protein	# PSMs	Master Prot	Positions in Mas	# Missed Cl	Theo. MH+ [
[K].AAAAAA	1xTMTpro [N	4.235E-05	4.225E-05	1	1	1	P37108	P37108 [108-136]	0	2672.4174
[R].EALLGV	1xTMTpro [K	2.358E-05	4.225E-05	1	1	1	Q14257	Q14257 [42-56]	0	2315.2626
[R].NRPLSD	1xTMTpro [K	4.095E-06	4.225E-05	1	1	2	O75533	O75533 [396-413	0	2719.3893
[K].EAILNDE	1xCarbamid	1.654E-05	4.225E-05	1	1	1	P35241	P35241 [108-133]	0	3486.8686
[K].NREPLM	1xTMTpro [K	2.358E-05	4.225E-05	1	1	2	O94826	O94826 [246-258	1	2165.2397
[R].NREEEW	1xTMTpro [K	2.635E-05	4.225E-05	1	1	2	Q9Y2W1	Q9Y2W1 [864-87	1	2301.1643
[R].NQYDND	1xTMTpro [N	1.782E-05	4.225E-05	1	1	2	P25786	P25786 [4-18]	0	2083.0164
[R].EALIQFL	1xTMTpro [K	2.697E-05	4.225E-05	1	1	1	P15169	P15169 [329-343]	0	2361.3786
[R].IAAGLPM	1xOxidation	2.009E-05	4.225E-05	1	1	2	Q9H061	Q9H061 [65-84]	0	2441.3433
[R].GPEEEH	1xTMTpro [N	1.526E-07	4.225E-05	1	1	3	P00450	P00450 [440-462]	0	2791.495
[R].LAALAD	1xTMTpro [K	3.508E-05	4.225E-05	1	1	3	Q13813	Q13813 [1633-16	0	2239.3094
[K].NQVAMN	1xOxidation	4.085E-05	4.225E-05	1	1	2	P11142	P11142 [57-71]	0	2274.2044
[K].NQVAMN	1xTMTpro [K	5.872E-06	4.225E-05	1	1	4	P11142	P11142 [57-71]	0	2258.2095
[R].LAALAL	1xCarbamid	6.183E-06	4.225E-05	1	1	2	O00567	O00567 [454-480	0	3807.9448
[K].EAINLLE	1xTMTpro [N	2.042E-05	4.225E-05	1	1	2	Q99460	Q99460 [667-684	0	2392.2502
[K].EAINLLE	1xOxidation	4.89E-05	4.225E-05	1	1	1	Q99460	Q99460 [667-684	0	2408.2451
[K].NQVALN	1xTMTpro [K	1.364E-05	4.225E-05	1	1	3	P0DMV9	P0DMV9 [57-71]	0	2267.264
[K].NQTLQN	1xTMTpro [N	5.86E-05	4.225E-05	1	1	1	P11277	P11277 [1520-15	0	1925.0524
[K].NQTAEK	2xTMTpro [K	9.5E-06	4.225E-05	1	1	4	P11142	P11142 [584-597]	1	2658.4304
[R].EALPAF	1xTMTpro [K	5.645E-05	4.225E-05	1	1	1	Q9UDR5	Q9UDR5 [746-76	0	2448.3895
[R].EALPAP	1xTMTpro [K	3.8E-05	4.225E-05	1	1	4	P07203	P07203 [131-148]	0	2451.2933
[K].LAALASD	1xTMTpro [K	8.154E-06	4.225E-05	1	1	4	Q8WUM0	Q8WUM0 [976-9	0	2376.2612
[R].IAAGLPM	1xTMTpro [N	8.245E-06	4.225E-05	1	1	1	Q9H061	Q9H061 [65-84]	0	2425.3484
[K].LAAGHE	1xTMTpro [N	1.966E-05	4.225E-05	1	1	1	P17302	P17302 [347-366]	0	2462.3799
[R].LAAGDQ	1xTMTpro [N	2.255E-05	4.225E-05	1	1	2	P55196	P55196 [1052-10	0	1618.9084
[K].NSDPLV	1xTMTpro [K	1.15E-05	4.225E-05	1	1	2	Q05707	Q05707 [1312-13	0	2106.205
[K].EAIEGTY	2xTMTpro [K	3.108E-05	4.225E-05	1	1	4	P62280	P62280 [49-59]	1	2179.279
[K].LAAAVSI	1xTMTpro [N	1.451E-05	4.225E-05	1	1	3	P36955	P36955 [54-67]	0	1863.9924
[K].NSLQEQ	1xTMTpro [N	1.862E-05	4.225E-05	1	1	2	P35580	P35580 [1346-13	0	2022.9535
[K].EALELTD	1xTMTpro [N	1.677E-06	4.225E-05	1	1	2	Q14204	Q14204 [1302-13	0	2124.0991
[R].EAIENAT	1xTMTpro [N	2.405E-05	4.225E-05	1	1	1	Q9BY43	Q9BY43 [91-104]	0	1834.983
[R].NSLQDQ	1xTMTpro [K	3.397E-06	4.225E-05	1	1	2	P35749	P35749 [1346-13	0	2258.1466

[K].EAIESAI(	1xTMTpro [K	1.634E-06	4.225E-05	1	1	2	P63172	P63172 [23-38]	0	2283.2225
[R].LAACGP	1xCarbamid(	4.401E-06	4.225E-05	1	1	1	P18065	P18065 [50-72]	0	2345.3083
[K].LAACLE(	2xCarbamid(	4.163E-06	4.225E-05	1	1	1	P00734	P00734 [100-117]	0	2273.0974
[K].NSLDCE	1xCarbamid(	5.132E-05	4.225E-05	1	1	6	Q01518	Q01518 [423-433]	0	1844.0079
[K].NQNSW(	1xTMTpro [K	2.345E-05	4.225E-05	1	1	2	P20700	P20700 [517-528]	0	1943.0114
[R].NSLCIEN	2xCarbamid(	3.064E-05	4.225E-05	1	1	1	Q15582	Q15582 [470-485]	1	2496.2943
[K].EALGGQ	1xTMTpro [K	4.491E-06	4.225E-05	1	1	2	Q9BUJ2	Q9BUJ2 [361-375	0	2203.3094
[K].NSIAYMI	2xTMTpro [K	2.635E-05	4.225E-05	1	1	2	P08123	P08123 [1274-128	1	2625.4374
[K].NSGQNL	1xTMTpro [K	2.86E-05	4.225E-05	1	1	1	Q9HAV7	Q9HAV7 [35-50]	0	2402.1749
[K].FVVDLSI	1xTMTpro [N	9.448E-06	4.225E-05	1	1	1	P35998	P35998 [121-139]	0	2425.224
[R].LAAFAD/	2xTMTpro [K	6.275E-06	4.225E-05	1	1	1	Q5SY16	Q5SY16 [471-495	1	3663.9363
[R].NSEQIVE	1xTMTpro [K	1.202E-05	4.225E-05	1	1	1	Q15006	Q15006 [29-47]	0	2759.4595
[K].EALKDE`	2xTMTpro [K	1.505E-05	4.225E-05	1	1	2	O00264	O00264 [133-163]	1	4536.2762
[R].FVTVQTI	1xTMTpro [N	5.047E-05	4.225E-05	1	1	4	P00505	P00505 [126-139]	0	1754.0132
[K].NSDVLQ	1xTMTpro [N	3.405E-05	4.225E-05	1	1	1	Q8NBJ5	Q8NBJ5 [606-619	0	1776.9411
[R].NSDVEIF	1xTMTpro [K	3.709E-05	4.225E-05	1	1	2	Q99715	Q99715 [566-577]	0	1886.0879
[K].FVVDVD	1xCarbamid(	1.051E-05	4.225E-05	1	1	2	P62195	P62195 [95-113]	1	2841.5061
[R].EALPMD	1xTMTpro [K	1.069E-06	4.225E-05	1	1	2	P43405	P43405 [339-361]	0	3287.6637
[K].NQLTSN	1xTMTpro [K	7.409E-06	4.225E-05	1	1	3	P11021	P11021 [82-96]	0	2286.2221
[R].NQLTAM	1xTMTpro [K	2.191E-05	4.225E-05	1	1	2	Q14152	Q14152 [521-532]	0	1871.0916
[R].NPVNYF	1xOxidation [	8.386E-07	4.225E-05	1	1	6	P04040	P04040 [321-349]	0	3800.8973
[R].NPVNYF	1xTMTpro [K	9.799E-07	4.225E-05	1	1	4	P04040	P04040 [321-349]	0	3784.9024
[K].NPTYGP	1xCarbamid(	5.41E-05	4.225E-05	1	1	1	P50281	P50281 [311-330]	0	2559.2291
[K].NPTDAY	1xCarbamid(	1.301E-07	4.225E-05	1	1	1	P53621	P53621 [1151-117	0	3709.7487
[K].NPSTNF(	1xTMTpro [K	1.855E-05	4.225E-05	1	1	2	P19623	P19623 [254-277]	0	3362.8022
[R].NPSSAG	1xTMTpro [N	8.124E-06	4.225E-05	1	1	3	P02671	P02671 [288-308]	0	2268.056
[K].NPSIVG\	2xCarbamid(	3.201E-06	4.225E-05	1	1	1	O43504	O43504 [15-34]	0	2464.2607
[K].NPSAMA	1xTMTpro [N	8.321E-06	4.225E-05	1	1	1	Q9BTV4	Q9BTV4 [169-189	0	2528.2961
[R].NPQNSS	1xTMTpro [N	1.572E-05	4.225E-05	1	1	2	Q01081	Q01081 [54-66]	0	1677.8475
[K].EAMVQA	1xTMTpro [N	8.681E-06	4.225E-05	1	1	2	O00567	O00567 [423-437]	0	1922.9813
[K].EAMEDG	1xOxidation [	1.051E-05	4.225E-05	1	1	1	P19338	P19338 [628-648]	1	3578.9517
[K].LAALTNY	1xTMTpro [N	4.106E-05	4.225E-05	1	1	1	Q6ICL3	Q6ICL3 [70-86]	0	2305.2624
[R].NPPGFA	1xTMTpro [N	5.775E-06	4.225E-05	1	1	1	P84103	P84103 [44-64]	1	2624.3064

[R].NPPGFA	1xTMTpro [N	2.989E-05	4.225E-05	2	2	6	Q16629; P84	Q16629 [45-58]; P	0	1925.9717
[R].IAAQDLL	1xTMTpro [N	5.34E-05	4.225E-05	1	1	2	Q96P70	Q96P70 [400-427	0	3090.6754
[K].EANLLNA	1xTMTpro [N	5.545E-05	4.225E-05	1	1	4	Q9UNZ2	Q9UNZ2 [357-368	0	1643.9764
[R].EANLQA	1xTMTpro [N	6.596E-06	4.225E-05	3	3	1	Q9UMX0; Q9	Q9UMX0 [563-58	0	2344.2792
[K].EANIQAV	1xTMTpro [K	6.048E-06	4.225E-05	1	1	1	Q9H2G2	Q9H2G2 [510-524	0	2182.2211
[K].NPNTSE	1xTMTpro [K	1.66E-05	4.225E-05	1	1	3	P05023	P05023 [495-508]	0	2216.2353
[K].LAAQEN	1xTMTpro [K	2.994E-05	4.225E-05	1	1	1	Q9BTW9	Q9BTW9 [635-65	0	3040.6671
[K].FVPYLIA	1xCarbamido	1.35E-06	4.225E-05	1	1	2	P12268	P12268 [456-474]	0	2725.4991
[R].NPNAVL	1xTMTpro [K	5.818E-06	4.225E-05	1	1	2	Q13576	Q13576 [239-257	0	2722.4907
[R].NPPGFA	1xTMTpro [N	7.83E-06	4.225E-05	1	1	3	Q16629	Q16629 [45-65]	1	2682.3119
[K].NSNILED	1xTMTpro [N	5.34E-05	4.225E-05	1	1	2	P48444	P48444 [73-84]	0	1720.94
[K].EAMEDG	3xTMTpro [K	2.636E-06	4.225E-05	1	1	1	P19338	P19338 [628-648]	1	3562.9568
[K].EAMEDG	1xTMTpro [K	1.654E-05	4.225E-05	1	1	4	P19338	P19338 [628-639]	0	1915.9563
[K].NQLNPIG	1xTMTpro [N	1.319E-05	4.225E-05	1	1	1	O75569	O75569 [123-141	0	2487.354
[K].NQILNLT	1xTMTpro [N	3.786E-07	4.225E-05	1	1	1	P13645	P13645 [208-228]	0	2671.4698
[K].IAAIDSLN	1xTMTpro [N	5.192E-05	4.225E-05	1	1	1	Q6YP21	Q6YP21 [88-99]	0	1668.924
[K].NQGYYD	1xTMTpro [K	3.135E-05	4.225E-05	1	1	1	Q02218	Q02218 [962-972	0	2011.0893
[R].NQGNTV	1xTMTpro [K	2.202E-05	4.225E-05	2	2	4	P01023; P20	P01023 [1035-104	0	2100.2097
[K].EAIQQQ	1xTMTpro [K	6.819E-06	4.225E-05	1	1	1	Q07157	Q07157 [788-804	0	2593.423
[R].NQGGYG	1xTMTpro [N	9.133E-07	4.225E-05	1	2	7	P09651	P09651 [353-370]	0	1998.9072
[K].NQGGGI	1xTMTpro [K	1.578E-06	4.225E-05	1	1	1	P07814	P07814 [992-1009	0	2196.15
[R].NQGFDV	1xTMTpro [N	2.316E-05	4.225E-05	1	1	2	P08240	P08240 [511-524]	0	1794.9669
[R].LAALEQI	1xTMTpro [N	4.242E-05	4.225E-05	1	1	1	O60437	O60437 [1427-14	0	1633.8716
[K].NPVTIFS	1xTMTpro [N	3.219E-05	4.225E-05	1	1	1	Q04837	Q04837 [52-66]	0	2083.0966
[K].LAALNPE	1xTMTpro [K	1.208E-06	4.225E-05	1	1	3	O00299	O00299 [96-113]	0	2453.3895
[K].NQALNT	1xTMTpro [N	1.569E-07	4.225E-05	1	1	5	Q13813	Q13813 [1253-12	0	2632.3399
[K].EALSTAT	1xTMTpro [N	4.057E-05	4.225E-05	1	1	1	Q709C8	Q709C8 [1727-17	0	1650.8982
[K].NQAFIEN	1xTMTpro [N	3.973E-07	4.225E-05	1	1	2	P26599	P26599 [95-122]	0	3510.7204
[R].EALTDAI	1xTMTpro [N	8.275E-06	4.225E-05	1	1	1	Q9NRY6	Q9NRY6 [246-265	0	2554.2996
[K].EALTYDG	1xTMTpro [N	4.106E-05	4.225E-05	1	1	3	Q15056	Q15056 [97-109]	0	1697.9029
[R].NPYYGG	1xTMTpro [K	6.037E-06	4.225E-05	1	1	1	P31150	P31150 [36-54]	0	2755.4322
[K].EALVDTI	1xTMTpro [N	3.597E-06	4.225E-05	1	1	1	Q96P70	Q96P70 [23-40]	0	2244.277
[K].EAIVTSE	1xTMTpro [K	9.799E-07	4.225E-05	1	1	1	Q13813	Q13813 [169-189	0	2974.6229

[R].NPYYGC	1xTMTpro [K	2.056E-06	4.225E-05	1	1	3	P50395	P50395 [36-54]	0	2725.4216
[R].EAMCPG	1xCarbamido	4.872E-08	4.225E-05	1	1	1	P14923	P14923 [87-115]	0	3365.6636
[K].EALSHSI	1xTMTpro [K	3.906E-05	4.225E-05	1	1	1	A1A4S6	A1A4S6 [371-393	0	3116.6984
[K].NPNAML	1xOxidation [	3.435E-05	4.225E-05	1	1	1	P46940	P46940 [242-266]	0	3459.8325
[R].NSNLVG	1xTMTpro [N	3.163E-07	4.225E-05	1	1	6	P02545	P02545 [281-296]	0	2057.0695
[K].NSNVDS	1xCarbamido	8.681E-06	4.225E-05	1	1	1	Q7Z2W4	Q7Z2W4 [630-64	0	2423.1468
[R].KYSNED	2xTMTpro [K	6.499E-06	4.225E-05	1	1	1	P26641	P26641 [296-316]	1	3501.8511
[K].NTIYEIQ	1xTMTpro [K	1.422E-05	4.225E-05	1	1	1	P41218	P41218 [336-355]	0	2736.4006
[R].KYTLPP	2xTMTpro [K	1.306E-06	4.225E-05	1	1	5	P04792	P04792 [141-171]	1	4139.2766
[R].KYTLPP	1xOxidation [	1.974E-06	4.225E-05	1	1	5	P04792	P04792 [141-171]	1	4155.2715
[R].NTLIQFE	1xTMTpro [N	9.712E-06	4.225E-05	1	1	1	P23368	P23368 [249-264]	0	2227.1215
[R].EAFQPQ	1xTMTpro [N	7.287E-06	4.225E-05	1	1	4	P18206	P18206 [833-853]	0	2751.3949
[K].EAFSLFD	2xTMTpro [K	5.188E-06	4.225E-05	1	1	5	P0DP25	P0DP25 [15-31]	1	2757.5127
[K].EAFTNKI	1xTMTpro [K	8.26E-06	4.225E-05	1	1	2	O75369	O75369 [1340-13	0	2331.3316
[K].FWEVISI	1xTMTpro [N	2.745E-06	4.225E-05	1	2	1	P68371	P68371 [20-46]	0	3420.6303
[R].NTIEETG	1xTMTpro [N	7.73E-06	4.225E-05	1	1	3	P11047	P11047 [1122-11	0	1849.9575
[R].EAGAGG	1xTMTpro [K	3.096E-06	4.225E-05	1	1	10	P21333	P21333 [2265-22	0	2035.1679
[R].EAGAGG	2xTMTpro [K	6.518E-08	4.225E-05	1	1	1	P21333	P21333 [2265-22	2	3414.9049
[R].EAGAGG	1xTMTpro [K	3.664E-06	4.225E-05	2	2	2	O75369; Q1	O75369 [2220-22	0	2051.1628
[K].FWEVISI	1xTMTpro [N	2.981E-07	4.225E-05	1	1	1	P07437	P07437 [20-46]	0	3406.6147
[R].NTGVIS\	1xTMTpro [N	5.572E-05	4.225E-05	1	1	1	P12830	P12830 [322-335]	0	1735.9873
[R].NTGTEA	1xTMTpro [K	3.059E-05	4.225E-05	1	1	2	Q13228	Q13228 [35-52]	0	2514.3219
[R].EAGDGV	1xCarbamido	1.959E-05	4.225E-05	1	1	2	Q14315	Q14315 [527-544]	0	2624.3198
[R].EAGDVG	1xCarbamido	4.177E-05	4.225E-05	1	1	4	Q07955	Q07955 [143-154]	0	1721.8124
[R].EAGDVG	1xCarbamido	4.765E-06	4.225E-05	1	1	1	Q07955	Q07955 [143-164]	1	2781.3473
[R].NTGIICT	1xCarbamido	5.85E-05	4.225E-05	1	1	2	P14618	P14618 [44-56]	0	1663.9121
[K].EAGEGG	1xTMTpro [K	5.112E-06	4.225E-05	1	1	1	P29966	P29966 [177-193]	0	2138.0857
[R].EAFLNTE	2xTMTpro [K	3.966E-07	4.225E-05	1	1	7	Q13813	Q13813 [1465-14	1	3206.7613
[K].KYSEIEE	2xTMTpro [K	2.488E-05	4.225E-05	1	1	1	Q53T59	Q53T59 [70-80]	1	2376.3267
[R].NTNAGA	1xTMTpro [N	3.322E-05	4.225E-05	1	1	1	Q02218	Q02218 [82-101]	0	2316.2267
[K].NTNDAN	1xCarbamido	5.475E-07	4.225E-05	2	2	2	P31150; P5C	P31150 [310-328]	0	2503.2643
[R].KYGGPF	2xTMTpro [K	1.888E-06	4.225E-05	1	1	3	O43390	O43390 [146-171]	1	3547.9617
[K].KYILGNF	3xTMTpro [K	1.473E-06	4.225E-05	1	1	1	P00352	P00352 [329-353]	2	4019.2958

[R].EAEPHY	1xTMTpro [K	5.638E-06	4.225E-05	1	1	2	Q13308	Q13308 [867-884]	0	2729.4352
[R].KYLLGV(	1xTMTpro [K	3.157E-05	4.225E-05	1	1	1	Q10713	Q10713 [269-288]	1	2796.554
[K].KYNILGT	2xTMTpro [K	1.448E-05	4.225E-05	1	1	1	Q9BUJ2	Q9BUJ2 [449-461	1	2393.4043
[R].FYALSAS	1xTMTpro [K	2.06E-05	4.225E-05	1	1	8	P27797	P27797 [74-87]	0	2216.1883
[K].NVCLPPI	1xCarbamido	1.242E-05	4.225E-05	1	1	1	P27816	P27816 [533-553]	0	2961.5921
[R].NVAQYN	1xTMTpro [N	2.825E-07	4.225E-05	1	1	1	Q14204	Q14204 [2468-24	0	2818.3902
[K].EAESCD	2xCarbamido	7.257E-07	4.225E-05	2	5	2	P07437; P68	P07437 [123-154]	0	3919.9484
[R].NVALVS(	1xTMTpro [K	3.594E-05	4.225E-05	1	1	3	Q16610	Q16610 [506-518]	0	1926.0788
[R].NTGDAD	1xCarbamido	1.15E-05	4.225E-05	1	1	1	Q12824	Q12824 [342-364]	1	3577.8334
[R].FWYGG(	1xCarbamido	2.726E-05	4.225E-05	1	1	2	P12111	P12111 [3140-315	0	1996.9831
[R].FWTFET	2xCarbamido	2.187E-05	4.225E-05	1	1	2	Q02809	Q02809 [260-275]	0	2282.0541
[R].NTVSQS	2xTMTpro [K	7.874E-06	4.225E-05	1	1	2	Q9BY44	Q9BY44 [521-536	1	2630.4817
[K].NTVLCN'	1xCarbamido	1.03E-05	4.225E-05	1	1	1	Q14258	Q14258 [66-83]	0	2394.277
[K].EAEVVL(	2xCarbamido	2.239E-06	4.225E-05	1	1	2	P42765	P42765 [110-130]	0	2647.2485
[K].KYPLLLD	1xCarbamido	5.018E-06	4.225E-05	1	1	1	P27487	P27487 [539-554]	1	2764.5888
[K].NTVANS	1xTMTpro [K	1.131E-05	4.225E-05	1	1	1	P61221	P61221 [542-557]	0	2267.2673
[K].KYPVAH	2xTMTpro [K	1.015E-05	4.225E-05	1	1	2	P05155	P05155 [329-341]	1	2472.4795
[K].FWQTYS	1xTMTpro [K	3.102E-05	4.225E-05	1	1	1	A0AVT1	A0AVT1 [659-672	0	2324.2418
[R].EAFDLFI	1xTMTpro [K	2.175E-05	4.225E-05	1	1	2	P41208	P41208 [35-51]	0	2422.2633
[K].NTSEQD	1xTMTpro [N	8.554E-06	4.225E-05	1	1	2	P20700	P20700 [458-473]	0	2183.0181
[K].KYNSQN	1xTMTpro [K	1.113E-05	4.225E-05	1	1	1	P01042	P01042 [43-58]	1	2611.3873
[R].NTGDAD	1xCarbamido	5.904E-06	4.225E-05	1	1	1	Q12824	Q12824 [342-363]	0	3145.5313
[R].EAGFLR	1xTMTpro [N	9.623E-06	4.225E-05	1	1	1	Q07157	Q07157 [640-658]	0	2333.3301
[K].EAGFPP	1xTMTpro [K	9.252E-07	4.225E-05	1	1	3	O94788	O94788 [228-258]	0	3612.0081
[R].NSYVAG	1xTMTpro [N	2.506E-05	4.225E-05	1	1	1	P11413	P11413 [105-120]	0	2111.9953
[K].EAGYEG	1xCarbamido	2.799E-05	4.225E-05	1	1	1	P12821	P12821 [547-561]	0	2112.014
[K].NSVVEA	1xTMTpro [K	1.706E-05	4.225E-05	1	1	3	Q04917	Q04917 [144-155]	0	1876.0308
[K].NSVTPD	1xTMTpro [K	5.132E-05	4.225E-05	1	1	2	P46777	P46777 [229-241]	0	2183.0678
[R].NSVTGG	1xCarbamido	3.597E-06	4.225E-05	1	1	1	P27708	P27708 [720-739]	0	2709.4049
[K].NSVSQIS	1xTMTpro [K	3.722E-05	4.225E-05	1	1	2	O15143	O15143 [327-339]	0	1884.1046
[R].NSVPCQ	2xCarbamido	1.169E-06	4.225E-05	1	1	4	Q8IZ83	Q8IZ83 [55-87]	0	3759.8237
[R].FVYTPAI	1xCarbamido	3.01E-05	4.225E-05	1	1	1	P01033	P01033 [83-98]	0	2268.0901
[K].IAAAGLD	1xTMTpro [K	4.194E-06	4.225E-05	1	1	2	Q9UBQ7	Q9UBQ7 [263-28(	0	3076.7902

[R].NSVDEL	1xTMTpro [K	7.23E-07	4.225E-05	1	1	11	P02675	P02675 [125-152]	0	3775.898
[R].NTAELQ	1xTMTpro [K	4.65E-05	4.225E-05	1	1	1	O15355	O15355 [509-520]	1	1938.09
[K].EAKPGA	1xTMTpro [K	5.495E-07	4.225E-05	1	1	1	Q6L8Q7	Q6L8Q7 [200-233	0	4108.049
[K].NSVAYM	1xTMTpro [K	2.479E-05	4.225E-05	1	1	1	P02452	P02452 [1372-138	0	2177.1516
[K].LAAAILG	2xTMTpro [K	1.415E-07	4.225E-05	1	1	6	P22087	P22087 [144-162]	0	2784.728
[K].LAAAILG	2xTMTpro [K	4.102E-06	4.225E-05	1	1	1	A6NHQ2	A6NHQ2 [156-172	0	2656.6694
[K].NSQSFF	1xTMTpro [K	3.67E-05	4.225E-05	1	1	2	P54920	P54920 [23-37]	0	2158.1424
[R].EALCGC	2xCarbamido	2.132E-05	4.225E-05	1	1	1	Q9UDY4	Q9UDY4 [260-275	0	2066.033
[R].EALCGC	2xCarbamido	1.045E-05	4.225E-05	1	1	2	P25685	P25685 [264-279]	0	2066.033
[R].EAIDSPV	1xTMTpro [N	4.295E-07	4.225E-05	1	1	1	Q92900	Q92900 [561-577]	0	2214.2202
[R].LAAAQA	1xTMTpro [N	2.312E-05	4.225E-05	1	1	1	Q6QNY0	Q6QNY0 [146-16	0	1782.0305
[K].NSPEDL	1xCarbamido	1.541E-05	4.225E-05	1	1	1	Q12888	Q12888 [499-514]	0	2301.1888
[R].EALEAES	1xCarbamido	2.56E-07	4.225E-05	1	1	3	Q15149	Q15149 [3812-38	0	3210.5737
[R].EALAEA	1xTMTpro [N	7.434E-07	4.225E-05	1	1	1	O14745	O14745 [271-287]	0	2097.1987
[K].NSNPALI	1xTMTpro [K	2.14E-05	4.225E-05	1	1	3	O00299	O00299 [120-131]	0	1937.0584
[K].NTAEWL	1xTMTpro [K	3.242E-05	4.225E-05	1	1	1	P00491	P00491 [12-22]	0	1908.0835
[R].EAGVEM	1xTMTpro [K	4.142E-05	4.225E-05	1	1	2	P14868	P14868 [357-374]	0	2544.2267
[K].EAGFPP	1xTMTpro [K	6.327E-07	4.225E-05	1	1	1	P05091	P05091 [227-257]	0	3627.9303
[R].NTFVGT	1xTMTpro [K	2.02E-05	4.225E-05	2	2	1	O00506; Q9	O00506 [173-188]	0	2445.3496
[K].EAGGGC	1xTMTpro [K	4.264E-05	4.225E-05	1	1	3	Q8NC51	Q8NC51 [40-52]	0	1621.9153
[R].NTFAEV	1xTMTpro [K	3.471E-05	4.225E-05	1	1	3	P02751	P02751 [959-976]	0	2602.4049
[K].EAGGNY	1xTMTpro [N	1.013E-05	4.225E-05	1	1	1	Q96ST3	Q96ST3 [344-364	0	2571.301
[R].NTEQEE	1xTMTpro [K	2.554E-06	4.225E-05	1	1	1	P00742	P00742 [292-310]	0	2704.4285
[K].NTEISFIL	1xTMTpro [K	4.827E-06	4.225E-05	1	1	1	P15090	P15090 [60-80]	1	3035.5817
[R].NTEILTG	1xTMTpro [K	5.514E-06	4.225E-05	1	1	2	P08603	P08603 [29-51]	0	3210.645
[R].EAGLAP	1xTMTpro [K	3.393E-05	4.225E-05	1	1	1	P06132	P06132 [250-263]	0	2065.2375
[K].EAGICYA	2xCarbamido	2.084E-08	4.225E-05	1	1	1	Q13126	Q13126 [207-235]	1	3984.8698
[R].NTCEHIY	1xCarbamido	4.194E-06	4.225E-05	1	1	1	A6NIH7	A6NIH7 [199-216]	0	2554.2567
[K].EAGIPEF	1xTMTpro [K	3.422E-06	4.225E-05	1	1	6	P00751	P00751 [566-581]	0	2451.3303
[K].NTELAVI	1xTMTpro [K	3.677E-05	4.225E-05	1	1	1	P41252	P41252 [1126-115	0	3410.7935
[R].EAGLVA	1xTMTpro [N	4.989E-05	4.225E-05	1	1	1	Q9BRJ6	Q9BRJ6 [84-95]	0	1549.877
[K].FWEVISI	1xTMTpro [N	2.212E-07	4.225E-05	1	1	1	Q9BUF5	Q9BUF5 [20-46]	0	3264.6132
[R].KYTYNY	1xTMTpro [K	2.537E-07	4.225E-05	1	1	2	P04114	P04114 [51-71]	1	2890.4351

[K].EAGNLE	1xTMTpro [K	6.621E-06	4.225E-05	1	1	1	Q9NXC5	Q9NXC5 [642-656	0	2137.2724
[R].NTDSDV	1xTMTpro [K	3.754E-05	4.225E-05	1	1	2	Q8N1G2	Q8N1G2 [472-487	0	2363.3678
[K].EAGQNF	1xTMTpro [N	3.645E-05	4.225E-05	1	1	1	Q6DKJ4	Q6DKJ4 [224-238	0	2000.0408
[K].FWEMIG	1xTMTpro [N	9.838E-06	4.225E-05	1	1	1	Q9H4B7	Q9H4B7 [20-37]	0	2366.1406
[R].EAGSQK	1xTMTpro [K	3.817E-06	4.225E-05	1	1	2	P02786	P02786 [156-174]	1	2819.4819
[R].LAAAEE	1xTMTpro [N	4.389E-05	4.225E-05	1	1	1	Q9BUH6	Q9BUH6 [139-150	0	1518.8447
[R].EAGISDY	1xTMTpro [K	4.657E-05	4.225E-05	1	1	1	Q9BTY2	Q9BTY2 [307-321	0	2288.2881
[K].NVDLLSI	1xTMTpro [K	4.432E-07	4.225E-05	1	1	4	P55209	P55209 [177-194]	0	2703.4519
[K].NPNAML	1xTMTpro [K	2.631E-06	4.225E-05	1	1	2	P46940	P46940 [242-266]	0	3443.8376
[K].NPLVWV	1xTMTpro [N	1.648E-05	4.225E-05	1	1	1	Q14624	Q14624 [789-805]	0	2244.2572
[R].NITFDEL	1xTMTpro [K	2.711E-05	4.225E-05	1	1	1	Q99707	Q99707 [166-181]	0	2506.3321
[R].FVIEENL	1xCarbamido	1.941E-05	4.225E-05	1	1	2	Q09161	Q09161 [312-323]	0	2123.2179
[R].NLTALGI	1xTMTpro [K	6.548E-06	4.225E-05	1	1	2	P31939	P31939 [23-39]	0	2208.3207
[K].FVLDHEI	1xTMTpro [K	3.39E-06	4.225E-05	1	1	3	Q14980	Q14980 [133-153]	0	3110.629
[K].EAVNLQ	1xTMTpro [N	1.422E-05	4.225E-05	1	1	2	O95394	O95394 [113-127]	0	1963.0932
[K].NLSSNE	1xTMTpro [N	6.869E-06	4.225E-05	1	1	2	P78527	P78527 [839-852]	0	1879.0092
[K].NISSAQI	2xTMTpro [K	1.332E-05	4.225E-05	1	1	1	P53990	P53990 [291-309]	0	2763.6185
[K].EAVQLVI	1xCarbamido	2.21E-05	4.225E-05	1	1	1	P54886	P54886 [531-547]	1	2364.2148
[K].NLSPVVS	1xTMTpro [K	5.266E-07	4.225E-05	1	1	2	Q8WXF1	Q8WXF1 [162-18	0	3140.7123
[R].EAVTEIL	1xTMTpro [K	1.368E-06	4.225E-05	1	1	1	Q15631	Q15631 [117-131]	1	2307.3052
[K].NISFTVW	1xTMTpro [K	5.617E-06	4.225E-05	1	2	1	P61204	P61204 [60-73]	0	2174.1737
[R].NISANEF	1xTMTpro [K	1.763E-05	4.225E-05	1	1	1	Q00013	Q00013 [342-362]	0	2962.4901
[R].NIQVDEA	1xTMTpro [K	6.852E-07	4.225E-05	1	1	3	P68036	P68036 [24-48]	0	3460.8608
[R].IADPEHD	1xTMTpro [N	5.766E-05	4.225E-05	1	1	2	P27361	P27361 [190-208]	0	2476.2428
[R].NLQPDT	1xTMTpro [N	1.117E-05	4.225E-05	1	1	2	Q99715	Q99715 [1902-19	0	2772.4011
[R].NLQPAS	1xTMTpro [K	9.838E-06	4.225E-05	1	1	4	P02751	P02751 [1055-10	0	2341.3623
[R].NLQNNA	1xTMTpro [N	1.105E-05	4.225E-05	1	1	1	P04114	P04114 [4107-41	0	2080.0895
[R].NIQEYLS	1xTMTpro [K	1.045E-05	4.225E-05	1	1	2	P04114	P04114 [4447-44	0	2314.2786
[R].FVHSEN	1xTMTpro [K	1.754E-05	4.225E-05	1	2	1	P68400	P68400 [284-303]	0	2933.5653
[K].FVHLLD	1xTMTpro [K	1.231E-06	4.225E-05	1	1	2	P46940	P46940 [872-891]	0	3058.5687
[R].NLQEQT	1xTMTpro [N	4.33E-05	4.225E-05	1	1	2	P12270	P12270 [1513-15	0	2077.1209
[K].LADIDKD	2xTMTpro [K	2.573E-06	4.225E-05	1	1	2	Q9NZN3	Q9NZN3 [487-50	1	3484.8814
[K].NITLNFG	1xTMTpro [N	5.766E-05	4.225E-05	1	1	1	O75306	O75306 [79-96]	0	2246.2477

[R].NLTNPN	1xTMTpro [K	4.634E-05	4.225E-05	1	1	3	P61106	P61106 [111-125]	0	2232.3571
[K].NLTQYS	1xTMTpro [N	3.429E-05	4.225E-05	1	1	3	Q9UIJ7	Q9UIJ7 [81-94]	0	2014.0717
[K].NMDPLN	1xTMTpro [K	4.077E-07	4.225E-05	1	1	1	P35579	P35579 [588-606]	0	2734.4326
[K].NMAEQII	1xOxidation	4.936E-06	4.225E-05	1	1	3	P29401	P29401 [265-281]	0	2647.4257
[K].NMAEQII	1xTMTpro [K	8.199E-06	4.225E-05	1	1	1	P29401	P29401 [265-281]	0	2631.4308
[K].NIYVLQE	1xTMTpro [K	2.57E-05	4.225E-05	1	1	2	P12956	P12956 [101-114]	0	2182.2363
[K].FVINYDY	1xTMTpro [N	3.514E-05	4.225E-05	1	1	4	Q92841	Q92841 [489-505]	0	2422.1635
[K].FVINYDY	1xTMTpro [N	4.849E-05	4.225E-05	1	1	2	P17844	P17844 [412-428]	0	2436.1791
[K].IADFLNS	1xCarbamido	4.898E-05	4.225E-05	1	1	1	Q8WUW1	Q8WUW1 [32-44]	0	1879.9002
[R].NLVVGD	1xTMTpro [N	5.252E-05	4.225E-05	1	1	4	Q05707	Q05707 [740-752]	0	1694.9244
[K].NLVTGD	1xTMTpro [N	3.741E-05	4.225E-05	1	1	1	O43768	O43768 [90-106]	0	2205.1947
[K].FVLINWT	1xTMTpro [N	1.587E-05	4.225E-05	1	1	2	Q9UJU6	Q9UJU6 [79-93]	0	2023.0932
[K].NLQEEID	1xTMTpro [N	4.127E-05	4.225E-05	1	1	1	Q9NQP4	Q9NQP4 [93-104]	0	1720.9037
[K].LADFWG	1xTMTpro [K	2.654E-05	4.225E-05	1	1	1	Q6ICL3	Q6ICL3 [36-56]	0	2960.4955
[K].NLVPGE	1xTMTpro [K	3.839E-05	4.225E-05	1	1	3	P22087	P22087 [110-121]	0	1900.0672
[K].NIVHCDI	1xCarbamido	3.951E-07	4.225E-05	2	2	1	Q9BZL6; Q1	Q9BZL6 [669-692	0	3617.0341
[R].EAVELPI	1xTMTpro [K	1.462E-05	4.225E-05	1	1	1	P43686	P43686 [179-192]	0	2297.3037
[K].NLVELAE	1xTMTpro [K	5.636E-05	4.225E-05	1	1	3	Q9UNH7	Q9UNH7 [376-38	0	1879.1396
[R].NLVDSY	1xTMTpro [K	5.261E-05	4.225E-05	1	1	2	P50570	P50570 [658-669]	0	1957.1614
[K].NLVDPF	1xTMTpro [K	5.183E-05	4.225E-05	2	2	1	Q9NZM1; O7	Q9NZM1 [393-40	0	2130.2091
[K].NLVDFLT	1xCarbamido	4.963E-07	4.225E-05	1	1	1	Q9H9J2	Q9H9J2 [155-171	0	2262.1872
[R].EAVFPF	1xCarbamido	2.74E-05	4.225E-05	1	1	3	P09382	P09382 [75-100]	0	3475.8427
[R].FVIGGP	1xTMTpro [N	2.453E-05	4.225E-05	1	1	3	P31153	P31153 [250-264]	0	1748.9615
[K].NLTVILS	1xTMTpro [K	1E-06	4.225E-05	1	1	1	Q9H0D6	Q9H0D6 [190-207	0	2446.3433
[R].EAVCIVL	2xCarbamido	7.287E-06	4.225E-05	1	1	3	P55072	P55072 [66-81]	0	2449.2082
[K].EAYMGN	1xTMTpro [N	2.026E-06	4.225E-05	1	1	2	P24752	P24752 [88-105]	0	2182.0882
[R].IADQFLG	1xTMTpro [N	2.994E-05	4.225E-05	1	1	1	O60341	O60341 [825-838]	0	1900.0322
[R].EAYPEE	1xTMTpro [K	4.279E-05	4.225E-05	1	1	1	P53396	P53396 [245-259]	0	2306.2048
[R].IAECSS	1xCarbamido	5.112E-07	4.225E-05	1	1	1	P35580	P35580 [1008-10	1	2734.4749
[R].LAECVS	1xCarbamido	1.47E-06	4.225E-05	1	1	1	Q99704	Q99704 [67-90]	0	2802.4667
[R].LAEDAPI	1xTMTpro [K	3.525E-06	4.225E-05	1	1	3	P78344	P78344 [139-158]	0	2620.3499
[K].NLLQEQ	1xTMTpro [N	1.61E-06	4.225E-05	1	1	3	P35749	P35749 [890-910]	0	2812.3994
[R].NILPMTF	1xTMTpro [K	5.841E-05	4.225E-05	1	1	1	O95487	O95487 [532-551]	0	2811.6087

[K].NLIPFDQ	1xOxidation [	5.252E-05	4.225E-05	1	1	1	O75947	O75947 [124-144]	0	3089.5997
[K].NLIPFDQ	1xTMTpro [K	3.664E-06	4.225E-05	1	1	4	O75947	O75947 [124-144]	0	3073.6048
[R].NILLTNE	1xTMTpro [N	3.152E-05	4.225E-05	1	1	4	Q9H9B4	Q9H9B4 [36-48]	0	1805.0088
[R].NLLLSGA	1xTMTpro [N	2.89E-05	4.225E-05	1	1	2	Q9BWM7	Q9BWM7 [35-47]	0	1675.9662
[R].NLILKPG	2xTMTpro [K	5.226E-05	4.225E-05	1	1	1	Q9BYT8	Q9BYT8 [667-687	0	3225.8308
[K].NLLTGET	1xTMTpro [K	1.339E-05	4.225E-05	1	1	1	O96005	O96005 [210-224]	0	2269.2241
[R].ECPEGH	1xCarbamido	3.423E-05	4.225E-05	1	1	2	Q14315	Q14315 [2554-257	0	3168.6717
[R].ECPGIEF	2xCarbamido	9.157E-06	4.225E-05	1	1	1	Q7Z4W1	Q7Z4W1 [50-69]	0	2636.2292
[R].NILHQGC	1xTMTpro [N	2.012E-05	4.225E-05	1	1	1	Q96C86	Q96C86 [243-255	0	1824.0411
[R].ECPSDE	2xCarbamido	1.236E-07	4.225E-05	1	1	2	P62979	P62979 [120-138]	0	2476.0651
[R].ECPSDE	2xCarbamido	4.473E-07	4.225E-05	1	1	2	P62979	P62979 [120-138]	0	2492.06
[K].LAEENPI	1xTMTpro [K	4.337E-05	4.225E-05	1	1	1	Q8TB36	Q8TB36 [174-188	0	2312.263
[K].FVESDAI	1xTMTpro [K	1.247E-06	4.225E-05	1	1	2	Q9GZP4	Q9GZP4 [67-87]	0	2992.5799
[R].IAEFTTN	1xTMTpro [K	1.667E-05	4.225E-05	1	1	4	P35579	P35579 [1001-10	0	2262.1997
[R].NILGGTV	1xCarbamido	4.51E-05	4.225E-05	1	1	2	P48735	P48735 [141-155]	1	2325.3608
[R].FVEQLG	1xTMTpro [K	2.117E-05	4.225E-05	1	1	1	Q9Y3D3	Q9Y3D3 [47-64]	0	2625.3804
[K].ECSIYLI(	1xCarbamido	4.533E-05	4.225E-05	1	1	1	Q9NQR4	Q9NQR4 [75-92]	0	2546.3304
[K].NIILEEGI	2xTMTpro [K	7.379E-07	4.225E-05	1	1	8	P23528	P23528 [46-73]	1	3975.2147
[K].NMDPLN	1xTMTpro [K	6.195E-06	4.225E-05	1	1	3	P35749	P35749 [595-613]	0	2656.3744
[R].NIITHAPI	1xTMTpro [N	3.931E-06	4.225E-05	1	1	1	Q9UN36	Q9UN36 [219-240	0	2978.508
[K].NLMNAV	1xTMTpro [K	5.654E-05	4.225E-05	1	2	2	P35221	P35221 [824-834]	0	1825.0861
[R].EAYPGD	1xTMTpro [N	2.238E-05	4.225E-05	1	1	2	P25705	P25705 [335-347]	0	1857.9455
[K].NLQDAE	1xTMTpro [K	6.806E-06	4.225E-05	1	1	2	P11277	P11277 [738-756]	0	2823.4081
[R].FVGTDP	1xTMTpro [K	4.587E-05	4.225E-05	1	1	1	Q92538	Q92538 [121-135]	0	2216.2128
[K].NLQAHY	1xTMTpro [N	1.882E-05	4.225E-05	1	1	1	Q9UPN3	Q9UPN3 [992-100	0	2040.0106
[K].NIPTVNE	1xTMTpro [K	1.404E-06	4.225E-05	1	1	10	P51884	P51884 [268-288]	0	3144.6709
[R].ECAPGY	2xCarbamido	2.706E-05	4.225E-05	1	1	4	O15230	O15230 [2101-21	0	2083.9649
[K].NIPQMSI	1xTMTpro [N	2.11E-06	4.225E-05	1	1	1	Q9GZP4	Q9GZP4 [110-131	1	2944.4066
[R].NIPPYFV	1xTMTpro [K	2.105E-05	4.225E-05	1	1	4	P12956	P12956 [405-424]	0	2952.585
[R].ECDTDG	2xCarbamido	1.77E-05	4.225E-05	1	1	2	P08603	P08603 [128-145]	0	2759.3512
[K].NLPIYSE	1xTMTpro [N	2.3E-05	4.225E-05	1	1	1	P35580	P35580 [130-143]	0	2059.0853
[R].NIIVFYG	1xTMTpro [N	1.427E-05	4.225E-05	1	1	1	P16435	P16435 [79-97]	0	2421.237
[K].NLPIYSE	1xTMTpro [K	1.508E-05	4.225E-05	1	1	3	P35579	P35579 [126-139]	0	2336.2703

[R].NIPGITLI	1xTMTpro [K	2.721E-05	4.225E-05	1	1	6	P36578	P36578 [223-234]	0	1877.1716
[K].IADSIGC	1xCarbamido	2.375E-05	4.225E-05	1	1	1	Q9UHY7	Q9UHY7 [196-215	0	2514.3193
[R].LADSPPI	1xTMTpro [K	2.246E-05	4.225E-05	1	1	1	P11441	P11441 [87-101]	0	2287.3306
[K].NLNYDTI	1xTMTpro [N	1.882E-07	4.225E-05	1	1	2	Q8IZ83	Q8IZ83 [500-535]	0	3946.071
[R].NLNPED	1xTMTpro [N	3.422E-06	4.225E-05	1	1	1	P33991	P33991 [273-291]	0	2445.3342
[R].NINENF(	1xOxidation [	4.61E-05	4.225E-05	1	1	1	Q9ULA0	Q9ULA0 [184-211	0	3789.0024
[R].FVFSLVI	1xTMTpro [K	2.687E-05	4.225E-05	1	1	3	P40926	P40926 [258-269]	0	1936.0858
[K].NLNEKD	1xCarbamido	5.872E-06	4.225E-05	1	1	4	P02787	P02787 [572-587]	1	2561.3525
[K].LADVDKI	2xTMTpro [K	8.703E-07	4.225E-05	1	1	2	Q9H4M9	Q9H4M9 [487-509	1	3466.925
[K].LADVFYS	1xTMTpro [N	3.124E-05	4.225E-05	1	1	1	Q9NYU1	Q9NYU1 [502-514	0	1850.0132
[R].NLPGLV(	1xTMTpro [K	1.03E-05	4.225E-05	1	1	2	Q7KZF4	Q7KZF4 [566-586	0	2914.5694
[R].EANSIIIT	1xCarbamido	2.379E-05	4.225E-05	1	1	1	Q13423	Q13423 [923-939]	0	2386.3296
[R].EATTDF	1xTMTpro [K	1.177E-06	4.225E-05	1	1	3	O75369	O75369 [1246-12	0	2995.598
[K].EATSILE	1xCarbamido	4.012E-06	4.225E-05	1	1	1	P48506	P48506 [130-149]	0	2584.3248
[R].EAQGEV	1xTMTpro [K	4.737E-05	4.225E-05	1	1	2	O95436	O95436 [673-684]	0	1825.9787
[R].IAAQTLL	1xTMTpro [K	5.09E-05	4.225E-05	1	1	2	Q53H96	Q53H96 [208-218	0	1695.066
[K].LAASEA	1xTMTpro [N	1.939E-07	4.225E-05	1	1	1	P16219	P16219 [350-380]	0	3490.7993
[K].EAQLFHI	1xTMTpro [N	4.135E-05	4.225E-05	1	1	1	Q8WWI1	Q8WWI1 [139-15	0	2144.1056
[K].NPDGTM	1xCarbamido	7.758E-06	4.225E-05	1	1	1	P63279	P63279 [31-49]	1	3088.6198
[K].LAASGE(	1xTMTpro [K	4.668E-07	4.225E-05	1	1	2	Q16643	Q16643 [43-62]	0	2680.4186
[K].LAASIAP	1xTMTpro [K	3.619E-05	4.225E-05	1	1	1	P33993	P33993 [336-351]	0	2321.2997
[R].NPDGDV	1xCarbamido	2.663E-05	4.225E-05	1	1	2	P00747	P00747 [533-549]	0	2210.0256
[K].LAATNAI	1xTMTpro [K	2.023E-05	4.225E-05	1	1	2	Q14974	Q14974 [192-206]	0	2214.2989
[R].NPDELA	1xTMTpro [N	5.175E-05	4.225E-05	1	1	1	O14908	O14908 [296-307]	0	1675.8458
[K].EAQNTC	1xCarbamido	3.114E-06	4.225E-05	1	1	1	P36957	P36957 [240-263]	0	3151.4488
[K].EAQPGQ	1xTMTpro [K	4.55E-06	4.225E-05	1	1	2	Q14766	Q14766 [513-530]	0	2552.3964
[R].EAQQAL	1xTMTpro [K	3.577E-06	4.225E-05	1	1	1	P11047	P11047 [1407-142	0	2240.2014
[R].EAQQQ(	1xTMTpro [K	5.064E-05	4.225E-05	1	1	1	Q8IY31	Q8IY31 [87-100]	0	2206.2687
[R].NPDDIT(	1xTMTpro [K	6.794E-06	4.225E-05	1	1	4	P08238	P08238 [292-306]	0	2456.2113
[R].NPDDITI	1xTMTpro [K	3.359E-06	4.225E-05	1	1	4	P07900	P07900 [300-314]	0	2442.1957
[R].EAQQYS	1xTMTpro [N	2.87E-05	4.225E-05	1	1	3	Q9H4G4	Q9H4G4 [38-50]	0	1757.8989
[R].EAQSFIS	1xTMTpro [K	2.635E-05	4.225E-05	1	1	2	Q8WXA9	Q8WXA9 [168-18	0	2272.2317
[R].FVPAEM	1xTMTpro [K	3.185E-05	4.225E-05	1	1	2	P21333	P21333 [2202-22	0	2111.1815

[R].NPDADK	1xCarbamido	2.371E-05	4.225E-05	1	1	3	P00747	P00747 [428-445]	1	2671.343
[R].NPCTDG	2xCarbamido	5.246E-06	4.225E-05	1	1	4	P07996	P07996 [648-659]	0	2026.9566
[R].NPDNDP	1xCarbamido	2.682E-05	4.225E-05	1	1	1	P00747	P00747 [154-171]	0	2742.2961
[R].NPDNDP	1xCarbamido	1.163E-05	4.225E-05	1	1	1	P00747	P00747 [154-172]	1	2898.3972
[R].NPDSST	1xCarbamido	1.389E-05	4.225E-05	1	1	1	P00734	P00734 [159-177]	0	2459.1468
[R].NPEILAI	1xTMTpro [N	4.898E-05	4.225E-05	1	1	1	Q92616	Q92616 [1571-15	0	2422.3876
[R].FVPQEM	1xTMTpro [K	2.444E-05	4.225E-05	1	1	1	O75369	O75369 [2157-21	0	2166.2237
[K].EAPAEG	1xTMTpro [K	7.19E-07	4.225E-05	1	1	2	P29966	P29966 [106-137]	0	3523.7168
[K].NPILWN	1xTMTpro [K	2.388E-05	4.225E-05	1	1	3	P04844	P04844 [492-504]	0	2089.2665
[R].FVPQEM	1xTMTpro [K	2.538E-05	4.225E-05	1	1	2	Q14315	Q14315 [2278-22	0	2148.2131
[R].EAPATQ	1xTMTpro [K	3.477E-07	4.225E-05	1	1	2	Q9UNF1	Q9UNF1 [77-100]	0	3083.6352
[K].NPHLITN	1xTMTpro [N	3.692E-06	4.225E-05	1	1	2	Q9UH65	Q9UH65 [554-57	0	2699.3748
[R].EAPEDI	1xTMTpro [N	5.786E-06	4.225E-05	1	1	1	Q9NUV9	Q9NUV9 [170-18	0	2168.0501
[K].NPGVWL	1xCarbamido	5.728E-05	4.225E-05	1	1	1	O95983	O95983 [203-216	0	2236.2404
[R].NPGNQA	1xOxidation	2.427E-05	4.225E-05	1	1	2	P18206	P18206 [685-699]	0	2361.1789
[R].NPGNQA	1xTMTpro [K	7.01E-06	4.225E-05	1	1	3	P18206	P18206 [685-699]	0	2345.184
[R].NPCQDF	1xCarbamido	5.627E-05	4.225E-05	1	1	2	Q12805	Q12805 [363-376	0	2021.0081
[K].NPGFEII	1xTMTpro [N	8.507E-06	4.225E-05	1	1	1	Q9NSD9	Q9NSD9 [505-51	0	1784.9978
[K].NPFTLGI	1xTMTpro [N	5.022E-05	4.225E-05	1	1	1	O75312	O75312 [368-383	0	2024.0004
[K].NPFGNA	1xTMTpro [K	2.902E-06	4.225E-05	1	1	2	P49748	P49748 [493-507]	0	2066.189
[K].NPEVGL	2xTMTpro [K	5.132E-05	4.225E-05	1	1	2	P07814	P07814 [536-549]	0	2526.49
[R].NPETDT	1xCarbamido	7.45E-06	4.225E-05	1	1	1	P00488	P00488 [176-200]	0	3669.7551
[R].NPELST	1xTMTpro [N	3.079E-06	4.225E-05	1	1	1	Q9NXF1	Q9NXF1 [566-58	0	2267.2679
[R].NPEISHI	1xTMTpro [N	6.701E-07	4.225E-05	1	1	1	Q9NRR5	Q9NRR5 [232-24	0	2099.0697
[R].NPEISHI	1xTMTpro [N	9.124E-06	4.225E-05	1	1	1	Q9UMX0	Q9UMX0 [222-23	0	2085.054
[R].EAQASG	1xTMTpro [N	7.888E-07	4.225E-05	1	1	1	Q02388	Q02388 [1179-12	0	2661.3659
[R].NPEISHL	1xTMTpro [N	6.452E-06	4.225E-05	1	1	1	Q9UHD9	Q9UHD9 [218-23	0	2067.0976
[K].EAQAVP	1xTMTpro [K	2.016E-05	4.225E-05	1	1	4	Q15149	Q15149 [1251-12	0	2276.2993
[K].NPFVLG	1xTMTpro [N	5.822E-05	4.225E-05	1	1	2	Q8IUX7	Q8IUX7 [754-767	0	1761.9567
[R].NPAVLS	1xTMTpro [N	5.794E-05	4.225E-05	1	1	1	O94979	O94979 [315-327	0	1608.8665
[K].NNYSED	1xTMTpro [K	1.595E-05	4.225E-05	1	1	1	P19174	P19174 [1194-12	0	2330.3099
[K].LAAVDA	1xTMTpro [N	5.464E-06	4.225E-05	1	1	3	Q15084	Q15084 [217-231	0	1832.0561
[K].EASSVSI	1xTMTpro [N	1.61E-05	4.225E-05	1	1	1	P41218	P41218 [282-298]	0	2244.0852

[K].NMQNVE	1xTMTpro [N	1.987E-05	4.225E-05	1	1	2	P20618	P20618 [185-198]	0	1956.0292
[R].NMPLLE	2xCarbamido	1.934E-06	4.225E-05	1	1	1	Q9NR99	Q9NR99 [205-226	0	3058.4214
[K].NMPFQD	1xTMTpro [K	1.189E-05	4.225E-05	1	1	1	Q05315	Q05315 [80-99]	0	2902.5152
[R].NMNCIEI	1xCarbamido	5.305E-05	4.225E-05	1	1	2	P21810	P21810 [184-209]	0	3438.626
[R].NMNCIEI	1xCarbamido	1.21E-06	4.225E-05	1	1	3	P21810	P21810 [184-209]	0	3422.6311
[R].NMNCIEI	1xCarbamido	2.053E-06	4.225E-05	1	1	3	P21810	P21810 [184-209]	0	3406.6362
[K].NMITGTS	1xCarbamido	2.515E-05	4.225E-05	1	3	1	P68104	P68104 [101-129]	0	3533.8475
[K].NMITGTS	1xCarbamido	1.854E-06	4.225E-05	1	3	2	P68104	P68104 [101-129]	0	3517.8526
[K].EATDVIII	1xTMTpro [K	4.029E-05	4.225E-05	1	1	1	P15144	P15144 [115-125]	0	1834.093
[K].NMQNVE	1xOxidation [	5.175E-05	4.225E-05	1	1	3	P20618	P20618 [185-198]	0	1972.0241
[K].NMITGTA	1xCarbamido	1.569E-07	4.225E-05	1	1	2	P49411	P49411 [139-165]	0	3116.5862
[K].NMLFSG	1xTMTpro [K	8.729E-06	4.225E-05	1	2	2	P16615	P16615 [206-218]	0	1932.0868
[R].NMGGPY	1xOxidation [	2.444E-07	4.225E-05	1	1	2	P22626	P22626 [326-350]	0	2510.1074
[R].NMGGPY	1xTMTpro [N	1.171E-07	4.225E-05	1	1	1	P22626	P22626 [326-350]	0	2494.1125
[K].NMFLVG	1xTMTpro [K	2.906E-05	4.225E-05	1	1	1	O75475	O75475 [425-442	0	2572.43
[K].EATIPGH	1xTMTpro [K	6.041E-05	4.225E-05	1	1	2	P02751	P02751 [656-669]	0	2152.2258
[R].EATLQD	1xCarbamido	1.03E-05	4.225E-05	1	1	2	O43866	O43866 [212-225	0	2154.1145
[R].EATNDD	1xTMTpro [K	8.352E-06	4.225E-05	1	1	1	Q14677	Q14677 [30-49]	0	2724.3795
[R].EATNPP	2xTMTpro [K	7.023E-06	4.225E-05	1	1	8	P30101	P30101 [483-496]	0	2492.4541
[K].NMFHPM	1xTMTpro [N	9.623E-06	4.225E-05	1	1	1	O43719	O43719 [270-288	0	2636.2808
[R].NMEQFQ	1xOxidation [	2.779E-05	4.225E-05	1	1	1	Q14624	Q14624 [124-139	0	2373.2728
[K].EATEAQ	1xCarbamido	3.276E-05	4.225E-05	1	2	1	Q96PC5	Q96PC5 [728-741	0	2175.1095
[K].NMDPLN	1xOxidation [	6.093E-06	4.225E-05	1	1	2	P35749	P35749 [595-613]	0	2672.3693
[K].NMSGSL	1xTMTpro [N	5.888E-05	4.225E-05	1	1	1	Q08945	Q08945 [305-316	0	1677.8259
[K].NMTIPED	1xTMTpro [N	4.618E-05	4.225E-05	1	1	1	P46734	P46734 [158-175]	0	2274.2698
[K].NNVNVA	1xTMTpro [N	5.2E-05	4.225E-05	1	1	1	Q6P2Q9	Q6P2Q9 [1995-20	0	1849.0099
[K].EAQVQY	1xTMTpro [N	2.171E-05	4.225E-05	1	1	1	P08243	P08243 [276-300]	0	3067.573
[R].NNVILQF	1xTMTpro [K	2.57E-05	4.225E-05	1	1	1	Q99715	Q99715 [2078-20	0	2348.347
[K].NNVEQV	3xCarbamido	1.279E-05	4.225E-05	1	1	2	P12109	P12109 [241-256]	0	2302.0334
[K].IAAYLQS	1xCarbamido	3.502E-05	4.225E-05	1	1	3	P21266	P21266 [198-209]	0	2052.108
[R].LACCVV	3xCarbamido	1.324E-05	4.225E-05	1	1	1	P08294	P08294 [205-220]	0	2137.0676
[K].FVNLFPE	1xTMTpro [K	1.538E-05	4.225E-05	1	1	1	O94973	O94973 [547-563	0	2623.5467
[R].LACESAS	1xCarbamido	3.042E-05	4.225E-05	1	1	1	P11215	P11215 [845-860]	0	2218.1881

[K].EASDIIL1	1xTMTpro [K	1.451E-05	4.225E-05	2	3	2	P20020; P23	P20020 [824-840]	0	2489.3631
[R].LACLSEI	1xCarbamido	3.236E-05	4.225E-05	1	1	1	Q9Y2L1	Q9Y2L1 [211-225	0	2244.1673
[K].FVLSGAI	1xCarbamido	7.617E-06	4.225E-05	1	2	4	Q9ULC4	Q9ULC4 [104-122	0	2528.3861
[K].NNQFQA	1xTMTpro [K	8.232E-07	4.225E-05	1	1	1	P26599	P26599 [219-238]	0	2851.5346
[K].IACVLPV	1xCarbamido	1.848E-05	4.225E-05	1	1	4	P10643	P10643 [629-654]	0	3840.1372
[K].NNLSFIE	1xTMTpro [K	1.913E-06	4.225E-05	1	1	4	Q15907	Q15907 [146-166]	0	2937.5337
[K].LADDVD	1xCarbamido	1.975E-08	4.225E-05	1	1	4	P55072	P55072 [390-424]	0	3976.9994
[K].NNLCPS	1xCarbamido	5.296E-05	4.225E-05	1	1	3	P60033	P60033 [172-187]	0	2386.3044
[K].LADEQLS	1xTMTpro [N	7.533E-06	4.225E-05	1	1	1	Q8NFV4	Q8NFV4 [201-216	0	2079.1075
[K].EASMVIT	1xTMTpro [N	9.929E-06	4.225E-05	1	1	2	P27105	P27105 [236-251]	0	2020.1068
[K].NNKDSH	1xTMTpro [K	3.833E-05	4.225E-05	1	1	2	P02671	P02671 [98-114]	1	2594.4216
[K].NNEGTY	1xTMTpro [N	4.634E-05	4.225E-05	1	1	2	P00450	P00450 [485-500]	0	2208.0277
[K].NNASTD	1xTMTpro [K	2.479E-05	4.225E-05	1	1	2	P39023	P39023 [301-312]	0	1950.99
[K].NNALNQ	1xTMTpro [K	5.305E-05	4.225E-05	1	1	3	P61009	P61009 [97-108]	0	2022.1628
[R].NNQFNQ	1xTMTpro [K	4.36E-05	4.225E-05	1	1	1	O95379	O95379 [88-101]	0	2302.1993
[R].IAEFTTN	2xTMTpro [K	4.944E-07	4.225E-05	1	1	2	P35579	P35579 [1001-101	1	2781.5338
[R].KYGGPF	2xTMTpro [K	2.641E-06	4.225E-05	1	1	3	O60506	O60506 [143-168]	1	3606.9624
[R].EAENFH	1xTMTpro [N	1.049E-05	4.225E-05	1	1	1	Q0ZGT2	Q0ZGT2 [485-501	0	2332.1125
[K].QDLPNA	1xOxidation [	3.375E-05	4.225E-05	1	2	1	P61204	P61204 [128-142]	0	2255.1833
[K].QDLPNA	1xTMTpro [K	6.334E-06	4.225E-05	1	2	3	P61204	P61204 [128-142]	0	2239.1884
[K].QDLPNA	1xOxidation [	4.249E-05	4.225E-05	1	1	3	P18085	P18085 [128-142]	0	2288.1758
[K].QDLPNA	1xTMTpro [K	1.036E-05	4.225E-05	1	1	4	P18085	P18085 [128-142]	0	2272.1809
[R].KVIAVAD	1xCarbamido	5.428E-05	4.225E-05	1	1	1	Q9HC35	Q9HC35 [802-812	1	2178.2773
[R].DWHGVI	1xTMTpro [N	3.23E-05	4.225E-05	1	1	3	P04004	P04004 [338-353]	0	1970.9826
[R].DWHGVI	1xOxidation [	4.518E-05	4.225E-05	1	1	2	P04004	P04004 [338-353]	0	1986.9775
[K].QDLPALI	1xTMTpro [K	5.27E-05	4.225E-05	1	1	2	P34932	P34932 [186-196]	0	1904.1097
[R].QDLMNI/	1xTMTpro [K	1.04E-05	4.225E-05	1	1	1	P78371	P78371 [157-170]	0	2087.1662
[R].QDILDDS	1xTMTpro [K	4.931E-05	4.225E-05	1	1	2	Q9BW30	Q9BW30 [152-165	0	2182.1523
[K].DWILPSI	1xTMTpro [N	4.156E-05	4.225E-05	1	1	1	O43852	O43852 [256-271]	0	2192.0579
[R].QDLGSP	1xTMTpro [N	5.722E-06	4.225E-05	1	1	2	P14543	P14543 [1018-103	0	1968.047
[K].QDLEAE	1xTMTpro [K	2.159E-05	4.225E-05	1	1	1	O60610	O60610 [535-550]	0	2325.2793
[R].QDIAVIS	1xTMTpro [N	3.037E-05	4.225E-05	1	1	1	P11047	P11047 [545-557]	0	1814.9608
[R].QDLADV	1xCarbamido	3.886E-05	4.225E-05	1	1	2	Q14204	Q14204 [4429-44	0	2069.1193

[R].GAAAAA	1xTMTpro [K	4.125E-06	4.225E-05	1	1	3	Q13263	Q13263 [525-554]	0	3180.7695
[R].QDGHLV	1xCarbamido	5.073E-05	4.225E-05	1	1	3	P02751	P02751 [380-397]	0	2805.3394
[K].KVIEASD	1xTMTpro [K	1.766E-05	4.225E-05	1	1	1	Q9BVP2	Q9BVP2 [136-151	1	2364.3994
[K].QDFDQM	1xTMTpro [K	5.794E-05	4.225E-05	1	1	1	Q6PML9	Q6PML9 [509-522	0	2359.2459
[K].KVLEDV	2xTMTpro [K	1.741E-05	4.225E-05	1	1	1	Q9BZZ5	Q9BZZ5 [181-196	1	2796.6038
[K].DWVLTA	1xCarbamido	1.587E-05	4.225E-05	1	1	2	P12544	P12544 [62-74]	0	2150.1672
[K].KVGTFF	2xTMTpro [K	2.799E-07	4.225E-05	1	1	2	P54886	P54886 [348-371]	1	3505.9293
[K].DVYVVT	1xOxidation [	1.322E-05	4.225E-05	1	1	3	Q14956	Q14956 [228-245]	0	2753.5079
[K].DVYVVT	1xTMTpro [K	3.059E-05	4.225E-05	1	1	2	Q14956	Q14956 [228-245]	0	2737.513
[K].GAAAHP	1xTMTpro [N	2.799E-05	4.225E-05	1	1	4	Q9Y490	Q9Y490 [876-889	0	1827.8905
[R].DVVLTT	1xTMTpro [K	1.195E-05	4.225E-05	1	1	3	P02748	P02748 [312-324]	0	2074.1927
[R].QEALELI	1xTMTpro [K	4.618E-05	4.225E-05	1	1	1	P43251	P43251 [80-103]	0	3416.7686
[K].QEAGGL	1xTMTpro [K	5.879E-05	4.225E-05	1	1	1	Q13425	Q13425 [121-131]	0	1681.014
[R].QEAFLE	1xTMTpro [K	6.701E-07	4.225E-05	1	1	5	P02549	P02549 [495-517]	0	3084.6345
[K].QEAATL	1xTMTpro [N	1.812E-05	4.225E-05	1	1	1	Q14980	Q14980 [420-435]	0	2004.0793
[R].DVVQIT	1xCarbamido	1.966E-05	4.225E-05	1	1	2	P09871	P09871 [315-331]	0	2240.1552
[K].QDVSPF	1xTMTpro [K	1E-06	4.225E-05	1	1	1	Q93099	Q93099 [258-276]	0	2830.4808
[K].KVESTG	3xTMTpro [K	4.874E-05	4.225E-05	1	1	2	Q6YN16	Q6YN16 [284-298	2	2894.698
[R].KVFANP	1xCarbamido	2.715E-06	4.225E-05	1	1	2	P10620	P10620 [42-55]	1	2494.3944
[R].DVWGIE	1xTMTpro [N	4.235E-05	4.225E-05	1	1	4	P04004	P04004 [198-212]	0	1951.0244
[R].KVIEEQL	2xTMTpro [K	8.713E-06	4.225E-05	1	1	1	P42704	P42704 [1224-123	1	2424.453
[K].QDVDDE	1xTMTpro [N	3.933E-05	4.225E-05	1	1	1	P51532	P51532 [712-726]	0	1969.9786
[K].DVYDKV	2xTMTpro [K	2.308E-05	4.225E-05	1	1	1	Q14254	Q14254 [156-169]	1	2514.4272
[K].QDSVITD	1xTMTpro [K	3.893E-05	4.225E-05	1	1	1	P48506	P48506 [589-604]	0	2477.3453
[R].DVYEELI	1xTMTpro [K	1.642E-05	4.225E-05	1	1	2	P46940	P46940 [285-302]	0	2685.4591
[R].QDSAAV	1xTMTpro [K	4.199E-05	4.225E-05	1	1	3	Q14247	Q14247 [280-290]	0	1808.9674
[K].QDPSVL	1xTMTpro [N	1.161E-05	4.225E-05	1	2	2	P50502	P50502 [18-29]	0	1745.8812
[R].QDPPSV	1xTMTpro [K	4.308E-05	4.225E-05	1	1	1	P25311	P25311 [204-220]	0	2384.3065
[R].QDPGDN	1xTMTpro [K	2.493E-05	4.225E-05	1	1	1	Q9UKY7	Q9UKY7 [118-136	0	2485.1545
[KR].DVYIVC	1xTMTpro [K	2.611E-05	4.225E-05	2	2	8	P28482; P27	P28482 [100-114]	0	2453.3129
[K].DVYQMS	1xTMTpro [K	2.187E-05	4.225E-05	1	1	1	Q14194	Q14194 [172-189]	0	2793.4301
[K].QDMEQA	1xTMTpro [K	1.978E-06	4.225E-05	1	1	1	P40616	P40616 [128-149]	0	2960.5023
[K].QDTLDA	1xTMTpro [N	3.417E-05	4.225E-05	1	1	2	P02549	P02549 [1970-198	0	2070.0423

[R].QDAVHA	1xTMTpro [N	1.773E-05	4.225E-05	1	1	1	Q5JPI3	Q5JPI3 [44-62]	0	2418.2948
[R].DYAAYN	1xTMTpro [N	2.234E-05	4.225E-05	1	1	1	Q86SX6	Q86SX6 [84-97]	0	1957.9826
[R].QDAQQE	1xTMTpro [N	2.823E-06	4.225E-05	1	1	1	Q14653	Q14653 [44-68]	0	3059.4818
[R].QAVQILD	1xTMTpro [K	1.773E-05	4.225E-05	1	1	2	Q99961	Q99961 [228-239]	0	1965.1512
[R].QAVLGA	1xCarbamido	3.322E-06	4.225E-05	1	1	2	P24752	P24752 [106-124]	0	2549.4617
[R].QAVDTA	1xTMTpro [N	1.344E-05	4.225E-05	1	1	4	P19827	P19827 [42-54]	0	1694.9397
[K].QAVDFLS	1xTMTpro [K	7.888E-06	4.225E-05	1	1	1	P15927	P15927 [244-265]	0	3145.5722
[R].KVPDGL	1xTMTpro [K	2.513E-08	4.225E-05	1	1	1	Q06828	Q06828 [237-265]	1	3991.0555
[K].QATVGD	1xOxidation [	3.502E-05	4.225E-05	1	1	2	P07108	P07108 [34-53]	0	2774.4639
[K].QATVGD	1xTMTpro [K	1.141E-06	4.225E-05	1	1	2	P07108	P07108 [34-53]	0	2758.4689
[R].DYFEEY	1xTMTpro [K	1.492E-06	4.225E-05	1	1	2	P22626	P22626 [130-147]	1	2828.485
[R].QATTIIAI	1xTMTpro [K	1.351E-05	4.225E-05	1	1	1	Q04837	Q04837 [128-145]	0	2600.4791
[R].QATLEG	1xTMTpro [N	2.263E-05	4.225E-05	1	1	3	Q9Y6C2	Q9Y6C2 [529-541	0	1703.9611
[K].QAVSMF	1xTMTpro [K	2.587E-06	4.225E-05	1	1	2	P49419	P49419 [376-389]	0	2088.1655
[K].QASVFS	1xTMTpro [N	4.642E-05	4.225E-05	1	1	1	P12111	P12111 [371-386]	0	1901.02
[R].QASQGN	1xTMTpro [N	3.459E-05	4.225E-05	1	1	2	Q9Y230	Q9Y230 [41-53]	0	1620.8811
[K].KVPEFQ	2xTMTpro [K	2.253E-07	4.225E-05	1	1	1	P34949	P34949 [163-180]	1	2955.7124
[R].KVPELM	2xTMTpro [K	8.275E-06	4.225E-05	1	1	2	O43747	O43747 [167-180]	1	2546.4906
[R].KVPGEG	2xTMTpro [K	4.801E-05	4.225E-05	1	1	2	P25685	P25685 [296-306]	1	2047.3096
[R].KVPGVT	1xTMTpro [K	4.643E-06	4.225E-05	1	1	1	P51114	P51114 [246-263]	1	2556.4165
[R].KVPILVA	2xTMTpro [K	1.471E-07	4.225E-05	1	1	1	Q9H7Z7	Q9H7Z7 [147-173	1	3766.1782
[R].DYGVLLI	1xTMTpro [N	8.957E-06	4.225E-05	1	1	3	P30048	P30048 [171-184]	0	1766.9972
[R].FYQVPV	1xTMTpro [N	5.09E-05	4.225E-05	1	1	1	Q9Y2R9	Q9Y2R9 [167-178	1	1791.0237
[K].DYGVYL	1xTMTpro [N	3.417E-05	4.225E-05	1	1	2	Q13162	Q13162 [187-200]	0	1928.9673
[K].QAQYLG	1xCarbamido	3.754E-06	4.225E-05	1	1	1	P23526	P23526 [413-431]	0	2878.426
[K].FYTEDG	1xTMTpro [N	1.22E-05	4.225E-05	1	1	1	P04040	P04040 [136-156]	0	2822.4109
[R].DVVLSIV	1xCarbamido	4.21E-06	4.225E-05	1	1	10	P12111	P12111 [2423-244	0	2419.2822
[K].QAVSMF	1xOxidation [	2.21E-05	4.225E-05	1	1	1	P49419	P49419 [376-389]	0	2104.1604
[K].KVNVTVI	1xTMTpro [K	1.646E-06	4.225E-05	1	1	1	Q7KZF4	Q7KZF4 [414-438	1	3355.8506
[R].QDAQDL	2xTMTpro [K	3.961E-05	4.225E-05	1	1	1	P09525	P09525 [173-185]	1	2407.3285
[K].QDACQG	1xCarbamido	1.848E-05	4.225E-05	1	1	1	P00736	P00736 [647-661]	0	1870.9037
[R].QCVPTE	4xCarbamido	1.113E-05	4.225E-05	1	1	1	P02748	P02748 [87-111]	0	3251.2941
[K].QCVENA	1xCarbamido	1.324E-05	4.225E-05	1	1	1	Q99460	Q99460 [111-129]	1	3138.7034

[K].KVLGAF	2xTMTpro [K	1.417E-07	4.225E-05	2	2	36	P02042; P68	P02042 [67-83]; F	1	2710.6072
[K].DYAGVF	1xTMTpro [N	1.662E-06	4.225E-05	1	1	3	P01024	P01024 [634-657]	0	2798.3552
[K].QCSPSQ	4xCarbamido	4.099E-05	4.225E-05	1	1	1	O14672	O14672 [502-518]	0	2595.2068
[K].QCQDLG	2xCarbamido	1.407E-05	4.225E-05	1	1	3	P01024	P01024 [1645-166	0	2480.1215
[K].QCQDLG	2xCarbamido	4.665E-05	4.225E-05	1	1	3	P01024	P01024 [1645-166	0	2464.1266
[R].KVIGIEC	1xCarbamido	1.19E-06	4.225E-05	1	1	2	Q99873	Q99873 [113-128]	1	2681.5364
[K].QAVSMF	2xTMTpro [K	1.816E-06	4.225E-05	1	1	2	P49419	P49419 [376-390]	1	2520.4676
[K].DYAVST	1xTMTpro [K	2.75E-06	4.225E-05	1	1	2	O94760	O94760 [160-175]	0	2293.3048
[K].QCLEDS	1xCarbamido	4.218E-06	4.225E-05	1	1	1	P46063	P46063 [48-70]	0	3123.4457
[K].KVLTSLC	2xTMTpro [K	2.287E-05	4.225E-05	2	2	3	P69891; P69	P69891 [67-77]; F	1	2057.315
[K].KVNESS	2xTMTpro [K	3.137E-06	4.225E-05	1	1	2	Q15417	Q15417 [72-91]	1	3242.8354
[K].QCANLQ	1xCarbamido	1.142E-05	4.225E-05	1	1	2	P13647	P13647 [406-420]	0	2006.0045
[K].QCANLQ	1xCarbamido	4.286E-05	4.225E-05	2	2	1	P04259; P02	P04259 [401-415]	0	1962.9986
[R].QAYTGN	1xTMTpro [K	1.467E-05	4.225E-05	1	1	2	O94826	O94826 [448-463]	0	2320.2211
[K].DYDSLA	1xTMTpro [N	5.926E-05	4.225E-05	1	1	4	P12110	P12110 [1001-10	0	1834.8931
[K].QAWFIE	1xTMTpro [K	1.098E-05	4.225E-05	1	1	5	P04083	P04083 [10-26]	0	2749.4329
[R].DYEDGII	1xTMTpro [K	1.934E-05	4.225E-05	1	1	1	Q9UKK3	Q9UKK3 [1135-11	0	2655.2852
[R].QAVTPP	1xTMTpro [K	3.405E-05	4.225E-05	1	1	1	O95394	O95394 [470-486]	0	2401.3946
[K].QCLNEV	2xCarbamido	4.106E-05	4.225E-05	1	1	2	P55001	P55001 [116-127]	0	1892.9318
[R].DYKPQV	2xTMTpro [K	1.558E-05	4.225E-05	1	1	2	Q99715	Q99715 [2603-26	0	2516.4541
[K].DVVIQDI	1xCarbamido	2.401E-05	4.225E-05	1	2	2	P17252	P17252 [373-388]	0	2503.2552
[R].KVESLQ	3xTMTpro [K	8.169E-06	4.225E-05	1	1	2	P08670	P08670 [223-236]	2	2878.7758
[R].QEQIQA	1xTMTpro [N	1.587E-05	4.225E-05	1	1	2	Q15149	Q15149 [1367-13	0	2089.1031
[K].QEQIINT	1xTMTpro [N	4.022E-05	4.225E-05	1	1	1	P12270	P12270 [756-768]	0	1894.0023
[R].QEQHEA	1xTMTpro [N	1.495E-06	4.225E-05	1	1	1	Q14980	Q14980 [664-681]	0	2424.2802
[K].QEQAPV	1xTMTpro [N	2.645E-06	4.225E-05	1	1	2	Q02952	Q02952 [823-857]	0	3973.8746
[K].DVNLAS	1xCarbamido	2.484E-05	4.225E-05	1	1	4	O43172	O43172 [293-306]	0	2015.0723
[K].DVNLRP	1xCarbamido	3.014E-08	4.225E-05	1	1	1	P09110	P09110 [75-102]	0	3267.7261
[R].GACAGS	1xCarbamido	3.582E-05	4.225E-05	1	1	2	Q13813	Q13813 [1620-16	0	1672.882
[R].QEPSQC	1xTMTpro [N	2.202E-06	4.225E-05	1	1	4	P01876	P01876 [283-299]	0	2140.1569
[R].QEPQPC	1xTMTpro [N	3.441E-06	4.225E-05	1	1	1	P00390	P00390 [48-81]	0	3542.8199
[K].KVEAQL	2xTMTpro [K	1.265E-05	4.225E-05	1	1	5	P35579	P35579 [1249-126	1	2325.4322
[K].QEPLGS	1xCarbamido	2.675E-06	4.225E-05	1	1	1	Q96FW1	Q96FW1 [11-36]	0	3200.4795

[R].DVPIDHS	1xTMTpro [K	5.831E-05	4.225E-05	1	1	2	Q7Z460	Q7Z460 [1295-13	0	2258.2888
[K].QEPGSN	2xTMTpro [K	1.124E-06	4.225E-05	1	1	2	P36969	P36969 [108-126]	1	3022.6302
[R].KVEDLFI	2xTMTpro [K	1.706E-05	4.225E-05	1	1	5	Q13813	Q13813 [2088-20	1	2223.3569
[K].QEPERN	1xCarbamido	8.801E-08	4.225E-05	1	1	8	P02768	P02768 [118-138]	2	3244.6413
[R].DVPNET	1xTMTpro [K	7.744E-06	4.225E-05	1	1	1	O95373	O95373 [239-259]	0	3203.6505
[K].DVPPLSI	2xTMTpro [K	6.633E-06	4.225E-05	1	1	2	P27816	P27816 [575-599]	1	3650.0179
[K].QEPERN	1xCarbamido	4.587E-05	4.225E-05	1	1	16	P02768	P02768 [118-130]	1	2323.2109
[K].KVEEAEI	2xTMTpro [K	2.583E-06	4.225E-05	1	1	3	Q13185	Q13185 [21-34]	1	2574.4483
[K].GAASEQ	1xTMTpro [N	4.863E-07	4.225E-05	1	1	2	Q14005	Q14005 [1269-12	0	3248.6388
[K].QEMLLS	1xTMTpro [K	4.235E-05	4.225E-05	1	1	1	P33241	P33241 [61-91]	0	4225.1003
[R].DVNECL	2xCarbamido	4.601E-06	4.225E-05	1	1	1	O95967	O95967 [54-70]	1	2906.5242
[R].QEQVTA	1xTMTpro [K	9.965E-08	4.225E-05	1	1	1	Q8IWX8	Q8IWX8 [128-145	0	2604.4059
[R].KVEAEV	1xTMTpro [K	3.438E-07	4.225E-05	1	1	1	Q9NT22	Q9NT22 [625-643	1	2681.4714
[R].QESGYL	1xTMTpro [N	1.156E-05	4.225E-05	1	1	1	Q92888	Q92888 [483-499]	0	2209.2035
[K].QFCLQN	1xCarbamido	6.652E-07	4.225E-05	1	2	1	P63218	P63218 [37-63]	0	3678.9306
[R].QFASQA	1xTMTpro [K	1.951E-05	4.225E-05	1	1	3	O43707	O43707 [653-668]	0	2382.3425
[K].QFAPIHA	1xOxidation [	1.238E-05	4.225E-05	1	1	6	P06576	P06576 [162-188]	0	3668.92
[K].QFAPIHA	1xTMTpro [K	2.037E-06	4.225E-05	1	1	1	P06576	P06576 [162-188]	0	3652.9251
[K].DVLQAE	2xCarbamido	4.785E-05	4.225E-05	1	1	2	Q9UNH7	Q9UNH7 [336-35	0	2416.2456
[K].QFAEEN	1xTMTpro [K	2.397E-05	4.225E-05	1	1	1	P61106	P61106 [141-156]	0	2375.3102
[K].KVAPAP	2xTMTpro [K	4.177E-05	4.225E-05	1	1	3	P62424	P62424 [11-20]	1	1892.2513
[R].QEYVLT	1xTMTpro [K	4.73E-06	4.225E-05	1	1	1	P21980	P21980 [157-173]	0	2568.3954
[K].GADFLV	1xTMTpro [K	1.152E-05	4.225E-05	1	1	5	P14618	P14618 [189-206]	0	2388.2902
[K].KVDDDL	2xTMTpro [K	5.701E-06	4.225E-05	1	1	2	P35580	P35580 [1379-13	1	2674.4967
[R].GAAPTL	1xTMTpro [N	2.568E-06	4.225E-05	1	1	1	Q14005	Q14005 [890-915]	0	2852.5073
[R].QEVNPV	1xTMTpro [K	3.715E-05	4.225E-05	1	1	1	P13611	P13611 [2101-21	1	3467.7633
[R].QEVGVA	1xTMTpro [N	2.057E-05	4.225E-05	1	1	1	O15230	O15230 [1122-11	0	1624.909
[K].QEVEEL	1xTMTpro [K	4.923E-05	4.225E-05	1	1	2	Q9UBG0	Q9UBG0 [435-44	0	2294.2888
[R].GADCCV	2xCarbamido	2.916E-05	4.225E-05	1	1	3	P51149	P51149 [80-97]	0	2622.3552
[K].QETFDA	1xTMTpro [K	1.158E-06	4.225E-05	1	1	3	Q13813	Q13813 [2018-20	0	3101.6763
[K].QETELSI	1xTMTpro [N	1.381E-05	4.225E-05	1	1	2	Q9Y285	Q9Y285 [188-203	0	2141.0504
[K].DVMQGT	1xCarbamido	9.327E-06	4.225E-05	1	1	3	P01871	P01871 [77-89]	0	2126.0866
[R].KVDVDE	1xCarbamido	3.951E-09	4.225E-05	1	1	1	O15511	O15511 [13-47]	2	4800.2523

[R].DVMQNI	1xCarbamido	1.026E-07	4.225E-05	1	1	1	P11413	P11413 [258-288]	1	4500.3175
[R].DVMQQ(	1xTMTpro [K	5.112E-06	4.225E-05	1	1	3	Q03252	Q03252 [365-381]	0	2658.4304
[R].DVMQQ(	1xOxidation	7.26E-06	4.225E-05	1	1	1	Q03252	Q03252 [365-381]	0	2674.4253
[R].KVDIIAD.	3xTMTpro [K	7.787E-07	4.225E-05	1	1	2	Q6YN16	Q6YN16 [220-236	1	3123.8923
[R].DVPWG\	1xTMTpro [N	6.012E-05	4.225E-05	1	1	1	Q16658	Q16658 [168-185]	0	2364.2519
[R].QEIVSLF	1xTMTpro [N	5.784E-05	4.225E-05	1	1	2	Q96HE7	Q96HE7 [438-449	0	1684.9342
[R].QELSHA	1xCarbamido	3.954E-05	4.225E-05	1	1	3	Q9UMS4	Q9UMS4 [101-115	0	2103.0361
[R].DVTFSP.	1xTMTpro [K	2.007E-06	4.225E-05	1	1	3	P55145	P55145 [48-62]	0	2285.2884
[R].KVEPPTI	2xTMTpro [K	2.799E-05	4.225E-05	1	1	2	Q6WCQ1	Q6WCQ1 [161-17	1	2387.4115
[R].QEEPQS	1xTMTpro [N	3.317E-05	4.225E-05	1	1	1	P29590	P29590 [361-372]	0	1659.8985
[R].GAAGAL	1xTMTpro [N	4.345E-05	4.225E-05	1	1	2	P61106	P61106 [83-95]	0	1641.8953
[R].QEELES	1xTMTpro [N	4.185E-07	4.225E-05	1	1	1	Q9NZN4	Q9NZN4 [399-425	0	3224.5489
[R].QEEEQD	2xTMTpro [K	2.329E-05	4.225E-05	1	1	1	P84157	P84157 [114-142]	1	4097.9404
[R].KVEQLQ	2xTMTpro [K	7.234E-06	4.225E-05	1	1	1	Q14258	Q14258 [237-249]	1	2566.4367
[K].DVTPPPI	1xTMTpro [K	3.774E-05	4.225E-05	1	1	2	P27816	P27816 [519-532]	0	2145.2662
[R].QEEELQ	2xTMTpro [K	4.48E-05	4.225E-05	1	1	3	P35580	P35580 [850-863]	1	2614.4756
[R].QEEDSY	1xCarbamido	1.084E-05	4.225E-05	1	1	1	Q96HS1	Q96HS1 [219-235	0	2427.1934
[K].QEFEQL	1xTMTpro [N	1.356E-07	4.225E-05	1	1	1	Q0ZGT2	Q0ZGT2 [406-429	1	3248.4973
[K].DVTVLQI	1xTMTpro [K	2.227E-06	4.225E-05	1	1	3	P02788	P02788 [566-583]	0	2597.3451
[R].DVTVTAI	1xOxidation	5.474E-06	4.225E-05	1	1	3	P12110	P12110 [751-766]	0	2357.2667
[R].KVEQLQ	1xOxidation	5.322E-05	4.225E-05	1	1	1	Q14258	Q14258 [237-249]	1	2582.4316
[R].KVESELI	2xTMTpro [K	4.642E-05	4.225E-05	1	1	2	P07225	P07225 [433-445]	1	2435.5166
[K].DVVFLLI	1xTMTpro [N	1.561E-05	4.225E-05	1	1	7	P12111	P12111 [1029-104	0	1709.9393
[R].DVVFLID	1xTMTpro [N	9.673E-07	4.225E-05	1	1	6	P12111	P12111 [1233-125	0	2531.3101
[R].DVVGND	1xTMTpro [N	4.434E-05	4.225E-05	1	1	2	Q9Y6W5	Q9Y6W5 [463-475	0	1662.9346
[K].QECCCA	5xCarbamido	2.929E-06	4.225E-05	1	1	1	P35555	P35555 [2081-210	1	3979.7673
[R].DVVICPI	1xCarbamido	2.167E-05	4.225E-05	1	1	2	Q99497	Q99497 [49-62]	0	2140.1451
[R].DVVICPI	1xCarbamido	9.659E-06	4.225E-05	1	1	6	Q99497	Q99497 [49-63]	1	2572.4473
[R].KVESLQ	2xTMTpro [K	8.649E-06	4.225E-05	1	1	3	P08670	P08670 [223-235]	1	2446.4737
[R].DVTVTAI	1xTMTpro [K	4.226E-06	4.225E-05	1	1	2	P12110	P12110 [751-766]	0	2341.2717
[R].DVVLPT\	1xTMTpro [N	2.76E-05	4.225E-05	1	1	1	Q7Z4H8	Q7Z4H8 [270-287	0	2395.2321
[R].KVENED	3xTMTpro [K	5.482E-05	4.225E-05	1	1	2	Q15019	Q15019 [310-325]	2	3162.8185
[K].KVEGTA	1xTMTpro [K	7.573E-07	4.225E-05	1	1	3	P01024	P01024 [264-281]	1	2602.4485

[K].KVEELE(	2xTMTpro [K	2.159E-05	4.225E-05	1	1	3	Q10589	Q10589 [112-126]	1	2652.5388
[R].DVQDFW	1xCarbamido	5.572E-05	4.225E-05	1	1	1	P35052	P35052 [388-404]	0	2603.3671
[K].DVQDSL	1xTMTpro [K	5.246E-06	4.225E-05	1	1	2	P52907	P52907 [211-226]	0	2314.2382
[R].QELNTL/	1xTMTpro [K	5.428E-05	4.225E-05	1	1	1	Q9NZD4	Q9NZD4 [72-84]	0	2067.2094
[R].DVQGTD	1xTMTpro [N	2.711E-05	4.225E-05	1	1	1	Q9UJZ1	Q9UJZ1 [338-352	0	1966.9525
[R].QEIIQDL	1xTMTpro [N	4.705E-05	4.225E-05	1	1	2	Q9UKV8	Q9UKV8 [636-647	0	1690.9481
[K].GAAPNV	1xTMTpro [K	1.272E-05	4.225E-05	1	1	4	Q16630	Q16630 [67-79]	0	1949.0988
[K].DVSALFI	1xCarbamido	1.202E-05	4.225E-05	1	1	2	Q10567	Q10567 [46-66]	0	3016.5615
[K].DVSALFI	1xCarbamido	7.274E-06	4.225E-05	1	1	2	Q10567	Q10567 [46-67]	1	3448.8636
[R].QELIECV	1xCarbamido	1.384E-05	4.225E-05	1	1	1	Q96RP9	Q96RP9 [251-272	0	3219.6045
[K].DVTCDV	1xCarbamido	5.209E-05	4.225E-05	1	1	1	Q99719	Q99719 [297-308]	0	1874.8662
[R].DVSELT(	1xTMTpro [N	5.006E-05	4.225E-05	1	1	1	P31939	P31939 [50-64]	0	1911.9805
[K].DVSGPM	1xTMTpro [N	4.419E-05	4.225E-05	1	1	1	P26640	P26640 [291-303]	0	1711.8281
[R].QEIFQE(	1xTMTpro [N	8.005E-06	4.225E-05	1	1	2	Q9Y678	Q9Y678 [610-624	0	2109.13
[R].QELEQH	1xTMTpro [K	1.427E-05	4.225E-05	1	1	2	Q14204	Q14204 [1151-11	0	3605.883
[R].QEIECQI	1xCarbamido	2.804E-05	4.225E-05	1	1	1	P35527	P35527 [428-444]	0	2703.4519
[R].QELAVF(	1xCarbamido	1.171E-05	4.225E-05	1	1	1	Q9Y490	Q9Y490 [2155-21	0	2181.187
[K].QEIAEA(	1xCarbamido	1.735E-05	4.225E-05	1	1	2	P53621	P53621 [1045-10	0	1977.0758
[K].QEKPAE	2xTMTpro [K	3.295E-07	4.225E-05	1	1	2	P54727	P54727 [145-173]	0	3858.0185
[K].DVSSLFI	1xCarbamido	2.593E-05	4.225E-05	1	1	2	P63010	P63010 [46-66]	0	3032.5564
[K].DVSSLFI	1xCarbamido	1.063E-05	4.225E-05	1	1	2	P63010	P63010 [46-67]	1	3464.8585
[K].KVEGFD	2xTMTpro [K	2.045E-05	4.225E-05	1	1	1	Q99541	Q99541 [204-219]	1	2840.6491
[R].QEIGNLI	1xTMTpro [K	1.121E-05	4.225E-05	1	1	1	O15270	O15270 [208-225]	1	2730.4918
[K].EAENPE	2xTMTpro [K	2.463E-08	4.225E-05	1	1	1	P51858	P51858 [171-197]	1	3966.0834
[R].QAQVNL	1xCarbamido	7.489E-07	4.225E-05	1	1	1	Q15746	Q15746 [1320-13	0	3257.7444
[R].QAQQEF	1xTMTpro [K	1.718E-06	4.225E-05	1	1	2	P35579	P35579 [1698-17	1	2697.3935
[K].KWENPT	1xTMTpro [K	1.222E-05	4.225E-05	1	1	1	P22694	P22694 [30-46]	1	2656.3611
[K].EAASILQ	1xTMTpro [K	9.911E-06	4.225E-05	1	1	2	O00232	O00232 [161-179]	0	2734.4465
[K].EAASILQ	1xOxidation	5.576E-06	4.225E-05	1	1	2	O00232	O00232 [161-179]	0	2750.4414
[K].EAASLSI	1xTMTpro [K	5.775E-06	4.225E-05	1	1	3	P46939	P46939 [1552-15	0	2700.4588
[K].EAATLE\	1xTMTpro [K	1.073E-06	4.225E-05	1	1	4	P51858	P51858 [181-197]	0	2549.414
[R].EAATQA	1xTMTpro [K	1.48E-05	4.225E-05	1	1	2	P47897	P47897 [35-50]	0	2270.2484
[K].KWESPA	1xTMTpro [K	3.866E-06	4.225E-05	1	1	2	P17612	P17612 [30-46]	1	2665.3978

[R].KWLLLN	2xTMTpro [K	1.628E-05	4.225E-05	1	1	1	Q9NZM1	Q9NZM1 [298-31	1	2702.5181
[K].NVQDAI	1xTMTpro [K	4.257E-05	4.225E-05	1	1	1	P35908	P35908 [419-436]	1	2573.3927
[K].EAAWAI	1xTMTpro [N	3.059E-05	4.225E-05	2	2	2	O00629; O0	O00629 [387-400	0	1792.9877
[K].EAAWAI	1xTMTpro [N	1.004E-05	4.225E-05	1	2	1	O60684	O60684 [397-415	0	2277.1794
[K].NVQDAI	1xTMTpro [N	5.984E-05	4.225E-05	1	1	2	P35908	P35908 [419-430]	0	1633.8465
[R].NVQALE	1xTMTpro [K	8.352E-06	4.225E-05	1	1	2	P13645	P13645 [371-386]	0	2405.4259
[K].EACDQH	1xCarbamido	3.613E-05	4.225E-05	1	1	1	P36551	P36551 [302-314]	0	2138.0832
[R].NVQAEE	1xOxidation	4.05E-05	4.225E-05	1	1	2	P25705	P25705 [89-103]	0	2292.2037
[R].NVQAEE	1xTMTpro [K	2.151E-05	4.225E-05	1	1	19	P25705	P25705 [89-103]	0	2276.2088
[R].EACPELI	2xCarbamido	1.359E-05	4.225E-05	1	1	1	P49327	P49327 [2008-20	0	2526.1964
[R].NVPQEE	1xTMTpro [K	1.67E-05	4.225E-05	1	1	1	O00505	O00505 [50-67]	0	2645.3074
[R].FYGAEI	1xTMTpro [K	3.761E-06	4.225E-05	1	1	2	P31749	P31749 [252-268]	0	2550.3736
[K].NVPLPEI	1xTMTpro [K	2.942E-05	4.225E-05	1	1	1	P14735	P14735 [282-299]	0	2795.5012
[R].NVPEIAV	1xTMTpro [K	3.113E-05	4.225E-05	1	1	1	Q15012	Q15012 [194-220	0	3644.957
[R].NVQVYN	1xTMTpro [N	5.039E-05	4.225E-05	1	1	2	Q99715	Q99715 [1939-19	0	2020.0783
[K].NVSINTV	1xTMTpro [N	3.796E-06	4.225E-05	1	1	1	Q9UGI8	Q9UGI8 [103-124	0	2775.4749
[R].NVSNLK	2xTMTpro [K	3.588E-05	4.225E-05	1	1	4	Q8NBX0	Q8NBX0 [215-22	0	2388.5159
[K].NVSSILG	1xTMTpro [N	2.182E-06	4.225E-05	1	1	1	Q9P0J1	Q9P0J1 [105-126	0	2661.3803
[K].NWQWG	1xCarbamido	4.192E-05	4.225E-05	1	1	1	Q86VB7	Q86VB7 [454-469	0	2602.264
[K].EAAGTT	1xTMTpro [N	1.404E-06	4.225E-05	1	1	1	O43290	O43290 [12-31]	0	2118.0746
[R].NWIEEV	1xOxidation	4.65E-05	4.225E-05	1	1	2	Q15417	Q15417 [34-53]	0	2857.5414
[K].NWGLSV	2xTMTpro [K	3.747E-06	4.225E-05	1	1	4	P02763	P02763 [139-153]	0	2621.4755
[R].KVYFHTI	2xTMTpro [K	5.803E-05	4.225E-05	1	1	1	Q9Y697	Q9Y697 [226-239	1	2447.4227
[R].KWDPYK	3xTMTpro [K	1.204E-05	4.225E-05	1	1	3	P02675	P02675 [295-313]	2	3342.8587
[K].FYGPQG	1xTMTpro [K	5.618E-05	4.225E-05	1	1	1	P62256	P62256 [38-52]	0	2294.2101
[R].NWAEVF	1xTMTpro [K	2.371E-05	4.225E-05	1	1	1	Q9UPU5	Q9UPU5 [254-27	0	3053.5687
[R].NVYTEQ	1xTMTpro [K	5.738E-05	4.225E-05	1	1	1	Q86WI1	Q86WI1 [483-501	0	2773.45
[K].EAALSTA	1xTMTpro [K	5.841E-05	4.225E-05	1	1	4	P02545	P02545 [145-155]	0	1728.0035
[K].NVPCGT	1xCarbamido	2.079E-05	4.225E-05	1	1	2	O94905	O94905 [73-88]	0	2077.0166
[K].EAALSY	2xCarbamido	4.32E-06	4.225E-05	1	1	1	P14555	P14555 [36-53]	0	2265.05
[K].EAALTSE	1xTMTpro [K	1.282E-06	4.225E-05	1	1	2	Q13813	Q13813 [1111-11	0	2866.5541
[R].NVVIAAD	1xTMTpro [K	3.981E-05	4.225E-05	1	1	2	Q9NYL2	Q9NYL2 [138-14	0	1707.066
[R].EAALWE	1xTMTpro [N	9.82E-06	4.225E-05	1	1	1	O94804	O94804 [740-754	0	2203.0773

[K].FYGNSL	1xTMTpro [N	3.532E-05	4.225E-05	1	1	1	Q9BZF1	Q9BZF1 [542-556	0	1931.0194
[K].NVTELNI	1xTMTpro [N	6.032E-05	4.225E-05	1	1	2	P61981	P61981 [29-42]	0	1947.9943
[K].NVTDVV	1xCarbamido	4.102E-06	4.225E-05	1	1	2	Q9Y3I0	Q9Y3I0 [477-492]	0	2338.2317
[R].NVSTGD	2xOxidation [	2.242E-05	4.225E-05	1	1	1	P13645	P13645 [296-322]	0	3208.5897
[K].EAAPDA	1xTMTpro [K	3.677E-05	4.225E-05	1	1	1	Q8N163	Q8N163 [288-309	0	2771.3867
[R].NVSTGD	1xOxidation [	1.073E-06	4.225E-05	1	1	1	P13645	P13645 [296-322]	0	3192.5948
[K].EAAQALI	1xTMTpro [K	5.898E-05	4.225E-05	1	1	1	Q9Y6W3	Q9Y6W3 [35-56]	0	2974.5687
[K].NVVLPTE	1xTMTpro [K	1.906E-05	4.225E-05	1	1	2	P27816	P27816 [333-346]	0	2076.2196
[R].EADECIE	1xCarbamido	5.566E-06	4.225E-05	1	1	1	Q6UWY5	Q6UWY5 [107-13	1	3851.9234
[R].EADECIE	1xCarbamido	6.511E-06	4.225E-05	1	1	2	Q6UWY5	Q6UWY5 [107-13	2	4284.2256
[K].EADEMH	1xCarbamido	3.886E-05	4.225E-05	1	1	1	Q86UP2	Q86UP2 [1124-11	0	2454.25
[R].EAEDTQ	1xTMTpro [N	5.056E-05	4.225E-05	1	1	1	Q96B36	Q96B36 [229-243	0	2034.0575
[K].NVIDGDI	1xCarbamido	1.215E-05	4.225E-05	1	1	2	Q15393	Q15393 [1172-11	0	2718.3359
[R].NVLAPY	1xTMTpro [N	1.217E-05	4.225E-05	1	1	1	Q4G176	Q4G176 [539-558	0	2512.4346
[R].EAEEHQ	1xCarbamido	5.149E-05	4.225E-05	1	1	2	Q99969	Q99969 [126-137	0	1833.872
[K].NVHMND	1xTMTpro [N	1.459E-05	4.225E-05	1	1	1	Q13630	Q13630 [90-107]	0	2314.1682
[R].EAEETQ	1xCarbamido	5.607E-06	4.225E-05	1	1	3	Q9P2E9	Q9P2E9 [1116-11	0	2362.0788
[K].NVHLAP	1xTMTpro [K	5.917E-05	4.225E-05	1	1	2	Q14204	Q14204 [4098-41	0	2244.2819
[K].EAEFGQ	1xTMTpro [K	5.334E-06	4.225E-05	1	1	2	Q86VB7	Q86VB7 [981-997	0	2483.3426
[R].NVGVEV	1xTMTpro [K	2.921E-05	4.225E-05	1	1	3	Q99715	Q99715 [266-277	0	1870.1294
[R].FYEGPE	1xTMTpro [N	9.276E-06	4.225E-05	1	1	1	P49747	P49747 [699-718]	0	2560.2924
[K].NVIFEISI	1xTMTpro [K	3.8E-05	4.225E-05	1	1	2	P46940	P46940 [1587-16	0	2660.4315
[K].EAEGAP	1xTMTpro [K	4.603E-05	4.225E-05	1	1	1	Q05682	Q05682 [523-535	1	1950.09
[K].NVFIIGA	1xTMTpro [N	6.104E-06	4.225E-05	1	1	5	P55072	P55072 [616-638]	0	2822.6324
[K].NVFDEA	1xTMTpro [K	1.148E-05	4.225E-05	1	1	5	P60953	P60953 [167-183]	0	2461.3834
[R].KYEMFA	1xTMTpro [K	2.844E-05	4.225E-05	1	1	2	P55072	P55072 [754-766]	1	2238.2196
[K].EAELDVI	2xTMTpro [K	5.046E-06	4.225E-05	1	1	2	P43034	P43034 [34-46]	1	2444.37
[K].KYEQGF	2xTMTpro [K	5.572E-05	4.225E-05	1	1	1	P12268	P12268 [109-124]	1	2733.6007
[R].NVELQC	1xCarbamido	1.123E-05	4.225E-05	1	1	6	P02730	P02730 [880-892]	0	2099.0934
[R].NVEGVE	1xTMTpro [N	2.32E-05	4.225E-05	1	1	1	Q9Y6M5	Q9Y6M5 [362-381	0	2592.349
[R].NVEGQD	1xTMTpro [K	1.03E-05	4.225E-05	2	2	2	Q10567; P63	Q10567 [889-901	0	2133.1506
[R].NVDMLS	1xOxidation [	1.703E-05	4.225E-05	1	1	2	Q99733	Q99733 [169-186	0	2759.4669
[R].NVDMLS	1xTMTpro [K	3.726E-06	4.225E-05	1	1	3	Q99733	Q99733 [169-186	0	2743.472

[K].NVGLDIE	1xTMTpro [K	2.968E-05	4.225E-05	1	1	3	P50990	P50990 [477-490]	0	2062.204
[K].NWRDPD	1xTMTpro [N	3.184E-06	4.225E-05	1	1	1	P06396	P06396 [395-419]	1	3147.5779
[K].NVIGGFE	5xCarbamido	3.496E-07	4.225E-05	1	1	1	P35555	P35555 [2183-222	0	4757.0538
[K].NVLGHM	1xTMTpro [N	3.525E-06	4.225E-05	1	1	1	Q01813	Q01813 [667-683	0	2115.0725
[K].EADESLI	1xTMTpro [K	2.206E-05	4.225E-05	1	1	3	Q9Y490	Q9Y490 [2334-23	0	2544.3325
[R].NVNLVLI	1xCarbamido	5.073E-05	4.225E-05	1	1	1	Q96KR1	Q96KR1 [805-818	0	2513.4941
[R].EADFYQ	1xTMTpro [K	4.442E-05	4.225E-05	1	1	1	Q4G0X4	Q4G0X4 [83-99]	0	2629.4369
[R].EADGSE	1xTMTpro [K	2.855E-05	4.225E-05	1	1	2	Q7KZF4	Q7KZF4 [234-249	0	2257.148
[K].NVMSAF	1xOxidation [	3.786E-07	4.225E-05	1	1	1	Q92734	Q92734 [180-203	0	2781.3321
[K].EADLAA	1xTMTpro [K	1.249E-05	4.225E-05	1	1	2	Q9Y3B7	Q9Y3B7 [180-191	0	1854.01
[K].EADLAA	2xTMTpro [K	6.026E-06	4.225E-05	1	1	1	Q9Y3B7	Q9Y3B7 [180-192	1	2286.3121
[K].NVMSAF	1xTMTpro [N	4.115E-07	4.225E-05	1	1	2	Q92734	Q92734 [180-203	0	2765.3371
[R].EADIDGI	1xTMTpro [K	9.133E-07	4.225E-05	1	1	42	P0DP25	P0DP25 [128-149	0	3098.4942
[R].EADIDGI	1xOxidation [	2.091E-06	4.225E-05	1	1	8	P0DP25	P0DP25 [128-149	0	3114.4891
[K].NVIGGFE	5xCarbamido	1.779E-05	4.225E-05	1	1	4	P35555	P35555 [2183-222	0	4773.0487
[K].NVMLLPY	1xOxidation [	6.241E-06	4.225E-05	1	1	4	Q96KP4	Q96KP4 [431-450	0	2706.4013
[K].KYAPTEA	2xTMTpro [K	8.371E-08	4.225E-05	1	1	3	P13010	P13010 [443-465]	1	3361.8857
[K].KYDAFLA	2xTMTpro [K	3.632E-05	4.225E-05	1	1	4	P62906	P62906 [106-118]	1	2397.421
[R].NVITEPI	1xTMTpro [N	9.14E-06	4.225E-05	1	1	1	Q13362	Q13362 [85-105]	0	2810.4693
[R].EADVME	1xCarbamido	6.183E-06	4.225E-05	1	1	1	P48651	P48651 [143-160]	0	2526.2076
[K].NVIIWGN	1xTMTpro [K	5.586E-06	4.225E-05	1	1	1	P40925	P40925 [180-199]	0	2888.5299
[K].NVLIVED	1xTMTpro [K	1.154E-05	4.225E-05	1	1	4	P00492	P00492 [129-141]	0	2037.2087
[R].EAEAAIY	1xTMTpro [N	1.149E-06	4.225E-05	1	1	14	P14618	P14618 [384-399]	0	2236.1933
[R].EAEAAIY	1xTMTpro [N	1.543E-06	4.225E-05	1	1	2	P14618	P14618 [384-400]	1	2392.2944
[R].FYEQMN	1xTMTpro [N	3.042E-05	4.225E-05	1	1	1	P29692	P29692 [25-38]	0	1830.9128
[K].EAEAMA	1xTMTpro [N	4.271E-05	4.225E-05	1	1	2	P54920	P54920 [7-19]	0	1707.8907
[K].NVMLLPY	1xTMTpro [K	2.899E-07	4.225E-05	1	1	5	Q96KP4	Q96KP4 [431-450	0	2690.4063
[K].QAQVAT	1xTMTpro [N	3.635E-07	4.225E-05	1	1	2	Q92945	Q92945 [655-683	0	3256.5619
[K].NWVVTG	1xTMTpro [N	5.348E-05	4.225E-05	1	1	1	P35606	P35606 [70-82]	0	1808.9284
[K].NYDGDV	1xCarbamido	1.034E-05	4.225E-05	1	1	1	O75874	O75874 [271-301	0	3874.8429
[R].KVQHQD	1xTMTpro [K	1.167E-05	4.225E-05	1	1	1	Q14974	Q14974 [594-613	1	2859.637
[K].QAGPAS	2xTMTpro [K	5.636E-05	4.225E-05	1	1	2	P30101	P30101 [131-147]	2	2799.6185
[K].DYNVYT	1xCarbamido	4.192E-05	4.225E-05	1	1	1	P49961	P49961 [245-258]	0	2375.1986

[R].DYPDFS	1xTMTpro [K	4.704E-06	4.225E-05	1	1	3	P12429	P12429 [14-29]	0	2390.2371
[R].KVQSGN	2xTMTpro [K	4.213E-05	4.225E-05	1	1	2	P49368	P49368 [21-31]	1	2042.2538
[R].QAGLGN	1xTMTpro [N	4.665E-05	4.225E-05	1	1	1	Q15582	Q15582 [397-409	0	1629.8628
[K].QAGGFL	1xTMTpro [K	5.719E-05	4.225E-05	1	1	1	Q9UM00	Q9UM00 [224-237	0	1918.1042
[R].FYPGQA	1xTMTpro [N	5.756E-05	4.225E-05	1	1	1	Q9Y276	Q9Y276 [368-386	0	2450.2788
[K].FYPEDV	1xTMTpro [N	4.863E-06	4.225E-05	1	1	3	P26038	P26038 [84-100]	0	2386.2097
[K].QAFTDV	1xCarbamido	9.946E-08	4.225E-05	1	1	2	P29401	P29401 [115-140]	0	3140.6
[K].DYPVVS	1xTMTpro [K	1.327E-05	4.225E-05	1	1	5	P06733	P06733 [286-306]	0	3118.529
[K].QAFSTA	1xTMTpro [K	2.497E-05	4.225E-05	1	1	1	O60343	O60343 [435-447	0	1902.094
[K].QAFDDA	1xTMTpro [K	3.525E-06	4.225E-05	1	1	2	Q04917	Q04917 [199-217	0	2766.3965
[K].FYPEDV	1xTMTpro [K	1.679E-05	4.225E-05	1	1	2	P15311	P15311 [84-100]	0	2646.4158
[K].QAEVAN	3xTMTpro [K	3.276E-05	4.225E-05	1	1	1	P05114	P05114 [62-95]	2	4848.4651
[K].KVQVPN	1xCarbamido	1.529E-06	4.225E-05	1	1	1	P53621	P53621 [447-467]	1	3015.6581
[K].QAESAS	2xTMTpro [K	1.088E-05	4.225E-05	1	1	1	P51572	P51572 [139-149]	1	2032.1855
[R].QAEMLD	1xTMTpro [K	5.09E-05	4.225E-05	1	1	1	P54819	P54819 [107-117]	0	1931.011
[R].DYQGPL	1xTMTpro [K	3.532E-05	4.225E-05	1	1	1	P53396	P53396 [363-378]	1	2539.4164
[R].QAEMEG	1xOxidation [	9.057E-06	4.225E-05	1	1	3	Q6NZI2	Q6NZI2 [79-95]	0	2429.2838
[R].QAEMEG	1xTMTpro [K	1.179E-06	4.225E-05	1	1	3	Q6NZI2	Q6NZI2 [79-95]	0	2413.2888
[R].QAHLCV	2xCarbamido	2.011E-06	4.225E-05	1	1	2	P25398	P25398 [46-63]	0	2743.3861
[R].QAHLCV	2xCarbamido	1.796E-06	4.225E-05	1	1	3	P25398	P25398 [46-63]	0	2759.3811
[K].KVQGGA	2xTMTpro [K	1.251E-05	4.225E-05	1	1	2	Q99832	Q99832 [199-217	1	2829.6654
[K].KVQAFL	2xCarbamido	3.803E-06	4.225E-05	1	1	1	Q99933	Q99933 [314-335	1	3276.6484
[R].DYLDFLI	1xTMTpro [K	2.246E-05	4.225E-05	1	1	1	P25205	P25205 [18-35]	0	2801.3649
[R].DYLDLT	1xTMTpro [N	5.473E-05	4.225E-05	1	1	1	Q9NYU2	Q9NYU2 [705-72	0	2443.1737
[R].QAQIEV	1xTMTpro [K	8.891E-06	4.225E-05	1	1	3	P30050	P30050 [68-83]	0	2275.3881
[K].QAQLAQ	1xTMTpro [N	4.661E-06	4.225E-05	1	1	2	Q14980	Q14980 [532-550	0	2430.302
[R].QAQGLQ	1xTMTpro [N	1.754E-05	4.225E-05	1	1	1	Q9H0H0	Q9H0H0 [695-712	0	2223.2529
[K].FYQTSVI	1xTMTpro [N	5.475E-07	4.225E-05	1	1	2	P29508	P29508 [126-144]	0	2480.1901
[R].DYLGDF	1xTMTpro [N	2.565E-07	4.225E-05	1	1	5	Q00577	Q00577 [112-140	0	3574.741
[K].QAQDQV	1xTMTpro [N	1.08E-05	4.225E-05	1	1	1	Q9BRF8	Q9BRF8 [179-193	0	2105.0947
[K].FYQTSVI	1xTMTpro [N	6.781E-06	4.225E-05	1	1	1	P48594	P48594 [126-144]	0	2482.1693
[R].QAPSIAT	1xCarbamido	6.041E-05	4.225E-05	1	1	1	Q14315	Q14315 [2143-215	0	2397.3303
[R].DYQTLP	1xTMTpro [N	4.65E-05	4.225E-05	1	1	1	O14639	O14639 [687-700	0	1935.9376

[K].FYQPYS	1xTMTpro [N	2.392E-05	4.225E-05	1	1	2	Q92572	Q92572 [19-33]	0	2220.1256
[K].KVPQVS	1xTMTpro [K	1.719E-05	4.225E-05	1	1	51	P02768	P02768 [438-452]	1	2248.352
[K].KVPTSN	1xCarbamido	7.141E-06	4.225E-05	1	1	1	Q5T4S7	Q5T4S7 [5109-51	1	2863.4904
[K].QALQES	1xTMTpro [N	5.68E-06	4.225E-05	1	1	1	P08240	P08240 [387-401]	0	2055.1882
[K].KVPTVSI	2xTMTpro [K	5.261E-05	4.225E-05	1	1	2	O75396	O75396 [102-121]	1	3314.8969
[R].QALQDL	1xTMTpro [N	1.111E-05	4.225E-05	1	1	2	P35221	P35221 [342-357]	0	2070.0609
[R].QAIPLDE	1xTMTpro [K	2.457E-05	4.225E-05	1	1	2	Q14764	Q14764 [378-394]	0	2539.3899
[K].DYIPVDC	1xTMTpro [K	3.545E-05	4.225E-05	1	1	1	Q14204	Q14204 [2880-28	1	2490.3372
[R].DYIPVEC	1xTMTpro [K	2.148E-05	4.225E-05	1	1	1	P16144	P16144 [1038-105	0	2699.4576
[K].FYQAEM	1xTMTpro [K	1.587E-05	4.225E-05	1	1	3	P35237	P35237 [113-129]	0	2657.3664
[K].QAIDDD	1xCarbamido	2.607E-06	4.225E-05	1	1	2	P38117	P38117 [125-159]	0	4453.1208
[K].QAMFHY	1xTMTpro [N	4.257E-05	4.225E-05	1	1	1	Q9UJ70	Q9UJ70 [186-197	0	1842.928
[K].DYQTSP	1xTMTpro [N	4.106E-05	4.225E-05	1	1	1	Q9Y6U3	Q9Y6U3 [598-615	0	2401.1955
[R].KVSASV.	1xTMTpro [K	1.818E-05	4.225E-05	1	1	1	Q27J81	Q27J81 [853-868]	1	2432.3277
[R].QAEELIQ	1xTMTpro [N	4.854E-07	4.225E-05	1	1	1	O14879	O14879 [76-92]	0	2312.1802
[K].QAAAAA	1xTMTpro [K	6.682E-06	4.225E-05	1	1	1	Q9Y4G6	Q9Y4G6 [926-946	0	2595.4346
[K].FYLQGP	1xTMTpro [N	3.927E-05	4.225E-05	1	1	1	O15230	O15230 [2764-27	0	2224.0841
[R].NYQQNY	1xTMTpro [K	1.428E-06	4.225E-05	1	1	1	P67809	P67809 [157-185]	1	3865.8105
[R].EAAAAL	1xTMTpro [N	4.179E-06	4.225E-05	1	1	1	O75934	O75934 [30-42]	1	1748.9462
[R].KVVDYS	1xTMTpro [K	8.99E-06	4.225E-05	1	1	1	Q9H1E3	Q9H1E3 [9-28]	1	2974.4198
[K].EAAASG	1xTMTpro [K	2.854E-06	4.225E-05	1	1	3	O95881	O95881 [49-63]	0	2158.2914
[K].NYNVPY	1xTMTpro [K	2.844E-05	4.225E-05	1	1	2	P53990	P53990 [172-205]	0	4304.1452
[R].NYNFGG	1xTMTpro [N	2.23E-05	4.225E-05	1	1	2	Q09161	Q09161 [97-110]	0	1950.9703
[K].NYIQGIN	1xTMTpro [K	2.75E-05	4.225E-05	1	1	2	P52815	P52815 [151-162]	0	1969.1726
[R].NYIMDF	1xOxidation	3.696E-05	4.225E-05	1	1	2	P09455	P09455 [60-82]	2	3690.9141
[K].KVTHAV	1xTMTpro [K	3.172E-06	4.225E-05	1	1	1	P11021	P11021 [164-181]	1	2624.4804
[R].NYIMDF	2xTMTpro [K	9.167E-08	4.225E-05	1	1	4	P09455	P09455 [60-82]	2	3674.9192
[K].EAADAID	1xTMTpro [N	1.412E-06	4.225E-05	1	1	2	P14923	P14923 [616-637]	0	2571.3044
[R].KVVETEI	2xTMTpro [K	3.657E-05	4.225E-05	1	1	1	Q15286	Q15286 [128-137]	1	2094.2263
[R].KVVLEAI	2xTMTpro [K	3.947E-05	4.225E-05	1	1	2	Q6GMV3	Q6GMV3 [80-92]	1	2355.4315
[R].NYILDQT	1xTMTpro [N	8.761E-06	4.225E-05	1	1	1	Q9BUJ2	Q9BUJ2 [501-515	0	2046.0575
[K].NYLAWY	2xTMTpro [K	2.144E-05	4.225E-05	1	1	4	P06312	P06312 [57-71]	0	2730.5548
[R].NYGILAD	1xTMTpro [K	5.169E-08	4.225E-05	1	1	2	Q9BZZ5	Q9BZZ5 [10-25]	0	2352.2803

[R].NYEASV	1xTMTpro [K	7.948E-08	4.225E-05	1	1	2	P21980	P21980 [49-74]	0	3263.6927
[K].KVVVEQ	2xTMTpro [K	1.941E-05	4.225E-05	1	1	3	P34949	P34949 [207-218]	1	2294.4992
[K].KVVYEN.	1xTMTpro [K	1.127E-05	4.225E-05	1	1	1	Q16881	Q16881 [274-289]	1	2486.38
[K].NYDIGA/	1xTMTpro [K	2.414E-05	4.225E-05	1	1	1	Q13501	Q13501 [421-435]	0	2280.2367
[K].EAADAE	1xTMTpro [N	6.1E-05	4.225E-05	1	1	1	Q08378	Q08378 [1018-10	0	1476.7977
[K].NYDGDV	1xCarbamid	5.557E-07	4.225E-05	1	1	1	O75874	O75874 [271-301]	0	3858.848
[K].QAAASA	1xTMTpro [K	8.34E-08	4.225E-05	1	1	2	Q9Y490	Q9Y490 [923-943	0	2603.4397
[K].QAADMII	1xOxidation [	3.499E-06	4.225E-05	1	4	1	P05023	P05023 [744-766]	0	2784.4045
[K].QAEAFLI	1xTMTpro [K	7.177E-07	4.225E-05	1	1	3	Q01082	Q01082 [1180-12	0	3898.0188
[R].DYSHYY	1xTMTpro [N	2.947E-05	4.225E-05	1	1	2	P08727	P08727 [126-138]	0	1978.983
[K].QADNPH	1xTMTpro [N	2.77E-05	4.225E-05	1	1	1	Q9Y4L1	Q9Y4L1 [107-119	0	1786.952
[K].QADLYIS	1xTMTpro [N	5.384E-05	4.225E-05	2	2	2	P40227; Q9	P40227 [105-117]	0	1802.972
[K].QADLEK	1xTMTpro [K	3.441E-06	4.225E-05	1	1	2	P35749	P35749 [1705-17	1	2783.4918
[R].QADFEA	1xTMTpro [N	4.857E-05	4.225E-05	1	1	2	P49915	P49915 [590-600]	0	1617.8668
[K].QADETLI	1xTMTpro [K	2.159E-05	4.225E-05	1	1	1	Q9Y4G6	Q9Y4G6 [2335-23	0	2558.3481
[K].DYTGED	1xTMTpro [N	2.838E-06	4.225E-05	1	1	3	Q99538	Q99538 [102-118]	0	2242.1675
[K].QADEEF	1xTMTpro [N	2.312E-05	4.225E-05	1	1	4	Q9H0U3	Q9H0U3 [92-105]	0	2011.0204
[K].DYTSGA	1xTMTpro [K	1.023E-05	4.225E-05	1	1	2	P23381	P23381 [419-431]	0	1994.076
[K].QAADMII	1xTMTpro [N	1.118E-06	4.225E-05	1	4	3	P05023	P05023 [744-766]	0	2768.4096
[K].DYTSGA	2xTMTpro [K	3.16E-06	4.225E-05	1	1	2	P23381	P23381 [419-432]	1	2426.3781
[R].DYVAPT.	1xTMTpro [K	4.572E-05	4.225E-05	1	1	3	Q7KZF4	Q7KZF4 [328-339	0	1943.073
[R].QAASQE	1xTMTpro [N	6.339E-07	4.225E-05	1	1	2	Q63ZY3	Q63ZY3 [460-480	0	2260.1125
[K].QAASGL	1xTMTpro [N	3.423E-05	4.225E-05	1	1	2	Q9Y265	Q9Y265 [34-46]	0	1604.8676
[K].QAAPPIE	1xTMTpro [N	1.508E-05	4.225E-05	1	1	2	O75400	O75400 [756-771]	0	2127.1405
[R].QAAPCV	1xCarbamid	1.438E-05	4.225E-05	1	1	1	P55072	P55072 [568-584]	0	2532.3664
[K].QAAGVIS	1xTMTpro [K	4.737E-05	4.225E-05	1	1	1	Q8NDT2	Q8NDT2 [832-845	0	1892.1461
[K].DYVPDT	1xTMTpro [N	7.409E-06	4.225E-05	1	1	1	Q9NQ66	Q9NQ66 [797-815	0	2455.2676
[R].KVTEEN	2xTMTpro [K	1.26E-05	4.225E-05	1	1	3	P07996	P07996 [300-313]	2	2585.5079
[R].DYVSQF	1xTMTpro [K	1.885E-05	4.225E-05	1	1	5	P02647	P02647 [52-64]	0	2009.0835
[R].QAAECG	1xCarbamid	4.518E-05	4.225E-05	1	1	1	Q9HBH5	Q9HBH5 [93-112]	0	2329.2141
[R].QAASSL	1xTMTpro [K	2.979E-05	4.225E-05	1	1	2	P38646	P38646 [635-646]	0	1840.0784
[K].QFDIQLL	1xTMTpro [K	1.043E-05	4.225E-05	1	1	2	P48507	P48507 [206-218]	0	2177.221
[R].NLIDEDC	1xTMTpro [K	4.51E-05	4.225E-05	1	1	1	O00410	O00410 [119-134]	0	2450.2807

[K].NILAEQL	1xOxidation [	9.107E-06	4.225E-05	1	1	1	P35580	P35580 [890-910]	0	2755.3779
[K].MQQNIQ	1xTMTpro [N	1.289E-06	4.225E-05	1	1	4	P35579	P35579 [941-959]	0	2637.2633
[K].MQQLEQ	1xTMTpro [N	3.826E-05	4.225E-05	1	1	2	P40763	P40763 [200-214]	0	2140.0884
[K].MQQENN	1xTMTpro [K	2.65E-06	4.225E-05	1	1	2	P22087	P22087 [286-304]	0	3000.5415
[K].IAPNTPQ	1xCarbamido	3.774E-05	4.225E-05	1	1	1	Q5JPE7	Q5JPE7 [393-413	0	2491.3298
[K].EFQDAG	1xTMTpro [K	4.21E-06	4.225E-05	1	1	1	P31937	P31937 [77-95]	0	2614.3492
[K].EFQLFSS	1xTMTpro [K	3.981E-05	4.225E-05	1	1	2	P02787	P02787 [300-310]	0	1885.0464
[R].MQNDTA	1xOxidation [	4.012E-06	4.225E-05	1	1	2	Q05682	Q05682 [137-152]	1	2825.4291
[R].MQNDTA	2xTMTpro [K	9.303E-07	4.225E-05	1	1	2	Q05682	Q05682 [137-152]	1	2809.4342
[K].EFQQYL	1xTMTpro [K	1.208E-05	4.225E-05	1	1	2	O00410	O00410 [618-632]	0	2388.3315
[K].EFQTVPI	1xTMTpro [K	3.411E-05	4.225E-05	1	1	2	Q9HB40	Q9HB40 [155-171	0	2607.3627
[R].IAPPEAF	1xTMTpro [K	4.572E-05	4.225E-05	1	1	2	P09874	P09874 [879-893]	0	2186.2175
[R].MQMPQ(	1xTMTpro [N	1.8E-06	4.225E-05	1	1	1	Q9Y5X2	Q9Y5X2 [54-74]	0	2694.4754
[K].MQMLED	2xTMTpro [K	4.416E-07	4.225E-05	1	1	1	Q14204	Q14204 [4346-43	1	3071.5733
[K].MQMLED	1xTMTpro [K	2.779E-05	4.225E-05	1	1	1	Q14204	Q14204 [4346-43	0	2639.2712
[-].MQLTHQI	1xCarbamido	5.149E-05	4.225E-05	1	1	1	Q9Y3C4	Q9Y3C4 [1-14]	0	2092.0639
[R].EFSGPS	1xTMTpro [K	1.404E-06	4.225E-05	1	1	3	Q09666	Q09666 [5788-58	0	3120.5869
[R].EFSGYV	1xTMTpro [K	5.936E-05	4.225E-05	1	1	2	P20073	P20073 [398-408]	0	1824.0035
[R].MQLSAE	1xTMTpro [K	1.927E-05	4.225E-05	1	1	2	O75643	O75643 [1902-19	0	2530.3566
[K].MQLQQE	1xTMTpro [K	3.037E-05	4.225E-05	1	1	1	Q7L7X3	Q7L7X3 [803-818	0	2544.3987
[K].LAPPLVT	1xTMTpro [N	1.488E-05	4.225E-05	1	1	2	Q10567	Q10567 [284-304]	0	2597.5237
[R].EFSITDV	1xTMTpro [N	4.352E-05	4.225E-05	1	1	3	P34932	P34932 [391-405]	0	2040.1337
[K].MQQNIQ	1xOxidation [	5.334E-06	4.225E-05	1	1	1	P35579	P35579 [941-959]	0	2653.2582
[K].EFPGFLI	2xTMTpro [K	1.394E-05	4.225E-05	1	1	3	P60903	P60903 [38-54]	1	2859.6073
[R].MQQQLD	1xTMTpro [K	1.569E-05	4.225E-05	1	1	9	P02545	P02545 [352-366]	0	2502.3405
[R].EFPDVLI	1xCarbamido	2.573E-06	4.225E-05	1	1	2	P48507	P48507 [65-80]	0	2468.2987
[R].MSDSAD	1xCarbamido	3.108E-05	4.225E-05	1	1	1	P28347	P28347 [19-55]	0	4717.2682
[R].IAPLAEG	1xTMTpro [N	5.188E-06	4.225E-05	1	1	1	O75746	O75746 [292-309]	0	2243.2719
[R].IAPLEEG	1xTMTpro [N	4.312E-06	4.225E-05	1	1	2	Q9UJS0	Q9UJS0 [293-310	0	2273.2461
[K].MSATFIC	1xOxidation [	9.73E-06	4.225E-05	2	3	3	Q13885; P68	Q13885 [363-379]	0	2482.3507
[K].MSATFIC	1xTMTpro [K	2.398E-06	4.225E-05	2	3	5	Q13885; P68	Q13885 [363-379]	0	2466.3558
[R].MSASQL	1xCarbamido	1.444E-07	4.225E-05	1	1	3	P35221	P35221 [452-478]	0	3739.1067
[R].MSASQL	1xCarbamido	1.231E-07	4.225E-05	1	1	4	P35221	P35221 [452-478]	0	3723.1117

[K].EFMPPG	3xTMTpro [K	1.433E-05	4.225E-05	1	1	1	P49591	P49591 [458-483]	1	4166.408
[K].MSASDP	1xOxidation [	2.187E-05	4.225E-05	1	1	1	P23381	P23381 [350-366]	0	2409.2463
[K].MSASDP	1xTMTpro [K	3.075E-05	4.225E-05	1	1	1	P23381	P23381 [350-366]	0	2393.2514
[K].MQILEGI	1xTMTpro [K	3.405E-05	4.225E-05	1	1	2	P05154	P05154 [89-101]	0	2065.2335
[R].LAPILCD	1xCarbamido	1.27E-06	4.225E-05	1	1	2	O43264	O43264 [563-582]	0	2466.3386
[K].EFNAETI	1xCarbamido	4.399E-07	4.225E-05	1	1	154	P02768	P02768 [525-543]	0	2868.437
[K].EFNAETI	1xCarbamido	1.737E-06	4.225E-05	1	1	3	P02768	P02768 [525-545]	1	3153.5807
[K].MRPSTD	1xOxidation [	3.683E-05	4.225E-05	2	2	2	P0C0L4; P0(	P0C0L4 [167-185]	0	2464.2607
[K].MRPSTD	1xTMTpro [N	2.023E-05	4.225E-05	2	2	2	P0C0L4; P0(	P0C0L4 [167-185]	0	2448.2658
[K].MREEYD	2xTMTpro [K	2.644E-05	4.225E-05	1	1	1	Q16666	Q16666 [39-55]	2	3053.6468
[R].MREEEN	1xTMTpro [N	3.213E-05	4.225E-05	1	1	1	Q93034	Q93034 [699-711]	1	1906.9976
[R].MREDYD	1xTMTpro [N	1.906E-05	4.225E-05	1	1	1	Q9Y5S9	Q9Y5S9 [50-68]	1	2527.1326
[K].MQVDQE	2xTMTpro [K	4.635E-06	4.225E-05	1	1	1	P34932	P34932 [522-557]	1	4999.3989
[K].MQTDKF	2xTMTpro [K	2.68E-06	4.225E-05	1	1	1	Q15417	Q15417 [193-212]	0	3196.7162
[R].MQQQLD	1xOxidation [	3.016E-05	4.225E-05	1	1	3	P02545	P02545 [352-366]	0	2518.3355
[R].LAPITSD	1xTMTpro [K	2.179E-07	4.225E-05	1	1	14	P14618	P14618 [401-422]	0	2783.5322
[R].IAPVHID	1xTMTpro [K	1.322E-05	4.225E-05	1	1	1	Q9NTI5	Q9NTI5 [608-623]	0	2315.383
[K].MQLEEQ	1xTMTpro [K	5.375E-05	4.225E-05	1	1	1	Q6NUQ1	Q6NUQ1 [86-101]	0	2453.3817
[R].MQKEIT/	2xTMTpro [K	3.886E-05	4.225E-05	3	6	2	P62736; P68	P62736 [315-328]	1	2461.4339
[K].MPYECG	2xCarbamido	4.33E-05	4.225E-05	1	1	1	P10643	P10643 [717-733]	0	2331.0375
[-].MPVTEKD	1xMet-loss [N	4.184E-05	4.225E-05	1	1	1	O75369	O75369 [1-15]	1	2511.4275
[R].MPVIKPD	2xTMTpro [K	3.551E-05	4.225E-05	1	1	2	P05165	P05165 [549-564]	0	2752.6252
[K].LAQDDLI	1xTMTpro [N	1.601E-05	4.225E-05	1	1	2	Q9BYD3	Q9BYD3 [182-210	0	3658.8382
[K].EFYGEN	1xTMTpro [K	1.078E-05	4.225E-05	1	1	1	P51648	P51648 [267-281]	1	2484.2538
[-].MPTGDFI	1xCarbamido	3.961E-05	4.225E-05	1	1	1	O75821	O75821 [1-32]	1	4728.3979
[K].MPTFES	2xTMTpro [K	6.003E-05	4.225E-05	1	1	1	Q13033	Q13033 [148-173]	1	3878.9938
[R].EFYQCD	2xCarbamido	5.738E-05	4.225E-05	1	1	2	P12081	P12081 [170-193]	0	3503.6453
[R].MPSESA	1xTMTpro [K	1.2E-05	4.225E-05	1	1	1	O43660	O43660 [117-135]	0	2550.4457
[K].FSQQQS	1xTMTpro [N	5.188E-06	4.225E-05	1	1	1	Q15642	Q15642 [67-87]	0	2773.4229
[R].FSSAVG	2xCarbamido	4.558E-06	4.225E-05	1	1	1	P53985	P53985 [387-411]	0	2958.648
[K].MPPYDE	1xTMTpro [N	1.812E-05	4.225E-05	1	1	1	P14314	P14314 [356-374]	0	2485.1989
[R].EGAAHA	1xTMTpro [K	3.249E-06	4.225E-05	1	1	3	P47755	P47755 [48-66]	0	2715.4386
[R].EGAAHA	1xTMTpro [K	3.968E-06	4.225E-05	1	1	2	P52907	P52907 [48-66]	0	2733.395

[R].EGADML	1xTMTpro [K	9.89E-07	4.225E-05	1	1	3	P13716	P13716 [244-262]	0	2743.4841
[K].MPNVPN	2xTMTpro [K	1.569E-05	4.225E-05	1	1	1	O15126	O15126 [53-78]	0	3819.0753
[-].MPNSEP/	1xMet-loss [I	1.587E-06	4.225E-05	1	1	1	P23381	P23381 [1-24]	0	2789.5004
[K].EGAFSN	1xTMTpro [K	6.022E-05	4.225E-05	2	2	1	Q9NR30; Q9	Q9NR30 [185-199	0	2277.2258
[R].EGAKDII	2xTMTpro [K	2.418E-05	4.225E-05	1	1	1	Q09666	Q09666 [168-181]	1	2448.3802
[R].MPGQTE	1xTMTpro [N	2.849E-05	4.225E-05	1	1	2	Q9Y4W2	Q9Y4W2 [655-68	0	3843.845
[-].MPGPTPS	1xMet-loss [I	2.529E-05	4.225E-05	1	1	2	P60468	P60468 [1-16]	0	1674.873
[K].EGAIHRE	1xTMTpro [N	1.648E-05	4.225E-05	2	2	2	P0C0L4; P0(	P0C0L4 [936-954]	1	2594.3646
[-].MPPPQG	1xMet-loss [I	1.902E-06	4.225E-05	1	1	1	Q8WZA9	Q8WZA9 [1-20]	0	2469.4361
[K].MSEEDE	1xCarbamido	2.687E-05	4.225E-05	1	1	1	Q9UKS6	Q9UKS6 [393-410	0	2429.0781
[K].MQAHIQ	1xTMTpro [N	2.707E-07	4.225E-05	1	1	5	P35580	P35580 [948-966]	0	2545.216
[R].FSSCGG	1xCarbamido	9.53E-07	4.225E-05	1	1	2	P04264	P04264 [46-65]	0	2069.9419
[K].MQHNVL	1xTMTpro [K	2.102E-05	4.225E-05	1	1	2	Q13618	Q13618 [717-731]	0	2346.3459
[K].LAPVPSI	2xTMTpro [K	2.275E-05	4.225E-05	1	1	1	Q96JM3	Q96JM3 [199-219	0	3066.8019
[K].MQHLNP	1xCarbamido	7.366E-07	4.225E-05	1	1	1	Q96AC1	Q96AC1 [498-525	0	3562.8205
[K].EFTEAFI	1xCarbamido	7.802E-06	4.225E-05	1	1	1	Q96PD5	Q96PD5 [371-385	0	2049.0547
[K].MQHLNP	1xCarbamido	1.417E-06	4.225E-05	1	1	2	Q96AC1	Q96AC1 [498-525	0	3546.8256
[R].MQEVYN	1xOxidation [	2.218E-05	4.225E-05	1	1	2	P09960	P09960 [521-536]	0	2262.1144
[K].LAQAAQ	1xTMTpro [N	1.009E-05	4.225E-05	1	1	3	Q9Y490	Q9Y490 [2044-20	0	1720.9877
[K].FSSGYY	1xTMTpro [N	1.334E-05	4.225E-05	1	1	2	P04844	P04844 [341-357]	0	2301.0995
[K].MQELLM	1xTMTpro [K	1.872E-05	4.225E-05	1	1	1	Q9NZN4	Q9NZN4 [359-370	0	2072.1164
[K].LAQANG	1xTMTpro [N	2.295E-05	4.225E-05	1	1	3	P06733	P06733 [359-372]	0	1829.9764
[K].MQAHIQ	1xOxidation [	1.843E-06	4.225E-05	1	1	3	P35580	P35580 [948-966]	0	2561.2109
[K].EFTPEV(	1xTMTpro [K	3.893E-05	4.225E-05	2	2	6	P69891; P69	P69891 [122-133]	0	2058.1152
[K].EFTPQM	1xTMTpro [K	4.595E-05	4.225E-05	1	1	2	P02042	P02042 [122-133]	0	2050.0923
[K].MQEEEE	1xTMTpro [K	3.163E-05	4.225E-05	1	1	1	P24928	P24928 [1269-128	1	2835.4036
[R].EFTQGV	1xTMTpro [K	2.159E-05	4.225E-05	1	1	1	Q8WWC4	Q8WWC4 [270-28	0	2256.2632
[R].FSSDFD	1xTMTpro [K	5.581E-05	4.225E-05	1	1	2	P02549	P02549 [375-389]	0	2400.1673
[R].MQEAMT	1xOxidation [	2.341E-06	4.225E-05	1	1	4	P18206	P18206 [587-607]	0	2982.4932
[R].EFVAVT(	1xTMTpro [N	5.226E-05	4.225E-05	1	1	1	Q9UNZ2	Q9UNZ2 [11-22]	0	1626.8294
[R].MQEAMT	1xTMTpro [K	4.125E-06	4.225E-05	1	1	2	P18206	P18206 [587-607]	0	2966.4982
[R].MQAQM(	1xTMTpro [N	5.959E-06	4.225E-05	1	1	2	Q15019	Q15019 [339-361]	0	2688.207
[R].MQAQAN	1xTMTpro [N	1.28E-06	4.225E-05	1	1	1	Q6NUK1	Q6NUK1 [413-432	0	2525.2998

[R].EFVGQL	1xTMTpro [N	6.127E-06	4.225E-05	1	1	2	Q6PCB0	Q6PCB0 [55-72]	0	2142.2606
[K].EFTPPV(	1xTMTpro [K	4.071E-05	4.225E-05	1	1	117	P68871	P68871 [122-133]	0	1987.1144
[K].IAQDLEN	1xTMTpro [K	7.845E-06	4.225E-05	1	1	4	P26038	P26038 [194-209]	0	2499.3449
[-].MSETAPA	1xMet-loss [M	2.388E-05	4.225E-05	1	1	2	P16403	P16403 [1-17]	0	2087.1628
[K].MSGGW	1xTMTpro [K	5.455E-05	4.225E-05	1	1	1	Q07021	Q07021 [105-119]	0	2230.1669
[K].MVEEGH	1xTMTpro [N	1.642E-05	4.225E-05	1	1	1	P02549	P02549 [859-873]	0	1951.9503
[-].MVDYYE\	1xMet-loss [M	5.047E-05	4.225E-05	1	1	1	O75190	O75190 [1-12]	0	1644.8916
[R].MVDVVE	1xTMTpro [K	3.304E-06	4.225E-05	1	1	1	P33993	P33993 [621-635]	1	2354.2881
[R].EFDGIGA	1xTMTpro [N	1.443E-05	4.225E-05	1	1	2	P54803	P54803 [54-69]	0	1784.9098
[R].EFDLTKE	1xTMTpro [K	5.504E-06	4.225E-05	1	1	1	O60313	O60313 [347-360]	1	2258.2524
[K].EFDPTIT	1xTMTpro [N	4.739E-06	4.225E-05	1	1	3	Q05682	Q05682 [120-135]	0	2053.0773
[K].FTAVAQ	2xTMTpro [K	1.224E-05	4.225E-05	1	1	2	P07093	P07093 [296-309]	1	2473.4846
[R].MVDENC	1xCarbamido	1.435E-06	4.225E-05	1	1	1	P27708	P27708 [782-809]	1	4162.1075
[R].EFDSSTI	1xTMTpro [N	3.981E-05	4.225E-05	1	1	1	Q96EC8	Q96EC8 [48-59]	0	1687.8458
[R].EFEDAFI	1xTMTpro [N	2.471E-05	4.225E-05	1	1	1	P41252	P41252 [547-565]	0	2475.1999
[K].MVCGPS	1xCarbamido	5.278E-05	4.225E-05	1	1	1	P26927	P26927 [560-573]	0	2097.2056
[K].FTASAGI	1xTMTpro [K	1.394E-06	4.225E-05	1	1	5	P06733	P06733 [307-326]	0	2641.4693
[K].FTALSSE	1xTMTpro [K	9.09E-06	4.225E-05	1	1	2	Q92538	Q92538 [984-1004	0	2832.5275
[R].MVAAAT	1xCarbamido	1.318E-06	4.225E-05	1	1	1	Q9Y490	Q9Y490 [2399-24	0	3164.6072
[K].LAMDLP(	1xTMTpro [N	3.042E-05	4.225E-05	1	1	1	Q9NVX2	Q9NVX2 [448-470	0	2846.3739
[R].MTVDES	1xCarbamido	2.275E-05	4.225E-05	1	1	1	O75083	O75083 [371-389]	0	2464.1325
[R].MTVDES	1xCarbamido	1.529E-06	4.225E-05	1	1	2	O75083	O75083 [371-389]	0	2448.1376
[K].LAMQEF	1xTMTpro [N	1.121E-05	4.225E-05	1	1	5	P06733	P06733 [163-179]	0	2212.1942
[K].MTTETA	1xTMTpro [K	1.462E-05	4.225E-05	1	1	1	Q92616	Q92616 [1853-18	0	2655.27
[K].LAMQEF	1xOxidation [	1.581E-05	4.225E-05	1	1	15	P06733	P06733 [163-179]	0	2228.1891
[K].LAMQEF	2xOxidation [	5.27E-05	4.225E-05	1	1	3	P06733	P06733 [163-179]	0	2244.184
[R].FTAVED(	2xCarbamido	4.947E-05	4.225E-05	1	1	5	Q13642	Q13642 [200-214]	0	2569.2235
[-].MVEKEEA	1xMet-loss [M	1.171E-06	4.225E-05	1	1	1	Q9UBE0	Q9UBE0 [1-21]	1	2775.3929
[R].FTAVYDA	1xCarbamido	8.924E-06	4.225E-05	1	1	1	P24043	P24043 [3008-302	0	2866.459
[R].MVEPEN	1xTMTpro [N	2.776E-06	4.225E-05	1	1	1	Q14160	Q14160 [816-837]	0	2833.4361
[R].MVIPGG	1xTMTpro [K	7.191E-08	4.225E-05	1	1	1	Q14194	Q14194 [64-94]	0	3966.0385
[K].MVIPGGI	1xTMTpro [K	3.728E-05	4.225E-05	1	1	3	Q14195	Q14195 [64-81]	0	2678.4442
[-].MVLLESE	1xMet-loss [M	4.525E-05	4.225E-05	1	1	2	P37108	P37108 [1-15]	0	1982.1129

[K].EEVGEE	1xTMTpro [K	2.547E-05	4.225E-05	1	1	1	P35579	P35579 [48-63]	0	2352.279
[R].FTCEMF	1xCarbamido	1.098E-05	4.225E-05	1	1	1	P60604	P60604 [73-87]	0	2188.0275
[R].MVLAAA	1xOxidation [	4.926E-06	4.225E-05	1	1	3	P31930	P31930 [229-248]	0	2716.5311
[R].MVLAAA	1xTMTpro [K	1.245E-07	4.225E-05	1	1	1	P31930	P31930 [229-248]	0	2700.5362
[-].MVKLTAE	1xMet-loss [N	1.381E-06	4.225E-05	1	1	1	P09661	P09661 [1-20]	1	2726.5696
[K].MVHNGII	1xCarbamido	1.93E-05	4.225E-05	1	1	1	P52209	P52209 [185-206]	0	3260.622
[K].EEVVTVI	1xTMTpro [K	9.344E-06	4.225E-05	1	1	20	P09211	P09211 [31-45]	0	2342.2735
[K].MTNPAIC	1xTMTpro [N	4.556E-05	4.225E-05	1	1	2	Q9NUQ9	Q9NUQ9 [147-16	0	2023.9867
[-].MVHLTPE	1xMet-loss [N	4.367E-07	4.225E-05	1	1	1	P68871	P68871 [1-31]	2	4074.2804
[K].MVGVPA	1xTMTpro [N	1.107E-05	4.225E-05	1	1	3	P40939	P40939 [191-205]	0	1865.9971
[-].MVGVKPY	1xMet-loss [N	3.936E-07	4.225E-05	1	1	3	O43396	O43396 [1-23]	0	2807.5183
[K].EEYNGV	1xTMTpro [K	1.799E-05	4.225E-05	1	1	2	Q9H4A4	Q9H4A4 [261-276	0	2436.279
[R].EEYQPA	2xTMTpro [K	3.824E-06	4.225E-05	1	1	1	Q9BVK6	Q9BVK6 [74-95]	1	3391.7912
[-].MVGQLSI	1xMet-loss [N	1.425E-05	4.225E-05	1	1	1	P27694	P27694 [1-16]	0	2124.2342
[K].EFADSLC	1xTMTpro [K	5.322E-05	4.225E-05	2	3	1	P62820; Q9H	P62820 [141-156]	0	2333.2884
[K].MVGGIAC	1xTMTpro [N	9.14E-06	4.225E-05	1	1	1	Q9Y490	Q9Y490 [2494-25	0	2134.1684
[-].MVGGEA	1xMet-loss [N	2.984E-06	4.225E-05	1	1	1	Q96HQ2	Q96HQ2 [1-18]	0	1918.0565
[K].MVGDVT	1xOxidation [	1.142E-05	4.225E-05	1	3	2	Q01629	Q01629 [88-103]	0	2194.1669
[R].MVEYEE	1xTMTpro [K	6.048E-06	4.225E-05	1	1	1	P61020	P61020 [142-165]	0	3363.6256
[K].MVGVPA	1xOxidation [	5.419E-05	4.225E-05	1	1	3	P40939	P40939 [191-205]	0	1881.992
[K].MTLVASI	1xOxidation [	9.431E-06	4.225E-05	1	1	2	Q13813	Q13813 [1890-19	0	2833.5513
[K].MTLSNP	1xOxidation [	1.272E-05	4.225E-05	1	1	1	P23434	P23434 [147-166]	0	2940.435
[K].IANDNSL	1xTMTpro [N	2.64E-05	4.225E-05	1	1	1	P17174	P17174 [61-81]	0	2703.4425
[R].MSVEAD	1xTMTpro [N	3.037E-05	4.225E-05	2	2	2	P02533; P08	P02533 [212-223]	1	1664.9073
[K].IAPALCC	2xCarbamido	3.551E-06	4.225E-05	1	1	7	O94788	O94788 [197-227	0	4212.3779
[K].MSVDNL	1xTMTpro [N	3.305E-05	4.225E-05	1	1	2	P42331	P42331 [307-322]	0	2019.1592
[K].IAPAVVH	1xTMTpro [N	1.969E-05	4.225E-05	1	1	1	Q92743	Q92743 [179-190	0	1669.012
[-].MSTGTFV	1xTMTpro [N	1.747E-05	4.225E-05	1	1	2	P49189	P49189 [1-15]	0	2004.0544
[R].LAPDYD	1xTMTpro [K	9.659E-06	4.225E-05	1	1	7	P62750	P62750 [140-152]	0	2013.1148
[K].FSVCVLC	2xCarbamido	2.636E-06	4.225E-05	1	1	3	P62906	P62906 [63-78]	0	2501.2409
[K].MSSTFIG	1xTMTpro [K	2.578E-06	4.225E-05	1	1	2	Q13509	Q13509 [363-379	0	2482.3507
[K].MSSEVV	1xTMTpro [N	5.6E-05	4.225E-05	1	1	1	Q9GZZ9	Q9GZZ9 [43-55]	0	1774.8601
[K].MSQYLE	1xTMTpro [N	4.721E-06	4.225E-05	1	1	2	P00387	P00387 [127-143]	0	2352.1535

[K].MSVQPT	1xTMTpro [N	2.382E-07	4.225E-05	1	1	3	P06748	P06748 [81-101]	0	2531.4227
[K].MSQVAP	1xOxidation [	6.334E-06	4.225E-05	1	1	1	O00567	O00567 [289-307	0	2177.1919
[K].MSNYDT	1xOxidation [	4.737E-05	4.225E-05	1	1	1	P49588	P49588 [255-272]	0	2805.4301
[K].MSNYDT	1xTMTpro [K	2.625E-05	4.225E-05	1	1	2	P49588	P49588 [255-272]	0	2789.4352
[-].MSNPFAI	1xMet-loss+/	5.037E-06	4.225E-05	1	1	1	P21399	P21399 [1-19]	0	2567.4113
[K].MSINAEE	1xOxidation [	3.538E-05	4.225E-05	1	1	1	P05023	P05023 [178-194]	0	2455.361
[K].MSINAEE	1xTMTpro [K	1.004E-05	4.225E-05	1	1	4	P05023	P05023 [178-194]	0	2439.366
[K].EFLLSLD	1xTMTpro [K	2.636E-06	4.225E-05	1	1	1	Q9BXP5	Q9BXP5 [154-171	1	2674.4431
[-].MSLHQFI	1xCarbamido	8.139E-06	4.225E-05	1	1	1	Q92747	Q92747 [1-18]	0	2468.2828
[R].FSTVAG	1xTMTpro [N	1.024E-05	4.225E-05	1	1	2	P04040	P04040 [113-127]	0	1787.9095
[R].FSTMPF	2xCarbamido	2.371E-05	4.225E-05	1	1	2	P08572	P08572 [1541-15	0	2752.2529
[K].MSGGW	1xOxidation [	2.388E-05	4.225E-05	1	1	1	Q07021	Q07021 [105-119	0	2246.1619
[K].MSQVAP	1xTMTpro [N	7.075E-06	4.225E-05	1	1	2	O00567	O00567 [289-307	0	2161.197
[-].MSETAP/	1xMet-loss [N	4.417E-06	4.225E-05	1	1	1	P10412	P10412 [1-17]	0	2087.1628
[K].MSVQPT	1xOxidation [	1.026E-06	4.225E-05	1	1	6	P06748	P06748 [81-101]	0	2547.4176
[K].EFKDED	1xTMTpro [K	3.13E-05	4.225E-05	1	1	1	Q04446	Q04446 [449-467	1	2954.485
[R].MTLDTL	2xTMTpro [K	1.038E-05	4.225E-05	1	1	2	P55884	P55884 [441-460]	1	3168.7716
[R].MTLDTL	1xTMTpro [K	1E-05	4.225E-05	1	1	2	P55884	P55884 [441-459]	0	2736.4695
[K].MTIAGT	1xTMTpro [N	8.111E-07	4.225E-05	1	1	1	P43304	P43304 [349-380]	0	3867.9394
[R].LANIDEE	1xTMTpro [K	5.545E-05	4.225E-05	1	1	4	P54136	P54136 [547-557]	0	1912.0705
[R].MTGSEF	1xOxidation [	2.002E-05	4.225E-05	1	1	4	P50395	P50395 [424-436]	1	2231.0968
[R].MTGSEF	1xTMTpro [K	8.761E-06	4.225E-05	1	1	1	P50395	P50395 [424-436]	1	2215.1019
[K].EFGDTG	1xTMTpro [N	5.266E-08	4.225E-05	1	1	1	Q02818	Q02818 [325-348	0	3116.4114
[K].EFGDTG	1xTMTpro [N	6.857E-06	4.225E-05	1	1	1	Q02818	Q02818 [325-349	1	3272.5125
[R].MTGSEF	1xTMTpro [K	3.168E-05	4.225E-05	1	1	1	P50395	P50395 [424-435]	0	2059.0008
[K].MTGLVD	1xTMTpro [K	3.853E-05	4.225E-05	1	1	2	P18206	P18206 [709-720]	0	1901.0545
[K].EFKDPLI	2xTMTpro [K	6.1E-05	4.225E-05	1	1	1	Q5BJF2	Q5BJF2 [53-67]	1	2757.5796
[K].EFGGGH	2xTMTpro [K	5.141E-05	4.225E-05	1	1	2	Q6IBS0	Q6IBS0 [111-126]	1	2646.5072
[R].IANLQTD	1xTMTpro [N	2.109E-05	4.225E-05	1	1	5	P21333	P21333 [64-76]	0	1719.956
[R].MTELAD	1xTMTpro [K	2.072E-06	4.225E-05	1	1	1	Q12768	Q12768 [450-465	0	2372.3503
[R].MTEEPL	2xCarbamido	3.719E-06	4.225E-05	1	1	1	P11310	P11310 [149-173]	0	3294.601
[R].MTDQEA	1xOxidation [	5.59E-05	4.225E-05	1	1	6	P06748	P06748 [278-291]	0	2140.0453
[R].MTDQEA	1xTMTpro [N	1.944E-05	4.225E-05	1	1	3	P06748	P06748 [278-291]	0	2124.0503

[K].MTANSV	1xCarbamido	2.083E-05	4.225E-05	1	1	4	P10155	P10155 [288-307]	0	2772.4403
[R].EFHGLG	1xCarbamido	1.601E-05	4.225E-05	1	1	2	P12235	P12235 [153-163]	0	1897.0497
[R].MTAEEM	1xTMTpro [K	1.879E-05	4.225E-05	1	1	1	P23193	P23193 [223-234]	0	1963.0008
[K].EFHQAG	2xCarbamido	1.215E-06	4.225E-05	1	2	2	A0A0B4J2D5	A0A0B4J2D5 [165	0	3192.8206
[K].LAPALAT	1xTMTpro [K	6.334E-06	4.225E-05	1	1	3	P30837	P30837 [196-209]	0	1994.1964
[K].IANLLKP	2xTMTpro [K	1.272E-05	4.225E-05	1	1	2	P09455	P09455 [33-53]	1	3330.9388
[R].FTDDTFI	1xTMTpro [K	6.015E-06	4.225E-05	1	1	2	Q9NP72	Q9NP72 [28-46]	0	2710.4107
[-].MPGIVEL	1xMet-loss [N	7.631E-06	4.225E-05	1	1	2	P51970	P51970 [1-19]	1	2920.7427
[-].MPGLVDS	1xMet-loss [N	2.819E-05	4.225E-05	1	1	1	Q14061	Q14061 [1-17]	0	2273.2269
[K].LAQSEY	1xTMTpro [K	2.23E-05	4.225E-05	1	1	1	Q9UPT5	Q9UPT5 [374-392	0	2841.5755
[K].EGITTYF	1xCarbamido	1.049E-05	4.225E-05	1	1	1	Q9NY33	Q9NY33 [166-182	0	2532.2242
[R].IAQWQS	1xTMTpro [K	3.719E-06	4.225E-05	1	1	4	P01023	P01023 [175-188]	0	2213.2574
[K].LASAFTE	1xTMTpro [N	2.89E-05	4.225E-05	1	1	1	Q9UHB9	Q9UHB9 [221-236	0	2144.1307
[K].MIINEELI	1xTMTpro [N	2.644E-05	4.225E-05	1	2	1	Q99613	Q99613 [825-846]	0	2860.469
[R].EGIVTAT	1xTMTpro [K	3.601E-05	4.225E-05	1	1	2	Q12931	Q12931 [450-461]	0	1912.0883
[K].MILIQDG	1xOxidation [	2.146E-06	4.225E-05	1	1	2	Q92945	Q92945 [267-284]	0	2666.4791
[K].MLLLEILI	1xTMTpro [K	3.619E-05	4.225E-05	1	1	1	Q96HE7	Q96HE7 [342-352	0	1960.2161
[R].EGMAAL	1xTMTpro [N	2.542E-05	4.225E-05	1	1	2	P49748	P49748 [616-632]	0	2326.1457
[R].EGMAAL	1xOxidation [	1.934E-05	4.225E-05	1	1	1	P49748	P49748 [616-632]	0	2342.1406
[K].MLLDPM	1xOxidation [	2.797E-06	4.225E-05	1	1	2	P49368	P49368 [49-68]	0	2451.2729
[K].MLLDPM	1xTMTpro [N	4.557E-07	4.225E-05	1	1	3	P49368	P49368 [49-68]	0	2435.278
[R].MLIDAQ	1xTMTpro [K	1.47E-05	4.225E-05	1	1	1	P52306	P52306 [428-442]	0	2196.219
[R].MLLCEA	1xCarbamido	3.52E-05	4.225E-05	1	1	2	P54136	P54136 [635-647]	0	2031.1296
[R].MLLCEA	1xCarbamido	2.789E-05	4.225E-05	1	1	2	P54136	P54136 [635-647]	0	2015.1347
[R].MLLADQ	1xOxidation [	2.597E-06	4.225E-05	1	1	5	P09211	P09211 [20-45]	1	3920.0932
[R].MLLADQ	2xTMTpro [K	1.353E-06	4.225E-05	1	1	9	P09211	P09211 [20-45]	1	3904.0983
[K].EGMQLIS	2xTMTpro [K	1.937E-05	4.225E-05	1	1	1	Q01082	Q01082 [1336-135	0	2800.5946
[R].MLLADQ	1xTMTpro [K	4.549E-05	4.225E-05	1	1	4	P09211	P09211 [20-30]	0	1885.0497
[K].MLGQMT	1xOxidation [	2.64E-05	4.225E-05	1	1	7	P18206	P18206 [327-339]	0	1797.9158
[K].MLGQMT	1xTMTpro [N	1.872E-05	4.225E-05	1	1	3	P18206	P18206 [327-339]	0	1781.9209
[K].FSLDEEA	1xCarbamido	1.533E-05	4.225E-05	1	1	1	Q9H0D6	Q9H0D6 [722-743	0	2833.4799
[K].EGITGPF	1xCarbamido	4.016E-05	4.225E-05	1	1	1	P20810	P20810 [213-249]	0	4515.3017
[K].MIPCDFL	1xCarbamido	5.545E-05	4.225E-05	1	1	1	P06744	P06744 [401-417]	0	2385.2742

[K].FSLDEE/	1xCarbamid	1.663E-05	4.225E-05	1	1	1	Q9H0D6	Q9H0D6 [722-74	0	2849.4748
[R].FSLSCSI	1xCarbamid	4.635E-06	4.225E-05	1	1	1	Q9Y2T3	Q9Y2T3 [214-231	0	2565.3548
[K].EGIESGI	1xTMTpro [N	2.584E-05	4.225E-05	1	1	1	Q12797	Q12797 [513-526]	0	1708.7945
[K].MISEIDK	2xTMTpro [K	5.691E-05	4.225E-05	1	1	1	P41208	P41208 [72-83]	1	2220.2726
[R].MISDLIA	1xOxidation	8.291E-06	4.225E-05	1	1	4	O43708	O43708 [101-120]	0	2734.6032
[R].MLSAVS	1xCarbamid	3.255E-06	4.225E-05	1	1	2	Q14204	Q14204 [1967-19	0	2265.2014
[K].EGILGQI	1xTMTpro [N	1.53E-05	4.225E-05	1	1	2	P17301	P17301 [534-553]	0	2528.3064
[K].EGILNDI	1xCarbamid	4.265E-06	4.225E-05	1	1	2	P26038	P26038 [108-133]	0	3474.8322
[K].MIQTPD/	1xTMTpro [K	7.355E-06	4.225E-05	1	1	1	Q13155	Q13155 [79-114]	0	4449.3566
[K].EGILSDE	1xCarbamid	1.401E-06	4.225E-05	1	1	3	P15311	P15311 [108-133]	0	3431.8264
[R].MIQMEP	1xCarbamid	3.8E-05	4.225E-05	1	1	1	Q9HCB6	Q9HCB6 [694-71	0	2480.2055
[R].FSHQGV	1xCarbamid	2.057E-05	4.225E-05	1	1	1	P55884	P55884 [371-386]	0	2224.114
[R].EGINIFLI	1xTMTpro [N	2.588E-05	4.225E-05	1	1	2	P61221	P61221 [307-323]	0	2254.2039
[K].EGIPALI	1xTMTpro [K	4.61E-05	4.225E-05	1	1	2	P13639	P13639 [846-857]	0	1940.0985
[R].FSLFGL(	1xTMTpro [N	2.338E-07	4.225E-05	1	1	1	Q9NX62	Q9NX62 [36-64]	0	2892.4811
[K].MLQFVLI	1xTMTpro [K	1.076E-05	4.225E-05	1	1	1	Q99622	Q99622 [50-67]	0	2693.592
[K].MIQALEI	1xTMTpro [N	5.654E-05	4.225E-05	1	1	1	Q7Z406	Q7Z406 [767-779]	0	1880.0271
[K].LAQQQA	1xTMTpro [K	3.857E-08	4.225E-05	1	1	1	Q9BZK7	Q9BZK7 [103-12	0	2663.472
[R].FSLENNI	1xTMTpro [N	5.374E-06	4.225E-05	1	1	1	P42224	P42224 [71-84]	0	2049.1201
[K].LAQQSQ	1xTMTpro [N	2.766E-06	4.225E-05	1	1	1	P19474	P19474 [218-235]	1	2402.3323
[K].EGLPVN	1xTMTpro [K	2.462E-05	4.225E-05	1	1	1	Q9Y2X0	Q9Y2X0 [314-332	0	2662.506
[K].LAQQYY	1xTMTpro [N	2.146E-06	4.225E-05	1	1	2	P25787	P25787 [93-113]	0	2825.5521
[R].IAQSDYI	1xTMTpro [N	2.697E-05	4.225E-05	1	1	6	P04899	P04899 [163-177]	0	2051.1092
[K].EGLNSV	2xTMTpro [K	2.167E-07	4.225E-05	1	1	4	P28289	P28289 [159-189]	0	4426.3446
[K].FSHEEIA	1xOxidation	5.081E-05	4.225E-05	1	1	2	P04075	P04075 [244-258]	0	1996.0493
[R].EGNDLY	1xTMTpro [K	3.273E-06	4.225E-05	1	1	4	P06576	P06576 [242-259]	0	2669.41
[R].EGNDLY	1xOxidation	1.899E-05	4.225E-05	1	1	2	P06576	P06576 [242-259]	0	2685.4049
[K].IASLPQE	1xTMTpro [K	2.136E-05	4.225E-05	1	1	2	Q9NQG5	Q9NQG5 [201-21	0	2377.3834
[R].MLAEDE	1xTMTpro [K	3.787E-05	4.225E-05	1	2	1	P61204	P61204 [110-127]	1	2655.5035
[R].MLADAP	2xCarbamid	1.569E-05	4.225E-05	1	1	2	P19823	P19823 [639-660]	0	3012.4397
[R].MLADAP	2xCarbamid	2.916E-05	4.225E-05	1	1	2	P19823	P19823 [639-660]	0	2996.4448
[K].EGQWD(	2xCarbamid	4.76E-05	4.225E-05	1	1	2	P49792	P49792 [1607-16	0	1812.8692
[R].EGRGES	1xTMTpro [N	2.973E-05	4.225E-05	1	1	1	Q08170	Q08170 [426-441]	1	2038.9709

[R].FSGSGS	1xTMTpro [N	2.631E-06	4.225E-05	2	2	4	P01619; A0A	P01619 [83-98]; A	0	1936.9935
[R].FSGSGS	1xTMTpro [K	9.929E-06	4.225E-05	1	7	4	P06310	P06310 [87-99]	0	1912.0308
[R].EGRPSG	1xTMTpro [K	2.045E-06	4.225E-05	1	1	2	P31943	P31943 [50-68]	0	2715.3969
[R].EGRPSG	1xTMTpro [K	7.932E-07	4.225E-05	1	1	1	P55795	P55795 [50-68]	0	2729.4125
[R].MIAGQV	1xTMTpro [K	6.984E-06	4.225E-05	1	1	9	P07910	P07910 [74-89]	0	2291.3289
[R].FSGSGA	1xTMTpro [K	1.14E-05	4.225E-05	1	2	2	A0A075B6R9	A0A075B6R9 [87-	0	1896.0359
[K].MKQELE	2xTMTpro [K	4.956E-05	4.225E-05	1	1	1	Q9Y5X3	Q9Y5X3 [124-137	1	2611.4986
[K].EGSDGF	2xTMTpro [K	1.401E-05	4.225E-05	1	1	1	P08240	P08240 [175-191]	0	2595.481
[K].MKQELE	2xTMTpro [K	4.273E-06	4.225E-05	1	2	2	Q9UNH7	Q9UNH7 [125-138	1	2625.5142
[R].MKPWFE	2xTMTpro [K	1.188E-06	4.225E-05	1	1	2	O60763	O60763 [259-276]	0	3008.5756
[K].LASLTPC	1xCarbamido	6.744E-06	4.225E-05	1	1	1	Q9Y4W6	Q9Y4W6 [509-534	0	2892.5209
[R].LASIVEQ	1xTMTpro [N	5.628E-06	4.225E-05	1	1	2	P54886	P54886 [95-110]	0	2045.1674
[R].LASPSG	1xCarbamido	8.356E-08	4.225E-05	1	1	2	Q13263	Q13263 [592-629]	0	3862.8936
[K].MKPLMG	2xTMTpro [K	2.21E-05	4.225E-05	1	1	4	P22061	P22061 [205-219]	0	2617.5641
[K].MKPLDG	1xOxidation	5.491E-05	4.225E-05	1	1	3	P12111	P12111 [515-533]	0	2665.4515
[R].EGSGSS	1xTMTpro [K	3.097E-05	4.225E-05	1	1	1	P38646	P38646 [660-671]	0	1731.9005
[K].EGSCPQ	2xCarbamido	3.939E-06	4.225E-05	1	1	3	Q14508	Q14508 [77-94]	0	2393.2025
[K].FSLVGIG	1xTMTpro [N	3.733E-06	4.225E-05	1	1	3	P13796	P13796 [473-488]	0	1980.047
[K].EGQMES	1xTMTpro [K	3.423E-05	4.225E-05	1	1	1	P05543	P05543 [274-287]	0	2092.0546
[R].IASLEVE	1xTMTpro [N	4.177E-05	4.225E-05	1	1	4	P29692	P29692 [84-95]	0	1662.9346
[K].FSHEEIA	1xTMTpro [N	1.725E-05	4.225E-05	1	1	14	P04075	P04075 [244-258]	0	1980.0544
[R].LASEAKI	2xTMTpro [K	2.005E-08	4.225E-05	1	1	4	Q9Y490	Q9Y490 [1896-19	0	3147.783
[R].MLEGGE	1xTMTpro [N	3.381E-05	4.225E-05	1	1	1	Q96S55	Q96S55 [525-537	0	1753.9114
[K].EGNNPA	1xTMTpro [K	5.581E-05	4.225E-05	1	1	2	P05204	P05204 [65-76]	0	1823.9379
[K].EGNPEE	1xTMTpro [K	2.287E-05	4.225E-05	1	1	3	P40121	P40121 [232-243]	0	1925.9948
[R].EGNQEV	1xTMTpro [K	1.158E-05	4.225E-05	1	1	1	P42704	P42704 [1002-102	0	2931.4544
[R].EGNSPS	1xTMTpro [K	8.306E-06	4.225E-05	1	1	1	Q9HCE1	Q9HCE1 [788-808	0	2896.486
[R].MLDNDS	1xTMTpro [N	2.414E-05	4.225E-05	1	1	1	O75175	O75175 [189-201]	0	1778.9642
[R].IASFSQN	1xCarbamido	1.799E-05	4.225E-05	1	1	3	P01042	P01042 [241-262]	1	3424.8391
[R].EGPIDLC	1xCarbamido	3.422E-06	4.225E-05	1	1	1	Q9Y5K5	Q9Y5K5 [183-206	0	3290.7335
[R].MIAGQV	1xOxidation	6.972E-06	4.225E-05	1	1	2	P07910	P07910 [74-89]	0	2307.3238
[R].MLDGLD	1xTMTpro [K	5.006E-05	4.225E-05	1	1	2	Q02880	Q02880 [900-914]	0	2349.2591
[K].EGPPPA	1xTMTpro [K	4.235E-05	4.225E-05	1	1	1	Q3LXA3	Q3LXA3 [421-434	0	2000.1672

[K].LASGVE(	1xTMTpro [K	7.234E-06	4.225E-05	1	1	2	O95747	O95747 [503-517]	0	2068.1054
[K].MLDAED	1xOxidation [	1.613E-05	4.225E-05	1	1	5	P12814	P12814 [221-236]	0	2384.2623
[K].EGPYDV	1xTMTpro [K	2.068E-06	4.225E-05	1	1	5	Q99497	Q99497 [64-89]	0	3192.7396
[R].EGPYSIS	1xTMTpro [N	4.102E-06	4.225E-05	1	1	4	P21333	P21333 [1516-153	0	2214.1249
[K].MLDAED	1xTMTpro [K	1.075E-05	4.225E-05	1	1	4	P12814	P12814 [221-236]	0	2368.2674
[R].FSGSNS	1xTMTpro [N	2.215E-06	4.225E-05	1	2	4	P80748	P80748 [80-95]	0	1916.9997
[K].MIATDPI	1xOxidation [	5.671E-07	4.225E-05	1	1	4	Q99715	Q99715 [1345-13	0	2788.3055
[K].MIATDPI	1xTMTpro [N	2.138E-06	4.225E-05	1	1	2	Q99715	Q99715 [1345-13	0	2772.3106
[R].MLAMPII	1xTMTpro [N	3.048E-05	4.225E-05	1	1	2	Q7Z7F7	Q7Z7F7 [74-89]	0	2135.1047
[K].MIDAGD	1xTMTpro [K	1.808E-05	4.225E-05	1	1	1	Q9P2J5	Q9P2J5 [498-512]	0	2304.2111
[R].EGAPIEF	1xTMTpro [K	9.748E-06	4.225E-05	1	1	1	Q96HS1	Q96HS1 [177-203	0	3645.8469
[K].EGLELPI	3xTMTpro [K	5.691E-05	4.225E-05	2	4	2	P07900; P08	P07900 [547-560]	2	2889.6562
[K].EGLELPI	1xTMTpro [K	3.459E-05	4.225E-05	2	5	6	P07900; P08	P07900 [547-558]	0	2025.052
[K].FSPNTS(	1xTMTpro [N	2.611E-06	4.225E-05	1	1	1	O43684	O43684 [22-40]	0	2475.2839
[K].MNSVVA	1xTMTpro [K	4.022E-05	4.225E-05	1	1	1	P14543	P14543 [1170-118	0	1969.1648
[K].MNSEEE	1xTMTpro [N	5.188E-06	4.225E-05	1	1	1	Q9NR28	Q9NR28 [124-140	0	2309.1403
[R].EGDVLT	1xTMTpro [N	4.533E-05	4.225E-05	1	1	4	P62857	P62857 [52-63]	0	1664.9026
[R].EGDYVL	1xTMTpro [K	2.144E-05	4.225E-05	1	1	2	P53396	P53396 [128-148]	0	2866.4503
[K].MNQVEI	1xCarbamido	1.667E-05	4.225E-05	1	1	2	Q14683	Q14683 [769-782]	0	2135.9697
[K].EGEAVV	1xTMTpro [N	6.003E-05	4.225E-05	1	1	1	P18827	P18827 [101-117]	0	2070.1402
[K].MNPGDL	1xTMTpro [N	6.061E-05	4.225E-05	1	1	1	O00625	O00625 [85-97]	0	1780.8794
[K].EGEEAG	1xTMTpro [K	2.994E-05	4.225E-05	1	1	2	Q16543	Q16543 [353-369]	0	2316.2579
[R].LAQEGS	1xCarbamido	7.338E-07	4.225E-05	1	1	1	Q6XQN6	Q6XQN6 [366-39	0	3796.0446
[K].MNLSEE	1xTMTpro [K	3.331E-08	4.225E-05	1	1	1	P43686	P43686 [352-369]	0	2731.4104
[R].FSPLTTI	1xTMTpro [N	1.67E-05	4.225E-05	1	1	1	P48047	P48047 [101-117]	0	2177.2249
[R].MNEDTG	1xOxidation [	3.152E-05	4.225E-05	1	1	1	Q8N8N7	Q8N8N7 [56-86]	0	3976.9982
[R].MNEDTG	1xTMTpro [K	1.68E-06	4.225E-05	1	1	4	Q8N8N7	Q8N8N7 [56-86]	0	3961.0032
[K].MMVDKI	2xTMTpro [K	1.079E-07	4.225E-05	1	1	1	P48643	P48643 [60-89]	1	4163.1313
[R].MMITSQ	1xTMTpro [K	2.466E-06	4.225E-05	1	1	2	P00403	P00403 [152-171]	0	2835.5757
[R].MMILYC	1xCarbamido	4.337E-05	4.225E-05	1	1	1	O75643	O75643 [326-345]	1	2952.5488
[R].IAQFLSI	1xTMTpro [N	3.902E-06	4.225E-05	1	1	1	P09110	P09110 [103-121]	0	2404.3407
[R].FSPDGE	1xTMTpro [N	7.128E-06	4.225E-05	1	1	2	Q9Y3F4	Q9Y3F4 [273-290	0	2205.0631
[K].EGETCG	2xCarbamido	5.535E-06	4.225E-05	1	1	1	P98095	P98095 [498-516]	0	2713.2577

[K].EGETITE	1xTMTpro [K	2.503E-06	4.225E-05	1	1	2	Q15063	Q15063 [716-731]	0	2373.3521
[R].MNYSDA	1xTMTpro [K	5.509E-05	4.225E-05	1	1	3	O43776	O43776 [391-401]	0	1948.0858
[K].EGDQTS	1xTMTpro [K	1.951E-05	4.225E-05	1	1	2	P25685	P25685 [223-240]	0	2568.4165
[R].LAQEGI	1xTMTpro [N	3.032E-05	4.225E-05	1	1	1	Q08J23	Q08J23 [603-618]	0	2189.1926
[R].MPCAED	2xCarbamido	1.709E-06	4.225E-05	1	1	11	P02768	P02768 [470-490]	0	3126.6282
[R].EGAQYL	1xTMTpro [N	2.333E-05	4.225E-05	1	1	1	P55011	P55011 [865-878]	0	1768.9335
[-].MPGIDKL	1xMet-loss [M	3.538E-06	4.225E-05	1	1	2	Q9UKG1	Q9UKG1 [1-20]	1	2746.5118
[-].MPGHLQ	1xCarbamido	5.786E-06	4.225E-05	1	1	1	Q8NC51	Q8NC51 [1-16]	0	1975.0139
[-].MPGGLLL	1xMet-loss [M	3.125E-06	4.225E-05	1	1	1	P30041	P30041 [1-22]	0	2402.2999
[K].MPGAPE	1xTMTpro [N	4.419E-05	4.225E-05	1	1	2	O75116	O75116 [10-26]	0	1845.9084
[K].EGCEAI	1xCarbamido	8.639E-08	4.225E-05	1	1	3	P07339	P07339 [288-309]	0	2638.3023
[K].EGCEAI	1xCarbamido	1.683E-06	4.225E-05	1	1	2	P07339	P07339 [288-309]	0	2654.2973
[R].MPFPVN	1xTMTpro [K	1.541E-05	4.225E-05	1	1	1	P09936	P09936 [179-195]	0	2451.3198
[K].FSQLAE	1xTMTpro [K	3.607E-05	4.225E-05	1	1	1	Q96EY1	Q96EY1 [135-150	0	2436.3154
[R].EGDAHF	1xTMTpro [N	1.979E-07	4.225E-05	1	1	1	Q5T653	Q5T653 [178-202	0	2846.4968
[K].EGETYF	1xTMTpro [N	1.828E-05	4.225E-05	1	1	2	Q9NYU2	Q9NYU2 [982-998	0	2200.1457
[R].EGDALE	1xCarbamido	1.497E-05	4.225E-05	1	1	1	Q9BY67	Q9BY67 [259-282	0	3307.7674
[-].MPEFLED	1xMet-loss [M	5.123E-05	4.225E-05	2	2	4	P42167; P42	P42167 [1-13]; P4	0	1983.1294
[K].IAQDLEM	1xOxidation	2.136E-05	4.225E-05	1	1	1	P26038	P26038 [194-209]	0	2515.3398
[R].LAQDPF	1xTMTpro [K	2.132E-05	4.225E-05	1	1	3	Q14764	Q14764 [92-107]	0	2424.367
[R].EGDFGC	1xCarbamido	5.166E-05	4.225E-05	1	1	2	P23634	P23634 [19-30]	0	1717.8209
[K].MPCQLH	1xCarbamido	2.692E-05	4.225E-05	1	1	2	P17655	P17655 [638-649]	0	1755.9681
[R].MPCAED	2xCarbamido	5.056E-06	4.225E-05	1	1	1	P02768	P02768 [470-499]	2	4414.3753
[R].MPCAED	2xCarbamido	2.124E-05	4.225E-05	1	1	1	P02768	P02768 [470-496]	1	3797.952
[R].MPCAED	2xCarbamido	8.719E-07	4.225E-05	1	1	2	P02768	P02768 [470-496]	1	3781.9571
[K].EGDLITL	1xTMTpro [N	5.964E-05	4.225E-05	1	1	1	Q9UQB8	Q9UQB8 [398-41	0	1730.0019
[R].MPCAED	2xCarbamido	5.189E-08	4.225E-05	1	1	15	P02768	P02768 [470-490]	0	3142.6231
[R].MPEVPG	2xCarbamido	3E-05	4.225E-05	1	1	1	Q9H269	Q9H269 [167-187	0	2735.3274
[K].MMDYLQ	1xOxidation	3.168E-05	4.225E-05	1	1	2	Q14697	Q14697 [338-354]	0	2248.0545
[K].MMDYLQ	1xTMTpro [N	3.604E-06	4.225E-05	1	1	2	Q14697	Q14697 [338-354]	0	2232.0596
[K].EGFGHL	1xTMTpro [K	7.071E-07	4.225E-05	1	1	2	P02679	P02679 [239-258]	0	2815.4547
[R].IAQLEEE	1xTMTpro [N	3.111E-07	4.225E-05	1	1	7	P35579	P35579 [1731-175	0	2776.3808
[R].IAQLEEE	1xTMTpro [N	1.468E-06	4.225E-05	1	1	2	P35580	P35580 [1738-175	0	2836.3842

[R].IAQLEEE	1xOxidation	1.578E-05	4.225E-05	1	1	2	P35580	P35580 [1738-175	0	2852.3791
[R].EGGWD:	1xTMTpro [K	1.438E-05	4.225E-05	1	1	1	P28288	P28288 [558-576]	0	2703.3216
[K].MLVLDE.	1xTMTpro [K	7.395E-06	4.225E-05	1	1	2	P38919	P38919 [183-195]	0	2129.1478
[K].FSMPGF	2xTMTpro [K	4.997E-05	4.225E-05	1	1	1	Q09666	Q09666 [3030-30	1	2946.6215
[K].MIVDPVE	1xTMTpro [K	2.453E-05	4.225E-05	1	1	3	Q13263	Q13263 [378-390]	0	2090.127
[K].EGHQVA	1xTMTpro [N	1.117E-05	4.225E-05	1	2	1	Q9UMR2	Q9UMR2 [356-37	0	2218.1279
[K].MLVDDI(	1xOxidation	3.441E-06	4.225E-05	1	1	2	P17987	P17987 [44-63]	0	2728.4934
[K].MLVDDI(	1xTMTpro [K	1.473E-06	4.225E-05	1	1	4	P17987	P17987 [44-63]	0	2712.4985
[R].IAQLEEE	1xOxidation	5E-06	4.225E-05	1	1	3	P35749	P35749 [1738-175	0	2771.2671
[K].IAQLEEQ	1xTMTpro [K	1.184E-05	4.225E-05	1	1	5	P35579	P35579 [1816-18	0	2139.1789
[K].EGKPTIV	2xTMTpro [K	3.983E-06	4.225E-05	1	1	3	O75937	O75937 [144-159]	0	2758.5331
[K].IAQLEEQ	1xTMTpro [N	2.849E-05	4.225E-05	1	1	2	P35749	P35749 [1823-18	0	1846.983
[R].LAQIHIQ	1xCarbamid	1.662E-06	4.225E-05	1	1	2	O75935	O75935 [129-148]	0	3005.5858
[K].EGICALC	1xCarbamid	1.396E-05	4.225E-05	1	1	1	P13797	P13797 [101-126]	0	3393.6248
[K].MLTESG	1xTMTpro [N	1.478E-05	4.225E-05	1	2	1	P07919	P07919 [9-34]	0	3309.5375
[R].MLTATQ	1xOxidation	1.464E-05	4.225E-05	1	1	2	Q6UX71	Q6UX71 [192-212	0	2646.3591
[R].MLTATQ	1xTMTpro [N	1.394E-05	4.225E-05	1	1	1	Q6UX71	Q6UX71 [192-212	0	2630.3641
[R].EGLEDK	1xTMTpro [K	3.146E-05	4.225E-05	1	1	1	Q9NWS0	Q9NWS0 [148-16	1	2612.3964
[R].FSLVPHI	1xTMTpro [K	1.561E-05	4.225E-05	1	1	1	Q96TA1	Q96TA1 [89-104]	0	2501.4048
[K].MLSSGV	1xCarbamid	2.958E-05	4.225E-05	1	1	1	Q8TC12	Q8TC12 [27-42]	0	2273.2489
[K].IAQLEEQ	1xTMTpro [K	1.46E-06	4.225E-05	1	1	3	P35579	P35579 [1816-18	1	2424.3226
[K].EGLELPE	2xTMTpro [K	9.276E-06	4.225E-05	2	5	2	P07900; P08	P07900 [547-559]	1	2457.3541
[K].EGGQYC	1xCarbamid	9.748E-06	4.225E-05	1	1	1	P55084	P55084 [448-474]	0	3329.6902
[R].EGGQVY	1xTMTpro [N	1.48E-05	4.225E-05	1	1	1	P28065	P28065 [127-141]	0	1842.9703
[R].FSPATHI	1xCarbamid	1.284E-05	4.225E-05	1	1	2	P00747	P00747 [137-153]	0	2298.078
[-].MMCGAP	1xAcetyl [N-	1.927E-05	4.225E-05	1	1	4	P04080	P04080 [1-24]	0	2917.3926
[R].MMAIAPI	1xTMTpro [N	1.903E-05	4.225E-05	1	1	1	Q9Y2X3	Q9Y2X3 [279-297	0	2263.2296
[K].MMADEA	2xOxidation	4.721E-05	4.225E-05	1	1	2	Q13011	Q13011 [232-245]	0	1772.8842
[K].MMADEA	1xOxidation	3.594E-05	4.225E-05	1	1	2	Q13011	Q13011 [232-245]	0	1756.8893
[K].MMADEA	1xTMTpro [N	1.92E-05	4.225E-05	1	1	1	Q13011	Q13011 [232-245]	0	1740.8944
[RK].EGFVE	1xTMTpro [K	3.508E-05	4.225E-05	2	2	3	O60518; O0	O60518 [702-713]	0	2036.1196
[R].FSNTGE	1xTMTpro [K	4.705E-05	4.225E-05	1	1	1	Q15393	Q15393 [896-911]	0	2393.2997
[K].EGFYDL:	1xCarbamid	1.535E-05	4.225E-05	1	1	3	P07942	P07942 [442-456]	0	2359.1408

[K].FSNQET	1xTMTpro [N	9.606E-06	4.225E-05	1	1	1	P11908	P11908 [35-49]	0	1986.0099
[K].EGGQYG	1xCarbamido	5.756E-07	4.225E-05	1	1	1	P55084	P55084 [448-474]	0	3313.6953
[K].MLYVPV	1xOxidation [	5.464E-05	4.225E-05	1	1	2	A0AVT1	A0AVT1 [992-100	0	1967.1102
[K].FSNQET	1xCarbamido	3.016E-05	4.225E-05	1	1	1	P60891	P60891 [35-49]	0	2059.0085
[K].MLYDME	1xTMTpro [N	1.845E-05	4.225E-05	1	1	2	Q93052	Q93052 [400-415]	0	2252.0323
[K].MIVVLGE	1xTMTpro [K	3.715E-05	4.225E-05	1	1	1	P53396	P53396 [712-726]	0	2246.2598
[K].LAQGEY	1xTMTpro [K	1.505E-05	4.225E-05	1	1	1	Q9ULC5	Q9ULC5 [547-564	1	2729.5118
[R].MLVTFD	1xTMTpro [N	5.883E-06	4.225E-05	1	1	1	Q13200	Q13200 [829-845]	0	2305.2909
[K].EGGLGP	1xTMTpro [N	1.449E-06	4.225E-05	1	1	4	P32119	P32119 [93-109]	0	2039.182
[R].EGGPLL	1xTMTpro [K	3.995E-05	4.225E-05	1	1	1	Q32P28	Q32P28 [448-464	0	2417.3242
[R].EGGPSQ	1xTMTpro [N	5.098E-05	4.225E-05	1	1	1	P04275	P04275 [1764-17	0	1878.004
[K].EGGQLP	1xTMTpro [N	1.633E-05	4.225E-05	1	1	1	P98160	P98160 [3436-34	0	2050.1001
[R].IAQLEEE	1xTMTpro [N	6.566E-07	4.225E-05	1	1	3	P35749	P35749 [1738-17	0	2755.2722
[R].EGGDFV	2xCarbamido	2.542E-05	4.225E-05	1	1	2	Q2TAY7	Q2TAY7 [441-453	0	1771.8427
[K].IAEGAQ	1xTMTpro [N	3.846E-05	4.225E-05	1	1	1	Q9UJ70	Q9UJ70 [220-232	0	1645.8829
[K].MVMIQD	1xTMTpro [K	3.225E-06	4.225E-05	1	1	1	Q96I24	Q96I24 [196-213]	0	2547.4283
[K].LALQQD	1xTMTpro [N	1.338E-06	4.225E-05	1	1	3	O94905	O94905 [150-169]	0	2428.3917
[K].NHAVVC	2xCarbamido	1.435E-05	4.225E-05	1	1	1	Q9UGI8	Q9UGI8 [356-374	0	2508.2155
[K].NGVQAM	1xTMTpro [N	2.023E-05	4.225E-05	1	1	2	P14866	P14866 [230-246]	0	2170.0882
[R].EDMEED	1xTMTpro [N	4.752E-05	4.225E-05	1	1	1	Q92544	Q92544 [192-203]	0	1885.8193
[R].NGSGTL	1xTMTpro [N	5.384E-05	4.225E-05	1	1	2	Q13938	Q13938 [158-170]	0	1754.9244
[K].NGSEAD	1xTMTpro [N	1.848E-05	4.225E-05	1	1	1	P22314	P22314 [44-57]	0	1829.8837
[K].NGQHVA	1xTMTpro [N	8.794E-06	4.225E-05	1	1	2	P21333	P21333 [2026-20	0	2752.4549
[K].NGPVEG	1xTMTpro [K	5.6E-05	4.225E-05	1	1	1	P07858	P07858 [246-263]	0	2614.4049
[R].NGKPVT	1xTMTpro [K	1.575E-05	4.225E-05	1	1	1	P01903	P01903 [149-165]	0	2410.395
[K].NGGLGH	1xTMTpro [K	3.968E-06	4.225E-05	1	1	1	P30048	P30048 [150-166]	0	2362.3408
[R].NGFDQC	1xCarbamido	1.483E-05	4.225E-05	1	1	2	P13611	P13611 [289-305]	0	2294.0467
[K].EDPANIL	1xTMTpro [K	8.23E-06	4.225E-05	1	1	1	Q96EB1	Q96EB1 [120-137	0	2599.4475
[R].EDPAYLI	1xTMTpro [N	5.853E-07	4.225E-05	1	1	2	P08567	P08567 [270-293]	0	2987.4859
[K].NGETGP	1xTMTpro [K	4.881E-06	4.225E-05	1	1	1	P02461	P02461 [611-629]	0	2328.2076
[K].NGEDLG	1xTMTpro [K	4.657E-05	4.225E-05	1	1	1	Q1KMD3	Q1KMD3 [379-39	0	2044.1359
[R].IAEVGGY	1xTMTpro [K	5.535E-06	4.225E-05	1	1	2	Q9H0W9	Q9H0W9 [59-75]	0	2418.4616
[K].EDPLLTF	1xTMTpro [N	3.042E-05	4.225E-05	1	1	2	P59768	P59768 [47-62]	0	2086.114

[R].NGDGV\	1xTMTpro [N	5.869E-05	4.225E-05	1	1	1	Q6NUK1	Q6NUK1 [34-49]	0	1975.0416
[K].LAFAYLE	1xTMTpro [K	3.85E-07	4.225E-05	1	1	2	O75396	O75396 [83-101]	0	2857.4652
[K].NFYNEH	1xTMTpro [N	1.818E-05	4.225E-05	1	1	1	Q86XP3	Q86XP3 [216-236	0	2891.4859
[R].NFTTEQ	1xTMTpro [K	1.202E-05	4.225E-05	1	1	2	P34932	P34932 [111-124]	0	2191.2288
[K].EDPQTF	1xTMTpro [K	3.886E-05	4.225E-05	1	1	4	P02787	P02787 [108-121]	0	2238.2302
[R].EDMAAL	1xTMTpro [K	9.293E-06	4.225E-05	1	2	7	Q71U36	Q71U36 [423-451	1	3844.725
[R].NHDGEC	1xCarbamido	1.728E-05	4.225E-05	1	1	1	Q12769	Q12769 [1161-11	0	1746.8149
[K].EDIYSGC	1xTMTpro [N	2.35E-05	4.225E-05	1	1	1	Q13151	Q13151 [177-189	0	1515.7359
[K].NHEEEV	2xTMTpro [K	1.443E-06	4.225E-05	1	1	1	P05783	P05783 [216-241]	1	3662.0217
[R].NKEVTW	2xTMTpro [K	4.681E-05	4.225E-05	1	1	1	P05455	P05455 [298-312]	1	2701.5229
[K].NKETLIIE	2xTMTpro [K	5.366E-05	4.225E-05	1	1	1	O14974	O14974 [319-329	1	2226.3525
[R].NKESPV	1xTMTpro [K	2.312E-05	4.225E-05	1	1	1	P09601	P09601 [68-85]	1	2767.5063
[R].IAEQEH	1xTMTpro [N	9.874E-06	4.225E-05	1	1	1	Q9UPN3	Q9UPN3 [1091-1	0	2142.111
[K].NKEDQY	2xTMTpro [K	5.775E-05	4.225E-05	1	1	1	P34932	P34932 [718-733]	1	2806.4498
[K].NKDQGT	1xTMTpro [K	8.974E-06	4.225E-05	1	1	5	P60660	P60660 [80-94]	1	2395.2385
[R].NHYNFN	1xCarbamido	1.023E-05	4.225E-05	1	1	1	P12544	P12544 [183-202]	0	2598.2699
[R].EDLQLD	2xTMTpro [K	3.813E-05	4.225E-05	1	1	2	P78347	P78347 [444-456]	0	2312.3642
[R].NHVQPY	1xTMTpro [N	1.358E-07	4.225E-05	1	1	2	Q96TA1	Q96TA1 [337-362	0	3230.7203
[K].NHQSAA	1xOxidation [	4.964E-05	4.225E-05	1	1	4	Q14195	Q14195 [424-440	0	2329.1202
[R].NFTKPQ	2xTMTpro [K	3.483E-05	4.225E-05	1	1	1	Q9Y3Z3	Q9Y3Z3 [577-595	0	2994.7444
[K].NHQSAA	1xTMTpro [N	1.284E-05	4.225E-05	1	1	4	Q14195	Q14195 [424-440	0	2313.1253
[R].NHQDVA	1xTMTpro [K	2.649E-05	4.225E-05	1	1	1	Q5VWZ2	Q5VWZ2 [137-15	0	2455.3589
[R].FTYTVL\	1xCarbamido	4.673E-05	4.225E-05	1	1	2	P08123	P08123 [1308-13	0	1998.0862
[K].NHLVEIF	1xTMTpro [N	2.812E-06	4.225E-05	1	1	5	P21810	P21810 [148-165]	0	2331.3356
[K].IAESLGC	1xTMTpro [N	1.026E-05	4.225E-05	1	1	2	Q14156	Q14156 [665-678	0	1728.9087
[R].EDITQSA	1xTMTpro [N	5.348E-05	4.225E-05	1	1	2	Q12906	Q12906 [312-323	0	1672.8938
[R].EDITSSA	1xTMTpro [N	5.926E-05	4.225E-05	1	1	1	Q96KR1	Q96KR1 [1008-10	0	1641.8767
[R].LAESLLA	1xTMTpro [K	6.022E-05	4.225E-05	1	1	1	Q9BVJ6	Q9BVJ6 [6-24]	0	2676.5315
[K].NHGVVM	1xTMTpro [K	2.981E-07	4.225E-05	1	1	2	Q02252	Q02252 [288-316	1	3587.8997
[R].LAETQE	1xTMTpro [K	1.478E-05	4.225E-05	1	1	1	Q9Y383	Q9Y383 [108-122	0	2197.2208
[K].EDIYAVE	1xTMTpro [N	2.242E-05	4.225E-05	1	1	2	P34932	P34932 [333-346]	0	1796.9713
[R].EDLRLPI	2xTMTpro [K	2.128E-05	4.225E-05	1	1	2	P63241	P63241 [110-126]	2	2881.6451
[K].NFTEVHI	1xTMTpro [K	2.906E-05	4.225E-05	1	1	1	P04040	P04040 [481-499]	0	2792.4863

[K].EDQFQL	1xTMTpro [N	2.677E-05	4.225E-05	1	1	2	P53618	P53618 [686-702]	0	2226.1508
[K].EDQLLC	2xCarbamid	4.078E-05	4.225E-05	1	1	3	Q14192	Q14192 [84-100]	0	2720.2675
[R].EDTGTY	1xCarbamid	2.031E-05	4.225E-05	1	1	1	Q9Y624	Q9Y624 [102-123	0	3021.3949
[K].NETLGG	2xCarbamid	4.81E-06	4.225E-05	1	1	1	P09622	P09622 [73-89]	0	2428.282
[K].NETGGG	1xTMTpro [K	8.433E-07	4.225E-05	1	1	1	Q00169	Q00169 [32-50]	0	2628.3649
[R].NESCSE	1xCarbamid	2.403E-06	4.225E-05	1	1	1	Q01813	Q01813 [638-659	0	3285.5389
[R].NENTFLI	1xTMTpro [K	4.935E-07	4.225E-05	1	1	2	Q16851	Q16851 [134-151	0	2764.5125
[K].EDTPNS	1xTMTpro [K	3.483E-05	4.225E-05	1	1	2	P09871	P09871 [297-308]	0	1981.0522
[R].NENQLII	1xTMTpro [N	1.349E-05	4.225E-05	1	1	2	Q15393	Q15393 [1017-10	0	2113.0885
[R].EDTVQS	2xTMTpro [K	1.399E-05	4.225E-05	1	1	1	Q9H3G5	Q9H3G5 [361-37	0	3207.754
[K].LAGFLDI	1xTMTpro [N	2.547E-05	4.225E-05	1	1	2	Q9Y262	Q9Y262 [475-487	0	1842.9921
[K].NENEEIL	1xTMTpro [N	2.429E-06	4.225E-05	1	1	1	Q9UHB6	Q9UHB6 [469-48	0	2271.2012
[K].EDTESLI	1xTMTpro [N	9.242E-06	4.225E-05	1	1	3	Q9BS26	Q9BS26 [271-285	0	2084.0467
[K].FTSSPGI	1xTMTpro [K	5.907E-05	4.225E-05	1	1	1	O14936	O14936 [48-61]	1	2146.2
[K].NEILDEV	1xTMTpro [K	5.581E-05	4.225E-05	1	1	1	O60313	O60313 [599-614	0	2393.3783
[KR].NELHN	2xTMTpro [K	4.567E-06	4.225E-05	2	2	2	Q96BM9; Q9	Q96BM9 [109-131	0	3465.0773
[R].FTSLRPI	1xTMTpro [N	8.617E-06	4.225E-05	1	1	1	Q8IXM6	Q8IXM6 [28-47]	0	2344.2944
[R].NEGVAT	1xTMTpro [N	1.075E-05	4.225E-05	1	1	2	P35222	P35222 [648-661]	0	1785.9819
[R].LAGGND	1xTMTpro [K	5.475E-07	4.225E-05	1	1	1	Q9UDY2	Q9UDY2 [525-55	0	3952.0795
[R].EDVTFEI	1xTMTpro [N	5.403E-06	4.225E-05	1	1	1	Q9NVG8	Q9NVG8 [93-109]	0	2286.0958
[K].NEGCQV	1xCarbamid	6.041E-05	4.225E-05	1	1	1	Q9Y282	Q9Y282 [196-209	0	2265.1829
[R].LAGLDA	1xTMTpro [N	1.561E-05	4.225E-05	1	1	2	Q9Y240	Q9Y240 [115-128	0	1804.0513
[K].NEEDEG	1xTMTpro [N	3.059E-05	4.225E-05	1	1	2	Q14103	Q14103 [73-85]	0	1761.7959
[R].NEEDAA	1xTMTpro [N	1.065E-06	4.225E-05	1	1	2	P22314	P22314 [351-368]	0	2188.1529
[K].NELMYQ	1xTMTpro [N	6.446E-08	4.225E-05	1	1	1	Q08211	Q08211 [366-385	0	2790.4052
[K].EDLNCQ	1xCarbamid	3.76E-05	4.225E-05	1	1	2	O75821	O75821 [135-148	0	2359.1038
[K].NEVDMQ	1xTMTpro [K	1.226E-05	4.225E-05	1	1	2	P47897	P47897 [167-180]	0	2201.2608
[K].NFAMIGI	1xCarbamid	7.247E-06	4.225E-05	1	1	1	P22314	P22314 [486-510]	0	3278.6602
[K].NFSGPV	1xTMTpro [K	1.008E-05	4.225E-05	1	1	1	Q9GZZ9	Q9GZZ9 [356-37	0	2823.5788
[K].LAFDMA	1xCarbamid	2.57E-05	4.225E-05	1	1	1	Q92882	Q92882 [175-190	0	2305.254
[R].EDQSILC	1xCarbamid	2.007E-06	4.225E-05	4	4	3	P35580; P35	P35580 [170-184]	0	2160.1098
[R].NFPPSQ	1xCarbamid	2.144E-05	4.225E-05	1	2	1	P0DOX2	P0DOX2 [167-19	0	3804.8882
[R].NFPPSQ	1xCarbamid	5.598E-07	4.225E-05	1	1	7	P01876	P01876 [52-81]	0	3776.9297

[K].NFPNAIE	1xTMTpro [N	1.394E-05	4.225E-05	1	1	1	P22314	P22314 [636-649]	0	2001.0626
[R].LAFLNV(	1xTMTpro [N	1.173E-05	4.225E-05	1	1	2	P43304	P43304 [558-572]	0	1946.103
[K].NFINSPV	1xTMTpro [K	8.094E-06	4.225E-05	1	1	2	P35749	P35749 [19-33]	0	2240.2319
[K].NFINNPL	1xTMTpro [K	1.115E-05	4.225E-05	1	1	3	P35579	P35579 [15-29]	0	2281.2585
[K].EDSAPS	1xTMTpro [K	3.359E-06	4.225E-05	1	1	2	Q15459	Q15459 [31-50]	0	2758.5635
[K].LAGEEL/	1xTMTpro [K	2.607E-05	4.225E-05	1	1	1	Q7Z4V5	Q7Z4V5 [575-589	0	2179.1738
[R].NFILDQT	1xTMTpro [N	1.903E-05	4.225E-05	1	1	3	Q00839	Q00839 [576-590]	0	1952.0521
[K].EDSCQL	2xCarbamido	1.204E-06	4.225E-05	1	1	2	P02760	P02760 [228-246]	0	2411.0419
[K].EDSHPF	1xTMTpro [K	2.824E-05	4.225E-05	1	1	1	P35244	P35244 [89-104]	0	2442.2797
[K].LAGANP.	1xCarbamido	1.968E-07	4.225E-05	1	1	2	P78527	P78527 [4051-40	0	2729.5152
[R].LAGASP.	1xTMTpro [K	4.417E-06	4.225E-05	1	1	1	Q13045	Q13045 [402-421]	0	2247.2953
[R].NFGKEF	2xTMTpro [K	6.819E-06	4.225E-05	1	1	3	P02042	P02042 [118-133]	1	2800.5272
[K].NFESLSI	1xTMTpro [N	3.807E-07	4.225E-05	1	1	1	P04844	P04844 [245-266]	0	2611.3435
[K].LAGAWA	2xTMTpro [K	3.747E-06	4.225E-05	1	1	1	O14548	O14548 [12-44]	0	4310.4257
[R].IAGDYVS	1xTMTpro [N	2.009E-05	4.225E-05	2	2	2	O95782; O9	O95782 [452-464]	0	1890.9557
[K].NFASVQ	1xTMTpro [N	1.549E-06	4.225E-05	1	1	2	Q99715	Q99715 [2533-25	0	2657.3167
[R].FTTDAIA	1xTMTpro [N	5.907E-05	4.225E-05	1	1	2	P28065	P28065 [174-185]	0	1600.8688
[R].NFILDQ(	1xCarbamido	3.619E-05	4.225E-05	1	1	2	Q1KMD3	Q1KMD3 [532-54	0	2132.0514
[R].EDYIEFA	1xTMTpro [N	3.13E-05	4.225E-05	1	1	2	Q07954	Q07954 [3210-32	0	1919.9306
[R].EDLMGL	1xTMTpro [N	6.142E-08	4.225E-05	2	2	1	Q06787; P51	Q06787 [227-245]	0	2299.2148
[R].NKHEMV	1xCarbamido	6.322E-06	4.225E-05	1	1	1	Q9Y678	Q9Y678 [261-283	1	3400.8537
[R].EDCSPD	1xCarbamido	4.603E-05	4.225E-05	1	1	1	P55039	P55039 [221-236]	0	2169.0453
[K].IAELLSP	1xTMTpro [N	3.091E-05	4.225E-05	1	1	1	P35237	P35237 [146-160]	0	1872.0761
[K].EDDLNS	1xTMTpro [K	2.151E-05	4.225E-05	1	1	3	P09960	P09960 [481-493]	0	2090.0897
[K].NLEWIA(	1xTMTpro [K	1.842E-05	4.225E-05	1	1	3	P12110	P12110 [693-708]	0	2352.3207
[R].NLEVSS(	1xCarbamido	2.804E-05	4.225E-05	1	1	1	Q9C0C2	Q9C0C2 [1169-11	0	1999.9674
[R].NIEVMNS	1xTMTpro [K	5.018E-07	4.225E-05	1	1	2	Q7L5N1	Q7L5N1 [80-97]	0	2727.4519
[R].EDDVGT	1xTMTpro [K	2.329E-05	4.225E-05	1	1	1	P49368	P49368 [340-353]	0	2025.136
[R].EDEEES	1xCarbamido	1.197E-05	4.225E-05	1	1	2	P55072	P55072 [192-210]	0	2490.0898
[K].EDEKGD	2xTMTpro [K	2.298E-06	4.225E-05	1	1	1	Q02952	Q02952 [1697-17	1	3918.9145
[K].EDEIPET	1xTMTpro [K	1.578E-05	4.225E-05	1	1	1	Q96A26	Q96A26 [80-96]	0	2498.3191
[K].NLEVLNI	1xTMTpro [K	2.88E-05	4.225E-05	1	1	1	Q15404	Q15404 [64-87]	0	3426.8764
[R].NIETIINT	1xTMTpro [K	1.098E-05	4.225E-05	1	1	5	P06702	P06702 [11-25]	0	2415.3528

[R].EDEMEE	1xTMTpro [K	9.802E-06	4.225E-05	1	1	1	O00161	O00161 [149-167]	0	2727.4366
[K].NLEPEW	2xTMTpro [K	9.602E-08	4.225E-05	1	1	2	Q15084	Q15084 [195-212]	1	2913.6138
[K].NLEPEW	1xTMTpro [K	2.994E-05	4.225E-05	1	1	2	Q15084	Q15084 [195-208]	0	2123.1628
[R].NLENDV	2xTMTpro [K	2.911E-05	4.225E-05	1	1	1	O75116	O75116 [528-544]	1	2899.6193
[K].IAELSAT	1xTMTpro [K	1.213E-05	4.225E-05	1	1	2	P04114	P04114 [4466-447	0	1995.1982
[R].EDFDVE	1xCarbamido	7.701E-07	4.225E-05	1	1	1	P09871	P09871 [221-245]	0	2976.3852
[R].NIELICQ	1xCarbamido	1.76E-05	4.225E-05	1	1	2	Q15691	Q15691 [223-241]	0	2574.2789
[R].LAEISDV	1xTMTpro [K	3.276E-05	4.225E-05	1	1	5	Q01082	Q01082 [1037-10	0	2058.1073
[R].NLEHLYI	1xTMTpro [K	1.713E-05	4.225E-05	1	1	1	Q99983	Q99983 [301-314]	0	2365.3007
[R].EDCSPA	1xCarbamido	5.227E-06	4.225E-05	1	1	1	Q96DG6	Q96DG6 [220-234	0	2374.184
[R].EDCPGL	1xCarbamido	3.135E-05	4.225E-05	1	1	1	Q15274	Q15274 [25-49]	0	3154.6487
[K].NIFESFV	2xTMTpro [K	8.02E-06	4.225E-05	1	1	1	Q93009	Q93009 [359-391]	1	4642.3405
[K].IAELLEN	1xTMTpro [K	4.417E-06	4.225E-05	1	1	2	Q14694	Q14694 [396-415]	0	2878.7374
[K].NILAEQL	1xTMTpro [N	7.098E-07	4.225E-05	1	1	1	P35580	P35580 [890-910]	0	2739.383
[R].FVEGVC	2xCarbamido	5.322E-05	4.225E-05	1	1	2	P56192	P56192 [400-414]	0	2123.985
[R].ECVGFE	1xCarbamido	9.133E-07	4.225E-05	2	2	2	P0C0L4; P0	P0C0L4 [1534-15	0	3804.875
[R].IAEGFSD	1xTMTpro [K	3.709E-05	4.225E-05	1	1	1	O43402	O43402 [115-131]	0	2433.3191
[K].NLKPIKP	2xTMTpro [K	2.561E-05	4.225E-05	1	1	3	P18669	P18669 [223-240]	0	3027.7481
[R].ECYCPP	3xCarbamido	2.76E-05	4.225E-05	1	1	2	P51888	P51888 [76-92]	0	2441.0531
[R].ECYFDT	2xCarbamido	6.241E-06	4.225E-05	1	1	2	Q8N2S1	Q8N2S1 [1182-11	0	2406.1025
[R].NLHVVF	1xTMTpro [K	5.879E-05	4.225E-05	1	1	1	Q14204	Q14204 [3061-30	1	2652.4423
[R].NLHVVF	1xTMTpro [K	5.064E-05	4.225E-05	1	1	2	Q14204	Q14204 [3061-30	0	2381.3143
[K].NIGWGT	1xTMTpro [K	5.525E-06	4.225E-05	1	1	1	Q9Y5U9	Q9Y5U9 [30-49]	0	2640.3913
[K].NLEGYV	1xTMTpro [N	2.277E-06	4.225E-05	1	1	3	Q16181	Q16181 [26-42]	0	2258.1889
[R].EDAGTY	1xCarbamido	5.563E-05	4.225E-05	1	1	1	P13688	P13688 [389-404]	0	2524.2674
[R].LAELEEI	1xTMTpro [N	4.177E-08	4.225E-05	1	1	3	Q00610	Q00610 [1183-12	0	2768.4287
[R].EDAMAM	1xCarbamido	1.224E-05	4.225E-05	1	1	1	P43243	P43243 [543-554]	0	2028.0208
[R].NLGIPLG	1xTMTpro [K	3.259E-05	4.225E-05	1	1	4	Q6NUK1	Q6NUK1 [50-62]	0	1992.1257
[K].NLGLEEI	1xTMTpro [N	1.628E-05	4.225E-05	1	1	2	P09622	P09622 [321-334]	0	1872.0398
[R].EDAPIGI	1xTMTpro [N	2.521E-06	4.225E-05	1	1	1	Q14152	Q14152 [503-520]	0	2309.1879
[R].EDAQVA	1xTMTpro [K	1.112E-06	4.225E-05	1	1	4	P08133	P08133 [521-540]	0	2680.4173
[R].EDAQVA	1xTMTpro [K	2.594E-07	4.225E-05	1	1	1	P08133	P08133 [521-546]	1	3367.7724
[K].NLGFSPI	1xTMTpro [N	3.508E-05	4.225E-05	1	1	1	O43252	O43252 [98-111]	1	1965.9949

[K].NIGDINQ	1xTMTpro [K	4.012E-06	4.225E-05	1	1	2	P23229	P23229 [422-445]	0	3128.6032
[K].LAEIGAF	1xTMTpro [N	2.697E-05	4.225E-05	1	1	1	Q13435	Q13435 [36-53]	1	2298.2737
[R].NLGSINT	1xTMTpro [N	6.322E-06	4.225E-05	1	1	1	O75396	O75396 [134-147	0	1891.0204
[R].IAEITHL(	1xTMTpro [N	2.162E-06	4.225E-05	1	1	1	Q15811	Q15811 [521-540	0	2655.4208
[K].NIEDVIA	1xTMTpro [K	4.626E-05	4.225E-05	1	1	3	P05387	P05387 [50-61]	0	1865.0988
[K].NLEAVE	1xCarbamid	8.586E-06	4.225E-05	1	4	2	P05023	P05023 [360-377]	0	2533.3311
[K].EDKESN	2xTMTpro [K	1.321E-06	4.225E-05	1	1	1	P24821	P24821 [970-989]	1	3056.6568
[K].EDKGTG	1xTMTpro [K	1.744E-06	4.225E-05	1	1	1	Q9P2J5	Q9P2J5 [382-404	1	2937.5661
[R].NLATTV	1xTMTpro [K	1.04E-05	4.225E-05	1	1	4	O43390	O43390 [347-359	0	2069.1986
[K].EDKLEC	1xCarbamid	8.719E-07	4.225E-05	1	2	4	Q00610	Q00610 [454-468	1	2676.4582
[R].EDKSPIA	1xTMTpro [K	3.157E-05	4.225E-05	1	1	1	P51398	P51398 [277-294]	1	2639.5012
[K].IAENNDL	1xTMTpro [K	4.558E-06	4.225E-05	1	1	1	O00499	O00499 [103-117	0	2498.2994
[R].NLANTV	1xTMTpro [K	1.604E-05	4.225E-05	1	1	4	O60506	O60506 [344-356	0	2082.1938
[K].EDLAAF	1xTMTpro [K	2.016E-07	4.225E-05	1	1	2	Q02880	Q02880 [1187-12	1	2715.4333
[K].NIANPTA	1xCarbamid	8.915E-07	4.225E-05	1	1	1	P51553	P51553 [321-340]	0	2822.5222
[R].NLAMGV	1xTMTpro [K	2.086E-05	4.225E-05	1	1	2	P12004	P12004 [65-77]	0	1974.1008
[K].NLCDLG	2xCarbamid	4.072E-06	4.225E-05	1	1	1	P50579	P50579 [434-451]	0	2710.444
[K].EDLDDA	2xTMTpro [K	5.491E-05	4.225E-05	1	1	2	Q9HBL0	Q9HBL0 [408-424	2	2972.5458
[R].NLALCP	1xCarbamid	4.375E-07	4.225E-05	1	1	2	P14923	P14923 [507-526]	0	2459.3512
[R].NIAFFST	1xCarbamid	3.119E-05	4.225E-05	1	1	1	P05023	P05023 [241-255]	0	1990.9976
[K].NLAEALI	1xTMTpro [K	1.991E-05	4.225E-05	1	1	1	Q96TA2	Q96TA2 [745-759	0	2273.2884
[K].NIADLMT	2xTMTpro [K	7.15E-07	4.225E-05	1	1	1	O75506	O75506 [51-75]	1	3641.9876
[K].FVAPEE	1xTMTpro [K	2.574E-05	4.225E-05	1	1	2	P49770	P49770 [288-305]	0	2641.462
[KR].LAEQA	1xTMTpro [K	4.874E-05	4.225E-05	2	2	1	P31946; P61	P31946 [14-29]; F	1	2421.2398
[R].NKSNED	2xTMTpro [K	2.69E-06	4.225E-05	1	1	1	P02545	P02545 [456-470]	1	2691.4341
[R].NKLNDL	2xTMTpro [K	4.21E-06	4.225E-05	1	1	1	P35908	P35908 [440-458]	2	3110.7626
[R].NKLNDL	2xTMTpro [K	1.399E-05	4.225E-05	1	1	2	P35908	P35908 [440-453]	1	2526.4708
[K].NKLNDLI	2xTMTpro [K	3.237E-06	4.225E-05	1	1	3	P04264	P04264 [442-460]	2	3096.7469
[K].EDLDKVI	2xTMTpro [K	9.057E-06	4.225E-05	1	1	2	Q14204	Q14204 [3280-32	1	2880.6499
[K].EDLIWEI	1xTMTpro [K	2.316E-05	4.225E-05	1	1	2	P02787	P02787 [279-295]	0	2678.4434
[R].FVATQA	1xTMTpro [N	1.449E-06	4.225E-05	1	1	1	Q92696	Q92696 [151-173	0	2751.4888
[K].NICFTVV	1xCarbamid	2.625E-05	4.225E-05	1	1	2	P18085	P18085 [60-73]	0	1970.9714
[R].NLEALAI	1xOxidation	8.761E-06	4.225E-05	1	1	5	P12956	P12956 [489-510]	0	3047.683

[R].FVDHVF	1xTMTpro [K	4.125E-06	4.225E-05	1	1	2	P04843	P04843 [355-371]	0	2599.4263
[K].NLDSTTY	1xCarbamido	2.902E-06	4.225E-05	1	1	9	P21291	P21291 [43-59]	0	2544.326
[K].NLDSTTY	1xCarbamido	2.677E-05	4.225E-05	1	1	1	Q16527	Q16527 [43-59]	0	2616.3471
[K].FVDGLM	1xOxidation [	4.171E-06	4.225E-05	1	1	1	P23396	P23396 [152-173]	0	2788.3935
[K].NLDQEQ	1xTMTpro [N	1.646E-06	4.225E-05	1	1	4	P13861	P13861 [142-158]	0	2340.1825
[K].FVDGLM	1xTMTpro [N	1.057E-06	4.225E-05	1	1	2	P23396	P23396 [152-173]	0	2772.3986
[R].NIDNPAI	1xTMTpro [K	1.566E-06	4.225E-05	1	1	1	O75083	O75083 [44-65]	0	3026.6555
[R].EDGHLW	1xCarbamido	2.602E-05	4.225E-05	1	1	1	Q9UBG0	Q9UBG0 [207-224	1	2789.3445
[R].FVCYCE	2xCarbamido	2.621E-05	4.225E-05	1	1	4	Q9BUH6	Q9BUH6 [20-34]	0	2097.8813
[K].NICFTVV	1xCarbamido	4.323E-05	4.225E-05	1	1	1	P84085	P84085 [60-73]	0	2247.1724
[R].EDGLPT	1xTMTpro [K	1.366E-05	4.225E-05	1	1	1	Q96AY3	Q96AY3 [486-499	0	2270.2465
[K].NLDLAVI	1xTMTpro [K	7.478E-06	4.225E-05	1	1	1	P78527	P78527 [1574-159	0	2598.4304
[K].NLDKEYI	1xCarbamido	1.03E-05	4.225E-05	1	1	3	P00505	P00505 [91-107]	1	2879.6157
[R].LAEMPA	1xTMTpro [N	5.093E-06	4.225E-05	1	1	3	P38606	P38606 [365-381]	0	2086.0598
[R].LAENFCY	2xCarbamido	1.518E-06	4.225E-05	1	1	3	P54819	P54819 [35-51]	0	2311.1316
[R].LAENFCY	2xCarbamido	2.148E-05	4.225E-05	1	1	1	P54819	P54819 [35-51]	0	2327.1266
[R].FVCMLV	1xCarbamido	1.409E-05	4.225E-05	1	1	3	Q13740	Q13740 [111-129	0	2820.5171
[K].NIDDGTS	1xTMTpro [N	6.475E-06	4.225E-05	1	1	4	P61353	P61353 [28-48]	0	2576.3024
[K].EDGTFY	1xTMTpro [N	3.632E-05	4.225E-05	1	1	1	O43776	O43776 [408-425	0	2405.1104
[R].FVAVGD	1xTMTpro [N	1.942E-06	4.225E-05	1	1	3	P13686	P13686 [28-46]	0	2302.2052
[R].EDGYSD	1xCarbamido	9.157E-06	4.225E-05	1	1	1	Q8N2S1	Q8N2S1 [277-291	0	2034.8823
[R].NLDLDSI	1xTMTpro [K	3.709E-05	4.225E-05	4	7	2	P35908; P04	P35908 [342-353]	0	1938.1403
[-].MVNPTVF	1xMet-loss [N	7.45E-06	4.225E-05	1	1	2	P62937	P62937 [1-19]	0	2250.209
[K].NEDSLVI	1xTMTpro [K	3.8E-05	4.225E-05	1	1	2	P01023	P01023 [124-135]	0	2003.0941
[K].NEDLWD	1xCarbamido	3.76E-05	4.225E-05	1	1	1	Q9NZ08	Q9NZ08 [475-492	0	2646.3035
[K].EEISATQ	1xCarbamido	2.337E-05	4.225E-05	1	1	2	O75643	O75643 [578-592	0	2326.282
[K].NADMSE	1xCarbamido	1.162E-06	4.225E-05	1	1	4	P63167	P63167 [10-31]	0	3122.4572
[R].EELTAFI	1xTMTpro [N	2.701E-05	4.225E-05	1	1	3	Q96D15	Q96D15 [186-201	0	2287.1501
[K].EEITKDA	1xTMTpro [K	3.19E-06	4.225E-05	1	1	1	P23634	P23634 [1067-108	1	3051.5436
[R].NADMQE	1xTMTpro [K	1.747E-05	4.225E-05	1	1	1	Q7Z3K3	Q7Z3K3 [1342-13	0	2569.3675
[K].NADLQV	1xTMTpro [K	2.17E-06	4.225E-05	1	1	1	P01833	P01833 [232-250]	0	2849.5904
[K].NADGFII	1xTMTpro [K	7.341E-06	4.225E-05	1	1	2	O43852	O43852 [203-231	0	3967.8464
[K].EEIVDKY	1xTMTpro [K	1.391E-07	4.225E-05	1	1	4	O43852	O43852 [288-311	1	3309.7498

[R].NAAQVL	1xTMTpro [K	2.009E-05	4.225E-05	1	1	1	Q9BT78	Q9BT78 [122-137	0	2246.3364
[R].NAALNTI	1xTMTpro [K	5.71E-05	4.225E-05	1	1	1	Q14008	Q14008 [1384-14	0	2811.5649
[R].NAAHAL	1xTMTpro [N	5.34E-05	4.225E-05	1	1	1	Q8NBJ5	Q8NBJ5 [62-76]	0	1839.0408
[K].MYYSAV	2xTMTpro [K	4.393E-06	4.225E-05	1	1	4	P00450	P00450 [538-558]	1	3259.7927
[R].MYSYVT	1xTMTpro [N	1.854E-06	4.225E-05	1	1	2	P10768	P10768 [120-142]	0	3028.5409
[K].MYSTDD	1xOxidation [	5.638E-06	4.225E-05	1	1	1	Q15029	Q15029 [481-495	0	2050.9612
[K].MYSTDD	1xTMTpro [N	2.644E-05	4.225E-05	1	1	1	Q15029	Q15029 [481-495	0	2034.9663
[K].MYLGYE	1xTMTpro [N	6.08E-05	4.225E-05	1	1	13	P02787	P02787 [332-343]	0	1782.942
[K].MYHDDD	1xOxidation [	3.293E-05	4.225E-05	1	1	1	P27816	P27816 [143-173]	0	3978.9563
[K].MYHDDD	1xTMTpro [K	1.204E-06	4.225E-05	1	1	1	P27816	P27816 [143-173]	0	3962.9614
[K].MYGISLC	1xCarbamido	1.356E-05	4.225E-05	1	1	2	P04083	P04083 [318-332]	0	2366.2591
[K].EEMQSN	1xTMTpro [N	1.639E-05	4.225E-05	1	1	1	Q92597	Q92597 [199-212	0	2025.0031
[K].MYGISLC	1xCarbamido	4.872E-06	4.225E-05	1	1	2	P04083	P04083 [318-332]	0	2350.2642
[R].EEIQSSI	1xTMTpro [N	3.057E-06	4.225E-05	1	1	3	P46940	P46940 [723-738]	0	2029.0521
[R].FTEQVE	1xTMTpro [N	4.077E-08	4.225E-05	1	1	4	P35221	P35221 [587-616]	0	3600.6971
[K].EELQSG	1xTMTpro [N	3.8E-05	4.225E-05	1	1	1	P46940	P46940 [369-387]	0	2369.1653
[R].EEIQPGI	1xTMTpro [N	1.462E-05	4.225E-05	1	1	3	Q13126	Q13126 [100-116	0	2304.2406
[K].NAIANAS	1xTMTpro [N	1.389E-05	4.225E-05	1	1	2	P09661	P09661 [206-219]	0	1762.9618
[K].LALELPD	2xTMTpro [K	5.784E-05	4.225E-05	1	1	1	Q96TC7	Q96TC7 [443-459	1	2808.6903
[R].NAGPIAN	1xTMTpro [N	4.575E-06	4.225E-05	1	1	1	Q08AF3	Q08AF3 [714-730	0	2207.1562
[K].EEILENW	1xTMTpro [K	7.874E-07	4.225E-05	1	1	3	Q15293	Q15293 [303-326	0	3396.6913
[K].EEILENW	1xOxidation [	4.308E-05	4.225E-05	1	1	1	Q15293	Q15293 [303-326	0	3412.6863
[R].NAGNEQ	1xTMTpro [K	5.006E-05	4.225E-05	1	1	2	P35221	P35221 [166-178]	0	2058.1111
[K].NAGNCL	1xCarbamido	5.554E-05	4.225E-05	1	1	1	O43175	O43175 [365-380	0	2234.3186
[K].NAFYIGS	1xCarbamido	2.218E-05	4.225E-05	1	1	1	O14579	O14579 [24-40]	0	2366.1518
[K].FTFHLEF	1xTMTpro [N	2.799E-05	4.225E-05	1	1	1	O60826	O60826 [299-320	0	2734.412
[R].LALETAL	1xTMTpro [K	2.329E-05	4.225E-05	1	1	2	P00488	P00488 [493-504]	0	1889.1062
[R].EENEGV	1xTMTpro [N	4.618E-05	4.225E-05	1	1	1	P49419	P49419 [49-62]	0	1857.8687
[R].EEIIPVA	1xTMTpro [K	2.401E-05	4.225E-05	1	1	1	P11310	P11310 [58-69]	0	1985.1087
[R].NAFACF	1xCarbamido	5.796E-06	4.225E-05	1	1	3	P24844	P24844 [105-124]	0	2669.2374
[K].LALETTV	1xTMTpro [N	2.802E-06	4.225E-05	1	1	1	P61160	P61160 [233-250]	0	2281.261
[R].EEINALV	1xTMTpro [N	5.756E-05	4.225E-05	1	1	1	Q8WWX9	Q8WWX9 [103-1	0	1985.0663
[R].NAENAIE	2xTMTpro [K	1.105E-05	4.225E-05	1	2	2	P16615	P16615 [111-128]	1	2948.5855

[R].EEIQDAS	1xTMTpro [K	3.236E-05	4.225E-05	1	1	2	Q9H7B4	Q9H7B4 [157-177	0	2974.5694
[K].EEIQETQ	1xTMTpro [N	5.437E-05	4.225E-05	1	1	2	Q9BYG3	Q9BYG3 [272-284	0	1859.9418
[K].LALFNSA	1xTMTpro [K	2.658E-05	4.225E-05	1	1	1	Q6ZXV5	Q6ZXV5 [633-649	0	2444.3715
[R].FTEQVE.	1xOxidation [	2.973E-05	4.225E-05	1	1	1	P35221	P35221 [587-616]	0	3616.692
[R].EELQLLC	1xTMTpro [N	1.592E-05	4.225E-05	1	1	1	P62195	P62195 [61-78]	0	2380.2679
[R].EELQNLI	1xTMTpro [K	2.105E-05	4.225E-05	1	1	1	Q9NX08	Q9NX08 [163-177	0	2308.3004
[R].NAFACF	1xCarbamido	3.786E-07	4.225E-05	1	2	5	O14950	O14950 [105-124]	0	2654.2364
[K].MYGAQE	1xCarbamido	8.859E-06	4.225E-05	1	1	1	Q8NF37	Q8NF37 [427-446	0	2737.3668
[K].IAILDIEP	1xTMTpro [K	5.664E-05	4.225E-05	1	1	1	Q00013	Q00013 [375-386]	0	1962.2131
[K].MYDSVL.	1xTMTpro [N	4.849E-05	4.225E-05	1	1	1	O95471	O95471 [66-81]	0	2014.0962
[R].MVSHLA	1xTMTpro [K	5.926E-05	4.225E-05	1	1	2	P54652	P54652 [240-250]	1	1955.1028
[K].MVSGMY	1xTMTpro [N	4.503E-05	4.225E-05	1	1	2	P19367	P19367 [296-307]	0	1658.8929
[K].MVSDINI	1xTMTpro [K	1.816E-06	4.225E-05	1	1	1	O43707	O43707 [379-395]	0	2607.3481
[R].MVSDINI	1xCarbamido	1.191E-05	4.225E-05	1	1	3	P12814	P12814 [360-376]	0	2617.3246
[R].MVSDINI	1xCarbamido	1.211E-05	4.225E-05	1	1	6	P12814	P12814 [360-376]	0	2601.3297
[R].EESLEKI	1xTMTpro [K	3.026E-05	4.225E-05	1	1	1	Q8WUM0	Q8WUM0 [753-77	1	3140.6396
[R].MVRPNQ	1xCarbamido	3.974E-05	4.225E-05	1	1	2	P43034	P43034 [239-259]	0	2682.3259
[R].EESPLLI	1xTMTpro [N	2.29E-06	4.225E-05	1	1	4	P02751	P02751 [1526-154	0	2259.2152
[R].EESPYC	6xCarbamido	8.202E-07	4.225E-05	1	1	1	Q14192	Q14192 [23-52]	0	4572.114
[R].MVRPNQ	1xCarbamido	1.145E-06	4.225E-05	1	1	1	P43034	P43034 [239-259]	0	2666.331
[K].EESDDE	3xTMTpro [K	2.854E-06	4.225E-05	1	1	1	P14625	P14625 [304-322]	1	3436.796
[R].MVQVHE	1xCarbamido	3.435E-05	4.225E-05	1	1	1	Q15436	Q15436 [172-185]	0	2211.1757
[K].MVQQSD	1xTMTpro [N	4.213E-05	4.225E-05	1	1	2	Q9H553	Q9H553 [287-301	0	2089.1071
[R].EESTSS	1xTMTpro [N	3.465E-05	4.225E-05	1	1	5	P23193	P23193 [105-116]	0	1570.7628
[R].MVQEQC	3xCarbamido	3.291E-06	4.225E-05	1	1	1	P23142	P23142 [63-90]	0	3663.6579
[R].MVQEQC	3xCarbamido	6.714E-08	4.225E-05	1	1	2	P23142	P23142 [63-90]	0	3647.663
[R].FTDEYQ	1xTMTpro [K	3.259E-05	4.225E-05	1	1	3	Q13557	Q13557 [10-22]	0	2227.1778
[K].MVPVSV	1xOxidation [	2.329E-05	4.225E-05	1	1	2	Q8WUM4	Q8WUM4 [358-37	0	2111.1238
[K].MVPVSV	1xTMTpro [N	3.576E-05	4.225E-05	1	1	1	Q8WUM4	Q8WUM4 [358-37	0	2095.1289
[R].IAIPGLA	1xTMTpro [N	5.485E-07	4.225E-05	1	1	4	P26599	P26599 [352-374]	0	2579.484
[K].LALQLHI	1xTMTpro [K	3.447E-05	4.225E-05	1	1	1	Q9UBS4	Q9UBS4 [48-66]	1	2793.5139
[R].MVNSNL	1xTMTpro [K	4.115E-07	4.225E-05	1	1	1	P08559	P08559 [324-343]	1	2882.5789
[R].MVQVHE	1xCarbamido	1.121E-05	4.225E-05	1	1	2	Q15436	Q15436 [172-185]	0	2195.1808

[R].EELLAEI	1xTMTpro [N	1.098E-05	4.225E-05	1	1	1	P58107	P58107 [2551-256	0	2336.2669
[R].MVSSYV	1xTMTpro [N	1.628E-05	4.225E-05	1	1	2	P55809	P55809 [111-124]	0	1921.9285
[-].MVSVINT	1xMet-loss+	1.69E-07	4.225E-05	1	1	4	P55735	P55735 [1-27]	0	3343.553
[R].MYAPAW	1xOxidation	4.809E-05	4.225E-05	1	1	1	Q13418	Q13418 [350-363	0	2199.2128
[R].MYAPAW	1xTMTpro [K	4.293E-05	4.225E-05	1	1	1	Q13418	Q13418 [350-363	0	2183.2179
[K].MWSIEN	1xOxidation	1.716E-05	4.225E-05	1	1	1	P43490	P43490 [372-389]	0	2532.3776
[K].MWSIEN	1xTMTpro [K	1.344E-05	4.225E-05	1	1	3	P43490	P43490 [372-389]	0	2516.3827
[K].EENVGL	1xCarbamido	5.898E-05	4.225E-05	1	1	2	P67936	P67936 [229-248]	0	2644.3208
[K].EEPFFPI	1xTMTpro [N	2.273E-06	4.225E-05	1	1	3	Q1KMD3	Q1KMD3 [418-43	0	2945.5045
[K].EEPGSD	1xTMTpro [N	2.431E-07	4.225E-05	1	1	2	O15355	O15355 [321-337	0	2006.0725
[R].LAILGIHI	1xTMTpro [K	4.744E-05	4.225E-05	1	1	6	P12814	P12814 [566-577]	0	1902.1668
[K].MWDPHI	1xTMTpro [K	1.524E-05	4.225E-05	1	1	5	P08621	P08621 [88-103]	0	2451.2007
[K].MWDLSS	1xTMTpro [K	5.915E-06	4.225E-05	1	1	1	P78406	P78406 [112-131]	0	2860.5271
[R].EESAAD	1xTMTpro [K	1.165E-05	4.225E-05	1	1	1	Q16643	Q16643 [23-42]	0	2886.3813
[K].MWCATT	1xCarbamido	7.274E-06	4.225E-05	1	1	2	P08253	P08253 [373-386]	1	2355.1353
[K].MVYWTD	1xOxidation	2.721E-05	4.225E-05	1	1	4	P14543	P14543 [991-100	0	1988.0118
[R].MVVTGD	1xTMTpro [K	1.709E-05	4.225E-05	1	1	1	Q92466	Q92466 [215-233	0	2597.4287
[R].LALLNVA	1xTMTpro [N	1.252E-06	4.225E-05	1	1	1	Q9H7D7	Q9H7D7 [540-558	0	2466.3788
[K].FTDKDQ	3xTMTpro [K	3.014E-07	4.225E-05	1	1	2	Q9NXG2	Q9NXG2 [78-101]	2	3797.9822
[R].IALLPLL(	1xTMTpro [N	1.916E-05	4.225E-05	1	1	2	Q9P0J0	Q9P0J0 [69-82]	1	1913.1503
[R].MVVEPN	1xTMTpro [N	1.505E-05	4.225E-05	1	1	1	A2RRP1	A2RRP1 [809-831	0	2995.5406
[R].LALNVM.	1xTMTpro [N	2.3E-05	4.225E-05	1	1	1	Q96KA5	Q96KA5 [174-196	0	2792.4361
[K].EEQTILP	1xCarbamido	2.113E-05	4.225E-05	1	1	1	Q6UVY6	Q6UVY6 [429-44	0	2192.1188
[K].LALPGH.	1xTMTpro [N	1.619E-06	4.225E-05	1	1	1	Q14137	Q14137 [305-327	0	2915.4746
[R].MVTEEE	1xTMTpro [K	3.954E-05	4.225E-05	1	1	1	P34913	P34913 [441-455]	0	2527.3398
[K].EEPPRP	1xTMTpro [N	2.735E-06	4.225E-05	1	1	2	P22105	P22105 [1251-12	0	3423.8331
[R].EEAAAA	1xTMTpro [N	1.332E-05	4.225E-05	1	1	1	Q5TZA2	Q5TZA2 [1125-11	0	1585.8254
[R].NALANP	1xCarbamido	2.453E-05	4.225E-05	1	1	1	P22695	P22695 [184-196]	0	1870.9441
[K].LALEGLT	2xTMTpro [K	4.33E-05	4.225E-05	1	1	1	Q14789	Q14789 [1574-15	1	2258.3424
[K].NDNDNII	1xOxidation	9.007E-06	4.225E-05	1	1	2	P01008	P01008 [103-123]	0	2923.5367
[K].LAGPQL	1xTMTpro [K	2.963E-05	4.225E-05	1	1	2	P17980	P17980 [251-266]	0	2253.2921
[R].NDLSPT	1xTMTpro [N	4.923E-05	4.225E-05	1	1	3	P30043	P30043 [79-92]	0	1781.9023
[K].NDLSICG	1xCarbamido	2.973E-05	4.225E-05	1	1	2	Q9Y333	Q9Y333 [21-39]	0	2798.5002

[R].NDIASHF	1xTMTpro [N	2.668E-05	4.225E-05	1	1	1	Q7KZF4	Q7KZF4 [716-731	0	2014.0313
[K].EEDATLS	1xTMTpro [N	2.655E-06	4.225E-05	1	1	2	Q02252	Q02252 [518-535]	0	2195.1007
[K].EEDKEP	2xTMTpro [K	4.906E-05	4.225E-05	1	1	2	Q9Y696	Q9Y696 [12-24]	1	2501.4683
[K].FTPSPVI	1xTMTpro [K	1.93E-05	4.225E-05	1	1	1	O14972	O14972 [150-168]	0	2742.521
[R].LAGSLLT	1xTMTpro [N	1.443E-06	4.225E-05	1	1	2	Q13724	Q13724 [352-369]	0	2204.1995
[K].FTPPPQI	2xCarbamido	1.738E-05	4.225E-05	1	1	2	Q13308	Q13308 [504-529]	1	3587.7577
[R].NDGAAII	1xTMTpro [K	1.032E-05	4.225E-05	1	1	1	P28331	P28331 [484-499]	0	2137.2472
[K].NDFTEEI	1xTMTpro [N	3.514E-05	4.225E-05	1	1	2	P63208	P63208 [143-154]	0	1770.8465
[R].NDFQLIC	1xTMTpro [N	9.548E-07	4.225E-05	1	1	1	P63241	P63241 [87-109]	0	2884.5012
[R].EEEAIQL	1xTMTpro [N	3.114E-06	4.225E-05	1	1	1	P14625	P14625 [52-67]	0	2090.1049
[R].EEECHF	1xCarbamido	1.176E-05	4.225E-05	1	1	1	Q13162	Q13162 [48-66]	0	2490.1315
[R].EEEEFNT	1xTMTpro [K	1.987E-05	4.225E-05	1	1	2	P62316	P62316 [20-37]	0	2615.406
[R].NDEELN	2xTMTpro [K	4.457E-07	4.225E-05	2	3	1	Q8IUE6; P16	Q8IUE6 [90-119];	1	4026.3783
[R].NDANPE	1xTMTpro [K	7.177E-08	4.225E-05	1	1	4	Q99798	Q99798 [480-506]	0	3388.8608
[RK].IAHFDL	2xTMTpro [K	3.048E-05	4.225E-05	1	2	1	O43293	O43293 [135-150]	0	2809.6466
[K].NCVDINE	4xCarbamido	5.597E-06	4.225E-05	1	1	1	P35555	P35555 [762-782]	0	2857.302
[R].LAHLGV(	1xTMTpro [K	2.122E-06	4.225E-05	1	1	1	P04843	P04843 [81-101]	1	2986.6089
[R].NDNDSV	1xTMTpro [K	3.607E-05	4.225E-05	1	1	1	Q8WVV9	Q8WVV9 [252-26	0	2452.2389
[R].FTQAGS	1xTMTpro [N	5.2E-05	4.225E-05	1	1	1	P06576	P06576 [311-324]	0	1739.9611
[K].NDPEILA	1xTMTpro [K	2.452E-06	4.225E-05	1	1	3	P61201	P61201 [304-328]	0	3477.7703
[R].EEAVQC	1xTMTpro [K	2.23E-05	4.225E-05	1	3	1	Q6VEQ5	Q6VEQ5 [27-42]	0	2473.3252
[R].NEDITEF	1xTMTpro [K	6.857E-06	4.225E-05	1	1	6	Q9Y2Q3	Q9Y2Q3 [129-144	0	2337.2793
[K].FTQVTPT	1xTMTpro [N	1.268E-06	4.225E-05	1	1	5	P02751	P02751 [1821-184	0	2996.5801
[R].EEAAGP	1xTMTpro [N	3.34E-05	4.225E-05	1	1	1	P51178	P51178 [243-257]	0	1844.0448
[K].EEAATG	1xTMTpro [K	2.588E-05	4.225E-05	1	1	1	O43242	O43242 [39-54]	0	2045.0279
[K].NEDFAA	1xCarbamido	2.287E-05	4.225E-05	1	1	1	Q02952	Q02952 [1461-14	0	2934.4184
[R].NECLEA	1xCarbamido	1.555E-05	4.225E-05	1	1	2	P17655	P17655 [37-61]	0	3317.7372
[R].NECFLQ	1xCarbamido	4.11E-06	4.225E-05	1	1	4	P02768	P02768 [123-138]	1	2605.3437
[R].EEAENT	1xTMTpro [N	1.265E-05	4.225E-05	1	1	2	P08670	P08670 [197-217]	1	2697.3399
[K].NEAIQA	1xCarbamido	5.295E-07	4.225E-05	1	1	3	P09936	P09936 [136-153]	0	2272.106
[R].NDYATM	1xOxidation [	2.994E-05	4.225E-05	1	1	1	P12110	P12110 [214-233]	0	2632.2368
[K].NCTVYC	2xCarbamido	2.121E-05	4.225E-05	1	1	1	Q01085	Q01085 [204-222]	0	2420.1692
[R].NDYATM	1xTMTpro [N	3.397E-06	4.225E-05	1	1	3	P12110	P12110 [214-233]	0	2616.2418

[R].NDSLAG	1xTMTpro [N	2.814E-05	4.225E-05	1	1	1	O15498	O15498 [72-88]	0	2124.0892
[K].EEAPDIL	1xCarbamid	2.045E-05	4.225E-05	1	1	4	P27695	P27695 [86-98]	0	2154.1608
[K].NDQDTV	1xTMTpro [K	3.71E-08	4.225E-05	1	1	2	P14866	P14866 [278-303]	1	3498.6766
[R].EEAPSLI	1xTMTpro [N	5.926E-06	4.225E-05	1	1	5	P02675	P02675 [54-72]	0	2255.2103
[K].NDQDTV	1xTMTpro [K	1.032E-05	4.225E-05	1	1	1	P14866	P14866 [278-302]	0	3342.5755
[K].EEASDY	1xTMTpro [K	2.308E-05	4.225E-05	1	1	4	P14625	P14625 [253-265]	0	2134.1411
[K].EEASGS	2xTMTpro [K	1.117E-05	4.225E-05	1	1	2	P13010	P13010 [689-703]	1	2435.3446
[R].FTQDTQ	1xTMTpro [N	1.334E-05	4.225E-05	1	1	1	Q14204	Q14204 [2784-27	0	2057.0412
[K].NDPHLC	1xCarbamid	2.649E-05	4.225E-05	1	1	4	P02794	P02794 [126-144]	0	2980.5119
[R].EEAVPE	1xTMTpro [K	4.695E-06	4.225E-05	1	1	3	O43491	O43491 [791-822	0	3860.0785
[R].NDTPCG	1xCarbamid	1.566E-05	4.225E-05	1	1	1	Q9ULA0	Q9ULA0 [419-434	0	1977.0394
[R].IAHVELA	1xTMTpro [N	5.158E-05	4.225E-05	1	1	2	P98160	P98160 [3542-35	0	1746.9458
[R].IAKEEIF	1xTMTpro [K	3.174E-05	4.225E-05	1	1	1	O94788	O94788 [413-427	1	2350.399
[R].IAKEEIF	2xTMTpro [K	9.107E-06	4.225E-05	1	1	2	P30837	P30837 [412-426]	1	2628.5945
[K].EELALLD	1xTMTpro [K	4.345E-05	4.225E-05	1	1	2	O15212	O15212 [45-58]	0	2142.2302
[K].NASEMII	1xTMTpro [K	1.213E-05	4.225E-05	1	1	1	P36542	P36542 [263-277]	1	2361.3092
[K].FTLDCTI	1xCarbamid	1.103E-06	4.225E-05	1	1	3	P35268	P35268 [21-46]	0	3387.5945
[K].NAQAIEI	1xTMTpro [K	7.672E-09	4.225E-05	1	1	2	Q99460	Q99460 [525-543	0	2770.3962
[K].IAIANIIDI	1xTMTpro [K	3.091E-05	4.225E-05	1	1	1	P04114	P04114 [2196-22	0	2063.2608
[K].NAPCILF	1xCarbamid	3.879E-05	4.225E-05	1	1	1	Q9Y4W6	Q9Y4W6 [399-41	0	2107.1177
[R].EELDKY	1xTMTpro [K	5.084E-08	4.225E-05	1	1	2	P05783	P05783 [265-300]	1	4651.3758
[K].NAPAIIFI	1xTMTpro [K	7.83E-06	4.225E-05	1	1	3	P55072	P55072 [296-312]	0	2419.4092
[K].NANKPLI	1xTMTpro [K	2.799E-05	4.225E-05	1	1	1	Q9H3P7	Q9H3P7 [470-484	0	2349.3786
[R].NALWHT	1xTMTpro [N	3.906E-05	4.225E-05	2	2	2	P07996; P35	P07996 [1078-10	0	1854.9894
[K].NATNVE	1xTMTpro [K	1.044E-06	4.225E-05	1	2	3	Q9H0U4	Q9H0U4 [154-17	0	2477.3024
[R].NALVSH	1xCarbamid	5.711E-06	4.225E-05	1	1	1	P31930	P31930 [397-415]	0	2358.2043
[R].NALQQE	1xTMTpro [K	2.597E-05	4.225E-05	1	1	1	Q9GZT3	Q9GZT3 [75-88]	0	2187.2377
[K].NALNIGI	1xOxidation	1.247E-05	4.225E-05	1	1	1	Q9NTX5	Q9NTX5 [222-24	0	2701.421
[K].NALNIGI	1xTMTpro [K	3.564E-06	4.225E-05	1	1	2	Q9NTX5	Q9NTX5 [222-24	0	2685.4261
[K].NAINIEEI	1xTMTpro [N	5.473E-05	4.225E-05	1	1	2	Q13636	Q13636 [151-164	0	1908.051
[K].EEIFGPV	1xTMTpro [K	2.502E-05	4.225E-05	1	1	2	P00352	P00352 [399-410]	0	2027.1491
[R].NALKQE	2xTMTpro [K	5.009E-06	4.225E-05	1	1	2	P51149	P51149 [172-191]	1	3303.8293
[R].FTGSLQ	1xTMTpro [N	1.137E-05	4.225E-05	1	1	2	P39059	P39059 [215-229]	0	2000.0884

[K].EELGDE	1xTMTpro [K	2.475E-05	4.225E-05	1	1	1	Q9Y315	Q9Y315 [274-287	0	2369.2997
[K].NAIDDG	1xCarbamid	4.626E-06	4.225E-05	1	1	2	P40227	P40227 [400-424]	0	3094.6408
[R].EELGQG	1xTMTpro [K	1.812E-05	4.225E-05	1	1	2	Q07065	Q07065 [149-161	0	2023.1315
[K].EELEQA	1xTMTpro [N	3.18E-05	4.225E-05	1	1	1	Q14980	Q14980 [483-495	0	1729.8788
[R].EEIHKDL	1xTMTpro [K	1.761E-07	4.225E-05	1	1	2	P49755	P49755 [46-71]	1	3323.7727
[R].LAKVDA	1xTMTpro [K	4.697E-05	4.225E-05	1	1	1	P07237	P07237 [79-97]	1	2701.4652
[K].NAVITVF	1xTMTpro [N	4.785E-05	4.225E-05	1	1	2	P38646	P38646 [188-202]	0	1999.0568
[K].EEEVSL	1xTMTpro [N	5.192E-05	4.225E-05	1	1	2	Q9Y6W5	Q9Y6W5 [86-97]	0	1678.8931
[K].FTIYNNM	1xCarbamid	5.357E-05	4.225E-05	1	1	1	P00740	P00740 [388-404]	0	2325.0864
[R].EEFAST	1xCarbamid	3.208E-05	4.225E-05	1	1	4	O00299	O00299 [217-238	0	3181.5379
[R].FTLWGL	1xTMTpro [K	3.806E-05	4.225E-05	1	1	1	Q8TDP1	Q8TDP1 [129-14	0	2253.2775
[K].NCLTNFI	1xCarbamid	3.34E-05	4.225E-05	1	1	1	P61247	P61247 [95-107]	0	1898.9172
[K].EEFPFAI	1xCarbamid	3.526E-05	4.225E-05	1	1	1	P01023	P01023 [1337-13	0	2914.5152
[K].NCLTNFI	1xCarbamid	1.549E-05	4.225E-05	1	1	3	P61247	P61247 [95-107]	0	1882.9223
[R].EEFRPTI	1xTMTpro [K	5.064E-05	4.225E-05	1	1	1	Q14344	Q14344 [82-94]	0	2204.2571
[R].EEFTAFI	1xTMTpro [K	6.334E-06	4.225E-05	1	1	5	Q15293	Q15293 [189-204	0	2629.3252
[R].IAKEEIF	2xTMTpro [K	5.295E-06	4.225E-05	1	1	3	P00352	P00352 [396-410]	1	2643.5724
[K].NAVLEHI	1xTMTpro [N	2.73E-05	4.225E-05	1	1	1	O95466	O95466 [395-414	0	2656.3935
[K].EEFTAFL	1xTMTpro [K	3.441E-05	4.225E-05	1	1	2	O43852	O43852 [174-189	0	2657.3089
[K].EEFTSG	1xTMTpro [K	4.05E-05	4.225E-05	1	1	1	P43034	P43034 [77-88]	0	1858.0202
[R].FTLTQSI	1xTMTpro [K	5.63E-07	4.225E-05	1	1	1	Q14166	Q14166 [323-342	0	2939.5435
[K].NCASPS	1xCarbamid	2.53E-06	4.225E-05	1	1	1	O94855	O94855 [823-840	0	2498.3239
[K].FTITPST	1xTMTpro [K	4.533E-06	4.225E-05	1	1	2	P47755	P47755 [179-193]	0	2213.3401
[R].NAYAVL	1xTMTpro [K	4.106E-05	4.225E-05	1	1	2	Q06323	Q06323 [221-232	0	2004.2025
[K].FTITPPT	1xTMTpro [K	1.589E-05	4.225E-05	1	1	3	P52907	P52907 [179-193]	0	2179.3346
[R].EEGPYE	2xTMTpro [K	4.146E-08	4.225E-05	1	1	5	P21333	P21333 [1037-10	0	4398.3577
[K].NAVVQG	1xTMTpro [N	4.665E-05	4.225E-05	1	1	1	Q07954	Q07954 [1695-17	0	2367.358
[R].EEGYDS	1xTMTpro [N	4.434E-05	4.225E-05	1	1	2	Q8NFW8	Q8NFW8 [163-17	0	1690.8607
[R].FTLNPNI	1xTMTpro [N	3.317E-05	4.225E-05	1	1	1	P45985	P45985 [59-75]	0	2238.195
[K].EEFTAFI	1xOxidation	4.626E-05	4.225E-05	1	1	1	O43852	O43852 [174-189	0	2673.3038
[K].QFEHLD	1xTMTpro [N	2.75E-05	4.225E-05	1	1	1	Q02818	Q02818 [137-152	0	2301.1332
[R].QFFMED	1xTMTpro [K	8.46E-06	4.225E-05	1	1	2	P49959	P49959 [306-327]	0	3209.6585
[K].QFGAQA	1xTMTpro [K	5.313E-05	4.225E-05	1	1	5	P12814	P12814 [634-649]	0	2366.3476

[K].DLVDYIT	1xTMTpro [K	3.146E-05	4.225E-05	1	1	1	O75439	O75439 [225-235]	0	1976.0985
[K].KMGLVD	2xTMTpro [K	1.441E-07	4.225E-05	1	1	2	P40939	P40939 [214-235]	1	3314.9326
[R].KMKPLD	2xTMTpro [K	6.041E-05	4.225E-05	1	1	1	P12111	P12111 [514-533]	1	3081.7587
[R].RTDIFGV	1xTMTpro [K	2.371E-05	4.225E-05	1	1	2	Q15459	Q15459 [473-486]	1	2144.2207
[K].RTDEEG	2xTMTpro [K	3.702E-05	4.225E-05	1	1	3	Q9BUJ2	Q9BUJ2 [545-562	2	2981.6182
[R].GDADQA	1xTMTpro [N	9.53E-07	4.225E-05	1	1	3	P43243	P43243 [103-120]	0	2097.0896
[K].DIVEMLF	1xOxidation [	2.027E-05	4.225E-05	1	1	1	Q92882	Q92882 [120-138]	0	2898.5527
[K].KMQAHI	1xTMTpro [K	5.648E-06	4.225E-05	1	1	1	P35580	P35580 [947-966]	1	2977.5181
[R].GDACEG	1xCarbamido	9.09E-06	4.225E-05	1	1	1	P00734	P00734 [561-575]	0	2151.0342
[R].GDACEG	1xCarbamido	4.179E-06	4.225E-05	1	1	2	P00734	P00734 [561-575]	0	2135.0393
[R].DIVFLVD	1xTMTpro [N	2.007E-06	4.225E-05	1	1	3	P12111	P12111 [445-465]	0	2496.3781
[K].GDACDH	2xCarbamido	1.135E-05	4.225E-05	1	1	1	P07996	P07996 [891-911]	1	3027.2936
[R].DLVGPL	1xTMTpro [N	3.981E-05	4.225E-05	1	1	1	Q9NTG7	Q9NTG7 [346-35	0	1564.8919
[R].KMQPDQ	1xCarbamido	2.625E-05	4.225E-05	1	1	1	P50552	P50552 [53-68]	1	2507.4082
[R].GDAAVR	1xTMTpro [N	4.099E-05	4.225E-05	1	1	1	Q15008	Q15008 [33-46]	1	1777.955
[K].RQEVISE	1xTMTpro [N	1.527E-05	4.225E-05	1	1	1	Q92888	Q92888 [417-433]	1	2254.2838
[K].DLVHTE	1xTMTpro [N	1.396E-06	4.225E-05	1	1	1	Q9Y547	Q9Y547 [92-116]	0	3099.5666
[R].DIVIAETI	1xTMTpro [N	3.696E-05	4.225E-05	1	1	2	Q96D15	Q96D15 [202-214	0	1805.9816
[R].DIVIEME	2xTMTpro [K	4.673E-05	4.225E-05	1	1	1	Q9NZM1	Q9NZM1 [1427-1	0	2714.583
[K].DLVLGPS	1xTMTpro [K	2.388E-05	4.225E-05	1	1	2	Q49A26	Q49A26 [339-355	0	2314.4102
[R].RQEQPS	1xTMTpro [N	5.305E-05	4.225E-05	1	1	1	Q5T5P2	Q5T5P2 [1640-16	1	2019.079
[R].RTPDGT	1xTMTpro [N	9.355E-07	4.225E-05	1	1	1	P19367	P19367 [518-539]	1	2670.3443
[K].RTPMGI	1xTMTpro [N	2.429E-06	4.225E-05	1	1	1	O75915	O75915 [159-178]	1	2573.3677
[R].RVDIDEF	1xTMTpro [K	1.872E-09	4.225E-05	1	1	2	Q9BPX5	Q9BPX5 [12-49]	2	4752.3408
[K].RVEAAQ	1xTMTpro [K	2.466E-06	4.225E-05	1	1	2	Q14203	Q14203 [502-518]	1	2558.407
[K].DITPLQV	1xTMTpro [K	1.242E-05	4.225E-05	1	1	1	Q14764	Q14764 [108-124]	0	2480.5096
[K].KMEDSV	1xCarbamido	1.932E-07	4.225E-05	1	1	4	P35579	P35579 [1372-138	1	2737.4568
[R].RYPNVF	1xCarbamido	8.681E-06	4.225E-05	1	1	2	Q9Y6N5	Q9Y6N5 [327-344	1	2647.4158
[K].KMEDSV	1xCarbamido	1.951E-05	4.225E-05	1	1	3	P35579	P35579 [1372-138	2	2893.5579
[K].RYEEELI	1xTMTpro [N	1E-05	4.225E-05	1	1	1	Q7L014	Q7L014 [931-946]	1	2314.1635
[K].DITSDSE	1xTMTpro [N	5.107E-05	4.225E-05	1	1	1	P25445	P25445 [317-328]	0	1688.8047
[K].RYDQDL	1xCarbamido	1.055E-05	4.225E-05	1	1	1	Q15029	Q15029 [160-177]	1	2669.2837
[K].KMEDSV	1xCarbamido	1.486E-05	4.225E-05	1	1	3	P35579	P35579 [1372-138	2	2909.5529

[K].RVIISAPS	1xTMTpro [K	1.488E-05	4.225E-05	1	1	1	P04406	P04406 [118-139]	1	2977.6247
[K].RVESPV	1xTMTpro [N	3.696E-05	4.225E-05	1	2	1	Q99717	Q99717 [130-143]	1	1862.1547
[R].RQEQIQ.	1xTMTpro [N	1.589E-06	4.225E-05	1	1	1	Q15149	Q15149 [1366-13	1	2245.2042
[K].KMEEEIL	2xTMTpro [K	6.608E-06	4.225E-05	1	1	3	P35580	P35580 [982-996]	1	2731.5368
[R].RVEPTV	1xTMTpro [N	3.146E-05	4.225E-05	1	1	2	P01920	P01920 [126-137]	1	1645.9556
[R].DLTTAG.	1xCarbamido	3.447E-05	4.225E-05	1	1	2	Q02543	Q02543 [99-111]	0	1759.8968
[R].DLTTGYI	2xTMTpro [K	4.323E-05	4.225E-05	1	1	2	Q96G03	Q96G03 [520-533	1	2495.3446
[R].RVEPGS	1xTMTpro [N	2.016E-05	4.225E-05	1	1	1	Q15599	Q15599 [38-51]	1	1727.9724
[K].RVEIQMI	1xTMTpro [K	5.236E-06	4.225E-05	1	1	1	Q9UHD8	Q9UHD8 [187-22	1	4463.3848
[K].RVELLQI	1xTMTpro [N	4.618E-05	4.225E-05	1	1	1	Q6ZMI0	Q6ZMI0 [72-86]	1	2072.1671
[K].RVEGPG	1xTMTpro [N	4.465E-05	4.225E-05	1	1	1	Q12972	Q12972 [237-252]	1	1934.0262
[K].GDAEKP	1xTMTpro [K	1.672E-07	4.225E-05	1	1	2	Q9NS69	Q9NS69 [23-47]	0	3529.6321
[R].RVEDEV	1xTMTpro [K	4.093E-08	4.225E-05	1	1	1	L0R6Q1	L0R6Q1 [27-49]	1	3041.6399
[K].DLVASVI	1xTMTpro [K	1.578E-05	4.225E-05	1	1	1	Q14919	Q14919 [85-106]	0	2953.3799
[K].KMEGVT	2xTMTpro [K	1.948E-05	4.225E-05	1	1	1	Q9C0B1	Q9C0B1 [409-422	1	2467.4523
[R].RQEEAA	1xTMTpro [K	8.842E-06	4.225E-05	1	1	2	P12821	P12821 [82-100]	1	2799.4921
[R].RQECSII	2xCarbamido	2.828E-06	4.225E-05	1	1	2	P00734	P00734 [178-198]	1	2752.35
[R].RQECSII	2xCarbamido	1.535E-07	4.225E-05	1	1	4	P00734	P00734 [178-198]	1	2736.3551
[K].DLYANN	1xOxidation [	3.747E-06	4.225E-05	1	3	3	P62736	P62736 [294-314]	0	2548.2673
[R].GCVNPM	1xCarbamido	1.109E-05	4.225E-05	1	1	2	Q12996	Q12996 [175-188]	0	2034.0584
[K].DLYANT	1xTMTpro [N	1.004E-07	4.225E-05	1	2	31	P63261	P63261 [292-312]	0	2519.2771
[K].DLYANT	1xOxidation [	4.062E-07	4.225E-05	1	2	14	P63261	P63261 [292-312]	0	2535.272
[K].RPPESP	1xTMTpro [N	8.094E-06	4.225E-05	1	1	1	Q9BTL3	Q9BTL3 [32-46]	0	2097.1048
[K].RPPEPT	1xTMTpro [K	6.416E-06	4.225E-05	1	1	1	Q9NX14	Q9NX14 [47-69]	0	3404.6778
[R].RPNVVL	2xTMTpro [K	7.531E-07	4.225E-05	1	1	3	P15586	P15586 [46-69]	1	3564.0651
[R].KNAPCIL	1xCarbamido	1.379E-05	4.225E-05	1	1	1	Q9Y4W6	Q9Y4W6 [398-41	1	2539.4198
[K].GCVEYE	1xCarbamido	3.582E-05	4.225E-05	1	1	2	P36269	P36269 [528-542]	0	2146.0194
[R].KNCASP	1xCarbamido	1.825E-05	4.225E-05	1	1	1	O94855	O94855 [822-840]	1	2930.626
[K].DLYANN	1xTMTpro [N	7.859E-06	4.225E-05	1	3	1	P62736	P62736 [294-314]	0	2532.2724
[R].RPLRPQ	1xTMTpro [K	5.047E-05	4.225E-05	1	1	3	Q9HDC9	Q9HDC9 [11-29]	0	2700.4925
[K].RPILQSL	1xTMTpro [K	1.555E-05	4.225E-05	1	1	2	Q8N766	Q8N766 [621-638	0	2708.5665
[R].RPLIDQ\	1xTMTpro [K	2.337E-05	4.225E-05	1	1	1	Q00610	Q00610 [968-993]	0	3489.9296
[K].KNDKEA	3xTMTpro [K	2.086E-05	4.225E-05	1	1	2	P27695	P27695 [32-52]	2	3488.9014

[K].DLYSGLI	1xCarbamido	4.029E-05	4.225E-05	1	1	4	P00450	P00450 [888-901]	0	1880.0635
[R].RPLHSA	1xTMTpro [N	5.728E-05	4.225E-05	1	1	2	Q9H4A4	Q9H4A4 [15-32]	0	2230.2012
[K].DIYYNNN	1xCarbamido	2.771E-06	4.225E-05	1	1	3	Q6DD88	Q6DD88 [361-386	0	4018.0282
[R].RPGGEF	1xTMTpro [N	2.915E-07	4.225E-05	1	1	3	P02751	P02751 [2426-244	0	2700.2933
[R].RPEQVT	1xTMTpro [N	5.566E-06	4.225E-05	1	1	1	O60826	O60826 [321-338]	0	2474.2959
[K].GCSNEP	3xCarbamido	4.785E-05	4.225E-05	1	1	1	Q68CZ2	Q68CZ2 [1250-12	0	3728.9094
[K].KNEPLAS	1xTMTpro [K	1.289E-07	4.225E-05	1	1	1	P56199	P56199 [428-458]	1	3851.0471
[K].RPLMVL	1xTMTpro [K	9.766E-06	4.225E-05	1	1	2	A1L0T0	A1L0T0 [285-303]	0	2606.5195
[R].KMDPAE	2xTMTpro [K	5.937E-06	4.225E-05	1	1	2	P34741	P34741 [120-134]	1	2682.4113
[K].DLWYLE	1xTMTpro [K	4.825E-05	4.225E-05	1	1	1	P51610	P51610 [355-369]	0	2420.347
[K].GCWDSI	1xCarbamido	3.839E-05	4.225E-05	1	1	2	P47756	P47756 [146-159]	0	2294.2095
[R].RQDQLQ	1xTMTpro [N	1.148E-05	4.225E-05	1	1	1	Q8N3D4	Q8N3D4 [1421-14	1	2348.2199
[K].DIVPGDI	1xTMTpro [K	5.046E-09	4.225E-05	1	1	4	P16615	P16615 [144-164]	1	2799.6112
[R].RQDLPS	1xTMTpro [N	3.213E-06	4.225E-05	1	1	1	P49593	P49593 [436-453]	1	2322.2373
[R].DIVPVDA	1xTMTpro [K	2.77E-05	4.225E-05	1	1	2	O43252	O43252 [224-235]	0	1943.0981
[R].RQDLED	1xTMTpro [N	2.652E-07	4.225E-05	1	1	1	Q13813	Q13813 [883-907]	1	3305.5452
[K].RQDEDY	1xTMTpro [K	8.924E-06	4.225E-05	1	1	1	O00410	O00410 [815-841]	1	3911.8438
[K].DLVQLPY	1xTMTpro [N	5.192E-05	4.225E-05	1	1	1	O95486	O95486 [416-430]	0	1931.1496
[R].GDAAAG	1xTMTpro [N	5.711E-06	4.225E-05	1	1	1	Q6DKJ4	Q6DKJ4 [62-83]	0	2151.075
[K].DIVTNNC	1xTMTpro [K	1.572E-06	4.225E-05	1	1	2	Q15063	Q15063 [350-371]	0	2982.7119
[K].RPWQG	1xTMTpro [K	2.968E-05	4.225E-05	1	1	1	P07902	P07902 [51-63]	0	2187.2894
[K].KMQQEN	2xTMTpro [K	6.844E-06	4.225E-05	1	1	1	P22087	P22087 [285-304]	1	3432.8436
[R].RPWNVA	1xTMTpro [K	2.023E-05	4.225E-05	1	1	5	P02748	P02748 [195-207]	0	2185.2625
[K].RPTELLS	1xTMTpro [N	2.412E-06	4.225E-05	1	1	1	P07384	P07384 [87-104]	0	2318.2788
[K].DLVVTEY	1xTMTpro [N	3E-06	4.225E-05	1	1	1	P24821	P24821 [628-648]	0	2767.3779
[K].RPTEICA	1xCarbamido	4.516E-06	4.225E-05	1	1	1	P17655	P17655 [77-94]	0	2306.2246
[K].RPSTSQ	1xTMTpro [K	3.859E-06	4.225E-05	1	1	1	P12270	P12270 [644-669]	0	3303.8292
[K].RPSSGIS	1xTMTpro [K	1.024E-05	4.225E-05	1	1	3	Q9UMS6	Q9UMS6 [86-102]	0	2439.3335
[R].RPSQEQ	1xTMTpro [N	2.461E-06	4.225E-05	1	1	1	Q5T1M5	Q5T1M5 [954-974	0	2500.2823
[K].RPSPDE	1xTMTpro [K	5.926E-05	4.225E-05	1	1	2	Q9UHD1	Q9UHD1 [108-12	0	2235.2299
[K].RPSENL	1xTMTpro [N	2.57E-05	4.225E-05	1	1	2	Q99805	Q99805 [91-104]	0	1906.0466
[K].GCYTVD	1xCarbamido	1.126E-06	4.225E-05	1	1	1	O14498	O14498 [368-392]	0	3128.5529
[R].RPQYSN	1xTMTpro [N	1.754E-07	4.225E-05	1	1	1	P67809	P67809 [205-234]	0	3527.7369

[R].RPTPQD	1xTMTpro [N	2.461E-06	4.225E-05	1	1	1	P49327	P49327 [1405-142	0	2492.3105
[K].DMAIATC	1xTMTpro [K	8.34E-08	4.225E-05	1	1	23	P10809	P10809 [315-344]	0	3705.9289
[K].SAADAE	1xTMTpro [K	9.566E-07	4.225E-05	1	1	1	O15460	O15460 [72-88]	0	2385.2694
[R].DITLFHA	1xTMTpro [K	5.984E-05	4.225E-05	1	1	1	Q13330	Q13330 [248-260]	0	2150.1924
[R].GDEGIFI	1xTMTpro [N	1.984E-05	4.225E-05	1	1	2	Q9UMY4	Q9UMY4 [106-11	0	1960.9459
[R].DISEASV	1xTMTpro [K	3.638E-05	4.225E-05	1	1	4	P62854	P62854 [52-66]	0	2262.2513
[K].KLVEGLS	2xTMTpro [K	6.421E-07	4.225E-05	1	1	2	P19971	P19971 [222-235]	1	2382.5152
[R].KIVEGIS	1xTMTpro [K	1.04E-06	4.225E-05	1	1	1	Q15126	Q15126 [94-110]	1	2549.4947
[R].SAQFEH	1xCarbamido	7.547E-06	4.225E-05	1	1	1	P53582	P53582 [354-373]	0	2595.3408
[K].GDEEGV	1xTMTpro [N	1.242E-05	4.225E-05	1	1	2	P13796	P13796 [310-326]	0	2048.0653
[K].SAPSIPK	1xCarbamido	2.551E-05	4.225E-05	1	1	1	P40925	P40925 [143-157]	1	2315.2673
[K].GDECQL	1xCarbamido	8.337E-06	4.225E-05	1	1	2	Q15149	Q15149 [962-977]	0	2417.2527
[K].KLVESLF	2xTMTpro [K	2.21E-05	4.225E-05	1	1	2	P52815	P52815 [163-173]	1	2196.3784
[R].SANYET	1xTMTpro [K	4.549E-05	4.225E-05	1	1	1	Q9H9G7	Q9H9G7 [386-40	0	2558.3059
[K].SAMQSV	1xTMTpro [K	3.995E-05	4.225E-05	2	2	1	Q8WVM7; Q	Q8WVM7 [96-110	0	2366.2194
[K].DLSEVSI	1xTMTpro [K	4.33E-05	4.225E-05	1	1	1	P41227	P41227 [211-225]	0	2248.1688
[R].SAMPFT	1xTMTpro [N	2.362E-05	4.225E-05	1	1	2	O00151	O00151 [123-138]	0	1886.9601
[K].DLSFSEY	1xTMTpro [K	2.291E-05	4.225E-05	1	1	2	Q99715	Q99715 [637-649]	0	2088.1145
[R].SALYYVI	1xTMTpro [K	4.213E-05	4.225E-05	1	1	2	P13798	P13798 [280-291]	0	1907.1134
[R].KLVIIESI	1xTMTpro [K	2.279E-05	4.225E-05	1	1	2	P09493	P09493 [168-182]	2	2408.4004
[K].SALVQIY	1xTMTpro [K	3.08E-05	4.225E-05	1	1	1	P54619	P54619 [101-113]	0	2167.2254
[K].SAIVHLII	1xTMTpro [N	3.243E-06	4.225E-05	1	1	2	P14923	P14923 [125-142]	0	2333.242
[K].SALTVQI	1xTMTpro [K	5.09E-05	4.225E-05	1	1	1	P61225	P61225 [17-31]	0	2235.288
[R].DISILQC	2xCarbamido	3.961E-06	4.225E-05	1	1	2	O75608	O75608 [163-188]	0	3552.8396
[K].SALTVQI	1xTMTpro [K	1.889E-05	4.225E-05	2	3	4	P61224; P62	P61224 [17-31]; F	0	2274.3353
[K].DISDIEG	1xTMTpro [K	5.803E-05	4.225E-05	1	1	1	P52789	P52789 [338-350]	1	2055.1214
[K].SASDLSI	1xTMTpro [K	4.833E-05	4.225E-05	1	1	2	Q92541	Q92541 [650-660]	0	1819.9933
[R].GDELAD	1xTMTpro [K	1.906E-05	4.225E-05	1	1	4	Q15582	Q15582 [643-655]	0	2016.1145
[K].DLSAGA	1xCarbamido	4.5E-06	4.225E-05	1	1	1	Q7L266	Q7L266 [86-103]	0	2422.362
[R].SAYQTII	1xTMTpro [N	8.05E-06	4.225E-05	1	1	1	Q6RW13	Q6RW13 [131-15	0	2596.285
[-].DIQMTQS	1xTMTpro [N	3.806E-05	4.225E-05	1	1	6	P0DOX7	P0DOX7 [1-18]	0	2197.109
[-].DIQMTQS	1xOxidation [	4.12E-05	4.225E-05	1	1	3	P0DOX7	P0DOX7 [1-18]	0	2213.1039
[K].KISSIQSI	1xTMTpro [K	4.508E-06	4.225E-05	1	1	2	P10809	P10809 [250-268]	1	2655.5801

[K].SAYEFSI	1xTMTpro [K	1.017E-05	4.225E-05	1	1	2	P09960	P09960 [231-243]	0	2130.092
[K].GDFFPP	1xTMTpro [K	6.199E-08	4.225E-05	1	1	2	P52272	P52272 [286-322]	0	4427.3327
[K].KLTAGE	1xCarbamido	1.659E-07	4.225E-05	1	1	1	O75521	O75521 [305-328]	1	3509.913
[K].SAWLSG	1xTMTpro [N	1.872E-05	4.225E-05	1	1	2	P13674	P13674 [383-396]	0	1868.9826
[K].SAVTAL	1xTMTpro [K	6.007E-08	4.225E-05	1	1	2	P68871	P68871 [10-31]	1	2836.5813
[R].KLTALDY	1xCarbamido	1.76E-05	4.225E-05	1	1	1	Q9Y224	Q9Y224 [5-26]	2	3538.8569
[K].SALTIQL	1xTMTpro [N	3.07E-07	4.225E-05	2	3	4	P01111; P01	P01111 [17-41]; F	0	3271.6442
[R].KLTATPT	1xTMTpro [K	3.604E-06	4.225E-05	1	1	1	O75533	O75533 [430-451]	1	2997.5782
[R].DLQTHG	1xTMTpro [K	1.776E-05	4.225E-05	1	1	1	Q7Z478	Q7Z478 [1264-12	0	2239.2367
[K].KITESVA	2xTMTpro [K	3.113E-05	4.225E-05	1	1	2	Q96A49	Q96A49 [71-84]	1	2431.4588
[K].SAVCIAD	1xCarbamido	1.341E-05	4.225E-05	1	1	1	Q99575	Q99575 [355-370]	0	2321.2667
[K].GDEVQC	1xCarbamido	5.831E-05	4.225E-05	1	2	1	Q96GK7	Q96GK7 [296-312	0	2553.3726
[K].SATLSST	1xTMTpro [K	5.179E-06	4.225E-05	1	1	2	Q02952	Q02952 [640-658]	0	2655.2985
[R].GDEVMV	2xTMTpro [K	8.215E-06	4.225E-05	1	1	1	P35580	P35580 [55-68]	1	2431.3683
[R].KLTMEQ	2xTMTpro [K	3.954E-05	4.225E-05	1	1	1	P24928	P24928 [1225-12	1	2103.2664
[K].KLTPVQ	2xTMTpro [K	3.881E-06	4.225E-05	1	1	2	Q9BX66	Q9BX66 [860-875	1	2671.6215
[R].SASPDD	1xTMTpro [N	1.504E-06	4.225E-05	1	1	2	O00193	O00193 [15-37]	0	2739.2301
[K].SASNYS	1xTMTpro [K	1.184E-05	4.225E-05	1	1	1	Q0JRZ9	Q0JRZ9 [59-77]	0	2725.4481
[R].SAVLSEI	1xTMTpro [K	3.338E-07	4.225E-05	1	1	2	Q6P587	Q6P587 [36-66]	0	3914.1633
[K].GDDITM	2xTMTpro [K	6.694E-06	4.225E-05	1	1	4	P01008	P01008 [308-322]	0	2581.5455
[K].SALSGH	1xTMTpro [K	3.514E-05	4.225E-05	1	2	4	P07355	P07355 [89-104]	0	2259.3932
[R].DISMEID	1xTMTpro [N	3.954E-05	4.225E-05	1	1	1	Q16204	Q16204 [247-260]	0	1971.9145
[K].SADVNV	1xTMTpro [K	2.677E-05	4.225E-05	1	1	1	P12111	P12111 [167-186]	0	2628.4012
[R].SADVES	1xTMTpro [N	3.657E-05	4.225E-05	1	1	2	P30038	P30038 [326-338]	0	1623.8873
[K].SADGVI	1xTMTpro [K	5.014E-05	4.225E-05	1	1	1	Q9UNH7	Q9UNH7 [194-21	1	2929.5187
[R].SADGLT	3xCarbamido	3.782E-06	4.225E-05	1	1	2	O00468	O00468 [262-277]	0	2043.9581
[R].DLTATE	1xTMTpro [N	2.804E-08	4.225E-05	1	1	2	P24821	P24821 [1892-19	0	2588.4003
[R].DLTDEEI	2xTMTpro [K	2.956E-06	4.225E-05	1	1	1	Q969G3	Q969G3 [106-123	1	3158.6197
[K].DITDTSI	1xTMTpro [N	2.956E-06	4.225E-05	1	1	1	Q99715	Q99715 [915-933]	0	2345.1767
[R].SADFVV	1xTMTpro [K	7.368E-06	4.225E-05	1	1	1	O75369	O75369 [567-593]	0	3289.7448
[K].SADFVVI	1xTMTpro [K	2.27E-07	4.225E-05	1	1	19	P21333	P21333 [594-620]	0	3303.724
[K].KLYHSE	2xTMTpro [K	2.823E-06	4.225E-05	1	1	2	P01009	P01009 [160-178]	1	3098.6615
[R].GDAVCT	2xCarbamido	1.734E-06	4.225E-05	1	1	4	P08603	P08603 [247-265]	0	2786.3733

[K].SADESG	1xTMTpro [N	2.546E-07	4.225E-05	1	1	1	Q13813	Q13813 [419-439]	0	2451.2071
[K].KLYHSE/	3xTMTpro [K	1.764E-07	4.225E-05	1	1	6	P01009	P01009 [160-179]	2	3530.9636
[R].KMDETD	2xTMTpro [K	7.274E-06	4.225E-05	1	1	3	Q13148	Q13148 [84-95]	1	2194.2205
[R].GDARPA	1xTMTpro [K	3.086E-05	4.225E-05	1	1	2	P49821	P49821 [402-417]	0	2395.3113
[R].SAAPSTI	1xTMTpro [K	4.328E-06	4.225E-05	1	1	2	Q9Y5M8	Q9Y5M8 [209-227	0	2397.3117
[R].SAAPGS	1xTMTpro [N	8.05E-06	4.225E-05	1	1	1	Q12931	Q12931 [221-248]	0	3115.5656
[K].SAAMLG	1xTMTpro [N	3.016E-05	4.225E-05	1	1	1	O60749	O60749 [347-362]	0	1963.9827
[R].SAALQS'	2xTMTpro [K	1.249E-05	4.225E-05	1	1	3	Q96BJ3	Q96BJ3 [74-92]	1	3064.6983
[K].SAALPIF	1xTMTpro [K	7.124E-07	4.225E-05	1	1	3	O76021	O76021 [258-277]	1	2834.5333
[K].DLTLDQ/	1xTMTpro [K	3.577E-06	4.225E-05	1	1	1	P23381	P23381 [205-220]	0	2409.2793
[R].SAALAAI	1xTMTpro [N	3.94E-05	4.225E-05	1	1	1	P15428	P15428 [164-177]	0	1678.923
[R].SADEPM	2xCarbamido	1.903E-05	4.225E-05	1	1	1	P23193	P23193 [280-297]	0	2391.0698
[R].SAADSIS	1xTMTpro [K	1.409E-05	4.225E-05	1	1	2	P45974	P45974 [779-793]	0	2052.1469
[K].SAEAGI/	1xTMTpro [K	2.405E-05	4.225E-05	1	1	1	Q7Z478	Q7Z478 [27-39]	0	1827.0104
[K].GDCTVL	1xCarbamido	2.921E-05	4.225E-05	1	1	2	O60488	O60488 [361-380]	0	2825.5219
[R].SALQSIN	2xTMTpro [K	6.776E-08	4.225E-05	1	1	2	P50454	P50454 [168-194]	2	3899.1331
[R].SALQSIN	2xTMTpro [K	3.723E-08	4.225E-05	1	1	3	P50454	P50454 [168-190]	1	3399.894
[R].SALQSIN	1xTMTpro [K	8.891E-06	4.225E-05	1	1	4	P50454	P50454 [168-184]	0	2428.2964
[K].KLVIVGD	1xCarbamido	1.651E-05	4.225E-05	2	2	3	P08134; P61	P08134 [7-18]; P6	1	2129.2933
[K].SAIDLTP	2xTMTpro [K	3E-06	4.225E-05	1	1	4	Q14839	Q14839 [1648-16	1	2698.5695
[R].GDDGIFI	1xTMTpro [N	3.005E-05	4.225E-05	1	1	3	O60493	O60493 [105-118]	0	1945.9099
[K].SAKPTKI	2xTMTpro [K	1.703E-05	4.225E-05	1	1	2	Q05682	Q05682 [691-710]	0	2903.7135
[R].SAGWNI	1xCarbamido	9.2E-07	4.225E-05	1	1	5	P02787	P02787 [144-162]	0	2475.3025
[R].SAGVQC	1xCarbamido	6.404E-06	4.225E-05	1	1	1	P22102	P22102 [88-107]	0	2646.3689
[R].DLSSAE/	1xTMTpro [K	4.125E-06	4.225E-05	1	1	2	Q9P000	Q9P000 [75-95]	0	2965.6279
[R].KLVSQE	1xTMTpro [K	5.183E-05	4.225E-05	1	1	1	P82909	P82909 [87-99]	1	2245.2506
[K].DISSDAF	2xTMTpro [K	4.73E-06	4.225E-05	1	1	2	P98082	P98082 [631-649]	1	2947.6444
[K].SAGTVE	1xTMTpro [K	1.512E-06	4.225E-05	1	1	1	P07384	P07384 [518-548]	0	4084.0378
[R].SAGQGE	1xTMTpro [K	4.954E-08	4.225E-05	1	1	4	P21333	P21333 [310-331]	0	2921.5136
[R].KLVPLLL	2xTMTpro [K	5.495E-07	4.225E-05	1	1	2	Q14204	Q14204 [36-59]	1	3388.9759
[K].SAGPQS	1xTMTpro [K	2.21E-05	4.225E-05	1	1	1	P35612	P35612 [512-527]	0	2207.2891
[R].SAGDGT	1xCarbamido	4.697E-05	4.225E-05	1	1	1	O43815	O43815 [303-331]	1	4192.0783
[R].SAGACT	1xCarbamido	3.293E-05	4.225E-05	1	1	4	P28838	P28838 [458-469]	0	1775.997

[K].KLVQTY!	2xTMTpro [K	3.334E-06	4.225E-05	1	1	1	P23258	P23258 [164-193]	1	4368.3981
[K].SAEPSP	1xTMTpro [N	1.899E-05	4.225E-05	1	1	1	P49023	P49023 [126-147]	0	2582.2939
[R].SAELPD,	1xTMTpro [K	2.947E-05	4.225E-05	1	1	2	Q96PU8	Q96PU8 [69-85]	0	2402.3786
[K].GDCVEC	3xCarbamido	3.119E-05	4.225E-05	1	1	2	P34949	P34949 [281-295]	0	2077.8731
[K].DLSSHQ	1xTMTpro [N	1.281E-05	4.225E-05	1	1	1	P09960	P09960 [494-510]	0	2304.2267
[K].GDFIALD	1xTMTpro [N	3.411E-05	4.225E-05	1	1	2	P19367	P19367 [78-91]	0	1758.9346
[K].DMAIATC	1xOxidation [	1.425E-07	4.225E-05	1	1	3	P10809	P10809 [315-344]	0	3721.9239
[K].GCQQGC	1xCarbamido	8.309E-07	4.225E-05	1	1	1	P35442	P35442 [225-245]	0	2580.2643
[R].RDNYVP	1xTMTpro [K	2.263E-05	4.225E-05	1	1	1	P08708	P08708 [81-103]	1	3253.7084
[K].KPAGPS	2xTMTpro [K	4.449E-05	4.225E-05	1	1	1	Q02539	Q02539 [37-55]	0	2768.6338
[K].DNHLLG	1xTMTpro [N	1.91E-06	4.225E-05	1	1	8	P11021	P11021 [475-492]	0	2238.2202
[K].RDETLQ	2xCarbamido	3.974E-05	4.225E-05	1	1	1	P04275	P04275 [2663-267	1	2490.1997
[K].DNKETF	1xTMTpro [K	3.178E-06	4.225E-05	1	1	1	P84157	P84157 [182-200]	1	2806.3809
[R].DNIACVI	1xCarbamido	2.707E-07	4.225E-05	1	1	1	P11413	P11413 [228-246]	1	2775.4995
[R].RDDGTG	1xTMTpro [N	3.317E-05	4.225E-05	1	1	1	Q15149	Q15149 [4003-40	1	2031.1154
[R].RDDDGA	1xCarbamido	4.765E-06	4.225E-05	1	1	1	P08294	P08294 [53-77]	1	2967.4662
[K].GCDNNF	1xCarbamido	1.842E-05	4.225E-05	1	1	1	O95487	O95487 [1174-11	0	3110.5749
[R].RCESCA	2xCarbamido	1.051E-05	4.225E-05	1	1	2	P98160	P98160 [850-868]	1	2685.3369
[K].KPDCDD	2xCarbamido	4.1E-08	4.225E-05	1	1	4	P02794	P02794 [88-109]	0	3530.7534
[K].RCELCD	2xCarbamido	4.592E-06	4.225E-05	1	1	1	P11047	P11047 [803-818]	1	2234.029
[K].KPDCDD	2xCarbamido	8.602E-06	4.225E-05	1	1	2	P02794	P02794 [88-109]	0	3546.7483
[R].KPDDLP,	1xTMTpro [K	8.908E-06	4.225E-05	1	1	1	Q8IWA5	Q8IWA5 [481-496	0	2386.3415
[R].GCALQC	2xCarbamido	5.41E-05	4.225E-05	3	3	2	O95757; P3	O95757 [375-388]	0	2144.1852
[R].KPDQVF	3xTMTpro [K	1.086E-05	4.225E-05	1	1	1	Q96K76	Q96K76 [701-717	0	3196.8545
[R].DNIQGIT	1xTMTpro [K	4.177E-05	4.225E-05	1	1	10	P62805	P62805 [25-36]	0	1934.1679
[R].RAPEEE	1xTMTpro [N	4.634E-05	4.225E-05	1	1	1	Q8IY67	Q8IY67 [30-45]	1	2194.1675
[R].KPDVSH	2xTMTpro [K	1.687E-06	4.225E-05	1	1	1	P04003	P04003 [241-260]	0	3137.691
[R].DNLTLW	1xTMTpro [K	1.135E-05	4.225E-05	1	1	5	P62258	P62258 [226-244]	0	2789.3544
[K].RAEISNA	1xTMTpro [K	5.516E-07	4.225E-05	1	1	2	P31939	P31939 [484-507]	1	3258.7349
[K].RDPEFV	1xTMTpro [K	2.984E-05	4.225E-05	1	1	1	Q969V3	Q969V3 [500-511	1	2165.1887
[K].DNFTAIF	1xTMTpro [N	3.352E-05	4.225E-05	1	1	2	Q14195	Q14195 [346-361]	0	2053.0157
[R].RDPPQY	1xTMTpro [N	3.195E-06	4.225E-05	1	1	3	P10153	P10153 [145-159]	1	2092.1623
[R].RDQDNI	1xTMTpro [N	3.961E-05	4.225E-05	1	1	1	P33176	P33176 [465-476]	1	1793.8884

[K].REPGYT	1xTMTpro [K	1.038E-05	4.225E-05	1	1	1	Q93052	Q93052 [328-352]	1	3190.6599
[K].DNCAPE	1xCarbamido	7.744E-06	4.225E-05	1	1	2	P05106	P05106 [73-88]	0	2125.0191
[R].REPFLLS	1xTMTpro [K	1.691E-05	4.225E-05	1	1	1	Q9BQ67	Q9BQ67 [319-333	1	2183.2316
[R].REPETP	1xTMTpro [N	4.057E-05	4.225E-05	1	1	1	Q02388	Q02388 [593-605]	1	1739.0135
[K].RELHGQ	1xCarbamido	5.813E-05	4.225E-05	1	1	1	Q16630	Q16630 [147-161]	1	2357.3004
[R].REGGDC	1xTMTpro [N	5.322E-05	4.225E-05	1	1	1	Q8N1G4	Q8N1G4 [291-306	1	1950.9072
[R].KNWYLF	1xTMTpro [K	4.427E-05	4.225E-05	1	1	1	Q7Z2K6	Q7Z2K6 [758-770	1	2165.2363
[R].GCFSDL	1xCarbamido	4.523E-07	4.225E-05	1	1	1	Q9BW27	Q9BW27 [514-534	0	2644.274
[K].KPAAAA	2xTMTpro [K	3.453E-05	4.225E-05	1	1	2	P50454	P50454 [23-35]	0	2095.2692
[R].KPAAGL	1xTMTpro [K	2.179E-07	4.225E-05	1	1	3	Q9NQC3	Q9NQC3 [58-91]	0	3880.1075
[R].DNLTLW	1xTMTpro [K	9.659E-06	4.225E-05	1	1	2	P62258	P62258 [226-255]	1	4059.8857
[R].REEFWL	1xTMTpro [K	1.825E-05	4.225E-05	1	1	3	P24821	P24821 [2051-206	1	2242.2476
[K].REEAPS	1xTMTpro [N	9.694E-06	4.225E-05	1	1	1	P02675	P02675 [53-72]	1	2411.3115
[R].REDSGL	1xCarbamido	5.813E-05	4.225E-05	1	1	1	P12314	P12314 [252-271]	1	2921.4595
[K].DNENVV	1xTMTpro [K	2.231E-06	4.225E-05	1	1	7	P02675	P02675 [164-178]	0	2377.2015
[K].DNENVV	1xTMTpro [K	4.401E-06	4.225E-05	1	1	2	P02675	P02675 [164-196]	1	4485.2665
[K].REDLVV	1xTMTpro [K	3.384E-06	4.225E-05	1	1	2	P12268	P12268 [182-195]	1	2090.2829
[K].KPADDQ	1xCarbamido	6.386E-07	4.225E-05	1	1	1	P20810	P20810 [643-673]	0	4190.0863
[K].DNFADV	1xCarbamido	5.733E-06	4.225E-05	1	1	1	Q9H0W9	Q9H0W9 [26-43]	0	2630.3627
[R].KPADLQ	2xTMTpro [K	7.395E-06	4.225E-05	1	1	2	Q9Y696	Q9Y696 [62-85]	0	3533.9937
[R].DNFGGC	1xTMTpro [K	2.295E-05	4.225E-05	1	1	1	Q96HD1	Q96HD1 [66-82]	0	2476.2236
[K].DNFHGL	1xTMTpro [N	9.909E-07	4.225E-05	1	1	4	Q12907	Q12907 [152-172]	0	2772.3436
[K].REEEEFI	1xTMTpro [K	1.193E-06	4.225E-05	1	1	4	P62316	P62316 [19-37]	1	2771.5071
[R].RAEDGS	1xTMTpro [N	1.158E-06	4.225E-05	1	1	1	P07355	P07355 [179-196]	1	2369.1904
[K].RADTVG	1xCarbamido	2.677E-05	4.225E-05	1	1	1	O75165	O75165 [2102-21	1	1849.9874
[R].KPDVSH	1xOxidation	5.545E-06	4.225E-05	1	1	1	P04003	P04003 [241-260]	0	3153.6859
[R].QYEQQT	1xTMTpro [K	2.916E-05	4.225E-05	1	1	2	Q9Y262	Q9Y262 [39-53]	0	2474.3786
[R].QYELVV	1xTMTpro [K	3.242E-05	4.225E-05	1	1	1	P16435	P16435 [244-257]	0	2210.2313
[K].QYEDAL	1xTMTpro [N	4.457E-05	4.225E-05	1	1	1	Q6UW02	Q6UW02 [223-23	0	1999.0489
[R].DNTAGP	1xCarbamido	2.175E-05	4.225E-05	1	1	1	P11047	P11047 [744-753]	0	1736.8881
[K].GAVYSF	1xTMTpro [N	4.595E-05	4.225E-05	1	1	2	P20618	P20618 [147-160]	0	1849.9404
[R].KPEYPK	2xTMTpro [K	1.98E-05	4.225E-05	1	1	1	Q12996	Q12996 [578-595]	0	3112.7434
[K].KPGHILV	2xTMTpro [K	7.491E-06	4.225E-05	1	1	1	P32455	P32455 [88-106]	0	2945.7492

[K].DNTIEHL	1xTMTpro [K	1.154E-05	4.225E-05	1	1	2	P30153	P30153 [359-374]	0	2473.4674
[R].QYAGLD	1xTMTpro [N	9.659E-06	4.225E-05	1	1	2	Q99720	Q99720 [48-60]	0	1810.9407
[R].QWYESI	1xTMTpro [N	3.202E-05	4.225E-05	1	1	2	P62241	P62241 [111-123]	0	1924.0037
[R].KPEVLRI	1xTMTpro [K	3.879E-05	4.225E-05	1	1	1	P55211	P55211 [117-146]	0	3757.0892
[R].DNTQLLI	1xTMTpro [N	3.525E-06	4.225E-05	1	1	1	Q15050	Q15050 [67-83]	0	2386.305
[R].QWQPD\	1xTMTpro [K	1.438E-06	4.225E-05	1	1	2	O75306	O75306 [36-56]	0	3060.5533
[K].DNTTLLT	1xTMTpro [N	1.776E-05	4.225E-05	1	1	2	Q13561	Q13561 [366-379]	0	1926.0285
[R].QWQCD	2xCarbamido	6.012E-05	4.225E-05	1	1	1	Q9GZM7	Q9GZM7 [132-14	0	2770.3494
[K].KPGMFF	1xTMTpro [K	7.032E-07	4.225E-05	1	1	1	P11413	P11413 [408-427]	0	2952.5057
[R].QWNNC/	1xCarbamido	1.163E-05	4.225E-05	1	2	2	P61224	P61224 [137-149]	0	2163.1148
[K].KPGMFF	1xOxidation [	9.31E-06	4.225E-05	1	1	2	P11413	P11413 [408-427]	0	2968.5006
[K].KPGNFQ	2xTMTpro [K	5.563E-05	4.225E-05	1	1	1	Q8TBP6	Q8TBP6 [80-93]	0	2482.4274
[K].GAVSAE	1xTMTpro [N	9.82E-06	4.225E-05	1	1	2	Q9UHV9	Q9UHV9 [19-32]	0	1722.9458
[K].QVYQIVI	2xCarbamido	6.701E-07	4.225E-05	1	1	1	Q9NR45	Q9NR45 [167-19	0	4460.3391
[R].DNWSSS	2xTMTpro [K	5.509E-05	4.225E-05	1	1	1	Q8WUM0	Q8WUM0 [1081-1	1	2791.4566
[R].QWVDTI	1xTMTpro [K	3.208E-05	4.225E-05	1	1	1	P15927	P15927 [106-127]	0	3118.5712
[R].GCGEQT	1xCarbamido	9.518E-06	4.225E-05	2	2	1	P0C0L4; P0	P0C0L4 [1009-10	0	2243.1483
[K].KPESFF	3xCarbamido	2.064E-06	4.225E-05	1	1	1	Q9UBE0	Q9UBE0 [118-13	0	2961.4553
[K].DNSNLY	1xTMTpro [N	6.04E-07	4.225E-05	2	2	1	P17612; P2	P17612 [113-134]	0	2893.3642
[R].DNLTLW	1xCarbamido	4.642E-05	4.225E-05	1	1	5	P27348	P27348 [223-245]	0	2759.1909
[R].DNLTLW	1xTMTpro [N	1.379E-05	4.225E-05	1	1	9	P63104	P63104 [223-245]	0	2713.2032
[K].RAAEDD	1xTMTpro [K	2.079E-05	4.225E-05	1	1	2	P06454	P06454 [90-103]	1	2202.0654
[K].KPEASG	1xCarbamido	8.697E-06	4.225E-05	1	1	1	A4D1P6	A4D1P6 [355-379	0	3378.7495
[K].QYVTSLI	1xTMTpro [N	5.031E-05	4.225E-05	1	1	2	P42226	P42226 [545-562]	0	2383.3192
[R].KPEDVLI	2xTMTpro [K	4.783E-06	4.225E-05	1	1	1	P35613	P35613 [350-366]	0	2697.4763
[R].QYTSPE	1xTMTpro [K	2.155E-05	4.225E-05	1	1	4	Q13442	Q13442 [16-31]	0	2458.2957
[K].DNNQFA	1xTMTpro [N	4.76E-05	4.225E-05	1	1	2	P35606	P35606 [154-165]	0	1641.8152
[K].GAYPLSI	1xTMTpro [N	5.527E-05	4.225E-05	1	1	2	P00450	P00450 [469-481]	0	1675.9702
[K].KPEDWI	2xTMTpro [K	1.12E-06	4.225E-05	1	1	3	P27797	P27797 [249-272]	0	3872.9145
[R].DNSQVN	1xTMTpro [K	8.245E-06	4.225E-05	1	1	3	O95810	O95810 [49-64]	0	2338.3473
[R].DNPHFQ	1xTMTpro [K	1.589E-05	4.225E-05	1	1	1	Q07157	Q07157 [43-61]	0	2694.4594
[R].QYPWG\	1xCarbamido	5.335E-08	4.225E-05	1	1	2	Q16181	Q16181 [266-286]	0	2824.3684
[R].QYPWG	1xCarbamido	6.07E-05	4.225E-05	1	1	1	Q14141	Q14141 [255-273]	0	2915.4642

[K].KPEDWI	1xOxidation	1.296E-06	4.225E-05	1	1	4	P27797	P27797 [249-272]	0	3888.9094
[K].DNPTED	2xCarbamido	2.945E-06	4.225E-05	1	1	1	P16284	P16284 [466-486]	0	3097.4235
[R].DNQHQI	1xTMTpro [N	4.776E-05	4.225E-05	1	1	1	O00461	O00461 [509-523]	0	1985.8868
[K].KPELVIS	2xTMTpro [K	5.701E-05	4.225E-05	1	1	3	P78347	P78347 [645-660]	0	2642.5771
[R].DNSDFD	1xTMTpro [N	1.164E-06	4.225E-05	1	1	2	O15371	O15371 [282-310]	0	3499.6056
[K].QYLVTY	1xTMTpro [N	4.898E-05	4.225E-05	1	1	1	Q99715	Q99715 [843-862]	0	2515.3363
[R].QYLSGE	1xTMTpro [N	9.09E-06	4.225E-05	1	1	1	P55809	P55809 [125-144]	0	2537.3418
[R].KPELWS	2xTMTpro [K	4.642E-05	4.225E-05	1	1	1	Q13043	Q13043 [246-259]	0	2638.4697
[R].QYPWG	1xCarbamido	3.331E-08	4.225E-05	3	3	2	Q9P0V9; Q9	Q9P0V9 [279-297	0	2956.4907
[K].DMALNI	1xTMTpro [K	2.607E-05	4.225E-05	1	1	2	O00161	O00161 [168-185]	0	2592.3947
[R].KNWVV	1xTMTpro [K	5.794E-05	4.225E-05	1	1	1	P35606	P35606 [69-82]	1	2241.2305
[K].KNVSIN	1xTMTpro [K	1.49E-06	4.225E-05	1	1	3	Q9UGI8	Q9UGI8 [102-124	1	3207.777
[K].KNLDST	1xCarbamido	4.057E-06	4.225E-05	1	1	2	P21291	P21291 [42-59]	1	2976.6281
[R].KNILEES	1xCarbamido	4.179E-06	4.225E-05	1	1	3	P78527	P78527 [2334-23	1	2558.5044
[K].KNIILEE	3xTMTpro [K	6.933E-06	4.225E-05	1	1	2	P23528	P23528 [45-73]	2	4407.5168
[R].DMGEM	1xTMTpro [N	7.478E-06	4.225E-05	1	1	1	Q01082	Q01082 [1123-11	0	2525.1794
[K].RIPIEDG	1xTMTpro [N	3.006E-06	4.225E-05	1	1	3	P01024	P01024 [290-304]	1	1931.0881
[K].DMGFQY	1xCarbamido	5.959E-06	4.225E-05	1	1	1	Q10588	Q10588 [252-267]	0	2601.3085
[K].KNLQYY	2xTMTpro [K	2.379E-05	4.225E-05	1	1	2	P62826	P62826 [142-152]	1	2255.3216
[R].RLLEDG	1xTMTpro [K	3.403E-06	4.225E-05	1	1	1	P05783	P05783 [382-407]	1	3504.7772
[K].RLETVAI	1xTMTpro [N	3.184E-06	4.225E-05	1	1	1	Q9UPN3	Q9UPN3 [4771-4	1	2384.3468
[R].KNLVEL	2xTMTpro [K	1.125E-05	4.225E-05	1	1	1	Q9UNH7	Q9UNH7 [375-38	1	2311.4417
[K].DMGSCE	1xCarbamido	2.405E-05	4.225E-05	1	1	1	P35606	P35606 [347-365]	0	2521.1883
[R].RLEPVV	1xTMTpro [K	4.704E-06	4.225E-05	1	1	2	P19838	P19838 [230-243]	1	2200.2469
[K].GCPAVL	1xCarbamido	4.608E-08	4.225E-05	1	1	3	Q15063	Q15063 [91-115]	0	2900.5623
[R].RIEESAI	1xTMTpro [K	1.332E-05	4.225E-05	1	1	1	O60256	O60256 [309-330]	1	3114.7291
[K].GCPAAL	1xCarbamido	3.425E-07	4.225E-05	1	1	2	Q15582	Q15582 [96-127]	1	4064.1506
[R].RLEEGT	1xTMTpro [K	1.182E-06	4.225E-05	1	1	1	Q08378	Q08378 [798-812]	1	2359.2484
[R].RLEASD	1xTMTpro [K	7.845E-08	4.225E-05	1	1	2	Q9Y285	Q9Y285 [13-42]	1	3631.9245
[K].RLEAEL	1xTMTpro [K	5.037E-06	4.225E-05	1	1	1	Q13813	Q13813 [1808-18	1	2826.5969
[R].DMIDNLI	1xOxidation	2.979E-05	4.225E-05	1	1	1	P07093	P07093 [163-180]	0	2320.2389
[R].RLDYQE	1xCarbamido	3.459E-05	4.225E-05	1	1	2	Q14624	Q14624 [911-930]	1	2638.3142
[K].RLDPDA	1xTMTpro [N	2.649E-05	4.225E-05	1	1	2	P53992	P53992 [320-340]	2	2737.4552

[K].RLPVPE!	1xTMTpro [N	2.931E-05	4.225E-05	1	1	1	Q8N5K1	Q8N5K1 [20-32]	1	1747.0186
[R].RLPWAT	1xTMTpro [K	3.664E-05	4.225E-05	1	1	3	Q9ULR3	Q9ULR3 [73-88]	1	2354.3476
[K].DMEYFF	1xCarbamid	3.514E-05	4.225E-05	1	1	2	P30519	P30519 [112-129]	0	2944.3777
[K].RIPYAPS	1xTMTpro [K	5.803E-05	4.225E-05	1	1	4	O60701	O60701 [470-481]	1	1936.1512
[R].RPDVVE	1xTMTpro [K	2.037E-06	4.225E-05	1	1	1	Q9NZL9	Q9NZL9 [98-122]	0	3216.7104
[K].DMAQLP	1xTMTpro [K	4.915E-05	4.225E-05	1	1	1	P27816	P27816 [477-490]	0	2122.171
[K].KNFESL!	1xTMTpro [K	2.171E-07	4.225E-05	1	1	2	P04844	P04844 [244-266]	1	3043.6457
[R].GCQLLV	1xCarbamid	4.055E-07	4.225E-05	1	1	1	Q9NQR4	Q9NQR4 [169-19	0	3145.6576
[R].DMASPN	1xTMTpro [N	3.677E-05	4.225E-05	1	1	2	Q15814	Q15814 [327-341]	0	2078.0184
[R].RPDLLTI	1xTMTpro [N	5.296E-05	4.225E-05	1	1	2	Q15121	Q15121 [72-83]	0	1781.9903
[R].RPDGEG	3xCarbamid	5.767E-07	4.225E-05	1	1	1	P35555	P35555 [2283-23	0	3385.5815
[R].RPDGCG	3xCarbamid	2.968E-05	4.225E-05	1	1	1	Q6UY14	Q6UY14 [470-485	0	2072.8867
[K].RPDDTA	1xTMTpro [N	2.159E-05	4.225E-05	1	1	1	O94901	O94901 [749-761]	0	1864.0248
[R].RPCFSA	1xCarbamid	1.934E-05	4.225E-05	1	1	177	P02768	P02768 [509-524]	0	2519.346
[R].RLDLVH	1xTMTpro [K	4.367E-05	4.225E-05	1	1	2	O75643	O75643 [953-966]	1	2204.3081
[R].KNGVQA	1xTMTpro [K	2.583E-06	4.225E-05	1	1	1	P14866	P14866 [229-246]	1	2602.3903
[K].RPAEDM	1xTMTpro [K	3.075E-05	4.225E-05	1	1	2	P61978	P61978 [22-34]	0	2188.12
[K].RPACTLI	3xCarbamid	5.149E-05	4.225E-05	1	1	2	P32320	P32320 [5-26]	0	3527.9317
[R].DMEALN	1xTMTpro [N	1.486E-05	4.225E-05	1	1	1	P49589	P49589 [226-240]	0	1987.0853
[K].RMPCAE	2xCarbamid	3.839E-05	4.225E-05	1	1	1	P02768	P02768 [469-496]	2	3954.0531
[K].RMPCAE	2xCarbamid	1.622E-06	4.225E-05	1	1	18	P02768	P02768 [469-490]	1	3298.7242
[K].RMPCAE	2xCarbamid	1.017E-07	4.225E-05	1	1	30	P02768	P02768 [469-490]	1	3282.7293
[K].KNHEEE	3xTMTpro [K	1.235E-05	4.225E-05	1	1	1	P05783	P05783 [215-241]	2	4094.3238
[R].RLTVSSI	1xTMTpro [K	5.192E-05	4.225E-05	1	1	2	P21333	P21333 [2334-23	1	2026.2152
[R].KNLDST	1xCarbamid	1.381E-05	4.225E-05	1	1	2	Q16527	Q16527 [42-59]	1	3048.6492
[R].DMETIGI	1xTMTpro [K	3.319E-07	4.225E-05	1	1	3	P07384	P07384 [437-461]	0	3347.8205
[K].DMDNQI	1xTMTpro [K	3.334E-05	4.225E-05	1	1	1	Q8NI08	Q8NI08 [837-852]	0	2463.2622
[R].DMLFQV	1xTMTpro [N	4.595E-05	4.225E-05	1	1	1	P35270	P35270 [178-192]	0	2078.1275
[K].RLDLTY!	2xCarbamid	1.388E-06	4.225E-05	1	1	1	P98160	P98160 [4086-41	1	3213.5264
[K].GCPAAL	1xCarbamid	1.807E-07	4.225E-05	1	1	4	Q15582	Q15582 [96-124]	0	3401.7582
[K].GCIELDI	1xCarbamid	1.93E-05	4.225E-05	1	1	1	Q16787	Q16787 [2560-25	0	2864.4996
[K].RGEQCV	3xCarbamid	5.974E-05	4.225E-05	1	1	1	Q12805	Q12805 [208-227]	1	2837.3088
[K].RGEENT	1xTMTpro [N	1.994E-05	4.225E-05	1	1	1	P18433	P18433 [581-598]	1	2410.1594

[K].DMSIQEI	1xTMTpro [K	2.593E-05	4.225E-05	1	1	1	O60437	O60437 [1742-17	0	2226.2659
[R].DMSISN	1xTMTpro [N	3.032E-05	4.225E-05	1	1	2	Q13618	Q13618 [476-488	0	1850.8584
[R].KNPLPP	2xTMTpro [K	4.689E-05	4.225E-05	1	1	2	Q8NC51	Q8NC51 [79-91]	1	2262.4002
[R].RGDVLA	1xTMTpro [K	5.419E-05	4.225E-05	1	1	1	P43304	P43304 [391-409]	1	2659.5539
[R].RGDIIGV	1xTMTpro [K	2.271E-05	4.225E-05	1	1	2	Q15046	Q15046 [178-190	1	1919.1318
[K].RGDACE	1xCarbamid	1.137E-05	4.225E-05	1	1	2	P00734	P00734 [560-575]	1	2307.1353
[K].RGDACE	1xCarbamid	2.66E-06	4.225E-05	1	1	2	P00734	P00734 [560-575]	1	2291.1404
[R].DMSGTM	1xTMTpro [K	3.67E-05	4.225E-05	1	1	2	P30626	P30626 [85-96]	0	1971.98
[K].KNPMVE	2xTMTpro [K	1.601E-05	4.225E-05	1	1	6	Q06124	Q06124 [199-213	1	2583.5724
[K].KNPQAV	2xTMTpro [K	5.682E-05	4.225E-05	1	2	3	Q13177	Q13177 [118-128	1	2137.3525
[K].RFEQQL	1xTMTpro [N	5.395E-07	4.225E-05	1	1	1	Q9BY43	Q9BY43 [71-90]	1	2672.3963
[K].DMTVPIL	2xTMTpro [K	4.148E-06	4.225E-05	1	1	2	P11182	P11182 [234-250]	0	2741.6456
[K].KNQWVN	2xTMTpro [K	4.315E-05	4.225E-05	1	1	1	O15027	O15027 [2137-21	1	2669.4715
[R].RFDEILE	1xOxidation	1.165E-05	4.225E-05	1	1	9	P14618	P14618 [279-294]	1	2142.1184
[R].RFDEILE	1xTMTpro [N	2.091E-06	4.225E-05	1	1	18	P14618	P14618 [279-294]	1	2126.1235
[K].DMVTEL	1xTMTpro [N	1.642E-05	4.225E-05	1	1	2	Q9BXV9	Q9BXV9 [42-59]	0	2417.2454
[K].DMVTEL	1xOxidation	8.554E-06	4.225E-05	1	1	2	Q9BXV9	Q9BXV9 [42-59]	0	2433.2403
[R].KNSLISS	1xTMTpro [K	2.134E-06	4.225E-05	1	1	2	Q14980	Q14980 [1223-12	1	2539.4587
[K].DMYSFL	1xTMTpro [K	2.151E-05	4.225E-05	1	1	4	P01023	P01023 [665-676]	0	2057.0579
[K].RFPELES	1xTMTpro [N	1.48E-05	4.225E-05	1	1	1	Q8WWY3	Q8WWY3 [121-1	1	2336.2934
[R].KNWCAL	1xCarbamid	3.34E-05	4.225E-05	1	1	2	Q92888	Q92888 [749-763	1	2604.5
[K].KNPEVP	2xTMTpro [K	1.976E-05	4.225E-05	1	1	2	O15347	O15347 [30-43]	1	2518.4486
[R].KNPDSQ	2xTMTpro [K	1.799E-05	4.225E-05	1	1	2	P30085	P30085 [43-55]	1	2433.3806
[R].RLDFTG	1xTMTpro [K	2.334E-07	4.225E-05	1	1	8	P20774	P20774 [147-165]	1	2778.4805
[K].RLDEYT	1xTMTpro [N	2.179E-05	4.225E-05	1	1	1	Q9BYD1	Q9BYD1 [154-16	1	2186.1049
[R].DMLLAN	1xTMTpro [K	3.514E-05	4.225E-05	1	2	1	P05388	P05388 [100-112]	1	1978.1763
[K].DMLLEL	1xTMTpro [N	4.142E-05	4.225E-05	1	1	2	Q32MZ4	Q32MZ4 [197-21	0	1980.0279
[K].GCNIEN	3xCarbamid	1.235E-05	4.225E-05	1	1	2	P32320	P32320 [52-68]	0	2301.0745
[K].RLDEEE	1xTMTpro [N	2.512E-06	4.225E-05	1	1	1	Q99613	Q99613 [288-303	1	2296.0285
[R].DMIILPE	1xTMTpro [K	2.175E-05	4.225E-05	1	1	1	P62841	P62841 [82-100]	0	2661.431
[K].RLDDYL	1xTMTpro [N	2.338E-07	4.225E-05	1	1	1	O15247	O15247 [145-170	1	3273.6446
[R].RKPEDV	2xTMTpro [K	8.586E-06	4.225E-05	1	1	1	P35613	P35613 [349-366]	1	2853.5774
[R].KNNTDT	1xCarbamid	2.833E-06	4.225E-05	1	1	1	Q9Y3P9	Q9Y3P9 [465-483	1	2894.4697

[K].DMQPSM	1xTMTpro [K	1.098E-05	4.225E-05	1	1	2	P27816	P27816 [291-304]	0	2190.11
[K].RKPADL(	2xTMTpro [K	1.75E-05	4.225E-05	1	1	4	Q9Y696	Q9Y696 [61-85]	1	3690.0948
[R].RHPDYS	1xTMTpro [N	4.776E-05	4.225E-05	1	1	21	P02768	P02768 [361-372]	1	1772.0502
[R].RGYQLS	3xCarbamido	4.836E-07	4.225E-05	1	1	1	P23142	P23142 [467-497]	1	3847.7645
[K].RGTGGV	1xTMTpro [N	2.697E-07	4.225E-05	1	1	3	P12277	P12277 [320-341]	1	2425.2391
[R].RGTELG	1xTMTpro [N	9.588E-06	4.225E-05	1	1	1	Q9Y6C2	Q9Y6C2 [383-406	1	2601.3453
[R].RGTDEC	1xCarbamido	1.49E-06	4.225E-05	1	1	2	P53634	P53634 [443-462]	1	2707.4944
[R].GCITIIG(	3xCarbamido	1.705E-06	4.225E-05	1	1	4	P00558	P00558 [366-382]	0	2363.2013
[R].RGPQLV	1xCarbamido	3.744E-07	4.225E-05	1	1	1	Q96DI7	Q96DI7 [162-177]	1	2298.2368
[R].KNPALW	2xTMTpro [K	3.375E-05	4.225E-05	1	1	2	Q9NQT8	Q9NQT8 [961-97	1	2479.5217
[K].KNPASLI	2xTMTpro [K	9.606E-06	4.225E-05	1	1	1	Q13428	Q13428 [1065-10	1	2364.4795
[R].RGPAPQ	1xTMTpro [N	5.545E-06	4.225E-05	1	1	3	O96005	O96005 [75-90]	1	1835.9796
[R].GCIVDAI	1xCarbamido	1.435E-05	4.225E-05	1	1	3	P62753	P62753 [99-115]	0	2435.4551
[K].QVYEEE	1xTMTpro [N	3.878E-07	4.225E-05	1	1	8	P08758	P08758 [127-151]	0	3192.4452
[K].KISIPVDI	2xTMTpro [K	1.949E-06	4.225E-05	1	1	2	P49368	P49368 [139-163]	1	3648.042
[R].KISGTTA	2xTMTpro [K	1.311E-06	4.225E-05	2	2	1	P30622; Q9U	P30622 [346-358]	1	2272.4056
[K].KLFVGG	2xTMTpro [K	1.391E-05	4.225E-05	2	4	5	P22626; P09	P22626 [113-129]	2	2920.6825
[R].DLGGIVI	2xCarbamido	4.465E-07	4.225E-05	1	1	2	Q99798	Q99798 [438-457	0	2476.2396
[K].SFSKEEI	1xOxidation	1.631E-05	4.225E-05	1	1	1	O00567	O00567 [511-533	1	3401.7814
[K].KIFVGGI	2xTMTpro [K	6.059E-06	4.225E-05	1	1	1	P51991	P51991 [127-143]	2	2923.6709
[R].GDNLET	1xTMTpro [K	5.331E-05	4.225E-05	1	1	1	Q66K74	Q66K74 [82-96]	0	2236.268
[K].KIFVGGL	2xTMTpro [K	6.89E-07	4.225E-05	1	1	2	Q14103	Q14103 [183-197	1	2529.4745
[K].DLGLSES	1xTMTpro [K	6.499E-06	4.225E-05	1	1	2	P13667	P13667 [464-484]	0	2727.418
[K].DLGLSES	2xTMTpro [K	3.528E-09	4.225E-05	1	1	5	P13667	P13667 [464-485]	1	3159.7201
[K].SFPVNSI	1xTMTpro [K	5.563E-05	4.225E-05	1	1	2	P48444	P48444 [352-363]	0	1870.093
[R].SFPDFP	1xTMTpro [N	6.003E-05	4.225E-05	1	1	4	P07741	P07741 [15-27]	0	1769.9546
[R].SFLVASV	1xTMTpro [N	4.257E-05	4.225E-05	1	1	1	Q7Z3K3	Q7Z3K3 [1322-13	0	2345.2784
[K].KIGDEYF	1xCarbamido	2.875E-05	4.225E-05	1	1	1	P49368	P49368 [354-367]	1	2649.4414
[R].SFLLDLL	1xTMTpro [K	3.097E-05	4.225E-05	1	1	2	O00571	O00571 [429-440	0	1900.1399
[K].SFIKDYF	2xTMTpro [K	3E-06	4.225E-05	1	1	3	P06733	P06733 [282-306]	1	3898.0156
[R].DLGPHH	1xCarbamido	4.442E-06	4.225E-05	1	1	1	Q96SL4	Q96SL4 [73-98]	0	3604.7887
[R].DLGPHH	1xCarbamido	3.102E-06	4.225E-05	1	1	1	Q96SL4	Q96SL4 [73-105]	1	4437.1966
[K].KIGDEYF	1xCarbamido	4.194E-06	4.225E-05	1	1	2	P49368	P49368 [354-370]	2	3293.8233

[R].GDMMC\	2xCarbamid(	2.171E-05	4.225E-05	1	1	1	Q9UBX5	Q9UBX5 [55-71]	0	2289.0568
[K].SFKPDF(	1xTMTpro [K	3.761E-06	4.225E-05	1	1	2	P35606	P35606 [423-442]	0	2768.5267
[R].SFGGGI	1xTMTpro [K	4.419E-05	4.225E-05	1	1	1	O75439	O75439 [326-336]	0	1692.9235
[K].KLGEMW	2xTMTpro [K	1.315E-05	4.225E-05	1	2	2	P09429	P09429 [128-141]	1	2505.3588
[R].SFSTAS/	1xTMTpro [N	3.387E-05	4.225E-05	1	1	3	P13647	P13647 [16-29]	0	1714.9295
[R].SFSTFDI	1xCarbamid(	4.185E-07	4.225E-05	1	1	3	P24821	P24821 [2121-214	1	3283.6973
[R].DLGELS(	1xTMTpro [N	4.51E-05	4.225E-05	1	1	2	Q8TD55	Q8TD55 [463-475	0	1688.9138
[K].SFTASCI	1xCarbamid(	6.051E-05	4.225E-05	1	1	2	Q6P587	Q6P587 [124-137	0	2106.1549
[K].SGDAAI\	1xCarbamid(	1.156E-06	4.225E-05	1	2	8	P68104	P68104 [396-423]	0	3619.809
[K].SGDAAI\	1xCarbamid(	2.224E-07	4.225E-05	1	2	3	P68104	P68104 [396-423]	0	3603.8141
[K].GDPEAA	1xTMTpro [N	1.053E-05	4.225E-05	1	1	1	Q9P2N5	Q9P2N5 [636-652	0	2194.1311
[R].SGCNEV	1xCarbamid(	2.682E-05	4.225E-05	1	1	3	Q6FHJ7	Q6FHJ7 [209-221	0	2016.0927
[K].SGAQAT	1xTMTpro [K	1.038E-05	4.225E-05	1	1	4	P02790	P02790 [387-402]	0	2446.3011
[R].SGAQAS	1xTMTpro [N	4.964E-05	4.225E-05	1	1	1	P02545	P02545 [12-25]	0	1663.8934
[R].SGAGTE	1xCarbamid(	1.446E-06	4.225E-05	1	1	3	P00450	P00450 [865-887]	0	3113.5381
[R].KLEWPL	2xTMTpro [K	2.498E-06	4.225E-05	1	1	2	Q9HC38	Q9HC38 [117-139	1	3462.8977
[R].SGAGP(	1xTMTpro [N	4.397E-05	4.225E-05	1	1	2	Q13144	Q13144 [20-33]	0	1348.6889
[R].KLEYGN	2xTMTpro [K	5.786E-06	4.225E-05	1	1	1	Q06278	Q06278 [722-733]	1	2325.3271
[R].SFEVEE'	1xTMTpro [N	2.016E-05	4.225E-05	1	1	1	Q9UHD8	Q9UHD8 [30-45]	0	2122.0623
[R].GDNVAV	1xTMTpro [N	5.365E-07	4.225E-05	1	1	1	O95777	O95777 [64-87]	0	2819.423
[R].KLFGQE	2xTMTpro [K	2.71E-06	4.225E-05	1	1	1	Q9BWH2	Q9BWH2 [68-80]	1	2290.3223
[R].DIGAIAQ	1xTMTpro [N	2.012E-08	4.225E-05	1	1	2	Q16555	Q16555 [190-211]	0	2681.3814
[R].DLGAPQ	1xTMTpro [N	4.792E-06	4.225E-05	1	1	1	O15230	O15230 [2330-23	0	2057.0947
[K].SFYPEE'	1xTMTpro [K	6.241E-06	4.225E-05	1	1	3	P11142	P11142 [113-126]	0	2225.2019
[R].KLFIGGL	1xTMTpro [K	5.86E-05	4.225E-05	1	1	1	P51991	P51991 [36-52]	1	2507.4001
[R].KLFIGGL	2xTMTpro [K	9.293E-06	4.225E-05	1	1	1	P51991	P51991 [36-57]	2	3481.9147
[K].SFVASNI	1xTMTpro [N	1.304E-07	4.225E-05	1	1	1	O15031	O15031 [142-166]	0	2683.3494
[R].KLFIGGL	1xTMTpro [K	1.123E-05	4.225E-05	1	1	2	P22626	P22626 [22-38]	1	2535.4314
[K].KLFSLTF	1xTMTpro [K	1.607E-05	4.225E-05	1	1	1	Q15036	Q15036 [63-75]	1	2126.2465
[R].DIGEGNI	1xTMTpro [K	3.772E-07	4.225E-05	1	1	1	Q8TAQ2	Q8TAQ2 [852-872	0	2493.4168
[K].SGAEAQ	1xTMTpro [N	3.933E-05	4.225E-05	1	1	3	O43493	O43493 [140-153]	0	1762.8527
[K].SFESTV(	1xTMTpro [N	3.9E-05	4.225E-05	1	1	1	P20645	P20645 [59-76]	0	2374.1886
[K].SFENSL(	1xTMTpro [N	5.27E-05	4.225E-05	1	1	1	Q16851	Q16851 [378-389]	0	1636.8978

[K].DLHEEM	1xTMTpro [K	7.816E-06	4.225E-05	1	1	2	P49354	P49354 [147-164]	0	2781.4625
[R].KLGINSL	2xTMTpro [K	1.973E-05	4.225E-05	1	1	2	P55327	P55327 [110-120]	1	2155.3631
[R].SEVELA	1xTMTpro [K	8.02E-06	4.225E-05	1	1	1	Q03252	Q03252 [159-171]	1	1997.1523
[R].SEVAAG	1xTMTpro [N	3.119E-05	4.225E-05	1	1	1	Q66K74	Q66K74 [313-325	0	1610.773
[K].KLGNNC	1xCarbamido	2.44E-05	4.225E-05	1	1	2	Q99714	Q99714 [53-69]	1	2762.5327
[K].SETSGS	1xTMTpro [K	1.358E-06	4.225E-05	1	1	4	P09525	P09525 [226-241]	0	2275.2677
[K].KLGQSL	1xCarbamido	1.942E-06	4.225E-05	1	1	3	P01042	P01042 [344-362]	1	3147.7329
[R].DLLAVV	1xTMTpro [K	4.587E-05	4.225E-05	1	1	3	P12956	P12956 [81-92]	0	1963.1396
[R].SETITEE	1xTMTpro [K	3.172E-06	4.225E-05	1	1	4	P16152	P16152 [160-174]	0	2301.2503
[K].GDLSTA	1xCarbamido	1.703E-05	4.225E-05	1	1	2	P42704	P42704 [836-851]	0	2392.2562
[R].DLLDDL	2xTMTpro [K	2.345E-05	4.225E-05	1	1	3	P08758	P08758 [64-76]	1	2359.3901
[R].SEVKPT	2xTMTpro [K	1.401E-05	4.225E-05	1	1	1	P48060	P48060 [46-69]	0	3578.9419
[R].DLLDDL	3xTMTpro [K	3.826E-05	4.225E-05	1	1	3	P08758	P08758 [64-79]	2	3067.8032
[R].SESNLS	1xCarbamido	8.065E-06	4.225E-05	1	1	1	Q9UKG1	Q9UKG1 [595-61	0	2901.4068
[K].SESEVE	1xTMTpro [N	5.405E-08	4.225E-05	1	1	2	Q16643	Q16643 [272-291]	0	2486.2806
[R].SESELID	1xCarbamido	1.326E-07	4.225E-05	1	1	1	P20810	P20810 [364-382]	1	2896.4014
[R].DLLDTV	1xTMTpro [K	1.882E-05	4.225E-05	1	1	2	Q86VP6	Q86VP6 [1043-10	0	2379.3415
[R].SESELID	1xTMTpro [N	1.288E-05	4.225E-05	1	1	3	P20810	P20810 [364-378]	0	2087.994
[R].SEREDE	1xTMTpro [N	4.389E-05	4.225E-05	1	2	1	Q8NFP7	Q8NFP7 [27-40]	1	1910.015
[R].SEQEFQ	1xTMTpro [N	5.455E-05	4.225E-05	1	1	1	Q96RQ3	Q96RQ3 [220-232	0	1884.9259
[R].SEQEDE	1xTMTpro [N	3.157E-05	4.225E-05	1	2	1	Q9NZJ9	Q9NZJ9 [28-41]	0	1881.9725
[R].DILELGG	1xTMTpro [K	5.654E-05	4.225E-05	1	1	1	Q9NPF5	Q9NPF5 [9-27]	0	2438.327
[K].DLIETCC	4xCarbamido	3.857E-07	4.225E-05	1	1	1	P98095	P98095 [440-473]	0	4272.8749
[K].KIGTTPD	1xTMTpro [K	1.312E-05	4.225E-05	1	1	2	Q10570	Q10570 [1421-14	1	2636.5002
[K].SGDAAI	1xCarbamido	6.781E-06	4.225E-05	1	2	4	P68104	P68104 [396-423]	0	3635.804
[R].SEVLLVS	1xTMTpro [K	4.689E-05	4.225E-05	1	1	1	Q9UJ70	Q9UJ70 [15-25]	0	1784.0297
[K].SEVSSD	1xTMTpro [N	4.228E-05	4.225E-05	1	1	3	P40939	P40939 [665-676]	0	1715.8407
[K].GDMEIPI	1xTMTpro [N	3.191E-05	4.225E-05	1	1	1	O76024	O76024 [82-94]	0	1867.9431
[K].KLGEMW	3xTMTpro [K	5.192E-05	4.225E-05	1	1	4	P09429	P09429 [128-146]	2	3454.8781
[R].SFEGLG	1xTMTpro [K	1.065E-05	4.225E-05	1	1	1	P35858	P35858 [333-354]	0	3105.7076
[R].SFDVNK	1xCarbamido	2.608E-07	4.225E-05	1	2	2	P41091	P41091 [261-285]	1	3779.1154
[K].KLGEMW	1xOxidation [	5.691E-05	4.225E-05	1	1	3	P09429	P09429 [128-146]	2	3470.8731
[R].SFDSMIS	1xCarbamido	2.849E-06	4.225E-05	1	1	3	P00390	P00390 [269-291]	0	3181.5889

[K].GDMDQI	1xCarbamido	2.654E-05	4.225E-05	1	1	1	Q8WXX5	Q8WXX5 [139-15	0	2704.2588
[R].DLKPEN	2xTMTpro [K	2.002E-05	4.225E-05	2	4	3	Q13557; Q1	Q13557 [136-147	0	2253.3998
[R].DLKPEN	2xTMTpro [K	5.374E-06	4.225E-05	2	3	1	P51812; Q1	P51812 [193-208]	0	2775.6073
[R].DLKPEN	2xTMTpro [K	3.27E-07	4.225E-05	1	1	2	P22694	P22694 [167-190]	0	3674.041
[R].GDIYFCT	1xCarbamido	6.416E-06	4.225E-05	1	1	2	Q9NRV9	Q9NRV9 [163-18	0	2745.3661
[K].SFCSMA	1xCarbamido	9.57E-06	4.225E-05	1	1	1	O94760	O94760 [176-196	0	2763.4301
[K].SFCPGG	1xCarbamido	1.826E-06	4.225E-05	1	1	1	Q9C0C9	Q9C0C9 [312-33	0	2806.4001
[K].DLKPETE	2xTMTpro [K	1.019E-05	4.225E-05	1	1	1	Q99715	Q99715 [1443-14	0	3857.0564
[K].DLKPETE	3xTMTpro [K	1.383E-06	4.225E-05	1	1	3	Q99715	Q99715 [1443-14	1	4576.4703
[R].KLGGSQ	2xTMTpro [K	2.449E-05	4.225E-05	1	1	1	P37837	P37837 [71-81]	1	2115.259
[K].DIKPQLE	2xTMTpro [K	1.741E-05	4.225E-05	1	1	2	P46821	P46821 [853-866]	0	2611.5011
[K].KLGGTID	1xCarbamido	1.47E-06	4.225E-05	1	1	1	P50991	P50991 [213-232]	1	3100.7744
[R].SEWSDL	1xTMTpro [K	5.682E-05	4.225E-05	1	1	1	P08237	P08237 [130-141]	0	2029.1097
[R].DLKPSN	1xCarbamido	4.854E-06	4.225E-05	1	1	1	Q15418	Q15418 [535-554	0	2927.5428
[K].SEWQSN	1xTMTpro [K	2.405E-05	4.225E-05	1	1	2	Q9UH99	Q9UH99 [393-40	0	2626.2951
[R].SEWGSL	1xTMTpro [K	1.443E-05	4.225E-05	1	1	2	P17858	P17858 [130-144]	0	2255.2415
[R].DLKPEN	2xTMTpro [K	4.73E-06	4.225E-05	1	2	2	P17612	P17612 [167-190]	0	3665.0407
[K].KLGVQT	1xTMTpro [K	1.158E-05	4.225E-05	1	1	1	Q96RQ3	Q96RQ3 [70-84]	1	2244.2844
[R].GDPECH	1xCarbamido	4.793E-05	4.225E-05	1	1	1	P18065	P18065 [302-317]	0	2297.0576
[R].KLEVFLC	2xCarbamido	4.226E-06	4.225E-05	1	1	2	O75695	O75695 [124-144	1	3349.868
[K].DIEAMDI	1xTMTpro [K	5.794E-05	4.225E-05	1	1	2	P46459	P46459 [151-161]	0	1840.0382
[K].SGQVYS	1xCarbamido	1.598E-05	4.225E-05	1	1	2	P18754	P18754 [85-101]	0	2120.999
[K].GDQFVF	1xTMTpro [K	3.664E-05	4.225E-05	1	1	2	P13798	P13798 [203-219]	0	2659.2994
[K].GDQENV	1xOxidation [	2.132E-05	4.225E-05	1	1	1	Q14974	Q14974 [778-793	0	2154.0933
[K].SGQSED	1xTMTpro [K	1.021E-05	4.225E-05	1	1	2	P01024	P01024 [567-584]	1	2594.3852
[K].GDQENV	1xTMTpro [N	8.23E-06	4.225E-05	1	1	1	Q14974	Q14974 [778-793	0	2138.0984
[K].KLEGELT	2xTMTpro [K	2.859E-06	4.225E-05	1	1	3	Q14683	Q14683 [454-467	1	2518.4255
[R].KLELDIL	2xTMTpro [K	3.604E-06	4.225E-05	1	1	2	P12270	P12270 [1299-13	1	3065.7914
[R].DLEDETI	1xTMTpro [N	5.618E-05	4.225E-05	1	1	2	P11277	P11277 [1486-14	0	1837.9139
[R].DLEDKE	2xTMTpro [K	4.249E-06	4.225E-05	1	1	2	Q13409	Q13409 [251-264	1	2415.3547
[K].SGPVED	1xTMTpro [N	2.376E-06	4.225E-05	1	1	1	P98160	P98160 [3973-39	0	2451.2662
[R].KIEIDNG	2xTMTpro [K	8.356E-08	4.225E-05	1	1	2	Q12792	Q12792 [294-315	1	3487.8777
[K].KIEIGDG	2xTMTpro [K	9.133E-08	4.225E-05	1	1	3	Q6IBS0	Q6IBS0 [294-315]	1	3386.8664

[K].SGPPAP	1xTMTpro [N	3.657E-05	4.225E-05	1	1	1	Q9NTI5	Q9NTI5 [1319-13	0	1888.8732
[R].SGPKPF	3xTMTpro [K	2.52E-05	4.225E-05	1	1	2	Q01518	Q01518 [295-312	0	3054.8093
[R].SGPFGQ	1xTMTpro [K	1.465E-08	4.225E-05	3	5	4	Q13885; P07	Q13885 [78-103];	0	3406.7577
[R].SGPEPL	1xTMTpro [K	2.206E-05	4.225E-05	1	1	2	P57737	P57737 [668-680]	0	1901.0624
[K].DLEEDH	1xCarbamido	1.969E-05	4.225E-05	1	1	7	P13639	P13639 [560-572]	1	2480.3999
[K].SGNTFR	1xCarbamido	1.924E-07	4.225E-05	2	3	2	P01876; P0D	P01876 [222-253]	0	3878.0805
[K].KLELQVI	2xTMTpro [K	2.167E-05	4.225E-05	1	1	1	Q96CV9	Q96CV9 [360-37	1	2559.5248
[R].KLELSDI	2xTMTpro [K	7.476E-07	4.225E-05	1	1	2	Q9BTT0	Q9BTT0 [68-86]	1	2940.7438
[R].KLEGDS	2xTMTpro [K	3.419E-08	4.225E-05	1	1	4	P35579	P35579 [1052-10	1	3527.9625
[K].SGSDEV	1xTMTpro [N	4.206E-05	4.225E-05	1	1	2	P01024	P01024 [1571-15	0	1593.8152
[K].KLEGDLI	3xTMTpro [K	4.915E-05	4.225E-05	1	1	2	P35749	P35749 [1621-16	2	3202.9039
[K].SGSGTM	1xTMTpro [N	8.109E-06	4.225E-05	1	1	3	P47756	P47756 [182-195]	0	1641.8549
[K].SHCFVT	1xCarbamido	1.407E-05	4.225E-05	1	1	1	Q9UKV3	Q9UKV3 [1050-1	0	2261.1192
[K].KLEEVV	2xTMTpro [K	2.529E-05	4.225E-05	1	1	2	Q9BY32	Q9BY32 [19-30]	1	2283.4104
[R].SHAIAC	1xCarbamido	2.926E-05	4.225E-05	1	1	1	O14787	O14787 [190-203	0	1965.9958
[K].KLEEVV	1xCarbamido	1.504E-07	4.225E-05	1	1	1	Q9BY32	Q9BY32 [19-39]	2	3632.1602
[K].SGYHQS	1xTMTpro [N	3.705E-06	4.225E-05	1	1	1	P10768	P10768 [65-86]	0	2612.3388
[K].SGYAIQ	1xTMTpro [N	2.079E-05	4.225E-05	1	1	1	Q7Z3B4	Q7Z3B4 [399-411	0	1783.9146
[K].SGVYQH	1xTMTpro [N	9.174E-06	4.225E-05	1	1	2	P07858	P07858 [264-281]	0	2234.113
[R].DLDQAS	1xTMTpro [N	3.617E-06	4.225E-05	1	1	2	Q9Y490	Q9Y490 [1674-16	0	2087.1416
[K].SGVPVL	1xTMTpro [K	4.689E-05	4.225E-05	1	1	1	Q53EL6	Q53EL6 [198-212	0	2048.2611
[K].SGVDAD	1xTMTpro [K	5.218E-05	4.225E-05	1	1	2	Q9Y394	Q9Y394 [322-332	0	1783.9358
[K].KLEMTEI	1xTMTpro [K	1.607E-05	4.225E-05	1	1	1	Q08379	Q08379 [341-355	1	2431.3874
[K].KIEFVVV	1xTMTpro [K	6.972E-06	4.225E-05	1	1	1	P22102	P22102 [66-87]	1	2857.7159
[K].SGTIFDN	1xTMTpro [K	1.484E-06	4.225E-05	1	1	5	P27797	P27797 [323-351]	0	3876.91
[R].SGTLAL	1xTMTpro [N	2.202E-05	4.225E-05	1	1	1	Q6SZW1	Q6SZW1 [290-30	0	2099.2031
[K].SGTGIA	1xTMTpro [K	4.125E-06	4.225E-05	1	1	3	P27449	P27449 [37-54]	0	2515.369
[R].DLDVVV	1xTMTpro [N	2.76E-05	4.225E-05	1	1	3	Q6DD88	Q6DD88 [57-70]	0	1751.0023
[K].SGTALEI	1xTMTpro [K	5.784E-05	4.225E-05	1	1	1	O95352	O95352 [118-130	0	1964.1672
[R].GDQPAA	1xTMTpro [N	4.809E-07	4.225E-05	1	1	2	O00264	O00264 [48-67]	0	2340.0911
[R].DLEADII	1xTMTpro [K	4.432E-07	4.225E-05	1	1	3	P08133	P08133 [141-156]	0	2354.2484
[R].DLEAEH	1xTMTpro [N	1.041E-05	4.225E-05	1	1	3	Q9H3K6	Q9H3K6 [15-30]	0	2174.0896
[R].SGSLSL	1xTMTpro [K	1.169E-05	4.225E-05	1	1	1	P15924	P15924 [2549-25	0	2306.2921

[R].KLEGDA	2xTMTpro [K	1.949E-06	4.225E-05	1	1	2	P35749	P35749 [1059-108	1	3581.9631
[K].SGTSAS	1xTMTpro [N	4.882E-05	4.225E-05	1	1	2	P01700	P01700 [87-99]	0	1523.8712
[R].SGNCYL	1xCarbamido	6.061E-05	4.225E-05	1	1	2	P35555	P35555 [1531-154	0	1654.8654
[R].SGMSHE	1xTMTpro [N	6.003E-06	4.225E-05	1	1	1	Q6P2Q9	Q6P2Q9 [1355-13	0	2193.0929
[K].DLEGEN	2xTMTpro [K	1.197E-06	4.225E-05	1	1	3	O60506	O60506 [395-412]	0	2954.6655
[K].DIESLMS	1xTMTpro [K	2.834E-05	4.225E-05	1	1	1	Q9NQT8	Q9NQT8 [196-206	0	1830.9763
[K].SGGELD	1xTMTpro [K	2.009E-05	4.225E-05	1	1	1	Q96M27	Q96M27 [254-266	0	1927.0992
[R].SGFTYV	2xCarbamido	1.572E-05	4.225E-05	1	1	1	P04275	P04275 [2479-249	0	2075.9598
[R].SGFHTV	2xCarbamido	3.514E-05	4.225E-05	1	1	1	Q07954	Q07954 [3811-38	0	2576.2305
[R].DLEVVA	1xTMTpro [N	2.307E-06	4.225E-05	1	1	3	P02751	P02751 [1544-156	0	2828.5729
[K].SGFEPA	3xTMTpro [K	3.16E-06	4.225E-05	1	1	10	P35579	P35579 [39-64]	2	4005.2537
[K].SGFEPA	2xTMTpro [K	6.218E-06	4.225E-05	1	1	3	P35579	P35579 [39-63]	1	3572.9516
[K].SGEYWI	1xCarbamido	2.968E-05	4.225E-05	1	1	6	P02452	P02452 [1271-128	0	2688.3583
[R].SGEWTE	1xTMTpro [N	2.367E-05	4.225E-05	1	1	2	Q13938	Q13938 [218-229]	1	1780.9149
[R].SGETED	1xCarbamido	1.899E-06	4.225E-05	2	3	9	P09104; P06	P09104 [373-394]	0	2961.5735
[R].SGGGG(	1xCarbamido	6.362E-07	4.225E-05	1	1	1	P13645	P13645 [16-35]	0	1853.8843
[K].DIFAMDI	2xTMTpro [K	6.457E-07	4.225E-05	1	1	2	Q96I99	Q96I99 [272-291]	1	3178.6394
[R].SGELQG	1xTMTpro [K	3.833E-05	4.225E-05	1	1	1	Q96A65	Q96A65 [468-486	0	2452.2811
[R].SGELAQ	1xTMTpro [K	3.18E-05	4.225E-05	1	1	1	Q14683	Q14683 [161-171]	1	1904.0369
[R].DLFEDEI	1xTMTpro [K	3.866E-05	4.225E-05	1	1	3	O60506	O60506 [172-184]	0	2202.219
[K].DLFGDK	2xTMTpro [K	1.555E-05	4.225E-05	1	1	2	P38571	P38571 [226-241]	1	2767.5851
[K].SGEEDF	1xCarbamido	8.337E-06	4.225E-05	1	1	1	Q13526	Q13526 [98-117]	0	2789.3067
[R].SGDSEV	1xTMTpro [K	6.694E-06	4.225E-05	1	1	2	Q04837	Q04837 [67-81]	0	2220.164
[R].DIFLDD(	1xTMTpro [K	1.336E-05	4.225E-05	1	1	1	P52655	P52655 [27-43]	0	2587.3821
[K].SGDRGE	1xTMTpro [N	3.451E-08	4.225E-05	1	1	5	P02452	P02452 [1063-108	1	2266.1859
[R].SGDPMII	1xCarbamido	2.269E-06	4.225E-05	1	1	2	Q92896	Q92896 [520-537]	0	2748.3902
[R].SGDITY(	1xTMTpro [N	1.184E-05	4.225E-05	1	1	1	P19174	P19174 [205-218]	0	1922.9931
[R].KLEVEAI	1xTMTpro [K	2.163E-05	4.225E-05	1	1	3	P47756	P47756 [95-108]	1	2305.2432
[R].SGDGVV	1xTMTpro [N	5.192E-05	4.225E-05	1	1	3	Q13938	Q13938 [194-205]	0	1536.8189
[K].KLETAVI	1xTMTpro [K	1.8E-06	4.225E-05	1	1	4	P21796	P21796 [201-218]	1	2554.4233
[R].SGGGG(	1xTMTpro [N	2.345E-06	4.225E-05	1	1	2	P35527	P35527 [14-29]	0	1536.805
[K].SGLTPLI	1xTMTpro [N	5.964E-05	4.225E-05	1	1	1	Q12955	Q12955 [696-709]	0	1811.9935
[R].SGLSTG	1xTMTpro [K	3.075E-05	4.225E-05	1	1	2	P04217	P04217 [95-106]	0	1873.0675

[K].SGLQGY	1xTMTpro [N	2.25E-05	4.225E-05	1	1	2	Q13492	Q13492 [113-125]	0	1778.9066
[K].SGINPFD	2xTMTpro [K	2.584E-05	4.225E-05	1	1	1	P61201	P61201 [289-303]	0	2593.4442
[R].DLEHSL(	1xTMTpro [N	5.132E-05	4.225E-05	1	1	1	Q9Y5S2	Q9Y5S2 [433-445	0	1924.9394
[R].DLEKPFI	1xTMTpro [K	3.178E-06	4.225E-05	1	1	2	P49411	P49411 [256-274]	0	2737.5784
[R].DLELAVI	1xTMTpro [N	2.755E-05	4.225E-05	1	1	2	P42345	P42345 [2135-215	0	2315.2566
[R].DLEIERF	2xCarbamido	4.525E-05	4.225E-05	1	1	1	Q92597	Q92597 [236-275]	0	4929.5292
[K].DLELFSN	1xTMTpro [K	3.242E-05	4.225E-05	1	1	1	Q7L775	Q7L775 [484-504]	0	3091.6748
[K].SGLGSQ	1xOxidation [	9.748E-06	4.225E-05	1	1	2	P05997	P05997 [202-221]	0	2172.1766
[R].DIEQVP(	1xTMTpro [N	1.852E-05	4.225E-05	1	1	2	P62993	P62993 [150-178]	0	3685.7981
[K].KIENCNY	1xCarbamido	1.499E-05	4.225E-05	3	3	3	Q14651; P13	Q14651 [457-469]	1	2450.3893
[K].SGIEILSI	1xTMTpro [K	4.558E-06	4.225E-05	1	1	2	Q9ULC5	Q9ULC5 [226-241	0	2330.3099
[K].SGLDVM	1xTMTpro [N	1.371E-05	4.225E-05	1	1	1	Q9Y6F6	Q9Y6F6 [491-505	0	1933.0748
[K].GDPLGG	1xTMTpro [K	1.15E-05	4.225E-05	1	1	1	O43795	O43795 [178-192]	0	2183.2567
[R].SGIDITD	1xTMTpro [K	5.682E-05	4.225E-05	1	1	1	P49790	P49790 [343-353]	0	1803.0144
[R].SGGTEG	1xTMTpro [K	4.48E-05	4.225E-05	1	1	4	Q9UHB9	Q9UHB9 [267-27	0	1669.9616
[K].KIENSEL	2xTMTpro [K	6.003E-05	4.225E-05	1	1	1	Q7Z2W4	Q7Z2W4 [761-77(	1	2101.2685
[R].SGGSG(	2xCarbamido	3.696E-07	4.225E-05	1	1	4	Q15005	Q15005 [11-32]	0	2161.927
[K].KLEPIWI	2xTMTpro [K	3.13E-05	4.225E-05	1	1	2	Q96JJ7	Q96JJ7 [58-71]	1	2598.5145
[R].SGGNLE	1xTMTpro [K	3.767E-05	4.225E-05	1	1	1	Q92905	Q92905 [71-84]	0	2014.1321
[R].SGGLGG	1xTMTpro [N	5.597E-06	4.225E-05	1	1	1	P42704	P42704 [115-128]	0	1654.9924
[R].SGLGEL	1xTMTpro [K	7.023E-06	4.225E-05	1	1	3	P07954	P07954 [351-371]	0	2733.4988
[K].DLQLCH	2xCarbamido	4.055E-07	4.225E-05	1	1	2	P20908	P20908 [1641-16(	0	3209.4376
[K].DLLEVAI	1xTMTpro [K	5.581E-05	4.225E-05	1	1	3	Q9HAV7	Q9HAV7 [110-12(	0	1852.0923
[R].SEPTQA	1xTMTpro [K	1.249E-08	4.225E-05	1	1	2	O43396	O43396 [212-234]	1	3176.7182
[K].DLNSQA	1xTMTpro [K	7.519E-06	4.225E-05	1	1	2	Q13813	Q13813 [1716-17	0	2853.4432
[K].SDKLPEI	2xTMTpro [K	1.868E-05	4.225E-05	1	1	1	P43121	P43121 [614-632]	2	2959.6339
[R].GDGQFF	1xCarbamido	3.954E-05	4.225E-05	1	1	1	O95163	O95163 [202-219]	0	2201.098
[K].GDGPYS	1xTMTpro [K	1.665E-07	4.225E-05	1	1	3	Q12860	Q12860 [791-818]	0	3379.7877
[K].GDGPVQ	1xTMTpro [K	1.673E-05	4.225E-05	1	1	3	P00441	P00441 [11-24]	0	2110.1788
[K].DINWDS	1xTMTpro [N	8.974E-06	4.225E-05	1	1	1	Q92900	Q92900 [221-236]	0	2305.1532
[K].SDGAPA	2xTMTpro [K	7.379E-07	4.225E-05	1	1	4	P80723	P80723 [164-184]	0	2845.4996
[R].SDGALLI	1xTMTpro [N	1.951E-05	4.225E-05	1	1	2	Q9BQA1	Q9BQA1 [38-52]	0	1707.956
[R].SDFYDIV	1xTMTpro [N	1.105E-05	4.225E-05	1	1	2	Q9UHG3	Q9UHG3 [305-31	0	2027.1133

[K].SDFEEN(	1xCarbamido	5.645E-05	4.225E-05	1	1	1	P21281	P21281 [246-271]	0	3304.5421
[R].KLQATV(	2xTMTpro [K	2.435E-05	4.225E-05	1	1	3	P31146	P31146 [439-449]	1	2198.3689
[K].KLQDFA:	2xTMTpro [K	9.982E-07	4.225E-05	1	1	1	P35749	P35749 [1379-139	1	2677.5229
[K].SDENED	1xTMTpro [K	5.047E-05	4.225E-05	1	1	2	Q9NZM1	Q9NZM1 [1508-15	0	2160.0952
[K].DLPDVQ	1xTMTpro [N	1.927E-05	4.225E-05	1	1	2	Q9UHB9	Q9UHB9 [509-52	0	1830.0292
[R].KLQEEE	1xTMTpro [K	3.091E-05	4.225E-05	1	1	1	Q8IVM0	Q8IVM0 [181-195	1	2411.346
[K].SDDSVI(	1xTMTpro [N	3.696E-05	4.225E-05	1	1	1	Q9BY67	Q9BY67 [69-81]	0	1774.9618
[R].SDDKSS	2xTMTpro [K	1.601E-05	4.225E-05	1	1	2	P02549	P02549 [1420-14	1	2551.4105
[R].DLPEPV	1xCarbamido	5.71E-05	4.225E-05	1	1	1	P42331	P42331 [241-268]	0	3784.9388
[K].SDDEVD	1xTMTpro [K	8.275E-06	4.225E-05	1	1	6	P12111	P12111 [1348-136	0	2040.0629
[K].DLPETLI	1xTMTpro [K	6.063E-07	4.225E-05	1	1	5	P21810	P21810 [226-240]	0	2396.3065
[R].SDATAD	1xTMTpro [N	1.625E-05	4.225E-05	1	1	1	Q9Y295	Q9Y295 [223-238	0	1993.9997
[K].KIQASTI	2xTMTpro [K	3.119E-05	4.225E-05	1	1	4	P37802	P37802 [79-88]	1	2037.2347
[R].SDLAVP:	1xTMTpro [K	1.862E-05	4.225E-05	1	1	2	Q08380	Q08380 [311-323	0	1964.1924
[K].DINQEV	1xTMTpro [K	1.277E-05	4.225E-05	1	1	3	Q99798	Q99798 [145-160	0	2362.2898
[K].SDIDEIVI	1xTMTpro [N	2.497E-05	4.225E-05	1	1	4	P11021	P11021 [354-367]	0	1764.9663
[K].DLMVGD	1xTMTpro [N	5.056E-05	4.225E-05	1	1	4	P61160	P61160 [54-65]	0	1638.8328
[R].SDPLLI(	1xTMTpro [K	6.012E-05	4.225E-05	1	1	2	Q9UBI6	Q9UBI6 [49-64]	0	2336.3357
[K].SDPLCVI	1xCarbamido	1.509E-06	4.225E-05	1	1	1	O75131	O75131 [27-47]	0	2845.415
[R].SDPGLL	1xTMTpro [K	1.581E-05	4.225E-05	1	1	2	P78527	P78527 [3993-40(	0	2245.2394
[K].SDPFLEI	2xTMTpro [K	2.444E-05	4.225E-05	1	1	1	Q96FN4	Q96FN4 [175-189	0	2627.4173
[K].SDNVLL(	1xTMTpro [K	1.927E-05	4.225E-05	1	1	1	Q13177	Q13177 [371-383	0	1957.092
[K].DINAYN(	1xCarbamido	1.284E-05	4.225E-05	1	1	2	P30041	P30041 [85-97]	0	2191.0833
[K].DINAYN(	1xCarbamido	1.177E-06	4.225E-05	1	1	1	P30041	P30041 [85-106]	1	3257.6644
[R].DLNCVP	1xCarbamido	7.464E-06	4.225E-05	1	1	1	O14744	O14744 [19-35]	0	2394.3194
[K].DINECET	2xCarbamido	3.423E-05	4.225E-05	1	1	2	P35555	P35555 [613-627]	0	2069.9374
[K].SDAPTG	1xTMTpro [K	6.428E-06	4.225E-05	1	1	2	Q9H4A6	Q9H4A6 [124-138	0	2152.1993
[K].SDNCED	1xCarbamido	9.157E-06	4.225E-05	1	1	1	P02787	P02787 [434-453]	1	3112.6441
[R].KLPIDVT	2xTMTpro [K	1.343E-06	4.225E-05	1	1	5	P26599	P26599 [65-84]	1	3024.8165
[K].KLPVDS	2xTMTpro [K	7.687E-06	4.225E-05	1	1	1	P49756	P49756 [688-708]	2	3363.7889
[K].SDLEMQ	2xOxidation [	1.592E-05	4.225E-05	1	1	1	P35527	P35527 [272-290]	1	3243.7309
[K].SDLEMQ	1xOxidation [	6.44E-06	4.225E-05	1	1	2	P35527	P35527 [272-290]	1	3227.736
[K].SDLEMQ	2xTMTpro [K	1.391E-05	4.225E-05	1	1	1	P35527	P35527 [272-290]	1	3211.7411

[K].SDLEMQ	2xOxidation [	2.942E-05	4.225E-05	1	1	3	P35527	P35527 [272-289]	0	2811.4288
[K].SDLEMQ	1xOxidation [	1.595E-05	4.225E-05	1	1	8	P35527	P35527 [272-289]	0	2795.4339
[K].SDLEMQ	1xTMTpro [K	1.604E-05	4.225E-05	1	1	3	P35527	P35527 [272-289]	0	2779.4389
[K].DLNLMA	1xTMTpro [N	3.709E-05	4.225E-05	1	1	2	O75477	O75477 [157-171]	0	1916.0958
[R].KIQALQ(	1xTMTpro [K	2.023E-05	4.225E-05	1	1	6	P67936	P67936 [13-27]	1	2351.2811
[K].SDNCED	1xCarbamido	2.461E-06	4.225E-05	1	1	14	P02787	P02787 [434-452]	0	2680.342
[K].KLQEESI	2xTMTpro [K	8.139E-06	4.225E-05	1	1	1	O75822	O75822 [122-133]	1	2315.3638
[R].DLPGALI	2xTMTpro [K	1.858E-05	4.225E-05	1	1	2	Q96BM9	Q96BM9 [133-146	1	2482.4585
[K].SDALETI	1xTMTpro [K	7.058E-07	4.225E-05	1	1	4	P14866	P14866 [553-568]	0	2475.3198
[K].DLQAIC(	2xCarbamido	1.589E-06	4.225E-05	1	1	3	P07996	P07996 [265-285]	0	2713.353
[K].KISELDA	3xTMTpro [K	9.709E-07	4.225E-05	1	1	2	Q06323	Q06323 [36-58]	2	3774.2158
[K].KLSENTI	2xTMTpro [K	2.561E-05	4.225E-05	1	1	1	Q14515	Q14515 [133-159]	1	3856.0432
[R].DIQAVAT	1xTMTpro [N	5.904E-06	4.225E-05	1	1	1	Q9NUL5	Q9NUL5 [76-93]	0	2229.2774
[R].SCGSST	1xCarbamido	3.626E-05	4.225E-05	1	1	1	P41091	P41091 [104-121]	0	2504.247
[R].DLQDAV	1xTMTpro [K	2.931E-05	4.225E-05	1	1	2	Q9BQS8	Q9BQS8 [16-26]	0	1827.0355
[K].DLQDEL.	1xTMTpro [N	3.502E-05	4.225E-05	1	1	1	Q9UQE7	Q9UQE7 [311-323	0	1778.884
[K].DIQDSLT	1xTMTpro [K	7.73E-06	4.225E-05	1	1	1	P47755	P47755 [211-226]	0	2356.2851
[K].SCGKPV	1xCarbamido	9.677E-06	4.225E-05	1	1	2	Q96A00	Q96A00 [99-114]	0	2746.563
[K].SCETDA	1xCarbamido	4.016E-05	4.225E-05	1	1	1	O94855	O94855 [779-791]	0	2124.1291
[K].GDGAEL	1xTMTpro [K	4.263E-07	4.225E-05	1	1	2	Q9UBT2	Q9UBT2 [337-362	1	3402.7131
[K].DLQEEV.	1xTMTpro [N	3.563E-05	4.225E-05	1	1	2	O94874	O94874 [539-552]	0	2011.0416
[K].DLQEIMI	1xTMTpro [K	1.021E-05	4.225E-05	1	1	4	Q86UU1	Q86UU1 [196-214	0	2623.4144
[R].SCDVTS	2xCarbamido	3.499E-06	4.225E-05	1	1	2	P35442	P35442 [407-423]	0	2200.0657
[R].SCDVES	1xCarbamido	8.633E-06	4.225E-05	1	1	1	P04278	P04278 [216-238]	0	2852.4208
[K].KLSEQN	2xTMTpro [K	8.761E-06	4.225E-05	1	1	1	Q0JRZ9	Q0JRZ9 [641-658	1	2951.6658
[K].SCDKTH	3xCarbamido	1.544E-05	4.225E-05	1	2	2	P0DOX5	P0DOX5 [221-250	1	4551.4707
[R].SCDGAA	1xCarbamido	1.795E-05	4.225E-05	1	1	1	Q63ZY3	Q63ZY3 [411-442	0	3749.8671
[K].KISGGS\	1xTMTpro [K	5.433E-06	4.225E-05	1	1	1	O75874	O75874 [4-20]	1	2432.2769
[K].SCCSCC	5xCarbamido	2.593E-05	4.225E-05	2	5	2	P80297; P04	P80297 [32-43]; F	0	2053.9242
[K].SCCDYA	2xCarbamido	7.194E-06	4.225E-05	1	1	2	Q14195	Q14195 [131-149]	0	2928.4264
[K].KISGGS\	1xOxidation [	4.923E-05	4.225E-05	1	1	1	O75874	O75874 [4-20]	1	2448.2718
[K].SCESNS	3xCarbamido	7.26E-06	4.225E-05	1	1	5	P02774	P02774 [95-114]	0	2873.3512
[R].SDPNRE	1xTMTpro [N	2.797E-06	4.225E-05	1	1	2	Q16531	Q16531 [415-434]	1	2554.3068

[R].SCLLPQI	1xCarbamido	1.628E-05	4.225E-05	1	1	1	Q92696	Q92696 [185-202]	0	2291.1886
[R].KISEEDE	1xTMTpro [K	1.327E-05	4.225E-05	1	1	1	Q4ZHG4	Q4ZHG4 [253-269	1	2567.3696
[K].DIPGQAS	1xTMTpro [K	6.494E-08	4.225E-05	1	1	2	O95302	O95302 [237-257]	0	2870.6272
[K].SDAEED	2xTMTpro [K	5.475E-07	4.225E-05	1	1	1	P27824	P27824 [554-573]	1	3118.5956
[K].SCVLFN(	2xCarbamido	3.359E-06	4.225E-05	1	1	1	Q8WUM4	Q8WUM4 [121-14	0	3559.8016
[R].SCVGET	2xCarbamido	8.507E-06	4.225E-05	1	1	1	P10643	P10643 [534-554]	0	2813.2525
[K].SCVEEP	1xCarbamido	3.981E-05	4.225E-05	1	1	1	P51858	P51858 [107-126]	1	3085.5452
[R].SCVDVN	3xCarbamido	3.154E-06	4.225E-05	1	1	2	O95967	O95967 [200-216]	0	2330.9793
[R].SCTVLN\	1xCarbamido	7.845E-06	4.225E-05	1	1	1	Q15758	Q15758 [466-488]	0	2768.4208
[R].SCTTLEI	1xCarbamido	5.784E-05	4.225E-05	1	1	1	O60462	O60462 [901-917]	0	2675.3518
[K].DIPNENE	1xTMTpro [N	3.01E-05	4.225E-05	1	1	2	Q10567	Q10567 [852-864]	0	1877.9677
[K].DIPNENE	1xTMTpro [K	1.102E-05	4.225E-05	1	1	5	P63010	P63010 [843-855]	0	2196.2156
[K].KISELDA	2xTMTpro [K	5.209E-05	4.225E-05	1	1	2	Q06323	Q06323 [36-45]	1	2076.2885
[K].DLPPDT	1xTMTpro [K	2.341E-05	4.225E-05	1	1	12	P07585	P07585 [78-92]	0	2305.2895
[R].DLPSAG	1xTMTpro [N	3.833E-05	4.225E-05	1	1	1	P46199	P46199 [423-439]	0	2174.1148
[R].SCTEETI	2xCarbamido	3.644E-06	4.225E-05	1	1	1	Q9UBG0	Q9UBG0 [1097-1	0	2205.0924
[R].SCTDIDE	4xCarbamido	1.899E-06	4.225E-05	1	1	2	P35555	P35555 [1235-126	0	3593.4844
[R].DIPTNSF	1xTMTpro [K	3.076E-07	4.225E-05	1	1	4	P01042	P01042 [270-288]	0	2747.4959
[R].KIQINNA	2xTMTpro [K	2.784E-05	4.225E-05	1	1	2	Q16363	Q16363 [336-347]	1	2316.3526
[K].KLQSQY	1xTMTpro [K	2.194E-05	4.225E-05	1	1	1	Q15075	Q15075 [257-273]	1	2518.4121
[R].SCQMNL	1xCarbamido	2.73E-06	4.225E-05	1	1	1	Q13158	Q13158 [167-184]	0	2393.2236
[K].KLSDDN	2xTMTpro [K	5.654E-05	4.225E-05	1	1	1	Q13813	Q13813 [1829-18	2	2786.5828
[K].DLPVTE/	1xTMTpro [N	6.731E-06	4.225E-05	1	1	2	P42704	P42704 [227-244]	0	2187.2093
[R].SCNLDE	1xCarbamido	6.676E-07	4.225E-05	1	1	2	P08575	P08575 [609-625]	1	2715.3387
[R].DLPPVS(	1xTMTpro [K	1.279E-05	4.225E-05	1	1	2	Q14204	Q14204 [640-652]	0	1991.1821
[K].SEPVKEI	1xTMTpro [K	1.486E-05	4.225E-05	2	2	1	Q15424; Q1	Q15424 [227-252]	1	3456.7262
[R].SDPTSY.	2xTMTpro [K	2.57E-07	4.225E-05	1	1	2	P54709	P54709 [98-112]	1	2599.4435
[K].SDQNLQ	1xTMTpro [N	2.083E-05	4.225E-05	1	1	2	O95373	O95373 [490-502]	0	1792.9724
[R].SELHIEN	1xTMTpro [N	4.057E-06	4.225E-05	1	1	2	P35613	P35613 [283-300]	0	2420.1835
[R].GDINILLI	1xTMTpro [K	4.071E-05	4.225E-05	1	1	2	P25205	P25205 [337-351]	0	2133.2775
[K].GDINIEG	1xTMTpro [K	2.19E-06	4.225E-05	1	1	2	Q09666	Q09666 [3501-35	0	3359.7285
[K].SELEEQI	1xTMTpro [N	1.263E-05	4.225E-05	1	1	2	P02649	P02649 [94-108]	0	2035.0514
[R].SELECV	1xCarbamido	9.588E-06	4.225E-05	1	1	2	Q9Y6W5	Q9Y6W5 [23-38]	0	2136.1654

[R].SELEAIA	1xTMTpro [N	7.948E-08	4.225E-05	1	1	2	Q99715	Q99715 [582-607]	0	3210.5914
[K].DLLLNTN	1xTMTpro [K	2.779E-05	4.225E-05	1	1	2	P78527	P78527 [3814-382	0	2029.1131
[K].SEHPGL	1xTMTpro [K	2.471E-05	4.225E-05	1	1	3	P26583	P26583 [115-127]	0	1920.0682
[R].SEGSSV	1xCarbamido	1.882E-05	4.225E-05	1	1	2	P00734	P00734 [199-217]	0	2375.1832
[K].KLLDLVQ	1xCarbamido	4.657E-05	4.225E-05	1	1	2	P55769	P55769 [21-33]	1	2521.4628
[K].KILDSVG	1xTMTpro [K	3.613E-05	4.225E-05	1	1	2	P05387	P05387 [25-38]	1	2154.1898
[K].KLLEAHE	2xTMTpro [K	6.819E-06	4.225E-05	1	1	1	P54920	P54920 [246-264]	1	3131.7041
[R].SEFPES	2xTMTpro [K	2.745E-05	4.225E-05	1	1	2	P01024	P01024 [765-783]	1	3242.7554
[R].SEFPES	1xTMTpro [K	5.064E-05	4.225E-05	1	1	1	P01024	P01024 [765-779]	0	2487.3052
[K].SEEWAD	1xTMTpro [N	9.911E-06	4.225E-05	1	1	1	O60664	O60664 [196-213]	0	2371.1849
[R].SEETKE	2xTMTpro [K	7.83E-07	4.225E-05	1	1	4	P01024	P01024 [1321-133	1	2768.477
[K].SEEQLK	2xTMTpro [K	2.345E-05	4.225E-05	1	1	2	P09622	P09622 [405-417]	1	2494.3857
[K].DILLVND	1xTMTpro [N	3.276E-05	4.225E-05	1	1	1	Q9NTI5	Q9NTI5 [381-394]	0	1985.1503
[K].SEEMQT	1xOxidation [	2.389E-06	4.225E-05	1	1	4	Q15149	Q15149 [2610-263	0	3804.9457
[K].SEEMQT	1xTMTpro [K	1.693E-06	4.225E-05	1	1	5	Q15149	Q15149 [2610-263	0	3788.9508
[K].SEEITTG	1xTMTpro [K	1.549E-07	4.225E-05	1	1	2	Q15121	Q15121 [36-54]	0	2778.423
[R].KIIATVLN	2xTMTpro [K	1.433E-05	4.225E-05	1	1	1	Q04637	Q04637 [714-726]	1	2387.4764
[K].SEIIPMF	1xTMTpro [N	2.154E-06	4.225E-05	1	1	4	P30153	P30153 [203-221]	0	2442.2142
[R].GDINVCI	1xCarbamido	4.603E-05	4.225E-05	1	1	1	Q14566	Q14566 [388-402]	0	2154.172
[R].GDLPDP	1xCarbamido	6.689E-07	4.225E-05	1	1	2	Q14573	Q14573 [1704-17	0	2830.4001
[K].SEPIPES	1xTMTpro [K	1.852E-05	4.225E-05	1	1	3	P30101	P30101 [367-379]	0	1977.0784
[K].SEPDKA	2xCarbamido	1.558E-05	4.225E-05	1	1	3	Q99733	Q99733 [229-255]	1	4026.9557
[R].SEPDDS	2xCarbamido	2.716E-05	4.225E-05	1	1	2	P55209	P55209 [237-263]	0	3698.6581
[R].DIIFLLDC	1xTMTpro [K	1.193E-05	4.225E-05	1	1	7	P12111	P12111 [639-652]	0	2070.2091
[K].SENQEF	1xTMTpro [K	3.638E-05	4.225E-05	1	1	2	O60506	O60506 [608-623]	0	2643.2971
[K].DILFPYIE	1xTMTpro [K	3.52E-05	4.225E-05	1	1	2	Q9UHY7	Q9UHY7 [29-40]	0	2088.1873
[K].SENGLE	1xTMTpro [K	1.828E-05	4.225E-05	1	1	1	P21796	P21796 [35-53]	0	2568.2921
[R].KLGVYE	1xTMTpro [K	1.868E-05	4.225E-05	1	1	1	Q12884	Q12884 [591-605]	1	2328.3419
[R].SELVVD	1xTMTpro [K	1.088E-05	4.225E-05	1	1	1	Q12769	Q12769 [175-192]	0	2605.4039
[K].SELVANI	1xTMTpro [N	1.057E-05	4.225E-05	2	2	3	P42167; P42	P42167 [18-33]; F	0	2002.0888
[R].KLLEMEI	1xTMTpro [K	7.366E-07	4.225E-05	1	1	2	Q16853	Q16853 [567-585]	1	2698.5093
[K].DLIGIDN	1xTMTpro [N	4.931E-05	4.225E-05	1	1	1	Q9Y4F1	Q9Y4F1 [746-758	0	1684.9917
[K].SELVAMI	1xTMTpro [N	2.457E-05	4.225E-05	1	1	2	P40616	P40616 [105-117]	0	1851.9693

[K].SELTQQ	1xTMTpro [K	2.804E-05	4.225E-05	1	1	2	P06727	P06727 [52-65]	0	2243.2527
[R].DLLGTV	1xTMTpro [K	8.321E-06	4.225E-05	1	1	1	Q9NTX5	Q9NTX5 [284-301	0	2433.3997
[K].KIGYNP	2xTMTpro [K	3.237E-06	4.225E-05	1	2	4	P68104	P68104 [180-212]	1	4608.3876
[K].SEISENT	1xTMTpro [K	3.683E-05	4.225E-05	1	1	2	Q9UHB6	Q9UHB6 [175-18	0	1845.9686
[K].SEIQTLK	2xTMTpro [K	4.102E-06	4.225E-05	1	1	2	Q14258	Q14258 [286-301	1	2788.6124
[R].SEIQFLI	1xCarbamid	3.378E-06	4.225E-05	1	1	1	Q14126	Q14126 [566-583	0	2707.3893
[K].SELPLD	1xTMTpro [K	1.315E-05	4.225E-05	1	1	4	Q15758	Q15758 [503-522	0	2766.5785
[K].KLHEEE	2xTMTpro [K	4.118E-06	4.225E-05	1	1	5	P08670	P08670 [236-270]	1	4963.7309
[K].DLLIAYY	1xTMTpro [K	1.845E-05	4.225E-05	1	1	7	P30101	P30101 [259-271]	0	2228.1982
[K].SELVAM	1xTMTpro [K	1.813E-06	4.225E-05	1	1	3	P40616	P40616 [105-118]	1	2284.2714
[K].KLIGEVS	1xTMTpro [K	3.835E-07	4.225E-05	1	1	1	P55008	P55008 [88-108]	1	2897.554
[R].KILGQE	2xTMTpro [K	4.673E-05	4.225E-05	1	1	1	Q12905	Q12905 [328-356	1	4006.1841
[K].DIINEEE	1xTMTpro [K	4.964E-05	4.225E-05	1	1	3	P49588	P49588 [373-384]	0	2085.1723
[K].SDYDMV	1xTMTpro [N	1.868E-05	4.225E-05	1	1	1	Q14974	Q14974 [750-762	0	1936.9282
[K].SDYAQL	1xTMTpro [N	8.476E-06	4.225E-05	1	1	2	P38606	P38606 [599-613]	0	2105.0293
[K].KLNECL	1xCarbamid	4.257E-05	4.225E-05	1	1	1	O00499	O00499 [81-97]	1	2741.4213
[K].KLNECL	1xCarbamid	9.31E-06	4.225E-05	1	1	2	O00499	O00499 [81-102]	2	3602.8729
[K].SDVGFL	1xTMTpro [K	9.293E-06	4.225E-05	1	1	2	P43652	P43652 [131-147]	0	2496.3518
[K].SDVEIPA	1xTMTpro [K	1.84E-06	4.225E-05	1	1	2	Q9UEY8	Q9UEY8 [402-42	0	3286.759
[R].SDVCTP	1xCarbamid	6.04E-07	4.225E-05	1	1	1	Q53H96	Q53H96 [232-254	0	2706.384
[K].KINESTQ	2xTMTpro [K	1.27E-06	4.225E-05	1	1	3	P51911	P51911 [74-93]	1	3325.8473
[K].DILVLPL	1xTMTpro [K	1.682E-05	4.225E-05	1	1	1	Q9Y394	Q9Y394 [104-123	0	2717.5217
[R].DLLVQQ	1xCarbamid	1.1E-05	4.225E-05	1	1	2	Q1KMD3	Q1KMD3 [509-52	0	2098.1822
[R].SDYLNT	1xTMTpro [K	4.228E-05	4.225E-05	1	1	4	O75874	O75874 [389-400	0	2118.0709
[K].LGHYAT	1xTMTpro [N	5.907E-05	4.225E-05	1	1	1	P51692	P51692 [87-100]	0	1984.0208
[R].SDTPAM	2xCarbamid	3.191E-05	4.225E-05	1	1	1	Q9NZB2	Q9NZB2 [697-72	0	3013.5415
[K].SDTALE	1xTMTpro [K	7.816E-06	4.225E-05	1	1	1	Q86TP1	Q86TP1 [113-133	0	2927.597
[R].GDKVDI	2xTMTpro [K	3.352E-05	4.225E-05	1	1	1	Q9Y5B9	Q9Y5B9 [694-705	1	2304.3743
[R].SDSYM	2xTMTpro [K	4.744E-05	4.225E-05	1	1	1	Q9UGP8	Q9UGP8 [700-71	0	2507.436
[K].KLNNLT	1xTMTpro [K	1.351E-05	4.225E-05	1	1	1	P20774	P20774 [211-237]	1	3732.098
[K].KLNQAL	1xTMTpro [K	4.687E-06	4.225E-05	1	1	4	P02792	P02792 [106-121]	1	2328.4007
[K].GDKDGS	1xOxidation	1.578E-05	4.225E-05	1	1	1	P36776	P36776 [794-811]	1	2791.5328
[K].GDKDGS	2xTMTpro [K	1.289E-06	4.225E-05	1	1	2	P36776	P36776 [794-811]	1	2775.5379

[K].SDRIEPL	1xTMTpro [N	1.462E-05	4.225E-05	1	1	1	P22413	P22413 [504-527]	1	3192.6649
[K].SDQVNG	1xTMTpro [K	3.94E-05	4.225E-05	1	1	2	Q6NZI2	Q6NZI2 [46-60]	0	2208.3095
[K].SDTSGD	1xTMTpro [K	3.242E-05	4.225E-05	1	1	2	P12429	P12429 [305-317]	0	2050.12
[K].SDQIGLF	1xTMTpro [N	7.631E-06	4.225E-05	1	1	1	P15144	P15144 [341-363]	0	2858.4102
[R].DIISIAED	1xTMTpro [N	3.657E-05	4.225E-05	1	1	2	P12956	P12956 [219-230]	0	1692.8975
[K].KLMSAN.	1xCarbamido	6.299E-06	4.225E-05	1	1	1	P34932	P34932 [275-303]	1	3823.8983
[K].SEDSTKI	2xTMTpro [K	7.194E-06	4.225E-05	1	1	1	O60841	O60841 [9-33]	1	3417.8053
[R].SEDPDQ	1xTMTpro [N	1.26E-05	4.225E-05	1	1	2	Q96QK1	Q96QK1 [500-514	0	2067.0678
[R].KLLLDPS	2xTMTpro [K	2.561E-05	4.225E-05	1	1	2	Q6IAA8	Q6IAA8 [20-31]	1	2208.3784
[K].KLLMMA	1xCarbamido	5.894E-06	4.225E-05	1	1	1	P15880	P15880 [212-227]	1	2453.2846
[K].GDLEEY	1xTMTpro [K	1.521E-05	4.225E-05	1	1	2	P43490	P43490 [448-463]	0	2472.3266
[K].KLLMMA	1xCarbamido	3.225E-05	4.225E-05	1	1	2	P15880	P15880 [212-227]	1	2469.2795
[K].DILPCLD	1xCarbamido	4.705E-05	4.225E-05	1	1	4	P78527	P78527 [791-801]	0	1915.0854
[R].SEDFGV	1xTMTpro [N	1.316E-06	4.225E-05	1	1	6	P04083	P04083 [189-204]	0	2043.9426
[K].SEDEVG	1xTMTpro [N	2.834E-05	4.225E-05	1	1	2	P23229	P23229 [861-874]	0	1976.9772
[K].DLLPSDI	1xTMTpro [N	4.827E-09	4.225E-05	1	1	2	Q15149	Q15149 [3184-32	0	3237.7108
[R].DLLSHEI	1xTMTpro [K	1.117E-05	4.225E-05	1	1	1	Q8NE86	Q8NE86 [166-180	0	2248.2429
[K].GDLDIAC	1xTMTpro [K	1.648E-05	4.225E-05	1	1	1	Q09666	Q09666 [3431-34	0	2169.1683
[K].KIMADS(	2xTMTpro [K	2.697E-05	4.225E-05	1	1	1	P07996	P07996 [1130-114	1	2250.2984
[K].SECLNN	1xCarbamido	1.544E-05	4.225E-05	1	1	3	Q92973	Q92973 [101-115]	0	1979.0187
[R].SEASEW	1xTMTpro [N	3.651E-05	4.225E-05	1	1	1	Q16832	Q16832 [308-332]	0	3047.5281
[R].SEALPTI	1xTMTpro [K	1.944E-05	4.225E-05	1	1	1	P14314	P14314 [282-301]	0	2657.4529
[K].SEALGV	1xTMTpro [K	6.032E-05	4.225E-05	1	1	1	Q9NZU5	Q9NZU5 [191-209	0	2387.379
[R].KLMEQY	2xTMTpro [K	1.16E-06	4.225E-05	1	1	1	P05164	P05164 [629-653]	2	3789.0648
[R].GDICKDI	2xCarbamido	2.136E-05	4.225E-05	1	1	1	P35442	P35442 [929-960]	1	4336.9742
[K].GDLCEH	2xCarbamido	1.722E-05	4.225E-05	1	1	3	P98160	P98160 [4137-415	0	2060.9449
[K].DIISDTS	1xTMTpro [K	3.387E-05	4.225E-05	1	2	3	P07355	P07355 [158-169]	1	1962.0788
[R].DLISHDE	1xTMTpro [K	1.53E-05	4.225E-05	1	1	2	P13693	P13693 [6-19]	0	2321.1979
[K].SEDCFIL	1xCarbamido	4.367E-05	4.225E-05	1	1	6	P06396	P06396 [328-338]	0	1929.0032
[R].QVYDGF	1xTMTpro [K	7.075E-06	4.225E-05	1	1	1	Q99598	Q99598 [223-241]	0	2716.4114
[R].GAVPWY	1xTMTpro [K	3.075E-05	4.225E-05	1	1	2	Q13510	Q13510 [45-60]	0	2455.3881
[R].DNYVPE	1xTMTpro [K	5.159E-06	4.225E-05	1	1	2	P08708	P08708 [82-103]	0	3097.6073
[K].DTDIVDE	1xTMTpro [K	2.916E-05	4.225E-05	1	1	4	O15145	O15145 [38-50]	0	2200.1669

[R].DTDMDG	2xCarbamido	1.875E-08	4.225E-05	1	1	2	P07996	P07996 [823-851]	0	3624.4716
[R].DTDMDG	2xCarbamido	1.179E-06	4.225E-05	1	1	2	P07996	P07996 [823-851]	0	3640.4665
[K].DTDPDS	2xTMTpro [K	9.677E-06	4.225E-05	2	2	1	Q14141; Q9I	Q14141 [310-327]	0	2983.5717
[R].QIILEKEI	2xTMTpro [K	7.589E-06	4.225E-05	1	1	2	P12956	P12956 [326-339]	2	2670.5858
[K].GAGTGG	1xCarbamido	1.118E-06	4.225E-05	2	2	4	O75369; Q14	O75369 [1067-10	0	2282.2306
[K].QLIIEDPY	1xCarbamido	3.975E-06	4.225E-05	1	1	1	P24666	P24666 [125-148]	0	3256.5428
[R].GAGTGG	1xTMTpro [K	9.338E-07	4.225E-05	1	1	3	P21333	P21333 [1382-13	0	2179.2214
[R].GAGTGG	1xTMTpro [K	6.694E-06	4.225E-05	1	1	1	Q14315	Q14315 [1377-13	0	2193.2371
[R].DTDSLQ	1xTMTpro [N	3.119E-05	4.225E-05	1	1	2	Q9UPN3	Q9UPN3 [5156-51	0	1809.915
[R].QLLEDES	1xTMTpro [N	1.454E-05	4.225E-05	1	1	1	P06401	P06401 [322-343]	0	2526.2432
[R].DTEDEE	1xTMTpro [K	1.532E-06	4.225E-05	1	1	2	P02549	P02549 [801-823]	0	3208.5665
[K].QILEDAA	1xTMTpro [K	8.46E-06	4.225E-05	1	1	1	Q96ST3	Q96ST3 [785-799	0	2279.3731
[K].DTEEED	1xTMTpro [K	2.578E-06	4.225E-05	1	1	13	P01009	P01009 [226-241]	0	2500.2699
[R].QILDWH	1xTMTpro [K	2.593E-05	4.225E-05	1	1	2	O60341	O60341 [527-550]	0	3337.844
[K].GAGTDE	1xCarbamido	6.003E-06	4.225E-05	1	1	3	P09525	P09525 [101-116]	0	1966.0235
[R].QLLDQV	1xTMTpro [K	3.617E-06	4.225E-05	1	1	1	Q7Z7H5	Q7Z7H5 [162-178	1	2769.5027
[R].QLLDQV	1xTMTpro [K	4.3E-05	4.225E-05	1	1	3	Q7Z7H5	Q7Z7H5 [162-172	0	1950.1516
[R].KTCTTV	1xCarbamido	9.322E-08	4.225E-05	1	1	2	P62424	P62424 [197-217]	2	3485.9779
[R].QLLDLDI	1xCarbamido	3.069E-05	4.225E-05	1	1	2	Q9H4A6	Q9H4A6 [270-282	0	2180.2128
[R].QLLDETS	1xTMTpro [K	1.513E-05	4.225E-05	1	1	1	O14776	O14776 [963-978]	0	2415.3627
[R].KSTAALI	2xTMTpro [K	1.91E-05	4.225E-05	2	2	1	Q15052; Q14	Q15052 [648-661]	1	2429.4432
[R].DTDIASL	2xTMTpro [K	1.238E-05	4.225E-05	1	1	2	Q14BN4	Q14BN4 [599-611	1	2402.3959
[R].QILLGPN	1xTMTpro [K	7.314E-06	4.225E-05	1	1	3	Q9NS69	Q9NS69 [118-142	1	2996.7462
[R].QLIIVHG	2xCarbamido	1.359E-05	4.225E-05	1	1	1	Q9P2I0	Q9P2I0 [560-579]	0	2597.3135
[K].QIIVDPLS	1xTMTpro [N	5.464E-05	4.225E-05	1	1	4	P35221	P35221 [288-300]	0	1837.0026
[R].QLLTLSS	1xTMTpro [N	2.588E-05	4.225E-05	1	1	2	P15311	P15311 [530-542]	0	1750.003
[R].QLLTDF(	1xCarbamido	5.484E-06	4.225E-05	1	1	2	Q9BT78	Q9BT78 [64-82]	0	2779.4944
[R].DSYYMT	1xTMTpro [N	4.541E-05	4.225E-05	1	2	1	P01880	P01880 [61-78]	0	2495.2196
[K].KSQIFST	2xTMTpro [K	9.964E-07	4.225E-05	1	1	4	P11021	P11021 [447-464]	1	2877.6502
[K].QILSLNY	1xTMTpro [N	3.741E-05	4.225E-05	1	1	1	Q12768	Q12768 [482-496]	0	1928.0044
[R].DTADGIL	1xTMTpro [K	2.73E-05	4.225E-05	1	1	1	P78539	P78539 [210-222]	0	1982.1665
[R].DTAEGV	1xTMTpro [N	5.345E-07	4.225E-05	1	1	1	O95747	O95747 [459-477]	0	2221.1631
[K].QLLQTVI	1xTMTpro [K	4.752E-05	4.225E-05	1	1	2	Q02952	Q02952 [1373-13	0	2217.3462

[R].QLIQGG'	1xCarbamid	3.191E-05	4.225E-05	1	1	1	P22307	P22307 [106-123]	0	2540.4402
[R].QLIDEGI	1xTMTpro [K	3.557E-05	4.225E-05	1	1	1	Q86WB0	Q86WB0 [34-50]	0	2349.2793
[R].DTANWL	1xTMTpro [N	6.09E-05	4.225E-05	1	1	1	P12830	P12830 [528-545]	0	2278.1635
[R].QLLQAN	1xTMTpro [K	4.689E-05	4.225E-05	1	1	3	P35580	P35580 [217-232]	0	2351.3579
[KR].QLLQA	1xTMTpro [K	5.839E-06	4.225E-05	3	3	5	P35749; P35	P35749 [217-232]	0	2335.3629
[R].GAGTVF	1xTMTpro [N	1.24E-06	4.225E-05	1	1	1	P06737	P06737 [322-352]	0	3611.8851
[R].QLIPIFTI	1xTMTpro [K	4.264E-05	4.225E-05	1	1	1	Q9UKN8	Q9UKN8 [449-46(	0	1966.2233
[K].DTCFEG	1xCarbamid	1.374E-05	4.225E-05	1	1	1	P03951	P03951 [27-44]	0	2554.2991
[K].QLLPCEI	2xCarbamid	4.127E-05	4.225E-05	1	1	1	Q9NR45	Q9NR45 [279-29(	0	2101.0736
[K].KSSGEI\	1xCarbamid	2.755E-06	4.225E-05	1	1	1	Q02543	Q02543 [56-70]	1	2643.4632
[K].DTDAAV	1xTMTpro [N	2.511E-05	4.225E-05	1	1	1	O15144	O15144 [211-230]	0	2488.3043
[K].GAGTNE	1xTMTpro [N	1.786E-06	4.225E-05	1	1	4	P12429	P12429 [105-120]	0	1978.0776
[R].QLIMANI	1xTMTpro [N	5.775E-05	4.225E-05	2	2	1	Q9UMX0; Q!	Q9UMX0 [207-22	0	2116.169
[K].DTAPVLI	1xCarbamid	1.969E-05	4.225E-05	1	1	1	O95202	O95202 [527-554]	2	4349.379
[R].DTFLQN	1xTMTpro [K	3.67E-05	4.225E-05	1	1	1	Q9Y6Q1	Q9Y6Q1 [379-397	0	2857.5016
[R].QLLDDH	1xTMTpro [K	7.561E-06	4.225E-05	1	1	1	O14874	O14874 [157-174]	1	2643.5325
[R].GAGSYT	1xOxidation	3.975E-06	4.225E-05	1	1	1	P21333	P21333 [842-863]	0	2616.3663
[K].QIGGDG	1xTMTpro [K	3.754E-06	4.225E-05	1	1	4	P00488	P00488 [469-483]	0	2253.1387
[K].QIGENLI	1xTMTpro [K	5.235E-05	4.225E-05	2	2	4	Q16555; Q1	Q16555 [44-56]; (	0	1932.1774
[R].QLGENF	2xCarbamid	5.295E-07	4.225E-05	1	1	1	P13798	P13798 [351-375]	0	3162.5308
[K].QIGDNLI	1xTMTpro [K	4.22E-05	4.225E-05	1	1	3	Q14195	Q14195 [44-56]	0	1918.1617
[R].QIGDALI	1xCarbamid	1.889E-05	4.225E-05	1	1	1	Q13740	Q13740 [345-360]	0	1979.0551
[K].DTMSDQ	1xTMTpro [N	3.403E-06	4.225E-05	1	1	1	P20810	P20810 [284-301]	0	2170.0981
[K].DTMSDQ	1xOxidation	4.419E-05	4.225E-05	1	1	1	P20810	P20810 [284-301]	0	2186.093
[R].DTMVED	1xTMTpro [N	3.873E-05	4.225E-05	1	1	2	P07358	P07358 [417-428]	0	1692.9525
[R].KTDGYS	1xTMTpro [K	6.1E-05	4.225E-05	1	1	1	O75351	O75351 [332-346]	1	2203.2578
[R].QLGCGC	2xCarbamid	4.257E-06	4.225E-05	1	1	1	A1L4H1	A1L4H1 [60-90]	0	3380.6839
[R].DTLVQG	1xTMTpro [K	1.865E-05	4.225E-05	1	1	1	Q9Y6E2	Q9Y6E2 [32-50]	0	2566.3856
[K].DTNGEN	1xTMTpro [N	5.555E-06	4.225E-05	1	1	1	Q7KZF4	Q7KZF4 [117-135	0	2264.1689
[K].DTNKYE	1xCarbamid	2.565E-05	4.225E-05	1	1	1	Q92520	Q92520 [205-227]	2	3664.8443
[R].DTNRPL	1xTMTpro [K	2.87E-05	4.225E-05	1	1	1	P55287	P55287 [134-148]	0	2350.3262
[K].QLGAGS	1xCarbamid	1.414E-05	4.225E-05	1	1	3	P00747	P00747 [40-52]	0	1942.0559
[R].QLFVLA(	1xOxidation	6.428E-06	4.225E-05	1	1	2	P08754	P08754 [106-128]	0	2983.667

[R].DTPENN	1xTMTpro [K	2.692E-05	4.225E-05	1	1	1	P19404	P19404 [43-61]	0	2849.3761
[R].DTPENN	1xTMTpro [K	4.926E-07	4.225E-05	1	1	2	P19404	P19404 [43-62]	1	3005.4773
[R].QLFVLA(	1xTMTpro [K	4.359E-07	4.225E-05	1	1	3	P08754	P08754 [106-128]	0	2967.6721
[R].QLFHPE	1xTMTpro [K	1.78E-06	4.225E-05	3	6	3	Q71U36; Q9	Q71U36 [85-105];	1	3023.6194
[R].QIFHPE(	1xTMTpro [K	4.271E-05	4.225E-05	3	8	6	Q71U36; Q9	Q71U36 [85-96]; (	0	2019.1883
[K].DTPTQE	1xTMTpro [N	1.184E-06	4.225E-05	1	1	2	Q9NQW7	Q9NQW7 [99-116	0	2333.1944
[K].DTNGSQ	1xTMTpro [K	1.619E-05	4.225E-05	1	1	2	P23284	P23284 [146-158]	0	2066.1414
[K].DSYLILE	1xTMTpro [N	1.051E-05	4.225E-05	1	1	2	P17980	P17980 [156-171]	0	2219.1403
[R].GAGQQ(	1xTMTpro [N	2.222E-05	4.225E-05	1	1	1	Q15435	Q15435 [6-20]	0	1997.934
[K].QLGGGY	1xTMTpro [N	1.094E-05	4.225E-05	1	1	1	Q9Y3Z3	Q9Y3Z3 [149-164	0	2027.0418
[R].GAGSYT	1xTMTpro [N	1.151E-06	4.225E-05	1	1	1	P21333	P21333 [842-863]	0	2600.3713
[R].DTGDEV	5xCarbamido	1.015E-07	4.225E-05	1	1	1	P23142	P23142 [192-228]	0	4515.9427
[K].QLLAGPI	1xTMTpro [N	5.188E-06	4.225E-05	1	1	1	Q8WUA7	Q8WUA7 [203-21	1	2029.1361
[R].QLLAGG	1xTMTpro [N	2.226E-05	4.225E-05	1	1	2	Q6NUK1	Q6NUK1 [197-20	0	1516.913
[R].DTGIFLD	1xTMTpro [K	4.308E-05	4.225E-05	1	1	3	P12956	P12956 [195-206]	0	2011.1542
[R].GAGSIA(	1xTMTpro [K	2.984E-05	4.225E-05	1	1	1	P15924	P15924 [2014-202	0	1681.9729
[R].QLKEHA	1xCarbamido	2.906E-05	4.225E-05	1	1	1	P02765	P02765 [104-120]	1	2942.6226
[R].DTGTYG	1xTMTpro [N	5.393E-05	4.225E-05	1	1	2	Q96IY4	Q96IY4 [388-399]	0	1672.8866
[R].QIHVIDM	1xTMTpro [N	2.449E-05	4.225E-05	1	1	1	Q8TBC4	Q8TBC4 [95-111]	0	2287.2035
[R].QLHEYE	1xTMTpro [K	3.512E-06	4.225E-05	1	1	1	P35749	P35749 [1597-16	1	2427.2647
[K].DTLTSRI	1xTMTpro [N	1.147E-06	4.225E-05	1	1	2	P02751	P02751 [1888-19	1	2798.5444
[K].DTLDYS	1xTMTpro [K	1.148E-05	4.225E-05	1	1	1	P49589	P49589 [440-457]	0	2703.3315
[K].QLHEQA	1xOxidation	1.032E-05	4.225E-05	1	1	1	Q13561	Q13561 [338-365	0	3849.0283
[K].QLHEQA	1xTMTpro [K	1.008E-06	4.225E-05	1	1	2	Q13561	Q13561 [338-365	0	3833.0334
[R].QLGVPL	1xTMTpro [N	1.327E-05	4.225E-05	1	1	1	O94952	O94952 [314-331	0	2367.3508
[R].QIGVEH	1xTMTpro [K	2.113E-05	4.225E-05	1	1	3	P49411	P49411 [173-185]	0	2092.241
[R].KTDFFIG	2xTMTpro [K	1.728E-05	4.225E-05	1	1	1	Q9Y266	Q9Y266 [39-53]	1	2571.3945
[K].QIGNVA	1xTMTpro [N	2.625E-05	4.225E-05	1	1	2	Q9Y3I0	Q9Y3I0 [68-81]	0	1749.0455
[K].QLGISAT	1xTMTpro [K	3.495E-05	4.225E-05	1	1	1	P46063	P46063 [153-167]	0	2116.1928
[R].QLGLLLI	1xTMTpro [N	1.211E-05	4.225E-05	1	1	1	P46939	P46939 [2990-30	0	1891.1448
[K].DTIQHQ	1xTMTpro [K	5.627E-05	4.225E-05	1	1	1	Q9UDY2	Q9UDY2 [873-88	0	2520.3338
[K].QLGGSV	1xTMTpro [K	1.173E-05	4.225E-05	1	1	2	Q96KP4	Q96KP4 [54-66]	0	1923.1407
[R].QLHEYE	1xTMTpro [N	2.735E-05	4.225E-05	1	1	4	P35749	P35749 [1597-16	0	1994.9626

[R].QIFDEYE	1xCarbamido	2.132E-05	4.225E-05	1	1	1	Q9Y3A3	Q9Y3A3 [176-190	0	2305.0879
[K].DSYIEVL	1xTMTpro [N	5.081E-05	4.225E-05	1	1	2	Q9Y305	Q9Y305 [85-101]	0	2234.2239
[R].QLIYNYF	1xTMTpro [N	2.147E-07	4.225E-05	1	1	1	P78371	P78371 [294-322]	0	3513.7796
[R].DSNPDE	1xTMTpro [K	2.549E-06	4.225E-05	1	1	2	Q15059	Q15059 [608-627	0	2941.565
[K].QITSYGE	1xCarbamido	3.352E-05	4.225E-05	1	1	1	P50990	P50990 [422-439]	0	2666.3991
[R].QITQVY(	1xCarbamido	3.664E-05	4.225E-05	3	3	2	P62714; P67	P62714 [122-135]	0	2096.0442
[R].GALDQL	2xCarbamido	1.76E-05	4.225E-05	1	1	2	O15230	O15230 [638-651	0	1751.8852
[R].KSELPQ	2xTMTpro [K	2.329E-05	4.225E-05	1	1	2	Q14738	Q14738 [572-583	1	2333.3897
[K].QLTLSCI	3xCarbamido	4.729E-05	4.225E-05	1	1	1	P22830	P22830 [398-412]	0	2121.0938
[K].KSEIQTL	3xTMTpro [K	1.186E-05	4.225E-05	1	1	2	Q14258	Q14258 [285-301	2	3220.9145
[R].QLTLLG(	1xTMTpro [N	1.518E-06	4.225E-05	1	1	5	P12111	P12111 [1097-11	0	2472.4145
[R].DSPIQCI	1xCarbamido	2.049E-05	4.225E-05	1	1	2	P02788	P02788 [59-72]	0	1918.9976
[K].QITIIDSF	1xTMTpro [N	1.566E-06	4.225E-05	1	1	2	Q9BVP2	Q9BVP2 [300-32	0	2833.5994
[R].KSEYLLF	3xTMTpro [K	3.393E-05	4.225E-05	1	1	1	Q15746	Q15746 [608-633	1	4029.4145
[K].DSPVLID	1xTMTpro [N	1.401E-05	4.225E-05	1	1	4	P04196	P04196 [140-153]	0	1986.998
[R].KSEYTQ	1xCarbamido	1.105E-06	4.225E-05	1	1	2	Q86XP3	Q86XP3 [270-291	1	3024.6432
[K].QLSSSV	1xCarbamido	1.885E-06	4.225E-05	1	1	1	P46940	P46940 [478-496]	0	2469.2211
[K].QLSSSG	1xTMTpro [K	1.414E-06	4.225E-05	1	1	1	Q9Y6N5	Q9Y6N5 [95-115]	0	2807.5911
[R].KSGSFP	2xTMTpro [K	4.964E-05	4.225E-05	1	1	1	P56199	P56199 [991-100	1	2133.2558
[R].KSGYQD	1xTMTpro [K	4.697E-05	4.225E-05	1	1	1	Q15436	Q15436 [669-682	1	2372.1837
[R].KSLPEEI	3xTMTpro [K	6.633E-06	4.225E-05	1	1	1	O60832	O60832 [20-43]	1	3983.2482
[K].DSQEEE	2xTMTpro [K	1.067E-05	4.225E-05	1	1	4	P06396	P06396 [714-728]	1	2578.4028
[K].QISQAYE	1xTMTpro [K	3.265E-05	4.225E-05	1	1	3	P31689	P31689 [47-59]	0	2060.1519
[K].QLSPTE/	1xTMTpro [N	3.258E-07	4.225E-05	1	1	2	O96008	O96008 [140-173	0	3865.0237
[K].DSNNLC	1xCarbamido	5.022E-05	4.225E-05	1	1	5	P09382	P09382 [38-49]	0	1790.8927
[R].GALEALI	2xCarbamido	5.504E-06	4.225E-05	1	1	3	Q8WU39	Q8WU39 [164-18	0	2426.2663
[R].KSEIEYY	2xTMTpro [K	1.892E-05	4.225E-05	1	1	2	P62888	P62888 [57-68]	1	2358.3559
[R].QLTVQM	1xTMTpro [N	7.83E-06	4.225E-05	1	1	1	P55209	P55209 [37-55]	0	2516.3648
[K].KSEDEE	1xCarbamido	1.648E-05	4.225E-05	1	1	1	O00442	O00442 [340-366	2	3953.9929
[R].QLYEEE	1xOxidation	5.829E-06	4.225E-05	1	1	3	P05787	P05787 [226-252]	1	3489.7338
[R].QLYEEE	1xTMTpro [N	7.918E-08	4.225E-05	1	1	4	P05787	P05787 [226-252]	1	3473.7389
[K].QLYAWN	1xTMTpro [K	4.705E-05	4.225E-05	1	1	1	Q6UWY5	Q6UWY5 [379-39	0	2348.2894
[K].DSITTWI	1xTMTpro [K	5.664E-05	4.225E-05	1	1	1	P01024	P01024 [797-812]	0	2404.2925

[K].QIWTLE(	1xCarbamido	3.31E-06	4.225E-05	1	1	3	Q16658	Q16658 [44-63]	0	2545.304
[K].DSITTWI	2xTMTpro [K	7.464E-06	4.225E-05	1	1	1	P01024	P01024 [797-813]	1	2836.5946
[K].QLWGLL	1xTMTpro [K	3.789E-06	4.225E-05	1	1	1	Q01082	Q01082 [1572-15	1	2223.2993
[R].DSIVAEL	1xTMTpro [N	3.747E-05	4.225E-05	1	1	1	O14579	O14579 [98-110]	1	1824.9445
[K].KSEEVF(	2xTMTpro [K	2.255E-05	4.225E-05	1	1	2	Q14789	Q14789 [1335-13	1	2632.5126
[R].DSQLYA	1xTMTpro [N	1.139E-06	4.225E-05	1	1	1	P82930	P82930 [30-48]	0	2522.2846
[K].QIVLTGI	1xCarbamido	1.295E-05	4.225E-05	1	1	2	Q96QK1	Q96QK1 [240-25	0	2046.1701
[R].QLVHELI	1xTMTpro [N	4.541E-05	4.225E-05	1	1	4	Q06323	Q06323 [199-210	0	1805.9353
[R].GAIETY(	1xTMTpro [K	6.633E-06	4.225E-05	1	1	4	Q12797	Q12797 [400-420	0	2837.5792
[K].QIVEILT/	1xTMTpro [N	5.158E-05	4.225E-05	1	1	2	Q16891	Q16891 [736-758	0	2692.4728
[R].QLVDTT	1xTMTpro [K	3.352E-05	4.225E-05	1	1	2	O75533	O75533 [832-843	0	1939.1356
[K].QLVDEF(	1xTMTpro [N	5.673E-05	4.225E-05	1	1	1	P43155	P43155 [68-82]	0	1895.9782
[R].DSLYVD	1xCarbamido	1.115E-05	4.225E-05	1	1	2	P35080	P35080 [76-89]	0	1963.906
[K].QLVARP	1xTMTpro [K	5.006E-05	4.225E-05	1	1	1	Q13435	Q13435 [467-486	0	2856.5533
[K].QLVALLI	1xTMTpro [K	5.86E-05	4.225E-05	1	1	1	O95208	O95208 [129-139	1	1922.1566
[K].DSMIWD	2xCarbamido	2.042E-05	4.225E-05	1	1	2	P02751	P02751 [117-130]	0	1945.889
[R].GALEMV	1xTMTpro [K	4.768E-05	4.225E-05	1	1	1	O43617	O43617 [137-149	0	1985.1055
[K].QLVKPE(	2xTMTpro [K	1.374E-05	4.225E-05	1	1	1	Q6UXV4	Q6UXV4 [34-52]	0	3062.8434
[K].QISNLQ(	1xTMTpro [K	7.787E-06	4.225E-05	1	1	2	P04264	P04264 [418-438]	1	2937.5885
[K].QISNLQ(	1xTMTpro [N	8.445E-06	4.225E-05	1	1	3	P04264	P04264 [418-432]	0	2021.0583
[K].QLSLPE	1xTMTpro [K	4.427E-05	4.225E-05	1	1	1	P14780	P14780 [77-92]	0	2310.3048
[R].DSTVAV	1xTMTpro [K	3.845E-06	4.225E-05	1	1	1	P20340	P20340 [85-106]	0	3037.6338
[K].DSTYSL	1xTMTpro [K	2.479E-06	4.225E-05	2	2	56	P01834; P0D	P01834 [63-76]; F	0	2111.1727
[R].QLPFRG	1xTMTpro [N	2.824E-05	4.225E-05	1	1	1	Q9UMY4	Q9UMY4 [101-11	1	2602.3109
[K].QLPDCI	1xCarbamido	1.815E-05	4.225E-05	1	1	1	Q9NXE4	Q9NXE4 [694-71	0	2370.3022
[K].QIPAITC	1xCarbamido	5.278E-05	4.225E-05	1	1	2	P46940	P46940 [775-787]	0	1905.0336
[K].QLPAHD	1xTMTpro [K	2.556E-05	4.225E-05	1	1	1	Q9UGI8	Q9UGI8 [153-163	0	1844.0158
[R].GAHGIIV	1xTMTpro [K	4.625E-07	4.225E-05	1	1	2	P62820	P62820 [83-103]	0	2913.5602
[R].QIPAAQI	1xTMTpro [K	4.972E-05	4.225E-05	1	1	1	P17301	P17301 [805-821]	0	2520.4066
[R].QLNYTI(	1xTMTpro [N	1.204E-05	4.225E-05	1	1	2	O60762	O60762 [219-234	0	2155.1718
[R].DSVPKP	1xTMTpro [K	1.096E-05	4.225E-05	1	1	1	Q9H553	Q9H553 [10-31]	0	2898.6333
[K].QLPTAAI	1xTMTpro [K	4.308E-05	4.225E-05	1	1	2	P56211	P56211 [76-101]	1	3433.8571
[R].QINVGN/	1xTMTpro [N	3.311E-05	4.225E-05	1	1	4	P12111	P12111 [1309-13	0	1766.972

[K].QLNNLAI	1xCarbamido	5.218E-05	4.225E-05	2	3	1	Q9NSK0; Q0	Q9NSK0 [339-352	0	2222.2571
[R].DSVSAA	1xTMTpro [N	1.896E-05	4.225E-05	1	1	2	Q96D15	Q96D15 [116-129	0	1905.8786
[R].DSVSAA	1xTMTpro [N	6.381E-06	4.225E-05	1	1	1	Q96D15	Q96D15 [116-132	1	2234.0281
[K].QLNIIQN	1xCarbamido	1.697E-07	4.225E-05	1	1	1	P80404	P80404 [61-81]	0	2886.4164
[K].DSVVAG	1xTMTpro [K	3.27E-05	4.225E-05	1	1	2	P13639	P13639 [677-688]	0	1917.0726
[K].QLNETG	2xTMTpro [K	5.475E-07	4.225E-05	1	1	1	P11532	P11532 [2269-229	1	3705.0527
[R].DSWLDN	1xTMTpro [K	5.022E-05	4.225E-05	1	1	1	O95400	O95400 [117-127	0	1999.0781
[R].DSWNA(	1xTMTpro [K	2.973E-06	4.225E-05	1	1	2	Q5XKP0	Q5XKP0 [82-101]	0	2673.4236
[R].DSWNA(	1xOxidation	8.337E-06	4.225E-05	1	1	3	Q5XKP0	Q5XKP0 [82-101]	0	2689.4185
[R].QLMETP	1xTMTpro [N	5.141E-05	4.225E-05	1	1	2	P28838	P28838 [201-214]	0	1922.9635
[R].DSVPIQ(	1xTMTpro [N	2.926E-05	4.225E-05	1	1	1	Q14934	Q14934 [869-887	0	2359.304
[K].QILVGDI	1xTMTpro [K	4.609E-06	4.225E-05	1	1	2	Q9Y281	Q9Y281 [54-73]	0	2804.5214
[K].QLPTSE/	1xTMTpro [N	4.319E-07	4.225E-05	1	1	2	O94901	O94901 [571-596	0	2833.4862
[R].QIQEEIT	1xTMTpro [N	3.08E-05	4.225E-05	1	1	1	Q9NVD7	Q9NVD7 [229-24	0	2050.0736
[R].DSQVPG	5xCarbamido	1.549E-05	4.225E-05	1	1	1	P98160	P98160 [1266-129	0	3975.7644
[R].QLSILVH	1xTMTpro [K	1.361E-05	4.225E-05	1	1	1	O75937	O75937 [79-95]	1	2572.3975
[R].QISEGVI	1xTMTpro [K	4.228E-05	4.225E-05	1	1	1	Q15746	Q15746 [1568-15	0	1911.0831
[R].DSSCGT	2xCarbamido	6.032E-05	4.225E-05	1	1	2	P23142	P23142 [245-261]	0	2531.1522
[R].QISDGEI	1xTMTpro [N	3.683E-05	4.225E-05	1	1	2	O95816	O95816 [71-86]	1	2149.1168
[K].KSPCPS	3xCarbamido	2.38E-06	4.225E-05	1	1	1	O00468	O00468 [199-225	1	3606.7482
[R].QLSAQG	1xCarbamido	4.171E-06	4.225E-05	1	1	1	Q9UNS2	Q9UNS2 [14-30]	0	2570.3926
[K].QLSAFG	1xTMTpro [K	2.921E-05	4.225E-05	1	1	1	O75489	O75489 [57-71]	0	2273.3037
[R].KSPQEL	1xCarbamido	1.171E-05	4.225E-05	1	1	3	P00734	P00734 [384-399]	1	2382.3307
[K].KSPSDV	2xTMTpro [K	6.032E-05	4.225E-05	1	1	2	P02724	P02724 [120-150]	1	4220.2278
[K].QLQALS	1xTMTpro [N	4.249E-05	4.225E-05	1	1	2	P35241	P35241 [527-539]	0	1718.972
[K].QIQSLCI	1xCarbamido	3.651E-05	4.225E-05	1	1	1	Q9BT78	Q9BT78 [373-387	0	2442.367
[R].DSSTCP	1xCarbamido	1.107E-05	4.225E-05	1	1	2	P46109	P46109 [40-57]	0	2277.0624
[K].GAHPSG	1xTMTpro [K	2.438E-06	4.225E-05	1	1	1	Q07065	Q07065 [22-34]	0	1789.9688
[K].QLQNISI	1xTMTpro [K	1.595E-05	4.225E-05	1	1	1	Q14204	Q14204 [4488-45	0	2037.1948
[K].QLQGPE	2xTMTpro [K	1.786E-05	4.225E-05	1	1	2	O60664	O60664 [308-325	1	2988.6934
[R].DSSTWL	1xTMTpro [K	2.984E-05	4.225E-05	1	1	2	P0C0L4	P0C0L4 [1073-10	0	1976.1348
[R].QIQESV	1xTMTpro [N	4.572E-05	4.225E-05	1	1	1	Q01484	Q01484 [1306-13	0	1960.0459
[R].KSQGPL	2xTMTpro [K	5.735E-08	4.225E-05	1	1	2	Q9P0M6	Q9P0M6 [250-271	1	3040.7459

[R].GAHGIIV	1xTMTpro [K	2.096E-07	4.225E-05	1	1	4	Q9H0U4	Q9H0U4 [80-100]		0	2886.5493
[K].QIQELVE	1xTMTpro [K	2.71E-06	4.225E-05	1	1	2	P17980	P17980 [194-209]		0	2470.4347
[K].QLQEET	1xTMTpro [N	6.31E-06	4.225E-05	1	1	1	Q9Y6M9	Q9Y6M9 [139-158		0	2405.2996
[K].DSSSTD	1xTMTpro [K	1.322E-05	4.225E-05	1	1	1	Q9NUQ6	Q9NUQ6 [114-13(		0	2396.2913
[K].QLYPAS/	1xTMTpro [K	2.155E-05	4.225E-05	1	1	2	P20908	P20908 [89-107]		0	2735.5151
[R].QLFALS(	1xCarbamido	1.006E-06	4.225E-05	1	1	2	P04899	P04899 [106-129]		0	2922.5202
[K].GAGPGC	1xCarbamido	4.17E-05	4.225E-05	1	1	1	P29144	P29144 [962-977]		0	2160.1978
[K].GAEADQ	1xTMTpro [K	3.069E-05	4.225E-05	1	1	4	Q12904	Q12904 [15-26]		0	1958.109
[R].QGPVNL	1xTMTpro [N	2.935E-06	4.225E-05	1	1	1	Q14624	Q14624 [754-775]		0	2719.3858
[R].QGNMT/	1xTMTpro [K	1.77E-05	4.225E-05	1	1	3	O15511	O15511 [48-60]		0	1925.1134
[R].DVEETLI	1xCarbamido	4.61E-05	4.225E-05	1	1	1	Q96BW9	Q96BW9 [271-29(		0	2605.2524
[R].QGMDLS	2xTMTpro [K	3.806E-05	4.225E-05	1	1	1	Q16658	Q16658 [277-304]		2	4197.1074
[K].GAEAAN	1xTMTpro [K	1.178E-05	4.225E-05	1	1	2	P67809	P67809 [119-137]		0	2304.2803
[K].GAEAAN	1xTMTpro [N	6.852E-07	4.225E-05	1	1	2	P16989	P16989 [151-169]		0	2087.0688
[R].DVEHYV	1xTMTpro [K	3.538E-05	4.225E-05	1	1	1	Q9UPP1	Q9UPP1 [182-20(		1	2797.4864
[K].QGLQSQ	1xTMTpro [N	3.502E-05	4.225E-05	1	1	1	P48681	P48681 [272-285]		0	1831.0357
[K].QGLNNA	1xTMTpro [N	6.908E-06	4.225E-05	1	1	1	Q07954	Q07954 [3054-30(		0	1899.9884
[K].DVEIPLD	2xTMTpro [K	5.937E-06	4.225E-05	1	1	2	Q16363	Q16363 [914-935]		0	3389.9177
[K].QGLLPL	1xTMTpro [K	2.23E-05	4.225E-05	1	1	3	Q99798	Q99798 [702-717]		0	2371.3153
[R].QGIFPVS	1xTMTpro [K	1.959E-05	4.225E-05	1	1	1	O94875	O94875 [986-998]		0	2073.224
[R].QGIFPIT	1xTMTpro [K	2.151E-05	4.225E-05	1	1	1	Q9BX66	Q9BX66 [915-927		0	2101.2553
[K].DVFDFIF	1xCarbamido	5.435E-09	4.225E-05	1	1	5	P12109	P12109 [758-790]		0	4123.2142
[R].KTTLQVI	1xTMTpro [K	1.642E-05	4.225E-05	1	1	2	P35580	P35580 [1761-17		1	2481.4168
[R].GADTAP	1xTMTpro [N	4.273E-06	4.225E-05	1	1	1	Q8IZL8	Q8IZL8 [1019-103		0	2413.253
[K].DVFFYQ	1xTMTpro [N	2.616E-05	4.225E-05	1	2	1	P23258	P23258 [49-63]		0	2219.0729
[K].QGGPTY	1xTMTpro [N	4.681E-05	4.225E-05	1	1	1	O96019	O96019 [63-76]		0	1872.9775
[K].QGGLGF	1xTMTpro [K	2.453E-05	4.225E-05	1	1	2	Q06830	Q06830 [94-109]		0	2231.2713
[K].QGGGG(	1xTMTpro [N	3.062E-06	4.225E-05	1	1	2	P52272	P52272 [389-403]		0	1588.8363
[R].QGQETA	2xTMTpro [K	9.566E-07	4.225E-05	1	1	2	Q15691	Q15691 [131-150]		0	2931.7448
[K].DVEDFLS	1xTMTpro [K	4.841E-05	4.225E-05	1	1	2	Q13405	Q13405 [123-134]		0	1941.1189
[R].GAEDDL	1xTMTpro [K	4.312E-06	4.225E-05	1	2	1	O14925	O14925 [150-169]		0	2666.3661
[R].QGQIIYN	1xTMTpro [N	2.291E-07	4.225E-05	1	1	3	P06396	P06396 [486-525]		0	4577.3079
[R].QHIVNDI	1xTMTpro [N	7.062E-06	4.225E-05	1	1	1	Q92597	Q92597 [213-234]		0	2871.4643

[R].QHGPN\	1xCarbamido	2.809E-05	4.225E-05	1	1	4	Q15582	Q15582 [43-53]	0	1846.0249
[R].QHFPAT	1xTMTpro [K	9.766E-06	4.225E-05	1	1	1	P53396	P53396 [996-1012	0	2579.4365
[R].QHFIQEI	1xCarbamido	8.543E-07	4.225E-05	1	1	2	P41250	P41250 [167-190]	0	3519.8723
[R].QHEADA	1xTMTpro [K	2.968E-05	4.225E-05	1	1	1	P55809	P55809 [356-368]	0	1990.0849
[K].KTSFDQ	1xTMTpro [K	3.372E-06	4.225E-05	1	1	1	Q02880	Q02880 [1574-15	1	3545.7932
[K].GAEILEV	1xTMTpro [N	1.256E-05	4.225E-05	1	1	1	Q15008	Q15008 [243-257	0	1908.1238
[K].QHAAVL	1xTMTpro [K	2.538E-05	4.225E-05	1	1	2	O95340	O95340 [461-474	0	2114.2101
[R].DVDIIDH	1xTMTpro [K	1.832E-05	4.225E-05	1	1	8	P21333	P21333 [922-936]	0	2393.2593
[R].QGYQLA	3xCarbamido	3.014E-08	4.225E-05	1	1	2	P98095	P98095 [969-997]	0	3590.6269
[K].QGGFLG	1xTMTpro [K	5.673E-05	4.225E-05	1	1	2	P15941	P15941 [1083-10	0	1742.0456
[K].KTSFVNI	1xCarbamido	5.831E-05	4.225E-05	1	1	2	P20042	P20042 [216-227]	1	2372.3464
[R].KTSLEDI	1xTMTpro [K	2.906E-05	4.225E-05	1	1	1	P36955	P36955 [225-237]	1	2253.1895
[K].DVDPDV	1xTMTpro [K	2.242E-05	4.225E-05	1	1	1	P46939	P46939 [1465-14	0	2103.1578
[K].KTTDTA	2xTMTpro [K	1.753E-06	4.225E-05	1	1	1	Q9ULV4	Q9ULV4 [428-440	1	2305.318
[K].QGTAVE	2xTMTpro [K	6.206E-06	4.225E-05	1	1	1	P49321	P49321 [289-317]	0	3937.0746
[K].GAEEME	1xOxidation	4.434E-05	4.225E-05	1	1	4	Q99497	Q99497 [13-27]	0	1996.005
[K].GAEEME	1xTMTpro [N	1E-05	4.225E-05	1	1	4	Q99497	Q99497 [13-27]	0	1980.0101
[R].GAEEEP	1xTMTpro [N	6.229E-06	4.225E-05	1	1	1	Q13144	Q13144 [34-54]	0	2541.3156
[K].QGQYSF	1xOxidation	2.68E-06	4.225E-05	1	1	1	P25705	P25705 [473-493]	0	2629.3615
[K].DVDYVC	1xCarbamido	3.859E-05	4.225E-05	1	1	4	Q96KP4	Q96KP4 [187-201	0	2455.2459
[K].QGQYSF	1xTMTpro [N	5.355E-07	4.225E-05	1	1	2	P25705	P25705 [473-493]	0	2613.3666
[R].GAEIEY	1xTMTpro [K	4.793E-05	4.225E-05	1	1	2	Q969H8	Q969H8 [114-125	0	1941.0283
[R].QGGDVL	1xTMTpro [N	1.038E-07	4.225E-05	1	1	1	P39748	P39748 [48-70]	0	2903.3623
[K].QGFWEE	1xCarbamido	2.144E-05	4.225E-05	1	1	2	Q06124	Q06124 [245-260	0	2695.3318
[R].KTVLGTI	1xTMTpro [K	9.032E-08	4.225E-05	1	1	2	P40939	P40939 [166-187]	1	2743.6326
[R].QFVPIK\	1xTMTpro [K	7.673E-06	4.225E-05	1	1	1	Q16881	Q16881 [400-416	1	2477.4372
[R].DVKPSN	2xTMTpro [K	4.963E-06	4.225E-05	1	1	2	P52564	P52564 [179-194]	0	2607.6014
[K].QFTYYPI	1xTMTpro [K	5.446E-05	4.225E-05	1	1	1	Q00653	Q00653 [322-337	1	2652.4165
[K].QFSSAD	2xTMTpro [K	7.888E-07	4.225E-05	1	1	2	Q9HCC0	Q9HCC0 [496-51	1	2659.5487
[R].DVIDEPI	1xTMTpro [N	3.091E-05	4.225E-05	1	1	4	O60216	O60216 [438-450	0	1815.9659
[K].QFSFPL	1xTMTpro [K	1.731E-05	4.225E-05	1	1	2	P01023	P01023 [189-204]	0	2457.2946
[K].QFRPPM	1xCarbamido	1.042E-06	4.225E-05	1	1	1	Q9UKK9	Q9UKK9 [82-111]	0	3582.768
[R].QFQSQL	1xTMTpro [K	2.994E-05	4.225E-05	1	1	1	Q5BJF6	Q5BJF6 [588-604	0	2593.4845

[R].QFQDAC	2xTMTpro [K	5.093E-06	4.225E-05	1	1	2	Q13813	Q13813 [743-757]	1	2660.4613
[R].QFQDAC	1xTMTpro [K	5.618E-05	4.225E-05	1	1	1	Q13813	Q13813 [743-756]	0	2228.1592
[KR].QFVTP.	1xTMTpro [K	6.572E-06	4.225E-05	2	2	3	Q14651; P13	Q14651 [349-362]	0	2067.173
[R].KVAETA	2xTMTpro [K	9.553E-06	4.225E-05	1	1	1	P62495	P62495 [204-218]	1	2518.5061
[R].QFNLTPI	1xCarbamido	7.561E-06	4.225E-05	1	1	1	Q13303	Q13303 [204-222]	0	2713.3186
[K].QFLQAA	1xTMTpro [K	5.285E-06	4.225E-05	1	1	3	P07237	P07237 [171-195]	0	3321.7498
[K].DVLGMA	1xTMTpro [K	9.023E-06	4.225E-05	1	1	1	P52209	P52209 [207-225]	0	2806.3672
[R].GADKVN	2xTMTpro [K	4.76E-05	4.225E-05	1	1	1	O43681	O43681 [327-348]	1	3258.8554
[K].QFIDSNF	1xTMTpro [K	5.484E-06	4.225E-05	1	1	2	P49588	P49588 [847-869]	0	3096.6531
[R].QFLDCS	2xCarbamido	2.631E-06	4.225E-05	1	1	1	Q8WYQ3	Q8WYQ3 [108-13	0	3244.5998
[R].QFLAPW	1xTMTpro [K	1.351E-05	4.225E-05	1	1	4	P40763	P40763 [32-49]	0	2719.4376
[R].QFGFIVI	1xTMTpro [N	5.853E-07	4.225E-05	1	1	1	P62244	P62244 [98-117]	0	2526.2982
[K].DVLLLEC	1xTMTpro [N	4.057E-05	4.225E-05	1	1	1	Q8IVL6	Q8IVL6 [445-459]	0	1963.1031
[K].QFGEPL.	1xTMTpro [N	2.823E-06	4.225E-05	1	1	1	Q9UKU7	Q9UKU7 [304-325	0	2805.4201
[K].QFNYTH	1xCarbamido	1.053E-05	4.225E-05	1	1	2	P13804	P13804 [102-117]	0	2380.2364
[R].QHPQPY	1xTMTpro [N	4.012E-06	4.225E-05	1	1	2	Q9Y6M9	Q9Y6M9 [75-93]	0	2480.2166
[K].GADIMY	1xCarbamido	2.171E-05	4.225E-05	2	2	2	P12236; P12	P12236 [246-259]	0	1948.9216
[K].QFYDQA	1xTMTpro [K	2.951E-06	4.225E-05	1	1	4	P60033	P60033 [125-144]	0	2861.4561
[K].QGFQCC	3xCarbamido	6.731E-06	4.225E-05	1	2	1	P17252	P17252 [63-76]	0	2405.2172
[K].QGFGNV	1xTMTpro [K	2.75E-05	4.225E-05	1	1	2	P02675	P02675 [301-313]	0	1917.0322
[K].KTVQGP	1xTMTpro [K	5.747E-05	4.225E-05	1	1	1	P04181	P04181 [32-46]	1	2298.2585
[R].DVFSGS	1xCarbamido	4.825E-05	4.225E-05	1	1	1	O60568	O60568 [480-495]	0	2400.1343
[R].QGFGEL	1xTMTpro [N	4.981E-06	4.225E-05	1	1	2	P27695	P27695 [238-254]	0	2152.1722
[R].KTVTAM	2xTMTpro [K	5.494E-06	4.225E-05	1	1	5	P62805	P62805 [80-92]	1	2351.4189
[K].QGFEAA	2xTMTpro [K	4.601E-06	4.225E-05	1	1	1	P35749	P35749 [43-54]	1	2249.2958
[K].QGFDFL	1xCarbamido	3.826E-05	4.225E-05	1	1	1	O14744	O14744 [36-49]	0	2055.0264
[K].DVGFGS	1xTMTpro [K	4.135E-05	4.225E-05	1	1	1	Q9UPN6	Q9UPN6 [729-762	0	3837.1042
[K].DVGHNI	1xTMTpro [N	3.619E-05	4.225E-05	1	1	1	Q14571	Q14571 [2103-21	0	2024.1361
[K].KVADAL	1xOxidation [	8.559E-07	4.225E-05	1	1	5	P69905	P69905 [62-91]	1	4053.2008
[K].QGEEED	1xTMTpro [K	2.515E-05	4.225E-05	1	1	1	Q9UHX1	Q9UHX1 [503-514	0	1968.0781
[R].KTVTAM	1xOxidation [	4.634E-05	4.225E-05	1	1	5	P62805	P62805 [80-92]	1	2367.4138
[K].GADPFK	2xTMTpro [K	2.182E-06	4.225E-05	1	1	1	Q9UBC2	Q9UBC2 [633-664	1	4371.1662
[K].KTYITDP	1xCarbamido	2.675E-06	4.225E-05	1	1	2	Q93052	Q93052 [353-371]	1	2995.7107

[K].QGAPTS	1xTMTpro [K	3.824E-06	4.225E-05	1	1	2	Q9BUJ2	Q9BUJ2 [147-165	0	2647.4699
[R].QGAPGC	1xTMTpro [N	1.882E-07	4.225E-05	1	1	2	Q9UH99	Q9UH99 [312-338	0	2880.5135
[R].QGAIVA\	1xTMTpro [K	4.687E-06	4.225E-05	1	3	2	P05023	P05023 [708-726]	0	2420.3641
[R].KTYTLTI	2xTMTpro [K	5.081E-05	4.225E-05	1	1	1	P27487	P27487 [41-50]	1	2158.294
[R].DVHNIY(	1xTMTpro [N	5.423E-06	4.225E-05	1	1	2	Q14697	Q14697 [570-588]	0	2449.2617
[R].GADINY/	1xCarbamid	1.249E-06	4.225E-05	1	1	1	O14974	O14974 [63-90]	0	3640.8231
[K].KVADAL	2xTMTpro [K	1.328E-09	4.225E-05	1	1	48	P69905	P69905 [62-91]	1	4037.2058
[R].QGDMVL	1xOxidation	3.363E-05	4.225E-05	1	1	1	Q9Y4W6	Q9Y4W6 [680-69	0	2419.2783
[R].QLEVYT	1xTMTpro [N	2.834E-05	4.225E-05	1	1	2	Q9Y262	Q9Y262 [243-262	0	2418.1744
[R].DVDEIE/	1xTMTpro [K	3.363E-05	4.225E-05	1	1	1	Q13813	Q13813 [1559-15	0	2042.0938
[R].KTQTVC	1xCarbamid	1.188E-06	4.225E-05	1	1	1	Q01469	Q01469 [82-103]	1	3474.8256
[K].KTEAPA/	2xTMTpro [K	2.72E-06	4.225E-05	1	1	3	P80723	P80723 [150-163]	1	2325.3594
[R].GAGGQ	1xTMTpro [N	5.046E-06	4.225E-05	1	1	1	Q14767	Q14767 [1390-14	0	2299.1056
[R].KTEPAT	2xTMTpro [K	2.132E-05	4.225E-05	1	1	1	Q16531	Q16531 [1081-11	1	3561.9985
[R].KTFPTVI	1xCarbamid	8.999E-08	4.225E-05	1	1	1	P05091	P05091 [52-78]	2	4180.2588
[K].KTFSYA	2xTMTpro [K	7.368E-06	4.225E-05	1	1	2	Q99832	Q99832 [218-230]	1	2446.3621
[R].DTTPLN	1xCarbamid	2.015E-06	4.225E-05	1	1	5	P12109	P12109 [737-757]	0	2805.604
[K].QLEAID(	1xTMTpro [K	6.707E-06	4.225E-05	1	1	4	O43707	O43707 [522-535]	0	2279.2891
[R].QLEAEA	1xTMTpro [N	1.538E-05	4.225E-05	1	1	1	Q7Z6Z7	Q7Z6Z7 [3767-37	0	1891.0278
[K].DTVATQ	1xTMTpro [N	5.628E-06	4.225E-05	1	1	2	O60664	O60664 [141-155]	0	1880.9885
[K].KTGQGD	2xTMTpro [K	8.309E-07	4.225E-05	1	1	1	O94874	O94874 [745-769]	2	3720.9497
[R].QLEADIL	1xTMTpro [K	2.435E-05	4.225E-05	1	1	1	Q86Y82	Q86Y82 [190-203	0	2254.2939
[R].GAGAYI	1xCarbamid	3.997E-06	4.225E-05	1	1	1	P49821	P49821 [200-219]	0	2676.4046
[R].QLDQCS	1xCarbamid	4.687E-06	4.225E-05	1	1	1	P46939	P46939 [1055-10	0	2919.5265
[K].QLDQCE	1xCarbamid	1.646E-06	4.225E-05	1	1	1	Q7Z4H3	Q7Z4H3 [141-163	0	3397.7012
[R].DTVEVA	1xTMTpro [K	6.464E-06	4.225E-05	1	1	1	Q14315	Q14315 [422-437]	1	2390.2695
[K].KTHIQDI	1xTMTpro [K	1.549E-07	4.225E-05	1	1	3	P21333	P21333 [1593-16	1	3195.6678
[K].GAGAFG	1xTMTpro [K	6.218E-06	4.225E-05	1	1	3	P04040	P04040 [78-93]	0	2321.2422
[K].DTVIKPL	2xTMTpro [K	3.8E-05	4.225E-05	1	1	4	P01023	P01023 [897-912]	0	2692.6317
[K].KTKPYIQ	3xTMTpro [K	4.618E-06	4.225E-05	1	1	1	P11021	P11021 [123-138]	1	2949.7878
[K].QLDDLK	1xTMTpro [K	3.417E-05	4.225E-05	1	1	1	P42766	P42766 [20-32]	1	2165.2785
[K].DTVQIHI	1xTMTpro [K	4.939E-05	4.225E-05	1	1	3	P02679	P02679 [167-177]	0	1835.0518
[K].QLEEDL	1xTMTpro [K	4.503E-05	4.225E-05	1	1	1	Q9HBI1	Q9HBI1 [110-123]	0	2286.2473

[K].DTSSST\	1xTMTpro [N	4.156E-05	4.225E-05	1	1	2	P31483	P31483 [90-101]	0	1571.8196
[K].QLEEEQ	1xTMTpro [K	5.572E-05	4.225E-05	1	1	3	P07951	P07951 [38-48]	0	1952.0944
[K].QLEEEQ	2xTMTpro [K	3.146E-05	4.225E-05	1	1	4	P07951	P07951 [38-49]	1	2384.3965
[K].QLEVLVS	2xCarbamido	4.642E-05	4.225E-05	1	1	1	Q9NTI5	Q9NTI5 [562-574]	0	2129.159
[K].QLETVLI	1xTMTpro [N	1.43E-05	4.225E-05	1	1	1	Q9NYL9	Q9NYL9 [31-51]	0	2630.3997
[R].QIETLQC	1xTMTpro [K	2.215E-06	4.225E-05	1	1	2	Q9UKU9	Q9UKU9 [102-12	0	2792.5901
[R].DTQSNE	1xTMTpro [K	1.315E-05	4.225E-05	1	1	1	Q07092	Q07092 [257-273	0	2510.323
[R].KTDPPS.	1xTMTpro [K	1.272E-05	4.225E-05	1	1	2	Q5SZK8	Q5SZK8 [2049-20	1	2458.3069
[R].GAGMPC	1xOxidation	4.491E-06	4.225E-05	1	1	4	P49257	P49257 [374-394]	0	2818.5013
[K].QLENVLI	1xTMTpro [N	2.74E-05	4.225E-05	1	1	1	Q9NZR1	Q9NZR1 [31-51]	0	2634.3404
[R].QLEMILN	2xTMTpro [K	2.547E-05	4.225E-05	2	3	2	Q04828; P42	Q04828 [172-183	0	2296.4243
[K].QLELQIL	1xTMTpro [K	4.184E-05	4.225E-05	1	1	1	Q15052	Q15052 [426-445	0	2947.6272
[R].DTSAST.	1xTMTpro [N	5.103E-06	4.225E-05	1	1	2	A0A0C4DH2	A0A0C4DH29 [92	0	1935.9653
[K].DTVTISC	1xTMTpro [N	4.689E-05	4.225E-05	1	1	1	P49327	P49327 [647-666]	0	2537.3571
[R].DTSCVN	1xCarbamido	8.875E-06	4.225E-05	1	1	3	P08603	P08603 [1045-10	0	2325.1828
[R].DTSFSG	1xTMTpro [K	5.166E-05	4.225E-05	1	1	1	Q9BRT2	Q9BRT2 [77-89]	0	2084.1043
[K].QIEINTIS	1xTMTpro [N	1.389E-05	4.225E-05	1	1	2	P48637	P48637 [142-158]	0	2068.1358
[R].QIEILELE	1xCarbamido	4.457E-05	4.225E-05	1	1	1	Q12769	Q12769 [1174-11	1	2796.5309
[R].DTSISTA	1xTMTpro [N	4.228E-05	4.225E-05	1	1	1	P23083	P23083 [92-104]	0	1777.8961
[R].QLELENI	1xTMTpro [N	3.689E-05	4.225E-05	1	1	2	P18031	P18031 [157-169]	0	1879.0092
[R].GAGIGG	1xTMTpro [K	3.845E-06	4.225E-05	1	1	2	O75369	O75369 [1355-13	0	2237.2633
[R].GAGLAW	2xCarbamido	2.235E-06	4.225E-05	1	1	1	Q8N2S1	Q8N2S1 [314-330	0	2220.0609
[K].QIEGHTI	1xCarbamido	5.996E-07	4.225E-05	1	1	1	P49821	P49821 [418-440]	0	2766.468
[R].QLEESV	1xTMTpro [N	3.148E-06	4.225E-05	1	1	3	P33176	P33176 [659-675]	0	2262.2148
[K].QIEELKC	2xTMTpro [K	5.719E-05	4.225E-05	1	1	1	P09936	P09936 [66-78]	1	2397.4169
[K].DTSEDIE	1xTMTpro [K	2.224E-07	4.225E-05	1	1	2	Q99460	Q99460 [916-934	0	2644.3962
[K].GAFTFQ	1xTMTpro [K	4.367E-05	4.225E-05	1	1	1	Q8N0X4	Q8N0X4 [310-325	0	2364.2951
[R].KTLLSNI	2xTMTpro [K	1.379E-05	4.225E-05	1	1	1	P10909	P10909 [68-78]	1	2158.3263
[K].QIDAASI	1xTMTpro [K	2.498E-06	4.225E-05	1	1	1	Q8NBX0	Q8NBX0 [330-35	0	3148.6083
[R].QKQEEE	2xTMTpro [K	2.608E-07	4.225E-05	1	1	3	Q05682	Q05682 [52-80]	1	4141.1353
[R].DVAWAI	1xCarbamido	6.357E-06	4.225E-05	1	1	1	P55735	P55735 [217-239]	0	2693.3524
[R].QKPSNT	2xTMTpro [K	2.295E-05	4.225E-05	1	1	3	P46063	P46063 [292-306]	0	2675.5072
[R].GAFFMD	1xTMTpro [K	1.154E-05	4.225E-05	1	1	1	P55081	P55081 [345-358]	1	2344.1775

[K].QKPDGV	1xOxidation [	3.505E-06	4.225E-05	1	1	1	P01024	P01024 [1112-113	0	3188.7018
[R].DVCISLE	1xCarbamido	4.442E-05	4.225E-05	1	1	1	O75822	O75822 [186-198]	1	2460.42
[K].QKPDGV	1xTMTpro [K	1.081E-07	4.225E-05	1	1	4	P01024	P01024 [1112-113	0	3172.7069
[R].KTPVKEI	3xTMTpro [K	3.716E-07	4.225E-05	1	1	1	P61026	P61026 [173-196]	2	3703.0807
[R].DVCTELI	1xCarbamido	5.244E-05	4.225E-05	1	1	1	P16152	P16152 [120-134]	0	2347.3663
[K].QKLPDG	1xTMTpro [K	3.108E-06	4.225E-05	1	1	1	Q96KP4	Q96KP4 [67-84]	1	2551.5467
[R].DVATSP	1xTMTpro [N	7.932E-06	4.225E-05	1	1	1	Q13153	Q13153 [216-237]	0	2601.3327
[K].GAFCDL	1xCarbamido	1.151E-06	4.225E-05	1	1	1	O00743	O00743 [189-210]	0	2840.3521
[R].QKHSQA	2xTMTpro [K	1.391E-05	4.225E-05	1	1	2	P35579	P35579 [1192-120	1	3008.6833
[K].DVDAAY	2xTMTpro [K	3.202E-05	4.225E-05	2	4	2	P04259; P02	P04259 [273-287]	1	2607.4632
[K].QKGADF	2xTMTpro [K	2.721E-05	4.225E-05	1	1	1	P14618	P14618 [187-206]	1	2948.6509
[R].QKEVNE	1xTMTpro [K	7.63E-08	4.225E-05	1	1	2	O75380	O75380 [60-83]	1	3366.8037
[K].KTQEQL	1xTMTpro [K	1.541E-05	4.225E-05	1	1	2	P26038	P26038 [412-427]	1	2440.3725
[K].GAEVAH	1xTMTpro [N	5.6E-05	4.225E-05	1	1	2	P21589	P21589 [98-109]	0	1619.8647
[R].QKDYET	2xTMTpro [K	1.622E-05	4.225E-05	1	1	3	P12814	P12814 [419-431]	1	2438.3959
[K].KTQEQL	1xOxidation [	4.657E-05	4.225E-05	1	1	3	P26038	P26038 [412-427]	1	2456.3674
[K].DVDEAY	1xTMTpro [K	1.258E-06	4.225E-05	1	1	4	P05787	P05787 [199-213]	1	2406.2466
[R].KTQPYD	1xTMTpro [K	2.745E-06	4.225E-05	1	1	1	O75306	O75306 [304-323]	1	2950.5442
[K].DVDAAY	2xTMTpro [K	3.113E-05	4.225E-05	1	2	5	P13647	P13647 [278-292]	1	2608.4473
[K].QHVVSV	1xTMTpro [K	7.802E-06	4.225E-05	1	1	3	P07996	P07996 [138-154]	0	2503.4164
[K].DVASLS	1xTMTpro [N	6.852E-08	4.225E-05	1	1	3	P35749	P35749 [1309-132	0	2694.3753
[K].QKSDAE	1xTMTpro [K	1.78E-06	4.225E-05	1	1	2	P27824	P27824 [552-570]	1	2717.2993
[K].DTYFLS	1xCarbamido	4.106E-05	4.225E-05	1	1	1	Q8TAT6	Q8TAT6 [332-350	0	2833.3846
[R].QICPYG	3xCarbamido	6.481E-07	4.225E-05	1	1	3	P35555	P35555 [2109-213	1	4283.0796
[R].QICPYG	3xCarbamido	1.067E-07	4.225E-05	1	1	4	P35555	P35555 [2109-213	1	4267.0847
[K].KTLSGLE	1xTMTpro [K	5.926E-06	4.225E-05	1	1	1	P00390	P00390 [300-316]	1	2353.3769
[K].QIATLHA	1xTMTpro [K	8.554E-06	4.225E-05	1	1	4	P35579	P35579 [1358-137	0	2034.1662
[K].QLAQIDC	1xTMTpro [N	2.968E-05	4.225E-05	1	1	1	Q9H444	Q9H444 [78-93]	0	2124.162
[K].DVAAIAC	1xTMTpro [K	1.017E-05	4.225E-05	1	1	1	P28331	P28331 [337-355]	0	2404.4307
[K].QLAQDT	1xTMTpro [N	1.059E-05	4.225E-05	1	4	1	O00470	O00470 [307-327]	0	2719.4851
[K].GAFNFD	1xTMTpro [N	8.202E-07	4.225E-05	1	1	1	Q9NY33	Q9NY33 [467-490	0	3133.5186
[K].QIANSQI	1xOxidation [	3.305E-05	4.225E-05	1	1	1	P10155	P10155 [15-32]	0	2445.1788
[K].QKQQEA	3xTMTpro [K	3.577E-06	4.225E-05	1	1	2	Q05682	Q05682 [556-570]	2	3001.8038

[R].DVACGA	1xCarbamido	5.807E-06	4.225E-05	1	1	2	Q9P258	Q9P258 [334-349	0	2336.2524
[K].DVAEEIA	1xTMTpro [N	3.924E-06	4.225E-05	1	1	1	Q01082	Q01082 [788-821]	0	4106.0386
[R].QLALETI	2xTMTpro [K	2.379E-05	4.225E-05	1	1	2	Q15428	Q15428 [32-48]	1	2951.6732
[R].KTNLSA\	2xTMTpro [K	1.783E-06	4.225E-05	1	1	1	O76094	O76094 [148-163]	1	2658.5395
[R].DVAFTV(	1xTMTpro [K	1.725E-05	4.225E-05	1	1	3	Q13451	Q13451 [185-204]	0	2735.4383
[R].QLAGED	1xTMTpro [K	3.607E-05	4.225E-05	1	1	2	P07384	P07384 [553-564]	0	1928.0654
[R].KTPESFI	2xTMTpro [K	6.781E-06	4.225E-05	1	1	1	Q9Y6I3	Q9Y6I3 [469-499]	1	4044.295
[K].QKVEGT	2xTMTpro [K	3.473E-06	4.225E-05	1	1	3	P19338	P19338 [296-318]	1	3480.9307
[K].DVAIKEF	2xTMTpro [K	2.584E-05	4.225E-05	1	1	2	Q99873	Q99873 [229-243]	1	2549.5371
[R].DVAPQA	1xTMTpro [K	9.677E-06	4.225E-05	1	1	1	Q9BX68	Q9BX68 [80-94]	0	2239.3458
[K].QKVDSL	2xTMTpro [K	3.607E-05	4.225E-05	1	4	2	P07910	P07910 [205-216]	1	2328.3955
[K].QIANSQI	1xTMTpro [N	3.657E-05	4.225E-05	1	1	1	P10155	P10155 [15-32]	0	2429.1839
[K].KSEAAVI	2xTMTpro [K	1.093E-06	4.225E-05	1	1	1	Q96A49	Q96A49 [119-148	2	4265.3234
[R].QLYRPE	1xTMTpro [N	2.621E-05	4.225E-05	1	1	1	Q96JQ0	Q96JQ0 [475-494	0	2571.4618
[R].QLYVLG	1xTMTpro [K	5.71E-05	4.225E-05	1	1	1	P22314	P22314 [58-68]	0	1897.0861
[K].QTMQVD	1xTMTpro [K	1.754E-05	4.225E-05	1	1	2	P42765	P42765 [215-234]	0	2989.6021
[R].KPVEEL	2xTMTpro [K	3.293E-05	4.225E-05	1	1	1	Q9Y2R9	Q9Y2R9 [53-63]	0	2243.2951
[R].DQDLTQ	1xTMTpro [K	2.516E-06	4.225E-05	1	1	1	Q9ULX6	Q9ULX6 [463-477	0	2412.2725
[R].DQDQLQ	1xTMTpro [K	3.508E-05	4.225E-05	1	1	1	Q16222	Q16222 [83-102]	0	2972.5245
[R].KPVEEY.	1xCarbamido	6.09E-05	4.225E-05	1	1	2	P02787	P02787 [588-600]	0	2195.1887
[K].QTLQSE	1xTMTpro [N	4.525E-05	4.225E-05	1	1	4	P35998	P35998 [85-97]	0	1802.0091
[R].GASIVEI	1xTMTpro [K	3.288E-05	4.225E-05	1	1	1	P00367	P00367 [77-90]	1	2152.2469
[R].QTLGPG	1xTMTpro [N	7.977E-07	4.225E-05	1	1	1	Q5JTZ9	Q5JTZ9 [644-663]	0	2494.2969
[K].QTIGNS(	1xCarbamido	1.552E-06	4.225E-05	1	1	2	P09936	P09936 [84-105]	0	2919.5602
[K].QTLENEI	1xTMTpro [K	5.433E-06	4.225E-05	1	1	3	P35579	P35579 [1220-123	1	2338.2858
[K].QTIDNS(	1xTMTpro [K	1.789E-05	4.225E-05	1	1	2	P27348	P27348 [140-157]	0	2623.3495
[R].KPVLGQ	1xTMTpro [K	1.592E-06	4.225E-05	1	1	1	Q9NTJ4	Q9NTJ4 [750-768	0	2432.4481
[R].DQEKPY	1xTMTpro [K	5.766E-05	4.225E-05	1	1	1	O15504	O15504 [74-91]	0	2644.3135
[R].DQELYF	1xCarbamido	3.459E-05	4.225E-05	1	1	2	P26639	P26639 [329-348]	0	3069.5676
[R].KPVPPS	1xCarbamido	2.238E-05	4.225E-05	1	1	1	Q9ULC5	Q9ULC5 [246-269	0	3441.8756
[K].KPVTDC	1xCarbamido	2.665E-06	4.225E-05	1	1	2	Q92598	Q92598 [135-153]	0	2776.4835
[K].KPVVDC	2xCarbamido	7.298E-07	4.225E-05	1	1	2	P34932	P34932 [135-153]	0	2849.4821
[K].KPVVNII	1xTMTpro [K	4.768E-05	4.225E-05	1	1	1	Q16719	Q16719 [385-399]	0	2326.3738

[K].DQFGFI	1xTMTpro [K	4.689E-05	4.225E-05	1	1	1	O75534	O75534 [683-696	0	2227.1527
[K].GASGPL	1xTMTpro [K	5.907E-05	4.225E-05	1	1	1	Q9UMS6	Q9UMS6 [210-22	0	1875.1195
[R].DQFNLI	1xTMTpro [K	6.119E-07	4.225E-05	1	1	4	Q14624	Q14624 [308-336	0	3870.0682
[R].QTNLEN	1xTMTpro [N	5.959E-06	4.225E-05	1	1	2	Q15149	Q15149 [350-365	0	2139.1001
[R].QTNPSA	1xTMTpro [N	2.053E-06	4.225E-05	1	1	2	P55072	P55072 [714-732]	0	2460.1884
[R].KPVDEY	1xCarbamid	2.602E-06	4.225E-05	1	1	7	P02787	P02787 [252-273]	1	3461.9144
[R].DQCQV	2xCarbamid	2.658E-05	4.225E-05	1	1	3	Q14508	Q14508 [95-108]	0	2289.0917
[K].DPYQEE	1xTMTpro [K	3.741E-05	4.225E-05	1	1	1	P11117	P11117 [54-70]	0	2660.3488
[K].KPTETQ	1xTMTpro [K	2.334E-07	4.225E-05	1	1	1	Q10567	Q10567 [495-522	1	3805.0223
[R].DPYTEQ	1xTMTpro [K	5.217E-06	4.225E-05	1	1	3	Q15149	Q15149 [3910-39	0	2380.2714
[KR].GASQA	1xTMTpro [N	7.903E-06	4.225E-05	1	2	2	P37802	P37802 [183-196]	0	1687.8215
[K].DPYTGQ	1xTMTpro [K	2.64E-05	4.225E-05	1	1	1	Q15149	Q15149 [2916-29	0	2463.3198
[R].DPYTGQ	1xTMTpro [K	9.157E-06	4.225E-05	1	1	2	Q15149	Q15149 [3244-32	0	2262.2626
[R].QTVSQ	1xTMTpro [N	6.941E-07	4.225E-05	1	1	2	Q9UK41	Q9UK41 [178-202	0	3085.5067
[R].QTVLTA	1xTMTpro [N	4.409E-06	4.225E-05	1	1	1	Q9Y4G6	Q9Y4G6 [638-65	0	2162.21
[K].QTTVSN	1xTMTpro [K	3.148E-06	4.225E-05	1	1	3	P31946	P31946 [141-159]	0	2767.4394
[K].GASQAG	1xTMTpro [K	3.465E-05	4.225E-05	1	1	2	P51911	P51911 [213-225]	0	1784.982
[K].QTELESI	1xTMTpro [K	5.218E-05	4.225E-05	1	1	2	Q13439	Q13439 [683-698	0	2400.3365
[R].QTSPVS	1xTMTpro [K	1.735E-05	4.225E-05	1	1	4	Q12805	Q12805 [441-453	0	1981.2011
[K].QTRPVQ	1xCarbamid	5.295E-07	4.225E-05	1	1	2	Q86VP6	Q86VP6 [404-437	0	4505.3122
[K].QTQVSV	1xTMTpro [K	1.399E-05	4.225E-05	1	1	4	P06396	P06396 [374-390]	0	2438.3786
[K].QTQTFT	1xTMTpro [N	2.178E-06	4.225E-05	1	1	2	P11142	P11142 [424-447]	0	3078.5339
[K].DQAPDE	1xTMTpro [K	4.785E-05	4.225E-05	1	1	1	Q9Y6D6	Q9Y6D6 [1085-11	0	2724.4489
[K].DQAVEN	1xTMTpro [K	1.105E-06	4.225E-05	1	1	4	P50454	P50454 [61-86]	0	3159.8484
[R].DQAVFP	1xTMTpro [N	4.05E-06	4.225E-05	1	1	1	Q14764	Q14764 [624-649	0	3116.6183
[R].DQAVMI	1xTMTpro [K	7.382E-06	4.225E-05	1	1	2	O00159	O00159 [133-146	0	1958.0509
[K].QTQIFTT	1xTMTpro [N	6.275E-06	4.225E-05	1	2	1	P0DMV9	P0DMV9 [424-44	0	3090.5703
[K].QTQAIP	1xCarbamid	5.627E-05	4.225E-05	1	1	1	Q9UKF6	Q9UKF6 [513-53	0	2991.6258
[K].QTPSGP	2xTMTpro [K	1.199E-07	4.225E-05	1	1	1	P29536	P29536 [294-324]	1	4206.2386
[R].QTSGG	1xTMTpro [N	6.381E-06	4.225E-05	1	1	1	P18859	P18859 [55-73]	0	2385.1489
[K].QTDVLQ	1xTMTpro [K	2.973E-05	4.225E-05	1	1	2	Q14204	Q14204 [1850-18	0	2396.3463
[R].KQADLE	2xTMTpro [K	5.354E-06	4.225E-05	1	1	1	P35749	P35749 [1704-17	2	3215.7939
[R].QTASVT	1xTMTpro [K	2.737E-07	4.225E-05	1	1	2	Q16891	Q16891 [230-257	0	3440.9469

[K].KQELEEI	1xCarbamido	5.85E-06	4.225E-05	1	1	4	P35579	P35579 [910-923]	1	2378.263
[K].KQELEEI	1xTMTpro [K	3.13E-05	4.225E-05	1	1	7	P35580	P35580 [917-930]	1	2347.3113
[R].DQLSVLI	1xTMTpro [N	5.295E-06	4.225E-05	1	1	1	Q92499	Q92499 [331-349]	0	2272.2468
[K].KQELEEI	1xTMTpro [K	2.794E-05	4.225E-05	1	1	5	P35749	P35749 [917-930]	1	2363.2885
[R].QSLLDM	1xTMTpro [N	3.124E-05	4.225E-05	1	1	2	O15382	O15382 [299-312]	0	1986.0074
[R].GAQGVI	1xTMTpro [N	3.833E-05	4.225E-05	1	1	2	Q9NP72	Q9NP72 [80-92]	0	1694.976
[K].QSLGELI	1xTMTpro [K	6.523E-06	4.225E-05	1	1	2	P60174	P60174 [20-33]	0	2023.2043
[R].GAQGPA	1xTMTpro [N	4.917E-08	4.225E-05	1	1	1	P12111	P12111 [2228-225	0	2445.3169
[K].QSLETIC	1xCarbamido	5.2E-05	4.225E-05	2	3	1	P62136; P62	P62136 [99-111];	0	2160.2594
[K].QSLEASI	1xTMTpro [N	2.794E-05	4.225E-05	1	1	4	P13645	P13645 [387-399]	0	1694.888
[K].QSNNEA	1xTMTpro [K	3.282E-05	4.225E-05	1	1	1	Q8WUM4	Q8WUM4 [641-65	1	2252.249
[K].QSIAIDD	2xCarbamido	1.114E-06	4.225E-05	1	1	3	Q96CW1	Q96CW1 [239-25	0	2154.0391
[R].DQMLQI	1xTMTpro [K	2.89E-05	4.225E-05	1	1	2	Q5H9R7	Q5H9R7 [224-244	0	2992.6157
[R].DQMQQ	1xTMTpro [K	8.215E-06	4.225E-05	1	1	3	P20700	P20700 [351-367]	0	2716.4108
[R].DQMQQ	1xOxidation [	2.87E-05	4.225E-05	1	1	1	P20700	P20700 [351-367]	0	2732.4057
[K].QSGVVP	1xTMTpro [K	2.911E-05	4.225E-05	1	1	1	P12111	P12111 [965-976]	0	1929.1453
[R].QSGQTD	1xTMTpro [K	2.608E-08	4.225E-05	1	1	2	P46940	P46940 [359-387]	1	3755.908
[R].QSGGTT	1xCarbamido	2.963E-05	4.225E-05	1	1	1	Q9H9J2	Q9H9J2 [260-278	0	2698.4406
[R].DQNFVIL	1xTMTpro [N	3.174E-05	4.225E-05	1	1	1	P16284	P16284 [185-200]	0	2282.1624
[R].QSGEAF	1xTMTpro [K	2.761E-06	4.225E-05	1	1	3	P52597	P52597 [53-68]	0	2318.2007
[R].KQELELY	1xTMTpro [K	2.515E-05	4.225E-05	1	1	1	Q7Z406	Q7Z406 [934-947]	1	2251.3153
[K].QSFTMV	1xTMTpro [N	2.74E-05	4.225E-05	1	1	4	Q14847	Q14847 [60-73]	0	1912.9758
[K].QSKPVT	3xTMTpro [K	3.81E-06	4.225E-05	1	1	3	P10809	P10809 [158-191]	1	4772.6497
[R].QTYFLPY	1xTMTpro [K	8.05E-06	4.225E-05	1	1	2	P17980	P17980 [130-144]	0	2301.335
[K].KQEDSV	2xTMTpro [K	1.472E-05	4.225E-05	1	1	1	Q9GZZ9	Q9GZZ9 [377-400	1	3565.8611
[K].KQEDFM	2xTMTpro [K	8.126E-07	4.225E-05	1	1	2	Q01082	Q01082 [1210-12	1	2729.3942
[R].QSVFPF	2xTMTpro [K	1.722E-05	4.225E-05	1	1	3	P17931	P17931 [187-199]	0	2410.3951
[R].GASGAG	1xTMTpro [N	3.67E-05	4.225E-05	1	1	2	Q96LD4	Q96LD4 [41-53]	0	1289.6881
[R].KQALQD	2xTMTpro [K	5.664E-05	4.225E-05	1	1	2	Q01082	Q01082 [842-853]	1	2304.4107
[R].QSVEAD	1xTMTpro [N	5.115E-05	4.225E-05	2	2	2	P13645; P13	P13645 [246-257]	1	1661.9254
[R].KQCDWI	1xCarbamido	4.412E-05	4.225E-05	1	1	2	P12111	P12111 [2834-284	1	2550.3591
[R].DQGSCC	2xCarbamido	1.389E-07	4.225E-05	1	1	5	P07858	P07858 [101-120]	0	2477.1145
[K].DQGSFT	1xTMTpro [K	1.587E-05	4.225E-05	1	1	1	P11717	P11717 [837-854]	0	2491.3358

[K].DQGTYE	1xTMTpro [N	3.961E-05	4.225E-05	1	1	2	P60660	P60660 [82-94]	0	1848.8935
[K].QSTWEK	3xTMTpro [K	1.927E-05	4.225E-05	1	1	1	O75400	O75400 [165-184]	1	3531.0211
[K].QSTSFL\	1xTMTpro [K	4.135E-05	4.225E-05	1	1	3	O00151	O00151 [212-228]	0	2588.4315
[K].DQLIYNL	2xTMTpro [K	2.952E-05	4.225E-05	1	1	3	P00338	P00338 [6-22]	1	2986.7029
[K].QSTQVM	1xTMTpro [K	1.592E-05	4.225E-05	1	1	1	Q92734	Q92734 [156-172]	0	2420.2809
[K].QSTFTLI	1xTMTpro [K	2.151E-05	4.225E-05	1	1	2	Q14767	Q14767 [455-475]	0	2852.6377
[R].QSTDLP	1xTMTpro [N	1.76E-05	4.225E-05	1	1	1	Q9HAU0	Q9HAU0 [54-72]	0	2462.1795
[K].KQDTVE	2xTMTpro [K	5.287E-05	4.225E-05	1	1	2	Q5JSL3	Q5JSL3 [747-764]	1	2940.7379
[K].QSSVAE	1xTMTpro [N	1.292E-06	4.225E-05	1	1	1	Q9UH99	Q9UH99 [432-450	0	2356.3155
[R].QSSGEN	1xCarbamido	4.549E-05	4.225E-05	1	1	2	P01833	P01833 [273-288]	0	2315.2157
[K].DQLLLGI	1xTMTpro [K	3.927E-05	4.225E-05	1	1	4	P55084	P55084 [349-361]	0	2025.1876
[R].QSSGAS	1xTMTpro [N	3.833E-05	4.225E-05	1	1	1	O00571	O00571 [604-617]	0	1665.7999
[R].QSSFEIF	1xTMTpro [K	4.825E-05	4.225E-05	1	1	1	P46937	P46937 [162-181]	0	2825.4675
[R].GAQVEE	1xTMTpro [K	1.197E-05	4.225E-05	1	1	1	Q9Y5K5	Q9Y5K5 [29-45]	0	2613.3692
[R].QSSATS	1xTMTpro [N	2.484E-06	4.225E-05	1	1	2	P08727	P08727 [8-24]	0	1858.9578
[K].DQLDET	1xTMTpro [K	1.93E-05	4.225E-05	1	1	1	Q14203	Q14203 [709-722]	0	2209.2208
[K].QSEPLEI	1xTMTpro [N	2.042E-05	4.225E-05	1	1	2	P24821	P24821 [1660-167	0	1900.0711
[K].KPTETQI	1xTMTpro [K	3.581E-07	4.225E-05	1	1	2	Q10567	Q10567 [495-520]	0	3533.8943
[R].KPTDGA	1xCarbamido	4.756E-06	4.225E-05	1	1	1	P49321	P49321 [698-717]	0	2752.4544
[R].QVQSLT	1xCarbamido	4.818E-09	4.225E-05	1	1	6	P08670	P08670 [322-342]	1	2985.5807
[R].QVQSLT	1xCarbamido	1.549E-05	4.225E-05	1	1	9	P08670	P08670 [322-334]	0	2099.1662
[R].DPEEIEK	2xTMTpro [K	5.926E-05	4.225E-05	1	1	1	P08865	P08865 [206-220]	1	2628.4185
[R].QVQGSE	1xCarbamido	2.136E-05	4.225E-05	1	1	2	Q9UK41	Q9UK41 [83-97]	0	2059.0085
[K].DPEGFV	1xTMTpro [K	3.954E-05	4.225E-05	1	1	1	P13674	P13674 [73-86]	0	2122.1577
[R].QVQFHQ	2xTMTpro [K	3.826E-05	4.225E-05	1	1	1	Q12965	Q12965 [896-914]	0	3025.7403
[K].QVQEFL	2xTMTpro [K	2.409E-05	4.225E-05	1	1	1	P13667	P13667 [295-313]	1	3075.791
[R].DPEIYTD	1xTMTpro [K	4.465E-05	4.225E-05	1	1	2	Q16647	Q16647 [394-405]	0	2061.1036
[R].DPENFP	1xTMTpro [K	4.249E-05	4.225E-05	1	1	4	P51149	P51149 [114-126]	0	2084.1672
[K].QVQEAI/	1xTMTpro [K	6.627E-07	4.225E-05	1	1	1	P26232	P26232 [246-268]	0	2908.5508
[R].KPLLES(	2xTMTpro [K	3.728E-05	4.225E-05	1	1	4	P22314	P22314 [593-604]	0	2156.3471
[K].QVPNES	1xTMTpro [K	5.27E-05	4.225E-05	1	1	2	Q99733	Q99733 [283-297]	0	2436.3207
[R].GAVGAL	1xTMTpro [K	1.184E-05	4.225E-05	1	2	5	Q15907	Q15907 [83-95]	0	1898.1607
[R].DPGGIT/	1xTMTpro [K	3.538E-05	4.225E-05	1	1	1	Q8WUZ0	Q8WUZ0 [148-16	0	2395.2671

[K].QVPGG(	1xTMTpro [N	7.111E-08	4.225E-05	1	1	2	Q9UHB9	Q9UHB9 [6-31]	0	2148.0098
[R].QVMVVF	1xCarbamid(	1.396E-05	4.225E-05	1	1	1	P26639	P26639 [620-636]	0	2545.3286
[R].KPLLPYT	2xTMTpro [K	1.059E-06	4.225E-05	1	1	3	Q08257	Q08257 [62-88]	0	3629.0407
[R].KPLVLC(	1xCarbamid(	4.2E-07	4.225E-05	1	1	1	P27695	P27695 [203-221]	0	2799.5683
[K].QVMADS	1xTMTpro [N	3.782E-06	4.225E-05	1	1	4	P35442	P35442 [1132-114	0	2233.0879
[R].KPLVIIAE	1xTMTpro [K	1.777E-07	4.225E-05	1	1	11	P10809	P10809 [269-290]	0	2973.748
[R].GAVEAA	1xTMTpro [N	3.108E-06	4.225E-05	1	1	1	Q8IZ83	Q8IZ83 [568-588]	0	2369.207
[R].DPDQTD	1xTMTpro [N	1.021E-06	4.225E-05	1	1	1	P06396	P06396 [398-419]	0	2691.3545
[R].KPIDYTII	2xTMTpro [K	1.148E-05	4.225E-05	1	1	1	Q9NYB9	Q9NYB9 [139-154	0	2696.5803
[R].DPDNEL	1xTMTpro [K	4.939E-05	4.225E-05	1	1	1	Q63HM9	Q63HM9 [105-120	0	2432.2742
[K].GAVLFG	1xTMTpro [K	2.109E-05	4.225E-05	1	1	2	O43301	O43301 [517-529]	0	1908.1814
[R].DNYVPE	2xTMTpro [K	4.963E-06	4.225E-05	1	1	1	P08708	P08708 [82-107]	1	3903.0765
[K].QVVSVV	1xTMTpro [N	7.976E-06	4.225E-05	1	1	1	Q96TA1	Q96TA1 [621-654	0	3865.9667
[K].QVVNQV	1xTMTpro [K	8.491E-06	4.225E-05	1	1	1	Q7Z6Z7	Q7Z6Z7 [1483-14	0	2320.352
[R].GAVNMF	1xTMTpro [K	4.247E-09	4.225E-05	1	1	2	Q16762	Q16762 [207-230]	1	3337.6729
[R].DPAEAL(	1xTMTpro [N	3.346E-05	4.225E-05	1	1	4	Q9Y285	Q9Y285 [281-295	0	2050.0598
[R].DPAFTYI	2xTMTpro [K	4.603E-05	4.225E-05	1	1	1	Q9Y673	Q9Y673 [98-118]	1	3226.73
[R].KPGSVV	2xTMTpro [K	1.253E-05	4.225E-05	1	1	2	P51608	P51608 [271-284]	0	2182.3376
[R].DPAGMF	2xTMTpro [K	6.032E-05	4.225E-05	1	1	1	O43252	O43252 [523-544]	1	3246.7034
[K].QVVHLN	1xCarbamid(	1.872E-05	4.225E-05	1	1	1	O00186	O00186 [344-357]	0	2279.2132
[K].QVVESA	1xTMTpro [K	3.638E-05	4.225E-05	1	1	6	P00338	P00338 [233-243]	0	1873.0926
[K].QVLNQD	1xTMTpro [K	4.81E-06	4.225E-05	1	1	1	Q5VWZ2	Q5VWZ2 [43-55]	0	2192.2683
[R].DPALCC	3xCarbamid(	7.83E-06	4.225E-05	1	1	2	Q16610	Q16610 [482-505]	0	3222.4978
[R].KPKPNP	2xTMTpro [K	1.978E-06	4.225E-05	1	1	1	P12694	P12694 [400-421]	0	3497.9759
[K].QVTQTY	1xTMTpro [N	3.514E-05	4.225E-05	1	1	2	P35442	P35442 [1044-105	0	1955.9782
[K].QVTQSY	1xTMTpro [N	3.405E-05	4.225E-05	1	1	2	P07996	P07996 [1042-105	0	1899.952
[K].KPIAGQV	2xTMTpro [K	2.775E-05	4.225E-05	1	1	3	P49023	P49023 [363-375]	0	2212.3668
[R].GAVLPH	1xTMTpro [K	1.412E-05	4.225E-05	1	1	1	Q08AM6	Q08AM6 [126-141	0	2322.3466
[R].DPDAGII	1xTMTpro [K	3.689E-07	4.225E-05	1	1	4	P08758	P08758 [162-186]	0	3266.6672
[R].DPDAQF	1xTMTpro [K	1.546E-06	4.225E-05	1	1	5	P07339	P07339 [236-253]	0	2396.2259
[R].DPDAQF	1xOxidation [	2.35E-05	4.225E-05	1	1	2	P07339	P07339 [236-253]	0	2412.2208
[R].QVSSEC	1xCarbamid(	8.429E-06	4.225E-05	1	1	3	Q92896	Q92896 [414-427]	0	2005.9278
[R].DPDEPV	1xTMTpro [K	2.038E-05	4.225E-05	1	1	1	P14550	P14550 [222-240]	0	2684.5254

[R].QVVDCQ	1xCarbamido	2.375E-05	4.225E-05	1	1	1	P28838	P28838 [441-455]	0	2281.2466
[K].QVLMGF	1xCarbamido	2.023E-05	4.225E-05	1	1	2	Q9H3P7	Q9H3P7 [118-142	0	3144.509
[R].QVLLSEI	1xTMTpro [N	3.124E-05	4.225E-05	1	1	3	P13798	P13798 [4-18]	0	1993.0925
[R].QVLLSA	1xTMTpro [N	9.023E-06	4.225E-05	1	1	2	P78371	P78371 [502-516]	0	1887.1234
[R].GATLAL	1xTMTpro [N	1.184E-05	4.225E-05	1	1	3	P43121	P43121 [98-112]	0	1904.0408
[K].DPSEEA	1xTMTpro [K	1.013E-05	4.225E-05	1	1	2	O95260	O95260 [491-508	0	2513.3743
[K].QVELALV	1xTMTpro [N	3.761E-06	4.225E-05	3	3	3	P08134; P62	P08134 [52-68]; F	0	2313.1318
[K].GATLAFI	1xTMTpro [K	5.198E-06	4.225E-05	1	1	2	O95825	O95825 [279-299	0	2912.5762
[K].QVEELYI	1xTMTpro [K	2.337E-05	4.225E-05	1	1	1	Q13813	Q13813 [1068-10	0	2395.3365
[K].KPSLVSI	2xTMTpro [K	2.712E-07	4.225E-05	1	1	1	Q13425	Q13425 [206-235	0	3957.0698
[R].QVDQLT	1xTMTpro [K	5.149E-05	4.225E-05	1	1	1	P08670	P08670 [160-170]	1	1896.0794
[K].DPTFDTI	1xTMTpro [K	6.499E-06	4.225E-05	1	1	2	Q9NZU5	Q9NZU5 [110-12	0	2774.4533
[K].QVDFWF	1xTMTpro [K	1.09E-05	4.225E-05	1	1	2	Q4G0J3	Q4G0J3 [41-53]	0	2185.1686
[R].QVCEQL	1xCarbamido	6.051E-05	4.225E-05	1	1	2	Q99715	Q99715 [2910-29	0	1866.9485
[R].DPSASP	1xTMTpro [N	7.154E-06	4.225E-05	1	1	4	P18206	P18206 [286-300]	0	1774.8891
[K].DPTGMD	1xTMTpro [K	1.396E-05	4.225E-05	1	1	2	Q15005	Q15005 [147-164	0	2613.3362
[R].DPTPSF	1xTMTpro [N	2.984E-05	4.225E-05	1	1	2	Q14137	Q14137 [275-295	0	2695.3323
[R].DPTVHD	1xTMTpro [N	2.865E-06	4.225E-05	1	1	1	P78527	P78527 [2073-20	0	2444.1934
[R].KPSTFNI	1xTMTpro [K	2.516E-06	4.225E-05	1	1	1	P35475	P35475 [598-619]	0	2985.5966
[K].QVAVAE	1xTMTpro [K	6.433E-07	4.225E-05	1	1	1	O76024	O76024 [194-221	0	3460.868
[K].DPVQEA	1xTMTpro [N	3.813E-05	4.225E-05	1	1	1	P14618	P14618 [476-489]	0	1946.9779
[R].DPVQLN	1xTMTpro [N	3.645E-05	4.225E-05	2	2	2	Q9Y4G6; Q9	Q9Y4G6 [210-222	0	1833.0554
[K].QVATAL	1xTMTpro [K	5.047E-05	4.225E-05	1	1	2	P18206	P18206 [465-476]	0	1923.1519
[R].DPVTIDC	2xCarbamido	5.235E-05	4.225E-05	1	1	2	Q12899	Q12899 [25-37]	0	1879.9114
[R].QVASMT	2xTMTpro [K	1.969E-05	4.225E-05	1	1	2	P05413	P05413 [32-45]	0	2459.4723
[K].DPVYTF	1xTMTpro [N	2.304E-05	4.225E-05	1	1	1	Q8TAT6	Q8TAT6 [459-476	0	2428.2468
[R].GATELTI	1xTMTpro [K	9.929E-06	4.225E-05	1	1	2	P17980	P17980 [411-430]	0	2842.4788
[K].QVAAST	1xCarbamido	6.56E-06	4.225E-05	2	2	3	Q9Y4G6; Q9	Q9Y4G6 [2431-24	0	2068.208
[K].QVEVDA	1xCarbamido	1.465E-07	4.225E-05	1	1	4	P61224	P61224 [43-68]	0	3288.587
[K].QVEVDC	2xCarbamido	2.727E-07	4.225E-05	1	1	4	P62834	P62834 [43-68]	0	3377.5805
[K].KPMVLG	2xTMTpro [K	4.947E-05	4.225E-05	1	1	1	Q00796	Q00796 [64-78]	0	2495.4472
[R].KPNVGC	1xCarbamido	2.631E-06	4.225E-05	1	1	2	A0AVT1	A0AVT1 [342-356	0	2657.4749
[K].QVLGQM	1xTMTpro [N	2.867E-07	4.225E-05	1	1	1	P28070	P28070 [110-131]	0	2747.3993

[K].KPPLLNI	2xTMTpro [K	5.813E-05	4.225E-05	1	1	1	P09874	P09874 [748-761]	0	2407.4489
[K].KPPPVP/	2xTMTpro [K	4.665E-05	4.225E-05	1	1	2	Q14258	Q14258 [392-402]	0	2043.3146
[R].QVLDLEI	1xTMTpro [K	5.875E-08	4.225E-05	1	1	1	O75643	O75643 [407-426]	0	2900.5472
[R].QVICVYT	1xCarbamido	5.031E-05	4.225E-05	1	1	1	Q9H3H3	Q9H3H3 [211-223	0	1935.9441
[K].QVLALQ	2xTMTpro [K	5.951E-07	4.225E-05	1	1	2	P35580	P35580 [1365-137	1	2455.5064
[K].QVKPYV	1xTMTpro [K	5.014E-05	4.225E-05	1	1	2	Q99832	Q99832 [107-123]	0	2627.5529
[K].QVKDNE	2xTMTpro [K	6.093E-06	4.225E-05	1	1	2	P02675	P02675 [161-178]	1	3036.6306
[K].QVEVDA	1xCarbamido	1.036E-06	4.225E-05	1	1	5	P61224	P61224 [43-68]	0	3304.5819
[K].KPSETQ	1xTMTpro [K	3.082E-07	4.225E-05	1	1	2	P63010	P63010 [495-520]	0	3519.8786
[K].QVGCFL	2xCarbamido	2.259E-05	4.225E-05	1	1	1	O14495	O14495 [129-148]	0	2857.4872
[K].KPSETQ	1xTMTpro [K	4.874E-05	4.225E-05	1	1	1	P63010	P63010 [495-522]	1	3791.0067
[R].DPPDPY	1xTMTpro [K	1.703E-05	4.225E-05	1	1	1	Q9BSJ8	Q9BSJ8 [1004-10	0	2290.319
[R].DPPHNN	1xTMTpro [K	2.529E-05	4.225E-05	1	1	2	Q9UBE0	Q9UBE0 [322-335	0	2321.1669
[K].DPPLAAY	1xTMTpro [N	1.009E-05	4.225E-05	1	1	2	Q15477	Q15477 [955-970]	0	1998.1555
[R].QVFGEA	2xTMTpro [K	3.988E-05	4.225E-05	1	1	2	P07339	P07339 [177-194]	1	2818.6647
[R].DPPQYP	1xTMTpro [N	3.08E-05	4.225E-05	1	1	2	P10153	P10153 [146-159]	0	1936.0612
[R].DPPSWS	1xTMTpro [N	3.988E-05	4.225E-05	1	1	1	Q9Y490	Q9Y490 [1626-16	0	1712.9039
[K].DPQALS	1xTMTpro [K	2.392E-05	4.225E-05	1	1	1	P31948	P31948 [514-523]	0	1746.0042
[R].QVFAEN	1xTMTpro [K	8.021E-07	4.225E-05	1	1	1	P13010	P13010 [45-81]	1	4670.3982
[K].QVGSGV	1xTMTpro [K	1.213E-06	4.225E-05	2	2	3	P0DOX6; P0	P0DOX6 [277-292	0	2226.2221
[K].GAQAMC	1xTMTpro [K	1.427E-05	4.225E-05	1	1	1	Q7L0Y3	Q7L0Y3 [195-216	0	3147.5597
[R].QSEDLG	1xTMTpro [K	1.224E-05	4.225E-05	1	1	1	O60763	O60763 [105-119]	0	2350.2786
[R].QSDVMIY	1xTMTpro [K	7.888E-06	4.225E-05	1	1	2	O75251	O75251 [116-129]	0	2085.1869
[K].QPAENV	1xTMTpro [K	1.782E-05	4.225E-05	1	1	6	P22314	P22314 [658-671]	0	2225.2058
[R].QNYVDL	1xTMTpro [N	4.872E-07	4.225E-05	1	1	1	O75521	O75521 [109-134]	0	3098.5449
[R].QNYHQD	1xTMTpro [N	5.235E-05	4.225E-05	1	1	4	P02794	P02794 [11-23]	0	1849.9112
[R].QNWED/	2xCarbamido	9.327E-06	4.225E-05	1	1	1	Q9NW64	Q9NW64 [13-35]	0	3143.4886
[K].DSELASC	1xCarbamido	1.747E-06	4.225E-05	1	1	1	Q9NTI5	Q9NTI5 [304-321]	0	2773.4587
[K].KQTALVI	2xTMTpro [K	3.495E-05	4.225E-05	1	1	17	P02768	P02768 [549-558]	1	2041.3201
[R].KQTIDNS	2xTMTpro [K	5.963E-07	4.225E-05	1	1	3	P27348	P27348 [139-157]	1	3055.6516
[R].QNVPVII	1xTMTpro [K	1.061E-05	4.225E-05	1	1	1	Q12929	Q12929 [714-731]	0	2640.4376
[K].QNVIMFY	1xTMTpro [K	3.477E-05	4.225E-05	1	1	1	P61011	P61011 [101-114]	0	2086.1975
[R].KQTIDNS	3xTMTpro [K	5.985E-07	4.225E-05	1	1	2	P27348	P27348 [139-158]	2	3487.9538

[R].QNVAYE	1xCarbamidc	2.273E-06	4.225E-05	1	1	1	P46940	P46940 [37-52]	1	2631.3845
[K].DSESAD	1xTMTpro [N	3.119E-05	4.225E-05	1	1	1	Q9Y5X1	Q9Y5X1 [180-191	0	1467.6995
[R].QNVAYE	1xCarbamidc	1.289E-06	4.225E-05	1	1	1	P46940	P46940 [37-51]	0	2475.2834
[R].KQVEVD	1xCarbamidc	1.465E-06	4.225E-05	1	1	1	P61224	P61224 [42-68]	1	3720.8891
[K].DSETVD	1xTMTpro [K	1.019E-05	4.225E-05	1	1	1	Q14690	Q14690 [1009-10	0	2870.465
[R].GALVTV	1xCarbamidc	3.124E-05	4.225E-05	1	1	1	Q9UBX3	Q9UBX3 [171-186	0	2318.267
[K].QNQQD	1xTMTpro [N	7.478E-06	4.225E-05	1	1	2	Q9NP74	Q9NP74 [67-84]	0	2509.3442
[R].KQVEVD	1xCarbamidc	8.761E-06	4.225E-05	1	1	1	P61224	P61224 [42-68]	1	3736.884
[R].KQYDTY	2xTMTpro [K	7.603E-06	4.225E-05	1	1	1	Q9UBS4	Q9UBS4 [84-95]	1	2343.3012
[R].KSAAFQ	1xCarbamidc	1.989E-06	4.225E-05	1	1	3	Q99715	Q99715 [2697-27	1	3035.6268
[R].QNLLSQ	1xTMTpro [N	1.892E-06	4.225E-05	1	1	2	Q01082	Q01082 [1162-11	0	2137.1474
[K].QPAIISQ	1xTMTpro [N	1.722E-05	4.225E-05	1	1	6	Q08211	Q08211 [1117-11	0	1884.051
[K].QPAIMP	1xCarbamidc	3.545E-05	4.225E-05	1	1	2	P14866	P14866 [456-475]	0	2796.3828
[K].QPAIMP	1xCarbamidc	3.119E-05	4.225E-05	1	1	2	P14866	P14866 [456-475]	0	2812.3777
[R].GAMGIM	1xTMTpro [K	2.371E-05	4.225E-05	2	2	1	P61006; Q9	P61006 [80-94]; C	0	2263.2322
[K].KQQDVL	2xTMTpro [K	3.826E-05	4.225E-05	1	1	2	O75368	O75368 [19-31]	1	2402.4224
[R].KQQMAE	1xTMTpro [K	1.581E-05	4.225E-05	1	1	1	Q15393	Q15393 [816-832	1	2559.2675
[R].QPQGHL	1xTMTpro [K	2.479E-05	4.225E-05	1	1	1	Q14204	Q14204 [2928-29	0	2183.3156
[R].QPPWCI	1xCarbamidc	3.135E-05	4.225E-05	1	1	1	Q92530	Q92530 [181-204	0	2956.4623
[R].DSCKGD	2xCarbamidc	1.288E-05	4.225E-05	1	1	1	P00746	P00746 [202-227]	1	3158.6066
[R].DSCNGD	2xCarbamidc	5.098E-05	4.225E-05	1	1	1	P12544	P12544 [206-223]	0	2274.045
[K].GANIQLL	1xTMTpro [K	5.246E-06	4.225E-05	1	1	1	P55039	P55039 [108-127]	0	2586.5111
[R].KQQMA\	2xTMTpro [K	5.869E-05	4.225E-05	1	1	1	P48728	P48728 [383-391]	1	1931.1928
[R].DSCQGD	2xCarbamidc	3.702E-05	4.225E-05	1	1	3	Q15661	Q15661 [218-231	0	2088.0345
[K].KQQSIA	2xTMTpro [K	3.551E-05	4.225E-05	1	1	1	Q12904	Q12904 [137-154	1	2799.6145
[R].GAISAE\	1xTMTpro [N	3.119E-05	4.225E-05	1	1	1	P10644	P10644 [98-115]	0	2235.11
[R].DSDAGS	1xTMTpro [N	2.865E-05	4.225E-05	1	1	3	P51532	P51532 [1416-142	0	1686.8102
[K].KQSEPLI	1xTMTpro [K	3.037E-05	4.225E-05	1	1	1	P24821	P24821 [1659-167	1	2332.3732
[K].QPGGEG	1xTMTpro [K	5.509E-05	4.225E-05	1	1	2	Q93052	Q93052 [60-100]	0	4831.4638
[K].DSDKEG	2xTMTpro [K	8.34E-08	4.225E-05	1	1	3	Q9P0M6	Q9P0M6 [164-189	1	3543.7755
[R].QPEYLD	1xTMTpro [N	8.57E-06	4.225E-05	1	1	1	Q14839	Q14839 [715-738	0	3093.5423
[R].KQSLGE	2xTMTpro [K	4.416E-07	4.225E-05	1	1	2	P60174	P60174 [19-33]	1	2455.5064
[K].QPDVILE	1xTMTpro [N	4.972E-05	4.225E-05	1	1	1	P46939	P46939 [1931-194	0	2140.1933

[R].KQSTDE	2xTMTpro [K	7.45E-06	4.225E-05	1	1	2	P23193	P23193 [55-67]	1	2348.3489
[K].QPCQPF	2xCarbamido	3.715E-05	4.225E-05	1	5	1	P22532	P22532 [10-22]	0	2114.1382
[K].QPCPSE	1xCarbamido	1.433E-05	4.225E-05	1	1	1	O75475	O75475 [202-216]	0	2356.1834
[K].DSDWPF	1xCarbamido	6.719E-06	4.225E-05	1	1	10	P02671	P02671 [49-63]	0	2572.1582
[K].QPPIQST	1xTMTpro [N	4.442E-05	4.225E-05	1	1	2	P55081	P55081 [10-23]	0	1724.9978
[R].QNLEPLI	1xTMTpro [N	1.81E-06	4.225E-05	3	4	1	P04259; P02	P04259 [208-223]	1	2351.2791
[R].QNLEPLI	1xTMTpro [N	2.267E-05	4.225E-05	3	4	2	P04259; P02	P04259 [208-222]	0	2195.178
[R].QNIAYEY	1xCarbamido	8.794E-06	4.225E-05	1	1	1	Q13576	Q13576 [34-49]	1	2645.4001
[K].KSCVEE	1xCarbamido	4.549E-05	4.225E-05	1	1	1	P51858	P51858 [106-125]	1	3085.5452
[R].QMLEAL	1xTMTpro [K	2.819E-05	4.225E-05	1	1	1	O94804	O94804 [140-151]	0	2039.1603
[R].KSDGIYI	2xTMTpro [K	5.261E-05	4.225E-05	1	1	3	P08865	P08865 [42-52]	1	2176.3522
[K].QMGFPL	1xOxidation [	1.722E-05	4.225E-05	1	1	1	P55786	P55786 [500-517]	0	2459.2084
[K].DSLDQLY	1xTMTpro [K	3.243E-06	4.225E-05	1	1	1	P01031	P01031 [377-408]	0	3977.0847
[R].DSLEDC	1xCarbamido	1.497E-05	4.225E-05	1	1	1	Q9UBG0	Q9UBG0 [476-49	0	2037.9871
[K].DSIEKEH	2xTMTpro [K	4.45E-06	4.225E-05	1	1	1	Q9Y265	Q9Y265 [423-441	1	3193.7085
[K].QMGFPL	1xTMTpro [N	4.678E-06	4.225E-05	1	1	1	P55786	P55786 [500-517]	0	2443.2134
[K].KSDIDEI	1xTMTpro [K	1.049E-05	4.225E-05	1	1	5	P11021	P11021 [353-367]	1	2197.2684
[R].KSDLFQ	2xTMTpro [K	3.184E-06	4.225E-05	1	1	3	Q9ULV4	Q9ULV4 [353-381	1	4184.1168
[K].KSASDLT	2xTMTpro [K	3.995E-05	4.225E-05	1	1	3	P02787	P02787 [453-464]	1	2290.3223
[K].QMFGNA	1xTMTpro [K	3.358E-05	4.225E-05	1	1	3	P18859	P18859 [80-94]	0	2357.1914
[R].DSLGDF	1xTMTpro [N	1.199E-06	4.225E-05	1	1	3	Q96QR8	Q96QR8 [85-116]	0	3559.726
[R].KSDLFQ	1xTMTpro [K	1.169E-07	4.225E-05	1	1	1	Q9BR76	Q9BR76 [355-383	1	3817.9052
[R].KSDLFQ	1xTMTpro [K	3.163E-07	4.225E-05	1	1	6	P31146	P31146 [355-383]	1	3781.9569
[K].QMEQIS	1xOxidation [	2.565E-05	4.225E-05	1	1	3	P37802	P37802 [89-102]	0	1999.0238
[K].QMEQIS	1xTMTpro [N	2.308E-05	4.225E-05	1	1	3	P37802	P37802 [89-102]	0	1983.0289
[R].DSLLQD	1xTMTpro [N	1.115E-05	4.225E-05	1	1	8	P07737	P07737 [76-89]	0	1929.9547
[R].QMEKDE	1xCarbamido	3.31E-06	4.225E-05	1	1	1	P47756	P47756 [196-215]	1	2895.4584
[R].QMAEIAY	1xTMTpro [N	3.544E-06	4.225E-05	1	1	1	P48643	P48643 [184-201]	0	2265.1902
[K].DSLPVCI	2xCarbamido	1.024E-06	4.225E-05	1	1	2	P41252	P41252 [345-371]	0	3571.758
[K].DSLQCT	2xCarbamido	6.404E-06	4.225E-05	1	1	1	Q96HF1	Q96HF1 [241-263	0	3324.5864
[R].QMFEPV	1xCarbamido	1.622E-05	4.225E-05	1	1	2	O95571	O95571 [27-43]	0	2368.1637
[K].KQQAIEI	1xTMTpro [K	5.65E-07	4.225E-05	1	1	3	O60216	O60216 [605-627]	1	3192.7396
[R].QMQLEN	1xTMTpro [N	5.597E-06	4.225E-05	2	2	2	P21333; O75	P21333 [101-116]	0	2196.1654

[K].DSHSLT	1xTMTpro [N	1.206E-05	4.225E-05	1	1	4	P02671	P02671 [101-114]	0	1934.0336
[K].DSGACF	1xCarbamido	4.361E-06	4.225E-05	1	1	1	Q8NB37	Q8NB37 [175-194	0	2436.1785
[R].QNLAMT	1xTMTpro [K	5.784E-05	4.225E-05	1	1	1	P36776	P36776 [875-888]	0	2057.1556
[K].QNKDDE	1xTMTpro [K	7.491E-06	4.225E-05	1	1	2	Q9UGP8	Q9UGP8 [605-62	1	2853.4623
[R].QNFTEP	1xTMTpro [K	3.988E-07	4.225E-05	1	1	2	P17844	P17844 [112-144]	0	3967.0879
[R].GALQNII	1xTMTpro [K	1.766E-05	4.225E-05	1	1	9	P04406	P04406 [201-215]	0	2020.2046
[R].DSGGRE	1xCarbamido	3.793E-05	4.225E-05	1	1	1	P29323	P29323 [349-363]	1	2347.2572
[K].QNDQVS	1xTMTpro [K	3.219E-05	4.225E-05	1	1	1	Q86UP2	Q86UP2 [786-800	0	2315.2739
[K].DSGLFC	1xCarbamido	3.108E-05	4.225E-05	1	1	1	Q8N392	Q8N392 [318-335	0	2366.2345
[K].DSGLPS	1xTMTpro [K	3.67E-05	4.225E-05	1	1	2	Q12959	Q12959 [597-608]	0	1871.0518
[K].QNCFDD	2xCarbamido	1.374E-05	4.225E-05	1	1	1	P48147	P48147 [524-539]	0	2630.2875
[R].QMQLEN	1xOxidation [	1.67E-05	4.225E-05	2	2	1	P21333; O75	P21333 [101-116]	0	2212.1603
[K].QNCELF	1xCarbamido	3.496E-08	4.225E-05	1	1	4	P02768	P02768 [414-434]	1	3207.7116
[K].DSGNKP	1xTMTpro [K	5.322E-05	4.225E-05	1	1	1	Q9Y2Q3	Q9Y2Q3 [50-61]	0	1859.0995
[R].QMVVLE	1xTMTpro [K	1.515E-06	4.225E-05	1	1	1	O60234	O60234 [41-58]	0	2770.4829
[R].DSGPPP	1xTMTpro [K	3.219E-06	4.225E-05	1	1	1	Q8N684	Q8N684 [324-342	0	2644.3308
[K].KSADTL	2xTMTpro [K	3.265E-05	4.225E-05	1	1	3	P00338	P00338 [318-328]	1	2159.3005
[K].DSGRDY	1xTMTpro [K	2.457E-06	4.225E-05	1	1	4	P02647	P02647 [48-64]	1	2424.2651
[K].DSGSDE	1xTMTpro [K	7.128E-06	4.225E-05	1	1	1	Q9H1E3	Q9H1E3 [129-150	0	3037.2872
[R].GAIQFV	1xTMTpro [N	5.888E-05	4.225E-05	1	1	1	Q15436	Q15436 [477-492]	0	2111.0953
[R].GALQAV	1xCarbamido	3.486E-06	4.225E-05	1	1	2	A1L0T0	A1L0T0 [158-174]	0	2524.4565
[K].DSHDPL	1xTMTpro [K	2.009E-05	4.225E-05	1	1	1	Q96AQ6	Q96AQ6 [566-581	0	2469.3746
[K].QMQVLL	1xTMTpro [N	2.3E-05	4.225E-05	1	1	1	Q9H5N1	Q9H5N1 [330-346	0	2302.2872
[K].QNCELF	1xCarbamido	9.73E-06	4.225E-05	1	1	87	P02768	P02768 [414-426]	0	2266.1669
[R].QPSSAF	1xTMTpro [K	5.926E-05	4.225E-05	1	1	4	P01024	P01024 [1061-107	0	1761.0191
[R].QPSWDI	1xTMTpro [K	2.593E-05	4.225E-05	1	1	3	Q9NQC3	Q9NQC3 [105-13	0	3355.8029
[K].QPTEEE	2xTMTpro [K	3.846E-05	4.225E-05	1	1	1	Q15459	Q15459 [21-50]	1	4149.2536
[K].DQVEEV	1xTMTpro [K	5.401E-05	4.225E-05	1	1	1	P50749	P50749 [263-277]	0	2408.2841
[K].DQVEYL	1xCarbamido	2.466E-05	4.225E-05	1	1	1	O00505	O00505 [402-423]	0	3211.7681
[R].QQQVEA	1xTMTpro [K	1.955E-05	4.225E-05	1	1	2	Q86UP2	Q86UP2 [1062-10	0	1980.1257
[K].QQQQQ	1xTMTpro [K	3.853E-05	4.225E-05	1	1	1	Q7Z478	Q7Z478 [501-514]	1	2403.2733
[K].QQQQQ	1xTMTpro [N	1.359E-05	4.225E-05	1	1	2	Q6Y7W6	Q6Y7W6 [1016-1	0	1980.0079
[K].KQELLEA	2xTMTpro [K	4.525E-05	4.225E-05	1	1	3	P12956	P12956 [596-605]	1	2085.31

[K].QQQQQI	2xCarbamido	1.558E-05	4.225E-05	1	1	1	P24468	P24468 [71-85]	0	2465.2521
[R].KQEPSL	1xCarbamido	1.793E-06	4.225E-05	1	1	3	O00585	O00585 [50-67]	1	2634.5297
[K].DQVTAQ	1xTMTpro [K	5.208E-07	4.225E-05	1	1	2	P13010	P13010 [546-565]	0	2851.4354
[R].GAPNPW	1xTMTpro [N	1.513E-05	4.225E-05	1	1	1	Q9UNK0	Q9UNK0 [119-13	0	2076.0357
[K].GAPTTSI	1xTMTpro [K	3.881E-06	4.225E-05	1	1	2	P49419	P49419 [219-232]	0	1953.1876
[K].QQQLEQ	1xTMTpro [N	2.891E-06	4.225E-05	1	1	1	O95630	O95630 [154-170	0	2495.2308
[K].QQQEIL/	2xTMTpro [K	1.339E-05	4.225E-05	1	1	2	Q92905	Q92905 [35-47]	0	2453.4696
[K].QQQDFS	1xTMTpro [N	4.148E-06	4.225E-05	1	1	1	O43290	O43290 [616-636	0	2682.3542
[R].QQQDE/	1xTMTpro [N	5.869E-05	4.225E-05	1	1	2	Q96CS3	Q96CS3 [291-302	0	1725.9091
[K].QQPTQF	1xTMTpro [N	1.858E-07	4.225E-05	1	1	2	Q15019	Q15019 [4-29]	0	3256.6823
[R].DRAEW/	1xTMTpro [K	5.617E-06	4.225E-05	1	1	1	P49354	P49354 [68-95]	1	3759.9474
[K].DRDDFP	1xTMTpro [K	1.533E-05	4.225E-05	1	1	1	P10301	P10301 [131-143]	1	2082.1839
[R].QQPMAL	1xTMTpro [K	4.177E-05	4.225E-05	1	1	1	P10253	P10253 [838-849]	0	1879.1331
[K].QQPLFV	1xTMTpro [K	1.779E-05	4.225E-05	1	1	2	P53621	P53621 [62-74]	0	2062.1101
[R].QQPGIEI	1xTMTpro [N	1.628E-05	4.225E-05	1	1	1	Q96ME7	Q96ME7 [508-522	0	2014.114
[R].QQNDEL	1xTMTpro [K	4.123E-07	4.225E-05	1	1	2	Q86VS8	Q86VS8 [284-307	1	3352.7728
[K].QQQFEN	1xTMTpro [N	4.264E-05	4.225E-05	1	1	1	O60749	O60749 [310-321	0	1850.968
[K].DRDGQF	1xTMTpro [N	3.231E-06	4.225E-05	1	1	1	P22105	P22105 [1613-162	1	2055.0902
[R].DQTVLE	2xCarbamido	2.625E-05	4.225E-05	1	1	1	Q9NR99	Q9NR99 [488-504	0	2513.2984
[K].QQSLET	1xTMTpro [N	3.163E-05	4.225E-05	1	1	4	P12111	P12111 [2484-249	0	1771.9332
[K].GAQAIFI	1xCarbamido	4.882E-05	4.225E-05	1	1	1	Q9UDR5	Q9UDR5 [230-249	0	2952.5421
[K].QRPPDL	1xTMTpro [N	1.057E-05	4.225E-05	1	1	2	P00736	P00736 [271-296]	0	3186.551
[R].QRPGAS	1xTMTpro [N	4.287E-07	4.225E-05	1	1	1	Q9UPT8	Q9UPT8 [1025-10	0	2851.4505
[K].DQQEAA	1xTMTpro [N	3.522E-07	4.225E-05	1	1	4	P09211	P09211 [83-101]	0	2421.1887
[K].DQQEAA	1xOxidation	4.601E-06	4.225E-05	1	1	5	P09211	P09211 [83-101]	0	2437.1836
[R].QRPEVF	1xCarbamido	8.109E-06	4.225E-05	1	1	1	Q9NZP8	Q9NZP8 [410-428	0	2619.2251
[K].QREEYQ	1xOxidation	4.503E-05	4.225E-05	1	1	1	Q9BVK6	Q9BVK6 [72-95]	2	3691.9458
[R].DQQLEA	1xTMTpro [K	2.016E-05	4.225E-05	1	1	2	Q08378	Q08378 [709-724	0	2547.3732
[K].QREEYQ	2xTMTpro [K	2.287E-05	4.225E-05	1	1	1	Q9BVK6	Q9BVK6 [72-95]	2	3675.9509
[R].QREELG	1xTMTpro [K	1.656E-06	4.225E-05	1	1	2	Q07065	Q07065 [147-161	1	2307.2912
[K].QQSEED	1xTMTpro [N	1.197E-05	4.225E-05	1	1	3	Q9UNL2	Q9UNL2 [9-22]	0	2012.0256
[R].QQWRP	1xTMTpro [K	5.527E-05	4.225E-05	1	1	1	Q13425	Q13425 [343-355	0	2194.2662
[R].GAQAAI	1xTMTpro [N	1.449E-06	4.225E-05	1	1	4	P51148	P51148 [93-111]	0	2330.2311

[K].QQVEGT	1xTMTpro [K	1.343E-08	4.225E-05	1	1	4	Q15847	Q15847 [11-41]	0	3649.8913
[R].QQVAPT	2xTMTpro [K	3.594E-05	4.225E-05	1	1	2	Q13813	Q13813 [958-981]	1	3665.973
[R].QQVAFY	1xTMTpro [N	1.369E-05	4.225E-05	1	1	1	Q96I24	Q96I24 [553-572]	0	2523.2547
[R].GAQAAI	1xTMTpro [N	1.783E-06	4.225E-05	1	1	2	P61020	P61020 [92-110]	0	2371.2577
[R].QQTPDK	2xTMTpro [K	4.945E-06	4.225E-05	1	1	1	P55290	P55290 [288-319]	1	4483.4363
[R].GAQAAI	1xTMTpro [N	3.378E-06	4.225E-05	1	1	2	P20339	P20339 [92-110]	0	2358.2261
[R].DQTDDQ	1xTMTpro [K	3.146E-05	4.225E-05	1	1	2	P48735	P48735 [90-106]	0	2457.2964
[K].GAPVGG	1xTMTpro [K	3.657E-05	4.225E-05	1	1	3	O00159	O00159 [210-224]	0	2162.2829
[K].QQTDIAV	1xTMTpro [K	6.869E-06	4.225E-05	1	1	1	Q13547	Q13547 [127-143]	0	2454.3497
[K].QQWQQ	1xTMTpro [K	4.76E-05	4.225E-05	1	1	1	Q7L2H7	Q7L2H7 [345-358	0	2430.3062
[R].EGSGST	1xTMTpro [K	4.657E-05	4.225E-05	1	1	1	P78524	P78524 [355-373]	0	2439.272
[K].QQNAQQ	1xTMTpro [K	9.709E-09	4.225E-05	1	1	2	P01023	P01023 [1241-126	0	2995.6093
[R].QQMQDI	1xTMTpro [K	4.923E-05	4.225E-05	1	1	1	Q9BXP5	Q9BXP5 [182-198	1	2863.4481
[R].QQEGEA	1xTMTpro [K	2.154E-06	4.225E-05	1	1	1	Q14315	Q14315 [2240-22	0	2774.3758
[R].QQEELL	1xTMTpro [N	4.3E-05	4.225E-05	1	1	1	Q15149	Q15149 [2719-27	0	1790.9204
[K].QQEAAL	2xTMTpro [K	3.331E-07	4.225E-05	1	1	4	Q05682	Q05682 [558-570]	1	2441.4432
[R].DRPMEE	1xTMTpro [N	1.754E-05	4.225E-05	1	1	1	P47897	P47897 [377-391]	0	2141.1054
[K].QQEAAL	1xTMTpro [K	1.645E-05	4.225E-05	1	1	4	Q05682	Q05682 [558-569]	0	2009.141
[R].KQLLLAI	2xTMTpro [K	5.056E-05	4.225E-05	1	1	2	P80365	P80365 [280-297]	1	2952.8066
[K].DRPSFS	1xTMTpro [K	1.151E-06	4.225E-05	1	1	4	Q13308	Q13308 [1050-10	0	2787.4656
[K].KQISNLQ	1xTMTpro [K	4.328E-06	4.225E-05	1	1	1	P04264	P04264 [417-432]	1	2453.3604
[K].DRPVSQ	1xTMTpro [K	3.259E-05	4.225E-05	1	1	1	Q9H4A3	Q9H4A3 [163-175	0	1978.1577
[K].QQDHFF	1xTMTpro [N	2.371E-05	4.225E-05	1	1	2	P08567	P08567 [76-89]	0	2070.0364
[K].QQEKPE	2xCarbamido	5.14E-06	4.225E-05	1	1	1	Q9NZU5	Q9NZU5 [214-247	1	4973.5501
[R].DRTDIQ	2xCarbamido	2.655E-06	4.225E-05	1	1	1	P23381	P23381 [299-318]	1	2800.3718
[K].KQIWTLI	1xCarbamido	1.806E-06	4.225E-05	1	1	2	Q16658	Q16658 [43-63]	1	2977.6061
[R].DRVTSA	1xTMTpro [N	9.856E-06	4.225E-05	1	1	1	P45974	P45974 [145-162]	1	2152.1529
[R].QQADFD	1xCarbamido	7.436E-06	4.225E-05	1	1	1	Q13459	Q13459 [144-164]	0	3041.5745
[K].KQPALD	2xTMTpro [K	6.334E-06	4.225E-05	1	1	2	Q14152	Q14152 [24-36]	1	2432.4403
[K].DSADIES	1xTMTpro [N	2.202E-05	4.225E-05	1	1	2	Q12882	Q12882 [8-21]	0	1817.9928
[R].QPVTVT	1xTMTpro [N	1.171E-05	4.225E-05	1	1	1	P15924	P15924 [2215-22	0	4121.1209
[R].GAPAED	1xTMTpro [N	1.049E-05	4.225E-05	1	1	1	Q9UFG5	Q9UFG5 [29-50]	0	2639.404
[R].QPTVTS	1xCarbamido	3.114E-06	4.225E-05	1	1	2	Q96HC4	Q96HC4 [206-225	0	2511.2316

[R].DSALETI	1xTMTpro [K	4.587E-05	4.225E-05	1	1	1	Q14980	Q14980 [1161-11	0	2169.1895
[K].DSAIQQ(	1xTMTpro [K	3.305E-05	4.225E-05	1	1	2	Q14204	Q14204 [1229-12	0	2182.2146
[R].QQDAQE	1xOxidation	3.317E-05	4.225E-05	1	1	1	P45974	P45974 [433-449]	0	2438.2457
[K].QQMQDI	1xTMTpro [K	2.859E-06	4.225E-05	1	1	1	P46939	P46939 [2490-25(	0	2752.422
[K].QQEMM/	1xTMTpro [K	1.716E-05	4.225E-05	1	1	1	Q92616	Q92616 [1415-14	0	2301.2074
[R].QQFEGY	1xTMTpro [K	5.97E-06	4.225E-05	1	1	1	Q96EY4	Q96EY4 [122-141	0	2857.5591
[K].GAPLVKI	2xTMTpro [K	3.253E-05	4.225E-05	1	1	1	Q9H3S7	Q9H3S7 [335-35	0	3427.0181
[K].DRDPEA	1xTMTpro [N	2.973E-06	4.225E-05	1	1	1	O14744	O14744 [491-505	1	2156.0766
[R].QQLVEL	1xCarbamid	1.164E-07	4.225E-05	1	1	2	Q9BZZ5	Q9BZZ5 [211-237	0	3420.6548
[R].DRDQPN	1xCarbamid	1.053E-05	4.225E-05	1	1	2	O75339	O75339 [995-100	1	2358.2368
[K].DRDVTF	1xTMTpro [K	1.76E-05	4.225E-05	1	1	1	P55145	P55145 [46-62]	1	2556.4165
[R].KQEVPH	1xCarbamid	6.018E-07	4.225E-05	1	1	1	P60510	P60510 [183-211]	1	3917.9082
[K].DREDYV	1xTMTpro [K	4.427E-05	4.225E-05	1	1	1	Q9NYL9	Q9NYL9 [82-93]	1	2080.0843
[K].QQLSAE	1xTMTpro [N	5.64E-07	4.225E-05	1	1	1	Q86V81	Q86V81 [236-253	0	2339.1798
[K].QQLQQ/	1xTMTpro [K	5.384E-05	4.225E-05	1	1	2	Q9Y6K9	Q9Y6K9 [265-277	0	2064.1945
[R].QQLPTF	1xTMTpro [N	2.802E-06	4.225E-05	1	1	1	Q9UMX0	Q9UMX0 [436-45	0	2949.4882
[K].QQEVVV	1xTMTpro [K	9.748E-06	4.225E-05	1	1	1	Q9H3P7	Q9H3P7 [307-322	0	2225.2633
[K].QQLNNM	1xTMTpro [K	3.069E-05	4.225E-05	1	1	1	P40763	P40763 [643-658]	0	2495.3282
[K].KQGGLG	1xOxidation	1.706E-05	4.225E-05	1	1	4	Q06830	Q06830 [93-109]	1	2679.5684
[R].DREVGII	1xTMTpro [K	6.67E-06	4.225E-05	1	1	1	P61158	P61158 [210-225]	1	2377.3219
[R].QQLDSF	1xTMTpro [N	7.991E-06	4.225E-05	1	1	1	P30876	P30876 [42-57]	0	2275.1348
[R].QQIACIG	2xCarbamid	1.22E-05	4.225E-05	1	1	1	P40763	P40763 [247-262]	0	2116.0962
[R].DRGPMY	1xTMTpro [N	9.838E-06	4.225E-05	1	1	1	P51608	P51608 [90-106]	1	2310.1144
[K].KQLADE	2xTMTpro [K	4.931E-05	4.225E-05	1	1	4	P20700	P20700 [182-191]	1	2071.2943
[R].DRHPEE	1xTMTpro [K	4.043E-05	4.225E-05	1	1	1	Q96DZ9	Q96DZ9 [9-27]	1	2704.455
[R].GAPEGT	2xCarbamid	5.638E-06	4.225E-05	1	1	1	O00468	O00468 [278-300	0	2713.2517
[R].DRLPDA	1xTMTpro [N	2.765E-05	4.225E-05	1	1	1	Q53LP3	Q53LP3 [116-132	1	2054.095
[K].QQFVEY	1xTMTpro [K	7.194E-06	4.225E-05	1	1	1	Q14257	Q14257 [104-129	1	3910.8362
[K].KQGGLG	2xTMTpro [K	1.111E-05	4.225E-05	1	1	3	Q06830	Q06830 [93-109]	1	2663.5735
[K].SHCIAE\	1xCarbamid	3.331E-08	4.225E-05	1	1	117	P02768	P02768 [311-337]	0	3582.7588
[K].MKPLDG	1xTMTpro [K	7.167E-06	4.225E-05	1	1	3	P12111	P12111 [515-533]	0	2649.4566
[K].MKPDIV(	1xCarbamid	8.539E-06	4.225E-05	1	1	1	Q9NR50	Q9NR50 [71-91]	0	2928.4761
[R].ILYLDSS	2xCarbamid	2.568E-06	4.225E-05	1	1	1	Q9BSJ8	Q9BSJ8 [595-624	0	3726.7739

[R].IIYGGSV	1xCarbamid	1.414E-05	4.225E-05	1	1	4	P60174	P60174 [207-219]	0	1935.0865
[R].IIYFQDE	1xTMTpro [K	2.279E-05	4.225E-05	1	1	2	Q9HCB6	Q9HCB6 [168-17	0	2022.1403
[K].LETTSN	1xTMTpro [K	2.911E-05	4.225E-05	1	1	2	P23229	P23229 [812-827]	0	2324.2953
[R].ETENDD	1xTMTpro [K	2.367E-05	4.225E-05	1	1	2	O95373	O95373 [552-564	0	2127.1425
[K].LLYDLAD	1xTMTpro [N	5.325E-07	4.225E-05	1	1	1	P13804	P13804 [233-249]	0	2117.1674
[R].FKVDLTI	2xTMTpro [K	2.206E-05	4.225E-05	1	1	2	O43708	O43708 [176-191	1	2735.6164
[R].IIWCPFII	3xCarbamid	3.961E-05	4.225E-05	1	1	1	Q6P2E9	Q6P2E9 [235-263	0	3822.762
[K].LLVYEAL	1xTMTpro [K	3.607E-05	4.225E-05	1	1	2	Q96DB5	Q96DB5 [141-151	0	1920.1338
[K].IETVNES	1xTMTpro [K	2.171E-05	4.225E-05	1	1	1	P21399	P21399 [616-632]	0	2483.3273
[K].IIVVMDA	1xTMTpro [N	3.879E-05	4.225E-05	1	1	2	P33121	P33121 [223-237]	0	1998.0901
[R].FKPVVP	1xTMTpro [K	5.617E-06	4.225E-05	1	1	1	Q86SF2	Q86SF2 [66-86]	0	3040.7116
[K].LEVAPIS	1xTMTpro [K	2.049E-05	4.225E-05	1	1	5	P13804	P13804 [127-139]	0	1990.2444
[K].ETFDPEI	2xTMTpro [K	3.502E-05	4.225E-05	1	1	1	P53621	P53621 [776-793]	1	2971.608
[K].ETFDVTI	1xTMTpro [K	1.987E-05	4.225E-05	1	1	2	P00488	P00488 [572-584]	0	2134.1927
[R].ILVTGGS	1xTMTpro [K	3.124E-05	4.225E-05	1	1	2	Q13630	Q13630 [10-21]	0	1709.0817
[K].ETFGVN	1xTMTpro [N	2.484E-05	4.225E-05	1	1	1	O75822	O75822 [134-153	0	2446.1992
[K].ETFGVN	1xOxidation	3.702E-05	4.225E-05	1	1	1	O75822	O75822 [134-153	0	2462.1941
[K].LLVSGA	1xTMTpro [K	2.76E-05	4.225E-05	1	1	2	Q9Y4G6	Q9Y4G6 [2033-2	0	1767.0508
[K].IEVEKPF	2xTMTpro [K	5.527E-05	4.225E-05	1	1	6	P23284	P23284 [205-215]	0	2157.3463
[K].FKNEEE	2xTMTpro [K	1.757E-05	4.225E-05	1	1	2	P49419	P49419 [438-452]	1	2795.5184
[R].LETQVW	1xTMTpro [K	2.895E-05	4.225E-05	1	1	2	Q13330	Q13330 [194-208	0	2389.3007
[R].FIADQLD	1xTMTpro [K	3.048E-05	4.225E-05	1	1	1	Q9BQC6	Q9BQC6 [87-99]	0	2122.2152
[K].FLADQQ	1xTMTpro [K	1.944E-05	4.225E-05	1	1	3	P12270	P12270 [28-40]	0	2072.1519
[R].IETQNQI	1xTMTpro [N	4.293E-05	4.225E-05	1	1	1	O75923	O75923 [672-684	0	1774.9982
[K].LMCPQE	1xCarbamid	1.313E-06	4.225E-05	1	1	4	O14561	O14561 [138-152	1	2735.5292
[K].LMAVPN	1xTMTpro [K	3.021E-05	4.225E-05	1	1	1	O94903	O94903 [105-121	0	2515.416
[K].IMALGNI	1xTMTpro [K	1.61E-06	4.225E-05	1	1	1	Q8TEQ6	Q8TEQ6 [550-56	0	2810.5618
[K].LETGAPI	1xTMTpro [N	3.435E-06	4.225E-05	1	1	1	Q9BXS5	Q9BXS5 [142-16	0	2327.2791
[R].ETDCGV	1xCarbamid	3.213E-06	4.225E-05	2	2	2	O94808; Q0	O94808 [458-477	0	2663.3954
[K].IMADNP	1xTMTpro [K	2.677E-05	4.225E-05	1	1	2	Q6P2Q9	Q6P2Q9 [2199-22	0	1971.0137
[K].IIYVGSA	1xTMTpro [N	4.192E-07	4.225E-05	1	1	2	Q9Y294	Q9Y294 [42-69]	0	3266.7116
[K].FLAITTD	1xCarbamid	3.705E-06	4.225E-05	1	1	1	P35222	P35222 [293-312]	0	2893.5625
[K].ETDLLLD	1xTMTpro [N	5.872E-06	4.225E-05	1	1	4	P30044	P30044 [160-176]	0	2211.1828

[R].IIYSPTV(	1xTMTpro [N	5.536E-05	4.225E-05	1	1	1	Q99715	Q99715 [2057-20	1	2653.4157
[R].IEVEQAL	1xTMTpro [K	8.291E-06	4.225E-05	1	1	1	P28482	P28482 [302-330]	0	3971.0246
[R].ETDLQE	1xTMTpro [N	3.146E-05	4.225E-05	1	1	1	O75821	O75821 [252-267	0	2199.1729
[R].IETMLGI	1xTMTpro [K	7.772E-07	4.225E-05	1	1	3	P26640	P26640 [538-552]	0	2188.2655
[K].LLYPAD1	1xTMTpro [K	1.709E-05	4.225E-05	1	1	5	Q15063	Q15063 [627-646	0	2834.6159
[R].ETDQML	1xTMTpro [K	3.429E-05	4.225E-05	1	1	2	P46939	P46939 [1341-135	0	2684.4308
[K].IETNENN	1xTMTpro [K	1.852E-05	4.225E-05	1	1	2	Q07065	Q07065 [567-578	0	1970.0686
[R].ILYMTDE	1xOxidation		2.561E-05	4.225E-05	1	1	1	P00403	P00403 [83-98]	0	2476.3501
[R].ETEDVN	1xTMTpro [K	2.037E-06	4.225E-05	1	1	2	P29083	P29083 [193-215]	0	3221.7325
[R].LLYMAID	1xTMTpro [N	5.813E-05	4.225E-05	1	1	2	Q9H0D6	Q9H0D6 [97-108]	0	1622.9259
[K].IIYIVHDE	2xTMTpro [K	9.588E-06	4.225E-05	1	1	3	P25788	P25788 [197-208]	1	2384.4369
[K].ETEEITS	1xTMTpro [N	3.81E-06	4.225E-05	1	1	1	Q14696	Q14696 [128-149	0	2904.4335
[R].ETEGGT	1xTMTpro [K	9.023E-06	4.225E-05	1	1	1	Q9UQE7	Q9UQE7 [852-87	0	2672.4486
[R].IIYSPTV(	1xTMTpro [N	9.909E-07	4.225E-05	1	1	5	Q99715	Q99715 [2057-20	0	2497.3145
[K].LIVPVLLI	1xTMTpro [K	5.393E-05	4.225E-05	1	1	1	Q15833	Q15833 [388-403	0	2305.4027
[K].ETGANL	1xCarbamido	1.061E-08	4.225E-05	1	1	3	P48643	P48643 [294-323]	0	3683.8559
[K].LLVPSPE	1xTMTpro [N	3.873E-05	4.225E-05	1	1	2	Q86UX7	Q86UX7 [424-438	0	2008.1108
[R].FKKEDE	3xTMTpro [K	3.927E-05	4.225E-05	1	1	1	Q96A26	Q96A26 [77-96]	2	3509.9918
[K].LLTSLFQ	1xTMTpro [K	5.554E-05	4.225E-05	1	1	1	Q5T4S7	Q5T4S7 [483-498	0	2463.4467
[R].ETKDTD	2xTMTpro [K	2.29E-06	4.225E-05	1	1	5	O15145	O15145 [35-50]	1	2862.5593
[K].LLTQHEI	2xTMTpro [K	3.288E-07	4.225E-05	1	1	2	Q01082	Q01082 [1100-11	1	3548.8689
[K].ILTQDTP	1xTMTpro [K	6.487E-06	4.225E-05	1	1	2	Q8TDL5	Q8TDL5 [340-356	0	2568.3954
[R].LLTQDE(	1xTMTpro [N	5.098E-05	4.225E-05	1	1	1	Q5T440	Q5T440 [198-212	0	1827.0295
[K].ILTPLVSI	1xTMTpro [K	4.228E-05	4.225E-05	1	1	2	Q9HC38	Q9HC38 [240-252	0	1962.2131
[K].LLTPITTI	1xTMTpro [K	4.818E-06	4.225E-05	1	1	5	P12111	P12111 [1404-14	0	2294.3827
[K].ETLDTMI	1xTMTpro [N	4.163E-05	4.225E-05	1	1	2	Q9P000	Q9P000 [176-187	0	1624.8535
[K].ETIEDVE	1xTMTpro [N	6.827E-07	4.225E-05	1	1	2	Q9UNM6	Q9UNM6 [133-15	0	2773.3997
[K].LITSNTD	1xTMTpro [K	1.544E-05	4.225E-05	1	1	1	Q14789	Q14789 [1125-11	0	2414.327
[R].ETIELVH	1xTMTpro [N	9.191E-06	4.225E-05	1	1	1	Q6IBS0	Q6IBS0 [210-228]	0	2439.305
[R].IITMLPT!	1xTMTpro [K	4.228E-05	4.225E-05	1	1	1	P31937	P31937 [99-121]	0	2998.7143
[K].ETLEQL!	2xTMTpro [K	2.573E-06	4.225E-05	1	1	2	Q8WZA0	Q8WZA0 [48-62]	1	2693.5178
[R].IITITGTQ	1xTMTpro [K	7.42E-07	4.225E-05	1	1	4	P61978	P61978 [434-456]	0	3197.8026
[K].FKGPFTI	2xTMTpro [K	4.315E-05	4.225E-05	1	1	4	Q9P0L0	Q9P0L0 [25-38]	1	2479.4741

[R].LLTLEVF	1xTMTpro [N	3.097E-05	4.225E-05	1	1	1	Q9HCE1	Q9HCE1 [376-393	0	2270.3403
[R].LLTLEQF	1xCarbamido	3.219E-05	4.225E-05	1	1	1	Q86VU5	Q86VU5 [74-99]	0	3235.6444
[K].IITLEEGI	1xTMTpro [K	2.584E-05	4.225E-05	1	1	1	Q6P587	Q6P587 [179-194	0	2321.3823
[K].IEVPLYS	1xTMTpro [K	2.908E-06	4.225E-05	1	1	1	Q92598	Q92598 [317-331]	0	2391.4143
[R].LEVSVD(	1xTMTpro [N	2.274E-07	4.225E-05	1	1	1	Q9H3P7	Q9H3P7 [10-49]	0	4299.2832
[K].LLTIDQD	1xTMTpro [K	3.053E-05	4.225E-05	1	1	2	P13798	P13798 [412-434]	0	3136.7823
[R].ILTNWGI	1xCarbamido	1.792E-05	4.225E-05	1	1	1	Q9UH99	Q9UH99 [694-708	0	2227.1289
[K].LMCPQE	1xCarbamido	3.453E-05	4.225E-05	1	1	1	O14561	O14561 [138-152]	1	2751.5241
[K].LLTSQC(	1xCarbamido	3.607E-05	4.225E-05	1	1	1	P82933	P82933 [228-243]	0	2142.082
[K].ILTTNTW	1xTMTpro [K	2.834E-05	4.225E-05	1	1	2	O60701	O60701 [208-220]	0	2088.1832
[K].LLVPCAI	3xCarbamido	9.694E-06	4.225E-05	1	1	1	Q9UBT2	Q9UBT2 [428-453	0	3577.8825
[K].ETGEHL'	1xTMTpro [K	5.166E-05	4.225E-05	1	1	7	P21333	P21333 [2015-202	0	1757.0201
[K].ILVNQLS	2xCarbamido	1.373E-06	4.225E-05	1	1	2	P14923	P14923 [395-424]	0	3872.0389
[R].FKLPPGI	1xCarbamido	2.412E-06	4.225E-05	1	1	2	P17655	P17655 [475-500]	2	3952.1499
[R].LEVLGC:	1xCarbamido	9.015E-07	4.225E-05	1	1	2	Q8IUX7	Q8IUX7 [535-563]	0	3597.7742
[K].LLVLEDE	2xTMTpro [K	1.969E-05	4.225E-05	1	1	1	Q13616	Q13616 [648-670]	0	3522.0134
[R].LLVLEAF	1xCarbamido	2.031E-05	4.225E-05	1	1	1	O43708	O43708 [193-206]	0	1973.0962
[K].ETGLSHI	2xCarbamido	5.853E-07	4.225E-05	1	1	3	Q9Y678	Q9Y678 [433-454	0	2931.4188
[K].IEVIKPGI	2xTMTpro [K	2.166E-06	4.225E-05	1	1	2	P38117	P38117 [206-221]	0	2596.5742
[K].ILVGPSE	1xTMTpro [K	5.375E-05	4.225E-05	1	1	1	Q14315	Q14315 [2031-204	1	2149.2836
[K].LLTSQD'	1xTMTpro [N	4.465E-05	4.225E-05	1	1	3	P11277	P11277 [1294-130	0	1800.9299
[R].LLVGND	1xTMTpro [N	4.744E-05	4.225E-05	1	1	1	Q7Z6Z7	Q7Z6Z7 [2606-26	0	1739.0135
[K].ETGTLE:	1xTMTpro [K	2.859E-06	4.225E-05	1	1	1	P02549	P02549 [2188-220	1	2184.2116
[K].IEVLQQH	1xTMTpro [K	5.313E-05	4.225E-05	1	1	1	O00505	O00505 [462-475]	0	2366.2847
[K].IEVLYGD	1xTMTpro [K	1.805E-05	4.225E-05	1	1	2	Q7Z4H8	Q7Z4H8 [116-133	0	2682.4998
[K].LLVDVDI	1xTMTpro [K	4.583E-08	4.225E-05	1	1	1	O75533	O75533 [478-495]	1	2695.4646
[R].LIVDHNI	1xTMTpro [K	4.681E-05	4.225E-05	1	1	2	Q6P2Q9	Q6P2Q9 [999-101	0	2212.2291
[K].IIVDELK(	2xTMTpro [K	2.745E-06	4.225E-05	1	1	2	P63244	P63244 [265-280]	1	2701.6168
[R].LIVDEAII	1xTMTpro [K	7.701E-06	4.225E-05	1	1	3	P55072	P55072 [26-45]	0	2778.5381
[K].LLVCEDI	3xCarbamido	2.214E-05	4.225E-05	1	1	1	P35555	P35555 [1803-182	0	2509.1804
[R].LLVALGT	1xTMTpro [K	1.235E-06	4.225E-05	1	1	1	Q9Y263	Q9Y263 [745-764	0	2649.5682
[R].ILVAGDS	1xTMTpro [K	2.479E-05	4.225E-05	1	1	2	O95782	O95782 [154-165]	0	1843.0491
[R].LLVFGGI	1xTMTpro [K	2.538E-05	4.225E-05	1	1	2	P51610	P51610 [94-105]	0	1921.1113

[R].IEVVNFL	1xTMTpro [K	4.65E-05	4.225E-05	1	1	2	O94905	O94905 [89-105]	0	2540.4984
[R].IESSSPT	1xCarbamido	2.931E-07	4.225E-05	1	1	4	P98160	P98160 [2634-265	0	2678.399
[R].FIAVGYV	1xTMTpro [N	1.017E-05	4.225E-05	1	1	2	P04439	P04439 [46-59]	0	1934.0343
[K].IMQSSSE	1xOxidation [	1.059E-06	4.225E-05	1	1	6	P10809	P10809 [494-516]	0	3132.5183
[K].IMQSSSE	1xTMTpro [K	2.662E-07	4.225E-05	1	1	2	P10809	P10809 [494-516]	0	3116.5234
[K].IMQNTDI	1xCarbamido	4.249E-06	4.225E-05	1	2	2	Q99613	Q99613 [458-485]	1	3962.0032
[R].LEQPDP	1xTMTpro [N	5.039E-05	4.225E-05	1	1	1	Q3LXA3	Q3LXA3 [552-567	0	1896.0874
[R].LEQQVP	1xTMTpro [K	4.827E-06	4.225E-05	1	1	2	P39023	P39023 [201-224]	0	3294.7899
[R].LEQQVP	1xOxidation [	1.133E-05	4.225E-05	1	1	4	P39023	P39023 [201-224]	0	3310.7849
[R].ESTEFL(	1xTMTpro [K	3.793E-05	4.225E-05	1	1	1	Q13616	Q13616 [247-262]	0	2552.3198
[R].ESTGAQ	1xTMTpro [N	3.464E-07	4.225E-05	3	3	2	P57721; Q15	P57721 [157-176]	0	2394.189
[R].ESTGAQ	1xOxidation [	4.963E-06	4.225E-05	3	3	2	P57721; Q15	P57721 [157-176]	0	2410.1839
[K].ESTGTS	1xTMTpro [K	5.509E-05	4.225E-05	1	1	1	Q9H9Q4	Q9H9Q4 [273-288	0	2294.296
[K].ESTLEAI	1xCarbamido	2.75E-06	4.225E-05	1	1	2	Q14974	Q14974 [148-169]	0	3173.6168
[R].LEQYTS/	1xTMTpro [K	3.947E-05	4.225E-05	1	1	2	Q05682	Q05682 [679-690]	0	1948.0883
[R].LMNETT/	1xTMTpro [K	8.649E-06	4.225E-05	2	2	2	O95757; P34	O95757 [170-185]	0	2366.3285
[K].LMNESLI	1xOxidation [	1.119E-05	4.225E-05	1	1	1	P07954	P07954 [448-467]	0	2883.5604
[K].LMNESLI	1xTMTpro [K	1.038E-06	4.225E-05	1	1	1	P07954	P07954 [448-467]	0	2867.5655
[R].LMNDMT	1xTMTpro [K	3.613E-05	4.225E-05	1	1	1	Q92598	Q92598 [170-185]	0	2425.3115
[R].IMMFIGC	1xOxidation [	3.152E-05	4.225E-05	1	1	1	Q15436	Q15436 [286-307]	0	2872.5267
[K].FLDGNE	1xCarbamido	3.021E-05	4.225E-05	1	1	1	Q9Y696	Q9Y696 [178-194	0	2559.3443
[R].LMLWDT	1xOxidation [	3.168E-05	4.225E-05	1	1	1	Q9ULC3	Q9ULC3 [60-76]	0	2592.3511
[R].LMLWDT	1xTMTpro [K	2.849E-05	4.225E-05	1	1	2	Q9ULC3	Q9ULC3 [60-76]	0	2576.3562
[K].IMLNTPE	1xOxidation [	5.831E-05	4.225E-05	1	1	1	O00231	O00231 [259-274]	0	2339.3136
[K].ESSTSE(	1xTMTpro [K	4.556E-05	4.225E-05	2	2	3	Q15424; Q14	Q15424 [371-381]	0	1746.9001
[K].IMQVNG	1xTMTpro [N	1.089E-06	4.225E-05	1	1	1	O14907	O14907 [77-94]	0	2437.1746
[R].IMRPTD\	1xCarbamido	1.532E-06	4.225E-05	1	1	1	P62140	P62140 [188-220]	1	4422.199
[K].IEQLQNI	1xTMTpro [K	1.899E-05	4.225E-05	1	1	1	O00629	O00629 [462-475]	0	2381.2593
[K].ESQFLKI	1xTMTpro [K	6.523E-06	4.225E-05	1	1	2	P35221	P35221 [82-98]	1	2570.4321
[K].ESQIFPF	1xCarbamido	1.934E-05	4.225E-05	1	1	1	P53384	P53384 [241-266]	0	3162.6134
[R].INAWNS	1xTMTpro [K	2.658E-05	4.225E-05	1	1	2	O60701	O60701 [42-58]	0	2521.431
[K].ESQISAE	1xTMTpro [K	3.021E-05	4.225E-05	1	1	1	Q96CW1	Q96CW1 [363-37	0	2395.3212
[K].LNATNIE	1xTMTpro [K	5.648E-06	4.225E-05	1	1	1	P28066	P28066 [210-231]	0	3082.6639

[K].FIDVPTL	1xCarbamido	9.588E-06	4.225E-05	1	1	2	P34949	P34949 [302-320]	0	2783.4491
[R].LEQELFS	1xTMTpro [K	4.997E-05	4.225E-05	1	2	2	O00571	O00571 [146-162]	0	2491.3324
[R].INALTAA	1xCarbamido	2.422E-07	4.225E-05	1	1	1	Q99832	Q99832 [500-524]	1	3264.8117
[R].INALTAA	1xCarbamido	3.422E-06	4.225E-05	1	1	3	Q99832	Q99832 [500-521]	0	2897.6149
[R].ESQSVE	2xTMTpro [K	1.906E-05	4.225E-05	1	1	3	P49915	P49915 [279-289]	1	2160.2692
[R].ESTVFEI	1xCarbamido	8.387E-08	4.225E-05	1	1	3	P02788	P02788 [230-255]	1	3436.5658
[R].LEQEIQT	1xTMTpro [K	5.348E-05	4.225E-05	1	1	1	Q8IXS6	Q8IXS6 [89-106]	0	2670.4329
[K].LEQGEN	1xTMTpro [K	1.063E-05	4.225E-05	1	1	5	P02746	P02746 [216-229]	0	2200.2105
[R].LEQGQA	1xTMTpro [K	1.242E-05	4.225E-05	1	1	6	P12277	P12277 [367-381]	0	2265.2404
[K].IMVDMLI	1xTMTpro [K	1.994E-05	4.225E-05	1	1	2	P17655	P17655 [579-591]	0	1976.0324
[R].LEQGQA	1xOxidation [	2.819E-05	4.225E-05	1	1	4	P12277	P12277 [367-381]	0	2281.2354
[R].FLDPLPT	1xTMTpro [K	2.57E-05	4.225E-05	1	1	2	Q5T440	Q5T440 [284-309]	0	3136.7749
[K].LMTQAQ	1xTMTpro [N	4.057E-05	4.225E-05	1	1	1	P82650	P82650 [105-116]	0	1694.9066
[K].LMTIMDS	1xTMTpro [K	6.997E-06	4.225E-05	1	1	1	P61011	P61011 [375-394]	0	2823.3706
[K].LEQLFQI	1xTMTpro [K	4.05E-05	4.225E-05	1	1	2	Q15149	Q15149 [2653-26	0	1928.0985
[R].ESSLILA	1xTMTpro [K	1.021E-05	4.225E-05	1	1	2	P50570	P50570 [167-188]	0	2881.5836
[K].ESSLTEI	1xTMTpro [K	7.917E-06	4.225E-05	1	1	2	Q14738	Q14738 [354-367]	0	2093.2713
[K].ESQVQE	1xTMTpro [K	5.654E-05	4.225E-05	1	1	1	Q8NHV1	Q8NHV1 [179-19	0	2152.2357
[K].ESVDGQ	1xCarbamido	5.384E-06	4.225E-05	1	1	2	P55735	P55735 [291-305]	0	2344.2099
[K].LESALTE	1xTMTpro [N	4.956E-05	4.225E-05	1	1	2	P12270	P12270 [583-594]	0	1705.9655
[K].ESVELPI	2xTMTpro [K	8.448E-07	4.225E-05	1	1	3	P62191	P62191 [199-220]	0	3498.9011
[K].LMEQYG	1xTMTpro [K	2.392E-05	4.225E-05	1	1	1	P05164	P05164 [630-653]	1	3356.7627
[K].LMEQYG	1xTMTpro [K	2.163E-05	4.225E-05	1	1	1	P05164	P05164 [630-652]	0	3200.6616
[R].IMEIVDA	1xTMTpro [N	4.027E-06	4.225E-05	1	1	1	P08237	P08237 [185-201]	0	2218.1821
[R].IMEGPAI	1xOxidation [	2.183E-05	4.225E-05	1	1	1	P11177	P11177 [309-324]	0	2068.0857
[R].ETADTD	1xTMTpro [K	1.584E-06	4.225E-05	1	1	6	P12814	P12814 [819-834]	0	2338.1728
[R].ETADTD	1xOxidation [	2.711E-05	4.225E-05	1	1	2	P12814	P12814 [819-834]	0	2354.1677
[R].LESIISG	1xCarbamido	1.304E-06	4.225E-05	2	2	2	P35221; P26	P35221 [309-326]	0	2186.1116
[K].LMEADIA	1xTMTpro [K	2.194E-05	4.225E-05	1	1	3	P11277	P11277 [572-583]	0	1912.0705
[K].LMDSMV	1xTMTpro [K	4.642E-05	4.225E-05	1	1	2	P40123	P40123 [52-62]	0	1892.0517
[K].ETAENYI	1xTMTpro [K	5.509E-05	4.225E-05	1	1	3	P38646	P38646 [176-187]	0	1942.0526
[R].IMEVIDA	1xTMTpro [N	3.213E-06	4.225E-05	1	1	1	P17858	P17858 [185-201]	0	2218.1821
[K].IMDQAIT	1xTMTpro [N	1.587E-06	4.225E-05	1	1	1	P05166	P05166 [144-165]	0	2459.3247

[R].IMDPNIV	1xTMTpro [N	1.332E-05	4.225E-05	1	1	1	P06576	P06576 [407-422]	0	2120.0766
[K].ETAIELG	2xTMTpro [K	8.232E-07	4.225E-05	1	1	1	P07954	P07954 [484-504]	0	3379.8606
[R].IESLNEE	1xTMTpro [K	5.09E-05	4.225E-05	1	1	2	P17661	P17661 [229-240]	0	2014.1716
[K].ETAPGG	1xTMTpro [N	4.071E-05	4.225E-05	1	1	1	Q8N392	Q8N392 [183-195	0	1636.8461
[K].FLCADY/	1xCarbamido	1.93E-05	4.225E-05	1	1	1	Q9UKV3	Q9UKV3 [1081-10	0	2349.0777
[R].LESLSY(	1xTMTpro [K	8.554E-06	4.225E-05	1	1	2	Q03252	Q03252 [314-326]	0	2074.204
[K].LMDLDVI	1xCarbamido	4.369E-06	4.225E-05	1	1	3	P12004	P12004 [118-138]	0	3089.603
[R].ETAVDG	1xTMTpro [K	9.893E-06	4.225E-05	1	1	2	P19823	P19823 [266-280]	0	2258.2775
[R].ETAVPG	1xTMTpro [K	5.535E-06	4.225E-05	1	1	2	Q12888	Q12888 [1413-14	0	2872.5435
[K].LMDLDVI	1xCarbamido	6.756E-06	4.225E-05	1	1	3	P12004	P12004 [118-138]	0	3073.6081
[R].IMDPNIV	1xOxidation [	4.61E-05	4.225E-05	1	1	1	P06576	P06576 [407-422]	0	2136.0715
[K].ETCAWF	1xCarbamido	2.994E-05	4.225E-05	1	1	2	Q13630	Q13630 [307-320]	0	2094.951
[R].IMEVIDA	1xOxidation [	3.016E-05	4.225E-05	1	1	1	P17858	P17858 [185-201]	0	2234.177
[R].FLCTGG	2xCarbamido	1.015E-05	4.225E-05	1	1	2	P00751	P00751 [680-696]	0	2219.0544
[K].LESALTE	1xTMTpro [K	7.154E-06	4.225E-05	1	1	1	P12270	P12270 [583-595]	1	2138.2676
[K].IMIMTDQ	1xOxidation [	9.964E-07	4.225E-05	2	2	2	P11388; Q02	P11388 [536-550]	0	2356.2132
[K].LMLLLEV	1xOxidation [	5.491E-05	4.225E-05	3	4	4	P12814; P35	P12814 [65-76]; F	0	1692.9889
[K].IESDVQE	1xTMTpro [K	1.521E-05	4.225E-05	1	1	2	P20042	P20042 [103-124]	0	3095.5586
[K].LMLGWE	1xTMTpro [K	5.175E-05	4.225E-05	1	1	1	Q8NBZ7	Q8NBZ7 [370-386	0	2532.4391
[K].ESVNAA	1xTMTpro [K	5.563E-05	4.225E-05	1	1	3	P30084	P30084 [242-257]	0	2322.2143
[K].LESDVS/	1xCarbamido	2.194E-05	4.225E-05	1	1	19	P02675	P02675 [212-224]	0	1891.8849
[R].IMLFTNE	1xOxidation [	9.09E-06	4.225E-05	1	1	2	P12956	P12956 [166-182]	0	2527.2743
[R].ESVNYL'	1xTMTpro [K	1.384E-05	4.225E-05	1	1	1	Q9BS92	Q9BS92 [225-247	0	3186.7728
[R].ESVNYL'	1xTMTpro [K	6.304E-07	4.225E-05	1	1	2	Q9UFN0	Q9UFN0 [225-247	0	3216.7834
[R].ESYVETI	1xTMTpro [K	3.526E-05	4.225E-05	1	1	3	Q00610	Q00610 [1166-11	0	2221.2612
[R].IMLFTG(	1xTMTpro [K	4.669E-06	4.225E-05	1	1	2	Q15437	Q15437 [288-309]	0	2852.5546
[R].LESEETI	1xTMTpro [K	2.69E-06	4.225E-05	1	1	2	P01024	P01024 [36-65]	0	3858.9715
[K].IMLEDGI	1xOxidation [	3.902E-06	4.225E-05	1	1	2	Q14195	Q14195 [452-467]	0	2031.0612
[K].IMLEDGI	1xTMTpro [N	1.065E-06	4.225E-05	1	1	2	Q14195	Q14195 [452-467]	0	2015.0663
[K].LMLDTW	1xTMTpro [K	4.874E-05	4.225E-05	1	1	1	Q93034	Q93034 [138-152]	0	2419.3187
[R].LESEETI	1xOxidation [	3.042E-07	4.225E-05	1	1	1	P01024	P01024 [36-65]	0	3874.9665
[K].LMHQAA	1xTMTpro [N	7.533E-06	4.225E-05	1	1	1	Q16881	Q16881 [219-234]	0	2056.1293
[R].LESEETI	2xTMTpro [K	6.131E-08	4.225E-05	1	1	2	P01024	P01024 [36-66]	1	4291.2737

[K].IMGNLNI	1xTMTpro [K	1.874E-06	4.225E-05	1	1	2	O95571	O95571 [225-246]	0	3075.7091
[K].IESEGLL	1xTMTpro [K	4.618E-05	4.225E-05	1	1	2	Q92552	Q92552 [347-361]	0	2239.3405
[R].LESGMQ	1xTMTpro [K	1.253E-05	4.225E-05	1	1	2	P05787	P05787 [402-414]	0	2084.1124
[K].LESEEDY	3xTMTpro [K	7.274E-06	4.225E-05	1	1	1	Q15746	Q15746 [1786-18	0	4423.4178
[R].ESQDTS	1xTMTpro [N	3.709E-05	4.225E-05	1	1	2	Q06210	Q06210 [164-176]	0	1816.9248
[R].FKEPPAY	1xCarbamido	1.966E-05	4.225E-05	1	1	1	Q08209	Q08209 [218-243]	1	3968.0067
[K].ILTFDQL	1xTMTpro [K	2.187E-05	4.225E-05	1	1	3	Q07020	Q07020 [120-132]	0	2069.2138
[K].FHPDTD	1xTMTpro [K	2.995E-06	4.225E-05	1	1	2	Q02818	Q02818 [422-439]	0	2514.312
[R].LFFSQQ	1xTMTpro [N	2.911E-05	4.225E-05	1	1	4	O95479	O95479 [520-547]	0	3414.784
[K].ILPVFDE	1xTMTpro [K	9.116E-08	4.225E-05	1	1	2	Q9NYL9	Q9NYL9 [173-192	1	2902.617
[K].ILPVFDE	1xTMTpro [K	2.916E-05	4.225E-05	1	1	1	Q9NYL9	Q9NYL9 [173-191	0	2746.5159
[K].IIPTLEEC	1xCarbamido	1.156E-06	4.225E-05	1	1	3	P61978	P61978 [104-139]	0	4663.4461
[K].ILPTLEAY	1xTMTpro [K	2.635E-05	4.225E-05	1	1	1	Q12905	Q12905 [128-141]	0	2018.2505
[R].LLPSFVS	1xTMTpro [N	8.849E-07	4.225E-05	1	1	3	P12111	P12111 [2815-283	0	2459.3141
[R].EVDDLG	1xTMTpro [K	2.148E-05	4.225E-05	1	1	1	O43143	O43143 [372-384]	0	1994.0938
[R].IIPPGSG	1xTMTpro [N	2.745E-06	4.225E-05	1	1	1	P21399	P21399 [169-187]	0	2394.3828
[R].ILPPGLL	1xTMTpro [K	9.606E-06	4.225E-05	1	1	2	O75165	O75165 [655-677]	1	3119.7484
[K].IFFYDSE	1xTMTpro [N	1.47E-05	4.225E-05	1	1	2	P27169	P27169 [291-306]	0	2188.1246
[R].LLPPAQI	1xTMTpro [N	2.239E-06	4.225E-05	1	1	3	Q8WZA9	Q8WZA9 [178-19	0	2499.3778
[K].ILPNKVD	2xTMTpro [K	1.815E-05	4.225E-05	1	1	2	P62195	P62195 [126-142]	1	2838.7017
[K].EVDEQM	1xTMTpro [K	2.222E-05	4.225E-05	3	4	5	Q13885; P07	Q13885 [325-336]	0	2055.1036
[R].LLPIDGA	1xTMTpro [K	2.797E-06	4.225E-05	1	1	1	O60502	O60502 [692-713]	0	2989.6894
[R].LLPHANI	1xTMTpro [K	2.238E-05	4.225E-05	1	1	1	P06727	P06727 [113-123]	0	1844.0886
[R].LFGAVEI	1xTMTpro [N	2.25E-05	4.225E-05	1	1	2	Q9BU23	Q9BU23 [439-456	0	2339.2831
[K].FHMVDG	1xOxidation [	2.69E-06	4.225E-05	1	1	2	O95433	O95433 [250-268]	0	2745.4049
[K].LIPFSPA	1xTMTpro [N	5.894E-06	4.225E-05	1	1	1	O95425	O95425 [868-895]	0	3168.6934
[K].ILPEYLS	1xTMTpro [K	1.622E-05	4.225E-05	1	1	3	O95831	O95831 [343-355]	0	2232.223
[R].LLPETPS	1xTMTpro [N	1.235E-05	4.225E-05	1	1	3	Q99715	Q99715 [2585-26	0	2403.2879
[R].LLPVLLS	1xTMTpro [N	2.064E-05	4.225E-05	1	1	1	Q8TEX9	Q8TEX9 [900-916	0	2155.2294
[K].EVCPVLI	2xCarbamido	2.838E-06	4.225E-05	1	1	1	Q9NY27	Q9NY27 [20-34]	0	2423.3071
[R].LLQAGF	2xTMTpro [K	2.75E-06	4.225E-05	1	1	2	Q9ULA0	Q9ULA0 [52-65]	1	2505.4745
[K].LLQALAC	1xTMTpro [N	1.454E-05	4.225E-05	1	1	1	Q9BQG0	Q9BQG0 [162-17	0	2354.29
[R].ILQLCMC	1xCarbamido	7.287E-06	4.225E-05	1	1	1	P15311	P15311 [280-293]	0	2082.0618

[R].LLQFYAI	1xCarbamido	5.691E-05	4.225E-05	1	1	3	O00159	O00159 [153-166]	0	1999.0278
[K].LLQFQDI	1xCarbamido	1.117E-05	4.225E-05	1	1	1	Q96CS3	Q96CS3 [17-33]	0	2358.1753
[K].LIQEQHI	1xTMTpro [K	5.822E-05	4.225E-05	1	1	4	Q13813	Q13813 [215-227]	0	2214.2626
[K].IIQEQDA	1xTMTpro [N	3.102E-06	4.225E-05	1	1	1	Q9UNK0	Q9UNK0 [147-164	0	2233.2359
[R].EVAGHT	1xTMTpro [N	3.551E-05	4.225E-05	1	1	3	P08727	P08727 [281-293]	0	1789.9186
[K].LFEIDPT	1xTMTpro [K	3.225E-05	4.225E-05	1	1	1	O60245	O60245 [791-806]	0	2269.3299
[R].LLQEGQ	1xCarbamido	5.821E-07	4.225E-05	1	1	3	P00751	P00751 [51-74]	0	3119.5831
[R].EVAKEFI	1xTMTpro [K	5.5E-05	4.225E-05	1	1	1	Q96D15	Q96D15 [63-79]	1	2585.3703
[K].EVAKPSI	2xTMTpro [K	1.505E-05	4.225E-05	1	1	2	Q53FA7	Q53FA7 [19-33]	0	2465.4795
[R].EVDLKD	2xTMTpro [K	5.869E-05	4.225E-05	1	1	4	P02671	P02671 [191-202]	1	2422.3282
[K].LFEMVL(	1xTMTpro [K	4.618E-05	4.225E-05	1	1	4	Q16891	Q16891 [85-101]	0	2467.4278
[R].ILQEAG/	1xTMTpro [K	5.244E-05	4.225E-05	1	1	2	Q96BW5	Q96BW5 [215-22!	0	1753.0351
[R].LLQDSVI	1xTMTpro [K	2.19E-06	4.225E-05	1	1	77	P08670	P08670 [79-97]	0	2734.4795
[K].LLQDSG.	1xCarbamido	5.305E-07	4.225E-05	1	1	1	Q02252	Q02252 [224-255]	0	4133.137
[K].LIQDQQI	1xTMTpro [K	1.584E-05	4.225E-05	1	1	2	Q92878	Q92878 [852-864]	0	2229.2847
[R].LLQDHP	2xTMTpro [K	3.946E-06	4.225E-05	1	1	2	P53396	P53396 [43-64]	0	3510.1028
[K].LLQDEFI	1xCarbamido	1.24E-06	4.225E-05	1	1	6	P02790	P02790 [131-151]	0	2668.3724
[R].LFENQL	1xTMTpro [N	7.124E-08	4.225E-05	1	1	1	Q9HDC9	Q9HDC9 [94-120]	0	3178.6162
[R].EVATNS	1xTMTpro [K	1.451E-05	4.225E-05	2	2	2	P02533; Q0	P02533 [316-328]	0	1970.105
[K].EVAYLGI	1xCarbamido	1.47E-05	4.225E-05	1	1	1	Q8NFW8	Q8NFW8 [367-38	1	2619.358
[R].LLQAME	1xTMTpro [N	3.879E-05	4.225E-05	1	1	2	Q16401	Q16401 [71-82]	0	1667.9586
[K].LIQEAPK	1xCarbamido	3.334E-05	4.225E-05	1	1	1	P20851	P20851 [193-204]	0	2354.357
[R].ILPDTPC	1xTMTpro [K	1.459E-05	4.225E-05	1	1	1	Q05707	Q05707 [1294-13	0	2732.5519
[R].LIPDTLY	1xTMTpro [N	6.433E-07	4.225E-05	1	1	2	Q99715	Q99715 [1994-20	0	3250.6915
[K].LFGEVTS	1xTMTpro [K	6.959E-06	4.225E-05	1	1	1	P00736	P00736 [25-55]	0	4094.2134
[K].LLNDEDI	1xTMTpro [K	3.933E-05	4.225E-05	1	1	2	P14923	P14923 [150-161]	0	1950.1403
[R].LLNCSDI	1xCarbamido	5.384E-05	4.225E-05	1	1	2	Q9H267	Q9H267 [543-556	0	2271.1645
[R].LINANMI	1xTMTpro [N	3.538E-06	4.225E-05	1	1	1	O00487	O00487 [162-175]	0	1899.0264
[R].FHLATAI	1xTMTpro [N	1.882E-05	4.225E-05	1	1	1	Q14197	Q14197 [103-116]	0	1944.0663
[R].EVENLIL	1xTMTpro [K	9.874E-06	4.225E-05	1	1	4	O60271	O60271 [409-424]	0	2494.426
[R].EVENVA	1xTMTpro [K	4.673E-05	4.225E-05	1	1	2	P17540	P17540 [187-200]	0	2094.2302
[K].EVEPEP	1xTMTpro [K	1.428E-06	4.225E-05	1	1	4	P20042	P20042 [43-62]	1	2954.4246
[K].LIMVQH\	1xCarbamido	3.135E-05	4.225E-05	1	1	3	O00584	O00584 [37-50]	0	2378.2856

[R].LIMVLAG	1xTMTpro [K	4.457E-05	4.225E-05	1	1	2	Q9UGP8	Q9UGP8 [246-262	0	2504.3715
[K].EVESPYI	1xTMTpro [N	3.447E-05	4.225E-05	1	1	1	Q6Y7W6	Q6Y7W6 [1163-1	0	1939.9721
[K].EVEGDD	1xTMTpro [K	3.594E-05	4.225E-05	1	1	1	Q1KMD3	Q1KMD3 [578-59	0	2429.2435
[K].LLMMAG	1xCarbamido	3.619E-05	4.225E-05	1	1	2	P15880	P15880 [213-227]	0	2036.9774
[R].EVEVEV	1xTMTpro [K	5.803E-05	4.225E-05	1	1	2	Q7KZF4	Q7KZF4 [593-603	0	1902.0022
[R].EVEVVA	1xTMTpro [N	2.547E-05	4.225E-05	1	1	1	Q63ZY3	Q63ZY3 [338-353	0	1860.0146
[R].ILMLGLD	1xTMTpro [K	4.412E-05	4.225E-05	1	1	3	P62330	P62330 [16-26]	0	1710.0479
[R].EVFEFRI	1xTMTpro [N	3.301E-07	4.225E-05	1	1	1	Q8WU90	Q8WU90 [293-31	0	2844.3131
[R].ILMAAPC	1xOxidation [	2.849E-05	4.225E-05	1	1	1	Q9H9B4	Q9H9B4 [234-253	0	2782.5565
[R].ILMAAPC	1xTMTpro [K	2.497E-05	4.225E-05	1	1	1	Q9H9B4	Q9H9B4 [234-253	0	2766.5616
[R].EVFGSG	2xCarbamido	6.059E-06	4.225E-05	1	1	1	O15382	O15382 [334-349]	0	2108.0336
[R].FHDIISD	1xTMTpro [K	5.261E-05	4.225E-05	1	1	3	P13674	P13674 [340-353]	0	2237.2673
[R].LLIYAVLI	1xTMTpro [K	1.148E-05	4.225E-05	1	1	4	P01023	P01023 [540-557]	0	2453.4511
[K].IFLAQKP	2xTMTpro [K	3.097E-05	4.225E-05	1	1	4	O00203	O00203 [597-612]	0	2711.668
[K].LLMMAG	1xCarbamido	1.555E-05	4.225E-05	1	1	2	P15880	P15880 [213-227]	0	2020.9825
[K].EVAENQ	1xTMTpro [K	5.028E-08	4.225E-05	1	1	1	O43491	O43491 [29-60]	1	4061.978
[K].EVEERP	1xTMTpro [K	3.113E-05	4.225E-05	1	1	3	O15143	O15143 [175-188]	0	2191.2003
[K].EVEELIL	1xTMTpro [K	5.945E-05	4.225E-05	1	1	2	Q9BZZ5	Q9BZZ5 [170-180	0	1898.0978
[R].EVDMVD	1xTMTpro [K	5.244E-05	4.225E-05	1	1	1	Q9BV86	Q9BV86 [86-101]	0	2460.3188
[K].LLPDIYG	1xTMTpro [K	3.793E-05	4.225E-05	1	1	1	P08473	P08473 [160-177]	0	2767.4951
[R].LLPDITLI	1xTMTpro [N	4.05E-06	4.225E-05	1	1	2	O15160	O15160 [235-255]	0	2598.4197
[R].LLPASFF	1xCarbamido	7.519E-06	4.225E-05	1	1	1	O14841	O14841 [401-424]	0	2888.4936
[R].LLPALAS	1xTMTpro [N	6.09E-05	4.225E-05	1	1	2	Q03154	Q03154 [394-408]	0	1755.0223
[K].LLNQEE	1xTMTpro [N	2.155E-07	4.225E-05	1	1	1	P17252	P17252 [277-302]	0	3341.5803
[R].LFGGNF	1xTMTpro [N	7.478E-06	4.225E-05	1	1	2	O14773	O14773 [246-259]	0	1778.9621
[R].LLNNWD	1xCarbamido	3.939E-06	4.225E-05	1	1	3	P02753	P02753 [81-103]	0	3220.5786
[K].ILNNSGL	1xTMTpro [K	1.067E-05	4.225E-05	1	1	1	Q96I99	Q96I99 [404-423]	0	2663.5111
[K].EVDVGL	1xTMTpro [N	7.22E-06	4.225E-05	1	1	2	Q13011	Q13011 [197-211]	0	1847.0194
[K].EVEEISL	1xTMTpro [K	1.008E-05	4.225E-05	1	1	6	O00567	O00567 [21-41]	0	2961.664
[R].ILNNGH	1xTMTpro [K	2.47E-06	4.225E-05	1	1	4	P00918	P00918 [59-76]	0	2671.3608
[R].EVDYLE	1xCarbamido	5.597E-06	4.225E-05	1	1	2	Q9NRN5	Q9NRN5 [93-112]	0	3004.5105
[R].IINEPTA	1xTMTpro [N	1.852E-05	4.225E-05	2	2	2	P17066; P0D	P17066 [174-189]	0	1992.1085
[R].IINEPTA	1xTMTpro [K	4.102E-06	4.225E-05	1	1	2	P11021	P11021 [198-214]	1	2424.4106

[R].IINEPTA,	2xTMTpro [K	5.445E-08	4.225E-05	2	2	3	P54652; P11	P54652 [173-189]	1	2700.6116
[K].EVEAVIF	1xCarbamid	1.639E-05	4.225E-05	1	1	1	P40939	P40939 [461-489]	0	3676.9752
[R].IINEPTA,	1xTMTpro [K	4.21E-06	4.225E-05	3	4	3	P54652; P11	P54652 [173-188]	0	2268.3095
[R].ILNEDGS	2xTMTpro [K	5.14E-06	4.225E-05	1	1	2	Q9UMX5	Q9UMX5 [147-17	1	3972.0483
[K].LFGIESS	1xTMTpro [K	6.032E-05	4.225E-05	1	1	2	Q8N766	Q8N766 [545-558	0	2146.2404
[R].EVEEEL,	1xTMTpro [N	7.816E-07	4.225E-05	1	2	1	Q9UPN3	Q9UPN3 [5580-5	0	3049.5034
[K].LLNDED	1xTMTpro [K	2.214E-05	4.225E-05	1	1	1	P35222	P35222 [159-170]	0	1994.1414
[R].LLNLDG	1xCarbamid	1.378E-06	4.225E-05	1	1	2	P02753	P02753 [140-157]	0	2369.1767
[R].LLTFMGI	1xTMTpro [K	1.991E-05	4.225E-05	1	1	1	Q7L2H7	Q7L2H7 [279-290	0	1962.1048
[K].EVAELE,	2xCarbamid	4.17E-05	4.225E-05	1	1	1	Q969M7	Q969M7 [40-53]	0	2242.1703
[K].LFECDRI	1xCarbamid	2.136E-05	4.225E-05	1	1	1	P42704	P42704 [949-963]	1	2616.3446
[R].LLSRPQI	1xTMTpro [N	3.105E-07	4.225E-05	1	1	2	Q9NVI7	Q9NVI7 [355-375	0	2554.4524
[K].LLSQQEI	1xTMTpro [K	4.48E-05	4.225E-05	1	1	1	Q8IWJ2	Q8IWJ2 [547-562	0	2462.4362
[R].IFAQYM	1xTMTpro [N	8.891E-06	4.225E-05	1	1	1	Q9NTI5	Q9NTI5 [458-473]	0	2253.1657
[K].ETPFLSI	1xTMTpro [K	1.845E-05	4.225E-05	1	1	5	P21589	P21589 [180-206]	0	3611.8976
[K].ETPFLSI	2xTMTpro [K	6.374E-07	4.225E-05	1	1	3	P21589	P21589 [180-208]	1	4157.2838
[K].IFASQAS	1xTMTpro [K	3.27E-07	4.225E-05	1	1	6	Q05707	Q05707 [899-916	0	2438.3245
[K].IFASQAS	1xOxidation	7.247E-06	4.225E-05	1	1	2	Q05707	Q05707 [899-916	0	2454.3194
[R].ETPLKPI	1xCarbamid	1.948E-05	4.225E-05	1	1	2	O60437	O60437 [396-419	0	3306.7899
[R].ETPLPID	1xTMTpro [K	3.78E-05	4.225E-05	1	2	1	P21127	P21127 [725-741]	0	2535.3813
[R].ETPPDA	1xTMTpro [N	1.359E-05	4.225E-05	1	1	2	Q8N2K0	Q8N2K0 [263-27	0	2217.2086
[K].IISNASC	2xCarbamid	1.558E-05	4.225E-05	1	1	8	P04406	P04406 [146-162]	0	2442.334
[R].LISLTDE	1xTMTpro [K	8.065E-06	4.225E-05	1	1	98	P14625	P14625 [117-135]	0	2654.4744
[K].ILSIGGG	1xTMTpro [K	2.958E-05	4.225E-05	1	1	1	P50135	P50135 [56-73]	0	2392.4307
[K].ETQFAQ	1xTMTpro [K	5.937E-06	4.225E-05	1	1	3	Q9NTI5	Q9NTI5 [741-753]	0	2196.2309
[K].FKDDVM	1xCarbamid	1.333E-06	4.225E-05	1	1	2	Q9HD45	Q9HD45 [98-116]	2	3533.9012
[K].ETQPPD	1xCarbamid	6.487E-06	4.225E-05	1	1	3	Q9UBG0	Q9UBG0 [958-98	0	3393.7645
[K].IFCCHG	2xCarbamid	1.091E-06	4.225E-05	2	3	2	P62136; P62	P62136 [169-187]	0	2552.2379
[R].IISLDPSI	2xCarbamid	2.3E-05	4.225E-05	1	1	2	Q15393	Q15393 [616-650	0	4436.2717
[R].ILSKPIE	1xTMTpro [K	6.003E-05	4.225E-05	1	1	1	Q7L014	Q7L014 [574-586	0	2004.2461
[K].LLSHCLI	1xCarbamid	7.787E-07	4.225E-05	1	1	4	P69905	P69905 [101-128]	0	3633.0482
[R].IFCGDL	1xCarbamid	3.712E-06	4.225E-05	1	1	2	Q9BTD8	Q9BTD8 [383-39	0	2225.1192
[R].LLSSAFI	1xTMTpro [K	3.544E-06	4.225E-05	1	1	1	O00410	O00410 [69-90]	0	2972.6476

[K].ILSSDDY	1xTMTpro [K	1.454E-05	4.225E-05	1	1	1	Q01082	Q01082 [660-675]	1	2408.2987
[K].ETPAATI	1xTMTpro [K	8.761E-06	4.225E-05	1	1	3	P80723	P80723 [185-198]	0	1995.089
[K].IISSDRD	1xTMTpro [K	2.568E-06	4.225E-05	1	1	2	P12956	P12956 [75-92]	1	2634.4998
[R].LLTETED	1xTMTpro [K	1.845E-05	4.225E-05	1	1	1	Q92598	Q92598 [668-685]	0	2777.4013
[K].IEWLESH	1xTMTpro [K	2.26E-06	4.225E-05	1	1	7	P11021	P11021 [602-617]	0	2583.3223
[K].IEWLESH	2xTMTpro [K	1.059E-05	4.225E-05	1	1	3	P11021	P11021 [602-619]	1	3086.6615
[R].ETLPAEC	1xTMTpro [K	6.032E-05	4.225E-05	1	1	1	P25786	P25786 [197-208]	0	1954.0988
[K].ETLPDSC	1xTMTpro [K	2.503E-06	4.225E-05	1	1	2	P18583	P18583 [1714-174	0	3561.9296
[K].ETIPLQE	1xTMTpro [N	8.681E-06	4.225E-05	1	1	2	P50897	P50897 [254-268]	0	2098.0987
[R].ILTEAEID	1xTMTpro [N	1.575E-06	4.225E-05	1	1	2	P60900	P60900 [229-246]	1	2283.2515
[R].ILTDYGF	1xTMTpro [N	2.23E-05	4.225E-05	1	1	2	O75489	O75489 [187-199]	0	1855.9662
[R].ETLVQD	1xTMTpro [N	1.42E-06	4.225E-05	1	1	3	Q07954	Q07954 [4037-405	0	2902.4655
[R].ETLVYLT	1xTMTpro [N	6.844E-06	4.225E-05	1	1	1	O14980	O14980 [459-474]	0	2271.1828
[R].IFDANTK	2xTMTpro [K	4.383E-07	4.225E-05	1	1	4	O60701	O60701 [143-170]	0	3970.247
[K].IITDVIGN	1xTMTpro [N	4.48E-05	4.225E-05	1	1	1	Q92925	Q92925 [481-493]	0	1788.9663
[K].FKDVAG	1xCarbamido	4.016E-05	4.225E-05	1	1	1	Q9Y4W6	Q9Y4W6 [307-31	1	2166.2045
[K].ILTATVD	1xTMTpro [N	3.243E-06	4.225E-05	1	1	1	Q04695	Q04695 [145-163]	0	2372.3468
[K].IEYDDFV	1xCarbamido	3.27E-05	4.225E-05	1	1	3	O43920	O43920 [58-69]	0	1875.9482
[K].LLSYVDA	1xTMTpro [N	4.801E-06	4.225E-05	1	1	2	P20908	P20908 [1712-173	0	2612.4077
[K].LISWYDN	1xTMTpro [N	2.989E-05	4.225E-05	1	1	7	P04406	P04406 [310-323]	0	2068.0095
[K].LISVDTE	1xTMTpro [N	1.793E-06	4.225E-05	1	1	1	P00450	P00450 [44-62]	0	2475.2799
[K].LISSVDP	1xTMTpro [K	5.728E-05	4.225E-05	1	1	3	Q9BVG4	Q9BVG4 [53-63]	0	1855.1185
[R].ETNLDSI	1xTMTpro [K	1.195E-06	4.225E-05	1	1	20	P08670	P08670 [425-439]	0	2277.2582
[R].ETNLDSI	1xTMTpro [K	4.029E-05	4.225E-05	1	1	2	P08670	P08670 [425-440]	1	2433.3593
[R].IFANTPD	1xCarbamido	3.689E-05	4.225E-05	1	1	1	P08237	P08237 [700-714]	0	1941.9846
[K].ETMLSD	1xTMTpro [N	4.936E-06	4.225E-05	1	1	1	P15291	P15291 [364-383]	0	2586.3404
[K].LLSGGSY	1xTMTpro [K	3.594E-05	4.225E-05	1	1	2	O95747	O95747 [94-105]	0	1823.1498
[R].LLSGEDY	1xTMTpro [N	1.356E-05	4.225E-05	1	1	2	P11277	P11277 [765-779]	0	1850.9415
[R].LISETTS	1xCarbamido	5.285E-06	4.225E-05	1	1	1	Q06136	Q06136 [237-252]	0	2702.5579
[K].IFDLDGD	1xCarbamido	2.091E-06	4.225E-05	1	1	3	Q8IYU8	Q8IYU8 [373-392]	0	2944.5258
[K].LLQTDDI	1xTMTpro [K	9.362E-06	4.225E-05	1	1	1	P55060	P55060 [252-268]	0	2537.4206
[R].LLQSNPY	1xTMTpro [K	4.721E-05	4.225E-05	1	1	1	O00159	O00159 [174-188]	0	2209.2836
[K].ETVQTTI	1xTMTpro [K	4.495E-05	4.225E-05	1	1	2	P18206	P18206 [60-71]	0	2013.136

[K].ETVQTTI	1xTMTpro [K	2.735E-06	4.225E-05	1	1	1	P18206	P18206 [60-72]	1	2169.2371
[R].LFDPINL	1xTMTpro [N	4.113E-05	4.225E-05	1	1	3	Q9UP83	Q9UP83 [493-506	0	1846.0546
[R].LIQQVA(	1xCarbamid(	3.311E-05	4.225E-05	1	1	1	Q9UG63	Q9UG63 [575-588	0	2352.3241
[K].ETVSQR	1xTMTpro [N	6.869E-06	4.225E-05	1	1	1	Q14203	Q14203 [1209-12	0	2986.5594
[K].ETVTILP	1xTMTpro [N	1.564E-05	4.225E-05	1	1	1	P55809	P55809 [369-390]	0	2709.3765
[K].LFDSDPI	1xTMTpro [N	2.259E-05	4.225E-05	1	1	1	P10909	P10909 [409-425]	0	2178.1977
[K].LLQVASI	1xTMTpro [N	2.497E-05	4.225E-05	1	1	2	O75694	O75694 [1052-10	0	2023.2024
[R].ETVVEVI	1xTMTpro [K	6.536E-06	4.225E-05	1	1	4	P55072	P55072 [466-486]	0	2950.5905
[K].ETVYCLI	1xCarbamid(	1.154E-07	4.225E-05	1	1	3	P13010	P13010 [292-315]	1	3509.7602
[R].IFDSEEII	1xTMTpro [K	1.776E-05	4.225E-05	1	1	2	O43776	O43776 [479-490]	0	1993.1138
[K].LIQNNHY	1xTMTpro [N	8.99E-06	4.225E-05	1	1	1	Q13813	Q13813 [533-547]	0	2079.0612
[K].LIQNLDA	1xTMTpro [N	3.734E-05	4.225E-05	1	1	3	Q96FQ6	Q96FQ6 [62-73]	0	1669.8941
[R].ETYGEM	2xCarbamid(	2.435E-05	4.225E-05	1	1	29	P02768	P02768 [106-117]	0	2042.9477
[R].LLQMDL	1xTMTpro [K	2.295E-05	4.225E-05	1	1	1	P80365	P80365 [134-147]	0	2195.2713
[R].ETYNAL	1xTMTpro [N	5.71E-05	4.225E-05	1	1	2	P20338	P20338 [99-112]	0	1972.0095
[R].EVAAFA(	1xTMTpro [N	2.778E-07	4.225E-05	1	1	3	P25705	P25705 [442-463]	0	2642.3745
[K].LFEAEE(	1xTMTpro [K	4.956E-05	4.225E-05	1	1	2	Q9H4M9	Q9H4M9 [270-28(	0	1977.0825
[K].FHTEALT	1xTMTpro [K	4.939E-05	4.225E-05	1	1	2	P62495	P62495 [131-145]	0	2256.2367
[R].ETVVEVI	1xTMTpro [K	2.417E-08	4.225E-05	1	1	5	P55072	P55072 [466-487]	1	3106.6916
[R].EVAEAA	1xTMTpro [K	5.775E-06	4.225E-05	1	1	3	Q99536	Q99536 [6-22]	0	2264.1902
[R].ETVGDL	1xTMTpro [N	4.08E-06	4.225E-05	1	1	1	Q14203	Q14203 [459-479]	0	2712.2412
[R].LLQVVEI	1xTMTpro [N	1.15E-05	4.225E-05	1	1	1	Q9BQ67	Q9BQ67 [183-198	0	2101.234
[K].FHYLPFI	1xCarbamid(	5.563E-05	4.225E-05	1	1	1	P01903	P01903 [173-189]	0	2463.1974
[K].LLSDTV/	1xTMTpro [K	6.523E-06	4.225E-05	1	1	2	Q9UPN3	Q9UPN3 [4025-4(	0	2794.5694
[K].LLSDTS\	1xTMTpro [K	4.345E-05	4.225E-05	1	1	1	Q7Z3J2	Q7Z3J2 [217-230]	0	2206.2615
[R].ETSSDV.	1xTMTpro [N	3.522E-07	4.225E-05	1	1	1	O00273	O00273 [230-246]	0	2088.162
[K].ETSSTGI	1xTMTpro [N	6.381E-06	4.225E-05	1	1	1	Q9H6R4	Q9H6R4 [650-664	0	1823.967
[K].LLSDPN	1xTMTpro [K	2.083E-05	4.225E-05	1	1	1	Q9BTV4	Q9BTV4 [100-114	0	2231.3044
[R].ETSVTPS	1xTMTpro [N	2.075E-05	4.225E-05	1	1	1	Q9H8Y8	Q9H8Y8 [81-101]	0	2475.3527
[K].ETSYEE/	1xTMTpro [N	5.252E-05	4.225E-05	1	1	2	Q9P2E9	Q9P2E9 [35-46]	0	1714.8567
[R].LISDFED	1xTMTpro [N	1.213E-05	4.225E-05	1	1	1	Q9UDY2	Q9UDY2 [918-95	0	3988.863
[R].LLSDAT\	2xTMTpro [K	9.327E-06	4.225E-05	1	1	1	Q14320	Q14320 [290-305]	1	2612.4712
[R].LLQVTP/	1xCarbamid(	5.879E-05	4.225E-05	1	1	2	P98160	P98160 [2490-25(	0	2012.0442

[K].ILSCGEV	1xCarbamido	5.34E-05	4.225E-05	1	1	1	Q9H2U2	Q9H2U2 [177-187	0	1863.1018
[R].LFDFQG	1xTMTpro [N	1.061E-05	4.225E-05	1	1	1	P45954	P45954 [326-347]	0	2783.4912
[K].LLSALCI	1xCarbamido	2.916E-05	4.225E-05	1	1	1	O60610	O60610 [262-278]	0	2303.2059
[K].LLSAEFL	1xTMTpro [N	3.689E-05	4.225E-05	1	1	1	Q9Y376	Q9Y376 [197-209	0	1925.0088
[K].ETTFNSI	1xCarbamido	7.45E-06	4.225E-05	1	1	2	P01023	P01023 [913-934]	0	3005.5633
[K].IISAASE	1xTMTpro [K	3.291E-06	4.225E-05	1	1	1	P14868	P14868 [223-241]	0	2569.4158
[K].ETTVGA	1xTMTpro [K	2.57E-05	4.225E-05	1	1	1	Q9Y5L0	Q9Y5L0 [882-893	0	1851.0831
[R].ETTVSQ	1xTMTpro [N	7.561E-06	4.225E-05	1	1	1	Q96J84	Q96J84 [179-196]	0	2289.2621
[K].LIRDEGL	1xTMTpro [K	4.05E-05	4.225E-05	1	1	1	Q12797	Q12797 [596-607]	1	1968.1444
[R].ETVAYL	1xTMTpro [N	3.716E-07	4.225E-05	1	1	1	Q07960	Q07960 [265-282]	0	2324.257
[K].LLQYNC	1xCarbamido	3.293E-05	4.225E-05	1	1	1	Q9BZF9	Q9BZF9 [120-135	0	2219.1198
[R].ETTDTD	1xTMTpro [K	5.324E-06	4.225E-05	1	1	4	O43707	O43707 [838-853]	0	2350.227
[K].EVFQIAS	1xTMTpro [N	3.525E-06	4.225E-05	1	1	1	Q9UKG1	Q9UKG1 [131-14	0	2104.0743
[R].IEQELAA	2xTMTpro [K	4.817E-05	4.225E-05	1	1	1	Q99715	Q99715 [620-629]	1	2055.2994
[R].INCLEGT	1xCarbamido	1.885E-05	4.225E-05	1	1	1	Q9BZV1	Q9BZV1 [208-225	0	2748.4311
[KR].IPMPVI	1xTMTpro [N	1.2E-05	4.225E-05	1	2	2	P22059	P22059 [434-449]	0	2190.1734
[R].LEGNTV	1xTMTpro [N	3.526E-05	4.225E-05	1	1	1	P46060	P46060 [56-67]	0	1519.8399
[R].FLLEAG	1xTMTpro [K	1.835E-05	4.225E-05	2	2	1	Q8IWZ3; O7	Q8IWZ3 [390-401	0	1966.089
[R].LEGPFN	1xTMTpro [K	5.628E-06	4.225E-05	2	2	3	O75487; Q9	O75487 [302-318]	0	2511.366
[K].LPIVNED	1xTMTpro [N	6.322E-06	4.225E-05	1	1	4	P12268	P12268 [209-224]	0	2084.1922
[K].LPLTPVF	1xTMTpro [K	4.106E-05	4.225E-05	1	1	2	Q12769	Q12769 [1237-12	0	2040.2389
[K].IPLSQEE	1xTMTpro [N	6.211E-07	4.225E-05	1	1	1	Q96RQ3	Q96RQ3 [368-385	0	2343.2628
[K].IPISIEFV	1xTMTpro [N	4.156E-05	4.225E-05	1	1	1	Q13724	Q13724 [328-351]	0	2792.4902
[R].LPLQLDI	1xTMTpro [N	6.165E-07	4.225E-05	1	1	1	P14868	P14868 [152-171]	0	2527.2959
[R].IPLPGAE	1xOxidation [	3.8E-05	4.225E-05	1	1	1	P23511	P23511 [252-270]	0	2737.4978
[R].EQPEKE	1xTMTpro [K	3.347E-06	4.225E-05	1	1	2	Q14152	Q14152 [416-438]	1	3402.8513
[R].IPLPGAE	1xTMTpro [K	1.082E-05	4.225E-05	1	1	1	P23511	P23511 [252-270]	0	2721.5029
[K].EQPFGE	1xTMTpro [K	5.121E-06	4.225E-05	1	1	2	Q8TBC4	Q8TBC4 [261-284	0	3389.7298
[R].LPIPESQ	1xTMTpro [K	2.802E-06	4.225E-05	1	1	4	O95336	O95336 [103-128]	0	3473.8911
[K].FLLDHQ	1xTMTpro [N	3.551E-05	4.225E-05	1	1	4	Q07960	Q07960 [422-439]	0	2273.1773
[K].EQPLDE	1xTMTpro [K	7.114E-06	4.225E-05	1	1	2	P05023	P05023 [527-551]	1	3442.7986
[R].LEGQLE	1xTMTpro [K	2.692E-05	4.225E-05	1	1	1	Q08378	Q08378 [417-433]	0	2408.3892
[K].LPIIGVVI	1xCarbamido	3.888E-06	4.225E-05	1	1	2	P53384	P53384 [221-237]	0	2482.4057

[K].LEGQMG	1xTMTpro [K	3.005E-05	4.225E-05	1	1	2	P13591	P13591 [617-629]		0	1986.0458
[R].IPIHNED	1xTMTpro [N	7.547E-06	4.225E-05	1	1	3	Q92734	Q92734 [24-42]		0	2634.3591
[K].LPIGDVA	1xCarbamido	1.549E-05	4.225E-05	1	1	1	Q99832	Q99832 [293-313]		1	2707.3292
[K].LPNFGFY	1xTMTpro [K	6.345E-06	4.225E-05	1	1	4	Q13283	Q13283 [377-393]		0	2546.3786
[R].EQMIQV	1xTMTpro [K	4.48E-05	4.225E-05	1	1	2	Q9UPN7	Q9UPN7 [224-244		0	3020.6106
[R].LEGLTDI	1xTMTpro [N	5.401E-05	4.225E-05	1	1	5	P05787	P05787 [214-225]		0	1723.955
[K].EQMAQ(	1xTMTpro [N	1.879E-05	4.225E-05	1	1	1	Q15149	Q15149 [2488-25		0	2198.0831
[R].IPPYHYII	1xTMTpro [N	1.422E-05	4.225E-05	1	1	2	Q14764	Q14764 [10-27]		0	2456.3006
[K].IPPVNTN	1xTMTpro [N	1.648E-05	4.225E-05	1	1	2	Q06828	Q06828 [308-324]		0	2259.2417
[R].EQLQAP	1xTMTpro [K	2.726E-05	4.225E-05	1	1	1	Q96AQ6	Q96AQ6 [251-274		0	3197.7736
[K].EQLQDM	1xTMTpro [K	5.807E-06	4.225E-05	1	1	3	P01008	P01008 [365-380]		0	2457.3191
[K].EQLQDM	1xOxidation [	1.312E-05	4.225E-05	1	1	3	P01008	P01008 [365-380]		0	2473.314
[K].LPPLNSI	1xTMTpro [N	3.471E-05	4.225E-05	1	1	2	Q6GYQ0	Q6GYQ0 [821-84		0	3061.5471
[R].LPPKVES	1xTMTpro [K	5.563E-05	4.225E-05	1	1	1	Q14980	Q14980 [1763-17		1	2765.6097
[R].LEGLGS	1xTMTpro [N	9.065E-07	4.225E-05	1	1	2	Q07065	Q07065 [455-473]		0	2209.1267
[R].LPPGVD	1xTMTpro [K	7.816E-06	4.225E-05	1	1	2	O14558	O14558 [123-160]		0	4126.3044
[R].EQISENI	1xTMTpro [N	4.149E-05	4.225E-05	1	1	1	Q05209	Q05209 [748-765]		0	2282.122
[R].EQQAQV	2xTMTpro [K	5.464E-05	4.225E-05	1	1	1	Q13435	Q13435 [850-870]		1	3302.7143
[K].LEGLLLG	1xTMTpro [K	7.547E-06	4.225E-05	1	1	1	Q7Z7G0	Q7Z7G0 [56-80]		0	3271.7858
[R].LPPGEY	1xTMTpro [K	3.913E-05	4.225E-05	1	1	1	P07384	P07384 [489-505]		0	2481.3885
[K].EQIVGPI	1xTMTpro [K	7.114E-06	4.225E-05	1	1	6	Q9Y3Z3	Q9Y3Z3 [270-288		0	2794.5159
[K].IPPDSEA	1xTMTpro [N	1.682E-05	4.225E-05	1	1	5	P02730	P02730 [220-233]		0	1771.0285
[K].LPNQTH	1xTMTpro [N	1.319E-05	4.225E-05	1	1	1	Q9NP81	Q9NP81 [170-187		0	2264.159
[R].EQIVVDI	1xTMTpro [N	6.942E-08	4.225E-05	1	1	2	O60701	O60701 [374-393]		0	2513.2803
[K].IPNPDFF	1xTMTpro [N	5.491E-05	4.225E-05	1	1	5	P27824	P27824 [402-415]		0	2040.0398
[K].IPNIYAIG	1xOxidation [	5.775E-06	4.225E-05	1	1	2	P09622	P09622 [347-365]		0	2603.4875
[K].IPNIYAIG	1xTMTpro [K	3.542E-08	4.225E-05	1	1	3	P09622	P09622 [347-365]		0	2587.4926
[K].LPNLTHI	1xTMTpro [K	3.67E-05	4.225E-05	1	1	3	Q92688	Q92688 [87-99]		0	2029.205
[R].LPNLSSI	1xTMTpro [N	4.881E-06	4.225E-05	1	1	1	O60784	O60784 [457-478]		0	2386.2686
[R].LPPGEY	1xTMTpro [K	2.207E-07	4.225E-05	1	1	1	P07384	P07384 [489-512]		1	3297.8015
[K].LPIGDVA	1xTMTpro [N	2.175E-05	4.225E-05	1	1	2	Q99832	Q99832 [293-306]		0	1870.003
[R].LPLEHLV	1xTMTpro [K	1.286E-05	4.225E-05	1	1	1	Q96I15	Q96I15 [150-170]		0	2913.6945
[R].IPIEDGS	1xTMTpro [N	2.167E-05	4.225E-05	1	1	3	P01024	P01024 [291-304]		0	1774.987

[K].EQSLPS\	1xTMTpro [K	1.903E-05	4.225E-05	1	1	2	Q9NZ32	Q9NZ32 [192-211	0	2722.4829
[K].LPGLLGI	1xTMTpro [K	2.515E-05	4.225E-05	1	1	4	Q13510	Q13510 [101-117]	0	2483.35
[K].IPGIFEL(	1xTMTpro [N	1.998E-05	4.225E-05	1	1	1	P07358	P07358 [260-274]	0	1923.0507
[K].FLHQDID	1xTMTpro [N	4.525E-05	4.225E-05	1	1	1	P11717	P11717 [1053-106	0	1789.9516
[K].IPGGIIED	1xCarbamido	3.563E-05	4.225E-05	1	1	3	P49368	P49368 [204-216]	0	1732.9587
[K].EQTADG	1xTMTpro [N	2.079E-05	4.225E-05	1	1	1	Q9UKK9	Q9UKK9 [56-70]	0	1900.0823
[K].EQTFSG	1xTMTpro [K	5.287E-05	4.225E-05	1	1	1	Q9Y2W1	Q9Y2W1 [203-21!	0	1994.0322
[R].EQTGLE	1xTMTpro [K	3.754E-05	4.225E-05	1	1	1	P48449	P48449 [47-61]	0	2217.2258
[K].LPGGELI	1xTMTpro [K	1.391E-05	4.225E-05	1	1	1	O43809	O43809 [106-123]	1	2517.3804
[K].IEGYPDF	1xTMTpro [K	1.2E-05	4.225E-05	1	1	1	Q15746	Q15746 [1832-18!	1	2901.5278
[K].EQSILEL	1xTMTpro [K	6.997E-06	4.225E-05	1	1	3	O00231	O00231 [47-59]	0	2009.2138
[K].IPGDQV\	1xTMTpro [K	2.524E-05	4.225E-05	1	1	4	P98160	P98160 [126-138]	0	2009.2291
[R].IPFYGSY	1xTMTpro [K	7.962E-07	4.225E-05	1	1	2	P61011	P61011 [157-178]	0	3053.6037
[R].LPFLDS(	1xCarbamido	5.073E-05	4.225E-05	1	1	1	Q92785	Q92785 [39-59]	0	3095.5826
[R].IPFDLMD	1xTMTpro [K	4.857E-05	4.225E-05	1	1	1	Q9NRY4	Q9NRY4 [402-42:	0	3067.6306
[R].LPEYTLS	1xTMTpro [K	2.117E-05	4.225E-05	1	1	1	O75569	O75569 [142-156]	0	2235.2265
[K].LPETNLF	1xTMTpro [K	1.348E-06	4.225E-05	1	1	3	Q15046	Q15046 [408-421]	1	2315.2739
[R].FLHNPD.	1xCarbamido	1.468E-07	4.225E-05	1	1	2	Q13228	Q13228 [255-276]	0	2693.3789
[R].IPESGGI	1xTMTpro [N	6.627E-07	4.225E-05	1	1	3	P07996	P07996 [21-41]	0	2499.2687
[R].LEHDVN	2xTMTpro [K	7.434E-07	4.225E-05	1	1	2	Q00341	Q00341 [1080-11(	1	4270.2084
[R].LEHENP.	1xTMTpro [K	2.906E-05	4.225E-05	1	1	2	Q5JRX3	Q5JRX3 [184-199	0	2459.3426
[R].LEHLYLN	1xTMTpro [K	3.411E-05	4.225E-05	1	1	2	P51888	P51888 [313-325]	0	2195.2316
[R].LPGDIQ\	1xTMTpro [N	1.22E-05	4.225E-05	1	1	1	P04275	P04275 [1619-164	0	2718.5109
[K].IEGHQD.	1xTMTpro [K	1.247E-05	4.225E-05	1	1	1	Q8IWB7	Q8IWB7 [20-35]	0	2284.352
[R].EQSHAE	1xCarbamido	1.137E-06	4.225E-05	1	1	1	Q02952	Q02952 [246-272]	1	3896.9276
[K].EQSELV.	1xTMTpro [K	2.533E-05	4.225E-05	1	1	2	O75165	O75165 [2120-21:	0	1824.0722
[K].EQQEAIE	1xTMTpro [N	5.908E-07	4.225E-05	1	2	1	Q01105	Q01105 [40-57]	0	2499.2282
[K].EQQEAIE	1xTMTpro [K	4.239E-09	4.225E-05	1	1	2	Q01105	Q01105 [40-68]	1	4058.081
[K].LPIDVTE	1xTMTpro [K	1.462E-05	4.225E-05	1	1	1	P26599	P26599 [66-84]	0	2592.5144
[K].IPLDMV/	1xTMTpro [K	2.654E-05	4.225E-05	1	1	2	Q8TDL5	Q8TDL5 [112-126	0	2223.3067
[R].LEGSDV	1xTMTpro [N	1.826E-06	4.225E-05	1	1	4	Q9NY33	Q9NY33 [679-698	0	2438.3098
[R].EQQHVN	1xTMTpro [N	2.921E-05	4.225E-05	1	1	1	P78527	P78527 [1769-178	0	2199.0824
[R].EQQHVN	1xOxidation	3.715E-05	4.225E-05	1	1	1	P78527	P78527 [1769-178	0	2215.0773

[R].IEGSNK\	1xTMTpro [K	3.017E-06	4.225E-05	1	1	1	P06396	P06396 [452-481]	1	4008.0675
[R].LPLAQS/	1xTMTpro [K	8.496E-07	4.225E-05	1	1	2	P11277	P11277 [1498-15	0	2946.6254
[K].LPHSVLI	1xTMTpro [K	6.051E-05	4.225E-05	1	1	2	Q9Y617	Q9Y617 [17-27]	0	1885.1766
[K].LPGLLGI	1xOxidation	5.357E-05	4.225E-05	1	1	2	Q13510	Q13510 [101-117	0	2499.3449
[R].EQQLSA	1xTMTpro [N	3.774E-05	4.225E-05	1	1	2	O60687	O60687 [394-406	0	1847.0194
[R].LEGVEG	1xTMTpro [K	1.369E-05	4.225E-05	1	1	2	Q14204	Q14204 [2244-22	0	2085.22
[R].EQQIVIQ	2xTMTpro [K	5.714E-08	4.225E-05	1	1	3	P38646	P38646 [542-563]	1	3330.8395
[K].LEGVLAI	1xTMTpro [N	1.213E-07	4.225E-05	1	1	2	P46940	P46940 [568-585]	0	2359.2941
[R].LPGPTG	1xTMTpro [K	3.8E-05	4.225E-05	1	1	2	Q7Z434	Q7Z434 [240-270	0	3372.7779
[R].LEGWQ	1xTMTpro [K	1.892E-05	4.225E-05	1	1	1	Q86SF2	Q86SF2 [444-463	0	2748.5216
[R].IPGMLIID	1xTMTpro [N	5.455E-05	4.225E-05	1	1	1	O60841	O60841 [695-713	0	2401.2869
[K].IEGYED	1xTMTpro [N	2.846E-07	4.225E-05	1	1	1	P52907	P52907 [67-86]	0	2549.2803
[K].IEGYPDI	1xTMTpro [K	1.962E-05	4.225E-05	1	1	4	Q15746	Q15746 [1832-18	0	2187.1982
[R].LPGISET	1xTMTpro [K	3E-05	4.225E-05	1	1	1	P31040	P31040 [380-397]	0	2457.3919
[K].EQSELD	1xTMTpro [K	5.915E-06	4.225E-05	1	1	2	P55209	P55209 [8-32]	0	3532.6318
[K].LPHLPGI	1xTMTpro [K	2.556E-07	4.225E-05	1	1	3	Q16795	Q16795 [329-348	0	2762.6312
[K].IPEISIQD	1xTMTpro [K	6.769E-06	4.225E-05	1	1	2	P21333	P21333 [2166-21	0	2610.4304
[R].LPQFVQ	1xTMTpro [N	4.713E-05	4.225E-05	1	1	1	O14617	O14617 [557-571	0	2063.1092
[R].LEGGSG	1xTMTpro [N	8.891E-06	4.225E-05	1	1	7	P62195	P62195 [259-271]	0	1594.7992
[K].LQAAVD	2xTMTpro [K	1.7E-05	4.225E-05	1	1	4	P51572	P51572 [233-246]	2	2443.3683
[R].IEEVPEL	1xTMTpro [K	9.023E-06	4.225E-05	1	1	4	P36578	P36578 [144-157]	0	2217.2874
[K].LQAAVD	1xTMTpro [K	5.166E-05	4.225E-05	1	1	2	P51572	P51572 [233-243]	0	1752.981
[R].IEEVPEL	2xTMTpro [K	2.342E-07	4.225E-05	1	1	6	P36578	P36578 [144-162]	1	3097.7853
[R].IEEVPEL	3xTMTpro [K	1.446E-06	4.225E-05	1	1	2	P36578	P36578 [144-163]	2	3530.0874
[K].IEEVSDT	1xTMTpro [K	3.01E-05	4.225E-05	1	1	1	Q9H6T3	Q9H6T3 [499-515	0	2440.3427
[K].LQAAATI	2xTMTpro [K	4.981E-05	4.225E-05	1	1	1	P51610	P51610 [1775-18	0	3826.2146
[R].IPYYNIH	1xTMTpro [N	1.994E-05	4.225E-05	1	1	2	O95479	O95479 [646-661	0	2310.25
[K].LPYVTM	1xTMTpro [K	2.324E-06	4.225E-05	1	1	2	Q99436	Q99436 [165-184	0	2695.4331
[R].IEFDND/	2xTMTpro [K	2.75E-06	4.225E-05	1	1	1	P23229	P23229 [97-127]	1	4265.1125
[K].EQFLDG	1xTMTpro [N	5.964E-05	4.225E-05	1	1	6	P27797	P27797 [25-36]	0	1714.8356
[K].LEFENL\	1xTMTpro [N	3.086E-05	4.225E-05	1	1	3	Q9Y625	Q9Y625 [81-95]	0	2153.1198
[K].LPYNTSI	1xTMTpro [K	1.475E-05	4.225E-05	1	1	1	Q9Y263	Q9Y263 [418-436	0	2894.522
[K].IPYLPITN	1xTMTpro [K	2.102E-05	4.225E-05	1	1	2	O75369	O75369 [153-169	0	2656.4379

[K].LPYGYG	1xTMTpro [K	3.016E-05	4.225E-05	1	1	1	P15502	P15502 [226-241]	0	2043.1519
[K].LPYDVTI	1xTMTpro [K	1.687E-06	4.225E-05	1	1	2	P46940	P46940 [1112-11	0	2547.395
[R].IPYDDYI	1xTMTpro [N	4.695E-06	4.225E-05	1	1	1	Q5EBL8	Q5EBL8 [5-30]	0	3399.7373
[R].LPVVVPI	1xTMTpro [K	7.773E-06	4.225E-05	1	1	1	O76031	O76031 [500-512	0	2054.2505
[R].LPVVIGC	1xCarbamido	2.926E-05	4.225E-05	1	1	2	Q00610	Q00610 [812-830	0	2649.5029
[R].LPVQEC	3xCarbamido	3.78E-05	4.225E-05	1	1	1	O15031	O15031 [464-479	0	2316.1106
[K].IEFLQSH	1xTMTpro [K	6.061E-05	4.225E-05	2	3	2	O60684; P52	O60684 [479-493	0	2514.3484
[K].EQELQQ	1xTMTpro [N	1.625E-05	4.225E-05	1	1	3	Q15149	Q15149 [2199-22	0	2617.3752
[K].IEEVIGA	1xCarbamido	1.467E-05	4.225E-05	2	2	2	P54760; P54	P54760 [617-631]	0	1969.002
[R].LQAEAQ	1xTMTpro [N	1.903E-05	4.225E-05	1	1	2	Q8TAE8	Q8TAE8 [156-171	0	2134.1464
[R].LEESLSI	1xTMTpro [K	3.242E-05	4.225E-05	1	1	2	Q6ZMI0	Q6ZMI0 [215-225	0	1883.0981
[K].EQALQE	1xTMTpro [N	2.995E-06	4.225E-05	1	1	1	Q9UH65	Q9UH65 [493-510	0	2491.2669
[K].EQAPHC	2xCarbamido	4.345E-05	4.225E-05	1	1	1	P01023	P01023 [842-853]	0	1716.8229
[R].FINPAIV	1xTMTpro [K	5.455E-05	4.225E-05	1	1	1	P21359	P21359 [1413-14	0	2455.4092
[R].IQCAAG	4xCarbamido	7.098E-08	4.225E-05	1	1	1	Q12805	Q12805 [157-185	0	3727.5913
[R].LQAYHT	1xTMTpro [N	3.939E-06	4.225E-05	1	1	1	P54819	P54819 [187-202]	0	2301.2199
[K].LQAVEV	1xTMTpro [K	7.559E-07	4.225E-05	1	1	2	Q9Y2V2	Q9Y2V2 [121-136	0	2284.3884
[R].EQCTAG	3xCarbamido	3.353E-06	4.225E-05	1	1	3	P24821	P24821 [138-154]	0	2140.9857
[K].EQCTGG	3xCarbamido	3.334E-06	4.225E-05	1	1	1	P22105	P22105 [119-140]	0	2615.1568
[K].LQATLDI	1xTMTpro [K	5.014E-05	4.225E-05	1	1	1	O00231	O00231 [206-223	0	2563.4045
[R].EQDAPV	1xTMTpro [N	3.108E-05	4.225E-05	1	1	1	Q14767	Q14767 [74-87]	0	1812.9775
[K].EQGPYE	1xTMTpro [K	3.322E-05	4.225E-05	1	1	1	P23921	P23921 [549-563]	0	2279.1687
[K].EQDDLL	1xTMTpro [K	5.627E-05	4.225E-05	1	1	1	O60763	O60763 [905-919	0	2351.295
[K].IEENSLK	3xTMTpro [K	4.618E-05	4.225E-05	1	1	1	Q14839	Q14839 [1566-15	2	3435.9211
[K].IQALQQ	1xTMTpro [N	1.676E-05	4.225E-05	1	1	2	P67936	P67936 [14-27]	0	1918.979
[R].IQAINEI	1xTMTpro [K	2.471E-05	4.225E-05	1	1	1	Q53EP0	Q53EP0 [935-951	0	2441.4048
[K].EQDYLC	1xCarbamido	3.069E-05	4.225E-05	1	1	2	O15498	O15498 [61-71]	0	1785.8913
[R].LQALDT	1xTMTpro [K	4.704E-06	4.225E-05	1	1	2	Q01082	Q01082 [1144-11	0	2133.1948
[K].LQALANI	1xTMTpro [K	6.942E-08	4.225E-05	1	1	4	Q86UP2	Q86UP2 [654-670	0	2415.3487
[K].IQAGEGI	1xTMTpro [K	2.11E-06	4.225E-05	1	1	2	Q15075	Q15075 [553-569	0	2436.359
[R].EQEELL	1xTMTpro [N	9.677E-06	4.225E-05	1	1	2	P19971	P19971 [377-393]	0	2173.1671
[K].LEESLLE	1xTMTpro [K	1.412E-05	4.225E-05	1	1	2	Q8NCM8	Q8NCM8 [3436-3	0	2810.5643
[K].LQAFGF	1xCarbamido	2.312E-07	4.225E-05	1	1	2	P49756	P49756 [126-142]	1	2683.4045

[K].IEENSLK	2xTMTpro [K	1.008E-05	4.225E-05	1	1	1	Q14839	Q14839 [1566-15	1	2775.508
[K].FLLQHQ.	1xTMTpro [K	3.741E-05	4.225E-05	1	1	3	P16157	P16157 [719-730]	0	1992.1522
[R].LPVIDPE	1xTMTpro [K	9.91E-08	4.225E-05	1	1	1	P54619	P54619 [153-170]	0	2618.5049
[K].LPVGSQ	1xCarbamid	7.023E-06	4.225E-05	1	1	2	P24821	P24821 [58-75]	0	2471.2943
[R].LPSGSG	1xTMTpro [N	4.32E-06	4.225E-05	1	1	2	Q09666	Q09666 [208-225	0	1947.0466
[R].LPSGLP	1xTMTpro [K	1.799E-05	4.225E-05	1	1	7	P51884	P51884 [199-216]	0	2565.5147
[R].LPSEGP	1xTMTpro [K	1.515E-07	4.225E-05	1	1	3	P30043	P30043 [40-63]	0	3077.7239
[K].IPSDTFY	2xTMTpro [K	4.981E-05	4.225E-05	1	1	1	Q14118	Q14118 [518-535	1	3038.6139
[K].IPSDTFY	1xTMTpro [K	2.068E-05	4.225E-05	1	1	1	Q14118	Q14118 [518-533	0	2493.2277
[R].IPSAVG	2xOxidation	1.657E-05	4.225E-05	1	1	2	P06576	P06576 [325-345]	0	2602.2812
[R].IPSAVG	1xOxidation	2.524E-05	4.225E-05	1	1	4	P06576	P06576 [325-345]	0	2586.2863
[K].EQIEWLI	1xTMTpro [K	4.257E-05	4.225E-05	1	1	1	Q93034	Q93034 [755-764	0	1933.1039
[R].EQLFAV	1xTMTpro [K	4.057E-05	4.225E-05	1	1	1	Q5JRX3	Q5JRX3 [991-100	0	1782.0042
[K].EQLGEF	2xCarbamid	3.845E-06	4.225E-05	1	1	3	P19652	P19652 [154-170]	0	2417.18
[K].EQLDSS	1xTMTpro [K	2.475E-05	4.225E-05	1	1	2	O60763	O60763 [876-892	0	2485.3641
[K].EQLGEF	1xCarbamid	2.711E-05	4.225E-05	1	1	8	P02763	P02763 [154-167]	0	2047.0126
[R].EQLGPV	1xTMTpro [K	1.394E-09	4.225E-05	1	1	8	P02647	P02647 [86-107]	1	3226.6876
[R].EQLGPV	2xTMTpro [K	3.192E-07	4.225E-05	1	1	1	P02647	P02647 [86-112]	2	4134.1634
[R].IPSAVG	1xTMTpro [N	2.134E-06	4.225E-05	1	1	3	P06576	P06576 [325-345]	0	2570.2914
[K].IEGEMQ	1xTMTpro [N	4.817E-05	4.225E-05	1	1	2	Q09666	Q09666 [1092-11	0	1804.9434
[R].LPRPPP	1xTMTpro [K	5.756E-05	4.225E-05	1	1	3	Q00325	Q00325 [342-355	0	2196.2706
[R].LPRPLP	1xTMTpro [N	3.645E-05	4.225E-05	1	1	1	P54802	P54802 [110-128]	0	2332.3308
[R].LPQVSP.	1xCarbamid	5.073E-05	4.225E-05	1	1	4	P98160	P98160 [2104-21	0	1981.9972
[K].LPQQAN	1xTMTpro [N	1.427E-05	4.225E-05	1	1	2	P04114	P04114 [2118-21	0	2299.1427
[K].EQLLQS	1xTMTpro [K	2.493E-05	4.225E-05	1	2	1	O43795	O43795 [140-156	0	2466.3848
[K].FIIHAPP	1xTMTpro [N	2.834E-05	4.225E-05	1	1	2	P47755	P47755 [20-37]	0	2405.2573
[R].EQLGPV	1xTMTpro [K	4.739E-06	4.225E-05	1	1	13	P02647	P02647 [86-101]	0	2541.3481
[K].LPQGYH	1xTMTpro [K	4.487E-05	4.225E-05	1	1	1	Q9UPN3	Q9UPN3 [392-40	0	2506.2858
[R].EQLDPD	1xTMTpro [K	1.892E-06	4.225E-05	1	1	1	Q9NYU2	Q9NYU2 [644-65	0	2407.2671
[K].LPSGVF	1xTMTpro [K	2.789E-05	4.225E-05	1	1	1	Q02750	Q02750 [325-340	0	2435.3466
[K].IPVDTYN	1xTMTpro [K	3.709E-05	4.225E-05	1	1	2	Q96QK1	Q96QK1 [404-41	0	2211.3244
[K].LEGDLKI	2xTMTpro [K	1.959E-05	4.225E-05	1	1	3	P35749	P35749 [1622-16	1	2770.6018
[K].EQGVLT	1xTMTpro [K	4.287E-07	4.225E-05	1	1	2	O00203	O00203 [996-102	0	3799.0596

[K].EQGVTF	1xTMTpro [K	5.645E-05	4.225E-05	1	1	1	Q92783	Q92783 [137-154]	0	2440.3328
[K].LEGDLK(	2xTMTpro [K	4.584E-06	4.225E-05	1	1	1	Q09666	Q09666 [2411-24	1	3485.8944
[K].EQGYDV	1xTMTpro [K	6.161E-06	4.225E-05	1	1	2	P00966	P00966 [26-41]	0	2390.3211
[WRQ].LEGI	2xTMTpro [K	9.588E-06	4.225E-05	1	1	1	Q8IVF2	Q8IVF2 [980-994]	1	2487.4486
[R].EQHLAQ	1xTMTpro [K	1.776E-05	4.225E-05	1	1	1	P49750	P49750 [55-70]	0	2596.4273
[R].EQHLYY	1xTMTpro [N	1.628E-05	4.225E-05	1	2	2	Q15661	Q15661 [92-106]	0	2193.1623
[K].LEGDLT(	1xCarbamido	3.131E-06	4.225E-05	1	1	3	Q09666	Q09666 [2142-21	0	3291.6798
[K].EQLDNQ	1xTMTpro [K	4.817E-05	4.225E-05	1	1	4	Q9Y3Y2	Q9Y3Y2 [214-226	0	2163.1247
[K].LEGDLT(	1xCarbamido	1.825E-05	4.225E-05	1	1	2	Q09666	Q09666 [1880-19	0	3233.6743
[K].LPTGQIS	1xTMTpro [K	5.563E-05	4.225E-05	1	1	4	Q09666	Q09666 [5513-55	0	1848.1086
[R].LEGDMA	1xTMTpro [N	1.126E-06	4.225E-05	1	1	1	P38606	P38606 [57-81]	0	2969.5097
[R].IEGDMIV	1xCarbamido	4.177E-07	4.225E-05	1	1	3	P46777	P46777 [69-85]	0	2525.3388
[R].LPTDTL(	1xCarbamido	3.782E-06	4.225E-05	1	1	1	Q96PP9	Q96PP9 [359-376	0	2385.2403
[R].LPTDASI	1xTMTpro [N	3.334E-06	4.225E-05	1	1	1	O60826	O60826 [108-128]	0	2459.2585
[R].LPTAEVI	1xCarbamido	6.719E-06	4.225E-05	1	1	1	O14908	O14908 [92-106]	0	2370.2805
[K].LPSVPT\	1xTMTpro [N	1.071E-05	4.225E-05	1	1	1	P50851	P50851 [1777-17	0	2762.4201
[R].EQICEVI	1xCarbamido	1.555E-05	4.225E-05	1	1	1	P47895	P47895 [58-84]	1	3973.0793
[R].IEGEGS\	1xTMTpro [K	5.491E-05	4.225E-05	1	1	3	Q14764	Q14764 [735-745]	0	1739.0195
[R].LPSLGA(	1xTMTpro [K	5.393E-05	4.225E-05	1	1	1	Q8IZL8	Q8IZL8 [243-255]	0	1883.1246
[R].IPTPLNT	1xCarbamido	5.437E-05	4.225E-05	1	1	3	Q96CW1	Q96CW1 [324-33	0	2366.3431
[K].IEPSTVV	1xTMTpro [K	3.692E-06	4.225E-05	1	1	1	Q5JRX3	Q5JRX3 [291-307	0	2529.4321
[R].EQVPSL	1xCarbamido	4.6E-08	4.225E-05	1	1	1	A1L0T0	A1L0T0 [557-577]	0	2917.501
[K].EQVQSC	1xCarbamido	3.997E-06	4.225E-05	1	1	1	P08603	P08603 [625-642]	0	2574.3841
[R].ESGMNL	1xTMTpro [N	1.425E-05	4.225E-05	1	1	2	Q96E11	Q96E11 [164-179	0	2063.0762
[K].LENFPIH	1xTMTpro [K	2.263E-05	4.225E-05	1	1	1	Q13459	Q13459 [1759-17	0	2160.3036
[K].INISQVIA	1xTMTpro [K	1.113E-05	4.225E-05	1	1	1	P24928	P24928 [779-796]	0	2504.4692
[K].LNISFPA	1xCarbamido	2.471E-05	4.225E-05	1	1	5	P62753	P62753 [3-14]	0	1944.0868
[K].LNIQPSE	1xTMTpro [N	4.525E-05	4.225E-05	1	1	2	Q9NYU2	Q9NYU2 [440-45	0	2008.067
[K].LENLLLL	1xTMTpro [N	1.889E-05	4.225E-05	1	1	4	P51888	P51888 [194-206]	0	1895.1034
[K].LNLPINII	1xCarbamido	7.816E-06	4.225E-05	1	1	2	P28838	P28838 [322-342]	0	2888.6233
[K].LNLPINII	1xCarbamido	3.359E-06	4.225E-05	1	1	2	P28838	P28838 [322-342]	0	2872.6284
[K].IENLSNL	1xTMTpro [N	5.179E-06	4.225E-05	1	1	1	Q15435	Q15435 [179-197]	0	2513.3465
[R].ESGVGQ	1xTMTpro [K	1.028E-06	4.225E-05	1	1	1	Q9C0C2	Q9C0C2 [1252-12	0	2604.3437

[R].INLPAPN	1xTMTpro [K	1.028E-05	4.225E-05	1	1	2	Q9UJS0	Q9UJS0 [627-641	0	2200.237
[K].LNINPED	1xTMTpro [K	6.819E-06	4.225E-05	1	1	1	Q99459	Q99459 [448-466]	0	2736.3682
[R].ESGYMH	1xCarbamido	2.867E-07	4.225E-05	1	1	1	P07099	P07099 [296-328]	0	4477.3563
[K].ESHPGL	1xTMTpro [K	9.781E-07	4.225E-05	1	1	2	P14543	P14543 [91-119]	0	3578.9027
[R].LNIISNLI	1xCarbamido	1.601E-06	4.225E-05	2	2	2	P30154; P30	P30154 [394-410]	0	2246.2498
[R].INLLSHD	1xTMTpro [N	3.091E-06	4.225E-05	1	1	1	Q5EB52	Q5EB52 [141-159	0	2536.3731
[R].ESKPLTA	2xTMTpro [K	2.109E-05	4.225E-05	1	1	2	P02751	P02751 [985-996]	0	2244.338
[R].ESLAEEI	1xTMTpro [N	3.334E-05	4.225E-05	1	1	1	Q10570	Q10570 [167-182]	0	2044.0266
[R].LNIIIVAE	2xTMTpro [K	5.256E-06	4.225E-05	1	1	1	Q01813	Q01813 [266-287]	0	3279.9344
[K].INIILSET	1xTMTpro [N	5.123E-05	4.225E-05	1	1	1	Q9GZT8	Q9GZT8 [362-377	1	2129.2137
[K].ESLCQA	1xCarbamido	1.673E-05	4.225E-05	1	1	3	Q96RQ3	Q96RQ3 [506-518	0	2024.207
[R].LNMQTG	1xCarbamido	2.158E-06	4.225E-05	1	1	1	Q8IUX7	Q8IUX7 [417-440]	0	3071.2954
[R].INNAAFE	1xTMTpro [K	2.802E-06	4.225E-05	1	1	2	P11908	P11908 [261-280]	0	2781.5278
[R].INNACFE	1xCarbamido	9.166E-07	4.225E-05	1	1	2	P60891	P60891 [261-280]	0	2870.5214
[R].ESGCSF	1xCarbamido	2.784E-05	4.225E-05	1	1	2	P07384	P07384 [414-426]	0	2078.127
[R].ESEQGV	1xCarbamido	4.516E-06	4.225E-05	1	1	2	P00736	P00736 [421-436]	0	2465.2626
[K].LEISDEF	1xTMTpro [N	3.597E-06	4.225E-05	1	1	4	Q9NZN4	Q9NZN4 [194-208	0	1954.0452
[K].FLESGG	1xOxidation [	1.903E-05	4.225E-05	1	1	1	P07237	P07237 [469-502]	0	4381.8553
[K].FLESGG	1xTMTpro [K	3.279E-06	4.225E-05	1	1	2	P07237	P07237 [469-502]	0	4365.8604
[R].FLEQQN	1xTMTpro [K	5.491E-05	4.225E-05	3	3	5	Q7Z794; P35	Q7Z794 [184-195]	0	2084.1996
[K].LELSGCI	1xCarbamido	8.554E-06	4.225E-05	1	1	2	P16455	P16455 [19-32]	0	2221.2142
[K].LEMNYV	1xTMTpro [N	4.427E-05	4.225E-05	1	1	3	P23142	P23142 [676-689]	0	1864.007
[R].FLEQQN	1xTMTpro [N	9.31E-06	4.225E-05	1	1	4	P17661	P17661 [128-142]	0	1978.0677
[K].INQDPQY	1xOxidation [	3.569E-05	4.225E-05	1	1	2	P36871	P36871 [528-545]	0	2588.5341
[K].LNQALLI	1xTMTpro [N	3.119E-06	4.225E-05	1	1	2	P02792	P02792 [107-121]	0	1896.0986
[R].LNLLDLI	1xTMTpro [K	4.092E-05	4.225E-05	1	1	2	P12111	P12111 [2008-202	0	2940.6061
[K].LNPVTTI	1xTMTpro [N	1.812E-05	4.225E-05	1	1	1	Q8WUA2	Q8WUA2 [247-26	0	2166.1613
[R].LNPTWN	1xTMTpro [K	6.511E-06	4.225E-05	1	1	4	Q9HB07	Q9HB07 [202-218	0	2578.3182
[R].LENDGA	1xTMTpro [N	1.412E-05	4.225E-05	1	1	1	P46060	P46060 [192-205]	0	1781.9353
[K].INPSSMI	1xTMTpro [K	5.756E-05	4.225E-05	1	1	1	P11216	P11216 [558-569]	0	1982.1025
[K].LNPNFLV	1xTMTpro [K	1.313E-06	4.225E-05	1	1	2	Q13228	Q13228 [438-460]	1	3155.7861
[R].FLEQELI	1xTMTpro [N	5.926E-05	4.225E-05	1	1	2	P55058	P55058 [41-55]	0	2121.1763
[K].ESFESQ	1xTMTpro [N	4.665E-05	4.225E-05	1	1	1	Q13616	Q13616 [230-242]	0	1862.9091

[R].INPDGS(	1xTMTpro [N	3.353E-06	4.225E-05	1	1	6	Q9Y2B0	Q9Y2B0 [58-73]	0	2035.0779
[K].LNPADAI	1xTMTpro [K	1.987E-05	4.225E-05	1	1	1	O94776	O94776 [596-611]	0	2261.3149
[R].INNMHLI	1xTMTpro [N	1.399E-05	4.225E-05	1	1	1	Q9H0Q0	Q9H0Q0 [167-185	0	2574.236
[R].INNEIDQ	1xTMTpro [N	7.207E-06	4.225E-05	1	1	1	Q9UQE7	Q9UQE7 [707-72(	0	2778.3834
[K].ESEVTV(	1xTMTpro [K	3.453E-05	4.225E-05	1	1	1	P22105	P22105 [1422-143	0	2047.1315
[R].LNLLDD(	1xCarbamido	5.218E-05	4.225E-05	1	1	1	Q92626	Q92626 [294-320]	0	3721.8731
[K].INIHEYYI	1xTMTpro [K	5.384E-05	4.225E-05	1	1	2	Q96DZ1	Q96DZ1 [144-157	0	2287.2764
[R].INLGFNS	1xTMTpro [K	1.532E-06	4.225E-05	1	1	2	Q9NR99	Q9NR99 [60-80]	0	2819.5799
[K].ESLVSFA	1xTMTpro [N	3.52E-05	4.225E-05	1	1	1	Q8IXB1	Q8IXB1 [223-234]	0	1707.9171
[R].LNEESG	2xCarbamido	1.944E-05	4.225E-05	1	1	1	P53992	P53992 [799-820]	0	2758.3823
[R].LNEELKY	2xTMTpro [K	2.524E-05	4.225E-05	1	1	1	Q9BRK5	Q9BRK5 [183-201	1	3184.7769
[K].LNEDMA	1xCarbamido	1.353E-06	4.225E-05	1	1	1	P25789	P25789 [68-91]	0	2913.4253
[K].LNEDMA	1xCarbamido	1.213E-07	4.225E-05	1	1	3	P25789	P25789 [68-91]	0	2897.4304
[K].ESMTDA	1xTMTpro [K	1.7E-05	4.225E-05	1	1	1	P32455	P32455 [469-485]	0	2532.3359
[R].ESNAEY	1xCarbamido	4.323E-05	4.225E-05	1	1	1	Q658P3	Q658P3 [127-144	0	2637.409
[R].INEANQI	1xCarbamido	3.146E-05	4.225E-05	1	1	1	Q86SF2	Q86SF2 [573-588	0	2577.3297
[K].LNEADH	1xTMTpro [N	1.045E-05	4.225E-05	1	1	1	Q9P2R3	Q9P2R3 [249-266	0	2241.1794
[R].LNEAAA(	1xTMTpro [N	5.056E-07	4.225E-05	1	1	4	Q9Y490	Q9Y490 [1242-12	0	2331.2588
[K].INEGLEH	1xTMTpro [K	5.636E-05	4.225E-05	1	1	1	Q99747	Q99747 [6-15]	0	1732.0249
[K].LNDLNSY	1xCarbamido	1.09E-05	4.225E-05	1	1	1	P04114	P04114 [3737-376	0	3579.9199
[K].LNDLEDA	1xTMTpro [K	4.533E-06	4.225E-05	1	1	1	P04264	P04264 [444-460]	1	2550.4019
[K].LNDLEDA	1xTMTpro [K	4.634E-05	4.225E-05	1	1	3	P04264	P04264 [444-455]	0	1966.1101
[K].LNDHFPI	1xTMTpro [N	4.881E-06	4.225E-05	1	1	1	P07954	P07954 [132-160]	0	3574.8032
[R].ESPLVSA	1xTMTpro [K	2.588E-05	4.225E-05	1	1	1	O95479	O95479 [552-566]	0	2283.2728
[K].LNDGSQ	1xTMTpro [K	4.626E-05	4.225E-05	1	1	3	O95831	O95831 [245-255]	0	1876.0308
[K].LNDGHF	1xTMTpro [N	1.229E-06	4.225E-05	1	1	2	P42330	P42330 [10-31]	0	2718.4033
[R].INDDLISI	1xTMTpro [K	5.831E-05	4.225E-05	2	2	2	Q12959; Q1!	Q12959 [727-738]	0	2014.0988
[R].INDALSC	2xCarbamido	3.988E-05	4.225E-05	1	1	2	Q52LJ0	Q52LJ0 [210-221]	0	1833.8431
[K].ESPVFAI	1xTMTpro [N	1.391E-05	4.225E-05	1	1	3	P09601	P09601 [70-85]	0	2221.1613
[R].FIECYIAI	2xCarbamido	5.393E-06	4.225E-05	1	1	1	P29401	P29401 [359-379]	0	2736.3478
[K].LEPQWII	1xCarbamido	1.488E-05	4.225E-05	1	2	1	P31995	P31995 [54-72]	0	2605.3615
[R].LNQQEQ	1xTMTpro [K	1.882E-05	4.225E-05	1	1	1	P42566	P42566 [874-890]	0	2550.4271
[K].LNEGVV	1xTMTpro [K	2.057E-05	4.225E-05	1	1	1	Q13243	Q13243 [144-158]	0	2249.2309

[K].LNELAD(	1xTMTpro [N	2.083E-05	4.225E-05	1	1	2	Q9C040	Q9C040 [281-294	0	1969.0462
[R].LENYPIF	1xTMTpro [N	2.371E-05	4.225E-05	1	1	4	Q00796	Q00796 [22-38]	0	2254.2403
[R].IEPADAH	1xTMTpro [K	5.296E-05	4.225E-05	1	1	1	Q9GZZ1	Q9GZZ1 [142-152	0	1829.0777
[K].ESLKELS	1xTMTpro [K	2.65E-06	4.225E-05	1	1	1	P13489	P13489 [284-302]	1	2596.4074
[K].FLEGTS(	1xCarbamido	9.856E-06	4.225E-05	1	1	1	Q15149	Q15149 [4065-40	0	2423.3136
[R].LNLEAIN	1xOxidation [	4.315E-05	4.225E-05	1	1	5	P09382	P09382 [113-128]	0	2409.2616
[R].LNLEAIN	1xTMTpro [K	1.299E-06	4.225E-05	1	1	36	P09382	P09382 [113-128]	0	2393.2667
[R].INLAEDV	1xTMTpro [N	5.084E-06	4.225E-05	1	1	1	Q969V3	Q969V3 [361-374	0	1999.0568
[K].IEPAQAV	1xTMTpro [N	8.527E-07	4.225E-05	1	1	1	Q9UJW0	Q9UJW0 [216-24	0	4118.1253
[K].FIEGGD(	1xTMTpro [K	1.244E-05	4.225E-05	1	1	1	Q15063	Q15063 [790-806]	1	2599.3648
[R].FLEFTTL	1xTMTpro [N	7.744E-07	4.225E-05	1	1	2	Q14699	Q14699 [40-58]	0	2300.2457
[K].LNEHFLN	1xTMTpro [K	1.549E-05	4.225E-05	1	1	2	P48735	P48735 [427-442]	0	2529.3845
[K].IEPEPFE	1xCarbamido	2.755E-05	4.225E-05	1	1	1	P48637	P48637 [401-418]	0	2386.2508
[R].ESLNASI	1xCarbamido	1.306E-06	4.225E-05	1	1	1	Q9UJZ1	Q9UJZ1 [151-171	0	2607.3156
[K].INETFVD	1xTMTpro [K	4.242E-05	4.225E-05	1	1	1	Q8WUA2	Q8WUA2 [139-15	1	2707.4587
[K].INESTQN	1xTMTpro [K	1.753E-06	4.225E-05	1	1	4	P51911	P51911 [75-93]	0	2893.5452
[K].LNESTFI	1xTMTpro [K	2.362E-05	4.225E-05	1	1	2	P78527	P78527 [1858-18	0	2005.1097
[K].ESLPVIA	1xTMTpro [K	5.295E-06	4.225E-05	1	1	2	Q9H3P7	Q9H3P7 [364-381	0	2632.514
[K].LEPGGG	1xTMTpro [N	2.117E-05	4.225E-05	1	1	2	Q14315	Q14315 [1015-10	0	1558.8508
[R].LNEQEG	1xTMTpro [N	2.371E-05	4.225E-05	1	1	1	Q96CS3	Q96CS3 [51-77]	0	3302.7201
[K].IEPGTLG	1xCarbamido	7.545E-07	4.225E-05	1	1	2	Q6UWY5	Q6UWY5 [297-31	0	2258.1195
[K].LEPISND	1xTMTpro [K	9.225E-06	4.225E-05	1	1	2	Q9NZM1	Q9NZM1 [554-56	0	2192.267
[K].ESISVSS	1xTMTpro [N	6.984E-06	4.225E-05	1	1	56	P00915	P00915 [215-228]	0	1884.9986
[K].LNFNGE	1xTMTpro [K	1.506E-06	4.225E-05	1	1	1	P00918	P00918 [228-251]	0	3391.76
[K].IPELLAS	1xOxidation [	4.064E-05	4.225E-05	1	1	1	P30740	P30740 [144-158]	0	2243.2271
[K].LNQTAL(	1xTMTpro [K	1.131E-05	4.225E-05	1	1	1	Q8NEU8	Q8NEU8 [390-40	0	2397.3997
[K].ESELTDI	1xTMTpro [N	4.264E-05	4.225E-05	1	1	1	P28370	P28370 [681-695]	0	2068.0617
[K].LPDMLN	1xTMTpro [K	2.206E-05	4.225E-05	1	1	1	O75643	O75643 [897-914]	0	2547.446
[R].LELGDV	1xTMTpro [K	1.472E-05	4.225E-05	1	1	1	Q5T160	Q5T160 [230-243]	0	2208.2884
[K].IPDHDP	1xTMTpro [N	3.086E-05	4.225E-05	1	1	1	Q9P2E9	Q9P2E9 [83-97]	0	1961.1139
[R].LEIGVQS	1xTMTpro [N	1.7E-05	4.225E-05	1	1	1	Q9H9T3	Q9H9T3 [243-256	0	1882.0241
[R].ERLEQQ	1xTMTpro [K	1.069E-06	4.225E-05	1	1	1	P39023	P39023 [199-224]	1	3579.9336
[R].LPDGSS	1xTMTpro [N	6.093E-06	4.225E-05	1	1	1	Q92575	Q92575 [326-346]	0	2582.2694

[K].LPDGSE	1xTMTpro [N	1.682E-05	4.225E-05	1	1	4	Q96KP4	Q96KP4 [69-84]	0	1991.186
[K].ERPDLPI	1xCarbamido	1.744E-06	4.225E-05	1	1	2	Q15436	Q15436 [47-63]	0	2373.2556
[K].IPDEFDN	1xTMTpro [N	2.23E-05	4.225E-05	1	1	1	Q3ZCQ8	Q3ZCQ8 [98-113]	0	2216.1882
[R].ERPGEQ	1xTMTpro [N	6.689E-07	4.225E-05	1	1	2	Q96SB3	Q96SB3 [583-603	0	2772.4447
[R].ERPGLG	1xCarbamido	1.376E-05	4.225E-05	1	1	1	Q08379	Q08379 [975-990]	0	2214.1449
[R].LPDDDP	1xCarbamido	9.784E-06	4.225E-05	1	1	1	Q9UBF2	Q9UBF2 [709-726	0	2520.2606
[R].LPCIYLV	1xCarbamido	3.124E-05	4.225E-05	1	1	1	Q9HCC0	Q9HCC0 [165-180	0	2098.1326
[K].FLGDIEV	1xTMTpro [K	3.947E-05	4.225E-05	1	1	3	P26639	P26639 [505-517]	0	2158.1676
[R].ERPQDR	1xTMTpro [N	3.722E-05	4.225E-05	1	1	1	Q01780	Q01780 [222-245]	0	3146.6302
[R].IPAYFVT	1xTMTpro [N	2.526E-06	4.225E-05	1	1	3	P26640	P26640 [731-752]	0	2603.3313
[R].ERPQVE	1xTMTpro [N	5.955E-05	4.225E-05	1	1	1	Q9NQ50	Q9NQ50 [100-113	0	2067.0678
[R].LPAVSSY	1xCarbamido	3.441E-05	4.225E-05	1	1	1	P18754	P18754 [344-362]	0	2445.3667
[R].LPAVEPT	1xCarbamido	4.495E-05	4.225E-05	1	1	2	P98160	P98160 [1920-193	0	2065.0707
[K].FLGDEQ	1xTMTpro [N	4.434E-05	4.225E-05	1	1	2	Q7Z6Z7	Q7Z6Z7 [418-431	0	1973.0299
[K].ERPTPSI	1xCarbamido	3.662E-07	4.225E-05	1	1	2	P07814	P07814 [734-756]	0	2984.4703
[K].IPDPEAV	2xTMTpro [K	2.124E-05	4.225E-05	1	1	3	P27824	P27824 [293-311]	0	3020.6033
[K].LELFLPE	1xTMTpro [K	4.36E-05	4.225E-05	1	1	3	P61088	P61088 [54-68]	0	2356.3118
[R].EREEQE	1xTMTpro [K	4.078E-05	4.225E-05	1	1	1	P12270	P12270 [565-575]	1	1932.0166
[R].IEIESFYE	1xTMTpro [N	3.131E-06	4.225E-05	1	1	11	P11021	P11021 [307-324]	0	2469.1992
[K].IPELLAS	1xTMTpro [K	3.18E-05	4.225E-05	1	1	1	P30740	P30740 [144-158]	0	2227.2322
[K].LPELKPV	3xTMTpro [K	4.33E-05	4.225E-05	1	1	1	Q9NUQ8	Q9NUQ8 [464-479	1	3057.8374
[K].IPEGTILT	1xTMTpro [K	3.677E-05	4.225E-05	1	1	4	Q9NR45	Q9NR45 [301-315	0	2270.2995
[R].LEKEIET	2xTMTpro [K	1.879E-05	4.225E-05	1	1	2	P35527	P35527 [447-472]	1	3792.9748
[K].EQVTNV	1xTMTpro [K	7.673E-06	4.225E-05	1	1	1	P37840	P37840 [61-80]	0	2536.459
[R].LPEEWS	1xTMTpro [N	4.255E-08	4.225E-05	1	1	4	P04792	P04792 [38-75]	0	4397.2678
[R].LEICNLT	1xCarbamido	2.358E-05	4.225E-05	1	1	2	P17655	P17655 [338-354]	0	2592.3722
[K].EQWLNN	1xTMTpro [K	4.972E-06	4.225E-05	1	1	1	Q01082	Q01082 [869-890]	1	3370.8073
[R].FLGTEPI	1xTMTpro [N	2.388E-05	4.225E-05	1	1	1	Q16762	Q16762 [188-206]	0	2314.1998
[R].LEICNLT	1xCarbamido	1.37E-06	4.225E-05	1	1	2	P17655	P17655 [338-355]	1	3024.6744
[R].LELLELD	1xTMTpro [N	4.997E-05	4.225E-05	1	1	1	Q8TD16	Q8TD16 [796-807	0	1799.9822
[R].FLGSND	1xOxidation		4.106E-05	4.225E-05	1	1	2	P20908	P20908 [1766-178	0	2484.1308
[K].IPEDEIW	1xTMTpro [N	2.11E-06	4.225E-05	1	1	3	P27338	P27338 [457-484]	0	3530.7862
[K].LPECEAI	2xCarbamido	1.152E-05	4.225E-05	1	1	1	P00738	P00738 [83-108]	0	3570.7601

[R].FLGSND	1xTMTpro [N	6.445E-07	4.225E-05	1	1	2	P20908	P20908 [1766-178	0	2468.1359
[R].IPEAPAG	2xTMTpro [K	9.124E-06	4.225E-05	1	1	1	Q9Y490	Q9Y490 [36-58]	1	3325.8136
[R].IPEAPAG	1xTMTpro [K	3.946E-06	4.225E-05	1	1	5	Q9Y490	Q9Y490 [36-57]	0	2893.5115
[K].LPEADD	1xTMTpro [N	1.087E-06	4.225E-05	1	1	3	P48147	P48147 [622-643]	0	2832.4045
[R].ERDFTS	1xTMTpro [N	4.163E-05	4.225E-05	1	1	1	Q07065	Q07065 [227-240]	1	2029.0157
[R].LELELRF	1xTMTpro [N	2.053E-06	4.225E-05	1	1	2	P04114	P04114 [1001-102	0	3128.6435
[R].IPDWTP	1xTMTpro [N	1.716E-05	4.225E-05	1	1	2	P51688	P51688 [207-233]	0	3359.7635
[R].ERDVYE	1xTMTpro [K	2.222E-05	4.225E-05	1	1	1	P46940	P46940 [283-302]	1	2970.6028
[K].EQYAQE	1xTMTpro [N	2.045E-05	4.225E-05	1	1	2	Q15126	Q15126 [49-61]	0	1923.9632
[K].FLFVDKI	2xTMTpro [K	3.253E-05	4.225E-05	1	1	2	P35749	P35749 [13-33]	1	3293.8503
[K].IPANQLA	1xTMTpro [K	5.822E-05	4.225E-05	1	1	2	Q9NZM1	Q9NZM1 [682-69	0	2004.2138
[R].ESAAAD	1xTMTpro [K	1.838E-05	4.225E-05	1	1	1	P52756	P52756 [575-588]	0	2035.0992
[K].ESDQGA	1xCarbamido	1.499E-05	4.225E-05	1	1	2	P98160	P98160 [471-485]	0	2006.8867
[K].ESDTGL	1xTMTpro [K	1.446E-05	4.225E-05	1	1	4	P35998	P35998 [67-84]	0	2478.3372
[R].LNVDEA	1xTMTpro [N	5.348E-05	4.225E-05	1	1	2	P10301	P10301 [177-188]	0	1736.9502
[K].LNVAEV	1xTMTpro [K	3.489E-05	4.225E-05	1	1	1	Q9HBI1	Q9HBI1 [133-146]	0	2124.2156
[K].LNVAEV	1xTMTpro [K	4.404E-05	4.225E-05	1	1	3	Q9NVD7	Q9NVD7 [141-154	0	2138.2313
[R].FLFASVI	1xTMTpro [K	1.152E-05	4.225E-05	1	1	1	Q99523	Q99523 [312-325]	1	2210.2135
[R].ESDVPLI	2xTMTpro [K	3.866E-05	4.225E-05	1	1	2	Q969H8	Q969H8 [131-145	1	2663.496
[R].LNTHVKI	2xTMTpro [K	3.242E-05	4.225E-05	1	1	2	Q9BXS5	Q9BXS5 [269-285	0	2931.7852
[R].LNTFGD	1xTMTpro [K	3.545E-05	4.225E-05	1	1	4	P11047	P11047 [256-268]	0	2104.1206
[K].LELNSM	1xTMTpro [K	4.228E-05	4.225E-05	1	1	1	Q15075	Q15075 [931-948]	0	2709.5101
[R].LNVDFAI	1xTMTpro [K	5.888E-05	4.225E-05	1	1	1	P60891	P60891 [185-194]	0	1778.082
[K].INSWVE	1xTMTpro [K	3.113E-05	4.225E-05	2	2	1	P48594; P29	P48594 [147-158]	0	2043.1002
[K].LNSSGS	1xTMTpro [N	6.694E-06	4.225E-05	1	1	2	Q96CV9	Q96CV9 [168-182	0	1930.9677
[K].LNSNTQ	2xTMTpro [K	1.813E-06	4.225E-05	1	1	1	P60842	P60842 [203-226]	1	3500.0226
[K].LNSNTQ	1xTMTpro [K	5.495E-07	4.225E-05	1	1	4	P60842	P60842 [203-225]	0	3067.7205
[K].LNSNDE	1xTMTpro [N	1.186E-05	4.225E-05	1	1	1	P04424	P04424 [81-94]	0	1931.9378
[R].FLEVQYI	1xTMTpro [N	8.051E-07	4.225E-05	1	1	2	Q15149	Q15149 [4524-45	0	2409.2985
[K].FLEVIKP	1xCarbamido	5.084E-06	4.225E-05	1	1	2	P61619	P61619 [5-24]	0	3367.9996
[K].INSENAD	1xTMTpro [N	3.99E-06	4.225E-05	1	1	1	Q8IWV7	Q8IWV7 [1218-12	0	2117.1886
[K].IEIPLFHV	1xTMTpro [N	5.572E-05	4.225E-05	1	2	2	Q8IZ07	Q8IZ07 [411-422]	0	1726.0335
[R].LNSAVE	1xCarbamido	2.031E-05	4.225E-05	1	1	1	O95396	O95396 [148-171]	0	2906.5617

[K].INQYPES	1xTMTpro [K	6.578E-07	4.225E-05	1	1	1	Q687X5	Q687X5 [110-132	0	3157.729
[R].INSVEVY	1xTMTpro [N	2.308E-05	4.225E-05	1	1	1	Q13308	Q13308 [468-480]	0	1905.9666
[K].LNQVCF	1xCarbamido	4.016E-05	4.225E-05	1	1	2	Q8N1F7	Q8N1F7 [418-434	0	2258.0315
[R].FIFDCVS	1xCarbamido	3.069E-05	4.225E-05	1	1	1	A6NDG6	A6NDG6 [239-254	0	2278.1133
[K].FLFDSVS	1xTMTpro [N	8.065E-06	4.225E-05	1	1	2	O00410	O00410 [135-148]	0	1873.0139
[R].ESAAPA	1xTMTpro [N	2.092E-07	4.225E-05	1	1	3	Q92841	Q92841 [36-68]	0	3550.9189
[K].ESADFW	3xCarbamido	1.555E-05	4.225E-05	1	1	1	P29590	P29590 [134-150]	0	2801.3229
[R].IELLHNII	1xCarbamido	9.535E-06	4.225E-05	1	1	1	P15848	P15848 [389-407]	0	2527.2934
[R].LPAGAE	1xTMTpro [K	1.631E-05	4.225E-05	1	1	1	Q86UV5	Q86UV5 [479-496	0	2685.4784
[K].LPAELQE	1xTMTpro [K	5.936E-05	4.225E-05	1	1	1	P27695	P27695 [104-136]	1	4193.2163
[K].LPAELQE	1xTMTpro [K	1E-05	4.225E-05	1	1	2	P27695	P27695 [104-125]	0	3074.6443
[R].LPACVVI	2xCarbamido	4.087E-06	4.225E-05	1	1	3	P61158	P61158 [5-18]	0	2149.1277
[R].FIFPFDY	1xCarbamido	3.657E-05	4.225E-05	1	1	1	O75923	O75923 [1871-18	0	2767.5025
[K].IPAANIL(	1xTMTpro [K	7.194E-06	4.225E-05	1	1	2	P26440	P26440 [263-274]	0	1885.1151
[R].ESAIQGS	1xTMTpro [N	2.109E-05	4.225E-05	1	1	1	Q9BQS8	Q9BQS8 [653-67	0	2264.2053
[K].LNVEGE	2xCarbamido	3.178E-06	4.225E-05	1	1	1	P07942	P07942 [430-441]	0	2068.0447
[R].LPAAGV	1xTMTpro [K	3.576E-05	4.225E-05	1	1	1	P62829	P62829 [52-66]	0	2068.179
[K].LNWNPS	1xTMTpro [K	4.142E-05	4.225E-05	1	1	3	Q99715	Q99715 [352-365]	0	2168.1996
[R].ESATAD	1xTMTpro [K	3.645E-05	4.225E-05	1	1	1	P98175	P98175 [686-699]	0	2047.1203
[R].LNWEAF	1xTMTpro [N	4.768E-05	4.225E-05	1	2	4	P22105	P22105 [3673-368	0	2137.1401
[R].INVYYNE	1xTMTpro [K	4.057E-05	4.225E-05	1	1	2	Q9BUF5	Q9BUF5 [47-58]	0	2040.0893
[R].INVYYNE	1xTMTpro [K	2.668E-05	4.225E-05	1	1	4	P68371	P68371 [47-58]	0	1937.0624
[K].LEILQQC	1xTMTpro [N	5.098E-05	4.225E-05	1	1	1	Q14980	Q14980 [617-631]	0	2057.1674
[R].INVLPLG	1xTMTpro [N	3.079E-06	4.225E-05	1	1	2	P04424	P04424 [194-213]	0	2237.2937
[R].INVLAAC	1xTMTpro [K	1.213E-05	4.225E-05	1	1	2	O00429	O00429 [308-329]	0	3045.6388
[K].ESDLPS/	1xTMTpro [K	2.973E-05	4.225E-05	1	1	1	Q99459	Q99459 [272-290]	0	2617.4216
[R].IEINFPAI	2xTMTpro [K	4.382E-05	4.225E-05	1	1	1	P68036	P68036 [53-67]	0	2702.5738
[K].ESAPFA'	1xTMTpro [K	6.07E-05	4.225E-05	1	1	2	Q99719	Q99719 [240-256]	0	2327.3102
[K].LQCENV	1xCarbamido	5.701E-06	4.225E-05	1	1	1	Q13443	Q13443 [573-596]	0	3012.6511
[K].IILWDHD	1xTMTpro [N	4.793E-05	4.225E-05	1	1	2	Q9HC35	Q9HC35 [533-544	0	1824.9928
[R].LLIVSTTI	2xTMTpro [K	3.588E-05	4.225E-05	1	1	2	Q9Y230	Q9Y230 [354-368	1	2607.5425
[R].FASWAL	1xTMTpro [K	1.973E-05	4.225E-05	1	1	4	Q13263	Q13263 [348-365]	0	2588.4216
[K].LKEAELL	1xTMTpro [K	4.89E-05	4.225E-05	1	1	2	P53618	P53618 [127-141]	1	2331.3965

[R].FFSASC\	1xCarbamido	3.411E-05	4.225E-05	1	1	4	P02788	P02788 [171-182]	0	1894.0024
[K].FATDGE	1xCarbamido	1.628E-05	4.225E-05	1	1	2	Q01082	Q01082 [594-610]	0	2561.3314
[K].LKEADEI	1xCarbamido	5.59E-05	4.225E-05	1	1	2	Q86UP2	Q86UP2 [1122-11	1	2999.6362
[K].LGDTPG	1xTMTpro [N	4.092E-05	4.225E-05	1	1	1	Q9H269	Q9H269 [511-525	0	1825.9615
[K].LGDVGM	1xCarbamido	2.248E-06	4.225E-05	1	1	1	P13489	P13489 [239-257]	0	2313.2014
[R].IGDVLVN	1xTMTpro [N	1.284E-05	4.225E-05	1	1	1	Q12802	Q12802 [2077-20	0	2052.0681
[R].LKDTVLS	1xTMTpro [K	1.186E-05	4.225E-05	1	1	1	Q96HP0	Q96HP0 [743-758	1	2438.3746
[K].FFQPTEI	1xTMTpro [N	1.125E-05	4.225E-05	1	1	2	O94973	O94973 [826-840]	0	2183.0551
[K].LKDISTL	3xTMTpro [K	6.104E-06	4.225E-05	2	3	6	P39687; Q92	P39687 [100-111]	2	2601.6696
[K].FATVEV	1xTMTpro [K	1.193E-06	4.225E-05	1	1	4	Q9NRV9	Q9NRV9 [41-56]	0	2398.3473
[K].LKDISTL	2xTMTpro [K	3.846E-05	4.225E-05	2	4	3	P39687; Q92	P39687 [100-110]	1	2169.3675
[R].LKDLNS(	2xTMTpro [K	9.513E-07	4.225E-05	1	1	1	Q13813	Q13813 [1714-17	1	3398.8293
[K].LGDVYV	1xTMTpro [N	4.226E-06	4.225E-05	2	2	5	P07205; P0C	P07205 [157-171]	0	1938.9993
[K].LKDLGHI	2xTMTpro [K	2.463E-07	4.225E-05	1	1	2	O60763	O60763 [927-962]	2	4990.3541
[K].FAVQDV	1xTMTpro [K	1.119E-05	4.225E-05	1	1	1	P35658	P35658 [823-843]	0	3149.6399
[R].LKDLEAI	2xTMTpro [K	5.047E-05	4.225E-05	1	1	3	P02545	P02545 [134-144]	1	2156.3471
[R].LKDLDLI	1xTMTpro [K	3.715E-05	4.225E-05	1	1	1	Q7KZ85	Q7KZ85 [1099-11	1	2385.3157
[R].LKDIALG	1xTMTpro [K	1.065E-05	4.225E-05	1	1	1	O95721	O95721 [216-236]	1	3053.632
[K].LKDHML	2xTMTpro [K	6.608E-06	4.225E-05	1	1	1	O94760	O94760 [252-267]	1	2782.6027
[K].FASQQG	1xTMTpro [N	1.903E-05	4.225E-05	1	1	3	P51911	P51911 [173-185]	0	1721.86
[K].LKECCEI	2xCarbamido	2.475E-05	4.225E-05	1	1	19	P02768	P02768 [299-310]	1	2763.6253
[R].IGDQEFI	1xTMTpro [K	4.618E-05	4.225E-05	1	1	1	P46108	P46108 [89-105]	0	2593.4045
[K].FFSEAVI	1xTMTpro [K	3.545E-05	4.225E-05	1	1	1	Q8IYQ7	Q8IYQ7 [250-270]	0	2762.526
[R].FAPPLVI	1xTMTpro [K	3.369E-05	4.225E-05	1	1	1	P04181	P04181 [414-426]	1	2135.272
[R].LKESVAI	1xCarbamido	7.301E-06	4.225E-05	1	1	1	Q9NPQ8	Q9NPQ8 [327-34	1	2480.4402
[K].FAPVCSI	2xCarbamido	2.524E-05	4.225E-05	1	1	2	Q8WUP2	Q8WUP2 [299-31	0	2077.0894
[K].IKESLPII	1xTMTpro [K	9.659E-06	4.225E-05	1	1	1	Q8N573	Q8N573 [282-296	1	2292.3419
[R].LKESAEI	2xTMTpro [K	6.392E-06	4.225E-05	1	1	2	P12270	P12270 [994-100	1	2463.4275
[R].FAQGSS	1xTMTpro [N	1.102E-05	4.225E-05	1	1	1	Q13308	Q13308 [79-92]	0	1759.9298
[R].LKEQGQ	2xTMTpro [K	9.023E-06	4.225E-05	1	1	1	P84095	P84095 [129-147]	1	2918.7244
[R].FAQHGT	1xTMTpro [N	2.673E-05	4.225E-05	1	1	1	P23246	P23246 [480-493]	0	2066.9891
[R].FFTGQIT	1xTMTpro [K	4.981E-05	4.225E-05	1	1	2	Q13423	Q13423 [214-224]	0	1749.0191
[K].LKEIYEN	2xTMTpro [K	5.331E-05	4.225E-05	1	1	1	Q13200	Q13200 [106-120]	2	2704.5272

[K].FFQPTEI	1xTMTpro [N	3.9E-05	4.225E-05	1	1	2	O95782	O95782 [864-878]	0	2167.0602
[K].IGDLHPC	1xTMTpro [K	3.086E-05	4.225E-05	1	1	8	P12111	P12111 [1002-10	0	2068.2774
[R].FAQPGT	1xTMTpro [N	2.52E-05	4.225E-05	1	1	2	Q8WXF1	Q8WXF1 [265-27	0	1953.9666
[K].FAQVVL	2xTMTpro [K	3.005E-05	4.225E-05	1	1	1	Q0VDF9	Q0VDF9 [458-469	1	2316.4107
[R].LKEGLDI	2xTMTpro [K	3.213E-06	4.225E-05	1	1	1	P55011	P55011 [922-943]	1	3393.9046
[R].FASEAS	1xTMTpro [N	7.167E-06	4.225E-05	1	1	4	P17661	P17661 [356-369]	0	1832.9098
[R].FASEIAC	1xTMTpro [N	4.035E-06	4.225E-05	1	1	3	P28331	P28331 [185-200]	0	1912.9935
[R].LKEGDT	2xTMTpro [K	2.29E-06	4.225E-05	1	1	1	Q14914	Q14914 [57-70]	1	2448.3771
[R].FASFEA	1xTMTpro [K	4.592E-06	4.225E-05	1	1	1	P01903	P01903 [76-92]	0	2360.3106
[R].LKEFLEI	2xTMTpro [K	2.163E-05	4.225E-05	1	1	1	P49756	P49756 [496-511]	2	2925.5298
[R].IKEETEII	2xTMTpro [K	3.568E-08	4.225E-05	1	1	2	Q9Y230	Q9Y230 [131-157	1	3854.1579
[K].IGDQEFI	1xTMTpro [K	3.225E-05	4.225E-05	1	1	2	P46109	P46109 [73-89]	0	2643.4314
[R].FAQPGS	1xTMTpro [N	4.768E-05	4.225E-05	1	1	4	Q15233	Q15233 [257-270]	0	1999.9543
[R].IKDFLQC	1xCarbamido	1.927E-05	4.225E-05	1	1	1	P15924	P15924 [2250-226	1	3157.7383
[K].FCAGNP	1xCarbamido	4.361E-06	4.225E-05	1	1	1	Q14573	Q14573 [1235-12	0	2135.1676
[R].IGDYVQ	1xTMTpro [K	4.064E-05	4.225E-05	1	1	1	P12081	P12081 [267-288]	0	3031.6708
[R].LHSLLGI	1xTMTpro [K	1.625E-05	4.225E-05	1	1	2	Q9Y262	Q9Y262 [280-292	0	2129.225
[R].FCQVNT	1xCarbamido	1.015E-05	4.225E-05	1	1	1	Q7Z3D6	Q7Z3D6 [55-73]	0	2722.473
[K].FCSEAQ	2xCarbamido	5.728E-05	4.225E-05	1	1	1	Q07954	Q07954 [2733-27	0	2016.8975
[K].LGEGQF	1xCarbamido	2.766E-06	4.225E-05	1	1	1	Q16832	Q16832 [569-587]	0	2756.3879
[R].FFPFGL	1xTMTpro [K	4.626E-05	4.225E-05	1	1	1	Q9HAT2	Q9HAT2 [315-329	0	2293.3088
[R].FCTGLT	1xCarbamido	1.667E-05	4.225E-05	1	1	2	P12277	P12277 [253-265]	0	2166.2124
[K].FCTTGIE	1xCarbamido	1.825E-05	4.225E-05	1	1	1	Q92747	Q92747 [343-358]	0	2471.2595
[R].LHSDIDS	1xTMTpro [K	3.893E-05	4.225E-05	1	1	1	P55287	P55287 [77-89]	0	1979.0689
[K].FFPEDV	1xTMTpro [N	1.735E-05	4.225E-05	1	1	1	P35241	P35241 [84-100]	0	2384.2305
[K].FCVSDTI	1xCarbamido	4.567E-06	4.225E-05	1	1	1	P42892	P42892 [434-453]	0	2854.4763
[R].FCPAGV	1xCarbamido	5.028E-06	4.225E-05	1	1	1	Q16134	Q16134 [560-578]	0	2478.2083
[R].LHQMSV	1xCarbamido	3.01E-05	4.225E-05	1	1	3	P98160	P98160 [2683-269	0	2055.9911
[R].IHQIGPC	2xCarbamido	2.493E-06	4.225E-05	1	1	1	P31689	P31689 [162-186]	0	3183.5239
[K].LHQEDN	1xTMTpro [K	4.404E-05	4.225E-05	1	1	1	P18031	P18031 [59-73]	0	2381.2956
[R].LHPVILA	1xTMTpro [N	5.831E-05	4.225E-05	1	1	1	O00303	O00303 [94-108]	0	2016.1449
[K].IGEHMEI	1xTMTpro [K	1.706E-05	4.225E-05	1	1	2	Q16881	Q16881 [386-396]	0	1888.0242
[K].LHNMIVI	1xTMTpro [K	1.667E-05	4.225E-05	1	1	1	Q16891	Q16891 [331-343]	0	2118.2237

[R].IHNLALN	2xCarbamido	7.194E-06	4.225E-05	1	1	1	P53992	P53992 [824-840]	0	2379.2232
[K].IGEHTPS	1xTMTpro [N	3.108E-06	4.225E-05	1	1	5	P04075	P04075 [154-173]	0	2411.3036
[R].FDCSSA	2xCarbamido	1.524E-05	4.225E-05	1	1	2	Q8IXB1	Q8IXB1 [401-424]	0	3373.7092
[K].IHMPAAL	1xCarbamido	3.27E-05	4.225E-05	1	1	1	Q8NE62	Q8NE62 [88-102]	0	2000.0377
[K].FDDDKV	1xTMTpro [K	3.375E-05	4.225E-05	1	1	3	P54578	P54578 [450-465]	1	2470.3685
[R].FDAERP	1xCarbamido	1.324E-05	4.225E-05	1	1	1	Q9HCD5	Q9HCD5 [192-20	0	2456.3463
[R].FFTINPE	1xTMTpro [K	5.572E-05	4.225E-05	1	1	3	P55287	P55287 [430-441]	0	2036.1348
[R].FCNPGF	2xCarbamido	1.98E-05	4.225E-05	1	1	2	Q99805	Q99805 [173-187	0	2397.2227
[K].IGEEEIQ	2xTMTpro [K	5.917E-05	4.225E-05	1	1	2	Q15459	Q15459 [488-499	0	2341.3431
[K].LKDEGV	2xTMTpro [K	5.545E-06	4.225E-05	1	1	4	Q99715	Q99715 [1323-13	1	2444.4945
[R].LKDEEIS	2xTMTpro [K	1.034E-05	4.225E-05	1	1	2	O75116	O75116 [1006-10	1	2200.3369
[R].FFQGMF	1xTMTpro [K	4.345E-05	4.225E-05	1	1	1	P43246	P43246 [22-35]	0	2247.2451
[R].LKDALLI	1xCarbamido	9.242E-06	4.225E-05	1	1	1	Q8NI27	Q8NI27 [729-750]	1	3155.7929
[K].FCEIGSI	2xCarbamido	7.45E-06	4.225E-05	1	1	2	Q14520	Q14520 [186-203	0	2467.0501
[R].LKCDEW	3xCarbamido	3.114E-06	4.225E-05	1	1	1	P02787	P02787 [372-399]	2	4445.3143
[R].LKCDEW	1xCarbamido	2.21E-05	4.225E-05	1	1	6	P02787	P02787 [372-384]	1	2434.358
[R].IKAEYEC	1xTMTpro [K	5.046E-07	4.225E-05	1	1	1	P22234	P22234 [312-331]	1	2700.5216
[R].LKAEFQI	2xTMTpro [K	5.907E-05	4.225E-05	1	1	1	P15924	P15924 [1330-13	1	2105.2423
[K].FCFSNEI	1xCarbamido	2.431E-05	4.225E-05	1	1	1	Q9Y3B3	Q9Y3B3 [108-120	0	2260.1352
[K].IGEEQSI	1xTMTpro [K	1.446E-06	4.225E-05	1	1	2	Q09028	Q09028 [350-376	0	3481.7731
[R].LKAEAEI	2xTMTpro [K	1.818E-05	4.225E-05	1	1	3	Q15149	Q15149 [2462-24	1	2311.4165
[R].FCGQLG	1xCarbamido	2.799E-05	4.225E-05	1	1	2	P00736	P00736 [88-102]	0	2137.172
[R].FCGQLG	1xCarbamido	9.641E-06	4.225E-05	1	1	1	P00736	P00736 [88-103]	1	2569.4741
[R].LGEDLN	1xTMTpro [K	4.177E-05	4.225E-05	1	1	1	Q14204	Q14204 [1072-10	1	2504.4845
[K].IHVSDQE	1xTMTpro [K	6.707E-06	4.225E-05	1	1	2	P22314	P22314 [807-830]	1	3291.7312
[R].IHVLEAC	1xTMTpro [K	3.317E-05	4.225E-05	1	1	2	Q9BSJ8	Q9BSJ8 [651-662	0	1958.193
[K].LGEDIVI	1xTMTpro [K	2.71E-06	4.225E-05	1	1	1	Q9NPJ3	Q9NPJ3 [96-108]	0	2016.2349
[R].FCLEGM	1xCarbamido	5.767E-07	4.225E-05	1	1	3	Q96KP4	Q96KP4 [160-180	0	2693.2758
[R].LHTLEEE	2xTMTpro [K	6.264E-06	4.225E-05	1	1	3	Q9UQE7	Q9UQE7 [200-21	1	2900.6186
[K].LHTEAQI	1xTMTpro [N	2.425E-06	4.225E-05	1	1	5	P01023	P01023 [320-338]	0	2414.2846
[K].LHTDDEI	1xTMTpro [N	1.446E-05	4.225E-05	1	1	2	Q9Y490	Q9Y490 [165-178	0	2025.0109
[R].FCGLPQ	1xCarbamido	1.109E-05	4.225E-05	1	1	2	P05164	P05164 [605-622]	0	2276.2392
[K].FFNANQ	1xTMTpro [K	4.923E-05	4.225E-05	1	1	1	Q9BTY2	Q9BTY2 [111-127	0	2523.3276

[K].FAPEMD	1xCarbamido	3.512E-06	4.225E-05	1	1	1	Q9BQG0	Q9BQG0 [348-36	0	2895.2772
[R].LKETIDN	1xTMTpro [K	7.298E-07	4.225E-05	1	1	2	P60228	P60228 [192-211]	1	2865.5926
[R].EYYFAE	1xTMTpro [K	3.079E-06	4.225E-05	1	1	3	P05546	P05546 [242-261]	0	2920.4901
[R].LIADDFF	1xTMTpro [K	5.209E-05	4.225E-05	1	1	2	P09238	P09238 [424-435]	0	1909.0926
[K].ILADAAA	1xTMTpro [N	4.352E-05	4.225E-05	1	1	2	Q5BJH7	Q5BJH7 [271-283	0	1585.9233
[K].ILACDDL	1xCarbamido	3.967E-05	4.225E-05	1	1	2	Q9P2R7	Q9P2R7 [427-438	0	1665.8437
[K].IIAATIEN	1xTMTpro [N	3.712E-06	4.225E-05	1	1	1	P08779	P08779 [178-196]	0	2368.3519
[K].LIAALSTI	1xCarbamido	5.158E-05	4.225E-05	1	1	3	Q92616	Q92616 [1344-13	0	3526.021
[R].FAADIIS	1xOxidation	6.09E-05	4.225E-05	1	1	2	Q13200	Q13200 [121-137	0	2132.1051
[K].LLAALLE	1xTMTpro [K	1.564E-08	4.225E-05	1	1	3	Q9Y490	Q9Y490 [593-613	0	2730.6009
[K].FAAEEFI	2xTMTpro [K	1.097E-07	4.225E-05	1	1	4	P61960	P61960 [35-69]	1	4488.4959
[R].ILAAAFP	1xTMTpro [N	4.465E-05	4.225E-05	1	1	1	Q63HR2	Q63HR2 [140-153	0	1859.0459
[K].IKWGDA	2xTMTpro [K	2.956E-06	4.225E-05	1	1	6	P04406	P04406 [85-107]	1	3430.8385
[R].LKVPPAI	1xTMTpro [K	4.665E-05	4.225E-05	1	1	4	P62424	P62424 [74-89]	1	2419.4317
[R].FGDEDL	1xTMTpro [K	1.356E-05	4.225E-05	1	1	1	Q9H425	Q9H425 [108-128	0	3133.5035
[R].FAATTVI	1xTMTpro [K	1.374E-05	4.225E-05	1	1	2	Q9C0C2	Q9C0C2 [156-167	0	1901.1239
[K].IKVDEFV	2xTMTpro [K	7.687E-06	4.225E-05	1	1	1	P11766	P11766 [340-357]	1	3060.7186
[R].LKTEPEI	2xTMTpro [K	3.779E-07	4.225E-05	1	1	2	Q16666	Q16666 [560-578	1	3030.6663
[R].LKTDNA	2xTMTpro [K	1.099E-07	4.225E-05	1	1	1	P14866	P14866 [61-97]	1	4135.003
[K].FACNGT	1xCarbamido	5.853E-08	4.225E-05	1	2	1	P41567	P41567 [67-90]	0	3064.5118
[K].LKSELV	1xTMTpro [K	1.657E-05	4.225E-05	2	2	1	P42167; P42	P42167 [16-33]; F	1	2547.475
[R].FADDTY	1xTMTpro [K	2.113E-05	4.225E-05	2	2	8	P62820; Q9I	P62820 [31-49]; Q	0	2780.4162
[R].FGAVWT	1xTMTpro [K	6.789E-07	4.225E-05	1	1	2	Q14697	Q14697 [611-627	0	2555.3174
[R].FGDLLNI	1xTMTpro [K	2.984E-05	4.225E-05	1	1	2	Q9BX66	Q9BX66 [777-788	0	1930.0777
[R].LIADLGS	1xTMTpro [N	2.186E-06	4.225E-05	1	1	1	Q92520	Q92520 [164-179	0	1982.1242
[R].FGDPEC	1xCarbamido	3.879E-05	4.225E-05	1	1	1	P22102	P22102 [293-306]	0	2237.2859
[R].EYWMDI	1xTMTpro [K	5.85E-05	4.225E-05	1	1	2	P53999	P53999 [87-100]	0	2333.155
[K].LLALNPD	1xTMTpro [K	4.801E-05	4.225E-05	1	1	2	Q9BSL1	Q9BSL1 [166-178	0	2051.2396
[K].LIAINANI	1xTMTpro [K	6.061E-05	4.225E-05	1	1	1	Q9H2U2	Q9H2U2 [204-216	0	1964.1308
[R].ILAIGLIN	1xTMTpro [K	8.607E-07	4.225E-05	1	1	5	P46940	P46940 [539-556]	0	2491.4263
[R].IGAFGYI	1xCarbamido	2.611E-05	4.225E-05	1	1	4	P61586	P61586 [151-162]	0	1942.0058
[R].LLALDPN	1xTMTpro [N	3.069E-05	4.225E-05	1	1	1	Q13043	Q13043 [448-463	0	2234.1732
[R].ILALCMC	1xCarbamido	6.003E-05	4.225E-05	2	2	3	P35241; P26	P35241 [280-293]	0	2041.0352

[R].EYTVINE	2xCarbamido	1.757E-05	4.225E-05	1	1	2	Q6UXH9	Q6UXH9 [25-43]	0	2617.2168
[R].ILALCMC	1xCarbamido	3E-06	4.225E-05	2	2	2	P35241; P26	P35241 [280-293]	0	2025.0403
[R].EYTVTEI	1xTMTpro [N	1.208E-05	4.225E-05	1	1	1	P14543	P14543 [588-603]	1	2098.0372
[R].ILAGDVE	1xOxidation [	8.139E-06	4.225E-05	1	1	1	P40123	P40123 [66-86]	0	2630.3316
[K].LKSEGE	2xTMTpro [K	1.202E-05	4.225E-05	1	1	4	P48444	P48444 [242-256]	1	2497.3822
[R].ILAGDVE	1xTMTpro [N	2.692E-05	4.225E-05	1	1	1	P40123	P40123 [66-86]	0	2614.3367
[R].LLAGDH	1xTMTpro [N	1.161E-05	4.225E-05	1	1	1	Q14515	Q14515 [567-579]	0	1750.0546
[K].EYVEPEI	1xTMTpro [N	4.495E-05	4.225E-05	1	1	1	Q7KZ85	Q7KZ85 [382-395	0	2117.0987
[K].LLAESVT	1xTMTpro [K	4.071E-05	4.225E-05	1	1	1	Q14204	Q14204 [1716-17	0	2143.2506
[R].LLAENNI	1xTMTpro [N	4.419E-05	4.225E-05	1	1	2	Q16706	Q16706 [61-73]	0	1803.0295
[R].LGAGPD	1xTMTpro [K	1.735E-05	4.225E-05	1	1	2	P51812	P51812 [311-323]	1	1949.1311
[K].EYVLPSI	1xTMTpro [K	1.204E-05	4.225E-05	1	1	4	P01024	P01024 [226-241]	0	2487.3878
[K].EYVNAS	1xTMTpro [K	1.676E-05	4.225E-05	1	1	1	O15231	O15231 [580-596]	0	2388.3015
[K].IIAEGAN	1xTMTpro [K	8.941E-06	4.225E-05	1	2	1	P00367	P00367 [400-420]	1	2850.5857
[K].IIAEGAN	1xTMTpro [K	5.509E-05	4.225E-05	1	2	2	P00367	P00367 [400-415]	0	2192.2054
[R].LLAEDDI	2xCarbamido	1.451E-05	4.225E-05	1	1	1	P56937	P56937 [22-36]	0	2132.0799
[K].ILAGDKN	1xTMTpro [K	3.495E-05	4.225E-05	1	1	1	P12814	P12814 [835-849]	1	2360.3139
[R].IKQESNC	2xTMTpro [K	2.584E-07	4.225E-05	1	1	2	Q12955	Q12955 [1140-11	1	4409.4748
[K].LKQEMG	1xTMTpro [K	4.213E-05	4.225E-05	1	1	1	P27701	P27701 [113-126]	1	2195.3077
[K].LGAGYP	1xOxidation [	4.785E-05	4.225E-05	1	1	2	Q16836	Q16836 [250-271]	0	2981.5826
[K].LKGPQIT	2xTMTpro [K	3.664E-06	4.225E-05	1	1	3	Q09666	Q09666 [370-387]	1	2735.6487
[R].LKGIEEE	2xCarbamido	4.583E-07	4.225E-05	1	1	1	P15170	P15170 [366-391]	1	3618.8904
[R].FAHYVV	1xTMTpro [N	1.287E-06	4.225E-05	1	1	3	P19827	P19827 [66-83]	0	2310.2162
[R].FAKPVYI	2xTMTpro [K	2.906E-05	4.225E-05	1	1	2	P51659	P51659 [563-579]	0	2936.6524
[R].LGCHPQ	1xCarbamido	4.737E-05	4.225E-05	1	1	1	P41743	P41743 [505-523]	0	2489.2468
[K].LKGEMM	2xTMTpro [K	1.951E-05	4.225E-05	1	1	1	P07195	P07195 [59-77]	1	3085.7358
[K].LKGEIDA	1xTMTpro [K	7.049E-06	4.225E-05	1	1	2	Q09666	Q09666 [1795-18	1	2449.3794
[K].IKGEHPC	2xTMTpro [K	5.179E-06	4.225E-05	1	2	5	P09429	P09429 [113-127]	1	2433.4646
[K].FALGIFA	1xTMTpro [K	5.028E-06	4.225E-05	1	1	2	P46940	P46940 [623-641]	0	2545.4158
[K].LKGEAT	2xTMTpro [K	4.695E-06	4.225E-05	1	1	3	P35637	P35637 [333-348]	1	2574.4595
[R].FAGYVT	1xTMTpro [K	1.319E-05	4.225E-05	1	1	1	O75533	O75533 [51-80]	0	3806.8628
[K].IKGDVPS	2xTMTpro [K	1.169E-07	4.225E-05	1	1	6	Q09666	Q09666 [5208-52	1	3232.8245
[K].IKGDVD\	2xTMTpro [K	3.99E-06	4.225E-05	1	1	3	Q09666	Q09666 [2467-24	1	2483.4537

[K].LKGDVD	1xOxidation [	1.376E-06	4.225E-05	1	1	4	Q09666	Q09666 [1337-13!	1	2773.5474
[K].LKGDVD	2xTMTpro [K	3.651E-05	4.225E-05	1	1	2	Q09666	Q09666 [1337-13!	1	2757.5524
[K].FAMEPE	1xTMTpro [N	1.341E-05	4.225E-05	1	1	3	P13667	P13667 [486-499]	0	1990.9387
[K].LKGDDL	3xTMTpro [K	4.783E-06	4.225E-05	1	1	3	P07910	P07910 [188-198]	2	2445.5545
[K].FFVAVP	1xTMTpro [K	1.14E-05	4.225E-05	1	1	2	O60271	O60271 [1228-12!	0	3132.6709
[K].LKFPEP	3xTMTpro [K	5.305E-05	4.225E-05	1	1	1	Q8IZQ5	Q8IZQ5 [105-119]	2	3029.8392
[K].IKFETNP	2xTMTpro [K	8.398E-06	4.225E-05	1	1	1	Q709C8	Q709C8 [610-639	1	4287.3216
[R].FFVADT	1xTMTpro [K	5.974E-05	4.225E-05	1	1	1	Q96I99	Q96I99 [59-73]	0	2205.2047
[R].IGDELDS	1xTMTpro [N	2.191E-05	4.225E-05	1	1	1	Q07812	Q07812 [66-78]	0	1823.9128
[R].FAIQDIS	1xTMTpro [K	5.839E-06	4.225E-05	3	4	8	P12814; P35	P12814 [134-147]	0	2146.1887
[K].LKETCVS	1xCarbamido	2.71E-06	4.225E-05	1	1	2	Q02952	Q02952 [466-487]	1	3288.7086
[K].LKGTEDI	3xTMTpro [K	1.167E-05	4.225E-05	2	2	3	P09493; P06	P09493 [50-65]; F	2	3055.7668
[K].FAGQMG	1xTMTpro [K	3.709E-05	4.225E-05	1	1	1	Q15286	Q15286 [138-152]	0	2236.2291
[K].FGAVFL	1xTMTpro [K	1.854E-08	4.225E-05	1	1	1	O75083	O75083 [127-147]	0	2816.4863
[K].FADTPIY	1xTMTpro [N	3.74E-06	4.225E-05	1	1	1	P35475	P35475 [290-313]	0	3091.5485
[K].FADTRP	1xTMTpro [K	1.663E-05	4.225E-05	1	1	1	Q9BZF1	Q9BZF1 [745-762	0	2831.4682
[K].IKPSTLQ	2xTMTpro [K	2.431E-07	4.225E-05	1	1	2	Q96F85	Q96F85 [52-74]	0	3360.0698
[R].LKPPFP	2xCarbamido	5.455E-07	4.225E-05	1	1	2	P49821	P49821 [225-256]	0	4008.1582
[K].IKPHLMS	1xOxidation [	2.775E-05	4.225E-05	1	1	1	P07237	P07237 [351-370]	1	3651.0721
[K].IKPHLMS	3xTMTpro [K	2.124E-05	4.225E-05	1	1	2	P07237	P07237 [351-370]	1	3635.0772
[K].LGAPLS	1xCarbamido	8.337E-06	4.225E-05	1	1	1	P50479	P50479 [242-257]	0	1973.0809
[K].LKPFLGS	1xCarbamido	2.105E-05	4.225E-05	1	1	1	Q9BZF1	Q9BZF1 [608-624	0	2762.5691
[K].LKPEDIT	1xTMTpro [K	7.141E-06	4.225E-05	1	1	2	P05556	P05556 [106-122]	0	2627.574
[K].FFYLLEF	1xCarbamido	5.86E-05	4.225E-05	1	1	1	Q9Y5Y6	Q9Y5Y6 [384-399	0	2404.323
[K].LKPDTP	1xTMTpro [K	4.263E-07	4.225E-05	1	1	3	Q99715	Q99715 [1813-18	0	2974.6017
[K].LKPAFIK	1xOxidation [	1.04E-06	4.225E-05	1	1	1	P55084	P55084 [292-325]	0	4805.6614
[K].LKPAFIK	3xTMTpro [K	2.472E-08	4.225E-05	1	1	4	P55084	P55084 [292-325]	0	4789.6665
[K].LKNSLFE	2xTMTpro [K	5.482E-05	4.225E-05	1	1	1	P02671	P02671 [88-97]	1	2182.3052
[K].FGAGAA	1xTMTpro [K	1.111E-05	4.225E-05	1	1	2	P15502	P15502 [266-312]	0	4371.4796
[R].LKNDIEE	2xTMTpro [K	1.709E-06	4.225E-05	1	1	1	Q92878	Q92878 [764-786]	1	3529.9127
[R].FGAFHS	2xTMTpro [K	4.549E-08	4.225E-05	1	1	2	Q9NZN4	Q9NZN4 [42-71]	0	4068.1357
[R].FAFVEF	1xTMTpro [N	1.812E-05	4.225E-05	1	1	1	Q8WXA9	Q8WXA9 [108-12	0	1930.9982
[K].LKLEAEL	2xTMTpro [K	2.016E-07	4.225E-05	1	1	4	P05787	P05787 [159-176]	1	2946.679

[K].LKGTVG	1xTMTpro [K	2.151E-05	4.225E-05	1	1	1	Q14204	Q14204 [3892-39	1	2816.5227
[R].IKGTQCI	3xCarbamido	1.932E-07	4.225E-05	1	1	6	P35555	P35555 [903-922]	1	3296.6781
[R].IGASTLL	1xTMTpro [N	5.209E-05	4.225E-05	1	1	2	Q9Y315	Q9Y315 [288-299	0	1578.9022
[K].LIALSIDS	1xTMTpro [K	8.816E-07	4.225E-05	1	1	3	P30041	P30041 [68-84]	0	2505.4208
[R].LHLVSPA	1xCarbamido	5.287E-05	4.225E-05	1	1	3	P98160	P98160 [2978-29	0	2007.0289
[K].FDDGAV	1xCarbamido	1.555E-06	4.225E-05	1	1	2	O14974	O14974 [38-58]	0	2825.4159
[R].IGPEPTT	1xTMTpro [K	9.14E-06	4.225E-05	1	1	3	Q9H223	Q9H223 [91-122]	0	3901.0073
[R].IGPEPTT	1xTMTpro [N	4.6E-07	4.225E-05	1	1	2	Q9H4M9	Q9H4M9 [88-119]	0	3576.8691
[K].LGPDDV	1xTMTpro [N	4.776E-05	4.225E-05	1	1	1	Q05086	Q05086 [227-240	0	1788.9663
[R].IGPAPAF	1xTMTpro [K	4.863E-07	4.225E-05	1	1	3	P98095	P98095 [1106-11	0	2420.4409
[K].IGPALSC	1xCarbamido	8.559E-07	4.225E-05	1	1	3	P00352	P00352 [180-210]	0	4097.3977
[K].LGPALA	1xTMTpro [K	8.035E-06	4.225E-05	1	1	2	P05091	P05091 [196-209]	0	1978.2015
[K].LGNYAG	1xCarbamido	3.569E-05	4.225E-05	1	1	1	O43765	O43765 [138-150	0	1756.8608
[R].LGGLTQ	1xTMTpro [K	2.855E-05	4.225E-05	1	1	1	P26368	P26368 [175-195]	0	2741.5805
[K].LGNTISS	1xTMTpro [K	2.027E-05	4.225E-05	1	1	1	Q9Y4L1	Q9Y4L1 [577-594	0	2344.3004
[R].IGGNEG	1xTMTpro [N	1.483E-05	4.225E-05	1	1	2	Q96AE4	Q96AE4 [272-284	0	1640.9291
[K].LGNNCV	1xCarbamido	1.631E-05	4.225E-05	1	1	1	Q99714	Q99714 [54-79]	1	3645.0138
[K].LGNNCV	1xCarbamido	5.509E-05	4.225E-05	1	1	1	Q99714	Q99714 [54-69]	0	2330.2306
[R].IGNLQTI	1xTMTpro [N	4.149E-05	4.225E-05	1	1	2	O75369	O75369 [37-49]	0	1705.9404
[K].LGNDFM	1xTMTpro [N	4.713E-06	4.225E-05	1	1	2	Q9HC38	Q9HC38 [97-116]	0	2356.225
[R].FEEPAPI	1xTMTpro [N	2.179E-05	4.225E-05	1	1	2	Q07157	Q07157 [1132-11	0	1968.0146
[K].LGMPQF	1xTMTpro [N	3.152E-05	4.225E-05	1	1	1	P51812	P51812 [285-300]	0	2091.1592
[K].LGGNVV	1xTMTpro [K	2.475E-05	4.225E-05	1	1	1	Q9Y281	Q9Y281 [153-166	0	1990.2192
[R].FEETGQ	1xTMTpro [K	1.575E-05	4.225E-05	1	1	1	P36405	P36405 [100-115]	0	2502.3107
[K].LGGNYG	1xTMTpro [K	1.247E-05	4.225E-05	1	1	1	O15382	O15382 [230-246	0	2395.3841
[K].LGIYDAI	1xTMTpro [K	1.446E-05	4.225E-05	1	1	2	Q12797	Q12797 [87-104]	0	2509.2226
[K].LGIWGG	1xTMTpro [K	3.08E-05	4.225E-05	1	1	1	Q709C8	Q709C8 [29-44]	0	2319.368
[R].LGPGGL	1xTMTpro [K	3.435E-06	4.225E-05	1	1	6	Q16543	Q16543 [287-307	0	2877.5741
[R].LGPHVT	1xTMTpro [N	8.215E-06	4.225E-05	1	1	2	P35442	P35442 [246-261]	0	2033.0623
[K].FFDHSG	1xTMTpro [N	6.381E-06	4.225E-05	1	1	2	Q15005	Q15005 [196-214	0	2518.2243
[R].LGPLLDI	1xTMTpro [N	3.091E-05	4.225E-05	1	1	2	Q13724	Q13724 [709-720	0	1586.9437
[R].LGQSDP	1xTMTpro [K	1.881E-06	4.225E-05	1	1	2	P29401	P29401 [187-204]	0	2677.4263
[R].IGQPTLL	1xTMTpro [N	2.937E-05	4.225E-05	1	1	1	P00568	P00568 [109-128]	0	2507.3499

[K].LGQPLLI	1xTMTpro [K	3.208E-05	4.225E-05	1	1	1	Q6NZY4	Q6NZY4 [183-202	0	2870.5908
[R].IGQNCSI	2xCarbamido	2.645E-06	4.225E-05	1	1	1	Q9Y5P6	Q9Y5P6 [262-287	0	3000.5566
[K].FEAAETI	1xTMTpro [N	2.682E-05	4.225E-05	1	1	1	Q07866	Q07866 [480-492]	0	1771.8856
[K].FEAASLI	1xCarbamido	7.301E-06	4.225E-05	1	1	1	Q9Y6X3	Q9Y6X3 [107-131	0	3432.8329
[R].LGQMVL	1xTMTpro [K	5.586E-06	4.225E-05	1	1	2	O60664	O60664 [181-195]	0	2125.2546
[K].FEAHPN	1xTMTpro [N	5.159E-06	4.225E-05	1	1	2	P78347	P78347 [495-514]	0	2661.3632
[R].LGQLEG	2xCarbamido	2.422E-09	4.225E-05	1	1	3	Q9Y6C2	Q9Y6C2 [567-594	0	3214.554
[R].LGQHVV	1xTMTpro [K	2.102E-05	4.225E-05	1	1	1	P43897	P43897 [256-282]	0	3301.723
[K].IGLVRPG	1xTMTpro [N	2.144E-05	4.225E-05	1	1	1	P15924	P15924 [2280-230	0	3424.9487
[R].FFDSFG	1xOxidation [	7.328E-06	4.225E-05	2	2	2	P69891; P69	P69891 [42-60]; P	0	2614.3467
[R].FFDSFG	1xTMTpro [K	1.425E-06	4.225E-05	2	2	4	P69891; P69	P69891 [42-60]; P	0	2598.3518
[R].FEDEEL(	1xTMTpro [K	3.375E-05	4.225E-05	1	1	1	O15514	O15514 [122-137]	0	2572.3638
[K].FFDLQM	2xTMTpro [K	5.993E-05	4.225E-05	1	1	1	A1X283	A1X283 [48-66]	1	3202.6919
[K].LGPVDM	1xCarbamido	3.545E-05	4.225E-05	1	1	1	Q06136	Q06136 [112-129]	0	2427.3054
[K].LGPQDS	1xCarbamido	8.402E-07	4.225E-05	1	1	1	Q27J81	Q27J81 [18-40]	0	2831.3688
[R].LGPLSAI	1xTMTpro [N	2.959E-08	4.225E-05	1	2	1	P22105	P22105 [3633-365	0	2786.4604
[R].LGPISAD	1xTMTpro [K	2.76E-05	4.225E-05	1	1	1	P22105	P22105 [3432-344	0	2108.2095
[R].LGPLLE(	1xTMTpro [N	3.967E-05	4.225E-05	1	1	1	Q8WWQ0	Q8WWQ0 [82-105	0	2776.5892
[K].FEDLLSF	1xTMTpro [N	7.314E-06	4.225E-05	1	1	1	Q9GZS1	Q9GZS1 [216-236	0	2703.3473
[K].IGPILDN	2xTMTpro [K	6.048E-06	4.225E-05	1	1	2	P30153	P30153 [547-566]	0	3106.8544
[R].IGQGHC	1xCarbamido	3.873E-05	4.225E-05	1	1	2	P35555	P35555 [2681-269	0	1750.847
[K].FEGGVV	1xTMTpro [N	2.239E-06	4.225E-05	1	1	1	P28070	P28070 [61-80]	0	2317.2181
[K].LGLVLD(	1xCarbamido	3.492E-06	4.225E-05	1	1	6	Q13938	Q13938 [139-153]	0	1934.0336
[K].LGLVGVI	1xTMTpro [K	5.287E-05	4.225E-05	1	1	1	P53396	P53396 [69-82]	0	2006.2505
[K].LGGTIDI	1xCarbamido	1.43E-06	4.225E-05	1	1	2	P50991	P50991 [214-232]	0	2668.4723
[K].LGLGSV	1xTMTpro [N	1.604E-05	4.225E-05	1	1	1	Q7LBC6	Q7LBC6 [129-143	0	1934.1282
[R].LGIGMD	1xCarbamido	2.809E-05	4.225E-05	1	1	2	P49903	P49903 [64-76]	0	1748.9722
[K].FENAFLS	1xTMTpro [N	1.501E-06	4.225E-05	1	1	3	P25705	P25705 [507-527]	0	2671.4639
[K].LGLGIDE	1xTMTpro [N	3.951E-08	4.225E-05	1	1	8	P08238	P08238 [686-719]	0	3836.8521
[K].LGLGIDE	1xTMTpro [N	8.434E-08	4.225E-05	1	1	4	P07900	P07900 [694-727]	0	3851.746
[R].LGHAET	1xTMTpro [K	1.242E-05	4.225E-05	1	1	2	Q5VYK3	Q5VYK3 [23-38]	0	2404.3328
[K].FEPIHGN	1xTMTpro [K	1.114E-06	4.225E-05	1	1	1	O43172	O43172 [457-475]	0	2716.459
[R].FEPSSQ	1xTMTpro [K	1.76E-05	4.225E-05	1	3	2	Q5JPE7	Q5JPE7 [935-951	0	2572.3573

[R].IGIFGQD	1xTMTpro [K	1.336E-05	4.225E-05	1	1	4	P16615	P16615 [638-650]	0	2017.1097
[K].FELSEN(	1xCarbamidc	5.384E-05	4.225E-05	1	1	1	Q14203	Q14203 [630-643]	0	1998.0034
[K].LGHPDT	1xTMTpro [K	2.202E-05	4.225E-05	1	1	6	P06702	P06702 [26-38]	0	2064.137
[R].LGLEFFI	1xTMTpro [N	1.113E-05	4.225E-05	1	1	1	P29372	P29372 [86-100]	0	1977.1129
[R].IGLDLPA	1xTMTpro [N	4.199E-05	4.225E-05	1	1	2	P05164	P05164 [579-590]	0	1644.9426
[K].LGLDFPI	1xTMTpro [K	1.008E-06	4.225E-05	1	1	2	P28161	P28161 [53-69]	0	2521.431
[R].FESLEPI	1xTMTpro [N	5.775E-05	4.225E-05	1	1	4	Q01082	Q01082 [891-904]	0	1955.9452
[K].LGLDFPI	1xTMTpro [K	8.965E-07	4.225E-05	2	3	1	Q03013; P4(	Q03013 [53-69]; F	0	2491.4205
[K].LGLAPQI	1xTMTpro [K	4.657E-05	4.225E-05	1	1	2	P46940	P46940 [162-174]	0	2024.2036
[K].IGKVDCT	2xCarbamidc	3.9E-05	4.225E-05	1	1	1	Q8NBS9	Q8NBS9 [242-26(	1	2872.469
[K].LGKDPN	2xTMTpro [K	3.213E-06	4.225E-05	1	1	2	Q16531	Q16531 [821-844]	1	3581.9382
[K].LGKDAV	2xTMTpro [K	2.271E-05	4.225E-05	1	1	2	P81605	P81605 [83-96]	1	2372.3853
[K].IGHYILG	1xTMTpro [K	1.178E-05	4.225E-05	1	1	2	Q13131	Q13131 [24-40]	0	2356.352
[R].FEQAFY	1xTMTpro [N	1.52E-06	4.225E-05	1	3	2	Q5JPE7	Q5JPE7 [644-665	0	2828.4677
[R].LGQSDP	1xOxidation [	1.409E-05	4.225E-05	1	1	4	P29401	P29401 [187-204]	0	2693.4213
[K].FEVNNTI	1xCarbamidc	3.261E-06	4.225E-05	1	1	1	P12955	P12955 [169-184]	0	2274.1872
[K].LGGTCV	2xCarbamidc	3.988E-05	4.225E-05	1	1	2	P00390	P00390 [98-110]	0	1969.0855
[R].LGGPEA	1xTMTpro [N	1.176E-05	4.225E-05	1	1	4	P02792	P02792 [155-169]	0	1912.0135
[R].IGITNYE	1xTMTpro [K	6.172E-06	4.225E-05	1	1	2	Q9Y4G6	Q9Y4G6 [121-139	0	2880.5374
[K].FEGNCA	1xCarbamidc	3.961E-05	4.225E-05	1	1	1	P02679	P02679 [348-364]	0	2624.2154
[R].LGLTDIN	1xTMTpro [K	4.36E-05	4.225E-05	1	1	1	Q9Y4P1	Q9Y4P1 [230-244	0	2287.3041
[R].IGLSVSE	1xCarbamidc	2.819E-05	4.225E-05	1	1	1	P50990	P50990 [121-137]	0	2199.165
[R].FEGPYT	1xTMTpro [K	5.393E-05	4.225E-05	1	1	1	Q99543	Q99543 [544-560]	0	2684.3376
[K].LGGSAV	1xTMTpro [K	5.2E-05	4.225E-05	1	1	7	P23528	P23528 [153-166]	0	1949.1927
[K].LGLSPGI	1xTMTpro [N	4.296E-06	4.225E-05	1	1	2	Q6NYC8	Q6NYC8 [51-89]	0	4219.2206
[K].LGISDVA	1xTMTpro [K	6.56E-06	4.225E-05	1	1	1	P61964	P61964 [88-109]	0	2887.5181
[K].LGGSLAI	1xTMTpro [K	8.974E-06	4.225E-05	1	1	2	P78371	P78371 [205-222]	0	2521.3681
[K].FEIPYFT	1xTMTpro [N	3.299E-05	4.225E-05	1	1	1	Q9BXS5	Q9BXS5 [380-393	0	1962.0656
[R].FEHSLG	1xCarbamidc	1.208E-05	4.225E-05	1	1	1	Q9Y3Z3	Q9Y3Z3 [165-185	0	2865.5577
[K].FELACLA	2xCarbamidc	5.334E-06	4.225E-05	1	1	1	O15514	O15514 [95-112]	0	2690.3661
[R].LGLQND	1xTMTpro [N	6.09E-05	4.225E-05	1	1	2	P55786	P55786 [616-627]	0	1650.9498
[K].LGGSPT	1xTMTpro [K	9.287E-08	4.225E-05	1	1	2	P14314	P14314 [439-459]	0	2776.455
[K].LGLMRD	1xTMTpro [K	7.088E-06	4.225E-05	1	1	1	P14927	P14927 [30-49]	2	2989.5796

[K].IGLIHGH	1xTMTpro [N	1.564E-05	4.225E-05	1	1	2	Q9UBQ0	Q9UBQ0 [82-104]	0	2829.5881
[K].LGLLGL/	1xTMTpro [N	5.112E-06	4.225E-05	1	1	4	P51659	P51659 [169-183]	0	1801.0867
[K].IGIIGGT(	1xTMTpro [N	1.888E-06	4.225E-05	1	1	2	Q13126	Q13126 [12-29]	0	2129.1773
[R].FEIGEGE	1xTMTpro [N	3.571E-06	4.225E-05	1	1	2	Q02790	Q02790 [190-206]	0	2255.1515
[R].LGLLAA(	1xTMTpro [N	4.542E-06	4.225E-05	1	1	1	Q9NT22	Q9NT22 [703-721	0	2293.3563
[K].LGIKPSII	2xTMTpro [K	6.844E-06	4.225E-05	1	1	2	O00584	O00584 [154-169]	0	2768.6167
[K].LGIQTDI	2xTMTpro [K	3.747E-05	4.225E-05	1	1	4	P00390	P00390 [347-368]	1	3396.8943
[R].FDDGAG	1xTMTpro [N	1.792E-05	4.225E-05	1	1	2	P35998	P35998 [285-297]	0	1683.7894
[R].IGGGDT	1xTMTpro [K	2.952E-05	4.225E-05	1	1	1	O14497	O14497 [1846-18	0	2465.2916
[K].LGQSVT	1xTMTpro [K	4.841E-05	4.225E-05	1	1	2	P62330	P62330 [35-55]	0	2862.6108
[K].FDEYFS(	1xCarbamido	3.619E-05	4.225E-05	1	1	6	P02788	P02788 [504-519]	0	2166.9722
[K].LGEMWI	2xTMTpro [K	1.693E-06	4.225E-05	1	1	6	P09429	P09429 [129-146]	1	3022.576
[R].IHEGCEI	1xCarbamido	9.39E-07	4.225E-05	1	1	3	Q00610	Q00610 [866-881]	0	2385.2477
[K].LGEMWI	1xOxidation	3.826E-05	4.225E-05	1	1	5	P09429	P09429 [129-146]	1	3038.5709
[K].LHEEEIQ	1xTMTpro [K	2.449E-07	4.225E-05	1	1	3	P08670	P08670 [237-270]	0	4531.4288
[R].FDGALN	1xTMTpro [N	1.662E-07	4.225E-05	3	7	3	Q71U36; Q9	Q71U36 [244-264	0	2713.4157
[R].LHEATD	1xTMTpro [K	2.435E-05	4.225E-05	1	1	2	P11047	P11047 [627-646]	0	3054.6333
[K].FDGGEE	1xTMTpro [K	2.967E-06	4.225E-05	1	1	1	Q16698	Q16698 [299-316]	1	2633.4067
[K].LHDVNS	1xTMTpro [K	2.186E-06	4.225E-05	1	1	2	P80303	P80303 [252-272]	0	3028.5759
[K].LGEPDY	1xTMTpro [N	3.569E-05	4.225E-05	1	1	2	Q14344	Q14344 [184-200]	0	2232.2195
[R].FFHPEE	1xTMTpro [K	1.374E-05	4.225E-05	1	1	1	P04066	P04066 [114-130]	0	2572.348
[K].LGESVQ	1xTMTpro [K	1.032E-05	4.225E-05	1	1	1	Q9UI12	Q9UI12 [342-360]	0	2741.4013
[K].LHDINA(	1xOxidation	1.488E-05	4.225E-05	1	1	1	P63010	P63010 [148-166]	0	2521.2676
[R].IHDGEAI	1xOxidation	5.917E-05	4.225E-05	1	1	2	P08253	P08253 [162-175]	0	1907.9605
[R].IHDGEAI	1xTMTpro [N	2.045E-05	4.225E-05	1	1	1	P08253	P08253 [162-175]	0	1891.9656
[K].LHDETLT	1xTMTpro [N	7.519E-06	4.225E-05	2	2	2	Q12800; Q9I	Q12800 [81-98]; (	0	2484.269
[K].LHCFQL	1xCarbamido	5.861E-06	4.225E-05	1	1	2	Q969M7	Q969M7 [62-80]	0	2821.4475
[R].IHAYSQI	1xTMTpro [N	4.503E-05	4.225E-05	1	1	1	Q15008	Q15008 [301-312]	0	1783.9662
[K].LGETYKI	2xTMTpro [K	2.488E-05	4.225E-05	1	1	3	P07237	P07237 [410-424]	1	2642.5334
[K].LHAVNAI	1xCarbamido	9.535E-06	4.225E-05	1	1	2	O75521	O75521 [360-374]	0	2014.0459
[K].LGEVALI	1xCarbamido	3.624E-06	4.225E-05	1	1	2	P53621	P53621 [677-696]	0	2662.3764
[K].LHEWTK	1xTMTpro [K	2.68E-06	4.225E-05	1	1	1	P30740	P30740 [255-275]	0	3144.7337
[K].FDETEQ	1xTMTpro [N	4.825E-05	4.225E-05	1	1	1	Q7L014	Q7L014 [780-791]	0	1726.8567

[R].LGELWV	1xTMTpro [N	1.828E-05	4.225E-05	1	1	2	P22105	P22105 [1677-16	0	2003.0769
[R].FFLHGV	1xTMTpro [N	1.483E-05	4.225E-05	1	1	1	O15230	O15230 [1182-12	0	2893.5459
[K].LHIIEVG	1xTMTpro [K	8.523E-06	4.225E-05	1	1	3	Q00610	Q00610 [228-245	0	2553.4685
[K].IGEHTPS	1xOxidation	4.792E-06	4.225E-05	1	1	3	P04075	P04075 [154-173]	0	2427.2985
[K].LHLGTT	1xTMTpro [N	3.78E-05	4.225E-05	1	1	1	O75351	O75351 [312-327	0	2107.1103
[R].LHLFPD	1xTMTpro [K	3.767E-05	4.225E-05	1	1	1	Q9BYD1	Q9BYD1 [125-13	0	2450.3827
[R].LGELQA	1xTMTpro [K	3.787E-05	4.225E-05	1	1	1	P11532	P11532 [979-100	0	3492.9081
[R].LHLETP	1xTMTpro [N	1.491E-05	4.225E-05	1	1	1	O14613	O14613 [133-152	0	2550.3272
[R].FFLSSGI	2xTMTpro [K	1.057E-05	4.225E-05	1	1	1	Q9BTV4	Q9BTV4 [190-204	1	2642.5374
[R].IGEIVAE	1xCarbamido	8.383E-06	4.225E-05	1	1	1	Q5SRE7	Q5SRE7 [36-50]	0	2043.0686
[R].IHIDPEI	1xTMTpro [N	2.556E-05	4.225E-05	1	1	1	P50479	P50479 [102-117]	0	2070.0787
[K].IHIDLPNI	1xTMTpro [N	5.366E-05	4.225E-05	1	1	2	P62333	P62333 [299-309]	0	1609.8981
[K].LGYVTAI	1xCarbamido	2.554E-06	4.225E-05	1	1	3	P08603	P08603 [479-496]	0	2424.2572
[R].FDEFFSI	1xCarbamido	2.506E-05	4.225E-05	1	1	5	P02787	P02787 [495-508]	0	2186.072
[R].FFLQGI	1xTMTpro [N	5.256E-06	4.225E-05	1	1	1	P11279	P11279 [299-314]	0	2150.2293
[R].FDEHQS	2xTMTpro [K	2.238E-05	4.225E-05	1	1	6	P12111	P12111 [890-904]	0	2709.5756
[K].IHGQNVI	1xTMTpro [K	4.525E-05	4.225E-05	1	1	1	O94985	O94985 [89-103]	0	2246.2789
[R].FDEILEA	1xTMTpro [N	9.259E-06	4.225E-05	1	1	3	P14618	P14618 [280-294]	0	1970.0224
[K].LGEIVTT	1xTMTpro [K	2.74E-05	4.225E-05	3	4	2	P61204; P18	P61204 [39-59]; F	0	2931.6575
[K].LHGGTP	1xTMTpro [K	6.908E-06	4.225E-05	1	1	2	Q9P2R7	Q9P2R7 [337-362	0	3231.7406
[K].IHGFTV	1xTMTpro [K	4.009E-05	4.225E-05	1	1	1	Q5JRX3	Q5JRX3 [46-66]	0	2908.6792
[R].IHFPLAT	1xTMTpro [K	9.673E-07	4.225E-05	3	6	7	Q71U36; Q9	Q71U36 [265-280	0	2365.3775
[R].LGELVV	1xTMTpro [N	1.335E-06	4.225E-05	1	1	5	Q99715	Q99715 [1130-11	0	2419.3404
[K].FDENDV	1xCarbamido	1.642E-05	4.225E-05	1	1	1	Q00839	Q00839 [400-423	0	3450.6543
[R].FDEFFSI	1xCarbamido	3.143E-06	4.225E-05	1	1	2	P02787	P02787 [495-509]	1	2618.3741
[K].LGYTPLI	1xCarbamido	5.822E-05	4.225E-05	1	1	1	Q01484	Q01484 [727-742	0	2413.3557
[K].LGEVNT	1xTMTpro [K	3.375E-05	4.225E-05	1	1	2	P06727	P06727 [66-78]	0	2016.1257
[K].LGEVVN	1xTMTpro [K	3.838E-06	4.225E-05	1	1	1	P30419	P30419 [128-146]	1	2697.4816
[R].FDSNCI	1xCarbamido	4.939E-05	4.225E-05	1	1	1	Q9H0D6	Q9H0D6 [155-172	0	2668.3242
[K].FDSNEE	1xTMTpro [K	2.593E-05	4.225E-05	1	1	1	Q02880	Q02880 [1464-14	0	2671.3383
[R].LGFGSF	1xTMTpro [K	1.895E-06	4.225E-05	1	1	2	P18084	P18084 [177-196]	1	2812.5165
[R].FFESFGI	1xTMTpro [K	1.825E-05	4.225E-05	1	1	7	P02042	P02042 [42-60]	0	2653.3464
[K].LGSTAP	1xTMTpro [K	1.924E-06	4.225E-05	1	1	1	Q9UNZ2	Q9UNZ2 [260-282	0	2922.5664

[K].LGSSSEI	1xTMTpro [K	4.657E-05	4.225E-05	1	1	2	Q6UXV4	Q6UXV4 [201-212	0	1825.0563
[K].IGFPETT	1xCarbamido	2.797E-06	4.225E-05	1	1	2	Q9Y3F4	Q9Y3F4 [320-349	0	3961.9396
[K].FDTGNL	1xCarbamido	4.945E-06	4.225E-05	1	1	2	P62701	P62701 [175-191]	0	2087.0333
[R].IGSSELQ	1xCarbamido	9.157E-06	4.225E-05	1	1	1	Q15043	Q15043 [109-128]	0	2625.3513
[R].FDTGSG	1xTMTpro [N	1.837E-06	4.225E-05	1	1	1	O00468	O00468 [1434-14	0	2712.4065
[K].IGTAEPD	1xTMTpro [N	3.632E-05	4.225E-05	1	1	1	P19174	P19174 [764-778]	0	1915.9721
[R].LGSPDY	1xTMTpro [N	8.247E-07	4.225E-05	1	1	2	Q14980	Q14980 [1860-18	0	2639.4112
[K].FDTTCKI	1xCarbamido	1.648E-05	4.225E-05	1	1	1	Q9NZM1	Q9NZM1 [606-62	0	2484.3048
[K].IGSLIDVI	1xTMTpro [K	1.024E-06	4.225E-05	1	2	2	P61326	P61326 [104-120]	1	2449.3906
[R].LGSLGA	1xCarbamido	3.551E-05	4.225E-05	1	1	1	P07357	P07357 [521-537]	0	2329.2313
[K].FDVNTS	1xTMTpro [N	1.011E-05	4.225E-05	1	1	1	Q00610	Q00610 [1075-10	0	2544.3741
[K].LGSIAIQ	1xTMTpro [K	4.213E-05	4.225E-05	1	1	2	P24752	P24752 [67-78]	0	1808.1137
[R].LGGEVS	1xCarbamido	2.049E-05	4.225E-05	1	1	2	P13804	P13804 [47-59]	0	1899.0865
[K].IGSCTQ	1xCarbamido	6.903E-07	4.225E-05	1	1	1	P14868	P14868 [127-141]	0	2350.2681
[R].IGRPSET	1xCarbamido	3.31E-06	4.225E-05	1	1	1	Q16531	Q16531 [112-129]	0	2287.2399
[K].IGRIEDV	1xTMTpro [N	3.191E-05	4.225E-05	1	1	1	P62263	P62263 [126-141]	1	2060.1307
[R].FFEEVNI	2xTMTpro [K	4.963E-06	4.225E-05	1	1	4	P49748	P49748 [112-137]	1	3953.0459
[R].LGGAVL	1xTMTpro [K	2.579E-05	4.225E-05	1	1	1	P27708	P27708 [1987-20	0	2289.2946
[K].LGQSLD	1xCarbamido	4.179E-06	4.225E-05	1	1	2	P01042	P01042 [345-362]	0	2715.4308
[R].FFESFGI	1xTMTpro [K	4.801E-06	4.225E-05	1	1	138	P68871	P68871 [42-60]	0	2667.362
[R].FFESFGI	1xOxidation	3.288E-05	4.225E-05	1	1	87	P68871	P68871 [42-60]	0	2683.3569
[K].IGYNPDT	1xTMTpro [K	1.404E-05	4.225E-05	1	2	3	P68104	P68104 [181-212]	0	4176.0855
[R].IGEWELI	1xTMTpro [K	2.846E-07	4.225E-05	1	1	2	P45974	P45974 [304-329]	0	3464.9437
[K].LGYAGN	1xCarbamido	3.591E-06	4.225E-05	1	1	3	P61158	P61158 [19-38]	0	2800.5563
[K].FFGNLA	1xCarbamido	2.849E-06	4.225E-05	1	1	1	Q16401	Q16401 [276-292]	0	2316.1436
[R].IGWPNA	1xTMTpro [N	4.194E-06	4.225E-05	1	1	1	P04275	P04275 [1642-16	0	2384.3297
[R].LGWDPK	1xTMTpro [K	4.22E-05	4.225E-05	1	1	2	P55786	P55786 [690-706]	0	2482.4062
[K].LGVYEVI	1xTMTpro [N	2.654E-05	4.225E-05	1	1	1	Q12884	Q12884 [592-605]	0	1896.0398
[K].FFGLPQ	1xTMTpro [N	1.98E-05	4.225E-05	1	1	1	P08253	P08253 [80-98]	0	2487.2509
[R].IGVVGG	1xCarbamido	9.183E-07	4.225E-05	1	1	2	Q8IW45	Q8IW45 [76-98]	0	2980.6098
[K].IGVSSDS	1xTMTpro [K	1.868E-05	4.225E-05	1	1	1	P49790	P49790 [934-951]	0	2420.2623
[K].FDSEPS	1xTMTpro [N	2.071E-05	4.225E-05	1	1	2	P18583	P18583 [158-172]	0	1936.0347
[K].LGFAGL	1xTMTpro [K	1.613E-05	4.225E-05	1	1	3	P48643	P48643 [353-368]	0	2276.3146

[R].LGFDISE	1xCarbamido	2.076E-06	4.225E-05	1	1	1	Q9H008	Q9H008 [71-92]	0	2966.6153
[R].FDPETN	1xCarbamido	8.633E-06	4.225E-05	1	1	1	Q9UHD1	Q9UHD1 [14-38]	0	3440.6713
[R].LGVNNN	1xTMTpro [K	7.617E-06	4.225E-05	1	1	1	Q9P2R3	Q9P2R3 [1070-10	0	2701.4917
[K].LGVNNIS	1xTMTpro [K	4.295E-08	4.225E-05	1	1	2	P20290	P20290 [95-122]	0	3694.9507
[K].FFEVILID	1xTMTpro [K	1.607E-05	4.225E-05	1	1	2	P61313	P61313 [129-140]	0	2113.2342
[K].LGVEFDI	1xTMTpro [N	1.697E-05	4.225E-05	1	1	2	P05413	P05413 [67-79]	0	1771.8669
[R].FDQLFD	1xTMTpro [K	6.744E-06	4.225E-05	1	1	4	Q8NC51	Q8NC51 [17-32]	0	2552.3052
[K].IGFFNQ(	1xTMTpro [N	2.677E-05	4.225E-05	1	1	2	Q8NE71	Q8NE71 [690-702	0	1918.0142
[K].IGTDGT(	1xOxidation [	2.124E-05	4.225E-05	1	1	3	Q05707	Q05707 [1065-10	0	2271.1246
[K].IGTDGT(	1xTMTpro [N	8.448E-07	4.225E-05	1	1	2	Q05707	Q05707 [1065-10	0	2255.1297
[K].LGVQVV	1xTMTpro [K	6.012E-05	4.225E-05	1	1	1	P17987	P17987 [248-264]	1	2531.5052
[R].LFLASLA	1xTMTpro [K	6.138E-06	4.225E-05	1	1	2	Q7Z6Z7	Q7Z6Z7 [2134-21	0	2953.6854
[K].EYSVLN	1xTMTpro [N	4.457E-05	4.225E-05	1	1	4	P46939	P46939 [2613-262	0	1668.8876
[K].LIAMYND	1xTMTpro [K	3.526E-05	4.225E-05	1	1	1	Q9BZH6	Q9BZH6 [766-78(	0	2334.2295
[R].IIGLDQV	1xTMTpro [K	1.27E-06	4.225E-05	1	1	5	P35579	P35579 [618-637]	0	2626.477
[QR].FGQGS	1xTMTpro [N	5.648E-06	4.225E-05	1	1	1	Q9UGM3	Q9UGM3 [289-30	0	1763.9611
[R].FGQGG	1xTMTpro [N	3.032E-05	4.225E-05	1	1	2	P23246	P23246 [667-681]	0	1645.873
[K].LIGLAVD	2xTMTpro [K	1.162E-06	4.225E-05	1	1	1	Q6P1A2	Q6P1A2 [152-175	1	3524.9053
[K].LLGHEVI	1xTMTpro [K	3.664E-05	4.225E-05	1	1	2	Q92900	Q92900 [456-467	0	1973.1927
[R].ILGGVIS	1xTMTpro [N	5.046E-08	4.225E-05	1	1	2	P04632	P04632 [61-84]	0	2751.5
[R].FGQDIIS	1xTMTpro [K	1.483E-05	4.225E-05	1	1	2	Q8WVM8	Q8WVM8 [51-63]	0	2025.224
[R].IFQNLD(	1xTMTpro [K	4.206E-05	4.225E-05	1	1	1	Q02809	Q02809 [206-220	0	2282.3252
[R].LIGGES(	1xTMTpro [K	1.156E-05	4.225E-05	1	1	1	Q9NWU1	Q9NWU1 [66-82]	0	2358.3412
[R].EVQTND	2xTMTpro [K	5.123E-05	4.225E-05	1	1	1	P61247	P61247 [175-187]	1	2428.4228
[K].LLGFLD\	1xCarbamido	5.636E-05	4.225E-05	1	1	2	Q5RI15	Q5RI15 [18-31]	0	1909.0173
[K].IIGFGSA	1xTMTpro [K	1.083E-06	4.225E-05	1	1	2	O94973	O94973 [870-897	0	3487.9445
[R].LLGEVG	1xTMTpro [N	3.387E-05	4.225E-05	1	1	2	P09619	P09619 [318-332]	0	1987.1296
[R].FGQDFS	1xOxidation [	1.578E-06	4.225E-05	1	1	2	Q9Y490	Q9Y490 [1274-12	0	3037.4168
[R].FGQDFS	1xTMTpro [N	8.639E-08	4.225E-05	1	1	2	Q9Y490	Q9Y490 [1274-12	0	3021.4219
[R].FGQAAT	1xTMTpro [N	3.282E-07	4.225E-05	1	1	2	Q15233	Q15233 [435-456	0	2467.2723
[K].IIGAVDQ	1xTMTpro [N	3.267E-06	4.225E-05	1	1	3	Q6NZI2	Q6NZI2 [61-78]	0	2329.3046
[R].EVQYIVL	1xTMTpro [N	9.659E-06	4.225E-05	1	1	1	O00203	O00203 [348-364	0	2310.2811
[R].IIGATDS	1xOxidation [	1.595E-05	4.225E-05	2	2	3	Q13185; P83	Q13185 [126-141	0	2337.269

[R].IIGATDS	1xTMTpro [K	3.499E-06	4.225E-05	2	2	6	Q13185; P83	Q13185 [126-141]	0	2321.2741
[K].IIGATDS	1xCarbamido	3.185E-05	4.225E-05	1	1	2	P45973	P45973 [126-141]	0	2380.257
[-].EVQLVET	1xTMTpro [N	3.225E-05	4.225E-05	1	1	2	P0DOX2	P0DOX2 [1-19]	0	2214.2413
[R].IIGLDQV	1xOxidation [	9.355E-07	4.225E-05	1	1	4	P35579	P35579 [618-637]	0	2642.4719
[R].ILGLGDL	1xCarbamido	2.849E-05	4.225E-05	1	1	1	Q16798	Q16798 [191-208]	0	2428.3588
[R].ILGLGDL	1xTMTpro [K	1.699E-06	4.225E-05	1	1	1	P23368	P23368 [166-183]	0	2367.3965
[K].LFPNSLD	1xCarbamido	5.904E-06	4.225E-05	1	1	1	P27797	P27797 [112-142]	0	4096.9222
[R].EVPFAD	1xTMTpro [K	4.748E-06	4.225E-05	1	1	3	Q13418	Q13418 [389-403]	0	2289.2114
[R].LLGSDG	1xTMTpro [N	7.177E-07	4.225E-05	1	1	4	Q6XQN6	Q6XQN6 [426-45	0	3298.6982
[R].ILGSASF	1xTMTpro [K	3.131E-06	4.225E-05	1	1	2	Q7Z422	Q7Z422 [102-122]	0	2973.6501
[R].EVPLLQS	1xTMTpro [N	8.981E-07	4.225E-05	1	1	2	O14498	O14498 [72-87]	0	2222.2617
[K].LIGQVEI	1xTMTpro [K	5.536E-05	4.225E-05	1	1	1	Q8WXH0	Q8WXH0 [742-75	0	2094.2091
[R].LLGQTD	1xTMTpro [K	2.379E-05	4.225E-05	1	1	1	Q6PCB7	Q6PCB7 [531-550	0	2580.4893
[R].EVPLPA	1xTMTpro [K	5.747E-05	4.225E-05	1	1	1	P18074	P18074 [167-181]	0	2265.2986
[K].ILGQEGI	1xTMTpro [K	1.11E-07	4.225E-05	1	1	6	Q12905	Q12905 [329-356]	0	3573.882
[R].LLGPSA	1xTMTpro [N	5.256E-07	4.225E-05	1	1	2	Q7Z6Z7	Q7Z6Z7 [2580-26	0	2541.4571
[R].ILGADTS	1xTMTpro [N	7.903E-06	4.225E-05	1	1	3	P25705	P25705 [59-73]	0	1879.9932
[R].FGSAIAF	2xTMTpro [K	5.331E-05	4.225E-05	1	1	1	P06756	P06756 [370-400]	1	4078.1589
[K].LLGPNAS	1xTMTpro [N	1.934E-05	4.225E-05	1	1	2	P42224	P42224 [526-541]	0	2011.1296
[K].LLGPDA	1xTMTpro [K	1.244E-05	4.225E-05	1	1	2	Q13561	Q13561 [157-175]	0	2473.4158
[K].EVPVIFII	1xTMTpro [N	8.924E-06	4.225E-05	1	1	1	Q86X10	Q86X10 [1388-14	0	2156.2551
[R].FGSAIAF	1xTMTpro [K	3.074E-06	4.225E-05	1	1	3	P06756	P06756 [370-399]	0	3645.8568
[R].LLGNVLV	1xCarbamido	1.191E-05	4.225E-05	1	1	38	P68871	P68871 [106-121]	0	2385.4085
[K].LLGNQA	1xTMTpro [N	9.929E-06	4.225E-05	1	1	2	P16157	P16157 [1121-113	0	2146.2191
[R].ILGIPVIV	1xTMTpro [K	5.098E-05	4.225E-05	1	1	4	Q96CN7	Q96CN7 [149-162	0	2178.3394
[R].EVQGFE	1xTMTpro [K	1.523E-06	4.225E-05	1	1	21	P06396	P06396 [148-162]	0	2331.2517
[R].EVQGNE	1xTMTpro [N	3.017E-06	4.225E-05	1	1	2	P40121	P40121 [97-112]	0	2223.0711
[R].EVQGNE	1xOxidation [	1.934E-05	4.225E-05	1	1	2	P40121	P40121 [97-112]	0	2239.0661
[R].LIGPNCF	2xCarbamido	1.086E-05	4.225E-05	1	1	3	P53597	P53597 [167-182]	0	2333.2601
[R].LLGADS	1xTMTpro [K	2.231E-06	4.225E-05	1	1	2	Q8WX93	Q8WX93 [936-96	0	3404.7353
[R].FGPYYT	1xTMTpro [K	2.418E-05	4.225E-05	1	1	2	P49720	P49720 [100-115]	0	2375.3143
[K].ILFVSQG	1xTMTpro [N	3.01E-05	4.225E-05	1	1	1	Q9NUQ7	Q9NUQ7 [370-38	0	1896.051
[K].LIETLKPI	1xTMTpro [K	1.687E-06	4.225E-05	1	1	2	P51003	P51003 [42-60]	0	2914.6057

[R].FGNQAD	1xTMTpro [K	1.969E-05	4.225E-05	1	1	1	Q9H488	Q9H488 [44-59]	0	2331.2741
[K].LLETHIH	1xTMTpro [K	5.974E-05	4.225E-05	1	1	1	Q13616	Q13616 [339-354]	0	2395.3953
[K].LFSLELV	1xTMTpro [N	2.479E-05	4.225E-05	1	1	5	P27694	P27694 [221-234]	0	1911.0394
[R].LLESSLS	1xTMTpro [K	1.008E-05	4.225E-05	1	1	1	P08648	P08648 [120-137]	0	2591.3584
[K].LLESLQE	1xTMTpro [N	6.428E-06	4.225E-05	1	1	1	P49903	P49903 [41-63]	0	3034.5263
[R].FGNDGL	1xTMTpro [K	3.886E-05	4.225E-05	1	1	1	Q9Y606	Q9Y606 [347-364	0	2650.3393
[R].LLEQWE	2xTMTpro [K	2.715E-06	4.225E-05	1	1	1	Q14696	Q14696 [66-85]	1	3350.7572
[R].ILEQEEE	2xTMTpro [K	1.709E-05	4.225E-05	1	1	2	Q9BXP5	Q9BXP5 [267-285	0	3039.6302
[K].EVTWEV	1xTMTpro [K	2.071E-05	4.225E-05	1	1	2	P05455	P05455 [300-312]	0	2155.1778
[K].IFSGPSS	1xTMTpro [K	2.23E-05	4.225E-05	1	1	2	P17301	P17301 [39-59]	0	2952.5751
[K].LLEQALV	1xTMTpro [N	3.913E-05	4.225E-05	2	3	2	Q12873; Q1	Q12873 [1799-18	0	1858.0969
[K].EVVCTN	2xCarbamido	1.191E-05	4.225E-05	1	1	3	P11047	P11047 [789-802]	0	2132.0971
[K].LLEPSVC	1xTMTpro [K	2.32E-05	4.225E-05	2	2	2	Q9Y4E1; Q6	Q9Y4E1 [870-892	0	3064.5494
[R].LLEPHCI	2xCarbamido	2.649E-05	4.225E-05	1	1	1	P10643	P10643 [555-578]	0	3357.8235
[R].ILEPGLN	1xTMTpro [N	2.799E-05	4.225E-05	1	1	1	Q9UJZ1	Q9UJZ1 [58-72]	0	1979.2224
[R].ILEPDDF	1xTMTpro [K	1.256E-05	4.225E-05	1	1	1	Q92785	Q92785 [157-178]	0	3249.5342
[R].LFTSDLC	2xTMTpro [K	3.747E-05	4.225E-05	1	1	1	Q8NFH4	Q8NFH4 [106-118	1	2513.4068
[K].LLENISP	1xTMTpro [N	2.885E-05	4.225E-05	1	1	2	Q92538	Q92538 [1600-16	0	2135.0974
[R].FGMLDA	1xTMTpro [N	3.604E-06	4.225E-05	1	1	2	Q6WCQ1	Q6WCQ1 [596-61	0	2141.0504
[R].EVVETPI	1xTMTpro [N	1.238E-05	4.225E-05	1	1	2	P35998	P35998 [189-200]	0	1722.971
[K].EVVHTVS	1xTMTpro [N	1.667E-05	4.225E-05	1	1	1	Q9Y230	Q9Y230 [237-253	0	2251.2366
[R].EVVADS	1xTMTpro [K	2.963E-05	4.225E-05	1	1	6	P01024	P01024 [545-556]	0	1954.1141
[K].IIGVHQE	1xCarbamido	3.255E-06	4.225E-05	1	1	1	Q93009	Q93009 [949-968]	0	2571.3408
[K].IFRDGEE	1xTMTpro [N	7.395E-06	4.225E-05	1	1	3	P30101	P30101 [105-119]	1	1956.9735
[K].LIEVANL	1xCarbamido	3.954E-05	4.225E-05	1	1	2	P35221	P35221 [430-448]	0	2668.4471
[R].EVSEAV	1xTMTpro [K	2.298E-06	4.225E-05	1	1	2	P98160	P98160 [110-125]	0	2419.31
[R].LLFSGAS	1xTMTpro [N	3.78E-05	4.225E-05	1	1	1	Q8IWB7	Q8IWB7 [213-231	0	2356.229
[K].LLFSADN	1xTMTpro [N	4.087E-06	4.225E-05	1	1	3	Q92629	Q92629 [147-162]	0	2037.0936
[K].ILFQPQT	1xTMTpro [K	2.942E-05	4.225E-05	1	1	1	O94855	O94855 [609-623]	0	2345.3361
[R].IFQNMSI	1xTMTpro [N	2.206E-05	4.225E-05	1	1	1	Q9UI26	Q9UI26 [688-704]	0	2295.2338
[R].LLFPAVD	1xTMTpro [K	5.841E-05	4.225E-05	1	1	2	Q02880	Q02880 [837-848]	0	1966.1869
[R].LFQQIYS	1xTMTpro [K	2.457E-06	4.225E-05	1	1	1	Q9Y2Z0	Q9Y2Z0 [312-326	1	2393.2956
[K].ILFHGEC	1xTMTpro [K	7.716E-06	4.225E-05	1	1	2	Q8WW22	Q8WW22 [223-24	0	3398.8339

[R].EVSQPD	1xTMTpro [K	3.334E-05	4.225E-05	1	1	3	P13667	P13667 [166-184]	0	2744.5366
[R].EVSQVD	2xCarbamido	5.056E-05	4.225E-05	1	1	1	Q8WUM0	Q8WUM0 [692-71	0	2890.4246
[K].EVTKEV'	1xCarbamido	1.197E-06	4.225E-05	1	1	1	P02671	P02671 [477-510]	1	4194.0714
[R].ILFEITAC	1xTMTpro [K	2.21E-05	4.225E-05	1	1	1	Q9Y3E7	Q9Y3E7 [168-179	0	1841.1392
[R].IIEVVDAI	1xTMTpro [N	2.554E-06	4.225E-05	1	1	1	Q01813	Q01813 [194-210]	0	2216.2028
[K].LLEVSDI	1xTMTpro [N	1.184E-07	4.225E-05	1	1	1	Q9UI12	Q9UI12 [397-419]	0	2799.4848
[R].LLEVPVI	1xTMTpro [K	1.147E-07	4.225E-05	1	1	1	Q96AB3	Q96AB3 [50-82]	0	4154.3431
[R].ILEVNHV	1xTMTpro [K	2.865E-05	4.225E-05	1	1	1	Q96L92	Q96L92 [101-115]	0	2268.2956
[K].LLEVLSC	2xTMTpro [K	6.127E-06	4.225E-05	1	1	3	P11277	P11277 [91-105]	0	2567.5662
[R].LFQVEY.	1xTMTpro [K	1.361E-05	4.225E-05	1	1	2	P28066	P28066 [21-32]	0	2032.1974
[K].EVTDMN	1xTMTpro [K	1.379E-05	4.225E-05	1	1	1	Q96TA1	Q96TA1 [368-384	0	2469.3151
[K].IIEVEEE(	1xTMTpro [N	1.055E-05	4.225E-05	1	1	3	P23142	P23142 [164-179]	0	2295.1312
[R].LIEVDKD	1xTMTpro [K	6.115E-06	4.225E-05	1	1	1	O15382	O15382 [151-187]	1	4600.5271
[R].IIEVAPQ	1xTMTpro [N	4.713E-05	4.225E-05	1	1	1	P49903	P49903 [372-392]	0	2411.3101
[R].EVSSWT	1xTMTpro [N	1.795E-05	4.225E-05	1	1	1	P53582	P53582 [63-82]	0	2586.2432
[R].ILENEKD	2xTMTpro [K	6.026E-06	4.225E-05	1	1	2	O75347	O75347 [81-98]	2	3120.6881
[R].ILHCLGL	1xCarbamido	1.361E-05	4.225E-05	1	1	1	P53004	P53004 [278-290]	0	2132.2393
[R].LLHDQV	1xTMTpro [K	4.793E-05	4.225E-05	1	1	2	P53621	P53621 [942-956]	0	2322.3466
[K].LFIGGLP	1xTMTpro [K	4.603E-05	4.225E-05	1	1	3	P26368	P26368 [261-276]	0	2414.3575
[K].LFIGGLS	1xTMTpro [N	6.475E-06	4.225E-05	1	1	4	P51991	P51991 [37-52]	0	2075.098
[K].LIINELSI	1xTMTpro [N	1.527E-05	4.225E-05	1	1	2	Q96P70	Q96P70 [920-935	0	2062.1286
[K].ILLMDLN	1xOxidation	3.119E-05	4.225E-05	1	1	1	P27169	P27169 [85-106]	0	3024.667
[K].LFIGGLS	1xTMTpro [N	2.38E-06	4.225E-05	1	2	9	P09651	P09651 [16-31]	0	2089.1137
[R].EVLEGLI	1xTMTpro [K	1.552E-05	4.225E-05	1	1	1	O95747	O95747 [129-139]	0	1938.1192
[R].ILILGLD(	1xTMTpro [K	5.654E-05	4.225E-05	1	1	4	P40616	P40616 [20-30]	0	1678.0759
[K].ILIIGESC	1xTMTpro [K	3.471E-05	4.225E-05	1	1	1	Q9NP72	Q9NP72 [11-21]	0	1694.0708
[KR].LLLIGD	1xTMTpro [K	5.098E-05	4.225E-05	7	8	4	P61026; P62	P61026 [12-22]; F	0	1680.0551
[R].EVIELPL	1xTMTpro [N	1.719E-05	4.225E-05	1	1	3	P62333	P62333 [147-161]	0	2102.1817
[KR].ILLLGA	1xTMTpro [K	4.286E-05	4.225E-05	7	12	5	P04899; P09	P04899 [36-46]; F	0	1666.0395
[K].EVIELPV	2xTMTpro [K	5.037E-06	4.225E-05	1	1	2	P62195	P62195 [163-184]	1	3369.9966
[R].FGWDCI	1xCarbamido	2.214E-05	4.225E-05	1	1	1	P41252	P41252 [83-98]	0	2573.299
[K].FGVVLDI	1xTMTpro [K	4.694E-07	4.225E-05	1	1	2	O14579	O14579 [66-85]	0	2763.59
[K].EVLEYL(	1xTMTpro [N	3.174E-05	4.225E-05	1	1	1	Q9Y296	Q9Y296 [81-97]	0	2238.209

[K].LLILEEA(	1xTMTpro [N	1.969E-05	4.225E-05	1	1	1	H7BZ55	H7BZ55 [1114-11	0	2371.3516
[K].LFIGGLS	1xTMTpro [N	1.578E-05	4.225E-05	1	1	13	P22626	P22626 [23-38]	0	2103.1293
[R].LLILADM	1xTMTpro [K	2.952E-05	4.225E-05	1	1	1	P35221	P35221 [138-149]	0	1973.1637
[K].EVIFSVE	1xTMTpro [K	4.177E-05	4.225E-05	1	1	2	O95834	O95834 [11-22]	0	1917.0825
[K].LLLGYSI	1xTMTpro [K	3.611E-06	4.225E-05	1	1	2	Q9HC78	Q9HC78 [133-153	0	2882.6734
[R].LILGLMN	1xOxidation [	2.556E-05	4.225E-05	1	1	8	P08133	P08133 [428-442]	0	2294.2896
[K].LLIPERD	1xTMTpro [K	2.005E-05	4.225E-05	1	1	1	Q68CZ2	Q68CZ2 [1278-13	1	3714.0457
[K].EVICQVL	1xCarbamido	1.023E-05	4.225E-05	1	1	1	P41217	P41217 [211-226]	0	2467.3874
[K].EVIAVSC	2xCarbamido	6.241E-06	4.225E-05	1	1	2	P38117	P38117 [60-76]	0	2222.1229
[K].LFIGGLN	1xTMTpro [N	9.929E-06	4.225E-05	1	1	4	Q13151	Q13151 [9-24]	0	1995.1194
[R].EVFSSC-	1xCarbamido	2.584E-05	4.225E-05	1	1	1	Q09666	Q09666 [103-123]	0	2726.2422
[K].LLLTYAD	1xTMTpro [N	5.775E-05	4.225E-05	1	1	2	Q96IV0	Q96IV0 [34-44]	0	1608.9644
[K].IIITVVEE	1xTMTpro [K	2.449E-05	4.225E-05	1	1	2	Q15149	Q15149 [3095-31(	0	2037.2087
[R].LFLDGD(	1xCarbamido	1.989E-06	4.225E-05	1	1	1	O15247	O15247 [172-189]	0	2627.461
[R].ILISLATC	1xTMTpro [N	2.246E-05	4.225E-05	1	1	1	P08133	P08133 [500-520]	1	2583.381
[K].ILISGLEF	1xTMTpro [N	5.446E-05	4.225E-05	1	2	2	P22105	P22105 [3609-362	0	1750.007
[R].LLLQQV!	1xOxidation [	3.34E-05	4.225E-05	1	1	2	P01023	P01023 [1298-131	0	2669.5079
[K].EVGPTN	1xCarbamido	9.929E-06	4.225E-05	1	1	5	P00450	P00450 [524-537]	0	2079.14
[R].LLLQQV!	1xTMTpro [K	1.224E-05	4.225E-05	1	1	6	P01023	P01023 [1298-131	0	2653.513
[K].LLLQMD!	1xTMTpro [K	1.75E-05	4.225E-05	1	1	1	P04114	P04114 [1146-116	0	2480.3562
[R].LILGLMN	1xTMTpro [K	4.817E-05	4.225E-05	1	1	3	P08133	P08133 [428-442]	0	2278.2947
[K].EVGTPH	1xCarbamido	4.085E-07	4.225E-05	1	1	4	P02790	P02790 [348-371]	0	2802.4415
[R].ILLQGTF	1xOxidation [	1.361E-05	4.225E-05	1	1	4	P01024	P01024 [980-999]	0	2477.2877
[R].ILLQGTF	1xTMTpro [N	1.833E-06	4.225E-05	1	1	6	P01024	P01024 [980-999]	0	2461.2928
[K].EVGVYE	2xTMTpro [K	8.745E-06	4.225E-05	1	1	2	P40925	P40925 [206-220]	1	2664.5065
[K].LLLQGLI	1xTMTpro [K	5.175E-05	4.225E-05	1	1	2	Q5VYK3	Q5VYK3 [880-892	0	2025.191
[K].FGYVDF	1xTMTpro [K	1.524E-05	4.225E-05	1	1	4	P19338	P19338 [349-362]	0	2257.152
[K].LLLQCQ'	1xCarbamido	2.187E-05	4.225E-05	1	1	1	Q15746	Q15746 [1115-11:	0	3062.7204
[K].LFIGGLN	1xTMTpro [K	1.855E-05	4.225E-05	2	2	4	P38159; Q9(	P38159 [10-22]; Q	0	2044.157
[R].LILPVGP	2xTMTpro [K	1.129E-05	4.225E-05	1	1	1	P22061	P22061 [179-204]	1	3697.0815
[R].EVIAEEE	1xTMTpro [N	2.132E-05	4.225E-05	1	1	4	Q02952	Q02952 [940-958]	0	2453.2731
[R].LILPVGP	1xTMTpro [K	3.933E-05	4.225E-05	1	1	3	P22061	P22061 [179-197]	0	2651.4722
[R].EVGTVL!	1xTMTpro [K	5.175E-05	4.225E-05	1	1	2	P04114	P04114 [4223-42:	0	1918.1141

[K].FGVSSS	1xTMTpro [K	6.08E-05	4.225E-05	1	1	1	P49790	P49790 [905-925]	0	2684.4023
[R].LLLFSDC	1xTMTpro [K	1.19E-06	4.225E-05	1	1	6	P12109	P12109 [939-957]	0	2540.4216
[R].LLLEYTD	1xTMTpro [K	1.217E-05	4.225E-05	2	3	3	Q03013; P28	Q03013 [19-31]; F	0	2198.1724
[K].IFNDNSL	1xTMTpro [N	1.808E-05	4.225E-05	1	1	1	P42226	P42226 [481-495]	0	2113.0456
[R].LILADAL	1xCarbamido	6.682E-06	4.225E-05	1	1	3	P28838	P28838 [369-384]	0	2455.3663
[R].LLKEYQI	2xTMTpro [K	3.202E-05	4.225E-05	2	3	1	P04259; P02	P04259 [444-455]	1	2420.4403
[K].LLKEGEI	2xTMTpro [K	1.728E-06	4.225E-05	1	1	2	O00264	O00264 [170-192]	2	3562.8581
[R].FGSYCP	2xCarbamido	9.748E-06	4.225E-05	1	1	12	P02679	P02679 [41-61]	0	3025.4931
[K].LFNESH	1xTMTpro [N	3.317E-05	4.225E-05	1	1	1	Q9BW27	Q9BW27 [71-84]	0	1935.0772
[R].LLHVAVS	1xTMTpro [N	4.106E-05	4.225E-05	1	1	1	Q99460	Q99460 [602-616]	1	2012.1208
[K].FGSTAD	1xTMTpro [N	2.283E-05	4.225E-05	1	1	1	Q5JRA6	Q5JRA6 [279-294	0	1988.9732
[K].LLHTLEG	1xTMTpro [N	1.757E-05	4.225E-05	1	1	1	Q9GZS3	Q9GZS3 [182-194	0	1792.0223
[K].EVMQEV	1xTMTpro [K	2.682E-05	4.225E-05	1	1	1	P49591	P49591 [232-249]	0	2794.4465
[K].EVLSMD	1xTMTpro [K	5.437E-05	4.225E-05	1	1	3	Q06210	Q06210 [537-547]	0	1915.0338
[R].LFPHEVA	1xTMTpro [K	1.822E-05	4.225E-05	1	1	1	Q9BTW9	Q9BTW9 [121-14	0	3081.748
[R].FGSHCG	2xCarbamido	2.027E-05	4.225E-05	1	1	1	Q9NZM1	Q9NZM1 [1649-16	0	2659.2353
[R].ILHIEGG	1xTMTpro [N	4.264E-05	4.225E-05	1	1	1	Q9Y223	Q9Y223 [130-147	0	2215.1889
[R].LLHEVQI	1xTMTpro [K	3.806E-05	4.225E-05	1	1	2	Q13561	Q13561 [106-119]	0	2276.2993
[K].LFPNSLI	1xCarbamido	9.927E-07	4.225E-05	1	1	3	P27797	P27797 [112-142]	0	4064.9324
[K].EVNKFQ	1xTMTpro [K	6.032E-05	4.225E-05	1	1	1	P37108	P37108 [75-88]	1	2321.2931
[K].LFPNSLI	1xCarbamido	6.789E-07	4.225E-05	1	1	2	P27797	P27797 [112-142]	0	4080.9273
[K].IIHEDGY	1xCarbamido	2.644E-05	4.225E-05	1	1	2	P08754	P08754 [55-67]	0	2203.0833
[K].EVNVSP	2xCarbamido	4.016E-05	4.225E-05	1	1	4	P61916	P61916 [36-51]	0	2452.282
[K].LLHDSGI	1xTMTpro [N	2.973E-06	4.225E-05	1	1	1	P27338	P27338 [22-36]	0	1939.1296
[K].EVPAES	1xTMTpro [K	6.481E-09	4.225E-05	1	1	2	Q9NX62	Q9NX62 [163-188	1	3612.8929
[K].EVMVLN	1xTMTpro [K	2.529E-05	4.225E-05	1	1	1	Q16666	Q16666 [579-594]	0	2464.3289
[K].IIHDFPQ	1xTMTpro [N	8.924E-06	4.225E-05	1	1	2	P35244	P35244 [105-121]	0	2343.2457
[K].ILLAELE	2xTMTpro [K	2.277E-06	4.225E-05	1	1	6	P08670	P08670 [130-143]	1	2452.5319
[R].LLIALLES	1xTMTpro [K	4.213E-05	4.225E-05	1	1	3	Q9NP81	Q9NP81 [460-471	0	1978.2192
[K].EVLLEAC	1xTMTpro [N	4.825E-05	4.225E-05	1	1	2	Q9NWU5	Q9NWU5 [113-12	0	1677.9165
[K].LLLENNA	1xTMTpro [N	3.362E-07	4.225E-05	1	1	1	P16157	P16157 [488-512]	0	2926.6182
[R].IFLLGLA	1xTMTpro [K	4.065E-06	4.225E-05	1	1	1	Q9UHB9	Q9UHB9 [312-333	0	2969.6327
[R].IFLLGLA	1xTMTpro [K	1.417E-06	4.225E-05	1	1	1	Q9UHB9	Q9UHB9 [312-335	1	3254.7764

[R].LLLEFTD	1xTMTpro [K	3.447E-05	4.225E-05	1	1	1	P21266	P21266 [23-36]	1	2352.2943
[R].LLLEFTD	1xTMTpro [K	6.475E-06	4.225E-05	1	1	5	P21266	P21266 [23-35]	0	2196.1931
[K].EVLLLM(	1xTMTpro [K	2.105E-05	4.225E-05	1	2	1	Q9NUQ3	Q9NUQ3 [138-15	0	2637.5029
[K].FGVEQD	1xOxidation		2.726E-05	4.225E-05	1	1	1	P14618	P14618 [231-246]	0	2180.1017
[R].LLLEAQ/	1xTMTpro [K	4.169E-08	4.225E-05	1	1	2	Q15149	Q15149 [3859-38	1	2749.6108
[K].FGVEQD	1xTMTpro [N	1.427E-05	4.225E-05	1	1	1	P14618	P14618 [231-246]	0	2164.1068
[R].IFLQDIKI	2xCarbamid	2.345E-05	4.225E-05	1	1	1	P02794	P02794 [81-109]	1	4692.4617
[K].EVLNDT\	1xTMTpro [K	3.859E-06	4.225E-05	1	1	1	Q96ST3	Q96ST3 [579-601	0	3255.6341
[K].IILDLISE	1xTMTpro [K	5.993E-05	4.225E-05	1	1	3	P61978	P61978 [208-219]	0	1949.2178
[R].FGTVLTI	1xTMTpro [N	1.878E-06	4.225E-05	1	1	1	O43598	O43598 [49-65]	0	2046.1303
[K].EVLPLAF	1xTMTpro [K	2.597E-06	4.225E-05	1	1	2	Q5VYK3	Q5VYK3 [1480-14	0	2649.4453
[K].LFLNET(	1xTMTpro [K	5.628E-06	4.225E-05	1	1	2	P41252	P41252 [1227-12	0	2726.5108
[K].EVLQALI	1xTMTpro [K	5.984E-05	4.225E-05	1	3	1	P33176	P33176 [490-505]	0	2470.3685
[K].IFINNEW	1xTMTpro [K	4.271E-05	4.225E-05	1	1	2	P00352	P00352 [23-36]	0	2254.2112
[R].LLLATMI	1xTMTpro [K	5.654E-05	4.225E-05	1	1	2	P00352	P00352 [101-113]	0	1973.1055
[K].EVIQEW	1xTMTpro [K	5.183E-05	4.225E-05	1	1	2	Q9Y490	Q9Y490 [346-357	0	2095.2043
[K].ILIANTGI	2xTMTpro [K	2.363E-06	4.225E-05	1	1	1	P78371	P78371 [237-250]	1	2445.4567
[K].ILIANTGI	1xTMTpro [K	5.491E-05	4.225E-05	1	1	2	P78371	P78371 [237-248]	0	1900.0705
[K].IILDLISE	1xTMTpro [K	1.412E-05	4.225E-05	1	1	2	P61978	P61978 [208-221]	1	2162.3404
[R].LLAITQE	1xTMTpro [N	4.661E-06	4.225E-05	1	1	1	Q14BN4	Q14BN4 [192-212	0	2679.3797
[K].EVVKPVI	2xTMTpro [K	4.89E-05	4.225E-05	1	1	2	Q96HC4	Q96HC4 [102-116	0	2463.5367
[K].IIENAENI	1xTMTpro [K	1.942E-06	4.225E-05	1	1	3	P52907	P52907 [231-256]	0	3648.7871
[R].EYFGGF	1xTMTpro [K	4.442E-06	4.225E-05	1	1	7	Q14103	Q14103 [200-218	0	2769.3937
[R].EYFGGF	1xOxidation		2.66E-06	4.225E-05	1	1	6	Q14103	Q14103 [200-218	0	2785.3886
[R].ILDETQE	1xTMTpro [N	3.453E-05	4.225E-05	1	1	2	Q96FZ7	Q96FZ7 [126-138	0	1897.9826
[R].EYFSWE	1xTMTpro [K	8.908E-06	4.225E-05	1	1	4	P26641	P26641 [415-428]	0	2293.1897
[R].LFYHGP	1xTMTpro [K	6.357E-06	4.225E-05	1	1	2	Q6BCY4	Q6BCY4 [124-143	0	2834.5445
[K].IIDELNVI	1xTMTpro [K	3.124E-05	4.225E-05	1	1	3	P12111	P12111 [862-874]	0	2092.2258
[K].ILDELLQ	3xCarbamid	9.166E-07	4.225E-05	1	1	1	P04275	P04275 [1182-12(	0	2992.4385
[R].ILDEIEEI	1xTMTpro [K	1.607E-05	4.225E-05	1	1	5	Q15019	Q15019 [199-209	0	1961.1199
[R].LLDEEE/	1xTMTpro [N	2.453E-05	4.225E-05	1	1	2	Q8WUM4	Q8WUM4 [457-46	0	1836.9146
[K].EYGGLD	1xTMTpro [K	4.442E-06	4.225E-05	1	1	3	P16152	P16152 [80-96]	0	2388.3419
[K].EYGGLN	1xTMTpro [K	4.293E-05	4.225E-05	1	1	1	O75828	O75828 [80-96]	0	2387.3579

[K].IFYNQQI	1xTMTpro [K	1.991E-05	4.225E-05	1	1	1	Q15848	Q15848 [135-149]	0	2380.2177
[R].IFYPEIEI	1xTMTpro [N	7.701E-06	4.225E-05	1	1	1	P33316	P33316 [225-241]	0	2371.1988
[R].IIDDSEIT	1xTMTpro [K	1.786E-06	4.225E-05	1	2	2	P61326	P61326 [63-82]	1	2933.5388
[K].LLDDNG	1xTMTpro [K	2.989E-05	4.225E-05	1	1	2	O60613	O60613 [133-148]	0	2365.347
[K].LFYSTFA	1xTMTpro [K	3.657E-05	4.225E-05	1	1	3	P30084	P30084 [262-273]	1	2072.1308
[K].FGFCPM	2xCarbamido	2.136E-05	4.225E-05	1	1	7	P02751	P02751 [458-479]	0	2956.3057
[R].LIDDHFL	1xCarbamido	6.794E-06	4.225E-05	1	1	1	P17540	P17540 [221-243]	0	3224.7456
[R].ILDDDTII	1xTMTpro [K	7.83E-07	4.225E-05	1	1	3	Q14204	Q14204 [3760-37	1	2481.442
[K].LLDDAM	2xTMTpro [K	1.417E-05	4.225E-05	1	1	1	Q9HC38	Q9HC38 [289-305	1	2838.5164
[K].EYLDNV	1xTMTpro [N	2.246E-05	4.225E-05	1	1	1	P28288	P28288 [544-557]	0	2003.0881
[R].EYFGEF	1xOxidation [	2.202E-06	4.225E-05	1	1	3	Q99729	Q99729 [172-190]	0	2838.4403
[R].EYFGEF	1xTMTpro [K	8.096E-07	4.225E-05	1	1	4	Q99729	Q99729 [172-190]	0	2822.4454
[K].EYFGAF	1xOxidation [	6.037E-06	4.225E-05	1	1	3	O14979	O14979 [251-269]	0	2827.4356
[K].EYFGAF	1xTMTpro [K	2.675E-06	4.225E-05	1	1	4	O14979	O14979 [251-269]	0	2811.4407
[K].EYDELAI	1xTMTpro [K	5.175E-05	4.225E-05	1	1	1	O60341	O60341 [493-503]	0	1890.994
[K].IFVGNVS	1xCarbamido	8.079E-06	4.225E-05	1	1	2	Q96PK6	Q96PK6 [81-96]	0	2056.0816
[R].LIDLHTN	1xTMTpro [K	2.829E-05	4.225E-05	1	1	1	Q8WVM8	Q8WVM8 [398-41	0	2495.4841
[R].FGGNPG	1xTMTpro [N	1.956E-06	4.225E-05	1	1	2	Q13148	Q13148 [276-293]	0	2030.9752
[K].EYDQAL	2xTMTpro [K	3.417E-05	4.225E-05	1	1	1	Q08752	Q08752 [322-332]	1	2206.2899
[R].ILDLGIT	1xTMTpro [N	2.867E-07	4.225E-05	1	1	2	Q16555	Q16555 [212-238]	0	3204.7184
[K].LIDIFYP	1xTMTpro [K	4.849E-05	4.225E-05	1	1	4	P54886	P54886 [283-300]	0	2637.442
[R].LLDIESQ	1xTMTpro [N	4.102E-06	4.225E-05	1	1	1	Q9H6S3	Q9H6S3 [103-122	0	2675.4099
[R].LLDLENI	1xTMTpro [K	1.359E-05	4.225E-05	1	1	1	Q96JJ3	Q96JJ3 [691-708]	0	2605.546
[R].LLDLENI	1xTMTpro [K	2.405E-05	4.225E-05	1	1	1	Q92556	Q92556 [698-715]	0	2591.5304
[K].LLDAVD	1xTMTpro [N	4.374E-05	4.225E-05	1	1	2	P49411	P49411 [242-255]	0	1847.0598
[R].FGGGNF	1xTMTpro [K	1.682E-05	4.225E-05	1	1	2	P40939	P40939 [611-625]	0	2186.2135
[K].LLDIACW	1xCarbamido	1.036E-05	4.225E-05	1	1	7	P07339	P07339 [112-122]	0	2014.1552
[R].FGGFGG	1xTMTpro [K	3.267E-06	4.225E-05	1	1	1	P35908	P35908 [123-166]	0	4700.4869
[R].FGGEHV	1xTMTpro [N	1.656E-08	4.225E-05	1	1	4	P21333	P21333 [1726-175	0	3249.6976
[R].EYEIPSN	1xTMTpro [N	5.34E-05	4.225E-05	1	1	2	Q8WUM0	Q8WUM0 [676-69	0	2202.1402
[K].IIDGVPV	1xTMTpro [K	2.279E-05	4.225E-05	1	1	4	Q15063	Q15063 [736-747]	0	1921.1502
[R].LFVSDG	1xCarbamido	4.057E-06	4.225E-05	1	1	2	P56192	P56192 [3-21]	0	2203.2228
[K].LFVYDPI	1xTMTpro [N	4.809E-05	4.225E-05	1	1	2	Q15165	Q15165 [290-305]	0	2151.1405

[K].LFYAPA/	1xTMTpro [N	3.479E-06	4.225E-05	1	1	2	Q99715	Q99715 [1876-19(	0	3047.6525
[K].LLDFGSI	1xOxidation [	2.828E-06	4.225E-05	1	1	4	P08708	P08708 [108-129]	0	3033.6575
[K].LLDFGSI	1xTMTpro [K	4.257E-06	4.225E-05	1	1	2	P08708	P08708 [108-129]	0	3017.6626
[R].FGGGGI	1xOxidation [	8.294E-08	4.225E-05	1	1	2	P20908	P20908 [526-554]	1	3470.7942
[R].LLDAQLS	1xTMTpro [K	8.429E-06	4.225E-05	1	1	1	Q15149	Q15149 [3270-32	0	2222.2888
[K].EYLGAIC	3xCarbamido	6.416E-06	4.225E-05	1	1	2	P02751	P02751 [2402-24	0	2183.0003
[R].LIDAGHS	1xTMTpro [K	6.037E-06	4.225E-05	1	1	4	P11277	P11277 [1757-17	0	2320.2793
[R].LIASYCN	1xCarbamido	2.75E-05	4.225E-05	1	1	1	P42704	P42704 [203-218]	0	2305.2354
[R].ILASTQF	1xTMTpro [N	4.964E-05	4.225E-05	1	1	1	Q9NZ08	Q9NZ08 [176-188	0	1708.9553
[R].LIASSLV	1xTMTpro [K	4.705E-05	4.225E-05	1	1	2	Q14C86	Q14C86 [109-119	0	1696.05
[R].LLASSDI	1xTMTpro [K	2.194E-06	4.225E-05	1	1	1	P34949	P34949 [30-56]	0	3617.9282
[K].LGADAV	1xTMTpro [N	3.096E-06	4.225E-05	1	1	2	P00491	P00491 [212-229]	0	2089.1646
[K].ILAQVVG	1xTMTpro [N	3.696E-05	4.225E-05	1	1	3	Q15029	Q15029 [380-394]	0	1887.087
[R].ILAQITG	1xTMTpro [K	2.332E-06	4.225E-05	1	1	1	Q96HC4	Q96HC4 [285-296	0	1932.1774
[K].LLAQLLG	1xTMTpro [K	3.988E-05	4.225E-05	1	1	2	Q9HB07	Q9HB07 [143-163	0	2891.5567
[R].IGAEVYH	1xTMTpro [K	5.636E-05	4.225E-05	1	1	6	P06733	P06733 [184-193]	0	1752.03
[R].EYQLND	1xTMTpro [N	1.15E-05	4.225E-05	1	1	1	P63096	P63096 [145-161]	0	2367.1424
[K].LLATEQE	1xTMTpro [K	1.267E-05	4.225E-05	1	1	3	Q9P2E9	Q9P2E9 [708-720	0	1967.1305
[R].EYQLND	1xTMTpro [N	2.66E-06	4.225E-05	1	1	3	P04899	P04899 [146-162]	0	2381.158
[R].ILAQATS	1xTMTpro [K	5.265E-06	4.225E-05	1	1	2	Q9Y490	Q9Y490 [828-841	0	2065.2513
[K].EYQPHV	1xTMTpro [N	3.613E-05	4.225E-05	1	1	1	P35611	P35611 [549-570]	0	2777.4429
[K].LIAPVAE	1xTMTpro [K	8.079E-06	4.225E-05	1	1	3	P07195	P07195 [8-23]	0	2303.3102
[K].EYQVET	1xCarbamido	1.825E-05	4.225E-05	1	1	2	Q86VP6	Q86VP6 [81-99]	0	2867.4662
[K].EYQVET	1xCarbamido	1.971E-08	4.225E-05	1	1	1	Q86VP6	Q86VP6 [81-103]	1	3393.7526
[K].ILANLEQ	1xTMTpro [N	1.124E-06	4.225E-05	1	1	1	Q8WVT3	Q8WVT3 [511-53	0	2679.3579
[K].LIANDDN	1xTMTpro [K	3.369E-05	4.225E-05	1	1	1	Q86TV6	Q86TV6 [37-52]	0	2340.2976
[R].EYSGTI/	1xTMTpro [K	3.305E-05	4.225E-05	1	1	1	P04114	P04114 [3508-35	0	2456.2801
[R].IIANALSS	1xCarbamido	7.904E-08	4.225E-05	1	1	4	P78527	P78527 [3336-33	1	3008.6218
[R].IIANALSS	1xCarbamido	4.409E-06	4.225E-05	1	1	2	P78527	P78527 [3336-33	0	2781.4836
[R].EYQLND	1xTMTpro [N	3.678E-06	4.225E-05	1	1	2	P08754	P08754 [145-161]	0	2383.1373
[R].FGGPVH	1xTMTpro [N	3.489E-05	4.225E-05	1	1	2	Q15717	Q15717 [207-217]	0	1537.8307
[K].LIATFPD	1xTMTpro [N	3.961E-05	4.225E-05	1	1	1	P23229	P23229 [739-753]	0	2036.1024
[K].ILATPPQ	1xTMTpro [N	7.787E-06	4.225E-05	1	1	5	P29401	P29401 [284-302]	0	2324.2781

[K].LIDAETT	1xTMTpro [N	3.664E-06	4.225E-05	1	1	1	Q3LXA3	Q3LXA3 [326-347	0	2531.3789
[K].FGFCPM	2xCarbamido	1.388E-06	4.225E-05	1	1	9	P02751	P02751 [458-479]	0	2940.3108
[K].EYLLGST	1xTMTpro [K	4.947E-05	4.225E-05	1	1	2	O43324	O43324 [58-68]	0	1848.0246
[R].LLCSVIG	1xCarbamido	9.073E-06	4.225E-05	1	1	1	P16157	P16157 [1172-119	0	3001.5796
[K].EYLLSGI	1xCarbamido	1.443E-06	4.225E-05	1	1	1	Q53EL6	Q53EL6 [337-352	0	2472.2936
[K].FGFCPM	2xCarbamido	2.351E-07	4.225E-05	1	1	2	P02751	P02751 [458-479]	0	2924.3159
[R].IICSAGL	1xCarbamido	3.532E-05	4.225E-05	1	1	1	Q9BV86	Q9BV86 [193-206	0	1836.022
[K].EYIPTVF	1xTMTpro [N	4.917E-06	4.225E-05	1	1	3	P84095	P84095 [31-49]	0	2436.2002
[K].ILCPGGE	3xCarbamido	1.263E-06	4.225E-05	1	1	5	P35555	P35555 [1587-162	0	4490.3014
[R].LGAAPE	1xTMTpro [K	4.206E-05	4.225E-05	1	1	1	Q9UNZ2	Q9UNZ2 [157-172	0	2229.1895
[K].ILATGAN	1xCarbamido	2.611E-06	4.225E-05	1	1	4	P17987	P17987 [278-298]	0	2784.5495
[R].EYLSEEY	1xTMTpro [K	4.449E-05	4.225E-05	1	1	1	Q53GS9	Q53GS9 [482-493	0	2040.1257
[R].LICGFMD	2xCarbamido	4.022E-05	4.225E-05	1	1	2	Q92896	Q92896 [233-249]	1	2981.6079
[K].LGAASL(	1xTMTpro [K	9.023E-06	4.225E-05	1	1	1	Q9Y4G6	Q9Y4G6 [2065-20	0	2819.601
[R].IICDNTG	1xCarbamido	1.156E-05	4.225E-05	1	1	2	P05164	P05164 [702-714]	0	2030.1448
[K].LICCDILI	2xCarbamido	5.227E-06	4.225E-05	1	1	2	P62258	P62258 [95-118]	1	3608.0008
[R].LIAYQEF	1xTMTpro [N	7.247E-06	4.225E-05	1	1	1	Q86WV6	Q86WV6 [311-33	0	2672.3738
[R].EYNEVE	1xTMTpro [K	1.119E-05	4.225E-05	1	1	1	Q13630	Q13630 [233-253]	0	2999.5956
[R].IIAVSFPS	1xTMTpro [N	5.536E-05	4.225E-05	1	1	2	Q9HBL0	Q9HBL0 [147-162	0	2099.1092
[K].LLAVNNV	1xCarbamido	1.889E-05	4.225E-05	1	1	2	Q12959	Q12959 [371-392]	0	3060.6895
[K].LLAVAAT	1xTMTpro [N	1.316E-06	4.225E-05	1	1	4	P18206	P18206 [608-629]	1	2685.4167
[R].EYPDAQ	1xTMTpro [N	5.252E-05	4.225E-05	1	1	1	Q15059	Q15059 [369-381]	0	1742.8669
[K].LGAASL(	1xTMTpro [K	4.035E-06	4.225E-05	1	1	4	Q9Y490	Q9Y490 [2064-20	0	2803.6061
[R].ILENEKD	2xTMTpro [K	1.045E-05	4.225E-05	1	1	4	O75347	O75347 [81-95]	1	2764.5073
[K].EYCISSD	2xCarbamido	2.885E-05	4.225E-05	1	1	1	Q16881	Q16881 [325-342]	0	2759.3552
[K].LLDLVQ(	1xCarbamido	3.358E-05	4.225E-05	1	1	4	P55769	P55769 [22-33]	0	2089.1607
[R].LLEGDG	1xCarbamido	8.433E-07	4.225E-05	1	1	2	P22102	P22102 [221-249]	0	3552.8322
[K].LFVGGLI	1xTMTpro [N	1.334E-05	4.225E-05	1	1	2	Q96EP5	Q96EP5 [12-27]	0	2127.1405
[R].LIEESSV	1xTMTpro [K	9.495E-07	4.225E-05	1	1	2	Q14558	Q14558 [296-317]	0	3059.6756
[K].IFVGGIK	1xTMTpro [K	9.694E-06	4.225E-05	2	4	9	P22626; P09	P22626 [114-129]	1	2488.3804
[K].IFVGGIK	1xTMTpro [K	2.94E-06	4.225E-05	1	1	1	P51991	P51991 [128-143]	1	2491.3688
[K].IFVGGLS	1xTMTpro [K	4.029E-05	4.225E-05	1	1	2	Q96DH6	Q96DH6 [112-127	0	2256.3095
[K].EVYTFAS	1xTMTpro [K	5.473E-05	4.225E-05	1	1	2	P12111	P12111 [2782-279	0	2401.2571

[K].LFVGGL:	1xTMTpro [K	1.683E-06	4.225E-05	1	1	3	Q14011	Q14011 [8-28]	0	2953.5803
[R].LLEEEIIA	1xTMTpro [K	5.545E-05	4.225E-05	1	1	1	Q9P0V9	Q9P0V9 [412-422	0	1900.1287
[K].IFVGGLS	1xTMTpro [K	2.663E-05	4.225E-05	1	1	6	Q14103	Q14103 [184-197	0	2097.1723
[R].FGLGSV	1xTMTpro [K	1.015E-07	4.225E-05	1	1	3	Q9UJS0	Q9UJS0 [332-353	0	2713.5784
[K].FGIGDGI	1xTMTpro [K	1.206E-05	4.225E-05	1	1	1	P30419	P30419 [467-482]	0	2559.3528
[R].ILEDQEE	1xTMTpro [K	8.433E-07	4.225E-05	1	1	5	O95336	O95336 [215-235	0	2907.6071
[R].LLEDGEI	1xOxidation	2.045E-06	4.225E-05	1	1	2	P05783	P05783 [383-407]	0	3364.671
[R].LLEDGEI	1xTMTpro [K	3.291E-06	4.225E-05	1	1	5	P05783	P05783 [383-407]	0	3348.6761
[R].LLEDFGI	1xOxidation	5.794E-05	4.225E-05	1	1	1	Q13573	Q13573 [54-78]	0	3067.5154
[R].LLEDFGI	1xTMTpro [N	1.662E-07	4.225E-05	1	1	2	Q13573	Q13573 [54-78]	0	3051.5205
[R].LLEAVG	1xCarbamid	2.427E-05	4.225E-05	1	1	1	Q8WU76	Q8WU76 [45-57]	0	1740.9386
[K].EWDLLN	1xTMTpro [K	2.329E-05	4.225E-05	1	1	2	O00232	O00232 [79-92]	0	2326.2972
[R].LLEAQIA	1xTMTpro [N	2.121E-05	4.225E-05	2	2	2	P58107; Q1	P58107 [1680-16	0	2331.3104
[R].LLEAQIA	1xTMTpro [K	9.483E-06	4.225E-05	1	1	1	P15924	P15924 [2367-23	0	2147.2931
[R].LLEGDG	1xCarbamid	2.885E-05	4.225E-05	1	1	1	P22102	P22102 [221-249]	0	3568.8271
[K].LLEGIGL	1xTMTpro [K	5.047E-05	4.225E-05	1	1	1	O75165	O75165 [2140-21	0	2349.3521
[R].LIEGLSH	1xCarbamid	2.162E-06	4.225E-05	1	1	1	Q9P258	Q9P258 [195-211	0	2115.1551
[K].EVYEGE	1xCarbamid	1.024E-05	4.225E-05	1	1	1	Q9Y265	Q9Y265 [129-152	0	3297.5787
[R].ILELSGS	1xTMTpro [K	4.369E-06	4.225E-05	1	1	2	P78527	P78527 [3359-33	0	2089.1156
[K].LLELQE\	1xTMTpro [N	5.384E-05	4.225E-05	1	1	2	P16144	P16144 [1061-10	0	1732.0176
[K].EVVSLTE	3xCarbamid	1.787E-07	4.225E-05	1	1	4	P02774	P02774 [66-87]	0	2822.2238
[R].EVVTDQ	2xCarbamid	1.748E-07	4.225E-05	1	1	2	P06681	P06681 [657-687]	1	4039.9509
[K].ILELIQS(	1xTMTpro [K	4.377E-06	4.225E-05	1	1	4	O94788	O94788 [371-384	0	2036.2247
[K].EVVTSEI	1xCarbamid	1.067E-07	4.225E-05	1	1	6	P02671	P02671 [481-510]	0	3432.6106
[K].EVVTSEI	1xCarbamid	8.021E-07	4.225E-05	1	1	4	P02671	P02671 [481-510]	0	3448.6055
[K].EVVVAE:	1xTMTpro [K	4.833E-05	4.225E-05	1	1	1	Q9BVM2	Q9BVM2 [175-18	0	1839.0719
[R].EVVVDL,	1xTMTpro [N	3.265E-05	4.225E-05	1	1	2	Q16647	Q16647 [360-373	0	1806.9413
[K].EVVVYLI	1xTMTpro [K	4.07E-07	4.225E-05	1	1	2	P52948	P52948 [798-817]	0	2849.5653
[R].EWEGG\	1xTMTpro [N	3.696E-05	4.225E-05	1	1	1	Q9Y240	Q9Y240 [28-41]	0	1923.8792
[K].LFVEAL(	1xTMTpro [K	1.407E-05	4.225E-05	1	1	2	P27708	P27708 [1435-14	0	2258.3768
[K].EVWEEL	1xTMTpro [N	4.271E-05	4.225E-05	1	1	2	Q02818	Q02818 [230-242	0	1875.9408
[R].LLELFTD	1xCarbamid	3.734E-05	4.225E-05	1	1	1	Q5VW32	Q5VW32 [43-58]	0	2549.3309
[R].IFVEESI\	1xTMTpro [N	2.493E-05	4.225E-05	1	1	4	P00352	P00352 [309-321]	0	1950.018

[R].EVWFFG	1xTMTpro [K	3.626E-05	4.225E-05	1	1	4	P26038	P26038 [41-53]	0	2269.2149
[R].LLEIDPY	1xTMTpro [K	1.991E-05	4.225E-05	1	1	2	Q04446	Q04446 [31-47]	0	2688.5256
[R].EVWLDF	1xTMTpro [N	2.701E-05	4.225E-05	1	1	1	Q5SZK8	Q5SZK8 [86-104]	0	2533.3734
[R].IFVEESI	1xTMTpro [N	5.366E-05	4.225E-05	1	1	2	O94788	O94788 [326-338]	0	1964.0336
[R].EVWNTY	1xTMTpro [K	4.472E-05	4.225E-05	1	1	2	Q14204	Q14204 [1468-14	0	2636.3852
[K].ILEHMPV	2xTMTpro [K	1.865E-05	4.225E-05	1	1	1	Q9BUQ8	Q9BUQ8 [566-58	0	3434.793
[K].FGLNTVI	1xTMTpro [N	5.247E-07	4.225E-05	1	1	2	Q9UHG3	Q9UHG3 [365-39	0	3096.69
[R].LLELGPI	1xTMTpro [K	1.766E-05	4.225E-05	1	1	1	P53621	P53621 [1128-114	0	2287.3629
[R].EWEGG	1xTMTpro [N	2.994E-05	4.225E-05	1	1	1	Q9Y240	Q9Y240 [28-43]	1	2209.0229
[R].LLEAQIA	1xTMTpro [N	2.593E-05	4.225E-05	1	1	1	Q15149	Q15149 [4179-41	0	2353.2683
[R].LLEAQIA	1xTMTpro [K	4.669E-06	4.225E-05	1	1	2	P15924	P15924 [2124-214	0	2861.6632
[K].LLDSWF	1xTMTpro [K	2.515E-05	4.225E-05	1	1	2	Q8N5M9	Q8N5M9 [169-18	0	2063.1305
[K].ILDSVGII	1xTMTpro [K	2.034E-06	4.225E-05	1	1	3	P05387	P05387 [26-41]	1	2381.3168
[K].ILDSVGII	1xTMTpro [N	2.21E-05	4.225E-05	1	1	2	P05387	P05387 [26-38]	0	1721.8877
[K].LLDSST	1xTMTpro [K	8.842E-06	4.225E-05	1	1	1	P60900	P60900 [60-71]	0	1969.1614
[R].EWTDGI	1xTMTpro [N	2.337E-05	4.225E-05	1	1	1	Q14204	Q14204 [2274-22	0	1777.9556
[R].IIDSLFN	2xTMTpro [K	1.145E-06	4.225E-05	1	1	1	O60701	O60701 [318-330]	1	2406.4424
[K].EWTTTT	1xTMTpro [K	6.003E-05	4.225E-05	1	1	1	Q8WVQ1	Q8WVQ1 [232-24	0	2713.3965
[R].LLDSLEF	1xTMTpro [N	3.143E-06	4.225E-05	1	1	1	Q92734	Q92734 [119-144]	0	3023.5129
[K].ILDSFAA	1xTMTpro [K	2.009E-05	4.225E-05	1	1	1	P18583	P18583 [241-258]	0	2465.4875
[K].ILDQGEI	1xTMTpro [N	1.744E-05	4.225E-05	1	1	4	P30040	P30040 [209-223]	0	2012.9918
[R].LFVGNLI	1xTMTpro [N	2.012E-05	4.225E-05	1	1	10	Q15233	Q15233 [76-91]	0	2164.1279
[K].ILDPNTG	1xTMTpro [K	6.523E-06	4.225E-05	1	1	1	P46060	P46060 [414-445]	0	3899.0974
[K].LLDPED	2xTMTpro [K	3.638E-05	4.225E-05	1	1	1	P46939	P46939 [227-241]	1	2592.5429
[K].LLDNWD	1xTMTpro [K	1.558E-05	4.225E-05	1	1	10	P02647	P02647 [70-83]	0	2221.1996
[K].FGGTICS	3xCarbamido	1.487E-06	4.225E-05	1	1	1	P07357	P07357 [69-92]	0	2956.3195
[K].EYACTA	1xCarbamido	4.009E-05	4.225E-05	1	1	1	P43304	P43304 [543-554]	0	1719.8365
[K].EYAEDD	1xTMTpro [K	2.187E-05	4.225E-05	1	1	3	Q96FW1	Q96FW1 [60-71]	0	2124.0741
[K].EYAESIG	1xTMTpro [K	1.443E-05	4.225E-05	1	1	1	Q13636	Q13636 [135-150]	0	2275.2677
[R].FGGSGS	1xTMTpro [N	3.495E-05	4.225E-05	1	1	1	Q13200	Q13200 [358-369]	0	1471.746
[R].LLDMAD	1xTMTpro [N	1.346E-05	4.225E-05	1	1	1	Q6P1M0	Q6P1M0 [528-547	0	2395.2498
[K].EYAQNI	1xTMTpro [K	6.09E-05	4.225E-05	1	1	1	P06737	P06737 [820-834]	0	2414.2847
[K].EYATEVI	1xTMTpro [N	3.947E-05	4.225E-05	2	2	4	Q10567; P63	Q10567 [360-371]	0	1746.887

[R].LFVGNLI	1xTMTpro [K	4.345E-05	4.225E-05	1	1	1	Q8WXF1	Q8WXF1 [84-100]	1	2602.4372
[R].EYCGVP	1xCarbamido	1.396E-05	4.225E-05	1	1	2	P02760	P02760 [335-349]	0	2012.9554
[R].IIDTSLTF	1xTMTpro [K	5.617E-06	4.225E-05	1	1	1	Q9NYL4	Q9NYL4 [73-90]	1	2619.5577
[K].EWNDST	1xTMTpro [N	3.471E-05	4.225E-05	1	1	2	Q9Y3Z3	Q9Y3Z3 [597-609	0	1837.9
[R].LLEAQA	1xCarbamido	1.861E-06	4.225E-05	1	1	1	Q15149	Q15149 [4448-44	1	3567.8973
[R].LLEAQA	1xCarbamido	6.639E-07	4.225E-05	1	1	3	Q15149	Q15149 [4448-44	0	2292.1825
[R].LFVGNLI	1xTMTpro [K	2.829E-05	4.225E-05	1	1	4	P23246	P23246 [299-314]	0	2416.3256
[R].EWFWDI	1xTMTpro [K	2.952E-05	4.225E-05	1	1	4	P02790	P02790 [167-178]	0	2093.1022
[R].FGIDDQI	1xTMTpro [N	4.17E-05	4.225E-05	1	1	1	P78356	P78356 [110-123]	0	1961.9524
[R].ILEAHQN	1xTMTpro [K	4.525E-06	4.225E-05	1	1	3	Q96AC1	Q96AC1 [539-555	0	2503.4198
[R].ILEAHQN	1xTMTpro [N	2.475E-06	4.225E-05	1	1	2	Q86UX7	Q86UX7 [524-542	0	2408.3581
[K].LLEAHEE	1xTMTpro [K	3.322E-06	4.225E-05	1	1	2	P54920	P54920 [247-264]	0	2699.402
[K].EWIECAI	1xCarbamido	3.728E-05	4.225E-05	1	1	1	O43920	O43920 [39-51]	0	1895.9393
[K].LLEAGNI	1xCarbamido	2.117E-05	4.225E-05	1	1	3	P35914	P35914 [299-312]	0	1923.0441
[K].LLDTVDI	1xTMTpro [N	5.722E-06	4.225E-05	1	1	2	Q9H4M9	Q9H4M9 [378-392	0	1979.0439
[K].LIEAEDN	1xCarbamido	4.249E-05	4.225E-05	1	1	1	P62318	P62318 [32-51]	0	2738.2755
[R].LLDYVAT	1xTMTpro [K	4.235E-05	4.225E-05	1	1	1	Q9NR09	Q9NR09 [2178-21	0	2287.2677
[R].FGIDDQI	1xTMTpro [N	2.075E-05	4.225E-05	1	1	1	Q8TBX8	Q8TBX8 [115-128	0	1946.019
[R].LFVGNLI	1xTMTpro [K	7.244E-07	4.225E-05	1	1	2	P23246	P23246 [299-315]	1	2572.4267
[R].LIDWGL	1xTMTpro [N	1.208E-06	4.225E-05	1	2	1	P68400	P68400 [173-191]	0	2611.3264
[R].LIDWGL	1xTMTpro [N	2.198E-06	4.225E-05	1	1	1	P19784	P19784 [174-192]	0	2625.3421
[R].IIDVVYN	1xTMTpro [N	2.132E-05	4.225E-05	1	1	2	P62241	P62241 [78-92]	0	2023.1143
[K].IIDVVQD	1xTMTpro [N	4.265E-06	4.225E-05	1	1	1	Q8NC56	Q8NC56 [418-435	0	2594.2516
[K].EWIQNT	1xTMTpro [K	4.308E-05	4.225E-05	1	1	1	O00116	O00116 [130-143]	0	2275.269
[R].LIDVPDF	1xTMTpro [K	6.022E-05	4.225E-05	2	2	1	Q13948; P39	Q13948 [96-115];	0	2780.6417
[K].FGKPDP	3xTMTpro [K	5.022E-05	4.225E-05	1	1	1	Q9NZM1	Q9NZM1 [17-32]	1	3035.8286
[K].LIEAEDN	1xCarbamido	5.829E-06	4.225E-05	1	1	4	P62318	P62318 [32-51]	0	2722.2806
[K].IEELLPG	1xTMTpro [N	3.08E-05	4.225E-05	1	1	2	P50453	P50453 [144-158]	0	1934.0402
[R].IQCPNQ	2xCarbamido	6.475E-06	4.225E-05	1	1	2	Q96ME7	Q96ME7 [406-424	0	2705.3882
[R].IQCSFD	1xCarbamido	7.382E-06	4.225E-05	1	1	2	Q9NRN5	Q9NRN5 [345-359	0	1985.9922
[R].IVSLEPA	1xTMTpro [K	4.33E-05	4.225E-05	1	1	2	Q14126	Q14126 [200-215]	0	2458.4241
[K].EIEGEEI	2xTMTpro [K	2.655E-06	4.225E-05	1	1	2	O43390	O43390 [398-414]	0	2821.6379
[R].ELEGHIS	1xTMTpro [N	5.861E-06	4.225E-05	1	1	3	P35749	P35749 [1115-113	0	2289.1166

[R].EIEGQW	1xTMTpro [K	8.554E-06	4.225E-05	1	1	1	Q96CW5	Q96CW5 [814-83	1	2812.4245
[R].EIEGSQ\	1xTMTpro [K	3.988E-05	4.225E-05	1	1	1	Q01459	Q01459 [170-185]	0	2401.2895
[R].IVSGIITF	1xTMTpro [K	2.53E-06	4.225E-05	1	1	2	Q92530	Q92530 [134-148]	0	2358.3677
[K].IVSGEAE	1xTMTpro [K	9.692E-08	4.225E-05	1	1	4	P36776	P36776 [727-755]	0	3784.03
[R].EIEIAEQ	1xTMTpro [N	3.459E-05	4.225E-05	1	1	2	O43865	O43865 [119-134]	0	2122.1385
[K].LDAENAI	1xTMTpro [N	1.247E-06	4.225E-05	1	1	1	Q01085	Q01085 [149-166]	0	2228.1565
[K].LVSELW	1xTMTpro [K	5.384E-05	4.225E-05	1	1	2	P12081	P12081 [427-437]	0	1839.0872
[R].LVSELDI	1xTMTpro [N	3.399E-05	4.225E-05	1	1	1	P46939	P46939 [1693-17(	0	2118.1362
[K].EIEIDIEP	1xTMTpro [K	6.464E-06	4.225E-05	1	1	3	Q15843	Q15843 [12-25]	1	2294.2735
[R].EIEIDYT(	1xCarbamid(	1.845E-05	4.225E-05	1	1	1	Q9NV96	Q9NV96 [77-93]	0	2548.2733
[K].IDAFHY\	1xTMTpro [N	4.906E-05	4.225E-05	1	1	1	Q9H857	Q9H857 [133-146	0	1958.0455
[K].LDAFIEA	1xTMTpro [K	2.616E-05	4.225E-05	1	1	1	O60313	O60313 [949-960]	0	2022.1516
[K].LVSDGQ	1xTMTpro [K	8.445E-06	4.225E-05	1	1	1	Q12931	Q12931 [131-151]	0	2931.5741
[R].EIELLCQ	1xCarbamid(	1.608E-07	4.225E-05	1	1	1	Q15555	Q15555 [274-292]	0	2629.2847
[K].EIELIGS(	1xTMTpro [K	4.135E-05	4.225E-05	1	1	1	P19525	P19525 [269-283]	0	2189.2462
[K].LVSDEM	1xTMTpro [K	1.078E-05	4.225E-05	1	1	4	P54819	P54819 [73-85]	0	2112.2118
[K].LVSASTI	1xCarbamid(	3.499E-06	4.225E-05	1	1	1	Q15291	Q15291 [81-103]	0	2869.3957
[K].LVRPPV(	1xTMTpro [N	5.482E-05	4.225E-05	1	1	2	P48047	P48047 [27-40]	0	1887.1135
[K].IVSLFAE	1xTMTpro [K	3.062E-06	4.225E-05	1	2	2	P41091	P41091 [318-342]	0	3178.7756
[K].LVSIGAE	1xTMTpro [K	2.457E-06	4.225E-05	1	1	4	O43707	O43707 [126-140]	0	2123.2204
[K].LVSIGAE	1xTMTpro [K	2.103E-06	4.225E-05	2	2	20	P12814; P35	P12814 [107-121]	0	2151.2517
[K].LDADKYI	2xTMTpro [K	4.776E-05	4.225E-05	1	1	2	Q9BV57	Q9BV57 [41-54]	1	2591.4021
[K].LCYVGY	1xCarbamid(	3.086E-05	4.225E-05	1	1	3	P61160	P61160 [220-232]	0	2252.1877
[R].LVTLEEF	1xTMTpro [N	3.846E-05	4.225E-05	1	1	2	Q02818	Q02818 [311-323]	0	1811.0234
[R].EIEDPEI	2xTMTpro [K	5.85E-05	4.225E-05	1	1	3	P27824	P27824 [275-292]	1	3195.6738
[K].LVTGGG	1xTMTpro [N	1.015E-07	4.225E-05	1	1	1	Q6DKJ4	Q6DKJ4 [13-29]	0	2000.0732
[R].ELEDVTI	1xTMTpro [N	1.944E-05	4.225E-05	1	1	2	Q7Z406	Q7Z406 [1923-19	0	1913.9082
[K].LVTENLI	2xTMTpro [K	8.859E-06	4.225E-05	1	1	4	Q9NW15	Q9NW15 [642-65	1	2616.4735
[K].LVTEMG	1xTMTpro [K	4.047E-09	4.225E-05	1	1	3	P53618	P53618 [514-534]	0	2836.537
[R].IVSWSQ	1xTMTpro [K	1.76E-05	4.225E-05	1	1	1	Q9BWD1	Q9BWD1 [280-30	0	2986.7295
[K].IVSWLE\	1xTMTpro [K	3.886E-05	4.225E-05	1	1	3	Q02790	Q02790 [296-313]	0	2754.4118
[R].LVSSPC(	2xCarbamid(	8.745E-06	4.225E-05	1	1	1	P08238	P08238 [584-604]	0	2752.3064
[R].LVRPEVI	1xCarbamid(	5.628E-06	4.225E-05	1	1	29	P02768	P02768 [139-161]	1	3690.9804

[R].LVSSPC(	2xCarbamid(	2.231E-06	4.225E-05	1	1	4	P08238	P08238 [584-604]	0	2736.3115
[K].IVSSAME	1xTMTpro [N	1.024E-05	4.225E-05	1	1	2	P08603	P08603 [157-175]	1	2496.2625
[K].IDAAQN\	1xTMTpro [N	5.314E-06	4.225E-05	1	1	2	P18206	P18206 [388-409]	0	2684.3235
[K].LVSQEEI	1xTMTpro [N	4.308E-05	4.225E-05	1	1	2	P82909	P82909 [88-99]	0	1812.9485
[K].EIEELQS	1xTMTpro [K	7.505E-06	4.225E-05	1	1	4	Q01082	Q01082 [1435-14!	0	2496.3437
[R].LVSQDN	1xTMTpro [N	3.853E-05	4.225E-05	1	1	1	O15020	O15020 [447-465]	0	2306.2675
[K].ELEEIVQ	1xTMTpro [K	4.882E-05	4.225E-05	1	1	4	P11021	P11021 [622-633]	0	2006.2029
[R].LVSQDN	1xTMTpro [K	1.805E-05	4.225E-05	1	1	1	Q01082	Q01082 [444-462]	0	2630.4321
[R].LVSPGS.	1xTMTpro [N	2.72E-06	4.225E-05	1	1	1	O75369	O75369 [2476-24!	0	2350.2785
[K].LVSPETE	1xTMTpro [K	4.21E-06	4.225E-05	1	1	1	Q12888	Q12888 [991-101!	0	3369.767
[K].LVSLSA(	1xCarbamid(	4.857E-05	4.225E-05	1	1	1	P25774	P25774 [159-174]	0	2372.2987
[K].IVSSAME	1xOxidation [	2.479E-05	4.225E-05	1	1	3	P08603	P08603 [157-175]	1	2512.2574
[R].LVRPEVI	1xCarbamid(	4.836E-07	4.225E-05	1	1	76	P02768	P02768 [139-160]	0	3274.6732
[K].EIEMASE	1xTMTpro [K	2.556E-05	4.225E-05	1	1	2	P27816	P27816 [225-246]	0	3081.6278
[R].LVRPEVI	1xCarbamid(	8.704E-08	4.225E-05	1	1	132	P02768	P02768 [139-160]	0	3258.6783
[R].ELETLQ(	1xTMTpro [K	4.635E-06	4.225E-05	1	1	1	Q4V328	Q4V328 [500-522	0	3192.6702
[K].LVQAAQ	1xTMTpro [N	1.913E-06	4.225E-05	1	1	5	P18206	P18206 [88-105]	0	2278.2185
[K].ELETQLI	1xCarbamid(	2.692E-05	4.225E-05	1	1	1	Q9NZ32	Q9NZ32 [174-191	0	2672.4308
[R].ELETVA/	1xTMTpro [N	2.789E-05	4.225E-05	1	1	1	Q969V3	Q969V3 [340-353	0	1930.0353
[R].LVPNPG	1xCarbamid(	5.064E-05	4.225E-05	1	1	1	P49747	P49747 [486-512]	1	3555.6789
[R].LVPNMT	1xOxidation [	5.107E-05	4.225E-05	1	1	1	Q96KP4	Q96KP4 [344-363	0	2829.5564
[K].EIETYHN	1xTMTpro [K	6.35E-07	4.225E-05	1	1	4	P35527	P35527 [450-472]	0	3118.5461
[K].LDAQVQ	1xTMTpro [K	1.417E-05	4.225E-05	1	1	4	P35580	P35580 [1257-12(	0	1860.0835
[K].LVPLVQ/	1xTMTpro [K	1.776E-05	4.225E-05	1	1	2	P16157	P16157 [1087-11(	0	2335.3881
[K].LVPLLDT	1xTMTpro [N	1.462E-06	4.225E-05	1	1	3	P52209	P52209 [88-107]	0	2464.3254
[K].LVQAAQ	1xOxidation [	8.908E-06	4.225E-05	1	1	1	P18206	P18206 [88-105]	0	2294.2134
[K].EIEVDSS	1xTMTpro [N	1.087E-07	4.225E-05	1	3	2	P61225	P61225 [43-68]	0	3128.5628
[K].IDASKNE	1xTMTpro [K	1.082E-05	4.225E-05	1	1	1	Q14103	Q14103 [68-85]	1	2580.2782
[R].LVPGGG	1xTMTpro [K	4.827E-06	4.225E-05	1	1	2	P50990	P50990 [408-421]	0	1963.172
[R].LVPFDH/	1xTMTpro [N	3.677E-05	4.225E-05	1	1	1	O95336	O95336 [82-96]	0	2072.0772
[R].LVPELDT	1xTMTpro [K	4.744E-05	4.225E-05	1	1	2	P05166	P05166 [298-312]	0	2262.3452
[R].LVNWSC	1xCarbamid(	2.547E-05	4.225E-05	1	1	1	P26640	P26640 [474-493]	1	3204.7755
[K].IDATQVE	1xCarbamid(	1.026E-06	4.225E-05	1	1	2	Q96I99	Q96I99 [235-259]	0	3358.7121

[K].LVNSLYF	1xOxidation [	4.603E-05	4.225E-05	1	1	6	Q01995	Q01995 [65-89]	1	3975.277
[K].LVNSLYF	3xTMTpro [K	6.252E-06	4.225E-05	1	1	4	Q01995	Q01995 [65-89]	1	3959.2821
[K].LVNSLYF	2xTMTpro [K	4.972E-05	4.225E-05	1	1	7	Q01995	Q01995 [65-78]	0	2429.4584
[K].IDATSAS	1xTMTpro [N	4.833E-05	4.225E-05	1	1	1	P13667	P13667 [120-131]	0	1494.8447
[K].IVPIAIQL	1xTMTpro [K	7.01E-06	4.225E-05	1	1	2	P09917	P09917 [321-345]	0	3310.8906
[R].LVTMQIV	1xTMTpro [N	3.242E-05	4.225E-05	1	1	4	P51149	P51149 [56-69]	0	1952.0231
[K].IVQAEGE	1xTMTpro [K	3.322E-05	4.225E-05	1	1	2	Q99623	Q99623 [225-236]	0	1824.0359
[R].LVQAEY	1xTMTpro [K	8.826E-06	4.225E-05	1	1	1	P50897	P50897 [180-196]	1	2769.4856
[R].IVQVVTA	1xTMTpro [K	4.389E-05	4.225E-05	1	1	1	P11137	P11137 [94-107]	0	2048.2975
[K].IVQTYHV	1xTMTpro [K	1.319E-05	4.225E-05	1	1	1	P12814	P12814 [578-603]	0	3498.8547
[R].IVQQWN	1xTMTpro [K	3.441E-05	4.225E-05	1	1	1	O00469	O00469 [179-197]	0	2991.5344
[K].ELENANI	1xTMTpro [K	3.576E-05	4.225E-05	1	1	4	P12270	P12270 [352-364]	0	2026.1312
[K].ELENEEI	1xTMTpro [K	1.36E-06	4.225E-05	1	1	2	Q8IWE2	Q8IWE2 [346-367	1	3244.5737
[K].EIENLTQ	1xTMTpro [K	4.427E-05	4.225E-05	1	1	3	P35749	P35749 [1400-141	0	2132.1367
[K].IVQIPFC	2xCarbamido	2.06E-05	4.225E-05	1	1	2	P07814	P07814 [1442-145	0	2730.4127
[R].EIENMIL	1xTMTpro [N	9.817E-07	4.225E-05	1	1	1	Q15067	Q15067 [35-56]	0	3007.4791
[R].EIENYPH	1xTMTpro [N	1.143E-06	4.225E-05	1	1	1	Q9H0Q0	Q9H0Q0 [11-32]	0	2986.4178
[R].FQEHLQ	1xTMTpro [K	2.096E-07	4.225E-05	1	1	1	Q00610	Q00610 [9-36]	0	3753.9766
[R].ELESVCY	2xCarbamido	7.918E-07	4.225E-05	1	1	1	Q6L8Q7	Q6L8Q7 [263-282	0	2567.2189
[K].ELEPEMI	1xCarbamido	4.981E-05	4.225E-05	1	1	1	Q9H307	Q9H307 [424-440	1	3064.5675
[K].LVQEQG	1xTMTpro [K	1.61E-05	4.225E-05	1	1	2	P35580	P35580 [553-562]	0	1720.9838
[R].ELEPITT	1xTMTpro [N	2.206E-05	4.225E-05	1	1	1	Q8IYB8	Q8IYB8 [455-470]	0	2031.1405
[K].EIEQEAA	1xTMTpro [N	3.582E-05	4.225E-05	1	1	1	P47985	P47985 [183-196]	0	1919.0405
[R].LVQDYG	1xTMTpro [K	1.015E-07	4.225E-05	1	1	2	P43304	P43304 [483-504]	0	3015.5919
[K].LVQDVA	1xTMTpro [N	1.617E-08	4.225E-05	1	1	5	P10809	P10809 [97-121]	0	2864.4557
[K].ELEQVC	1xCarbamido	5.832E-08	4.225E-05	1	1	3	P0DMV9	P0DMV9 [598-628	0	3663.9085
[R].LVQDLPI	1xTMTpro [K	2.994E-05	4.225E-05	1	1	2	Q9H4Z3	Q9H4Z3 [36-51]	0	2471.379
[K].IDAQAIE	1xTMTpro [K	6.104E-06	4.225E-05	1	2	1	P62068	P62068 [335-352]	0	2608.4002
[K].LVQAIFP	1xTMTpro [K	5.261E-05	4.225E-05	2	2	1	Q09472; Q9	Q09472 [587-601]	0	2189.319
[R].ELESQIS	1xTMTpro [N	1.435E-06	4.225E-05	1	1	4	P35579	P35579 [1108-112	0	2338.1581
[K].ELEPGD	1xCarbamido	1.013E-05	4.225E-05	1	1	4	Q86XP3	Q86XP3 [320-336	0	2127.1439
[R].FQDVGF	1xTMTpro [K	1.341E-05	4.225E-05	1	1	1	Q9UJU6	Q9UJU6 [149-164	0	2328.2844
[R].LVTPGE	1xTMTpro [N	3.108E-05	4.225E-05	1	1	1	P55268	P55268 [585-601]	0	2095.1143

[R].EIEDKP\	1xTMTpro [K	5.926E-05	4.225E-05	1	1	1	Q8NF91	Q8NF91 [5760-57	0	2977.645
[R].FQIQDIS	2xTMTpro [K	3.744E-07	4.225E-05	2	2	4	Q01082; O1!	Q01082 [157-172]	1	2835.5556
[R].LYDEVQ	1xTMTpro [N	1.646E-06	4.225E-05	1	1	1	Q0VDG4	Q0VDG4 [30-51]	0	2895.4848
[R].ELDDATI	1xTMTpro [N	9.606E-06	4.225E-05	1	1	2	P35580	P35580 [1906-19	0	1823.8942
[K].EIDDEAN	1xTMTpro [N	3.626E-05	4.225E-05	1	1	2	A5YKK6	A5YKK6 [843-854	0	1790.8516
[K].ELDDISQ	1xTMTpro [N	3.34E-05	4.225E-05	1	1	1	Q9UBC2	Q9UBC2 [385-398	0	1962.0463
[R].FQIQDIS	1xTMTpro [K	3.813E-05	4.225E-05	2	2	2	Q01082; O1!	Q01082 [157-170]	0	2274.2109
[R].IYDDDFF	1xTMTpro [N	7.382E-06	4.225E-05	1	1	4	P22314	P22314 [559-581]	0	2904.3971
[R].IYDAVSC	1xTMTpro [K	4.695E-06	4.225E-05	1	1	1	Q16363	Q16363 [651-666]	0	2444.3681
[R].EIDDTYII	1xTMTpro [N	2.239E-06	4.225E-05	1	1	1	Q99459	Q99459 [506-521]	0	2115.0049
[R].ELDEATI	1xTMTpro [N	4.865E-05	4.225E-05	1	1	2	P35749	P35749 [1906-19	0	1855.8663
[R].LCQCSD	3xCarbamid	8.05E-06	4.225E-05	1	1	2	P11047	P11047 [827-844]	0	2411.0821
[R].ELDECF	1xCarbamid	1.516E-05	4.225E-05	1	1	1	P15924	P15924 [106-126]	0	2814.3609
[R].LYAYEP	1xOxidation	2.484E-05	4.225E-05	1	1	1	O95994	O95994 [149-165]	0	2565.3766
[R].LYAYEP	1xTMTpro [K	6.997E-06	4.225E-05	1	1	4	O95994	O95994 [149-165]	0	2549.3817
[R].LCQDYD	1xCarbamid	3.471E-05	4.225E-05	1	1	1	P16157	P16157 [1069-10	0	2361.2252
[K].LCQIPVH	1xCarbamid	5.964E-05	4.225E-05	1	1	1	Q14766	Q14766 [429-441]	0	2014.1763
[K].IYAMEIS	1xTMTpro [N	7.715E-07	4.225E-05	1	1	3	Q8IUX7	Q8IUX7 [599-619]	0	2737.3099
[R].ELDFTEI	1xTMTpro [N	5.209E-05	4.225E-05	1	1	1	Q01484	Q01484 [3590-36	0	1962.0252
[R].LCSDLM	2xCarbamid	3.826E-05	4.225E-05	1	1	1	Q9UNF0	Q9UNF0 [37-48]	0	1851.8835
[K].ICSEDLE	1xCarbamid	2.942E-05	4.225E-05	1	1	2	Q6UXG2	Q6UXG2 [359-36	0	1828.997
[R].LWYCDL	1xCarbamid	6.138E-06	4.225E-05	1	1	2	P48147	P48147 [261-278]	0	2689.4515
[R].ELDALD	1xTMTpro [N	1.15E-05	4.225E-05	1	1	2	Q08211	Q08211 [838-853]	0	2046.0674
[K].LYDNLLE	1xTMTpro [N	3.709E-05	4.225E-05	1	1	1	O00231	O00231 [326-337]	0	1808.0237
[R].IYDPQE(	1xTMTpro [N	1.008E-05	4.225E-05	1	1	1	Q9NXF1	Q9NXF1 [599-617	0	2389.2683
[K].LYDYCD	3xCarbamid	7.154E-06	4.225E-05	1	1	2	P00747	P00747 [551-575]	0	3856.9165
[K].EIAQGD	1xTMTpro [K	3.564E-06	4.225E-05	1	1	2	Q7L5N7	Q7L5N7 [474-487	0	2224.1629
[R].LYGPSS	1xTMTpro [N	7.314E-06	4.225E-05	1	1	1	P50454	P50454 [134-148]	0	1964.0085
[R].LCNSPV	1xCarbamid	2.88E-05	4.225E-05	1	1	2	P35442	P35442 [463-474]	0	1896.0327
[R].LYGIQGI	1xTMTpro [N	5.236E-06	4.225E-05	1	1	1	Q6DKJ4	Q6DKJ4 [269-290	0	2731.5387
[R].ELAQQV	1xCarbamid	1.173E-05	4.225E-05	1	1	2	P17844	P17844 [178-192]	0	2097.0718
[R].ELAQQV	1xTMTpro [K	5.535E-06	4.225E-05	1	1	4	Q92841	Q92841 [255-269]	0	2300.2378
[R].IYGLGSI	1xTMTpro [K	3.754E-05	4.225E-05	1	1	1	P36542	P36542 [68-79]	0	1935.1447

[K].LCPGGQ	1xCarbamido	4.792E-06	4.225E-05	1	1	2	O00299	O00299 [58-79]	0	3068.6371
[K].ELASQPI	2xTMTpro [K	3.413E-09	4.225E-05	1	1	6	P60174	P60174 [220-248]	0	3942.2044
[K].IYGAPGV	1xTMTpro [N	4.833E-05	4.225E-05	1	1	1	Q15334	Q15334 [60-73]	0	1820.9978
[R].LCSGPG	1xCarbamido	1.622E-06	4.225E-05	1	1	2	Q9Y5P6	Q9Y5P6 [244-261	0	2115.1551
[K].IYGADDI	1xTMTpro [K	1.055E-06	4.225E-05	1	1	2	P11586	P11586 [833-848]	0	2420.3317
[R].ELATNLN	1xTMTpro [K	5.227E-06	4.225E-05	2	2	1	Q8NFD5; O1	Q8NFD5 [1194-12	0	2458.3645
[K].IYFGSNI	1xCarbamido	1.22E-05	4.225E-05	1	1	1	O75477	O75477 [297-315	0	2771.4392
[K].LYESLLV	1xTMTpro [K	4.009E-05	4.225E-05	1	1	1	P13807	P13807 [404-418]	0	2287.2863
[R].EIAVGDS	1xTMTpro [N	9.016E-08	4.225E-05	1	1	1	Q13409	Q13409 [584-607	0	2861.5216
[R].FQLTDC	1xCarbamido	5.403E-06	4.225E-05	1	1	1	Q16555	Q16555 [174-189	0	2288.228
[R].IYELAAG	1xTMTpro [N	2.597E-06	4.225E-05	1	1	3	P07954	P07954 [269-286]	0	2068.1358
[K].EICNAYT	1xCarbamido	9.57E-06	4.225E-05	1	1	2	Q15046	Q15046 [494-507	0	2029.9642
[R].EICPAGI	1xCarbamido	4.626E-06	4.225E-05	1	1	1	Q14767	Q14767 [722-738	0	2201.0616
[K].IYEFPET	2xTMTpro [K	3.946E-06	4.225E-05	1	1	2	Q16181	Q16181 [222-238	1	3010.6077
[R].LYEEDD	1xTMTpro [N	4.271E-07	4.225E-05	1	1	1	Q86W92	Q86W92 [950-970	1	2846.3321
[R].ICPQEY	3xCarbamido	3.08E-05	4.225E-05	1	1	1	Q9Y625	Q9Y625 [59-74]	0	2673.216
[R].LCSGVL	1xCarbamido	8.891E-06	4.225E-05	1	1	1	Q9Y6C9	Q9Y6C9 [78-90]	0	1935.1341
[K].LCSLFY	1xCarbamido	1.604E-05	4.225E-05	1	1	2	P43243	P43243 [805-817]	0	2182.171
[K].IWSTVP	1xTMTpro [N	5.872E-06	4.225E-05	1	1	1	Q9GZL7	Q9GZL7 [215-235	0	2825.3219
[R].ELDTVTI	1xTMTpro [K	2.77E-05	4.225E-05	1	1	3	O43242	O43242 [66-76]	0	1884.0821
[R].ELDTVTI	2xTMTpro [K	6.051E-05	4.225E-05	1	1	1	O43242	O43242 [66-80]	1	2681.5542
[K].LCYVALD	1xCarbamido	7.338E-07	4.225E-05	2	4	77	P62736; P68	P62736 [218-240]	0	3144.5725
[RK].LVVVG	1xTMTpro [K	5.794E-05	4.225E-05	2	2	2	P62070; P10	P62070 [17-27]; F	0	1549.9921
[K].FQGLDLI	1xTMTpro [K	3.961E-06	4.225E-05	1	1	2	P98160	P98160 [3787-380	0	3180.6749
[R].LVVVDEI	1xTMTpro [K	4.989E-05	4.225E-05	1	1	1	P54619	P54619 [300-310]	0	1837.0926
[K].LVVTQEI	1xOxidation [	5.265E-06	4.225E-05	1	1	1	P49903	P49903 [226-246]	0	2761.353
[K].LCYVALD	1xCarbamido	1.023E-06	4.225E-05	2	4	22	P62736; P68	P62736 [218-240]	0	3160.5674
[R].LVVPAT	1xCarbamido	9.327E-06	4.225E-05	1	1	3	Q15365	Q15365 [102-115	0	2051.2179
[R].LVVPAS	1xCarbamido	6.138E-06	4.225E-05	2	2	4	P57721; Q15	P57721 [134-147]	0	2037.2022
[K].IVYGHLD	1xTMTpro [N	4.296E-06	4.225E-05	1	1	1	Q99798	Q99798 [69-84]	0	2146.11
[K].IVVLMLT	1xOxidation [	1.516E-05	4.225E-05	1	1	1	P12111	P12111 [2738-275	0	2602.4081
[R].LVVLDEI	1xTMTpro [K	9.782E-09	4.225E-05	1	1	3	P60983	P60983 [42-64]	1	3245.7648
[R].FQGEW	2xCarbamido	1.09E-05	4.225E-05	1	1	2	Q86VB7	Q86VB7 [283-305	0	3259.4956

[K].ELEASEI	1xCarbamido	2.621E-05	4.225E-05	1	1	1	O76003	O76003 [218-231]	0	2242.1768
[K].LCYVALI	1xCarbamido	6.566E-07	4.225E-05	1	3	133	P63261	P63261 [216-238]	0	3158.5881
[R].ELEDATI	1xTMTpro [N	9.09E-06	4.225E-05	1	1	8	P35579	P35579 [1899-191	0	1869.8819
[K].LVVECVI	2xCarbamido	1.09E-05	4.225E-05	1	1	4	Q01469	Q01469 [116-129]	0	1999.0094
[K].LVVDLTI	1xTMTpro [K	8.26E-06	4.225E-05	1	1	5	P09960	P09960 [366-386]	0	2946.5843
[R].LVTSPC(	2xCarbamido	5.354E-06	4.225E-05	1	1	2	P07900	P07900 [592-612]	0	2766.322
[R].LVTSPC(	2xCarbamido	4.591E-07	4.225E-05	1	1	2	P07900	P07900 [592-612]	0	2750.3271
[K].LVTSIGD	1xTMTpro [N	1.888E-06	4.225E-05	1	1	1	P11216	P11216 [623-640]	0	2196.1944
[K].LVVLGS(	1xTMTpro [K	4.33E-05	4.225E-05	2	2	5	P61224; P62	P61224 [6-16]; P6	0	1594.0184
[K].LCYVALI	1xCarbamido	1.823E-06	4.225E-05	1	3	78	P63261	P63261 [216-238]	0	3174.583
[R].IVYNNAI	1xTMTpro [K	5.16E-07	4.225E-05	1	1	12	Q05707	Q05707 [567-591]	0	3399.8179
[R].LWADH(	1xCarbamido	3.657E-05	4.225E-05	1	1	1	P04899	P04899 [131-143]	0	1820.9185
[K].LCSLLDS	2xCarbamido	5.433E-06	4.225E-05	1	1	2	Q92973	Q92973 [141-162]	0	3099.5259
[K].EIDKNDI	2xTMTpro [K	1.446E-05	4.225E-05	1	1	2	Q9UNF1	Q9UNF1 [338-364	1	3883.1884
[K].IWNVIYE	1xCarbamido	1.962E-05	4.225E-05	1	1	1	Q96HE7	Q96HE7 [199-215	0	3094.7216
[K].EIDLLLG	1xTMTpro [N	4.564E-05	4.225E-05	1	1	2	O75643	O75643 [156-168]	0	1792.9612
[R].LWNTTE	1xTMTpro [K	9.694E-06	4.225E-05	1	1	1	O43815	O43815 [595-612]	0	2614.4372
[R].LWLESP	1xTMTpro [N	4.21E-06	4.225E-05	1	1	2	Q96T60	Q96T60 [10-35]	0	2991.6627
[K].FQLAVE/	1xTMTpro [K	2.414E-05	4.225E-05	1	1	1	P48681	P48681 [260-271]	0	2013.1512
[K].ELDLSD/	1xTMTpro [N	9.641E-06	4.225E-05	1	1	2	Q9P2R3	Q9P2R3 [103-119	0	2239.1269
[R].ELDLSNI	1xCarbamido	5.897E-08	4.225E-05	1	1	4	P13489	P13489 [402-423]	0	2719.4256
[K].LWIDQS(	1xTMTpro [K	4.257E-06	4.225E-05	1	1	1	P22059	P22059 [590-604]	0	2375.3215
[K].FQGQTC	4xCarbamido	3.947E-05	4.225E-05	1	1	1	P05556	P05556 [625-644]	0	3052.4063
[R].LCTLDES	1xCarbamido	5.607E-06	4.225E-05	1	1	2	Q9GZT8	Q9GZT8 [253-268	0	2128.0949
[R].LWGTQI	1xTMTpro [K	1.286E-05	4.225E-05	1	2	2	P11169	P11169 [229-248]	1	2944.4966
[K].ELDPTNI	1xTMTpro [K	2.908E-06	4.225E-05	1	1	2	P31948	P31948 [253-272]	0	2956.5258
[R].LWELTD	1xTMTpro [N	4.503E-05	4.225E-05	1	1	1	O94921	O94921 [426-442]	0	2312.228
[R].IWDTAS(	1xCarbamido	5.149E-05	4.225E-05	1	1	2	P61964	P61964 [197-207]	0	1887.029
[K].IWDPTPS	1xTMTpro [N	5.158E-05	4.225E-05	1	1	1	O75533	O75533 [253-270]	0	2136.1157
[R].FQGTSY	1xCarbamido	2.819E-05	4.225E-05	1	1	1	P08253	P08253 [297-310]	0	1912.8666
[K].IWCFGP	1xCarbamido	2.462E-05	4.225E-05	1	1	4	P13639	P13639 [649-667]	0	2713.4515
[K].ELDSGL/	1xTMTpro [N	4.651E-07	4.225E-05	1	1	2	P53990	P53990 [91-109]	0	2319.2515
[R].LWAHVY	1xTMTpro [K	9.73E-06	4.225E-05	1	1	1	P61086	P61086 [147-164]	0	2584.4055

[K].FQGQTC	4xCarbamido	3.247E-05	4.225E-05	1	1	1	P05556	P05556 [625-644]	0	3068.4012
[R].LCTSATI	1xCarbamido	5.85E-05	4.225E-05	1	1	2	P31930	P31930 [379-390]	0	1627.8281
[K].LYGPTNI	1xTMTpro [N	1.633E-05	4.225E-05	1	1	2	O75131	O75131 [391-406]	0	2103.167
[R].EIFDIAFI	1xTMTpro [N	1.513E-05	4.225E-05	1	1	1	P36776	P36776 [941-959]	0	2467.2676
[K].ELFEPY(	1xTMTpro [N	1.015E-05	4.225E-05	1	1	1	O95319	O95319 [58-73]	0	2215.2082
[K].LDEYLNS	1xTMTpro [K	6.452E-06	4.225E-05	1	1	4	Q9Y696	Q9Y696 [151-172	0	3187.5848
[K].IDFEDVI/	1xTMTpro [K	6.827E-07	4.225E-05	1	1	1	Q03135	Q03135 [66-86]	0	3013.5439
[R].LVECLET	1xCarbamido	3.954E-05	4.225E-05	1	1	2	O95782	O95782 [280-290]	0	1926.1226
[R].LDFGDV	1xTMTpro [N	4.65E-05	4.225E-05	1	1	1	P14222	P14222 [465-479]	0	1848.055
[K].LVEALC/	1xCarbamido	4.047E-07	4.225E-05	1	1	3	P25398	P25398 [64-78]	0	2359.3663
[K].IVEAAEN	1xTMTpro [K	1.625E-06	4.225E-05	1	1	3	P47755	P47755 [231-256]	0	3591.7656
[K].ELLELEA	1xTMTpro [K	1.135E-05	4.225E-05	1	1	2	Q14764	Q14764 [705-723]	0	2600.4348
[K].ELLELEA	1xOxidation [	7.075E-06	4.225E-05	1	1	4	Q14764	Q14764 [705-723]	0	2616.4298
[K].IVDRIDN	1xTMTpro [K	5.425E-07	4.225E-05	1	1	2	Q15293	Q15293 [87-105]	1	2744.4598
[K].LVDQNIF	1xTMTpro [N	4.503E-05	4.225E-05	1	1	3	P07339	P07339 [223-235]	0	1906.0394
[R].ELLENPV	1xCarbamido	4.575E-06	4.225E-05	1	1	3	Q9Y490	Q9Y490 [1375-14	0	3737.8356
[R].ELLENPV	1xCarbamido	3.097E-05	4.225E-05	1	1	2	Q9Y490	Q9Y490 [1375-14	0	3753.8306
[R].IDFSSIA\	1xTMTpro [N	3.135E-05	4.225E-05	1	1	2	Q5TDH0	Q5TDH0 [94-108]	0	1837.9979
[K].ELIEPVLT	1xCarbamido	5.244E-05	4.225E-05	1	1	1	Q08AM6	Q08AM6 [93-108]	0	2183.0974
[R].ELLESNI	1xTMTpro [K	1.697E-05	4.225E-05	1	1	1	Q96A33	Q96A33 [173-189	0	2460.3114
[K].IVDIIDTT	1xTMTpro [K	1.502E-05	4.225E-05	1	1	2	P29144	P29144 [64-85]	0	2882.549
[R].IVDKPGI	2xTMTpro [K	1.021E-05	4.225E-05	1	1	1	Q96Q11	Q96Q11 [208-228	0	3174.7463
[K].LVDKFLE	3xTMTpro [K	1.48E-05	4.225E-05	1	1	3	P01009	P01009 [150-160]	2	2550.6012
[K].FPGFAKI	2xTMTpro [K	5.039E-05	4.225E-05	1	1	2	Q9Y5X1	Q9Y5X1 [208-223	0	2679.569
[K].IVDFYPE	1xTMTpro [N	1.026E-05	4.225E-05	1	1	1	Q86WQ0	Q86WQ0 [89-112	0	3054.538
[K].ELLFLSN	1xTMTpro [N	2.493E-05	4.225E-05	1	1	1	P51398	P51398 [379-393]	0	2020.1398
[R].LVEEGIV	1xTMTpro [N	4.289E-06	4.225E-05	1	1	2	Q9Y2S2	Q9Y2S2 [209-231	0	2750.4275
[R].FPLDYYS	1xTMTpro [N	7.154E-06	4.225E-05	1	1	3	Q14157	Q14157 [830-849]	0	2587.3767
[R].LVEGILH	1xTMTpro [K	4.036E-05	4.225E-05	1	1	1	Q13148	Q13148 [56-79]	0	3232.8014
[R].IVEGSD/	1xTMTpro [N	2.899E-07	4.225E-05	1	1	4	P00734	P00734 [364-383]	0	2569.3114
[K].ELKDQLS	2xTMTpro [K	4.163E-05	4.225E-05	1	1	1	Q9BZF9	Q9BZF9 [1004-10	1	2359.3649
[K].FPPLGG	1xTMTpro [N	4.782E-07	4.225E-05	1	1	2	P23246	P23246 [631-663]	0	3499.6438
[K].ELKEHG	2xTMTpro [K	4.603E-05	4.225E-05	1	1	1	Q13636	Q13636 [102-119]	1	2862.6328

[K].ELKEQL(	2xTMTpro [K	2.89E-05	4.225E-05	1	1	1	Q9NR30	Q9NR30 [656-668	1	2430.3908
[R].ELKEQQ	3xTMTpro [K	1.713E-05	4.225E-05	1	1	1	P08697	P08697 [443-461]	2	3420.9479
[K].LDEIYVA	1xTMTpro [N	2.481E-07	4.225E-05	1	1	2	O60506	O60506 [43-60]	0	2319.2152
[R].IVESDV(	1xTMTpro [N	2.471E-05	4.225E-05	1	1	1	Q07157	Q07157 [510-522]	0	1803.9448
[K].ELLALDS	1xTMTpro [N	4.915E-05	4.225E-05	1	1	2	O95817	O95817 [461-473]	0	1717.9291
[K].LVEQLDI	1xTMTpro [K	5.473E-05	4.225E-05	1	1	1	Q92552	Q92552 [313-326]	0	2269.2419
[R].IVEQEN/	1xTMTpro [N	2.9E-05	4.225E-05	1	1	2	Q9Y5X2	Q9Y5X2 [376-389	0	1978.0598
[K].LVDELMI	1xTMTpro [N	1.065E-05	4.225E-05	1	1	2	P48960	P48960 [304-323]	0	2425.2968
[R].IVEPYIA\	1xTMTpro [K	2.3E-05	4.225E-05	1	1	4	P18124	P18124 [135-148]	0	2271.3033
[R].IVENSD/	1xTMTpro [K	6.003E-06	4.225E-05	1	1	3	Q16401	Q16401 [110-128]	0	2693.5217
[R].IVELPAP	2xTMTpro [K	5.638E-06	4.225E-05	1	1	1	Q6P2E9	Q6P2E9 [572-591	0	3054.7907
[R].LVELLMI	1xTMTpro [K	4.618E-05	4.225E-05	2	3	2	O60684; P52	O60684 [299-309]	0	1983.1229
[R].IVEIGDE	1xTMTpro [N	6.726E-07	4.225E-05	1	1	3	O95865	O95865 [114-134]	0	2539.3211
[K].EILDKFT	2xTMTpro [K	6.022E-05	4.225E-05	1	1	2	P49189	P49189 [299-310]	1	2362.405
[K].IVEIDAHI	1xCarbamido	2.497E-05	4.225E-05	1	1	2	P28066	P28066 [67-86]	0	2692.4658
[K].IDESSLT	1xTMTpro [K	1.526E-06	4.225E-05	2	2	1	P20020; P23	P20020 [238-251]	0	2125.1269
[R].LDETDD	1xTMTpro [N	5.287E-05	4.225E-05	1	1	2	P07384	P07384 [401-413]	0	1829.7997
[K].LDETGN	1xTMTpro [N	6.946E-06	4.225E-05	1	1	1	Q13813	Q13813 [102-121]	0	2524.2673
[R].FPLGGD	1xTMTpro [K	9.623E-06	4.225E-05	1	1	1	Q9UKG1	Q9UKG1 [73-91]	0	2694.394
[R].LVEPVTI	1xTMTpro [N	2.87E-06	4.225E-05	1	1	1	P09619	P09619 [257-273]	0	2362.28
[R].IVDDWA	1xTMTpro [K	1.782E-05	4.225E-05	1	1	3	P27824	P27824 [446-458]	0	2097.1261
[RK].IDFVAH	1xTMTpro [K	9.659E-06	4.225E-05	2	2	2	P49585; Q9Y	P49585 [163-183]	0	2922.4653
[R].IVDDCG(	1xCarbamido	2.63E-05	4.225E-05	1	1	1	Q99595	Q99595 [13-35]	0	2936.5506
[R].ELLNALF	1xCarbamido	2.735E-06	4.225E-05	1	1	1	Q9H074	Q9H074 [302-321	0	2885.5397
[R].ITYQPST	1xTMTpro [N	2.973E-06	4.225E-05	1	1	2	Q99715	Q99715 [1785-18(	0	2414.2119
[R].ITYGET(	1xTMTpro [K	3.961E-06	4.225E-05	1	1	3	P02751	P02751 [1571-159	0	2776.4649
[R].LTYAYF/	1xTMTpro [K	1.048E-06	4.225E-05	1	1	8	P02679	P02679 [302-328]	0	3442.5883
[R].ELLNPV\	1xCarbamido	9.048E-07	4.225E-05	1	1	1	P78527	P78527 [2453-247	0	2389.2505
[R].FPEELT(	1xCarbamido	4.199E-05	4.225E-05	1	1	3	P26641	P26641 [328-348]	0	2870.3846
[R].FPEELT(	1xCarbamido	4.695E-06	4.225E-05	1	1	1	P26641	P26641 [328-348]	0	2854.3897
[R].EIINTYTI	1xTMTpro [K	2.711E-05	4.225E-05	1	1	1	Q9HCS7	Q9HCS7 [373-389	0	2576.4103
[K].EIINVGH	1xTMTpro [N	4.359E-07	4.225E-05	1	1	6	P00915	P00915 [59-77]	0	2560.25
[R].ITWDEL/	1xCarbamido	1.657E-05	4.225E-05	1	1	2	Q9NRG7	Q9NRG7 [39-68]	0	3484.8065

[R].ITYVPIT(	1xTMTpro [K	1.021E-05	4.225E-05	1	1	1	P24821	P24821 [1742-176	0	2645.4716
[K].LTVYTTL	1xTMTpro [K	3.202E-05	4.225E-05	1	1	3	Q13510	Q13510 [367-378]	0	1975.1971
[K].FPEEEAI	1xTMTpro [K	1.006E-05	4.225E-05	1	1	2	P04844	P04844 [505-523]	0	2742.4846
[R].FPEDGP	1xTMTpro [N	6.831E-06	4.225E-05	1	1	1	Q9NY33	Q9NY33 [704-724	0	2619.3473
[K].LTVTSQI	1xTMTpro [N	1.994E-05	4.225E-05	1	1	2	P04233	P04233 [81-94]	0	1933.1038
[K].IDGDKD(	2xTMTpro [K	1.815E-05	4.225E-05	1	1	2	O43852	O43852 [80-94]	1	2563.4435
[K].EIIQDVTI	2xTMTpro [K	4.009E-05	4.225E-05	1	1	1	Q9Y265	Q9Y265 [233-267	0	4774.5874
[K].ELLQEFI	1xTMTpro [K	5.074E-06	4.225E-05	1	1	4	O75556	O75556 [44-61]	0	2546.3304
[K].ELLQEFI	1xOxidation		3.526E-05	4.225E-05	1	1	1	O75556	O75556 [44-61]	0	2562.3253
[K].LTVTMPI	1xTMTpro [N	1.015E-05	4.225E-05	1	1	1	Q5SZK8	Q5SZK8 [2542-25	0	2285.2681
[R].LTVSSL(	1xTMTpro [K	2.687E-05	4.225E-05	1	1	3	P21333	P21333 [2335-234	0	1870.1141
[R].ITVPLVS	1xTMTpro [N	3.8E-05	4.225E-05	1	1	2	A0FGR8	A0FGR8 [365-379	0	1970.1969
[R].LTVVDTI	1xCarbamido	1.31E-05	4.225E-05	1	1	3	Q15019	Q15019 [97-112]	0	2055.05
[R].LVEWIIV	1xCarbamido	6.464E-06	4.225E-05	1	1	5	Q01995	Q01995 [30-47]	0	2413.2981
[K].IVADKDY	2xTMTpro [K	4.65E-05	4.225E-05	1	1	3	P07195	P07195 [78-91]	1	2423.3962
[K].IVAERPG	1xTMTpro [N	1.169E-05	4.225E-05	1	1	2	Q13263	Q13263 [408-427]	0	2323.2512
[R].LVDAGF	1xTMTpro [K	2.809E-05	4.225E-05	1	1	1	Q96D46	Q96D46 [95-108]	0	2194.2152
[K].LVCSGLI	1xCarbamido	5.766E-05	4.225E-05	1	1	2	Q9UHG3	Q9UHG3 [256-26	0	1784.0596
[K].LVCGMV	1xCarbamido	3.747E-05	4.225E-05	1	1	1	Q15067	Q15067 [447-462]	0	2156.1163
[K].EILGTAQ	1xCarbamido	3.968E-06	4.225E-05	1	1	7	P30050	P30050 [131-146]	0	1980.014
[K].FPEVED	1xTMTpro [N	3.52E-05	4.225E-05	1	1	1	P05198	P05198 [13-25]	0	1836.9849
[R].LVARPEI	1xTMTpro [N	3.276E-05	4.225E-05	1	1	3	Q16658	Q16658 [202-217]	0	2124.1773
[K].IVAQVW	1xTMTpro [K	4.697E-05	4.225E-05	1	1	2	Q9BSJ8	Q9BSJ8 [145-159	0	2417.3547
[R].LVAQDN	1xTMTpro [K	3.902E-06	4.225E-05	1	1	1	P11277	P11277 [444-461]	0	2503.3688
[K].FPEIVAP	1xCarbamido	5.384E-05	4.225E-05	1	1	1	Q03426	Q03426 [257-277]	0	2677.4442
[K].IVAPISD	1xTMTpro [K	4.036E-05	4.225E-05	1	1	1	O95336	O95336 [171-185]	0	2210.3153
[K].LVAEGN	1xTMTpro [K	5.158E-05	4.225E-05	1	1	2	P11277	P11277 [1234-124	0	1817.03
[K].LVAIVDV	1xTMTpro [N	4.293E-05	4.225E-05	1	1	5	P50914	P50914 [24-35]	0	1658.976
[R].IDFYFDE	1xTMTpro [K	3.213E-05	4.225E-05	1	2	4	Q01105	Q01105 [137-150]	0	2449.2208
[R].LVAGSTI	1xTMTpro [N	3.457E-07	4.225E-05	1	1	2	Q14137	Q14137 [490-514]	0	2936.6052
[R].LVAGEM	1xTMTpro [N	7.088E-06	4.225E-05	1	1	1	Q99714	Q99714 [131-147]	0	2090.0256
[R].ELLLGPS	1xCarbamido	5.103E-06	4.225E-05	1	1	1	Q96JB2	Q96JB2 [340-360]	0	2590.383
[K].LVAGEH	1xTMTpro [N	4.503E-05	4.225E-05	1	1	1	O43847	O43847 [752-762]	0	1481.9123

[R].LVAFCPI	1xCarbamido	2.757E-07	4.225E-05	1	1	2	O00567	O00567 [48-77]	0	3533.8018
[R].IVAFADA	1xOxidation [	8.005E-06	4.225E-05	1	1	1	P24752	P24752 [312-338]	0	3442.9192
[K].LVAEWE	1xTMTpro [K	1.855E-05	4.225E-05	1	1	3	Q02809	Q02809 [167-185]	0	2839.4282
[R].ELLLPNV	1xTMTpro [N	1.454E-06	4.225E-05	1	1	1	Q09666	Q09666 [9-28]	0	2481.3533
[R].FPEHEL	1xTMTpro [N	4.681E-05	4.225E-05	1	1	3	Q9Y4L1	Q9Y4L1 [120-131	0	1819.9298
[R].IVALSSS	1xTMTpro [K	5.348E-05	4.225E-05	1	1	2	O75643	O75643 [1487-14	0	1798.093
[K].LVNQQL	1xTMTpro [K	1.186E-05	4.225E-05	1	1	3	P28331	P28331 [674-692]	0	2708.657
[R].LVEYDID	1xTMTpro [K	3.304E-06	4.225E-05	1	1	2	P52788	P52788 [131-151]	0	3154.5964
[K].FPPLGG	1xOxidation [	7.547E-06	4.225E-05	1	1	1	P23246	P23246 [631-663]	0	3515.6387
[K].LVINTEE	1xTMTpro [K	1.982E-06	4.225E-05	1	1	1	P78527	P78527 [2149-216	0	2287.3306
[R].EIFTNLA	1xTMTpro [K	3.152E-05	4.225E-05	1	2	1	Q8TCE6	Q8TCE6 [307-324	0	2562.3583
[K].LVINGNF	1xTMTpro [K	7.758E-08	4.225E-05	1	1	2	P04406	P04406 [67-84]	1	2649.522
[K].LVINGNF	1xTMTpro [N	4.48E-05	4.225E-05	1	1	3	P04406	P04406 [67-80]	0	1918.1081
[K].LDDKDLI	1xTMTpro [K	5.428E-05	4.225E-05	1	1	1	Q96RN5	Q96RN5 [710-739	1	3955.1348
[R].LVILDNY	1xTMTpro [K	5.59E-05	4.225E-05	1	1	1	Q8TDQ7	Q8TDQ7 [3-18]	0	2429.3572
[K].ELGAFG	1xCarbamido	2.363E-06	4.225E-05	1	1	2	P49748	P49748 [138-162]	0	2940.5209
[K].IVILDEAI	1xTMTpro [N	7.932E-06	4.225E-05	1	1	1	P35249	P35249 [146-164]	0	2279.2236
[K].LVILDEA	1xTMTpro [N	4.801E-06	4.225E-05	1	1	1	P40937	P40937 [121-139]	0	2391.2509
[K].IVIGYQS	1xTMTpro [K	2.275E-05	4.225E-05	1	1	1	P06730	P06730 [193-206]	0	2112.1945
[K].IVLGQEC	1xTMTpro [K	1.244E-05	4.225E-05	1	1	3	P02743	P02743 [150-162]	0	2002.1101
[R].ELGCGA	1xCarbamido	5.112E-06	4.225E-05	1	1	4	O43866	O43866 [64-85]	0	2812.4683
[R].ELGCGG	1xCarbamido	8.26E-06	4.225E-05	1	1	1	A1L4H1	A1L4H1 [344-372]	0	3121.5948
[K].LDDIEEF	1xTMTpro [K	4.915E-05	4.225E-05	1	1	1	Q8NHZ8	Q8NHZ8 [13-24]	1	2129.1734
[K].LVLEQV	1xTMTpro [K	2.079E-05	4.225E-05	1	1	2	O00410	O00410 [507-526]	0	2710.537
[K].IDDILSAI	1xTMTpro [K	5.984E-05	4.225E-05	1	1	1	Q9P291	Q9P291 [198-209	0	1936.1247
[K].IVLDSDA	1xTMTpro [N	7.575E-06	4.225E-05	1	1	1	Q04446	Q04446 [648-662]	0	1934.9891
[R].IDDLQM	1xTMTpro [N	3.032E-05	4.225E-05	1	1	1	Q93050	Q93050 [265-279]	0	2131.0773
[R].IVLANDF	1xTMTpro [K	1.93E-05	4.225E-05	1	1	3	Q96G03	Q96G03 [317-333	1	2418.3848
[R].LVKPGN	2xTMTpro [K	1.067E-05	4.225E-05	1	1	2	Q9UQ80	Q9UQ80 [156-172	0	2839.6246
[R].ELGEYA	1xTMTpro [K	4.801E-05	4.225E-05	1	1	2	P47895	P47895 [488-501]	0	2223.204
[R].LDCSQG	1xCarbamido	1.27E-05	4.225E-05	1	1	2	Q9BVG4	Q9BVG4 [123-139	0	2305.109
[R].IVITGDG	1xTMTpro [N	2.265E-07	4.225E-05	1	1	1	O43491	O43491 [958-977]	0	2425.3006
[R].ELFSNL	1xTMTpro [K	1.589E-05	4.225E-05	1	1	4	Q9H8H3	Q9H8H3 [57-71]	0	2232.2156

[R].LDCGAS	2xCarbamido	2.038E-07	4.225E-05	1	1	1	Q8IVL6	Q8IVL6 [75-112]	0	4051.9449
[K].LVNMLD.	1xTMTpro [K	2.559E-06	4.225E-05	1	1	2	O95810	O95810 [65-78]	0	2247.2411
[K].FQDTAE.	1xTMTpro [K	2.443E-06	4.225E-05	1	1	1	Q9Y2X3	Q9Y2X3 [50-67]	0	2522.3456
[K].EIFEQPE	1xTMTpro [N	5.664E-05	4.225E-05	1	1	2	Q06210	Q06210 [346-359]	0	1983.0176
[R].LDAVNTI	1xTMTpro [N	4.199E-05	4.225E-05	1	1	2	Q9NZM1	Q9NZM1 [654-66(	0	1720.9587
[K].IVNGWQ	1xTMTpro [N	8.005E-06	4.225E-05	1	1	1	P11216	P11216 [171-185]	0	2134.0888
[K].FQDLGA	1xTMTpro [K	1.038E-06	4.225E-05	1	1	1	Q9UBS4	Q9UBS4 [67-83]	1	2536.3539
[K].EIFFNILV	1xTMTpro [K	4.064E-05	4.225E-05	1	1	1	Q13438	Q13438 [620-635]	0	2413.3623
[R].EIFGLYG	1xTMTpro [K	2.462E-05	4.225E-05	1	1	4	Q9NR31	Q9NR31 [149-160	0	1922.0879
[K].FQDLGA	1xTMTpro [K	9.553E-06	4.225E-05	1	1	5	Q9UBS4	Q9UBS4 [67-82]	0	2380.2528
[KR].IVNDD(	1xOxidation [	1.812E-05	4.225E-05	2	2	2	Q16555; Q1	Q16555 [24-43]; (	0	2973.5047
[R].ELGEYG	1xTMTpro [K	2.952E-05	4.225E-05	1	1	4	P00352	P00352 [477-490]	0	2309.1945
[KR].IVNDD(	1xTMTpro [K	3.74E-06	4.225E-05	2	2	4	Q16555; Q1	Q16555 [24-43]; (	0	2957.5098
[R].EIFLSQF	1xTMTpro [K	3.846E-05	4.225E-05	2	3	1	P62136; P62	P62136 [44-60]; F	0	2561.545
[K].FQDGDL	1xTMTpro [N	4.872E-06	4.225E-05	1	1	1	P09211	P09211 [56-71]	0	2188.1569
[K].EIFMQVE	1xTMTpro [N	6.003E-05	4.225E-05	1	1	1	P35580	P35580 [1853-18(	1	1755.9019
[K].IDCDNLE	1xCarbamido	1.006E-05	4.225E-05	1	1	1	Q92499	Q92499 [517-536]	1	3237.703
[R].IDCFSEV	1xCarbamido	3.607E-05	4.225E-05	1	1	2	O00567	O00567 [382-396]	0	2323.2136
[K].ELFPIAA	2xTMTpro [K	4.635E-06	4.225E-05	1	1	1	P16219	P16219 [52-72]	1	3263.8972
[K].ELFPIQM	1xTMTpro [K	5.803E-05	4.225E-05	1	1	1	O96008	O96008 [92-102]	0	1899.0905
[R].ELFPVFT	1xTMTpro [K	3.551E-05	4.225E-05	1	1	1	Q6UWU4	Q6UWU4 [244-25	0	2180.2764
[K].LVLVSPT	1xTMTpro [N	1.941E-05	4.225E-05	1	1	1	O00178	O00178 [65-80]	0	2124.1872
[K].LVLVGD(	1xTMTpro [K	4.947E-05	4.225E-05	1	1	5	P62826	P62826 [13-23]	0	1623.9925
[K].IDCDNLE	1xCarbamido	8.398E-06	4.225E-05	1	1	1	Q92499	Q92499 [517-535]	0	2805.4009
[R].ELGEYG	1xTMTpro [K	1.3E-05	4.225E-05	1	1	4	P05091	P05091 [493-506]	0	2208.2044
[R].LVHVEEI	1xTMTpro [N	1.486E-05	4.225E-05	1	1	5	P01023	P01023 [720-732]	0	1850.0091
[K].LVHSNLE	1xTMTpro [N	2.506E-05	4.225E-05	1	1	1	Q9H7X7	Q9H7X7 [145-158	0	1970.015
[R].ELGSLP(	1xTMTpro [K	1.272E-05	4.225E-05	1	1	2	Q9UQE7	Q9UQE7 [945-95(	0	1956.0934
[K].LDDQSE	1xTMTpro [K	1.888E-06	4.225E-05	1	1	1	Q96AC1	Q96AC1 [155-187	0	4076.0731
[R].LVGGPM	1xTMTpro [N	2.54E-06	4.225E-05	1	1	1	P01034	P01034 [35-51]	1	2105.098
[R].LVGGKD	1xTMTpro [K	3.78E-05	4.225E-05	1	1	1	O00151	O00151 [18-32]	1	2238.3102
[R].LDDTVH	1xTMTpro [N	2.561E-05	4.225E-05	1	1	1	P26196	P26196 [212-225]	0	1797.019
[R].IVGELEC	1xOxidation [	3.741E-05	4.225E-05	1	1	1	Q00341	Q00341 [1120-11:	0	2386.2243

[K].EIGWTD'	1xTMTpro [K	6.299E-06	4.225E-05	1	1	2	Q01813	Q01813 [460-480]	0	2727.4598
[R].IVGELEQ	1xTMTpro [N	2.367E-05	4.225E-05	1	1	3	Q00341	Q00341 [1120-11:	0	2370.2294
[K].ELGYVTI	1xCarbamido	2.76E-05	4.225E-05	1	1	1	Q9HD20	Q9HD20 [854-873	0	2721.4083
[K].ELGYVTI	1xCarbamido	5.664E-05	4.225E-05	1	1	1	Q9HD20	Q9HD20 [854-873	0	2737.4032
[R].IVGLDQV	1xTMTpro [K	1.381E-07	4.225E-05	1	1	3	P35580	P35580 [625-644]	0	2696.4461
[R].ELHGQN	1xCarbamido	1.53E-05	4.225E-05	1	1	1	Q16630	Q16630 [148-161]	0	2201.1992
[K].IVGAPMI	1xCarbamido	5.261E-05	4.225E-05	1	1	2	P11586	P11586 [176-198]	0	3186.7048
[K].LVGAIVY	1xTMTpro [N	8.554E-06	4.225E-05	1	1	1	P43304	P43304 [212-227]	0	2095.0892
[K].LVFQQFI	1xCarbamido	2.246E-05	4.225E-05	1	1	2	P00736	P00736 [58-77]	0	3092.5571
[R].ELHLLGV	1xTMTpro [N	9.495E-07	4.225E-05	1	1	1	P11277	P11277 [1844-186	0	2385.321
[K].IVFPHGI'	1xTMTpro [N	1.467E-05	4.225E-05	1	1	1	Q07954	Q07954 [366-379]	0	1871.1074
[R].LVFNPD(	1xTMTpro [N	7.154E-06	4.225E-05	1	1	2	P35442	P35442 [914-928]	0	1993.9786
[R].LDEHDF	1xCarbamido	4.199E-05	4.225E-05	1	1	1	Q96FN4	Q96FN4 [105-125	0	2948.5319
[R].IVFENPD	1xTMTpro [K	4.689E-05	4.225E-05	1	1	2	Q96C86	Q96C86 [189-207	0	2723.5151
[K].IVFDSIDI	1xTMTpro [K	7.032E-08	4.225E-05	1	1	3	Q9UJ70	Q9UJ70 [166-185	0	2824.5377
[K].ELHSEFS	1xTMTpro [N	2.099E-06	4.225E-05	1	1	2	P40939	P40939 [67-86]	0	2724.3258
[K].LVGDVD	1xTMTpro [N	5.545E-05	4.225E-05	1	1	1	P13995	P13995 [289-299]	0	1509.8232
[K].EIKDAIS(	2xTMTpro [K	1.694E-05	4.225E-05	1	1	2	P08133	P08133 [100-113]	1	2388.3802
[K].IDDPTDS	3xTMTpro [K	2.63E-05	4.225E-05	1	1	3	P27797	P27797 [225-248]	0	3977.0921
[R].ELGQTL	1xTMTpro [K	1.075E-05	4.225E-05	1	1	5	P46939	P46939 [1292-13:	0	2637.4842
[R].IVHSLDY	1xCarbamido	6.954E-07	4.225E-05	1	1	1	Q9BXP5	Q9BXP5 [618-639	0	3063.3784
[K].ELGGES	1xTMTpro [K	4.289E-06	4.225E-05	1	1	1	Q99961	Q99961 [103-122]	0	2648.3369
[R].ELGGIPI	1xTMTpro [K	5.536E-05	4.225E-05	1	1	2	Q8N2F6	Q8N2F6 [136-146	0	1719.066
[R].IVHELNT	1xTMTpro [K	4.078E-05	4.225E-05	1	1	1	Q4VC31	Q4VC31 [30-46]	0	2393.3684
[K].ELGLDE(	1xTMTpro [K	2.809E-05	4.225E-05	1	1	1	Q8WXX5	Q8WXX5 [206-21	0	1883.0617
[K].LVHDKEI	1xOxidation [	4.257E-05	4.225E-05	1	1	1	O43237	O43237 [348-365]	1	3043.6954
[K].IVHAFDN	1xTMTpro [K	4.981E-05	4.225E-05	1	1	4	Q9NZ45	Q9NZ45 [56-68]	0	2098.1135
[R].IVGYFVS	1xCarbamido	2.056E-06	4.225E-05	1	1	1	P42765	P42765 [279-305]	0	3313.8184
[K].FPVFNP/	1xCarbamido	6.28E-07	4.225E-05	1	1	1	P00352	P00352 [38-62]	1	3836.9357
[R].EIGLLSE	1xTMTpro [K	2.551E-05	4.225E-05	1	1	1	P11586	P11586 [337-352]	0	2417.3307
[RG].ELGQV	2xCarbamido	8.094E-06	4.225E-05	1	1	1	Q9HC84	Q9HC84 [2372-23	0	2285.1589
[K].LVGTPNI	1xTMTpro [N	2.755E-05	4.225E-05	1	1	1	Q93062	Q93062 [110-130]	0	2523.4254
[R].LVGSQE	1xTMTpro [N	1.217E-06	4.225E-05	1	1	1	Q13200	Q13200 [144-160]	0	2264.1631

[K].ELGLVD(	1xTMTpro [K	2.662E-07	4.225E-05	1	1	2	Q8TBC4	Q8TBC4 [435-458	0	3151.7746
[R].LVGSGL	1xTMTpro [N	4.475E-06	4.225E-05	1	1	2	Q9Y6C2	Q9Y6C2 [513-528	0	1871.0306
[R].LVGQGA	1xTMTpro [K	7.257E-07	4.225E-05	1	1	3	Q99714	Q99714 [30-52]	0	2803.5809
[R].EIGNLLH	1xTMTpro [K	5.897E-07	4.225E-05	1	1	1	P49591	P49591 [134-157]	1	3297.7571
[K].IVGPSGA	1xCarbamido	4.465E-08	4.225E-05	1	1	2	P21333	P21333 [1008-103	1	3057.7011
[R].EIGPND(	1xCarbamido	4.956E-05	4.225E-05	1	1	1	P51452	P51452 [159-176]	0	2364.1937
[R].ELGPQD	1xTMTpro [N	8.671E-07	4.225E-05	1	1	1	Q8N0W3	Q8N0W3 [499-52	0	2826.4052
[K].IVGPSGA	1xCarbamido	5.464E-05	4.225E-05	1	1	6	P21333	P21333 [1008-10	0	1764.0334
[K].LVGPEE.	1xTMTpro [N	5.545E-05	4.225E-05	1	1	3	O60568	O60568 [359-372	0	1728.9451
[K].FPVFNM	1xCarbamido	3.403E-06	4.225E-05	1	1	2	P53621	P53621 [363-382]	0	2647.3332
[K].FPDAGE	1xTMTpro [K	5.803E-05	4.225E-05	1	1	3	P46940	P46940 [1175-118	0	1842.0141
[K].LYGSAG	1xTMTpro [K	9.465E-06	4.225E-05	1	1	5	P11021	P11021 [634-654]	1	2784.4071
[K].ELAPYDI	1xTMTpro [N	4.689E-05	4.225E-05	1	1	4	P39019	P39019 [44-56]	0	2007.9772
[R].EHIVDLE	1xTMTpro [N	5.437E-05	4.225E-05	1	1	1	Q12805	Q12805 [460-477	0	2351.26
[K].MFGIDRI	1xTMTpro [N	4.009E-05	4.225E-05	1	1	2	P29401	P29401 [604-617]	1	1867.0179
[R].MFFEDE	2xTMTpro [K	2.484E-05	4.225E-05	1	1	2	Q14008	Q14008 [792-811	0	3299.769
[R].LATYGIT	1xCarbamido	2.778E-08	4.225E-05	1	1	2	O75643	O75643 [559-577	0	2698.473
[R].EHNFQP	1xCarbamido	1.462E-05	4.225E-05	1	1	1	O00267	O00267 [417-439	0	3234.6345
[R].MFDYTD	1xTMTpro [N	1.206E-06	4.225E-05	1	1	1	Q09161	Q09161 [291-311	0	2670.2135
[R].IAVAQYS	1xTMTpro [K	5.609E-05	4.225E-05	1	1	4	P12111	P12111 [875-885]	0	1817.03
[K].MFAEEE.	1xTMTpro [K	3.18E-05	4.225E-05	1	1	1	Q9H173	Q9H173 [344-360	0	2607.2814
[R].IAVAQYS	1xTMTpro [K	4.426E-06	4.225E-05	1	1	3	P12111	P12111 [875-889]	1	2288.2742
[R].MEVQEQ	1xTMTpro [N	8.094E-06	4.225E-05	1	1	2	P78527	P78527 [3218-323	0	2110.0657
[K].FSAVLVE	1xOxidation [	5.09E-05	4.225E-05	1	1	1	Q13263	Q13263 [805-835	0	3270.6523
[K].EHPVLAI	1xTMTpro [K	1.885E-06	4.225E-05	1	1	2	Q9H488	Q9H488 [186-210	0	3381.9291
[R].METEYG	1xTMTpro [N	2.132E-05	4.225E-05	1	1	1	Q9NR99	Q9NR99 [2713-27	0	2723.4649
[R].EHPWEV	2xTMTpro [K	3.813E-05	4.225E-05	1	1	1	P08865	P08865 [192-220]	2	4491.2635
[R].MESPIPI	1xOxidation [	7.194E-06	4.225E-05	1	1	1	Q99719	Q99719 [325-343	0	2702.4818
[K].LAVDDPI	1xTMTpro [K	3.761E-06	4.225E-05	1	1	2	Q5SZK8	Q5SZK8 [2974-29	0	2806.5482
[K].LAVEALS	1xTMTpro [N	6.794E-06	4.225E-05	1	1	7	P12277	P12277 [157-172]	0	1891.0456
[K].MENQSE	1xTMTpro [K	1.881E-06	4.225E-05	1	1	1	Q99816	Q99816 [305-326	0	3141.627
[R].MENILS(	1xTMTpro [K	1.85E-06	4.225E-05	1	1	2	Q9P013	Q9P013 [160-183	0	3191.7378
[R].EHQNIQI	1xTMTpro [K	1.032E-05	4.225E-05	1	1	1	Q9BZF9	Q9BZF9 [292-309	1	2860.4932

[R].EHQPFS	1xCarbamido	6.061E-05	4.225E-05	1	1	1	P08648	P08648 [949-962]	0	2344.2251
[R].EHLPAP(	2xCarbamido	2.479E-06	4.225E-05	1	1	1	Q9UHQ9	Q9UHQ9 [264-29	0	4011.115
[R].EHINLG(	1xCarbamido	8.291E-06	4.225E-05	1	1	3	P21796	P21796 [121-139]	0	2480.1869
[R].EHINLG(	1xCarbamido	9.929E-06	4.225E-05	1	1	5	P21796	P21796 [121-139]	0	2464.192
[K].EHIMQM	1xTMTpro [K	1.906E-05	4.225E-05	1	1	1	O00410	O00410 [361-373	0	2278.1968
[K].MFVLDE.	1xOxidation [	3.651E-05	4.225E-05	2	2	5	P60842; Q14	P60842 [178-190]	0	1875.9152
[K].MFVLDE.	1xTMTpro [N	2.547E-05	4.225E-05	2	2	3	P60842; Q14	P60842 [178-190]	0	1859.9202
[K].FSDLFSI	2xTMTpro [K	9.518E-06	4.225E-05	1	1	1	Q68E01	Q68E01 [967-986	0	3202.6976
[K].MFVEEV	1xCarbamido	2.579E-05	4.225E-05	1	1	1	P49588	P49588 [514-531]	0	2635.3603
[K].LATPTY(	1xTMTpro [N	5.208E-07	4.225E-05	1	1	2	Q13509	Q13509 [217-241	0	2909.5362
[R].MFTTAP	2xTMTpro [K	1.549E-06	4.225E-05	1	1	3	P00450	P00450 [599-619]	1	3386.6878
[R].FSDEPP	1xTMTpro [K	2.267E-05	4.225E-05	1	1	1	Q5JTV8	Q5JTV8 [59-76]	0	2647.3787
[K].MFTAGII	1xTMTpro [K	1.427E-05	4.225E-05	1	1	1	Q13011	Q13011 [113-131	0	2705.4208
[K].MFSEAD	1xCarbamido	3.152E-05	4.225E-05	1	1	1	Q01082	Q01082 [854-868	0	2468.1969
[K].MFSEAD	1xCarbamido	4.177E-05	4.225E-05	1	1	1	Q01082	Q01082 [854-868	0	2452.202
[K].MENAEL	1xOxidation [	2.775E-05	4.225E-05	1	1	2	P07996	P07996 [174-189]	0	2169.1181
[K].MFSDEIL	1xTMTpro [N	5.6E-05	4.225E-05	1	1	1	Q7Z4H8	Q7Z4H8 [216-227	0	1728.9525
[R].IATSLDG	1xTMTpro [N	3.231E-06	4.225E-05	1	1	2	O60664	O60664 [214-230	0	2139.1365
[K].FSDASY(	1xTMTpro [N	1.854E-06	4.225E-05	1	1	1	P01031	P01031 [518-539]	0	2745.3837
[R].LATVGEI	1xTMTpro [N	2.9E-05	4.225E-05	1	1	3	P13611	P13611 [277-288]	0	1618.9236
[K].EHLIDEL	1xTMTpro [K	7.114E-06	4.225E-05	1	1	1	Q13107	Q13107 [85-103]	0	2892.5639
[R].EHLLMA	1xTMTpro [N	3.866E-05	4.225E-05	1	1	1	P98160	P98160 [1075-109	0	2083.2269
[R].EHLLMA	1xOxidation [	4.923E-05	4.225E-05	1	1	2	P98160	P98160 [1075-109	0	2099.2218
[K].MFIGGLS	2xTMTpro [K	1.959E-05	4.225E-05	1	1	1	Q14103	Q14103 [99-111]	1	2396.3828
[K].MFIGGLS	1xTMTpro [K	3.146E-05	4.225E-05	1	1	4	Q14103	Q14103 [99-110]	0	1964.0807
[K].MFIGGLS	2xTMTpro [K	2.113E-05	4.225E-05	1	1	1	O14979	O14979 [150-162	1	2382.3672
[K].MFIGGLS	1xTMTpro [K	5.627E-05	4.225E-05	1	1	2	O14979	O14979 [150-161	0	1950.065
[K].LATQSNI	1xTMTpro [N	9.157E-06	4.225E-05	1	1	2	P04792	P04792 [172-188]	0	2210.1988
[K].MENAEL	1xTMTpro [N	5.084E-06	4.225E-05	1	1	1	P07996	P07996 [174-189]	0	2153.1232
[K].MEMEMI	1xTMTpro [K	4.503E-05	4.225E-05	1	1	2	Q16181	Q16181 [353-364	0	2170.0548
[K].MELNEA	1xTMTpro [N	3.563E-05	4.225E-05	1	1	2	P02549	P02549 [1265-12	0	1894.9288
[R].FSAEDE.	1xTMTpro [N	3.317E-05	4.225E-05	1	1	1	Q9Y608	Q9Y608 [17-29]	0	1726.8931
[R].IAVSKPS	1xTMTpro [K	1.637E-06	4.225E-05	1	1	1	Q8IV08	Q8IV08 [163-188]	0	3267.8669

[R].MDYVEII	1xOxidation [	1.228E-05	4.225E-05	1	1	2	Q00341	Q00341 [435-445]	0	2001.0607
[K].EKANED	1xCarbamido	1.779E-05	4.225E-05	1	1	1	Q9C0B1	Q9C0B1 [161-178	1	2658.3511
[K].EKAPSIIF	2xTMTpro [K	3.341E-06	4.225E-05	1	1	2	P17980	P17980 [277-294]	1	2872.6852
[R].MDYVEII	1xTMTpro [K	9.659E-06	4.225E-05	1	1	3	Q00341	Q00341 [435-445]	0	1985.0658
[R].LAVTTH(	1xCarbamido	4.704E-06	4.225E-05	1	1	4	P00734	P00734 [225-243]	0	2603.4623
[K].MDVVGT	3xCarbamido	6.657E-06	4.225E-05	1	1	1	Q16666	Q16666 [343-368]	1	3718.8094
[K].MDVVGT	2xCarbamido	1.315E-05	4.225E-05	1	1	1	Q16666	Q16666 [343-361]	0	2754.3141
[K].MDVNVG	1xOxidation [	1.115E-05	4.225E-05	1	1	1	Q09666	Q09666 [1620-163	0	2312.1936
[K].EKAEAQ	2xTMTpro [K	2.038E-05	4.225E-05	1	1	2	Q14624	Q14624 [97-111]	1	2477.418
[K].MDVNVG	1xTMTpro [K	2.804E-05	4.225E-05	1	1	3	Q09666	Q09666 [1620-163	0	2296.1986
[R].EKDILTA	1xCarbamido	2.443E-06	4.225E-05	1	1	2	P51648	P51648 [38-51]	1	2473.4516
[K].EKDILVL	2xTMTpro [K	2.367E-06	4.225E-05	1	1	2	Q9Y394	Q9Y394 [102-123	1	3278.8664
[K].MDSTAN	1xOxidation [	4.029E-05	4.225E-05	1	1	2	P07237	P07237 [425-436]	0	1918.0083
[K].MDSTAN	1xTMTpro [K	4.809E-05	4.225E-05	1	1	2	P07237	P07237 [425-436]	0	1902.0134
[-].MDSSSSS	1xAcetyl [N-T	1.889E-05	4.225E-05	1	1	1	O60220	O60220 [1-29]	0	3669.8562
[R].EKDRDP	1xTMTpro [K	3.702E-05	4.225E-05	1	1	2	O14744	O14744 [489-505]	2	2717.4213
[R].MDSFDE	1xTMTpro [N	1.971E-06	4.225E-05	1	1	2	Q9Y2W1	Q9Y2W1 [573-59	0	2454.2254
[R].MDQITA(	2xTMTpro [K	2.475E-05	4.225E-05	1	1	2	Q9UMS6	Q9UMS6 [502-51	1	2348.2948
[R].LAYINPD	1xTMTpro [K	1.984E-05	4.225E-05	1	1	3	P31948	P31948 [352-364]	0	2097.2087
[R].MDLLPLI	1xTMTpro [K	2.906E-05	4.225E-05	1	1	5	P21980	P21980 [652-663]	0	2013.1633
[R].MDTDLE	1xTMTpro [N	6.56E-06	4.225E-05	1	1	1	O75643	O75643 [387-406]	0	2482.1761
[R].MGAGLG	1xOxidation [	5.536E-05	4.225E-05	1	1	3	P52272	P52272 [419-429]	0	1421.6949
[K].MEDSVG	1xCarbamido	5.384E-06	4.225E-05	1	1	2	P35579	P35579 [1373-138	0	2305.1547
[K].MEDSVG	1xCarbamido	7.758E-08	4.225E-05	1	1	3	P35579	P35579 [1373-138	1	2461.2558
[K].MEIDLKD	2xTMTpro [K	9.766E-06	4.225E-05	1	1	2	P35580	P35580 [1622-163	1	2843.6005
[K].MEKPPA	2xTMTpro [K	9.413E-06	4.225E-05	1	1	2	Q15428	Q15428 [214-231]	0	2682.5833
[R].FSAICQ(	1xCarbamido	1.472E-05	4.225E-05	1	1	4	P04003	P04003 [405-418]	0	1885.9186
[K].MEGNAE	1xCarbamido	1.959E-05	4.225E-05	1	1	1	Q00610	Q00610 [206-221]	0	2181.0276
[K].MEGGGA	1xTMTpro [N	6.59E-07	4.225E-05	1	1	1	Q09666	Q09666 [5248-52	0	2370.2043
[K].MEGDLK	2xTMTpro [K	3.429E-05	4.225E-05	1	1	4	Q09666	Q09666 [3770-37	1	2343.341
[K].MEEVEA	1xOxidation [	5.107E-05	4.225E-05	1	1	1	P01011	P01011 [284-297]	0	2257.2315
[K].MEEVEA	1xTMTpro [K	3.981E-05	4.225E-05	1	1	3	P01011	P01011 [284-297]	0	2241.2366
[K].MEEQGL	1xTMTpro [K	2.159E-05	4.225E-05	1	1	2	Q9UBB4	Q9UBB4 [439-451	0	2013.1182

[K].EHVEEIS	1xTMTpro [K	2.031E-05	4.225E-05	1	1	1	Q9Y265	Q9Y265 [428-441	0	2317.2208
[K].MEDSVG	1xCarbamido	5.794E-05	4.225E-05	1	1	1	P35579	P35579 [1373-138	0	2321.1496
[K].MEENLS	1xCarbamido	1.268E-06	4.225E-05	1	1	1	P02549	P02549 [2062-207	0	2460.2182
[R].IAVGSDA	1xTMTpro [K	6.475E-06	4.225E-05	1	1	6	Q16555	Q16555 [401-418]	0	2508.3841
[R].MEEGEV	1xTMTpro [K	1.589E-05	4.225E-05	1	1	2	P50579	P50579 [355-371]	0	2456.2511
[K].MEEFKD	1xCarbamido	2.027E-05	4.225E-05	1	1	3	P38646	P38646 [596-610]	1	2766.4259
[R].EHVPQL	1xTMTpro [N	1.236E-07	4.225E-05	1	1	2	P12111	P12111 [741-768]	0	3355.8181
[K].MEEEILL	1xTMTpro [K	1.782E-05	4.225E-05	1	1	2	P35580	P35580 [983-996]	0	2299.2347
[-].MEEEIAA	1xCarbamido	2.677E-05	4.225E-05	1	1	1	P63261	P63261 [1-18]	0	2444.2657
[K].MEEDGS	1xTMTpro [K	2.388E-05	4.225E-05	1	1	1	O75410	O75410 [563-580]	0	2503.3093
[R].MEEADA	1xCarbamido	2.602E-05	4.225E-05	1	1	2	Q99460	Q99460 [560-572]	0	1840.9104
[K].MEDTLE	2xTMTpro [K	1.404E-05	4.225E-05	1	1	3	Q02952	Q02952 [1239-12	1	3402.8494
[K].LAVQKYI	1xTMTpro [K	3.741E-05	4.225E-05	1	1	1	P54920	P54920 [223-239]	1	2608.4267
[R].MEEIVEC	1xCarbamido	2.959E-07	4.225E-05	2	2	1	P14923; P35	P14923 [556-572]	0	2203.1534
[K].LAYLQIL	1xTMTpro [K	3.893E-05	4.225E-05	1	1	1	P19525	P19525 [160-173]	0	2202.2877
[K].MGAMAK	2xCarbamido	3.722E-05	4.225E-05	1	1	1	Q01469	Q01469 [35-50]	0	2680.4111
[K].MGAPES	1xTMTpro [K	1.341E-05	4.225E-05	1	1	4	P02794	P02794 [159-173]	0	2236.1815
[R].MHEDINI	1xTMTpro [K	2.947E-05	4.225E-05	1	1	1	P28331	P28331 [277-289]	0	2254.1306
[K].EGTQAS	1xCarbamido	1.813E-06	4.225E-05	1	1	1	Q16643	Q16643 [589-615]	0	3677.7045
[K].EGTQEE	1xTMTpro [K	3.975E-06	4.225E-05	1	1	1	Q8N2G8	Q8N2G8 [328-347	0	2682.4442
[K].EGTSNS	1xTMTpro [K	4.271E-05	4.225E-05	1	1	1	Q9P0M6	Q9P0M6 [168-189	0	2794.3874
[K].MHDLNT	1xTMTpro [N	4.845E-07	4.225E-05	1	1	2	O43684	O43684 [81-100]	0	2580.2796
[R].MGYAEE	1xTMTpro [K	2.084E-08	4.225E-05	1	1	2	P22061	P22061 [145-178]	0	4113.2087
[K].LASVPAC	1xTMTpro [K	1.323E-08	4.225E-05	1	1	5	P05387	P05387 [62-94]	0	3382.8462
[K].EGVFPD	3xTMTpro [K	8.005E-06	4.225E-05	1	1	1	Q9Y5K6	Q9Y5K6 [317-339	1	3864.1688
[-].MGVQVET	1xMet-loss [N	1.916E-05	4.225E-05	1	1	3	P62942	P62942 [1-14]	0	1618.872
[R].LASVPG-	1xTMTpro [K	6.682E-06	4.225E-05	1	1	2	O00592	O00592 [399-411]	0	1893.1665
[K].IASWADI	1xTMTpro [K	1.83E-06	4.225E-05	1	1	1	P11172	P11172 [333-352]	0	2627.5165
[K].EGVIGVT	1xTMTpro [K	4.296E-06	4.225E-05	1	1	4	Q9UH99	Q9UH99 [518-533	0	2382.3637
[K].EGVLKEI	2xCarbamido	1.709E-06	4.225E-05	1	1	2	Q9GZN8	Q9GZN8 [113-133	1	2899.5513
[K].EGVILTN	1xTMTpro [N	1.741E-06	4.225E-05	1	1	1	Q08J23	Q08J23 [713-737]	0	2892.4142
[K].MGSNLT	1xTMTpro [N	9.191E-06	4.225E-05	1	1	2	Q9NSA3	Q9NSA3 [29-42]	0	1887.9441
[-].MGQNDL	1xOxidation [	2.947E-05	4.225E-05	1	1	3	O15260	O15260 [1-19]	0	2348.1148

[R].EGVMEF	3xCarbamid	7.062E-06	4.225E-05	1	1	1	P28331	P28331 [114-150]	0	4603.0086
[-].MGQNDL	1xMet-loss [I	2.019E-06	4.225E-05	1	1	5	O15260	O15260 [1-19]	0	2332.1199
[R].MGQMAI	1xTMTpro [N	3.702E-05	4.225E-05	1	1	1	Q15233	Q15233 [384-398]	0	1842.8766
[K].EGVPGA	1xCarbamid	2.159E-05	4.225E-05	1	1	1	Q9H1B7	Q9H1B7 [487-516	0	3489.7751
[K].MGQAVF	1xTMTpro [N	1.247E-06	4.225E-05	1	1	2	Q96AE4	Q96AE4 [593-620	0	3212.5431
[R].EGTINVI	1xTMTpro [K	1.15E-05	4.225E-05	1	1	2	P15941	P15941 [1109-112	0	2630.3706
[R].EGTIGDI	1xTMTpro [K	1.555E-06	4.225E-05	1	1	1	P78371	P78371 [482-500]	0	2617.4403
[R].FSFCCS	3xCarbamid	1.861E-06	4.225E-05	1	1	1	Q13501	Q13501 [23-46]	0	2930.2714
[K].EGTGST	1xTMTpro [K	3.265E-05	4.225E-05	1	1	1	O15372	O15372 [6-24]	0	2236.1549
[-].MKPDETI	2xTMTpro [K	2.63E-05	4.225E-05	1	1	1	Q96EK6	Q96EK6 [1-15]	0	2661.4812
[K].EGSMEN	1xTMTpro [K	1.27E-05	4.225E-05	1	1	1	Q14012	Q14012 [60-72]	0	2065.1243
[R].LASQAN	1xTMTpro [K	3.518E-06	4.225E-05	2	2	3	Q10567; P63	Q10567 [345-359]	0	2177.3149
[K].MKIPFTD	1xOxidation	4.22E-05	4.225E-05	1	1	1	Q16363	Q16363 [1166-11	1	2970.6618
[R].EGSPDA	3xCarbamid	4.148E-06	4.225E-05	1	1	1	P14543	P14543 [662-690]	0	3475.5861
[R].EGSPVL	1xCarbamid	4.634E-05	4.225E-05	1	1	2	P46977	P46977 [629-641]	0	2132.1739
[K].MKLDYIL	2xTMTpro [K	4.776E-05	4.225E-05	1	1	3	P46781	P46781 [92-101]	1	2106.3177
[K].MKGEVD	1xOxidation	2.259E-05	4.225E-05	1	1	1	Q09666	Q09666 [3233-32	1	2732.532
[R].MKGEAE	2xTMTpro [K	7.194E-06	4.225E-05	1	1	4	Q9HBL7	Q9HBL7 [106-118	1	2405.3414
[K].EGSRGE	1xTMTpro [N	2.819E-05	4.225E-05	1	1	1	Q7Z6Z7	Q7Z6Z7 [1296-13	1	2083.9448
[R].EGVQKE	1xTMTpro [K	3.35E-07	4.225E-05	1	1	5	P01024	P01024 [955-979]	1	3363.7047
[R].EGSRTD	1xTMTpro [N	1.386E-07	4.225E-05	1	1	1	P14625	P14625 [40-67]	2	3461.7428
[K].MKETAE	2xTMTpro [K	3.141E-05	4.225E-05	1	1	6	P11021	P11021 [153-163]	1	2153.2456
[K].MKEIAE	1xTMTpro [K	1.099E-07	4.225E-05	1	1	2	P0DMV9	P0DMV9 [127-15	1	3869.0439
[R].IASQVA	1xTMTpro [K	2.042E-07	4.225E-05	1	1	2	P28331	P28331 [519-538]	0	2679.5689
[R].EGSVML	1xTMTpro [K	2.658E-05	4.225E-05	1	1	3	Q13228	Q13228 [420-433]	0	2128.1815
[R].LASTLVI	1xTMTpro [N	3.71E-07	4.225E-05	1	1	2	Q00610	Q00610 [1227-12	0	2275.2366
[K].MKEEVP	1xOxidation	5.393E-05	4.225E-05	1	1	1	Q8TAQ2	Q8TAQ2 [703-717	1	2333.3143
[R].EGTCPE	2xCarbamid	8.602E-06	4.225E-05	1	1	6	P02787	P02787 [347-362]	0	2730.4259
[K].EGTDSS	1xCarbamid	5.64E-07	4.225E-05	1	1	2	Q99832	Q99832 [11-37]	0	3133.6119
[K].MHSMN(	1xCarbamid	6.369E-06	4.225E-05	1	1	2	P00450	P00450 [620-638]	0	2812.3357
[R].LASVLG	1xTMTpro [K	2.593E-05	4.225E-05	1	1	1	Q9NY33	Q9NY33 [216-233	0	2427.3474
[K].MKETAE	3xTMTpro [K	4.449E-05	4.225E-05	1	1	4	P11021	P11021 [153-164]	2	2585.5477
[K].MGPTVS	1xCarbamid	1.875E-05	4.225E-05	1	1	1	P09871	P09871 [541-572]	0	3670.7875

[R].LATALQI	2xTMTpro [K	2.559E-06	4.225E-05	4	4	2	P67936; P09	P67936 [70-89]; F	2	3114.7463
[R].MGPTEL	3xTMTpro [K	1.233E-05	4.225E-05	1	1	1	P02675	P02675 [335-353]	2	3435.9372
[K].EHASGS	1xTMTpro [N	4.972E-05	4.225E-05	1	1	1	Q14980	Q14980 [1100-11	0	1718.8377
[K].EHAVEG	1xCarbamido	7.701E-06	4.225E-05	1	1	6	P02765	P02765 [107-120]	0	2269.1778
[R].MGLAMC	1xOxidation [	5.822E-05	4.225E-05	1	1	2	P52272	P52272 [607-621]	0	1703.8164
[R].MGLAMC	1xTMTpro [N	1.069E-05	4.225E-05	1	1	3	P52272	P52272 [607-621]	0	1687.8215
[K].EHDPVG	1xOxidation [	5.756E-05	4.225E-05	1	1	1	P55060	P55060 [913-925]	0	2089.0992
[R].LATGSD	1xCarbamido	8.02E-06	4.225E-05	1	1	3	O75083	O75083 [162-180]	0	2652.326
[K].FSDTDLI	1xCarbamido	2.511E-05	4.225E-05	1	1	1	Q9BXD5	Q9BXD5 [174-190	0	2334.0991
[R].FSDTCFI	3xCarbamido	4.783E-06	4.225E-05	1	1	1	P98160	P98160 [825-849]	0	3090.3199
[-].MGHFTEI	1xMet-loss [N	3.645E-05	4.225E-05	2	2	1	P69891; P69	P69891 [1-18]; P6	1	2832.5712
[R].LATLETE	1xTMTpro [N	2.702E-07	4.225E-05	1	1	2	Q6ZMI0	Q6ZMI0 [144-163]	0	2427.3163
[R].EHAPSIII	1xTMTpro [N	8.065E-06	4.225E-05	1	1	1	P62195	P62195 [240-258]	0	2408.2087
[K].MGGMEC	2xOxidation [	3.069E-05	4.225E-05	1	1	1	P52272	P52272 [346-362]	0	2050.8774
[R].FSDSEIC	1xTMTpro [N	9.208E-06	4.225E-05	1	1	1	P42226	P42226 [563-578]	0	2019.1194
[K].MGGMEC	1xTMTpro [N	1.04E-05	4.225E-05	1	1	2	P52272	P52272 [346-362]	0	2018.8876
[R].MGGGSI	1xTMTpro [N	4.61E-05	4.225E-05	1	1	1	Q96I24	Q96I24 [251-262]	0	1492.8113
[R].IATLGYL	1xTMTpro [N	5.974E-05	4.225E-05	1	1	2	P29992	P29992 [167-181]	0	1992.1449
[R].MGGEAF	1xTMTpro [K	3.125E-06	4.225E-05	1	1	2	Q07812	Q07812 [38-57]	0	2634.394
[K].LATPELL	1xTMTpro [N	5.756E-05	4.225E-05	1	1	3	Q8N6R0	Q8N6R0 [660-674	0	1959.1082
[K].MGAVFM	1xTMTpro [N	5.218E-05	4.225E-05	1	1	1	P31937	P31937 [150-167]	0	1997.0268
[R].EHGGPE	1xTMTpro [K	1.906E-06	4.225E-05	1	1	2	Q14240	Q14240 [11-41]	0	4081.9039
[K].MGAPES	1xTMTpro [K	2.453E-05	4.225E-05	1	1	1	P02794	P02794 [159-183]	1	3291.5971
[K].MGAPES	1xOxidation [	3.135E-05	4.225E-05	1	1	3	P02794	P02794 [159-173]	0	2252.1764
[R].EHEHVV	1xCarbamido	9.984E-06	4.225E-05	1	1	1	P43034	P43034 [274-302]	0	3844.8467
[R].EHGQCA	2xCarbamido	6.831E-06	4.225E-05	1	1	1	Q6UXH1	Q6UXH1 [284-299	0	2456.1677
[K].IATGHNI	1xCarbamido	4.874E-05	4.225E-05	1	1	2	Q16563	Q16563 [180-194]	0	2286.2772
[K].EHALAQ	1xTMTpro [K	5.701E-05	4.225E-05	1	1	1	O15126	O15126 [79-89]	0	1831.0933
[R].MGPTEL	1xOxidation [	7.947E-06	4.225E-05	1	1	6	P02675	P02675 [335-351]	1	2920.5616
[R].MGPTEL	2xTMTpro [K	2.763E-07	4.225E-05	1	1	4	P02675	P02675 [335-351]	1	2904.5667
[R].MGPTEL	1xOxidation [	3.594E-05	4.225E-05	1	1	13	P02675	P02675 [335-348]	0	2316.2111
[K].EGVYEL	2xTMTpro [K	2.885E-05	4.225E-05	1	1	1	Q9UFN0	Q9UFN0 [143-166	0	3524.9068
[R].MGPTEL	1xTMTpro [K	5.68E-06	4.225E-05	1	1	6	P02675	P02675 [335-348]	0	2300.2162

[R].MGPSG(	1xTMTpro [N	1.566E-05	4.225E-05	1	1	2	Q14847	Q14847 [131-144]	1	1793.8594
[K].LATAMQ	1xTMTpro [K	2.865E-06	4.225E-05	1	1	1	Q14203	Q14203 [903-927]	0	3389.8019
[K].LATDKNI	1xCarbamido	6.608E-06	4.225E-05	1	1	3	P02794	P02794 [121-144]	1	3813.0098
[R].MGPGAT	1xTMTpro [K	3.734E-05	4.225E-05	1	1	3	P62820	P62820 [176-187]	0	1654.9078
[R].FSELPT(	1xTMTpro [K	9.893E-06	4.225E-05	1	1	1	Q9Y312	Q9Y312 [208-227	0	2802.4879
[R].EHALLA'	1xTMTpro [K	1.601E-05	4.225E-05	1	3	11	P68104	P68104 [135-146]	0	1923.1559
[K].EGWPSS	1xTMTpro [K	2.484E-05	4.225E-05	1	1	2	P16152	P16152 [187-198]	0	1890.0253
[R].EGYCFT	2xCarbamido	1.21E-06	4.225E-05	1	1	3	P35555	P35555 [2336-235	0	2697.239
[R].EGYCFT	2xCarbamido	4.377E-06	4.225E-05	1	1	3	P35555	P35555 [2336-235	0	2713.2339
[R].MGPAM(	1xTMTpro [N	2.829E-05	4.225E-05	1	1	2	P52272	P52272 [592-606]	0	1731.9205
[K].MGMEA\	1xCarbamido	2.212E-07	4.225E-05	1	1	2	P17858	P17858 [316-345]	0	3452.7217
[K].MGLVDQ	1xOxidation [	8.321E-06	4.225E-05	1	1	1	P40939	P40939 [215-235]	0	2898.6254
[K].MGLVDQ	1xTMTpro [K	3.07E-07	4.225E-05	1	1	3	P40939	P40939 [215-235]	0	2882.6305
[R].MGIGLG	1xCarbamido	1.773E-05	4.225E-05	1	1	1	Q9UNF1	Q9UNF1 [503-528	0	3324.6273
[K].EGYVLT/	1xTMTpro [K	1.725E-05	4.225E-05	1	3	1	Q5JPE7	Q5JPE7 [856-871	0	2307.2728
[K].MGLGHE	1xCarbamido	5.455E-05	4.225E-05	1	1	1	Q9UGI8	Q9UGI8 [10-24]	0	2226.1655
[K].MGLGHE	1xCarbamido	2.466E-05	4.225E-05	1	1	1	Q9UGI8	Q9UGI8 [10-24]	0	2210.1706
[R].IATETDQ	1xTMTpro [N	1.115E-05	4.225E-05	1	1	1	Q9UEU0	Q9UEU0 [152-17	0	2535.3109
[R].LYGSEA	1xTMTpro [K	9.184E-08	4.225E-05	1	1	8	P01011	P01011 [160-177]	0	2500.2851
[K].MDIDVPI	1xTMTpro [K	1.924E-06	4.225E-05	1	1	2	Q09666	Q09666 [859-876]	0	2701.4151
[K].MDIDAPI	1xOxidation [	1.842E-05	4.225E-05	1	1	3	Q09666	Q09666 [1236-125	0	2689.3787
[R].LYQTDP	1xTMTpro [K	8.367E-06	4.225E-05	1	2	1	O14818	O14818 [144-157]	0	2275.2003
[K].EKYDTD	2xTMTpro [K	1.538E-05	4.225E-05	1	1	3	P14868	P14868 [382-393]	1	2462.3635
[K].EKYIDQE	2xTMTpro [K	5.41E-05	4.225E-05	2	4	2	P07900; P08	P07900 [282-292]	1	2321.3169
[K].LCHLLGI	1xCarbamido	4.382E-05	4.225E-05	1	1	3	P35580	P35580 [381-394]	0	2041.0352
[K].EKYQEE	2xTMTpro [K	7.701E-06	4.225E-05	1	1	3	P49257	P49257 [287-302]	1	3039.588
[R].FQPVHD	1xTMTpro [N	2.542E-05	4.225E-05	2	2	2	Q8WUD1; P	Q8WUD1 [31-46];	0	2090.1354
[K].LYQPEY(	1xTMTpro [K	1.751E-08	4.225E-05	1	1	1	Q9Y4L1	Q9Y4L1 [754-774	1	3150.6087
[K].LYQPEY(	1xTMTpro [N	5.357E-05	4.225E-05	1	1	1	Q9Y4L1	Q9Y4L1 [754-768	0	2203.0838
[K].ELAAEVI	1xTMTpro [K	1.371E-05	4.225E-05	1	1	1	P08574	P08574 [147-170]	0	3256.611
[K].IYQNIQD	1xTMTpro [K	2.399E-07	4.225E-05	1	1	1	P61106	P61106 [172-201]	0	3758.9706
[R].LYQGIN(	1xTMTpro [K	3.853E-05	4.225E-05	1	1	1	P43246	P43246 [407-423]	0	2549.4947
[K].ELAAQLI	1xTMTpro [K	4.989E-05	4.225E-05	1	1	1	Q9Y5B9	Q9Y5B9 [480-491	1	1980.137

[K].LYQGGP	1xTMTpro [N	7.874E-06	4.225E-05	1	1	1	P54652	P54652 [613-639]	0	2624.2395
[K].IYQGEDI	1xTMTpro [K	1.572E-05	4.225E-05	1	1	2	Q6DD88	Q6DD88 [331-341	0	1905.0726
[K].LYQEVEI	1xTMTpro [N	1.053E-05	4.225E-05	1	1	4	Q92900	Q92900 [828-843]	0	2129.1198
[K].IYQEEEN	1xTMTpro [N	2.784E-05	4.225E-05	1	1	1	P35269	P35269 [56-73]	0	2377.0937
[R].LYQASP.	1xCarbamido	2.839E-05	4.225E-05	1	1	4	P98160	P98160 [2394-240	0	2019.9765
[R].EIADGLC	1xCarbamido	1.251E-05	4.225E-05	1	1	3	P13693	P13693 [22-34]	0	2041.1131
[R].EIADLGE	1xTMTpro [K	1.882E-05	4.225E-05	1	1	4	P49588	P49588 [794-812]	0	2661.5107
[R].IYPVLGT	1xCarbamido	3.767E-05	4.225E-05	1	1	2	Q9GZM7	Q9GZM7 [111-12	0	2075.034
[R].ELADYLC	1xCarbamido	5.682E-05	4.225E-05	1	1	2	Q27J81	Q27J81 [892-914]	0	3315.6256
[K].EKVWAH	2xTMTpro [K	4.177E-07	4.225E-05	1	1	1	Q7KZF4	Q7KZF4 [655-676	1	3610.9283
[R].FQQDDA	1xCarbamido	3.028E-06	4.225E-05	1	1	2	P26639	P26639 [458-477]	0	3075.5047
[R].LYQYPN	1xTMTpro [K	3.866E-06	4.225E-05	1	1	2	Q9BY44	Q9BY44 [200-216	0	2483.3691
[K].ICGDIHG	1xCarbamido	2.316E-05	4.225E-05	1	2	1	P62136	P62136 [61-74]	0	2027.034
[K].IYVDDGI	1xTMTpro [K	1.006E-05	4.225E-05	1	1	2	P14618	P14618 [174-186]	0	2071.2295
[K].LYTVCDY	2xCarbamido	4.65E-05	4.225E-05	1	1	1	Q29RF7	Q29RF7 [1075-10	0	2363.2958
[K].EKPYFPI	1xTMTpro [K	1.462E-06	4.225E-05	1	1	5	Q00839	Q00839 [463-484]	0	3332.7699
[R].FQSADE	1xTMTpro [N	8.481E-08	4.225E-05	1	1	1	P02549	P02549 [426-448]	0	2836.3305
[R].FQQLE	1xTMTpro [N	1.885E-05	4.225E-05	1	1	1	Q9UMX0	Q9UMX0 [547-56	0	2214.166
[K].LYTLVLT	1xTMTpro [K	7.561E-06	4.225E-05	1	1	1	P30086	P30086 [63-77]	1	2297.3361
[K].LYTLVLT	1xTMTpro [N	2.02E-05	4.225E-05	1	1	4	P30086	P30086 [63-76]	0	1865.0339
[K].LYTGPG	1xCarbamido	9.023E-06	4.225E-05	1	1	1	O43670	O43670 [44-59]	0	2433.3391
[R].LCENIAC	1xCarbamido	2.354E-05	4.225E-05	1	1	2	P04040	P04040 [459-468]	0	1763.0129
[K].LYTDFDI	1xTMTpro [N	1.396E-05	4.225E-05	1	1	1	O00429	O00429 [100-117]	1	2604.2749
[R].ELAEAM.	1xCarbamido	9.478E-08	4.225E-05	1	1	1	Q14980	Q14980 [788-808]	0	2953.4891
[R].EKQPMV	1xTMTpro [K	2.275E-05	4.225E-05	1	1	1	P33993	P33993 [568-589]	1	3149.6507
[K].LYSNAYI	1xCarbamido	1.268E-06	4.225E-05	1	1	1	P00352	P00352 [114-128]	0	2323.2612
[R].EKQSDD	2xTMTpro [K	5.907E-05	4.225E-05	1	1	2	Q15459	Q15459 [448-467]	1	3135.6878
[R].EKRPEA	3xTMTpro [K	1.075E-05	4.225E-05	1	1	1	P49589	P49589 [117-139]	1	3784.2477
[K].ICGDIHG	1xCarbamido	1.969E-05	4.225E-05	1	1	2	P62140	P62140 [60-73]	0	1965.0183
[R].LYSILGT	2xTMTpro [K	3.538E-06	4.225E-05	1	1	4	O75083	O75083 [471-484]	1	2450.4686
[R].EKTPLID	1xCarbamido	4.377E-06	4.225E-05	1	1	2	Q9NZB2	Q9NZB2 [817-83	1	2885.5859
[R].IYSHDG	1xTMTpro [K	1.117E-05	4.225E-05	1	1	2	Q9NYU2	Q9NYU2 [1178-1	0	3253.6872
[R].EKVEVQ	2xTMTpro [K	5.039E-05	4.225E-05	1	1	1	Q13303	Q13303 [223-235]	1	2508.5006

[R].EKVGEQ	1xTMTpro [K	1.985E-06	4.225E-05	1	1	2	Q00610	Q00610 [42-63]	1	3060.6643
[K].EKVGEV	2xTMTpro [K	5.59E-05	4.225E-05	1	1	1	P52272	P52272 [93-110]	1	2922.6314
[K].LYSPSQI	1xOxidation [	5.59E-05	4.225E-05	1	1	2	P38646	P38646 [160-173]	0	2178.2488
[K].ICLEDNV	1xCarbamido	2.053E-05	4.225E-05	1	1	2	Q92520	Q92520 [85-97]	0	2086.1532
[R].FQPGET	1xTMTpro [N	6.764E-08	4.225E-05	1	1	3	P46940	P46940 [1398-142	0	3145.5609
[K].IYPTVNC	1xCarbamido	3.754E-05	4.225E-05	1	1	4	P01042	P01042 [364-380]	0	2573.4149
[K].IYIGDDN	1xTMTpro [K	3.202E-05	4.225E-05	1	1	2	P06756	P06756 [647-660]	0	2182.2979
[R].ELAILLG	2xTMTpro [K	8.948E-07	4.225E-05	1	1	3	P30041	P30041 [109-125]	1	2797.6201
[R].ELAILLG	1xOxidation [	1.906E-05	4.225E-05	1	1	2	P30041	P30041 [109-125]	1	2813.615
[R].LYIFQAS	1xCarbamido	1.595E-05	4.225E-05	1	1	2	P98160	P98160 [2296-23	0	2263.1501
[R].FQNIDF/	1xTMTpro [N	3.514E-05	4.225E-05	1	1	2	Q9Y2T3	Q9Y2T3 [114-126	0	1935.9772
[R].LYLEQQ	1xTMTpro [K	2.098E-05	4.225E-05	1	1	1	Q9Y5X1	Q9Y5X1 [569-584	0	2610.4311
[R].LYLEDDI	1xTMTpro [N	4.085E-05	4.225E-05	1	1	1	Q9BT78	Q9BT78 [154-170	0	2328.1679
[K].FQNDRT	1xTMTpro [K	8.005E-06	4.225E-05	1	1	1	Q9Y376	Q9Y376 [309-323	1	2523.2243
[R].FQMTQE	2xCarbamido	4.989E-05	4.225E-05	1	1	1	Q9UBS4	Q9UBS4 [185-20(	0	2608.2701
[K].IYKVPST	2xTMTpro [K	4.071E-05	4.225E-05	1	1	2	P31150	P31150 [116-137]	1	3371.8411
[R].LCNGVQ	3xCarbamido	1.51E-05	4.225E-05	1	1	1	Q07954	Q07954 [91-109]	0	2511.044
[R].FQMTQE	2xCarbamido	1.987E-05	4.225E-05	1	1	2	Q9UBS4	Q9UBS4 [185-20(	0	2592.2752
[R].LYKLELE	2xTMTpro [K	3.833E-05	4.225E-05	1	1	2	Q03252	Q03252 [273-285	1	2539.4952
[R].LYKEELE	2xTMTpro [K	5.454E-06	4.225E-05	1	1	2	P20700	P20700 [259-271]	1	2564.4541
[K].LYHSEAI	2xTMTpro [K	4.087E-06	4.225E-05	1	1	61	P01009	P01009 [161-179]	1	3098.6615
[K].LYHSEAI	1xTMTpro [K	5.839E-06	4.225E-05	1	1	81	P01009	P01009 [161-178]	0	2666.3594
[K].LYHPECI	3xCarbamido	4.972E-06	4.225E-05	1	1	2	P50479	P50479 [273-289]	0	2752.3211
[R].LCNSPS	2xCarbamido	1.114E-06	4.225E-05	1	1	1	P07996	P07996 [461-479]	0	2740.3461
[K].IYHLPDA	1xTMTpro [K	2.984E-05	4.225E-05	1	1	1	Q15019	Q15019 [210-229	1	3045.4933
[R].FQMPDQ	1xOxidation [	3.322E-05	4.225E-05	1	1	4	Q16555	Q16555 [76-94]	0	2775.325
[R].FQMPDQ	1xTMTpro [K	3.927E-05	4.225E-05	1	1	2	Q16555	Q16555 [76-94]	0	2759.3301
[K].LYGSGD	1xTMTpro [K	1.776E-05	4.225E-05	1	1	4	P28838	P28838 [177-188]	0	1990.0526
[K].LYKQEFI	2xTMTpro [K	5.955E-05	4.225E-05	1	1	1	P46939	P46939 [985-996]	1	2382.3485
[K].LYVMEM	1xTMTpro [K	1.028E-05	4.225E-05	1	1	1	Q96SM3	Q96SM3 [334-35	0	3053.5568
[R].LYIGLAG	1xTMTpro [N	2.781E-06	4.225E-05	1	1	3	P49720	P49720 [49-66]	0	2193.2562
[R].ELAKVEI	2xTMTpro [K	4.947E-05	4.225E-05	1	1	1	P55327	P55327 [82-100]	1	3011.7809
[K].EIAEAYL	1xTMTpro [N	2.924E-06	4.225E-05	1	1	2	P0DMV9	P0DMV9 [129-15	0	3305.7013

[K].EIAEDEL	1xTMTpro [K	1.154E-05	4.225E-05	1	1	1	P24043	P24043 [1738-175	0	2222.2775
[R].ELAEDG	1xTMTpro [N	4.206E-05	4.225E-05	1	1	2	P23396	P23396 [28-40]	0	1727.8771
[R].IYPLPDD	1xTMTpro [N	4.113E-05	4.225E-05	1	1	3	Q9NZM1	Q9NZM1 [1526-15	0	1938.0656
[K].LYPIANG	1xTMTpro [K	2.593E-05	4.225E-05	1	1	2	P00915	P00915 [20-35]	0	2351.3215
[R].EIAEEYG	1xTMTpro [N	4.233E-06	4.225E-05	1	1	4	Q00341	Q00341 [825-839]	0	1988.0118
[K].LYPFQCI	1xCarbamido	2.117E-05	4.225E-05	1	1	1	Q8WUA4	Q8WUA4 [603-61	0	2169.1234
[R].ELAELSA	1xTMTpro [K	6.404E-06	4.225E-05	1	1	1	O60504	O60504 [278-291]	1	2210.2524
[K].ELAEITLI	1xCarbamido	3.293E-05	4.225E-05	1	2	2	Q96LR5	Q96LR5 [63-80]	0	2517.3514
[R].LYPELSC	1xTMTpro [N	6.92E-06	4.225E-05	1	1	1	O00560	O00560 [49-67]	0	2588.3237
[K].IYLGQLE	1xCarbamido	1.731E-05	4.225E-05	1	1	1	O60547	O60547 [230-246]	0	2549.3929
[R].IYPEEMI	1xTMTpro [N	2.154E-06	4.225E-05	1	1	1	P21281	P21281 [164-185]	0	2713.386
[R].LYPAFMI	1xTMTpro [K	4.713E-05	4.225E-05	1	1	1	Q12979	Q12979 [737-753]	0	2402.3285
[K].ELAEQLC	1xTMTpro [K	1.488E-05	4.225E-05	1	1	4	O75821	O75821 [181-193]	0	1983.1254
[K].LYNTCAY	1xCarbamido	3.381E-05	4.225E-05	1	1	2	Q9NQR4	Q9NQR4 [93-109]	0	2448.3452
[R].LYNFNW	1xCarbamido	2.009E-05	4.225E-05	1	2	1	P14207	P14207 [75-85]	0	2062.046
[R].IYNEIPD	1xTMTpro [N	2.26E-06	4.225E-05	1	1	1	Q53EL6	Q53EL6 [408-428	0	2790.4633
[K].IYNCELE	1xCarbamido	7.22E-06	4.225E-05	1	1	2	Q9NZM1	Q9NZM1 [1480-15	0	3349.643
[K].LCMGSG	2xCarbamido	1.167E-05	4.225E-05	1	1	5	P02787	P02787 [516-530]	0	2315.1802
[K].LCMGSG	2xCarbamido	1.719E-05	4.225E-05	1	1	6	P02787	P02787 [516-530]	0	2331.1751
[R].LYLSHNC	1xTMTpro [K	1.438E-05	4.225E-05	1	1	1	Q9BXN1	Q9BXN1 [155-171	0	2587.5103
[K].LCMGSG	2xCarbamido	8.817E-08	4.225E-05	1	1	1	P02787	P02787 [516-541]	1	3579.7315
[R].LCLISTF	1xCarbamido	2.409E-05	4.225E-05	1	1	1	O15260	O15260 [31-43]	0	1841.0162
[K].EKEDDV	2xTMTpro [K	3.259E-05	4.225E-05	1	1	1	P28838	P28838 [44-61]	1	2968.5356
[K].EKPSYDT	2xTMTpro [K	4.185E-07	4.225E-05	1	1	1	Q9HB71	Q9HB71 [177-196	0	3165.6806
[K].ICEDSAE	1xCarbamido	7.062E-06	4.225E-05	1	1	1	Q92973	Q92973 [163-186]	0	3364.7988
[R].MCTLIDK	1xCarbamido	5.907E-05	4.225E-05	1	1	1	P53621	P53621 [40-55]	1	2557.3035
[R].EKEPIVD	2xTMTpro [K	2.404E-07	4.225E-05	1	1	5	P02549	P02549 [917-939]	1	3359.8151
[R].EKEPIVG	3xTMTpro [K	1.27E-07	4.225E-05	1	1	1	Q13813	Q13813 [908-930]	2	3711.0481
[K].MCPQLQ	1xCarbamido	6.061E-05	4.225E-05	1	1	1	P01023	P01023 [688-704]	0	2410.1273
[K].MCPQLQ	1xCarbamido	1.336E-05	4.225E-05	1	1	3	P01023	P01023 [688-704]	0	2394.1324
[K].EKEQLM	1xTMTpro [K	5.888E-05	4.225E-05	1	1	2	Q13813	Q13813 [273-285]	1	2134.1094
[K].MCPQLQ	1xCarbamido	2.243E-06	4.225E-05	1	1	1	P01023	P01023 [688-704]	0	2378.1375
[R].MCPIFQI	1xCarbamido	7.888E-06	4.225E-05	1	1	1	O00178	O00178 [359-377]	0	2800.5233

[K].ICAVQEI	2xCarbamido	5.252E-05	4.225E-05	1	1	1	Q9UL15	Q9UL15 [156-168	0	2217.1573
[K].MCPGLS	1xCarbamido	3.906E-05	4.225E-05	1	1	4	Q96AM1	Q96AM1 [17-30]	0	1913.942
[R].MCLAAD	1xCarbamido	2.807E-06	4.225E-05	1	1	2	Q9UBT2	Q9UBT2 [128-152	0	3216.7504
[K].LCCPAG	6xCarbamido	3.542E-07	4.225E-05	1	1	1	Q96HD1	Q96HD1 [141-175	0	4106.7665
[K].EKETLAV	2xTMTpro [K	1.339E-05	4.225E-05	1	1	1	Q16204	Q16204 [126-147]	2	3583.9312
[K].MAYQEY	1xCarbamido	3.741E-05	4.225E-05	1	1	4	P27816	P27816 [108-142]	0	4871.2889
[K].MAVTFIG	1xOxidation [	1.3E-05	4.225E-05	1	1	6	P07437	P07437 [363-379]	0	2494.3871
[K].MAVTFIG	1xTMTpro [K	7.274E-06	4.225E-05	1	1	9	P07437	P07437 [363-379]	0	2478.3922
[K].EKFEGV	2xTMTpro [K	1.067E-06	4.225E-05	1	1	2	P54578	P54578 [13-30]	1	3021.6423
[K].EKFEGV	1xOxidation [	5.829E-06	4.225E-05	1	1	1	P54578	P54578 [13-30]	1	3037.6372
[K].EKFENL(	2xTMTpro [K	2.745E-05	4.225E-05	1	1	2	P17980	P17980 [210-221]	1	2312.3794
[K].LCDCPG	2xCarbamido	4.817E-05	4.225E-05	1	1	2	P36915	P36915 [409-423]	0	2062.1149
[K].MAVDGG	1xCarbamido	1.93E-05	4.225E-05	1	1	2	Q8TBC4	Q8TBC4 [22-37]	0	1986.8605
[R].MDACCC	5xCarbamido	4.73E-06	4.225E-05	1	1	2	P35555	P35555 [977-999]	0	3070.1921
[K].EKEPEEI	2xTMTpro [K	1.998E-05	4.225E-05	1	1	2	Q86UX7	Q86UX7 [154-166	1	2521.3854
[R].MDACCC	5xCarbamido	2.891E-06	4.225E-05	1	1	2	P35555	P35555 [977-999]	0	3086.187
[R].MDACCC	5xCarbamido	4.483E-06	4.225E-05	1	1	1	P35555	P35555 [977-999]	0	3102.1819
[K].EKEDLL(	1xCarbamido	4.367E-05	4.225E-05	1	1	2	P11717	P11717 [469-481]	1	2348.2948
[R].EKEDPE	2xTMTpro [K	5.617E-06	4.225E-05	1	1	2	Q9UHG3	Q9UHG3 [391-40	1	2793.4763
[R].EKEDSL	2xTMTpro [K	4.947E-05	4.225E-05	1	1	2	Q9Y6D9	Q9Y6D9 [147-163	1	2729.5501
[K].MDIDAPI	1xOxidation [	8.617E-06	4.225E-05	1	1	1	Q09666	Q09666 [2030-20	0	2684.3634
[K].MDIDAPI	1xTMTpro [K	2.109E-05	4.225E-05	1	1	2	Q09666	Q09666 [2030-20	0	2668.3685
[K].EKEEFAV	2xTMTpro [K	1.336E-05	4.225E-05	2	2	4	Q9UMX0; Q9	Q9UMX0 [46-62];	1	2908.5873
[R].MDLADL	1xTMTpro [N	9.379E-06	4.225E-05	1	1	1	O14933	O14933 [123-137]	0	2080.1068
[-].MDHYDS(	1xAcetyl [N-T	1.472E-05	4.225E-05	1	1	2	P12814	P12814 [1-31]	1	4534.0735
[K].EKEELIE	2xTMTpro [K	1.885E-05	4.225E-05	1	1	2	O15269	O15269 [56-75]	1	3298.8755
[K].MDFILSH	1xCarbamido	5.794E-05	4.225E-05	1	1	1	Q9NYU2	Q9NYU2 [870-881	0	1815.9205
[K].EKGEIQI	2xTMTpro [K	2.979E-05	4.225E-05	1	1	1	P03952	P03952 [527-537]	1	2212.3481
[K].MDENQF	1xOxidation [	3.021E-05	4.225E-05	3	3	1	Q16555; Q1	Q16555 [375-390]	0	2350.2204
[K].MDENEF	1xTMTpro [K	1.955E-05	4.225E-05	1	1	2	O14531	O14531 [375-390]	0	2335.2095
[R].LAYLQP	1xTMTpro [N	3.502E-05	4.225E-05	1	1	2	P35858	P35858 [206-221]	0	2066.1969
[K].MDATAN	1xOxidation [	5.331E-05	4.225E-05	1	1	5	P30101	P30101 [434-448]	0	1984.9605
[R].EKEFGD	3xTMTpro [K	2.916E-05	4.225E-05	1	1	2	Q9BRX8	Q9BRX8 [200-217	2	3206.9141

[K].MDATAN	1xTMTpro [N	1.334E-05	4.225E-05	1	1	4	P30101	P30101 [434-448]	0	1968.9656
[K].IAYQLAV	1xTMTpro [K	3.18E-05	4.225E-05	1	1	4	P35606	P35606 [656-669]	0	2187.2153
[K].LAYVAA(	1xTMTpro [K	8.527E-07	4.225E-05	1	1	2	P22314	P22314 [386-411]	0	3211.8045
[K].LAYVAA(	1xOxidation [	4.608E-07	4.225E-05	1	1	2	P22314	P22314 [386-411]	0	3227.7994
[R].LCAAAA:	1xCarbamido	1.552E-06	4.225E-05	1	2	4	P30046	P30046 [23-37]	0	2122.2298
[K].ICANHYI	1xCarbamido	5.69E-06	4.225E-05	1	1	1	P43487	P43487 [98-118]	0	3026.5506
[K].MDENQF	1xTMTpro [K	5.638E-06	4.225E-05	3	3	3	Q16555; Q1	Q16555 [375-390]	0	2334.2255
[K].MATYLT(	1xTMTpro [K	2.148E-05	4.225E-05	1	1	1	Q6QNY1	Q6QNY1 [48-64]	0	2502.2929
[R].MATNAA	1xTMTpro [K	4.947E-05	4.225E-05	1	1	2	Q9Y490	Q9Y490 [899-910	0	1812.0293
[R].MATEVA	1xOxidation [	1.676E-05	4.225E-05	1	1	2	P62753	P62753 [32-46]	0	2245.1666
[R].MAHAMN	1xCarbamido	9.362E-06	4.225E-05	1	1	1	Q96GX9	Q96GX9 [177-193	0	2284.0844
[R].MAHAMN	1xCarbamido	8.697E-06	4.225E-05	1	1	1	Q96GX9	Q96GX9 [177-193	0	2268.0894
[-].MAGPESI	1xMet-loss [N	3.085E-06	4.225E-05	1	1	3	Q96IX5	Q96IX5 [1-16]	0	2220.1792
[R].EKLQEE	1xTMTpro [K	3.086E-05	4.225E-05	1	1	3	P08670	P08670 [187-207]	2	3216.6814
[K].ICDPGLT	1xCarbamido	2.462E-05	4.225E-05	1	1	1	Q13555	Q13555 [452-476]	0	3384.71
[K].ICDPYAV	1xCarbamido	1.628E-05	4.225E-05	1	1	1	P48147	P48147 [24-40]	0	2675.3155
[K].ICDQWD	1xCarbamido	1.706E-05	4.225E-05	1	1	1	O43707	O43707 [498-512]	0	2063.0299
[R].MAEIGEI	1xTMTpro [N	3.628E-07	4.225E-05	1	1	1	P27708	P27708 [522-547]	0	2984.4703
[R].MAEGG\	1xTMTpro [N	2.973E-05	4.225E-05	1	1	1	P54252	P54252 [48-59]	0	1618.7702
[K].MAEDEA	1xCarbamido	6.908E-06	4.225E-05	1	1	1	O00629	O00629 [441-461]	0	2932.4411
[R].MAKPEE	1xTMTpro [K	1.231E-05	4.225E-05	1	1	2	O14980	O14980 [424-442]	0	2749.505
[K].MAEDEA	1xCarbamido	1.899E-05	4.225E-05	1	1	2	O00629	O00629 [441-461]	0	2916.4462
[R].EKPAGG	2xTMTpro [K	2.087E-06	4.225E-05	1	1	3	Q8TDL5	Q8TDL5 [64-82]	0	2804.743
[R].EKPDDP	1xCarbamido	6.676E-07	4.225E-05	1	1	2	Q86Y39	Q86Y39 [82-104]	0	3029.601
[K].ICDVYN/	1xCarbamido	5.898E-05	4.225E-05	1	1	1	Q9Y5B9	Q9Y5B9 [322-334	0	2134.1532
[K].MADKDG	2xTMTpro [K	5.719E-05	4.225E-05	1	1	2	O43852	O43852 [162-173]	1	2190.262
[R].FQSVES	1xTMTpro [N	4.687E-06	4.225E-05	1	1	4	Q9NXG2	Q9NXG2 [117-13	0	1971.0619
[R].MADAIIL	1xTMTpro [N	1.284E-05	4.225E-05	1	1	2	O94979	O94979 [595-612]	0	2130.2276
[K].FQSPYE	1xTMTpro [N	4.981E-05	4.225E-05	1	1	1	O00461	O00461 [403-415]	0	1985.9888
[-].MACGLV/	2xCarbamido	1.659E-07	4.225E-05	1	1	2	P09382	P09382 [1-19]	0	2580.4246
[K].LCEAICF	2xCarbamido	1.324E-05	4.225E-05	1	1	1	O00217	O00217 [116-133]	0	2346.2117
[K].LYYHVL(	1xCarbamido	9.893E-06	4.225E-05	1	1	1	P48147	P48147 [210-233]	0	3447.7274
[K].ICDQWD	1xCarbamido	2.375E-05	4.225E-05	1	1	3	P12814	P12814 [479-492]	0	2270.2095

[K].ICEDSSE	1xCarbamido	3.933E-05	4.225E-05	1	1	1	O14787	O14787 [153-176]	0	3351.7784
[K].EKIPIIVG	1xCarbamido	2.426E-07	4.225E-05	1	1	1	Q9Y5X1	Q9Y5X1 [230-258	1	3934.0415
[K].MALDGQ	2xCarbamido	3.363E-05	4.225E-05	1	1	1	O95758	O95758 [237-252]	0	2204.0581
[R].MATEVA	1xTMTpro [K	1.924E-06	4.225E-05	1	1	3	P62753	P62753 [32-46]	0	2229.1717
[K].MAQQMI	1xOxidation [	1.996E-06	4.225E-05	1	1	3	P35749	P35749 [948-966]	0	2583.2238
[K].MAQQMI	1xTMTpro [N	1.428E-06	4.225E-05	1	1	2	P35749	P35749 [948-966]	0	2567.2288
[R].MAQEVL	1xTMTpro [K	6.051E-05	4.225E-05	1	1	1	O14980	O14980 [45-54]	0	1778.049
[R].MAPYQG	1xOxidation [	4.857E-05	4.225E-05	1	1	2	O43707	O43707 [883-899]	0	2417.2667
[K].MAPVPL	1xTMTpro [K	4.036E-05	4.225E-05	1	1	1	Q9NYF8	Q9NYF8 [551-567	0	2420.3463
[K].EKLCYVA	1xCarbamido	1.764E-07	4.225E-05	2	4	3	P62736; P68	P62736 [216-240]	1	3705.9172
[K].ICDFENA	1xCarbamido	5.756E-05	4.225E-05	1	1	2	P51659	P51659 [276-301]	0	3793.051
[K].EKLCYVA	1xCarbamido	3.85E-08	4.225E-05	1	3	11	P63261	P63261 [214-238]	1	3719.9328
[K].EKLCYVA	1xCarbamido	1.607E-06	4.225E-05	1	3	2	P63261	P63261 [214-238]	1	3735.9277
[K].EKLPGEI	2xTMTpro [K	1.153E-06	4.225E-05	1	1	3	O75821	O75821 [194-209]	1	2693.5654
[-].MAPPSVF	1xMet-loss [N	4.228E-05	4.225E-05	1	1	1	P17174	P17174 [1-20]	0	2632.5358
[-].MAPPAPC	1xMet-loss [N	2.506E-05	4.225E-05	1	1	2	O14579	O14579 [1-23]	0	2705.4278
[K].EKLDSVI	1xTMTpro [K	5.107E-05	4.225E-05	1	1	2	P54819	P54819 [120-137]	1	2682.5573
[R].FQVDPS	1xTMTpro [K	2.829E-05	4.225E-05	1	1	1	Q9HB07	Q9HB07 [254-267	0	2140.2145
[K].LCDFGV	1xCarbamido	6.714E-07	4.225E-05	2	2	2	P36507; Q02	P36507 [210-231]	0	2678.3237
[R].EKLEAYC	2xTMTpro [K	1.589E-06	4.225E-05	1	1	2	P46940	P46940 [967-988]	1	3596.0345
[-].MAPEINL	1xMet-loss [N	5.5E-05	4.225E-05	1	1	1	P32456	P32456 [1-18]	0	2418.3558
[K].MALELLT	1xTMTpro [N	3.032E-05	4.225E-05	1	1	2	P49588	P49588 [117-132]	0	2164.2007
[K].ICDPGLT	1xCarbamido	1.878E-06	4.225E-05	1	1	3	Q13557	Q13557 [372-396]	0	3092.5141
[K].EKLIAPV	2xTMTpro [K	6.07E-06	4.225E-05	1	1	3	P07195	P07195 [6-23]	1	2864.6549
[K].ICDPGLT	1xCarbamido	2.515E-05	4.225E-05	1	1	1	Q13557	Q13557 [372-396]	0	3108.509
[R].EKIDLEN	1xTMTpro [K	1.301E-06	4.225E-05	1	1	2	Q16204	Q16204 [198-216]	1	2879.5606
[K].LTVPDDI	1xCarbamido	2.926E-05	4.225E-05	1	1	3	O94760	O94760 [212-230]	0	2752.5199
[R].ELLQSFI	1xTMTpro [K	5.869E-05	4.225E-05	1	1	1	P49792	P49792 [257-270]	0	2173.236
[R].ELIQTSA	1xTMTpro [N	2.475E-05	4.225E-05	1	1	3	Q9Y371	Q9Y371 [134-148	0	2020.1762
[K].LEAGINC	1xTMTpro [N	1.456E-05	4.225E-05	1	1	1	P20742	P20742 [182-203]	0	2710.4371
[R].IQTLVGN	1xTMTpro [K	4.002E-05	4.225E-05	1	1	1	Q9HC78	Q9HC78 [320-330	0	1844.1613
[R].ENAEQL	1xCarbamido	9.802E-06	4.225E-05	1	1	1	Q96MM6	Q96MM6 [232-25	0	2580.3299
[R].IQTLPNC	1xTMTpro [K	5.984E-05	4.225E-05	1	1	1	Q8IX12	Q8IX12 [205-221]	0	2559.475

[R].ENAEVD	1xTMTpro [K	3.363E-05	4.225E-05	1	1	1	P05198	P05198 [296-312]	0	2475.1325
[R].LEALASI	1xTMTpro [K	3.845E-06	4.225E-05	1	1	1	P83436	P83436 [184-205]	0	2882.6119
[K].LQTASDI	2xTMTpro [K	1.401E-05	4.225E-05	1	1	2	Q13813	Q13813 [1571-15	1	2937.5985
[K].LQSYVD.	1xTMTpro [N	2.158E-06	4.225E-05	1	1	1	Q9NUQ2	Q9NUQ2 [156-17	0	2661.3917
[K].LEAIEDD	2xTMTpro [K	1.036E-05	4.225E-05	1	1	2	P54578	P54578 [215-238]	1	3350.7631
[K].ENALVQ	1xTMTpro [K	5.014E-05	4.225E-05	1	1	1	P26599	P26599 [401-424]	0	3185.644
[K].ENAPAIII	1xTMTpro [K	6.287E-06	4.225E-05	1	1	3	P43686	P43686 [256-273]	0	2552.4467
[K].ENAYDLI	1xTMTpro [K	4.078E-05	4.225E-05	1	1	2	Q9UBQ5	Q9UBQ5 [39-52]	0	2171.2204
[R].IEALGGI	1xCarbamid	2.214E-05	4.225E-05	1	1	1	P49593	P49593 [303-317]	0	2082.022
[K].ENCMYQ	3xCarbamid	1.483E-05	4.225E-05	1	1	1	P27694	P27694 [474-488]	0	2527.133
[K].ENDAVT	1xTMTpro [K	5.444E-06	4.225E-05	1	1	1	Q96QK1	Q96QK1 [734-74	0	2434.4161
[R].LQSIGTE	1xTMTpro [N	1.244E-05	4.225E-05	1	1	3	P04075	P04075 [44-57]	1	1951.0164
[R].LQSIGTE	1xTMTpro [N	2.819E-05	4.225E-05	1	1	1	P04075	P04075 [44-56]	0	1794.9153
[K].ENDPSS	1xTMTpro [K	2.654E-05	4.225E-05	1	1	1	Q9BZG1	Q9BZG1 [153-167	0	2213.2673
[K].LQSLCAS	3xCarbamid	1.428E-06	4.225E-05	1	1	1	Q96IG2	Q96IG2 [249-274]	0	3137.5461
[R].FISGSM	1xTMTpro [K	1.793E-06	4.225E-05	1	1	2	Q6NUK1	Q6NUK1 [292-31	0	2988.5529
[R].ENEFSFI	1xTMTpro [N	5.278E-05	4.225E-05	1	2	1	Q14839	Q14839 [818-829]	0	1774.8567
[K].ENAAYF	1xTMTpro [K	4.516E-06	4.225E-05	1	1	2	P15924	P15924 [374-393]	0	2994.5017
[R].EMYLQS	1xCarbamid	5.375E-05	4.225E-05	1	1	1	Q12769	Q12769 [455-474]	0	2985.5346
[R].LQTQAS	1xTMTpro [K	3.754E-05	4.225E-05	1	1	2	Q00341	Q00341 [147-159]	0	1936.1723
[R].LQTSSVI	1xTMTpro [N	5.682E-05	4.225E-05	1	1	5	P22314	P22314 [70-81]	0	1563.9389
[K].IRDEMV	1xTMTpro [N	5.296E-05	4.225E-05	1	1	1	P16615	P16615 [235-246]	1	1780.9183
[R].IRCEEEI	1xCarbamid	7.802E-06	4.225E-05	1	1	1	Q9Y230	Q9Y230 [373-392	1	2749.298
[R].LQYTEH	1xTMTpro [K	3.141E-05	4.225E-05	1	1	1	Q9NZI7	Q9NZI7 [126-138]	0	2268.2268
[R].IQYQLVI	1xTMTpro [N	1.326E-06	4.225E-05	1	1	2	Q9H299	Q9H299 [33-51]	1	2611.3469
[R].IQYQLVI	1xTMTpro [N	2.561E-05	4.225E-05	1	1	1	Q9H299	Q9H299 [33-47]	0	2080.1358
[R].LQWDLC	1xTMTpro [N	3.82E-05	4.225E-05	1	1	1	Q5JTH9	Q5JTH9 [404-415	0	1708.9818
[R].IQVVVLS	1xTMTpro [K	5.5E-05	4.225E-05	1	1	1	P98160	P98160 [2038-20	0	2315.383
[R].LQVTNV	1xTMTpro [K	1.852E-05	4.225E-05	1	1	3	P04844	P04844 [290-306]	0	2448.4681
[K].LQVSQQ	1xTMTpro [K	4.036E-05	4.225E-05	1	1	4	P29218	P29218 [146-156]	0	1897.0886
[K].IQVQSSI	1xTMTpro [K	2.828E-06	4.225E-05	1	1	1	Q02952	Q02952 [1410-14	0	2747.4595
[K].LQSGTH	1xCarbamid	7.903E-06	4.225E-05	1	1	1	P01033	P01033 [162-180]	0	2809.4798
[K].LEAEGVI	1xTMTpro [K	5.545E-05	4.225E-05	1	1	3	O76003	O76003 [68-79]	0	1895.0617

[R].EMVEML	1xTMTpro [K	5.474E-06	4.225E-05	1	1	3	O60684	O60684 [84-103]	0	2909.4768
[R].FISNPNS	1xTMTpro [K	6.275E-06	4.225E-05	1	1	1	O00429	O00429 [173-198]	0	3300.8005
[R].LQVGEV	1xTMTpro [K	1.401E-05	4.225E-05	1	1	1	P40616	P40616 [37-59]	0	3116.7739
[K].LQVEPA	1xCarbamido	3.145E-07	4.225E-05	1	1	5	P21333	P21333 [1247-127	0	3067.5478
[R].LQVEHP	1xCarbamido	4.917E-06	4.225E-05	1	1	1	P05165	P05165 [354-375]	0	2884.5232
[K].LEAELGI	1xTMTpro [K	4.005E-06	4.225E-05	1	1	3	P05787	P05787 [161-176]	0	2401.2929
[K].LEAELGI	1xOxidation [	4.681E-05	4.225E-05	1	1	1	P05787	P05787 [161-176]	0	2417.2878
[R].IQTYLQS	1xOxidation [	6.012E-05	4.225E-05	1	1	1	P30085	P30085 [152-171]	0	3298.8425
[R].IQTYLQS	2xTMTpro [K	7.744E-06	4.225E-05	1	1	2	P30085	P30085 [152-171]	0	3282.8476
[R].LEAGDH	1xTMTpro [N	2.921E-05	4.225E-05	1	1	1	P09486	P09486 [206-218]	0	1723.9662
[K].IQVIDISN	1xTMTpro [N	4.029E-05	4.225E-05	2	2	1	P11908; P60	P11908 [287-301]	0	1989.1738
[R].LQSGAW	7xCarbamido	2.162E-06	4.225E-05	1	1	3	P28799	P28799 [299-332]	0	4667.0407
[R].ENEITGA	1xCarbamido	1.384E-05	4.225E-05	1	1	2	Q9Y3Z3	Q9Y3Z3 [70-85]	0	2121.0817
[K].FLSESVL	1xTMTpro [N	9.107E-06	4.225E-05	1	1	1	Q9NWT1	Q9NWT1 [212-22	0	2138.1664
[R].LQQELD	1xTMTpro [N	3.441E-06	4.225E-05	1	1	4	P35749	P35749 [1425-144	0	2217.1682
[R].LQQELD	1xTMTpro [N	4.635E-06	4.225E-05	1	1	1	P35580	P35580 [1425-144	0	2242.1635
[R].LQQELD	1xTMTpro [N	4.087E-06	4.225E-05	1	1	4	P35579	P35579 [1418-143	0	2254.1998
[R].IQQEIAV	1xTMTpro [N	3.202E-05	4.225E-05	1	1	1	Q96FW1	Q96FW1 [37-51]	0	2028.1409
[K].ENIIAVAT	1xTMTpro [K	8.842E-06	4.225E-05	1	1	2	P63151	P63151 [428-445]	0	2675.49
[R].LQQEET	1xTMTpro [N	1.026E-05	4.225E-05	1	1	1	Q9UBC2	Q9UBC2 [508-52	0	2162.1372
[R].LQQEAT	1xTMTpro [N	3.219E-06	4.225E-05	1	1	1	Q9Y6C2	Q9Y6C2 [692-706	0	2062.0008
[K].LQQDSE	2xTMTpro [K	7.01E-06	4.225E-05	1	1	1	Q92643	Q92643 [319-338]	1	3345.6647
[K].IQQDADS	1xTMTpro [N	2.621E-05	4.225E-05	1	1	2	Q9H3P7	Q9H3P7 [387-399	0	1705.9404
[R].ENLISAL	1xTMTpro [K	6.003E-05	4.225E-05	1	1	5	P22102	P22102 [394-404]	0	1825.0563
[K].LQQELEI	2xTMTpro [K	1.206E-05	4.225E-05	1	1	2	P43686	P43686 [47-66]	1	3450.8824
[R].LQPVLQ	1xTMTpro [K	9.518E-06	4.225E-05	1	1	4	P12111	P12111 [1216-123	0	2195.3407
[R].LQPTWN	2xTMTpro [K	5.514E-06	4.225E-05	1	1	2	Q8NBS9	Q8NBS9 [95-113]	1	3137.6394
[K].FISADQS	1xTMTpro [K	2.677E-05	4.225E-05	1	1	1	O43301	O43301 [571-585]	0	2185.2724
[K].IQPSGGT	1xTMTpro [N	1.516E-05	4.225E-05	1	1	1	P19823	P19823 [380-394]	0	1887.0619
[R].LQPQTT	1xTMTpro [K	3.13E-05	4.225E-05	1	1	2	Q99715	Q99715 [1055-10	0	2501.4511
[R].IEDANLII	1xTMTpro [K	2.389E-06	4.225E-05	1	1	4	P07996	P07996 [61-83]	1	3187.7495
[R].FLRPDG	2xCarbamido	4.226E-06	4.225E-05	1	1	1	P14222	P14222 [66-87]	0	2883.4412
[R].IQPGNPI	1xTMTpro [K	2.09E-05	4.225E-05	1	1	2	P63010	P63010 [905-917]	0	2053.1937

[R].ENLWLN	1xCarbamido	2.755E-05	4.225E-05	1	1	1	P45974	P45974 [206-221]	0	2165.1344
[R].FLREPV\	1xTMTpro [N	1.324E-05	4.225E-05	1	1	1	Q66K74	Q66K74 [453-469	1	2214.2202
[K].LQPFATI	1xTMTpro [N	3.113E-05	4.225E-05	1	1	2	P33992	P33992 [628-642]	0	1993.0561
[K].ENLQLN	2xTMTpro [K	1.015E-05	4.225E-05	1	1	1	Q96BW5	Q96BW5 [69-88]	1	3364.882
[K].EMTDGC	1xTMTpro [N	5.252E-05	4.225E-05	1	3	1	P00325	P00325 [257-272]	0	2063.0075
[R].LQQETA	1xTMTpro [K	2.584E-05	4.225E-05	1	1	1	P42566	P42566 [457-472]	0	2385.2641
[K].LECSEEI	1xCarbamido	3.387E-05	4.225E-05	1	2	2	Q00610	Q00610 [457-468]	0	2000.0866
[R].IQSALEE	1xTMTpro [K	1.032E-06	4.225E-05	1	1	2	P57740	P57740 [230-250]	0	2830.533
[K].IQRPPEI	1xTMTpro [K	1.598E-05	4.225E-05	1	1	3	Q16851	Q16851 [78-91]	0	2308.2793
[R].IQQYFPS	1xTMTpro [K	1.407E-05	4.225E-05	1	1	2	P16885	P16885 [815-837]	0	3373.6971
[R].LQQVLQ	1xTMTpro [N	1.089E-06	4.225E-05	1	1	1	Q14974	Q14974 [558-574]	0	2304.1937
[K].LQQTYA	1xTMTpro [K	5.85E-05	4.225E-05	1	1	2	P46940	P46940 [1506-15	0	1845.0726
[R].LQQTQN	1xTMTpro [N	4.963E-06	4.225E-05	1	1	1	Q15836	Q15836 [15-30]	0	2220.1614
[R].LQQTQA	1xTMTpro [N	1.592E-06	4.225E-05	1	1	2	P63027	P63027 [32-47]	0	2177.1555
[K].ENFMTV	1xTMTpro [N	3.881E-06	4.225E-05	1	1	1	P28676	P28676 [126-145]	0	2605.2272
[R].LQQSHP	1xTMTpro [K	3.074E-06	4.225E-05	1	1	1	Q13813	Q13813 [321-335]	0	2286.3425
[R].LQQQLT	1xTMTpro [K	4.382E-05	4.225E-05	1	1	1	Q9Y4P3	Q9Y4P3 [428-440	0	2137.2472
[K].IECVSAE	2xCarbamido	2.443E-06	4.225E-05	1	1	8	P02787	P02787 [385-399]	0	2334.1813
[K].IQQLTAL	1xTMTpro [K	1.906E-05	4.225E-05	1	1	2	Q99471	Q99471 [139-153]	0	2093.2574
[K].LQQLFNI	1xOxidation	1.835E-05	4.225E-05	3	3	3	P35580; P35	P35580 [483-501]	0	2787.4095
[K].ENGTVT	1xTMTpro [K	5.334E-06	4.225E-05	1	1	2	P24752	P24752 [274-302]	0	3369.7816
[R].ENGVHS	1xTMTpro [N	3.363E-05	4.225E-05	1	1	1	Q8WX93	Q8WX93 [1187-1	0	1955.0881
[K].FISDKDA	2xTMTpro [K	9.566E-07	4.225E-05	1	1	2	P30101	P30101 [148-173]	1	3850.9996
[K].LEATLQE	1xTMTpro [N	1.361E-05	4.225E-05	1	1	1	O95202	O95202 [466-481]	0	2170.1423
[R].ENKPSII	1xCarbamido	2.592E-06	4.225E-05	1	1	2	O75351	O75351 [225-243]	0	2801.4999
[K].IECDDK	2xCarbamido	2.295E-05	4.225E-05	2	2	2	P21333; Q1	P21333 [621-634]	1	2234.0673
[R].LQQHIQI	1xTMTpro [N	2.038E-05	4.225E-05	1	1	1	Q7Z406	Q7Z406 [965-983]	0	2505.3017
[R].ENLATVI	1xTMTpro [N	8.05E-06	4.225E-05	1	1	2	Q13561	Q13561 [380-395]	0	2069.047
[K].LQQGYN	1xTMTpro [N	3.558E-06	4.225E-05	1	1	2	P50897	P50897 [105-122]	0	2306.1671
[R].LQQLGE	1xTMTpro [N	7.462E-07	4.225E-05	1	1	1	Q14980	Q14980 [771-786]	0	2126.1525
[R].IEDDMD	1xCarbamido	2.206E-05	4.225E-05	1	1	1	P23229	P23229 [163-178]	0	2178.9028
[R].LRDPEV	1xTMTpro [N	8.291E-06	4.225E-05	1	1	1	P06132	P06132 [121-142]	1	2781.547
[K].IREDLPN	1xTMTpro [K	9.356E-08	4.225E-05	1	1	3	Q96QK1	Q96QK1 [750-768	1	2852.5133

[R].EMGEAF	1xTMTpro [N	5.132E-05	4.225E-05	1	1	1	O95782	O95782 [142-153]	0	1610.8168
[R].LDVEFKI	2xTMTpro [K	2.602E-05	4.225E-05	1	1	3	P98160	P98160 [3953-397	0	3014.7747
[K].IDVESLS	1xTMTpro [N	5.518E-05	4.225E-05	1	1	1	Q6NXG1	Q6NXG1 [70-86]	0	2136.1467
[K].LDVGEA	1xTMTpro [N	1.673E-05	4.225E-05	1	1	2	Q8IZL8	Q8IZL8 [499-511]	0	1683.8808
[K].EMGFTN	1xTMTpro [K	9.659E-06	4.225E-05	1	1	1	Q9NVP1	Q9NVP1 [196-208	0	2174.123
[R].FLVEDYI	1xTMTpro [K	5.719E-05	4.225E-05	1	1	4	P51178	P51178 [702-712]	0	1882.009
[RK].ISEIED	1xTMTpro [N	4.89E-05	4.225E-05	2	2	3	O75477; O9	O75477 [244-255]	0	1638.9022
[K].EMGLPV	1xTMTpro [K	1.053E-05	4.225E-05	1	1	1	Q9BQ52	Q9BQ52 [261-279	0	2430.465
[K].EMGSEV	2xTMTpro [K	9.057E-06	4.225E-05	1	1	2	Q8IWJ2	Q8IWJ2 [765-792	1	3891.0361
[K].IDVGFVY	1xTMTpro [N	7.932E-06	4.225E-05	1	1	3	Q9NZM1	Q9NZM1 [282-297	0	2109.0758
[K].LSELDDI	1xTMTpro [K	1.153E-06	4.225E-05	2	2	3	Q15836; P63	Q15836 [43-66]; P	1	3102.6199
[K].ISELDAF	2xTMTpro [K	5.103E-07	4.225E-05	1	1	2	Q06323	Q06323 [37-58]	1	3341.9137
[K].LSEGSQ	1xTMTpro [N	6.645E-06	4.225E-05	1	1	1	Q9UKV3	Q9UKV3 [239-257	0	2422.1177
[K].FIVDGW	1xOxidation	3.247E-05	4.225E-05	1	1	1	Q16836	Q16836 [272-295]	0	3385.7131
[R].LSEGEA	2xTMTpro [K	1.818E-05	4.225E-05	1	1	1	Q5JRA6	Q5JRA6 [677-695	1	3159.6415
[K].FIVDGW	1xTMTpro [K	8.948E-07	4.225E-05	1	1	2	Q16836	Q16836 [272-295]	0	3369.7182
[R].LSEELQ	1xTMTpro [N	9.984E-06	4.225E-05	1	1	1	P22415	P22415 [261-279]	0	2502.3371
[K].EMKPPT	1xTMTpro [K	1.137E-05	4.225E-05	1	1	1	Q7KZN9	Q7KZN9 [107-123	0	2730.3689
[K].IDVGFVY	1xOxidation	2.079E-05	4.225E-05	1	1	2	Q9NZM1	Q9NZM1 [282-297	0	2125.0707
[K].LSEEEIL	1xTMTpro [N	2.735E-06	4.225E-05	1	1	2	Q14257	Q14257 [283-305]	0	2945.4587
[R].LSEDYG	1xTMTpro [K	3.172E-06	4.225E-05	1	1	1	P32119	P32119 [111-127]	1	2537.3743
[R].EMFSQL	1xTMTpro [N	4.575E-06	4.225E-05	1	1	1	Q9UPN3	Q9UPN3 [5135-51	0	2587.2339
[K].IDVDAPD	1xTMTpro [K	6.176E-07	4.225E-05	1	1	4	Q09666	Q09666 [3260-32	0	2299.2426
[K].LSENNIQ	1xTMTpro [K	4.065E-06	4.225E-05	1	1	3	P05556	P05556 [326-346]	0	3078.6643
[K].EMFPYE	1xCarbamido	6.756E-06	4.225E-05	1	1	2	P42167	P42167 [347-364]	0	2324.0858
[R].EMEENF	1xOxidation	1.942E-06	4.225E-05	1	1	7	P08670	P08670 [346-364]	0	2507.168
[R].LSGGIDI	1xTMTpro [N	4.133E-06	4.225E-05	1	1	2	Q53GG5	Q53GG5 [17-31]	0	1934.103
[R].ISGETIFY	1xTMTpro [N	3.561E-07	4.225E-05	1	1	1	Q00610	Q00610 [298-320]	0	2657.4582
[K].LSGAYLY	1xCarbamido	2.091E-06	4.225E-05	1	1	1	P11717	P11717 [192-213]	0	2761.3674
[R].LSGAQA	1xTMTpro [N	2.294E-06	4.225E-05	1	1	1	O95571	O95571 [105-121]	0	2101.0845
[K].IDTHNIIV	1xCarbamido	8.523E-06	4.225E-05	1	1	3	O43681	O43681 [270-290]	0	3389.9071
[K].ISGADIN	1xCarbamido	2.405E-05	4.225E-05	1	1	1	P43686	P43686 [370-388]	0	2341.1811
[R].LDTHPAI	1xTMTpro [N	2.075E-05	4.225E-05	1	1	1	Q12931	Q12931 [603-619]	0	2116.1214

[R].LDTLAQI	1xTMTpro [K	2.271E-05	4.225E-05	1	1	4	P05452	P05452 [51-62]	0	1922.1818
[R].LDTLAQI	1xCarbamido	2.962E-06	4.225E-05	1	1	1	P05452	P05452 [51-73]	1	3525.0542
[R].LDVGNF	2xCarbamido	1.206E-05	4.225E-05	1	1	2	P62241	P62241 [60-74]	0	2091.9547
[K].ISFLENN	1xTMTpro [K	4.279E-05	4.225E-05	1	3	3	P33176	P33176 [832-844]	0	2157.2411
[K].LSFGDTI	1xTMTpro [N	9.396E-06	4.225E-05	1	1	2	Q969X5	Q969X5 [153-176	0	2805.4603
[R].EMFEVT	1xTMTpro [N	8.124E-06	4.225E-05	1	1	1	Q9BWS9	Q9BWS9 [119-13	0	2354.1229
[R].EMFEVT	1xOxidation [	2.371E-05	4.225E-05	1	1	1	Q9BWS9	Q9BWS9 [119-13	0	2370.1178
[K].ISFDLAE	1xTMTpro [N	5.474E-06	4.225E-05	1	1	1	O60547	O60547 [116-134]	0	2346.2148
[K].LSEVLQA	1xTMTpro [N	6.518E-08	4.225E-05	1	1	2	Q93009	Q93009 [524-543]	0	2594.4109
[R].EMFGSG	2xCarbamido	3.154E-06	4.225E-05	1	1	1	P54687	P54687 [327-346]	0	2841.4481
[R].ISETSLP	1xCarbamido	4.345E-05	4.225E-05	1	1	12	P51884	P51884 [316-330]	0	2115.0421
[R].LSEQELI	1xTMTpro [N	4.665E-05	4.225E-05	1	1	1	P52756	P52756 [684-695]	0	1733.9604
[R].LSEPDPS	1xTMTpro [N	5.618E-05	4.225E-05	1	1	1	Q9H3U7	Q9H3U7 [338-350	0	1813.9251
[K].EMFPYE	1xCarbamido	9.327E-06	4.225E-05	1	1	2	P42167	P42167 [347-364]	0	2308.0909
[K].LSFGLED	1xTMTpro [K	6.997E-06	4.225E-05	1	1	1	O94905	O94905 [323-337]	0	2258.2412
[K].IDVSIEA	1xTMTpro [K	2.687E-05	4.225E-05	1	1	2	P09622	P09622 [289-300]	0	1755.0144
[K].LSDVDR	1xOxidation [	3.473E-06	4.225E-05	1	1	1	Q9NZN4	Q9NZN4 [492-516	1	3413.739
[K].LSDVDR	1xTMTpro [K	8.367E-06	4.225E-05	1	1	2	Q9NZN4	Q9NZN4 [492-516	1	3397.7441
[K].LRTEGD	1xTMTpro [K	8.05E-06	4.225E-05	1	1	1	P00738	P00738 [117-131]	1	2317.2643
[R].LRQEEP	1xTMTpro [N	1.564E-05	4.225E-05	1	1	2	P29590	P29590 [359-372]	1	1929.0837
[R].FISVGYV	1xTMTpro [N	2.238E-05	4.225E-05	4	4	1	P01889; P01	P01889 [46-59]; F	0	1950.0292
[R].LRPQTY	1xTMTpro [K	5.822E-05	4.225E-05	1	1	1	Q92599	Q92599 [83-98]	0	2540.4328
[R].LRPNFLA	1xCarbamido	2.994E-05	4.225E-05	1	1	1	O43688	O43688 [123-137]	0	2150.1136
[K].LRPNDIE	2xCarbamido	4.495E-05	4.225E-05	1	1	1	Q6ZMP0	Q6ZMP0 [780-795	0	2498.2446
[R].EMQDVD	2xTMTpro [K	6.222E-07	4.225E-05	1	1	3	Q92597	Q92597 [4-19]	0	2755.5732
[R].EMQDVD	1xOxidation [	4.012E-06	4.225E-05	1	1	4	Q92597	Q92597 [4-19]	0	2771.5681
[K].LRPLYDI	1xTMTpro [N	3.747E-05	4.225E-05	1	1	1	Q7KZF4	Q7KZF4 [390-403	0	2088.1271
[K].IRPLTSL	1xTMTpro [N	8.794E-06	4.225E-05	1	1	1	Q9HCB6	Q9HCB6 [376-402	0	3285.7187
[R].LDYFLLS	1xCarbamido	4.713E-06	4.225E-05	1	1	4	P27695	P27695 [282-299]	0	2700.4926
[K].LRPIPITA	1xTMTpro [N	1.557E-06	4.225E-05	1	1	1	P23229	P23229 [616-633]	0	2297.3148
[K].LEAAEDI	1xTMTpro [N	3.053E-05	4.225E-05	1	1	1	P35232	P35232 [241-253]	0	1782.9557
[K].IRPGEQE	1xTMTpro [K	4.417E-06	4.225E-05	1	1	2	P11171	P11171 [451-466]	0	2490.3484
[K].IRLESEE	1xTMTpro [N	1.738E-05	4.225E-05	1	1	1	P06493	P06493 [35-50]	1	2090.1413

[R].FLSQPF(	1xOxidation [	8.915E-07	4.225E-05	1	1	3	P06576	P06576 [463-480]	0	2647.4198
[K].EMSCIAE	1xCarbamido	9.588E-06	4.225E-05	1	1	1	Q9Y678	Q9Y678 [94-111]	0	2604.412
[R].EMSGDL	1xTMTpro [K	6.657E-06	4.225E-05	1	1	2	P50995	P50995 [413-427]	0	2216.1798
[R].EMSGDL	1xOxidation [	2.611E-05	4.225E-05	1	1	1	P50995	P50995 [413-427]	0	2232.1747
[R].LRGDAA	1xTMTpro [N	3.803E-06	4.225E-05	1	1	1	Q6DKJ4	Q6DKJ4 [60-83]	1	2420.2602
[R].EMSGSF	1xTMTpro [K	1.133E-05	4.225E-05	2	2	3	P0C0L4; P0(	P0C0L4 [392-404	0	1868.0443
[R].IREPLTA	1xTMTpro [N	8.139E-06	4.225E-05	1	1	1	Q6P1N0	Q6P1N0 [790-805	1	2190.2049
[R].LDYLGV:	1xTMTpro [N	4.106E-05	4.225E-05	1	1	2	Q9H0A0	Q9H0A0 [706-718	0	1757.9757
[R].FLSQPF(	1xTMTpro [K	3.828E-07	4.225E-05	1	1	4	P06576	P06576 [463-480]	0	2631.4249
[R].LSADSG	1xTMTpro [K	4.072E-06	4.225E-05	1	1	2	P16234	P16234 [1012-10:	1	3446.855
[K].FLTALAQ	1xTMTpro [N	1.412E-06	4.225E-05	1	1	3	Q8WUM4	Q8WUM4 [584-60	0	2808.495
[R].ISDLTTN	2xTMTpro [K	4.156E-06	4.225E-05	1	1	2	P35749	P35749 [1008-10:	1	2703.5233
[R].ISDLTTN	1xTMTpro [K	6.523E-06	4.225E-05	1	1	3	P35749	P35749 [1008-10:	0	2200.184
[K].LSDLLAF	1xTMTpro [K	2.444E-05	4.225E-05	1	1	3	Q01518	Q01518 [101-113	0	2035.2295
[K].IDVTAPD	1xTMTpro [K	7.659E-06	4.225E-05	1	1	3	Q09666	Q09666 [785-800	0	2307.2575
[R].LSDGELI	1xCarbamido	2.26E-06	4.225E-05	1	1	1	Q13685	Q13685 [121-134	0	2184.1615
[K].LSDFNIID	1xTMTpro [N	1.217E-05	4.225E-05	1	1	1	Q13976	Q13976 [357-373	0	2085.13
[K].LSDDNTI	1xTMTpro [K	3.31E-06	4.225E-05	1	1	2	Q13813	Q13813 [1830-18	1	2354.2807
[R].LSDAGQ	1xCarbamido	2.216E-08	4.225E-05	1	1	3	P01833	P01833 [212-231]	1	3083.5884
[K].LSDAGIT	1xTMTpro [N	4.817E-05	4.225E-05	1	1	4	P12111	P12111 [2533-25	0	1707.9964
[K].LSCQPM	1xCarbamido	8.096E-07	4.225E-05	1	1	1	O75607	O75607 [103-123	0	2796.4384
[R].LSAEDL\	1xTMTpro [N	1.558E-05	4.225E-05	1	1	4	P11047	P11047 [590-603]	0	1746.9921
[R].EMIQLP(	1xTMTpro [K	5.6E-05	4.225E-05	1	1	1	P22570	P22570 [243-265]	0	3173.7888
[K].FITHAPP	1xTMTpro [N	3.722E-05	4.225E-05	1	1	3	P52907	P52907 [20-37]	0	2393.2209
[R].EMLTAD	1xTMTpro [N	6.032E-05	4.225E-05	1	1	1	Q96PZ0	Q96PZ0 [549-562	0	1954.9533
[R].LSCFAQ	1xCarbamido	3.048E-05	4.225E-05	1	1	3	Q16658	Q16658 [119-131	0	2046.1185
[K].LSCAYS(	1xCarbamido	3.213E-05	4.225E-05	1	1	1	Q9Y624	Q9Y624 [48-59]	0	1635.812
[R].LSCAAS(	1xCarbamido	6.1E-05	4.225E-05	1	1	2	P0DOX5	P0DOX5 [20-31]	0	1607.8171
[R].LSASSSI	1xTMTpro [N	3.236E-05	4.225E-05	1	1	1	Q9NP61	Q9NP61 [450-466	0	2100.078
[K].EMNPAL	1xCarbamido	8.34E-07	4.225E-05	1	1	2	P48643	P48643 [484-496]	0	2106.1331
[K].EMNPAL	1xCarbamido	6.142E-07	4.225E-05	1	1	1	P48643	P48643 [484-496]	0	2122.1281
[R].ISAFGYL	1xCarbamido	2.588E-05	4.225E-05	1	1	3	P08134	P08134 [151-162]	0	1954.06
[K].LDYDED.	1xTMTpro [K	3.075E-05	4.225E-05	1	2	4	P50502	P50502 [211-222]	0	1979.0287

[R].LSCQNL	1xCarbamido	2.053E-06	4.225E-05	1	1	1	Q14690	Q14690 [359-378]	0	2815.5308
[R].EMEENF	1xTMTpro [N	2.65E-06	4.225E-05	1	1	53	P08670	P08670 [346-364]	0	2491.173
[K].LQNTGV	1xTMTpro [N	3.259E-05	4.225E-05	1	1	3	Q8WX93	Q8WX93 [1240-1	0	1693.9193
[R].IQNSGD	1xTMTpro [K	5.618E-05	4.225E-05	1	1	2	P29350	P29350 [54-68]	0	2314.1847
[K].LQELNA	1xCarbamido	3.143E-06	4.225E-05	1	1	1	Q16513	Q16513 [100-120]	0	2725.3786
[R].IQEIIEQL	2xTMTpro [K	9.15E-08	4.225E-05	1	1	2	P10809	P10809 [371-389]	1	3207.7817
[R].IQEIIEQL	1xTMTpro [K	7.45E-06	4.225E-05	1	1	6	P10809	P10809 [371-387]	0	2646.437
[R].IQELGD	1xTMTpro [N	2.471E-05	4.225E-05	1	1	1	Q9NWS0	Q9NWS0 [187-20	0	1834.003
[K].LQELES	1xOxidation [	3.82E-05	4.225E-05	1	1	1	P11047	P11047 [1049-10	0	3214.5745
[R].EPLPPIQ	1xTMTpro [K	2.076E-06	4.225E-05	1	1	4	P13010	P13010 [503-525]	0	3261.795
[R].EPLPPIQ	1xOxidation [	1.98E-05	4.225E-05	1	1	2	P13010	P13010 [503-525]	0	3277.7899
[R].LEEEED	1xTMTpro [N	1.483E-05	4.225E-05	1	1	2	P35749	P35749 [931-945]	1	2134.0696
[R].EPIPSLE	1xTMTpro [K	2.3E-05	4.225E-05	1	1	1	Q15833	Q15833 [66-82]	0	2508.4457
[R].IQELENL	1xTMTpro [N	2.814E-05	4.225E-05	1	1	2	P51532	P51532 [382-397]	0	2029.1249
[K].EPISVSS	1xTMTpro [K	3.774E-05	4.225E-05	1	1	5	P00918	P00918 [213-224]	0	1924.1247
[R].EPLTADI	1xTMTpro [K	5.888E-05	4.225E-05	1	1	1	Q9NRL2	Q9NRL2 [768-784	1	2631.387
[R].LQELEG	1xTMTpro [N	3.927E-05	4.225E-05	1	1	1	P55268	P55268 [1752-17	0	1913.9412
[K].LQEIEGF	1xOxidation [	2.63E-05	4.225E-05	1	1	8	Q05707	Q05707 [991-100	0	2184.2077
[K].LQEIEGF	1xTMTpro [K	4.972E-06	4.225E-05	1	1	4	Q05707	Q05707 [991-100	0	2168.2128
[K].IQELAHE	1xTMTpro [K	1.77E-05	4.225E-05	1	1	1	O75381	O75381 [184-195]	0	1902.1304
[R].LQEKED	1xTMTpro [K	3.677E-05	4.225E-05	1	1	4	P02545	P02545 [29-41]	1	2238.2221
[K].IQEGVFI	1xTMTpro [K	1.491E-05	4.225E-05	1	1	2	P61019	P61019 [171-186]	0	2369.2956
[R].LQEGQT	1xTMTpro [K	1.319E-05	4.225E-05	1	1	1	P55268	P55268 [602-617]	0	2367.3779
[K].LQEESD	1xTMTpro [K	5.627E-05	4.225E-05	1	1	3	O75822	O75822 [123-133]	0	1883.0617
[R].EPNAEV	1xTMTpro [N	1.094E-05	4.225E-05	1	1	1	Q9NX40	Q9NX40 [9-32]	0	3059.5757
[K].LEEEDEI	1xCarbamido	1.399E-06	4.225E-05	1	1	4	P17655	P17655 [391-414]	0	3349.6125
[K].IQENALF	1xTMTpro [K	6.608E-06	4.225E-05	1	1	1	Q9UMR2	Q9UMR2 [118-14	0	3456.9016
[K].EPLGPAI	1xTMTpro [N	2.354E-05	4.225E-05	1	1	2	Q13228	Q13228 [449-460]	0	1606.9236
[R].IQEQES	1xTMTpro [N	2.206E-05	4.225E-05	1	2	1	P41236	P41236 [116-134]	0	2468.1232
[R].EPFFHG	1xTMTpro [N	5.701E-06	4.225E-05	1	1	1	P19784	P19784 [231-245]	0	2169.0684
[K].IQFHNVI	2xTMTpro [K	6.264E-06	4.225E-05	1	1	1	Q9BPW8	Q9BPW8 [74-97]	0	3702.0723
[K].LQFHNV	1xCarbamido	1.184E-05	4.225E-05	1	1	1	O75323	O75323 [76-91]	0	2902.6065
[R].EPFLSC	2xCarbamido	5.419E-05	4.225E-05	2	2	2	P0C0L4; P0	P0C0L4 [730-743]	0	2047.9901

[R].LQFHDV	1xCarbamido	2.475E-06	4.225E-05	1	1	3	P11413	P11413 [371-386]	0	2551.3371
[K].LQFGSQ	1xTMTpro [K	2.259E-05	4.225E-05	1	1	2	Q96ST3	Q96ST3 [135-152	0	2751.4671
[R].LEEALQ	1xCarbamido	7.409E-06	4.225E-05	1	1	1	Q8IVL6	Q8IVL6 [239-254]	0	2126.0541
[R].EPGCGC	5xCarbamido	1.034E-06	4.225E-05	1	1	1	P17936	P17936 [64-87]	0	3079.3371
[K].EPGEAD	1xTMTpro [N	3.279E-06	4.225E-05	1	1	1	Q9BTW9	Q9BTW9 [744-76	0	2789.4528
[R].LQEVPH	1xCarbamido	9.461E-08	4.225E-05	2	2	2	P62714; P67	P62714 [186-206]	0	2813.3194
[K].LQEEML	1xTMTpro [N	9.874E-06	4.225E-05	1	1	1	P08670	P08670 [189-217]	2	3724.852
[K].EPGEYA	1xCarbamido	1.394E-06	4.225E-05	1	1	1	O75369	O75369 [612-649	1	4931.361
[K].LQEVGQ	1xTMTpro [N	5.27E-05	4.225E-05	1	1	2	P06756	P06756 [339-351]	0	1747.0033
[R].LQEVFG	1xTMTpro [K	4.972E-05	4.225E-05	1	1	2	P54136	P54136 [80-89]	0	1750.0507
[K].IEEAMD	1xTMTpro [K	3.207E-06	4.225E-05	1	1	1	Q13435	Q13435 [771-790	0	2842.504
[R].EPGLES	1xTMTpro [K	4.882E-05	4.225E-05	1	1	1	Q6WCQ1	Q6WCQ1 [238-25	1	2489.2321
[K].LEEDINS	1xTMTpro [N	3.163E-07	4.225E-05	1	1	3	Q15365	Q15365 [79-101]	0	2793.4372
[K].FIPLSEP	1xTMTpro [N	5.046E-06	4.225E-05	1	1	1	Q99627	Q99627 [179-199	0	2617.4673
[K].LQETSN	1xCarbamido	9.548E-07	4.225E-05	1	1	3	P0C0L4	P0C0L4 [1100-11	0	3436.6763
[K].LEEDISS	1xTMTpro [N	8.081E-07	4.225E-05	1	1	1	Q15366	Q15366 [79-101]	0	2766.4263
[K].LQETEM	1xTMTpro [N	2.179E-05	4.225E-05	1	1	2	Q92896	Q92896 [866-882	0	2419.1514
[R].LQESCS	2xCarbamido	6.252E-06	4.225E-05	1	1	2	Q9Y6C2	Q9Y6C2 [298-313	0	2116.0486
[K].LQEVLD	1xTMTpro [K	2.295E-05	4.225E-05	1	1	1	Q8TBC4	Q8TBC4 [382-398	0	2561.414
[K].EPNPPID	1xTMTpro [N	4.947E-05	4.225E-05	1	1	1	P52294	P52294 [113-130]	0	2194.2039
[K].LEEEQII	1xCarbamido	4.801E-05	4.225E-05	1	1	2	P35579	P35579 [976-989]	0	2369.2514
[K].EPNSEN	1xTMTpro [K	5.081E-05	4.225E-05	1	1	1	P61026	P61026 [178-196]	0	2541.3077
[R].EPVGTC	1xCarbamido	2.593E-05	4.225E-05	1	1	1	P06756	P06756 [153-165]	0	2060.0978
[R].LQDLSS	1xCarbamido	1.595E-05	4.225E-05	1	1	1	P29590	P29590 [383-394]	0	1958.0872
[R].LQDISEL	1xTMTpro [N	5.189E-08	4.225E-05	1	1	1	Q9NQ55	Q9NQ55 [224-260	0	4108.0641
[R].EPVQTL	1xTMTpro [K	1.344E-05	4.225E-05	1	1	1	Q9UHY1	Q9UHY1 [376-392	0	2526.3947
[R].EPVQTL	1xTMTpro [K	1.378E-06	4.225E-05	1	1	1	Q9UHY1	Q9UHY1 [376-398	1	3285.7862
[R].IQDLLAE	1xTMTpro [N	3.525E-06	4.225E-05	1	1	2	Q96HY6	Q96HY6 [249-265	0	2133.1722
[K].FINQLLG	1xTMTpro [K	6.195E-06	4.225E-05	1	1	2	Q14CX7	Q14CX7 [384-401	0	2579.494
[K].IQDKEGI	1xTMTpro [K	5.993E-05	4.225E-05	2	4	10	P62987; P62	P62987 [30-42]; F	1	2132.1955
[K].EQAADS	1xTMTpro [K	4.404E-05	4.225E-05	1	1	1	Q75QN2	Q75QN2 [193-207	0	2179.283
[K].LQDFSD	1xTMTpro [K	3.069E-05	4.225E-05	1	1	2	P04114	P04114 [4499-451	0	2359.1949
[K].EPVGEG	1xTMTpro [N	1.339E-05	4.225E-05	1	1	1	Q96FV2	Q96FV2 [104-119	0	2003.0802

[K].LQDFAS	1xTMTpro [K	1.433E-06	4.225E-05	1	1	9	P35749	P35749 [1380-139	0	2245.2208
[K].EQADFA	1xTMTpro [K	4.002E-05	4.225E-05	2	2	2	P35580; P35	P35580 [415-426]	0	1900.0672
[R].EQADYC	1xCarbamido	1.561E-05	4.225E-05	1	1	2	Q13813	Q13813 [2436-24	0	2939.5212
[R].LQDEIQ	1xTMTpro [K	2.7E-06	4.225E-05	1	1	4	P08670	P08670 [365-378]	1	2343.2292
[R].LQDDSV	1xTMTpro [K	1.135E-05	4.225E-05	1	1	1	P49356	P49356 [35-50]	0	2371.2848
[K].LQDAYY	1xTMTpro [K	4.865E-05	4.225E-05	1	1	1	O14579	O14579 [198-211	0	2343.2186
[R].LQDAFS	1xTMTpro [K	7.287E-06	4.225E-05	1	1	1	Q05193	Q05193 [16-44]	0	3491.899
[K].EQAGEA	1xTMTpro [N	3.914E-07	4.225E-05	1	1	1	O14617	O14617 [538-556	0	2307.157
[R].EQAGGI	1xTMTpro [N	2.325E-07	4.225E-05	1	1	1	P00167	P00167 [53-73]	0	2510.1351
[K].EQAHNL	1xTMTpro [K	6.051E-05	4.225E-05	1	1	1	Q9BZF9	Q9BZF9 [731-741	0	1922.0661
[R].IQCSVS	1xCarbamido	1.002E-05	4.225E-05	1	1	1	P13727	P13727 [145-162]	0	2277.2093
[K].EQADFA	1xTMTpro [K	3.113E-05	4.225E-05	2	2	15	P35579; Q7	P35579 [408-419]	0	1914.0828
[K].EPFFHG	1xTMTpro [N	5.149E-05	4.225E-05	1	1	1	P68400	P68400 [230-244]	0	2178.0688
[K].EPTQAV	1xTMTpro [K	3.432E-08	4.225E-05	1	1	2	O43491	O43491 [101-123	1	3300.7455
[R].LQDPVL	3xCarbamido	2.26E-06	4.225E-05	1	1	2	P08514	P08514 [903-928]	0	3295.575
[K].LQEEML	1xTMTpro [N	3.201E-06	4.225E-05	1	1	4	P08670	P08670 [189-207]	1	2655.3367
[R].EPNVLIC	1xCarbamido	5.974E-05	4.225E-05	1	1	3	P01903	P01903 [126-136]	0	1956.112
[R].LQEELS	1xTMTpro [K	9.57E-06	4.225E-05	1	1	1	Q14203	Q14203 [412-427	0	2441.3267
[K].LQEEIQL	1xTMTpro [K	5.875E-07	4.225E-05	1	1	4	P17661	P17661 [194-212]	1	2853.5602
[K].LEEGEVI	1xTMTpro [N	4.651E-07	4.225E-05	1	1	3	Q8IYM9	Q8IYM9 [205-227	0	2829.4913
[R].LQEDKE	1xTMTpro [K	2.354E-05	4.225E-05	1	1	1	Q15149	Q15149 [2483-25	1	3115.5974
[K].EPPLLLG	1xTMTpro [K	5.357E-05	4.225E-05	1	1	1	Q9HDC9	Q9HDC9 [74-87]	0	2136.3036
[K].EPPPEFI	1xTMTpro [K	1.023E-05	4.225E-05	1	1	3	Q15459	Q15459 [145-168	0	3267.7321
[K].EPPPGM	1xTMTpro [K	5.393E-05	4.225E-05	1	1	1	Q9H832	Q9H832 [114-130	0	2468.3061
[R].EPPPVY	1xTMTpro [K	1.691E-05	4.225E-05	1	1	1	A8MXV4	A8MXV4 [223-25	0	3917.9981
[K].EPTPSIA	1xTMTpro [N	8.035E-06	4.225E-05	1	1	3	A0FGR8	A0FGR8 [751-77	0	2442.3411
[K].EPPTDV	1xTMTpro [N	1.589E-05	4.225E-05	1	1	1	P49915	P49915 [570-589]	0	2449.3509
[R].LQEALD	1xCarbamido	1.654E-05	4.225E-05	1	1	1	Q1KMD3	Q1KMD3 [33-58]	0	3111.5218
[R].EPQATV	1xTMTpro [N	6.984E-06	4.225E-05	1	1	1	O15230	O15230 [1282-12	0	2157.1987
[R].IQEADNI	1xTMTpro [N	5.236E-06	4.225E-05	1	1	1	P55287	P55287 [725-748]	0	3016.4495
[R].LQEAAE	1xTMTpro [N	1.268E-06	4.225E-05	1	1	5	P02730	P02730 [247-263]	0	2181.245
[R].EPQVYT	1xTMTpro [K	5.563E-05	4.225E-05	1	1	5	P01861	P01861 [225-240]	0	2485.314
[R].EPQVYT	1xTMTpro [K	1.847E-06	4.225E-05	1	2	30	P0DOX5	P0DOX5 [347-36	1	2481.3845

[R].EPQVYT	1xTMTpro [K	1.475E-05	4.225E-05	2	2	19	P01859; P01	P01859 [224-239]	1	2513.3565
[K].IQDVGL\	1xOxidation [	5.331E-05	4.225E-05	1	1	3	P52815	P52815 [92-125]	0	3958.1161
[K].IQDVGL\	1xTMTpro [K	3.231E-06	4.225E-05	1	1	4	P52815	P52815 [92-125]	0	3942.1212
[R].LQDRVD	1xTMTpro [N	1.569E-05	4.225E-05	1	1	1	Q9Y6C2	Q9Y6C2 [542-559	1	2382.222
[R].IEEGVP(	1xTMTpro [K	3.185E-05	4.225E-05	1	1	5	P12111	P12111 [1332-134	0	2324.3721
[K].IQNTGD\	1xTMTpro [K	4.673E-05	4.225E-05	1	1	1	Q06124	Q06124 [56-70]	0	2344.1953
[R].EPEVLST	1xTMTpro [K	1.279E-05	4.225E-05	1	1	3	O14980	O14980 [797-810]	0	2152.2543
[R].LQFIADH	1xCarbamido	4.65E-05	4.225E-05	1	1	1	Q13098	Q13098 [60-71]	0	1774.9593
[R].ENSASQ	1xTMTpro [K	9.09E-06	4.225E-05	2	2	1	Q13948; P39	Q13948 [329-345]	0	2469.3441
[K].ENSEEL(	1xTMTpro [N	3.893E-05	4.225E-05	1	1	1	Q0VF96	Q0VF96 [96-115]	0	2615.3272
[K].ENSETV'	1xTMTpro [K	1.808E-05	4.225E-05	1	1	1	Q9GZS3	Q9GZS3 [30-45]	0	2314.2633
[R].IEDGNNI	1xTMTpro [K	4.009E-05	4.225E-05	1	1	3	Q06323	Q06323 [142-155]	0	2128.153
[R].ENSIEILS	2xTMTpro [K	1.277E-05	4.225E-05	1	1	2	Q9NWV4	Q9NWV4 [73-95]	0	3567.8999
[K].IQIVSQM	1xTMTpro [N	2.64E-05	4.225E-05	1	1	1	Q9UK53	Q9UK53 [232-245	0	1962.1013
[K].LEDGTEI	1xTMTpro [K	9.259E-06	4.225E-05	1	1	3	P26885	P26885 [60-87]	0	3661.9245
[K].LQIVEMF	1xTMTpro [K	8.99E-06	4.225E-05	1	1	4	P50454	P50454 [253-263]	0	1887.1381
[R].IQLVEEE	1xTMTpro [N	2.607E-06	4.225E-05	4	4	1	P67936; P09	P67936 [56-69]; F	1	2032.0994
[K].LQISHEA	1xCarbamido	5.586E-06	4.225E-05	1	1	3	Q08211	Q08211 [1090-11(	0	1944.0656
[R].ENTEFTI	1xTMTpro [K	2.677E-05	4.225E-05	1	1	3	P37235	P37235 [18-32]	0	2577.2753
[R].ENTEGE	1xTMTpro [K	2.732E-07	4.225E-05	1	1	1	P50213	P50213 [147-169]	0	3110.6501
[R].LQIRPAD	1xTMTpro [N	4.744E-05	4.225E-05	1	1	1	Q96FN9	Q96FN9 [22-40]	0	2455.3377
[K].LQLQEQ	1xCarbamido	1.268E-06	4.225E-05	1	1	26	P35579	P35579 [883-903]	0	2805.426
[K].ENTLNQ	1xTMTpro [N	3.037E-05	4.225E-05	1	1	3	Q02252	Q02252 [299-316]	0	2121.1372
[R].IEDGVP(	1xTMTpro [K	6.933E-06	4.225E-05	1	1	5	P12111	P12111 [1536-155	0	2282.3728
[K].LQLNIVE	1xTMTpro [K	1.797E-07	4.225E-05	1	1	2	O94760	O94760 [112-136]	1	3356.8016
[R].FLQNSL(	2xCarbamido	2.665E-06	4.225E-05	1	1	1	P32929	P32929 [238-257]	0	2628.3233
[K].ENVDHI(	1xTMTpro [N	2.427E-05	4.225E-05	1	1	1	Q86VW0	Q86VW0 [463-478	0	2218.0916
[K].IEDLGAA	1xTMTpro [K	3.846E-05	4.225E-05	1	1	1	Q13813	Q13813 [2265-228	0	2453.3453
[K].LEDILES	1xTMTpro [K	3.626E-05	4.225E-05	1	1	2	Q9Y3A6	Q9Y3A6 [153-164	0	1982.1665
[K].LQMEAG	1xCarbamido	1.83E-06	4.225E-05	1	1	1	Q15149	Q15149 [538-561]	0	3035.5097
[K].LQMEAP	1xTMTpro [N	4.027E-06	4.225E-05	1	1	2	P60842	P60842 [147-161]	0	1923.0805
[K].LQMEAP	1xOxidation [	5.813E-05	4.225E-05	1	1	1	P60842	P60842 [147-161]	0	1939.0754
[K].IEDGNDI	1xTMTpro [K	4.344E-06	4.225E-05	1	1	2	Q9UL46	Q9UL46 [132-145	0	2143.1527

[R].ENNAVY	1xTMTpro [K	1.142E-05	4.225E-05	1	1	2	O75368	O75368 [87-104]	0	2457.3633
[K].LQNQLQ	1xTMTpro [K	2.279E-05	4.225E-05	1	1	2	O75351	O75351 [116-132	0	2528.5168
[K].ENNQVP	1xTMTpro [K	3.005E-05	4.225E-05	1	1	1	P14543	P14543 [211-231]	0	2885.5805
[R].LQNNEN	1xCarbamido	5.278E-05	4.225E-05	2	2	1	P36980; Q0:	P36980 [120-132]	0	1893.9408
[R].IQNLDQ:	2xCarbamido	2.094E-05	4.225E-05	1	1	1	P98160	P98160 [3453-34	0	3169.5803
[K].LQNLDAI	1xOxidation	9.413E-06	4.225E-05	1	1	1	Q15435	Q15435 [223-241	0	2451.3196
[K].ENPGFD	1xTMTpro [K	6.794E-06	4.225E-05	1	1	1	Q8WVJ2	Q8WVJ2 [130-14	0	2541.2753
[K].ENPGLT	1xOxidation	4.752E-05	4.225E-05	1	1	1	Q9BUL8	Q9BUL8 [54-65]	0	1983.1076
[K].LQNEVE	1xOxidation	5.936E-05	4.225E-05	1	1	4	P35749	P35749 [1285-13	0	2572.342
[K].LQNEVE	1xTMTpro [K	1.581E-06	4.225E-05	1	1	5	P35749	P35749 [1285-13	0	2556.3471
[R].ENVEYIE	1xTMTpro [N	3.967E-05	4.225E-05	1	1	1	O95218	O95218 [110-129	1	2769.2447
[K].LQNELDI	2xTMTpro [K	6.398E-08	4.225E-05	1	1	3	P35580	P35580 [1285-13	1	2985.6924
[K].ENPQLLI	1xTMTpro [K	3.477E-05	4.225E-05	1	1	1	Q7Z4H8	Q7Z4H8 [331-349	0	2825.5006
[K].ENPSATI	1xTMTpro [K	1.229E-06	4.225E-05	1	1	1	Q92900	Q92900 [271-295	0	3423.8251
[R].ENPSEE	1xTMTpro [N	1.783E-06	4.225E-05	1	1	1	Q12874	Q12874 [118-138	0	2717.2498
[K].ENPVLD	1xTMTpro [N	1.013E-05	4.225E-05	1	1	1	Q12888	Q12888 [55-73]	0	2387.201
[K].LQNELDI	1xTMTpro [K	1.667E-05	4.225E-05	1	1	4	P35580	P35580 [1285-13	0	2553.3903
[K].LQNDPQ	1xTMTpro [N	3.768E-06	4.225E-05	1	1	1	O60437	O60437 [1060-10	0	2786.3665
[R].FLQTED:	1xTMTpro [N	3.208E-05	4.225E-05	1	1	1	Q8N5L8	Q8N5L8 [89-111]	0	2848.4688
[R].ENQELN	1xTMTpro [N	5.504E-06	4.225E-05	1	1	1	Q6WCQ1	Q6WCQ1 [854-86	0	2127.0498
[K].FIQQTYF	1xCarbamido	1.265E-05	4.225E-05	1	1	2	Q8WUM4	Q8WUM4 [24-41]	0	2466.1679
[K].IQMLDD	1xTMTpro [N	6.206E-06	4.225E-05	1	1	4	Q9Y4F1	Q9Y4F1 [44-59]	0	2224.1239
[R].ENPPVE	1xTMTpro [K	2.973E-06	4.225E-05	1	1	1	Q8TCJ2	Q8TCJ2 [491-505	1	2340.1447
[R].IQIFPVD	1xTMTpro [K	1.456E-05	4.225E-05	1	1	1	Q6ZMI0	Q6ZMI0 [281-299	0	2707.5526
[R].LQLFGQ	1xTMTpro [N	5.701E-05	4.225E-05	1	1	2	Q92619	Q92619 [756-768	0	1793.9618
[R].LQLEYVI	1xTMTpro [N	3.719E-06	4.225E-05	1	1	1	Q13303	Q13303 [147-171	0	3236.6581
[R].EPAVGE	1xTMTpro [N	1.855E-05	4.225E-05	1	1	3	P05362	P05362 [160-176]	0	2072.1559
[R].LEDSSN	1xTMTpro [K	5.183E-05	4.225E-05	1	1	3	P46939	P46939 [628-641]	0	2098.1636
[K].EPCGGL	1xCarbamido	1.184E-05	4.225E-05	1	1	2	P12109	P12109 [167-180]	0	2097.0778
[R].IEDSTGI	1xTMTpro [N	1.679E-05	4.225E-05	1	1	1	Q9BQ70	Q9BQ70 [157-17	0	2171.174
[K].EPCLHPI	1xCarbamido	8.445E-06	4.225E-05	1	1	1	Q9UIQ6	Q9UIQ6 [33-49]	0	2413.1665
[K].LQGSGV	1xTMTpro [K	4.713E-05	4.225E-05	1	1	2	Q09666	Q09666 [5848-58	0	1654.9983
[K].FLQDTIE	1xTMTpro [K	1.818E-05	4.225E-05	1	1	2	O95202	O95202 [263-274	0	2046.1437

[K].EPCVES	1xCarbamid	1.731E-06	4.225E-05	1	1	6	P02652	P02652 [27-46]	0	2958.505
[K].LEDTEN	1xTMTpro [K	1.425E-05	4.225E-05	1	1	3	P34932	P34932 [652-668]	0	2689.3125
[R].IQGLTVE	1xTMTpro [N	6.082E-06	4.225E-05	1	1	4	Q14204	Q14204 [3924-39	0	1817.0452
[K].IEDPNQI	1xTMTpro [K	2.973E-05	4.225E-05	1	1	2	Q9UEY8	Q9UEY8 [485-50	0	2918.5755
[R].LQGINC	1xCarbamid	1.186E-05	4.225E-05	1	1	2	Q04637	Q04637 [657-675	0	2368.2039
[R].IQGGYE	1xTMTpro [N	5.266E-07	4.225E-05	1	1	2	Q02809	Q02809 [589-610	0	2835.4419
[K].IEDVGSI	2xTMTpro [K	2.009E-05	4.225E-05	1	2	1	P08238	P08238 [250-265]	1	2650.3512
[R].LQGEVV	1xTMTpro [K	5.331E-05	4.225E-05	1	1	1	Q3MHD2	Q3MHD2 [25-37]	0	2092.157
[K].EPEAFD	1xCarbamid	6.657E-06	4.225E-05	1	1	3	P40261	P40261 [101-121]	0	2787.3255
[K].IEDVGSI	3xTMTpro [K	9.82E-06	4.225E-05	1	2	1	P08238	P08238 [250-266]	2	3082.6533
[R].LQGDAN	1xTMTpro [N	9.46E-07	4.225E-05	1	1	1	P52434	P52434 [125-140]	0	2076.043
[K].LQGAVC	1xCarbamid	2.098E-05	4.225E-05	1	1	2	Q9UBG0	Q9UBG0 [1238-1	0	1883.0128
[K].EPEKEIF	2xTMTpro [K	5.366E-05	4.225E-05	1	1	6	P50395	P50395 [361-379]	1	3173.8602
[K].EPELFQ	1xTMTpro [N	5.509E-05	4.225E-05	1	1	4	Q9H4M9	Q9H4M9 [14-26]	0	1792.9764
[R].IQFVGA	1xCarbamid	1.123E-05	4.225E-05	1	1	2	Q14204	Q14204 [2706-27	0	1932.9921
[K].LEDVADI	1xCarbamid	3.023E-06	4.225E-05	1	1	1	Q9ULC5	Q9ULC5 [476-49	0	3068.52
[K].EPESILQ	1xTMTpro [K	2.09E-05	4.225E-05	1	1	1	P17174	P17174 [277-290]	0	2239.2499
[R].LEDMEQ	1xTMTpro [K	3.947E-05	4.225E-05	1	1	3	P05155	P05155 [367-380]	0	2202.1972
[K].LQHLENI	1xTMTpro [K	1.085E-06	4.225E-05	1	1	72	P01009	P01009 [284-298]	0	2412.3742
[K].LQLEYC	2xCarbamid	1.501E-06	4.225E-05	1	1	3	P13489	P13489 [147-167]	0	2671.3754
[R].ENVIMS	1xCarbamid	4.618E-05	4.225E-05	1	1	2	P30153	P30153 [319-331]	0	2153.2318
[R].ENVLVE	1xTMTpro [K	1.83E-06	4.225E-05	1	1	2	P27338	P27338 [243-258]	0	2527.3358
[R].LEDLLQI	2xTMTpro [K	8.899E-07	4.225E-05	1	1	2	Q15149	Q15149 [897-915	1	3233.7358
[K].LQLEDD	1xTMTpro [K	1.208E-05	4.225E-05	1	1	1	P02549	P02549 [1922-19	0	2554.311
[K].LQLDSPI	1xTMTpro [K	2.242E-05	4.225E-05	1	1	1	O43242	O43242 [426-440	0	2283.2728
[R].ENVPEN	1xTMTpro [N	4.677E-07	4.225E-05	1	1	4	O75368	O75368 [51-76]	0	3307.6667
[R].LEDLSES	1xOxidation	2.973E-05	4.225E-05	1	1	1	P49755	P49755 [154-169]	0	2498.298
[K].IQIASES	1xCarbamid	9.929E-06	4.225E-05	1	1	1	Q96I24	Q96I24 [111-138]	0	3605.934
[K].LQIASDE	2xTMTpro [K	9.784E-06	4.225E-05	1	1	3	Q13813	Q13813 [64-81]	1	2946.6353
[R].EPAPCA	1xCarbamid	4.409E-06	4.225E-05	1	1	2	Q9BTY7	Q9BTY7 [134-157	0	2686.35
[K].ENYAELI	1xTMTpro [K	1.636E-05	4.225E-05	1	1	7	P12109	P12109 [791-803]	0	2163.1829
[R].FLQESN	1xTMTpro [N	9.174E-06	4.225E-05	1	1	1	P40763	P40763 [71-84]	0	2065.115
[K].IQKLESD	1xCarbamid	5.719E-05	4.225E-05	1	1	1	P02675	P02675 [209-224]	1	2565.3297

[R].LQKEEE	1xTMTpro [K	3.27E-05	4.225E-05	1	1	1	P41252	P41252 [670-686]	1	2762.4856
[R].EPAAEIE	1xTMTpro [N	7.859E-06	4.225E-05	1	1	2	Q12765	Q12765 [99-114]	0	2031.1115
[R].LQHVED	1xOxidation	4.273E-06	4.225E-05	1	1	1	Q07065	Q07065 [413-429]	0	2160.1402
[R].LQHVED	1xTMTpro [N	2.407E-06	4.225E-05	1	1	3	Q07065	Q07065 [413-429]	0	2144.1453
[K].IEDLSQ(	1xTMTpro [K	2.007E-06	4.225E-05	1	2	6	Q13765	Q13765 [128-142]	0	2223.2476
[K].EPAFEDI	1xTMTpro [N	2.663E-05	4.225E-05	1	1	2	O75400	O75400 [777-789]	0	1839.9295
[K].IQHILCT	2xCarbamido	5.287E-05	4.225E-05	1	1	2	Q9UBQ0	Q9UBQ0 [31-43]	0	2166.2019
[K].EPALNE	1xTMTpro [K	5.491E-05	4.225E-05	1	1	1	Q06323	Q06323 [46-58]	0	2021.1523
[R].FIQFEPE	1xTMTpro [K	5.572E-05	4.225E-05	1	1	1	Q9NUQ7	Q9NUQ7 [87-98]	1	2159.1992
[R].LDTDILL	1xCarbamido	4.135E-05	4.225E-05	1	1	1	A6NDG6	A6NDG6 [262-275	0	2098.2074
[K].ISGGSV	1xTMTpro [N	2.641E-06	4.225E-05	1	1	4	O75874	O75874 [5-20]	0	1999.9748
[R].ISGGYEI	1xTMTpro [K	3.613E-05	4.225E-05	1	1	2	O00469	O00469 [599-614]	0	2384.2412
[K].ITGEAFV	1xTMTpro [K	4.401E-06	4.225E-05	1	1	3	P52597	P52597 [151-167]	0	2476.3579
[R].ELQAGT	1xTMTpro [N	1.259E-07	4.225E-05	1	1	1	P46952	P46952 [193-223]	0	3591.8138
[K].ELQAHG	1xTMTpro [K	2.466E-05	4.225E-05	1	1	3	O15144	O15144 [66-77]	0	1932.1046
[K].ELQAHG	1xTMTpro [K	3.465E-05	4.225E-05	1	1	2	O15144	O15144 [66-78]	1	2088.2057
[K].LTGDVEI	2xTMTpro [K	2.159E-05	4.225E-05	1	1	3	Q9UKF6	Q9UKF6 [533-549	0	2824.6488
[R].LTGADG	1xTMTpro [K	4.177E-05	4.225E-05	1	1	1	Q02978	Q02978 [109-122]	0	1995.177
[R].ELQAQI	1xTMTpro [K	1.008E-05	4.225E-05	1	1	3	P35580	P35580 [1115-113	0	2615.3696
[R].ELQAQL	1xTMTpro [K	2.399E-07	4.225E-05	1	1	1	P17661	P17661 [249-275]	0	3707.9592
[R].ITFTGEA	1xTMTpro [K	1.484E-06	4.225E-05	1	1	2	O60884	O60884 [231-255]	0	3248.791
[K].LTFSCL(	1xCarbamido	5.628E-06	4.225E-05	1	1	2	Q15185	Q15185 [36-48]	0	2054.0872
[K].ITFHGE(	1xTMTpro [K	2.194E-06	4.225E-05	1	1	2	P31689	P31689 [222-246]	0	3328.792
[K].LTFDTTF	2xTMTpro [K	1.438E-05	4.225E-05	1	1	2	P45880	P45880 [108-121]	1	2469.4169
[K].LTFDTTF	1xTMTpro [K	4.323E-05	4.225E-05	1	1	3	P45880	P45880 [108-120]	0	2037.1148
[K].LTFDSSI	2xTMTpro [K	4.936E-06	4.225E-05	1	1	4	P21796	P21796 [97-110]	1	2441.3856
[KR].ELQDQ	1xTMTpro [K	1.7E-05	4.225E-05	2	2	2	O75116; Q1	O75116 [861-877]	0	2713.4216
[K].LTFDSSI	1xTMTpro [K	5.098E-05	4.225E-05	1	1	2	P21796	P21796 [97-109]	0	2009.0835
[K].ITFDDYI	2xCarbamido	3E-05	4.225E-05	1	1	2	P30626	P30626 [154-165]	0	2113.0954
[R].ELQEGT	1xCarbamido	9.548E-07	4.225E-05	1	1	3	P00491	P00491 [186-207]	0	2777.3445
[R].ELQEGT	1xCarbamido	4.592E-06	4.225E-05	1	1	1	P00491	P00491 [186-207]	0	2793.3395
[R].ITFDAFP	2xTMTpro [K	1.822E-05	4.225E-05	1	1	2	Q12904	Q12904 [251-267]	1	2830.5807
[K].ELQELNI	2xTMTpro [K	6.731E-06	4.225E-05	1	1	2	Q8WU90	Q8WU90 [77-93]	0	2868.7015

[K].LTGEDVI	1xTMTpro [K	5.566E-06	4.225E-05	1	1	2	Q9Y2Z4	Q9Y2Z4 [261-281	0	2728.5628
[R].LTGEEVI	1xCarbamido	1.502E-05	4.225E-05	1	1	1	P49589	P49589 [174-191]	0	2627.4094
[K].FNPETD	1xTMTpro [K	7.301E-06	4.225E-05	1	1	2	Q99798	Q99798 [507-520	0	2166.121
[K].FNPETD	2xTMTpro [K	2.535E-06	4.225E-05	1	1	2	Q99798	Q99798 [507-521	1	2598.4231
[K].ITINPGC	1xCarbamido	9.157E-06	4.225E-05	1	1	1	P78347	P78347 [897-916]	0	2695.4807
[R].FNQMLN	1xTMTpro [N	2.462E-05	4.225E-05	1	1	2	Q99715	Q99715 [2923-29	0	2259.1147
[K].LTLHGL(	1xTMTpro [K	3.219E-05	4.225E-05	2	2	4	O00148; Q1	O00148 [256-267	0	2071.2196
[K].ELPPHL(	1xTMTpro [N	1.878E-06	4.225E-05	1	1	1	P22105	P22105 [3447-34	0	2469.3156
[R].LTIGEG(	1xTMTpro [K	7.328E-06	4.225E-05	1	1	3	P02679	P02679 [418-432]	0	2154.2275
[K].ELPQFA	1xTMTpro [N	2.916E-05	4.225E-05	1	1	2	Q9BZZ5	Q9BZZ5 [85-97]	0	1775.9611
[R].LTIFDEE	1xTMTpro [N	2.711E-05	4.225E-05	1	1	2	Q53T59	Q53T59 [228-246	0	2410.2097
[K].ITLETDE	1xTMTpro [N	4.541E-05	4.225E-05	1	1	2	Q15393	Q15393 [313-326	0	1969.0119
[K].ITLEGPT	1xTMTpro [K	5.227E-06	4.225E-05	1	1	1	Q00341	Q00341 [408-429	0	3008.6106
[R].ITLEDVIS	1xTMTpro [K	1.488E-05	4.225E-05	1	1	1	Q13576	Q13576 [840-850	0	1850.0879
[R].ELQELQ	1xTMTpro [N	6.061E-05	4.225E-05	1	1	2	P12270	P12270 [1234-12	0	1848.9622
[K].FNPNAI(	1xTMTpro [K	4.556E-05	4.225E-05	1	1	1	O75339	O75339 [842-856	0	2280.2996
[K].ITKPGSII	1xTMTpro [K	7.164E-07	4.225E-05	1	1	1	Q04637	Q04637 [1072-10	0	2580.439
[K].LTKEEIV	2xTMTpro [K	1.145E-06	4.225E-05	1	1	2	O43852	O43852 [285-311	2	3956.1837
[K].LTKEEILI	2xTMTpro [K	1.702E-06	4.225E-05	1	1	1	Q15293	Q15293 [300-326	1	4043.1252
[K].ITHSPLT	1xCarbamido	1.009E-05	4.225E-05	1	1	2	P04899	P04899 [279-296]	0	2657.4253
[K].ITHPVYS	1xTMTpro [K	1.862E-05	4.225E-05	1	1	1	Q9UGP8	Q9UGP8 [634-64	0	2331.288
[R].ELPTAFI	1xTMTpro [N	2.973E-05	4.225E-05	1	1	4	Q13813	Q13813 [2455-24	0	1891.9761
[K].ITGTMPI	1xTMTpro [N	1.338E-06	4.225E-05	1	1	1	P08571	P08571 [128-148]	0	2442.3961
[R].LTGSYN	1xTMTpro [K	6.252E-06	4.225E-05	1	1	1	Q9Y512	Q9Y512 [128-148	0	2793.4771
[R].ELPVLW	1xCarbamido	2.418E-05	4.225E-05	1	1	2	Q13895	Q13895 [373-388	0	2309.3123
[R].LTGPGL	1xTMTpro [N	4.352E-06	4.225E-05	1	1	1	Q8WU39	Q8WU39 [113-13	0	2926.5304
[K].FNPLNTI	1xTMTpro [N	6.037E-06	4.225E-05	1	1	1	Q92466	Q92466 [164-182	0	2482.1992
[K].LTEVPVE	1xTMTpro [K	4.721E-06	4.225E-05	1	1	2	Q9H307	Q9H307 [537-553	0	2482.4413
[R].ELQELV(	1xTMTpro [K	1.475E-05	4.225E-05	1	1	4	P55072	P55072 [488-502]	0	2432.3317
[K].ELQEMD	2xTMTpro [K	5.107E-05	4.225E-05	1	1	2	P52566	P52566 [34-47]	1	2605.4211
[R].ITDPQYI	1xTMTpro [K	3.405E-05	4.225E-05	1	1	2	Q5T013	Q5T013 [156-175	0	2869.5955
[R].ELQQAV	1xTMTpro [N	2.64E-05	4.225E-05	1	1	3	Q02818	Q02818 [388-399	0	1785.9601
[R].LTDPIPT	1xTMTpro [N	3.747E-05	4.225E-05	1	1	2	Q2M2I8	Q2M2I8 [353-367	0	1916.066

[R].ITDNMF(	1xCarbamido	1.773E-06	4.225E-05	1	1	1	P00734	P00734 [544-560]	1	2914.5371
[K].LTDISVT	2xTMTpro [K	4.3E-05	4.225E-05	1	1	1	Q9Y333	Q9Y333 [40-58]	1	3085.7424
[K].ELQREP	1xTMTpro [N	4.142E-05	4.225E-05	1	1	1	Q6FI81	Q6FI81 [129-144]	1	2214.2049
[K].EIQSAFV	1xTMTpro [K	3.733E-06	4.225E-05	1	1	1	Q5VYK3	Q5VYK3 [985-100	0	3103.6291
[K].ITDLANL	1xTMTpro [K	9.874E-06	4.225E-05	1	2	1	A0A0B4J2D5	A0A0B4J2D5 [117	0	3050.6555
[K].LTDFGF(	1xCarbamido	2.529E-05	4.225E-05	2	3	2	Q13153; Q1	Q13153 [405-420]	0	2450.2881
[R].ELQSQIS	1xTMTpro [N	6.339E-07	4.225E-05	1	1	3	P05787	P05787 [234-252]	0	2413.22
[K].FNHPEIV	1xTMTpro [N	1.129E-05	4.225E-05	1	1	2	Q7Z4H3	Q7Z4H3 [175-191	0	2314.2362
[R].ELQSQIS	1xOxidation [	8.005E-06	4.225E-05	1	1	1	P05787	P05787 [234-252]	0	2429.2149
[K].LDINLLD	1xCarbamido	3.786E-08	4.225E-05	1	1	2	O14980	O14980 [23-44]	0	2845.4586
[R].ITCTEEG	1xCarbamido	1.561E-05	4.225E-05	1	2	2	Q03591	Q03591 [70-82]	0	2114.1084
[K].EIQTTTG	1xTMTpro [N	5.366E-05	4.225E-05	1	1	4	Q8TC12	Q8TC12 [84-97]	0	1891.0568
[K].ELQTVLS	1xTMTpro [K	5.617E-06	4.225E-05	1	1	1	Q14155	Q14155 [289-306]	0	2714.5584
[R].ELQVGIF	1xTMTpro [N	3.453E-05	4.225E-05	1	1	1	Q9BWM7	Q9BWM7 [199-21	0	1886.0303
[R].ELQVLD(	1xTMTpro [K	6.633E-06	4.225E-05	1	1	2	Q9NV70	Q9NV70 [195-212	0	2554.4042
[R].IDINMSG	1xTMTpro [K	7.164E-07	4.225E-05	1	1	3	P13797	P13797 [321-336]	1	2476.2997
[K].IDLPAEN	1xTMTpro [K	8.617E-06	4.225E-05	1	1	2	Q00341	Q00341 [615-632]	0	2523.4162
[K].LTAVESI	2xTMTpro [K	3.293E-05	4.225E-05	1	1	1	Q5KU26	Q5KU26 [83-92]	1	2016.2521
[K].EIRDEYV	1xTMTpro [K	4.657E-05	4.225E-05	1	1	1	Q8N1B4	Q8N1B4 [283-294	1	2090.1625
[K].FNHDGE	1xTMTpro [N	1.3E-05	4.225E-05	1	1	1	Q6PJT7	Q6PJT7 [308-324	0	2346.9554
[K].ITLPVDF	1xTMTpro [K	2.844E-05	4.225E-05	1	1	3	P00558	P00558 [280-291]	0	1927.1396
[K].LDLITVE	1xTMTpro [K	1.589E-06	4.225E-05	1	1	1	O95456	O95456 [242-257]	0	2424.4722
[K].ITDYPCS	1xCarbamido	3.276E-08	4.225E-05	1	1	2	O14786	O14786 [426-455]	0	3493.6568
[K].LTEVLTD	1xTMTpro [K	2.179E-05	4.225E-05	1	1	1	Q92616	Q92616 [1543-15	0	1850.0879
[R].LDLFEIS	1xTMTpro [N	5.098E-05	4.225E-05	1	1	2	P55196	P55196 [25-44]	0	2680.3612
[K].EIQEPLE	1xTMTpro [N	2.083E-05	4.225E-05	1	1	1	P48681	P48681 [866-882]	0	2351.205
[R].LTESVDY	1xTMTpro [N	2.631E-06	4.225E-05	1	1	2	O43776	O43776 [458-478]	0	2507.3169
[R].ELQETN.	1xTMTpro [N	4.964E-05	4.225E-05	1	1	2	P49747	P49747 [37-49]	0	1790.9568
[R].LTESPCA	1xCarbamido	1.515E-06	4.225E-05	1	1	1	P14625	P14625 [640-660]	0	2660.2768
[R].LDLGEDT	1xTMTpro [K	4.487E-05	4.225E-05	1	1	1	O43143	O43143 [7-17]	0	1801.9828
[K].EIQGFFS	1xTMTpro [N	2.475E-05	4.225E-05	1	1	2	Q14558	Q14558 [139-152]	0	1973.0452
[K].IDLLDDS	1xCarbamido	7.816E-07	4.225E-05	1	1	2	Q9ULC3	Q9ULC3 [123-141	1	3059.6751
[K].ITENIGCT	1xCarbamido	8.414E-06	4.225E-05	1	1	2	P60900	P60900 [72-88]	0	2160.0418

[R].LTDWIL(	1xTMTpro [N	1.497E-05	4.225E-05	1	1	2	Q03518	Q03518 [211-226]	0	2143.0991
[R].LTEMETI	2xOxidation	5.627E-05	4.225E-05	1	1	2	P35579	P35579 [868-882]	0	2392.2595
[R].LTEMETI	1xTMTpro [K	2.645E-06	4.225E-05	1	1	3	P35579	P35579 [868-882]	0	2360.2697
[R].ITEIYEG	1xTMTpro [N	4.249E-05	4.225E-05	1	1	2	P16219	P16219 [387-399]	0	1842.9768
[R].LDILDTA	1xTMTpro [N	9.023E-06	4.225E-05	2	2	3	P62070; P10	P62070 [64-79]; F	0	2070.0497
[R].LDIISEDI	1xTMTpro [K	3.253E-05	4.225E-05	1	1	3	Q7Z3B4	Q7Z3B4 [351-363	0	2111.2091
[R].ITEGVP(	1xTMTpro [N	2.159E-05	4.225E-05	1	1	3	P12111	P12111 [1128-114	0	2042.2181
[R].LTEEET\	1xCarbamido	1.361E-05	4.225E-05	1	1	1	P15924	P15924 [791-808]	1	2791.4679
[K].LDIISPQI	1xCarbamido	2.105E-05	4.225E-05	1	1	1	Q15643	Q15643 [1309-13	0	2803.5195
[R].LTEDLE\	1xTMTpro [N	3.689E-05	4.225E-05	1	1	1	P22059	P22059 [450-462]	0	1950.0139
[K].LTEDKAI	1xTMTpro [K	3.225E-06	4.225E-05	1	1	1	Q13263	Q13263 [775-790]	1	2394.3848
[K].ELQNTA\	1xCarbamido	5.545E-06	4.225E-05	1	1	1	Q8WX93	Q8WX93 [446-46	0	2319.2298
[R].LTEMETI	1xOxidation	3.196E-05	4.225E-05	1	1	6	P35579	P35579 [868-882]	0	2376.2646
[K].FNGGGI	1xTMTpro [K	1.52E-06	4.225E-05	1	1	1	P04179	P04179 [90-114]	0	3245.6736
[K].ITLPVDF	2xTMTpro [K	2.037E-06	4.225E-05	1	1	3	P00558	P00558 [280-297]	1	2935.6597
[R].ELPLGP/	1xTMTpro [K	2.178E-06	4.225E-05	1	1	1	Q8N201	Q8N201 [664-681	0	2527.4086
[R].EILTLLQ	1xTMTpro [K	1.051E-06	4.225E-05	1	1	2	Q15631	Q15631 [37-56]	0	2772.5904
[R].EILTLLQ	1xTMTpro [K	6.04E-07	4.225E-05	1	1	1	Q15631	Q15631 [37-57]	1	2928.6915
[K].ELLTQP(	1xTMTpro [K	7.645E-06	4.225E-05	1	1	3	Q15063	Q15063 [523-542]	0	2900.569
[K].ITSLTEV	2xCarbamido	4.768E-05	4.225E-05	1	1	1	Q03405	Q03405 [85-105]	0	2597.2983
[R].IDGNLV\	2xTMTpro [K	7.561E-06	4.225E-05	1	1	2	P00387	P00387 [85-111]	1	3888.1939
[R].FPAEDE	1xTMTpro [K	1.937E-05	4.225E-05	1	1	3	P12277	P12277 [14-32]	0	2778.3801
[R].ELLTTM(	1xTMTpro [N	1.91E-05	4.225E-05	1	1	1	P24844	P24844 [125-144]	1	2754.2922
[R].ITSGPFE	2xTMTpro [K	4.587E-05	4.225E-05	1	1	1	Q9UQ80	Q9UQ80 [333-35	1	3538.8807
[R].LDGNYL	1xCarbamido	1.587E-05	4.225E-05	1	1	1	P51888	P51888 [357-375]	0	2901.5685
[R].ITSEPQI	1xCarbamido	2.583E-06	4.225E-05	1	1	1	Q92626	Q92626 [248-268]	0	2665.2735
[R].ITSEGQI	1xTMTpro [K	8.924E-06	4.225E-05	1	1	1	Q76M96	Q76M96 [203-221	0	2686.5523
[K].FNYGFE	1xTMTpro [K	1.896E-05	4.225E-05	1	1	1	Q14204	Q14204 [1866-18	0	2188.157
[K].EILVGDV	1xTMTpro [K	2.626E-06	4.225E-05	1	1	10	P23528	P23528 [54-73]	0	2774.5108
[K].EILVGDV	1xTMTpro [K	2.86E-05	4.225E-05	1	1	1	P60981	P60981 [54-69]	0	2311.3405
[K].ELLVPLT	1xTMTpro [K	3.311E-05	4.225E-05	1	1	1	Q99471	Q99471 [61-75]	0	2242.3013
[K].ELLVSAI	1xTMTpro [K	3.124E-05	4.225E-05	1	1	2	Q8WTW3	Q8WTW3 [443-46	0	2841.5337
[R].ITSEAED	1xTMTpro [K	1.598E-05	4.225E-05	1	1	2	P61289	P61289 [22-36]	0	2289.2622

[K].LTSDPTI	1xCarbamido	7.617E-06	4.225E-05	1	1	1	O43504	O43504 [55-78]	0	3267.7096
[K].FNWYVD	1xTMTpro [K	2.636E-06	4.225E-05	1	2	46	P0DOX5	P0DOX5 [277-290	0	2286.2163
[R].EIMDAAI	1xTMTpro [K	3.826E-05	4.225E-05	1	1	1	P14868	P14868 [16-26]	0	1863.9654
[R].ITSAPDN	1xTMTpro [K	3.8E-05	4.225E-05	1	1	1	Q99598	Q99598 [77-93]	0	2500.3348
[R].LTSNPLA	1xTMTpro [K	8.713E-06	4.225E-05	1	1	1	Q969X5	Q969X5 [177-190	0	2180.2571
[K].LTTFMEI	1xTMTpro [N	4.293E-05	4.225E-05	1	1	1	O43310	O43310 [393-415]	0	2933.4192
[R].ELISWG/	1xTMTpro [N	3.873E-05	4.225E-05	1	1	2	Q03518	Q03518 [115-129]	0	1850.9568
[R].EILSVDC	1xCarbamido	1.078E-05	4.225E-05	1	1	2	P10909	P10909 [307-322]	0	2371.2419
[R].EILQTYG	1xTMTpro [K	9.259E-06	4.225E-05	1	1	1	Q96AX1	Q96AX1 [431-449	0	2922.6221
[K].ITVNEVE	1xTMTpro [K	1.578E-05	4.225E-05	1	1	2	Q9UNM6	Q9UNM6 [302-31	0	1996.2008
[R].ELIRDEC	1xTMTpro [K	4.3E-05	4.225E-05	1	1	2	O43772	O43772 [255-267]	1	2131.2254
[R].LTVMTD	2xTMTpro [K	2.706E-05	4.225E-05	1	1	2	Q12907	Q12907 [224-237]	1	2634.4629
[R].LTVMSL(	1xTMTpro [K	5.775E-05	4.225E-05	1	1	1	O75369	O75369 [2290-23(	0	1914.1226
[R].LTVLPPI	1xTMTpro [K	4.865E-05	4.225E-05	1	1	5	Q15404	Q15404 [192-208]	0	2416.4307
[K].LTVIAEQ	1xTMTpro [N	3.154E-06	4.225E-05	1	1	1	Q9BV19	Q9BV19 [78-93]	0	2181.2311
[R].ITVDPTT	1xTMTpro [K	4.085E-05	4.225E-05	1	1	6	Q99715	Q99715 [57-68]	0	1853.0512
[K].LTVAENI	1xTMTpro [K	4.556E-05	4.225E-05	1	1	1	P07814	P07814 [1390-14(	0	1813.0199
[R].LDGLVE	1xTMTpro [N	5.121E-06	4.225E-05	1	1	4	P55209	P55209 [56-72]	0	2164.1821
[R].LTSADAI	1xTMTpro [N	5.894E-06	4.225E-05	1	1	2	Q92616	Q92616 [2293-23	0	2370.404
[K].ELISLVE	3xCarbamido	7.992E-07	4.225E-05	1	1	2	P43652	P43652 [254-279]	0	3320.5404
[R].ELISNAS	1xTMTpro [K	3.381E-05	4.225E-05	2	2	8	P14625; P08	P14625 [103-114]	0	1884.057
[R].ELISNAS	1xTMTpro [K	2E-06	4.225E-05	2	2	2	P14625; P08	P14625 [103-116]	1	2153.2422
[R].ELISNAS	1xTMTpro [K	1.265E-05	4.225E-05	1	1	2	Q12931	Q12931 [115-126]	0	1898.0726
[K].LTTPTY(	1xTMTpro [N	2.859E-06	4.225E-05	1	1	2	Q9BUF5	Q9BUF5 [217-241	0	2939.5467
[R].ELISNSS	1xTMTpro [K	3.67E-05	4.225E-05	1	2	4	P07900	P07900 [47-58]	0	1900.0519
[K].ELLSNVI	1xTMTpro [K	4.016E-05	4.225E-05	1	1	1	Q92974	Q92974 [424-438]	0	2358.3048
[K].LTTPTY(	1xCarbamido	7.83E-06	4.225E-05	3	5	2	Q13885; P07	Q13885 [217-241]	0	3028.5403
[K].LTTPTY(	1xCarbamido	8.171E-07	4.225E-05	3	5	2	Q13885; P07	Q13885 [217-241]	0	3012.5454
[K].LTTLPSI	1xCarbamido	1.533E-05	4.225E-05	1	1	2	Q96AG4	Q96AG4 [51-66]	0	2410.366
[R].LTTINCE	1xCarbamido	5.014E-05	4.225E-05	1	1	2	Q16666	Q16666 [722-732]	0	1887.9978
[R].LTTVCP	1xCarbamido	1.478E-05	4.225E-05	1	1	2	P12931	P12931 [244-260]	0	2742.6004
[R].LTRPGS	1xTMTpro [K	4.567E-06	4.225E-05	1	1	1	O75937	O75937 [46-74]	0	3959.0934
[R].LTQYMD	1xTMTpro [K	4.89E-05	4.225E-05	1	1	2	Q13557	Q13557 [434-445]	0	1936.0164

[K].LTQVLNT	1xTMTpro [N	2.079E-05	4.225E-05	2	2	1	P51532; P51	P51532 [893-905]	0	1816.04
[R].LDKDLTI	2xTMTpro [K	3.813E-05	4.225E-05	1	1	2	Q9Y570	Q9Y570 [326-338	1	2359.3835
[K].ITPAHDQ	1xTMTpro [N	7.274E-06	4.225E-05	1	1	1	P26640	P26640 [592-606]	0	2047.0164
[R].LTNVAAT	1xTMTpro [N	5.261E-05	4.225E-05	1	1	1	Q32P28	Q32P28 [489-501	0	1628.8563
[K].ITNSLTV	1xCarbamido	5.305E-05	4.225E-05	1	1	1	O75822	O75822 [199-210]	0	1973.1233
[K].LDKDNLS	1xTMTpro [K	8.957E-06	4.225E-05	1	1	1	Q15121	Q15121 [55-71]	1	2700.4852
[K].EIPAWVI	1xTMTpro [K	2.916E-05	4.225E-05	1	1	5	P25311	P25311 [150-165]	0	2391.3568
[K].FNVEDG	1xTMTpro [N	1.948E-05	4.225E-05	1	1	2	Q8N766	Q8N766 [202-214	0	1836.9775
[K].LTNLEEF	1xCarbamido	7.587E-07	4.225E-05	1	1	1	Q13045	Q13045 [316-338]	0	2981.5032
[K].LDKGQIC	1xTMTpro [K	2.87E-05	4.225E-05	1	1	1	P54652	P54652 [329-345]	1	2421.4321
[R].ITNDARE	1xTMTpro [K	6.844E-06	4.225E-05	1	1	4	O00161	O00161 [143-167]	1	3397.7765
[K].EINSDQA	1xTMTpro [N	2.384E-05	4.225E-05	1	1	1	Q29RF7	Q29RF7 [1220-12	0	2038.9961
[K].ELPEFVV	1xTMTpro [K	2.542E-05	4.225E-05	1	1	1	Q8IY37	Q8IY37 [975-990]	0	2532.4093
[R].FNSVTEI	1xCarbamido	2.291E-05	4.225E-05	1	1	2	Q8N4A0	Q8N4A0 [496-510	0	2359.2459
[K].LTIVDTV	1xTMTpro [K	3.82E-05	4.225E-05	1	1	2	Q9NVA2	Q9NVA2 [96-110]	0	2228.2782
[R].ELPEPLN	1xTMTpro [N	4.776E-05	4.225E-05	1	1	1	Q13459	Q13459 [1777-179	0	2331.2014
[K].LTIVDAV	1xTMTpro [K	3.174E-05	4.225E-05	1	1	1	Q92599	Q92599 [99-113]	0	2198.2676
[R].LTLVCES	1xCarbamido	4.148E-06	4.225E-05	1	1	1	P52566	P52566 [72-96]	1	3601.0049
[R].LTLVCES	1xCarbamido	2.921E-07	4.225E-05	1	1	4	P52566	P52566 [72-96]	1	3585.01
[R].FNSPTTC	1xCarbamido	2.398E-06	4.225E-05	1	1	2	P41221	P41221 [291-311]	0	2791.3568
[K].FNQVLD	1xTMTpro [K	5.664E-05	4.225E-05	1	1	2	O43242	O43242 [375-385]	0	1933.0675
[K].LTLQPVI	1xTMTpro [K	1.425E-05	4.225E-05	1	1	1	Q15417	Q15417 [233-251]	0	2668.4835
[R].ELPLAAI	1xTMTpro [K	1.135E-05	4.225E-05	1	1	1	Q9Y446	Q9Y446 [579-594	0	2283.3092
[R].FNTAND	1xTMTpro [N	1.98E-05	4.225E-05	1	1	4	P04040	P04040 [432-444]	0	1797.9051
[K].ITLQDVV	1xTMTpro [K	2.621E-05	4.225E-05	1	1	1	P46940	P46940 [925-935]	0	1835.0882
[K].ELNQVM	1xTMTpro [N	1.868E-05	4.225E-05	1	1	1	Q14683	Q14683 [476-488]	0	1806.9339
[K].EINNAHA	1xTMTpro [K	1.454E-05	4.225E-05	1	1	2	Q9H3Z4	Q9H3Z4 [59-72]	0	2119.2003
[K].ELMGEE	1xTMTpro [K	9.327E-06	4.225E-05	1	1	1	Q9P2J5	Q9P2J5 [351-370]	0	2717.4927
[R].ITQTAEC	1xTMTpro [K	3.767E-05	4.225E-05	1	1	3	Q14112	Q14112 [629-645]	0	2500.379
[R].ELMLHA	1xOxidation [	1.776E-05	4.225E-05	1	1	1	P12270	P12270 [1085-110	0	2334.2983
[K].LTQEQV	1xTMTpro [N	1.513E-05	4.225E-05	1	1	2	Q9P0I2	Q9P0I2 [44-57]	0	1920.0721
[R].IDGPVIS	1xTMTpro [N	3.696E-08	4.225E-05	1	1	1	P42167	P42167 [270-297]	0	3233.6894
[K].ITQDIFQ	1xTMTpro [K	5.756E-05	4.225E-05	1	1	1	P56192	P56192 [365-375]	0	1955.1821

[R].ITQAVLC	1xCarbamido	2.36E-08	4.225E-05	1	1	1	O95834	O95834 [285-304]	0	2359.2876
[K].LTQAQIF	1xTMTpro [N	2.407E-06	4.225E-05	1	1	3	P00491	P00491 [42-58]	0	2313.2199
[R].ELNEDK	3xTMTpro [K	5.491E-05	4.225E-05	1	1	1	O00567	O00567 [222-240]	2	3421.9241
[K].ELNELVS	1xTMTpro [K	6.357E-06	4.225E-05	1	1	2	Q96G03	Q96G03 [587-604	0	2766.4958
[K].LTPEEEE	2xTMTpro [K	3.52E-05	4.225E-05	1	1	2	P62241	P62241 [129-140]	1	2355.3951
[K].FNVTGTI	1xTMTpro [N	1.3E-05	4.225E-05	1	1	2	Q9UI09	Q9UI09 [115-131]	0	2264.1518
[R].LTPRPTA	1xTMTpro [K	1.312E-05	4.225E-05	1	1	2	Q9BSJ8	Q9BSJ8 [768-791	0	3286.8866
[K].ITPNLAE	1xTMTpro [N	3.064E-05	4.225E-05	1	1	6	P01009	P01009 [50-63]	0	1946.0707
[K].ITPMNAD	1xTMTpro [K	3.265E-05	4.225E-05	1	1	1	Q8N4X5	Q8N4X5 [241-256	0	2307.3602
[R].LDKACE	1xCarbamido	2.47E-06	4.225E-05	1	1	2	P01024	P01024 [1533-154	1	2569.4152
[K].EINLAPD	1xTMTpro [K	1.76E-06	4.225E-05	1	1	4	P02751	P02751 [1858-187	0	2725.5301
[K].EINLAPD	1xOxidation [	3.358E-05	4.225E-05	1	1	2	P02751	P02751 [1858-187	0	2741.5251
[K].ELNIQVD	1xTMTpro [N	5.287E-05	4.225E-05	1	1	1	P51178	P51178 [171-182]	0	1726.8931
[K].ITPGKPY	1xTMTpro [K	2.568E-06	4.225E-05	2	2	1	P0C0L4; P0(	P0C0L4 [245-269]	0	3413.9111
[K].LDKAQIH	1xTMTpro [K	3.601E-05	4.225E-05	1	1	3	P0DMV9	P0DMV9 [326-342	1	2430.4324
[K].ELNMIIM	1xTMTpro [N	3.305E-05	4.225E-05	1	1	1	P35237	P35237 [227-242]	0	2208.1575
[R].ELNFSVI	1xTMTpro [N	5.879E-05	4.225E-05	1	1	2	Q12955	Q12955 [4111-41	0	1881.0037
[K].ITAQSIEI	1xCarbamido	4.703E-07	4.225E-05	1	1	3	P78527	P78527 [1423-144	0	2866.4576
[K].EIRPALE	1xTMTpro [K	1.004E-05	4.225E-05	1	1	2	P50395	P50395 [365-379]	0	2386.4201
[K].LTALDYH	1xCarbamido	2.804E-05	4.225E-05	1	1	1	Q9Y224	Q9Y224 [6-26]	1	3106.5548
[K].FMPVEG	2xCarbamido	2.409E-05	4.225E-05	1	1	3	Q9UGI8	Q9UGI8 [403-417	0	2428.2029
[K].LDQEDA	1xCarbamido	4.458E-06	4.225E-05	1	1	2	Q99426	Q99426 [67-84]	0	2327.1145
[R].ELVPSSI	1xTMTpro [K	1.217E-05	4.225E-05	1	1	2	Q92979	Q92979 [188-207]	0	2677.5209
[K].ISNAQLC	1xTMTpro [K	9.157E-06	4.225E-05	1	1	1	Q93034	Q93034 [729-743]	0	2307.3779
[R].ISMSHEI	1xTMTpro [N	6.596E-06	4.225E-05	1	1	1	Q9Y5P6	Q9Y5P6 [75-94]	0	2383.2611
[K].ISMQDVI	1xTMTpro [K	1.467E-05	4.225E-05	1	1	2	Q09666	Q09666 [500-513]	0	2098.171
[R].LDQEVA	2xTMTpro [K	1.291E-05	4.225E-05	1	1	1	Q99816	Q99816 [277-292]	1	2768.6226
[K].IDQLEGI	1xTMTpro [K	4.125E-06	4.225E-05	1	1	6	P12814	P12814 [685-704]	0	2947.6021
[K].ISMPDVN	1xTMTpro [K	5.5E-05	4.225E-05	1	1	1	Q09666	Q09666 [2184-219	0	1852.0858
[K].ELVSDAI	1xTMTpro [K	1.295E-05	4.225E-05	1	1	1	P30153	P30153 [332-342]	0	1848.0471
[R].EIVSDVII	1xTMTpro [N	2.113E-05	4.225E-05	1	1	1	Q32P44	Q32P44 [671-687	0	2176.178
[R].LDQLIYII	1xTMTpro [K	4.199E-05	4.225E-05	1	1	5	P55072	P55072 [639-651]	0	2165.2713
[K].ISMPDVI	1xTMTpro [K	1.167E-05	4.225E-05	1	1	1	Q09666	Q09666 [697-707]	0	1862.0701

[R].ELVTGV	1xCarbamido	1.631E-05	4.225E-05	1	1	1	Q99569	Q99569 [633-649]	0	2499.3773
[K].ISMPDID	1xTMTpro [K	1.763E-05	4.225E-05	1	1	3	Q09666	Q09666 [2835-284	0	2122.2074
[K].ISMPDLD	2xTMTpro [K	4.286E-05	4.225E-05	1	1	1	Q09666	Q09666 [2707-27	1	2453.4618
[R].IDQVNQ	1xTMTpro [K	1.27E-05	4.225E-05	1	1	1	P61201	P61201 [402-415]	0	2301.3058
[K].EIVVLET	1xTMTpro [K	1.962E-05	4.225E-05	1	1	1	Q15293	Q15293 [205-217]	0	2124.2295
[K].EIVVIHQ	1xTMTpro [K	3.441E-05	4.225E-05	1	1	1	P41252	P41252 [849-861]	0	2099.2356
[K].ISIVEALT	1xTMTpro [K	1.822E-05	4.225E-05	1	1	3	Q9P032	Q9P032 [114-127	0	2173.32
[K].EIVVTDY	1xTMTpro [K	1.169E-05	4.225E-05	1	1	3	P40261	P40261 [80-96]	0	2631.4009
[R].ELVPES	1xTMTpro [N	1.685E-05	4.225E-05	3	3	2	Q6STE5; Q9	Q6STE5 [114-130	0	2315.1913
[K].LDQALV	1xTMTpro [K	4.149E-05	4.225E-05	1	1	1	Q7LDG7	Q7LDG7 [418-429	0	2002.1829
[K].IDPTVTM	1xCarbamido	1.368E-06	4.225E-05	1	1	1	P35998	P35998 [157-181]	0	3727.9049
[K].FMQDPM	1xTMTpro [K	2.87E-05	4.225E-05	1	1	3	Q13838	Q13838 [242-256]	0	2453.2224
[R].ISQAEEE	1xTMTpro [K	4.677E-07	4.225E-05	1	1	3	Q9BX68	Q9BX68 [100-119	0	2842.617
[K].LDPQTLI	1xCarbamido	9.31E-06	4.225E-05	1	1	4	Q9NRN5	Q9NRN5 [301-318	0	2504.2047
[R].LSPVTA	1xCarbamido	1.719E-05	4.225E-05	1	1	1	Q8NBF2	Q8NBF2 [603-617	0	2229.2557
[K].LSPPYSS	1xTMTpro [N	2.958E-05	4.225E-05	1	1	2	Q13263	Q13263 [751-767]	0	2182.11
[R].ELVEPLT	1xTMTpro [N	8.367E-06	4.225E-05	1	1	2	P00533	P00533 [687-705]	0	2338.2937
[R].LSPPSSS	1xTMTpro [N	4.926E-06	4.225E-05	1	1	1	P50402	P50402 [48-68]	0	2465.2115
[K].LSPPFKF	1xTMTpro [K	4.071E-05	4.225E-05	1	1	1	P31749	P31749 [421-436]	0	2411.3426
[K].EIVEVKE	2xTMTpro [K	1.329E-05	4.225E-05	1	1	1	P82970	P82970 [96-111]	1	2787.5444
[R].ELVFKEI	2xTMTpro [K	1.355E-06	4.225E-05	1	2	2	P47813	P47813 [25-40]	1	2808.5964
[K].FMQTFV	1xTMTpro [K	4.857E-05	4.225E-05	1	1	1	Q9UN86	Q9UN86 [108-123	0	2373.3132
[K].IDQWLE	1xTMTpro [N	1.651E-05	4.225E-05	1	1	1	P33241	P33241 [227-243]	0	2326.1998
[K].ELVGPPI	1xTMTpro [K	2.175E-05	4.225E-05	1	1	1	P49792	P49792 [1384-139	0	2206.2979
[R].EIVHLQA	1xCarbamido	4.678E-06	4.225E-05	4	6	2	Q13885; Q1	Q13885 [3-19]; Q	0	2431.3371
[R].LSPGGY	1xTMTpro [N	5.006E-05	4.225E-05	1	1	1	O43148	O43148 [308-326]	0	2373.2774
[R].FMQTFV	1xOxidation	5.287E-05	4.225E-05	1	1	1	Q13283	Q13283 [108-123]	0	2363.2925
[K].EIVLADV	1xTMTpro [N	2.484E-05	4.225E-05	1	1	3	P22234	P22234 [202-215]	0	1949.0299
[R].FMQTFV	1xTMTpro [K	4.345E-05	4.225E-05	1	1	1	Q13283	Q13283 [108-123]	0	2347.2976
[R].LSPEPW	1xTMTpro [K	2.542E-05	4.225E-05	2	2	1	O60488; O9	O60488 [673-690]	0	2596.4154
[R].LSPEIVE	1xTMTpro [N	1.295E-05	4.225E-05	1	1	1	Q9NXG6	Q9NXG6 [303-318	0	2112.2235
[K].ISNYGW	1xTMTpro [K	5.446E-05	4.225E-05	1	1	2	Q9HB71	Q9HB71 [75-85]	0	1920.9947
[R].ISNSATY	1xTMTpro [N	1.235E-06	4.225E-05	1	1	1	Q96HC4	Q96HC4 [376-407	0	3567.7846

[K].IDPTVTM	1xCarbamido	2.171E-07	4.225E-05	1	1	1	P35998	P35998 [157-181]	0	3711.91
[K].ISPLMNL	1xTMTpro [K	3.347E-06	4.225E-05	1	1	3	Q9H223	Q9H223 [393-434	0	4978.4912
[K].ISLSPEY	1xTMTpro [N	3.709E-05	4.225E-05	1	1	4	P12111	P12111 [1381-139	0	2036.1024
[K].LSLQDA\	1xTMTpro [N	8.275E-06	4.225E-05	1	1	1	P15924	P15924 [2669-268	0	2351.2196
[K].ELWFSD	1xTMTpro [K	3.358E-05	4.225E-05	1	1	4	O95831	O95831 [178-189]	0	2059.0992
[R].EMACTT	1xCarbamido	3.913E-05	4.225E-05	1	1	1	Q9ULA0	Q9ULA0 [454-469	0	2421.3377
[K].LSLDGQ	2xCarbamido	1.073E-05	4.225E-05	1	1	2	P26599	P26599 [239-254]	0	2202.0966
[K].LSLCGEI	1xCarbamido	2.198E-05	4.225E-05	1	1	1	P36871	P36871 [371-387]	0	2169.0566
[R].LDSHPC	1xCarbamido	4.579E-05	4.225E-05	1	1	1	Q9H4A4	Q9H4A4 [80-94]	0	1957.0496
[R].IDSLSAQ	1xTMTpro [K	1.61E-05	4.225E-05	1	1	6	P02545	P02545 [299-311]	0	2039.1992
[K].ISHVVVE	1xTMTpro [K	3.664E-05	4.225E-05	1	1	1	Q7Z5K2	Q7Z5K2 [119-132	0	2135.2204
[K].IDSNLDE	1xTMTpro [N	5.888E-05	4.225E-05	1	1	2	O95721	O95721 [202-215]	0	1823.9492
[R].ISHLVLP	1xTMTpro [K	5.132E-05	4.225E-05	1	1	1	Q5JTV8	Q5JTV8 [564-578	0	2266.3779
[R].LSHLKPS	2xTMTpro [K	4.419E-05	4.225E-05	1	1	2	P10155	P10155 [185-200]	0	2592.5905
[R].LDSSAVI	1xTMTpro [K	5.313E-05	4.225E-05	1	1	1	P42704	P42704 [740-750]	0	1713.9878
[R].EMAAMC	1xCarbamido	6.92E-06	4.225E-05	1	1	1	Q9Y2X3	Q9Y2X3 [134-148	0	1950.9883
[K].LSHEGP	1xTMTpro [N	4.163E-06	4.225E-05	1	1	1	Q14203	Q14203 [1134-115	0	2058.0939
[K].LSGVQD	1xTMTpro [N	6.639E-07	4.225E-05	1	1	1	P39060	P39060 [540-569]	0	3460.7416
[R].LSGVQA	1xTMTpro [N	1.125E-05	4.225E-05	1	1	2	Q5TZA2	Q5TZA2 [1574-15	0	2033.1198
[K].LDSSDIY	1xTMTpro [K	5.822E-05	4.225E-05	1	1	1	P29144	P29144 [1036-104	0	1905.0461
[K].LSGSNP	1xTMTpro [K	1.013E-05	4.225E-05	1	1	4	O43707	O43707 [605-622]	0	2528.4216
[K].EMDPVT	1xTMTpro [K	1.356E-05	4.225E-05	1	1	2	Q13740	Q13740 [191-208]	0	2758.4175
[K].EMDPVT	1xOxidation [	2.574E-05	4.225E-05	1	1	1	Q13740	Q13740 [191-208]	0	2774.4124
[R].LDSVES\	1xCarbamido	4.156E-06	4.225E-05	1	1	3	Q99805	Q99805 [67-90]	0	3363.6586
[K].FLVQNIH	1xTMTpro [K	5.936E-05	4.225E-05	1	1	2	Q8TDN6	Q8TDN6 [141-153	0	2134.288
[R].LDTDDLI	1xTMTpro [K	5.331E-05	4.225E-05	1	2	2	Q9UMR2	Q9UMR2 [466-47	0	1914.0199
[R].EMEAEL	1xTMTpro [K	1.962E-05	4.225E-05	1	1	1	P35579	P35579 [1593-160	1	1987.0298
[R].LSGWEE	1xTMTpro [K	5.993E-05	4.225E-05	1	1	1	Q9P270	Q9P270 [120-136	0	2664.3325
[K].ELVEFA(	1xTMTpro [N	2.668E-05	4.225E-05	1	1	1	Q14677	Q14677 [134-146]	1	1909.9939
[K].FIYEGSS	1xCarbamido	7.773E-06	4.225E-05	1	1	1	P51659	P51659 [363-384]	0	3073.62
[K].EIYTHFT	1xCarbamido	1.722E-05	4.225E-05	3	3	1	P04899; P08	P04899 [319-331]	0	2195.1298
[R].LSIPVMV	1xTMTpro [N	5.85E-05	4.225E-05	1	1	1	Q4ZIN3	Q4ZIN3 [230-243]	0	1845.0839
[R].IDQYQG.	1xTMTpro [K	2.367E-05	4.225E-05	1	1	1	O43396	O43396 [88-102]	0	2244.2004

[K].LSLPQNI	1xTMTpro [K	3.18E-05	4.225E-05	1	1	2	Q9HC35	Q9HC35 [868-883	0	2339.3314
[K].ISLPGQN	1xTMTpro [K	3.906E-05	4.225E-05	1	1	1	Q9H8Y8	Q9H8Y8 [213-228	0	2232.3281
[R].FMCNLD	3xCarbamido	1.667E-05	4.225E-05	1	1	1	P06280	P06280 [50-67]	0	2870.2961
[R].FMCAQL	1xCarbamido	9.5E-06	4.225E-05	1	1	1	Q9H4M9	Q9H4M9 [136-162	0	3523.9036
[K].IDRIEESI	1xTMTpro [N	5.764E-06	4.225E-05	1	1	1	Q9BUQ8	Q9BUQ8 [459-478	1	2561.3894
[R].ELYERPI	1xTMTpro [K	8.924E-06	4.225E-05	1	1	1	O94832	O94832 [70-87]	0	2712.5005
[K].ISIPDVDI	1xTMTpro [K	5.563E-05	4.225E-05	1	1	2	Q09666	Q09666 [4849-485	0	1836.0974
[K].ISLNSLC	1xCarbamido	4.308E-05	4.225E-05	1	1	2	O75131	O75131 [196-208	0	2124.0961
[K].LSLEGDI	1xTMTpro [K	1.578E-05	4.225E-05	1	2	3	P07355	P07355 [11-28]	0	2453.3168
[R].FMATND	1xTMTpro [K	1.962E-05	4.225E-05	1	1	2	Q86VP6	Q86VP6 [24-36]	0	2150.1481
[R].LDSGDL	1xTMTpro [N	1.189E-05	4.225E-05	1	1	1	Q6XQN6	Q6XQN6 [319-332	0	1890.0252
[K].FLYGCQ	2xCarbamido	3.288E-05	4.225E-05	1	1	1	Q16531	Q16531 [169-188	0	2724.3233
[K].EIYNQVN	1xTMTpro [K	6.07E-05	4.225E-05	1	1	1	Q9NUQ9	Q9NUQ9 [34-44]	0	1927.1508
[R].ISLGMPV	1xTMTpro [K	1.51E-05	4.225E-05	1	1	1	Q13435	Q13435 [615-627	0	1929.1236
[R].ISLGLPV	1xCarbamido	9.966E-06	4.225E-05	1	1	4	P62829	P62829 [16-35]	0	2578.4518
[R].ISIGGGS	1xCarbamido	5.009E-06	4.225E-05	2	3	1	P04259; P02	P04259 [70-86]; F	0	1902.9663
[R].IDSGLYL	1xTMTpro [N	5.756E-07	4.225E-05	1	1	2	P02788	P02788 [333-351]	0	2392.2832
[K].LSLESLT	1xTMTpro [K	1.966E-05	4.225E-05	1	1	3	P04114	P04114 [3481-349	0	2500.3678
[K].LSLEPLF	1xCarbamido	4.149E-05	4.225E-05	1	1	2	Q96RS6	Q96RS6 [25-45]	0	2966.6404
[K].LSLEIEQ	1xTMTpro [N	3.886E-05	4.225E-05	1	1	5	P04003	P04003 [574-585]	0	1775.0234
[K].LSLLEVG	1xCarbamido	3.435E-06	4.225E-05	1	1	3	Q9H8H3	Q9H8H3 [72-86]	0	2174.2135
[K].EIVDSYL	1xTMTpro [K	1.657E-05	4.225E-05	1	1	1	P07602	P07602 [108-122]	0	2338.4129
[K].EIVDPLY	1xTMTpro [K	1.388E-06	4.225E-05	1	1	2	Q68EM7	Q68EM7 [118-136	0	2748.5679
[R].LSQALG	1xTMTpro [K	1.401E-05	4.225E-05	1	1	2	Q8IW45	Q8IW45 [234-246	0	1965.1988
[R].ISVNNVL	1xTMTpro [K	2.726E-05	4.225E-05	1	1	5	P07339	P07339 [206-222]	0	2567.4511
[R].ELSFSGI	1xCarbamido	5.627E-05	4.225E-05	1	1	1	Q9NVG8	Q9NVG8 [28-41]	0	1825.9438
[R].ISVMGG	1xTMTpro [K	7.26E-06	4.225E-05	1	1	1	Q9HCC0	Q9HCC0 [470-487	0	2411.3824
[R].LSVLDLV	1xCarbamido	9.023E-06	4.225E-05	1	1	1	Q5JTH9	Q5JTH9 [751-771	0	2763.5822
[K].LSVLAVD	1xCarbamido	2.565E-05	4.225E-05	1	1	1	Q8IVH4	Q8IVH4 [174-194	0	2671.4468
[R].ELSGTIK	1xCarbamido	1.28E-06	4.225E-05	1	1	1	P30050	P30050 [124-146]	1	3012.628
[R].ISVGSDS	1xTMTpro [K	8.109E-06	4.225E-05	1	1	8	Q14195	Q14195 [401-418	0	2524.379
[K].ELSLAGI	1xTMTpro [N	5.065E-06	4.225E-05	1	1	4	P13489	P13489 [288-302]	0	1834.9466
[K].EISLWFK	2xTMTpro [K	1.336E-05	4.225E-05	1	1	3	P22392	P22392 [129-143]	0	2808.6004

[R].ISVFATY	1xTMTpro [K	1.441E-06	4.225E-05	1	1	2	Q99715	Q99715 [973-100	0	3498.7619
[R].IDNDGD	1xTMTpro [K	1.36E-06	4.225E-05	1	1	3	Q15293	Q15293 [91-105]	0	2261.1793
[K].FNEEHIF	1xTMTpro [N	7.802E-06	4.225E-05	1	1	6	P21333	P21333 [2311-23	0	2771.396
[R].ELSNTA	1xTMTpro [N	5.261E-05	4.225E-05	1	1	4	Q9HAT2	Q9HAT2 [142-15	0	1642.872
[R].LDNMLL	1xTMTpro [K	1.544E-05	4.225E-05	1	2	1	P40424	P40424 [108-122]	0	2165.2132
[K].EISPDTT	1xTMTpro [N	1.773E-06	4.225E-05	1	1	3	P21810	P21810 [87-106]	0	2590.3531
[K].EISPDTT	1xTMTpro [K	2.027E-07	4.225E-05	1	1	5	P21810	P21810 [87-107]	1	3022.6552
[R].LDNMLL	1xOxidation	1.855E-05	4.225E-05	1	2	1	P40424	P40424 [108-122]	0	2181.2081
[K].ISTSLPV	1xTMTpro [K	1.208E-06	4.225E-05	1	1	3	P13796	P13796 [546-570]	0	3306.9056
[K].LSTPPPI	1xTMTpro [N	5.536E-05	4.225E-05	1	1	2	P16157	P16157 [959-974]	0	1971.0718
[K].IDNSQVI	1xTMTpro [K	6.127E-06	4.225E-05	1	1	3	P27797	P27797 [186-206]	0	2999.513
[R].ELSQGL	1xTMTpro [N	2.52E-05	4.225E-05	1	1	1	P98175	P98175 [264-305]	0	4724.4702
[K].ELSQIEA	1xCarbamid	3.728E-05	4.225E-05	1	1	1	Q96P70	Q96P70 [632-646	0	2082.0101
[R].LSVEEA	1xTMTpro [K	1.077E-06	4.225E-05	1	1	1	P58107	P58107 [2281-22	0	2493.4056
[K].IDNSQVI	2xTMTpro [K	1.718E-06	4.225E-05	1	1	8	P27797	P27797 [186-207]	1	3431.8151
[K].EISEVFP	1xTMTpro [K	5.266E-07	4.225E-05	1	1	2	P07686	P07686 [339-359]	0	3043.5908
[R].ELSEPM	1xTMTpro [N	1.121E-05	4.225E-05	1	1	1	Q04206	Q04206 [279-295	0	2389.1189
[K].LDLPNRI	1xCarbamid	1.069E-05	4.225E-05	1	1	1	Q9NZM1	Q9NZM1 [1996-2	0	2943.5683
[K].LTALADI	1xTMTpro [K	5.006E-05	4.225E-05	1	1	2	Q9H269	Q9H269 [731-745	0	2383.2888
[K].FNFLNPI	1xTMTpro [N	2.392E-05	4.225E-05	1	1	2	Q15459	Q15459 [81-95]	0	2235.0943
[K].ELSAIGS	1xTMTpro [K	5.256E-06	4.225E-05	1	1	1	Q9Y5X2	Q9Y5X2 [259-285	0	3397.8135
[K].LTAGEA	1xCarbamid	2.425E-06	4.225E-05	1	1	2	O75521	O75521 [306-328	0	3077.6109
[K].LTAEFEI	1xCarbamid	2.109E-05	4.225E-05	1	1	2	Q9P2E9	Q9P2E9 [1311-13	0	1916.9343
[R].ITADLLS	2xTMTpro [K	6.44E-06	4.225E-05	1	1	2	Q9UHD8	Q9UHD8 [461-49	2	4365.2918
[R].ITADLLS	1xTMTpro [K	1.677E-06	4.225E-05	1	1	2	Q9UHD8	Q9UHD8 [461-48	1	3477.7517
[R].FNFLAPI	1xTMTpro [N	1.009E-05	4.225E-05	1	1	2	P55084	P55084 [204-229]	0	3144.5267
[R].LDLQEIN	1xTMTpro [K	3.213E-05	4.225E-05	1	1	4	P36955	P36955 [175-189]	0	2438.3357
[R].LDLWNL	1xTMTpro [N	2.307E-06	4.225E-05	1	1	1	Q13409	Q13409 [549-575	0	3256.6769
[R].LSYQND	1xTMTpro [N	4.213E-05	4.225E-05	1	1	1	Q02487	Q02487 [635-653	0	2456.3145
[K].LDISDEF	1xTMTpro [K	2.74E-05	4.225E-05	2	2	2	Q9NZN3; Q	Q9NZN3 [194-20	0	2003.1192
[R].LSWTAD	1xTMTpro [K	2.602E-05	4.225E-05	1	1	4	P24821	P24821 [1638-16	0	2449.3259
[K].ELSDPA	1xTMTpro [N	3.01E-05	4.225E-05	1	1	2	O15067	O15067 [528-541	0	1796.9713
[K].ELSDQA	1xTMTpro [N	3.94E-05	4.225E-05	1	1	1	O43301	O43301 [183-198	0	2086.9848

[R].ISVYYNE	1xTMTpro [K	5.437E-05	4.225E-05	1	1	3	P07437	P07437 [47-58]	0	1910.0515
[R].ISVYSIM	1xTMTpro [N	2.958E-05	4.225E-05	1	1	2	O94979	O94979 [328-342]	0	1859.9856
[K].LDITTLT(	1xTMTpro [K	1.369E-05	4.225E-05	1	1	1	O43181	O43181 [59-73]	0	2274.3201
[K].EISEITG(	1xTMTpro [K	3.933E-05	4.225E-05	1	1	2	O95822	O95822 [362-376]	0	2209.2571
[R].ELSELV\	1xTMTpro [N	5.27E-05	4.225E-05	1	1	2	Q8WU39	Q8WU39 [81-93]	0	1855.9972
[R].LSVQSA	1xTMTpro [K	4.397E-05	4.225E-05	1	1	1	Q9UPN3	Q9UPN3 [4711-47	0	2166.2626
[R].ISYFTFI(	1xTMTpro [K	2.74E-05	4.225E-05	1	1	2	O43396	O43396 [260-279]	0	2902.5305
[K].EGSLGD	1xTMTpro [N	6.475E-06	4.225E-05	1	1	2	Q96CX2	Q96CX2 [149-184	0	3959.9681
[K].LDNVML	1xTMTpro [K	2.341E-05	4.225E-05	1	2	2	P17252	P17252 [466-478]	0	2079.14
[K].ELSSFID	1xCarbamido	5.009E-06	4.225E-05	1	1	1	P02774	P02774 [395-420]	2	4319.2534
[K].FNALFA(	1xTMTpro [K	4.882E-05	4.225E-05	1	1	1	Q00610	Q00610 [368-382]	0	2239.2003
[R].FNAHGD	1xCarbamido	1.472E-05	4.225E-05	1	1	9	P09382	P09382 [50-64]	0	2256.1687
[K].FNAESQ	1xCarbamido	5.305E-05	4.225E-05	1	1	1	Q9Y4K0	Q9Y4K0 [407-423	0	2224.0008
[K].LSSLIILM	1xTMTpro [N	1.358E-06	4.225E-05	1	1	8	P50454	P50454 [264-280]	0	2288.312
[K].ISSLLEE	1xTMTpro [K	3.236E-05	4.225E-05	1	1	3	P62241	P62241 [158-170]	0	2115.1941
[K].LDPGSE	1xTMTpro [N	2.745E-05	4.225E-05	1	1	2	P26641	P26641 [402-414]	0	1748.935
[R].LSSEMN	1xTMTpro [N	1.48E-05	4.225E-05	1	1	4	P20700	P20700 [277-290]	0	1800.9081
[K].FNADEFI	1xTMTpro [K	1.613E-05	4.225E-05	1	1	4	P27635	P27635 [176-188]	0	2153.0716
[R].FNACFE.	1xCarbamido	3.557E-05	4.225E-05	1	1	1	Q14683	Q14683 [1070-10	0	2629.3464
[K].LSQSGE	1xTMTpro [K	3.939E-06	4.225E-05	1	1	3	Q86UX7	Q86UX7 [304-333	0	3633.8991
[R].LSSLNQI	1xTMTpro [K	5.563E-05	4.225E-05	1	1	1	Q15643	Q15643 [404-419]	0	2369.2804
[R].LSQLSV	1xTMTpro [N	1.055E-05	4.225E-05	1	2	1	P22105	P22105 [3658-367	0	1911.0718
[K].LSQERP	1xCarbamido	2.462E-05	4.225E-05	1	1	1	Q14166	Q14166 [347-368]	0	3163.7793
[R].LSQEQV	1xTMTpro [N	9.57E-06	4.225E-05	1	1	1	Q16891	Q16891 [527-545]	0	2502.2796
[K].ELTVSNI	1xTMTpro [N	2.779E-05	4.225E-05	1	1	2	P13489	P13489 [174-188]	0	1935.0103
[K].FMVQTIF	1xTMTpro [K	2.584E-05	4.225E-05	1	1	2	Q9P0L0	Q9P0L0 [95-114]	0	2920.5233
[R].LSQELE\	1xTMTpro [K	6.138E-06	4.225E-05	1	1	2	P12270	P12270 [134-147]	1	2331.3052
[K].ELTYQN	2xTMTpro [K	5.669E-06	4.225E-05	1	1	2	Q32MZ4	Q32MZ4 [594-612	1	3208.7405
[K].FMVQSM	1xTMTpro [K	2.405E-05	4.225E-05	1	1	2	O95292	O95292 [88-107]	0	2929.443
[R].IDPNVLL	2xTMTpro [K	2.711E-05	4.225E-05	1	1	1	Q8N7H5	Q8N7H5 [94-119]	1	3777.099
[R].LSQDILC	2xCarbamido	5.018E-06	4.225E-05	1	1	2	Q9BSF0	Q9BSF0 [65-83]	0	2898.5098
[K].FMTPVIC	1xCarbamido	1.753E-06	4.225E-05	1	1	2	O15371	O15371 [4-26]	0	2937.4347
[K].ELTTEID	1xTMTpro [K	1.344E-05	4.225E-05	1	1	6	P13645	P13645 [346-362]	0	2605.3853

[K].ELSSFID	1xCarbamid	8.875E-06	4.225E-05	1	1	3	P02774	P02774 [395-419]	1	3886.9513
[K].ELTGYN	1xCarbamid	2.227E-06	4.225E-05	1	1	2	Q6L8Q7	Q6L8Q7 [338-354	0	2299.1559
[R].ISSMVVI	1xTMTpro [K	3.767E-05	4.225E-05	1	1	1	P46063	P46063 [426-439]	0	2158.1856
[K].ELSSTE	1xTMTpro [N	1.854E-07	4.225E-05	1	1	2	Q7Z406	Q7Z406 [1326-13	0	2731.3706
[K].ELSSYEI	1xTMTpro [N	3.734E-05	4.225E-05	1	1	1	Q9UIQ6	Q9UIQ6 [504-515	0	1748.8662
[K].LDPAQV	1xTMTpro [K	1.364E-05	4.225E-05	1	1	1	Q9H0R3	Q9H0R3 [108-133	0	3442.7047
[K].LSTIALAI	1xTMTpro [N	5.945E-05	4.225E-05	2	2	3	P30154; P30	P30154 [47-58]; F	0	1546.9488
[K].IDPAYFD	1xTMTpro [N	5.822E-05	4.225E-05	1	1	1	Q86VS8	Q86VS8 [48-60]	0	1956.9775
[R].ELSVPQ	2xCarbamid	4.642E-05	4.225E-05	1	1	1	P42226	P42226 [323-361]	0	4636.3519
[K].ISTEVGI	2xTMTpro [K	2.084E-07	4.225E-05	1	1	4	P04844	P04844 [369-392]	1	3442.9461
[R].LSTANDI	1xTMTpro [K	2.06E-05	4.225E-05	1	1	1	Q96DM3	Q96DM3 [558-572	0	2239.3041
[K].FNDEHIF	1xTMTpro [N	1.934E-06	4.225E-05	1	1	2	O75369	O75369 [2266-22	0	2885.4641
[R].ISTAIDDI	1xTMTpro [K	1.934E-05	4.225E-05	1	1	3	Q9Y3Z3	Q9Y3Z3 [409-421	0	2066.0971
[K].ISSIQSIV	1xTMTpro [N	3.23E-05	4.225E-05	1	1	6	P10809	P10809 [251-268]	0	2223.278
[K].LSSVTA	1xCarbamid	4.042E-06	4.225E-05	1	9	2	P06331	P06331 [108-123]	0	2052.0391
[K].LSSVGSI	1xCarbamid	2.345E-05	4.225E-05	1	1	1	P56705	P56705 [31-45]	0	2263.1619
[K].LSSTWE	1xTMTpro [K	3.322E-05	4.225E-05	1	1	2	P37837	P37837 [143-154]	0	1885.0675
[K].ISSTLYQ	1xTMTpro [K	1.77E-05	4.225E-05	1	1	1	Q8N6L1	Q8N6L1 [111-127	0	2339.383
[K].IDPENAE	1xCarbamid	4.434E-05	4.225E-05	1	1	2	Q13325	Q13325 [464-479	0	2195.1337
[R].LSSSSP	1xTMTpro [K	5.183E-05	4.225E-05	1	1	1	Q8NI08	Q8NI08 [169-197]	1	3531.7946
[R].LSSSQG	1xTMTpro [N	2.316E-05	4.225E-05	1	1	1	Q9UHD2	Q9UHD2 [508-525	0	2241.153
[K].LSSSFGI	1xTMTpro [K	6.299E-06	4.225E-05	1	1	4	O94979	O94979 [349-384	0	4434.4681
[K].LDPFQE	1xTMTpro [N	2.462E-05	4.225E-05	1	1	1	Q96K76	Q96K76 [1239-12	0	2366.241
[R].LSSQEA	1xTMTpro [N	1.488E-05	4.225E-05	1	1	2	P05165	P05165 [244-257]	0	1773.8574
[R].EITENLM	1xTMTpro [N	2.337E-06	4.225E-05	1	1	4	P13796	P13796 [52-68]	0	2182.0617
[K].FNDEHIF	1xTMTpro [N	3.838E-06	4.225E-05	1	1	2	Q14315	Q14315 [2387-24	0	2831.4171
[K].SHCIAE\	1xCarbamid	1.46E-07	4.225E-05	1	1	38	P02768	P02768 [311-337]	0	3598.7537
[K].DLLVTG	1xTMTpro [N	4.273E-06	4.225E-05	1	1	8	P49755	P49755 [51-71]	0	2383.2424
[K].SHCIAE\	2xCarbamid	3.444E-07	4.225E-05	1	1	2	P02768	P02768 [311-341]	1	4405.1818
[R].GLEYLYI	1xTMTpro [N	1.889E-05	4.225E-05	1	1	2	Q9Y6G9	Q9Y6G9 [98-116]	1	2655.297
[R].GWTQW	1xTMTpro [N	1.896E-05	4.225E-05	1	1	2	P09455	P09455 [106-121]	0	2304.1402
[R].VLPPVP	1xTMTpro [N	1.448E-05	4.225E-05	1	1	1	Q99943	Q99943 [233-253	0	2476.3618
[R].GWTQW	1xOxidation	9.802E-06	4.225E-05	1	1	3	P09455	P09455 [106-121]	0	2320.1351

[K].ALIEVLQ	1xTMTpro [N	1.962E-05	4.225E-05	1	1	1	P23381	P23381 [433-448]	0	2105.2402
[K].VLPMNT	1xCarbamido	2.031E-05	4.225E-05	1	1	1	P04181	P04181 [136-151]	0	2285.2125
[K].VLPLEAL	1xTMTpro [K	8.263E-07	4.225E-05	1	1	2	Q92616	Q92616 [617-635]	0	2520.4416
[R].GWVLGI	2xTMTpro [K	1.202E-06	4.225E-05	1	1	2	P48556	P48556 [307-337]	0	4382.3125
[K].AILFSQP	1xCarbamido	1.038E-06	4.225E-05	1	1	1	Q8NBF2	Q8NBF2 [700-723	0	3002.6304
[R].VLPGSS	1xCarbamido	1.244E-05	4.225E-05	1	1	4	Q96AB3	Q96AB3 [11-26]	0	2431.3221
[R].GYAFNH	1xTMTpro [N	4.135E-05	4.225E-05	1	1	3	P61160	P61160 [201-214]	0	1917.9414
[K].GLEWM	1xTMTpro [N	2.132E-05	4.225E-05	1	1	1	A0A0C4DH3	A0A0C4DH38 [63	0	2114.0548
[K].GYAFVH	1xTMTpro [K	1.244E-05	4.225E-05	1	1	2	Q13310	Q13310 [139-153]	0	2321.2058
[K].VLPFFEI	1xTMTpro [K	1.645E-05	4.225E-05	1	1	2	Q96HE7	Q96HE7 [318-334	0	2663.4841
[R].VLPEGG	2xCarbamido	2.388E-05	4.225E-05	1	1	2	O00468	O00468 [1605-16	0	1946.9747
[K].VLPAVGI	1xTMTpro [K	2.844E-05	4.225E-05	1	1	3	Q6NUK1	Q6NUK1 [454-469	0	2390.3649
[K].VIPATDL	1xOxidation	3.607E-05	4.225E-05	1	1	1	P06737	P06737 [657-681]	0	3102.612
[K].AIIKPQY	2xTMTpro [K	2.547E-05	4.225E-05	1	1	2	P61221	P61221 [166-178]	0	2425.4999
[K].VIPATDL	1xTMTpro [K	3.119E-06	4.225E-05	1	1	2	P06737	P06737 [657-681]	0	3086.6171
[R].VLPAQA	1xTMTpro [N	1.8E-07	4.225E-05	1	1	1	Q7KZF4	Q7KZF4 [788-819	1	3811.9462
[K].VIPACLP	1xCarbamido	2.345E-05	4.225E-05	1	1	2	P00747	P00747 [681-696]	0	2075.1279
[R].VLPTPQ	1xTMTpro [K	4.503E-05	4.225E-05	1	1	1	P16452	P16452 [227-244]	0	2517.4532
[K].VLPVGD	1xTMTpro [K	4.273E-06	4.225E-05	1	1	2	P21589	P21589 [163-179]	0	2340.367
[K].VLPWLS	1xTMTpro [K	1.773E-05	4.225E-05	1	1	1	Q7RTN6	Q7RTN6 [240-259	0	2906.6272
[R].VLQALG	1xTMTpro [K	7.603E-06	4.225E-05	1	1	3	O00159	O00159 [910-927]	0	2519.5093
[R].VIQVAA	1xTMTpro [K	4.603E-05	4.225E-05	1	1	2	P05091	P05091 [269-280]	0	1795.0933
[K].VLQTADI	1xTMTpro [K	2.635E-05	4.225E-05	1	1	2	Q8WXH0	Q8WXH0 [5940-5	0	2327.3024
[R].ALLDLQ	1xTMTpro [N	3.594E-05	4.225E-05	1	1	1	Q9BXF6	Q9BXF6 [320-333	0	1783.9986
[R].GWGPH	2xCarbamido	1.1E-05	4.225E-05	1	1	4	P35555	P35555 [2370-238	0	2699.3718
[K].VLQQLL	1xTMTpro [K	2.397E-05	4.225E-05	1	1	1	Q92903	Q92903 [426-443]	0	2775.6628
[K].GWLRDI	1xTMTpro [N	7.355E-06	4.225E-05	1	1	2	P18206	P18206 [282-300]	1	2287.175
[R].VLQIVTN	1xTMTpro [K	1.186E-06	4.225E-05	1	1	1	O95782	O95782 [465-481]	1	2498.4222
[K].VLQLIND	1xTMTpro [N	2.973E-06	4.225E-05	1	1	4	Q9Y4L1	Q9Y4L1 [199-217	0	2399.3254
[K].VLQHYQ	2xTMTpro [K	4.457E-07	4.225E-05	1	1	1	Q9Y6C9	Q9Y6C9 [91-111]	1	3267.779
[K].VIQGQTI	1xTMTpro [N	1.581E-05	4.225E-05	1	1	2	P11277	P11277 [1127-114	0	2134.1827
[K].VINQYQ	1xTMTpro [K	2.312E-05	4.225E-05	1	1	1	Q14694	Q14694 [772-785]	0	2253.3211
[R].VLQGM	1xTMTpro [N	6.345E-06	4.225E-05	1	1	1	Q53GG5	Q53GG5 [255-272	0	2193.0889

[R].VIQGDG	1xTMTpro [K	1.468E-06	4.225E-05	1	1	2	P43490	P43490 [350-369]	0	2766.5203
[R].VIQESEA	1xTMTpro [N	6.806E-06	4.225E-05	1	1	1	Q9UMS6	Q9UMS6 [270-28	0	1745.9353
[R].GIFEALF	1xTMTpro [N	2.178E-06	4.225E-05	1	1	2	Q14204	Q14204 [2805-28	0	2427.4295
[R].VIQEGLE	1xTMTpro [K	1.779E-05	4.225E-05	1	1	2	P49916	P49916 [649-660]	0	1906.1869
[R].GLFDEE	1xTMTpro [K	9.784E-06	4.225E-05	1	1	4	Q15149	Q15149 [4207-42	0	2777.3683
[R].GWTGQ	1xTMTpro [N	5.15E-06	4.225E-05	1	1	2	P34897	P34897 [42-62]	0	2768.3157
[R].ALLELQL	1xTMTpro [N	7.45E-06	4.225E-05	1	1	3	P13639	P13639 [163-180]	0	2524.3618
[K].VIQCFAE	1xCarbamido	1.478E-05	4.225E-05	1	1	2	Q00610	Q00610 [488-500	0	2116.1716
[K].VLQATV	1xTMTpro [K	7.673E-06	4.225E-05	1	1	7	P61604	P61604 [41-54]	0	1924.1723
[K].VLQASVI	1xTMTpro [N	1.637E-06	4.225E-05	1	1	1	Q9BSJ8	Q9BSJ8 [423-445	0	2867.5487
[R].VIQGDG	1xOxidation	4.713E-06	4.225E-05	1	1	1	P43490	P43490 [350-369]	0	2782.5152
[K].ALLDASE	1xTMTpro [N	6.07E-05	4.225E-05	1	1	2	Q14258	Q14258 [250-261	0	1568.8451
[R].VINQILTI	1xOxidation	2.422E-05	4.225E-05	1	1	3	P55072	P55072 [600-614]	0	2304.2435
[R].AILLLQT.	1xTMTpro [N	4.092E-05	4.225E-05	1	1	1	Q6PGP7	Q6PGP7 [1006-1	0	2451.3414
[R].VILQDFT	1xOxidation	3.569E-05	4.225E-05	1	1	1	P21399	P21399 [83-101]	0	2369.2858
[R].AILQQLC	1xCarbamido	8.617E-06	4.225E-05	1	1	3	P19367	P19367 [802-820]	0	2696.5148
[R].ALLQQQ	1xTMTpro [K	1.508E-05	4.225E-05	1	1	1	Q9UHB9	Q9UHB9 [405-41	1	2136.1905
[R].ALIQYFS	1xTMTpro [N	4.865E-05	4.225E-05	1	1	1	Q13098	Q13098 [373-388	0	2202.1337
[R].VLLPTRF	1xTMTpro [N	3.931E-06	4.225E-05	1	1	1	Q9UFG5	Q9UFG5 [9-28]	0	2547.4829
[R].VLLPPEA	1xTMTpro [N	1.425E-05	4.225E-05	1	1	2	Q9NY15	Q9NY15 [1707-17	0	2469.3461
[K].VLLPAQI	1xTMTpro [N	1.414E-06	4.225E-05	1	1	2	Q9BZV1	Q9BZV1 [226-255	0	3749.9081
[R].VLLNDG	1xTMTpro [N	2.215E-06	4.225E-05	1	1	2	Q9Y6C2	Q9Y6C2 [892-914	0	2729.4106
[R].VILITPTF	3xCarbamido	1.023E-05	4.225E-05	1	1	3	Q2TAA2	Q2TAA2 [128-151	0	3467.8232
[K].VLLLSSF	1xTMTpro [N	4.177E-05	4.225E-05	1	1	2	Q8N163	Q8N163 [310-323	0	1893.1016
[R].GLESNV	1xTMTpro [K	1.322E-05	4.225E-05	1	1	1	Q15063	Q15063 [152-173	1	3096.7232
[R].GLESNV	1xTMTpro [K	2.602E-06	4.225E-05	1	1	1	Q15063	Q15063 [152-172	0	2940.6221
[R].VLLIESL.	1xTMTpro [K	5.207E-06	4.225E-05	1	1	2	Q5VYK3	Q5VYK3 [1813-18	0	3006.6677
[K].VILLDGS	1xCarbamido	2.807E-06	4.225E-05	1	1	2	Q9Y2J2	Q9Y2J2 [114-129	1	2505.3514
[R].AILVDLE	1xTMTpro [N	3.08E-05	4.225E-05	3	4	5	Q13885; Q1	Q13885 [63-77]; (	0	1920.0431
[R].VLILDEA	1xCarbamido	1.024E-06	4.225E-05	1	1	2	Q03519	Q03519 [627-651	0	3178.601
[R].ALLVEP\	1xTMTpro [N	1.286E-05	4.225E-05	1	1	4	P21980	P21980 [565-580]	0	2104.2337
[K].GLESLPI	1xTMTpro [N	2.359E-06	4.225E-05	1	1	2	P56192	P56192 [243-264]	0	2671.5215
[K].VILHLKE	1xTMTpro [K	3.492E-06	4.225E-05	2	4	6	P07900; P0	P07900 [186-201]	1	2623.4587

[R].VILGSEA	1xTMTpro [N	2.199E-07	4.225E-05	1	1	1	Q9NY33	Q9NY33 [126-141	0	2067.1154
[K].VLLGQD	1xTMTpro [K	9.183E-07	4.225E-05	1	1	1	Q9BT73	Q9BT73 [66-80]	0	2287.367
[R].VILQPD\	1xCarbamido	3.806E-05	4.225E-05	1	1	1	Q9UH99	Q9UH99 [590-614	0	3096.6161
[K].VLIQVGY	1xTMTpro [N	3.722E-05	4.225E-05	1	1	1	Q9Y6C9	Q9Y6C9 [26-41]	0	2056.2126
[R].VIISAPS/	1xTMTpro [K	5.247E-08	4.225E-05	1	1	9	P04406	P04406 [119-139]	0	2821.5236
[R].VIISAPS/	1xOxidation [	1.625E-06	4.225E-05	1	1	8	P04406	P04406 [119-139]	0	2837.5185
[K].VLNNME	1xOxidation [	4.634E-05	4.225E-05	1	1	2	P00558	P00558 [247-264]	0	2591.3518
[K].AILLPDL:	1xTMTpro [N	1.03E-05	4.225E-05	1	1	2	Q8WYP5	Q8WYP5 [2104-2	0	2909.558
[K].VLNNME	1xTMTpro [K	4.319E-07	4.225E-05	1	1	3	P00558	P00558 [247-264]	0	2575.3569
[R].VINLPLD	1xTMTpro [N	1.263E-06	4.225E-05	1	1	1	Q96PD5	Q96PD5 [137-166	0	3513.8259
[R].VINEPTA	2xTMTpro [K	7.758E-06	4.225E-05	1	1	1	P38646	P38646 [219-238]	1	3017.6975
[K].ALLLVGI	1xTMTpro [N	8.998E-07	4.225E-05	1	1	1	Q9H269	Q9H269 [787-805	0	2265.2886
[K].VINDPIH	1xTMTpro [N	3.154E-06	4.225E-05	1	1	3	Q9Y3Z3	Q9Y3Z3 [117-134	0	2376.3835
[K].ALINADE	1xTMTpro [N	3.98E-07	4.225E-05	1	1	3	Q13813	Q13813 [382-402	0	2431.3
[R].GLEVTA	1xTMTpro [N	2.287E-05	4.225E-05	1	1	1	P14550	P14550 [204-218]	0	1855.9721
[K].VIMVGS(	1xTMTpro [K	5.464E-05	4.225E-05	2	2	2	P11233; P11	P11233 [17-27]; F	0	1611.9748
[R].VINQILTI	1xTMTpro [K	3.291E-06	4.225E-05	1	1	4	P55072	P55072 [600-614]	0	2288.2486
[R].VIMGEE	1xTMTpro [K	4.908E-06	4.225E-05	1	1	1	P27708	P27708 [1241-126	0	2795.5542
[R].GYAVNV	1xTMTpro [N	5.883E-06	4.225E-05	1	1	1	Q15738	Q15738 [61-79]	0	2471.2638
[R].GLETSL/	1xCarbamido	9.893E-06	4.225E-05	1	1	1	P05156	P05156 [188-200]	0	2065.1131
[R].VLMAQH	1xCarbamido	4.643E-06	4.225E-05	1	1	2	P46063	P46063 [456-473]	0	2786.3886
[R].GIETPQ(	3xCarbamido	2.053E-06	4.225E-05	1	1	2	P07942	P07942 [1138-116	0	2967.3566
[R].VLIVSED	1xTMTpro [K	3.163E-07	4.225E-05	1	1	2	O95831	O95831 [159-177	0	2791.5923
[K].GYAYTFI	1xTMTpro [N	4.833E-05	4.225E-05	1	1	3	Q7L014	Q7L014 [717-729	0	1838.9244
[K].GLESTTI	1xCarbamido	3.148E-06	4.225E-05	1	1	8	P21291	P21291 [152-168]	1	2812.5219
[K].ALLQEM	1xTMTpro [N	4.518E-05	4.225E-05	1	1	4	P10155	P10155 [271-283]	0	1773.0627
[R].VIISAPS/	2xTMTpro [K	6.269E-07	4.225E-05	1	1	2	P04406	P04406 [119-145]	1	3846.075
[R].VIISAPS/	2xOxidation [	8.974E-06	4.225E-05	1	1	4	P04406	P04406 [119-139]	0	2853.5134
[K].VIMELFD	1xTMTpro [K	2.295E-07	4.225E-05	1	1	3	Q9NZM1	Q9NZM1 [1211-1:	1	2833.505
[R].ALLVTAS	1xCarbamido	3.19E-06	4.225E-05	1	1	4	Q01518	Q01518 [85-100]	0	2366.2994
[R].GWGHQ	2xCarbamido	5.037E-06	4.225E-05	1	1	1	P35556	P35556 [2417-24:	0	2710.3725
[K].VIRPLDQ	1xTMTpro [K	3.422E-06	4.225E-05	1	1	2	Q86VP6	Q86VP6 [549-566	0	2655.5002
[R].ALGFTDI	2xCarbamido	3.267E-06	4.225E-05	1	1	1	P05107	P05107 [430-448]	0	2510.3066

[K].GVVEVT	1xTMTpro [K	4.61E-05	4.225E-05	1	1	5	P50454	P50454 [309-319]		0	1833.0726
[R].AIGGGLS	1xTMTpro [K	3.467E-06	4.225E-05	1	2	2	P02538	P02538 [534-550]		0	2056.1894
[K].ALGHGV	1xTMTpro [N	8.794E-06	4.225E-05	1	1	1	P23219	P23219 [222-239]		0	2240.1743
[K].GVVFSV	1xTMTpro [K	5.738E-05	4.225E-05	1	1	1	Q9Y696	Q9Y696 [49-60]		0	1873.129
[K].GVVIGT(	1xTMTpro [K	2.784E-05	4.225E-05	1	1	1	P98194	P98194 [230-246]		0	2377.2895
[R].GLGGEV	1xTMTpro [N	9.641E-06	4.225E-05	1	1	2	Q6UW68	Q6UW68 [125-14		0	2027.0266
[R].VLVEPD.	1xOxidation	2.431E-05	4.225E-05	1	1	3	P42126	P42126 [47-61]		0	2080.1968
[K].GVVPLA(	1xTMTpro [N	2.135E-07	4.225E-05	1	1	5	P09972	P09972 [112-134]		0	2577.3327
[K].GVVPLA(	1xTMTpro [N	5.735E-07	4.225E-05	1	1	2	P04075	P04075 [112-134]		0	2576.3487
[K].ALGISPF	1xTMTpro [N	8.199E-06	4.225E-05	1	1	1	P02766	P02766 [101-123]		0	2755.4123
[R].VLVEDD	1xTMTpro [N	1.951E-05	4.225E-05	1	1	3	P09466	P09466 [143-156]		0	1968.0431
[K].GLGEISA	1xTMTpro [K	3.532E-05	4.225E-05	1	1	1	P13591	P13591 [593-604]		0	1817.03
[R].GVVQEL	1xTMTpro [K	4.427E-05	4.225E-05	1	1	3	P29692	P29692 [96-107]		0	1908.141
[R].VLVDGE	1xTMTpro [K	1.519E-05	4.225E-05	1	1	1	Q9NPA0	Q9NPA0 [67-79]		0	2050.1829
[K].VLVALAS	1xTMTpro [K	1.679E-05	4.225E-05	1	1	2	A0FGR8	A0FGR8 [891-902		0	1851.1447
[K].VLVAGD	1xTMTpro [K	2.094E-05	4.225E-05	1	1	3	O94973	O94973 [154-165]		0	1843.0491
[R].ALGQAF	3xCarbamido	9.191E-06	4.225E-05	1	1	1	Q8WUP2	Q8WUP2 [257-27		0	2387.1378
[R].ALGSAA.	1xTMTpro [N	5.813E-05	4.225E-05	1	1	1	Q13753	Q13753 [996-100		0	1334.7347
[R].ALGSAIE	1xTMTpro [N	1.938E-06	4.225E-05	1	1	2	P12111	P12111 [2714-273		0	2582.3422
[K].GVVTIE(	1xTMTpro [N	1.341E-05	4.225E-05	1	1	1	P09917	P09917 [581-595]		0	1959.1082
[R].GVVDSE	1xTMTpro [N	9.174E-06	4.225E-05	3	3	5	P07900; P08	P07900 [387-400]		0	1817.9928
[K].VLVNVLT	1xTMTpro [K	2.079E-06	4.225E-05	1	1	4	Q15036	Q15036 [129-149]		0	2822.6007
[K].VLVPEHI	1xCarbamido	2.574E-05	4.225E-05	1	1	1	P48147	P48147 [328-345]		1	2800.5675
[K].GVVDSD	1xTMTpro [N	1.065E-05	4.225E-05	1	1	5	P14625	P14625 [435-448]		0	1789.9615
[R].AIFLADG	1xTMTpro [N	7.062E-06	4.225E-05	1	1	4	Q9ULV4	Q9ULV4 [224-239		0	2020.0823
[K].AIFLHPE	1xTMTpro [K	2.616E-05	4.225E-05	1	1	3	Q9ULC5	Q9ULC5 [640-660		0	2945.6996
[R].VLYAYG	1xTMTpro [K	4.881E-06	4.225E-05	1	1	3	O14880	O14880 [98-112]		1	2375.2891
[RK].ALFPGI	1xTMTpro [N	2.911E-05	4.225E-05	1	2	1	P24941	P24941 [201-214]		0	1912.0135
[R].VLYAYG	1xTMTpro [K	1.587E-05	4.225E-05	1	1	2	O14880	O14880 [98-111]		0	2219.188
[R].VIYAMAE	1xTMTpro [K	4.634E-05	4.225E-05	1	2	1	P30825	P30825 [361-373]		0	2078.1852
[R].VLWLAD	2xCarbamido	1.085E-07	4.225E-05	1	1	2	P13489	P13489 [374-401]		0	3365.6789
[K].GVTSVS	1xTMTpro [N	1.76E-06	4.225E-05	1	1	3	P05155	P05155 [217-233]		0	2131.1831
[R].VLVVHD	1xTMTpro [K	4.016E-05	4.225E-05	1	1	2	P08237	P08237 [433-445]		0	1992.1774

[K].VIVVGNF	1xCarbamido	2.015E-06	4.225E-05	1	1	3	P40925	P40925 [126-142]	0	2366.3357
[R].AIGSASE	1xTMTpro [K	1.496E-08	4.225E-05	1	1	3	P28066	P28066 [169-187]	0	2570.3706
[R].VLVVGA	1xCarbamido	2.484E-05	4.225E-05	1	1	3	Q9UBT2	Q9UBT2 [20-34]	0	2093.2648
[R].VLVSLSA	1xTMTpro [N	2.897E-06	4.225E-05	1	1	2	P09525	P09525 [151-172]	1	2623.4011
[K].ALGALVI	2xCarbamido	1.156E-06	4.225E-05	1	1	4	Q14315	Q14315 [190-219]	0	3639.728
[R].ALGALVI	2xCarbamido	1.572E-07	4.225E-05	1	1	1	P21333	P21333 [197-226]	0	3837.9184
[K].ALGALVI	2xCarbamido	2.701E-05	4.225E-05	1	1	1	O75369	O75369 [170-199]	0	3932.9555
[K].AIGAVPL	1xCarbamido	5.354E-06	4.225E-05	1	1	3	P07339	P07339 [314-331]	0	2597.4327
[K].AIGAVPL	1xCarbamido	1.305E-05	4.225E-05	1	1	3	P07339	P07339 [314-331]	0	2613.4276
[R].VIVSDVC	1xTMTpro [N	1.547E-05	4.225E-05	1	1	2	Q15393	Q15393 [1000-10	0	1981.1078
[K].VLVQNA	1xTMTpro [K	3.859E-05	4.225E-05	1	1	2	Q9Y490	Q9Y490 [2032-20	0	1852.0784
[R].AIGDVFC	1xTMTpro [K	1.071E-06	4.225E-05	1	1	1	P49593	P49593 [327-346]	0	2689.4441
[R].ALGEPIT	1xTMTpro [N	2.401E-05	4.225E-05	1	1	1	O43172	O43172 [114-129]	0	1961.0663
[K].VLVTVEE	1xTMTpro [K	8.186E-07	4.225E-05	1	1	2	Q9NR45	Q9NR45 [335-355	0	2993.5633
[K].GIFGFTD	1xCarbamido	2.958E-05	4.225E-05	1	1	1	P23381	P23381 [265-277]	0	2025.0607
[K].GVWGSV	2xCarbamido	2.839E-05	4.225E-05	1	1	1	O43866	O43866 [263-283]	1	3379.6867
[K].GIGAGG	1xTMTpro [K	1.129E-05	4.225E-05	1	1	1	Q92466	Q92466 [152-163]	0	1639.0034
[K].AIHVLDV	1xTMTpro [N	4.915E-05	4.225E-05	1	1	1	Q9H6Y2	Q9H6Y2 [107-120	0	1911.0619
[K].GLFLPEI	1xTMTpro [K	2.875E-05	4.225E-05	1	1	1	Q12797	Q12797 [610-622]	1	2168.2207
[R].VLSLETS	1xTMTpro [K	8.908E-06	4.225E-05	1	1	1	Q15075	Q15075 [616-634]	0	2712.4911
[K].AIHYALN	3xCarbamido	1.37E-06	4.225E-05	1	1	2	P21926	P21926 [145-169]	0	3401.73
[R].ALKDEEI	3xTMTpro [K	1.211E-05	4.225E-05	1	1	1	P06753	P06753 [135-150]	2	3161.8596
[K].VLSIAQA	1xCarbamido	4.005E-06	4.225E-05	1	1	1	P08571	P08571 [174-191]	0	2304.209
[K].VLSKPPE	1xOxidation [	4.906E-05	4.225E-05	1	1	1	Q02952	Q02952 [488-508]	0	2924.5894
[R].ALKDPN	3xTMTpro [K	5.305E-05	4.225E-05	1	1	1	Q7L2H7	Q7L2H7 [211-230	1	3497.14
[K].VLSKPPE	1xTMTpro [K	1.529E-06	4.225E-05	1	1	1	Q02952	Q02952 [488-508]	0	2908.5945
[R].VLSHQD	1xTMTpro [K	4.673E-05	4.225E-05	1	1	1	Q93034	Q93034 [80-91]	0	1948.1359
[R].GLFIIDPI	1xTMTpro [K	5.974E-05	4.225E-05	1	1	4	P30048	P30048 [185-196]	0	1894.1657
[R].GWEEGY	1xTMTpro [N	1.991E-05	4.225E-05	1	1	4	P62942	P62942 [59-72]	0	1837.9186
[R].GWEEGY	1xOxidation [	5.331E-05	4.225E-05	1	1	4	P62942	P62942 [59-72]	0	1853.9135
[R].VISFEEC	1xTMTpro [N	4.673E-05	4.225E-05	1	1	1	Q9BT78	Q9BT78 [96-107]	0	1681.9444
[K].AIKVEQA	2xTMTpro [K	2.275E-05	4.225E-05	1	1	1	P38159	P38159 [78-93]	1	2660.5552
[K].ALLACEA	3xCarbamido	7.63E-08	4.225E-05	1	1	2	Q63ZY3	Q63ZY3 [757-785	0	3795.769

[K].GLFIKPT	1xTMTpro [K	9.856E-06	4.225E-05	1	1	1	P47895	P47895 [390-406]	0	2562.4246
[K].VLSASQ.	1xTMTpro [N	6.041E-05	4.225E-05	1	1	1	Q9NUQ2	Q9NUQ2 [184-19	0	1552.8767
[R].GWFDAE	1xTMTpro [K	6.31E-06	4.225E-05	1	1	2	P27694	P27694 [413-431]	0	2616.3801
[R].VLRPQV	1xTMTpro [K	9.694E-06	4.225E-05	1	1	1	Q14204	Q14204 [483-506]	0	3270.776
[R].VLRPPG	1xTMTpro [N	5.084E-06	4.225E-05	1	1	1	Q9UK76	Q9UK76 [20-43]	0	2860.4913
[R].GWDLVD	1xTMTpro [N	1.252E-06	4.225E-05	1	1	1	Q27J81	Q27J81 [1046-10	0	3460.8032
[K].AIHTAPV	1xCarbamido	7.63E-07	4.225E-05	1	1	1	Q12788	Q12788 [106-134]	0	3205.6305
[R].GIFPVLC	1xCarbamido	5.074E-07	4.225E-05	1	1	2	P14618	P14618 [468-489]	1	3165.6898
[R].VLSQLTS	1xTMTpro [K	2.834E-05	4.225E-05	1	1	1	P16144	P16144 [159-173]	0	2237.2673
[K].AIGTFLF	1xTMTpro [K	1.619E-06	4.225E-05	1	1	4	O14494	O14494 [101-120]	0	2590.4736
[R].GLGAGA	1xTMTpro [N	2.095E-06	4.225E-05	1	1	1	O15173	O15173 [79-97]	0	2002.0525
[R].VLTPPM	1xTMTpro [K	4.626E-05	4.225E-05	1	1	1	Q15582	Q15582 [497-510]	0	2109.2307
[K].AIGVLTS	1xTMTpro [N	2.195E-07	4.225E-05	2	2	2	Q01813; P17	Q01813 [26-44]; F	0	2092.114
[R].VITNQYN	1xTMTpro [K	1.019E-05	4.225E-05	1	1	2	O00151	O00151 [139-166]	0	3709.8953
[R].VLTLSEF	1xTMTpro [N	3.417E-05	4.225E-05	1	1	1	Q9GZY8	Q9GZY8 [119-14	0	3482.8814
[R].VLTILEQ	1xTMTpro [K	3.495E-05	4.225E-05	1	1	2	Q8NHP8	Q8NHP8 [397-41	0	2433.4646
[R].GVYSSD	1xOxidation	9.82E-06	4.225E-05	1	1	2	P00450	P00450 [1009-10	0	2989.4613
[K].VLTFALS	1xTMTpro [K	1.184E-07	4.225E-05	1	1	1	P55786	P55786 [795-821]	0	3324.8659
[R].VLTEDEI	1xOxidation	3.163E-05	4.225E-05	1	1	2	P14324	P14324 [93-110]	0	2273.1403
[R].GVYSSD	1xTMTpro [N	4.981E-06	4.225E-05	1	1	1	P00450	P00450 [1009-10	0	2973.4664
[R].VLTEDEI	1xTMTpro [N	2.667E-07	4.225E-05	1	1	2	P14324	P14324 [93-110]	0	2257.1454
[K].GVYVLM	1xTMTpro [N	3.097E-05	4.225E-05	1	1	2	P26440	P26440 [275-287]	0	1755.9634
[K].VLTCTDI	1xCarbamido	7.234E-06	4.225E-05	1	1	3	Q9NUQ9	Q9NUQ9 [7-33]	0	3737.85
[K].VLSVPES	1xTMTpro [K	1.584E-05	4.225E-05	1	1	3	P61960	P61960 [20-34]	0	2196.3135
[K].VLSVDES	2xTMTpro [K	1.616E-05	4.225E-05	1	1	1	Q4KMQ2	Q4KMQ2 [167-18	0	3465.9337
[K].VLSVCPS	1xCarbamido	1.913E-05	4.225E-05	1	1	2	P14923	P14923 [337-359]	0	3238.8472
[K].VLSTTKF	2xTMTpro [K	2.475E-05	4.225E-05	1	1	1	Q16666	Q16666 [209-225]	0	2941.6049
[K].VLSSSGS	1xCarbamido	8.85E-08	4.225E-05	1	1	1	Q15149	Q15149 [978-100	0	3530.812
[R].VISSGSV	1xTMTpro [N	8.655E-07	4.225E-05	1	1	2	P98160	P98160 [168-187]	0	2421.2733
[R].VLSRPN.	1xTMTpro [N	6.051E-05	4.225E-05	1	1	1	Q99623	Q99623 [108-123]	0	2193.1769
[R].VLSQQA	1xTMTpro [K	5.775E-05	4.225E-05	1	1	3	P30566	P30566 [405-415]	0	1738.0718
[R].VITDVYY	1xTMTpro [N	2.435E-05	4.225E-05	1	1	1	Q12888	Q12888 [1253-12	0	2062.0664
[R].GYCLIIN	1xCarbamido	3.465E-05	4.225E-05	1	1	1	Q14790	Q14790 [234-246]	0	2172.188

[R].VLIGGDI	1xTMTpro [N	4.352E-05	4.225E-05	1	1	2	O60701	O60701 [178-190]	0	1674.8982
[K].VILGAHC	1xTMTpro [N	1.572E-06	4.225E-05	1	1	3	P00747	P00747 [635-656]	0	2800.5277
[R].GIEDNPI	1xCarbamido	3.8E-05	4.225E-05	1	1	2	P26640	P26640 [652-665]	0	1917.9846
[R].ALQPLEI	1xTMTpro [K	2.308E-05	4.225E-05	1	1	1	Q14764	Q14764 [336-360]	1	3494.816
[K].ALQPIVS	1xTMTpro [K	6.362E-07	4.225E-05	1	1	3	P43155	P43155 [53-67]	0	2346.2949
[R].GYGPDC	1xOxidation [	4.981E-05	4.225E-05	1	1	1	Q9UJU6	Q9UJU6 [411-430	0	2533.2029
[K].GIEDDLN	1xTMTpro [K	5.047E-05	4.225E-05	1	1	2	Q9BRF8	Q9BRF8 [302-312	0	1870.0487
[K].VLEDSDI	2xTMTpro [K	1.215E-05	4.225E-05	1	1	1	P11021	P11021 [345-367]	2	3400.9355
[R].ALQQVIC	1xTMTpro [N	1.535E-05	4.225E-05	1	1	1	Q9UI10	Q9UI10 [232-250]	0	2506.3109
[K].GYGYGC	1xTMTpro [N	8.429E-06	4.225E-05	1	1	1	Q16527	Q16527 [70-84]	0	1863.8979
[R].ALQQYT	2xTMTpro [K	4.817E-05	4.225E-05	1	1	1	Q9Y678	Q9Y678 [561-577	0	2919.6648
[R].ALQSGC	1xCarbamido	1.529E-06	4.225E-05	1	1	1	O43175	O43175 [248-270]	1	2792.3957
[K].VLEDNKI	2xCarbamido	7.859E-06	4.225E-05	1	1	2	P49419	P49419 [237-263]	1	3674.9736
[K].GYGYGC	1xTMTpro [K	4.765E-06	4.225E-05	1	1	2	P21291	P21291 [70-84]	0	2083.0952
[K].ALQSVG	1xTMTpro [K	2.573E-06	4.225E-05	1	1	2	P62333	P62333 [49-62]	0	2049.2564
[R].AIQTLGY	1xTMTpro [K	4.412E-05	4.225E-05	1	1	1	Q5VYK3	Q5VYK3 [861-879	0	2698.4485
[K].GYGYGC	2xTMTpro [K	6.603E-09	4.225E-05	1	1	4	P21291	P21291 [70-91]	1	3071.6829
[R].AIQTVSC	2xCarbamido	1.021E-05	4.225E-05	1	1	1	Q9H3U1	Q9H3U1 [414-431	0	2264.1447
[K].GYKPPD	2xTMTpro [K	1.903E-05	4.225E-05	1	1	2	Q92905	Q92905 [192-210]	0	3045.6713
[R].VIDVFAN	1xTMTpro [K	9.14E-06	4.225E-05	1	1	2	O00487	O00487 [69-94]	0	3299.7841
[K].VLDSTPV	1xTMTpro [N	2.429E-06	4.225E-05	1	1	2	Q16647	Q16647 [334-350]	0	2134.1926
[K].VIDSSDV	1xTMTpro [N	4.071E-05	4.225E-05	1	1	2	Q13823	Q13823 [213-227]	0	1919.0769
[K].VLDSGA	1xTMTpro [K	9.909E-07	4.225E-05	1	1	2	P06576	P06576 [125-143]	1	2527.5103
[K].VIEGLDT	1xCarbamido	1.808E-05	4.225E-05	1	1	2	Q96AY3	Q96AY3 [435-451	0	2154.0854
[K].VLEGMV	1xTMTpro [N	2.031E-05	4.225E-05	1	1	1	Q14126	Q14126 [276-292]	0	2249.1767
[R].GYGPDC	1xTMTpro [N	5.039E-05	4.225E-05	1	1	5	Q9UJU6	Q9UJU6 [411-430	0	2517.208
[K].AIQLTYN	1xOxidation [	5.743E-06	4.225E-05	1	1	8	P02679	P02679 [89-111]	0	3448.8814
[R].GYFNEE	1xTMTpro [K	8.523E-06	4.225E-05	1	1	1	P15924	P15924 [2395-241	0	2767.3806
[K].GYFVQP	1xTMTpro [N	1.043E-05	4.225E-05	1	1	2	P00352	P00352 [379-395]	0	2294.1446
[K].VIESTQD	1xTMTpro [N	3.334E-06	4.225E-05	1	1	4	Q01082	Q01082 [977-996]	0	2434.2931
[R].GLEEGC	1xCarbamido	9.727E-07	4.225E-05	1	1	3	Q9Y6C2	Q9Y6C2 [709-726	0	2191.0733
[K].VIESEDY	2xCarbamido	1.49E-06	4.225E-05	1	1	4	Q9Y3A5	Q9Y3A5 [196-218	0	3044.5028
[K].ALQEGA	1xCarbamido	3.163E-05	4.225E-05	1	1	1	Q86XP3	Q86XP3 [372-386	0	1904.0231

[K].ALQEGE	1xTMTpro [N	2.533E-05	4.225E-05	1	1	2	P49589	P49589 [296-310]	0	1864.9572
[K].VLEQPV	1xTMTpro [N	1.636E-05	4.225E-05	1	1	2	Q9BZE1	Q9BZE1 [335-350	0	1987.1143
[K].VLEQLT	1xTMTpro [K	5.581E-05	4.225E-05	1	1	3	P62913	P62913 [39-52]	0	2155.2618
[K].GIEEEEI	2xCarbamido	3.222E-07	4.225E-05	1	1	2	P15170	P15170 [368-391]	0	3073.5042
[R].ALRDPA	1xTMTpro [N	1.75E-05	4.225E-05	1	1	1	Q15628	Q15628 [243-258	1	2186.1413
[K].VLEPSE	1xTMTpro [N	6.714E-07	4.225E-05	1	1	1	P18583	P18583 [299-338]	0	4630.2816
[K].ALQGAS	1xTMTpro [N	5.296E-05	4.225E-05	1	1	2	O00429	O00429 [719-731	0	1673.9869
[K].GYGFVH	1xTMTpro [N	9.465E-06	4.225E-05	1	1	2	P11940	P11940 [139-153]	0	2045.0048
[K].VIEINPYI	2xCarbamido	4.791E-07	4.225E-05	1	1	2	P28062	P28062 [106-129]	0	3150.5018
[K].GLEDNQ	1xTMTpro [N	3.839E-05	4.225E-05	1	1	1	Q8IXB1	Q8IXB1 [103-119]	0	2383.091
[R].ALQLLDI	1xTMTpro [N	4.825E-05	4.225E-05	1	1	1	Q13485	Q13485 [532-552	0	2633.418
[K].VLELEGI	2xTMTpro [K	3.588E-05	4.225E-05	1	1	2	P61201	P61201 [57-70]	1	2533.4482
[K].VLELEDI	1xTMTpro [N	4.793E-05	4.225E-05	1	1	2	Q08378	Q08378 [1083-10	0	1763.9346
[K].VIEHIME	1xOxidation	2.926E-05	4.225E-05	1	1	6	P06702	P06702 [58-72]	0	2367.2358
[K].VIEHIME	1xTMTpro [K	7.874E-06	4.225E-05	1	1	23	P06702	P06702 [58-72]	0	2351.2408
[K].AIQLTYN	2xTMTpro [K	1.409E-07	4.225E-05	1	1	6	P02679	P02679 [89-111]	0	3432.8865
[R].VLENVEI	1xTMTpro [K	5.149E-05	4.225E-05	1	1	3	Q9H553	Q9H553 [274-285	0	2109.1836
[R].GYFIQPT	1xTMTpro [K	9.242E-06	4.225E-05	1	1	4	P05091	P05091 [395-414]	0	2795.4934
[K].GYIFLEY	1xTMTpro [K	1.27E-05	4.225E-05	1	1	1	P55884	P55884 [231-248]	0	2559.4103
[R].VLDPAIF	1xTMTpro [N	2.362E-05	4.225E-05	1	1	2	P55268	P55268 [221-235]	0	1944.0761
[K].VLAVNQ	1xOxidation	3.091E-05	4.225E-05	1	1	2	P12814	P12814 [265-281]	0	2676.3682
[K].VLAVNQ	1xTMTpro [K	8.46E-06	4.225E-05	1	1	5	P12814	P12814 [265-281]	0	2660.3733
[K].VLAVNQ	1xOxidation	6.206E-06	4.225E-05	1	1	4	O43707	O43707 [284-300	0	2685.3686
[K].VLAVNQ	1xTMTpro [K	5.495E-07	4.225E-05	1	1	3	O43707	O43707 [284-300	0	2669.3736
[K].GYNDDY	1xTMTpro [N	8.383E-06	4.225E-05	1	1	1	P50402	P50402 [89-103]	0	2226.9787
[K].VLATVTI	2xTMTpro [K	3.08E-05	4.225E-05	1	1	2	Q02878	Q02878 [88-100]	0	2197.3736
[K].GYNPAE	1xTMTpro [K	3.626E-05	4.225E-05	1	1	1	Q13144	Q13144 [482-493	0	1741.9729
[K].GIDPFSL	1xTMTpro [K	5.393E-05	4.225E-05	1	1	3	P40227	P40227 [296-307]	0	1871.077
[R].VLASDA	1xTMTpro [K	5.609E-05	4.225E-05	1	1	2	P42566	P42566 [34-44]	0	1714.0395
[R].GYNPGL	1xTMTpro [N	9.945E-07	4.225E-05	1	1	1	P19838	P19838 [164-186]	0	2716.4014
[R].ALSVAEI	1xTMTpro [N	2.401E-05	4.225E-05	1	1	2	Q9H4B7	Q9H4B7 [283-297	0	1984.0493
[R].ALSVEFI	1xTMTpro [K	1.906E-05	4.225E-05	1	1	2	P06401	P06401 [900-919]	0	2798.5328
[K].VLAQNS	1xTMTpro [K	4.33E-05	4.225E-05	1	1	1	P40227	P40227 [450-465]	0	2370.3524

[K].ALSVGN	1xCarbamido	3.096E-06	4.225E-05	1	1	2	P31948	P31948 [14-32]	0	2675.4206
[K].VIAPVDE	1xTMTpro [K	5.509E-05	4.225E-05	1	1	1	P16284	P16284 [496-511]	0	2306.3827
[K].VLAPQIS	1xTMTpro [N	1.028E-05	4.225E-05	1	1	1	P16083	P16083 [184-201]	0	2290.225
[R].VLAPAS	1xTMTpro [N	6.369E-06	4.225E-05	1	1	1	Q14126	Q14126 [1050-10	0	2769.4412
[K].VLANNSI	1xTMTpro [K	4.61E-05	4.225E-05	1	1	2	O76094	O76094 [74-84]	0	1830.0617
[R].VLALSVE	1xTMTpro [K	1.962E-05	4.225E-05	1	1	3	P05388	P05388 [248-264]	0	2504.4144
[K].VIAINVDI	1xTMTpro [K	2.347E-08	4.225E-05	1	1	2	Q15181	Q15181 [156-177	1	3052.6195
[K].VIAINVDI	1xTMTpro [K	2.839E-05	4.225E-05	1	1	3	Q15181	Q15181 [156-176	0	2896.5184
[K].VLCDDV	2xCarbamido	1.505E-05	4.225E-05	1	1	3	P02452	P02452 [68-79]	0	2075.0645
[K].GYIWNY	1xTMTpro [K	2.425E-06	4.225E-05	1	1	2	Q15181	Q15181 [89-109]	0	3069.535
[K].VLCGGD	1xCarbamido	2.136E-05	4.225E-05	1	1	4	P49189	P49189 [353-366]	0	2170.171
[K].VLCPIIQ	1xCarbamido	4.027E-06	4.225E-05	2	2	2	Q7Z460; O7	Q7Z460 [1426-14	0	2721.5869
[K].VLDNYL	1xTMTpro [N	1.399E-06	4.225E-05	1	1	3	O00299	O00299 [139-165	0	3296.5977
[K].ALSAIAD	1xTMTpro [N	2.324E-06	4.225E-05	1	1	2	O60716	O60716 [711-725	0	1957.0674
[K].GLDWVK	1xCarbamido	3.457E-07	4.225E-05	1	1	4	P27695	P27695 [80-98]	1	3156.7431
[R].GIDVQQ	1xTMTpro [N	1.391E-06	4.225E-05	2	2	3	P60842; Q14	P60842 [335-353]	0	2448.3418
[R].VLDLSGI	1xTMTpro [N	5.929E-07	4.225E-05	1	1	1	Q8N1G4	Q8N1G4 [103-140	0	4094.2205
[K].VIDLPGC	1xTMTpro [K	3.675E-08	4.225E-05	1	1	1	Q9H479	Q9H479 [76-92]	0	2362.3448
[K].GYIGTHC	3xCarbamido	2.277E-06	4.225E-05	1	1	2	P35555	P35555 [139-159]	0	2583.1822
[R].AISESGV	1xTMTpro [K	3.208E-05	4.225E-05	2	2	1	Q9UKY3; P2	Q9UKY3 [244-258	0	2082.2666
[K].GLDVDS	2xTMTpro [K	1.098E-05	4.225E-05	1	1	2	P18621	P18621 [106-124]	1	2987.771
[K].VLDHAM	1xCarbamido	6.895E-06	4.225E-05	1	1	3	Q8WYA6	Q8WYA6 [356-37	0	2502.2725
[K].VIDPATA	1xTMTpro [N	3.387E-05	4.225E-05	1	1	2	P50991	P50991 [194-206]	0	1661.9393
[K].VIDGLET	2xTMTpro [K	2.493E-05	4.225E-05	1	1	4	Q9H2H8	Q9H2H8 [121-139	1	3066.7754
[R].GYISPYF	1xTMTpro [K	2.775E-05	4.225E-05	1	1	4	P10809	P10809 [222-233]	0	1998.1192
[K].VLDFEHI	1xTMTpro [K	1.226E-05	4.225E-05	1	1	8	P60660	P60660 [64-79]	0	2496.418
[R].VIDFDEN	1xTMTpro [N	6.003E-05	4.225E-05	1	1	1	Q14257	Q14257 [130-148	0	2519.1745
[R].VLDENLA	1xTMTpro [K	2.031E-05	4.225E-05	1	1	2	P29590	P29590 [595-616]	0	3152.7123
[K].GYLTDLI	2xTMTpro [K	8.899E-07	4.225E-05	1	1	1	O94906	O94906 [272-293	1	3314.8235
[R].GYLVTQ	1xTMTpro [K	3.091E-05	4.225E-05	1	1	1	P19388	P19388 [25-41]	0	2636.3951
[R].ALSNLES	1xTMTpro [N	3.288E-05	4.225E-05	2	2	3	Q9UMX0; Q9	Q9UMX0 [258-27	0	1979.0881
[K].VLDAPG	1xOxidation	8.414E-06	4.225E-05	1	1	1	Q9HA64	Q9HA64 [76-94]	0	2334.1575
[R].GIDQCIF	1xCarbamido	5.85E-05	4.225E-05	1	1	2	O95373	O95373 [753-768	0	2135.149

[R].VLDALFF	2xCarbamido	3.201E-06	4.225E-05	1	1	2	P38606	P38606 [233-256]	0	3044.6193
[K].GLDVDS	1xTMTpro [K	2.725E-06	4.225E-05	1	1	1	P18621	P18621 [106-121]	0	2387.379
[R].GYFEYIE	1xTMTpro [K	3.168E-05	4.225E-05	1	1	1	Q00839	Q00839 [256-268]	1	2306.1949
[K].ALQDQL	1xTMTpro [K	5.384E-05	4.225E-05	1	1	2	P01019	P01019 [83-94]	0	1877.1716
[K].AIQDMFF	1xTMTpro [N	3.588E-05	4.225E-05	1	1	2	Q9H0E2	Q9H0E2 [236-250	0	2111.0585
[R].VIHLQFN	1xCarbamido	4.483E-06	4.225E-05	1	1	3	P20774	P20774 [238-256]	0	2844.521
[K].VLHGEQ	1xTMTpro [K	1.446E-05	4.225E-05	1	1	1	P51659	P51659 [404-419]	0	2563.4892
[K].VLHFDQ	1xTMTpro [K	1.105E-05	4.225E-05	1	1	2	P29508	P29508 [56-69]	0	2197.2109
[K].VLHFDQ	1xTMTpro [K	5.554E-05	4.225E-05	1	1	2	P48594	P48594 [56-69]	0	2269.232
[K].VLHEAE	1xCarbamido	3.637E-06	4.225E-05	1	1	1	P62318	P62318 [9-29]	0	2718.3476
[K].VIHDNFC	1xOxidation [	3.951E-07	4.225E-05	1	1	18	P04406	P04406 [163-186]	0	3219.7691
[K].VIHDNFC	1xTMTpro [K	2.937E-09	4.225E-05	1	1	9	P04406	P04406 [163-186]	0	3203.7742
[R].VLHDEIL	2xTMTpro [K	2.358E-05	4.225E-05	1	1	1	Q9Y2D2	Q9Y2D2 [72-86]	0	2692.5888
[R].ALNLFQ	1xTMTpro [K	4.503E-05	4.225E-05	1	1	1	P43246	P43246 [309-332]	0	3085.7025
[K].ALNLGY	1xTMTpro [N	2.895E-05	4.225E-05	1	1	6	P12111	P12111 [913-925]	0	1771.9662
[R].GLEPGQ	1xTMTpro [K	7.247E-06	4.225E-05	1	1	3	P24821	P24821 [1042-105	0	2369.3208
[R].VLGVPII	1xTMTpro [K	1.845E-05	4.225E-05	1	1	1	Q14498	Q14498 [218-232]	0	2160.3248
[R].GYDQSA	1xTMTpro [K	5.756E-07	4.225E-05	1	1	1	P10321	P10321 [136-155]	1	2914.4714
[R].GYDSAG	1xCarbamido	4.503E-05	4.225E-05	1	1	1	Q06210	Q06210 [34-56]	1	3345.6262
[K].AINQGGI	1xTMTpro [N	4.51E-05	4.225E-05	1	1	4	P60900	P60900 [31-43]	0	1589.9294
[K].VLGTAFI	1xTMTpro [K	5.491E-05	4.225E-05	1	1	2	Q92598	Q92598 [222-234]	0	1930.1294
[R].VLGSSV	1xTMTpro [N	3.866E-05	4.225E-05	1	1	1	P98160	P98160 [2409-24	0	3419.9684
[K].VLGSQE	1xTMTpro [K	7.478E-06	4.225E-05	1	1	2	P21397	P21397 [380-395]	0	2444.3429
[K].ALNSIID	1xTMTpro [K	5.446E-05	4.225E-05	1	1	6	P05109	P05109 [8-18]	0	1881.109
[K].VLGSLE	1xTMTpro [K	1.967E-06	4.225E-05	1	1	3	P27338	P27338 [371-386]	0	2421.3521
[K].VLGSGA	1xTMTpro [K	2.005E-05	4.225E-05	2	3	2	P04626; P0	P04626 [725-736]	0	1807.0609
[R].AINEAYK	2xTMTpro [K	5.464E-05	4.225E-05	1	1	4	P08133	P08133 [472-483]	1	2393.3281
[K].GYDFES	1xTMTpro [K	3.429E-05	4.225E-05	1	1	2	Q06210	Q06210 [140-154]	0	2314.1582
[K].VLHPLE	1xTMTpro [K	1.389E-05	4.225E-05	1	1	1	Q13740	Q13740 [176-189]	0	2143.3499
[K].AINCATS	2xCarbamido	3.108E-05	4.225E-05	1	1	1	P49327	P49327 [1445-14	0	2108.1275
[K].ALLYLPI	1xTMTpro [K	2.295E-05	4.225E-05	1	1	2	Q15291	Q15291 [398-436]	0	4777.3938
[K].GLEQLL	2xTMTpro [K	5.464E-05	4.225E-05	1	1	1	P12109	P12109 [136-151]	1	2634.5759
[K].VLIFQEE	1xTMTpro [K	4.442E-05	4.225E-05	1	1	1	O95810	O95810 [157-173]	0	2570.4726

[K].VLIFDDS	1xTMTpro [N	3.768E-06	4.225E-05	1	1	1	Q12797	Q12797 [716-735]	0	2731.3323
[K].GLEQLLY	1xTMTpro [K	2.9E-05	4.225E-05	1	1	3	P12109	P12109 [136-148]	0	1959.1883
[R].GIEQAV(	1xTMTpro [N	1.323E-06	4.225E-05	1	1	1	Q16891	Q16891 [548-564]	0	2128.0954
[R].VLLEAGI	1xTMTpro [N	4.07E-08	4.225E-05	1	1	3	Q9NY33	Q9NY33 [578-602	0	2829.5277
[K].VILDYNT	1xTMTpro [K	1.966E-05	4.225E-05	1	1	1	Q14258	Q14258 [459-469]	0	1896.0835
[R].AIMEMP:	1xTMTpro [N	5.2E-05	4.225E-05	1	1	1	P55265	P55265 [475-489]	0	2040.0366
[K].ALMGLY	1xCarbamido	2.038E-05	4.225E-05	1	1	4	P40939	P40939 [338-350]	0	2098.1797
[K].ALNVEPI	1xCarbamido	2.64E-05	4.225E-05	1	1	3	P30044	P30044 [192-214]	0	2687.4245
[R].VLLDQL(	1xTMTpro [N	3.607E-05	4.225E-05	1	1	2	P04114	P04114 [2296-23(	0	1896.0761
[K].GYDCSD	3xCarbamido	2.779E-05	4.225E-05	1	1	1	P24821	P24821 [462-479]	0	2515.9767
[R].VLLDAGI	1xTMTpro [K	1.43E-05	4.225E-05	1	1	2	P54289	P54289 [705-722]	0	2705.4794
[K].VILALGD	2xCarbamido	1.584E-07	4.225E-05	1	1	2	Q14240	Q14240 [120-142]	0	2753.3856
[R].ALMLQG	1xTMTpro [K	6.315E-07	4.225E-05	1	1	6	P10809	P10809 [38-58]	0	2721.5539
[R].ALMLQG	1xOxidation [	2.967E-06	4.225E-05	1	1	4	P10809	P10809 [38-58]	0	2737.5488
[R].ALMLQG	2xOxidation [	5.68E-06	4.225E-05	1	1	2	P10809	P10809 [38-58]	0	2753.5437
[R].VIKDFMI	1xTMTpro [K	3.866E-05	4.225E-05	1	1	3	P23284	P23284 [96-109]	1	2235.2451
[K].VLHYGD	1xTMTpro [K	7.409E-06	4.225E-05	1	1	1	Q96JJ3	Q96JJ3 [578-600]	0	3241.6509
[R].VIHVVTS	1xTMTpro [K	5.037E-07	4.225E-05	1	1	3	P35221	P35221 [552-571]	0	2918.5101
[R].VLHVSEI	1xTMTpro [N	4.923E-05	4.225E-05	1	1	1	Q8N1G4	Q8N1G4 [311-324	0	1864.0976
[R].VLLDDVI	1xTMTpro [N	1.685E-05	4.225E-05	1	1	2	Q96JQ0	Q96JQ0 [882-917	0	4137.1715
[K].GIEILTDI	1xTMTpro [K	1.564E-05	4.225E-05	1	1	2	P50991	P50991 [144-174]	1	3999.1564
[K].VLGQLTI	1xTMTpro [K	7.395E-06	4.225E-05	1	1	1	Q96EK6	Q96EK6 [56-73]	0	2571.4348
[R].ALPAVQ	1xTMTpro [N	5.037E-06	4.225E-05	1	1	4	P22314	P22314 [369-384]	0	2113.1573
[K].AIPVSPS	1xTMTpro [N	2.799E-08	4.225E-05	1	1	2	Q07157	Q07157 [1613-16:	0	2898.4288
[K].VIFPYEA	1xTMTpro [K	4.817E-05	4.225E-05	1	1	2	Q96B97	Q96B97 [274-289	0	2503.3576
[R].ALQAAL,	1xTMTpro [K	3.411E-05	4.225E-05	1	1	1	Q15628	Q15628 [44-63]	0	2607.4671
[R].ALQAIQ	1xTMTpro [K	3.261E-06	4.225E-05	1	1	2	Q92616	Q92616 [69-87]	0	2560.459
[K].ALQAAY(	1xTMTpro [N	1.348E-06	4.225E-05	1	1	3	O43488	O43488 [279-297]	0	2109.1623
[R].VLFPGN.	1xTMTpro [K	6.032E-05	4.225E-05	1	1	1	P46821	P46821 [487-504]	0	2630.4685
[R].VLFPGC'	2xCarbamido	2.449E-05	4.225E-05	1	1	1	Q66K74	Q66K74 [430-447	0	2289.2419
[R].VIFPAAE	2xTMTpro [K	3.353E-06	4.225E-05	1	1	2	Q00341	Q00341 [761-779]	1	2998.7645
[R].ALQALQ	1xTMTpro [K	3.174E-05	4.225E-05	1	1	1	Q9BRG1	Q9BRG1 [152-16:	0	1774.0467
[K].GYEVIYL	1xCarbamido	7.816E-08	4.225E-05	1	1	5	P14625	P14625 [562-587]	1	3712.9064

[R].ALPVFCI	1xCarbamido	1.812E-05	4.225E-05	1	1	1	O75636	O75636 [116-135]	0	2602.2753
[K].VLFLLGA	1xCarbamido	2.538E-05	4.225E-05	1	1	6	P28331	P28331 [544-557]	0	1796.006
[K].VIFHPEF	1xTMTpro [N	2.034E-06	4.225E-05	1	1	1	P13807	P13807 [475-498]	0	3125.6519
[K].VLFEDPI	1xTMTpro [K	3.13E-05	4.225E-05	1	1	2	O15484	O15484 [25-42]	0	2741.457
[R].GYEYVL	1xTMTpro [N	1.123E-05	4.225E-05	1	1	1	Q96I51	Q96I51 [154-175]	0	2849.5004
[K].AIQDGTI	1xTMTpro [K	7.744E-06	4.225E-05	1	1	1	Q92520	Q92520 [138-156]	0	2577.3726
[K].AIQDGTI	1xOxidation [	1.096E-05	4.225E-05	1	1	1	Q92520	Q92520 [138-156]	0	2593.3675
[K].VLFASQI	1xTMTpro [N	2.435E-05	4.225E-05	1	1	4	O75369	O75369 [823-836]	0	1866.0445
[R].VIEVGNI	1xTMTpro [N	3.037E-05	4.225E-05	1	3	1	Q5JPE7	Q5JPE7 [1035-10	0	2348.2781
[K].ALQDLEI	1xTMTpro [N	5.295E-06	4.225E-05	1	1	3	Q9NQG5	Q9NQG5 [183-19	0	1935.0103
[R].VLEVGA	1xTMTpro [N	3.328E-05	4.225E-05	1	1	1	O95602	O95602 [126-140]	0	1993.1289
[K].VLEVDPI	1xTMTpro [N	1.119E-05	4.225E-05	1	1	1	P09914	P09914 [167-187]	0	2571.2898
[R].VIFLEDD	1xTMTpro [N	3.365E-06	4.225E-05	1	1	2	Q06210	Q06210 [291-306]	0	2037.0824
[K].VLYNDL(	1xTMTpro [K	4.315E-05	4.225E-05	1	1	1	P46939	P46939 [1523-153	0	2115.1941
[K].GYEVIYL	1xCarbamido	7.18E-06	4.225E-05	1	1	3	P14625	P14625 [562-586]	0	3556.8053
[K].VLGAFSI	1xTMTpro [K	1.162E-07	4.225E-05	2	2	124	P02042; P68	P02042 [68-83]; F	0	2278.3051
[R].ALPAVQ	1xTMTpro [K	7.314E-06	4.225E-05	1	1	2	P22314	P22314 [369-385]	1	2545.4594
[K].VLGPYTI	1xCarbamido	5.287E-05	4.225E-05	1	1	1	P22314	P22314 [269-288]	0	2659.3033
[R].VLGPLAI	1xTMTpro [N	3.352E-05	4.225E-05	1	1	2	Q02127	Q02127 [200-221]	0	2559.4466
[R].ALPDME	1xTMTpro [K	1.019E-05	4.225E-05	1	1	2	Q14204	Q14204 [2611-263	0	3053.7088
[R].GYDYGF	1xTMTpro [N	5.028E-07	4.225E-05	1	1	2	P49327	P49327 [998-1019	0	2672.2912
[K].VIGNQSI	1xTMTpro [N	3.817E-06	4.225E-05	1	1	2	Q13011	Q13011 [215-230]	0	2036.146
[K].GYEDGC	1xCarbamido	3.18E-05	4.225E-05	1	2	1	O00478	O00478 [156-167]	0	1709.8236
[R].GLELIAS	1xCarbamido	3.657E-05	4.225E-05	1	1	2	P34897	P34897 [70-82]	0	1799.9281
[K].ALPGQLI	2xTMTpro [K	3.166E-06	4.225E-05	1	1	3	P09211	P09211 [122-141]	0	3038.7819
[K].GIELGDI	1xTMTpro [K	2.819E-05	4.225E-05	1	1	1	Q9BZF1	Q9BZF1 [824-838	0	2197.2571
[K].GLEHVT'	1xTMTpro [K	2.071E-05	4.225E-05	1	1	1	Q9NPA8	Q9NPA8 [58-74]	0	2444.3892
[K].ALPLEAE	1xTMTpro [K	4.142E-05	4.225E-05	1	1	2	P27816	P27816 [465-476]	0	1845.1341
[K].VLGITDM	1xTMTpro [K	3.974E-05	4.225E-05	1	1	2	P07093	P07093 [310-321]	0	1921.0596
[K].ALPLIVG	1xTMTpro [K	1.498E-07	4.225E-05	1	1	3	Q5SSJ5	Q5SSJ5 [15-29]	0	2167.3458
[R].VIGLSSD	1xTMTpro [N	2.412E-06	4.225E-05	1	1	1	O00303	O00303 [262-278]	0	1962.0939
[R].GLELEP(	2xTMTpro [K	3.037E-05	4.225E-05	1	1	1	P39060	P39060 [609-639]	1	4010.2419
[K].VLGETAI	1xTMTpro [K	2.707E-07	4.225E-05	1	1	2	Q02952	Q02952 [1430-145	0	3441.8972

[K].VLGEAM	1xTMTpro [K	2.198E-05	4.225E-05	1	1	2	Q9Y490	Q9Y490 [1402-14	0	2027.1451
[K].VLGDVIE	1xTMTpro [K	2.128E-05	4.225E-05	1	1	1	P02511	P02511 [93-103]	0	1774.0718
[R].GIEKPPF	2xTMTpro [K	5.831E-05	4.225E-05	1	1	2	Q13435	Q13435 [516-529]	0	2542.5101
[R].VLGATLI	1xTMTpro [K	4.465E-05	4.225E-05	1	1	2	P55786	P55786 [782-794]	0	1989.2604
[R].VLGAHIL	1xCarbamido	9.304E-08	4.225E-05	1	1	2	P09622	P09622 [450-482]	0	3658.8528
[K].ALPLEAE	2xTMTpro [K	8.035E-06	4.225E-05	1	1	2	P27816	P27816 [465-490]	1	3644.0801
[K].VLYQVE	1xTMTpro [K	1.368E-07	4.225E-05	1	1	1	O43795	O43795 [519-536]	1	2793.5431
[K].ALFEEVF	2xTMTpro [K	8.154E-06	4.225E-05	1	1	3	P31939	P31939 [510-525]	1	2758.6059
[K].ALFEEVF	1xTMTpro [K	2.958E-05	4.225E-05	1	1	1	P31939	P31939 [510-524]	0	2326.3038
[K].VQDGTL	2xCarbamido	4.071E-05	4.225E-05	1	1	1	Q6UY14	Q6UY14 [437-455	0	2318.1552
[R].VQDDEV	1xTMTpro [N	3.641E-08	4.225E-05	1	1	4	P78371	P78371 [90-111]	0	2592.3688
[R].VQCPAL	1xCarbamido	1.144E-05	4.225E-05	1	1	1	Q86WA6	Q86WA6 [232-24	1	2636.5202
[K].VQAQYP	1xCarbamido	1.191E-05	4.225E-05	1	1	4	P50135	P50135 [74-97]	0	3251.7226
[K].VQAQLN	1xTMTpro [K	4.587E-05	4.225E-05	1	1	1	Q96QD9	Q96QD9 [199-215	0	2492.4216
[R].ALAEIAK	1xTMTpro [K	2.551E-05	4.225E-05	1	1	1	P23246	P23246 [343-358]	1	2352.3089
[K].VQANLG	1xTMTpro [K	1.231E-05	4.225E-05	1	1	5	Q09666	Q09666 [5164-51	0	2446.3797
[R].AIAELGI	1xTMTpro [N	1.978E-06	4.225E-05	1	1	3	P06576	P06576 [388-406]	0	2292.2406
[K].VQALEE	1xTMTpro [K	4.713E-05	4.225E-05	1	1	2	P35527	P35527 [171-184]	0	2195.18
[R].VQAHAA	1xCarbamido	5.169E-06	4.225E-05	1	1	2	O00410	O00410 [458-475]	0	2564.3787
[K].ALAESW	1xTMTpro [K	2.799E-05	4.225E-05	1	1	1	Q15382	Q15382 [136-151]	0	2303.2527
[K].ALAETGI	1xTMTpro [K	1.391E-05	4.225E-05	1	1	1	Q7L4I2	Q7L4I2 [306-327]	0	2885.5727
[K].GVIPSSL	1xTMTpro [K	2.902E-06	4.225E-05	1	1	1	O76024	O76024 [257-277]	0	2871.4755
[K].GLLGNP	1xCarbamido	5.183E-05	4.225E-05	1	1	1	P23219	P23219 [532-559]	0	3979.1608
[R].AIAFTQY	1xCarbamido	1.978E-06	4.225E-05	1	1	1	P22830	P22830 [185-208]	0	3003.4841
[K].ALAGCD	1xCarbamido	2.053E-05	4.225E-05	1	1	3	P37837	P37837 [246-258]	0	2001.1335
[R].VQAALV	1xTMTpro [N	1.364E-05	4.225E-05	1	1	1	Q86VR2	Q86VR2 [56-67]	0	1699.9927
[R].VPYTDD	1xTMTpro [N	1.422E-05	4.225E-05	1	1	2	P35250	P35250 [211-230]	0	2516.2662
[K].VPVPED	3xTMTpro [K	6.984E-06	4.225E-05	1	1	1	O75947	O75947 [79-99]	2	3606.0055
[K].VPVLQLI	1xCarbamido	1.179E-06	4.225E-05	1	1	1	P56192	P56192 [48-67]	0	2544.3451
[R].AIAIASDI	2xCarbamido	1.47E-05	4.225E-05	1	1	1	P32320	P32320 [84-103]	0	2502.1746
[R].VQDLSA	1xTMTpro [N	9.023E-06	4.225E-05	1	1	1	P40222	P40222 [485-503]	0	2178.0958
[K].VQDTEIS	1xTMTpro [N	7.036E-06	4.225E-05	1	1	2	Q8WXH0	Q8WXH0 [2635-2	0	2496.349
[K].ALADVEY	1xTMTpro [N	4.956E-05	4.225E-05	1	1	1	P52888	P52888 [67-79]	0	1768.9764

[R].GVILTSD	1xCarbamido	5.244E-05	4.225E-05	1	1	1	P42126	P42126 [93-116]	0	2855.4715
[K].AKVDEFI	1xCarbamido	1.794E-07	4.225E-05	1	1	3	P27635	P27635 [41-69]	1	3889.9232
[R].GVGWSI	1xCarbamido	1.7E-05	4.225E-05	1	1	3	P04003	P04003 [160-174]	0	2315.2826
[K].VQGSDS	1xTMTpro [N	3.651E-06	4.225E-05	1	1	3	P26640	P26640 [522-537]	0	1996.0154
[K].GVLFGV	1xCarbamido	1.713E-05	4.225E-05	1	1	6	P30044	P30044 [87-102]	0	2202.2237
[R].VQGIDG	1xTMTpro [N	1.694E-05	4.225E-05	1	1	1	Q0VF96	Q0VF96 [46-62]	0	2229.1947
[K].ALAAAG	1xTMTpro [K	5.756E-05	4.225E-05	4	5	8	P16402; P10	P16402 [66-76]; F	0	1715.9824
[K].ALAAAG	1xTMTpro [K	1.287E-06	4.225E-05	4	5	1	P16402; P10	P16402 [66-80]; F	1	2187.2014
[K].GVLFYG	1xCarbamido	5.784E-05	4.225E-05	1	1	4	P55072	P55072 [513-524]	0	1860.0334
[K].VQFVPFI	1xTMTpro [N	3.833E-05	4.225E-05	1	1	1	Q8N2K0	Q8N2K0 [359-371	0	1868.9978
[R].GVLHQV	1xTMTpro [N	4.681E-05	4.225E-05	1	1	1	Q9NVH1	Q9NVH1 [533-547	0	1971.1017
[K].GVIQVYI	1xTMTpro [N	2.418E-05	4.225E-05	1	1	1	O75694	O75694 [298-314]	0	2127.0824
[K].VQEVQV	1xTMTpro [K	9.007E-06	4.225E-05	1	1	1	Q96CM8	Q96CM8 [535-547	1	2079.2054
[R].GVLIAVL	2xTMTpro [K	9.781E-07	4.225E-05	1	1	2	P29144	P29144 [37-63]	0	3549.0178
[R].AIAANEA	2xTMTpro [K	2.717E-08	4.225E-05	1	1	3	P02787	P02787 [70-107]	0	4866.6386
[R].ALAAQG	1xCarbamido	2.94E-06	4.225E-05	1	1	1	P10746	P10746 [231-256]	0	2884.5522
[K].VQEQVH	1xTMTpro [N	3.114E-06	4.225E-05	1	1	3	Q07065	Q07065 [506-523]	0	2326.2434
[R].ALAAQN	1xTMTpro [N	1.369E-05	4.225E-05	1	1	1	Q16222	Q16222 [229-242]	0	1891.9867
[R].VQEQLS	1xTMTpro [N	2.298E-06	4.225E-05	1	1	1	O15230	O15230 [2352-23	0	2635.3647
[R].VQEQLH	1xTMTpro [K	2.462E-05	4.225E-05	1	1	2	Q9NQ48	Q9NQ48 [250-259	0	1821.0184
[K].GIIIMGEI	1xTMTpro [N	4.043E-05	4.225E-05	1	1	1	Q9GZP4	Q9GZP4 [90-106]	0	2206.0439
[K].GVLLEIE	1xTMTpro [K	5.813E-05	4.225E-05	1	1	1	P46940	P46940 [1572-158	0	2353.3623
[R].VQELGH	1xCarbamido	1.371E-05	4.225E-05	1	1	1	Q9Y490	Q9Y490 [1920-19	0	2091.1876
[K].VQETGD	1xTMTpro [K	2.08E-07	4.225E-05	1	1	4	P51659	P51659 [590-614]	0	3144.692
[R].AKSPQP	2xCarbamido	1.006E-06	4.225E-05	1	1	1	Q00839	Q00839 [269-300]	1	4747.3112
[K].VPVGDQ	1xTMTpro [K	4.833E-05	4.225E-05	1	1	2	P50570	P50570 [143-157]	0	2306.2888
[R].VPVAVQ	1xTMTpro [K	2.287E-07	4.225E-05	1	1	10	P01024	P01024 [387-408]	0	2806.5442
[K].VPLSQLI	1xTMTpro [N	1.896E-05	4.225E-05	1	1	1	A0FGR8	A0FGR8 [618-634	0	2208.1865
[K].VPLQQN	1xTMTpro [K	2.779E-05	4.225E-05	1	1	4	P80188	P80188 [36-50]	0	2399.2963
[K].VPLPSLS	1xTMTpro [N	3.119E-05	4.225E-05	1	1	2	P10515	P10515 [94-110]	0	2043.1592
[K].VPLPSAI	2xTMTpro [K	3.225E-05	4.225E-05	1	1	1	Q8NEN9	Q8NEN9 [599-621	0	3355.8929
[R].VPINESF	1xTMTpro [N	5.244E-05	4.225E-05	1	1	1	Q12768	Q12768 [215-228]	0	1866.0478
[R].VPLFIRP	1xTMTpro [K	2.206E-06	4.225E-05	1	1	1	P16144	P16144 [946-976]	1	3986.2094

[R].GVMLAV	2xTMTpro [K	2.717E-08	4.225E-05	1	1	2	P10809	P10809 [143-157]	1	2469.5295
[R].VPLASQ	1xTMTpro [K	9.766E-06	4.225E-05	1	1	3	P05161	P05161 [58-77]	0	2558.5413
[R].VPHNAA	1xTMTpro [N	1.998E-05	4.225E-05	1	1	1	Q14764	Q14764 [462-474]	0	1835.9724
[K].VPHDPV	1xTMTpro [N	1.881E-06	4.225E-05	1	1	1	Q9UH65	Q9UH65 [45-75]	1	3900.8571
[R].VPGTST	1xTMTpro [N	2.167E-05	4.225E-05	1	1	2	P02751	P02751 [2241-225	0	1765.9979
[R].GVMLAV	1xOxidation [	2.682E-05	4.225E-05	1	1	1	P10809	P10809 [143-157]	1	2485.5244
[R].VPGHED	1xTMTpro [K	1.102E-05	4.225E-05	1	1	1	P22105	P22105 [2240-225	0	2495.3386
[R].GLLEES	1xTMTpro [K	4.793E-05	4.225E-05	1	1	1	Q13601	Q13601 [53-66]	0	2147.2244
[K].VPGFAD	1xCarbamido	5.581E-05	4.225E-05	1	1	2	Q9P2B2	Q9P2B2 [417-430	0	1851.923
[R].ALCDRD	1xCarbamido	7.587E-07	4.225E-05	1	1	1	P12110	P12110 [746-766]	1	2956.5516
[R].VPGDQT	1xTMTpro [N	2.413E-07	4.225E-05	1	1	3	P24821	P24821 [670-691]	0	2795.4786
[R].ALCDVG	2xCarbamido	3.13E-05	4.225E-05	1	1	1	Q9BQB6	Q9BQB6 [41-53]	0	1713.8583
[R].ALCELES	1xCarbamido	1.865E-05	4.225E-05	1	1	1	Q5TDH0	Q5TDH0 [32-59]	0	3443.7548
[K].ALCGFTI	1xCarbamido	2.672E-08	4.225E-05	1	1	3	Q9Y490	Q9Y490 [1432-14	0	4227.11
[R].GLLEEQ	1xTMTpro [K	4.626E-05	4.225E-05	1	1	1	Q13464	Q13464 [907-921]	0	2422.2997
[R].GLIEIISN	1xTMTpro [N	1.017E-05	4.225E-05	1	1	1	O75643	O75643 [1844-18	0	2319.2879
[R].GVMLAV	1xTMTpro [K	4.89E-05	4.225E-05	1	1	2	P10809	P10809 [143-156]	0	2037.2274
[R].VPIWDQ	1xTMTpro [K	5.955E-05	4.225E-05	1	1	1	Q6RFH5	Q6RFH5 [182-197	0	2479.3841
[R].VPMGSG	1xTMTpro [K	4.352E-06	4.225E-05	1	1	3	Q9H3S7	Q9H3S7 [82-103]	0	2899.5519
[K].GILFVGS	1xTMTpro [N	2.94E-06	4.225E-05	1	1	3	P52209	P52209 [120-136]	0	1896.0146
[K].VPTTEKI	1xTMTpro [K	3E-05	4.225E-05	1	1	1	Q04637	Q04637 [827-840]	0	2197.2836
[K].AIALQPE	1xTMTpro [N	9.157E-06	4.225E-05	1	1	1	Q96TC7	Q96TC7 [355-371	0	2182.2126
[R].VPTANV	1xCarbamido	2.692E-05	4.225E-05	1	1	8	P04406	P04406 [235-248]	0	1835.0016
[R].VPTAIHQ	2xTMTpro [K	1.071E-05	4.225E-05	1	1	1	P51888	P51888 [240-263]	1	3674.0774
[R].VPTAIHQ	1xTMTpro [K	5.654E-05	4.225E-05	1	1	3	P51888	P51888 [240-253]	0	2207.268
[K].VPTADLI	1xTMTpro [K	1.075E-05	4.225E-05	1	1	4	P02774	P02774 [243-264]	0	2974.6844
[K].AIANECQ	1xCarbamido	1.379E-05	4.225E-05	1	1	3	P55072	P55072 [530-543]	0	2187.2087
[R].VPSYTLI	1xTMTpro [N	3.774E-05	4.225E-05	1	1	2	P04114	P04114 [3292-331	0	2547.5234
[R].ALANSL	1xCarbamido	4.849E-05	4.225E-05	1	1	3	P04075	P04075 [332-342]	0	1740.9922
[K].GLLGLPI	1xTMTpro [K	1.916E-05	4.225E-05	1	1	1	Q12972	Q12972 [152-175]	0	3292.7193
[K].ALANVNI	1xCarbamido	2.364E-08	4.225E-05	1	1	4	P05386	P05386 [50-92]	0	4417.343
[K].VPSTETI	1xTMTpro [K	1.934E-05	4.225E-05	1	1	3	P31150	P31150 [119-137]	0	2663.3916
[K].VPSTEAI	1xOxidation [	7.128E-06	4.225E-05	1	1	1	P50395	P50395 [119-137]	0	2604.4086

[K].VPSTEAI	1xTMTpro [K	9.677E-06	4.225E-05	1	1	1	P50395	P50395 [119-137]	0	2588.4137
[R].ALASGG	2xCarbamido	1.188E-06	4.225E-05	1	1	1	P20933	P20933 [46-66]	0	2416.1226
[R].GVLSQA	1xCarbamido	3.152E-05	4.225E-05	1	1	1	Q12904	Q12904 [220-235]	0	2303.2053
[K].VPQIEVE	1xTMTpro [K	2.068E-05	4.225E-05	1	1	3	Q14204	Q14204 [2552-25	0	1788.0511
[R].ALASPE	1xTMTpro [N	4.071E-05	4.225E-05	1	1	1	Q9Y3Q8	Q9Y3Q8 [367-384	0	2125.1936
[K].GILEQG	1xTMTpro [N	2.045E-05	4.225E-05	1	1	3	Q99627	Q99627 [147-160]	0	1865.9677
[R].GVMEHL	1xTMTpro [N	4.704E-06	4.225E-05	1	1	1	P11277	P11277 [797-814]	0	2436.1937
[R].GLLEMM	1xTMTpro [K	3.097E-05	4.225E-05	1	1	1	Q86W92	Q86W92 [57-71]	1	2359.2493
[R].VPSVAE.	1xTMTpro [K	4.678E-06	4.225E-05	1	1	1	Q63ZY3	Q63ZY3 [538-556	0	2442.4324
[K].VQIAVAN	1xTMTpro [N	1.613E-05	4.225E-05	1	1	2	Q9Y5L4	Q9Y5L4 [28-41]	0	1857.0877
[K].AKQTLEI	2xTMTpro [K	3.375E-05	4.225E-05	1	1	2	P35579	P35579 [1218-123	2	2841.625
[K].VQLDLAI	1xTMTpro [N	4.11E-06	4.225E-05	1	1	2	Q9BSJ8	Q9BSJ8 [1076-10	0	2019.1042
[R].VRPCVV	1xCarbamido	5.963E-07	4.225E-05	1	2	1	O00571	O00571 [295-311]	0	2192.1929
[K].AHYGGF	1xTMTpro [K	2.86E-05	4.225E-05	1	1	2	P02675	P02675 [354-367]	0	2144.138
[K].AHYGGF	2xTMTpro [K	4.756E-06	4.225E-05	1	1	2	P02675	P02675 [354-374]	1	3280.7895
[R].GIISALL\	1xTMTpro [K	6.041E-05	4.225E-05	1	1	2	P04114	P04114 [159-174]	0	2275.3405
[R].VRGEES	1xCarbamido	8.355E-07	4.225E-05	1	1	1	P22105	P22105 [2998-30	1	2511.3369
[K].VREEVV	1xCarbamido	7.573E-10	4.225E-05	1	1	1	P02751	P02751 [2274-23	1	4974.3084
[R].VREEVP	1xTMTpro [K	1.384E-05	4.225E-05	1	1	3	Q92922	Q92922 [725-739]	1	2355.3892
[K].AKDDSE	1xCarbamido	2.844E-06	4.225E-05	1	1	6	Q92896	Q92896 [957-972]	1	2704.5007
[K].VQYTLPI	1xTMTpro [N	5.098E-05	4.225E-05	1	1	1	P42025	P42025 [239-255]	0	2093.1198
[K].AKDEQS	2xTMTpro [K	1.815E-05	4.225E-05	1	1	2	Q01082	Q01082 [1621-16	1	2219.2886
[R].VQYTET	1xTMTpro [N	4.235E-05	4.225E-05	1	1	1	Q86VM9	Q86VM9 [390-402	0	2003.9782
[R].AKDFSLI	1xTMTpro [K	9.518E-06	4.225E-05	1	1	1	Q99623	Q99623 [172-191]	1	2848.5952
[K].AKDLIVT	2xTMTpro [K	2.885E-05	4.225E-05	1	1	1	P54687	P54687 [27-39]	1	2295.4832
[R].AKDINQI	2xTMTpro [K	2.716E-05	4.225E-05	1	1	2	Q99798	Q99798 [143-160]	1	2865.6291
[K].AKDLQD	1xTMTpro [K	2.449E-05	4.225E-05	1	1	1	Q9UQE7	Q9UQE7 [309-32	1	2282.2232
[K].AKDIVPC	2xTMTpro [K	6.161E-06	4.225E-05	1	1	2	P16615	P16615 [142-158]	1	2651.58
[R].VQVQVC	1xTMTpro [N	3.968E-06	4.225E-05	1	1	1	Q96JQ0	Q96JQ0 [1422-14	0	2242.1536
[R].VQVQDN	1xCarbamido	2.72E-06	4.225E-05	1	1	4	P21333	P21333 [709-724]	0	2393.299
[K].VQVPNC	1xCarbamido	6.115E-06	4.225E-05	1	1	1	P53621	P53621 [448-467]	0	2583.356
[R].AKDQNE	2xTMTpro [K	3.124E-05	4.225E-05	1	1	2	Q96AC1	Q96AC1 [660-674	1	2759.4378
[K].VQVLTA	1xTMTpro [N	1.004E-06	4.225E-05	1	1	1	P39210	P39210 [18-40]	0	2731.5347

[K].VSAFENI	1xTMTpro [K	4.874E-05	4.225E-05	1	1	1	P50148	P50148 [121-133]	0	2061.1512
[K].VSALDLA	1xTMTpro [N	1.015E-05	4.225E-05	1	1	3	Q13561	Q13561 [268-282]	0	1903.082
[K].GLLSDSI	1xTMTpro [N	8.217E-07	4.225E-05	1	1	4	O43399	O43399 [16-34]	0	2208.1501
[K].VSAQGIT	1xTMTpro [N	1.799E-05	4.225E-05	3	3	2	Q63HR2; Q9	Q63HR2 [1318-13	0	1706.9356
[K].VSELKEE	3xTMTpro [K	4.849E-05	4.225E-05	1	1	2	Q00839	Q00839 [13-22]	2	2419.5277
[R].AHQILVL	1xTMTpro [K	5.719E-05	4.225E-05	1	1	1	Q03519	Q03519 [669-679]	0	1844.1249
[R].VSEGGF	1xTMTpro [K	2.146E-06	4.225E-05	1	1	5	O14907	O14907 [60-76]	0	2249.2633
[R].AHQLVM	1xCarbamido	5.907E-05	4.225E-05	2	2	1	P62714; P67	P62714 [240-254]	0	2220.0398
[K].AHQPYG	2xCarbamido	1.277E-06	4.225E-05	1	1	2	Q6UXG2	Q6UXG2 [629-64	0	2535.3092
[K].VSEEQT	1xTMTpro [N	6.769E-06	4.225E-05	1	1	1	Q16666	Q16666 [144-165]	0	2493.2033
[R].AHQVVE	1xTMTpro [K	5.065E-06	4.225E-05	2	3	3	P62136; P62	P62136 [247-260]	0	2248.1894
[K].VSEALLD	1xCarbamido	1.123E-05	4.225E-05	1	1	1	Q6N069	Q6N069 [804-824	0	2715.2527
[K].VSEADS	1xTMTpro [K	5.444E-06	4.225E-05	1	1	2	P00751	P00751 [324-337]	0	2117.1006
[R].AHSSAG	1xTMTpro [N	3.774E-05	4.225E-05	1	1	4	P98160	P98160 [1935-194	0	1415.7674
[K].VQVEAIE	1xTMTpro [K	2.984E-05	4.225E-05	1	1	1	Q9H173	Q9H173 [260-272	0	1950.1516
[K].VSDSISA	1xTMTpro [N	1.924E-09	4.225E-05	1	1	4	P31040	P31040 [47-75]	0	3219.6605
[K].AHTMTD	1xTMTpro [K	4.389E-05	4.225E-05	1	1	2	Q00610	Q00610 [101-112]	0	2060.0767
[K].AHTMTD	1xOxidation [	4.776E-05	4.225E-05	1	1	2	Q00610	Q00610 [101-112]	0	2076.0716
[R].VSCLGV	1xCarbamido	3.273E-06	4.225E-05	2	2	1	P62873; P62	P62873 [315-337]	0	3040.5251
[R].VSCLGV	1xCarbamido	1.381E-06	4.225E-05	2	2	7	P62873; P62	P62873 [315-337]	0	3024.5302
[R].VSCEAP	1xCarbamido	8.23E-06	4.225E-05	1	1	2	Q9BXV9	Q9BXV9 [19-41]	0	2971.5149
[R].GVFEAIV	1xTMTpro [K	5.926E-06	4.225E-05	1	1	2	Q14684	Q14684 [215-236]	0	3117.5946
[K].GIISILDE	1xCarbamido	1.1E-05	4.225E-05	1	1	3	O00159	O00159 [489-512]	0	3327.8002
[R].AHVIVMA	1xTMTpro [N	4.673E-05	4.225E-05	1	1	2	P55072	P55072 [339-358]	0	2450.3621
[K].VSASPLI	1xTMTpro [K	8.215E-06	4.225E-05	1	1	2	P02786	P02786 [496-508]	0	2042.2393
[R].GILSGTS	1xTMTpro [N	1.238E-06	4.225E-05	1	1	4	P18206	P18206 [114-132]	0	2340.2618
[R].AHSSMV	1xOxidation [	4.533E-05	4.225E-05	1	1	1	P00558	P00558 [172-184]	0	1992.1192
[R].AKEAAE	2xTMTpro [K	5.898E-05	4.225E-05	1	1	1	P26373	P26373 [199-209]	1	2130.2223
[R].VQVALE	1xTMTpro [K	2.475E-05	4.225E-05	1	1	3	Q14204	Q14204 [1319-13	0	1993.1825
[K].AKEALEI	1xTMTpro [K	1.078E-05	4.225E-05	1	1	2	Q14204	Q14204 [1300-13	1	2627.4383
[K].VQQELS	1xTMTpro [N	4.087E-06	4.225E-05	1	1	1	P48637	P48637 [307-330]	0	2953.5923
[R].AKLEAAI	1xTMTpro [K	1.994E-05	4.225E-05	1	1	5	P08729	P08729 [330-342]	1	2009.1523
[R].VQQEAQ	1xTMTpro [K	6.908E-06	4.225E-05	1	1	1	H7BZ55	H7BZ55 [969-992	0	3254.6996

[R].VQPVLFI	1xTMTpro [N	6.082E-06	4.225E-05	1	1	1	Q96PE3	Q96PE3 [794-812	0	2459.3577
[K].AKNEILD	2xTMTpro [K	1.607E-06	4.225E-05	1	1	3	O60313	O60313 [597-614]	1	2896.7175
[K].GVGIISE	1xTMTpro [N	1.985E-06	4.225E-05	1	3	1	P05023	P05023 [630-647]	0	2134.1311
[R].GLLPGC	1xCarbamido	2.155E-05	4.225E-05	1	3	1	Q5JPE7	Q5JPE7 [1007-10	0	2092.2233
[R].VQNMAL	1xTMTpro [K	2.071E-05	4.225E-05	1	1	2	P51571	P51571 [61-73]	0	1974.0974
[R].VQNATL	1xTMTpro [N	2.443E-06	4.225E-05	1	1	2	P27105	P27105 [126-144]	0	2234.206
[R].AKPEGA	1xCarbamido	2.809E-07	4.225E-05	1	1	3	P16422	P16422 [82-106]	0	3665.8574
[K].GVGLGP	1xTMTpro [N	3.8E-05	4.225E-05	1	1	2	P08123	P08123 [85-99]	0	1699.9307
[R].VQLYDL	1xTMTpro [K	1.038E-05	4.225E-05	1	1	1	P18428	P18428 [456-467]	0	2035.2196
[K].AKPPPG	2xTMTpro [K	4.765E-06	4.225E-05	1	1	1	O43242	O43242 [15-38]	0	3362.8235
[K].GLLPEEI	1xTMTpro [K	1.327E-05	4.225E-05	1	1	2	P13804	P13804 [86-101]	0	2344.4347
[R].GVGQAD	1xTMTpro [N	1.832E-05	4.225E-05	1	1	2	Q9C0C2	Q9C0C2 [1275-12	0	1788.9564
[R].AKPTCT	1xCarbamido	6.115E-06	4.225E-05	1	1	1	Q6P9B6	Q6P9B6 [383-405	0	3587.862
[K].VQLPTE	1xTMTpro [N	1.959E-05	4.225E-05	2	2	3	P32456; P32	P32456 [341-356]	0	2209.2512
[K].VQLNLM	1xTMTpro [K	3.435E-06	4.225E-05	1	1	1	Q13751	Q13751 [189-207]	0	2650.5093
[K].GVGTDE	1xCarbamido	2.583E-06	4.225E-05	1	1	4	P50995	P50995 [287-302]	0	2008.0704
[K].AKPYEG	1xCarbamido	4.747E-07	4.225E-05	1	1	3	P00367	P00367 [364-386]	0	3402.8647
[R].GVGVFG	1xTMTpro [N	3.04E-06	4.225E-05	1	1	1	P56192	P56192 [599-618]	0	2409.2192
[R].AKQEEL	1xTMTpro [K	3.225E-05	4.225E-05	1	1	4	P08729	P08729 [352-363]	1	1994.1526
[K].VQLVGLI	1xCarbamido	6.487E-06	4.225E-05	1	1	2	P61962	P61962 [47-62]	0	2185.113
[R].GVNAIV	1xTMTpro [K	1.584E-06	4.225E-05	1	1	2	Q9NVJ2	Q9NVJ2 [88-103]	1	2373.3092
[K].VQQLEE	1xTMTpro [K	2.362E-05	4.225E-05	1	1	3	Q9Y6C2	Q9Y6C2 [178-190	0	2138.2313
[R].GVGGS	2xTMTpro [K	1.409E-06	4.225E-05	1	1	1	P15144	P15144 [141-167]	1	3833.1517
[K].VQTLSN	1xTMTpro [K	6.003E-05	4.225E-05	1	1	2	Q8N163	Q8N163 [113-123	0	1849.1403
[K].GIIRPGT	1xTMTpro [K	8.433E-07	4.225E-05	1	1	1	Q15149	Q15149 [4093-41	0	3351.9172
[K].VQTLEA	1xTMTpro [N	1.544E-05	4.225E-05	1	1	1	P28907	P28907 [235-247]	0	1769.9982
[R].VQTDAF	1xCarbamido	1.288E-05	4.225E-05	1	1	1	Q9UI10	Q9UI10 [447-466]	0	2917.4381
[K].AKEEEAI	1xTMTpro [K	4.383E-07	4.225E-05	1	1	1	Q6DKJ4	Q6DKJ4 [361-383	1	3191.6426
[K].AKEETYI	2xTMTpro [K	2.597E-05	4.225E-05	1	2	1	Q9UPN3	Q9UPN3 [6384-63	1	2364.3591
[R].GVGDD	1xTMTpro [N	4.199E-05	4.225E-05	1	1	1	P20645	P20645 [257-277]	1	2645.232
[R].VQSTEL	3xCarbamido	2.249E-08	4.225E-05	1	1	3	P00747	P00747 [739-769]	0	3845.8388
[R].VQSTAF	1xTMTpro [K	9.553E-06	4.225E-05	1	1	1	Q5TBB1	Q5TBB1 [189-210	1	3102.5511
[K].GVGDGT	1xTMTpro [N	5.883E-06	4.225E-05	2	2	2	Q13404; Q15	Q13404 [27-47]; Q	0	2557.2047

[R].GLLPQLI	1xTMTpro [K	3.8E-05	4.225E-05	1	1	1	Q9UJS0	Q9UJS0 [393-405	0	1943.2185
[K].VQSNCP	1xCarbamido	4.665E-05	4.225E-05	1	1	1	O00534	O00534 [195-211]	0	2545.31
[K].VQSLQA	1xTMTpro [N	5.576E-06	4.225E-05	1	1	2	Q07065	Q07065 [162-177]	0	2101.1613
[K].AKESWE	2xTMTpro [K	3E-05	4.225E-05	1	1	1	Q02790	Q02790 [255-266]	1	2380.2635
[K].GVGDGT	1xOxidation [	2.895E-05	4.225E-05	2	2	1	Q13404; Q1	Q13404 [27-47]; (	0	2573.1996
[K].VQSGSE	1xTMTpro [N	2.54E-06	4.225E-05	1	1	3	Q15149	Q15149 [1442-14	0	2113.1096
[K].VQSFLAS	2xTMTpro [K	2.69E-06	4.225E-05	1	1	2	Q8IWE2	Q8IWE2 [316-332	1	2802.6797
[K].VQSDGQ	1xTMTpro [K	8.124E-06	4.225E-05	1	1	2	Q08211	Q08211 [1075-10	0	2323.3153
[R].AKFEEY	1xCarbamido	1.351E-05	4.225E-05	1	2	1	Q9BWF3	Q9BWF3 [96-111]	1	2809.5626
[R].VQRPED	1xTMTpro [K	1.966E-05	4.225E-05	1	1	2	Q9UI08	Q9UI08 [235-252]	0	2355.2396
[R].AKFYPEI	1xTMTpro [K	4.08E-06	4.225E-05	1	1	4	P26038	P26038 [82-100]	1	2889.549
[K].AKGEVD	2xTMTpro [K	5.123E-05	4.225E-05	1	1	3	Q09666	Q09666 [2400-24	1	2069.2423
[K].AKENDE	1xCarbamido	1.879E-05	4.225E-05	1	1	1	P49756	P49756 [76-97]	1	3322.7405
[R].GIDIQDV	1xTMTpro [K	1.26E-05	4.225E-05	1	1	1	Q9BUQ8	Q9BUQ8 [727-74	0	2506.3177
[R].GLIDVKF	2xTMTpro [K	2.952E-05	4.225E-05	1	1	3	Q8WVY7	Q8WVY7 [225-23	0	2407.5257
[R].VPFDAA	1xTMTpro [K	7.18E-06	4.225E-05	1	1	1	P06396	P06396 [420-447]	0	3481.6972
[R].VNDVPE	1xTMTpro [N	4.192E-05	4.225E-05	1	1	2	O95486	O95486 [458-472]	0	2110.114
[K].VNDTIQI	1xTMTpro [K	1.346E-05	4.225E-05	1	1	6	P62701	P62701 [156-168]	0	2054.1625
[R].VNDGVC	5xCarbamido	8.941E-06	4.225E-05	1	1	3	P14314	P14314 [92-117]	0	3649.5465
[R].GLGWV(	1xTMTpro [N	2.721E-05	4.225E-05	1	1	1	Q9GZT3	Q9GZT3 [61-74]	0	1868.9826
[K].ALELEEF	1xTMTpro [K	5.85E-05	4.225E-05	1	1	1	P43246	P43246 [848-871]	0	3339.695
[K].VNCTAE	1xCarbamido	3.387E-05	4.225E-05	1	1	1	Q9BS40	Q9BS40 [68-97]	0	3883.9192
[K].GVSTFM	1xTMTpro [N	1.815E-05	4.225E-05	1	1	1	P43246	P43246 [721-737]	0	2160.1364
[R].GLGVGF	1xTMTpro [K	3.131E-06	4.225E-05	1	1	1	P13647	P13647 [560-576]	0	2048.1268
[K].GVTFNV	1xTMTpro [K	3.465E-05	4.225E-05	1	1	2	O00299	O00299 [38-49]	0	1890.0828
[R].ALEILQE	1xTMTpro [N	3.814E-07	4.225E-05	2	2	2	P0C0L4; P0	P0C0L4 [757-775]	0	2529.3255
[K].AIELLQE	1xTMTpro [K	4.149E-05	4.225E-05	1	1	1	O76094	O76094 [357-376]	0	2890.5442
[K].GLGTGT	1xTMTpro [N	1.48E-05	4.225E-05	1	1	1	P54105	P54105 [31-43]	0	1641.9131
[K].AIELNPA	1xCarbamido	4.849E-05	4.225E-05	1	1	1	O43765	O43765 [117-131]	0	2056.0605
[R].AIEINPD	1xTMTpro [K	2.649E-05	4.225E-05	1	3	4	P50502	P50502 [174-186]	0	2054.1414
[R].VNAGDQ	1xTMTpro [K	1.718E-06	4.225E-05	1	1	2	Q02252	Q02252 [345-364]	0	2570.4434
[K].GVTGHE	1xCarbamido	1.451E-05	4.225E-05	1	1	1	Q9Y5P4	Q9Y5P4 [452-468	0	2356.1464
[K].GLGTDE	1xTMTpro [N	5.504E-06	4.225E-05	1	1	6	P08758	P08758 [30-45]	0	2009.1086

[R].AIELQP	1xCarbamid	3.16E-06	4.225E-05	1	1	1	O15294	O15294 [285-304	0	2893.5526
[K].GLGTDE	1xTMTpro [N	1.549E-06	4.225E-05	1	1	6	P04083	P04083 [129-144]	0	2007.0929
[K].GLGTDE	1xTMTpro [N	1.206E-06	4.225E-05	1	1	4	P08133	P08133 [378-393]	0	2073.1147
[K].VMVLDF	1xTMTpro [N	2.159E-05	4.225E-05	1	1	2	Q16881	Q16881 [188-202	0	1950.1053
[K].VNEAAP	1xTMTpro [K	3.775E-06	4.225E-05	1	1	2	Q5T4S7	Q5T4S7 [3767-37	0	3353.7065
[K].VNEASG	1xTMTpro [K	3.131E-06	4.225E-05	1	1	1	Q96C86	Q96C86 [68-86]	0	2525.3227
[R].GVSSPI	1xTMTpro [N	3.954E-05	4.225E-05	1	1	1	Q9UMS6	Q9UMS6 [635-66	0	3708.8406
[K].VNEMIIG	1xOxidation	2.312E-05	4.225E-05	1	1	2	P00558	P00558 [231-246]	0	2352.2951
[R].GVQDIV	1xCarbamid	9.407E-07	4.225E-05	1	1	2	P35232	P35232 [44-70]	0	3732.0591
[K].AIEEELQ	1xTMTpro [K	4.099E-05	4.225E-05	1	1	1	O00339	O00339 [797-812	0	2409.3005
[K].GVQGIIV	1xTMTpro [K	2.779E-05	4.225E-05	1	1	3	Q9NP97	Q9NP97 [16-31]	0	2245.3775
[K].AIEEGV	1xTMTpro [N	3.997E-06	4.225E-05	2	2	1	P46926; Q8	P46926 [215-234]	0	2641.3265
[R].VNLEES	1xTMTpro [K	4.131E-08	4.225E-05	1	1	2	Q8WVM8	Q8WVM8 [292-31	0	2549.3815
[R].VNLDAL	1xTMTpro [K	6.138E-06	4.225E-05	1	1	2	Q8NFV4	Q8NFV4 [234-24	0	1975.1468
[K].ALEEPA	1xTMTpro [K	4.271E-05	4.225E-05	1	1	1	Q96AP7	Q96AP7 [277-293	1	2460.359
[K].ALEEPA	1xTMTpro [N	3.076E-07	4.225E-05	1	1	1	P20591	P20591 [464-481]	0	2331.2008
[R].VNIAFN	1xOxidation	2.105E-05	4.225E-05	1	1	1	Q13838	Q13838 [356-374	0	2620.2309
[R].ALEESN	1xTMTpro [K	3.253E-05	4.225E-05	1	1	4	P13645	P13645 [166-177]	0	1990.0625
[K].GLGTDE	1xCarbamid	2.146E-06	4.225E-05	1	2	4	P07355	P07355 [120-135]	0	2082.0708
[R].VNIAFN	1xTMTpro [N	6.381E-06	4.225E-05	1	1	3	Q13838	Q13838 [356-374	0	2604.236
[K].VNKDDE	2xTMTpro [K	1.763E-05	4.225E-05	1	1	2	P00450	P00450 [945-957]	1	2479.3496
[R].VNHEPE	1xTMTpro [K	4.08E-06	4.225E-05	1	1	2	O00499	O00499 [312-330	0	2451.3487
[K].AIEHAD	1xTMTpro [N	3.096E-06	4.225E-05	1	1	2	P48637	P48637 [453-474]	0	2439.2839
[K].ALEHAF	1xTMTpro [N	2.329E-05	4.225E-05	1	1	1	Q14204	Q14204 [2436-24	0	2228.1817
[K].VNFLPEI	1xTMTpro [K	3.9E-05	4.225E-05	1	1	1	Q14204	Q14204 [737-748	0	1982.2182
[R].GVSAIV	1xTMTpro [K	1.195E-05	4.225E-05	1	1	2	Q96BM9	Q96BM9 [88-103]	0	2304.2401
[R].VNFDDY	1xTMTpro [K	5.607E-06	4.225E-05	1	1	3	Q06210	Q06210 [362-375	0	2163.1941
[K].GVSEAA	2xTMTpro [K	1.854E-08	4.225E-05	1	1	1	O95104	O95104 [1112-11	1	4508.3824
[K].VNESSLI	1xTMTpro [K	2.963E-05	4.225E-05	1	1	3	Q15417	Q15417 [73-91]	0	2810.5333
[R].GVSFYE	1xTMTpro [N	1.506E-06	4.225E-05	1	1	1	O00159	O00159 [106-125	0	2571.3567
[R].VNKEES	2xTMTpro [K	5.536E-05	4.225E-05	1	1	1	Q86UP2	Q86UP2 [561-582	1	3498.9294
[R].ALEEAN	1xTMTpro [K	1.882E-05	4.225E-05	1	1	2	Q04695	Q04695 [104-115	0	1954.0988
[K].VMVELL	1xTMTpro [N	5.659E-06	4.225E-05	1	1	2	Q7L2H7	Q7L2H7 [184-201	0	2287.1559

[K].VMVCQL	1xCarbamido	1.971E-06	4.225E-05	1	1	1	Q9H477	Q9H477 [148-168	0	2638.3937
[K].VMIPQDI	1xTMTpro [N	9.57E-06	4.225E-05	1	1	1	Q15637	Q15637 [140-160]	0	2704.4703
[R].ALESSIA	1xTMTpro [N	1.374E-05	4.225E-05	1	1	1	Q9Y265	Q9Y265 [318-333	0	1992.1449
[K].VMLPGQ	1xCarbamido	8.445E-06	4.225E-05	1	1	1	O43414	O43414 [252-270]	0	2807.512
[R].VMLGET	1xOxidation	2.13E-06	4.225E-05	2	3	3	P15531; P22	P15531 [89-105];	0	2410.3256
[R].VMLGET	1xTMTpro [K	4.809E-07	4.225E-05	2	3	3	P15531; P22	P15531 [89-105];	0	2394.3307
[K].GIGMGN	1xOxidation	4.241E-06	4.225E-05	1	1	2	P52272	P52272 [323-345]	0	2802.4596
[K].GIGMGN	1xTMTpro [K	1.075E-06	4.225E-05	1	1	2	P52272	P52272 [323-345]	0	2786.4647
[K].GIGMGM	1xTMTpro [K	1.668E-06	4.225E-05	1	1	2	Q9NRV9	Q9NRV9 [73-96]	0	3103.6452
[K].VMEMYQ	1xCarbamido	5.081E-05	4.225E-05	1	1	1	Q92769	Q92769 [249-271]	0	2833.349
[K].ALEVAEY	1xTMTpro [K	3.947E-05	4.225E-05	1	1	2	Q9NT62	Q9NT62 [12-24]	0	2054.2393
[K].VMEMYQ	1xCarbamido	1.364E-05	4.225E-05	1	1	1	Q92769	Q92769 [249-271]	0	2817.3541
[K].ALEVIKP	2xTMTpro [K	1.744E-05	4.225E-05	1	1	1	Q14152	Q14152 [533-546]	0	2501.5635
[K].VMEMFQ	1xCarbamido	8.035E-06	4.225E-05	1	1	1	Q13547	Q13547 [248-270]	0	2817.3541
[K].VMEGTV	1xTMTpro [N	1.622E-05	4.225E-05	1	1	2	P41240	P41240 [172-185]	0	1919.9492
[R].ALFARPI	1xTMTpro [N	7.73E-06	4.225E-05	1	1	2	Q9NUQ8	Q9NUQ8 [341-36	0	2716.5027
[R].VMAGAL	1xTMTpro [K	1.163E-05	4.225E-05	2	2	4	P61163; P42	P61163 [47-61]; P	0	2126.2175
[R].ALFCGV	2xCarbamido	1.03E-06	4.225E-05	1	1	2	Q9NQT4	Q9NQT4 [150-17	0	3018.5772
[R].VLYVQD	1xTMTpro [N	5.175E-05	4.225E-05	1	1	2	P48147	P48147 [99-111]	0	1782.9557
[K].GVTSEE	1xTMTpro [K	4.908E-06	4.225E-05	1	1	2	O60784	O60784 [437-450]	1	2294.216
[K].VLYSNM	1xTMTpro [N	7.088E-06	4.225E-05	1	1	2	Q00325	Q00325 [146-161]	0	2292.1654
[K].AIFDTPD	1xTMTpro [N	4.972E-06	4.225E-05	1	1	1	Q9GZP9	Q9GZP9 [207-236	0	3678.742
[K].ALESPEI	1xTMTpro [K	2.049E-06	4.225E-05	1	1	4	P00558	P00558 [200-216]	0	2377.4099
[K].GIGMNE	1xCarbamido	5.324E-06	4.225E-05	1	1	2	O00233	O00233 [49-64]	0	2111.0221
[R].VMLVNS	1xTMTpro [K	5.964E-05	4.225E-05	1	1	4	P18206	P18206 [189-199]	0	1844.0629
[R].VMPICLF	1xCarbamido	3.678E-06	4.225E-05	1	1	4	P00738	P00738 [262-277]	1	2443.3333
[R].VMTIPYC	1xCarbamido	7.088E-06	4.225E-05	1	1	4	Q15365	Q15365 [178-200]	0	2795.385
[R].ALENELI	1xTMTpro [K	2.924E-06	4.225E-05	1	1	1	Q0VF96	Q0VF96 [896-916	0	2910.53
[R].VMTIPYC	1xCarbamido	1.716E-05	4.225E-05	1	1	1	Q15365	Q15365 [178-200]	0	2779.39
[R].AIENIDTI	1xTMTpro [K	1.601E-05	4.225E-05	1	1	1	Q15435	Q15435 [200-217]	0	2599.4838
[R].VMTIAPC	1xOxidation	1.167E-05	4.225E-05	1	1	6	Q99714	Q99714 [193-212]	0	2709.5756
[R].VMTIAPC	1xTMTpro [K	1.966E-05	4.225E-05	1	1	2	Q99714	Q99714 [193-212]	0	2693.5807
[R].VMSSQS	1xTMTpro [K	1.845E-05	4.225E-05	1	1	1	Q4ZHG4	Q4ZHG4 [270-287	0	2611.4661

[K].VMSQEI(	1xOxidation [	2.362E-05	4.225E-05	1	1	1	Q14204	Q14204 [3255-32(	0	2094.1509
[K].AIEPPPL	1xTMTpro [N	1.294E-06	4.225E-05	1	1	1	Q08211	Q08211 [820-837]	0	2275.2617
[K].VMQVLN	1xTMTpro [K	3.747E-05	4.225E-05	1	1	1	Q7KZF4	Q7KZF4 [347-359	0	2008.212
[K].GLGTDE	1xTMTpro [N	1.041E-05	4.225E-05	1	1	3	P09525	P09525 [29-44]	0	1997.0874
[K].VMQQQ(	1xTMTpro [K	1.055E-05	4.225E-05	1	1	3	Q15459	Q15459 [116-131]	0	2550.3954
[R].VMQPQI	1xCarbamido	4.989E-05	4.225E-05	1	1	1	Q14166	Q14166 [598-614]	0	2409.1862
[K].AIEQADL	1xTMTpro [N	4.48E-05	4.225E-05	1	1	1	P61966	P61966 [134-149]	0	2147.0787
[K].GVTIASC	1xTMTpro [N	4.22E-05	4.225E-05	1	1	1	Q9P0M6	Q9P0M6 [97-108]	0	1430.865
[K].VMQEQ(	1xOxidation [	4.874E-05	4.225E-05	1	1	6	P35579	P35579 [546-555]	0	1778.9715
[K].VMQEEII	1xOxidation [	3.514E-05	4.225E-05	1	1	2	P51648	P51648 [327-343]	0	2533.4959
[K].GLGSTV	1xTMTpro [K	2.789E-05	4.225E-05	1	1	2	Q96CN7	Q96CN7 [163-177	0	2125.236
[R].GLGQEC	1xCarbamido	4.247E-07	4.225E-05	1	1	1	P78527	P78527 [37-61]	0	2923.5882
[K].VMPNAI\	1xTMTpro [K	2.542E-05	4.225E-05	1	1	1	O95831	O95831 [363-378]	0	2181.2557
[K].GVTIPYF	1xTMTpro [K	2.198E-05	4.225E-05	1	1	2	Q15366	Q15366 [177-199]	0	3037.7079
[R].ALESDM	1xTMTpro [N	8.383E-06	4.225E-05	1	1	2	Q9Y230	Q9Y230 [315-330	0	2036.084
[K].GVTIASC	1xTMTpro [K	8.051E-07	4.225E-05	1	1	3	O75367	O75367 [97-116]	0	2594.5525
[R].ALEEAN/	1xTMTpro [K	3.102E-05	4.225E-05	4	4	1	P02533; P19	P02533 [135-146]	0	1910.0726
[R].ALEEAM	1xTMTpro [K	1.796E-06	4.225E-05	1	1	1	P35579	P35579 [1484-149	1	2255.2197
[K].GVPVSE	1xCarbamido	2.279E-05	4.225E-05	2	2	2	Q5VT66; Q9	Q5VT66 [71-85]; (	0	1880.9529
[R].VPANLEI	2xTMTpro [K	1.637E-06	4.225E-05	1	1	5	P13671	P13671 [232-256]	1	3754.9996
[R].ALDKLD(	1xTMTpro [K	5.518E-05	4.225E-05	1	1	1	Q13247	Q13247 [162-174]	1	2010.1475
[K].ALDIAEN	1xTMTpro [N	3.793E-05	4.225E-05	1	1	2	P23526	P23526 [21-34]	0	1863.9628
[K].GVNTGA	1xTMTpro [K	9.945E-07	4.225E-05	1	1	2	P52943	P52943 [187-205]	1	2606.3706
[R].GVNWA/	1xTMTpro [N	3.185E-05	4.225E-05	1	1	1	P53621	P53621 [207-227]	0	2558.3145
[R].ALDIDLD	2xTMTpro [K	3.974E-05	4.225E-05	1	1	5	O14617	O14617 [801-815]	0	2555.4748
[R].VPAGSA	1xCarbamido	1.235E-05	4.225E-05	1	1	2	P98160	P98160 [3586-36]	0	3186.6789
[R].ALDLFSI	1xTMTpro [K	2.095E-06	4.225E-05	1	1	6	Q15084	Q15084 [265-288]	0	3245.7801
[R].VPAGNV	1xTMTpro [K	1.173E-05	4.225E-05	1	1	1	Q15029	Q15029 [544-561]	0	2542.4889
[R].VPAFLS/	1xTMTpro [N	5.244E-05	4.225E-05	1	1	1	Q14894	Q14894 [4-18]	0	1972.0823
[K].VPAFEG	1xCarbamido	9.927E-07	4.225E-05	1	1	2	P26641	P26641 [58-85]	0	3502.6432
[R].VPAEGL	1xTMTpro [K	3.237E-06	4.225E-05	1	1	2	P57737	P57737 [574-596]	0	3058.6804
[K].VPADTE\	1xCarbamido	2.32E-06	4.225E-05	1	1	10	P60174	P60174 [34-53]	0	2496.2764
[R].VPADLG	1xTMTpro [N	1.734E-06	4.225E-05	1	1	2	P35270	P35270 [66-84]	0	2196.2671

[K].VPAATS/	1xTMTpro [K	2.964E-07	4.225E-05	1	1	3	P61960	P61960 [42-69]	0	3361.8975
[K].VNWEEE	2xTMTpro [K	2.175E-05	4.225E-05	1	1	1	P04114	P04114 [4056-40	1	3212.7983
[K].VNWEEE	1xTMTpro [K	1.065E-05	4.225E-05	1	1	2	P04114	P04114 [4056-40	0	2355.3052
[K].GIIAILDD	1xCarbamido	2.027E-05	4.225E-05	1	1	2	O94832	O94832 [451-465	0	2198.2169
[K].AIDIYEQ	1xTMTpro [K	1.113E-05	4.225E-05	1	1	2	P54920	P54920 [181-199]	0	2686.4617
[K].AIDIYEQ	1xOxidation [	8.124E-06	4.225E-05	1	1	1	P54920	P54920 [181-199]	0	2702.4566
[R].VNVVAP	1xTMTpro [K	5.945E-05	4.225E-05	1	1	1	Q8N4T8	Q8N4T8 [173-187	0	2223.2451
[R].AIDGLNL	1xTMTpro [N	3.01E-05	4.225E-05	1	1	1	P34059	P34059 [362-376]	0	1898.1394
[R].GLLCGL	2xCarbamido	1.017E-07	4.225E-05	1	1	1	P53007	P53007 [124-147]	0	3051.69
[K].VPASMT	1xTMTpro [N	5.894E-06	4.225E-05	1	1	1	Q96P48	Q96P48 [1193-12	0	2192.1804
[R].VPATLQ	1xTMTpro [N	1.399E-06	4.225E-05	1	1	4	Q07960	Q07960 [350-369	0	2615.4728
[R].GVNECD	1xCarbamido	5.664E-05	4.225E-05	1	1	1	Q9GZM7	Q9GZM7 [442-45	0	2241.1293
[R].VPELQG	1xTMTpro [K	1.113E-05	4.225E-05	1	1	1	Q63HN8	Q63HN8 [3941-39	0	2545.4885
[K].VPEIEVT	1xTMTpro [N	2.294E-06	4.225E-05	1	1	1	P78347	P78347 [681-702]	0	2668.3861
[K].VPEGVA	1xTMTpro [N	2.231E-06	4.225E-05	1	1	1	A0AV96	A0AV96 [22-41]	0	2312.2603
[R].ALDAVA	1xTMTpro [N	2.89E-05	4.225E-05	1	1	2	P06401	P06401 [650-672]	0	2664.464
[R].VPEGPG	1xTMTpro [N	9.048E-07	4.225E-05	1	1	1	P22105	P22105 [997-101	0	2062.0888
[R].VPEEEK	1xTMTpro [K	2.924E-06	4.225E-05	1	1	1	O14744	O14744 [349-368	1	2792.5472
[K].VPEANS	1xTMTpro [N	2.721E-05	4.225E-05	1	1	2	P10515	P10515 [491-504]	0	1908.9809
[K].VPEANIL	1xTMTpro [K	1.665E-06	4.225E-05	1	1	1	P45954	P45954 [270-284]	0	2204.2683
[R].GVNEDT	1xCarbamido	4.618E-06	4.225E-05	1	1	1	Q9H936	Q9H936 [259-273	0	1973.9558
[K].VNVTVD	1xTMTpro [N	3.048E-07	4.225E-05	1	1	1	Q7KZF4	Q7KZF4 [415-438	0	2923.5485
[K].GVNIGG/	1xTMTpro [K	1.611E-07	4.225E-05	1	1	2	P52943	P52943 [66-97]	0	3819.0936
[K].VPDNYG	1xTMTpro [N	4.634E-05	4.225E-05	1	1	1	Q92900	Q92900 [394-407	0	1908.0034
[K].GLIDEVN	1xTMTpro [N	2.89E-05	4.225E-05	1	1	8	P02671	P02671 [72-84]	0	1824.9411
[R].ALDEGD	1xTMTpro [K	6.022E-05	4.225E-05	1	1	2	P35998	P35998 [24-34]	0	1766.0555
[R].ALDEGIF	1xTMTpro [N	1.258E-05	4.225E-05	1	1	1	Q5T6V5	Q5T6V5 [109-131	0	2787.4736
[K].GVNLPG	1xTMTpro [K	4.575E-06	4.225E-05	1	1	2	P14618	P14618 [208-224]	0	2245.3048
[K].ALDELMI	1xTMTpro [K	1.892E-05	4.225E-05	1	1	1	Q93009	Q93009 [756-770	0	2315.2812
[K].ALDELMI	2xTMTpro [K	5E-06	4.225E-05	1	1	1	Q93009	Q93009 [756-784	1	4145.1416
[K].GVNLPG	2xTMTpro [K	3.14E-07	4.225E-05	1	1	2	P14618	P14618 [208-230]	1	3261.8875
[R].GVNQAT	1xTMTpro [K	8.57E-06	4.225E-05	1	1	1	O00291	O00291 [941-957	0	2168.2531
[K].VPCNFS	2xCarbamido	1.133E-05	4.225E-05	1	1	1	Q9UK59	Q9UK59 [337-362	0	3656.8631

[R].AIDEDCS	1xCarbamidc	2.444E-05	4.225E-05	1	1	2	P07357	P07357 [135-151]	0	2545.2736
[R].VNVPVIC	1xTMTpro [K	1.76E-05	4.225E-05	1	1	6	P40926	P40926 [192-203]	0	1756.0725
[K].GILADED	1xTMTpro [K	2.67E-06	4.225E-05	1	1	1	P49327	P49327 [1430-144	0	2294.3
[R].VNVGEG	1xTMTpro [N	4.177E-05	4.225E-05	1	1	1	Q14315	Q14315 [756-766]	0	1484.7777
[K].VNNSSLI	1xTMTpro [K	2.851E-09	4.225E-05	1	1	5	P45880	P45880 [248-267]	0	2711.57
[K].VNNSSLI	2xTMTpro [K	4.747E-07	4.225E-05	1	1	5	P21796	P21796 [237-256]	0	3015.8023
[K].ALEDAFI	1xTMTpro [K	5.766E-05	4.225E-05	1	1	1	O15355	O15355 [93-104]	0	1885.0926
[R].GVPGAI\	1xCarbamidc	3.866E-05	4.225E-05	1	1	2	Q7Z4W1	Q7Z4W1 [126-141	0	1963.035
[K].AIEDDLM	1xTMTpro [K	1.137E-06	4.225E-05	1	1	1	Q4L180	Q4L180 [644-661]	1	2808.4105
[K].AIEDEGC	1xTMTpro [K	7.314E-06	4.225E-05	1	1	1	Q15424	Q15424 [64-83]	0	2725.366
[K].AIEDEGC	2xTMTpro [K	4.017E-08	4.225E-05	1	1	2	Q15424	Q15424 [64-84]	1	3157.6681
[R].AIEDGNI	1xTMTpro [N	9.276E-06	4.225E-05	1	1	1	P51531	P51531 [1328-134	0	2066.9871
[R].VNMPPA	1xTMTpro [N	4.849E-05	4.225E-05	1	1	1	Q9BYN8	Q9BYN8 [42-59]	0	2366.2208
[R].ALEDLAC	2xTMTpro [K	7.071E-07	4.225E-05	1	1	3	P11277	P11277 [480-497]	1	3085.6622
[K].VNNVVW	1xCarbamidc	9.166E-07	4.225E-05	1	1	4	P26639	P26639 [122-143]	0	3278.7586
[K].VNLQNN	1xOxidation		4.397E-05	4.225E-05	1	1	2	P51572	P51572 [80-95]	0	2491.283
[K].GLKDGT	1xCarbamidc	5.031E-05	4.225E-05	1	1	2	Q99439	Q99439 [53-66]	1	2476.4448
[R].GVPGPF	1xTMTpro [K	3.576E-05	4.225E-05	1	1	3	P02452	P02452 [599-612]	0	1769.0565
[R].VNLLSFT	1xTMTpro [K	1.3E-05	4.225E-05	1	1	2	P49419	P49419 [267-280]	0	2059.2043
[K].GVPIIFAI	2xTMTpro [K	6.149E-06	4.225E-05	1	1	1	Q14118	Q14118 [795-823]	0	4065.2286
[R].VNIHCPC	3xCarbamidc	1.073E-05	4.225E-05	1	1	1	Q96EM0	Q96EM0 [133-152	0	2579.2488
[R].ALEEAAY	1xTMTpro [N	1.206E-05	4.225E-05	1	1	1	Q96II8	Q96II8 [52-67]	0	1877.0663
[K].GLHVVET	1xTMTpro [K	5.305E-07	4.225E-05	1	1	2	O75369	O75369 [1300-13	0	2834.5584
[R].ALEEALE	1xTMTpro [K	3E-06	4.225E-05	1	1	1	P35580	P35580 [1491-150	1	2272.2317
[R].ALEEALE	1xTMTpro [K	6.959E-06	4.225E-05	1	1	1	P35749	P35749 [1491-150	1	2238.2473
[K].VNIFGVE	2xTMTpro [K	2.316E-05	4.225E-05	1	1	2	Q9GZS3	Q9GZS3 [130-140	1	2090.279
[K].VNLQNN	1xTMTpro [K	2.234E-05	4.225E-05	1	1	2	P51572	P51572 [80-95]	0	2475.2881
[K].GVNCIDY	1xCarbamidc	4.532E-08	4.225E-05	1	1	2	P35606	P35606 [187-209]	0	3143.5599
[K].GLKPGV	1xTMTpro [K	2.851E-07	4.225E-05	1	1	3	P02751	P02751 [670-694]	0	3407.8931
[K].GIKPVTL	2xTMTpro [K	2.191E-05	4.225E-05	1	1	4	P49189	P49189 [247-258]	0	2124.3572
[R].VNVGAG	1xTMTpro [K	2.77E-05	4.225E-05	1	1	2	P21333	P21333 [761-771]	0	1687.9735
[R].GVPESL	1xTMTpro [K	1.725E-06	4.225E-05	1	1	3	O95865	O95865 [18-40]	0	2688.5064
[K].VNVEDA	1xTMTpro [N	2.259E-05	4.225E-05	2	2	3	P69891; P69	P69891 [19-31]; F	0	1620.8512

[R].ALDQYLI	1xCarbamido	6.051E-05	4.225E-05	1	1	2	P07357	P07357 [486-498]	0	1934.9424
[K].VNVEAP	1xTMTpro [K	1.095E-06	4.225E-05	1	1	3	Q09666	Q09666 [664-679]	0	2219.2527
[R].ALDTMN	1xTMTpro [K	5.766E-05	4.225E-05	2	2	2	Q13310; P11	Q13310 [68-78]; F	0	1875.0541
[K].ALDTVLF	1xTMTpro [N	4.825E-05	4.225E-05	1	1	2	Q13586	Q13586 [194-207]	0	1817.0856
[K].VNVDIIN	1xTMTpro [K	6.536E-06	4.225E-05	1	2	2	P55036	P55036 [136-152]	0	2557.3641
[K].ALDVGS	1xCarbamido	6.719E-06	4.225E-05	1	1	1	P22061	P22061 [82-98]	0	1999.0602
[K].VNVDEV	1xTMTpro [N	1.533E-05	4.225E-05	1	1	75	P68871	P68871 [19-31]	0	1618.872
[K].VNPDTG	1xTMTpro [N	6.744E-06	4.225E-05	1	1	1	P34896	P34896 [174-191]	0	2415.1748
[K].VNVDAV	1xTMTpro [N	1.966E-05	4.225E-05	1	1	6	P02042	P02042 [19-31]	0	1560.8665
[K].VNTFSAI	1xTMTpro [K	3.967E-05	4.225E-05	1	1	1	O43681	O43681 [331-348]	0	2583.4678
[R].VNSININ	1xTMTpro [N	7.645E-06	4.225E-05	1	1	2	O00159	O00159 [1013-10	0	2206.1899
[K].VNQWTT	1xTMTpro [K	1.951E-05	4.225E-05	1	1	1	P63172	P63172 [39-56]	0	2697.5067
[K].VNQPAS	1xTMTpro [K	1.835E-05	4.225E-05	1	1	2	P21333	P21333 [2347-23	0	2111.2105
[K].ALEAAH	1xTMTpro [K	4.382E-05	4.225E-05	1	1	2	Q15750	Q15750 [295-319]	0	3191.6651
[K].VNQIGS	1xCarbamido	4.458E-06	4.225E-05	1	2	12	P06733	P06733 [344-358]	0	2242.2357
[K].VNQIGS	1xCarbamido	1.376E-05	4.225E-05	1	1	1	P09104	P09104 [344-358]	0	2226.2408
[R].ALEAAN	1xTMTpro [K	3.435E-05	4.225E-05	1	1	4	P08727	P08727 [100-111]	0	1852.0672
[K].ALEAAQI	1xTMTpro [K	6.1E-05	4.225E-05	1	1	4	Q08623	Q08623 [54-71]	0	2561.5232
[K].GILAADE	1xTMTpro [K	6.806E-06	4.225E-05	1	1	2	P04075	P04075 [29-42]	0	1941.1148
[K].GILAADE	1xOxidation	2.506E-05	4.225E-05	1	1	1	P09972	P09972 [29-42]	0	1973.0869
[R].VLAGQT	1xOxidation	1.667E-05	4.225E-05	1	1	1	Q9UKM9	Q9UKM9 [79-99]	0	3178.8074
[R].VLAGQT	2xTMTpro [K	3.19E-06	4.225E-05	1	1	2	Q9UKM9	Q9UKM9 [79-99]	0	3162.8125
[K].VLAGETI	1xTMTpro [N	3.062E-06	4.225E-05	1	1	3	Q12906	Q12906 [183-200]	0	2214.1937
[K].APVDFG	1xTMTpro [N	1.326E-06	4.225E-05	1	1	2	Q9UHD8	Q9UHD8 [272-28	0	2314.2072
[K].APVDFG	1xOxidation	4.681E-05	4.225E-05	1	1	1	Q9UHD8	Q9UHD8 [272-28	0	2330.2022
[R].VDQSAV	1xTMTpro [K	5.322E-05	4.225E-05	1	1	2	Q14247	Q14247 [132-144]	0	2036.0944
[R].VDQMEC	1xTMTpro [N	1.061E-05	4.225E-05	1	1	1	O94901	O94901 [397-412]	0	1835.9241
[K].APVIDIG	1xTMTpro [K	4.449E-05	4.225E-05	1	1	1	Q9Y4P3	Q9Y4P3 [189-201	0	1877.1352
[R].VDQLTA	1xTMTpro [N	1.773E-05	4.225E-05	1	1	2	P18206	P18206 [548-561]	0	1789.0139
[K].VDQIQEI	1xTMTpro [K	1.158E-05	4.225E-05	1	1	2	P46940	P46940 [1038-10	0	2362.3837
[R].APVPAS	1xTMTpro [N	1.178E-05	4.225E-05	1	1	3	Q15149	Q15149 [3447-34	0	1871.0921
[K].APVPGT	1xTMTpro [N	4.264E-05	4.225E-05	1	1	2	Q9UJZ1	Q9UJZ1 [322-337	0	1818.9517
[K].APVPTG	1xTMTpro [N	2.834E-05	4.225E-05	1	1	4	P27824	P27824 [62-77]	0	2075.0405

[K].VDQETL	1xTMTpro [K	1.787E-07	4.225E-05	1	1	1	Q9NS86	Q9NS86 [280-297	0	2731.4832
[R].APWIEQ	1xTMTpro [N	2.086E-05	4.225E-05	1	1	1	P04439	P04439 [73-89]	0	2438.1584
[R].APWIEQ	1xTMTpro [N	3.23E-05	4.225E-05	1	1	5	P01889	P01889 [73-86]	0	2080.0095
[K].VDPLVSI	1xTMTpro [K	4.367E-05	4.225E-05	1	1	2	P62195	P62195 [131-142]	0	1969.1358
[R].APWVEQ	1xTMTpro [N	4.036E-05	4.225E-05	1	1	3	P10321	P10321 [73-86]	0	2065.9939
[K].VDPALFI	1xTMTpro [N	3.124E-05	4.225E-05	1	1	2	Q13724	Q13724 [424-441	0	2227.281
[K].VDNSSL	1xTMTpro [N	6.819E-06	4.225E-05	1	4	3	P05023	P05023 [213-227]	0	1923.9579
[K].VDNIQA	1xTMTpro [N	8.367E-06	4.225E-05	1	1	2	Q08945	Q08945 [40-54]	0	2005.0674
[K].VDNDEN	1xTMTpro [N	5.993E-05	4.225E-05	1	1	2	P06748	P06748 [33-45]	0	1872.9371
[K].VDNALQ	2xTMTpro [K	1.386E-06	4.225E-05	2	2	7	P01834; P0D	P01834 [43-76]; F	1	4532.3308
[K].VDNALQ	1xTMTpro [K	1.075E-07	4.225E-05	2	2	8	P01834; P0D	P01834 [43-62]; F	0	2744.383
[R].APVAIG	1xTMTpro [K	2.865E-06	4.225E-05	1	1	1	P41214	P41214 [142-162]	0	2626.4804
[R].APVAGT	1xCarbamido	6.241E-06	4.225E-05	1	1	7	P30086	P30086 [162-179]	0	2678.3416
[R].HGVQEL	1xTMTpro [K	2.88E-05	4.225E-05	1	1	1	P35527	P35527 [375-390]	0	2446.3797
[R].VDSAAT	1xTMTpro [N	1.799E-05	4.225E-05	1	1	1	P24666	P24666 [42-59]	0	2216.0791
[K].VDVSAP	1xTMTpro [K	2.942E-05	4.225E-05	1	1	1	Q09666	Q09666 [610-627	0	2571.3699
[K].HGNRGE	1xTMTpro [N	1.851E-07	4.225E-05	1	1	4	P08123	P08123 [975-996]	1	2331.2237
[K].VDVNAP	1xTMTpro [K	2.545E-06	4.225E-05	1	1	2	Q09666	Q09666 [2494-25	0	2412.3167
[R].VDVMQ	1xCarbamido	4.939E-05	4.225E-05	1	1	1	O60504	O60504 [639-655	0	2318.0865
[R].VDVMQ	1xCarbamido	5.756E-05	4.225E-05	1	1	1	O60504	O60504 [639-655	0	2302.0916
[K].GKDGVG	1xTMTpro [K	5.081E-05	4.225E-05	1	1	1	Q96RP9	Q96RP9 [81-96]	1	2302.2027
[R].VDVIPVN	1xTMTpro [N	2.644E-05	4.225E-05	1	1	4	P02751	P02751 [939-953]	0	1934.0779
[R].APSTYG	1xTMTpro [N	1.934E-05	4.225E-05	1	1	2	P08779	P08779 [42-55]	0	1642.872
[R].APSTYG	1xTMTpro [N	1.535E-05	4.225E-05	1	1	1	P02533	P02533 [42-56]	0	1729.904
[R].GKDGSN	1xTMTpro [K	4.681E-05	4.225E-05	1	1	1	Q9NZM1	Q9NZM1 [1273-1	1	2100.2421
[R].AQAALQ	1xTMTpro [N	1.215E-07	4.225E-05	1	1	1	O95758	O95758 [147-183	0	3822.068
[K].VDVGGS	1xTMTpro [K	2.466E-06	4.225E-05	1	1	1	Q9NQT5	Q9NQT5 [131-15	0	2722.4431
[K].VDVEGP	1xTMTpro [K	4.917E-06	4.225E-05	1	1	2	Q09666	Q09666 [2930-29	0	2262.2109
[K].VDVECP	1xCarbamido	2.114E-06	4.225E-05	1	1	4	Q09666	Q09666 [2802-28	0	2365.2201
[K].HGTCAA	1xCarbamido	1.872E-05	4.225E-05	1	1	2	O00584	O00584 [118-132	0	2208.1687
[K].SHCIAE	2xCarbamido	1.584E-08	4.225E-05	1	1	3	P02768	P02768 [311-341]	1	4389.1869
[R].VDVAVN	1xCarbamido	3.352E-05	4.225E-05	1	1	2	Q99714	Q99714 [85-99]	0	2082.1873
[K].VDTNAP	1xTMTpro [K	8.974E-06	4.225E-05	1	1	3	Q09666	Q09666 [1034-10	0	2250.2109

[K].VDTDGN	1xTMTpro [K	3.207E-06	4.225E-05	1	1	2	P13796	P13796 [21-39]	0	2769.4227
[K].HGTDDG	1xTMTpro [K	2.706E-05	4.225E-05	1	1	10	P02675	P02675 [459-471]	0	2153.1094
[R].HGVGLD	1xTMTpro [N	4.793E-05	4.225E-05	1	1	1	Q08AF3	Q08AF3 [72-83]	0	1610.9338
[K].VDSDDQ	1xCarbamido	9.27E-08	4.225E-05	1	1	4	P35613	P35613 [192-222]	0	3819.8066
[K].VDVEVP	1xTMTpro [K	8.941E-06	4.225E-05	1	1	4	Q09666	Q09666 [1290-13	0	2277.247
[K].APSEED	2xTMTpro [K	1.825E-05	4.225E-05	1	1	2	Q9Y676	Q9Y676 [36-57]	1	3373.7984
[K].AQAALV	1xTMTpro [N	1.558E-05	4.225E-05	1	1	1	Q9ULN7	Q9ULN7 [62-80]	0	2354.2788
[K].VDMTFE	1xTMTpro [K	4.163E-05	4.225E-05	1	1	2	Q14318	Q14318 [257-271	0	2402.3133
[R].VDIETPN	1xTMTpro [N	2.701E-05	4.225E-05	1	1	2	Q09666	Q09666 [541-556	0	2016.0933
[R].AQAVLE	1xTMTpro [K	4.954E-06	4.225E-05	1	1	2	P36776	P36776 [483-498]	0	2442.2466
[R].AQAVSE	1xTMTpro [N	1.184E-05	4.225E-05	1	1	3	P55884	P55884 [121-134]	0	1664.8159
[K].AQAVTQ	2xTMTpro [K	2.471E-05	4.225E-05	1	1	1	P49750	P49750 [938-961]	0	3388.9773
[K].VDIEGPD	1xTMTpro [K	7.395E-06	4.225E-05	1	1	6	Q09666	Q09666 [2546-25	0	2276.2266
[K].VDIEAPD	1xTMTpro [K	5.638E-06	4.225E-05	1	1	4	Q09666	Q09666 [1162-11	0	2263.2313
[K].VDIDVPD	1xTMTpro [K	4.319E-07	4.225E-05	1	1	2	Q09666	Q09666 [2876-28	0	2654.4434
[R].GHVFEE	1xTMTpro [K	1.075E-05	4.225E-05	1	1	2	P13639	P13639 [768-785]	0	2570.3933
[K].VDIDVPD	1xTMTpro [K	4.665E-05	4.225E-05	1	1	4	Q09666	Q09666 [4224-42	0	2304.2579
[R].AQDAPL	1xTMTpro [N	1.706E-05	4.225E-05	1	1	1	P48681	P48681 [407-421]	0	1859.0306
[K].VDIDVPD	1xTMTpro [K	3.113E-05	4.225E-05	1	1	4	Q09666	Q09666 [1546-15	0	2318.2735
[K].VDIDVPD	1xTMTpro [K	5.226E-05	4.225E-05	1	1	2	Q09666	Q09666 [4170-41	0	2655.4274
[K].AQDEAF	1xTMTpro [N	5.722E-06	4.225E-05	1	1	3	Q9Y3B7	Q9Y3B7 [125-142	0	2249.2097
[K].VDIDTPD	1xTMTpro [K	4.434E-05	4.225E-05	1	1	2	Q09666	Q09666 [4096-41	0	2329.2531
[K].AQDEGL	1xTMTpro [K	1.394E-05	4.225E-05	1	1	3	P55084	P55084 [255-268]	0	2126.1989
[K].AQDEGL	2xTMTpro [K	9.518E-06	4.225E-05	1	1	1	P55084	P55084 [255-272]	1	2811.6436
[K].AQDEIPA	1xTMTpro [N	1.143E-07	4.225E-05	1	1	2	P43686	P43686 [11-43]	0	3934.0041
[K].VDIDAPD	1xTMTpro [K	3.459E-05	4.225E-05	1	1	2	Q09666	Q09666 [4352-43	0	2249.2157
[K].VDIDAPD	1xTMTpro [K	3.671E-06	4.225E-05	1	1	7	Q09666	Q09666 [1694-17	0	2708.4176
[K].AQDIEAC	1xCarbamido	2.431E-07	4.225E-05	1	1	5	P50991	P50991 [97-122]	0	3229.7117
[K].VDIDAPD	1xTMTpro [K	1.899E-05	4.225E-05	1	1	4	Q09666	Q09666 [731-748	0	2636.3964
[K].VDLGGF	1xTMTpro [K	3.879E-05	4.225E-05	1	1	2	P22102	P22102 [468-480]	0	1960.1399
[R].VDIIENQ	1xTMTpro [N	4.092E-05	4.225E-05	1	1	4	P21266	P21266 [101-112]	0	1782.9379
[R].GHVFEE	1xOxidation [	3E-06	4.225E-05	1	1	1	P13639	P13639 [768-785]	0	2586.3882
[K].HHSLGG	1xTMTpro [K	3.471E-05	4.225E-05	1	1	1	Q15084	Q15084 [86-102]	0	2434.3487

[K].GHYTEG	1xTMTpro [N	6.764E-07	4.225E-05	4	5	5	Q13885; Q1:	Q13885 [104-121]	0	2263.1889
[K].HGYEKP	1xTMTpro [K	6.018E-07	4.225E-05	1	1	2	Q7L014	Q7L014 [390-410]	0	2903.6057
[R].AQAEAQ	1xTMTpro [N	1.544E-05	4.225E-05	1	1	1	Q15149	Q15149 [1274-12:	0	1849.9727
[R].AQAEAQ	1xTMTpro [N	1.359E-05	4.225E-05	1	1	1	Q15149	Q15149 [1274-12!	1	2363.2275
[K].HGYIGEI	1xTMTpro [N	2.079E-05	4.225E-05	1	1	4	P62244	P62244 [44-57]	0	2005.0099
[R].AQAELE	1xTMTpro [K	1.722E-05	4.225E-05	1	1	2	Q7Z406	Q7Z406 [1302-13	0	2468.3124
[K].AQAELQ	1xTMTpro [N	3.664E-05	4.225E-05	1	1	1	P41229	P41229 [969-982]	0	1889.0663
[K].AQAELV	1xTMTpro [N	9.057E-06	4.225E-05	1	1	4	P30049	P30049 [137-150]	0	1735.9146
[K].HGYPLII	1xCarbamid	1.051E-05	4.225E-05	1	1	2	P31937	P31937 [61-76]	0	2516.3503
[K].VDLSFSI	1xTMTpro [N	1.555E-05	4.225E-05	1	1	1	P01023	P01023 [568-586]	0	2353.2584
[K].VDMTFE	1xOxidation	3.853E-05	4.225E-05	1	1	1	Q14318	Q14318 [257-271]	0	2418.3082
[K].VDISAPD	1xTMTpro [K	1.491E-05	4.225E-05	1	1	5	Q09666	Q09666 [1364-13:	0	2608.4015
[K].VDINTPD	1xTMTpro [K	1.923E-05	4.225E-05	1	1	6	Q09666	Q09666 [4762-47	0	2665.423
[K].AQAGEG	1xTMTpro [N	9.344E-06	4.225E-05	1	1	2	O00764	O00764 [276-292]	0	2143.1613
[K].VDINTED	1xCarbamid	1.533E-05	4.225E-05	1	1	2	P21333	P21333 [2090-21(	0	1940.9191
[K].HHAAYV	1xTMTpro [K	1.142E-05	4.225E-05	1	1	6	P04179	P04179 [54-68]	0	2347.265
[K].AQAHAE	1xCarbamid	1.141E-06	4.225E-05	1	1	1	P46940	P46940 [506-538]	0	4220.0175
[K].AQALAIE	1xTMTpro [N	6.719E-06	4.225E-05	1	1	3	Q14764	Q14764 [748-761]	0	1847.0194
[K].VDINAPD	1xTMTpro [K	3.416E-06	4.225E-05	1	1	2	Q09666	Q09666 [2620-26:	0	2568.4066
[K].VDINAPD	1xTMTpro [K	2.042E-07	4.225E-05	1	1	6	Q09666	Q09666 [1492-15(	0	2649.4281
[K].VDINAPD	1xTMTpro [K	4.161E-07	4.225E-05	1	1	4	Q09666	Q09666 [3132-31	0	2626.4121
[K].VDINAPD	1xTMTpro [K	3.236E-05	4.225E-05	1	1	4	Q09666	Q09666 [3584-36(	0	2635.4124
[K].VDIQTED	1xCarbamid	7.991E-06	4.225E-05	1	1	1	O75369	O75369 [2045-20!	0	2231.1357
[K].VDVSAP	1xTMTpro [K	1.802E-05	4.225E-05	1	1	3	Q09666	Q09666 [980-997]	0	2608.3573
[K].VDVSAP	1xOxidation	4.541E-05	4.225E-05	1	1	2	Q09666	Q09666 [980-997]	0	2624.3522
[R].HGIVED\	1xTMTpro [N	5.464E-05	4.225E-05	1	1	3	P61158	P61158 [80-91]	0	1803.9019
[K].GKGGEI	2xTMTpro [K	4.367E-05	4.225E-05	1	1	2	P61604	P61604 [55-66]	1	2111.3005
[K].HGEVCP	1xCarbamid	4.012E-06	4.225E-05	1	1	2	Q13162	Q13162 [241-263]	0	3315.7976
[K].VEGDLK	2xTMTpro [K	3.886E-05	4.225E-05	1	1	2	Q09666	Q09666 [4746-47!	1	2283.3376
[R].GKGEITI	2xTMTpro [K	4.465E-05	4.225E-05	1	1	2	Q15532	Q15532 [12-23]	1	2125.3161
[R].VEGAET	1xTMTpro [N	9.518E-06	4.225E-05	1	1	1	Q8IUR7	Q8IUR7 [298-316]	0	2449.3145
[R].APHPDQ	1xTMTpro [N	5.948E-06	4.225E-05	1	1	3	Q05707	Q05707 [1771-17!	0	3193.5471
[R].APHPDQ	1xOxidation	1.98E-05	4.225E-05	1	1	2	Q05707	Q05707 [1771-17!	0	3209.5421

[K].APHYPG	1xTMTpro [N	4.21E-06	4.225E-05	1	1	1	O94875	O94875 [69-88]	0	2351.2679
[K].APKPDG	1xTMTpro [K	1.913E-05	4.225E-05	1	1	3	P35637	P35637 [449-472]	0	2861.3881
[K].VEFLLNA	1xTMTpro [K	2.11E-06	4.225E-05	1	1	3	P12111	P12111 [1282-13(	1	2892.5823
[K].VEFLLNA	1xTMTpro [K	3.839E-05	4.225E-05	1	1	1	P12111	P12111 [1282-129	0	1853.0777
[K].GKFPVQ	1xTMTpro [K	2.641E-06	4.225E-05	1	1	1	Q92542	Q92542 [350-371]	1	3053.7279
[R].VEFATL(	1xTMTpro [K	3.477E-07	4.225E-05	1	1	2	Q14980	Q14980 [1043-10	0	2479.4052
[K].VEEVLEI	1xTMTpro [K	3.157E-05	4.225E-05	1	1	1	P83916	P83916 [10-25]	0	2589.3315
[K].APLATGI	1xTMTpro [K	2.952E-05	4.225E-05	1	1	1	P20290	P20290 [178-206]	1	3727.759
[R].APIDHGI	1xTMTpro [K	3.591E-06	4.225E-05	1	1	2	P52630	P52630 [249-267]	0	2692.459
[R].APLDIPII	3xTMTpro [K	4.404E-05	4.225E-05	1	1	1	Q9UL46	Q9UL46 [62-85]	2	3935.1464
[R].VEESTQ	1xTMTpro [N	2.277E-06	4.225E-05	1	1	2	Q14315	Q14315 [2207-22	0	2628.3265
[K].VEESSW	1xTMTpro [K	1.832E-05	4.225E-05	1	1	2	Q9Y266	Q9Y266 [228-239	0	2000.0832
[K].VEEQEP	1xTMTpro [K	3.699E-06	4.225E-05	1	1	3	Q07021	Q07021 [155-174]	0	2895.5847
[K].VEELSQ	1xTMTpro [K	4.525E-05	4.225E-05	1	1	2	Q14789	Q14789 [861-871]	0	1840.0672
[K].GKGGVT	2xTMTpro [K	1.202E-05	4.225E-05	1	1	3	Q09666	Q09666 [5724-57	1	2431.3973
[R].APGPGL	1xTMTpro [N	2.252E-06	4.225E-05	1	1	2	Q8N1G4	Q8N1G4 [65-83]	0	2228.3198
[K].HGEVCP	1xCarbamido	3.141E-05	4.225E-05	1	1	3	Q06830	Q06830 [169-190]	0	3623.0156
[R].VEGDPD	1xTMTpro [K	3.13E-05	4.225E-05	1	1	2	Q9HCB6	Q9HCB6 [65-79]	0	2339.2163
[R].VELSDV(	1xTMTpro [K	2.212E-07	4.225E-05	1	1	2	Q9P035	Q9P035 [23-43]	0	2961.6541
[K].VELNALI	1xOxidation [	5.2E-05	4.225E-05	1	1	1	Q9NR31	Q9NR31 [113-135	0	3121.7674
[K].HGEIDYI	1xTMTpro [K	4.135E-05	4.225E-05	1	1	1	P62333	P62333 [323-333]	0	1882.0566
[R].APFPLY	1xTMTpro [K	1.53E-05	4.225E-05	1	1	2	Q9HCU5	Q9HCU5 [12-38]	0	3163.8011
[R].VELLHNI	1xCarbamido	4.705E-05	4.225E-05	1	1	2	P01024	P01024 [864-879]	0	2409.3456
[K].GKIEEDS	2xTMTpro [K	6.012E-05	4.225E-05	1	1	1	O60888	O60888 [111-124]	1	2534.439
[R].APGAEE	1xCarbamido	7.207E-06	4.225E-05	1	1	1	Q9BVM4	Q9BVM4 [102-12	0	2451.2298
[K].VELFHY(	1xTMTpro [N	3.76E-05	4.225E-05	1	1	1	Q14554	Q14554 [108-124]	0	2430.1798
[K].APGAEE	2xTMTpro [K	2.986E-07	4.225E-05	1	1	1	P09543	P09543 [262-276]	1	2559.4599
[K].VELFFKI	2xTMTpro [K	8.094E-06	4.225E-05	1	1	3	P22234	P22234 [126-149]	1	3719.9737
[K].VEEILEQ	1xTMTpro [K	9.242E-06	4.225E-05	1	1	2	Q96DB5	Q96DB5 [83-101]	0	2853.4537
[R].VELEDW	1xTMTpro [N	1.98E-05	4.225E-05	1	1	1	P02671	P02671 [726-743]	0	2503.1849
[K].VELAQL(	1xTMTpro [K	1.506E-06	4.225E-05	1	1	1	Q9H307	Q9H307 [213-228	0	2546.3495
[K].APGEQT	1xTMTpro [N	5.295E-06	4.225E-05	1	1	3	Q9Y5B9	Q9Y5B9 [607-623	0	2130.1627
[K].GKLDVQ	2xTMTpro [K	1.115E-05	4.225E-05	1	1	3	P21333	P21333 [905-916]	1	2205.3423

[R].VEHWGI	1xTMTpro [K	6.041E-05	4.225E-05	1	1	1	P20036	P20036 [196-207]	0	2043.1883
[RK].VEHWC	1xTMTpro [K	2.163E-05	4.225E-05	2	2	2	P01903; P01	P01903 [190-201]	0	2044.1723
[K].VEGTEP	1xTMTpro [K	2.781E-06	4.225E-05	1	1	5	P19338	P19338 [298-318]	0	2920.57
[R].GKKPIED	3xTMTpro [K	3.01E-05	4.225E-05	1	1	1	O60716	O60716 [632-648]	1	3087.7831
[R].VEGGFL	1xTMTpro [N	2.597E-05	4.225E-05	1	1	1	Q9H6I2	Q9H6I2 [151-173]	0	2537.3432
[K].APGIILLD	1xTMTpro [N	1.217E-05	4.225E-05	1	1	1	Q9NP58	Q9NP58 [744-763	0	2390.2734
[K].VEGFPTI	1xTMTpro [K	7.961E-06	4.225E-05	1	1	5	P13667	P13667 [597-611]	0	2236.2145
[K].GKLEAII	2xTMTpro [K	4.099E-05	4.225E-05	1	1	4	O75367	O75367 [122-134]	1	2247.4257
[R].HGGEDY	1xCarbamido	1.61E-05	4.225E-05	1	1	1	P08574	P08574 [205-228]	0	2958.4627
[K].GKENQL	1xCarbamido	2.333E-05	4.225E-05	1	1	2	P54578	P54578 [268-284]	1	2947.6491
[K].APLNVQ	1xTMTpro [K	3.13E-05	4.225E-05	1	1	1	O75369	O75369 [878-894]	0	2375.3579
[R].HGLEVS	1xTMTpro [N	5.344E-06	4.225E-05	1	1	1	Q15393	Q15393 [440-463]	0	2896.4946
[K].APPHEL	1xTMTpro [K	2.77E-05	4.225E-05	1	1	2	Q13409	Q13409 [203-213]	0	1888.0308
[R].APPNAT	1xTMTpro [K	3.219E-05	4.225E-05	1	1	1	Q96G23	Q96G23 [78-93]	0	2354.3
[K].APPPSL	1xCarbamido	4.939E-05	4.225E-05	1	1	1	Q9NZZ3	Q9NZZ3 [12-27]	0	1990.0235
[K].VEALTKE	1xTMTpro [K	3.146E-05	4.225E-05	1	1	1	Q9Y5B9	Q9Y5B9 [787-802	1	2544.4205
[R].VEAGDV	1xTMTpro [K	8.154E-06	4.225E-05	1	1	1	Q9Y265	Q9Y265 [185-201	0	2343.3052
[K].VEAEQN	1xTMTpro [K	3.8E-05	4.225E-05	1	1	1	Q8IY31	Q8IY31 [117-132]	0	2593.3794
[R].VEAEGL	1xCarbamido	5.701E-05	4.225E-05	1	1	1	Q96T76	Q96T76 [317-334]	0	2202.1872
[K].APPTAC	1xCarbamido	5.112E-06	4.225E-05	1	1	1	P17676	P17676 [243-260]	0	2365.283
[R].HGLLVPI	1xTMTpro [N	1.879E-05	4.225E-05	1	1	1	P56537	P56537 [68-85]	0	2389.2907
[K].HGLEVIY	1xCarbamido	2.631E-06	4.225E-05	1	1	2	P07900	P07900 [514-534]	0	3201.682
[K].VEAANV	1xTMTpro [K	6.041E-05	4.225E-05	1	1	3	P55287	P55287 [347-357]	0	1801.0464
[R].APQGCS	2xCarbamido	5.701E-05	4.225E-05	1	1	2	Q96AQ6	Q96AQ6 [584-596	0	1710.7859
[R].APQLLIA	1xTMTpro [K	1.645E-05	4.225E-05	1	1	1	O15460	O15460 [319-340]	1	3198.7443
[K].VEAAEIV	1xTMTpro [N	1.753E-06	4.225E-05	1	1	2	Q14315	Q14315 [2261-22	0	2128.0729
[K].VEAADH	1xTMTpro [N	4.713E-05	4.225E-05	1	1	1	P02549	P02549 [1017-10	0	2028.1197
[R].VDYSGG	1xTMTpro [N	4.374E-05	4.225E-05	1	1	1	P49959	P49959 [367-380]	0	1876.9764
[K].HGLTEAI	1xTMTpro [K	3.582E-05	4.225E-05	1	1	1	Q96PZ0	Q96PZ0 [107-118	0	1849.0675
[K].APQVTE	1xCarbamido	1.222E-05	4.225E-05	1	1	1	Q02952	Q02952 [1123-11	0	3356.7751
[R].VDYIMQI	2xTMTpro [K	1.713E-05	4.225E-05	1	1	1	P45974	P45974 [483-508]	1	3993.1421
[R].VDYIMQI	1xTMTpro [K	4.721E-05	4.225E-05	1	1	1	P45974	P45974 [483-499]	0	2526.3956
[K].GKDLSM	2xTMTpro [K	1.713E-05	4.225E-05	2	2	2	P48594; P29	P48594 [238-256]	1	2995.7682

[K].APQETY.	1xTMTpro [K	9.911E-06	4.225E-05	1	1	2	P62191	P62191 [179-198]	0	2811.502
[R].HKEDAG	1xCarbamido	4.3E-05	4.225E-05	1	1	1	Q86VB7	Q86VB7 [810-825	1	2503.2929
[R].VEAVNM	1xTMTpro [K	1.538E-05	4.225E-05	1	1	1	P38646	P38646 [579-595]	0	2465.3201
[R].GKEDPW	1xTMTpro [K	8.186E-07	4.225E-05	1	1	1	O60830	O60830 [85-106]	1	2822.5656
[R].HGGGG(	1xTMTpro [N	1.032E-05	4.225E-05	1	1	1	P35908	P35908 [46-61]	0	1624.79
[R].HGGPGF	1xTMTpro [N	8.586E-06	4.225E-05	1	1	1	Q8WUF5	Q8WUF5 [554-57	0	2532.265
[R].APLQVA'	1xTMTpro [N	4.118E-06	4.225E-05	1	1	1	Q14315	Q14315 [1475-14	0	2306.3403
[K].APIRPDI'	1xTMTpro [N	3.582E-05	4.225E-05	1	1	4	P36578	P36578 [30-45]	0	2166.2467
[R].VEEGVP	1xTMTpro [N	1.895E-06	4.225E-05	1	1	3	P12111	P12111 [342-363]	0	2598.431
[R].VEEEEE	1xTMTpro [K	8.035E-06	4.225E-05	1	1	3	P35580	P35580 [931-945]	1	2495.3233
[K].VEEDDY	1xTMTpro [K	4.874E-05	4.225E-05	1	1	1	Q5JRA6	Q5JRA6 [720-740	0	3030.4923
[R].VEDYEP	1xTMTpro [K	4.931E-05	4.225E-05	1	1	1	O95169	O95169 [59-77]	0	2818.305
[K].APLYDL/	1xTMTpro [K	4.11E-06	4.225E-05	1	1	2	Q9GZL7	Q9GZL7 [377-388	0	1951.0781
[R].VEDYDA	1xTMTpro [K	5.039E-05	4.225E-05	1	1	1	P50895	P50895 [339-352]	0	2176.1265
[K].GKEDEG	1xTMTpro [K	2.475E-06	4.225E-05	1	1	1	Q14204	Q14204 [2400-24	1	2596.3532
[K].GKEESLI	1xTMTpro [K	2.538E-05	4.225E-05	1	1	1	P02775	P02775 [48-62]	1	2333.248
[K].GKEEEL(	1xTMTpro [K	1.913E-05	4.225E-05	1	1	1	Q4V328	Q4V328 [560-579	2	3037.5682
[K].VEDMAE	1xCarbamido	1.011E-05	4.225E-05	1	1	1	P35749	P35749 [87-106]	0	2634.3187
[K].VEDMAE	1xCarbamido	1.097E-07	4.225E-05	1	1	3	P35749	P35749 [87-106]	0	2618.3237
[K].VEDMAE	1xCarbamido	5.445E-08	4.225E-05	2	2	3	P35580; P35	P35580 [87-106];	0	2894.5247
[K].APNYSC	1xCarbamido	3.242E-05	4.225E-05	1	1	2	P78406	P78406 [138-151]	0	2224.1022
[K].HGLEVIY	1xCarbamido	6.003E-06	4.225E-05	1	1	2	P07900	P07900 [514-534]	0	3185.6871
[R].VEDAYIL	1xCarbamido	1.98E-05	4.225E-05	1	1	4	P40227	P40227 [225-241]	0	2654.3879
[K].VECYDY	1xCarbamido	3.032E-05	4.225E-05	1	1	1	O75131	O75131 [212-235	0	3434.6012
[K].VECYDY	1xCarbamido	4.064E-05	4.225E-05	1	1	2	O75131	O75131 [212-235	0	3418.6063
[K].VECTYIS	1xCarbamido	2.064E-05	4.225E-05	1	1	1	Q12765	Q12765 [52-64]	0	1883.9856
[K].HGGTIPI	1xTMTpro [N	3.78E-05	4.225E-05	1	1	1	P00367	P00367 [481-496]	0	2042.099
[R].VELSEQ	1xTMTpro [K	1.648E-05	4.225E-05	1	1	1	P09543	P09543 [300-316]	0	2622.4271
[K].VDKGVV	1xTMTpro [K	5.682E-08	4.225E-05	1	1	2	P04075	P04075 [109-134]	1	3222.7462
[R].AQDPSE	1xTMTpro [K	4.651E-07	4.225E-05	1	1	3	Q12905	Q12905 [148-174	0	3478.7755
[K].AQNLYQ	1xTMTpro [K	3.249E-06	4.225E-05	1	1	1	P04114	P04114 [4148-41	0	2974.613
[R].VAVEEV	1xTMTpro [K	2.122E-06	4.225E-05	1	1	2	P02545	P02545 [440-453]	1	2214.2262
[K].AQNVTLI	1xTMTpro [N	5.516E-08	4.225E-05	1	1	3	O00505	O00505 [68-96]	0	3325.8035

[K].HLFYAEI	1xTMTpro [K	2.034E-05	4.225E-05	1	1	1	O00339	O00339 [813-829]	0	2670.3253
[K].AQPAQP	1xTMTpro [K	3.119E-06	4.225E-05	1	1	2	P25786	P25786 [244-263]	1	2769.3645
[K].AQPARP	1xTMTpro [K	1.95E-08	4.225E-05	1	1	3	O94875	O94875 [926-946]	0	2703.5438
[K].HLGGIPV	1xCarbamido	5.365E-07	4.225E-05	1	1	1	P31930	P31930 [249-269]	0	2658.3669
[K].VAVAIPN	1xTMTpro [N	3.195E-06	4.225E-05	1	1	2	P51659	P51659 [480-506]	0	3170.7493
[R].VAVAGC	2xCarbamido	2.175E-05	4.225E-05	1	1	1	Q9UK59	Q9UK59 [3-15]	0	2024.0549
[R].GHFWV(	1xCarbamido	3.119E-05	4.225E-05	1	1	1	Q96JY6	Q96JY6 [324-336]	0	2248.1352
[R].AQPFGQ	1xTMTpro [N	2.475E-06	4.225E-05	1	1	2	Q08345	Q08345 [842-862]	0	2700.3589
[R].VATTEM	1xTMTpro [N	3.839E-05	4.225E-05	1	1	1	Q7L775	Q7L775 [586-600]	0	2023.0126
[R].VATSSLI	1xTMTpro [K	4.65E-05	4.225E-05	1	1	2	Q9BV38	Q9BV38 [189-199	0	1757.03
[K].AQPPEA	1xTMTpro [N	6.404E-06	4.225E-05	1	1	1	A0FGR8	A0FGR8 [720-735	0	1947.0367
[R].VATQAV	1xTMTpro [K	1.613E-05	4.225E-05	1	1	1	Q4G0N4	Q4G0N4 [326-339	0	2079.2305
[R].VATPVD'	3xTMTpro [K	1.837E-06	4.225E-05	1	1	2	P30041	P30041 [175-200]	2	4071.2829
[R].VATPVD'	1xOxidation [	5.103E-06	4.225E-05	1	1	1	P30041	P30041 [175-199]	1	3654.9757
[R].VATPVD'	2xTMTpro [K	3.617E-06	4.225E-05	1	1	2	P30041	P30041 [175-199]	1	3638.9808
[R].VATNAA	1xTMTpro [K	2.295E-05	4.225E-05	1	1	2	Q9Y4G6	Q9Y4G6 [902-913	0	1780.0573
[K].AQPTTL'	1xTMTpro [N	9.31E-06	4.225E-05	1	1	1	Q6P2C8	Q6P2C8 [150-168	0	2416.3407
[R].AQPVQV	1xTMTpro [K	6.827E-07	4.225E-05	1	1	10	P06396	P06396 [627-648]	0	2880.5023
[K].AQNKPF	2xTMTpro [K	3.393E-05	4.225E-05	1	1	2	Q14232	Q14232 [221-234]	0	2540.4693
[K].VAVGQE	1xTMTpro [N	3.265E-05	4.225E-05	1	1	2	Q14315	Q14315 [978-991]	0	1809.9778
[R].GHGLCE	4xCarbamido	1.028E-06	4.225E-05	1	1	1	P22105	P22105 [534-557]	0	2965.214
[R].VAVLISG	1xTMTpro [N	9.82E-06	4.225E-05	1	1	1	P22102	P22102 [810-829]	0	2319.3203
[K].GHIIVDE	1xTMTpro [K	1.078E-05	4.225E-05	1	1	2	P00390	P00390 [355-368]	0	2222.2425
[K].HLEINPD	1xTMTpro [N	6.726E-07	4.225E-05	1	1	11	P07900	P07900 [633-647]	0	2091.1518
[R].VCDSCY	2xCarbamido	1.835E-05	4.225E-05	1	1	1	Q8IWB7	Q8IWB7 [343-356	1	2370.1197
[R].GHLFLQ'	1xTMTpro [N	2.227E-06	4.225E-05	2	2	2	P0C0L4; P0(	P0C0L4 [139-155]	0	2288.2107
[K].AQLNIGN	2xCarbamido	2.737E-08	4.225E-05	1	1	3	P62917	P62917 [94-123]	0	3876.0313
[R].VCDATY	1xCarbamido	5.936E-05	4.225E-05	1	1	1	Q12974	Q12974 [45-57]	1	2408.3312
[R].GHLENN	1xTMTpro [K	5.984E-05	4.225E-05	1	2	3	P05388	P05388 [67-77]	0	1830.0365
[R].GHLDPT	1xTMTpro [N	5.984E-05	4.225E-05	1	1	1	Q9Y2E5	Q9Y2E5 [396-411	0	2208.2209
[R].VAYVSF(	1xTMTpro [K	3.683E-05	4.225E-05	1	1	2	P50914	P50914 [12-23]	0	1841.0565
[K].AQIQQFI	1xTMTpro [K	1.529E-09	4.225E-05	1	1	1	Q86UP2	Q86UP2 [583-607	0	3343.8254
[K].VATGQE	1xTMTpro [K	2.579E-05	4.225E-05	1	1	1	Q8IYU8	Q8IYU8 [356-372]	0	2457.3845

[R].AQLSDY	2xTMTpro [K	2.545E-06	4.225E-05	1	1	1	O60216	O60216 [339-359]	1	3188.8235
[K].VAYTEAI	1xTMTpro [N	2.443E-06	4.225E-05	1	1	2	P13674	P13674 [173-192]	0	2793.3513
[K].VAWIQD	1xTMTpro [N	3.435E-05	4.225E-05	1	1	1	P56202	P56202 [234-249]	0	2318.2035
[K].GHLDAE	1xTMTpro [N	2.053E-05	4.225E-05	1	1	1	Q9Y3Y2	Q9Y3Y2 [229-248	0	2510.1312
[R].VAVVTYI	1xTMTpro [N	6.933E-06	4.225E-05	1	1	29	P12111	P12111 [2445-245	0	2012.0983
[R].VAVVQY	1xTMTpro [N	1.208E-05	4.225E-05	1	1	7	P12109	P12109 [873-888]	0	2079.1266
[R].AQLVPLI	1xCarbamido	1.98E-05	4.225E-05	1	1	17	P02765	P02765 [188-211]	0	3187.7205
[K].VAVVLMI	1xTMTpro [K	1.032E-05	4.225E-05	1	1	2	Q6DD88	Q6DD88 [136-154	0	2604.4199
[K].VAVVAG	1xTMTpro [K	2.721E-05	4.225E-05	1	1	2	P23526	P23526 [215-226]	0	1743.0296
[R].HIEWES	1xCarbamido	5.904E-06	4.225E-05	1	1	1	O60716	O60716 [520-535]	0	2190.1297
[R].GHHVAQ	1xTMTpro [K	5.037E-08	4.225E-05	1	1	2	Q02218	Q02218 [141-171]	0	3808.026
[R].AQLSVV	1xTMTpro [N	2.427E-05	4.225E-05	1	1	1	Q6UVK1	Q6UVK1 [1854-18	0	2606.3269
[R].AQILVAD	1xTMTpro [K	5.813E-05	4.225E-05	1	1	2	Q5T6V5	Q5T6V5 [209-223	0	2238.2989
[K].VATAQD	1xTMTpro [K	8.719E-07	4.225E-05	1	1	4	P40227	P40227 [80-104]	0	3152.7546
[R].VASVSQ	1xTMTpro [N	2.3E-05	4.225E-05	1	1	1	O75694	O75694 [315-332]	0	2032.147
[R].VAPQQD	1xTMTpro [N	5.084E-06	4.225E-05	1	1	1	P24043	P24043 [554-575]	0	2686.3603
[K].VAPGYY	1xTMTpro [N	2.151E-05	4.225E-05	1	1	1	P16144	P16144 [916-930]	0	1944.9986
[R].VAPEEH	1xTMTpro [K	1.049E-06	4.225E-05	1	2	85	P63261	P63261 [96-113]	0	2562.4787
[R].VAPEEH	1xTMTpro [K	1.107E-05	4.225E-05	2	4	9	P62736; P68	P62736 [98-115];	0	2564.458
[K].VAPAQP	1xTMTpro [K	4.752E-05	4.225E-05	1	1	1	P23588	P23588 [512-525]	1	2031.1479
[R].AQSELA	1xTMTpro [K	5.192E-05	4.225E-05	1	1	3	P25786	P25786 [52-61]	0	1690.9732
[K].VAPAPA	1xOxidation [	1.427E-05	4.225E-05	1	1	1	P10515	P10515 [397-429]	0	3529.9412
[K].VAPAPA	1xTMTpro [N	1.592E-06	4.225E-05	1	1	4	P10515	P10515 [397-429]	0	3513.9463
[K].VAPADG	1xTMTpro [K	2.838E-06	4.225E-05	1	1	1	Q92896	Q92896 [1125-11	0	2660.3919
[K].AQSSQD	1xTMTpro [N	9.2E-07	4.225E-05	1	1	1	Q9UHX1	Q9UHX1 [277-298	0	2691.3367
[R].GHFEAF	1xCarbamido	5.735E-07	4.225E-05	1	1	3	Q13151	Q13151 [25-45]	0	2927.5581
[R].VANPSG	1xTMTpro [N	6.869E-06	4.225E-05	1	1	3	P21333	P21333 [1297-13	0	2068.063
[R].VANILHD	1xCarbamido	2.642E-07	4.225E-05	1	1	2	Q9BS26	Q9BS26 [181-203	0	3169.6596
[K].VANEPV	1xTMTpro [N	1.391E-05	4.225E-05	1	1	3	P30038	P30038 [32-47]	0	2019.083
[K].VANDNA	1xTMTpro [K	6.584E-06	4.225E-05	1	1	2	Q9UHA4	Q9UHA4 [35-62]	0	3535.8789
[K].VANAES	1xTMTpro [K	2.429E-06	4.225E-05	1	1	1	P02786	P02786 [268-287]	0	2758.5305
[R].AQTEGII	1xTMTpro [K	1.721E-06	4.225E-05	1	1	2	Q9Y265	Q9Y265 [379-400	0	2932.5871
[R].VAMQDA	1xOxidation [	3.372E-06	4.225E-05	1	1	3	Q99798	Q99798 [96-117]	0	2952.5488

[R].AQTGAP	1xCarbamido	4.235E-05	4.225E-05	1	1	2	Q9BQ67	Q9BQ67 [60-73]	0	1838.9754
[R].VAMFLTI	1xTMTpro [K	1.991E-05	4.225E-05	1	1	3	Q15046	Q15046 [554-565]	0	1961.1022
[R].AQTLPTS	1xTMTpro [K	4.417E-06	4.225E-05	1	1	2	Q01082	Q01082 [2326-234	0	2511.4162
[R].VAQCSQ	3xCarbamido	1.654E-05	4.225E-05	1	1	2	P04275	P04275 [2465-247	0	2333.1292
[K].VAQEQG	1xTMTpro [K	4.564E-05	4.225E-05	1	1	2	Q7Z406	Q7Z406 [572-581]	0	1658.947
[K].VAQGVS	1xTMTpro [K	4.449E-05	4.225E-05	1	1	2	P12268	P12268 [439-450]	0	1767.0256
[K].AQQVSQ	1xTMTpro [K	7.73E-06	4.225E-05	1	1	3	P18206	P18206 [353-366]	0	2066.2101
[R].AQQEDA	1xTMTpro [N	5.861E-06	4.225E-05	1	1	1	Q13951	Q13951 [132-147]	0	2109.0532
[K].AQQEFA	1xTMTpro [N	1.808E-05	4.225E-05	1	1	2	O14828	O14828 [314-329]	0	1996.0571
[K].VASTEN	1xTMTpro [N	8.496E-07	4.225E-05	1	1	1	P53618	P53618 [791-814]	0	2759.4171
[K].VASSSS	1xTMTpro [N	4.686E-07	4.225E-05	1	1	2	Q14980	Q14980 [187-217]	0	3515.7218
[K].AQQEQE	1xTMTpro [K	1.435E-05	4.225E-05	1	1	2	P14314	P14314 [207-219]	0	2057.1159
[K].VASSDLY	1xOxidation [	5.348E-05	4.225E-05	1	1	1	O75955	O75955 [134-152]	0	2607.4559
[R].AQQEVL	1xTMTpro [N	5.839E-06	4.225E-05	1	1	2	Q9UPN3	Q9UPN3 [5059-50	0	2304.2519
[R].HLIATQL	1xTMTpro [N	1.589E-05	4.225E-05	1	1	1	P48960	P48960 [324-339]	0	2171.2177
[R].VASGPS	1xTMTpro [N	7.339E-08	4.225E-05	1	1	2	Q14764	Q14764 [862-893]	0	3423.794
[R].VASEQS	1xCarbamido	3.322E-05	4.225E-05	1	1	1	P31930	P31930 [59-80]	0	2667.3367
[K].AQQATP	1xTMTpro [N	4.997E-05	4.225E-05	1	1	2	Q9BX68	Q9BX68 [43-58]	0	1877.02
[K].GHFGPI	1xTMTpro [K	3.477E-05	4.225E-05	1	1	1	Q13347	Q13347 [283-298]	0	2288.2432
[K].VAQVAE	1xTMTpro [K	3.514E-05	4.225E-05	1	1	3	O94905	O94905 [221-232]	0	1915.1144
[K].VAQTDG	1xTMTpro [K	7.83E-06	4.225E-05	1	1	1	P17302	P17302 [115-128]	0	2135.1774
[K].AQQMHT	1xCarbamido	1.401E-05	4.225E-05	1	1	2	P78406	P78406 [81-100]	0	2839.3999
[K].VAQPTIT	1xTMTpro [K	1.679E-05	4.225E-05	1	1	2	P21333	P21333 [1815-183	1	2423.375
[K].AQQNNV	2xTMTpro [K	3.91E-06	4.225E-05	1	1	1	P62081	P62081 [161-178]	1	2990.6516
[K].VAQLEA	3xCarbamido	4.458E-06	4.225E-05	1	1	2	P02679	P02679 [154-185]	2	4929.565
[K].VAQLEA	2xCarbamido	2.687E-08	4.225E-05	1	1	11	P02679	P02679 [154-177]	1	3680.9556
[K].VAQLEA	2xCarbamido	3.715E-05	4.225E-05	1	1	3	P02679	P02679 [154-166]	0	2169.1288
[K].GHFFVE	1xCarbamido	3.168E-05	4.225E-05	1	1	6	O00151	O00151 [297-309]	0	2280.1615
[R].AQQVAV	1xTMTpro [N	2.804E-05	4.225E-05	1	1	1	O75955	O75955 [262-274]	0	1773.9778
[R].AQQIIQL	1xTMTpro [K	3.92E-05	4.225E-05	1	1	1	Q9BX59	Q9BX59 [292-305	0	2160.2996
[R].VCEDGF	1xCarbamido	3.76E-05	4.225E-05	1	1	1	O00584	O00584 [240-253]	1	2545.4305
[K].VCEDLD	2xCarbamido	2.019E-06	4.225E-05	1	1	2	P45880	P45880 [209-229]	0	2703.2673
[K].HLEINPD	1xTMTpro [N	1.414E-06	4.225E-05	1	1	7	P08238	P08238 [625-639]	0	2087.1569

[K].AQEQQ(	1xTMTpro [K	3.747E-06	4.225E-05	1	1	2	Q9P2E9	Q9P2E9 [908-921	0	2264.1949
[K].AQEQQ(	1xOxidation	1.284E-05	4.225E-05	1	1	1	Q9P2E9	Q9P2E9 [908-921	0	2280.1898
[R].VDELTTI	1xTMTpro [K	2.87E-05	4.225E-05	1	1	1	Q14203	Q14203 [332-343]	0	1997.1662
[K].HLCQQL	1xCarbamido	7.716E-06	4.225E-05	1	1	2	Q14980	Q14980 [1365-13	0	2333.2527
[K].VDELSLY	1xTMTpro [K	4.352E-05	4.225E-05	1	1	2	Q9BUR5	Q9BUR5 [37-51]	0	2259.2364
[K].VDEFVTI	1xTMTpro [K	5.056E-06	4.225E-05	1	1	3	P11766	P11766 [342-357]	0	2515.3324
[K].VDEFPL(	1xCarbamido	2.476E-07	4.225E-05	1	1	3	P27635	P27635 [43-69]	0	3386.584
[K].AQFAQP	1xTMTpro [N	4.558E-06	4.225E-05	1	1	2	P30084	P30084 [158-178]	0	2429.3472
[R].VDEEPT	1xTMTpro [K	5.545E-05	4.225E-05	2	2	1	Q9Y4E1; Q6	Q9Y4E1 [305-320	0	2334.2797
[R].HLDACE	2xCarbamido	2.372E-06	4.225E-05	2	4	3	P20020; P23	P20020 [459-476]	0	2602.2555
[R].AQFEYD	1xCarbamido	4.729E-05	4.225E-05	1	1	3	O14936	O14936 [619-634]	1	2822.5215
[K].AQGAIAI	1xTMTpro [K	5.107E-05	4.225E-05	1	1	2	Q5VYK3	Q5VYK3 [1545-15	0	1740.0333
[K].VDDSSE	2xTMTpro [K	4.48E-05	4.225E-05	1	1	1	P49588	P49588 [551-564]	1	2512.3599
[R].AQGFLLI	1xTMTpro [N	3.604E-06	4.225E-05	1	1	1	Q02388	Q02388 [893-910]	0	2372.2067
[K].AQGGD(	1xTMTpro [N	2.075E-05	4.225E-05	1	1	1	Q13753	Q13753 [637-652]	0	1903.9681
[R].VDDPQD	1xTMTpro [K	2.779E-05	4.225E-05	1	1	1	O94901	O94901 [332-345]	0	2213.2058
[R].GHQLLE	1xTMTpro [N	1.404E-06	4.225E-05	1	1	1	Q8IY81	Q8IY81 [428-452]	0	3034.5111
[K].VDDDLG	1xTMTpro [K	3.728E-05	4.225E-05	1	1	1	P35580	P35580 [1380-13	0	2242.1946
[K].VDCTQH	2xCarbamido	5.494E-06	4.225E-05	1	1	2	Q8NBS9	Q8NBS9 [245-26(	0	2270.0613
[K].AQGLEE	1xTMTpro [K	9.641E-06	4.225E-05	1	1	4	Q13308	Q13308 [815-830]	0	2264.3357
[R].AQGLLS.	1xTMTpro [N	1.564E-07	4.225E-05	1	1	1	P11277	P11277 [1647-16	0	2080.147
[K].HLCGDT	1xCarbamido	1.071E-05	4.225E-05	1	1	1	P05166	P05166 [446-466]	0	2913.4883
[R].HLCEPG	1xCarbamido	2.274E-07	4.225E-05	1	2	1	Q14839	Q14839 [1466-14	0	2303.1046
[R].AQEPES	1xTMTpro [K	6.345E-06	4.225E-05	1	1	2	P78527	P78527 [4091-41(	0	2240.1902
[K].AQELSAI	1xCarbamido	5.669E-06	4.225E-05	1	1	1	Q9BXW7	Q9BXW7 [100-12	0	3319.7522
[R].AQDPSE	1xOxidation	1.156E-06	4.225E-05	1	1	2	Q12905	Q12905 [148-174]	0	3494.7704
[R].AQDVDA	1xCarbamido	5.869E-05	4.225E-05	1	1	1	O00139	O00139 [185-202]	0	2445.1532
[K].AQDVLV	1xTMTpro [K	3.645E-05	4.225E-05	1	1	2	Q99459	Q99459 [632-644]	0	2096.1917
[K].AQEACG	1xCarbamido	7.772E-07	4.225E-05	1	1	4	Q9Y490	Q9Y490 [1041-10	0	2997.5517
[K].AQEACG	1xCarbamido	1.625E-06	4.225E-05	1	1	3	Q9Y490	Q9Y490 [1041-10	0	3013.5466
[K].HKLDVT	2xTMTpro [K	4.127E-05	4.225E-05	1	1	2	P48643	P48643 [264-275]	1	2346.3485
[K].AQEALD	1xTMTpro [N	3.253E-05	4.225E-05	1	1	2	P60484	P60484 [148-159]	0	1701.8767
[R].AQEAPG	1xTMTpro [N	1.247E-06	4.225E-05	1	1	2	O14745	O14745 [113-138]	0	2892.4043

[R].HIADLAC	1xTMTpro [K	5.475E-08	4.225E-05	1	1	2	P06733	P06733 [133-162]	0	3619.984
[K].VDGVAQ	1xTMTpro [K	9.379E-06	4.225E-05	1	1	1	Q9P2C4	Q9P2C4 [237-252	0	2357.2415
[K].VDCTAN	2xCarbamido	3.23E-05	4.225E-05	1	1	6	P30101	P30101 [83-94]	0	2005.9927
[R].VDGVAA	1xTMTpro [N	5.313E-05	4.225E-05	1	1	2	P41226	P41226 [532-545]	0	1723.9298
[K].HLAGLG	1xTMTpro [K	8.713E-06	4.225E-05	1	1	2	P50454	P50454 [320-332]	0	1946.1566
[K].AQEGGC	1xTMTpro [K	2.155E-05	4.225E-05	1	1	1	Q8NCA5	Q8NCA5 [134-147	0	2087.1265
[R].AQEGLR	1xCarbamido	1.226E-05	4.225E-05	1	1	2	P08311	P08311 [132-149]	0	2233.1831
[K].VDFTEEI	1xTMTpro [K	2.444E-05	4.225E-05	1	1	2	P46940	P46940 [175-186]	0	2077.0767
[K].HLAGLG	2xTMTpro [K	1.086E-05	4.225E-05	1	1	1	P50454	P50454 [320-334]	1	2492.5017
[K].VDFPQD	1xTMTpro [N	3.113E-05	4.225E-05	1	1	1	P40926	P40926 [216-229]	0	1865.0088
[K].GHTGIGI	1xTMTpro [K	2.21E-05	4.225E-05	1	1	1	P26232	P26232 [269-286]	0	2465.328
[K].AQEIQDI	1xTMTpro [K	9.548E-07	4.225E-05	1	1	1	P46940	P46940 [591-615]	0	3465.8146
[K].AQEIQDI	2xTMTpro [K	8.544E-08	4.225E-05	1	1	4	P46940	P46940 [591-622]	1	4570.3882
[K].VDFEQLT	1xTMTpro [N	8.429E-06	4.225E-05	1	1	2	Q9Y613	Q9Y613 [890-904	0	2095.0991
[R].VDGAEW	1xTMTpro [N	1.038E-06	4.225E-05	1	1	4	Q15149	Q15149 [676-696]	0	2526.2908
[K].AQGPAA	2xTMTpro [K	1.008E-05	4.225E-05	1	1	2	P80723	P80723 [199-226]	0	3676.001
[K].AQGPAA	2xTMTpro [K	3.737E-07	4.225E-05	1	1	6	P80723	P80723 [199-227]	1	3805.0436
[R].AQGPDC	1xTMTpro [N	7.918E-07	4.225E-05	1	1	1	Q6XQN6	Q6XQN6 [173-203	0	3393.6882
[R].AQLEAH	1xTMTpro [N	5.275E-06	4.225E-05	1	1	1	Q08379	Q08379 [373-387]	0	1972.0605
[K].HIDIDPN	1xCarbamido	3.47E-07	4.225E-05	1	1	2	Q8TDQ7	Q8TDQ7 [96-124]	0	3828.8743
[R].VCSEVL	1xCarbamido	1.491E-05	4.225E-05	1	1	2	Q13217	Q13217 [328-346]	1	2825.4781
[K].VCNYGL	1xCarbamido	5.401E-05	4.225E-05	1	1	3	Q9Y277	Q9Y277 [64-74]	0	1939.0603
[K].VCNEVV	2xCarbamido	5.869E-05	4.225E-05	1	1	1	P35813	P35813 [267-278]	0	2108.1012
[R].AQLELYI	1xTMTpro [K	5.554E-05	4.225E-05	1	1	1	Q9BYI3	Q9BYI3 [267-283]	0	2490.4827
[K].VCNDEQ	1xCarbamido	5.803E-05	4.225E-05	1	1	2	Q7Z6Z7	Q7Z6Z7 [28-42]	0	2451.3773
[K].VCLLGC	2xCarbamido	7.084E-07	4.225E-05	1	1	5	P11766	P11766 [169-188]	0	2620.4083
[K].GHNGW	1xTMTpro [N	9.709E-07	4.225E-05	1	1	1	P63244	P63244 [13-36]	0	2931.5106
[K].HLEEAF	1xTMTpro [N	5.141E-05	4.225E-05	1	1	1	Q8TCJ2	Q8TCJ2 [755-768	0	2058.0728
[R].VCTLAIID	1xCarbamido	2.06E-05	4.225E-05	1	1	2	P62888	P62888 [91-106]	0	2062.1174
[K].VCHLMG	1xCarbamido	1.566E-05	4.225E-05	1	1	3	P35749	P35749 [381-394]	0	1967.0162
[K].VCHLGD	1xCarbamido	1.032E-05	4.225E-05	1	1	2	O00471	O00471 [110-124]	0	1999.035
[K].VCGSDG	2xCarbamido	5.701E-05	4.225E-05	1	1	2	O00468	O00468 [936-951]	0	2395.1878
[R].VCGQTL	1xCarbamido	5.313E-05	4.225E-05	1	1	1	Q96FS4	Q96FS4 [731-749	0	2384.3403

[R].VCGLCG	2xCarbamido	3.902E-06	4.225E-05	1	1	2	Q9HC84	Q9HC84 [1022-10	0	2463.1352
[R].AQLGVQ	1xTMTpro [K	1.91E-05	4.225E-05	1	1	1	P40227	P40227 [433-449]	0	2376.451
[K].AQIHDLV	1xTMTpro [N	2.226E-05	4.225E-05	1	1	3	P0DMV9	P0DMV9 [329-342	0	1770.0193
[R].AQIHEQI	1xTMTpro [K	3.895E-06	4.225E-05	1	1	2	Q9BWM7	Q9BWM7 [303-31	0	2626.4121
[R].HLEEIVH	1xTMTpro [N	4.595E-05	4.225E-05	1	1	2	Q01433	Q01433 [441-452]	0	1749.9567
[K].VCELDEI	4xCarbamido	1E-05	4.225E-05	1	1	1	P09486	P09486 [82-110]	0	4005.8293
[R].VCEEIAII	1xCarbamido	2.87E-05	4.225E-05	1	1	2	P08708	P08708 [34-45]	1	2299.3876
[R].AQLGDL	1xTMTpro [K	3.13E-05	4.225E-05	1	1	2	P05156	P05156 [346-358]	0	2047.2196
[K].GHVDILA	1xTMTpro [K	2.345E-06	4.225E-05	1	1	2	Q92499	Q92499 [703-720]	0	2512.463
[R].VCTTAG	1xCarbamido	1.301E-06	4.225E-05	1	1	1	Q02388	Q02388 [215-236]	0	2545.3
[K].VCVETVI	1xCarbamido	4.882E-05	4.225E-05	1	1	4	P48735	P48735 [401-413]	0	2035.0695
[K].AQGPQQ	1xTMTpro [K	1.892E-05	4.225E-05	1	1	1	Q3ZCQ8	Q3ZCQ8 [48-64]	0	2338.2283
[K].VDCTAH	2xCarbamido	6.621E-06	4.225E-05	1	1	2	Q8NBS9	Q8NBS9 [119-134	0	2065.9714
[R].HIDALED	1xTMTpro [K	2.198E-05	4.225E-05	1	1	2	P49959	P49959 [489-503]	2	2446.3546
[K].VDCEAN	1xCarbamido	1.433E-05	4.225E-05	1	1	1	Q92791	Q92791 [273-290]	0	2606.3416
[R].VDCDQH	1xCarbamido	3.806E-05	4.225E-05	1	1	1	Q9BS26	Q9BS26 [90-101]	0	1747.8353
[R].AQGWAI	2xTMTpro [K	2.358E-05	4.225E-05	1	1	3	Q99829	Q99829 [515-529]	0	2473.5111
[K].VDATEES	1xTMTpro [N	3.045E-06	4.225E-05	1	1	5	P07237	P07237 [82-97]	0	2085.0419
[R].HIDCAAI	1xCarbamido	2.755E-06	4.225E-05	1	1	2	P14550	P14550 [43-61]	0	2708.4209
[R].HIDCAAI	1xCarbamido	5.056E-07	4.225E-05	1	1	3	P14550	P14550 [43-68]	1	3694.9567
[R].HIDCAHV	1xCarbamido	1.381E-06	4.225E-05	1	1	3	P15121	P15121 [42-62]	0	3061.5657
[K].VCVETVI	1xCarbamido	8.491E-06	4.225E-05	1	1	5	P48735	P48735 [401-413]	0	2019.0746
[R].AQHEDQ	2xTMTpro [K	2.784E-05	4.225E-05	1	1	2	P02545	P02545 [250-261]	1	2415.3448
[K].VDALND	1xTMTpro [N	5.974E-05	4.225E-05	1	1	6	P08729	P08729 [215-226]	0	1722.9346
[R].HLDHVA	1xTMTpro [N	2.561E-05	4.225E-05	1	1	2	P11216	P11216 [411-425]	0	1966.0466
[K].AQHTHD	1xTMTpro [K	1.998E-05	4.225E-05	1	1	2	Q9Y4G6	Q9Y4G6 [1790-18	0	2373.284
[R].AQHVFQ	2xTMTpro [K	2.194E-05	4.225E-05	1	1	1	Q13867	Q13867 [49-68]	0	3169.8051
[R].VDAETG	1xTMTpro [N	3.747E-05	4.225E-05	1	1	1	P33151	P33151 [95-107]	0	1725.8978
[R].AQKDEE	3xTMTpro [K	3.435E-05	4.225E-05	1	1	2	P09493	P09493 [134-149]	2	3176.8342
[K].GHPDLQ	1xTMTpro [N	8.908E-06	4.225E-05	1	1	2	P49257	P49257 [310-329]	0	2485.2279
[R].AQLADS	1xTMTpro [N	3.747E-06	4.225E-05	1	1	3	Q13813	Q13813 [567-580]	0	2012.0673
[R].AQLCED	7xCarbamido	2.123E-07	4.225E-05	1	1	1	Q9HC84	Q9HC84 [352-392	0	4958.1625
[K].AQLDAA	1xTMTpro [N	1.544E-05	4.225E-05	1	1	2	P15289	P15289 [464-479]	0	1935.0619

[R].VDAQDE	1xTMTpro [N	1.738E-05	4.225E-05	1	1	2	Q9Y6C2	Q9Y6C2 [546-559	0	1869.9513
[K].VSEPLLV	1xTMTpro [K	2.587E-06	4.225E-05	1	1	1	Q15427	Q15427 [24-46]	0	3213.799
[K].VEITYTP	1xTMTpro [K	2.094E-05	4.225E-05	1	1	4	O75874	O75874 [152-164]	0	2047.1203
[K].APESVS	1xTMTpro [K	5.107E-05	4.225E-05	1	1	1	Q8WUU5	Q8WUU5 [129-14	0	2464.3831
[K].VGVNDF	1xCarbamid	1.154E-05	4.225E-05	1	1	3	P21810	P21810 [315-328]	0	2135.1273
[K].VGVEVP	1xTMTpro [K	3.146E-05	4.225E-05	1	1	6	Q09666	Q09666 [913-928]	0	2246.2524
[R].AMADPE	1xTMTpro [N	1.667E-05	4.225E-05	1	1	3	P31948	P31948 [489-505]	0	2194.0626
[K].HDAATC	1xCarbamid	4.3E-05	4.225E-05	1	1	1	P54920	P54920 [79-93]	0	2232.1363
[R].VGTQVE	1xTMTpro [N	1.709E-05	4.225E-05	1	1	1	Q9BRZ2	Q9BRZ2 [251-264	0	1761.9666
[K].VGTGEP	2xCarbamid	1.928E-07	4.225E-05	1	1	2	P52209	P52209 [164-184]	0	2885.3842
[K].VGTFFSI	1xTMTpro [K	1.463E-07	4.225E-05	1	1	1	P54886	P54886 [349-371]	0	3073.6272
[K].GICEALE	1xCarbamid	1.971E-07	4.225E-05	1	1	1	O00339	O00339 [833-854]	1	2952.4864
[K].VGSLDN	1xTMTpro [K	1.516E-05	4.225E-05	1	1	2	P27816	P27816 [1071-108	0	2199.2741
[K].VGSFSQ	1xTMTpro [K	1.766E-05	4.225E-05	1	1	1	P42575	P42575 [78-93]	0	2366.3939
[K].VGSFCL	1xCarbamid	1.298E-05	4.225E-05	1	1	1	P45954	P45954 [171-189]	0	2511.3045
[R].VGSALF	1xTMTpro [K	4.228E-05	4.225E-05	1	1	2	P29622	P29622 [139-150]	0	1894.1406
[K].AMDQEI	1xTMTpro [K	2.345E-05	4.225E-05	1	1	3	Q9UNS2	Q9UNS2 [392-406	0	2356.2826
[K].VGSAAD	1xCarbamid	2.809E-05	4.225E-05	1	1	1	P21333	P21333 [1965-200	2	4527.4988
[K].AMDSDV	1xTMTpro [N	3.174E-05	4.225E-05	1	1	4	P55084	P55084 [392-405]	0	1996.8853
[K].VGSAAD	1xTMTpro [N	2.274E-09	4.225E-05	1	1	4	P21333	P21333 [1965-199	0	3197.7588
[K].VGRPSN	1xTMTpro [N	2.147E-07	4.225E-05	1	1	1	Q9UHX1	Q9UHX1 [202-223	0	2666.4545
[R].VGQAVD	2xTMTpro [K	2.064E-05	4.225E-05	1	1	2	Q13200	Q13200 [846-860]	0	2365.4383
[R].VGPVSA	1xTMTpro [K	1.462E-06	4.225E-05	1	1	4	P22105	P22105 [2172-219	0	2828.5537
[R].HDKVED	2xTMTpro [K	4.071E-05	4.225E-05	1	1	2	P42704	P42704 [724-739]	2	2870.5828
[R].AMEAEA	1xTMTpro [N	6.003E-05	4.225E-05	1	1	1	Q7Z406	Q7Z406 [1357-13	1	2449.2312
[R].HCQLEP	1xCarbamid	4.737E-05	4.225E-05	1	1	1	Q9Y5L0	Q9Y5L0 [379-400	0	2947.3124
[R].HCNNGI	1xCarbamid	3.168E-05	4.225E-05	1	1	1	Q96TA1	Q96TA1 [193-214	1	3035.55
[K].GLCGSC	2xCarbamid	1.934E-06	4.225E-05	1	1	4	O43294	O43294 [226-245]	0	2666.4726
[R].VGVVQF	1xTMTpro [K	2.09E-05	4.225E-05	1	1	5	P12111	P12111 [1475-149	0	2596.4307
[K].VHPEIIN	1xTMTpro [K	5.872E-06	4.225E-05	1	1	2	O95881	O95881 [124-138]	0	2319.2589
[R].VHNYCV	2xCarbamid	1.93E-05	4.225E-05	1	1	1	P11277	P11277 [956-968]	0	2249.0822
[K].VHNDAQ	1xTMTpro [K	1.744E-06	4.225E-05	1	1	2	O43852	O43852 [38-59]	0	3087.494
[K].VHLVGID	1xTMTpro [K	6.051E-05	4.225E-05	2	3	3	Q9GZV4; P6	Q9GZV4 [56-67];	0	1907.161

[R].VHIPNDI	1xCarbamido	5.959E-06	4.225E-05	1	1	1	Q16576	Q16576 [83-113]	1	4207.0607
[K].ALYDTFS	1xCarbamido	1.167E-05	4.225E-05	2	2	2	Q13310; P11	Q13310 [114-129]	0	2415.2874
[RK].VHLEN	1xTMTpro [N	7.01E-06	4.225E-05	3	3	1	P11277; Q01	P11277 [128-144]	0	2234.1056
[R].ALYDYE	1xTMTpro [K	1.119E-05	4.225E-05	1	1	2	Q9UNF0	Q9UNF0 [433-448	0	2552.2801
[R].VHLDIQV	1xTMTpro [K	4.55E-06	4.225E-05	1	1	2	Q9BVK6	Q9BVK6 [139-158	0	2801.5078
[K].VHKEDD	1xCarbamido	4.863E-07	4.225E-05	1	1	1	Q9BY67	Q9BY67 [209-235	1	3634.9255
[R].VGPQYQ	1xTMTpro [K	4.206E-05	4.225E-05	1	1	1	Q9UKL0	Q9UKL0 [107-122	0	2339.2891
[R].VHGPGI	2xTMTpro [K	1.394E-05	4.225E-05	1	1	2	P21333	P21333 [1360-137	0	2547.4823
[K].HAVVNLI	1xTMTpro [N	4.257E-06	4.225E-05	1	1	2	P35222	P35222 [134-151]	0	2346.2373
[R].HCDEVG	1xCarbamido	1.03E-05	4.225E-05	1	1	1	P51808	P51808 [7-23]	0	2577.3011
[R].ALYLSDI	1xTMTpro [K	3.683E-05	4.225E-05	1	1	5	Q15404	Q15404 [138-155]	0	2628.4416
[K].VHAPGL	1xTMTpro [K	2.35E-05	4.225E-05	1	1	1	Q09666	Q09666 [5324-53	0	1914.1417
[K].VHAIQCI	1xCarbamido	4.626E-06	4.225E-05	1	1	1	Q16698	Q16698 [111-133]	1	3291.7321
[K].VHAIQCI	1xCarbamido	1.435E-05	4.225E-05	1	1	1	Q16698	Q16698 [111-133]	1	3275.7372
[K].GICSSDI	1xCarbamido	5.437E-05	4.225E-05	1	1	1	Q63HN8	Q63HN8 [2916-29	0	1666.8753
[R].VGVVQY	1xTMTpro [K	9.167E-09	4.225E-05	1	1	2	P12110	P12110 [660-688]	1	3878.0104
[R].VGVVQY	1xTMTpro [N	6.327E-08	4.225E-05	1	1	16	P12110	P12110 [660-680]	0	2696.3487
[R].ALYYLQI	1xTMTpro [N	2.397E-05	4.225E-05	1	1	3	Q16531	Q16531 [515-527]	0	1948.0976
[K].VHEYNV	1xTMTpro [N	2.574E-05	4.225E-05	1	1	1	P30533	P30533 [183-195]	0	1877.0452
[R].GLDAHL	1xTMTpro [N	2.766E-06	4.225E-05	1	1	1	P11277	P11277 [688-706]	0	2426.257
[K].AMEALA	1xCarbamido	4.099E-05	4.225E-05	1	1	3	Q9P2E9	Q9P2E9 [1027-10	0	2002.0593
[K].VGPMPV	1xTMTpro [K	2.35E-05	4.225E-05	1	1	1	P22830	P22830 [305-320]	0	2363.2925
[K].AMGIMN	1xOxidation [	3.601E-05	4.225E-05	6	14	13	Q5QNW6; Q	Q5QNW6 [59-73]	0	2064.0214
[K].AMGIMN	2xOxidation [	5.609E-05	4.225E-05	6	14	8	Q5QNW6; Q	Q5QNW6 [59-73]	0	2080.0163
[K].VGINYQI	1xTMTpro [K	7.991E-06	4.225E-05	3	7	8	Q71U36; Q9	Q71U36 [353-370	0	2433.3997
[K].AMGNLQ	1xTMTpro [N	3.163E-05	4.225E-05	1	1	2	P04899	P04899 [87-100]	0	1838.939
[R].VGLNAQ	1xCarbamido	3.346E-05	4.225E-05	1	1	1	P48681	P48681 [149-160]	0	1531.8334
[K].GLATSL	1xTMTpro [K	4.36E-05	4.225E-05	1	1	2	Q8N0X7	Q8N0X7 [98-112]	0	2221.2684
[R].AMGPEN	1xOxidation [	5.296E-05	4.225E-05	1	1	1	P20592	P20592 [82-96]	0	2396.2048
[K].AMGPLV	1xTMTpro [K	2.457E-05	4.225E-05	1	1	2	P55010	P55010 [274-288]	0	2269.3122
[K].AMGVVV	1xTMTpro [K	1.998E-05	4.225E-05	1	1	1	P16615	P16615 [219-234]	0	2154.2448
[R].VGLIGS	1xCarbamido	5.27E-05	4.225E-05	1	1	1	Q99798	Q99798 [379-395]	0	2166.0126
[R].VGLIGS	1xCarbamido	1.055E-06	4.225E-05	1	1	2	Q99798	Q99798 [379-395]	0	2150.0177

[R].GLATFCI	1xCarbamido	5.743E-06	4.225E-05	1	1	1	O15173	O15173 [154-184]	2	4184.0771
[K].VGLGLG	1xTMTpro [K	4.279E-05	4.225E-05	1	1	2	Q13045	Q13045 [731-746]	0	2385.4037
[R].VGIGAFI	1xTMTpro [N	2.259E-05	4.225E-05	1	1	1	P30520	P30520 [304-324]	0	2591.3636
[R].GLATFCI	1xCarbamido	1.543E-07	4.225E-05	1	1	3	O15173	O15173 [154-184]	2	4168.0822
[R].VGIFPIS	1xTMTpro [K	4.556E-05	4.225E-05	1	1	1	O94875	O94875 [909-919]	0	1860.1126
[K].VGIEAAE	1xTMTpro [N	2.053E-05	4.225E-05	1	1	1	O00442	O00442 [273-286]	0	1804.0322
[K].VGIDTPI	1xTMTpro [K	1.119E-05	4.225E-05	1	1	2	Q09666	Q09666 [4560-45	0	2271.2476
[R].AMLEAQ	1xTMTpro [K	1.017E-05	4.225E-05	1	1	1	Q9NQ50	Q9NQ50 [138-156	0	2765.4887
[R].VGLADLI	1xTMTpro [N	1.215E-05	4.225E-05	1	1	1	Q9BYD3	Q9BYD3 [77-92]	0	1983.0983
[R].VGKETV	2xTMTpro [K	7.478E-06	4.225E-05	1	1	1	P18206	P18206 [57-71]	1	2601.5279
[K].AMGIMN	1xTMTpro [N	5.494E-06	4.225E-05	6	14	5	Q5QNW6; Q	Q5QNW6 [59-73]	0	2048.0264
[K].VGIPQEI	1xTMTpro [K	3.551E-06	4.225E-05	1	1	1	P98082	P98082 [690-710]	0	2961.5198
[K].VGLSDA	1xTMTpro [N	3.34E-05	4.225E-05	1	1	1	Q6UX71	Q6UX71 [278-289	0	1602.9287
[R].VGLTHTI	1xTMTpro [K	4.465E-05	4.225E-05	1	1	1	P55039	P55039 [347-363]	0	2557.4304
[K].GLAYVE	1xTMTpro [K	4.21E-06	4.225E-05	1	1	2	Q15020	Q15020 [842-860]	0	2725.3999
[R].VGPEPT	1xCarbamido	3.131E-06	4.225E-05	1	1	2	Q9NZN4	Q9NZN4 [88-124]	1	4865.5422
[R].VGPEPT	1xCarbamido	1E-07	4.225E-05	1	1	2	Q9NZN4	Q9NZN4 [88-123]	0	4433.2401
[K].AMEFVD	1xTMTpro [N	3.696E-05	4.225E-05	1	1	2	Q8NB37	Q8NB37 [50-62]	0	1772.8808
[R].HDIAFVE	1xTMTpro [N	4.249E-06	4.225E-05	1	1	3	P09012	P09012 [244-261]	0	2293.1532
[R].VGPAAC	2xCarbamido	1.822E-05	4.225E-05	1	1	1	Q8ND94	Q8ND94 [66-87]	0	2548.2971
[K].VGNVEY	1xTMTpro [K	5.324E-06	4.225E-05	1	1	2	P05026	P05026 [224-248]	0	3437.7702
[R].AMEGQI	1xCarbamido	3.381E-05	4.225E-05	1	1	1	P06401	P06401 [594-606]	0	1810.8648
[R].VGNPWI	1xTMTpro [K	6.694E-06	4.225E-05	1	1	2	P49419	P49419 [359-375]	0	2515.3953
[K].VGNPFE	1xTMTpro [K	1.368E-06	4.225E-05	1	1	1	P30837	P30837 [348-369]	1	3169.641
[K].AMEALA	1xCarbamido	3.8E-05	4.225E-05	1	1	1	Q9P2E9	Q9P2E9 [1027-10	0	2018.0542
[K].GLAYCE	3xCarbamido	7.325E-07	4.225E-05	1	1	1	P48059	P48059 [236-257]	0	3065.3776
[R].HDIAFVE	1xTMTpro [N	8.57E-06	4.225E-05	1	1	1	P08579	P08579 [187-204]	0	2251.1063
[R].VGNLGL	1xTMTpro [N	3.447E-05	4.225E-05	1	1	2	O00571	O00571 [535-548]	0	1828.9877
[K].AMEVDE	1xTMTpro [K	3.154E-06	4.225E-05	1	1	2	P17980	P17980 [174-193]	0	2861.4483
[K].VGNIEIK	1xTMTpro [K	4.133E-06	4.225E-05	1	1	1	P61160	P61160 [47-65]	1	2696.4784
[K].AMFESQ	1xCarbamido	1.691E-05	4.225E-05	1	1	2	P12956	P12956 [47-74]	0	3891.8986
[R].AMFLQP	1xTMTpro [K	4.17E-05	4.225E-05	1	1	1	Q9BQG0	Q9BQG0 [412-43	0	2891.5105
[K].AMGAAQ	1xTMTpro [N	5.764E-06	4.225E-05	1	1	3	Q00796	Q00796 [194-209]	0	1894.0387

[K].AMGAAQ	1xOxidation [	1.991E-05	4.225E-05	1	1	2	Q00796	Q00796 [194-209]	0	1910.0336
[K].VGMEGC	1xCarbamido	9.007E-06	4.225E-05	1	1	1	Q9UKK3	Q9UKK3 [160-177	0	2252.1334
[R].VGLVQF	1xTMTpro [K	3.322E-06	4.225E-05	1	1	3	P12111	P12111 [678-699]	0	3082.6592
[K].AMESHK	2xTMTpro [K	8.908E-06	4.225E-05	1	1	2	Q9Y316	Q9Y316 [143-165	1	3413.917
[K].AIWNVIN	1xTMTpro [N	2.435E-05	4.225E-05	1	1	4	P04179	P04179 [203-216]	0	2048.0884
[K].HAVSDP	1xTMTpro [N	4.712E-07	4.225E-05	1	1	1	P05198	P05198 [155-182]	1	3494.8046
[K].VHQYFN	1xTMTpro [K	1.295E-05	4.225E-05	1	1	4	P01024	P01024 [1463-147	0	2450.4051
[K].VKNPED	2xTMTpro [K	2.14E-05	4.225E-05	1	1	2	Q9Y570	Q9Y570 [120-133	1	2445.3839
[R].VKLESPT	2xTMTpro [K	1.776E-06	4.225E-05	1	1	2	Q96C36	Q96C36 [290-307	1	2740.6277
[R].ALTVPEI	1xTMTpro [K	7.423E-06	4.225E-05	2	3	5	Q13509; P68	Q13509 [283-297]	0	2300.2816
[R].GYTSDS	1xTMTpro [K	4.337E-05	4.225E-05	1	1	1	Q92538	Q92538 [1315-133	0	2477.2552
[R].ALTVPEI	1xTMTpro [N	5.296E-05	4.225E-05	1	1	2	Q9BUF5	Q9BUF5 [283-297	0	2024.0806
[R].ALTVPEI	1xTMTpro [K	3.601E-05	4.225E-05	1	2	2	Q13885	Q13885 [283-297]	0	2316.2765
[R].ALTVPEI	1xTMTpro [K	4.184E-05	4.225E-05	1	1	6	P07437	P07437 [283-297]	0	2268.3095
[K].VKLEGH	2xTMTpro [K	3.358E-05	4.225E-05	1	1	1	Q9NZN3	Q9NZN3 [510-530	1	3230.9081
[K].AIVAGDC	1xTMTpro [K	3.219E-05	4.225E-05	1	1	4	Q13642	Q13642 [107-118]	0	1915.0781
[K].VKLEGH	2xTMTpro [K	1.607E-05	4.225E-05	1	1	1	Q9H4M9	Q9H4M9 [510-530	1	3185.8867
[K].GYVSLEI	2xTMTpro [K	5.031E-05	4.225E-05	1	1	1	P02549	P02549 [2332-235	1	3346.8239
[K].GLDIAMT	1xCarbamido	1.272E-05	4.225E-05	1	1	5	Q9Y680	Q9Y680 [95-108]	0	2146.0838
[R].AIVAIENI	1xTMTpro [N	2.11E-06	4.225E-05	1	1	4	P08865	P08865 [64-80]	0	2045.1562
[R].ALVAIGT	1xTMTpro [K	9.407E-07	4.225E-05	1	1	1	Q9NSD9	Q9NSD9 [155-176	0	2899.6061
[R].GYYEVT	1xTMTpro [K	4.601E-06	4.225E-05	1	1	2	O43776	O43776 [264-286]	0	3106.6956
[K].VKGDVD	2xTMTpro [K	5.636E-05	4.225E-05	1	1	3	Q09666	Q09666 [4735-474	1	2083.2943
[K].GYYSPY	1xTMTpro [N	1.679E-05	4.225E-05	1	1	1	Q15149	Q15149 [4610-462	0	2087.0001
[R].AIVDALP	2xCarbamido	2.755E-05	4.225E-05	1	1	2	Q15181	Q15181 [261-282]	0	2963.535
[R].AIVDCIIS	1xCarbamido	3.253E-05	4.225E-05	1	1	1	Q9UBF2	Q9UBF2 [416-432	0	2524.3824
[R].HAAEEE	1xTMTpro [K	2.88E-05	4.225E-05	1	1	1	Q9Y2D4	Q9Y2D4 [604-617	0	2268.2116
[K].GIDGVSY	1xTMTpro [N	5.59E-05	4.225E-05	1	1	1	Q9GZT8	Q9GZT8 [188-200	0	1599.8662
[R].VKPAPD	2xTMTpro [K	5.5E-05	4.225E-05	1	1	1	Q12905	Q12905 [44-60]	1	2800.6389
[R].VKPFMT	2xTMTpro [K	6.334E-06	4.225E-05	1	2	2	P13693	P13693 [111-123]	0	2332.3879
[K].ALTSQLT	1xTMTpro [N	7.464E-06	4.225E-05	1	1	2	P23368	P23368 [497-511]	0	1936.0307
[K].AITSGGI	3xCarbamido	9.659E-06	4.225E-05	1	1	2	Q13642	Q13642 [168-193]	0	3609.742
[R].VLAEHG	1xTMTpro [N	3.518E-06	4.225E-05	1	1	1	Q9H6R3	Q9H6R3 [381-398	0	2141.2402

[K].GYPHLC	2xCarbamido	1.713E-05	4.225E-05	1	1	1	P43243	P43243 [288-304]	0	2605.3511
[R].AITGASL	1xOxidation	5.527E-05	4.225E-05	1	1	3	P83731	P83731 [81-93]	0	1886.0913
[K].ALTGHLI	1xTMTpro [K	1.937E-05	4.225E-05	1	1	3	P04083	P04083 [99-113]	0	2214.3717
[R].VIACDG	1xCarbamido	1.015E-06	4.225E-05	1	1	1	O75380	O75380 [84-98]	0	2017.1145
[R].AITHLNN	1xTMTpro [K	4.085E-05	4.225E-05	1	1	2	P14866	P14866 [435-448]	0	2243.2251
[K].VIAALLQ	1xTMTpro [N	3.734E-05	4.225E-05	1	1	2	O00410	O00410 [442-457	0	2091.1188
[R].AITIAGIF	1xCarbamido	5.226E-05	4.225E-05	1	1	2	Q15366	Q15366 [145-160	0	2321.3758
[R].AITIAGVI	1xCarbamido	4.367E-05	4.225E-05	1	1	4	Q15365	Q15365 [145-160	0	2281.3081
[R].AITIANQ	1xCarbamido	9.57E-06	4.225E-05	1	1	2	Q16555	Q16555 [239-254	0	2429.3718
[R].GLDGIPI	1xCarbamido	2.444E-05	4.225E-05	1	1	1	Q15654	Q15654 [371-393	0	2932.4583
[R].AITIASQ	1xCarbamido	1.406E-06	4.225E-05	1	1	3	Q14195	Q14195 [239-254	0	2388.3452
[K].GYPPED	1xTMTpro [K	5.645E-05	4.225E-05	1	1	2	Q9NR45	Q9NR45 [321-333	0	2057.1563
[R].AITPPHF	1xTMTpro [N	3.065E-07	4.225E-05	1	1	1	P23142	P23142 [620-642]	0	2779.5062
[R].GYQLSD	3xCarbamido	1.474E-07	4.225E-05	1	1	2	P23142	P23142 [468-497]	0	3691.6634
[K].VKVEEEI	3xTMTpro [K	6.908E-06	4.225E-05	1	1	1	Q9Y2X3	Q9Y2X3 [466-485	2	3565.9477
[K].GLDLNG	1xTMTpro [N	4.293E-05	4.225E-05	1	1	1	P02730	P02730 [361-384]	0	2771.4647
[R].VKQENL	1xCarbamido	3.285E-06	4.225E-05	1	1	2	P49247	P49247 [119-136]	1	2725.5315
[R].VKQELLI	3xTMTpro [K	2.064E-05	4.225E-05	1	1	3	P50552	P50552 [347-357]	2	2559.6226
[R].ALTQTG	1xTMTpro [K	4.05E-05	4.225E-05	1	1	4	P21333	P21333 [1284-129	0	1717.0252
[K].VKQDMF	2xTMTpro [K	8.065E-06	4.225E-05	1	1	1	Q9P0J0	Q9P0J0 [6-22]	1	2774.5367
[R].GYSYDL	1xTMTpro [N	3.968E-06	4.225E-05	1	1	2	P28074	P28074 [201-216]	0	2196.078
[K].ALTLDQ	1xTMTpro [K	2.755E-05	4.225E-05	1	1	1	Q9HBL8	Q9HBL8 [282-293	0	2090.189
[K].ALVDILS	1xTMTpro [K	5.822E-05	4.225E-05	1	1	2	Q9BQG0	Q9BQG0 [180-19	0	1782.0868
[K].VKGDLD	2xTMTpro [K	3.261E-06	4.225E-05	1	1	2	Q09666	Q09666 [3429-34	1	2700.5388
[K].ALVDPG	1xCarbamido	6.241E-06	4.225E-05	1	1	1	P46100	P46100 [1707-17	0	2689.4515
[K].VKADRD	1xTMTpro [K	3.571E-06	4.225E-05	1	1	3	P62263	P62263 [62-84]	2	3100.6341
[K].VHYENN	1xOxidation	2.057E-05	4.225E-05	1	1	1	P04843	P04843 [216-232]	0	2330.177
[K].VHYENN	1xTMTpro [N	3.322E-05	4.225E-05	1	1	1	P04843	P04843 [216-232]	0	2314.1821
[R].VHVSEE	2xTMTpro [K	3.282E-07	4.225E-05	1	1	1	P06396	P06396 [258-295]	1	4870.5713
[R].VHVSEE	2xTMTpro [K	7.504E-08	4.225E-05	1	1	1	P06396	P06396 [258-289]	0	4214.2835
[R].ALVSAQ	1xTMTpro [N	4.078E-05	4.225E-05	1	1	2	P25325	P25325 [9-21]	0	1717.992
[K].ALVSEE	2xTMTpro [K	5.861E-06	4.225E-05	1	1	2	Q13618	Q13618 [327-349	1	3399.9191
[K].VHVGDE	1xTMTpro [N	3.722E-05	4.225E-05	1	1	3	P04080	P04080 [57-68]	0	1726.9196

[R].GIDDNLI	1xCarbamido	1.124E-06	4.225E-05	1	1	1	Q9Y6Y8	Q9Y6Y8 [427-460	0	4075.0224
[R].ALVSQC	1xCarbamido	3.191E-05	4.225E-05	1	1	1	P49589	P49589 [520-532]	0	1800.942
[R].AIVQDQ	1xCarbamido	1.34E-06	4.225E-05	1	1	2	Q9NVT9	Q9NVT9 [33-64]	0	3796.0725
[K].ALVTGYI	1xTMTpro [N	1.178E-05	4.225E-05	1	1	2	O43143	O43143 [692-706]	0	2039.1067
[K].VHTECC	4xCarbamido	2.179E-05	4.225E-05	1	1	1	P02768	P02768 [265-298]	2	4922.3634
[K].VHTECC	3xCarbamido	4.359E-09	4.225E-05	1	1	35	P02768	P02768 [265-286]	1	3193.532
[K].VHTECC	3xCarbamido	4.146E-07	4.225E-05	1	1	14	P02768	P02768 [265-281]	0	2391.0447
[R].VHTDFTI	1xTMTpro [K	2.457E-05	4.225E-05	1	2	1	O94885	O94885 [561-576]	0	2431.2637
[K].VHSPSG	1xCarbamido	2.057E-07	4.225E-05	1	1	3	P21333	P21333 [2368-239	1	3374.7182
[K].VHSPSG	1xCarbamido	7.204E-07	4.225E-05	1	1	4	P21333	P21333 [2368-238	0	2885.4483
[K].HAQAVE	1xTMTpro [K	1.03E-05	4.225E-05	1	1	4	P35749	P35749 [1201-12	0	2508.359
[R].HATALEI	1xTMTpro [K	1.14E-05	4.225E-05	1	1	1	P35580	P35580 [1201-12	0	2405.3168
[K].VHSAVIT	1xTMTpro [N	5.609E-05	4.225E-05	1	1	1	P54652	P54652 [140-156]	0	2208.1732
[K].GIDASD	1xTMTpro [K	1.467E-05	4.225E-05	1	1	2	P48147	P48147 [429-445]	0	2540.3528
[R].VHTVMT	1xCarbamido	2.768E-07	4.225E-05	1	1	2	Q12905	Q12905 [298-319]	0	2927.4748
[K].VGKDEL	1xCarbamido	6.211E-09	4.225E-05	1	1	3	Q16658	Q16658 [248-271]	1	3283.76
[K].VKDAVE	2xTMTpro [K	5.09E-05	4.225E-05	1	1	1	Q9NX58	Q9NX58 [174-185	1	2242.3223
[R].VKDLAT	2xTMTpro [K	4.906E-05	4.225E-05	1	1	1	P02647	P02647 [35-51]	2	2790.6545
[R].AIVDVHF	1xTMTpro [N	2.89E-05	4.225E-05	1	1	1	O00468	O00468 [1202-12	0	1893.019
[K].VKEYQV	1xCarbamido	1.294E-06	4.225E-05	1	1	1	Q86VP6	Q86VP6 [79-103]	2	3925.1231
[K].VKEYQV	1xCarbamido	3.696E-05	4.225E-05	1	1	1	Q86VP6	Q86VP6 [79-99]	1	3398.8367
[K].ALVEGVI	1xTMTpro [K	2.392E-05	4.225E-05	1	1	1	P01031	P01031 [1441-145	0	2447.3678
[R].ALVENC	1xCarbamido	3.18E-05	4.225E-05	1	1	2	Q15020	Q15020 [336-349]	0	2002.1115
[K].HADVGV	1xTMTpro [N	2.002E-05	4.225E-05	1	1	1	Q9HD20	Q9HD20 [874-888	0	1837.0251
[K].VKEQLE	2xTMTpro [K	1.141E-06	4.225E-05	1	1	1	Q8WUD4	Q8WUD4 [86-110	1	3671.12
[K].VKEPSV	2xTMTpro [K	2.182E-06	4.225E-05	1	1	2	Q8IYB3	Q8IYB3 [230-246]	1	2744.5862
[K].AIVHAVG	1xTMTpro [K	7.631E-06	4.225E-05	1	1	1	P27708	P27708 [1184-120	0	3054.7959
[R].AIVHDNI	2xTMTpro [K	4.442E-05	4.225E-05	1	1	1	O75369	O75369 [1566-15	1	3032.6873
[R].VKDLAT	2xTMTpro [K	2.034E-05	4.225E-05	1	1	2	P02647	P02647 [35-47]	1	2375.473
[K].VKEESQ	1xTMTpro [K	7.531E-07	4.225E-05	1	1	2	Q02952	Q02952 [1050-10	1	2971.6344
[R].ALVLELC	2xCarbamido	2.886E-06	4.225E-05	1	1	1	P22314	P22314 [1033-105	0	2870.3871
[K].ALVIGGE	1xCarbamido	6.041E-05	4.225E-05	1	1	1	P06865	P06865 [450-472]	0	2868.4344
[K].ALVLIAF	1xCarbamido	4.249E-05	4.225E-05	1	1	1	P02768	P02768 [45-65]	0	3098.6993

[R].VKEDEN	2xTMTpro [K	1.279E-05	4.225E-05	1	1	3	P11233	P11233 [114-128]	1	2613.5432
[R].HAIYDKL	1xTMTpro [K	1.258E-05	4.225E-05	1	1	1	P30876	P30876 [842-859]	1	2576.4328
[R].VKDTED	2xTMTpro [K	6.172E-06	4.225E-05	1	1	2	P62834	P62834 [103-117]	1	2570.5044
[K].HAPGLIA	1xTMTpro [K	1.456E-05	4.225E-05	1	1	1	Q5VW32	Q5VW32 [204-22	0	2686.4848
[K].HAPVLEI	1xTMTpro [K	4.541E-05	4.225E-05	1	1	1	P40123	P40123 [321-330]	0	1701.0191
[R].VKDTDD	2xTMTpro [K	1.009E-05	4.225E-05	1	2	2	P61224	P61224 [103-117]	1	2556.4887
[K].VKDQEE	2xTMTpro [K	5.747E-05	4.225E-05	1	1	1	Q92614	Q92614 [1551-15	1	3444.8348
[K].ALVLDCI	1xCarbamido	3.246E-08	4.225E-05	1	1	2	Q07021	Q07021 [181-207	0	3440.6123
[K].HGDIFTI	1xTMTpro [N	5.609E-05	4.225E-05	1	1	2	Q16647	Q16647 [61-72]	0	1588.913
[R].VGHSEL	1xTMTpro [N	1.569E-05	4.225E-05	1	1	3	P38606	P38606 [45-56]	0	1612.9342
[R].VGGVQS	1xTMTpro [N	6.694E-06	4.225E-05	1	1	2	P17174	P17174 [101-114]	0	1575.9138
[R].VFDKEG	1xTMTpro [K	2.121E-05	4.225E-05	2	3	3	P60660; P05	P60660 [95-110];	1	2331.2622
[K].ANQQFL	1xCarbamido	3.924E-06	4.225E-05	1	1	4	P02679	P02679 [200-222]	0	3269.6545
[K].HESENL	1xCarbamido	2.904E-07	4.225E-05	1	1	3	P12110	P12110 [767-784]	0	2782.4438
[K].VFDEFKI	2xTMTpro [K	1.311E-06	4.225E-05	1	1	18	P02768	P02768 [397-413]	0	2957.7168
[K].VFCNME	2xCarbamido	1.396E-06	4.225E-05	1	1	2	P02452	P02452 [1289-13	0	3170.5452
[K].VFCNME	2xCarbamido	4.02E-06	4.225E-05	1	1	1	P02452	P02452 [1289-13	0	3154.5503
[R].VFCNFQ	1xCarbamido	4.549E-05	4.225E-05	1	1	1	Q14139	Q14139 [708-724	0	2620.4453
[R].VFCESG	1xCarbamido	3.632E-05	4.225E-05	1	1	1	Q8IYQ7	Q8IYQ7 [201-215	0	2233.1302
[R].VFASELI	1xTMTpro [K	2.621E-05	4.225E-05	1	1	2	Q9P2J5	Q9P2J5 [796-807	0	1870.1294
[K].VFAQNE	1xTMTpro [K	5.305E-05	4.225E-05	1	1	1	Q8TD06	Q8TD06 [80-94]	0	2387.2521
[K].HFADLLI	1xCarbamido	4.012E-06	4.225E-05	1	1	3	O00410	O00410 [213-238	0	3574.8132
[R].VFAECN	1xCarbamido	2.963E-05	4.225E-05	1	1	2	Q9NX40	Q9NX40 [34-46]	0	2010.9339
[K].VFADYE	1xCarbamido	1.165E-05	4.225E-05	1	1	1	P11216	P11216 [774-793]	0	2802.3187
[R].VEYVPL	1xTMTpro [K	3.927E-05	4.225E-05	1	1	1	Q9H6R3	Q9H6R3 [232-243	0	1997.1815
[K].VEYTPYI	1xCarbamido	1.177E-07	4.225E-05	1	1	4	P21333	P21333 [1319-135	0	4601.1625
[R].ANSTDY	1xTMTpro [K	4.449E-05	4.225E-05	1	1	1	P47895	P47895 [432-446]	0	2167.1891
[K].VEYTLGI	1xTMTpro [N	3.924E-06	4.225E-05	1	1	2	Q04637	Q04637 [1422-14	0	1968.9834
[R].VEYQED	1xTMTpro [K	3.201E-06	4.225E-05	1	1	1	P40123	P40123 [334-351]	1	2788.486
[R].VEYELSI	1xTMTpro [K	8.591E-08	4.225E-05	1	1	7	P02751	P02751 [752-785]	1	4383.2739
[R].ANTVQE	1xTMTpro [K	3.34E-05	4.225E-05	1	1	2	Q14624	Q14624 [61-75]	0	2315.2561
[R].VEYELSI	1xTMTpro [N	4.115E-07	4.225E-05	1	1	3	P02751	P02751 [752-784]	0	3950.9718
[K].ANQGDE	2xCarbamido	2.002E-05	4.225E-05	1	1	1	P29323	P29323 [279-294]	0	2133.9725

[K].HESCGÇ	3xCarbamid	3.67E-05	4.225E-05	1	1	1	P49821	P49821 [376-386]	0	1695.7321
[R].ANQAIQI	2xCarbamid	3.774E-05	4.225E-05	1	1	1	Q9Y490	Q9Y490 [1470-15	0	3824.9953
[K].VFEEDAI	1xTMTpro [K	2.526E-06	4.225E-05	1	1	1	Q14204	Q14204 [1515-15	1	2459.3062
[K].VFHLPTT	1xTMTpro [N	1.082E-05	4.225E-05	1	1	1	Q02218	Q02218 [185-204]	0	2488.3883
[R].VFHLLGY	1xTMTpro [K	3.568E-07	4.225E-05	1	1	6	P00491	P00491 [102-123]	0	2843.6639
[R].ANNSDF	1xTMTpro [K	2.66E-06	4.225E-05	1	1	2	O94788	O94788 [438-456]	0	2618.407
[K].ANNSQE	1xTMTpro [N	2.401E-05	4.225E-05	1	1	1	Q96HC4	Q96HC4 [306-324	0	2248.1489
[R].ANNSTY	1xTMTpro [K	1.152E-05	4.225E-05	1	1	2	P05091	P05091 [437-451]	0	2136.1945
[R].ANNTFY	1xTMTpro [K	2.015E-06	4.225E-05	1	1	2	P00352	P00352 [421-435]	0	2198.2101
[K].ANNYNE	1xTMTpro [N	4.809E-05	4.225E-05	1	1	1	Q09161	Q09161 [118-129]	0	1729.9193
[R].VFHAGF	1xTMTpro [K	2.574E-05	4.225E-05	1	1	1	P54136	P54136 [436-449]	0	2083.1832
[R].VFGETTI	1xTMTpro [N	2.232E-07	4.225E-05	1	1	2	Q99715	Q99715 [2033-20	0	2981.4713
[R].ANPDPN	2xCarbamid	9.5E-06	4.225E-05	1	1	1	P62995	P62995 [112-132]	0	2688.3081
[K].GKVEEV	2xTMTpro [K	3.299E-05	4.225E-05	1	2	2	Q99873	Q99873 [144-155]	1	2268.3631
[K].ANPFGG	1xTMTpro [K	4.241E-06	4.225E-05	1	1	2	P62266	P62266 [38-48]	0	1664.9364
[R].VFFVHE	1xTMTpro [K	6.67E-06	4.225E-05	1	1	1	P08237	P08237 [48-66]	0	2725.4594
[K].HEPLVLF	3xCarbamid	6.657E-06	4.225E-05	1	1	3	Q13263	Q13263 [214-230]	0	2441.1583
[R].VFEVSL	1xTMTpro [N	3.403E-06	4.225E-05	1	1	25	P61247	P61247 [66-82]	0	2256.1831
[K].VFEPNEI	1xTMTpro [K	2.657E-07	4.225E-05	1	1	1	O43615	O43615 [223-237]	0	2289.3098
[R].ANPQLG	1xTMTpro [N	1.454E-05	4.225E-05	1	1	1	Q9BQE5	Q9BQE5 [232-248	0	2062.1517
[R].ANPQVG	1xTMTpro [K	7.154E-06	4.225E-05	1	1	2	P13010	P13010 [401-413]	0	1986.178
[K].VFELMTS	1xTMTpro [K	2.561E-05	4.225E-05	1	1	1	Q06323	Q06323 [156-166]	0	1914.1014
[K].ANPTVTI	1xTMTpro [K	2.462E-05	4.225E-05	1	3	3	P0DOX8	P0DOX8 [115-133	0	2651.4536
[K].HEQILVL	1xTMTpro [K	3.196E-05	4.225E-05	1	1	3	Q9P0L0	Q9P0L0 [11-24]	0	2226.2989
[K].HEQNID	1xCarbamid	1.731E-05	4.225E-05	1	1	4	P27797	P27797 [99-111]	0	2085.0679
[K].VFGAPN	1xTMTpro [K	1.356E-05	4.225E-05	1	1	2	Q8TAT6	Q8TAT6 [99-116]	0	2631.4162
[R].ANNLHS	1xTMTpro [N	6.392E-06	4.225E-05	1	1	1	Q9BZF1	Q9BZF1 [300-318	0	2477.1976
[K].VEVITGE	1xCarbamid	3.174E-05	4.225E-05	1	1	1	Q9H6Z4	Q9H6Z4 [389-407	0	2772.4403
[R].VEVERD	1xTMTpro [N	2.279E-05	4.225E-05	1	1	6	P08670	P08670 [171-184]	1	1993.0344
[K].VEPQLAI	1xTMTpro [K	5.169E-07	4.225E-05	1	2	2	Q9UPN3	Q9UPN3 [6680-67	0	3522.8216
[K].VEPLYEI	1xTMTpro [N	4.035E-06	4.225E-05	1	1	1	P07358	P07358 [467-486]	0	2565.3408
[R].HGAEVII	1xTMTpro [K	1.414E-05	4.225E-05	1	1	1	P12081	P12081 [87-100]	0	2163.2305
[K].VEPGLG	1xTMTpro [N	1.842E-05	4.225E-05	1	1	6	P21333	P21333 [1020-10	0	1616.8927

[K].VEPAVS:	1xTMTpro [K	9.766E-06	4.225E-05	1	1	2	Q9P2E9	Q9P2E9 [149-166	0	2465.4471
[R].APEAWD	1xTMTpro [N	1.619E-06	4.225E-05	1	1	4	P00387	P00387 [242-259]	0	2416.1563
[R].APEAWD	1xOxidation		1.516E-05	4.225E-05	1	1	1	P00387	P00387 [242-259]	0	2432.1512
[K].APEDAG	1xTMTpro [N	2.136E-05	4.225E-05	1	1	4	Q9Y5Z4	Q9Y5Z4 [27-41]	0	1890.9517
[R].VENQEN	1xTMTpro [K	7.961E-06	4.225E-05	1	1	3	Q01518	Q01518 [331-348]	0	2681.4489
[R].VENPNS	1xTMTpro [K	2.128E-05	4.225E-05	1	1	1	Q12955	Q12955 [4124-41	0	2428.3765
[K].APEEPN	1xTMTpro [K	2.895E-05	4.225E-05	1	1	1	P43121	P43121 [135-153]	0	2624.4903
[K].APEEPN	1xCarbamido	1.499E-05	4.225E-05	1	1	2	P43121	P43121 [135-164]	1	3852.0457
[R].GKLPGP	2xTMTpro [K	2.833E-06	4.225E-05	1	1	1	P07741	P07741 [90-107]	1	2879.6487
[K].APEGEE	1xTMTpro [N	1.989E-06	4.225E-05	1	1	1	Q9H3Z4	Q9H3Z4 [140-164	0	3172.4765
[R].VENLQA	1xTMTpro [K	1.578E-05	4.225E-05	1	1	1	Q9HCU5	Q9HCU5 [141-158	0	2627.4172
[K].VENFEA	1xTMTpro [K	1.766E-05	4.225E-05	1	1	1	O43765	O43765 [104-116]	0	2119.1468
[K].APELLIY	1xTMTpro [N	5.046E-06	4.225E-05	1	1	2	A0A087WSZ	A0A087WSZ0 [65	0	2277.2774
[K].HGDEIYI	1xTMTpro [K	3.995E-05	4.225E-05	1	1	2	Q96GX9	Q96GX9 [52-65]	0	2122.1788
[K].VEMGTS	1xOxidation		1.951E-05	4.225E-05	1	1	1	P43307	P43307 [241-266]	0	3588.7806
[K].VEMGTS	1xTMTpro [K	4.352E-06	4.225E-05	1	1	1	P43307	P43307 [241-266]	0	3572.7857
[K].APEPHV	1xTMTpro [K	1.384E-05	4.225E-05	1	1	2	P52566	P52566 [5-21]	0	2548.2182
[K].APDVDV	1xTMTpro [K	6.682E-06	4.225E-05	1	1	2	Q09666	Q09666 [5057-50	0	2174.2313
[R].VEPVGN	1xTMTpro [K	2.592E-06	4.225E-05	1	1	1	Q14669	Q14669 [1207-12	0	2165.3302
[K].APDMSS	1xOxidation		2.453E-05	4.225E-05	1	1	2	Q00341	Q00341 [1241-12	0	2706.3577
[K].VEQATK	1xTMTpro [K	5.728E-05	4.225E-05	1	1	1	P38159	P38159 [81-93]	0	2044.1319
[K].HFDGED	1xCarbamido	1.208E-05	4.225E-05	1	1	1	Q02880	Q02880 [229-246]	0	2781.3686
[R].VETGVLI	1xOxidation		1.098E-05	4.225E-05	1	2	5	P68104	P68104 [267-290]	0	3444.0004
[R].VETGVLI	2xTMTpro [K	3.413E-07	4.225E-05	1	2	30	P68104	P68104 [267-290]	0	3428.0055
[R].HFGLQIL	1xTMTpro [K	4.744E-05	4.225E-05	1	1	1	Q9HAV4	Q9HAV4 [65-76]	0	2028.225
[R].APAALP	1xCarbamido	1.505E-05	4.225E-05	1	1	1	Q8TEX9	Q8TEX9 [34-54]	0	2438.3396
[R].APAFCD	2xCarbamido	6.115E-06	4.225E-05	1	1	1	Q9BZL6	Q9BZL6 [148-164	0	2284.0996
[K].HFIDVGA	1xTMTpro [N	1.09E-05	4.225E-05	1	1	2	P33316	P33316 [180-194]	0	2010.0252
[K].VESTDVS	1xTMTpro [N	1.705E-06	4.225E-05	1	1	2	P28062	P28062 [259-272]	0	1966.0201
[R].VESLSQ	1xTMTpro [N	2.053E-06	4.225E-05	1	1	1	P07942	P07942 [1551-15	0	2469.3632
[R].HFLENL	1xTMTpro [K	1.319E-05	4.225E-05	1	1	3	Q05707	Q05707 [179-194]	0	2414.3211
[K].HFCPNV	1xCarbamido	2.644E-05	4.225E-05	2	2	3	P08134; P61	P08134 [105-118]	0	2216.2869
[K].VESDLK	2xTMTpro [K	2.978E-06	4.225E-05	1	1	4	Q09666	Q09666 [4484-45	1	2810.5604

[R].HFIMQV\	2xCarbamido	2.918E-06	4.225E-05	1	1	1	Q14974	Q14974 [216-232]	0	2396.148
[K].VEQVPI\	1xTMTpro [K	2.775E-05	4.225E-05	1	1	1	P20908	P20908 [1807-182	0	2874.5203
[R].APASEE	1xTMTpro [N	5.794E-05	4.225E-05	1	1	3	P29590	P29590 [45-56]	0	1727.8924
[K].VEQVLSI	1xTMTpro [K	3.316E-06	4.225E-05	1	1	3	O95292	O95292 [4-17]	0	2257.3048
[R].APAVAV.	1xTMTpro [K	2.275E-05	4.225E-05	1	1	1	Q9P2E9	Q9P2E9 [102-131	0	3568.0832
[R].HFIPADY	1xTMTpro [N	2.263E-05	4.225E-05	1	1	2	Q9BX66	Q9BX66 [716-731	0	2271.1617
[R].HFNALG	1xTMTpro [K	4.695E-06	4.225E-05	1	1	1	P09960	P09960 [340-355]	0	2365.2908
[K].APDAGG	3xCarbamido	6.814E-07	4.225E-05	1	1	2	P02675	P02675 [89-124]	0	4260.1283
[R].VEQIAAL	1xTMTpro [N	1.105E-06	4.225E-05	1	1	3	P12814	P12814 [451-475]	0	3112.5758
[R].VEQIAAL	1xTMTpro [N	2.042E-08	4.225E-05	1	1	6	O43707	O43707 [470-494]	0	3209.6034
[R].APAPAS.	1xTMTpro [N	1.923E-05	4.225E-05	1	1	4	Q9Y6C2	Q9Y6C2 [276-292	0	1964.0772
[R].HEMPPH	1xOxidation [	7.154E-06	4.225E-05	1	1	6	P35579	P35579 [144-159]	0	2235.1188
[K].VFLDCCI	2xCarbamido	3.767E-05	4.225E-05	1	1	1	P01024	P01024 [723-735]	0	2006.9999
[K].ANIVHLM	1xTMTpro [K	5.898E-05	4.225E-05	1	1	1	P55060	P55060 [94-109]	0	2416.3878
[K].VGDQVV	1xTMTpro [N	2.885E-05	4.225E-05	1	1	2	P49588	P49588 [585-597]	0	1878.0081
[K].GLAITFV	1xTMTpro [K	4.872E-06	4.225E-05	2	2	2	O00148; Q1	O00148 [384-397]	0	2088.1469
[K].HEELMLI	1xCarbamido	5.313E-05	4.225E-05	1	1	1	P07814	P07814 [651-662]	0	2050.0957
[R].HEEQPA	1xTMTpro [N	4.249E-05	4.225E-05	1	1	3	Q07157	Q07157 [1157-11	0	1897.9112
[K].HEFSVD	2xCarbamido	9.362E-06	4.225E-05	1	1	1	O00244	O00244 [4-21]	0	2317.0331
[K].VGDDVE	1xTMTpro [N	4.184E-05	4.225E-05	1	1	1	O75534	O75534 [65-77]	0	1757.8513
[K].VGDAIPA	2xTMTpro [K	6.07E-06	4.225E-05	1	1	1	P30044	P30044 [58-83]	1	3655.0563
[K].VGDAIPA	1xTMTpro [K	2.641E-06	4.225E-05	1	1	4	P30044	P30044 [58-75]	0	2436.3266
[K].HEKEDG	1xTMTpro [K	2.979E-05	4.225E-05	1	1	2	P50990	P50990 [365-378]	1	2176.2581
[K].VGASAS	1xCarbamido	2.239E-06	4.225E-05	2	2	1	O60907; Q9I	O60907 [561-576]	0	1909.9972
[K].VGAIPAN	1xTMTpro [N	3.307E-07	4.225E-05	1	1	1	Q9Y490	Q9Y490 [2376-23	0	2614.3908
[K].VGAHAG	1xTMTpro [N	2.918E-06	4.225E-05	1	1	127	P69905	P69905 [18-32]	0	1833.9414
[K].VGAEDA	1xTMTpro [N	6.487E-06	4.225E-05	1	1	1	P49588	P49588 [283-296]	0	1786.8601
[R].AMYQYF	1xTMTpro [N	3.447E-05	4.225E-05	1	1	1	O60504	O60504 [619-634]	0	2359.1672
[R].GLALALI	1xTMTpro [K	5.41E-05	4.225E-05	1	1	1	P49736	P49736 [494-505]	0	1781.0817
[K].AMYSIDI	3xCarbamido	5.904E-06	4.225E-05	1	1	1	P04275	P04275 [2758-27	0	2824.233
[K].HELQAN	1xCarbamido	4.817E-05	4.225E-05	1	1	1	P23528	P23528 [133-144]	0	2128.0989
[R].VFYELQI	2xTMTpro [K	5.636E-05	4.225E-05	1	1	1	Q93009	Q93009 [263-277]	0	2660.5228
[K].HELQAN	1xCarbamido	4.359E-07	4.225E-05	1	1	5	P23528	P23528 [133-146]	1	2399.2269

[K].VFVGGL	1xTMTpro [K	9.124E-06	4.225E-05	1	1	3	O14979	O14979 [235-250]	0	2314.2786
[R].VFVGEE	1xTMTpro [N	2.198E-05	4.225E-05	1	1	1	Q86UX7	Q86UX7 [20-35]	0	2081.0722
[K].AMSQDG	1xTMTpro [N	9.623E-06	4.225E-05	1	1	1	Q16851	Q16851 [11-25]	0	1970.9925
[K].VGDTSLI	1xTMTpro [N	9.107E-06	4.225E-05	1	1	1	P51608	P51608 [145-162]	0	2260.1053
[K].VGEATE	1xCarbamido	1.073E-06	4.225E-05	2	2	4	P16615; Q93	P16615 [437-451]	0	2229.2292
[K].HEDFEE	1xTMTpro [K	4.374E-05	4.225E-05	1	1	1	P02549	P02549 [518-531]	0	2318.1432
[R].VGGVGF	1xTMTpro [N	3.07E-07	4.225E-05	1	1	1	Q6WCQ1	Q6WCQ1 [560-58	0	2816.4498
[K].VGGTSD	2xTMTpro [K	1.215E-05	4.225E-05	1	1	3	P10809	P10809 [406-418]	1	2274.3121
[K].VGGSGV	1xTMTpro [K	6.051E-05	4.225E-05	1	1	2	Q09666	Q09666 [253-263]	0	1609.9517
[R].VGGNIEY	1xTMTpro [N	5.073E-05	4.225E-05	1	2	1	Q14839	Q14839 [1407-14	0	1649.9294
[R].HEALLYT	1xCarbamido	4.491E-06	4.225E-05	1	1	1	Q14204	Q14204 [2577-26	0	3686.0709
[K].AMITDPE	1xTMTpro [K	2.086E-05	4.225E-05	1	1	2	Q709C8	Q709C8 [2107-21	0	2329.3333
[R].VGGEES	1xTMTpro [K	1.154E-08	4.225E-05	1	1	2	P22105	P22105 [2141-215	1	2408.3277
[R].VGGEES	1xTMTpro [N	8.559E-07	4.225E-05	1	1	2	P22105	P22105 [2141-215	0	1976.0256
[R].VGGDVF	1xTMTpro [N	7.234E-06	4.225E-05	1	1	2	P60510	P60510 [68-86]	0	2435.1872
[R].GLAPGQ	1xTMTpro [N	4.397E-05	4.225E-05	1	1	2	P22105	P22105 [809-821]	0	1721.9506
[R].VFVEEQ	1xTMTpro [N	5.183E-05	4.225E-05	1	1	4	P47895	P47895 [320-332]	0	1935.0183
[K].VGEVTY	1xTMTpro [K	2.049E-05	4.225E-05	1	1	8	P52272	P52272 [95-110]	0	2361.2867
[K].AMQAAG	1xOxidation [	1.703E-05	4.225E-05	1	1	1	P26368	P26368 [109-146]	0	4109.1768
[K].VGEQAQ	1xOxidation [	1.047E-05	4.225E-05	1	1	2	Q00610	Q00610 [44-63]	0	2515.3146
[K].VGEQAQ	1xTMTpro [N	4.748E-06	4.225E-05	1	1	2	Q00610	Q00610 [44-63]	0	2499.3196
[R].VGEPGQ	1xTMTpro [N	1.971E-06	4.225E-05	1	1	1	O75369	O75369 [2375-24	0	4001.0212
[K].AMQEQL	1xOxidation [	5.073E-05	4.225E-05	1	1	1	Q9H223	Q9H223 [361-373	0	2241.1353
[K].VGELNE	1xTMTpro [K	2.804E-05	4.225E-05	1	1	1	Q9UPU7	Q9UPU7 [433-45	0	2612.4283
[K].VGEIFSA	1xTMTpro [K	2.198E-05	4.225E-05	1	1	2	Q8IXM2	Q8IXM2 [8-21]	0	1977.1301
[K].GLAPDLI	1xTMTpro [K	2.745E-05	4.225E-05	1	1	1	P62277	P62277 [79-93]	0	2302.3302
[R].VGEGPG	1xCarbamido	6.895E-06	4.225E-05	1	1	1	Q9H4A4	Q9H4A4 [144-161	0	2464.315
[K].HECQAN	1xCarbamido	1.08E-05	4.225E-05	1	1	5	P60981	P60981 [133-145]	0	1843.8676
[K].AMQAAG	1xTMTpro [N	1.501E-07	4.225E-05	1	1	3	P26368	P26368 [109-146]	0	4093.1819
[R].VFTTQEI	1xTMTpro [N	2.589E-08	4.225E-05	1	1	1	O95433	O95433 [225-246]	0	2749.448
[R].HELTEIS	1xTMTpro [K	4.678E-06	4.225E-05	1	1	3	Q09666	Q09666 [187-203]	0	2494.3281
[K].VFSVAIT	1xTMTpro [N	5.607E-06	4.225E-05	1	1	6	P12109	P12109 [186-199]	0	1885.0503
[K].VFNTTPI	1xTMTpro [K	1.409E-06	4.225E-05	1	1	1	Q9Y3I0	Q9Y3I0 [335-357]	0	3234.7291

[R].HEMPPH	1xTMTpro [N	1.019E-05	4.225E-05	1	1	4	P35749	P35749 [148-163]	0	2189.1133
[R].VFNPYTI	1xTMTpro [N	5.719E-05	4.225E-05	1	1	1	Q9BUP0	Q9BUP0 [77-89]	0	1936.9764
[R].ANHGPG	1xTMTpro [N	7.658E-07	4.225E-05	1	1	1	Q9UNF0	Q9UNF0 [288-310	0	2962.3684
[R].VFIWTCI	1xCarbamido	2.511E-05	4.225E-05	1	1	3	P55735	P55735 [240-256]	0	2622.3154
[R].ANHSAF	1xTMTpro [N	7.533E-06	4.225E-05	1	1	2	Q9NTJ4	Q9NTJ4 [452-475	0	2757.3374
[K].GLAFTDY	1xTMTpro [K	1.486E-05	4.225E-05	1	1	1	P02751	P02751 [1728-174	0	1988.1196
[R].HEMPPH	1xOxidation [	5.803E-05	4.225E-05	1	1	3	P35749	P35749 [148-163]	0	2205.1082
[R].VFITDDF	1xTMTpro [K	1.722E-05	4.225E-05	1	1	2	P14625	P14625 [416-428]	0	2204.1376
[K].GLAEVQ	1xTMTpro [K	5.175E-05	4.225E-05	1	1	1	Q02952	Q02952 [582-602]	0	2728.3405
[R].VFNVFC	1xCarbamido	2.814E-05	4.225E-05	1	1	4	P14866	P14866 [399-411]	0	2197.1971
[R].HEMPPH	1xOxidation [	4.533E-05	4.225E-05	1	1	1	P35580	P35580 [148-163]	0	2221.1031
[K].ANLLNNI	1xTMTpro [K	4.315E-05	4.225E-05	1	1	1	P46063	P46063 [576-592]	0	2476.3838
[R].ANLLQEI	1xTMTpro [N	2.034E-05	4.225E-05	1	1	1	Q6ZMZ3	Q6ZMZ3 [793-811	0	2519.2697
[K].ANLISED	1xTMTpro [K	4.503E-05	4.225E-05	1	1	1	Q12929	Q12929 [173-188]	0	2300.2477
[K].ANINVEN	1xTMTpro [N	2.316E-05	4.225E-05	1	1	2	P61006	P61006 [154-167]	0	1884.0299
[R].ANLPQS	1xTMTpro [K	1.462E-05	4.225E-05	1	1	6	P21333	P21333 [1465-147	0	2043.1366
[K].ANLPVLF	1xTMTpro [N	6.416E-06	4.225E-05	1	1	1	P23743	P23743 [448-469]	0	2501.4775
[R].HEMPPH	1xTMTpro [N	6.857E-06	4.225E-05	1	1	4	P35579	P35579 [144-159]	0	2219.1238
[R].ANLQELI	1xTMTpro [K	2.735E-05	4.225E-05	1	1	1	Q9Y312	Q9Y312 [113-131	0	2789.5369
[K].ANLQIDC	1xTMTpro [N	3.76E-05	4.225E-05	1	1	6	P35579	P35579 [1755-177	0	2174.1736
[R].VFLDPNI	1xTMTpro [N	1.786E-05	4.225E-05	1	1	1	P48147	P48147 [112-128]	0	2149.1824
[K].VFLLGEE	1xTMTpro [K	5.446E-05	4.225E-05	1	1	2	P11177	P11177 [53-68]	0	2410.315
[K].VGGYILC	1xTMTpro [N	5.69E-06	4.225E-05	2	2	3	O95782; O9	O95782 [499-516]	0	2152.1722
[K].VFPGST	1xTMTpro [N	1.19E-06	4.225E-05	1	1	4	Q13263	Q13263 [508-524]	0	2257.2035
[R].VFPVYN	1xCarbamido	3.273E-06	4.225E-05	1	1	2	O94788	O94788 [54-74]	0	2988.5268
[K].VFSNGA	2xTMTpro [K	2.73E-06	4.225E-05	1	1	2	P01009	P01009 [335-355]	1	3074.7554
[K].ANCIDST	1xCarbamido	2.354E-06	4.225E-05	1	1	1	P22087	P22087 [266-284]	0	2578.3314
[K].ANCIDST	1xCarbamido	4.08E-06	4.225E-05	1	1	2	P22087	P22087 [266-285]	1	3010.6335
[K].VFSNGA	1xTMTpro [K	6.481E-07	4.225E-05	1	1	5	P01009	P01009 [335-352]	0	2442.3372
[K].ANCPYIV	1xCarbamido	1.535E-05	4.225E-05	1	1	1	O75884	O75884 [125-144]	0	2963.5257
[K].ANCSDN	1xCarbamido	8.784E-07	4.225E-05	1	1	1	Q16891	Q16891 [601-623]	0	2810.395
[K].ANDDIIV	1xTMTpro [N	5.581E-05	4.225E-05	1	1	4	P13797	P13797 [519-530]	0	1732.9302
[K].ANDGEG	1xTMTpro [N	1.376E-06	4.225E-05	1	1	1	Q9H3Q1	Q9H3Q1 [147-165	0	2207.0019

[K].ANDTQE	1xTMTpro [N	1.538E-05	4.225E-05	1	1	3	P51452	P51452 [90-104]	0	2109.0208
[R].ANDTTF	1xTMTpro [N	4.289E-06	4.225E-05	1	1	2	P49189	P49189 [412-426]	0	1844.9826
[R].HEMLPA	1xTMTpro [N	4.76E-05	4.225E-05	1	1	1	P52209	P52209 [435-447]	0	1797.9965
[K].ANDYAN	1xTMTpro [N	1.247E-05	4.225E-05	1	1	1	Q53GS9	Q53GS9 [232-250	0	2330.2788
[R].VFSDIIY	1xCarbamido	7.859E-06	4.225E-05	1	1	1	Q8NI27	Q8NI27 [1020-103	0	2466.2142
[K].VFSDEV	1xTMTpro [N	4.981E-06	4.225E-05	1	1	3	Q9Y262	Q9Y262 [446-461	0	2153.1522
[K].ANEGTV	1xTMTpro [N	2.206E-05	4.225E-05	1	1	1	P05186	P05186 [123-136]	0	1665.8727
[K].VFSATL	1xTMTpro [K	2.89E-05	4.225E-05	1	1	2	P09874	P09874 [552-564]	0	1970.2182
[R].VFQTEA	1xTMTpro [K	1.032E-05	4.225E-05	1	1	2	Q92878	Q92878 [687-704]	0	2672.4638
[K].ANELPQ	1xTMTpro [K	6.051E-05	4.225E-05	1	1	1	P78347	P78347 [327-343]	0	2337.3058
[R].ANENSN	1xTMTpro [N	3.495E-05	4.225E-05	1	1	1	Q6UN15	Q6UN15 [332-344	0	1777.9364
[R].VFQSST	1xCarbamido	3.747E-05	4.225E-05	1	1	1	P33176	P33176 [51-67]	0	2599.2954
[R].VFQSLP	2xTMTpro [K	1.063E-06	4.225E-05	1	1	1	P04080	P04080 [69-89]	0	3370.8939
[K].VFQGTD	1xTMTpro [N	2.616E-05	4.225E-05	1	1	1	Q92878	Q92878 [296-313]	0	2518.2394
[R].GLAGLG	1xTMTpro [N	4.235E-05	4.225E-05	1	1	4	P11216	P11216 [18-29]	0	1460.8392
[K].AHQANQ	1xTMTpro [N	4.669E-06	4.225E-05	1	1	1	Q14204	Q14204 [768-786]	0	2461.3523
[R].AHPVFY	1xTMTpro [K	2.503E-06	4.225E-05	1	1	1	Q96CS3	Q96CS3 [150-167	0	2618.3859
[R].VSFELF	2xTMTpro [K	3.08E-05	4.225E-05	1	1	2	P62937	P62937 [20-31]	1	2292.3784
[K].GQLTTD	1xTMTpro [K	3.702E-05	4.225E-05	1	1	2	P49748	P49748 [80-103]	0	3390.8077
[K].ADAEGE	1xTMTpro [N	5.945E-05	4.225E-05	1	1	2	Q9Y490	Q9Y490 [842-854	0	1696.7945
[K].GNAMVE	1xTMTpro [K	7.772E-07	4.225E-05	1	1	2	Q13813	Q13813 [847-862]	0	2312.1837
[K].GQLVPL	1xTMTpro [N	1.595E-05	4.225E-05	1	1	4	P00568	P00568 [64-77]	0	1888.0897
[RK].YHDMF	1xTMTpro [N	5.822E-05	4.225E-05	1	2	1	Q8WWQ0	Q8WWQ0 [1042-1	0	2037.0799
[R].YHDEAD	1xTMTpro [N	4.809E-05	4.225E-05	1	1	1	Q12789	Q12789 [1246-125	0	1736.8523
[R].YHALLIP	1xCarbamido	3.259E-05	4.225E-05	1	1	1	Q8NB37	Q8NB37 [86-109]	0	2774.483
[R].GNAGQS	1xTMTpro [N	4.042E-06	4.225E-05	1	1	2	P49327	P49327 [2027-204	0	2077.9681
[K].GNAGGI	1xTMTpro [K	1.973E-05	4.225E-05	1	1	2	Q8NCW5	Q8NCW5 [228-24	0	2373.3997
[R].ADALQA	1xTMTpro [K	1.088E-05	4.225E-05	2	2	2	Q15836; P63	Q15836 [50-66]; F	0	2274.2221
[K].ADAIYNM	1xTMTpro [K	4.199E-05	4.225E-05	1	1	1	P42892	P42892 [497-514]	0	2681.3963
[K].GQPAIID	1xTMTpro [K	2.568E-06	4.225E-05	1	1	2	Q9Y266	Q9Y266 [213-227	0	2254.2575
[R].ADAVTLI	1xTMTpro [K	3.473E-06	4.225E-05	1	1	7	P02788	P02788 [73-92]	0	2679.4525
[K].ADCASG	1xCarbamido	1.08E-05	4.225E-05	1	1	1	O43747	O43747 [398-410]	0	1961.0658
[K].GQPDVV	2xTMTpro [K	2.453E-05	4.225E-05	1	1	1	P24752	P24752 [244-257]	1	2547.4122

[R].ADCEGF	2xCarbamido	4.359E-07	4.225E-05	1	1	1	Q8IVL6	Q8IVL6 [255-294]	0	4692.1174
[K].YGVKPH	2xTMTpro [K	4.05E-05	4.225E-05	1	1	1	P51911	P51911 [98-132]	0	4815.5596
[R].GQPFEV	1xTMTpro [K	2.045E-05	4.225E-05	1	1	1	P47929	P47929 [84-99]	0	2344.3044
[R].GMYDGF	1xTMTpro [K	4.192E-05	4.225E-05	1	1	1	Q14194	Q14194 [497-511]	0	2232.1866
[K].YGSGSG	1xTMTpro [N	1.414E-06	4.225E-05	1	1	1	Q9HCN8	Q9HCN8 [60-83]	0	2825.2934
[K].YGQTIRF	2xCarbamido	3.607E-05	4.225E-05	1	1	2	P00751	P00751 [588-605]	0	2427.2444
[K].ADAASS	1xTMTpro [K	1.202E-05	4.225E-05	1	1	2	Q9H8Y8	Q9H8Y8 [404-420	0	2252.263
[K].GQIQEIV	1xTMTpro [N	1.98E-05	4.225E-05	1	1	2	P54652	P54652 [332-345]	0	1761.019
[R].YHTSQS	1xTMTpro [N	4.255E-07	4.225E-05	1	1	2	P08238	P08238 [457-475]	0	2481.1523
[R].ACYLSIN	1xCarbamido	1.828E-05	4.225E-05	1	1	2	P61163	P61163 [221-238]	1	3051.6489
[R].ACPSHQ	1xCarbamido	3.276E-05	4.225E-05	1	1	1	Q16610	Q16610 [283-310]	0	3594.9122
[R].ACQAAT	1xCarbamido	4.825E-05	4.225E-05	1	1	1	Q9Y4G6	Q9Y4G6 [756-768	0	2016.0927
[K].GQLITGN	1xCarbamido	1.101E-06	4.225E-05	1	1	1	O95302	O95302 [88-106]	0	2396.2055
[K].YKPAVN	1xCarbamido	1.892E-05	4.225E-05	1	1	2	P15121	P15121 [178-195]	0	3130.7176
[K].GNDISS	1xTMTpro [K	2.413E-07	4.225E-05	1	1	4	P30086	P30086 [94-113]	0	2558.3594
[K].ACQSIYF	1xCarbamido	2.885E-05	4.225E-05	1	1	2	P61247	P61247 [200-213]	0	2009.0598
[K].YKGQHV	2xTMTpro [K	3.617E-06	4.225E-05	1	1	1	P21333	P21333 [2216-224	1	3507.9317
[K].YKGQEA	1xTMTpro [K	3.967E-05	4.225E-05	1	1	1	O43570	O43570 [195-214]	1	2944.6064
[K].YKETDLI	2xTMTpro [K	2.89E-05	4.225E-05	1	1	2	P05455	P05455 [175-185]	1	2295.4144
[R].YKEEYA	2xTMTpro [K	5.354E-06	4.225E-05	1	1	1	Q14204	Q14204 [3447-34	1	2767.6062
[R].YGQSTG	1xTMTpro [N	6.074E-07	4.225E-05	1	1	1	P21589	P21589 [442-463]	0	2729.4218
[K].ACTAQS	1xCarbamido	4.768E-05	4.225E-05	1	1	1	Q16854	Q16854 [86-101]	0	2148.0571
[K].GQLITGN	1xCarbamido	1.688E-05	4.225E-05	1	1	1	O95302	O95302 [88-106]	0	2412.2005
[K].YKDLDEI	2xTMTpro [K	1.575E-05	4.225E-05	1	1	2	Q9NYL9	Q9NYL9 [12-30]	1	3136.7082
[R].GQIPPLV	1xCarbamido	3.644E-06	4.225E-05	1	1	1	O94855	O94855 [289-310]	0	2702.3561
[R].ACVCQT	2xCarbamido	2.551E-05	4.225E-05	1	1	1	A6NHR9	A6NHR9 [58-71]	0	2200.1597
[K].GNDDHW	1xCarbamido	1.816E-06	4.225E-05	1	1	1	P02753	P02753 [118-139]	0	2997.328
[R].YKAAFTI	3xCarbamido	1.991E-05	4.225E-05	1	1	1	P02768	P02768 [185-205]	2	3632.9744
[R].YKAAFTI	2xCarbamido	1.073E-05	4.225E-05	1	1	2	P02768	P02768 [185-198]	1	2575.3465
[K].ACYALEI	1xCarbamido	4.556E-05	4.225E-05	1	1	2	Q8TEX9	Q8TEX9 [458-472	0	2333.2455
[R].YHVPVV	1xTMTpro [N	1.885E-06	4.225E-05	1	1	2	P04844	P04844 [267-289]	0	2807.5011
[R].ACYLSIN	1xCarbamido	1.619E-05	4.225E-05	1	1	2	P42025	P42025 [221-238]	1	3021.6383
[R].ACTEAE	3xCarbamido	4.04E-07	4.225E-05	1	1	1	P98160	P98160 [198-218]	0	2884.266

[K].YKPVCN	2xCarbamid	4.235E-05	4.225E-05	1	1	1	P42330	P42330 [184-199]	0	2719.3729
[K].ADDKET	1xCarbamid	1.913E-05	4.225E-05	1	1	5	P02768	P02768 [585-597]	1	2412.2533
[K].GQPLGP	1xTMTpro [N	5.073E-05	4.225E-05	1	3	1	Q5JPE7	Q5JPE7 [139-152	0	1682.9873
[R].YGGTFQ	1xTMTpro [K	5.236E-06	4.225E-05	1	1	1	O94973	O94973 [808-825	0	2587.4739
[K].GMTTVD	1xTMTpro [K	2.295E-05	4.225E-05	1	1	2	Q14195	Q14195 [82-94]	0	2055.0713
[K].YGGPYH	1xTMTpro [K	5.964E-05	4.225E-05	1	1	6	P21333	P21333 [2501-25	0	1988.0886
[K].YGGPPP	1xTMTpro [K	4.148E-06	4.225E-05	1	1	2	O60506	O60506 [144-168	0	3174.6603
[R].YGGLHF	1xTMTpro [N	5.009E-06	4.225E-05	1	1	4	P12110	P12110 [96-115]	0	2498.2271
[K].GMTSSE	2xOxidation [	6.051E-05	4.225E-05	1	1	3	Q99715	Q99715 [409-421	0	2050.0692
[K].ADFDNT	1xTMTpro [N	4.185E-07	4.225E-05	1	1	1	P00390	P00390 [502-522]	0	2619.3585
[R].YGGEEE	1xTMTpro [K	5.2E-05	4.225E-05	1	1	1	Q9UMX5	Q9UMX5 [55-69]	0	2319.2364
[R].ADFLEQ	1xTMTpro [N	2.187E-05	4.225E-05	1	1	2	P02730	P02730 [234-246]	0	1795.0073
[K].GMTSSE	1xOxidation [	3.135E-05	4.225E-05	1	1	8	Q99715	Q99715 [409-421	0	2034.0743
[K].ADFPAG	1xCarbamid	3.947E-05	4.225E-05	1	1	2	P26640	P26640 [908-923]	0	1993.9972
[R].YGGAPQ	1xTMTpro [K	4.533E-05	4.225E-05	1	1	2	O95782	O95782 [846-856	0	1727.0347
[R].YGFIEGI	1xTMTpro [N	2.214E-05	4.225E-05	1	1	4	P16070	P16070 [79-90]	0	1690.96
[K].YGFCPH	2xCarbamid	7.177E-07	4.225E-05	1	1	2	P08253	P08253 [271-292]	0	3079.472
[K].YGFCPE	2xCarbamid	3.135E-05	4.225E-05	1	1	1	P08253	P08253 [329-359]	0	3950.9047
[K].GMTSSE	1xTMTpro [K	2.9E-05	4.225E-05	1	1	2	Q99715	Q99715 [409-421	0	2018.0794
[K].YGFCEA	1xCarbamid	5.735E-07	4.225E-05	1	1	2	P07360	P07360 [185-201]	0	2360.1301
[K].YGEVEE	1xCarbamid	1.949E-06	4.225E-05	1	1	2	Q01081	Q01081 [93-116]	0	3361.6688
[K].YGEGHQ	1xTMTpro [K	3.545E-05	4.225E-05	1	1	2	P49903	P49903 [345-359]	0	2308.2945
[R].YGEGDS	1xTMTpro [K	3.236E-05	4.225E-05	1	1	2	Q15043	Q15043 [51-63]	0	2060.1519
[K].ADGATS	1xTMTpro [N	5.554E-05	4.225E-05	1	1	1	Q07157	Q07157 [805-819	0	1919.8902
[K].YGHAAC	2xCarbamid	5.831E-05	4.225E-05	1	1	1	P52888	P52888 [422-436]	0	2010.9961
[K].YGKDAT	2xTMTpro [K	6.176E-07	4.225E-05	1	1	1	P06733	P06733 [200-221]	1	3221.7259
[K].YGKDAT	3xTMTpro [K	1.013E-06	4.225E-05	1	1	1	P06733	P06733 [200-228]	2	4308.3868
[R].ADELNQ	1xTMTpro [K	1.741E-05	4.225E-05	1	1	1	P46939	P46939 [745-761]	0	2482.3467
[R].ADDKVY	1xCarbamid	6.701E-07	4.225E-05	1	1	2	P00450	P00450 [145-177]	1	4302.0462
[R].YGPQYG	1xTMTpro [K	8.109E-06	4.225E-05	1	1	1	P14866	P14866 [359-393]	0	4393.2247
[K].YGPPCP	2xCarbamid	3.299E-05	4.225E-05	1	1	8	P01861	P01861 [102-128]	0	3856.0786
[R].YGPLTAI	1xTMTpro [N	1.879E-05	4.225E-05	1	1	1	P22105	P22105 [1236-124	0	1752.9087
[R].YGPLSG	1xTMTpro [N	3.806E-05	4.225E-05	1	1	4	Q13595	Q13595 [142-155	0	1870.9982

[K].ADDPSS	1xTMTpro [K	4.845E-07	4.225E-05	1	1	4	Q00610	Q00610 [1131-11	0	3348.655
[K].ADDPSS	1xOxidation	4.475E-06	4.225E-05	1	1	5	Q00610	Q00610 [1131-11	0	3364.6499
[K].YGPIAD\	1xTMTpro [N	6.104E-06	4.225E-05	1	1	3	P62995	P62995 [141-156]	0	2115.1042
[R].GMVFGI	1xOxidation	4.801E-05	4.225E-05	1	1	3	P98160	P98160 [486-502]	0	2147.1854
[R].ADDVRP	1xTMTpro [N	4.271E-05	4.225E-05	1	1	1	Q9P2B2	Q9P2B2 [306-319	0	1969.0099
[K].ADDKET	1xCarbamido	2.603E-07	4.225E-05	1	1	9	P02768	P02768 [585-598]	2	2844.5554
[K].YGNLGH	1xTMTpro [K	1.478E-05	4.225E-05	1	1	1	Q96MW5	Q96MW5 [537-55	0	2972.7217
[R].GMVFGI	1xTMTpro [N	6.31E-06	4.225E-05	1	1	3	P98160	P98160 [486-502]	0	2131.1905
[K].GQPSVL	1xCarbamido	4.224E-08	4.225E-05	1	1	5	P00734	P00734 [518-537]	0	2840.6676
[K].YGIVDYI	1xTMTpro [K	1.676E-05	4.225E-05	1	1	4	P13667	P13667 [273-288]	0	2392.2714
[R].YGISSMI	1xTMTpro [K	2.715E-06	4.225E-05	1	1	2	P14868	P14868 [29-43]	0	2347.2572
[K].ADEDPIN	1xTMTpro [K	1.448E-05	4.225E-05	1	1	2	P61970	P61970 [91-106]	0	2500.3224
[R].YGINTTD	1xTMTpro [K	2.865E-06	4.225E-05	1	1	5	P37802	P37802 [103-120]	0	2708.4427
[K].GQPVAP	1xTMTpro [K	3.423E-05	4.225E-05	1	1	1	O94992	O94992 [197-221	0	3496.7186
[K].GQQELL	1xTMTpro [K	5.917E-05	4.225E-05	1	1	1	A5YKK6	A5YKK6 [1228-12	0	2199.3033
[R].GQQLVE	1xCarbamido	5.103E-06	4.225E-05	1	1	1	P11277	P11277 [1541-15	0	2407.173
[K].ADEHVID	1xTMTpro [N	3.651E-06	4.225E-05	1	1	2	P13861	P13861 [162-181]	0	2611.2232
[K].ADDYIEI	1xTMTpro [K	1.215E-05	4.225E-05	1	1	4	Q9Y3Z3	Q9Y3Z3 [393-405	0	1918.0413
[K].YKPYEE	1xTMTpro [K	4.213E-05	4.225E-05	1	1	1	Q15020	Q15020 [295-309	0	2386.3262
[R].ACNQLG	1xCarbamido	5.673E-05	4.225E-05	2	2	1	O95782; O9	O95782 [330-341	0	1775.9294
[K].GQILMPI	2xTMTpro [K	7.491E-06	4.225E-05	1	1	3	P62910	P62910 [51-65]	1	2532.4788
[K].YLILPVH	1xTMTpro [K	3.259E-05	4.225E-05	1	1	1	Q7L2E3	Q7L2E3 [699-714	0	2507.401
[R].GQESAG	1xTMTpro [K	5.964E-05	4.225E-05	1	1	1	Q06203	Q06203 [44-62]	0	2475.3223
[K].GQETST	1xTMTpro [N	3.125E-06	4.225E-05	1	1	2	O75874	O75874 [322-338	0	2183.1416
[K].YILGNPL	2xTMTpro [K	7.217E-07	4.225E-05	1	1	3	P00352	P00352 [330-353]	1	3586.9937
[R].AAVVTSI	1xTMTpro [K	9.535E-06	4.225E-05	1	1	1	P35611	P35611 [7-21]	0	2082.2203
[K].YILGNPL	1xTMTpro [K	3.305E-05	4.225E-05	1	1	1	P00352	P00352 [330-348]	0	2619.5002
[R].YLIFPNP	1xCarbamido	3.405E-05	4.225E-05	1	1	2	P55268	P55268 [674-690]	0	2618.4548
[K].AAVYHH	1xTMTpro [N	5.226E-05	4.225E-05	1	1	2	P01024	P01024 [914-926]	0	1775.9512
[K].YLLETSC	1xTMTpro [K	1E-05	4.225E-05	1	1	3	P43490	P43490 [175-189]	0	2323.2677
[K].GQFAEH	1xTMTpro [N	5.894E-06	4.225E-05	1	1	1	Q9H2U1	Q9H2U1 [799-814	0	1980.0622
[R].GNFGGS	1xTMTpro [N	3.864E-08	4.225E-05	1	1	2	P52272	P52272 [628-650]	0	2339.16
[K].YLIANAT	1xTMTpro [K	2.124E-05	4.225E-05	1	1	4	P27348	P27348 [104-115]	0	1929.0937

[R].AAWYTA	1xCarbamido	6.664E-07	4.225E-05	1	1	2	Q9H3K2	Q9H3K2 [218-240	0	2948.5506
[K].YLHVGY	1xTMTpro [K	2.711E-05	4.225E-05	1	1	1	O60749	O60749 [198-211]	0	2191.2771
[R].YLHLLD(	1xCarbamido	2.68E-06	4.225E-05	1	1	1	Q8N1G4	Q8N1G4 [438-469	0	4214.1247
[R].GQFSTD	1xTMTpro [K	9.379E-06	4.225E-05	1	2	2	Q00610	Q00610 [838-851]	0	2160.168
[R].YIGTEG(	1xTMTpro [K	1.569E-05	4.225E-05	1	1	1	Q01415	Q01415 [181-203]	0	2969.5058
[R].GQFSTD	1xTMTpro [K	2.072E-06	4.225E-05	1	2	1	Q00610	Q00610 [838-852]	1	2316.2691
[K].YLGPQY	1xTMTpro [K	1.882E-05	4.225E-05	1	1	4	P02788	P02788 [681-694]	0	2145.2564
[R].YLGPAC	1xCarbamido	4.449E-05	4.225E-05	1	1	1	Q9UIS9	Q9UIS9 [52-65]	0	2268.223
[R].YLGNTV	1xTMTpro [N	3.042E-05	4.225E-05	1	1	1	Q9UBB5	Q9UBB5 [196-209	0	1937.9928
[K].AAVQVLI	1xTMTpro [K	4.465E-05	4.225E-05	1	1	2	P22033	P22033 [731-741]	0	1809.0613
[R].AAVPSG	1xTMTpro [K	1.224E-06	4.225E-05	1	1	1	P06733	P06733 [33-56]	2	3142.6988
[R].AAVPSG	1xTMTpro [K	1.101E-06	4.225E-05	1	1	2	P06733	P06733 [33-54]	1	2885.55
[R].AAVPSG	1xTMTpro [N	2.984E-06	4.225E-05	2	3	20	P09104; P06	P09104 [33-50]; F	0	2109.1511
[R].AAVETL(	1xCarbamido	1.071E-05	4.225E-05	1	1	1	A1L0T0	A1L0T0 [306-322]	0	2053.0894
[K].GNKEPN	2xTMTpro [K	1.067E-05	4.225E-05	1	1	1	Q9BSJ8	Q9BSJ8 [495-515	1	3254.7871
[K].AAVEWF	2xTMTpro [K	5.047E-05	4.225E-05	1	1	2	Q01844	Q01844 [425-439]	1	2597.418
[K].GQDMET	1xOxidation [	7.631E-06	4.225E-05	1	1	3	Q15363	Q15363 [118-145]	1	4059.1017
[K].GQEEG(	1xTMTpro [K	2.371E-05	4.225E-05	1	1	1	P53621	P53621 [873-912]	0	4720.3074
[R].YINENLI'	1xTMTpro [N	1.446E-05	4.225E-05	1	1	2	P17987	P17987 [131-145]	0	2067.1042
[R].YIMVPS(	1xOxidation [	1.589E-05	4.225E-05	1	1	2	Q9P003	Q9P003 [85-104]	0	2597.2811
[R].YIMVPS(	1xTMTpro [N	3.82E-05	4.225E-05	1	1	2	Q9P003	Q9P003 [85-104]	0	2581.2862
[R].YLMEED	1xTMTpro [K	2.814E-05	4.225E-05	1	1	2	P46777	P46777 [210-220]	0	2013.9971
[R].GQELAF	1xTMTpro [N	6.037E-06	4.225E-05	1	1	2	P26641	P26641 [379-400]	0	2991.4484
[K].AAYLQE'	2xTMTpro [K	2.064E-05	4.225E-05	1	1	3	P04083	P04083 [82-97]	0	2689.5592
[K].YLIVVTD	1xCarbamido	2.697E-05	4.225E-05	1	1	2	P12109	P12109 [152-180]	1	4086.1674
[K].YLLSQS	1xTMTpro [N	7.314E-06	4.225E-05	1	1	3	Q12792	Q12792 [137-155]	0	2379.2727
[K].YILSDSS	1xTMTpro [N	5.701E-06	4.225E-05	1	1	1	P23786	P23786 [275-296]	0	2774.4208
[K].GQELCA	1xCarbamido	6.844E-06	4.225E-05	1	1	3	P02774	P02774 [403-419]	0	2663.2791
[K].GQELCA	1xCarbamido	2.745E-06	4.225E-05	1	1	3	P02774	P02774 [403-420]	1	3095.5812
[R].AAVLQE	1xTMTpro [N	2.273E-06	4.225E-05	1	1	1	Q96KN1	Q96KN1 [263-287	0	2958.524
[R].GQELIY/	1xTMTpro [K	3.52E-05	4.225E-05	1	1	1	P53396	P53396 [849-864]	0	2404.3442
[R].AAVMHD	1xTMTpro [K	4.665E-05	4.225E-05	1	1	2	Q6P2Q9	Q6P2Q9 [687-702	0	2379.273
[R].AAVMVY	2xTMTpro [K	1.566E-05	4.225E-05	1	1	1	Q8N8S7	Q8N8S7 [11-22]	1	2237.278

[K].GNFTLPI	1xCarbamido	1.96E-07	4.225E-05	1	1	5	Q00839	Q00839 [638-664]	1	4100.1354
[R].GQENQL	1xTMTpro [N	3.601E-05	4.225E-05	1	1	1	Q9NUM4	Q9NUM4 [73-88]	0	2135.1416
[K].YLIVVTD	1xTMTpro [K	5.074E-06	4.225E-05	1	1	2	P12109	P12109 [152-166]	0	2312.3146
[K].AAYLQE	3xTMTpro [K	7.947E-07	4.225E-05	1	1	3	P04083	P04083 [82-98]	1	3121.8614
[R].GQFYQC	1xCarbamido	1.892E-05	4.225E-05	1	1	1	Q9H7D7	Q9H7D7 [468-487	0	2676.2683
[K].GQGTIC	2xCarbamido	5.121E-06	4.225E-05	1	1	1	Q14554	Q14554 [76-91]	0	2114.9555
[K].YICENQI	1xCarbamido	1.639E-05	4.225E-05	1	1	38	P02768	P02768 [287-298]	0	2052.0563
[R].YLAPSG	1xTMTpro [K	3.947E-05	4.225E-05	1	1	2	Q16658	Q16658 [230-241]	0	1799.0559
[K].YIALDEV	1xCarbamido	4.89E-05	4.225E-05	1	1	1	P09486	P09486 [280-293]	0	2251.2077
[K].YIAIAST	2xTMTpro [K	5.209E-05	4.225E-05	1	1	2	P31150	P31150 [349-379]	1	4355.3941
[K].ACGDST	1xCarbamido	2.883E-07	4.225E-05	1	1	2	P62879	P62879 [24-42]	0	2236.1563
[R].GNEFFC	1xCarbamido	3.893E-05	4.225E-05	1	1	1	P67870	P67870 [18-33]	0	2616.242
[R].GQLEAL	1xTMTpro [N	9.838E-06	4.225E-05	1	1	1	P08729	P08729 [150-167]	1	2258.2424
[R].YLAEVA	2xTMTpro [K	2.663E-05	4.225E-05	1	1	4	P63104	P63104 [128-139]	1	2192.2743
[R].ACGVSR	2xCarbamido	2.255E-05	4.225E-05	1	1	1	P55769	P55769 [92-107]	0	2326.3231
[K].ACLDYP	2xCarbamido	9.057E-06	4.225E-05	1	1	1	P53801	P53801 [65-82]	0	2599.4119
[K].YLDEDTI	1xTMTpro [N	1.412E-05	4.225E-05	1	1	1	P31153	P31153 [235-249]	0	2111.0729
[R].YLAEKYI	1xTMTpro [K	2.916E-05	4.225E-05	1	1	1	P13639	P13639 [634-647]	1	2351.2527
[K].ACLIFFD	1xCarbamido	2.644E-05	4.225E-05	1	1	1	P35998	P35998 [269-284]	0	2072.0806
[K].ACLISLG	1xCarbamido	1.721E-06	4.225E-05	1	1	1	P12814	P12814 [773-794]	0	2700.3258
[K].ACLISLG	1xCarbamido	1.154E-05	4.225E-05	1	1	3	O43707	O43707 [792-805]	0	1928.9707
[K].ACLISLG	1xCarbamido	1.128E-06	4.225E-05	1	1	2	O43707	O43707 [792-813]	1	2860.3855
[R].YKVEYY	2xTMTpro [K	5.287E-05	4.225E-05	1	1	2	Q99715	Q99715 [1415-14	1	2302.3263
[K].YKVEYPI	1xCarbamido	8.169E-06	4.225E-05	1	1	1	O14880	O14880 [36-59]	1	3582.8005
[R].YKVEGF	2xTMTpro [K	6.206E-06	4.225E-05	1	1	2	P13667	P13667 [595-611]	1	2831.58
[K].GQILMPI	1xTMTpro [K	4.681E-05	4.225E-05	1	1	2	P62910	P62910 [51-64]	0	2100.1767
[R].YKTPEEI	2xTMTpro [K	4.681E-05	4.225E-05	1	1	1	Q99715	Q99715 [1600-16	2	2748.5236
[K].ACNDAT	1xCarbamido	4.336E-06	4.225E-05	1	1	1	Q9HAV0	Q9HAV0 [24-42]	0	2356.1556
[R].YLAADKI	1xCarbamido	2.128E-05	4.225E-05	1	1	2	Q16658	Q16658 [69-82]	1	2220.1574
[K].ADGESC	1xCarbamido	1.213E-05	4.225E-05	1	1	3	P01008	P01008 [274-289]	0	2359.086
[KR].GQHVF	1xTMTpro [K	7.164E-07	4.225E-05	2	2	1	P21333; Q1	P21333 [2218-22	0	2912.5663
[R].ACEPIIQ	3xCarbamido	7.271E-07	4.225E-05	1	1	2	Q92896	Q92896 [695-725]	0	4256.0376
[R].YLGDLS	1xTMTpro [K	2.23E-05	4.225E-05	1	1	2	P09601	P09601 [137-148]	0	1858.093

[R].YLGDAS	1xTMTpro [N	3.075E-05	4.225E-05	1	1	3	P17568	P17568 [9-34]	0	3242.5675
[R].GNEIVLS	1xTMTpro [N	5.869E-05	4.225E-05	1	1	2	Q14204	Q14204 [3911-39	0	1604.8927
[R].AAYNVT	1xTMTpro [K	3.441E-05	4.225E-05	1	1	2	Q9C0B1	Q9C0B1 [197-211	0	2333.2819
[K].YLFPGE	2xCarbamido	1.169E-05	4.225E-05	1	1	1	P04275	P04275 [883-906]	0	3174.4984
[K].YLFNQLI	1xTMTpro [N	5.648E-06	4.225E-05	1	1	1	Q9UBT2	Q9UBT2 [191-225	1	4299.9914
[K].YLFNQLI	1xTMTpro [N	2.083E-05	4.225E-05	1	1	1	Q9UBT2	Q9UBT2 [191-210	0	2676.2749
[K].YLEVVLN	1xTMTpro [K	1.642E-05	4.225E-05	1	1	1	Q14974	Q14974 [731-749	0	2755.5486
[R].YLEVSEI	2xCarbamido	2.533E-05	4.225E-05	1	1	1	O15371	O15371 [184-204	0	3189.5252
[K].ACANPA	1xCarbamido	1.219E-06	4.225E-05	1	1	3	P00558	P00558 [107-123]	0	2073.1446
[K].YLDQME	1xTMTpro [K	4.765E-06	4.225E-05	1	1	3	O43681	O43681 [302-317	0	2666.3668
[K].GQGTLS	1xTMTpro [K	8.617E-06	4.225E-05	1	1	3	P01024	P01024 [1338-135	0	2100.1767
[K].ACDELVI	1xCarbamido	3.775E-06	4.225E-05	1	1	1	O00469	O00469 [575-588	0	2318.1288
[K].ACDGINI	4xCarbamido	3.879E-05	4.225E-05	1	1	2	P05156	P05156 [240-257]	0	2725.1818
[R].YIELFLN	1xTMTpro [N	1.294E-06	4.225E-05	1	1	1	P31942	P31942 [262-287]	0	2826.351
[R].ACDLPA	1xCarbamido	2.273E-06	4.225E-05	1	1	1	A0FGR8	A0FGR8 [180-194	0	2118.0398
[R].ACDSVT	1xCarbamido	2.54E-06	4.225E-05	1	1	1	Q96T76	Q96T76 [385-403	0	2779.5308
[K].GQGVYL	1xCarbamido	3.441E-06	4.225E-05	1	1	2	P30040	P30040 [147-170]	0	2903.4755
[K].ACEDIDE	4xCarbamido	1.804E-07	4.225E-05	1	1	2	P35555	P35555 [1443-14	0	3498.6234
[K].ACEFGG	1xCarbamido	1.657E-05	4.225E-05	1	1	1	Q9Y4G6	Q9Y4G6 [237-256	0	2895.4856
[R].YIDTEHC	1xTMTpro [N	5.192E-05	4.225E-05	1	1	1	Q15436	Q15436 [705-716	0	1637.8203
[K].YIDSADL	1xTMTpro [N	2.839E-05	4.225E-05	1	1	1	P17812	P17812 [336-353]	0	2366.2047
[K].GQGVYL	1xCarbamido	1.84E-06	4.225E-05	1	1	1	P30040	P30040 [147-170]	0	2887.4806
[R].AAVESL	1xTMTpro [N	5.936E-05	4.225E-05	1	1	2	Q99460	Q99460 [617-628	0	1626.9538
[R].YGEAGE	1xTMTpro [N	9.73E-06	4.225E-05	1	1	1	P26640	P26640 [24-39]	0	1901.9214
[R].ADGEYW	1xOxidation	3.259E-05	4.225E-05	1	1	1	P55083	P55083 [106-123]	0	2726.4831
[R].YDAMAL	1xTMTpro [K	8.761E-06	4.225E-05	1	1	1	P21589	P21589 [110-133]	0	3253.7059
[K].ADVIPVH	1xTMTpro [K	1.31E-05	4.225E-05	1	1	1	P29144	P29144 [984-999]	0	2431.4245
[K].ADVIQAT	1xCarbamido	5.384E-06	4.225E-05	1	1	1	Q08945	Q08945 [189-203	0	1954.0387
[R].GMGGAI	2xTMTpro [K	6.548E-06	4.225E-05	1	1	2	P12236	P12236 [281-296]	1	2652.5615
[K].ADVLTT	1xTMTpro [K	1.971E-06	4.225E-05	1	1	5	P04040	P04040 [24-38]	0	2023.1315
[R].YDAFGE	1xTMTpro [N	4.81E-06	4.225E-05	1	1	2	Q9Y2X3	Q9Y2X3 [372-388	0	2138.962
[K].YCYLQN	1xCarbamido	5.563E-05	4.225E-05	1	1	1	P15924	P15924 [739-752]	0	2417.2819
[K].ADVQAH	1xCarbamido	2.035E-07	4.225E-05	1	1	3	P19827	P19827 [525-549]	1	3762.9135

[K].YCPQGT	2xCarbamido	5.455E-05	4.225E-05	1	1	3	Q99439	Q99439 [273-309]	0	4297.845
[K].ADVVEA	1xTMTpro [K	4.286E-05	4.225E-05	1	1	2	P02549	P02549 [1937-194	0	1825.0351
[R].YCNTWF	1xCarbamido	3.981E-05	4.225E-05	1	1	2	Q00796	Q00796 [300-314]	0	2349.2591
[R].YCKDINE	3xCarbamido	2.484E-05	4.225E-05	1	1	1	P35555	P35555 [610-627]	1	2825.3334
[K].ADVVISA	1xTMTpro [K	7.673E-06	4.225E-05	1	1	1	Q12882	Q12882 [431-446]	0	2213.2673
[R].YCFSEM	2xCarbamido	8.527E-07	4.225E-05	1	1	2	Q9UBE0	Q9UBE0 [294-316	0	3149.6693
[R].YCELCA	2xCarbamido	4.689E-05	4.225E-05	1	1	1	P24043	P24043 [897-913]	0	2562.2136
[R].YAYVVD	2xCarbamido	3.954E-05	4.225E-05	1	1	1	Q9HC84	Q9HC84 [701-713	0	1906.9111
[K].AEAAAS	1xTMTpro [N	4.533E-06	4.225E-05	1	1	2	O43633	O43633 [198-215]	0	2093.0318
[K].YAWQG	1xTMTpro [N	3.411E-05	4.225E-05	1	1	2	Q9H0D6	Q9H0D6 [642-656	0	2068.1187
[K].GSEKPLI	1xTMTpro [K	7.124E-07	4.225E-05	1	1	2	Q9NTJ5	Q9NTJ5 [312-334	0	3052.5761
[R].GSEIEDI	1xTMTpro [K	1.013E-05	4.225E-05	1	1	1	Q9NXV6	Q9NXV6 [551-574	0	3241.8175
[K].GMFEVL	1xTMTpro [N	1.607E-05	4.225E-05	1	1	1	P42345	P42345 [2046-206	0	2094.0505
[R].ADVGDA	1xTMTpro [N	2.375E-05	4.225E-05	1	1	1	Q9P2E5	Q9P2E5 [401-416	0	2019.0678
[R].YDDMLV	1xTMTpro [K	3.601E-05	4.225E-05	1	1	1	Q96GX9	Q96GX9 [155-171	0	2613.3977
[R].GSDVIIM	1xTMTpro [K	4.043E-05	4.225E-05	1	2	2	P20340	P20340 [116-127]	0	1854.1014
[K].GSDHSA	1xTMTpro [N	9.31E-06	4.225E-05	1	1	1	Q9NR45	Q9NR45 [247-264	0	2171.1263
[K].YEASILT	1xTMTpro [N	5.183E-05	4.225E-05	1	1	3	P02679	P02679 [122-134]	0	1795.9509
[K].ADSPSID	1xTMTpro [K	3.588E-07	4.225E-05	1	1	1	Q08AM6	Q08AM6 [741-757	0	2571.3586
[K].ADSQDA	2xTMTpro [K	7.874E-07	4.225E-05	1	1	1	Q02952	Q02952 [1585-16	1	4160.0571
[R].YDYEEV	1xTMTpro [K	6.44E-06	4.225E-05	1	1	2	P36871	P36871 [428-440]	0	2125.0581
[R].YDYCDIL	4xCarbamido	3.219E-06	4.225E-05	1	1	2	P00747	P00747 [173-196]	0	3684.5189
[R].GSAVVG	1xTMTpro [N	4.836E-06	4.225E-05	1	1	3	O95479	O95479 [306-321]	0	2062.0525
[R].YDSIPVS	1xTMTpro [N	2.134E-06	4.225E-05	1	1	1	Q5JS54	Q5JS54 [58-82]	0	2889.4648
[R].YDQDLC	1xCarbamido	2.625E-05	4.225E-05	1	1	1	Q15029	Q15029 [161-177]	0	2513.1825
[K].YDPSLKI	1xTMTpro [K	9.623E-06	4.225E-05	1	1	2	P00918	P00918 [40-58]	0	2748.5064
[R].GSCSTE	1xCarbamido	1.488E-05	4.225E-05	1	1	1	O75348	O75348 [67-80]	1	2524.3381
[R].YASICQ	1xCarbamido	7.701E-07	4.225E-05	2	2	3	P04075; P09	P04075 [174-201]	1	3785.0188
[R].GSCYPA	1xCarbamido	1.422E-05	4.225E-05	1	1	2	P55268	P55268 [45-58]	0	1769.9175
[K].YDMVTD	1xCarbamido	2.511E-05	4.225E-05	1	1	2	Q9NRN5	Q9NRN5 [129-143	0	2112.0061
[K].GSDCGI	1xCarbamido	2.206E-06	4.225E-05	1	1	2	P49419	P49419 [475-500]	0	3114.6021
[K].YDILASE	1xTMTpro [N	1.81E-06	4.225E-05	1	1	1	O95400	O95400 [63-86]	0	2810.4015
[K].ADTLTPE	1xCarbamido	5.296E-05	4.225E-05	1	1	2	Q16181	Q16181 [196-209]	1	2607.4269

[R].GMGLSM	1xCarbamido	4.595E-05	4.225E-05	1	1	1	P28062	P28062 [164-178]	0	2238.1035
[K].YDGSTIV	1xCarbamido	1.208E-06	4.225E-05	1	1	1	Q14019	Q14019 [31-57]	0	3417.5977
[R].YDGQVA	1xTMTpro [K	3.359E-06	4.225E-05	1	1	6	P22314	P22314 [451-465]	0	2264.2054
[K].YDGNHII	1xTMTpro [N	3.632E-05	4.225E-05	1	1	1	Q14315	Q14315 [1840-18	0	2667.3486
[K].GSDGAF	1xTMTpro [K	2.951E-06	4.225E-05	1	1	4	Q9NZ08	Q9NZ08 [552-573	0	2970.5857
[R].YDFGIYD	1xTMTpro [N	8.169E-06	4.225E-05	1	1	1	Q8IXB1	Q8IXB1 [120-136]	0	2378.1723
[K].YDMVTD	1xCarbamido	3.747E-05	4.225E-05	1	1	2	Q9NRN5	Q9NRN5 [129-14	0	2128.001
[R].ADSGPT	1xTMTpro [K	1.544E-05	4.225E-05	1	1	1	O15027	O15027 [2071-20	0	2501.3743
[R].YASICQ	1xCarbamido	1.874E-06	4.225E-05	2	2	1	P04075; P0	P04075 [174-200]	0	3628.9177
[K].YASEDA	1xTMTpro [K	5.984E-05	4.225E-05	1	1	1	Q6IQ22	Q6IQ22 [143-156]	0	2130.1938
[K].YAEQLP	2xTMTpro [K	2.295E-05	4.225E-05	1	1	2	Q92643	Q92643 [345-360]	0	2775.6701
[R].YAEEQC	1xTMTpro [N	1.505E-05	4.225E-05	1	2	2	O00203	O00203 [105-122]	0	2416.2679
[R].GSFQCG	3xCarbamido	1.837E-06	4.225E-05	1	1	2	P49747	P49747 [202-223]	0	2704.1986
[R].YADLTEI	1xCarbamido	2.953E-08	4.225E-05	1	1	6	P18669	P18669 [142-162]	1	3033.5694
[R].YADLTEI	1xCarbamido	6.596E-06	4.225E-05	1	1	3	P18669	P18669 [142-157]	0	2477.2725
[K].GSFSDT	1xTMTpro [K	3.913E-05	4.225E-05	1	1	3	Q9Y2W1	Q9Y2W1 [376-38	0	1748.9311
[K].GSFSEQ	1xTMTpro [N	1.645E-05	4.225E-05	1	1	4	Q15084	Q15084 [374-386]	0	1787.9247
[R].YAAVTQ	1xTMTpro [N	3.696E-05	4.225E-05	1	2	1	P55786	P55786 [173-185]	0	1746.8982
[K].YAATSQ	1xCarbamido	1.139E-06	4.225E-05	1	1	2	P01871	P01871 [65-89]	1	3688.9859
[K].YAATSQ	1xTMTpro [K	5.348E-05	4.225E-05	2	2	4	P0DOX6; P0	P0DOX6 [190-20	0	1886.1243
[K].YAASSYI	1xTMTpro [K	6.831E-06	4.225E-05	3	7	15	A0M8Q6; P0	A0M8Q6 [66-80];	0	2352.2731
[R].YAALSD	1xTMTpro [K	2.14E-05	4.225E-05	1	1	1	Q8WX93	Q8WX93 [1348-1	0	1902.0828
[K].GSFTYF	1xTMTpro [N	2.929E-06	4.225E-05	1	1	2	Q15063	Q15063 [130-150]	0	2709.2752
[R].YAACNA	1xCarbamido	2.174E-06	4.225E-05	1	1	1	O00410	O00410 [417-436]	0	2755.3828
[K].GSFTYF	1xTMTpro [N	4.54E-07	4.225E-05	1	1	2	Q15063	Q15063 [130-151]	1	2865.3763
[R].AEDGSV	1xTMTpro [N	5.693E-07	4.225E-05	1	1	10	P07355	P07355 [180-196]	0	2213.0893
[K].WYVDG	1xTMTpro [K	2.187E-05	4.225E-05	1	1	4	P01860	P01860 [207-218]	0	2025.1049
[R].WYQMG	1xCarbamido	2.132E-05	4.225E-05	1	1	2	P00734	P00734 [582-599]	1	2752.3467
[R].WYQMG	1xCarbamido	1.682E-05	4.225E-05	1	1	1	P00734	P00734 [582-596]	0	2147.9962
[R].AEDLEQ	1xTMTpro [N	1.81E-06	4.225E-05	1	1	1	B7ZBB8	B7ZBB8 [171-196	0	2792.5477
[K].AEDLFVI	1xTMTpro [K	1.173E-05	4.225E-05	1	1	1	P02549	P02549 [2041-20	0	1914.0617
[R].YAESGD	1xTMTpro [N	5.226E-05	4.225E-05	1	1	1	P24557	P24557 [169-180]	0	1675.8247
[R].YAFGQE	1xTMTpro [N	5.807E-06	4.225E-05	1	1	1	O43678	O43678 [69-89]	0	2675.3385

[K].AECEILM	1xCarbamido	1.685E-05	4.225E-05	1	1	1	Q9BUJ2	Q9BUJ2 [419-434	0	2298.2515
[R].AEATTLH	1xTMTpro [N	2.162E-06	4.225E-05	1	1	1	P07360	P07360 [70-90]	0	2463.2985
[R].YAQTPA	1xCarbamido	2.911E-05	4.225E-05	1	1	2	P10153	P10153 [125-141]	0	2354.1229
[R].YAQTPA	1xCarbamido	1.384E-05	4.225E-05	1	1	1	P10153	P10153 [125-141]	0	2338.1279
[K].YAQDFG	2xCarbamido	6.199E-07	4.225E-05	1	1	5	P53634	P53634 [310-332]	0	3263.5521
[R].YAPTEV	1xTMTpro [K	3.399E-05	4.225E-05	1	1	1	O75369	O75369 [1788-18	0	2233.2295
[K].AEAGAG	1xTMTpro [N	1.67E-05	4.225E-05	1	2	2	P46783	P46783 [140-153]	0	1745.8778
[K].AEAGEA	1xCarbamido	4.836E-06	4.225E-05	1	1	2	O95671	O95671 [260-276	0	2061.9579
[K].YAPSGF	1xTMTpro [K	3.079E-06	4.225E-05	1	1	4	O75083	O75083 [66-81]	0	2227.1891
[K].AEAGPE	1xTMTpro [K	8.476E-06	4.225E-05	1	1	2	Q9Y4L1	Q9Y4L1 [670-685	0	2117.137
[R].YAPSEA	1xTMTpro [N	4.45E-06	4.225E-05	1	1	3	P21333	P21333 [1832-184	0	1892.9496
[R].AEAGVP	1xTMTpro [N	2.493E-05	4.225E-05	1	1	4	P21333	P21333 [2251-226	0	1837.9768
[K].YASFQV	1xTMTpro [N	4.817E-05	4.225E-05	1	1	2	P53992	P53992 [723-734]	0	1789.8676
[K].YAPISG	1xTMTpro [K	9.606E-06	4.225E-05	1	1	2	P24821	P24821 [927-942]	0	2263.2214
[R].GSFACQ	2xCarbamido	2.275E-05	4.225E-05	1	1	4	Q12805	Q12805 [195-207	0	2108.0549
[K].GSFEEL	1xCarbamido	2.198E-05	4.225E-05	1	1	2	Q9ULC5	Q9ULC5 [604-616	0	1869.9448
[K].GMDQGI	1xCarbamido	2.308E-05	4.225E-05	1	1	2	Q96AY3	Q96AY3 [213-226	0	1824.8726
[R].GSFEFP	1xTMTpro [K	1.709E-05	4.225E-05	2	2	2	P0C0L4; P0	P0C0L4 [917-929	0	1948.0672
[K].AEAQAQ	1xTMTpro [K	1.76E-05	4.225E-05	1	1	3	Q14624	Q14624 [99-111]	0	1916.0733
[R].GSFGEL	1xTMTpro [N	3.113E-05	4.225E-05	2	2	2	P31323; P13	P31323 [219-232]	0	1859.9645
[K].YAGKDG	2xTMTpro [K	4.427E-05	4.225E-05	1	1	1	P31949	P31949 [24-36]	1	2392.3329
[K].AEASIQA	1xTMTpro [K	7.366E-07	4.225E-05	1	1	1	Q09666	Q09666 [5687-57	0	2671.3343
[K].YAGEPV	1xTMTpro [N	8.554E-06	4.225E-05	1	1	2	P42704	P42704 [1365-138	0	3018.5208
[R].YAFSGV	1xTMTpro [K	1.786E-05	4.225E-05	1	1	2	P23919	P23919 [98-109]	0	1827.0296
[K].GSEVGF	1xTMTpro [K	4.337E-05	4.225E-05	1	1	1	Q09666	Q09666 [5529-55	0	2179.2003
[R].GMLDM	1xCarbamido	8.816E-07	4.225E-05	1	1	1	P10155	P10155 [515-531]	0	2175.0568
[R].YECVAA	1xCarbamido	4.705E-05	4.225E-05	1	1	2	Q9NR99	Q9NR99 [2120-21	0	1713.8913
[K].ADRDQY	1xCarbamido	2.144E-05	4.225E-05	1	1	5	P02787	P02787 [237-251]	1	2186.0831
[R].GQVEQA	1xTMTpro [K	2.897E-06	4.225E-05	1	1	2	P55268	P55268 [1503-152	0	2649.4703
[K].YFLQATI	1xTMTpro [N	4.677E-07	4.225E-05	1	1	1	Q6ZXV5	Q6ZXV5 [468-488	0	2673.3527
[R].YFLNLVL	1xTMTpro [N	7.314E-06	4.225E-05	1	1	1	O75436	O75436 [272-285	1	2085.13
[R].YFILPDS	1xTMTpro [K	8.26E-06	4.225E-05	1	1	2	P62314	P62314 [67-86]	0	2895.6615
[K].ADIDVSC	1xTMTpro [K	2.52E-05	4.225E-05	1	1	1	Q09666	Q09666 [1739-17	0	3120.5716

[R].GQVGGI	1xOxidation [	1.766E-05	4.225E-05	1	1	1	P02533	P02533 [262-282]	0	2406.189
[K].YFKPGM	1xOxidation [	4.022E-05	4.225E-05	1	1	1	P01024	P01024 [363-386]	0	3373.7245
[K].YFKPGM	1xTMTpro [K	1.709E-06	4.225E-05	1	1	1	P01024	P01024 [363-386]	0	3357.7296
[R].YFHVVIA	1xTMTpro [K	1.589E-05	4.225E-05	1	1	1	P61088	P61088 [34-53]	0	2804.4903
[R].GQVGGI	1xTMTpro [N	6.59E-07	4.225E-05	1	1	1	Q04695	Q04695 [231-251]	0	2418.2254
[R].YFEITDE	1xTMTpro [K	4.36E-05	4.225E-05	1	1	2	Q9ULV4	Q9ULV4 [292-311	0	3048.5486
[K].YFEIQFS	1xTMTpro [K	5.401E-05	4.225E-05	1	1	1	Q12965	Q12965 [173-188]	0	2336.2054
[K].YFDTVPY	1xCarbamido	1.061E-05	4.225E-05	1	1	3	Q15393	Q15393 [329-343]	0	2293.258
[R].ADIEVAC	1xCarbamido	2.206E-05	4.225E-05	1	1	3	P05198	P05198 [193-209]	0	2481.2827
[K].YFDLGLI	1xTMTpro [K	3.02E-07	4.225E-05	1	1	2	P48735	P48735 [81-106]	1	3532.8415
[R].YFAYDC	1xCarbamido	1.247E-05	4.225E-05	1	1	1	P07942	P07942 [165-183]	0	2765.3446
[R].ADLFYDY	1xTMTpro [K	1.521E-05	4.225E-05	1	1	4	P04196	P04196 [220-235]	0	2433.3045
[R].YFAYDC	1xCarbamido	9.057E-06	4.225E-05	1	1	1	P07942	P07942 [165-183]	0	2749.3497
[K].ADIGCTF	1xCarbamido	1.471E-07	4.225E-05	1	1	1	P01024	P01024 [623-657]	1	4146.033
[R].YFAGTM	1xTMTpro [N	1.636E-05	4.225E-05	1	1	1	Q15493	Q15493 [113-129]	0	2160.0966
[K].YFAEAL(	1xTMTpro [K	4.043E-05	4.225E-05	1	2	1	P50225	P50225 [23-48]	0	3548.9285
[K].GQVCLP	1xCarbamido	3.362E-07	4.225E-05	1	2	3	P68036	P68036 [83-100]	0	2910.6691
[K].YFLVGA(	1xCarbamido	9.673E-07	4.225E-05	1	1	3	P22314	P22314 [471-485]	0	2219.2754
[R].ADLAEEY	1xTMTpro [K	3.358E-05	4.225E-05	1	1	1	P68036	P68036 [123-133]	1	1905.0209
[R].YFPAGN	2xTMTpro [K	3.316E-06	4.225E-05	1	1	2	Q9H4G4	Q9H4G4 [133-154	1	3407.882
[R].ADGFGD	1xCarbamido	4.135E-05	4.225E-05	1	1	1	Q96F10	Q96F10 [43-65]	0	3096.6109
[R].ADGGPE	1xTMTpro [N	1.13E-06	4.225E-05	1	1	2	Q86UU1	Q86UU1 [627-651	0	2598.3694
[K].YGDSGE	1xTMTpro [K	2.057E-05	4.225E-05	1	1	2	P10619	P10619 [430-442]	0	1979.073
[K].YGDLLP/	1xTMTpro [K	1.808E-05	4.225E-05	2	2	1	P20020; P23	P20020 [220-237]	0	2523.4314
[K].ADGLAV	1xTMTpro [K	3.328E-05	4.225E-05	1	1	12	P00915	P00915 [139-150]	0	1795.1007
[K].ADGLAV	1xOxidation [	4.412E-05	4.225E-05	1	1	3	P00915	P00915 [139-150]	0	1811.0956
[R].ADGNQL	1xTMTpro [N	5.618E-05	4.225E-05	1	1	1	Q6YHK3	Q6YHK3 [375-385	0	1549.8141
[K].GQSVQ(	1xTMTpro [K	4.997E-05	4.225E-05	1	1	1	P13533	P13533 [415-430]	0	2320.3157
[K].GMTRPL	1xCarbamido	4.954E-07	4.225E-05	1	1	3	P04114	P04114 [230-252]	0	3152.6575
[R].ADGSSL	1xTMTpro [N	7.587E-07	4.225E-05	1	1	1	Q13308	Q13308 [539-559]	0	2548.2751
[R].GQVGG(	1xTMTpro [K	1.328E-06	4.225E-05	1	1	11	P08727	P08727 [227-247]	0	2622.423
[K].YGAFVK	1xTMTpro [K	4.923E-05	4.225E-05	1	1	2	Q99426	Q99426 [220-244]	0	3338.7328
[R].YGAAMA	2xCarbamido	1.648E-05	4.225E-05	1	1	1	Q99460	Q99460 [650-666]	0	2323.1853

[K].YFYVSAI	1xTMTpro [K	6.09E-05	4.225E-05	1	1	1	O95881	O95881 [139-152]	0	2257.2182
[R].YFYTGA	1xTMTpro [K	2.14E-05	4.225E-05	1	1	1	P11277	P11277 [1802-18	0	2340.2983
[K].GQTLVV	1xTMTpro [K	5.71E-05	4.225E-05	1	1	5	P27797	P27797 [88-98]	0	1828.1188
[R].ADHDFV	1xTMTpro [K	4.906E-05	4.225E-05	1	1	3	P62333	P62333 [357-369]	0	2189.1193
[K].YFVQGM	1xTMTpro [N	1.098E-05	4.225E-05	1	1	3	Q92597	Q92597 [307-322]	0	2130.0142
[R].ADHQPL	1xCarbamido	2.431E-07	4.225E-05	1	1	5	P08865	P08865 [129-155]	0	3300.6854
[K].ADKDQA	1xTMTpro [K	1.073E-07	4.225E-05	1	1	1	A0FGR8	A0FGR8 [524-546	1	3057.6712
[R].GQTQEE	1xTMTpro [K	1.741E-05	4.225E-05	1	1	1	O15234	O15234 [195-205]	0	1909.0635
[K].YFQFQE	1xTMTpro [K	6.252E-06	4.225E-05	1	1	2	O15144	O15144 [128-141]	1	2369.2017
[R].YGAATA	1xTMTpro [K	1.144E-05	4.225E-05	1	1	1	P43304	P43304 [239-254]	0	2338.2972
[K].GQVLNS	1xTMTpro [N	2.332E-06	4.225E-05	1	1	3	O00764	O00764 [54-70]	0	2267.1839
[R].GMQVIN	1xTMTpro [N	1.353E-06	4.225E-05	1	1	1	Q15031	Q15031 [119-142]	0	2877.4347
[K].ADLINNL	1xTMTpro [K	2.649E-05	4.225E-05	2	4	6	P07900; P08	P07900 [101-112]	0	1851.1195
[R].YEGILYT	1xTMTpro [K	1.862E-05	4.225E-05	1	1	2	Q8ND56	Q8ND56 [22-40]	0	2709.4842
[K].ADPAALI	1xTMTpro [K	4.427E-05	4.225E-05	1	1	1	O94788	O94788 [14-36]	0	3007.7146
[R].YEGILYT	1xTMTpro [K	5.235E-05	4.225E-05	1	1	1	Q9BX40	Q9BX40 [24-42]	0	2695.4686
[R].ADPEAA	1xTMTpro [N	5.393E-05	4.225E-05	1	1	1	Q9UBT2	Q9UBT2 [211-225	0	1946.9415
[K].ADPIVGS	1xTMTpro [K	1.137E-05	4.225E-05	1	1	1	Q96KP1	Q96KP1 [768-787	0	2806.4947
[R].ADPNHL	1xCarbamido	6.831E-06	4.225E-05	1	1	1	O75351	O75351 [371-403]	0	4172.027
[R].GRPITMI	1xTMTpro [N	8.826E-06	4.225E-05	1	1	1	Q9HC35	Q9HC35 [233-251	0	2528.2774
[K].YEFGAC	1xCarbamido	4.257E-06	4.225E-05	1	1	1	Q7Z3D6	Q7Z3D6 [95-115]	0	2989.5109
[K].GMMIDE	1xTMTpro [K	3.253E-05	4.225E-05	1	2	1	Q9UL03	Q9UL03 [625-641	0	2460.2395
[R].YEEIVKE	2xTMTpro [K	2.701E-05	4.225E-05	1	2	2	P68104	P68104 [167-179]	1	2513.4683
[K].ADNIEVF	1xTMTpro [K	1.806E-06	4.225E-05	1	1	1	P29144	P29144 [489-506]	0	2562.4172
[R].ADQELLI	1xCarbamido	2.444E-07	4.225E-05	1	1	2	P62879	P62879 [257-280]	0	3044.5141
[R].ADQELM	1xCarbamido	3.557E-05	4.225E-05	1	1	1	P62873	P62873 [257-280]	0	3340.6685
[K].YEDICPS	1xCarbamido	1.3E-05	4.225E-05	2	3	2	Q9GZV4; P6	Q9GZV4 [69-85];	0	2641.3246
[R].YEDGTL	1xTMTpro [K	4.21E-06	4.225E-05	1	1	1	P04114	P04114 [1537-155	0	2736.4799
[R].GSAADS	1xTMTpro [N	1.587E-06	4.225E-05	1	1	1	P13716	P13716 [91-109]	0	2270.1948
[K].GSAAST	1xCarbamido	3.465E-05	4.225E-05	1	1	1	Q0ZGT2	Q0ZGT2 [661-674	0	2046.1397
[K].GMLDNP	1xCarbamido	2.287E-05	4.225E-05	1	1	2	Q9Y4G6	Q9Y4G6 [1377-14	0	3698.752
[R].ADQQYE	1xCarbamido	9.677E-06	4.225E-05	1	1	1	Q00534	Q00534 [9-26]	0	2596.2845
[R].YEDEEC	1xCarbamido	5.331E-05	4.225E-05	1	1	2	P35555	P35555 [962-974]	0	1856.9019

[K].ADRDES	1xTMTpro [N	3.716E-07	4.225E-05	1	1	1	P62263	P62263 [64-84]	1	2569.2636
[K].ADRDES	1xOxidation [	1.828E-05	4.225E-05	1	1	2	P62263	P62263 [64-84]	1	2585.2585
[R].ADQELM	1xCarbamido	4.73E-06	4.225E-05	1	1	1	P62873	P62873 [257-280]	0	3324.6736
[R].ADGEYW	1xTMTpro [K	3.886E-07	4.225E-05	1	1	2	P55083	P55083 [106-123]	0	2710.4882
[K].ADNFEY	1xTMTpro [N	1.156E-05	4.225E-05	1	1	2	P36871	P36871 [471-486]	0	2075.9841
[R].YEHALS	1xCarbamido	3.545E-05	4.225E-05	1	1	1	Q14203	Q14203 [670-683	0	2307.1935
[K].YEVSVIF	1xTMTpro [N	3.406E-07	4.225E-05	1	1	1	Q99715	Q99715 [788-814	0	3187.6118
[R].GQVPPL	1xTMTpro [K	5.244E-05	4.225E-05	1	1	1	P53992	P53992 [351-364]	0	2121.2927
[R].ADLNQG	1xTMTpro [N	5.098E-05	4.225E-05	1	1	1	Q96C19	Q96C19 [63-77]	0	1872.9735
[K].GQVQEV	1xTMTpro [N	4.312E-06	4.225E-05	1	1	1	P17858	P17858 [445-461]	0	2198.1426
[R].YESSALI	1xTMTpro [N	6.946E-06	4.225E-05	1	1	1	Q12931	Q12931 [470-489	0	2450.237
[K].ADIQMPI	1xCarbamido	1.673E-05	4.225E-05	1	1	1	Q13740	Q13740 [212-231	0	2858.4349
[R].ADLQNH	1xTMTpro [K	1.19E-06	4.225E-05	1	1	1	Q15075	Q15075 [663-679	0	2503.3396
[R].YESHPV	1xCarbamido	3.317E-05	4.225E-05	1	1	2	Q9NX63	Q9NX63 [177-189	0	2126.1196
[K].YESDMV	2xCarbamido	7.673E-06	4.225E-05	1	1	1	P42704	P42704 [705-723]	0	2497.1845
[K].YEQGTG	1xCarbamido	2.88E-05	4.225E-05	1	1	4	P14314	P14314 [465-477]	0	1856.8669
[R].YEGSYA	1xTMTpro [N	2.682E-05	4.225E-05	1	1	2	O95810	O95810 [395-408	0	1908.9146
[K].YEQETLI	1xTMTpro [K	9.483E-06	4.225E-05	1	1	1	Q9NW15	Q9NW15 [59-76]	0	2707.4434
[R].ADLTAEI	1xTMTpro [N	5.926E-05	4.225E-05	1	1	1	Q9H3Q1	Q9H3Q1 [20-35]	0	2011.0489
[K].YEPFSF/	1xCarbamido	1.036E-05	4.225E-05	1	1	1	P54578	P54578 [401-429]	0	3641.6926
[K].GMQELG	1xCarbamido	4.66E-07	4.225E-05	1	1	2	P42704	P42704 [464-492]	0	3632.6957
[K].GQWGT	2xCarbamido	5.252E-05	4.225E-05	1	1	1	O43866	O43866 [43-63]	1	3058.537
[R].GQWGT	2xCarbamido	2.011E-06	4.225E-05	1	1	1	Q08380	Q08380 [43-63]	0	2756.3092
[K].YEIMDG/	1xTMTpro [K	2.781E-06	4.225E-05	1	1	1	O75436	O75436 [233-249	1	2483.3824
[RK].GQYFG	1xTMTpro [K	6.941E-07	4.225E-05	2	2	2	P31323; P13	P31323 [347-361]	0	2301.3211
[R].GQYQGI	1xTMTpro [K	1.173E-06	4.225E-05	1	1	2	Q9NYU2	Q9NYU2 [1436-14	0	4072.1054
[R].GQYQGI	1xOxidation [	1.595E-05	4.225E-05	1	1	1	Q9NYU2	Q9NYU2 [1436-14	0	4088.1003
[K].GRPGPQ	1xCarbamido	3.803E-06	4.225E-05	1	1	2	P00748	P00748 [65-84]	0	2632.2646
[K].GQWGT	1xCarbamido	2.979E-05	4.225E-05	1	1	3	O43866	O43866 [43-56]	0	2245.1203
[K].GSGDSS	1xTMTpro [N	1.519E-05	4.225E-05	1	1	1	P05106	P05106 [99-113]	0	1836.9371
[R].AAVENLI	1xTMTpro [N	4.487E-05	4.225E-05	1	1	2	Q14974	Q14974 [28-42]	0	1963.1183
[R].AAVEEG	1xCarbamido	2.695E-06	4.225E-05	1	1	5	P10809	P10809 [430-446]	0	1989.1122
[R].GPMTGC	1xTMTpro [N	4.194E-06	4.225E-05	1	1	1	Q9H0L4	Q9H0L4 [469-493	0	2587.3734

[R].GNWGH	1xTMTpro [N	4.33E-05	4.225E-05	1	1	2	P23141	P23141 [187-199]	0	1740.9465
[R].YTAILNQ	1xTMTpro [N	2.183E-05	4.225E-05	1	1	1	Q05707	Q05707 [1635-16	0	2091.1518
[R].AAEWQL	1xTMTpro [N	4.106E-05	4.225E-05	1	1	2	Q9NVZ3	Q9NVZ3 [32-45]	0	1934.968
[R].YTAGIHC	1xTMTpro [N	5.464E-05	4.225E-05	1	1	1	Q8IUX7	Q8IUX7 [620-632	0	1690.9196
[K].AAFDDA	1xTMTpro [K	1.509E-06	4.225E-05	1	1	7	P62258	P62258 [197-215]	0	2696.3798
[K].YTAESSI	1xCarbamido	3.489E-05	4.225E-05	1	1	2	Q9NSE4	Q9NSE4 [993-10(	0	1703.823
[R].YSVTGP	1xTMTpro [K	2.597E-06	4.225E-05	1	1	1	P19022	P19022 [195-227]	0	4047.241
[K].AAFGEE	1xTMTpro [N	5.305E-06	4.225E-05	1	1	1	O00273	O00273 [214-229	0	1940.9885
[R].AAFGISD	1xTMTpro [N	6.946E-06	4.225E-05	1	1	1	Q9UQ35	Q9UQ35 [137-15	0	2323.1162
[R].AAFGLSI	1xCarbamido	1.131E-05	4.225E-05	1	1	2	P31040	P31040 [76-92]	0	2352.2513
[R].AAFGQG	2xCarbamido	8.052E-08	4.225E-05	1	1	2	Q08380	Q08380 [77-104]	0	3521.7206
[R].AAFGQG	2xCarbamido	6.252E-06	4.225E-05	1	1	2	Q08380	Q08380 [77-104]	0	3537.7155
[K].YSVSEEI	2xTMTpro [K	5.149E-05	4.225E-05	1	1	1	Q9BZF9	Q9BZF9 [1016-10	1	2110.2212
[R].YSVQLL	1xTMTpro [K	3.853E-05	4.225E-05	1	1	6	Q9Y265	Q9Y265 [405-418	0	2139.3033
[R].YSTSGS	1xTMTpro [K	3.168E-05	4.225E-05	1	1	1	Q16891	Q16891 [33-45]	0	1854.01
[K].YSTIGLA	1xTMTpro [N	1.467E-05	4.225E-05	1	1	2	P02549	P02549 [2233-22	0	2557.3734
[K].GPNVVG	1xTMTpro [K	2.287E-05	4.225E-05	1	1	1	P03886	P03886 [36-54]	0	2582.4296
[R].AAFQSLI	1xTMTpro [K	7.045E-08	4.225E-05	1	1	2	Q9UBI1	Q9UBI1 [31-52]	0	2975.597
[K].GPPAPT	2xTMTpro [K	4.213E-05	4.225E-05	1	1	1	Q9UL03	Q9UL03 [681-701	0	3131.8761
[R].YSSQDA	1xTMTpro [K	4.729E-05	4.225E-05	1	1	1	Q9C0C2	Q9C0C2 [918-932	0	2484.1811
[R].YTCQGN	2xCarbamido	1.238E-05	4.225E-05	1	1	1	P13671	P13671 [744-763]	0	2951.4523
[R].GPLTSG	1xCarbamido	2.503E-06	4.225E-05	1	1	2	O43237	O43237 [199-233	0	4237.3034
[K].AAESLAI	1xTMTpro [K	3.499E-06	4.225E-05	1	1	4	P35606	P35606 [784-802]	0	2673.4267
[R].GPITSA	1xTMTpro [N	7.505E-06	4.225E-05	1	1	1	P17813	P17813 [154-171]	0	2199.2304
[K].GPAFVN	1xTMTpro [N	4.55E-06	4.225E-05	1	1	2	Q9Y305	Q9Y305 [212-231	0	2574.3411
[K].YTPVQQ	1xTMTpro [K	1.495E-06	4.225E-05	1	1	4	P21333	P21333 [937-958]	0	2894.5908
[R].AAEEED	1xTMTpro [K	1.835E-05	4.225E-05	1	1	4	P20962	P20962 [82-93]	1	1968.0166
[K].YTPTQQ	1xTMTpro [K	1.51E-05	4.225E-05	1	1	2	O75369	O75369 [910-931	0	3015.6105
[K].YTPEEIA	1xTMTpro [N	3.689E-05	4.225E-05	1	1	2	P09972	P09972 [244-258]	0	1970.0588
[R].YTNLSYI	1xCarbamido	2.194E-06	4.225E-05	1	1	1	P28482	P28482 [25-48]	0	3297.6052
[R].YTLSYT	1xOxidation	7.22E-06	4.225E-05	1	1	1	P98160	P98160 [1417-14	0	4506.2782
[K].YTLPPG	1xOxidation	5.841E-05	4.225E-05	1	1	2	P04792	P04792 [142-171]	0	3722.9694
[K].YTLPPG	1xTMTpro [K	5.115E-05	4.225E-05	1	1	2	P04792	P04792 [142-171]	0	3706.9745

[R].AAEGAE	1xTMTpro [N	4.01E-08	4.225E-05	1	1	1	Q9P253	Q9P253 [235-259	0	2911.4334
[K].GNVQVV	1xTMTpro [K	1.315E-05	4.225E-05	1	1	2	P22314	P22314 [605-627]	0	3129.66
[R].YTLNVLE	1xTMTpro [K	1.002E-05	4.225E-05	1	1	2	P13797	P13797 [505-518]	0	2173.1996
[K].AAELEM	1xTMTpro [K	4.389E-05	4.225E-05	1	1	1	Q9UHV9	Q9UHV9 [44-62]	0	2763.5094
[K].AAELEPI	1xTMTpro [K	1.041E-05	4.225E-05	1	1	1	Q6PGP7	Q6PGP7 [66-86]	0	2980.6275
[K].AAELIAN	1xTMTpro [N	1.121E-05	4.225E-05	1	1	3	P35232	P35232 [220-239]	0	2302.2937
[R].GPLLVQ	1xTMTpro [N	4.012E-06	4.225E-05	1	1	5	P04040	P04040 [48-66]	0	2493.2767
[R].YTIAALL	1xTMTpro [K	2.144E-05	4.225E-05	1	1	4	P02766	P02766 [125-147]	1	3097.6953
[R].YTKKVP	2xTMTpro [K	5.226E-05	4.225E-05	1	1	2	P02768	P02768 [435-452]	2	2944.7652
[K].YTGYGN	1xTMTpro [N	2.829E-05	4.225E-05	1	2	1	Q9NPQ8	Q9NPQ8 [410-42	0	1701.9243
[R].GPLLVQ	1xOxidation	9.057E-06	4.225E-05	1	1	1	P04040	P04040 [48-66]	0	2509.2716
[K].AAEMLS	1xTMTpro [N	4.271E-05	4.225E-05	1	1	2	P78318	P78318 [52-64]	0	1784.9536
[R].GPLLVQ	1xTMTpro [N	1.009E-05	4.225E-05	1	1	3	P04040	P04040 [48-68]	1	2778.4204
[R].YTLNILE	1xTMTpro [K	5.894E-06	4.225E-05	1	1	3	P13796	P13796 [502-515]	0	2143.2254
[K].YTVQDE	1xCarbamido	4.516E-06	4.225E-05	1	1	3	P63244	P63244 [140-155]	0	2286.078
[R].AAFTAFI	1xTMTpro [N	1.587E-05	4.225E-05	1	1	2	Q96CT7	Q96CT7 [171-183	0	1754.9397
[K].YSSGFV	1xTMTpro [K	3.927E-05	4.225E-05	1	1	2	Q6BCY4	Q6BCY4 [223-23	0	1927.0491
[R].AAGSSG	1xTMTpro [N	2.54E-06	4.225E-05	1	1	1	Q5T013	Q5T013 [26-50]	0	2883.4484
[K].GNVAYA	1xTMTpro [K	1.76E-06	4.225E-05	1	1	2	Q7L266	Q7L266 [178-192	0	2060.1632
[R].AAGTDG	1xTMTpro [N	4.113E-05	4.225E-05	1	1	1	Q8N5M9	Q8N5M9 [9-20]	0	1565.7628
[K].AAGVNV	1xTMTpro [K	1.462E-05	4.225E-05	1	1	4	P05386	P05386 [34-49]	0	2311.3094
[R].YSGAYG	1xTMTpro [K	3.852E-06	4.225E-05	1	1	1	Q9NX63	Q9NX63 [49-64]	1	2340.2327
[K].YSFCTDI	1xCarbamido	3.657E-05	4.225E-05	1	1	6	P02751	P02751 [398-411]	0	2031.0289
[K].YSEVFE	1xTMTpro [K	5.455E-05	4.225E-05	1	2	1	P68400	P68400 [50-64]	0	2379.2688
[R].GPPQEE	1xTMTpro [K	1.864E-06	4.225E-05	1	1	1	P51858	P51858 [209-235]	1	3650.6961
[K].AAHEAL	2xCarbamido	3.108E-05	4.225E-05	1	1	1	Q8TEX9	Q8TEX9 [716-730	0	2321.2139
[R].GPPQPL	1xTMTpro [K	2.408E-07	4.225E-05	1	1	1	Q9Y2D5	Q9Y2D5 [655-683	0	3718.9395
[K].YSEEAN	1xCarbamido	4.65E-05	4.225E-05	1	1	1	Q96HE7	Q96HE7 [120-136	0	2388.0945
[K].YSDVEV	1xCarbamido	4.04E-07	4.225E-05	1	1	2	P35611	P35611 [407-434]	0	3228.4962
[K].YSDMIV	1xOxidation	4.89E-05	4.225E-05	1	1	4	P07305	P07305 [28-40]	0	2063.1339
[R].AAHSEG	1xTMTpro [N	7.519E-06	4.225E-05	1	1	1	P78371	P78371 [467-481]	0	1834.9037
[K].YSDMIV	1xTMTpro [K	2.009E-05	4.225E-05	1	1	5	P07305	P07305 [28-40]	0	2047.1389
[R].AAIAAPII	1xTMTpro [K	5.618E-05	4.225E-05	1	1	1	P36269	P36269 [515-527]	0	1913.1828

[R].YSDIEPS	1xTMTpro [N	4.991E-06	4.225E-05	1	1	2	P07942	P07942 [194-208]	0	2046.0351
[K].AALAGG	1xTMTpro [K	1.363E-07	4.225E-05	1	1	9	Q14195	Q14195 [95-122]	0	3537.8642
[K].AALAGG	1xOxidation		2.407E-06	4.225E-05	1	1	4	Q14195	Q14195 [95-122]	0	3553.8591
[K].AALAGG	1xOxidation		1.77E-05	4.225E-05	1	1	1	Q16555	Q16555 [95-124]	0	3457.8109
[R].YSDGDL	1xCarbamido	1.27E-05	4.225E-05	1	1	1	P11717	P11717 [405-426]	0	2791.284
[R].AAGSPS	1xTMTpro [K	2.74E-05	4.225E-05	1	1	1	Q9BZE9	Q9BZE9 [497-512	0	2081.1887
[K].GNVFSS	1xTMTpro [K	1.564E-05	4.225E-05	1	1	6	Q05682	Q05682 [719-733]	0	2056.1319
[R].AAGLVS	1xTMTpro [N	8.908E-06	4.225E-05	1	1	1	Q9BW92	Q9BW92 [638-65	0	2065.1096
[K].GNVFSS	2xTMTpro [K	1.602E-07	4.225E-05	1	1	3	Q05682	Q05682 [719-739]	1	2959.6669
[R].GNVPSS	1xTMTpro [K	5.638E-06	4.225E-05	1	1	1	O00422	O00422 [49-67]	0	2761.4726
[K].YSSDYF	1xTMTpro [N	5.132E-05	4.225E-05	1	1	4	Q08380	Q08380 [442-454]	0	1902.8829
[K].YSSDED	2xTMTpro [K	1.561E-05	4.225E-05	1	1	2	P55008	P55008 [37-51]	1	2627.4385
[K].AAGCNII	1xCarbamido	4.062E-07	4.225E-05	1	1	2	Q9Y315	Q9Y315 [115-137	0	2845.5638
[R].AAGDGD	1xCarbamido	1.332E-05	4.225E-05	1	1	4	Q3LXA3	Q3LXA3 [398-410	0	1608.7356
[K].GPPDFS	1xTMTpro [N	4.065E-06	4.225E-05	2	2	1	P42167; P42	P42167 [61-86]; F	1	2874.4189
[K].AAGDVD	1xTMTpro [N	3.486E-06	4.225E-05	1	1	1	P09758	P09758 [213-228]	0	2036.9885
[R].YSQTGN	1xTMTpro [N	8.245E-06	4.225E-05	1	1	2	P39656	P39656 [283-297]	0	1976.0408
[R].AAGEVL	1xTMTpro [K	2.277E-06	4.225E-05	1	1	1	Q9Y5K8	Q9Y5K8 [225-247	1	3123.6593
[K].AAGFKD	2xTMTpro [K	4.426E-06	4.225E-05	1	1	2	P22102	P22102 [481-499]	1	2717.5654
[K].AAFTEC	2xCarbamido	2.259E-05	4.225E-05	1	1	44	P02768	P02768 [187-198]	0	1979.9811
[R].GPPGPN	1xTMTpro [N	3.409E-06	4.225E-05	1	1	1	P02452	P02452 [995-101	0	2089.082
[K].YSQKED	3xTMTpro [K	2.113E-05	4.225E-05	1	1	5	P06753	P06753 [215-227]	2	2905.63
[R].AAGGAV	2xCarbamido	1.051E-05	4.225E-05	1	1	2	Q9HC84	Q9HC84 [2346-23	0	1991.9962
[K].YSQAVP	1xTMTpro [K	1.024E-05	4.225E-05	1	1	1	P07339	P07339 [55-72]	0	2506.4413
[R].YSPSFQ	1xTMTpro [K	3.346E-05	4.225E-05	1	1	1	A0A0C4DH3	A0A0C4DH38 [79	0	2236.2105
[R].AAGGGA	1xTMTpro [N	3.255E-06	4.225E-05	1	1	3	Q9H4A6	Q9H4A6 [28-43]	0	1739.8116
[R].YSNVSS	1xTMTpro [K	3.557E-05	4.225E-05	1	1	2	O15231	O15231 [597-611]	0	2315.1721
[K].AAGGIEI	1xTMTpro [N	2.493E-06	4.225E-05	1	1	1	P46926	P46926 [127-156]	0	3228.6972
[R].YSILSES	1xTMTpro [K	1.899E-05	4.225E-05	1	1	3	P16157	P16157 [1386-14	0	2823.4578
[K].AAGLATI	1xTMTpro [N	2.407E-06	4.225E-05	1	1	1	O75533	O75533 [603-625]	0	2874.4119
[K].AAGIDE	1xTMTpro [K	1.142E-05	4.225E-05	1	1	1	P28838	P28838 [105-118]	0	2192.1228
[R].GPPGPN	1xOxidation		2.994E-05	4.225E-05	1	1	4	P02452	P02452 [995-101	0	2105.0769
[K].AAEDYG	2xTMTpro [K	8.974E-06	4.225E-05	1	1	3	Q01995	Q01995 [100-121]	1	3389.8119

[K].YVANMH	1xTMTpro [N	2.427E-05	4.225E-05	1	1	1	P14384	P14384 [78-90]	0	1752.8658
[K].GPLLED(	1xTMTpro [K	4.304E-06	4.225E-05	1	1	3	Q8NCA5	Q8NCA5 [21-44]	0	2940.581
[K].YYGLQIL	1xTMTpro [K	3.113E-05	4.225E-05	1	1	1	O14980	O14980 [77-88]	0	2061.224
[R].YYGGTE	1xCarbamido	1.616E-05	4.225E-05	1	1	4	P34896	P34896 [82-98]	0	2674.3566
[K].AAAPAPI	1xCarbamido	6.908E-06	4.225E-05	1	1	3	P26641	P26641 [254-275]	0	2965.4415
[R].YYGGAE	1xCarbamido	3.219E-06	4.225E-05	1	1	1	P34897	P34897 [105-121]	0	2318.1658
[R].YYEVLG.	1xTMTpro [N	1.592E-05	4.225E-05	1	1	1	P46379	P46379 [297-321]	1	3219.4898
[R].YYEGYY	1xTMTpro [N	4.064E-05	4.225E-05	1	1	2	Q93052	Q93052 [296-310]	0	1947.9196
[R].AAAPLF	1xTMTpro [N	2.049E-05	4.225E-05	1	1	2	Q8NDA8	Q8NDA8 [1585-16	0	2130.2031
[R].AAAPQA	1xTMTpro [N	1.075E-06	4.225E-05	1	1	1	Q16643	Q16643 [391-412]	0	2575.3047
[K].AAAQCY	1xCarbamido	3.393E-05	4.225E-05	1	1	2	P53618	P53618 [280-291]	0	1987.1542
[K].AAAQLL(	1xTMTpro [K	1.051E-05	4.225E-05	1	1	1	Q9NVA2	Q9NVA2 [400-418	0	2565.424
[K].YYEAAD	1xTMTpro [N	9.431E-06	4.225E-05	1	1	2	Q8TAQ2	Q8TAQ2 [14-28]	0	2096.0256
[K].GPCVSE	2xCarbamido	1.709E-05	4.225E-05	1	1	3	Q15436	Q15436 [430-447]	0	2588.2729
[R].GPGGNS	1xCarbamido	4.367E-07	4.225E-05	1	1	4	Q05707	Q05707 [1405-14	0	3536.7434
[K].YYDEIIN.	1xTMTpro [K	6.07E-05	4.225E-05	1	1	1	O15031	O15031 [1801-18	0	2590.3532
[K].AAASMG	1xTMTpro [N	8.139E-06	4.225E-05	1	1	1	Q96P48	Q96P48 [952-976	0	2940.4944
[R].GPGLAS	1xTMTpro [N	2.502E-05	4.225E-05	1	1	2	Q92785	Q92785 [63-77]	0	1840.9877
[R].GPCIIYN	1xCarbamido	5.384E-05	4.225E-05	1	1	2	P36578	P36578 [206-219]	0	2214.2084
[K].YYAELC.	1xCarbamido	4.817E-05	4.225E-05	1	1	1	Q96G03	Q96G03 [568-586	1	3092.6543
[K].YYADGE	1xTMTpro [K	4.915E-05	4.225E-05	1	2	2	P41227	P41227 [137-148]	0	2004.9869
[K].YWSQQI	1xTMTpro [N	6.108E-08	4.225E-05	1	1	3	P05783	P05783 [270-300]	0	3732.8775
[R].YWPQE.	1xCarbamido	7.574E-08	4.225E-05	1	1	1	P21333	P21333 [635-655]	0	2840.3633
[R].GPDAAP	1xTMTpro [N	1.996E-06	4.225E-05	1	1	1	Q5T440	Q5T440 [63-93]	0	3269.8217
[R].AAAITSD	1xTMTpro [N	1.519E-05	4.225E-05	1	1	4	Q15063	Q15063 [252-265]	0	1704.9815
[K].GPGDLE	1xTMTpro [N	1.994E-05	4.225E-05	1	1	3	Q99715	Q99715 [1381-13	0	1958.0514
[K].YYLQDG	1xTMTpro [K	1.017E-05	4.225E-05	1	1	1	P11717	P11717 [2215-22	0	2515.3212
[K].AAAAAA.	1xTMTpro [K	3.988E-05	4.225E-05	1	1	4	P36578	P36578 [354-364]	0	1564.9667
[R].AAAAAA	1xTMTpro [N	2.231E-06	4.225E-05	1	1	2	Q8NCE2	Q8NCE2 [6-38]	0	3477.7417
[R].YYWGG(	1xTMTpro [K	2.789E-05	4.225E-05	1	1	12	P02675	P02675 [446-458]	0	2277.1294
[K].AAAANL(	1xCarbamido	1.647E-07	4.225E-05	1	1	4	Q53GG5	Q53GG5 [38-68]	0	3491.6854
[R].GPEGPQ	1xCarbamido	3.359E-06	4.225E-05	1	1	3	P12109	P12109 [575-602]	0	3530.7903
[K].GPELLTI	1xTMTpro [N	2.561E-05	4.225E-05	1	1	2	P55072	P55072 [544-560]	0	2240.1341

[K].GPELLTI	1xOxidation		5.064E-05	4.225E-05	1	1	3	P55072	P55072 [544-560]	0	2256.129
[K].AAADLM.	1xCarbamido	7.787E-06	4.225E-05	1	1	1	P59768	P59768 [33-46]	0	2130.0968
[R].GPEPGF	1xTMTpro [K	1.92E-05	4.225E-05	1	1	1	Q52LJ0	Q52LJ0 [3-26]	0	3122.6211
[R].YYTSAS	1xTMTpro [K	5.701E-06	4.225E-05	1	1	2	P07900	P07900 [465-478]	0	2159.1186
[R].YWLCAA	1xCarbamido	6.051E-05	4.225E-05	1	1	3	P63244	P63244 [246-257]	0	1975.0967
[R].GPEVQL	1xTMTpro [K	1.595E-05	4.225E-05	2	2	3	P0C0L4; P0(	P0C0L4 [105-118]	0	2169.2676
[K].GPDGLT	1xTMTpro [K	1.549E-06	4.225E-05	1	1	2	P58546	P58546 [98-114]	0	2356.264
[R].AAAEVN	1xTMTpro [K	1.096E-05	4.225E-05	1	1	3	P07954	P07954 [102-115]	0	2099.1265
[K].AAAFEE	1xTMTpro [K	8.491E-06	4.225E-05	1	1	3	Q9Y490	Q9Y490 [2477-24	0	2272.2317
[K].AAAGEF.	1xCarbamido	4.923E-05	4.225E-05	1	1	1	P35221	P35221 [106-120]	0	2133.0778
[K].AAAGEL	1xCarbamido	1.546E-06	4.225E-05	1	1	2	Q96C19	Q96C19 [160-176	0	2064.0715
[K].AAAGEL	1xTMTpro [K	3.097E-05	4.225E-05	1	1	1	Q9BUP0	Q9BUP0 [158-174	0	2283.251
[K].GPDDILL	1xTMTpro [K	1.508E-05	4.225E-05	1	1	2	Q8IX05	Q8IX05 [85-103]	0	2728.3672
[K].AAAGSLI	1xTMTpro [N	2.44E-05	4.225E-05	1	1	1	Q9BQS8	Q9BQS8 [970-988	0	2184.2056
[K].YYPTAEI	2xTMTpro [K	1.021E-05	4.225E-05	1	1	2	Q15029	Q15029 [65-98]	0	4817.5845
[K].GPFVEA	1xCarbamido	3.545E-05	4.225E-05	1	1	2	Q09666	Q09666 [1819-18	0	2695.3781
[K].AAAEDV	1xTMTpro [K	5.974E-05	4.225E-05	1	1	1	Q9NQP4	Q9NQP4 [8-22]	0	2273.1905
[K].YVYIAEL	1xTMTpro [K	3.768E-06	4.225E-05	1	1	1	P27169	P27169 [234-244]	0	1928.1501
[K].YVYDSA	1xTMTpro [K	2.502E-05	4.225E-05	1	1	1	Q9BWM7	Q9BWM7 [72-85]	0	2237.137
[R].YVWLVY	1xCarbamido	2.929E-06	4.225E-05	1	1	1	P30086	P30086 [120-141]	1	3401.8172
[K].YVLNFLE	1xTMTpro [K	2.711E-05	4.225E-05	1	1	3	Q08AF3	Q08AF3 [276-286	0	1985.1352
[K].GPGPLG	1xTMTpro [K	4.689E-05	4.225E-05	1	1	2	Q16799	Q16799 [569-586]	0	2350.4142
[K].YVLINWY	1xTMTpro [N	1.879E-05	4.225E-05	1	1	1	Q16643	Q16643 [80-94]	0	2050.0929
[R].GPGTSF	1xTMTpro [K	1.625E-05	4.225E-05	1	1	2	Q99497	Q99497 [157-175]	0	2529.4208
[K].AADLNG	1xTMTpro [N	5.132E-05	4.225E-05	1	1	3	Q15293	Q15293 [177-188]	0	1521.8192
[K].AADPPA	1xTMTpro [N	2.916E-05	4.225E-05	1	1	3	P67809	P67809 [305-324]	0	2202.0118
[R].YVGGQE	1xTMTpro [N	1.065E-06	4.225E-05	1	1	9	P02763	P02763 [109-123]	0	2057.1615
[K].YVGETQ	1xTMTpro [K	1.872E-05	4.225E-05	1	1	2	Q9Y3Z3	Q9Y3Z3 [456-467	0	1929.0937
[K].AAEAAA	2xTMTpro [K	9.235E-09	4.225E-05	1	1	3	P80723	P80723 [122-149]	1	3548.8536
[K].GPHLVQ	1xTMTpro [N	1.381E-07	4.225E-05	1	1	3	P49257	P49257 [54-81]	0	3261.6724
[R].GPGNPV	1xTMTpro [K	4.785E-05	4.225E-05	1	1	1	Q6P2Q9	Q6P2Q9 [9-29]	0	2773.4726
[K].AAEAAPI	1xTMTpro [N	2.275E-05	4.225E-05	1	1	1	Q8N163	Q8N163 [447-479	0	3684.7432
[K].YVDSEG	1xTMTpro [N	2.246E-05	4.225E-05	1	1	2	Q03135	Q03135 [6-19]	0	1953.0401

[K].GPHVDV	1xTMTpro [K	7.462E-07	4.225E-05	1	1	3	Q09666	Q09666 [2417-24	0	2526.3332
[K].YVDLGG	1xTMTpro [N	2.77E-05	4.225E-05	1	1	1	P27338	P27338 [53-67]	0	1929.999
[K].AAEAGG	1xTMTpro [K	5.423E-06	4.225E-05	1	1	1	P46821	P46821 [1052-10	0	2549.3379
[K].GPLASQ	1xTMTpro [K	2.312E-05	4.225E-05	1	1	3	P21589	P21589 [148-162]	0	2215.2982
[R].AAECNI\	1xCarbamido	5.609E-05	4.225E-05	1	1	4	Q08211	Q08211 [435-446	0	1661.8964
[K].YVAVMP	1xOxidation	2.745E-06	4.225E-05	1	1	2	P30043	P30043 [146-170]	0	2991.5569
[K].YVAVMP	1xTMTpro [N	4.566E-08	4.225E-05	1	1	2	P30043	P30043 [146-170]	0	2975.562
[R].AAEDDE	1xTMTpro [K	5.235E-05	4.225E-05	1	1	3	P06454	P06454 [91-103]	0	2045.9643
[R].AAEDDE	2xTMTpro [K	8.02E-06	4.225E-05	1	1	4	P06454	P06454 [91-104]	1	2478.2664
[K].YVDTPF	2xTMTpro [K	5.246E-06	4.225E-05	1	1	2	Q13126	Q13126 [33-49]	0	2733.6007
[R].AALAHS	1xTMTpro [K	1.238E-06	4.225E-05	1	1	3	Q15149	Q15149 [2744-27	0	2392.3328
[K].YVMLPV.	1xCarbamido	2.952E-05	4.225E-05	1	1	2	P00738	P00738 [298-311]	0	2012.0264
[R].AADEEA	1xTMTpro [N	3.953E-06	4.225E-05	1	1	2	P55060	P55060 [356-371]	0	2191.9951
[R].YVWLVY	1xTMTpro [K	6.061E-05	4.225E-05	1	1	4	P30086	P30086 [120-132]	0	2317.32
[K].AAAVSL	1xTMTpro [K	3.358E-05	4.225E-05	2	2	1	Q8IVF7; Q9	Q8IVF7 [836-849]	0	2050.2404
[K].YVVVTG	1xTMTpro [K	3.282E-05	4.225E-05	1	1	1	P11586	P11586 [371-386]	0	2239.3193
[R].YVVISQ	1xTMTpro [K	1.3E-05	4.225E-05	1	1	2	Q07954	Q07954 [2000-20	0	1983.1883
[R].GPGLGS	1xTMTpro [N	7.686E-07	4.225E-05	1	1	2	Q00577	Q00577 [177-199	0	2615.4476
[R].AAAYNL	1xCarbamido	1.868E-08	4.225E-05	1	1	5	P17858	P17858 [100-129]	0	3406.7861
[K].YVTSAP	1xTMTpro [N	3.92E-05	4.225E-05	2	2	5	P0DOX6; P0	P0DOX6 [500-51	0	1904.986
[R].AACAQL	2xCarbamido	5.41E-05	4.225E-05	2	2	3	P0C0L4; P0	P0C0L4 [1725-17	0	2577.2033
[R].YVSSLT	1xTMTpro [K	1.023E-05	4.225E-05	1	1	2	P49257	P49257 [362-373]	1	2020.157
[R].AACEQL	1xCarbamido	1.796E-06	4.225E-05	1	1	1	Q00577	Q00577 [290-322	0	4043.8695
[R].YVPGSS	1xTMTpro [N	5.628E-06	4.225E-05	1	1	1	Q9Y263	Q9Y263 [477-499	0	2543.2697
[R].AACEQ	1xCarbamido	3.787E-05	4.225E-05	1	1	1	Q9BUH6	Q9BUH6 [78-93]	0	2107.0773
[K].GPAVGI	1xCarbamido	7.701E-07	4.225E-05	1	1	1	P11142	P11142 [4-25]	0	2871.5319
[K].YVRPGG	1xTMTpro [K	2.593E-05	4.225E-05	1	1	4	P22352	P22352 [121-137]	0	2563.4317
[K].YVRPGG	1xTMTpro [K	3.715E-05	4.225E-05	1	1	2	P07203	P07203 [98-114]	0	2596.3878
[R].AACQEA	1xCarbamido	3.931E-06	4.225E-05	1	1	1	Q53H12	Q53H12 [41-61]	0	2891.5693
[R].YVQLPA	1xTMTpro [N	4.385E-06	4.225E-05	1	1	3	Q9Y333	Q9Y333 [69-87]	0	2449.2894
[K].YVQGDA	1xTMTpro [K	5.287E-05	4.225E-05	1	1	1	P23743	P23743 [42-58]	0	2571.3739
[R].AADCEV	1xCarbamido	9.073E-06	4.225E-05	1	1	2	Q9UBQ7	Q9UBQ7 [26-43]	0	2668.3056
[R].AADCEV	1xCarbamido	5.265E-06	4.225E-05	1	1	1	Q9UBQ7	Q9UBQ7 [26-47]	1	3195.576

[K].YVQELPI	1xTMTpro [N	5.41E-05	4.225E-05	1	1	2	O00534	O00534 [129-142]	0	1878.0292
[K].AADDTW	1xTMTpro [K	1.028E-05	4.225E-05	1	1	4	P02766	P02766 [56-68]	0	2003.0366
[R].YVSSLTE	1xTMTpro [K	4.981E-05	4.225E-05	1	1	2	P49257	P49257 [362-372]	0	1864.0559
[K].YLNWES	1xCarbamido	7.423E-06	4.225E-05	1	1	1	Q9UBG0	Q9UBG0 [319-339	0	2826.296
[K].AALANLC	1xCarbamido	3.857E-08	4.225E-05	1	1	2	O00151	O00151 [39-69]	0	3616.8019
[R].AAIAQAL	1xTMTpro [N	6.719E-06	4.225E-05	1	1	1	Q2TAY7	Q2TAY7 [143-160	0	2040.1773
[K].YMNSGF	1xOxidation [	5.454E-06	4.225E-05	1	2	5	P22392	P22392 [67-85]	0	2717.465
[R].AASAATA	1xTMTpro [K	3.119E-06	4.225E-05	1	1	2	O00116	O00116 [63-85]	0	2691.4809
[K].AASADS	1xTMTpro [K	3.195E-06	4.225E-05	1	1	8	O75367	O75367 [168-189]	0	2735.4231
[K].YMNSGF	1xTMTpro [K	1.483E-05	4.225E-05	1	2	3	P22392	P22392 [67-85]	0	2701.4701
[R].AASAGQ	1xTMTpro [N	7.601E-07	4.225E-05	1	1	1	Q96HY6	Q96HY6 [28-46]	0	2182.1172
[R].YMNPAI	1xTMTpro [N	2.256E-06	4.225E-05	1	1	2	Q13576	Q13576 [1108-11	0	3313.6152
[R].YMNPAI	1xOxidation [	2.079E-06	4.225E-05	1	1	2	P46940	P46940 [1195-122	0	3312.6741
[R].YMNPAI	1xTMTpro [N	1.363E-06	4.225E-05	1	1	3	P46940	P46940 [1195-122	0	3296.6792
[R].YMMIVDI	1xOxidation [	1.779E-05	4.225E-05	1	1	2	P10253	P10253 [438-463]	0	3152.5352
[K].AASDIAM	1xTMTpro [N	4.917E-06	4.225E-05	1	1	12	P62258	P62258 [154-170]	0	2124.1442
[K].AASDIAM	1xOxidation [	1.303E-05	4.225E-05	1	1	9	P62258	P62258 [154-170]	0	2140.1392
[K].AASDIWI	1xTMTpro [K	1.389E-05	4.225E-05	1	1	1	Q96JM3	Q96JM3 [510-526	0	2506.4161
[R].YMMIVDI	1xTMTpro [N	3.526E-05	4.225E-05	1	1	1	P10253	P10253 [438-463]	0	3136.5403
[K].YMLLPN	1xOxidation [	7.561E-06	4.225E-05	1	1	2	O14980	O14980 [657-674]	0	2772.525
[R].YMIGVT	1xTMTpro [N	7.561E-06	4.225E-05	1	1	2	O75369	O75369 [1588-16	0	2321.1807
[K].YMHSGF	1xOxidation [	1.142E-05	4.225E-05	1	1	2	P15531	P15531 [67-85]	0	2740.481
[K].AASGFN	1xTMTpro [N	7.589E-06	4.225E-05	1	1	3	P09525	P09525 [10-24]	0	1885.9397
[K].YMHSGF	1xTMTpro [K	1.85E-06	4.225E-05	1	1	2	P15531	P15531 [67-85]	0	2724.4861
[R].GPVYIGI	1xTMTpro [N	5.813E-05	4.225E-05	1	1	2	Q9H0E2	Q9H0E2 [10-23]	0	1908.055
[R].YMGDLS	1xTMTpro [K	2.329E-05	4.225E-05	1	1	2	P30519	P30519 [157-168]	0	1876.0494
[R].AASGYP	2xTMTpro [K	2.02E-05	4.225E-05	1	1	2	P26640	P26640 [1223-124	1	3013.7026
[R].AASAAF	1xTMTpro [N	3.722E-05	4.225E-05	1	1	1	Q9BTW9	Q9BTW9 [509-52	0	1524.809
[K].YMTISGI	1xTMTpro [N	6.572E-06	4.225E-05	1	1	3	P08758	P08758 [213-227]	0	2107.0701
[K].YMTISGI	1xOxidation [	1.581E-05	4.225E-05	1	1	4	P08758	P08758 [213-227]	0	2123.065
[R].AAQVAQ	1xTMTpro [N	3.927E-05	4.225E-05	1	1	1	Q8IVM0	Q8IVM0 [196-207	0	1604.8563
[K].GPVGTV	1xTMTpro [N	3.988E-05	4.225E-05	1	1	2	Q9NZM1	Q9NZM1 [168-18	0	1588.8978
[K].YNNPAM	1xOxidation [	2.397E-05	4.225E-05	1	1	1	Q9UNS2	Q9UNS2 [367-38	0	2555.3242

[K].YNMGLP	1xTMTpro [K	1.422E-06	4.225E-05	1	1	2	Q07960	Q07960 [297-319]	0	3297.7837
[R].YNLSPSI	2xCarbamido	2.29E-06	4.225E-05	1	1	2	Q00796	Q00796 [111-131]	0	2748.3081
[R].GPVLPP	1xTMTpro [K	5.324E-06	4.225E-05	1	1	1	Q15113	Q15113 [409-434]	0	3506.9979
[R].GPVSGT	1xTMTpro [N	7.088E-06	4.225E-05	1	1	1	Q14247	Q14247 [435-454]	0	2459.172
[K].YNILGTN	1xTMTpro [K	1.969E-05	4.225E-05	1	1	4	Q00839	Q00839 [525-536]	0	1991.1127
[R].GPVSGT	1xOxidation [	2.682E-05	4.225E-05	1	1	2	Q14247	Q14247 [435-454]	0	2475.1669
[K].GPVTDV	1xCarbamido	3.425E-09	4.225E-05	1	1	2	O75083	O75083 [490-511]	0	2888.4744
[K].GPVTIPY	1xTMTpro [N	3.597E-06	4.225E-05	1	1	1	P78347	P78347 [381-409]	0	3573.8953
[K].AASLEYI	1xTMTpro [N	4.689E-05	4.225E-05	1	1	2	Q96N66	Q96N66 [289-300	0	1734.887
[K].YNHAAC	2xCarbamido	5.073E-05	4.225E-05	1	1	1	Q9BYT8	Q9BYT8 [446-465	0	2537.2349
[R].YNFPVE	1xTMTpro [N	5.581E-05	4.225E-05	1	1	2	P78527	P78527 [1988-20	0	1912.9798
[R].YNFPNP	1xOxidation [	2.167E-05	4.225E-05	1	1	2	O15371	O15371 [335-359]	1	3569.7503
[R].AAQLIGT	1xCarbamido	3.048E-07	4.225E-05	1	1	3	Q9NZL4	Q9NZL4 [165-190	0	2985.6475
[R].YNFPNP	1xTMTpro [K	1.546E-06	4.225E-05	1	1	2	O15371	O15371 [335-359]	1	3553.7553
[K].AAQNSG	1xTMTpro [K	1.206E-05	4.225E-05	1	1	1	P07942	P07942 [1662-167	0	2018.0686
[R].YNEETF	2xTMTpro [K	7.247E-06	4.225E-05	1	1	1	Q14165	Q14165 [104-126]	1	3646.9712
[K].YNDGLW	1xTMTpro [N	2.533E-05	4.225E-05	1	1	1	Q16363	Q16363 [1548-15	0	1952.035
[R].YNDFLH	1xCarbamido	5.322E-05	4.225E-05	1	1	1	Q8NHP8	Q8NHP8 [481-49	0	2230.1822
[K].AAQTAE	1xTMTpro [K	2.989E-05	4.225E-05	1	1	1	P30622	P30622 [1084-11	0	2505.2643
[K].GPVYHE	2xCarbamido	4.872E-06	4.225E-05	1	1	1	Q6UW63	Q6UW63 [130-14	0	2692.2567
[R].AAQLCG	1xCarbamido	1.188E-06	4.225E-05	1	1	2	P19367	P19367 [830-844]	0	2070.1331
[K].YNPPDH	1xTMTpro [N	3.607E-05	4.225E-05	2	2	1	Q15059; O6	Q15059 [390-402]	0	1802.9179
[K].AASLGLI	1xCarbamido	2.538E-05	4.225E-05	1	1	1	P11182	P11182 [314-339]	0	3487.8751
[R].YMEEMA	1xTMTpro [K	8.681E-06	4.225E-05	1	1	1	Q14203	Q14203 [293-311]	0	2756.3324
[R].AATITAT	1xTMTpro [N	7.888E-06	4.225E-05	1	1	1	P31323	P31323 [233-249]	0	2005.1038
[R].YLSAQK	1xTMTpro [K	4.009E-05	4.225E-05	1	1	1	P23786	P23786 [64-78]	0	2358.3425
[R].AATMSA	1xCarbamido	3.689E-05	4.225E-05	1	1	1	Q53H96	Q53H96 [255-267	0	1642.8212
[R].YLSAGP	1xTMTpro [K	4.042E-06	4.225E-05	1	1	1	Q8TAA9	Q8TAA9 [444-457	1	2235.2265
[R].YLQTLT	1xTMTpro [K	3.27E-05	4.225E-05	1	1	2	P27105	P27105 [252-263]	0	1960.1611
[K].GNLEVLI	1xTMTpro [K	4.673E-05	4.225E-05	2	2	3	Q01082; O1	Q01082 [360-372]	0	2070.2455
[K].GQDMET	2xTMTpro [K	1.173E-06	4.225E-05	1	1	2	Q15363	Q15363 [118-145]	1	4043.1068
[K].AATTEPT	1xTMTpro [K	1.333E-06	4.225E-05	1	1	2	Q9BXK5	Q9BXK5 [387-412	0	3391.8227
[R].YIQPWE	1xTMTpro [N	1.937E-05	4.225E-05	1	1	4	Q6P2Q9	Q6P2Q9 [1371-13	0	2102.0514

[R].YLQGML	1xTMTpro [N	9.442E-07	4.225E-05	1	1	2	P50479	P50479 [213-233]	0	2448.1937
[R].YLQEIYN	1xTMTpro [K	3.813E-05	4.225E-05	1	1	8	P02679	P02679 [135-146]	0	2122.1424
[R].YLQEFAI	1xTMTpro [N	1.364E-05	4.225E-05	1	1	1	Q96IJ6	Q96IJ6 [82-99]	0	2330.2253
[K].YIPQQD/	1xTMTpro [K	5.6E-05	4.225E-05	1	1	1	Q9Y4F1	Q9Y4F1 [185-195	0	1928.0621
[K].YIPPCLD	1xCarbamidc	5.252E-05	4.225E-05	1	1	2	P09486	P09486 [151-166]	0	2254.1749
[R].AAVATFI	1xTMTpro [K	3.657E-05	4.225E-05	1	1	7	P22314	P22314 [785-802]	0	2541.4572
[K].AAVAWE	2xTMTpro [K	2.425E-06	4.225E-05	1	1	3	P11766	P11766 [10-31]	0	3217.8653
[R].AAVDAG	1xOxidation	2.524E-05	4.225E-05	1	1	1	P13804	P13804 [250-268]	0	2529.3263
[R].YLPPAT(	1xTMTpro [K	3.604E-06	4.225E-05	1	1	2	P38919	P38919 [207-231]	0	3386.9253
[R].YLPEIMN	1xTMTpro [N	1.691E-05	4.225E-05	1	1	1	Q9Y625	Q9Y625 [431-461	0	3906.9867
[R].AAVEDN	1xTMTpro [K	1.822E-05	4.225E-05	1	1	2	Q8TBN0	Q8TBN0 [269-29(	0	2905.6378
[R].YLPDTLI	1xCarbamidc	5.85E-05	4.225E-05	1	1	1	P21964	P21964 [197-211]	0	2109.1585
[K].YISLIYTN	2xTMTpro [K	3.73E-08	4.225E-05	1	1	5	P09211	P09211 [104-121]	1	3067.6808
[R].YLSPDAT	1xTMTpro [N	4.697E-05	4.225E-05	1	1	3	Q8IUX7	Q8IUX7 [733-745	0	1741.9291
[R].AATHGL	1xTMTpro [N	3.369E-08	4.225E-05	1	1	1	Q96GQ5	Q96GQ5 [381-40	0	2863.5307
[K].AATGEE	1xCarbamidc	4.872E-07	4.225E-05	1	1	2	Q9HCC0	Q9HCC0 [249-26	0	2362.1264
[R].YMEDME	1xCarbamidc	3.92E-05	4.225E-05	1	1	1	Q9UKS6	Q9UKS6 [220-23	0	2413.043
[R].GQAAVQ	1xTMTpro [N	3.447E-06	4.225E-05	2	2	2	O75828; P16	O75828 [43-58]; F	0	1957.0786
[R].GQAGVN	1xTMTpro [K	6.08E-05	4.225E-05	1	1	1	P02452	P02452 [575-586]	0	1753.9915
[R].GQATDI/	1xTMTpro [K	3.557E-05	4.225E-05	1	1	1	Q16740	Q16740 [186-200	0	2226.2295
[K].AASPSEI	1xCarbamidc	3.347E-06	4.225E-05	1	1	1	Q9NZM1	Q9NZM1 [961-98	0	3356.5125
[R].YMAFAH	1xTMTpro [N	3.514E-05	4.225E-05	1	1	1	P15289	P15289 [201-214]	0	1943.9427
[R].GNLNFT	1xCarbamidc	3.353E-06	4.225E-05	1	1	1	Q15269	Q15269 [18-36]	0	2325.1577
[R].GQCDLE	2xCarbamidc	3.417E-05	4.225E-05	1	1	1	Q00610	Q00610 [924-941	0	2761.4145
[K].YLYHGQ	1xTMTpro [K	6.511E-06	4.225E-05	1	1	3	Q15582	Q15582 [448-461	0	2188.2258
[K].YIYDQCI	1xCarbamidc	4.273E-06	4.225E-05	1	1	2	P31930	P31930 [448-470]	0	3006.4627
[R].YMEEMA	1xTMTpro [K	2E-06	4.225E-05	1	1	1	Q14203	Q14203 [293-317	1	3501.6389
[R].GNLLQC	2xCarbamidc	5.41E-05	4.225E-05	1	1	2	P02751	P02751 [253-265]	0	1766.8961
[R].GQCVNT	2xCarbamidc	4.809E-05	4.225E-05	1	1	1	P35555	P35555 [1084-10	0	2120.0396
[R].YLTMDD	1xTMTpro [N	7.18E-06	4.225E-05	1	1	2	P54687	P54687 [310-324]	0	2017.0231
[R].GNILIPG	1xTMTpro [K	1.373E-07	4.225E-05	1	1	6	Q96KP4	Q96KP4 [255-275	0	2812.57
[R].YITGDQI	1xTMTpro [N	5.474E-06	4.225E-05	1	1	1	P09104	P09104 [270-285]	0	2163.1405
[R].GQDEAS	1xTMTpro [K	3.322E-06	4.225E-05	1	1	2	Q96M27	Q96M27 [204-21	0	2144.1632

[R].YLTEHPI	1xTMTpro [K	4.713E-05	4.225E-05	1	1	1	Q6P2Q9	Q6P2Q9 [1113-11	0	2839.4506
[R].YITDVLT	1xTMTpro [K	1.258E-05	4.225E-05	1	1	2	Q7L5Y1	Q7L5Y1 [154-170	0	2601.4519
[R].GQDHC(	1xCarbamido	5.335E-07	4.225E-05	1	1	3	P07858	P07858 [315-331]	0	2128.0776
[K].GNLGMN	1xCarbamido	2.72E-06	4.225E-05	1	1	1	Q92896	Q92896 [474-498]	0	3113.4951
[K].AATGEC	1xCarbamido	8.665E-06	4.225E-05	1	1	1	P01042	P01042 [102-114]	1	1930.0672
[R].YLVALGI	2xCarbamido	2.031E-05	4.225E-05	1	1	2	Q9NR12	Q9NR12 [293-313	0	3073.552
[R].AAQAPS	1xTMTpro [K	3.626E-05	4.225E-05	1	1	2	P11586	P11586 [805-819]	0	2260.2833
[R].GNPEPP	2xCarbamido	1.604E-06	4.225E-05	1	1	4	P35555	P35555 [2695-271	0	3390.542
[R].AAPVTG	1xTMTpro [N	3.276E-05	4.225E-05	1	1	2	P18583	P18583 [2301-231	0	1634.9041
[R].YQSHDY	1xTMTpro [K	4.374E-05	4.225E-05	1	1	1	Q9UHG3	Q9UHG3 [169-18	0	2169.1108
[R].GNSRPG	1xTMTpro [N	1.226E-05	4.225E-05	1	1	2	P35269	P35269 [383-402]	0	2223.1285
[R].YQQAGL	1xTMTpro [K	1.356E-05	4.225E-05	1	1	3	P21399	P21399 [759-772]	0	2079.2822
[K].YQPCAW	2xCarbamido	3.059E-05	4.225E-05	1	1	3	Q9Y6C2	Q9Y6C2 [82-99]	0	2590.196
[K].YQPCAW	2xCarbamido	6.487E-06	4.225E-05	1	1	1	Q9Y6C2	Q9Y6C2 [82-99]	0	2574.2011
[K].YQNLLPI	1xTMTpro [K	2.921E-05	4.225E-05	1	1	2	Q7Z7H8	Q7Z7H8 [140-160	0	3049.704
[R].AALMES	1xTMTpro [N	2.354E-05	4.225E-05	1	1	2	Q14980	Q14980 [986-999]	0	1908.9405
[K].GPQVSS	1xTMTpro [K	8.875E-06	4.225E-05	1	1	2	Q09666	Q09666 [5599-56	0	2025.1472
[R].GPSAGG	1xTMTpro [N	9.655E-07	4.225E-05	1	1	1	Q7Z406	Q7Z406 [33-53]	0	2260.1277
[K].YQLNLPI	1xCarbamido	5.287E-05	4.225E-05	1	1	1	Q8NCW5	Q8NCW5 [271-28	0	2332.1603
[K].YQLEKEI	1xTMTpro [K	8.602E-06	4.225E-05	1	1	1	P22033	P22033 [492-511]	1	2900.5861
[K].YQLDPT.	1xTMTpro [K	5.014E-05	4.225E-05	1	1	3	P45880	P45880 [236-247]	0	1902.0828
[K].YQIDPD/	1xCarbamido	4.825E-05	4.225E-05	1	1	2	P21796	P21796 [225-236]	0	2023.045
[K].YQLDKD	2xTMTpro [K	3.683E-05	4.225E-05	1	1	2	P07237	P07237 [196-207]	1	2337.3998
[K].GPSGCV	2xCarbamido	2.77E-05	4.225E-05	1	1	2	P47897	P47897 [653-666]	0	1854.9009
[K].YQGDGI	1xTMTpro [K	2.016E-05	4.225E-05	1	1	1	Q9NYF8	Q9NYF8 [892-911	0	2966.3705
[R].AALSGA	1xTMTpro [K	7.802E-06	4.225E-05	1	1	3	Q07954	Q07954 [2361-23	0	2008.2298
[K].YQEVTN	1xTMTpro [K	1.799E-05	4.225E-05	1	1	4	Q14444	Q14444 [99-110]	0	2064.1257
[R].AALSPL/	1xTMTpro [N	1.564E-05	4.225E-05	1	1	1	Q6B0K9	Q6B0K9 [78-92]	0	1864.1339
[R].GPSGQ(	1xTMTpro [K	8.745E-06	4.225E-05	1	1	2	Q13724	Q13724 [310-327]	0	2494.4273
[K].GPSLDID	1xTMTpro [K	1.927E-05	4.225E-05	1	1	1	Q09666	Q09666 [4423-44	0	2561.359
[R].YQVELE	1xTMTpro [K	3.626E-05	4.225E-05	1	1	1	P82094	P82094 [866-879]	1	2406.316
[K].YQVEQC	1xCarbamido	5.691E-05	4.225E-05	1	1	1	Q96SI9	Q96SI9 [137-151]	0	2126.1235
[R].YQVGVH	1xTMTpro [K	2.105E-05	4.225E-05	1	1	2	Q14204	Q14204 [1004-10	0	2332.2317

[K].YQVLDG	1xTMTpro [N	1.92E-06	4.225E-05	1	1	1	P23219	P23219 [253-276]	0	3124.5443
[R].YSDESG	1xCarbamido	1.116E-06	4.225E-05	1	1	1	P04632	P04632 [217-235]	0	2573.1608
[K].AALCHF	2xCarbamido	9.748E-06	4.225E-05	1	1	2	P54920	P54920 [209-222]	0	2272.1896
[K].AALDEA	1xTMTpro [N	1.139E-07	4.225E-05	1	1	1	O75533	O75533 [23-50]	0	3241.5092
[K].AALDGTI	1xTMTpro [K	1.228E-05	4.225E-05	1	1	1	P09417	P09417 [139-154]	0	2161.1641
[K].GNTATQ	1xTMTpro [N	2.75E-06	4.225E-05	1	1	2	P50453	P50453 [48-68]	0	2604.3007
[K].YSAEAQ	3xCarbamido	4.397E-05	4.225E-05	1	1	1	Q9HC84	Q9HC84 [5691-57	0	2578.0862
[K].AAIDWFI	2xTMTpro [K	2.009E-05	4.225E-05	1	1	1	P35637	P35637 [349-365]	1	2807.5548
[K].YSAEAQ	3xCarbamido	4.686E-07	4.225E-05	1	1	1	Q9HC84	Q9HC84 [5691-57	0	2562.0913
[R].YRPYDG	1xTMTpro [N	5.917E-05	4.225E-05	1	1	1	O15027	O15027 [1199-12	0	2170.0637
[K].GNTAAQ	1xTMTpro [K	2.984E-05	4.225E-05	1	1	1	P35237	P35237 [47-61]	0	2202.2196
[K].AALTELS	1xTMTpro [K	1.289E-06	4.225E-05	1	1	2	P04114	P04114 [1703-172	0	2846.5829
[R].YRGDQD	1xTMTpro [K	7.601E-07	4.225E-05	1	1	2	P01024	P01024 [1392-14	1	3715.8149
[K].GPQLFH	1xCarbamido	8.109E-06	4.225E-05	1	1	2	P28066	P28066 [150-168]	0	2467.1818
[R].YRDTVLI	1xTMTpro [K	3.363E-05	4.225E-05	1	1	1	Q9NSE4	Q9NSE4 [60-74]	1	2404.3554
[R].AALEEVI	1xTMTpro [K	1.119E-05	4.225E-05	1	1	1	Q15057	Q15057 [19-34]	0	2323.2888
[K].YRDLDE	1xTMTpro [N	1.587E-07	4.225E-05	1	1	1	P28289	P28289 [10-28]	1	2583.3109
[R].AALEEV	1xTMTpro [N	2.625E-05	4.225E-05	1	1	2	Q9H0D6	Q9H0D6 [660-675	0	2137.0984
[R].AALEEV	1xTMTpro [N	6.131E-07	4.225E-05	1	1	1	Q9H0D6	Q9H0D6 [660-676	1	2293.1995
[K].AAIEHEL	1xTMTpro [K	5.945E-05	4.225E-05	1	1	2	Q6ZMI0	Q6ZMI0 [449-461	0	2017.1574
[K].AALEQD	1xTMTpro [N	3.352E-05	4.225E-05	1	1	1	P09601	P09601 [87-100]	0	1941.019
[R].YQVSWS	1xTMTpro [K	5.384E-05	4.225E-05	1	1	2	P51571	P51571 [86-95]	0	1871.0307
[K].AALEYLE	1xTMTpro [K	1.773E-05	4.225E-05	1	1	6	Q9BRX8	Q9BRX8 [44-55]	0	2001.14
[K].AALEDTI	1xTMTpro [N	3.52E-05	4.225E-05	1	1	4	P08727	P08727 [318-330]	0	1693.8928
[K].YQEEFE	1xTMTpro [K	1.319E-05	4.225E-05	1	1	2	P49257	P49257 [289-302]	0	2478.2433
[R].YQEALH	1xTMTpro [N	2.87E-05	4.225E-05	1	1	1	O00231	O00231 [143-155	0	1832.035
[K].GNRPVIL	1xTMTpro [K	5.6E-05	4.225E-05	1	1	1	Q9UGV2	Q9UGV2 [52-68]	0	2555.4702
[K].YPIEHGI	1xTMTpro [K	6.026E-06	4.225E-05	1	2	2	P63261	P63261 [69-84]	0	2555.3096
[K].YPIEHGI	1xOxidation	5.287E-05	4.225E-05	2	4	51	P62736; P68	P62736 [71-86]; F	0	2585.3201
[K].GPTEQL	1xTMTpro [N	5.074E-06	4.225E-05	1	1	1	P35813	P35813 [209-226]	0	2336.2053
[R].YPLAAA	1xTMTpro [N	4.412E-05	4.225E-05	1	1	1	Q7Z5L9	Q7Z5L9 [117-128	0	1615.9239
[RK].YPHLP	1xCarbamido	2.502E-05	4.225E-05	3	4	1	Q9UKV8; Q9	Q9UKV8 [322-335	0	2305.2618
[R].YPGVSN	1xTMTpro [K	5.5E-05	4.225E-05	1	1	1	Q13564	Q13564 [442-456	0	2306.216

[R].GNPTVE	1xTMTpro [K	2.179E-05	4.225E-05	1	1	3	P06733	P06733 [16-28]	0	2015.1305
[R].AAPEAS	1xTMTpro [N	1.651E-05	4.225E-05	1	1	2	P31146	P31146 [417-432]	0	1806.9153
[K].GPTVGG	1xTMTpro [K	2.783E-07	4.225E-05	1	1	3	Q09666	Q09666 [5473-54	0	3054.7265
[K].GPVAGW	1xTMTpro [K	2.57E-05	4.225E-05	1	1	1	Q96KP4	Q96KP4 [135-149	0	2225.2574
[K].YPIEHGI	1xOxidation [	2.607E-05	4.225E-05	1	2	4	P63261	P63261 [69-84]	0	2571.3045
[R].YPEAQS	1xTMTpro [N	5.682E-05	4.225E-05	1	1	2	Q99615	Q99615 [192-204	0	1752.9451
[R].YPCDVE	3xCarbamido	4.206E-05	4.225E-05	1	1	2	Q9BZ67	Q9BZ67 [161-177	0	2331.0739
[K].YPASTV	1xTMTpro [K	1.547E-05	4.225E-05	1	1	2	O00567	O00567 [321-333	0	1985.1563
[K].YPALTKI	1xTMTpro [K	2.65E-06	4.225E-05	1	1	2	P13797	P13797 [377-398]	0	3197.6974
[R].AAPLQG	1xOxidation [	1.959E-05	4.225E-05	1	1	5	P12111	P12111 [610-625]	0	1938.153
[R].YPALHKI	1xTMTpro [K	2.17E-06	4.225E-05	1	1	4	P13796	P13796 [374-395]	0	3147.6355
[R].YNVYGY	1xTMTpro [N	1.137E-05	4.225E-05	1	1	1	Q13523	Q13523 [687-703	0	2227.1467
[K].GPVEGY	1xTMTpro [N	1.544E-05	4.225E-05	1	1	2	Q9UI30	Q9UI30 [69-82]	0	1971.9619
[R].YNVLGA	1xTMTpro [N	5.6E-05	4.225E-05	1	1	1	Q1KMD3	Q1KMD3 [481-49	0	1912.0282
[K].AAPSVTI	1xTMTpro [K	3.141E-05	4.225E-05	2	4	5	A0M8Q6; P0	A0M8Q6 [5-23]; P	0	2594.4321
[R].YNQMDS	1xTMTpro [K	1.281E-05	4.225E-05	1	1	1	Q99805	Q99805 [333-349	0	2686.2587
[R].YPDQWI	1xTMTpro [N	1.949E-06	4.225E-05	1	2	1	Q9NZJ9	Q9NZJ9 [42-65]	0	2845.3786
[K].AALANL	1xCarbamido	8.293E-07	4.225E-05	1	1	1	O00151	O00151 [39-69]	0	3632.7968
[K].YPLESG	1xTMTpro [K	1.167E-05	4.225E-05	1	1	1	Q9NQ66	Q9NQ66 [444-46	0	2645.4214
[K].AANSLE	1xTMTpro [K	2.616E-05	4.225E-05	1	1	2	Q9Y4L1	Q9Y4L1 [739-753	0	2292.2367
[R].AALVAQ	1xTMTpro [N	5.084E-06	4.225E-05	1	1	2	Q9BS40	Q9BS40 [13-30]	0	2348.243
[R].AALVDLI	1xTMTpro [N	5.841E-05	4.225E-05	1	1	2	Q9BUF5	Q9BUF5 [63-77]	0	1877.9962
[R].AALVGG	1xTMTpro [K	2.781E-06	4.225E-05	1	1	1	Q14194	Q14194 [95-122]	0	3477.9159
[K].GPSSEG	1xTMTpro [K	2.027E-05	4.225E-05	1	1	1	P84157	P84157 [125-142]	0	2493.1913
[R].YQCGFC	3xCarbamido	2.799E-05	4.225E-05	1	1	1	O94855	O94855 [383-407	0	3447.5781
[K].YQCACN	2xCarbamido	1.808E-05	4.225E-05	1	1	1	P35555	P35555 [1178-11	0	2129.9452
[K].YQAVTA	1xTMTpro [K	5.545E-05	4.225E-05	1	2	4	P40429	P40429 [149-159]	0	1861.0563
[R].GNQPVC	1xTMTpro [K	3.328E-05	4.225E-05	1	1	2	P35442	P35442 [628-640]	0	1848.0835
[R].YQAVLA	2xCarbamido	1.006E-05	4.225E-05	1	1	1	Q8WU79	Q8WU79 [12-34]	1	3641.9124
[R].AAMEAL	1xTMTpro [K	3.983E-06	4.225E-05	1	1	1	Q08211	Q08211 [1105-11	1	3429.9271
[R].GPTCNE	3xCarbamido	2.911E-05	4.225E-05	1	1	1	P55268	P55268 [1104-11	0	2027.8805
[R].YPVCGS	2xCarbamido	1.024E-05	4.225E-05	1	1	1	Q16270	Q16270 [117-134	0	2322.0814
[R].AAMIWI	1xTMTpro [N	3.059E-05	4.225E-05	2	2	1	Q10567; P6	Q10567 [446-458	0	1812.9638

[K].AANAAE	1xTMTpro [N	5.494E-06	4.225E-05	1	1	1	Q8NE71	Q8NE71 [276-294	0	2289.0737
[R].YPSPPM	1xTMTpro [N	6.048E-06	4.225E-05	1	1	2	P46109	P46109 [105-129]	0	3008.4995
[K].AANDAG	1xTMTpro [K	4.467E-06	4.225E-05	1	1	3	P42765	P42765 [192-209]	0	2563.2994
[R].GNQHD\	1xTMTpro [K	3.372E-06	4.225E-05	1	1	1	P55212	P55212 [165-184]	0	2825.5289
[R].AANILVG	1xCarbamido	3.961E-05	4.225E-05	2	2	1	P07947; P12	P07947 [399-411]	0	2009.1709
[K].YPPPTEI	1xTMTpro [N	3.569E-05	4.225E-05	1	1	2	O75643	O75643 [1295-13	0	2423.3869
[R].YPPPAV	1xTMTpro [N	1.971E-07	4.225E-05	1	1	8	P12111	P12111 [1425-145	0	3744.9193
[R].YPNIELS	1xTMTpro [K	1.423E-07	4.225E-05	1	1	1	O75643	O75643 [2014-20	1	2648.4427
[K].YPNKYE	1xCarbamido	4.609E-06	4.225E-05	1	1	2	Q10567	Q10567 [421-445	1	3534.7918
[K].GPSVFP	1xCarbamido	5.822E-05	4.225E-05	1	2	2	P0DOX5	P0DOX5 [124-149	1	3401.9283
[R].AQTLPT	1xTMTpro [K	1.774E-08	4.225E-05	1	1	3	Q01082	Q01082 [2326-23	1	2667.5173
[K].WWDLD	1xCarbamido	5.115E-05	4.225E-05	1	1	1	Q9UNX4	Q9UNX4 [175-185	0	2144.0879
[R].AEDIPQI	1xTMTpro [K	5.627E-05	4.225E-05	1	1	1	Q9NZM1	Q9NZM1 [367-38	0	2403.2358
[R].VVAQGV	1xTMTpro [N	8.665E-06	4.225E-05	1	1	2	P02760	P02760 [167-185]	0	2309.2131
[R].VVAQEA	2xCarbamido	8.263E-08	4.225E-05	1	1	2	Q96LJ7	Q96LJ7 [46-72]	0	3222.569
[K].VVAPTIS	1xCarbamido	3.479E-06	4.225E-05	1	1	3	Q9Y490	Q9Y490 [722-741	0	2444.3138
[K].VVALIGV	1xTMTpro [K	1.738E-05	4.225E-05	1	1	2	Q9NUJ1	Q9NUJ1 [169-185	0	2250.3928
[K].GTSIVVF	2xTMTpro [K	2.275E-05	4.225E-05	1	1	2	Q9NUQ7	Q9NUQ7 [190-20	1	2844.7531
[K].VVAGVA	1xTMTpro [K	1.279E-05	4.225E-05	2	2	12	P02042; P68	P02042 [134-147]	1	2058.2104
[K].VVAGVA	1xTMTpro [K	2.281E-06	4.225E-05	2	2	136	P02042; P68	P02042 [134-145]	0	1758.0882
[K].AGGAAV	1xTMTpro [K	1.852E-05	4.225E-05	1	1	1	Q9P258	Q9P258 [78-92]	0	2088.1945
[R].AGGADA	1xTMTpro [N	9.191E-06	4.225E-05	1	1	1	Q9H223	Q9H223 [15-29]	0	1676.9251
[K].VVAGDE	1xTMTpro [N	1.075E-06	4.225E-05	1	1	2	Q14764	Q14764 [137-154	0	2310.209
[R].AGGAG\	1xTMTpro [K	9.338E-07	4.225E-05	1	1	2	P55809	P55809 [147-173]	0	3203.7232
[R].VVAELQ	1xCarbamido	4.991E-06	4.225E-05	1	1	1	P49189	P49189 [434-461]	0	3694.9435
[K].VVAEEA	1xTMTpro [K	4.89E-05	4.225E-05	1	1	1	Q9Y4E8	Q9Y4E8 [417-428	0	2033.1311
[K].VVACTS	1xCarbamido	3.52E-05	4.225E-05	1	1	3	P30043	P30043 [106-120]	0	2316.2917
[K].AGGFAP	1xTMTpro [K	3.728E-05	4.225E-05	1	1	1	P02461	P02461 [1213-12	0	2373.1717
[K].VVAACA	1xCarbamido	5.728E-05	4.225E-05	1	1	2	P28331	P28331 [88-98]	0	1785.0081
[R].VTYTPM	1xOxidation	6.032E-05	4.225E-05	1	1	2	P21333	P21333 [2485-25	0	2365.3333
[R].VTYTPM	1xTMTpro [K	3.293E-05	4.225E-05	1	1	4	P21333	P21333 [2485-25	0	2349.3384
[R].AGGGA	1xTMTpro [N	2.695E-06	4.225E-05	1	1	1	Q8TD19	Q8TD19 [40-56]	0	2115.1154
[K].VTYLVHI	1xOxidation	1.754E-05	4.225E-05	1	1	1	O75874	O75874 [165-187	0	3183.5735

[R].AGGGPT	1xCarbamido	1.412E-05	4.225E-05	1	1	2	Q96N66	Q96N66 [272-288	0	2288.22
[K].AGFFGT	1xTMTpro [N	3.208E-05	4.225E-05	1	1	1	Q9NVH1	Q9NVH1 [328-341	0	1806.9346
[R].VVAQGV	1xOxidation [	2.726E-05	4.225E-05	1	1	1	P02760	P02760 [167-185]	0	2325.208
[R].VVAQST	1xTMTpro [N	9.746E-08	4.225E-05	1	1	2	Q16881	Q16881 [419-443]	0	3027.5628
[R].GTSFDA	1xTMTpro [K	7.874E-06	4.225E-05	1	1	1	P13804	P13804 [170-187]	0	2239.1334
[K].VVEIAPA	1xTMTpro [N	5.883E-06	4.225E-05	1	1	1	P11498	P11498 [274-288]	0	1933.119
[R].AGEAGK	1xTMTpro [K	3.557E-05	4.225E-05	1	1	2	Q9BR76	Q9BR76 [447-461	1	2268.2513
[R].VVECVM	2xCarbamido	5.081E-05	4.225E-05	1	1	1	P33121	P33121 [329-340]	0	2011.0783
[K].VVDYSQ	1xTMTpro [N	1.855E-05	4.225E-05	1	1	2	Q9H1E3	Q9H1E3 [10-28]	0	2542.1177
[K].AGEIITVI	1xTMTpro [K	4.642E-05	4.225E-05	1	1	2	Q92783	Q92783 [233-249]	0	2567.3637
[K].GTQCED	3xCarbamido	2.725E-06	4.225E-05	1	1	3	P35555	P35555 [905-922]	0	2751.292
[K].GTQEQD	1xTMTpro [N	8.215E-06	4.225E-05	1	1	2	P05154	P05154 [217-232]	0	2163.0889
[K].GTQGVV	1xTMTpro [N	5.509E-05	4.225E-05	1	1	1	P55884	P55884 [536-548]	0	1771.9662
[R].VVDLMA	1xTMTpro [K	8.291E-06	4.225E-05	1	1	7	P04406	P04406 [324-334]	0	1810.0211
[R].VVDLLN	1xTMTpro [K	2.989E-05	4.225E-05	1	1	2	Q92797	Q92797 [36-51]	0	2322.3524
[K].VTYHPD	1xTMTpro [N	1.646E-06	4.225E-05	1	1	2	Q15046	Q15046 [371-392]	0	2812.3538
[R].GTQLLS	1xTMTpro [K	2.098E-05	4.225E-05	1	1	1	Q12788	Q12788 [575-588]	0	1970.1414
[K].VVDLLA	1xCarbamido	3.299E-05	4.225E-05	1	1	5	P30520	P30520 [46-59]	0	1891.0278
[K].VVDLGTI	2xCarbamido	3.253E-05	4.225E-05	1	1	1	Q96PE1	Q96PE1 [184-199	0	2239.117
[K].VVDKIDV	1xCarbamido	3.514E-05	4.225E-05	1	1	1	P49747	P49747 [513-532]	1	2928.5632
[R].VVDGAV	1xTMTpro [N	2.179E-05	4.225E-05	1	1	2	P10515	P10515 [622-636]	0	1922.0455
[R].VVDALG	2xTMTpro [K	1.674E-06	4.225E-05	1	1	2	P25705	P25705 [150-167]	1	2623.5599
[R].VVDALG	1xTMTpro [K	1.744E-05	4.225E-05	1	1	2	P25705	P25705 [150-161]	0	1780.046
[K].AGEVIN	1xTMTpro [N	5.022E-05	4.225E-05	1	1	2	P18206	P18206 [890-903]	0	1822.9297
[R].VVCSSG	1xCarbamido	1.443E-05	4.225E-05	1	1	1	P16455	P16455 [148-165]	0	2333.2779
[K].GTQTYS	1xTMTpro [K	2.144E-05	4.225E-05	1	1	1	P52701	P52701 [520-537]	0	2583.307
[K].GTQVSG	1xTMTpro [N	3.866E-05	4.225E-05	1	1	1	P49746	P49746 [350-362]	0	1626.8771
[R].AGEQLA	1xTMTpro [N	2.275E-05	4.225E-05	1	1	1	H7BZ55	H7BZ55 [668-683	0	2059.1103
[K].VVENLQ	1xTMTpro [K	2.401E-05	4.225E-05	1	1	2	P40763	P40763 [164-177]	0	2354.216
[R].AGGIETI	1xTMTpro [N	1.872E-05	4.225E-05	1	1	1	P34932	P34932 [20-33]	0	1799.9095
[R].GTSVVLI	1xCarbamido	4.556E-05	4.225E-05	1	1	3	Q9UK45	Q9UK45 [78-103]	0	3132.5665
[K].VTSEDLI	1xCarbamido	1.354E-05	4.225E-05	1	1	1	Q7Z2W9	Q7Z2W9 [108-12	0	2521.3139
[R].VTSAVE	1xTMTpro [N	6.633E-06	4.225E-05	1	1	2	P45974	P45974 [147-162]	0	1881.0248

[R].AGIEGPI	1xTMTpro [N	4.518E-05	4.225E-05	1	1	2	P18583	P18583 [1750-176	0	1658.9397
[R].AGLELG.	1xTMTpro [N	1.224E-07	4.225E-05	1	1	4	Q14112	Q14112 [1169-11	0	3275.7806
[R].GLPVLVI	1xTMTpro [K	6.1E-05	4.225E-05	1	1	1	O00192	O00192 [710-724]	0	2249.3248
[K].AGLENT	1xCarbamido	2.885E-05	4.225E-05	1	1	1	P13646	P13646 [343-355]	0	1753.871
[K].VTQSAY.	1xTMTpro [K	2.551E-05	4.225E-05	1	1	1	Q9Y4W6	Q9Y4W6 [653-66	0	2522.3569
[R].GTTITLV	2xTMTpro [K	3.253E-05	4.225E-05	1	1	1	P14625	P14625 [244-265]	1	3364.9283
[K].VTQLDPI	1xTMTpro [K	2.897E-06	4.225E-05	1	1	2	Q9Y6W5	Q9Y6W5 [79-97]	1	2764.5336
[R].VTQGLV	1xCarbamido	3.751E-07	4.225E-05	1	1	3	P22749	P22749 [118-136]	0	2392.2461
[R].VTQDSL	1xTMTpro [N	1.028E-05	4.225E-05	1	1	2	Q12884	Q12884 [403-421]	0	2572.2163
[K].VTQDELI	1xTMTpro [K	7.098E-07	4.225E-05	1	1	2	P19338	P19338 [404-420]	1	2600.4063
[R].VTPVEVI	1xTMTpro [K	6.003E-05	4.225E-05	1	1	1	Q8N7H5	Q8N7H5 [199-212	0	2213.2536
[K].VTPSTVI	1xCarbamido	5.888E-05	4.225E-05	1	1	1	Q9BZE9	Q9BZE9 [26-41]	0	2135.1701
[R].VTPPEG	1xTMTpro [K	4.163E-05	4.225E-05	1	1	1	O00151	O00151 [315-329]	0	2270.2928
[K].GTTVTV	1xTMTpro [K	1.694E-05	4.225E-05	1	1	2	P0DOX2	P0DOX2 [108-122	0	2028.1469
[K].GTVAAP	1xTMTpro [K	5.207E-06	4.225E-05	1	1	1	P0DOX7	P0DOX7 [108-126	0	2611.4627
[R].VTPNLM	1xCarbamido	9.966E-06	4.225E-05	1	1	1	Q16610	Q16610 [417-430]	0	1900.9805
[K].VTPGST	2xCarbamido	2.295E-07	4.225E-05	1	2	4	P00325	P00325 [190-213]	0	2933.5543
[R].AGLNEM	1xTMTpro [N	9.518E-06	4.225E-05	1	1	1	Q14738	Q14738 [147-160]	0	1923.9918
[K].AGINQNI	1xTMTpro [K	1.706E-05	4.225E-05	1	1	1	Q9UPN3	Q9UPN3 [4990-50	0	2440.2997
[R].GTTLQS	1xTMTpro [N	4.149E-05	4.225E-05	1	1	1	Q9BZE9	Q9BZE9 [153-169	0	1937.0987
[R].AGLAAS	1xTMTpro [N	4.8E-07	4.225E-05	1	1	1	P08294	P08294 [161-176]	0	1781.0353
[K].VTSLTA	1xCarbamido	1.242E-05	4.225E-05	1	1	2	P04216	P04216 [22-35]	0	1867.0278
[K].GTTGTQ	2xTMTpro [K	1.338E-06	4.225E-05	1	1	1	P30566	P30566 [200-224]	1	3676.9639
[K].GLQAQI	1xTMTpro [K	1.718E-06	4.225E-05	1	1	3	P05783	P05783 [223-241]	0	2492.4216
[R].VTWDSS	1xCarbamido	1.832E-05	4.225E-05	1	1	2	Q9ULV4	Q9ULV4 [32-44]	0	1842.9128
[R].VTWAPF	1xTMTpro [N	4.857E-05	4.225E-05	1	1	5	P02751	P02751 [1376-13	0	2230.2555
[R].VTWAHF	1xCarbamido	1.76E-05	4.225E-05	1	1	1	Q96EE3	Q96EE3 [63-81]	0	2511.241
[R].VTVVCE	1xCarbamido	6.844E-06	4.225E-05	1	1	1	P31939	P31939 [142-164]	0	3210.6042
[K].VTVTPIY	1xTMTpro [K	3.931E-06	4.225E-05	1	1	3	Q05707	Q05707 [806-825]	0	2585.4318
[K].AGGSAS	1xTMTpro [K	1.773E-05	4.225E-05	1	1	2	P20618	P20618 [165-184]	0	2612.4362
[K].VTVNTNI	1xTMTpro [K	2.554E-06	4.225E-05	1	1	1	P53618	P53618 [853-871]	0	2838.5679
[R].GLPWSC	1xCarbamido	2.392E-05	4.225E-05	1	1	2	P31943	P31943 [17-29]	0	1808.8921
[K].VTVLFAC	1xTMTpro [K	2.916E-05	4.225E-05	1	1	5	P21333	P21333 [356-367]	0	1892.1613

[R].VTYGTE	1xTMTpro [K	9.379E-06	4.225E-05	1	1	1	Q99996	Q99996 [183-201]	0	2733.4955
[K].AGGVFT	1xTMTpro [K	5.879E-05	4.225E-05	1	1	3	Q8NBX0	Q8NBX0 [395-407	0	1818.0405
[R].AGHDQV	1xTMTpro [N	3.253E-05	4.225E-05	1	1	1	Q9BQA9	Q9BQA9 [95-108]	0	1862.0567
[K].VTVAGL	1xCarbamido	1.195E-05	4.225E-05	1	1	3	Q99497	Q99497 [33-48]	1	2266.2833
[R].GLPWSC	1xCarbamido	2.497E-05	4.225E-05	1	1	2	P55795	P55795 [17-29]	0	1811.874
[K].VTTVVAT	1xTMTpro [K	3.213E-05	4.225E-05	1	1	1	P49841	P49841 [37-60]	0	3154.6876
[R].VTTMDA	1xOxidation [	8.035E-06	4.225E-05	3	3	4	P35241; P26	P35241 [9-27]; P2	0	2690.4203
[R].VTTMDA	1xTMTpro [K	2.354E-05	4.225E-05	3	3	4	P35241; P26	P35241 [9-27]; P2	0	2674.4253
[R].VTTLPGI	1xTMTpro [K	1.011E-06	4.225E-05	1	1	2	O14617	O14617 [511-528]	0	2574.5263
[R].AGKPVIC	1xCarbamido	3.648E-07	4.225E-05	1	1	8	P14618	P14618 [320-336]	0	2789.5908
[R].AGKPVIC	1xCarbamido	4.035E-06	4.225E-05	1	1	6	P14618	P14618 [320-336]	0	2805.5857
[R].GTTEIGN	2xTMTpro [K	1.391E-05	4.225E-05	1	1	1	Q9HAU0	Q9HAU0 [780-796	0	2665.5415
[R].VTVGDIT	1xCarbamido	3.505E-06	4.225E-05	1	1	3	O75569	O75569 [70-84]	0	2133.1353
[R].AGDRED	1xCarbamido	1.137E-05	4.225E-05	1	1	1	P35222	P35222 [454-469]	1	2091.0824
[R].VVEQMC	1xCarbamido	5.85E-05	4.225E-05	1	1	3	P04156	P04156 [209-220]	0	1859.9315
[K].VVEREE	1xTMTpro [K	3.772E-07	4.225E-05	1	1	3	O43491	O43491 [787-822]	1	4343.359
[K].AFVDFLS	1xTMTpro [K	8.778E-06	4.225E-05	1	1	6	Q07021	Q07021 [81-94]	1	2306.2524
[K].VVLLGEC	1xCarbamido	3.064E-05	4.225E-05	1	1	2	Q9UL25	Q9UL25 [22-32]	0	1739.0381
[K].AFVDSG	1xCarbamido	3.551E-05	4.225E-05	1	2	1	Q5TDH0	Q5TDH0 [249-267	0	2377.127
[R].VVLLGEI	2xCarbamido	2.012E-05	4.225E-05	1	1	3	Q9NY12	Q9NY12 [70-87]	0	2751.4705
[R].AFVHWY	1xTMTpro [N	1.515E-06	4.225E-05	3	6	45	Q71U36; Q9	Q71U36 [403-422	0	2634.2254
[R].AFVHWY	1xOxidation [	1.167E-05	4.225E-05	3	6	5	Q71U36; Q9	Q71U36 [403-422	0	2650.2203
[K].AFVLSSV	1xTMTpro [N	9.82E-06	4.225E-05	1	1	2	P04275	P04275 [1437-145	0	1925.0299
[R].GISDPLT	1xTMTpro [N	3.796E-06	4.225E-05	1	1	3	O00116	O00116 [587-603]	0	2109.1147
[R].VVIIESVI	1xCarbamido	2.057E-05	4.225E-05	1	1	1	Q9NZ32	Q9NZ32 [89-102]	0	1960.1009
[R].AFVSMV	1xTMTpro [N	5.474E-06	4.225E-05	1	1	2	Q14683	Q14683 [78-92]	0	1993.9496
[K].AFVTQD	1xTMTpro [K	7.287E-06	4.225E-05	1	1	1	Q8WWX9	Q8WWX9 [58-73]	0	2531.3976
[R].AFVVETC	1xCarbamido	5.822E-05	4.225E-05	1	1	2	P52630	P52630 [315-335]	0	3070.719
[R].VVILDDL	1xTMTpro [K	2.692E-05	4.225E-05	1	1	1	Q5SZK8	Q5SZK8 [2081-20	0	2417.4147
[K].VVLHPN	1xTMTpro [K	9.893E-06	4.225E-05	1	1	1	P00738	P00738 [236-251]	0	2403.4255
[K].VVLHPN	1xTMTpro [K	4.029E-05	4.225E-05	1	1	1	P00739	P00739 [178-193]	0	2453.4524
[K].AFYGDT	1xTMTpro [N	4.397E-05	4.225E-05	1	1	1	Q9HCC0	Q9HCC0 [349-36	0	1721.9182
[K].VVIHFTD	1xTMTpro [N	8.842E-06	4.225E-05	1	1	2	P12111	P12111 [1947-196	0	2255.174

[R].VVLHDG	1xTMTpro [K	3.699E-06	4.225E-05	1	1	1	Q06124	Q06124 [290-317]	0	3764.9337
[R].GTIEILSI	1xTMTpro [K	2.384E-05	4.225E-05	1	2	1	P05388	P05388 [150-162]	0	2037.2451
[R].AFYNNV	1xTMTpro [K	5.244E-05	4.225E-05	1	1	1	P04181	P04181 [114-129]	0	2561.3419
[K].AFYPEEI	1xTMTpro [K	9.073E-06	4.225E-05	1	2	3	P0DMV9	P0DMV9 [113-126	0	2223.2227
[K].GLSEDT	1xTMTpro [K	9.584E-08	4.225E-05	1	1	4	P19338	P19338 [578-597]	1	2808.4395
[K].VVLNMN	1xTMTpro [K	2.198E-05	4.225E-05	1	1	1	Q9Y2T2	Q9Y2T2 [317-337	0	2933.5938
[R].VVLPCS	1xCarbamid	6.127E-06	4.225E-05	1	1	1	P48739	P48739 [9-31]	0	3162.7001
[R].AFTMDC	1xCarbamid	4.48E-05	4.225E-05	1	1	1	Q9UQE7	Q9UQE7 [645-66	0	2300.097
[R].AFQGLL	1xTMTpro [N	1.98E-05	4.225E-05	1	1	1	Q6XQN6	Q6XQN6 [282-29	0	1859.9975
[R].AFQPFF	1xTMTpro [N	2.267E-05	4.225E-05	2	2	2	P01023; P20	P01023 [788-804]	0	2349.2636
[K].VVNNSP	1xTMTpro [K	1.043E-05	4.225E-05	1	1	3	P19823	P19823 [94-112]	0	2730.5434
[R].AFQTVV	1xTMTpro [N	1.604E-06	4.225E-05	1	1	2	P49747	P49747 [533-558]	0	3185.655
[R].GLSIEQL	1xTMTpro [K	2.471E-05	4.225E-05	1	1	2	P40763	P40763 [519-531]	0	2011.1931
[K].AFQYVE	1xCarbamid	3.74E-06	4.225E-05	1	1	1	P30048	P30048 [218-248]	0	4298.2372
[R].VVMITGI	1xCarbamid	8.649E-06	4.225E-05	1	1	1	Q9HD20	Q9HD20 [743-766	0	3330.7834
[R].AFREEF	1xTMTpro [N	2.642E-07	4.225E-05	1	1	2	P51570	P51570 [19-37]	1	2337.2158
[K].AFSAVD	1xTMTpro [K	5.198E-06	4.225E-05	1	1	1	Q9NZT1	Q9NZT1 [16-38]	0	2871.5344
[R].AFSEVPI	1xTMTpro [N	6.051E-05	4.225E-05	1	1	1	Q4V9L6	Q4V9L6 [149-170	1	2735.3556
[R].AFYSLGI	1xTMTpro [K	1.948E-05	4.225E-05	1	1	1	Q5EBM0	Q5EBM0 [305-32	0	2354.3252
[K].VVMALG	2xCarbamid	2.046E-09	4.225E-05	1	1	2	P60842	P60842 [119-141]	0	2743.3108
[R].GTGALI	1xTMTpro [N	3.931E-06	4.225E-05	1	1	1	Q9HB90	Q9HB90 [136-156	0	2619.3295
[R].VVLVTG	1xTMTpro [N	4.199E-05	4.225E-05	1	1	4	P51659	P51659 [11-23]	0	1473.9072
[R].GTGGVD	1xTMTpro [N	3.489E-07	4.225E-05	1	1	3	P12277	P12277 [321-341]	0	2269.138
[R].VVIVDPE	1xTMTpro [K	4.102E-06	4.225E-05	1	1	1	Q15149	Q15149 [4278-42	1	2793.4988
[R].GLSFSE	1xTMTpro [K	7.114E-06	4.225E-05	1	1	2	Q9UEY8	Q9UEY8 [178-191	0	2032.157
[R].GTGLQP	1xTMTpro [K	1.48E-05	4.225E-05	1	1	3	P45974	P45974 [604-628]	0	3207.728
[R].AFTELF	2xCarbamid	2.545E-06	4.225E-05	1	1	1	Q14166	Q14166 [554-578]	0	3432.794
[K].AFTGFIV	1xTMTpro [N	4.315E-05	4.225E-05	1	1	1	P11310	P11310 [218-235]	0	2196.1984
[K].AFTHTA	1xTMTpro [N	5.85E-06	4.225E-05	1	1	2	P31939	P31939 [178-194]	0	2339.1263
[K].AFTKPEI	1xCarbamid	8.842E-06	4.225E-05	1	1	2	P22102	P22102 [126-148]	0	3452.932
[K].AFSGYL	1xTMTpro [K	4.768E-05	4.225E-05	1	1	2	P10768	P10768 [187-198]	0	1882.0202
[K].VVIGMD	1xTMTpro [N	1.657E-05	4.225E-05	1	1	14	P06733	P06733 [240-253]	0	1844.99
[K].AGAAAG	2xCarbamid	1.18E-05	4.225E-05	1	1	1	Q16270	Q16270 [98-114]	0	2126.1342

[R].AGAAGG	2xTMTpro [K	1.592E-05	4.225E-05	1	1	1	Q8WW12	Q8WW12 [17-32]	0	2452.3864
[K].GIRPFPS	1xTMTpro [N	3.213E-06	4.225E-05	1	1	1	P52735	P52735 [125-143]	0	2544.2173
[R].GTLSDE	1xTMTpro [K	4.81E-06	4.225E-05	1	1	1	O43504	O43504 [35-54]	0	2603.4648
[K].GTLSVM	1xCarbamido	2.091E-06	4.225E-05	1	1	1	P50895	P50895 [157-175]	0	2373.1345
[K].VVGAWD	1xTMTpro [N	4.022E-05	4.225E-05	1	1	1	Q96SL4	Q96SL4 [155-177	0	2825.5844
[K].AGCECL	3xCarbamido	4.634E-07	4.225E-05	1	1	1	O43396	O43396 [133-151]	0	2587.0931
[R].AGCHFY	3xCarbamido	1.838E-05	4.225E-05	1	1	2	Q9HC84	Q9HC84 [4965-49	0	2427.0712
[R].AGCLEY	2xCarbamido	9.007E-06	4.225E-05	1	1	1	Q8IVN8	Q8IVN8 [119-148]	0	3928.913
[K].AGCQVV	1xCarbamido	5.022E-05	4.225E-05	1	1	2	P13716	P13716 [160-174]	0	1897.889
[R].GTITDAF	1xTMTpro [N	4.737E-05	4.225E-05	1	1	2	P50995	P50995 [192-210]	1	2347.2577
[K].GTIVLAS	2xTMTpro [K	1.715E-06	4.225E-05	1	1	2	Q14914	Q14914 [82-104]	1	3324.862
[R].AGAVGA	1xTMTpro [K	1.045E-05	4.225E-05	1	1	1	Q9BY50	Q9BY50 [4-23]	0	2517.4321
[R].VVFGPE	1xTMTpro [K	3.454E-06	4.225E-05	1	1	1	Q14764	Q14764 [480-506]	0	3464.9649
[K].AGDFLE	1xTMTpro [N	1.113E-05	4.225E-05	1	1	2	P01024	P01024 [1172-118	0	1945.9761
[R].VVEVNQ	1xCarbamido	1.725E-06	4.225E-05	1	1	3	P02549	P02549 [214-238]	0	3504.7925
[R].AGDGDC	1xTMTpro [N	1.828E-05	4.225E-05	1	1	2	Q96D15	Q96D15 [90-103]	0	1749.9091
[R].AGDKDD	1xCarbamido	3.741E-05	4.225E-05	1	1	1	P14923	P14923 [445-460]	1	2339.2521
[K].AGDKPH	2xTMTpro [K	5.322E-05	4.225E-05	1	1	1	P21912	P21912 [52-67]	0	2758.5014
[K].VVETDPS	2xCarbamido	3.99E-06	4.225E-05	1	1	2	P55072	P55072 [165-191]	1	3689.9163
[K].VVETDPS	2xCarbamido	1.483E-05	4.225E-05	1	1	1	P55072	P55072 [165-190]	0	3533.8152
[K].VVESPD	2xTMTpro [K	1.628E-05	4.225E-05	1	1	1	Q9Y6Y8	Q9Y6Y8 [923-938	1	2740.4861
[R].AGDMEN	1xTMTpro [N	1.494E-05	4.225E-05	1	1	2	P42704	P42704 [245-259]	0	1967.985
[R].GLQQQN	1xTMTpro [K	4.022E-05	4.225E-05	1	1	2	P46940	P46940 [337-348]	0	2088.137
[K].VVFAAD	1xTMTpro [K	2.54E-06	4.225E-05	1	1	1	O00469	O00469 [136-149]	1	2195.2832
[K].VTNGAF	1xOxidation	3.34E-05	4.225E-05	1	1	1	P60174	P60174 [70-85]	0	2246.2346
[R].VVGFHV	1xTMTpro [K	6.252E-06	4.225E-05	1	1	2	Q16881	Q16881 [585-607]	0	2890.6435
[K].AGATLDI	1xTMTpro [N	1.024E-05	4.225E-05	1	1	2	P16278	P16278 [469-482]	0	1763.9645
[K].GTINFLH	1xCarbamido	3.563E-05	4.225E-05	1	1	1	Q9BS26	Q9BS26 [292-305	1	2302.2258
[R].VVIEGQT	1xCarbamido	3.453E-05	4.225E-05	1	1	3	Q92626	Q92626 [443-457]	0	2331.251
[K].AGAAPY	1xTMTpro [K	5.065E-07	4.225E-05	1	1	6	Q01518	Q01518 [38-60]	0	2959.6425
[K].GISCMNT	1xCarbamido	5.59E-05	4.225E-05	1	1	2	Q15181	Q15181 [239-253]	0	2296.1809
[K].GISCMNT	1xCarbamido	3.387E-05	4.225E-05	1	1	2	Q15181	Q15181 [239-253]	0	2280.186
[K].VVIEDEQ	1xTMTpro [N	1.361E-05	4.225E-05	1	1	1	Q6IBS0	Q6IBS0 [30-48]	0	2342.2887

[K].VVLEAPI	1xTMTpro [N	1.341E-05	4.225E-05	1	1	1	Q92797	Q92797 [667-683]	0	2142.2705
[K].VVLDDKI	1xTMTpro [K	5.813E-05	4.225E-05	1	1	4	P61604	P61604 [81-92]	1	2138.2142
[K].VVLAYEI	1xTMTpro [K	9.984E-06	4.225E-05	1	1	14	P60174	P60174 [161-175]	0	2211.3033
[K].AGAGSA	1xTMTpro [N	4.05E-06	4.225E-05	1	1	8	P40926	P40926 [242-257]	0	1758.9128
[R].VVGGFD	1xTMTpro [K	1.067E-05	4.225E-05	1	1	2	Q13356	Q13356 [400-418]	0	2615.3882
[K].AGAGSA	1xOxidation [	2.63E-05	4.225E-05	1	1	1	P40926	P40926 [242-257]	0	1774.9077
[R].VVKEEE.	2xTMTpro [K	3.045E-06	4.225E-05	1	1	3	P51159	P51159 [142-154]	1	2341.4159
[K].AGAKPD	1xTMTpro [K	1.204E-05	4.225E-05	1	1	1	Q92900	Q92900 [794-811]	0	2522.4222
[R].AGAIAPC	1xCarbamido	7.687E-06	4.225E-05	1	2	4	P05388	P05388 [113-134]	0	2788.5159
[R].VVGSSG	1xTMTpro [N	5.701E-06	4.225E-05	1	1	1	P98160	P98160 [2505-252	0	2263.2577
[R].VVGSPF	1xTMTpro [K	5.064E-05	4.225E-05	1	1	2	O94788	O94788 [348-365]	0	2523.3586
[K].AGALNS	1xTMTpro [K	1.298E-05	4.225E-05	1	1	5	P06396	P06396 [585-597]	0	1928.1097
[R].AGAISAS	1xTMTpro [K	1.179E-06	4.225E-05	1	1	1	Q09666	Q09666 [226-243]	0	2246.2385
[K].AGAQAL	1xTMTpro [K	4.135E-05	4.225E-05	1	1	1	A6NGB9	A6NGB9 [340-360	0	2651.5165
[K].GTIQVIT	1xTMTpro [K	2.593E-05	4.225E-05	1	1	2	O43175	O43175 [352-364]	0	1954.1829
[R].VVGICGT	1xCarbamido	4.382E-05	4.225E-05	1	1	1	Q8N8N7	Q8N8N7 [183-192	0	1707.9707
[K].VVLAEVI	1xTMTpro [N	5.964E-05	4.225E-05	1	1	1	Q99622	Q99622 [18-35]	0	2217.2562
[R].VVNSET	2xCarbamido	1.851E-07	4.225E-05	1	1	2	Q99757	Q99757 [74-94]	0	3037.552
[K].VTNGAF	1xTMTpro [K	5.433E-06	4.225E-05	1	1	4	P60174	P60174 [70-85]	0	2230.2397
[K].VTNEYN	1xTMTpro [K	2.88E-05	4.225E-05	1	1	1	Q4G0N4	Q4G0N4 [356-372	0	2620.3638
[K].GVDEAT	1xTMTpro [K	2.206E-05	4.225E-05	1	1	4	P04083	P04083 [59-71]	0	1996.1822
[K].GVDEAT	1xTMTpro [K	5.018E-06	4.225E-05	1	1	1	P04083	P04083 [59-72]	1	2152.2833
[R].AGYIIPL	1xTMTpro [N	5.535E-06	4.225E-05	1	1	2	P10253	P10253 [820-837]	0	2178.209
[R].AGYLTLY	1xTMTpro [N	1.486E-05	4.225E-05	1	1	2	Q16647	Q16647 [175-188]	0	1841.0492
[K].GVDEVT	1xTMTpro [N	9.553E-06	4.225E-05	1	1	6	P07355	P07355 [50-63]	0	1847.0557
[K].GIMGEDT	1xTMTpro [K	5.437E-05	4.225E-05	1	1	1	P09668	P09668 [198-210]	0	2067.0713
[R].GLMELH	1xTMTpro [K	7.991E-06	4.225E-05	1	1	1	Q92616	Q92616 [1105-11	0	2504.3384
[R].AHADDE	1xTMTpro [N	4.713E-05	4.225E-05	1	1	2	P48681	P48681 [54-64]	0	1485.7981
[R].VSSGYV	1xTMTpro [K	5.738E-05	4.225E-05	1	1	1	Q15942	Q15942 [168-184]	0	2328.3095
[K].GVDIVMI	1xTMTpro [K	1.651E-05	4.225E-05	1	1	5	Q99536	Q99536 [256-271]	0	2183.1873
[K].GVDIVMI	1xOxidation [	1.987E-05	4.225E-05	1	1	4	Q99536	Q99536 [256-271]	0	2199.1823
[R].VSQTTW	1xCarbamido	7.888E-06	4.225E-05	1	1	3	P31146	P31146 [30-45]	0	2405.2415
[K].VSQPIEC	1xTMTpro [K	2.665E-06	4.225E-05	1	1	1	Q00610	Q00610 [190-205]	0	2325.2847

[K].VSQMAQ	1xOxidation [	4.601E-06	4.225E-05	1	1	1	Q9Y490	Q9Y490 [1731-17	0	2806.5305
[K].VSQMAQ	1xTMTpro [K	1.069E-06	4.225E-05	1	1	4	Q9Y490	Q9Y490 [1731-17	0	2790.5356
[K].GIIYGTD	1xTMTpro [K	1.273E-06	4.225E-05	1	1	2	Q14790	Q14790 [321-344]	0	3209.7226
[R].AHEEQL	2xTMTpro [K	1.593E-07	4.225E-05	1	1	2	Q15149	Q15149 [1244-12	1	3415.9253
[K].AHEGEIE	1xTMTpro [K	7.049E-06	4.225E-05	1	1	2	Q9HCU5	Q9HCU5 [194-20	0	2259.2113
[K].AHEILPN	2xCarbamido	5.081E-05	4.225E-05	1	1	2	P50990	P50990 [139-152]	0	2220.2125
[K].GVEGLID	1xTMTpro [N	2.316E-05	4.225E-05	1	1	1	Q13442	Q13442 [76-88]	0	1729.9404
[K].AHEPTYI	1xCarbamido	5.506E-08	4.225E-05	1	1	36	P21333	P21333 [786-809]	0	3173.6069
[K].AGWTIIT	1xTMTpro [K	1.43E-05	4.225E-05	1	1	1	P50440	P50440 [333-356]	0	3281.7888
[R].AGVYPE	1xTMTpro [K	4.05E-06	4.225E-05	1	1	1	P50570	P50570 [623-651]	1	3876.8478
[K].AGVVGP	1xTMTpro [K	1.164E-06	4.225E-05	1	1	1	Q15149	Q15149 [3552-35	0	2385.3633
[R].VSTDQA	1xTMTpro [K	2.049E-06	4.225E-05	1	1	2	Q5T1M5	Q5T1M5 [784-808	0	3352.788
[R].GVCISPE	1xCarbamido	3.125E-06	4.225E-05	1	1	3	Q6FHJ7	Q6FHJ7 [134-151	0	2550.3981
[K].GVCTEA	1xCarbamido	4.427E-05	4.225E-05	1	1	2	P62195	P62195 [361-372]	0	1631.8205
[R].VSYALLF	1xTMTpro [N	6.104E-06	4.225E-05	1	1	1	Q9UBV2	Q9UBV2 [225-243	0	2443.3305
[K].GVDDLD	2xTMTpro [K	1.946E-08	4.225E-05	1	1	6	P61158	P61158 [54-75]	0	3356.8082
[R].AGVLAH	1xTMTpro [N	5.691E-05	4.225E-05	2	2	3	P35580; P35	P35580 [772-782]	0	1527.845
[R].VSWDPS	1xTMTpro [K	4.382E-05	4.225E-05	1	1	2	Q99715	Q99715 [2134-21	0	2140.1934
[K].VSVVEP	1xTMTpro [K	8.021E-07	4.225E-05	1	1	2	Q02338	Q02338 [233-258]	0	3299.8383
[R].VSVVAN	1xOxidation [	2.906E-05	4.225E-05	1	1	3	P12111	P12111 [1887-19	0	3622.8119
[R].VSVVAN	1xTMTpro [K	1.27E-06	4.225E-05	1	1	5	P12111	P12111 [1887-19	0	3606.8169
[K].AGVLSQ	1xCarbamido	9.218E-07	4.225E-05	1	1	3	P61421	P61421 [25-46]	0	3087.6528
[K].VSQGQL	1xTMTpro [K	4.36E-05	4.225E-05	1	1	3	P13667	P13667 [350-362]	0	2051.1815
[R].VSVQLE	1xTMTpro [K	1.332E-05	4.225E-05	1	1	3	P01023	P01023 [824-841]	0	2492.462
[R].AGVNTV	1xTMTpro [K	2.151E-05	4.225E-05	1	1	2	P62424	P62424 [138-150]	0	1954.1465
[R].AGVNTV	2xTMTpro [K	4.712E-07	4.225E-05	1	1	2	P62424	P62424 [138-151]	1	2386.4486
[K].AGVQSV	2xTMTpro [K	1.366E-05	4.225E-05	1	1	1	Q86VM9	Q86VM9 [169-18	1	2515.4548
[R].VSVPTH	1xTMTpro [K	3.888E-06	4.225E-05	1	1	5	P13611	P13611 [101-119]	0	2514.4423
[R].VSVPLIA	1xTMTpro [K	5.597E-06	4.225E-05	1	1	2	P11047	P11047 [604-622]	0	2598.4634
[R].VSVIQFL	1xTMTpro [K	6.275E-06	4.225E-05	1	1	2	Q14160	Q14160 [445-467]	0	3039.6018
[K].VSVLDYI	1xTMTpro [K	6.56E-06	4.225E-05	1	1	2	P13674	P13674 [205-223]	0	2784.4951
[K].VSVLCQ	2xCarbamido	1.059E-05	4.225E-05	1	1	2	P08603	P08603 [835-854]	0	3020.5564
[K].VSVGEF	1xTMTpro [K	1.706E-05	4.225E-05	1	1	1	O95793	O95793 [220-232]	0	1902.0464

[K].VSVGAP	1xTMTpro [K	3.768E-06	4.225E-05	1	1	6	Q09666	Q09666 [5385-54	0	2367.3263
[K].AGVNFS	1xTMTpro [K	2.844E-05	4.225E-05	1	1	3	O75340	O75340 [78-90]	0	2050.1253
[K].VSYGIGI	1xTMTpro [N	4.11E-06	4.225E-05	1	1	3	P27695	P27695 [142-156]	0	1994.9375
[K].GVEGTW	1xCarbamid	6.487E-06	4.225E-05	1	1	2	O60488	O60488 [631-651]	0	2954.5247
[K].AHFNLDI	1xTMTpro [N	1.741E-05	4.225E-05	1	1	2	Q9Y4L1	Q9Y4L1 [541-555	0	1977.0361
[R].VSGVDG	1xTMTpro [N	2.034E-05	4.225E-05	1	1	1	Q14566	Q14566 [270-282]	0	1685.8665
[K].AHILACA	2xCarbamid	8.05E-06	4.225E-05	1	1	1	Q9HCE1	Q9HCE1 [548-56	0	2358.1978
[R].VSGPWE	1xTMTpro [N	3.518E-06	4.225E-05	1	1	1	Q8N2S1	Q8N2S1 [240-254	0	1890.9517
[K].VSGPGV	1xTMTpro [N	4.404E-05	4.225E-05	1	1	1	Q14315	Q14315 [1255-12	0	1607.9189
[K].GVESVF	1xTMTpro [N	4.863E-06	4.225E-05	1	1	1	O75643	O75643 [1978-19	0	2219.0167
[R].VSGISMI	2xCarbamid	5.131E-06	4.225E-05	1	1	1	P98160	P98160 [1102-11	0	3781.7678
[K].AHLMEI(	1xTMTpro [K	3.305E-05	4.225E-05	1	1	1	P39023	P39023 [178-193]	0	2305.283
[K].GLLTIPM	1xTMTpro [N	1.754E-05	4.225E-05	1	1	2	Q92538	Q92538 [725-741]	0	2261.2283
[K].VSGLPTI	2xTMTpro [K	1.075E-05	4.225E-05	1	1	1	Q8WX93	Q8WX93 [1158-1	0	3613.0318
[K].VSGGTP	2xTMTpro [K	3.467E-06	4.225E-05	1	1	2	Q9UEY8	Q9UEY8 [477-50	1	3962.2055
[R].AHIVFDF	1xTMTpro [N	2.833E-06	4.225E-05	1	1	2	P30520	P30520 [133-151]	0	2514.2809
[K].VSGELF/	1xTMTpro [N	2.215E-06	4.225E-05	1	1	2	Q8NC96	Q8NC96 [64-90]	0	3154.6227
[K].VSGAQE	1xTMTpro [K	1.872E-05	4.225E-05	1	1	1	O60664	O60664 [129-140]	0	1801.9974
[K].VSFTGS'	1xTMTpro [K	2.73E-05	4.225E-05	1	1	2	P49189	P49189 [228-239]	0	1819.0279
[K].GVETIAN	1xTMTpro [K	5.357E-05	4.225E-05	1	1	2	P10515	P10515 [533-547]	0	2125.236
[K].AHNQDL	1xCarbamid	2.214E-05	4.225E-05	1	1	2	P08572	P08572 [1526-15	0	1886.9826
[R].AHNVPV	2xCarbamid	2.493E-05	4.225E-05	1	1	1	Q9UI10	Q9UI10 [429-442]	0	2298.223
[K].VSFLSAI	2xTMTpro [K	1.425E-05	4.225E-05	1	1	2	P02647	P02647 [251-263]	1	2427.4315
[K].VSFLSAI	1xTMTpro [K	2.607E-05	4.225E-05	1	1	17	P02647	P02647 [251-262]	0	1995.1294
[K].AHPEPG	1xTMTpro [K	4.192E-05	4.225E-05	1	1	1	P0DMN0	P0DMN0 [148-16	0	2222.1737
[K].VSFFQPI	1xCarbamid	1.388E-06	4.225E-05	1	1	1	O95825	O95825 [77-104]	0	3435.7313
[R].VSGVEC	1xCarbamid	2.167E-05	4.225E-05	1	1	1	Q9HCC0	Q9HCC0 [126-14	0	2315.2595
[K].VSHIDVI	1xTMTpro [K	5.141E-05	4.225E-05	1	1	1	Q9UQE7	Q9UQE7 [1195-1	0	2022.1549
[K].VSHLLGI	1xTMTpro [N	2.529E-05	4.225E-05	1	1	4	P35579	P35579 [374-387]	0	1876.0612
[R].GVESQG	1xCarbamid	7.287E-06	4.225E-05	1	1	1	P54577	P54577 [433-450]	0	2238.1542
[K].VSPNPE	1xTMTpro [K	3.538E-05	4.225E-05	1	1	1	Q9UMS6	Q9UMS6 [704-72	0	2433.3845
[R].AHFPLD'	1xTMTpro [N	3.874E-06	4.225E-05	1	1	3	P10253	P10253 [394-411]	0	2526.2043
[R].VSPGPS	1xTMTpro [N	2.607E-05	4.225E-05	1	1	2	Q9Y6N6	Q9Y6N6 [639-655	0	2014.1292

[K].VSPGAF	1xTMTpro [K	6.032E-05	4.225E-05	1	1	6	P07585	P07585 [120-130]	0	1724.0602
[K].AHGGYS	1xTMTpro [N	2.875E-05	4.225E-05	1	1	3	P06576	P06576 [226-239]	0	1710.8883
[K].VSPETVI	1xTMTpro [N	3.438E-07	4.225E-05	1	1	2	P42765	P42765 [46-71]	0	3055.5941
[R].VSPEEV	1xTMTpro [N	1.502E-05	4.225E-05	1	1	1	Q8TEM1	Q8TEM1 [897-915	0	2456.3105
[K].GIIVHTM	1xTMTpro [K	1.802E-05	4.225E-05	1	1	2	O95573	O95573 [250-265]	0	2189.2972
[R].VSNDNQ	1xTMTpro [K	1.226E-05	4.225E-05	1	1	4	P35442	P35442 [297-313]	0	2522.3899
[K].VSMFVL	1xTMTpro [N	1.024E-05	4.225E-05	1	1	2	Q9NZM1	Q9NZM1 [318-334	1	2191.1501
[R].VSPVGE	1xTMTpro [K	4.315E-05	4.225E-05	1	1	1	P58335	P58335 [112-125]	0	2137.2149
[R].AHGQDL	1xCarbamido	1.855E-05	4.225E-05	1	2	4	P02462	P02462 [1482-149	0	1746.8876
[K].VSMFVL	1xTMTpro [N	3.594E-05	4.225E-05	1	1	2	Q9NZM1	Q9NZM1 [318-333	0	2035.0489
[K].GVEITGF	1xTMTpro [K	1.446E-08	4.225E-05	1	1	1	P22102	P22102 [351-375]	0	3163.7647
[K].VSLLDD	1xCarbamido	3.174E-05	4.225E-05	1	1	1	P11171	P11171 [214-228]	0	2377.2816
[K].GLLTYTS	1xTMTpro [N	1.247E-05	4.225E-05	1	1	2	P28331	P28331 [312-325]	0	1916.0085
[K].AHIANPS	1xCarbamido	1.423E-07	4.225E-05	1	1	2	O75369	O75369 [1268-12	0	4127.9788
[R].VSLDVN	1xTMTpro [K	8.649E-06	4.225E-05	1	1	7	P04792	P04792 [97-112]	0	2392.3368
[R].VSLAGA	1xCarbamido	1.838E-05	4.225E-05	1	1	2	P13647	P13647 [49-63]	0	1714.8866
[R].VSIAELA	1xTMTpro [N	4.713E-06	4.225E-05	1	1	3	Q96HY6	Q96HY6 [289-306	0	2190.2202
[K].VSHVST	1xTMTpro [K	3.845E-06	4.225E-05	2	2	2	P07205; P00	P07205 [389-406]	0	2349.3269
[K].VSHSED	1xCarbamido	1.502E-05	4.225E-05	1	1	3	P01024	P01024 [1451-146	0	2016.0352
[K].GVEIEGF	1xTMTpro [N	5.115E-05	4.225E-05	1	1	2	P28070	P28070 [241-264]	0	2936.4307
[R].VSYPLCI	2xCarbamido	2.67E-06	4.225E-05	2	2	4	O60234; P60	O60234 [82-108];	0	4024.0661
[R].VSYPLCI	2xCarbamido	1.094E-05	4.225E-05	2	2	2	O60234; P60	O60234 [82-108];	0	4040.061
[K].VSYQLAI	1xTMTpro [N	1.563E-06	4.225E-05	1	1	2	P22105	P22105 [3788-380	0	2508.265
[R].VTGEAD	1xTMTpro [K	3.779E-08	4.225E-05	1	1	3	P52597	P52597 [327-347]	0	2800.4319
[R].VTGEAD	1xOxidation	5.897E-07	4.225E-05	1	1	2	P31943	P31943 [327-347]	0	2802.4111
[R].VTGEAD	1xTMTpro [K	1.807E-07	4.225E-05	1	1	15	P31943	P31943 [327-347]	0	2786.4162
[R].VTGEAD	1xTMTpro [K	1.042E-07	4.225E-05	1	1	4	P55795	P55795 [327-347]	0	2770.4213
[K].VTGDEW	1xTMTpro [N	2.833E-06	4.225E-05	1	1	2	P13807	P13807 [53-74]	0	2806.3643
[R].GTVQGQ	1xTMTpro [K	6.041E-05	4.225E-05	1	1	1	P07311	P07311 [46-58]	0	1921.1362
[R].AGPDLA	2xCarbamido	3.514E-05	4.225E-05	1	1	2	Q8N2S1	Q8N2S1 [952-966	0	1981.9278
[R].GTVTDF	1xTMTpro [N	4.549E-05	4.225E-05	1	1	4	P08758	P08758 [7-18]	0	1644.8189
[R].VTFLGL	1xTMTpro [N	9.483E-06	4.225E-05	1	1	2	Q08431	Q08431 [93-107]	0	2070.182
[R].AGPGTL	1xTMTpro [K	1.842E-05	4.225E-05	1	1	2	O75369	O75369 [2412-24	0	2022.1727

[K].AGPHCP	1xCarbamido	4.33E-05	4.225E-05	1	2	1	P02776	P02776 [63-77]	0	2186.2611
[R].GLPDNIS	1xTMTpro [K	4.618E-05	4.225E-05	1	1	2	Q16851	Q16851 [96-107]	0	1865.0988
[K].GIPDLYD	1xTMTpro [K	5.313E-05	4.225E-05	1	1	1	Q14534	Q14534 [437-451]	0	2246.22
[R].VTEYLVV	1xOxidation [	2.461E-06	4.225E-05	1	1	1	P24821	P24821 [649-669]	0	2789.4139
[K].VTEVALK	1xCarbamido	9.107E-06	4.225E-05	1	1	1	Q02952	Q02952 [1299-13	1	2762.5062
[R].VTETGLI	1xTMTpro [N	4.417E-06	4.225E-05	1	1	1	Q9NXK8	Q9NXK8 [189-206	0	2297.2308
[K].VTEQLIE	1xTMTpro [K	3.141E-05	4.225E-05	1	1	2	Q13557	Q13557 [353-371]	0	2763.4696
[K].VTEMLVI	1xCarbamido	2.493E-05	4.225E-05	1	1	2	Q08209	Q08209 [361-392]	0	3702.7434
[R].GTVTYIA	1xTMTpro [K	3.689E-05	4.225E-05	1	1	1	P38606	P38606 [174-200]	0	3521.8547
[R].VTELVQ	1xTMTpro [N	2.44E-05	4.225E-05	1	1	2	P41226	P41226 [971-988]	0	2197.226
[K].GTVVYG	1xTMTpro [K	7.559E-09	4.225E-05	1	1	2	Q16555	Q16555 [271-293]	0	3033.5813
[R].VTGEAD	1xOxidation [	1.446E-05	4.225E-05	1	1	2	P52597	P52597 [327-347]	0	2816.4268
[K].AGNNML	1xOxidation [	4.412E-05	4.225E-05	1	1	2	P21333	P21333 [2585-259	0	1754.9655
[K].AGNNML	1xTMTpro [N	2.524E-05	4.225E-05	1	1	4	P21333	P21333 [2585-259	0	1738.9706
[K].GLPEPSY	1xTMTpro [N	3.813E-05	4.225E-05	1	1	1	Q13308	Q13308 [348-362]	0	2024.0673
[K].GTVLDQ	1xTMTpro [K	3.282E-05	4.225E-05	1	2	1	Q9Y657	Q9Y657 [74-90]	0	2464.4671
[R].VTMTTD	1xTMTpro [N	3.538E-05	4.225E-05	1	1	2	A0A0C4DH3	A0A0C4DH31 [87	0	2212.0797
[K].VTMNDE	1xTMTpro [N	8.184E-06	4.225E-05	1	1	1	Q96A33	Q96A33 [249-266	0	2368.112
[K].VTLVVGA	1xTMTpro [K	2.06E-05	4.225E-05	1	1	2	P21964	P21964 [162-177]	0	2289.4037
[K].VTLVFEK	1xTMTpro [N	2.304E-05	4.225E-05	1	1	1	P11802	P11802 [89-101]	0	1875.0295
[K].VTLTCVA	1xCarbamido	1.875E-05	4.225E-05	1	1	4	P04217	P04217 [228-244]	0	2180.2068
[K].GIPIEVIF	1xTMTpro [N	2.87E-05	4.225E-05	1	1	1	P49247	P49247 [212-227]	0	2045.1788
[K].GTVLLAI	1xCarbamido	1.741E-06	4.225E-05	1	1	1	P21964	P21964 [213-234]	0	2639.4299
[R].GIPLDFS	1xTMTpro [K	4.882E-05	4.225E-05	1	1	2	Q9NZM1	Q9NZM1 [56-70]	0	2154.303
[K].GTVLLDI	1xTMTpro [N	3.644E-06	4.225E-05	1	1	1	P02730	P02730 [117-138]	0	2630.4684
[K].AGQCVI	1xCarbamido	6.183E-06	4.225E-05	1	1	2	Q99439	Q99439 [161-174]	0	2085.144
[K].GTVIIIAN	1xTMTpro [N	5.098E-05	4.225E-05	1	1	2	Q16851	Q16851 [474-485]	0	1569.9032
[R].GLPGDV	1xTMTpro [K	1.934E-06	4.225E-05	1	1	2	Q8IY67	Q8IY67 [65-85]	0	2950.5653
[K].AGMSAE	1xTMTpro [K	3.961E-05	4.225E-05	1	1	1	Q9Y2Q3	Q9Y2Q3 [145-158	0	2041.1243
[K].VTLENFY	1xTMTpro [N	2.897E-06	4.225E-05	1	1	1	Q15208	Q15208 [25-41]	0	2367.2264
[R].VTIAQGY	1xTMTpro [K	1.741E-06	4.225E-05	1	1	1	Q13423	Q13423 [183-202]	0	2681.4464
[K].VTHAVV	1xTMTpro [N	1.013E-05	4.225E-05	1	1	6	P11021	P11021 [165-181]	0	2192.1783
[K].AGNASK	2xTMTpro [K	3.514E-05	4.225E-05	1	1	3	P23381	P23381 [28-41]	1	2317.318

[K].VTGTQP	1xCarbamido	1.575E-05	4.225E-05	1	1	2	Q15746	Q15746 [1261-12	0	2131.1535
[K].GTVPDD	2xTMTpro [K	5.794E-05	4.225E-05	1	1	2	P20810	P20810 [427-444]	1	2698.5443
[R].VTGESH	1xTMTpro [K	2.692E-05	4.225E-05	1	1	1	Q86UX7	Q86UX7 [36-48]	0	1918.1617
[K].AGNLGG	1xTMTpro [N	4.657E-05	4.225E-05	1	1	2	P35268	P35268 [53-65]	0	1546.8872
[K].VTLLDG	1xCarbamido	5.581E-05	4.225E-05	1	1	1	O43491	O43491 [222-236	0	2351.2296
[R].GTWDH	1xTMTpro [N	4.681E-05	4.225E-05	1	1	2	Q99715	Q99715 [1673-16	0	1867.9258
[R].VTEGTIF	1xTMTpro [K	1.443E-06	4.225E-05	1	1	4	O43491	O43491 [762-786	1	3555.7946
[K].VTEGLVI	3xTMTpro [K	5.305E-05	4.225E-05	1	1	1	O43390	O43390 [269-287	2	3414.0149
[K].AGTEEA	1xTMTpro [K	5.192E-05	4.225E-05	1	1	1	Q9NQR4	Q9NQR4 [235-24	0	2232.2255
[K].VTAVDW	1xCarbamido	3.664E-05	4.225E-05	1	1	1	P27708	P27708 [1359-13	0	2646.2578
[K].VTATECI	1xCarbamido	1.789E-05	4.225E-05	1	1	1	P05107	P05107 [415-429]	0	2085.097
[R].AGTFQA	1xTMTpro [K	2.587E-06	4.225E-05	1	1	1	Q13813	Q13813 [1392-14	0	3483.8305
[K].VTASGP	1xTMTpro [N	5.799E-07	4.225E-05	1	1	1	O75369	O75369 [1520-15	0	2851.5161
[R].VTASDP	1xTMTpro [N	6.493E-09	4.225E-05	2	2	3	P0C0L4; P0	P0C0L4 [980-100	0	2787.5059
[R].AGTLSIT	1xTMTpro [N	2.005E-07	4.225E-05	1	1	2	Q15149	Q15149 [4361-43	0	2419.2247
[R].AGTLTVI	1xTMTpro [N	1.063E-06	4.225E-05	1	1	2	P58107	P58107 [2477-25	0	2817.5641
[K].AGTNMM	1xTMTpro [K	1.102E-05	4.225E-05	1	1	1	Q14315	Q14315 [2663-26	0	2038.0892
[K].VTAQGP	1xTMTpro [K	1.041E-05	4.225E-05	1	1	4	P21333	P21333 [384-400]	0	2261.2745
[R].VTCDGA	2xCarbamido	4.48E-05	4.225E-05	1	1	2	O00468	O00468 [419-434	0	2129.0187
[K].AGTQIE	1xTMTpro [N	1.667E-05	4.225E-05	1	1	2	O43707	O43707 [67-79]	0	1811.9095
[K].AGTQIE	1xTMTpro [N	2.083E-05	4.225E-05	2	3	4	P12814; P35	P12814 [48-60]; F	0	1825.9251
[K].AGTQIE	1xTMTpro [K	9.107E-06	4.225E-05	1	1	4	P12814	P12814 [48-64]	1	2543.3597
[K].VTAPDVI	1xTMTpro [K	1.879E-05	4.225E-05	1	1	2	Q09666	Q09666 [4916-49	0	1816.0824
[R].VTAHAE	1xTMTpro [K	4.487E-05	4.225E-05	1	1	2	Q8IUX7	Q8IUX7 [953-965	0	1940.0733
[K].VTAEVVI	1xTMTpro [N	1.296E-06	4.225E-05	1	1	1	P04843	P04843 [49-65]	0	1958.099
[K].GVAINM	1xTMTpro [K	9.276E-06	4.225E-05	1	1	3	P60842	P60842 [370-382]	1	2070.1509
[K].VTAEAD	1xTMTpro [K	3.026E-05	4.225E-05	1	1	1	A0MZ66	A0MZ66 [486-505	0	2559.3645
[R].GVCHIPI	2xCarbamido	4.264E-05	4.225E-05	1	1	2	Q13126	Q13126 [134-147	0	2251.1681
[K].AGVAAP	1xTMTpro [N	5.526E-07	4.225E-05	1	1	3	Q15149	Q15149 [631-650	0	2299.3053
[R].AGVAFF	1xTMTpro [N	2.462E-05	4.225E-05	1	1	1	Q8N5M4	Q8N5M4 [114-12	0	2042.0415
[R].VTAPPE	1xTMTpro [N	3.67E-05	4.225E-05	1	1	3	P08648	P08648 [695-708]	0	1792.9764
[K].AGLQDA	1xTMTpro [K	2.602E-06	4.225E-05	1	1	2	Q9UKK3	Q9UKK3 [611-62	0	2584.459
[K].GLNDLQ	1xCarbamido	9.242E-06	4.225E-05	1	1	1	Q9Y673	Q9Y673 [179-197	0	2432.2134

[R].GVAGAL	1xTMTpro [K	7.101E-06	4.225E-05	1	2	2	P47985	P47985 [21-46]	0	3248.9226
[K].VTEGLVI	1xTMTpro [K	2.154E-06	4.225E-05	1	1	4	O43390	O43390 [269-285]	0	2549.4107
[K].VTEGLTI	3xTMTpro [K	6.645E-06	4.225E-05	1	1	1	O60506	O60506 [266-284]	2	3415.9942
[K].VTEGLTI	1xTMTpro [K	9.379E-06	4.225E-05	1	1	1	O60506	O60506 [266-282]	0	2551.3899
[R].GLNYYLI	1xTMTpro [K	2.452E-06	4.225E-05	1	1	1	P02549	P02549 [2302-231	0	2671.3569
[K].VTEENKI	1xTMTpro [K	5.014E-05	4.225E-05	1	1	2	P07996	P07996 [301-313]	1	2153.2058
[K].AGQLSQ	1xCarbamido	3.023E-06	4.225E-05	1	1	2	Q9UBG0	Q9UBG0 [507-52	0	1989.9368
[R].VTEEDIV	2xCarbamido	2.963E-05	4.225E-05	1	1	1	Q9BY77	Q9BY77 [291-307	0	2590.3752
[R].AGQMVT	1xTMTpro [K	3.8E-05	4.225E-05	1	1	1	Q03252	Q03252 [525-549]	0	3129.7163
[K].GTWESH	1xTMTpro [N	1.344E-05	4.225E-05	1	1	1	P36915	P36915 [528-545]	0	2343.2264
[R].VTEDEN	1xCarbamido	1.735E-07	4.225E-05	1	1	6	Q13148	Q13148 [7-42]	0	4179.0246
[R].GVAGVF	1xTMTpro [N	1.472E-05	4.225E-05	1	1	2	Q14315	Q14315 [2327-23	0	1793.9869
[R].AGQSAA	1xTMTpro [N	1.364E-05	4.225E-05	1	1	3	P21333	P21333 [8-24]	0	1746.9054
[R].GTYEVG	1xTMTpro [K	6.608E-06	4.225E-05	1	1	2	Q14112	Q14112 [461-485]	0	3314.6672
[K].AGQTVT	1xTMTpro [K	8.974E-06	4.225E-05	1	1	1	P20700	P20700 [492-516]	0	3176.76
[K].VTDLLG	1xTMTpro [K	5.682E-05	4.225E-05	1	1	4	O43615	O43615 [283-293]	0	1758.0657
[K].AGQVVT	1xTMTpro [K	1.575E-09	4.225E-05	1	1	4	P02545	P02545 [491-515]	0	3141.7341
[K].VTDFGD	1xTMTpro [K	2.19E-06	4.225E-05	1	1	2	Q14204	Q14204 [229-251]	1	3187.6879
[K].GTYIPVF	1xTMTpro [K	1.805E-05	4.225E-05	1	1	3	P00488	P00488 [115-130]	0	2296.3408
[K].AGSDGE	1xCarbamido	2.621E-05	4.225E-05	1	1	2	Q9Y696	Q9Y696 [25-40]	0	1985.9306
[K].GVAAGS	1xTMTpro [K	5.491E-05	4.225E-05	1	1	1	P19544	P19544 [249-259]	0	1557.9092
[K].AGSMKD	1xTMTpro [K	2.218E-05	4.225E-05	1	1	2	Q9UM54	Q9UM54 [303-319	1	2495.3208
[R].GVAFVG	1xTMTpro [K	7.744E-06	4.225E-05	1	1	1	O15067	O15067 [1110-11	0	2353.3048
[K].AGQSVI	1xTMTpro [K	5.455E-05	4.225E-05	1	1	2	Q15417	Q15417 [159-172]	0	2012.1454
[R].WVVIGD	1xTMTpro [N	1.838E-05	4.225E-05	1	1	1	Q99798	Q99798 [657-671]	0	1971.9731
[K].GISLNPE	1xTMTpro [K	2.324E-05	4.225E-05	1	1	3	P53999	P53999 [102-114]	0	2108.1996
[K].GTFSQL	1xCarbamido	3.334E-05	4.225E-05	1	1	1	P02042	P02042 [84-105]	1	3237.6607
[K].WLAEME	1xTMTpro [N	4.057E-06	4.225E-05	1	1	2	P11277	P11277 [870-889]	0	2745.3547
[K].AEILGNV	1xTMTpro [N	2.7E-06	4.225E-05	1	1	1	Q96D15	Q96D15 [300-323	0	2990.4637
[K].WKSDEV	1xCarbamido	9.984E-06	4.225E-05	1	1	2	Q02952	Q02952 [1510-15	1	2862.5124
[R].GSQAQA	1xTMTpro [K	3.933E-05	4.225E-05	1	1	1	Q9Y4G6	Q9Y4G6 [974-100	0	3509.9095
[R].AELLVTE	1xTMTpro [K	3.502E-05	4.225E-05	1	1	1	P98160	P98160 [1759-17	0	2918.6694
[K].WKPDYD	2xTMTpro [K	5.149E-05	4.225E-05	1	1	2	Q99747	Q99747 [27-40]	0	2529.3442

[R].GSQAQF	1xTMTpro [K	1.738E-07	4.225E-05	1	1	3	Q9Y490	Q9Y490 [972-999	0	3362.82
[K].WKGDNS	1xCarbamido	3.409E-06	4.225E-05	1	1	1	Q15061	Q15061 [164-181]	1	2849.5284
[R].WKEATII	2xTMTpro [K	3.974E-05	4.225E-05	1	1	1	P02751	P02751 [654-669]	1	2770.6072
[K].WKDTDE	2xTMTpro [K	3.913E-05	4.225E-05	1	1	2	P45973	P45973 [142-154]	1	2416.3904
[K].WKDSDE	2xTMTpro [K	2.375E-05	4.225E-05	1	1	2	Q13185	Q13185 [142-154]	1	2402.3747
[R].GSQTQS	1xCarbamido	4.764E-07	4.225E-05	1	1	1	Q96PD5	Q96PD5 [234-256	0	2831.3338
[R].GSSCFE	2xCarbamido	1.286E-05	4.225E-05	1	1	1	P21964	P21964 [235-251]	0	2475.1029
[K].GSSGAS	1xTMTpro [N	4.642E-08	4.225E-05	1	1	4	Q14839	Q14839 [220-258]	0	3931.117
[K].GSSGAS	1xTMTpro [K	3.406E-07	4.225E-05	1	1	3	Q14839	Q14839 [220-259]	1	4363.4191
[K].WGTIEVI	1xCarbamido	5.084E-07	4.225E-05	1	1	1	Q9UHD8	Q9UHD8 [520-53	0	2530.2356
[K].WGTDEA	1xTMTpro [N	1.67E-05	4.225E-05	1	1	1	P08133	P08133 [192-206]	0	2087.0881
[K].AEISCTD	2xCarbamido	5.084E-07	4.225E-05	1	1	4	P21333	P21333 [1908-193	0	3909.9746
[R].GSSGGC	1xCarbamido	2.034E-08	4.225E-05	1	1	3	P13645	P13645 [60-86]	0	2647.1915
[R].WGLGGT	2xCarbamido	1.706E-05	4.225E-05	2	2	2	Q16881; Q9I	Q16881 [203-217]	0	2226.2019
[K].WGGQEY	1xTMTpro [K	5.334E-06	4.225E-05	1	1	2	Q8WVY7	Q8WVY7 [9-29]	0	2964.5334
[K].AELLDNE	2xTMTpro [K	8.665E-06	4.225E-05	1	1	2	Q9HB71	Q9HB71 [52-74]	0	3312.9235
[R].WLCTGD	2xCarbamido	9.293E-06	4.225E-05	1	1	2	O95573	O95573 [559-575]	0	2605.2922
[R].WLDESD	1xTMTpro [N	4.697E-05	4.225E-05	1	1	1	Q9P035	Q9P035 [110-121	0	1797.8648
[K].GSPLGE	1xTMTpro [N	4.841E-05	4.225E-05	1	1	3	P51398	P51398 [219-232]	0	1745.9717
[R].WLPAAA	1xCarbamido	3.604E-06	4.225E-05	1	1	1	P51649	P51649 [69-96]	0	3132.593
[R].WINDVE	1xTMTpro [N	5.576E-06	4.225E-05	1	1	2	P05023	P05023 [894-911]	0	2621.2228
[R].GSNPHL	1xTMTpro [N	4.673E-05	4.225E-05	1	1	1	Q9BSJ8	Q9BSJ8 [171-183	0	1809.9567
[R].GSNTIAS	2xTMTpro [K	1.277E-06	4.225E-05	1	1	5	P55060	P55060 [715-736]	1	3070.8081
[R].GSPAINV	1xTMTpro [N	1.502E-05	4.225E-05	1	1	3	P02766	P02766 [42-54]	0	1670.9661
[R].WLLLCN	1xCarbamido	3.399E-05	4.225E-05	1	1	1	P11216	P11216 [492-507]	0	2450.3973
[R].WLLEGT	1xTMTpro [N	2.87E-06	4.225E-05	1	1	1	O75781	O75781 [54-72]	1	2422.217
[K].WLKEDK	1xCarbamido	1.527E-05	4.225E-05	1	2	2	Q00610	Q00610 [451-468]	2	3407.9237
[R].WLHSMT	1xTMTpro [K	1.598E-05	4.225E-05	1	1	1	Q9UI09	Q9UI09 [89-106]	0	2675.4471
[R].GSPDGS	2xTMTpro [K	3.429E-05	4.225E-05	1	1	2	Q8IZL8	Q8IZL8 [480-495]	0	2439.4024
[R].GSSGGC	1xCarbamido	5.375E-05	4.225E-05	1	1	1	O00478	O00478 [212-223]	0	1453.7752
[R].AEIDYSD	1xTMTpro [N	1.092E-05	4.225E-05	1	1	2	O95155	O95155 [1221-124	1	3316.5673
[K].WLHGLE	1xTMTpro [K	4.442E-05	4.225E-05	1	1	2	Q01082	Q01082 [1395-14	0	2484.3015
[R].WLHEIN	2xCarbamido	4.22E-05	4.225E-05	1	1	1	Q15286	Q15286 [102-115]	0	2163.0031

[K].GSPFPE	1xTMTpro [N	8.414E-06	4.225E-05	1	1	2	Q8WVM8	Q8WVM8 [331-34	0	2456.2265
[K].AELEQH	1xTMTpro [K	2.911E-05	4.225E-05	1	1	1	Q16204	Q16204 [157-173	0	2707.4183
[R].WLEDVE	1xTMTpro [K	3.9E-05	4.225E-05	1	1	2	P02549	P02549 [702-717]	0	2592.3114
[K].AELFTQS	1xCarbamido	3.961E-05	4.225E-05	1	1	3	Q01082	Q01082 [1382-13	0	2106.1033
[K].GSPGAD	1xTMTpro [N	2.952E-05	4.225E-05	1	1	1	P02452	P02452 [935-958]	0	2377.218
[R].GLVLPV	1xTMTpro [K	5.348E-05	4.225E-05	1	1	1	Q71SY5	Q71SY5 [225-242	0	2242.3415
[K].WIDITPD	1xCarbamido	1.855E-05	4.225E-05	1	1	2	Q6FHJ7	Q6FHJ7 [152-171	0	3069.5703
[K].WLDLKD	2xTMTpro [K	1.76E-05	4.225E-05	1	1	3	Q96AG4	Q96AG4 [112-126	1	2649.5796
[R].WLHNED	1xTMTpro [K	2.524E-05	4.225E-05	1	1	5	P37837	P37837 [296-307]	0	2108.109
[R].WLPAEY	1xTMTpro [K	4.774E-06	4.225E-05	1	1	2	P05164	P05164 [198-218]	0	3020.569
[R].AEISNAI	1xTMTpro [K	3.023E-06	4.225E-05	1	1	3	P31939	P31939 [485-507]	0	3102.6338
[R].AEITDAE	1xTMTpro [K	3.242E-05	4.225E-05	1	1	1	Q14203	Q14203 [933-944	0	1825.0563
[K].GSTAAQ	1xCarbamido	3.322E-05	4.225E-05	1	1	1	P50452	P50452 [48-62]	0	2237.1914
[R].GSTAPV	1xTMTpro [N	1.004E-06	4.225E-05	1	1	3	Q15084	Q15084 [393-415	1	2660.3752
[K].AENGKL	2xTMTpro [K	1.04E-05	4.225E-05	1	1	1	P04406	P04406 [62-84]	2	3452.9682
[R].WEDVKE	1xTMTpro [K	2.218E-05	4.225E-05	1	1	1	P49137	P49137 [349-364]	1	2505.2973
[R].WEDTVE	1xTMTpro [K	9.909E-07	4.225E-05	1	1	1	P48681	P48681 [951-983]	1	4337.1011
[R].GSTDDK	2xTMTpro [K	3.601E-07	4.225E-05	1	1	3	Q96KP4	Q96KP4 [129-149	1	3132.7146
[K].AENNSE	1xTMTpro [N	4.954E-07	4.225E-05	1	1	2	Q08211	Q08211 [121-141	0	2467.1809
[K].WEAVDE	1xTMTpro [K	4.827E-06	4.225E-05	1	1	1	O15042	O15042 [761-778	0	2601.354
[R].GIVCPF	1xCarbamido	2.621E-05	4.225E-05	1	1	1	P19174	P19174 [1106-11	0	2792.4672
[R].WDVLIQ	1xCarbamido	3.526E-05	4.225E-05	1	1	2	Q9BUJ2	Q9BUJ2 [477-490	0	2049.0871
[K].GLVAVIT	1xTMTpro [N	3.307E-07	4.225E-05	1	1	4	Q99714	Q99714 [10-29]	0	2117.2249
[K].GSTLTSI	1xCarbamido	5.756E-05	4.225E-05	1	1	1	P08567	P08567 [52-65]	1	2162.152
[K].WDTCAF	1xCarbamido	2.045E-05	4.225E-05	1	1	1	O95861	O95861 [246-261	0	2361.2881
[K].AENSSLI	1xTMTpro [K	4.915E-05	4.225E-05	1	1	2	P46459	P46459 [188-198]	0	1754.0304
[K].GSTPYK	1xTMTpro [K	4.337E-05	4.225E-05	1	1	1	Q14160	Q14160 [747-762	1	2350.2534
[K].WDPYKC	2xTMTpro [K	4.027E-06	4.225E-05	1	1	2	P02675	P02675 [296-313]	1	2910.5566
[K].WDPTGI	1xCarbamido	3.813E-05	4.225E-05	1	1	1	Q9BZK7	Q9BZK7 [357-374	0	2632.3242
[K].GLTVPIA	1xTMTpro [K	7.83E-06	4.225E-05	1	1	2	Q8NCW5	Q8NCW5 [209-22	0	2605.4733
[K].GITTAAV	1xCarbamido	5.141E-05	4.225E-05	1	1	2	Q9Y315	Q9Y315 [93-105]	0	1682.9219
[R].GSTTATI	1xTMTpro [N	1.197E-05	4.225E-05	1	1	1	P11413	P11413 [331-348]	0	2232.1831
[R].AEPEDH	1xTMTpro [N	2.766E-06	4.225E-05	1	1	3	P61158	P61158 [103-123]	0	2786.3956

[K].GIVDQS(	1xTMTpro [K	5.94E-07	4.225E-05	1	1	22	P63104	P63104 [140-157]	0	2649.4016
[K].WEMPFI	1xTMTpro [N	5.366E-05	4.225E-05	1	1	1	P01011	P01011 [217-230]	0	2077.9721
[R].WEQGQ.	1xTMTpro [K	1.386E-05	4.225E-05	1	1	1	P46976	P46976 [325-342]	0	2683.2954
[K].GIVDQS(	2xTMTpro [K	2.194E-05	4.225E-05	1	1	1	P63104	P63104 [140-158]	1	3081.7037
[K].AEITFDD	1xTMTpro [K	5.86E-05	4.225E-05	1	1	1	O75369	O75369 [2236-22	0	1683.9198
[K].GLVEPVI	1xTMTpro [N	7.977E-08	4.225E-05	1	1	7	P21333	P21333 [1492-15	0	2848.4648
[K].GIVEFSC	1xTMTpro [K	5.039E-05	4.225E-05	1	1	1	Q15233	Q15233 [191-202	0	1840.0936
[K].WGDAG/	1xOxidation	9.407E-07	4.225E-05	1	1	61	P04406	P04406 [87-107]	0	2901.4472
[K].WGDAG/	1xTMTpro [K	3.635E-07	4.225E-05	1	1	111	P04406	P04406 [87-107]	0	2885.4523
[R].AEIVTEA	1xTMTpro [N	6.536E-06	4.225E-05	1	1	2	P24821	P24821 [1613-16	0	2991.5118
[R].AELYKEI	1xTMTpro [K	5.563E-05	4.225E-05	1	1	1	Q9H6R4	Q9H6R4 [58-71]	1	2314.2534
[R].GSSGVC	1xTMTpro [N	2.484E-05	4.225E-05	1	1	1	P33993	P33993 [408-420]	0	1491.8814
[R].GSSSGC	1xTMTpro [N	1.678E-07	4.225E-05	1	1	5	P35908	P35908 [525-544]	0	2044.9127
[R].WFNGQI	1xTMTpro [N	1.188E-06	4.225E-05	1	1	1	Q01081	Q01081 [134-151	0	2418.2525
[R].WGDSVI	1xTMTpro [N	2.001E-07	4.225E-05	1	1	4	Q9BYD3	Q9BYD3 [212-23	0	3116.5893
[K].AEMEDL	2xTMTpro [K	1.08E-05	4.225E-05	1	1	3	P35749	P35749 [1511-15	1	2535.3792
[R].GSSSYS	1xCarbamid	1.167E-05	4.225E-05	1	1	1	Q9UIA9	Q9UIA9 [54-69]	0	2295.251
[R].WFHPNI	1xTMTpro [N	1.967E-06	4.225E-05	1	1	1	Q06124	Q06124 [6-23]	0	2414.3151
[R].WFGFLE	1xTMTpro [K	1.184E-05	4.225E-05	1	1	2	P07814	P07814 [150-166]	0	2552.3793
[R].WFEEN/	1xTMTpro [K	4.345E-05	4.225E-05	1	1	2	Q12905	Q12905 [210-221	0	2034.0788
[R].WFCTGI	2xCarbamid	1.046E-06	4.225E-05	1	1	1	O60488	O60488 [550-570	0	2840.3455
[K].AEMIIEQ	1xTMTpro [K	9.327E-06	4.225E-05	1	1	1	Q9HB40	Q9HB40 [256-275	0	2921.5574
[R].GSSVDC	1xTMTpro [N	4.249E-05	4.225E-05	1	1	1	Q86U38	Q86U38 [311-324	0	1765.0179
[R].GSSVEM	1xTMTpro [K	1.795E-05	4.225E-05	1	1	1	Q8IYM9	Q8IYM9 [243-257	0	2259.222
[K].WESPA(	1xTMTpro [N	7.83E-06	4.225E-05	1	1	1	P17612	P17612 [31-46]	0	2233.0957
[K].WERPFE	2xTMTpro [K	9.431E-06	4.225E-05	1	1	7	P01009	P01009 [218-241]	1	3876.0272
[R].WFLNG(	1xTMTpro [N	1.815E-05	4.225E-05	1	1	1	P04440	P04440 [158-176]	0	2438.2999
[R].AELAGA	1xTMTpro [K	3.538E-05	4.225E-05	1	1	1	Q9H5N1	Q9H5N1 [45-58]	0	2073.1216
[R].WLPAEY	1xTMTpro [N	9.872E-07	4.225E-05	1	1	2	P11678	P11678 [170-189]	0	2625.3309
[K].WLPELV	1xTMTpro [K	1.409E-05	4.225E-05	1	1	1	Q02252	Q02252 [332-341	0	1830.0769
[R].WTDENI	1xTMTpro [K	2.551E-05	4.225E-05	1	1	4	Q14204	Q14204 [2845-28	0	2013.1148
[R].WTCTSS	2xCarbamid	2.978E-06	4.225E-05	1	1	1	Q7L576	Q7L576 [332-353	0	2950.3817
[R].GIYKDDI	2xTMTpro [K	1.735E-05	4.225E-05	1	1	1	O00267	O00267 [284-306	1	3508.9355

[R].WTCCAL	2xCarbamido	1.651E-05	4.225E-05	1	1	1	O15371	O15371 [435-448]	0	2280.2012
[R].WSTLVE	1xTMTpro [N	2.755E-05	4.225E-05	1	1	2	O00232	O00232 [317-329]	0	1902.9591
[K].WSTIIEN	1xTMTpro [K	1.885E-05	4.225E-05	1	1	1	P20591	P20591 [414-427]	0	2311.2327
[K].WSTDDV	1xTMTpro [K	7.101E-06	4.225E-05	1	1	3	P46782	P46782 [23-42]	0	2931.5595
[R].GSHTDA	1xCarbamido	3.726E-06	4.225E-05	1	1	1	Q9P2R3	Q9P2R3 [447-464	0	2218.0842
[K].GLYGGS	2xCarbamido	1.04E-06	4.225E-05	1	1	1	O94855	O94855 [1009-10	0	2570.3027
[R].GSLAEA	1xTMTpro [N	3.416E-06	4.225E-05	1	1	1	Q9Y6I3	Q9Y6I3 [446-468]	0	2476.3367
[K].AEENLLI	1xTMTpro [N	2.621E-05	4.225E-05	1	1	2	Q9H9E3	Q9H9E3 [247-262	0	2098.048
[R].WSQEDI	1xCarbamido	4.127E-05	4.225E-05	1	1	1	P17480	P17480 [22-35]	0	2392.2206
[R].WSPIAS	1xTMTpro [N	3.8E-05	4.225E-05	1	1	2	Q13561	Q13561 [319-332]	0	1901.0816
[K].WSPELP	2xCarbamido	5.619E-07	4.225E-05	1	1	4	P02749	P02749 [130-154]	0	3123.6694
[R].GSLCAT	2xCarbamido	5.384E-05	4.225E-05	1	1	2	O43294	O43294 [402-415]	0	1752.9056
[R].GSLCSG	2xCarbamido	2.068E-05	4.225E-05	1	1	1	P49023	P49023 [532-545]	0	2129.1451
[R].GLYDGP	1xCarbamido	8.199E-06	4.225E-05	1	1	2	Q16555	Q16555 [497-511]	0	2229.2081
[R].WSGQT	2xCarbamido	4.263E-07	4.225E-05	1	1	1	P00751	P00751 [151-175]	0	2956.4001
[R].GIYAYGI	1xTMTpro [K	2.65E-06	4.225E-05	3	3	3	P60842; P38	P60842 [46-61]; F	0	2436.3531
[K].GSLESP	1xTMTpro [K	1.662E-07	4.225E-05	1	1	1	O00232	O00232 [331-350]	1	2704.3921
[K].GLWMD	2xCarbamido	1.892E-05	4.225E-05	1	1	2	O95471	O95471 [49-65]	0	2600.2295
[R].WTELAG	1xCarbamido	5.563E-05	4.225E-05	1	1	2	Q12907	Q12907 [196-207]	0	1730.8491
[K].GSGVVII	1xTMTpro [K	4.002E-05	4.225E-05	1	1	1	P51659	P51659 [436-451]	0	2355.2762
[K].GSGTAE	2xTMTpro [K	5.244E-05	4.225E-05	1	1	2	P14618	P14618 [126-136]	1	2031.2266
[K].WTMLIT	1xTMTpro [K	3.689E-05	4.225E-05	1	1	1	Q8WXH0	Q8WXH0 [5037-5	0	2324.318
[R].WVTYFN	1xTMTpro [K	3.505E-06	4.225E-05	1	1	2	P20674	P20674 [56-71]	0	2661.4321
[R].GSGDVC	1xCarbamido	1.261E-06	4.225E-05	1	1	1	Q8WUY8	Q8WUY8 [103-12	0	2319.1682
[R].WVQTLS	1xTMTpro [N	9.893E-06	4.225E-05	1	1	1	P02649	P02649 [57-79]	0	3034.6016
[R].AEDNAD	1xTMTpro [K	2.797E-06	4.225E-05	1	1	2	P12004	P12004 [92-110]	0	2683.407
[R].GMAFLH	1xOxidation	5.536E-05	4.225E-05	1	1	1	Q13418	Q13418 [304-317]	0	1915.0795
[R].GSGGGS	1xTMTpro [N	4.293E-05	4.225E-05	1	1	6	P04264	P04264 [603-616]	0	1396.71
[K].WVPLQII	1xTMTpro [K	5.609E-05	4.225E-05	1	1	1	Q6PKG0	Q6PKG0 [252-265	0	2316.3394
[K].WVPFDC	1xTMTpro [N	2.045E-05	4.225E-05	1	1	2	P39656	P39656 [345-359]	0	2140.1034
[R].GSGGLG	1xCarbamido	6.07E-06	4.225E-05	2	3	3	P04259; P02	P04259 [43-59]; F	0	1728.8407
[R].GMADQI	1xOxidation	1.793E-06	4.225E-05	1	1	3	P19827	P19827 [613-640]	0	3923.0711
[K].AEFAEV	2xTMTpro [K	2.673E-05	4.225E-05	1	1	1	P02768	P02768 [250-264]	1	2563.5163

[R].GMADQI	2xTMTpro [K	2.163E-07	4.225E-05	1	1	2	P19827	P19827 [613-640]	0	3907.0761
[R].WVGGPI	1xTMTpro [N	7.787E-06	4.225E-05	1	1	2	P48643	P48643 [324-340]	0	2043.1558
[R].WVEQHI	1xTMTpro [N	2.745E-05	4.225E-05	1	1	1	Q8TCD5	Q8TCD5 [115-127	0	1929.0302
[R].GSGQGI	1xTMTpro [K	2.855E-05	4.225E-05	1	1	1	Q5J8M3	Q5J8M3 [35-50]	0	2264.2054
[K].GSGSAC	1xTMTpro [K	6.801E-07	4.225E-05	1	1	1	P18084	P18084 [98-123]	0	3334.7709
[K].WVAIQS	1xTMTpro [K	7.301E-06	4.225E-05	1	1	1	Q96KP4	Q96KP4 [25-38]	1	2265.2999
[K].WVAIQS	1xTMTpro [K	3.92E-05	4.225E-05	1	1	3	Q96KP4	Q96KP4 [25-37]	0	2109.1988
[R].GIYTGLS	1xTMTpro [N	5.226E-05	4.225E-05	1	1	2	Q02978	Q02978 [79-90]	0	1524.9069
[R].WTPYQ(	3xCarbamido	5.936E-05	4.225E-05	1	1	2	P04003	P04003 [354-369]	0	2604.2541
[K].AEEEHL(	1xTMTpro [K	3.255E-06	4.225E-05	1	1	1	P00450	P00450 [800-819]	0	2736.4812
[K].WTNQLN	1xTMTpro [K	2.444E-05	4.225E-05	1	1	1	P61201	P61201 [427-441]	0	2310.3062
[K].GMAAAC	1xTMTpro [N	2.701E-05	4.225E-05	1	1	3	Q9Y3I0	Q9Y3I0 [309-321]	0	1684.8549
[R].WQVVD	1xTMTpro [N	3.416E-06	4.225E-05	1	1	1	Q9BZE4	Q9BZE4 [217-233	0	2294.21
[K].GSIFVVF	1xTMTpro [K	1.026E-05	4.225E-05	1	1	4	P05455	P05455 [152-165]	0	2107.1931
[K].WQQHQ	1xTMTpro [N	4.233E-06	4.225E-05	1	1	1	Q2TAY7	Q2TAY7 [171-188	0	2441.3083
[K].GLVVDM	1xTMTpro [K	4.567E-06	4.225E-05	1	1	1	P49588	P49588 [433-446]	1	2232.1826
[K].GLVVDM	1xTMTpro [N	3.393E-05	4.225E-05	1	1	2	P49588	P49588 [433-445]	0	1799.8805
[R].AEGSDV	1xCarbamido	4.44E-07	4.225E-05	1	1	4	P14618	P14618 [343-367]	0	3102.5579
[R].AEGSDV	1xCarbamido	1.27E-06	4.225E-05	1	1	4	P14618	P14618 [343-367]	0	3118.5528
[R].AEGSDV	1xCarbamido	9.321E-09	4.225E-05	1	1	4	P14618	P14618 [343-376]	1	4103.0557
[R].GSLLWN	1xTMTpro [N	1.832E-05	4.225E-05	1	1	1	Q5UCC4	Q5UCC4 [72-88]	0	2207.174
[K].WMEEQ/	1xTMTpro [K	1.589E-05	4.225E-05	1	1	2	P09234	P09234 [41-52]	0	2086.1135
[K].AEGSEY	1xTMTpro [N	1.195E-05	4.225E-05	1	1	2	Q07954	Q07954 [3862-38	0	2389.1843
[K].AEGSST.	1xTMTpro [K	1.006E-05	4.225E-05	1	1	1	Q9NZB2	Q9NZB2 [503-519	0	2175.1385
[R].GLVSSD	1xTMTpro [K	3.276E-07	4.225E-05	1	1	2	Q13813	Q13813 [1360-13	1	2781.549
[R].WNDNCI	1xCarbamido	4.721E-05	4.225E-05	1	1	2	P17655	P17655 [297-312]	0	2337.0525
[R].WIVPGG	1xTMTpro [N	4.05E-05	4.225E-05	1	1	1	O95989	O95989 [46-65]	0	2414.2345
[R].GSLTFEI	1xTMTpro [K	2.109E-05	4.225E-05	1	1	2	Q9NQW7	Q9NQW7 [555-57	0	2352.4034
[R].GSLVDN	1xTMTpro [N	2.538E-05	4.225E-05	1	1	1	O43427	O43427 [153-168	0	2146.1576
[R].WLSTRF	1xCarbamido	9.276E-06	4.225E-05	1	1	1	Q8TEM1	Q8TEM1 [56-79]	0	3379.7812
[K].GSLVQA	1xTMTpro [N	3.432E-07	4.225E-05	1	1	2	P19827	P19827 [342-360]	0	2308.2216
[R].WLSDEC	1xCarbamido	2.947E-05	4.225E-05	1	1	2	O75521	O75521 [375-390	0	2215.1137
[R].WLSAEII	2xTMTpro [K	2.644E-05	4.225E-05	1	1	2	Q16795	Q16795 [361-373	0	2398.4162

[K].WIQGLQ	1xTMTpro [N	4.11E-06	4.225E-05	1	1	1	Q9BQ95	Q9BQ95 [373-392	0	2614.4676
[K].WIQEAE	1xTMTpro [N	1.279E-05	4.225E-05	1	1	1	P46939	P46939 [2458-247	0	2372.2529
[K].AEKDEP	2xTMTpro [K	2.312E-05	4.225E-05	1	1	2	Q14697	Q14697 [218-231]	1	2549.3704
[R].GSNNVA	1xTMTpro [K	1.35E-06	4.225E-05	1	1	3	P35606	P35606 [282-298]	0	2344.3004
[R].WLVIDP	1xTMTpro [K	1.034E-05	4.225E-05	1	1	1	Q6P1N0	Q6P1N0 [806-824	0	2566.5253
[K].AEPEKTI	2xTMTpro [K	1.61E-05	4.225E-05	1	1	3	P80723	P80723 [85-97]	1	2243.2699
[R].WNDVA(	1xTMTpro [K	4.449E-05	4.225E-05	1	1	1	Q9UN37	Q9UN37 [126-136	0	1767.9885
[R].WNGHTI	1xTMTpro [K	8.199E-06	4.225E-05	1	1	1	Q9UHG3	Q9UHG3 [485-50	0	2530.2528
[R].WQQGN	1xCarbamido	6.946E-06	4.225E-05	1	1	1	P01860	P01860 [347-365]	0	2618.2676
[R].WQPAIA	1xTMTpro [K	2.735E-06	4.225E-05	1	1	3	P24821	P24821 [1819-18	0	2736.474
[R].WQLSVA	1xTMTpro [N	5.796E-06	4.225E-05	1	1	1	Q9NR30	Q9NR30 [703-718	0	2144.1307
[R].AEFSAG	1xTMTpro [N	4.689E-05	4.225E-05	1	1	2	Q8N6S5	Q8N6S5 [57-68]	0	1611.8086
[K].GSLGG(	1xTMTpro [N	1.162E-07	4.225E-05	1	1	3	P13645	P13645 [41-59]	0	2011.9793
[R].WQCDA	1xCarbamido	2.471E-05	4.225E-05	1	1	2	P11215	P11215 [503-519]	0	2354.1307
[K].AEGAAT	1xTMTpro [K	2.649E-05	4.225E-05	1	1	3	P80723	P80723 [26-38]	0	1897.9999
[K].GLVYET	1xTMTpro [N	1.274E-05	4.225E-05	1	1	2	O75390	O75390 [77-92]	0	2067.0929
[R].GSIHDF	1xTMTpro [K	3.648E-07	4.225E-05	1	1	2	P08133	P08133 [12-34]	0	3132.5592
[K].WPYAG	1xTMTpro [K	5.183E-05	4.225E-05	1	2	2	A0A0B4J2D	A0A0B4J2D5 [20	0	1815.0296
[K].WNFYYS	2xTMTpro [K	7.22E-06	4.225E-05	1	1	1	P04114	P04114 [4022-40	1	2659.4337
[K].GSLLFTA	1xTMTpro [N	4.33E-05	4.225E-05	1	1	2	P53004	P53004 [148-161]	0	1822.987
[K].GSLLIDS	1xTMTpro [K	4.009E-05	4.225E-05	1	1	1	P31937	P31937 [126-141]	0	2211.2728
[R].GLVVLG	1xCarbamido	1.343E-06	4.225E-05	1	1	2	P07203	P07203 [70-88]	0	2723.4583
[K].WNTDN	1xTMTpro [N	8.703E-07	4.225E-05	1	1	4	P21796	P21796 [75-93]	0	2480.2588
[K].AEGGG(	1xTMTpro [K	3.299E-05	4.225E-05	1	1	3	Q00839	Q00839 [216-234	0	2147.1449
[K].WNTDN	1xTMTpro [K	1.601E-05	4.225E-05	1	1	2	Q9Y277	Q9Y277 [75-90]	0	2516.2913
[K].WNTDN	1xCarbamido	9.099E-07	4.225E-05	1	1	5	P45880	P45880 [86-107]	0	3127.6225
[R].WNSPAE	1xCarbamido	1.735E-05	4.225E-05	1	1	2	P34932	P34932 [406-422]	0	2537.211
[K].WNPFDI	1xCarbamido	2.511E-05	4.225E-05	1	1	1	Q92828	Q92828 [90-108]	0	2853.3897
[K].WNNTG(	1xCarbamido	1.076E-05	4.225E-05	1	1	1	P01833	P01833 [498-515]	0	2597.291
[R].AEGIPVE	1xTMTpro [K	2.21E-06	4.225E-05	1	1	2	Q3LXA3	Q3LXA3 [127-147	0	2798.5505
[K].GSLLGC	1xCarbamido	3.375E-07	4.225E-05	1	1	2	P17655	P17655 [235-257]	0	2992.5793
[K].VVNVAN	1xTMTpro [N	1.683E-06	4.225E-05	1	1	1	O00203	O00203 [1026-10	0	2250.2274
[K].AEPLAF	1xTMTpro [K	2.786E-06	4.225E-05	1	1	1	P04114	P04114 [1935-19	0	2134.1465

[R].WDNNY(	1xTMTpro [N	3.845E-06	4.225E-05	1	1	2	P21980	P21980 [241-262]	0	2785.3575
[R].VVYSPV	1xTMTpro [N	6.334E-06	4.225E-05	1	1	2	Q99715	Q99715 [1966-19	0	2633.4218
[R].VVYNVL	1xTMTpro [K	3.893E-05	4.225E-05	2	2	2	P23497; Q9I	P23497 [105-115]	0	1901.1239
[R].VVYGGG	2xCarbamid	4.032E-09	4.225E-05	1	1	1	P48643	P48643 [418-449]	1	4053.0375
[R].VVYEPL	1xTMTpro [K	5.192E-05	4.225E-05	1	1	1	Q7Z5N4	Q7Z5N4 [1837-18	0	2094.2455
[K].VVYENA	1xTMTpro [N	1.332E-05	4.225E-05	1	1	1	Q16881	Q16881 [275-289	0	2054.0779
[K].AFFESHI	1xTMTpro [N	4.579E-05	4.225E-05	1	1	2	P55786	P55786 [870-882]	0	1749.888
[K].GTDTVA	1xTMTpro [K	4.279E-05	4.225E-05	1	1	2	P43490	P43490 [217-228]	0	1767.0872
[R].VVWTAC	1xTMTpro [N	2.74E-05	4.225E-05	1	1	2	P22105	P22105 [979-994]	0	2246.1929
[K].GTDVNV	1xTMTpro [N	1.244E-05	4.225E-05	1	1	2	P04083	P04083 [215-228]	0	1855.0244
[R].VVVVDD	1xCarbamid	2.126E-06	4.225E-05	1	1	2	P07741	P07741 [123-145]	0	2694.4126
[R].AFGFSH	1xTMTpro [K	5.545E-05	4.225E-05	1	1	2	Q96FW1	Q96FW1 [95-109]	0	2258.2313
[R].AFGGPG	1xCarbamid	3.293E-05	4.225E-05	1	1	1	Q9H479	Q9H479 [16-29]	0	1639.8182
[R].VVVVDD	1xCarbamid	6.269E-07	4.225E-05	1	1	2	P07741	P07741 [123-145]	0	2678.4176
[R].VVVTVE	1xTMTpro [N	1.879E-05	4.225E-05	1	1	1	O15269	O15269 [446-459	0	1964.0507
[R].GISTKP\	2xTMTpro [K	6.67E-06	4.225E-05	1	1	1	Q14258	Q14258 [316-332	0	2836.6753
[R].GISSQPI	1xCarbamid	5.711E-06	4.225E-05	1	1	1	Q16401	Q16401 [401-416	0	2363.3037
[R].AFGPGL	1xTMTpro [K	3.075E-05	4.225E-05	1	1	2	Q14315	Q14315 [1355-13	0	1867.0933
[K].AFGPGL	1xCarbamid	4.171E-06	4.225E-05	1	1	1	Q14315	Q14315 [672-695	0	3169.6847
[K].AFGPGL	1xTMTpro [N	4.931E-05	4.225E-05	1	1	2	P21333	P21333 [1072-10	0	1733.9254
[K].VVVQVL	1xTMTpro [K	2.779E-05	4.225E-05	1	1	1	P49327	P49327 [1852-18	0	2231.3506
[R].AFGSPF	1xCarbamid	6.09E-05	4.225E-05	1	1	1	Q9BQ24	Q9BQ24 [29-48]	1	2957.5111
[R].VVYTGT	1xTMTpro [K	3.741E-05	4.225E-05	1	1	1	Q96F85	Q96F85 [83-98]	0	2339.2626
[R].AFENDV	1xCarbamid	2.337E-05	4.225E-05	1	1	1	P05166	P05166 [260-272]	0	1840.9183
[K].VWAHYE	1xTMTpro [K	5.853E-07	4.225E-05	1	1	2	Q7KZF4	Q7KZF4 [657-676	0	3049.5836
[K].VWAHYE	1xOxidation	3.734E-05	4.225E-05	1	1	1	Q7KZF4	Q7KZF4 [657-676	0	3065.5785
[K].VWTSGC	1xTMTpro [N	4.926E-06	4.225E-05	1	1	1	P29972	P29972 [244-263]	0	2616.2385
[K].AFDFLSE	1xTMTpro [N	4.192E-05	4.225E-05	1	1	1	O60271	O60271 [606-621	0	2077.0409
[K].GLTAVSI	1xTMTpro [N	5.915E-06	4.225E-05	1	1	1	P55809	P55809 [84-104]	0	2405.3472
[K].VWSEVN	1xTMTpro [N	5.419E-05	4.225E-05	1	1	2	Q9NWU2	Q9NWU2 [172-18	0	2126.0838
[K].AFDLIVD	1xTMTpro [N	8.005E-06	4.225E-05	1	1	1	O96000	O96000 [30-43]	0	1918.1445
[K].VWSDVT	1xTMTpro [N	4.981E-05	4.225E-05	1	1	1	P24347	P24347 [136-152]	0	2277.1835
[R].VWQIQS	2xCarbamid	9.166E-07	4.225E-05	1	1	2	P36405	P36405 [152-175]	0	3361.6623

[K].VWQLQI	1xTMTpro [N	3.253E-05	4.225E-05	1	1	2	Q9NYU2	Q9NYU2 [320-333	0	1967.067
[K].VWQLGS	1xTMTpro [K	2.628E-07	4.225E-05	1	1	2	P35606	P35606 [169-186]	0	2624.3964
[R].AFDQGA	1xTMTpro [K	7.023E-06	4.225E-05	1	1	1	Q9UDW1	Q9UDW1 [35-51]	0	2472.2651
[R].AFGYVC	1xCarbamido	1.955E-05	4.225E-05	1	1	1	P98082	P98082 [133-150]	0	2624.3575
[K].AFDSGII	1xTMTpro [K	1.359E-05	4.225E-05	1	1	3	P53396	P53396 [949-962]	0	2176.1968
[K].VWPHKI	2xTMTpro [K	5.031E-05	4.225E-05	1	1	2	P04040	P04040 [302-315]	1	2561.5424
[K].GTDSQA	1xTMTpro [K	3.644E-06	4.225E-05	1	1	1	Q13451	Q13451 [442-457	0	2352.1633
[K].VWLEVE	1xTMTpro [K	2.263E-05	4.225E-05	1	1	2	P43121	P43121 [252-263]	0	2008.1797
[K].VWLDPN	1xTMTpro [N	1.789E-05	4.225E-05	1	1	3	P84098	P84098 [22-38]	0	2247.1325
[R].VWGVGI	1xTMTpro [K	1.242E-06	4.225E-05	1	1	2	Q53GQ0	Q53GQ0 [36-64]	0	3377.7985
[K].VWGNV	1xTMTpro [K	1.112E-06	4.225E-05	1	1	5	O43390	O43390 [316-339	0	3304.7055
[K].VWGNV	1xTMTpro [K	8.703E-07	4.225E-05	1	1	3	O60506	O60506 [313-336	0	3262.6586
[K].GTDSQA	1xOxidation	2.515E-05	4.225E-05	1	1	1	Q13451	Q13451 [442-457	0	2368.1582
[R].VWDIAY	1xTMTpro [K	5.907E-05	4.225E-05	1	1	1	Q9UNX4	Q9UNX4 [217-235	0	2894.4955
[K].VWDLAQ	1xTMTpro [N	1.805E-05	4.225E-05	1	1	4	Q9BQA1	Q9BQA1 [151-164	0	1968.0874
[K].VWPQQI	1xCarbamido	1.854E-07	4.225E-05	1	1	3	P02765	P02765 [68-99]	0	3982.0227
[R].VWYVSN	1xTMTpro [K	1.857E-06	4.225E-05	1	1	1	P06744	P06744 [181-194]	0	2211.2418
[R].VVVLMG	1xCarbamido	3.893E-05	4.225E-05	1	1	1	P22234	P22234 [268-283]	0	2340.2547
[R].VVVDPV	1xTMTpro [K	1.078E-05	4.225E-05	1	1	1	Q8WWQ0	Q8WWQ0 [1511-	0	2422.3685
[K].AFIPAIDS	1xTMTpro [N	1.222E-05	4.225E-05	1	1	2	Q15029	Q15029 [873-888	0	2103.1082
[R].VVSGMV	1xCarbamido	7.257E-07	4.225E-05	1	1	3	P21980	P21980 [223-240]	0	2237.1338
[R].VVSEDF	1xTMTpro [K	1.222E-05	4.225E-05	1	1	3	P09874	P09874 [453-467]	0	2233.2208
[R].VVRPDS	1xTMTpro [K	1.569E-05	4.225E-05	1	1	1	Q15293	Q15293 [38-66]	0	3968.007
[R].AFLQGG	1xTMTpro [N	4.827E-06	4.225E-05	1	1	1	Q14203	Q14203 [769-785	0	2120.2035
[K].VVQLHE	1xTMTpro [K	2.658E-05	4.225E-05	1	1	1	Q14683	Q14683 [111-123	0	2211.2153
[R].VVQHSN	1xTMTpro [N	3.375E-05	4.225E-05	1	1	1	Q16795	Q16795 [119-132	0	1852.1088
[K].VVQHLG	1xTMTpro [K	4.184E-05	4.225E-05	1	1	1	O95487	O95487 [837-849	0	2083.1832
[R].VVQGDI	1xTMTpro [K	5.485E-07	4.225E-05	1	1	2	Q86XP3	Q86XP3 [459-484	0	3342.8037
[R].GTETTL	1xOxidation	4.6E-07	4.225E-05	1	1	5	Q05707	Q05707 [436-460	0	3061.5393
[R].GTETTL	2xOxidation	5.217E-06	4.225E-05	1	1	2	Q05707	Q05707 [436-460	0	3077.5342
[K].VVQDLIF	2xTMTpro [K	8.383E-06	4.225E-05	1	1	2	Q9HCB6	Q9HCB6 [346-375	0	4197.2536
[K].GTFAQL	1xCarbamido	2.148E-05	4.225E-05	2	2	2	P69891; P69	P69891 [84-96]; F	0	2114.1196
[K].GTFATLS	1xCarbamido	7.589E-06	4.225E-05	1	1	58	P68871	P68871 [84-96]	0	2087.1087

[R].VVQCSD	1xCarbamid	1.011E-05	4.225E-05	1	1	3	P07585	P07585 [64-77]	1	2470.4519
[R].GLSPAQ	1xTMTpro [K	3.276E-05	4.225E-05	1	1	2	O43491	O43491 [384-399]	0	2284.2429
[K].GTFATLS	1xCarbamid	7.177E-07	4.225E-05	1	1	18	P68871	P68871 [84-105]	1	3194.6548
[R].AFPALTS	1xTMTpro [N	5.131E-06	4.225E-05	1	1	1	P08571	P08571 [192-210]	0	2277.2046
[K].VVPSFLF	1xTMTpro [N	4.989E-05	4.225E-05	1	1	1	Q14126	Q14126 [668-684]	0	2031.1558
[K].VVPLADI	1xTMTpro [N	1.002E-06	4.225E-05	1	1	2	O00764	O00764 [140-160]	0	2586.4462
[K].GTFSQL	1xCarbamid	5.009E-06	4.225E-05	1	1	6	P02042	P02042 [84-96]	0	2130.1145
[R].AFINLEA	1xTMTpro [K	1.163E-05	4.225E-05	1	1	2	Q7Z2K6	Q7Z2K6 [273-285	0	1855.0933
[R].VVSGMV	1xCarbamid	5.444E-06	4.225E-05	1	1	4	P21980	P21980 [223-240]	0	2253.1287
[R].GTELGG	1xTMTpro [N	7.977E-07	4.225E-05	1	1	2	Q9Y6C2	Q9Y6C2 [384-406	0	2445.2442
[K].VVSLEAI	2xTMTpro [K	1.102E-05	4.225E-05	1	1	1	Q92614	Q92614 [1516-15	1	3488.9152
[K].GTEAAG	1xTMTpro [K	3.518E-06	4.225E-05	1	1	8	P01009	P01009 [368-389]	0	2867.5542
[K].GTEAAG	1xOxidation	9.483E-06	4.225E-05	1	1	9	P01009	P01009 [368-389]	0	2883.5492
[K].VVTPLSF	1xTMTpro [N	1.406E-06	4.225E-05	1	1	1	P02751	P02751 [1170-11	0	3346.7966
[K].AFLADPS	1xTMTpro [K	1.7E-07	4.225E-05	1	1	6	P05388	P05388 [267-297]	0	3360.8811
[R].VVTNYN	1xTMTpro [N	2.878E-07	4.225E-05	1	1	1	P21980	P21980 [297-317]	0	2801.4178
[K].GTEDELI	2xTMTpro [K	4.525E-05	4.225E-05	2	2	2	P09493; P06	P09493 [52-65]; F	1	2510.3806
[K].VVTDTDI	1xTMTpro [N	5.123E-05	4.225E-05	1	1	5	P05198	P05198 [277-288]	0	1652.8662
[K].GTEDELI	3xTMTpro [K	7.503E-07	4.225E-05	2	2	3	P09493; P06	P09493 [52-70]; F	2	3385.8479
[R].AFLDFH/	1xTMTpro [N	7.22E-06	4.225E-05	1	1	1	Q9H7Z7	Q9H7Z7 [118-136	0	2518.3777
[K].VVTATLV	1xTMTpro [K	1.059E-06	4.225E-05	1	1	1	P27694	P27694 [355-372]	1	2575.3648
[K].VVVLGS	1xTMTpro [K	5.41E-05	4.225E-05	1	3	2	P61225	P61225 [6-16]	0	1580.0027
[R].VVSVLTV	2xTMTpro [K	3.931E-06	4.225E-05	1	1	2	P01859	P01859 [181-199]	1	3126.8132
[R].VVSVLTV	2xTMTpro [K	1.317E-05	4.225E-05	3	4	2	P01861; P0D	P01861 [182-200]	1	3140.8288
[K].AFLEVNI	1xTMTpro [N	6.222E-07	4.225E-05	1	1	21	P01008	P01008 [403-425]	0	2595.3585
[R].VVSVLTV	1xTMTpro [K	1.233E-05	4.225E-05	3	4	5	P01861; P0D	P01861 [182-197]	0	2416.4208
[K].VVSQYS	1xOxidation	5.322E-05	4.225E-05	1	1	1	P50991	P50991 [175-193]	0	2664.4596
[K].VVSQYS	1xTMTpro [K	8.617E-06	4.225E-05	1	1	2	P50991	P50991 [175-193]	0	2648.4647
[K].VVSQYH	1xTMTpro [N	2.706E-05	4.225E-05	1	1	1	Q16891	Q16891 [354-366]	0	1832.035
[K].GTEDFIV	1xTMTpro [N	1.67E-05	4.225E-05	1	1	3	P43307	P43307 [111-125]	0	1990.0089
[K].GTEFAE	1xTMTpro [N	2.727E-07	4.225E-05	1	1	2	P04921	P04921 [98-123]	0	2883.3564
[K].VVSNFM	1xTMTpro [K	5.022E-05	4.225E-05	1	1	1	Q9H0P0	Q9H0P0 [226-240	0	2309.2343
[R].VVSMDE	1xTMTpro [K	2.844E-06	4.225E-05	1	1	4	P01023	P01023 [148-171]	0	3418.8576

[R].VVSVLT\	1xTMTpro [K	1.401E-05	4.225E-05	1	1	5	P01859	P01859 [181-196]	0	2402.4051
[K].VYACEV	1xCarbamido	1.099E-06	4.225E-05	2	2	6	P01834; P0D	P01834 [84-100];	0	2484.3412
[R].VYALPEI	1xTMTpro [K	3.381E-05	4.225E-05	1	1	3	P13796	P13796 [597-610]	0	2194.2615
[R].VYAPAS	1xTMTpro [N	6.59E-07	4.225E-05	1	1	1	Q14126	Q14126 [967-990]	0	2883.5059
[R].GSVTFH	1xCarbamido	6.183E-06	4.225E-05	1	1	1	P01833	P01833 [251-268]	0	2465.3466
[R].WAELLP	1xCarbamido	2.844E-05	4.225E-05	1	1	5	P13489	P13489 [20-34]	0	2157.2173
[R].GITNLC\	1xCarbamido	7.049E-06	4.225E-05	1	1	1	Q01813	Q01813 [118-138]	0	2439.2985
[K].GSVYIG(	1xTMTpro [N	3E-06	4.225E-05	1	1	1	P98160	P98160 [4330-434	0	1995.083
[K].GLTMLD	1xOxidation [	4.427E-05	4.225E-05	1	1	1	Q9UBE0	Q9UBE0 [62-83]	0	2787.4306
[R].AESLQQ	1xTMTpro [K	2.88E-05	4.225E-05	1	1	1	Q14203	Q14203 [318-329]	0	1953.1148
[K].GLTMLD	1xTMTpro [N	2.21E-06	4.225E-05	1	1	1	Q9UBE0	Q9UBE0 [62-83]	0	2771.4357
[R].WADAVL	1xTMTpro [K	2.295E-05	4.225E-05	1	1	1	Q4G0N4	Q4G0N4 [151-17(	0	2526.3882
[R].GSYGDL	1xTMTpro [K	1.317E-05	4.225E-05	1	1	3	P61978	P61978 [378-396]	0	2525.4471
[R].GTADVT	1xTMTpro [K	6.682E-06	4.225E-05	1	1	2	P11166	P11166 [233-249]	1	2554.3063
[K].VYYTAG	1xTMTpro [K	1.782E-05	4.225E-05	1	1	3	Q9Y5Z4	Q9Y5Z4 [173-184	0	1970.0879
[K].AESSFSI	2xTMTpro [K	7.088E-06	4.225E-05	1	1	1	Q9Y6G5	Q9Y6G5 [45-65]	1	3300.7303
[K].AESSSD	1xTMTpro [N	1.002E-05	4.225E-05	1	1	2	Q16363	Q16363 [683-696]	0	1728.8207
[R].AESTAN	1xTMTpro [N	5.482E-05	4.225E-05	1	1	1	O75179	O75179 [224-236]	0	1624.7846
[K].VYYDGT	1xTMTpro [K	5.454E-06	4.225E-05	1	1	1	Q7Z4R8	Q7Z4R8 [133-159	0	3464.689
[K].VYWSGF	1xCarbamido	2.511E-05	4.225E-05	1	1	1	O14896	O14896 [321-339]	0	2433.292
[K].GTATEPI	1xCarbamido	1.038E-05	4.225E-05	1	1	1	Q9P2B4	Q9P2B4 [299-321	0	2906.4058
[R].AETECQ	1xCarbamido	3.734E-05	4.225E-05	1	1	2	P13645	P13645 [423-439]	0	2691.3791
[R].VYVGSI\	1xTMTpro [N	2.875E-05	4.225E-05	1	1	1	Q9UHX1	Q9UHX1 [131-14(	0	2181.1399
[K].VYVGNL	1xTMTpro [K	4.756E-06	4.225E-05	1	1	1	Q16629	Q16629 [13-29]	1	2328.3167
[K].VYVGNL	1xTMTpro [K	1.757E-05	4.225E-05	1	1	4	Q16629	Q16629 [13-24]	0	1744.0249
[K].GLTPTGI	1xTMTpro [N	4.665E-05	4.225E-05	1	1	1	P16298	P16298 [434-450]	0	1888.0645
[R].AESAPLI	1xTMTpro [N	1.329E-05	4.225E-05	1	1	1	O95674	O95674 [39-57]	0	2285.1944
[K].GSVAIW	1xCarbamido	1.676E-05	4.225E-05	1	1	4	P15529	P15529 [145-159]	0	2541.4679
[K].AERPGL	1xTMTpro [N	4.48E-05	4.225E-05	1	1	1	Q6P1M3	Q6P1M3 [683-698	0	2113.1507
[K].AEPPKAI	2xTMTpro [K	9.174E-06	4.225E-05	1	1	3	P80723	P80723 [98-121]	1	3210.7575
[K].AEPPQC	1xCarbamido	4.694E-07	4.225E-05	1	1	3	P63244	P63244 [281-308]	0	3372.649
[K].WDISDS	1xTMTpro [N	4.564E-05	4.225E-05	1	1	4	Q05707	Q05707 [755-766]	0	1799.8883
[K].GSTVNT	1xCarbamido	8.778E-06	4.225E-05	1	1	1	O95602	O95602 [904-924]	0	2610.355

[R].AEPYCS	1xCarbamido	4.434E-06	4.225E-05	1	1	2	Q9BUJ2	Q9BUJ2 [387-408	0	2865.4929
[K].WDGEGS	1xTMTpro [K	4.442E-05	4.225E-05	1	1	1	O00764	O00764 [120-137	0	2706.3981
[K].GSVAEA	1xCarbamido	1.628E-05	4.225E-05	1	1	2	O15294	O15294 [307-321	0	1959.9401
[K].WDDSGI	1xTMTpro [K	1.238E-05	4.225E-05	2	2	2	P35221; P26	P35221 [705-717]	0	2054.1414
[R].AEQEEE	1xTMTpro [K	4.503E-05	4.225E-05	1	1	1	Q16204	Q16204 [105-118	0	2293.2208
[K].AEQEEY	1xTMTpro [N	3.906E-05	4.225E-05	2	2	2	Q12965; O0(	Q12965 [423-434	0	1754.888
[R].GTAYVV	1xTMTpro [K	2.234E-05	4.225E-05	1	1	3	Q9Y3B4	Q9Y3B4 [58-71]	0	2199.1829
[K].WDAWN	1xTMTpro [K	4.48E-05	4.225E-05	1	1	2	O75521	O75521 [93-104]	0	1966.1042
[R].WDAPDI	1xTMTpro [N	2.865E-05	4.225E-05	1	1	1	P11277	P11277 [23-38]	0	2123.9437
[K].AEQINQ	1xTMTpro [K	3.409E-06	4.225E-05	1	1	2	Q9UJZ1	Q9UJZ1 [234-250	0	2279.2851
[K].WDAENA	1xTMTpro [N	8.761E-06	4.225E-05	1	1	1	P31483	P31483 [158-175]	0	2321.1416
[K].WCGTTC	1xCarbamido	1.648E-05	4.225E-05	1	1	5	P02751	P02751 [445-457]	0	2195.0683
[K].GSVAGC	1xTMTpro [K	8.527E-07	4.225E-05	1	1	2	Q5XKP0	Q5XKP0 [15-36]	0	2846.5431
[R].AEQMAS	1xTMTpro [N	5.455E-05	4.225E-05	1	1	1	Q9UDY2	Q9UDY2 [665-67(	0	1636.8396
[R].WCDKSI	1xCarbamido	2.163E-05	4.225E-05	1	1	2	O00571	O00571 [127-145	1	3259.651
[R].AEQWN\	1xTMTpro [K	2.312E-05	4.225E-05	1	1	2	P11233	P11233 [146-159]	0	2247.1901
[R].WCAVSE	1xCarbamido	1.26E-05	4.225E-05	1	1	6	P02787	P02787 [27-37]	0	1926.0035
[K].WARPPI	1xTMTpro [K	6.218E-06	4.225E-05	1	1	4	Q96CW1	Q96CW1 [381-40	0	2852.5777
[K].WDAVPC	1xTMTpro [K	3.806E-05	4.225E-05	1	1	2	Q05707	Q05707 [463-488	0	3314.7804
[K].AETLASS	1xTMTpro [N	4.51E-05	4.225E-05	1	1	1	Q69YQ0	Q69YQ0 [619-634	0	2046.0787
[K].AETLMT\	1xTMTpro [N	1.071E-07	4.225E-05	1	1	1	Q96EE3	Q96EE3 [212-233	0	2672.4037
[K].VYVELQI	1xTMTpro [K	6.769E-06	4.225E-05	1	1	1	P02730	P02730 [57-74]	1	2843.5469
[R].VYIASSS	1xTMTpro [K	1.713E-05	4.225E-05	1	1	2	O75368	O75368 [5-17]	0	1892.0985
[K].VYGTAA	1xTMTpro [K	4.257E-05	4.225E-05	1	1	1	P26232	P26232 [893-907]	0	2186.2465
[R].VYFQSP	1xTMTpro [N	1.913E-05	4.225E-05	1	1	1	Q96C90	Q96C90 [28-52]	1	2790.3766
[R].VYFQSP	1xTMTpro [N	9.677E-06	4.225E-05	1	1	1	Q96C90	Q96C90 [28-51]	0	2634.2755
[K].AEYEGD	1xTMTpro [N	2.565E-05	4.225E-05	1	1	2	P22234	P22234 [314-331]	0	2155.1355
[K].VYEVVN	1xCarbamido	4.841E-05	4.225E-05	1	1	1	Q9Y5T5	Q9Y5T5 [605-623	0	2531.2407
[K].VYEIQDI	1xTMTpro [K	4.817E-05	4.225E-05	1	1	2	Q9Y262	Q9Y262 [88-101]	0	2396.263
[K].VYEEVLS	1xTMTpro [K	1.805E-05	4.225E-05	1	1	1	Q12797	Q12797 [476-491	0	2376.2943
[K].VYDYYE	1xCarbamido	2.429E-06	4.225E-05	1	1	2	P01023	P01023 [1449-14(	0	3156.5004
[K].VYDTQLI	1xCarbamido	2.829E-05	4.225E-05	1	1	1	O75923	O75923 [1504-15:	0	3334.6433
[R].AEWADI	1xTMTpro [K	3.429E-05	4.225E-05	1	1	4	P49354	P49354 [70-95]	0	3488.8193

[R].AFADALI	1xTMTpro [N	1.971E-07	4.225E-05	1	1	1	P48643	P48643 [450-478]	0	3439.7231
[R].AFADAM	1xOxidation [	9.548E-07	4.225E-05	1	1	1	P50991	P50991 [453-481]	0	3350.7473
[R].VYDQVG	1xTMTpro [K	5.86E-05	4.225E-05	1	1	1	P52888	P52888 [47-66]	0	2945.4912
[K].VYDESIQ	1xTMTpro [K	2.602E-05	4.225E-05	1	1	2	Q07954	Q07954 [1835-184	0	1955.073
[K].VYCNME	2xCarbamido	7.801E-07	4.225E-05	1	1	1	P05997	P05997 [1326-134	0	2676.2387
[R].VYCDMN	1xCarbamido	1.679E-05	4.225E-05	1	1	7	P02675	P02675 [268-285]	0	2477.1509
[R].VYCDMN	1xCarbamido	3.441E-06	4.225E-05	1	1	3	P02675	P02675 [268-285]	0	2461.1559
[K].VYCDFS	2xCarbamido	2.973E-05	4.225E-05	1	1	6	P08123	P08123 [1193-120	0	1911.89
[K].VYAYYN	1xCarbamido	5.926E-05	4.225E-05	1	1	4	P01024	P01024 [1479-149	0	1971.9441
[K].AFASPVI	2xCarbamido	1.75E-05	4.225E-05	1	1	1	P16671	P16671 [299-316]	0	2757.3144
[K].AFAVGV	1xTMTpro [K	2.124E-05	4.225E-05	1	1	4	O95373	O95373 [295-306]	0	1881.1817
[R].VYDQVV	1xTMTpro [N	4.279E-05	4.225E-05	1	1	2	Q9NP77	Q9NP77 [115-126	0	1740.9087
[R].WDNNY(	1xOxidation [	1.376E-05	4.225E-05	1	1	1	P21980	P21980 [241-262]	0	2801.3524
[K].GTDIMY	1xCarbamido	2.989E-05	4.225E-05	1	1	2	P05141	P05141 [246-259]	0	1992.9479
[K].AEVVET	1xTMTpro [N	9.252E-07	4.225E-05	1	1	1	Q15149	Q15149 [3686-37	1	2344.1952
[K].AETSEG	1xTMTpro [K	3.532E-05	4.225E-05	1	1	1	P51608	P51608 [62-82]	0	2538.3179
[K].VYVELQ	1xTMTpro [K	2.279E-05	4.225E-05	1	1	6	P02730	P02730 [57-69]	0	2203.2176
[R].AETTCH	3xCarbamido	7.18E-06	4.225E-05	1	1	1	Q9HD89	Q9HD89 [85-102]	0	2416.0222
[K].VYTVVD	1xTMTpro [N	2.479E-05	4.225E-05	1	1	2	P53680	P53680 [110-124]	0	2046.0901
[K].AETVQA	1xTMTpro [N	2.171E-05	4.225E-05	1	1	2	P55268	P55268 [1604-16	0	1719.9196
[R].GITGVDI	1xTMTpro [K	1.731E-05	4.225E-05	1	1	2	P53621	P53621 [183-198]	0	2201.2673
[K].AEVAAE	1xCarbamido	2.775E-05	4.225E-05	1	1	2	Q8TBC4	Q8TBC4 [125-147	1	3248.713
[R].GTCQCS	2xCarbamido	3.219E-05	4.225E-05	1	1	1	Q6UX06	Q6UX06 [81-98]	0	2344.1233
[K].GITFDSC	1xTMTpro [K	3.053E-05	4.225E-05	1	1	2	P28838	P28838 [283-294]	0	1803.0508
[R].VYQSLC	1xCarbamido	7.859E-06	4.225E-05	1	1	2	P14854	P14854 [60-77]	0	2574.2254
[R].VYIGSFV	1xTMTpro [N	4.865E-05	4.225E-05	1	1	1	Q9H223	Q9H223 [255-271	0	2313.1947
[K].AEVEVAI	1xTMTpro [K	1.845E-05	4.225E-05	1	1	1	Q7L5D6	Q7L5D6 [96-110]	0	2251.2677
[K].VYPGEQ	1xCarbamido	5.355E-07	4.225E-05	1	1	2	P00450	P00450 [149-177]	0	3568.6531
[R].VYNVTQ	1xTMTpro [K	7.26E-06	4.225E-05	1	1	2	P46778	P46778 [64-78]	0	2249.3262
[K].AEVLSEI	1xTMTpro [K	5.645E-05	4.225E-05	1	1	2	Q7L1Q6	Q7L1Q6 [369-379	0	1836.0974
[R].VYNLDFI	1xTMTpro [K	3.116E-07	4.225E-05	1	1	1	P27144	P27144 [135-170]	0	4593.3505
[K].GITEPPF	1xTMTpro [K	4.603E-05	4.225E-05	1	1	2	Q14126	Q14126 [93-106]	0	2174.2505
[K].VYMNQV	1xCarbamido	2.687E-05	4.225E-05	1	1	1	Q9UH62	Q9UH62 [202-215	0	2005.9642

[R].GTDECA	1xCarbamido	2.824E-05	4.225E-05	1	1	1	P53634	P53634 [444-462]	0	2551.3933
[R].VYIGSFV	1xTMTpro [N	3.631E-06	4.225E-05	1	1	2	Q9NZN4	Q9NZN4 [252-268	0	2295.2457
[K].AEVTEE	2xTMTpro [K	1.088E-05	4.225E-05	1	1	2	Q9H2G2	Q9H2G2 [309-337	1	4145.0601
[K].AEVVET	1xTMTpro [N	4.776E-05	4.225E-05	1	1	1	Q15149	Q15149 [3686-37	0	2188.094
[K].VYPGEQ	1xCarbamido	1.83E-06	4.225E-05	1	1	1	P00450	P00450 [149-177]	0	3584.648
[K].VAMANI	2xOxidation [	4.106E-05	4.225E-05	1	1	2	P18206	P18206 [739-758]	0	2378.2855
[KN].VDVDV	1xTMTpro [K	2.512E-06	4.225E-05	1	1	5	Q09666	Q09666 [3186-32	0	2290.2422
[R].CITDPQ	2xCarbamido	1.725E-05	4.225E-05	1	1	3	Q15149	Q15149 [4245-42	0	2337.3166
[R].SVLAVE	1xTMTpro [N	4.043E-05	4.225E-05	1	1	1	Q9NQT8	Q9NQT8 [1346-13	0	1747.0285
[K].SVLAETE	1xTMTpro [K	1.91E-05	4.225E-05	1	1	2	Q86UP2	Q86UP2 [1141-11	0	1896.1298
[K].DAGEGC	1xTMTpro [K	2.607E-06	4.225E-05	1	1	4	P21333	P21333 [1892-19	0	2109.1683
[K].DAGEGL	2xTMTpro [K	6.865E-08	4.225E-05	1	1	5	P21333	P21333 [1574-15	0	2850.6393
[R].DAGEGL	2xTMTpro [K	4.375E-08	4.225E-05	1	1	2	O75369	O75369 [1546-15	0	2881.6451
[K].SVKEDS	2xTMTpro [K	7.395E-06	4.225E-05	1	1	4	P46940	P46940 [1443-14	1	2403.3911
[R].SVGYKP	2xTMTpro [K	1.688E-05	4.225E-05	1	1	2	P00492	P00492 [171-186]	0	2710.5272
[R].DAGIEP	1xTMTpro [K	1.069E-05	4.225E-05	1	1	3	P42704	P42704 [260-280]	0	2829.5166
[K].SVGSQV	2xCarbamido	2.163E-05	4.225E-05	1	1	2	P04275	P04275 [2516-25	0	2607.2615
[R].SVGNTI	1xTMTpro [K	2.484E-05	4.225E-05	1	1	1	P46821	P46821 [401-414]	0	2139.2669
[R].DAGNDS	1xTMTpro [N	3.358E-05	4.225E-05	1	1	1	Q8N573	Q8N573 [318-329	0	1465.7202
[K].SVGMIA	1xOxidation [	1.792E-05	4.225E-05	1	1	1	P00387	P00387 [174-192]	0	2221.2368
[K].DAGQCI	1xCarbamido	3.135E-05	4.225E-05	1	1	1	Q96BJ3	Q96BJ3 [180-194	0	2274.2295
[K].DAGQIS	1xTMTpro [N	4.572E-05	4.225E-05	1	1	1	P38646	P38646 [207-218]	0	1546.8872
[R].DAGSGF	1xTMTpro [K	2.607E-06	4.225E-05	1	1	1	Q86VY4	Q86VY4 [190-211	0	2825.4483
[K].DAGTIA	1xTMTpro [N	5.141E-05	4.225E-05	1	1	4	P11142	P11142 [160-171]	0	1503.8814
[R].SVGLAN	1xTMTpro [N	1.086E-05	4.225E-05	1	1	1	P78540	P78540 [91-105]	0	1876.0459
[R].SVGGSC	1xTMTpro [N	1.82E-06	4.225E-05	1	1	4	P02545	P02545 [628-644]	0	1870.9578
[R].SVGELP	1xTMTpro [K	7.787E-06	4.225E-05	1	1	3	Q07065	Q07065 [492-505	0	2082.1938
[K].DAGVIA	1xTMTpro [N	5.41E-05	4.225E-05	1	2	3	P0DMV9	P0DMV9 [160-17	0	1501.9021
[R].SVGDGE	2xTMTpro [K	8.325E-08	4.225E-05	1	1	2	P67809	P67809 [102-137]	1	4385.2929
[R].SVIDPVF	1xTMTpro [K	1.643E-06	4.225E-05	1	1	2	Q13177	Q13177 [197-216	0	2539.4012
[R].SVIEQG	1xTMTpro [K	2.475E-05	4.225E-05	1	1	1	Q8WUM4	Q8WUM4 [423-43	0	2336.3681
[K].SVILEAF	1xTMTpro [K	8.859E-06	4.225E-05	1	1	2	Q86VP6	Q86VP6 [859-873	0	2230.2462
[K].DAFDRN	1xTMTpro [K	4.065E-06	4.225E-05	1	1	4	P52209	P52209 [378-396]	1	2918.5544

[K].KEEDVS	2xTMTpro [K	5.413E-06	4.225E-05	1	1	3	Q14789	Q14789 [1404-14	1	2624.4599
[K].SVPEDD	1xTMTpro [N	1.569E-05	4.225E-05	1	1	3	Q02952	Q02952 [1674-16	0	1812.9411
[R].SVPAGL	1xTMTpro [K	3.334E-05	4.225E-05	1	1	1	Q9HAU0	Q9HAU0 [596-61	0	2505.4532
[R].DAEEAIS	1xTMTpro [K	2.179E-05	4.225E-05	1	1	3	P12109	P12109 [223-240]	0	2600.3985
[R].SVPADV	1xTMTpro [N	2.908E-06	4.225E-05	1	1	1	O95967	O95967 [355-379]	0	3046.5957
[R].SVPADIF	1xTMTpro [N	3.886E-05	4.225E-05	1	1	4	Q9UBX5	Q9UBX5 [360-37	0	1868.986
[K].SVNGQII	1xTMTpro [N	5.278E-05	4.225E-05	1	1	1	P02461	P02461 [1240-12	0	1963.0416
[K].SVNEVM	1xTMTpro [K	2.354E-05	4.225E-05	1	1	1	Q92598	Q92598 [754-771]	0	2703.3548
[K].SVNESLI	1xTMTpro [N	6.029E-07	4.225E-05	1	1	2	Q00610	Q00610 [1462-14	0	2659.3534
[K].DAEGLD	1xTMTpro [N	2.737E-07	4.225E-05	1	1	2	P23634	P23634 [1072-10	0	2147.0246
[K].DAGYEY	2xCarbamid	5.841E-05	4.225E-05	1	1	1	P06280	P06280 [83-100]	0	2567.1324
[K].DAEGILE	1xTMTpro [N	2.068E-05	4.225E-05	1	1	3	Q9NUQ9	Q9NUQ9 [45-57]	0	1812.9299
[K].SVNDQP	2xTMTpro [K	1.069E-05	4.225E-05	1	1	2	O75533	O75533 [455-477]	0	3549.9046
[R].SVMTEE	1xTMTpro [K	1.228E-05	4.225E-05	1	1	1	Q96AE4	Q96AE4 [99-118]	1	2836.5233
[R].DAEMPA	2xTMTpro [K	2.87E-06	4.225E-05	1	1	4	P21333	P21333 [25-42]	1	2929.5433
[R].KEEEAV	1xTMTpro [K	3.27E-07	4.225E-05	1	1	2	Q8N163	Q8N163 [353-382	1	3842.0468
[R].DAEMPA	3xTMTpro [K	3.881E-06	4.225E-05	1	1	4	P21333	P21333 [25-43]	2	3361.8454
[R].SVMDAT	1xCarbamid	6.404E-06	4.225E-05	1	1	2	P34932	P34932 [155-169]	0	1953.0217
[R].KEEECQ	1xCarbamid	2.414E-05	4.225E-05	1	1	1	Q96JB5	Q96JB5 [146-158]	1	2117.0611
[K].SVLTGG	1xTMTpro [K	2.379E-05	4.225E-05	1	1	2	Q8IWE2	Q8IWE2 [222-236	0	2128.2509
[R].SVLLLAY	1xTMTpro [K	5.619E-07	4.225E-05	1	1	2	Q16531	Q16531 [929-947]	0	2788.5563
[R].DAFCVF	1xCarbamid	3.613E-05	4.225E-05	1	1	1	Q16853	Q16853 [427-442]	1	2254.1722
[K].DAELAG	1xTMTpro [N	9.947E-06	4.225E-05	1	1	2	Q53T59	Q53T59 [122-138]	0	2122.1351
[R].DAGYGC	1xTMTpro [K	1.076E-05	4.225E-05	1	1	1	O75369	O75369 [2029-20	0	2129.1734
[R].SVGDGE	1xTMTpro [K	1.721E-06	4.225E-05	2	3	2	P16989; P67	P16989 [134-150]	0	2404.2375
[R].DAGYGC	1xCarbamid	1.153E-06	4.225E-05	1	1	2	P21333	P21333 [2074-21	1	3776.878
[R].GEVQTV	1xTMTpro [K	1.08E-05	4.225E-05	1	1	5	Q99715	Q99715 [2628-26	0	2190.1786
[K].DALLVGY	1xTMTpro [N	3.393E-05	4.225E-05	1	1	2	P50151	P50151 [46-63]	1	2199.2205
[R].SVAQYT	1xTMTpro [K	4.997E-05	4.225E-05	1	1	2	Q10713	Q10713 [289-299]	0	1702.9983
[K].DALNLAC	1xTMTpro [K	9.659E-06	4.225E-05	1	2	2	Q9NVI7	Q9NVI7 [73-92]	0	2924.5643
[R].GEVQAM	1xTMTpro [N	1.094E-05	4.225E-05	1	1	3	P02649	P02649 [138-152]	0	1952.0078
[R].DALNQA	1xTMTpro [K	2.431E-05	4.225E-05	1	1	5	Q9P2E9	Q9P2E9 [807-819	0	1999.0952
[R].DALPELI	1xTMTpro [N	6.022E-05	4.225E-05	1	1	1	Q9NSI2	Q9NSI2 [149-162]	0	1744.9764

[R].STYLGD	1xTMTpro [K	1.587E-05	4.225E-05	1	1	2	Q99538	Q99538 [226-248]	0	3361.6066
[R].KEFSPF	2xTMTpro [K	1.516E-05	4.225E-05	1	1	2	P11940	P11940 [312-324]	1	2325.3634
[R].STVLTPI	1xTMTpro [N	2.158E-06	4.225E-05	1	1	1	P78527	P78527 [2599-26	0	2599.3721
[K].DALLSAI	1xCarbamid	5.074E-07	4.225E-05	1	1	2	Q01082	Q01082 [949-968]	0	2927.5428
[K].STVISYE	2xCarbamid	2.658E-05	4.225E-05	1	1	5	Q15582	Q15582 [77-90]	0	2301.1387
[R].GEVITTY	1xCarbamid	1.657E-05	4.225E-05	1	1	3	P49419	P49419 [63-79]	0	2209.1242
[K].STVEELI	1xTMTpro [N	8.445E-06	4.225E-05	1	1	1	Q9GZX9	Q9GZX9 [91-105]	0	2058.1191
[K].DALRDE	1xTMTpro [N	3.24E-07	4.225E-05	1	1	1	O15173	O15173 [163-184]	1	2858.3797
[R].DALRPP	1xTMTpro [K	5.218E-05	4.225E-05	1	1	2	Q14008	Q14008 [197-212]	0	2414.4375
[R].DALSDL	1xTMTpro [K	1.195E-05	4.225E-05	1	1	2	P50991	P50991 [307-319]	0	2065.1937
[K].STVAALI	1xTMTpro [K	9.5E-06	4.225E-05	1	1	4	Q03518	Q03518 [545-575]	0	4029.2417
[K].STTSVSI	1xTMTpro [N	2.921E-05	4.225E-05	1	1	1	Q96IZ0	Q96IZ0 [228-240]	0	1687.8306
[R].STTSTIE	1xTMTpro [K	7.673E-06	4.225E-05	1	1	2	O95232	O95232 [174-187]	0	2108.1367
[R].STTQVA	1xTMTpro [N	5.278E-05	4.225E-05	1	1	2	Q96A65	Q96A65 [314-325	0	1616.8199
[K].DALSSV	1xTMTpro [N	1.506E-06	4.225E-05	1	1	4	P02656	P02656 [45-60]	0	2021.0583
[R].GEVPCT	1xCarbamid	2.438E-06	4.225E-05	1	1	4	P48047	P48047 [137-158]	0	2926.5575
[K].KEDLVFI	2xTMTpro [K	4.065E-06	4.225E-05	2	2	5	P23528; Q9	P23528 [96-112];	1	2902.6899
[K].SVASVG	1xTMTpro [K	1.646E-06	4.225E-05	1	1	2	P47989	P47989 [344-371]	0	3311.8165
[R].SVCTEA	1xCarbamid	6.051E-05	4.225E-05	1	1	2	P35998	P35998 [387-398]	0	1645.8361
[R].SVFDIPII	1xTMTpro [K	1.213E-05	4.225E-05	1	1	1	Q9NP66	Q9NP66 [214-230	0	2631.4314
[R].KEELGE	2xTMTpro [K	3.591E-06	4.225E-05	1	1	3	P11277	P11277 [1432-14	1	3790.9877
[K].SVEVQG	1xTMTpro [K	4.235E-05	4.225E-05	1	1	1	O60763	O60763 [828-842]	0	2213.2521
[K].KEELVAI	2xTMTpro [K	2.515E-05	4.225E-05	1	1	2	Q7L1Q6	Q7L1Q6 [309-319	1	2170.3263
[R].SVEFEEI	1xTMTpro [N	1.948E-05	4.225E-05	1	1	1	Q14376	Q14376 [59-75]	0	2284.145
[K].DAHSQG	1xCarbamid	1.109E-05	4.225E-05	1	1	1	Q92896	Q92896 [259-272]	0	2167.1309
[K].SVEEVA	1xTMTpro [N	3.016E-05	4.225E-05	1	1	3	P15924	P15924 [2000-20	0	1908.0398
[K].SVEELLE	1xTMTpro [K	3.265E-05	4.225E-05	1	1	5	Q86UP2	Q86UP2 [812-823	0	1981.1713
[K].KEENLAI	2xTMTpro [K	4.609E-06	4.225E-05	1	1	1	P07814	P07814 [1020-10	1	2736.5388
[K].SVEDAQ	1xTMTpro [N	1.491E-05	4.225E-05	1	1	4	P12111	P12111 [1755-17	0	1936.0307
[K].KEFGPV	2xTMTpro [K	8.633E-06	4.225E-05	1	1	1	Q14204	Q14204 [1100-11	1	2264.3471
[R].KEEPPR	1xTMTpro [K	1.384E-05	4.225E-05	1	1	1	P22105	P22105 [1250-12	1	3856.1352
[R].DALDGP	1xTMTpro [N	9.218E-07	4.225E-05	1	1	1	Q15149	Q15149 [2768-27	0	2401.1591
[R].GEVTYT	1xTMTpro [K	3.317E-05	4.225E-05	1	1	2	P17813	P17813 [40-51]	0	1908.057

[K].DALECLI	1xCarbamido	2.608E-07	4.225E-05	1	1	1	Q13325	Q13325 [69-92]	1	3506.7565
[K].SVDPEN	1xTMTpro [N	1.825E-05	4.225E-05	1	1	3	Q9Y5L0	Q9Y5L0 [450-468	0	2370.2836
[K].DALENID	1xCarbamido	4.863E-06	4.225E-05	1	1	1	P05546	P05546 [276-296]	0	3108.6064
[R].SVDPDS	1xTMTpro [N	4.33E-05	4.225E-05	1	1	1	O14745	O14745 [181-194]	0	1704.8724
[R].KEEQEV	2xTMTpro [K	1.021E-06	4.225E-05	1	1	1	Q08211	Q08211 [152-182]	1	4394.2932
[R].SVDETT	1xTMTpro [K	6.633E-06	4.225E-05	1	1	1	P26368	P26368 [204-225]	0	2994.5738
[K].GEVTEM	2xTMTpro [K	9.781E-07	4.225E-05	1	1	2	Q8TCT9	Q8TCT9 [338-361	1	3557.8138
[K].KEESEE	1xTMTpro [K	3.967E-05	4.225E-05	2	2	2	P05386; P05	P05386 [98-114];	1	2558.1736
[K].DAIAQAE	1xTMTpro [K	3.119E-05	4.225E-05	1	1	1	Q8NE86	Q8NE86 [322-332	0	1813.0021
[K].GEVLPLI	1xTMTpro [K	7.167E-06	4.225E-05	1	1	2	P04196	P04196 [467-486]	0	2834.5849
[R].SVPSDIF	1xTMTpro [N	2.026E-06	4.225E-05	1	1	4	Q12805	Q12805 [405-427]	0	2904.5426
[R].DAEDAM	1xTMTpro [N	9.781E-07	4.225E-05	1	1	6	Q01130	Q01130 [67-83]	0	2055.9282
[R].SYDVPP	1xOxidation [	5.937E-06	4.225E-05	1	2	5	P18669	P18669 [118-138]	0	3041.521
[R].SYDVPP	1xTMTpro [K	1.847E-06	4.225E-05	1	2	5	P18669	P18669 [118-138]	0	3025.5261
[K].GFDQTII	1xTMTpro [N	4.397E-05	4.225E-05	1	1	1	O95777	O95777 [29-44]	0	2191.1314
[R].KDSAIQ	2xTMTpro [K	5.691E-05	4.225E-05	1	1	1	Q14204	Q14204 [1228-12	1	2614.5167
[K].SYCAEIA	1xCarbamido	3.626E-05	4.225E-05	1	1	1	P62910	P62910 [94-106]	0	2074.0883
[R].SYAVVG	1xTMTpro [K	4.673E-05	4.225E-05	1	1	4	P09110	P09110 [306-331]	0	3234.8456
[R].CVSTLLI	1xCarbamido	2.984E-05	4.225E-05	2	2	1	Q10567; P63	Q10567 [391-402]	0	1999.1753
[K].SWVNQI	1xTMTpro [K	2.277E-06	4.225E-05	1	1	1	P46940	P46940 [1097-11	0	2290.1629
[K].SWTDVS	1xTMTpro [K	1.195E-07	4.225E-05	1	1	1	Q9Y6K5	Q9Y6K5 [539-568	0	3829.0416
[K].CVVTGE	1xCarbamido	4.972E-05	4.225E-05	1	1	1	P13591	P13591 [96-113]	0	2489.2685
[K].KDSSQL	2xTMTpro [K	5.064E-05	4.225E-05	1	1	1	O43491	O43491 [13-24]	1	2163.2437
[R].SWQDEI	1xTMTpro [N	3.037E-05	4.225E-05	1	1	1	Q86UE4	Q86UE4 [5-20]	0	2109.0168
[R].CWSPG	2xCarbamido	3.219E-05	4.225E-05	1	1	2	P35555	P35555 [365-380]	0	2164.0673
[K].GFDDNN	1xCarbamido	3.304E-06	4.225E-05	1	1	2	Q9NW15	Q9NW15 [110-12	0	3001.4567
[K].CYASDQ	1xCarbamido	1.878E-06	4.225E-05	1	1	2	P29590	P29590 [338-353]	0	2245.0701
[K].GFCYVE	1xCarbamido	6.946E-06	4.225E-05	1	1	2	Q15056	Q15056 [83-96]	0	2316.1714
[R].CYGFVT	1xCarbamido	4.634E-05	4.225E-05	1	1	1	Q15424	Q15424 [448-462]	0	2303.1543
[R].KDTVIKF	3xTMTpro [K	3.141E-05	4.225E-05	1	1	3	P01023	P01023 [896-912]	1	3124.9338
[K].SWIEEQ	1xTMTpro [K	1.494E-05	4.225E-05	1	1	2	P28838	P28838 [238-253]	0	2391.2874
[K].CYLFGG	1xCarbamido	3.613E-05	4.225E-05	1	1	1	P51610	P51610 [149-168]	1	2888.4857
[R].GFCHLC	4xCarbamido	3.483E-08	4.225E-05	1	1	1	P08559	P08559 [89-119]	0	3838.7729

[K].SYDWSQ	1xTMTpro [K	3.538E-05	4.225E-05	1	1	1	Q01459	Q01459 [72-86]	0	2312.2418
[K].SYDYQS	1xCarbamido	5.917E-05	4.225E-05	1	1	1	P54289	P54289 [897-911]	0	2280.1462
[R].KDQVEY	1xCarbamido	3.178E-06	4.225E-05	1	1	1	O00505	O00505 [401-423]	1	3644.0702
[K].CVNHYG	2xCarbamido	1.061E-05	4.225E-05	1	1	4	Q12805	Q12805 [61-73]	0	2189.1491
[K].CVASNA	1xCarbamido	1.336E-05	4.225E-05	1	1	1	Q9NR99	Q9NR99 [2025-20	0	1779.9343
[R].SYNVTP	1xTMTpro [N	9.583E-07	4.225E-05	1	1	1	P07738	P07738 [118-140]	0	3115.4968
[K].SYNPFD	1xTMTpro [N	7.18E-06	4.225E-05	1	1	1	O95721	O95721 [7-26]	0	2598.1816
[K].GFEFSVI	1xTMTpro [K	3.893E-05	4.225E-05	1	1	3	P22105	P22105 [4220-423	0	2027.0804
[R].SYLVAG.	1xTMTpro [N	4.367E-07	4.225E-05	1	1	1	P06401	P06401 [403-428]	0	2962.615
[R].CVDVDE	3xCarbamido	3.157E-05	4.225E-05	1	1	3	P23142	P23142 [354-369]	0	2412.1489
[R].KDLYAN	1xTMTpro [K	2.513E-07	4.225E-05	1	3	3	P62736	P62736 [293-314]	1	2964.5745
[R].CVEDPE	2xCarbamido	1.286E-05	4.225E-05	1	1	1	Q15149	Q15149 [3008-30	0	2568.3545
[K].SYLSMV	3xCarbamido	4.45E-06	4.225E-05	1	1	3	P02774	P02774 [181-201]	0	2979.458
[K].SYLSMV	3xCarbamido	3.712E-06	4.225E-05	1	1	3	P02774	P02774 [181-201]	0	2963.4631
[K].CYVDMS	1xCarbamido	4.695E-06	4.225E-05	1	1	1	Q49A26	Q49A26 [356-378	0	2876.4341
[R].GFEESE	1xTMTpro [N	7.874E-07	4.225E-05	1	1	1	P22105	P22105 [3830-385	0	2795.4423
[R].SYLMTN	1xTMTpro [K	1.312E-05	4.225E-05	1	1	1	O75223	O75223 [124-140]	0	2584.3249
[R].KDLYAN	1xOxidation [	3.951E-07	4.225E-05	1	3	5	P62736	P62736 [293-314]	1	2980.5694
[K].SYIEGYV	1xCarbamido	4.651E-08	4.225E-05	1	1	4	P24534	P24534 [23-54]	0	3749.8804
[R].KDLYAN	1xTMTpro [K	6.108E-08	4.225E-05	1	2	29	P63261	P63261 [291-312]	1	2951.5792
[K].SYFSSF	1xTMTpro [K	2.682E-05	4.225E-05	1	1	1	O94826	O94826 [259-275]	0	2587.361
[R].SYFSQY	1xCarbamido	2.75E-05	4.225E-05	1	1	1	Q96EP5	Q96EP5 [28-43]	0	2533.2962
[K].SYFSEE	1xTMTpro [N	2.388E-05	4.225E-05	1	1	2	P04062	P04062 [146-159]	0	1952.0085
[R].KDLYAN	1xOxidation [	1.412E-07	4.225E-05	1	2	8	P63261	P63261 [291-312]	1	2967.5741
[R].KDPELW	1xTMTpro [K	3.053E-05	4.225E-05	1	1	1	Q00610	Q00610 [951-967]	1	2611.4376
[KR].SYELPI	1xTMTpro [N	5.14E-06	4.225E-05	5	10	26	A5A3E0; Q9	A5A3E0 [939-954	0	2095.0991
[K].SYLNMD	1xTMTpro [K	3.613E-05	4.225E-05	1	1	1	P05165	P05165 [120-132]	0	2107.1423
[R].GFCFITF	1xCarbamido	4.308E-05	4.225E-05	1	1	2	Q14103	Q14103 [224-238]	2	3075.7694
[K].DAAAEY	1xTMTpro [N	7.075E-06	4.225E-05	1	1	2	Q9UJW0	Q9UJW0 [371-39	0	3158.4761
[R].GFCFLE	1xCarbamido	3.196E-05	4.225E-05	2	2	5	O43390; O6	O43390 [290-300]	0	2053.0345
[R].SVTHAN	1xTMTpro [K	3.974E-05	4.225E-05	1	1	1	P02730	P02730 [731-743]	0	1937.1134
[K].SVTGTD	1xTMTpro [K	1.483E-05	4.225E-05	1	1	2	Q9BW30	Q9BW30 [49-61]	0	1976.1196
[K].SVTEQG	1xTMTpro [N	1.346E-05	4.225E-05	1	1	11	P63104	P63104 [28-41]	0	1852.9208

[R].DADADA	1xTMTpro [N	5.056E-05	4.225E-05	1	1	1	Q14657	Q14657 [3-18]	0	1680.7381
[K].SVTDKD.	1xCarbamido	1.317E-05	4.225E-05	1	1	2	Q9NR99	Q9NR99 [2210-22	1	2278.1993
[K].KEAGIPE	2xTMTpro [K	1.065E-06	4.225E-05	1	1	2	P00751	P00751 [565-581]	1	2883.6324
[K].SVSLPSI	1xTMTpro [K	5.545E-05	4.225E-05	1	1	3	P04114	P04114 [642-654]	0	1880.0985
[K].KEDEPP	2xTMTpro [K	3.944E-07	4.225E-05	1	1	1	P11171	P11171 [598-615]	1	3022.5826
[K].SVSGTD	1xCarbamido	3.826E-05	4.225E-05	1	1	3	P49321	P49321 [244-255]	0	1670.7975
[K].SVSDGIA	1xTMTpro [K	2.362E-05	4.225E-05	1	1	1	P04114	P04114 [3830-384	0	2266.2898
[K].SVTTSAS	1xTMTpro [N	3.737E-07	4.225E-05	1	1	1	P0CG08	P0CG08 [233-259	0	3181.6395
[K].SVSAFAI	2xCarbamido	1.242E-05	4.225E-05	1	1	5	P10768	P10768 [168-185]	0	2611.402
[K].KEDLISA	1xCarbamido	9.15E-08	4.225E-05	1	1	1	Q9Y3A5	Q9Y3A5 [68-85]	1	2982.591
[R].DADSITL	1xTMTpro [K	1.27E-05	4.225E-05	1	1	4	P53621	P53621 [468-480]	0	2088.1469
[K].DADSQN	1xTMTpro [K	5.654E-05	4.225E-05	1	1	2	P53621	P53621 [399-411]	0	1951.9853
[R].DADVQF	1xTMTpro [N	1.91E-05	4.225E-05	1	1	2	Q6XQN6	Q6XQN6 [74-97]	0	3004.5376
[R].DADVQN	1xTMTpro [K	2.045E-05	4.225E-05	1	1	3	P42126	P42126 [271-283]	0	2078.1414
[RK].SVQTT	1xTMTpro [K	4.776E-05	4.225E-05	2	2	2	O75477; O9	O75477 [63-74]; (	0	1957.1097
[K].SVQNLA	1xTMTpro [K	4.142E-05	4.225E-05	1	1	1	P20936	P20936 [936-949]	0	2098.2152
[R].SVQETV	2xTMTpro [K	5.401E-05	4.225E-05	1	1	1	P51608	P51608 [295-305]	1	2154.3678
[K].GEYQGV	1xCarbamido	2.206E-05	4.225E-05	1	1	1	Q9UBX3	Q9UBX3 [232-246	0	2304.1938
[R].SVPTST	1xTMTpro [K	6.744E-06	4.225E-05	1	1	5	P29401	P29401 [439-456]	0	2493.3368
[K].SVQYDD	1xTMTpro [K	5.2E-05	4.225E-05	1	1	1	Q13740	Q13740 [77-89]	1	2222.1585
[R].DAEDAM	1xOxidation	2.911E-05	4.225E-05	1	1	8	Q01130	Q01130 [67-83]	0	2071.9232
[R].SVVALHI	1xTMTpro [K	5.831E-05	4.225E-05	1	1	2	P51665	P51665 [268-279]	0	1930.173
[R].KEAAEN	2xTMTpro [K	7.673E-06	4.225E-05	1	1	1	P62258	P62258 [142-153]	1	2235.3165
[R].DAAAPA	1xTMTpro [N	5.682E-05	4.225E-05	1	1	2	O76024	O76024 [56-69]	0	1766.9105
[R].DAADLL	1xTMTpro [N	2.113E-05	4.225E-05	1	1	1	Q14728	Q14728 [241-253	0	1671.9964
[R].DAADQN	1xTMTpro [K	4.689E-05	4.225E-05	1	1	1	O95716	O95716 [13-24]	0	2073.0243
[R].GFAYVQ	1xTMTpro [N	3.042E-05	4.225E-05	1	1	1	O75494	O75494 [52-73]	1	2884.4185
[R].SVYGGE	1xTMTpro [K	3.508E-05	4.225E-05	1	1	2	Q9NQG5	Q9NQG5 [115-12	0	1977.1301
[K].KDVIELT	2xTMTpro [K	5.265E-06	4.225E-05	1	1	4	Q15084	Q15084 [160-172	1	2437.3642
[K].SVVSQS	1xCarbamido	5.313E-05	4.225E-05	1	1	2	Q9Y2T2	Q9Y2T2 [22-38]	0	2631.3256
[K].DAAGIAN	1xTMTpro [N	1.379E-05	4.225E-05	1	1	2	P08240	P08240 [497-510]	0	1710.9168
[K].DAALATA	1xTMTpro [K	4.533E-05	4.225E-05	1	1	2	P20700	P20700 [146-156]	0	1653.9667
[K].DAALATA	2xTMTpro [K	8.586E-06	4.225E-05	1	1	4	P20700	P20700 [146-157]	1	2086.2688

[R].KEAESC	2xCarbamido	4.442E-06	4.225E-05	2	5	2	P07437; P68	P07437 [122-154]		1	4352.2505
[K].SVVSGS	1xTMTpro [N	2.105E-05	4.225E-05	1	1	2	Q99541	Q99541 [154-167		0	1679.9611
[R].DAANCW	1xCarbamido	3.705E-06	4.225E-05	1	1	1	Q8WVJ2	Q8WVJ2 [95-120]		0	3388.5534
[K].SVVLMS	2xTMTpro [K	1.219E-06	4.225E-05	1	1	2	P00558	P00558 [57-86]		1	4176.3381
[K].KDYSQY	2xTMTpro [K	2.979E-05	4.225E-05	1	1	1	Q9C075	Q9C075 [117-137		1	3449.8369
[K].SVVLMS	1xTMTpro [K	1.566E-05	4.225E-05	1	1	3	P00558	P00558 [57-75]		0	2643.4606
[R].GFAFVTI	1xTMTpro [K	2.466E-05	4.225E-05	2	4	3	P38159; Q96	P38159 [50-63]; Q		0	2095.1356
[K].DAASLES	1xTMTpro [N	4.131E-07	4.225E-05	1	1	3	P35580	P35580 [1309-132		0	2708.3546
[R].GFAFVTI	1xTMTpro [K	4.005E-06	4.225E-05	1	1	1	P51991	P51991 [168-182]		0	2322.1898
[R].SVVDSLI	1xTMTpro [K	2.435E-05	4.225E-05	1	1	1	P53041	P53041 [160-180]		0	2922.4786
[R].SVVCSD	1xCarbamido	5.345E-07	4.225E-05	1	1	2	Q7L014	Q7L014 [587-605		1	3130.7486
[R].DAATVT	1xTMTpro [N	2.166E-06	4.225E-05	1	1	1	Q15334	Q15334 [74-89]		0	2037.0507
[K].SVVPGG	1xTMTpro [N	2.476E-07	4.225E-05	1	1	2	P17987	P17987 [407-433]		0	3003.5417
[R].STTELGI	1xTMTpro [N	3.582E-05	4.225E-05	1	1	1	P46939	P46939 [2111-212		0	1793.9564
[K].STSQGF	2xCarbamido	1.119E-05	4.225E-05	1	1	2	Q9NVA2	Q9NVA2 [35-54]		0	2783.4352
[KR].STSGG	1xCarbamido	3.953E-06	4.225E-05	2	3	11	P0DOX5; P0	P0DOX5 [136-149		0	1930.0923
[R].SSPSLTI	1xTMTpro [K	2.958E-05	4.225E-05	1	1	2	P30038	P30038 [409-420]		0	1739.0559
[R].SSPQFG	1xTMTpro [N	7.976E-06	4.225E-05	1	1	1	O75746	O75746 [587-603		0	2242.2403
[K].DCNCLH	5xCarbamido	9.303E-07	4.225E-05	1	1	1	Q9P0T7	Q9P0T7 [52-79]		0	3733.6683
[R].SSPQFG	1xTMTpro [N	9.208E-06	4.225E-05	1	1	2	Q9UJS0	Q9UJS0 [589-605		0	2256.2559
[R].DCPDGS	2xCarbamido	4.434E-06	4.225E-05	1	1	2	Q07954	Q07954 [52-68]		0	2513.178
[R].SSPGQT	1xTMTpro [N	2.143E-08	4.225E-05	1	1	1	Q14166	Q14166 [15-40]		0	2909.5077
[K].GESGPS	1xTMTpro [N	1.361E-05	4.225E-05	1	1	4	P02452	P02452 [782-796]		0	1601.8203
[K].KFAMEP	1xTMTpro [K	1.903E-05	4.225E-05	1	1	2	P13667	P13667 [485-499]		1	2423.2408
[K].SSPEPV	1xTMTpro [N	2.277E-06	4.225E-05	1	1	2	Q9Y678	Q9Y678 [632-649		0	2311.1988
[R].DCPLNA	1xCarbamido	3.582E-05	4.225E-05	1	1	2	Q10567	Q10567 [865-876		0	1870.9824
[R].DCPQEC	4xCarbamido	9.984E-06	4.225E-05	1	1	2	Q06828	Q06828 [75-95]		0	2951.1846
[R].DCPQEC	4xCarbamido	2.362E-05	4.225E-05	1	1	1	Q06828	Q06828 [75-95]		0	2967.1795
[R].SSPDLT	1xTMTpro [N	7.154E-06	4.225E-05	1	1	4	O60784	O60784 [130-145		0	2069.1086
[R].DCPVPL	1xCarbamido	3.961E-05	4.225E-05	1	1	1	Q8N4Q0	Q8N4Q0 [54-68]		0	1927.0278
[R].KFAPVC	2xCarbamido	1.244E-05	4.225E-05	1	1	1	Q8WUP2	Q8WUP2 [298-31		1	2509.3915
[R].SSNVLSI	1xCarbamido	2.958E-05	4.225E-05	1	1	1	P21283	P21283 [212-231]		0	2651.2942
[R].GESENA	1xTMTpro [N	2.21E-05	4.225E-05	1	1	2	Q08170	Q08170 [429-441		0	1696.8057

[K].SSNLIILE	1xTMTpro [K	2.475E-05	4.225E-05	1	1	2	P08603	P08603 [755-766]	0	2004.1985
[R].DCQLNA	1xCarbamido	6.517E-07	4.225E-05	1	1	4	Q13263	Q13263 [231-250]	1	3095.5613
[K].SSMSVT	1xOxidation [	1.272E-05	4.225E-05	1	1	1	Q9H9B4	Q9H9B4 [291-305	0	2205.1928
[K].SSMSET	1xCarbamido	3.645E-05	4.225E-05	1	1	1	Q86VB7	Q86VB7 [853-865	0	1716.858
[R].SSPVEFI	1xCarbamido	3.016E-05	4.225E-05	1	1	3	O75131	O75131 [242-253]	0	2047.0662
[R].SSPVVID	1xTMTpro [N	4.651E-07	4.225E-05	1	1	3	P02751	P02751 [1983-200	0	2217.2046
[K].KFAEAL(	2xTMTpro [K	3.626E-05	4.225E-05	1	1	1	P78347	P78347 [477-488]	1	2164.2794
[K].DCLQFID	1xCarbamido	5.384E-05	4.225E-05	1	1	2	Q9NUY8	Q9NUY8 [85-99]	0	2429.2878
[K].SSSSVT	1xCarbamido	6.08E-05	4.225E-05	1	1	1	P50552	P50552 [322-346]	0	3011.3707
[R].DCEQAE	1xCarbamido	5.627E-05	4.225E-05	1	1	2	Q13813	Q13813 [1453-14	0	1784.8015
[R].KEVEGD	2xTMTpro [K	7.084E-07	4.225E-05	1	1	3	Q1KMD3	Q1KMD3 [577-59	1	2861.5456
[K].DCFIIYQ	1xCarbamido	5.315E-07	4.225E-05	1	1	2	P28331	P28331 [563-592]	0	3835.9973
[K].SSSQPS	2xCarbamido	2.904E-07	4.225E-05	1	1	1	Q13501	Q13501 [282-312]	0	3847.8141
[K].DCGATW	1xCarbamido	1.374E-05	4.225E-05	1	1	5	P60174	P60174 [86-99]	0	1890.9452
[R].KEYGGL	2xTMTpro [K	6.751E-07	4.225E-05	1	1	3	P16152	P16152 [79-96]	1	2820.644
[R].DCGGAA	1xCarbamido	1.581E-05	4.225E-05	1	1	2	Q8NDH3	Q8NDH3 [283-29	0	1568.8174
[K].SSSPAP	1xTMTpro [N	6.138E-06	4.225E-05	1	1	1	Q13283	Q13283 [230-247]	0	2189.1369
[R].KEYGGL	2xTMTpro [K	4.765E-06	4.225E-05	1	1	1	O75828	O75828 [79-96]	1	2819.66
[K].GESDPA	1xTMTpro [K	7.83E-06	4.225E-05	1	1	1	P13667	P13667 [314-335]	1	3183.5475
[R].SSSFSD	1xTMTpro [K	3.637E-06	4.225E-05	1	1	2	P15924	P15924 [2606-26	0	3325.6819
[R].SSSAEE	1xTMTpro [K	1.98E-05	4.225E-05	1	1	1	Q14789	Q14789 [537-555]	0	2651.3292
[K].DCHLAQ	1xCarbamido	1.293E-05	4.225E-05	1	1	2	P02787	P02787 [259-273]	0	1994.0561
[K].SSQSVL	1xTMTpro [K	4.184E-05	4.225E-05	1	1	2	P06312	P06312 [45-56]	0	1922.0475
[K].DCKDVD	4xCarbamido	4.954E-06	4.225E-05	1	1	2	P07225	P07225 [198-218]	1	3658.9054
[R].SSQSSS	1xTMTpro [N	1.872E-05	4.225E-05	1	1	1	Q92841	Q92841 [671-684]	0	1759.8894
[K].DCIGGC	2xCarbamido	1.649E-06	4.225E-05	1	1	3	P35754	P35754 [78-98]	0	2612.2979
[R].DCLIPM(	1xCarbamido	4.673E-05	4.225E-05	1	1	2	P09110	P09110 [176-191]	0	2109.0639
[K].GESLAD	1xCarbamido	5.454E-06	4.225E-05	1	1	1	P27708	P27708 [2004-20	0	2604.2969
[K].GESGYV	1xTMTpro [K	3.657E-05	4.225E-05	1	1	2	Q15113	Q15113 [43-61]	0	2634.3695
[R].DCLQAP	1xCarbamido	2.316E-05	4.225E-05	1	1	1	P31270	P31270 [92-109]	0	2377.3041
[K].SSSEGG	1xTMTpro [N	5.006E-05	4.225E-05	1	1	1	Q9Y3E1	Q9Y3E1 [178-190	0	1556.7108
[K].SSMNVD	1xTMTpro [N	1.417E-05	4.225E-05	1	1	4	P51153	P51153 [154-167]	0	1817.9023
[K].GESDPA	1xTMTpro [N	2.526E-06	4.225E-05	1	1	2	P13667	P13667 [314-331]	0	2344.1125

[R].SSLVGV	1xTMTpro [K	2.324E-05	4.225E-05	1	1	2	Q9ULC5	Q9ULC5 [580-599	0	2609.5046
[K].SSGNSS	1xTMTpro [N	3.641E-07	4.225E-05	1	1	3	Q12797	Q12797 [9-33]	0	2407.0889
[R].GEQGFN	1xTMTpro [N	4.419E-05	4.225E-05	1	1	1	P08572	P08572 [1350-136	0	2054.9885
[R].GEQCVD	3xCarbamido	4.687E-06	4.225E-05	1	1	2	Q12805	Q12805 [209-227]	0	2681.2077
[K].KFDGPC	2xCarbamido	3.249E-06	4.225E-05	1	1	1	O00468	O00468 [312-329]	1	2625.3045
[R].KFDLNSI	1xTMTpro [K	3.607E-05	4.225E-05	1	1	1	O75489	O75489 [232-246]	1	2477.3473
[K].SSGGRE	1xTMTpro [N	6.1E-05	4.225E-05	1	1	1	Q9UK76	Q9UK76 [70-84]	1	1881.9586
[K].SSGEIVY	1xCarbamido	9.893E-06	4.225E-05	1	1	3	Q02543	Q02543 [57-70]	0	2211.1611
[KR].KFDQL	2xTMTpro [K	4.337E-05	4.225E-05	4	4	1	P35580; P35	P35580 [1452-146	1	2133.2736
[R].SSFLQVI	1xTMTpro [N	2.924E-06	4.225E-05	1	1	2	Q15436	Q15436 [587-604]	0	2444.1689
[K].DDFDFG	1xTMTpro [N	3.709E-05	4.225E-05	1	1	2	Q13423	Q13423 [412-424]	0	1813.8862
[K].DDEENY	1xTMTpro [K	1.208E-05	4.225E-05	1	1	1	Q07666	Q07666 [140-152]	0	2233.1269
[R].GEPPLP	1xTMTpro [K	5.014E-05	4.225E-05	1	1	2	O15372	O15372 [292-303]	0	1919.0617
[R].SSFDEM	1xTMTpro [N	5.375E-05	4.225E-05	1	1	1	Q02218	Q02218 [870-883]	0	1955.9605
[K].SSFADIS	1xTMTpro [N	2.963E-05	4.225E-05	1	1	1	Q15785	Q15785 [279-293]	0	1994.0878
[R].KFDVNT	1xTMTpro [K	2.179E-07	4.225E-05	1	1	4	Q00610	Q00610 [1074-109	1	2976.6762
[R].SSEMPE	1xTMTpro [N	8.875E-06	4.225E-05	1	1	1	P05164	P05164 [406-421]	0	2149.1316
[K].SSEMNV	1xOxidation [	2.333E-05	4.225E-05	1	1	5	Q01518	Q01518 [434-458]	0	3435.7274
[R].DDGSAV	1xTMTpro [K	5.784E-05	4.225E-05	1	1	2	Q14019	Q14019 [19-30]	0	1946.0879
[K].SSEMNV	1xTMTpro [K	2.079E-05	4.225E-05	1	1	7	Q01518	Q01518 [434-458]	0	3419.7325
[R].DDGTGQ	1xTMTpro [N	1.422E-05	4.225E-05	1	1	2	Q15149	Q15149 [4004-40	0	1875.0143
[K].KFEDEYS	2xTMTpro [K	1.541E-05	4.225E-05	1	1	6	Q9H2H8	Q9H2H8 [70-80]	1	2363.2951
[R].DDGVFV	1xTMTpro [N	4.304E-06	4.225E-05	1	1	2	Q09666	Q09666 [29-45]	0	2137.0845
[R].DDFLGQ	1xTMTpro [N	4.872E-06	4.225E-05	1	1	2	P46934	P46934 [519-538]	0	2589.352
[R].SSSVGS	1xTMTpro [N	1.224E-05	4.225E-05	1	1	1	Q15149	Q15149 [4384-44	0	2059.0627
[K].SSGPTS	1xTMTpro [N	2.328E-06	4.225E-05	1	1	4	Q00839	Q00839 [187-204]	0	2019.1194
[R].SSGPYG	1xTMTpro [K	1.804E-07	4.225E-05	1	1	1	P09651	P09651 [337-352]	0	2237.1959
[K].SSLTIQF	1xTMTpro [K	2.023E-05	4.225E-05	1	1	1	Q15382	Q15382 [20-45]	0	3574.8449
[K].DCVGDV	2xCarbamido	2.304E-05	4.225E-05	1	1	2	P07996	P07996 [530-543]	0	2260.1193
[K].SSLSVPY	2xTMTpro [K	7.436E-06	4.225E-05	1	1	1	P01024	P01024 [892-913]	1	3297.9854
[K].KFAYGY	2xTMTpro [K	2.202E-05	4.225E-05	1	1	1	P30740	P30740 [203-213]	1	2259.3205
[R].SSLSSA(	1xTMTpro [N	1.143E-06	4.225E-05	1	1	2	Q13813	Q13813 [2138-215	0	2256.1427
[K].SSLSCSI	1xCarbamido	9.966E-06	4.225E-05	1	1	1	P52701	P52701 [611-632]	0	3005.5534

[R].KFCTTG	1xCarbamido	4.525E-06	4.225E-05	1	1	1	Q92747	Q92747 [342-358]	1	2903.5616
[K].GEQLFLI	1xTMTpro [N	5.34E-05	4.225E-05	1	1	2	Q9NXE4	Q9NXE4 [524-538	0	2081.1714
[K].SSILLDV	1xCarbamido	4.73E-06	4.225E-05	1	1	3	P29692	P29692 [196-219]	0	3399.742
[K].SSILLDV	1xOxidation [	2.602E-05	4.225E-05	1	1	2	P24534	P24534 [140-157]	0	2991.6165
[R].DDDYAG	1xTMTpro [N	1.916E-05	4.225E-05	2	2	3	P07996; P35	P07996 [1019-103	0	2117.9735
[K].SSILLDV	2xTMTpro [K	6.92E-06	4.225E-05	1	1	2	P24534	P24534 [140-157]	0	2975.6216
[R].KFDFDA	1xCarbamido	5.894E-06	4.225E-05	2	2	2	Q15424; Q1	Q15424 [356-370]	1	2647.437
[K].SSLESIP	1xTMTpro [N	1.638E-07	4.225E-05	1	1	3	Q8IYI6	Q8IYI6 [96-130]	0	3340.7668
[K].SSLELE\	1xTMTpro [K	6.07E-05	4.225E-05	1	1	1	Q709C8	Q709C8 [2878-28	0	2672.3944
[R].SSLDNIE	1xTMTpro [N	5.6E-05	4.225E-05	1	1	2	Q8N1F7	Q8N1F7 [176-187	0	1673.8488
[K].SSGVPV	1xTMTpro [N	3.601E-05	4.225E-05	1	1	2	Q9BSD7	Q9BSD7 [28-42]	0	1945.9826
[R].DDDKNN	2xTMTpro [K	9.856E-06	4.225E-05	1	1	3	Q8NI22	Q8NI22 [127-143]	1	2905.4672
[R].SSGSPY	1xTMTpro [N	4.668E-07	4.225E-05	1	1	2	P51991	P51991 [355-376]	0	2214.9971
[K].GEQGQY	1xCarbamido	2.913E-06	4.225E-05	1	1	1	P54578	P54578 [190-209]	0	2789.2765
[K].SSGSAL	1xTMTpro [N	4.575E-06	4.225E-05	1	1	1	Q13630	Q13630 [200-214]	0	1793.9465
[R].GEQGFM	1xOxidation [	2.175E-05	4.225E-05	1	1	2	P08572	P08572 [1350-136	0	2070.9834
[K].SSLLDDI	1xTMTpro [N	9.174E-06	4.225E-05	1	1	2	O00429	O00429 [693-709]	0	2227.1083
[R].SSTDFSI	1xTMTpro [N	2.829E-05	4.225E-05	1	1	1	Q86V48	Q86V48 [956-967	0	1699.8458
[K].DCDLQE	3xCarbamido	3.086E-05	4.225E-05	1	1	2	P62633	P62633 [66-79]	0	2079.8489
[R].SSTGPE	1xTMTpro [N	3.191E-05	4.225E-05	1	1	1	Q9UQ35	Q9UQ35 [357-373	0	2024.0983
[R].STMVGT	1xTMTpro [N	4.989E-05	4.225E-05	2	3	1	Q13153; Q1	Q13153 [422-438]	0	2229.1367
[K].DAQGQF	1xTMTpro [K	8.882E-07	4.225E-05	1	1	2	P22105	P22105 [1297-132	1	3510.832
[R].DAQGQF	1xTMTpro [N	8.99E-06	4.225E-05	1	1	2	P22105	P22105 [3298-331	0	1942.0313
[R].STIYPVG	3xCarbamido	1.12E-06	4.225E-05	1	1	1	P04275	P04275 [2435-246	0	3830.68
[K].STLVAGS	1xCarbamido	9.174E-06	4.225E-05	1	1	1	O00203	O00203 [200-218]	0	2371.1957
[K].STITLDG	1xTMTpro [K	5.423E-06	4.225E-05	1	1	2	P15090	P15090 [83-97]	0	2175.2993
[R].KENCPA	2xCarbamido	4.417E-06	4.225E-05	1	1	1	Q8TBC4	Q8TBC4 [361-381	1	3316.7598
[R].STLQTM	1xTMTpro [N	8.891E-06	4.225E-05	1	1	2	Q07065	Q07065 [312-326]	0	2077.0443
[R].GETYCL	2xCarbamido	9.31E-06	4.225E-05	1	1	1	Q0ZGT2	Q0ZGT2 [634-655	0	3267.518
[R].DAQQSS	1xTMTpro [N	3.208E-05	4.225E-05	1	1	1	Q14139	Q14139 [545-558]	0	1763.8843
[K].STNCFG	2xCarbamido	2.885E-05	4.225E-05	1	1	2	Q9H2U2	Q9H2U2 [158-176	0	2736.3101
[K].STLINSL	1xTMTpro [N	6.007E-07	4.225E-05	1	1	1	Q16181	Q16181 [64-86]	0	2911.5161
[K].STLINSL	1xTMTpro [N	4.352E-06	4.225E-05	1	1	1	Q15019	Q15019 [51-66]	0	2186.2028

[R].DASGAT	1xTMTpro [K	4.002E-05	4.225E-05	1	2	3	P0DOX2	P0DOX2 [256-270	0	2121.1108
[R].STLLELF	1xTMTpro [N	5.218E-05	4.225E-05	1	1	2	Q9Y2I8	Q9Y2I8 [56-67]	0	1709.9757
[R].DASGVT	1xTMTpro [K	1.868E-05	4.225E-05	1	1	5	P01876	P01876 [154-168]	0	2149.1421
[K].STIGGQI	1xTMTpro [K	3.393E-05	4.225E-05	1	1	1	P15170	P15170 [88-103]	0	2322.2693
[K].STLAEIE	1xTMTpro [K	3.441E-05	4.225E-05	1	1	3	Q9NZM1	Q9NZM1 [749-760	0	2028.1145
[K].STGSIVC	1xTMTpro [N	4.981E-05	4.225E-05	1	1	1	P30038	P30038 [510-524]	0	1765.9516
[K].DASIVGF	1xTMTpro [K	1.061E-06	4.225E-05	1	1	7	P30101	P30101 [153-173]	0	2956.486
[K].STGKPTI	1xCarbamido	2.529E-05	4.225E-05	2	2	1	P0DOX6; P0	P0DOX6 [555-576	0	2973.5193
[K].DASSPV	1xTMTpro [N	4.61E-05	4.225E-05	1	1	1	O94875	O94875 [163-183]	0	2513.4312
[R].DASALLI	1xCarbamido	2.979E-05	4.225E-05	1	1	1	Q9BTE3	Q9BTE3 [277-295	0	2456.124
[R].GETTLW	1xCarbamido	5.27E-05	4.225E-05	1	1	2	P26640	P26640 [372-394]	0	3090.6174
[K].STNGDT	1xTMTpro [N	8.491E-06	4.225E-05	1	1	1	P38646	P38646 [266-284]	0	2360.1689
[R].STPAAV	1xTMTpro [K	4.681E-05	4.225E-05	1	1	2	Q9UGI8	Q9UGI8 [214-225	0	1784.9708
[R].DAIWTEI	1xCarbamido	5.014E-05	4.225E-05	1	1	2	Q7Z7G0	Q7Z7G0 [939-956	0	2813.3696
[R].STSFNV	1xTMTpro [K	3.931E-06	4.225E-05	1	1	2	Q15746	Q15746 [1386-140	0	2501.3168
[R].STSEST/	1xCarbamido	3.961E-06	4.225E-05	2	2	9	P01861; P01	P01861 [17-30]; P	0	2032.124
[K].STSEGLI	1xTMTpro [K	8.554E-06	4.225E-05	1	1	2	P46939	P46939 [1571-158	0	2530.3532
[K].GEVLGD	1xTMTpro [K	8.035E-06	4.225E-05	1	1	3	Q14980	Q14980 [406-419]	0	2122.2615
[R].KEGGLG	1xTMTpro [K	1.358E-06	4.225E-05	1	1	8	P32119	P32119 [92-109]	1	2471.4841
[K].KEGKPT	3xTMTpro [K	4.14E-06	4.225E-05	1	1	1	O75937	O75937 [143-159]	1	3190.8352
[R].STQAPLI	1xTMTpro [K	1.217E-06	4.225E-05	1	1	4	P43490	P43490 [303-323]	0	2843.6486
[R].DANVSG	1xTMTpro [K	1.855E-05	4.225E-05	1	1	1	Q96IZ0	Q96IZ0 [255-270]	0	2216.2266
[R].STQAAT	1xTMTpro [N	1.224E-05	4.225E-05	1	1	1	O75439	O75439 [44-59]	0	2018.1201
[R].STNLNC	1xCarbamido	1.462E-05	4.225E-05	1	1	1	Q96EL3	Q96EL3 [44-56]	0	1752.9234
[R].KEGTGS	2xTMTpro [K	3.423E-05	4.225E-05	1	1	1	O15372	O15372 [5-24]	1	2668.457
[R].DAPEGG	1xCarbamido	6.416E-06	4.225E-05	1	1	1	P05106	P05106 [243-261]	0	2662.2984
[R].DAPEGG	1xCarbamido	1.034E-05	4.225E-05	1	1	1	P18084	P18084 [244-260]	0	2356.2463
[R].KELEQIF	1xCarbamido	2.677E-05	4.225E-05	1	1	1	Q02218	Q02218 [324-336]	1	2584.4261
[K].STPVTS/	1xTMTpro [K	2.479E-05	4.225E-05	1	1	1	Q96KR1	Q96KR1 [528-541	0	2064.2196
[R].KEIEVDS	1xTMTpro [K	5.764E-06	4.225E-05	1	3	1	P61225	P61225 [42-68]	1	3560.865
[R].KELLELE	2xTMTpro [K	8.367E-06	4.225E-05	1	1	2	Q14764	Q14764 [704-723]	1	3032.737
[R].STPAITL	1xTMTpro [K	3.555E-07	4.225E-05	1	1	3	P00387	P00387 [30-46]	1	2509.4521
[R].STPAITL	1xTMTpro [K	3.086E-05	4.225E-05	1	1	2	P00387	P00387 [30-42]	0	1980.1509

[K].KELIINEI	2xTMTpro [K	2.238E-05	4.225E-05	1	1	2	Q13177	Q13177 [288-299]	1	2355.4865
[R].DAQDVQ	1xTMTpro [N	2.124E-05	4.225E-05	1	1	2	Q9P2E9	Q9P2E9 [971-988	0	2293.0976
[K].DAPDHQ	1xTMTpro [N	9.966E-06	4.225E-05	1	1	2	P01024	P01024 [1285-130	0	2451.2799
[R].SYQDPS	1xTMTpro [N	1.519E-05	4.225E-05	1	1	4	Q9UNZ2	Q9UNZ2 [200-214	0	2059.0416
[K].STGGIS\	1xTMTpro [N	1.041E-05	4.225E-05	1	1	5	P02452	P02452 [171-187]	0	1857.9812
[K].STGGAP	1xTMTpro [K	4.187E-06	4.225E-05	1	1	5	P07737	P07737 [92-105]	0	1988.1308
[K].STAAMS	1xTMTpro [K	1.294E-06	4.225E-05	1	1	1	P02655	P02655 [78-98]	0	2841.5564
[R].DAVSGM	1xTMTpro [K	6.048E-06	4.225E-05	1	1	1	P49720	P49720 [178-192]	0	2176.2655
[R].GETGPA	1xTMTpro [N	5.985E-07	4.225E-05	1	1	7	P02452	P02452 [1067-108	0	1851.0044
[K].DAVSTG	1xTMTpro [K	1.786E-05	4.225E-05	1	1	1	Q96Q06	Q96Q06 [882-896	0	2025.1836
[R].SSYDVF	1xTMTpro [K	4.264E-05	4.225E-05	1	1	1	Q16658	Q16658 [409-426]	0	2718.3907
[R].DAVTYTI	1xTMTpro [K	2.409E-05	4.225E-05	1	1	13	P62805	P62805 [69-78]	0	1742.9569
[K].SSWLEE	1xTMTpro [N	8.124E-06	4.225E-05	1	1	2	O15460	O15460 [384-397]	0	1921.9463
[R].KEPFTLE	2xTMTpro [K	2.507E-06	4.225E-05	1	1	1	P34932	P34932 [437-466]	1	4342.3104
[K].SSVLIAQ	1xTMTpro [K	1.616E-05	4.225E-05	1	1	1	P46060	P46060 [453-468]	0	2327.2586
[K].KEPTQA	2xTMTpro [K	6.12E-08	4.225E-05	1	1	1	O43491	O43491 [100-123]	2	3733.0476
[R].STAEPEI	2xCarbamido	2.981E-07	4.225E-05	1	1	2	Q8WX93	Q8WX93 [496-524	0	3518.699
[K].SSVLEAI	1xTMTpro [N	5.331E-05	4.225E-05	2	2	2	P20591; P20	P20591 [84-97]; F	0	1703.0023
[R].SSTTSM	2xTMTpro [K	2.677E-05	4.225E-05	1	1	1	Q13200	Q13200 [84-97]	0	2376.3988
[R].DAYELQ	1xCarbamido	1.378E-06	4.225E-05	1	1	1	Q9UEW8	Q9UEW8 [61-85]	0	3254.7699
[R].GETGDD	1xTMTpro [N	2.849E-05	4.225E-05	1	1	3	P12111	P12111 [2291-230	1	1867.8701
[R].SSTSGD	1xTMTpro [K	6.074E-07	4.225E-05	1	1	1	O15231	O15231 [152-175]	0	3282.5153
[K].KEQVEE	2xTMTpro [K	6.794E-06	4.225E-05	1	1	2	Q14789	Q14789 [946-960]	1	2603.4344
[R].DAYSGG	1xTMTpro [N	6.997E-06	4.225E-05	1	1	1	P28074	P28074 [226-239]	0	1825.9516
[R].SSTPLPT	1xTMTpro [N	3.328E-05	4.225E-05	2	2	3	P42167; P42	P42167 [158-173]	0	1952.0256
[K].DCAVIEC	2xCarbamido	1.682E-05	4.225E-05	1	1	1	Q15746	Q15746 [522-538]	0	2300.1334
[K].SSTKPA	2xTMTpro [K	1.456E-05	4.225E-05	1	1	2	Q15942	Q15942 [185-201]	0	2578.5173
[K].DCDGGY	5xCarbamido	1.345E-06	4.225E-05	1	1	1	Q14767	Q14767 [1119-115	0	4458.8657
[R].KEQTAD	1xTMTpro [K	1.793E-06	4.225E-05	1	1	1	Q9UKK9	Q9UKK9 [55-70]	1	2332.3844
[R].DASVAE	1xTMTpro [N	1.28E-06	4.225E-05	1	1	2	Q01082	Q01082 [2025-204	0	2396.2417
[K].STAGDT	1xTMTpro [N	3.644E-06	4.225E-05	2	2	5	P54652; P11	P54652 [224-239]	0	1995.9327
[K].STAPETA	1xCarbamido	1.244E-05	4.225E-05	1	1	1	Q14839	Q14839 [1585-160	0	2911.4487
[K].STGEPL(	1xTMTpro [N	4.697E-05	4.225E-05	1	1	1	Q93052	Q93052 [11-22]	0	1524.8454

[R].STGEAF	1xTMTpro [K	1.696E-06	4.225E-05	2	2	5	P31943; P55	P31943 [151-167]	0	2450.3059
[R].STGEAF	1xTMTpro [K	3.102E-05	4.225E-05	1	1	3	P31942	P31942 [56-67]	0	1880.0409
[K].DATNVA	1xTMTpro [N	1.425E-05	4.225E-05	1	1	2	P51151	P51151 [157-170]	0	1767.9196
[K].DATNVG	1xTMTpro [K	4.827E-06	4.225E-05	1	1	6	P06733	P06733 [203-221]	0	2569.339
[K].DATNVG	2xTMTpro [K	1.479E-06	4.225E-05	1	1	5	P06733	P06733 [203-228]	1	3655.9999
[K].DATNVG	1xTMTpro [K	1.54E-06	4.225E-05	1	1	2	P09104	P09104 [203-228]	0	3310.7298
[R].DATSLL	1xTMTpro [N	3.843E-08	4.225E-05	1	1	2	Q9P270	Q9P270 [93-119]	0	3169.6548
[K].DATSRP	1xTMTpro [N	5.384E-05	4.225E-05	1	1	1	Q9UGP8	Q9UGP8 [263-27	0	2227.273
[K].GETLLSI	1xCarbamid	1.924E-07	4.225E-05	1	1	1	Q07157	Q07157 [1690-17	0	2417.354
[K].DAVITVF	1xTMTpro [N	2.167E-05	4.225E-05	1	1	1	Q9Y4L1	Q9Y4L1 [171-185	0	2010.0979
[K].STGDIA	1xTMTpro [K	5.208E-07	4.225E-05	1	1	2	Q9UDY2	Q9UDY2 [461-47	1	2479.3648
[R].DAVEEG	1xCarbamid	2.434E-06	4.225E-05	1	1	1	P21980	P21980 [81-116]	0	4252.1039
[K].STFFNVI	1xCarbamid	1.05E-07	4.225E-05	1	1	2	Q9NTK5	Q9NTK5 [36-63]	0	3497.6603
[K].KEPAVLI	3xTMTpro [K	1.524E-05	4.225E-05	1	1	1	Q01518	Q01518 [317-328	2	2557.607
[R].GETGPS	1xTMTpro [N	2.867E-07	4.225E-05	1	1	10	P08123	P08123 [979-996]	0	1866.9993
[R].STDQVI	2xTMTpro [K	2.574E-05	4.225E-05	1	1	2	O60507	O60507 [286-301	0	2568.5541
[R].STDQTV	1xTMTpro [K	2.341E-05	4.225E-05	1	1	1	Q96S59	Q96S59 [392-405	0	2142.2149
[K].STDFAPI	2xTMTpro [K	3.495E-05	4.225E-05	1	1	1	P46821	P46821 [1462-14	1	2624.4388
[K].KEPEAFI	1xCarbamid	4.062E-07	4.225E-05	1	1	3	P40261	P40261 [100-121]	1	3219.6276
[R].STAYED	1xTMTpro [N	8.554E-06	4.225E-05	1	1	1	O43390	O43390 [428-441	0	2062.983
[K].STATISG	1xTMTpro [K	4.077E-07	4.225E-05	1	1	3	P02751	P02751 [1592-16	0	3078.7331
[K].STFVLDI	1xTMTpro [K	4.857E-05	4.225E-05	1	1	2	P26641	P26641 [286-295]	1	1850.0668
[K].KDLLPSI	1xTMTpro [K	4.991E-08	4.225E-05	1	1	1	Q15149	Q15149 [3183-32	1	3670.013
[RK].SYSCQ	1xCarbamid	2.786E-06	4.225E-05	2	6	4	P0DOY2; P0	P0DOY2 [84-98];	0	2320.1735
[K].SYSMIVI	2xTMTpro [K	3.131E-06	4.225E-05	1	1	1	Q9HB71	Q9HB71 [124-143	0	3078.7689
[R].TELLPGI	1xTMTpro [N	4.495E-05	4.225E-05	1	1	3	Q16270	Q16270 [206-221	1	2116.1682
[R].TELGLDI	1xTMTpro [K	1.776E-05	4.225E-05	1	1	2	Q86UE4	Q86UE4 [34-45]	0	1893.1189
[K].TELEDTI	1xTMTpro [N	3.154E-06	4.225E-05	1	1	1	P35580	P35580 [1153-11	0	2238.1421
[K].TELEDTI	1xTMTpro [N	6.252E-06	4.225E-05	1	1	2	P35749	P35749 [1153-11	0	2254.137
[K].TELEDTI	1xTMTpro [N	3.678E-06	4.225E-05	1	1	3	P35579	P35579 [1146-11	0	2224.1264
[R].TEIDKPS	1xOxidation	3.92E-05	4.225E-05	1	1	7	P02751	P02751 [1631-16	0	3390.8243
[K].CGVCG	3xCarbamid	1.822E-05	4.225E-05	1	1	1	Q6ZMP0	Q6ZMP0 [401-41	0	2609.2766
[R].TEIDKPS	2xTMTpro [K	5.557E-07	4.225E-05	1	1	7	P02751	P02751 [1631-16	0	3374.8293

[K].CGVMAS	3xCarbamido	9.124E-06	4.225E-05	1	1	1	P55001	P55001 [160-178]	0	2276.0575
[K].TELDAHI	1xTMTpro [N	2.246E-05	4.225E-05	1	1	1	Q9NR46	Q9NR46 [33-45]	0	1832.9826
[R].TELATYI	1xCarbamido	1.258E-05	4.225E-05	1	1	2	Q9Y394	Q9Y394 [219-252	0	4179.2503
[K].GFLNSSI	1xTMTpro [N	3.961E-06	4.225E-05	1	1	1	P43897	P43897 [162-178]	0	2021.0623
[K].CHAGHL	1xCarbamido	2.75E-05	4.225E-05	1	1	1	P02679	P02679 [365-382]	0	2620.3222
[R].TEHPFT	1xTMTpro [K	3.174E-05	4.225E-05	1	1	4	P01023	P01023 [215-228]	0	2281.2724
[K].KASYSG	1xTMTpro [K	3.325E-08	4.225E-05	1	1	7	P07585	P07585 [321-345]	1	3499.8506
[R].TEGSLEI	1xTMTpro [K	1.251E-05	4.225E-05	1	1	2	Q15020	Q15020 [555-568	0	2199.1789
[R].TEGISTS	1xTMTpro [N	3.747E-05	4.225E-05	2	2	1	P49585; Q9	P49585 [197-208]	0	1596.8764
[R].TEGDGV	1xTMTpro [K	1.587E-05	4.225E-05	1	1	3	P00738	P00738 [119-131]	0	2048.0792
[R].CKDDEF	1xCarbamido	1.99E-07	4.225E-05	1	1	3	P27797	P27797 [163-185]	1	3769.0087
[R].TEGDGV	2xTMTpro [K	4.022E-05	4.225E-05	2	2	2	P00739; P00	P00739 [61-73]; F	1	2352.3227
[R].TEGDGV	1xTMTpro [K	3.532E-05	4.225E-05	2	2	2	P00739; P00	P00739 [61-72]; F	0	1920.0206
[K].TEILSLEI	1xTMTpro [K	4.793E-05	4.225E-05	1	1	1	Q9GZT8	Q9GZT8 [235-252	0	2616.5403
[K].TEIPFEF	1xTMTpro [K	1.448E-05	4.225E-05	1	1	1	O14972	O14972 [89-100]	0	2079.2134
[K].TELQGLI	1xTMTpro [K	1.298E-05	4.225E-05	1	1	2	Q9UL15	Q9UL15 [308-324	0	2480.4103
[R].TELRPG	1xTMTpro [N	2.218E-05	4.225E-05	1	1	1	P01024	P01024 [463-478]	0	2176.2409
[R].TEPTAQ	1xTMTpro [K	1.002E-05	4.225E-05	1	2	1	Q99613	Q99613 [847-862	0	2363.3426
[K].TEPPQG	1xCarbamido	4.367E-05	4.225E-05	1	1	1	A1X283	A1X283 [652-667	0	2175.0671
[R].TEPMAW	1xTMTpro [N	4.625E-07	4.225E-05	1	1	1	P35222	P35222 [685-710]	0	3108.5267
[K].TEPFEEI	1xTMTpro [K	2.109E-05	4.225E-05	1	1	2	Q9H9Q4	Q9H9Q4 [181-197	0	2726.3879
[R].TEPELL\	1xTMTpro [N	4.818E-06	4.225E-05	1	1	2	P09601	P09601 [124-136]	0	1803.9924
[RK].CFIEEII	1xCarbamido	4.022E-05	4.225E-05	2	2	2	Q7Z7H5; Q9	Q7Z7H5 [41-57]; (	0	2390.1691
[RK].CFIEEII	1xCarbamido	5.41E-05	4.225E-05	2	2	1	Q7Z7H5; Q9	Q7Z7H5 [41-57]; (	0	2406.1641
[K].CFIVGAI	1xCarbamido	3.242E-05	4.225E-05	1	2	4	P05388	P05388 [27-38]	0	1875.029
[K].KAQDEG	3xTMTpro [K	6.744E-06	4.225E-05	1	1	1	P55084	P55084 [254-272]	2	3243.9458
[K].KAQDLT	2xTMTpro [K	5.446E-05	4.225E-05	1	1	1	Q8WXH0	Q8WXH0 [2191-2	1	2029.2837
[K].TEGDEE	1xTMTpro [K	4.533E-06	4.225E-05	1	1	2	P12956	P12956 [10-31]	0	3109.4101
[K].CFSIDNI	1xCarbamido	1.633E-05	4.225E-05	1	1	2	O75083	O75083 [438-470	0	3701.8189
[R].CFYNSD	1xCarbamido	1.828E-05	4.225E-05	1	1	1	Q93099	Q93099 [146-160	0	2411.2561
[K].TEMSEV	1xTMTpro [N	4.776E-05	4.225E-05	1	1	2	O43615	O43615 [294-305	0	1724.9424
[R].KAQPAC	2xTMTpro [K	4.737E-05	4.225E-05	1	1	2	P25786	P25786 [243-256]	1	2392.3652
[K].CGDLEE	1xCarbamido	6.608E-06	4.225E-05	1	1	4	P67936	P67936 [154-169]	1	2788.5331

[R].GFLWDE	1xTMTpro [N	1.15E-05	4.225E-05	1	1	1	Q13724	Q13724 [447-461]	0	2135.1357
[R].GFLTERI	2xCarbamido	3.405E-05	4.225E-05	1	1	4	Q14192	Q14192 [262-279]	2	2732.3879
[R].CGEEIAV	1xCarbamido	4.931E-05	4.225E-05	1	1	1	O43175	O43175 [295-308]	0	2233.1852
[R].GFLTERI	2xCarbamido	2.611E-06	4.225E-05	1	1	2	Q14192	Q14192 [262-277]	1	2504.2769
[R].KASGPP	2xTMTpro [K	3.939E-06	4.225E-05	3	3	10	P16402; P10	P16402 [35-47]; P	1	2239.3842
[K].TELSQSI	1xTMTpro [N	4.825E-05	4.225E-05	1	1	2	P62495	P62495 [234-245]	0	1760.8444
[K].KAQLNIG	2xCarbamido	1.195E-08	4.225E-05	1	1	3	P62917	P62917 [93-123]	1	4308.3334
[R].CKLEEM	1xCarbamido	1.075E-05	4.225E-05	1	1	1	Q92599	Q92599 [303-329]	1	4051.9721
[K].KATDAE	1xTMTpro [K	1.713E-05	4.225E-05	2	2	6	P09493; P07	P09493 [77-90]; P	1	2069.1483
[K].KATEDE	2xTMTpro [K	1.361E-05	4.225E-05	1	1	1	P01008	P01008 [61-78]	2	2915.589
[R].CLCYDG	2xCarbamido	5.926E-05	4.225E-05	1	1	7	P35555	P35555 [1263-127	0	2351.0672
[R].TECAEP	1xCarbamido	1.757E-05	4.225E-05	1	1	1	P53041	P53041 [9-26]	1	2561.3161
[K].KAVDVFI	2xTMTpro [K	1.985E-06	4.225E-05	1	1	1	Q00610	Q00610 [246-269]	1	3619.9651
[K].TEAVAS	1xTMTpro [N	1.443E-05	4.225E-05	1	1	2	P16278	P16278 [286-299]	0	1813.0026
[R].TEAPSA	1xTMTpro [N	5.403E-06	4.225E-05	1	1	2	P39060	P39060 [1711-172	0	1907.0153
[K].CIDLEPD	1xCarbamido	4.002E-05	4.225E-05	1	1	2	O94826	O94826 [502-516]	0	2384.2412
[K].TEAPAAI	1xTMTpro [K	5.869E-05	4.225E-05	1	1	1	P80723	P80723 [151-163]	0	1893.0573
[K].KAVLFCI	1xCarbamido	5.484E-06	4.225E-05	1	1	3	P23528	P23528 [34-45]	2	2654.6056
[K].TEAEIAH	1xTMTpro [N	1.197E-05	4.225E-05	1	1	1	O75955	O75955 [92-109]	0	2322.2373
[K].TEAASDI	1xTMTpro [N	1.51E-05	4.225E-05	1	1	1	Q99536	Q99536 [23-49]	0	2861.4713
[K].TECALL	1xCarbamido	3.048E-05	4.225E-05	1	1	2	P23634	P23634 [535-547]	0	2075.1702
[R].KAVQGA	1xTMTpro [K	4.541E-05	4.225E-05	1	1	1	Q13045	Q13045 [1046-10	1	2295.3429
[R].KAVQLT	2xTMTpro [K	2.844E-06	4.225E-05	1	1	3	O94979	O94979 [729-749]	1	3057.8162
[K].CIENLEE	1xCarbamido	4.009E-05	4.225E-05	1	1	2	Q15435	Q15435 [112-123]	0	1807.9543
[K].GFHQVP	1xTMTpro [N	4.89E-05	4.225E-05	1	1	2	P47897	P47897 [602-616]	0	2061.1605
[K].KAYCEP	4xCarbamido	1.428E-07	4.225E-05	1	1	1	Q93052	Q93052 [462-486]	1	4076.0352
[K].TDTLEDI	1xTMTpro [K	5.563E-05	4.225E-05	1	1	2	P13010	P13010 [470-481]	0	1989.1036
[K].TDTESEI	1xTMTpro [N	6.032E-05	4.225E-05	1	1	1	P11586	P11586 [761-772]	0	1682.8768
[R].CIGDESI	2xCarbamido	3.641E-07	4.225E-05	1	1	1	Q8WUP2	Q8WUP2 [275-29	0	3062.3831
[K].GFGTDE	1xTMTpro [N	2.54E-06	4.225E-05	1	1	2	P20073	P20073 [200-215]	0	1995.0466
[R].TDSRED	1xTMTpro [K	3.219E-06	4.225E-05	1	1	1	P10644	P10644 [75-92]	1	2585.4067
[R].TDSEIAL	1xTMTpro [K	1.519E-05	4.225E-05	1	1	1	P61923	P61923 [55-70]	0	2359.3616
[R].TDYNAS	1xTMTpro [N	4.936E-06	4.225E-05	1	1	4	P61978	P61978 [70-86]	0	2085.0056

[R].TEQDET	2xTMTpro [K	8.337E-06	4.225E-05	1	1	1	Q12882	Q12882 [411-430]	1	3344.6885
[R].TECFITG	1xCarbamido	5.85E-06	4.225E-05	1	1	3	P00747	P00747 [697-717]	0	2881.505
[R].TEDNQS	1xTMTpro [K	3.34E-05	4.225E-05	1	1	1	Q9Y6X4	Q9Y6X4 [293-309	0	2547.3104
[K].TEFLSFN	1xTMTpro [K	5.796E-06	4.225E-05	1	1	2	P31949	P31949 [37-52]	0	2458.3184
[R].KATEDFI	1xTMTpro [K	1.638E-07	4.225E-05	1	1	1	Q9UH99	Q9UH99 [122-154	1	4161.914
[R].TEETATI	1xTMTpro [K	4.601E-06	4.225E-05	1	1	1	Q02952	Q02952 [1564-15	0	2968.5793
[K].TEESPAS	1xTMTpro [K	8.124E-06	4.225E-05	1	1	2	P05114	P05114 [83-95]	0	1957.9846
[R].CLAENA	1xCarbamido	6.149E-06	4.225E-05	1	1	1	P02788	P02788 [553-565]	0	2002.0923
[R].TEEQKT	2xTMTpro [K	9.659E-06	4.225E-05	1	1	1	Q02952	Q02952 [428-461]	1	4613.312
[R].CLAFHD	1xCarbamido	1.332E-05	4.225E-05	1	1	2	P49773	P49773 [38-57]	0	2899.6148
[R].CIALAQL	1xCarbamido	2.219E-06	4.225E-05	1	1	3	Q13838	Q13838 [300-319]	0	2581.4608
[K].TEELNKI	1xTMTpro [K	2.967E-06	4.225E-05	1	1	1	P08779	P08779 [312-330]	1	2728.4609
[K].GFIGPGI	1xOxidation [	4.603E-05	4.225E-05	1	1	2	P00367	P00367 [213-231]	0	2236.1239
[R].TECYGY	1xCarbamido	2.799E-05	4.225E-05	1	1	1	P53041	P53041 [75-87]	0	1780.8495
[K].GFIGPGI	1xTMTpro [N	1.963E-06	4.225E-05	1	1	2	P00367	P00367 [213-231]	0	2220.129
[K].CIAVGES	1xCarbamido	1.985E-06	4.225E-05	1	2	1	P00367	P00367 [327-346]	0	2738.3951
[R].KATQQA	1xTMTpro [K	2.563E-06	4.225E-05	1	1	3	P35749	P35749 [1761-17	1	2525.3815
[K].KAVAFSI	2xTMTpro [K	2.337E-05	4.225E-05	1	1	1	P17858	P17858 [715-726]	1	2202.3678
[R].TEDSGL	2xCarbamido	8.418E-08	4.225E-05	1	1	2	Q9Y490	Q9Y490 [693-721	0	3660.9068
[R].KAVDEA	2xTMTpro [K	1.156E-05	4.225E-05	1	1	2	Q16891	Q16891 [286-297]	1	2156.3107
[R].CLCLPS	4xCarbamido	2.523E-07	4.225E-05	1	1	2	P13611	P13611 [3151-317	0	3670.693
[K].CICNSG	2xCarbamido	1.346E-05	4.225E-05	1	1	2	P35555	P35555 [748-761]	0	2198.0713
[K].TEDPDLI	1xTMTpro [N	2.142E-06	4.225E-05	1	1	3	Q6P2Q9	Q6P2Q9 [342-362	0	2761.4157
[K].CLCPEG	2xCarbamido	5.936E-05	4.225E-05	1	1	4	P35555	P35555 [2038-205	0	1860.8903
[R].CICPPG	2xCarbamido	5.218E-05	4.225E-05	1	1	2	P35555	P35555 [1998-201	0	2174.123
[R].KATGPP	2xTMTpro [K	1.361E-05	4.225E-05	1	1	3	P16401	P16401 [37-49]	1	2253.3998
[R].CFCQVS	4xCarbamido	2.929E-06	4.225E-05	1	1	1	Q96HE7	Q96HE7 [35-55]	0	2917.2432
[R].KAPPPL	1xTMTpro [K	2.36E-07	4.225E-05	1	1	2	P49593	P49593 [70-96]	1	3481.9663
[R].CFCLEEI	2xCarbamido	5.813E-05	4.225E-05	1	1	1	Q07092	Q07092 [278-296]	0	2557.1982
[K].TFTTQE	1xTMTpro [K	1.547E-05	4.225E-05	1	1	5	P06744	P06744 [212-226]	0	2264.2266
[K].TFTDCFI	2xCarbamido	1.491E-05	4.225E-05	2	3	3	P62136; P62	P62136 [151-168]	0	2722.4076
[K].TFTCTA	1xCarbamido	6.032E-05	4.225E-05	1	1	1	P01876	P01876 [201-212]	0	1984.0341
[K].GFPTDA	2xTMTpro [K	2.506E-05	4.225E-05	1	1	2	P05455	P05455 [117-134]	1	3005.6288

[R].TFSVWY	1xTMTpro [K	4.142E-05	4.225E-05	1	1	3	P21333	P21333 [341-355]	0	2359.2942
[K].TFSHELS	1xTMTpro [N	9.928E-08	4.225E-05	1	1	5	P30101	P30101 [306-329]	0	2879.511
[K].TFSDEPI	1xTMTpro [N	2.238E-05	4.225E-05	1	1	2	Q9NQW7	Q9NQW7 [508-53	0	3383.5949
[K].TFPTVNI	1xTMTpro [N	2.064E-06	4.225E-05	1	1	2	P30837	P30837 [53-73]	0	2501.2955
[K].TFPTVNI	1xCarbamido	1.56E-06	4.225E-05	1	1	1	P05091	P05091 [53-78]	1	3747.9567
[R].CDLCQE	2xCarbamido	1.519E-05	4.225E-05	1	1	2	P48059	P48059 [97-111]	0	2375.2595
[K].TFTTQE	2xTMTpro [K	4.739E-06	4.225E-05	1	1	1	P06744	P06744 [212-234]	1	3541.9359
[K].TFPLDV(	1xTMTpro [K	1.049E-05	4.225E-05	1	1	1	P48147	P48147 [374-389]	0	2276.2782
[K].TFPGFFS	1xOxidation [	2.475E-05	4.225E-05	1	1	5	P02671	P02671 [528-547]	0	2585.2553
[K].CDMEVS	4xCarbamido	2.529E-05	4.225E-05	1	1	2	P28799	P28799 [284-298]	0	2213.8713
[K].TFPGFFS	1xTMTpro [N	3.467E-06	4.225E-05	1	1	3	P02671	P02671 [528-547]	0	2569.2604
[R].TFNTST(	1xTMTpro [K	7.491E-06	4.225E-05	1	1	1	P12956	P12956 [302-317]	0	2260.268
[K].CDNSSM	1xCarbamido	2.265E-07	4.225E-05	1	1	1	Q99439	Q99439 [240-266]	0	3197.4733
[K].GFPTDA	1xTMTpro [K	5.841E-05	4.225E-05	1	1	2	P05455	P05455 [117-128]	0	1901.0512
[K].CDPHEA	2xCarbamido	4.22E-05	4.225E-05	1	1	2	P02751	P02751 [2379-239	0	2175.9931
[K].CDPVDQ	2xCarbamido	3.584E-06	4.225E-05	1	1	3	P02751	P02751 [555-578]	0	3473.5757
[K].GFPQPIL	1xTMTpro [N	5.287E-05	4.225E-05	1	1	2	Q9NZM1	Q9NZM1 [1685-1(	0	1706.9033
[R].CDSGFA	1xCarbamido	4.249E-05	4.225E-05	1	1	2	P35555	P35555 [1055-10(	0	1689.7709
[R].TFPLCFI	1xCarbamido	2.09E-05	4.225E-05	1	1	1	Q12824	Q12824 [163-190]	0	3520.7854
[R].CDTYAT	1xCarbamido	2.002E-05	4.225E-05	1	1	1	Q9Y265	Q9Y265 [206-225	0	2998.4887
[K].CDEDED	1xCarbamido	1.488E-05	4.225E-05	1	1	1	P11717	P11717 [1957-197	0	2355.1206
[K].KALIEVL	1xTMTpro [K	5.86E-05	4.225E-05	1	1	1	P23381	P23381 [432-448]	1	2537.5423
[K].KALAAA(	2xTMTpro [K	1.109E-05	4.225E-05	4	5	3	P16402; P10	P16402 [65-76]; F	1	2148.2845
[K].CCAAAD	3xCarbamido	2.371E-05	4.225E-05	1	1	16	P02768	P02768 [384-396]	0	2161.012
[R].GFQFVS	1xCarbamido	1.2E-05	4.225E-05	1	1	2	P62633	P62633 [42-56]	0	2080.0493
[R].CCDLPF	4xCarbamido	3.76E-05	4.225E-05	1	1	1	Q16610	Q16610 [449-465]	0	2751.2378
[R].TGEAIVI	1xTMTpro [N	9.431E-06	4.225E-05	1	1	3	Q15084	Q15084 [119-132]	0	1690.9659
[K].TGCTDIN	3xCarbamido	7.128E-06	4.225E-05	1	1	2	P35555	P35555 [1318-13:	0	2644.2409
[K].CCESAS	3xCarbamido	2.625E-05	4.225E-05	1	1	2	P02774	P02774 [265-276]	0	2055.9099
[R].KALDIAE	1xTMTpro [K	5.384E-06	4.225E-05	1	1	1	P23526	P23526 [20-34]	1	2296.2649
[R].TGAVGD	1xTMTpro [N	5.437E-05	4.225E-05	1	1	1	Q5JTZ9	Q5JTZ9 [757-769]	0	1589.9182
[K].GFQALG	1xTMTpro [N	3.086E-05	4.225E-05	1	1	6	P01033	P01033 [71-82]	0	1537.8294
[R].GFPTIYF	1xTMTpro [K	3.059E-05	4.225E-05	1	1	4	P30101	P30101 [449-460]	0	1950.0981

[K].CCLTYCI	3xCarbamido	1.307E-05	4.225E-05	1	1	1	P62979	P62979 [144-156]	0	2647.3499
[R].TGACQD	5xCarbamido	1.938E-06	4.225E-05	1	1	1	P35555	P35555 [241-272]	0	4153.9001
[R].TFYGLH	1xTMTpro [K	1.184E-05	4.225E-05	1	1	2	P28838	P28838 [85-103]	0	2685.526
[K].TFYEPGI	1xCarbamido	3.737E-07	4.225E-05	1	1	3	P02749	P02749 [39-58]	0	2991.5054
[R].TFVSGA	1xCarbamido	3.886E-05	4.225E-05	1	1	2	P62879	P62879 [198-209]	0	1864.013
[K].CCSAGC	4xCarbamido	4.228E-05	4.225E-05	1	1	1	Q14508	Q14508 [61-76]	0	2456.1322
[K].CCSGAII	2xCarbamido	4.286E-05	4.225E-05	1	1	3	P14618	P14618 [423-433]	0	1830.0473
[R].TFVQED	1xTMTpro [N	1.055E-05	4.225E-05	1	1	3	P05091	P05091 [325-338]	0	2094.0351
[K].CCVECP	4xCarbamido	2.138E-06	4.225E-05	1	1	6	P01859	P01859 [102-127]	0	3821.0231
[K].TFVNITP	1xTMTpro [K	1.308E-06	4.225E-05	1	1	3	P07737	P07737 [39-56]	1	2523.479
[K].TFVNITP	1xTMTpro [K	1.04E-05	4.225E-05	1	1	4	P07737	P07737 [39-54]	0	2252.351
[R].KALEGL	2xTMTpro [K	7.301E-06	4.225E-05	1	1	4	P14543	P14543 [1145-116	1	2784.6116
[K].TDSCDV	2xCarbamido	2.484E-06	4.225E-05	1	1	1	O43252	O43252 [204-223]	0	2711.2936
[R].TFLRPSI	1xTMTpro [K	3.184E-06	4.225E-05	1	1	2	Q9UDY2	Q9UDY2 [494-51	0	2643.4274
[K].CDYENV	2xCarbamido	1.182E-05	4.225E-05	1	1	2	Q16881	Q16881 [515-539]	0	3487.6893
[K].TFAPEEI	1xTMTpro [K	8.507E-06	4.225E-05	1	1	3	P11021	P11021 [139-152]	0	2145.2121
[R].TFAEVTI	1xTMTpro [K	9.208E-06	4.225E-05	1	1	2	P11047	P11047 [1450-146	0	2561.3413
[R].GFNCES	2xCarbamido	3.917E-06	4.225E-05	1	1	1	P02751	P02751 [84-107]	1	3815.8461
[R].TEWTPA	1xTMTpro [N	4.89E-06	4.225E-05	1	1	2	P22105	P22105 [767-797]	0	3650.8338
[R].CELCDD	2xCarbamido	1.818E-05	4.225E-05	1	1	2	P11047	P11047 [804-818]	0	2077.9278
[K].TEVVHEI	1xTMTpro [K	5.673E-05	4.225E-05	1	1	1	Q9Y6Q2	Q9Y6Q2 [364-377	0	2276.2452
[K].CELLSDI	1xCarbamido	1.115E-05	4.225E-05	1	1	2	P13798	P13798 [292-306]	0	1952.9918
[R].GFNCES	2xCarbamido	1.327E-05	4.225E-05	1	1	3	P02751	P02751 [84-100]	0	2960.4586
[K].CELSSSY	1xCarbamido	4.095E-06	4.225E-05	1	1	2	P38646	P38646 [317-340]	0	3250.6467
[R].CENTDP	3xCarbamido	5.305E-05	4.225E-05	1	1	1	P07996	P07996 [608-623]	0	2254.001
[K].TFAPEEI	1xOxidation	5.322E-05	4.225E-05	1	1	2	P11021	P11021 [139-152]	0	2161.207
[K].TEVPGS	1xTMTpro [K	4.33E-05	4.225E-05	1	1	2	P34949	P34949 [350-360]	0	1818.0141
[R].CEQCQF	2xCarbamido	5.047E-05	4.225E-05	1	1	1	P98160	P98160 [1187-120	0	2035.8921
[R].TEVEVEI	1xTMTpro [N	3.879E-05	4.225E-05	1	1	1	Q9P2X3	Q9P2X3 [163-183	1	2719.4586
[K].TEVEETA	1xTMTpro [K	2.354E-05	4.225E-05	1	1	1	Q02952	Q02952 [433-461]	0	3693.8185
[R].CEVEAP	1xCarbamido	5.598E-07	4.225E-05	1	1	1	Q13308	Q13308 [53-77]	0	3141.5635
[K].CEVNGA	1xCarbamido	3.359E-06	4.225E-05	1	1	1	P07203	P07203 [115-130]	0	2063.0816
[K].TETQEKI	3xTMTpro [K	2.109E-05	4.225E-05	1	1	2	P62328	P62328 [21-39]	2	3445.9531

[K].KAPPLVI	1xTMTpro [K	5.252E-05	4.225E-05	1	1	1	O14908	O14908 [10-24]	1	2244.248
[K].TETGQE	2xCarbamido	6.903E-09	4.225E-05	1	1	1	Q92896	Q92896 [651-672]	0	2963.4046
[K].GFMVQT	1xTMTpro [N	2.607E-05	4.225E-05	1	1	1	Q9H2H8	Q9H2H8 [48-61]	0	1727.8706
[R].CEYLME	2xCarbamido	3.023E-06	4.225E-05	1	1	2	P14314	P14314 [500-528]	0	3661.6014
[R].KAPPDG	2xTMTpro [K	1.409E-05	4.225E-05	1	1	1	P41223	P41223 [9-28]	1	3206.7765
[R].KANDITD	1xTMTpro [K	4.557E-07	4.225E-05	1	1	2	Q16787	Q16787 [2294-23	1	3063.6454
[R].CEFEEV	1xCarbamido	8.859E-06	4.225E-05	1	1	1	Q9NX20	Q9NX20 [167-182	0	2544.3048
[K].GFNEGL	1xTMTpro [K	3.664E-05	4.225E-05	1	1	1	O75475	O75475 [76-89]	0	2241.1796
[K].CDYMDE	1xCarbamido	7.575E-06	4.225E-05	1	1	3	Q1KMD3	Q1KMD3 [602-624	1	3659.8641
[R].CEAFGW	2xCarbamido	4.5E-06	4.225E-05	1	1	1	P29401	P29401 [206-226]	0	3065.5469
[K].GFPIKED	3xTMTpro [K	4.336E-06	4.225E-05	1	1	4	P08697	P08697 [171-196]	1	4082.3319
[K].KANLQID	1xTMTpro [K	3.023E-06	4.225E-05	1	1	1	P35579	P35579 [1754-17	1	2606.4757
[R].TFGSGE	1xCarbamido	2.324E-06	4.225E-05	1	1	2	P00734	P00734 [328-344]	0	2492.3099
[R].CECDDG	3xCarbamido	9.659E-06	4.225E-05	1	1	2	P24821	P24821 [392-407]	0	2442.0867
[R].CECDMG	2xCarbamido	4.939E-05	4.225E-05	1	1	1	P35555	P35555 [1429-144	0	2181.027
[R].TFFPEY	1xTMTpro [K	2.551E-05	4.225E-05	1	1	2	O14929	O14929 [50-70]	0	3121.5439
[K].TFFPEEI	1xTMTpro [K	9.929E-06	4.225E-05	1	1	3	P54652	P54652 [114-127]	0	2237.2383
[R].CECFPG	2xCarbamido	4.898E-05	4.225E-05	1	1	3	P35555	P35555 [637-650]	0	1854.9162
[K].TFAYTNI	1xTMTpro [N	2.77E-05	4.225E-05	1	1	1	P06737	P06737 [372-387]	0	2166.1514
[R].CECPVG	2xCarbamido	3.265E-05	4.225E-05	1	1	2	P35555	P35555 [1791-180	0	2144.0437
[K].CEDCGK	4xCarbamido	7.284E-07	4.225E-05	1	1	1	Q15942	Q15942 [537-563]	0	3742.7577
[K].CEDIDEC	5xCarbamido	8.554E-06	4.225E-05	1	1	3	P35555	P35555 [286-314]	0	3996.7561
[K].TFFEELA	1xTMTpro [K	4.89E-05	4.225E-05	1	1	2	Q8NE71	Q8NE71 [40-50]	0	1936.0559
[R].KAPDAG	3xCarbamido	8.096E-09	4.225E-05	1	1	8	P02675	P02675 [88-124]	1	4692.4304
[R].CEEEDV	1xCarbamido	5.175E-05	4.225E-05	1	1	1	Q9Y230	Q9Y230 [375-392	0	2480.1128
[K].TFEEDPA	1xTMTpro [K	9.31E-06	4.225E-05	1	1	1	P30084	P30084 [75-92]	0	2427.3263
[K].TFDTFCI	1xCarbamido	1.438E-05	4.225E-05	1	2	1	Q96GK7	Q96GK7 [210-224	0	2275.2652
[R].GFPEISI	1xTMTpro [K	1.169E-05	4.225E-05	1	1	1	P14543	P14543 [141-173]	1	4214.169
[K].TFDQLTI	1xTMTpro [K	1.725E-05	4.225E-05	1	1	2	Q15293	Q15293 [71-83]	1	2174.1585
[K].KAPGFG	3xTMTpro [K	2.997E-07	4.225E-05	1	1	1	P48444	P48444 [179-211]	1	4429.443
[R].CEDCGC	4xCarbamido	3.71E-07	4.225E-05	1	1	2	Q93052	Q93052 [569-594]	0	3575.6519
[K].KFEEDLI	2xTMTpro [K	5.766E-05	4.225E-05	1	1	2	Q7Z406	Q7Z406 [999-101	1	2733.5491
[K].TDQVIQS	1xTMTpro [N	1.141E-07	4.225E-05	1	2	4	P68036	P68036 [101-122]	0	2787.5324

[R].TDPVPE	1xTMTpro [N	2.183E-05	4.225E-05	1	1	1	P55327	P55327 [51-81]	1	3734.8051
[R].TALPTS(	1xTMTpro [N	1.465E-06	4.225E-05	1	1	1	Q9Y6I3	Q9Y6I3 [412-434]	0	2530.3684
[K].TALPAQ:	1xTMTpro [N	2.87E-05	4.225E-05	1	1	2	Q01082	Q01082 [2178-21!	0	1671.9713
[R].GFFICD(	2xCarbamid(	4.035E-06	4.225E-05	1	1	2	P07814	P07814 [676-693]	0	2802.3763
[R].TALINST	1xTMTpro [N	1.61E-05	4.225E-05	1	1	5	P02545	P02545 [528-541]	0	1795.9543
[R].TALLDA/	2xTMTpro [K	1.509E-07	4.225E-05	1	1	3	P10809	P10809 [527-554]	1	3781.203
[R].TALLDA/	1xTMTpro [K	5.093E-06	4.225E-05	1	1	2	P10809	P10809 [527-551]	0	3090.8157
[R].CQVFEE	1xCarbamid(	4.729E-05	4.225E-05	1	1	2	Q99832	Q99832 [345-357]	0	1856.9132
[K].TALKED(	3xCarbamid(	9.225E-06	4.225E-05	1	1	1	P19623	P19623 [195-218]	1	3799.0161
[K].TAIHTAA	1xCarbamid(	7.15E-07	4.225E-05	1	1	2	Q6YN16	Q6YN16 [199-21!	0	2520.2328
[R].KDGLAL(	2xTMTpro [K	2.401E-05	4.225E-05	1	1	1	Q9H0R4	Q9H0R4 [152-17:	1	3176.8023
[K].TALALAL	1xTMTpro [K	6.345E-06	4.225E-05	1	1	2	Q9Y265	Q9Y265 [77-90]	0	1994.2142
[R].TAKPFP(	1xTMTpro [K	7.533E-06	4.225E-05	1	1	2	Q9UMS6	Q9UMS6 [588-60'	0	2708.5016
[K].TAHLDEI	1xOxidation		3.588E-05	4.225E-05	1	1	2	P11586	P11586 [199-223]	1	3621.9978
[R].KDGLSY	1xTMTpro [K	1.404E-06	4.225E-05	1	1	1	Q53QV2	Q53QV2 [37-53]	1	2608.3539
[K].TAGYPN	1xTMTpro [N	9.023E-06	4.225E-05	1	1	2	Q01082	Q01082 [187-203]	0	2282.1637
[R].CSATGS	1xCarbamid(	3.479E-06	4.225E-05	1	1	2	P98160	P98160 [1887-19:	0	2978.5537
[K].TAGYPH	1xTMTpro [K	1.61E-05	4.225E-05	1	1	1	P11277	P11277 [187-203]	0	2517.3382
[R].CSCASG	2xCarbamid(	5.993E-05	4.225E-05	1	1	1	P98095	P98095 [926-939]	0	2065.0702
[R].TAGWNI	1xTMTpro [K	6.1E-05	4.225E-05	1	1	3	P02787	P02787 [476-489]	0	2186.2288
[R].TAGTICL	1xCarbamid(	4.549E-05	4.225E-05	1	2	2	P15170	P15170 [459-469]	0	1849.0385
[R].TAGPLE:	1xTMTpro [K	1.872E-05	4.225E-05	1	1	2	Q9P2E9	Q9P2E9 [1334-13	0	2385.2641
[R].GFFIKPT	1xTMTpro [K	1.98E-05	4.225E-05	1	1	2	P30837	P30837 [395-411]	0	2522.3721
[R].TALVAN'	1xTMTpro [N	2.316E-05	4.225E-05	1	1	2	P38606	P38606 [309-323]	0	1820.0019
[K].TAMLLAI	1xTMTpro [N	2.271E-05	4.225E-05	1	1	1	Q8NFV4	Q8NFV4 [146-16(	0	2044.1908
[K].TAMNVN	1xTMTpro [K	2.316E-05	4.225E-05	1	1	3	P61020	P61020 [166-179]	0	2129.2284
[K].TAVDGP	1xTMTpro [N	2.388E-05	4.225E-05	1	1	2	Q14318	Q14318 [202-217]	0	2036.0289
[R].CPTGYY	1xCarbamid(	5.974E-05	4.225E-05	1	1	3	P35555	P35555 [1633-16	0	1792.8495
[R].KDAEAW	2xTMTpro [K	5.115E-05	4.225E-05	1	2	1	P13645	P13645 [334-343]	1	2150.2062
[R].TATSEY(	1xTMTpro [N	1.941E-05	4.225E-05	1	1	1	P00734	P00734 [315-327]	0	1865.9353
[K].TATPQQ	1xTMTpro [K	1.592E-05	4.225E-05	1	1	3	P60174	P60174 [176-188]	0	2075.1377
[R].TATESF/	1xTMTpro [K	6.554E-07	4.225E-05	1	1	3	P14618	P14618 [93-115]	0	3073.7065
[K].TATDEA'	2xTMTpro [K	1.113E-05	4.225E-05	1	1	6	Q13813	Q13813 [595-610]	1	2650.4504

[K].KDCEVV	1xCarbamido	1.812E-05	4.225E-05	1	1	2	Q00839	Q00839 [495-510]	1	2617.4696
[K].CQCDEL	6xCarbamido	3.958E-07	4.225E-05	1	1	1	P04004	P04004 [38-64]	0	4090.7663
[K].TASNVEI	1xTMTpro [K	6.334E-06	4.225E-05	1	1	2	P61019	P61019 [152-165]	0	2103.1578
[R].CSEGSF	1xCarbamido	1.013E-05	4.225E-05	1	1	2	Q15233	Q15233 [208-239]	0	4245.1227
[K].CQCPSG	2xCarbamido	1.349E-05	4.225E-05	1	1	4	P35555	P35555 [935-948]	0	1857.8577
[K].KDDEEN	2xTMTpro [K	3.358E-05	4.225E-05	1	1	3	Q07666	Q07666 [139-152]	1	2665.429
[K].TASESIS	1xTMTpro [K	4.016E-05	4.225E-05	1	1	1	P30622	P30622 [191-206]	0	2202.2109
[K].CQEVISV	1xCarbamido	1.422E-05	4.225E-05	1	1	2	P0DMV9	P0DMV9 [574-595	1	3574.8668
[K].TASEMV	1xOxidation [	4.465E-07	4.225E-05	1	2	1	P16615	P16615 [728-750]	0	2745.3572
[K].TASEMV	1xTMTpro [N	5.578E-07	4.225E-05	1	2	3	P16615	P16615 [728-750]	0	2729.3623
[R].TAPSTNS	1xTMTpro [N	1.813E-06	4.225E-05	1	1	2	Q7Z6Z7	Q7Z6Z7 [2997-30	0	4547.3953
[R].KDDLGD	1xTMTpro [K	4.249E-05	4.225E-05	1	1	3	Q9NUV9	Q9NUV9 [156-169	1	2283.2225
[R].KDDPLTI	2xTMTpro [K	1.069E-06	4.225E-05	1	1	5	P12814	P12814 [198-214]	1	2803.5658
[R].TANDAV	1xTMTpro [K	2.175E-05	4.225E-05	1	1	2	Q9P2B2	Q9P2B2 [97-107]	0	1819.0569
[R].KDDPVT	2xTMTpro [K	5.996E-07	4.225E-05	1	1	4	O43707	O43707 [217-233]	1	2816.561
[R].TASGSS	1xTMTpro [N	1.043E-05	4.225E-05	1	1	1	Q92597	Q92597 [328-341]	0	1642.8567
[R].CSEGVF	1xCarbamido	2.715E-06	4.225E-05	1	1	2	P23246	P23246 [431-462]	0	4205.2183
[K].TAFQEAI	1xTMTpro [K	1.916E-05	4.225E-05	1	1	7	P10599	P10599 [9-21]	0	1945.0522
[K].GFFDPN	1xTMTpro [N	9.174E-06	4.225E-05	1	1	1	P58107	P58107 [2385-240	0	2468.2893
[R].TAAENE	2xTMTpro [K	1.796E-06	4.225E-05	1	2	2	P08729	P08729 [188-199]	1	2261.3685
[R].CTDDVR	2xCarbamido	2.155E-05	4.225E-05	1	2	1	P26927	P26927 [361-379]	0	2631.1636
[R].TAAENE	1xTMTpro [K	4.673E-05	4.225E-05	1	2	4	P08729	P08729 [188-198]	0	1829.0664
[K].CTEEGK	3xCarbamido	4.32E-06	4.225E-05	1	1	1	P02749	P02749 [124-154]	1	4132.1565
[K].GFESPS	1xOxidation [	8.414E-06	4.225E-05	1	1	2	O15511	O15511 [113-131]	0	2787.3904
[K].GFESPS	1xTMTpro [K	5.028E-07	4.225E-05	1	1	3	O15511	O15511 [113-131]	0	2771.3954
[R].TAAEND	2xTMTpro [K	2.556E-05	4.225E-05	1	1	3	P35908	P35908 [276-287]	1	2249.3321
[K].GFEPTLI	1xTMTpro [K	4.3E-05	4.225E-05	1	1	2	P04114	P04114 [696-707]	0	1917.0977
[R].CTGGEV	1xCarbamido	2.087E-06	4.225E-05	1	1	1	P30050	P30050 [17-31]	0	2027.1087
[R].TAADELI	1xTMTpro [N	1.497E-05	4.225E-05	1	1	2	Q3YEC7	Q3YEC7 [702-719	0	1993.031
[R].TAALGPI	1xTMTpro [N	3.417E-05	4.225E-05	1	1	2	O95070	O95070 [251-265]	0	1729.9226
[K].TAAAVA	1xTMTpro [N	6.07E-05	4.225E-05	1	1	1	Q9Y6N5	Q9Y6N5 [345-358	0	1647.9349
[K].SYYYAIS	1xTMTpro [N	5.098E-05	4.225E-05	1	1	2	P11047	P11047 [272-285]	0	1872.9451
[K].CTITGLT	2xCarbamido	1.43E-05	4.225E-05	1	1	1	P17813	P17813 [382-399]	0	2462.1287

[K].SYYTVHI	1xTMTpro [N	3.839E-05	4.225E-05	1	1	1	P17706	P17706 [152-170]	0	2541.3268
[K].SYWLST	2xOxidation [	1.008E-05	4.225E-05	1	1	2	P08572	P08572 [1564-158	0	3635.8985
[R].SYTITGL	1xTMTpro [K	4.809E-05	4.225E-05	1	1	6	P02751	P02751 [1958-197	0	2152.1782
[R].CTTPPP:	2xCarbamido	1.475E-05	4.225E-05	1	1	1	P00747	P00747 [262-277]	0	2402.234
[R].KDLEEA(	2xTMTpro [K	1.921E-07	4.225E-05	1	1	4	P48681	P48681 [1142-116	1	2889.6026
[K].SYSSGG	1xTMTpro [N	4.776E-05	4.225E-05	1	1	1	Q13347	Q13347 [299-310]	0	1580.7512
[K].KDIHFMI	1xCarbamido	1.263E-05	4.225E-05	1	2	2	P15170	P15170 [254-270]	1	2801.5622
[K].GFEKPTI	2xTMTpro [K	2.952E-05	4.225E-05	1	1	1	Q9BPX5	Q9BPX5 [115-133	0	3112.7247
[K].TAAAAAI	1xTMTpro [N	3.879E-05	4.225E-05	1	1	2	O43242	O43242 [55-65]	0	1416.7515
[R].TAVDSL\	1xTMTpro [K	2.538E-05	4.225E-05	1	1	2	Q07065	Q07065 [555-566]	0	1861.0926
[K].KDGTHV	2xTMTpro [K	4.472E-05	4.225E-05	1	1	1	P00488	P00488 [447-463]	1	2732.5511
[R].CTCDPA	4xCarbamido	1.932E-07	4.225E-05	1	1	2	P07942	P07942 [398-427]	0	3644.5681
[K].TAFDEAI	1xTMTpro [K	9.362E-06	4.225E-05	1	1	3	P63104	P63104 [194-212]	0	2740.406
[K].TAFDEAI	1xTMTpro [K	1.055E-06	4.225E-05	1	1	7	P31946	P31946 [196-214]	0	2767.4169
[K].TAFDEAI	1xTMTpro [K	1.487E-06	4.225E-05	1	1	5	P27348	P27348 [194-212]	0	2753.4013
[K].TAFDDAI	1xTMTpro [K	1.868E-06	4.225E-05	1	1	7	P61981	P61981 [199-217]	0	2739.3856
[K].CSLPAEI	1xCarbamido	9.362E-06	4.225E-05	1	1	2	P27816	P27816 [635-647]	0	2085.1029
[K].TAEYDV.	1xTMTpro [N	9.057E-06	4.225E-05	1	1	4	P12109	P12109 [991-1005	0	2062.0201
[K].GFFDPN	1xTMTpro [N	4.427E-05	4.225E-05	1	1	2	Q15149	Q15149 [3648-36	0	2014.9942
[K].GFFDPN	1xTMTpro [N	7.903E-06	4.225E-05	2	2	1	P58107; Q15	P58107 [766-784]	0	2611.3476
[R].TAELSPI	1xTMTpro [K	2.636E-06	4.225E-05	1	1	2	Q00013	Q00013 [390-417]	0	3683.0188
[K].TAEHFQ	1xTMTpro [K	2.128E-05	4.225E-05	1	1	1	Q9BRK5	Q9BRK5 [131-143	0	2145.0778
[R].KDGSCG	1xCarbamido	2.702E-07	4.225E-05	1	1	6	P21333	P21333 [2289-231	1	3298.7446
[R].TAEHEA.	1xTMTpro [K	3.754E-05	4.225E-05	1	1	1	Q86UP2	Q86UP2 [508-521	0	2164.149
[K].GFFDPN	1xOxidation [	2.716E-05	4.225E-05	1	1	1	Q15149	Q15149 [4226-424	0	2637.2826
[R].CSVCKE	2xCarbamido	3.974E-05	4.225E-05	1	1	2	Q93052	Q93052 [536-554]	1	2796.4338
[R].CSVCSE	2xCarbamido	1.284E-05	4.225E-05	1	1	1	Q15942	Q15942 [504-522]	1	2523.1961
[K].GFFDPN	1xTMTpro [N	8.26E-06	4.225E-05	1	1	2	Q15149	Q15149 [4226-424	0	2621.2877
[R].TADTAV	1xTMTpro [K	1.086E-05	4.225E-05	1	1	1	P39059	P39059 [1344-136	0	2198.2524
[R].KDGNAS	1xCarbamido	5.266E-08	4.225E-05	1	2	5	P68104	P68104 [219-247]	1	4062.2838
[K].CSYTED.	2xCarbamido	1.663E-05	4.225E-05	1	1	3	P02749	P02749 [307-324]	0	2694.3246
[R].GFEVVY	1xCarbamido	1.642E-05	4.225E-05	1	1	4	P08238	P08238 [507-526]	0	3072.5554
[K].TACNVE	1xCarbamido	4.681E-05	4.225E-05	1	1	1	Q8WUD1	Q8WUD1 [152-16	0	2176.1564

[R].GFEVVY	1xCarbamid	1.204E-05	4.225E-05	1	1	2	P08238	P08238 [507-526]	0	3056.5605
[K].CSTSPLI	2xCarbamid	4.057E-05	4.225E-05	1	1	1	P02788	P02788 [696-709]	0	1986.9948
[R].GFGEPF	1xTMTpro [N	9.984E-06	4.225E-05	1	1	2	O15230	O15230 [984-999	0	2065.119
[R].KCVEEA	1xCarbamid	4.963E-06	4.225E-05	1	1	2	Q9NXG2	Q9NXG2 [56-77]	1	3522.7589
[K].TAVVVG	1xTMTpro [N	3.886E-05	4.225E-05	1	1	5	Q07020	Q07020 [79-91]	0	1649.9393
[R].TDLEMQ	2xTMTpro [K	1.374E-05	4.225E-05	1	1	1	P08727	P08727 [198-215]	1	3035.7155
[R].TDIELEI	1xTMTpro [K	1.098E-05	4.225E-05	1	1	1	O43670	O43670 [73-93]	1	3091.5459
[K].VAMANI	1xOxidation	1.425E-05	4.225E-05	1	1	4	P18206	P18206 [739-758]	0	2362.2906
[R].CLTQSG	2xCarbamid	1.328E-07	4.225E-05	1	1	2	P30626	P30626 [57-76]	0	2880.4992
[K].TDLEKDI	2xTMTpro [K	9.201E-08	4.225E-05	1	2	10	P07355	P07355 [153-169]	2	2852.5822
[K].TDLEKDI	1xTMTpro [K	8.908E-06	4.225E-05	1	2	4	P07355	P07355 [153-168]	1	2420.2801
[K].TDKTLVI	2xTMTpro [K	4.849E-05	4.225E-05	1	1	4	P07737	P07737 [106-116]	1	2131.3341
[K].TDKTDEI	2xTMTpro [K	5.209E-05	4.225E-05	1	1	1	Q9HBL0	Q9HBL0 [540-562	1	3212.7467
[K].TDKELA	1xCarbamid	2.687E-05	4.225E-05	1	1	1	P08253	P08253 [45-62]	1	3059.6692
[R].CLYASVI	1xCarbamid	5.183E-05	4.225E-05	1	1	4	P13639	P13639 [728-739]	0	1682.9219
[R].TDLEMQ	3xTMTpro [K	1.378E-07	4.225E-05	1	1	1	P08727	P08727 [198-216]	2	3468.0176
[R].CIYNLLC	1xCarbamid	5.955E-05	4.225E-05	1	1	2	P53618	P53618 [248-261]	0	2188.2291
[R].TDGILAL	1xCarbamid	2.295E-05	4.225E-05	1	1	2	Q9UBX3	Q9UBX3 [54-70]	0	2101.1283
[R].GFGFVT	1xTMTpro [K	7.23E-07	4.225E-05	1	2	3	P09651	P09651 [56-78]	0	3118.6276
[R].KCMTTV	2xCarbamid	3.946E-06	4.225E-05	1	1	1	P09455	P09455 [82-99]	2	3400.8532
[R].TDGALL	2xTMTpro [K	2.535E-06	4.225E-05	1	1	3	P50454	P50454 [195-213]	0	3131.7168
[K].GFGFVT	1xTMTpro [N	2.345E-05	4.225E-05	1	2	1	O43251	O43251 [161-175	0	1936.936
[K].TDFFIGC	1xTMTpro [K	2.238E-05	4.225E-05	1	1	2	Q9Y266	Q9Y266 [40-53]	0	2139.0924
[K].TDELPQI	1xTMTpro [K	1.261E-06	4.225E-05	1	1	2	P02751	P02751 [2082-21	0	3796.114
[K].TDEFQLI	1xTMTpro [K	4.351E-08	4.225E-05	1	1	4	P21796	P21796 [175-197]	0	3208.6042
[R].GFGFVT	2xTMTpro [K	9.73E-06	4.225E-05	1	1	1	Q14011	Q14011 [48-70]	1	3421.7411
[R].TDDYLD	1xCarbamid	2.89E-05	4.225E-05	1	1	2	P50395	P50395 [194-208]	0	2207.0246
[K].CIYNTAC	2xCarbamid	5.86E-05	4.225E-05	1	1	2	P11047	P11047 [852-863]	0	1843.8427
[R].CMSSTP	1xCarbamid	3.582E-05	4.225E-05	1	1	3	Q13308	Q13308 [481-495	0	1869.9118
[R].TDLEMQ	1xOxidation	1.446E-05	4.225E-05	1	1	1	P08727	P08727 [198-216]	2	3484.0125
[R].GFGFVT	2xCarbamid	7.589E-06	4.225E-05	1	1	1	P51991	P51991 [77-99]	0	3239.5932
[K].CLHPLAI	1xCarbamid	2.706E-05	4.225E-05	1	1	1	Q13642	Q13642 [71-83]	0	2108.1818
[K].TDPTTLT	1xTMTpro [N	2.171E-05	4.225E-05	1	1	3	P11586	P11586 [505-517]	0	1808.9197

[K].CLKDGA	1xCarbamido	4.558E-06	4.225E-05	1	1	2	P02787	P02787 [213-225]		1	2292.3202
[K].KCAGCT	2xCarbamido	2.809E-05	4.225E-05	1	1	1	Q14192	Q14192 [220-235]		1	2533.4047
[K].CLIHDGA	1xCarbamido	1.032E-05	4.225E-05	1	1	1	P98160	P98160 [3043-305		0	2318.2822
[R].TDPHLC	1xCarbamido	4.003E-07	4.225E-05	1	1	6	P02792	P02792 [122-140]		0	2953.4897
[K].TDNAGD	1xTMTpro [K	8.527E-10	4.225E-05	1	1	5	P14866	P14866 [63-97]		0	3589.6168
[K].TDMFQT	1xOxidation [	4.016E-05	4.225E-05	1	1	8	Q01995	Q01995 [109-121]		0	2155.1237
[K].TDMFQT	1xTMTpro [K	2.191E-05	4.225E-05	1	1	6	Q01995	Q01995 [109-121]		0	2139.1288
[R].KCCVEC	4xCarbamido	4.337E-05	4.225E-05	1	1	3	P01859	P01859 [101-127]		1	4253.3252
[RK].TDLEM	1xTMTpro [K	1.217E-05	4.225E-05	1	2	2	P13646	P13646 [223-240]		0	2765.4233
[K].CLMDQA	1xCarbamido	2.206E-05	4.225E-05	1	1	2	P78527	P78527 [4106-41		0	1907.9638
[K].TDLVPAI	1xTMTpro [K	3.381E-05	4.225E-05	1	1	2	P30153	P30153 [281-292]		0	1985.1385
[R].CLNALEI	1xCarbamido	3.917E-06	4.225E-05	1	1	1	Q7Z4V5	Q7Z4V5 [496-515		0	2866.6204
[K].CINHYG(	2xCarbamido	5.098E-05	4.225E-05	1	1	2	O95967	O95967 [71-83]		0	1926.9638
[K].KCEVLC:	2xCarbamido	5.514E-06	4.225E-05	1	1	1	Q92544	Q92544 [99-119]		1	3375.8545
[R].GFGHIGI	1xCarbamido	1.435E-05	4.225E-05	1	1	1	Q04760	Q04760 [124-140]		0	2399.3037
[R].CIPALDS	1xCarbamido	1.502E-05	4.225E-05	1	1	3	P10809	P10809 [447-462]		0	2380.2674
[K].KCFIEEII	1xCarbamido	5.525E-06	4.225E-05	1	1	1	Q9BVK6	Q9BVK6 [48-65]		1	2822.4712
[K].TDLNPDI	1xTMTpro [N	4.45E-06	4.225E-05	1	1	2	P12277	P12277 [108-130]		0	2822.3764
[R].KCFSIDN	1xCarbamido	1.368E-09	4.225E-05	1	1	4	O75083	O75083 [437-470]		1	4134.121
[R].GFGFVY	1xTMTpro [K	7.976E-06	4.225E-05	1	1	2	Q13151	Q13151 [140-154]		0	2324.1955
[K].TDMDNQ	1xTMTpro [N	6.997E-06	4.225E-05	1	1	2	P50991	P50991 [258-274]		0	2305.076
[R].CLHMFL(	1xCarbamido	3.411E-05	4.225E-05	1	1	1	O60493	O60493 [140-152]		0	2270.2169
[R].TDDYLD(	1xCarbamido	2.673E-05	4.225E-05	1	1	3	P31150	P31150 [194-208]		0	2143.0297
[R].TDDEVV	1xTMTpro [N	1.498E-08	4.225E-05	1	1	2	P14625	P14625 [44-67]		1	3032.5456
[R].TCPKPD	1xCarbamido	4.609E-06	4.225E-05	1	1	4	P02749	P02749 [22-38]		0	2826.6256
[K].TCNVDY	2xCarbamido	3.624E-06	4.225E-05	1	1	2	Q8IUX7	Q8IUX7 [966-984]		0	2535.2291
[K].TCNTNP	2xCarbamido	5.807E-06	4.225E-05	1	1	1	Q9NZB2	Q9NZB2 [1087-11		0	2336.1042
[K].KCSTSPI	2xCarbamido	4.849E-05	4.225E-05	1	1	1	P02788	P02788 [695-709]		1	2419.2969
[R].KCSTSS	2xCarbamido	5.175E-05	4.225E-05	1	1	2	P02787	P02787 [683-696]		1	2268.1972
[R].CPENAF	1xCarbamido	4.12E-05	4.225E-05	1	1	1	O00159	O00159 [802-813]		0	1808.9073
[R].CPETLF(	1xCarbamido	9.448E-06	4.225E-05	2	4	1	P62736; P68	P62736 [259-286]		0	3796.888
[K].CPFGAL:	1xCarbamido	1.882E-05	4.225E-05	1	1	1	P61221	P61221 [65-81]		0	2467.3874
[K].TCGFDF	1xCarbamido	5.018E-06	4.225E-05	1	1	3	P00505	P00505 [186-200]		0	2255.151

[R].TCGFDF	1xCarbamid	3.657E-05	4.225E-05	1	1	1	Q13347	Q13347 [98-114]	0	2518.2602
[R].CPAPELI	1xCarbamid	3.154E-06	4.225E-05	1	1	6	P01860	P01860 [159-178]	0	3063.7885
[K].KCSYTEI	2xCarbamid	2.74E-06	4.225E-05	1	1	6	P02749	P02749 [306-324]	1	3126.6268
[K].TCFVDC	2xCarbamid	3.747E-06	4.225E-05	1	1	1	Q15029	Q15029 [143-158]	0	2322.1542
[R].CPINCLL	4xCarbamid	8.337E-06	4.225E-05	1	1	1	P13671	P13671 [78-99]	0	3261.5333
[K].GFGFVD	1xTMTpro [K	1.094E-05	4.225E-05	1	1	5	P19338	P19338 [611-624]	0	2170.0948
[K].TCESDTI	1xCarbamid	2.74E-06	4.225E-05	1	1	1	Q9NYU2	Q9NYU2 [158-181	0	3645.0753
[K].GFGFVC	1xCarbamid	2.457E-05	4.225E-05	3	4	3	Q9H361; Q1	Q9H361 [334-348	0	2271.1611
[KR].TCAYTI	1xCarbamid	2.735E-05	4.225E-05	2	2	2	P11216; P11	P11216 [372-387]	0	2179.1137
[R].GFGFPQ	1xCarbamid	9.619E-07	4.225E-05	1	1	1	Q06278	Q06278 [922-942]	0	2818.5305
[R].TCADVD	3xCarbamid	1.694E-05	4.225E-05	1	1	1	Q07954	Q07954 [2979-29	0	2338.0069
[R].GFGFITF	1xTMTpro [N	2.75E-05	4.225E-05	1	1	1	P98179	P98179 [48-65]	0	2286.166
[K].TAWGQ(	1xTMTpro [N	3.16E-06	4.225E-05	1	1	2	Q9UNM6	Q9UNM6 [253-27	0	2286.2162
[R].CPGESS	2xCarbamid	3.961E-05	4.225E-05	1	1	2	P50897	P50897 [152-164]	0	1881.8907
[R].GFGFVT	1xTMTpro [K	6.67E-06	4.225E-05	1	1	3	Q14011	Q14011 [48-61]	0	2168.1519
[R].TCQDINI	3xCarbamid	2.171E-05	4.225E-05	1	1	3	Q12805	Q12805 [331-345]	0	2233.9443
[R].TCQDINI	4xCarbamid	1.66E-05	4.225E-05	1	1	1	Q12805	Q12805 [331-358]	1	3931.5794
[K].CMTTVS	2xCarbamid	3.259E-05	4.225E-05	1	1	4	P09455	P09455 [83-99]	1	2968.5511
[R].GFGFVT	1xTMTpro [K	4.705E-05	4.225E-05	1	1	2	Q96DH6	Q96DH6 [152-166	0	2325.2258
[K].TDAVEAI	1xTMTpro [N	3.893E-07	4.225E-05	1	1	1	Q8WVV9	Q8WVV9 [507-52	0	2119.1467
[R].TDASSA	1xTMTpro [N	1.139E-06	4.225E-05	1	1	1	Q14498	Q14498 [330-346]	0	2134.0107
[K].TDAGGE	1xTMTpro [N	1.892E-05	4.225E-05	1	1	2	Q9NT62	Q9NT62 [186-198	0	1650.8618
[R].GFGFVT	2xTMTpro [K	5.907E-05	4.225E-05	1	1	2	P22626	P22626 [154-173]	1	3189.7764
[R].GFGFVT	1xTMTpro [K	4.308E-05	4.225E-05	1	1	9	P22626	P22626 [154-168]	0	2304.1792
[R].TCVHTFI	1xCarbamid	5.654E-05	4.225E-05	1	1	1	Q9GZS3	Q9GZS3 [265-281	0	2712.3848
[R].CNEPAV	1xCarbamid	2.259E-05	4.225E-05	1	1	2	Q00610	Q00610 [1102-11	0	2011.0927
[K].TCVDVN	4xCarbamid	7.903E-06	4.225E-05	1	1	2	P35555	P35555 [1277-130	0	3421.5988
[R].TCQDINI	4xCarbamid	2.452E-06	4.225E-05	1	1	1	Q12805	Q12805 [331-358]	1	3915.5845
[K].CNFYDN	2xCarbamid	1.299E-06	4.225E-05	1	1	1	P10619	P10619 [246-265]	1	3096.5374
[K].CNLEAAI	1xCarbamid	1.499E-05	4.225E-05	1	1	1	Q5W0V3	Q5W0V3 [624-637	0	2291.2138
[K].TCVADE	2xCarbamid	1.336E-05	4.225E-05	1	1	2	P02768	P02768 [76-97]	1	3409.7184
[K].TCVADE	2xCarbamid	1.795E-05	4.225E-05	1	1	9	P02768	P02768 [76-88]	0	2106.9927
[K].TCTTVAI	1xCarbamid	6.165E-08	4.225E-05	1	1	2	P62424	P62424 [198-217]	1	3053.6757

[K].CNQIANI	3xCarbamido	8.184E-06	4.225E-05	1	1	1	P48506	P48506 [605-624]	0	2685.2754
[K].TCTTVAI	1xCarbamido	4.61E-05	4.225E-05	1	1	2	P62424	P62424 [198-212]	0	2309.1939
[K].KCPFGA	1xCarbamido	5.617E-06	4.225E-05	1	1	2	P61221	P61221 [64-81]	1	2899.6895
[K].TCSPASI	1xCarbamido	2.277E-06	4.225E-05	1	1	1	Q14258	Q14258 [185-203]	0	2282.1617
[R].TCSECQ	3xCarbamido	3.322E-05	4.225E-05	1	1	1	P07942	P07942 [1113-113	0	2592.1124
[R].TCRPEG	1xCarbamido	1.045E-05	4.225E-05	1	1	1	P98095	P98095 [643-662]	0	2847.5067
[R].TCVDINE	2xCarbamido	1.317E-05	4.225E-05	1	1	5	P35555	P35555 [1970-198	0	1922.9635
[K].SSEHINE	1xCarbamido	4.906E-05	4.225E-05	1	1	1	P35613	P35613 [228-243]	0	2413.2347
[K].KFEEEG	1xTMTpro [K	4.882E-05	4.225E-05	1	1	2	Q9HCC0	Q9HCC0 [512-525	1	2285.1694
[K].SSEATSA	1xTMTpro [N	6.085E-07	4.225E-05	1	1	1	Q9P2L0	Q9P2L0 [997-101	0	2583.332
[K].SLLLTTII	1xTMTpro [K	6.032E-05	4.225E-05	1	1	1	Q13126	Q13126 [249-271]	0	3189.8015
[K].KKLEAQY	3xTMTpro [K	4.487E-05	4.225E-05	1	1	1	P35749	P35749 [1255-126	2	2745.6979
[R].SLILLDLS	1xTMTpro [N	4.972E-05	4.225E-05	1	1	1	Q06828	Q06828 [224-236]	0	1861.0867
[R].SILIDEST	1xCarbamido	1.156E-05	4.225E-05	1	1	2	Q92900	Q92900 [643-666]	0	3195.7137
[R].SLIIDEGI	1xTMTpro [N	3.163E-05	4.225E-05	1	1	2	O14618	O14618 [197-209]	0	1735.9033
[K].GEADAM	1xOxidation [	2.588E-05	4.225E-05	1	1	1	P02788	P02788 [406-423]	0	2429.215
[K].GEADAM	1xTMTpro [K	5.495E-07	4.225E-05	1	1	3	P02788	P02788 [406-423]	0	2413.2201
[R].KKLVIVG	1xCarbamido	3.196E-05	4.225E-05	2	2	1	P08134; P61	P08134 [6-18]; P6	2	2561.5954
[R].SLLETNE	1xCarbamido	2.06E-06	4.225E-05	1	1	1	Q96S55	Q96S55 [254-274	0	2889.604
[R].DGWPAI	1xTMTpro [K	1.206E-05	4.225E-05	1	1	1	P17844	P17844 [364-375]	0	1891.998
[K].DGWQDI	1xTMTpro [N	3.236E-05	4.225E-05	1	1	1	P23229	P23229 [367-382]	0	2184.1045
[K].SLLEQYI	1xTMTpro [K	1.208E-05	4.225E-05	1	1	2	P08236	P08236 [564-576]	0	2152.2258
[K].DGYADIV	1xTMTpro [K	3.744E-07	4.225E-05	1	2	2	Q86TX2	Q86TX2 [287-306	0	2753.4489
[R].SLLEGQ	1xTMTpro [K	3.242E-05	4.225E-05	1	1	4	P08727	P08727 [382-398]	0	2513.3128
[R].SLLEGE(	1xTMTpro [N	2.002E-05	4.225E-05	1	1	2	P13645	P13645 [451-464]	0	1566.8043
[R].KKNPEP	3xTMTpro [K	4.61E-05	4.225E-05	1	1	1	Q9C002	Q9C002 [45-61]	2	3209.8675
[R].SILDVSM	1xTMTpro [K	1.281E-05	4.225E-05	1	1	1	Q03169	Q03169 [632-652]	0	2896.6462
[K].SLLDIISD	1xTMTpro [K	3.576E-05	4.225E-05	1	1	1	Q8WVM8	Q8WVM8 [437-45	0	2394.2896
[R].SLLDGN	1xTMTpro [N	4.495E-05	4.225E-05	1	1	1	Q96ST3	Q96ST3 [968-984	0	2216.1002
[R].GDYTLN	1xCarbamido	5.813E-05	4.225E-05	1	1	1	P07358	P07358 [368-380]	0	2071.0886
[K].SLLAYDI	1xTMTpro [K	3.086E-05	4.225E-05	1	1	3	P41218	P41218 [27-38]	0	1903.1396
[K].SLLNEIE	1xTMTpro [N	3.664E-05	4.225E-05	1	1	2	Q96ST3	Q96ST3 [748-760	0	1884.9874
[K].SILNSLS	1xTMTpro [K	4.89E-05	4.225E-05	1	1	2	Q9NRY5	Q9NRY5 [266-280	0	2241.2834

[K].KKHEAIE	2xTMTpro [K	4.833E-05	4.225E-05	1	1	1	P11277	P11277 [462-477]	2	2803.5406
[K].KKGEPG	3xTMTpro [K	2.259E-05	4.225E-05	1	1	2	Q9P2E9	Q9P2E9 [633-651	2	3258.8879
[K].DGTHVV	1xTMTpro [K	2.584E-05	4.225E-05	1	1	3	P00488	P00488 [448-463]	0	2300.249
[R].DGTIDFT	1xTMTpro [K	5.09E-05	4.225E-05	1	1	1	O00159	O00159 [1032-10	0	2315.299
[K].SINDNIA	1xTMTpro [K	4.61E-05	4.225E-05	1	1	1	Q07065	Q07065 [247-260]	0	2200.2469
[R].DGTSVQ	1xTMTpro [K	1.441E-06	4.225E-05	1	1	1	Q9Y6K5	Q9Y6K5 [733-757	0	3325.727
[K].SIMGLE(	1xOxidation [	1.69E-06	4.225E-05	1	1	1	Q8WVM8	Q8WVM8 [360-38	0	2892.4462
[K].DGTVLC	1xCarbamido	3.19E-06	4.225E-05	1	1	4	P37802	P37802 [58-78]	0	2895.5782
[K].DGTVLC	1xCarbamido	4.399E-07	4.225E-05	1	1	6	P37802	P37802 [58-79]	1	3327.8803
[K].SIMGLE(	1xTMTpro [K	1.446E-06	4.225E-05	1	1	2	Q8WVM8	Q8WVM8 [360-38	0	2876.4513
[R].SLMEQD	1xTMTpro [K	1.92E-06	4.225E-05	1	1	2	Q96AC1	Q96AC1 [258-273	1	2496.3987
[K].DGTYAV	1xTMTpro [K	9.124E-06	4.225E-05	1	1	2	O75369	O75369 [1187-12	0	2904.5383
[R].DHAVVV	1xTMTpro [K	4.042E-06	4.225E-05	1	1	2	P22087	P22087 [305-318]	0	2142.2679
[K].SIMAAA(	1xCarbamido	3.538E-05	4.225E-05	1	1	1	Q96RQ3	Q96RQ3 [168-192	0	3269.6426
[R].GEAGAA	1xTMTpro [N	8.81E-06	4.225E-05	1	1	5	P08123	P08123 [694-708]	0	1539.8199
[R].SLLTEAD	1xTMTpro [N	1.191E-08	4.225E-05	1	1	1	Q9NZM1	Q9NZM1 [918-94	0	2916.3818
[K].SILSSAS	1xTMTpro [K	4.255E-07	4.225E-05	1	1	2	Q9NRY5	Q9NRY5 [91-113]	0	2840.6225
[R].SLLSNVE	1xOxidation [	2.228E-07	4.225E-05	1	1	3	P00915	P00915 [229-253]	0	3383.7985
[R].SLLSNVE	1xTMTpro [K	4.981E-09	4.225E-05	1	1	5	P00915	P00915 [229-253]	0	3367.8036
[K].DGVLTL/	1xTMTpro [K	2.654E-05	4.225E-05	1	1	2	P27816	P27816 [561-574]	0	2021.1887
[R].KKEPELI	2xTMTpro [K	3.601E-05	4.225E-05	1	1	1	Q9H4M9	Q9H4M9 [12-26]	2	2657.5806
[K].SLLSDVE	1xTMTpro [K	9.201E-09	4.225E-05	1	1	2	Q16363	Q16363 [348-365]	1	2654.4492
[R].DGVNKD	1xTMTpro [K	4.065E-06	4.225E-05	1	1	1	Q13123	Q13123 [108-127]	1	2926.4926
[K].DGVPEG	1xTMTpro [N	2.692E-05	4.225E-05	1	1	1	P16989	P16989 [269-283]	0	1863.9996
[R].SLLVIPN	1xTMTpro [K	1.235E-06	4.225E-05	1	1	3	P17987	P17987 [444-466]	0	2961.7116
[R].DHCQW(	2xCarbamido	1.365E-06	4.225E-05	1	1	1	Q96F85	Q96F85 [132-153]	0	3347.6069
[K].KKPAGP	3xTMTpro [K	2.275E-05	4.225E-05	1	1	3	Q02539	Q02539 [36-55]	1	3200.9359
[R].KKPSEE	2xTMTpro [K	2.973E-05	4.225E-05	1	1	1	P13498	P13498 [165-195]	1	3952.1848
[R].SLGPGT	1xCarbamido	5.627E-05	4.225E-05	1	1	3	Q14767	Q14767 [677-690]	0	1774.9805
[K].SLGNILQ	2xTMTpro [K	5.645E-05	4.225E-05	1	1	3	Q13428	Q13428 [571-586]	0	2524.5279
[R].SLGLSLS	1xTMTpro [N	1.871E-06	4.225E-05	1	1	1	Q9UJ70	Q9UJ70 [70-85]	0	1865.9524
[K].SLGLSDI	1xTMTpro [K	5.014E-05	4.225E-05	1	1	2	Q9P2J5	Q9P2J5 [163-173]	0	1798.0454
[K].DHQYQF	1xTMTpro [N	4.412E-05	4.225E-05	1	1	1	Q13263	Q13263 [239-250]	0	1824.92

[K].SLGLPEI	1xCarbamido	3.886E-05	4.225E-05	1	1	1	P80188	P80188 [178-198]	0	2624.3713
[K].SLGLFGI	1xTMTpro [N	2.155E-05	4.225E-05	1	1	1	Q9H845	Q9H845 [103-127	0	3026.5253
[K].GDVTAQ	1xTMTpro [K	1.835E-05	4.225E-05	1	1	8	P04179	P04179 [76-89]	0	2033.225
[R].SLGETQ	1xTMTpro [K	2.084E-07	4.225E-05	1	1	3	Q07065	Q07065 [474-491]	1	2657.5006
[R].SLGETQ	1xTMTpro [K	2.057E-05	4.225E-05	1	1	1	Q07065	Q07065 [474-490]	0	2501.3994
[K].SLGPNS	2xCarbamido	3.344E-07	4.225E-05	1	1	3	P02790	P02790 [412-441]	0	3890.9337
[K].GDVPSV	1xTMTpro [K	1.763E-05	4.225E-05	1	1	2	Q09666	Q09666 [5210-52	0	2687.4383
[R].SLGEIPI	1xTMTpro [K	1.697E-05	4.225E-05	1	1	4	P53618	P53618 [482-494]	0	2022.1978
[K].SLGECC	3xCarbamido	3.902E-06	4.225E-05	1	1	3	P02774	P02774 [371-388]	0	2701.2399
[K].SLGDDIS	1xTMTpro [K	1.861E-06	4.225E-05	1	1	1	P12429	P12429 [139-154]	1	2322.2069
[K].SLGDDIS	1xTMTpro [N	9.124E-06	4.225E-05	1	1	1	P12429	P12429 [139-153]	0	1889.9048
[R].KIANLLK	3xTMTpro [K	2.179E-05	4.225E-05	1	1	2	P09455	P09455 [32-53]	2	3763.2409
[K].SLGAEPI	1xTMTpro [K	2.654E-05	4.225E-05	1	1	1	Q13423	Q13423 [268-279]	0	1879.1032
[R].SIFYVPD	1xTMTpro [K	2.819E-05	4.225E-05	1	1	1	Q12931	Q12931 [341-357]	0	2627.3857
[K].SLFWEN	1xTMTpro [N	7.341E-06	4.225E-05	1	1	1	Q10588	Q10588 [106-124]	0	2554.301
[K].DKADFC	1xCarbamido	1.744E-05	4.225E-05	2	2	2	P35580; P35	P35580 [571-583]	1	2450.3682
[R].SLFTDLE	1xCarbamido	7.032E-07	4.225E-05	1	1	3	P01023	P01023 [72-93]	0	3083.6367
[R].SLGEIPI	2xTMTpro [K	8.602E-06	4.225E-05	1	1	2	P53618	P53618 [482-495]	1	2454.4999
[K].SLNNQF	1xTMTpro [K	4.744E-05	4.225E-05	1	1	2	P04264	P04264 [186-197]	0	1992.1046
[R].KLAGAN	1xCarbamido	1.678E-08	4.225E-05	1	1	2	P78527	P78527 [4050-40	1	3161.8173
[K].GDVTIT	1xTMTpro [K	5.883E-06	4.225E-05	1	1	2	P50991	P50991 [66-79]	0	2026.1676
[R].KKTDELI	3xTMTpro [K	2.271E-05	4.225E-05	1	1	1	P02751	P02751 [2080-21	2	4660.7183
[K].SLKELQE	3xTMTpro [K	3.146E-05	4.225E-05	1	1	1	P52566	P52566 [31-47]	2	3237.8393
[R].KLADEQ	1xTMTpro [K	3.747E-06	4.225E-05	1	1	1	Q8NFV4	Q8NFV4 [200-216	1	2511.4096
[R].KIAELEE	2xTMTpro [K	7.645E-06	4.225E-05	1	1	1	P46821	P46821 [327-347]	1	3323.7245
[R].KIAELMF	2xTMTpro [K	1.799E-05	4.225E-05	1	1	1	P62195	P62195 [346-360]	1	2413.4305
[K].SLHQAIE	1xTMTpro [K	7.26E-06	4.225E-05	1	1	5	P08133	P08133 [648-663]	0	2326.2534
[R].GDWSV	1xTMTpro [K	4.495E-05	4.225E-05	1	1	2	Q96I24	Q96I24 [344-365]	0	2855.5071
[K].DHGGAL	1xTMTpro [K	4.487E-05	4.225E-05	1	1	1	O43707	O43707 [780-791]	0	1865.0049
[R].SLHDAL	1xCarbamido	2.002E-05	4.225E-05	1	1	2	P78371	P78371 [389-402]	0	2163.2451
[R].SLGYDLI	1xTMTpro [N	4.703E-07	4.225E-05	1	1	2	Q13813	Q13813 [2354-23	0	3551.7059
[R].SLGPPQ	1xTMTpro [N	3.702E-05	4.225E-05	1	1	2	P78527	P78527 [2107-21	0	1771.9146
[R].SLGYAY	1xTMTpro [N	5.285E-06	4.225E-05	2	2	2	Q13310; P1	Q13310 [51-67]; F	0	2233.1209

[R].SLGVGY	2xTMTpro [K	1.677E-06	4.225E-05	1	1	1	Q13423	Q13423 [1044-10	0	3738.0757
[K].SLGTIQ(	3xCarbamid(	2.279E-05	4.225E-05	1	1	1	P78527	P78527 [1120-11	0	2351.1225
[R].SIGTGGI	1xTMTpro [K	5.123E-05	4.225E-05	1	1	2	O00471	O00471 [378-388]	0	1697.0089
[K].SLGTGA	1xTMTpro [K	7.874E-06	4.225E-05	1	1	1	P33240	P33240 [103-130]	0	3439.7976
[R].GDVVN(	1xTMTpro [K	1.505E-05	4.225E-05	1	1	2	Q9UBQ7	Q9UBQ7 [246-26	0	2401.2855
[K].SIGTANF	1xTMTpro [N	4.389E-05	4.225E-05	1	1	2	Q12906	Q12906 [258-273]	0	1905.0295
[K].DHPAFA	1xTMTpro [N	3.906E-05	4.225E-05	1	1	1	P30519	P30519 [90-105]	0	2245.1547
[K].DHPAVA	1xTMTpro [K	8.26E-06	4.225E-05	2	2	2	Q07866; Q9I	Q07866 [292-309]	0	2475.4215
[R].SLGTAD	1xTMTpro [N	4.057E-05	4.225E-05	1	1	3	Q86V81	Q86V81 [145-155	0	1535.8137
[K].DHPFGF	1xTMTpro [K	3.146E-05	4.225E-05	1	1	1	P63279	P63279 [19-30]	0	1923.0984
[R].KLAEVTI	1xTMTpro [K	1.626E-07	4.225E-05	1	1	2	O94906	O94906 [148-167]	1	2879.5646
[K].SINPDEA	1xTMTpro [K	6.481E-07	4.225E-05	1	1	2	P54652	P54652 [365-387]	0	2894.6119
[K].DGTFPLI	1xTMTpro [N	1.792E-05	4.225E-05	1	1	1	P05362	P05362 [434-449]	0	1993.0925
[K].SINPDEA	1xTMTpro [K	4.177E-07	4.225E-05	1	2	9	P0DMV9	P0DMV9 [362-38	0	2912.5683
[K].SISMTTF	1xTMTpro [K	2.136E-05	4.225E-05	1	1	1	P52594	P52594 [67-83]	0	2639.4246
[K].KHFVGY	2xTMTpro [K	2.324E-05	4.225E-05	1	1	1	Q14914	Q14914 [11-25]	1	2694.5072
[K].GEDFYC	2xCarbamid(	1.719E-05	4.225E-05	1	1	2	Q13642	Q13642 [145-157]	0	2254.0764
[R].SLSELES	2xTMTpro [K	3.651E-05	4.225E-05	1	1	1	P22059	P22059 [238-254]	1	2786.5968
[R].SLSEGD	3xCarbamid(	8.507E-06	4.225E-05	1	1	1	P98088	P98088 [723-745]	0	3119.5707
[K].SLSDALI	1xTMTpro [N	1.738E-05	4.225E-05	1	1	2	Q9Y2A7	Q9Y2A7 [202-216	0	1997.1061
[R].DGMAFN	1xTMTpro [K	4.51E-05	4.225E-05	1	1	1	Q01082	Q01082 [204-214]	0	1825.0286
[R].DGMAFN	1xOxidation [	6.041E-05	4.225E-05	1	1	1	Q01082	Q01082 [204-214]	0	1841.0235
[R].DGMDN(	1xTMTpro [N	1.728E-05	4.225E-05	1	1	1	P31942	P31942 [288-301]	0	1716.7931
[R].DGMEYF	1xTMTpro [N	7.019E-08	4.225E-05	1	1	2	Q9UBS4	Q9UBS4 [218-24(	0	2820.3106
[R].SLSNATI	1xTMTpro [K	4.273E-06	4.225E-05	1	1	2	P61011	P61011 [16-33]	0	2568.4562
[R].DGMLDD	1xTMTpro [K	1.425E-06	4.225E-05	1	1	1	Q9NZN4	Q9NZN4 [498-51	0	2712.4046
[K].DGNASG	1xCarbamid(	5.093E-08	4.225E-05	1	2	7	P68104	P68104 [220-247]	0	3629.9817
[K].DGNASG	1xCarbamid(	1.53E-05	4.225E-05	1	2	4	P68104	P68104 [220-255]	1	4890.7219
[R].DGNDYS	1xTMTpro [K	3.526E-05	4.225E-05	1	1	1	Q9HCM4	Q9HCM4 [241-26	0	2906.5279
[K].DGNGYI:	1xTMTpro [N	3.375E-05	4.225E-05	1	1	1	P0DP25	P0DP25 [96-107]	0	1569.8192
[R].SLRDQD	1xCarbamid(	1.709E-05	4.225E-05	1	1	1	O43795	O43795 [94-114]	2	3203.751
[R].DGNIQE	1xTMTpro [K	4.323E-05	4.225E-05	1	1	2	Q07157	Q07157 [220-231]	0	1895.073
[K].KHLEINF	1xTMTpro [K	8.247E-07	4.225E-05	1	1	2	P08238	P08238 [624-639]	1	2519.459

[K].SLQWMA	1xOxidation [	7.154E-06	4.225E-05	1	1	1	P12109	P12109 [693-711]	0	2437.2141
[K].SLQWMA	1xTMTpro [N	9.407E-07	4.225E-05	1	1	8	P12109	P12109 [693-711]	0	2421.2192
[K].DGPGET	1xTMTpro [K	2.124E-05	4.225E-05	1	1	1	O94925	O94925 [107-121]	0	2089.033
[R].KHGLEV	1xCarbamido	1.79E-06	4.225E-05	1	1	2	P07900	P07900 [513-534]	1	3617.9892
[K].DGPLGE	3xCarbamido	1.7E-05	4.225E-05	1	1	1	Q92900	Q92900 [173-189]	0	2304.0378
[R].SLSPDC	1xCarbamido	3.696E-05	4.225E-05	1	1	1	Q8NI36	Q8NI36 [861-878]	0	2563.3392
[R].SLSQEG	1xTMTpro [K	3.813E-05	4.225E-05	1	1	1	Q9NRW3	Q9NRW3 [137-15	0	2668.3672
[KR].KHEAF	1xTMTpro [K	9.007E-06	4.225E-05	3	3	3	P12814; P35	P12814 [436-450]	1	2362.2395
[K].SLVDLTA	1xTMTpro [N	2.3E-05	4.225E-05	1	1	2	O75489	O75489 [110-122]	0	1689.9706
[R].DGIDDES	2xTMTpro [K	1.487E-06	4.225E-05	1	1	1	Q13547	Q13547 [230-247]	0	2956.5794
[R].DGIDDES	1xOxidation [	1.295E-05	4.225E-05	1	1	1	Q13547	Q13547 [230-247]	0	2972.5743
[R].GEDVPL	1xTMTpro [K	4.603E-05	4.225E-05	1	1	1	P61923	P61923 [150-168]	0	2637.4591
[R].DGLEDP	1xTMTpro [N	1.112E-07	4.225E-05	1	1	1	Q9UIA9	Q9UIA9 [427-451]	0	3044.5707
[K].SLTYPGI	1xTMTpro [N	5.609E-05	4.225E-05	1	1	2	Q4ZHG4	Q4ZHG4 [410-430	0	2481.2792
[K].SLTTSQY	1xTMTpro [K	2.658E-05	4.225E-05	1	1	1	Q9NZB2	Q9NZB2 [211-224	0	2216.2241
[R].DGIEPGI	1xTMTpro [K	7.462E-07	4.225E-05	1	1	2	P25325	P25325 [198-224]	0	3518.8663
[K].SLTPAVF	3xTMTpro [K	2.279E-05	4.225E-05	1	1	2	P20810	P20810 [133-150]	0	3053.8351
[K].GEDLTEI	1xTMTpro [N	2.547E-05	4.225E-05	1	1	1	Q02790	Q02790 [139-152]	0	1836.9146
[K].DGLGFC	1xCarbamido	5.123E-05	4.225E-05	1	1	2	P12814	P12814 [175-185]	0	1562.8433
[R].DGLLEN	1xCarbamido	8.633E-06	4.225E-05	1	1	1	P49327	P49327 [1977-199	0	3177.707
[K].SLTNDW	1xTMTpro [K	2.388E-05	4.225E-05	2	2	12	P07900; P08	P07900 [315-327]	0	2136.1581
[K].GEDQSE	1xTMTpro [K	2.471E-05	4.225E-05	1	1	2	Q8NC56	Q8NC56 [340-352	0	2029.0945
[K].KHEAIET	1xTMTpro [K	1.7E-05	4.225E-05	1	1	2	Q01082	Q01082 [463-477]	1	2383.2749
[R].SLTFSPI	1xTMTpro [K	1.802E-05	4.225E-05	1	1	1	Q9GZS3	Q9GZS3 [195-215	0	2865.5377
[R].SITCIHG	2xCarbamido	8.215E-06	4.225E-05	1	1	2	P08603	P08603 [730-749]	0	2934.5825
[R].SLSVLSS	1xTMTpro [K	6.908E-06	4.225E-05	1	1	1	P27694	P27694 [390-410]	0	2813.5792
[K].SISTSLP	1xTMTpro [N	2.789E-05	4.225E-05	1	1	2	Q14651	Q14651 [546-566]	0	2469.4248
[K].SLSSSLI	2xTMTpro [K	2.804E-05	4.225E-05	1	1	3	O95292	O95292 [156-168]	1	2321.338
[K].KHEALM	1xTMTpro [K	7.874E-06	4.225E-05	1	1	1	Q13813	Q13813 [931-950]	1	2785.5162
[K].DGLLAH	1xTMTpro [N	1.678E-07	4.225E-05	1	1	2	P08253	P08253 [188-222]	0	3942.9218
[R].GDVGSA	1xTMTpro [K	1.067E-05	4.225E-05	1	1	1	Q9Y5M8	Q9Y5M8 [253-265	0	1955.0577
[K].DGPLNM	1xTMTpro [K	5.345E-07	4.225E-05	1	1	3	P23526	P23526 [122-142]	0	2860.537
[R].SIQQVLC	1xTMTpro [N	1.407E-05	4.225E-05	1	1	1	Q8IZL8	Q8IZL8 [134-156]	0	2812.5562

[R].SIPLDEG	1xTMTpro [N	2.175E-05	4.225E-05	1	1	2	Q14204	Q14204 [2384-23	0	1762.8778
[R].DGSDYE	1xCarbamid	1.383E-06	4.225E-05	1	1	3	Q14697	Q14697 [494-518	0	3170.358
[R].DGSDYE	1xCarbamid	3.611E-06	4.225E-05	1	1	2	Q14697	Q14697 [494-518	0	3186.3529
[R].DGSEAS	1xTMTpro [N	2.706E-05	4.225E-05	1	1	2	P11047	P11047 [532-544]	0	1756.8309
[R].GEASED	1xCarbamid	2.71E-06	4.225E-05	1	1	1	Q8N163	Q8N163 [637-656	0	2578.3064
[R].SLPGES	1xTMTpro [K	2.337E-06	4.225E-05	1	1	2	P19823	P19823 [55-76]	0	3172.5562
[K].KHSQFIC	2xTMTpro [K	2.625E-05	4.225E-05	1	2	1	P07900	P07900 [209-224]	1	2819.664
[R].SIPAYLA	1xOxidation	1.973E-05	4.225E-05	1	1	5	P08758	P08758 [246-260]	0	2358.2911
[R].DGSLIVS	1xCarbamid	1.601E-05	4.225E-05	1	1	3	P61964	P61964 [182-196]	0	1932.9656
[R].SIPAYLA	1xTMTpro [K	3.545E-05	4.225E-05	1	1	2	P08758	P08758 [246-260]	0	2342.2962
[K].DGSCGV	1xCarbamid	4.542E-06	4.225E-05	1	1	2	Q14315	Q14315 [2366-23	0	2896.453
[K].DGSPIAI	1xTMTpro [K	4.518E-05	4.225E-05	1	1	1	Q9BYT8	Q9BYT8 [551-562	0	1881.0461
[K].DGSVAI	1xTMTpro [K	1.868E-05	4.225E-05	1	1	2	P55809	P55809 [177-187]	0	1709.0201
[R].SLNYCG	2xCarbamid	5.784E-05	4.225E-05	1	1	1	Q93009	Q93009 [698-712	0	2421.255
[K].SLNWSS	1xTMTpro [K	2.654E-05	4.225E-05	1	1	1	Q9UHB6	Q9UHB6 [704-72	0	3201.5984
[K].SINSILD	1xTMTpro [K	5.313E-05	4.225E-05	1	1	2	P61201	P61201 [111-123]	0	2049.1723
[R].GEAGVF	1xTMTpro [N	1.619E-05	4.225E-05	1	1	2	O75369	O75369 [2206-22	0	1823.9611
[K].SINPLGC	1xTMTpro [K	1.36E-06	4.225E-05	1	1	2	P39023	P39023 [313-334]	0	3045.6363
[K].SINPDEA	1xTMTpro [K	2.147E-07	4.225E-05	1	1	6	P11142	P11142 [362-384]	0	2868.5599
[K].DGTCTV	1xCarbamid	2.984E-05	4.225E-05	1	1	1	Q14315	Q14315 [1911-19	0	2589.319
[K].DGTCTV	1xCarbamid	3.967E-05	4.225E-05	1	1	3	O75369	O75369 [1873-18	0	2921.5826
[K].SINPDEA	1xOxidation	1.034E-06	4.225E-05	1	2	4	P0DMV9	P0DMV9 [362-38	0	2928.5632
[K].SLPADIL	1xCarbamid	6.984E-06	4.225E-05	1	1	2	Q9BX68	Q9BX68 [63-79]	0	2371.2287
[K].DGPLNM	1xOxidation	1.366E-07	4.225E-05	1	1	3	P23526	P23526 [122-142]	0	2876.5319
[K].DGSCGV	1xCarbamid	2.056E-06	4.225E-05	1	1	6	P21333	P21333 [2290-23	0	2866.4424
[R].GEAVDE	1xCarbamid	1.213E-06	4.225E-05	1	1	4	Q14204	Q14204 [2119-21	0	3936.0114
[K].DGPSLL	1xTMTpro [N	4.556E-05	4.225E-05	1	1	5	Q8N1G4	Q8N1G4 [543-55	0	1615.9338
[R].GEDEDE	1xTMTpro [N	1.213E-06	4.225E-05	1	1	1	Q8IUX7	Q8IUX7 [794-813	0	2578.1864
[R].SIQLDGI	1xTMTpro [K	5.554E-05	4.225E-05	1	1	3	P29692	P29692 [220-232]	0	1982.1566
[K].DGQAML	1xTMTpro [K	4.085E-05	4.225E-05	1	1	2	P63244	P63244 [213-225]	0	2085.093
[K].SLQGDK	2xTMTpro [K	4.099E-05	4.225E-05	1	1	2	Q15582	Q15582 [591-602	1	2229.3634
[R].SIQFVDV	1xCarbamid	5.527E-05	4.225E-05	1	2	7	P68366	P68366 [340-352]	0	2193.1658
[R].GECVPG	1xCarbamid	2.849E-06	4.225E-05	1	1	2	P02760	P02760 [186-202]	0	2224.1603

[K].SIQEIQE	2xTMTpro [K	5.081E-05	4.225E-05	1	1	2	P52565	P52565 [34-50]	2	2958.6564
[K].SIQEIQE	1xTMTpro [K	1.529E-06	4.225E-05	1	1	4	P52565	P52565 [34-49]	1	2526.3543
[R].SLQEEH	1xTMTpro [N	3.423E-05	4.225E-05	1	1	1	Q15149	Q15149 [1732-17	0	1783.9986
[K].GEATVS	1xTMTpro [K	4.825E-05	4.225E-05	2	2	4	P35637; Q92	P35637 [335-348]	0	2029.0734
[K].DGQMLF	1xTMTpro [K	1.182E-05	4.225E-05	1	1	2	Q15637	Q15637 [192-216]	0	3317.7001
[K].SLQALGI	1xTMTpro [N	1.799E-05	4.225E-05	1	1	2	Q9Y285	Q9Y285 [43-56]	0	1832.0448
[R].GECQAE	1xCarbamido	1.131E-05	4.225E-05	1	1	4	P02790	P02790 [152-166]	0	2016.9768
[K].SLQADT	1xTMTpro [K	1.484E-06	4.225E-05	1	1	3	Q8IUD2	Q8IUD2 [603-625]	0	3044.6131
[R].SIQADGI	1xTMTpro [K	2.14E-05	4.225E-05	1	1	3	P24534	P24534 [164-176]	0	1956.1046
[R].GECQAC	3xCarbamido	2.677E-05	4.225E-05	1	1	1	Q96H79	Q96H79 [97-109]	0	1984.8747
[K].DGRPLC	2xCarbamido	2.132E-05	4.225E-05	1	1	1	Q8WUP2	Q8WUP2 [227-24	0	2313.0923
[R].SLPVSD	1xTMTpro [N	1.927E-05	4.225E-05	1	1	2	P07360	P07360 [154-168]	0	1925.0299
[R].KHPDAD	1xTMTpro [K	1.193E-05	4.225E-05	1	1	1	Q12904	Q12904 [167-187]	1	2947.5657
[R].GEAVGV	1xCarbamido	5.907E-05	4.225E-05	1	1	1	Q9BVJ7	Q9BVJ7 [88-101]	0	1733.9076
[R].SLPSVE	2xCarbamido	2.57E-05	4.225E-05	2	3	1	P16615; Q93	P16615 [335-352]	0	2561.3446
[R].GECQC	4xCarbamido	7.73E-06	4.225E-05	1	1	1	P16144	P16144 [516-530]	0	2132.869
[K].KGYEVI	1xCarbamido	1.449E-06	4.225E-05	1	1	3	P14625	P14625 [561-586]	1	3989.1074
[R].DKAPGQ	2xCarbamido	5.963E-07	4.225E-05	1	1	2	Q9Y490	Q9Y490 [1653-16	1	2925.5418
[R].KLAQQY	1xTMTpro [K	2.804E-07	4.225E-05	1	1	2	P25787	P25787 [92-113]	1	3257.8542
[K].DIALGVF	1xCarbamido	1.744E-05	4.225E-05	1	1	1	Q12888	Q12888 [1911-19	0	2910.6142
[R].SKVDEA	2xTMTpro [K	7.45E-06	4.225E-05	2	2	1	Q9H361; P1	Q9H361 [600-615	1	2606.5446
[K].DLAILQC	1xCarbamido	8.035E-06	4.225E-05	1	1	1	O95372	O95372 [165-182]	0	2367.2484
[K].DLAILQC	1xCarbamido	4.315E-05	4.225E-05	1	1	2	O95372	O95372 [165-182]	0	2383.2433
[R].DLALVNI	1xTMTpro [N	2.77E-05	4.225E-05	1	1	2	Q29RF7	Q29RF7 [391-404	0	1877.0816
[K].KIEDLGA	2xTMTpro [K	7.729E-07	4.225E-05	1	1	3	Q13813	Q13813 [2264-22	1	2885.6474
[K].GDSGGF	1xCarbamido	8.112E-08	4.225E-05	1	1	3	P08311	P08311 [199-219]	0	2723.4431
[R].DLANDIN	1xTMTpro [K	6.07E-05	4.225E-05	1	1	1	O14617	O14617 [146-163]	0	2727.4818
[R].SKQLED	2xTMTpro [K	6.694E-06	4.225E-05	1	1	2	P09493	P09493 [36-48]	1	2429.4432
[K].SKQEALI	3xTMTpro [K	5.107E-05	4.225E-05	1	1	1	P47756	P47756 [253-267]	2	2902.7718
[K].GDSGGF	1xCarbamido	2.19E-06	4.225E-05	1	1	1	P23946	P23946 [201-221]	0	2338.2144
[K].DIAPTLT	1xTMTpro [K	3.619E-05	4.225E-05	2	2	5	P00739; P00	P00739 [158-169]	0	1899.1447
[K].DIAPTLT	2xTMTpro [K	2.865E-06	4.225E-05	2	2	3	P00739; P00	P00739 [158-170]	1	2331.4468
[K].SKPTVS	2xTMTpro [K	5.473E-05	4.225E-05	1	1	2	P04114	P04114 [3450-34	0	2240.2777

[K].KIEDNNT	2xTMTpro [K	3.793E-05	4.225E-05	1	1	7	P62750	P62750 [89-103]	1	2659.5851
[R].SKPGCS	1xCarbamido	3.108E-06	4.225E-05	1	1	2	Q2PZI1	Q2PZI1 [623-643]	0	3200.6536
[R].DIAQQL(	1xCarbamido	4.827E-06	4.225E-05	1	1	1	O60664	O60664 [332-356]	0	3238.7597
[K].SKPGAA	1xOxidation [	3.334E-05	4.225E-05	1	1	6	P14866	P14866 [417-434]	0	2492.2769
[K].SKPGAA	1xTMTpro [K	7.247E-06	4.225E-05	1	1	1	P14866	P14866 [417-434]	0	2476.282
[K].DIASGLI(	1xCarbamido	5.028E-06	4.225E-05	1	1	5	P00450	P00450 [188-201]	0	2078.2539
[K].SKLTFS(	1xCarbamido	8.713E-06	4.225E-05	1	1	2	Q15185	Q15185 [34-48]	1	2573.4214
[K].SKVDQV	2xTMTpro [K	1.639E-05	4.225E-05	1	1	1	Q13576	Q13576 [949-966]	1	2853.6866
[K].SKYDEIF	2xTMTpro [K	4.491E-06	4.225E-05	1	1	4	Q9NZN4	Q9NZN4 [451-466	1	2743.5123
[K].KLEDEIL	2xTMTpro [K	2.315E-06	4.225E-05	1	1	5	P35749	P35749 [982-996]	1	2716.5007
[K].GDSLAS	1xTMTpro [K	6.852E-07	4.225E-05	1	1	2	Q9NUY8	Q9NUY8 [64-84]	0	2917.5551
[K].DKYPNL(	2xTMTpro [K	1.261E-06	4.225E-05	1	1	3	P12268	P12268 [292-311]	1	2998.7506
[R].KLDYGQ	1xTMTpro [K	7.917E-06	4.225E-05	1	1	2	P38919	P38919 [152-166]	1	2206.2588
[R].SIAQYW	1xCarbamido	3.01E-05	4.225E-05	1	1	2	P04004	P04004 [464-478]	0	1974.0227
[R].SLAPYA(	1xTMTpro [K	3.76E-05	4.225E-05	1	1	3	P06727	P06727 [222-233]	0	1959.0679
[K].KLEAQV(	2xTMTpro [K	8.02E-06	4.225E-05	1	1	3	P35749	P35749 [1256-126	1	2313.3958
[K].SIALLLTI	1xTMTpro [K	1.038E-05	4.225E-05	1	1	1	Q9UMS6	Q9UMS6 [378-39	0	2162.2676
[R].DLAALGI	1xTMTpro [K	6.026E-06	4.225E-05	1	1	1	Q13813	Q13813 [1281-12	1	2467.3648
[K].SLALDID	1xTMTpro [N	6.012E-05	4.225E-05	1	1	1	Q9NYM9	Q9NYM9 [37-51]	1	2035.0375
[R].KLEATII(	2xTMTpro [K	1.544E-05	4.225E-05	1	1	1	Q9UHL4	Q9UHL4 [463-474	1	2299.4206
[K].SLAESLI	1xTMTpro [N	1.219E-06	4.225E-05	1	1	1	Q9Y2L1	Q9Y2L1 [720-741	0	2769.463
[R].SKIQKLE	1xCarbamido	5.617E-06	4.225E-05	1	1	1	P02675	P02675 [207-224]	2	3084.6638
[K].DLADEL/	1xTMTpro [K	1.665E-06	4.225E-05	1	1	7	P00338	P00338 [43-57]	0	2266.2674
[K].SLAELG(	1xTMTpro [N	3.014E-07	4.225E-05	1	1	1	P06727	P06727 [288-304]	0	2232.158
[K].KIEDELIT	2xCarbamido	6.234E-07	4.225E-05	1	1	3	Q05707	Q05707 [1200-12	1	3559.932
[K].DLAEDL	1xTMTpro [K	1.272E-05	4.225E-05	1	1	4	Q9NVD7	Q9NVD7 [118-13	0	2215.2102
[K].DLAEIGI/	1xTMTpro [K	4.525E-05	4.225E-05	1	1	1	P33176	P33176 [561-575]	0	2136.2156
[R].GDSPAS	1xTMTpro [K	1.91E-05	4.225E-05	1	1	3	P02751	P02751 [1616-163	0	2200.2218
[K].DLAEVG	1xTMTpro [N	1.127E-05	4.225E-05	1	1	1	Q9C0C2	Q9C0C2 [1237-12	0	1786.9003
[K].SLADEL/	2xTMTpro [K	1.019E-07	4.225E-05	1	1	1	P07195	P07195 [44-60]	1	2783.6586
[K].DLAGCIH	1xCarbamido	3.27E-05	4.225E-05	1	1	1	P48735	P48735 [414-426]	0	1992.1192
[K].SLADEL/	1xTMTpro [K	2.26E-06	4.225E-05	1	1	3	P07195	P07195 [44-58]	0	2238.2725
[R].GDSIDQ(	1xCarbamido	3.906E-05	4.225E-05	1	1	3	P12111	P12111 [2371-238	0	2156.1877

[K].SLAELG(	1xTMTpro [N	1.123E-05	4.225E-05	1	1	1	P06727	P06727 [288-305]	1	2388.2591
[K].SKLPGIV	2xTMTpro [K	1.47E-05	4.225E-05	1	1	2	P01008	P01008 [381-402]	2	3329.8674
[K].KLEEEGI	2xTMTpro [K	3.059E-05	4.225E-05	1	1	4	P22307	P22307 [443-453]	1	2248.3005
[R].SKLEDIA	2xTMTpro [K	1.18E-07	4.225E-05	1	1	3	Q8WVM8	Q8WVM8 [125-14	1	2983.7608
[K].SKAECE	1xCarbamido	5.514E-06	4.225E-05	1	1	2	Q9BUJ2	Q9BUJ2 [417-434	1	2817.5857
[K].SHVEDG	1xTMTpro [K	3.381E-08	4.225E-05	1	1	4	Q9P2E9	Q9P2E9 [1263-12	0	3520.8051
[R].SHTLAQ	1xCarbamido	8.665E-06	4.225E-05	1	1	2	P11277	P11277 [1161-11	0	2402.2782
[R].SHTEED	1xCarbamido	4.774E-06	4.225E-05	1	1	1	P07919	P07919 [61-78]	0	2540.1683
[R].KLEEVLS	3xTMTpro [K	4.533E-05	4.225E-05	1	1	2	O15355	O15355 [521-540	2	3393.8854
[R].SHPEVI(	1xTMTpro [K	4.579E-05	4.225E-05	1	1	1	Q9BVV7	Q9BVV7 [151-164	0	2093.1887
[R].SHMMD\	1xOxidation [	1.446E-05	4.225E-05	1	1	1	P13667	P13667 [371-391]	1	3265.7377
[R].SHMMD\	2xTMTpro [K	9.712E-06	4.225E-05	1	1	1	P13667	P13667 [371-391]	1	3249.7428
[R].SHMMD\	1xOxidation [	2.994E-05	4.225E-05	1	1	2	P13667	P13667 [371-386]	0	2359.1878
[R].SHMMD\	1xTMTpro [K	8.124E-06	4.225E-05	1	1	3	P13667	P13667 [371-386]	0	2343.1928
[R].SKAEEL(	1xTMTpro [K	1.731E-05	4.225E-05	1	1	1	P09110	P09110 [291-305]	1	2203.367
[R].DLDFAN	1xTMTpro [K	3.453E-05	4.225E-05	1	1	1	Q14573	Q14573 [441-458	0	2492.2834
[R].SHLYGIT	1xCarbamido	1.002E-05	4.225E-05	1	1	1	Q14112	Q14112 [1358-13	0	2259.1551
[K].SHIMPAE	1xCarbamido	9.007E-06	4.225E-05	1	1	2	Q9H0W9	Q9H0W9 [216-23	0	2999.4734
[K].SHLLNC(	2xCarbamido	3.715E-05	4.225E-05	1	1	1	Q9Y383	Q9Y383 [38-53]	0	2170.0817
[R].SHFEQW	1xCarbamido	3.747E-06	4.225E-05	1	2	2	P09651	P09651 [32-52]	1	3129.5741
[K].DIDIEDLI	1xTMTpro [K	8.321E-06	4.225E-05	1	1	2	Q14152	Q14152 [655-672	0	2729.4087
[R].SHFEQW	1xCarbamido	5.618E-05	4.225E-05	1	2	1	P09651	P09651 [32-47]	0	2286.0966
[R].SHFEQW	1xCarbamido	1.763E-05	4.225E-05	1	2	11	P09651	P09651 [32-47]	0	2270.1017
[K].GDRSED	1xTMTpro [N	9.927E-07	4.225E-05	1	1	2	P04083	P04083 [186-204]	1	2372.0921
[K].SHDDGN	1xTMTpro [K	8.554E-06	4.225E-05	1	1	1	P18583	P18583 [142-157]	0	2400.2175
[K].DLDIIDN	1xTMTpro [K	2.929E-06	4.225E-05	1	1	2	O75369	O75369 [895-909	0	2419.2637
[R].DIDFLKE	1xCarbamido	4.389E-05	4.225E-05	1	1	1	P12268	P12268 [162-181]	1	3453.7738
[R].GDSVSD	1xTMTpro [N	1.604E-05	4.225E-05	1	1	1	Q53EL6	Q53EL6 [74-86]	0	1569.7676
[R].SKAEEW	2xTMTpro [K	1.213E-05	4.225E-05	1	1	2	P11234	P11234 [145-160]	1	2724.5025
[K].DIDDDLE	1xCarbamido	2.255E-05	4.225E-05	1	1	1	Q9UHX1	Q9UHX1 [474-48	0	2432.163
[K].DLATVAI	1xCarbamido	2.416E-06	4.225E-05	1	1	2	P56545	P56545 [53-71]	0	2771.4166
[K].DLATVY\	1xTMTpro [K	5.907E-05	4.225E-05	1	1	1	P02647	P02647 [37-47]	0	1844.1025
[R].DLAVAG	1xTMTpro [K	6.061E-05	4.225E-05	1	1	4	Q14764	Q14764 [809-820	0	1866.065

[K].GDSGAA	1xCarbamido	9.465E-06	4.225E-05	1	1	1	Q12888	Q12888 [973-988]	1	2297.2051
[R].SKESVP	2xTMTpro [K	3.042E-05	4.225E-05	1	1	2	P16949	P16949 [28-41]	1	2454.4424
[K].SKESMT	2xTMTpro [K	1.592E-06	4.225E-05	1	1	2	P32455	P32455 [467-485]	1	3051.67
[K].GDSGAA	1xTMTpro [K	1.491E-05	4.225E-05	1	1	2	Q12888	Q12888 [973-984]	0	1754.9052
[K].SKEQEV	2xTMTpro [K	7.423E-06	4.225E-05	1	1	1	Q9P0K7	Q9P0K7 [844-855	1	2357.3856
[K].KLEEEQI	1xCarbamido	7.436E-06	4.225E-05	1	1	5	P35579	P35579 [975-989]	1	2801.5535
[K].SKELTTE	2xTMTpro [K	4.065E-06	4.225E-05	1	1	3	P13645	P13645 [344-362]	1	3124.7194
[K].SKAELM	2xTMTpro [K	1.558E-05	4.225E-05	1	1	4	P05455	P05455 [75-86]	1	2292.2937
[K].SKEFQLI	2xTMTpro [K	4.071E-05	4.225E-05	1	1	2	P02787	P02787 [298-310]	1	2404.3805
[K].KLEELEL	3xTMTpro [K	2.401E-05	4.225E-05	1	1	1	P36507	P36507 [40-52]	2	2846.698
[K].SKEEAFI	1xCarbamido	2.766E-06	4.225E-05	1	1	1	Q9BW30	Q9BW30 [91-106]	1	2678.5004
[K].DICEEQV	1xCarbamido	2.765E-05	4.225E-05	1	1	3	P01024	P01024 [1156-117	0	2398.2779
[R].DLCGCY	3xCarbamido	1.036E-06	4.225E-05	1	1	5	P01876	P01876 [178-200]	0	3201.5411
[K].SKEDTIY	1xTMTpro [K	5.917E-05	4.225E-05	1	1	1	P56199	P56199 [710-723]	1	2339.2375
[K].SKDYEFI	1xCarbamido	2.563E-06	4.225E-05	1	1	2	P12277	P12277 [266-292]	1	3777.9377
[R].KIEEIKDI	2xTMTpro [K	9.073E-06	4.225E-05	1	1	2	P63173	P63173 [4-16]	2	2488.5319
[K].SKDDQV	2xTMTpro [K	5.189E-08	4.225E-05	1	1	3	P29401	P29401 [498-523]	1	3562.0672
[K].DLDADR	1xTMTpro [N	5.115E-05	4.225E-05	1	1	1	P20338	P20338 [129-143]	1	2041.0521
[K].SKAELM	3xTMTpro [K	3.191E-05	4.225E-05	1	1	2	P05455	P05455 [75-88]	2	2825.6435
[K].KLEEGG	2xTMTpro [K	7.73E-06	4.225E-05	1	1	1	O95202	O95202 [133-150]	1	2810.6484
[R].DKYEPA	3xTMTpro [K	8.745E-06	4.225E-05	1	1	1	P05023	P05023 [8-22]	2	2881.6412
[R].DKYEPA	2xTMTpro [K	2.687E-05	4.225E-05	1	1	1	P05023	P05023 [8-21]	1	2449.3391
[R].SLATTRI	1xTMTpro [K	2.179E-05	4.225E-05	1	1	1	Q9UN37	Q9UN37 [410-425	0	2323.3477
[R].DKFPEV	2xTMTpro [K	3.012E-06	4.225E-05	1	1	2	Q00341	Q00341 [535-550]	1	2770.596
[R].SLEGTE.	1xTMTpro [K	4.434E-05	4.225E-05	1	1	1	Q13045	Q13045 [822-832]	0	1817.03
[K].SLEGAD	1xTMTpro [K	1.129E-05	4.225E-05	1	1	1	P35249	P35249 [65-84]	0	2654.4685
[K].KIDAAQI	1xTMTpro [K	2.717E-07	4.225E-05	1	1	4	P18206	P18206 [387-409]	1	3116.6256
[K].SIEEQLC	2xTMTpro [K	8.784E-07	4.225E-05	1	1	2	P26196	P26196 [448-466]	0	3023.7445
[K].KLDAGN	2xTMTpro [K	4.148E-06	4.225E-05	1	1	1	Q92616	Q92616 [2222-22	1	2704.6178
[R].SLEELLL	1xTMTpro [N	1.875E-05	4.225E-05	2	2	2	Q14160; Q9I	Q14160 [37-49]; (	0	1818.0292
[R].SLEEGE	1xTMTpro [N	1.002E-05	4.225E-05	1	1	1	Q7L014	Q7L014 [439-455]	0	2104.1279
[R].KLDDAIE	1xCarbamido	9.929E-06	4.225E-05	1	1	2	Q99615	Q99615 [309-322]	1	2504.3847
[R].DKHPPE	1xCarbamido	5.261E-05	4.225E-05	1	1	1	Q96EY7	Q96EY7 [538-556	1	3037.6597

[K].SIEIPRP\	1xCarbamido	5.504E-06	4.225E-05	1	1	2	P26368	P26368 [414-431]	0	2517.3991
[R].DKLAAC	2xCarbamido	3.662E-07	4.225E-05	1	1	2	P00734	P00734 [98-117]	1	2820.4264
[R].SLEEEG	1xTMTpro [N	1.855E-05	4.225E-05	1	1	2	P48681	P48681 [934-950]	0	2219.1111
[R].DKLDGN	2xTMTpro [K	7.339E-08	4.225E-05	1	1	5	Q96AG4	Q96AG4 [13-33]	1	3225.8035
[K].KLDDQS	2xTMTpro [K	9.14E-06	4.225E-05	1	1	1	Q96AC1	Q96AC1 [154-187	1	4508.3752
[K].KLDEAV/	2xTMTpro [K	1.053E-05	4.225E-05	1	1	2	P23526	P23526 [389-401]	1	2293.3696
[K].KIDGAEF	3xTMTpro [K	9.966E-06	4.225E-05	1	1	2	P28370	P28370 [834-851]	2	3259.8414
[K].DKLELEI	2xTMTpro [K	4.036E-05	4.225E-05	1	1	2	O60763	O60763 [893-903	1	2203.3002
[K].DKLEQA	2xTMTpro [K	2.89E-05	4.225E-05	1	1	1	Q15007	Q15007 [133-146	1	2572.4551
[K].DKLESEI	1xTMTpro [K	8.433E-07	4.225E-05	1	1	4	P23246	P23246 [517-536]	1	3036.5341
[K].SLEDLQI	1xTMTpro [K	2.506E-05	4.225E-05	1	1	1	P42224	P42224 [162-173]	0	2110.0836
[K].SLEDLIF	1xTMTpro [K	8.761E-06	4.225E-05	1	1	1	O95260	O95260 [253-269	0	2537.3743
[R].SIEEEQE	1xTMTpro [K	2.269E-06	4.225E-05	1	1	2	Q9NR99	Q9NR99 [291-310	0	2898.4712
[R].KLDGET	2xTMTpro [K	6.131E-08	4.225E-05	1	1	4	P35580	P35580 [1059-108	1	3626.9945
[K].GDVDVS	1xTMTpro [K	1.789E-05	4.225E-05	1	1	2	Q09666	Q09666 [2469-24	0	1938.0675
[K].DKEWQE	2xTMTpro [K	1.697E-05	4.225E-05	1	1	2	Q9UFN0	Q9UFN0 [106-123	1	3112.7863
[K].DKCPCC	4xCarbamido	1.182E-06	4.225E-05	1	1	2	P12111	P12111 [2385-242	1	4759.2432
[K].SLFEAS(	1xTMTpro [K	2.755E-05	4.225E-05	1	1	1	A6NHR9	A6NHR9 [1642-16	0	2246.2346
[K].DKDDDG	2xCarbamido	3.07E-09	4.225E-05	1	1	1	Q08211	Q08211 [595-621	1	3653.5736
[R].KIAVAAA	3xTMTpro [K	2.625E-05	4.225E-05	1	1	1	P29373	P29373 [31-45]	1	2712.7492
[R].SIEYSPQ	1xTMTpro [N	5.879E-05	4.225E-05	1	1	2	P98160	P98160 [93-106]	0	1855.9357
[K].DKDGQF	1xTMTpro [K	1.845E-05	4.225E-05	1	1	1	P22105	P22105 [1510-152	1	2331.2912
[R].SLEYLLL	1xTMTpro [N	3.429E-05	4.225E-05	1	1	1	Q7Z5L7	Q7Z5L7 [334-346	0	1890.0768
[K].SLEYLDI	1xTMTpro [N	2.079E-05	4.225E-05	1	1	4	P51884	P51884 [185-198]	0	1973.0663
[K].DKDQEV	2xTMTpro [K	5.009E-07	4.225E-05	1	1	3	P04114	P04114 [538-558]	2	3302.8048
[K].SLEYCS	1xCarbamido	3.597E-06	4.225E-05	1	1	2	P78527	P78527 [3010-302	0	2848.4166
[K].DKFEPG	2xTMTpro [K	3.265E-05	4.225E-05	1	1	2	Q8N8N7	Q8N8N7 [294-308	1	2748.5905
[R].SLETENS	1xTMTpro [N	3.617E-06	4.225E-05	1	1	4	P20700	P20700 [52-67]	0	2122.1311
[R].SLETCM	1xCarbamido	5.047E-05	4.225E-05	1	1	1	Q14204	Q14204 [863-872	0	1891.9538
[R].DKEETLI	1xTMTpro [K	4.179E-06	4.225E-05	1	1	2	P07099	P07099 [22-39]	1	2694.4019
[R].KICALDD	2xCarbamido	1.62E-07	4.225E-05	1	1	4	O14818	O14818 [61-81]	1	2920.4975
[K].GDVEED	1xTMTpro [N	2.611E-06	4.225E-05	1	1	1	Q92973	Q92973 [328-346	0	2504.2072
[K].SLESLDT	1xTMTpro [K	4.89E-05	4.225E-05	1	1	1	Q12874	Q12874 [292-303	0	1919.0981

[R].SLERPM	1xTMTpro [N	5.34E-05	4.225E-05	1	1	1	O94875	O94875 [239-254]	0	2076.0173
[R].SLEQNI(	1xTMTpro [N	2.692E-05	4.225E-05	1	1	1	P33993	P33993 [500-514]	0	1957.1401
[R].DKEVGN	1xTMTpro [K	4.177E-05	4.225E-05	1	1	2	Q9Y3Z3	Q9Y3Z3 [353-366	1	2333.2204
[R].KICYQE\	2xCarbamido	2.083E-06	4.225E-05	1	1	1	Q16531	Q16531 [723-739]	1	2669.4035
[K].DKEVQQ	2xTMTpro [K	1.263E-06	4.225E-05	1	1	1	Q14789	Q14789 [2170-21!	1	3518.9522
[K].SLETEIL	1xTMTpro [K	5.09E-05	4.225E-05	1	1	2	P48681	P48681 [820-830]	0	1870.1029
[K].KLAPPL\	1xTMTpro [K	8.202E-07	4.225E-05	1	1	3	P63010	P63010 [283-304]	1	3001.7946
[K].SIEDFIS(	2xCarbamido	3.124E-05	4.225E-05	1	1	2	O94874	O94874 [634-654]	0	2982.4754
[R].SLEAQV.	1xTMTpro [N	2.607E-06	4.225E-05	1	1	1	Q14980	Q14980 [1682-16!	0	1870.0102
[R].SLDLDG	1xTMTpro [K	3.429E-05	4.225E-05	1	1	4	P08729	P08729 [254-265]	0	1881.1189
[K].SLDKEIS	1xTMTpro [K	3.933E-05	4.225E-05	1	1	2	Q9BXK5	Q9BXK5 [69-85]	1	2511.3223
[R].SLDGIPF	1xCarbamido	2.057E-05	4.225E-05	1	1	1	Q93052	Q93052 [508-531]	1	3581.9606
[R].SLDFTEI	1xTMTpro [K	2.016E-05	4.225E-05	1	1	2	P01019	P01019 [238-250]	0	2046.1251
[R].KIDQEGI	3xTMTpro [K	5.71E-05	4.225E-05	1	1	1	Q9H845	Q9H845 [86-100]	2	2959.7457
[K].SLDEISQ	1xTMTpro [K	4.179E-06	4.225E-05	1	1	2	P12111	P12111 [561-574]	1	2222.2636
[K].KLDVEEI	1xTMTpro [K	4.917E-06	4.225E-05	1	1	1	Q14980	Q14980 [1822-18:	1	2653.3601
[R].SLDDSC	2xCarbamido	3.601E-05	4.225E-05	1	1	1	P13807	P13807 [559-579]	0	2833.3092
[R].DKVAGH	2xTMTpro [K	2.031E-05	4.225E-05	1	1	1	Q15717	Q15717 [54-72]	1	2938.6607
[K].KLDVLSI	2xTMTpro [K	1.24E-05	4.225E-05	1	1	2	P09467	P09467 [73-87]	1	2645.555
[R].SLDLDSI	1xTMTpro [K	4.374E-05	4.225E-05	1	1	3	P04264	P04264 [344-355]	0	1911.1294
[R].DKVLTD:	2xTMTpro [K	5.413E-06	4.225E-05	1	1	1	Q86UE4	Q86UE4 [208-245	1	4835.6234
[R].SLDAQS	1xTMTpro [K	8.539E-06	4.225E-05	1	1	1	Q9H488	Q9H488 [300-324	0	3425.7972
[R].GDTGGE	1xTMTpro [N	3.053E-05	4.225E-05	1	1	2	Q9H773	Q9H773 [10-22]	0	1507.7308
[K].DKVQAG	2xTMTpro [K	1.741E-05	4.225E-05	1	1	2	Q9Y230	Q9Y230 [185-197	1	2314.3798
[K].SLCNTVI	1xCarbamido	2.337E-05	4.225E-05	1	1	1	Q8IWV7	Q8IWV7 [1202-12	0	2429.4446
[K].SLCNLEI	1xCarbamido	2.658E-05	4.225E-05	1	1	2	Q52LJ0	Q52LJ0 [61-74]	0	1840.9394
[K].DKVTLV\	2xTMTpro [K	6.041E-05	4.225E-05	1	1	1	P21964	P21964 [160-177]	1	2836.7328
[K].SLCIPFK	2xCarbamido	4.849E-05	4.225E-05	1	1	2	P07814	P07814 [1478-14!	0	2870.6452
[K].DKVVLD'	1xCarbamido	1.291E-05	4.225E-05	1	1	1	Q99873	Q99873 [89-108]	1	2993.6984
[K].GDTFSC	1xCarbamido	7.744E-07	4.225E-05	1	1	2	P01876	P01876 [308-327]	0	2817.4559
[R].KLDVYFI	2xTMTpro [K	4.434E-05	4.225E-05	1	1	1	Q8WVM8	Q8WVM8 [417-42	1	2375.3315
[K].SLDDDLI	1xTMTpro [K	1.725E-05	4.225E-05	1	1	1	O15042	O15042 [731-748]	0	2513.275
[K].KIDGQQ'	1xCarbamido	1.023E-06	4.225E-05	1	1	1	P47755	P47755 [147-166]	1	3227.8027

[K].DKQNKP	2xTMTpro [K	4.397E-05	4.225E-05	1	1	1	P78406	P78406 [223-239]	1	2730.5719
[K].SLDLFN(	1xCarbamid(	4.882E-05	4.225E-05	1	1	5	Q9BTT0	Q9BTT0 [117-132	0	2306.1294
[K].SLEAEIL	1xTMTpro [N	1.501E-06	4.225E-05	1	1	4	P35580	P35580 [1684-17(	0	2349.2469
[K].SLEADLI	1xOxidation [	9.57E-06	4.225E-05	1	1	3	P35749	P35749 [1684-17(	0	2323.1771
[K].SLEADLI	1xTMTpro [N	9.362E-06	4.225E-05	1	1	1	P35749	P35749 [1684-17(	0	2307.1821
[K].DKLVGA	1xTMTpro [K	6.908E-06	4.225E-05	1	1	1	P43304	P43304 [210-227]	1	2642.4182
[R].DKLYHP	3xCarbamid(	4.964E-05	4.225E-05	1	1	1	P50479	P50479 [271-289]	1	3299.6501
[K].SLDVNQ	1xTMTpro [K	5.209E-05	4.225E-05	1	1	4	Q99584	Q99584 [62-72]	0	1856.0257
[K].SIDVACI	1xCarbamid(	3.048E-05	4.225E-05	1	1	2	P08603	P08603 [411-424]	0	2136.1767
[K].DKNEQA	1xTMTpro [K	9.518E-06	4.225E-05	1	1	2	P27694	P27694 [552-568]	1	2694.3768
[K].DKNFQW	2xTMTpro [K	3.406E-07	4.225E-05	1	1	1	P49792	P49792 [2867-28	1	3582.9121
[K].DKNQDL	1xCarbamid(	1.572E-05	4.225E-05	1	1	1	Q14554	Q14554 [459-471]	1	2488.3646
[R].DKQLYA	2xTMTpro [K	5.617E-06	4.225E-05	1	1	4	Q6UWY5	Q6UWY5 [377-39	1	2895.6185
[K].KLDLEA\	1xTMTpro [K	1.716E-05	4.225E-05	1	1	1	P49591	P49591 [376-390]	1	2302.284
[K].SLDQEIA	1xTMTpro [K	2.367E-06	4.225E-05	1	1	1	P48681	P48681 [790-808]	0	2881.5551
[K].SLDPSRI	1xTMTpro [K	1.186E-06	4.225E-05	1	1	2	P08236	P08236 [472-491]	0	2776.4761
[R].DKPEEQ	2xTMTpro [K	9.874E-06	4.225E-05	1	1	2	P46108	P46108 [163-179]	1	3016.5581
[K].DKPELQ	1xCarbamid(	1.646E-06	4.225E-05	1	1	3	P09543	P09543 [28-50]	0	3648.9651
[K].KLDPDSI	1xTMTpro [K	1.18E-05	4.225E-05	1	1	1	O94855	O94855 [258-275]	1	2644.4802
[R].DKPLKD'	1xCarbamid(	6.357E-06	4.225E-05	1	1	6	P23284	P23284 [191-204]	1	2788.6747
[K].SIDNGIF	1xTMTpro [N	2.064E-06	4.225E-05	1	1	1	O00560	O00560 [131-153]	0	2702.516
[R].DKPMGF	2xTMTpro [K	9.5E-06	4.225E-05	1	1	3	P08253	P08253 [501-519]	0	3080.7675
[R].SLDMDS	1xTMTpro [K	4.713E-05	4.225E-05	1	1	2	P05787	P05787 [253-264]	0	1929.0858
[K].SLDLFN(	1xCarbamid(	8.429E-06	4.225E-05	2	2	2	P39687; Q9:	P39687 [117-132]	0	2277.1141
[K].DKNTPS	1xTMTpro [K	1.394E-06	4.225E-05	1	1	2	P48506	P48506 [210-229]	1	2907.4504
[R].CAVDAA	1xCarbamid(	1.24E-05	4.225E-05	1	1	1	Q16787	Q16787 [2171-21	0	2445.3303
[K].DGLAFN.	1xTMTpro [K	4.022E-05	4.225E-05	1	1	2	P11277	P11277 [204-214]	0	1807.0722
[R].SLVEAS:	1xCarbamid(	1.105E-05	4.225E-05	1	1	2	Q9UQ80	Q9UQ80 [34-51]	0	2460.3511
[R].SQAGVS	1xTMTpro [N	3.92E-05	4.225E-05	1	1	2	Q14980	Q14980 [1887-18	0	1474.7933
[K].KGDTFS	1xCarbamid(	9.838E-06	4.225E-05	1	1	2	P01876	P01876 [307-327]	1	3265.753
[K].KGDVKP	1xCarbamid(	1.272E-05	4.225E-05	1	1	1	P23141	P23141 [258-275]	1	3115.8654
[K].KGDVLTI	2xTMTpro [K	1.639E-05	4.225E-05	1	1	4	P02549	P02549 [1000-10	1	2300.4369
[R].SPYTVT'	2xCarbamid(	6.633E-06	4.225E-05	1	1	2	P21333	P21333 [468-484]	0	2172.0497

[K].KGDVVH	1xCarbamido	2.495E-08	4.225E-05	1	1	2	Q00688	Q00688 [127-154]	1	4054.116
[K].SPYQEF	1xTMTpro [K	3.924E-06	4.225E-05	1	1	2	P15880	P15880 [264-275]	0	2072.1308
[K].SPYLYPI	1xTMTpro [N	1.833E-06	4.225E-05	2	2	2	P31150; P50	P31150 [222-240]	0	2445.3138
[K].KGEDED	2xCarbamido	9.174E-06	4.225E-05	1	1	1	Q06323	Q06323 [91-109]	2	3346.7512
[K].SPWNFL	1xTMTpro [K	1.049E-05	4.225E-05	1	1	1	O96005	O96005 [325-346]	0	3223.5563
[K].GEIGDA	1xTMTpro [K	2.341E-05	4.225E-05	1	1	1	P30876	P30876 [994-1010	0	2383.2749
[R].SPVTLLA	1xTMTpro [K	2.839E-05	4.225E-05	1	1	2	P18428	P18428 [258-275]	0	2544.4351
[K].SPVPPG	1xTMTpro [N	1.607E-05	4.225E-05	1	1	3	Q16853	Q16853 [304-322]	0	2306.2617
[R].DEVQEV	1xTMTpro [N	5.188E-06	4.225E-05	1	1	1	Q96FV2	Q96FV2 [38-56]	0	2329.2107
[R].DEVQEV	1xTMTpro [K	2.407E-06	4.225E-05	1	1	1	Q12765	Q12765 [33-51]	0	2787.3969
[R].SPVISIDI	1xTMTpro [K	1.047E-05	4.225E-05	1	1	4	P98160	P98160 [3021-304	0	2896.5548
[K].SPVGVQ	1xCarbamido	9.031E-07	4.225E-05	1	1	2	P00738	P00738 [326-345]	0	2796.4668
[K].SPVGVQ	1xCarbamido	1.222E-07	4.225E-05	1	1	2	P00738	P00738 [326-345]	0	2780.4719
[R].SPVEVA	1xTMTpro [K	3.78E-05	4.225E-05	1	1	2	Q6IAN0	Q6IAN0 [268-282]	0	2091.2305
[K].GEIGAV	1xTMTpro [N	2.223E-06	4.225E-05	1	1	4	P08123	P08123 [265-282]	0	1851.9996
[R].SPVDES	1xTMTpro [K	1.513E-05	4.225E-05	1	1	2	Q8WX93	Q8WX93 [979-10	0	3681.7584
[R].DESTNV	1xTMTpro [N	2.814E-05	4.225E-05	1	1	2	P50995	P50995 [347-359]	0	1769.8659
[R].SQALNA	1xTMTpro [K	1.468E-06	4.225E-05	1	1	2	Q13217	Q13217 [159-179]	0	2768.4751
[R].SQAPLE	1xTMTpro [N	2.959E-07	4.225E-05	1	1	1	Q14980	Q14980 [1782-18	0	2497.2741
[K].SQCCSS	5xCarbamido	1.728E-06	4.225E-05	1	1	5	P35555	P35555 [873-898]	0	3507.6443
[K].KGDILTL	2xTMTpro [K	1.984E-05	4.225E-05	1	1	4	Q13813	Q13813 [990-100	1	2329.4271
[K].SQEEPK	2xTMTpro [K	1.09E-05	4.225E-05	1	1	1	Q9NYF8	Q9NYF8 [183-204	1	3520.7536
[R].SQEDPY	1xTMTpro [K	8.891E-06	4.225E-05	1	1	3	P34949	P34949 [329-349]	0	3063.588
[K].DENATLI	1xTMTpro [N	6.819E-06	4.225E-05	1	1	1	O94760	O94760 [121-136]	0	1983.9943
[R].KGDSNA	1xCarbamido	2.563E-06	4.225E-05	1	1	1	Q8IZL8	Q8IZL8 [512-527]	1	2257.1851
[R].DENQSII	1xTMTpro [N	5.888E-05	4.225E-05	1	1	2	P20073	P20073 [332-348]	0	2318.0751
[R].GEILIGG	1xTMTpro [K	2.963E-05	4.225E-05	1	1	1	O95573	O95573 [526-541]	0	2324.2816
[R].DENSVE	1xTMTpro [K	4.057E-06	4.225E-05	1	1	3	Q14697	Q14697 [134-148]	0	2291.1721
[R].DENSVE	1xOxidation [	3.23E-05	4.225E-05	1	1	1	Q14697	Q14697 [134-148]	0	2307.167
[R].SQEATE	1xCarbamido	4.027E-06	4.225E-05	1	1	2	Q9UHD8	Q9UHD8 [238-25	0	2397.0982
[R].GELFWD	1xTMTpro [N	4.51E-05	4.225E-05	1	1	1	P10253	P10253 [855-870]	0	2198.0936
[K].DEPDWE	1xTMTpro [N	6.041E-05	4.225E-05	1	1	5	P49915	P49915 [536-548]	0	1894.987
[K].SQDSGIA	1xTMTpro [K	2.102E-05	4.225E-05	1	1	1	Q96FC7	Q96FC7 [39-57]	0	2702.4315

[R].DEPSVA	1xTMTpro [K	8.57E-06	4.225E-05	1	1	1	P53396	P53396 [522-538]	0	2472.2725
[R].DEPSVA	1xOxidation [	1.941E-05	4.225E-05	1	1	1	P53396	P53396 [522-538]	0	2488.2674
[R].DEPTGE	1xTMTpro [K	3.502E-05	4.225E-05	1	1	4	O75643	O75643 [34-46]	0	1952.1196
[K].SQDILLS	1xTMTpro [N	3.069E-05	4.225E-05	1	1	2	P07225	P07225 [547-561]	0	2054.1453
[K].SQDFGN	1xTMTpro [K	2.379E-05	4.225E-05	1	1	1	Q02880	Q02880 [1441-14	0	2460.2691
[K].GELLGC	1xCarbamido	6.933E-06	4.225E-05	1	1	1	Q92947	Q92947 [171-194	0	2845.3303
[K].SQDAEV	1xTMTpro [K	8.998E-07	4.225E-05	1	1	3	Q99832	Q99832 [85-106]	0	2860.5435
[R].SQCTYS	3xCarbamido	3.384E-06	4.225E-05	1	1	1	P22792	P22792 [479-500]	0	2848.2983
[R].SQCLED	2xCarbamido	1.315E-05	4.225E-05	1	1	1	P20851	P20851 [119-135]	0	2694.3664
[K].SQDVEL	1xTMTpro [K	2.906E-05	4.225E-05	1	1	2	Q9UHB6	Q9UHB6 [726-73	0	2126.1625
[R].SPTGAV	1xTMTpro [N	1.179E-06	4.225E-05	1	1	2	Q5ZPR3	Q5ZPR3 [242-267	0	2924.59
[K].DFAIFGF	1xTMTpro [K	1.148E-05	4.225E-05	1	1	1	Q9UH99	Q9UH99 [645-664	0	2851.5009
[K].SPTDWA	1xTMTpro [N	1.043E-05	4.225E-05	1	1	1	Q9UJU6	Q9UJU6 [24-41]	0	2390.1584
[K].KGEKEQ	2xTMTpro [K	1.589E-05	4.225E-05	1	2	1	Q01105	Q01105 [36-57]	2	3549.8965
[K].KGEICHI	2xCarbamido	2.3E-05	4.225E-05	1	1	1	Q13451	Q13451 [99-121]	1	3795.1078
[K].SPNIIFAI	2xCarbamido	3.21E-08	4.225E-05	1	1	1	O94788	O94788 [291-325	0	4190.9619
[R].GEIAGPI	1xTMTpro [N	3.411E-05	4.225E-05	1	1	2	P61086	P61086 [41-55]	0	1819.9146
[K].SPLVMD	1xTMTpro [N	2.148E-05	4.225E-05	1	1	1	Q14204	Q14204 [1585-15	0	1973.1173
[K].KGEPVS	2xTMTpro [K	1.046E-06	4.225E-05	1	1	2	P11586	P11586 [596-616]	1	3013.7424
[K].SPITGNI	1xTMTpro [K	2.379E-05	4.225E-05	1	1	2	P41250	P41250 [265-283]	0	2672.4613
[K].DFGNYL	1xTMTpro [K	5.34E-05	4.225E-05	1	1	1	Q96A49	Q96A49 [56-70]	0	2274.205
[R].SPISVAA	1xTMTpro [K	8.729E-06	4.225E-05	1	1	1	Q00403	Q00403 [249-268	0	2574.4093
[K].GEHSIV	2xTMTpro [K	1.51E-05	4.225E-05	1	1	2	Q02790	Q02790 [214-232	0	2951.6811
[R].SPPTVL	2xCarbamido	2.655E-06	4.225E-05	1	1	1	Q8NCW5	Q8NCW5 [107-12	0	2614.3401
[K].GEHPGL	1xTMTpro [K	3.225E-05	4.225E-05	1	2	2	P09429	P09429 [115-127]	0	1888.0784
[R].SPGVVIS	1xCarbamido	1.36E-06	4.225E-05	1	1	2	P00492	P00492 [5-34]	0	3739.7757
[K].DFLAGG	1xTMTpro [K	1.057E-05	4.225E-05	1	1	4	P12236	P12236 [11-23]	0	1842.0981
[K].DFLAGG	1xTMTpro [K	1.256E-05	4.225E-05	1	1	3	P05141	P05141 [11-23]	0	1828.0824
[R].SPGSPV	1xTMTpro [K	1.709E-05	4.225E-05	1	1	1	P35611	P35611 [355-369]	0	1962.0788
[K].DFLDGV	1xTMTpro [N	2.171E-05	4.225E-05	1	1	1	O75369	O75369 [505-522	0	2401.1671
[R].GEGYAK	2xTMTpro [K	1.293E-05	4.225E-05	1	1	1	Q02790	Q02790 [158-179	0	3269.7874
[R].DFIDNQI	1xTMTpro [N	3.866E-05	4.225E-05	1	1	1	Q8TE77	Q8TE77 [313-326	0	1980.0908
[K].GEGWGI	2xCarbamido	2.049E-06	4.225E-05	1	1	3	P35555	P35555 [2089-21	0	2626.1509

[K].SPGALE	1xTMTpro [K	1.086E-05	4.225E-05	1	1	1	Q9Y2D5	Q9Y2D5 [393-414	0	2624.3771
[K].GEGVEI	1xTMTpro [K	3.196E-05	4.225E-05	1	1	1	Q86W92	Q86W92 [293-307	1	2294.2847
[K].SPIAPEE	1xTMTpro [N	3.346E-05	4.225E-05	1	1	1	P51398	P51398 [280-294]	0	1963.1296
[R].SQEGRF	1xTMTpro [K	2.358E-05	4.225E-05	1	1	2	Q7L5N1	Q7L5N1 [60-75]	0	2260.3633
[R].SPQENL	1xCarbamido	2.148E-05	4.225E-05	1	1	2	Q9HD20	Q9HD20 [328-342	0	2057.1384
[K].SPQPPV	2xCarbamido	9.53E-07	4.225E-05	1	1	1	Q00839	Q00839 [271-300]	0	4243.972
[R].DFALLSI	2xTMTpro [K	2.547E-05	4.225E-05	2	2	2	P0C0L4; P0(	P0C0L4 [81-95]; F	1	2570.5738
[R].DFAISVV	1xTMTpro [K	4.833E-05	4.225E-05	1	1	1	P39059	P39059 [90-103]	0	2114.2465
[R].KGEELE	2xTMTpro [K	4.991E-06	4.225E-05	1	1	3	Q8IUX7	Q8IUX7 [369-382]	1	2617.4177
[K].KGEFSG	2xTMTpro [K	5.974E-07	4.225E-05	1	1	1	Q16363	Q16363 [963-992]	1	4160.1532
[R].DFAVST	1xTMTpro [N	1.3E-05	4.225E-05	1	1	1	O95865	O95865 [158-173]	0	1973.0775
[K].SPSIVLA	4xCarbamido	5.714E-08	4.225E-05	1	1	1	P30837	P30837 [290-324]	0	4301.8924
[K].KGEGAP	2xTMTpro [K	7.659E-06	4.225E-05	1	1	1	Q9P2E9	Q9P2E9 [539-548	1	1897.1687
[R].DFDDGV	1xCarbamido	9.709E-07	4.225E-05	1	1	2	O14672	O14672 [322-346]	0	3102.5698
[K].SPSLDV	1xTMTpro [K	7.816E-06	4.225E-05	1	1	2	Q09666	Q09666 [4993-50	0	3189.7386
[R].SPSKPLI	3xTMTpro [K	4.549E-05	4.225E-05	1	1	2	P05455	P05455 [92-109]	1	3262.8676
[K].SPQLILV	1xCarbamido	4.533E-06	4.225E-05	1	1	1	Q96E11	Q96E11 [140-160	0	2869.5811
[R].KGEGYS	1xTMTpro [K	2.929E-06	4.225E-05	1	1	2	Q13451	Q13451 [155-175]	1	2865.4987
[R].DFDHNL	1xCarbamido	6.09E-05	4.225E-05	1	1	1	P23946	P23946 [180-193]	0	1988.9932
[R].DFDPAV	1xTMTpro [N	5.252E-05	4.225E-05	1	1	2	P09467	P09467 [219-230]	0	1757.9029
[K].SPSDVK	1xTMTpro [K	4.337E-05	4.225E-05	1	1	1	P02724	P02724 [121-150]	0	3787.9257
[R].DFEADD	2xCarbamido	3.761E-06	4.225E-05	1	1	1	Q9NWX6	Q9NWX6 [40-56]	0	2397.1287
[R].SPSDSS	1xTMTpro [N	2.414E-05	4.225E-05	1	1	1	Q16643	Q16643 [337-354]	0	2166.0845
[K].DFEELK	2xTMTpro [K	3.886E-05	4.225E-05	1	1	1	P46821	P46821 [839-852]	1	2564.4276
[R].GEIAVLE	1xTMTpro [N	3.988E-05	4.225E-05	1	1	1	Q15075	Q15075 [1276-12	0	2190.1322
[R].SPRPTS	1xTMTpro [K	1.712E-06	4.225E-05	1	1	1	P11171	P11171 [555-580]	0	3116.7195
[K].SPQTPE	1xTMTpro [N	3.846E-05	4.225E-05	1	1	2	Q9NZB2	Q9NZB2 [652-665	0	1862.0343
[R].SPQSLS	1xTMTpro [K	2.071E-05	4.225E-05	1	1	2	P50281	P50281 [57-70]	0	2011.1502
[R].GELESIL	1xTMTpro [K	2.68E-06	4.225E-05	1	1	2	Q16647	Q16647 [311-333]	0	3191.7444
[K].SQENKG	2xTMTpro [K	5.554E-05	4.225E-05	1	1	1	P57081	P57081 [47-71]	1	3462.8533
[K].SQEPLP	1xTMTpro [K	4.65E-05	4.225E-05	1	1	1	Q6P2Q9	Q6P2Q9 [367-389	0	3357.691
[K].DEILPTT	1xTMTpro [K	1.969E-05	4.225E-05	1	1	3	P23396	P23396 [215-227]	0	2079.1829
[R].KFMQDF	2xTMTpro [K	1.835E-05	4.225E-05	1	1	2	Q13838	Q13838 [241-256]	1	2885.5245

[K].DDPLTNI	1xTMTpro [K	2.038E-05	4.225E-05	1	1	2	P12814	P12814 [199-214]	0	2371.2637
[R].KFNALF/	2xTMTpro [K	2.761E-06	4.225E-05	1	1	2	Q00610	Q00610 [367-382]	1	2671.5024
[K].DDPVTN	1xTMTpro [K	1.329E-05	4.225E-05	1	1	2	O43707	O43707 [218-233]	0	2384.2589
[K].GEMMDI	1xTMTpro [N	4.257E-05	4.225E-05	1	1	2	P00338	P00338 [60-73]	0	1937.9897
[K].DDQFLLI	1xCarbamid	3.782E-06	4.225E-05	1	1	4	P10768	P10768 [225-246]	0	3131.6215
[R].SQVVFS	1xTMTpro [N	8.778E-06	4.225E-05	1	1	1	Q02809	Q02809 [122-137]	1	2213.1886
[R].DDQYVF	2xCarbamid	3.213E-06	4.225E-05	1	1	4	Q14112	Q14112 [1036-10	0	3250.5905
[R].SQVVFS	1xTMTpro [N	1.651E-05	4.225E-05	1	1	3	Q02809	Q02809 [122-136]	0	2057.0874
[K].SQVVAG	1xTMTpro [K	5.473E-05	4.225E-05	1	1	3	P04080	P04080 [45-56]	0	1935.1195
[R].KFIEMGI	2xTMTpro [K	3.375E-05	4.225E-05	1	1	1	Q12884	Q12884 [606-616]	1	2269.3082
[K].SQVNAL	1xTMTpro [N	4.556E-05	4.225E-05	1	1	1	O75146	O75146 [386-399]	0	1905.9837
[K].SQVLDD	1xTMTpro [K	5.993E-05	4.225E-05	1	1	1	Q9UGP8	Q9UGP8 [451-47	0	3053.6862
[K].SQVFST.	1xTMTpro [K	5.217E-06	4.225E-05	1	1	4	P38646	P38646 [469-485]	0	2417.3168
[R].KFNEIV/	1xCarbamid	4.704E-06	4.225E-05	1	1	1	Q9Y5S2	Q9Y5S2 [1366-13	1	2609.5093
[R].SQTVSA	1xTMTpro [K	4.642E-05	4.225E-05	1	1	1	P24821	P24821 [1699-17	0	2158.2033
[R].KFPSGT	2xTMTpro [K	1.456E-05	4.225E-05	1	1	4	P02774	P02774 [51-65]	1	2607.5326
[R].SQTEAV	1xTMTpro [N	1.293E-05	4.225E-05	1	1	1	Q9Y2S7	Q9Y2S7 [150-165	0	2095.0375
[R].SQSVRP	1xCarbamid	6.744E-06	4.225E-05	1	1	1	P98160	P98160 [1780-17	0	2445.3416
[R].DDVFGY	2xTMTpro [K	6.183E-06	4.225E-05	1	1	1	Q93008	Q93008 [1615-16	0	2996.6186
[K].KFPVFNI	1xCarbamid	1.996E-06	4.225E-05	1	1	2	P00352	P00352 [37-62]	2	4269.2378
[R].SQSAAV	1xTMTpro [N	1.084E-05	4.225E-05	1	1	3	Q16186	Q16186 [211-226]	0	1871.963
[R].SQVMDE	1xTMTpro [N	1.951E-05	4.225E-05	1	1	3	Q15149	Q15149 [4037-40	0	1765.9438
[K].SQQSSD	1xCarbamid	1.54E-06	4.225E-05	1	1	2	P02788	P02788 [436-461]	0	3162.5809
[R].SQYEQL	1xTMTpro [K	8.81E-06	4.225E-05	1	1	3	P13645	P13645 [323-334]	1	2102.1486
[R].SQYEVM	1xTMTpro [K	1.354E-05	4.225E-05	1	1	1	P08727	P08727 [254-265]	1	2091.1148
[K].SSDTNIF	1xTMTpro [K	2.159E-05	4.225E-05	1	1	1	Q6PJT7	Q6PJT7 [85-99]	0	2233.1592
[K].GEPGPP	1xTMTpro [K	3.995E-05	4.225E-05	1	1	1	Q9P2E9	Q9P2E9 [635-651	0	2394.2837
[K].SSDEIEN	1xTMTpro [K	4.419E-05	4.225E-05	1	1	3	P02549	P02549 [2353-23	0	2317.2167
[K].SSCSLIA	1xCarbamid	2.902E-06	4.225E-05	1	1	1	P57737	P57737 [40-67]	0	3552.8976
[R].KFGDPV	2xTMTpro [K	4.737E-05	4.225E-05	1	1	3	P0DMV9	P0DMV9 [77-88]	1	2263.2937
[R].DDLESF	1xTMTpro [K	4.556E-05	4.225E-05	1	1	1	Q52LJ0	Q52LJ0 [75-89]	0	2349.2834
[R].SSAYES	1xTMTpro [K	2.697E-05	4.225E-05	1	1	1	Q14974	Q14974 [526-537]	0	1965.0858
[R].SSAVVE	1xTMTpro [N	2.855E-05	4.225E-05	1	1	1	Q4G0J3	Q4G0J3 [96-108]	0	1679.9135

[R].GEPETF	2xTMTpro [K	4.713E-06	4.225E-05	1	1	4	Q14683	Q14683 [565-584]	0	3232.7809
[R].KFGEAI(	2xTMTpro [K	1.916E-05	4.225E-05	1	1	3	P78347	P78347 [879-891]	1	2293.3559
[R].KFLDGN	1xCarbamid(	7.434E-07	4.225E-05	1	1	3	Q9Y696	Q9Y696 [177-194	1	2991.6464
[R].KFGVVLI	2xTMTpro [K	2.931E-05	4.225E-05	1	1	1	O14579	O14579 [65-85]	1	3195.8921
[K].SSALQW	1xTMTpro [K	4.397E-05	4.225E-05	1	1	1	P09960	P09960 [113-127]	0	2241.2371
[R].KFGYVD	2xTMTpro [K	1.202E-06	4.225E-05	1	1	5	P19338	P19338 [348-362]	1	2689.4541
[K].DDITASL	1xTMTpro [K	2.394E-06	4.225E-05	1	1	1	Q9H3S7	Q9H3S7 [584-599	0	2371.2307
[R].KFHYLPI	1xCarbamid(	2.392E-05	4.225E-05	1	1	1	P01903	P01903 [172-189]	1	2895.4995
[K].SSACFTI	1xCarbamid(	5.926E-05	4.225E-05	1	1	2	P46060	P46060 [138-148]	0	1892.0443
[R].KFIAYQF	2xTMTpro [K	4.273E-06	4.225E-05	1	1	2	O00267	O00267 [199-213]	1	2725.6109
[R].GENLVS	1xTMTpro [K	9.62E-08	4.225E-05	1	2	2	P14678	P14678 [74-94]	1	2776.5159
[R].GENLVS	1xTMTpro [K	2.136E-05	4.225E-05	1	2	2	P14678	P14678 [74-88]	0	2163.1975
[R].KFLDGIY	2xTMTpro [K	5.518E-05	4.225E-05	1	1	2	P32969	P32969 [174-184]	1	2211.3205
[R].KFLDGN	1xCarbamid(	1.888E-06	4.225E-05	1	1	3	O00299	O00299 [166-183]	1	2973.6899
[K].GEPAAA	1xTMTpro [K	6.633E-06	4.225E-05	1	1	2	P29966	P29966 [88-105]	0	2231.2163
[K].SPFVVQ	2xCarbamid(	6.404E-06	4.225E-05	1	1	2	O75369	O75369 [440-456]	0	2209.0813
[R].DDVSSF	1xTMTpro [K	1.69E-06	4.225E-05	1	1	2	Q5VT79	Q5VT79 [168-192	0	3246.6662
[K].KFSNQE	1xCarbamid(	8.745E-06	4.225E-05	1	1	2	P60891	P60891 [34-49]	1	2491.3106
[R].DEFEGL	2xTMTpro [K	2.556E-05	4.225E-05	1	1	1	P22314	P22314 [650-671]	1	3494.8624
[K].SQIDVAL	1xTMTpro [N	5.322E-05	4.225E-05	1	1	1	Q92900	Q92900 [1100-11	0	2201.1005
[R].KGAAYG	2xTMTpro [K	1.676E-05	4.225E-05	1	1	1	Q92616	Q92616 [1395-14(	1	2060.3048
[K].SQLDIIIF	1xTMTpro [K	5.701E-05	4.225E-05	1	1	2	P13010	P13010 [145-155]	0	1875.1559
[R].DEFTNT(	1xCarbamid(	8.607E-07	4.225E-05	1	1	3	Q9Y696	Q9Y696 [228-249	1	3430.7504
[R].DEFWDT	1xTMTpro [N	5.107E-05	4.225E-05	1	2	1	Q8WUN7	Q8WUN7 [55-67]	0	1844.8774
[R].SQHPYV	1xTMTpro [K	2.784E-05	4.225E-05	1	1	4	P12111	P12111 [1919-19:	0	2139.1941
[R].KGADIM	1xCarbamid(	1.184E-05	4.225E-05	2	2	1	P12236; P12	P12236 [245-259]	1	2381.2237
[R].DEGWL/	1xTMTpro [K	3.532E-05	4.225E-05	1	1	1	Q16822	Q16822 [275-294]	0	2746.5093
[R].SQGKPII	2xTMTpro [K	1.667E-05	4.225E-05	1	1	2	Q16537	Q16537 [38-52]	0	2546.6102
[R].SQLELLI	2xCarbamid(	3.417E-05	4.225E-05	1	1	1	Q29RF7	Q29RF7 [571-584	0	2244.2223
[R].DELADE	1xTMTpro [K	8.681E-06	4.225E-05	1	1	3	P35579	P35579 [1704-17:	0	1957.037
[K].KGCQQ(	1xCarbamid(	1.184E-06	4.225E-05	1	1	2	P35442	P35442 [224-245]	1	3012.5664
[K].SQETGD	1xTMTpro [K	2.91E-07	4.225E-05	1	1	1	P23142	P23142 [147-179]	1	4372.1448
[K].SQETGD	1xTMTpro [K	8.899E-07	4.225E-05	1	1	6	P23142	P23142 [147-163]	0	2400.2386

[K].SQETEC	1xCarbamido	2.191E-05	4.225E-05	1	1	3	P40926	P40926 [280-296]	0	2582.3668
[K].DELHIVE	1xTMTpro [K	3.751E-07	4.225E-05	1	1	3	P06748	P06748 [55-73]	0	2753.4312
[K].DELHIVE	1xOxidation [	3.558E-06	4.225E-05	1	1	3	P06748	P06748 [55-73]	0	2769.4261
[K].SQEQLA	1xTMTpro [K	7.194E-06	4.225E-05	1	1	4	P15311	P15311 [413-427]	0	2260.2317
[K].DELLFPS	1xTMTpro [K	6.381E-06	4.225E-05	1	1	3	P39060	P39060 [1647-167	0	3211.7283
[K].KGDEVQ	1xCarbamido	6.428E-06	4.225E-05	1	2	1	Q96GK7	Q96GK7 [295-312	1	2985.6747
[K].DELILEG	1xTMTpro [K	6.814E-07	4.225E-05	1	1	1	P32969	P32969 [142-168]	0	3492.9293
[R].SQGCSE	1xCarbamido	5.993E-05	4.225E-05	1	1	1	P78527	P78527 [3290-330	0	2057.1556
[R].DDVSSF	1xTMTpro [K	1.043E-05	4.225E-05	1	1	1	P13928	P13928 [168-192]	0	3232.6505
[R].DEEVHA	1xTMTpro [N	4.242E-05	4.225E-05	1	1	4	P50454	P50454 [104-116]	0	1741.9404
[R].DEELSC	1xCarbamido	3.839E-05	4.225E-05	1	1	2	P01011	P01011 [256-267]	0	2030.0971
[K].SQQATA	1xTMTpro [N	2.259E-05	4.225E-05	1	1	1	A6NGB9	A6NGB9 [392-409	0	2259.1801
[K].KFSNQE	1xTMTpro [K	1.541E-05	4.225E-05	1	1	2	P11908	P11908 [34-49]	1	2418.312
[R].SQPELIE	1xCarbamido	2.765E-05	4.225E-05	1	1	1	Q9BTW9	Q9BTW9 [889-90	0	1873.9397
[K].SQPDMA	1xTMTpro [K	1.598E-05	4.225E-05	1	1	2	P63010	P63010 [79-93]	0	2246.2169
[K].KFVTDSI	2xTMTpro [K	2.214E-05	4.225E-05	1	1	1	Q14008	Q14008 [55-67]	1	2361.4322
[R].SQNVMA	1xTMTpro [K	1.779E-05	4.225E-05	1	1	1	P17987	P17987 [19-33]	0	2125.2295
[R].KFYGPE	1xTMTpro [K	2.435E-05	4.225E-05	1	1	2	O00264	O00264 [105-119]	1	2253.2312
[R].SQNIVTI	1xTMTpro [N	3.895E-06	4.225E-05	1	1	2	Q01082	Q01082 [1548-156	0	2082.0998
[K].SQMTQE	1xTMTpro [K	4.827E-07	4.225E-05	1	1	2	Q9P0K7	Q9P0K7 [617-636	1	2909.3571
[K].SQMTQE	1xTMTpro [K	5.563E-05	4.225E-05	1	1	1	Q9P0K7	Q9P0K7 [617-631	0	2308.0929
[K].DEESGG	1xTMTpro [K	7.505E-06	4.225E-05	1	1	1	Q9Y678	Q9Y678 [9-24]	0	2339.1759
[R].SQMESM	1xCarbamido	5E-07	4.225E-05	1	1	2	P17987	P17987 [204-227]	0	3195.6166
[R].SQMDGI	1xTMTpro [N	5.175E-05	4.225E-05	1	1	2	Q9Y2X3	Q9Y2X3 [121-133	0	1702.9117
[K].SQLSCV	1xCarbamido	2.345E-05	4.225E-05	1	1	2	P46459	P46459 [595-607]	0	1823.9492
[R].SQLPTLI	1xTMTpro [N	3.939E-06	4.225E-05	1	1	1	P46937	P46937 [350-375]	0	3060.5227
[K].SQLNSQ	1xTMTpro [K	2.839E-05	4.225E-05	1	1	2	O60763	O60763 [799-810]	0	1942.1101
[K].KFYPLEI	2xTMTpro [K	1.265E-06	4.225E-05	1	1	2	P09874	P09874 [637-653]	1	2956.6124
[R].DEDPYC	2xCarbamido	1.464E-05	4.225E-05	1	1	3	Q13643	Q13643 [204-220]	0	2626.2813
[K].KFYPLEI	3xTMTpro [K	4.293E-05	4.225E-05	1	1	1	P09874	P09874 [637-654]	2	3388.9145
[K].SQIHDIV	1xTMTpro [N	3.059E-05	4.225E-05	1	1	4	P11142	P11142 [329-342]	0	1786.0142
[R].DEELEKI	2xTMTpro [K	3.259E-05	4.225E-05	1	1	1	Q8WU90	Q8WU90 [142-15	1	2809.4018
[K].SQIFSTA	1xTMTpro [K	4.202E-06	4.225E-05	1	1	3	P11021	P11021 [448-464]	0	2445.3481

[R].DEATAA'	1xTMTpro [N	2.867E-07	4.225E-05	1	1	2	Q9NTZ6	Q9NTZ6 [908-925	0	2217.1794
[R].SIVDYKF	2xTMTpro [K	6.827E-07	4.225E-05	1	1	3	O43707	O43707 [696-723]	0	4237.3689
[R].KGFFIEF	1xTMTpro [K	8.476E-06	4.225E-05	1	1	1	O94788	O94788 [395-412]	1	2711.4358
[K].SPFSVA'	1xTMTpro [K	1.667E-05	4.225E-05	1	1	6	P21333	P21333 [959-973]	0	2142.2302
[K].KGSITSV	1xTMTpro [K	1.501E-08	4.225E-05	1	1	2	P06576	P06576 [351-387]	1	4451.3954
[R].SMVAVM	1xOxidation [	4.737E-05	4.225E-05	1	1	3	P04632	P04632 [146-158]	0	1966.0117
[R].SMVAVM	1xTMTpro [K	1.805E-05	4.225E-05	1	1	3	P04632	P04632 [146-158]	0	1950.0168
[R].SMTPAQ	1xTMTpro [K	7.26E-06	4.225E-05	1	1	2	P11171	P11171 [376-391]	0	2373.2616
[K].DGDSVM	1xTMTpro [K	1.731E-05	4.225E-05	1	1	3	P30041	P30041 [183-199]	0	2438.298
[K].SMTEAE	1xOxidation [	8.171E-07	4.225E-05	1	1	4	P12277	P12277 [178-209]	0	4212.1965
[K].DGDSVM	2xTMTpro [K	5.69E-06	4.225E-05	1	1	2	P30041	P30041 [183-200]	1	2870.6001
[K].SMTEAE	1xTMTpro [K	2.386E-09	4.225E-05	1	1	2	P12277	P12277 [178-209]	0	4196.2016
[R].SMQNWI	1xOxidation [	2.668E-05	4.225E-05	1	1	1	Q99439	Q99439 [78-93]	0	2613.3739
[K].DGEAGA	1xTMTpro [N	2.47E-06	4.225E-05	1	1	6	P02452	P02452 [613-631]	0	1994.9851
[K].DGEAVW	1xCarbamido	4.36E-05	4.225E-05	1	1	4	Q13867	Q13867 [318-330]	0	2048.0403
[R].DGEDQT	1xTMTpro [K	5.81E-08	4.225E-05	3	3	3	P01893; P04	P01893 [244-267]	0	3245.6054
[R].DGEEAG	1xTMTpro [N	4.874E-05	4.225E-05	1	1	3	P30101	P30101 [108-119]	0	1540.7199
[R].SMQNWI	1xTMTpro [K	1.006E-05	4.225E-05	1	1	3	Q99439	Q99439 [78-93]	0	2597.379
[R].KGTDIM'	1xCarbamido	2.071E-05	4.225E-05	1	1	1	P05141	P05141 [245-259]	1	2425.25
[K].DGEHVII	1xTMTpro [N	8.245E-06	4.225E-05	1	1	1	P31323	P31323 [177-196]	0	2583.1919
[R].DGELPV	1xTMTpro [K	2.812E-06	4.225E-05	1	1	3	Q15084	Q15084 [416-440]	1	3366.6819
[R].SMMQSI	1xTMTpro [N	2.05E-07	4.225E-05	1	1	1	Q9UMX0	Q9UMX0 [402-435	0	4137.9972
[R].DGENYV	1xTMTpro [N	7.341E-06	4.225E-05	1	3	4	Q5JPE7	Q5JPE7 [1106-11	0	1995.0718
[K].SMMGG(	1xTMTpro [K	9.606E-06	4.225E-05	1	1	1	P51659	P51659 [385-403]	0	2501.3752
[K].DGEQIE(	2xTMTpro [K	3.225E-06	4.225E-05	1	1	1	P13591	P13591 [250-275]	1	3973.9858
[K].SMVPVQ	1xTMTpro [K	5.784E-05	4.225E-05	1	1	1	P18754	P18754 [162-176]	0	2246.3438
[K].DGDDVII	1xTMTpro [K	4.503E-05	4.225E-05	1	1	2	P13667	P13667 [302-313]	0	1899.1083
[K].KGSLLID	2xTMTpro [K	7.274E-06	4.225E-05	1	1	2	P31937	P31937 [125-141]	1	2643.5749
[R].KGSLES	2xTMTpro [K	3.14E-07	4.225E-05	1	1	2	O00232	O00232 [330-350]	2	3136.6942
[K].KGQPSS	1xTMTpro [K	4.525E-05	4.225E-05	1	1	1	Q03169	Q03169 [54-69]	1	2195.1548
[K].SNFAEAI	1xTMTpro [K	3.276E-05	4.225E-05	1	1	2	P07237	P07237 [32-42]	0	1767.0045
[R].SNEYQL	1xCarbamido	3.13E-05	4.225E-05	1	2	1	P63092	P63092 [166-181]	0	2615.3307
[K].SNEILTA	1xTMTpro [N	3.846E-05	4.225E-05	1	1	2	Q14974	Q14974 [170-182]	0	1749.9852

[R].SNELGD	2xCarbamido	1.323E-07	4.225E-05	1	1	3	P13489	P13489 [65-86]	0	3006.5632
[R].SNEFDY	1xTMTpro [K	4.434E-05	4.225E-05	1	1	1	P05107	P05107 [296-310]	0	2300.2167
[K].SNEDVN	1xTMTpro [K	3.172E-06	4.225E-05	1	1	1	Q99614	Q99614 [83-102]	0	2963.4865
[R].DGAAYF	1xTMTpro [K	7.917E-06	4.225E-05	1	1	1	Q86U86	Q86U86 [1186-12	0	3256.6447
[K].GEFGGF	1xTMTpro [K	1.955E-05	4.225E-05	1	1	1	Q09028	Q09028 [103-114]	0	1736.9463
[K].SNEDQS	1xTMTpro [K	1.541E-05	4.225E-05	1	1	4	P02545	P02545 [458-470]	0	2145.089
[R].SMLQDF	1xCarbamido	3.871E-07	4.225E-05	2	2	2	P35749; Q7Z	P35749 [164-184]	1	2890.453
[K].GEFGDV	1xTMTpro [N	5.27E-05	4.225E-05	1	1	1	P41240	P41240 [204-215]	0	1662.8117
[K].KGQTPG	2xTMTpro [K	8.99E-06	4.225E-05	1	1	1	Q13616	Q13616 [74-90]	1	2705.5806
[R].DGALTQ	1xTMTpro [N	2.457E-05	4.225E-05	1	1	2	P16435	P16435 [585-597]	0	1695.9349
[R].SNDPQM	1xOxidation [	7.874E-06	4.225E-05	1	1	1	O14980	O14980 [766-790]	0	3164.6257
[R].SNDPQM	1xTMTpro [N	3.835E-07	4.225E-05	1	1	1	O14980	O14980 [766-790]	0	3148.6308
[K].DGAPVA	1xTMTpro [N	2.705E-06	4.225E-05	1	1	1	Q96S52	Q96S52 [342-355	0	1743.9098
[K].SNCKPS	1xCarbamido	1.792E-05	4.225E-05	1	1	2	Q99460	Q99460 [804-821]	0	2918.6266
[R].SNASLTI	1xTMTpro [K	4.776E-05	4.225E-05	1	1	1	P12270	P12270 [1385-140	0	2353.3331
[K].GEFDPG	1xTMTpro [K	3.774E-05	4.225E-05	1	1	1	Q99798	Q99798 [535-549]	0	2324.1803
[K].SNAHYN	1xTMTpro [K	8.932E-07	4.225E-05	1	1	1	Q01082	Q01082 [228-249]	0	3091.6569
[K].SNAAAY	1xCarbamido	9.465E-06	4.225E-05	1	1	1	O60716	O60716 [384-396]	0	1870.9553
[K].DGAGNS	1xTMTpro [N	1.494E-05	4.225E-05	1	1	1	P11717	P11717 [1373-138	0	1729.8676
[R].SMLEVN	1xTMTpro [N	3.645E-05	4.225E-05	1	1	2	P61160	P61160 [66-80]	0	2056.0527
[K].KGTVAC	1xCarbamido	1.057E-05	4.225E-05	1	1	4	P13611	P13611 [3291-330	1	2567.4796
[K].GEETPV	1xCarbamido	3.406E-08	4.225E-05	1	1	3	P49411	P49411 [213-237]	1	3218.7586
[R].KGVAINI	2xTMTpro [K	5.322E-05	4.225E-05	1	1	1	P60842	P60842 [369-382]	2	2502.453
[R].DGGNPF	1xTMTpro [N	4.05E-06	4.225E-05	1	1	1	O14828	O14828 [6-33]	0	3394.6735
[K].SLYDEV	1xTMTpro [N	1.061E-05	4.225E-05	1	1	2	P07814	P07814 [825-838]	0	1839.9772
[K].SLYDDVI	1xTMTpro [K	5.945E-05	4.225E-05	1	1	1	Q99715	Q99715 [941-951]	0	1849.9675
[R].KGVCEE	1xCarbamido	1.213E-05	4.225E-05	1	1	2	P02760	P02760 [88-100]	1	2370.2791
[R].SLYASSI	1xTMTpro [N	3.702E-05	4.225E-05	1	1	5	P08670	P08670 [51-64]	0	1732.9189
[R].DGGTFIS	1xTMTpro [K	3.288E-05	4.225E-05	1	1	2	Q96ST2	Q96ST2 [548-566	0	2548.346
[R].DGHADA	1xTMTpro [N	3.163E-07	4.225E-05	1	1	1	Q6PJG6	Q6PJG6 [622-644	0	2705.345
[K].SLWNDF	1xCarbamido	2.493E-05	4.225E-05	1	1	1	P50148	P50148 [134-147]	0	2056.9718
[K].KGVNLP	2xTMTpro [K	8.263E-07	4.225E-05	1	1	5	P14618	P14618 [207-224]	1	2677.6069
[R].DGGLQN	1xTMTpro [N	9.431E-06	4.225E-05	1	1	1	P48444	P48444 [289-304]	0	2119.0782

[R].SIWAFG	1xTMTpro [K	8.617E-06	4.225E-05	1	1	4	Q15029	Q15029 [731-756]	0	3365.7761
[K].KGVNLP	3xTMTpro [K	6.967E-07	4.225E-05	1	1	1	P14618	P14618 [207-230]	2	3694.1896
[K].DGHVILC	1xCarbamido	7.22E-06	4.225E-05	1	1	1	P01031	P01031 [1457-147	0	2574.3669
[R].SIVSELA	1xTMTpro [K	5.455E-05	4.225E-05	1	1	1	P40763	P40763 [216-233]	0	2546.4395
[R].SLVQGE	1xTMTpro [K	1.799E-05	4.225E-05	1	1	2	P35611	P35611 [532-543]	0	1840.1035
[R].GEEGHD	1xTMTpro [K	2.312E-05	4.225E-05	1	1	2	P51991	P51991 [22-35]	1	2229.1755
[R].DGKLVS	2xTMTpro [K	4.397E-05	4.225E-05	1	1	1	P05787	P05787 [470-483]	1	2386.401
[K].SIVGMTI	1xTMTpro [K	3.662E-07	4.225E-05	1	1	1	Q9Y2A7	Q9Y2A7 [719-744	0	3475.8784
[K].KGVSYLI	1xTMTpro [K	4.361E-06	4.225E-05	1	1	1	Q15113	Q15113 [394-408]	1	2331.2986
[K].KGVTEC	2xCarbamido	4.642E-05	4.225E-05	1	1	1	Q9UBT2	Q9UBT2 [153-168	1	2902.5484
[R].SIVEEIEI	1xTMTpro [N	5.192E-05	4.225E-05	1	1	4	P04844	P04844 [179-190]	0	1676.939
[R].SIVVSPII	1xTMTpro [N	2.711E-05	4.225E-05	1	1	2	P55290	P55290 [139-152]	0	1869.1129
[R].SNFLVLC	1xCarbamido	3.435E-06	4.225E-05	1	1	1	P48147	P48147 [346-358]	0	2216.2393
[K].SIYGEKF	2xTMTpro [K	1.766E-05	4.225E-05	1	1	4	P62937	P62937 [77-91]	1	2744.5327
[R].SIYVGN\	1xCarbamido	3.917E-06	4.225E-05	1	1	1	Q86U42	Q86U42 [173-200	0	3356.5925
[R].DGFDISC	1xCarbamido	2.122E-06	4.225E-05	1	1	2	P02750	P02750 [292-312]	0	2789.316
[K].DGFFGL	1xCarbamido	1.725E-05	4.225E-05	1	1	2	O15230	O15230 [813-827]	0	2071.9503
[R].SMGGKE	1xOxidation [	3.789E-06	4.225E-05	1	1	2	P02787	P02787 [274-295]	1	3458.8558
[R].SMGGKE	2xTMTpro [K	3.709E-05	4.225E-05	1	1	2	P02787	P02787 [274-295]	1	3442.8609
[K].GEETPV	1xCarbamido	1.899E-06	4.225E-05	1	1	2	P49411	P49411 [213-230]	0	2162.1446
[K].GEETGID	1xTMTpro [K	2.422E-07	4.225E-05	1	1	3	Q09666	Q09666 [446-482]	0	4189.2259
[R].DGFIDKE	2xTMTpro [K	3.172E-06	4.225E-05	2	3	8	P24844; O14	P24844 [46-63]; C	1	2916.5957
[GR].GEESE	1xTMTpro [K	2.779E-05	4.225E-05	1	1	1	P22105	P22105 [2143-215	1	2252.2378
[GR].GEESE	1xTMTpro [N	2.277E-06	4.225E-05	1	1	1	P22105	P22105 [2143-215	0	1819.9357
[K].SMEFAP	2xTMTpro [K	7.128E-06	4.225E-05	2	2	1	Q13948; P39	Q13948 [374-399]	0	3644.0073
[R].SLYGGF	1xTMTpro [K	2.094E-05	4.225E-05	1	1	1	P53602	P53602 [162-179]	0	2610.3154
[K].SMEAEM	1xOxidation [	9.208E-06	4.225E-05	1	1	3	P35579	P35579 [1677-169	0	2369.1648
[R].SMDELN	1xTMTpro [N	9.911E-06	4.225E-05	1	1	1	Q14671	Q14671 [124-140]	0	2250.1144
[R].SMDAET	1xTMTpro [N	5.719E-05	4.225E-05	1	1	2	P55196	P55196 [465-476]	0	1689.8073
[R].DGFSCS	3xCarbamido	2.908E-06	4.225E-05	1	1	2	O95967	O95967 [237-257]	0	2916.2082
[R].SMCGYC	1xCarbamido	2.384E-05	4.225E-05	1	1	2	P15586	P15586 [138-149]	0	2005.0167
[K].DGFVQD	1xTMTpro [K	3.417E-05	4.225E-05	1	1	2	P10643	P10643 [579-593]	0	2235.1611
[R].GEESEV	1xCarbamido	2.789E-05	4.225E-05	1	1	1	P22105	P22105 [3000-30	0	2256.1673

[K].KGTVEG	2xTMTpro [K	7.075E-06	4.225E-05	1	1	3	P37108	P37108 [43-55]	1	2304.3016
[R].KGTVLL/	1xCarbamido	5.617E-06	4.225E-05	1	1	1	P21964	P21964 [212-234]	1	3071.732
[K].GEELLSI	1xTMTpro [N	1.906E-06	4.225E-05	1	1	2	O00159	O00159 [362-378]	0	2178.1726
[R].KGVAINI	2xTMTpro [K	4.713E-05	4.225E-05	1	1	2	Q14240	Q14240 [370-383]	2	2518.4809
[K].SMEAEM	1xTMTpro [N	7.006E-07	4.225E-05	1	1	4	P35579	P35579 [1677-169	0	2353.1699
[K].KGPSTV	2xTMTpro [K	6.09E-05	4.225E-05	1	1	1	O94973	O94973 [619-631]	1	2303.3275
[R].KGPNVV	2xTMTpro [K	5.743E-06	4.225E-05	1	1	1	P03886	P03886 [35-54]	1	3014.7317
[K].SNLAYDI	1xTMTpro [K	9.073E-06	4.225E-05	1	1	3	P34932	P34932 [85-102]	0	2511.4678
[K].KGGSWI	2xTMTpro [K	3.551E-05	4.225E-05	1	1	1	P78527	P78527 [4023-40:	1	2471.4438
[R].DFNHIN\	1xTMTpro [K	6.07E-05	4.225E-05	1	1	2	P32969	P32969 [37-50]	0	2207.268
[R].DFNLDG	1xCarbamido	6.719E-06	4.225E-05	1	1	2	Q9NYU2	Q9NYU2 [1372-1:	0	2399.0933
[K].DFNLLE(	1xCarbamido	5.037E-07	4.225E-05	1	1	1	Q16363	Q16363 [1211-12:	0	3117.537
[K].DFNMPL	1xTMTpro [K	2.726E-05	4.225E-05	1	1	2	Q96HC4	Q96HC4 [23-34]	0	1974.1226
[R].DFNVGD	1xTMTpro [N	5.984E-05	4.225E-05	1	1	1	P06737	P06737 [257-270]	0	1929.0037
[K].KGHQLQ	1xCarbamido	4.849E-05	4.225E-05	1	1	1	Q14515	Q14515 [497-510]	1	2577.4428
[R].DFPAMV	1xTMTpro [N	4.296E-06	4.225E-05	1	1	1	P10253	P10253 [423-436]	0	1888.9659
[K].SPASDT	1xTMTpro [K	1.2E-05	4.225E-05	1	2	4	Q13765	Q13765 [114-127]	0	2093.141
[R].KGKEDE	2xTMTpro [K	1.725E-05	4.225E-05	1	1	1	Q14204	Q14204 [2399-24.	2	3028.6553
[R].DFNDPS	1xTMTpro [K	2.194E-05	4.225E-05	1	1	2	P21399	P21399 [345-364]	0	2930.4915
[K].DFPEYTI	1xTMTpro [K	9.929E-06	4.225E-05	1	1	4	P13667	P13667 [444-463]	0	2917.4275
[K].DFPGAA	1xTMTpro [N	1.79E-06	4.225E-05	1	1	1	O43491	O43491 [566-593]	0	3022.5441
[K].DFPLNDI	1xTMTpro [K	6.657E-06	4.225E-05	1	1	2	Q14204	Q14204 [338-352]	0	2299.2677
[K].KGLEQLI	2xTMTpro [K	2.425E-06	4.225E-05	1	1	3	P12109	P12109 [135-148]	1	2391.4904
[R].GEGGTT	1xTMTpro [K	9.712E-06	4.225E-05	1	1	1	Q96FW1	Q96FW1 [239-25(	0	2462.2807
[R].GEGGILI	1xTMTpro [N	4.127E-05	4.225E-05	1	1	2	P31040	P31040 [313-325]	0	1633.8829
[R].DFPYEE	1xTMTpro [N	5.047E-05	4.225E-05	1	1	1	Q96I25	Q96I25 [191-201]	0	1714.8356
[R].SPAGPA	1xTMTpro [N	3.384E-06	4.225E-05	1	1	1	Q13428	Q13428 [967-985]	0	2054.095
[R].DFQEYV	1xTMTpro [N	5.654E-05	4.225E-05	1	1	1	Q9Y6G9	Q9Y6G9 [193-21(	0	2415.1424
[R].DFQPVL	1xTMTpro [N	7.533E-06	4.225E-05	1	1	3	P39060	P39060 [1576-159	0	2455.3451
[R].DFQPVL	1xOxidation		2.574E-05	4.225E-05	1	1	3	P39060	P39060 [1576-159	0	2471.34
[K].KGLDPY	2xTMTpro [K	7.355E-06	4.225E-05	1	1	2	P10606	P10606 [57-68]	1	2227.3631
[K].SPAGLQ	1xTMTpro [K	2.658E-05	4.225E-05	1	1	3	P24534	P24534 [8-22]	0	2212.2469
[R].DFMYVS	2xTMTpro [K	1.934E-05	4.225E-05	1	1	1	P55084	P55084 [339-361]	1	3539.9276

[K].DFMIQG	1xTMTpro [N	3.253E-05	4.225E-05	1	1	2	Q9Y3C6	Q9Y3C6 [59-72]	0	1755.8655
[R].SPFLQVI	1xTMTpro [N	4.689E-05	4.225E-05	1	1	2	Q15437	Q15437 [589-606]	0	2454.1897
[R].SPEYLSI	1xTMTpro [K	4.092E-05	4.225E-05	1	1	2	Q13618	Q13618 [384-395]	0	2035.1243
[K].KGFIGP(	1xTMTpro [K	4.171E-06	4.225E-05	1	1	2	P00367	P00367 [212-231]	1	2652.4311
[K].KGFNPP	1xTMTpro [K	5.348E-05	4.225E-05	1	1	1	Q9Y2R4	Q9Y2R4 [409-424	1	2438.4415
[R].DFIIQTG	1xTMTpro [N	5.031E-05	4.225E-05	1	1	1	Q8WUA2	Q8WUA2 [48-61]	0	1781.9353
[K].SPEQQE	1xTMTpro [N	1.09E-05	4.225E-05	1	1	3	Q14624	Q14624 [225-240]	0	2116.1569
[K].SPEPEVI	1xTMTpro [K	3.417E-05	4.225E-05	1	1	1	Q12888	Q12888 [294-312]	0	2771.4231
[K].DFINFISI	2xTMTpro [K	3.152E-05	4.225E-05	1	1	1	Q9H3N1	Q9H3N1 [122-133	1	2454.3849
[K].DFLNQE	1xTMTpro [N	1.741E-05	4.225E-05	1	1	1	P55268	P55268 [1521-153	0	2412.1672
[R].KGFSEG	2xTMTpro [K	2.354E-06	4.225E-05	1	1	1	P51858	P51858 [80-96]	1	2860.6025
[K].GEGPEF	1xTMTpro [K	3.594E-05	4.225E-05	1	1	2	Q09666	Q09666 [1013-10	0	1900.0308
[K].SPELAA(	1xTMTpro [K	2.219E-06	4.225E-05	1	1	2	Q8NBN7	Q8NBN7 [263-28	0	3054.6491
[R].KGGEAD	2xTMTpro [K	3.893E-05	4.225E-05	1	1	2	O60216	O60216 [406-418]	1	2348.3278
[K].SPEEGA	1xTMTpro [K	3.369E-08	4.225E-05	1	1	3	P16152	P16152 [243-272]	0	3772.9565
[R].KGGETS	2xTMTpro [K	3.213E-06	4.225E-05	1	1	5	P02675	P02675 [247-267]	1	3297.7969
[R].SPEACC	2xCarbamid	2.511E-05	4.225E-05	1	1	2	P06132	P06132 [61-74]	0	1978.0057
[R].DFLTLH(	1xTMTpro [K	1.691E-05	4.225E-05	1	1	2	P51178	P51178 [6-24]	0	2792.5326
[R].SPDVISS	1xTMTpro [N	4.005E-06	4.225E-05	1	1	1	Q6P2E9	Q6P2E9 [729-754	0	2948.5383
[R].SPDVGL	1xCarbamid	3.522E-07	4.225E-05	1	1	2	P46940	P46940 [648-672]	0	3315.6699
[K].SPDTNY	1xTMTpro [N	1.754E-05	4.225E-05	2	2	2	P62714; P67	P62714 [75-89]; F	0	2096.9918
[R].KGGETS	1xOxidation	3E-05	4.225E-05	1	1	2	P02675	P02675 [247-267]	1	3313.7918
[K].SPDLLM	1xOxidation	2.584E-05	4.225E-05	1	1	1	P82909	P82909 [61-78]	0	2612.4137
[K].KGGAPA	2xTMTpro [K	5.86E-05	4.225E-05	1	1	1	P29536	P29536 [527-551]	1	3383.0105
[R].KGFFIEF	1xOxidation	1.587E-05	4.225E-05	1	1	1	O94788	O94788 [395-412]	1	2727.4308
[R].SPAAEC	1xCarbamid	9.327E-06	4.225E-05	1	1	2	Q12931	Q12931 [568-587]	1	2976.4761
[R].KGIQAEI	2xTMTpro [K	5.764E-06	4.225E-05	1	2	2	P32455	P32455 [453-466]	1	2546.5374
[K].DFTEGTI	1xTMTpro [K	4.442E-05	4.225E-05	1	1	2	Q93034	Q93034 [659-676]	0	2683.4475
[R].SNMDNI	1xOxidation	4.009E-05	4.225E-05	1	1	6	P05787	P05787 [134-148]	0	2168.0072
[R].SNMDNI	1xTMTpro [N	9.396E-06	4.225E-05	1	1	4	P05787	P05787 [134-148]	0	2152.0122
[K].KGNTALI	1xTMTpro [K	5.898E-05	4.225E-05	1	1	1	P16157	P16157 [77-95]	1	2571.4862
[R].SNIWVA	1xCarbamid	5.883E-06	4.225E-05	1	1	1	O95831	O95831 [431-446]	0	2438.267
[R].KGPAPE	2xTMTpro [K	6.664E-07	4.225E-05	1	1	2	Q13435	Q13435 [815-843]	1	4004.1904

[R].DFTSLEI	1xTMTpro [N	3.981E-05	4.225E-05	1	1	2	Q07065	Q07065 [229-240]	0	1743.872
[R].SNLVGM	1xOxidation	1.54E-06	4.225E-05	1	1	2	P21399	P21399 [806-834]	0	3333.732
[R].SNLVGM	1xTMTpro [N	7.311E-07	4.225E-05	1	1	2	P21399	P21399 [806-834]	0	3317.7371
[R].DFTVSAI	1xTMTpro [K	7.274E-06	4.225E-05	1	1	2	P60842	P60842 [296-309]	0	2190.0815
[R].SNMDNN	1xTMTpro [N	4.575E-06	4.225E-05	1	1	1	P05787	P05787 [134-149]	1	2308.1134
[R].DFVAEP	2xTMTpro [K	9.167E-08	4.225E-05	1	1	3	O75531	O75531 [9-32]	0	3312.8694
[R].DFVDHID	1xTMTpro [K	2.218E-05	4.225E-05	1	1	1	P49407	P49407 [26-49]	0	3319.7957
[K].SNLIQQV	1xTMTpro [K	7.947E-06	4.225E-05	1	1	1	Q6YHK3	Q6YHK3 [173-191	0	2752.5489
[K].KGPGEV	2xTMTpro [K	5.509E-05	4.225E-05	1	1	1	Q86U44	Q86U44 [164-177	1	2241.3383
[R].DFVMNL	1xTMTpro [N	2.655E-06	4.225E-05	1	1	1	P12111	P12111 [660-677]	0	2353.2141
[R].DFVMNL	1xOxidation	4.286E-05	4.225E-05	1	1	2	P12111	P12111 [660-677]	0	2369.209
[R].DFVNCS	1xCarbamido	4.163E-05	4.225E-05	1	1	1	P05164	P05164 [726-742]	0	2268.1766
[R].SNLDPS	1xCarbamido	6.052E-07	4.225E-05	1	1	2	P04844	P04844 [77-109]	0	4125.0578
[R].SNLDED	1xTMTpro [N	5.69E-06	4.225E-05	1	1	3	P01024	P01024 [749-764]	0	2121.0995
[K].DFVSEQ	2xTMTpro [K	6.362E-07	4.225E-05	1	1	1	P54136	P54136 [169-193]	1	3612.0829
[R].SNLCAL	2xCarbamido	4.016E-05	4.225E-05	1	1	2	P02788	P02788 [520-535]	0	2416.2092
[R].GEFTYY	1xTMTpro [K	2.214E-05	4.225E-05	1	1	3	Q16666	Q16666 [700-713]	0	2273.1582
[K].KGIIRPG	2xTMTpro [K	1.193E-05	4.225E-05	1	1	2	Q15149	Q15149 [4092-41	1	3784.2193
[K].DFTATDI	1xTMTpro [K	1.581E-05	4.225E-05	1	1	3	P42765	P42765 [26-38]	0	2024.0832
[K].SNNSET	1xTMTpro [K	3.163E-05	4.225E-05	1	5	1	P49792	P49792 [2555-25	0	2307.1556
[R].SNVDMD	1xTMTpro [K	4.567E-06	4.225E-05	1	1	3	P00488	P00488 [517-532]	0	2375.2408
[K].DFSALES	1xTMTpro [N	2.628E-07	4.225E-05	1	1	5	P35579	P35579 [1302-13	0	2797.3811
[K].SNTSPEI	1xTMTpro [N	1.899E-05	4.225E-05	1	1	1	Q9Y490	Q9Y490 [1875-18	0	2553.2752
[R].DFSAPTI	1xTMTpro [K	6.933E-06	4.225E-05	1	1	2	P55081	P55081 [359-371]	0	2129.1159
[R].SNTPILV	2xTMTpro [K	1.329E-05	4.225E-05	1	1	2	P06744	P06744 [107-124]	1	2868.6321
[R].GEGEPC	1xCarbamido	5.926E-05	4.225E-05	1	1	3	Q16270	Q16270 [66-78]	0	1464.6821
[R].SNTGGQ	1xCarbamido	3.772E-07	4.225E-05	1	1	1	P13639	P13639 [802-839]	0	4549.1914
[R].GEGEGG	1xTMTpro [K	4.882E-05	4.225E-05	1	1	2	P48681	P48681 [495-507]	0	2010.1152
[R].KGISLNF	2xTMTpro [K	1.319E-05	4.225E-05	1	1	2	P53999	P53999 [101-114]	1	2540.5017
[K].DFSHPC	1xCarbamido	2.027E-05	4.225E-05	1	1	1	Q9BSH5	Q9BSH5 [104-12	0	2551.2207
[K].DFSWSF	1xTMTpro [K	4.549E-05	4.225E-05	1	1	1	P55884	P55884 [469-492]	1	3325.7501
[R].KGIVLLE	2xTMTpro [K	2.471E-05	4.225E-05	1	1	1	Q9Y3D6	Q9Y3D6 [53-64]	1	2264.4774
[R].SNQQLE	1xTMTpro [K	3.826E-05	4.225E-05	1	1	3	P46940	P46940 [902-916]	0	2383.2783

[K].SNPSEV	1xCarbamido	2.121E-05	4.225E-05	1	1	1	Q9Y2H6	Q9Y2H6 [549-573	0	3929.2199
[K].SNPSEN	2xTMTpro [K	1.731E-05	4.225E-05	1	1	1	Q13217	Q13217 [134-150]	1	2843.5567
[R].SNPENN	1xCarbamido	3.07E-08	4.225E-05	1	1	2	P55036	P55036 [43-70]	0	3318.6807
[R].SNPEDQ	1xTMTpro [N	3.645E-05	4.225E-05	1	1	4	Q14165	Q14165 [91-103]	0	1896.9622
[K].SNPAIQ/	1xCarbamido	7.355E-06	4.225E-05	1	1	1	Q9Y619	Q9Y619 [3-34]	0	3755.9592
[K].DFSSVE	1xCarbamido	1.16E-06	4.225E-05	1	1	3	Q05707	Q05707 [1242-12	0	3483.7209
[K].DFSSVE	1xCarbamido	3.403E-06	4.225E-05	1	1	1	Q05707	Q05707 [1242-12	0	3499.7158
[K].GEGAIG	1xTMTpro [N	5.618E-05	4.225E-05	1	1	1	Q9Y385	Q9Y385 [123-137	0	1897.9463
[K].KGNKEP	3xTMTpro [K	7.947E-06	4.225E-05	1	1	2	Q9BSJ8	Q9BSJ8 [494-515	2	3687.0892
[R].SNQQLE	1xOxidation	5.898E-05	4.225E-05	1	1	2	P46940	P46940 [902-916]	0	2399.2732
[R].TGEGFL	1xCarbamido	5.322E-05	4.225E-05	1	2	1	P01112	P01112 [74-88]	0	2279.235
[K].LGHVVM	1xTMTpro [K	1.572E-06	4.225E-05	1	1	1	P60228	P60228 [388-407]	0	2791.542
[K].HSQAVE	1xTMTpro [K	4.07E-08	4.225E-05	1	1	3	P35579	P35579 [1194-12	1	2604.4237
[R].GGGPGI	1xTMTpro [K	5.115E-05	4.225E-05	1	1	1	P10301	P10301 [16-31]	0	2095.1064
[R].GGSSEI/	1xTMTpro [N	4.61E-05	4.225E-05	1	1	1	Q6NUM9	Q6NUM9 [294-30	0	2016.1197
[R].HNIAYFI	1xTMTpro [N	3.059E-05	4.225E-05	1	1	2	Q96AB3	Q96AB3 [29-43]	0	1990.1194
[R].ASPNSD	1xTMTpro [K	2.358E-05	4.225E-05	1	1	1	Q9UH62	Q9UH62 [109-125	0	2437.3066
[K].AVEHMF	1xTMTpro [K	2.711E-05	4.225E-05	1	1	1	Q9Y657	Q9Y657 [136-152	1	2674.3063
[K].TVSEWI	1xTMTpro [N	1.009E-05	4.225E-05	1	1	1	Q7Z6Z7	Q7Z6Z7 [1509-15	0	2608.3724
[R].TVSGNT	1xTMTpro [N	5.485E-08	4.225E-05	1	1	3	P24821	P24821 [1844-18	0	2847.4583
[R].HTLEQH	1xTMTpro [N	1.22E-05	4.225E-05	1	1	1	Q96CS3	Q96CS3 [34-50]	0	2365.1968
[R].TPDGTE	1xTMTpro [N	4.022E-05	4.225E-05	1	1	1	P19367	P19367 [519-539]	0	2514.2432
[R].TPCTVS	3xCarbamido	5.93E-08	4.225E-05	1	1	2	P02675	P02675 [225-246]	1	3156.6347
[R].TPCTVS	2xCarbamido	1.855E-05	4.225E-05	1	1	5	P02675	P02675 [225-239]	0	2227.207
[K].AVEILAQ	1xTMTpro [K	1.575E-05	4.225E-05	1	1	1	P22307	P22307 [251-270]	0	2876.4917
[K].AVELLGI	1xCarbamido	1.721E-06	4.225E-05	1	1	6	P31930	P31930 [143-163]	0	2968.5793
[K].AVELLGI	1xCarbamido	1.226E-06	4.225E-05	1	1	2	P31930	P31930 [143-165]	1	3253.723
[K].ASPESQ	1xTMTpro [N	1.061E-05	4.225E-05	1	1	1	Q5SRE5	Q5SRE5 [1728-17	0	2316.2883
[K].TVSGVN	1xTMTpro [K	2.644E-05	4.225E-05	1	1	1	P21281	P21281 [49-64]	0	2256.3571
[R].ASPFLL(	1xTMTpro [N	2.547E-05	4.225E-05	1	1	1	Q14558	Q14558 [153-169]	0	2385.2774
[R].TVSKVD	2xTMTpro [K	3.741E-05	4.225E-05	1	1	1	P52209	P52209 [35-48]	1	2449.4119
[R].HTLDGA	1xCarbamido	1.334E-05	4.225E-05	1	1	1	Q9Y3A3	Q9Y3A3 [127-140	0	2122.157
[K].AVEGCV	1xCarbamido	3.157E-07	4.225E-05	1	1	2	Q9Y490	Q9Y490 [746-765	0	2366.1941

[R].TPGAAA	1xTMTpro [N	6.941E-07	4.225E-05	1	1	2	P04217	P04217 [448-469]	0	2600.3904
[K].HTGCCG	3xCarbamido	6.566E-07	4.225E-05	1	1	3	Q15181	Q15181 [110-128]	0	2742.2777
[K].HTGPGIL	1xCarbamido	1.321E-06	4.225E-05	1	1	1	P62937	P62937 [92-118]	0	3399.7433
[K].TVQTAA.	1xTMTpro [K	5.992E-06	4.225E-05	1	1	1	O15126	O15126 [312-334]	0	2760.4772
[K].HTGPGIL	1xCarbamido	4.065E-06	4.225E-05	1	1	1	P62937	P62937 [92-118]	0	3415.7383
[R].TPEVTC'	1xCarbamido	1.263E-06	4.225E-05	1	1	27	P01859	P01859 [135-167]	0	4406.1895
[R].TPEVTC'	1xCarbamido	1.061E-06	4.225E-05	1	1	5	P01860	P01860 [186-206]	0	3022.5687
[K].AVEGLLI	1xTMTpro [N	5.807E-06	4.225E-05	1	1	1	A5YKK6	A5YKK6 [1665-16	0	2204.2093
[R].TPEVTC'	1xCarbamido	1.764E-07	4.225E-05	1	2	22	P0DOX5	P0DOX5 [258-276	0	2747.4417
[K].HTGPNS	1xTMTpro [N	1.709E-05	4.225E-05	2	2	4	P31943; P55	P31943 [99-114];	0	1988.9745
[K].ASPPFPI	2xTMTpro [K	1.253E-05	4.225E-05	1	1	1	Q8N3R9	Q8N3R9 [176-203	0	3977.2793
[R].TPEQVA	1xTMTpro [N	1.384E-05	4.225E-05	1	1	1	Q9NPF5	Q9NPF5 [237-252	0	2251.1777
[K].HTHIDKF	1xCarbamido	1.923E-05	4.225E-05	1	1	1	Q99805	Q99805 [242-260]	0	3061.6015
[K].TPELNLI	1xTMTpro [K	7.575E-06	4.225E-05	1	1	7	P27824	P27824 [171-182]	0	2065.121
[R].AVEFQE	1xTMTpro [K	4.005E-06	4.225E-05	1	1	2	P51148	P51148 [143-166]	0	3286.6433
[R].HTLADN	1xTMTpro [N	2.829E-05	4.225E-05	1	1	1	P20645	P20645 [148-161]	0	1932.9735
[K].AVEEED	2xTMTpro [K	4.036E-05	4.225E-05	1	1	1	O00487	O00487 [258-273]	1	2743.5368
[K].AVEIYAS	1xTMTpro [K	1.142E-05	4.225E-05	1	1	1	P11047	P11047 [1264-129	0	3526.8772
[K].AVENPT.	1xCarbamido	2.535E-06	4.225E-05	1	1	2	P09132	P09132 [40-64]	0	3312.7641
[K].AVENSS'	1xTMTpro [N	4.029E-05	4.225E-05	1	1	2	P25788	P25788 [30-41]	0	1521.8556
[R].GGSSSG	1xTMTpro [K	1.202E-06	4.225E-05	1	1	1	P35908	P35908 [604-623]	0	2197.0977
[K].ASNTAE'	1xTMTpro [N	3.334E-05	4.225E-05	1	1	6	P49748	P49748 [304-316]	0	1716.8876
[K].ASNPFLI	1xTMTpro [N	2.242E-05	4.225E-05	1	1	1	Q9Y6I3	Q9Y6I3 [500-534]	0	3790.0247
[R].TNVLYEI	1xTMTpro [N	1.857E-06	4.225E-05	1	1	2	P16435	P16435 [383-403]	0	2757.4266
[R].TVSYNG	1xCarbamido	1.22E-05	4.225E-05	1	1	2	P21980	P21980 [513-527]	0	2204.1877
[K].TNTPAD'	1xTMTpro [N	8.655E-07	4.225E-05	1	1	1	P10155	P10155 [457-484]	0	3277.7176
[R].TNTNVN	2xCarbamido	4.703E-07	4.225E-05	1	1	3	P02751	P02751 [2452-247	2	3414.5684
[K].TPADCP'	1xCarbamido	4.689E-05	4.225E-05	1	1	2	Q9BS26	Q9BS26 [314-327	0	1865.9751
[R].AVFQAN	1xTMTpro [K	4.382E-05	4.225E-05	1	1	3	P05023	P05023 [431-444]	0	2193.2887
[R].TNTNVN	2xCarbamido	8.154E-06	4.225E-05	1	1	2	P02751	P02751 [2452-247	0	2798.3231
[R].GGGGP(	1xTMTpro [N	1.65E-07	4.225E-05	1	1	2	Q92945	Q92945 [41-70]	0	2574.2728
[R].ASNLEN.	1xTMTpro [K	4.533E-05	4.225E-05	1	1	1	P53621	P53621 [383-398]	0	2437.3106
[R].GGGGNI	1xTMTpro [N	1.92E-05	4.225E-05	1	1	5	P22626	P22626 [214-228]	0	1681.8366

[R].AVFVDLI	1xTMTpro [N	1.169E-05	4.225E-05	2	3	6	Q71U36; Q9	Q71U36 [65-79]; (	0	2006.1129
[K].TNQNTG	1xTMTpro [K	5.039E-05	4.225E-05	1	1	1	O95786	O95786 [667-679]	0	2012.109
[K].TNQLNL(	1xTMTpro [K	4.518E-05	4.225E-05	1	1	2	Q05707	Q05707 [72-83]	0	1954.1213
[K].HNGPND	1xTMTpro [N	3.709E-05	4.225E-05	1	1	2	P31942	P31942 [7-19]	0	1643.8057
[K].GGGGYT	1xCarbamido	3.27E-05	4.225E-05	1	1	1	P15924	P15924 [168-185]	0	2502.1851
[R].HTSVQT	1xTMTpro [N	2.308E-05	4.225E-05	1	1	1	P02751	P02751 [273-290]	0	2168.0903
[R].AVFDNLI	1xTMTpro [K	3.839E-05	4.225E-05	1	1	1	Q9UHY1	Q9UHY1 [116-131	0	2474.4627
[R].AVENTV	1xTMTpro [N	1.09E-05	4.225E-05	1	1	1	Q5JTZ9	Q5JTZ9 [679-711]	0	3830.9805
[R].TPCNAG	1xCarbamido	5.636E-05	4.225E-05	1	1	2	O43684	O43684 [127-139]	0	2045.0617
[R].TVSLGA(	2xTMTpro [K	3.27E-07	4.225E-05	1	1	5	P06748	P06748 [46-73]	1	3842.0826
[R].TVSLGA(	1xOxidation [	2.584E-07	4.225E-05	1	1	2	P06748	P06748 [46-73]	1	3858.0775
[R].TPAVEG	1xTMTpro [N	3.696E-05	4.225E-05	1	1	1	O43399	O43399 [35-50]	0	2077.062
[R].GGGNQ\	1xTMTpro [K	2.63E-05	4.225E-05	1	1	1	Q14839	Q14839 [1001-10	0	2252.2928
[K].TVSLLDE	1xTMTpro [K	5.747E-05	4.225E-05	1	1	1	P78527	P78527 [3303-331	0	2377.3106
[K].ASPDGY	1xCarbamido	2.443E-06	4.225E-05	1	1	3	Q14914	Q14914 [206-231]	0	3479.6379
[R].TPALVFE	1xTMTpro [K	2.942E-05	4.225E-05	1	2	2	P68400	P68400 [108-122]	0	2340.2844
[R].ASPDGL	1xCarbamido	2.809E-05	4.225E-05	1	1	2	O15230	O15230 [3166-31	0	1919.9928
[K].AVEYFQ	1xTMTpro [K	5.636E-05	4.225E-05	1	1	1	Q9UPT5	Q9UPT5 [122-138	0	2561.3015
[K].TPALIVY	1xTMTpro [K	3.213E-06	4.225E-05	1	1	2	Q96IU4	Q96IU4 [152-173]	0	3055.6054
[R].ASPATQ	1xTMTpro [K	2.635E-05	4.225E-05	1	1	3	Q07666	Q07666 [57-96]	0	4335.4096
[K].TVSQIISI	1xTMTpro [K	4.882E-05	4.225E-05	1	1	2	O76021	O76021 [125-136]	0	1939.2083
[R].AVEYLLN	1xTMTpro [N	4.689E-05	4.225E-05	1	1	3	P54727	P54727 [221-233]	0	1737.9529
[K].TPAILEA	1xCarbamido	2.891E-06	4.225E-05	1	1	1	O60716	O60716 [677-695]	0	2217.1981
[R].HTPLVEI	1xTMTpro [K	6.92E-06	4.225E-05	1	1	1	Q96RQ3	Q96RQ3 [708-722	1	2453.2804
[K].TPGDFS	1xTMTpro [K	5.354E-06	4.225E-05	1	1	1	P46821	P46821 [1864-188	0	2598.3332
[R].TPGTGS	1xTMTpro [N	4.135E-05	4.225E-05	1	1	1	Q96GD0	Q96GD0 [190-206	0	1849.9939
[K].TPHCFL	1xCarbamido	1.137E-07	4.225E-05	1	1	3	Q7L266	Q7L266 [112-138]	0	3451.7634
[R].HNIMDF	1xTMTpro [K	1.427E-05	4.225E-05	1	1	1	Q00610	Q00610 [1589-16	0	2593.3625
[R].TVLVNAI	1xTMTpro [N	5.861E-06	4.225E-05	1	1	3	Q03252	Q03252 [562-575]	0	1807.9543
[R].AVCMLS	1xCarbamido	6.161E-06	4.225E-05	1	1	2	Q9BQE3	Q9BQE3 [374-39	0	2155.0959
[R].GGHFAA	1xTMTpro [N	1.587E-06	4.225E-05	1	1	3	P07099	P07099 [429-446]	0	2304.1944
[K].AVCVEA	1xCarbamido	2.88E-05	4.225E-05	1	1	2	P17980	P17980 [398-409]	0	1593.8776
[K].HNQLPL	1xTMTpro [K	7.73E-06	4.225E-05	1	1	2	P07237	P07237 [231-247]	0	2573.4583

[K].GGSGTA	1xTMTpro [N	4.549E-05	4.225E-05	1	1	1	P98172	P98172 [290-307]	0	2045.1198
[K].TPQQLW	1xTMTpro [K	4.898E-05	4.225E-05	1	1	2	P17480	P17480 [201-211]	0	2039.1206
[R].AVCMLS	1xCarbamido	2.206E-05	4.225E-05	2	6	7	Q71U36; P68	Q71U36 [374-390	0	2185.1065
[K].TPQMGD	1xTMTpro [N	1.868E-06	4.225E-05	1	1	1	Q15642	Q15642 [434-456]	0	2801.431
[K].TPQICVV	1xCarbamido	8.794E-06	4.225E-05	1	1	2	P22570	P22570 [38-59]	0	2952.6261
[K].AVDDGY	1xTMTpro [N	2.397E-05	4.225E-05	1	1	1	P51687	P51687 [510-529]	0	2547.3163
[R].HTCILITF	3xCarbamido	4.908E-07	4.225E-05	1	1	1	Q14112	Q14112 [840-866]	0	3235.5366
[K].TVMPYIS	1xTMTpro [K	6.09E-05	4.225E-05	1	1	1	P05556	P05556 [191-202]	0	1917.1011
[R].TPPPELI	1xTMTpro [K	4.467E-06	4.225E-05	1	1	2	P42858	P42858 [386-408]	0	2884.7003
[R].ASQSVS	1xTMTpro [K	1.042E-07	4.225E-05	1	1	3	P01619	P01619 [45-66]	0	3047.6194
[R].ASQSVS	1xTMTpro [K	1.504E-06	4.225E-05	1	2	1	P01624	P01624 [45-65]	0	2911.567
[K].ASQTQC	1xTMTpro [K	6.048E-06	4.225E-05	1	1	2	Q8N2G8	Q8N2G8 [102-140	0	4629.4139
[R].AVCMLS	1xCarbamido	3.881E-06	4.225E-05	2	6	15	Q71U36; P68	Q71U36 [374-390	0	2169.1116
[R].TPSALAI	1xTMTpro [N	2.88E-05	4.225E-05	1	1	1	P09972	P09972 [158-173]	0	1957.1401
[R].AVCEAD	1xCarbamido	2.117E-05	4.225E-05	1	1	1	O14545	O14545 [265-277]	0	1687.8141
[K].HSTIFEN	1xTMTpro [K	5.014E-05	4.225E-05	1	1	12	P02787	P02787 [226-236]	0	1882.0678
[K].HNYECL	1xCarbamido	2.989E-05	4.225E-05	1	1	1	P13010	P13010 [414-431]	0	2630.3066
[R].AVASLN	1xTMTpro [K	3.866E-06	4.225E-05	1	1	2	P34913	P34913 [354-376]	0	3019.6782
[K].TPVGSC	1xCarbamido	2.049E-05	4.225E-05	1	1	1	P08514	P08514 [156-170]	0	1909.9761
[K].TPVGFIG	1xTMTpro [K	1.667E-05	4.225E-05	1	1	3	P31937	P31937 [40-56]	0	2312.2751
[R].AVASQL	1xCarbamido	2.471E-05	4.225E-05	1	1	3	P62333	P62333 [186-197]	0	1974.0974
[R].AVASQP	1xTMTpro [N	4.642E-05	4.225E-05	1	1	2	Q9NVE7	Q9NVE7 [463-476	0	1719.8833
[K].TPVEEVI	1xTMTpro [N	3.961E-05	4.225E-05	1	1	3	Q15393	Q15393 [943-958]	0	1986.0979
[K].TVLSPGI	1xCarbamido	1.934E-05	4.225E-05	1	1	1	O95985	O95985 [446-472]	0	3392.719
[K].ASSEGG	1xTMTpro [K	2.502E-05	4.225E-05	1	1	1	O15143	O15143 [309-326]	0	2237.2017
[R].TPTQEQ	1xOxidation [	3.696E-05	4.225E-05	1	1	2	Q8IUX7	Q8IUX7 [778-793]	0	1963.0602
[R].TPTQEQ	1xTMTpro [N	3.053E-05	4.225E-05	1	1	2	Q8IUX7	Q8IUX7 [778-793]	0	1947.0653
[R].TVITPDF	1xTMTpro [N	2.098E-05	4.225E-05	1	1	1	P24928	P24928 [365-382]	0	2225.2461
[R].AVAVVV	1xTMTpro [K	2.677E-05	4.225E-05	1	1	2	O00487	O00487 [140-152]	0	1933.1978
[R].TPSSYG	1xTMTpro [N	4.391E-07	4.225E-05	1	1	2	P17661	P17661 [79-105]	0	3206.6177
[K].TPSFGIS	1xTMTpro [K	2.312E-05	4.225E-05	1	1	3	Q09666	Q09666 [5184-520	0	2791.585
[K].TVIVHGF	1xTMTpro [K	5.09E-05	4.225E-05	1	1	2	Q9NSE4	Q9NSE4 [650-661	0	1909.1403
[R].TPPLLEN	1xCarbamido	5.85E-05	4.225E-05	1	1	1	Q8NEW0	Q8NEW0 [297-31	0	2120.1129

[K].TNPFPLI	1xTMTpro [K	2.865E-05	4.225E-05	2	2	1	Q9Y4E1; Q6	Q9Y4E1 [1175-11	0	2745.4115
[R].HTDDEM	1xTMTpro [N	3.557E-05	4.225E-05	1	1	1	Q16539	Q16539 [174-186]	0	1799.8553
[R].TVMVNIE	1xOxidation	5.581E-05	4.225E-05	1	1	4	P01024	P01024 [162-176]	0	2264.2816
[R].TPLQGIL	2xTMTpro [K	3.34E-05	4.225E-05	1	1	2	O75146	O75146 [755-770]	0	2675.664
[R].ASQGISI	2xTMTpro [K	1.572E-05	4.225E-05	1	1	2	A0A075B6S5	A0A075B6S5 [47-	0	2951.6559
[R].HTDVQF	1xTMTpro [K	1.107E-06	4.225E-05	1	1	2	Q9Y5B9	Q9Y5B9 [735-753	0	2761.454
[R].TVPPAV	1xTMTpro [K	5.103E-07	4.225E-05	1	1	32	P04075	P04075 [260-289]	0	3665.9882
[K].HTEAPL	2xTMTpro [K	4.393E-06	4.225E-05	1	1	1	Q13308	Q13308 [674-700]	0	3832.0625
[K].TPLIDLC	1xCarbamido	3.526E-05	4.225E-05	1	1	1	Q9NZB2	Q9NZB2 [819-834	0	2324.2412
[R].TPLLDEE	2xTMTpro [K	2.438E-06	4.225E-05	1	1	2	P23634	P23634 [1181-119	1	2856.5295
[R].ASQGISS	2xTMTpro [K	5.719E-05	4.225E-05	1	2	1	A0A0C4DH6	A0A0C4DH69 [47	0	2924.645
[K].TVPVPD	1xTMTpro [K	1.336E-05	4.225E-05	1	1	2	Q9BW83	Q9BW83 [48-68]	0	2802.5057
[K].AVDVFFI	1xTMTpro [K	1.937E-05	4.225E-05	1	1	5	Q00610	Q00610 [247-269]	0	3187.6629
[R].TVQIEAS	1xTMTpro [N	3.157E-05	4.225E-05	1	1	2	Q15019	Q15019 [78-91]	0	1908.0245
[R].GGGTGC	1xTMTpro [N	3.981E-05	4.225E-05	1	1	1	P48634	P48634 [1067-108	0	1575.8311
[K].ASQAGQ	1xTMTpro [N	2.791E-06	4.225E-05	2	2	1	Q14678; Q6	Q14678 [1230-124	0	2002.0823
[R].TPLAVEI	1xTMTpro [N	1.099E-06	4.225E-05	1	2	1	Q86TX2	Q86TX2 [100-118	0	2334.2261
[K].TVQLTSS	1xTMTpro [K	2.405E-05	4.225E-05	1	1	2	P42704	P42704 [656-672]	0	2487.4049
[R].TVQSLEI	1xTMTpro [N	3.242E-05	4.225E-05	1	1	2	P05783	P05783 [302-314]	0	1810.954
[K].TPLGDT	1xCarbamido	4.412E-05	4.225E-05	1	1	6	P01860	P01860 [102-113]	0	1659.8444
[R].TPLSEAI	1xTMTpro [N	1.616E-05	4.225E-05	1	1	2	Q16630	Q16630 [407-420]	0	1969.986
[R].AVDQSV	2xTMTpro [K	7.992E-07	4.225E-05	1	1	3	P01023	P01023 [599-625]	0	3830.1441
[K].TVPEELV	2xTMTpro [K	3.375E-05	4.225E-05	1	1	6	Q9UMS4	Q9UMS4 [193-20	0	2510.4898
[K].TPPGLQ	1xTMTpro [N	1.137E-05	4.225E-05	1	1	1	Q9BVL2	Q9BVL2 [331-347	0	2237.131
[R].TPPEAIA	1xCarbamido	5.446E-05	4.225E-05	1	1	1	P49840	P49840 [372-391]	0	2496.2975
[K].GGSISG	1xTMTpro [K	2.65E-06	4.225E-05	1	1	2	P35908	P35908 [581-595]	0	1805.9637
[K].TVNELQI	1xTMTpro [N	9.606E-06	4.225E-05	1	1	1	Q9Y6M1	Q9Y6M1 [531-547	0	2187.2304
[R].TPMGIVI	1xOxidation	2.67E-06	4.225E-05	1	1	4	O75915	O75915 [160-178]	0	2433.2615
[R].TPMGIVI	1xTMTpro [N	1.913E-06	4.225E-05	1	1	4	O75915	O75915 [160-178]	0	2417.2665
[K].AVDIPHN	1xTMTpro [K	1.417E-05	4.225E-05	1	1	3	P62906	P62906 [79-91]	0	2060.1706
[R].HTDNVIC	1xTMTpro [K	2.376E-06	4.225E-05	1	1	3	P46940	P46940 [112-130]	0	2802.5104
[K].HNIQFSS	1xTMTpro [N	3.886E-05	4.225E-05	1	1	2	O76003	O76003 [172-188]	0	2374.1635
[K].AVDIPHN	2xTMTpro [K	3.023E-06	4.225E-05	1	1	3	P62906	P62906 [79-92]	1	2492.4727

[R].TVNTFS(	1xTMTpro [N	1.407E-05	4.225E-05	1	1	1	Q8WY22	Q8WY22 [49-67]	0	2391.2111
[K].TPMENI(	1xTMTpro [N	5.869E-05	4.225E-05	1	1	1	P25205	P25205 [464-478]	0	1978.0598
[R].AVDNQV	1xTMTpro [N	1.799E-05	4.225E-05	1	1	2	Q9NQR4	Q9NQR4 [196-21(	0	1866.004
[K].AVDPTS(	1xTMTpro [N	4.744E-05	4.225E-05	1	1	1	Q8TEM1	Q8TEM1 [1259-12	0	1751.9611
[R].HTDTTYI	2xCarbamido	2.654E-05	4.225E-05	1	1	1	Q9NVH6	Q9NVH6 [242-261	0	3089.5469
[R].ASQKPQ	2xTMTpro [K	5.714E-07	4.225E-05	1	1	2	O60763	O60763 [593-615]	0	3650.9134
[R].GGSPGE	1xTMTpro [N	4.323E-05	4.225E-05	1	1	1	Q6P9B9	Q6P9B9 [454-469	0	1774.9771
[R].AVDFQE	1xTMTpro [K	3.544E-06	4.225E-05	1	1	2	P20339	P20339 [142-165]	0	3288.6226
[K].TNIVTAS	1xTMTpro [K	1.177E-06	4.225E-05	1	1	1	O00154	O00154 [268-283]	0	2353.3007
[R].ASNGDA	1xTMTpro [K	3.393E-05	4.225E-05	1	1	1	P38646	P38646 [147-159]	0	1950.0325
[R].TNISDEE	1xOxidation [	1.677E-06	4.225E-05	1	1	3	P18206	P18206 [1077-110	0	3670.8184
[K].HMVFLG	1xTMTpro [K	1.491E-05	4.225E-05	1	1	1	P61160	P61160 [352-366]	0	2210.2685
[R].HVFIVDI	1xTMTpro [K	9.641E-06	4.225E-05	1	1	4	Q99715	Q99715 [2479-24	0	2220.1832
[R].TWNDPS	1xTMTpro [K	2.515E-05	4.225E-05	1	1	8	P11021	P11021 [102-113]	0	2039.1053
[R].TIVITSHI	1xTMTpro [K	2.989E-05	4.225E-05	1	1	1	P31689	P31689 [284-296]	0	2001.2352
[K].TLVLSNI	1xTMTpro [K	2.17E-06	4.225E-05	1	1	4	P19338	P19338 [487-508]	0	3109.68
[K].ASLGSLI	1xTMTpro [K	2.931E-05	4.225E-05	1	1	1	Q09666	Q09666 [5748-57(	0	2341.2379
[R].HVGDLG	1xCarbamido	2.49E-08	4.225E-05	1	1	2	P00441	P00441 [81-116]	1	4329.2277
[K].TLVNPAI	1xTMTpro [K	5.132E-05	4.225E-05	1	1	2	O75608	O75608 [191-201]	0	1812.0875
[K].GGEYGF	1xTMTpro [N	2.087E-06	4.225E-05	1	1	1	Q15124	Q15124 [283-298]	0	1908.8683
[K].TLTTVQ(	2xTMTpro [K	6.572E-06	4.225E-05	1	2	2	P41567	P41567 [43-57]	1	2580.4701
[K].GGETVM	1xTMTpro [K	9.355E-07	4.225E-05	1	1	3	Q14914	Q14914 [141-163]	0	2694.5104
[K].TITSVAM	1xTMTpro [K	3.594E-05	4.225E-05	1	1	1	Q99541	Q99541 [56-71]	0	2282.3649
[K].HMMAFII	2xTMTpro [K	1.04E-05	4.225E-05	1	2	1	P36543	P36543 [14-33]	1	3246.6955
[K].TITSQWI	1xTMTpro [K	5.63E-07	4.225E-05	1	1	1	Q02252	Q02252 [511-535]	1	3343.7522
[K].TITSEDV	1xTMTpro [K	6.003E-06	4.225E-05	1	1	1	P48426	P48426 [146-160]	0	2309.2667
[R].TLTSDV/	2xTMTpro [K	5.365E-07	4.225E-05	1	1	2	O75116	O75116 [977-995]	1	3015.6779
[K].TLTYFNY	1xTMTpro [N	2.597E-06	4.225E-05	1	1	2	Q05707	Q05707 [1327-13	0	3132.5121
[R].TWIEGL	1xTMTpro [K	1.79E-06	4.225E-05	1	1	5	Q99439	Q99439 [36-52]	0	2470.3837
[K].TLVQQL	1xCarbamido	5.813E-05	4.225E-05	1	1	1	Q8NE86	Q8NE86 [181-199	0	2938.6316
[K].TWGPE(	1xCarbamido	6.022E-05	4.225E-05	1	1	3	Q9Y3Z3	Q9Y3Z3 [44-55]	0	1783.9121
[K].TLYVEE\	1xTMTpro [N	1.516E-05	4.225E-05	1	1	1	P41250	P41250 [564-583]	0	2522.3826
[K].TTPPVLI	1xTMTpro [N	4.133E-06	4.225E-05	1	1	2	P01861	P01861 [273-289]	0	2206.1351

[R].TVYVGN	1xTMTpro [K	3.334E-06	4.225E-05	1	1	1	Q8WXA9	Q8WXA9 [67-88]	0	3097.6701
[R].AVIGIGIE	1xTMTpro [K	2.255E-05	4.225E-05	1	1	1	O94906	O94906 [528-540]	1	2037.1836
[K].AVIGMTA	1xTMTpro [N	4.3E-05	4.225E-05	1	1	1	Q02978	Q02978 [123-146]	0	2577.4394
[R].AVIGSTY	1xTMTpro [K	8.649E-06	4.225E-05	1	1	2	Q14764	Q14764 [400-417]	0	2691.4559
[K].TWELAN	1xTMTpro [K	2.602E-05	4.225E-05	1	1	2	Q92905	Q92905 [12-30]	0	2891.4741
[R].TIYAGNA	1xCarbamido	4.956E-05	4.225E-05	1	1	1	P13804	P13804 [147-158]	0	1919.0916
[R].TLWIWE	1xCarbamido	3E-05	4.225E-05	1	1	2	P13489	P13489 [260-271]	0	2086.1651
[K].HMVFLG	1xOxidation [	5.841E-05	4.225E-05	1	1	1	P61160	P61160 [352-366]	0	2226.2634
[K].TLWEDP	1xCarbamido	2.64E-05	4.225E-05	1	1	2	P29992	P29992 [134-147]	0	2085.9871
[K].TIVYLDG	1xCarbamido	2.911E-05	4.225E-05	1	1	2	P21589	P21589 [342-354]	0	1789.9074
[R].ASLLQNI	1xTMTpro [K	1.238E-06	4.225E-05	1	1	1	Q9BT78	Q9BT78 [171-188	0	2724.4812
[R].TWGADV	1xTMTpro [K	1.247E-05	4.225E-05	1	1	1	Q13126	Q13126 [188-206]	0	2652.4926
[R].HVFGES	1xTMTpro [K	3.806E-05	4.225E-05	1	1	6	P60174	P60174 [101-113]	0	2067.1366
[K].TLVTQNS	1xTMTpro [N	7.407E-07	4.225E-05	1	1	1	P14923	P14923 [427-444]	0	2239.3093
[K].TLVSTVC	1xTMTpro [N	8.217E-07	4.225E-05	1	1	2	Q9P2E9	Q9P2E9 [652-669	0	2229.1505
[K].TLTPIIQE	1xTMTpro [N	6.339E-09	4.225E-05	1	1	2	Q53EL6	Q53EL6 [167-189	0	3010.5151
[K].ASLNGA	2xCarbamido	7.395E-06	4.225E-05	1	1	3	P14866	P14866 [249-264]	0	2338.2027
[R].TYADYE	2xCarbamido	2.194E-05	4.225E-05	1	1	2	P84090	P84090 [18-34]	0	2663.2283
[R].TLTPAGI	1xTMTpro [N	6.499E-06	4.225E-05	1	1	2	Q5JRX3	Q5JRX3 [729-747	0	2370.193
[K].TYCSEPI	1xCarbamido	5.384E-05	4.225E-05	1	1	1	P00450	P00450 [238-258]	2	3502.7213
[K].TYDAAS	1xCarbamido	9.893E-06	4.225E-05	1	1	1	P42166	P42166 [621-637]	0	2561.2725
[R].TYDATTI	1xCarbamido	4.752E-05	4.225E-05	1	1	2	P50395	P50395 [403-418]	0	2526.2314
[K].HVLFGTY	1xTMTpro [N	7.659E-06	4.225E-05	1	1	4	P04216	P04216 [61-75]	0	2016.0986
[K].TYDLPGI	1xTMTpro [N	4.972E-05	4.225E-05	1	1	1	O94874	O94874 [145-160]	0	2142.1514
[R].HVLLQEI	1xTMTpro [N	1.56E-06	4.225E-05	1	1	2	Q6P4E1	Q6P4E1 [39-55]	0	2280.2631
[R].TLSPTPS	1xTMTpro [N	4.857E-05	4.225E-05	1	1	1	O14639	O14639 [429-443]	0	1924.9935
[R].TLTAAAV	1xTMTpro [K	2.362E-05	4.225E-05	1	1	1	O60664	O60664 [69-84]	0	2136.2884
[K].TLSPGDS	1xCarbamido	1.249E-05	4.225E-05	1	1	1	Q9NQR4	Q9NQR4 [131-14	0	2255.061
[R].AVLVDLE	1xTMTpro [N	2.593E-05	4.225E-05	1	3	4	P68371	P68371 [63-77]	0	1906.0275
[R].TLSGTPI	1xTMTpro [K	3.747E-05	4.225E-05	1	1	1	P12111	P12111 [626-637]	0	1878.0577
[K].AVIVLVE	1xTMTpro [K	4.956E-05	4.225E-05	1	1	2	Q96FQ6	Q96FQ6 [11-21]	0	1903.1548
[K].AVLVTG	2xCarbamido	4.005E-06	4.225E-05	1	1	1	P37059	P37059 [84-100]	0	2336.271
[K].GGELVY	1xTMTpro [N	5.664E-05	4.225E-05	1	1	2	Q15149	Q15149 [3028-30	0	1600.8138

[R].AVIYNPA	1xTMTpro [K	1.178E-05	4.225E-05	1	1	3	P35580	P35580 [19-33]	0	2257.2472
[K].TLSGLEV	1xTMTpro [N	2.25E-05	4.225E-05	1	1	3	P00390	P00390 [301-316]	0	1921.0748
[R].TLSLDEV	1xTMTpro [K	3.563E-05	4.225E-05	1	1	2	Q9NQW7	Q9NQW7 [405-42	0	2523.3838
[R].ASLEAAI	1xTMTpro [N	1.969E-05	4.225E-05	1	1	5	P05787	P05787 [329-341]	0	1648.8825
[R].AVLTFAL	1xTMTpro [N	5.664E-05	4.225E-05	1	1	1	Q9NQT4	Q9NQT4 [179-19(	0	1624.9229
[R].AVLTCSI	1xCarbamido	2.529E-05	4.225E-05	1	1	2	Q9Y624	Q9Y624 [149-168	0	2910.4475
[R].AVLPLLD	1xCarbamido	7.141E-06	4.225E-05	1	1	3	Q6IBS0	Q6IBS0 [56-72]	0	2337.296
[R].TLTLVDT	1xOxidation [	4.427E-05	4.225E-05	2	3	6	P07900; P08	P07900 [88-100];	0	1974.1437
[R].TLTLVDT	1xTMTpro [K	1.381E-05	4.225E-05	2	3	3	P07900; P08	P07900 [88-100];	0	1958.1488
[K].TITLEVE	1xTMTpro [K	1.823E-06	4.225E-05	2	4	3	P62987; P62	P62987 [12-27]; F	0	2396.3416
[K].GGETSE	1xOxidation [	1.417E-06	4.225E-05	1	1	4	P02675	P02675 [248-267]	0	2881.4897
[R].TLTGTA/	1xTMTpro [N	1.722E-05	4.225E-05	1	1	1	P09960	P09960 [34-51]	0	2222.1948
[K].GGETSE	1xTMTpro [K	2.369E-07	4.225E-05	1	1	3	P02675	P02675 [248-267]	0	2865.4948
[R].AVLQNT.	1xTMTpro [N	5.545E-05	4.225E-05	1	1	1	P22670	P22670 [763-780]	0	2333.2566
[R].HVGMAV	1xTMTpro [N	5.764E-06	4.225E-05	1	1	2	P25788	P25788 [73-86]	0	1684.9488
[R].HVGMAV	1xOxidation [	3.651E-05	4.225E-05	1	1	2	P25788	P25788 [73-86]	0	1700.9437
[R].GGESIY(	1xTMTpro [K	2.23E-05	4.225E-05	1	1	2	Q13427	Q13427 [86-103]	0	2547.2899
[K].ASLEGN	1xTMTpro [N	1.547E-05	4.225E-05	1	1	2	Q04695	Q04695 [322-334]	0	1707.8833
[K].AVLSAE(	1xTMTpro [N	2.856E-07	4.225E-05	1	1	8	P50454	P50454 [95-116]	1	2709.4855
[R].ASLEDAI	1xTMTpro [N	2.267E-05	4.225E-05	1	1	2	Q7Z2W4	Q7Z2W4 [283-29!	0	1705.8928
[K].TITAEVL.	1xTMTpro [K	4.184E-05	4.225E-05	1	1	1	P32926	P32926 [461-475]	0	2232.2619
[R].ASLEAAI	2xTMTpro [K	4.426E-06	4.225E-05	1	1	1	P05787	P05787 [329-352]	2	3367.9001
[R].ASLEAAI	1xTMTpro [K	2.053E-05	4.225E-05	1	1	1	P05787	P05787 [329-347]	1	2564.4539
[K].ASLENSI	1xTMTpro [K	4.518E-05	4.225E-05	2	2	2	P02533; P08	P02533 [353-365]	1	2042.1374
[K].TVLSLFD	2xTMTpro [K	7.141E-06	4.225E-05	2	2	1	Q9Y4E1; Q6	Q9Y4E1 [799-822	1	3732.9822
[K].HNAVFD	1xTMTpro [K	3.31E-06	4.225E-05	1	1	3	O14936	O14936 [716-733]	0	2727.4849
[R].AVIFEDC	1xCarbamido	5.738E-05	4.225E-05	1	1	1	Q9UKU7	Q9UKU7 [245-25{	0	1864.991
[K].HNELTG	1xTMTpro [K	1.034E-05	4.225E-05	1	1	2	Q8N5K1	Q8N5K1 [117-131	0	2228.2894
[R].AVGPSS	1xTMTpro [N	4.634E-05	4.225E-05	1	1	2	Q7L576	Q7L576 [551-563]	0	1712.9325
[R].ASMQQ(	1xTMTpro [N	6.1E-05	4.225E-05	1	1	1	Q9Y3Y2	Q9Y3Y2 [39-51]	0	1750.9189
[K].TNDDQT	2xCarbamido	1.26E-05	4.225E-05	1	1	3	P35555	P35555 [1882-18!	0	2318.0018
[R].GGGGF(	1xTMTpro [N	6.131E-09	4.225E-05	1	1	2	Q96QR8	Q96QR8 [153-18:	0	3171.687
[R].TNAENE	2xTMTpro [K	1.191E-05	4.225E-05	1	1	1	P04264	P04264 [278-289]	1	2306.3536

[R].TNAENE	1xTMTpro [K	5.984E-05	4.225E-05	1	1	6	P04264	P04264 [278-288]	0	1874.0515
[R].ASNAMN	1xTMTpro [N	4.713E-05	4.225E-05	1	1	2	P28289	P28289 [328-340]	0	1753.8645
[R].AVGTQT	1xTMTpro [K	4.89E-05	4.225E-05	1	1	1	Q13596	Q13596 [273-286]	0	1924.1723
[R].ASMQPI	1xTMTpro [N	9.677E-06	4.225E-05	1	1	2	Q14980	Q14980 [1968-19	0	2078.1235
[K].TMTVEV	1xTMTpro [K	6.153E-07	4.225E-05	1	1	1	Q02952	Q02952 [1641-16	0	4028.0149
[R].AVHANP	1xTMTpro [N	1.024E-05	4.225E-05	1	1	1	Q13724	Q13724 [507-535]	0	3425.8575
[R].TMTIHN	1xTMTpro [N	2.963E-05	4.225E-05	1	1	1	P02675	P02675 [396-410]	0	2125.0166
[R].TMTGLD	1xTMTpro [K	3.168E-05	4.225E-05	1	1	1	Q8N573	Q8N573 [755-768	0	2127.2413
[K].TVTNAV	1xTMTpro [N	5.701E-06	4.225E-05	1	1	3	P11142	P11142 [138-155]	0	2286.2049
[R].HVAAGT	1xTMTpro [N	1.133E-05	4.225E-05	1	1	1	P14923	P14923 [541-555]	0	1903.9945
[R].GGGGDI	1xTMTpro [K	3.789E-06	4.225E-05	1	1	1	P08621	P08621 [311-346]	0	3981.8064
[R].GGSTSY	1xTMTpro [N	3.81E-06	4.225E-05	1	1	3	P02671	P02671 [272-287]	0	1876.8844
[K].HTYLPLE	1xCarbamido	9.465E-06	4.225E-05	3	4	1	Q9UKV8; Q9	Q9UKV8 [336-351	0	2174.1711
[R].GGGGG	1xCarbamido	7.257E-07	4.225E-05	1	1	1	P42356	P42356 [8-28]	0	1886.833
[R].TNISDEE	1xTMTpro [K	6.877E-09	4.225E-05	1	1	5	P18206	P18206 [1077-110	0	3654.8235
[K].AVGDKL	2xCarbamido	3.145E-07	4.225E-05	1	1	3	P00738	P00738 [78-108]	1	4345.2162
[R].GGGGGI	1xCarbamido	4.099E-05	4.225E-05	1	1	2	Q96QR8	Q96QR8 [11-24]	0	1608.7872
[R].AVGEPM	3xCarbamido	1.321E-06	4.225E-05	1	1	1	Q8TEX9	Q8TEX9 [554-584	1	3873.8675
[K].TVTAMD	1xTMTpro [K	3.502E-05	4.225E-05	1	1	54	P62805	P62805 [81-92]	0	1919.1167
[R].TNLIVNY	1xTMTpro [N	6.241E-06	4.225E-05	1	1	2	Q15717	Q15717 [20-37]	0	2466.2982
[R].GGGGG	1xTMTpro [N	2.245E-08	4.225E-05	1	1	3	P04264	P04264 [519-549]	0	2688.159
[R].TNLDESI	2xTMTpro [K	2.11E-06	4.225E-05	1	1	3	Q9Y3I0	Q9Y3I0 [129-158]	1	4165.242
[K].GGGGG	1xOxidation [	4.862E-10	4.225E-05	1	1	2	P04632	P04632 [10-60]	0	3901.8627
[K].TVTAMD	1xOxidation [	5.41E-05	4.225E-05	1	1	12	P62805	P62805 [81-92]	0	1935.1117
[K].AVGLISH	1xTMTpro [N	1.982E-06	4.225E-05	1	1	1	Q13825	Q13825 [250-269]	0	2474.2959
[R].TNEKVE	1xTMTpro [K	3.526E-05	4.225E-05	2	2	4	P17661; P08	P17661 [106-118]	1	2196.2116
[K].TVTAMD	1xTMTpro [K	1.109E-05	4.225E-05	1	1	4	P62805	P62805 [81-93]	1	2075.2179
[R].AVGMHS	1xTMTpro [N	9.984E-06	4.225E-05	1	1	1	Q9BSH5	Q9BSH5 [203-222	0	2396.3443
[K].GGGGG	1xTMTpro [N	5.803E-13	4.225E-05	1	1	3	P04632	P04632 [10-60]	0	3885.8678
[R].AVGNIVT	1xCarbamido	1.969E-05	4.225E-05	1	1	1	O00629	O00629 [307-339]	0	4154.22
[K].TNDQDG	1xTMTpro [N	3.563E-05	4.225E-05	1	1	2	Q8TC07	Q8TC07 [27-39]	0	1705.9404
[K].HNEETG	1xTMTpro [K	4.273E-06	4.225E-05	1	1	2	Q9NZ45	Q9NZ45 [90-104]	0	2244.248
[K].TMADSS	1xTMTpro [K	5.121E-06	4.225E-05	1	1	1	Q96AC1	Q96AC1 [479-497	0	2781.4988

[K].HNDDEQ	1xTMTpro [N	3.243E-06	4.225E-05	2	3	6	P07900; P08	P07900 [154-173]	0	2560.166
[K].TVTSFYI	1xTMTpro [K	1.543E-08	4.225E-05	1	1	1	O14874	O14874 [49-69]	0	2863.5445
[K].TVWDWI	2xTMTpro [K	8.386E-07	4.225E-05	1	1	2	P14625	P14625 [329-348]	0	3454.9126
[K].HVEDVP	1xTMTpro [N	3.036E-07	4.225E-05	1	1	2	P25311	P25311 [40-60]	0	2707.4163
[R].ASLPYS(	1xTMTpro [N	7.18E-06	4.225E-05	1	1	2	Q96S52	Q96S52 [44-60]	0	2135.2144
[K].AVLEEG	1xTMTpro [K	1.154E-05	4.225E-05	1	1	1	Q9NSE4	Q9NSE4 [434-445	0	1881.1189
[K].TVYFAEI	1xCarbamido	4.677E-07	4.225E-05	1	1	3	P21291	P21291 [16-32]	0	2653.3212
[R].TMGYSV	1xTMTpro [N	9.766E-06	4.225E-05	1	1	1	O15305	O15305 [226-239	1	1887.9554
[K].AVLEQEI	1xTMTpro [N	4.947E-05	4.225E-05	1	1	2	Q9Y2J2	Q9Y2J2 [912-924	0	1678.8931
[K].TMLEDL(	1xTMTpro [K	2.587E-06	4.225E-05	1	1	5	P63208	P63208 [29-56]	0	3605.821
[R].HVEMYQ	1xTMTpro [N	2.779E-05	4.225E-05	1	1	1	Q9BTV4	Q9BTV4 [118-131	0	2127.0136
[K].AVIFCLS	1xCarbamido	3.27E-05	4.225E-05	1	1	2	P60981	P60981 [35-45]	1	2164.298
[R].TVYGGG	1xCarbamido	3.108E-06	4.225E-05	1	1	2	P78371	P78371 [406-427]	0	2670.3121
[K].ASIPFSV	1xTMTpro [K	1.667E-05	4.225E-05	1	1	2	Q15019	Q15019 [233-249	0	2368.3732
[R].TVYGGG	1xCarbamido	3.975E-06	4.225E-05	1	1	1	P78371	P78371 [406-427]	0	2686.307
[R].GGGAFV	1xTMTpro [N	5.115E-05	4.225E-05	1	1	2	P33176	P33176 [942-956]	0	1790.9833
[K].GGFVLLI	1xTMTpro [K	9.659E-06	4.225E-05	1	1	4	Q13228	Q13228 [161-174	0	2119.1931
[K].AVLFCLS	1xCarbamido	6.07E-05	4.225E-05	1	1	4	P23528	P23528 [35-45]	1	2222.3035
[R].GGGAGI	1xTMTpro [K	6.003E-05	4.225E-05	1	1	1	P10643	P10643 [379-400]	0	2745.4703
[R].TVVTGIE	1xTMTpro [K	4.695E-06	4.225E-05	1	1	2	P49411	P49411 [304-314]	0	1870.0752
[K].AVLDVFI	1xTMTpro [K	6.04E-07	4.225E-05	1	1	16	P01011	P01011 [361-379]	0	2516.374
[K].TMLEDL(	1xOxidation	5.226E-05	4.225E-05	1	1	2	P63208	P63208 [29-56]	0	3621.816
[K].TMQGSE	1xTMTpro [K	4.518E-05	4.225E-05	1	1	2	Q8WUM4	Q8WUM4 [542-55	0	1913.1022
[R].GGGFG(	1xTMTpro [N	1.764E-07	4.225E-05	1	1	1	P35908	P35908 [93-122]	0	2703.2256
[K].TVVDLSI	1xTMTpro [K	3.247E-05	4.225E-05	1	1	1	Q567U6	Q567U6 [168-179	0	2014.1829
[R].GGGEY(	1xTMTpro [K	5.527E-05	4.225E-05	1	1	2	P48147	P48147 [506-516]	0	1828.9474
[R].TMQALE	1xTMTpro [K	2.466E-05	4.225E-05	1	1	1	Q04695	Q04695 [306-321	0	2458.3541
[K].TVVGQI	1xTMTpro [N	2.829E-05	4.225E-05	1	1	1	O75390	O75390 [58-73]	0	2062.0455
[K].TVVLGDI	1xTMTpro [K	1.226E-05	4.225E-05	1	1	2	Q8TCD5	Q8TCD5 [135-150	1	2380.3943
[R].TMMACC	1xCarbamido	1.224E-06	4.225E-05	1	1	1	Q99832	Q99832 [322-344	0	2643.3224
[R].TVVNISS	1xCarbamido	3.8E-05	4.225E-05	1	1	1	P35270	P35270 [151-165]	0	2285.3183
[R].HVDLLE	1xTMTpro [K	2.418E-05	4.225E-05	1	1	2	Q8NBU5	Q8NBU5 [281-30(	0	2768.4598
[R].TVVQPS	1xCarbamido	5.179E-07	4.225E-05	1	1	1	P02765	P02765 [341-361]	0	2320.2767

[R].TMLITHM	2xOxidation [	6.287E-06	4.225E-05	1	1	1	Q15019	Q15019 [278-300]	0	3198.5519
[R].TMLITHM	1xOxidation [	3.372E-06	4.225E-05	1	1	2	Q15019	Q15019 [278-300]	0	3182.557
[R].TMLITHM	1xTMTpro [N	4.583E-07	4.225E-05	1	1	1	Q15019	Q15019 [278-300]	0	3166.5621
[R].HVDNIMI	1xTMTpro [N	2.947E-05	4.225E-05	1	1	1	Q8TAT6	Q8TAT6 [222-236	0	2102.0408
[K].TMLESA(	1xTMTpro [N	1.994E-05	4.225E-05	1	1	2	Q9Y490	Q9Y490 [1605-16	0	1751.9645
[R].TVVSGL'	1xTMTpro [N	8.602E-06	4.225E-05	1	1	1	Q12904	Q12904 [188-205]	0	2325.2668
[R].HVAEVLI	1xTMTpro [K	9.259E-06	4.225E-05	1	1	1	P05198	P05198 [114-133]	1	3042.6392
[R].ASSIIDEI	1xTMTpro [N	6.09E-05	4.225E-05	1	1	4	P10909	P10909 [183-194]	0	1697.9029
[K].HSSVYP'	1xTMTpro [N	5.354E-06	4.225E-05	1	1	1	Q12906	Q12906 [18-40]	0	2975.4488
[R].GGHMDI	1xTMTpro [N	1.854E-06	4.225E-05	2	2	2	P38159; Q96	P38159 [126-144]	0	2353.9919
[K].TSTILGD	1xTMTpro [K	2.493E-05	4.225E-05	1	1	2	Q96AC1	Q96AC1 [362-380	0	2658.5097
[R].HQLTEV	1xTMTpro [N	3.059E-05	4.225E-05	1	1	1	Q8NEZ5	Q8NEZ5 [204-218	0	2038.1252
[K].ATAVVD(	2xTMTpro [K	1.291E-05	4.225E-05	1	1	2	P32119	P32119 [17-29]	1	2247.3529
[R].ATISLSD	1xTMTpro [N	5.691E-05	4.225E-05	1	1	1	Q7L2E3	Q7L2E3 [1116-11	0	1594.8971
[K].TVDFTQI	1xTMTpro [K	1.899E-05	4.225E-05	1	1	3	Q9Y3F4	Q9Y3F4 [105-122	0	2610.3543
[R].ATISNPI'	1xTMTpro [N	2.616E-05	4.225E-05	1	1	1	P13674	P13674 [363-379]	0	2191.1678
[R].TSTDLD'	2xTMTpro [K	4.092E-05	4.225E-05	1	1	1	Q14766	Q14766 [1331-13	1	2731.4818
[R].TSTSAVI	1xTMTpro [K	1.581E-05	4.225E-05	1	1	2	P35611	P35611 [480-497]	0	2508.4317
[K].ATLVESS	1xTMTpro [N	5.671E-07	4.225E-05	1	1	1	P49321	P49321 [672-697]	0	2747.3841
[R].HPHDIID	1xCarbamido	2.042E-05	4.225E-05	1	1	2	P30050	P30050 [147-165]	0	2351.1257
[R].HQIVEV/	1xTMTpro [K	4.817E-05	4.225E-05	1	1	2	Q07960	Q07960 [70-83]	1	2195.2064
[K].ATATSCI	3xCarbamido	1.015E-05	4.225E-05	1	1	1	P98160	P98160 [806-823]	0	2387.1225
[K].HQLYIDE	1xTMTpro [N	6.234E-07	4.225E-05	1	1	21	P02675	P02675 [179-196]	0	2431.2901
[K].TSSAETI	1xTMTpro [K	9.553E-06	4.225E-05	1	1	2	Q16891	Q16891 [582-600]	0	2480.4103
[R].TSRPEN	1xTMTpro [K	2.537E-07	4.225E-05	1	1	6	P29401	P29401 [472-493]	0	3116.6256
[K].TSQTVA'	1xTMTpro [K	2.259E-05	4.225E-05	1	1	1	P52888	P52888 [287-301]	0	2260.268
[R].TVDGMQ	1xTMTpro [K	7.194E-06	4.225E-05	1	1	2	P22102	P22102 [258-281]	0	3195.6925
[K].TSTSNVI	1xTMTpro [K	4.016E-05	4.225E-05	1	1	1	P12270	P12270 [957-978]	0	3067.5749
[R].ATLQAAI	1xCarbamido	2.341E-06	4.225E-05	1	1	1	P59998	P59998 [14-32]	0	2440.2825
[K].ATCAPQ	1xCarbamido	1.95E-07	4.225E-05	1	1	5	P21333	P21333 [2541-255	0	2398.2429
[R].TVASQP	1xTMTpro [K	3.411E-05	4.225E-05	1	1	1	Q5T4S7	Q5T4S7 [205-234	0	3742.9114
[R].GGQLQE	1xTMTpro [N	5.598E-08	4.225E-05	1	1	1	Q6P2E9	Q6P2E9 [1016-10	0	2674.4443
[K].ATDCVG	1xCarbamido	1.7E-05	4.225E-05	1	1	1	P19367	P19367 [625-638]	0	1859.9969

[K].HPLKPDI	1xCarbamido	1.527E-05	4.225E-05	1	1	2	P04196	P04196 [487-507]	0	3261.7506
[K].GGNTLT	1xTMTpro [N	5.701E-05	4.225E-05	1	1	2	Q99715	Q99715 [1273-12	0	1768.9699
[K].ATDAEAI	1xTMTpro [N	3.174E-05	4.225E-05	2	2	4	P09493; P07	P09493 [78-90]; F	0	1636.8461
[K].TSWAGA	1xTMTpro [K	1.98E-05	4.225E-05	1	1	2	P48681	P48681 [1589-160	0	2405.3585
[R].TSVQTE	1xTMTpro [N	4.296E-06	4.225E-05	2	2	2	P35221; P26	P35221 [654-670]	0	2123.09
[K].GGNTLT	1xTMTpro [K	8.496E-10	4.225E-05	1	1	2	Q05707	Q05707 [232-256]	0	3264.7508
[K].TSVPVLI	1xTMTpro [K	4.064E-05	4.225E-05	1	1	1	O95163	O95163 [1305-13	0	2234.3292
[K].TVCDWE	1xCarbamido	9.623E-06	4.225E-05	1	1	2	Q7L576	Q7L576 [517-534]	0	2448.1573
[K].ATCIGNI	1xCarbamido	6.275E-06	4.225E-05	1	1	3	P25789	P25789 [161-176]	0	2216.2023
[K].HQLEIFD	1xCarbamido	9.641E-06	4.225E-05	1	1	1	Q9UPN3	Q9UPN3 [5036-50	0	2526.3266
[K].TSTVDLI	1xTMTpro [N	1.378E-06	4.225E-05	1	1	2	P34932	P34932 [574-591]	0	2445.3309
[K].HQILEQA	1xTMTpro [K	3.768E-06	4.225E-05	1	1	1	Q01082	Q01082 [1634-16	0	2946.5817
[R].HQIQSY	1xCarbamido	1.48E-05	4.225E-05	1	1	1	P17661	P17661 [326-339]	0	2314.2357
[K].ATLPSPI	2xTMTpro [K	5.252E-05	4.225E-05	1	1	4	P22314	P22314 [831-843]	1	2283.3893
[R].ATATQLI	1xTMTpro [N	4.404E-05	4.225E-05	1	1	1	Q13043	Q13043 [270-282]	0	1786.0295
[K].TVASPG	1xTMTpro [N	1.587E-06	4.225E-05	1	1	1	P31939	P31939 [109-133]	0	2857.5842
[R].ATMNLA	1xCarbamido	7.489E-07	4.225E-05	1	1	1	Q9HCU5	Q9HCU5 [71-94]	0	2827.4878
[R].TSQENIS	2xTMTpro [K	2.968E-05	4.225E-05	1	1	1	Q13510	Q13510 [344-366]	0	3556.9389
[R].ATPSRP	1xCarbamido	8.671E-08	4.225E-05	1	1	2	P06401	P06401 [429-464]	0	4090.1258
[K].HRPQVA	1xCarbamido	2.045E-05	4.225E-05	1	1	2	P00491	P00491 [23-41]	0	2587.4634
[K].ATPTTCI	1xCarbamido	5.166E-05	4.225E-05	1	1	1	P56202	P56202 [279-302]	0	3149.716
[K].TVEEAEI	1xTMTpro [N	5.454E-06	4.225E-05	1	1	2	O76070	O76070 [81-96]	0	1978.0776
[R].TSIFWNI	1xTMTpro [K	8.924E-06	4.225E-05	1	1	2	P55884	P55884 [316-332]	1	2643.4274
[K].TSIEDQD	1xTMTpro [N	5.842E-08	4.225E-05	1	1	2	P56537	P56537 [165-188]	0	2888.5536
[R].GGMGS	1xTMTpro [K	9.712E-06	4.225E-05	1	1	1	P05783	P05783 [56-90]	1	3943.992
[K].TVEEAAI	1xOxidation	4.389E-05	4.225E-05	1	1	3	Q7L266	Q7L266 [243-256]	0	2151.1499
[R].TSIDAYD	1xTMTpro [N	2.913E-06	4.225E-05	1	1	2	Q00610	Q00610 [1482-14	0	2247.1213
[K].TSIAIDTI	1xTMTpro [K	2.167E-05	4.225E-05	1	1	2	P25705	P25705 [219-230]	0	1925.1563
[R].ATQFTG	1xTMTpro [K	1.1E-05	4.225E-05	1	1	1	Q14126	Q14126 [729-746]	0	2438.3092
[R].TVEEIEA	2xCarbamido	3.265E-05	4.225E-05	1	1	1	P12955	P12955 [471-484]	0	2221.0795
[R].TSHYPY.	1xCarbamido	4.785E-05	4.225E-05	1	1	1	P08236	P08236 [383-398]	0	2321.032
[K].ATAGDT	1xTMTpro [N	2.015E-06	4.225E-05	2	4	3	P17066; P0D	P17066 [223-238]	0	1979.9378
[K].GGQTHL	1xTMTpro [K	4.449E-05	4.225E-05	1	1	2	P53597	P53597 [91-105]	0	2176.2734

[R].TVEGVLI	1xTMTpro [N	2.875E-05	4.225E-05	1	1	3	O43809	O43809 [79-90]	0	1692.9716
[R].GGMGS(	1xOxidation [	3.966E-08	4.225E-05	1	1	4	P05783	P05783 [56-81]	0	2885.5105
[K].TVEEAAI	1xTMTpro [K	5.841E-05	4.225E-05	1	1	2	Q7L266	Q7L266 [243-256]	0	2135.155
[K].TVEAEA/	1xTMTpro [N	5.305E-06	4.225E-05	1	1	1	O75874	O75874 [302-314]	0	1645.8829
[K].TSLQQS	1xTMTpro [K	5.86E-05	4.225E-05	1	1	1	Q8WXH0	Q8WXH0 [4654-4	0	2896.5871
[K].HQPQEF	1xCarbamid(	1.044E-06	4.225E-05	1	1	3	P02774	P02774 [128-149]	0	3011.4165
[K].ATNESEI	1xTMTpro [K	3.626E-05	4.225E-05	1	1	2	O76021	O76021 [357-373]	0	2448.3477
[K].ATASSS/	1xTMTpro [N	2.814E-05	4.225E-05	1	1	1	Q9H9Q2	Q9H9Q2 [222-238	0	2141.0464
[K].TVDNFV/	1xTMTpro [K	1.848E-05	4.225E-05	1	1	5	P23284	P23284 [72-84]	0	1973.1199
[R].TSQDFLI	1xTMTpro [K	1.182E-05	4.225E-05	1	1	1	Q92621	Q92621 [1086-11(	0	2771.49
[K].TSPVEG	1xTMTpro [K	1.885E-05	4.225E-05	1	1	1	P23634	P23634 [60-75]	0	2222.216
[K].ATAPVSI	1xTMTpro [K	3.838E-06	4.225E-05	1	1	2	Q5VW32	Q5VW32 [12-31]	0	2624.4216
[K].TSPVAD/	1xTMTpro [K	1.646E-06	4.225E-05	1	1	1	Q9Y6N5	Q9Y6N5 [306-326	1	2903.5507
[R].HQVEYL	1xTMTpro [N	5.031E-05	4.225E-05	1	1	1	O94832	O94832 [608-620]	0	1874.0455
[K].TVDSQG	1xCarbamid(	5.719E-05	4.225E-05	1	1	1	Q9BYG3	Q9BYG3 [227-24	0	2309.1767
[K].TSMNVN	1xTMTpro [K	5.419E-05	4.225E-05	1	1	2	P20339	P20339 [166-179]	0	2177.1954
[R].TSMGGT	1xTMTpro [N	1.847E-06	4.225E-05	1	1	2	P35222	P35222 [551-565]	0	1928.9819
[R].GGNIGD	1xTMTpro [N	2.023E-05	4.225E-05	1	1	3	P55072	P55072 [587-599]	0	1420.71
[K].ATALQEI	2xCarbamid(	2.484E-06	4.225E-05	1	1	1	Q9Y6X3	Q9Y6X3 [354-372	0	2521.2822
[K].TSLSSPI	1xTMTpro [N	1.862E-05	4.225E-05	1	1	2	Q7Z2W9	Q7Z2W9 [54-74]	0	2639.3888
[K].HRDFVA	2xTMTpro [K	1.009E-05	4.225E-05	1	1	1	O75531	O75531 [7-32]	1	3606.0294
[R].HRHPDE	1xTMTpro [K	5.446E-05	4.225E-05	1	1	2	P02671	P02671 [511-527]	1	2495.2923
[R].TSQNSE	2xTMTpro [K	2.667E-07	4.225E-05	1	1	3	P35908	P35908 [248-266]	1	3168.6663
[K].TSGTLIS	1xTMTpro [K	5.295E-06	4.225E-05	1	1	1	Q13423	Q13423 [147-166]	0	2814.5897
[R].ATDKDIT	2xTMTpro [K	3.307E-07	4.225E-05	1	1	1	P16083	P16083 [51-77]	1	3912.0483
[R].TTAENEI	2xTMTpro [K	4.21E-06	4.225E-05	1	1	4	P13647	P13647 [266-277]	1	2323.3512
[R].ATFYLN\	1xTMTpro [K	4.981E-05	4.225E-05	1	1	1	Q9Y678	Q9Y678 [528-538	0	1934.1243
[R].TTITTTT	1xTMTpro [N	4.426E-06	4.225E-05	1	1	3	Q96S97	Q96S97 [8-30]	0	2556.351
[K].TTQVPQ	1xTMTpro [N	1.561E-05	4.225E-05	1	1	3	Q08211	Q08211 [418-434]	0	2354.2311
[R].ATENDI\	1xTMTpro [N	3.577E-06	4.225E-05	1	1	2	P55795	P55795 [300-316]	0	2333.1555
[R].ATENDI/	1xTMTpro [N	1.222E-05	4.225E-05	1	1	1	P31942	P31942 [206-222]	0	2223.1729
[K].ATEMVE	1xTMTpro [N	1.504E-06	4.225E-05	1	1	3	Q6NZI2	Q6NZI2 [338-355]	0	2154.978
[R].HQEGEII	2xTMTpro [K	1.735E-05	4.225E-05	1	1	2	Q02878	Q02878 [227-239]	1	2502.3656

[K].ATFYGE(	1xTMTpro [K	4.981E-05	4.225E-05	1	1	2	P46940	P46940 [1517-152	0	2092.0883
[K].TTLQVD1	1xTMTpro [N	2.475E-06	4.225E-05	1	1	2	P35580	P35580 [1762-177	0	2049.1147
[R].TTTDDEI	1xTMTpro [K	8.916E-08	4.225E-05	1	1	1	P32856	P32856 [158-188]	0	4067.051
[K].TTIPEEE	1xTMTpro [N	4.239E-09	4.225E-05	1	1	5	P20700	P20700 [548-577]	0	3715.7782
[R].ATGEAD	1xTMTpro [K	7.244E-08	4.225E-05	1	1	4	P31942	P31942 [233-253]	0	2786.4162
[R].TTTGDV	1xTMTpro [K	4.774E-06	4.225E-05	1	1	1	P11717	P11717 [2049-206	0	2305.3371
[K].HQAFEA	1xTMTpro [N	1.786E-05	4.225E-05	1	1	2	Q13813	Q13813 [614-627	0	1891.9582
[R].ATEGIAN	1xCarbamido	2.375E-05	4.225E-05	1	1	1	Q96ST3	Q96ST3 [538-552	0	2267.1543
[K].TTTTNT(	1xTMTpro [N	4.729E-07	4.225E-05	1	1	4	Q05682	Q05682 [81-99]	0	2402.1642
[K].TTTAAA\	1xTMTpro [N	2.973E-05	4.225E-05	1	1	2	P27816	P27816 [810-824]	0	1681.904
[R].ATFSSVI	1xTMTpro [N	4.179E-06	4.225E-05	1	1	2	P98160	P98160 [1495-152	0	3213.7551
[R].TTIVICC	2xCarbamido	4.257E-05	4.225E-05	1	1	1	P33176	P33176 [296-313]	0	2684.3403
[R].TTNFAG	1xTMTpro [N	4.487E-05	4.225E-05	1	1	4	P09874	P09874 [866-878]	0	1681.9556
[K].ATESGA	1xTMTpro [K	2.755E-05	4.225E-05	1	1	1	Q02790	Q02790 [8-28]	0	2693.4312
[R].ATFAGL\	1xTMTpro [N	6.764E-08	4.225E-05	1	1	6	P48637	P48637 [333-360]	0	3126.5914
[K].TTPPVLI	1xTMTpro [K	1.643E-06	4.225E-05	1	2	49	P0DOX5	P0DOX5 [395-411	0	2482.3361
[K].TTPPMLI	1xOxidation	2.655E-06	4.225E-05	1	1	7	P01859	P01859 [272-288]	0	2530.3031
[K].TTPPMLI	1xTMTpro [K	1.095E-06	4.225E-05	1	1	35	P01859	P01859 [272-288]	0	2514.3082
[K].TTPLKPS	2xTMTpro [K	3.265E-05	4.225E-05	1	1	2	Q7Z6Z7	Q7Z6Z7 [1927-19	0	2729.6997
[K].ATFGCH	2xCarbamido	4.845E-07	4.225E-05	1	1	2	P02749	P02749 [230-250]	0	2994.4105
[R].HQEAEM	1xTMTpro [N	2.697E-05	4.225E-05	1	1	1	P35222	P35222 [475-486]	0	1687.8505
[K].ATFMVG	1xTMTpro [K	4.587E-05	4.225E-05	1	1	3	P52566	P52566 [139-164]	0	3552.8216
[K].ATFMVG	1xOxidation	4.465E-05	4.225E-05	1	1	2	P52566	P52566 [139-164]	0	3568.8165
[R].TTNIQGI	1xTMTpro [N	1.987E-05	4.225E-05	2	2	4	P0C0L4; P0(	P0C0L4 [124-137	0	1868.0561
[K].HQALQA	1xTMTpro [N	3.961E-05	4.225E-05	1	1	1	Q13813	Q13813 [826-839	0	1861
[K].TTPYQIA	1xCarbamido	2.493E-06	4.225E-05	1	1	2	P26639	P26639 [100-121]	0	2929.5949
[K].ATFQTPI	1xTMTpro [N	1.685E-05	4.225E-05	1	1	4	P04114	P04114 [2610-262	0	2138.1817
[K].TTNIELQ	1xTMTpro [K	2.663E-05	4.225E-05	1	1	1	Q15291	Q15291 [454-474	0	2881.6279
[R].GGPPFA	1xTMTpro [N	1.406E-06	4.225E-05	1	1	2	Q07955	Q07955 [52-74]	1	2845.3752
[RK].ATEND	1xTMTpro [N	5.084E-06	4.225E-05	2	2	3	P31943; P52	P31943 [300-316]	0	2301.1835
[R].HPYFYA	1xTMTpro [K	1.2E-05	4.225E-05	1	1	9	P02768	P02768 [170-183]	0	2351.3084
[K].HPPAPA	1xTMTpro [K	3.594E-05	4.225E-05	1	1	2	Q9P2E9	Q9P2E9 [1099-11	0	2112.1581
[R].ATGEAD	1xOxidation	5.509E-05	4.225E-05	1	1	1	P31942	P31942 [233-253]	0	2802.4111

[K].GGPGSA	1xTMTpro [K	5.084E-08	4.225E-05	1	1	6	P04083	P04083 [30-53]	0	2964.5711
[K].GGPVQV	1xTMTpro [K	3.435E-05	4.225E-05	1	1	2	Q13228	Q13228 [358-370]	0	2021.141
[K].ATDSSD	1xTMTpro [K	3.005E-05	4.225E-05	1	1	1	Q96Q11	Q96Q11 [336-354	0	2776.4649
[K].TTFDEAI	1xTMTpro [K	6.07E-06	4.225E-05	1	1	2	P31947	P31947 [196-214]	0	2782.3737
[K].TVAGGA	1xTMTpro [K	4.642E-05	4.225E-05	1	1	3	P61513	P61513 [63-80]	0	2435.3426
[K].TTESQEI	1xTMTpro [K	3.873E-05	4.225E-05	1	1	2	Q05682	Q05682 [216-229]	0	2190.1819
[R].TVAGPV	1xCarbamido	7.062E-06	4.225E-05	1	1	1	Q9UGN4	Q9UGN4 [23-40]	0	2457.3303
[K].GGPAEG	1xTMTpro [N	2.027E-05	4.225E-05	1	1	4	Q07157	Q07157 [62-74]	0	1674.8366
[K].HQGPCD	4xCarbamido	6.241E-06	4.225E-05	1	1	1	O00468	O00468 [457-482]	0	3424.6514
[K].GGNVGY	1xTMTpro [K	4.426E-06	4.225E-05	1	1	1	Q6YN16	Q6YN16 [353-378	0	3327.6005
[R].TTDFSDI	1xCarbamido	2.493E-05	4.225E-05	1	1	1	Q9UHD1	Q9UHD1 [47-61]	0	2299.2136
[K].ATLENIT	1xTMTpro [N	2.171E-05	4.225E-05	1	1	1	Q9NWV4	Q9NWV4 [10-26]	0	2249.2209
[K].GGNTMT	1xOxidation [	1.709E-05	4.225E-05	1	1	3	Q99715	Q99715 [214-228]	0	2179.156
[K].TTCSCSI	2xCarbamido	5.166E-05	4.225E-05	1	1	1	O43681	O43681 [51-63]	0	2063.1121
[R].HPQLDY	1xTMTpro [K	3.974E-05	4.225E-05	1	1	2	P46939	P46939 [2280-229	0	2395.3629
[K].GGNTMT	1xTMTpro [K	4.634E-05	4.225E-05	1	1	1	Q99715	Q99715 [214-228]	0	2163.1611
[R].TTCHDF	2xCarbamido	5.2E-05	4.225E-05	1	1	1	P98095	P98095 [1071-108	0	2139.9871
[K].TVAGQD	1xTMTpro [N	7.947E-06	4.225E-05	1	1	4	P30043	P30043 [64-78]	0	1817.0816
[K].TTGFGM	1xTMTpro [K	1.861E-06	4.225E-05	1	1	2	P62847	P62847 [69-83]	0	2290.1921
[K].HPSAVT	1xCarbamido	5.861E-06	4.225E-05	1	1	1	Q9Y678	Q9Y678 [318-338	0	2630.3163
[R].TTGIVLD	1xTMTpro [N	2.783E-08	4.225E-05	2	4	2	P62736; P68	P62736 [150-179]	0	3500.8167
[K].ATGGPY	1xTMTpro [K	2.579E-05	4.225E-05	1	1	2	O43301	O43301 [341-357]	0	2398.2422
[K].TTVLLAD	1xTMTpro [K	1.231E-05	4.225E-05	1	1	2	P52758	P52758 [79-97]	0	2791.5373
[R].TTVYQG	1xCarbamido	4.556E-05	4.225E-05	1	1	1	Q13308	Q13308 [601-626]	0	3895.1356
[R].TVAAGA	1xTMTpro [K	8.481E-08	4.225E-05	1	1	1	O14498	O14498 [88-101]	0	1947.1883
[R].ATEDGE	1xTMTpro [K	1.293E-05	4.225E-05	1	1	2	P49736	P49736 [157-178]	0	3146.5066
[R].TTIDLTE	1xTMTpro [K	1.195E-06	4.225E-05	1	1	1	P24821	P24821 [847-876]	0	4052.0843
[K].TTHFVE	1xTMTpro [N	1.08E-05	4.225E-05	1	1	3	P18124	P18124 [224-242]	1	2420.1874
[R].TTGSPE	1xTMTpro [K	1.532E-06	4.225E-05	1	1	1	Q7Z2K6	Q7Z2K6 [126-145	0	2893.6166
[R].TVAAPS	1xTMTpro [K	1.096E-05	4.225E-05	1	1	44	P01834	P01834 [2-19]	0	2554.4413
[K].ATGPPV	1xTMTpro [K	2.159E-05	4.225E-05	1	1	5	P16401	P16401 [38-49]	0	1821.0977
[K].ATGQCL	3xCarbamido	5.5E-05	4.225E-05	1	1	1	P07942	P07942 [1050-106	0	2380.1491
[R].TTGIVMI	1xOxidation [	3.192E-08	4.225E-05	1	2	7	P63261	P63261 [148-177]	0	3503.8164

[R].TTGIVMI	1xTMTpro [N	3.723E-08	4.225E-05	1	2	9	P63261	P63261 [148-177]	0	3487.8215
[K].ATEDFL	1xTMTpro [N	2.042E-05	4.225E-05	1	1	1	Q9UH99	Q9UH99 [123-15	0	3729.6119
[K].ATGSPV	2xTMTpro [K	3.435E-05	4.225E-05	1	1	1	Q96KG9	Q96KG9 [38-62]	0	3533.9672
[K].ATGVLYI	1xTMTpro [K	5.993E-05	4.225E-05	1	1	2	P04114	P04114 [4077-40	0	1851.0508
[R].TTGIVLD	1xOxidation	5.075E-08	4.225E-05	2	4	2	P62736; P6	P62736 [150-179]	0	3516.8116
[R].TTVLAA	1xTMTpro [K	5.039E-05	4.225E-05	1	1	1	Q765P7	Q765P7 [45-59]	0	2203.2982
[R].TLSFGSI	1xTMTpro [N	1.284E-05	4.225E-05	1	1	1	Q14152	Q14152 [490-502	0	1748.9138
[K].ATQMPE	1xTMTpro [K	3.9E-05	4.225E-05	1	1	1	P78527	P78527 [945-963]	0	2675.3817
[K].TVEHGF	1xTMTpro [N	4.956E-05	4.225E-05	1	1	1	Q15334	Q15334 [28-47]	0	2529.3057
[K].TVIGDH	1xTMTpro [K	1.454E-06	4.225E-05	1	1	1	P23141	P23141 [463-482]	0	2757.5107
[K].TVIGELP	1xCarbamid	2.88E-05	4.225E-05	1	1	1	Q86VP6	Q86VP6 [112-132	0	2650.473
[K].TQLEWT	1xTMTpro [N	1.008E-05	4.225E-05	1	1	1	Q9P2E9	Q9P2E9 [1292-13	0	2394.2108
[RK].TQLEE	1xTMTpro [K	3.658E-06	4.225E-05	3	3	6	P35580; P35	P35580 [1546-15	0	2570.3329
[R].TQLASW	1xTMTpro [K	7.476E-07	4.225E-05	1	1	2	Q15149	Q15149 [4402-44	0	3496.819
[K].ASTEQA	1xTMTpro [N	4.591E-07	4.225E-05	1	1	1	P24821	P24821 [1069-10	0	2819.4382
[R].TQIAICP	1xCarbamid	2.214E-05	4.225E-05	1	1	1	O15143	O15143 [21-37]	0	2674.4267
[R].GGSGG	1xTMTpro [N	4.419E-05	4.225E-05	1	1	1	O43823	O43823 [110-122	0	1450.7206
[R].TQKEEN	2xTMTpro [K	1.572E-05	4.225E-05	1	1	1	Q9NQT8	Q9NQT8 [134-14	1	2409.3078
[R].TQGSVA	1xCarbamid	9.82E-06	4.225E-05	1	1	1	P46952	P46952 [267-282]	0	2270.2306
[R].TVLGQQ	1xTMTpro [K	2.885E-05	4.225E-05	1	1	1	Q99959	Q99959 [15-37]	0	2963.6909
[K].TQGNVF	1xCarbamid	1.907E-07	4.225E-05	1	1	2	O15371	O15371 [241-260	0	2474.2702
[R].ASTALSI	1xTMTpro [N	5.123E-05	4.225E-05	1	1	2	P12270	P12270 [1054-10	0	1579.8359
[R].TVLGQV	1xTMTpro [N	9.929E-06	4.225E-05	1	1	1	Q9NQT5	Q9NQT5 [22-58]	0	4157.2413
[R].AVAEAV	1xTMTpro [N	1.948E-05	4.225E-05	1	1	2	Q9NPQ8	Q9NPQ8 [5-23]	0	2336.1975
[K].KAHVLA	2xTMTpro [K	3.961E-06	4.225E-05	1	1	1	P35221	P35221 [57-75]	1	2997.7189
[K].HSQAVE	1xTMTpro [K	1.566E-06	4.225E-05	1	1	8	P35579	P35579 [1194-12	0	2448.3226
[R].TQLGAI	1xCarbamid	1.275E-06	4.225E-05	1	1	2	P61970	P61970 [27-49]	0	3222.6749
[R].ASTNAM	1xTMTpro [K	5.227E-06	4.225E-05	1	1	1	O43143	O43143 [63-78]	0	2192.2968
[K].ASTNIQ	1xCarbamid	9.728E-08	4.225E-05	1	1	1	O75832	O75832 [163-184	0	2775.3175
[R].TQPDGT	1xTMTpro [N	2.87E-05	4.225E-05	1	1	1	Q14980	Q14980 [1744-17	0	2228.1478
[R].TVHAGE	1xTMTpro [K	3.502E-05	4.225E-05	1	1	1	P00813	P00813 [212-225]	0	1991.1417
[K].TVHYLPI	1xTMTpro [N	4.729E-05	4.225E-05	1	1	1	Q96KA5	Q96KA5 [206-221	0	2233.2664
[R].GGIMSL	1xCarbamid	4.135E-05	4.225E-05	1	1	1	Q86VN1	Q86VN1 [261-27	0	2016.0577

[R].TQNPMV	1xOxidation [	3.423E-05	4.225E-05	1	1	1	P28070	P28070 [46-60]	0	2156.2241
[R].TQNPMV	1xTMTpro [K	7.917E-06	4.225E-05	1	1	2	P28070	P28070 [46-60]	0	2140.2291
[K].ASVDELI	1xTMTpro [N	5.27E-05	4.225E-05	1	1	2	P61225	P61225 [151-162]	0	1652.9179
[K].TQNDVD	1xTMTpro [K	1.425E-05	4.225E-05	1	1	4	P16422	P16422 [203-218]	0	2499.2899
[K].HPASEA	1xTMTpro [N	2.138E-06	4.225E-05	1	1	2	Q13308	Q13308 [133-149]	0	2209.1896
[K].TQMLDQ	1xTMTpro [N	1.43E-05	4.225E-05	1	1	1	P33176	P33176 [451-464]	0	1939.0126
[R].HSNLML	2xTMTpro [K	6.381E-06	4.225E-05	1	1	2	O43491	O43491 [626-643]	1	3038.7012
[K].TQIQSVE	1xTMTpro [K	6.022E-05	4.225E-05	1	1	2	P40763	P40763 [632-642]	0	1902.0828
[R].TVLEHY/	1xTMTpro [K	6.1E-05	4.225E-05	1	1	1	Q3ZCQ8	Q3ZCQ8 [296-312	0	2540.3892
[K].ASTPGA	1xTMTpro [K	7.932E-06	4.225E-05	1	1	3	P02730	P02730 [744-757]	0	1979.1417
[K].TVLELMI	1xTMTpro [K	1.663E-05	4.225E-05	1	1	1	P46940	P46940 [407-431]	0	3486.8838
[K].ASTNIQD	1xCarbamido	1.777E-07	4.225E-05	1	1	1	O75832	O75832 [163-189]	1	3635.8103
[K].AVAAAA	1xTMTpro [K	3.099E-07	4.225E-05	1	1	3	Q8IYB3	Q8IYB3 [836-869]	0	3723.0824
[R].TQGFLA	1xTMTpro [K	1.766E-05	4.225E-05	1	1	1	Q9Y230	Q9Y230 [254-269	0	2292.2731
[K].TQPNLD	1xCarbamido	6.719E-06	4.225E-05	1	1	2	P01034	P01034 [102-118]	0	2669.375
[R].ASSVTTI	1xCarbamido	5.331E-05	4.225E-05	1	1	1	P52943	P52943 [113-128]	0	2028.998
[R].GGSGG(	1xTMTpro [N	3.689E-05	4.225E-05	1	1	3	P35637	P35637 [219-234]	0	1527.722
[R].TQDHDE	2xCarbamido	3.299E-05	4.225E-05	1	1	2	O14787	O14787 [263-288]	0	3725.8435
[K].HSQTDQ	1xCarbamido	6.56E-06	4.225E-05	1	1	1	P08572	P08572 [1495-151	0	2483.1973
[K].TQDDIET	1xTMTpro [N	3.774E-05	4.225E-05	1	1	2	Q63ZY3	Q63ZY3 [715-727	0	1882.0241
[R].GGSGGS	1xTMTpro [N	4.549E-09	4.225E-05	1	1	5	P35527	P35527 [491-513]	0	2095.9349
[R].TQCAAD	1xCarbamido	7.845E-08	4.225E-05	1	1	2	Q32P28	Q32P28 [77-105]	0	3419.6286
[K].TQAYQD	1xTMTpro [K	5.073E-05	4.225E-05	1	1	1	P36871	P36871 [9-23]	0	2258.2385
[R].GGKPDE	2xTMTpro [K	4.591E-07	4.225E-05	1	1	3	Q05707	Q05707 [392-410]	0	2799.6284
[R].GGSGG(	1xTMTpro [N	4.343E-09	4.225E-05	1	1	3	P35908	P35908 [550-572]	0	2045.9556
[R].HSSLAG	1xCarbamido	1.716E-05	4.225E-05	1	1	2	Q00610	Q00610 [145-157]	0	1822.9553
[K].TVIPGMI	1xTMTpro [N	4.308E-05	4.225E-05	1	1	4	Q15637	Q15637 [31-46]	0	1953.1526
[R].AVAQAG	1xTMTpro [K	2.952E-05	4.225E-05	1	1	2	Q3LXA3	Q3LXA3 [95-109]	0	2049.2927
[R].GGHVDL	1xTMTpro [K	1.242E-05	4.225E-05	1	1	1	P55809	P55809 [391-406]	0	2252.2387
[K].TVLQIDD	1xTMTpro [N	6.608E-06	4.225E-05	1	1	1	Q15785	Q15785 [110-127]	0	2248.2104
[K].TPVTQVI	1xTMTpro [N	6.07E-05	4.225E-05	1	1	2	Q13405	Q13405 [135-148]	0	1819.0244
[R].GGHPPA	1xTMTpro [N	1.399E-06	4.225E-05	1	1	2	Q9HCD5	Q9HCD5 [336-353	0	2190.2314
[K].AVASAA	1xTMTpro [K	3.715E-05	4.225E-05	1	1	3	Q9Y490	Q9Y490 [674-685	0	1693.0868

[K].TVLPFVN	1xTMTpro [K	2.295E-05	4.225E-05	1	1	1	P05107	P05107 [175-186]	0	1976.1461
[K].HSQTDQ	1xCarbamido	6.682E-06	4.225E-05	1	1	1	P08572	P08572 [1495-151	0	2467.2024
[R].GGLGGG	1xTMTpro [N	1.258E-06	4.225E-05	1	1	2	P05787	P05787 [48-88]	0	4229.2559
[R].GGLGGG	1xOxidation [	1.067E-07	4.225E-05	1	1	2	P05787	P05787 [48-88]	0	4245.2508
[K].ASSTSPY	1xTMTpro [K	8.035E-06	4.225E-05	1	1	3	P13804	P13804 [188-203]	0	2385.2793
[R].TQFPSLI	1xTMTpro [K	3.944E-07	4.225E-05	1	1	1	Q96AT9	Q96AT9 [165-185	0	2819.5071
[K].HSQEIAC	1xTMTpro [N	1.137E-05	4.225E-05	1	1	1	Q08378	Q08378 [1247-12	0	2132.1056
[K].AVAEQIF	1xTMTpro [N	5.563E-05	4.225E-05	1	1	3	Q9Y490	Q9Y490 [958-971	0	1797.0917
[K].HSQFIGY	1xTMTpro [K	2.979E-05	4.225E-05	1	2	4	P07900	P07900 [210-224]	0	2387.3619
[K].AVAFQN	1xTMTpro [N	1.091E-06	4.225E-05	1	1	2	P22695	P22695 [163-183]	0	2654.4122
[R].GGSGGG	1xTMTpro [N	1.272E-05	4.225E-05	1	1	4	P04264	P04264 [589-602]	0	1310.6368
[K].HSQFIGY	1xTMTpro [K	1.76E-05	4.225E-05	1	3	2	P08238	P08238 [205-219]	0	2417.3724
[K].TVLIMEL	1xTMTpro [K	3.363E-05	4.225E-05	1	1	5	P06576	P06576 [213-225]	0	2066.2539
[R].TQETPS.	1xTMTpro [N	2.937E-05	4.225E-05	1	1	6	Q01082	Q01082 [2192-22	0	2013.0031
[K].ASSSILID	1xTMTpro [N	6.572E-06	4.225E-05	1	1	2	Q9UNZ2	Q9UNZ2 [283-301	0	2378.2734
[K].AVAFSPY	2xTMTpro [K	2.701E-05	4.225E-05	1	1	3	P17858	P17858 [716-727]	1	2202.3678
[R].AVAGDA	1xTMTpro [K	6.781E-06	4.225E-05	1	1	4	P05023	P05023 [446-458]	0	1840.0672
[K].AVAGNIS	1xTMTpro [K	2.493E-05	4.225E-05	1	1	3	P18206	P18206 [803-815]	0	1878.094
[K].TQEQLA	1xTMTpro [N	2.799E-05	4.225E-05	1	1	3	P26038	P26038 [413-427]	0	2008.0704
[K].TVLIMEL	1xOxidation [	4.085E-05	4.225E-05	1	1	5	P06576	P06576 [213-225]	0	2082.2488
[R].AVAIDLF	1xTMTpro [K	5.296E-05	4.225E-05	1	1	2	Q96IU4	Q96IU4 [64-76]	0	1886.1355
[K].TVLGTPI	1xTMTpro [N	3.065E-07	4.225E-05	1	1	2	P40939	P40939 [167-187]	0	2311.3305
[K].ATQPET	1xTMTpro [K	2.287E-05	4.225E-05	1	1	1	A0MZ66	A0MZ66 [393-407	1	2326.2746
[K].TQPPED	1xCarbamido	4.009E-05	4.225E-05	1	1	4	O94979	O94979 [165-178]	0	1990.9976
[R].TQQQVE	1xTMTpro [K	2.663E-05	4.225E-05	1	1	1	Q9H9Q2	Q9H9Q2 [205-217	0	2096.1843
[R].TVESEA.	1xTMTpro [N	3.569E-05	4.225E-05	1	1	1	P61289	P61289 [167-181]	0	1973.0147
[R].HPECYV	3xCarbamido	4.029E-05	4.225E-05	1	1	2	O00151	O00151 [280-294]	0	2462.1758
[K].TSEVPY.	1xTMTpro [K	8.623E-07	4.225E-05	1	1	1	O60841	O60841 [1000-10	0	2290.3051
[K].GGLVGT	1xTMTpro [K	3.185E-05	4.225E-05	1	1	1	Q14315	Q14315 [1074-10	0	2068.1934
[R].TSESLC	1xCarbamido	2.621E-05	4.225E-05	1	1	1	O14980	O14980 [159-172]	0	2245.2176
[R].ATSNVF.	1xTMTpro [K	2.053E-06	4.225E-05	2	3	8	P24844; O14	P24844 [18-35]; C	0	2699.3995
[R].ATSNVF.	1xOxidation [	1.026E-05	4.225E-05	2	3	2	P24844; O14	P24844 [18-35]; C	0	2715.3944
[R].TSEVQD	1xTMTpro [N	2.405E-05	4.225E-05	1	1	2	P42566	P42566 [367-379]	0	1850.9415

[K].TVESLEI	2xTMTpro [K	2.397E-05	4.225E-05	1	1	3	Q14914	Q14914 [195-205]	1	2189.3209
[R].GGSDDS	2xTMTpro [K	1.725E-05	4.225E-05	1	1	2	P09874	P09874 [780-796]	1	2738.4301
[R].HPECFV	3xCarbamido	9.448E-06	4.225E-05	1	1	2	Q53GG5	Q53GG5 [314-328	0	2485.2282
[R].ATSSSS	1xTMTpro [N	2.23E-05	4.225E-05	1	1	3	Q03252	Q03252 [417-430]	0	1572.8148
[K].TVFEAL(	1xCarbamido	1.702E-06	4.225E-05	1	1	1	O94973	O94973 [481-497]	0	2535.3885
[K].TVFEAL(	1xCarbamido	1.015E-06	4.225E-05	1	1	2	O95782	O95782 [482-498]	0	2553.3449
[K].TVFLSGI	1xTMTpro [N	6.392E-06	4.225E-05	1	1	1	Q9NP92	Q9NP92 [112-143	0	3679.943
[R].HSFATE(	1xTMTpro [N	3.651E-05	4.225E-05	1	1	1	Q6ZRV2	Q6ZRV2 [410-427	0	2110.0637
[K].ASYGVE	1xTMTpro [N	1.662E-06	4.225E-05	1	1	1	Q9UJZ1	Q9UJZ1 [115-135	0	2634.3041
[K].ATSHVG	1xTMTpro [N	3.671E-06	4.225E-05	1	1	1	P30622	P30622 [381-396]	0	2028.1045
[R].ATSGTE	1xCarbamido	3.754E-05	4.225E-05	1	1	2	Q15113	Q15113 [150-162]	0	1711.8141
[K].ASYSGV	1xTMTpro [N	7.476E-07	4.225E-05	1	1	4	P07585	P07585 [322-345]	0	3067.5485
[R].GGMDY/	1xTMTpro [N	2.182E-06	4.225E-05	1	1	1	Q93052	Q93052 [269-295]	0	3169.5372
[K].ATQQAE	1xTMTpro [N	6.334E-06	4.225E-05	1	1	2	P35749	P35749 [1762-17	0	2093.0794
[K].ATAAPA(	1xTMTpro [K	5.305E-05	4.225E-05	1	1	1	P22695	P22695 [24-42]	0	2518.3797
[R].ATQQH	1xTMTpro [N	1.407E-05	4.225E-05	1	1	1	P78527	P78527 [2637-265	0	2222.1121
[K].TVEIPDF	1xTMTpro [K	6.31E-06	4.225E-05	1	1	4	P21980	P21980 [635-649]	0	2220.2255
[K].HSATTVI	1xCarbamido	3.169E-07	4.225E-05	1	1	1	Q9NZM1	Q9NZM1 [1116-1	0	2498.2417
[R].ATAAFIL	1xTMTpro [K	1.006E-05	4.225E-05	1	1	2	O00410	O00410 [196-212]	0	2438.4051
[K].TSFTPV(	1xOxidation [	1.569E-05	4.225E-05	1	1	2	P04844	P04844 [323-340]	0	2669.414
[K].TVEIPDF	1xTMTpro [K	3.137E-06	4.225E-05	1	1	1	P21980	P21980 [635-651]	1	2475.395
[K].TSFTPV(	1xTMTpro [K	3.817E-06	4.225E-05	1	1	1	P04844	P04844 [323-340]	0	2653.4191
[R].TSFPED	1xTMTpro [K	5.6E-05	4.225E-05	1	1	2	P08174	P08174 [53-64]	0	2009.1087
[K].TSFIQYL	1xTMTpro [N	1.66E-05	4.225E-05	1	1	1	Q9NZN4	Q9NZN4 [72-87]	0	2171.1668
[K].TVELPLN	1xTMTpro [K	3.334E-05	4.225E-05	1	1	1	Q9BVJ6	Q9BVJ6 [115-127	1	2178.2626
[K].ATSETL/	1xTMTpro [K	7.164E-07	4.225E-05	1	1	1	Q9HBL0	Q9HBL0 [1725-17	0	2649.4743
[K].HPELFE/	1xTMTpro [K	4.705E-05	4.225E-05	1	1	1	P62195	P62195 [171-184]	0	2158.2516
[R].TVENVT	1xTMTpro [K	1.279E-05	4.225E-05	1	1	2	Q99536	Q99536 [211-225]	0	2119.1891
[R].ATSFLL/	1xTMTpro [N	2.885E-05	4.225E-05	1	1	1	P04843	P04843 [66-80]	0	1964.1024
[K].TVFPGA	1xTMTpro [K	1.131E-05	4.225E-05	1	1	2	Q9UBC2	Q9UBC2 [217-233	0	2282.3768
[K].TQPYDV	1xTMTpro [N	2.819E-05	4.225E-05	1	1	1	O75306	O75306 [305-323]	0	2518.2421
[R].ATSTATS	1xTMTpro [K	2.855E-05	4.225E-05	1	1	2	P51659	P51659 [316-331]	0	2076.1581
[R].ATTAALI	1xTMTpro [N	1.304E-06	4.225E-05	1	1	6	Q15149	Q15149 [3519-354	0	2532.4357

[R].GGLQSQ	1xTMTpro [K	6.07E-05	4.225E-05	1	1	1	Q99081	Q99081 [504-519]	0	2213.2633
[R].TQVSEG	1xTMTpro [N	1.75E-05	4.225E-05	1	1	1	Q4ZHG4	Q4ZHG4 [775-799	1	2822.238
[K].TVGMLS	1xTMTpro [N	4.809E-05	4.225E-05	1	1	1	P38606	P38606 [537-552]	0	2124.0611
[K].HSLDAS	1xTMTpro [N	1.472E-05	4.225E-05	1	1	1	O00330	O00330 [195-208]	0	1701.8839
[K].HPDASV	1xTMTpro [K	5.401E-05	4.225E-05	1	1	2	P09429	P09429 [31-43]	0	2073.0897
[R].TQVELLI	1xTMTpro [K	8.859E-06	4.225E-05	1	1	1	P13807	P13807 [75-89]	1	2274.3677
[K].HSLECTI	1xCarbamido	1.041E-05	4.225E-05	1	1	1	Q02880	Q02880 [472-487]	0	2382.283
[K].HSHAGE	1xTMTpro [K	1.197E-07	4.225E-05	1	1	4	P02730	P02730 [161-180]	0	2650.5284
[RK].TQTVC	1xCarbamido	4.801E-06	4.225E-05	1	2	1	Q01469	Q01469 [83-109]	1	3729.8786
[K].ASVGFG	1xCarbamido	2.89E-05	4.225E-05	1	1	2	O60701	O60701 [268-279]	0	1852.9871
[R].TQTAIAS	1xTMTpro [K	7.328E-06	4.225E-05	1	1	3	Q01082	Q01082 [1081-10	0	2658.3788
[R].TVGTPIA	2xTMTpro [K	7.301E-06	4.225E-05	1	1	1	Q99460	Q99460 [270-302]	1	4221.1537
[R].TQSNLP	1xCarbamido	8.052E-08	4.225E-05	1	1	1	Q13045	Q13045 [211-237]	0	3237.6228
[R].ATVNTF	1xTMTpro [K	5.654E-05	4.225E-05	1	1	2	O75533	O75533 [1076-10	0	1793.0453
[R].ATVPLQ	1xTMTpro [K	3.016E-05	4.225E-05	1	1	1	O75165	O75165 [953-969]	0	2392.3402
[R].GGLPLE	1xTMTpro [N	6.082E-06	4.225E-05	1	1	4	P15289	P15289 [98-114]	0	2028.166
[RK].TQTVC	1xCarbamido	6.993E-07	4.225E-05	1	2	1	Q01469	Q01469 [83-103]	0	3042.5235
[R].TQYSSA	1xTMTpro [N	8.697E-06	4.225E-05	1	1	2	P55786	P55786 [591-606]	0	2070.1224
[K].ASVITQV	1xTMTpro [N	5.514E-06	4.225E-05	1	1	1	Q00341	Q00341 [73-87]	0	2029.1401
[R].TREDGP	1xCarbamido	7.888E-06	4.225E-05	1	1	2	P11047	P11047 [166-188]	1	3409.6291
[K].TSAPITC	1xCarbamido	3.76E-05	4.225E-05	1	1	1	Q14204	Q14204 [1993-20	0	1955.1127
[R].ASVTVG	1xTMTpro [N	1.189E-05	4.225E-05	1	1	2	Q8TEA8	Q8TEA8 [11-25]	0	1748.9826
[K].TVGAALI	1xCarbamido	2.9E-05	4.225E-05	1	1	2	P16278	P16278 [384-399]	0	2220.2917
[R].ASVTGY	1xTMTpro [K	2.358E-05	4.225E-05	1	1	1	P24821	P24821 [1912-19	0	2509.4045
[K].TSALQEI	1xTMTpro [N	1.391E-05	4.225E-05	1	1	1	Q8WXH0	Q8WXH0 [2952-2	0	1751.9095
[R].TVGALQ	1xTMTpro [K	1.316E-06	4.225E-05	1	1	1	P78527	P78527 [1275-12	0	2423.4365
[K].TVGIDDI	1xTMTpro [N	4.592E-06	4.225E-05	1	1	4	Q9UIJ7	Q9UIJ7 [147-161]	0	1931.0769
[K].HSGFCL	1xCarbamido	2.838E-06	4.225E-05	1	1	1	Q96C23	Q96C23 [301-319	0	2560.2322
[K].TRPVILD	1xTMTpro [N	1.322E-05	4.225E-05	1	1	1	P29728	P29728 [631-650]	0	2326.2322
[R].GGSFEN	1xTMTpro [K	5.926E-05	4.225E-05	1	1	2	P17858	P17858 [375-386]	0	2037.0685
[K].ASVPTIQ	1xCarbamido	1.179E-06	4.225E-05	1	1	2	Q9Y490	Q9Y490 [1006-10	0	2742.441
[K].HSGQFT	1xTMTpro [N	4.397E-05	4.225E-05	1	1	1	Q14112	Q14112 [1344-13	0	2010.984
[K].ATTPVIM	1xTMTpro [N	1.449E-06	4.225E-05	1	1	2	P16435	P16435 [524-547]	0	2762.5234

[R].TREEEC	1xCarbamido	1.113E-05	4.225E-05	1	1	1	Q13162	Q13162 [46-66]	1	2747.2803
[R].ATTVTG	1xCarbamido	4.374E-05	4.225E-05	1	1	3	P00747	P00747 [494-512]	0	2430.1791
[R].ATVAPE	1xCarbamido	1.169E-10	4.225E-05	1	1	2	Q9BWD1	Q9BWD1 [45-71]	0	3097.606
[K].ATVAVY	1xTMTpro [K	5.618E-05	4.225E-05	1	1	1	P04114	P04114 [2475-248	0	2146.2251
[K].TSAQVV	1xTMTpro [K	4.556E-05	4.225E-05	1	1	1	Q14008	Q14008 [677-689]	0	1953.1512
[K].AVMDDF	1xTMTpro [K	2.187E-05	4.225E-05	1	1	112	P02768	P02768 [570-581]	0	1951.0491
[R].GGTFTD	1xCarbamido	1.102E-05	4.225E-05	1	1	1	O14841	O14841 [15-31]	0	2110.0459
[R].HQAQID	1xTMTpro [K	2.479E-05	4.225E-05	1	1	4	Q9NQC3	Q9NQC3 [1158-1	0	2216.2432
[K].VADDLL	1xTMTpro [N	4.917E-07	4.225E-05	1	1	2	P02549	P02549 [1140-115	0	2361.2985
[K].VADALT	1xOxidation	7.744E-08	4.225E-05	1	1	10	P69905	P69905 [63-91]	0	3620.8987
[R].HYQLLT	1xTMTpro [N	6.997E-06	4.225E-05	1	1	2	P52630	P52630 [647-662]	0	2270.21
[R].HYPLNT	1xCarbamido	4.849E-05	4.225E-05	1	1	1	Q9HBL0	Q9HBL0 [1764-17	0	2409.1828
[K].AQYYLP	1xTMTpro [N	3.311E-05	4.225E-05	1	1	2	P61163	P61163 [239-255]	0	2171.1304
[R].TKDDIIC	1xCarbamido	2.69E-06	4.225E-05	1	1	2	Q9Y512	Q9Y512 [58-72]	1	2678.5255
[K].AVVILVT	1xTMTpro [N	1.386E-06	4.225E-05	1	1	1	P04275	P04275 [1795-18	0	2461.3469
[R].GGAEVC	1xTMTpro [K	1.019E-05	4.225E-05	1	2	2	A0A0B4J2D	A0A0B4J2D5 [73-	0	2997.6112
[R].VAEGTQ	1xTMTpro [K	2.735E-05	4.225E-05	1	1	2	P01008	P01008 [295-307]	0	2039.2033
[R].GFSSGS	1xTMTpro [N	2.083E-05	4.225E-05	1	1	1	P35908	P35908 [21-34]	0	1558.8145
[K].TGVAVN	2xTMTpro [K	1.495E-06	4.225E-05	1	1	5	P21333	P21333 [685-700]	0	2559.4963
[K].VAGHPN	1xTMTpro [N	1.158E-06	4.225E-05	1	1	2	Q16698	Q16698 [134-155]	0	2595.3963
[K].TIADLEL	1xTMTpro [N	3.644E-06	4.225E-05	1	1	1	P15924	P15924 [579-592]	0	2052.1085
[K].KAEGEP	2xTMTpro [K	1.47E-05	4.225E-05	1	1	1	Q9NYF8	Q9NYF8 [168-18	1	2354.3384
[R].KAEADP	2xTMTpro [K	4.603E-05	4.225E-05	1	1	1	P51608	P51608 [256-266]	1	2080.2583
[K].AVVHGIL	1xCarbamido	5.775E-06	4.225E-05	1	1	3	P61916	P61916 [72-94]	0	3053.6812
[K].VADALT	1xTMTpro [K	1.206E-10	4.225E-05	1	1	105	P69905	P69905 [63-91]	0	3604.9037
[K].VAGHSL	1xTMTpro [K	4.442E-06	4.225E-05	1	1	2	Q15717	Q15717 [56-72]	0	2391.3316
[K].KAEDLF	1xTMTpro [K	2.452E-06	4.225E-05	1	1	4	O95671	O95671 [409-425]	1	2669.3703
[K].VAGIESH	1xTMTpro [N	8.794E-06	4.225E-05	1	1	1	Q14980	Q14980 [856-869]	0	1830.004
[K].AVVHGIL	1xCarbamido	1.896E-07	4.225E-05	1	1	6	P61916	P61916 [72-94]	0	3037.6863
[R].AYPDVA	1xTMTpro [N	1.967E-06	4.225E-05	1	1	2	O14773	O14773 [448-465]	0	2287.1678
[K].VACITEC	1xCarbamido	4.906E-05	4.225E-05	1	1	2	P04843	P04843 [475-488]	0	2196.2917
[R].HIVAVLF	1xTMTpro [K	9.379E-06	4.225E-05	1	1	2	Q9UJ70	Q9UJ70 [245-261	0	2483.4881
[K].VAASVL	1xTMTpro [K	5.86E-05	4.225E-05	1	1	3	P48047	P48047 [74-84]	0	1769.0817

[K].TIFSALE	1xTMTpro [N	4.48E-05	4.225E-05	1	1	2	O15254	O15254 [50-63]	0	1898.0343
[K].HLVDPID	1xTMTpro [K	7.049E-06	4.225E-05	1	1	2	Q7Z3D6	Q7Z3D6 [455-468	0	2175.2669
[R].HIIVACE	1xCarbamido	3.683E-05	4.225E-05	1	1	1	P07998	P07998 [133-156]	0	2933.431
[K].AVVLAAI	1xTMTpro [N	4.603E-05	4.225E-05	1	1	3	Q13423	Q13423 [203-213	0	1458.8501
[K].GHDLNE	1xTMTpro [K	7.802E-06	4.225E-05	1	1	2	O43852	O43852 [115-130	0	2499.2647
[R].KAAAGE	1xCarbamido	5.141E-07	4.225E-05	1	1	1	Q96C19	Q96C19 [159-176	1	2496.3736
[K].AYMQNF	1xCarbamido	5.555E-06	4.225E-05	1	1	1	O60313	O60313 [424-445	0	2798.3772
[K].VAFITGC	1xTMTpro [K	3.453E-05	4.225E-05	1	1	2	Q16698	Q16698 [61-73]	0	1786.0718
[K].TIEEVYM	1xTMTpro [N	2.602E-05	4.225E-05	1	1	1	Q8IWE2	Q8IWE2 [439-457	0	2432.255
[K].ASASGS	1xTMTpro [N	1.872E-07	4.225E-05	1	1	2	P02545	P02545 [598-624]	0	2669.3661
[K].GFYPSD	1xTMTpro [K	5.433E-06	4.225E-05	2	3	8	P01861; P0D	P01861 [251-272]	0	3152.5457
[R].GHDDLG	1xCarbamido	4.125E-06	4.225E-05	1	1	2	Q13098	Q13098 [164-183	0	2823.3863
[K].AYHEQL	1xCarbamido	1.43E-07	4.225E-05	2	5	12	Q71U36; P6	Q71U36 [281-304	0	3358.7056
[R].AYAPGG	1xTMTpro [K	1.504E-06	4.225E-05	1	1	2	Q13057	Q13057 [395-417	0	3009.633
[K].TIEEYAV	1xCarbamido	5.006E-05	4.225E-05	1	1	3	P36871	P36871 [153-164]	0	2046.1073
[R].VADGMV	2xCarbamido	6.499E-06	4.225E-05	1	1	2	P09874	P09874 [283-305]	0	3135.6173
[K].KAALDE	1xTMTpro [K	8.325E-08	4.225E-05	1	1	1	O75533	O75533 [22-50]	1	3673.8113
[R].VADGLP	1xOxidation	7.436E-06	4.225E-05	1	1	1	O75396	O75396 [10-28]	0	2294.1254
[R].VADGLP	1xTMTpro [N	7.947E-07	4.225E-05	1	1	2	O75396	O75396 [10-28]	0	2278.1304
[R].AVVVNA	1xTMTpro [K	2.064E-05	4.225E-05	1	1	3	Q02978	Q02978 [191-206	0	2244.2844
[K].GGAAPE	2xTMTpro [K	2.68E-06	4.225E-05	1	1	1	P49321	P49321 [215-243]	0	3863.9603
[R].VAGGPQ	1xTMTpro [K	5.536E-05	4.225E-05	1	1	1	Q13228	Q13228 [383-397	0	2122.2186
[R].TLEIPGN	1xTMTpro [N	1.065E-05	4.225E-05	2	2	1	P0C0L4; P0	P0C0L4 [957-979	0	2855.3841
[R].TLEIPGN	1xOxidation	2.337E-05	4.225E-05	2	2	1	P0C0L4; P0	P0C0L4 [957-979	0	2871.379
[K].KAEGAQ	2xTMTpro [K	2.308E-05	4.225E-05	1	1	2	Q9P2E9	Q9P2E9 [501-511	1	2071.2076
[R].ARDDLIT	1xTMTpro [K	2.037E-06	4.225E-05	1	1	2	P36543	P36543 [86-99]	1	2195.2527
[K].KAAAPAI	1xCarbamido	9.184E-08	4.225E-05	1	1	3	P26641	P26641 [253-275]	1	3397.7436
[K].TIELSDD	2xCarbamido	2.049E-06	4.225E-05	1	1	3	O75131	O75131 [86-109]	0	3324.6471
[R].TLENELI	1xTMTpro [N	2.252E-06	4.225E-05	1	1	1	P28289	P28289 [29-49]	0	2612.3738
[K].TLEPVKI	1xTMTpro [K	4.328E-06	4.225E-05	1	1	1	Q8IZP0	Q8IZP0 [200-219]	0	2821.5414
[R].TLADLTL	1xTMTpro [K	8.109E-06	4.225E-05	1	1	3	P04114	P04114 [2013-202	0	2008.2186
[R].VADEGS	1xCarbamido	3.174E-05	4.225E-05	1	1	2	Q5ZPR3	Q5ZPR3 [114-127	0	1891.9543
[R].TLESTW	2xTMTpro [K	2.706E-05	4.225E-05	1	1	2	O14776	O14776 [452-464	0	2501.4432

[R].VAGGPQ	1xTMTpro [K	4.257E-06	4.225E-05	1	1	2	Q13228	Q13228 [383-398]	1	2278.3197
[R].TLESVDI	1xTMTpro [N	1.242E-06	4.225E-05	1	1	1	O00429	O00429 [377-397]	0	2515.4302
[R].VADGGG	1xTMTpro [N	7.83E-06	4.225E-05	1	1	1	Q9Y5M8	Q9Y5M8 [9-27]	0	2199.1365
[K].HIMGQN	1xTMTpro [N	3.873E-05	4.225E-05	1	1	4	P46777	P46777 [198-209]	0	1738.8688
[R].HLLQAPY	1xTMTpro [N	1.235E-06	4.225E-05	1	1	4	Q15436	Q15436 [683-699]	0	2246.2212
[K].TLGDFA	1xTMTpro [K	5.581E-05	4.225E-05	1	1	2	P09874	P09874 [109-119]	0	1793.9929
[K].AVTPPM	1xTMTpro [K	1.328E-06	4.225E-05	1	1	7	Q9UHX1	Q9UHX1 [302-332	0	3402.9678
[R].AYDGTT	1xTMTpro [K	5.682E-05	4.225E-05	1	1	1	Q53GS9	Q53GS9 [214-231	0	2517.4208
[R].GFSLDQ	2xCarbamido	8.386E-07	4.225E-05	1	1	4	P35555	P35555 [2555-257	0	2803.1855
[K].GGVNDN	1xTMTpro [N	3.39E-06	4.225E-05	1	1	2	P07996	P07996 [202-216]	0	1921.0211
[K].HYGPGW	1xTMTpro [K	2.367E-05	4.225E-05	1	1	5	P23284	P23284 [132-145]	0	2083.1039
[K].TLHPAVI	1xTMTpro [N	5.609E-05	4.225E-05	1	1	1	P31939	P31939 [67-79]	0	1659.9978
[R].ASDVVL	1xTMTpro [K	9.5E-06	4.225E-05	1	1	2	Q96C23	Q96C23 [52-68]	0	2447.3678
[K].GFSLADI	1xTMTpro [N	1.09E-05	4.225E-05	1	1	1	Q9Y625	Q9Y625 [41-58]	0	2320.2257
[R].VAAALDI	1xTMTpro [N	2.045E-05	4.225E-05	1	1	1	P19971	P19971 [330-342]	0	1519.8399
[K].ASEAGE	1xTMTpro [N	3.381E-05	4.225E-05	1	1	2	Q7L5N1	Q7L5N1 [244-258	0	1973.0412
[R].HILLAVA	1xTMTpro [K	6.814E-07	4.225E-05	2	2	4	O75367; Q9I	O75367 [80-96]; (	0	2541.4896
[K].HLNEIDL	1xCarbamido	4.426E-06	4.225E-05	1	1	6	Q15185	Q15185 [49-65]	0	2675.3743
[R].VAAAES	1xCarbamido	3.76E-05	4.225E-05	1	1	1	O00410	O00410 [721-735]	0	1935.0363
[K].AVTPPM	1xOxidation [	4.833E-05	4.225E-05	1	1	2	Q9UHX1	Q9UHX1 [302-332	0	3418.9628
[K].GFTMIGI	2xCarbamido	2.518E-08	4.225E-05	1	1	2	P08174	P08174 [256-284]	0	3643.6211
[R].TYVTPP	1xTMTpro [N	5.879E-05	4.225E-05	1	1	1	Q9NPF4	Q9NPF4 [31-47]	0	2054.0878
[R].TKTETIT	1xTMTpro [K	6.677E-08	4.225E-05	1	1	2	P02751	P02751 [1927-195	1	3180.7508
[K].HYGNKP	2xTMTpro [K	1.291E-05	4.225E-05	1	1	2	Q9H0W9	Q9H0W9 [195-21	0	2773.6003
[R].ASEDTT	1xTMTpro [K	5.287E-05	4.225E-05	1	1	1	Q9BZZ5	Q9BZZ5 [456-467	0	1784.9522
[K].TYTVGC	4xCarbamido	1.319E-05	4.225E-05	1	1	1	P01033	P01033 [142-161]	0	3029.4736
[K].HINMDG	1xTMTpro [K	6.041E-05	4.225E-05	1	1	1	O75643	O75643 [524-537]	0	2227.1673
[K].TGSGGV	1xTMTpro [N	3.67E-05	4.225E-05	1	1	2	Q15428	Q15428 [11-24]	0	1599.7894
[K].AVTNFG	1xTMTpro [K	1.959E-05	4.225E-05	1	1	4	Q9NPI1	Q9NPI1 [523-544]	0	3063.617
[K].TYTDELT	1xTMTpro [K	1.776E-05	4.225E-05	1	1	3	O75489	O75489 [145-161]	0	2508.3729
[K].ASEIMVI	1xTMTpro [N	2.687E-07	4.225E-05	1	1	1	Q8IY81	Q8IY81 [270-291]	0	2774.3474
[K].AYIATQG	1xTMTpro [N	5.728E-05	4.225E-05	1	2	1	Q13332	Q13332 [1749-17	0	2373.2046
[K].AVTLHD	1xTMTpro [N	3.772E-07	4.225E-05	1	1	2	P22314	P22314 [98-119]	0	2811.4385

[K].TKSENG	2xTMTpro [K	3.255E-06	4.225E-05	1	1	2	P21796	P21796 [33-53]	1	3101.6419
[K].HYGNKP	1xOxidation [	3.447E-05	4.225E-05	1	1	2	Q9H0W9	Q9H0W9 [195-21	0	2789.5953
[K].AYCEPC	4xCarbamido	4.601E-06	4.225E-05	1	1	1	Q93052	Q93052 [463-486]	0	3659.728
[R].GGAGTS	1xTMTpro [K	4.673E-05	4.225E-05	1	1	2	Q9UBU9	Q9UBU9 [106-11	0	1673.895
[K].TLGTMIA	1xTMTpro [N	1.786E-05	4.225E-05	1	1	1	P20073	P20073 [465-479]	0	1861.9285
[R].TLGDQL	1xTMTpro [N	4.564E-05	4.225E-05	1	1	2	Q9UBG0	Q9UBG0 [125-13	0	1660.9917
[R].VAAQVD	1xCarbamido	3.114E-06	4.225E-05	1	1	1	O95782	O95782 [812-832]	0	2572.3836
[K].TGTLTTI	1xCarbamido	2.341E-05	4.225E-05	2	2	2	P16615; Q9	P16615 [353-365]	0	1772.8954
[R].VAGSVT	1xTMTpro [K	3.247E-05	4.225E-05	1	1	3	Q9Y490	Q9Y490 [2276-22	0	2226.2659
[K].TLGECG	1xCarbamido	8.575E-08	4.225E-05	1	1	3	Q15370	Q15370 [56-80]	0	2940.5321
[K].TIGGGDI	1xTMTpro [K	8.687E-07	4.225E-05	2	5	16	Q71U36; Q9	Q71U36 [41-60];	0	2616.3073
[R].TIGILTKI	3xTMTpro [K	5.455E-05	4.225E-05	1	1	1	P20591	P20591 [242-259]	1	3159.8981
[K].VAAQCS	1xCarbamido	2.375E-05	4.225E-05	1	1	1	Q9Y3E5	Q9Y3E5 [82-95]	0	2071.125
[K].HLIPAAN	1xTMTpro [K	5.366E-05	4.225E-05	1	1	3	P62258	P62258 [107-118]	0	1846.0678
[K].TGTITTF	1xTMTpro [N	5.123E-05	4.225E-05	1	1	2	P13639	P13639 [482-495]	0	1919.9717
[K].KAEIGIAI	2xTMTpro [K	6.603E-07	4.225E-05	2	3	3	P16615; Q9	P16615 [712-727]	1	2416.4414
[K].KAELLDI	3xTMTpro [K	2.667E-07	4.225E-05	1	1	1	Q9HB71	Q9HB71 [51-74]	1	3745.2256
[R].TLGLYGI	1xTMTpro [K	1.848E-07	4.225E-05	1	1	2	P09211	P09211 [76-101]	1	3457.8129
[K].GGAGGT	1xTMTpro [N	8.957E-06	4.225E-05	1	1	1	Q9UDY4	Q9UDY4 [74-87]	0	1541.7628
[R].GGVQVF	1xTMTpro [N	3.782E-06	4.225E-05	1	1	2	Q09666	Q09666 [271-287]	0	1903.0568
[K].TGTAEM	1xTMTpro [N	1.92E-05	4.225E-05	1	1	3	P25705	P25705 [46-58]	0	1727.8805
[R].AVTTVT	1xTMTpro [N	9.53E-07	4.225E-05	1	1	1	P51610	P51610 [1483-15	0	3228.7435
[R].VAGTGE	1xTMTpro [K	3.678E-06	4.225E-05	1	1	3	Q9UJU6	Q9UJU6 [42-61]	0	2614.3526
[R].TKENDA	2xTMTpro [K	5.861E-06	4.225E-05	1	1	2	Q9NYL9	Q9NYL9 [193-20	1	2863.6458
[R].HLVGVC	1xCarbamido	3.607E-05	4.225E-05	1	1	2	P08574	P08574 [134-146]	0	2129.1193
[K].VAAIEAL	1xTMTpro [K	7.859E-06	4.225E-05	1	2	8	P50502	P50502 [119-132]	0	2079.1941
[K].HYGPGV	1xOxidation [	3.208E-05	4.225E-05	1	1	5	P23284	P23284 [132-145]	0	2099.0988
[R].GGAGG	1xTMTpro [N	9.293E-06	4.225E-05	1	1	6	Q6VY07	Q6VY07 [5-21]	0	1560.7798
[K].TKVEAF	2xTMTpro [K	5.209E-05	4.225E-05	1	1	2	Q9UL46	Q9UL46 [155-166	1	2265.3634
[R].VAAENA	1xTMTpro [N	1.679E-05	4.225E-05	1	1	2	Q14789	Q14789 [3165-31	0	1904.0408
[R].TIGTGL\	1xTMTpro [K	8.154E-06	4.225E-05	1	1	2	P49411	P49411 [433-450]	0	2516.3773
[R].VAAALT	1xTMTpro [K	5.344E-06	4.225E-05	1	1	2	P19971	P19971 [266-279]	0	2022.2025
[R].TIGTGL\	1xOxidation [	2.488E-05	4.225E-05	1	1	2	P49411	P49411 [433-450]	0	2532.3722

[R].AYCHILL	2xCarbamidc	4.704E-06	4.225E-05	1	1	1	Q9Y2Z0	Q9Y2Z0 [52-69]	0	2646.4028
[K].AYSDPS	1xTMTpro [N	2.42E-06	4.225E-05	1	1	3	Q15149	Q15149 [3317-33	0	2374.1482
[RK].TLEELY	1xTMTpro [K	1.719E-05	4.225E-05	1	2	1	Q96RT1	Q96RT1 [47-63]	0	2626.4471
[K].AREEEE	2xTMTpro [K	1.067E-05	4.225E-05	1	1	2	Q13442	Q13442 [34-52]	2	2885.5057
[K].KADIGV/	2xTMTpro [K	1.842E-05	4.225E-05	1	2	1	P05023	P05023 [727-743]	1	2531.4683
[R].TLATGY	1xTMTpro [K	3.016E-05	4.225E-05	1	1	1	Q9NRG7	Q9NRG7 [272-28	0	2538.4099
[R].TLAEINA	1xTMTpro [N	1.096E-05	4.225E-05	1	1	1	Q13409	Q13409 [617-635	1	2349.1966
[K].AYHEQL	1xCarbamidc	1.211E-07	4.225E-05	1	1	3	Q9BQE3	Q9BQE3 [281-30	0	3388.7161
[R].GGYTCV	2xCarbamidc	5.504E-06	4.225E-05	1	1	2	Q8N2S1	Q8N2S1 [377-39	0	2208.0385
[R].GFVLQD	1xTMTpro [N	5.34E-05	4.225E-05	1	1	2	P56192	P56192 [377-388]	0	1708.9553
[R].ASAILPC	2xTMTpro [K	9.748E-06	4.225E-05	1	1	2	Q9BZQ8	Q9BZQ8 [601-63	1	4712.5338
[K].AWLTEK	2xTMTpro [K	4.192E-05	4.225E-05	1	1	2	P46939	P46939 [543-554]	1	2344.3693
[K].HIQETEV	1xTMTpro [K	3.702E-05	4.225E-05	1	1	1	Q14515	Q14515 [287-299	0	2208.1541
[K].KADCTIT	1xCarbamidc	1.95E-07	4.225E-05	1	1	2	P22307	P22307 [492-511]	1	3101.6501
[K].AYHEQL	1xCarbamidc	2.08E-07	4.225E-05	1	1	3	Q9BQE3	Q9BQE3 [281-30	0	3372.7212
[K].ARPFPD	1xTMTpro [K	1.613E-06	4.225E-05	1	1	3	P13639	P13639 [606-625]	1	2751.4921
[K].THLPLAI	1xTMTpro [K	4.043E-05	4.225E-05	1	2	1	P50225	P50225 [107-122]	0	2409.4725
[K].TLAEMLI	1xTMTpro [K	6.138E-06	4.225E-05	1	1	2	Q6UWY5	Q6UWY5 [118-13	0	2242.2132
[K].TLCECA	4xCarbamidc	4.609E-06	4.225E-05	1	1	1	P04275	P04275 [253-273]	0	2718.2679
[R].AYLESE	1xTMTpro [K	6.206E-06	4.225E-05	1	1	9	Q9NQC3	Q9NQC3 [1075-1	0	2416.3467
[R].KACNDA	1xCarbamidc	3.662E-07	4.225E-05	1	1	2	Q9HAV0	Q9HAV0 [23-42]	1	2788.4577
[K].KAEAAA	1xTMTpro [K	1.519E-05	4.225E-05	1	1	1	O43633	O43633 [197-215	1	2525.3339
[K].TLCSME	1xCarbamidc	6.051E-05	4.225E-05	1	1	1	Q9HD89	Q9HD89 [20-31]	0	1756.8529
[R].VADQDC	1xTMTpro [N	4.092E-05	4.225E-05	1	1	6	Q96D15	Q96D15 [174-185	0	1569.7498
[K].TLDEFTI	1xTMTpro [N	4.45E-06	4.225E-05	1	1	2	P00736	P00736 [314-331]	0	2558.3574
[K].TLDEILE	2xTMTpro [K	1.792E-05	4.225E-05	1	1	2	P46939	P46939 [952-966]	0	2661.5756
[K].VADPVV	1xCarbamidc	1.015E-05	4.225E-05	1	1	2	Q15029	Q15029 [655-673	0	2689.4614
[R].KACGDS	1xCarbamidc	2.707E-07	4.225E-05	1	1	1	P62879	P62879 [23-42]	1	2668.4584
[R].AWGPGI	1xTMTpro [N	4.931E-05	4.225E-05	1	1	3	O75369	O75369 [554-566	0	1580.8981
[R].AYADEV	1xTMTpro [N	2.784E-05	4.225E-05	1	1	1	Q9Y4D7	Q9Y4D7 [1145-11	0	2522.2581
[K].TIAECLA	1xCarbamidc	1.818E-05	4.225E-05	1	1	2	P46782	P46782 [168-182]	0	2240.2452
[R].AWGPGI	1xTMTpro [K	1.802E-05	4.225E-05	1	1	4	P21333	P21333 [581-593]	0	1835.0671
[K].VAELILH	2xTMTpro [K	1.032E-06	4.225E-05	1	1	4	Q8IWE2	Q8IWE2 [465-484	0	3145.7673

[K].TLDGGLI	1xTMTpro [K	7.301E-06	4.225E-05	1	1	2	Q16851	Q16851 [358-377]	0	2591.5264
[K].GFVEPD	1xTMTpro [N	5.031E-05	4.225E-05	1	1	1	P02786	P02786 [395-409]	0	1977.0513
[K].TIAQGNI	1xTMTpro [K	1.125E-05	4.225E-05	1	1	2	P22695	P22695 [360-375]	0	2239.2538
[K].TLAEMLI	1xOxidation [	9.802E-06	4.225E-05	1	1	2	Q6UWY5	Q6UWY5 [118-13	1	2690.5102
[K].TLAESAI	1xTMTpro [K	4.315E-05	4.225E-05	1	1	2	Q9Y490	Q9Y490 [1767-17	0	2130.2666
[K].TLAESAI	1xTMTpro [K	5.098E-05	4.225E-05	1	1	2	Q9Y4G6	Q9Y4G6 [1769-17	0	2118.2124
[R].THNDIIH	1xTMTpro [N	2.263E-05	4.225E-05	1	1	2	P15311	P15311 [548-559]	0	1797.8985
[K].HLQEAL	1xTMTpro [N	1.533E-05	4.225E-05	1	1	1	Q9Y240	Q9Y240 [50-61]	0	1565.9083
[K].KADVGF	2xTMTpro [K	1.782E-05	4.225E-05	2	4	1	P20020; P23	P20020 [807-823]	1	2563.4734
[R].THPVEIF	1xTMTpro [N	1.26E-05	4.225E-05	1	1	1	O43143	O43143 [316-337]	1	2950.527
[R].KADTTTI	1xTMTpro [K	4.487E-05	4.225E-05	1	1	1	Q15059	Q15059 [245-260]	1	2230.2534
[R].TIAIIAEG	1xTMTpro [N	2.159E-05	4.225E-05	1	1	2	P53396	P53396 [593-607]	0	1872.1125
[K].THTCPP	2xCarbamido	7.845E-06	4.225E-05	1	2	15	P0DOX5	P0DOX5 [225-250	0	3757.079
[R].TIAMDGT	1xTMTpro [N	5.473E-05	4.225E-05	1	1	5	P06576	P06576 [110-121]	0	1566.8481
[K].ASAHAIT	1xTMTpro [N	7.19E-07	4.225E-05	1	1	3	Q05707	Q05707 [347-367]	0	2426.321
[K].TLAMDTI	1xTMTpro [K	5.636E-05	4.225E-05	1	1	2	Q8TDQ7	Q8TDQ7 [161-172	0	1870.0963
[K].TLAQLNI	1xTMTpro [K	3.143E-06	4.225E-05	1	1	3	P06744	P06744 [195-211]	0	2440.4307
[R].VAEGTQ	3xTMTpro [K	1.639E-05	4.225E-05	1	1	1	P01008	P01008 [295-322]	1	4297.5238
[K].THMVVA	1xOxidation [	2.427E-05	4.225E-05	1	1	1	P07814	P07814 [1422-143	0	2275.1706
[K].KADMVN	2xTMTpro [K	4.532E-07	4.225E-05	1	1	1	Q9NYU2	Q9NYU2 [1212-12	1	3833.8996
[K].VAEKLDI	1xTMTpro [K	3.204E-07	4.225E-05	1	1	2	O60506	O60506 [39-60]	1	3050.6654
[K].ARPTDY	1xTMTpro [N	2.392E-05	4.225E-05	1	1	1	Q9Y259	Q9Y259 [298-314	0	2404.2693
[K].AWRPAL	2xCarbamido	9.355E-07	4.225E-05	1	1	1	O00391	O00391 [89-111]	0	2941.462
[K].GFTGIDS	2xTMTpro [K	1.962E-05	4.225E-05	1	1	3	O43252	O43252 [184-203]	0	3134.7441
[K].KADVIPV	2xTMTpro [K	1.364E-05	4.225E-05	1	1	1	P29144	P29144 [983-999]	1	2863.7266
[R].AWRDPI	1xTMTpro [K	5.767E-07	4.225E-05	1	1	5	P14550	P14550 [219-240]	1	3097.7429
[R].VADYCE	1xCarbamido	8.875E-06	4.225E-05	1	1	2	Q8IZP0	Q8IZP0 [29-44]	1	2568.3008
[K].VADWTG	1xTMTpro [K	3.417E-05	4.225E-05	1	1	2	O15371	O15371 [42-53]	0	1963.0417
[K].THIQDNI	1xTMTpro [N	1.356E-07	4.225E-05	1	1	2	P21333	P21333 [1594-161	0	2763.3657
[R].THYSNIE	1xTMTpro [N	4.833E-05	4.225E-05	1	1	1	P04632	P04632 [85-99]	0	2082.0059
[R].KADLNTI	1xTMTpro [K	5.853E-08	4.225E-05	1	1	2	P46939	P46939 [1598-162	1	3649.978
[K].KAEEQL	2xTMTpro [K	5.209E-05	4.225E-05	1	1	2	P19878	P19878 [139-151]	1	2332.3726
[R].TGVSVE	1xTMTpro [N	3E-05	4.225E-05	1	1	1	Q00341	Q00341 [325-344]	0	2403.3302

[R].AYLFAH/	1xTMTpro [K	4.542E-06	4.225E-05	1	1	1	Q2NL82	Q2NL82 [251-273	0	3142.7069
[K].TIDGQQ	1xCarbamid	2.502E-05	4.225E-05	1	1	1	P52907	P52907 [147-166]	0	2922.5639
[K].KAASGE	3xTMTpro [K	2.692E-05	4.225E-05	3	3	3	P16401; P10	P16401 [113-122]	1	2203.3915
[R].TIEDAIA	1xTMTpro [N	1.412E-05	4.225E-05	1	1	1	Q96CT7	Q96CT7 [146-167	1	2696.4174
[R].KAAQNS	2xTMTpro [K	9.407E-07	4.225E-05	1	1	3	P07942	P07942 [1661-16	1	2450.3707
[K].VAEQTP	1xTMTpro [K	1.006E-05	4.225E-05	1	1	2	P05091	P05091 [210-226]	0	2452.4671
[R].HLMLPD	1xTMTpro [K	8.745E-06	4.225E-05	1	1	2	P13010	P13010 [82-97]	0	2523.366
[K].AVYTCN	1xCarbamid	1.993E-06	4.225E-05	1	1	3	P08603	P08603 [110-127]	0	2467.2247
[K].TIEDEDL	2xTMTpro [K	3.282E-05	4.225E-05	1	1	2	Q9ULR3	Q9ULR3 [362-378	1	2879.6222
[R].AVYQDA	1xTMTpro [N	7.207E-06	4.225E-05	1	1	1	O15439	O15439 [548-571]	0	2942.4954
[K].TLADVL	1xTMTpro [K	1.315E-05	4.225E-05	1	1	2	P49441	P49441 [51-62]	0	1936.1974
[K].KAALSAI	2xTMTpro [K	1.587E-05	4.225E-05	1	1	3	P78527	P78527 [310-321]	1	2190.3678
[R].VADLTE	1xTMTpro [N	6.115E-06	4.225E-05	1	1	2	Q9BWF3	Q9BWF3 [183-19	0	2160.0892
[R].AVYLGE	1xTMTpro [N	1.741E-07	4.225E-05	1	1	1	Q9NYU2	Q9NYU2 [669-69	0	3299.7054
[K].ASASDG	1xTMTpro [N	2.35E-05	4.225E-05	1	1	1	Q9H4G4	Q9H4G4 [120-13	0	1557.8192
[K].TIECISLI	1xCarbamid	5.115E-05	4.225E-05	1	1	1	O00410	O00410 [557-570]	0	2082.2488
[K].KAAIISAI	2xTMTpro [K	3.947E-05	4.225E-05	1	1	1	P35232	P35232 [208-219]	1	2102.2637
[R].TIEDLDE	2xTMTpro [K	5.296E-05	4.225E-05	1	1	4	Q9H993	Q9H993 [168-183	1	2896.576
[R].AVYEDR	1xTMTpro [N	5.509E-05	4.225E-05	1	1	1	Q9H1Z4	Q9H1Z4 [126-146	0	2463.2799
[K].HIMGQN	1xOxidation	4.841E-05	4.225E-05	1	1	4	P46777	P46777 [198-209]	0	1754.8637
[R].KAALEQ	1xTMTpro [K	5.14E-06	4.225E-05	1	1	1	P09601	P09601 [86-100]	1	2373.3211
[K].TIEECE	1xCarbamid	6.206E-06	4.225E-05	1	1	1	Q1KMD3	Q1KMD3 [448-46	0	2540.396
[K].TLEEDE	1xTMTpro [K	4.923E-05	4.225E-05	1	1	2	P43487	P43487 [40-50]	0	1990.0512
[K].TGYTPD	4xCarbamid	2.755E-06	4.225E-05	1	1	2	P35555	P35555 [2431-24	0	4373.1491
[K].GGVTGS	1xTMTpro [K	2.817E-06	4.225E-05	1	1	2	Q09666	Q09666 [5726-57	0	1942.0737
[K].TLEEEAI	1xTMTpro [K	3.253E-05	4.225E-05	1	1	1	P35579	P35579 [1175-11	1	2651.3954
[R].TLEEGH	1xTMTpro [K	2.206E-06	4.225E-05	1	1	3	Q9Y5Y2	Q9Y5Y2 [235-261	0	3512.8557
[K].GGVTEIS	1xTMTpro [K	4.854E-06	4.225E-05	1	1	1	Q9NQW7	Q9NQW7 [355-37	1	2288.2378
[K].ASAGPQ	1xCarbamid	9.259E-06	4.225E-05	1	1	2	Q9Y490	Q9Y490 [944-957	0	2064.1767
[K].AYHEQL	1xCarbamid	3.128E-07	4.225E-05	2	5	9	Q71U36; P6	Q71U36 [281-304	0	3374.7005
[K].VAETANI	2xTMTpro [K	5.518E-05	4.225E-05	1	1	2	P61221	P61221 [333-344]	1	2259.3012
[K].TLDGPE	1xTMTpro [K	1.935E-07	4.225E-05	1	1	2	Q14699	Q14699 [257-276]	0	2756.4598
[K].VAEQTP	1xTMTpro [K	1.679E-05	4.225E-05	1	1	2	P30837	P30837 [210-226]	0	2425.4562

[K].THAAED	1xTMTpro [N	1.792E-05	4.225E-05	1	1	1	Q53EP0	Q53EP0 [985-100	0	2178.1362
[K].VADPTPI	1xTMTpro [K	2.649E-05	4.225E-05	1	1	1	P16152	P16152 [97-112]	0	2392.319
[K].THLPGF'	1xTMTpro [K	2.701E-05	4.225E-05	1	1	2	P30044	P30044 [103-116]	0	2148.2309
[R].KACADA	1xCarbamido	3.644E-06	4.225E-05	1	1	1	P62873	P62873 [23-42]	1	2752.4907
[K].GHAVGD	1xTMTpro [N	5.728E-05	4.225E-05	1	1	2	P62266	P62266 [109-119]	0	1381.7871
[K].GFTGIDS	1xTMTpro [K	4.249E-06	4.225E-05	1	1	2	O95340	O95340 [174-190]	0	2549.3015
[K].TIDLCNN	1xCarbamido	5.261E-05	4.225E-05	1	1	2	Q9NYU2	Q9NYU2 [1489-14	0	1915.0273
[R].TLDLGEI	1xTMTpro [N	1.307E-05	4.225E-05	1	1	1	P02750	P02750 [192-209]	0	2341.2934
[K].GHAVNL	1xTMTpro [N	3.032E-05	4.225E-05	1	1	1	O00429	O00429 [620-634]	0	1861.0979
[K].THIDTVII	1xTMTpro [K	4.036E-05	4.225E-05	1	1	2	O95782	O95782 [368-378]	0	1833.109
[K].TLDLSNI	1xCarbamido	6.857E-06	4.225E-05	1	1	2	Q8N1G4	Q8N1G4 [206-226	0	2936.5531
[R].AWDQE/	1xTMTpro [N	2.234E-05	4.225E-05	1	1	2	Q8IZ83	Q8IZ83 [774-789]	0	2003.0153
[R].AYLLQNI	2xTMTpro [K	1.096E-05	4.225E-05	1	1	2	Q5T4S7	Q5T4S7 [1175-11	0	2981.64
[R].THEDLYI	1xCarbamido	2.911E-05	4.225E-05	1	1	2	P22692	P22692 [192-206]	0	2160.1079
[K].TIDWVAI	1xTMTpro [K	4.556E-05	4.225E-05	1	1	2	O75947	O75947 [10-25]	0	2481.3997
[K].ARPEDV	1xTMTpro [K	5.064E-05	4.225E-05	1	1	1	Q9HBI1	Q9HBI1 [336-347]	0	1977.1624
[K].ASKPGW	1xTMTpro [K	2.716E-05	4.225E-05	1	1	1	P12259	P12259 [1876-18	0	2693.4655
[K].TLDQVLI	2xCarbamido	1.524E-05	4.225E-05	1	1	1	O75431	O75431 [178-196]	0	2582.251
[R].KAASQP	2xTMTpro [K	4.434E-05	4.225E-05	1	1	1	Q96N66	Q96N66 [456-468	1	2240.343
[K].TLDSWR	1xTMTpro [N	1.206E-05	4.225E-05	1	1	2	P51149	P51149 [98-113]	1	2238.1838
[R].VAEQAC	1xTMTpro [N	1.716E-05	4.225E-05	1	1	1	Q5TZA2	Q5TZA2 [810-826	0	2228.2206
[K].TLDTDLI	1xTMTpro [K	4.029E-05	4.225E-05	1	1	1	P07384	P07384 [691-706]	0	2407.3252
[K].GFTDAD	1xCarbamido	2.182E-06	4.225E-05	1	1	5	Q13185	Q13185 [53-81]	0	3990.9199
[K].AWCVSC	4xCarbamido	3.495E-05	4.225E-05	1	1	1	Q7Z4I7	Q7Z4I7 [275-289]	0	2462.1217
[R].VADPDH	1xTMTpro [N	2.136E-05	4.225E-05	1	1	3	P28482	P28482 [173-191]	0	2448.2115
[K].AYLPVNI	1xTMTpro [N	1.247E-05	4.225E-05	1	1	3	P13639	P13639 [786-801]	0	2104.1034
[K].KAASITS	2xTMTpro [K	2.121E-05	4.225E-05	1	1	1	Q9Y5B9	Q9Y5B9 [185-196	1	2207.3216
[R].AYIQEFC	1xTMTpro [K	3.645E-05	4.225E-05	1	1	2	Q6UWY5	Q6UWY5 [55-65]	0	1998.0828
[K].THEAEIV	1xCarbamido	2.088E-07	4.225E-05	1	1	2	P21333	P21333 [2185-220	0	2362.1417
[R].HLMLPD	1xOxidation		4.936E-06	4.225E-05	1	1	6	P13010	P13010 [82-97]	0	2539.361
[R].TGSESS	1xTMTpro [N	1.088E-05	4.225E-05	1	1	2	P23588	P23588 [420-435]	0	1877.9008
[RK].HYFQN	1xTMTpro [N	3.441E-06	4.225E-05	3	4	3	P61204; P18	P61204 [80-99]; F	1	2742.3919
[K].AQWGSI	1xTMTpro [N	2.488E-05	4.225E-05	1	1	1	P46821	P46821 [2410-243	0	2704.3506

[R].TIPIDGN	1xTMTpro [N	5.627E-05	4.225E-05	1	1	4	P00352	P00352 [144-156]	0	1848.9815
[R].AYFAVFI	1xTMTpro [N	1.498E-06	4.225E-05	1	1	1	P49593	P49593 [192-207]	0	1956.9887
[R].TGIAGGI	1xTMTpro [K	3.613E-05	4.225E-05	1	1	1	P22897	P22897 [605-616]	0	1838.1031
[K].TIPLTDN	1xTMTpro [K	1.653E-06	4.225E-05	1	1	1	Q6DKI1	Q6DKI1 [173-188]	0	2388.363
[R].ASGNYA	2xTMTpro [K	2.367E-05	4.225E-05	1	1	1	P62917	P62917 [129-145]	1	2729.5402
[R].AVQELV	1xTMTpro [N	3.774E-05	4.225E-05	1	1	2	A5YKK6	A5YKK6 [1400-14	0	1665.9607
[K].TYGESM	1xTMTpro [N	3.651E-05	4.225E-05	1	1	1	Q9Y305	Q9Y305 [408-420	0	1836.9121
[K].TIPMDG(	1xCarbamido	3.689E-05	4.225E-05	1	1	1	P30837	P30837 [160-172]	0	1913.9321
[K].ASGPGL	1xTMTpro [K	5.864E-07	4.225E-05	1	1	4	P21333	P21333 [1550-157	0	2950.6381
[K].GGDISV(	1xCarbamido	5.71E-05	4.225E-05	1	1	4	P14854	P14854 [48-59]	0	1773.8549
[R].HIPGAAI	1xCarbamido	5.285E-06	4.225E-05	1	1	2	P25325	P25325 [53-68]	0	2153.0405
[R].GFSAIS(	1xCarbamido	8.337E-06	4.225E-05	1	1	1	Q96EY5	Q96EY5 [27-44]	0	2394.2619
[K].ASGPPV	1xTMTpro [K	2.324E-05	4.225E-05	3	3	7	P16402; P10	P16402 [36-47]; F	0	1807.0821
[R].TKIDCDI	1xCarbamido	1.13E-06	4.225E-05	1	1	1	Q92499	Q92499 [515-535]	1	3338.7507
[R].ASGQAF	1xTMTpro [N	3.747E-05	4.225E-05	1	1	4	P16949	P16949 [15-27]	0	1692.9604
[K].TKGVDE	1xTMTpro [K	2.968E-05	4.225E-05	1	1	2	P07355	P07355 [48-63]	1	2380.4055
[R].KAGNFY	2xTMTpro [K	3.92E-05	4.225E-05	1	1	2	P18124	P18124 [77-88]	1	2233.3161
[K].AVQAQ(	1xTMTpro [N	2.053E-05	4.225E-05	1	1	1	Q14980	Q14980 [1574-15	0	1890.9589
[R].AVQALC	1xCarbamido	8.021E-07	4.225E-05	1	1	1	O60701	O60701 [191-205]	0	2103.1129
[K].TIPSWA	1xTMTpro [N	1.792E-05	4.225E-05	1	1	4	Q5SSJ5	Q5SSJ5 [132-146	0	1906.0717
[K].ASGSEN	1xTMTpro [N	5.766E-05	4.225E-05	1	1	1	Q02880	Q02880 [1549-15	0	1756.8057
[K].CAIGVTI	1xCarbamido	1.92E-05	4.225E-05	1	1	2	Q92896	Q92896 [734-747]	0	2240.2539
[R].KAGTQII	2xTMTpro [K	2.211E-08	4.225E-05	1	1	5	O43707	O43707 [66-83]	2	2961.6462
[R].KAEEEH	2xTMTpro [K	1.358E-07	4.225E-05	1	1	7	P00450	P00450 [799-819]	1	3168.7833
[R].ASGTVY	1xTMTpro [K	4.689E-05	4.225E-05	1	1	1	O95470	O95470 [145-155]	0	1735.9358
[K].TYGADL	1xTMTpro [N	4.449E-07	4.225E-05	1	1	1	P30740	P30740 [111-129]	0	2357.1329
[R].AQVGDL	1xTMTpro [N	3.961E-05	4.225E-05	1	1	1	Q96A19	Q96A19 [380-391	0	1575.8662
[R].VAEEHA	1xTMTpro [K	1.345E-06	4.225E-05	1	1	4	P62191	P62191 [273-293]	0	2862.5744
[K].HLYTLD(	1xCarbamido	1.854E-07	4.225E-05	1	1	2	P63244	P63244 [226-245]	0	2580.32
[R].GGDPHS	1xTMTpro [K	5.296E-05	4.225E-05	1	1	1	Q8WXH0	Q8WXH0 [1061-1	0	1820.9787
[K].CAGGHI	1xCarbamido	4.776E-05	4.225E-05	1	1	1	P49916	P49916 [729-740]	0	1547.7556
[K].AVQGG(	1xTMTpro [N	9.513E-07	4.225E-05	1	1	1	P09012	P09012 [124-152]	0	3031.614
[R].TKLDDL	2xTMTpro [K	1.959E-05	4.225E-05	1	1	2	Q13523	Q13523 [98-111]	1	2528.5003

[K].AVTLEC\	1xCarbamido	1.93E-05	4.225E-05	1	1	2	P98160	P98160 [3129-314	0	1692.891
[R].AVSDSF	1xTMTpro [N	3.981E-05	4.225E-05	1	1	1	Q969X1	Q969X1 [79-92]	0	1841.8625
[R].GGDGIN	1xTMTpro [K	5.428E-05	4.225E-05	1	1	1	O00469	O00469 [77-89]	0	1767.9845
[K].HLVQNY	1xTMTpro [N	2.214E-05	4.225E-05	1	1	1	Q96L93	Q96L93 [190-209]	0	2591.2843
[K].TYLFLQE	2xTMTpro [K	3.384E-06	4.225E-05	1	1	1	P29508	P29508 [112-125]	1	2657.5734
[R].HVYGEL	1xTMTpro [N	2.745E-05	4.225E-05	1	1	3	P00488	P00488 [717-728]	0	1760.9615
[K].ASAFAL(	1xTMTpro [K	2.554E-06	4.225E-05	1	1	2	P36957	P36957 [287-307]	0	2825.554
[R].HVVSLEI	1xTMTpro [K	5.617E-06	4.225E-05	1	1	2	P35442	P35442 [135-150]	0	2391.3164
[R].TLNQLG	1xTMTpro [N	3.119E-05	4.225E-05	1	1	2	O15400	O15400 [35-49]	0	1973.0623
[R].KAFDICF	1xCarbamido	3.423E-05	4.225E-05	1	1	2	P01031	P01031 [1154-116	1	2103.2816
[R].TGIIVTT	2xTMTpro [K	9.425E-07	4.225E-05	1	1	1	Q96CN7	Q96CN7 [255-277	1	3360.9923
[R].TLNQPD	1xTMTpro [K	1.147E-06	4.225E-05	1	1	6	P01009	P01009 [126-149]	0	3182.7553
[K].AVQGFF	1xTMTpro [N	3.299E-05	4.225E-05	1	1	2	O43242	O43242 [116-128]	0	1716.8989
[R].AQVLGH	1xTMTpro [K	4.776E-05	4.225E-05	1	1	1	Q86UU1	Q86UU1 [721-731	0	1830.1093
[K].GGTYDT	1xTMTpro [K	4.095E-06	4.225E-05	1	1	2	Q96AY3	Q96AY3 [198-212	0	2225.2098
[R].TIPEASA	1xTMTpro [K	6.048E-06	4.225E-05	1	1	1	Q504Q3	Q504Q3 [586-606	0	2666.4744
[R].CACTYG	3xCarbamido	4.776E-05	4.225E-05	1	1	2	P35555	P35555 [166-179]	0	2010.8791
[K].GFSDED	1xCarbamido	6.984E-06	4.225E-05	1	1	2	P83916	P83916 [44-72]	0	4034.9097
[K].AVQLYQ	1xTMTpro [N	5.926E-06	4.225E-05	1	1	2	Q99747	Q99747 [133-149]	0	2343.19
[K].AVQLTQ	1xTMTpro [K	7.234E-06	4.225E-05	1	1	2	O94979	O94979 [730-749]	0	2625.5141
[R].TLPETLI	1xTMTpro [N	2.64E-05	4.225E-05	1	1	2	O95168	O95168 [13-30]	0	2350.2097
[R].TGIILNNI	1xCarbamido	1.892E-05	4.225E-05	1	1	3	P36269	P36269 [420-434]	0	2077.1283
[K].TGLIDYN	1xTMTpro [N	1.376E-05	4.225E-05	1	1	2	P34897	P34897 [201-214]	0	1853.0452
[K].TGIEQG	1xCarbamido	1.716E-05	4.225E-05	1	1	1	P40939	P40939 [310-326]	0	2451.2317
[K].TLPGGN	2xCarbamido	3.053E-05	4.225E-05	2	2	2	P35555; P35	P35555 [73-86]; F	0	1889.0056
[K].VALSSE	1xTMTpro [N	2.375E-05	4.225E-05	1	1	3	P27816	P27816 [436-448]	0	1649.9393
[K].CAFSSQ	1xCarbamido	9.606E-06	4.225E-05	1	1	2	P02788	P02788 [200-216]	0	2584.2674
[R].VALSPA(	1xTMTpro [K	1.572E-05	4.225E-05	1	1	3	Q13423	Q13423 [72-84]	0	1904.1825
[R].TGKPFN	1xTMTpro [K	5.917E-05	4.225E-05	1	1	1	Q9H1P3	Q9H1P3 [148-164	0	2556.4681
[R].TIPWLEI	1xTMTpro [K	1.812E-05	4.225E-05	1	1	1	O43707	O43707 [312-323]	1	2090.2254
[R].HVPVVY	1xTMTpro [N	4.108E-07	4.225E-05	1	1	1	Q14204	Q14204 [2730-275	0	2997.5794
[K].TKEQAN	2xTMTpro [K	1.119E-05	4.225E-05	1	1	1	O76070	O76070 [59-80]	1	3145.7885
[K].AQTTVT(	1xCarbamido	1.561E-05	4.225E-05	1	1	1	P08603	P08603 [425-441]	0	2240.0759

[K].TGGAYG	1xCarbamid	3.617E-06	4.225E-05	1	1	1	P49327	P49327 [2450-24		0	2898.4071
[K].HVLVILT	1xTMTpro [N	1.616E-05	4.225E-05	1	2	1	P21281	P21281 [292-308]		0	2222.2174
[R].KAHEILF	2xCarbamid	2.819E-05	4.225E-05	1	1	2	P50990	P50990 [138-152]		1	2652.5146
[R].AVNTQA	1xTMTpro [N	6.041E-05	4.225E-05	1	1	2	O60749	O60749 [270-283		0	1674.9822
[K].VALVYG	1xTMTpro [N	8.842E-06	4.225E-05	1	1	4	P06576	P06576 [265-279]		0	1906.0176
[R].GGEGT	1xTMTpro [N	9.448E-06	4.225E-05	1	1	2	P04196	P04196 [188-201]		0	1794.8982
[K].TYEAAL	1xTMTpro [K	5.738E-05	4.225E-05	1	1	2	O00231	O00231 [396-409		0	2207.1873
[K].TKDVPF	1xTMTpro [K	1.215E-05	4.225E-05	1	1	1	Q02952	Q02952 [1275-12		1	2976.6174
[R].ASHPED	1xTMTpro [N	2.963E-05	4.225E-05	1	1	1	Q9UHL4	Q9UHL4 [449-462		0	1768.9149
[K].CASQAG	1xCarbamid	3.293E-05	4.225E-05	1	1	1	Q15417	Q15417 [173-185		0	1677.8008
[K].CASQVG	1xCarbamid	4.634E-05	4.225E-05	1	1	1	Q99439	Q99439 [215-227		0	1639.8215
[R].KAEEEH	3xTMTpro [K	1.456E-05	4.225E-05	1	1	1	P00450	P00450 [799-821]		2	3700.1538
[K].TYDTYV	1xTMTpro [K	1.043E-05	4.225E-05	1	1	1	O95302	O95302 [192-208		0	2537.3606
[K].TKEEVA	2xTMTpro [K	1.049E-06	4.225E-05	1	1	1	Q0JRZ9	Q0JRZ9 [115-138		1	3499.0199
[R].TIQNDIM	1xTMTpro [N	2.88E-05	4.225E-05	1	1	2	P08311	P08311 [104-116]		0	1849.0536
[R].HVIPMN	1xCarbamid	1.169E-05	4.225E-05	1	1	2	P13796	P13796 [142-165]		0	3262.7208
[R].HVIPMN	1xCarbamid	1.683E-06	4.225E-05	1	1	3	P13796	P13796 [142-165]		0	3246.7259
[KR].HVINFD	1xTMTpro [N	1.315E-05	4.225E-05	1	2	2	O00571	O00571 [512-528		0	2387.2315
[R].TISAQN	1xTMTpro [K	5.037E-06	4.225E-05	1	1	3	P17050	P17050 [288-304]		0	2538.4279
[K].TYDPSG	1xCarbamid	3.399E-05	4.225E-05	1	1	3	Q9Y2X3	Q9Y2X3 [427-441		0	2237.1251
[R].TISDGTI	1xTMTpro [K	3.322E-05	4.225E-05	1	1	1	Q5T0N5	Q5T0N5 [293-303		0	1687.9722
[K].ASKPGE	1xCarbamid	2.968E-05	4.225E-05	1	1	1	P49589	P49589 [325-339]		0	2596.4162
[K].TLSDVE	1xTMTpro [K	1.417E-06	4.225E-05	1	1	1	Q5T4S7	Q5T4S7 [166-185		1	2821.5439
[K].VAMANI	1xTMTpro [N	1.773E-06	4.225E-05	1	1	6	P18206	P18206 [739-758]		0	2346.2957
[R].TGEPDE	1xTMTpro [N	3.793E-05	4.225E-05	1	1	1	O00168	O00168 [70-81]		0	1670.7829
[K].AVMDDF	1xOxidation	5.183E-05	4.225E-05	1	1	79	P02768	P02768 [570-581]		0	1967.044
[K].GFQQIL	1xOxidation	2.948E-07	4.225E-05	1	1	3	P06576	P06576 [490-519]		0	3975.0494
[R].TLQGLQ	1xTMTpro [N	5.883E-06	4.225E-05	1	1	1	Q13751	Q13751 [661-680		0	2569.4408
[R].TYEEGL	2xTMTpro [K	3.722E-05	4.225E-05	1	1	2	P31948	P31948 [94-109]		1	2783.5508
[R].TLQGLE	1xOxidation	1.984E-05	4.225E-05	2	2	5	P08727; P13	P08727 [302-317]		0	2442.3769
[R].HVPGAS	1xCarbamid	2.611E-05	4.225E-05	1	1	2	Q16762	Q16762 [52-65]		0	1967.9605
[R].VALTGL	1xTMTpro [N	3.399E-05	4.225E-05	1	1	3	P06576	P06576 [282-294]		0	1743.9964
[R].TIQEAD	1xTMTpro [K	3.881E-06	4.225E-05	1	1	1	Q99653	Q99653 [159-178		0	2809.4387

[R].HVPEAV	1xTMTpro [N	7.084E-07	4.225E-05	1	1	2	O15230	O15230 [1680-17	0	3172.6063
[K].ASGVEG	1xTMTpro [K	4.442E-05	4.225E-05	1	1	2	P19367	P19367 [177-187]	0	1639.9511
[K].TGGLEID	1xTMTpro [N	2.409E-05	4.225E-05	1	1	2	O95831	O95831 [409-422]	0	1774.8931
[K].CAPGVV	1xCarbamido	3.671E-06	4.225E-05	1	1	3	P21333	P21333 [810-828]	0	2319.1974
[K].CAPGVV	1xCarbamido	7.573E-07	4.225E-05	1	1	1	P21333	P21333 [810-837]	1	3657.8714
[R].CAPGYY	2xCarbamido	1.244E-05	4.225E-05	1	1	1	Q16363	Q16363 [169-185]	0	2479.2969
[R].AVPPNN	1xTMTpro [N	6.018E-07	4.225E-05	1	1	3	P00488	P00488 [14-38]	0	2906.5179
[K].AVPMIEC	1xTMTpro [N	4.005E-06	4.225E-05	1	1	1	Q9H3U1	Q9H3U1 [776-792	0	2386.1565
[R].CAPGYY	2xCarbamido	1.117E-05	4.225E-05	1	1	1	P98160	P98160 [1249-126	0	2244.0245
[R].KAGTQIE	2xTMTpro [K	9.82E-06	4.225E-05	1	1	3	P12814	P12814 [47-64]	2	2975.6618
[K].TYFPHFI	1xTMTpro [K	4.608E-07	4.225E-05	1	1	48	P69905	P69905 [42-57]	0	2442.3062
[K].AQVEIVT	2xTMTpro [K	3.131E-06	4.225E-05	1	1	2	P40121	P40121 [206-231]	0	3675.0859
[K].TKEYELL	2xTMTpro [K	5.331E-05	4.225E-05	1	1	2	P33176	P33176 [513-526]	1	2622.4807
[R].VALVEQ	1xTMTpro [N	4.776E-05	4.225E-05	1	1	2	P06401	P06401 [280-293]	0	1757.9539
[K].VAIVKPG	2xTMTpro [K	3.696E-05	4.225E-05	1	1	4	Q9Y4L1	Q9Y4L1 [47-62]	0	2619.6452
[R].GGTPAF	1xTMTpro [N	3.632E-05	4.225E-05	1	1	1	Q96JY6	Q96JY6 [255-275]	0	2361.2733
[K].AVPLQPI	1xTMTpro [K	9.191E-06	4.225E-05	1	1	1	O15230	O15230 [3011-30	0	2111.272
[R].VAEELAI	1xTMTpro [K	4.022E-05	4.225E-05	1	1	2	Q9NX63	Q9NX63 [66-76]	0	1809.0613
[K].TLQEVTC	1xTMTpro [N	1E-05	4.225E-05	1	1	4	P40939	P40939 [112-125]	0	1935.0466
[R].AVPLAVI	1xTMTpro [N	2.031E-05	4.225E-05	1	1	1	Q86W42	Q86W42 [4-22]	0	2341.3563
[R].HMAAPL	1xTMTpro [N	5.2E-05	4.225E-05	1	1	1	P24043	P24043 [1290-130	0	1611.9324
[K].TKEQVTI	2xTMTpro [K	3.594E-08	4.225E-05	1	1	2	P37840	P37840 [59-80]	1	3069.8088
[R].AVPGGA	1xTMTpro [N	2.658E-05	4.225E-05	1	1	2	Q96TC7	Q96TC7 [68-84]	0	1868.0561
[R].TLQGLEI	1xTMTpro [K	1.293E-05	4.225E-05	2	2	5	P08727; P13	P08727 [302-317]	0	2426.382
[K].TGGFGA	1xTMTpro [K	5.701E-05	4.225E-05	1	1	1	P35658	P35658 [1967-20	0	4564.3433
[K].AQTYELC	1xTMTpro [K	3.393E-05	4.225E-05	1	1	3	Q9P0V9	Q9P0V9 [107-120	0	2259.2476
[R].TLNETEI	1xOxidation [	5.724E-07	4.225E-05	1	1	2	P08729	P08729 [227-253]	0	3330.6542
[R].TLNETEI	1xTMTpro [N	1.24E-07	4.225E-05	1	1	2	P08729	P08729 [227-253]	0	3314.6593
[K].ASGGPY	1xCarbamido	2.824E-05	4.225E-05	1	1	1	Q96MM6	Q96MM6 [346-36	0	2484.2876
[K].TLNDELE	1xOxidation [	5.563E-05	4.225E-05	1	1	1	P10809	P10809 [206-221]	1	2547.362
[K].TLLGDGI	1xTMTpro [K	3.399E-05	4.225E-05	1	1	4	P52566	P52566 [51-63]	0	1920.1298
[R].AVTELGI	1xTMTpro [K	1.948E-05	4.225E-05	2	2	1	P01911; Q3C	P01911 [78-94]; Q	0	2572.3651
[R].VAECST	1xCarbamido	2.74E-05	4.225E-05	1	1	1	Q9C0B1	Q9C0B1 [323-337	0	2030.0548

[R].AVTDSIN	1xCarbamido	1.021E-05	4.225E-05	1	1	3	Q9Y490	Q9Y490 [1341-13	0	2928.5415
[R].AVTDSIN	1xCarbamido	1.075E-06	4.225E-05	1	1	2	Q9Y490	Q9Y490 [1341-13	0	2912.5465
[R].GGVDTA	1xTMTpro [N	1.504E-06	4.225E-05	1	1	1	Q14657	Q14657 [25-47]	0	2256.1804
[R].VAHEPV	2xTMTpro [K	1.456E-05	4.225E-05	1	1	1	O95674	O95674 [8-38]	2	4195.1095
[R].TYMSEN	1xTMTpro [N	1.366E-05	4.225E-05	1	1	1	O43292	O43292 [48-69]	0	2715.202
[K].ASAFNSY	1xTMTpro [N	1.419E-05	4.225E-05	1	1	3	Q13813	Q13813 [2100-21	0	2602.2381
[R].KAEVTLI	2xTMTpro [K	1.092E-05	4.225E-05	1	1	2	P52948	P52948 [818-831]	1	2471.4326
[K].TYMLAFI	3xTMTpro [K	2.444E-05	4.225E-05	1	1	1	P02763	P02763 [127-153]	1	4352.3265
[R].VAHMEF	2xCarbamido	7.15E-07	4.225E-05	1	1	1	Q01082	Q01082 [613-630]	0	2559.2114
[R].AYTASL(	1xTMTpro [K	4.954E-06	4.225E-05	1	1	1	Q99567	Q99567 [229-251]	0	2904.6003
[K].HLVMGD	1xTMTpro [K	7.961E-06	4.225E-05	1	1	1	P49321	P49321 [53-75]	0	2960.6523
[K].TILGTMF	1xTMTpro [K	1.152E-05	4.225E-05	1	1	2	P04114	P04114 [2592-26(	0	2555.4763
[K].TILLSTTI	1xTMTpro [K	1.061E-05	4.225E-05	1	1	2	P55196	P55196 [265-284]	0	2713.5155
[K].TLINAED	1xTMTpro [N	5.728E-05	4.225E-05	1	1	1	P46940	P46940 [857-870]	0	1858.0427
[K].VAKPVVI	1xTMTpro [K	3.879E-05	4.225E-05	1	1	2	P48735	P48735 [46-60]	0	2285.2125
[K].TILPAAA	1xTMTpro [N	4.228E-05	4.225E-05	1	1	2	P04843	P04843 [282-294]	0	1784.9866
[K].TGQEVV	1xTMTpro [K	4.427E-05	4.225E-05	1	1	2	P04844	P04844 [443-456]	0	2141.1734
[R].VAIAALE	1xTMTpro [K	6.621E-06	4.225E-05	1	1	3	P04181	P04181 [332-351]	0	2749.5115
[K].TYMLAFI	1xTMTpro [K	4.113E-05	4.225E-05	1	1	1	P02763	P02763 [127-138]	0	2054.076
[K].HLLEVEI	1xTMTpro [K	5.879E-05	4.225E-05	1	1	2	P11277	P11277 [559-569]	0	1945.1614
[R].TGLYNY	2xTMTpro [K	1.217E-05	4.225E-05	1	1	6	P50454	P50454 [240-252]	1	2550.3544
[R].GGCVAL	1xCarbamido	2.994E-05	4.225E-05	1	1	1	Q9UBG0	Q9UBG0 [616-63	0	2415.302
[K].AVSSAIA	1xTMTpro [N	9.252E-07	4.225E-05	1	1	2	Q9Y490	Q9Y490 [1097-11	0	2877.539
[K].TILQGNY	1xTMTpro [N	1.506E-06	4.225E-05	1	1	1	Q03701	Q03701 [733-754]	0	2877.4776
[R].ASFITPV	1xTMTpro [K	4.737E-05	4.225E-05	1	1	2	P11586	P11586 [265-292]	0	3414.8331
[K].AYSPELI	1xTMTpro [K	4.003E-07	4.225E-05	1	1	2	Q8IW45	Q8IW45 [118-140	0	3151.7283
[K].TKPVTGI	2xTMTpro [K	2.027E-05	4.225E-05	1	1	2	Q14204	Q14204 [1264-12	0	3440.9933
[R].AYEAAA	1xTMTpro [K	1.023E-05	4.225E-05	1	1	1	P42166	P42166 [572-591]	0	2642.4798
[K].TYTCNVI	1xCarbamido	4.817E-05	4.225E-05	1	1	1	P01861	P01861 [80-93]	0	2577.3911
[K].HLLGVEI	1xTMTpro [K	5.437E-05	4.225E-05	1	1	4	Q01082	Q01082 [559-569]	0	1873.1403
[K].TLKPGTI	2xTMTpro [K	4.595E-05	4.225E-05	1	1	2	P07948	P07948 [276-295]	0	3120.7617
[R].HLVLLDT	1xTMTpro [N	1.307E-05	4.225E-05	1	1	1	O00754	O00754 [842-857]	0	1948.1048
[K].KAENTG	2xTMTpro [K	1.709E-06	4.225E-05	1	1	3	P02549	P02549 [1102-11	1	2886.6029

[RK].HYFQN	1xTMTpro [N	4.05E-05	4.225E-05	3	4	1	P61204; P18	P61204 [80-97]; F		0	2457.2482
[R].AVTGYT	1xTMTpro [K	1.349E-05	4.225E-05	1	1	2	P58107	P58107 [687-708]		0	3055.6054
[R].AVTGYN	1xTMTpro [K	3.416E-06	4.225E-05	1	1	2	P15924	P15924 [2335-235		0	2991.5741
[R].TLLATVD	1xTMTpro [N	2.148E-05	4.225E-05	1	1	1	Q05397	Q05397 [963-981]		0	2352.3458
[R].AVTGYK	2xTMTpro [K	9.09E-06	4.225E-05	1	1	3	Q15149	Q15149 [2910-29		1	3386.8599
[K].TYQGLE	1xCarbamido	5.074E-06	4.225E-05	1	1	1	Q9UHD1	Q9UHD1 [165-19		0	3617.8264
[R].TYQELLY	1xTMTpro [N	3.378E-06	4.225E-05	1	1	1	Q9NX24	Q9NX24 [23-41]		0	2459.3577
[K].ASEVMG	1xTMTpro [N	1.349E-05	4.225E-05	1	1	1	Q8WWY3	Q8WWY3 [77-91]		0	1909.9649
[K].AYEDQT	2xTMTpro [K	3.048E-05	4.225E-05	1	1	2	Q9UIJ7	Q9UIJ7 [175-189]		0	2787.5385
[K].TYNTNA	1xCarbamido	1.154E-05	4.225E-05	1	1	1	P05186	P05186 [100-122]		0	3010.5436
[K].TGQYSG	1xCarbamido	4.857E-05	4.225E-05	1	1	2	Q6NUK1	Q6NUK1 [321-332		0	1971.0137
[K].KAEVNTI	2xTMTpro [K	5.115E-05	4.225E-05	1	1	1	P05165	P05165 [186-200]		1	2486.4799
[R].TIIDNTDI	1xOxidation [	1.131E-05	4.225E-05	1	1	1	Q96AG3	Q96AG3 [348-370		0	2975.4991
[K].ASFATPY	1xTMTpro [K	1.578E-05	4.225E-05	1	1	1	O95671	O95671 [49-63]		0	2216.1553
[K].AVTEQG	1xTMTpro [N	7.368E-06	4.225E-05	1	1	5	P31946	P31946 [30-43]		0	1902.9477
[R].ASFENN	2xCarbamido	9.007E-06	4.225E-05	1	1	2	P56537	P56537 [5-18]		0	2255.108
[K].AVTEQG	1xTMTpro [N	1.154E-05	4.225E-05	1	1	10	P27348	P27348 [28-41]		0	1836.9259
[R].GFSELS	1xTMTpro [N	2.183E-05	4.225E-05	1	1	1	Q96FX7	Q96FX7 [224-241		0	2355.2879
[K].HILGFDT	1xTMTpro [N	2.921E-07	4.225E-05	1	1	2	Q9Y224	Q9Y224 [186-205		0	2457.3421
[K].TKDEYLI	2xTMTpro [K	3.148E-06	4.225E-05	1	1	1	Q9NSE4	Q9NSE4 [539-555		1	2931.6608
[K].TKPYIQV	2xTMTpro [K	5.374E-06	4.225E-05	1	1	6	P11021	P11021 [124-138]		0	2517.4857
[K].AVTELNI	1xTMTpro [N	2.942E-05	4.225E-05	1	1	2	Q04917	Q04917 [29-42]		0	1890.9728
[R].AQVTELI	1xTMTpro [K	1.059E-05	4.225E-05	1	1	1	Q7Z406	Q7Z406 [1563-15		0	2441.2903
[K].TYNFLPE	1xTMTpro [K	4.572E-05	4.225E-05	1	1	3	P30740	P30740 [97-110]		0	2295.288
[R].TLLQGS	1xCarbamido	8.941E-06	4.225E-05	1	1	1	Q15149	Q15149 [3486-35		0	2547.3984
[K].TIIPLISQ	1xCarbamido	4.989E-05	4.225E-05	1	1	2	P40926	P40926 [204-215]		0	1979.1855
[R].GGCVDS	1xCarbamido	1.256E-06	4.225E-05	1	1	1	Q9Y2P8	Q9Y2P8 [290-314		0	3243.7209
[R].GGDGGI	1xTMTpro [N	2.789E-05	4.225E-05	1	1	1	Q8N8J7	Q8N8J7 [10-26]		1	2022.9284
[R].VAILVDD	2xCarbamido	1.675E-07	4.225E-05	2	2	2	P11908; P60	P11908 [215-235]		0	2884.4499
[K].HIVNHA\	1xTMTpro [N	3.702E-05	4.225E-05	1	1	1	Q9NQT8	Q9NQT8 [352-36		0	1989.0585
[K].AVSIIECI	1xCarbamido	1.244E-05	4.225E-05	1	1	2	Q92890	Q92890 [179-202		1	3376.7049
[R].TGPLPP	1xTMTpro [N	3.012E-06	4.225E-05	1	1	1	Q14914	Q14914 [248-266		0	2377.3451
[R].HWILPQ	1xTMTpro [N	3.881E-06	4.225E-05	1	1	4	Q15293	Q15293 [271-286		0	2254.1324

[R].AQVPFE	1xTMTpro [K	2.379E-05	4.225E-05	1	1	1	Q99828	Q99828 [51-65]	0	2320.3772
[K].TKPIVKP	2xTMTpro [K	1.059E-05	4.225E-05	1	1	1	O95793	O95793 [269-290]	0	3322.9303
[K].GHELVL/	1xCarbamido	6.458E-08	4.225E-05	1	1	1	Q13308	Q13308 [373-400]	0	3241.6343
[K].ASGDLIF	1xTMTpro [N	3.447E-06	4.225E-05	1	1	1	Q9H0A0	Q9H0A0 [600-625	0	3040.4971
[K].ASGDSA	1xTMTpro [N	5.764E-06	4.225E-05	1	1	1	Q9UJS0	Q9UJS0 [313-331	0	2294.2424
[K].AYVSTLI	1xTMTpro [N	3.76E-05	4.225E-05	1	1	3	O00303	O00303 [215-226]	0	1554.8633
[R].ASGEMA	1xTMTpro [N	3.051E-06	4.225E-05	1	1	2	P54578	P54578 [142-157]	0	1944.018
[K].TKPMLE	1xTMTpro [K	3.393E-05	4.225E-05	1	1	1	Q9HAV4	Q9HAV4 [1182-12	0	3055.6153
[K].TLMFGS	1xTMTpro [K	1.399E-05	4.225E-05	1	1	2	P19652	P19652 [127-138]	0	2027.0651
[K].TKLEAQ/	1xTMTpro [K	4.665E-05	4.225E-05	1	1	1	Q9UHB9	Q9UHB9 [182-198	1	2524.4089
[R].AYYENS	1xTMTpro [K	7.888E-06	4.225E-05	1	1	3	P01024	P01024 [208-225]	0	2774.4169
[K].HWLDSF	1xTMTpro [N	4.17E-05	4.225E-05	1	1	1	Q02218	Q02218 [583-600]	0	2460.242
[R].TYLLDG:	1xCarbamido	3.509E-07	4.225E-05	1	1	2	Q13813	Q13813 [2226-22	0	3269.6413
[R].TGPCFT	3xCarbamido	1.006E-05	4.225E-05	1	1	2	P35555	P35555 [183-206]	0	3310.6548
[K].TLNDELE	1xTMTpro [K	1.587E-05	4.225E-05	1	1	3	P10809	P10809 [206-218]	0	2113.1706
[R].TGPCFT	3xCarbamido	7.393E-07	4.225E-05	1	1	3	P35555	P35555 [183-206]	0	3294.6599
[R].AYYNGIS	1xTMTpro [N	5.261E-05	4.225E-05	1	1	1	P21810	P21810 [330-353]	0	3150.6008
[K].VALSEC	1xCarbamido	4.673E-05	4.225E-05	1	1	1	Q14258	Q14258 [470-494]	0	3207.5999
[R].TGPAAT	1xTMTpro [K	7.341E-06	4.225E-05	1	1	8	P07237	P07237 [133-162]	0	3543.9078
[R].KAEEEL(	2xTMTpro [K	8.46E-06	4.225E-05	1	1	2	Q9UQE7	Q9UQE7 [684-695	1	2258.306
[R].TYLGNA	2xCarbamido	1.206E-05	4.225E-05	1	1	4	P02751	P02751 [68-83]	0	2096.0224
[K].TLNDELE	1xOxidation [	5.384E-05	4.225E-05	1	1	2	P10809	P10809 [206-218]	0	2129.1655
[K].VAIQLND	1xTMTpro [N	4.899E-07	4.225E-05	1	1	1	P11216	P11216 [334-352]	0	2422.3447
[R].TKPPEG	2xTMTpro [K	1.722E-05	4.225E-05	1	1	2	Q53GG5	Q53GG5 [349-363	0	2621.5007
[K].TGPIQDI	1xTMTpro [K	8.591E-07	4.225E-05	1	1	2	O14786	O14786 [680-702]	0	3143.5525
[K].HWPFM\	1xTMTpro [K	3.824E-06	4.225E-05	1	1	3	P11142	P11142 [89-102]	0	2262.2461
[K].ASADLM	1xCarbamido	5.527E-05	4.225E-05	1	1	1	Q9UBI6	Q9UBI6 [35-48]	0	1943.8911
[K].TYLVSG(	1xTMTpro [K	8.306E-06	4.225E-05	1	1	2	Q9H7Z7	Q9H7Z7 [174-193	0	2924.5975
[R].TYIVEET	1xTMTpro [K	7.505E-06	4.225E-05	1	1	2	Q5SWX8	Q5SWX8 [4-23]	0	2877.5853
[R].TGQCEC	3xCarbamido	2.493E-06	4.225E-05	1	1	1	P11047	P11047 [951-967]	0	2322.0345
[K].ASFVDEI	1xCarbamido	4.817E-05	4.225E-05	1	1	1	Q9NNW7	Q9NNW7 [159-17	0	2128.1352
[K].AYTNFD/	1xTMTpro [K	1.14E-05	4.225E-05	1	2	2	P07355	P07355 [29-47]	1	2763.4809
[K].AVSPLLI	1xTMTpro [N	5.516E-07	4.225E-05	1	1	2	P19525	P19525 [81-107]	0	3125.6836

[K].TGQATV	1xCarbamido	1.404E-06	4.225E-05	1	1	1	P00558	P00558 [298-323]	1	3517.8599
[R].TYLTITD	1xTMTpro [K	2.584E-05	4.225E-05	1	1	1	Q5JTH9	Q5JTH9 [719-735	0	2560.4729
[K].HWVFDE	1xTMTpro [K	1.472E-05	4.225E-05	1	1	1	P50281	P50281 [387-401]	0	2383.2578
[K].GFSEGL	1xTMTpro [K	2.083E-05	4.225E-05	1	1	2	P51858	P51858 [81-96]	0	2428.3004
[K].TILSNQT	1xTMTpro [K	2.14E-05	4.225E-05	1	1	2	P32969	P32969 [3-21]	0	2721.553
[K].TGQAPG	1xTMTpro [K	1.224E-05	4.225E-05	1	1	2	P99999	P99999 [41-54]	0	2037.0897
[K].TKPSPS	1xTMTpro [K	1.802E-05	4.225E-05	1	1	1	Q8TCS8	Q8TCS8 [88-103]	0	2473.3769
[R].AYVAVD	1xTMTpro [N	4.419E-05	4.225E-05	1	1	2	P16278	P16278 [443-457]	0	1891.0608
[K].HWPFQ\	2xTMTpro [K	2.902E-06	4.225E-05	1	1	5	P0DMV9	P0DMV9 [89-102]	0	2593.4707
[K].TKDGQII	1xTMTpro [K	2.64E-05	4.225E-05	1	1	1	Q9P258	Q9P258 [319-333	1	2243.3731
[R].AVSIQT(	1xTMTpro [K	3.293E-05	4.225E-05	1	1	1	Q9Y365	Q9Y365 [164-179	0	2271.3204
[R].TYLSEGI	1xTMTpro [K	3.651E-05	4.225E-05	1	1	1	Q16401	Q16401 [483-504]	0	2969.5639
[K].TYITDPV	1xCarbamido	1.073E-05	4.225E-05	1	1	2	Q93052	Q93052 [354-371]	0	2563.4086
[K].TGQAAE	1xTMTpro [K	5.428E-05	4.225E-05	1	1	3	O00231	O00231 [60-71]	0	1766.0668
[R].AWVWN	1xCarbamido	5.074E-07	4.225E-05	1	1	1	P43487	P43487 [119-141]	0	3347.7491
[K].AVSMPS	1xTMTpro [N	4.705E-05	4.225E-05	1	1	2	P04114	P04114 [3277-32	0	1870.0064
[R].AVSMLE	1xTMTpro [N	1.305E-05	4.225E-05	1	1	1	Q99959	Q99959 [356-369]	0	1860.9631
[R].KIYFTDS	2xTMTpro [K	0.0001684	7.389E-05	1	1	1	Q9HDC9	Q9HDC9 [211-22	1	2088.2157
[K].KLEDEIL	1xOxidation	0.0001121	7.389E-05	1	1	1	P35749	P35749 [982-996]	1	2732.4957
[R].HLPFEG	1xTMTpro [N	0.0001075	7.389E-05	1	1	1	Q9NY15	Q9NY15 [1849-18	0	2824.5317
[R].KIWCFG	1xCarbamido	8.171E-05	7.389E-05	1	1	2	P13639	P13639 [648-667]	1	3145.7536
[R].HPDVEV	1xTMTpro [N	7.428E-05	7.389E-05	1	1	2	P09874	P09874 [66-78]	0	1803.9196
[R].GQLQEA	1xTMTpro [N	8.084E-05	7.389E-05	1	1	2	O15294	O15294 [103-113]	0	1647.8774
[K].KLESFYI	2xTMTpro [K	0.0001279	7.389E-05	1	1	2	P07686	P07686 [382-390]	1	2068.2623
[R].HGESAW	1xTMTpro [N	7.169E-05	7.389E-05	1	1	7	P18669	P18669 [11-21]	0	1616.81
[K].KLYDIDV	2xTMTpro [K	0.0001729	7.389E-05	1	1	3	P62750	P62750 [115-123]	1	1977.2201
[K].IAPGAV\	1xCarbamido	8.298E-05	7.389E-05	1	1	2	O00399	O00399 [11-24]	0	1803.9958
[K].GPGVDL	1xTMTpro [K	7.337E-05	7.389E-05	1	1	2	Q09666	Q09666 [5282-52	0	2119.2077
[R].LGGLDV	1xTMTpro [K	0.0001793	7.389E-05	1	1	1	Q5SGD2	Q5SGD2 [72-84]	0	1986.1403
[R].LAPEYE	1xTMTpro [N	0.0001914	7.389E-05	1	1	4	P30101	P30101 [63-73]	0	1495.8076
[R].HPEELSI	1xTMTpro [K	0.0001918	7.389E-05	1	1	1	Q96AC1	Q96AC1 [134-142	0	1674.0082
[R].HPDYSV	1xTMTpro [N	6.297E-05	7.389E-05	1	1	4	P02768	P02768 [362-372]	0	1615.9491
[K].HFVGML	1xCarbamido	0.0001306	7.389E-05	1	1	1	P60981	P60981 [70-81]	1	2097.1229

[K].IFVEFTS	1xCarbamido	0.0001435	7.389E-05	1	1	1	P26368	P26368 [432-444]	0	2228.1917
[R].GPGAGF	1xTMTpro [N	0.0001442	7.389E-05	1	1	2	Q9Y6C2	Q9Y6C2 [611-623	0	1369.7507
[K].HFKDED	1xTMTpro [K	7.791E-05	7.389E-05	1	1	1	O95365	O95365 [537-555]	1	2739.3353
[R].KGPELP	2xTMTpro [K	8.453E-05	7.389E-05	1	1	2	Q96DI7	Q96DI7 [8-18]	1	2089.3565
[K].IAFGGET	1xTMTpro [N	0.000157	7.389E-05	1	1	1	P51648	P51648 [300-311]	0	1570.8032
[K].LAYVYQI	1xTMTpro [K	0.0001971	7.389E-05	1	1	1	Q12884	Q12884 [162-173]	0	2110.2192
[K].GPGAGS	1xTMTpro [N	9.587E-05	7.389E-05	1	1	1	O95782	O95782 [621-632]	0	1376.7089
[K].KIEEQLT	2xTMTpro [K	6.307E-05	7.389E-05	1	1	2	P30740	P30740 [245-254]	1	2143.3154
[R].HFIEQGI	2xCarbamido	0.0001392	7.389E-05	1	1	1	P00505	P00505 [263-279]	0	2675.3929
[R].IAFSSTS	1xTMTpro [K	0.0001368	7.389E-05	1	1	2	Q9Y305	Q9Y305 [241-250	0	1675.0286
[K].IFQTLNG	1xTMTpro [K	0.0001656	7.389E-05	1	1	1	O00469	O00469 [218-232]	0	2254.3302
[K].LGGSQE	1xTMTpro [K	0.0001789	7.389E-05	1	1	2	P37837	P37837 [72-81]	0	1682.9569
[K].GQSEDF	1xTMTpro [N	0.0001817	7.389E-05	1	1	1	P08195	P08195 [511-524]	0	1809.9666
[K].LAQANG	1xOxidation [	9.394E-05	7.389E-05	1	1	2	P06733	P06733 [359-372]	0	1845.9713
[R].HPNIITLI	1xTMTpro [K	0.0001136	7.389E-05	1	1	1	O43293	O43293 [73-86]	0	2299.3054
[K].IAFTGST	1xTMTpro [K	7.508E-05	7.389E-05	2	2	4	P47895; O94	P47895 [253-263]	0	1717.998
[R].HLLIAQE	1xTMTpro [N	0.0001331	7.389E-05	1	1	1	Q86YR5	Q86YR5 [315-325	0	1582.9236
[R].LGGSPF	1xTMTpro [K	0.0001824	7.389E-05	1	1	2	Q14204	Q14204 [1900-19	0	1754.0092
[K].HLIEDLII	1xTMTpro [K	8.479E-05	7.389E-05	1	1	1	Q08495	Q08495 [161-172]	0	2005.1825
[K].HFSGLEI	1xTMTpro [N	6.633E-05	7.389E-05	1	1	2	P50990	P50990 [21-31]	0	1611.845
[K].GQSGIIY	1xCarbamido	0.000165	7.389E-05	1	1	1	P46063	P46063 [314-325]	0	1996.0818
[K].ICANHYI	1xCarbamido	8.427E-05	7.389E-05	1	1	1	P43487	P43487 [98-118]	0	3042.5455
[R].ICDECN	2xCarbamido	8.638E-05	7.389E-05	1	1	1	Q7RTV0	Q7RTV0 [45-57]	0	1925.8441
[K].LFQLMVI	1xTMTpro [K	9.035E-05	7.389E-05	1	1	2	Q12907	Q12907 [273-291]	0	2926.5152
[K].LAPPLVT	1xTMTpro [N	6.79E-05	7.389E-05	1	1	2	P63010	P63010 [284-304]	0	2569.4924
[K].HEWQVN	1xTMTpro [K	8.705E-05	7.389E-05	1	1	1	Q9Y281	Q9Y281 [133-146	1	2333.2494
[R].GQTSTY	1xTMTpro [K	0.0001522	7.389E-05	1	1	2	Q96T37	Q96T37 [411-420]	0	1709.9718
[R].HILEMNI	1xTMTpro [K	0.0001672	7.389E-05	1	1	1	O95785	O95785 [1622-16	0	1726.9806
[R].HLIEQDF	1xTMTpro [N	8.009E-05	7.389E-05	1	1	2	Q9H4M9	Q9H4M9 [77-87]	0	1646.8644
[R].ICDDELII	1xCarbamido	0.000196	7.389E-05	1	1	6	P17987	P17987 [356-365]	0	1840.0745
[R].KIEFPPF	1xTMTpro [K	0.0002015	7.389E-05	1	1	1	P62195	P62195 [314-325]	1	2035.1468
[K].HQAFMA	1xOxidation [	6.885E-05	7.389E-05	1	1	1	Q01082	Q01082 [1316-13	0	1971.0614
[K].HQAFMA	1xTMTpro [K	9.234E-05	7.389E-05	1	1	2	Q01082	Q01082 [1316-13	0	1955.0664

[RK].GPFTD	1xTMTpro [K	8.059E-05	7.389E-05	2	2	2	O95292; Q9I	O95292 [20-31]; (	0	1900.1035
[R].LGHELQ	1xTMTpro [K	6.357E-05	7.389E-05	1	1	1	Q14980	Q14980 [1655-16(	0	1802.078
[R].HIIEDPC	1xCarbamido	0.00018	7.389E-05	1	1	2	Q7Z6Z7	Q7Z6Z7 [1826-18	0	1557.8378
[K].GQSSW(	1xTMTpro [N	7.393E-05	7.389E-05	1	1	2	Q03252	Q03252 [550-561]	0	1602.7832
[R].HFCGGA	1xCarbamido	0.0001078	7.389E-05	1	1	5	P20160	P20160 [50-60]	0	1542.8283
[R].HFEELE	1xTMTpro [N	7.004E-05	7.389E-05	2	2	2	Q8TAQ2; Q9	Q8TAQ2 [905-915	0	1723.8644
[R].KGPAPE	1xOxidation [	8.009E-05	7.389E-05	1	1	1	Q13435	Q13435 [815-843]	1	4020.1854
[R].LGGIGQI	1xTMTpro [K	0.0001392	7.389E-05	1	1	4	Q08211	Q08211 [810-819]	0	1612.0078
[R].KLEHLIT	1xTMTpro [K	7.416E-05	7.389E-05	1	1	2	Q14204	Q14204 [1807-18.	1	2224.3421
[K].HFSVEG	1xTMTpro [N	9.091E-05	7.389E-05	2	5	1	P07900; P08	P07900 [328-338]	0	1652.8716
[K].LGGINNI	1xTMTpro [N	6.748E-05	7.389E-05	1	1	2	Q9UL18	Q9UL18 [569-581	0	1735.0298
[K].KIENLCA	1xCarbamido	9.587E-05	7.389E-05	1	1	1	P61086	P61086 [165-176]	1	2062.1069
[K].GQPDEE	2xTMTpro [K	0.0001964	7.389E-05	1	1	2	P54577	P54577 [475-484]	0	2053.211
[R].HLMTLVI	1xTMTpro [N	0.0001025	7.389E-05	1	1	1	P30566	P30566 [310-329]	0	2706.4067
[R].KLENMY	2xTMTpro [K	0.0001322	7.389E-05	1	1	2	P11277	P11277 [1685-169	1	2476.4566
[K].HGDGTT	1xTMTpro [K	0.0001053	7.389E-05	1	1	1	P55786	P55786 [754-765]	0	1909.0709
[K].LAPGTIV	1xTMTpro [K	7.202E-05	7.389E-05	1	1	3	P07686	P07686 [415-425]	0	1821.113
[K].GQNVTE	1xCarbamido	8.584E-05	7.389E-05	1	1	1	Q9NZD2	Q9NZD2 [168-179	0	2044.0512
[R].HPFPGP	1xTMTpro [N	0.0001564	7.389E-05	1	1	1	P49915	P49915 [436-446]	0	1465.8599
[R].LAVQQV	1xTMTpro [N	0.0001315	7.389E-05	1	1	2	O00461	O00461 [416-428]	0	1816.0248
[K].HPESNT.	1xTMTpro [K	0.0001746	7.389E-05	1	1	1	Q9Y696	Q9Y696 [111-124	0	2126.1196
[R].KGTDVN	1xTMTpro [K	6.509E-05	7.389E-05	1	1	1	P04083	P04083 [214-228]	1	2287.3266
[R].GPGLSQ	1xTMTpro [K	8.298E-05	7.389E-05	1	1	2	Q14315	Q14315 [2642-265	0	1797.0514
[K].LAQECC	2xCarbamido	0.0001241	7.389E-05	1	1	1	Q9NVH2	Q9NVH2 [347-357	0	1768.8178
[K].KGTPWE	2xTMTpro [K	9.894E-05	7.389E-05	1	1	3	P63279	P63279 [49-59]	1	2132.2684
[R].LAGLGL(	1xTMTpro [N	0.0001762	7.389E-05	1	1	1	Q6EMK4	Q6EMK4 [199-214	0	2021.1351
[R].GQLQSH	1xTMTpro [N	7.451E-05	7.389E-05	1	1	1	P98175	P98175 [147-157]	0	1484.8253
[K].IAVTGTG	1xTMTpro [K	6.54E-05	7.389E-05	1	1	1	Q96SL4	Q96SL4 [118-130	0	1878.1093
[K].LAPITYP	1xTMTpro [K	0.0001482	7.389E-05	1	1	2	P15153	P15153 [134-147]	0	2064.2713
[K].LGGNEIV	1xTMTpro [K	0.0001528	7.389E-05	1	1	1	O60762	O60762 [241-251]	0	1785.0766
[R].IFTVAQV	1xTMTpro [K	7.269E-05	7.389E-05	1	1	1	Q9Y666	Q9Y666 [878-892	0	2317.2717
[R].KMGAPE	2xTMTpro [K	0.0001023	7.389E-05	1	1	1	P02794	P02794 [158-173]	1	2668.4836
[K].IFSPNVV	1xTMTpro [K	7.091E-05	7.389E-05	1	1	2	O00429	O00429 [134-152]	0	2666.5447

[K].HLLDKPI	1xTMTpro [K	0.0001968	7.389E-05	1	1	1	P09917	P09917 [36-48]	0	2302.2476
[R].LFSQDE	1xCarbamido	0.0001662	7.389E-05	1	1	2	Q6P6C2	Q6P6C2 [94-102]	0	1705.9075
[R].LAGCTV	1xCarbamido	0.0001178	7.389E-05	1	1	2	Q6YN16	Q6YN16 [8-20]	0	1656.9062
[K].LFTAESL	1xTMTpro [K	7.213E-05	7.389E-05	1	1	4	P48444	P48444 [337-347]	0	1800.1126
[K].LFTEVE(	1xCarbamido	0.0001803	7.389E-05	1	1	1	P62487	P62487 [30-41]	0	1950.0498
[K].KMEMEM	1xOxidation [	0.0001918	7.389E-05	1	1	2	Q16181	Q16181 [352-364]	1	2618.3518
[R].IAWTHIT	1xTMTpro [N	0.0001803	7.389E-05	1	1	1	Q9H0E2	Q9H0E2 [131-143	0	1841.0604
[R].GPGIFEI	1xTMTpro [K	7.026E-05	7.389E-05	1	1	1	Q9H1E5	Q9H1E5 [124-138	0	2344.3044
[K].KGRPMV	2xTMTpro [K	7.815E-05	7.389E-05	1	1	1	Q9NR45	Q9NR45 [149-166	1	2896.5697
[R].GQPIYIQ	1xTMTpro [K	6.653E-05	7.389E-05	1	1	2	P26599	P26599 [123-134]	0	2040.1522
[K].IAWESP	1xTMTpro [N	0.0001752	7.389E-05	1	1	2	P02751	P02751 [1741-175	0	1661.893
[R].GQPLPE	1xTMTpro [K	0.0001202	7.389E-05	1	1	1	P51531	P51531 [195-209]	0	2187.2993
[K].HIGLVYS	1xTMTpro [N	0.0001118	7.389E-05	1	1	1	P25787	P25787 [71-84]	0	1868.9648
[K].HPFVDS	1xTMTpro [N	6.337E-05	7.389E-05	1	1	1	Q8TB45	Q8TB45 [206-218	0	1940.035
[R].IFTSIGEI	1xTMTpro [N	8.718E-05	7.389E-05	1	1	1	P35232	P35232 [106-117]	0	1748.8662
[K].IAVYSCF	1xCarbamido	9.754E-05	7.389E-05	1	1	1	P50990	P50990 [239-254]	0	2440.2748
[R].HGESEF	1xTMTpro [K	0.0001075	7.389E-05	1	1	1	O60825	O60825 [257-267]	0	1839.0256
[K].KISIPVDI	1xOxidation [	0.0001306	7.389E-05	1	1	1	P49368	P49368 [139-163]	1	3664.0369
[R].HPDEAA	1xTMTpro [K	0.0001166	7.389E-05	1	1	4	P02671	P02671 [513-527]	0	2202.1323
[R].LAQPYV	1xTMTpro [K	6.769E-05	7.389E-05	1	1	3	P36776	P36776 [159-169]	0	1843.1337
[R].LGAGPQ	1xTMTpro [N	0.0001885	7.389E-05	1	1	1	O75676	O75676 [285-296]	0	1486.8297
[R].LALTTAE	1xCarbamido	6.601E-05	7.389E-05	1	2	2	P21281	P21281 [277-291]	0	2366.2921
[K].HNFCFM	1xCarbamido	0.0001061	7.389E-05	1	1	1	Q96RQ3	Q96RQ3 [329-339	0	1790.8096
[R].LASTSDI	1xTMTpro [K	0.0001051	7.389E-05	1	1	1	O14974	O14974 [505-514]	0	1700.9562
[K].KIPGIYVI	2xTMTpro [K	8.492E-05	7.389E-05	1	1	3	P50897	P50897 [61-74]	1	2442.5516
[K].LGAGQT	1xTMTpro [K	9.587E-05	7.389E-05	1	1	1	Q96RT7	Q96RT7 [1543-15	0	2828.6741
[R].LAQQISI	1xTMTpro [N	0.000111	7.389E-05	1	1	1	Q93008	Q93008 [1221-12	0	1521.8192
[R].KLPDSFI	3xTMTpro [K	0.0001166	7.389E-05	1	1	2	P46937	P46937 [90-102]	1	2746.6648
[K].LGEGEG	1xTMTpro [K	0.0001789	7.389E-05	2	2	2	Q9UNH7; Q	Q9UNH7 [110-11	0	1616.8809
[R].GPPSTS	1xTMTpro [N	0.0002023	7.389E-05	1	1	1	O00160	O00160 [983-993]	0	1333.7395
[R].KICLDLQ	1xCarbamido	7.911E-05	7.389E-05	1	1	2	P02776	P02776 [81-92]	1	2374.4348
[R].IALTDNA	1xTMTpro [N	0.0001082	7.389E-05	1	1	2	P18124	P18124 [167-177]	0	1474.8912
[K].LGAGYP	1xTMTpro [K	9.063E-05	7.389E-05	1	1	1	Q16836	Q16836 [250-271]	0	2965.5877

[K].KICAVGI	1xCarbamido	0.0001036	7.389E-05	1	1	1	P55060	P55060 [840-848]	1	1902.2027
[K].GPVYHE	2xCarbamido	0.0001123	7.389E-05	1	1	1	Q7Z4H8	Q7Z4H8 [134-152	0	3001.4104
[K].GPQEVA	1xTMTpro [K	7.508E-05	7.389E-05	1	1	1	Q15075	Q15075 [279-292]	0	2196.252
[K].KKDDET	3xTMTpro [K	0.0001228	7.389E-05	1	1	1	Q9UJX2	Q9UJX2 [129-143	2	2890.6878
[R].HGYFCF	1xCarbamido	0.0001647	7.389E-05	1	1	1	Q9BZQ8	Q9BZQ8 [173-185	0	2218.0995
[K].LGEEAFI	1xCarbamido	0.0001228	7.389E-05	1	1	1	Q9P016	Q9P016 [107-128	1	3410.8057
[R].LALGDD	1xTMTpro [K	6.407E-05	7.389E-05	1	1	2	P17174	P17174 [87-97]	0	1708.0137
[K].LALQWH	2xTMTpro [K	9.133E-05	7.389E-05	1	1	1	Q13217	Q13217 [417-433]	1	3038.6516
[R].GPVVLA	1xCarbamido	9.617E-05	7.389E-05	1	1	1	P21281	P21281 [142-163]	0	2773.4336
[K].LALQWH	1xTMTpro [K	0.0001218	7.389E-05	1	1	2	Q13217	Q13217 [417-432]	0	2606.3495
[R].LALQTIP	1xTMTpro [K	6.822E-05	7.389E-05	1	2	1	P51003	P51003 [173-191]	1	2776.5952
[R].LGAPEN	1xTMTpro [N	0.0001131	7.389E-05	1	1	1	Q9Y4F1	Q9Y4F1 [14-27]	0	1747.9509
[K].KLNVTE	2xTMTpro [K	8.571E-05	7.389E-05	1	1	4	P06733	P06733 [81-89]	1	2001.2161
[K].HLVQAL	1xTMTpro [K	9.119E-05	7.389E-05	1	1	1	Q08378	Q08378 [1172-118	0	1945.1726
[K].LALGQIN	1xTMTpro [K	8.046E-05	7.389E-05	1	1	1	Q9BZE4	Q9BZE4 [94-103]	0	1649.0606
[R].KIPNPDF	1xTMTpro [K	6.643E-05	7.389E-05	1	1	3	P27824	P27824 [401-415]	1	2472.3419
[K].IGEDAE\	1xTMTpro [K	0.0001442	7.389E-05	1	1	1	Q14185	Q14185 [236-253]	0	2603.377
[K].KLPSVE	1xTMTpro [K	7.258E-05	7.389E-05	1	1	1	Q9UHA4	Q9UHA4 [12-27]	1	2328.3895
[R].IAQPNYI	1xTMTpro [N	9.291E-05	7.389E-05	1	1	1	P63096	P63096 [162-176]	0	2060.146
[K].KHPDAS	2xTMTpro [K	0.0001614	7.389E-05	1	1	2	P09429	P09429 [30-43]	1	2505.3918
[K].KHPDAS	3xTMTpro [K	0.0001508	7.389E-05	1	1	1	P09429	P09429 [30-44]	2	2937.6939
[R].KHPEVD	1xTMTpro [K	9.482E-05	7.389E-05	1	1	1	P53396	P53396 [562-576]	1	2346.3789
[R].KIQDHQ	1xCarbamido	7.439E-05	7.389E-05	1	1	1	Q8N8S7	Q8N8S7 [52-66]	1	2675.5847
[R].HGVPIS\	1xTMTpro [K	6.591E-05	7.389E-05	1	1	1	O75955	O75955 [59-72]	0	2014.2305
[R].GPPNPN	1xTMTpro [K	0.0001196	7.389E-05	1	1	1	Q9NY27	Q9NY27 [69-84]	0	2455.2823
[K].KLGEMW	2xTMTpro [K	7.102E-05	7.389E-05	1	1	2	P26583	P26583 [128-139]	1	2306.2995
[K].KHSQFI	2xTMTpro [K	6.57E-05	7.389E-05	1	2	1	P08238	P08238 [204-219]	1	2849.6746
[R].GQCLIT	1xCarbamido	0.0001012	7.389E-05	1	1	2	Q14520	Q14520 [123-135]	0	1886.9754
[R].GQCIKPI	1xCarbamido	0.0001564	7.389E-05	1	1	2	P35555	P35555 [668-680]	0	2331.4039
[R].HLDLAYI	1xTMTpro [N	0.0001125	7.389E-05	1	1	1	Q8N1F8	Q8N1F8 [258-270	0	1855.0146
[R].LATALQI	2xTMTpro [K	9.262E-05	7.389E-05	4	4	2	P67936; P09	P67936 [70-82]; P	1	2356.4268
[R].GPPPEL	1xTMTpro [N	9.557E-05	7.389E-05	1	1	1	O15230	O15230 [3229-324	0	1900.0248
[R].GPPPTD	1xTMTpro [N	7.202E-05	7.389E-05	1	1	2	Q16630	Q16630 [377-392]	0	2086.0677

[K].KLGEMW	1xOxidation [	0.0001587	7.389E-05	1	1	2	P26583	P26583 [128-139]	1	2322.2944
[K].LGELPS\	1xTMTpro [N	9.739E-05	7.389E-05	1	1	3	P56134	P56134 [23-33]	0	1618.931
[R].HIVLKPF	2xTMTpro [K	8.479E-05	7.389E-05	1	1	2	P12111	P12111 [426-443]	0	2940.843
[K].KLDDFVI	1xTMTpro [K	7.18E-05	7.389E-05	1	1	2	Q14108	Q14108 [391-402]	1	2016.1257
[R].KIGLETV	2xTMTpro [K	6.208E-05	7.389E-05	1	1	1	Q16881	Q16881 [449-458]	1	1956.2674
[K].GPVYVA	3xCarbamido	7.462E-05	7.389E-05	1	1	2	P54709	P54709 [138-174]	0	4711.3313
[R].LAQMISI	1xTMTpro [K	6.168E-05	7.389E-05	1	1	1	Q14157	Q14157 [38-53]	0	2471.2368
[R].KLDALCI	1xCarbamido	0.0001075	7.389E-05	1	1	3	P53621	P53621 [517-529]	1	2204.2465
[R].LAQMSI(	1xCarbamido	7.578E-05	7.389E-05	1	1	1	O95861	O95861 [53-64]	0	1640.8783
[K].HGVYNP	1xTMTpro [K	0.0001703	7.389E-05	1	1	1	P40926	P40926 [158-176]	1	2751.5801
[R].LALFAHI	1xTMTpro [K	0.0001842	7.389E-05	1	1	1	Q96QK1	Q96QK1 [306-323	1	2528.4481
[K].IGEGTY(	1xTMTpro [K	9.049E-05	7.389E-05	2	3	1	P24941; P06	P24941 [10-20]; F	0	1794.0293
[R].LALFGEI	1xTMTpro [N	6.178E-05	7.389E-05	1	1	1	Q9BWH6	Q9BWH6 [1246-1	0	1586.9338
[K].KHVLFG	1xTMTpro [K	6.217E-05	7.389E-05	1	1	2	P04216	P04216 [60-75]	1	2448.4007
[R].KKDASD	3xTMTpro [K	6.367E-05	7.389E-05	1	1	1	P20042	P20042 [63-79]	2	3229.7846
[K].LGEGSY	1xTMTpro [K	7.755E-05	7.389E-05	1	1	2	Q13188	Q13188 [33-43]	0	1751.9824
[K].HNCDHA	2xCarbamido	0.0001849	7.389E-05	1	1	1	Q86VB7	Q86VB7 [246-260	0	2321.1258
[R].IGAPGVI	1xTMTpro [K	0.0001415	7.389E-05	1	1	2	Q15208	Q15208 [368-377]	0	1620.9816
[R].LGAPQQ	1xTMTpro [N	0.0001043	7.389E-05	1	1	1	Q96CX2	Q96CX2 [129-143	0	1759.9774
[R].GPSGLL	1xTMTpro [K	0.0001044	7.389E-05	1	1	2	Q9UHQ9	Q9UHQ9 [148-15	0	1702.0031
[R].KIANLLK	3xTMTpro [K	0.0001991	7.389E-05	1	1	2	P09455	P09455 [32-41]	1	2356.5433
[K].HKDFLEI	1xTMTpro [K	0.0001342	7.389E-05	1	1	2	P11277	P11277 [785-796]	1	2140.153
[R].LALIICNI	1xCarbamido	0.0001041	7.389E-05	1	1	1	P29466	P29466 [164-178]	0	2094.1224
[K].LGDDLL(	1xCarbamido	8.868E-05	7.389E-05	1	1	1	P04070	P04070 [168-180]	0	2074.1611
[R].HMEASL	1xOxidation [	0.0001929	7.389E-05	1	1	1	P24593	P24593 [195-204]	0	1810.0024
[K].LGDGLFI	2xCarbamido	0.000162	7.389E-05	1	1	1	O43837	O43837 [224-242]	0	2849.4709
[R].GPSGLL	1xTMTpro [K	8.965E-05	7.389E-05	1	1	1	P00387	P00387 [144-154]	0	1727.0347
[K].IGDGMN	1xTMTpro [K	0.0001732	7.389E-05	1	1	1	Q13596	Q13596 [155-166]	0	1910.0337
[K].HIAEDAI	1xTMTpro [K	0.0001313	7.389E-05	1	1	1	P09493	P09493 [153-161]	1	1662.9419
[K].HIYYITGI	1xTMTpro [K	0.0001109	7.389E-05	1	2	4	P07900	P07900 [490-499]	0	1833.0402
[R].KLAEQE	1xTMTpro [K	0.0001723	7.389E-05	1	2	1	P13533	P13533 [1702-17	1	2154.2262
[K].LGDTALI	1xTMTpro [K	0.0001103	7.389E-05	1	1	2	Q92882	Q92882 [139-150]	0	1862.078
[K].LASEEPI	1xTMTpro [N	0.0001239	7.389E-05	1	1	2	Q9UBB4	Q9UBB4 [289-305	0	2160.0991

[R].KLIDEM\	2xTMTpro [K	0.0001466	7.389E-05	1	1	1	O75695	O75695 [252-260]	1	1945.1972
[R].GPSLQG	1xTMTpro [K	7.672E-05	7.389E-05	1	1	1	Q09666	Q09666 [5367-53	0	2065.1785
[K].HMDLCL	1xCarbamido	8.059E-05	7.389E-05	1	1	1	Q10471	Q10471 [492-502]	0	1662.8627
[R].LASEQQ	1xTMTpro [K	7.779E-05	7.389E-05	1	1	1	Q9UBV2	Q9UBV2 [656-676	0	3028.5265
[K].GPSYGL	1xTMTpro [K	6.217E-05	7.389E-05	2	2	2	Q15417; Q9	Q15417 [7-17]; Q	0	1715.9824
[R].IALLPLL(	1xTMTpro [N	0.0001324	7.389E-05	1	1	1	Q9P0J0	Q9P0J0 [69-81]	0	1757.0492
[K].KLIELQA	2xTMTpro [K	6.591E-05	7.389E-05	1	1	2	P14314	P14314 [158-166]	1	1912.2411
[K].KLAFAYL	2xTMTpro [K	0.0002031	7.389E-05	1	1	4	O75396	O75396 [82-101]	1	3289.7673
[K].GPSVFP	1xTMTpro [K	0.0001224	7.389E-05	1	2	12	P0DOX5	P0DOX5 [124-135	0	1795.0609
[K].GPSVFP	1xCarbamido	0.00012	7.389E-05	3	3	8	P01861; P01	P01861 [5-16]; P0	0	1591.8586
[R].IASFSQN	1xCarbamido	0.0001033	7.389E-05	1	1	3	P01042	P01042 [241-254]	0	2208.1615
[R].LGDLLIS	1xCarbamido	7.743E-05	7.389E-05	1	1	1	Q92974	Q92974 [318-336]	0	2689.4185
[K].IGDPLLE	1xTMTpro [N	0.000165	7.389E-05	1	1	2	P49189	P49189 [317-326]	0	1432.7967
[K].IAILTCPF	1xCarbamido	0.0001237	7.389E-05	1	1	3	P48643	P48643 [248-261]	0	2523.5189
[R].LGDLWT	1xTMTpro [K	0.0001684	7.389E-05	1	1	1	P51610	P51610 [229-255]	0	3587.0129
[R].KKVEAQ	3xTMTpro [K	0.0001328	7.389E-05	1	1	2	P35579	P35579 [1248-126	2	2757.7343
[K].LASDLLE	1xTMTpro [N	0.0002003	7.389E-05	2	2	1	P12814; O43	P12814 [282-291]	0	1519.8803
[K].IALLEEA	2xTMTpro [K	0.0001779	7.389E-05	1	1	1	P35241	P35241 [428-436]	1	1927.2408
[K].KIHTEPQ	1xTMTpro [K	9.482E-05	7.389E-05	1	1	1	P47897	P47897 [80-95]	1	2463.4215
[K].LGAPQT	1xTMTpro [K	7.393E-05	7.389E-05	2	2	1	Q9BUP0; Q9	Q9BUP0 [122-132	0	1743.0773
[K].GPQITGI	1xTMTpro [K	9.724E-05	7.389E-05	1	1	1	Q09666	Q09666 [372-387]	0	2190.2626
[K].KKHPDA	3xTMTpro [K	0.0001605	7.389E-05	1	1	1	P09429	P09429 [29-43]	2	2937.6939
[R].LGAQAL	1xTMTpro [K	9.512E-05	7.389E-05	1	1	2	P32322	P32322 [205-215]	0	1621.0293
[K].LGAQLAI	1xTMTpro [K	9.205E-05	7.389E-05	1	1	1	Q9HA64	Q9HA64 [103-115	0	2016.1733
[K].LALQEFS	1xTMTpro [N	9.438E-05	7.389E-05	1	1	1	Q9Y5S2	Q9Y5S2 [572-583	0	1752.9451
[R].LALGSF\	1xTMTpro [K	9.572E-05	7.389E-05	1	1	1	P61962	P61962 [34-46]	0	2092.157
[R].HHLQIPI	1xTMTpro [K	0.0001635	7.389E-05	1	1	1	Q9Y2Q3	Q9Y2Q3 [75-85]	0	1975.1885
[R].GPVGPS	1xTMTpro [K	0.0001002	7.389E-05	1	1	3	P02461	P02461 [1140-115	0	1557.9245
[R].LGAVDE	1xTMTpro [K	0.0001196	7.389E-05	1	1	2	Q96HE7	Q96HE7 [137-150	0	2114.1473
[R].LASCSAI	1xCarbamido	7.169E-05	7.389E-05	1	1	2	Q9NRL3	Q9NRL3 [559-569	0	1440.7436
[K].HLANTD\	1xCarbamido	0.0001378	7.389E-05	1	1	1	O14638	O14638 [768-788]	0	3020.6523
[K].KINELTG	2xTMTpro [K	0.0001899	7.389E-05	1	1	1	P35998	P35998 [58-66]	1	1928.2361
[K].LGAVEN.	1xTMTpro [K	0.0001995	7.389E-05	1	1	1	Q8IY21	Q8IY21 [1136-115	0	2244.2731

[R].GQDIFII(	1xTMTpro [N	0.0001249	7.389E-05	1	1	1	Q14558	Q14558 [57-68]	0	1704.9968
[K].HHPEDV	1xTMTpro [N	0.0001852	7.389E-05	1	1	2	P14550	P14550 [86-96]	0	1603.8512
[K].GPVFAP	1xTMTpro [K	6.961E-05	7.389E-05	1	1	1	P11387	P11387 [224-239]	0	2362.3302
[R].GPQLVC	1xCarbamid	0.0001082	7.389E-05	1	1	1	Q96DI7	Q96DI7 [163-177]	0	2142.1356
[R].HLAISNM	1xTMTpro [K	0.0001087	7.389E-05	1	1	1	Q9NZ08	Q9NZ08 [213-223	0	1831.1119
[K].HLWDYT	1xTMTpro [K	7.719E-05	7.389E-05	1	1	5	P61160	P61160 [87-97]	0	2001.0726
[R].KINDQYI	2xTMTpro [K	0.000114	7.389E-05	1	1	1	Q9H0V1	Q9H0V1 [558-573	1	2715.6225
[R].GPQVQ(	1xTMTpro [N	7.875E-05	7.389E-05	1	1	4	Q15436	Q15436 [217-228]	0	1608.8777
[K].KKNPEV	3xTMTpro [K	0.0002047	7.389E-05	1	1	1	O15347	O15347 [29-43]	2	2950.7507
[R].LGDAVI\	1xTMTpro [N	8.479E-05	7.389E-05	1	1	1	Q12769	Q12769 [744-765]	0	2770.496
[K].LAILDQE	1xTMTpro [K	6.529E-05	7.389E-05	1	1	1	Q12888	Q12888 [731-741]	0	1917.1301
[R].LASDGA	1xTMTpro [K	0.000126	7.389E-05	1	1	1	Q8NHP8	Q8NHP8 [355-367	0	2003.1094
[K].GPSFPE	1xTMTpro [N	0.000139	7.389E-05	1	1	1	P06132	P06132 [105-120]	1	2203.1314
[K].KKPRPP	3xTMTpro [K	0.0001304	7.389E-05	1	1	1	Q01105	Q01105 [14-35]	1	3445.0683
[R].KLMEQY	2xTMTpro [K	8.558E-05	7.389E-05	1	1	1	P05164	P05164 [629-652]	1	3632.9637
[K].HMNLIL(	2xCarbamid	6.347E-05	7.389E-05	1	2	3	P14678	P14678 [37-49]	0	2026.9468
[R].HLVYESI	1xTMTpro [K	9.335E-05	7.389E-05	1	1	3	O43852	O43852 [272-281]	0	1841.0049
[K].IALYETP	1xTMTpro [K	0.0001995	7.389E-05	1	1	4	P36871	P36871 [350-360]	0	1887.0872
[R].LALEAE\	1xTMTpro [N	0.0001188	7.389E-05	1	1	1	Q7Z406	Q7Z406 [1244-12	0	1533.8807
[K].LATALSL	1xTMTpro [K	0.000106	7.389E-05	1	1	2	P04114	P04114 [3395-34(	0	1626.0082
[R].KLVILEG	1xTMTpro [K	0.0001824	7.389E-05	1	1	1	P67936	P67936 [132-146]	2	2392.4055
[K].LAQENM	1xTMTpro [K	0.0001536	7.389E-05	1	1	1	P35221	P35221 [479-488]	0	1817.0123
[R].HGFEAA	1xTMTpro [K	8.854E-05	7.389E-05	1	1	1	P35580	P35580 [43-54]	1	1982.0951
[R].GQGTYE	1xTMTpro [N	0.0001508	7.389E-05	1	1	1	P14649	P14649 [139-151]	0	1838.888
[R].KLEAELI	1xTMTpro [K	0.0001349	7.389E-05	1	1	2	Q969G3	Q969G3 [240-251	1	2079.2305
[R].KLEVEYI	2xTMTpro [K	9.277E-05	7.389E-05	1	1	1	Q9Y2W2	Q9Y2W2 [109-11	1	2078.2314
[K].LGGDLG	1xTMTpro [K	0.0001842	7.389E-05	1	1	1	Q16595	Q16595 [136-147]	0	1858.093
[R].KLVAIVD	2xTMTpro [K	7.428E-05	7.389E-05	1	1	3	Q14697	Q14697 [462-472]	1	2145.394
[K].KLEADSI	2xTMTpro [K	6.612E-05	7.389E-05	1	1	1	Q15075	Q15075 [734-743]	1	2044.247
[K].KHEAFE	2xTMTpro [K	6.653E-05	7.389E-05	1	1	1	Q13813	Q13813 [1911-19	1	2501.3969
[R].HLQELV(	1xTMTpro [N	7.191E-05	7.389E-05	1	1	1	Q8WX92	Q8WX92 [321-33	0	1824.0299
[K].GPLLEE(	1xTMTpro [K	8.022E-05	7.389E-05	1	1	2	Q52LJ0	Q52LJ0 [27-37]	0	1807.0821
[R].LAGTQP	1xTMTpro [N	0.0001581	7.389E-05	1	1	1	P22314	P22314 [679-693]	0	1928.1136

[R].IAPAFSS	1xTMTpro [K	7.091E-05	7.389E-05	1	1	2	O43396	O43396 [40-50]	0	1760.9861
[K].IAGYVTH	1xTMTpro [K	9.602E-05	7.389E-05	1	1	5	P08708	P08708 [50-59]	0	1741.0326
[R].HLEVLN	1xTMTpro [K	0.0001703	7.389E-05	1	1	1	Q15813	Q15813 [180-190	0	1890.094
[K].IANPVEG	1xTMTpro [N	0.0001533	7.389E-05	1	1	3	Q15366	Q15366 [323-333	0	1462.7821
[K].KHEAFLI	2xTMTpro [K	9.678E-05	7.389E-05	1	1	1	P02549	P02549 [940-956]	1	2864.5797
[K].LAHGLLE	1xTMTpro [K	6.832E-05	7.389E-05	1	1	1	Q92616	Q92616 [600-609	0	1731.066
[R].LAQFVEI	1xTMTpro [K	8.638E-05	7.389E-05	1	1	1	P02549	P02549 [1794-18	0	1895.0671
[K].LFVIDAL	1xTMTpro [K	0.0001956	7.389E-05	1	1	1	Q8IUX1	Q8IUX1 [124-137	0	2206.2615
[K].KLTELGT	2xTMTpro [K	7.695E-05	7.389E-05	1	1	3	P46940	P46940 [1465-14	1	2113.3049
[K].LATQLTC	1xTMTpro [N	8.598E-05	7.389E-05	1	1	5	P26373	P26373 [146-158]	0	1686.9896
[K].KLEVNEA	1xTMTpro [K	0.0001129	7.389E-05	1	1	2	Q6NZI2	Q6NZI2 [137-147]	1	1922.1566
[K].LFVTNDA	1xTMTpro [N	9.32E-05	7.389E-05	1	1	1	P50990	P50990 [63-74]	0	1637.9546
[R].LAQFVEI	1xTMTpro [K	9.277E-05	7.389E-05	1	1	1	Q13813	Q13813 [1845-18	0	1766.0245
[R].KLDVTDI	1xTMTpro [K	7.997E-05	7.389E-05	1	1	1	Q86SK9	Q86SK9 [173-186	1	2162.304
[K].LANPPPI	1xTMTpro [N	0.0001178	7.389E-05	1	1	2	Q12955	Q12955 [1030-10	0	1942.0387
[R].HLQFIPV	1xTMTpro [N	0.000119	7.389E-05	1	1	2	Q14118	Q14118 [715-725	0	1606.9753
[R].KGVSQT	1xCarbamido	0.000146	7.389E-05	1	1	1	Q12888	Q12888 [1365-13	1	2854.486
[K].HPAKPD	1xCarbamido	9.847E-05	7.389E-05	1	1	1	Q16576	Q16576 [156-171	0	2398.2429
[R].IAGQVAA	1xTMTpro [K	9.133E-05	7.389E-05	1	1	4	P39019	P39019 [134-143]	0	1550.951
[R].KLVILEG	1xTMTpro [K	7.08E-05	7.389E-05	1	1	1	P07951	P07951 [168-182]	2	2408.4004
[K].KLEVDEI	2xTMTpro [K	8.034E-05	7.389E-05	1	1	1	Q12884	Q12884 [510-521	1	2449.4523
[K].HLFTGPY	1xTMTpro [K	0.0001544	7.389E-05	1	1	1	P16930	P16930 [48-57]	0	1707.0449
[R].KGVAINI	2xTMTpro [K	0.0001623	7.389E-05	1	1	4	P38919	P38919 [374-382]	1	1888.22
[K].IAVEPVN	1xTMTpro [K	8.882E-05	7.389E-05	1	1	3	Q15029	Q15029 [590-602	0	2001.1876
[K].GQIIGNF	1xTMTpro [N	0.00011	7.389E-05	1	1	1	Q14126	Q14126 [407-427	0	2561.3068
[R].LAGPPG	1xTMTpro [K	0.0001579	7.389E-05	1	1	1	Q9BQS8	Q9BQS8 [556-57	0	2040.1985
[R].IGGGIDV	1xTMTpro [N	0.000141	7.389E-05	1	1	2	Q92945	Q92945 [321-331	0	1383.8279
[K].KLVPFAT	1xTMTpro [K	0.000126	7.389E-05	1	1	2	P06727	P06727 [79-90]	1	2048.2148
[K].KIVNSAQ	2xTMTpro [K	7.314E-05	7.389E-05	1	1	1	O75964	O75964 [55-66]	1	2192.3219
[K].LAVATFA	1xTMTpro [K	6.779E-05	7.389E-05	1	1	1	Q9UJS0	Q9UJS0 [642-653	0	1842.0981
[R].GPLEYV	1xTMTpro [K	0.0001813	7.389E-05	1	1	2	Q14764	Q14764 [361-370	0	1668.9816
[R].KLECLPI	1xCarbamido	7.755E-05	7.389E-05	1	1	1	Q96CS3	Q96CS3 [346-363	1	2949.6059
[K].GQLESIV	1xTMTpro [N	9.049E-05	7.389E-05	1	1	2	P17858	P17858 [475-485]	0	1561.8869

[R].KGVEGL	1xTMTpro [K	0.0001506	7.389E-05	1	1	1	Q13442	Q13442 [75-88]	1	2162.2425
[R].GQFPEL	1xTMTpro [N	0.0001235	7.389E-05	1	1	1	Q6ZS17	Q6ZS17 [810-824	0	2035.0779
[K].HIFENVI(	1xTMTpro [K	0.000121	7.389E-05	1	1	1	P33527	P33527 [805-814]	0	1762.0507
[K].GQIEEA(	1xTMTpro [K	0.0001063	7.389E-05	1	1	1	P08237	P08237 [446-466]	0	2762.403
[R].HGFCGII	1xCarbamid(	8.222E-05	7.389E-05	1	1	1	P12268	P12268 [137-149]	0	1734.8917
[K].LAVASV	1xTMTpro [K	0.0002055	7.389E-05	1	1	1	O75165	O75165 [1621-16	0	1752.1126
[K].LAPALC(	2xCarbamid(	8.584E-05	7.389E-05	1	1	4	P47895	P47895 [191-221]	0	4272.399
[R].GQLEAL	1xTMTpro [N	6.726E-05	7.389E-05	1	1	2	P08729	P08729 [150-161]	0	1546.8508
[K].GQLCEL	2xCarbamid(	6.287E-05	7.389E-05	1	1	3	Q99873	Q99873 [357-369]	0	1892.8802
[K].HPDADS	1xTMTpro [K	9.739E-05	7.389E-05	1	1	1	P54577	P54577 [381-391]	0	1882.0202
[R].GQKPPF	2xTMTpro [K	0.0001656	7.389E-05	1	1	1	Q9Y4F1	Q9Y4F1 [28-38]	0	2006.2215
[K].HPCNAS	2xCarbamid(	0.000182	7.389E-05	1	1	1	Q9Y3S2	Q9Y3S2 [34-44]	0	1956.9222
[K].LGGFDN	1xTMTpro [K	8.611E-05	7.389E-05	1	1	1	Q4LE39	Q4LE39 [350-363	0	2101.1574
[R].KLVIPSE	1xTMTpro [K	6.853E-05	7.389E-05	1	1	1	P26885	P26885 [103-115]	1	2069.225
[K].IAVAAQI	1xCarbamid(	0.000141	7.389E-05	1	1	5	P60174	P60174 [60-69]	0	1745.9864
[K].IAVAAAS	2xTMTpro [K	8.718E-05	7.389E-05	1	1	2	P29373	P29373 [32-45]	0	2280.4471
[R].LFVGNLI	1xOxidation	7.827E-05	7.389E-05	1	1	2	Q15233	Q15233 [76-91]	0	2180.1228
[R].HLQEAE	1xTMTpro [K	0.0001533	7.389E-05	1	1	1	Q96P70	Q96P70 [428-436	0	1691.9572
[K].LAGLHD	1xTMTpro [N	0.0001892	7.389E-05	1	1	2	O75027	O75027 [584-593]	0	1382.8439
[R].HGLEAIS	1xTMTpro [N	7.743E-05	7.389E-05	1	1	2	P26640	P26640 [607-618]	0	1632.8699
[K].IGFEEKD	2xTMTpro [K	0.0001268	7.389E-05	1	1	2	O75368	O75368 [32-47]	2	2775.5457
[R].LALADA(	1xTMTpro [N	6.716E-05	7.389E-05	1	1	1	Q9P2J5	Q9P2J5 [741-766]	0	2952.4944
[R].LAQHEA	1xTMTpro [N	9.663E-05	7.389E-05	1	1	1	O15230	O15230 [2374-23	0	1657.9015
[R].KLDPEIV	2xTMTpro [K	0.0001363	7.389E-05	1	1	1	O43148	O43148 [83-93]	1	2209.326
[K].HNIQDEI	1xTMTpro [K	8.718E-05	7.389E-05	1	1	1	O15031	O15031 [1668-16	0	2257.2221
[R].GPPAPA	1xTMTpro [N	8.584E-05	7.389E-05	1	1	1	P48681	P48681 [167-180]	0	1736.9502
[K].LALASL(	1xTMTpro [K	0.0001061	7.389E-05	1	1	3	Q8WUM4	Q8WUM4 [111-12	0	1673.0129
[R].KHNMC(	1xCarbamid(	6.822E-05	7.389E-05	1	1	1	P21266	P21266 [87-98]	1	2404.2417
[K].KLFYSTI	1xTMTpro [K	0.0001635	7.389E-05	1	1	1	P30084	P30084 [261-272]	1	2072.1308
[R].KIQGIEE	3xTMTpro [K	0.0001776	7.389E-05	1	1	1	Q9Y6G9	Q9Y6G9 [86-95]	2	2452.528
[R].LALDGE	1xTMTpro [N	0.0001463	7.389E-05	1	1	1	Q14160	Q14160 [1416-14	0	3372.6039
[R].KHNMC(	1xCarbamid(	9.394E-05	7.389E-05	1	1	1	P21266	P21266 [87-98]	1	2420.2366
[R].HNIMDF	1xOxidation	7.325E-05	7.389E-05	1	1	3	Q00610	Q00610 [1589-16	0	2609.3574

[K].LALDMEI	1xTMTpro [N	6.591E-05	7.389E-05	1	1	3	P02545	P02545 [367-377]	0	1635.8848
[R].IGESIEL	1xCarbamido	0.000181	7.389E-05	1	1	1	P13671	P13671 [724-734]	0	1855.0491
[K].LATEGS	1xTMTpro [K	0.0001373	7.389E-05	1	1	1	Q9NW68	Q9NW68 [62-73]	0	1758.9729
[R].IATEDDL	1xTMTpro [K	0.0001044	7.389E-05	1	1	1	Q8IWA4	Q8IWA4 [48-61]	0	2220.1714
[K].HLSVND	1xTMTpro [N	9.708E-05	7.389E-05	1	1	2	P30048	P30048 [197-207]	0	1510.8661
[R].LGADES	1xTMTpro [N	8.651E-05	7.389E-05	1	1	1	O43847	O43847 [81-91]	0	1495.7196
[K].LGENVA	1xTMTpro [K	7.695E-05	7.389E-05	1	1	2	Q9Y3I1	Q9Y3I1 [290-299]	0	1729.014
[R].LGADME	1xCarbamido	0.0001264	7.389E-05	1	1	1	P02649	P02649 [122-132]	0	1526.7262
[K].KLGEMW	2xTMTpro [K	8.134E-05	7.389E-05	1	1	2	O15347	O15347 [126-139]	1	2590.4115
[R].HIDLVEC	1xTMTpro [N	0.0001514	7.389E-05	1	1	2	P19367	P19367 [244-254]	0	1543.8036
[R].KLQEQE	2xTMTpro [K	0.0001116	7.389E-05	1	1	1	Q9H2G2	Q9H2G2 [1196-12	1	2208.3209
[R].GPPASS	1xTMTpro [K	0.0001517	7.389E-05	1	1	2	Q15942	Q15942 [254-265]	0	1684.9878
[R].HGPLTA	1xTMTpro [N	6.779E-05	7.389E-05	1	1	1	P22105	P22105 [1750-176	0	1629.8516
[K].LGEMWN	1xTMTpro [K	0.0001216	7.389E-05	1	1	1	O15347	O15347 [127-139]	0	2158.1094
[K].HLVESTI	1xTMTpro [K	0.0001387	7.389E-05	1	1	1	Q9NYU2	Q9NYU2 [307-319	0	2077.1607
[K].LALDVEI	1xTMTpro [N	0.0001845	7.389E-05	4	9	1	P35908; P04	P35908 [471-481]	0	1567.9015
[R].GQDLGC	1xTMTpro [K	9.527E-05	7.389E-05	1	1	2	O15230	O15230 [2669-26	0	2633.439
[R].IGAADY	1xTMTpro [N	0.0001039	7.389E-05	1	1	1	P09471	P09471 [163-177]	0	1994.0514
[K].LGAAAG	1xTMTpro [K	0.0001422	7.389E-05	1	1	2	Q53FA7	Q53FA7 [184-193	0	1619.9401
[R].LAQGHT	1xTMTpro [N	0.0001729	7.389E-05	1	1	1	Q15149	Q15149 [2809-28	0	1714.9407
[R].KHIMGQ	1xTMTpro [K	9.35E-05	7.389E-05	1	1	2	P46777	P46777 [197-209]	1	2171.1709
[R].HIEIQVL	1xTMTpro [K	0.000196	7.389E-05	1	1	1	P05165	P05165 [269-278]	0	1760.0562
[K].LFYGMS	1xTMTpro [K	0.0001707	7.389E-05	1	1	1	Q9P2T1	Q9P2T1 [265-276	0	2003.0296
[R].GPLLVQ	1xOxidation	0.0001196	7.389E-05	1	1	2	P04040	P04040 [48-68]	1	2794.4153
[R].GPLPAA	1xTMTpro [N	0.000138	7.389E-05	1	1	2	Q9NQC3	Q9NQC3 [92-104]	0	1575.9178
[R].KHFVCE	2xCarbamido	6.737E-05	7.389E-05	1	1	1	Q9NZU5	Q9NZU5 [331-345	1	2428.2721
[K].LGFEDG	1xTMTpro [K	0.0001202	7.389E-05	1	1	2	P09936	P09936 [106-115]	0	1672.9765
[K].GPLPAG	1xTMTpro [K	0.0001087	7.389E-05	1	1	2	P51610	P51610 [714-723]	0	1575.0125
[K].GPLQSV	1xTMTpro [N	0.0001952	7.389E-05	1	1	2	P62249	P62249 [5-15]	0	1491.8603
[K].GQEVET	1xTMTpro [N	6.674E-05	7.389E-05	1	1	2	O43169	O43169 [17-28]	0	1735.8822
[K].LAQGEY	1xTMTpro [K	9.364E-05	7.389E-05	2	2	2	Q9ULC5; P3	Q9ULC5 [547-557	0	1827.0508
[K].LGFDKEI	1xTMTpro [K	0.0001742	7.389E-05	1	1	1	O60264	O60264 [962-974]	1	2206.2
[K].LANILFT	1xTMTpro [N	0.0001129	7.389E-05	1	1	3	Q8TC12	Q8TC12 [207-218	0	1692.9968

[K].HLQINQ1	1xTMTpro [N	0.0001243	7.389E-05	2	2	1	P51692; P42	P51692 [141-152]		0	1831.9986
[R].IFYLVAD	1xTMTpro [K	0.0001779	7.389E-05	1	1	1	Q13480	Q13480 [93-106]		0	2296.2027
[R].HLQLAIF	1xTMTpro [K	0.0001678	7.389E-05	4	12	2	Q16777; Q7I	Q16777 [83-96]; (		1	2301.3171
[R].HISDLYE	1xTMTpro [N	0.0001415	7.389E-05	1	1	1	Q15149	Q15149 [199-208]		0	1564.829
[K].LATLNIH	1xTMTpro [K	0.000122	7.389E-05	1	1	1	Q9H1A4	Q9H1A4 [1188-11		0	2010.1879
[K].LFYSTFA	1xTMTpro [N	0.000171	7.389E-05	1	1	1	P30084	P30084 [262-272]		0	1639.8287
[R].HGLIGEI	1xTMTpro [K	8.979E-05	7.389E-05	1	1	1	Q9UPN3	Q9UPN3 [2529-25		0	1790.0304
[K].LGAAAAI	1xTMTpro [N	7.531E-05	7.389E-05	1	1	1	Q9H4A3	Q9H4A3 [39-50]		0	1376.7817
[K].HNNLYL\	1xTMTpro [K	0.0001856	7.389E-05	1	1	1	Q9BXS5	Q9BXS5 [62-72]		0	1868.0886
[R].HNNLDL	1xTMTpro [N	0.0001273	7.389E-05	1	1	1	O43837	O43837 [155-164]		0	1510.9025
[R].IAKPNVS	1xTMTpro [K	6.779E-05	7.389E-05	1	1	1	Q13315	Q13315 [77-90]		0	2038.1901
[R].HLEIIYAI	1xTMTpro [N	0.0001138	7.389E-05	1	1	1	P11216	P11216 [400-410]		0	1673.9658
[R].HLEFSHI	1xTMTpro [N	0.0001723	7.389E-05	1	1	2	Q9NR45	Q9NR45 [87-96]		0	1635.8199
[K].GPNSAL	1xTMTpro [K	0.000187	7.389E-05	1	1	2	Q5H9R7	Q5H9R7 [475-486		0	1876.1512
[R].KLDPGS	1xTMTpro [K	9.35E-05	7.389E-05	1	1	4	P26641	P26641 [401-414]		1	2181.2371
[R].LAKTYET	2xTMTpro [K	0.0001046	7.389E-05	1	1	2	P02768	P02768 [373-383]		1	2209.326
[K].HNMCGE	1xCarbamido	0.0001796	7.389E-05	1	1	1	P21266	P21266 [88-98]		0	1971.9396
[R].HISTLNI(	1xTMTpro [K	0.0001015	7.389E-05	1	1	1	P35749	P35749 [1365-137		0	2064.1945
[K].LATNAA\	1xTMTpro [N	0.00015	7.389E-05	1	1	3	P50990	P50990 [510-520]		0	1432.8807
[R].LGDLYEI	1xTMTpro [N	0.0001576	7.389E-05	1	1	8	P08670	P08670 [146-155]		0	1558.7742
[R].KVFDPV	2xTMTpro [K	0.0001072	7.389E-05	1	1	3	Q08211	Q08211 [697-708]		1	2198.3729
[R].IFQILYAI	1xTMTpro [K	0.0001239	7.389E-05	1	1	1	O75116	O75116 [1227-12:		0	2120.1883
[K].LEGDSE	1xTMTpro [K	0.0001519	7.389E-05	1	1	1	Q92629	Q92629 [78-91]		0	2218.2251
[R].IEGDPQ(	1xTMTpro [K	0.0001659	7.389E-05	1	1	1	Q00341	Q00341 [483-494]		0	1878.0577
[R].GYAFVT	1xCarbamido	0.000108	7.389E-05	1	1	2	O60506	O60506 [204-213]		0	1801.9803
[K].IDGSLEV	1xTMTpro [N	0.0001769	7.389E-05	1	1	1	Q9NVM9	Q9NVM9 [437-44		0	1531.8651
[R].GTVIDVT	1xTMTpro [K	6.896E-05	7.389E-05	1	1	2	P02748	P02748 [447-472]		0	3397.8499
[K].GTVIKPF	2xTMTpro [K	9.262E-05	7.389E-05	1	1	3	Q9P0K7	Q9P0K7 [566-580		0	2661.499
[R].LDHKFD	2xTMTpro [K	8.336E-05	7.389E-05	3	7	3	Q71U36; Q9	Q71U36 [391-401		1	2293.3195
[R].IDHLSFG	1xTMTpro [K	0.0001432	7.389E-05	1	1	1	Q96RQ1	Q96RQ1 [217-238		0	2986.6745
[R].HYCTVA	1xCarbamido	9.617E-05	7.389E-05	1	1	1	Q14764	Q14764 [57-67]		0	1607.8283
[K].KYGETA	1xCarbamido	0.0001579	7.389E-05	1	1	2	P49321	P49321 [76-93]		1	3030.5488
[K].KYFAAT(	1xTMTpro [K	0.000149	7.389E-05	1	1	1	Q9UIQ6	Q9UIQ6 [287-300		1	2221.2625

[K].IEGDEEI	1xCarbamido	0.0001968	7.389E-05	1	1	1	P08603	P08603 [184-200]	0	2650.2045
[K].LEGALG.	1xTMTpro [K	7.936E-05	7.389E-05	1	1	1	Q9NZM1	Q9NZM1 [1094-1	0	2229.1378
[K].GTVIVQF	2xTMTpro [K	0.0001599	7.389E-05	1	1	1	P46100	P46100 [124-147]	2	3641.9995
[K].GTVPDD	1xTMTpro [K	7.803E-05	7.389E-05	1	1	4	P20810	P20810 [427-443]	0	2266.2422
[K].KYETVQ	2xTMTpro [K	9.512E-05	7.389E-05	1	1	1	Q02880	Q02880 [1032-10	1	2149.3049
[K].GYAFVQ	1xTMTpro [N	0.0002015	7.389E-05	1	1	2	Q9UKM9	Q9UKM9 [56-66]	0	1637.8243
[R].LEFESLE	1xTMTpro [K	0.0001178	7.389E-05	1	1	2	Q9NW15	Q9NW15 [627-63	0	1787.0446
[K].HYDGSY	1xTMTpro [N	8.453E-05	7.389E-05	1	1	1	P01023	P01023 [1020-10	0	1722.8043
[R].KYNLQE	2xTMTpro [K	0.0001576	7.389E-05	1	1	1	Q9NXG2	Q9NXG2 [261-26	1	2033.2575
[K].LEGELK	2xTMTpro [K	9.909E-05	7.389E-05	1	1	2	Q09666	Q09666 [2337-23	1	2339.4002
[R].GTVLAEI	1xTMTpro [K	9.049E-05	7.389E-05	1	1	1	Q96H20	Q96H20 [25-38]	0	2099.1662
[R].KDHPFG	2xTMTpro [K	0.000119	7.389E-05	1	1	2	P63279	P63279 [18-30]	1	2355.4005
[R].LDGNLL	1xTMTpro [N	0.0001277	7.389E-05	1	1	1	Q9BTW9	Q9BTW9 [170-18	0	1686.9458
[R].KDPNQV	2xTMTpro [K	0.0001034	7.389E-05	1	1	2	O43278	O43278 [203-214	1	2339.3903
[R].GTSWGI	1xTMTpro [K	0.0001769	7.389E-05	1	1	1	P22105	P22105 [825-834]	0	1611.935
[R].IDGNLVV	1xTMTpro [K	0.0001262	7.389E-05	1	1	1	P00387	P00387 [85-103]	0	2712.47
[K].HVVFGH	1xTMTpro [K	0.0001933	7.389E-05	1	1	2	P30405	P30405 [168-175]	0	1530.94
[K].LEGLED.	1xTMTpro [K	0.0001678	7.389E-05	2	3	1	P04259; P02	P04259 [427-436]	0	1724.0086
[K].HVVFGQ	1xTMTpro [K	0.0001121	7.389E-05	1	1	1	Q08752	Q08752 [146-154	0	1635.0238
[R].GYCAPG	2xCarbamido	7.18E-05	7.389E-05	1	1	2	Q16270	Q16270 [79-89]	0	1879.936
[K].GYAYVE	1xTMTpro [K	0.000149	7.389E-05	1	1	2	Q15287	Q15287 [204-218	0	2369.1793
[K].KYEDICF	1xCarbamido	0.0001328	7.389E-05	2	3	3	Q9GZV4; P6	Q9GZV4 [68-85];	1	3073.6267
[R].HVVLGA	1xTMTpro [K	0.0001573	7.389E-05	1	1	1	P41227	P41227 [170-179]	0	1688.0351
[K].HWELTA	1xTMTpro [N	0.0001271	7.389E-05	1	1	1	Q9Y285	Q9Y285 [60-72]	0	1844.9462
[R].KDLPPLI	2xTMTpro [K	0.0001089	7.389E-05	1	1	1	Q9H0A0	Q9H0A0 [689-697	1	1949.2979
[K].GTTPLAI	1xTMTpro [K	0.0001638	7.389E-05	1	1	1	Q96D71	Q96D71 [692-704	0	1939.1984
[R].KSQGTS	1xTMTpro [K	0.0002019	7.389E-05	1	1	1	Q08378	Q08378 [106-117	1	1815.0216
[K].GTTQIDI	1xTMTpro [N	9.863E-05	7.389E-05	1	1	2	P35442	P35442 [971-982]	0	1703.94
[R].LDGNYL	1xCarbamido	0.0001533	7.389E-05	1	1	1	P51888	P51888 [357-375]	0	2917.5634
[K].KDLHDA	1xTMTpro [K	0.0001245	7.389E-05	1	1	1	P53985	P53985 [224-236]	1	2076.1693
[K].GTVACG	1xCarbamido	6.458E-05	7.389E-05	1	1	2	P13611	P13611 [3292-33	0	2135.1774
[R].KDHSGQ	2xTMTpro [K	0.000149	7.389E-05	1	1	2	P07996	P07996 [110-124]	1	2501.4292
[K].LEGFHT	1xTMTpro [K	0.0001319	7.389E-05	1	1	3	Q06323	Q06323 [167-176	0	1768.0249

[K].IDKLMIEI	2xTMTpro [K	8.505E-05	7.389E-05	1	1	2	P06733	P06733 [90-103]		1	2549.4135
[R].GYAFIEY	1xTMTpro [N	0.0001432	7.389E-05	1	1	2	P08621	P08621 [145-155]		0	1717.8505
[R].LEEYLGS	1xTMTpro [K	0.0001093	7.389E-05	1	1	2	Q9UPT5	Q9UPT5 [109-118		0	1748.9748
[R].LDLDLTA	1xTMTpro [K	0.0001468	7.389E-05	1	1	2	Q13263	Q13263 [493-507		0	2267.2779
[R].GWPLEL	1xCarbamido	0.000155	7.389E-05	1	1	1	Q12906	Q12906 [248-257		0	1853.0487
[R].HYGALIC	1xTMTpro [N	0.0001479	7.389E-05	1	1	2	P07738	P07738 [91-100]		0	1417.8235
[K].KDFESC	1xCarbamido	0.0001968	7.389E-05	1	1	1	P21399	P21399 [387-396]		1	2067.1725
[K].GWLYYE	1xTMTpro [N	0.000171	7.389E-05	1	1	3	Q15149	Q15149 [4514-45		0	1546.7973
[R].KYAVLY(	2xTMTpro [K	0.0001453	7.389E-05	1	1	1	P55209	P55209 [105-116]		1	2397.4362
[K].KDEGSY	1xTMTpro [K	0.0001944	7.389E-05	1	1	1	P34741	P34741 [174-184]		1	1876.9896
[K].GTYQGL	1xTMTpro [K	7.428E-05	7.389E-05	1	1	2	P53007	P53007 [179-190]		0	1860.1086
[K].GTYTDC	1xCarbamido	0.0001313	7.389E-05	1	1	3	P12109	P12109 [126-135]		1	2069.1881
[K].KTDELP(	2xTMTpro [K	8.26E-05	7.389E-05	1	1	1	P02751	P02751 [2081-21		1	4228.4162
[K].IDIDNSG	1xTMTpro [K	7.863E-05	7.389E-05	1	1	2	Q14651	Q14651 [23-41]		0	2842.4642
[R].GVAAST	1xTMTpro [N	6.95E-05	7.389E-05	1	1	1	Q9Y4G6	Q9Y4G6 [1139-11		0	2217.1253
[K].KDDSHS	3xTMTpro [K	0.0001437	7.389E-05	1	1	1	Q9H1E3	Q9H1E3 [70-87]		2	3317.6874
[K].IDIDPEE	1xTMTpro [K	0.000111	7.389E-05	1	1	4	P54727	P54727 [15-24]		0	1767.0032
[R].KYAALY(	2xTMTpro [K	0.0001114	7.389E-05	1	1	1	Q99733	Q99733 [94-105]		1	2369.4049
[K].LEELKES	2xTMTpro [K	7.625E-05	7.389E-05	1	1	1	O75832	O75832 [19-30]		1	2300.3893
[K].LEEIKEN	1xTMTpro [K	0.0001288	7.389E-05	1	1	1	Q8NFQ8	Q8NFQ8 [196-208		1	2185.1778
[R].LDIDPET	1xTMTpro [N	6.237E-05	7.389E-05	1	1	6	P11586	P11586 [521-532]		0	1790.9608
[K].IEEIKDFI	1xTMTpro [K	7.815E-05	7.389E-05	1	1	2	P63173	P63173 [5-16]		1	2056.2298
[K].KDCEVV	1xCarbamido	6.387E-05	7.389E-05	1	1	2	Q00839	Q00839 [495-510		1	2633.4645
[R].GVAALT	1xTMTpro [K	7.359E-05	7.389E-05	1	1	2	Q9Y490	Q9Y490 [1137-11		0	3077.7226
[K].LDGNELI	1xTMTpro [K	0.0001084	7.389E-05	1	1	3	Q96AG4	Q96AG4 [15-33]		0	2678.4744
[K].LEEIQQ(	1xTMTpro [K	0.000149	7.389E-05	1	1	1	Q8WXH0	Q8WXH0 [1783-1		0	2106.2414
[K].LDLDIPV	1xTMTpro [K	0.0001172	7.389E-05	1	1	1	P83111	P83111 [183-199]		0	2647.4627
[K].IDKLMIEI	1xOxidation	0.0001007	7.389E-05	1	1	2	P06733	P06733 [90-103]		1	2565.4084
[R].GWVTD(	1xTMTpro [K	7.303E-05	7.389E-05	1	1	2	P02656	P02656 [61-71]		0	1805.0089
[K].GTVRPA	1xTMTpro [K	8.965E-05	7.389E-05	1	1	3	P08133	P08133 [355-370]		0	2296.2177
[K].IEEVLSP	1xTMTpro [K	0.000144	7.389E-05	1	1	2	Q9UEY8	Q9UEY8 [668-68(		0	1980.1145
[K].KTANDM	1xTMTpro [K	0.0001458	7.389E-05	1	1	1	P26038	P26038 [538-550]		1	2139.1295
[K].LEESYTI	1xTMTpro [N	0.0001333	7.389E-05	1	1	2	P30740	P30740 [278-290]		0	1814.9455

[R].HYEGST	1xTMTpro [K	0.0001729	7.389E-05	1	1	3	P00738	P00738 [312-321]	0	1754.9569
[K].LEESYDI	1xTMTpro [N	6.337E-05	7.389E-05	1	1	1	P35237	P35237 [276-287]	0	1774.8852
[R].KTCTTV/	1xCarbamido	0.0001277	7.389E-05	1	1	1	P62424	P62424 [197-212]	1	2741.496
[K].LEELWLI	1xCarbamido	0.0001054	7.389E-05	1	1	2	Q9UBU9	Q9UBU9 [317-33.	0	2272.1239
[R].GTVTDF	1xTMTpro [N	0.0001385	7.389E-05	1	1	1	P08758	P08758 [7-25]	1	2401.1955
[K].LEEQLQI	1xTMTpro [K	0.0001749	7.389E-05	1	1	1	Q96T23	Q96T23 [947-959	0	2122.2251
[K].LDKSQIH	1xTMTpro [K	8.362E-05	7.389E-05	1	1	2	P11142	P11142 [326-342]	1	2446.4273
[K].IDKTDYN	1xTMTpro [K	0.000181	7.389E-05	1	1	2	P52565	P52565 [139-152]	1	2210.1771
[K].IEEQFAN	1xTMTpro [K	7.015E-05	7.389E-05	1	1	1	Q96FK6	Q96FK6 [4-15]	0	2049.1988
[R].LEEQAA	1xTMTpro [K	8.362E-05	7.389E-05	1	1	1	Q15149	Q15149 [2017-20.	0	1661.9466
[K].KYDYYY	2xTMTpro [K	7.303E-05	7.389E-05	1	1	1	Q13576	Q13576 [608-618	1	2372.259
[K].KYDVDT	2xTMTpro [K	0.0001094	7.389E-05	1	1	1	P21964	P21964 [179-194]	1	2937.6001
[K].LEENLPI	1xTMTpro [K	9.133E-05	7.389E-05	1	1	1	O60664	O60664 [103-116	0	2260.3044
[R].LDLAGR	1xTMTpro [K	0.0001331	7.389E-05	3	7	2	P62736; P68	P62736 [180-193]	1	2232.2554
[K].LEESAV	1xOxidation	0.0001602	7.389E-05	1	1	1	Q9NZL9	Q9NZL9 [192-203	0	1993.0808
[R].LEELHVI	1xTMTpro [K	0.0001146	7.389E-05	1	1	2	Q16531	Q16531 [159-168	0	1803.0872
[R].GTSVVLI	1xCarbamido	0.0001522	7.389E-05	1	1	1	Q9UK45	Q9UK45 [78-103]	0	3148.5614
[K].HVVFGE	1xTMTpro [N	0.000123	7.389E-05	1	1	1	Q9UNP9	Q9UNP9 [262-27	0	1974.0979
[K].KSADDF	2xTMTpro [K	0.0001672	7.389E-05	1	1	1	Q9Y2A7	Q9Y2A7 [395-403	1	1951.1317
[K].KEEEEEI	2xTMTpro [K	0.0001867	7.389E-05	1	1	1	Q14165	Q14165 [230-246	1	2998.4833
[R].LDFTGN	1xTMTpro [K	9.077E-05	7.389E-05	1	1	2	P20774	P20774 [148-165]	0	2622.3794
[R].LELHVD	1xTMTpro [N	6.337E-05	7.389E-05	1	1	3	P04217	P04217 [392-406]	0	2028.1674
[R].LAADTVI	1xTMTpro [K	0.0001326	7.389E-05	1	1	1	Q86VY4	Q86VY4 [116-133	0	2368.3844
[R].HVGDLG	1xTMTpro [K	7.672E-05	7.389E-05	1	1	1	P00441	P00441 [81-92]	0	1834.0314
[R].LDFTVEI	1xTMTpro [K	9.77E-05	7.389E-05	1	3	1	Q5JPE7	Q5JPE7 [309-319	0	1912.0672
[R].IELGDVT	1xTMTpro [K	0.0001335	7.389E-05	1	1	1	Q9GZZ1	Q9GZZ1 [6-17]	0	1944.141
[K].GYEEWL	1xTMTpro [N	9.205E-05	7.389E-05	3	3	5	P12814; P35	P12814 [377-387]	0	1725.9131
[K].GTLQHA	1xTMTpro [K	0.0001703	7.389E-05	1	1	1	P32929	P32929 [320-330]	0	1865.114
[R].KSAPAT	2xTMTpro [K	9.693E-05	7.389E-05	1	4	2	Q5TEC6	Q5TEC6 [28-37]	1	1828.1472
[K].LELEQT	1xTMTpro [K	0.0001511	7.389E-05	1	1	4	Q03252	Q03252 [276-285	0	1831.0457
[R].LEIEPEW	1xTMTpro [K	6.267E-05	7.389E-05	1	1	2	Q00688	Q00688 [190-200	0	1943.077
[R].LDFVCSI	1xCarbamido	0.0001608	7.389E-05	1	1	2	Q14980	Q14980 [61-70]	0	1865.0487
[R].KEDWNI	2xTMTpro [K	0.000138	7.389E-05	1	1	4	Q10471	Q10471 [42-52]	1	2299.3114

[K].IDFVGEL	1xTMTpro [K	0.0001482	7.389E-05	1	2	2	P55786	P55786 [146-155]	0	1757.9929
[K].KSDFQV	1xTMTpro [K	0.000143	7.389E-05	1	1	1	O60716	O60716 [846-858]	1	2101.1646
[R].IAAAILN	1xTMTpro [N	0.0001392	7.389E-05	1	1	3	P00505	P00505 [326-337]	0	1571.944
[R].LEICNLT	1xCarbamido	8.375E-05	7.389E-05	1	1	3	P07384	P07384 [348-359]	0	1995.144
[R].IDFSDIM	2xTMTpro [K	6.581E-05	7.389E-05	1	1	1	P55011	P55011 [1081-109	0	2818.6205
[K].KEEEES	3xTMTpro [K	9.925E-05	7.389E-05	1	1	2	O75400	O75400 [376-386]	2	2519.4822
[KR].KEEEL	1xTMTpro [K	0.0001058	7.389E-05	3	3	4	P35749; P35	P35749 [1088-109	1	1866.094
[K].GTGVVT	1xTMTpro [N	0.000139	7.389E-05	1	1	1	Q9P2J5	Q9P2J5 [385-404]	0	2261.1944
[R].LAAIAES	1xTMTpro [N	0.0001498	7.389E-05	1	1	4	P28072	P28072 [210-220]	0	1419.8127
[K].KEEESA	2xTMTpro [K	7.213E-05	7.389E-05	1	1	1	P61081	P61081 [12-21]	1	1948.1167
[K].LDENSA	1xTMTpro [K	7.648E-05	7.389E-05	1	1	3	Q13813	Q13813 [1972-19	0	2220.1945
[R].KQSTTE	2xTMTpro [K	0.0001656	7.389E-05	1	1	1	Q14980	Q14980 [2024-20	1	1933.1535
[R].IAAGQA	1xTMTpro [K	7.911E-05	7.389E-05	1	1	1	O14981	O14981 [60-71]	0	1778.1031
[R].GYFEYIE	1xTMTpro [K	9.878E-05	7.389E-05	1	1	8	Q00839	Q00839 [256-265]	0	1899.9984
[R].HVEMYQ	1xOxidation [	0.0001138	7.389E-05	1	1	1	Q9BTV4	Q9BTV4 [118-131	0	2143.0085
[K].GTGLFC	1xCarbamido	0.0001487	7.389E-05	1	1	1	Q16706	Q16706 [1083-10	0	1865.0083
[K].LEILTNL	1xTMTpro [N	0.0001304	7.389E-05	1	1	1	O00203	O00203 [390-407]	0	2302.3301
[R].GTLSGW	1xTMTpro [K	0.0001031	7.389E-05	1	1	6	P27824	P27824 [78-87]	0	1670.0133
[K].HVFAIFN	1xTMTpro [N	0.0001085	7.389E-05	1	1	1	P50570	P50570 [584-594]	0	1665.9032
[R].GTGIVS	1xTMTpro [K	0.0001852	7.389E-05	1	1	4	P15880	P15880 [201-211]	0	1634.0133
[R].IDEYDYS	2xTMTpro [K	6.737E-05	7.389E-05	1	1	4	P31040	P31040 [601-615]	0	2724.5025
[R].HVFFVD	2xTMTpro [K	7.96E-05	7.389E-05	1	1	1	Q05707	Q05707 [1188-12	1	2527.4165
[K].LELLMIH	1xTMTpro [N	7.048E-05	7.389E-05	1	1	1	Q9NR99	Q9NR99 [81-100]	0	2492.3866
[R].LAAFGQ	1xTMTpro [K	0.0002003	7.389E-05	1	1	3	Q12906	Q12906 [324-332]	0	1592.9768
[K].LDFIESD	1xCarbamido	8.827E-05	7.389E-05	1	1	1	O43432	O43432 [1402-14	0	2493.2674
[K].LDFLGE	1xTMTpro [K	0.0001487	7.389E-05	1	1	1	P50613	P50613 [15-28]	0	2196.2196
[K].LDFNTDI	1xTMTpro [K	0.0001944	7.389E-05	1	1	1	P42285	P42285 [409-417]	0	1718.9093
[K].KRDPPS	1xTMTpro [K	0.0001157	7.389E-05	1	1	1	P08575	P08575 [934-950]	2	2591.4073
[R].LELLPDF	1xTMTpro [K	9.467E-05	7.389E-05	1	1	1	Q9Y618	Q9Y618 [110-137	0	3550.0388
[R].HVLDTLI	1xTMTpro [K	0.0001147	7.389E-05	1	1	2	Q7Z6Z7	Q7Z6Z7 [3335-33	0	1859.161
[R].GTITDAF	1xTMTpro [N	6.109E-05	7.389E-05	1	1	6	P50995	P50995 [192-204]	0	1663.8975
[K].KEDFSE	2xTMTpro [K	0.0001235	7.389E-05	1	1	2	Q53FA7	Q53FA7 [194-203	1	2080.2106
[R].GTSHVF	1xCarbamido	9.816E-05	7.389E-05	1	1	1	Q9H6U6	Q9H6U6 [427-444	0	2290.1722

[K].HVMDVV	1xTMTpro [K	0.0001999	7.389E-05	1	1	1	P36776	P36776 [433-444]	0	2009.0869
[K].LEGSAP	1xTMTpro [K	9.724E-05	7.389E-05	1	1	2	Q86UX7	Q86UX7 [334-353	0	2707.5261
[K].KDVDEA	2xTMTpro [K	6.993E-05	7.389E-05	1	1	1	P05787	P05787 [198-207]	1	2125.178
[K].KDVDAA	3xTMTpro [K	0.0001315	7.389E-05	1	2	1	P13647	P13647 [277-292]	2	3040.7494
[K].HVMDVV	1xOxidation [	9.785E-05	7.389E-05	1	1	1	P36776	P36776 [433-444]	0	2025.0818
[K].GTSQND	1xTMTpro [N	0.0001153	7.389E-05	1	1	6	P07996	P07996 [969-980]	0	1676.8676
[R].LDGLDL	1xTMTpro [K	0.0001228	7.389E-05	1	1	2	P05186	P05186 [247-257]	0	1883.077
[K].KDTSITG	2xTMTpro [K	7.135E-05	7.389E-05	1	1	1	P35749	P35749 [206-216]	1	2133.2583
[R].KDVFFY	1xTMTpro [K	0.0001567	7.389E-05	1	2	1	P23258	P23258 [48-63]	1	2651.375
[R].KDTELL	2xTMTpro [K	0.0001241	7.389E-05	1	1	1	Q8IUD2	Q8IUD2 [478-488	1	2172.342
[K].LEGQGD	1xTMTpro [K	7.566E-05	7.389E-05	1	1	2	Q9Y2Z0	Q9Y2Z0 [257-267	0	1749.0038
[R].KSMYEE	1xTMTpro [K	0.0001694	7.389E-05	1	1	1	P20700	P20700 [209-220]	1	2137.1091
[K].HVNPVQ	1xTMTpro [K	9.423E-05	7.389E-05	1	1	1	P35221	P35221 [890-901]	0	1977.1413
[R].KSNQQN	2xTMTpro [K	6.367E-05	7.389E-05	1	1	1	P23921	P23921 [414-424]	1	2143.3015
[K].KDSGFQ	1xTMTpro [K	0.0001813	7.389E-05	1	1	3	P02787	P02787 [122-132]	1	1932.0617
[K].IEGNLIFI	1xTMTpro [K	0.0001533	7.389E-05	1	1	2	P04114	P04114 [655-669]	0	2355.3204
[K].KDSFVEI	1xTMTpro [K	0.0001373	7.389E-05	1	1	1	Q14571	Q14571 [1685-16	1	2003.1053
[R].HVVAVV	1xTMTpro [N	0.000107	7.389E-05	1	1	1	Q9BT22	Q9BT22 [34-45]	0	1524.9181
[K].HVVEDLI	1xTMTpro [N	9.234E-05	7.389E-05	1	1	2	P08473	P08473 [438-448]	0	1596.9393
[R].GTSSTT	1xTMTpro [K	0.0001036	7.389E-05	1	1	1	P00747	P00747 [387-396]	0	1548.8725
[K].KYTMGD	1xTMTpro [K	0.0002039	7.389E-05	2	3	1	Q03013; P28	Q03013 [32-43]; F	1	2040.0352
[R].HVLVTL(	1xTMTpro [K	0.0001834	7.389E-05	1	1	4	P60660	P60660 [111-119]	0	1604.0027
[K].HVLTGS	1xCarbamido	0.0001842	7.389E-05	1	1	1	Q13347	Q13347 [66-77]	0	1620.8083
[K].GYDVIA(	1xTMTpro [K	0.0001025	7.389E-05	2	2	1	P60842; Q14	P60842 [69-82]; Q	0	2003.1053
[K].GTLTEAI	1xTMTpro [K	0.0001694	7.389E-05	1	1	1	P29144	P29144 [716-728]	0	1898.1243
[K].KSDVETI	2xTMTpro [K	0.000138	7.389E-05	1	1	3	Q9UKM9	Q9UKM9 [35-44]	1	2066.2314
[R].LEHAFC	1xCarbamido	8.691E-05	7.389E-05	1	1	1	Q9BV20	Q9BV20 [194-210	0	2354.1631
[K].KSDVGF	2xTMTpro [K	0.0001	7.389E-05	1	1	1	P43652	P43652 [130-147]	1	2928.6539
[K].GTMVTIE	1xTMTpro [N	0.0001863	7.389E-05	1	1	2	Q13126	Q13126 [167-176	0	1364.7527
[K].KEAVTFI	2xTMTpro [K	6.208E-05	7.389E-05	1	1	1	Q9Y263	Q9Y263 [538-554	1	2772.6076
[R].KEATDP	1xCarbamido	0.0001054	7.389E-05	1	1	1	P47755	P47755 [103-121]	1	2881.4758
[K].HVIGLQN	1xTMTpro [N	0.0001208	7.389E-05	1	1	3	Q01995	Q01995 [173-183	0	1515.8385
[R].IEGSGD(	1xTMTpro [N	7.439E-05	7.389E-05	1	1	1	Q05193	Q05193 [344-361	0	2172.074

[K].GTPAPQ	1xTMTpro [N	0.000157	7.389E-05	1	1	2	Q9NR99	Q9NR99 [1976-19	0	1889.0241
[K].GTPEPG	1xTMTpro [N	0.0001856	7.389E-05	1	1	3	Q9ULA0	Q9ULA0 [212-225	0	1755.9196
[R].KYWNDC	1xCarbamido	0.0001073	7.389E-05	1	1	1	P04196	P04196 [84-95]	1	2175.0785
[K].GTPWEC	1xTMTpro [K	0.0001249	7.389E-05	1	1	1	P63279	P63279 [50-59]	0	1699.9663
[R].KEAADP	2xTMTpro [K	7.291E-05	7.389E-05	1	1	1	P53618	P53618 [703-712]	1	1942.1789
[K].KYVLPNI	2xTMTpro [K	7.887E-05	7.389E-05	2	2	3	P0C0L4; P0(	P0C0L4 [235-244]	1	2149.3201
[R].LDGCTA	1xCarbamido	0.0001121	7.389E-05	1	1	1	O43252	O43252 [320-333]	0	1907.9315
[K].KDVSGP	1xTMTpro [K	7.405E-05	7.389E-05	1	1	1	P26640	P26640 [290-303]	1	2144.1302
[K].IEGTPLE	1xTMTpro [K	8.745E-05	7.389E-05	1	1	2	O43676	O43676 [24-34]	0	1837.0926
[K].KDVLETI	2xTMTpro [K	7.543E-05	7.389E-05	1	1	4	P21926	P21926 [170-179]	1	2092.2834
[R].LDFYQH	1xTMTpro [N	0.0001605	7.389E-05	1	1	1	Q9HB07	Q9HB07 [232-243	0	1836.9716
[R].LEELENI	1xTMTpro [N	8.022E-05	7.389E-05	1	1	2	P24821	P24821 [126-137]	0	1705.9655
[K].KYAAELI	2xTMTpro [K	0.0001405	7.389E-05	1	1	1	P00918	P00918 [113-126]	1	2622.5336
[R].LEELDAI	1xTMTpro [K	0.0001065	7.389E-05	1	1	1	Q9UG63	Q9UG63 [196-207	1	2028.0927
[K].KVAVATI	2xTMTpro [K	0.0001806	7.389E-05	1	1	2	P19338	P19338 [71-79]	1	1797.1778
[K].GVLLFGI	1xTMTpro [K	0.0001295	7.389E-05	1	1	4	P35998	P35998 [211-222]	0	1751.0711
[R].GVSAFS	1xTMTpro [K	0.0001371	7.389E-05	1	2	1	O14776	O14776 [674-683]	0	1719.9561
[K].KAEGAQ	2xTMTpro [K	0.0001561	7.389E-05	1	1	5	Q9P2E9	Q9P2E9 [261-270	1	1943.149
[R].GVILTSD	1xCarbamido	0.0001842	7.389E-05	1	1	2	P42126	P42126 [93-116]	0	2871.4664
[K].LDPTQT:	1xTMTpro [K	0.0001479	7.389E-05	1	1	4	Q9UN36	Q9UN36 [278-287	0	1758.0293
[K].KVDLSQ	1xCarbamido	7.96E-05	7.389E-05	1	1	2	Q16762	Q16762 [237-249]	1	2109.2346
[R].GVQVNY	1xTMTpro [K	0.0001561	7.389E-05	1	1	1	Q9UBV2	Q9UBV2 [462-472	0	1828.0824
[K].GVILYGI	1xTMTpro [K	9.394E-05	7.389E-05	1	1	4	P62191	P62191 [221-232]	0	1767.066
[K].GVLMYG	1xTMTpro [K	0.000102	7.389E-05	1	1	1	P17980	P17980 [222-233]	0	1785.0225
[K].LEAEDIA	1xTMTpro [K	0.0001427	7.389E-05	1	1	1	Q9HBI0	Q9HBI0 [90-103]	0	2098.1887
[K].KVDTTPI	2xTMTpro [K	0.0001867	7.389E-05	1	1	2	Q9P2E9	Q9P2E9 [271-280	1	2000.1957
[R].LEAATQ(	1xTMTpro [N	6.178E-05	7.389E-05	1	1	1	Q08379	Q08379 [675-686]	0	1703.936
[K].LEAAIAE	1xTMTpro [K	0.0001012	7.389E-05	1	1	1	P08729	P08729 [332-348]	1	2421.3845
[K].KAEGGC	2xTMTpro [K	0.0001127	7.389E-05	1	1	1	Q00839	Q00839 [215-234]	1	2579.447
[R].KVPAES	1xTMTpro [K	7.135E-05	7.389E-05	1	1	1	Q7LBC6	Q7LBC6 [592-607	1	2350.3448
[R].LDQELD	1xTMTpro [K	0.0001964	7.389E-05	1	1	1	P37198	P37198 [389-401]	0	2185.236
[K].GVQLQT	1xTMTpro [K	0.0001641	7.389E-05	1	1	1	P48444	P48444 [324-335]	0	1944.1158
[RK].KAEGT	2xTMTpro [K	8.414E-05	7.389E-05	1	1	3	Q9P2E9	Q9P2E9 [221-230	1	1942.1538

[K].LEAGDY.	1xTMTpro [K	0.0001412	7.389E-05	1	1	3	Q14008	Q14008 [312-322]	0	1802.0191
[R].KVAQVP	1xCarbamido	0.0001331	7.389E-05	1	1	1	P35052	P35052 [236-248]	1	2049.1771
[K].KVPDPV	1xTMTpro [K	6.317E-05	7.389E-05	1	1	1	O60437	O60437 [940-955]	1	2521.427
[R].GVSSES	1xTMTpro [N	0.0001856	7.389E-05	1	1	5	Q13435	Q13435 [341-350]	0	1284.6351
[R].IDPNPAD	1xTMTpro [K	0.0001759	7.389E-05	1	1	1	O75663	O75663 [258-267]	0	1692.9412
[K].GVGISVI	1xTMTpro [N	9.007E-05	7.389E-05	1	1	1	Q9Y617	Q9Y617 [34-45]	0	1588.88
[K].KAEEIAN	2xTMTpro [K	0.0001468	7.389E-05	1	1	1	P49588	P49588 [651-664]	1	2459.3996
[K].LEAQVQ	1xTMTpro [K	9.708E-05	7.389E-05	1	1	3	P35749	P35749 [1257-126	0	1881.0937
[R].KAQQYL	1xCarbamido	0.0001544	7.389E-05	1	1	1	Q5T0N5	Q5T0N5 [121-131	1	2383.3083
[K].LEAQETI	1xTMTpro [K	0.0001623	7.389E-05	1	1	3	O00461	O00461 [94-102]	0	1653.9667
[K].GVSYLLI	1xTMTpro [N	0.000119	7.389E-05	1	1	2	Q15113	Q15113 [395-408]	0	1898.9965
[R].LEANHG	1xTMTpro [N	0.0001536	7.389E-05	1	1	1	Q969G5	Q969G5 [109-119	0	1496.8868
[R].KVAFCG	1xCarbamido	0.0001544	7.389E-05	1	1	1	Q8IZ83	Q8IZ83 [245-256]	1	1929.0508
[R].GVLPYV	1xTMTpro [K	7.625E-05	7.389E-05	1	1	2	Q5T4S7	Q5T4S7 [4204-42	0	1882.1657
[R].KVAHSD	2xTMTpro [K	6.347E-05	7.389E-05	1	1	1	Q9P0L0	Q9P0L0 [205-221	1	2688.5249
[K].LEAIPNS	1xTMTpro [N	0.0001268	7.389E-05	1	1	2	Q8WWI5	Q8WWI5 [76-89]	0	1872.9557
[R].GVIDMG	1xTMTpro [N	6.875E-05	7.389E-05	1	1	2	Q13813	Q13813 [1608-16	0	1607.8746
[K].GVLFAS	1xTMTpro [N	6.297E-05	7.389E-05	1	1	3	P28838	P28838 [189-200]	0	1536.8817
[K].LEAIITPF	1xTMTpro [K	0.0001567	7.389E-05	1	1	2	O75367	O75367 [124-134]	0	1758.1021
[K].LDPSIFE	1xTMTpro [K	7.359E-05	7.389E-05	1	1	2	Q9Y265	Q9Y265 [172-182	0	1885.0926
[R].KAPSDL	2xTMTpro [K	9.632E-05	7.389E-05	1	1	2	P07384	P07384 [229-240]	1	2301.4362
[K].KAPPPPI	2xTMTpro [K	0.0001053	7.389E-05	1	1	1	O15027	O15027 [2151-21	1	2160.3031
[R].LEALAAE	1xTMTpro [K	0.0001776	7.389E-05	1	1	1	Q9UH99	Q9UH99 [287-300	0	2202.205
[K].IEAGYIQ	1xTMTpro [N	0.0001564	7.389E-05	1	1	1	Q9Y450	Q9Y450 [509-519	0	1526.8134
[R].LEALPNS	1xTMTpro [N	0.0001004	7.389E-05	1	1	1	P35858	P35858 [494-507]	0	1768.0652
[R].LDQETA	1xTMTpro [N	0.0001877	7.389E-05	1	1	1	Q96G03	Q96G03 [15-24]	0	1563.845
[K].KVNQQF	2xTMTpro [K	0.0001796	7.389E-05	1	1	1	P54578	P54578 [381-391]	1	2171.26
[K].KAPIQTY	2xTMTpro [K	0.0001319	7.389E-05	1	1	1	Q69YN2	Q69YN2 [62-79]	1	2886.6869
[K].LDVSNV.	1xTMTpro [N	0.0001319	7.389E-05	1	1	1	P50851	P50851 [1244-125	0	1623.8509
[K].KVELFH	1xTMTpro [K	6.208E-05	7.389E-05	1	1	1	Q14554	Q14554 [107-124]	1	2862.4819
[R].KVEITTE	2xTMTpro [K	0.0001002	7.389E-05	1	1	1	Q92643	Q92643 [309-318]	1	2074.294
[K].KVEQDP	1xTMTpro [K	0.0001306	7.389E-05	1	1	1	Q92817	Q92817 [1141-115	1	2194.2323
[R].KAGIFQS	2xTMTpro [K	0.0001929	7.389E-05	1	1	2	P09669	P09669 [67-75]	1	1890.1993

[R].KAIVICP	1xCarbamido	6.706E-05	7.389E-05	1	1	1	Q9BUJ2	Q9BUJ2 [527-541	2	2685.5426
[K].LDSLGL(	2xCarbamido	0.0001178	7.389E-05	1	1	1	Q9BSH4	Q9BSH4 [246-265	0	2818.4975
[R].KVGWIF	1xTMTpro [K	0.0001138	7.389E-05	1	1	1	Q8TAT6	Q8TAT6 [307-321	1	2374.3262
[R].IDSQWT	1xTMTpro [K	0.0001466	7.389E-05	1	1	1	Q8NF91	Q8NF91 [6884-69	0	2679.4849
[K].KVEGAQ	2xTMTpro [K	9.148E-05	7.389E-05	1	1	3	Q9P2E9	Q9P2E9 [301-310	1	1971.1803
[K].LDSSET	1xTMTpro [K	0.000159	7.389E-05	1	1	2	Q9UBE0	Q9UBE0 [199-208	0	1718.949
[R].LDTVAG	1xTMTpro [N	6.664E-05	7.389E-05	1	1	2	Q9Y6C2	Q9Y6C2 [737-748	0	1503.8814
[R].GVNTDS	1xCarbamido	7.997E-05	7.389E-05	1	1	2	P12955	P12955 [149-159]	0	1455.7181
[K].LDTSQW	1xTMTpro [K	0.0001371	7.389E-05	1	1	2	O60832	O60832 [47-57]	0	1922.1607
[K].LDSTDF	1xTMTpro [K	8.009E-05	7.389E-05	1	1	1	P02786	P02786 [135-145]	0	1806.0141
[R].KALEEA(	2xTMTpro [K	7.497E-05	7.389E-05	1	1	1	P17655	P17655 [629-637]	1	1905.1626
[K].KVFLDC(	2xCarbamido	0.0001479	7.389E-05	1	1	1	P01024	P01024 [722-735]	1	2439.302
[R].LDSVLLL	1xTMTpro [N	0.0001297	7.389E-05	1	1	1	Q13868	Q13868 [104-121	0	2218.2436
[R].IDSVYLD	1xTMTpro [N	0.0001118	7.389E-05	1	1	1	O60443	O60443 [250-261	0	1740.9855
[R].KALDDM	2xTMTpro [K	8.745E-05	7.389E-05	1	1	1	P49368	P49368 [128-138]	1	2147.2926
[R].KVGNPF	2xTMTpro [K	0.0001684	7.389E-05	1	1	1	P30837	P30837 [347-369]	2	3601.9431
[K].IDPLAPL	2xCarbamido	6.726E-05	7.389E-05	1	1	1	P11766	P11766 [160-188]	1	3887.1591
[R].KVEFLEI	2xTMTpro [K	6.695E-05	7.389E-05	1	1	1	Q86UA1	Q86UA1 [573-586	1	2539.4224
[K].LDVTSVI	1xTMTpro [K	0.0001533	7.389E-05	1	1	2	P48643	P48643 [266-275]	0	1776.9875
[K].IDQLQEI	1xTMTpro [K	7.695E-05	7.389E-05	1	1	1	O60313	O60313 [215-228	0	2316.3419
[K].LEAAIAE	1xTMTpro [N	8.171E-05	7.389E-05	1	1	2	P08729	P08729 [332-342]	0	1505.8131
[K].IDQISIEI	1xTMTpro [K	0.0001241	7.389E-05	1	1	1	Q9Y295	Q9Y295 [250-263	0	2300.3245
[R].IDYGTGI	3xCarbamido	0.0001125	7.389E-05	1	1	2	Q15257	Q15257 [184-203	0	2912.4389
[K].IDQISME	1xTMTpro [N	0.0001914	7.389E-05	1	1	1	P55039	P55039 [248-258]	0	1638.8328
[R].LDQPMT	1xTMTpro [N	0.0001015	7.389E-05	1	1	4	P22314	P22314 [1011-102	0	1592.8637
[R].LDQVEQ	1xTMTpro [N	9.754E-05	7.389E-05	1	1	2	Q9UQE7	Q9UQE7 [840-851	0	1789.9615
[R].KVLIIGG	1xTMTpro [K	0.0001877	7.389E-05	1	1	1	P19623	P19623 [96-109]	1	1962.2356
[R].LDYFQP	1xTMTpro [N	0.0001614	7.389E-05	1	1	1	Q9NQY0	Q9NQY0 [191-203	0	1919.0234
[R].IDSDISG	1xTMTpro [K	0.000102	7.389E-05	1	1	2	Q9Y2B0	Q9Y2B0 [125-134	0	1656.9664
[K].LDQVVE	1xTMTpro [N	7.997E-05	7.389E-05	1	1	2	P16157	P16157 [850-861]	0	1627.9338
[K].GVIQVYI	1xOxidation	6.188E-05	7.389E-05	1	1	1	O75694	O75694 [298-314	0	2143.0773
[K].GVLTLSI	1xTMTpro [K	0.0001749	7.389E-05	1	1	1	Q96G03	Q96G03 [301-312	0	1843.1185
[R].IDVYGFS	1xTMTpro [K	6.653E-05	7.389E-05	1	1	4	Q9UBV8	Q9UBV8 [171-181	0	1907.0922

[K].KVEEVV	1xTMTpro [K	0.0001729	7.389E-05	1	1	3	Q15631	Q15631 [204-215]	1	2058.2091
[R].IDVVVNI	1xTMTpro [N	0.0001458	7.389E-05	1	1	2	P51659	P51659 [93-104]	0	1586.9549
[R].GVIVES/	1xTMTpro [K	9.482E-05	7.389E-05	1	1	1	Q15172	Q15172 [113-125]	0	1988.156
[R].LDVVAG	1xTMTpro [N	9.957E-05	7.389E-05	1	1	1	Q9Y6C2	Q9Y6C2 [369-382	0	1676.9866
[K].KANLCTI	1xCarbamid	0.0001138	7.389E-05	1	1	1	P25445	P25445 [300-309]	1	2060.2354
[R].KVLFYTI	1xTMTpro [K	9.423E-05	7.389E-05	1	1	2	Q14112	Q14112 [1221-12	1	2073.2352
[R].KVLLDG	1xTMTpro [K	0.0001995	7.389E-05	1	1	3	P01024	P01024 [305-315]	1	1847.1358
[R].LEASLC	3xCarbamid	0.0001155	7.389E-05	1	1	1	O14638	O14638 [108-122]	0	2117.9585
[R].LDPHLDI	1xCarbamid	0.0001163	7.389E-05	1	1	1	Q9BRZ2	Q9BRZ2 [194-210	0	2257.2082
[K].KASSVE	3xTMTpro [K	8.362E-05	7.389E-05	1	1	1	Q02539	Q02539 [113-125]	1	2506.5345
[K].GVDLVLI	1xTMTpro [K	8.44E-05	7.389E-05	1	1	2	P49327	P49327 [1740-175	0	1995.1618
[R].LDILDTA	1xOxidation	0.0001514	7.389E-05	2	2	2	P62070; P1C	P62070 [64-79]; F	0	2086.0446
[R].LEEDQE	1xTMTpro [K	0.0001911	7.389E-05	1	1	1	Q92614	Q92614 [1748-17	0	2232.102
[R].IDLLELL	1xTMTpro [K	0.0001266	7.389E-05	1	1	1	Q96RW7	Q96RW7 [359-37	0	2196.3347
[K].KTGCNV	1xCarbamid	0.0001736	7.389E-05	1	1	3	P50991	P50991 [292-302]	1	2186.3511
[K].GVEAVG	1xTMTpro [N	0.0001315	7.389E-05	1	1	2	Q12996	Q12996 [152-164]	0	1683.8621
[K].KWDTC/	1xCarbamid	7.59E-05	7.389E-05	1	1	1	O95861	O95861 [245-261]	1	2793.5902
[R].LDILVNI	1xTMTpro [K	8.209E-05	7.389E-05	1	1	1	P15428	P15428 [85-98]	0	2121.2159
[K].LEEAEK/	1xTMTpro [K	7.66E-05	7.389E-05	4	4	1	P67936; P0	P67936 [77-89]; F	1	2085.0956
[K].IEEDSEV	1xTMTpro [K	0.0001297	7.389E-05	1	1	1	O60888	O60888 [113-124]	0	2045.1154
[R].KTLLSNI	3xTMTpro [K	0.0001644	7.389E-05	1	1	1	P10909	P10909 [68-79]	2	2590.6284
[K].LDLMDA	1xTMTpro [N	6.972E-05	7.389E-05	1	1	1	O00429	O00429 [217-233]	0	2128.0408
[K].KVYENY	1xTMTpro [K	6.982E-05	7.389E-05	1	1	1	P35659	P35659 [349-362]	1	2399.2739
[R].GVVQLF	1xTMTpro [K	0.0001742	7.389E-05	1	1	1	Q9Y3B9	Q9Y3B9 [169-179	0	1811.1035
[K].KAATALI	3xTMTpro [K	0.0001533	7.389E-05	1	1	1	Q15084	Q15084 [67-77]	2	2360.5382
[R].KVVSCL	2xCarbamid	9.306E-05	7.389E-05	1	1	1	Q13630	Q13630 [108-121]	1	2566.4553
[K].KVVNPLI	2xTMTpro [K	0.000111	7.389E-05	1	1	4	P62424	P62424 [26-34]	1	1986.2568
[R].KCGETA	3xCarbamid	6.674E-05	7.389E-05	1	1	1	P22234	P22234 [80-101]	1	3303.6642
[R].KTPPTM	2xTMTpro [K	9.754E-05	7.389E-05	1	1	1	Q8N183	Q8N183 [80-90]	1	2199.3239
[K].GVVFDV	1xTMTpro [K	0.0001295	7.389E-05	1	1	2	Q9UMX5	Q9UMX5 [70-79]	0	1616.9503
[R].IEDVTPII	1xTMTpro [N	9.408E-05	7.389E-05	1	1	4	P62263	P62263 [129-141]	0	1733.9241
[K].IDLPEYQ	1xTMTpro [K	7.28E-05	7.389E-05	1	1	2	Q9BY32	Q9BY32 [40-56]	0	2582.3845
[R].KCPQEA	1xCarbamid	0.000139	7.389E-05	1	1	1	P24588	P24588 [61-76]	1	2379.2218

[R].IDILDSAI	1xTMTpro [N	0.0001335	7.389E-05	1	1	1	P62195	P62195 [298-310]	0	1743.0448
[K].GVALHR	1xTMTpro [N	8.638E-05	7.389E-05	2	2	4	P0DOX6; P0	P0DOX6 [446-461	0	2078.2194
[R].LEEIAEL	1xTMTpro [N	6.843E-05	7.389E-05	1	1	1	Q6IN85	Q6IN85 [140-156]	0	2128.2185
[K].HYLDINT	1xTMTpro [K	0.000155	7.389E-05	1	1	1	Q63HN8	Q63HN8 [3172-31	0	1953.1301
[K].GWEFHS	1xTMTpro [N	0.0001824	7.389E-05	1	1	2	Q6UXG2	Q6UXG2 [227-23	0	1677.8668
[R].LEEGPP	1xTMTpro [N	0.0001224	7.389E-05	1	1	2	P08559	P08559 [46-58]	0	1715.9863
[R].KWNTTL	1xTMTpro [K	0.0001157	7.389E-05	1	1	1	P07384	P07384 [365-376]	1	2163.1842
[R].GVCLIDE	1xCarbamido	0.0001176	7.389E-05	1	1	2	P49736	P49736 [582-591]	0	1803.9806
[K].IDLENTL	1xTMTpro [N	0.0001611	7.389E-05	1	1	1	Q16204	Q16204 [200-216	0	2318.2159
[K].LEEGGP	1xTMTpro [K	7.337E-05	7.389E-05	1	1	1	O95202	O95202 [134-150	0	2378.3463
[K].KTEKEES	3xTMTpro [K	0.0001653	7.389E-05	1	1	1	P20810	P20810 [250-261]	2	2637.5816
[R].KWISDW	2xTMTpro [K	0.0001049	7.389E-05	1	1	1	Q7L2H7	Q7L2H7 [149-160	1	2433.3958
[K].HYQINQ	1xTMTpro [N	0.0001896	7.389E-05	1	1	4	P02751	P02751 [58-67]	0	1705.873
[R].GWDWT	1xTMTpro [K	7.015E-05	7.389E-05	1	1	2	P14207	P14207 [153-162]	0	1757.9466
[R].LDIFAHN	1xTMTpro [N	6.653E-05	7.389E-05	1	1	1	P56202	P56202 [63-75]	0	1801.004
[R].KTDPWS	1xTMTpro [K	0.0001602	7.389E-05	1	1	1	Q15477	Q15477 [84-106]	1	3070.7545
[K].GWDQG	1xCarbamido	0.0001445	7.389E-05	1	1	3	P26885	P26885 [88-101]	0	2204.0971
[R].KAAALEA	3xTMTpro [K	7.707E-05	7.389E-05	1	1	1	P31948	P31948 [434-446]	2	2656.5849
[R].LDLIAQG	1xTMTpro [N	7.96E-05	7.389E-05	1	1	1	P36543	P36543 [200-212]	0	1848.0042
[R].KAADDT	2xTMTpro [K	8.209E-05	7.389E-05	1	1	1	P02766	P02766 [55-68]	1	2435.3387
[R].GWDEAI	1xTMTpro [K	6.939E-05	7.389E-05	1	1	2	Q00688	Q00688 [174-184	0	1859.0228
[K].IEEFLEA	1xCarbamido	0.0001031	7.389E-05	1	1	3	O00299	O00299 [80-92]	0	1877.0162
[K].GTFTLHY	1xTMTpro [N	0.0001218	7.389E-05	1	1	1	O75339	O75339 [524-536	0	1804.9513
[R].KVVEQG	2xTMTpro [K	7.048E-05	7.389E-05	1	1	1	Q9Y4E8	Q9Y4E8 [116-125	1	2077.266
[R].IDIPPGA	1xTMTpro [K	0.000187	7.389E-05	1	1	2	Q16851	Q16851 [486-497	0	1874.1243
[K].GVTQIDN	1xTMTpro [K	0.0001536	7.389E-05	1	1	2	P21283	P21283 [128-137]	0	1710.9882
[R].KVSEIKD	3xTMTpro [K	8.937E-05	7.389E-05	1	1	1	P07942	P07942 [1226-124	2	3253.9876
[R].GVGAEF	1xTMTpro [N	9.105E-05	7.389E-05	1	1	2	Q9H0D6	Q9H0D6 [884-895	0	1612.913
[K].KTTIENI	1xTMTpro [K	0.0001021	7.389E-05	1	1	1	P33991	P33991 [628-643]	1	2472.4794
[K].IEDGEDI	1xTMTpro [K	6.417E-05	7.389E-05	1	1	1	Q13459	Q13459 [371-383	1	2211.1537
[K].KAVAISL	2xTMTpro [K	0.0001349	7.389E-05	1	1	6	P50454	P50454 [300-308]	1	1839.2248
[R].GVTNDQ	1xTMTpro [K	0.0001262	7.389E-05	1	1	1	Q9Y2P8	Q9Y2P8 [119-133	0	2194.2211
[K].LDPDSIF	1xTMTpro [N	0.0001849	7.389E-05	1	1	1	O94855	O94855 [259-275	0	2212.178

[K].IEDAQV(	1xTMTpro [N	0.0001144	7.389E-05	1	1	1	Q96RW7	Q96RW7 [2161-2	0	1535.7985
[R].GVFNVQ	1xTMTpro [K	0.0001896	7.389E-05	1	1	2	P05198	P05198 [267-276]	0	1756.9912
[R].KAEEDA	2xTMTpro [K	0.0001852	7.389E-05	1	1	2	Q13428	Q13428 [64-74]	1	2086.2324
[R].LDPGAV	1xTMTpro [K	0.0001036	7.389E-05	1	1	1	P42356	P42356 [1578-15!	0	2019.1618
[K].LDPGLIM	1xTMTpro [K	0.0001742	7.389E-05	1	1	2	Q9Y5L4	Q9Y5L4 [17-27]	0	1851.0905
[K].KVQDGL	2xTMTpro [K	0.0001373	7.389E-05	1	1	1	P46100	P46100 [956-967]	1	2215.3114
[R].GVGGAF	1xTMTpro [N	0.0001266	7.389E-05	1	1	3	Q07954	Q07954 [771-783]	0	1541.9334
[K].GVGGGS	1xTMTpro [N	0.0001112	7.389E-05	1	1	1	O15211	O15211 [199-209]	0	1305.7446
[K].KVQAAQ	2xTMTpro [K	0.0001237	7.389E-05	1	1	1	Q16891	Q16891 [344-353]	1	1972.2007
[K].IDPHAPN	1xOxidation [	8.813E-05	7.389E-05	1	1	1	O43813	O43813 [143-155]	0	1832.9284
[K].GVGLGP	1xOxidation [	0.0001345	7.389E-05	1	1	7	P08123	P08123 [85-99]	0	1715.9256
[K].LECGGG	1xCarbamido	6.139E-05	7.389E-05	1	1	3	P00352	P00352 [368-378]	0	1782.9453
[R].IEDANLII	1xTMTpro [K	0.0001458	7.389E-05	1	1	2	P07996	P07996 [61-74]	0	2144.2095
[K].KTPSKPI	3xTMTpro [K	6.529E-05	7.389E-05	1	1	1	P18887	P18887 [256-271]	1	2878.7871
[K].KAVIFCL	1xCarbamido	8.965E-05	7.389E-05	1	1	2	P60981	P60981 [34-45]	2	2596.6001
[K].LDNLVAI	1xTMTpro [N	7.972E-05	7.389E-05	1	1	2	P29401	P29401 [175-186]	0	1672.9917
[R].LEDLVCI	1xCarbamido	0.0001542	7.389E-05	1	1	2	O43776	O43776 [354-364]	0	1636.8535
[K].KVVEDIE	2xTMTpro [K	0.0001226	7.389E-05	1	1	1	O95299	O95299 [268-277]	1	2148.3096
[K].KVTQLDI	2xTMTpro [K	0.0001576	7.389E-05	1	1	2	Q13442	Q13442 [95-105]	1	2126.3001
[K].KTQDQIS	2xTMTpro [K	0.0001004	7.389E-05	1	1	2	Q14847	Q14847 [112-121]	1	2087.2641
[K].KADAAA	2xTMTpro [K	7.827E-05	7.389E-05	1	1	1	Q9P2E9	Q9P2E9 [589-598	1	1886.1276
[K].KVTNDV	1xCarbamido	0.0001691	7.389E-05	1	1	1	Q14767	Q14767 [1589-16	1	2026.1247
[R].GVVCIDI	1xCarbamido	0.0001476	7.389E-05	1	1	1	P25205	P25205 [404-413]	0	1789.965
[R].GVEVDP	1xTMTpro [K	0.0001437	7.389E-05	1	1	2	O60313	O60313 [825-834]	0	1665.0078
[R].LEDLLQI	2xTMTpro [K	0.0001188	7.389E-05	1	1	1	Q9UPN3	Q9UPN3 [824-842	1	3174.7384
[K].GVTQYY	1xTMTpro [N	0.0001273	7.389E-05	1	1	2	P26196	P26196 [308-319]	0	1753.908
[R].LDMMDE	1xTMTpro [N	0.0001007	7.389E-05	1	1	2	P35237	P35237 [259-273]	0	2096.0211
[K].KCADSS	1xCarbamido	0.0001284	7.389E-05	1	1	1	Q9HC84	Q9HC84 [454-467	1	2205.2193
[R].LEDLATI	1xTMTpro [K	0.000142	7.389E-05	1	1	2	O43795	O43795 [704-713]	0	1752.0763
[K].KTSEVDI	3xTMTpro [K	0.0002043	7.389E-05	1	1	1	Q8WUM4	Q8WUM4 [11-23]	1	2644.6754
[K].LEDHEYI	2xTMTpro [K	0.0001599	7.389E-05	1	1	2	Q8IWX8	Q8IWX8 [661-672	0	2396.3642
[R].GVEVDP	1xTMTpro [K	0.0001304	7.389E-05	1	1	1	O60313	O60313 [825-842]	1	2750.5121
[R].GVFCND	1xCarbamido	0.0001136	7.389E-05	1	1	2	O14494	O14494 [37-46]	0	1776.9446

[K].LLDNLLLD	1xTMTpro [K	0.0001087	7.389E-05	1	1	1	Q16512	Q16512 [743-755]	0	2101.2036
[R].GVTSFG	1xTMTpro [K	0.0002007	7.389E-05	1	1	2	P12544	P12544 [224-233]	0	1659.9561
[R].KVSSAE	2xTMTpro [K	6.737E-05	7.389E-05	1	1	1	P05114	P05114 [5-14]	1	1860.1371
[K].KTSATV	2xTMTpro [K	0.0001726	7.389E-05	1	1	3	P16104	P16104 [120-128]	1	1801.1363
[R].IDEMPE	1xTMTpro [K	0.0001375	7.389E-05	1	1	2	P51858	P51858 [30-39]	0	1710.9592
[R].KQQTIIL	2xTMTpro [K	6.158E-05	7.389E-05	2	2	1	P51692; P42	P51692 [242-256]	1	2783.6487
[R].KQQTLE	2xTMTpro [K	9.453E-05	7.389E-05	1	1	2	Q16643	Q16643 [238-248]	1	2187.2801
[K].LFEVGG	1xTMTpro [N	9.602E-05	7.389E-05	2	2	2	P16298; Q08	P16298 [110-121]	0	1551.845
[R].HSEAAT	1xTMTpro [K	0.0001281	7.389E-05	1	1	1	Q14103	Q14103 [86-98]	1	2151.1438
[K].HDTSLKI	2xTMTpro [K	9.77E-05	7.389E-05	1	1	3	P00915	P00915 [41-58]	0	2841.6291
[R].HSEAFE	1xTMTpro [K	9.754E-05	7.389E-05	1	1	2	Q07065	Q07065 [395-405]	0	1896.0471
[K].LFEMVL	1xOxidation [	6.982E-05	7.389E-05	1	1	3	Q16891	Q16891 [85-101]	0	2483.4228
[R].KPEDPE	1xCarbamido	0.0001691	7.389E-05	1	1	1	Q9GZU8	Q9GZU8 [37-52]	0	2597.2685
[K].LAEGFPI	1xTMTpro [K	0.0001116	7.389E-05	1	1	1	P18428	P18428 [444-454]	0	1806.1385
[K].GSGNLE	1xTMTpro [K	8.518E-05	7.389E-05	1	1	2	P78371	P78371 [192-203]	0	1860.1199
[K].GSGPPP	1xTMTpro [N	8.937E-05	7.389E-05	1	1	1	Q9UPT8	Q9UPT8 [917-930	0	1774.8415
[R].IFEGTND	1xTMTpro [N	0.0001995	7.389E-05	1	1	1	P49748	P49748 [460-469]	0	1481.8283
[R].LFEFAG	1xTMTpro [N	0.0001068	7.389E-05	1	1	3	P54136	P54136 [224-234]	0	1633.8909
[R].HSEDNP	1xTMTpro [N	7.146E-05	7.389E-05	1	1	1	P11532	P11532 [1315-132	0	1513.7678
[K].HSELSEI	1xTMTpro [K	7.863E-05	7.389E-05	2	2	1	O75886; Q9	O75886 [344-353]	0	1764.0147
[K].LFEDLEF	1xTMTpro [N	0.000101	7.389E-05	1	1	2	Q86UU1	Q86UU1 [804-815	0	1870.987
[R].GSGSG	1xTMTpro [N	9.572E-05	7.389E-05	1	1	1	Q86YZ3	Q86YZ3 [897-908	0	1443.7147
[HR].GSGSS	1xTMTpro [N	7.258E-05	7.389E-05	1	1	2	Q86YZ3	Q86YZ3 [1106-11	0	1433.694
[K].HSEVQC	1xTMTpro [K	0.0001752	7.389E-05	1	1	3	P35606	P35606 [319-328]	0	1762.0103
[K].LFEDDEI	1xCarbamido	0.0001005	7.389E-05	1	1	1	Q9Y2D5	Q9Y2D5 [193-207	1	2619.3005
[K].KGCPPD	1xCarbamido	0.0001838	7.389E-05	1	1	2	P05556	P05556 [73-84]	1	2006.0621
[K].KGDPVY	2xTMTpro [K	0.0001782	7.389E-05	1	1	1	Q5JRA6	Q5JRA6 [68-77]	1	2144.2572
[R].LFFAGEI	1xTMTpro [N	0.0001194	7.389E-05	1	1	1	O60341	O60341 [796-805]	0	1494.8388
[R].IFFAGTE	1xTMTpro [K	6.591E-05	7.389E-05	1	1	2	P21397	P21397 [430-440]	0	1794.0293
[R].IFFDDYV	2xCarbamido	0.0001078	7.389E-05	1	1	2	P28676	P28676 [173-184]	0	2145.1004
[R].ICISGDS	1xCarbamido	0.0001896	7.389E-05	1	1	1	Q6PIU2	Q6PIU2 [185-211]	0	3301.7342
[R].IFGGLDN	1xTMTpro [K	6.519E-05	7.389E-05	1	1	3	Q92688	Q92688 [76-86]	0	1802.0378
[K].HEAYGE	1xCarbamido	0.0001703	7.389E-05	1	1	2	P12110	P12110 [240-248]	0	1764.8871

[K].KPAEDE	3xTMTpro [K	8.298E-05	7.389E-05	1	1	3	P02792	P02792 [84-98]	1	2919.6391
[R].GSFMGS	1xTMTpro [K	0.0001116	7.389E-05	1	1	1	P50281	P50281 [465-479]	0	2386.1921
[K].HEAMITD	1xTMTpro [N	7.997E-05	7.389E-05	1	1	2	P35579	P35579 [1025-103	0	1647.8331
[R].IFGESIM	1xCarbamido	0.0001542	7.389E-05	1	1	1	Q8NFH5	Q8NFH5 [231-246	0	2719.5707
[K].KGDTFS	1xCarbamido	0.0001089	7.389E-05	1	1	2	P01876	P01876 [307-327]	1	3249.758
[K].KPALGF	1xTMTpro [K	6.307E-05	7.389E-05	1	1	1	Q99459	Q99459 [219-240]	0	3158.5926
[R].KPEEEE	2xTMTpro [K	0.0001827	7.389E-05	1	1	1	O43818	O43818 [62-78]	0	2988.5353
[K].LFGALTF	1xTMTpro [N	0.0001103	7.389E-05	1	1	2	O14617	O14617 [244-254]	0	1517.9011
[R].KPASFM	1xCarbamido	0.0001732	7.389E-05	1	1	1	P53396	P53396 [836-848]	0	2150.123
[K].LAEIGAF	1xTMTpro [N	0.0001641	7.389E-05	1	1	1	Q13435	Q13435 [36-47]	0	1542.8923
[R].LCNNPT	1xCarbamido	8.311E-05	7.389E-05	1	1	3	P07996	P07996 [518-529]	0	1941.0508
[K].LAELEEA	1xTMTpro [K	0.0001046	7.389E-05	1	1	4	P13647	P13647 [432-441]	0	1752.0399
[K].KGDSSA	2xTMTpro [K	6.317E-05	7.389E-05	1	1	7	P26373	P26373 [136-145]	1	1976.148
[KR].HEAFE	1xTMTpro [N	0.0001239	7.389E-05	3	4	1	P12814; P35	P12814 [437-450]	0	1929.9374
[K].IAEIEAE	1xTMTpro [N	0.0001881	7.389E-05	1	1	1	Q9Y295	Q9Y295 [8-17]	0	1436.7738
[K].LAEKEE	1xTMTpro [K	0.0001268	7.389E-05	1	1	2	P21980	P21980 [465-476]	1	1974.0644
[K].HDVGAY	1xTMTpro [K	8.571E-05	7.389E-05	1	1	2	P78527	P78527 [3850-386	0	1936.0316
[R].KPAISLA	1xTMTpro [K	8.492E-05	7.389E-05	1	1	1	Q5VSL9	Q5VSL9 [205-217	0	1995.173
[R].LFEAEA	1xTMTpro [N	8.937E-05	7.389E-05	1	1	4	Q9H223	Q9H223 [273-283	0	1642.876
[R].GSGSVV	1xTMTpro [K	8.937E-05	7.389E-05	1	1	2	O75746	O75746 [359-370]	0	1835.0228
[R].KGADIM	1xCarbamido	0.0001659	7.389E-05	2	2	1	P12236; P12	P12236 [245-259]	1	2397.2187
[K].LADQSG	1xTMTpro [N	0.00014	7.389E-05	1	1	1	Q8N983	Q8N983 [104-114	0	1490.8498
[R].LFCDPTI	1xCarbamido	6.674E-05	7.389E-05	1	1	1	Q9Y6Q1	Q9Y6Q1 [26-43]	0	2552.2451
[R].HSNLML	1xTMTpro [K	0.0001328	7.389E-05	1	1	1	O43491	O43491 [626-636]	0	1937.0658
[R].GSLLSEI	1xTMTpro [N	9.678E-05	7.389E-05	1	1	2	Q9NQT8	Q9NQT8 [731-742	0	1573.9233
[R].ICSIYTQ	1xCarbamido	7.566E-05	7.389E-05	1	1	1	Q9Y4W2	Q9Y4W2 [503-52	0	3262.6757
[R].LFAVAPI	1xTMTpro [K	0.0001746	7.389E-05	1	1	3	P12110	P12110 [187-197]	0	1823.1035
[K].LADQIFA	1xTMTpro [K	0.0001573	7.389E-05	1	2	2	Q7L576	Q7L576 [706-715]	0	1840.05
[K].ICSWNV	1xCarbamido	0.0001983	7.389E-05	1	1	1	P27695	P27695 [64-73]	0	1523.796
[K].KPIDFVL	2xTMTpro [K	0.0001417	7.389E-05	1	1	1	Q2TB10	Q2TB10 [563-571	0	1986.2568
[R].IFDAVGF	1xTMTpro [N	0.0001342	7.389E-05	1	1	2	Q9UBB6	Q9UBB6 [76-87]	0	1687.9127
[R].HDFSTV	1xTMTpro [N	0.00018	7.389E-05	1	1	1	Q9UPT5	Q9UPT5 [424-437	0	1949.118
[K].LCTEQG	1xCarbamido	0.00012	7.389E-05	1	1	3	P35749	P35749 [553-562]	0	1764.9558

[K].IFAQLDS	1xTMTpro [N	8.171E-05	7.389E-05	1	1	3	Q9Y2S2	Q9Y2S2 [105-116	0	1709.9393
[K].KPLKPC(	3xCarbamido	0.0001366	7.389E-05	1	1	1	Q14061	Q14061 [18-30]	0	2805.593
[R].GSIQVD(	1xTMTpro [N	0.0002003	7.389E-05	1	1	1	P98160	P98160 [4305-43	0	1749.9302
[K].HSPTED	1xTMTpro [K	9.708E-05	7.389E-05	1	1	1	P46821	P46821 [969-979]	0	1837.9423
[K].IFAQDGE	1xTMTpro [N	0.0001834	7.389E-05	1	1	5	O75369	O75369 [682-691]	0	1424.7453
[R].LCTSVTI	1xCarbamido	0.00012	7.389E-05	1	1	1	O75439	O75439 [388-399]	0	1655.8594
[K].LCVPAM	1xCarbamido	0.0001034	7.389E-05	2	2	1	O43865; Q9	O43865 [271-284]	0	2156.1699
[K].LCWFLD	1xCarbamido	0.000122	7.389E-05	1	1	2	O95336	O95336 [236-246]	0	1655.8535
[R].GSLQILD	1xTMTpro [K	8.134E-05	7.389E-05	1	1	2	Q9BV20	Q9BV20 [11-23]	0	2046.2818
[R].KPAAVLI	3xTMTpro [K	0.0001632	7.389E-05	1	1	2	Q9H0D6	Q9H0D6 [776-788	0	2685.6444
[K].LFDDCT(	1xCarbamido	0.0001506	7.389E-05	1	1	2	Q14738	Q14738 [480-489]	0	1925.9923
[R].KFVADG	2xTMTpro [K	0.0001149	7.389E-05	1	1	4	P23396	P23396 [10-18]	1	1937.204
[K].LFDYFPI	1xTMTpro [K	0.0001772	7.389E-05	1	1	1	P08574	P08574 [171-186]	0	2523.3528
[K].KPEMFE	2xTMTpro [K	0.0001455	7.389E-05	1	1	1	P78347	P78347 [807-816]	0	2106.2449
[K].LCPQLM	1xCarbamido	0.0001769	7.389E-05	1	1	1	Q8TEX9	Q8TEX9 [349-362	0	2005.0604
[R].HDSGD(	1xTMTpro [K	8.298E-05	7.389E-05	1	1	1	P21589	P21589 [518-534]	0	2486.3019
[R].ICPVEFN	1xCarbamido	0.0001361	7.389E-05	1	1	1	Q9UI30	Q9UI30 [32-44]	0	1866.9856
[K].HSGEAT	1xTMTpro [K	8.923E-05	7.389E-05	1	1	1	A5YKK6	A5YKK6 [1949-19	0	1752.9736
[R].KGAAPA	2xTMTpro [K	0.0001375	7.389E-05	1	1	1	Q13428	Q13428 [384-393]	1	1806.1418
[K].ICPVETL	2xCarbamido	9.379E-05	7.389E-05	1	1	1	P30084	P30084 [212-228]	0	2597.381
[K].KPEVPFI	2xTMTpro [K	0.0001482	7.389E-05	1	1	1	Q2PPJ7	Q2PPJ7 [1787-18	0	2917.7008
[R].HSNLML	1xTMTpro [K	0.0001277	7.389E-05	1	1	1	P11171	P11171 [663-673]	0	1923.0501
[K].LAEDILN	1xTMTpro [K	8.827E-05	7.389E-05	1	1	1	Q13045	Q13045 [1116-11	0	2456.2875
[K].GSLEVLI	2xTMTpro [K	7.113E-05	7.389E-05	1	1	2	Q9Y3A5	Q9Y3A5 [232-250	2	3062.6714
[K].GSLFPM	1xTMTpro [N	7.102E-05	7.389E-05	1	1	1	O00142	O00142 [223-243]	0	2624.3648
[K].LAEAQIE	1xTMTpro [N	0.0001553	7.389E-05	1	1	2	Q9NR28	Q9NR28 [208-217	0	1475.8389
[R].KPGINV/	1xTMTpro [K	0.0001405	7.389E-05	1	1	1	Q14697	Q14697 [930-944]	0	2301.3323
[R].IADYEA/	1xTMTpro [N	0.0001025	7.389E-05	1	1	1	Q9Y5J6	Q9Y5J6 [79-103]	0	2634.3218
[K].GSLGAT	1xTMTpro [K	0.0001435	7.389E-05	1	1	2	Q09666	Q09666 [5463-54	0	1540.919
[K].KPGPGE	2xTMTpro [K	7.405E-05	7.389E-05	1	1	3	O15382	O15382 [48-59]	0	2138.3154
[R].KFVTETI	1xTMTpro [K	0.0001371	7.389E-05	1	1	1	Q9NXF1	Q9NXF1 [462-473	1	1990.1101
[K].IFDEILVN	1xTMTpro [K	0.0001427	7.389E-05	2	2	2	P11388; Q0	P11388 [84-96]; Q	0	2070.1727
[R].HDMLAW	1xTMTpro [K	0.0001168	7.389E-05	1	1	1	Q15691	Q15691 [18-34]	0	2634.4569

[K].HRPEDV	1xTMTpro [K	0.0001249	7.389E-05	1	1	1	Q53T59	Q53T59 [57-69]	0	2162.2577
[K].HEEAPG	1xTMTpro [K	6.287E-05	7.389E-05	1	1	5	P21291	P21291 [92-108]	0	2451.2984
[K].GSETDS	1xTMTpro [K	0.0001034	7.389E-05	1	1	1	P30419	P30419 [68-80]	0	1970.0322
[K].HQEPVY	1xTMTpro [N	0.0001361	7.389E-05	1	1	1	Q9BZK7	Q9BZK7 [441-455	0	1993.0099
[K].LFIYNPT	1xTMTpro [N	8.109E-05	7.389E-05	1	1	2	P54709	P54709 [18-31]	0	1932.055
[K].LFLSYDY	1xTMTpro [K	0.0001476	7.389E-05	1	1	4	Q9UHG3	Q9UHG3 [421-43(	0	1827.0548
[R].GQYCYE	1xCarbamido	0.0001793	7.389E-05	1	1	3	P04004	P04004 [177-186]	0	1912.9606
[K].LAEQEAI	1xTMTpro [N	0.0001146	7.389E-05	1	1	1	Q9NUV9	Q9NUV9 [283-293	0	1590.8559
[K].HQFLLT(	1xTMTpro [N	0.0001789	7.389E-05	1	1	3	P04217	P04217 [79-90]	0	1676.9039
[R].GRDPAL	1xTMTpro [K	6.737E-05	7.389E-05	1	1	3	Q13724	Q13724 [564-576]	1	2000.2148
[R].GREEGF	1xTMTpro [N	0.0001354	7.389E-05	1	1	1	Q02388	Q02388 [670-682]	1	1628.9403
[K].HQGMF(	1xTMTpro [K	7.924E-05	7.389E-05	1	1	2	Q3KQZ1	Q3KQZ1 [146-159	0	2196.2091
[K].KGGPSP	2xTMTpro [K	7.985E-05	7.389E-05	1	1	1	P09661	P09661 [193-205]	1	2167.2903
[K].LAEQAEI	1xTMTpro [K	0.0001762	7.389E-05	1	1	1	P62258	P62258 [13-28]	1	2537.2871
[R].IFLLTNN	1xTMTpro [K	6.317E-05	7.389E-05	1	1	1	O43795	O43795 [1007-10	0	2338.399
[K].LAENNIN	1xTMTpro [K	7.28E-05	7.389E-05	1	1	1	P18084	P18084 [326-339]	0	2168.2935
[R].GRPITMI	1xOxidation [	8.453E-05	7.389E-05	1	1	1	Q9HC35	Q9HC35 [233-251	0	2544.2724
[K].GRPMVIS	1xTMTpro [K	0.0001125	7.389E-05	1	1	2	Q9NR45	Q9NR45 [150-166	0	2464.2676
[K].IFLHQDL	1xTMTpro [N	0.0001806	7.389E-05	1	1	1	Q9BX69	Q9BX69 [485-498	0	1978.2173
[K].GSAAAP	1xTMTpro [K	0.0001842	7.389E-05	1	1	1	Q9UGM6	Q9UGM6 [22-31]	0	1521.9245
[R].GSAAAV	1xTMTpro [K	9.063E-05	7.389E-05	1	1	2	Q13636	Q13636 [77-89]	0	1916.1348
[R].HQGVM\	1xTMTpro [K	7.497E-05	7.389E-05	3	7	12	P62736; P68	P62736 [42-52]; F	0	1779.9854
[K].LCEGFN	1xCarbamido	0.0001684	7.389E-05	1	1	1	Q9Y6H1	Q9Y6H1 [133-142	0	1817.0123
[K].LFLNEDI	2xTMTpro [K	9.248E-05	7.389E-05	1	1	1	O94925	O94925 [321-346]	0	3748.1288
[R].HQGVM\	1xOxidation [	6.737E-05	7.389E-05	3	7	16	P62736; P68	P62736 [42-52]; F	0	1795.9803
[R].KGGPVT	1xTMTpro [K	7.497E-05	7.389E-05	1	1	1	O14841	O14841 [385-400]	1	2205.3211
[K].LFNEHIE	1xTMTpro [K	8.349E-05	7.389E-05	1	1	2	O14776	O14776 [945-955]	0	1923.1195
[R].LCDELT(	3xCarbamido	0.0001573	7.389E-05	1	1	2	O15230	O15230 [1490-15(	0	2022.973
[K].GQVFDQ	1xTMTpro [N	6.458E-05	7.389E-05	1	1	1	P14384	P14384 [317-337]	0	2642.3857
[R].LAEVAL/	1xTMTpro [K	0.0001983	7.389E-05	1	1	2	P13716	P13716 [150-159]	0	1657.018
[K].KGLEQLI	3xTMTpro [K	7.695E-05	7.389E-05	1	1	1	P12109	P12109 [135-151]	2	3066.878
[K].KNEFQG	2xTMTpro [K	0.0001544	7.389E-05	1	1	2	Q8NBJ4	Q8NBJ4 [67-76]	1	2134.2324
[K].LFQEDN	1xTMTpro [K	0.0001085	7.389E-05	1	1	1	P24310	P24310 [32-44]	0	2216.2458

[K].LFQECC	2xCarbamido	6.811E-05	7.389E-05	1	1	2	P61978	P61978 [180-191]	0	1853.8594
[R].LFPPWII	2xTMTpro [K	6.337E-05	7.389E-05	1	1	2	Q6P2Q9	Q6P2Q9 [935-954	0	3233.9158
[R].ICDGVQI	1xCarbamido	9.972E-05	7.389E-05	1	1	1	Q9Y512	Q9Y512 [456-467	0	1596.8487
[K].ICDQISD	1xCarbamido	0.000119	7.389E-05	1	1	1	P31153	P31153 [33-53]	0	2960.5279
[R].LCDLLSI	1xCarbamido	0.0001395	7.389E-05	1	1	2	Q96LW7	Q96LW7 [62-71]	0	1558.8695
[K].IAEQVEI	1xTMTpro [K	7.348E-05	7.389E-05	1	1	3	P26232	P26232 [683-694]	0	2079.173
[R].HQEGEII	1xTMTpro [K	0.0001632	7.389E-05	1	1	1	Q02878	Q02878 [227-237	0	1941.0209
[R].KGHAVG	1xTMTpro [K	0.0001407	7.389E-05	1	1	1	P62266	P62266 [108-119]	1	1814.0892
[R].KNGGLG	2xTMTpro [K	0.0001881	7.389E-05	1	1	1	P30048	P30048 [149-166]	1	2794.6429
[K].IAEQVAS	1xTMTpro [K	0.000119	7.389E-05	1	1	2	P35221	P35221 [684-695]	0	1987.0992
[K].LFPDTPI	1xTMTpro [K	9.248E-05	7.389E-05	1	1	2	Q12906	Q12906 [588-600	0	2023.172
[R].GQVTSS	1xCarbamido	8.362E-05	7.389E-05	1	1	4	Q6PCE3	Q6PCE3 [119-129	0	1500.7396
[R].HENIIGII	1xTMTpro [N	7.66E-05	7.389E-05	1	1	1	P28482	P28482 [80-91]	0	1710.9822
[KR].KNHEE	2xTMTpro [K	0.0001888	7.389E-05	3	6	2	P13645; P19	P13645 [285-292]	1	1957.0993
[K].GQVPEN	1xTMTpro [K	7.911E-05	7.389E-05	1	1	1	P12830	P12830 [382-397]	0	2320.3368
[K].GSLVSP	1xTMTpro [K	0.0002011	7.389E-05	1	1	1	Q460N5	Q460N5 [1006-10	0	2107.2805
[R].IAENLGD	1xTMTpro [K	0.0001387	7.389E-05	1	1	1	Q9H078	Q9H078 [521-533	0	2010.1363
[K].LCESEE	1xCarbamido	7.613E-05	7.389E-05	1	1	1	Q8N584	Q8N584 [96-109]	0	2186.187
[K].ICLDLQA	1xCarbamido	0.0001672	7.389E-05	1	1	2	P02776	P02776 [82-92]	0	1942.1327
[R].HQIVEV	1xTMTpro [K	0.0001161	7.389E-05	1	1	1	Q07960	Q07960 [70-80]	0	1819.0205
[K].GSEEEL	1xTMTpro [K	0.0001867	7.389E-05	1	1	1	Q8WXX5	Q8WXX5 [122-13	0	1698.9406
[R].LFLAGYI	1xTMTpro [N	6.217E-05	7.389E-05	1	1	1	Q4G0F5	Q4G0F5 [248-260	0	1815.9998
[R].LFLAEF	1xTMTpro [N	0.0001127	7.389E-05	1	1	1	P08575	P08575 [652-662]	0	1624.9382
[K].LFHVRP	1xTMTpro [N	6.832E-05	7.389E-05	1	1	1	O75116	O75116 [1205-12	0	2035.1408
[R].IFGYPV	1xTMTpro [K	0.0001216	7.389E-05	1	1	1	Q9HCC0	Q9HCC0 [362-38	0	2776.5529
[R].KNPVTS	2xTMTpro [K	0.0001466	7.389E-05	1	1	1	Q13131	Q13131 [448-457	1	2037.2161
[K].LFGYEM	1xTMTpro [K	0.0001471	7.389E-05	1	1	2	Q9UKD2	Q9UKD2 [192-20	0	1842.9956
[R].KNPLPP	3xTMTpro [K	0.0001852	7.389E-05	1	1	1	Q8NC51	Q8NC51 [79-92]	2	2694.7023
[K].GSEENL	1xTMTpro [N	0.0001647	7.389E-05	1	1	1	P35611	P35611 [585-594]	0	1423.6984
[R].LFGSPL	1xTMTpro [N	6.601E-05	7.389E-05	1	1	2	O95479	O95479 [438-453	0	2139.1082
[K].LFGQES	1xTMTpro [K	0.0001166	7.389E-05	1	1	2	Q9BWH2	Q9BWH2 [69-80]	0	1858.0202
[R].GSELVG	1xTMTpro [N	0.0001178	7.389E-05	1	1	4	Q96SM3	Q96SM3 [415-425	0	1464.7766
[K].HEGLGA	1xTMTpro [K	0.0001123	7.389E-05	1	1	1	Q6NUK1	Q6NUK1 [337-345	0	1629.9245

[R].LCLGTC'	2xCarbamido	6.468E-05	7.389E-05	1	1	1	Q9HC84	Q9HC84 [890-910	0	2678.2662
[K].HEGALE'	1xTMTpro [N	0.0001153	7.389E-05	1	1	1	Q8WUM4	Q8WUM4 [61-70]	0	1442.8286
[R].GSESGII	1xTMTpro [K	8.009E-05	7.389E-05	1	1	2	P02671	P02671 [548-558]	0	1748.9674
[R].HQYYNQ	1xTMTpro [N	9.32E-05	7.389E-05	1	1	1	P22314	P22314 [945-957]	0	2143.0317
[R].IFGQAN'	1xTMTpro [K	0.0001256	7.389E-05	1	1	2	P33121	P33121 [377-386]	0	1701.0191
[K].IFGVTTL	1xTMTpro [N	0.0001038	7.389E-05	1	1	5	P40926	P40926 [166-176]	0	1537.9273
[R].HEIVQTL	1xTMTpro [K	0.0001479	7.389E-05	1	1	5	Q13228	Q13228 [236-245]	0	1776.0875
[K].KGEKDIF	2xTMTpro [K	0.0001417	7.389E-05	1	1	1	P62753	P62753 [116-131]	2	2639.5548
[K].IFLDESA	1xTMTpro [N	0.0001479	7.389E-05	1	1	1	Q9ULT8	Q9ULT8 [60-76]	0	2193.1722
[K].GSAFST	1xTMTpro [K	0.0001471	7.389E-05	1	1	1	Q9Y285	Q9Y285 [178-187	0	1592.9139
[R].GSALAA	1xTMTpro [K	0.0001107	7.389E-05	1	1	1	Q5EBL4	Q5EBL4 [6-17]	0	1755.0144
[R].GSAIDEI	1xTMTpro [N	6.928E-05	7.389E-05	1	1	1	O14657	O14657 [296-306]	0	1479.7974
[R].GSAIDPI	1xTMTpro [N	0.0001859	7.389E-05	1	1	1	Q9NXW9	Q9NXW9 [163-17	0	2242.1212
[K].KGFDQE	2xTMTpro [K	6.347E-05	7.389E-05	1	1	1	P30876	P30876 [821-834]	1	2622.4708
[K].KGEYVF	2xTMTpro [K	0.0001831	7.389E-05	1	1	1	Q9HCE1	Q9HCE1 [602-610	1	1951.1833
[K].GSAPPG	1xTMTpro [N	0.0001075	7.389E-05	1	1	3	P78417	P78417 [12-25]	0	1624.8978
[R].HQGVM\	2xOxidation [	0.0001194	7.389E-05	3	7	13	P62736; P68	P62736 [42-52]; F	0	1811.9752
[K].LAENIDA	1xTMTpro [K	0.0001178	7.389E-05	1	1	2	P37198	P37198 [436-445]	0	1723.0246
[K].LCHLYQI	1xCarbamido	6.811E-05	7.389E-05	1	1	1	P41221	P41221 [82-98]	0	2671.3616
[R].KNPAAY	2xTMTpro [K	6.468E-05	7.389E-05	1	1	1	O14949	O14949 [73-82]	1	2062.1749
[K].KGESQT	1xTMTpro [K	9.453E-05	7.389E-05	1	1	1	P49368	P49368 [249-266]	2	2762.4452
[R].GSCIAN/	1xCarbamido	7.048E-05	7.389E-05	1	1	1	P29323	P29323 [224-238]	0	2210.1982
[K].KGENSV	2xTMTpro [K	0.0001859	7.389E-05	1	1	1	O14617	O14617 [1122-11	1	2032.1855
[K].LCGDTS	1xCarbamido	7.048E-05	7.389E-05	1	1	1	O00410	O00410 [265-276]	0	1712.8379
[K].GSDGAA	1xTMTpro [K	8.571E-05	7.389E-05	1	1	3	Q14203	Q14203 [350-361]	0	1791.9733
[K].LCHLLGI	1xCarbamido	6.287E-05	7.389E-05	1	1	2	P35580	P35580 [381-394]	0	2025.0403
[K].LFIFHSS	1xTMTpro [K	0.0001827	7.389E-05	1	1	1	O94855	O94855 [577-592]	0	2323.3306
[K].LFLEGMI	1xTMTpro [K	0.0001956	7.389E-05	1	1	1	Q9UJ41	Q9UJ41 [162-170	0	1745.9904
[R].IAELVAT	1xTMTpro [N	0.0001342	7.389E-05	1	1	1	O76074	O76074 [778-792]	0	2015.0405
[K].GSCGIG	1xCarbamido	6.188E-05	7.389E-05	2	2	1	P02533; P08	P02533 [16-30]; F	0	1582.7927
[R].LAEVEA/	1xTMTpro [K	0.0001746	7.389E-05	1	1	4	Q15149	Q15149 [1499-15	0	1681.0028
[K].LFADAEI	1xTMTpro [N	0.000123	7.389E-05	1	1	1	P46459	P46459 [305-314]	0	1511.7661
[R].IEYDTFG	1xTMTpro [K	0.0001161	7.389E-05	1	1	1	P07954	P07954 [52-61]	0	1823.0082

[K].GTDIMY	1xCarbamido	0.0001226	7.389E-05	1	1	2	P05141	P05141 [246-259]	0	2008.9428
[K].LAAQSC	1xCarbamido	8.909E-05	7.389E-05	1	1	2	Q08211	Q08211 [237-248]	0	1592.9113
[R].LDCTQC	2xCarbamido	0.0001058	7.389E-05	1	1	1	Q15746	Q15746 [1708-17	0	2438.2638
[R].LDDAIH\	1xTMTpro [N	0.0001463	7.389E-05	2	2	1	P15884; Q9	P15884 [393-401]	0	1355.7966
[K].KENSET	2xTMTpro [K	0.0001114	7.389E-05	1	1	1	Q9GZS3	Q9GZS3 [29-45]	1	2746.5655
[K].HTLSYVI	1xTMTpro [K	8.466E-05	7.389E-05	1	1	1	P31040	P31040 [625-636]	0	1885.0675
[K].HTNYTM	1xTMTpro [N	0.0001468	7.389E-05	3	3	4	P12814; P35	P12814 [705-714]	0	1605.8127
[K].LDDFLDI	1xTMTpro [K	0.0001284	7.389E-05	1	1	1	Q8WVY7	Q8WVY7 [297-30	0	1838.0304
[R].KENQW(	1xCarbamido	0.0001073	7.389E-05	1	1	2	P63208	P63208 [155-163]	1	2163.1685
[K].GYMDLM	1xTMTpro [K	0.0001279	7.389E-05	1	1	1	O43396	O43396 [122-132]	0	1937.052
[R].IEPSSSC	1xCarbamido	0.0001127	7.389E-05	1	1	1	P98160	P98160 [2345-23	0	4111.1163
[R].LEPQSM	1xTMTpro [N	8.691E-05	7.389E-05	1	1	1	P11532	P11532 [3287-33(	0	2009.1326
[R].KENEEE	2xTMTpro [K	6.643E-05	7.389E-05	1	1	1	Q15170	Q15170 [6-17]	1	2298.2758
[K].LDDIFEP	1xTMTpro [K	9.191E-05	7.389E-05	1	1	3	Q14008	Q14008 [1510-15	0	2233.2975
[K].IEPPDTG	1xTMTpro [K	0.0001838	7.389E-05	1	1	1	P80303	P80303 [45-59]	0	2424.283
[K].IDDIWNL	1xTMTpro [K	0.0001752	7.389E-05	1	1	2	P04114	P04114 [3538-35	0	1853.0664
[R].LEPNAQ	1xTMTpro [N	0.0001131	7.389E-05	1	1	1	O95782	O95782 [945-955]	0	1624.8436
[K].LEPIVNL	1xTMTpro [K	0.0001759	7.389E-05	1	1	1	Q96DM3	Q96DM3 [363-37	0	1859.1498
[K].GYLISGS	1xTMTpro [N	8.531E-05	7.389E-05	1	1	1	Q86VP6	Q86VP6 [972-982	0	1477.797
[K].GYPEVA	1xTMTpro [K	7.337E-05	7.389E-05	1	1	1	P53621	P53621 [633-643]	0	1868.0926
[K].HTLNQID	1xTMTpro [K	0.00014	7.389E-05	1	1	4	P02765	P02765 [58-67]	0	1805.0413
[R].LEQGGT	1xTMTpro [N	0.0001118	7.389E-05	1	1	1	O43290	O43290 [351-361]	0	1420.7715
[K].KEPAVLE	2xTMTpro [K	8.146E-05	7.389E-05	1	1	2	Q01518	Q01518 [317-327]	1	2125.3049
[R].GYQVCF	2xCarbamido	6.367E-05	7.389E-05	1	1	2	Q9HC84	Q9HC84 [1368-13	0	1983.9951
[K].IDASQTE	1xTMTpro [K	7.731E-05	7.389E-05	1	1	2	O94979	O94979 [462-471]	0	1775.9671
[K].GTCEQG	1xCarbamido	6.277E-05	7.389E-05	1	1	1	P28799	P28799 [333-347]	0	2392.2033
[K].GTCEQG	1xCarbamido	7.314E-05	7.389E-05	1	1	1	Q16270	Q16270 [154-167]	0	2079.14
[K].IESALLD	1xTMTpro [N	0.0001838	7.389E-05	1	1	2	Q14112	Q14112 [1210-12	0	1493.8131
[R].HTGYVIE	1xTMTpro [K	0.0001121	7.389E-05	1	1	1	P47897	P47897 [640-652]	0	2131.2519
[K].IEQVDKE	2xTMTpro [K	0.0001455	7.389E-05	1	1	1	Q9Y2X3	Q9Y2X3 [445-457	1	2488.3963
[K].GTCSGF	1xCarbamido	0.0001208	7.389E-05	1	1	1	Q9NZU5	Q9NZU5 [33-44]	0	1724.8134
[K].GTCWQ	1xCarbamido	0.0001084	7.389E-05	1	1	1	P35555	P35555 [851-861]	0	1596.8123
[R].LEPKPQ	1xTMTpro [K	0.0001053	7.389E-05	1	1	1	Q9UHD8	Q9UHD8 [223-23	0	2238.3102

[K].LEQMME	1xTMTpro [N	0.00018	7.389E-05	1	1	1	Q709C8	Q709C8 [2143-21	0	1497.7725
[K].KQEIEKF	3xTMTpro [K	7.672E-05	7.389E-05	1	1	1	P78346	P78346 [45-67]	1	3771.2777
[K].GTDDSM	1xTMTpro [K	7.371E-05	7.389E-05	1	1	2	O00422	O00422 [115-126]	0	1919.0036
[R].GYPGDE	1xTMTpro [N	8.109E-05	7.389E-05	1	1	6	P12111	P12111 [2062-207	0	1494.7144
[R].IEQLSPF	1xTMTpro [K	0.0001144	7.389E-05	1	1	3	Q02218	Q02218 [930-943]	0	2268.3499
[R].HTLGEN	1xTMTpro [K	8.222E-05	7.389E-05	1	1	1	P42892	P42892 [663-676]	0	2047.1428
[K].GYPEVA	2xTMTpro [K	0.0001007	7.389E-05	1	1	1	P53621	P53621 [633-646]	1	2544.4642
[R].GTDELL(	1xTMTpro [K	0.0001466	7.389E-05	1	1	1	O75600	O75600 [245-265]	0	2812.5622
[K].LEQLDL(	1xTMTpro [N	0.0001542	7.389E-05	1	1	1	Q14160	Q14160 [176-200]	0	2979.6322
[R].LEQIAAE	1xTMTpro [K	0.0001971	7.389E-05	1	1	2	Q9UKM9	Q9UKM9 [208-21(	0	1637.9718
[K].KQELEEI	1xOxidation [	0.0001295	7.389E-05	1	1	2	P35749	P35749 [917-930]	1	2379.2834
[K].IEPGVDF	1xTMTpro [K	0.0001066	7.389E-05	1	1	1	Q9H4A4	Q9H4A4 [399-416	0	2690.3329
[K].IDDMTA/	1xTMTpro [N	0.0001528	7.389E-05	1	1	2	P15927	P15927 [94-105]	0	1638.815
[R].LEPGQQ	1xTMTpro [N	0.0001756	7.389E-05	1	1	1	P04180	P04180 [172-182]	0	1715.856
[R].KQMVID'	2xTMTpro [K	0.0001432	7.389E-05	1	1	3	P62847	P62847 [21-32]	1	2277.3933
[K].LDEFGE(	1xTMTpro [K	0.0001415	7.389E-05	1	2	4	P16615	P16615 [253-262]	0	1773.9878
[K].GTEGLS	1xCarbamido	0.0001525	7.389E-05	1	1	1	Q12802	Q12802 [411-421]	0	1441.7025
[K].LENDQIE	1xTMTpro [N	0.0001587	7.389E-05	1	1	2	P56192	P56192 [805-814]	0	1520.824
[K].LDEGW\	1xTMTpro [K	7.601E-05	7.389E-05	1	1	1	P05455	P05455 [42-54]	0	2151.2379
[R].HVAVTN	1xTMTpro [N	0.0001275	7.389E-05	1	3	1	P33176	P33176 [191-203]	0	1785.8985
[R].KQPPVS	2xTMTpro [K	6.612E-05	7.389E-05	1	1	2	P17096	P17096 [31-46]	1	2506.5173
[K].KEFPGL/	2xTMTpro [K	0.0001299	7.389E-05	1	1	1	Q8NBS9	Q8NBS9 [366-375	1	1958.2255
[R].KQPPVS	3xTMTpro [K	0.0001487	7.389E-05	1	1	1	P17096	P17096 [31-55]	2	3775.211
[R].LDEDLA/	1xCarbamido	0.00018	7.389E-05	1	1	3	P09543	P09543 [103-112]	0	1529.7589
[R].LDELEEI	1xTMTpro [N	8.678E-05	7.389E-05	1	1	4	O75306	O75306 [255-266]	0	1762.9506
[K].HVEAVY	1xTMTpro [N	0.0001016	7.389E-05	1	1	1	Q00169	Q00169 [135-146]	0	1704.924
[R].HVEDGN	1xTMTpro [N	0.0001004	7.389E-05	1	1	1	P52306	P52306 [369-386]	0	2221.2008
[R].IDELPEG	2xTMTpro [K	8.993E-05	7.389E-05	1	1	3	Q9Y3E1	Q9Y3E1 [29-43]	0	2490.4748
[K].GYFVQP	1xOxidation [	0.0001153	7.389E-05	1	1	2	P00352	P00352 [379-395]	0	2310.1396
[R].IELQACN	1xCarbamido	0.000112	7.389E-05	1	1	1	P06213	P06213 [820-833]	0	2006.9772
[K].KQQLS	2xTMTpro [K	0.0001281	7.389E-05	1	1	1	P35580	P35580 [809-817]	1	1956.2422
[K].LDELQAS	1xTMTpro [K	0.0001694	7.389E-05	1	1	1	P12270	P12270 [166-177]	0	1912.0883
[K].GTFQWT	1xTMTpro [N	0.0001517	7.389E-05	1	1	1	P22897	P22897 [574-585]	0	1798.9295

[K].GTFSIQF	1xTMTpro [N	0.0001239	7.389E-05	1	1	2	Q96JQ0	Q96JQ0 [2113-21	0	1839.0295
[R].GYGAG\	1xTMTpro [K	0.0001366	7.389E-05	1	1	1	Q9NX46	Q9NX46 [115-125	0	1706.0133
[K].GTAYTFI	1xTMTpro [K	6.55E-05	7.389E-05	1	1	2	Q92841	Q92841 [516-528]	0	2025.1301
[K].GTEDEV	2xTMTpro [K	7.004E-05	7.389E-05	1	1	2	P07951	P07951 [52-65]	1	2512.3599
[R].IENFDVS	1xTMTpro [N	0.000162	7.389E-05	1	1	2	Q9NUQ8	Q9NUQ8 [180-19	0	1602.8083
[R].KQEQAN	2xTMTpro [K	6.247E-05	7.389E-05	1	1	2	O14617	O14617 [673-684]	1	2392.3805
[R].IEPFTTY	1xTMTpro [K	0.0001517	7.389E-05	1	1	2	P50851	P50851 [2318-233	0	2336.3146
[R].HTVDQT	1xTMTpro [N	8.786E-05	7.389E-05	1	1	1	Q07954	Q07954 [2310-23:	0	1817.9578
[K].GYLGPE	1xCarbamido	0.0001023	7.389E-05	1	1	3	P40926	P40926 [79-91]	0	2098.1498
[R].KQFGHF	1xTMTpro [K	0.0001719	7.389E-05	1	1	1	P11277	P11277 [707-717]	1	1919.1107
[R].GYLAPS	1xTMTpro [N	0.0002031	7.389E-05	1	1	2	Q8N2S1	Q8N2S1 [1240-12	0	1552.8654
[K].KEGGTV	2xTMTpro [K	0.0001827	7.389E-05	1	1	1	P49419	P49419 [390-400]	1	2007.2055
[R].LENWITS	1xTMTpro [N	0.000131	7.389E-05	1	1	1	P40763	P40763 [263-278]	0	2193.1835
[R].KQGLEQ	2xTMTpro [K	8.732E-05	7.389E-05	1	1	2	O60493	O60493 [119-128]	1	2117.2899
[R].LENEGD	3xCarbamido	0.000162	7.389E-05	1	1	1	O94979	O94979 [676-700]	0	3493.7257
[K].LDDNEA	1xTMTpro [K	6.158E-05	7.389E-05	1	1	2	P61011	P61011 [312-321]	0	1767.9984
[K].KQIDQF/	1xTMTpro [K	0.0001453	7.389E-05	1	1	1	Q9BUT1	Q9BUT1 [63-74]	1	2085.1584
[R].LAALSIE	1xTMTpro [K	0.0001684	7.389E-05	1	1	2	Q16363	Q16363 [286-295]	0	1638.9922
[R].GTDVRE	1xTMTpro [N	0.0001766	7.389E-05	1	1	3	Q9UBG0	Q9UBG0 [813-82	1	1832.9058
[R].IENVVLV	1xTMTpro [K	0.0001752	7.389E-05	1	1	2	Q9NQW7	Q9NQW7 [536-54	0	1817.1756
[R].IENVVLD	1xCarbamido	6.758E-05	7.389E-05	1	1	2	Q9H074	Q9H074 [349-360	0	1693.8862
[R].IDDVAAL	2xTMTpro [K	0.0001297	7.389E-05	1	1	1	P06737	P06737 [716-725]	1	2000.2208
[R].LENLGIF	1xTMTpro [N	7.815E-05	7.389E-05	1	1	1	Q01658	Q01658 [108-120]	0	1829.0339
[R].LAALPEI	1xTMTpro [K	6.853E-05	7.389E-05	1	1	3	O75947	O75947 [42-58]	0	2540.4045
[K].IDEAILIT	1xTMTpro [K	0.000157	7.389E-05	1	1	2	P19367	P19367 [163-173]	0	1911.1447
[K].KEGETV	2xTMTpro [K	0.000171	7.389E-05	1	1	1	Q08211	Q08211 [266-275]	1	2092.2106
[R].GTASAD	1xTMTpro [N	7.59E-05	7.389E-05	1	1	1	Q92889	Q92889 [659-670]	0	1484.7512
[R].KESYSIY	2xTMTpro [K	0.0001308	7.389E-05	2	7	6	P33778; Q8I	P33778 [35-44]; Q	1	2192.2783
[R].GYSFSL	1xTMTpro [K	7.291E-05	7.389E-05	1	1	2	P25787	P25787 [5-18]	0	2087.1305
[R].KFEDFL(	1xTMTpro [K	0.0001144	7.389E-05	1	1	1	P11277	P11277 [1209-12	1	2195.1411
[K].GSPNAN	1xTMTpro [K	6.499E-05	7.389E-05	1	1	1	P28838	P28838 [268-282]	0	2134.2152
[R].GSPPVP	1xTMTpro [N	9.863E-05	7.389E-05	1	1	1	Q9H4L4	Q9H4L4 [211-227	0	2026.0235
[R].LEVNLQ.	1xTMTpro [K	0.0001863	7.389E-05	1	1	4	P35579	P35579 [1558-15	0	1653.9853

[K].GSQFGQ	2xCarbamido	0.0001564	7.389E-05	1	1	2	P07814	P07814 [329-339]	0	1603.764
[K].GSQPPP	1xTMTpro [N	7.091E-05	7.389E-05	1	1	2	O43491	O43491 [46-60]	0	1815.952
[R].LADICKE	1xCarbamido	0.0001163	7.389E-05	1	1	1	Q9P2K8	Q9P2K8 [450-463	1	2332.2463
[K].KPSISIT	2xTMTpro [K	8.071E-05	7.389E-05	1	1	3	Q9UKV3	Q9UKV3 [861-872	0	2216.3682
[R].LADGPG	1xCarbamido	9.8E-05	7.389E-05	1	1	1	O43866	O43866 [140-148	0	1562.8605
[K].KPPTDE	3xTMTpro [K	7.059E-05	7.389E-05	1	1	1	P35659	P35659 [318-330]	1	2744.655
[R].LADGGA	1xTMTpro [N	0.000159	7.389E-05	1	1	3	Q08380	Q08380 [26-36]	0	1363.7249
[R].LEVGTE	1xTMTpro [K	0.0001779	7.389E-05	1	2	1	Q01581	Q01581 [90-100]	0	1826.0767
[K].IEVGLVV	1xTMTpro [K	0.0001118	7.389E-05	1	1	1	P46459	P46459 [172-187]	0	2297.3361
[K].GSSGLG	1xTMTpro [N	0.00015	7.389E-05	1	1	1	Q04695	Q04695 [16-26]	0	1225.6456
[R].KPSPQD	1xTMTpro [K	0.0001892	7.389E-05	1	1	1	Q63HN8	Q63HN8 [2973-29	0	2031.2206
[K].IEVEKPF	2xTMTpro [K	8.009E-05	7.389E-05	1	1	3	P23284	P23284 [205-216]	1	2286.3889
[K].LADFGV	1xTMTpro [K	0.0001361	7.389E-05	2	3	1	O00506; Q9	O00506 [156-171	0	2285.2997
[K].LEVEANI	1xTMTpro [N	6.864E-05	7.389E-05	1	1	2	P47756	P47756 [96-108]	0	1872.9411
[K].IETWHKI	2xTMTpro [K	7.948E-05	7.389E-05	1	1	1	P48739	P48739 [111-134]	0	3713.0267
[R].HSVATA	1xTMTpro [K	7.767E-05	7.389E-05	1	1	1	Q5VYK3	Q5VYK3 [274-285	0	1863.0831
[K].HSSLITP	1xTMTpro [N	0.0001964	7.389E-05	1	1	1	P78527	P78527 [2787-28	0	1825.0615
[R].HAQAQY	1xTMTpro [N	0.0001025	7.389E-05	1	1	1	Q14112	Q14112 [946-957	0	1636.8515
[K].GSPMEI	1xTMTpro [K	6.109E-05	7.389E-05	1	1	2	P09960	P09960 [84-97]	0	2051.2066
[K].HAVTEA	1xTMTpro [K	7.767E-05	7.389E-05	1	1	2	Q96SB3	Q96SB3 [678-689	0	1975.1468
[K].GSIVWQ	1xTMTpro [K	6.327E-05	7.389E-05	1	1	2	P07686	P07686 [401-412]	0	2031.1043
[K].LADQFLI	2xTMTpro [K	0.0001138	7.389E-05	1	1	1	Q6P4E1	Q6P4E1 [183-197	1	2747.5396
[R].HCQLLE	1xCarbamido	6.519E-05	7.389E-05	1	1	1	P52948	P52948 [1589-16	0	2207.1774
[R].GSMCN	1xCarbamido	0.0001944	7.389E-05	1	1	1	Q9NUU6	Q9NUU6 [223-23	0	2457.2649
[R].GSMGTS	1xCarbamido	6.972E-05	7.389E-05	1	1	2	P98160	P98160 [883-893]	0	1416.6531
[R].LEYCEAI	1xCarbamido	8.611E-05	7.389E-05	1	1	2	P14868	P14868 [346-356]	0	1672.8722
[R].HCLLTCI	3xCarbamido	0.0001726	7.389E-05	1	1	1	Q56VL3	Q56VL3 [129-138	0	1957.9789
[K].IADPTLA	1xTMTpro [K	6.726E-05	7.389E-05	1	2	1	Q6P996	Q6P996 [8-18]	0	1754.0014
[R].LEWLTLI	1xTMTpro [K	8.531E-05	7.389E-05	1	1	2	A0FGR8	A0FGR8 [503-517	0	2353.3081
[K].KFESLFF	2xTMTpro [K	9.863E-05	7.389E-05	1	1	2	Q13045	Q13045 [1007-10	1	1965.1989
[R].HCGDFE	1xCarbamido	0.000108	7.389E-05	1	1	1	P83436	P83436 [481-492]	0	1778.8563
[K].LADMAL	1xTMTpro [N	6.377E-05	7.389E-05	1	1	2	P16219	P16219 [314-325]	0	1564.8688
[R].IEWHLN	1xTMTpro [K	0.0001614	7.389E-05	1	1	2	Q05707	Q05707 [208-218	0	1954.1042

[R].KPIVPQK	3xTMTpro [K	8.745E-05	7.389E-05	1	1	1	Q86U86	Q86U86 [1293-13	1	2919.8388
[K].HCEDGI(	2xCarbamido	0.0001599	7.389E-05	1	1	1	P19838	P19838 [117-130]	0	2153.0975
[R].LADLTGI	1xTMTpro [K	0.0001228	7.389E-05	1	1	1	P28289	P28289 [344-354]	0	1746.1021
[K].KPMVLG	1xOxidation [	0.0001852	7.389E-05	1	1	1	Q00796	Q00796 [64-78]	0	2511.4421
[R].IADLPPA	1xTMTpro [N	0.0001058	7.389E-05	1	1	1	O75746	O75746 [625-640]	0	2010.0728
[K].KFFDAN'	2xTMTpro [K	6.822E-05	7.389E-05	1	1	1	Q15691	Q15691 [113-130]	2	3004.5985
[K].KPPCCV	2xCarbamido	0.0001766	7.389E-05	2	2	2	Q12857; Q1	Q12857 [99-111];	0	2455.3617
[R].HCFYGL	1xCarbamido	9.119E-05	7.389E-05	1	1	2	Q05707	Q05707 [972-984]	0	2252.1302
[R].LFAAYNI	1xTMTpro [K	6.822E-05	7.389E-05	1	1	1	Q13492	Q13492 [207-221]	0	2316.3459
[K].HSVPLP'	1xTMTpro [K	0.0001103	7.389E-05	1	1	1	Q6UXV4	Q6UXV4 [219-232	0	2103.1941
[R].HSVVAG	1xTMTpro [N	0.0001506	7.389E-05	1	1	1	Q6PKG0	Q6PKG0 [1039-10	0	1443.7624
[K].LADFALL	1xCarbamido	6.622E-05	7.389E-05	1	1	4	P02788	P02788 [587-598]	0	1944.112
[R].LADDLG	1xTMTpro [K	7.972E-05	7.389E-05	1	1	2	O95716	O95716 [153-167]	0	2268.2044
[K].LADDED'	2xTMTpro [K	6.149E-05	7.389E-05	1	1	1	P02549	P02549 [604-617]	1	2592.4337
[K].LDAQAS	1xTMTpro [K	0.0001653	7.389E-05	1	1	2	P19827	P19827 [205-214]	0	1698.0082
[R].KQALEQ	2xTMTpro [K	6.417E-05	7.389E-05	1	1	1	Q9UH65	Q9UH65 [511-521	1	2277.3271
[R].LESLGQ	1xTMTpro [N	7.997E-05	7.389E-05	1	1	2	Q9NZD8	Q9NZD8 [188-19	0	1593.8767
[K].GSVSCP	2xCarbamido	0.0001983	7.389E-05	1	1	2	Q96ME7	Q96ME7 [167-17	0	1682.8273
[R].GSVSDE	1xTMTpro [N	8.923E-05	7.389E-05	1	1	2	P13796	P13796 [4-15]	0	1686.7998
[K].GSVVNV	1xTMTpro [N	0.0001188	7.389E-05	1	1	1	O95819	O95819 [899-919]	0	2585.3603
[R].IDAMHG'	1xTMTpro [K	7.28E-05	7.389E-05	1	1	2	P36871	P36871 [222-234]	0	1994.1389
[R].LESGMQ	1xOxidation [	8.505E-05	7.389E-05	1	1	1	P05787	P05787 [402-414]	0	2100.1073
[R].KEVEETI	1xTMTpro [K	9.863E-05	7.389E-05	1	1	1	P31689	P31689 [350-372]	1	3402.6503
[K].KETGAA	1xCarbamido	0.0001687	7.389E-05	1	1	1	P29144	P29144 [19-29]	1	1848.0293
[R].LESEETI	1xOxidation [	0.0001054	7.389E-05	1	1	1	P01024	P01024 [36-66]	1	4307.2686
[K].LDAQSLI	1xTMTpro [N	6.488E-05	7.389E-05	1	1	2	Q9UJ41	Q9UJ41 [369-383	0	2055.0314
[R].KESYSV'	2xTMTpro [K	6.832E-05	7.389E-05	4	9	7	Q5QNW6; Q	Q5QNW6 [35-44]	1	2178.2627
[K].HTEDVG	1xCarbamido	0.0001647	7.389E-05	1	1	1	Q9Y4K0	Q9Y4K0 [150-162	1	2110.1207
[K].GYTLVE'	1xTMTpro [K	0.0001479	7.389E-05	1	1	3	Q9Y5S9	Q9Y5S9 [115-125	0	1974.0716
[R].HTFLNIA	1xTMTpro [K	0.0001161	7.389E-05	1	1	1	Q96KP1	Q96KP1 [724-735	0	2094.1628
[K].LESDVS/	1xCarbamido	0.0001508	7.389E-05	1	1	2	P02675	P02675 [212-224]	0	1907.8798
[K].LESEMEI	1xTMTpro [N	8.759E-05	7.389E-05	1	1	1	P23246	P23246 [519-536]	0	2489.205
[R].LDADIHT	1xCarbamido	0.0001672	7.389E-05	1	1	1	P57772	P57772 [433-443]	0	1619.8131

[K].GYVVW(	1xTMTpro [K	0.000162	7.389E-05	1	1	2	P06865	P06865 [369-380]		0	2092.1359
[K].KPVIIIFD	1xTMTpro [K	0.0001258	7.389E-05	1	1	1	Q5T5P2	Q5T5P2 [1441-14		0	2294.3438
[K].IETSSLD	1xTMTpro [N	7.779E-05	7.389E-05	1	1	1	Q14112	Q14112 [1255-12		0	1524.7825
[R].LETRPG	1xTMTpro [N	7.202E-05	7.389E-05	1	1	1	Q96JQ0	Q96JQ0 [193-215		0	2751.4848
[K].HALLDV	1xTMTpro [K	0.000196	7.389E-05	1	1	1	Q9UDY2	Q9UDY2 [788-79		0	1601.9871
[K].HAEATL(	1xTMTpro [N	8.26E-05	7.389E-05	1	2	1	Q7L9L4	Q7L9L4 [31-42]		0	1529.8355
[K].LDAFLVL	1xTMTpro [N	0.0001082	7.389E-05	1	1	2	P35749	P35749 [690-700]		0	1620.9644
[R].IDAHSGI	1xCarbamid	0.000159	7.389E-05	1	1	1	Q96JQ0	Q96JQ0 [631-642		0	1635.808
[K].IETQDIQ	1xTMTpro [N	0.0001317	7.389E-05	1	1	2	P23368	P23368 [58-67]		0	1490.8498
[R].HSWPEN	1xCarbamid	0.0001324	7.389E-05	1	1	1	Q92765	Q92765 [128-144		0	2419.1672
[R].LDAIEDL	1xTMTpro [K	9.482E-05	7.389E-05	1	1	2	P52701	P52701 [792-804]		0	2066.1699
[R].KFADIGN	2xTMTpro [K	0.000118	7.389E-05	1	1	1	P11172	P11172 [314-323]		1	2005.2262
[R].KPVAGA	2xTMTpro [K	0.0001084	7.389E-05	1	1	2	Q99627	Q99627 [166-178		0	2258.3689
[K].IADFELP	1xTMTpro [K	0.0001528	7.389E-05	1	1	1	P04114	P04114 [3847-38		0	3074.7885
[K].LDAILTD	1xTMTpro [K	0.0001581	7.389E-05	1	1	1	Q8IUZ5	Q8IUZ5 [429-439]		0	1886.0436
[K].IETISHEI	1xTMTpro [N	0.0001726	7.389E-05	1	1	1	P07225	P07225 [592-602]		0	1644.8876
[R].IETLDPA	1xTMTpro [N	7.291E-05	7.389E-05	1	1	1	P62191	P62191 [334-346]		0	1755.0448
[R].IESVLSS	1xTMTpro [K	9.909E-05	7.389E-05	1	1	1	P17900	P17900 [170-179]		0	1614.9558
[K].IDALREE	1xTMTpro [N	0.0001015	7.389E-05	1	1	1	Q9BXX0	Q9BXX0 [322-335		1	1982.0006
[K].HADIVTT	1xTMTpro [K	0.0001392	7.389E-05	1	1	1	P34897	P34897 [270-280]		0	1832.0522
[K].LESPTVS	1xTMTpro [K	8.909E-05	7.389E-05	1	1	1	Q96C36	Q96C36 [292-307		0	2209.2571
[R].IDAMHG	1xTMTpro [N	8.993E-05	7.389E-05	1	1	1	Q15124	Q15124 [227-239		0	1749.91
[R].HTAAPTI	1xTMTpro [N	0.0001479	7.389E-05	1	1	1	Q04941	Q04941 [140-152		0	1552.7927
[K].IAPQLST	1xTMTpro [K	0.0001202	7.389E-05	1	1	1	P43652	P43652 [438-453]		0	2322.3412
[K].NQVSLT(	1xCarbamid	0.0001352	7.389E-05	4	5	12	P01861; P01	P01861 [241-250]		0	1770.0439
[R].IGLFYMI	1xTMTpro [N	6.129E-05	7.389E-05	1	1	3	Q15008	Q15008 [147-159		0	1875.0005
[R].TICAILEI	1xCarbamid	7.96E-05	7.389E-05	1	1	1	P49591	P49591 [436-448]		0	2191.1924
[R].TICSHV(	1xCarbamid	0.0001558	7.389E-05	1	1	2	P32969	P32969 [72-82]		0	1939.0749
[R].TICSHV(	1xCarbamid	0.000141	7.389E-05	1	1	1	P32969	P32969 [72-82]		0	1955.0698
[K].TLDGELI	1xTMTpro [K	0.0001729	7.389E-05	1	1	2	P07942	P07942 [1689-16		0	1627.9034
[R].TLDKVSI	1xTMTpro [K	0.0001425	7.389E-05	1	1	1	Q9BRP8	Q9BRP8 [112-132		1	2835.5708
[K].TLDNVAI	1xTMTpro [K	0.000102	7.389E-05	1	1	1	Q9C0C9	Q9C0C9 [867-878		0	1968.1145
[K].TIDSWM	1xTMTpro [K	0.0001564	7.389E-05	1	1	2	P07093	P07093 [276-287]		0	2016.1154

[K].TLDVFNI	1xTMTpro [N	9.816E-05	7.389E-05	1	1	2	Q709C8	Q709C8 [435-445	0	1578.9538
[R].TIEDAIA\	1xTMTpro [N	6.139E-05	7.389E-05	1	1	1	Q96CT7	Q96CT7 [146-163	0	2177.1621
[K].TIEDDLV	1xTMTpro [N	0.0001779	7.389E-05	1	1	1	Q9Y606	Q9Y606 [111-122	0	1634.9284
[R].TLEDEEI	1xTMTpro [N	0.0001626	7.389E-05	1	1	1	O00515	O00515 [19-28]	0	1581.7563
[R].TLEDEEI	1xTMTpro [N	0.0001536	7.389E-05	1	1	1	O00515	O00515 [19-30]	1	1866.9
[K].TIEDTLM	1xTMTpro [K	0.0001194	7.389E-05	1	1	2	P53367	P53367 [249-258]	0	1759.0167
[K].TIEEYAI(	1xCarbamido	0.0001043	7.389E-05	1	1	2	Q15124	Q15124 [158-169]	0	1783.922
[R].TLEGELI	1xTMTpro [K	7.613E-05	7.389E-05	1	1	1	P02545	P02545 [157-171]	1	2274.3062
[K].TIEIESLN	1xTMTpro [K	0.0001402	7.389E-05	1	1	1	Q13439	Q13439 [1538-15	0	1996.1822
[K].TIEIQATI	1xTMTpro [K	8.311E-05	7.389E-05	1	1	1	Q9NQ48	Q9NQ48 [170-182	0	2054.1625
[K].TLEQHEI	1xTMTpro [N	6.779E-05	7.389E-05	1	1	1	P16157	P16157 [1269-128	0	1875.9884
[R].TLEVEIE	1xTMTpro [N	0.0001813	7.389E-05	1	1	2	Q9UBS4	Q9UBS4 [207-217	0	1545.8807
[R].TIEYLEE	1xTMTpro [K	0.0001319	7.389E-05	1	1	1	P40939	P40939 [236-249]	0	2235.2768
[K].TLFATED	1xTMTpro [N	6.843E-05	7.389E-05	1	1	2	O00159	O00159 [722-733]	0	1668.9128
[R].TLFAYE(	1xTMTpro [K	9.162E-05	7.389E-05	1	1	2	Q9Y5K6	Q9Y5K6 [276-291	0	2486.2946
[K].TLFLGVT	1xTMTpro [K	7.382E-05	7.389E-05	1	1	2	Q05707	Q05707 [917-928]	0	1913.1716
[K].TLFQPQ	1xTMTpro [K	6.843E-05	7.389E-05	1	1	2	P53992	P53992 [671-685]	0	2275.2942
[R].TIFVCDE	1xCarbamido	8.813E-05	7.389E-05	1	1	1	Q8TDQ7	Q8TDQ7 [235-248	0	1986.0173
[R].TLGQAE	1xTMTpro [K	9.423E-05	7.389E-05	1	1	5	P27144	P27144 [93-102]	0	1653.9667
[R].TLGSAN	1xTMTpro [K	0.0001458	7.389E-05	1	1	2	P12821	P12821 [126-136]	0	1693.0504
[R].TLAYIITE	1xTMTpro [N	8.531E-05	7.389E-05	1	1	1	Q9Y5K8	Q9Y5K8 [183-194	0	1740.9703
[R].TLATDIL	1xTMTpro [K	9.542E-05	7.389E-05	1	1	1	O43143	O43143 [266-277]	0	1883.1531
[K].TIAQMIE	1xTMTpro [N	0.000101	7.389E-05	2	2	1	O95359; O7!	O95359 [2792-28(	0	1637.8488
[R].TIAQDY(	1xTMTpro [K	0.0001672	7.389E-05	1	1	4	Q06830	Q06830 [111-120]	0	1716.0187
[R].TGSAVA	1xTMTpro [N	6.601E-05	7.389E-05	1	1	1	Q8IZL8	Q8IZL8 [49-61]	0	1606.8985
[K].TGSGLN	1xTMTpro [N	0.0001085	7.389E-05	1	1	1	P49792	P49792 [585-596]	0	1648.825
[R].TGSQG(	1xCarbamido	0.0001474	7.389E-05	1	1	4	P62857	P62857 [21-31]	0	1525.7712
[K].TGTCGY	2xCarbamido	0.0001073	7.389E-05	1	1	1	O75380	O75380 [109-120]	0	1723.8215
[R].TGTIIVID	1xTMTpro [K	0.0001987	7.389E-05	1	1	1	P29350	P29350 [460-476]	0	2487.4058
[K].TGTIVVE	1xTMTpro [N	0.0001232	7.389E-05	1	1	1	P41252	P41252 [930-946]	0	2224.1529
[R].TGTVLIP	1xTMTpro [K	0.000196	7.389E-05	1	1	1	Q53FZ2	Q53FZ2 [143-157	0	2166.2989
[R].TGVELG	2xTMTpro [K	7.28E-05	7.389E-05	1	1	2	P21333	P21333 [885-900]	0	2611.5388
[R].TGVLAH	1xTMTpro [N	9.663E-05	7.389E-05	1	1	2	P35749	P35749 [772-782]	0	1557.8556

[R].TGVLKP(	2xTMTpro [K	9.527E-05	7.389E-05	1	1	1	P23946	P23946 [152-166]	0	2484.4854
[R].TGYTLD'	1xTMTpro [N	7.972E-05	7.389E-05	2	2	2	O43390; O6(	O43390 [134-145]	0	1615.8611
[K].THEAQI(	1xTMTpro [N	0.0001726	7.389E-05	1	1	3	P35579	P35579 [1182-119	0	1546.7967
[R].THLPEVI	1xTMTpro [K	0.000153	7.389E-05	1	1	3	P02774	P02774 [354-363]	0	1779.066
[K].TLGTMIA	1xOxidation [	7.911E-05	7.389E-05	1	1	2	P20073	P20073 [465-479]	0	1877.9234
[K].THMVVA	1xTMTpro [K	0.0001405	7.389E-05	1	1	2	P07814	P07814 [1422-143	0	2259.1757
[K].TKDDLE(	2xTMTpro [K	0.0001813	7.389E-05	1	1	1	Q13277	Q13277 [73-85]	1	2446.4221
[R].TKDEIIT	1xTMTpro [K	9.364E-05	7.389E-05	1	1	1	O95870	O95870 [428-445]	1	2671.4468
[R].TKDIED\	2xTMTpro [K	6.168E-05	7.389E-05	1	1	2	Q07955	Q07955 [29-38]	1	2170.2576
[R].TKEQLG	1xTMTpro [K	0.0002007	7.389E-05	1	1	1	P14735	P14735 [825-838]	1	2203.2731
[K].TKGDFIL	1xTMTpro [K	0.0001617	7.389E-05	1	1	1	Q16531	Q16531 [916-928]	1	2073.2022
[K].TKPSDEI	2xTMTpro [K	0.0001253	7.389E-05	1	1	1	P07108	P07108 [18-33]	0	2870.5579
[K].TLAALVD	1xCarbamido	8.184E-05	7.389E-05	1	1	1	Q96CW5	Q96CW5 [380-39	0	1644.8811
[K].TLADAE(	1xTMTpro [N	0.0001043	7.389E-05	1	1	2	Q02252	Q02252 [130-140]	0	1497.7868
[K].TIAEIFGI	1xTMTpro [N	0.0001021	7.389E-05	1	1	2	Q16401	Q16401 [468-480]	0	1811.9975
[K].TLAEMLI	2xTMTpro [K	8.298E-05	7.389E-05	1	1	2	Q6UWY5	Q6UWY5 [118-13	1	2674.5153
[R].TLAGAV:	1xTMTpro [K	8.184E-05	7.389E-05	1	1	2	Q9Y4G6	Q9Y4G6 [617-627	0	1696.05
[K].TLAGDVI	1xTMTpro [N	8.109E-05	7.389E-05	1	1	1	Q9UBQ0	Q9UBQ0 [51-60]	0	1384.8232
[K].TLAPTW	2xTMTpro [K	0.0001968	7.389E-05	1	1	1	Q8NBS9	Q8NBS9 [355-366	1	2315.3791
[K].THVMLG	1xTMTpro [K	8.272E-05	7.389E-05	1	5	1	Q6VEQ5	Q6VEQ5 [199-21(	0	1953.0607
[K].TLGVSF'	1xCarbamido	0.0001987	7.389E-05	1	1	1	O60826	O60826 [357-370]	0	1886.9601
[K].TIHGLIYI	1xTMTpro [K	0.0001614	7.389E-05	2	2	2	Q14738; Q1:	Q14738 [461-471]	0	1851.1348
[R].TLHSDD	1xTMTpro [N	0.0001004	7.389E-05	1	1	1	O00170	O00170 [40-54]	0	1963.9528
[R].TLQGIP(	1xTMTpro [N	0.0001192	7.389E-05	1	1	1	P04114	P04114 [805-818]	0	1859.0744
[K].TLQLALD	1xTMTpro [N	6.843E-05	7.389E-05	1	1	1	P53618	P53618 [340-351]	0	1619.9651
[K].TLQLDNI	1xTMTpro [K	6.158E-05	7.389E-05	1	1	2	Q9UMS4	Q9UMS4 [429-43!	0	1929.0937
[K].TLQIFNIE	1xTMTpro [K	0.0001834	7.389E-05	1	2	3	Q00610	Q00610 [87-96]	0	1845.08
[R].TLQILNA	1xTMTpro [N	0.0001075	7.389E-05	1	1	1	Q96RW7	Q96RW7 [2630-2	0	1846.9942
[K].TIQVDNT	1xTMTpro [N	0.0001082	7.389E-05	1	1	1	P28838	P28838 [357-368]	0	1622.8305
[R].TLQVSPI	1xTMTpro [N	8.571E-05	7.389E-05	1	1	2	P29350	P29350 [394-407]	0	1845.0401
[R].TIRPMDI	1xTMTpro [K	0.0001856	7.389E-05	1	1	1	P11177	P11177 [270-285]	0	2460.3156
[R].TLSGME	1xCarbamido	0.0001271	7.389E-05	1	1	1	P50991	P50991 [442-452]	0	1606.7888
[K].TISQEFL	1xTMTpro [K	0.0001235	7.389E-05	1	1	1	Q0ZGT2	Q0ZGT2 [363-373	0	1829.0664

[K].TLSQWI/	1xTMTpro [K	6.853E-05	7.389E-05	1	1	1	P34949	P34949 [70-85]	0	2385.2906
[K].TISTSDP	1xTMTpro [K	6.695E-05	7.389E-05	1	1	1	Q86UE4	Q86UE4 [492-504	0	1968.1509
[R].TLTAEE/	1xTMTpro [N	0.0001732	7.389E-05	1	1	1	P35269	P35269 [154-166]	0	1896.9146
[R].TIQFVDV	1xCarbamido	0.000122	7.389E-05	2	5	3	Q71U36; Q9	Q71U36 [340-352	0	2207.1815
[K].TLTDEEI	1xTMTpro [K	6.811E-05	7.389E-05	1	1	2	P40763	P40763 [234-244]	0	1929.0461
[R].TLTELILI	1xTMTpro [K	0.0001983	7.389E-05	1	1	1	P17480	P17480 [84-97]	0	2218.2939
[R].TLTEPCI	1xCarbamido	0.0001292	7.389E-05	1	1	1	Q969N2	Q969N2 [267-279	0	1764.9121
[K].TLTIDCV	1xCarbamido	0.0001144	7.389E-05	1	1	2	P23786	P23786 [440-450]	0	1684.9012
[K].TITTMID(	1xTMTpro [N	8.159E-05	7.389E-05	1	1	2	P22105	P22105 [835-847]	0	1764.9485
[K].TIVEAPS	1xTMTpro [K	9.988E-05	7.389E-05	1	1	1	Q2PZI1	Q2PZI1 [73-91]	0	2773.4913
[R].TIVEEVQ	1xTMTpro [K	0.0001094	7.389E-05	1	1	2	Q04695	Q04695 [410-419]	0	1725.9878
[K].TLVGSEI	1xTMTpro [N	7.497E-05	7.389E-05	1	1	2	Q13576	Q13576 [772-785]	0	1800.055
[K].TIVMND(	1xCarbamido	0.0001859	7.389E-05	1	1	1	Q9BTE1	Q9BTE1 [40-49]	0	1538.8354
[R].TIVTTLQ	1xTMTpro [N	0.0001075	7.389E-05	1	1	3	P07996	P07996 [289-299]	0	1550.9073
[R].TLYDFP(	1xTMTpro [K	7.875E-05	7.389E-05	1	1	3	P46109	P46109 [130-145]	0	2450.2735
[R].TLYDFP(	2xTMTpro [K	0.0001146	7.389E-05	1	1	2	P46109	P46109 [130-146]	1	2882.5756
[R].TMAAPQ	1xTMTpro [K	0.0001345	7.389E-05	1	1	1	Q9BX79	Q9BX79 [598-621	0	3253.6688
[K].TMADSS	1xOxidation [	0.0001579	7.389E-05	1	1	1	Q96AC1	Q96AC1 [479-497	0	2797.4938
[K].TLTDEL/	1xTMTpro [K	8.362E-05	7.389E-05	1	1	1	Q8NBQ5	Q8NBQ5 [198-21	0	2181.2986
[K].TGQQAE	1xTMTpro [K	9.617E-05	7.389E-05	1	1	2	P98082	P98082 [151-163]	0	2006.1778
[K].TLQEVTI	1xTMTpro [K	0.0001251	7.389E-05	1	1	1	P58107	P58107 [2742-275	0	1988.0866
[K].TLQALQI	1xTMTpro [K	0.000145	7.389E-05	1	1	3	P46940	P46940 [557-567]	0	1762.1082
[R].TLHYECI	1xCarbamido	0.0001558	7.389E-05	1	1	1	Q9UKK9	Q9UKK9 [71-81]	0	1983.1593
[K].TLKPGTI	2xTMTpro [K	0.0001322	7.389E-05	1	1	1	P07947	P07947 [306-325]	0	3176.7702
[K].TLKPGTI	2xTMTpro [K	8.121E-05	7.389E-05	1	1	1	P12931	P12931 [299-318]	0	3118.7461
[K].TLKVDEI	1xTMTpro [K	8.453E-05	7.389E-05	1	1	1	Q99715	Q99715 [1001-10	1	2174.1619
[K].TLLENT/	1xTMTpro [N	8.336E-05	7.389E-05	1	1	3	Q92973	Q92973 [774-785]	0	1605.9495
[K].TLINAED	1xOxidation [	0.0001417	7.389E-05	1	1	2	P46940	P46940 [857-870]	0	1874.0377
[K].TLLPMP(	2xTMTpro [K	0.0001796	7.389E-05	1	1	1	O15254	O15254 [235-250]	1	2568.5437
[R].TLIQNC(	1xCarbamido	0.0001656	7.389E-05	1	1	2	P49368	P49368 [450-461]	0	1637.8964
[K].TLLSNLE	1xTMTpro [K	0.0001407	7.389E-05	1	1	1	P10909	P10909 [69-78]	0	1726.0242
[K].TLLSNLE	2xTMTpro [K	9.754E-05	7.389E-05	1	1	1	P10909	P10909 [69-79]	1	2158.3263
[K].TIISYIDE	1xTMTpro [N	7.08E-05	7.389E-05	1	1	7	Q15019	Q15019 [117-128]	0	1817.9604

[K].TILTLTG	1xTMTpro [K	0.0001091	7.389E-05	1	1	1	A6NDG6	A6NDG6 [276-29(	0	2126.2928
[K].TILTYAE	1xTMTpro [N	6.149E-05	7.389E-05	1	1	1	Q96HP0	Q96HP0 [1531-15	0	1815.9482
[K].TLQEEVI	1xTMTpro [K	7.887E-05	7.389E-05	1	1	1	Q96A35	Q96A35 [193-205	0	2087.1372
[R].TLIVTHS	1xTMTpro [K	0.0001564	7.389E-05	1	1	1	O60306	O60306 [849-865]	0	2564.4692
[R].TINEVEN	1xTMTpro [N	7.048E-05	7.389E-05	2	2	3	P12814; O43	P12814 [727-738]	0	1733.9717
[K].TLNICEV	1xCarbamido	0.0002023	7.389E-05	1	1	1	P13671	P13671 [908-918]	0	1579.8797
[R].TLNISGN	1xTMTpro [N	0.0002047	7.389E-05	1	1	1	Q6UWE0	Q6UWE0 [154-16	0	1548.8665
[R].TINLYPL	1xTMTpro [K	8.453E-05	7.389E-05	1	1	3	O43809	O43809 [36-50]	0	2354.3252
[R].TLNWGC	1xTMTpro [K	0.0001672	7.389E-05	1	2	1	O75912	O75912 [470-484]	0	2232.1792
[R].TLPGSD	1xTMTpro [N	7.405E-05	7.389E-05	1	1	1	P56192	P56192 [468-483]	0	2108.1096
[K].TLPGWN	1xTMTpro [N	0.000114	7.389E-05	1	1	2	P30520	P30520 [404-416]	0	1748.9251
[K].TIPNDPC	1xTMTpro [N	7.648E-05	7.389E-05	1	1	1	Q93009	Q93009 [802-816]	0	1934.0666
[K].TLPSGCI	1xCarbamido	0.0001495	7.389E-05	1	1	1	P11047	P11047 [1595-16(	0	2256.219
[K].TLPSGLI	1xTMTpro [N	0.0001736	7.389E-05	1	1	1	Q13724	Q13724 [577-587]	0	1537.8181
[K].TIPTDDN	1xCarbamido	9.453E-05	7.389E-05	1	1	4	P47895	P47895 [155-167]	0	1841.9387
[K].TLPVDF\	1xCarbamido	0.0001632	7.389E-05	1	1	1	Q96PY5	Q96PY5 [727-738	0	1783.9406
[K].TLQALEI	1xTMTpro [N	0.000101	7.389E-05	1	1	1	Q08380	Q08380 [377-393]	0	2288.3086
[R].TLMNLG	1xOxidation [	0.0001089	7.389E-05	1	1	2	P37802	P37802 [128-139]	0	1535.8899
[R].TGQPMI	3xTMTpro [K	0.0001918	7.389E-05	1	1	1	Q01844	Q01844 [393-410]	1	3272.9182
[R].TGQPMI	1xTMTpro [K	7.314E-05	7.389E-05	1	1	1	Q01844	Q01844 [393-404]	0	2024.1494
[K].TGPNLH	1xTMTpro [N	0.0001402	7.389E-05	1	1	3	P99999	P99999 [29-39]	0	1472.8293
[K].TCIDVNE	4xCarbamido	0.000123	7.389E-05	1	1	1	P35556	P35556 [1322-134	0	3531.5814
[R].TCLLNE	1xCarbamido	0.0001567	7.389E-05	1	1	1	P15104	P15104 [358-372]	0	2423.2408
[K].TCSQMA	1xCarbamido	0.0001178	7.389E-05	1	1	1	Q96ER9	Q96ER9 [330-342	0	2029.143
[R].TCTDIKF	1xCarbamido	0.0001553	7.389E-05	1	1	2	O43143	O43143 [749-760]	0	2402.3934
[K].TCWLQL	1xCarbamido	0.00014	7.389E-05	1	1	1	P41221	P41221 [251-260]	0	1613.8429
[K].TDAETLI	1xTMTpro [K	0.0001306	7.389E-05	1	1	1	O43252	O43252 [249-258]	0	1666.9871
[R].TDCPGD	1xCarbamido	6.726E-05	7.389E-05	1	1	2	Q96PD5	Q96PD5 [528-54(	0	1796.9172
[R].TDDIPV\	1xTMTpro [K	0.000107	7.389E-05	1	1	2	P63208	P63208 [82-94]	0	2214.1938
[R].TDEEGK	2xTMTpro [K	0.0001072	7.389E-05	1	1	1	Q9BUJ2	Q9BUJ2 [546-562	1	2825.5171
[R].TDFKEEI	2xTMTpro [K	0.0001662	7.389E-05	1	1	1	P47897	P47897 [617-628]	1	2336.2954
[R].TDGALL	1xOxidation [	0.0001687	7.389E-05	1	1	1	P50454	P50454 [195-213]	0	3147.7117
[K].TDGAMC	1xOxidation [	0.0001208	7.389E-05	1	1	1	Q5TZA2	Q5TZA2 [577-589	0	1749.8145

[R].TDLEELS	1xTMTpro [N	0.0001333	7.389E-05	1	1	1	P53365	P53365 [240-250]	0	1533.8444
[K].TCFYAE(	1xCarbamido	7.213E-05	7.389E-05	1	1	1	P49588	P49588 [532-550]	0	2849.4312
[RK].TDLEM	1xOxidation [	8.414E-05	7.389E-05	1	2	1	P13646	P13646 [223-240]	0	2781.4182
[K].TDLLLEF	1xTMTpro [K	0.00017	7.389E-05	1	1	2	P35579	P35579 [290-299]	0	1814.0555
[R].TDIQCLI	2xCarbamido	0.0001553	7.389E-05	1	1	1	P23381	P23381 [301-318]	0	2529.2437
[K].TDIQIALI	1xCarbamido	6.633E-05	7.389E-05	1	1	1	P33316	P33316 [156-169]	0	1854.9703
[K].TDLSNV(	1xTMTpro [K	7.303E-05	7.389E-05	1	1	1	Q8NBL1	Q8NBL1 [316-329	0	2242.2939
[R].TDMDQII	1xTMTpro [K	0.0001933	7.389E-05	1	1	3	Q96TA1	Q96TA1 [307-316	0	1759.9756
[K].TDPSILG	1xTMTpro [N	0.0001065	7.389E-05	1	1	2	P48047	P48047 [177-188]	0	1562.8895
[K].TDPWSL	1xTMTpro [N	0.000123	7.389E-05	1	1	1	Q15477	Q15477 [85-106]	0	2638.4524
[K].TDSTTE'	1xTMTpro [N	0.0001769	7.389E-05	1	1	2	Q8NEU8	Q8NEU8 [506-518	0	1819.9067
[K].TDVLILP	1xTMTpro [K	8.375E-05	7.389E-05	1	1	2	Q9NZ08	Q9NZ08 [586-599	0	2292.3347
[K].TDVNKIE	2xTMTpro [K	7.803E-05	7.389E-05	1	1	1	Q96NY7	Q96NY7 [538-550	1	2506.4221
[K].TDYMVG	1xTMTpro [N	8.545E-05	7.389E-05	1	1	2	P52565	P52565 [142-152]	0	1549.764
[R].TEAAET	1xTMTpro [K	9.482E-05	7.389E-05	1	1	1	Q8TB61	Q8TB61 [98-111]	0	2185.1819
[R].TEAESW	1xTMTpro [K	0.0001638	7.389E-05	1	1	6	P13647	P13647 [355-364]	0	1850.978
[K].TDLGAA	1xTMTpro [K	0.0002047	7.389E-05	1	1	1	Q9NX24	Q9NX24 [112-121	0	1498.8721
[R].TEALSVI	1xTMTpro [K	0.0001157	7.389E-05	1	1	1	Q5VYK3	Q5VYK3 [1785-17	0	1937.2178
[R].TCESLG.	1xCarbamido	8.492E-05	7.389E-05	1	1	3	P98160	P98160 [1627-163	0	1474.7279
[R].TCAQLP	2xCarbamido	0.000157	7.389E-05	1	1	2	P11678	P11678 [256-267]	0	2040.1266
[R].TAAENE	2xTMTpro [K	7.224E-05	7.389E-05	2	4	1	P04259; P02	P04259 [261-272]	1	2263.3478
[R].TAATLAT	1xTMTpro [N	0.0001397	7.389E-05	1	1	1	Q8N1G4	Q8N1G4 [371-381	0	1487.8501
[K].TAAVANS	1xTMTpro [K	0.0001021	7.389E-05	1	1	2	Q9NYU2	Q9NYU2 [732-744	0	1992.108
[R].TAAVWE	1xTMTpro [K	0.0001732	7.389E-05	1	1	1	Q96EE3	Q96EE3 [82-96]	0	2256.2004
[K].TADDPS	1xTMTpro [K	7.485E-05	7.389E-05	1	1	4	P39656	P39656 [78-88]	0	1768.0348
[K].TADMDV	1xTMTpro [N	0.0001567	7.389E-05	1	1	1	Q86WR0	Q86WR0 [101-11	0	1750.8866
[K].TAEAQL	1xTMTpro [N	8.518E-05	7.389E-05	1	1	1	Q14254	Q14254 [234-247]	0	1824.9775
[K].TAEEVA	1xTMTpro [N	0.0001892	7.389E-05	1	1	3	Q6P2Q9	Q6P2Q9 [1841-18	0	1376.8068
[K].TAENATS	1xTMTpro [N	0.0001803	7.389E-05	1	1	2	Q9UQ80	Q9UQ80 [377-394	0	2141.9642
[K].TAESQTI	1xTMTpro [K	7.291E-05	7.389E-05	1	1	1	P55265	P55265 [596-613]	0	2452.2851
[K].TAFHQE	1xTMTpro [K	0.000182	7.389E-05	1	1	1	P51580	P51580 [38-50]	0	2145.206
[K].TAHLDEI	2xTMTpro [K	9.617E-05	7.389E-05	1	1	1	P11586	P11586 [199-223]	1	3606.0029
[R].TALASG(	1xTMTpro [N	6.427E-05	7.389E-05	1	1	1	O43505	O43505 [65-80]	0	1856.9673

[R].TCDALD	1xCarbamido	8.505E-05	7.389E-05	1	1	1	Q8N4A0	Q8N4A0 [564-578	1	2767.4905
[R].TALINST	1xOxidation [	6.247E-05	7.389E-05	1	1	3	P02545	P02545 [528-541]	0	1811.9492
[K].TAITVEH	1xTMTpro [K	0.0001317	7.389E-05	1	1	1	Q9NR30	Q9NR30 [407-417	0	1804.1188
[R].TALTYYI	1xTMTpro [N	0.0001264	7.389E-05	1	1	1	P16435	P16435 [369-382]	0	1942.0605
[K].TAMNVN	1xTMTpro [K	6.885E-05	7.389E-05	1	1	2	P51148	P51148 [167-180]	0	2161.2005
[K].TAMNVN	1xOxidation [	0.000111	7.389E-05	1	1	3	P51148	P51148 [167-180]	0	2177.1954
[K].TANKDH	2xTMTpro [K	0.0001178	7.389E-05	1	1	1	Q00688	Q00688 [53-70]	1	3014.688
[K].TAPAVV	1xTMTpro [N	9.941E-05	7.389E-05	1	1	1	O43464	O43464 [157-169	0	1764.0227
[K].TASGDY	1xTMTpro [N	7.451E-05	7.389E-05	1	1	2	Q86UX7	Q86UX7 [6-19]	0	1903.9357
[K].TASTNNI	1xTMTpro [N	0.0001144	7.389E-05	1	1	2	Q9UBI6	Q9UBI6 [5-15]	0	1450.7933
[K].TATAGP	1xTMTpro [K	0.0001729	7.389E-05	1	1	2	P46821	P46821 [2334-234	0	1512.8877
[R].TAVCIEN	2xCarbamido	0.0001476	7.389E-05	1	1	2	Q15063	Q15063 [464-475	0	2066.0212
[K].TAVETA	1xTMTpro [N	0.00014	7.389E-05	1	1	3	P49368	P49368 [508-518]	0	1489.9273
[K].TAVNALV	1xTMTpro [K	0.000155	7.389E-05	1	1	5	P02042	P02042 [10-18]	0	1567.9452
[K].TAYSYF	1xTMTpro [K	6.297E-05	7.389E-05	1	1	1	O00231	O00231 [227-246	0	2961.4326
[K].TALNNTI	1xTMTpro [N	7.555E-05	7.389E-05	1	1	1	Q12768	Q12768 [312-324	0	1734.9669
[R].TMATMG	1xTMTpro [N	9.007E-05	7.389E-05	1	1	1	O15027	O15027 [1722-17	0	1558.7888
[R].TEAWVL	1xTMTpro [N	0.0001644	7.389E-05	1	1	1	Q96FV2	Q96FV2 [167-177	0	1536.8341
[K].TEDSLM	1xTMTpro [N	8.298E-05	7.389E-05	1	1	3	Q15459	Q15459 [687-699	0	1899.9329
[R].TFEMSD	1xTMTpro [N	7.224E-05	7.389E-05	1	1	2	O75369	O75369 [2017-20	0	1764.8798
[R].TFHDLE	1xTMTpro [K	0.0001206	7.389E-05	1	1	2	Q7Z2K6	Q7Z2K6 [694-704	0	1839.0256
[K].TFHEASI	1xCarbamido	0.0001576	7.389E-05	1	1	2	P05452	P05452 [90-101]	0	1755.8291
[K].TFHFNT	1xTMTpro [N	0.0001605	7.389E-05	1	1	2	P30740	P30740 [57-69]	0	1906.9731
[R].TFHTIGF	1xCarbamido	0.0001153	7.389E-05	2	2	1	Q07352; P47	Q07352 [160-172	0	1856.9437
[K].TFLTLSL	1xTMTpro [N	7.291E-05	7.389E-05	1	1	2	Q9UNS2	Q9UNS2 [313-325	0	1786.9692
[R].TFITSDF	1xTMTpro [K	7.191E-05	7.389E-05	1	1	1	P49915	P49915 [639-664]	0	3384.862
[K].TFSYAGI	1xTMTpro [K	0.00014	7.389E-05	1	1	4	Q99832	Q99832 [219-230	0	2014.06
[R].TFTEMD	1xTMTpro [K	6.695E-05	7.389E-05	1	1	2	P48444	P48444 [132-142]	0	1961.977
[K].TFVDFFS	1xCarbamido	6.158E-05	7.389E-05	1	1	1	Q53GQ0	Q53GQ0 [207-22	0	2282.0871
[R].TFVEESI	1xTMTpro [N	0.0001338	7.389E-05	1	1	2	P30837	P30837 [325-338]	0	2080.0558
[K].TGAESIS	1xCarbamido	8.453E-05	7.389E-05	1	1	2	O60216	O60216 [574-586	0	1752.9485
[K].TGAFSIF	1xTMTpro [K	0.0001484	7.389E-05	1	1	2	Q92499	Q92499 [53-69]	0	2487.4718
[K].TFDSIVM	1xTMTpro [K	0.0001881	7.389E-05	1	1	2	P13667	P13667 [534-543]	0	1760.9748

[K].TGASWT	1xTMTpro [K	0.000143	7.389E-05	1	1	1	Q7Z6K5	Q7Z6K5 [191-203	0	2031.0825
[K].TGEAYV	1xTMTpro [K	7.531E-05	7.389E-05	1	1	1	Q12849	Q12849 [290-309]	0	2876.4996
[K].TGEEDE	1xCarbamido	9.248E-05	7.389E-05	1	1	1	P49792	P49792 [1186-119	0	1965.8456
[K].TGEEVG	1xTMTpro [K	0.0002003	7.389E-05	1	1	2	O75369	O75369 [1630-164	0	1859.0406
[K].TGENVE	1xTMTpro [K	6.427E-05	7.389E-05	1	1	1	P61106	P61106 [157-170]	0	2102.1261
[R].TGESVE	1xCarbamido	9.957E-05	7.389E-05	1	1	4	P08603	P08603 [1193-120	0	1763.9493
[K].TGFQAV	1xTMTpro [K	0.0002007	7.389E-05	1	1	1	P20042	P20042 [316-324]	0	1516.8979
[K].TGGLDLI	1xTMTpro [K	7.124E-05	7.389E-05	1	1	1	Q15113	Q15113 [366-381]	0	2119.2254
[R].TGKPIPD	2xTMTpro [K	0.0001123	7.389E-05	1	1	1	P46952	P46952 [156-166]	0	2122.3416
[K].TGKPMIN	2xTMTpro [K	0.0001264	7.389E-05	1	1	1	Q92804	Q92804 [266-277]	0	2293.3406
[K].TGKPMIN	4xTMTpro [K	6.478E-05	7.389E-05	1	1	1	Q92804	Q92804 [266-283]	1	3528.0937
[R].TGLCYLI	1xCarbamido	7.08E-05	7.389E-05	1	1	1	Q13045	Q13045 [43-57]	0	2314.2972
[R].TGLLVE(	1xTMTpro [K	0.0001121	7.389E-05	1	1	1	Q9HC84	Q9HC84 [162-174	0	2031.1618
[R].TGISPLA	1xTMTpro [K	0.0001896	7.389E-05	1	1	2	P04114	P04114 [220-229]	0	1621.0544
[K].TGCNVL	1xCarbamido	0.0001736	7.389E-05	1	1	1	P50991	P50991 [293-302]	0	1754.049
[R].TEDPLLS	1xTMTpro [K	0.0001383	7.389E-05	1	1	2	P52630	P52630 [531-543]	0	2131.1567
[K].TFDQLTI	1xTMTpro [K	0.0001299	7.389E-05	1	1	2	Q15293	Q15293 [71-81]	0	1889.0148
[R].TFAQGL	1xTMTpro [K	7.985E-05	7.389E-05	1	1	1	O75487	O75487 [220-234]	0	2071.2043
[K].TEDTAV	1xCarbamido	0.0001665	7.389E-05	1	2	1	A0A0A0MS1	A0A0A0MS15 [10	0	1682.8015
[K].TEEAIIYS	1xTMTpro [K	0.0001581	7.389E-05	1	1	1	O15269	O15269 [158-180]	0	3074.6582
[K].TEEELQ	1xTMTpro [K	9.453E-05	7.389E-05	1	1	2	Q9NQ55	Q9NQ55 [304-315	0	1982.1301
[R].TEEIFYY	1xTMTpro [K	0.0001874	7.389E-05	1	1	1	Q6UX06	Q6UX06 [450-463	0	2352.1891
[K].TEELKP(	2xTMTpro [K	0.000155	7.389E-05	1	1	1	Q9UN86	Q9UN86 [206-216	0	2242.3111
[R].TEFGMA	1xTMTpro [K	0.000155	7.389E-05	1	1	1	Q99523	Q99523 [168-181]	0	2046.0822
[K].TEFLSFN	1xOxidation	0.0001058	7.389E-05	1	1	2	P31949	P31949 [37-52]	0	2474.3133
[K].TEGAEA	1xTMTpro [K	7.303E-05	7.389E-05	1	1	2	Q16666	Q16666 [117-128]	0	1767.9733
[K].TEGTQE	1xTMTpro [K	9.176E-05	7.389E-05	1	1	2	Q02952	Q02952 [1261-12	0	2193.0803
[R].TEHYEE	1xTMTpro [K	6.307E-05	7.389E-05	1	1	2	P02748	P02748 [214-225]	0	2132.1156
[K].TEIDEVV	1xTMTpro [N	6.79E-05	7.389E-05	1	1	1	P48723	P48723 [397-410]	0	1778.9819
[R].TEMEDL	1xTMTpro [K	0.0001089	7.389E-05	1	1	4	P35579	P35579 [1504-151	0	1778.916
[R].TEMENE	1xTMTpro [K	8.979E-05	7.389E-05	1	1	4	P05787	P05787 [187-197]	0	1961.0909
[R].TFCQLIL	1xCarbamido	9.364E-05	7.389E-05	1	1	2	P13639	P13639 [288-299]	0	2103.2168
[R].TEMENE	1xOxidation	0.0001647	7.389E-05	1	1	2	P05787	P05787 [187-197]	0	1977.0858

[K].TEMQDN	1xTMTpro [N	8.298E-05	7.389E-05	1	1	1	O43143	O43143 [492-504]	0	1913.9598
[K].TEMQNM	1xTMTpro [N	0.0001422	7.389E-05	1	1	1	Q9NTZ6	Q9NTZ6 [77-87]	0	1655.8416
[K].TEMVTIS	1xTMTpro [N	8.362E-05	7.389E-05	1	1	2	O43491	O43491 [875-886]	0	1641.8437
[K].TENLNDI	2xTMTpro [K	0.0001151	7.389E-05	1	1	2	P13796	P13796 [571-584]	1	2530.3929
[K].TENPGD	1xTMTpro [N	8.678E-05	7.389E-05	1	1	1	O60437	O60437 [493-505]	0	1663.8207
[K].TENPLIL	1xTMTpro [K	7.672E-05	7.389E-05	1	1	1	P36776	P36776 [582-594]	0	2107.2142
[K].TENYLG	1xTMTpro [N	0.0001271	7.389E-05	1	1	1	Q01432	Q01432 [408-419]	0	1723.8611
[K].TESSTE	2xTMTpro [K	0.0001328	7.389E-05	1	1	2	P43243	P43243 [618-630]	1	2394.2816
[R].TESTPIT	1xTMTpro [K	0.0001749	7.389E-05	1	1	1	Q9Y678	Q9Y678 [591-600	0	1654.9871
[R].TETPPPI	1xTMTpro [K	6.982E-05	7.389E-05	1	1	1	Q3MHD2	Q3MHD2 [73-86]	0	2062.204
[K].TEVLSEI	1xTMTpro [N	8.009E-05	7.389E-05	1	1	1	Q68EM7	Q68EM7 [22-34]	0	1849.0238
[K].TEVSLTL	1xTMTpro [K	0.0001295	7.389E-05	1	1	3	P46940	P46940 [1359-13	0	1714.0242
[K].TFAEAQI	1xCarbamido	0.0001359	7.389E-05	1	1	2	Q9Y2Y8	Q9Y2Y8 [120-130	0	1586.7916
[R].TEMENE	1xOxidation	0.0001166	7.389E-05	1	1	1	P05787	P05787 [187-198]	1	2409.3879
[R].TAAENE	1xTMTpro [K	8.813E-05	7.389E-05	2	4	3	P04259; P02	P04259 [261-271]	0	1831.0457
[R].TMDNMT	1xTMTpro [N	9.724E-05	7.389E-05	1	1	1	Q9Y580	Q9Y580 [120-133	0	1899.9588
[R].TMKEDL	1xTMTpro [K	0.0001907	7.389E-05	1	1	2	Q03169	Q03169 [223-234]	1	2028.1291
[R].TVIIEQS	1xTMTpro [K	7.555E-05	7.389E-05	1	1	2	P10809	P10809 [61-72]	0	1953.1301
[K].TVLLLAD	1xTMTpro [N	9.277E-05	7.389E-05	1	2	2	P48730	P48730 [104-115]	0	1663.9736
[K].TVLITGA	1xTMTpro [N	7.247E-05	7.389E-05	1	1	1	Q9HBH5	Q9HBH5 [45-57]	0	1562.9185
[R].TVLPFS	1xTMTpro [N	0.0001995	7.389E-05	1	1	1	Q7L576	Q7L576 [867-877]	0	1655.9076
[K].TVLSNV	1xTMTpro [N	6.79E-05	7.389E-05	1	1	2	O43237	O43237 [463-474]	0	1706.9244
[R].TVLVNAI	1xOxidation	0.0001608	7.389E-05	1	1	1	Q03252	Q03252 [562-575]	0	1823.9492
[K].TVIVNM	1xTMTpro [K	0.0001222	7.389E-05	1	1	4	P55010	P55010 [34-44]	0	1797.08
[K].TVLWPN	1xTMTpro [N	0.000182	7.389E-05	1	1	1	Q07954	Q07954 [697-712]	0	2013.1452
[R].TVMLGT	1xTMTpro [K	9.708E-05	7.389E-05	1	1	1	P57735	P57735 [53-62]	0	1598.9795
[R].TVMVNIE	1xTMTpro [K	6.438E-05	7.389E-05	1	1	3	P01024	P01024 [162-176]	0	2248.2867
[K].TVNAVVI	1xTMTpro [K	8.732E-05	7.389E-05	1	1	1	Q709C8	Q709C8 [640-652	0	2091.173
[K].TVNQQT	1xTMTpro [K	0.0001402	7.389E-05	1	1	1	Q9Y520	Q9Y520 [1121-11	0	1995.1553
[K].TVNVVQ	1xTMTpro [K	0.0001247	7.389E-05	1	1	4	P41250	P41250 [491-501]	0	1856.0773
[R].TVPFLPI	1xCarbamido	7.636E-05	7.389E-05	1	1	1	Q7Z7H8	Q7Z7H8 [170-188	0	2391.3277
[K].TVQGPP	1xTMTpro [N	7.382E-05	7.389E-05	1	1	2	P04181	P04181 [33-46]	0	1865.9564
[K].TVQSNS	1xTMTpro [K	0.0001691	7.389E-05	1	1	1	O95292	O95292 [201-216]	0	2179.2578

[K].TVQTDH	1xTMTpro [N	0.0001058	7.389E-05	1	1	2	O43747	O43747 [335-345]	0	1572.8413
[K].TVSEALL	1xTMTpro [N	7.743E-05	7.389E-05	1	1	1	Q14515	Q14515 [315-333]	0	2351.172
[R].TVSSSVI	1xTMTpro [N	6.468E-05	7.389E-05	2	2	2	P24468; P10	P24468 [372-385]	0	1916.0812
[K].TVTAEV(	1xCarbamid(	0.0001306	7.389E-05	1	1	2	Q32P28	Q32P28 [636-647	0	1649.86
[K].TVTAMD	1xOxidation [	0.0001093	7.389E-05	1	1	3	P62805	P62805 [81-93]	1	2091.2128
[K].TVTATF(	1xTMTpro [N	9.724E-05	7.389E-05	1	1	2	Q16563	Q16563 [73-83]	0	1563.849
[K].TVTDMLI	1xCarbamid(	9.423E-05	7.389E-05	1	1	2	Q9Y490	Q9Y490 [107-118	0	1715.8814
[R].TVTPALS	1xTMTpro [N	0.0001944	7.389E-05	1	1	2	Q9C0E8	Q9C0E8 [211-223	0	1658.976
[R].TVTQLV/	1xTMTpro [N	0.00015	7.389E-05	1	1	1	Q9H4I3	Q9H4I3 [63-74]	0	1579.8611
[R].TVVSGL\	1xTMTpro [K	7.648E-05	7.389E-05	1	1	3	P54577	P54577 [401-412]	0	1882.1657
[R].TVYHAE	1xCarbamid(	8.414E-05	7.389E-05	1	1	2	Q16527	Q16527 [16-28]	0	1867.8928
[R].TVIFVAT	1xTMTpro [K	6.357E-05	7.389E-05	1	1	3	O95336	O95336 [198-208]	0	1730.0344
[K].TVLDQQ	1xTMTpro [N	0.0001881	7.389E-05	1	1	2	P42704	P42704 [1129-113	0	1576.8614
[R].TVLDLQ	1xTMTpro [K	0.0001239	7.389E-05	1	1	1	Q9BXX0	Q9BXX0 [197-213	0	2382.3736
[R].TVLDLIV	2xTMTpro [K	0.0001123	7.389E-05	1	1	1	Q69YN4	Q69YN4 [1198-12	1	3074.7289
[R].TTQIINIT	1xTMTpro [K	6.438E-05	7.389E-05	1	1	2	Q14980	Q14980 [1811-18	0	1872.112
[R].TTSAGT(	1xTMTpro [N	0.0001005	7.389E-05	1	1	2	P60468	P60468 [47-57]	0	1428.7225
[K].TTSSTD(	1xTMTpro [K	0.0002051	7.389E-05	1	1	1	Q9NZN8	Q9NZN8 [308-316	0	1501.8354
[K].TTTLSG	1xTMTpro [N	6.297E-05	7.389E-05	1	1	1	P27816	P27816 [872-888]	0	1890.0616
[R].TTTVES	1xTMTpro [N	0.0001796	7.389E-05	1	1	1	P53814	P53814 [684-693]	0	1430.781
[R].TVAATNI	1xTMTpro [N	0.0001885	7.389E-05	1	2	1	Q9NQT8	Q9NQT8 [209-22	0	1713.8397
[R].TVAEVQ	1xTMTpro [N	6.811E-05	7.389E-05	1	1	1	Q8TEQ6	Q8TEQ6 [1373-13	0	1894.0275
[K].TVAMHE	1xCarbamid(	0.0001796	7.389E-05	1	1	2	Q9UNH7	Q9UNH7 [140-15(	0	1666.8728
[K].TVAPMP	1xOxidation [	0.0001838	7.389E-05	1	1	1	P22102	P22102 [208-219]	0	1916.0555
[R].TVATLSI	1xTMTpro [N	0.0001008	7.389E-05	1	1	2	Q9H3U1	Q9H3U1 [230-24(	0	1435.8803
[K].TVAVWD	1xTMTpro [N	6.875E-05	7.389E-05	1	1	2	Q96DI7	Q96DI7 [132-143]	0	1653.8403
[R].TVCAHE	1xCarbamid(	0.0001553	7.389E-05	1	1	3	P55001	P55001 [140-149]	0	1531.8222
[R].TVDLSSI	1xTMTpro [K	0.0001395	7.389E-05	1	1	1	P04843	P04843 [39-48]	0	1678.9983
[R].TVYNLAI	1xCarbamid(	0.0001345	7.389E-05	1	1	1	P41221	P41221 [229-239]	0	1862.0337
[K].TVDSCF(	1xCarbamid(	0.0001458	7.389E-05	1	1	2	Q9NQ48	Q9NQ48 [31-40]	0	1820.9708
[K].TVELLS(	1xTMTpro [K	0.0001313	7.389E-05	1	1	1	Q9UJS0	Q9UJS0 [57-69]	0	1997.1774
[K].TVEINNT	1xTMTpro [N	0.0001005	7.389E-05	1	1	1	Q8NDH3	Q8NDH3 [335-34(	0	1622.8305
[K].TVENFV/	1xTMTpro [K	8.895E-05	7.389E-05	1	1	1	P45877	P45877 [66-78]	0	1987.1356

[K].TVEQTA	1xTMTpro [K	8.979E-05	7.389E-05	1	1	3	P08579	P08579 [112-122]	0	1802.0151
[K].TVESLEI	1xTMTpro [K	0.0001678	7.389E-05	1	1	2	Q14914	Q14914 [195-204]	0	1757.0188
[R].TVETEA	1xTMTpro [K	0.000134	7.389E-05	1	1	2	Q14738	Q14738 [550-560]	0	1857.0647
[K].TVETRD	1xTMTpro [N	8.009E-05	7.389E-05	1	1	1	P08670	P08670 [446-466]	1	2727.3141
[K].TVEVPFI	1xTMTpro [K	9.941E-05	7.389E-05	1	1	1	Q9P2T1	Q9P2T1 [292-306	1	2335.3266
[R].TVFVCD	1xCarbamido	9.754E-05	7.389E-05	1	1	2	P46926	P46926 [235-248]	0	2248.2027
[R].TVFYTNI	1xTMTpro [K	0.0001144	7.389E-05	1	1	1	Q9UPU7	Q9UPU7 [246-262	0	2690.4209
[R].TVGLFL	1xTMTpro [K	0.00018	7.389E-05	1	1	2	P43304	P43304 [464-473]	0	1628.0027
[K].TVGSDT	1xTMTpro [K	9.119E-05	7.389E-05	1	1	2	P01042	P01042 [65-75]	0	1860.0035
[K].TVGVEP.	1xTMTpro [K	0.0002015	7.389E-05	1	1	5	P46779	P46779 [48-58]	0	1651.9511
[K].TVEELLE	1xTMTpro [K	0.0001054	7.389E-05	1	1	1	Q86UP2	Q86UP2 [746-761	0	2352.3882
[R].TWEQQ	1xTMTpro [N	0.0001089	7.389E-05	1	1	2	Q9UJU6	Q9UJU6 [236-246	0	1693.8829
[K].TWIEVS	1xTMTpro [K	6.664E-05	7.389E-05	1	2	2	P61619	P61619 [378-388]	0	1773.0038
[K].TWQDCE	1xCarbamido	0.0001306	7.389E-05	1	1	1	P01042	P01042 [90-101]	1	2427.2795
[K].VADWTG	1xTMTpro [K	8.545E-05	7.389E-05	1	1	1	O15371	O15371 [42-54]	1	2119.1428
[R].VADYCE	1xCarbamido	0.0001479	7.389E-05	1	1	1	Q8IZP0	Q8IZP0 [29-43]	0	2412.1997
[K].VAEAHE	1xTMTpro [K	0.0001112	7.389E-05	1	1	1	Q08257	Q08257 [308-324]	0	2299.265
[K].VAEAVA	1xTMTpro [N	0.000187	7.389E-05	1	1	2	Q9Y570	Q9Y570 [359-369	0	1493.9011
[K].VAEDEA	1xTMTpro [K	0.0001182	7.389E-05	1	1	2	P08195	P08195 [148-160]	0	1854.01
[R].VAEELV	1xTMTpro [N	0.0001859	7.389E-05	1	1	1	P49593	P49593 [389-398]	0	1332.7806
[K].VAEEWA	1xTMTpro [K	0.0001659	7.389E-05	1	1	2	P04424	P04424 [70-80]	0	1874.0304
[R].VAEGQT	1xTMTpro [K	0.0001466	7.389E-05	1	1	2	P98160	P98160 [2838-284	0	1681.998
[K].VAEIEHA	2xTMTpro [K	0.0001368	7.389E-05	1	1	2	P78371	P78371 [264-274]	1	2195.2852
[R].VAELSSI	1xTMTpro [N	6.287E-05	7.389E-05	1	1	1	Q92896	Q92896 [298-310]	0	1807.9146
[K].VAEITEL	1xTMTpro [K	0.0001284	7.389E-05	1	1	2	Q06265	Q06265 [258-267]	0	1737.1017
[R].VAELYEI	1xTMTpro [N	0.0001299	7.389E-05	1	1	3	P17661	P17661 [151-160]	0	1554.8335
[K].VAEQVLI	1xTMTpro [K	0.0001838	7.389E-05	1	1	2	Q9HB40	Q9HB40 [233-243	0	1793.0777
[R].VADTFQ	1xCarbamido	6.458E-05	7.389E-05	1	1	1	Q9Y2T2	Q9Y2T2 [86-103]	0	2659.3205
[K].VAETAV	1xTMTpro [K	6.317E-05	7.389E-05	1	1	1	P62495	P62495 [205-218]	0	2086.204
[K].VAFTGS	1xTMTpro [K	9.335E-05	7.389E-05	1	1	3	P00352	P00352 [242-252]	0	1703.9824
[R].VAGLLEI	1xTMTpro [N	6.407E-05	7.389E-05	1	1	2	P35858	P35858 [278-293]	0	1975.1547
[K].VAGMDV	1xTMTpro [N	8.624E-05	7.389E-05	1	1	3	P62258	P62258 [30-42]	0	1751.9169
[R].VAGMLQ	1xTMTpro [K	7.158E-05	7.389E-05	1	1	1	P28331	P28331 [326-336]	0	1774.0177

[R].VAGSVT	1xOxidation [	8.545E-05	7.389E-05	1	1	2	Q9Y490	Q9Y490 [2276-22	0	2242.2608
[R].VAGTQA	1xCarbamido	0.0001262	7.389E-05	1	1	1	Q9BRR6	Q9BRR6 [409-424	0	1984.9929
[K].VAGYAA	1xTMTpro [K	7.791E-05	7.389E-05	1	1	1	P54920	P54920 [168-180]	0	2062.1829
[K].VAHALAI	1xCarbamido	9.816E-05	7.389E-05	1	1	1	P60174	P60174 [114-131]	0	2416.3878
[R].VAHEPV.	1xTMTpro [K	0.0001672	7.389E-05	1	1	1	O95674	O95674 [8-19]	0	1897.0675
[R].VAIHYIN	1xTMTpro [K	6.227E-05	7.389E-05	1	1	1	P78406	P78406 [240-252]	0	2042.2043
[K].VALNVS(	1xCarbamido	8.388E-05	7.389E-05	1	1	1	O75131	O75131 [9-21]	0	2025.1658
[K].VALVTG/	1xTMTpro [N	0.0001361	7.389E-05	1	1	1	P15428	P15428 [7-19]	0	1516.913
[K].VAMSHF	1xTMTpro [K	6.377E-05	7.389E-05	1	1	1	Q99798	Q99798 [32-50]	0	2943.5206
[R].VAEVLNI	1xTMTpro [K	0.0001849	7.389E-05	1	1	2	Q07866	Q07866 [506-518]	0	2096.1189
[R].TTPSYV/	1xTMTpro [N	0.0001041	7.389E-05	4	6	4	P17066; P54	P17066 [39-51]; F	0	1791.9084
[K].VADPVD	1xCarbamido	9.105E-05	7.389E-05	1	1	1	Q12888	Q12888 [89-116]	0	3343.6759
[R].VADPAY	1xTMTpro [N	7.625E-05	7.389E-05	1	1	2	P50148	P50148 [167-181]	0	1990.0929
[K].TWTDFE	1xTMTpro [K	6.664E-05	7.389E-05	1	1	2	Q7Z2W4	Q7Z2W4 [545-557	0	2275.156
[R].TYAEPL	1xOxidation [	8.362E-05	7.389E-05	1	1	1	Q14204	Q14204 [2764-27	0	2658.2751
[K].TYEDIKE	1xTMTpro [K	0.0001096	7.389E-05	1	1	1	P25325	P25325 [165-176]	1	2105.137
[K].TYEEIAS	1xTMTpro [K	9.497E-05	7.389E-05	1	1	2	Q9Y5X1	Q9Y5X1 [470-483	0	2186.22
[K].TYEQVLI	1xTMTpro [K	0.000101	7.389E-05	1	1	3	Q16762	Q16762 [164-175]	0	2061.136
[K].TYEWSS	2xTMTpro [K	0.000101	7.389E-05	1	1	2	Q8WXF7	Q8WXF7 [18-31]	1	2653.4177
[R].TYGEPE	1xTMTpro [N	8.196E-05	7.389E-05	1	1	1	P50402	P50402 [104-115]	0	1554.7719
[K].TYGGCE	1xCarbamido	7.743E-05	7.389E-05	1	1	4	Q15369	Q15369 [7-20]	0	2156.0648
[K].TYGGCE	1xCarbamido	0.0001015	7.389E-05	1	1	3	Q15369	Q15369 [7-20]	0	2172.0597
[K].TYHVGE	1xTMTpro [K	0.0001068	7.389E-05	1	1	1	P02751	P02751 [2392-240	0	1884.026
[R].TYLGDP	1xTMTpro [K	0.000121	7.389E-05	1	1	1	Q96MH6	Q96MH6 [276-296	0	2971.5796
[R].TYINPFV	1xTMTpro [N	0.0001975	7.389E-05	2	2	2	O95486; O9	O95486 [436-448]	0	1904.0237
[R].TYIQCIA	1xCarbamido	0.0001678	7.389E-05	1	1	1	Q86VP6	Q86VP6 [233-243	0	1599.8848
[K].VADPTPI	1xOxidation [	6.695E-05	7.389E-05	1	1	1	P16152	P16152 [97-112]	0	2408.3139
[K].TYMLAFI	1xOxidation [	0.000141	7.389E-05	1	1	2	P02763	P02763 [127-138]	0	2070.0709
[K].TYQIGW	1xCarbamido	0.0001352	7.389E-05	1	1	2	Q99715	Q99715 [2212-22	0	2126.1236
[K].TYSYLTI	1xTMTpro [K	7.247E-05	7.389E-05	1	1	5	P15880	P15880 [247-257]	0	1995.1083
[R].VAAALP(	1xTMTpro [N	0.0001023	7.389E-05	1	1	2	P20340	P20340 [170-183]	0	1749.9125
[R].VAALQN	1xTMTpro [K	0.0001849	7.389E-05	1	1	2	Q14974	Q14974 [235-243]	0	1564.0078
[R].VAALQT.	1xTMTpro [K	0.0001892	7.389E-05	1	1	1	Q9H269	Q9H269 [648-660	0	1977.1301

[K].VAAPDV	1xTMTpro [N	6.448E-05	7.389E-05	1	1	4	Q14204	Q14204 [2562-25	0	1856.0812
[R].VAASNIV	2xTMTpro [K	0.0001033	7.389E-05	1	1	1	P49721	P49721 [20-34]	1	2583.4381
[K].VACAEE	1xCarbamido	8.059E-05	7.389E-05	1	1	1	O75663	O75663 [85-95]	0	1668.7971
[K].VADALSI	1xTMTpro [N	0.0001405	7.389E-05	1	1	1	P09105	P09105 [63-73]	0	1447.844
[K].VADKIQL	2xTMTpro [K	9.35E-05	7.389E-05	1	1	2	P00558	P00558 [217-230]	1	2527.5098
[K].VADIGLA	1xTMTpro [N	8.222E-05	7.389E-05	1	1	2	P23526	P23526 [9-19]	0	1432.8232
[R].VADISGI	1xTMTpro [K	0.0001395	7.389E-05	1	1	2	Q7KZF4	Q7KZF4 [504-513	0	1641.9303
[K].VADMAL	1xTMTpro [K	0.0001268	7.389E-05	1	1	3	P50990	P50990 [297-307]	0	1841.0235
[R].TYNASIT	1xTMTpro [K	0.0002043	7.389E-05	1	1	1	P11387	P11387 [591-603]	0	2116.2258
[R].TTPQTLS	1xCarbamido	0.000146	7.389E-05	1	1	1	Q9UKV8	Q9UKV8 [555-566	0	1984.1393
[K].TTPDVIF	1xTMTpro [N	0.0001547	7.389E-05	1	1	3	P62847	P62847 [50-61]	0	1702.9488
[K].TTLTGLF	1xTMTpro [K	6.417E-05	7.389E-05	1	1	1	P24821	P24821 [950-969]	0	2628.5216
[K].TPENYPI	1xCarbamido	6.347E-05	7.389E-05	1	1	2	P00747	P00747 [412-427]	0	2205.0388
[K].TPENYPI	1xCarbamido	8.923E-05	7.389E-05	1	1	2	P00747	P00747 [412-427]	0	2221.0337
[R].TPESQLI	1xTMTpro [N	7.015E-05	7.389E-05	1	1	1	P51178	P51178 [61-76]	0	2195.1515
[R].TPFGAY	1xTMTpro [K	9.754E-05	7.389E-05	1	1	2	P42765	P42765 [15-25]	0	1732.0289
[R].TPFLLSC	1xTMTpro [K	0.0001517	7.389E-05	1	1	2	P55084	P55084 [62-72]	0	1822.0606
[K].TPFLLVC	1xTMTpro [N	0.0001662	7.389E-05	1	1	2	P60953	P60953 [108-120]	0	1777.0543
[R].TPLEQEI	1xTMTpro [K	9.77E-05	7.389E-05	1	2	1	Q9BVJ6	Q9BVJ6 [182-194	0	2190.2778
[K].TPIESLF	1xTMTpro [K	0.000119	7.389E-05	1	1	1	Q13459	Q13459 [1530-15	0	2086.1927
[R].TPLFDQI	1xTMTpro [N	0.0001678	7.389E-05	1	1	2	P54886	P54886 [627-638]	0	1765.9842
[K].TPLLLML	1xTMTpro [N	0.0001914	7.389E-05	1	1	2	P13798	P13798 [665-676]	0	1689.9529
[K].TPLLLML	1xTMTpro [N	9.219E-05	7.389E-05	1	1	1	P13798	P13798 [665-677]	1	1846.054
[R].TPLWIGI	1xTMTpro [N	7.048E-05	7.389E-05	1	1	1	Q9UBG0	Q9UBG0 [1181-1	0	1789.9768
[R].TPMGLL	1xTMTpro [N	0.0001629	7.389E-05	1	1	2	O60831	O60831 [161-178	0	2148.1178
[K].TPEEVIE	1xTMTpro [N	6.726E-05	7.389E-05	1	1	1	O60264	O60264 [876-890	0	2159.098
[K].TPPGVS	1xCarbamido	8.022E-05	7.389E-05	1	1	1	P29558	P29558 [208-222]	0	2175.2339
[R].TPQAPA	1xTMTpro [N	8.598E-05	7.389E-05	1	1	1	Q9UQ35	Q9UQ35 [2329-23	0	1682.9509
[R].TPQMPF	1xTMTpro [K	0.0001295	7.389E-05	1	1	2	Q16666	Q16666 [428-443	0	2362.2608
[K].TPSAAYI	1xTMTpro [K	8.134E-05	7.389E-05	1	1	1	P06396	P06396 [598-615]	0	2446.311
[K].TPSQDS	1xTMTpro [K	0.0001239	7.389E-05	1	1	1	Q05209	Q05209 [81-95]	0	2321.2269
[R].TPTAPA	1xTMTpro [N	7.158E-05	7.389E-05	1	1	2	Q9UQ35	Q9UQ35 [2289-23	0	1542.8923
[K].TPVCHQ	2xCarbamido	0.0001987	7.389E-05	1	1	3	Q9NR12	Q9NR12 [279-287	0	1774.9337

[K].TPVKEPI	2xTMTpro [K	0.0001049	7.389E-05	1	1	1	P61026	P61026 [174-196]	1	3270.7786
[K].TPVLFDI	1xTMTpro [K	6.158E-05	7.389E-05	1	1	2	P50995	P50995 [272-282]	0	1946.1494
[R].TPVQPN	1xTMTpro [K	0.0001681	7.389E-05	1	1	1	O96000	O96000 [17-29]	0	2126.1998
[K].TPVTDP/	1xTMTpro [K	0.000119	7.389E-05	1	1	2	P49748	P49748 [265-276]	0	1765.0351
[R].TPYGAS	1xTMTpro [K	0.0001085	7.389E-05	1	4	2	Q04724	Q04724 [727-737]	0	1807.0246
[K].TPYTIMF	1xTMTpro [K	0.0001178	7.389E-05	1	1	2	P27824	P27824 [183-193]	0	1878.0327
[K].TQAIVC(	1xCarbamido	7.191E-05	7.389E-05	1	1	1	P61221	P61221 [196-210]	0	2376.3565
[K].TPPPVAI	2xTMTpro [K	8.937E-05	7.389E-05	1	1	1	Q9UMS6	Q9UMS6 [755-76	0	2114.3518
[K].TQASSS	1xTMTpro [K	0.0001043	7.389E-05	1	1	1	P46087	P46087 [670-685]	0	2234.1545
[K].TPEDGD	1xTMTpro [K	7.899E-05	7.389E-05	1	1	1	P46821	P46821 [1932-194	0	2267.1575
[K].TPEAGL/	1xTMTpro [N	0.0001279	7.389E-05	1	1	1	Q8WUP2	Q8WUP2 [51-68]	0	2155.1579
[R].TMLELLI	1xTMTpro [N	9.021E-05	7.389E-05	1	1	2	P62191	P62191 [308-322]	0	2056.0704
[R].TMLELLI	1xTMTpro [K	0.0002047	7.389E-05	1	1	2	P17980	P17980 [309-327]	0	2797.5414
[K].TMLETEI	1xTMTpro [N	0.0001284	7.389E-05	1	1	1	O60610	O60610 [233-246]	0	1921.0999
[R].TMIQQQ	1xTMTpro [K	0.0001109	7.389E-05	1	1	2	Q8N4T8	Q8N4T8 [121-140	0	2637.5253
[K].TMNVLA	1xTMTpro [K	0.0001153	7.389E-05	1	1	1	Q8IWE2	Q8IWE2 [238-250	0	2017.092
[K].TMQFEP	1xCarbamido	0.0001576	7.389E-05	1	1	1	Q9Y490	Q9Y490 [16-30]	0	2139.9833
[K].TMQFEP	1xCarbamido	0.0001417	7.389E-05	1	1	1	Q9Y490	Q9Y490 [16-30]	0	2155.9782
[R].TMQNTN	1xTMTpro [N	8.545E-05	7.389E-05	1	1	1	P35222	P35222 [201-212]	0	1683.8291
[K].TMTELEI	1xTMTpro [N	6.939E-05	7.389E-05	1	1	2	P45974	P45974 [292-303]	0	1784.8842
[K].TNADTD	1xTMTpro [K	0.0001378	7.389E-05	1	1	2	P09622	P09622 [431-440]	0	1659.8867
[K].TNANAE	2xTMTpro [K	7.508E-05	7.389E-05	1	1	1	Q9UMR2	Q9UMR2 [41-56]	2	2791.4526
[K].TNDDQT	2xCarbamido	6.129E-05	7.389E-05	1	1	5	P35555	P35555 [1882-18	0	2333.9967
[K].TNDEVIH	1xTMTpro [K	8.272E-05	7.389E-05	1	1	1	Q9Y5L0	Q9Y5L0 [356-366	0	1881.0726
[R].TPEDFVI	1xTMTpro [N	7.135E-05	7.389E-05	1	1	2	O95376	O95376 [192-207]	0	2220.1872
[K].TNEAQA	1xTMTpro [N	0.00015	7.389E-05	1	2	3	P61088	P61088 [131-141]	0	1507.8036
[R].TNHIYVS	1xTMTpro [K	0.0001131	7.389E-05	1	1	1	Q6P2Q9	Q6P2Q9 [2087-20	0	2000.0944
[K].TNLESIL	1xTMTpro [K	9.335E-05	7.389E-05	1	1	2	P05155	P05155 [191-201]	0	1873.0926
[R].TNLIVNY	1xOxidation [	0.0001827	7.389E-05	1	1	1	Q15717	Q15717 [20-37]	0	2482.2931
[K].TNQVNS	1xTMTpro [N	9.739E-05	7.389E-05	1	1	1	P02747	P02747 [199-210]	0	1561.8981
[R].TNSTFN	1xTMTpro [K	6.327E-05	7.389E-05	1	1	2	Q07020	Q07020 [39-49]	0	1859.0882
[R].TPAEVV	2xCarbamido	7.015E-05	7.389E-05	1	1	1	Q49A26	Q49A26 [316-335	0	2805.4294
[K].TPAFAES	1xTMTpro [N	6.685E-05	7.389E-05	1	1	3	P36957	P36957 [75-88]	0	1782.9193

[R].TPAGNF	1xTMTpro [K	0.0001182	7.389E-05	1	1	1	P43490	P43490 [435-447]	0	1971.1043
[K].TPAQFD.	1xTMTpro [N	9.847E-05	7.389E-05	1	1	4	P04083	P04083 [114-124]	0	1566.8083
[R].TPAQSG	1xTMTpro [N	6.685E-05	7.389E-05	1	1	1	O00267	O00267 [814-831]	0	2214.0859
[K].TPAQYD	1xTMTpro [K	9.467E-05	7.389E-05	1	2	5	P07355	P07355 [105-115]	0	1831.0093
[R].TPCQQE	1xCarbamido	0.0001142	7.389E-05	1	1	2	P18065	P18065 [225-237]	0	1919.9816
[K].TPDKVN	2xTMTpro [K	0.0001134	7.389E-05	1	1	1	P09668	P09668 [275-291]	1	2710.5708
[R].TNGKEP	1xTMTpro [K	0.0001216	7.389E-05	1	1	1	P46778	P46778 [143-160]	1	2686.4294
[R].TMFSLD	1xTMTpro [K	6.875E-05	7.389E-05	1	1	3	P12111	P12111 [292-302]	0	1902.0174
[R].TQDECIL	1xCarbamido	0.0001352	7.389E-05	2	2	1	Q8TAQ2; Q9	Q8TAQ2 [631-64	0	1735.9121
[K].TQEFPQ	1xTMTpro [N	0.0001038	7.389E-05	1	1	2	Q9NZ08	Q9NZ08 [829-841	0	1820.0601
[K].TSIRDPC	3xCarbamido	0.0001153	7.389E-05	1	1	1	P54289	P54289 [828-842]	1	2344.1703
[K].TSLSALT	1xTMTpro [K	8.937E-05	7.389E-05	1	1	3	O00186	O00186 [322-332]	0	1801.0749
[R].TSIYPFL	1xTMTpro [N	0.0001479	7.389E-05	1	1	1	P43251	P43251 [123-139]	0	2301.2272
[K].TSNFNA	1xTMTpro [K	8.745E-05	7.389E-05	1	1	2	P02748	P02748 [232-242]	0	1774.0355
[R].TSPDLVI	1xTMTpro [N	0.0001132	7.389E-05	1	1	1	O94973	O94973 [178-191]	0	1865.9387
[K].TSQEGS	1xTMTpro [N	6.119E-05	7.389E-05	1	1	2	P46100	P46100 [870-881]	0	1585.7261
[R].TSSPAQ	1xTMTpro [N	7.803E-05	7.389E-05	1	1	1	Q9Y342	Q9Y342 [12-34]	0	2620.4014
[R].TSTADY.	1xTMTpro [K	0.0001301	7.389E-05	1	1	9	P02679	P02679 [283-292]	0	1742.9279
[R].TSTDVM	1xTMTpro [K	0.0001593	7.389E-05	1	1	1	Q08AF3	Q08AF3 [123-138	0	2364.2612
[K].TSTGAP.	1xTMTpro [K	0.0001315	7.389E-05	1	1	2	P40121	P40121 [128-137]	0	1524.9241
[R].TSTSSS	1xTMTpro [N	7.247E-05	7.389E-05	1	1	3	Q9UKV3	Q9UKV3 [697-707	0	1414.7457
[K].TSTTSSI	1xTMTpro [N	9.647E-05	7.389E-05	1	2	1	P41236	P41236 [19-33]	0	1856.9343
[K].TSVLEMI	1xTMTpro [N	9.021E-05	7.389E-05	1	1	2	O60313	O60313 [302-312]	0	1522.8582
[R].TSIIEAQ	1xTMTpro [N	0.0001719	7.389E-05	1	1	1	Q14690	Q14690 [437-446]	0	1497.8596
[R].TSVQPT	1xTMTpro [N	0.0001599	7.389E-05	1	1	1	Q5T4S7	Q5T4S7 [3845-38	0	1789.9404
[R].TTAENEI	1xTMTpro [K	7.202E-05	7.389E-05	1	1	2	P13647	P13647 [266-276]	0	1891.0491
[K].TTCMSS	1xCarbamido	0.0001759	7.389E-05	1	1	1	Q9P0V9	Q9P0V9 [20-35]	1	2451.2099
[R].TTDDLTI	1xTMTpro [K	8.937E-05	7.389E-05	1	1	3	O60234	O60234 [125-137]	0	2158.1523
[R].TTDLLTD	1xTMTpro [K	0.0001817	7.389E-05	1	1	2	Q14204	Q14204 [1254-12	0	1830.0141
[R].TTEEDA	1xCarbamido	0.0001258	7.389E-05	1	1	1	O00533	O00533 [484-502]	0	2725.3271
[K].TTEMETI	1xTMTpro [K	0.0001258	7.389E-05	1	1	4	Q9Y230	Q9Y230 [165-177	0	2110.1233
[R].TTFNSS	1xTMTpro [N	0.0001458	7.389E-05	1	2	1	P54821	P54821 [99-111]	0	1798.9618
[K].TTGEEN	1xTMTpro [K	8.298E-05	7.389E-05	1	1	1	O00429	O00429 [78-92]	0	2244.1276

[K].TTHFVE(	1xTMTpro [N	0.0001442	7.389E-05	1	1	1	P18124	P18124 [224-236]	0	1664.8312
[R].TTLLAGI	1xTMTpro [N	9.453E-05	7.389E-05	1	1	2	Q07954	Q07954 [1379-13!	0	1626.9134
[K].TTLLEGF	1xTMTpro [N	7.303E-05	7.389E-05	1	1	2	P35658	P35658 [770-783]	0	1796.9713
[K].TTIIWDA	1xTMTpro [K	6.95E-05	7.389E-05	2	3	1	O60907; Q9I	O60907 [352-364]	0	2051.1417
[K].TTITEPH	1xTMTpro [K	6.57E-05	7.389E-05	1	1	1	Q6P2Q9	Q6P2Q9 [1959-19	0	3055.6384
[R].TTAAMW	1xTMTpro [K	0.0001297	7.389E-05	1	1	2	Q6UW68	Q6UW68 [110-12	0	2058.1549
[K].TQDQQS	1xTMTpro [N	8.26E-05	7.389E-05	1	1	1	P06401	P06401 [74-89]	0	2088.0165
[R].TSLGNV	1xTMTpro [K	0.000171	7.389E-05	1	1	2	O60231	O60231 [758-768]	0	1765.1443
[R].TSLATIL	1xTMTpro [K	6.438E-05	7.389E-05	1	1	1	Q8IXH7	Q8IXH7 [207-222]	0	2298.2684
[R].TQEQCV	1xCarbamido	7.66E-05	7.389E-05	1	1	1	O60313	O60313 [782-797]	1	2899.54
[R].TQEVLQ	1xTMTpro [K	0.0001852	7.389E-05	1	1	1	Q02952	Q02952 [1039-10	0	1824.0722
[R].TQFEDC	1xCarbamido	0.0001144	7.389E-05	1	1	1	P33527	P33527 [1474-14	0	1995.0289
[R].TQFNSL	1xTMTpro [K	0.0001224	7.389E-05	1	1	1	P12931	P12931 [221-235]	0	2398.3262
[R].TQGLVP	1xTMTpro [N	0.00011	7.389E-05	1	1	1	Q9NZM1	Q9NZM1 [1734-1	0	1669.9193
[R].TQHIGN	1xTMTpro [K	7.015E-05	7.389E-05	1	1	1	P08575	P08575 [959-969]	0	1906.0274
[K].TQIGMTI	1xTMTpro [K	0.0001644	7.389E-05	1	1	1	Q8WXH0	Q8WXH0 [1759-1	0	1829.0698
[K].TQMDGN	1xTMTpro [N	0.0001084	7.389E-05	1	1	1	P15586	P15586 [389-401]	0	1778.9828
[K].TQPAVA	1xTMTpro [K	0.0001463	7.389E-05	1	1	1	P51608	P51608 [436-449]	0	1938.1152
[K].TQPILDII	1xTMTpro [K	0.0001723	7.389E-05	1	1	1	Q9Y376	Q9Y376 [288-297	0	1762.1334
[R].TQQYDD	1xTMTpro [K	7.439E-05	7.389E-05	1	1	1	P23368	P23368 [228-240]	0	2254.1557
[K].TQSSLVI	1xTMTpro [N	0.000131	7.389E-05	1	1	3	O60888	O60888 [125-138]	0	1838.0343
[K].TQVAGG	1xTMTpro [K	0.0001896	7.389E-05	1	1	1	P46060	P46060 [16-26]	0	1744.0249
[K].TSLDWW	1xTMTpro [K	0.0001584	7.389E-05	1	1	2	Q14108	Q14108 [235-244]	0	1873.0351
[R].TQVELV	1xTMTpro [N	0.0001675	7.389E-05	1	1	2	Q96T60	Q96T60 [49-60]	0	1661.9029
[K].TQVTVQ	1xTMTpro [N	0.0001596	7.389E-05	1	1	2	P31153	P31153 [182-192]	0	1672.8648
[R].TRPEFYI	1xTMTpro [K	0.0001359	7.389E-05	1	1	2	P12111	P12111 [1076-10	0	2271.2087
[K].TSAALST	1xTMTpro [N	9.408E-05	7.389E-05	1	1	4	O43399	O43399 [140-153]	0	1624.9189
[R].TSACGL	2xCarbamido	9.119E-05	7.389E-05	2	2	4	P30154; P30	P30154 [157-169]	0	1821.8947
[K].TSATWL	1xTMTpro [N	0.0001929	7.389E-05	1	1	3	P05023	P05023 [414-423]	0	1409.8072
[K].TSDLIVL	1xTMTpro [K	0.0001049	7.389E-05	1	1	3	Q13148	Q13148 [103-114]	0	1950.192
[K].TSDQIHF	1xTMTpro [K	0.0001742	7.389E-05	1	1	3	P01008	P01008 [147-157]	0	1949.0777
[R].TSEEISL	1xTMTpro [K	7.18E-05	7.389E-05	1	1	1	Q14789	Q14789 [648-662]	0	2213.2157
[K].TSEVDL	2xTMTpro [K	6.581E-05	7.389E-05	1	1	2	Q8WUM4	Q8WUM4 [12-23]	0	2212.3733

[K].TSEVQE	1xTMTpro [K	0.0001136	7.389E-05	1	1	1	Q9UBC2	Q9UBC2 [418-442	1	3498.8055
[R].TSFDKPI	2xTMTpro [K	0.0001933	7.389E-05	1	1	1	Q9Y2A7	Q9Y2A7 [882-895	0	2511.4097
[R].TSGGAG	1xTMTpro [N	0.0001101	7.389E-05	1	1	2	P17661	P17661 [59-70]	0	1336.7504
[R].TSGSVY	1xTMTpro [K	0.0001647	7.389E-05	1	1	2	P37108	P37108 [22-31]	0	1677.0078
[R].TQVSTQ	1xTMTpro [N	0.0001085	7.389E-05	1	1	2	P06727	P06727 [144-154]	0	1564.8614
[K].VANQIKE	2xTMTpro [K	0.0001602	7.389E-05	1	1	1	Q9Y6Y8	Q9Y6Y8 [905-915	1	2229.2907
[R].TAAEND	1xTMTpro [K	6.427E-05	7.389E-05	1	1	1	P35908	P35908 [276-286]	0	1817.03
[K].SYSVGA	1xTMTpro [N	6.129E-05	7.389E-05	1	1	1	Q8N684	Q8N684 [407-418	0	1448.7301
[K].SLFPENI	2xTMTpro [K	9.693E-05	7.389E-05	1	1	1	Q12965	Q12965 [544-555]	1	2302.3587
[R].SLFSEE/	1xTMTpro [K	0.0001301	7.389E-05	1	1	1	Q9HD15	Q9HD15 [195-206	0	1962.0312
[R].SLGAWN	1xTMTpro [N	0.000153	7.389E-05	1	1	1	P48681	P48681 [894-904]	0	1576.8767
[R].SLGEISA	1xTMTpro [K	7.359E-05	7.389E-05	1	1	2	P18206	P18206 [434-444]	0	1714.0242
[R].SLGFCD	1xCarbamido	0.0001026	7.389E-05	1	1	1	P02745	P02745 [186-195]	0	1750.9289
[K].SLGGIEC	1xCarbamido	0.0001028	7.389E-05	1	1	2	Q5R3I4	Q5R3I4 [46-56]	0	1728.981
[K].SLGPPG	1xTMTpro [N	0.0002015	7.389E-05	1	1	1	Q9NZM1	Q9NZM1 [1770-17	0	1753.992
[K].SLGQILE	1xTMTpro [N	0.0001184	7.389E-05	1	1	1	Q8NDA8	Q8NDA8 [286-300	0	1791.0295
[K].SIGTCNF	1xCarbamido	8.505E-05	7.389E-05	1	1	1	Q96SI9	Q96SI9 [250-265]	0	1976.0667
[K].SLGTDLI	1xTMTpro [N	8.336E-05	7.389E-05	1	1	1	P05556	P05556 [164-174]	0	1570.7888
[R].SLGWVE	1xTMTpro [K	8.059E-05	7.389E-05	1	1	1	Q92870	Q92870 [423-438]	0	2340.2401
[R].SLHDAL(	1xCarbamido	0.0001354	7.389E-05	2	2	3	P50991; P48	P50991 [404-413]	0	1487.8324
[R].SLHDAIN	1xTMTpro [N	0.0001653	7.389E-05	1	1	1	Q99832	Q99832 [388-397]	0	1458.8422
[R].SLHSELI	2xCarbamido	7.985E-05	7.389E-05	2	2	1	Q99496; Q0	Q99496 [44-59]; C	0	2555.335
[K].SLKDED'	2xTMTpro [K	0.0001458	7.389E-05	1	1	2	Q9NZ01	Q9NZ01 [58-67]	1	2087.2528
[K].SIKDTICI	1xCarbamido	0.0001279	7.389E-05	1	1	1	Q9ULV4	Q9ULV4 [450-461	1	2087.1047
[K].SLKEES'	2xTMTpro [K	8.388E-05	7.389E-05	1	1	1	P48681	P48681 [809-819]	1	2131.279
[R].SLKPWD	2xTMTpro [K	0.0001492	7.389E-05	1	1	1	O95436	O95436 [599-609]	0	2142.3103
[R].SILAETE	1xTMTpro [N	0.0001514	7.389E-05	1	1	2	Q9P2E9	Q9P2E9 [1133-11	0	1523.8422
[K].SILALVD	2xTMTpro [K	0.0001031	7.389E-05	1	1	1	Q9NYK5	Q9NYK5 [103-121	0	3087.7733
[R].SLIANLA	1xCarbamido	0.000119	7.389E-05	1	1	1	P55263	P55263 [150-162]	0	2017.1396
[K].SLLDASE	2xTMTpro [K	0.0001995	7.389E-05	1	1	1	P18206	P18206 [721-732]	1	2216.3318
[K].SILDWTS	1xTMTpro [N	0.00012	7.389E-05	1	1	2	P06280	P06280 [241-252]	0	1799.9247
[K].SLIEEGC	1xTMTpro [N	8.492E-05	7.389E-05	1	1	1	Q15008	Q15008 [166-177]	1	1736.8887
[K].SLLGKD'	2xTMTpro [K	6.674E-05	7.389E-05	2	2	1	P07205; P0C	P07205 [87-97]; F	1	2145.3827

[R].SILGTLT	1xTMTpro [N	0.0001468	7.389E-05	1	1	2	Q14254	Q14254 [113-127]	0	2040.1296
[R].SILGTLT	1xTMTpro [K	0.0001165	7.389E-05	1	1	1	Q14254	Q14254 [113-132]	1	2933.6228
[K].SLFIDTY	1xTMTpro [N	0.000146	7.389E-05	1	1	1	Q9UK99	Q9UK99 [77-88]	0	1676.8815
[K].SIFDDDN	1xTMTpro [K	6.95E-05	7.389E-05	1	1	1	Q9Y4E1	Q9Y4E1 [1268-12	0	2626.3202
[R].SLESVYS	1xTMTpro [N	0.0001922	7.389E-05	1	1	1	Q13315	Q13315 [2162-21	0	1919.0445
[K].SLESTTL	1xTMTpro [K	0.0001542	7.389E-05	1	1	6	Q16527	Q16527 [152-161]	0	1716.9875
[K].SLANAES	1xTMTpro [N	0.0001629	7.389E-05	1	1	1	Q9HCN4	Q9HCN4 [279-289	0	1535.8097
[R].SLASVPA	1xTMTpro [K	0.0001638	7.389E-05	1	1	1	Q9NR99	Q9NR99 [45-55]	0	1622.0133
[K].SLATGG	1xTMTpro [K	0.0001273	7.389E-05	1	1	1	Q8N3P4	Q8N3P4 [454-471	0	2323.3477
[R].SIAVLEL	1xCarbamido	6.529E-05	7.389E-05	1	1	1	Q9UKN8	Q9UKN8 [85-106]	0	2802.5256
[K].SLAVSGI	1xTMTpro [N	0.0001716	7.389E-05	1	1	1	Q02880	Q02880 [488-499]	0	1432.8807
[K].SLCLGPA	1xCarbamido	8.664E-05	7.389E-05	1	1	1	Q99643	Q99643 [105-117]	0	1989.1811
[R].SICTTVL	1xCarbamido	9.8E-05	7.389E-05	1	1	2	P27348	P27348 [92-103]	0	2000.1593
[K].SLDDEVI	1xTMTpro [K	9.453E-05	7.389E-05	1	1	2	Q14141	Q14141 [388-397]	0	1745.9565
[K].SLDEISQ	1xTMTpro [K	6.347E-05	7.389E-05	1	1	4	P12111	P12111 [561-573]	0	2066.1625
[K].SLDEQC	1xCarbamido	0.0001508	7.389E-05	1	1	2	O15162	O15162 [249-258]	0	1742.9602
[R].SLDGIPF	1xCarbamido	6.843E-05	7.389E-05	1	1	1	Q93052	Q93052 [508-530]	0	3149.6585
[R].SLDMDS	1xOxidation [	6.928E-05	7.389E-05	1	1	4	P05787	P05787 [253-264]	0	1945.0808
[R].SLDPAD	1xTMTpro [K	0.0001373	7.389E-05	1	1	1	Q8WXI9	Q8WXI9 [17-30]	1	2152.1741
[R].SLLINAV	1xCarbamido	0.0001363	7.389E-05	2	2	2	Q96C36; P3	Q96C36 [252-264	0	1749.9852
[K].SIDSNPY	1xTMTpro [K	6.832E-05	7.389E-05	1	1	2	P46459	P46459 [106-116]	0	1862.9627
[K].SIEDFISC	2xCarbamido	0.0001075	7.389E-05	1	1	1	O94874	O94874 [634-654]	0	2998.4703
[K].SLEDLQI	1xTMTpro [K	7.169E-05	7.389E-05	1	1	4	P51884	P51884 [138-148]	0	1906.089
[K].SIEDMTE	1xTMTpro [K	0.0001056	7.389E-05	1	1	1	P14735	P14735 [873-884]	0	2036.0502
[K].SLEEECI	2xCarbamido	0.0002027	7.389E-05	1	1	1	Q6ZMI0	Q6ZMI0 [363-378]	0	2175.0229
[K].SLEEEEI	1xTMTpro [K	0.0001484	7.389E-05	1	1	1	Q7Z478	Q7Z478 [251-263]	1	2230.1483
[K].SLEEFMA	1xCarbamido	9.542E-05	7.389E-05	1	1	1	Q9C0E2	Q9C0E2 [1136-11	0	2375.2958
[K].SLEELRL	1xTMTpro [N	0.0001511	7.389E-05	1	1	1	P52948	P52948 [199-212]	1	2040.0681
[K].SLEEIYL	1xTMTpro [K	0.0001028	7.389E-05	1	1	2	P15880	P15880 [77-89]	0	2160.2812
[K].SIEEQQI	1xTMTpro [K	9.77E-05	7.389E-05	1	1	1	Q9UKV8	Q9UKV8 [242-255	0	2267.2487
[R].SLENYH	1xTMTpro [K	0.0001147	7.389E-05	1	1	1	Q14677	Q14677 [110-122]	0	2183.1377
[R].SLEPLPS	1xTMTpro [K	9.925E-05	7.389E-05	1	1	2	Q9Y512	Q9Y512 [8-36]	0	3624.8453
[K].SLEQQLA	1xTMTpro [N	0.000144	7.389E-05	1	1	1	Q9BZF9	Q9BZF9 [1365-13	0	1549.8141

[K].SLESQVI	1xTMTpro [K	0.000123	7.389E-05	1	1	4	P12270	P12270 [1492-150	0	1883.073
[K].SLEDALS	1xTMTpro [N	9.32E-05	7.389E-05	1	1	1	P08133	P08133 [484-498]	0	1925.9524
[R].SILLSVP	1xTMTpro [K	0.0001186	7.389E-05	1	1	2	P53621	P53621 [1031-104	0	2118.3394
[K].SLLQALN	1xTMTpro [K	0.0001877	7.389E-05	1	1	2	Q14204	Q14204 [3748-375	0	1723.0609
[K].SLLSAEE	1xTMTpro [K	0.0001453	7.389E-05	1	1	2	Q7KZF4	Q7KZF4 [642-651	0	1626.9558
[K].SLTGLLS	1xTMTpro [K	6.706E-05	7.389E-05	1	1	1	Q5T4S7	Q5T4S7 [4240-42	0	2317.351
[R].SITNTTV	1xCarbamido	0.0001237	7.389E-05	1	1	2	Q15637	Q15637 [272-281]	0	1732.9759
[K].SLVALLV	1xTMTpro [K	9.894E-05	7.389E-05	2	2	2	Q8TAQ2; Q9	Q8TAQ2 [886-897	0	1940.1746
[K].SLVALLV	1xOxidation [	0.000118	7.389E-05	2	2	1	Q8TAQ2; Q9	Q8TAQ2 [886-897	0	1956.1695
[R].SIVDNW	1xTMTpro [K	0.0001522	7.389E-05	1	1	1	P23368	P23368 [145-156]	0	2046.1264
[R].SLVDYEI	1xTMTpro [K	0.0002007	7.389E-05	1	1	4	Q9UNH7	Q9UNH7 [316-325	0	1760.9674
[K].SLVEIAD	1xTMTpro [K	9.379E-05	7.389E-05	1	1	2	O00410	O00410 [239-249]	0	1780.0712
[R].SLVEIIEI	1xTMTpro [K	6.581E-05	7.389E-05	1	1	2	O75533	O75533 [685-700]	0	2445.3845
[R].SLVESVS	2xTMTpro [K	0.0001007	7.389E-05	1	1	1	Q9H2U2	Q9H2U2 [309-334	1	3859.033
[R].SLVPAAI	1xTMTpro [N	0.0001132	7.389E-05	1	1	2	Q15149	Q15149 [3116-31	0	1588.9229
[K].SIVTAEV	1xCarbamido	0.0001392	7.389E-05	1	1	1	Q86SZ2	Q86SZ2 [137-150	0	2088.1325
[K].SIWDEFI	1xTMTpro [K	0.0001678	7.389E-05	1	1	1	Q9NY35	Q9NY35 [49-62]	0	2292.1527
[R].SIYAVFE	1xTMTpro [K	0.0001147	7.389E-05	1	1	1	P16671	P16671 [274-286]	0	2093.1774
[K].SITGEEN	1xTMTpro [K	0.0001235	7.389E-05	1	1	1	Q9NZM1	Q9NZM1 [1806-18	0	2080.1128
[R].SIYDDIS	1xTMTpro [N	7.303E-05	7.389E-05	1	1	1	Q8NFH5	Q8NFH5 [93-111]	0	2270.1835
[K].SIYYITGI	1xTMTpro [K	8.84E-05	7.389E-05	1	2	4	P08238	P08238 [482-491]	0	1768.9977
[K].SLYYYIQ	1xTMTpro [K	7.936E-05	7.389E-05	1	2	6	P07355	P07355 [314-324]	0	2030.109
[R].SMAEDT	1xTMTpro [K	0.000121	7.389E-05	1	1	1	P43304	P43304 [442-453]	0	1858.0236
[R].SMAEDT	1xOxidation [	0.000159	7.389E-05	1	1	1	P43304	P43304 [442-453]	0	1874.0185
[K].SMAEQIE	1xTMTpro [N	0.0001326	7.389E-05	1	1	1	O00178	O00178 [122-135]	0	1892.0482
[R].SMANPP	1xTMTpro [K	0.0001161	7.389E-05	1	1	2	Q14204	Q14204 [3309-33	0	1593.9278
[K].SMEMLV	1xTMTpro [N	0.0001726	7.389E-05	1	1	1	Q9UNM6	Q9UNM6 [362-37	0	2058.0319
[K].SMENYY	1xTMTpro [N	0.0001331	7.389E-05	1	1	2	P46063	P46063 [394-404]	0	1667.7655
[R].SMGFIGI	1xTMTpro [K	8.401E-05	7.389E-05	1	1	1	P53396	P53396 [1066-107	0	2004.0868
[R].SMGGA/	1xTMTpro [K	7.743E-05	7.389E-05	1	1	1	Q96I25	Q96I25 [169-184]	0	2137.2182
[K].SMGLPT	1xTMTpro [K	7.96E-05	7.389E-05	1	1	2	Q9Y266	Q9Y266 [298-308	0	1800.9657
[K].SMLDQL	1xTMTpro [K	6.208E-05	7.389E-05	1	1	4	Q9BRX8	Q9BRX8 [100-114	0	2241.3172
[R].SMLQDR	1xCarbamido	8.479E-05	7.389E-05	2	2	1	P35749; Q7Z	P35749 [164-184]	1	2906.4479

[K].SLYESF\	1xTMTpro [N	0.0001322	7.389E-05	1	1	1	P18615	P18615 [131-142]	0	1680.84
[K].SLAMLG	1xOxidation	6.347E-05	7.389E-05	1	1	2	Q13596	Q13596 [350-365]	0	1971.9976
[K].SLSSPTI	1xTMTpro [N	8.492E-05	7.389E-05	1	1	2	Q9Y2X7	Q9Y2X7 [359-372	0	1836.0034
[R].SLSNYPI	1xTMTpro [N	9.162E-05	7.389E-05	1	1	2	P49961	P49961 [155-167]	0	1805.9142
[K].SLLSNLI	2xTMTpro [K	9.077E-05	7.389E-05	1	1	1	Q8WUM4	Q8WUM4 [554-56	1	2158.3263
[R].SILSPGC	1xCarbamido	7.779E-05	7.389E-05	1	1	2	P78347	P78347 [207-219]	0	1881.0759
[K].SIITYVSS	1xTMTpro [N	7.247E-05	7.389E-05	1	1	3	Q15149	Q15149 [387-401]	0	2020.0744
[K].SLNEEA\	2xTMTpro [K	0.0002047	7.389E-05	1	1	1	P01023	P01023 [1169-117	1	1930.1789
[K].SINNDTT	1xCarbamido	0.0001647	7.389E-05	1	1	1	Q53H82	Q53H82 [92-102]	0	1937.0294
[K].SLNQSL/	1xTMTpro [K	0.0001116	7.389E-05	1	1	4	P54709	P54709 [8-17]	0	1784.0198
[R].SIPAGAE	1xTMTpro [K	0.0001262	7.389E-05	1	1	1	Q9H910	Q9H910 [132-142	0	1663.9511
[R].SLPEVPI	1xTMTpro [N	0.0001153	7.389E-05	1	1	2	P98160	P98160 [4222-42	0	1915.0707
[K].SLPFGA(	1xTMTpro [N	0.0001007	7.389E-05	1	1	1	Q9UHL4	Q9UHL4 [113-12	0	1495.8188
[K].SLPGAEI	1xTMTpro [K	0.0001258	7.389E-05	1	1	2	O75718	O75718 [190-199]	0	1700.9715
[K].SLPGQN	1xTMTpro [K	7.314E-05	7.389E-05	1	1	1	P01040	P01040 [72-89]	0	2584.4114
[K].SIPLAMA	1xTMTpro [K	8.324E-05	7.389E-05	1	1	2	Q9UBF2	Q9UBF2 [578-590	0	2039.1855
[K].SLPQEW	2xCarbamido	0.0001723	7.389E-05	1	1	1	Q9NYU2	Q9NYU2 [1467-14	0	2919.3937
[K].SLSPQD	1xCarbamido	0.000129	7.389E-05	1	1	1	Q63HN8	Q63HN8 [1126-11	0	2437.1625
[R].SLPSTSS	1xTMTpro [K	6.601E-05	7.389E-05	1	1	2	Q12857	Q12857 [265-276]	0	1790.9991
[R].SLPVPQ	1xTMTpro [K	0.0001206	7.389E-05	1	1	1	O00154	O00154 [341-355]	0	2289.2834
[R].SIPYDQS	1xTMTpro [K	0.0001817	7.389E-05	1	1	1	Q9H3K2	Q9H3K2 [179-189	0	1797.0038
[K].SLQADT	1xTMTpro [K	9.091E-05	7.389E-05	1	1	1	Q8IUD2	Q8IUD2 [603-627]	1	3329.7568
[K].SIQCLTV	1xCarbamido	0.0001144	7.389E-05	1	1	1	O75083	O75083 [322-330]	0	1693.9915
[R].SIQEAP\	1xTMTpro [N	7.555E-05	7.389E-05	1	1	3	Q5JTV8	Q5JTV8 [179-192	0	1860.0398
[K].SLQEEP	1xTMTpro [N	9.663E-05	7.389E-05	1	1	1	Q712K3	Q712K3 [19-29]	0	1594.8396
[K].SLQETIC	1xTMTpro [K	0.0001273	7.389E-05	1	1	1	Q6NUQ4	Q6NUQ4 [435-44	0	1755.0508
[R].SLQLTGI	1xTMTpro [K	0.0001237	7.389E-05	1	1	1	Q9H270	Q9H270 [74-84]	0	1864.0824
[R].SLQTLAI	1xTMTpro [N	0.0001168	7.389E-05	1	1	1	Q8N766	Q8N766 [244-257	0	1993.0925
[R].SLQYGE	1xCarbamido	7.875E-05	7.389E-05	1	1	1	O75165	O75165 [2169-21	0	2013.0394
[K].SISCQEI	1xCarbamido	8.965E-05	7.389E-05	1	1	3	P27918	P27918 [334-346]	0	1793.9135
[R].SLSLCNI	1xCarbamido	7.258E-05	7.389E-05	1	1	1	Q9Y2A7	Q9Y2A7 [595-608	0	2267.173
[R].SISLYYT	1xTMTpro [K	9.863E-05	7.389E-05	1	1	3	P19338	P19338 [458-467]	0	1768.9977
[K].SLPTPA\	1xTMTpro [K	0.0001335	7.389E-05	1	1	1	Q8WX93	Q8WX93 [632-64	0	1932.2025

[K].SLAMLG	1xTMTpro [N	0.0001445	7.389E-05	1	1	1	Q13596	Q13596 [350-365]	0	1956.0027
[K].SIAFPAI(	1xTMTpro [K	0.0001558	7.389E-05	1	1	1	Q460N5	Q460N5 [1126-11	0	2198.2829
[K].SLAEGYI	1xTMTpro [N	0.0001075	7.389E-05	1	1	2	O43813	O43813 [16-27]	0	1560.7977
[R].SELNQV	1xCarbamido	8.531E-05	7.389E-05	1	1	1	P14780	P14780 [686-707]	0	2889.3783
[R].SELPIAE	1xTMTpro [N	0.0001528	7.389E-05	1	1	1	Q9H7D7	Q9H7D7 [602-614	0	1727.9611
[R].SEISLLP	1xTMTpro [N	0.0001299	7.389E-05	1	1	1	P43304	P43304 [616-627]	0	1648.9077
[R].SENEEF	1xTMTpro [N	7.839E-05	7.389E-05	1	1	1	P10644	P10644 [307-317]	0	1598.7981
[K].SEPVSEI	1xTMTpro [K	0.0001789	7.389E-05	1	1	1	Q9HAU0	Q9HAU0 [828-840	0	2012.1407
[K].SESTSG.	1xOxidation	0.0001605	7.389E-05	1	1	1	Q6UXV4	Q6UXV4 [235-249	0	2207.1146
[K].SESVPP	1xTMTpro [K	6.227E-05	7.389E-05	1	1	3	P35613	P35613 [244-257]	0	2273.2098
[R].SETITEE	1xOxidation	6.758E-05	7.389E-05	1	1	1	P16152	P16152 [160-174]	0	2317.2453
[K].SETVLT(	1xCarbamido	0.0001539	7.389E-05	1	1	1	P26006	P26006 [898-908]	0	1498.7855
[K].SEVEVD	1xCarbamido	6.129E-05	7.389E-05	1	1	1	P04275	P04275 [2741-275	0	2172.1251
[K].SEVPLVI	1xTMTpro [K	9.647E-05	7.389E-05	1	1	2	O43795	O43795 [1027-10	0	1695.0184
[R].SEVTFL/	1xTMTpro [K	9.941E-05	7.389E-05	1	1	1	Q96GK7	Q96GK7 [94-107]	0	2168.2571
[R].SEYDSLI	1xTMTpro [N	7.026E-05	7.389E-05	1	1	1	P23368	P23368 [560-584]	0	3114.5002
[K].SELLSQI	1xTMTpro [N	9.693E-05	7.389E-05	1	1	1	Q92665	Q92665 [175-189]	0	2117.0794
[R].SEYGAA	1xTMTpro [K	0.0001007	7.389E-05	1	1	2	P13671	P13671 [449-459]	0	1833.0038
[K].SFDCEE	3xCarbamido	0.0001281	7.389E-05	1	1	1	P24043	P24043 [976-994]	0	2840.2934
[R].SFDGFS	1xTMTpro [K	7.416E-05	7.389E-05	1	1	2	Q9Y5Z4	Q9Y5Z4 [141-150	0	1681.9041
[R].SFDVNK	1xCarbamido	8.427E-05	7.389E-05	1	2	4	P41091	P41091 [261-275]	0	2635.4218
[R].SFEGMV	1xTMTpro [N	6.57E-05	7.389E-05	1	1	2	Q13641	Q13641 [194-207]	0	1724.9688
[K].SFEGNN	1xTMTpro [N	9.291E-05	7.389E-05	1	1	2	O14786	O14786 [539-552]	0	1959.9368
[R].SFGGEV	1xTMTpro [K	0.0001363	7.389E-05	1	1	2	O00541	O00541 [349-358]	0	1719.9198
[K].SFLAMV	1xOxidation	7.314E-05	7.389E-05	1	1	1	O00764	O00764 [89-102]	0	2216.2856
[R].SFLESID	1xTMTpro [K	0.0001178	7.389E-05	1	1	2	Q15750	Q15750 [116-128]	0	2046.1251
[K].SFIGQQI	1xTMTpro [K	0.0001132	7.389E-05	1	1	1	Q15124	Q15124 [458-475]	0	2533.4059
[K].SFLSGL	1xTMTpro [K	0.000144	7.389E-05	1	1	1	Q9UJA5	Q9UJA5 [249-261	0	2123.2033
[K].SFLSQG	1xTMTpro [K	0.0001116	7.389E-05	1	1	4	P48047	P48047 [163-172]	0	1715.0347
[K].SFQEILQ	1xTMTpro [N	7.113E-05	7.389E-05	1	1	4	Q7Z3U7	Q7Z3U7 [1168-11	0	1820.0601
[K].SFQGEII	1xTMTpro [K	0.0001212	7.389E-05	2	2	1	Q13948; P39	Q13948 [70-80]; F	0	1803.0144
[R].SEYQAD	1xTMTpro [N	0.000134	7.389E-05	1	1	2	P04114	P04114 [1600-16	0	1664.8087
[K].SFSKEEI	2xTMTpro [K	7.743E-05	7.389E-05	1	1	1	O00567	O00567 [511-533]	1	3385.7865

[K].SEIIPMF	1xOxidation [	8.651E-05	7.389E-05	1	1	2	P30153	P30153 [203-221]	0	2458.2091
[K].SELGNQ	1xTMTpro [N	6.297E-05	7.389E-05	1	1	2	Q5JTV8	Q5JTV8 [309-321	0	1653.8363
[K].SDSELN	1xTMTpro [N	8.965E-05	7.389E-05	1	1	2	Q53TN4	Q53TN4 [260-271	0	1581.8039
[R].SDSWSL	1xTMTpro [K	9.693E-05	7.389E-05	1	1	1	Q9P2R3	Q9P2R3 [90-102]	0	2004.0893
[R].SDSYVE	1xTMTpro [N	0.0001684	7.389E-05	1	1	2	P52298	P52298 [11-21]	0	1650.8294
[R].SDTSLT	1xTMTpro [K	0.0001165	7.389E-05	1	1	2	P02788	P02788 [463-473]	0	1846.0202
[R].SDVLVE	1xTMTpro [N	8.965E-05	7.389E-05	1	1	2	P15586	P15586 [410-421]	0	1655.856
[R].SDVQIH	1xTMTpro [N	0.0001157	7.389E-05	1	1	1	O75569	O75569 [57-69]	0	1851.0084
[K].SDVYCE	2xCarbamido	6.811E-05	7.389E-05	1	1	1	P07602	P07602 [311-323]	0	2256.1536
[R].SDYLNT	1xOxidation [	0.0001941	7.389E-05	1	1	3	O75874	O75874 [389-400	0	2134.0658
[R].SEAACL	1xCarbamido	8.324E-05	7.389E-05	1	1	1	O75882	O75882 [653-665	0	1576.8436
[K].SEAAGS	1xTMTpro [N	0.0001687	7.389E-05	1	1	1	Q96S66	Q96S66 [519-535	0	1984.9531
[R].SEAGAG	1xTMTpro [K	0.0001127	7.389E-05	1	1	2	Q9GZP8	Q9GZP8 [37-46]	0	1478.8459
[K].SEALGV	1xTMTpro [K	0.0001103	7.389E-05	1	1	3	Q9UGI8	Q9UGI8 [184-193	0	1582.9296
[R].SEATFQ	1xTMTpro [N	0.0001239	7.389E-05	1	1	3	Q93009	Q93009 [67-77]	0	1618.8396
[K].SELLPAC	1xTMTpro [K	0.0001275	7.389E-05	1	1	1	Q92530	Q92530 [51-62]	0	1951.0893
[R].SEDCAA	1xCarbamido	0.0001275	7.389E-05	1	1	1	Q13797	Q13797 [622-634	0	2043.0672
[K].SEDFQN	1xTMTpro [K	0.000126	7.389E-05	1	1	1	Q7Z2Z2	Q7Z2Z2 [840-854	0	2289.1643
[K].SEDFSLI	1xTMTpro [N	0.0001271	7.389E-05	1	1	2	P13073	P13073 [30-41]	0	1734.8328
[R].SEDGEI	1xTMTpro [N	0.0001487	7.389E-05	1	1	1	Q12769	Q12769 [949-959	0	1493.7767
[R].SEDLLD	1xTMTpro [N	0.0001383	7.389E-05	1	1	2	P04843	P04843 [194-204]	0	1615.8287
[R].SEDTAV	1xCarbamido	0.0001471	7.389E-05	3	5	4	P01742; A0A	P01742 [107-117]	0	1638.7753
[R].SEDYVE	1xTMTpro [N	0.0001849	7.389E-05	1	1	1	O60568	O60568 [441-450	0	1541.8131
[K].SEEEQS	1xTMTpro [K	7.827E-05	7.389E-05	1	1	2	P07910	P07910 [233-243]	0	1804.942
[K].SEEIKDT	2xTMTpro [K	0.0002035	7.389E-05	1	1	1	Q9H2G2	Q9H2G2 [488-50	1	3374.8723
[K].SEGALE	1xTMTpro [K	0.0001134	7.389E-05	1	1	1	Q08J23	Q08J23 [339-356]	0	2450.3634
[K].SEGETIN	1xTMTpro [K	7.451E-05	7.389E-05	1	1	3	P48444	P48444 [244-256]	0	1951.996
[K].SEGGFI	1xCarbamido	0.0001752	7.389E-05	1	1	2	O75874	O75874 [261-270	0	1762.9442
[R].SELDVG	1xTMTpro [N	7.719E-05	7.389E-05	1	1	1	O75676	O75676 [324-337	0	1917.9513
[R].SEIFGTC	1xCarbamido	0.0001669	7.389E-05	1	2	1	Q9UPN3	Q9UPN3 [5736-57	0	1993.0192
[K].SEDCFIL	1xCarbamido	0.0001058	7.389E-05	1	1	3	P06396	P06396 [328-341]	1	2533.3537
[K].SMPWN	1xTMTpro [K	0.0001593	7.389E-05	1	1	2	Q16543	Q16543 [111-121	0	1886.0337
[K].SFSLGDI	1xTMTpro [K	0.0001971	7.389E-05	1	1	1	P11717	P11717 [2147-21	0	1785.0078

[K].SFTSEDI	1xTMTpro [K	0.0001232	7.389E-05	1	1	2	O00203	O00203 [524-533]	0	1748.9562
[K].SGSALEI	1xTMTpro [K	8.096E-05	7.389E-05	1	1	1	O75688	O75688 [91-103]	0	1941.1148
[K].SGSEAQ	1xTMTpro [K	0.0001944	7.389E-05	1	1	3	O43493	O43493 [112-120]	0	1516.8463
[K].SGSLAVI	1xTMTpro [K	8.664E-05	7.389E-05	1	1	1	P35573	P35573 [876-889]	0	2008.157
[K].SGSMSA	1xTMTpro [N	0.0001926	7.389E-05	1	1	2	P07384	P07384 [632-641]	0	1422.6677
[R].SGTTPK	1xTMTpro [K	0.0001659	7.389E-05	1	1	1	Q99459	Q99459 [427-441]	0	2120.2319
[R].SGVSLA	2xTMTpro [K	8.558E-05	7.389E-05	3	3	6	P16402; P10	P16402 [56-65]; F	1	1886.2255
[R].SGYIEAI	1xTMTpro [K	0.0001277	7.389E-05	1	1	3	P22676	P22676 [124-133]	0	1731.9773
[R].SHCFTQ	1xCarbamido	7.169E-05	7.389E-05	1	1	1	Q9UBM1	Q9UBM1 [68-80]	0	1866.9274
[R].SHEAQV	1xTMTpro [N	0.0001983	7.389E-05	1	1	2	P35749	P35749 [1189-119	0	1518.7654
[R].SHPLDP	1xTMTpro [N	0.0001361	7.389E-05	1	1	1	P47897	P47897 [96-109]	0	1944.9986
[R].SHYQEA	1xTMTpro [K	0.0001656	7.389E-05	1	1	1	A0AVF1	A0AVF1 [165-175	0	1975.0781
[R].SKDVVE	1xTMTpro [K	0.0001313	7.389E-05	1	1	2	P26640	P26640 [666-681]	1	2593.511
[K].SKEESS	1xTMTpro [K	9.162E-05	7.389E-05	1	1	1	Q96AC1	Q96AC1 [409-424	1	2380.2535
[R].SGQVLE	1xTMTpro [K	0.0001299	7.389E-05	1	1	2	P21281	P21281 [83-93]	0	1698.9882
[K].SKEFAQ	2xTMTpro [K	0.000143	7.389E-05	1	1	3	P07954	P07954 [222-230]	1	1976.2361
[K].SKEQLTI	2xTMTpro [K	8.071E-05	7.389E-05	1	1	7	P02652	P02652 [68-77]	1	2069.315
[K].SKGIAYII	2xTMTpro [K	0.0001539	7.389E-05	1	1	1	P19338	P19338 [428-437]	1	2068.2623
[R].SKLPDG	2xTMTpro [K	6.843E-05	7.389E-05	1	1	1	Q12888	Q12888 [1749-17	1	3688.9414
[K].SKPIPIM	2xTMTpro [K	0.0001176	7.389E-05	1	1	1	O00429	O00429 [607-619]	0	2306.4086
[K].SKPYLTV	2xTMTpro [K	7.325E-05	7.389E-05	1	1	1	Q9NQ66	Q9NQ66 [236-252	0	2955.6504
[R].SKSDPIN	2xTMTpro [K	0.0001762	7.389E-05	1	1	1	P08559	P08559 [312-321]	1	2044.2656
[R].SKVDEA	2xTMTpro [K	0.0001206	7.389E-05	1	1	1	Q13310	Q13310 [614-629]	1	2615.5449
[R].SKVELIA	2xTMTpro [K	0.0001178	7.389E-05	1	1	1	P51608	P51608 [134-144]	1	2239.3518
[K].SIAAATS	1xTMTpro [K	8.401E-05	7.389E-05	2	2	2	Q9Y4G6; Q9	Q9Y4G6 [2352-23	0	1640.0238
[K].SLAAEEI	1xTMTpro [N	0.0001885	7.389E-05	1	1	2	Q15149	Q15149 [2130-21	0	1350.7184
[R].SLAASS	1xTMTpro [N	9.364E-05	7.389E-05	1	1	2	Q13724	Q13724 [730-741]	0	1577.8243
[R].SLAEDF	1xTMTpro [K	8.799E-05	7.389E-05	1	1	2	Q9Y4L1	Q9Y4L1 [159-170	0	1956.0934
[R].SIAEEQY	1xTMTpro [K	0.0001979	7.389E-05	1	1	1	O75116	O75116 [930-941]	0	2020.073
[R].SKEPQL	2xTMTpro [K	9.988E-05	7.389E-05	1	1	1	P19634	P19634 [568-579]	1	2373.4111
[R].SFTETM	2xTMTpro [K	8.951E-05	7.389E-05	1	1	1	Q9HB07	Q9HB07 [111-128	0	2924.6008
[K].SGQSED	1xOxidation	0.0001044	7.389E-05	1	1	1	P01024	P01024 [567-584]	1	2610.3801
[K].SGQAPV	1xTMTpro [K	0.0001084	7.389E-05	1	1	1	A0A075B6K4	A0A075B6K4 [58-	0	2114.1989

[R].SFVDAE	1xTMTpro [N	0.000171	7.389E-05	1	1	1	Q6P1J9	Q6P1J9 [212-222]	0	1541.8131
[R].SFVEFIL	1xTMTpro [K	0.0001373	7.389E-05	1	1	1	Q15029	Q15029 [368-379]	0	2093.2178
[K].SFYPEE	1xOxidation	8.518E-05	7.389E-05	1	1	2	P11142	P11142 [113-126]	0	2241.1968
[K].SGAEEQ	1xTMTpro [K	0.0001544	7.389E-05	1	1	1	O43493	O43493 [224-237]	0	1980.053
[R].SGAELA	1xCarbamido	0.0001453	7.389E-05	1	1	1	Q9BRJ6	Q9BRJ6 [97-108]	0	1671.8695
[R].SGAGSS	1xTMTpro [K	0.00018	7.389E-05	1	1	1	Q9UQ35	Q9UQ35 [1214-12	0	1528.8463
[R].SGATAG	1xTMTpro [N	0.0001301	7.389E-05	1	1	2	P50991	P50991 [9-19]	0	1179.6401
[K].SGCTSL	2xCarbamido	8.272E-05	7.389E-05	1	1	1	Q9NXR7	Q9NXR7 [42-55]	0	1825.8492
[K].SGDGEG	1xTMTpro [N	9.587E-05	7.389E-05	1	1	2	Q7L576	Q7L576 [1228-124	0	1643.8308
[R].SGDLGD	1xTMTpro [K	0.0001224	7.389E-05	1	1	2	O60716	O60716 [920-930]	0	1769.9599
[K].SGDLYV	1xTMTpro [K	7.497E-05	7.389E-05	1	1	2	Q9NSE4	Q9NSE4 [326-336	0	1760.0086
[K].SGESQV	1xTMTpro [N	8.518E-05	7.389E-05	1	1	1	P48723	P48723 [354-367]	0	1885.9826
[K].SGFNLE	1xTMTpro [K	0.0001581	7.389E-05	1	1	2	Q9BVP2	Q9BVP2 [425-434	0	1773.9878
[R].SGQGAF	1xCarbamido	0.0001096	7.389E-05	1	1	1	P36578	P36578 [87-97]	0	1488.7007
[K].SGGGG(	1xTMTpro [N	0.0001301	7.389E-05	1	1	1	Q13151	Q13151 [270-284]	0	1496.7161
[R].SGGPVV	1xTMTpro [N	8.993E-05	7.389E-05	1	1	1	O75643	O75643 [2031-204	0	1656.9968
[R].SGGSG(	1xTMTpro [N	7.291E-05	7.389E-05	1	1	1	O43290	O43290 [42-52]	0	1211.5936
[R].SGKYDL	2xTMTpro [K	0.0001112	7.389E-05	1	1	1	P06733	P06733 [254-269]	2	2739.477
[K].SGLDSV	1xTMTpro [K	0.0001746	7.389E-05	1	1	1	Q6PD74	Q6PD74 [90-103]	0	2068.1934
[R].SGLELE	1xTMTpro [N	0.0001116	7.389E-05	1	1	1	Q92888	Q92888 [386-399]	0	1899.9772
[R].SGLGEL	1xOxidation	7.428E-05	7.389E-05	1	1	1	P07954	P07954 [351-371]	0	2749.4938
[R].SGNEEV	1xTMTpro [N	6.499E-05	7.389E-05	1	1	1	Q6IA69	Q6IA69 [382-392]	0	1492.7927
[K].SGNIINM	1xTMTpro [K	9.077E-05	7.389E-05	1	1	1	Q9BUT1	Q9BUT1 [125-139	0	2102.1771
[K].SGNLTE	2xTMTpro [K	9.863E-05	7.389E-05	1	1	1	P13797	P13797 [574-587]	1	2455.3357
[R].SGPPPS	1xTMTpro [N	0.0001476	7.389E-05	1	1	2	Q9NTZ6	Q9NTZ6 [105-116	0	1450.7279
[K].SGPSVP	1xTMTpro [K	9.176E-05	7.389E-05	1	1	2	Q9Y2W2	Q9Y2W2 [615-62	0	1794.0617
[K].SGPVILA	1xTMTpro [K	0.0001328	7.389E-05	1	1	2	P53367	P53367 [108-118]	0	1750.0606
[R].SGPYGF	1xTMTpro [K	0.0001425	7.389E-05	1	1	1	O75165	O75165 [986-100	0	2418.2408
[R].SGGMEF	1xTMTpro [N	0.0001466	7.389E-05	1	1	2	Q14697	Q14697 [591-602]	0	1623.896
[R].SYTIAVA	1xTMTpro [K	0.000153	7.389E-05	1	1	1	P06280	P06280 [364-374]	0	1718.0344
[R].SMQDVV	1xTMTpro [K	0.0001599	7.389E-05	1	1	2	P08729	P08729 [168-177]	0	1805.9599
[K].SMYEEE	1xTMTpro [N	7.924E-05	7.389E-05	1	1	5	P20700	P20700 [210-220]	0	1704.807
[K].STGCDF	1xCarbamido	9.632E-05	7.389E-05	1	1	2	P55809	P55809 [501-511]	0	1776.9446

[K].STGDPW	1xTMTpro [N	0.0001635	7.389E-05	1	1	1	O15230	O15230 [2934-29	0	2420.1689
[K].STGFETI	1xTMTpro [K	6.716E-05	7.389E-05	1	1	1	O75521	O75521 [136-152	0	2392.3103
[K].STGGIS\	1xOxidation	9.708E-05	7.389E-05	1	1	6	P02452	P02452 [171-187]	0	1873.9761
[R].STGSFV	1xTMTpro [K	0.0001007	7.389E-05	1	1	2	Q9UJS0	Q9UJS0 [361-372	0	1927.0491
[K].STLINSL	1xTMTpro [N	9.063E-05	7.389E-05	1	1	1	Q8WYJ6	Q8WYJ6 [44-59]	0	2203.193
[K].STLMDTI	1xTMTpro [K	9.306E-05	7.389E-05	2	2	2	Q14141; Q9I	Q14141 [56-66]; (	0	1879.0491
[K].STLTDSI	1xCarbamido	0.0001286	7.389E-05	1	1	2	P13639	P13639 [33-42]	0	1731.9806
[R].STPAED	1xTMTpro [K	0.0001378	7.389E-05	1	1	1	Q9UHB6	Q9UHB6 [336-34	1	2143.1123
[K].STPKEE	1xTMTpro [K	7.66E-05	7.389E-05	1	1	1	O00567	O00567 [537-553	1	2504.2873
[K].STPVSQ	1xTMTpro [K	8.923E-05	7.389E-05	1	1	1	Q9UBC2	Q9UBC2 [796-82	0	3997.9951
[K].STPVTV	1xTMTpro [K	0.0001397	7.389E-05	1	1	4	Q15029	Q15029 [183-194	0	1865.1239
[K].STPYEC	1xCarbamido	0.0001888	7.389E-05	1	1	2	P03897	P03897 [34-48]	0	2018.9271
[K].STQDEI	1xTMTpro [N	0.000153	7.389E-05	1	1	2	Q9UBC2	Q9UBC2 [535-54	0	1465.7566
[K].STQFEY	1xCarbamido	9.133E-05	7.389E-05	1	1	2	Q9Y3D6	Q9Y3D6 [33-44]	0	1863.9383
[K].STQPGV	1xTMTpro [N	0.0001025	7.389E-05	1	1	1	Q9Y5S2	Q9Y5S2 [1136-11	0	1888.0823
[K].STQYQE	1xTMTpro [K	0.0001375	7.389E-05	1	1	1	Q9UPN3	Q9UPN3 [4690-4	0	2290.2422
[K].STSLDVI	1xTMTpro [N	6.664E-05	7.389E-05	1	1	1	O43815	O43815 [501-513	0	1831.9761
[R].STTLDA	1xTMTpro [K	0.0001134	7.389E-05	1	1	1	O75400	O75400 [709-718	0	1627.9511
[K].STVEGI	1xTMTpro [K	6.227E-05	7.389E-05	1	1	2	P18206	P18206 [656-666]	0	1727.0195
[R].STVLSLI	1xCarbamido	0.0001739	7.389E-05	2	2	1	Q92747; O1	Q92747 [143-165	0	3152.6694
[K].STYEQV	1xTMTpro [K	0.0002007	7.389E-05	1	1	1	Q9NR30	Q9NR30 [392-40	0	1861.0563
[K].SVAADFI	1xTMTpro [N	8.401E-05	7.389E-05	1	1	3	Q9BUT1	Q9BUT1 [160-171	0	1608.9029
[R].SVASSQ	2xTMTpro [K	0.0001849	7.389E-05	1	1	2	Q07157	Q07157 [175-186	0	2113.2797
[R].SVATITP	1xCarbamido	0.000149	7.389E-05	1	1	1	Q92626	Q92626 [232-247	0	2176.1351
[K].SVAWAF	1xCarbamido	0.0001466	7.389E-05	1	1	1	O76071	O76071 [110-125	0	1994.0449
[K].SVDCFLI	1xCarbamido	0.0001723	7.389E-05	1	1	1	O00186	O00186 [87-98]	0	2034.061
[K].STESLQ	1xTMTpro [N	8.222E-05	7.389E-05	1	1	4	P26373	P26373 [106-116]	0	1536.8301
[R].STDYGIF	1xTMTpro [N	8.159E-05	7.389E-05	1	1	3	P61626	P61626 [69-80]	0	1704.8876
[K].STCIYG	1xCarbamido	0.0001611	7.389E-05	2	2	4	Q92841; P1	Q92841 [275-284	0	1661.9177
[K].STAALEI	1xTMTpro [K	0.0001222	7.389E-05	2	2	1	Q15052; Q1	Q15052 [649-661	0	1997.141
[K].SSGQW	1xTMTpro [N	0.0002051	7.389E-05	1	1	1	P06681	P06681 [66-77]	0	1579.8148
[R].SSGSLLI	1xTMTpro [K	9.262E-05	7.389E-05	2	2	2	P01023; P2	P01023 [1082-10	0	1712.0198
[R].SSLEWN	1xCarbamido	0.0001053	7.389E-05	1	7	1	P49792	P49792 [199-212]	0	2259.2299

[R].SSIMAF/	1xTMTpro [K	6.129E-05	7.389E-05	1	1	4	P12111	P12111 [575-585]	0	1747.0068
[R].SSLQPIT	1xTMTpro [K	6.853E-05	7.389E-05	1	1	3	Q13308	Q13308 [794-804]	0	1753.0715
[K].SSLSVP\`	1xTMTpro [K	0.0001021	7.389E-05	1	1	3	P01024	P01024 [892-904]	0	2010.2495
[R].SSLYEG	1xTMTpro [K	0.0002031	7.389E-05	1	2	1	P20020	P20020 [1126-11:	0	2103.1578
[K].SSMNVD	1xOxidation [	0.0001241	7.389E-05	1	1	1	P51153	P51153 [154-167]	0	1833.8972
[R].SSNTYTI	1xTMTpro [N	8.895E-05	7.389E-05	1	1	2	Q13740	Q13740 [294-304]	0	1560.8189
[R].SSPAAFI	1xTMTpro [K	6.674E-05	7.389E-05	1	1	1	Q9BY77	Q9BY77 [126-144	0	2489.4623
[K].SSPEVS:	1xTMTpro [K	6.188E-05	7.389E-05	1	1	1	Q9NRG0	Q9NRG0 [30-47]	0	2481.4056
[K].SSPGAV	1xTMTpro [N	0.0001345	7.389E-05	1	1	1	Q9BWG4	Q9BWG4 [341-35	0	1842.9993
[K].SSPVVN	1xTMTpro [N	0.0001971	7.389E-05	1	1	1	Q12849	Q12849 [243-253]	0	1432.8079
[K].SVDDVL:	1xTMTpro [N	9.972E-05	7.389E-05	1	1	1	Q86Y56	Q86Y56 [242-253	0	1677.888
[R].SSQEQC	1xTMTpro [N	9.602E-05	7.389E-05	1	1	1	O75146	O75146 [591-601]	0	1522.7781
[R].SSQESY	1xTMTpro [K	7.803E-05	7.389E-05	1	1	1	P08603	P08603 [886-896]	0	1802.9529
[K].SSSNEE	1xOxidation [	0.0001085	7.389E-05	1	1	1	P01023	P01023 [94-108]	0	2322.2507
[K].SSSPAPI	1xTMTpro [K	6.277E-05	7.389E-05	1	1	1	Q9Y4F1	Q9Y4F1 [861-877	0	2339.2375
[K].SSSQVP	1xTMTpro [K	0.0001251	7.389E-05	1	1	1	Q86UE4	Q86UE4 [532-545	0	2137.1996
[K].SSSSDE	1xTMTpro [K	0.000196	7.389E-05	1	1	1	Q8N4X5	Q8N4X5 [47-59]	0	2161.0615
[K].SSSSGS	1xTMTpro [K	6.961E-05	7.389E-05	1	1	4	P13645	P13645 [568-580]	0	1793.9373
[K].SSSSTT	1xTMTpro [K	9.482E-05	7.389E-05	1	1	1	Q8NFD5	Q8NFD5 [1117-11	0	1592.8623
[K].SSTITVD	1xTMTpro [K	0.0001519	7.389E-05	1	1	2	Q53EL6	Q53EL6 [393-402	0	1717.965
[K].SSTPPG	1xTMTpro [K	0.0002035	7.389E-05	1	1	1	Q9UQ35	Q9UQ35 [1041-1(	0	2492.3528
[K].SSTSNAI	1xCarbamid(	7.815E-05	7.389E-05	1	1	1	Q13576	Q13576 [563-578]	0	2330.179
[R].SSVFAAI	1xTMTpro [N	7.578E-05	7.389E-05	1	1	1	Q9UMX3	Q9UMX3 [7-20]	0	1862.9278
[K].SSVLENI	1xTMTpro [N	0.0001944	7.389E-05	3	3	1	Q05193; P5(	Q05193 [45-54]; F	0	1411.7864
[K].SSVNCP	1xCarbamid(	7.566E-05	7.389E-05	1	1	2	Q08211	Q08211 [1025-10:	0	2095.0444
[K].SSQESI\	1xCarbamid(	0.000141	7.389E-05	1	1	1	Q9NXV6	Q9NXV6 [496-50(	0	1611.8331
[K].SVDFLD/	1xTMTpro [K	7.543E-05	7.389E-05	1	1	1	O15254	O15254 [485-499]	0	2289.2622
[K].SVDPTL/	1xTMTpro [N	0.000139	7.389E-05	1	1	2	Q00610	Q00610 [469-481]	0	1738.007
[K].SVEAAAI	1xTMTpro [K	8.545E-05	7.389E-05	1	1	19	P20962	P20962 [5-15]	0	1683.9773
[K].SWDVCS	1xCarbamid(	9.219E-05	7.389E-05	1	1	1	A2RRP1	A2RRP1 [1315-13	0	2604.2319
[K].SWEEQI	1xTMTpro [N	0.0001297	7.389E-05	1	1	2	Q00059	Q00059 [217-227]	0	1667.8382
[K].SWFEPL	1xTMTpro [N	6.488E-05	7.389E-05	1	1	1	P02649	P02649 [281-292]	0	1840.9223
[K].SWLPQI	1xCarbamid(	6.387E-05	7.389E-05	1	1	2	P22234	P22234 [179-191]	0	2200.175

[K].SWMEGI	1xCarbamido	0.0001268	7.389E-05	1	1	1	O00487	O00487 [224-239]	0	2592.2718
[R].SWPFNL	1xTMTpro [K	0.0002055	7.389E-05	1	1	2	O00584	O00584 [78-87]	0	1871.0559
[R].SWPGC(	3xCarbamido	0.0001198	7.389E-05	1	1	2	P11047	P11047 [1024-103	0	1974.858
[R].SWPVDF	1xTMTpro [N	6.612E-05	7.389E-05	1	1	1	O95870	O95870 [155-168]	0	2062.9942
[R].SWTAAD	1xTMTpro [K	7.508E-05	7.389E-05	2	2	1	P01893; P04	P01893 [156-168]	0	2002.0923
[K].SWVNQI	1xOxidation [	8.071E-05	7.389E-05	1	1	2	P46940	P46940 [1097-11	0	2306.1578
[K].SWVQC(	1xCarbamido	0.0001127	7.389E-05	1	1	2	Q7L014	Q7L014 [373-382]	0	1803.9741
[K].SYCNDQ	1xCarbamido	6.208E-05	7.389E-05	1	1	1	P00492	P00492 [104-115]	0	1995.9937
[R].SYDLSD	1xTMTpro [N	0.0001863	7.389E-05	1	1	1	O75427	O75427 [64-77]	0	1887.9619
[K].SWCQEI	1xCarbamido	0.000171	7.389E-05	1	1	1	Q92616	Q92616 [937-948]	0	2044.1029
[K].SYEAYVI	1xTMTpro [N	9.133E-05	7.389E-05	1	1	3	P05026	P05026 [97-107]	0	1630.9124
[R].SYELQE	1xTMTpro [N	0.0001769	7.389E-05	1	1	1	Q9NVA2	Q9NVA2 [84-93]	0	1528.7927
[K].SYEPLEI	1xTMTpro [K	9.941E-05	7.389E-05	1	1	2	P37837	P37837 [205-215]	0	1842.0141
[R].SYGATA	1xTMTpro [N	0.0001859	7.389E-05	1	1	1	Q8TB61	Q8TB61 [137-148	0	1500.7614
[K].SYLENP/	1xTMTpro [K	6.198E-05	7.389E-05	1	1	2	P11182	P11182 [469-482]	0	2262.27
[K].SYLSGM	1xCarbamido	0.0001498	7.389E-05	1	1	2	Q96CW1	Q96CW1 [204-21	0	1779.9265
[R].SYLTPVI	1xTMTpro [N	0.0001544	7.389E-05	1	1	1	O14974	O14974 [668-682]	1	2100.0528
[R].SYNDEL	1xTMTpro [K	6.643E-05	7.389E-05	1	1	3	Q9Y3I0	Q9Y3I0 [4-14]	0	1994.0726
[R].SYPGLL'	1xTMTpro [N	9.191E-05	7.389E-05	1	1	1	Q86WG5	Q86WG5 [1152-1	0	2330.3039
[R].SYQSEL	1xTMTpro [N	0.000142	7.389E-05	1	1	1	O60437	O60437 [1211-12	0	1499.8025
[R].SYSPTLI	1xTMTpro [N	0.0001956	7.389E-05	1	1	1	Q14139	Q14139 [938-949]	0	1673.9182
[K].SYSPYD	1xTMTpro [K	0.0001831	7.389E-05	1	2	1	P07355	P07355 [234-246]	1	2197.1819
[R].SYSSIVL	2xTMTpro [K	0.0001849	7.389E-05	1	1	1	O94769	O94769 [687-699]	0	2389.4635
[R].SYSSTL	1xTMTpro [N	0.0001044	7.389E-05	1	1	1	O94875	O94875 [841-851]	0	1521.7538
[K].SYEECIII	1xCarbamido	0.0001544	7.389E-05	1	1	1	P11717	P11717 [1913-192	0	1589.78
[R].SSGIVSI	1xTMTpro [N	0.0001258	7.389E-05	1	1	2	P12111	P12111 [1565-15	0	1450.8185
[K].SWCPD(	2xCarbamido	0.0001075	7.389E-05	1	1	2	Q9BRA2	Q9BRA2 [41-54]	0	2006.9747
[K].SVYQLL	1xTMTpro [N	0.0001352	7.389E-05	1	1	1	Q96FV9	Q96FV9 [359-373	0	2008.0307
[K].SVEEEE	1xTMTpro [N	0.0001723	7.389E-05	1	1	3	Q9P2E9	Q9P2E9 [1148-11	0	1594.8032
[R].SVEPQD	1xTMTpro [N	0.0001212	7.389E-05	1	1	1	Q9NZT2	Q9NZT2 [349-36(	0	1601.8454
[R].SVFALTI	1xTMTpro [K	8.272E-05	7.389E-05	1	1	1	Q02878	Q02878 [273-285]	0	2055.1883
[K].SVFPEQ	1xTMTpro [N	0.0001859	7.389E-05	1	1	2	O43242	O43242 [274-287]	0	1965.9738
[K].SVLGQL	1xTMTpro [K	0.0001335	7.389E-05	1	1	7	P01009	P01009 [325-334]	0	1624.0289

[R].SVIGSS(	1xCarbamido	0.0001626	7.389E-05	1	1	2	Q9NR50	Q9NR50 [373-382	0	1671.9959
[K].SVIPSDG	2xCarbamido	0.0001012	7.389E-05	1	1	1	P02787	P02787 [47-69]	2	3434.8956
[K].SVLVDFI	1xTMTpro [K	0.0001856	7.389E-05	1	1	1	Q9NPH2	Q9NPH2 [313-325	0	1956.2025
[K].SVMLYA	1xTMTpro [K	0.0001995	7.389E-05	1	1	1	P09132	P09132 [103-114]	0	1961.1096
[K].SVPKEIS	2xTMTpro [K	0.0001675	7.389E-05	1	1	1	P21810	P21810 [83-107]	2	3738.1105
[K].SVPLCIL	1xCarbamido	0.0001056	7.389E-05	1	1	2	P21980	P21980 [541-550]	0	1830.0691
[K].SVPNQP	1xTMTpro [K	9.925E-05	7.389E-05	1	1	1	Q5VYK3	Q5VYK3 [1606-16	0	2446.4161
[R].SVQETV	1xTMTpro [K	0.0001039	7.389E-05	1	1	1	P51608	P51608 [295-304]	0	1722.0657
[K].SVYWEN	1xTMTpro [K	0.0001859	7.389E-05	1	1	1	P57740	P57740 [294-305]	0	2099.1781
[R].SVQWCA	1xCarbamido	0.0001155	7.389E-05	1	1	2	P02788	P02788 [24-37]	0	2199.1724
[R].SVSIGYL	1xTMTpro [K	0.0001101	7.389E-05	1	1	6	P08572	P08572 [1485-149	0	1687.065
[K].SVSPSAS	1xCarbamido	0.00015	7.389E-05	1	1	1	Q12841	Q12841 [103-118]	0	2042.0296
[R].SVSSSLS	1xTMTpro [N	0.0001983	7.389E-05	1	1	1	O95487	O95487 [1030-10	0	1312.7028
[R].SVSVTA	1xCarbamido	7.803E-05	7.389E-05	1	1	1	O00291	O00291 [211-221]	0	1439.7596
[R].SVVAND	1xTMTpro [N	0.0001796	7.389E-05	1	1	1	Q9NXH9	Q9NXH9 [179-188	0	1323.7188
[R].SVVCQE	1xCarbamido	6.347E-05	7.389E-05	1	1	1	Q9NS86	Q9NS86 [184-200	0	2284.145
[R].SVVGDA	1xTMTpro [K	7.96E-05	7.389E-05	1	1	1	Q9HC84	Q9HC84 [1043-10	0	2117.1523
[R].SVVGDV	1xTMTpro [K	8.598E-05	7.389E-05	1	1	1	P98088	P98088 [1053-10	0	2145.1836
[R].SVVGTP	1xTMTpro [N	0.0001016	7.389E-05	1	2	1	Q15139	Q15139 [742-756]	0	1876.0863
[K].SVVLMS	1xOxidation [	0.0001005	7.389E-05	1	1	2	P00558	P00558 [57-75]	0	2659.4555
[K].SVVPGG	1xOxidation [	8.584E-05	7.389E-05	1	1	1	P17987	P17987 [407-433]	0	3019.5366
[K].SVVTGG	1xTMTpro [N	0.000107	7.389E-05	1	1	1	O60664	O60664 [167-180]	0	1667.907
[R].SVYFKPS	1xTMTpro [K	8.034E-05	7.389E-05	1	1	1	P49790	P49790 [380-394]	0	2323.2942
[K].SVSFSDY	1xTMTpro [K	0.0001547	7.389E-05	1	1	2	O43676	O43676 [60-69]	0	1770.9922
[R].SSGLTA	1xTMTpro [N	0.00017	7.389E-05	1	1	1	P53621	P53621 [413-423]	0	1450.8337
[R].SSGLPN	1xTMTpro [N	8.096E-05	7.389E-05	1	1	3	P02786	P02786 [326-339]	0	1773.019
[R].SSGLES	1xTMTpro [N	0.0001368	7.389E-05	1	1	1	Q8N6H7	Q8N6H7 [413-422	0	1326.682
[R].SPDEVT	1xTMTpro [N	0.0001093	7.389E-05	1	1	2	Q9UKS6	Q9UKS6 [319-332	0	1819.0132
[R].SPDFTN	1xTMTpro [N	8.388E-05	7.389E-05	1	1	2	P05023	P05023 [228-240]	0	1823.9095
[K].SPDLLM	1xTMTpro [K	7.485E-05	7.389E-05	1	1	2	P82909	P82909 [61-78]	0	2596.4188
[K].SPDSAT	1xOxidation [	0.000122	7.389E-05	1	1	1	Q6WCQ1	Q6WCQ1 [977-99	0	2095.0873
[R].SPEEAG	1xTMTpro [K	0.0001264	7.389E-05	1	1	1	Q8IV56	Q8IV56 [114-129]	1	2348.165
[K].SPEELES	1xTMTpro [K	0.0001347	7.389E-05	1	1	1	P46939	P46939 [1157-11	1	2242.1881

[R].SPEEVD	1xTMTpro [K	9.632E-05	7.389E-05	1	1	1	P48681	P48681 [905-915]	1	1912.0267
[K].SPELVQ	1xTMTpro [K	0.000121	7.389E-05	1	1	1	Q9UBB4	Q9UBB4 [227-237	0	1855.0643
[K].SPEQQL	1xTMTpro [K	0.0001579	7.389E-05	1	1	2	P52294	P52294 [95-105]	0	1825.0311
[R].SPEVLS	1xTMTpro [N	8.786E-05	7.389E-05	1	1	2	Q86W42	Q86W42 [180-193	0	1676.8774
[K].SPFTVG	1xTMTpro [K	6.297E-05	7.389E-05	1	1	1	O75369	O75369 [932-946	0	2110.2404
[K].SPFVVN	1xTMTpro [K	0.0001258	7.389E-05	1	1	2	Q14315	Q14315 [954-968	0	2191.2982
[K].SPGADL	1xTMTpro [K	0.0001142	7.389E-05	1	1	2	Q3LXA3	Q3LXA3 [511-522	0	1850.1243
[R].SPDEED	1xTMTpro [K	0.0001503	7.389E-05	1	1	1	P46821	P46821 [1881-189	0	2377.0851
[R].SPGAPG	1xTMTpro [K	0.0001863	7.389E-05	1	1	4	Q15942	Q15942 [344-354	0	1646.0133
[K].SPLAQM	1xTMTpro [N	0.0001029	7.389E-05	1	1	2	Q16181	Q16181 [334-344	1	1649.86
[K].SPIEFLE	1xTMTpro [K	0.0001903	7.389E-05	1	1	1	Q16850	Q16850 [86-97]	0	2048.1196
[R].SPLGEA	1xCarbamido	6.811E-05	7.389E-05	1	1	1	Q96B54	Q96B54 [99-112]	0	1739.907
[K].SPLIEST	1xTMTpro [K	9.972E-05	7.389E-05	1	1	1	O14974	O14974 [299-315	0	2485.2848
[K].SPLIIFSD	1xCarbamido	6.287E-05	7.389E-05	1	1	1	P49189	P49189 [259-274]	0	2432.2809
[R].SPLTQE	1xTMTpro [K	7.213E-05	7.389E-05	1	1	1	Q9UNE7	Q9UNE7 [273-287	0	2291.3289
[K].SPNELVI	1xTMTpro [K	0.0001503	7.389E-05	1	1	2	Q9UNZ2	Q9UNZ2 [114-124	0	1885.0563
[K].SPNLDL	1xTMTpro [K	0.0002019	7.389E-05	1	1	1	Q9H2M9	Q9H2M9 [568-579	0	1966.1352
[K].SPPGQV	1xTMTpro [K	0.0001264	7.389E-05	1	1	2	P15121	P15121 [23-33]	0	1721.0089
[R].SPPPGM	1xTMTpro [N	0.0001558	7.389E-05	1	1	2	P23246	P23246 [33-44]	0	1571.8283
[K].SPSAQLI	1xTMTpro [K	0.000107	7.389E-05	1	1	1	Q14203	Q14203 [1180-119	0	2138.2135
[R].SPSDEY	2xTMTpro [K	6.643E-05	7.389E-05	1	1	1	Q9UKD2	Q9UKD2 [80-94]	1	2659.4508
[R].SPSELF	1xTMTpro [K	8.134E-05	7.389E-05	1	1	1	Q9Y2W1	Q9Y2W1 [622-639	0	2650.5325
[R].SPGIISQ	1xTMTpro [N	0.0001407	7.389E-05	1	1	1	Q08495	Q08495 [105-116	0	1487.8501
[R].SPSKPLI	2xTMTpro [K	0.0001528	7.389E-05	1	1	1	P05455	P05455 [92-105]	0	2502.4272
[K].SPCYIDK	3xCarbamido	0.0001644	7.389E-05	1	1	1	P51648	P51648 [212-227]	1	2637.2967
[R].SPAQYQ	1xTMTpro [N	0.0001292	7.389E-05	1	1	2	Q6XQN6	Q6XQN6 [513-524	0	1680.924
[K].SMYEEE	1xTMTpro [N	0.0001036	7.389E-05	1	1	1	P20700	P20700 [210-221]	1	1860.9081
[R].SNADYQ	1xTMTpro [N	0.0001206	7.389E-05	1	1	1	Q9UIV1	Q9UIV1 [56-66]	0	1674.8771
[K].SNALDIIF	1xTMTpro [K	0.0001602	7.389E-05	1	1	1	P09871	P09871 [265-280]	0	2372.3317
[R].SNDPVA	1xTMTpro [K	6.885E-05	7.389E-05	1	1	2	P28289	P28289 [236-249]	0	2130.1761
[R].SNDVTQ	1xCarbamido	0.0001125	7.389E-05	1	1	1	Q6UXG2	Q6UXG2 [801-812	0	1601.7509
[K].SNEDQS	1xOxidation [	0.0001239	7.389E-05	1	1	9	P02545	P02545 [458-470]	0	2161.0839
[K].SNFKPSI	2xTMTpro [K	6.769E-05	7.389E-05	1	1	4	Q96CW1	Q96CW1 [309-31	0	2145.3212

[K].SNFSILQ	1xTMTpro [K	0.0001519	7.389E-05	1	1	1	Q96JG6	Q96JG6 [476-487	0	2071.1832
[K].SNKDGG	1xTMTpro [K	7.767E-05	7.389E-05	1	1	1	P13861	P13861 [316-329]	1	2125.1493
[R].SNLGSV	1xTMTpro [K	6.57E-05	7.389E-05	1	1	4	O43143	O43143 [505-515]	0	1766.1031
[K].SNILLLG	1xTMTpro [K	0.0001159	7.389E-05	1	1	1	O76031	O76031 [287-299]	0	1865.1352
[R].SNILTLM	1xCarbamido	8.505E-05	7.389E-05	1	1	1	Q92973	Q92973 [674-687]	0	2353.221
[K].SNLNSLI	1xTMTpro [K	0.0001151	7.389E-05	1	1	1	O75223	O75223 [89-101]	0	2041.1057
[K].SPCQPN	3xCarbamido	8.196E-05	7.389E-05	1	1	1	O00468	O00468 [1590-16	0	2012.9172
[R].SNIWLA	1xTMTpro [K	0.0001793	7.389E-05	1	1	3	Q96CW1	Q96CW1 [61-70]	0	1711.0398
[K].SNNSMA	1xTMTpro [K	0.0001479	7.389E-05	1	1	2	O43633	O43633 [95-104]	0	1689.8908
[K].SNNVEM	1xTMTpro [K	8.071E-05	7.389E-05	1	1	2	P31943	P31943 [88-98]	0	1943.0552
[K].SNQQLV	1xTMTpro [K	0.0001378	7.389E-05	1	1	2	P61289	P61289 [111-121]	0	1895.1094
[R].SNRDEL	1xTMTpro [N	7.269E-05	7.389E-05	1	1	1	Q14980	Q14980 [228-241]	1	1992.0317
[K].SNSVEM	1xTMTpro [K	7.382E-05	7.389E-05	1	1	2	P55795	P55795 [88-98]	0	1916.0443
[K].SNTLPIS	1xTMTpro [N	0.0001573	7.389E-05	1	1	2	P42704	P42704 [530-541]	0	1632.9604
[K].SNVSDA	1xTMTpro [N	7.291E-05	7.389E-05	1	1	5	P60174	P60174 [195-206]	0	1538.8094
[K].SNYNFE	1xTMTpro [K	0.0001983	7.389E-05	1	1	3	P62826	P62826 [153-166]	0	2393.3262
[K].SPAGSI	1xTMTpro [K	0.0001874	7.389E-05	1	1	1	O43776	O43776 [368-385]	0	2540.4117
[R].SPAIAA	1xTMTpro [K	9.647E-05	7.389E-05	1	1	1	O75439	O75439 [462-472]	0	1632.034
[K].SPAITAT	1xTMTpro [K	0.00014	7.389E-05	1	1	1	Q8TBC4	Q8TBC4 [399-40	0	1696.0137
[K].SPAPPLI	1xTMTpro [K	0.0001149	7.389E-05	1	1	1	Q8N163	Q8N163 [124-138	0	2107.2883
[R].SPAQILI	1xTMTpro [N	0.0001979	7.389E-05	1	1	1	P11532	P11532 [3490-35	0	1876.0347
[R].SNLYIAE	1xTMTpro [N	0.0001182	7.389E-05	1	1	1	Q9NWU5	Q9NWU5 [132-14	0	1601.8454
[R].SMVEDI	1xTMTpro [N	0.000162	7.389E-05	1	1	1	P15924	P15924 [2792-28	0	1424.7738
[R].SPSLGS	1xTMTpro [N	7.247E-05	7.389E-05	1	1	1	Q13425	Q13425 [393-405]	0	1655.8924
[K].SPSSPQ	1xTMTpro [K	0.0001308	7.389E-05	1	1	1	Q92793	Q92793 [2076-20	0	2403.3851
[R].SQVEEE	1xTMTpro [N	0.0001703	7.389E-05	1	1	4	Q15149	Q15149 [2361-23	0	1626.8658
[K].SQWNNI	1xTMTpro [K	0.0001956	7.389E-05	1	1	2	P05107	P05107 [745-755]	0	1971.058
[R].SQWSP	1xTMTpro [K	6.377E-05	7.389E-05	1	2	3	P61077	P61077 [91-101]	0	1826.0668
[R].SQWSP	1xTMTpro [K	0.0001371	7.389E-05	1	2	2	P51668	P51668 [91-101]	0	1812.0511
[R].SQYEQL	1xTMTpro [N	0.0001161	7.389E-05	1	1	2	P13645	P13645 [323-333]	0	1669.8465
[R].SQYEVM	1xTMTpro [N	0.0001948	7.389E-05	1	1	2	P08727	P08727 [254-264]	0	1658.8127
[K].SQYLLT	1xTMTpro [K	0.0001746	7.389E-05	1	1	1	Q8N474	Q8N474 [277-286	0	1782.0769
[K].SRPEFM	1xTMTpro [K	0.0001888	7.389E-05	1	1	1	P06737	P06737 [193-206]	0	2316.2819

[K].SRPELLE	1xTMTpro [K	0.0001208	7.389E-05	1	1	1	Q14258	Q14258 [448-458]	0	2019.177
[R].SRPQGL	1xTMTpro [N	9.119E-05	7.389E-05	1	1	1	Q00653	Q00653 [161-172]	0	1675.9047
[K].SRPVDP	1xTMTpro [K	9.602E-05	7.389E-05	1	1	1	P22570	P22570 [364-376]	0	2039.1417
[K].SSAEAQ	1xTMTpro [K	0.0001813	7.389E-05	1	1	1	O43493	O43493 [70-83]	0	2083.0799
[K].SSAEVIA	1xTMTpro [N	0.0001766	7.389E-05	1	1	2	Q16555	Q16555 [259-268]	0	1335.7551
[K].SQTTYY.	1xCarbamido	0.0001053	7.389E-05	1	1	1	Q9NR46	Q9NR46 [259-269	0	1744.8284
[R].SSALREI	1xTMTpro [N	0.0001979	7.389E-05	1	1	1	Q14980	Q14980 [1287-12	1	1678.9407
[K].SSASFN	1xTMTpro [K	0.0001647	7.389E-05	1	1	1	P06756	P06756 [952-964]	0	2097.1512
[R].SSASSG	1xTMTpro [K	0.000123	7.389E-05	1	1	1	P08648	P08648 [900-910]	0	1682.9933
[R].SSCPLAI	1xCarbamido	8.705E-05	7.389E-05	1	1	1	Q9BXY0	Q9BXY0 [42-55]	0	2148.1615
[K].SSDEAV	1xCarbamido	0.0001547	7.389E-05	1	1	3	Q9UNM6	Q9UNM6 [106-11	0	1729.965
[R].SSDPDY	1xTMTpro [K	0.0001013	7.389E-05	1	1	3	P16278	P16278 [149-160]	0	1889.0148
[R].SSDRVD	1xTMTpro [K	0.0001163	7.389E-05	1	1	2	P12111	P12111 [952-964]	1	1954.0849
[R].SSEDPN	1xTMTpro [N	7.613E-05	7.389E-05	1	1	2	P01591	P01591 [47-58]	0	1693.82
[K].SSEIEQA	1xTMTpro [N	7.566E-05	7.389E-05	1	1	3	Q9BPX5	Q9BPX5 [90-102]	0	1765.9251
[K].SSELEQ	1xTMTpro [N	0.0001641	7.389E-05	1	1	3	Q9UH65	Q9UH65 [409-418	0	1556.824
[R].SSELRP	1xTMTpro [N	0.0001395	7.389E-05	1	1	1	Q92743	Q92743 [270-302]	0	3783.0248
[K].SSEPVQ	1xTMTpro [N	6.478E-05	7.389E-05	1	1	1	Q9UBF2	Q9UBF2 [630-646	0	2289.157
[R].SSFESS	1xCarbamido	0.0001359	7.389E-05	1	1	1	Q5JTJ3	Q5JTJ3 [84-96]	0	2192.1302
[K].SSFNLG	1xTMTpro [K	8.146E-05	7.389E-05	1	1	2	P49790	P49790 [1046-105	0	1805.03
[K].SSASAPI	1xTMTpro [N	0.0001762	7.389E-05	1	1	3	Q8NC51	Q8NC51 [391-408	0	2064.0093
[R].SPSSMV	1xTMTpro [K	0.000119	7.389E-05	1	1	2	P36269	P36269 [467-479]	0	1981.1648
[K].SQSIDTF	1xTMTpro [N	0.0001842	7.389E-05	1	1	2	Q96ST3	Q96ST3 [156-167	0	1563.8662
[R].SQPVLQ	1xTMTpro [N	0.0001073	7.389E-05	1	1	1	Q9ULC5	Q9ULC5 [565-579	0	2014.1405
[R].SPTMAG	1xTMTpro [K	0.0001533	7.389E-05	1	2	1	Q8N4A0	Q8N4A0 [306-318	0	1874.0701
[K].SPVDIVT	1xTMTpro [N	0.0001659	7.389E-05	1	1	2	P50851	P50851 [1488-150	0	1700.9866
[K].SPWCD\	1xCarbamido	0.000182	7.389E-05	1	1	1	Q9UNW1	Q9UNW1 [299-31	0	2176.0876
[R].SPWSNI	1xTMTpro [K	6.307E-05	7.389E-05	1	1	1	P47756	P47756 [73-92]	1	2826.4376
[K].SPYQLVI	1xTMTpro [N	8.611E-05	7.389E-05	1	1	2	Q15582	Q15582 [28-38]	0	1631.9189
[K].SQAADC	1xTMTpro [K	0.0001952	7.389E-05	1	1	2	Q96I99	Q96I99 [219-227]	0	1569.9092
[K].SQAEPL	2xTMTpro [K	0.0002015	7.389E-05	1	1	1	P49750	P49750 [854-875]	1	3241.7481
[K].SQAFIEI	1xTMTpro [N	0.0001487	7.389E-05	1	1	3	P43243	P43243 [533-542]	0	1515.7796
[K].SQDADS	1xTMTpro [K	8.466E-05	7.389E-05	1	1	1	P55196	P55196 [1774-179	0	2516.276

[K].SQDEAP	1xTMTpro [K	8.531E-05	7.389E-05	1	1	1	Q86UX7	Q86UX7 [390-405	0	2361.2906
[R].SQDSMS	1xTMTpro [N	6.407E-05	7.389E-05	1	1	1	P25205	P25205 [599-609]	0	1488.692
[R].SQECDV	1xCarbamido	0.0001136	7.389E-05	1	1	1	O60437	O60437 [1690-170	0	2118.0669
[R].SQECYF	1xCarbamido	9.191E-05	7.389E-05	1	1	2	P11047	P11047 [348-359]	0	1910.8914
[K].SQQLSA	1xTMTpro [K	0.000126	7.389E-05	1	1	1	P30622	P30622 [1169-118	0	2082.1687
[K].SQEDTE	2xTMTpro [K	0.0001184	7.389E-05	1	1	1	Q9NYH9	Q9NYH9 [453-463	1	2194.2536
[K].SQEPVT	1xTMTpro [K	6.612E-05	7.389E-05	1	1	1	P54289	P54289 [553-571]	0	2784.4799
[K].SQFTITP	1xTMTpro [N	0.0001533	7.389E-05	1	1	1	Q99798	Q99798 [412-424]	0	1767.956
[K].SQGGEF	1xTMTpro [N	6.748E-05	7.389E-05	1	1	1	P35080	P35080 [92-105]	0	1738.9043
[K].SQGMAS	1xOxidation [	6.188E-05	7.389E-05	1	1	1	P35221	P35221 [846-860]	0	2213.2244
[K].SQGQDV	1xTMTpro [K	7.102E-05	7.389E-05	1	1	1	P15374	P15374 [75-88]	0	2185.1455
[K].SQLDIIIH	2xTMTpro [K	0.0001166	7.389E-05	1	1	1	P13010	P13010 [145-156]	1	2307.458
[R].SQLEES	1xTMTpro [N	9.572E-05	7.389E-05	1	1	3	Q9BUR5	Q9BUR5 [58-68]	0	1593.8767
[K].SQLLKDI	2xTMTpro [K	0.0001762	7.389E-05	1	3	1	P52435	P52435 [48-62]	1	2619.569
[R].SQILSGT	1xTMTpro [K	7.371E-05	7.389E-05	1	1	1	Q14185	Q14185 [130-143]	0	2137.236
[K].SQNTVW	1xTMTpro [K	0.0001525	7.389E-05	1	1	1	Q9Y619	Q9Y619 [149-158	0	1770.0405
[R].SQNVMA	1xOxidation [	0.0001378	7.389E-05	1	1	1	P17987	P17987 [19-33]	0	2141.2244
[R].SQPAILL	1xTMTpro [N	0.0001228	7.389E-05	1	1	4	Q5JTV8	Q5JTV8 [405-416	0	1557.9647
[K].SQPDMA	1xTMTpro [K	0.0001281	7.389E-05	1	1	1	Q10567	Q10567 [79-93]	0	2260.2325
[K].SQEGDS	1xTMTpro [N	0.0001726	7.389E-05	1	1	1	O75976	O75976 [614-625]	0	1551.8298
[K].VANVSLI	1xTMTpro [K	7.887E-05	7.389E-05	1	1	4	P62266	P62266 [125-135]	0	1799.1286
[K].VAPAQP	1xTMTpro [N	0.0001492	7.389E-05	1	1	2	P23588	P23588 [512-524]	0	1598.8458
[K].VAQLCD	1xCarbamido	0.0001385	7.389E-05	1	1	2	P09497	P09497 [195-204]	0	1799.997
[K].WGDEHI	1xTMTpro [N	0.0001968	7.389E-05	1	1	8	P21333	P21333 [2632-264	0	1717.8617
[K].WGDIEF	1xTMTpro [N	0.0001273	7.389E-05	1	1	1	P53396	P53396 [233-244]	0	1721.8971
[R].WGDYDS	1xTMTpro [N	8.705E-05	7.389E-05	1	1	1	Q6P2Q9	Q6P2Q9 [1668-16	0	1696.7886
[R].WGPNHA	1xTMTpro [N	0.0001018	7.389E-05	1	1	1	Q9BW91	Q9BW91 [165-17	0	1751.9512
[K].WGTLTD	1xCarbamido	9.632E-05	7.389E-05	1	1	4	P51991	P51991 [58-68]	0	1641.8412
[K].WGTLTD	1xCarbamido	7.169E-05	7.389E-05	1	1	1	P51991	P51991 [58-73]	1	2515.3293
[K].WGTPVF	1xTMTpro [K	0.0001182	7.389E-05	1	1	1	P56192	P56192 [501-513]	0	2083.1356
[R].WGTVCI	1xCarbamido	8.222E-05	7.389E-05	1	1	1	A1L4H1	A1L4H1 [41-52]; [	0	1783.8393
[R].WGVISA	1xTMTpro [N	0.0001495	7.389E-05	1	1	1	P48506	P48506 [297-307]	0	1508.8028
[R].WGYSST	1xTMTpro [N	0.0001617	7.389E-05	1	1	1	P10253	P10253 [376-385]	0	1445.7708

[R].WKNFPS	1xTMTpro [K	9.291E-05	7.389E-05	1	1	1	P02790	P02790 [90-102]	1	2143.1944
[K].WKPSSG	1xTMTpro [K	7.091E-05	7.389E-05	1	1	1	Q8TBF4	Q8TBF4 [185-199	0	2214.1646
[K].WKRPTE	1xTMTpro [K	0.0001103	7.389E-05	1	1	1	P07384	P07384 [85-104]	1	2936.6602
[K].WKYEKF	2xTMTpro [K	0.0001103	7.389E-05	1	1	1	Q13740	Q13740 [56-71]	1	2852.6279
[K].WLCNDQ	1xCarbamido	8.034E-05	7.389E-05	1	1	1	Q9H3U1	Q9H3U1 [533-544	0	1752.8658
[R].WIDETPI	1xTMTpro [N	6.57E-05	7.389E-05	1	1	4	Q15257	Q15257 [122-134]	0	1843.9509
[K].WLDTQV	1xTMTpro [K	0.0001361	7.389E-05	1	1	1	P11216	P11216 [216-235]	0	2901.5716
[K].WIEAGP	1xTMTpro [K	0.0001245	7.389E-05	1	1	3	Q13308	Q13308 [123-132]	0	1720.0653
[R].WLGLEE	1xCarbamido	0.0001087	7.389E-05	1	1	1	P50583	P50583 [116-129]	0	2301.2557
[R].WLHNED	1xOxidation [	0.000196	7.389E-05	1	1	3	P37837	P37837 [296-307]	0	2124.1039
[R].WLLLCN	1xCarbamido	7.625E-05	7.389E-05	1	1	1	P06737	P06737 [492-507]	0	2448.418
[K].WIIQADT	1xTMTpro [K	9.527E-05	7.389E-05	1	1	1	P11532	P11532 [1694-170	0	2269.2571
[R].WLISAAI	1xCarbamido	0.0001208	7.389E-05	1	1	1	Q8NI36	Q8NI36 [636-647]	0	1726.894
[R].WLPLEA	1xTMTpro [K	6.478E-05	7.389E-05	1	1	1	P15374	P15374 [6-21]	0	2507.4154
[K].WLQDLQ	1xTMTpro [K	0.0001026	7.389E-05	1	1	2	Q86UP2	Q86UP2 [896-908	0	2240.2054
[R].WLQDNA	1xTMTpro [K	0.0001056	7.389E-05	1	1	1	P07359	P07359 [235-247]	0	2273.221
[R].WLQGSC	1xTMTpro [N	0.0001766	7.389E-05	2	3	4	P01876; P0D	P01876 [264-273]	0	1517.8395
[R].WFYIDF	2xTMTpro [K	0.0001877	7.389E-05	1	1	1	Q9UJS0	Q9UJS0 [606-624	0	3066.6943
[K].WFVPW	1xCarbamido	8.651E-05	7.389E-05	1	1	1	Q5T9L3	Q5T9L3 [62-73]	0	2151.109
[R].WFVFDT	1xTMTpro [K	0.0001964	7.389E-05	1	1	1	O00423	O00423 [608-616]	0	1780.9765
[R].WFLLEQ	1xTMTpro [K	8.285E-05	7.389E-05	1	1	2	P08697	P08697 [277-293]	0	2737.5362
[K].WAAVVV	1xTMTpro [N	9.678E-05	7.389E-05	1	1	4	P10321	P10321 [268-280]	0	1730.9509
[K].WADNFN	1xCarbamido	8.664E-05	7.389E-05	1	1	1	Q8N357	Q8N357 [27-40]	0	2138.0291
[K].WADNFN	1xCarbamido	0.0001877	7.389E-05	1	1	1	Q8N357	Q8N357 [27-40]	0	2154.024
[K].WAEDNE	2xCarbamido	0.0001131	7.389E-05	1	1	1	Q15075	Q15075 [1349-13	0	2420.1289
[K].WAEKPA	2xTMTpro [K	6.227E-05	7.389E-05	1	1	1	Q68EM7	Q68EM7 [244-259	0	2765.5806
[R].WAESVF	1xTMTpro [K	9.972E-05	7.389E-05	1	1	1	Q8WUM0	Q8WUM0 [579-60	0	3518.8663
[K].WAEYHA	1xTMTpro [K	8.26E-05	7.389E-05	1	1	3	Q9NRX4	Q9NRX4 [49-59]	0	2019.0468
[K].WAIAQLI	1xTMTpro [K	6.961E-05	7.389E-05	1	1	1	Q14204	Q14204 [3395-34	0	2145.2022
[R].WALSQS	1xTMTpro [N	9.091E-05	7.389E-05	1	1	2	P55072	P55072 [454-465]	0	1633.8981
[R].WANFHL	1xTMTpro [K	9.234E-05	7.389E-05	1	1	1	P13797	P13797 [276-288]	0	2225.1747
[K].WAPFVQ	1xTMTpro [K	6.961E-05	7.389E-05	1	1	1	Q9NXE4	Q9NXE4 [469-482	0	2348.2968
[K].WCEYGI	1xCarbamido	7.113E-05	7.389E-05	1	1	2	P45880	P45880 [75-85]	0	2042.0549

[R].WDFDTC	1xTMTpro [K	7.235E-05	7.389E-05	1	1	1	Q13123	Q13123 [481-496]	0	2707.2478
[R].WLSCET	1xCarbamido	8.046E-05	7.389E-05	1	1	2	O75643	O75643 [1275-12	0	1927.0067
[K].WDGQET	1xTMTpro [N	0.0001359	7.389E-05	1	1	2	P05413	P05413 [98-107]	0	1508.8028
[R].WDPTAN	1xTMTpro [K	0.0001322	7.389E-05	1	1	1	Q14257	Q14257 [217-232]	0	2550.3372
[R].WDPTGE	1xTMTpro [K	6.95E-05	7.389E-05	1	1	2	P48681	P48681 [1313-132	0	2488.3076
[R].WDQTAD	1xTMTpro [K	0.0001176	7.389E-05	1	1	1	O75533	O75533 [200-213]	0	2124.1217
[K].WDTGEN	1xTMTpro [K	0.0001264	7.389E-05	1	1	4	P05556	P05556 [775-784]	0	1830.9882
[R].WDVDDV	1xTMTpro [N	6.347E-05	7.389E-05	1	1	2	Q01082	Q01082 [25-38]	0	2012.8905
[R].WEAAEC	1xTMTpro [K	7.791E-05	7.389E-05	1	1	1	O14579	O14579 [232-245]	0	2181.2047
[K].WEDAIPI	1xTMTpro [K	0.0001013	7.389E-05	1	1	2	P09960	P09960 [548-557]	0	1764.0551
[K].WEGLVY	1xTMTpro [K	0.0001253	7.389E-05	1	1	1	Q9Y2W1	Q9Y2W1 [471-48	0	1825.0504
[R].WEGVPF	1xTMTpro [K	0.0001522	7.389E-05	1	1	1	O95479	O95479 [349-360]	0	1972.1222
[K].WELLQC	1xTMTpro [N	9.678E-05	7.389E-05	1	1	3	P04264	P04264 [212-223]	0	1779.956
[R].WEQLLE	1xTMTpro [N	7.28E-05	7.389E-05	1	1	1	P02549	P02549 [2017-202	0	1742.9509
[R].WETGEV	1xTMTpro [K	0.0001769	7.389E-05	1	1	2	P33241	P33241 [267-279]	0	2013.0897
[K].WEYVPL	1xTMTpro [K	0.0002007	7.389E-05	1	1	2	P16930	P16930 [242-253]	0	2014.1657
[R].WDPDKA	3xTMTpro [K	0.0001237	7.389E-05	1	1	1	P21912	P21912 [47-67]	1	3703.9895
[K].WISDWN	1xTMTpro [K	6.918E-05	7.389E-05	1	1	2	Q7L2H7	Q7L2H7 [150-160	0	2001.0937
[R].WLTIQS	1xTMTpro [K	6.377E-05	7.389E-05	1	1	2	O43920	O43920 [17-28]	0	2058.1516
[R].WITVGN	1xCarbamido	0.0001944	7.389E-05	1	1	1	P17152	P17152 [74-83]	0	1836.0446
[K].WTSQDS	1xTMTpro [N	6.643E-05	7.389E-05	1	1	2	P49327	P49327 [1592-160	0	2017.9972
[K].WTSTSM	1xTMTpro [K	6.448E-05	7.389E-05	1	1	4	Q7L266	Q7L266 [278-289]	0	1945.0497
[K].WVDEVV	1xTMTpro [K	7.405E-05	7.389E-05	1	1	1	Q99447	Q99447 [85-104]	0	2854.5734
[K].WVDLTM	1xTMTpro [N	0.0001995	7.389E-05	1	1	2	Q00169	Q00169 [235-244]	0	1567.8109
[R].WVGPLF	1xTMTpro [K	0.0001072	7.389E-05	1	1	1	Q96BP3	Q96BP3 [50-62]	0	1989.2029
[K].WVGVILI	1xTMTpro [K	0.0001078	7.389E-05	1	1	1	Q14203	Q14203 [57-66]	0	1738.0395
[K].WVHAEN	1xTMTpro [K	0.0001053	7.389E-05	1	1	1	P46063	P46063 [172-180]	0	1721.9653
[K].WVINPS	1xTMTpro [K	7.314E-05	7.389E-05	1	1	3	P22307	P22307 [342-353]	0	1879.1297
[K].WVPAGC	1xTMTpro [N	0.0001492	7.389E-05	1	1	2	Q8N8S7	Q8N8S7 [23-34]	0	1525.8082
[K].WVPEITI	1xCarbamido	7.779E-05	7.389E-05	1	1	3	P60953	P60953 [97-107]	0	2012.1032
[R].WVPPLI	1xTMTpro [N	0.0001746	7.389E-05	1	1	3	Q9P0J0	Q9P0J0 [116-128]	0	1817.0645
[K].WVPVVD	1xTMTpro [N	0.0001301	7.389E-05	1	1	2	Q8WUZ0	Q8WUZ0 [48-59]	0	1786.9295
[R].WVTTAS	1xTMTpro [K	7.936E-05	7.389E-05	1	1	1	Q15393	Q15393 [1032-10	0	2534.3634

[R].WTQVN(	1xTMTpro [K	0.000149	7.389E-05	1	1	1	Q8WXH0	Q8WXH0 [5366-5	0	2251.269
[K].WYMQFI	1xTMTpro [K	0.0001407	7.389E-05	1	1	2	P53680	P53680 [19-28]	0	1984.9606
[K].YAAELHI	1xTMTpro [K	0.0001508	7.389E-05	1	1	1	P00918	P00918 [114-126]	0	2190.2315
[K].YADALQ	1xTMTpro [N	8.134E-05	7.389E-05	1	1	2	Q9Y6N5	Q9Y6N5 [242-253	0	1752.9451
[R].YADGPQ	1xTMTpro [N	7.169E-05	7.389E-05	1	1	3	Q96JJ3	Q96JJ3 [53-66]	0	1959.0143
[K].YAEEELI	1xTMTpro [N	0.0001736	7.389E-05	1	1	2	Q13586	Q13586 [316-325]	0	1569.808
[K].YAELLVS	1xTMTpro [K	0.000165	7.389E-05	1	1	1	Q02218	Q02218 [540-561]	0	3132.6596
[R].YAGPED	1xTMTpro [K	7.213E-05	7.389E-05	1	1	1	Q02880	Q02880 [661-676]	0	2277.2258
[K].YAGSQV	1xTMTpro [K	0.0001345	7.389E-05	1	1	1	P04275	P04275 [1349-136	0	2048.1519
[K].YALQSA	1xTMTpro [K	0.0001359	7.389E-05	1	1	1	Q9NTI5	Q9NTI5 [419-427]	0	1516.8979
[R].YALYDA	1xTMTpro [K	8.571E-05	7.389E-05	2	2	4	P23528; Q9Y	P23528 [82-92]; Q	0	1946.0403
[K].YANEVN	1xTMTpro [K	9.878E-05	7.389E-05	1	1	2	Q9UHB9	Q9UHB9 [492-504	0	1994.0475
[K].YAPLHL\	1xTMTpro [N	0.0001109	7.389E-05	1	2	2	Q7L576	Q7L576 [1054-10	0	1725.0382
[K].YAQAGF	1xTMTpro [K	0.000107	7.389E-05	1	1	2	O75643	O75643 [470-479]	0	1725.9456
[K].YAQGAD	1xTMTpro [N	6.247E-05	7.389E-05	3	3	2	Q9UKV8; Q9	Q9UKV8 [494-506	0	1774.8753
[K].WYYNAA	1xTMTpro [K	0.0001123	7.389E-05	1	1	2	P14927	P14927 [20-29]	0	1841.983
[K].WAASLE	1xTMTpro [K	7.485E-05	7.389E-05	1	1	1	O43665	O43665 [38-53]	0	2379.3052
[K].WTLLQE	1xTMTpro [K	0.0001608	7.389E-05	3	4	4	P04259; P02	P04259 [195-204]	0	1812.0511
[R].WTELGA	1xTMTpro [N	8.247E-05	7.389E-05	1	1	2	O75643	O75643 [841-855]	0	2020.1221
[R].WLVGEN	1xCarbamido	6.257E-05	7.389E-05	1	1	1	P10643	P10643 [619-628]	0	1896.0116
[R].WMDEA(	1xTMTpro [N	7.303E-05	7.389E-05	1	1	1	Q9BXJ9	Q9BXJ9 [430-442	0	1825.871
[K].WMDEA(	1xTMTpro [N	6.864E-05	7.389E-05	1	1	1	Q6N069	Q6N069 [430-442	0	1841.8659
[K].WMEEQA	2xTMTpro [K	0.0001956	7.389E-05	1	1	1	P09234	P09234 [41-61]	1	3322.7922
[K].WMEPAI	1xTMTpro [K	0.0001553	7.389E-05	1	1	2	Q8NBL1	Q8NBL1 [140-152	0	2147.1855
[K].WMGGA	1xTMTpro [N	9.8E-05	7.389E-05	1	1	2	P48147	P48147 [234-245]	0	1597.76
[K].WNEPFD	1xTMTpro [N	0.0001495	7.389E-05	1	1	1	P50453	P50453 [171-181]	0	1761.8403
[K].WNTDS\	1xTMTpro [K	9.557E-05	7.389E-05	1	1	2	O60613	O60613 [149-161]	0	2192.1367
[K].WPEVDI	2xTMTpro [K	0.0001176	7.389E-05	1	1	1	O43583	O43583 [182-198]	1	2886.5553
[K].WPQIMII	1xCarbamido	7.948E-05	7.389E-05	1	1	1	Q8NF37	Q8NF37 [200-214	0	2154.0795
[R].WPTETD	1xTMTpro [K	8.388E-05	7.389E-05	1	1	2	P27816	P27816 [322-332]	0	1828.9937
[R].WQAFQ	1xTMTpro [N	0.0001522	7.389E-05	1	1	3	P11277	P11277 [936-946]	0	1668.9029
[R].WQAVLA	1xTMTpro [N	0.0001914	7.389E-05	1	1	3	Q15149	Q15149 [1325-13	0	1590.8923
[R].WTEYGI	1xTMTpro [K	7.566E-05	7.389E-05	1	1	3	P21796	P21796 [64-74]	0	1983.0719

[R].WQEVIP	1xTMTpro [N	0.0001361	7.389E-05	1	1	2	P09601	P09601 [101-113]	0	1923.0118
[R].WQSIPL(	1xCarbamido	0.0001596	7.389E-05	1	1	2	P08603	P08603 [858-867]	0	1868.0596
[R].WSAGLT	1xTMTpro [K	0.000102	7.389E-05	1	1	1	P08185	P08185 [288-303]	0	2373.331
[K].WSDVAC	1xTMTpro [K	7.102E-05	7.389E-05	1	1	2	O75351	O75351 [133-143]	0	1740.9776
[K].WSEGP(	2xCarbamido	0.000112	7.389E-05	1	1	1	Q9NPY3	Q9NPY3 [160-181	0	2917.4104
[K].WSEPNE	1xTMTpro [K	0.0001326	7.389E-05	1	1	2	P39748	P39748 [298-308]	0	1982.0726
[R].WSGGEI	1xCarbamido	0.0001759	7.389E-05	1	1	1	O60687	O60687 [169-183]	0	2264.1877
[K].WSGYMI	1xTMTpro [N	0.0001786	7.389E-05	1	1	1	P21397	P21397 [441-454]	0	1845.8761
[R].WSMQNI	1xCarbamido	0.000134	7.389E-05	1	1	1	Q5SY16	Q5SY16 [210-228	0	2925.5611
[R].WSNAGI	1xTMTpro [N	9.364E-05	7.389E-05	1	1	1	P49770	P49770 [51-62]	0	1722.9168
[R].WSQLLA	1xTMTpro [N	0.0001944	7.389E-05	1	1	1	Q13813	Q13813 [2065-20	0	1520.8504
[R].WSSLAC	1xCarbamido	0.0002023	7.389E-05	1	1	1	P49368	P49368 [168-181]	0	2156.2029
[K].WSSPP(	2xCarbamido	0.0001402	7.389E-05	1	1	1	P08603	P08603 [920-932]	0	2154.0968
[R].WTCPC\	3xCarbamido	0.0001638	7.389E-05	1	1	1	P04275	P04275 [1944-195	0	1875.8471
[K].WQIGEQ	1xTMTpro [N	6.158E-05	7.389E-05	1	1	2	P35611	P35611 [370-382]	0	1940.986
[R].VYWND(	1xTMTpro [N	8.571E-05	7.389E-05	1	1	2	O43252	O43252 [387-397]	0	1732.8614
[K].VYVPTG	1xTMTpro [K	6.327E-05	7.389E-05	1	1	1	P07099	P07099 [392-411]	0	2887.589
[K].VYVIGSF	1xTMTpro [K	0.000119	7.389E-05	1	1	2	Q7L0Y3	Q7L0Y3 [306-315	0	1735.0286
[K].VTSIQDV	1xTMTpro [K	0.0001347	7.389E-05	1	1	5	P00738	P00738 [392-401]	0	1812.0511
[K].VTSVVVI	1xTMTpro [N	0.0001034	7.389E-05	1	1	2	P13798	P13798 [340-350]	0	1473.896
[K].VTTFEH(	1xTMTpro [K	0.0001779	7.389E-05	1	1	1	P29992	P29992 [121-133]	0	2131.2043
[K].VTVAGL/	1xTMTpro [K	0.0001769	7.389E-05	1	1	4	Q99497	Q99497 [33-41]	0	1423.9128
[R].VTVEDK	1xTMTpro [K	6.499E-05	7.389E-05	1	1	1	Q02487	Q02487 [376-390]	1	2368.3116
[K].VTVLFA(	1xTMTpro [N	6.237E-05	7.389E-05	1	1	1	Q14315	Q14315 [350-361]	0	1650.9498
[K].VTVNTNI	1xOxidation [	0.0001614	7.389E-05	1	1	1	P53618	P53618 [853-871]	0	2854.5628
[R].VTWEPA	1xTMTpro [K	0.0001506	7.389E-05	1	1	3	Q99715	Q99715 [1105-11	0	1821.0402
[R].VTYCPTI	1xCarbamido	0.0001066	7.389E-05	1	1	4	P21333	P21333 [2104-21	0	2490.3558
[K].VVAEVG	1xTMTpro [K	0.000171	7.389E-05	1	1	2	Q9ULC3	Q9ULC3 [107-122	0	2289.3674
[R].VVAGQII	1xTMTpro [K	7.613E-05	7.389E-05	1	1	1	Q9UBV2	Q9UBV2 [54-80]	0	3618.8406
[K].VVAIGE(	1xCarbamido	6.217E-05	7.389E-05	1	1	1	Q6P1N9	Q6P1N9 [107-119	0	1754.9066
[R].VVASDK	1xTMTpro [K	9.176E-05	7.389E-05	1	1	1	Q6P5R6	Q6P5R6 [96-107]	1	2018.1414
[R].VTQSNF	1xTMTpro [K	8.022E-05	7.389E-05	1	1	3	P21796	P21796 [164-174]	0	1822.0355
[R].VVATAP(	3xCarbamido	6.591E-05	7.389E-05	2	2	1	P42356; A4(	P42356 [1886-190	0	2466.211

[K].VVCSCT	2xCarbamido	0.0001888	7.389E-05	1	1	2	P00740	P00740 [153-162]	0	1534.7313
[K].VVDLLAT	1xTMTpro [N	0.0001192	7.389E-05	1	1	2	Q8N142	Q8N142 [49-62]	0	1805.0339
[R].VVDLMA	1xOxidation [	0.0001665	7.389E-05	1	1	4	P04406	P04406 [324-334]	0	1826.016
[R].VVDLMA	1xTMTpro [K	9.32E-05	7.389E-05	1	1	3	P04406	P04406 [324-335]	1	1939.0637
[K].VVDMFG	3xCarbamido	7.791E-05	7.389E-05	1	1	1	Q9BT22	Q9BT22 [379-395	0	2624.3353
[R].VVDSME	1xTMTpro [N	8.664E-05	7.389E-05	1	1	2	Q9Y285	Q9Y285 [128-138	0	1610.8015
[R].VVEEYF	1xTMTpro [K	0.0001806	7.389E-05	1	1	1	O43716	O43716 [110-126	0	2467.4092
[K].VVESGG	1xTMTpro [K	0.0001317	7.389E-05	1	1	1	Q9H583	Q9H583 [1534-15	0	1736.045
[K].VVETEDA	1xTMTpro [K	0.0001415	7.389E-05	1	1	1	Q15286	Q15286 [129-137	0	1661.9242
[K].VVFFAN	1xTMTpro [K	0.0002027	7.389E-05	1	1	1	O95786	O95786 [293-307	0	2418.3677
[R].VVFTCQ	1xCarbamido	0.0001063	7.389E-05	1	1	1	Q96J84	Q96J84 [240-256]	0	2243.1814
[R].VVGAMQ	1xTMTpro [N	0.0001186	7.389E-05	1	2	2	Q00610	Q00610 [177-188	0	1641.8953
[R].VVGAMQ	1xTMTpro [K	0.0001004	7.389E-05	1	2	1	Q00610	Q00610 [177-189	1	2074.1975
[R].VVAVTP	1xTMTpro [N	7.015E-05	7.389E-05	1	1	1	P22105	P22105 [848-869]	0	2754.5473
[K].VVGAVG	1xTMTpro [K	8.518E-05	7.389E-05	1	1	3	Q14914	Q14914 [169-178	0	1568.9139
[R].VTQILQS	1xCarbamido	0.0001354	7.389E-05	1	1	1	Q9UEY8	Q9UEY8 [58-80]	1	3384.8151
[K].VTPQSD	1xTMTpro [K	9.077E-05	7.389E-05	1	1	2	P34932	P34932 [467-477]	0	1700.9311
[K].VTEDTS	1xTMTpro [N	0.0001944	7.389E-05	1	1	1	P05165	P05165 [654-663]	0	1410.7759
[K].VTEEPM	1xTMTpro [K	0.0001397	7.389E-05	1	1	1	Q00013	Q00013 [77-87]	0	1826.0589
[K].VTEKDIV	2xTMTpro [K	8.786E-05	7.389E-05	1	1	2	P21397	P21397 [466-479]	1	2600.4752
[R].VTELNPI	1xCarbamido	0.000129	7.389E-05	1	1	2	Q9Y6N5	Q9Y6N5 [118-135	1	3055.5822
[K].VTEIWQI	1xTMTpro [N	0.0002023	7.389E-05	1	1	2	P08294	P08294 [42-52]	0	1722.9168
[R].VTEPISA	1xTMTpro [N	0.0001584	7.389E-05	1	1	1	O14791	O14791 [306-320	0	1934.999
[R].VTFEADI	1xTMTpro [K	7.543E-05	7.389E-05	1	1	1	Q9NX63	Q9NX63 [10-24]	0	2316.2579
[R].VTFEEG	1xCarbamido	0.0001511	7.389E-05	1	1	1	Q96SM3	Q96SM3 [687-702	0	2493.3343
[K].VTFQLTY	1xTMTpro [K	8.492E-05	7.389E-05	1	1	1	P19827	P19827 [158-169]	0	2078.2029
[K].VTFSCA	1xCarbamido	8.759E-05	7.389E-05	1	1	2	Q14203	Q14203 [1248-125	0	1604.8174
[R].VTGCPP	1xCarbamido	8.196E-05	7.389E-05	1	1	1	P04275	P04275 [2690-270	0	1894.9977
[K].VTGEGC	1xCarbamido	6.664E-05	7.389E-05	1	1	2	P01023	P01023 [1316-132	0	2163.1975
[K].VTGEVLI	1xTMTpro [N	0.0001112	7.389E-05	1	1	2	O75955	O75955 [393-403	0	1519.9015
[K].VTQLDLI	1xTMTpro [K	0.0001547	7.389E-05	1	1	2	Q13442	Q13442 [96-105]	0	1693.998
[K].VTLAVSI	1xTMTpro [K	0.000119	7.389E-05	1	1	2	Q9HC38	Q9HC38 [156-165	0	1682.0344
[K].VTIKPAP	2xTMTpro [K	0.0001581	7.389E-05	1	1	5	P01024	P01024 [1365-137	0	2125.3049

[K].VTIMWTI	1xTMTpro [N	8.44E-05	7.389E-05	1	1	2	P02751	P02751 [923-938]	0	2112.1119
[K].VTIMWTI	1xOxidation [	7.887E-05	7.389E-05	1	1	4	P02751	P02751 [923-938]	0	2128.1068
[R].VTLNPP(	1xTMTpro [K	8.059E-05	7.389E-05	1	1	2	Q86Y39	Q86Y39 [41-55]	0	2151.2669
[R].VTLPAGI	1xTMTpro [N	0.0001911	7.389E-05	1	1	1	Q969L2	Q969L2 [23-33]	0	1455.8854
[K].VTLSFPS	1xTMTpro [K	6.149E-05	7.389E-05	1	2	2	P55786	P55786 [129-145]	0	2359.3728
[R].VTISGLE	1xTMTpro [K	0.0001093	7.389E-05	1	1	2	P22105	P22105 [2356-236	0	1781.03
[K].VTLVQTI	1xTMTpro [N	9.785E-05	7.389E-05	1	1	2	Q15149	Q15149 [2565-257	0	1603.9702
[K].VTNDVC	1xCarbamido	0.000112	7.389E-05	1	1	1	Q14767	Q14767 [1590-16(	0	1593.8226
[K].VTNSTEI	1xTMTpro [K	0.0001005	7.389E-05	1	1	2	Q15075	Q15075 [473-485]	0	2121.1796
[K].VTPNEG	1xTMTpro [K	7.224E-05	7.389E-05	1	1	1	Q14690	Q14690 [1240-125	0	2201.2462
[K].VTPNPD	1xTMTpro [K	8.691E-05	7.389E-05	1	1	1	Q8N3V7	Q8N3V7 [633-649	0	2476.379
[K].VTPPAV	1xTMTpro [N	8.732E-05	7.389E-05	1	1	2	Q99735	Q99735 [35-48]	0	1790.9608
[K].VTIDGT(	1xCarbamido	8.965E-05	7.389E-05	1	1	1	Q63HR2	Q63HR2 [51-61]	0	1468.7749
[R].YATALYS	1xTMTpro [K	9.394E-05	7.389E-05	1	1	1	P48047	P48047 [41-51]	0	1753.998
[R].VVGCEG	1xCarbamido	0.0001952	7.389E-05	1	1	1	Q709C8	Q709C8 [2922-29	0	2355.2411
[K].VVGDRE	1xTMTpro [K	6.499E-05	7.389E-05	1	1	1	O43399	O43399 [178-206]	1	3493.7691
[K].VVSPWN	1xTMTpro [K	0.00015	7.389E-05	1	1	2	P11177	P11177 [174-184]	0	1840.0096
[K].VVSSLQ	1xTMTpro [K	8.813E-05	7.389E-05	1	1	1	P48681	P48681 [515-532]	1	2810.518
[K].VVTDETS	1xTMTpro [K	7.291E-05	7.389E-05	1	1	1	P16157	P16157 [788-801]	0	2147.2091
[K].VVTLLSD	1xTMTpro [K	0.0001762	7.389E-05	1	1	1	Q99570	Q99570 [541-553]	0	2035.2295
[R].VVTTFAS	1xTMTpro [N	9.262E-05	7.389E-05	1	1	2	P16452	P16452 [288-301]	0	1655.9036
[K].VVTYGM	1xTMTpro [K	8.827E-05	7.389E-05	1	1	1	Q99536	Q99536 [282-295]	0	2072.207
[R].VVVEEV	1xTMTpro [K	0.0001852	7.389E-05	1	1	1	Q9C0C9	Q9C0C9 [294-305	0	1978.208
[R].VVVSGL	1xTMTpro [K	0.0001214	7.389E-05	1	1	3	Q07955	Q07955 [123-138]	0	2277.3098
[K].VVVTME	1xTMTpro [K	0.0001536	7.389E-05	1	1	1	P55809	P55809 [437-446]	0	1708.9912
[K].VVVVTG.	1xTMTpro [K	0.0002015	7.389E-05	1	1	1	Q8TC12	Q8TC12 [43-55]	0	1823.1246
[K].VVWVEE	1xTMTpro [K	0.000144	7.389E-05	1	1	1	O00571	O00571 [419-428]	1	1855.0569
[K].VWDLFP	1xTMTpro [K	8.895E-05	7.389E-05	1	2	1	Q99613	Q99613 [765-776]	1	2083.1832
[K].VWIKPG.	2xTMTpro [K	0.0001556	7.389E-05	1	1	1	Q9Y221	Q9Y221 [97-115]	0	3098.7971
[R].VVSNTIE	1xTMTpro [K	0.000144	7.389E-05	1	1	1	P78559	P78559 [179-192]	0	2206.3091
[K].VWSPLV	1xTMTpro [K	0.0001232	7.389E-05	1	1	1	O00151	O00151 [88-99]	1	2009.1675
[K].VYLEQN	1xTMTpro [N	8.624E-05	7.389E-05	1	1	2	Q9H3S7	Q9H3S7 [614-625	0	1723.9298
[R].VYIGSFV	1xTMTpro [N	0.0001352	7.389E-05	2	2	2	Q9NZN3; Q9	Q9NZN3 [252-268	0	2318.2617

[R].VYINYYD	1xTMTpro [N	0.0001448	7.389E-05	1	1	7	P14174	P14174 [95-115]	0	2731.2782
[K].VYIVHGV	1xTMTpro [N	0.000117	7.389E-05	1	1	1	Q15024	Q15024 [12-23]	0	1731.9713
[R].VYNASS	1xTMTpro [K	8.558E-05	7.389E-05	1	1	1	Q9NRG9	Q9NRG9 [177-18	0	1987.172
[K].VYQLLPI	1xTMTpro [K	9.847E-05	7.389E-05	1	1	1	Q00978	Q00978 [111-128]	0	2490.4576
[R].VYQVTE	1xTMTpro [K	0.000182	7.389E-05	1	1	1	Q5JRA6	Q5JRA6 [1208-12	0	2060.1519
[R].VYSLFLD	1xTMTpro [N	0.0001892	7.389E-05	1	1	1	Q9Y230	Q9Y230 [429-438	0	1532.828
[R].VYSLND	2xTMTpro [K	0.0001944	7.389E-05	1	1	1	P01031	P01031 [145-156]	0	2275.3478
[K].VYTAQN	1xTMTpro [K	0.000117	7.389E-05	1	1	1	P00750	P00750 [248-263]	0	2226.2738
[K].VYTVGP	1xTMTpro [N	7.791E-05	7.389E-05	1	2	1	O43314	O43314 [249-262]	0	1852.9513
[K].VYVELQI	1xOxidation [	8.854E-05	7.389E-05	1	1	5	P02730	P02730 [57-69]	0	2219.2125
[K].VYVGNL	1xTMTpro [K	7.707E-05	7.389E-05	1	1	1	P84103	P84103 [12-28]	1	2485.3655
[K].VYENYP	1xTMTpro [N	0.0001033	7.389E-05	1	1	2	P35659	P35659 [350-362]	0	1966.9717
[K].VVGCSC	2xCarbamido	0.0001983	7.389E-05	1	1	1	P25398	P25398 [103-112]	0	1714.984
[R].VVSMDE	1xOxidation [	8.854E-05	7.389E-05	1	1	1	P01023	P01023 [148-171]	0	3434.8525
[K].VVQETV	1xTMTpro [N	0.0001004	7.389E-05	1	1	1	Q9Y2J2	Q9Y2J2 [851-861]	0	1604.9179
[K].VVGEHV	1xTMTpro [N	0.0001536	7.389E-05	1	1	3	O14558	O14558 [92-102]	0	1535.8613
[K].VVGIHM	1xCarbamido	0.0001558	7.389E-05	1	1	1	P00390	P00390 [474-496]	0	3067.6387
[R].VVLAPQ	1xTMTpro [N	0.0001395	7.389E-05	1	1	2	Q13308	Q13308 [227-238]	0	1569.9647
[R].VVLEGG	1xTMTpro [N	0.0001288	7.389E-05	1	1	2	P05164	P05164 [537-548]	0	1584.9644
[K].VVLEGP	1xTMTpro [N	0.0001127	7.389E-05	1	1	4	Q9NR12	Q9NR12 [6-17]	0	1631.9229
[R].VVLGAH	1xTMTpro [N	0.0001373	7.389E-05	1	1	3	P08246	P08246 [82-91]	0	1369.8235
[K].VVIGMDY	1xOxidation [	0.0001186	7.389E-05	1	1	2	P06733	P06733 [240-253]	0	1860.9849
[K].VVLLEDI	1xTMTpro [N	9.105E-05	7.389E-05	1	1	1	Q96HY6	Q96HY6 [228-241	0	1816.0863
[K].VVLIGDS	1xTMTpro [K	6.993E-05	7.389E-05	1	2	3	Q15907	Q15907 [14-24]	0	1652.0238
[K].VVLIGES	1xTMTpro [K	0.0001681	7.389E-05	1	1	1	P57735	P57735 [15-25]	0	1666.0395
[R].VVIQPDI	1xCarbamido	9.035E-05	7.389E-05	1	1	1	O94901	O94901 [657-672]	0	2515.3663
[R].VVIQPEA	1xTMTpro [K	0.0001665	7.389E-05	1	1	1	P29590	P29590 [638-651]	0	2236.2873
[R].VVIQSNI	1xTMTpro [N	0.0001253	7.389E-05	1	1	1	Q93008	Q93008 [777-788]	0	1620.8876
[K].VVSKEE	1xTMTpro [K	0.0001165	7.389E-05	1	1	1	Q9NP61	Q9NP61 [264-276	1	2041.2149
[K].VVLSSQI	2xTMTpro [K	0.0001258	7.389E-05	1	3	2	Q5JPE7	Q5JPE7 [431-440	1	2031.2266
[R].VVLWGS	1xTMTpro [K	0.0001077	7.389E-05	1	1	2	O95834	O95834 [318-327]	0	1762.0031
[K].VVNLGV	1xTMTpro [K	0.0001053	7.389E-05	1	1	1	Q8NI36	Q8NI36 [815-824]	0	1649.0606
[K].VVNIVPV	1xTMTpro [K	0.0001995	7.389E-05	1	1	6	Q9UHD8	Q9UHD8 [436-44	0	1660.1017

[K].VVNTQC	1xCarbamido	8.171E-05	7.389E-05	1	1	1	Q8NG11	Q8NG11 [195-205	0	1614.8229
[R].VVNVSS	1xTMTpro [N	0.0001072	7.389E-05	1	1	3	P16152	P16152 [135-145]	0	1494.8633
[R].VVPGYG	1xTMTpro [N	7.731E-05	7.389E-05	1	1	3	O75390	O75390 [341-351]	0	1471.8705
[K].VVPIASL	1xTMTpro [K	7.462E-05	7.389E-05	1	1	2	P27694	P27694 [184-196]	0	2011.2083
[K].VVPQAV	1xTMTpro [K	7.314E-05	7.389E-05	1	1	1	Q5T447	Q5T447 [725-741]	0	2589.5147
[K].VVPTTDI	1xTMTpro [K	0.0001769	7.389E-05	1	1	1	O75663	O75663 [129-140]	0	1963.0992
[R].VVQCSD	1xCarbamido	8.651E-05	7.389E-05	1	1	2	P07585	P07585 [64-74]	0	1842.0287
[R].VVQCSD	1xCarbamido	0.0001556	7.389E-05	1	1	4	P21810	P21810 [73-82]	0	1727.0017
[K].VVQDAQ	1xTMTpro [K	0.0001849	7.389E-05	1	1	2	P17050	P17050 [138-149]	0	2030.1203
[K].VVQDTY	1xTMTpro [K	0.0002019	7.389E-05	1	1	2	O14964	O14964 [127-136]	0	1833.0436
[R].VVLVNN	1xTMTpro [N	0.0001039	7.389E-05	1	1	1	O95295	O95295 [92-105]	0	1914.1092
[R].VTEAEIV	1xTMTpro [K	0.0001842	7.389E-05	1	1	3	O75369	O75369 [2140-215	0	1782.0327
[K].YAVLYQI	1xTMTpro [K	0.0001271	7.389E-05	1	1	2	P55209	P55209 [106-117]	1	2121.2352
[R].YAVWFG	1xCarbamido	7.102E-05	7.389E-05	1	1	1	P61158	P61158 [375-397]	0	3287.6513
[R].YPGAYY	1xTMTpro [K	0.0001188	7.389E-05	1	1	4	Q9UBX5	Q9UBX5 [374-384	0	1971.1235
[R].YPGSTF	1xTMTpro [N	0.000142	7.389E-05	1	1	1	P53396	P53396 [692-703]	0	1726.8906
[K].YPIEHGI	1xTMTpro [K	8.965E-05	7.389E-05	2	4	73	P62736; P68	P62736 [71-86]; F	0	2569.3252
[R].YPLFEG	2xTMTpro [K	6.758E-05	7.389E-05	1	1	1	P41250	P41250 [723-734]	1	2309.3321
[K].YPQAGA	1xTMTpro [K	0.0001842	7.389E-05	1	1	1	Q07075	Q07075 [915-925]	0	1782.0154
[K].YPSPFF	1xTMTpro [K	0.0001806	7.389E-05	1	1	2	Q08211	Q08211 [1038-104	0	1926.0657
[R].YPVAGY	1xTMTpro [N	6.337E-05	7.389E-05	1	1	1	Q5XPI4	Q5XPI4 [256-274]	0	2418.3101
[R].YPVNSV	1xTMTpro [K	0.0001455	7.389E-05	1	1	2	P17987	P17987 [190-199]	0	1755.066
[R].YPYLML	1xTMTpro [K	0.0001034	7.389E-05	1	1	1	Q7Z4H8	Q7Z4H8 [382-394	0	2120.2321
[R].YQAVQE	1xTMTpro [N	0.0001653	7.389E-05	1	1	2	P46939	P46939 [886-896]	0	1611.8298
[R].YQCHEE	1xCarbamido	0.0001331	7.389E-05	1	1	2	P55268	P55268 [747-758]	0	2055.0825
[R].YQDGVY	1xTMTpro [N	0.000144	7.389E-05	1	2	1	Q9UKU9	Q9UKU9 [462-472	0	1737.8556
[K].YQDLYT	1xTMTpro [N	0.00012	7.389E-05	1	1	1	P54289	P54289 [64-76]	0	1886.9568
[K].YQEEFE	2xTMTpro [K	8.022E-05	7.389E-05	1	1	1	P49257	P49257 [289-303]	1	2910.5454
[K].YQEEVP	1xTMTpro [K	0.0001623	7.389E-05	1	1	1	O43264	O43264 [709-719]	0	1959.1083
[R].YQEQG	1xTMTpro [N	0.0001626	7.389E-05	1	1	2	Q9UJU6	Q9UJU6 [224-235	0	1653.8152
[K].YQFSLS	1xTMTpro [K	0.0001335	7.389E-05	1	1	2	Q9NZ08	Q9NZ08 [789-798	0	1797.9878
[R].YQIDDKI	1xTMTpro [K	7.531E-05	7.389E-05	1	1	1	Q9NP92	Q9NP92 [231-242	0	2112.1693
[R].YQILPLH	1xTMTpro [N	0.0001415	7.389E-05	1	1	1	Q08211	Q08211 [681-692]	0	1769.0393

[R].YQLPVY(	1xCarbamido	0.0001056	7.389E-05	1	1	1	Q8WUN7	Q8WUN7 [116-13	0	2786.5117
[K].YQIYQD(	1xTMTpro [K	7.875E-05	7.389E-05	1	1	1	Q9NR99	Q9NR99 [2395-24	0	2191.2618
[R].YQPCLP	1xCarbamido	0.0001342	7.389E-05	1	1	1	Q96RP9	Q96RP9 [720-734	0	2400.2725
[K].YQQGDF	1xCarbamido	0.000142	7.389E-05	1	1	1	P67870	P67870 [101-111]	0	1694.7916
[K].YQQVDE	1xTMTpro [N	0.0002055	7.389E-05	1	1	1	P09619	P09619 [970-979]	0	1630.8396
[K].YQYAIDE	1xTMTpro [N	0.000165	7.389E-05	1	1	2	Q8N128	Q8N128 [132-141	0	1687.8287
[R].YREDLD	1xTMTpro [N	0.0001944	7.389E-05	1	1	1	P33527	P33527 [1302-13	1	1629.892
[K].YRPEEV	1xTMTpro [K	0.0001075	7.389E-05	1	1	1	Q9HAU0	Q9HAU0 [628-638	0	1943.073
[K].YPFILPH	1xTMTpro [K	7.428E-05	7.389E-05	1	2	3	Q9ULC4	Q9ULC4 [88-99]	0	2093.2039
[R].YNVLGA	1xOxidation		6.939E-05	7.389E-05	1	1	2	Q1KMD3	Q1KMD3 [481-49	0	1928.0231
[K].YNQPFF	1xTMTpro [K	0.0001347	7.389E-05	1	1	1	P29144	P29144 [945-958]	0	2279.2204
[K].YNPTWH	1xCarbamido	0.0001662	7.389E-05	2	2	3	Q96FJ2; P63	Q96FJ2 [50-60]; F	0	1706.8756
[K].YIQQTKF	1xTMTpro [K	0.0001333	7.389E-05	1	1	2	O43809	O43809 [24-35]	0	2098.2516
[K].YISGFGN	1xCarbamido	0.000106	7.389E-05	1	1	1	Q93099	Q93099 [6-20]	0	2021.9194
[R].YISPDQL	1xTMTpro [K	9.205E-05	7.389E-05	1	1	2	P06733	P06733 [270-281]	0	2034.1403
[R].YLSYTLN	1xTMTpro [N	0.000123	7.389E-05	1	1	2	P15144	P15144 [844-855]	0	1771.9914
[K].YLTAEAF	1xTMTpro [K	8.745E-05	7.389E-05	1	1	2	Q16658	Q16658 [23-32]	0	1754.9973
[R].YLTESY(	1xTMTpro [N	0.0001407	7.389E-05	1	1	1	Q9UM54	Q9UM54 [167-18	0	2036.9732
[R].YLTLDGI	1xTMTpro [N	0.0001008	7.389E-05	1	1	2	Q7L576	Q7L576 [815-826	0	1752.895
[R].YLTTAVI	1xTMTpro [K	0.0001194	7.389E-05	1	1	2	P60228	P60228 [256-265]	0	1732.05
[R].YIVPMLT	1xTMTpro [K	0.0001681	7.389E-05	1	1	2	P14550	P14550 [298-308]	0	1844.0847
[K].YIYDSAF	1xTMTpro [K	0.0001361	7.389E-05	1	1	2	Q9H9B4	Q9H9B4 [73-86]	0	2251.1527
[K].YLYSSEI	1xTMTpro [K	9.831E-05	7.389E-05	1	1	3	Q13200	Q13200 [432-441	0	1889.0188
[R].YLYTLVI	2xTMTpro [K	0.0001087	7.389E-05	1	1	2	P63173	P63173 [41-52]	1	2398.4414
[K].YMADMD	1xTMTpro [K	0.0001445	7.389E-05	1	1	1	Q9UNE7	Q9UNE7 [207-22	0	2429.186
[R].YRVPDV	1xTMTpro [N	0.0001533	7.389E-05	1	1	2	Q14697	Q14697 [113-127	1	1985.1503
[K].YMASGP	1xOxidation		0.0001005	7.389E-05	1	1	1	Q13232	Q13232 [84-102]	0	2398.2582
[R].YMDGM	1xOxidation		0.0001026	7.389E-05	1	1	4	P23142	P23142 [651-661]	0	1547.7881
[K].YMEEND	2xTMTpro [K	0.0001533	7.389E-05	1	1	2	P51572	P51572 [150-159]	1	2210.2307
[R].YMGLPT	1xTMTpro [N	0.0001266	7.389E-05	1	1	2	P27487	P27487 [670-684]	0	2125.0344
[R].YMPQNF	1xCarbamido	7.936E-05	7.389E-05	1	1	2	Q09028	Q09028 [132-143	0	2044.1215
[R].YNAYCC	2xCarbamido	0.0001665	7.389E-05	1	1	4	P35555	P35555 [63-72]	0	1926.9486
[K].YNEQHV	1xTMTpro [N	8.827E-05	7.389E-05	1	1	3	P21333	P21333 [1938-19	0	1906.9731

[K].YNLDASI	1xTMTpro [K	0.0001684	7.389E-05	1	1	1	O95218	O95218 [183-195]	0	2122.0432
[R].YNLEEL\`	1xCarbamido	0.0001907	7.389E-05	1	1	1	Q13619	Q13619 [81-97]	0	2718.4053
[K].YNIEKDL	2xTMTpro [K	0.0001803	7.389E-05	1	1	1	P63167	P63167 [32-43]	1	2327.3903
[K].YNILGTN	1xOxidation	7.474E-05	7.389E-05	1	1	4	Q00839	Q00839 [525-536]	0	2007.1076
[R].YNIPHGI	1xTMTpro [N	8.745E-05	7.389E-05	1	1	1	P50402	P50402 [19-31]	0	1700.9403
[R].YNPENL	1xTMTpro [N	0.00012	7.389E-05	1	1	3	Q9UBQ5	Q9UBQ5 [21-31]	0	1623.8662
[K].YNPPDH	1xTMTpro [N	0.0001427	7.389E-05	1	1	1	P25440	P25440 [428-440]	0	1788.9022
[R].YMDGMT	1xTMTpro [N	0.0001536	7.389E-05	1	1	1	P23142	P23142 [651-661]	0	1531.7932
[R].YSALNV	1xTMTpro [K	0.0001013	7.389E-05	1	1	1	O00461	O00461 [111-122]	0	2040.1556
[R].YSCIDDS	1xCarbamido	9.364E-05	7.389E-05	1	1	1	Q9H4Z3	Q9H4Z3 [453-463	0	1666.7702
[K].YSELTT\	1xTMTpro [N	0.0001153	7.389E-05	1	1	1	O43592	O43592 [68-80]	0	1883.0557
[R].YVDGGF	1xTMTpro [K	0.0001522	7.389E-05	1	1	1	Q16853	Q16853 [384-393]	0	1638.8805
[R].YVDQVL	1xTMTpro [K	0.0001206	7.389E-05	1	1	1	P11717	P11717 [1583-159	0	1976.1712
[R].YVEMST	1xTMTpro [K	9.724E-05	7.389E-05	1	1	1	Q9Y376	Q9Y376 [172-189	0	2651.3559
[K].YVLENHI	1xTMTpro [K	8.059E-05	7.389E-05	1	1	1	Q5SSJ5	Q5SSJ5 [367-385	0	2866.5166
[R].YVLEPEI	1xTMTpro [K	7.731E-05	7.389E-05	1	1	1	Q9NYU2	Q9NYU2 [1039-10	0	2555.3525
[R].YVIIPTTI	1xTMTpro [N	8.44E-05	7.389E-05	1	1	1	O15484	O15484 [468-486]	0	2494.3665
[K].YVISAIPI	1xTMTpro [K	0.0001216	7.389E-05	1	1	4	P27338	P27338 [259-271]	0	1998.1953
[K].YVMLPV	1xCarbamido	0.0001094	7.389E-05	1	1	5	P00738	P00738 [298-311]	0	2028.0214
[R].YVSGYG	1xTMTpro [K	0.0001525	7.389E-05	1	1	2	Q969N2	Q969N2 [357-365	0	1622.9398
[K].YVTSAPI	1xOxidation	0.0001085	7.389E-05	2	2	5	P0DOX6; P0	P0DOX6 [500-514	0	1920.9809
[K].YWNDCI	1xCarbamido	0.000171	7.389E-05	1	1	2	P04196	P04196 [85-95]	0	1742.7764
[R].YYCFQG	1xCarbamido	0.0001907	7.389E-05	1	1	6	P02790	P02790 [198-208]	0	1799.8858
[K].YYDDTY	1xTMTpro [K	0.0001528	7.389E-05	1	1	4	P61923	P61923 [30-39]	0	1858.9719
[R].YVCEGP	1xCarbamido	0.000121	7.389E-05	1	1	1	P19838	P19838 [59-76]	0	2440.2422
[K].YYDQIIT	1xTMTpro [K	0.0001979	7.389E-05	1	1	1	O43157	O43157 [2098-21	0	2566.3532
[K].YYLCGF	2xCarbamido	9.542E-05	7.389E-05	1	1	1	O95232	O95232 [37-52]	0	2316.1112
[K].YYLHDD	1xTMTpro [K	0.0001713	7.389E-05	1	1	1	Q9Y2W1	Q9Y2W1 [880-89	1	2292.1388
[K].YYNAES	1xTMTpro [N	0.0001096	7.389E-05	1	1	1	Q9NP58	Q9NP58 [465-475	0	1726.8244
[K].YYNCNV	1xCarbamido	0.0001266	7.389E-05	1	1	1	Q6ZMP0	Q6ZMP0 [979-989	0	1689.8702
[K].YYNDYG	1xTMTpro [K	0.0001138	7.389E-05	2	2	1	Q8WVM7; Q	Q8WVM7 [896-90	0	1872.0035
[K].YYQENF	2xCarbamido	0.00015	7.389E-05	1	1	2	P13671	P13671 [55-67]	0	2377.1448
[K].YYSEEAI	1xTMTpro [N	0.0001172	7.389E-05	1	1	1	Q13769	Q13769 [36-46]	0	1677.8291

[R].YYSLDEI	1xTMTpro [K	0.0001253	7.389E-05	1	1	4	Q9BRF8	Q9BRF8 [292-301	0	1854.9981
[K].YYTLEEI	1xTMTpro [K	0.0001979	7.389E-05	1	1	4	P00167	P00167 [11-19]	0	1795.0133
[R].YYTSAS(	1xOxidation		0.0001072	7.389E-05	1	1	3	P07900	P07900 [465-478]	0	2175.1135
[R].YYVDVP	1xTMTpro [N	0.0001614	7.389E-05	1	1	1	Q5QJ74	Q5QJ74 [307-319	0	1945.0026
[K].YYVLLHI	1xTMTpro [N	0.0001147	7.389E-05	1	1	1	Q9Y2L5	Q9Y2L5 [192-206	0	2069.0623
[K].YYVTIID/	1xTMTpro [N	9.248E-05	7.389E-05	1	2	8	P68104	P68104 [85-96]	0	1708.9342
[R].YYDVMS	1xTMTpro [N	7.827E-05	7.389E-05	1	1	2	O15460	O15460 [341-352	0	1852.8594
[R].YLQQLA	1xTMTpro [K	7.911E-05	7.389E-05	1	1	1	Q01433	Q01433 [269-281	0	2197.2836
[K].YVATLG'	1xTMTpro [N	0.0001123	7.389E-05	1	1	2	P62826	P62826 [39-56]	0	2356.3097
[R].YTNWA/	1xTMTpro [K	9.988E-05	7.389E-05	1	1	1	P22897	P22897 [1176-118	0	1802.9569
[K].YSETDAI	1xTMTpro [N	0.000181	7.389E-05	1	1	1	Q86SK9	Q86SK9 [137-148	0	1679.7944
[R].YSGSYN	1xTMTpro [N	0.0001933	7.389E-05	1	1	1	Q96PK6	Q96PK6 [648-657	0	1541.7555
[R].YSKEEE	2xTMTpro [K	0.0001039	7.389E-05	1	1	1	Q9HD45	Q9HD45 [255-275	2	3523.6814
[K].YSIDQEI	1xTMTpro [K	0.000187	7.389E-05	1	1	1	Q9BYT8	Q9BYT8 [373-381	0	1750.9871
[K].YSLKPNI	2xTMTpro [K	0.0001216	7.389E-05	1	1	2	P55263	P55263 [48-61]	0	2546.4646
[R].YSLLEA(	1xTMTpro [N	0.0001602	7.389E-05	1	1	1	Q96JQ0	Q96JQ0 [2743-27	0	1649.8818
[K].YSPPAIS	1xTMTpro [K	0.0001874	7.389E-05	1	1	1	Q14694	Q14694 [364-376	0	1996.1611
[K].YSQKED	2xTMTpro [K	0.0001617	7.389E-05	1	1	3	P09493	P09493 [214-226]	2	2629.429
[K].YSQLVVI	1xTMTpro [N	0.0002023	7.389E-05	1	1	2	Q92522	Q92522 [48-57]	0	1511.8753
[K].YSQVLAI	1xTMTpro [K	0.0001085	7.389E-05	1	1	3	P62269	P62269 [95-106]	0	1930.089
[K].YSQYNA	1xTMTpro [N	0.0001614	7.389E-05	1	1	1	Q6ZMI0	Q6ZMI0 [233-246	0	1993.0575
[R].YSSEVA	1xTMTpro [K	0.0001149	7.389E-05	1	1	1	Q8WTT2	Q8WTT2 [779-79	0	2282.2048
[K].YSSLAE/	1xTMTpro [K	0.000149	7.389E-05	1	1	3	P00915	P00915 [129-138]	0	1634.9245
[R].YTTPED/	1xTMTpro [N	0.0001952	7.389E-05	1	2	2	P63092	P63092 [318-333]	0	2078.9838
[K].YSSSFLI	1xTMTpro [N	0.0001188	7.389E-05	1	1	1	Q6VY07	Q6VY07 [680-690	0	1608.7977
[R].YSTDVS	1xTMTpro [K	6.509E-05	7.389E-05	1	2	2	P00367	P00367 [152-162]	0	1850.0039
[R].YSYYDE	1xTMTpro [N	7.997E-05	7.389E-05	1	1	1	Q9Y316	Q9Y316 [199-211	0	1976.9197
[R].YTASGC	1xCarbamid	7.059E-05	7.389E-05	1	1	1	O60341	O60341 [595-608	0	1844.9496
[R].YTCHVQ	1xCarbamid	0.0001136	7.389E-05	2	2	1	P01889; P04	P01889 [281-297]	0	2657.4841
[K].YTDQLKI	1xTMTpro [K	7.863E-05	7.389E-05	1	1	1	Q9Y625	Q9Y625 [193-205	0	2144.1996
[R].YTEFYH'	1xTMTpro [K	9.693E-05	7.389E-05	1	1	1	Q96HC4	Q96HC4 [247-261	0	2390.2272
[K].YTEGVQ	1xTMTpro [K	9.647E-05	7.389E-05	1	1	2	Q9Y5B9	Q9Y5B9 [893-904	0	2034.1152
[K].YTFAAHI	1xTMTpro [K	0.0001471	7.389E-05	1	1	2	Q15363	Q15363 [80-91]	0	2013.0396

[R].YTFAPS\	1xTMTpro [K	9.785E-05	7.389E-05	1	1	3	Q9NR12	Q9NR12 [104-114	0	1835.0559
[R].YTGASL	1xTMTpro [K	0.0001354	7.389E-05	1	1	1	O15379	O15379 [107-121]	0	2174.2061
[R].YTGDKC	3xCarbamido	0.0001192	7.389E-05	1	1	2	Q07954	Q07954 [4226-42	1	2765.2726
[K].YTGEDF	1xTMTpro [N	0.0001347	7.389E-05	1	1	1	Q14204	Q14204 [2967-29	0	1663.7771
[R].YTLNFE/	1xTMTpro [K	0.0001239	7.389E-05	1	1	4	P13611	P13611 [161-170]	0	1793.0089
[K].YSSTEE\	1xTMTpro [K	0.0001084	7.389E-05	1	1	2	P56199	P56199 [227-238]	0	1905.0825
[R].YINTEH(	1xTMTpro [N	0.0001929	7.389E-05	1	1	2	Q15437	Q15437 [707-718]	0	1636.8363
[R].YLNSGG	1xTMTpro [N	0.000126	7.389E-05	1	1	1	O00469	O00469 [163-178]	0	2095.0932
[R].YLNIFGE	1xTMTpro [K	0.0001005	7.389E-05	1	1	1	Q9UJS0	Q9UJS0 [44-56]	0	2115.173
[R].YELGSG	1xTMTpro [N	9.542E-05	7.389E-05	1	1	2	P98160	P98160 [3993-40(	0	1481.8647
[K].YELISET	1xTMTpro [K	9.8E-05	7.389E-05	2	2	2	Q12906; Q9(	Q12906 [541-554]	1	2200.1854
[R].YEILTPN	1xTMTpro [K	0.0001331	7.389E-05	2	2	2	P35580; Q7	P35580 [728-738]	0	1867.1185
[R].YEILTPN	1xTMTpro [K	9.408E-05	7.389E-05	1	1	3	P35579	P35579 [721-731]	0	1883.1134
[K].YELQQL	1xTMTpro [N	0.0001359	7.389E-05	1	1	2	Q92878	Q92878 [462-473]	0	1728.8724
[K].YEITTIH	1xTMTpro [N	9.394E-05	7.389E-05	1	1	3	P05546	P05546 [193-203]	0	1710.9498
[K].YENYEL	1xTMTpro [K	0.0001506	7.389E-05	1	1	2	P04114	P04114 [1563-15	0	1780.9977
[R].YEPQLP	1xTMTpro [K	0.0001432	7.389E-05	1	1	2	P07942	P07942 [616-626]	0	2050.089
[R].YESLTDI	2xTMTpro [K	0.00011	7.389E-05	2	4	2	P07900; P0	P07900 [61-74]; F	1	2452.3751
[R].YESSSY	1xTMTpro [N	8.298E-05	7.389E-05	1	1	1	Q07157	Q07157 [1061-10	0	1773.8251
[R].YEVDQC	1xTMTpro [K	7.146E-05	7.389E-05	1	1	1	P04114	P04114 [2348-23(	0	2217.2081
[K].YEVTED\	1xTMTpro [N	0.0001739	7.389E-05	1	1	2	Q9UKG1	Q9UKG1 [161-17	0	1665.8291
[R].YEVTSG	1xTMTpro [N	8.518E-05	7.389E-05	1	1	1	P15924	P15924 [28-39]	0	1474.7457
[R].YEIEETE	1xTMTpro [K	9.049E-05	7.389E-05	1	1	3	Q05682	Q05682 [160-170]	0	1950.0563
[K].YEYEVD	2xTMTpro [K	0.0001084	7.389E-05	1	1	2	Q96RQ1	Q96RQ1 [62-73]	1	2422.2958
[K].YFGDIIS	1xTMTpro [N	0.0001212	7.389E-05	1	1	2	Q96CN7	Q96CN7 [132-142	0	1558.8549
[K].YFLGTP\	1xTMTpro [K	0.0001058	7.389E-05	1	1	2	Q13459	Q13459 [620-639]	0	2874.5872
[R].YFLNHID	1xTMTpro [N	6.227E-05	7.389E-05	1	1	1	P46937	P46937 [188-203]	0	2339.174
[R].YFLQMT	1xTMTpro [K	0.0002031	7.389E-05	1	1	1	Q9BX59	Q9BX59 [127-137	0	1969.096
[R].YFLSGS	1xTMTpro [K	0.0001782	7.389E-05	1	1	1	Q5JSH3	Q5JSH3 [662-671	0	1694.9609
[K].YFVEAG	1xTMTpro [N	9.148E-05	7.389E-05	1	1	2	P17987	P17987 [299-309]	0	1517.8106
[R].YFVISNT	1xTMTpro [N	9.894E-05	7.389E-05	1	1	1	P49354	P49354 [241-253]	0	1853.9353
[K].YFVQGM	1xOxidation	7.767E-05	7.389E-05	1	1	1	Q92597	Q92597 [307-322]	0	2146.0091
[K].YGAHNY	1xTMTpro [N	0.0001914	7.389E-05	1	1	1	P04181	P04181 [50-64]	0	2041.0939

[K].YGALVIC	1xCarbamido	0.000131	7.389E-05	1	1	1	P43897	P43897 [234-246]	0	2106.1397
[K].YGDLAN	1xTMTpro [K	0.0002011	7.389E-05	1	2	1	P55809	P55809 [407-418]	0	1973.081
[K].YGEPGE	1xTMTpro [K	7.531E-05	7.389E-05	1	1	2	P23246	P23246 [320-330]	0	1861.0351
[K].YGFCEA	1xCarbamido	6.811E-05	7.389E-05	1	1	1	P07360	P07360 [185-202]	1	2516.2312
[KR].YEYHW	1xTMTpro [K	0.0001999	7.389E-05	1	2	2	Q7L9L4	Q7L9L4 [93-104]	0	2105.0948
[K].YGGDEII	1xTMTpro [N	9.678E-05	7.389E-05	1	1	4	P21333	P21333 [1622-163	0	1704.8553
[K].YELDKAI	1xTMTpro [K	0.0001482	7.389E-05	1	1	1	P01024	P01024 [1432-144	1	1852.0096
[K].YEGIGPV	1xTMTpro [N	6.706E-05	7.389E-05	1	1	1	O60504	O60504 [121-137]	0	2092.0704
[R].YCAQDA	1xCarbamido	8.009E-05	7.389E-05	1	1	2	Q13011	Q13011 [186-196]	0	1985.0446
[R].YCVDVN	3xCarbamido	0.0001395	7.389E-05	1	1	1	Q9NS15	Q9NS15 [613-629	0	2316.0014
[R].YDAFGE	1xOxidation [	6.769E-05	7.389E-05	1	1	1	Q9Y2X3	Q9Y2X3 [372-388	0	2154.9569
[K].YDEAAS	1xTMTpro [K	0.0001036	7.389E-05	1	1	3	P04899	P04899 [297-307]	0	1883.0042
[R].YDEMVE	2xTMTpro [K	9.957E-05	7.389E-05	1	1	1	P62258	P62258 [20-29]	1	2172.1861
[R].YDGLVG	1xTMTpro [K	0.0001466	7.389E-05	2	2	3	P12081; P49	P12081 [363-373]	0	1850.0014
[K].YDHQAE	1xTMTpro [N	0.0001975	7.389E-05	1	1	5	Q15417	Q15417 [24-33]	0	1579.7672
[R].YDLGGL	1xTMTpro [K	0.000139	7.389E-05	1	1	2	Q15274	Q15274 [162-171]	0	1703.0057
[R].YDLLCLE	1xCarbamido	8.096E-05	7.389E-05	1	1	2	Q9NSD9	Q9NSD9 [72-82]	0	1654.9157
[K].YDPGAL	1xTMTpro [K	0.0001299	7.389E-05	1	1	1	Q9NTK5	Q9NTK5 [254-270	0	2398.3878
[K].YDPPLEI	1xTMTpro [N	8.518E-05	7.389E-05	1	1	1	P47756	P47756 [79-92]	0	1822.8965
[K].YDPTIED	1xTMTpro [K	0.0001065	7.389E-05	2	2	1	P61224; P62	P61224 [32-42]; P	1	1995.0679
[K].YDVENC	1xCarbamido	0.0001971	7.389E-05	1	1	5	P01023	P01023 [558-567]	0	1833.9661
[K].YEKPDG	1xTMTpro [K	0.0001397	7.389E-05	1	1	1	Q13740	Q13740 [58-71]	0	2234.2465
[K].YDVQHL	1xTMTpro [N	0.0001322	7.389E-05	1	1	1	O14653	O14653 [75-85]	0	1647.9138
[K].YDYVEV	1xTMTpro [N	0.0001277	7.389E-05	1	1	1	O14786	O14786 [84-100]	0	2394.0958
[R].YEALKEI	1xTMTpro [K	0.0001188	7.389E-05	1	1	2	Q13813	Q13813 [766-776]	1	1915.0967
[K].YEAIVEE	2xTMTpro [K	0.0001888	7.389E-05	1	1	1	Q99417	Q99417 [76-85]	1	2135.2528
[K].YEAPQA	1xTMTpro [N	7.004E-05	7.389E-05	1	1	1	O60784	O60784 [386-402]	0	2023.0416
[K].YEAWLA	1xTMTpro [N	0.0001322	7.389E-05	1	1	1	Q15642	Q15642 [464-474]	0	1628.824
[K].YEDFGP	1xTMTpro [K	0.0001392	7.389E-05	1	1	1	Q9BTY2	Q9BTY2 [100-110	0	1896.0399
[K].YEDFKEI	2xTMTpro [K	0.0001313	7.389E-05	1	1	3	Q9NTK5	Q9NTK5 [350-363	1	2557.3602
[R].YEDGIEV	1xTMTpro [K	7.695E-05	7.389E-05	1	1	1	O60832	O60832 [323-339]	0	2558.4209
[R].YEDLSH	1xTMTpro [K	0.0001558	7.389E-05	1	1	1	P46939	P46939 [1318-132	0	1900.0308
[K].YEEENF	1xTMTpro [K	7.66E-05	7.389E-05	1	1	2	P14927	P14927 [84-96]	0	2345.2197

[K].YEELFPA	1xTMTpro [N	0.0001094	7.389E-05	1	1	2	P54920	P54920 [228-239]		0	1764.8764
[K].YEELQSI	1xTMTpro [K	0.0001553	7.389E-05	1	1	2	P05787	P05787 [286-295]		0	1745.9929
[R].YEEVSV.	1xTMTpro [N	0.00014	7.389E-05	1	1	1	Q9BRA2	Q9BRA2 [4-17]		0	2018.9779
[K].YDYNSG	1xTMTpro [K	0.0001383	7.389E-05	1	1	1	Q9Y3F4	Q9Y3F4 [250-262		0	2205.0843
[K].YAVTTGI	1xTMTpro [N	0.0001432	7.389E-05	1	1	1	P53621	P53621 [551-562]		0	1606.8872
[K].YGGELV	1xTMTpro [N	0.000196	7.389E-05	1	1	1	O75369	O75369 [1208-12		0	1646.8974
[K].YGGQPV	1xTMTpro [K	9.925E-05	7.389E-05	1	1	3	P21333	P21333 [1235-124		0	1899.062
[R].YLAEVA	1xTMTpro [K	9.423E-05	7.389E-05	1	1	1	P63104	P63104 [128-138]		0	1759.9722
[R].YLAEVA(	1xCarbamido	0.0001354	7.389E-05	1	1	2	P27348	P27348 [128-138]		0	1572.7647
[R].YLAEVA(	1xCarbamido	0.0001091	7.389E-05	1	1	1	P27348	P27348 [128-139]		1	2005.0668
[R].YLAEVAS	1xTMTpro [K	0.0001849	7.389E-05	1	1	2	Q04917	Q04917 [133-142		0	1674.9558
[R].YLAEVAT	1xTMTpro [K	8.084E-05	7.389E-05	1	1	1	P31947	P31947 [130-140]		0	1789.9828
[R].YLAEVAT	1xTMTpro [K	0.0001561	7.389E-05	1	1	2	P61981	P61981 [133-142]		0	1688.9715
[K].YLDNPN.	1xTMTpro [N	0.0001656	7.389E-05	1	1	2	Q63ZY3	Q63ZY3 [604-614		0	1609.8505
[K].YIEGLSA	1xTMTpro [K	0.0001786	7.389E-05	1	1	1	Q86U86	Q86U86 [1613-16		0	2106.1574
[R].YLETADY	1xTMTpro [N	0.0001914	7.389E-05	1	1	3	O95782	O95782 [412-421		0	1518.8123
[K].YLETLLY	1xTMTpro [K	0.000146	7.389E-05	1	1	2	Q13596	Q13596 [496-509		0	2306.3252
[K].YIETSEL	1xCarbamido	0.0001286	7.389E-05	1	1	1	O00429	O00429 [354-365		0	1659.8331
[K].YLGAMQ	1xTMTpro [K	8.071E-05	7.389E-05	1	1	1	Q8WYA6	Q8WYA6 [448-45		0	1703.9646
[K].YLGGTD	1xTMTpro [K	0.00015	7.389E-05	1	1	4	P23141	P23141 [403-412]		0	1676.9351
[R].YLAEFAT	1xTMTpro [K	0.0001933	7.389E-05	1	1	1	P62258	P62258 [131-142]		1	1993.0998
[R].YLGIPGD	2xCarbamido	8.196E-05	7.389E-05	1	1	1	Q16881	Q16881 [317-342		1	3907.0114
[R].YLHDNIV	1xTMTpro [N	0.0001453	7.389E-05	1	1	1	Q8IUZ5	Q8IUZ5 [84-95]		0	1810.9407
[K].YIKDDVII	2xTMTpro [K	0.0001116	7.389E-05	1	1	1	Q9Y287	Q9Y287 [93-120]		1	4046.2154
[K].YILANVG	1xCarbamido	0.0001539	7.389E-05	1	1	1	Q7LBC6	Q7LBC6 [989-100		0	2723.4392
[K].YLIATSE	1xTMTpro [N	6.55E-05	7.389E-05	1	1	1	P49591	P49591 [267-281]		0	1987.1296
[R].YLLHGLE	1xCarbamido	0.0001719	7.389E-05	1	1	1	P55010	P55010 [313-332]		0	2934.6189
[K].YIIIGDM(	1xTMTpro [K	6.726E-05	7.389E-05	1	1	2	P61106	P61106 [14-24]		0	1774.0429
[K].YIIIGDTG	1xTMTpro [K	7.135E-05	7.389E-05	2	2	2	Q8WUD1; P	Q8WUD1 [9-19]; I		0	1744.05
[K].YLLLSDL	1xTMTpro [N	0.0002003	7.389E-05	1	1	1	Q99766	Q99766 [182-192		0	1549.9273
[K].YLLNLNL	1xTMTpro [K	8.979E-05	7.389E-05	1	1	4	P21980	P21980 [528-540]		0	2188.2509
[K].YLIPNAT	1xTMTpro [K	0.0001206	7.389E-05	1	1	3	P31946	P31946 [106-117]		0	1969.125
[R].YLLQETV	1xTMTpro [K	0.0001315	7.389E-05	1	1	2	Q96HE7	Q96HE7 [266-275		0	1931.1134

[R].YLLQYQ	2xCarbamido	0.0001789	7.389E-05	1	1	1	P25789	P25789 [97-118]	0	3302.766
[K].YLMDEG	1xTMTpro [K	8.247E-05	7.389E-05	1	1	2	O60701	O60701 [356-370]	0	2410.2721
[K].YLGQDY	1xTMTpro [N	0.0001796	7.389E-05	1	1	3	P07384	P07384 [37-46]	0	1588.829
[R].YGGQPL	1xTMTpro [K	0.0001354	7.389E-05	1	1	2	Q1KMD3	Q1KMD3 [264-27	0	1733.9718
[R].YKVEGF	3xTMTpro [K	0.0001852	7.389E-05	1	1	2	P13667	P13667 [595-612]	2	3263.8821
[R].YKQDVY	2xTMTpro [K	0.0001184	7.389E-05	2	2	2	O43865; Q9	O43865 [477-486]	1	2179.3307
[R].YGLDTE	1xTMTpro [N	0.0001484	7.389E-05	1	1	1	Q9NXC5	Q9NXC5 [402-41	0	1570.8185
[R].YGLFPA	1xTMTpro [N	0.00017	7.389E-05	1	1	1	Q14247	Q14247 [538-549]	0	1745.9546
[K].YGLLVG	1xTMTpro [N	0.0001275	7.389E-05	1	1	1	Q15274	Q15274 [150-161]	0	1504.8555
[K].YGIVDY	1xOxidation	0.0001536	7.389E-05	1	1	1	P13667	P13667 [273-288]	0	2408.2663
[R].YGLVTY	1xTMTpro [K	0.0001232	7.389E-05	1	1	2	P00751	P00751 [309-319]	0	1884.0763
[K].YGPFVA	2xTMTpro [K	0.0002003	7.389E-05	1	1	1	P24539	P24539 [116-131]	1	2754.5283
[R].YGPMEE	1xTMTpro [K	7.924E-05	7.389E-05	1	1	2	P36969	P36969 [180-191]	0	2013.1222
[K].YGQESE	1xCarbamido	0.0001049	7.389E-05	1	1	1	Q8N668	Q8N668 [152-167	0	2601.3038
[K].YGQISE	1xTMTpro [K	6.307E-05	7.389E-05	1	1	3	Q14011	Q14011 [29-39]	0	1829.1028
[K].YGSVAF	1xCarbamido	0.0001349	7.389E-05	1	1	1	O00116	O00116 [390-406]	0	2219.1238
[K].YGVEQV	1xTMTpro [N	0.0001232	7.389E-05	1	1	1	Q9UPT5	Q9UPT5 [718-72	0	1685.8488
[R].YGVIILD	1xTMTpro [N	0.0001888	7.389E-05	1	1	1	O43143	O43143 [254-265]	0	1718.9397
[R].YGVMDT	1xTMTpro [N	6.55E-05	7.389E-05	1	1	1	Q6IAN0	Q6IAN0 [256-267]	0	1603.8069
[R].YKVCDV	1xCarbamido	0.0001495	7.389E-05	1	1	1	P07738	P07738 [142-154]	1	2211.2451
[K].YGVNPG	1xTMTpro [N	7.247E-05	7.389E-05	2	2	2	P42167; P42	P42167 [127-139]	0	1634.9185
[K].YGVVVC	1xCarbamido	0.0001347	7.389E-05	1	1	2	Q9Y5P6	Q9Y5P6 [144-155	0	1629.8226
[K].YGYEIPV	1xCarbamido	0.0001659	7.389E-05	1	1	1	P60900	P60900 [105-116]	0	2096.1052
[K].YHLGAY	1xTMTpro [N	0.0001448	7.389E-05	1	1	5	Q15661	Q15661 [192-203]	0	1670.8458
[K].YHLMQF	1xTMTpro [N	0.0001617	7.389E-05	1	1	1	Q5JVF3	Q5JVF3 [274-284	0	1698.8957
[K].YHPDKN	2xTMTpro [K	7.743E-05	7.389E-05	1	1	1	Q9H3Z4	Q9H3Z4 [42-56]	1	2623.3932
[K].YHPDKN	3xTMTpro [K	0.0001579	7.389E-05	1	1	1	Q9H3Z4	Q9H3Z4 [42-58]	2	3202.7638
[K].YHQGIN	1xTMTpro [N	0.0001065	7.389E-05	1	1	1	P11277	P11277 [1944-19	0	1734.9094
[R].YHYVGT	1xTMTpro [K	0.0001165	7.389E-05	1	1	1	O95302	O95302 [59-70]	0	2020.0784
[K].YKDDDD	1xTMTpro [K	0.0001002	7.389E-05	1	1	1	O60568	O60568 [185-197]	1	2302.1483
[R].YKDGVV	1xCarbamido	9.617E-05	7.389E-05	1	1	2	P36915	P36915 [356-373]	1	2822.6055
[K].YKDNPF	1xTMTpro [K	8.453E-05	7.389E-05	1	1	1	Q8N6H7	Q8N6H7 [358-37	1	2312.2167
[R].YKGQSG	1xCarbamido	0.0001271	7.389E-05	1	1	1	P46063	P46063 [312-325]	1	2591.4472

[K].YKPVCN	2xCarbamido	0.0001023	7.389E-05	1	2	1	Q04828	Q04828 [184-200]	0	2847.4314
[K].YGVSGY	1xTMTpro [K	0.0001749	7.389E-05	1	1	3	P30101	P30101 [95-104]	0	1692.9816
[K].VTDETTI	1xTMTpro [K	0.0001161	7.389E-05	1	1	2	Q99715	Q99715 [731-740]	0	1750.9355
[K].VTDEMF	1xTMTpro [K	0.0001435	7.389E-05	1	1	2	O94832	O94832 [466-478]	0	2105.1444
[R].VTDEILH	1xTMTpro [N	7.543E-05	7.389E-05	1	2	1	P51003	P51003 [206-221]	0	2199.2093
[K].VGEVTY	1xOxidation [	0.0001078	7.389E-05	1	1	2	P52272	P52272 [95-110]	0	2377.2816
[R].VGFYES	1xTMTpro [N	0.0001121	7.389E-05	1	1	5	P01023	P01023 [705-715]	0	1563.7796
[K].VGGGGT	1xTMTpro [N	9.234E-05	7.389E-05	1	1	1	O75822	O75822 [28-38]	0	1207.635
[K].VGGTSD	1xTMTpro [K	9.105E-05	7.389E-05	1	1	2	P10809	P10809 [406-417]	0	1842.01
[R].VGHDYQ	1xTMTpro [K	7.997E-05	7.389E-05	1	1	1	P13611	P13611 [3219-323	0	2153.1635
[K].VGLDATI	1xCarbamido	0.0001786	7.389E-05	1	1	1	Q9NXR7	Q9NXR7 [27-36]	0	1422.7694
[K].VGLFTEI	1xCarbamido	6.928E-05	7.389E-05	1	1	1	P62487	P62487 [95-110]	0	2118.1047
[K].VGIGPG	1xCarbamido	0.0001719	7.389E-05	1	1	1	Q9P2T1	Q9P2T1 [178-189	0	1507.8222
[K].VGLLLEY	1xTMTpro [K	0.0001188	7.389E-05	1	1	1	Q8WW59	Q8WW59 [151-16	0	1871.1134
[R].VGLQAQ	1xTMTpro [K	6.853E-05	7.389E-05	1	1	2	Q12955	Q12955 [1177-11	0	2101.2513
[R].VGMVET	1xTMTpro [K	0.0001729	7.389E-05	1	1	3	Q9Y3C6	Q9Y3C6 [142-158	0	2497.3212
[K].VGNIIDT	1xTMTpro [K	0.0001018	7.389E-05	1	1	2	Q9Y3Z3	Q9Y3Z3 [378-392	0	2259.2914
[R].VGNLTV	2xTMTpro [K	0.000111	7.389E-05	1	1	1	P17174	P17174 [268-290]	1	3410.9749
[R].VGPDVV	1xTMTpro [N	0.000143	7.389E-05	1	1	1	Q9NV31	Q9NV31 [138-152	0	1890.0656
[R].VGPGAG	1xTMTpro [N	9.925E-05	7.389E-05	1	1	1	Q8TEQ6	Q8TEQ6 [41-55]	0	1729.9193
[R].VGPVAA	1xTMTpro [K	0.0001937	7.389E-05	1	1	1	Q9NPH2	Q9NPH2 [518-530	0	1969.1436
[K].VGSGDT	1xTMTpro [K	9.408E-05	7.389E-05	1	1	1	Q9NR45	Q9NR45 [132-145	0	2149.1421
[R].VGSVLQ	1xCarbamido	8.362E-05	7.389E-05	1	1	1	P31040	P31040 [528-538]	0	1741.9762
[K].VGTVTM	1xTMTpro [N	0.0001849	7.389E-05	1	1	1	Q9H944	Q9H944 [126-136	0	1379.7636
[R].VGVDYE	1xCarbamido	9.693E-05	7.389E-05	1	1	1	Q02809	Q02809 [671-681]	0	1472.7123
[K].VGVNDF	1xCarbamido	0.0001877	7.389E-05	1	1	3	P21810	P21810 [315-328]	0	2151.1222
[R].VGVPTV	1xTMTpro [N	8.311E-05	7.389E-05	1	1	2	O60306	O60306 [1126-11	0	1630.9084
[R].VGVTVA	1xCarbamido	0.0001249	7.389E-05	1	1	1	P47897	P47897 [539-558]	0	2531.3281
[R].VGVVQF	1xTMTpro [N	0.0001093	7.389E-05	1	1	5	P12111	P12111 [281-291]	0	1536.8341
[R].VGVVQY	1xTMTpro [N	0.0001458	7.389E-05	1	1	2	Q99715	Q99715 [179-189]	0	1514.8134
[R].VGWEQI	1xTMTpro [N	0.000149	7.389E-05	2	2	5	P12814; O43	P12814 [715-726]	0	1690.9811
[K].VGYTPD	1xTMTpro [N	0.0001723	7.389E-05	1	2	1	Q00610	Q00610 [508-519]	0	1784.0066
[K].VGETIIDI	1xTMTpro [N	0.0001281	7.389E-05	1	1	2	Q9NZM1	Q9NZM1 [1634-16	0	1562.8709

[K].VGEIEFE	1xTMTpro [N	7.28E-05	7.389E-05	1	1	2	Q9UEY8	Q9UEY8 [366-376	0	1583.8422
[R].VGEGDK	1xTMTpro [K	0.0002051	7.389E-05	1	1	1	Q8WXX5	Q8WXX5 [47-57]	1	1784.9634
[K].VGEFSG	2xTMTpro [K	0.0001726	7.389E-05	1	1	1	P10599	P10599 [86-96]	1	2078.2062
[K].VFDENK	1xTMTpro [K	7.779E-05	7.389E-05	1	1	1	O60879	O60879 [379-394]	1	2555.3233
[K].VFEEDAI	1xTMTpro [K	7.202E-05	7.389E-05	1	1	2	Q14204	Q14204 [1515-15	0	2076.0781
[R].VFEFGG	1xTMTpro [K	0.0001153	7.389E-05	1	1	2	Q08257	Q08257 [13-23]	0	1830.0657
[K].VFEGNR	1xTMTpro [K	6.217E-05	7.389E-05	1	1	2	P06744	P06744 [467-481]	0	2317.316
[K].VFFTDY(	1xTMTpro [K	8.786E-05	7.389E-05	1	1	3	Q07954	Q07954 [337-347]	0	1923.0872
[R].VFLADQ	1xTMTpro [N	7.566E-05	7.389E-05	1	1	1	P46939	P46939 [2625-263	0	2015.0769
[R].VFLALVE	1xTMTpro [N	6.633E-05	7.389E-05	1	1	2	Q9H3U1	Q9H3U1 [670-681	0	1722.9597
[R].VFIGNLN	1xTMTpro [K	0.000112	7.389E-05	1	2	4	Q9UKM9	Q9UKM9 [23-34]	0	1897.1766
[K].VFLIYEG	1xTMTpro [K	7.636E-05	7.389E-05	1	1	1	P17858	P17858 [48-66]	0	2737.4944
[R].VFIPHGL	1xTMTpro [N	0.0001749	7.389E-05	1	1	1	P00492	P00492 [35-45]	0	1601.9157
[R].VFITDDF	1xOxidation [	0.00012	7.389E-05	1	1	7	P14625	P14625 [416-428]	0	2220.1325
[K].VFLTVPS	1xTMTpro [K	0.0001849	7.389E-05	1	1	3	Q16363	Q16363 [945-959]	0	2216.267
[R].VFNTTEI	1xTMTpro [K	6.109E-05	7.389E-05	1	1	1	Q14571	Q14571 [2180-21	1	2119.1275
[K].VHDVND	1xTMTpro [N	0.0001635	7.389E-05	1	1	1	P33151	P33151 [141-154]	0	2040.0371
[K].VFPNQV	2xTMTpro [K	6.972E-05	7.389E-05	1	1	1	Q7Z7H8	Q7Z7H8 [125-139	0	2673.5796
[R].VFSLQW	1xTMTpro [K	0.0001065	7.389E-05	1	1	2	P05156	P05156 [481-490]	0	1801.0504
[K].VFTAIAN	1xTMTpro [K	0.0001448	7.389E-05	1	1	2	Q16401	Q16401 [417-429]	0	2082.1992
[R].VFVPLP(	1xCarbamido	0.0001742	7.389E-05	1	1	1	Q5SWX8	Q5SWX8 [346-36	0	2434.337
[R].VFYYLV(	1xTMTpro [K	0.0001249	7.389E-05	1	2	2	P61326	P61326 [121-130]	0	1896.1126
[K].VGAAEE	1xTMTpro [K	0.00014	7.389E-05	1	1	2	Q9P2E9	Q9P2E9 [1160-11	0	1681.9616
[R].VGASEA	1xTMTpro [K	0.0001306	7.389E-05	1	1	2	Q9UBC2	Q9UBC2 [34-44]	0	1714.0395
[K].VGAVDA	2xTMTpro [K	0.000101	7.389E-05	1	1	1	Q15084	Q15084 [78-102]	1	3493.9372
[K].VGDDPA	1xTMTpro [K	0.0001466	7.389E-05	1	1	2	Q9Y490	Q9Y490 [2105-21	0	1836.0511
[R].VGDGPQ	1xTMTpro [N	0.0001746	7.389E-05	1	1	1	Q96NY7	Q96NY7 [359-373	0	1945.9058
[K].VGDHQF	1xTMTpro [N	0.0001914	7.389E-05	1	1	1	Q8IYS2	Q8IYS2 [239-250]	0	1695.9138
[R].VGDIIQII	1xTMTpro [K	0.0001544	7.389E-05	1	1	2	O14936	O14936 [642-651]	0	1694.0708
[K].VGDITIL\	1xTMTpro [K	0.0001258	7.389E-05	1	1	1	O75911	O75911 [114-130]	0	2328.3895
[R].VGDSTP	1xTMTpro [K	8.979E-05	7.389E-05	1	1	1	Q9H8Y8	Q9H8Y8 [429-452	0	2922.5188
[R].VFPVYN	1xCarbamido	0.0001584	7.389E-05	1	1	1	O94788	O94788 [54-79]	1	3835.004
[KR].VHELN	1xTMTpro [K	0.0001051	7.389E-05	2	2	3	Q9NQ29; Q9	Q9NQ29 [126-135	0	1776.0147

[K].VHENEN	1xTMTpro [K	8.084E-05	7.389E-05	1	1	1	Q14515	Q14515 [390-408]	0	2727.383
[R].VHFEES	1xTMTpro [K	0.0001026	7.389E-05	1	1	1	P12956	P12956 [231-244]	1	2310.2949
[K].VLALLDV	1xTMTpro [K	0.0001964	7.389E-05	1	1	2	Q9NX62	Q9NX62 [273-282	0	1691.0599
[K].VLALPEF	1xTMTpro [N	6.8E-05	7.389E-05	1	1	4	Q15149	Q15149 [1187-12	0	1836.0914
[K].VIAQLSE	1xCarbamido	0.0001806	7.389E-05	1	1	2	Q92878	Q92878 [983-992]	0	1785.0072
[K].VLAQYY	1xTMTpro [N	0.0001681	7.389E-05	1	1	1	Q9NX58	Q9NX58 [336-349	0	2036.0521
[K].VLATAFD	1xTMTpro [N	0.0001046	7.389E-05	1	1	2	P34932	P34932 [222-234]	0	1625.9182
[K].VIATDINI	1xTMTpro [K	0.0001653	7.389E-05	1	1	2	Q9BUT1	Q9BUT1 [33-42]	0	1697.9929
[K].VIAVADD	1xCarbamido	0.0001008	7.389E-05	1	1	2	Q9HC35	Q9HC35 [803-812	0	1745.9752
[R].VICAEEF	2xCarbamido	6.643E-05	7.389E-05	1	1	1	P49915	P49915 [447-457]	0	1990.0633
[R].VLCDETI	2xCarbamido	0.0001109	7.389E-05	1	1	1	O94769	O94769 [131-142]	0	1849.8678
[R].VLDAIWS	1xTMTpro [N	0.0001222	7.389E-05	1	1	2	Q86U38	Q86U38 [564-575	0	1575.9178
[R].VLDASW	1xTMTpro [N	0.0001	7.389E-05	1	1	2	Q16762	Q16762 [31-42]	0	1655.8712
[R].VLDDGE	1xTMTpro [K	0.000143	7.389E-05	1	1	2	P46459	P46459 [517-529]	0	2066.1989
[K].VLDFEHI	1xOxidation [	8.336E-05	7.389E-05	1	1	3	P60660	P60660 [64-79]	0	2512.4129
[R].VLAHLAI	1xTMTpro [K	9.785E-05	7.389E-05	1	1	1	Q68E01	Q68E01 [469-481	0	2043.2247
[K].VLDFHNI	1xTMTpro [K	0.0001333	7.389E-05	1	1	3	P20908	P20908 [45-57]	0	2077.1937
[R].VIDIFDTI	1xTMTpro [K	0.00012	7.389E-05	1	1	1	P63098	P63098 [58-73]	0	2392.2528
[R].VLDLGSI	1xOxidation [	9.091E-05	7.389E-05	1	1	1	Q9ULA0	Q9ULA0 [439-453	0	1957.086
[R].VLDLVEY	1xTMTpro [K	0.0001146	7.389E-05	1	1	4	Q9BQG0	Q9BQG0 [828-83	0	1836.1702
[K].VLDNAIE	1xTMTpro [K	0.0001322	7.389E-05	1	1	4	Q01082	Q01082 [293-302]	0	1740.0035
[R].VLDPFTI	1xTMTpro [K	7.325E-05	7.389E-05	1	1	2	P29401	P29401 [531-542]	0	2022.2243
[K].VIDPVTG	1xCarbamido	0.000182	7.389E-05	1	1	1	Q9Y2R5	Q9Y2R5 [91-119]	0	3992.1718
[K].VLDSEM	1xTMTpro [K	0.0001054	7.389E-05	1	1	1	Q99985	Q99985 [661-673]	0	2014.1386
[R].VLDVNLI	1xTMTpro [N	7.291E-05	7.389E-05	1	1	2	Q99714	Q99714 [117-130]	0	1895.0744
[K].VLDVSDI	1xTMTpro [K	6.357E-05	7.389E-05	1	1	2	Q8TE77	Q8TE77 [282-294	0	2000.1407
[K].VIDVTVF	1xCarbamido	0.0001144	7.389E-05	1	1	2	Q9NVH1	Q9NVH1 [485-497	0	2092.2696
[R].VLEDNS	1xTMTpro [K	0.0001599	7.389E-05	1	1	1	P27487	P27487 [493-502]	0	1711.9722
[K].VLEEAN	1xTMTpro [K	0.0001407	7.389E-05	1	1	4	Q92841	Q92841 [536-547]	0	1934.1203
[K].VLEEGG	1xTMTpro [K	7.779E-05	7.389E-05	1	1	3	Q9NR12	Q9NR12 [314-324	0	1892.0297
[K].VLDLELK	2xTMTpro [K	6.633E-05	7.389E-05	1	1	2	P04083	P04083 [251-262]	1	2284.3944
[K].VFDAIMN	1xTMTpro [K	0.0001672	7.389E-05	1	1	4	P13639	P13639 [300-308]	0	1692.9639
[R].VLADPSI	1xTMTpro [K	0.0001856	7.389E-05	2	2	4	P58107; Q15	P58107 [2375-238	0	1668.93

[K].VKYEVTI	1xTMTpro [K	0.0001132	7.389E-05	1	1	1	Q9UKG1	Q9UKG1 [159-17	1	2197.1996
[R].VHPVST	1xTMTpro [K	0.0001519	7.389E-05	1	1	2	P00338	P00338 [270-278]	0	1619.9799
[K].VHQLYE	1xTMTpro [N	0.0001911	7.389E-05	1	1	4	Q13561	Q13561 [309-318]	0	1590.8923
[R].VHSADV	1xTMTpro [N	0.0001522	7.389E-05	1	1	2	Q16647	Q16647 [198-208]	0	1619.8613
[K].VHSDFT	1xTMTpro [N	0.0001049	7.389E-05	1	1	1	O75153	O75153 [385-395]	0	1479.7875
[R].VHSEVA	1xTMTpro [K	0.0001288	7.389E-05	1	1	2	Q9Y305	Q9Y305 [373-383	0	1835.0518
[R].VHVCAE	2xCarbamido	0.0001824	7.389E-05	1	2	1	P25490	P25490 [324-332]	0	1667.8853
[K].VKDILQN	2xTMTpro [K	0.0001033	7.389E-05	1	1	2	P46063	P46063 [79-88]	1	2116.331
[R].VKDTDD	1xOxidation	8.336E-05	7.389E-05	1	2	1	P61224	P61224 [103-117]	1	2572.4836
[R].VKDTDD	1xCarbamido	0.0001845	7.389E-05	1	2	1	P61224	P61224 [103-124]	2	3489.8385
[R].VKDTED	1xOxidation	0.0001005	7.389E-05	1	1	2	P62834	P62834 [103-117]	1	2586.4993
[K].VKEEIIE	1xTMTpro [K	8.375E-05	7.389E-05	1	1	2	P50552	P50552 [362-375]	1	2311.3517
[K].VKELVEI	1xTMTpro [K	9.364E-05	7.389E-05	1	1	1	Q14677	Q14677 [132-144]	1	2172.1792
[K].VKETQE	3xTMTpro [K	0.0001041	7.389E-05	1	1	1	O43491	O43491 [177-191]	2	2819.6619
[K].VIAAEGE	1xTMTpro [N	0.0001286	7.389E-05	1	1	3	P27105	P27105 [221-232]	0	1551.812
[K].VKGDMD	2xTMTpro [K	0.0001352	7.389E-05	1	1	1	Q09666	Q09666 [4607-46	1	2115.2664
[K].VKGDMD	2xTMTpro [K	8.772E-05	7.389E-05	1	1	1	Q09666	Q09666 [1667-16	1	2087.2351
[K].VKGDVD	2xTMTpro [K	7.985E-05	7.389E-05	1	1	2	Q09666	Q09666 [2977-29	1	2083.2943
[K].VKGDVD	2xTMTpro [K	0.0001326	7.389E-05	1	1	3	Q09666	Q09666 [2131-21	1	2069.2787
[R].VKGEEI	1xTMTpro [K	0.0001402	7.389E-05	1	1	1	Q96P70	Q96P70 [883-896	1	2234.1982
[K].VKGEYD	2xTMTpro [K	0.0001863	7.389E-05	1	1	1	Q09666	Q09666 [960-970]	1	2179.2613
[K].VKGEYD	2xTMTpro [K	0.0001112	7.389E-05	1	1	1	Q09666	Q09666 [711-721]	1	2147.2892
[R].VKKDDE	2xTMTpro [K	0.0001948	7.389E-05	1	1	1	Q9NYJ1	Q9NYJ1 [15-31]	2	2941.6298
[R].VKIEPG	2xTMTpro [K	6.257E-05	7.389E-05	1	1	1	Q9H4A4	Q9H4A4 [397-416	1	3221.7034
[R].VKPLQD	2xTMTpro [K	6.357E-05	7.389E-05	1	1	2	Q12874	Q12874 [200-212]	0	2428.438
[R].VKPPEG	2xTMTpro [K	6.438E-05	7.389E-05	1	1	2	P50479	P50479 [310-326]	0	2758.596
[R].VKQELLI	2xTMTpro [K	8.401E-05	7.389E-05	1	1	1	P50552	P50552 [347-356]	1	2127.3205
[K].VKSPEL	2xTMTpro [K	6.664E-05	7.389E-05	1	1	1	P02652	P02652 [52-62]	1	2112.2845
[K].VKTEPTI	2xTMTpro [K	7.743E-05	7.389E-05	1	1	1	P78347	P78347 [220-240]	1	3117.7533
[K].VKGDMD	2xTMTpro [K	7.791E-05	7.389E-05	1	1	3	Q09666	Q09666 [3105-31	1	2101.2507
[R].VFAFDA	2xTMTpro [K	6.758E-05	7.389E-05	1	1	2	Q9H1B7	Q9H1B7 [402-418	0	2829.5677
[K].VEVYQQ	1xTMTpro [K	0.0001275	7.389E-05	1	2	1	Q9UPN3	Q9UPN3 [6161-61	0	2132.1553
[RK].VEVTEI	1xTMTpro [K	7.887E-05	7.389E-05	1	2	3	Q01105	Q01105 [123-132]	0	1817.0188

[K].VDATAD	1xCarbamid	0.0001292	7.389E-05	1	1	2	P53396	P53396 [221-230]		0	1763.9493
[K].VDATAE	1xTMTpro [K	8.084E-05	7.389E-05	1	1	2	P13667	P13667 [235-245]		0	1741.9828
[K].VDCQAY	2xCarbamid	0.0001308	7.389E-05	1	1	1	Q8IXB1	Q8IXB1 [726-737]		0	2080.0447
[K].VDDFLAI	1xTMTpro [K	0.0001347	7.389E-05	1	1	2	P52209	P52209 [39-48]		0	1729.9616
[K].VDEAVA	1xTMTpro [K	8.414E-05	7.389E-05	2	2	1	Q9H361; P1	Q9H361 [602-615		0	2087.2105
[K].VDELTCI	1xCarbamid	7.578E-05	7.389E-05	1	1	1	O60271	O60271 [438-456]		1	3087.7064
[K].VDEIVVL	1xTMTpro [K	0.0001132	7.389E-05	1	1	2	Q14254	Q14254 [378-390]		0	1983.1254
[R].VDEMSQ	1xTMTpro [K	7.972E-05	7.389E-05	1	1	2	O15372	O15372 [250-259]		0	1771.9756
[K].VDENHS	1xTMTpro [N	8.171E-05	7.389E-05	1	1	1	O14497	O14497 [2244-22		0	2029.9786
[R].VDEVPD	2xTMTpro [K	0.0001442	7.389E-05	1	1	2	O75475	O75475 [25-39]		0	2478.4384
[R].VDFILQF	1xTMTpro [K	0.0002019	7.389E-05	1	1	1	Q66K79	Q66K79 [577-589		0	2023.1906
[K].VDFPTAI	1xTMTpro [N	0.0001216	7.389E-05	1	1	2	O96019	O96019 [35-47]		0	1737.9529
[K].VDFTEEI	1xTMTpro [N	9.091E-05	7.389E-05	1	1	1	Q13576	Q13576 [172-183]		0	1773.8648
[K].VDAQFG	1xTMTpro [N	0.000129	7.389E-05	1	1	1	P54577	P54577 [179-189]		0	1509.7981
[K].VDGETA	1xTMTpro [N	6.397E-05	7.389E-05	1	1	2	O95674	O95674 [27-38]		0	1556.7359
[K].VDHVQM	1xTMTpro [K	7.899E-05	7.389E-05	1	1	1	P26196	P26196 [237-250]		0	2220.219
[R].VDKDCA	2xCarbamid	0.0001096	7.389E-05	1	1	1	P16144	P16144 [44-57]		1	2418.1384
[K].VDKLDA	1xTMTpro [K	0.0001087	7.389E-05	1	1	1	Q00610	Q00610 [1610-16		1	1841.0624
[K].VDIDTPD	1xTMTpro [K	8.324E-05	7.389E-05	1	1	1	Q09666	Q09666 [3712-37		0	2310.232
[K].VDIDVPD	1xTMTpro [K	7.235E-05	7.389E-05	1	1	1	Q09666	Q09666 [3058-30		0	2304.2579
[R].VDILENQ	1xTMTpro [N	6.217E-05	7.389E-05	1	1	1	P46439	P46439 [97-113]		0	2359.2069
[K].VDLLNQI	1xTMTpro [K	0.0001176	7.389E-05	1	1	2	P35908	P35908 [303-314]		0	2069.2138
[R].VDIISWG	1xCarbamid	9.248E-05	7.389E-05	1	1	1	Q96C23	Q96C23 [31-46]		0	2413.3656
[K].VDINAPD	1xTMTpro [K	9.277E-05	7.389E-05	1	1	1	Q09666	Q09666 [3385-33		0	1992.1257
[K].VDINCED	2xCarbamid	0.0001511	7.389E-05	1	1	2	Q14315	Q14315 [2084-20		0	2294.0594
[K].VDLSTS	1xTMTpro [K	0.0001422	7.389E-05	1	1	1	Q9BQ69	Q9BQ69 [87-96]		0	1759.9722
[K].VDLTNP	1xTMTpro [K	6.832E-05	7.389E-05	1	1	1	Q9NXG2	Q9NXG2 [230-24		0	2439.4354
[K].VDLTPYI	1xTMTpro [K	7.648E-05	7.389E-05	1	1	1	O43708	O43708 [178-192]		1	2312.347
[K].VDHFGF	1xTMTpro [K	0.00018	7.389E-05	1	1	1	P42785	P42785 [57-66]		0	1771.9987
[K].VDLVDEI	1xTMTpro [N	0.0001105	7.389E-05	1	1	1	P61086	P61086 [29-40]		0	1753.9291
[K].VDALMD	1xOxidation	0.0001579	7.389E-05	1	1	2	P13647	P13647 [293-304]		0	2050.0845
[K].VDAAPG	1xTMTpro [K	0.0001732	7.389E-05	1	1	1	Q15814	Q15814 [152-172]		0	2709.5319
[R].VAQPGF	1xTMTpro [N	0.0001903	7.389E-05	1	1	2	Q96HY6	Q96HY6 [47-59]		0	1722.9346

[K].VASEEEI	1xTMTpro [K	6.208E-05	7.389E-05	1	1	1	Q8N3V7	Q8N3V7 [261-275	0	2312.3245
[K].VASIQPC	2xTMTpro [K	9.724E-05	7.389E-05	1	1	1	P37059	P37059 [257-278]	1	3231.8194
[K].VASSPVI	1xTMTpro [N	0.0001466	7.389E-05	1	1	1	P51610	P51610 [595-608]	0	1719.9383
[K].VATWVD	1xTMTpro [K	0.0001204	7.389E-05	1	1	1	O95071	O95071 [443-457	0	2201.2309
[K].VAVDGQ	1xTMTpro [N	0.0001161	7.389E-05	1	1	1	O00182	O00182 [319-332	0	2038.0466
[R].VAVEEV	1xTMTpro [K	7.037E-05	7.389E-05	1	1	3	P02545	P02545 [440-450]	0	1811.9882
[R].VAVEYLI	1xTMTpro [K	9.335E-05	7.389E-05	1	1	2	O43684	O43684 [203-216	0	2182.2251
[K].VAVLETC	1xTMTpro [K	0.0001626	7.389E-05	1	1	3	Q63ZY3	Q63ZY3 [289-297	0	1609.018
[R].VAVIQFS	2xTMTpro [K	0.0001834	7.389E-05	1	1	1	P12111	P12111 [1271-130	2	4396.4081
[K].VAVNDA	1xTMTpro [N	0.0001066	7.389E-05	1	1	3	P17931	P17931 [211-224]	0	1954.0578
[K].VAVQAV	1xTMTpro [N	8.209E-05	7.389E-05	1	1	2	Q16774	Q16774 [86-96]	0	1490.8433
[R].VAWHID	1xTMTpro [N	0.0001029	7.389E-05	1	1	1	O00754	O00754 [191-202	0	1725.9144
[K].VDALLSA	1xTMTpro [K	0.0001952	7.389E-05	1	1	2	Q9NYU2	Q9NYU2 [950-959	0	1650.0082
[K].VAYTTVI	1xTMTpro [N	6.56E-05	7.389E-05	1	1	2	Q63ZY3	Q63ZY3 [618-629	0	1783.0073
[R].VCCEGM	2xCarbamido	0.0001553	7.389E-05	1	1	1	Q96A33	Q96A33 [213-223	0	1682.8711
[K].VCDIAAE	1xCarbamido	0.0001687	7.389E-05	1	1	1	O00410	O00410 [109-118	0	1421.7742
[K].VCDVPLI	1xCarbamido	7.613E-05	7.389E-05	1	1	5	P07738	P07738 [144-154]	0	1615.8797
[R].VCEATY	1xCarbamido	0.0001903	7.389E-05	1	1	1	Q93096	Q93096 [48-60]	0	2137.1342
[R].VCEDGF	1xCarbamido	6.327E-05	7.389E-05	1	1	3	O00584	O00584 [240-252	0	2113.1284
[R].VCEEIAI	1xCarbamido	0.0001114	7.389E-05	1	1	3	P08708	P08708 [34-44]	0	1867.0854
[K].VCELDEI	4xCarbamido	0.0001053	7.389E-05	1	1	1	P09486	P09486 [82-110]	0	4021.8242
[R].VCENIPI	2xCarbamido	7.767E-05	7.389E-05	1	1	2	P62826	P62826 [111-123]	0	2124.1801
[R].VCEPCY	2xCarbamido	0.0001258	7.389E-05	1	1	1	O14964	O14964 [211-221	0	1771.8427
[K].VCHLMG	1xCarbamido	0.000153	7.389E-05	1	1	2	P35749	P35749 [381-394]	0	1983.0111
[K].VCLESA\	1xCarbamido	0.0001437	7.389E-05	1	1	1	P46736	P46736 [227-237]	0	1853.0698
[R].VCNLIDS	1xCarbamido	0.0001245	7.389E-05	1	1	2	Q02252	Q02252 [367-376	0	1714.9653
[R].VCSQYA	1xCarbamido	6.367E-05	7.389E-05	1	1	2	P02774	P02774 [219-229]	0	1883.9817
[K].VCALYIM	1xCarbamido	0.0001118	7.389E-05	1	1	2	Q14165	Q14165 [205-220	0	2360.2849
[K].VLEFGW	1xTMTpro [K	6.257E-05	7.389E-05	1	1	2	Q9HBL0	Q9HBL0 [199-214	0	2460.3783
[R].VDIVQA/	1xTMTpro [N	0.0001584	7.389E-05	1	1	2	P38435	P38435 [486-498]	0	1820.9978
[K].VDQSTT	1xTMTpro [K	0.0001118	7.389E-05	1	1	1	Q70UQ0	Q70UQ0 [157-166	0	1675.918
[K].VEGDLK	3xTMTpro [K	0.0001474	7.389E-05	1	1	2	Q09666	Q09666 [2604-26	2	2897.7453
[K].VEGDMC	1xTMTpro [K	6.982E-05	7.389E-05	1	1	2	Q09666	Q09666 [3898-39	0	2067.1288

[K].VEGFPTI	2xTMTpro [K	0.0001402	7.389E-05	1	1	1	P13667	P13667 [597-612]		1	2668.5167
[R].VEGIVHF	1xTMTpro [K	6.417E-05	7.389E-05	1	1	2	Q9P0M6	Q9P0M6 [212-226		0	2230.2939
[K].VEIAGPC	1xTMTpro [N	0.0001806	7.389E-05	1	1	1	P54136	P54136 [154-167]		0	1826.0608
[K].VELCSFS	1xCarbamido	0.0002035	7.389E-05	1	1	2	P83731	P83731 [3-12]		0	1797.9701
[K].VELEVPC	1xCarbamido	0.0001455	7.389E-05	1	1	1	P04114	P04114 [80-93]		0	2283.3278
[RK].VEILAN	1xTMTpro [N	0.0001002	7.389E-05	5	7	3	P17066; P54	P17066 [28-38]; F		0	1532.8352
[K].VELNALN	1xTMTpro [K	0.000165	7.389E-05	1	1	2	Q9NR31	Q9NR31 [113-135		0	3105.7725
[K].VELVFSA	1xTMTpro [K	0.000153	7.389E-05	1	1	2	Q68CZ2	Q68CZ2 [294-304		0	1828.0712
[K].VELVPPT	1xTMTpro [N	0.0001719	7.389E-05	1	1	3	O75964	O75964 [36-48]		0	1722.0121
[K].VEMQPT	1xTMTpro [K	9.816E-05	7.389E-05	1	1	2	O43491	O43491 [161-171]		0	1869.0647
[R].VENMVD	1xTMTpro [N	0.0001867	7.389E-05	1	1	2	P41252	P41252 [419-428]		0	1520.8426
[K].VEGDLK	2xTMTpro [K	6.267E-05	7.389E-05	1	1	8	Q09666	Q09666 [2604-26		1	2311.3689
[K].VEPLRAI	1xTMTpro [N	0.0001971	7.389E-05	1	1	4	P02647	P02647 [143-155]		1	1771.9986
[R].VEPYGE	1xTMTpro [K	0.000157	7.389E-05	1	1	4	P06727	P06727 [307-316]		0	1804.9725
[K].VEQATK	1xTMTpro [K	9.831E-05	7.389E-05	1	1	1	Q96E39	Q96E39 [81-91]		0	1900.0784
[K].VEQIEAC	1xTMTpro [N	0.0001338	7.389E-05	1	1	1	Q16881	Q16881 [406-416		0	1460.8028
[R].VEQTET	1xTMTpro [N	0.0001991	7.389E-05	1	1	1	P52701	P52701 [483-495]		0	1854.8897
[K].VESAQS	1xTMTpro [K	0.0002047	7.389E-05	1	1	1	Q09161	Q09161 [699-707		0	1613.899
[K].VESEIKV	2xTMTpro [K	9.557E-05	7.389E-05	1	1	2	Q09666	Q09666 [843-855		1	2397.4421
[K].VESLEQI	1xTMTpro [N	8.362E-05	7.389E-05	1	1	1	P05067	P05067 [439-450]		0	1678.8567
[R].VETAATI	1xTMTpro [K	0.0001914	7.389E-05	1	1	1	Q8WXH0	Q8WXH0 [2861-2		0	1770.0141
[R].VETCGC	2xCarbamido	9.878E-05	7.389E-05	1	1	1	O14972	O14972 [216-227		0	1676.7692
[R].VETFLGV	1xCarbamido	0.0001131	7.389E-05	1	1	2	Q9NRY6	Q9NRY6 [94-105]		0	1815.9019
[R].VETPVLI	1xTMTpro [N	0.000145	7.389E-05	2	3	2	Q15796; P84	Q15796 [170-182		0	1720.0692
[R].VETYLNI	1xTMTpro [N	0.0002051	7.389E-05	1	1	2	Q9UQE7	Q9UQE7 [828-83		0	1554.8447
[R].VEVGKD	1xTMTpro [K	0.0001245	7.389E-05	1	1	1	O75369	O75369 [956-969		1	2231.2163
[K].VEPSHD	1xTMTpro [K	0.000162	7.389E-05	1	1	4	P21333	P21333 [866-874]		0	1577.8779
[K].VDQEIIN	1xTMTpro [N	8.759E-05	7.389E-05	1	1	4	O96000	O96000 [92-103]		0	1777.9438
[K].VEGDLK	2xTMTpro [K	6.529E-05	7.389E-05	1	1	3	Q09666	Q09666 [4410-44		1	2325.3846
[K].VEFVFSY	1xTMTpro [K	9.816E-05	7.389E-05	1	1	2	Q9HBL0	Q9HBL0 [425-435		0	1910.0555
[R].VDSDNA	1xTMTpro [K	0.0001834	7.389E-05	1	1	1	P16234	P16234 [982-994]		0	2053.1097
[R].VDSGIQI	1xTMTpro [K	9.277E-05	7.389E-05	1	1	1	P05165	P05165 [431-451]		0	2893.5149
[K].VDSLLEN	1xTMTpro [K	0.0001662	7.389E-05	1	4	3	P07910	P07910 [207-216]		0	1768.0348

[K].VDVEALI	1xTMTpro [N	8.26E-05	7.389E-05	1	1	1	O76094	O76094 [516-531]	0	2012.062
[R].VDVERD	1xTMTpro [N	6.79E-05	7.389E-05	1	1	3	P17661	P17661 [176-189]	1	2004.0681
[K].VDVGKD	2xTMTpro [K	0.0001005	7.389E-05	1	1	5	P21333	P21333 [983-994]	1	2277.3271
[K].VDVIQEF	1xTMTpro [N	0.0001991	7.389E-05	1	1	2	Q9H1E5	Q9H1E5 [91-102]	0	1573.8869
[R].VDVQAG	1xCarbamido	8.584E-05	7.389E-05	2	2	2	P0C0L4; P0(	P0C0L4 [560-570]	0	1741.9398
[R].VDVSCE	1xCarbamido	8.414E-05	7.389E-05	1	1	2	Q12888	Q12888 [768-780]	0	2069.108
[K].VDVTEQ	1xTMTpro [N	0.0001212	7.389E-05	1	1	2	Q9H3N1	Q9H3N1 [83-94]	0	1561.8505
[K].VDYNAS	1xTMTpro [K	7.393E-05	7.389E-05	1	1	2	P35749	P35749 [584-594]	0	1876.046
[R].VEADKY	1xTMTpro [K	9.277E-05	7.389E-05	1	1	1	Q8IUL8	Q8IUL8 [902-915]	1	2337.2735
[K].VEAPSLI	1xTMTpro [K	6.822E-05	7.389E-05	1	1	1	Q09666	Q09666 [4949-49	0	3085.6007
[K].VEGAQN	1xTMTpro [K	0.0002023	7.389E-05	1	1	2	Q9P2E9	Q9P2E9 [302-310	0	1538.8782
[K].VEAQHIL	1xTMTpro [N	9.572E-05	7.389E-05	1	1	1	Q53H96	Q53H96 [33-46]	0	1811.9935
[K].VEAYQW	1xTMTpro [N	0.0001786	7.389E-05	1	1	1	Q9Y263	Q9Y263 [362-373	0	1714.872
[K].VECEPS	2xCarbamido	7.124E-05	7.389E-05	1	1	2	Q9UBG0	Q9UBG0 [380-39	0	2384.0871
[K].VEDIIEAI	1xTMTpro [N	8.868E-05	7.389E-05	1	1	1	Q99715	Q99715 [500-513]	0	1984.0711
[K].VEDLST(	1xCarbamido	0.00017	7.389E-05	1	1	1	P22059	P22059 [218-230]	0	2086.1346
[K].VEDPES	1xTMTpro [N	0.0001672	7.389E-05	1	1	2	P09871	P09871 [370-383]	0	1852.9976
[K].VEDVSA	1xTMTpro [N	6.643E-05	7.389E-05	1	1	1	Q92598	Q92598 [332-346]	0	1805.9928
[R].VEDVVV	1xCarbamido	0.000146	7.389E-05	1	1	1	Q96EK6	Q96EK6 [119-129	0	1610.8015
[R].VEEKEP	1xTMTpro [K	0.0001845	7.389E-05	1	1	1	Q8N6H7	Q8N6H7 [384-402	1	2806.5806
[K].VEEKPPI	2xTMTpro [K	0.0001284	7.389E-05	1	1	1	Q9Y520	Q9Y520 [2864-28	0	2728.6541
[R].VEELCPI	1xCarbamido	6.95E-05	7.389E-05	1	1	1	Q96HY7	Q96HY7 [835-852	0	2758.4287
[K].VEEIDAA	1xTMTpro [N	0.0001561	7.389E-05	1	1	1	Q03001	Q03001 [5820-58	0	1432.8331
[K].VEEISG\	1xTMTpro [K	0.0001138	7.389E-05	1	2	1	O94851	O94851 [260-273]	0	2189.2462
[R].VEFEEL(	1xCarbamido	8.121E-05	7.389E-05	1	1	2	Q9Y4L1	Q9Y4L1 [346-358	0	1960.9645
[K].VEAQLQ	1xTMTpro [K	6.217E-05	7.389E-05	1	1	3	P35579	P35579 [1250-12	0	1893.1301
[K].VLEGSEI	1xTMTpro [K	6.188E-05	7.389E-05	1	1	4	Q14980	Q14980 [87-97]	0	1796.0661
[K].VIELENW	1xTMTpro [K	7.497E-05	7.389E-05	1	1	2	Q9NTX5	Q9NTX5 [90-100]	0	1926.0828
[R].VLELVSI	1xTMTpro [K	0.0001204	7.389E-05	1	1	2	P34897	P34897 [399-409]	0	1795.1185
[K].VPGLPTI	1xTMTpro [N	0.0001061	7.389E-05	1	1	2	Q6P2Q9	Q6P2Q9 [728-741	0	1854.0842
[K].VPIDGPF	1xTMTpro [N	0.0001587	7.389E-05	1	1	2	P11717	P11717 [1715-17	0	1552.9018
[R].VPITAVI	1xTMTpro [K	0.0001159	7.389E-05	1	1	3	P29144	P29144 [629-638]	0	1591.0438
[K].VPLVAEI	3xTMTpro [K	0.0001453	7.389E-05	1	1	2	P41250	P41250 [478-490]	1	2664.7169

[R].VPMSVV	2xTMTpro [K	6.129E-05	7.389E-05	1	1	2	Q9BYD2	Q9BYD2 [233-244	0	2287.3664
[K].VPPAINQ	1xTMTpro [N	0.0001208	7.389E-05	1	1	2	P62424	P62424 [76-89]	0	1874.0455
[K].VPPFTFH	1xTMTpro [K	0.000149	7.389E-05	1	1	4	Q6NZI2	Q6NZI2 [318-326]	0	1680.0129
[K].VPQAQP	2xTMTpro [K	7.648E-05	7.389E-05	1	1	2	O95793	O95793 [356-367]	0	2190.379
[K].VPQVST	1xTMTpro [N	6.188E-05	7.389E-05	1	1	5	P02768	P02768 [439-452]	0	1816.0499
[R].VPSENV	1xTMTpro [K	0.0001147	7.389E-05	1	1	1	P49748	P49748 [317-331]	0	2127.1941
[R].VPSSAS.	1xTMTpro [N	0.0001182	7.389E-05	1	1	5	P22105	P22105 [798-808]	0	1484.7664
[K].VPTTLMI	1xTMTpro [K	8.545E-05	7.389E-05	1	1	1	Q7Z434	Q7Z434 [298-311]	0	2092.2696
[R].VPVFRP	1xTMTpro [N	7.169E-05	7.389E-05	1	1	1	P63151	P63151 [154-169]	0	2148.1629
[R].VPGGLA	1xTMTpro [K	0.0001107	7.389E-05	1	1	1	P07585	P07585 [284-292]	0	1515.9139
[K].VPVHDV	1xTMTpro [K	7.191E-05	7.389E-05	1	1	4	P21333	P21333 [1440-145	0	1776.0147
[R].VQAELD	1xTMTpro [N	0.0001789	7.389E-05	1	1	2	Q16678	Q16678 [356-366]	0	1517.8607
[K].VQAGDV	1xTMTpro [K	7.325E-05	7.389E-05	1	1	1	Q9Y230	Q9Y230 [187-197	0	1767.0508
[K].VQAQVI(	1xTMTpro [K	7.135E-05	7.389E-05	1	1	3	Q15459	Q15459 [132-144]	0	2061.2564
[R].VQCIPGI	4xCarbamido	0.0001877	7.389E-05	1	1	1	Q15057	Q15057 [405-423]	0	2454.0953
[K].VQDICFS	2xCarbamido	0.0001845	7.389E-05	1	1	2	Q9H6U6	Q9H6U6 [408-418	0	1740.8117
[K].VQDTSN	1xTMTpro [K	0.0001776	7.389E-05	1	1	1	Q8IWA0	Q8IWA0 [792-810	0	2663.4496
[R].VQEAVE	1xTMTpro [K	0.000149	7.389E-05	1	1	1	Q96C01	Q96C01 [9-18]	0	1727.9857
[K].VQENCII	1xCarbamido	0.0001979	7.389E-05	1	1	1	O75533	O75533 [1031-10	0	1606.8542
[K].VQEQLG	1xTMTpro [K	0.0002047	7.389E-05	1	1	1	O15305	O15305 [52-63]	0	1966.1101
[K].VQESTK	2xTMTpro [K	6.357E-05	7.389E-05	1	1	1	O43390	O43390 [115-126]	1	2201.2594
[K].VQFGAS	1xTMTpro [K	7.985E-05	7.389E-05	1	1	1	P55196	P55196 [489-498]	0	1728.0088
[R].VQGFQV	1xTMTpro [K	0.0001827	7.389E-05	1	1	1	O95793	O95793 [499-507]	0	1705.9769
[R].VQGLDT	1xTMTpro [K	0.0002011	7.389E-05	1	1	1	Q8IZQ1	Q8IZQ1 [2545-25	0	2185.2724
[K].VQAAVG	1xTMTpro [N	9.306E-05	7.389E-05	1	1	1	P02649	P02649 [301-317]	0	1925.0048
[K].VQGLEQ	2xTMTpro [K	6.55E-05	7.389E-05	1	1	1	O95817	O95817 [432-446]	1	2574.4708
[R].VPGATM	1xTMTpro [K	0.0001317	7.389E-05	1	1	4	Q02252	Q02252 [214-223]	0	1609.0003
[R].VPFDAA	2xOxidation	9.379E-05	7.389E-05	1	1	1	P06396	P06396 [420-447]	0	3513.687
[R].VNLAIWI	1xTMTpro [N	8.584E-05	7.389E-05	1	1	2	Q9UL25	Q9UL25 [68-80]	0	1776.9564
[K].VNIEGG.	1xCarbamido	0.0001929	7.389E-05	1	1	1	Q9BWD1	Q9BWD1 [342-36	0	2253.2093
[K].VNLEVIK	2xTMTpro [K	0.000146	7.389E-05	1	1	3	Q8IYB3	Q8IYB3 [43-54]	0	2352.4835
[K].VNIFGVE	1xTMTpro [K	0.0001779	7.389E-05	1	1	1	Q9GZS3	Q9GZS3 [130-139	0	1657.9769
[R].VNIGQG	1xTMTpro [K	0.0001779	7.389E-05	1	1	2	O75369	O75369 [734-744]	0	1773.0263

[K].VNIIGEV	1xTMTpro [K	7.636E-05	7.389E-05	1	1	1	Q9UEY8	Q9UEY8 [192-207	0	2294.3211
[K].VNLLSVL	1xTMTpro [K	8.196E-05	7.389E-05	1	1	2	Q9BRX8	Q9BRX8 [207-217	0	1765.1079
[K].VNLQMV	1xCarbamido	9.739E-05	7.389E-05	1	1	1	Q9Y285	Q9Y285 [482-494	0	1898.9788
[K].VNMVTA	1xTMTpro [K	0.0001971	7.389E-05	1	1	1	Q9UDR5	Q9UDR5 [571-584	0	2116.2332
[R].VNPALAI	1xTMTpro [N	9.077E-05	7.389E-05	1	1	2	P13489	P13489 [54-64]	0	1513.9021
[K].VNPIQGI	1xTMTpro [K	0.0001756	7.389E-05	1	1	2	P13073	P13073 [150-159]	0	1635.0085
[R].VNPVTA	1xTMTpro [K	8.545E-05	7.389E-05	1	1	1	Q9Y2D4	Q9Y2D4 [759-770	0	1906.1869
[R].VNVAEV	1xTMTpro [K	0.0001587	7.389E-05	1	1	1	Q12955	Q12955 [710-728]	0	2600.4652
[R].VPFLVLE	1xCarbamido	7.755E-05	7.389E-05	1	1	3	Q9NRP0	Q9NRP0 [7-18]	0	2037.2062
[K].VNVEGIH	1xCarbamido	0.0001681	7.389E-05	1	1	1	P24043	P24043 [1138-114	0	1502.7705
[R].VNVYHH	1xTMTpro [K	8.937E-05	7.389E-05	1	1	2	P36269	P36269 [316-327]	0	2060.2148
[K].VPALIQC	1xCarbamido	6.217E-05	7.389E-05	1	1	1	O15254	O15254 [103-120]	0	2545.4014
[K].VPAINVN	1xTMTpro [K	7.439E-05	7.389E-05	1	1	3	P23526	P23526 [175-186]	0	1865.0988
[K].VPANPA	1xTMTpro [K	0.0001987	7.389E-05	1	1	1	Q7Z434	Q7Z434 [312-325]	0	1962.1516
[K].VPDDPE	1xTMTpro [N	7.863E-05	7.389E-05	1	1	1	Q9Y2S2	Q9Y2S2 [288-298	0	1523.8137
[K].VPDEHM	1xOxidation [	0.0001956	7.389E-05	1	1	1	Q07954	Q07954 [547-562]	0	2225.1378
[K].VPDEHM	2xOxidation [	0.0001453	7.389E-05	1	1	1	Q07954	Q07954 [547-562]	0	2241.1327
[R].VPDLSG	1xTMTpro [K	0.0001013	7.389E-05	1	1	2	Q8IYM9	Q8IYM9 [280-291	0	1908.1484
[R].VPDITEC	2xCarbamido	0.0001004	7.389E-05	1	1	1	O96000	O96000 [65-87]	1	3847.8541
[K].VPDSTYI	1xTMTpro [K	8.222E-05	7.389E-05	1	1	2	P62195	P62195 [143-156]	0	2133.1393
[R].VPDVLV	1xTMTpro [N	0.0001402	7.389E-05	1	1	2	Q14697	Q14697 [115-127]	0	1665.9859
[K].VPENPP	1xTMTpro [K	0.0001874	7.389E-05	1	1	6	Q01995	Q01995 [79-89]	0	1853.0487
[R].VPFDAA	1xOxidation [	7.069E-05	7.389E-05	1	1	1	P06396	P06396 [420-447]	0	3497.6921
[K].VNVPGS	1xTMTpro [K	6.438E-05	7.389E-05	1	1	2	Q02818	Q02818 [219-229]	0	1749.0514
[R].VNFVAG	1xTMTpro [K	6.8E-05	7.389E-05	1	1	1	Q13488	Q13488 [167-178]	0	1876.0936
[K].VQHIQLL	1xTMTpro [K	0.0001762	7.389E-05	1	1	4	P02671	P02671 [149-157]	0	1715.0823
[K].VQIFEYN	1xTMTpro [N	0.0001448	7.389E-05	1	1	2	Q96EE3	Q96EE3 [197-207	0	1716.8876
[K].VSMFVL	1xOxidation [	0.0001432	7.389E-05	1	1	1	Q9NZM1	Q9NZM1 [318-333	0	2051.0439
[K].VSNIFDD	1xTMTpro [N	0.0001638	7.389E-05	2	2	1	Q9Y4E1; Q6	Q9Y4E1 [1306-13	0	1897.9615
[K].VSQFLVI	2xCarbamido	8.184E-05	7.389E-05	1	1	1	Q8N0X7	Q8N0X7 [490-507	0	2561.3711
[K].VSQLEM	1xTMTpro [N	0.0001082	7.389E-05	1	1	1	Q0VF96	Q0VF96 [1070-10	0	1795.9067
[K].VSQLME	1xTMTpro [K	0.000141	7.389E-05	1	1	4	Q9H0U3	Q9H0U3 [41-50]	0	1844.0232
[R].VSQTDN	1xTMTpro [N	6.664E-05	7.389E-05	1	1	2	P24821	P24821 [902-914]	0	1852.9724

[K].VSSAEG	2xTMTpro [K	8.311E-05	7.389E-05	1	1	2	P05114	P05114 [6-18]	1	2215.275
[R].VSSASS	1xTMTpro [N	0.000196	7.389E-05	1	1	2	P55196	P55196 [560-569]	0	1298.6871
[K].VSSLQA	1xTMTpro [N	6.653E-05	7.389E-05	1	1	2	Q9H2M9	Q9H2M9 [914-92	0	1500.8705
[R].VSSQNL	1xTMTpro [K	7.18E-05	7.389E-05	1	1	2	Q9Y2T2	Q9Y2T2 [264-277	0	2125.2877
[R].VSSTAT	1xTMTpro [K	0.0001304	7.389E-05	1	1	1	P55196	P55196 [62-78]	0	2401.3318
[K].VSSTTS	1xTMTpro [K	0.0001127	7.389E-05	1	1	1	Q14789	Q14789 [1325-13	0	1660.9613
[K].VSSVFK	1xTMTpro [K	0.0001987	7.389E-05	1	1	1	P46060	P46060 [476-487]	1	1946.1203
[R].VSISEG	1xTMTpro [K	9.894E-05	7.389E-05	1	1	2	P22087	P22087 [123-135]	1	2119.1527
[K].VSTAVL	1xTMTpro [K	8.678E-05	7.389E-05	1	1	2	Q99460	Q99460 [828-838	0	1698.0657
[K].VSYDMD	1xCarbamid	0.0001739	7.389E-05	1	1	1	Q12965	Q12965 [511-521	0	1682.7474
[K].VSYFPT	1xTMTpro [K	0.0001713	7.389E-05	1	1	4	O75369	O75369 [2059-20	0	2365.3663
[R].VTAAIAS	1xTMTpro [N	0.0001405	7.389E-05	1	1	1	Q9Y2Q5	Q9Y2Q5 [44-58]	0	1926.0404
[R].VTAAPQ	1xCarbamid	0.0001644	7.389E-05	1	1	3	P01023	P01023 [587-598]	0	1576.88
[K].VTAETQ	2xTMTpro [K	0.0001863	7.389E-05	1	1	1	Q92805	Q92805 [282-299	1	3010.6877
[K].VTAGISF	1xTMTpro [K	0.000138	7.389E-05	1	1	1	Q92743	Q92743 [347-359	0	1914.1192
[R].VTCIDP	1xCarbamid	0.0001315	7.389E-05	1	1	1	Q9H8H3	Q9H8H3 [94-105]	0	2042.0872
[K].VTCLSC	2xCarbamid	0.0001324	7.389E-05	1	1	2	P27487	P27487 [442-453]	0	1781.8845
[R].VTCPNH	1xCarbamid	0.0001264	7.389E-05	1	1	1	Q00403	Q00403 [13-28]	0	2203.1137
[K].VTCTNA	1xCarbamid	0.0001049	7.389E-05	1	1	2	P01031	P01031 [1604-16	0	1742.9966
[K].VTCVEM	1xCarbamid	0.0001991	7.389E-05	1	1	1	Q15262	Q15262 [1011-10	0	2099.0836
[R].VTDATE	1xTMTpro [N	7.66E-05	7.389E-05	1	1	4	P02751	P02751 [1913-19	0	1898.019
[K].VTDDLV	1xCarbamid	6.79E-05	7.389E-05	1	1	2	P49458	P49458 [42-52]	0	1933.096
[R].VSWSP	1xTMTpro [N	0.0001	7.389E-05	1	1	1	Q02388	Q02388 [526-538	0	1751.94
[R].VQIALA	1xTMTpro [N	9.191E-05	7.389E-05	1	1	2	Q99715	Q99715 [1236-12	0	1721.9506
[K].VSLEET	1xTMTpro [N	6.149E-05	7.389E-05	1	1	1	Q9BRP8	Q9BRP8 [116-13	0	2074.11
[K].VSGPDL	1xTMTpro [K	0.0001749	7.389E-05	1	1	2	Q09666	Q09666 [5297-53	0	1779.0508
[R].VQLGPY	1xCarbamid	0.0001395	7.389E-05	1	1	1	Q9BZ67	Q9BZ67 [178-194	0	2202.1773
[R].VQLSGP	1xTMTpro [K	7.224E-05	7.389E-05	1	1	3	Q9UNS2	Q9UNS2 [326-33	0	1794.0253
[K].VQISPD	1xTMTpro [N	0.0001635	7.389E-05	1	1	3	Q92945	Q92945 [178-190	0	1658.9033
[R].VQIYHN	1xTMTpro [N	0.000108	7.389E-05	1	1	1	P50552	P50552 [36-48]	0	1850.9833
[R].VQPICL	1xCarbamid	0.0001849	7.389E-05	1	1	1	Q6UXH9	Q6UXH9 [576-58	0	1418.8109
[R].VQPNEA	1xTMTpro [K	0.0001165	7.389E-05	1	1	2	P11413	P11413 [394-403]	0	1757.0089
[K].VQPYLD	1xTMTpro [K	0.0001159	7.389E-05	1	1	4	P02647	P02647 [121-130]	0	1861.0351

[K].VQQGEF	1xTMTpro [K	0.0001046	7.389E-05	1	1	1	O60749	O60749 [457-469]	1	2172.1905
[R].VQQQED	1xTMTpro [K	0.0001273	7.389E-05	1	1	1	Q9HC35	Q9HC35 [31-42]	0	2038.1676
[K].VQQTVQ	1xTMTpro [N	0.0001292	7.389E-05	1	1	3	P38646	P38646 [395-405]	0	1594.8872
[K].VQQVQF	1xTMTpro [N	0.0001101	7.389E-05	1	1	2	Q96TA1	Q96TA1 [294-306	0	1772.0172
[K].VQREPL	1xTMTpro [K	7.948E-05	7.389E-05	1	1	1	Q13200	Q13200 [179-189]	1	1904.2188
[K].VQSLCY	1xCarbamido	0.0001109	7.389E-05	1	1	2	Q8IWB7	Q8IWB7 [245-255	0	1684.9375
[K].VSHQAG	1xTMTpro [N	0.0001405	7.389E-05	1	1	1	Q14764	Q14764 [349-360]	0	1722.9359
[R].VQSSIAF	1xCarbamido	0.0001247	7.389E-05	1	1	1	P11717	P11717 [126-136]	0	1818.0439
[R].VQTLEEI	1xCarbamido	0.0001127	7.389E-05	1	1	1	Q9BVJ6	Q9BVJ6 [508-526	1	3235.7337
[R].VQWTAA	1xTMTpro [K	7.543E-05	7.389E-05	1	1	1	Q02388	Q02388 [250-263]	0	2073.1624
[R].VRGEES	1xTMTpro [K	0.0002051	7.389E-05	1	1	1	P22105	P22105 [2782-279	2	2507.4073
[K].VRPCIDI	1xCarbamido	8.059E-05	7.389E-05	2	2	1	P20591; P20	P20591 [49-60]; P	0	1761.0012
[R].VSAPDG	1xCarbamido	0.000162	7.389E-05	1	1	2	Q8N2S1	Q8N2S1 [337-350	0	1793.8812
[K].VSAQYLS	1xTMTpro [K	0.0001043	7.389E-05	1	1	1	O75955	O75955 [199-211]	0	2077.1495
[K].VSASTVI	1xTMTpro [N	0.0001482	7.389E-05	1	1	1	Q7Z434	Q7Z434 [372-384]	0	1567.8247
[K].VSATAAI	1xTMTpro [N	7.337E-05	7.389E-05	1	1	1	P31260	P31260 [181-193]	0	1633.9233
[K].VSDFYD	1xTMTpro [N	0.0001796	7.389E-05	1	1	1	Q15746	Q15746 [1460-14	0	1576.7814
[K].VSDLTQ	1xTMTpro [K	0.0001573	7.389E-05	1	1	1	P17661	P17661 [300-309]	0	1654.962
[R].VSEYVP	1xTMTpro [K	0.0001739	7.389E-05	1	1	1	P49589	P49589 [241-254]	0	2259.288
[K].VSFLWD	1xTMTpro [N	7.439E-05	7.389E-05	1	1	1	P24043	P24043 [2204-22	0	1682.9185
[R].VSGISFF	1xTMTpro [N	9.291E-05	7.389E-05	1	1	1	Q9BW92	Q9BW92 [276-28	0	1723.9914
[R].VQTAVT	1xTMTpro [K	0.000159	7.389E-05	1	1	2	Q7LBR1	Q7LBR1 [79-87]	0	1542.9169
[R].SDSEDIC	1xCarbamido	0.0001528	7.389E-05	1	1	1	O76021	O76021 [90-109]	1	3224.6449
[K].VNFHMF	1xTMTpro [N	0.0001749	7.389E-05	1	3	3	P63092	P63092 [217-228]	0	1710.8705
[R].VNEVGV	1xTMTpro [N	0.0001796	7.389E-05	1	1	1	Q7KZ85	Q7KZ85 [966-975	0	1404.7766
[K].VLIIGGG	1xTMTpro [N	0.0001492	7.389E-05	1	1	1	P19623	P19623 [97-109]	0	1529.9334
[K].VLLLIDD	1xTMTpro [K	0.0001482	7.389E-05	1	1	2	Q8N766	Q8N766 [639-648	0	1829.0916
[K].VLIPIHEA	1xTMTpro [N	0.0002055	7.389E-05	1	1	1	Q96N67	Q96N67 [1800-18	0	1465.881
[R].VLLQAFI	1xTMTpro [N	0.000142	7.389E-05	1	1	2	Q15750	Q15750 [105-115]	0	1592.9331
[R].VILSSST	1xCarbamido	0.0001739	7.389E-05	1	1	1	Q9Y2S2	Q9Y2S2 [117-130	0	2118.1794
[R].VIITNFPA	1xTMTpro [K	0.0001147	7.389E-05	1	1	2	P47897	P47897 [577-586]	0	1682.0497
[K].VLIWNLD	1xTMTpro [K	0.0001004	7.389E-05	1	1	1	Q9UQ03	Q9UQ03 [162-174	0	2090.2505
[K].VLMATN	1xTMTpro [N	0.0001049	7.389E-05	1	1	1	P35998	P35998 [317-336]	0	2528.3761

[K].VIMDYES	1xTMTpro [K	0.0001432	7.389E-05	1	1	2	P00491	P00491 [245-254]	0	1835.0116
[KR].VIMVTC	1xTMTpro [K	7.102E-05	7.389E-05	1	6	2	P05023	P05023 [613-625]	0	1990.1651
[KR].VIMVTC	1xOxidation	7.258E-05	7.389E-05	1	6	2	P05023	P05023 [613-625]	0	2006.16
[R].VINYEEF	2xTMTpro [K	0.000196	7.389E-05	1	1	1	Q9BW30	Q9BW30 [69-77]	1	2082.2415
[K].VLPGGD	1xTMTpro [N	0.0001769	7.389E-05	1	1	1	Q9H6X2	Q9H6X2 [112-126	0	2011.9867
[R].VLIKPEI(	2xTMTpro [K	0.0001659	7.389E-05	1	1	1	Q9HAU0	Q9HAU0 [298-309	0	2336.4482
[K].VLPGGD	1xOxidation	0.0001975	7.389E-05	1	1	1	Q9H6X2	Q9H6X2 [112-126	0	2027.9816
[R].VLPGHS	1xTMTpro [N	0.0001786	7.389E-05	1	1	2	P17813	P17813 [223-232]	0	1294.7551
[K].VLPGMH	2xTMTpro [K	0.000119	7.389E-05	1	1	1	Q92879	Q92879 [84-101]	0	2927.6416
[R].VLPGSS'	1xCarbamido	8.745E-05	7.389E-05	1	1	1	Q96AB3	Q96AB3 [11-26]	0	2447.317
[K].VLPMNT	1xCarbamido	9.617E-05	7.389E-05	1	1	1	P04181	P04181 [136-151]	0	2301.2074
[R].VLPSITT	1xTMTpro [K	8.638E-05	7.389E-05	1	1	2	P35232	P35232 [118-128]	0	1822.1545
[R].VLPSIVN	1xTMTpro [K	8.109E-05	7.389E-05	1	1	2	Q99623	Q99623 [132-142	0	1819.1548
[R].VLQDLVI	1xTMTpro [N	0.0001134	7.389E-05	1	1	1	P53618	P53618 [317-327]	0	1618.9521
[K].VLQDMG	1xTMTpro [N	6.601E-05	7.389E-05	1	1	2	Q12906	Q12906 [461-474	0	1747.9332
[K].VLQDMG	1xOxidation	8.691E-05	7.389E-05	1	1	2	Q12906	Q12906 [461-474	0	1763.9281
[K].VIQEIWF	1xTMTpro [K	6.864E-05	7.389E-05	1	1	1	Q9Y5L0	Q9Y5L0 [637-651	0	2377.3987
[K].VIQENEI	1xTMTpro [K	0.0001262	7.389E-05	1	1	1	Q9NTJ3	Q9NTJ3 [999-100	0	1917.1049
[R].VLQQFA	1xTMTpro [N	8.234E-05	7.389E-05	1	1	2	P51659	P51659 [544-555]	0	1695.8985
[R].VLQSFT'	1xTMTpro [K	8.638E-05	7.389E-05	1	1	2	O75369	O75369 [1439-14	0	1819.0457
[K].VLPGGIL	2xTMTpro [K	6.822E-05	7.389E-05	1	1	1	Q9Y6Q2	Q9Y6Q2 [301-320	0	3222.8417
[K].VLQVSIG	1xTMTpro [K	9.262E-05	7.389E-05	1	1	1	Q12965	Q12965 [915-926	0	1816.1552
[R].VLLGGP	1xTMTpro [K	0.0001899	7.389E-05	1	1	1	P06756	P06756 [199-224]	0	3399.8808
[K].VILESEV	1xTMTpro [K	8.772E-05	7.389E-05	1	1	1	O15226	O15226 [482-496	0	2164.3084
[K].VLEMET	1xTMTpro [K	0.0001204	7.389E-05	1	1	1	O75150	O75150 [272-288	0	2686.4141
[R].VLEQLIV	1xOxidation	0.0001208	7.389E-05	1	1	2	P78527	P78527 [1713-172	0	2088.1595
[R].VIEQLLV	1xTMTpro [K	0.0001299	7.389E-05	1	1	2	Q9BTW9	Q9BTW9 [361-37	0	1720.1228
[R].VLESEE(	1xTMTpro [N	7.135E-05	7.389E-05	1	1	1	Q13617	Q13617 [76-89]	0	2036.0806
[K].VLETAEI	1xTMTpro [N	0.0001463	7.389E-05	1	1	1	Q13813	Q13813 [8-18]	0	1606.8607
[K].VLETAEE	1xTMTpro [N	0.0001125	7.389E-05	1	1	2	P02549	P02549 [17-27]	0	1620.8764
[K].VLETTVE	1xTMTpro [K	6.509E-05	7.389E-05	1	1	2	Q96QK1	Q96QK1 [372-382	0	1901.1239
[K].VLETVG'	1xTMTpro [K	0.0001237	7.389E-05	1	1	1	Q9Y2S7	Q9Y2S7 [61-72]	0	1925.1603
[K].VLEVAKI	2xTMTpro [K	6.109E-05	7.389E-05	1	1	1	P13667	P13667 [438-463]	1	3861.0302

[R].VLFDPFI	1xTMTpro [N	0.0001153	7.389E-05	1	1	2	Q15269	Q15269 [734-749]	0	2097.1187
[R].VIFITEYI	1xTMTpro [K	0.0001317	7.389E-05	1	1	2	Q9UHY1	Q9UHY1 [146-15	0	2183.2278
[R].VIFTGMI	1xTMTpro [N	8.388E-05	7.389E-05	1	1	1	O95248	O95248 [937-955]	0	2361.3171
[K].VLFYTDI	1xTMTpro [N	7.767E-05	7.389E-05	1	1	2	Q14112	Q14112 [1222-12	0	1640.9331
[R].VLLFTDI	1xTMTpro [K	7.543E-05	7.389E-05	1	1	1	Q709C8	Q709C8 [3061-30	0	2028.2237
[K].VLGDLIF	1xTMTpro [N	0.000123	7.389E-05	1	1	2	Q8WVC6	Q8WVC6 [68-79]	0	1690.9447
[R].VLGHFE	1xCarbamido	8.558E-05	7.389E-05	1	1	1	Q8IY17	Q8IY17 [195-209]	0	2466.4187
[K].VLGMDP	1xTMTpro [K	0.0001525	7.389E-05	1	1	4	Q12906	Q12906 [333-342]	0	1664.9901
[K].VIGMHYI	1xTMTpro [K	9.831E-05	7.389E-05	1	1	2	P40939	P40939 [494-505]	0	2001.1123
[R].VIGNMG	1xTMTpro [K	7.672E-05	7.389E-05	1	1	2	P04114	P04114 [491-507]	0	2497.365
[R].VLGPCS	1xCarbamido	0.0001116	7.389E-05	1	1	2	P10644	P10644 [358-367]	0	1710.0116
[R].VLGQVS	1xCarbamido	0.0001002	7.389E-05	1	1	1	Q06265	Q06265 [55-67]	0	2024.1706
[R].VIGSGCI	1xCarbamido	0.0001626	7.389E-05	2	4	1	P07195; P0C	P07195 [159-170]	0	1552.8073
[K].VLGTEDI	1xTMTpro [K	9.957E-05	7.389E-05	1	1	2	P68400	P68400 [248-268]	1	3152.6647
[K].VIGVLEF	1xTMTpro [K	6.832E-05	7.389E-05	1	1	2	Q96RP9	Q96RP9 [552-566	0	2296.2932
[K].VLHDAQ	1xCarbamido	0.0001599	7.389E-05	1	1	1	A1L0T0	A1L0T0 [600-609]	0	1558.8079
[R].VLHNAQ	1xTMTpro [K	0.0001007	7.389E-05	1	1	1	P49916	P49916 [454-468]	1	2342.3072
[K].VLLDDT(	1xTMTpro [N	0.00018	7.389E-05	1	1	1	Q14789	Q14789 [2082-20	0	1621.8716
[K].VLIEGSII	1xTMTpro [N	0.0001053	7.389E-05	1	1	3	P59998	P59998 [61-71]	0	1490.8862
[K].VLGESM	1xTMTpro [K	0.0001212	7.389E-05	1	1	1	Q9Y4G6	Q9Y4G6 [1404-14	0	2013.1294
[R].VNFDDY	2xTMTpro [K	8.146E-05	7.389E-05	1	1	1	Q06210	Q06210 [362-379]	1	2960.6662
[K].VLQYHK	1xTMTpro [K	9.847E-05	7.389E-05	1	1	1	O95989	O95989 [128-144]	0	2687.5165
[K].VLSECSI	1xCarbamido	0.0001579	7.389E-05	1	1	1	P53618	P53618 [619-633]	0	2375.2595
[K].VLYSNM	1xOxidation	0.0001995	7.389E-05	1	1	1	Q00325	Q00325 [146-161]	0	2308.1603
[K].VMAAEN	1xTMTpro [K	0.0001752	7.389E-05	1	1	1	P42224	P42224 [653-665]	0	2034.1549
[R].VMEIVD/	1xTMTpro [K	8.732E-05	7.389E-05	1	1	2	Q9Y5B9	Q9Y5B9 [202-211	0	1756.9647
[R].VMFSSF	1xCarbamido	0.000159	7.389E-05	1	1	1	Q92879	Q92879 [126-138]	0	1893.9158
[K].VMGTFS	1xTMTpro [K	9.277E-05	7.389E-05	1	1	1	P08240	P08240 [374-386]	0	1966.1175
[K].VMKHEA	1xCarbamido	0.0001078	7.389E-05	1	1	1	P12110	P12110 [237-248]	1	2427.2981
[R].VMIPVP/	1xOxidation	0.0001596	7.389E-05	1	1	1	Q96EY1	Q96EY1 [304-319	0	1988.0806
[R].VMNTGS	1xTMTpro [K	0.0001412	7.389E-05	1	1	1	Q8WVM8	Q8WVM8 [523-53	0	2135.1485
[R].VMPICLI	1xCarbamido	0.0001867	7.389E-05	1	1	2	P00738	P00738 [262-277]	1	2459.3282
[K].VMPYVD	1xTMTpro [N	0.0001288	7.389E-05	1	1	1	P55263	P55263 [230-249]	0	2548.3077

[K].VMQEEII	1xTMTpro [K	0.0001378	7.389E-05	1	1	2	P51648	P51648 [327-343]	0	2517.501
[R].VMQHQY	1xTMTpro [N	0.000182	7.389E-05	1	1	1	P11215	P11215 [950-963]	0	1992.0404
[K].VMQQNL	1xTMTpro [N	6.706E-05	7.389E-05	1	1	1	O75718	O75718 [330-342	0	2046.055
[R].VLYPND	1xTMTpro [K	0.0001149	7.389E-05	3	3	2	P11216; P06	P11216 [279-290]	0	2051.1094
[R].VMVDAN	1xOxidation [	0.000149	7.389E-05	1	1	1	P50440	P50440 [370-381]	0	1967.1127
[K].VMVLDF	1xOxidation [	0.0001852	7.389E-05	1	1	2	Q16881	Q16881 [188-202	0	1966.1003
[K].VMVTNV	1xTMTpro [K	6.468E-05	7.389E-05	2	2	4	Q9Y4G6; Q9	Q9Y4G6 [2121-21	0	1813.1113
[K].VMVTNV	1xOxidation [	9.8E-05	7.389E-05	2	2	4	Q9Y4G6; Q9	Q9Y4G6 [2121-21	0	1829.1062
[K].VMYTPM	1xTMTpro [K	8.558E-05	7.389E-05	1	1	5	O75369	O75369 [2440-24	0	2392.3264
[R].VNAIHFL	1xTMTpro [K	0.0001046	7.389E-05	1	1	2	A1A4S6	A1A4S6 [492-501	0	1786.0983
[R].VNAIPFT	1xTMTpro [K	9.234E-05	7.389E-05	1	1	1	Q13421	Q13421 [339-353	0	2358.3565
[K].VNAMTF	1xTMTpro [K	0.0001025	7.389E-05	1	3	1	Q5JPE7	Q5JPE7 [540-554	0	2262.2448
[K].VNATGP	1xTMTpro [K	0.0001392	7.389E-05	1	1	1	Q4G0J3	Q4G0J3 [444-458	0	2124.2672
[R].VNCLAP	1xCarbamido	0.0001611	7.389E-05	1	2	2	Q9BTZ2	Q9BTZ2 [207-216	0	1693.0326
[K].VNDDIIV	1xTMTpro [N	7.815E-05	7.389E-05	1	1	1	P13796	P13796 [516-530]	0	2104.1358
[R].VNEFGM	1xTMTpro [N	6.307E-05	7.389E-05	1	1	1	O96005	O96005 [595-634	0	4173.0981
[R].VNEISVE	1xTMTpro [N	0.0001687	7.389E-05	1	1	1	P27708	P27708 [2188-220	0	1762.9142
[K].VNEMIIG	2xOxidation [	0.0001941	7.389E-05	1	1	1	P00558	P00558 [231-246]	0	2368.29
[R].VMVGDL	1xTMTpro [K	9.091E-05	7.389E-05	1	1	1	P11532	P11532 [2498-25	0	2327.2846
[R].VLSAPPI	1xTMTpro [N	0.0001896	7.389E-05	1	1	2	P26641	P26641 [31-45]	0	2012.0785
[R].VLWAFP	1xTMTpro [N	8.096E-05	7.389E-05	1	1	1	Q9NR99	Q9NR99 [2476-24	0	2452.3348
[K].VLVPGIF	2xTMTpro [K	0.0001397	7.389E-05	1	1	1	Q15061	Q15061 [403-428	0	3613.1046
[R].VLSEQL	1xTMTpro [N	7.314E-05	7.389E-05	1	1	1	Q9UPN3	Q9UPN3 [1311-13	0	2366.2887
[R].VLSFIPP	1xTMTpro [N	0.0002027	7.389E-05	1	1	1	Q9Y2T2	Q9Y2T2 [247-258	0	1665.9284
[R].VLSGDL	1xTMTpro [N	6.633E-05	7.389E-05	1	1	1	Q16822	Q16822 [32-45]	0	1730.0132
[R].VLSGGP	1xTMTpro [N	0.0001155	7.389E-05	1	1	1	Q02388	Q02388 [362-383	0	2525.4007
[K].VLSLAQI	1xTMTpro [K	6.695E-05	7.389E-05	2	2	2	P0C0L4; P0	P0C0L4 [1085-10	0	2150.2313
[R].VLSIGDC	1xTMTpro [N	0.0001561	7.389E-05	1	1	3	P25705	P25705 [74-83]	0	1304.7857
[K].VLSMAP	1xTMTpro [N	0.0001726	7.389E-05	1	1	1	O95340	O95340 [535-551	0	2134.2265
[K].VLSPVD	1xTMTpro [K	0.0001402	7.389E-05	1	1	1	P11277	P11277 [1224-12	0	1623.9561
[K].VISQIAM	1xTMTpro [K	9.617E-05	7.389E-05	1	1	2	O95436	O95436 [282-292	0	1856.0443
[K].VLSTTKI	3xTMTpro [K	8.84E-05	7.389E-05	1	1	1	Q16666	Q16666 [209-226	1	3373.907
[K].VLTDEQ	1xTMTpro [N	0.0001338	7.389E-05	1	1	1	Q9ULZ3	Q9ULZ3 [140-150	0	1625.8818

[R].VLTHSEI	1xTMTpro [N	0.0001356	7.389E-05	1	1	2	Q14515	Q14515 [607-617]	0	1539.9178
[R].VLTPPM	1xOxidation [	9.647E-05	7.389E-05	1	1	3	Q15582	Q15582 [497-510]	0	2125.2256
[R].VLVSPTI	1xCarbamido	0.0001482	7.389E-05	1	1	1	O60763	O60763 [289-305]	0	2351.2885
[K].VLTSEDI	1xTMTpro [N	0.0001896	7.389E-05	1	1	1	Q9BZQ8	Q9BZQ8 [125-138	0	1958.0038
[K].VLTSLGI	1xTMTpro [K	8.786E-05	7.389E-05	2	2	2	P69891; P69	P69891 [68-77]; P	0	1625.0129
[R].VLTVINQ	1xTMTpro [K	0.0001442	7.389E-05	1	1	2	P42766	P42766 [57-66]	0	1752.0875
[K].VLVALYE	2xTMTpro [K	7.202E-05	7.389E-05	1	1	3	Q99417	Q99417 [30-49]	0	3187.8435
[K].VLVDSLV	1xTMTpro [N	0.0001342	7.389E-05	1	1	1	Q92621	Q92621 [1524-153	0	1563.8549
[R].VLVEATY	1xTMTpro [K	7.863E-05	7.389E-05	1	1	1	Q8IX12	Q8IX12 [186-199]	0	2231.239
[R].VLVEDD	1xOxidation [	0.0001007	7.389E-05	1	1	4	P09466	P09466 [143-156]	0	1984.038
[R].VLVEPDA	1xTMTpro [K	6.129E-05	7.389E-05	1	1	1	P42126	P42126 [47-61]	0	2064.2019
[R].VIVESAS	1xTMTpro [K	8.159E-05	7.389E-05	1	1	3	Q9NZM1	Q9NZM1 [4-14]	0	1765.0715
[R].VIVGGSS	1xTMTpro [K	0.0001662	7.389E-05	1	1	1	Q14683	Q14683 [97-106]	0	1646.9609
[R].VLVLEMI	1xTMTpro [N	0.000107	7.389E-05	1	1	1	P55268	P55268 [724-740]	0	2082.1224
[K].VLVIGAG	1xCarbamido	6.387E-05	7.389E-05	1	1	1	Q8TBC4	Q8TBC4 [72-86]	0	2107.2805
[K].VLVLSQI	1xTMTpro [K	9.863E-05	7.389E-05	1	1	3	Q01082	Q01082 [549-558]	0	1730.0344
[R].VLVNEQ	1xTMTpro [K	0.0001101	7.389E-05	1	1	1	Q9BXJ0	Q9BXJ0 [128-142	0	2238.2374
[R].VLTSGIF	1xTMTpro [K	0.0001172	7.389E-05	1	2	3	P63092	P63092 [202-211]	0	1703.0235
[R].SDSEDIC	1xCarbamido	8.813E-05	7.389E-05	1	1	2	O76021	O76021 [90-100]	0	1923.0025
[R].SDSALLI	1xTMTpro [N	0.000157	7.389E-05	1	1	1	P42858	P42858 [2112-212	0	1777.9615
[K].SDPEGE	1xTMTpro [K	0.0001068	7.389E-05	1	1	1	Q6P996	Q6P996 [537-550	0	2088.1581
[K].LSDVTLV	1xCarbamido	0.0001385	7.389E-05	1	1	2	O95865	O95865 [252-267]	0	2384.3238
[R].LSEAEFI	1xTMTpro [K	0.0001232	7.389E-05	1	1	4	P04275	P04275 [1288-129	0	1773.029
[R].ISEAETL	1xCarbamido	0.000126	7.389E-05	1	1	1	Q6PGP7	Q6PGP7 [1342-13	0	1759.9756
[R].LSEEELI	1xTMTpro [K	0.0001703	7.389E-05	1	2	1	Q9Y3L3	Q9Y3L3 [130-140	0	1850.113
[R].LSEEPLC	1xTMTpro [N	7.625E-05	7.389E-05	1	1	1	Q9NZ08	Q9NZ08 [115-127	0	1851.0295
[R].LSEGFSI	1xTMTpro [N	0.0001796	7.389E-05	1	1	1	P20618	P20618 [57-66]	0	1450.7973
[K].LSELEAA	1xTMTpro [N	0.0001703	7.389E-05	1	1	4	P05787	P05787 [353-362]	0	1433.8283
[R].ISEILLDI	1xTMTpro [K	0.0001138	7.389E-05	1	1	1	P16157	P16157 [287-301]	0	2213.3149
[K].LSELLPE	1xTMTpro [K	7.169E-05	7.389E-05	1	1	2	Q9Y2X3	Q9Y2X3 [222-235	0	2193.251
[K].LSEISGII	1xTMTpro [K	0.0001827	7.389E-05	1	1	1	Q96K76	Q96K76 [1259-12	0	2355.3303
[R].ISEISME	1xTMTpro [N	0.0001	7.389E-05	1	1	2	P24043	P24043 [1354-136	0	1752.9121
[K].LSEMAD	1xTMTpro [K	0.0001172	7.389E-05	1	1	1	O94911	O94911 [547-558]	0	1958.076

[R].ISEYSEA	1xTMTpro [N	8.375E-05	7.389E-05	1	1	2	Q7L014	Q7L014 [958-969]	0	1656.9128
[R].LSFEDF\	1xTMTpro [N	7.416E-05	7.389E-05	1	1	1	Q9UBV8	Q9UBV8 [270-282	0	1807.922
[R].LSFPSIK	2xTMTpro [K	9.572E-05	7.389E-05	1	1	1	Q92785	Q92785 [93-107]	0	2602.5272
[R].LSFQHD	1xTMTpro [N	0.0001089	7.389E-05	1	1	2	Q14697	Q14697 [915-929]	0	2045.1351
[K].ISFTGST	1xTMTpro [K	9.467E-05	7.389E-05	1	1	2	P51649	P51649 [280-290]	0	1707.9773
[K].ISGAIGP	1xCarbamido	0.0001849	7.389E-05	1	1	1	Q15436	Q15436 [416-429]	0	2011.1502
[R].LSGGLG	1xCarbamido	0.0001605	7.389E-05	1	1	2	Q04695	Q04695 [31-41]	0	1338.7119
[R].ISGLGLT	1xTMTpro [K	0.0001046	7.389E-05	1	1	2	O95831	O95831 [99-109]	0	1751.0559
[R].ISGLIYEI	1xTMTpro [N	0.000122	7.389E-05	1	1	36	P62805	P62805 [47-56]	0	1484.828
[K].LSGLVFI	1xTMTpro [N	0.0001232	7.389E-05	1	1	1	Q9Y2C4	Q9Y2C4 [275-286	0	1704.9756
[K].LSGMPD	1xTMTpro [N	6.478E-05	7.389E-05	1	1	1	Q9Y2T2	Q9Y2T2 [212-226	0	1969.0206
[R].LSGNVL:	1xTMTpro [K	0.0001025	7.389E-05	1	1	1	P10643	P10643 [179-191]	0	2064.1985
[K].LSGPDD	1xTMTpro [K	0.0001852	7.389E-05	1	1	1	Q86TI2	Q86TI2 [577-587]	0	1801.994
[R].LSGTGS	1xTMTpro [N	9.941E-05	7.389E-05	1	1	1	P36871	P36871 [504-515]	0	1394.7923
[R].LSGVMV	1xTMTpro [N	8.121E-05	7.389E-05	1	1	2	Q9UBB4	Q9UBB4 [11-27]	0	2113.201
[R].LSDGVF	2xTMTpro [K	0.0001007	7.389E-05	1	1	2	P51888	P51888 [207-221]	0	2573.4908
[R].LSDETLI	1xTMTpro [N	0.0001112	7.389E-05	1	1	2	P19367	P19367 [31-42]	0	1710.9267
[K].LSCLGA:	1xCarbamido	9.708E-05	7.389E-05	1	1	1	Q9HC84	Q9HC84 [781-790	0	1684.9912
[RK].ISATSII	1xTMTpro [K	0.000139	7.389E-05	2	2	2	P34897; P34	P34897 [183-196]	0	2229.2121
[K].LQSSNIF	1xTMTpro [K	7.96E-05	7.389E-05	1	1	2	Q10567	Q10567 [877-887]	0	1816.0824
[K].IQSTVTQ	1xTMTpro [K	0.0001759	7.389E-05	1	3	1	Q5JPE7	Q5JPE7 [160-170	0	1724.0198
[R].IQTDSV\	1xTMTpro [K	9.816E-05	7.389E-05	1	1	2	P08397	P08397 [33-43]	0	1783.0821
[R].LQTELD\	1xTMTpro [N	0.0001968	7.389E-05	1	1	1	Q15276	Q15276 [807-819]	0	1848.9986
[K].LQTHDII	1xTMTpro [N	8.638E-05	7.389E-05	1	1	1	Q12768	Q12768 [644-655]	0	1725.9819
[R].LQTMKE	2xTMTpro [K	6.633E-05	7.389E-05	1	1	2	P02545	P02545 [197-208]	1	2422.3832
[R].IQTQPG\	1xTMTpro [N	0.0001885	7.389E-05	1	1	4	Q00325	Q00325 [190-201]	0	1665.9243
[R].IQTTVTE	1xTMTpro [K	7.359E-05	7.389E-05	1	1	1	Q15075	Q15075 [834-843]	0	1769.0664
[K].IQVDYD(	1xCarbamido	6.297E-05	7.389E-05	1	1	1	Q12841	Q12841 [90-99]	0	1842.9664
[K].LQVEEVI	1xTMTpro [N	0.0001863	7.389E-05	1	1	2	Q9NR28	Q9NR28 [192-202	0	1641.9243
[R].LQVGQE	1xTMTpro [K	6.822E-05	7.389E-05	1	1	1	Q7Z460	Q7Z460 [21-34]	0	2263.2466
[K].IQVLGSL	1xOxidation [	0.0001395	7.389E-05	1	1	1	P49419	P49419 [125-138]	0	2098.2437
[K].IQVLVEF	1xTMTpro [K	0.0001371	7.389E-05	1	1	4	P17931	P17931 [200-210]	0	1933.1403
[R].ISHGEVI	1xTMTpro [K	0.0001297	7.389E-05	1	1	2	Q9BXP5	Q9BXP5 [654-664	0	1934.0991

[K].LQWLLD	2xTMTpro [K	0.0001125	7.389E-05	1	1	2	Q9NZ08	Q9NZ08 [814-826	1	2491.4377
[R].LREDEN	1xTMTpro [K	6.928E-05	7.389E-05	1	1	2	Q16643	Q16643 [150-165]	1	2458.3069
[K].IRLDETD	1xTMTpro [N	0.000149	7.389E-05	1	1	1	P07384	P07384 [399-413]	1	2098.9848
[R].LRPGSIE	1xTMTpro [K	7.485E-05	7.389E-05	1	1	2	Q9BVJ7	Q9BVJ7 [128-140	0	2158.2
[R].LRPHFLI	1xCarbamido	0.0001093	7.389E-05	1	1	1	O14494	O14494 [126-140]	0	2493.3204
[R].IRPLNSE	1xCarbamido	0.0001046	7.389E-05	1	1	1	Q9Y5S2	Q9Y5S2 [1503-15	0	2497.3516
[R].IRPLPEE	1xTMTpro [N	0.000126	7.389E-05	1	1	1	Q01780	Q01780 [426-438]	0	1879.0431
[K].IRQEEIE	1xTMTpro [N	7.102E-05	7.389E-05	1	1	1	Q14254	Q14254 [252-264]	1	1945.1038
[RK].LRTEG	1xTMTpro [K	0.0001338	7.389E-05	2	2	1	P00739; P0C	P00739 [59-72]; P	1	2189.2058
[R].LSAAEDI	1xTMTpro [N	7.887E-05	7.389E-05	1	1	2	P48449	P48449 [248-260]	0	1702.9659
[R].LSAEERI	1xTMTpro [N	0.0001716	7.389E-05	1	1	1	P61457	P61457 [8-21]	1	1958.099
[R].ISAEGGI	1xTMTpro [N	0.0001759	7.389E-05	1	1	1	Q9BQE5	Q9BQE5 [249-259	0	1478.777
[R].LSALLQE	1xTMTpro [K	0.0001665	7.389E-05	1	1	2	O60763	O60763 [850-858]	0	1610.9973
[K].ISANENS	1xTMTpro [N	0.0001508	7.389E-05	1	1	2	Q9UHB6	Q9UHB6 [325-335	0	1477.8294
[R].LQYAIISI	1xTMTpro [N	0.0001885	7.389E-05	1	1	2	Q9Y2S2	Q9Y2S2 [198-208	0	1653.9284
[R].ISHLEQC	1xCarbamido	8.022E-05	7.389E-05	1	1	1	P11277	P11277 [612-629]	0	2396.1658
[R].ISHLVLP	1xCarbamido	7.863E-05	7.389E-05	1	1	1	Q8NFQ8	Q8NFQ8 [450-470	0	2656.4339
[K].LSKDPNI	2xTMTpro [K	0.0001782	7.389E-05	1	1	3	P30101	P30101 [423-433]	1	2110.3416
[K].LSPESAI	1xTMTpro [K	8.868E-05	7.389E-05	1	1	1	Q9HCS7	Q9HCS7 [175-188	0	2279.2303
[R].LSPGEA	1xTMTpro [K	0.0001656	7.389E-05	1	1	1	Q63HR2	Q63HR2 [940-956	0	2217.2735
[K].LSPIYNL	1xTMTpro [K	0.0001964	7.389E-05	1	1	4	P02748	P02748 [473-483]	0	1851.1599
[R].LSPQAV	1xTMTpro [K	6.622E-05	7.389E-05	1	1	3	P30626	P30626 [136-146]	0	1736.0562
[K].LSQADG	1xTMTpro [K	0.0001121	7.389E-05	1	1	2	P46939	P46939 [2046-205	0	1673.9718
[K].LSQAPLI	1xTMTpro [K	0.0001635	7.389E-05	1	1	2	Q92574	Q92574 [107-118]	0	1888.2127
[R].LSQEEIL	1xTMTpro [N	0.0001068	7.389E-05	1	1	1	Q9NSK0	Q9NSK0 [17-27]	0	1536.8553
[R].LSQELEY	1xTMTpro [K	9.379E-05	7.389E-05	1	1	1	P12270	P12270 [134-146]	0	2175.204
[R].ISQEIGN	1xTMTpro [K	0.0001155	7.389E-05	1	1	2	Q32P28	Q32P28 [417-426	0	1741.0174
[K].LSQGVT	1xTMTpro [N	8.624E-05	7.389E-05	1	1	1	P23919	P23919 [87-97]	0	1490.8862
[R].LSQLEGT	1xTMTpro [N	0.0001129	7.389E-05	1	1	3	Q96QK1	Q96QK1 [227-237	0	1547.8712
[K].LSQNNF.	1xTMTpro [K	6.149E-05	7.389E-05	1	1	2	Q9Y277	Q9Y277 [164-174	0	1863.062
[R].ISQTYQC	1xTMTpro [N	0.0001983	7.389E-05	1	1	2	P09525	P09525 [124-134]	0	1675.8723
[R].LSNTSPI	1xTMTpro [N	0.000162	7.389E-05	1	1	1	Q9NTJ5	Q9NTJ5 [146-161	0	2185.113
[K].LSSCDSI	2xCarbamido	8.786E-05	7.389E-05	1	1	1	P07814	P07814 [89-109]	0	2758.3459

[R].LSSEMN	1xOxidation [	0.0001012	7.389E-05	1	1	3	P20700	P20700 [277-290]	0	1816.903
[K].LSSLIILM	1xOxidation [	0.0001375	7.389E-05	1	1	2	P50454	P50454 [264-280]	0	2304.3069
[K].LSSLNLD	1xTMTpro [K	0.0001186	7.389E-05	1	1	1	P82673	P82673 [177-187]	0	1821.0362
[R].LSSLNLT	1xTMTpro [K	9.909E-05	7.389E-05	1	1	1	Q86YS7	Q86YS7 [733-745	0	2024.1996
[R].ISSINSIS	1xCarbamido	0.0001015	7.389E-05	1	1	1	O60701	O60701 [231-260]	0	3428.6844
[K].LSSPAVI	1xTMTpro [K	0.0001151	7.389E-05	1	1	2	P00747	P00747 [671-680]	0	1638.9922
[K].LSSQISA	1xTMTpro [K	6.317E-05	7.389E-05	1	1	1	Q86UE4	Q86UE4 [293-303	0	1756.9937
[R].LSSTDD	1xTMTpro [K	0.0001328	7.389E-05	1	1	1	Q96A57	Q96A57 [22-35]	0	2210.1836
[R].ISSVLAG	1xCarbamido	0.0001101	7.389E-05	2	2	1	P02533; P08	P02533 [31-41]; P	0	1410.7694
[R].ISSVPAII	1xTMTpro [N	0.000149	7.389E-05	1	1	4	P51888	P51888 [303-312]	0	1374.8024
[R].LSTAITLI	1xTMTpro [N	9.647E-05	7.389E-05	1	1	2	Q86UU1	Q86UU1 [50-63]	0	1817.0703
[R].ISTGGGI	2xTMTpro [K	0.0001762	7.389E-05	1	1	1	Q15063	Q15063 [765-778]	1	2362.3646
[R].LSTLCPS	1xCarbamido	0.0001075	7.389E-05	1	1	1	Q86VP6	Q86VP6 [1130-11	0	1648.9375
[K].ISSDLDG	1xTMTpro [K	0.0001198	7.389E-05	1	1	2	P29401	P29401 [103-114]	0	1873.0675
[R].LQSQVIS	1xCarbamido	0.0001239	7.389E-05	1	1	1	Q9BXX0	Q9BXX0 [677-689	0	2099.1662
[R].LSNRPA	1xTMTpro [K	0.0001417	7.389E-05	2	2	2	P35609; O43	P35609 [354-366]	0	2042.1349
[K].ISNPWQ	1xTMTpro [N	0.0001466	7.389E-05	1	1	1	Q13505	Q13505 [191-206]	0	2028.1197
[R].LSKEETY	1xTMTpro [K	6.357E-05	7.389E-05	1	1	1	P04844	P04844 [152-178]	1	3545.9783
[K].ISKPAPY	2xTMTpro [K	0.0001354	7.389E-05	1	1	1	Q13162	Q13162 [81-99]	0	3020.6913
[K].ISLEDIQ	1xTMTpro [K	9.277E-05	7.389E-05	1	1	2	Q8WXX5	Q8WXX5 [108-11	0	1901.0876
[K].LSLFDLQ	1xTMTpro [N	0.0001944	7.389E-05	1	1	1	Q96AY4	Q96AY4 [1162-11	0	2174.1777
[R].LSIIATDI	1xTMTpro [N	0.0001333	7.389E-05	1	1	5	P12109	P12109 [200-210]	0	1593.892
[K].LSLLEEL	1xTMTpro [K	7.543E-05	7.389E-05	1	1	4	P20774	P20774 [166-181]	0	2421.446
[K].ISLLPND	1xTMTpro [K	0.0001852	7.389E-05	1	1	1	Q7Z3T8	Q7Z3T8 [1012-10	0	2815.5948
[R].LSIIPPVI	1xTMTpro [K	0.0001204	7.389E-05	1	1	1	Q9ULL0	Q9ULL0 [313-327	0	2255.4234
[K].ISIPDVDI	2xTMTpro [K	0.0001463	7.389E-05	1	1	2	Q09666	Q09666 [4849-48	1	2422.4737
[K].ISIPDVG	1xTMTpro [K	7.371E-05	7.389E-05	1	1	2	Q09666	Q09666 [1451-14	0	1800.1239
[R].ISLPEAM	1xTMTpro [N	6.875E-05	7.389E-05	1	1	2	Q2NL82	Q2NL82 [94-106]	0	1818.0114
[R].LSIPTYG	1xCarbamido	8.453E-05	7.389E-05	1	1	2	P49327	P49327 [2264-22	0	1712.9325
[R].ISISTSG	1xTMTpro [N	0.0001587	7.389E-05	1	1	2	P13647	P13647 [74-84]	0	1415.7814
[K].ISNQFDV	1xTMTpro [N	8.209E-05	7.389E-05	1	1	1	P42704	P42704 [79-89]	0	1684.88
[K].LSITGTY	1xTMTpro [K	7.158E-05	7.389E-05	1	1	8	P01009	P01009 [315-324]	0	1719.0184
[K].ISMPDID	1xTMTpro [K	0.0001608	7.389E-05	1	1	2	Q09666	Q09666 [4385-43	0	1901.0698

[K].ISMPDLD	1xTMTpro [K	9.162E-05	7.389E-05	1	1	2	Q09666	Q09666 [2386-239	0	1890.1014
[K].ISMPDVD	1xTMTpro [K	7.875E-05	7.389E-05	1	1	2	Q09666	Q09666 [2312-232	0	1887.0541
[K].ISMPDVD	1xTMTpro [K	8.638E-05	7.389E-05	1	1	3	Q09666	Q09666 [818-828]	0	1876.0858
[K].ISMPDVD	1xOxidation [	9.724E-05	7.389E-05	1	1	3	Q09666	Q09666 [818-828]	0	1892.0807
[K].ISMPDVG	1xTMTpro [K	7.875E-05	7.389E-05	1	1	2	Q09666	Q09666 [1579-158	0	1795.0643
[K].ISMPEID	1xTMTpro [K	8.414E-05	7.389E-05	1	1	2	Q09666	Q09666 [3293-330	0	1881.1011
[K].ISMPEVD	1xTMTpro [K	8.923E-05	7.389E-05	1	1	2	Q09666	Q09666 [4129-413	0	1867.0854
[K].ISMPGFK	2xTMTpro [K	9.602E-05	7.389E-05	1	1	1	Q09666	Q09666 [2774-279	1	2912.6372
[K].ISNDPSF	1xTMTpro [K	0.0001331	7.389E-05	1	1	1	Q9NPF4	Q9NPF4 [170-185	0	2372.2412
[R].LSNLALV	2xTMTpro [K	0.0001025	7.389E-05	1	1	2	O14737	O14737 [56-66]	0	2124.3572
[R].ISNIPSLI	1xTMTpro [K	6.918E-05	7.389E-05	1	1	1	Q6DKJ4	Q6DKJ4 [129-144	0	2298.3565
[K].LSNNAQ	1xTMTpro [N	9.394E-05	7.389E-05	1	1	1	O43252	O43252 [13-24]	0	1722.8665
[K].ISMPDFD	1xTMTpro [K	0.000142	7.389E-05	1	1	3	Q09666	Q09666 [2579-258	0	1924.0858
[K].LQSLFDS	1xTMTpro [K	0.0001629	7.389E-05	1	1	2	P36955	P36955 [334-345]	0	1992.0934
[R].LQSLEAI	2xTMTpro [K	7.791E-05	7.389E-05	1	1	1	Q4L180	Q4L180 [592-604]	1	2446.4737
[R].IQSIAPS	1xTMTpro [K	0.0001881	7.389E-05	1	1	1	P42345	P42345 [2153-216	0	2093.2826
[R].LQAAGN	1xTMTpro [K	0.0001402	7.389E-05	2	2	3	Q9Y4G6; Q9	Q9Y4G6 [2457-24	0	1479.9139
[K].LQAANA	1xTMTpro [K	9.542E-05	7.389E-05	1	1	4	Q02818	Q02818 [72-81]	0	1680.9776
[R].LQAAND	1xCarbamido	0.0001929	7.389E-05	1	1	2	Q8TDH9	Q8TDH9 [109-118	0	1437.7439
[K].LQAAVD	1xOxidation [	0.0001155	7.389E-05	1	1	2	P51572	P51572 [233-243]	0	1768.9759
[R].LQAAYA	2xTMTpro [K	9.248E-05	7.389E-05	1	1	1	Q01082	Q01082 [1870-188	1	2519.4286
[K].LQAEAD	1xTMTpro [N	7.707E-05	7.389E-05	1	1	1	Q08378	Q08378 [1233-124	0	1575.8662
[K].LQAEAPI	1xTMTpro [N	8.979E-05	7.389E-05	1	1	1	Q14240	Q14240 [148-162]	0	1849.0615
[R].LQAEEV.	1xTMTpro [K	0.0001599	7.389E-05	1	1	2	Q15149	Q15149 [1783-179	0	1752.0147
[R].LQAEIEG	1xTMTpro [K	0.0002055	7.389E-05	1	1	8	P05787	P05787 [317-325]	0	1608.9816
[K].LQAFSA	1xCarbamido	7.474E-05	7.389E-05	1	1	1	Q02790	Q02790 [332-344]	0	2047.1138
[R].IQAGEVS	1xTMTpro [K	8.171E-05	7.389E-05	1	1	1	Q9BRP8	Q9BRP8 [171-181	0	1752.0147
[K].LQALAN	1xTMTpro [K	0.0001713	7.389E-05	1	1	1	Q9UQ90	Q9UQ90 [725-735	0	1792.1188
[K].IQAIELEI	1xTMTpro [N	0.000119	7.389E-05	1	1	3	P21810	P21810 [241-251]	0	1616.9542
[R].IPYYNIH	1xOxidation [	0.0001338	7.389E-05	1	1	2	O95479	O95479 [646-661]	0	2326.245
[K].IQALGLG	1xTMTpro [K	0.0001008	7.389E-05	1	1	1	Q02809	Q02809 [55-68]	0	2180.2207
[K].LQANTN	1xTMTpro [N	0.0001292	7.389E-05	1	1	1	Q7L775	Q7L775 [510-521]	0	1825.9516
[K].LQATVQ	1xTMTpro [K	0.000138	7.389E-05	1	1	2	P31146	P31146 [440-449]	0	1766.0668

[R].LQAVTD	1xTMTpro [N	0.0001806	7.389E-05	1	1	2	P30043	P30043 [125-134]	0	1471.8188
[R].IQAYDYL	1xCarbamido	0.0001903	7.389E-05	1	1	1	P62745	P62745 [151-162]	0	2069.0869
[K].IQCEENL	1xCarbamido	0.0001015	7.389E-05	1	1	1	O75718	O75718 [275-292]	0	2654.4355
[K].IQCFCFE	2xCarbamido	0.0002007	7.389E-05	1	1	1	Q9Y6N1	Q9Y6N1 [215-224	0	1720.8106
[R].IQDLETE	1xTMTpro [K	0.0001198	7.389E-05	1	1	1	Q9UBP9	Q9UBP9 [173-184	0	2071.1237
[R].LQDIVSA	1xTMTpro [N	0.0001842	7.389E-05	1	1	1	Q14643	Q14643 [1613-16	0	1562.8709
[K].LQDMLD	1xTMTpro [K	0.0001474	7.389E-05	1	1	2	P55010	P55010 [85-94]	0	1788.0221
[R].LQDNVR	1xTMTpro [K	7.405E-05	7.389E-05	1	1	1	Q9H7Z7	Q9H7Z7 [299-313	1	2387.2698
[R].LQDSGT	1xCarbamido	7.269E-05	7.389E-05	1	1	2	Q13308	Q13308 [93-104]	0	1685.86
[R].LQDTYN	1xTMTpro [K	7.146E-05	7.389E-05	1	1	3	P13674	P13674 [137-150]	0	2235.2
[R].IQDVME	1xTMTpro [K	0.0001672	7.389E-05	1	1	1	Q9HAU0	Q9HAU0 [765-774	0	1727.9857
[K].LQANFFI	1xTMTpro [K	0.0001226	7.389E-05	1	1	2	Q9UKV8	Q9UKV8 [40-51]	0	2061.1335
[R].IQEAGTE	1xTMTpro [K	8.26E-05	7.389E-05	1	1	3	P40926	P40926 [230-239]	0	1681.998
[R].LPYTIITT	2xTMTpro [K	0.0001968	7.389E-05	1	1	1	P04114	P04114 [3137-315	1	3174.8635
[K].LPYDVT	1xTMTpro [K	0.0002015	7.389E-05	1	1	1	Q13576	Q13576 [1025-10	0	2575.4151
[R].LPLVVEI	1xTMTpro [K	0.0001584	7.389E-05	1	1	2	Q8NB37	Q8NB37 [165-174	0	1767.0912
[K].LPLWQQ	1xCarbamido	0.0001579	7.389E-05	1	1	1	Q9UQB8	Q9UQB8 [223-23	0	2122.1611
[K].IPMPDFI	1xTMTpro [K	0.0001046	7.389E-05	1	1	3	Q09666	Q09666 [2963-29	0	1934.1065
[K].LPNFGF	1xTMTpro [N	0.0001271	7.389E-05	1	1	1	Q9UN86	Q9UN86 [371-387	0	2270.1777
[K].LPNGLVI	1xTMTpro [N	7.695E-05	7.389E-05	1	1	2	P22695	P22695 [43-60]	0	2233.2512
[R].LPPGFS	2xTMTpro [K	0.0001043	7.389E-05	1	1	1	O75694	O75694 [392-407	0	2544.4854
[K].LPPKSE	1xTMTpro [K	8.705E-05	7.389E-05	1	1	2	P40939	P40939 [661-676]	1	2455.3324
[R].LPPVLSI	1xTMTpro [N	6.198E-05	7.389E-05	1	1	2	Q04206	Q04206 [175-187	0	1809.0342
[K].IPQAIAQ	1xTMTpro [K	0.0001678	7.389E-05	1	1	3	Q16795	Q16795 [148-157	0	1677.0555
[R].IPQLAPF	1xTMTpro [K	0.000107	7.389E-05	1	1	1	Q16513	Q16513 [551-565	0	2133.2411
[K].LPQMET	2xTMTpro [K	6.972E-05	7.389E-05	1	1	1	Q5T4S7	Q5T4S7 [2596-26	1	2859.5048
[R].IPQSTLS	1xTMTpro [N	0.0001987	7.389E-05	1	1	1	P21281	P21281 [495-506]	0	1741.9444
[R].LPRPPPI	2xTMTpro [K	0.0001094	7.389E-05	1	1	2	Q00325	Q00325 [342-356	1	2628.5727
[R].LPYTIITT	1xTMTpro [K	0.0001556	7.389E-05	1	1	4	P04114	P04114 [3137-314	0	1965.228
[K].LPSNLP	1xTMTpro [K	0.0001123	7.389E-05	1	1	1	Q9NVU7	Q9NVU7 [9-21]	0	2086.288
[K].LPSSVF	1xTMTpro [K	7.684E-05	7.389E-05	1	1	1	Q96GA3	Q96GA3 [109-127	0	2689.458
[K].IPTACIT	1xCarbamido	0.0001292	7.389E-05	1	1	1	Q9Y646	Q9Y646 [229-244	0	2128.0408
[R].LPTEEE	1xTMTpro [N	0.00011	7.389E-05	1	1	1	Q8NBJ7	Q8NBJ7 [163-174	0	1781.9029

[K].LPTFGAI	1xTMTpro [K	0.0001093	7.389E-05	1	1	1	Q14258	Q14258 [403-416]	0	2136.256
[R].IPTPLNT	1xCarbamido	6.168E-05	7.389E-05	1	1	1	Q96CW1	Q96CW1 [324-33	0	2382.338
[K].LPTSVSL	1xTMTpro [K	9.248E-05	7.389E-05	1	1	1	Q9Y4E1	Q9Y4E1 [566-589	0	3178.5923
[K].LPTTGM	1xTMTpro [K	0.0001063	7.389E-05	1	1	2	Q14155	Q14155 [526-535]	0	1671.0007
[R].LPVANP	1xCarbamido	0.0001914	7.389E-05	1	1	1	P00736	P00736 [612-625]	0	1972.0394
[K].LPVDFSI	1xTMTpro [K	0.0001359	7.389E-05	1	1	1	Q9NXR7	Q9NXR7 [177-190	0	2228.3186
[K].IPVFDAG	1xTMTpro [N	0.0001	7.389E-05	1	1	1	Q9NPF5	Q9NPF5 [215-224	0	1444.7868
[K].LPVGPSI	1xTMTpro [K	0.0001903	7.389E-05	1	1	1	O95182	O95182 [41-48]	0	1442.8975
[K].IPVMALV	1xTMTpro [N	7.037E-05	7.389E-05	1	1	1	A1L0T0	A1L0T0 [539-556]	0	2232.213
[R].LPVPVVI	1xTMTpro [K	0.000171	7.389E-05	1	1	1	O75382	O75382 [374-382]	0	1612.0078
[R].LPSSSS	1xTMTpro [N	8.786E-05	7.389E-05	1	1	1	Q16585	Q16585 [268-284]	0	2052.0317
[K].LSTLVET	2xTMTpro [K	0.000181	7.389E-05	1	1	1	P53041	P53041 [223-236]	1	2504.5003
[R].LQEALD	1xCarbamido	7.451E-05	7.389E-05	1	1	1	Q1KMD3	Q1KMD3 [33-58]	0	3127.5168
[K].LQEAQLI	1xTMTpro [K	0.0001204	7.389E-05	1	1	3	O60237	O60237 [900-911]	0	1979.1669
[R].LQLLEEI	1xTMTpro [K	0.0001852	7.389E-05	1	1	1	Q9GZY8	Q9GZY8 [307-317	1	2009.1523
[K].IQLINNM	1xTMTpro [K	0.0001249	7.389E-05	1	1	1	P00558	P00558 [221-230]	0	1810.0752
[R].LQLLPES	1xCarbamido	0.0001442	7.389E-05	1	1	1	Q9Y3I1	Q9Y3I1 [277-287]	0	1956.1484
[K].IQIPRPD	1xTMTpro [K	8.209E-05	7.389E-05	1	1	2	Q00341	Q00341 [184-197]	0	2229.2847
[K].LQIVEMI	1xOxidation [	6.357E-05	7.389E-05	1	1	2	P50454	P50454 [253-263]	0	1903.1331
[R].IQIWDTA	1xTMTpro [N	0.0001468	7.389E-05	1	1	2	P59190	P59190 [59-69]	0	1620.8665
[K].LQMEQG	1xTMTpro [N	6.227E-05	7.389E-05	1	1	1	Q9NS69	Q9NS69 [106-117	0	1861.9873
[K].IQMSNLI	1xTMTpro [N	0.000153	7.389E-05	1	1	2	P36543	P36543 [70-80]	0	1609.8474
[R].IQNAGG	1xTMTpro [N	7.887E-05	7.389E-05	2	2	2	P35813; O75	P35813 [175-186]	0	1577.8753
[R].IQNILTEI	1xTMTpro [K	0.0001937	7.389E-05	1	1	2	P27169	P27169 [307-316]	0	1793.0664
[R].IQNVVTS	1xTMTpro [N	0.0001614	7.389E-05	1	1	5	P55268	P55268 [108-119]	0	1663.9451
[K].IQPEILEI	1xTMTpro [K	0.0001547	7.389E-05	2	2	2	Q92556; Q9	Q92556 [540-549]	0	1804.1439
[K].IQPFHFII	1xTMTpro [K	0.0001258	7.389E-05	1	1	2	Q8NEZ5	Q8NEZ5 [187-194	0	1638.0023
[K].LQLLEDI	1xTMTpro [K	6.853E-05	7.389E-05	1	1	5	P09874	P09874 [572-582]	1	1981.121
[K].LQPHEF	1xTMTpro [K	8.786E-05	7.389E-05	1	1	1	P10515	P10515 [553-574]	0	3030.6367
[R].LQPSST	1xTMTpro [K	0.0001849	7.389E-05	1	1	1	Q99700	Q99700 [806-821]	0	2386.2779
[R].LQPTWN	1xTMTpro [K	7.924E-05	7.389E-05	1	1	2	Q8NBS9	Q8NBS9 [95-105]	0	1895.0518
[R].IQQFDD	1xTMTpro [K	0.0001172	7.389E-05	1	1	1	Q5H9R7	Q5H9R7 [609-626	0	2748.3132
[K].IQQGALI	1xTMTpro [N	0.0001859	7.389E-05	1	1	1	Q92621	Q92621 [1566-15	0	1444.8807

[R].LQQIAA	1xTMTpro [K	0.0001471	7.389E-05	1	1	1	O15031	O15031 [1824-18	0	1807.0933
[R].LQQIAA	1xTMTpro [K	0.0001789	7.389E-05	1	1	1	O43157	O43157 [2121-21	0	1793.0777
[R].IQQIAEG	1xTMTpro [K	0.000121	7.389E-05	1	1	3	Q14254	Q14254 [297-305	0	1623.9561
[K].IQQLAIS	1xTMTpro [K	0.0001395	7.389E-05	1	2	2	Q9Y2T2	Q9Y2T2 [379-388	0	1679.0711
[R].IQQIETT	1xTMTpro [K	7.839E-05	7.389E-05	1	1	2	Q9Y3C0	Q9Y3C0 [59-72]	0	2208.3095
[R].LQSAMT	1xTMTpro [N	0.0001218	7.389E-05	1	1	1	Q9H0U6	Q9H0U6 [161-17	0	1664.9325
[R].LQSEVA	1xTMTpro [K	0.0001647	7.389E-05	1	1	4	P53990	P53990 [110-118]	0	1624.9765
[K].IQSFLGC	1xTMTpro [K	7.102E-05	7.389E-05	1	1	1	Q13421	Q13421 [504-517	0	2084.1883
[R].LQSGICI	1xCarbamid	0.0002043	7.389E-05	1	1	2	P14868	P14868 [198-207]	0	1534.8483
[R].LQPHLLI	1xOxidation	6.79E-05	7.389E-05	1	1	2	Q969N2	Q969N2 [423-441	0	2769.6192
[K].LQEAQE	1xTMTpro [N	8.965E-05	7.389E-05	1	1	1	Q9UDT6	Q9UDT6 [689-70	0	1788.9775
[K].IQLLDLP	1xTMTpro [K	7.416E-05	7.389E-05	1	1	1	Q9Y295	Q9Y295 [113-126	0	2088.2924
[R].LQLEETI	1xTMTpro [K	0.00017	7.389E-05	1	1	2	Q15149	Q15149 [2330-23	0	1849.0311
[K].LQEDYD	1xTMTpro [N	6.811E-05	7.389E-05	1	1	2	P50453	P50453 [274-285]	0	1801.8961
[K].IQEEILVI	1xTMTpro [N	0.0001726	7.389E-05	1	1	1	Q6P2E9	Q6P2E9 [213-222	0	1553.9334
[K].LQEEML	1xOxidation	6.267E-05	7.389E-05	1	1	3	P08670	P08670 [189-207]	1	2671.3317
[R].IQELENL	1xTMTpro [N	0.0001687	7.389E-05	1	1	1	P51531	P51531 [358-373]	0	2095.1718
[K].LQELPD	1xTMTpro [N	6.822E-05	7.389E-05	1	1	1	P33992	P33992 [229-243]	0	1993.0496
[R].LQEMMA	1xCarbamid	9.467E-05	7.389E-05	1	1	1	Q9NPF4	Q9NPF4 [269-28	0	1831.8494
[K].LQESVPI	1xCarbamid	8.247E-05	7.389E-05	1	1	2	Q96S82	Q96S82 [51-68]	0	2421.2655
[R].IQETQAI	1xTMTpro [N	0.000165	7.389E-05	1	1	1	Q14566	Q14566 [208-217	0	1488.8341
[R].IQEVADI	1xTMTpro [K	0.0001703	7.389E-05	1	1	1	P18085	P18085 [100-109]	0	1781.03
[R].IQEVAGS	1xTMTpro [N	0.0001363	7.389E-05	1	1	1	Q9HD89	Q9HD89 [32-42]	0	1536.9069
[K].LQEVEVI	1xTMTpro [N	0.0001749	7.389E-05	1	1	2	O95834	O95834 [328-341	0	1918.0241
[R].IQFDEGY	1xTMTpro [K	9.408E-05	7.389E-05	1	1	1	Q92665	Q92665 [226-240	0	2411.2011
[R].IQFKPDI	1xTMTpro [K	0.0001134	7.389E-05	1	1	1	Q96I24	Q96I24 [287-299]	0	2110.1788
[K].IQLEHHI	1xCarbamid	7.899E-05	7.389E-05	1	1	1	Q9NZN4	Q9NZN4 [342-35	0	2629.3688
[R].IQFKPDI	1xTMTpro [K	9.007E-05	7.389E-05	1	1	1	Q96AE4	Q96AE4 [309-321	0	2112.1581
[R].LQGGVL	1xTMTpro [K	6.643E-05	7.389E-05	1	1	1	P15309	P15309 [258-272]	0	2273.3295
[R].LQGLEQ	1xTMTpro [N	0.0001063	7.389E-05	1	1	2	Q9NZQ3	Q9NZQ3 [56-69]	0	1902.0616
[R].LQGLYC	1xCarbamid	0.000162	7.389E-05	1	1	1	Q96RE7	Q96RE7 [25-37]	0	2088.2019
[K].LQGPQT	1xTMTpro [N	0.0001425	7.389E-05	1	1	1	Q8N335	Q8N335 [299-310	0	1652.8927
[K].LQGYQL	1xTMTpro [K	7.048E-05	7.389E-05	1	1	1	Q15811	Q15811 [89-103]	0	2250.3176

[R].LQHAAE	1xTMTpro [K	0.0001838	7.389E-05	1	1	2	Q8WUM4	Q8WUM4 [277-28	0	1631.0136
[R].IQHAVQI	1xTMTpro [K	6.188E-05	7.389E-05	1	1	2	P49589	P49589 [155-168]	0	2185.2836
[K].LQHGSIL	1xTMTpro [K	0.0001172	7.389E-05	1	2	1	P00367	P00367 [353-363]	0	1805.0929
[K].LQHINPL	1xCarbamido	0.0002051	7.389E-05	1	1	1	Q9BTE3	Q9BTE3 [315-328	0	2239.3241
[R].LQHTLQ	1xTMTpro [N	6.317E-05	7.389E-05	1	1	1	O00273	O00273 [171-182]	0	1783.0146
[K].IQIADLM	1xTMTpro [K	0.0001536	7.389E-05	1	2	2	Q16666	Q16666 [46-55]	0	1798.0276
[K].IQIAPDS	1xTMTpro [N	0.0001511	7.389E-05	1	1	4	Q96AE4	Q96AE4 [134-146	0	1656.924
[K].LQLDNQ	1xTMTpro [K	0.0001675	7.389E-05	1	1	1	Q9UKY7	Q9UKY7 [238-251	0	2284.2793
[K].LQGEVL	1xTMTpro [N	6.529E-05	7.389E-05	1	1	1	Q86UU1	Q86UU1 [709-720	0	1689.9342
[R].LPLQMA	1xTMTpro [K	0.000182	7.389E-05	1	1	1	O15013	O15013 [589-604]	0	2408.3966
[K].ISTLYYQ	1xTMTpro [N	0.0001395	7.389E-05	1	1	1	Q13217	Q13217 [228-246]	0	2527.3363
[K].ISTVELE	1xTMTpro [K	8.909E-05	7.389E-05	1	1	1	Q9BXN1	Q9BXN1 [253-262	0	1789.0239
[K].IVQLLAG	1xTMTpro [K	0.0001245	7.389E-05	1	1	2	O75746	O75746 [56-68]	0	1964.2036
[R].IVQQVT	1xTMTpro [K	0.0001218	7.389E-05	1	1	1	Q8TAD4	Q8TAD4 [706-715	0	1707.1024
[R].LVQSPN	1xTMTpro [K	6.119E-05	7.389E-05	1	2	2	Q71UM5	Q71UM5 [24-36]	0	2136.1655
[R].LVRPEVI	1xCarbamido	0.0001026	7.389E-05	1	1	6	P02768	P02768 [139-161]	1	3706.9753
[K].LVSDEM	1xOxidation	0.0001528	7.389E-05	1	1	2	P54819	P54819 [73-85]	0	2128.2067
[R].LVSDGN	1xTMTpro [N	0.0001004	7.389E-05	1	1	3	Q01082	Q01082 [1235-12	0	1493.7879
[R].LVSEDPI	1xTMTpro [N	0.0001249	7.389E-05	1	1	1	P98160	P98160 [4282-42	0	1970.9891
[R].LVSEFPY	1xTMTpro [K	0.0001129	7.389E-05	1	1	1	P43304	P43304 [526-538]	0	2132.2135
[K].LVSESSI	1xTMTpro [K	7.303E-05	7.389E-05	1	1	2	P05787	P05787 [473-483]	0	1782.0504
[R].IVSGCPL	2xCarbamido	8.034E-05	7.389E-05	1	1	1	Q9H0A0	Q9H0A0 [495-512	0	2444.2273
[K].IVSLPEC	1xCarbamido	9.077E-05	7.389E-05	1	1	1	Q9NQR4	Q9NQR4 [38-52]	0	2290.2397
[R].IVSRPEE	1xTMTpro [K	9.335E-05	7.389E-05	1	1	5	P49368	P49368 [331-353]	1	3104.7447
[R].LVTLEEF	1xTMTpro [K	0.0001769	7.389E-05	1	1	3	P80303	P80303 [312-320]	0	1700.049
[R].IVTSEEV	1xTMTpro [N	0.0001759	7.389E-05	1	1	4	Q9Y639	Q9Y639 [150-159	0	1462.88
[R].IVVFQYS	1xTMTpro [K	0.000145	7.389E-05	1	1	2	Q16531	Q16531 [848-857]	0	1764.0187
[R].LVVLATF	1xTMTpro [N	9.527E-05	7.389E-05	1	1	2	P46976	P46976 [35-48]	0	1820.0271
[R].IVVPPHL	1xTMTpro [N	0.0001188	7.389E-05	1	1	1	O95302	O95302 [333-346]	0	1827.0084
[K].LVVVGA	1xTMTpro [K	7.069E-05	7.389E-05	2	3	2	P01111; P01	P01111 [6-16]; P0	0	1564.0078
[R].IVYEGGI	1xTMTpro [N	0.0001849	7.389E-05	1	1	2	P11678	P11678 [509-520]	0	1648.9593
[R].LVYVCD	1xCarbamido	7.348E-05	7.389E-05	1	1	1	O00764	O00764 [108-137]	1	4369.2956
[K].LWAEDG	1xTMTpro [N	0.0001094	7.389E-05	1	1	1	Q9Y2D5	Q9Y2D5 [430-441	0	1685.8454

[K].LWDFQC	1xCarbamido	0.0001147	7.389E-05	1	1	2	P43034	P43034 [176-186]		0	1774.8906
[K].LWEPLV	1xTMTpro [K	6.653E-05	7.389E-05	1	1	3	Q16718	Q16718 [99-113]		0	2445.331
[R].IWGLGF	1xTMTpro [K	8.584E-05	7.389E-05	1	1	1	Q8IXB1	Q8IXB1 [662-683]		0	3072.6901
[R].LWGLTE	1xTMTpro [N	0.0001297	7.389E-05	1	1	2	Q9NS69	Q9NS69 [48-58]		0	1682.8895
[K].LWKEEE	3xTMTpro [K	8.096E-05	7.389E-05	1	1	1	P55735	P55735 [193-207]		2	3178.7525
[K].IWLAAH\	1xTMTpro [K	0.0001239	7.389E-05	1	1	1	O60216	O60216 [17-25]		0	1748.0139
[R].LVQIHVD	1xTMTpro [K	0.0001288	7.389E-05	1	1	2	Q9Y3Z3	Q9Y3Z3 [107-116		0	1792.0647
[K].LVQEALI	1xTMTpro [N	0.0001208	7.389E-05	1	1	2	Q15269	Q15269 [774-784]		0	1528.9018
[R].IVQAVV\	1xCarbamido	6.56E-05	7.389E-05	1	1	3	Q9ULC5	Q9ULC5 [314-325		0	1626.8957
[R].LVQAFQ	1xTMTpro [K	0.0001322	7.389E-05	1	1	3	Q13162	Q13162 [231-240]		0	1821.0402
[K].LVGVFH	1xTMTpro [N	6.961E-05	7.389E-05	1	1	1	Q9UFN0	Q9UFN0 [180-193		0	1880.035
[K].LVHHILQ	1xTMTpro [K	7.875E-05	7.389E-05	1	1	2	Q9NXG2	Q9NXG2 [140-15(		0	2005.1549
[R].LVLAEA(	1xTMTpro [N	0.0001123	7.389E-05	1	1	1	P50895	P50895 [110-121]		0	1603.8975
[K].LVLDEDI	1xTMTpro [K	7.202E-05	7.389E-05	1	1	1	P46100	P46100 [1537-155		1	2757.5278
[R].LVLDTVI	1xTMTpro [N	0.0001361	7.389E-05	1	2	2	Q9UPN3	Q9UPN3 [5617-56		0	1548.8916
[K].IVLEDGT	1xTMTpro [N	7.015E-05	7.389E-05	1	1	1	Q16555	Q16555 [452-467]		0	1987.0779
[K].IVIGMDV	1xTMTpro [N	0.0001896	7.389E-05	1	1	1	P09104	P09104 [240-253]		0	1875.0005
[R].IVLLDSS	1xTMTpro [K	8.196E-05	7.389E-05	1	1	4	P49368	P49368 [238-248]		0	1888.1287
[K].IVILPDYI	1xTMTpro [N	0.0001991	7.389E-05	1	1	2	P45974	P45974 [122-133]		0	1719.0376
[K].LVLPAP(	1xTMTpro [K	6.337E-05	7.389E-05	1	1	2	Q99459	Q99459 [295-312]		0	2557.5097
[K].IVLQNG/	1xTMTpro [N	0.0001838	7.389E-05	1	1	1	Q08257	Q08257 [188-201]		0	1938.0629
[K].LVIQSTG	1xCarbamido	0.0001016	7.389E-05	1	1	1	Q5GLZ8	Q5GLZ8 [1007-10		0	3595.9326
[K].LVIVGDC	1xCarbamido	9.925E-05	7.389E-05	2	2	1	P08134; P61	P08134 [8-18]; P6		0	1696.9912
[K].LWIEGIE	1xTMTpro [K	0.0001176	7.389E-05	1	1	1	Q96A65	Q96A65 [114-122		0	1733.0242
[K].IVMEAIQ	1xTMTpro [K	0.0001952	7.389E-05	1	1	1	Q8TCS8	Q8TCS8 [570-582		0	1996.1757
[K].LVMMEQ	2xTMTpro [K	0.0001691	7.389E-05	1	1	1	Q96JB2	Q96JB2 [492-507]		1	2803.5878
[K].LVNANG	1xCarbamido	0.0001877	7.389E-05	1	1	1	P04040	P04040 [222-233]		0	1946.0661
[R].IVNEGGI	1xTMTpro [N	9.148E-05	7.389E-05	1	1	2	Q92626	Q92626 [1108-11		0	1599.9389
[K].LVNEVTI	1xTMTpro [K	6.633E-05	7.389E-05	1	1	95	P02768	P02768 [66-75]		0	1758.0293
[R].LVNHFVI	1xTMTpro [K	6.601E-05	7.389E-05	1	1	6	P0DMV9	P0DMV9 [237-247		1	2026.173
[R].LVNLQH	1xTMTpro [K	0.0001479	7.389E-05	1	1	3	Q96AG4	Q96AG4 [84-96]		0	2142.289
[K].IVNSAQ1	1xTMTpro [K	6.55E-05	7.389E-05	1	1	2	O75964	O75964 [56-66]		0	1760.0198
[K].IVNSFLC	1xCarbamido	0.0001196	7.389E-05	1	1	1	Q5W0V3	Q5W0V3 [568-58		0	2199.2339

[R].IVNTPFC	1xTMTpro [K	8.518E-05	7.389E-05	1	1	1	Q92544	Q92544 [85-98]	0	2228.2604
[R].LVNTPY\	1xTMTpro [K	7.235E-05	7.389E-05	1	1	2	P83111	P83111 [383-394]	0	2021.1199
[R].IVPGQFI	1xTMTpro [K	0.000131	7.389E-05	1	1	1	Q15393	Q15393 [115-126]	0	1892.1501
[R].LVPNMT	1xTMTpro [K	0.00015	7.389E-05	1	1	1	Q96KP4	Q96KP4 [344-363	0	2813.5614
[K].IVPNVLL	1xTMTpro [K	8.624E-05	7.389E-05	1	1	2	O75390	O75390 [383-393]	0	1818.1344
[K].LVMEEA	1xTMTpro [K	7.371E-05	7.389E-05	1	1	1	Q9Y3I0	Q9Y3I0 [466-476]	0	1904.0331
[K].LWIEQY(	1xTMTpro [K	0.0001736	7.389E-05	1	1	1	Q9BXW6	Q9BXW6 [720-73	0	2464.3844
[R].LWNFTE	1xTMTpro [N	9.091E-05	7.389E-05	1	1	2	Q5SWX8	Q5SWX8 [124-13	0	1842.9193
[K].LWSGYS	1xTMTpro [K	7.325E-05	7.389E-05	1	1	1	P08572	P08572 [1511-152	0	2428.3044
[K].IYPTATG	3xTMTpro [K	6.297E-05	7.389E-05	1	1	1	Q05209	Q05209 [47-61]	2	2923.7245
[K].IYPTVNC	1xCarbamido	0.0001018	7.389E-05	1	1	2	P01042	P01042 [364-380]	0	2589.4098
[K].IYPTVNC	1xCarbamido	7.269E-05	7.389E-05	1	1	1	P01042	P01042 [364-380]	0	2605.4047
[K].IYQIYEG	1xTMTpro [N	6.317E-05	7.389E-05	1	1	2	P11310	P11310 [396-408]	0	1903.0244
[R].LYQQHG	1xTMTpro [N	6.488E-05	7.389E-05	1	1	2	O14773	O14773 [507-520]	0	1909.0251
[K].LYQSAG	1xTMTpro [N	0.0001304	7.389E-05	1	1	5	P11142	P11142 [610-646]	0	3650.7062
[K].LYQSAG	1xOxidation [	0.0001096	7.389E-05	1	1	5	P11142	P11142 [610-646]	0	3666.7011
[K].LYQSAG	2xOxidation [	0.0001779	7.389E-05	1	1	2	P11142	P11142 [610-646]	0	3682.6961
[K].LYQVIFE	1xTMTpro [K	7.269E-05	7.389E-05	1	1	1	O14786	O14786 [776-787]	0	2004.1661
[K].IYQWINE	1xTMTpro [N	9.191E-05	7.389E-05	1	1	1	Q92600	Q92600 [22-35]	0	2040.0721
[K].IYSFNST	1xTMTpro [K	0.0001506	7.389E-05	1	1	1	Q7Z478	Q7Z478 [66-83]	0	2538.2968
[K].LYSILQG	1xTMTpro [K	0.0001352	7.389E-05	1	1	1	O15042	O15042 [477-488]	0	1930.1141
[K].IYSLNEG	1xTMTpro [K	8.558E-05	7.389E-05	1	1	1	O00757	O00757 [209-218]	0	1765.998
[R].LYPAVDI	1xTMTpro [N	9.277E-05	7.389E-05	1	1	1	Q96S59	Q96S59 [154-167	0	1932.0398
[K].LYTEGY(	1xTMTpro [K	6.896E-05	7.389E-05	1	1	1	O75306	O75306 [384-404]	0	2877.553
[R].LYTQNIE	1xTMTpro [N	0.0002019	7.389E-05	1	1	3	Q9NTG7	Q9NTG7 [225-235	0	1625.8818
[R].IYTSPTV	1xTMTpro [N	7.767E-05	7.389E-05	1	1	1	Q08380	Q08380 [408-426]	0	2466.2261
[R].IYVVDV(	1xTMTpro [N	7.508E-05	7.389E-05	1	1	2	Q13228	Q13228 [104-114]	0	1537.8545
[K].LYVYNTI	1xCarbamido	9.291E-05	7.389E-05	1	1	2	Q9H8Y8	Q9H8Y8 [163-174	0	1837.871
[-].MAASCVI	1xCarbamido	0.0001742	7.389E-05	1	1	2	P14550	P14550 [1-13]	0	1935.0977
[R].MADALD	1xTMTpro [N	9.162E-05	7.389E-05	1	1	2	P05165	P05165 [466-476]	0	1584.8375
[K].MADCG(	1xCarbamido	9.925E-05	7.389E-05	1	1	1	Q9UGV2	Q9UGV2 [287-30	0	2165.1703
[K].MADLHA	1xOxidation [	0.0001602	7.389E-05	1	1	4	Q14195	Q14195 [488-496]	0	1329.7268
[K].MADSG(	1xTMTpro [K	0.0001567	7.389E-05	1	1	2	Q9UN36	Q9UN36 [288-302	0	2123.1411

[R].MAENLG	1xTMTpro [K	8.505E-05	7.389E-05	1	1	2	Q96I99	Q96I99 [207-218]	0	1884.0909
[-].MAENTE	1xMet-loss+	0.0001051	7.389E-05	1	1	1	Q6PCE3	Q6PCE3 [1-34]	0	3992.9698
[R].MAEVLV	1xTMTpro [N	0.0001437	7.389E-05	1	1	2	Q8TB45	Q8TB45 [30-41]	0	1649.9216
[R].MAGAIPI	1xTMTpro [K	6.57E-05	7.389E-05	1	1	1	Q53FA7	Q53FA7 [163-176	0	1952.1494
[K].LYTLVLT	2xTMTpro [K	7.875E-05	7.389E-05	1	1	1	P30086	P30086 [63-80]	2	2941.7179
[K].LVGSVN	1xTMTpro [N	0.0001956	7.389E-05	1	1	1	Q9BQG0	Q9BQG0 [232-24	0	1950.0616
[R].IYNLGT	1xTMTpro [K	8.558E-05	7.389E-05	1	1	1	Q14376	Q14376 [266-286	0	2911.5553
[K].LYLVDL	1xTMTpro [K	0.0001013	7.389E-05	1	3	2	P33176	P33176 [227-237]	0	1816.0712
[K].LWSLNP	1xTMTpro [K	9.863E-05	7.389E-05	1	1	2	O75339	O75339 [698-715	0	2744.4063
[R].IWSVPN.	1xCarbamid	7.337E-05	7.389E-05	1	1	4	Q9UMS4	Q9UMS4 [290-30	0	1919.0492
[KR].LWTG	1xTMTpro [N	0.0001094	7.389E-05	1	4	1	Q04724	Q04724 [631-641	0	1535.8501
[K].LWWAD	1xTMTpro [K	0.0001395	7.389E-05	1	1	2	Q07954	Q07954 [1799-18	0	1870.0355
[R].LWYENF	1xTMTpro [K	0.0001163	7.389E-05	1	1	2	Q92626	Q92626 [1249-12	0	2699.5052
[R].IYADSLK	2xTMTpro [K	0.0001544	7.389E-05	1	1	1	P55196	P55196 [252-264]	0	2434.4526
[K].IYAPQGI	1xTMTpro [N	9.32E-05	7.389E-05	1	1	1	Q7Z5G4	Q7Z5G4 [104-11	0	2003.1496
[K].LYAQDA	1xCarbamid	7.069E-05	7.389E-05	1	1	2	Q14315	Q14315 [704-717	0	2187.1611
[K].IYDALDV	1xTMTpro [N	7.146E-05	7.389E-05	1	1	2	P54136	P54136 [322-333]	0	1710.9597
[K].LYDDIDF	1xTMTpro [K	8.466E-05	7.389E-05	1	1	4	Q96KP4	Q96KP4 [276-28	0	2341.2095
[K].LYDLDNI	1xTMTpro [N	0.0001593	7.389E-05	1	1	1	P62166	P62166 [107-118]	0	1761.8978
[K].IYEFPET	1xTMTpro [K	0.0001964	7.389E-05	1	1	1	Q16181	Q16181 [222-235	0	2366.1531
[K].LYEIGAG	1xTMTpro [N	6.8E-05	7.389E-05	1	1	4	P26440	P26440 [402-413]	0	1598.8709
[R].LYLVSDV	1xTMTpro [K	0.0001184	7.389E-05	1	1	1	O15042	O15042 [595-608	0	2180.2458
[K].LYEPDQ	1xTMTpro [K	8.196E-05	7.389E-05	1	1	5	Q9NZB2	Q9NZB2 [748-75	0	1984.1247
[R].LYFLQCI	2xCarbamid	0.0001999	7.389E-05	1	1	2	P20042	P20042 [297-308]	0	1917.9271
[R].IYGESAD	1xTMTpro [K	0.0001087	7.389E-05	1	1	3	P51114	P51114 [264-273]	0	1660.9402
[K].IYGNFFF	1xTMTpro [K	0.0001192	7.389E-05	1	1	2	P61601	P61601 [51-63]	0	2087.1094
[R].LYHDIAQ	1xTMTpro [K	8.786E-05	7.389E-05	1	1	1	Q14152	Q14152 [173-183	0	1942.1042
[R].LYHILTP	1xTMTpro [N	7.566E-05	7.389E-05	1	1	1	Q5SY16	Q5SY16 [620-633	0	1981.1442
[K].LYHNEVI	1xTMTpro [K	0.0001987	7.389E-05	1	1	1	Q14683	Q14683 [228-237	0	1882.0566
[K].IYKVPST	2xTMTpro [K	6.488E-05	7.389E-05	1	1	1	P50395	P50395 [116-137]	1	3296.8632
[K].IYIDCYEI	1xCarbamid	7.625E-05	7.389E-05	1	1	3	Q99715	Q99715 [2662-26	0	2067.1328
[R].LYLDELE	1xCarbamid	8.951E-05	7.389E-05	1	1	3	Q9HD26	Q9HD26 [393-40	0	2388.2361
[K].IYIDSNN	1xTMTpro [N	0.0001089	7.389E-05	1	1	4	Q00610	Q00610 [882-892	0	1638.8407

[R].LYLFGLE	1xTMTpro [K	0.0001277	7.389E-05	1	1	1	Q96P48	Q96P48 [827-843	0	2628.4317
[K].IYIGDQV	1xTMTpro [K	0.0001653	7.389E-05	1	1	2	Q9UIA9	Q9UIA9 [560-568]	0	1671.9925
[R].LYILLPLI	1xCarbamido	0.0002023	7.389E-05	1	1	1	Q86WV6	Q86WV6 [198-22(	0	2919.5279
[K].LYEQILA	1xTMTpro [K	6.685E-05	7.389E-05	1	1	5	O14974	O14974 [935-945]	0	1958.109
[K].IVGPEEN	1xTMTpro [N	6.478E-05	7.389E-05	1	1	2	O00469	O00469 [359-372]	0	1816.9724
[K].LVGLTGT	1xTMTpro [N	7.037E-05	7.389E-05	1	1	1	O75880	O75880 [224-239]	1	2047.1467
[R].IVGGWD	1xTMTpro [N	0.000155	7.389E-05	1	1	1	Q96RQ9	Q96RQ9 [262-271	0	1429.8487
[R].LTESLNI	1xTMTpro [N	9.379E-05	7.389E-05	1	1	1	Q14344	Q14344 [265-279]	0	2067.1405
[R].LTESYCI	1xCarbamido	0.0001937	7.389E-05	1	1	5	P39060	P39060 [1701-171	0	1648.796
[K].ITEVTSE	1xTMTpro [N	0.0001641	7.389E-05	1	1	5	Q99715	Q99715 [1663-167	0	1442.781
[R].LTFPEQS	1xTMTpro [N	0.00018	7.389E-05	1	1	1	Q9Y6K5	Q9Y6K5 [118-132	0	1994.103
[K].ITGEIMH	1xTMTpro [K	0.0001427	7.389E-05	1	1	1	Q9NR12	Q9NR12 [348-357	0	1721.0275
[K].LTGEVVA	2xTMTpro [K	0.0001694	7.389E-05	1	1	1	P24941	P24941 [25-34]	1	1970.283
[K].ITGNMGI	1xTMTpro [K	0.0001596	7.389E-05	1	1	1	P22307	P22307 [525-534]	0	1643.9468
[R].LTGSSG	1xTMTpro [K	8.171E-05	7.389E-05	1	1	1	O75882	O75882 [138-153]	0	2208.1792
[R].LTLAELN	1xTMTpro [N	8.046E-05	7.389E-05	1	1	1	P35573	P35573 [899-910]	0	1751.0386
[K].LTLDTIFY	1xTMTpro [K	0.000131	7.389E-05	1	1	3	Q9Y277	Q9Y277 [97-109]	0	2027.2033
[K].LTLDTIFY	2xTMTpro [K	6.367E-05	7.389E-05	1	1	2	Q9Y277	Q9Y277 [97-110]	1	2459.5054
[R].LTLFHIE	1xTMTpro [K	8.545E-05	7.389E-05	1	1	4	Q7L266	Q7L266 [233-242]	0	1794.0769
[K].LTLIDPE	1xTMTpro [N	0.0001142	7.389E-05	1	1	2	Q86VP6	Q86VP6 [958-969	0	1685.0168
[R].LTESLDI	1xTMTpro [N	6.843E-05	7.389E-05	1	1	3	P42345	P42345 [1142-115	0	1821.919
[K].ITIIPQDF	1xTMTpro [N	0.0001482	7.389E-05	1	1	1	P33527	P33527 [1370-138	0	2060.2075
[K].LTLSALII	1xTMTpro [K	0.0001556	7.389E-05	2	2	4	P21796; Q9Y	P21796 [257-266]	0	1639.0286
[K].LTLSALV	1xTMTpro [K	0.0001359	7.389E-05	1	1	2	P45880	P45880 [268-277]	0	1625.0129
[R].LTLSELD	1xTMTpro [K	8.598E-05	7.389E-05	1	1	2	Q16363	Q16363 [546-556]	0	1868.1236
[K].LTITSNP	1xTMTpro [N	0.0001564	7.389E-05	1	1	1	Q9H3U1	Q9H3U1 [715-729	0	1997.0333
[R].LTLYDIA	1xTMTpro [K	0.0001968	7.389E-05	1	1	1	P40926	P40926 [53-74]	0	2973.6541
[K].LTMVPPI	1xTMTpro [K	0.0001579	7.389E-05	1	1	2	Q15404	Q15404 [52-63]	0	1934.164
[K].LTNAEGY	1xTMTpro [K	9.957E-05	7.389E-05	1	1	1	O76094	O76094 [288-297]	0	1715.9824
[K].ITNDFYF	1xTMTpro [K	7.474E-05	7.389E-05	1	1	2	P55884	P55884 [217-228]	0	2036.0468
[K].ITNDFYF	2xTMTpro [K	0.0001354	7.389E-05	1	1	1	P55884	P55884 [217-230]	1	2569.3966
[K].ITNLTQQ	1xTMTpro [K	0.0001147	7.389E-05	1	1	1	P15924	P15924 [1612-162	0	2294.3575
[K].ITNNINVI	1xTMTpro [K	0.0001445	7.389E-05	1	1	2	Q9BZZ5	Q9BZZ5 [417-426	0	1750.1082

[K].LTPEEEE	1xTMTpro [K	0.0001926	7.389E-05	1	1	3	P62241	P62241 [129-139]	0	1923.093
[K].LTPEYLC	1xTMTpro [K	0.0001756	7.389E-05	1	1	2	O94905	O94905 [276-285]	0	1844.0847
[K].ITLLSAL'	1xTMTpro [N	8.895E-05	7.389E-05	1	1	6	P01011	P01011 [380-390]	0	1519.9379
[R].LTPLQF(	1xTMTpro [K	7.815E-05	7.389E-05	1	1	1	Q9HC84	Q9HC84 [226-236	0	1867.1297
[K].ITENIGC	1xCarbamido	0.000107	7.389E-05	1	1	1	P60900	P60900 [72-88]	0	2176.0367
[R].ITELTDE	1xTMTpro [K	9.191E-05	7.389E-05	1	1	1	P19387	P19387 [11-20]	0	1770.0141
[R].LSTVVD/	1xTMTpro [K	6.208E-05	7.389E-05	1	1	2	O75027	O75027 [667-683]	0	2423.3889
[R].LSVEADI	1xTMTpro [N	7.985E-05	7.389E-05	1	1	3	Q04695	Q04695 [181-191]	0	1490.8498
[K].LSVEESI	1xTMTpro [K	6.139E-05	7.389E-05	1	1	1	P49321	P49321 [427-442]	0	2215.1585
[K].LSVISVE	1xTMTpro [N	0.000196	7.389E-05	1	1	3	P38117	P38117 [222-233]	0	1643.9288
[R].ISVNNVL	1xOxidation	0.0001407	7.389E-05	1	1	2	P07339	P07339 [206-222]	0	2583.446
[R].LSVPYVI	1xTMTpro [N	8.638E-05	7.389E-05	1	1	1	Q14156	Q14156 [679-692]	0	1922.019
[K].LSVQGE	1xTMTpro [K	6.822E-05	7.389E-05	1	1	1	Q14165	Q14165 [178-190]	0	1961.1199
[K].LSVVEYI	1xTMTpro [K	0.0001004	7.389E-05	1	1	2	Q10570	Q10570 [103-116]	0	2181.2047
[R].LSWTVA	1xTMTpro [K	0.0001096	7.389E-05	1	1	1	P22105	P22105 [1279-129	0	2712.4351
[K].LTAAVLL	1xTMTpro [K	0.0001772	7.389E-05	1	1	4	Q6PCE3	Q6PCE3 [133-141	0	1508.0067
[R].ITADGAI	1xTMTpro [N	9.587E-05	7.389E-05	1	1	1	Q8IWX8	Q8IWX8 [201-211	0	1533.8345
[R].LTAEDLI	1xTMTpro [N	0.0001759	7.389E-05	1	1	3	Q15149	Q15149 [3784-379	0	1468.7967
[R].ITAHLVH	1xTMTpro [N	8.732E-05	7.389E-05	1	1	2	P42765	P42765 [361-370]	0	1492.8919
[R].ITENDIQ	1xTMTpro [K	8.375E-05	7.389E-05	1	1	1	P04114	P04114 [2151-216	0	2167.2102
[K].LTALDYI	1xCarbamido	7.069E-05	7.389E-05	1	1	1	Q9Y224	Q9Y224 [6-20]	0	2329.2255
[K].LTATPTI	1xTMTpro [N	6.188E-05	7.389E-05	1	1	1	O75533	O75533 [431-451]	0	2565.2761
[K].ITCLCQ\	2xCarbamido	8.272E-05	7.389E-05	1	1	2	P49588	P49588 [899-912]	0	1949.0016
[R].LTCLVAI	1xCarbamido	0.0001395	7.389E-05	1	1	1	Q9BQS8	Q9BQS8 [291-300	0	1783.0643
[K].LTDCVVI	1xCarbamido	0.0001157	7.389E-05	1	1	1	P22626	P22626 [47-59]	1	2100.1437
[K].ITDEEEL	1xTMTpro [K	6.875E-05	7.389E-05	1	1	2	Q9BZJ0	Q9BZJ0 [214-224	0	1977.0308
[R].LTDEQV.	1xTMTpro [N	0.0002019	7.389E-05	1	1	1	Q14137	Q14137 [196-205]	0	1447.844
[K].ITDSAGI	1xTMTpro [K	0.0001147	7.389E-05	1	1	2	P49755	P49755 [76-87]	0	1913.0988
[K].LTDSEDI	1xTMTpro [K	0.0001769	7.389E-05	1	1	1	Q9P0U3	Q9P0U3 [413-429	0	2650.2937
[K].ITDVIIGF	2xCarbamido	0.0001796	7.389E-05	1	1	3	P35579	P35579 [779-791]	0	1856.9682
[K].LTEDPEI	2xTMTpro [K	0.0001987	7.389E-05	1	1	1	Q6XQN6	Q6XQN6 [408-42	1	2455.4224
[K].LTEKEEI	2xTMTpro [K	0.0001561	7.389E-05	1	1	1	Q9NZM1	Q9NZM1 [1443-14	1	2704.5014
[R].ITELKEE	2xTMTpro [K	0.0001216	7.389E-05	1	1	2	Q9NQP4	Q9NQP4 [33-43]	1	2243.3679

[K].LTELSM(	1xTMTpro [K	0.0001707	7.389E-05	1	1	1	P62072	P62072 [69-81]	0	2175.1533
[R].LTAIIEE/	1xTMTpro [N	6.664E-05	7.389E-05	1	1	2	P58107	P58107 [2571-259	0	2868.5175
[K].LSTPDV'	1xTMTpro [N	9.306E-05	7.389E-05	1	1	1	Q9BTX1	Q9BTX1 [438-458	0	2499.2873
[K].LTPITYP	1xTMTpro [K	7.719E-05	7.389E-05	1	1	3	P63000	P63000 [134-147]	0	2112.2383
[R].ITPSYVA	1xTMTpro [N	6.267E-05	7.389E-05	1	1	4	P11021	P11021 [61-74]	0	1870.987
[K].LVDPVA(	1xTMTpro [N	0.0001023	7.389E-05	1	1	1	O95400	O95400 [27-40]	0	1572.8665
[R].LVDYLD'	1xTMTpro [N	0.0001899	7.389E-05	1	1	3	P12111	P12111 [1258-127	0	1817.9604
[K].LVEALA/	1xTMTpro [K	0.0002047	7.389E-05	1	1	1	Q9BRJ7	Q9BRJ7 [188-198	0	1724.045
[R].LVEALPY	1xTMTpro [K	9.408E-05	7.389E-05	1	1	1	P28289	P28289 [272-286]	0	2315.3176
[R].IVEANPL	1xTMTpro [K	9.863E-05	7.389E-05	1	1	1	Q9UM54	Q9UM54 [182-196	0	2194.2727
[K].IVEDAVE	1xTMTpro [K	9.35E-05	7.389E-05	1	1	1	Q7Z3D6	Q7Z3D6 [386-397	0	1908.1298
[R].LVEEEVI	1xTMTpro [N	0.0002051	7.389E-05	1	1	1	Q9NZL9	Q9NZL9 [19-30]	1	1771.9986
[K].LVEENAI	1xTMTpro [K	8.071E-05	7.389E-05	1	1	2	Q13201	Q13201 [1080-10	0	2041.1461
[K].LVEGPPI	1xTMTpro [K	0.0001159	7.389E-05	1	1	1	Q9NWU5	Q9NWU5 [172-18	0	1962.1556
[R].IVEGSD/	1xOxidation		6.139E-05	7.389E-05	1	1	1	P00734	P00734 [364-383]	0	2585.3063
[K].LVEHGLI	1xTMTpro [N	8.979E-05	7.389E-05	1	1	1	Q7Z3B4	Q7Z3B4 [490-502	0	1835.0459
[K].LVEKPSI	2xTMTpro [K	8.624E-05	7.389E-05	1	1	1	P53618	P53618 [768-786]	0	3002.7859
[K].LVELAH(	1xTMTpro [K	8.44E-05	7.389E-05	1	1	2	P04114	P04114 [2361-236	0	1709.0242
[R].LVDNYC	1xCarbamid(	0.000159	7.389E-05	1	1	2	P39059	P39059 [1334-134	0	1629.8014
[R].LVEMEQ	1xTMTpro [K	0.0001295	7.389E-05	1	1	2	O94911	O94911 [818-830]	0	2098.2074
[R].IVETHQI	1xTMTpro [N	6.307E-05	7.389E-05	1	1	1	Q9H9E3	Q9H9E3 [282-298	0	2263.2042
[R].IVEVFDI(	1xTMTpro [K	0.0001813	7.389E-05	1	1	4	Q99715	Q99715 [1225-12	0	1725.0442
[K].LVEVIEE	1xTMTpro [K	0.0001224	7.389E-05	1	1	1	A0MZ66	A0MZ66 [172-181	0	1780.0712
[R].LVEVNG	1xTMTpro [K	0.0001617	7.389E-05	1	1	1	O14745	O14745 [59-69]	0	1838.0515
[R].IVEYLVG	1xTMTpro [N	6.217E-05	7.389E-05	1	1	2	Q9C0E8	Q9C0E8 [259-271	0	1763.9611
[K].LVFLGE(	1xTMTpro [K	6.297E-05	7.389E-05	1	2	2	P20340	P20340 [16-26]	0	1785.0766
[K].LVFLGLI	1xTMTpro [K	7.004E-05	7.389E-05	2	2	3	Q9NR31; Q9	Q9NR31 [28-38];	0	1755.066
[K].LVFPQDI	1xTMTpro [K	0.000171	7.389E-05	1	1	1	Q8TCT9	Q8TCT9 [242-251	0	1810.097
[R].IVFSGNI	2xTMTpro [K	0.0001036	7.389E-05	1	1	1	Q96TA1	Q96TA1 [69-84]	1	2789.5766
[K].IVGCSVI	1xCarbamid(	0.0001275	7.389E-05	1	2	3	P07910	P07910 [43-50]	0	1507.891
[R].IVGEFLQ	1xTMTpro [K	0.0001838	7.389E-05	1	1	1	Q14534	Q14534 [162-176]	0	2265.3251
[R].LVGEIM(	1xTMTpro [N	7.258E-05	7.389E-05	1	1	2	Q00341	Q00341 [244-255]	0	1637.8852
[R].LVGGDN	1xCarbamid(	0.0001983	7.389E-05	1	1	1	O43866	O43866 [246-256]	0	1451.7596

[K].IVEQPTV	1xTMTpro [K	0.0001144	7.389E-05	1	1	1	Q15054	Q15054 [262-274]	0	2035.1931
[K].LTPITYP	1xOxidation [	0.000144	7.389E-05	1	1	3	P63000	P63000 [134-147]	0	2128.2332
[K].IVDLVSE	2xTMTpro [K	0.0001324	7.389E-05	1	1	2	P29622	P29622 [194-203]	1	2056.3198
[R].LVDGVT	1xCarbamido	0.000139	7.389E-05	1	1	3	Q86VB7	Q86VB7 [268-278	0	1496.7698
[R].LTPYEVI	1xTMTpro [N	0.0001514	7.389E-05	1	1	1	Q92841	Q92841 [144-154]	1	1694.9397
[K].LTQDQD	1xTMTpro [K	0.0001218	7.389E-05	1	1	2	P30153	P30153 [567-576]	0	1768.9937
[K].ITQSNAI	1xCarbamido	0.000111	7.389E-05	1	2	2	Q03013	Q03013 [70-82]	0	1827.0118
[K].LTSAVSS	1xTMTpro [K	6.178E-05	7.389E-05	1	1	3	Q8WVM8	Q8WVM8 [382-39	0	2095.2506
[K].ITSEALL	1xTMTpro [K	6.95E-05	7.389E-05	1	1	2	Q86VP6	Q86VP6 [535-548	0	2151.3244
[R].ITSGPFE	1xTMTpro [K	8.611E-05	7.389E-05	1	1	1	Q9UQ80	Q9UQ80 [333-34	0	1975.1032
[K].LTSLAAA	2xTMTpro [K	0.0002043	7.389E-05	1	1	1	O95202	O95202 [668-682]	1	2458.4333
[R].LTSIIFAE	1xTMTpro [N	7.731E-05	7.389E-05	1	1	1	Q8TEM1	Q8TEM1 [92-108]	0	2181.245
[R].ITSLTQL	1xTMTpro [K	7.371E-05	7.389E-05	1	1	1	Q13049	Q13049 [70-84]	0	2268.367
[R].LTSLVPF	1xTMTpro [N	9.77E-05	7.389E-05	1	1	2	Q14152	Q14152 [455-469]	0	2039.1496
[R].LTTDFGI	1xTMTpro [K	0.0001425	7.389E-05	1	1	2	P02786	P02786 [656-665]	0	1703.946
[R].LTVAAPI	1xTMTpro [N	9.587E-05	7.389E-05	2	2	5	P0C0L4; P0(	P0C0L4 [460-484]	0	2878.5858
[R].LTVADAI	1xTMTpro [K	0.0001533	7.389E-05	1	1	1	P10644	P10644 [265-281]	0	2468.3528
[R].IVDHLLA	1xTMTpro [N	0.0001387	7.389E-05	1	1	1	A2RRP1	A2RRP1 [2312-23	0	1754.0244
[K].LTVPFLL	1xTMTpro [N	9.894E-05	7.389E-05	1	1	3	Q99685	Q99685 [227-240]	0	1834.0758
[K].ITVTSEV	1xTMTpro [K	7.015E-05	7.389E-05	1	1	1	P35268	P35268 [70-81]	1	1972.1723
[K].ITVVDAL	1xTMTpro [K	9.364E-05	7.389E-05	1	1	2	Q16270	Q16270 [263-275]	0	2042.2505
[K].ITVVDAL	2xTMTpro [K	0.0001326	7.389E-05	1	1	1	Q16270	Q16270 [263-276]	1	2474.5526
[R].LTWHAY	2xTMTpro [K	8.209E-05	7.389E-05	1	1	1	P45973	P45973 [172-186]	1	2743.4871
[K].ITWPNG	1xTMTpro [N	0.0001466	7.389E-05	1	1	1	Q07954	Q07954 [3188-32	0	2082.1191
[K].LTYTAEV	1xTMTpro [K	8.558E-05	7.389E-05	1	1	3	P09960	P09960 [155-165]	0	1816.0712
[K].IVAERPG	1xOxidation [	0.0001239	7.389E-05	1	1	2	Q13263	Q13263 [408-427]	0	2339.2461
[K].LVALETC	1xTMTpro [K	0.0001021	7.389E-05	1	1	2	Q86UU1	Q86UU1 [780-789	0	1680.0551
[R].LVAPTEC	1xTMTpro [K	0.0001253	7.389E-05	1	1	1	Q9HAD4	Q9HAD4 [200-216	0	2507.4253
[R].LVASAEC	1xTMTpro [K	8.492E-05	7.389E-05	1	1	4	Q9NY33	Q9NY33 [346-355	0	1680.0551
[R].LVASAYS	1xTMTpro [K	6.716E-05	7.389E-05	1	1	2	O95861	O95861 [11-21]	0	1759.0609
[R].IVASTLS	1xTMTpro [K	0.0001742	7.389E-05	1	1	1	P17174	P17174 [306-325]	0	2842.5482
[R].IVDDLKD	1xTMTpro [K	6.397E-05	7.389E-05	1	1	1	O75533	O75533 [853-865]	1	2202.1898
[K].ITVTSEV	1xTMTpro [K	7.124E-05	7.389E-05	1	1	2	P35268	P35268 [70-80]	0	1816.0712

[R].LPLMEC	1xCarbamido	0.0001772	7.389E-05	1	1	1	P08237	P08237 [346-356]	0	1926.1048
[R].IPILYGE	1xTMTpro [K	0.0001098	7.389E-05	1	1	1	Q9NZL9	Q9NZL9 [182-191	0	1769.0704
[R].LPLLAV	1xCarbamido	0.0001087	7.389E-05	1	1	1	Q15149	Q15149 [944-953	0	1800.0585
[K].LIDLAYT	1xTMTpro [K	8.375E-05	7.389E-05	1	1	1	Q96SY0	Q96SY0 [204-217	0	2209.324
[K].LLDIEGL	1xTMTpro [K	0.0001012	7.389E-05	1	1	2	Q92621	Q92621 [1979-19	0	1759.0497
[R].LIDLHSP	1xTMTpro [K	7.224E-05	7.389E-05	1	1	3	P60866	P60866 [88-99]	0	1959.177
[K].ILDILGE	1xCarbamido	0.0001911	7.389E-05	2	2	2	Q15424; Q1	Q15424 [217-226	0	1770.0327
[R].ILDIVPP	2xCarbamido	0.0001146	7.389E-05	1	1	1	Q09161	Q09161 [465-486	0	3098.6914
[R].LLDMDG	1xTMTpro [K	7.135E-05	7.389E-05	1	1	2	Q14152	Q14152 [341-351	0	1854.0902
[K].LLDNLHI	1xTMTpro [K	0.0001172	7.389E-05	1	1	2	P06401	P06401 [876-885]	0	1788.0875
[R].LLDPED	1xTMTpro [K	0.0001299	7.389E-05	1	1	2	Q15149	Q15149 [372-386	0	2317.2419
[K].ILDPISS	1xTMTpro [N	0.0001726	7.389E-05	1	1	1	Q8N0U7	Q8N0U7 [204-22	0	2178.2453
[R].LLDTAFI	1xTMTpro [K	0.000117	7.389E-05	1	1	1	Q02218	Q02218 [1009-10	0	2005.1502
[K].ILDVIGL	1xTMTpro [N	9.35E-05	7.389E-05	1	1	1	Q00534	Q00534 [231-245	0	2013.0976
[R].ILDWHV	1xTMTpro [K	0.0001584	7.389E-05	1	1	2	P37837	P37837 [193-203]	0	1920.0835
[R].ILDWHV	2xTMTpro [K	0.0001508	7.389E-05	1	1	2	P37837	P37837 [193-204]	1	2352.3856
[K].ILEALAS	1xTMTpro [N	0.0001564	7.389E-05	1	1	1	Q9UIQ6	Q9UIQ6 [893-904	0	1606.8971
[R].LLEALQI	1xTMTpro [K	7.543E-05	7.389E-05	1	1	1	O43182	O43182 [377-392	0	2319.3779
[R].LIEDTED	1xTMTpro [N	7.113E-05	7.389E-05	1	1	2	Q96HC4	Q96HC4 [264-27	0	1733.9142
[K].ILEDTQF	1xTMTpro [K	0.0001929	7.389E-05	1	1	1	Q14C86	Q14C86 [76-87]	0	2036.1196
[K].IIEEAPA	1xTMTpro [K	7.863E-05	7.389E-05	1	1	2	Q96RQ3	Q96RQ3 [285-29	0	1746.0657
[R].LLEEEN	1xTMTpro [N	0.0001165	7.389E-05	1	1	2	P48681	P48681 [883-893]	0	1663.8822
[R].LLEELEE	1xTMTpro [K	9.248E-05	7.389E-05	2	2	3	Q13404; Q1	Q13404 [17-26];	0	1796.0297
[K].IIEELQD	1xTMTpro [K	0.0001053	7.389E-05	1	1	1	Q13438	Q13438 [272-288	0	2593.4369
[R].LLEEQL	1xTMTpro [K	9.105E-05	7.389E-05	1	1	3	Q86UP2	Q86UP2 [684-696	0	2189.2422
[K].LIEEVMI	1xTMTpro [K	0.000181	7.389E-05	1	1	1	P78371	P78371 [348-358]	0	1884.0644
[R].LLEFELA	1xTMTpro [K	0.0001284	7.389E-05	1	1	1	Q13464	Q13464 [820-830	0	1913.1603
[K].LLEHLP	1xTMTpro [K	9.988E-05	7.389E-05	1	1	1	O75616	O75616 [356-371	0	2543.4477
[R].LLELADI	1xTMTpro [N	0.0002023	7.389E-05	1	1	1	Q8WVM0	Q8WVM0 [280-29	0	1926.1343
[R].ILELDQE	1xTMTpro [K	0.0001232	7.389E-05	1	1	2	Q92878	Q92878 [474-483	0	1822.1181
[K].ILDKLED	2xTMTpro [K	0.0002055	7.389E-05	1	1	1	Q12769	Q12769 [1412-14	1	2305.3584
[K].ILDHQQ	1xTMTpro [K	0.0001253	7.389E-05	1	1	1	Q9Y4G6	Q9Y4G6 [1754-17	0	2406.3307
[R].LLDHMQ	1xTMTpro [K	6.664E-05	7.389E-05	1	1	1	Q9NVP1	Q9NVP1 [312-325	0	2303.2172

[K].LLDGPS	2xTMTpro [K	0.0002043	7.389E-05	1	1	1	P23193	P23193 [76-89]	1	2472.4013
[R].LLAAVE/	1xTMTpro [N	0.0001046	7.389E-05	1	1	1	Q8IWX8	Q8IWX8 [686-703	0	2330.2576
[R].IIADLEEI	1xTMTpro [N	0.0001599	7.389E-05	1	1	1	P46939	P46939 [3266-32	0	1519.8287
[R].LLADPT(	1xTMTpro [K	0.0001558	7.389E-05	1	1	2	P30044	P30044 [149-159]	0	1698.0082
[K].LIAELWD	1xTMTpro [K	0.0001968	7.389E-05	1	1	2	P49590	P49590 [428-438]	0	1853.1028
[K].IIALGHS(	1xTMTpro [K	8.651E-05	7.389E-05	1	1	2	P21589	P21589 [215-227]	0	2026.1287
[R].LLAQDQ	1xTMTpro [N	0.0001387	7.389E-05	1	1	3	Q15102	Q15102 [215-231]	0	2022.1191
[K].LLASCS/	1xCarbamido	0.0001146	7.389E-05	1	1	2	P43034	P43034 [164-175]	0	1918.0633
[K].LIASMSS	1xTMTpro [N	0.000118	7.389E-05	1	1	1	P00488	P00488 [706-716]	0	1483.8109
[R].ILASPVE	1xTMTpro [K	6.529E-05	7.389E-05	1	1	1	Q9NYU2	Q9NYU2 [334-34	0	2091.3107
[R].IICDNTG	1xCarbamido	7.803E-05	7.389E-05	1	1	2	P11678	P11678 [674-686]	0	1753.9438
[K].LLCGGG	1xCarbamido	0.0001466	7.389E-05	1	1	2	P05091	P05091 [384-394]	0	1406.7745
[R].ILCHMQI	1xCarbamido	9.572E-05	7.389E-05	1	1	1	O15321	O15321 [105-120]	0	2217.1803
[K].LICQATG	1xCarbamido	9.909E-05	7.389E-05	2	2	4	P0DOX6; P0	P0DOX6 [257-267	0	1553.8429
[K].ILELIESG	2xTMTpro [K	0.0001025	7.389E-05	1	1	1	P47895	P47895 [365-374]	1	2042.3041
[K].ILCQEEQ	1xCarbamido	9.394E-05	7.389E-05	1	1	1	P46736	P46736 [238-248]	0	1728.8546
[R].ILDAAG/	1xTMTpro [K	0.00017	7.389E-05	1	1	3	Q9UBQ7	Q9UBQ7 [67-76]	0	1593.982
[R].LIDDMV/	1xTMTpro [K	6.529E-05	7.389E-05	1	1	4	O75874	O75874 [250-260]	0	1843.0313
[R].LIDDMV/	1xOxidation [	0.0001647	7.389E-05	1	1	2	O75874	O75874 [250-260]	0	1859.0262
[R].LIDDMV/	1xTMTpro [K	0.0001034	7.389E-05	1	1	4	P48735	P48735 [289-299]	0	1853.1062
[K].IIDDSDS	1xTMTpro [K	0.0001564	7.389E-05	1	3	1	P49792	P49792 [721-733]	0	2013.136
[K].ILDDWG	1xCarbamido	0.0001482	7.389E-05	1	1	6	P55145	P55145 [143-152]	0	1844.9708
[K].LLDEAIQ	1xTMTpro [K	9.438E-05	7.389E-05	1	1	2	Q96QK1	Q96QK1 [15-24]	0	1708.05
[R].IIDEDFEI	1xTMTpro [N	0.0001395	7.389E-05	1	1	2	Q15746	Q15746 [1550-15	0	1683.8761
[R].LLDENKI	2xTMTpro [K	0.0001498	7.389E-05	1	1	1	Q66K14	Q66K14 [890-902	1	2461.4482
[K].LLDEVFF	1xTMTpro [K	0.0002019	7.389E-05	1	1	2	P25789	P25789 [55-64]	0	1835.0446
[R].LLDFSYE	1xTMTpro [K	8.146E-05	7.389E-05	1	1	1	Q969V3	Q969V3 [89-99]	0	2042.109
[R].LLDFTQ/	1xTMTpro [N	0.0001498	7.389E-05	1	1	1	A2RRP1	A2RRP1 [1639-16	0	1920.1238
[R].ILDGMF/	1xCarbamido	6.79E-05	7.389E-05	1	1	1	P12081	P12081 [216-230]	0	2221.1852
[K].LLCSNS/	1xCarbamido	0.0001442	7.389E-05	1	1	2	Q13564	Q13564 [382-391]	0	1484.8215
[K].LLELQSI	1xTMTpro [N	0.000123	7.389E-05	1	1	3	O95202	O95202 [332-345]	0	1922.103
[K].ILELVHD	1xTMTpro [K	0.0001536	7.389E-05	1	1	1	P52630	P52630 [560-569]	0	1825.1191
[K].IIENELE(	1xTMTpro [N	0.0001665	7.389E-05	1	1	3	Q9Y295	Q9Y295 [160-171	0	1693.9444

[R].ILHLLGQ	1xTMTpro [K	6.664E-05	7.389E-05	1	1	3	Q9Y678	Q9Y678 [455-465	0	1813.1191
[K].ILHQHLG	1xTMTpro [N	0.0001746	7.389E-05	1	1	2	Q9NZM1	Q9NZM1 [1715-17	0	1703.9512
[R].LLHSLDC	1xTMTpro [K	0.0001349	7.389E-05	1	1	2	O15230	O15230 [2443-245	0	1632.9929
[R].LLHVAVS	1xTMTpro [N	8.414E-05	7.389E-05	1	1	1	Q99460	Q99460 [602-615]	0	1856.0197
[R].LIKEDQE	2xTMTpro [K	0.0001881	7.389E-05	1	1	1	Q8IYM9	Q8IYM9 [151-166	2	2856.6135
[R].LIKEDQL	1xTMTpro [K	6.168E-05	7.389E-05	1	1	1	Q96BY6	Q96BY6 [2118-21	1	2414.3423
[K].LIKEELQ	2xTMTpro [K	0.0001547	7.389E-05	1	1	1	P29144	P29144 [169-186]	1	3059.7809
[K].LLIADDN	1xTMTpro [N	0.0001075	7.389E-05	1	1	1	Q13393	Q13393 [899-917]	0	2331.2839
[K].ILLAELE	1xTMTpro [K	8.096E-05	7.389E-05	1	1	41	P08670	P08670 [130-139]	0	1778.1283
[R].LIIAGTSA	1xTMTpro [N	0.0001127	7.389E-05	1	1	4	P34897	P34897 [220-230]	0	1439.8541
[K].LIIAGTSC	1xCarbamido	0.000165	7.389E-05	1	1	2	P34896	P34896 [197-207]	0	1544.8426
[R].LLLDGAI	1xTMTpro [K	6.519E-05	7.389E-05	1	1	1	Q9H0R4	Q9H0R4 [133-145	0	1982.2658
[R].LLIDGDC	1xTMTpro [N	0.0001632	7.389E-05	1	1	1	Q6I9Y2	Q6I9Y2 [14-26]	1	1676.8887
[R].LLHEMIL	1xCarbamido	7.146E-05	7.389E-05	1	1	1	P53618	P53618 [94-106]	0	1937.0308
[K].IIIDKEYY	2xTMTpro [K	0.0001995	7.389E-05	1	1	1	Q7L5N1	Q7L5N1 [98-108]	1	2361.3886
[K].ILLDPNS	2xTMTpro [K	6.188E-05	7.389E-05	1	1	1	P51531	P51531 [660-675]	1	2699.5283
[R].LILDVFC	1xCarbamido	0.0001166	7.389E-05	1	1	1	P11413	P11413 [440-454]	0	2126.121
[R].IIIEDLLE	1xTMTpro [N	0.0001237	7.389E-05	1	1	2	Q92616	Q92616 [2151-216	0	1589.9433
[R].LLLEIAD	1xTMTpro [K	8.096E-05	7.389E-05	1	1	2	Q8N8A2	Q8N8A2 [652-666	0	2247.3092
[R].ILLELLN	1xTMTpro [K	6.257E-05	7.389E-05	1	1	1	P43686	P43686 [288-306]	0	2811.557
[K].LLLETLP	1xTMTpro [K	0.000153	7.389E-05	1	1	1	Q96Q05	Q96Q05 [431-446	0	2367.4031
[R].LLLFSSA	1xTMTpro [N	0.0001782	7.389E-05	1	1	2	Q12788	Q12788 [166-178]	0	1681.9808
[R].LLLLDTV	1xTMTpro [N	0.0001584	7.389E-05	1	1	1	Q9Y678	Q9Y678 [846-859	0	1878.1053
[R].LLLLFAD	1xTMTpro [N	0.0001653	7.389E-05	1	1	1	Q7LBC6	Q7LBC6 [13-43]	0	3128.6911
[R].IILLFDAI	1xTMTpro [K	0.0001528	7.389E-05	3	3	1	Q9H223; Q9	Q9H223 [188-196	0	1678.0547
[R].ILLLGLD	1xTMTpro [K	7.048E-05	7.389E-05	1	1	1	P36405	P36405 [20-30]	0	1735.0973
[R].LLLNNDI	1xTMTpro [N	0.0002019	7.389E-05	2	2	4	P47755; P52	P47755 [38-47]; F	0	1501.9021
[K].ILIPALM	1xTMTpro [K	0.0001911	7.389E-05	1	1	2	P34913	P34913 [484-495]	0	1907.1895
[K].LLLDPEF	1xTMTpro [N	0.0001665	7.389E-05	1	1	2	Q9BZH6	Q9BZH6 [901-912	0	1762.0434
[R].LKYALTC	3xTMTpro [K	0.0001831	7.389E-05	1	3	1	P62701	P62701 [52-63]	2	2581.607
[K].IIHEDGY	1xCarbamido	7.997E-05	7.389E-05	1	1	2	P04899	P04899 [55-67]	0	1940.8979
[R].IIGVDINI	2xTMTpro [K	0.0001161	7.389E-05	1	1	2	P11766	P11766 [219-228]	1	2027.2681
[K].LLENMPI	1xTMTpro [N	0.0001149	7.389E-05	1	1	1	Q6P2Q9	Q6P2Q9 [102-114	0	1961.0308

[K].LLEPVV(	1xCarbamid(	0.0001073	7.389E-05	1	1	1	Q9UN37	Q9UN37 [397-409	0	1866.9811
[K].LLEPVVS	1xTMTpro [N	0.00017	7.389E-05	1	1	2	O75351	O75351 [404-416]	0	1793.9824
[K].LLEQSIQ	1xTMTpro [K	0.0001172	7.389E-05	1	1	2	P11532	P11532 [1364-137	0	2212.2317
[R].LLEQVLI	1xTMTpro [N	7.015E-05	7.389E-05	1	1	1	Q86U38	Q86U38 [331-343	0	1823.1325
[R].LLESLD(	1xTMTpro [N	0.000114	7.389E-05	1	1	3	O95816	O95816 [28-38]	0	1632.9492
[K].LLETGLV	1xTMTpro [N	9.925E-05	7.389E-05	1	1	1	Q7Z3U7	Q7Z3U7 [798-808	0	1586.9008
[K].LLETIDQ	1xTMTpro [K	0.0002003	7.389E-05	1	1	1	P12814	P12814 [503-516]	0	2320.3296
[K].IIETLTQ(	1xTMTpro [K	0.0001359	7.389E-05	1	1	3	Q9UHV9	Q9UHV9 [100-111	0	1994.2142
[K].LLETTDF	1xTMTpro [N	0.0001142	7.389E-05	1	1	2	Q14974	Q14974 [510-525]	0	2183.1488
[K].IIEVGDTI	1xTMTpro [K	0.0001522	7.389E-05	1	2	1	P61619	P61619 [99-109]	1	1851.0831
[R].LLEVLS(	2xTMTpro [K	0.0001361	7.389E-05	1	1	1	O15020	O15020 [94-108]	0	2549.6098
[R].LLFNDV(	1xTMTpro [K	7.972E-05	7.389E-05	1	1	3	Q15149	Q15149 [588-597]	0	1799.0922
[R].ILGVGPI	1xTMTpro [N	0.0001498	7.389E-05	1	1	1	P48449	P48449 [163-175]	0	1669.9444
[R].LLFQLA(	1xTMTpro [K	9.467E-05	7.389E-05	1	1	2	Q9Y6X3	Q9Y6X3 [147-159	0	2162.3193
[K].LLGELLQ	1xTMTpro [K	7.684E-05	7.389E-05	1	1	2	P37837	P37837 [259-269]	0	1822.093
[K].LLGELQI	1xTMTpro [K	0.0001941	7.389E-05	1	1	1	Q99996	Q99996 [358-369]	0	2006.2142
[R].LIGEMNS	1xTMTpro [N	0.000187	7.389E-05	1	1	1	Q9P2W9	Q9P2W9 [242-254	0	1826.9642
[R].ILGGHLI	1xTMTpro [K	0.0001877	7.389E-05	2	2	6	P00739; P0(	P00739 [104-112]	0	1531.9452
[R].IIGGSDA	1xTMTpro [K	0.0001687	7.389E-05	1	1	1	P09871	P09871 [438-447]	0	1596.9452
[R].ILGLCLL	2xCarbamid(	8.584E-05	7.389E-05	1	1	1	O95071	O95071 [2535-255	0	2444.3688
[K].LIGIDDVI	1xTMTpro [N	0.0001204	7.389E-05	1	1	2	P98082	P98082 [54-64]	0	1487.8389
[R].LLGIPLL(	1xTMTpro [N	9.262E-05	7.389E-05	1	1	1	Q5QJ74	Q5QJ74 [259-273	0	2047.1759
[K].LLGLTAF	1xTMTpro [K	9.423E-05	7.389E-05	1	1	1	Q9NR09	Q9NR09 [3298-33	0	3502.8925
[R].ILGPAES	1xTMTpro [N	8.772E-05	7.389E-05	1	1	2	Q5T4S7	Q5T4S7 [1337-13	0	1721.9393
[R].LLGPQW	1xCarbamid(	0.0001656	7.389E-05	1	1	1	Q6P9B6	Q6P9B6 [181-193	0	1954.9652
[K].ILGSEGE	1xTMTpro [N	0.0001907	7.389E-05	1	1	2	Q8TED1	Q8TED1 [142-152	0	1479.8127
[K].LIGTATV	1xTMTpro [K	0.0001324	7.389E-05	1	1	2	Q9NZM1	Q9NZM1 [80-89]	0	1595.0388
[K].ILFYCHF	1xCarbamid(	0.0001635	7.389E-05	1	1	2	Q9H553	Q9H553 [133-146	0	2388.3645
[K].LKVEPG	2xTMTpro [K	0.0001929	7.389E-05	1	1	1	P50281	P50281 [491-499]	1	1943.2146
[R].IKVDFVII	2xTMTpro [K	0.0002007	7.389E-05	1	1	2	O75534	O75534 [287-295]	1	1971.2823
[K].IKSEHP(	2xTMTpro [K	9.176E-05	7.389E-05	1	1	1	P26583	P26583 [113-127]	1	2465.4544
[R].LGTDES	1xCarbamid(	9.049E-05	7.389E-05	1	1	1	P20073	P20073 [357-371]	0	2032.0163
[R].LGTDES	1xCarbamid(	7.648E-05	7.389E-05	1	1	1	P20073	P20073 [357-371]	0	2048.0112

[R].LGTFEVI	1xTMTpro [N	9.219E-05	7.389E-05	1	1	1	P27487	P27487 [598-611]	0	1881.9877
[K].LGVCFD'	1xCarbamido	6.468E-05	7.389E-05	1	1	1	Q9NR30	Q9NR30 [679-696	0	2601.409
[K].LGVEFDI	1xTMTpro [K	7.636E-05	7.389E-05	1	1	2	P05413	P05413 [67-80]	1	2204.1691
[K].LGVIEDH	1xTMTpro [N	0.0001245	7.389E-05	1	2	5	P14625	P14625 [494-503]	0	1443.7875
[R].IGVPSAT	1xTMTpro [K	0.0001025	7.389E-05	1	1	2	Q9NR30	Q9NR30 [563-573	0	1736.0813
[R].LGVQDL	1xTMTpro [K	7.684E-05	7.389E-05	1	1	3	P30740	P30740 [291-301]	0	1816.046
[K].LGVRPS	1xTMTpro [N	0.0001317	7.389E-05	1	1	5	P02751	P02751 [1117-112	0	1627.9199
[K].IGVVGG	1xTMTpro [K	0.0001907	7.389E-05	1	1	1	O15305	O15305 [41-51]	0	1715.9824
[R].LGWSQF	1xTMTpro [K	7.707E-05	7.389E-05	1	1	1	Q9UBL3	Q9UBL3 [438-455	0	2565.4321
[K].LGYHFEI	1xTMTpro [N	0.0001333	7.389E-05	1	1	1	P29536	P29536 [450-460]	0	1563.8603
[K].IGYNPDT	1xOxidation [	9.617E-05	7.389E-05	1	2	6	P68104	P68104 [181-212]	0	4192.0804
[R].LGSTVF'	1xTMTpro [K	7.948E-05	7.389E-05	1	1	2	P52272	P52272 [202-214]	0	2035.172
[R].LGYSDIT	1xTMTpro [K	7.348E-05	7.389E-05	1	1	1	Q12882	Q12882 [209-219	0	1894.0817
[K].LHAVVE	1xTMTpro [N	0.0001766	7.389E-05	1	1	1	Q13596	Q13596 [326-337	0	1691.9876
[K].LHDMLG	1xTMTpro [N	0.0002007	7.389E-05	1	1	1	Q14839	Q14839 [946-956	0	1623.8783
[K].LHDPEG	1xTMTpro [N	7.863E-05	7.389E-05	1	1	2	P33176	P33176 [99-110]	0	1638.8957
[R].IHDPQLI	1xTMTpro [N	0.0001922	7.389E-05	1	1	1	P14868	P14868 [432-441]	0	1525.8658
[R].LHDVLM	1xTMTpro [K	0.0001827	7.389E-05	1	1	1	P46939	P46939 [415-424]	0	1834.0752
[K].IHEDIFDI	1xTMTpro [N	0.0001087	7.389E-05	1	2	2	P23258	P23258 [114-124]	0	1689.9131
[R].LHEENFI	1xTMTpro [N	0.0001867	7.389E-05	1	1	1	Q96Q11	Q96Q11 [115-126	0	1805.9717
[K].LHEMEG	1xTMTpro [K	0.0001222	7.389E-05	1	1	4	P35749	P35749 [1801-180	0	1621.9227
[K].LHEQLTI	1xCarbamido	8.272E-05	7.389E-05	1	1	2	Q9UJX2	Q9UJX2 [476-492	0	2656.3896
[K].LHFGIDF	1xTMTpro [N	0.0001517	7.389E-05	1	1	2	Q7L266	Q7L266 [293-308	0	2074.0664
[R].LHFLMT	1xTMTpro [N	7.405E-05	7.389E-05	1	2	1	P23258	P23258 [266-286]	0	2641.3979
[R].IHFPEAL	1xTMTpro [N	9.678E-05	7.389E-05	1	1	1	O00562	O00562 [466-478	0	1764.024
[R].LHGLPE	1xTMTpro [K	0.0001402	7.389E-05	1	1	1	Q02880	Q02880 [698-712	0	2283.2993
[K].LHAELSI	1xTMTpro [K	8.799E-05	7.389E-05	1	1	1	Q5JRA6	Q5JRA6 [1373-13	0	1776.0511
[K].IHLAVTE	1xTMTpro [K	0.0001987	7.389E-05	1	1	1	Q9Y2X7	Q9Y2X7 [678-691	0	2165.2648
[K].IGSTIDD	1xTMTpro [K	0.0001249	7.389E-05	1	1	2	Q14677	Q14677 [208-218	0	1758.0141
[R].LGSPDE	1xTMTpro [K	7.566E-05	7.389E-05	1	1	2	Q12846	Q12846 [34-44]	0	1914.0253
[R].LGLGEG	1xTMTpro [K	8.159E-05	7.389E-05	1	1	3	P18754	P18754 [326-335]	0	1610.9245
[K].LGIHEDS	1xTMTpro [N	0.0001573	7.389E-05	1	1	4	P08238	P08238 [439-448]	0	1445.7668
[R].LGLHQV	1xTMTpro [N	0.0001922	7.389E-05	1	1	1	O60610	O60610 [341-351	0	1595.9552

[K].LGLKPLE	2xTMTpro [K	0.0001849	7.389E-05	1	1	1	O43290	O43290 [130-141]	0	2207.4307
[R].LGLIPLIS	1xTMTpro [K	0.000121	7.389E-05	1	1	1	P19387	P19387 [68-81]	0	2119.287
[K].LGINSLQ	1xTMTpro [K	0.0001415	7.389E-05	1	1	1	P55327	P55327 [111-120]	0	1723.0609
[R].LGLQYS	1xTMTpro [N	8.518E-05	7.389E-05	1	1	1	Q9UI10	Q9UI10 [210-225]	0	1954.0677
[K].LGLSDNI	1xTMTpro [N	0.0001687	7.389E-05	2	2	1	Q14160; Q9I	Q14160 [64-73]; C	0	1448.8028
[R].LGLSTLC	1xTMTpro [K	0.0001002	7.389E-05	1	1	2	O43399	O43399 [81-90]	0	1639.0286
[K].IGIVGLPI	1xTMTpro [K	0.0001681	7.389E-05	1	1	2	Q9NTK5	Q9NTK5 [25-35]	0	1675.0762
[R].LGMFNIC	1xCarbamido	0.000162	7.389E-05	1	1	9	P01009	P01009 [248-257]	0	1872.0116
[K].LGMNLM	1xTMTpro [K	0.0001345	7.389E-05	1	1	2	Q16890	Q16890 [71-80]	0	1771.0102
[R].IGNNFMI	1xTMTpro [K	0.0001435	7.389E-05	1	1	2	P51911	P51911 [46-55]	0	1716.9599
[R].IGSSLYA	1xCarbamido	0.0001567	7.389E-05	1	1	1	Q9UNH7	Q9UNH7 [248-265	0	2537.3413
[K].LGNPIVF	1xTMTpro [N	0.0001914	7.389E-05	1	1	4	P78527	P78527 [2133-214	0	1509.9436
[K].IGPECVD	2xCarbamido	0.0001331	7.389E-05	1	1	2	O95967	O95967 [158-169]	0	1766.8372
[R].LGPLFSI	1xTMTpro [N	7.439E-05	7.389E-05	1	1	2	Q16401	Q16401 [46-58]	0	1814.0244
[R].LGPNYLI	1xCarbamido	8.759E-05	7.389E-05	1	1	3	P04040	P04040 [366-380]	0	2117.1285
[R].IGPYQPI	1xTMTpro [K	6.716E-05	7.389E-05	1	1	1	P43243	P43243 [781-798]	0	2577.4936
[K].LGQALTI	1xTMTpro [K	9.148E-05	7.389E-05	1	1	3	Q00341	Q00341 [350-360]	0	1801.0715
[R].IGQGHC	1xCarbamido	0.0001817	7.389E-05	1	1	2	P35555	P35555 [2681-269	0	1766.8419
[K].IGQLEEC	1xTMTpro [K	7.135E-05	7.389E-05	1	1	2	P35580	P35580 [1823-183	0	2123.184
[K].IGQQPQ	1xTMTpro [K	0.0001093	7.389E-05	1	1	1	Q92945	Q92945 [629-646]	0	2589.3917
[K].LGQSES	1xTMTpro [N	9.557E-05	7.389E-05	1	1	1	O00233	O00233 [124-134]	0	1459.7824
[K].IGQVHYA	1xTMTpro [K	7.124E-05	7.389E-05	1	1	1	Q9UIQ6	Q9UIQ6 [387-399	0	2067.2094
[R].LGRPVF	1xTMTpro [N	0.0001956	7.389E-05	1	1	1	P35475	P35475 [490-502]	0	1822.0295
[R].LGSIAEII	1xTMTpro [K	0.0001284	7.389E-05	1	1	1	Q9BXP5	Q9BXP5 [134-150	0	2344.3806
[K].LGSLTQI	1xCarbamido	0.0001075	7.389E-05	1	1	1	O75151	O75151 [357-375]	0	2858.4501
[K].LGNTTVI	1xCarbamido	0.0001295	7.389E-05	1	1	2	Q96B26	Q96B26 [53-63]	0	1770.0439
[K].LILPHVD	1xTMTpro [K	0.000121	7.389E-05	1	1	1	P48735	P48735 [70-80]	0	1897.213
[K].LHLDYIG	1xCarbamido	0.000187	7.389E-05	1	1	2	P09486	P09486 [141-150]	0	1824.0334
[R].IHIFAQQ	1xTMTpro [K	7.113E-05	7.389E-05	1	1	1	Q6IA86	Q6IA86 [187-199]	0	2225.2323
[R].LKELTLE	2xTMTpro [K	0.0001345	7.389E-05	1	1	2	P08571	P08571 [118-127]	1	2114.3253
[K].LKEIVTN	1xTMTpro [K	0.0001008	7.389E-05	1	1	4	P25705	P25705 [540-553]	1	2160.256
[K].IKELVVT	1xTMTpro [K	0.0001684	7.389E-05	1	1	1	Q01813	Q01813 [288-301]	1	2243.3255
[R].LKENLYI	1xTMTpro [K	9.291E-05	7.389E-05	1	1	1	Q9NRW7	Q9NRW7 [482-49	1	2372.3833

[K].LKETAES	2xTMTpro [K	0.0001224	7.389E-05	1	1	2	P34932	P34932 [125-134]	1	2030.2678
[K].IKETVDQ	1xTMTpro [K	0.000153	7.389E-05	1	1	1	Q14204	Q14204 [3208-32	1	2067.1941
[R].LKEVLEY	2xTMTpro [K	6.237E-05	7.389E-05	1	1	1	P14324	P14324 [111-123]	1	2346.4213
[K].LKEVLLV	2xTMTpro [K	7.59E-05	7.389E-05	1	1	4	Q93034	Q93034 [434-442]	1	1967.3449
[K].LKEYSSN	1xTMTpro [K	7.96E-05	7.389E-05	1	1	1	O95470	O95470 [130-144]	1	2455.2572
[R].LKFDFQ/	2xTMTpro [K	8.868E-05	7.389E-05	1	1	1	O60504	O60504 [387-397]	1	2221.3161
[R].IKGCTDN	1xCarbamido	8.109E-05	7.389E-05	1	1	1	O00151	O00151 [70-83]	1	2170.2509
[K].LKGELES	1xTMTpro [K	0.000131	7.389E-05	1	1	1	Q9P2E9	Q9P2E9 [1184-11	1	1969.121
[K].LKGEMM	1xTMTpro [K	0.0001245	7.389E-05	1	1	1	P00338	P00338 [58-73]	1	2483.3758
[K].LKELQNI	2xTMTpro [K	0.0001198	7.389E-05	1	1	2	Q86U42	Q86U42 [136-145	1	2142.295
[K].LKGTEDI	2xTMTpro [K	0.0001732	7.389E-05	1	1	5	P07951	P07951 [50-59]	1	2060.2055
[R].IKLPQGY	2xTMTpro [K	6.8E-05	7.389E-05	1	1	1	Q9UPN3	Q9UPN3 [390-40	1	3051.672
[K].LKLSYEC	2xTMTpro [K	0.0001644	7.389E-05	1	1	1	P36955	P36955 [317-327]	1	2179.3154
[R].IKNQADC	1xCarbamido	0.0001892	7.389E-05	1	1	1	P05164	P05164 [303-314]	1	2117.1821
[R].IKPATWI	1xTMTpro [K	9.133E-05	7.389E-05	1	1	1	O14786	O14786 [406-418]	0	2098.1975
[R].LKPDEG	3xTMTpro [K	0.0001335	7.389E-05	1	1	1	P61221	P61221 [396-415]	0	3414.0149
[K].LKPEGLI	2xTMTpro [K	0.0001184	7.389E-05	1	1	3	Q9BY32	Q9BY32 [95-110]	0	2707.5963
[K].LKPGDLY	2xTMTpro [K	0.0001576	7.389E-05	1	1	4	P17980	P17980 [145-155]	0	2052.2997
[K].LKPGTM	1xTMTpro [K	8.979E-05	7.389E-05	1	1	1	Q9BPW8	Q9BPW8 [192-20	0	2381.3044
[R].LKPGYLI	1xTMTpro [K	9.831E-05	7.389E-05	1	1	2	Q15181	Q15181 [178-191]	0	2303.3044
[K].IKPHLMS	1xOxidation [	8.854E-05	7.389E-05	1	1	1	P07237	P07237 [351-366]	0	2894.5902
[R].LKPTNP/	2xTMTpro [K	0.0001412	7.389E-05	1	1	1	P52948	P52948 [533-542]	0	1980.2422
[K].LKPYFLT	2xTMTpro [K	7.578E-05	7.389E-05	1	1	1	Q9BWD1	Q9BWD1 [234-26	0	4105.2824
[R].LKPYGA	2xTMTpro [K	0.0001605	7.389E-05	1	1	4	P12109	P12109 [52-61]	0	2016.2674
[K].LKGTEDI	3xTMTpro [K	9.467E-05	7.389E-05	1	1	1	P07951	P07951 [50-65]	2	3057.746
[R].LHIEALL	1xTMTpro [K	0.0001842	7.389E-05	1	1	1	P14735	P14735 [723-735]	0	2067.257
[K].LKEIDDE	2xTMTpro [K	0.0001345	7.389E-05	1	1	1	Q8WXF7	Q8WXF7 [286-29	1	2162.2889
[K].LKEEIGK	2xTMTpro [K	0.000162	7.389E-05	1	1	2	P06727	P06727 [98-110]	2	2498.501
[K].IHLFDIDY	1xTMTpro [K	7.497E-05	7.389E-05	1	1	1	Q9NQR4	Q9NQR4 [113-12	0	1862.1031
[K].LHLIAQE	1xTMTpro [N	6.56E-05	7.389E-05	1	1	1	O00471	O00471 [166-177]	0	1756.0077
[K].LHIVVSE	1xTMTpro [K	9.467E-05	7.389E-05	1	1	3	Q05707	Q05707 [1360-13	0	1846.1657
[K].LHNAAN/	1xTMTpro [K	8.84E-05	7.389E-05	1	1	1	O75175	O75175 [32-41]	0	1688.9688
[K].IHPMAYC	1xTMTpro [K	6.653E-05	7.389E-05	1	1	1	P67870	P67870 [192-208]	0	2568.4252

[K].IHPVDKL	2xTMTpro [K	9.379E-05	7.389E-05	1	1	1	Q99798	Q99798 [718-730]	1	2374.5002
[R].LHQLTE	1xTMTpro [K	7.037E-05	7.389E-05	1	1	1	Q8TBA6	Q8TBA6 [591-601	0	1932.1774
[R].LHQMSV	1xCarbamido	0.0001012	7.389E-05	1	1	1	P98160	P98160 [2683-26	0	2071.986
[K].LHSFLGI	1xTMTpro [N	0.000138	7.389E-05	1	1	2	P06737	P06737 [522-533]	0	1722.9498
[K].IHSLCYN	2xCarbamido	6.54E-05	7.389E-05	1	1	1	Q6UXG2	Q6UXG2 [650-66	0	1976.9278
[R].IHSLEQE	1xTMTpro [N	8.691E-05	7.389E-05	1	1	1	H7BZ55	H7BZ55 [1269-12	0	1956.047
[K].LHSLVKF	1xCarbamido	0.0001354	7.389E-05	1	1	1	O43813	O43813 [240-255]	0	2798.6419
[K].LHTDDD	1xTMTpro [N	0.0001286	7.389E-05	1	1	1	Q9Y4G6	Q9Y4G6 [167-180	0	2041.0058
[K].LKEELSE	2xTMTpro [K	0.0001669	7.389E-05	1	1	1	Q15075	Q15075 [377-387]	1	2217.3158
[R].IHTGEKF	2xCarbamido	6.779E-05	7.389E-05	1	1	1	P17026	P17026 [132-146]	0	2458.2099
[K].LHYCVS	2xCarbamido	8.492E-05	7.389E-05	1	2	1	P62854	P62854 [71-82]	0	2083.1073
[K].IHYLDTT	1xTMTpro [N	0.0001632	7.389E-05	1	1	1	P46108	P46108 [106-120]	0	2062.1504
[K].LKDDEV	2xTMTpro [K	0.0001178	7.389E-05	1	1	3	P07195	P07195 [309-318]	1	2071.2579
[R].LKDETL	1xTMTpro [K	6.488E-05	7.389E-05	1	1	1	Q9HCG8	Q9HCG8 [603-61	1	2511.4467
[R].LKDGSL	2xTMTpro [K	6.779E-05	7.389E-05	1	1	1	A0AVT1	A0AVT1 [728-739	1	2318.3689
[R].LKDIGPE	2xTMTpro [K	0.0001005	7.389E-05	1	1	1	P51659	P51659 [635-644]	1	2010.2779
[K].IKDPDAS	2xTMTpro [K	7.625E-05	7.389E-05	1	1	3	P27797	P27797 [208-222]	1	2713.4613
[K].LKDVLL	1xTMTpro [K	8.718E-05	7.389E-05	1	1	4	P35579	P35579 [1844-18	2	2207.3003
[K].LKDYAF	1xTMTpro [K	0.000145	7.389E-05	1	1	3	O43390	O43390 [373-384]	1	2148.1733
[R].IKDYLLM	1xTMTpro [K	7.269E-05	7.389E-05	1	1	1	P62191	P62191 [68-80]	1	2307.2914
[R].LKEAEE	2xTMTpro [K	8.196E-05	7.389E-05	1	1	1	Q4V328	Q4V328 [529-548	1	3298.7947
[K].LKEAVE	2xTMTpro [K	9.988E-05	7.389E-05	1	1	1	Q13620	Q13620 [220-234]	1	2543.5225
[R].LKEDAV	2xTMTpro [K	8.799E-05	7.389E-05	1	1	5	P51884	P51884 [171-181]	1	2091.263
[R].LHVAALI	1xTMTpro [K	9.542E-05	7.389E-05	1	1	1	Q9NR99	Q9NR99 [2040-20	0	2160.3149
[R].LLIQAE	1xCarbamido	8.121E-05	7.389E-05	1	1	1	P54289	P54289 [1030-10	0	2724.4192
[R].IIIQESAL	1xTMTpro [N	6.843E-05	7.389E-05	1	1	3	Q92896	Q92896 [990-100	0	1624.9229
[K].LLLQVQ	1xTMTpro [K	8.868E-05	7.389E-05	3	3	3	P12236; P05	P12236 [34-43]; P	0	1745.0929
[K].LNCQVI	2xCarbamido	0.0001849	7.389E-05	1	1	1	Q06830	Q06830 [69-92]	0	3361.7429
[R].LNDGNE	1xTMTpro [K	6.287E-05	7.389E-05	1	1	2	Q01082	Q01082 [2279-22	0	2020.0995
[K].LNDLEE	1xTMTpro [K	0.0001166	7.389E-05	1	1	1	P35908	P35908 [442-453]	0	1980.1257
[R].LNDYIFS	1xTMTpro [K	0.0001226	7.389E-05	1	1	1	P54136	P54136 [513-522]	0	1870.0242
[K].LNEAQP	1xTMTpro [N	0.0001036	7.389E-05	1	1	1	P56537	P56537 [224-237]	0	1822.9652
[K].INEELES	1xTMTpro [K	6.864E-05	7.389E-05	1	1	1	O00193	O00193 [76-91]	0	2537.2685

[R].INEFSIS	1xCarbamid	6.853E-05	7.389E-05	1	1	2	Q06828	Q06828 [325-344]	0	2900.536
[K].LNEIEGT	1xTMTpro [K	0.0001194	7.389E-05	1	1	2	P11047	P11047 [1535-154	0	1739.0195
[K].INEINTE	1xTMTpro [K	8.209E-05	7.389E-05	1	1	1	Q8WYA0	Q8WYA0 [316-32	0	2279.3102
[K].LNENHS	1xTMTpro [K	0.0001533	7.389E-05	1	1	3	Q13418	Q13418 [199-209]	0	1935.058
[R].INENTGS	1xTMTpro [N	8.786E-05	7.389E-05	1	1	2	P55290	P55290 [190-201]	0	1580.8563
[R].LNEQAS	1xTMTpro [K	0.0001023	7.389E-05	1	1	2	Q01105	Q01105 [58-68]	0	1882.0777
[K].LNEQQS	1xTMTpro [N	0.0002035	7.389E-05	1	1	2	Q14683	Q14683 [1010-10	0	1518.8559
[R].LNCEDIE	2xCarbamid	0.0001611	7.389E-05	1	1	7	Q12805	Q12805 [290-299]	0	1627.7375
[K].INEWLTL	1xTMTpro [K	0.0001297	7.389E-05	1	1	2	Q14204	Q14204 [1698-17	0	1853.1028
[K].LNHQLE	1xTMTpro [K	0.0001547	7.389E-05	1	1	2	P06727	P06727 [234-246]	0	2167.2189
[K].LNHQLE	1xOxidation	0.0001058	7.389E-05	1	1	1	P06727	P06727 [234-246]	0	2183.2138
[R].LNHVAIA	1xTMTpro [K	8.937E-05	7.389E-05	1	1	2	Q96PE7	Q96PE7 [48-60]	0	2027.2145
[R].INIEHAL	1xTMTpro [N	0.0001623	7.389E-05	1	1	1	Q8WXH0	Q8WXH0 [92-102	0	1630.96
[K].LNLEHIA	1xTMTpro [K	6.653E-05	7.389E-05	1	1	2	Q96QK1	Q96QK1 [383-396	0	2078.2101
[K].LNIGDLQ	1xTMTpro [K	0.0001202	7.389E-05	1	1	1	Q9UNM6	Q9UNM6 [123-13	0	1709.0453
[K].LNIGGG	1xTMTpro [N	0.0001268	7.389E-05	1	1	1	Q86XP3	Q86XP3 [651-661	0	1380.7919
[R].LNIISPDI	1xCarbamid	7.416E-05	7.389E-05	1	1	1	P17813	P17813 [572-585]	0	2113.1819
[R].LNLNNT	1xTMTpro [K	0.0001581	7.389E-05	1	1	2	P07814	P07814 [426-435]	0	1724.0562
[K].LNIPATN	1xTMTpro [N	0.0001971	7.389E-05	1	1	1	P78330	P78330 [123-134]	0	1633.9345
[R].LNIPVSQ	1xTMTpro [N	8.311E-05	7.389E-05	1	2	4	P05023	P05023 [648-658]	0	1540.913
[K].LNLSCIH	1xCarbamid	8.638E-05	7.389E-05	1	1	1	Q9Y2X3	Q9Y2X3 [102-117	0	2186.1745
[K].LNLTEVE	1xTMTpro [N	0.0001492	7.389E-05	1	1	2	Q969Y2	Q969Y2 [153-173	0	2626.4007
[R].LNFSTPT	1xCarbamid	0.0001397	7.389E-05	1	1	1	O15067	O15067 [94-109]	0	2100.1079
[R].INLWHLE	1xTMTpro [N	0.0001752	7.389E-05	2	2	1	Q66LE6; P6	Q66LE6 [206-216	0	1713.9607
[R].LNAYTG	1xTMTpro [N	0.0001361	7.389E-05	1	1	1	P98095	P98095 [1135-114	0	1700.9655
[R].LNAIYQN	1xTMTpro [K	7.695E-05	7.389E-05	1	1	3	P00390	P00390 [154-164]	0	1900.1148
[K].IMGVAG	1xOxidation	0.000114	7.389E-05	1	1	1	Q8WWY3	Q8WWY3 [231-24	0	1885.1072
[K].LMHVAY	1xTMTpro [K	9.291E-05	7.389E-05	1	1	2	Q14204	Q14204 [401-411]	0	2004.0756
[K].LMIEMD	1xTMTpro [K	7.531E-05	7.389E-05	1	1	4	P06733	P06733 [93-103]	0	1889.0004
[K].LMIEMD	1xOxidation	0.0001498	7.389E-05	1	1	19	P06733	P06733 [93-103]	0	1904.9953
[K].LMIETDA	1xCarbamid	0.000134	7.389E-05	1	1	1	Q6P1N9	Q6P1N9 [217-229	0	2128.1426
[K].IMLGDEA	1xOxidation	8.745E-05	7.389E-05	1	1	1	Q9BW27	Q9BW27 [286-29	0	2055.1652
[K].IMLLDEF	1xTMTpro [K	0.0001533	7.389E-05	1	1	2	O00186	O00186 [34-43]	0	1819.0531

[K].IMIMTDQ	2xOxidation [	6.885E-05	7.389E-05	2	2	1	P11388; Q02	P11388 [536-550]	0	2372.2082
[K].IMLNTPE	1xTMTpro [K	8.466E-05	7.389E-05	1	1	2	O00231	O00231 [259-274]	0	2323.3187
[R].LMMDPL	1xTMTpro [N	0.0001448	7.389E-05	1	1	3	O43390	O43390 [196-206]	0	1565.8099
[R].IMNEEDI	1xTMTpro [K	0.0001397	7.389E-05	1	1	1	Q96A33	Q96A33 [444-454	1	1997.0617
[R].IMNTFSV	1xTMTpro [K	0.0001576	7.389E-05	2	3	3	Q13885; Q9I	Q13885 [163-174]	0	1960.0892
[R].IMNTFSV	1xOxidation [	0.0001492	7.389E-05	2	3	3	Q13885; Q9I	Q13885 [163-174]	0	1976.0841
[R].LNATASI	1xTMTpro [N	0.0001281	7.389E-05	1	1	1	O76024	O76024 [27-37]	0	1535.8349
[R].IMNTFSV	1xTMTpro [K	0.0001665	7.389E-05	3	4	7	Q13509; P07	Q13509 [163-174]	0	1928.1171
[R].IMNVIGE	1xTMTpro [N	9.708E-05	7.389E-05	1	1	5	P06576	P06576 [144-155]	0	1689.9165
[K].LMQADL	1xTMTpro [N	6.928E-05	7.389E-05	1	1	1	O00534	O00534 [359-378]	0	2520.3815
[K].LMSSGN	1xTMTpro [N	7.851E-05	7.389E-05	1	1	1	P23786	P23786 [306-316]	0	1526.7804
[K].LMSSLPI	1xCarbamido	0.0001273	7.389E-05	1	1	1	Q96PU8	Q96PU8 [27-43]	0	2339.196
[K].LMSSLPI	1xCarbamido	9.105E-05	7.389E-05	1	1	1	Q96PU8	Q96PU8 [27-43]	0	2355.1909
[R].LMTDTIN	1xCarbamido	0.0001256	7.389E-05	1	1	1	O43776	O43776 [426-439]	0	2009.0731
[K].LMTTGN	1xTMTpro [N	0.0002019	7.389E-05	1	1	1	Q9H832	Q9H832 [167-176	0	1410.7694
[R].IMVANIE	1xTMTpro [N	6.509E-05	7.389E-05	1	1	4	O75396	O75396 [148-159]	0	1718.9794
[R].IMVANIE	1xOxidation [	0.00011	7.389E-05	1	1	2	O75396	O75396 [148-159]	0	1734.9743
[K].IMVDMLI	1xOxidation [	9.831E-05	7.389E-05	1	1	2	P17655	P17655 [579-591]	0	1992.0273
[K].LMWIPV	1xOxidation [	0.0001983	7.389E-05	1	1	2	Q05707	Q05707 [656-665]	0	1788.0374
[R].IMYTVFE	1xTMTpro [N	0.0001756	7.389E-05	1	1	1	P41250	P41250 [584-596]	0	1984.0434
[K].INAANYA	1xTMTpro [K	0.0001091	7.389E-05	1	1	2	Q96IU4	Q96IU4 [142-151]	0	1658.9721
[R].IMNTFSV	1xOxidation [	0.0001271	7.389E-05	3	4	8	Q13509; P07	Q13509 [163-174]	0	1944.112
[K].IMGVAG	1xTMTpro [K	8.545E-05	7.389E-05	1	1	2	Q8WWY3	Q8WWY3 [231-24	0	1869.1123
[K].LNMGITI	1xTMTpro [N	6.407E-05	7.389E-05	2	2	2	P0C0L4; P0C	P0C0L4 [326-337]	0	1634.9219
[K].LNPNFLV	1xTMTpro [K	9.878E-05	7.389E-05	1	1	2	Q13228	Q13228 [438-448]	0	1872.0875
[R].IPDQLVI	1xOxidation [	0.0001922	7.389E-05	1	1	2	P49755	P49755 [117-127]	0	1909.1324
[R].IPDSEMI	1xTMTpro [K	0.0001075	7.389E-05	1	1	1	Q9Y6X4	Q9Y6X4 [492-507	0	2405.1708
[K].LPDVYG	1xTMTpro [K	9.602E-05	7.389E-05	1	1	2	P39656	P39656 [381-391]	0	1921.1079
[K].LPDYKP	2xTMTpro [K	7.371E-05	7.389E-05	1	1	1	Q00535	Q00535 [233-254]	0	3405.9312
[R].LPEASQ	1xTMTpro [K	9.878E-05	7.389E-05	1	1	1	Q13330	Q13330 [516-527]	0	1890.1556
[K].IPEDQHA	1xTMTpro [K	0.0001635	7.389E-05	1	1	1	O94874	O94874 [720-730]	0	1871.1246
[R].LPEDQP	1xTMTpro [N	6.257E-05	7.389E-05	1	1	1	Q96JQ0	Q96JQ0 [1554-15	0	2071.1619
[R].LPEEHIF	1xTMTpro [N	6.267E-05	7.389E-05	1	1	1	Q5NDL2	Q5NDL2 [42-55]	0	2067.1095

[R].LPEEPVI	1xTMTpro [K	0.0001605	7.389E-05	1	1	1	Q15811	Q15811 [329-344]	0	2532.3689
[R].LPEFADV	1xTMTpro [K	7.026E-05	7.389E-05	1	1	1	P28067	P28067 [82-102]	0	2969.554
[R].LPEGDL	2xTMTpro [K	8.134E-05	7.389E-05	1	2	4	P63241	P63241 [114-126]	1	2368.3904
[K].IPEISIQD	1xOxidation [	7.948E-05	7.389E-05	1	1	2	P21333	P21333 [2166-218	0	2626.4253
[R].IPEPQKF	2xTMTpro [K	7.382E-05	7.389E-05	1	1	1	Q07157	Q07157 [1326-13	0	2896.744
[R].IPDQLVI	1xTMTpro [K	8.545E-05	7.389E-05	1	1	2	P49755	P49755 [117-127]	0	1893.1375
[R].IPEPVHL	1xTMTpro [N	0.0001021	7.389E-05	1	1	1	Q9P2A4	Q9P2A4 [188-201	0	1829.0604
[K].LPETNLF	1xTMTpro [N	6.653E-05	7.389E-05	1	1	2	Q15046	Q15046 [408-420]	0	1882.9717
[K].IPGEKDS	1xCarbamido	9.335E-05	7.389E-05	1	1	2	P08174	P08174 [72-83]	1	2271.3563
[K].LPGGVQ	1xTMTpro [N	7.428E-05	7.389E-05	1	1	4	Q7Z6Z7	Q7Z6Z7 [1226-12	0	1935.0772
[K].LPGLVFL	1xTMTpro [K	0.0001907	7.389E-05	1	1	3	P51888	P51888 [149-159]	0	1918.1368
[K].LPGLVFL	1xOxidation [	0.0001739	7.389E-05	1	1	7	P51888	P51888 [149-159]	0	1934.1317
[K].IPGNWF	1xTMTpro [N	0.0001366	7.389E-05	1	1	2	Q14315	Q14315 [2160-21	0	1967.0128
[K].IPGTCCD	4xCarbamido	8.786E-05	7.389E-05	1	1	1	P04275	P04275 [2711-27	0	2702.1486
[R].LPHLEAV	1xTMTpro [N	0.0001297	7.389E-05	1	1	1	Q7Z2E3	Q7Z2E3 [33-43]	0	1507.928
[K].IPKEEQE	1xTMTpro [K	0.0001659	7.389E-05	1	1	2	O94855	O94855 [472-484]	1	2138.1949
[R].LPLAVV	1xTMTpro [K	6.139E-05	7.389E-05	1	1	1	Q16181	Q16181 [244-260]	0	2332.4096
[R].LPIDENQ	1xTMTpro [K	6.918E-05	7.389E-05	1	1	2	P08473	P08473 [262-275]	0	2236.2503
[R].LPIDENQ	1xOxidation [	6.726E-05	7.389E-05	1	1	1	P08473	P08473 [262-275]	0	2252.2452
[K].LPLFPEF	1xTMTpro [N	0.0001138	7.389E-05	1	1	2	P26358	P26358 [1324-13	0	1937.1332
[K].IPEQSVL	1xCarbamido	6.695E-05	7.389E-05	1	1	1	P00505	P00505 [201-227]	0	3700.9877
[R].LNPINTT	1xTMTpro [K	0.0001511	7.389E-05	1	1	2	Q9UGV2	Q9UGV2 [277-28	0	1735.0973
[R].LPDIFEA	1xTMTpro [N	8.184E-05	7.389E-05	1	1	4	P08729	P08729 [137-149]	0	1747.0073
[R].LPDGTS	1xTMTpro [N	0.0001271	7.389E-05	1	1	2	Q04323	Q04323 [220-231]	0	1639.8975
[K].INPSSMF	1xTMTpro [K	0.0001707	7.389E-05	1	1	1	P06737	P06737 [558-569]	0	1973.1022
[K].INQEEQI	1xOxidation [	0.0001182	7.389E-05	1	1	1	P50453	P50453 [187-205]	0	2994.5309
[K].INQFYGA	1xTMTpro [N	0.0001922	7.389E-05	1	1	1	Q9NUB1	Q9NUB1 [381-39	0	1640.908
[K].INQLSEE	1xTMTpro [K	7.485E-05	7.389E-05	1	1	2	Q14980	Q14980 [313-326]	0	2202.1898
[K].INQMATA	1xTMTpro [N	9.572E-05	7.389E-05	1	1	1	Q9UQE7	Q9UQE7 [144-15	0	1635.8444
[K].LNQNLN	1xTMTpro [K	8.159E-05	7.389E-05	1	1	2	O00499	O00499 [259-272]	0	2177.2785
[K].LNQPPE	1xTMTpro [K	7.474E-05	7.389E-05	1	1	4	O43684	O43684 [9-21]	0	1992.1257
[K].INQQEEI	1xTMTpro [N	7.405E-05	7.389E-05	1	1	1	Q8TC07	Q8TC07 [289-302	0	1964.0408
[R].LNQVELI	1xTMTpro [N	0.000153	7.389E-05	1	1	1	Q9UG56	Q9UG56 [126-13	0	1708.9818

[K].INSDSEE	1xTMTpro [N	0.0001476	7.389E-05	1	1	1	P46939	P46939 [610-620]	0	1595.8196
[K].INSFYAF	1xTMTpro [K	0.0001184	7.389E-05	1	1	2	Q8TED1	Q8TED1 [44-53]	0	1826.0344
[K].INSITVDI	1xCarbamido	0.0001026	7.389E-05	1	1	2	Q01518	Q01518 [367-376]	0	1771.9868
[K].INTDEIM	1xTMTpro [K	6.417E-05	7.389E-05	1	1	2	P02461	P02461 [1229-123	0	1873.0596
[K].LPDGYE	1xTMTpro [K	0.0001556	7.389E-05	1	1	1	P09382	P09382 [101-112]	1	2091.1519
[K].LNTQEIF	1xTMTpro [N	6.56E-05	7.389E-05	1	1	2	Q14204	Q14204 [693-704]	0	1811.9247
[R].LNVFAKI	2xTMTpro [K	7.291E-05	7.389E-05	1	1	2	P50895	P50895 [141-156]	0	2656.549
[R].LNVFKNI	2xTMTpro [K	9.105E-05	7.389E-05	1	1	2	P14866	P14866 [273-303]	2	4404.2425
[K].LNVIDDD	2xTMTpro [K	7.337E-05	7.389E-05	1	1	1	O75886	O75886 [272-284]	1	2442.4272
[K].INVNEIFY	1xTMTpro [N	7.371E-05	7.389E-05	2	3	1	P61224; P62	P61224 [152-163]	0	1799.0023
[K].INVNVNS	1xTMTpro [K	7.439E-05	7.389E-05	1	1	1	O60687	O60687 [324-341]	0	2603.4325
[R].LNYGIPA	1xTMTpro [K	0.0001249	7.389E-05	1	1	2	P14923	P14923 [478-487]	0	1696.0653
[R].IPAASAA	1xTMTpro [N	9.176E-05	7.389E-05	1	1	1	Q9UQ35	Q9UQ35 [2232-22	0	1718.9543
[R].LPAGAIH	1xTMTpro [N	0.0001188	7.389E-05	1	1	1	Q13438	Q13438 [75-84]	0	1413.8286
[K].LPANQY	1xTMTpro [N	0.00015	7.389E-05	1	1	1	P29144	P29144 [402-412]	0	1626.8559
[K].LPAQLAV	1xTMTpro [N	8.799E-05	7.389E-05	1	1	1	Q70J99	Q70J99 [715-727]	0	1829.0241
[K].LPATEKI	2xTMTpro [K	0.0001842	7.389E-05	1	1	2	Q9Y4L1	Q9Y4L1 [878-889	0	2208.4148
[K].LPCEMD	1xCarbamido	6.939E-05	7.389E-05	1	1	3	Q9UGI8	Q9UGI8 [194-204	0	1853.9745
[K].LPDATPT	1xTMTpro [K	0.0002035	7.389E-05	1	1	3	P02774	P02774 [430-440]	0	1764.0399
[R].INVDIPE	1xTMTpro [N	9.091E-05	7.389E-05	1	1	1	Q9H9A6	Q9H9A6 [58-77]	0	2521.2854
[K].MALDIEL	1xTMTpro [N	9.234E-05	7.389E-05	1	2	10	P08670	P08670 [391-401]	0	1599.8735
[R].IMGIPEE	1xOxidation [	0.0001161	7.389E-05	1	1	7	P35579	P35579 [328-341]	0	1936.0203
[R].IMFVDPS	1xTMTpro [N	0.0001016	7.389E-05	2	2	3	O95994; Q8	O95994 [128-138]	0	1581.8994
[R].ILQDSLG	1xCarbamido	8.146E-05	7.389E-05	1	3	1	P33176	P33176 [285-295]	0	1536.8123
[R].ILQEAQN	1xTMTpro [K	0.0001635	7.389E-05	1	1	1	Q99459	Q99459 [362-380]	0	2720.5512
[R].ILQEHEC	1xTMTpro [K	0.0001033	7.389E-05	1	1	3	Q14152	Q14152 [624-632]	0	1746.0405
[R].LLQEIVD	1xCarbamido	0.000149	7.389E-05	1	1	1	O15269	O15269 [411-422]	0	1822.9475
[K].ILQEYITC	1xTMTpro [K	0.0002035	7.389E-05	1	1	1	Q9Y6Q5	Q9Y6Q5 [130-141	0	2073.1836
[K].ILQGGVC	1xTMTpro [N	9.035E-05	7.389E-05	1	1	1	P48729	P48729 [66-77]	0	1563.9654
[R].ILQLCMC	1xCarbamido	0.0001653	7.389E-05	1	1	2	P15311	P15311 [280-293]	0	2098.0567
[R].ILQILNTC	1xTMTpro [N	0.0001484	7.389E-05	1	1	3	Q96RW7	Q96RW7 [2927-2	0	1902.1343
[K].LIQLMEE	1xTMTpro [K	0.0001918	7.389E-05	1	1	1	Q92841	Q92841 [406-417]	0	2056.1678
[K].ILQINKDI	1xCarbamido	6.685E-05	7.389E-05	1	1	1	O76094	O76094 [37-51]	1	2680.5636

[R].LIQIPQLI	1xTMTpro [N	7.348E-05	7.389E-05	1	1	1	O00291	O00291 [285-300]		0	2193.2715
[K].LLQMQP	1xTMTpro [K	8.678E-05	7.389E-05	1	2	1	O00203	O00203 [468-481]		0	2214.2924
[K].LIQNNHY	1xOxidation [	0.000181	7.389E-05	1	1	1	Q13813	Q13813 [533-547]		0	2095.0562
[R].LLQCYPI	1xCarbamido	0.0001033	7.389E-05	1	1	1	O95782	O95782 [264-277]		0	2209.2183
[R].LLQQQN	1xTMTpro [K	0.0001845	7.389E-05	1	1	2	O15357	O15357 [247-267]		0	3016.7174
[K].IIQTPGLY	1xTMTpro [K	7.815E-05	7.389E-05	1	1	1	Q9NP72	Q9NP72 [169-183		0	2365.3007
[K].IISALSG(	1xTMTpro [N	0.000171	7.389E-05	1	1	1	A2RRP1	A2RRP1 [2044-20		0	1774.9982
[R].LLSDSLF	1xTMTpro [N	7.303E-05	7.389E-05	1	1	2	Q9Y490	Q9Y490 [1223-12		0	2338.221
[K].LLSELDQ	1xTMTpro [N	0.0001944	7.389E-05	1	1	1	O95793	O95793 [552-565]		0	1951.0126
[K].LLSESA(	1xTMTpro [K	0.000187	7.389E-05	1	1	1	Q9H3N1	Q9H3N1 [224-233		0	1694.0344
[K].ILSGDPY	1xCarbamido	0.0002003	7.389E-05	1	1	2	P43251	P43251 [364-373]		0	1789.965
[R].LLSGLQI	2xTMTpro [K	0.0001116	7.389E-05	1	1	1	Q92797	Q92797 [649-660]		0	2268.3743
[K].LLSGYLF	1xTMTpro [N	0.0001881	7.389E-05	1	1	1	Q8WUA7	Q8WUA7 [234-24		0	1621.9233
[R].LLSLDPS	1xTMTpro [N	0.0002035	7.389E-05	1	1	1	O15460	O15460 [233-242]		0	1470.8236
[R].IISLETYN	1xTMTpro [N	0.0001241	7.389E-05	1	1	3	Q15149	Q15149 [3794-38		0	1638.975
[K].IISIFSGT	1xTMTpro [K	0.0001288	7.389E-05	1	1	2	Q13177	Q13177 [53-62]		0	1703.0235
[R].ILSISADI	1xTMTpro [K	7.815E-05	7.389E-05	1	1	2	P61978	P61978 [87-102]		0	2323.398
[K].LLSNDEY	1xTMTpro [K	0.0001881	7.389E-05	1	1	2	Q92734	Q92734 [48-57]		0	1740.0399
[R].LLQQVS	1xTMTpro [N	0.0001556	7.389E-05	1	1	1	Q14152	Q14152 [439-454]		0	2243.2355
[R].LLSPFMI	1xTMTpro [N	0.0001476	7.389E-05	1	1	2	P26640	P26640 [1059-10		0	2258.2214
[R].LLQAQL(	1xTMTpro [K	0.000144	7.389E-05	1	1	1	Q96AQ6	Q96AQ6 [284-293		0	1749.0878
[R].LLPYLAE	1xTMTpro [N	0.0001713	7.389E-05	1	1	4	P13224	P13224 [128-138]		0	1635.9277
[K].LLLSEEE	1xTMTpro [K	0.0001012	7.389E-05	1	1	2	Q9HAB8	Q9HAB8 [278-287		0	1811.0658
[K].LLLTAQ(	1xTMTpro [K	8.611E-05	7.389E-05	1	1	1	Q16512	Q16512 [162-174]		0	2097.2233
[K].ILLTEPPI	1xTMTpro [K	0.0001716	7.389E-05	1	1	3	P61160	P61160 [107-118]		0	1962.1589
[K].LLLWAQ	1xTMTpro [K	0.0001759	7.389E-05	1	1	1	Q9NWU2	Q9NWU2 [198-20		0	2079.2094
[K].LIIWDSY	1xTMTpro [K	6.832E-05	7.389E-05	3	3	2	Q9HAV0; P6	Q9HAV0 [79-89];		0	1962.1192
[K].LLIYSNN	1xTMTpro [N	9.847E-05	7.389E-05	1	2	1	P01700	P01700 [66-81]		0	2133.1736
[K].LIMAMQ	1xOxidation [	0.000149	7.389E-05	1	1	1	Q8TEX9	Q8TEX9 [206-220		0	2311.3261
[K].ILMDKPE	2xTMTpro [K	9.364E-05	7.389E-05	1	1	1	P55786	P55786 [559-571]		0	2442.4644
[K].ILMDKPE	1xOxidation [	0.0001669	7.389E-05	1	1	1	P55786	P55786 [559-571]		0	2458.4593
[K].IIMLFTD(	1xTMTpro [N	0.0001397	7.389E-05	1	1	1	P54289	P54289 [357-368]		0	1684.8899
[R].ILMVGLD	1xTMTpro [K	0.0001161	7.389E-05	3	4	3	P61204; P18	P61204 [20-30]; F		0	1696.0323

[R].ILMVGLD	1xOxidation	8.558E-05	7.389E-05	3	4	3	P61204; P18	P61204 [20-30]; F	0	1712.0272
[K].LIMVQHV	1xCarbamido	0.0001281	7.389E-05	1	1	3	O00584	O00584 [37-50]	0	2394.2805
[R].LLQAGF:	1xTMTpro [K	0.0001072	7.389E-05	1	1	2	Q9ULA0	Q9ULA0 [52-61]	0	1714.0395
[K].IIMYDQN	1xTMTpro [N	6.653E-05	7.389E-05	1	1	1	Q6PD62	Q6PD62 [123-134	0	1776.975
[R].IINDLLQ:	1xTMTpro [N	0.0001528	7.389E-05	1	1	3	Q92945	Q92945 [385-394]	0	1488.9069
[K].ILNDLSS	1xTMTpro [N	0.0001277	7.389E-05	1	1	2	P16220	P16220 [123-136]	0	1757.9717
[K].IINPMGL	1xTMTpro [K	6.227E-05	7.389E-05	1	1	3	Q9H9J2	Q9H9J2 [234-246	0	2077.2587
[K].LLPAFNT	1xTMTpro [K	0.0001694	7.389E-05	1	1	2	Q9NR34	Q9NR34 [291-303	0	1977.2029
[K].LIPDSIGI	2xTMTpro [K	0.0001665	7.389E-05	1	1	2	P61247	P61247 [188-199]	1	2240.3682
[R].IIPGFMC	1xCarbamido	7.348E-05	7.389E-05	1	2	7	P62937	P62937 [56-69]	0	1902.9525
[K].LLPGLDI	1xTMTpro [K	9.119E-05	7.389E-05	1	1	1	P08473	P08473 [674-683]	0	1728.0664
[R].ILPGPHF	1xTMTpro [N	0.0001425	7.389E-05	1	1	2	Q96AX1	Q96AX1 [517-526	0	1498.8337
[K].LLPLVSD	1xTMTpro [N	6.832E-05	7.389E-05	1	1	4	P11216	P11216 [522-533]	0	1705.0219
[K].LLPPFKF	1xTMTpro [K	7.695E-05	7.389E-05	1	1	1	P31751	P31751 [422-437]	0	2435.4154
[K].ILPVLCG	1xCarbamido	6.407E-05	7.389E-05	1	1	1	Q96KG9	Q96KG9 [507-520	0	2162.275
[R].IIPVLLEH	1xTMTpro [N	8.965E-05	7.389E-05	1	1	1	P18858	P18858 [516-527]	0	1693.0332
[K].LLPYEQ:	1xTMTpro [K	0.0001239	7.389E-05	1	1	1	Q2M389	Q2M389 [68-81]	0	2254.3554
[K].IINADSEI	1xTMTpro [K	0.0001458	7.389E-05	1	1	1	P35998	P35998 [101-110]	0	1709.9565
[R].IMGIPEE	1xTMTpro [N	8.159E-05	7.389E-05	1	1	3	P35579	P35579 [328-341]	0	1920.0254
[K].LLSPVVF	1xTMTpro [K	0.0001463	7.389E-05	1	1	2	Q8N1F7	Q8N1F7 [645-660	0	2286.3677
[K].LLSSEDI	1xTMTpro [N	9.482E-05	7.389E-05	1	1	2	P53582	P53582 [130-140]	0	1553.8164
[R].LLVVDQI	1xTMTpro [N	0.0001031	7.389E-05	1	1	1	Q15599	Q15599 [85-97]	0	1863.003
[R].LLVYDTS	1xTMTpro [K	0.000142	7.389E-05	1	1	1	Q9HBG6	Q9HBG6 [35-50]	0	2384.3932
[K].LIYDFIEI	1xTMTpro [N	9.941E-05	7.389E-05	1	1	2	P42768	P42768 [289-304]	0	2142.1402
[R].LLYEALV	1xCarbamido	0.000182	7.389E-05	1	1	2	Q7L2H7	Q7L2H7 [167-176	0	1832.0483
[K].IIYGDPV	1xTMTpro [N	6.139E-05	7.389E-05	1	1	1	Q63HN8	Q63HN8 [4717-47	0	2042.1758
[K].LIYGQYC	3xCarbamido	6.367E-05	7.389E-05	1	1	1	P05107	P05107 [528-543]	0	2398.0797
[K].LLYLLES	1xTMTpro [N	0.0001253	7.389E-05	1	1	2	Q8N2F6	Q8N2F6 [101-116	0	2207.2494
[K].ILYLTPE	2xTMTpro [K	8.146E-05	7.389E-05	1	1	2	P62495	P62495 [343-354]	1	2389.4159
[R].ILYIVASI	1xTMTpro [N	9.878E-05	7.389E-05	1	1	1	Q9ULT8	Q9ULT8 [2006-20	0	1700.9542
[K].IIYIVHDE	1xTMTpro [K	9.863E-05	7.389E-05	1	1	3	P25788	P25788 [197-206]	0	1837.1079
[R].ILYMTDE	1xTMTpro [K	6.488E-05	7.389E-05	1	1	3	P00403	P00403 [83-98]	0	2460.3551
[K].LLYNNV:	1xTMTpro [N	0.000153	7.389E-05	1	1	4	Q00610	Q00610 [1216-12	0	1600.8767

[R].LLYPPET	1xTMTpro [N	0.0001044	7.389E-05	1	1	3	P41240	P41240 [95-107]	0	1822.0798
[R].LLVVDPI	1xTMTpro [K	0.0001222	7.389E-05	1	1	2	O14745	O14745 [88-101]	0	2235.2728
[K].ILYSQCC	1xCarbamido	0.0001789	7.389E-05	2	2	3	P60660; P14	P60660 [27-37]; P	0	1645.8361
[K].IMADSGI	1xOxidation	0.0001012	7.389E-05	1	1	3	P07996	P07996 [1131-114	0	1833.9912
[K].LMAEAL	1xTMTpro [N	0.0001448	7.389E-05	1	1	1	Q04759	Q04759 [287-302	0	2067.0898
[K].IMAISAG	1xTMTpro [K	8.965E-05	7.389E-05	1	1	1	Q99959	Q99959 [822-835	0	2020.1029
[K].IMDATNI	1xTMTpro [K	6.387E-05	7.389E-05	1	1	1	P40763	P40763 [659-679]	0	2983.7074
[K].LMDEVA	1xTMTpro [N	9.847E-05	7.389E-05	1	1	2	O00154	O00154 [253-264	0	1548.8739
[K].LMDHVG	1xTMTpro [K	0.0001168	7.389E-05	1	1	1	P04844	P04844 [218-229]	0	1935.0865
[R].IMDLLGI	1xTMTpro [K	0.0001284	7.389E-05	1	1	3	P21397	P21397 [230-239]	0	1740.0221
[R].LMDLLG	1xTMTpro [K	0.0001228	7.389E-05	1	1	2	P54136	P54136 [462-471]	0	1697.0163
[R].LMDLLG	1xTMTpro [K	0.0001136	7.389E-05	1	1	1	P54136	P54136 [462-472]	1	1853.1174
[R].IMEFTTT	1xTMTpro [K	7.18E-05	7.389E-05	1	1	2	P78527	P78527 [1341-135	0	2576.3926
[K].LMELHG	1xTMTpro [K	0.0001178	7.389E-05	1	1	1	P61247	P61247 [228-240]	0	1940.0403
[K].LMEWTS	1xTMTpro [N	0.0001956	7.389E-05	2	2	4	P48594; P29	P48594 [266-276]	0	1712.8783
[K].LMFNSP	1xTMTpro [N	6.499E-05	7.389E-05	1	1	2	Q16401	Q16401 [430-444	0	2077.0748
[K].IMADSGI	1xTMTpro [K	0.000145	7.389E-05	1	1	3	P07996	P07996 [1131-114	0	1817.9963
[R].ILSQCG	1xCarbamido	6.726E-05	7.389E-05	1	1	1	Q9UKK3	Q9UKK3 [1015-10	0	2412.2877
[K].LLVTPTS	1xTMTpro [K	0.0001852	7.389E-05	1	1	2	Q05707	Q05707 [62-71]	0	1580.9867
[RK].LLVSAS	1xTMTpro [K	0.0001803	7.389E-05	3	4	2	Q9HAV0; P6	Q9HAV0 [69-78];	0	1625.9718
[K].IISSNQE	1xTMTpro [N	0.0001165	7.389E-05	1	1	2	Q9UHD2	Q9UHD2 [345-357	0	1825.9979
[R].LLSTDP\	1xTMTpro [K	0.0001056	7.389E-05	1	1	3	P63010	P63010 [529-538]	0	1653.0078
[R].LLSTVA(	1xTMTpro [K	9.909E-05	7.389E-05	1	1	4	Q14554	Q14554 [66-75]	0	1638.0446
[R].ILSVQGT	1xTMTpro [K	0.0001299	7.389E-05	1	1	1	Q9H8H0	Q9H8H0 [112-125	0	2152.3237
[K].LITASET	1xTMTpro [N	0.0001605	7.389E-05	1	1	1	Q96AY4	Q96AY4 [1838-18	0	1822.0241
[K].ILTDIMW	1xTMTpro [K	0.0001232	7.389E-05	1	1	3	Q13057	Q13057 [439-450	0	2022.2317
[K].ILTDVLF	1xCarbamido	0.000106	7.389E-05	1	1	3	Q92609	Q92609 [185-195	0	1674.9208
[K].LLTECPI	1xCarbamido	6.653E-05	7.389E-05	1	1	1	P55060	P55060 [849-863]	0	2437.2309
[K].LLTECPI	1xCarbamido	0.0001471	7.389E-05	1	1	1	P55060	P55060 [849-863]	0	2453.2258
[R].IITEGFE	1xTMTpro [K	0.0001239	7.389E-05	1	1	2	P40227	P40227 [118-127]	0	1686.9922
[R].LLTFYNI	1xCarbamido	9.863E-05	7.389E-05	1	1	1	Q9H583	Q9H583 [1933-19	0	2279.2601
[R].LITGASD	1xTMTpro [N	0.000126	7.389E-05	1	1	1	Q9UNX4	Q9UNX4 [206-216	0	1465.8181
[K].ILTHVAE	1xTMTpro [K	0.0001948	7.389E-05	1	1	1	Q9UH99	Q9UH99 [492-502	0	1835.0705

[K].LLVSQG.	2xTMTpro [K	7.985E-05	7.389E-05	1	1	2	O75832	O75832 [190-206]		1	2833.6491
[R].LITLEEE	1xTMTpro [K	0.0001427	7.389E-05	1	1	6	P07237	P07237 [317-326]		0	1815.0429
[R].LLTIGDA	1xTMTpro [N	0.0001484	7.389E-05	1	1	1	Q86X55	Q86X55 [37-49]		0	1703.9611
[K].LLTSNDV	1xTMTpro [K	0.0001051	7.389E-05	1	1	1	O15229	O15229 [247-259]		0	2134.204
[R].LITTQQV	1xTMTpro [K	0.0001182	7.389E-05	1	1	4	P00846	P00846 [42-51]		0	1852.1552
[K].ILVEDEL	1xTMTpro [N	7.543E-05	7.389E-05	1	2	1	Q6P996	Q6P996 [379-393		0	2006.1493
[R].ILVELAT	1xTMTpro [K	0.0001359	7.389E-05	1	1	2	Q9NZM1	Q9NZM1 [537-54		0	1884.1702
[R].ILVLDDT	1xTMTpro [N	0.0001593	7.389E-05	1	1	1	P09543	P09543 [118-128]		0	1628.8927
[K].LLVLYSG	1xTMTpro [N	7.235E-05	7.389E-05	1	1	1	Q9H3U1	Q9H3U1 [822-836		0	2067.1293
[R].ILVNACC	2xCarbamido	0.0001313	7.389E-05	1	1	1	O75828	O75828 [221-232]		0	1936.1004
[K].LLVPAAE	1xTMTpro [K	0.0001417	7.389E-05	1	1	1	Q96PU8	Q96PU8 [178-190		0	1950.1403
[R].LLVPLVF	1xTMTpro [N	7.102E-05	7.389E-05	1	1	3	Q9BSJ8	Q9BSJ8 [308-323		0	2093.2653
[R].LLVPYLN	1xTMTpro [N	0.0001094	7.389E-05	1	1	2	Q16836	Q16836 [222-232]		0	1621.9671
[R].ILVQQFE	1xTMTpro [N	6.438E-05	7.389E-05	1	1	1	Q9BVL2	Q9BVL2 [348-361		0	2096.1823
[R].LLVQSIE	1xTMTpro [N	8.034E-05	7.389E-05	1	1	2	Q96JC1	Q96JC1 [274-285		0	1756.0764
[R].LITLEEE	1xOxidation [	0.0001338	7.389E-05	1	1	4	P07237	P07237 [317-326]		0	1831.0378
[K].IGLFGG/	1xTMTpro [K	7.875E-05	7.389E-05	1	1	2	P06576	P06576 [202-212]		0	1583.9765
[K].MALDIEL	1xOxidation [	0.0001533	7.389E-05	1	2	6	P08670	P08670 [391-401]		0	1615.8685
[K].MALVLE/	1xOxidation [	0.0001273	7.389E-05	1	1	1	Q14254	Q14254 [357-370]		0	2092.2696
[R].QLIMANI	1xOxidation [	9.831E-05	7.389E-05	2	2	1	Q9UMX0; Q9	Q9UMX0 [207-22		0	2132.1639
[R].QILMASC	1xTMTpro [K	0.0001361	7.389E-05	1	1	1	Q6P2Q9	Q6P2Q9 [1246-12		0	1993.1284
[K].QLIPVISI	1xTMTpro [K	0.0001468	7.389E-05	1	1	1	P42858	P42858 [2801-28		0	2324.4085
[R].QLLSPG	1xTMTpro [N	7.359E-05	7.389E-05	1	1	2	Q15149	Q15149 [2856-28		0	2997.7308
[K].QININNP	1xTMTpro [K	0.0001245	7.389E-05	1	1	1	Q9UKF6	Q9UKF6 [296-306		0	1942.1406
[R].QLPADS	1xTMTpro [N	0.0001793	7.389E-05	1	1	1	Q9Y3R5	Q9Y3R5 [2259-22		0	2561.3207
[K].QLQEML	1xTMTpro [K	0.0001445	7.389E-05	1	1	2	Q15436	Q15436 [199-208]		0	1755.033
[K].QIQESEI	1xTMTpro [K	0.0001249	7.389E-05	1	1	1	Q15746	Q15746 [1277-12		0	1737.9449
[R].QLQQEL	1xTMTpro [N	0.0001918	7.389E-05	1	1	2	Q9H3S7	Q9H3S7 [1173-11		0	1565.8607
[K].QIQVLQ/	1xTMTpro [N	0.0001044	7.389E-05	1	1	2	Q14BN4	Q14BN4 [544-554		0	1628.9767
[R].QIQVNDI	1xTMTpro [N	0.0001617	7.389E-05	1	1	1	Q9ULC3	Q9ULC3 [50-59]		0	1519.8036
[K].QLSDAIS	1xTMTpro [N	0.0001626	7.389E-05	1	1	1	P55060	P55060 [110-120]		0	1476.8705
[R].QLSGNN	1xTMTpro [K	0.0001204	7.389E-05	1	1	1	Q9NZM1	Q9NZM1 [210-22		0	1933.1839
[K].QLSNPQ	1xTMTpro [K	6.664E-05	7.389E-05	1	1	1	O94973	O94973 [843-856]		0	2281.2796

[K].QLSSDA	1xTMTpro [K	0.0002015	7.389E-05	1	1	1	Q12888	Q12888 [874-883]	0	1684.9361
[K].QISSFMS	1xTMTpro [K	7.359E-05	7.389E-05	1	1	2	Q9Y678	Q9Y678 [363-376	0	2256.1714
[K].QISSFVS	1xTMTpro [K	6.56E-05	7.389E-05	1	1	1	Q9UBF2	Q9UBF2 [363-376	0	2224.1993
[K].QLSVPEI	1xTMTpro [K	0.0001295	7.389E-05	1	1	1	Q9BRK5	Q9BRK5 [252-280	0	3864.0311
[K].QLSVVVI	1xTMTpro [K	0.0001834	7.389E-05	1	1	1	Q9Y673	Q9Y673 [66-78]	0	2100.1832
[R].QITLNDL	1xTMTpro [N	0.0002011	7.389E-05	1	1	2	Q13162	Q13162 [213-223]	0	1529.8971
[K].QLTQEM	1xTMTpro [K	7.911E-05	7.389E-05	1	1	2	Q86UP2	Q86UP2 [357-366	0	1846.9898
[K].QLTQPE	1xTMTpro [N	0.000146	7.389E-05	1	1	1	Q9UJU6	Q9UJU6 [289-299	0	1617.8668
[R].QLTSYV	1xTMTpro [N	6.188E-05	7.389E-05	1	1	1	P57740	P57740 [308-330]	0	2830.5634
[R].QITVNDI	1xTMTpro [N	0.000155	7.389E-05	2	2	2	Q06830; P32	Q06830 [141-151]	0	1515.8814
[K].QLVAEQ	1xTMTpro [N	0.0001638	7.389E-05	1	1	1	P49736	P49736 [838-848]	0	1638.9134
[R].QLVEQV	1xTMTpro [K	0.0001971	7.389E-05	1	1	2	Q9BVK6	Q9BVK6 [170-180	0	1950.1516
[K].QLVTNA	1xCarbamido	9.909E-05	7.389E-05	1	1	2	Q99873	Q99873 [244-253]	0	1768.0647
[R].QILLGPN	1xTMTpro [K	7.684E-05	7.389E-05	1	1	1	Q9NS69	Q9NS69 [118-141	0	2883.6622
[R].QIILEKEI	2xTMTpro [K	6.227E-05	7.389E-05	1	1	3	P12956	P12956 [326-338]	1	2514.4847
[K].QLLHLAE	1xTMTpro [K	0.000157	7.389E-05	1	1	1	Q00341	Q00341 [715-723]	0	1689.0191
[K].QLLHDAI	1xTMTpro [K	0.0001174	7.389E-05	1	1	1	P43353	P43353 [42-54]	0	2110.2264
[K].QIDLSTV	1xTMTpro [K	9.724E-05	7.389E-05	1	1	2	P55145	P55145 [124-133]	0	1740.0399
[R].QLDMILI	1xTMTpro [N	0.0001759	7.389E-05	1	1	2	P49257	P49257 [351-360]	0	1564.8324
[K].QIDQFAI	1xTMTpro [N	0.0001719	7.389E-05	1	1	2	Q9BUT1	Q9BUT1 [64-74]	0	1652.8563
[R].QLEADIN	1xTMTpro [K	0.0001806	7.389E-05	1	1	1	O15400	O15400 [177-190]	0	2287.2499
[R].QLEADIN	1xOxidation	6.267E-05	7.389E-05	1	1	1	O15400	O15400 [177-190]	0	2303.2449
[K].QLEALM	1xTMTpro [N	7.382E-05	7.389E-05	1	1	1	Q15154	Q15154 [842-852]	0	1629.8702
[K].QLEDCLI	1xCarbamido	0.0001395	7.389E-05	1	1	1	Q9H2M9	Q9H2M9 [892-906	0	2419.3874
[K].QLEDELY	1xTMTpro [K	7.291E-05	7.389E-05	1	1	5	P09493	P09493 [38-48]	0	1910.109
[R].QLEEAE	1xTMTpro [N	9.785E-05	7.389E-05	1	1	2	P35579	P35579 [1878-188	0	1635.8145
[R].QLEEAE	1xTMTpro [N	7.091E-05	7.389E-05	1	1	6	P35580	P35580 [1885-189	0	1608.8036
[R].QLEEAE	1xTMTpro [N	8.121E-05	7.389E-05	1	1	2	P35749	P35749 [1885-189	0	1651.8094
[K].QLEHGW	1xTMTpro [K	0.0001911	7.389E-05	1	1	1	Q8WXH0	Q8WXH0 [2602-2	0	2488.344
[R].QLELACI	1xCarbamido	0.0001153	7.389E-05	1	1	1	Q05193	Q05193 [602-617]	0	2573.3049
[R].QIYLTDN	1xTMTpro [K	7.543E-05	7.389E-05	1	1	1	Q8NEU8	Q8NEU8 [376-389	0	2183.2567
[R].QIEIQFA	1xTMTpro [N	0.000153	7.389E-05	1	2	2	O75494	O75494 [80-90]	0	1608.8665
[K].QLEPVYI	1xTMTpro [K	6.612E-05	7.389E-05	1	1	2	P13667	P13667 [560-570]	0	1870.093

[K].QLEVVH	1xTMTpro [K	8.923E-05	7.389E-05	1	1	1	O94874	O94874 [44-54]	0	1847.0882
[K].QLGCPT	1xCarbamid	0.0001048	7.389E-05	1	1	2	Q86VB7	Q86VB7 [306-318	0	1647.9171
[K].QIGGDG	1xOxidation	0.0001241	7.389E-05	1	1	2	P00488	P00488 [469-483]	0	2269.1336
[R].QLGTVQ	1xTMTpro [N	8.759E-05	7.389E-05	1	1	2	P12111	P12111 [1193-12	0	1661.9506
[K].QIKDDN	1xTMTpro [K	0.0001739	7.389E-05	1	1	1	Q96LB3	Q96LB3 [311-324	1	2285.2051
[K].QLLAEE	1xTMTpro [K	6.227E-05	7.389E-05	1	1	1	Q8IWA0	Q8IWA0 [683-701	0	2775.5828
[K].QLICDPS	1xCarbamid	8.993E-05	7.389E-05	1	1	4	P31150	P31150 [279-290]	0	1780.9223
[R].QLLDDE	1xTMTpro [K	8.882E-05	7.389E-05	1	1	3	Q9BSJ8	Q9BSJ8 [107-118	0	2011.1203
[K].QLLDLPI	1xTMTpro [K	0.0001599	7.389E-05	1	1	1	O43264	O43264 [306-320	0	2364.3154
[K].QIIDQDK	1xTMTpro [K	0.000196	7.389E-05	1	1	1	Q07157	Q07157 [696-715	1	2869.6027
[R].QLLDSE	1xTMTpro [K	0.0001476	7.389E-05	1	1	1	Q66K14	Q66K14 [251-262	0	2002.0988
[R].QLLDTTI	1xTMTpro [K	7.863E-05	7.389E-05	1	1	1	O43353	O43353 [489-503	0	2316.2579
[K].QLEPEF	1xTMTpro [K	0.0001116	7.389E-05	1	1	1	Q86X10	Q86X10 [1474-14	0	2636.4467
[R].QIYPPIN	1xTMTpro [N	6.367E-05	7.389E-05	1	2	3	P21281	P21281 [387-400]	0	1901.118
[R].QMAEIA	1xOxidation	0.0001638	7.389E-05	1	1	1	P48643	P48643 [184-201]	0	2281.1851
[K].QMEELIE	3xCarbamid	9.785E-05	7.389E-05	1	1	1	Q5T4S7	Q5T4S7 [4826-48	0	2442.1456
[R].QPPPDS	1xTMTpro [K	7.015E-05	7.389E-05	1	1	4	Q15257	Q15257 [7-27]	0	2871.5384
[K].QPPSNP	1xTMTpro [N	0.0001665	7.389E-05	1	1	1	P13498	P13498 [150-164]	0	1915.0469
[K].QPPTAA	1xTMTpro [N	6.612E-05	7.389E-05	1	1	1	Q9Y6G9	Q9Y6G9 [386-40	0	1824.0047
[R].QPQQVI	1xTMTpro [K	0.000153	7.389E-05	1	1	1	P26006	P26006 [412-421]	0	1772.031
[R].QPQVAE	1xTMTpro [N	6.287E-05	7.389E-05	1	1	4	P51570	P51570 [6-17]	0	1628.9291
[R].QPSQGF	1xTMTpro [K	9.542E-05	7.389E-05	1	1	2	P11586	P11586 [411-421]	0	1768.0249
[K].QPTHQG	1xTMTpro [N	6.758E-05	7.389E-05	1	1	1	Q15477	Q15477 [540-551	0	1595.8209
[K].QPTNPF	1xTMTpro [K	9.909E-05	7.389E-05	1	1	2	Q9NTI5	Q9NTI5 [592-602]	0	1926.1014
[K].QPVAGS	1xTMTpro [N	0.0001672	7.389E-05	1	1	1	Q9UGI8	Q9UGI8 [135-146	0	1566.8195
[R].QQAADD	1xCarbamid	6.337E-05	7.389E-05	1	1	1	O00291	O00291 [424-434	0	1656.7971
[K].QQAHDL	1xTMTpro [K	9.925E-05	7.389E-05	2	2	1	P51692; P42	P51692 [601-618]	0	2687.5488
[K].QQASSW	1xTMTpro [K	9.379E-05	7.389E-05	1	1	2	Q8N474	Q8N474 [92-103]	0	1979.157
[R].QQDIVP	1xTMTpro [K	9.847E-05	7.389E-05	1	1	1	Q709C8	Q709C8 [589-609	0	2780.5901
[R].QPPPCY	1xCarbamid	9.408E-05	7.389E-05	1	1	1	Q9HCU5	Q9HCU5 [276-29	0	2536.2978
[K].QQDSQF	1xTMTpro [K	0.0001266	7.389E-05	1	1	1	Q9H307	Q9H307 [378-397	0	2955.4935
[R].QQGSSL	1xTMTpro [K	9.785E-05	7.389E-05	1	1	1	Q14161	Q14161 [357-366	0	1586.8994
[K].QQHVIE	1xTMTpro [K	7.66E-05	7.389E-05	1	1	2	P48643	P48643 [503-513]	0	1874.1355

[R].QQICNQ	2xCarbamido	0.0001602	7.389E-05	1	1	2	Q7Z2W4	Q7Z2W4 [159-170	0	1819.8863
[R].QQLGTA	1xTMTpro [K	0.0001691	7.389E-05	1	1	1	Q9NYL9	Q9NYL9 [295-307	0	2026.1498
[K].QQLLIGA	1xTMTpro [K	0.0001363	7.389E-05	1	1	2	Q99832	Q99832 [431-440]	0	1713.0555
[K].QQIQSIQ	1xTMTpro [N	0.0001691	7.389E-05	1	1	2	P55769	P55769 [114-125]	0	1761.9778
[K].QQISLAT	1xTMTpro [N	0.000146	7.389E-05	1	1	2	P48643	P48643 [515-525]	0	1578.8957
[K].QQMLEN	1xTMTpro [N	0.0001304	7.389E-05	1	1	2	Q01082	Q01082 [1422-14	0	1709.8634
[K].QQMLEQ	1xOxidation [	0.0001511	7.389E-05	1	1	2	P40763	P40763 [141-152]	0	1844.9608
[K].QQQLLN	1xTMTpro [N	0.0001611	7.389E-05	1	2	1	Q9NVI7	Q9NVI7 [195-205]	0	1688.9251
[R].QQQPPF	1xTMTpro [K	0.0001769	7.389E-05	1	1	2	Q14203	Q14203 [545-556]	0	2070.1152
[K].QQQQQ	1xTMTpro [K	0.0001697	7.389E-05	1	1	1	Q02818	Q02818 [401-410]	0	1846.0175
[R].QQSDEV	1xTMTpro [K	9.234E-05	7.389E-05	1	1	5	Q9HCB6	Q9HCB6 [274-284	0	1868.0985
[R].QQGLAS	1xTMTpro [N	0.0001198	7.389E-05	1	1	2	Q15149	Q15149 [3771-378	0	1603.8399
[R].QLDETN	1xTMTpro [N	0.000144	7.389E-05	1	1	2	O75116	O75116 [563-572]	0	1476.8341
[R].QPNLPS	1xTMTpro [K	0.0001297	7.389E-05	1	1	1	Q03519	Q03519 [450-469]	0	2626.5424
[K].QPGNET	1xTMTpro [K	7.393E-05	7.389E-05	1	1	2	P61758	P61758 [40-51]	0	1881.0573
[K].QMEQTY	1xTMTpro [N	0.0001075	7.389E-05	1	1	2	P49747	P49747 [616-628]	0	2002.9765
[K].QMEQVA	1xTMTpro [K	0.0001043	7.389E-05	1	1	7	Q01995	Q01995 [90-99]	0	1830.0439
[K].QMFGNA	1xOxidation [	0.0001653	7.389E-05	1	1	2	P18859	P18859 [80-94]	0	2373.1863
[K].QMFLTQ	1xTMTpro [N	6.601E-05	7.389E-05	1	1	2	P78527	P78527 [394-406]	0	1831.8815
[K].QMGYQ	1xCarbamido	0.0001856	7.389E-05	1	1	1	Q13347	Q13347 [115-128]	0	2102.0159
[K].QMIAVAI	1xTMTpro [K	6.864E-05	7.389E-05	1	1	1	Q9BRK5	Q9BRK5 [321-341	0	3052.6017
[K].QMNAIID	1xTMTpro [K	0.0001903	7.389E-05	1	1	1	P51531	P51531 [1386-139	0	2131.2077
[R].QMQEAE	1xTMTpro [K	0.0001834	7.389E-05	1	1	1	Q9UBV2	Q9UBV2 [179-192	0	2314.1195
[R].QMSCLM	1xCarbamido	9.049E-05	7.389E-05	1	1	1	Q92896	Q92896 [1089-110	0	2063.0467
[K].QMTEAIC	1xTMTpro [N	0.0001004	7.389E-05	1	1	2	Q14764	Q14764 [797-808]	0	1607.8746
[K].QMVIDVI	1xTMTpro [K	8.799E-05	7.389E-05	1	1	4	P62847	P62847 [22-32]	0	1845.0912
[K].QMVIDVI	1xOxidation [	0.0001038	7.389E-05	1	1	2	P62847	P62847 [22-32]	0	1861.0861
[K].QNEKEE	2xTMTpro [K	0.0002027	7.389E-05	1	1	1	Q15075	Q15075 [949-965]	1	2954.6363
[R].QPKPFF	1xCarbamido	0.0001125	7.389E-05	1	1	1	Q9P2B2	Q9P2B2 [556-572	0	2886.5463
[K].QNKPTG	1xTMTpro [K	6.726E-05	7.389E-05	1	1	1	P78406	P78406 [225-239]	0	2183.2428
[R].QNLPLS	1xTMTpro [K	0.0001911	7.389E-05	1	1	1	P25325	P25325 [138-146]	0	1551.935
[R].QNISPDI	2xTMTpro [K	7.887E-05	7.389E-05	1	1	2	Q14204	Q14204 [4231-42	1	2509.4959
[R].QNLSQF	1xTMTpro [N	0.000144	7.389E-05	1	1	2	Q8N684	Q8N684 [163-173	0	1595.8461

[K].QNQHEE	1xTMTpro [N	7.069E-05	7.389E-05	1	1	1	Q8NHH9	Q8NHH9 [271-28	0	1698.8843
[K].QNVAIYN	1xTMTpro [N	8.923E-05	7.389E-05	1	1	1	Q15223	Q15223 [76-94]	0	2383.2763
[R].QNVAVN	1xCarbamido	0.0001599	7.389E-05	1	1	3	Q15942	Q15942 [376-386	0	1563.8232
[R].QNVDHS	1xTMTpro [K	0.0001542	7.389E-05	1	1	1	O00391	O00391 [421-431	0	1834.9903
[R].QNVQVF	1xTMTpro [K	0.000159	7.389E-05	1	1	1	P42330	P42330 [279-294]	0	2535.3409
[K].QNVVPT	1xOxidation [	0.0001726	7.389E-05	1	1	1	P12110	P12110 [967-993]	0	3148.6731
[R].QPCMPN	1xCarbamido	0.0001856	7.389E-05	1	1	1	P40763	P40763 [326-340]	0	2427.3319
[R].QPCMPT	1xCarbamido	0.0001611	7.389E-05	1	2	1	P42224	P42224 [322-336]	0	2410.3707
[R].QPDFISF	1xTMTpro [N	0.000106	7.389E-05	1	1	1	P98160	P98160 [3720-373	0	2226.1991
[R].QPEWLE	1xTMTpro [N	0.0001885	7.389E-05	1	1	2	Q14980	Q14980 [948-958	0	1703.8672
[R].QNLFQE	1xTMTpro [N	6.822E-05	7.389E-05	1	1	1	Q9UL46	Q9UL46 [22-34]	0	1991.0194
[K].QLDDKD	2xTMTpro [K	0.000111	7.389E-05	1	1	1	P33176	P33176 [431-448]	1	3071.6677
[R].QLDALLE	1xTMTpro [K	0.0001182	7.389E-05	1	1	4	P55268	P55268 [1662-167	0	1722.0657
[K].QIAVEAC	1xTMTpro [N	0.0001786	7.389E-05	1	1	1	Q14254	Q14254 [267-277	0	1573.9233
[K].QDLPDA	2xTMTpro [K	0.0001629	7.389E-05	1	1	2	P62330	P62330 [124-138]	0	2691.4956
[K].QDLPNA	2xOxidation [	9.647E-05	7.389E-05	1	1	2	P18085	P18085 [128-142]	0	2304.1707
[K].QDLQNA	1xTMTpro [N	8.965E-05	7.389E-05	1	1	1	Q9P0K7	Q9P0K7 [537-548	0	1735.8782
[R].QDLTTLI	1xTMTpro [K	0.0001881	7.389E-05	1	2	3	P41091	P41091 [18-27]	0	1742.0191
[K].QDMPNA	1xTMTpro [K	6.779E-05	7.389E-05	1	1	1	P84085	P84085 [128-142]	0	2284.1809
[K].QDNCVH	1xCarbamido	0.000129	7.389E-05	1	1	1	Q9BZK7	Q9BZK7 [380-392	0	2187.1221
[R].QDSINAY	1xTMTpro [K	7.839E-05	7.389E-05	1	1	1	O60264	O60264 [539-552	0	2189.133
[R].QDVDNA	1xTMTpro [N	0.0001929	7.389E-05	1	1	6	P08670	P08670 [208-217]	0	1392.7402
[K].QDVIITAI	1xTMTpro [N	6.337E-05	7.389E-05	1	1	2	A0AVT1	A0AVT1 [561-574	0	1861.035
[R].QEATLVY	1xTMTpro [N	8.584E-05	7.389E-05	1	1	1	P36871	P36871 [53-64]	0	1505.8243
[R].QEAVAL	1xTMTpro [N	0.000111	7.389E-05	1	1	2	P46108	P46108 [21-31]	0	1516.8767
[K].QEDDLA	1xTMTpro [K	7.439E-05	7.389E-05	1	1	3	P08697	P08697 [197-209]	0	2181.1796
[K].QEEASG	1xTMTpro [K	9.602E-05	7.389E-05	1	1	1	P50552	P50552 [241-252]	0	1779.9733
[R].QDHAQC	1xTMTpro [N	6.287E-05	7.389E-05	1	1	1	Q14980	Q14980 [579-592	0	1871.9531
[R].QEEEMN	1xOxidation [	0.0001402	7.389E-05	1	1	4	P35579	P35579 [843-856]	1	2651.4088
[R].QEELYS	1xTMTpro [N	0.0001144	7.389E-05	1	1	4	Q15149	Q15149 [3358-33	0	1669.8716
[K].QEESITV	1xTMTpro [N	7.863E-05	7.389E-05	1	1	1	Q12765	Q12765 [245-259	0	2085.0639
[R].QEFIDGI	1xTMTpro [K	0.0001892	7.389E-05	1	2	1	Q9UPN3	Q9UPN3 [7064-70	0	1829.0664
[R].QEGLDG	1xTMTpro [K	0.0001793	7.389E-05	1	1	1	Q8N163	Q8N163 [764-788	0	3212.7699

[K].QEGLES	1xTMTpro [K	0.0001892	7.389E-05	1	1	2	P14923	P14923 [386-394]	0	1610.9609
[R].QEGTDN	1xCarbamido	0.0001347	7.389E-05	1	1	1	Q5T5P2	Q5T5P2 [1426-14	0	2327.1681
[R].QEHCGC	2xCarbamido	0.0001405	7.389E-05	1	1	1	Q15746	Q15746 [1302-13	0	2359.203
[K].QELEAE	1xTMTpro [K	6.961E-05	7.389E-05	1	2	1	Q9UNH7	Q9UNH7 [127-138	0	2062.1716
[K].QELELLN	1xTMTpro [K	0.0001262	7.389E-05	1	1	2	Q9UKU9	Q9UKU9 [87-98]	0	2064.2196
[R].QELILSN	1xTMTpro [K	6.347E-05	7.389E-05	1	1	1	P53621	P53621 [261-271]	0	1884.057
[R].QELNTIA	1xTMTpro [K	0.0001066	7.389E-05	1	1	1	P11215	P11215 [298-309]	0	1962.1628
[K].QELSEAI	1xTMTpro [N	0.0001678	7.389E-05	1	1	2	P01024	P01024 [429-439]	0	1565.809
[K].QEMASA	1xTMTpro [N	6.139E-05	7.389E-05	1	3	1	P09651	P09651 [184-194]	0	1485.7287
[K].QEELQQ	1xTMTpro [N	0.000117	7.389E-05	1	1	2	Q15149	Q15149 [2707-27	0	1732.9149
[K].QEMLQT	1xCarbamido	0.0001584	7.389E-05	1	1	1	Q9UI12	Q9UI12 [63-75]	0	2118.0815
[K].QDFVQH	1xTMTpro [N	9.063E-05	7.389E-05	1	1	2	P14324	P14324 [81-92]	0	1807.9774
[R].QDAYDG	1xTMTpro [K	7.543E-05	7.389E-05	1	1	1	P04439	P04439 [139-155]	1	2607.3546
[R].QALAVM	1xCarbamido	0.000123	7.389E-05	1	1	1	P57737	P57737 [318-329]	0	1680.9096
[K].QAIELSF	1xTMTpro [K	7.755E-05	7.389E-05	1	1	1	O14879	O14879 [198-210	0	2113.1785
[K].QAIQASH	1xTMTpro [K	0.0001466	7.389E-05	1	1	1	P13671	P13671 [301-308]	0	1490.8935
[K].QALQDT	1xTMTpro [K	7.531E-05	7.389E-05	1	1	2	Q01082	Q01082 [843-853	0	1872.1086
[K].QALSEV	1xTMTpro [N	6.397E-05	7.389E-05	1	1	1	Q13586	Q13586 [414-424	0	1462.8549
[R].QALVEFI	1xTMTpro [K	8.146E-05	7.389E-05	1	1	1	Q8WVV9	Q8WVV9 [113-12	0	2072.1519
[R].QAMTSIV	1xTMTpro [K	7.371E-05	7.389E-05	1	1	1	Q5VYK3	Q5VYK3 [1140-11	0	2186.2135
[R].QAQCGC	2xCarbamido	0.0001361	7.389E-05	1	1	1	P07357	P07357 [93-103]	0	1977.9766
[R].QAQEYE	1xTMTpro [K	7.146E-05	7.389E-05	1	1	2	P05783	P05783 [359-370]	0	2028.1621
[R].QASALID	1xTMTpro [N	0.0001232	7.389E-05	1	1	1	Q9H4G0	Q9H4G0 [405-419	0	2022.1092
[K].QASCSG	1xCarbamido	0.0001736	7.389E-05	1	1	2	O95394	O95394 [197-206	0	1476.6708
[R].QASIELF	1xTMTpro [K	0.0001208	7.389E-05	1	1	1	P33241	P33241 [250-264]	0	2141.2132
[R].QASPNIV	1xTMTpro [K	8.121E-05	7.389E-05	1	1	3	P51148	P51148 [122-135]	0	2004.2097
[K].QDENDD	1xCarbamido	0.0001684	7.389E-05	1	1	1	Q14974	Q14974 [333-346	0	2374.0385
[R].QASPNIV	1xTMTpro [K	6.208E-05	7.389E-05	1	1	2	P20339	P20339 [121-134]	0	2020.2046
[R].QASTLID	1xTMTpro [N	7.648E-05	7.389E-05	1	1	1	O43491	O43491 [527-541	0	2042.1102
[R].QASVAD	1xTMTpro [K	0.0001322	7.389E-05	1	1	1	P49419	P49419 [82-93]	0	1948.0519
[K].QATVVLI	1xCarbamido	7.224E-05	7.389E-05	1	1	2	Q8NBX0	Q8NBX0 [91-103]	0	1780.9699
[K].QAVEQC	1xTMTpro [N	0.0001713	7.389E-05	1	1	1	P33176	P33176 [699-709]	0	1627.8835
[R].QAVTNP	1xTMTpro [K	0.0001911	7.389E-05	1	1	2	P38646	P38646 [108-121]	0	2177.1846

[K].QAWATD	1xTMTpro [K	0.0001713	7.389E-05	1	1	1	A5YKK6	A5YKK6 [1601-16	0	2232.1792
[K].QCCDCC	4xCarbamido	0.0001142	7.389E-05	1	1	2	P98095	P98095 [517-527]	0	1702.7453
[K].QCFHEE	1xCarbamido	0.0001506	7.389E-05	1	1	1	Q9Y3Q3	Q9Y3Q3 [39-50]	0	2098.0883
[K].QCIHQL(	2xCarbamido	6.519E-05	7.389E-05	1	1	2	Q13361	Q13361 [103-114]	0	1866.9638
[R].QCLPSLI	2xCarbamido	0.000138	7.389E-05	1	1	2	P78527	P78527 [1498-150	0	1929.0429
[R].QCNTLA	1xCarbamido	6.417E-05	7.389E-05	1	1	1	Q9NXC5	Q9NXC5 [115-135	0	2995.5592
[K].QCSWSS	1xCarbamido	0.0001112	7.389E-05	1	1	1	Q96CG8	Q96CG8 [108-122	0	2336.22
[R].QDAQDL	1xTMTpro [K	0.0001445	7.389E-05	1	1	3	P09525	P09525 [173-184]	0	1975.0264
[R].QASPSI\	1xTMTpro [K	7.405E-05	7.389E-05	1	1	2	P61020	P61020 [121-134]	0	1977.1988
[R].QQTDMA	1xTMTpro [K	7.887E-05	7.389E-05	1	1	1	Q92769	Q92769 [128-144]	0	2472.3062
[K].QEMVDII	1xTMTpro [N	7.416E-05	7.389E-05	1	1	2	P83876	P83876 [110-121]	0	1799.9533
[R].QEPLLIG	1xTMTpro [K	0.0001933	7.389E-05	1	1	1	P49327	P49327 [317-326]	0	1694.0344
[K].QGQDNL	1xTMTpro [K	0.0001762	7.389E-05	1	1	2	P30040	P30040 [183-192]	0	1683.9521
[R].QGQGQI	1xCarbamido	6.347E-05	7.389E-05	1	1	1	Q16531	Q16531 [370-383]	0	2089.1356
[R].QGRPVV	1xCarbamido	0.0001243	7.389E-05	1	1	2	Q06210	Q06210 [631-647]	1	2900.6332
[K].QGSATV	1xTMTpro [K	0.0001899	7.389E-05	1	1	2	P25786	P25786 [31-39]	0	1468.8979
[QR].QGSGS	1xTMTpro [N	0.0001987	7.389E-05	1	1	2	Q86YZ3	Q86YZ3 [652-662	0	1351.6885
[K].QGTIFLA	1xTMTpro [K	6.178E-05	7.389E-05	1	1	1	Q9HCC0	Q9HCC0 [236-248	0	1949.2079
[R].QGTLHV	1xTMTpro [N	0.0001506	7.389E-05	1	1	1	O14936	O14936 [527-537]	0	1528.8403
[K].QGTYYD	1xTMTpro [K	0.0001995	7.389E-05	1	1	1	Q14766	Q14766 [1454-14	0	1678.9296
[R].QGVDAD	1xTMTpro [N	6.612E-05	7.389E-05	1	1	1	P35527	P35527 [251-261]	0	1461.7981
[K].QHALCV	1xCarbamido	0.000118	7.389E-05	1	2	1	Q9ULC4	Q9ULC4 [140-149	0	1750.9952
[K].QHAMQ(	2xCarbamido	0.0001517	7.389E-05	1	1	1	Q14192	Q14192 [157-166]	0	1897.9691
[K].QHGIPIP	1xTMTpro [K	0.0001782	7.389E-05	1	1	1	P00966	P00966 [166-176]	0	1795.1086
[K].QHPVPP	1xTMTpro [N	7.497E-05	7.389E-05	1	1	1	Q15942	Q15942 [329-343]	0	2014.0902
[K].QGIVHLI	1xCarbamido	0.0001987	7.389E-05	1	1	1	Q15746	Q15746 [1579-15	0	3020.7205
[K].QHYNCE	1xCarbamido	6.387E-05	7.389E-05	1	1	6	P50454	P50454 [152-160]	0	1810.915
[K].QKDFLD	1xTMTpro [K	8.375E-05	7.389E-05	1	1	2	O75369	O75369 [503-522]	1	2961.5278
[R].QKEFDP	1xTMTpro [K	7.269E-05	7.389E-05	1	1	2	Q05682	Q05682 [118-135]	1	2613.438
[K].QKEFVIT	2xTMTpro [K	0.0001629	7.389E-05	1	1	1	Q9HD20	Q9HD20 [844-853	1	2105.315
[K].QKLEED.	2xTMTpro [K	0.00017	7.389E-05	1	1	2	P05455	P05455 [215-224]	1	2133.2042
[R].QKLHEL(	2xTMTpro [K	0.0001703	7.389E-05	1	1	2	P02647	P02647 [156-164]	1	2065.2586
[R].QKIVQAI	2xTMTpro [K	0.0001511	7.389E-05	1	1	1	Q99623	Q99623 [223-236]	1	2384.3965

[K].QKPITPE	2xTMTpro [K	0.0002043	7.389E-05	1	1	2	P60953	P60953 [134-144]	0	2154.295
[R].QKQEST	1xTMTpro [K	6.56E-05	7.389E-05	1	1	2	Q05707	Q05707 [959-971]	1	1957.0958
[K].QLADTM	1xTMTpro [N	0.0001375	7.389E-05	1	1	1	Q8NEU8	Q8NEU8 [106-119	0	1949.1213
[R].QLAEAH	1xTMTpro [K	8.466E-05	7.389E-05	1	1	2	Q15149	Q15149 [1511-152	0	1674.9783
[K].QLAGILQ	1xTMTpro [N	7.191E-05	7.389E-05	1	1	2	Q86VN1	Q86VN1 [248-260	0	1770.0445
[K].QLAPIWI	3xTMTpro [K	0.0001581	7.389E-05	1	1	1	P07237	P07237 [402-424]	2	3898.2583
[K].QIATLHA	2xTMTpro [K	0.0001041	7.389E-05	1	1	1	P35579	P35579 [1358-137	1	2466.4683
[R].QKAEAD	3xTMTpro [K	6.864E-05	7.389E-05	2	3	3	P07900; P08	P07900 [648-657]	2	2363.4035
[K].QEPGEN	1xTMTpro [K	0.0001026	7.389E-05	1	1	3	P22352	P22352 [107-120]	0	2163.2153
[K].QGLSSS	1xTMTpro [K	0.0001653	7.389E-05	1	1	2	P49419	P49419 [453-462]	0	1675.9874
[R].QGIFPAS	1xTMTpro [N	6.158E-05	7.389E-05	1	1	2	O60504	O60504 [502-514]	0	1755.9713
[R].QEQALL	1xTMTpro [N	0.0001061	7.389E-05	1	1	1	Q15149	Q15149 [1387-139	0	1661.9029
[K].QESEITY	1xCarbamido	0.0001132	7.389E-05	1	1	1	Q9UFN0	Q9UFN0 [124-136	0	2261.2132
[R].QESTSV	1xTMTpro [K	8.272E-05	7.389E-05	1	1	1	Q14126	Q14126 [549-561]	0	2085.1683
[K].QESTVG	1xTMTpro [N	7.213E-05	7.389E-05	1	1	1	Q05707	Q05707 [961-971]	0	1396.7351
[K].QETQLLI	1xTMTpro [N	6.843E-05	7.389E-05	1	1	1	Q9UKM7	Q9UKM7 [479-495	0	2296.1992
[R].QEVEEN	1xTMTpro [N	0.0001147	7.389E-05	1	1	1	P16144	P16144 [845-856]	0	1825.9251
[K].QEVMISI	1xTMTpro [N	8.993E-05	7.389E-05	1	1	1	P35580	P35580 [1032-104	0	1652.8485
[R].QEVQDL	2xTMTpro [K	8.196E-05	7.389E-05	1	1	1	Q15075	Q15075 [90-103]	1	2557.4653
[R].QEWISH	1xCarbamido	0.0001299	7.389E-05	1	1	1	Q96S99	Q96S99 [120-131	0	1889.9499
[K].QEYDEA	1xTMTpro [N	7.004E-05	7.389E-05	2	3	3	P62736; P68	P62736 [362-374]	0	1804.9149
[K].QEYDES	1xTMTpro [N	7.291E-05	7.389E-05	3	7	8	A5A3E0; Q9	A5A3E0 [1060-10	0	1820.9098
[K].QFAAATI	1xTMTpro [N	6.853E-05	7.389E-05	1	1	1	O00203	O00203 [419-430]	0	1580.908
[K].QFLECA(	1xCarbamido	0.0001995	7.389E-05	1	2	1	Q9Y6H1	Q9Y6H1 [120-132	0	2159.1411
[R].QGLLPV	1xTMTpro [K	7.359E-05	7.389E-05	1	1	6	P02647	P02647 [240-250]	0	1839.1235
[R].QFLESSI	1xTMTpro [N	0.0001647	7.389E-05	1	1	4	O60749	O60749 [260-269]	0	1509.8232
[K].QFPEDG	1xCarbamido	7.485E-05	7.389E-05	1	1	1	Q9HCB6	Q9HCB6 [530-547	0	2675.3366
[R].QFVYLES	1xTMTpro [K	7.069E-05	7.389E-05	1	1	1	Q12884	Q12884 [110-120]	0	1987.0668
[R].QFYDGE	1xCarbamido	0.0001192	7.389E-05	1	1	1	Q15047	Q15047 [1199-12	0	2446.2092
[K].QGAAIGI	1xTMTpro [N	0.000111	7.389E-05	1	1	1	Q08257	Q08257 [125-138]	0	1832.0026
[R].QGAGTY	1xTMTpro [K	0.0001144	7.389E-05	1	1	1	Q8N0W3	Q8N0W3 [449-46	0	2452.3157
[R].QGALAP	1xTMTpro [K	8.109E-05	7.389E-05	1	1	2	Q9H330	Q9H330 [419-432	0	2015.2297
[K].QGCDCE	3xCarbamido	0.0001176	7.389E-05	1	1	3	Q9NRX4	Q9NRX4 [67-78]	0	1672.7161

[R].QGCTM	1xCarbamido	0.0001834	7.389E-05	1	1	1	Q15124	Q15124 [58-69]	0	1570.7637
[R].QGDLPV	1xTMTpro [N	0.0001632	7.389E-05	1	1	1	Q7L775	Q7L775 [83-96]	0	1849.9615
[K].QGFEAA	3xTMTpro [K	8.772E-05	7.389E-05	1	1	3	P35749	P35749 [43-67]	2	3921.1962
[K].QGFGNL	1xCarbamido	6.267E-05	7.389E-05	1	1	2	P11586	P11586 [855-866]	0	1944.0691
[R].QGFVPA	1xTMTpro [K	0.000117	7.389E-05	1	1	4	Q13813	Q13813 [1013-10	0	1688.0027
[R].QGLDEM	1xTMTpro [K	0.000171	7.389E-05	1	1	1	Q96T51	Q96T51 [394-405	0	2079.0713
[R].QFLEVEI	1xTMTpro [N	0.0001212	7.389E-05	1	1	2	Q14980	Q14980 [1463-14	0	1651.8975
[R].QAKPAA	2xCarbamido	0.0001533	7.389E-05	1	1	1	Q8N488	Q8N488 [15-33]	0	2853.3944
[R].QQTHML	1xTMTpro [N	0.0001153	7.389E-05	1	1	1	P10909	P10909 [168-182]	0	2177.0551
[K].QQVPSG	1xTMTpro [N	7.113E-05	7.389E-05	1	1	1	P09874	P09874 [270-282]	0	1703.9247
[K].RQEYLD	1xTMTpro [K	0.0001448	7.389E-05	1	1	1	Q9NQT8	Q9NQT8 [1102-1	1	2057.1635
[K].RQLEEA	1xTMTpro [N	7.924E-05	7.389E-05	1	1	1	P35579	P35579 [1877-18	1	1791.9156
[K].RQNGDI	1xTMTpro [N	0.0001579	7.389E-05	1	1	2	P02545	P02545 [471-482]	1	1751.936
[R].RQQEEL	1xTMTpro [N	0.0001375	7.389E-05	1	1	1	Q15149	Q15149 [2718-27	1	1947.0215
[R].RQQEQ	1xTMTpro [K	0.0001114	7.389E-05	1	1	1	P49327	P49327 [1105-11	1	2104.237
[R].RQVDQL	1xTMTpro [K	0.0001903	7.389E-05	1	1	1	P08670	P08670 [159-168]	1	1825.0423
[K].RTDEAA	1xTMTpro [K	0.0001539	7.389E-05	1	1	2	P26447	P26447 [49-57]	1	1673.9466
[R].RTEDDS	1xTMTpro [K	0.0001107	7.389E-05	1	1	1	Q8TD19	Q8TD19 [71-81]	1	1956.1046
[K].RTEMEN	1xTMTpro [K	0.0001132	7.389E-05	1	1	1	P05787	P05787 [186-197]	1	2117.192
[K].RVDIIPG	1xTMTpro [N	9.77E-05	7.389E-05	1	1	1	Q9H4A4	Q9H4A4 [470-481	1	1767.9713
[R].RVEGIQ	1xTMTpro [K	9.32E-05	7.389E-05	1	1	1	O60870	O60870 [380-391	1	2045.1523
[R].RVENSIF	1xTMTpro [K	6.529E-05	7.389E-05	1	1	1	Q15020	Q15020 [646-669	1	2973.6249
[R].RVEPYG	1xTMTpro [K	0.0001926	7.389E-05	1	1	1	P06727	P06727 [306-316]	1	1961.0736
[R].RVESEE	2xTMTpro [K	0.0001182	7.389E-05	1	1	1	Q15435	Q15435 [21-34]	2	2491.3456
[R].RVTFEAI	1xTMTpro [K	0.0001222	7.389E-05	1	1	1	Q9NX63	Q9NX63 [9-24]	1	2472.359
[K].RYELPAI	1xTMTpro [K	0.0001556	7.389E-05	1	1	1	O75934	O75934 [86-97]	1	1941.1049
[K].RYTCGE	1xCarbamido	8.222E-05	7.389E-05	1	1	1	P21266	P21266 [36-47]	1	1806.84
[K].SAAAIAL	1xTMTpro [K	0.0001952	7.389E-05	1	1	1	P50851	P50851 [213-224]	0	1731.1024
[R].SAATVL	1xTMTpro [K	0.0001881	7.389E-05	1	1	2	Q8TE77	Q8TE77 [420-430	0	1734.0116
[K].SADCYN	1xCarbamido	7.985E-05	7.389E-05	1	1	1	Q8NBN3	Q8NBN3 [86-97]	0	2132.0614
[R].SADDSL	1xTMTpro [N	0.0001002	7.389E-05	1	1	1	Q15334	Q15334 [714-724	0	1409.7555
[K].SADEVLI	1xTMTpro [K	0.0001425	7.389E-05	1	1	2	Q9Y5X3	Q9Y5X3 [193-203	0	1774.0242
[R].SADFNP	1xTMTpro [K	0.0001716	7.389E-05	1	1	1	Q96GQ7	Q96GQ7 [79-91]	0	2125.1097

[R].SADIALV	1xTMTpro [N	0.0001073	7.389E-05	1	1	1	Q5T653	Q5T653 [136-147	0	1420.8079
[K].SADTLW	1xTMTpro [K	0.0001089	7.389E-05	1	1	3	P00338	P00338 [319-328]	0	1726.9983
[K].SAEAAAI	1xTMTpro [K	0.0001347	7.389E-05	1	1	2	Q3LXA3	Q3LXA3 [526-535	0	1556.8776
[R].SAEDSF	1xTMTpro [N	0.0001084	7.389E-05	1	1	1	Q6P1M3	Q6P1M3 [704-714	0	1519.7712
[R].RPYAPIN	1xTMTpro [K	8.571E-05	7.389E-05	1	1	1	O94776	O94776 [481-492	0	1936.1624
[R].RPSEIVI	1xCarbamido	0.0001352	7.389E-05	1	1	2	P04196	P04196 [96-106]	0	1895.1028
[R].RPQTDQ	1xTMTpro [N	6.581E-05	7.389E-05	1	1	1	Q8IYB5	Q8IYB5 [108-120]	0	1911.0408
[R].RPQGES	1xTMTpro [N	0.0001361	7.389E-05	1	1	1	Q7KZ85	Q7KZ85 [1178-11	0	1723.9047
[K].RKGDEV	2xTMTpro [K	0.0001163	7.389E-05	1	1	1	P09874	P09874 [208-221]	2	2429.3816
[R].RKPDTIE	2xTMTpro [K	0.0001629	7.389E-05	3	3	3	P35241; P26	P35241 [295-306]	1	2385.4104
[R].RKPDYE	2xTMTpro [K	0.0001926	7.389E-05	1	1	1	Q9NP61	Q9NP61 [404-419	1	2831.5356
[K].RLDEELI	1xTMTpro [K	0.0001034	7.389E-05	1	1	2	Q15008	Q15008 [83-93]	1	1955.0577
[R].RLDGES	1xTMTpro [N	8.084E-05	7.389E-05	1	1	1	Q7Z406	Q7Z406 [1076-10	1	2594.28
[K].RLEEMLI	1xTMTpro [N	0.0001271	7.389E-05	1	1	1	P13716	P13716 [60-74]	1	2144.2181
[R].RLEESD	1xTMTpro [N	0.0001114	7.389E-05	1	1	1	O43615	O43615 [90-101]	1	1748.9462
[R].RLESML	1xTMTpro [N	8.44E-05	7.389E-05	1	1	1	Q9BQS8	Q9BQS8 [339-355	1	2289.2556
[R].RIPADTF	1xTMTpro [K	0.0001786	7.389E-05	1	1	3	P46940	P46940 [231-241]	1	1811.1035
[K].RLPLEYS	1xTMTpro [N	0.0001058	7.389E-05	1	1	1	P07358	P07358 [323-334]	1	1849.9768
[R].RLPSSP	1xTMTpro [K	7.887E-05	7.389E-05	1	1	1	Q9H4G0	Q9H4G0 [537-548	1	1832.0886
[R].RLPVEVI	1xTMTpro [K	0.0001536	7.389E-05	1	1	1	Q9NX05	Q9NX05 [683-694	1	1958.2658
[R].RLTLEDI	1xTMTpro [N	0.0001587	7.389E-05	1	1	1	Q6P2Q9	Q6P2Q9 [1402-14	1	1952.0044
[K].SAEFLLH	1xTMTpro [K	0.000182	7.389E-05	1	1	2	P18621	P18621 [87-96]	0	1797.0588
[R].RMDAPA	1xTMTpro [N	0.0001262	7.389E-05	1	1	1	O43488	O43488 [50-61]	1	1519.8334
[K].RPAEDM	1xTMTpro [K	0.0001224	7.389E-05	1	1	1	P61978	P61978 [22-35]	1	2344.2211
[K].RPCPFW	2xCarbamido	0.0002035	7.389E-05	1	1	2	Q96HE7	Q96HE7 [83-96]	0	2097.0078
[R].RPDPDS	1xTMTpro [N	0.0002043	7.389E-05	1	1	1	Q96I25	Q96I25 [150-163]	0	2041.8906
[R].RPDQQL	1xTMTpro [K	6.297E-05	7.389E-05	1	1	3	Q8NC51	Q8NC51 [112-122	0	1864.0532
[K].RPEEVC	1xCarbamido	8.598E-05	7.389E-05	1	1	2	P98160	P98160 [363-374]	0	1779.9131
[R].RPEHGG	1xTMTpro [N	0.0001492	7.389E-05	1	1	1	O43709	O43709 [7-24]	0	2404.1853
[R].RPEIPTE	1xTMTpro [N	0.0001561	7.389E-05	1	1	2	Q8N766	Q8N766 [882-891	0	1516.8403
[K].RPEQAV	1xTMTpro [K	0.0001222	7.389E-05	1	1	1	Q15334	Q15334 [769-780	0	1905.1413
[K].RPLLAFA	1xCarbamido	0.0001983	7.389E-05	1	1	2	Q96J01	Q96J01 [319-332]	1	2518.4268
[R].RPISADS	1xTMTpro [K	6.864E-05	7.389E-05	1	1	3	Q00610	Q00610 [64-78]	0	2166.2196

[R].RPISADS	1xOxidation [	0.0001096	7.389E-05	1	1	2	Q00610	Q00610 [64-78]	0	2182.2146
[K].RPMEED	2xTMTpro [K	0.0001845	7.389E-05	1	1	1	Q12906	Q12906 [372-385]	1	2531.3592
[R].RPPQEC	2xCarbamido	7.474E-05	7.389E-05	1	1	3	Q9Y6C2	Q9Y6C2 [348-360	0	1889.9281
[K].RNEDED	1xTMTpro [K	0.0001232	7.389E-05	1	1	2	P62899	P62899 [92-101]	1	1811.9379
[K].SAELAA	1xTMTpro [N	7.113E-05	7.389E-05	1	1	1	Q9BSJ8	Q9BSJ8 [792-806	0	1942.0639
[K].SAFSSAI	1xTMTpro [K	8.691E-05	7.389E-05	1	1	2	Q96LJ7	Q96LJ7 [221-234]	0	2023.0839
[K].SAGQEN	1xTMTpro [K	7.135E-05	7.389E-05	1	1	1	P48681	P48681 [831-841]	0	1798.0202
[K].SCAVAE	1xCarbamido	6.674E-05	7.389E-05	2	2	2	P00739; P00	P00739 [322-333]	0	1954.06
[K].SCCTSV	3xCarbamido	0.0001511	7.389E-05	1	1	1	P53582	P53582 [192-208]	0	2308.0803
[K].SCDIPVF	1xCarbamido	0.0001732	7.389E-05	1	1	4	P08603	P08603 [508-518]	0	1613.8099
[K].SCLLHQI	1xCarbamido	0.0001716	7.389E-05	1	1	1	P20338	P20338 [27-36]	0	1883.0705
[K].SCLLLQF	1xCarbamido	9.617E-05	7.389E-05	2	2	3	Q8WUD1; P	Q8WUD1 [20-29];	0	1833.0436
[K].SCLLLQF	1xCarbamido	9.497E-05	7.389E-05	2	2	1	Q8WUD1; P	Q8WUD1 [20-30];	1	1989.1447
[K].SCLNVFI	1xCarbamido	0.0001322	7.389E-05	1	1	1	Q709C8	Q709C8 [2394-24	0	1888.0606
[K].SCMAGV	1xCarbamido	9.306E-05	7.389E-05	1	1	1	P98095	P98095 [488-497]	0	1601.8999
[R].SCQDINI	2xCarbamido	0.0001232	7.389E-05	1	1	3	Q9UBX5	Q9UBX5 [285-295	0	1751.776
[K].SCSGVE	1xCarbamido	7.851E-05	7.389E-05	1	1	2	P45880	P45880 [46-64]	0	2516.2066
[K].SCSPLAI	1xCarbamido	7.636E-05	7.389E-05	1	1	1	Q96EY5	Q96EY5 [230-245	0	2322.2659
[R].SCTDVT	1xCarbamido	0.0001166	7.389E-05	1	1	1	Q9H2D6	Q9H2D6 [1841-18	0	1732.8495
[R].SCYLSSI	1xCarbamido	0.0001371	7.389E-05	1	1	1	Q9BQ69	Q9BQ69 [245-258	0	2010.0649
[K].SCALSNI	2xCarbamido	0.0001016	7.389E-05	1	1	1	P03951	P03951 [192-202]	0	1568.8208
[R].SDALNS	1xTMTpro [K	0.0001328	7.389E-05	1	1	3	P35221	P35221 [361-370]	0	1641.9303
[R].SDEGQL	1xTMTpro [N	0.0001678	7.389E-05	1	1	1	Q15149	Q15149 [714-724]	0	1464.7614
[K].SDEVDE	1xCarbamido	6.519E-05	7.389E-05	1	1	1	Q02952	Q02952 [1512-15	0	2244.131
[K].SDFCWC	2xCarbamido	0.0001245	7.389E-05	1	1	2	Q14112	Q14112 [1058-10	1	2153.04
[R].SDGFLW	1xCarbamido	0.0001903	7.389E-05	1	1	1	P08253	P08253 [253-267]	0	2463.2146
[K].SDGGYT	1xTMTpro [K	0.0001105	7.389E-05	1	1	1	P54136	P54136 [378-393]	0	2285.1793
[K].SDGVYT	1xTMTpro [N	8.247E-05	7.389E-05	1	1	1	P30273	P30273 [61-71]	0	1459.7712
[K].SDKTEEI	2xTMTpro [K	0.0001008	7.389E-05	1	1	1	Q9NR30	Q9NR30 [38-54]	1	2893.5499
[K].SDLDTLI	1xTMTpro [K	0.0001533	7.389E-05	1	1	1	Q96JJ3	Q96JJ3 [677-688]	0	1991.0685
[K].SDLETQI	1xTMTpro [K	8.492E-05	7.389E-05	1	1	1	Q9BZF9	Q9BZF9 [1234-12	0	2072.1367
[K].SDLLEEI	1xTMTpro [N	0.0001226	7.389E-05	1	1	1	Q969Q5	Q969Q5 [122-130	1	1436.7664
[R].SDLREE	1xTMTpro [N	0.0001632	7.389E-05	1	1	1	Q96EY7	Q96EY7 [525-537	1	1881.0257

[K].SDMMEE	1xTMTpro [K	0.0001206	7.389E-05	1	1	1	P82970	P82970 [49-67]	0	2678.3145
[K].SDNILLG	1xTMTpro [K	0.00017	7.389E-05	1	2	1	Q13153	Q13153 [392-404]	0	1957.092
[R].SDDTAV	1xCarbamido	0.0001284	7.389E-05	2	2	3	P23083; A0A	P23083 [107-117]	0	1624.7596
[K].RGSIGEI	1xTMTpro [K	0.0001599	7.389E-05	1	1	1	Q05682	Q05682 [200-215]	1	2412.3048
[K].SCALAEI	1xCarbamido	9.162E-05	7.389E-05	1	1	2	Q8IX12	Q8IX12 [464-475]	0	1692.8546
[R].SAYQEA	1xTMTpro [K	0.0001614	7.389E-05	1	1	1	P31947	P31947 [149-159]	0	1850.9814
[K].SAIDLEE	1xTMTpro [K	9.119E-05	7.389E-05	1	1	1	P61011	P61011 [58-71]	0	2086.1346
[R].SAIELDT	1xTMTpro [K	0.0001611	7.389E-05	1	1	1	Q9BTE6	Q9BTE6 [137-161	0	3269.7067
[K].SALIAAL	1xCarbamido	0.0001317	7.389E-05	1	1	1	Q8WZA9	Q8WZA9 [21-50]	1	3775.0192
[R].SALIPPV	1xTMTpro [N	7.485E-05	7.389E-05	1	1	1	Q9H300	Q9H300 [77-96]	0	2579.408
[R].SAISLLA	1xTMTpro [K	0.0001736	7.389E-05	1	1	1	Q8TC71	Q8TC71 [156-171	0	2337.3521
[K].SAIYGFG	1xTMTpro [N	8.146E-05	7.389E-05	1	1	1	Q8NBX0	Q8NBX0 [199-211	0	1731.8985
[K].SAIYQLE	1xTMTpro [K	9.8E-05	7.389E-05	1	1	1	P15924	P15924 [246-260]	0	2450.331
[R].SAMPAD	1xCarbamido	8.388E-05	7.389E-05	1	1	2	Q06828	Q06828 [358-369]	0	1605.8412
[K].SANVNE	1xTMTpro [K	6.427E-05	7.389E-05	1	1	2	P55060	P55060 [471-481]	0	1826.0668
[R].SAPLAA	1xCarbamido	0.000187	7.389E-05	1	1	1	Q92743	Q92743 [30-40]	0	1418.7381
[R].SAPINSD	1xTMTpro [N	0.0001121	7.389E-05	1	1	1	P78356	P78356 [124-134]	0	1435.746
[R].SAPYEFI	1xTMTpro [N	6.188E-05	7.389E-05	1	1	1	Q01433	Q01433 [142-159]	0	2454.1996
[R].SAQPAS	1xTMTpro [N	0.0002007	7.389E-05	1	1	2	Q9Y490	Q9Y490 [625-634	0	1317.7082
[K].SCAEWV	1xCarbamido	8.923E-05	7.389E-05	1	1	2	O75947	O75947 [100-109]	0	1774.9653
[R].SAQPLP	2xTMTpro [K	0.0001392	7.389E-05	1	1	1	Q9UKV3	Q9UKV3 [525-540	1	2633.6422
[K].SASDDQ	1xTMTpro [K	0.0001691	7.389E-05	1	1	2	P13674	P13674 [261-270]	0	1688.8583
[K].SASDLTV	1xTMTpro [K	0.0001425	7.389E-05	1	1	4	P02787	P02787 [454-464]	0	1858.0202
[K].SATEQS	1xTMTpro [N	8.545E-05	7.389E-05	1	1	2	O95831	O95831 [519-529]	0	1410.7508
[K].SATGNS	1xTMTpro [N	0.0001999	7.389E-05	1	1	1	Q8WWP7	Q8WWP7 [41-51]	0	1407.7875
[K].SATLASI	1xTMTpro [K	6.267E-05	7.389E-05	1	1	1	P33176	P33176 [527-539]	0	1955.1305
[R].SATSSSY	1xTMTpro [K	0.0001782	7.389E-05	1	1	1	P42566	P42566 [741-754]	0	1988.1519
[K].SAVENC	1xCarbamido	9.8E-05	7.389E-05	1	1	2	P52209	P52209 [397-407]	0	1655.7767
[K].SAVGFD	1xTMTpro [K	0.0001319	7.389E-05	1	1	2	Q14247	Q14247 [172-181]	0	1679.9248
[K].SAVNVA	1xTMTpro [K	8.611E-05	7.389E-05	1	1	1	A2RRP1	A2RRP1 [564-577	0	2102.2101
[K].SAVTALV	1xTMTpro [K	0.0001903	7.389E-05	1	1	91	P68871	P68871 [10-18]	0	1540.9343
[K].SAYALCT	1xCarbamido	0.0001216	7.389E-05	1	1	2	Q9BY32	Q9BY32 [111-130	0	2445.2403
[R].SAYDST	1xTMTpro [N	8.009E-05	7.389E-05	1	1	2	P53396	P53396 [577-592]	0	2185.0225

[R].SAYGGF	1xTMTpro [N	0.0001999	7.389E-05	1	1	1	P08729	P08729 [53-64]	0	1408.7868
[K].SAQQES	1xTMTpro [K	0.0001678	7.389E-05	1	1	1	Q5JRA6	Q5JRA6 [961-973	0	2133.1142
[K].RGPGLY	1xTMTpro [N	7.291E-05	7.389E-05	1	1	1	P28074	P28074 [166-179]	1	1886.968
[K].RGPDED	1xTMTpro [N	6.674E-05	7.389E-05	1	1	1	Q9C0H2	Q9C0H2 [417-432	1	1985.9484
[R].RGPALE	3xCarbamido	6.208E-05	7.389E-05	1	1	1	P54826	P54826 [121-137]	1	2646.2202
[R].QTVSWA	1xTMTpro [K	0.0001956	7.389E-05	1	1	2	P01023	P01023 [854-863]	0	1725.0191
[R].QTYDDG	1xTMTpro [N	0.0001279	7.389E-05	1	1	1	Q6PJT7	Q6PJT7 [145-154	0	1401.6929
[K].QTYSTEI	1xTMTpro [K	0.0001151	7.389E-05	1	1	3	P46779	P46779 [23-33]	0	1903.0417
[K].QVAEAY	1xTMTpro [K	7.815E-05	7.389E-05	1	2	1	O75190	O75190 [48-60]	0	2031.1254
[K].QVCEIIE	1xCarbamido	6.55E-05	7.389E-05	1	1	1	Q7L5N1	Q7L5N1 [141-153	0	2184.2594
[K].QVCEPE	2xCarbamido	0.0001495	7.389E-05	1	1	2	P35442	P35442 [644-653]	0	1868.949
[K].QVCEPE	2xCarbamido	9.663E-05	7.389E-05	1	1	2	P35442	P35442 [644-655]	1	2416.2781
[K].QVEEALI	1xTMTpro [N	0.0001168	7.389E-05	1	1	2	O00233	O00233 [92-103]	0	1734.957
[K].QVEHPL	1xTMTpro [K	0.0001023	7.389E-05	1	1	1	P54577	P54577 [155-178]	0	3333.859
[K].QVENAG	1xTMTpro [N	7.048E-05	7.389E-05	1	1	1	Q9UHD8	Q9UHD8 [118-12	0	1502.8246
[R].QVETELI	1xCarbamido	0.000101	7.389E-05	1	3	4	O43488	O43488 [206-216	0	1695.9059
[K].QVFLYPI	2xTMTpro [K	8.479E-05	7.389E-05	1	1	2	P04114	P04114 [140-157]	1	3122.7958
[R].QVGSISI	1xTMTpro [K	8.159E-05	7.389E-05	1	1	1	Q10567	Q10567 [741-754	0	2142.2084
[R].QTSPVS	1xOxidation [	0.0001662	7.389E-05	1	1	1	Q12805	Q12805 [441-453	0	1997.1961
[K].QVHPDT	1xTMTpro [K	8.059E-05	7.389E-05	7	15	7	Q5QNW6; Q	Q5QNW6 [48-58]	0	1777.01
[K].QVIDVLE	1xTMTpro [K	0.0001352	7.389E-05	1	1	1	P80303	P80303 [60-69]	0	1768.0348
[R].QVLEGE	1xTMTpro [K	7.213E-05	7.389E-05	1	1	2	Q03252	Q03252 [506-516	0	1887.0719
[K].QVIGTGS	1xTMTpro [K	7.508E-05	7.389E-05	1	1	3	Q13642	Q13642 [134-144	0	1789.0504
[K].QVLLAQ	1xTMTpro [K	0.0001371	7.389E-05	1	1	2	Q14254	Q14254 [308-318	0	1808.0773
[R].QVILLHT	1xTMTpro [N	0.0001144	7.389E-05	1	1	1	Q8WVC6	Q8WVC6 [188-19	0	1654.9811
[K].QVIQGLE	1xTMTpro [K	0.0001672	7.389E-05	1	1	1	Q07866	Q07866 [30-39]	0	1707.066
[K].QVLQLQ	1xTMTpro [N	0.0001793	7.389E-05	1	1	1	Q9P2E9	Q9P2E9 [865-874	0	1483.8664
[R].QVITLLN	1xTMTpro [K	0.0001471	7.389E-05	1	1	2	P09110	P09110 [386-395]	0	1779.1235
[K].QVMMFS	1xTMTpro [K	7.247E-05	7.389E-05	1	1	2	Q13838	Q13838 [223-233	0	1851.0364
[R].QVMVVF	1xCarbamido	8.159E-05	7.389E-05	1	1	1	P26639	P26639 [620-636]	0	2529.3337
[R].QVPGGL	1xTMTpro [K	0.0001956	7.389E-05	1	1	1	Q8NFI3	Q8NFI3 [262-281]	0	2781.5795
[K].QVPVDV	1xTMTpro [K	0.0001159	7.389E-05	1	1	2	P55884	P55884 [553-562]	0	1752.0221
[K].QVQTLQ	1xTMTpro [K	0.0001665	7.389E-05	1	1	3	Q9UPN3	Q9UPN3 [3876-38	0	1938.1152

[R].QVLDNL	1xOxidation [	0.0002031	7.389E-05	1	1	4	P35527	P35527 [262-271]	0	1815.0178
[R].QVQVAL	1xTMTpro [N	0.0001581	7.389E-05	1	1	2	Q15149	Q15149 [1699-17	0	1546.8872
[K].QTPSGP	2xTMTpro [K	0.0001903	7.389E-05	1	1	2	P29536	P29536 [294-308]	0	2394.3809
[R].QTLSTP	1xTMTpro [K	9.587E-05	7.389E-05	1	1	1	Q9BSD7	Q9BSD7 [131-14	0	2444.4984
[K].QSCITE	1xCarbamid	0.0001662	7.389E-05	1	1	1	P54289	P54289 [972-988]	0	2719.3165
[R].QSEDST	1xTMTpro [N	8.651E-05	7.389E-05	1	1	3	P00450	P00450 [721-732]	0	1735.8458
[K].QSFTMV	1xOxidation [	7.474E-05	7.389E-05	1	1	3	Q14847	Q14847 [60-73]	0	1928.9707
[K].QSGESI	1xTMTpro [N	0.0001479	7.389E-05	1	1	1	Q16531	Q16531 [93-103]	0	1522.8396
[K].QSGEYW	1xTMTpro [K	9.512E-05	7.389E-05	1	1	1	P05997	P05997 [1307-13	0	2744.4023
[K].QSLEYL	1xTMTpro [N	0.0001511	7.389E-05	1	1	2	Q9BZE4	Q9BZE4 [146-15	0	1568.8603
[K].QSIGQF	1xTMTpro [K	0.0001249	7.389E-05	1	1	1	O95425	O95425 [1770-17	0	2286.2374
[K].QSLPPG	1xTMTpro [K	0.0001611	7.389E-05	1	1	4	P63173	P63173 [58-67]	0	1618.0184
[K].QSISNS	1xTMTpro [N	0.0002055	7.389E-05	1	1	1	P04839	P04839 [549-559]	0	1465.7566
[R].QSMAFS	1xTMTpro [K	0.000181	7.389E-05	1	1	1	Q14980	Q14980 [2046-20	0	1945.1072
[R].QSPVDI	1xTMTpro [K	0.0001025	7.389E-05	1	1	2	P00918	P00918 [28-39]	0	1920.0682
[R].QSQQLE	1xTMTpro [K	0.0001129	7.389E-05	1	1	1	Q9P0K7	Q9P0K7 [914-926	0	2136.2268
[R].QSSEAE	1xTMTpro [K	0.0001793	7.389E-05	1	1	2	Q15149	Q15149 [1564-15	0	1698.9518
[K].QTPMAS	1xTMTpro [K	0.0001432	7.389E-05	1	1	1	Q5SSJ5	Q5SSJ5 [150-160	0	1808.0344
[K].QSTCEA	1xCarbamid	7.695E-05	7.389E-05	1	3	1	P40424	P40424 [217-227]	0	1611.8518
[K].QSVEDI	2xTMTpro [K	0.0001354	7.389E-05	1	1	1	P36871	P36871 [407-419]	1	2538.4496
[R].QSVEND	1xTMTpro [N	0.0001224	7.389E-05	1	1	1	P05783	P05783 [176-186]	0	1571.8461
[R].QSVEND	1xTMTpro [K	0.0001665	7.389E-05	1	1	1	P05783	P05783 [176-187]	1	2004.1482
[K].QTDVGI	1xTMTpro [N	8.272E-05	7.389E-05	1	1	1	Q6P2Q9	Q6P2Q9 [1345-13	0	1477.8082
[R].QTESLE	1xTMTpro [K	0.0001237	7.389E-05	1	1	1	Q07065	Q07065 [430-440	0	1843.0668
[K].QTESTS	1xTMTpro [K	0.0001647	7.389E-05	1	1	1	P60900	P60900 [172-181]	0	1777.9828
[K].QTEVLL	1xTMTpro [N	0.0001212	7.389E-05	1	1	2	Q9Y371	Q9Y371 [62-74]	0	1783.9986
[R].QTEVLL	1xTMTpro [N	0.0001926	7.389E-05	1	1	2	Q9NR46	Q9NR46 [59-71]	0	1756.9877
[R].QTFSPF	1xTMTpro [N	0.0001038	7.389E-05	2	2	3	Q01085; P3	Q01085 [223-235	0	1857.9852
[R].QTGEG	1xTMTpro [K	0.0001066	7.389E-05	1	1	1	Q9P2J5	Q9P2J5 [254-268	0	2228.2418
[R].QTHQPF	1xTMTpro [N	8.882E-05	7.389E-05	1	1	2	P02747	P02747 [127-139]	0	1762.9883
[R].QTLMW	1xTMTpro [K	0.0001514	7.389E-05	2	2	2	Q92841; P1	Q92841 [351-361	0	1957.0861
[R].QTLSIY	1xTMTpro [K	9.878E-05	7.389E-05	1	1	5	Q15149	Q15149 [3839-38	0	1773.0766
[R].QSVEAD	1xTMTpro [N	0.0001025	7.389E-05	2	3	2	P13645; P1	P13645 [246-256]	0	1505.8243

[R].QQTQSA	1xTMTpro [N	0.000181	7.389E-05	1	1	1	Q86YS7	Q86YS7 [337-346	0	1492.8039
[K].QVSELS	1xTMTpro [K	8.171E-05	7.389E-05	1	1	2	Q9P0K7	Q9P0K7 [644-653	0	1803.0508
[R].QVTQEE	1xTMTpro [N	0.0001093	7.389E-05	1	1	1	P62070	P62070 [136-147]	0	1690.9043
[R].RAQEILS	1xTMTpro [K	0.0001948	7.389E-05	1	1	2	P35754	P35754 [28-39]	1	2004.2461
[R].RCEPIAN	1xCarbamido	0.000144	7.389E-05	1	1	1	P31146	P31146 [344-354]	1	1633.8837
[R].RDCAVK	2xCarbamido	0.0001196	7.389E-05	1	1	2	Q96HE7	Q96HE7 [97-114]	1	2986.5906
[K].RDEFWD	1xTMTpro [N	7.851E-05	7.389E-05	1	2	1	Q8WUN7	Q8WUN7 [54-67]	1	2000.9786
[K].RDELTLE	1xTMTpro [K	6.387E-05	7.389E-05	1	1	2	P38919	P38919 [243-252]	1	1782.0617
[R].RDEPTG	1xTMTpro [K	6.685E-05	7.389E-05	1	1	1	O75643	O75643 [33-46]	1	2108.2207
[K].RDHALLI	1xTMTpro [K	8.324E-05	7.389E-05	1	1	4	O43707	O43707 [633-643]	1	1934.0951
[K].RDPHLA	1xCarbamido	8.222E-05	7.389E-05	1	2	2	Q00610	Q00610 [912-923]	1	1790.9291
[R].RDTVEV	1xTMTpro [K	7.015E-05	7.389E-05	1	1	1	Q14315	Q14315 [421-431]	1	1883.073
[R].RECPSD	2xCarbamido	0.0001817	7.389E-05	1	1	1	P62979	P62979 [119-138]	1	2632.1662
[R].REEAAV	1xTMTpro [K	0.0001196	7.389E-05	1	1	1	Q15149	Q15149 [1541-15	1	1981.0958
[K].REELFIV	1xTMTpro [K	0.0001817	7.389E-05	1	1	2	P15121	P15121 [70-78]	1	1729.0504
[K].REELITN	1xTMTpro [N	6.864E-05	7.389E-05	1	1	5	Q13813	Q13813 [336-347]	1	1891.0357
[R].RAPSSA	1xTMTpro [K	9.021E-05	7.389E-05	1	1	1	P57737	P57737 [876-887]	1	1987.1104
[K].REELSN	1xTMTpro [N	0.000111	7.389E-05	1	1	1	Q9Y3U8	Q9Y3U8 [87-98]	1	1692.9386
[R].REPVSLI	1xTMTpro [K	0.0001587	7.389E-05	1	1	1	Q8TC07	Q8TC07 [314-324	1	1982.1203
[R].RFDAMP	1xTMTpro [N	0.0002019	7.389E-05	1	1	2	Q9UQ80	Q9UQ80 [272-281	1	1557.8531
[R].RFDDAV	1xTMTpro [K	7.247E-05	7.389E-05	1	1	6	P11142	P11142 [77-88]	1	2019.0825
[R].RFDDAV	1xOxidation	0.0001072	7.389E-05	1	1	7	P11142	P11142 [77-88]	1	2035.0774
[R].RFDEAA	1xTMTpro [K	0.0001564	7.389E-05	1	1	1	Q99747	Q99747 [169-179]	1	1886.0991
[SK].RFDVS	1xTMTpro [K	0.0001899	7.389E-05	1	1	2	P13667	P13667 [131-141]	1	1891.0933
[R].RFEEAL	1xTMTpro [N	7.672E-05	7.389E-05	1	1	1	O94832	O94832 [771-782]	1	1828.0037
[R].RFNTAN	1xTMTpro [N	6.716E-05	7.389E-05	1	1	2	P04040	P04040 [431-444]	1	1954.0062
[R].RGAAAS	1xTMTpro [N	0.0002039	7.389E-05	1	1	3	P84157	P84157 [29-39]	1	1386.7773
[R].RGEAHL	1xTMTpro [N	0.0001766	7.389E-05	1	1	1	Q02790	Q02790 [359-373]	1	2002.0789
[R].RGEAQL	1xTMTpro [K	0.0002035	7.389E-05	1	1	1	Q13451	Q13451 [357-371]	1	2331.2622
[R].RGETES	1xTMTpro [K	0.0002007	7.389E-05	1	1	2	Q05682	Q05682 [543-553]	1	1949.0108
[R].RGGPPF	1xTMTpro [N	8.479E-05	7.389E-05	1	1	1	Q07955	Q07955 [51-74]	2	3001.4764
[R].REGAAA	1xTMTpro [N	0.0001903	7.389E-05	1	1	1	Q7Z2K6	Q7Z2K6 [20-31]	1	1525.8406
[R].QVSSIPI	1xTMTpro [K	0.0001039	7.389E-05	1	1	1	Q709C8	Q709C8 [846-858	0	1895.1457

[R].RAPQTG	2xCarbamido	7.96E-05	7.389E-05	1	1	2	P05019	P05019 [85-98]	1	2012.9965
[K].RAGELTI	1xTMTpro [N	0.000142	7.389E-05	1	1	2	P62269	P62269 [55-66]	1	1707.8833
[K].QVTQLA	1xTMTpro [N	6.612E-05	7.389E-05	1	1	2	Q04637	Q04637 [782-793]	0	1706.9244
[R].QVVAVT	1xTMTpro [K	0.0001087	7.389E-05	2	4	1	P20020; P23	P20020 [790-806]	0	2250.2585
[R].QVVNFG	1xTMTpro [K	0.0001514	7.389E-05	1	1	2	Q9Y617	Q9Y617 [6-16]	0	1722.0194
[K].QVVNIPS	1xTMTpro [N	0.0001547	7.389E-05	1	1	4	P46781	P46781 [140-150]	0	1575.9542
[R].QVWGLI	1xTMTpro [K	0.0001425	7.389E-05	1	1	2	P50552	P50552 [87-96]	0	1744.0038
[R].QVYGLN	1xTMTpro [K	0.0001109	7.389E-05	1	1	2	Q8N8S7	Q8N8S7 [85-94]	0	1720.9878
[K].QWAEIG	1xTMTpro [K	9.191E-05	7.389E-05	1	1	3	Q8IZ81	Q8IZ81 [151-163]	0	2099.1053
[R].QWCNC	2xCarbamido	0.000146	7.389E-05	1	1	1	P62834	P62834 [137-149]	0	2209.1026
[R].QWLNS(	1xTMTpro [N	7.015E-05	7.389E-05	1	1	1	O14974	O14974 [183-194]	0	1742.9257
[R].QWNTLY	1xCarbamido	9.587E-05	7.389E-05	1	1	1	P56192	P56192 [302-321]	0	2959.4639
[R].QWPELI	1xTMTpro [K	6.357E-05	7.389E-05	1	1	1	Q9UI26	Q9UI26 [124-137]	0	2261.3401
[K].QYAGYD	1xTMTpro [N	7.497E-05	7.389E-05	1	1	1	Q969M3	Q969M3 [38-49]	0	1739.8308
[R].QYDIDD	1xTMTpro [K	0.0001168	7.389E-05	1	1	2	Q15149	Q15149 [4339-43	0	1759.9722
[R].RAPEEE	1xTMTpro [K	0.0001746	7.389E-05	1	1	1	Q8IY67	Q8IY67 [30-46]	2	2626.4696
[K].QYDSFT	1xTMTpro [K	6.55E-05	7.389E-05	1	1	1	Q9Y3B3	Q9Y3B3 [92-102]	0	1903.0093
[R].QYFPND	1xTMTpro [K	0.0001635	7.389E-05	1	1	1	P13674	P13674 [119-132]	0	2190.1323
[K].QYGNEV	1xTMTpro [K	0.0001114	7.389E-05	1	1	4	P50990	P50990 [172-181]	0	1777.014
[K].QYIISEE	1xTMTpro [K	9.557E-05	7.389E-05	1	1	1	Q9UKK9	Q9UKK9 [15-27]	0	2117.1986
[R].QYLQEV	1xTMTpro [K	7.555E-05	7.389E-05	1	1	1	O43815	O43815 [164-179]	0	2493.3732
[R].QYMEGF	1xTMTpro [K	7.213E-05	7.389E-05	1	1	2	Q16543	Q16543 [247-260]	0	2329.1666
[K].QYNDGL	1xTMTpro [N	9.572E-05	7.389E-05	1	1	1	Q16363	Q16363 [1306-13	0	2311.2002
[K].QYPISLV	1xTMTpro [N	8.745E-05	7.389E-05	1	2	2	O00571	O00571 [265-276]	0	1661.991
[R].QYQIDA	1xTMTpro [N	0.0001691	7.389E-05	2	2	2	Q13620; Q1	Q13620 [847-856]	0	1480.8443
[R].QYQLLF	2xTMTpro [K	8.44E-05	7.389E-05	1	1	1	Q8TCT9	Q8TCT9 [137-161	1	3907.0582
[K].QYVETH	1xCarbamido	0.0001222	7.389E-05	1	1	1	Q96P70	Q96P70 [83-95]	0	2274.1469
[K].QYYEGS	1xTMTpro [N	6.843E-05	7.389E-05	1	1	2	P19827	P19827 [502-514]	0	1774.9295
[R].QYYTLLI	1xTMTpro [N	8.868E-05	7.389E-05	1	1	1	Q13283	Q13283 [18-32]	0	2167.1289
[R].RADLNQ	1xTMTpro [N	0.0001136	7.389E-05	1	1	1	Q96C19	Q96C19 [62-77]	1	2029.0746
[K].QYECSL	1xCarbamido	6.198E-05	7.389E-05	1	1	1	Q8TE77	Q8TE77 [431-441	0	1700.8597
[R].QAHILLD	2xCarbamido	6.247E-05	7.389E-05	1	1	1	P06756	P06756 [619-635]	0	2909.5793
[K].QAGVFE	1xTMTpro [K	9.957E-05	7.389E-05	1	1	2	P17987	P17987 [500-510]	0	1797.0766

[K].QAGPAS	1xTMTpro [K	9.291E-05	7.389E-05	1	1	3	P30101	P30101 [131-146]	1	2367.3164
[R].MQILEDI	1xTMTpro [K	8.121E-05	7.389E-05	2	3	1	P46939; P11	P46939 [3332-334	0	1735.9657
[K].MQLLEII	1xTMTpro [K	0.0001385	7.389E-05	1	1	4	P40939	P40939 [506-516]	0	1927.143
[R].MQILQA\	1xTMTpro [K	0.0001911	7.389E-05	1	1	1	Q16513	Q16513 [192-218]	0	3647.0486
[R].MQNDTA	1xTMTpro [K	0.0001044	7.389E-05	1	1	1	Q05682	Q05682 [137-149]	0	2119.0469
[K].MQNWC	2xCarbamido	0.0001038	7.389E-05	1	1	2	Q9BY44	Q9BY44 [128-144	0	2474.0858
[K].MQTDKF	1xOxidation [	0.0001107	7.389E-05	1	1	2	Q15417	Q15417 [193-212]	0	3212.7111
[K].MSAEINI	1xTMTpro [N	0.0002003	7.389E-05	1	1	4	P18206	P18206 [237-246]	0	1479.816
[-].MSAHLQ\	1xMet-loss+/	0.0002039	7.389E-05	1	1	2	P46779	P46779 [1-11]	0	1572.864
[K].MSATFIG	1xTMTpro [K	6.327E-05	7.389E-05	2	3	2	Q13885; P68	Q13885 [363-380]	1	2622.4569
[-].MSDKPDI	1xMet-loss+/	9.467E-05	7.389E-05	1	1	1	P62328	P62328 [1-12]	0	2217.2253
[K].MSGSAS	1xTMTpro [N	0.0001405	7.389E-05	1	1	1	O43493	O43493 [346-355]	0	1329.6388
[-].MSHQTG	1xMet-loss+/	0.0001789	7.389E-05	1	1	1	Q12792	Q12792 [1-14]	0	2050.1061
[K].MSNALA	1xTMTpro [K	0.0001136	7.389E-05	1	1	1	Q13573	Q13573 [82-95]	0	2071.1349
[K].MSPNET	1xOxidation [	0.0001161	7.389E-05	1	2	1	Q9HB90	Q9HB90 [85-98]	0	2235.1823
[-].MSSSGTI	1xMet-loss+/	6.643E-05	7.389E-05	1	1	1	P00352	P00352 [1-17]	0	2293.3147
[K].MSSVFE	1xTMTpro [N	6.993E-05	7.389E-05	1	1	1	Q9P0J1	Q9P0J1 [469-484]	0	2123.0875
[K].MSSYAF	1xCarbamido	0.0001335	7.389E-05	2	3	2	P26583; P09	P26583 [13-24]; F	0	1816.8681
[R].MSVDAV	1xTMTpro [N	6.726E-05	7.389E-05	1	1	2	P78347	P78347 [102-113]	0	1666.9005
[R].MSVEAD	1xTMTpro [N	0.0001922	7.389E-05	2	2	2	P02533; P08	P02533 [212-222]	0	1508.8062
[R].MSVIEE(	1xCarbamido	0.0001004	7.389E-05	1	1	1	P11216	P11216 [429-439]	1	1932.0174
[-].MSVTLHT	1xMet-loss+/	7.936E-05	7.389E-05	1	1	1	Q9H2H8	Q9H2H8 [1-13]	0	1935.1043
[R].MSYLTA	1xCarbamido	0.0001023	7.389E-05	1	1	1	Q9UDY2	Q9UDY2 [901-917	0	2245.0258
[R].MTALAQ	1xTMTpro [N	0.000101	7.389E-05	1	1	1	P50416	P50416 [202-216]	0	1851.0118
[K].MTCVDV	2xCarbamido	0.0001425	7.389E-05	1	1	1	Q14766	Q14766 [1658-16	0	2172.9643
[K].MTELFQ	1xTMTpro [N	0.0001015	7.389E-05	1	1	1	P46939	P46939 [2845-285	0	2055.0864
[R].MTESHL	2xCarbamido	0.000181	7.389E-05	1	1	1	Q9UKG1	Q9UKG1 [540-553	0	2315.2053
[R].MTESWI	1xTMTpro [N	0.0001407	7.389E-05	1	1	1	Q16401	Q16401 [383-393]	0	1634.8168
[-].MQLKPMI	2xTMTpro [K	0.0001241	7.389E-05	1	1	1	P09936	P09936 [1-15]	0	2728.538
[R].MQHNLE	1xTMTpro [N	0.0001241	7.389E-05	1	1	1	Q13813	Q13813 [2304-23	0	1799.9506
[K].MQEVVA	1xTMTpro [K	0.0001094	7.389E-05	1	1	1	Q16531	Q16531 [1105-11	0	2493.2609
[K].MQELLM	1xOxidation [	8.059E-05	7.389E-05	1	1	2	Q9NZN4	Q9NZN4 [359-370	0	2088.1113
[R].MLVSGQ	1xTMTpro [N	8.184E-05	7.389E-05	1	1	1	Q9BXW7	Q9BXW7 [134-15	0	2295.1909

[R].MIVTPQS	1xTMTpro [N	0.0001039	7.389E-05	1	1	1	P24928	P24928 [524-546]	0	2830.5602
[K].MLYDME	1xOxidation [	0.0001235	7.389E-05	1	1	1	Q93052	Q93052 [400-415]	0	2268.0272
[K].MLYYGE	1xTMTpro [K	9.234E-05	7.389E-05	1	1	3	Q16363	Q16363 [402-414]	0	2204.1553
[K].MLYYGE	1xOxidation [	0.0001125	7.389E-05	1	1	2	Q16363	Q16363 [402-414]	0	2220.1502
[R].MMAIAPI	1xOxidation [	7.146E-05	7.389E-05	1	1	4	Q9Y2X3	Q9Y2X3 [279-297	0	2279.2245
[-].MMCGAP	1xAcetyl [N-T	0.0001061	7.389E-05	1	1	2	P04080	P04080 [1-24]	0	2933.3875
[K].MMEMFC	1xTMTpro [K	0.0001605	7.389E-05	1	1	2	Q05707	Q05707 [1232-12	0	1822.9761
[R].MMEVAA	1xTMTpro [K	0.0001435	7.389E-05	1	1	3	Q96KP4	Q96KP4 [44-53]	0	1672.9258
[R].MMEVAA	1xOxidation [	0.0001347	7.389E-05	1	1	4	Q96KP4	Q96KP4 [44-53]	0	1688.9207
[R].MMLLEN	1xCarbamido	0.0001944	7.389E-05	1	1	5	Q6FHJ7	Q6FHJ7 [265-275	0	2004.0824
[K].MNESDA	1xTMTpro [K	7.325E-05	7.389E-05	1	1	2	O60749	O60749 [299-309]	0	1993.9821
[K].MNFGDF	1xTMTpro [K	6.387E-05	7.389E-05	1	1	4	P41208	P41208 [84-96]	0	2140.1426
[R].MTLADD	1xOxidation [	8.993E-05	7.389E-05	1	1	2	P62191	P62191 [372-387]	0	2389.285
[K].MNLYGF	1xTMTpro [N	0.00012	7.389E-05	1	1	3	P22105	P22105 [2053-20	0	1583.8072
[R].MPAAET	1xCarbamido	0.0001567	7.389E-05	1	1	1	O00534	O00534 [507-519]	0	2031.0746
[K].MPCQLH	1xCarbamido	0.0001188	7.389E-05	1	1	1	P17655	P17655 [638-649]	0	1771.9631
[K].MPEDGL	1xTMTpro [K	0.0001306	7.389E-05	1	1	2	Q05682	Q05682 [622-631]	0	1714.8813
[K].MPEDGL	3xTMTpro [K	0.0001656	7.389E-05	1	1	3	Q05682	Q05682 [622-635]	1	2823.6067
[-].MPFLELD	1xMet-loss [M	0.0001073	7.389E-05	1	2	1	P30046	P30046 [1-14]	0	1803.9924
[R].MPGNPY	1xTMTpro [N	0.0001324	7.389E-05	1	1	1	Q5T4S7	Q5T4S7 [4367-43	0	2221.1065
[-].MPQYQT	1xMet-loss [M	0.0001387	7.389E-05	1	1	5	P49458	P49458 [1-12]	0	1774.872
[-].MPVDFTC	1xMet-loss [M	0.0001041	7.389E-05	1	1	4	P09455	P09455 [1-10]	0	1720.9554
[-].MPYQYP	1xMet-loss [M	7.439E-05	7.389E-05	1	1	2	P04075	P04075 [1-13]	0	2043.1407
[K].MQAGEE	1xTMTpro [N	9.248E-05	7.389E-05	1	1	2	Q5T8P6	Q5T8P6 [825-835	0	1566.8117
[R].MQALEP	1xTMTpro [K	0.0001121	7.389E-05	1	1	1	O43148	O43148 [415-428]	0	2173.1455
[K].MQEDLV	1xTMTpro [K	0.0002039	7.389E-05	1	1	2	P54289	P54289 [40-49]	0	1756.017
[K].MQELAL	1xTMTpro [N	0.000102	7.389E-05	1	1	1	O75165	O75165 [600-612]	0	1718.943
[R].MNTLQA	1xTMTpro [K	0.0001295	7.389E-05	1	1	1	Q12931	Q12931 [292-304]	0	2187.162
[-].MTLDVGF	1xMet-loss [M	8.518E-05	7.389E-05	1	1	1	P15735	P15735 [1-18]	0	2435.295
[K].MTNYDV	1xTMTpro [K	0.0001567	7.389E-05	1	1	2	P08133	P08133 [569-579]	0	1959.0501
[K].MTNYDV	1xOxidation [	9.542E-05	7.389E-05	1	1	4	P08133	P08133 [569-579]	0	1975.045
[K].MYLGYE	1xOxidation [	7.555E-05	7.389E-05	1	1	7	P02787	P02787 [332-343]	0	1798.9369
[K].NACGSG	1xCarbamido	0.000121	7.389E-05	1	1	1	P49821	P49821 [185-199]	0	2009.971

[K].NADMSE	1xCarbamidc	0.0001266	7.389E-05	1	1	1	Q96FJ2	Q96FJ2 [10-31]	0	3096.3874
[K].NADMSE	1xCarbamidc	6.622E-05	7.389E-05	1	1	1	P63167	P63167 [10-31]	0	3138.4521
[K].NADPILIS	1xTMTpro [K	0.0001644	7.389E-05	1	1	2	Q9ULV4	Q9ULV4 [382-391	0	1692.0551
[R].NAENAIE	1xTMTpro [K	0.000182	7.389E-05	1	2	4	P16615	P16615 [111-120]	0	1680.9776
[K].NAEQAA	1xTMTpro [K	0.0001392	7.389E-05	1	1	2	Q86UP2	Q86UP2 [478-487	0	1681.9729
[R].NAEQYK	2xTMTpro [K	8.772E-05	7.389E-05	1	1	1	P35579	P35579 [1857-18	1	2222.2233
[R].NAESAIE	1xTMTpro [K	0.0001732	7.389E-05	1	1	1	Q93084	Q93084 [111-120	0	1653.9667
[R].NAFEISC	1xTMTpro [N	9.632E-05	7.389E-05	1	1	1	Q14155	Q14155 [544-555	0	1657.8539
[R].NAFMML	1xTMTpro [N	7.439E-05	7.389E-05	1	1	1	P53618	P53618 [192-204]	0	1865.9321
[K].NAGVEG	1xTMTpro [K	7.258E-05	7.389E-05	1	1	1	P10809	P10809 [482-493]	0	1824.0722
[K].NAIDDG	1xCarbamidc	8.059E-05	7.389E-05	1	1	2	P40227	P40227 [400-424]	0	3078.6459
[K].MYEVVY	1xOxidation	0.0001166	7.389E-05	1	1	2	Q9UHG3	Q9UHG3 [292-30	0	1908.9696
[K].NALDPM	1xTMTpro [N	0.0001065	7.389E-05	1	1	1	P42785	P42785 [463-474]	0	1603.9161
[K].NALESY	1xTMTpro [K	7.96E-05	7.389E-05	1	1	2	P54652	P54652 [543-553]	0	1924.0672
[K].NALTTL	1xTMTpro [K	0.0001028	7.389E-05	1	1	1	Q9H3P2	Q9H3P2 [147-161	0	2101.2877
[R].NALWHT	1xTMTpro [N	0.0001933	7.389E-05	1	1	1	P49747	P49747 [652-665]	0	1917.9738
[R].NANAEP	1xTMTpro [N	0.0001968	7.389E-05	1	1	3	P14209	P14209 [171-180]	0	1373.7456
[R].NAPWTL	1xTMTpro [N	7.269E-05	7.389E-05	1	1	1	P49585	P49585 [148-162]	0	2086.1041
[R].NAQSNA	1xTMTpro [N	0.0001068	7.389E-05	1	1	1	P11717	P11717 [2437-24	0	1434.762
[R].NASGDS	1xTMTpro [N	8.466E-05	7.389E-05	1	1	1	Q12929	Q12929 [572-597	0	2985.5713
[K].NAVEEY	1xTMTpro [N	0.0001075	7.389E-05	1	1	2	P34932	P34932 [619-629]	0	1706.8379
[R].NAVIVAL	1xTMTpro [K	0.0001279	7.389E-05	1	1	2	P61086	P61086 [177-186]	0	1610.0133
[R].NAVTQE	1xTMTpro [N	7.258E-05	7.389E-05	1	1	1	Q99798	Q99798 [634-648	0	1905.999
[K].NAWADN	1xCarbamidc	0.0001249	7.389E-05	1	1	1	Q9NTJ5	Q9NTJ5 [436-447	0	1913.9784
[K].NAWNIIG	1xTMTpro [N	0.0001573	7.389E-05	1	1	2	O75954	O75954 [136-146	0	1649.8753
[R].NAYTPQ	1xTMTpro [K	9.739E-05	7.389E-05	1	1	1	P34059	P34059 [106-129]	0	3232.7961
[K].NALESY	1xTMTpro [K	6.907E-05	7.389E-05	1	2	1	P0DMV9	P0DMV9 [540-55	0	1896.0181
[K].MLVSGA	1xOxidation	0.0001198	7.389E-05	2	2	1	P40227; Q9	P40227 [46-55]; Q	0	1614.9381
[R].MYDAQ	1xOxidation	6.674E-05	7.389E-05	1	1	1	Q07954	Q07954 [794-806	0	2135.1047
[K].MWEVLE	1xTMTpro [K	6.509E-05	7.389E-05	1	1	2	Q01082	Q01082 [1361-13	0	2061.1182
[K].MTVCSP	1xCarbamidc	0.0001498	7.389E-05	1	1	2	P09758	P09758 [41-52]	0	1537.7422
[K].MTVTDQ	1xCarbamidc	0.0001029	7.389E-05	1	1	1	P05090	P05090 [177-187]	0	1917.0065
[K].MVAAVA	1xCarbamidc	0.000159	7.389E-05	1	1	2	Q9HCC0	Q9HCC0 [425-43	0	1853.0633

[K].MVADLV	1xTMTpro [K	8.196E-05	7.389E-05	1	1	1	Q00341	Q00341 [573-590]	0	2620.4552
[K].MVADLV	1xOxidation [	6.57E-05	7.389E-05	1	1	1	Q00341	Q00341 [573-590]	0	2636.4501
[K].MVDPEK	1xOxidation [	0.0001228	7.389E-05	1	1	1	P09874	P09874 [143-156]	0	2253.2227
[K].MVEEVF	1xCarbamido	8.453E-05	7.389E-05	1	1	1	P42285	P42285 [419-436]	0	2709.3243
[K].MVEINFL	1xCarbamido	6.716E-05	7.389E-05	1	2	1	P30419	P30419 [242-252]	0	1998.116
[-].MVKETTY	1xMet-loss [N	6.387E-05	7.389E-05	1	1	1	P31689	P31689 [1-24]	2	4174.4441
[-].MVLAELY	1xMet-loss [N	0.0001288	7.389E-05	1	1	2	O43776	O43776 [1-11]	0	1468.8331
[-].MVLESTM	1xCarbamido	7.037E-05	7.389E-05	1	1	1	P55036	P55036 [1-17]	0	2237.0571
[K].MVIPGGI	1xOxidation [	0.0001103	7.389E-05	1	1	5	Q14195	Q14195 [64-81]	0	2694.4392
[R].MVIPNQI	1xTMTpro [K	0.0001756	7.389E-05	1	1	2	Q93052	Q93052 [164-176]	0	2018.1964
[K].MWEVQT	1xCarbamido	6.519E-05	7.389E-05	1	1	1	P43034	P43034 [218-228]	0	2009.048
[-].MVLSPAD	1xMet-loss [N	0.0001091	7.389E-05	1	1	8	P69905	P69905 [1-12]	1	2084.2896
[R].MVNSNL	1xTMTpro [K	6.601E-05	7.389E-05	1	1	1	P08559	P08559 [324-336]	0	2042.1448
[R].MVNSNL	1xOxidation [	0.0001258	7.389E-05	1	1	1	P08559	P08559 [324-343]	1	2898.5738
[R].MVPAGM	1xTMTpro [N	8.518E-05	7.389E-05	1	1	2	P52272	P52272 [532-543]	0	1492.7935
[-].MVPPVQY	1xMet-loss [N	0.0001756	7.389E-05	1	1	2	P56385	P56385 [1-12]	0	1785.1494
[-].MVQAWY	1xMet-loss [N	0.0001277	7.389E-05	1	1	1	Q9BV57	Q9BV57 [1-15]	0	1924.9183
[K].MVTGVA	1xTMTpro [N	7.113E-05	7.389E-05	1	1	4	P69892	P69892 [134-145]	0	1482.8269
[K].MVTMVE	1xCarbamido	6.488E-05	7.389E-05	1	1	1	P52788	P52788 [215-230]	0	2477.2768
[K].MVTVEF	1xTMTpro [N	7.393E-05	7.389E-05	1	1	2	Q96AC1	Q96AC1 [623-633	0	1599.8372
[K].MVVESA	1xTMTpro [K	8.034E-05	7.389E-05	1	1	2	P07195	P07195 [234-244]	0	1876.0745
[K].MVVESA	1xOxidation [	7.555E-05	7.389E-05	1	1	4	P07195	P07195 [234-244]	0	1892.0695
[-].MVVGAFI	1xMet-loss [N	0.0001387	7.389E-05	1	1	2	Q9H6K4	Q9H6K4 [1-10]	0	1527.9213
[-].MVVSEVD	1xMet-loss [N	0.000106	7.389E-05	1	1	2	P20591	P20591 [1-10]	0	1567.9551
[K].MWDPHI	1xOxidation [	9.335E-05	7.389E-05	1	1	2	P08621	P08621 [88-103]	0	2467.1956
[-].MVMEKPS	1xMet-loss [N	6.885E-05	7.389E-05	2	2	1	Q13283; Q9	Q13283 [1-13]; Q	0	1934.1753
[K].MLVSGA	1xTMTpro [K	0.0001155	7.389E-05	2	2	3	P40227; Q9	P40227 [46-55]; Q	0	1598.9431
[K].MLVQPN	1xCarbamido	9.021E-05	7.389E-05	1	1	2	Q93099	Q93099 [172-184]	0	1904.0417
[K].MLVNEN	1xTMTpro [N	0.0001026	7.389E-05	1	1	3	P09455	P09455 [11-22]	0	1860.9485
[R].MESGIEI	1xTMTpro [N	9.021E-05	7.389E-05	1	1	1	Q9NWU2	Q9NWU2 [51-67]	0	2224.0974
[R].MESPIPI	1xTMTpro [K	7.314E-05	7.389E-05	1	1	1	Q99719	Q99719 [325-343]	0	2686.4869
[K].MEVDYS	1xTMTpro [N	0.0001016	7.389E-05	1	1	2	O00232	O00232 [16-27]	0	1717.8386
[R].MEVGPT	1xTMTpro [K	0.0001284	7.389E-05	1	1	1	P30047	P30047 [12-36]	0	3294.63

[K].MEVWA\	1xTMTpro [K	0.0001885	7.389E-05	1	1	1	Q6P9B6	Q6P9B6 [406-421	0	2397.2616
[R].MFDMGF	1xTMTpro [N	0.0001249	7.389E-05	1	1	2	Q7L014	Q7L014 [534-545]	0	1791.8551
[R].MFEGEM	1xTMTpro [K	0.0001046	7.389E-05	1	1	1	Q9HA64	Q9HA64 [51-64]	0	2149.1893
[K].MFESFIE	1xTMTpro [K	0.0001662	7.389E-05	1	1	1	P13861	P13861 [251-263]	0	2148.227
[R].MFFDAL	1xTMTpro [K	0.0001012	7.389E-05	1	1	1	Q04637	Q04637 [1544-15	0	2200.1492
[K].MFIGGLS	1xTMTpro [N	0.0001301	7.389E-05	1	1	1	Q96DH6	Q96DH6 [23-38]	0	2099.0915
[K].MFILSDC	1xTMTpro [K	0.0001842	7.389E-05	1	1	2	P35244	P35244 [40-49]	0	1704.9486
[-].MFLQYYL	1xTMTpro [N	7.601E-05	7.389E-05	1	1	1	Q9NPE3	Q9NPE3 [1-13]	0	1980.9809
[K].MFQQCL	1xCarbamido	0.0001226	7.389E-05	1	1	2	Q9Y2A7	Q9Y2A7 [552-564	0	1927.9689
[K].MEPLNN	1xOxidation		0.0001405	7.389E-05	2	2	1	Q10567; P63	Q10567 [806-817]	0	1980.1444
[R].MFTEDQ	1xTMTpro [N	8.247E-05	7.389E-05	1	1	1	P23497	P23497 [37-47]	0	1616.7546
[R].MFTTAP	1xOxidation		6.257E-05	7.389E-05	1	1	2	P00450	P00450 [599-619]	1	3402.6827
[K].MFVGGL	1xTMTpro [K	6.237E-05	7.389E-05	1	1	3	Q99729	Q99729 [71-82]	0	1936.0494
[R].MGDADY	1xTMTpro [N	0.0001558	7.389E-05	1	1	1	Q9UBN7	Q9UBN7 [710-72	0	1709.8389
[-].MGELPLD	1xOxidation		9.049E-05	7.389E-05	1	1	2	Q9BWM7	Q9BWM7 [1-14]	0	1945.0384
[R].MGESDD	1xTMTpro [N	0.0001803	7.389E-05	1	1	2	P63220	P63220 [62-71]	0	1426.7167
[R].MGGEAF	1xOxidation		9.909E-05	7.389E-05	1	1	1	Q07812	Q07812 [38-57]	0	2650.389
[K].MGGMEC	1xOxidation		9.542E-05	7.389E-05	1	1	2	P52272	P52272 [346-362]	0	2034.8825
[R].MGHAGA	1xTMTpro [K	7.648E-05	7.389E-05	1	1	3	P53597	P53597 [297-308]	0	1690.9918
[-].MGHFTEE	1xMet-loss+A	7.636E-05	7.389E-05	2	2	1	P69891; P69	P69891 [1-18]; P6	1	2874.5818
[-].MGKDYY	1xMet-loss [N	0.0001736	7.389E-05	1	1	1	P25685	P25685 [1-13]	1	1993.1362
[R].MGLALS	1xTMTpro [K	0.0001716	7.389E-05	1	1	1	O43765	O43765 [165-174]	0	1641.9853
[K].MGIGGLI	1xOxidation		6.247E-05	7.389E-05	1	1	1	P46459	P46459 [218-232]	1	2309.2455
[R].MGPAMC	1xOxidation		0.0001644	7.389E-05	1	1	2	P52272	P52272 [592-606]	0	1747.9154
[R].MFTQQC	1xTMTpro [N	0.0002055	7.389E-05	1	1	2	Q96CX2	Q96CX2 [63-74]	0	1780.9335
[R].MGPAMC	2xOxidation		7.158E-05	7.389E-05	1	1	1	P52272	P52272 [592-606]	0	1763.9103
[K].MEPLNN	1xTMTpro [K	8.937E-05	7.389E-05	2	2	2	Q10567; P63	Q10567 [806-817]	0	1964.1494
[R].MEELHN	1xTMTpro [K	0.0001363	7.389E-05	1	1	2	P23246	P23246 [549-559]	0	2025.0389
[-].MAPGQL	1xMet-loss [N	0.000145	7.389E-05	1	1	1	P31939	P31939 [1-14]	0	1941.1301
[-].MAPLDLD	1xMet-loss [N	0.0001118	7.389E-05	1	1	1	O00743	O00743 [1-14]	1	2111.2356
[-].MAPSVPA	1xMet-loss [N	0.0001863	7.389E-05	1	1	2	P54819	P54819 [1-14]	0	1964.0985
[R].MAPYQG	1xTMTpro [K	7.613E-05	7.389E-05	1	1	5	O43707	O43707 [883-899]	0	2401.2717
[K].MASGAA	1xTMTpro [K	0.0001239	7.389E-05	1	1	1	Q92520	Q92520 [73-84]	0	1709.9864

[R].MATQAS	1xTMTpro [K	7.382E-05	7.389E-05	1	1	1	Q13423	Q13423 [380-394]	0	2251.2248
[R].MAVQDA	1xTMTpro [K	9.32E-05	7.389E-05	1	1	1	P46060	P46060 [488-500]	0	2028.1113
[K].MCCCSY	3xCarbamido	0.0001918	7.389E-05	1	1	2	P35555	P35555 [1718-172	0	1624.7023
[K].MCPGLS	1xCarbamido	0.0001498	7.389E-05	1	1	2	Q96AM1	Q96AM1 [17-30]	0	1929.937
[R].MCTLIDK	1xCarbamido	9.453E-05	7.389E-05	1	1	1	P53621	P53621 [40-55]	1	2541.3085
[K].MDAEVP	1xTMTpro [K	0.000149	7.389E-05	1	1	2	Q09666	Q09666 [1418-14	0	2308.1986
[K].MDAEVP	1xOxidation [	8.965E-05	7.389E-05	1	1	1	Q09666	Q09666 [1418-14	0	2324.1936
[K].MDAILTE	1xTMTpro [K	0.0001859	7.389E-05	1	1	1	Q5SSJ5	Q5SSJ5 [161-170	0	1713.0112
[R].MEKGEH	3xTMTpro [K	7.462E-05	7.389E-05	1	1	1	Q02790	Q02790 [211-232]	1	3644.0663
[K].MDASLG	1xTMTpro [N	0.0001867	7.389E-05	1	1	1	Q92520	Q92520 [31-41]	0	1498.8007
[K].MDATAN	1xTMTpro [N	0.0001536	7.389E-05	1	1	2	P13667	P13667 [583-594]	0	1595.7655
[K].MDCQET	1xCarbamido	9.632E-05	7.389E-05	1	1	2	O75369	O75369 [2429-24	0	1965.9542
[K].MDIDVPI	1xOxidation [	6.247E-05	7.389E-05	1	1	2	Q09666	Q09666 [859-876]	0	2717.41
[R].MDLLPLI	1xOxidation [	0.0001172	7.389E-05	1	1	10	P21980	P21980 [652-663]	0	2029.1582
[K].MDSNEL	1xTMTpro [N	8.598E-05	7.389E-05	1	1	1	O14498	O14498 [129-139]	0	1626.8481
[K].MDSNEL	1xOxidation [	0.0002031	7.389E-05	1	1	1	O14498	O14498 [129-139]	0	1642.843
[R].MDSTNA	1xTMTpro [N	7.863E-05	7.389E-05	1	1	1	Q99615	Q99615 [205-216]	0	1659.8331
[R].MDWLVF	1xCarbamido	0.0001466	7.389E-05	1	1	4	P04004	P04004 [422-443]	0	3182.6186
[R].MDWLVF	1xCarbamido	0.0001732	7.389E-05	1	1	2	P04004	P04004 [422-443]	0	3198.6136
[K].MEAENL	1xTMTpro [K	0.0001023	7.389E-05	1	1	2	P11047	P11047 [1291-130	0	2169.1717
[K].MEDGEF	1xTMTpro [N	0.0001856	7.389E-05	1	1	1	P07384	P07384 [329-339]	0	1738.7888
[K].MEDIKES	1xTMTpro [K	0.0001703	7.389E-05	1	1	2	Q13445	Q13445 [151-162]	1	2090.1117
[R].MEDTEP	1xTMTpro [N	9.205E-05	7.389E-05	1	1	1	P09471	P09471 [114-130]	0	2262.0775
[R].MDASTG	1xTMTpro [K	7.247E-05	7.389E-05	1	1	4	Q96AC1	Q96AC1 [596-605	0	1616.8809
[K].NCEDMD	2xCarbamido	9.007E-05	7.389E-05	1	1	3	P35555	P35555 [569-579]	0	1732.726
[R].MGPGAA	1xTMTpro [K	6.769E-05	7.389E-05	1	1	3	Q9H0U4	Q9H0U4 [173-187	0	2050.1359
[R].MGPGAT	1xOxidation [	7.146E-05	7.389E-05	1	1	3	P62820	P62820 [176-187]	0	1670.9027
[K].MLGQMT	2xOxidation [	0.0001519	7.389E-05	1	1	1	P18206	P18206 [327-339]	0	1813.9107
[K].MLLDSVI	1xTMTpro [N	0.0001519	7.389E-05	1	1	1	Q96GC5	Q96GC5 [144-155	0	1718.943
[K].MIMDTA(	1xCarbamido	0.0001105	7.389E-05	1	1	1	P31483	P31483 [38-57]	0	2773.2492
[R].MIMTNPI	1xTMTpro [K	0.000159	7.389E-05	1	1	2	Q9NZU5	Q9NZU5 [99-109]	0	1785.0258
[K].MINLSVF	1xTMTpro [N	0.0001036	7.389E-05	2	2	4	P13796; P13	P13796 [166-178]	0	1806.9591
[K].MINLSVF	1xOxidation [	0.000111	7.389E-05	2	2	8	P13796; P13	P13796 [166-178]	0	1822.954

[K].MLPLLE\	1xTMTpro [K	0.0001224	7.389E-05	1	1	3	Q9NRN5	Q9NRN5 [62-71]	0	1751.0633
[K].MLPLLE\	1xOxidation		0.0001703	7.389E-05	1	1	2	Q9NRN5	Q9NRN5 [62-71]	0	1767.0582
[K].MIPSLCT	1xCarbamido	0.0001315	7.389E-05	1	1	2	P35555	P35555 [1034-104	0	1751.9792
[K].MLPVDE	1xTMTpro [K	9.119E-05	7.389E-05	1	1	1	Q8NBJ5	Q8NBJ5 [518-531	0	2289.2519
[K].MLPVDE	1xOxidation		0.0001442	7.389E-05	1	1	1	Q8NBJ5	Q8NBJ5 [518-531	0	2305.2468
[K].MLPVDE	2xTMTpro [K	0.0001345	7.389E-05	1	1	1	Q8NBJ5	Q8NBJ5 [518-538	1	3433.872
[K].MLQGES	1xOxidation		0.0001995	7.389E-05	1	1	1	Q9NYH9	Q9NYH9 [576-588	0	2005.092
[R].MIGGPIL	1xOxidation		0.0001476	7.389E-05	1	1	1	P49419	P49419 [163-173]	0	1489.8368
[K].MLQHAA	1xTMTpro [K	0.0002047	7.389E-05	1	1	1	P46940	P46940 [1231-123	0	1607.9183
[K].MLQPCG	1xCarbamido	0.0001165	7.389E-05	1	1	2	Q9BWJ5	Q9BWJ5 [72-86]	0	2291.1656
[R].MLREPA	1xTMTpro [K	8.678E-05	7.389E-05	1	1	1	P50479	P50479 [193-206]	1	2146.2186
[K].MISAEAF	1xTMTpro [K	8.705E-05	7.389E-05	1	1	2	Q13952	Q13952 [60-71]	0	1885.1113
[K].MISGMY	1xOxidation		9.219E-05	7.389E-05	3	3	2	P19367; P52	P19367 [744-755]	0	1688.9035
[R].MLSNKP	1xTMTpro [K	8.909E-05	7.389E-05	1	1	1	Q9NZM1	Q9NZM1 [191-202	0	2085.1771
[K].MLTAQD	1xTMTpro [N	6.864E-05	7.389E-05	1	1	1	Q01082	Q01082 [1295-130	0	1834.8635
[K].MITGDS(	1xTMTpro [N	0.0002047	7.389E-05	1	1	1	P98194	P98194 [568-583]	0	1954.0235
[K].MLTPEH	1xTMTpro [K	0.0001831	7.389E-05	1	1	1	P09871	P09871 [502-515]	0	2300.2869
[K].MLTPEH	1xOxidation		0.0001542	7.389E-05	1	1	2	P09871	P09871 [502-515]	0	2316.2819
[-].MITSAAG	1xOxidation		7.695E-05	7.389E-05	1	1	2	Q99460	Q99460 [1-20]	0	2768.5247
[K].MIVDPVE	1xOxidation		7.52E-05	7.389E-05	1	1	3	Q13263	Q13263 [378-390]	0	2106.1219
[K].MLVLDE/	1xOxidation		9.572E-05	7.389E-05	1	1	2	P38919	P38919 [183-195]	0	2145.1427
[-].MLVLVLG	1xMet-loss [M	7.755E-05	7.389E-05	1	1	1	Q9UBQ0	Q9UBQ0 [1-14]	0	1786.1022
[K].MLQIQPE	2xTMTpro [K	7.613E-05	7.389E-05	1	1	2	Q8NAV1	Q8NAV1 [77-92]	1	2856.7089
[R].MGPGA/	1xOxidation		0.0001161	7.389E-05	1	1	1	Q9H0U4	Q9H0U4 [173-187	0	2066.1308
[R].MIGGPIL	1xTMTpro [N	0.0001703	7.389E-05	1	1	1	P49419	P49419 [163-173]	0	1473.8419
[K].MLGEAL	1xTMTpro [K	7.213E-05	7.389E-05	1	1	1	Q9NWT6	Q9NWT6 [325-34	0	2833.5811
[R].MGPSG(	1xTMTpro [N	0.0001138	7.389E-05	1	1	2	Q14847	Q14847 [131-143]	0	1637.7583
[R].MGPTEL	2xOxidation		6.928E-05	7.389E-05	1	1	3	P02675	P02675 [335-348]	0	2332.206
[R].MGPTEL	1xOxidation		9.739E-05	7.389E-05	1	1	1	P02675	P02675 [335-353]	2	3451.9322
[-].MGSTWG	1xMet-loss [M	0.0001342	7.389E-05	1	1	1	Q9BQB6	Q9BQB6 [1-12]	0	1493.782
[K].MGVEW	1xCarbamido	0.0001995	7.389E-05	1	1	1	P06681	P06681 [631-646]	0	2448.2395
[-].MGVQPP	1xMet-loss [M	6.918E-05	7.389E-05	1	1	2	Q9BVJ7	Q9BVJ7 [1-15]	0	1858.0295
[R].MGVVPF	1xTMTpro [K	0.000122	7.389E-05	1	1	1	Q96NB2	Q96NB2 [55-71]	0	2438.3973

[K].MHLYGL	1xTMTpro [N	0.000108	7.389E-05	1	1	2	P22105	P22105 [1438-144		0	1644.86
[K].MHLYGL	1xTMTpro [N	0.0001885	7.389E-05	1	1	1	P22105	P22105 [2161-217		0	1516.8014
[R].MHMWV	1xTMTpro [K	6.811E-05	7.389E-05	1	1	1	Q9UHG3	Q9UHG3 [152-16		0	2011.0637
[R].MKEFLD	2xTMTpro [K	0.0001605	7.389E-05	1	1	1	Q2TAL8	Q2TAL8 [27-37]		1	2181.2769
[K].MKEIAEA	2xTMTpro [K	7.707E-05	7.389E-05	1	1	2	P11142	P11142 [127-137]		1	2165.282
[R].MKPALF	1xCarbamido	7.936E-05	7.389E-05	1	1	2	P40424	P40424 [73-85]		0	2475.4648
[K].MIGFPSS	1xTMTpro [N	0.0001013	7.389E-05	1	1	1	P13646	P13646 [416-429]		0	1696.9012
[K].MKPLMG	3xTMTpro [K	9.557E-05	7.389E-05	1	1	1	P22061	P22061 [205-221]		1	3178.9088
[K].MKPVPD	1xOxidation [	0.0001703	7.389E-05	1	1	7	P02671	P02671 [226-238]		0	2370.4035
[R].MIAAVD	1xTMTpro [N	0.00017	7.389E-05	1	1	1	Q07812	Q07812 [79-89]		0	1479.7796
[K].MLAAYL	1xTMTpro [K	0.0001013	7.389E-05	1	1	1	Q96KP4	Q96KP4 [462-475		1	2252.2492
[K].MLAAYL	1xOxidation [	9.119E-05	7.389E-05	1	1	3	Q96KP4	Q96KP4 [462-475		1	2268.2441
[R].MIAEVQI	1xCarbamido	0.0001417	7.389E-05	1	1	1	P52823	P52823 [120-131]		0	2095.0695
[R].MIAILTEI	1xTMTpro [K	0.0001544	7.389E-05	1	1	1	P26639	P26639 [600-611]		0	1918.0963
[K].MLCDTW	2xCarbamido	6.357E-05	7.389E-05	1	1	1	P49748	P49748 [601-613]		0	1900.9039
[K].MLDAED	1xOxidation [	0.0001087	7.389E-05	1	1	4	O43707	O43707 [240-255		0	2441.2838
[K].MLDKED	2xTMTpro [K	0.0001656	7.389E-05	1	1	2	P13807	P13807 [419-429]		1	2301.2473
[R].MIDMGF	1xOxidation [	0.0001983	7.389E-05	1	1	2	Q9BUQ8	Q9BUQ8 [554-56		0	2034.0532
[K].MIEEAG	1xTMTpro [N	7.224E-05	7.389E-05	1	1	2	O00154	O00154 [79-90]		0	1594.8794
[K].MLEENT	1xTMTpro [K	0.0001356	7.389E-05	1	1	2	Q9NYL9	Q9NYL9 [308-317		0	1813.0385
[R].MLFKDD	2xTMTpro [K	7.779E-05	7.389E-05	1	1	1	P63279	P63279 [62-74]		1	2437.3617
[K].MKPVPD	2xTMTpro [K	8.786E-05	7.389E-05	1	1	6	P02671	P02671 [226-238]		0	2354.4086
[K].NCISEQI	1xCarbamido	9.176E-05	7.389E-05	1	1	2	P17050	P17050 [48-61]		0	2047.957
[K].NCIVLID	1xCarbamido	8.518E-05	7.389E-05	1	1	2	P62241	P62241 [99-110]		0	1754.943
[K].NCMDID	3xCarbamido	7.485E-05	7.389E-05	1	1	1	P35555	P35555 [1110-112		1	2399.0895
[K].NSAQGN	1xTMTpro [K	0.0002047	7.389E-05	1	1	2	Q14498	Q14498 [468-477		0	1687.9623
[K].NSCPPT	1xCarbamido	0.0001354	7.389E-05	1	1	1	P22352	P22352 [154-168]		0	1937.9558
[K].NSDEAD	1xTMTpro [K	0.0001944	7.389E-05	1	1	2	P83916	P83916 [140-150]		0	1766.978
[R].NSDSILE	1xTMTpro [K	0.0001335	7.389E-05	1	1	1	P19404	P19404 [144-154]		0	1826.0515
[K].NSEEFA	1xTMTpro [N	0.0001319	7.389E-05	1	1	1	P04114	P04114 [118-128]		0	1516.7385
[K].NSETFP	1xTMTpro [K	8.336E-05	7.389E-05	1	1	1	O60610	O60610 [1253-12		0	2087.1516
[K].NSFREQ	2xTMTpro [K	0.0001503	7.389E-05	1	1	1	P35579	P35579 [1339-13		2	3271.7375
[K].NSIAYMI	1xTMTpro [K	0.0001979	7.389E-05	1	1	1	P02461	P02461 [1374-13		0	2106.1145

[R].NSLCIEN	2xCarbamido	9.572E-05	7.389E-05	1	1	1	Q15582	Q15582 [470-484]	0	2340.1932
[K].NSIEDIH.	1xTMTpro [N	0.0001856	7.389E-05	1	1	1	Q9NRY5	Q9NRY5 [369-380	0	1689.9243
[K].NSLESY/	1xTMTpro [K	8.159E-05	7.389E-05	1	1	4	P11142	P11142 [540-550]	0	1912.013
[K].NSMIALV	1xTMTpro [K	0.0001013	7.389E-05	1	1	1	O94832	O94832 [570-582]	0	1984.1393
[K].NSQEDS	2xTMTpro [K	7.839E-05	7.389E-05	1	1	2	Q9H1E3	Q9H1E3 [53-67]	1	2637.3308
[R].NQVVHV	1xTMTpro [K	7.827E-05	7.389E-05	1	1	1	Q9Y5S2	Q9Y5S2 [1223-12	0	2730.5686
[K].NSQGEE	1xTMTpro [N	0.0001752	7.389E-05	1	1	4	P20700	P20700 [533-542]	0	1421.7304
[K].NSTFSEI	2xTMTpro [K	0.0001085	7.389E-05	1	1	2	P29401	P29401 [344-353]	1	2113.2474
[R].NSVDEL	1xOxidation [	0.0001375	7.389E-05	1	1	1	P02675	P02675 [125-152]	0	3791.893
[K].NSVFSVI	1xTMTpro [K	7.291E-05	7.389E-05	1	1	1	Q9UPN3	Q9UPN3 [1211-12	0	2059.1567
[K].NTDEPM	1xTMTpro [K	6.427E-05	7.389E-05	1	1	2	O14936	O14936 [496-506]	0	1827.0178
[R].NTDQAS	1xTMTpro [K	0.000159	7.389E-05	2	2	1	P35749; P35	P35749 [366-380]	0	2200.116
[R].NTDQAS	1xTMTpro [K	0.0001445	7.389E-05	1	1	2	P35580	P35580 [366-380]	0	2242.1629
[R].NTDQAT	1xTMTpro [K	7.543E-05	7.389E-05	1	1	1	Q7Z406	Q7Z406 [385-399]	0	2214.1316
[R].NTDVAQ	1xTMTpro [K	0.0001012	7.389E-05	1	1	2	Q9P2E9	Q9P2E9 [609-620	0	1865.026
[R].NTEDLTI	1xTMTpro [N	0.0001186	7.389E-05	1	1	2	P60983	P60983 [125-135]	0	1709.8665
[R].NTLCAPI	1xCarbamido	6.357E-05	7.389E-05	1	1	2	Q96CS3	Q96CS3 [191-205	0	2005.1071
[R].NTLEWC	1xCarbamido	7.382E-05	7.389E-05	1	1	1	P48444	P48444 [436-448]	0	2167.2077
[R].NTLFNLS	1xTMTpro [K	0.0001349	7.389E-05	1	2	2	Q13492	Q13492 [101-112]	0	2034.1516
[R].NTLTNIA	1xTMTpro [N	0.0002047	7.389E-05	1	1	1	P33992	P33992 [177-195]	0	2391.3138
[K].NSSYFVI	1xTMTpro [K	9.572E-05	7.389E-05	5	7	6	Q13885; Q1:	Q13885 [337-350]	0	2305.2472
[K].NTLTQT	1xTMTpro [N	0.0001584	7.389E-05	1	1	1	P15924	P15924 [1414-142	0	1594.872
[K].NQVLVF	1xTMTpro [N	8.234E-05	7.389E-05	1	1	2	O75643	O75643 [719-728]	0	1502.8763
[R].NQVDPE	1xCarbamido	0.0002035	7.389E-05	1	2	1	Q8IUX4	Q8IUX4 [240-252]	0	1896.8942
[R].NNNTDL	1xTMTpro [K	0.0001635	7.389E-05	1	1	2	Q99460	Q99460 [345-354]	0	1784.0232
[K].NNPHYD	2xTMTpro [K	0.000146	7.389E-05	1	1	1	Q9P016	Q9P016 [143-157	1	2654.3991
[K].NNRPSE	1xTMTpro [N	0.000134	7.389E-05	1	1	3	Q00610	Q00610 [572-583]	0	1672.905
[K].NNSDKD	1xTMTpro [K	6.247E-05	7.389E-05	1	1	1	Q03252	Q03252 [490-502]	1	2142.1184
[R].NNSGEE	1xCarbamido	0.0001258	7.389E-05	1	1	1	Q08J23	Q08J23 [591-602]	0	1749.7822
[K].NNSGLS	1xTMTpro [N	0.0001442	7.389E-05	1	1	1	Q13618	Q13618 [48-59]	0	1732.8825
[K].NNTVGL	1xTMTpro [K	7.004E-05	7.389E-05	1	1	2	P30084	P30084 [44-56]	0	2075.2581
[K].NNWEVS	1xTMTpro [N	0.000196	7.389E-05	1	1	1	P78527	P78527 [811-820]	0	1479.7875
[R].NPDAVA	1xCarbamido	0.0001013	7.389E-05	1	1	2	P08519	P08519 [79-91]; [:	0	1801.8862

[R].NPDFLLI	1xTMTpro [N	9.693E-05	7.389E-05	1	1	1	Q8N201	Q8N201 [871-881	0	1669.9709
[R].NPDGSE	1xCarbamido	8.854E-05	7.389E-05	1	2	1	P26927	P26927 [242-260]	0	2625.2323
[K].NPDGTM	1xCarbamido	8.084E-05	7.389E-05	1	1	2	P63279	P63279 [31-48]	0	2656.3177
[R].NPDIFTE	2xCarbamido	0.0001299	7.389E-05	1	1	1	Q7Z6Z7	Q7Z6Z7 [1881-18	0	2012.9853
[R].NQVEDL	1xTMTpro [K	0.0001105	7.389E-05	1	1	1	Q92542	Q92542 [392-403]	0	1981.1461
[R].NPDNDII	1xCarbamido	0.00012	7.389E-05	1	1	1	P00748	P00748 [268-282]	0	2220.1303
[K].NPLLITE	1xTMTpro [K	9.724E-05	7.389E-05	1	1	1	Q9UH65	Q9UH65 [106-117	0	1998.1403
[K].NPLPSKI	2xTMTpro [K	0.0001315	7.389E-05	1	1	3	P62328	P62328 [27-39]	1	2425.4119
[K].NPNGPY	1xTMTpro [K	0.0001877	7.389E-05	1	1	2	P14866	P14866 [569-579]	0	1872.0511
[R].NPNLPP	1xTMTpro [K	0.0001066	7.389E-05	1	1	3	P05090	P05090 [152-164]	0	2032.157
[K].NPPDVD	1xTMTpro [N	0.0001482	7.389E-05	1	1	1	O60487	O60487 [127-139]	0	1684.9189
[R].NPPLAE	1xTMTpro [K	0.000181	7.389E-05	1	1	1	Q5TDH0	Q5TDH0 [156-170	0	2175.2517
[R].NPWGE\	1xTMTpro [N	0.0001356	7.389E-05	1	1	2	P17655	P17655 [286-296]	0	1634.8246
[R].NQDATV	1xTMTpro [K	0.0001075	7.389E-05	1	1	1	Q15427	Q15427 [10-23]	0	2117.137
[R].NQELLQ	1xTMTpro [K	0.0001347	7.389E-05	1	1	1	O60763	O60763 [745-756]	0	2039.1628
[K].NQEQLL	1xTMTpro [N	6.79E-05	7.389E-05	1	1	1	Q9Y5Z4	Q9Y5Z4 [151-163	0	1803.0659
[K].NQLEEV	1xTMTpro [N	9.394E-05	7.389E-05	1	1	3	P51888	P51888 [160-171]	0	1656.924
[R].NQSFCP	1xCarbamido	8.545E-05	7.389E-05	1	1	1	P46776	P46776 [66-87]	1	3244.728
[K].NQVAMN	1xTMTpro [K	7.887E-05	7.389E-05	1	1	1	P54652	P54652 [58-72]	0	2272.2251
[K].NPESLFI	1xTMTpro [N	0.0001703	7.389E-05	1	1	3	O00160	O00160 [678-689]	0	1749.9706
[R].NNNQQL	1xTMTpro [K	0.0002003	7.389E-05	1	1	1	Q9Y2A7	Q9Y2A7 [71-81]	0	1907.0954
[K].NTLYLQI	1xTMTpro [N	0.0001004	7.389E-05	2	7	2	P01768; A0A	P01768 [96-106];	0	1656.9062
[K].NTSEQD	1xOxidation [	0.0001345	7.389E-05	1	1	1	P20700	P20700 [458-473]	0	2199.013
[K].NVQLTEI	1xTMTpro [N	0.0001333	7.389E-05	1	1	1	P62136	P62136 [27-36]	0	1519.8399
[R].NVTHIDC	1xTMTpro [N	0.0001186	7.389E-05	1	1	1	Q5HYK3	Q5HYK3 [221-234	0	1936.032
[K].NVVLQTI	1xTMTpro [N	0.0001368	7.389E-05	1	1	1	Q14254	Q14254 [101-112]	0	1682.9873
[K].NWEAMI	1xTMTpro [K	0.0001437	7.389E-05	1	1	1	Q86UP2	Q86UP2 [1036-10	0	2088.0927
[K].NWETEI	1xTMTpro [K	7.069E-05	7.389E-05	1	1	1	P05452	P05452 [156-169]	0	2154.1323
[K].NWGLSF	2xTMTpro [K	0.0001729	7.389E-05	1	1	2	P19652	P19652 [139-153]	0	2669.4755
[R].NWYVQI	1xCarbamido	0.0001039	7.389E-05	1	1	2	P62330	P62330 [148-174]	0	3718.8343
[R].NYDPAL	1xTMTpro [N	6.109E-05	7.389E-05	1	1	1	Q9NV06	Q9NV06 [27-39]	0	1858.9771
[R].NYDVMK	1xTMTpro [K	9.262E-05	7.389E-05	1	1	2	P09669	P09669 [55-66]	1	2185.0913
[K].NYGENP	1xTMTpro [K	0.0001273	7.389E-05	1	1	1	Q9H3S7	Q9H3S7 [33-46]	0	2278.1483

[K].NYGFVH	1xTMTpro [K	6.217E-05	7.389E-05	1	2	1	Q9BWF3	Q9BWF3 [36-45]	0	1830.0042
[R].NYLLPA(	1xTMTpro [N	0.0001827	7.389E-05	1	1	1	Q12888	Q12888 [1845-18	0	1957.035
[R].NYIMDF(	1xTMTpro [K	0.0001867	7.389E-05	1	1	2	P09455	P09455 [60-69]	0	1823.0017
[R].NVQGILI	1xTMTpro [K	9.394E-05	7.389E-05	1	1	1	Q8IW45	Q8IW45 [163-172	0	1695.0296
[R].NYIMDF(	1xTMTpro [K	7.66E-05	7.389E-05	1	1	1	P09455	P09455 [60-81]	1	3242.6171
[K].NYPDPV	1xTMTpro [K	0.0001445	7.389E-05	1	1	2	P41252	P41252 [607-617]	0	1882.093
[R].NYPNTF	1xTMTpro [K	7.508E-05	7.389E-05	1	1	1	P17655	P17655 [376-390]	0	2537.3507
[K].NYSPYY	2xTMTpro [K	9.77E-05	7.389E-05	1	1	2	P35527	P35527 [200-224]	1	3815.0319
[K].NYTDEA	1xTMTpro [K	6.509E-05	7.389E-05	1	1	1	O14818	O14818 [175-189	0	2349.2317
[R].QAAAHT	1xTMTpro [N	0.0001268	7.389E-05	1	1	2	Q16891	Q16891 [486-495	0	1423.7725
[R].QAAEVA	1xTMTpro [K	0.0001983	7.389E-05	1	1	1	Q9NW64	Q9NW64 [278-28	0	1524.8877
[K].QAATMS	1xTMTpro [N	7.911E-05	7.389E-05	1	1	1	Q9UHB9	Q9UHB9 [289-29	0	1611.812
[K].QAEETY	1xTMTpro [K	9.191E-05	7.389E-05	1	1	1	O00410	O00410 [28-41]	0	2202.1534
[R].QAELEE	1xTMTpro [N	0.0001498	7.389E-05	1	1	1	O00461	O00461 [463-478	0	2252.1703
[K].QAEVAN	1xTMTpro [K	0.0002055	7.389E-05	1	1	2	P05114	P05114 [62-71]	0	1725.9627
[R].QAFAPF	1xTMTpro [K	0.0001445	7.389E-05	1	1	2	Q9UHX1	Q9UHX1 [147-15	0	1684.0078
[R].QAFTPS	1xTMTpro [K	9.925E-05	7.389E-05	1	1	1	Q14157	Q14157 [422-438	0	2582.349
[R].QAGAEA	1xTMTpro [N	7.555E-05	7.389E-05	1	1	1	Q92616	Q92616 [1177-11	0	1575.8774
[R].NYPATF	1xTMTpro [K	0.0001442	7.389E-05	1	1	3	P07384	P07384 [386-398]	0	2220.2097
[K].NTLYLQI	1xTMTpro [N	0.0001611	7.389E-05	1	1	1	A0A0J9YX35	A0A0J9YX35 [96-	0	1629.8953
[K].NVQGIIE	1xTMTpro [K	0.0001626	7.389E-05	1	1	2	P42704	P42704 [454-463]	0	1735.0973
[R].NVPQND	1xTMTpro [K	0.0001368	7.389E-05	1	1	1	P41218	P41218 [205-218]	0	2130.2778
[R].NTTGVT	1xTMTpro [K	6.407E-05	7.389E-05	1	1	2	Q13813	Q13813 [2316-23	0	1771.0093
[K].NTVELL	1xTMTpro [K	0.0001975	7.389E-05	1	1	2	O75369	O75369 [400-409	0	1768.0348
[R].NTVVLF	1xTMTpro [N	6.591E-05	7.389E-05	1	1	1	Q9UJZ1	Q9UJZ1 [35-51]	0	2318.2828
[R].NTVYLQ	1xTMTpro [N	0.0001514	7.389E-05	1	1	2	P0DOX2	P0DOX2 [76-86]	0	1642.8906
[R].NTWDC(	1xCarbamido	0.0001034	7.389E-05	1	1	2	P53007	P53007 [258-268]	0	1956.0868
[R].NVCTEA	1xCarbamido	0.0001525	7.389E-05	1	1	1	P62333	P62333 [345-356]	0	1672.847
[K].NVDDILK	1xTMTpro [K	0.0001653	7.389E-05	1	1	1	P49750	P49750 [1818-18	0	1832.0886
[K].NVDEAIN	1xTMTpro [N	8.171E-05	7.389E-05	1	1	2	P51648	P51648 [344-355]	0	1737.9091
[R].NVDFNY	1xTMTpro [K	0.0001952	7.389E-05	1	1	2	Q8WWM7	Q8WWM7 [197-2	0	1679.9248
[R].NVDGVN	1xTMTpro [N	6.178E-05	7.389E-05	1	1	1	Q9UBR2	Q9UBR2 [70-81]	0	1612.8614
[K].NVDILKD	2xTMTpro [K	0.0001218	7.389E-05	1	1	2	O14980	O14980 [675-686	1	2283.374

[K].NVDLSTI	1xTMTpro [N	0.0001503	7.389E-05	1	1	2	O94826	O94826 [149-159]	0	1660.8614
[R].NVEAMN	1xTMTpro [N	9.602E-05	7.389E-05	1	1	2	P36957	P36957 [334-345]	0	1712.8597
[K].NVPQVV	1xTMTpro [K	7.743E-05	7.389E-05	1	1	1	P35658	P35658 [1118-112	0	1975.1832
[K].NVEFIHS	1xTMTpro [N	0.0001611	7.389E-05	1	1	1	Q9Y3R5	Q9Y3R5 [1370-13	0	1659.9502
[R].NVELTEY	1xTMTpro [N	0.0001218	7.389E-05	1	1	1	Q96G21	Q96G21 [241-250	0	1417.797
[R].NVESGE	1xTMTpro [K	0.000108	7.389E-05	1	1	1	Q96HS1	Q96HS1 [77-88]	0	1900.0155
[K].NVGQFS	1xTMTpro [K	8.209E-05	7.389E-05	1	1	2	P35659	P35659 [126-137]	0	1965.0726
[K].NVHDNH	1xCarbamido	7.474E-05	7.389E-05	1	1	1	Q9UQ03	Q9UQ03 [39-51]	0	1887.9203
[K].NVLGHM	1xTMTpro [N	0.0001279	7.389E-05	1	1	1	P08237	P08237 [657-673]	0	2145.083
[K].NVIGLQN	1xTMTpro [N	0.0001397	7.389E-05	1	1	4	P37802	P37802 [172-182]	0	1506.8382
[K].NVLITDF	1xTMTpro [N	8.453E-05	7.389E-05	1	1	2	Q92643	Q92643 [297-308]	0	1671.9389
[R].NVITNQF	1xTMTpro [K	0.00011	7.389E-05	1	1	1	Q9BQS7	Q9BQS7 [45-63]	0	2669.5006
[K].NVLVTLY	1xTMTpro [K	0.000117	7.389E-05	1	1	2	P09486	P09486 [173-191]	1	2886.5704
[R].NVMEEG	1xTMTpro [K	0.0002027	7.389E-05	1	1	2	P35606	P35606 [834-846]	1	2145.1254
[K].NVNIQNI	1xTMTpro [K	7.684E-05	7.389E-05	1	1	1	P12814	P12814 [163-174]	0	2108.1897
[K].NVNVQN	1xTMTpro [K	9.988E-05	7.389E-05	1	1	1	O43707	O43707 [182-193]	0	2094.174
[K].NVPLPVI	1xTMTpro [K	8.951E-05	7.389E-05	2	2	3	P0DOX6; P0	P0DOX6 [225-237	0	1995.2498
[R].NVEGQD	1xOxidation [	7.124E-05	7.389E-05	2	2	1	Q10567; P63	Q10567 [889-901]	0	2149.1455
[R].MAIMVQ	1xTMTpro [K	0.0001849	7.389E-05	1	1	1	P09874	P09874 [35-47]	0	2063.0983
[K].NNIPYFE	1xTMTpro [K	6.317E-05	7.389E-05	1	1	4	P51149	P51149 [147-157]	0	1892.0409
[K].NNKDSH	1xOxidation [	8.272E-05	7.389E-05	1	1	1	P02671	P02671 [98-114]	1	2610.4165
[R].NFLVFQ	1xTMTpro [K	0.0001018	7.389E-05	1	1	1	Q14683	Q14683 [132-146]	0	2262.2812
[R].NFIVWLE	1xTMTpro [K	9.191E-05	7.389E-05	1	1	4	Q9Y224	Q9Y224 [27-36]	0	1900.0824
[R].NFSDNQ	1xTMTpro [K	9.063E-05	7.389E-05	1	1	5	P37802	P37802 [161-171]	0	1888.0056
[K].NFSGAE	1xTMTpro [N	0.0001482	7.389E-05	1	1	1	P46459	P46459 [435-446]	0	1595.8712
[K].NFYQEH	1xTMTpro [N	0.0001361	7.389E-05	1	1	1	P17844	P17844 [57-67]	0	1693.8617
[K].NGDLDE	2xTMTpro [K	0.0001146	7.389E-05	1	1	4	P58546	P58546 [12-24]	1	2378.3384
[K].NHDEES	2xCarbamido	7.325E-05	7.389E-05	2	2	1	O43432; Q0	O43432 [920-931]	0	1865.8441
[K].NHEEEM	1xTMTpro [K	0.0001425	7.389E-05	1	1	1	P13645	P13645 [286-295]	1	1909.0093
[R].NHEGDE	1xTMTpro [N	0.00014	7.389E-05	1	1	1	Q9UNE7	Q9UNE7 [183-194	0	1713.7748
[R].NHLLHVI	1xTMTpro [K	6.737E-05	7.389E-05	1	1	2	P09525	P09525 [203-213]	0	2023.1257
[R].NHIIKDQ	2xTMTpro [K	0.000165	7.389E-05	1	1	2	Q15582	Q15582 [437-447]	1	2179.3379
[R].NHPGLL	1xTMTpro [N	0.0001146	7.389E-05	1	1	1	P11498	P11498 [559-571]	0	1818.9856

[R].NKDHPA	1xTMTpro [K	8.882E-05	7.389E-05	1	1	1	P30519	P30519 [88-105]	1	2791.4998
[R].NFGSYV	1xTMTpro [K	0.0001776	7.389E-05	2	2	1	Q96FJ2; P63	Q96FJ2 [61-71]; P	0	1891.0205
[R].NKDTYF	1xCarbamido	0.000107	7.389E-05	1	1	1	Q8TAT6	Q8TAT6 [330-350	1	3379.7296
[K].NKHEAM	1xTMTpro [K	0.0001856	7.389E-05	1	1	2	P35579	P35579 [1023-103	1	2194.1782
[KR].NKYED	2xTMTpro [K	0.0001941	7.389E-05	5	7	2	P04264; P04	P04264 [268-276]	1	2065.1746
[R].NLAATL(	1xTMTpro [K	0.000181	7.389E-05	1	1	1	Q86UU1	Q86UU1 [1021-10	0	1925.1199
[R].NLADEIE	1xTMTpro [K	0.0001544	7.389E-05	1	1	1	Q5VZ89	Q5VZ89 [1231-12	0	2148.1502
[R].NLALDE/	1xTMTpro [N	0.000134	7.389E-05	1	1	2	Q53TN4	Q53TN4 [274-283	0	1390.761
[K].NLATAYI	1xTMTpro [K	7.091E-05	7.389E-05	1	1	1	Q8WUM4	Q8WUM4 [655-67	0	2503.4052
[R].NLAVSQ	1xTMTpro [K	0.0001087	7.389E-05	1	1	2	P01011	P01011 [351-360]	0	1703.046
[K].NLCEAM	1xCarbamido	8.388E-05	7.389E-05	1	1	1	P20851	P20851 [214-227]	0	2364.2006
[R].NLCWD\	2xCarbamido	8.718E-05	7.389E-05	1	1	1	O00754	O00754 [266-283]	0	2532.2546
[K].NLDCPE	1xCarbamido	0.0001	7.389E-05	1	1	1	P45973	P45973 [56-68]	0	2220.1536
[K].NLDDTIE	1xTMTpro [K	0.0001641	7.389E-05	1	2	1	Q13310	Q13310 [300-309]	0	1785.9362
[R].NLDFQD	1xTMTpro [K	0.0001776	7.389E-05	1	1	1	Q9Y3I0	Q9Y3I0 [442-451]	0	1815.0144
[K].NLDGAY	1xTMTpro [K	0.0001506	7.389E-05	1	1	1	Q4LE39	Q4LE39 [70-82]	0	2043.1366
[K].NKEEAA	2xTMTpro [K	0.0001536	7.389E-05	1	1	2	P62753	P62753 [202-211]	1	2065.1746
[K].NLDKDM	1xTMTpro [K	0.0001468	7.389E-05	1	1	1	Q13573	Q13573 [453-466]	1	2263.152
[R].NFGSVQ	1xTMTpro [K	0.0001498	7.389E-05	1	1	2	Q08431	Q08431 [309-320]	0	1955.0882
[K].NFEDVA	2xTMTpro [K	0.0001626	7.389E-05	1	1	3	P07237	P07237 [376-386]	1	2254.2536
[K].NCPHIV\	1xCarbamido	0.0001127	7.389E-05	1	1	3	Q13838	Q13838 [164-175]	0	1610.8756
[K].NCSETQ	1xCarbamido	0.0001322	7.389E-05	1	1	2	P61981	P61981 [111-120]	0	1853.9195
[R].NCTIVSF	1xCarbamido	0.0001729	7.389E-05	1	1	1	P60891	P60891 [164-176]	0	1898.0297
[K].NCVAIA/	1xCarbamido	8.349E-05	7.389E-05	1	1	1	P49720	P49720 [18-27]	1	1449.7916
[R].NCVDEN	2xCarbamido	8.349E-05	7.389E-05	1	1	1	Q14112	Q14112 [798-811]	0	2012.8874
[K].NCVLLSI	1xCarbamido	9.306E-05	7.389E-05	1	1	2	O94855	O94855 [852-866]	0	2093.098
[R].NDEELN	1xTMTpro [K	0.0001112	7.389E-05	1	3	1	Q7L7L0	Q7L7L0 [90-100]	1	1909.0998
[R].NDEVHQ	1xTMTpro [N	0.0001769	7.389E-05	1	1	1	Q8WUA7	Q8WUA7 [269-27	0	1580.7624
[R].NDGVLL	1xTMTpro [N	6.367E-05	7.389E-05	1	1	1	O60763	O60763 [184-195]	0	1616.9655
[K].NDHDGI	1xTMTpro [K	8.745E-05	7.389E-05	1	1	2	Q9Y680	Q9Y680 [201-212	0	1909.99
[K].NDIAINE	1xTMTpro [K	6.377E-05	7.389E-05	1	1	2	P51665	P51665 [104-113]	0	1769.0123
[K].NDLGVG	1xTMTpro [K	0.0001005	7.389E-05	1	1	1	Q12797	Q12797 [458-474]	0	2399.3062
[K].NDNDNII	1xTMTpro [K	0.0001166	7.389E-05	1	1	2	P01008	P01008 [103-123]	0	2907.5418

[K].NFGIGQI	1xTMTpro [K	9.693E-05	7.389E-05	1	1	2	P62424	P62424 [38-48]	0	1825.0464
[K].NDNLVYY	1xTMTpro [K	0.000121	7.389E-05	1	1	1	Q16363	Q16363 [901-913]	0	2121.1836
[K].NDPYHP	2xCarbamido	6.726E-05	7.389E-05	1	1	1	P48059	P48059 [151-166]	0	2554.1847
[K].NDVLAH	1xTMTpro [K	0.0002027	7.389E-05	1	2	1	Q9UPN3	Q9UPN3 [6271-62	0	2247.2589
[R].NEDLEE	1xTMTpro [K	0.0001956	7.389E-05	1	1	1	P78318	P78318 [65-77]	0	2085.1207
[K].NEEKPV	2xTMTpro [K	0.0001881	7.389E-05	1	1	2	P35237	P35237 [192-202]	0	2293.2865
[R].NEFGEIC	1xTMTpro [K	0.000153	7.389E-05	1	1	2	P21980	P21980 [318-327]	0	1744.9361
[K].NEGSES	1xTMTpro [N	6.119E-05	7.389E-05	1	1	2	P67809	P67809 [171-185]	0	1891.9065
[R].NELESY	1xTMTpro [K	8.453E-05	7.389E-05	1	1	2	P11021	P11021 [563-573]	0	1925.0512
[R].NELQLVI	1xTMTpro [N	0.000131	7.389E-05	1	1	1	Q9BZH6	Q9BZH6 [545-556	0	1658.9397
[K].NENLAAI	1xTMTpro [N	0.0001338	7.389E-05	1	1	2	P54727	P54727 [393-409]	0	2299.1162
[R].NFAEPG	1xTMTpro [N	0.0001442	7.389E-05	1	1	1	P35573	P35573 [470-481]	0	1685.8818
[R].NFAQIIE	1xTMTpro [N	6.591E-05	7.389E-05	1	1	1	Q7L523	Q7L523 [193-208]	0	2211.1981
[K].NFATSLY	1xTMTpro [K	6.811E-05	7.389E-05	1	1	5	P08758	P08758 [291-301]	0	1883.0592
[K].NFATSLY	1xOxidation	0.0001995	7.389E-05	1	1	7	P08758	P08758 [291-301]	0	1899.0541
[K].NDPQLS	1xTMTpro [K	0.000146	7.389E-05	1	1	1	Q92783	Q92783 [121-133]	0	2038.1862
[R].NNLAGA	1xTMTpro [N	6.695E-05	7.389E-05	1	1	2	Q00610	Q00610 [355-366]	0	1608.8665
[R].NLDIERF	1xTMTpro [N	6.928E-05	7.389E-05	3	7	5	Q71U36; Q9	Q71U36 [216-229	0	2023.0892
[R].NLDPLV	1xTMTpro [K	0.0001116	7.389E-05	1	1	2	Q9BSJ2	Q9BSJ2 [77-87]	0	1883.1498
[K].NLPEIMN	1xTMTpro [K	0.0001732	7.389E-05	1	1	1	Q9Y3C4	Q9Y3C4 [135-146	0	2009.1597
[K].NLPIYSE	1xOxidation	6.748E-05	7.389E-05	1	1	2	P35579	P35579 [126-139]	0	2352.2653
[R].NLPSVS	1xTMTpro [K	0.0001484	7.389E-05	1	1	2	P21397	P21397 [494-503]	0	1636.0289
[K].NLQCLVI	1xCarbamido	8.813E-05	7.389E-05	1	1	2	Q9NVP1	Q9NVP1 [326-337	0	1749.9125
[K].NLQEAE	1xTMTpro [K	0.0001479	7.389E-05	1	2	12	P08670	P08670 [283-292]	0	1918.0202
[R].NLQEILH	1xTMTpro [N	0.0001694	7.389E-05	1	1	1	P00533	P00533 [139-149]	0	1553.9083
[K].NIQEVFD	1xTMTpro [K	0.0001669	7.389E-05	1	1	1	Q53GL0	Q53GL0 [65-77]	0	2208.168
[R].NLQEVL	1xTMTpro [K	0.0001584	7.389E-05	1	1	2	P54577	P54577 [17-26]	0	1767.0144
[R].NIQFEAF	1xTMTpro [K	9.423E-05	7.389E-05	1	1	1	Q9Y376	Q9Y376 [269-279	0	1988.1249
[K].NIQSLEV	1xTMTpro [K	0.0001082	7.389E-05	1	1	2	P02775	P02775 [76-85]	0	1709.0453
[K].NLQYYD	1xTMTpro [K	0.0001	7.389E-05	1	1	4	P62826	P62826 [143-152]	0	1823.0195
[R].NISHYEE	1xTMTpro [K	6.706E-05	7.389E-05	1	1	2	P13861	P13861 [381-391]	0	1968.1046
[K].NLSPDG	1xTMTpro [N	0.000196	7.389E-05	1	1	1	Q8TD06	Q8TD06 [108-118	0	1549.8294
[K].NLPEDAI	1xTMTpro [K	9.133E-05	7.389E-05	1	1	1	Q99715	Q99715 [952-970]	0	2801.4999

[R].NLSSTTI	1xTMTpro [N	0.000107	7.389E-05	1	1	1	O00515	O00515 [36-47]	0	1609.7989
[K].NITLDDA	1xTMTpro [N	7.059E-05	7.389E-05	1	1	1	O75534	O75534 [759-769]	0	1476.7977
[R].NITYLPA	1xTMTpro [N	7.96E-05	7.389E-05	1	1	2	P35232	P35232 [256-272]	0	2159.2395
[R].NLVCTD	1xCarbamido	0.0001903	7.389E-05	1	1	2	P26196	P26196 [387-396]	0	1542.8269
[K].NIVHNYS	1xTMTpro [K	7.948E-05	7.389E-05	1	2	1	Q9Y6I3	Q9Y6I3 [12-23]	0	2025.1261
[K].NIVLSGG	1xTMTpro [N	0.000171	7.389E-05	1	1	2	P61158	P61158 [318-329]	0	1585.8691
[R].NLVPLLL	1xTMTpro [K	0.0001038	7.389E-05	1	1	1	Q8NHH9	Q8NHH9 [326-340	0	2270.3979
[R].NLVTAFS	1xTMTpro [K	6.488E-05	7.389E-05	1	1	1	P39059	P39059 [1133-114	0	2335.2282
[K].NIWVSG	1xTMTpro [K	0.0001295	7.389E-05	1	1	1	Q9NWH9	Q9NWH9 [385-39	0	1914.094
[R].NLWVSG	1xTMTpro [N	7.625E-05	7.389E-05	1	1	3	Q14151	Q14151 [408-419]	0	1624.8978
[R].NIYLMDT	1xTMTpro [K	0.0001317	7.389E-05	1	1	1	P27694	P27694 [345-354]	0	1749.9701
[K].NLYSDD	1xTMTpro [N	0.0001077	7.389E-05	1	1	4	P60228	P60228 [60-71]	0	1717.9193
[R].NMQDLV	1xTMTpro [K	0.0001378	7.389E-05	1	1	5	P13647	P13647 [246-255]	0	1846.9865
[R].NMSVIAI	1xTMTpro [K	0.0001297	7.389E-05	1	1	3	P13639	P13639 [21-32]	0	1916.0668
[R].NLTEDN	1xTMTpro [K	9.894E-05	7.389E-05	1	1	1	Q9UBB4	Q9UBB4 [424-438	0	2311.2385
[K].NIDINDV	1xCarbamido	0.0001239	7.389E-05	1	1	1	P62195	P62195 [102-113]	0	1734.8764
[R].NINENFG	1xTMTpro [K	7.135E-05	7.389E-05	1	1	1	Q9ULA0	Q9ULA0 [184-211	0	3773.0075
[K].NLMQLN	1xOxidation [	8.598E-05	7.389E-05	1	1	1	P51888	P51888 [222-234]	0	1870.0652
[R].NIDRTEL	1xTMTpro [N	0.000142	7.389E-05	1	1	1	P12111	P12111 [1577-159	1	2090.1161
[R].NLEALAI	1xTMTpro [K	6.149E-05	7.389E-05	1	1	4	P12956	P12956 [489-510]	0	3031.6881
[R].NIEELQG	1xTMTpro [N	0.0001161	7.389E-05	1	1	2	P12270	P12270 [542-552]	0	1703.8996
[K].NIEIDSP	1xTMTpro [N	7.743E-05	7.389E-05	1	1	3	P04843	P04843 [380-391]	0	1739.9135
[R].NIEMTQI	1xTMTpro [N	0.0001968	7.389E-05	1	1	2	O75643	O75643 [638-647]	0	1538.7804
[K].NLFASFI	1xTMTpro [K	0.0001352	7.389E-05	1	1	1	P49792	P49792 [2595-261	0	2703.4162
[K].NLFIEAG	1xCarbamido	0.0001306	7.389E-05	1	1	1	O95758	O95758 [493-503]	0	1846.0388
[R].NLFWTS	2xTMTpro [K	7.672E-05	7.389E-05	1	1	2	Q07954	Q07954 [1671-168	1	2429.3645
[K].NLGIPIIT	1xTMTpro [K	9.632E-05	7.389E-05	1	1	1	Q14008	Q14008 [941-954]	0	2048.2611
[K].NIGNLFE	1xTMTpro [K	8.046E-05	7.389E-05	1	1	2	P07942	P07942 [1245-125	0	1872.0359
[R].NLGQNL	1xTMTpro [N	0.0001036	7.389E-05	1	1	1	Q9P258	Q9P258 [131-141	0	1595.8726
[K].NIGVDNI	1xTMTpro [K	0.00014	7.389E-05	1	1	2	P78371	P78371 [73-82]	0	1606.9408
[K].NLHALIL	1xTMTpro [K	0.0001026	7.389E-05	1	1	5	P07585	P07585 [106-116]	0	1857.1566
[K].NINCSIE	1xCarbamido	0.0001136	7.389E-05	1	1	2	P35914	P35914 [138-149]	0	1800.887
[K].NLKPIKP	1xOxidation [	0.0001626	7.389E-05	1	1	2	P18669	P18669 [223-240]	0	3043.743

[R].NLIAFSE	1xTMTpro [N	0.0001182	7.389E-05	1	1	2	A0FGR8	A0FGR8 [812-826	0	1987.0092
[K].NLIDAG\	1xTMTpro [N	0.0001766	7.389E-05	1	1	2	P12268	P12268 [312-322]	0	1460.8392
[K].NLLFNDI	1xCarbamido	7.037E-05	7.389E-05	1	1	2	P02788	P02788 [659-671]	0	1883.9605
[K].NILFVITK	2xTMTpro [K	0.000123	7.389E-05	1	3	4	Q13765	Q13765 [101-113]	0	2462.5203
[R].NIIGLQM	1xTMTpro [K	7.048E-05	7.389E-05	1	1	2	P51911	P51911 [162-172]	0	1797.0548
[K].NLIGTYM	2xCarbamido	6.769E-05	7.389E-05	1	1	1	P35555	P35555 [2267-228	0	2223.068
[K].NLLHQD.	1xTMTpro [N	0.0001144	7.389E-05	1	1	1	P57105	P57105 [75-86]	0	1744.9665
[R].NILMYGI	1xTMTpro [K	0.0001899	7.389E-05	1	1	1	Q9NVI7	Q9NVI7 [395-406]	0	1856.0596
[K].NILQLHD	1xTMTpro [K	0.0001098	7.389E-05	1	1	2	P48147	P48147 [359-373]	0	2258.3728
[R].NLLQVD	1xTMTpro [K	0.0001445	7.389E-05	2	2	1	P17612; P22	P17612 [272-281]	1	1808.1249
[K].NLISPEL	1xTMTpro [K	8.732E-05	7.389E-05	1	1	1	P78559	P78559 [126-142]	0	2510.4514
[R].NLITDIC	2xCarbamido	0.0001479	7.389E-05	1	1	1	Q9Y2A7	Q9Y2A7 [612-631	0	2970.5772
[K].NILTSNN	1xTMTpro [K	9.306E-05	7.389E-05	1	1	2	P05090	P05090 [165-175]	0	1839.0831
[K].NLKPIKP	3xTMTpro [K	9.467E-05	7.389E-05	1	1	2	P18669	P18669 [223-241]	1	3460.0502
[R].GPEASV	1xCarbamido	6.149E-05	7.389E-05	1	1	2	Q02388	Q02388 [1037-10	0	1802.939
[R].LGLPGD	1xTMTpro [K	0.0001232	7.389E-05	1	1	3	P78527	P78527 [2705-27	0	1764.9987
[K].DSYVGD	1xTMTpro [K	7.015E-05	7.389E-05	3	6	14	P62736; P68	P62736 [53-63]; F	0	1806.9365
[R].ALWADS	1xTMTpro [N	8.298E-05	7.389E-05	1	1	2	Q14344	Q14344 [151-164]	0	1883.9571
[R].ALWEET	1xTMTpro [N	0.0001121	7.389E-05	1	1	2	O15067	O15067 [1009-10	0	1798.9295
[R].DPIDVDI	1xTMTpro [N	0.0001018	7.389E-05	1	1	3	P29590	P29590 [412-424]	0	1801.9139
[R].DPHLAC	1xCarbamido	0.0001188	7.389E-05	1	2	2	Q00610	Q00610 [913-923]	0	1634.828
[R].DPGVITY	1xTMTpro [K	6.896E-05	7.389E-05	1	1	4	P26640	P26640 [274-289]	0	2307.2728
[R].DPETLV(	1xCarbamido	8.401E-05	7.389E-05	1	1	1	P49327	P49327 [123-137]	0	2015.985
[K].DPEIQE/	1xTMTpro [K	7.303E-05	7.389E-05	1	1	1	Q02318	Q02318 [354-378]	0	3313.8036
[K].DPDPWT	1xTMTpro [N	0.000142	7.389E-05	1	1	2	Q8IVL6	Q8IVL6 [403-418]	0	2066.1242
[R].ALYDFE/	1xTMTpro [K	0.0001077	7.389E-05	1	1	1	O75886	O75886 [209-224]	0	2512.3103
[K].GEVDEE	1xTMTpro [N	6.961E-05	7.389E-05	1	1	1	Q9NPQ8	Q9NPQ8 [238-24	0	1670.8193
[K].AIYDIERI	1xTMTpro [K	0.0001021	7.389E-05	1	1	3	O14639	O14639 [394-417]	0	3492.7706
[R].DPIVMG	1xTMTpro [K	0.0001008	7.389E-05	1	1	1	O60841	O60841 [1106-11	0	2151.2339
[K].DPDDHT	1xCarbamido	7.625E-05	7.389E-05	1	1	1	P00387	P00387 [197-214]	0	2748.3907
[R].FDDAVV	1xTMTpro [K	6.737E-05	7.389E-05	1	1	4	P11142	P11142 [78-88]	0	1862.9814
[K].DPCHGV	3xCarbamido	9.557E-05	7.389E-05	1	1	1	Q9Y6R7	Q9Y6R7 [1643-16	0	2453.1979
[K].ALYESEI	1xTMTpro [N	0.000101	7.389E-05	1	1	2	Q03252	Q03252 [94-104]	0	1541.8131

[R].DPASDQ	1xTMTpro [K	6.407E-05	7.389E-05	1	1	4	P04040	P04040 [6-16]	0	1950.9988
[R].DPAGMF	1xTMTpro [K	0.0001593	7.389E-05	1	1	1	O95340	O95340 [513-523]	0	1787.9606
[R].DPAGMF	1xTMTpro [K	8.059E-05	7.389E-05	1	1	1	O43252	O43252 [523-534]	0	1844.982
[R].DPAAAP	1xTMTpro [K	7.601E-05	7.389E-05	1	1	2	Q02218	Q02218 [989-999]	0	1620.9201
[K].GETVND	1xCarbamido	0.0001085	7.389E-05	1	1	1	P55265	P55265 [903-916]	0	1904.9455
[K].ALYETEI	1xTMTpro [N	0.0001136	7.389E-05	1	1	2	P20700	P20700 [80-90]	0	1555.8287
[K].DNWVLS	1xTMTpro [N	0.0001498	7.389E-05	1	1	1	Q8WXI4	Q8WXI4 [402-414	0	1836.9775
[R].DNWTDA	1xTMTpro [N	0.0001756	7.389E-05	1	1	1	Q13523	Q13523 [666-676]	0	1693.7777
[R].AIYDTPC	1xCarbamido	7.997E-05	7.389E-05	1	1	2	O00232	O00232 [249-262]	0	2233.1666
[K].DNWSPA	1xTMTpro [K	0.0001579	7.389E-05	2	3	2	Q96LR5; P5	Q96LR5 [145-155	0	1840.046
[R].DPIYFTG	1xTMTpro [N	0.0001948	7.389E-05	1	1	1	Q99523	Q99523 [565-579]	0	1897.9979
[K].DPNNSL	1xTMTpro [K	0.0001165	7.389E-05	1	1	1	Q9H3U1	Q9H3U1 [330-345	0	2421.3633
[R].ALVDHH	1xTMTpro [K	0.0001315	7.389E-05	1	1	1	Q8N2F6	Q8N2F6 [323-333	0	1842.0365
[R].DQAVMI	1xOxidation	6.307E-05	7.389E-05	1	1	1	O00159	O00159 [133-146]	0	1974.0458
[R].GFAFVE	1xTMTpro [N	0.0001157	7.389E-05	1	1	1	P98175	P98175 [172-186]	0	2029.0462
[R].AIVFVVD	1xTMTpro [N	0.0001245	7.389E-05	1	1	2	Q9Y5M8	Q9Y5M8 [138-150	0	1726.9811
[K].DQAAEG	1xTMTpro [K	7.337E-05	7.389E-05	1	1	2	P82650	P82650 [319-329]	0	1780.046
[R].DPYTEQ	1xOxidation	0.0001564	7.389E-05	1	1	1	Q15149	Q15149 [3910-39	0	2396.2663
[R].DPYSGS	1xTMTpro [K	8.799E-05	7.389E-05	1	1	2	Q15149	Q15149 [3575-35	0	2381.2667
[K].ALVLIGN	1xTMTpro [K	0.0001859	7.389E-05	1	1	3	Q9ULC5	Q9ULC5 [191-200	0	1664.0602
[R].ALVMET	1xTMTpro [N	0.0001719	7.389E-05	1	1	2	Q96GQ5	Q96GQ5 [281-29	0	1536.8375
[K].AIVNVIG	1xTMTpro [K	0.000182	7.389E-05	1	1	1	O75533	O75533 [999-100	0	1690.033
[R].GEYNVY	1xTMTpro [K	0.0001026	7.389E-05	2	3	2	Q66LE6; P6	Q66LE6 [75-94];	0	3061.5075
[R].AIWAAL	1xTMTpro [K	6.79E-05	7.389E-05	1	1	1	Q9NYU2	Q9NYU2 [813-82	0	2182.2476
[K].FCHLPIP	1xCarbamido	0.0001331	7.389E-05	1	1	3	Q14767	Q14767 [426-441]	1	2220.1667
[K].DPTFIPA	1xTMTpro [K	7.672E-05	7.389E-05	1	1	3	P01019	P01019 [64-75]	0	1906.1294
[R].AIVPILLD	1xTMTpro [K	7.985E-05	7.389E-05	1	1	1	P61764	P61764 [389-404]	0	2340.367
[R].DPTEVT	1xTMTpro [K	6.407E-05	7.389E-05	1	1	1	P30613	P30613 [450-465]	0	2227.2466
[R].GEWTCI	1xCarbamido	0.0001134	7.389E-05	1	1	2	P02751	P02751 [504-515]	0	1787.907
[K].ALVQND	1xTMTpro [K	7.497E-05	7.389E-05	1	1	3	Q92522	Q92522 [95-106]	0	1950.1879
[K].GEWTCK	1xCarbamido	7.213E-05	7.389E-05	1	1	3	P02751	P02751 [175-185]	0	2231.2674
[K].DPPANLI	2xTMTpro [K	6.972E-05	7.389E-05	1	1	2	Q96AY3	Q96AY3 [500-527	1	4107.1089
[K].ALVTGYI	1xOxidation	0.0001087	7.389E-05	1	1	1	O43143	O43143 [692-706]	0	2055.1017

[K].DPPANLI	1xTMTpro [K	6.277E-05	7.389E-05	1	1	1	Q96AY3	Q96AY3 [500-513	0	2227.156
[K].ALVTYDI	1xTMTpro [N	6.56E-05	7.389E-05	1	1	1	Q13315	Q13315 [2045-20	0	2041.1137
[K].GEVVDA	1xTMTpro [K	8.059E-05	7.389E-05	1	1	1	P62487	P62487 [82-94]	0	1966.1465
[K].DPVSAA	1xTMTpro [N	0.0001644	7.389E-05	1	1	1	Q68EM7	Q68EM7 [581-592	0	1440.813
[K].ALYLQY	1xTMTpro [N	9.364E-05	7.389E-05	1	1	2	P00450	P00450 [70-81]	0	1823.9499
[R].GETLGII	1xTMTpro [N	0.0001184	7.389E-05	1	1	2	Q13363	Q13363 [174-184	0	1389.8385
[R].FDDPGL	1xOxidation	0.0001033	7.389E-05	1	1	1	P12956	P12956 [340-357]	0	2962.733
[K].GEQGQY	1xCarbamido	0.0001877	7.389E-05	1	1	1	P54578	P54578 [190-209]	0	2805.2714
[K].DNLIVPII	1xTMTpro [N	0.0002047	7.389E-05	1	1	1	Q9Y3A3	Q9Y3A3 [204-225	0	2675.3583
[K].AMEGAG	1xTMTpro [K	0.00015	7.389E-05	1	1	2	P08133	P08133 [447-456]	0	1616.8445
[K].AMEGIFN	2xTMTpro [K	0.0001573	7.389E-05	1	1	1	Q99470	Q99470 [190-203	0	2506.4593
[R].AMEGQH	1xCarbamido	8.691E-05	7.389E-05	1	1	1	P06401	P06401 [594-606]	0	1826.8597
[R].FDGAEG	1xTMTpro [K	0.0001806	7.389E-05	1	1	2	Q92888	Q92888 [500-510	0	1879.9834
[K].FDGGEE	1xTMTpro [N	0.0001245	7.389E-05	1	1	1	Q16698	Q16698 [299-315	0	2201.1045
[K].FDGILGN	1xTMTpro [N	0.0001707	7.389E-05	1	1	2	P07339	P07339 [195-205]	0	1543.8262
[R].DNLEFFI	1xTMTpro [N	0.0001638	7.389E-05	1	1	1	P49327	P49327 [791-802]	0	1655.9076
[R].AMGEQA	1xTMTpro [N	9.925E-05	7.389E-05	1	1	1	P05165	P05165 [315-325]	0	1420.7902
[R].DNLAEE	1xTMTpro [N	0.0001258	7.389E-05	1	1	2	P48681	P48681 [82-94]	0	1676.8774
[R].AMEELQ	1xTMTpro [K	8.272E-05	7.389E-05	1	1	1	Q9NZM1	Q9NZM1 [1041-1(	0	2991.5054
[R].FDHIFYT	1xTMTpro [K	8.854E-05	7.389E-05	1	1	1	P51648	P51648 [178-191]	0	2178.1839
[K].DNFTLIP	1xTMTpro [N	0.0001707	7.389E-05	1	1	1	Q16555	Q16555 [346-361	0	2097.0419
[K].DNFQDT	1xTMTpro [N	6.168E-05	7.389E-05	1	1	1	P42892	P42892 [218-232]	0	2111.0841
[K].AMGNLQ	1xOxidation	9.863E-05	7.389E-05	1	1	4	P04899	P04899 [87-100]	0	1854.9339
[K].AMGNLQ	1xTMTpro [N	0.0001004	7.389E-05	1	1	1	P04899	P04899 [87-105]	1	2367.1682
[R].GENVTV	1xTMTpro [N	0.0001948	7.389E-05	1	1	1	Q96A65	Q96A65 [326-336	0	1546.8145
[K].AMGYQF	1xTMTpro [N	7.948E-05	7.389E-05	1	1	1	Q15738	Q15738 [346-361	0	2132.0146
[R].GENVST	1xTMTpro [N	8.453E-05	7.389E-05	1	1	1	Q6PCB7	Q6PCB7 [516-530	0	1880.9885
[K].DNFNVD	1xTMTpro [K	0.0001345	7.389E-05	1	1	1	Q6IQ22	Q6IQ22 [189-199]	0	1962.0828
[R].FDLGLEI	1xTMTpro [K	0.0001638	7.389E-05	1	1	1	O60476	O60476 [66-82]	0	2446.3837
[R].AMLDQL	1xTMTpro [N	0.0001579	7.389E-05	1	1	2	Q9Y383	Q9Y383 [9-19]	0	1526.799
[R].GENLVH	1xTMTpro [N	0.0001288	7.389E-05	1	1	1	Q86VB7	Q86VB7 [1087-10	0	1660.909
[K].AMGLAE	1xTMTpro [N	0.0001838	7.389E-05	1	1	1	P48382	P48382 [248-260]	0	1729.8499
[K].AMEAVA	1xTMTpro [K	8.759E-05	7.389E-05	1	2	2	P18669	P18669 [242-251]	0	1583.9071

[K].DNINIEE.	1xTMTpro [N	0.0001759	7.389E-05	1	1	1	Q13637	Q13637 [177-186]	0	1448.7664
[K].DNIQAM'	1xTMTpro [N	9.863E-05	7.389E-05	1	1	2	P26196	P26196 [163-174]	0	1660.9375
[R].ALYYDLI	1xTMTpro [K	0.000171	7.389E-05	1	1	1	P36955	P36955 [107-123]	0	2564.3892
[K].DNTRPG	1xTMTpro [K	8.096E-05	7.389E-05	1	1	1	Q92499	Q92499 [473-490]	0	2611.343
[R].DNTNEI	1xTMTpro [K	0.0001749	7.389E-05	1	1	2	P05556	P05556 [542-551]	0	1748.9311
[K].DNTEPG	1xTMTpro [K	0.0001608	7.389E-05	1	1	1	Q9UPU7	Q9UPU7 [685-698	0	2236.2218
[R].FDEILEA	1xOxidation [	0.0001354	7.389E-05	1	1	2	P14618	P14618 [280-294]	0	1986.0173
[R].DNSTMG	1xTMTpro [K	8.375E-05	7.389E-05	1	2	2	P08238	P08238 [613-623]	0	1856.92
[R].GETESE	1xTMTpro [K	0.0001161	7.389E-05	1	1	3	Q05682	Q05682 [544-553]	0	1792.9096
[R].FDEISFV	1xTMTpro [N	0.000114	7.389E-05	1	1	2	Q08945	Q08945 [371-381]	0	1648.8654
[R].DNSLFVI	1xTMTpro [N	0.0001944	7.389E-05	1	1	2	Q5SRE7	Q5SRE7 [223-234	0	1676.9291
[R].DNSKNT	1xTMTpro [K	0.0001402	7.389E-05	1	6	1	P01768	P01768 [92-106]	1	2405.3103
[R].FDENSQ	1xTMTpro [N	0.0001605	7.389E-05	1	1	1	P09238	P09238 [410-423]	0	1975.9139
[K].AMAVED	1xTMTpro [N	0.000196	7.389E-05	1	1	1	P07357	P07357 [413-422]	0	1408.7789
[K].DNQNFC	2xCarbamido	0.0001453	7.389E-05	1	1	3	Q14192	Q14192 [145-156]	0	2182.0553
[R].DNQLSE	1xTMTpro [K	0.0001492	7.389E-05	1	1	1	Q14978	Q14978 [24-33]	0	1725.9627
[K].DNPLDP	1xTMTpro [K	0.0001448	7.389E-05	1	1	2	Q96AG4	Q96AG4 [117-126	0	1690.0031
[K].DNPGVV	1xCarbamido	9.049E-05	7.389E-05	1	1	2	P22314	P22314 [227-239]	0	1749.8761
[R].DNLTLW	1xTMTpro [N	7.985E-05	7.389E-05	1	1	5	P31946	P31946 [225-246]	0	2655.1613
[K].GESPVD	1xTMTpro [N	0.0001075	7.389E-05	1	1	3	Q15084	Q15084 [246-256]	0	1455.7035
[R].DNLTLW	1xTMTpro [N	0.0001665	7.389E-05	1	1	2	Q04917	Q04917 [228-246]	0	2426.0915
[R].DNLTLW	1xTMTpro [N	0.0001971	7.389E-05	1	1	4	P61981	P61981 [228-247]	0	2498.0875
[K].AMDSDV	1xOxidation [	0.0001899	7.389E-05	1	1	4	P55084	P55084 [392-405]	0	2012.8802
[R].DNLTLW	1xOxidation [	7.755E-05	7.389E-05	1	1	3	P62258	P62258 [226-244]	0	2805.3493
[R].FDFLCQ	1xCarbamido	0.0001373	7.389E-05	1	1	1	Q9NTK5	Q9NTK5 [71-83]	0	2553.4104
[R].DNLTLW	1xTMTpro [N	9.816E-05	7.389E-05	1	1	1	P31947	P31947 [225-248]	0	2833.3083
[K].DNLQLPI	1xTMTpro [N	0.0002039	7.389E-05	1	1	1	O15118	O15118 [85-96]	0	1748.0026
[R].DQCQVD	2xCarbamido	8.375E-05	7.389E-05	1	1	2	Q14508	Q14508 [95-108]	0	2305.0867
[R].DQDELN	1xTMTpro [N	0.0001237	7.389E-05	1	1	1	O43615	O43615 [428-439]	0	1781.8778
[R].DQDKDQ	1xCarbamido	0.000165	7.389E-05	1	1	1	O43795	O43795 [97-114]	1	2847.5338
[R].FCDDILS	1xCarbamido	0.0001831	7.389E-05	1	1	1	Q5HYI7	Q5HYI7 [232-242]	0	1726.843
[K].AIQESND	1xTMTpro [K	7.613E-05	7.389E-05	1	1	2	Q8TCD1	Q8TCD1 [40-49]	0	1727.942
[K].DRYDSF	1xTMTpro [K	0.0001018	7.389E-05	1	1	2	Q9NSD9	Q9NSD9 [134-145	1	2151.1578

[R].FAEFEDI	1xTMTpro [N	9.785E-05	7.389E-05	1	1	1	Q9Y4P1	Q9Y4P1 [13-31]	0	2573.2883
[R].FAEFLLF	1xTMTpro [K	7.543E-05	7.389E-05	1	1	2	Q96T76	Q96T76 [255-266]	0	2041.2593
[truncated: 563,645 more chars]
